# Supplementary material for: Completing the BASEL phage collection to unlock hidden diversity for systematic exploration of phage–host interactions
Source: PLoS Biol. 2025 Apr 7;23(4):e3003063. doi: 10.1371/journal.pbio.3003063 (PMC11990801; doi:10.1371/journal.pbio.3003063)
Supplement: S4 Table — (DOCX) [file pbio.3003063.s004.docx]

# S4 Table. List of all oligonucleotide primers used in this study

| ***Primer name*** | ***Sequence (5'-3')*** |
| --- | --- |
| prAH2151 | CATTCATCCGCTTATTATCACTTA |
| prAH2152 | GTAATGACCTCAGAACTCCATCT |
| prAS0001 | ATAAGTGATAATAAGCGGATGAATGTAAGGAGGAACAATATGGTATATATAATAATCGTTTCCCAC |
| prAS0002 | CCAGATGGAGTTCTGAGGTCATTACGCTTTATATTACGGGTGAAAAACT |
| prDP0079 | TTGAAACCAAAAAACGCCCGAAATACATCATCAAGAGAGTCAAAAAATGACGTAATTTTTTTAAGGCAGTTATTG |
| prDP0080 | TAGGTTGGTATTATAGCTTGTGCGCGCCATGATTGGCGCGCAATTTAAACCTTACTGTCCCTAGTGCTTGG |
| prDP0081 | AAAACGCCCGAAATACATCATCAAGAGAGTCAAAAAATGAGTTTAAATTGCGCGCCAATCATGGCGCGCACAAGCTATAA |
| prDP0082 | TTATAGCTTGTGCGCGCCATGATTGGCGCGCAATTTAAACTCATTTTTTGACTCTCTTGATGATGTATTTCGGGCGTTTT |
| prDP0083 | TTAGTGGTAGCCAGTGTAGC |
| prDP0084 | CATTTGATAGTCAATACCGC |
