# Supplementary material for: Completing the BASEL phage collection to unlock hidden diversity for systematic exploration of phage–host interactions
Source: PLoS Biol. 2025 Apr 7;23(4):e3003063. doi: 10.1371/journal.pbio.3003063 (PMC11990801; doi:10.1371/journal.pbio.3003063)
Supplement: S2 Data — (ZIP) [file pbio.3003063.s009.zip › entries/1.html]

FANPEZAQ\_CDS\_0001


Return to summary | Go to next

|  |  |
| --- | --- |
| FANPEZAQ\_CDS\_0001 Page creation date: 02 Sep 2024, 12:00  Project folder: n/a  Input sequences file: Escherichia\_virus\_HeidiAbel.gb | terminase small packaging dna nu1 phage hypothetical domain\_containing fragment helix\_turn\_helix duf1441 hth merr\_type dna\_packaging elements external origin putative dna\_binding transcriptional regulator |

### Sequence information

|  |  |
| --- | --- |
| Name | FANPEZAQ\_CDS\_0001  01\_FANPEZAQ\_CDS\_0001 (pipeline id) |
| Imported annotations | Escherichia\_virus\_HeidiAbel Bas97 |
| Protein sequence | MWRKDGCPVVTMGGRGKEWEFDTAEVANWLRDRDVRAATGDKVTDQEELKRRKLAAETEK AELELAKAKGEVAPLDQVERAVARAFAEVRAGMRNLPQRTVSMLIGETDERRFKSVMMGE IDEVLKSLVTASLLDGYDEGGDGDSEE |
| Number of residues | 147 |
| Molecular weight (Da) | 16389.26 |
| Output files | ../../query\_sequences/01\_FANPEZAQ\_CDS\_0001.fasta |

### Putative domain architecture and protein family

#### Search results (HHblits)1

|  |  |
| --- | --- |
| Domain family databases searched | Pfam, Ncbi-cd, Cath, Phrogs |
| Results, scheme(s)  (Top layers only; threshold 1.00e-03 (evalue)) | xml version="1.0" encoding="utf-8" standalone="no"?       2024-09-02T21:08:11.598965 image/svg+xml   Matplotlib v3.7.2, https://matplotlib.org/ |
| Results, table  (E-value ≤ 1.00e-03 (evalue)) | | db | id | prob | evalue | pvalue | score | cols | query | query\_len | template | template\_len | name | description | | --- | --- | --- | --- | --- | --- | --- | --- | --- | --- | --- | --- | --- | | pfam | PF07278 | 99.2 | 5.9e-16 | 1e-19 | 94.3 | 113 | (16, 133) | 147 | (33, 145) | 147 | DUF1441 | Protein of unknown function (DUF1441) | | pfam | PF07471 | 99.0 | 1.8e-14 | 3e-18 | 88.3 | 124 | (2, 127) | 147 | (22, 161) | 162 | Phage\_Nu1 | Phage DNA packaging protein Nu1 | | phrogs | 141 | 99.8 | 2.1e-24 | 2.5e-28 | 150.6 | 121 | (17, 145) | 147 | (41, 161) | 163 | terminase small subunit | terminase small subunit; Category: head and packaging; p69558 VI\_02299 | | phrogs | 57 | 99.7 | 9.1e-23 | 1.2e-26 | 143.9 | 128 | (2, 136) | 147 | (24, 154) | 173 | terminase small subunit | terminase small subunit; Category: head and packaging; p277333 VI\_02593 | | phrogs | 21782 | 99.6 | 1.2e-20 | 1.3e-24 | 121.2 | 126 | (17, 145) | 147 | (7, 132) | 136 | NA | NA; Category: unknown function; p302384 VI\_08027 | | phrogs | 1790 | 98.6 | 7.5e-12 | 8.4e-16 | 77.4 | 86 | (44, 131) | 147 | (12, 97) | 114 | terminase small subunit | terminase small subunit; Category: head and packaging; p410932 VI\_07810 | | phrogs | 5710 | 97.2 | 4.1e-07 | 4.7e-11 | 55.2 | 62 | (16, 79) | 147 | (41, 102) | 104 | NA | NA; Category: unknown function; p435585 VI\_08997 | | phrogs | 6039 | 97.1 | 5.1e-07 | 5.7e-11 | 48.2 | 54 | (91, 147) | 147 | (2, 55) | 55 | NA | NA; Category: unknown function; p386363 VI\_02030 | |
| Top keywords  (threshold 1.00e-03 (evalue)) | **packaging, terminase, small, head, and, DUF1441, Phage, DNA, Nu1, p69558** |
| Output files | ../../domain\_architecture/01\_FANPEZAQ\_CDS\_0001\_cath.hhr ../../domain\_architecture/01\_FANPEZAQ\_CDS\_0001\_merged.svg ../../domain\_architecture/01\_FANPEZAQ\_CDS\_0001\_ncbi-cd.hhr ../../domain\_architecture/01\_FANPEZAQ\_CDS\_0001\_pfam.hhr ../../domain\_architecture/01\_FANPEZAQ\_CDS\_0001\_phrogs.hhr |

### Identical protein sequences/structures

#### Search results

|  |  |
| --- | --- |
| Protein sequence databases searched | Pdb, Swissprot, Refseq |
| Identical proteins found | -- |
| Top keywords | -- |
| Output files | -- |

### Similar protein sequences/structures

#### Sequence similarity search results (HHblits)1

|  |  |
| --- | --- |
| Sequence databases searched | Uniclust, Pdb70 |
| Results, scheme(s)  (Top layers only, threshold 1.00e-03 (evalue)) | xml version="1.0" encoding="utf-8" standalone="no"?       2024-09-02T21:08:27.846833 image/svg+xml   Matplotlib v3.7.2, https://matplotlib.org/ |
| Results, table(s)  (threshold 1.00e-03 (evalue)) | | db | id | prob | evalue | pvalue | score | cols | query | query\_len | template | template\_len | name | description | | --- | --- | --- | --- | --- | --- | --- | --- | --- | --- | --- | --- | --- | | uniclust | UniRef100\_A0A017HCB4 | 99.9 | 1.7e-28 | 3.6e-34 | 182.6 | 142 | (1, 142) | 147 | (57, 198) | 231 | Phage DNA packaging Nu1 | Phage DNA packaging Nu1 | | uniclust | UniRef100\_A0A073IUP9 | 99.9 | 4.4e-25 | 9.4e-31 | 164.7 | 130 | (3, 137) | 147 | (59, 189) | 229 | Terminase small subunit | Terminase small subunit | | uniclust | UniRef100\_A0A103EHP4 | 99.8 | 2.6e-22 | 5e-28 | 138.7 | 142 | (1, 142) | 147 | (25, 166) | 175 | Terminase small subunit | Terminase small subunit | | uniclust | UniRef100\_A0A3B8QTC1 | 99.8 | 3.2e-22 | 6.2e-28 | 140.3 | 136 | (1, 139) | 147 | (27, 164) | 180 | DNA packaging protein | DNA packaging protein | | uniclust | UniRef100\_A0A192IJE2 | 99.8 | 5.8e-22 | 1.2e-27 | 144.2 | 125 | (2, 130) | 147 | (34, 161) | 189 | Terminase small subunit | Terminase small subunit | | uniclust | UniRef100\_A0A137SPY3 | 99.8 | 7.7e-22 | 1.5e-27 | 143.2 | 134 | (3, 141) | 147 | (35, 169) | 216 | Phage DNA packaging protein Nu1 | Phage DNA packaging protein Nu1 | | uniclust | UniRef100\_A0A0C9PQA6 | 99.8 | 1.2e-21 | 2.3e-27 | 139.5 | 130 | (1, 134) | 147 | (49, 178) | 188 | Uncharacterized protein | Uncharacterized protein | | uniclust | UniRef100\_A0A031G4Z9 | 99.7 | 3.1e-21 | 6.3e-27 | 139.8 | 127 | (1, 133) | 147 | (44, 172) | 208 | Phage DNA packaging protein Nu1 | Phage DNA packaging protein Nu1 | | uniclust | UniRef100\_A0A081RFK1 | 99.7 | 6.2e-21 | 1.3e-26 | 140.8 | 133 | (2, 141) | 147 | (61, 197) | 220 | Phage DNA packaging protein Nu1 | Phage DNA packaging protein Nu1 | | uniclust | UniRef100\_A0A0B1Q7B2 | 99.7 | 6.3e-21 | 1.3e-26 | 139.1 | 123 | (2, 130) | 147 | (37, 164) | 196 | Uncharacterized protein | Uncharacterized protein | | uniclust | UniRef100\_A0A061NPR6 | 99.7 | 1.2e-20 | 2.4e-26 | 140.5 | 134 | (2, 140) | 147 | (58, 197) | 229 | Uncharacterized protein | Uncharacterized protein | | uniclust | UniRef100\_A0A0R3MP30 | 99.7 | 1.2e-20 | 2.5e-26 | 138.4 | 129 | (1, 129) | 147 | (50, 180) | 212 | DNA packaging protein | DNA packaging protein | | uniclust | UniRef100\_A0A062V3Q6 | 99.7 | 2.3e-20 | 4.7e-26 | 131.5 | 124 | (2, 132) | 147 | (44, 169) | 172 | Phage DNA packaging protein Nu1 | Phage DNA packaging protein Nu1 | | uniclust | UniRef100\_A0A0A8K3F1 | 99.7 | 4.5e-20 | 9.4e-26 | 135.6 | 124 | (2, 132) | 147 | (54, 181) | 205 | Phage DNA packaging protein, Nu1 subunit of terminase | Phage DNA packaging protein, Nu1 subunit of terminase | | uniclust | UniRef100\_A0A1X7L0X5 | 99.7 | 5.4e-20 | 1e-25 | 127.1 | 131 | (1, 137) | 147 | (32, 165) | 168 | Phage DNA packaging protein, Nu1 subunit of terminase | Phage DNA packaging protein, Nu1 subunit of terminase | | uniclust | UniRef100\_A0A2D8RC70 | 99.7 | 6.6e-20 | 1.3e-25 | 129.5 | 141 | (2, 142) | 147 | (23, 165) | 174 | Terminase small subunit | Terminase small subunit | | uniclust | UniRef100\_A0A1M6SLK9 | 99.7 | 7.8e-20 | 1.6e-25 | 133.0 | 135 | (2, 142) | 147 | (34, 175) | 205 | Phage DNA packaging protein, Nu1 subunit of terminase | Phage DNA packaging protein, Nu1 subunit of terminase | | uniclust | UniRef100\_A0A069I596 | 99.7 | 2e-19 | 4e-25 | 128.0 | 130 | (1, 136) | 147 | (25, 155) | 178 | DNA packaging protein | DNA packaging protein | | uniclust | UniRef100\_A0A126NZA6 | 99.7 | 2.2e-19 | 4.3e-25 | 123.6 | 132 | (1, 133) | 147 | (28, 159) | 162 | Terminase small subunit | Terminase small subunit | | uniclust | UniRef100\_A0A1Q3WRG7 | 99.6 | 3.3e-19 | 6.6e-25 | 126.8 | 132 | (1, 139) | 147 | (35, 166) | 184 | Terminase small subunit | Terminase small subunit | | uniclust | UniRef100\_A0A1C3WPE9 | 99.6 | 4e-19 | 8e-25 | 129.9 | 126 | (3, 134) | 147 | (33, 160) | 215 | Phage DNA packaging protein, Nu1 subunit of terminase | Phage DNA packaging protein, Nu1 subunit of terminase | | uniclust | UniRef100\_A0A078MJZ8 | 99.6 | 4.7e-19 | 9.4e-25 | 130.7 | 130 | (3, 137) | 147 | (38, 169) | 224 | Phage DNA packaging protein Nu1 | Phage DNA packaging protein Nu1 | | uniclust | UniRef100\_A0A1H8QIJ8 | 99.6 | 5.5e-19 | 1.1e-24 | 125.0 | 130 | (2, 138) | 147 | (32, 163) | 187 | Uncharacterized protein | Uncharacterized protein | | uniclust | UniRef100\_A0A8J7MTI5 | 99.6 | 8.8e-19 | 1.7e-24 | 119.4 | 132 | (1, 133) | 147 | (22, 153) | 159 | Terminase small subunit | Terminase small subunit | | uniclust | UniRef100\_A0A2D5VNR8 | 99.6 | 2.6e-18 | 5e-24 | 119.6 | 133 | (2, 134) | 147 | (22, 160) | 169 | Terminase small subunit | Terminase small subunit | | uniclust | UniRef100\_A0A088C3V1 | 99.6 | 3.1e-18 | 6e-24 | 122.2 | 136 | (1, 139) | 147 | (44, 182) | 202 | Terminase small subunit | Terminase small subunit | | uniclust | UniRef100\_A0A0D0QHJ8 | 99.6 | 6.7e-18 | 1.4e-23 | 123.0 | 126 | (2, 135) | 147 | (49, 176) | 196 | Phage DNA packaging protein, Nu1 subunit of terminase | Phage DNA packaging protein, Nu1 subunit of terminase | | uniclust | UniRef100\_A0A1H5XMK5 | 99.6 | 9.8e-18 | 2e-23 | 122.1 | 128 | (2, 131) | 147 | (40, 170) | 201 | Phage DNA packaging protein Nu1 | Phage DNA packaging protein Nu1 | | uniclust | UniRef100\_UPI00174BBF1A | 99.5 | 1.4e-17 | 2.6e-23 | 117.6 | 135 | (1, 135) | 147 | (28, 165) | 200 | terminase small subunit | terminase small subunit | | uniclust | UniRef100\_A0A240TSP7 | 99.5 | 1.4e-17 | 2.8e-23 | 118.8 | 127 | (3, 135) | 147 | (51, 178) | 202 | Terminase small subunit, Nu1 | Terminase small subunit, Nu1 | | uniclust | UniRef100\_A0A1Q6PWQ4 | 99.5 | 1.6e-17 | 3.2e-23 | 117.1 | 130 | (7, 142) | 147 | (35, 164) | 176 | Terminase small subunit | Terminase small subunit | | uniclust | UniRef100\_A0A081NI16 | 99.5 | 1.5e-17 | 3.2e-23 | 124.8 | 131 | (3, 138) | 147 | (42, 195) | 216 | Terminase small subunit | Terminase small subunit | | uniclust | UniRef100\_A0A0Q4E400 | 99.5 | 6.7e-17 | 1.3e-22 | 115.4 | 128 | (2, 135) | 147 | (37, 168) | 175 | HTH merR-type domain-containing protein | HTH merR-type domain-containing protein | | uniclust | UniRef100\_A0A1G1HG69 | 99.5 | 9.5e-17 | 1.7e-22 | 110.4 | 132 | (1, 134) | 147 | (19, 150) | 185 | Uncharacterized protein | Uncharacterized protein | | uniclust | UniRef100\_A0A087M4C5 | 99.5 | 9.1e-17 | 1.9e-22 | 118.0 | 126 | (1, 133) | 147 | (42, 171) | 204 | DNA packaging protein | DNA packaging protein | | uniclust | UniRef100\_A0A165IZT2 | 99.5 | 9.8e-17 | 2e-22 | 119.7 | 131 | (1, 137) | 147 | (37, 192) | 219 | Terminase small subunit | Terminase small subunit | | uniclust | UniRef100\_A0A248UJZ5 | 99.5 | 1.4e-16 | 2.6e-22 | 119.8 | 129 | (2, 137) | 147 | (148, 279) | 309 | Homeo-like domain protein | Homeo-like domain protein | | uniclust | UniRef100\_A0A1U7MFY0 | 99.5 | 1.4e-16 | 2.9e-22 | 115.6 | 133 | (1, 141) | 147 | (42, 176) | 188 | Phage DNA packaging protein Nu1 | Phage DNA packaging protein Nu1 | | uniclust | UniRef100\_A0A7C6RL71 | 99.5 | 2.1e-16 | 4.1e-22 | 112.2 | 138 | (1, 138) | 147 | (23, 172) | 181 | Terminase small subunit | Terminase small subunit | | uniclust | UniRef100\_A0A7V5XCS4 | 99.5 | 2.2e-16 | 4.2e-22 | 112.6 | 124 | (1, 128) | 147 | (48, 171) | 202 | Terminase small subunit | Terminase small subunit | | uniclust | UniRef100\_A0A068SKV9 | 99.5 | 2.1e-16 | 4.2e-22 | 118.3 | 131 | (1, 132) | 147 | (94, 231) | 251 | Phage DNA packaging Nu1 | Phage DNA packaging Nu1 | | uniclust | UniRef100\_A0A0K1NDZ1 | 99.5 | 2.2e-16 | 4.5e-22 | 116.9 | 125 | (2, 131) | 147 | (38, 184) | 214 | Uncharacterized protein | Uncharacterized protein | | uniclust | UniRef100\_A0A1G1JJM4 | 99.4 | 2.9e-16 | 5.8e-22 | 116.2 | 125 | (1, 134) | 147 | (77, 201) | 230 | HTH merR-type domain-containing protein | HTH merR-type domain-containing protein | | uniclust | UniRef100\_A0A6L4BEY6 | 99.4 | 3.4e-16 | 6.3e-22 | 108.5 | 134 | (1, 136) | 147 | (44, 177) | 193 | Terminase small subunit | Terminase small subunit | | uniclust | UniRef100\_A0A127CGJ9 | 99.4 | 3.1e-16 | 6.4e-22 | 114.3 | 135 | (2, 142) | 147 | (45, 190) | 196 | DNA packaging protein | DNA packaging protein | | uniclust | UniRef100\_A0A086Y826 | 99.4 | 3.2e-16 | 6.5e-22 | 118.6 | 126 | (1, 129) | 147 | (74, 219) | 252 | DNA packaging protein | DNA packaging protein | | uniclust | UniRef100\_A0A0Q7XWC0 | 99.4 | 3.8e-16 | 7.4e-22 | 114.8 | 133 | (1, 134) | 147 | (73, 214) | 228 | Terminase small subunit | Terminase small subunit | | uniclust | UniRef100\_UPI0021CCE98E | 99.4 | 5.2e-16 | 9.6e-22 | 110.2 | 135 | (1, 135) | 147 | (29, 163) | 231 | terminase small subunit | terminase small subunit | | uniclust | UniRef100\_A0A073J8E6 | 99.4 | 6.3e-16 | 1.3e-21 | 118.0 | 106 | (1, 106) | 147 | (75, 201) | 261 | Phage DNA packaging protein Nu1 | Phage DNA packaging protein Nu1 | | uniclust | UniRef100\_A0A2R3Q9L1 | 99.4 | 7.5e-16 | 1.5e-21 | 111.0 | 123 | (2, 135) | 147 | (31, 183) | 196 | Terminase small subunit | Terminase small subunit | | uniclust | UniRef100\_A0A0H3ZUV8 | 99.4 | 1.1e-15 | 2.3e-21 | 111.6 | 128 | (1, 128) | 147 | (39, 172) | 211 | Phage DNA packaging | Phage DNA packaging | | uniclust | UniRef100\_A0A510E7W2 | 99.4 | 1.4e-15 | 2.7e-21 | 106.8 | 127 | (1, 134) | 147 | (24, 150) | 174 | Uncharacterized protein | Uncharacterized protein | | uniclust | UniRef100\_A0A2T5K719 | 99.4 | 1.6e-15 | 3e-21 | 109.9 | 134 | (1, 134) | 147 | (44, 180) | 215 | Phage terminase Nu1 subunit (DNA packaging protein) | Phage terminase Nu1 subunit (DNA packaging protein) | | uniclust | UniRef100\_A0A136M3Y9 | 99.4 | 1.7e-15 | 3.3e-21 | 98.9 | 91 | (43, 133) | 147 | (11, 101) | 109 | Tetrapyrrole biosynthesis glutamyl-tRNA reductase dimerisation domain-containing protein (Fragment) | Tetrapyrrole biosynthesis glutamyl-tRNA reductase dimerisation domain-containing protein (Fragment) | | uniclust | UniRef100\_A0A235H584 | 99.4 | 1.9e-15 | 3.8e-21 | 113.4 | 135 | (1, 136) | 147 | (82, 224) | 240 | Terminase small subunit (DNA packaging protein Nu1) | Terminase small subunit (DNA packaging protein Nu1) | | uniclust | UniRef100\_A0A2N2QIM9 | 99.4 | 2.9e-15 | 5.8e-21 | 109.5 | 89 | (44, 132) | 147 | (89, 177) | 209 | Terminase | Terminase | | uniclust | UniRef100\_A0A0E1N6N6 | 99.3 | 4.1e-15 | 7.9e-21 | 105.5 | 129 | (2, 135) | 147 | (27, 159) | 174 | Phage DNA packaging Nu1 family protein | Phage DNA packaging Nu1 family protein | | uniclust | UniRef100\_A0A0F9D3W0 | 99.3 | 3.9e-15 | 8e-21 | 108.3 | 136 | (1, 138) | 147 | (23, 173) | 190 | Terminase small subunit (Fragment) | Terminase small subunit (Fragment) | | uniclust | UniRef100\_A0A071LTT6 | 99.3 | 5.3e-15 | 1.1e-20 | 112.1 | 128 | (1, 133) | 147 | (63, 191) | 240 | Uncharacterized protein | Uncharacterized protein | | uniclust | UniRef100\_A0A318U841 | 99.3 | 6.4e-15 | 1.2e-20 | 107.4 | 121 | (4, 131) | 147 | (103, 228) | 230 | Phage terminase Nu1 subunit (DNA packaging protein) | Phage terminase Nu1 subunit (DNA packaging protein) | | uniclust | UniRef100\_A0A1M7CWV3 | 99.3 | 6.9e-15 | 1.4e-20 | 107.6 | 130 | (2, 137) | 147 | (31, 169) | 197 | Phage DNA packaging protein, Nu1 subunit of terminase | Phage DNA packaging protein, Nu1 subunit of terminase | | uniclust | UniRef100\_A0A1F9UST3 | 99.3 | 8.2e-15 | 1.5e-20 | 100.6 | 126 | (1, 131) | 147 | (47, 172) | 176 | Helix-turn-helix domain-containing protein | Helix-turn-helix domain-containing protein | | uniclust | UniRef100\_A0A1F9I5E8 | 99.3 | 8e-15 | 1.6e-20 | 106.7 | 94 | (44, 137) | 147 | (101, 194) | 200 | Terminase small subunit | Terminase small subunit | | uniclust | UniRef100\_A0A315EM69 | 99.3 | 1e-14 | 2e-20 | 102.3 | 135 | (1, 135) | 147 | (32, 166) | 182 | DNA-packaging protein | DNA-packaging protein | | uniclust | UniRef100\_A0A024HPJ0 | 99.3 | 1.1e-14 | 2.1e-20 | 107.8 | 127 | (2, 135) | 147 | (42, 182) | 213 | p21 prophage-derived terminase small subunit | p21 prophage-derived terminase small subunit | | uniclust | UniRef100\_A0A3N2E0T8 | 99.3 | 1.7e-14 | 3e-20 | 98.7 | 138 | (1, 138) | 147 | (27, 165) | 170 | Phage terminase Nu1 subunit (DNA packaging protein) | Phage terminase Nu1 subunit (DNA packaging protein) | | uniclust | UniRef100\_A0A0C2YAI7 | 99.3 | 1.8e-14 | 3.6e-20 | 103.8 | 133 | (2, 140) | 147 | (34, 179) | 186 | Uncharacterized protein | Uncharacterized protein | | uniclust | UniRef100\_A0A031IUW6 | 99.3 | 1.8e-14 | 3.9e-20 | 109.0 | 95 | (44, 138) | 147 | (99, 193) | 230 | Terminase small subunit | Terminase small subunit | | uniclust | UniRef100\_A0A4Y3TG37 | 99.3 | 2.2e-14 | 4e-20 | 100.7 | 132 | (1, 133) | 147 | (27, 160) | 203 | Terminase small subunit | Terminase small subunit | | uniclust | UniRef100\_A0A098F576 | 99.3 | 2.3e-14 | 4.7e-20 | 105.0 | 129 | (1, 135) | 147 | (50, 187) | 200 | Phage DNA packaging protein, Nu1 subunit of terminase | Phage DNA packaging protein, Nu1 subunit of terminase | | uniclust | UniRef100\_A0A7G8BY94 | 99.2 | 4.3e-14 | 8e-20 | 99.1 | 127 | (2, 131) | 147 | (28, 165) | 183 | Terminase small subunit | Terminase small subunit | | uniclust | UniRef100\_A0A3B1BSM9 | 99.2 | 4.7e-14 | 8.6e-20 | 92.3 | 109 | (21, 135) | 147 | (9, 117) | 126 | Uncharacterized protein | Uncharacterized protein | | uniclust | UniRef100\_A0A0E4BPR8 | 99.2 | 4.8e-14 | 1e-19 | 106.7 | 115 | (2, 131) | 147 | (71, 185) | 229 | Terminase small subunit | Terminase small subunit | | uniclust | UniRef100\_A0A0F8WMT9 | 99.2 | 5.7e-14 | 1.1e-19 | 99.0 | 128 | (3, 135) | 147 | (28, 158) | 196 | Uncharacterized protein | Uncharacterized protein | | uniclust | UniRef100\_A0A021X9M2 | 99.2 | 8.2e-14 | 1.7e-19 | 106.9 | 94 | (45, 138) | 147 | (119, 212) | 242 | Terminase small subunit | Terminase small subunit | | uniclust | UniRef100\_A0A957S2F1 | 99.2 | 1.5e-13 | 2.9e-19 | 98.6 | 131 | (3, 135) | 147 | (25, 186) | 192 | Uncharacterized protein | Uncharacterized protein | | uniclust | UniRef100\_A0A075KE40 | 99.2 | 1.6e-13 | 3.3e-19 | 100.0 | 127 | (1, 136) | 147 | (44, 173) | 183 | Terminase small subunit | Terminase small subunit | | uniclust | UniRef100\_A0A024E8L0 | 99.1 | 2.7e-13 | 5.6e-19 | 106.2 | 93 | (44, 136) | 147 | (136, 228) | 291 | Terminase small subunit | Terminase small subunit | | uniclust | UniRef100\_A0A5K8AH95 | 99.1 | 3.8e-13 | 7.2e-19 | 93.6 | 132 | (1, 132) | 147 | (19, 155) | 157 | Terminase small subunit | Terminase small subunit | | uniclust | UniRef100\_A0A1A8TAI5 | 99.1 | 4.1e-13 | 7.7e-19 | 93.8 | 128 | (1, 131) | 147 | (23, 156) | 166 | Phage DNA packaging protein Nu1 | Phage DNA packaging protein Nu1 | | uniclust | UniRef100\_E8X2N4 | 99.1 | 5.3e-13 | 9.7e-19 | 93.1 | 132 | (1, 139) | 147 | (31, 169) | 189 | Putative terminase small subunit, Nu1 | Putative terminase small subunit, Nu1 | | uniclust | UniRef100\_A0A0G3XF80 | 99.1 | 4.9e-13 | 1e-18 | 100.9 | 125 | (2, 129) | 147 | (46, 194) | 225 | Putative transcriptional regulator | Putative transcriptional regulator | | uniclust | UniRef100\_A0A0S6UUS0 | 99.1 | 7e-13 | 1.3e-18 | 86.3 | 87 | (44, 132) | 147 | (13, 99) | 102 | Uncharacterized protein | Uncharacterized protein | | uniclust | UniRef100\_A0A011QU19 | 99.1 | 7.5e-13 | 1.5e-18 | 100.3 | 95 | (43, 137) | 147 | (111, 207) | 233 | Elements of external origin | Elements of external origin | | uniclust | UniRef100\_A0A1M3NUS7 | 99.1 | 8.4e-13 | 1.7e-18 | 99.1 | 97 | (2, 106) | 147 | (53, 149) | 212 | Terminase small subunit | Terminase small subunit | | uniclust | UniRef100\_UPI0013FDE30D | 99.1 | 9.5e-13 | 1.7e-18 | 92.4 | 128 | (1, 129) | 147 | (37, 169) | 196 | terminase small subunit | terminase small subunit | | uniclust | UniRef100\_A0A066T0V2 | 99.1 | 1e-12 | 2e-18 | 101.9 | 131 | (1, 133) | 147 | (52, 198) | 313 | Terminase small subunit | Terminase small subunit | | uniclust | UniRef100\_UPI00177B5103 | 99.0 | 1.3e-12 | 2.4e-18 | 92.9 | 122 | (3, 131) | 147 | (89, 211) | 214 | terminase small subunit | terminase small subunit | | uniclust | UniRef100\_A0A3M2C0J2 | 99.0 | 1.6e-12 | 3e-18 | 87.0 | 124 | (3, 131) | 147 | (13, 139) | 141 | Terminase | Terminase | | uniclust | UniRef100\_A0A0A0F9C3 | 99.0 | 1.6e-12 | 3.1e-18 | 94.1 | 132 | (1, 142) | 147 | (32, 164) | 205 | Terminase small subunit | Terminase small subunit | | uniclust | UniRef100\_A0A1F2RY64 | 99.0 | 1.6e-12 | 3.2e-18 | 92.4 | 89 | (43, 131) | 147 | (80, 168) | 174 | Uncharacterized protein | Uncharacterized protein | | uniclust | UniRef100\_A0A0F8W5B6 | 99.0 | 1.7e-12 | 3.3e-18 | 93.9 | 132 | (1, 135) | 147 | (34, 166) | 198 | Terminase small subunit (Fragment) | Terminase small subunit (Fragment) | | uniclust | UniRef100\_A0A0E4FVD0 | 99.0 | 1.8e-12 | 3.5e-18 | 91.8 | 127 | (3, 135) | 147 | (26, 156) | 177 | Uncharacterized protein | Uncharacterized protein | | uniclust | UniRef100\_A0A1C3XV01 | 99.0 | 1.9e-12 | 3.5e-18 | 93.4 | 126 | (2, 133) | 147 | (43, 171) | 203 | Phage DNA packaging protein, Nu1 subunit of terminase | Phage DNA packaging protein, Nu1 subunit of terminase | | uniclust | UniRef100\_A0A081MYL8 | 99.0 | 2.4e-12 | 4.6e-18 | 93.2 | 136 | (1, 136) | 147 | (24, 171) | 188 | Terminase small subunit | Terminase small subunit | | uniclust | UniRef100\_A0A1S6TPB0 | 99.0 | 2.5e-12 | 5e-18 | 93.2 | 123 | (3, 135) | 147 | (25, 147) | 184 | Phage DNA packaging protein, Nu1 subunit of terminase | Phage DNA packaging protein, Nu1 subunit of terminase | | uniclust | UniRef100\_A0A9D5VVB4 | 99.0 | 3e-12 | 5.5e-18 | 87.2 | 125 | (1, 131) | 147 | (26, 151) | 157 | Uncharacterized protein | Uncharacterized protein | | uniclust | UniRef100\_A0A3A8WV56 | 99.0 | 3.4e-12 | 6.4e-18 | 88.2 | 132 | (3, 139) | 147 | (24, 156) | 164 | Terminase small subunit | Terminase small subunit | | uniclust | UniRef100\_A0A023XKN2 | 99.0 | 3.3e-12 | 6.5e-18 | 94.1 | 111 | (3, 129) | 147 | (54, 164) | 199 | Uncharacterized protein | Uncharacterized protein | | uniclust | UniRef100\_A0A1V1UM65 | 99.0 | 3.4e-12 | 6.7e-18 | 94.9 | 127 | (2, 133) | 147 | (44, 174) | 213 | Phage DNA packaging protein Nu1 | Phage DNA packaging protein Nu1 | | uniclust | UniRef100\_A0A133ZYV3 | 99.0 | 3.6e-12 | 7.1e-18 | 96.3 | 133 | (2, 134) | 147 | (84, 227) | 248 | Phage DNA packaging protein Nu1 | Phage DNA packaging protein Nu1 | | uniclust | UniRef100\_A0A1H5S2V5 | 99.0 | 4.1e-12 | 8.3e-18 | 93.5 | 119 | (23, 141) | 147 | (45, 166) | 184 | Phage DNA packaging protein, Nu1 subunit of terminase | Phage DNA packaging protein, Nu1 subunit of terminase | | uniclust | UniRef100\_A0A2E5KQ39 | 99.0 | 4.7e-12 | 8.9e-18 | 89.8 | 121 | (3, 135) | 147 | (27, 147) | 175 | Terminase small subunit, Nu1 | Terminase small subunit, Nu1 | | uniclust | UniRef100\_A0A1G1KXY1 | 99.0 | 5.1e-12 | 9.4e-18 | 87.6 | 120 | (1, 130) | 147 | (34, 153) | 176 | Terminase small subunit | Terminase small subunit | | uniclust | UniRef100\_K2J734 | 99.0 | 5.1e-12 | 9.4e-18 | 89.3 | 131 | (1, 134) | 147 | (32, 187) | 202 | Terminase small subunit | Terminase small subunit | | uniclust | UniRef100\_A0A962SUK3 | 99.0 | 5.2e-12 | 9.6e-18 | 87.8 | 126 | (1, 129) | 147 | (25, 155) | 166 | DUF1441 family protein | DUF1441 family protein | | uniclust | UniRef100\_A0A540V7P3 | 98.9 | 5.8e-12 | 1.1e-17 | 86.1 | 121 | (1, 130) | 147 | (26, 146) | 160 | Terminase small subunit | Terminase small subunit | | uniclust | UniRef100\_A0A0A1YW04 | 98.9 | 6e-12 | 1.2e-17 | 94.8 | 122 | (21, 142) | 147 | (61, 185) | 234 | Uncharacterized protein | Uncharacterized protein | | uniclust | UniRef100\_A0A176Z207 | 98.9 | 7.1e-12 | 1.4e-17 | 90.0 | 93 | (43, 135) | 147 | (74, 166) | 175 | Uncharacterized protein | Uncharacterized protein | | uniclust | UniRef100\_A0A175RTU2 | 98.9 | 7.2e-12 | 1.4e-17 | 95.1 | 128 | (1, 129) | 147 | (60, 198) | 248 | Terminase small subunit | Terminase small subunit | | uniclust | UniRef100\_A0A379CXL1 | 98.9 | 9.5e-12 | 1.8e-17 | 90.4 | 132 | (1, 140) | 147 | (40, 172) | 225 | Phage DNA packaging protein Nu1 | Phage DNA packaging protein Nu1 | | uniclust | UniRef100\_A0A088FV50 | 98.9 | 9.7e-12 | 1.8e-17 | 80.9 | 99 | (48, 146) | 147 | (6, 104) | 108 | Small terminase subunit | Small terminase subunit | | uniclust | UniRef100\_A0A928NFM1 | 98.9 | 1.1e-11 | 1.9e-17 | 79.7 | 90 | (43, 132) | 147 | (14, 103) | 107 | Terminase small subunit | Terminase small subunit | | uniclust | UniRef100\_A0A024L3J4 | 98.9 | 1.1e-11 | 2e-17 | 92.6 | 129 | (1, 131) | 147 | (36, 180) | 278 | DNA-packaging protein | DNA-packaging protein | | uniclust | UniRef100\_A0A1H7YHA6 | 98.9 | 1.1e-11 | 2.2e-17 | 90.6 | 130 | (1, 136) | 147 | (43, 184) | 192 | Phage DNA packaging protein, Nu1 subunit of terminase | Phage DNA packaging protein, Nu1 subunit of terminase | | uniclust | UniRef100\_A0A1B7L4T6 | 98.9 | 1.2e-11 | 2.4e-17 | 89.1 | 116 | (21, 136) | 147 | (41, 158) | 185 | Phage DNA packaging protein Nu1 | Phage DNA packaging protein Nu1 | | uniclust | UniRef100\_A0A946DB58 | 98.9 | 1.3e-11 | 2.5e-17 | 87.9 | 125 | (2, 131) | 147 | (32, 157) | 192 | Uncharacterized protein | Uncharacterized protein | | uniclust | UniRef100\_UPI0018E13DD1 | 98.9 | 1.5e-11 | 2.7e-17 | 89.6 | 124 | (5, 134) | 147 | (101, 225) | 251 | type IV toxin-antitoxin system AbiEi family antitoxin domain-containing protein | type IV toxin-antitoxin system AbiEi family antitoxin domain-containing protein | | uniclust | UniRef100\_A0A316SBE2 | 98.9 | 1.6e-11 | 2.9e-17 | 88.2 | 130 | (1, 137) | 147 | (74, 206) | 223 | Terminase small subunit | Terminase small subunit | | uniclust | UniRef100\_A0A933XTS0 | 98.9 | 1.7e-11 | 3e-17 | 81.8 | 92 | (42, 133) | 147 | (30, 121) | 134 | Uncharacterized protein | Uncharacterized protein | | uniclust | UniRef100\_A0A935WCC7 | 98.9 | 1.7e-11 | 3e-17 | 84.6 | 129 | (2, 136) | 147 | (23, 159) | 168 | Uncharacterized protein | Uncharacterized protein | | uniclust | UniRef100\_A0A2A5EK29 | 98.9 | 1.7e-11 | 3.1e-17 | 87.6 | 131 | (2, 132) | 147 | (44, 185) | 216 | Terminase small subunit | Terminase small subunit | | uniclust | UniRef100\_A0A0A8IL49 | 98.9 | 1.6e-11 | 3.3e-17 | 91.6 | 95 | (43, 137) | 147 | (91, 187) | 199 | Terminase small subunit | Terminase small subunit | | uniclust | UniRef100\_A0A0Q6MN60 | 98.9 | 1.7e-11 | 3.5e-17 | 93.0 | 96 | (45, 140) | 147 | (108, 204) | 231 | Uncharacterized protein | Uncharacterized protein | | uniclust | UniRef100\_UPI00041AA28B | 98.8 | 2.2e-11 | 4e-17 | 87.4 | 126 | (3, 134) | 147 | (51, 177) | 213 | terminase small subunit | terminase small subunit | | uniclust | UniRef100\_A0A158L615 | 98.8 | 2.2e-11 | 4.5e-17 | 91.8 | 88 | (44, 131) | 147 | (96, 186) | 213 | Putative bacteriophage--like protein | Putative bacteriophage--like protein | | uniclust | UniRef100\_A0A7L5ZY90 | 98.8 | 2.5e-11 | 4.7e-17 | 79.7 | 87 | (55, 141) | 147 | (4, 90) | 121 | Uncharacterized protein | Uncharacterized protein | | uniclust | UniRef100\_UPI00223107AD | 98.8 | 2.8e-11 | 5.2e-17 | 82.5 | 109 | (29, 137) | 147 | (3, 113) | 154 | terminase small subunit, Nu1 | terminase small subunit, Nu1 | | uniclust | UniRef100\_A0A0S4V076 | 98.8 | 2.8e-11 | 5.5e-17 | 80.9 | 94 | (43, 136) | 147 | (7, 102) | 107 | Bacteriophage-related protein | Bacteriophage-related protein | | uniclust | UniRef100\_A0A0J6WV29 | 98.8 | 3e-11 | 5.6e-17 | 85.1 | 133 | (2, 140) | 147 | (31, 163) | 173 | DNA-binding protein | DNA-binding protein | | uniclust | UniRef100\_A0A1L3ZRR3 | 98.8 | 2.9e-11 | 5.7e-17 | 92.0 | 126 | (1, 129) | 147 | (44, 193) | 244 | Terminase small subunit | Terminase small subunit | | uniclust | UniRef100\_A0A2X2UEZ9 | 98.8 | 3.5e-11 | 6.4e-17 | 87.0 | 120 | (18, 137) | 147 | (97, 216) | 233 | Phage DNA packaging protein, Nu1 subunit of terminase | Phage DNA packaging protein, Nu1 subunit of terminase | | uniclust | UniRef100\_H8YXV1 | 98.8 | 3.7e-11 | 6.8e-17 | 81.8 | 117 | (15, 134) | 147 | (32, 148) | 152 | Phage DNA packaging protein, Nu1 subunit of terminase | Phage DNA packaging protein, Nu1 subunit of terminase | | uniclust | UniRef100\_UPI0022B810E0 | 98.8 | 3.9e-11 | 7.1e-17 | 85.2 | 130 | (2, 137) | 147 | (55, 196) | 203 | hypothetical protein | hypothetical protein | | uniclust | UniRef100\_A0A7V9ZEA9 | 98.8 | 3.7e-11 | 7.2e-17 | 88.1 | 96 | (44, 141) | 147 | (100, 195) | 201 | Terminase small subunit | Terminase small subunit | | uniclust | UniRef100\_A0A0Q4ZGW7 | 98.8 | 3.8e-11 | 7.4e-17 | 89.3 | 109 | (1, 109) | 147 | (57, 173) | 218 | Uncharacterized protein | Uncharacterized protein | | uniclust | UniRef100\_A0A9D1S5U4 | 98.8 | 4.2e-11 | 7.7e-17 | 85.0 | 126 | (1, 132) | 147 | (32, 172) | 202 | Uncharacterized protein | Uncharacterized protein | | uniclust | UniRef100\_A0A0X8CI04 | 98.8 | 4.1e-11 | 7.9e-17 | 87.8 | 130 | (3, 132) | 147 | (64, 198) | 207 | Terminase small subunit | Terminase small subunit | | uniclust | UniRef100\_D1Y312 | 98.8 | 4.6e-11 | 8.5e-17 | 82.3 | 133 | (3, 140) | 147 | (10, 147) | 164 | Phage DNA packaging protein Nu1 | Phage DNA packaging protein Nu1 | | uniclust | UniRef100\_A0A014MCD6 | 98.8 | 5.3e-11 | 1.1e-16 | 88.8 | 114 | (2, 126) | 147 | (39, 156) | 197 | Terminase small subunit | Terminase small subunit | | uniclust | UniRef100\_A0A023E0R0 | 98.8 | 5.9e-11 | 1.1e-16 | 86.1 | 87 | (48, 134) | 147 | (103, 189) | 196 | Terminase small subunit | Terminase small subunit | | uniclust | UniRef100\_A0A6I6DNA2 | 98.7 | 7.9e-11 | 1.5e-16 | 85.4 | 128 | (2, 140) | 147 | (70, 198) | 231 | Terminase small subunit | Terminase small subunit | | uniclust | UniRef100\_A0A060BBC7 | 98.7 | 8.1e-11 | 1.7e-16 | 90.6 | 91 | (13, 105) | 147 | (76, 166) | 234 | Terminase small subunit | Terminase small subunit | | uniclust | UniRef100\_UPI0009DC06AE | 98.7 | 1e-10 | 2e-16 | 84.9 | 127 | (1, 134) | 147 | (68, 198) | 212 | terminase small subunit | terminase small subunit | | uniclust | UniRef100\_A0A7Z8R5N7 | 98.7 | 1e-10 | 2e-16 | 83.8 | 126 | (1, 126) | 147 | (22, 154) | 184 | Terminase small subunit | Terminase small subunit | | uniclust | UniRef100\_B0U9E3 | 98.7 | 1.1e-10 | 2.1e-16 | 76.1 | 89 | (44, 132) | 147 | (7, 95) | 115 | Uncharacterized protein | Uncharacterized protein | | uniclust | UniRef100\_A0A0H3ZKI4 | 98.7 | 1e-10 | 2.1e-16 | 86.4 | 131 | (2, 136) | 147 | (30, 166) | 190 | Terminase small subunit | Terminase small subunit | | uniclust | UniRef100\_UPI0006B47411 | 98.7 | 1.2e-10 | 2.2e-16 | 81.3 | 132 | (3, 139) | 147 | (37, 172) | 177 | hypothetical protein | hypothetical protein | | uniclust | UniRef100\_A0A017HTQ6 | 98.7 | 1.2e-10 | 2.2e-16 | 80.1 | 137 | (1, 137) | 147 | (10, 148) | 160 | Phage DNA packaging protein Nu1 | Phage DNA packaging protein Nu1 | | uniclust | UniRef100\_A0A1H8NQA9 | 98.7 | 1.2e-10 | 2.3e-16 | 84.8 | 87 | (46, 132) | 147 | (98, 184) | 190 | Phage DNA packaging protein, Nu1 subunit of terminase | Phage DNA packaging protein, Nu1 subunit of terminase | | uniclust | UniRef100\_A0A071M1J9 | 98.7 | 1.2e-10 | 2.4e-16 | 91.8 | 98 | (45, 142) | 147 | (154, 253) | 291 | Uncharacterized protein | Uncharacterized protein | | uniclust | UniRef100\_A0A8J6NMN9 | 98.7 | 1.4e-10 | 2.6e-16 | 79.0 | 123 | (2, 131) | 147 | (26, 148) | 151 | Uncharacterized protein | Uncharacterized protein | | uniclust | UniRef100\_A0A6N6MDM0 | 98.7 | 1.3e-10 | 2.7e-16 | 84.3 | 97 | (2, 106) | 147 | (36, 132) | 175 | Terminase small subunit, Nu1 | Terminase small subunit, Nu1 | | uniclust | UniRef100\_UPI0012E9DC02 | 98.7 | 1.5e-10 | 2.7e-16 | 82.8 | 122 | (2, 130) | 147 | (86, 207) | 209 | hypothetical protein | hypothetical protein | | uniclust | UniRef100\_A0A0Q7F4I9 | 98.7 | 1.4e-10 | 2.8e-16 | 84.4 | 119 | (6, 131) | 147 | (30, 170) | 185 | Terminase small subunit | Terminase small subunit | | uniclust | UniRef100\_A0A1V5Z7S9 | 98.7 | 1.6e-10 | 3e-16 | 80.4 | 91 | (43, 133) | 147 | (82, 172) | 174 | Phage DNA packaging protein Nu1 | Phage DNA packaging protein Nu1 | | uniclust | UniRef100\_A0A7T1HQ48 | 98.7 | 1.8e-10 | 3.3e-16 | 82.7 | 87 | (48, 134) | 147 | (87, 179) | 190 | Terminase small subunit | Terminase small subunit | | uniclust | UniRef100\_A0A0K6HIX8 | 98.7 | 1.7e-10 | 3.4e-16 | 86.8 | 96 | (2, 105) | 147 | (64, 159) | 221 | Phage DNA packaging protein, Nu1 subunit of terminase | Phage DNA packaging protein, Nu1 subunit of terminase | | uniclust | UniRef100\_A0A0F2PHE5 | 98.7 | 2.3e-10 | 4.4e-16 | 81.0 | 127 | (3, 136) | 147 | (32, 159) | 174 | HTH merR-type domain-containing protein | HTH merR-type domain-containing protein | | uniclust | UniRef100\_A0A0C5XPQ2 | 98.7 | 2.3e-10 | 4.5e-16 | 83.9 | 112 | (16, 134) | 147 | (62, 173) | 193 | Terminase | Terminase | | uniclust | UniRef100\_A0A6L9M419 | 98.7 | 2.5e-10 | 4.7e-16 | 79.3 | 91 | (44, 134) | 147 | (69, 159) | 170 | Terminase small subunit | Terminase small subunit | | uniclust | UniRef100\_A0A1G2ZKR4 | 98.6 | 2.8e-10 | 5.1e-16 | 84.4 | 130 | (2, 138) | 147 | (117, 246) | 276 | Helix-turn-helix domain-containing protein | Helix-turn-helix domain-containing protein | | uniclust | UniRef100\_A0A517Q5A6 | 98.6 | 3e-10 | 5.8e-16 | 82.4 | 127 | (1, 132) | 147 | (60, 189) | 195 | Phage DNA packaging protein Nu1 | Phage DNA packaging protein Nu1 | | uniclust | UniRef100\_A0A956GQK7 | 98.6 | 3.2e-10 | 5.9e-16 | 81.7 | 122 | (1, 129) | 147 | (37, 159) | 220 | Uncharacterized protein | Uncharacterized protein | | uniclust | UniRef100\_A0A059KUP0 | 98.6 | 3.2e-10 | 6.3e-16 | 86.5 | 129 | (4, 136) | 147 | (51, 185) | 237 | DNA packaging protein | DNA packaging protein | | uniclust | UniRef100\_A0A064A069 | 98.6 | 3.4e-10 | 6.4e-16 | 82.1 | 106 | (21, 136) | 147 | (65, 170) | 186 | Terminase | Terminase | | uniclust | UniRef100\_A0A099EVD2 | 98.6 | 3.2e-10 | 6.4e-16 | 86.1 | 96 | (43, 138) | 147 | (112, 209) | 225 | Elements of external origin | Elements of external origin | | uniclust | UniRef100\_A0L5S8 | 98.6 | 3.6e-10 | 6.6e-16 | 84.4 | 114 | (21, 134) | 147 | (167, 283) | 292 | Transcriptional regulator, Fis family | Transcriptional regulator, Fis family | | uniclust | UniRef100\_A0A0U2S671 | 98.6 | 4.6e-10 | 8.5e-16 | 79.8 | 126 | (1, 128) | 147 | (21, 162) | 181 | Terminase small subunit | Terminase small subunit | | uniclust | UniRef100\_A0A0M9GBF1 | 98.6 | 5.6e-10 | 1.1e-15 | 75.6 | 91 | (43, 133) | 147 | (22, 112) | 122 | Uncharacterized protein (Fragment) | Uncharacterized protein (Fragment) | | uniclust | UniRef100\_A0A064AK97 | 98.6 | 5.4e-10 | 1.1e-15 | 83.6 | 122 | (2, 142) | 147 | (78, 199) | 210 | Helix-turn-helix domain-containing protein | Helix-turn-helix domain-containing protein | | uniclust | UniRef100\_A0A076GEC0 | 98.6 | 6.1e-10 | 1.2e-15 | 80.6 | 133 | (1, 134) | 147 | (54, 195) | 200 | Small terminase subunit | Small terminase subunit | | uniclust | UniRef100\_UPI0020034B75 | 98.6 | 6.3e-10 | 1.2e-15 | 78.7 | 126 | (2, 131) | 147 | (63, 189) | 190 | hypothetical protein | hypothetical protein | | uniclust | UniRef100\_A0A8S7YJX2 | 98.6 | 6.5e-10 | 1.2e-15 | 87.7 | 127 | (1, 129) | 147 | (21, 163) | 473 | Phage tail collar domain-containing protein | Phage tail collar domain-containing protein | | uniclust | UniRef100\_E7C6A2 | 98.6 | 7.3e-10 | 1.3e-15 | 72.4 | 90 | (45, 134) | 147 | (3, 92) | 113 | Uncharacterized protein | Uncharacterized protein | | uniclust | UniRef100\_A0A1T4PVA8 | 98.6 | 7.4e-10 | 1.4e-15 | 80.8 | 126 | (3, 135) | 147 | (83, 208) | 213 | Phage DNA packaging protein, Nu1 subunit of terminase | Phage DNA packaging protein, Nu1 subunit of terminase | | uniclust | UniRef100\_A0A4R6UIY8 | 98.5 | 7.8e-10 | 1.5e-15 | 69.3 | 72 | (63, 134) | 147 | (2, 73) | 81 | Uncharacterized protein | Uncharacterized protein | | uniclust | UniRef100\_A0A945GZD5 | 98.5 | 8.3e-10 | 1.5e-15 | 71.4 | 89 | (44, 132) | 147 | (13, 101) | 106 | Uncharacterized protein | Uncharacterized protein | | uniclust | UniRef100\_UPI0020CD6DAF | 98.5 | 8.4e-10 | 1.6e-15 | 81.1 | 90 | (43, 132) | 147 | (128, 217) | 229 | hypothetical protein | hypothetical protein | | uniclust | UniRef100\_A0A1E7PYA9 | 98.5 | 8.9e-10 | 1.8e-15 | 79.5 | 91 | (43, 136) | 147 | (62, 152) | 162 | Terminase small subunit | Terminase small subunit | | uniclust | UniRef100\_UPI001EE2AD5A | 98.5 | 1e-09 | 1.8e-15 | 82.8 | 127 | (2, 134) | 147 | (23, 175) | 310 | hypothetical protein | hypothetical protein | | uniclust | UniRef100\_A0A151FHD5 | 98.5 | 1e-09 | 1.9e-15 | 78.7 | 92 | (43, 134) | 147 | (71, 162) | 172 | Terminase | Terminase | | uniclust | UniRef100\_A0A2T5IIL6 | 98.5 | 1e-09 | 2e-15 | 69.2 | 75 | (60, 134) | 147 | (2, 76) | 84 | Uncharacterized protein | Uncharacterized protein | | uniclust | UniRef100\_A0A1G7GZW8 | 98.5 | 1.3e-09 | 2.5e-15 | 79.4 | 115 | (2, 131) | 147 | (30, 144) | 168 | Uncharacterized protein | Uncharacterized protein | | uniclust | UniRef100\_E2CDD4 | 98.5 | 1.4e-09 | 2.7e-15 | 79.3 | 87 | (45, 131) | 147 | (118, 204) | 217 | Uncharacterized protein | Uncharacterized protein | | uniclust | UniRef100\_A0A6J5EMI8 | 98.5 | 1.5e-09 | 2.8e-15 | 72.1 | 93 | (44, 136) | 147 | (8, 102) | 106 | Uncharacterized protein | Uncharacterized protein | | uniclust | UniRef100\_UPI00223516E7 | 98.5 | 1.6e-09 | 2.9e-15 | 85.3 | 127 | (1, 129) | 147 | (21, 163) | 452 | terminase small subunit | terminase small subunit | | uniclust | UniRef100\_UPI001FB86660 | 98.5 | 1.7e-09 | 3.1e-15 | 73.4 | 122 | (6, 132) | 147 | (4, 126) | 141 | hypothetical protein | hypothetical protein | | uniclust | UniRef100\_D8F202 | 98.5 | 2e-09 | 3.6e-15 | 74.4 | 127 | (1, 132) | 147 | (22, 149) | 159 | Uncharacterized protein | Uncharacterized protein | | uniclust | UniRef100\_UPI001C52BE35 | 98.5 | 2e-09 | 3.7e-15 | 75.9 | 115 | (20, 134) | 147 | (46, 163) | 172 | hypothetical protein | hypothetical protein | | uniclust | UniRef100\_A0A6I1JC22 | 98.5 | 2e-09 | 3.8e-15 | 76.5 | 97 | (2, 106) | 147 | (29, 125) | 165 | Helix-turn-helix domain-containing protein | Helix-turn-helix domain-containing protein | | uniclust | UniRef100\_A0A3S0BZF6 | 98.4 | 2.2e-09 | 4.1e-15 | 75.6 | 129 | (3, 134) | 147 | (21, 152) | 164 | Terminase small subunit | Terminase small subunit | | uniclust | UniRef100\_UPI0012ED759B | 98.4 | 2.4e-09 | 4.4e-15 | 71.4 | 91 | (44, 134) | 147 | (32, 122) | 126 | hypothetical protein | hypothetical protein | | uniclust | UniRef100\_A0A094YII6 | 98.4 | 2.3e-09 | 4.5e-15 | 80.5 | 88 | (45, 132) | 147 | (102, 189) | 212 | Helix-turn-helix domain-containing protein | Helix-turn-helix domain-containing protein | | uniclust | UniRef100\_A0A167H541 | 98.4 | 2.5e-09 | 5e-15 | 79.4 | 127 | (1, 134) | 147 | (20, 161) | 189 | Uncharacterized protein | Uncharacterized protein | | uniclust | UniRef100\_A0A8I0KDZ5 | 98.4 | 3.1e-09 | 5.7e-15 | 64.0 | 64 | (66, 129) | 147 | (6, 69) | 70 | Uncharacterized protein | Uncharacterized protein | | uniclust | UniRef100\_A0A077NSP9 | 98.4 | 3.2e-09 | 6.2e-15 | 78.2 | 127 | (1, 132) | 147 | (22, 187) | 196 | Uncharacterized protein | Uncharacterized protein | | uniclust | UniRef100\_A0A9E8X8G8 | 98.4 | 3.4e-09 | 6.3e-15 | 76.0 | 116 | (8, 130) | 147 | (84, 202) | 205 | Uncharacterized protein | Uncharacterized protein | | uniclust | UniRef100\_A0A060HBH3 | 98.4 | 3.3e-09 | 6.5e-15 | 79.5 | 92 | (44, 135) | 147 | (100, 191) | 219 | Phage-related protein | Phage-related protein | | uniclust | UniRef100\_UPI0012F7E1C9 | 98.4 | 3.5e-09 | 6.5e-15 | 72.8 | 110 | (17, 130) | 147 | (40, 149) | 153 | DUF1441 family protein | DUF1441 family protein | | uniclust | UniRef100\_A0A929F7T2 | 98.4 | 3.6e-09 | 6.7e-15 | 75.3 | 90 | (43, 132) | 147 | (102, 191) | 193 | Uncharacterized protein | Uncharacterized protein | | uniclust | UniRef100\_UPI001688EBE8 | 98.4 | 3.9e-09 | 7.1e-15 | 73.7 | 125 | (2, 131) | 147 | (32, 163) | 169 | hypothetical protein | hypothetical protein | | uniclust | UniRef100\_H3RLL8 | 98.4 | 3.8e-09 | 7.1e-15 | 69.4 | 94 | (44, 137) | 147 | (3, 99) | 103 | DNA-directed RNA polymerase sigma subunit | DNA-directed RNA polymerase sigma subunit | | uniclust | UniRef100\_A0A7C3DJT8 | 98.4 | 3.8e-09 | 7.1e-15 | 68.2 | 84 | (45, 129) | 147 | (12, 95) | 98 | Tetrapyrrole biosynthesis glutamyl-tRNA reductase dimerisation domain-containing protein | Tetrapyrrole biosynthesis glutamyl-tRNA reductase dimerisation domain-containing protein | | uniclust | UniRef100\_A0A0J6K589 | 98.4 | 3.7e-09 | 7.1e-15 | 78.9 | 92 | (44, 135) | 147 | (114, 207) | 213 | Elements of external origin | Elements of external origin | | uniclust | UniRef100\_A0A8G2CJ81 | 98.4 | 4.4e-09 | 8.1e-15 | 76.2 | 126 | (2, 132) | 147 | (21, 152) | 219 | Phage DNA packaging protein, Nu1 subunit of terminase | Phage DNA packaging protein, Nu1 subunit of terminase | | uniclust | UniRef100\_A0A9E5R9F2 | 98.4 | 4.6e-09 | 8.4e-15 | 72.0 | 121 | (1, 131) | 147 | (19, 147) | 149 | Terminase small subunit | Terminase small subunit | | uniclust | UniRef100\_A0A0A1AKA0 | 98.4 | 4.5e-09 | 8.5e-15 | 76.0 | 123 | (4, 132) | 147 | (26, 178) | 187 | Terminase small subunit | Terminase small subunit | | uniclust | UniRef100\_A0A109J6S7 | 98.4 | 4.6e-09 | 8.6e-15 | 77.9 | 127 | (2, 134) | 147 | (84, 212) | 233 | Phage terminase Nu1 subunit (DNA packaging protein) | Phage terminase Nu1 subunit (DNA packaging protein) | | uniclust | UniRef100\_A0A6I6MLK3 | 98.4 | 4.7e-09 | 8.8e-15 | 71.1 | 85 | (46, 132) | 147 | (28, 112) | 125 | Uncharacterized protein | Uncharacterized protein | | uniclust | UniRef100\_UPI00135677F5 | 98.4 | 5.1e-09 | 9.4e-15 | 68.8 | 96 | (4, 104) | 147 | (17, 112) | 114 | hypothetical protein | hypothetical protein | | uniclust | UniRef100\_A0A562RXT0 | 98.4 | 4.9e-09 | 9.4e-15 | 78.9 | 125 | (3, 132) | 147 | (35, 204) | 228 | Phage terminase small subunit | Phage terminase small subunit | | uniclust | UniRef100\_A0A7Y3AH85 | 98.4 | 5.6e-09 | 1e-14 | 72.6 | 110 | (17, 134) | 147 | (39, 148) | 164 | DUF1441 family protein | DUF1441 family protein | | uniclust | UniRef100\_A0A7C4DTE2 | 98.4 | 5.6e-09 | 1e-14 | 73.0 | 123 | (3, 132) | 147 | (28, 160) | 169 | DNA packaging protein | DNA packaging protein | | uniclust | UniRef100\_A0A376VNK9 | 98.3 | 6e-09 | 1.1e-14 | 73.5 | 107 | (1, 107) | 147 | (25, 147) | 166 | DNA packaging protein | DNA packaging protein | | uniclust | UniRef100\_A0A1Q6UMH2 | 98.3 | 5.7e-09 | 1.1e-14 | 80.0 | 128 | (1, 138) | 147 | (82, 214) | 232 | Terminase small subunit | Terminase small subunit | | uniclust | UniRef100\_A0A974SWB6 | 98.3 | 6.1e-09 | 1.2e-14 | 75.7 | 117 | (14, 133) | 147 | (41, 157) | 179 | DUF1441 family protein | DUF1441 family protein | | uniclust | UniRef100\_UPI0020003892 | 98.3 | 7e-09 | 1.3e-14 | 69.3 | 95 | (1, 95) | 147 | (23, 125) | 126 | terminase small subunit | terminase small subunit | | uniclust | UniRef100\_UPI001FF8B96B | 98.3 | 6.8e-09 | 1.3e-14 | 64.3 | 70 | (61, 132) | 147 | (2, 71) | 73 | hypothetical protein | hypothetical protein | | uniclust | UniRef100\_A0A3D1NTE5 | 98.3 | 7.2e-09 | 1.3e-14 | 75.1 | 90 | (44, 133) | 147 | (106, 195) | 199 | Terminase small subunit | Terminase small subunit | | uniclust | UniRef100\_A0A7L6A869 | 98.3 | 8.7e-09 | 1.6e-14 | 71.6 | 103 | (28, 130) | 147 | (41, 143) | 162 | MarR family transcriptional regulator | MarR family transcriptional regulator | | uniclust | UniRef100\_A0A0P6VRG9 | 98.3 | 8.2e-09 | 1.6e-14 | 75.7 | 128 | (3, 135) | 147 | (25, 168) | 178 | Elements of external origin | Elements of external origin | | uniclust | UniRef100\_A0A2D2C217 | 98.3 | 8.7e-09 | 1.6e-14 | 76.6 | 121 | (12, 132) | 147 | (61, 203) | 230 | Terminase small subunit | Terminase small subunit | | uniclust | UniRef100\_A0A1J5SR61 | 98.3 | 8.4e-09 | 1.6e-14 | 76.8 | 124 | (16, 139) | 147 | (28, 155) | 207 | Uncharacterized protein | Uncharacterized protein | | uniclust | UniRef100\_A0A1G0DQX9 | 98.3 | 7.7e-09 | 1.6e-14 | 79.5 | 89 | (45, 133) | 147 | (109, 197) | 212 | Terminase small subunit | Terminase small subunit | | uniclust | UniRef100\_A0A165RXZ6 | 98.3 | 9.1e-09 | 1.7e-14 | 75.1 | 95 | (43, 137) | 147 | (96, 192) | 195 | Elements of external origin | Elements of external origin | | uniclust | UniRef100\_A0A3T0L2W5 | 98.3 | 9.2e-09 | 1.7e-14 | 76.0 | 88 | (44, 131) | 147 | (126, 213) | 217 | Terminase | Terminase | | uniclust | UniRef100\_A0A1I3TC35 | 98.3 | 1e-08 | 1.9e-14 | 73.6 | 123 | (2, 134) | 147 | (21, 147) | 177 | Uncharacterized protein | Uncharacterized protein | | uniclust | UniRef100\_UPI00137A5DD6 | 98.3 | 1.2e-08 | 2.1e-14 | 75.2 | 124 | (14, 137) | 147 | (86, 210) | 243 | terminase small subunit | terminase small subunit | | uniclust | UniRef100\_A0A7W6H5E1 | 98.3 | 1.1e-08 | 2.1e-14 | 68.5 | 83 | (44, 130) | 147 | (30, 112) | 113 | Phage terminase Nu1 subunit (DNA packaging protein) | Phage terminase Nu1 subunit (DNA packaging protein) | | uniclust | UniRef100\_UPI0020049ABE | 98.3 | 1.2e-08 | 2.3e-14 | 70.2 | 110 | (1, 118) | 147 | (24, 134) | 147 | terminase small subunit | terminase small subunit | | uniclust | UniRef100\_A0A2M8EDC0 | 98.3 | 1.3e-08 | 2.4e-14 | 71.5 | 94 | (45, 138) | 147 | (64, 157) | 172 | Terminase small subunit (Fragment) | Terminase small subunit (Fragment) | | uniclust | UniRef100\_A0A0A0FF05 | 98.3 | 1.3e-08 | 2.6e-14 | 71.3 | 89 | (1, 89) | 147 | (24, 128) | 136 | DNA-packaging protein | DNA-packaging protein | | uniclust | UniRef100\_UPI00036DAC41 | 98.2 | 1.5e-08 | 2.8e-14 | 73.0 | 129 | (1, 129) | 147 | (32, 184) | 205 | DUF1441 family protein | DUF1441 family protein | | uniclust | UniRef100\_UPI000A1E6997 | 98.2 | 1.5e-08 | 2.8e-14 | 67.5 | 96 | (1, 102) | 147 | (23, 120) | 122 | terminase small subunit | terminase small subunit | | uniclust | UniRef100\_A0A973GUH3 | 98.2 | 1.5e-08 | 2.9e-14 | 70.1 | 90 | (44, 134) | 147 | (32, 121) | 144 | Uncharacterized protein | Uncharacterized protein | | uniclust | UniRef100\_A0A1M7R7I9 | 98.2 | 1.4e-08 | 2.9e-14 | 75.5 | 93 | (44, 136) | 147 | (94, 186) | 192 | Phage terminase small subunit | Phage terminase small subunit | | uniclust | UniRef100\_UPI00045BE5E8 | 98.2 | 1.6e-08 | 3e-14 | 72.4 | 135 | (4, 140) | 147 | (33, 181) | 198 | hypothetical protein | hypothetical protein | | uniclust | UniRef100\_UPI001FAD9689 | 98.2 | 1.7e-08 | 3.1e-14 | 69.2 | 114 | (21, 134) | 147 | (5, 122) | 146 | hypothetical protein | hypothetical protein | | uniclust | UniRef100\_A0A418VWY4 | 98.2 | 1.7e-08 | 3.2e-14 | 69.7 | 92 | (43, 134) | 147 | (36, 127) | 133 | Uncharacterized protein | Uncharacterized protein | | uniclust | UniRef100\_A0A6M0K1R5 | 98.2 | 1.8e-08 | 3.3e-14 | 69.3 | 128 | (7, 134) | 147 | (1, 132) | 150 | Terminase small subunit | Terminase small subunit | | uniclust | UniRef100\_A0A661I0F5 | 98.2 | 1.9e-08 | 3.4e-14 | 71.6 | 90 | (44, 133) | 147 | (97, 186) | 188 | Uncharacterized protein | Uncharacterized protein | | uniclust | UniRef100\_A0A8S5NY96 | 98.2 | 2e-08 | 3.6e-14 | 73.1 | 100 | (42, 142) | 147 | (112, 211) | 220 | Uncharacterized protein | Uncharacterized protein | | uniclust | UniRef100\_A0A067ZIZ9 | 98.2 | 1.9e-08 | 3.7e-14 | 73.5 | 123 | (4, 133) | 147 | (26, 176) | 190 | Terminase small subunit | Terminase small subunit | | uniclust | UniRef100\_A0A952DRL5 | 98.2 | 2.1e-08 | 3.9e-14 | 73.0 | 87 | (46, 132) | 147 | (97, 183) | 187 | Uncharacterized protein | Uncharacterized protein | | uniclust | UniRef100\_UPI0015E45FFC | 98.2 | 2.2e-08 | 4e-14 | 74.1 | 105 | (1, 105) | 147 | (58, 183) | 249 | terminase small subunit | terminase small subunit | | uniclust | UniRef100\_A0A6H1ZN68 | 98.2 | 2.8e-08 | 5.1e-14 | 61.9 | 77 | (54, 130) | 147 | (2, 78) | 81 | Uncharacterized protein (Fragment) | Uncharacterized protein (Fragment) | | uniclust | UniRef100\_A0A0F7L3Q2 | 98.2 | 2.8e-08 | 5.1e-14 | 71.9 | 129 | (1, 132) | 147 | (36, 202) | 209 | MerR family transcriptional regulator | MerR family transcriptional regulator | | uniclust | UniRef100\_A0A560D0R5 | 98.2 | 2.6e-08 | 5.1e-14 | 75.7 | 125 | (1, 132) | 147 | (26, 208) | 237 | Terminase small subunit | Terminase small subunit | | uniclust | UniRef100\_A0A1F9DZH8 | 98.2 | 2.9e-08 | 5.3e-14 | 72.0 | 105 | (1, 107) | 147 | (23, 127) | 213 | HTH merR-type domain-containing protein | HTH merR-type domain-containing protein | | uniclust | UniRef100\_A0A2E1R925 | 98.2 | 2.8e-08 | 5.4e-14 | 73.6 | 132 | (4, 135) | 147 | (33, 190) | 202 | Terminase small subunit | Terminase small subunit | | uniclust | UniRef100\_A0A5E8GTT9 | 98.2 | 3.1e-08 | 5.7e-14 | 70.1 | 128 | (3, 134) | 147 | (37, 167) | 180 | Phage DNA packaging protein, Nu1 subunit of terminase | Phage DNA packaging protein, Nu1 subunit of terminase | | uniclust | UniRef100\_A0A0F9RRF6 | 98.2 | 3.1e-08 | 6e-14 | 74.7 | 127 | (4, 136) | 147 | (89, 218) | 236 | Helix-turn-helix domain-containing protein | Helix-turn-helix domain-containing protein | | uniclust | UniRef100\_F8KQH1 | 98.2 | 3.1e-08 | 6e-14 | 80.6 | 122 | (4, 137) | 147 | (234, 355) | 392 | Uncharacterized protein | Uncharacterized protein | | uniclust | UniRef100\_A0A0Q5ZNV5 | 98.2 | 3.1e-08 | 6e-14 | 69.6 | 92 | (43, 134) | 147 | (43, 136) | 140 | Elements of external origin | Elements of external origin | | uniclust | UniRef100\_X1GDY6 | 98.1 | 3.4e-08 | 6.3e-14 | 68.0 | 125 | (1, 130) | 147 | (24, 148) | 149 | Helix-turn-helix domain-containing protein (Fragment) | Helix-turn-helix domain-containing protein (Fragment) | | uniclust | UniRef100\_A0A2J0PYP4 | 98.1 | 4e-08 | 8e-14 | 70.1 | 63 | (44, 106) | 147 | (41, 103) | 141 | Terminase | Terminase | | uniclust | UniRef100\_A0A2D9DW54 | 98.1 | 4e-08 | 8.3e-14 | 73.8 | 119 | (24, 142) | 147 | (43, 165) | 181 | Terminase small subunit | Terminase small subunit | | uniclust | UniRef100\_UPI001032D246 | 98.1 | 4.6e-08 | 8.5e-14 | 71.3 | 126 | (1, 132) | 147 | (87, 217) | 220 | hypothetical protein | hypothetical protein | | uniclust | UniRef100\_A0A4P7WC83 | 98.1 | 4.5e-08 | 9e-14 | 74.8 | 105 | (1, 105) | 147 | (39, 165) | 216 | Terminase small subunit | Terminase small subunit | | uniclust | UniRef100\_UPI00186BB4F4 | 98.1 | 5e-08 | 9.2e-14 | 62.4 | 83 | (47, 131) | 147 | (10, 92) | 94 | hypothetical protein | hypothetical protein | | uniclust | UniRef100\_UPI0021AED1F4 | 98.1 | 5.4e-08 | 9.9e-14 | 63.9 | 102 | (28, 131) | 147 | (2, 106) | 109 | hypothetical protein | hypothetical protein | | uniclust | UniRef100\_A0A2I5AR94 | 98.1 | 5.6e-08 | 1.1e-13 | 71.2 | 87 | (44, 132) | 147 | (102, 188) | 191 | Terminase small subunit | Terminase small subunit | | uniclust | UniRef100\_UPI0021A78783 | 98.1 | 5.9e-08 | 1.1e-13 | 64.3 | 89 | (44, 132) | 147 | (18, 106) | 116 | hypothetical protein | hypothetical protein | | uniclust | UniRef100\_A0A7C7KJQ7 | 98.1 | 6.2e-08 | 1.1e-13 | 69.6 | 128 | (1, 131) | 147 | (32, 183) | 197 | Terminase small subunit | Terminase small subunit | | uniclust | UniRef100\_A0A0F8X8C5 | 98.1 | 6.2e-08 | 1.1e-13 | 66.1 | 97 | (3, 106) | 147 | (39, 135) | 138 | Uncharacterized protein | Uncharacterized protein | | uniclust | UniRef100\_A0A8J7C8B9 | 98.1 | 6.5e-08 | 1.2e-13 | 67.9 | 111 | (21, 135) | 147 | (54, 164) | 167 | Uncharacterized protein | Uncharacterized protein | | uniclust | UniRef100\_UPI0018C227CA | 98.1 | 6.5e-08 | 1.2e-13 | 71.1 | 90 | (46, 135) | 147 | (114, 203) | 208 | hypothetical protein | hypothetical protein | | uniclust | UniRef100\_UPI0012DF7B95 | 98.1 | 7e-08 | 1.3e-13 | 66.9 | 122 | (2, 128) | 147 | (23, 144) | 153 | hypothetical protein | hypothetical protein | | uniclust | UniRef100\_A0A2A4V1Q2 | 98.1 | 7e-08 | 1.3e-13 | 69.1 | 87 | (44, 130) | 147 | (98, 184) | 191 | Uncharacterized protein | Uncharacterized protein | | uniclust | UniRef100\_A0A166JZ29 | 98.1 | 6.7e-08 | 1.3e-13 | 72.4 | 123 | (4, 134) | 147 | (23, 163) | 196 | DNA packaging protein | DNA packaging protein | | uniclust | UniRef100\_UPI001C90BF93 | 98.1 | 7.5e-08 | 1.4e-13 | 70.6 | 131 | (1, 132) | 147 | (59, 198) | 209 | terminase small subunit | terminase small subunit | | uniclust | UniRef100\_F5R896 | 98.1 | 7.7e-08 | 1.4e-13 | 67.5 | 124 | (1, 132) | 147 | (25, 148) | 166 | Uncharacterized protein | Uncharacterized protein | | uniclust | UniRef100\_A0A6G5Y155 | 98.1 | 7.4e-08 | 1.4e-13 | 70.7 | 85 | (45, 129) | 147 | (101, 185) | 194 | Terminase small subunit | Terminase small subunit | | uniclust | UniRef100\_A0A0G9K8N3 | 98.0 | 7.4e-08 | 1.4e-13 | 73.2 | 89 | (45, 133) | 147 | (137, 225) | 230 | Uncharacterized protein | Uncharacterized protein | | uniclust | UniRef100\_A0A085EXP4 | 98.0 | 7.1e-08 | 1.5e-13 | 75.0 | 132 | (1, 133) | 147 | (43, 198) | 237 | MarR family protein | MarR family protein | | uniclust | UniRef100\_A0A3G8M604 | 98.0 | 7.7e-08 | 1.5e-13 | 70.9 | 89 | (46, 134) | 147 | (111, 199) | 207 | Uncharacterized protein | Uncharacterized protein | | uniclust | UniRef100\_A0A934VIF6 | 98.0 | 8.1e-08 | 1.5e-13 | 69.7 | 88 | (42, 129) | 147 | (118, 205) | 211 | Uncharacterized protein | Uncharacterized protein | | uniclust | UniRef100\_A0A934FD39 | 98.0 | 8.3e-08 | 1.5e-13 | 62.5 | 77 | (1, 83) | 147 | (22, 98) | 103 | Terminase small subunit (Fragment) | Terminase small subunit (Fragment) | | uniclust | UniRef100\_A0A258L5M1 | 98.0 | 8.6e-08 | 1.6e-13 | 68.7 | 114 | (4, 130) | 147 | (55, 168) | 193 | Terminase small subunit | Terminase small subunit | | uniclust | UniRef100\_A0A7V0QL40 | 98.0 | 8.6e-08 | 1.6e-13 | 69.6 | 124 | (1, 132) | 147 | (79, 203) | 211 | DNA-binding protein | DNA-binding protein | | uniclust | UniRef100\_A0A0F9MYQ4 | 98.0 | 9.7e-08 | 1.8e-13 | 67.8 | 126 | (1, 132) | 147 | (30, 160) | 180 | HTH merR-type domain-containing protein (Fragment) | HTH merR-type domain-containing protein (Fragment) | | uniclust | UniRef100\_UPI001E5479A9 | 98.0 | 9.8e-08 | 1.8e-13 | 67.9 | 81 | (47, 129) | 147 | (99, 179) | 181 | hypothetical protein | hypothetical protein | | uniclust | UniRef100\_A0A963KDJ3 | 98.0 | 1.1e-07 | 2e-13 | 66.8 | 94 | (44, 137) | 147 | (70, 163) | 167 | Uncharacterized protein | Uncharacterized protein | | uniclust | UniRef100\_D8F5N7 | 98.0 | 1.1e-07 | 2.1e-13 | 63.3 | 105 | (27, 132) | 147 | (3, 108) | 118 | Uncharacterized protein | Uncharacterized protein | | uniclust | UniRef100\_A0A071MGC1 | 98.0 | 1.3e-07 | 2.5e-13 | 73.1 | 102 | (5, 106) | 147 | (69, 203) | 250 | Uncharacterized protein | Uncharacterized protein | | uniclust | UniRef100\_UPI001F38D2C2 | 98.0 | 1.3e-07 | 2.5e-13 | 68.6 | 126 | (4, 129) | 147 | (72, 206) | 210 | terminase small subunit | terminase small subunit | | uniclust | UniRef100\_UPI000AA827A0 | 98.0 | 1.4e-07 | 2.5e-13 | 66.6 | 111 | (21, 131) | 147 | (45, 163) | 171 | hypothetical protein | hypothetical protein | | uniclust | UniRef100\_A0A951ZIQ2 | 98.0 | 1.5e-07 | 2.7e-13 | 67.1 | 121 | (2, 129) | 147 | (42, 162) | 182 | Uncharacterized protein | Uncharacterized protein | | uniclust | UniRef100\_A0A2V7CIN0 | 98.0 | 1.5e-07 | 2.7e-13 | 66.2 | 122 | (6, 133) | 147 | (34, 156) | 167 | Terminase small subunit | Terminase small subunit | | uniclust | UniRef100\_A0A1Q8Q2C1 | 98.0 | 1.5e-07 | 2.8e-13 | 65.8 | 87 | (48, 134) | 147 | (29, 118) | 128 | Uncharacterized protein | Uncharacterized protein | | uniclust | UniRef100\_A0A1L8CPS1 | 98.0 | 1.6e-07 | 3e-13 | 67.0 | 118 | (13, 136) | 147 | (33, 150) | 161 | Phage DNA packaging protein Nu1 | Phage DNA packaging protein Nu1 | | uniclust | UniRef100\_A0A501WV30 | 98.0 | 1.7e-07 | 3e-13 | 74.1 | 105 | (1, 105) | 147 | (224, 349) | 415 | Terminase small subunit | Terminase small subunit | | uniclust | UniRef100\_A0A1I3KS91 | 97.9 | 1.7e-07 | 3.1e-13 | 66.2 | 122 | (4, 130) | 147 | (9, 149) | 155 | Uncharacterized protein | Uncharacterized protein | | uniclust | UniRef100\_A0A973E0Z4 | 97.9 | 1.7e-07 | 3.2e-13 | 67.4 | 88 | (44, 131) | 147 | (74, 162) | 174 | Uncharacterized protein | Uncharacterized protein | | uniclust | UniRef100\_UPI001359000C | 97.9 | 1.9e-07 | 3.5e-13 | 59.9 | 75 | (58, 133) | 147 | (15, 89) | 94 | hypothetical protein | hypothetical protein | | uniclust | UniRef100\_UPI000BFF95C5 | 97.9 | 2e-07 | 3.6e-13 | 67.7 | 86 | (45, 130) | 147 | (117, 202) | 206 | hypothetical protein | hypothetical protein | | uniclust | UniRef100\_A0A835YX25 | 97.9 | 2.4e-07 | 4.4e-13 | 79.1 | 123 | (3, 133) | 147 | (258, 381) | 962 | Phage terminase large subunit-domain-containing protein | Phage terminase large subunit-domain-containing protein | | uniclust | UniRef100\_A0A254T9S7 | 97.9 | 2.4e-07 | 4.5e-13 | 66.0 | 105 | (24, 130) | 147 | (25, 132) | 159 | DNA packaging Nu1 | DNA packaging Nu1 | | uniclust | UniRef100\_UPI000F3E537D | 97.9 | 2.5e-07 | 4.6e-13 | 59.8 | 69 | (1, 75) | 147 | (23, 92) | 97 | terminase small subunit | terminase small subunit | | uniclust | UniRef100\_A0A5E4NWJ3 | 97.9 | 2.5e-07 | 4.6e-13 | 67.3 | 122 | (1, 132) | 147 | (55, 186) | 209 | Uncharacterized protein | Uncharacterized protein | | uniclust | UniRef100\_UPI00209DF2FA | 97.9 | 2.5e-07 | 4.6e-13 | 68.6 | 125 | (6, 135) | 147 | (82, 216) | 240 | hypothetical protein | hypothetical protein | | uniclust | UniRef100\_A0A1H5Z527 | 97.9 | 2.4e-07 | 4.7e-13 | 71.6 | 107 | (20, 128) | 147 | (99, 209) | 258 | DNA packaging protein | DNA packaging protein | | uniclust | UniRef100\_M5PQI4 | 97.9 | 2.9e-07 | 5.3e-13 | 65.7 | 91 | (44, 134) | 147 | (73, 163) | 181 | Terminase small subunit | Terminase small subunit | | uniclust | UniRef100\_UPI001AE9382A | 97.9 | 3e-07 | 5.5e-13 | 58.7 | 80 | (55, 134) | 147 | (3, 84) | 90 | hypothetical protein | hypothetical protein | | uniclust | UniRef100\_UPI001E317423 | 97.9 | 3.1e-07 | 5.6e-13 | 62.7 | 83 | (46, 131) | 147 | (51, 133) | 135 | terminase small subunit | terminase small subunit | | uniclust | UniRef100\_A0A0F9PXT5 | 97.9 | 2.9e-07 | 5.7e-13 | 69.9 | 91 | (45, 135) | 147 | (113, 203) | 222 | HTH merR-type domain-containing protein | HTH merR-type domain-containing protein | | uniclust | UniRef100\_A0A6H1ZMV7 | 97.9 | 3.2e-07 | 5.9e-13 | 64.0 | 119 | (5, 129) | 147 | (33, 152) | 155 | Putative terminase | Putative terminase | | uniclust | UniRef100\_A0A2X1LFL7 | 97.9 | 3.2e-07 | 6e-13 | 62.0 | 86 | (45, 131) | 147 | (32, 117) | 124 | Uncharacterized protein | Uncharacterized protein | | uniclust | UniRef100\_A0A327KXV4 | 97.9 | 3.4e-07 | 6.2e-13 | 62.9 | 91 | (45, 136) | 147 | (41, 131) | 140 | Tetrapyrrole biosynthesis glutamyl-tRNA reductase dimerisation domain-containing protein | Tetrapyrrole biosynthesis glutamyl-tRNA reductase dimerisation domain-containing protein | | uniclust | UniRef100\_UPI001FDEB81B | 97.9 | 3.5e-07 | 6.4e-13 | 64.6 | 102 | (4, 105) | 147 | (2, 123) | 168 | DUF1441 family protein | DUF1441 family protein | | uniclust | UniRef100\_A0A2X1K069 | 97.8 | 3.3e-07 | 6.5e-13 | 68.2 | 96 | (6, 103) | 147 | (49, 145) | 187 | Prophage protein | Prophage protein | | uniclust | UniRef100\_A0A2J7TM92 | 97.8 | 3.6e-07 | 6.7e-13 | 59.4 | 87 | (46, 133) | 147 | (3, 89) | 100 | Uncharacterized protein | Uncharacterized protein | | uniclust | UniRef100\_A0A1W1CCQ2 | 97.8 | 3.9e-07 | 7.1e-13 | 63.6 | 125 | (2, 131) | 147 | (22, 152) | 155 | HTH merR-type domain-containing protein | HTH merR-type domain-containing protein | | uniclust | UniRef100\_UPI0012EB806B | 97.8 | 3.9e-07 | 7.2e-13 | 62.3 | 90 | (45, 134) | 147 | (28, 119) | 136 | hypothetical protein | hypothetical protein | | uniclust | UniRef100\_A0A7C7JGL7 | 97.8 | 4.1e-07 | 7.5e-13 | 60.8 | 87 | (44, 131) | 147 | (28, 114) | 118 | Host attachment protein | Host attachment protein | | uniclust | UniRef100\_A0A256F2V5 | 97.8 | 4.1e-07 | 7.9e-13 | 66.4 | 113 | (3, 129) | 147 | (40, 154) | 173 | Terminase small subunit | Terminase small subunit | | uniclust | UniRef100\_A0A5E7Q645 | 97.8 | 4.3e-07 | 7.9e-13 | 65.5 | 87 | (45, 131) | 147 | (101, 187) | 195 | Terminase small subunit | Terminase small subunit | | uniclust | UniRef100\_UPI00235EB4F2 | 97.8 | 4.5e-07 | 8.3e-13 | 65.6 | 92 | (44, 135) | 147 | (104, 195) | 198 | hypothetical protein | hypothetical protein | | uniclust | UniRef100\_UPI001CD487CF | 97.8 | 4.4e-07 | 8.4e-13 | 68.1 | 116 | (2, 132) | 147 | (63, 178) | 220 | hypothetical protein | hypothetical protein | | uniclust | UniRef100\_A0A369R942 | 97.8 | 4.5e-07 | 8.4e-13 | 65.4 | 121 | (3, 132) | 147 | (36, 156) | 180 | DNA-binding protein | DNA-binding protein | | uniclust | UniRef100\_A0A0H4A1N2 | 97.8 | 4.3e-07 | 8.5e-13 | 68.5 | 130 | (1, 136) | 147 | (30, 172) | 202 | Phage protein | Phage protein | | uniclust | UniRef100\_UPI0011982A0B | 97.8 | 4.7e-07 | 8.7e-13 | 62.7 | 89 | (44, 132) | 147 | (37, 127) | 135 | hypothetical protein | hypothetical protein | | uniclust | UniRef100\_M5T875 | 97.8 | 4.8e-07 | 9e-13 | 64.8 | 122 | (1, 129) | 147 | (23, 153) | 165 | Host attachment protein | Host attachment protein | | uniclust | UniRef100\_UPI001FA8243C | 97.8 | 4.8e-07 | 9e-13 | 60.6 | 77 | (3, 84) | 147 | (26, 104) | 108 | hypothetical protein | hypothetical protein | | uniclust | UniRef100\_UPI001E589372 | 97.8 | 5e-07 | 9.2e-13 | 58.4 | 71 | (1, 74) | 147 | (26, 96) | 96 | terminase small subunit | terminase small subunit | | uniclust | UniRef100\_E3H7D0 | 97.8 | 5.5e-07 | 1e-12 | 63.1 | 118 | (20, 142) | 147 | (35, 152) | 159 | Uncharacterized protein | Uncharacterized protein | | uniclust | UniRef100\_A0A358JR36 | 97.8 | 5.5e-07 | 1e-12 | 61.9 | 88 | (44, 131) | 147 | (43, 130) | 139 | Uncharacterized protein | Uncharacterized protein | | uniclust | UniRef100\_A0A3D4L0I6 | 97.8 | 5.5e-07 | 1e-12 | 69.7 | 131 | (2, 134) | 147 | (171, 309) | 336 | Uncharacterized protein | Uncharacterized protein | | uniclust | UniRef100\_A0A1S1TKW3 | 97.8 | 5.7e-07 | 1.1e-12 | 66.9 | 88 | (50, 137) | 147 | (90, 179) | 187 | Uncharacterized protein | Uncharacterized protein | | uniclust | UniRef100\_A0A345DE68 | 97.8 | 6.6e-07 | 1.2e-12 | 64.6 | 124 | (2, 131) | 147 | (32, 162) | 193 | Terminase small subunit | Terminase small subunit | | uniclust | UniRef100\_UPI00098D0DFA | 97.8 | 6.7e-07 | 1.2e-12 | 61.3 | 124 | (7, 132) | 147 | (1, 126) | 137 | hypothetical protein | hypothetical protein | | uniclust | UniRef100\_A0A651HJ89 | 97.8 | 6.7e-07 | 1.2e-12 | 65.6 | 105 | (4, 111) | 147 | (53, 158) | 217 | Helix-turn-helix domain-containing protein | Helix-turn-helix domain-containing protein | | uniclust | UniRef100\_A0A369QU30 | 97.8 | 6.8e-07 | 1.2e-12 | 63.4 | 87 | (44, 130) | 147 | (77, 163) | 170 | Terminase small subunit | Terminase small subunit | | uniclust | UniRef100\_A0A5C7Q6H9 | 97.8 | 7.1e-07 | 1.3e-12 | 64.9 | 132 | (2, 133) | 147 | (25, 196) | 203 | Terminase small subunit | Terminase small subunit | | uniclust | UniRef100\_UPI000B983DC3 | 97.7 | 7.1e-07 | 1.3e-12 | 60.4 | 84 | (48, 131) | 147 | (40, 123) | 126 | hypothetical protein | hypothetical protein | | uniclust | UniRef100\_A0A0S9PYS1 | 97.7 | 7e-07 | 1.3e-12 | 66.7 | 125 | (2, 133) | 147 | (24, 192) | 195 | Uncharacterized protein | Uncharacterized protein | | uniclust | UniRef100\_A0A978BM29 | 97.7 | 7.7e-07 | 1.4e-12 | 61.8 | 121 | (1, 131) | 147 | (21, 141) | 148 | DNA packaging protein | DNA packaging protein | | uniclust | UniRef100\_A0A149VFT2 | 97.7 | 7.5e-07 | 1.4e-12 | 63.0 | 89 | (45, 133) | 147 | (48, 136) | 144 | Uncharacterized protein | Uncharacterized protein | | uniclust | UniRef100\_A0A7V8JF95 | 97.7 | 7.8e-07 | 1.5e-12 | 62.5 | 93 | (43, 135) | 147 | (36, 130) | 136 | Uncharacterized protein | Uncharacterized protein | | uniclust | UniRef100\_A0A812JEV6 | 97.7 | 8.1e-07 | 1.5e-12 | 82.6 | 90 | (43, 132) | 147 | (273, 362) | 4451 | Phage portal protein | Phage portal protein | | uniclust | UniRef100\_A0A2U3QE23 | 97.7 | 8.4e-07 | 1.5e-12 | 63.0 | 92 | (43, 134) | 147 | (62, 153) | 171 | Terminase small subunit | Terminase small subunit | | uniclust | UniRef100\_A0A272EML8 | 97.7 | 9.1e-07 | 1.7e-12 | 55.9 | 70 | (62, 131) | 147 | (6, 75) | 84 | Uncharacterized protein | Uncharacterized protein | | uniclust | UniRef100\_A0A3A0EF07 | 97.7 | 9.2e-07 | 1.7e-12 | 64.8 | 89 | (44, 132) | 147 | (83, 171) | 194 | Terminase small subunit | Terminase small subunit | | uniclust | UniRef100\_A0A965H8A5 | 97.7 | 9.5e-07 | 1.7e-12 | 56.3 | 84 | (46, 131) | 147 | (2, 85) | 88 | Uncharacterized protein | Uncharacterized protein | | uniclust | UniRef100\_A0A937TUA9 | 97.7 | 9.5e-07 | 1.7e-12 | 62.3 | 124 | (1, 133) | 147 | (29, 153) | 163 | Uncharacterized protein | Uncharacterized protein | | uniclust | UniRef100\_UPI0021F8C375 | 97.7 | 1e-06 | 1.9e-12 | 59.4 | 83 | (50, 132) | 147 | (37, 119) | 122 | hypothetical protein | hypothetical protein | | uniclust | UniRef100\_A0A3D9Z369 | 97.7 | 1.1e-06 | 1.9e-12 | 61.2 | 107 | (22, 128) | 147 | (2, 113) | 149 | Uncharacterized protein | Uncharacterized protein | | uniclust | UniRef100\_A0A176FBY6 | 97.7 | 1.1e-06 | 2.1e-12 | 64.4 | 113 | (3, 130) | 147 | (34, 146) | 170 | Uncharacterized protein | Uncharacterized protein | | uniclust | UniRef100\_A0A0F9KLK2 | 97.7 | 1.2e-06 | 2.2e-12 | 59.4 | 84 | (47, 130) | 147 | (34, 119) | 126 | Uncharacterized protein (Fragment) | Uncharacterized protein (Fragment) | | uniclust | UniRef100\_A0A8S0GWK0 | 97.7 | 1.2e-06 | 2.3e-12 | 56.8 | 71 | (63, 133) | 147 | (1, 71) | 91 | Uncharacterized protein | Uncharacterized protein | | uniclust | UniRef100\_A0A0F9I3X3 | 97.7 | 1.3e-06 | 2.3e-12 | 59.4 | 88 | (2, 96) | 147 | (34, 124) | 127 | DNA-binding protein (Fragment) | DNA-binding protein (Fragment) | | uniclust | UniRef100\_UPI0011E5ABD1 | 97.7 | 1.3e-06 | 2.4e-12 | 59.2 | 74 | (61, 134) | 147 | (20, 93) | 126 | hypothetical protein | hypothetical protein | | uniclust | UniRef100\_A0A0Q1ADF8 | 97.7 | 1.2e-06 | 2.4e-12 | 67.0 | 87 | (46, 132) | 147 | (124, 210) | 218 | Terminase | Terminase | | uniclust | UniRef100\_UPI0006948996 | 97.7 | 1.3e-06 | 2.5e-12 | 62.6 | 86 | (1, 86) | 147 | (48, 155) | 156 | hypothetical protein | hypothetical protein | | uniclust | UniRef100\_UPI002306CCEF | 97.7 | 1.3e-06 | 2.5e-12 | 64.9 | 96 | (1, 102) | 147 | (44, 139) | 238 | hypothetical protein | hypothetical protein | | uniclust | UniRef100\_UPI0010B5C855 | 97.7 | 1.4e-06 | 2.5e-12 | 65.1 | 115 | (3, 132) | 147 | (69, 183) | 225 | hypothetical protein | hypothetical protein | | uniclust | UniRef100\_A0A964PCS7 | 97.6 | 1.4e-06 | 2.6e-12 | 62.4 | 132 | (5, 139) | 147 | (45, 177) | 180 | Uncharacterized protein | Uncharacterized protein | | uniclust | UniRef100\_A0A179D3Q0 | 97.6 | 1.4e-06 | 2.7e-12 | 64.7 | 129 | (1, 135) | 147 | (27, 156) | 207 | HTH merR-type domain-containing protein | HTH merR-type domain-containing protein | | uniclust | UniRef100\_UPI0009E87574 | 97.6 | 1.5e-06 | 2.8e-12 | 56.5 | 88 | (48, 135) | 147 | (4, 91) | 98 | hypothetical protein | hypothetical protein | | uniclust | UniRef100\_R7UYV5 | 97.6 | 1.6e-06 | 2.8e-12 | 66.8 | 80 | (54, 133) | 147 | (3, 82) | 310 | Phage terminase large subunit GpA ATPase domain-containing protein (Fragment) | Phage terminase large subunit GpA ATPase domain-containing protein (Fragment) | | uniclust | UniRef100\_UPI0004D72F9B | 97.6 | 1.7e-06 | 3.1e-12 | 72.5 | 107 | (1, 107) | 147 | (278, 400) | 697 | terminase small subunit | terminase small subunit | | uniclust | UniRef100\_A0A124V088 | 97.6 | 1.7e-06 | 3.1e-12 | 55.0 | 70 | (63, 132) | 147 | (9, 78) | 86 | Uncharacterized protein | Uncharacterized protein | | uniclust | UniRef100\_A0A090R9J5 | 97.6 | 1.7e-06 | 3.3e-12 | 59.8 | 62 | (45, 106) | 147 | (15, 76) | 115 | Phage protein | Phage protein | | uniclust | UniRef100\_A0A935IH67 | 97.6 | 1.9e-06 | 3.5e-12 | 57.5 | 85 | (45, 129) | 147 | (28, 112) | 113 | Uncharacterized protein | Uncharacterized protein | | uniclust | UniRef100\_UPI0019149DFE | 97.6 | 1.9e-06 | 3.5e-12 | 60.3 | 84 | (46, 129) | 147 | (63, 146) | 152 | hypothetical protein | hypothetical protein | | uniclust | UniRef100\_A0A847LN12 | 97.6 | 2e-06 | 3.6e-12 | 61.6 | 125 | (1, 131) | 147 | (42, 169) | 178 | Terminase small subunit | Terminase small subunit | | uniclust | UniRef100\_A0A432RHB9 | 97.6 | 2e-06 | 3.6e-12 | 59.7 | 87 | (45, 132) | 147 | (50, 136) | 144 | Host attachment protein | Host attachment protein | | uniclust | UniRef100\_A0A351U3V4 | 97.6 | 2.1e-06 | 3.9e-12 | 60.4 | 115 | (1, 129) | 147 | (25, 139) | 149 | Helix-turn-helix domain-containing protein (Fragment) | Helix-turn-helix domain-containing protein (Fragment) | | uniclust | UniRef100\_UPI000C997E74 | 97.6 | 2.3e-06 | 4.1e-12 | 62.2 | 110 | (21, 131) | 147 | (59, 169) | 197 | hypothetical protein | hypothetical protein | | uniclust | UniRef100\_A0A3G2R721 | 97.6 | 2.4e-06 | 4.4e-12 | 53.6 | 59 | (74, 132) | 147 | (3, 61) | 79 | Uncharacterized protein | Uncharacterized protein | | uniclust | UniRef100\_A0A2E9M7Y8 | 97.6 | 2.3e-06 | 4.4e-12 | 61.1 | 107 | (14, 130) | 147 | (36, 143) | 144 | Uncharacterized protein | Uncharacterized protein | | uniclust | UniRef100\_A0A7J6YMH7 | 97.6 | 2.4e-06 | 4.5e-12 | 70.6 | 83 | (49, 131) | 147 | (447, 529) | 531 | ParB/Sulfiredoxin domain-containing protein | ParB/Sulfiredoxin domain-containing protein | | uniclust | UniRef100\_A0A090DYF1 | 97.6 | 2.3e-06 | 4.6e-12 | 65.6 | 93 | (3, 103) | 147 | (62, 154) | 199 | Uncharacterized protein | Uncharacterized protein | | uniclust | UniRef100\_UPI00125E4FA9 | 97.6 | 2.5e-06 | 4.7e-12 | 55.4 | 81 | (50, 130) | 147 | (9, 89) | 96 | hypothetical protein | hypothetical protein | | uniclust | UniRef100\_A0A849W6W6 | 97.6 | 2.6e-06 | 4.7e-12 | 58.7 | 128 | (4, 132) | 147 | (4, 135) | 137 | Uncharacterized protein | Uncharacterized protein | | uniclust | UniRef100\_UPI000F0FA4D5 | 97.6 | 2.5e-06 | 4.7e-12 | 59.8 | 80 | (21, 100) | 147 | (41, 121) | 131 | terminase small subunit | terminase small subunit | | uniclust | UniRef100\_A0A1F9BH28 | 97.6 | 2.6e-06 | 4.8e-12 | 62.1 | 130 | (1, 135) | 147 | (39, 182) | 201 | Helix-turn-helix domain-containing protein | Helix-turn-helix domain-containing protein | | uniclust | UniRef100\_A0A8X6HP51 | 97.5 | 2.7e-06 | 5e-12 | 66.6 | 80 | (50, 129) | 147 | (269, 348) | 350 | DNA methylase-like protein | DNA methylase-like protein | | uniclust | UniRef100\_A0A9D5VQR4 | 97.5 | 2.7e-06 | 5e-12 | 61.3 | 83 | (46, 129) | 147 | (103, 185) | 186 | Uncharacterized protein | Uncharacterized protein | | uniclust | UniRef100\_A0A850RI89 | 97.5 | 2.9e-06 | 5.3e-12 | 61.1 | 127 | (6, 133) | 147 | (43, 170) | 184 | Terminase small subunit | Terminase small subunit | | uniclust | UniRef100\_UPI001FBB6124 | 97.5 | 2.9e-06 | 5.3e-12 | 56.3 | 88 | (45, 132) | 147 | (5, 92) | 108 | hypothetical protein | hypothetical protein | | uniclust | UniRef100\_A0A0B0VTU2 | 97.5 | 2.9e-06 | 5.4e-12 | 61.8 | 128 | (1, 130) | 147 | (24, 167) | 185 | Terminase | Terminase | | uniclust | UniRef100\_A0A1G3M3Z5 | 97.5 | 3e-06 | 5.6e-12 | 59.9 | 83 | (44, 129) | 147 | (75, 157) | 162 | Uncharacterized protein | Uncharacterized protein | | uniclust | UniRef100\_UPI0018800AA8 | 97.5 | 3.2e-06 | 5.8e-12 | 60.3 | 88 | (44, 131) | 147 | (79, 166) | 172 | hypothetical protein | hypothetical protein | | uniclust | UniRef100\_A0A352L2D0 | 97.5 | 3.2e-06 | 5.9e-12 | 62.6 | 106 | (21, 130) | 147 | (71, 176) | 194 | Terminase small subunit | Terminase small subunit | | uniclust | UniRef100\_A0A965BIW1 | 97.5 | 3.3e-06 | 6.1e-12 | 55.6 | 87 | (46, 132) | 147 | (11, 97) | 103 | Uncharacterized protein | Uncharacterized protein | | uniclust | UniRef100\_A0A066Q1C8 | 97.5 | 3.2e-06 | 6.1e-12 | 64.2 | 124 | (4, 131) | 147 | (58, 186) | 209 | Prophage Qin DNA packaging protein NU1-like protein | Prophage Qin DNA packaging protein NU1-like protein | | uniclust | UniRef100\_A0A0F9B6S6 | 97.5 | 3.5e-06 | 6.3e-12 | 64.0 | 110 | (21, 131) | 147 | (130, 239) | 270 | Uncharacterized protein | Uncharacterized protein | | uniclust | UniRef100\_A0A953PU68 | 97.5 | 3.6e-06 | 6.6e-12 | 61.3 | 92 | (44, 137) | 147 | (99, 190) | 198 | Uncharacterized protein | Uncharacterized protein | | uniclust | UniRef100\_UPI0013EA2CF4 | 97.5 | 3.6e-06 | 6.7e-12 | 62.3 | 87 | (45, 131) | 147 | (126, 212) | 222 | hypothetical protein | hypothetical protein | | uniclust | UniRef100\_UPI00039DC22E | 97.5 | 3.7e-06 | 6.8e-12 | 59.8 | 88 | (44, 131) | 147 | (72, 159) | 168 | hypothetical protein | hypothetical protein | | uniclust | UniRef100\_A0A348YJM0 | 97.5 | 4.3e-06 | 8e-12 | 61.5 | 86 | (44, 130) | 147 | (103, 188) | 191 | Terminase small subunit | Terminase small subunit | | uniclust | UniRef100\_A0A286GNB3 | 97.5 | 4.4e-06 | 8.1e-12 | 58.5 | 118 | (6, 129) | 147 | (17, 144) | 150 | Uncharacterized protein | Uncharacterized protein | | uniclust | UniRef100\_A0A2T2PZJ4 | 97.5 | 4.6e-06 | 8.4e-12 | 62.6 | 94 | (2, 103) | 147 | (85, 178) | 233 | Uncharacterized protein | Uncharacterized protein | | uniclust | UniRef100\_A0A8I2ABK2 | 97.5 | 4.7e-06 | 8.8e-12 | 59.6 | 121 | (4, 132) | 147 | (27, 147) | 153 | Uncharacterized protein | Uncharacterized protein | | uniclust | UniRef100\_A0A5C8B3W1 | 97.5 | 4.8e-06 | 8.9e-12 | 59.9 | 87 | (45, 132) | 147 | (84, 170) | 181 | DNA packaging protein | DNA packaging protein | | uniclust | UniRef100\_UPI001421EE26 | 97.4 | 5.1e-06 | 9.4e-12 | 58.7 | 123 | (2, 130) | 147 | (24, 156) | 159 | terminase small subunit | terminase small subunit | | uniclust | UniRef100\_UPI001008D493 | 97.4 | 5.2e-06 | 9.6e-12 | 59.8 | 113 | (21, 133) | 147 | (27, 147) | 181 | hypothetical protein | hypothetical protein | | uniclust | UniRef100\_A0A177QEF8 | 97.4 | 5.3e-06 | 9.8e-12 | 52.4 | 67 | (66, 132) | 147 | (2, 68) | 81 | Uncharacterized protein | Uncharacterized protein | | uniclust | UniRef100\_D8JWB5 | 97.4 | 5.2e-06 | 9.8e-12 | 61.8 | 85 | (44, 133) | 147 | (111, 195) | 203 | Terminase small subunit | Terminase small subunit | | uniclust | UniRef100\_A0A4V2SN76 | 97.4 | 5.5e-06 | 1e-11 | 63.1 | 83 | (47, 129) | 147 | (187, 269) | 275 | Phage terminase Nu1 subunit (DNA packaging protein) (Fragment) | Phage terminase Nu1 subunit (DNA packaging protein) (Fragment) | | uniclust | UniRef100\_A0A2V9B9X4 | 97.4 | 5.5e-06 | 1e-11 | 54.6 | 85 | (25, 109) | 147 | (5, 96) | 103 | Uncharacterized protein | Uncharacterized protein | | uniclust | UniRef100\_A0A0J1LWF8 | 97.4 | 5.6e-06 | 1.1e-11 | 60.7 | 93 | (12, 106) | 147 | (41, 133) | 176 | Terminase | Terminase | | uniclust | UniRef100\_A0A969QZA3 | 97.4 | 5.8e-06 | 1.1e-11 | 59.1 | 84 | (47, 130) | 147 | (87, 170) | 171 | Uncharacterized protein | Uncharacterized protein | | uniclust | UniRef100\_A0A7C1MJZ5 | 97.4 | 5.8e-06 | 1.1e-11 | 60.6 | 113 | (17, 134) | 147 | (68, 180) | 204 | DUF1441 family protein | DUF1441 family protein | | uniclust | UniRef100\_A0A536XED3 | 97.4 | 5.8e-06 | 1.1e-11 | 57.0 | 86 | (44, 130) | 147 | (48, 133) | 135 | Uncharacterized protein | Uncharacterized protein | | uniclust | UniRef100\_A0A0D6PUD1 | 97.4 | 5.8e-06 | 1.1e-11 | 65.2 | 104 | (1, 104) | 147 | (77, 207) | 265 | Phage DNA packaging protein | Phage DNA packaging protein | | uniclust | UniRef100\_A0A437MJQ9 | 97.4 | 5.9e-06 | 1.1e-11 | 62.1 | 60 | (42, 101) | 147 | (85, 144) | 197 | DNA packaging protein | DNA packaging protein | | uniclust | UniRef100\_A0A5E4N3A7 | 97.4 | 6.6e-06 | 1.2e-11 | 64.8 | 84 | (50, 133) | 147 | (39, 122) | 359 | Ankyrin repeat-containing domain,Ankyrin repeat | Ankyrin repeat-containing domain,Ankyrin repeat | | uniclust | UniRef100\_A0A518LAV0 | 97.4 | 6.7e-06 | 1.3e-11 | 60.6 | 125 | (1, 130) | 147 | (27, 154) | 197 | Phage DNA packaging protein Nu1 | Phage DNA packaging protein Nu1 | | uniclust | UniRef100\_E6X1P9 | 97.4 | 7e-06 | 1.3e-11 | 59.6 | 84 | (48, 131) | 147 | (106, 189) | 191 | Terminase small subunit | Terminase small subunit | | uniclust | UniRef100\_A0A106BVR3 | 97.4 | 7.1e-06 | 1.3e-11 | 60.2 | 79 | (62, 140) | 147 | (98, 176) | 196 | Terminase small subunit | Terminase small subunit | | uniclust | UniRef100\_A0A6G5R4R9 | 97.4 | 7.5e-06 | 1.4e-11 | 55.0 | 81 | (46, 129) | 147 | (21, 101) | 115 | Uncharacterized protein | Uncharacterized protein | | uniclust | UniRef100\_UPI001EE44BAD | 97.4 | 7.5e-06 | 1.4e-11 | 52.9 | 79 | (47, 125) | 147 | (10, 88) | 91 | terminase small subunit | terminase small subunit | | uniclust | UniRef100\_A0A1V5JDJ1 | 97.4 | 7.6e-06 | 1.4e-11 | 59.0 | 79 | (1, 91) | 147 | (40, 118) | 181 | Phage DNA packaging protein Nu1 | Phage DNA packaging protein Nu1 | | uniclust | UniRef100\_A0A517V825 | 97.4 | 7.2e-06 | 1.4e-11 | 60.9 | 117 | (1, 133) | 147 | (33, 150) | 169 | Uncharacterized protein | Uncharacterized protein | | uniclust | UniRef100\_A0A354SKF5 | 97.4 | 7.8e-06 | 1.4e-11 | 61.1 | 81 | (51, 131) | 147 | (147, 227) | 234 | Uncharacterized protein | Uncharacterized protein | | uniclust | UniRef100\_A0A7W2H2Q2 | 97.4 | 8.4e-06 | 1.5e-11 | 53.5 | 74 | (62, 136) | 147 | (2, 75) | 99 | Uncharacterized protein | Uncharacterized protein | | uniclust | UniRef100\_A0A316FGX7 | 97.3 | 8.5e-06 | 1.7e-11 | 54.7 | 34 | (1, 34) | 147 | (50, 83) | 90 | Excisionase DUF1233 | Excisionase DUF1233 | | uniclust | UniRef100\_UPI0015923F61 | 97.3 | 9.3e-06 | 1.7e-11 | 56.4 | 90 | (45, 134) | 147 | (45, 136) | 140 | hypothetical protein | hypothetical protein | | uniclust | UniRef100\_A0A9E1EL47 | 97.3 | 9.4e-06 | 1.8e-11 | 57.9 | 97 | (3, 104) | 147 | (52, 149) | 153 | Uncharacterized protein (Fragment) | Uncharacterized protein (Fragment) | | uniclust | UniRef100\_A0A6J7X1I5 | 97.3 | 9.6e-06 | 1.8e-11 | 59.4 | 122 | (1, 129) | 147 | (41, 165) | 200 | Terminase small subunit | Terminase small subunit | | uniclust | UniRef100\_A0A1I3HJ39 | 97.3 | 9.1e-06 | 1.8e-11 | 61.3 | 90 | (44, 133) | 147 | (89, 178) | 191 | Homeodomain-like domain-containing protein | Homeodomain-like domain-containing protein | | uniclust | UniRef100\_A0A353ZI83 | 97.3 | 1e-05 | 1.9e-11 | 59.0 | 118 | (5, 134) | 147 | (59, 178) | 194 | Uncharacterized protein | Uncharacterized protein | | uniclust | UniRef100\_UPI000D30D27B | 97.3 | 1.1e-05 | 2e-11 | 54.4 | 86 | (45, 130) | 147 | (25, 110) | 116 | hypothetical protein | hypothetical protein | | uniclust | UniRef100\_A0A0S9S7I7 | 97.3 | 1.1e-05 | 2.1e-11 | 61.8 | 105 | (1, 106) | 147 | (52, 171) | 283 | HTH merR-type domain-containing protein | HTH merR-type domain-containing protein | | uniclust | UniRef100\_A0A177RGU4 | 97.3 | 1e-05 | 2.1e-11 | 63.9 | 91 | (6, 103) | 147 | (93, 191) | 242 | Terminase small subunit | Terminase small subunit | | uniclust | UniRef100\_A0A7X8UB87 | 97.3 | 1.2e-05 | 2.1e-11 | 57.3 | 122 | (1, 134) | 147 | (26, 147) | 164 | Terminase small subunit | Terminase small subunit | | uniclust | UniRef100\_UPI000D0BA657 | 97.3 | 1.2e-05 | 2.1e-11 | 55.5 | 107 | (25, 131) | 147 | (10, 118) | 133 | hypothetical protein | hypothetical protein | | uniclust | UniRef100\_A0A2Z6DY60 | 97.3 | 1.2e-05 | 2.1e-11 | 57.8 | 85 | (46, 131) | 147 | (87, 171) | 174 | Uncharacterized protein | Uncharacterized protein | | uniclust | UniRef100\_A0A5C7PCC4 | 97.3 | 1.2e-05 | 2.1e-11 | 59.5 | 101 | (1, 106) | 147 | (87, 193) | 213 | Terminase small subunit | Terminase small subunit | | uniclust | UniRef100\_UPI001FBB0D88 | 97.3 | 1.2e-05 | 2.2e-11 | 55.2 | 78 | (1, 78) | 147 | (29, 111) | 129 | terminase small subunit | terminase small subunit | | uniclust | UniRef100\_UPI00223AE246 | 97.3 | 1.2e-05 | 2.3e-11 | 59.8 | 82 | (50, 131) | 147 | (141, 222) | 225 | hypothetical protein | hypothetical protein | | uniclust | UniRef100\_A0A1X7JJP1 | 97.3 | 1.3e-05 | 2.4e-11 | 54.7 | 93 | (43, 135) | 147 | (19, 111) | 124 | Uncharacterized protein | Uncharacterized protein | | uniclust | UniRef100\_UPI001FB94040 | 97.3 | 1.3e-05 | 2.4e-11 | 59.4 | 89 | (45, 134) | 147 | (62, 150) | 217 | hypothetical protein | hypothetical protein | | uniclust | UniRef100\_A0A0Q5HEW5 | 97.3 | 1.4e-05 | 2.5e-11 | 57.2 | 104 | (1, 107) | 147 | (53, 162) | 169 | Terminase small subunit | Terminase small subunit | | uniclust | UniRef100\_A0A7V3AH88 | 97.3 | 1.4e-05 | 2.6e-11 | 57.9 | 95 | (6, 105) | 147 | (27, 127) | 183 | Elements of external origin | Elements of external origin | | uniclust | UniRef100\_UPI00211ECC39 | 97.3 | 1.4e-05 | 2.6e-11 | 56.0 | 108 | (21, 136) | 147 | (36, 143) | 147 | hypothetical protein | hypothetical protein | | uniclust | UniRef100\_A0A4Q3DN64 | 97.3 | 1.3e-05 | 2.6e-11 | 64.2 | 91 | (44, 135) | 147 | (74, 164) | 284 | Uncharacterized protein | Uncharacterized protein | | uniclust | UniRef100\_A0A1Y6C3X7 | 97.3 | 1.4e-05 | 2.6e-11 | 56.3 | 88 | (43, 130) | 147 | (60, 147) | 152 | Phage DNA packaging protein, Nu1 subunit of terminase | Phage DNA packaging protein, Nu1 subunit of terminase | | uniclust | UniRef100\_A0A356R6T8 | 97.3 | 1.5e-05 | 2.7e-11 | 56.2 | 111 | (16, 131) | 147 | (39, 149) | 152 | Terminase small subunit | Terminase small subunit | | uniclust | UniRef100\_A0A1Q8YAQ8 | 97.3 | 1.5e-05 | 2.7e-11 | 55.7 | 89 | (42, 130) | 147 | (37, 125) | 143 | Uncharacterized protein | Uncharacterized protein | | uniclust | UniRef100\_A0A966W431 | 97.3 | 1.5e-05 | 2.7e-11 | 53.4 | 107 | (21, 129) | 147 | (2, 108) | 111 | Uncharacterized protein | Uncharacterized protein | | uniclust | UniRef100\_A0A0P1F0B1 | 97.3 | 1.5e-05 | 2.7e-11 | 53.8 | 84 | (2, 90) | 147 | (21, 107) | 111 | Uncharacterized protein | Uncharacterized protein | | uniclust | UniRef100\_A0A9D2GWA4 | 97.2 | 1.4e-05 | 2.8e-11 | 61.8 | 123 | (3, 132) | 147 | (97, 221) | 235 | Uncharacterized protein | Uncharacterized protein | | uniclust | UniRef100\_UPI001F0AC2C8 | 97.2 | 1.6e-05 | 2.8e-11 | 65.2 | 96 | (46, 141) | 147 | (64, 159) | 506 | phage terminase large subunit family protein | phage terminase large subunit family protein | | uniclust | UniRef100\_A0A1I4VF06 | 97.2 | 1.6e-05 | 3e-11 | 58.9 | 111 | (21, 132) | 147 | (80, 193) | 217 | Phage DNA packaging protein, Nu1 subunit of terminase | Phage DNA packaging protein, Nu1 subunit of terminase | | uniclust | UniRef100\_A0A068YVF4 | 97.2 | 1.7e-05 | 3.1e-11 | 59.5 | 90 | (44, 133) | 147 | (101, 193) | 205 | RNA polymerase subunit sigma-70 | RNA polymerase subunit sigma-70 | | uniclust | UniRef100\_I6APC4 | 97.2 | 1.8e-05 | 3.2e-11 | 59.8 | 123 | (4, 131) | 147 | (86, 210) | 237 | Uncharacterized protein | Uncharacterized protein | | uniclust | UniRef100\_A0A2H0LPX9 | 97.2 | 1.8e-05 | 3.3e-11 | 56.7 | 122 | (2, 131) | 147 | (39, 160) | 170 | HTH merR-type domain-containing protein | HTH merR-type domain-containing protein | | uniclust | UniRef100\_UPI001AB0391E | 97.2 | 1.8e-05 | 3.3e-11 | 58.3 | 88 | (45, 132) | 147 | (103, 190) | 206 | hypothetical protein | hypothetical protein | | uniclust | UniRef100\_A0A3G6WFJ2 | 97.2 | 1.8e-05 | 3.3e-11 | 62.2 | 103 | (1, 103) | 147 | (47, 171) | 342 | Uncharacterized protein | Uncharacterized protein | | uniclust | UniRef100\_UPI002004F906 | 97.2 | 1.8e-05 | 3.4e-11 | 58.7 | 113 | (3, 116) | 147 | (53, 174) | 217 | hypothetical protein | hypothetical protein | | uniclust | UniRef100\_N9NDI8 | 97.2 | 1.9e-05 | 3.4e-11 | 54.9 | 81 | (50, 130) | 147 | (52, 132) | 139 | Uncharacterized protein | Uncharacterized protein | | uniclust | UniRef100\_A0A8S5U955 | 97.2 | 1.9e-05 | 3.5e-11 | 59.5 | 125 | (1, 131) | 147 | (99, 235) | 242 | DNA packaging protein | DNA packaging protein | | uniclust | UniRef100\_UPI00137449B1 | 97.2 | 2e-05 | 3.6e-11 | 59.5 | 115 | (17, 131) | 147 | (40, 181) | 244 | hypothetical protein | hypothetical protein | | uniclust | UniRef100\_A0A978BJL5 | 97.2 | 2e-05 | 3.7e-11 | 55.7 | 88 | (43, 130) | 147 | (65, 152) | 154 | Uncharacterized protein | Uncharacterized protein | | uniclust | UniRef100\_A0A2V5U841 | 97.2 | 2.2e-05 | 4e-11 | 56.2 | 122 | (5, 132) | 147 | (38, 159) | 167 | DUF4332 domain-containing protein | DUF4332 domain-containing protein | | uniclust | UniRef100\_UPI0018655FAC | 97.2 | 2.2e-05 | 4.1e-11 | 54.2 | 89 | (45, 133) | 147 | (23, 114) | 121 | hypothetical protein | hypothetical protein | | uniclust | UniRef100\_A0A4Q2XV87 | 97.2 | 2.3e-05 | 4.3e-11 | 52.5 | 58 | (43, 100) | 147 | (53, 110) | 110 | Uncharacterized protein | Uncharacterized protein | | uniclust | UniRef100\_UPI00142DB13B | 97.2 | 2.5e-05 | 4.6e-11 | 55.2 | 88 | (43, 132) | 147 | (25, 112) | 153 | terminase small subunit | terminase small subunit | | uniclust | UniRef100\_UPI00130115E7 | 97.1 | 2.6e-05 | 4.8e-11 | 54.2 | 85 | (44, 128) | 147 | (14, 98) | 137 | hypothetical protein | hypothetical protein | | uniclust | UniRef100\_A0A248JSB1 | 97.1 | 2.6e-05 | 5e-11 | 60.0 | 87 | (45, 131) | 147 | (105, 191) | 216 | Terminase small subunit | Terminase small subunit | | uniclust | UniRef100\_UPI00069BD42E | 97.1 | 2.8e-05 | 5.2e-11 | 56.4 | 118 | (5, 126) | 147 | (25, 146) | 182 | terminase small subunit | terminase small subunit | | uniclust | UniRef100\_UPI002240E942 | 97.1 | 2.8e-05 | 5.2e-11 | 56.7 | 121 | (2, 129) | 147 | (36, 157) | 189 | hypothetical protein | hypothetical protein | | uniclust | UniRef100\_A0A847HLK2 | 97.1 | 2.8e-05 | 5.2e-11 | 56.8 | 122 | (1, 132) | 147 | (34, 155) | 193 | Terminase small subunit | Terminase small subunit | | uniclust | UniRef100\_A0A930QBP3 | 97.1 | 2.8e-05 | 5.2e-11 | 52.4 | 86 | (48, 134) | 147 | (22, 110) | 114 | Uncharacterized protein | Uncharacterized protein | | uniclust | UniRef100\_A0A0Q7W4M6 | 97.1 | 2.8e-05 | 5.5e-11 | 60.9 | 85 | (50, 134) | 147 | (115, 199) | 234 | Terminase small subunit | Terminase small subunit | | uniclust | UniRef100\_A0A2T5K4X9 | 97.1 | 3e-05 | 5.5e-11 | 57.3 | 91 | (44, 134) | 147 | (103, 193) | 207 | Phage terminase Nu1 subunit (DNA packaging protein) | Phage terminase Nu1 subunit (DNA packaging protein) | | uniclust | UniRef100\_A0A379V2M5 | 97.1 | 3e-05 | 5.5e-11 | 54.1 | 74 | (1, 74) | 147 | (21, 110) | 140 | Terminase | Terminase | | uniclust | UniRef100\_A0A0F9I7J9 | 97.1 | 3.1e-05 | 5.7e-11 | 55.7 | 125 | (2, 132) | 147 | (38, 163) | 172 | Helix-turn-helix domain-containing protein | Helix-turn-helix domain-containing protein | | uniclust | UniRef100\_A0A376MST9 | 97.1 | 3.1e-05 | 5.7e-11 | 52.4 | 88 | (20, 107) | 147 | (2, 105) | 112 | Terminase small subunit | Terminase small subunit | | uniclust | UniRef100\_A0A929Y0V3 | 97.1 | 3.2e-05 | 5.8e-11 | 53.5 | 100 | (6, 110) | 147 | (27, 127) | 132 | DNA-packaging protein (Fragment) | DNA-packaging protein (Fragment) | | uniclust | UniRef100\_A0A936H996 | 97.1 | 3.1e-05 | 5.9e-11 | 53.3 | 83 | (43, 128) | 147 | (13, 95) | 116 | DUF1441 family protein | DUF1441 family protein | | uniclust | UniRef100\_A0A1D8IV69 | 97.1 | 3.3e-05 | 6e-11 | 52.6 | 81 | (48, 131) | 147 | (31, 111) | 120 | DUF1441 family protein | DUF1441 family protein | | uniclust | UniRef100\_UPI000BE6EFA5 | 97.1 | 3.3e-05 | 6e-11 | 49.4 | 35 | (1, 35) | 147 | (19, 53) | 83 | terminase small subunit | terminase small subunit | | uniclust | UniRef100\_UPI0002E51B9A | 97.1 | 3.4e-05 | 6.2e-11 | 56.5 | 92 | (45, 137) | 147 | (72, 166) | 193 | hypothetical protein | hypothetical protein | | uniclust | UniRef100\_A0A4Q3LZ14 | 97.1 | 3.5e-05 | 6.5e-11 | 55.2 | 106 | (29, 134) | 147 | (47, 159) | 166 | DUF892 family protein | DUF892 family protein | | uniclust | UniRef100\_A0A1V6E2G2 | 97.1 | 3.6e-05 | 6.6e-11 | 54.6 | 96 | (1, 106) | 147 | (40, 135) | 156 | Uncharacterized protein | Uncharacterized protein | | uniclust | UniRef100\_A0A0F9FRE9 | 97.1 | 3.8e-05 | 6.9e-11 | 55.3 | 116 | (1, 123) | 147 | (23, 140) | 171 | Terminase small subunit (Fragment) | Terminase small subunit (Fragment) | | uniclust | UniRef100\_A0A382JWV4 | 97.1 | 3.8e-05 | 7e-11 | 53.2 | 65 | (43, 107) | 147 | (57, 121) | 134 | Helix-turn-helix domain-containing protein | Helix-turn-helix domain-containing protein | | uniclust | UniRef100\_A0A934R2B7 | 97.1 | 3.9e-05 | 7.1e-11 | 54.7 | 110 | (21, 131) | 147 | (37, 147) | 160 | Uncharacterized protein | Uncharacterized protein | | uniclust | UniRef100\_UPI001CF1D285 | 97.1 | 4.1e-05 | 7.5e-11 | 49.3 | 36 | (1, 36) | 147 | (21, 56) | 86 | terminase small subunit | terminase small subunit | | uniclust | UniRef100\_UPI0013598FD9 | 97.1 | 4.1e-05 | 7.7e-11 | 57.8 | 112 | (16, 129) | 147 | (65, 182) | 203 | hypothetical protein | hypothetical protein | | uniclust | UniRef100\_A0A376PB86 | 97.1 | 4.1e-05 | 7.8e-11 | 51.9 | 35 | (1, 35) | 147 | (21, 55) | 98 | DNA packaging protein | DNA packaging protein | | uniclust | UniRef100\_A0A2M7YS23 | 97.0 | 4.7e-05 | 9.1e-11 | 55.5 | 57 | (43, 99) | 147 | (90, 146) | 146 | Uncharacterized protein (Fragment) | Uncharacterized protein (Fragment) | | uniclust | UniRef100\_UPI0005F41AB5 | 97.0 | 5e-05 | 9.2e-11 | 67.2 | 83 | (49, 131) | 147 | (289, 371) | 1163 | uncharacterized protein LOC105557561 | uncharacterized protein LOC105557561 | | uniclust | UniRef100\_A0A389MXY8 | 97.0 | 5e-05 | 9.2e-11 | 57.6 | 129 | (1, 130) | 147 | (63, 214) | 246 | Terminase small subunit | Terminase small subunit | | uniclust | UniRef100\_X1DAN0 | 97.0 | 5.1e-05 | 9.3e-11 | 50.1 | 86 | (45, 130) | 147 | (5, 90) | 99 | Uncharacterized protein (Fragment) | Uncharacterized protein (Fragment) | | uniclust | UniRef100\_A0A7X6FQV0 | 97.0 | 5.1e-05 | 9.4e-11 | 49.6 | 74 | (21, 98) | 147 | (19, 92) | 93 | Uncharacterized protein | Uncharacterized protein | | uniclust | UniRef100\_A0A376RGB0 | 97.0 | 5.2e-05 | 9.6e-11 | 50.8 | 35 | (1, 35) | 147 | (21, 55) | 106 | DNA packaging protein | DNA packaging protein | | uniclust | UniRef100\_A0A3D0I2E3 | 97.0 | 5.3e-05 | 9.7e-11 | 54.1 | 115 | (15, 134) | 147 | (34, 152) | 160 | Terminase small subunit | Terminase small subunit | | uniclust | UniRef100\_UPI002023B37C | 97.0 | 5.5e-05 | 1e-10 | 57.4 | 87 | (45, 131) | 147 | (148, 237) | 246 | hypothetical protein | hypothetical protein | | uniclust | UniRef100\_UPI0002DEF5FE | 97.0 | 5.8e-05 | 1.1e-10 | 52.0 | 93 | (44, 136) | 147 | (14, 106) | 127 | terminase small subunit | terminase small subunit | | uniclust | UniRef100\_A0A7X7H556 | 97.0 | 5.9e-05 | 1.1e-10 | 52.7 | 118 | (1, 127) | 147 | (8, 130) | 138 | Terminase small subunit | Terminase small subunit | | uniclust | UniRef100\_UPI00209FEA06 | 97.0 | 6.8e-05 | 1.2e-10 | 50.9 | 85 | (48, 132) | 147 | (24, 108) | 116 | hypothetical protein | hypothetical protein | | uniclust | UniRef100\_A0A517X1A7 | 97.0 | 6.8e-05 | 1.2e-10 | 53.3 | 125 | (1, 130) | 147 | (21, 148) | 155 | Phage DNA packaging protein Nu1 | Phage DNA packaging protein Nu1 | | uniclust | UniRef100\_UPI001BCF985F | 97.0 | 6.8e-05 | 1.3e-10 | 54.9 | 91 | (43, 133) | 147 | (70, 160) | 189 | hypothetical protein | hypothetical protein | | uniclust | UniRef100\_A0A426QG20 | 96.9 | 6.9e-05 | 1.3e-10 | 52.6 | 112 | (23, 134) | 147 | (12, 126) | 142 | Uncharacterized protein | Uncharacterized protein | |
| Top keywords  (threshold 1.00e-03 (evalue)) | **Terminase, small, DNA, packaging, hypothetical, Phage, Nu1, domain\_containing, Fragment, Helix\_turn\_helix** |
| Output files | ../../similar\_sequences/01\_FANPEZAQ\_CDS\_0001\_merged.svg ../../similar\_sequences/01\_FANPEZAQ\_CDS\_0001\_pdb70.a3m ../../similar\_sequences/01\_FANPEZAQ\_CDS\_0001\_pdb70.hhr ../../similar\_sequences/01\_FANPEZAQ\_CDS\_0001\_uniclust.a3m ../../similar\_sequences/01\_FANPEZAQ\_CDS\_0001\_uniclust.hhr |

#### Structure prediction (AlphaFold)2

|  |  |
| --- | --- |
| Stats | xml version="1.0" encoding="utf-8" standalone="no"?       2024-09-02T21:08:57.193908 image/svg+xml   Matplotlib v3.7.2, https://matplotlib.org/ |
| Predicted structure | **NGL Viewer Controls:**  - Center: *Left-Click* - Rotate: *Left-Click + Drag* - Translate: *Right-Click + Drag* - Zoom: *Shift + Left-Click + Drag* |
| Output files | ../../predicted\_structures/01\_FANPEZAQ\_CDS\_0001/features.pkl ../../predicted\_structures/01\_FANPEZAQ\_CDS\_0001/ranked\_0.pdb ../../predicted\_structures/01\_FANPEZAQ\_CDS\_0001/ranked\_0\_plots.svg ../../predicted\_structures/01\_FANPEZAQ\_CDS\_0001/result\_model\_1\_ptm\_pred\_0.pkl |

#### Structure similarity search results (Foldseek)3

|  |  |
| --- | --- |
| Structure databases searched | Pdb, Afdb-proteome, Afdb-uniprot50 |
| Results, scheme(s)  (Top layers only, threshold 1.00e-02 (evalue)) | xml version="1.0" encoding="utf-8" standalone="no"?       2024-09-02T21:10:02.435958 image/svg+xml   Matplotlib v3.7.2, https://matplotlib.org/ |
| Results, table  (threshold 1.00e-02 (evalue)) | | db | id | prob | evalue | bits | fident | alnlen | mismatch | gapopen | qstart | qend | tstart | tend | name | description | | --- | --- | --- | --- | --- | --- | --- | --- | --- | --- | --- | --- | --- | --- | --- | | afdb-proteome | AF-P31062-F1-MODEL\_V4 | 0.981 | 0.005043 | 79 | 0.194 | 159 | 92 | 5 | 2 | 130 | 22 | 174 | DNA-packaging protein NU1 homolog | DNA-packaging protein NU1 homolog | | afdb-uniprot50 | AF-A0A5Y3B0P0-F1-MODEL\_V4 | 1.0 | 6.8e-12 | 421 | 0.605 | 147 | 57 | 1 | 2 | 147 | 29 | 175 | Terminase small subunit | Terminase small subunit | | afdb-uniprot50 | AF-A0A2Z3I612-F1-MODEL\_V4 | 1.0 | 4.42e-12 | 419 | 0.567 | 141 | 61 | 0 | 2 | 142 | 26 | 166 | DNA-packaging protein | DNA-packaging protein | | afdb-uniprot50 | AF-A0A410UF08-F1-MODEL\_V4 | 1.0 | 7.975e-11 | 387 | 0.527 | 146 | 69 | 0 | 2 | 147 | 28 | 173 | Terminase small subunit | Terminase small subunit | | afdb-uniprot50 | AF-A0A0Q2LYU8-F1-MODEL\_V4 | 1.0 | 2.477e-11 | 381 | 0.554 | 146 | 62 | 1 | 2 | 147 | 30 | 172 | DNA-packaging protein | DNA-packaging protein | | afdb-uniprot50 | AF-A0A3G6WMJ0-F1-MODEL\_V4 | 1.0 | 4.752e-10 | 356 | 0.475 | 145 | 76 | 0 | 2 | 146 | 58 | 202 | Helix-turn-helix domain-containing protein | Helix-turn-helix domain-containing protein | | afdb-uniprot50 | AF-A0A149SVI6-F1-MODEL\_V4 | 1.0 | 5.572e-09 | 336 | 0.443 | 133 | 73 | 1 | 2 | 134 | 30 | 161 | Uncharacterized protein | Uncharacterized protein | | afdb-uniprot50 | AF-A0A6A4RCC3-F1-MODEL\_V4 | 1.0 | 3.951e-10 | 336 | 0.516 | 151 | 67 | 5 | 2 | 147 | 32 | 181 | Terminase small subunit | Terminase small subunit | | afdb-uniprot50 | AF-A0A1R7Q9M5-F1-MODEL\_V4 | 1.0 | 5.926e-09 | 332 | 0.496 | 131 | 66 | 0 | 2 | 132 | 29 | 159 | Phage DNA packaging protein Nu1 | Phage DNA packaging protein Nu1 | | afdb-uniprot50 | AF-A0A291LYW6-F1-MODEL\_V4 | 1.0 | 4.752e-10 | 327 | 0.493 | 148 | 72 | 3 | 2 | 147 | 30 | 176 | DNA-packaging protein | DNA-packaging protein | | afdb-uniprot50 | AF-A0A7J0BX75-F1-MODEL\_V4 | 1.0 | 1.166e-08 | 319 | 0.407 | 140 | 71 | 2 | 2 | 134 | 43 | 177 | Uncharacterized protein | Uncharacterized protein | | afdb-uniprot50 | AF-A0A8A6KGW4-F1-MODEL\_V4 | 1.0 | 6.302e-09 | 314 | 0.446 | 150 | 74 | 5 | 2 | 147 | 28 | 172 | Terminase small subunit | Terminase small subunit | | afdb-uniprot50 | AF-A0A1I1F6U7-F1-MODEL\_V4 | 1.0 | 2.354e-09 | 307 | 0.387 | 147 | 88 | 2 | 2 | 147 | 20 | 165 | Phage DNA packaging protein, Nu1 subunit of terminase | Phage DNA packaging protein, Nu1 subunit of terminase | | afdb-uniprot50 | AF-A0A844HQQ0-F1-MODEL\_V4 | 1.0 | 7.86e-08 | 297 | 0.41 | 146 | 85 | 1 | 2 | 147 | 27 | 171 | Terminase small subunit | Terminase small subunit | | afdb-uniprot50 | AF-A0A6I6JFF5-F1-MODEL\_V4 | 1.0 | 1.794e-08 | 292 | 0.386 | 150 | 82 | 3 | 2 | 147 | 29 | 172 | Terminase small subunit | Terminase small subunit | | afdb-uniprot50 | AF-A0A103EHP4-F1-MODEL\_V4 | 1.0 | 3.755e-08 | 290 | 0.449 | 138 | 75 | 1 | 11 | 147 | 3 | 140 | Uncharacterized protein | Uncharacterized protein | | afdb-uniprot50 | AF-A0A0A6SUS5-F1-MODEL\_V4 | 1.0 | 1.86e-07 | 274 | 0.321 | 146 | 97 | 2 | 2 | 147 | 33 | 176 | Uncharacterized protein | Uncharacterized protein | | afdb-uniprot50 | AF-A0A017HTQ6-F1-MODEL\_V4 | 1.0 | 2.104e-07 | 270 | 0.31 | 148 | 99 | 3 | 2 | 147 | 11 | 157 | Uncharacterized protein | Uncharacterized protein | | afdb-uniprot50 | AF-A0A2D7GUJ0-F1-MODEL\_V4 | 1.0 | 7.86e-08 | 265 | 0.392 | 130 | 78 | 1 | 2 | 130 | 22 | 151 | Uncharacterized protein | Uncharacterized protein | | afdb-uniprot50 | AF-A0A7C8HWC0-F1-MODEL\_V4 | 1.0 | 6.776e-07 | 261 | 0.359 | 142 | 84 | 3 | 2 | 138 | 40 | 179 | Uncharacterized protein | Uncharacterized protein | | afdb-uniprot50 | AF-A0A3N2E0T8-F1-MODEL\_V4 | 1.0 | 3.443e-07 | 259 | 0.356 | 146 | 88 | 3 | 2 | 144 | 28 | 170 | Phage terminase Nu1 subunit (DNA packaging protein) | Phage terminase Nu1 subunit (DNA packaging protein) | | afdb-uniprot50 | AF-A0A126NZA6-F1-MODEL\_V4 | 1.0 | 5.991e-07 | 252 | 0.333 | 132 | 85 | 3 | 2 | 132 | 9 | 138 | Uncharacterized protein | Uncharacterized protein | | afdb-uniprot50 | AF-A0A5P9IWW9-F1-MODEL\_V4 | 1.0 | 4.141e-07 | 252 | 0.342 | 140 | 81 | 1 | 2 | 130 | 22 | 161 | Phage DNA packaging protein Nu1 | Phage DNA packaging protein Nu1 | | afdb-uniprot50 | AF-A0A7W6S2H6-F1-MODEL\_V4 | 1.0 | 3.443e-07 | 239 | 0.328 | 149 | 95 | 3 | 2 | 146 | 25 | 172 | Phage terminase Nu1 subunit (DNA packaging protein) | Phage terminase Nu1 subunit (DNA packaging protein) | | afdb-uniprot50 | AF-A0A315EM69-F1-MODEL\_V4 | 1.0 | 3.994e-08 | 238 | 0.4 | 155 | 84 | 3 | 2 | 147 | 23 | 177 | Uncharacterized protein | Uncharacterized protein | | afdb-uniprot50 | AF-A0A1W1Z3G8-F1-MODEL\_V4 | 1.0 | 8.667e-07 | 237 | 0.324 | 148 | 96 | 4 | 2 | 147 | 24 | 169 | Phage DNA packaging protein Nu1 | Phage DNA packaging protein Nu1 | | afdb-uniprot50 | AF-A0A7V5XCS4-F1-MODEL\_V4 | 1.0 | 6.606e-06 | 223 | 0.273 | 146 | 98 | 3 | 2 | 143 | 49 | 190 | Uncharacterized protein | Uncharacterized protein | | afdb-uniprot50 | AF-A0A178LGQ7-F1-MODEL\_V4 | 1.0 | 1.47e-05 | 216 | 0.282 | 145 | 92 | 5 | 3 | 147 | 10 | 142 | Uncharacterized protein | Uncharacterized protein | | afdb-uniprot50 | AF-A0A2E0ENP5-F1-MODEL\_V4 | 1.0 | 1.222e-05 | 216 | 0.251 | 139 | 93 | 2 | 2 | 140 | 24 | 151 | Uncharacterized protein | Uncharacterized protein | | afdb-uniprot50 | AF-A0A6A8A3Z2-F1-MODEL\_V4 | 1.0 | 2.968e-06 | 216 | 0.298 | 151 | 101 | 2 | 2 | 147 | 32 | 182 | Uncharacterized protein | Uncharacterized protein | | afdb-uniprot50 | AF-A0A1G1HG69-F1-MODEL\_V4 | 1.0 | 1.47e-05 | 211 | 0.237 | 135 | 101 | 1 | 2 | 136 | 20 | 152 | Uncharacterized protein | Uncharacterized protein | | afdb-uniprot50 | AF-A0A386UIG2-F1-MODEL\_V4 | 1.0 | 5.492e-06 | 210 | 0.283 | 148 | 102 | 3 | 2 | 147 | 29 | 174 | Uncharacterized protein | Uncharacterized protein | | afdb-uniprot50 | AF-A0A165U8L6-F1-MODEL\_V4 | 1.0 | 2.721e-05 | 202 | 0.29 | 131 | 92 | 1 | 2 | 132 | 24 | 153 | Phage DNA packaging protein Nu1 | Phage DNA packaging protein Nu1 | | afdb-uniprot50 | AF-A0A7X2NZP6-F1-MODEL\_V4 | 1.0 | 8.761e-05 | 201 | 0.412 | 126 | 69 | 2 | 22 | 147 | 1 | 121 | Uncharacterized protein | Uncharacterized protein | | afdb-uniprot50 | AF-A0A7Y7QTY3-F1-MODEL\_V4 | 1.0 | 2.721e-05 | 200 | 0.291 | 144 | 95 | 2 | 4 | 147 | 33 | 169 | Terminase small subunit | Terminase small subunit | | afdb-uniprot50 | AF-A0A1Q5SRW0-F1-MODEL\_V4 | 1.0 | 2.558e-05 | 197 | 0.344 | 145 | 82 | 4 | 3 | 147 | 29 | 160 | Uncharacterized protein | Uncharacterized protein | | afdb-uniprot50 | AF-A0A2A5EK29-F1-MODEL\_V4 | 1.0 | 2e-05 | 197 | 0.212 | 165 | 111 | 3 | 2 | 147 | 44 | 208 | Uncharacterized protein | Uncharacterized protein | | afdb-uniprot50 | AF-A0A4R2YRS0-F1-MODEL\_V4 | 1.0 | 4.735e-05 | 195 | 0.289 | 145 | 91 | 6 | 3 | 147 | 34 | 166 | Uncharacterized protein | Uncharacterized protein | | afdb-uniprot50 | AF-A0A235H584-F1-MODEL\_V4 | 1.0 | 2.558e-05 | 194 | 0.258 | 155 | 106 | 4 | 2 | 147 | 25 | 179 | Uncharacterized protein | Uncharacterized protein | | afdb-uniprot50 | AF-A0A2U2N0D2-F1-MODEL\_V4 | 1.0 | 0.0001621 | 193 | 0.244 | 139 | 99 | 3 | 2 | 136 | 22 | 158 | Uncharacterized protein | Uncharacterized protein | | afdb-uniprot50 | AF-A0A433J1A9-F1-MODEL\_V4 | 1.0 | 4.452e-05 | 192 | 0.282 | 138 | 93 | 2 | 2 | 134 | 27 | 163 | Terminase small subunit (DNA packaging protein Nu1) | Terminase small subunit (DNA packaging protein Nu1) | | afdb-uniprot50 | AF-A0A1X7JJP1-F1-MODEL\_V4 | 1.0 | 0.0002074 | 191 | 0.401 | 107 | 61 | 2 | 22 | 128 | 1 | 104 | Uncharacterized protein | Uncharacterized protein | | afdb-uniprot50 | AF-A0A3M6EKX5-F1-MODEL\_V4 | 1.0 | 0.0003393 | 191 | 0.305 | 118 | 80 | 1 | 16 | 133 | 36 | 151 | Uncharacterized protein | Uncharacterized protein | | afdb-uniprot50 | AF-A0A2V5D2Z0-F1-MODEL\_V4 | 1.0 | 0.0001192 | 188 | 0.315 | 133 | 84 | 5 | 16 | 147 | 38 | 164 | Uncharacterized protein | Uncharacterized protein | | afdb-uniprot50 | AF-G4KQ86-F1-MODEL\_V4 | 1.0 | 0.0001348 | 187 | 0.21 | 147 | 109 | 5 | 3 | 147 | 23 | 164 | Putative terminase small subunit | Putative terminase small subunit | | afdb-uniprot50 | AF-A0A1G3BHK7-F1-MODEL\_V4 | 1.0 | 7.284e-05 | 186 | 0.224 | 129 | 94 | 2 | 2 | 130 | 23 | 145 | Uncharacterized protein | Uncharacterized protein | | afdb-uniprot50 | AF-A0A4S2H4R7-F1-MODEL\_V4 | 1.0 | 0.0001724 | 185 | 0.232 | 146 | 106 | 3 | 3 | 145 | 27 | 169 | Terminase small subunit, Nu1 | Terminase small subunit, Nu1 | | afdb-uniprot50 | AF-A0A8B2NVC2-F1-MODEL\_V4 | 1.0 | 3.48e-05 | 183 | 0.31 | 148 | 99 | 3 | 2 | 147 | 41 | 187 | Uncharacterized protein | Uncharacterized protein | | afdb-uniprot50 | AF-A9IRX2-F1-MODEL\_V4 | 1.0 | 2.721e-05 | 182 | 0.31 | 145 | 83 | 6 | 3 | 147 | 18 | 145 | Uncharacterized protein | Uncharacterized protein | | afdb-uniprot50 | AF-A0A2E1HAF3-F1-MODEL\_V4 | 1.0 | 0.0001434 | 181 | 0.234 | 141 | 97 | 3 | 10 | 147 | 27 | 159 | Uncharacterized protein | Uncharacterized protein | | afdb-uniprot50 | AF-A0A6L4BEY6-F1-MODEL\_V4 | 1.0 | 0.0001192 | 179 | 0.288 | 142 | 97 | 2 | 2 | 143 | 45 | 182 | Terminase small subunit | Terminase small subunit | | afdb-uniprot50 | AF-A0A6L3T3B7-F1-MODEL\_V4 | 1.0 | 0.0002821 | 177 | 0.281 | 135 | 90 | 2 | 2 | 130 | 30 | 163 | Terminase small subunit | Terminase small subunit | | afdb-uniprot50 | AF-A0A2D2C217-F1-MODEL\_V4 | 1.0 | 0.0001267 | 177 | 0.231 | 160 | 106 | 4 | 3 | 147 | 46 | 203 | Uncharacterized protein | Uncharacterized protein | | afdb-uniprot50 | AF-A0A2A5A3W5-F1-MODEL\_V4 | 1.0 | 7.284e-05 | 176 | 0.333 | 135 | 82 | 4 | 2 | 132 | 26 | 156 | Uncharacterized protein | Uncharacterized protein | | afdb-uniprot50 | AF-A0A1C3WPE9-F1-MODEL\_V4 | 1.0 | 0.0001054 | 176 | 0.285 | 147 | 91 | 4 | 10 | 147 | 27 | 168 | Phage DNA packaging protein, Nu1 subunit of terminase | Phage DNA packaging protein, Nu1 subunit of terminase | | afdb-uniprot50 | AF-G2IX52-F1-MODEL\_V4 | 1.0 | 0.0001054 | 173 | 0.205 | 146 | 104 | 2 | 2 | 147 | 24 | 157 | Phage terminase small subunit gpNu1 | Phage terminase small subunit gpNu1 | | afdb-uniprot50 | AF-A0A4Y3TG37-F1-MODEL\_V4 | 1.0 | 0.0004909 | 173 | 0.26 | 150 | 106 | 4 | 2 | 147 | 28 | 176 | Uncharacterized protein | Uncharacterized protein | | afdb-uniprot50 | AF-A0A2T7TXU6-F1-MODEL\_V4 | 1.0 | 0.0002653 | 170 | 0.22 | 136 | 99 | 2 | 2 | 130 | 27 | 162 | Terminase small subunit (DNA packaging protein Nu1) | Terminase small subunit (DNA packaging protein Nu1) | | afdb-uniprot50 | AF-X1HCJ8-F1-MODEL\_V4 | 1.0 | 0.0001724 | 170 | 0.246 | 154 | 102 | 4 | 2 | 147 | 23 | 170 | Uncharacterized protein | Uncharacterized protein | | afdb-uniprot50 | AF-A0A3U4W908-F1-MODEL\_V4 | 1.0 | 0.0003191 | 167 | 0.251 | 135 | 100 | 1 | 14 | 147 | 33 | 167 | Uncharacterized protein | Uncharacterized protein | | afdb-uniprot50 | AF-A0A1S7LLS5-F1-MODEL\_V4 | 1.0 | 0.002022 | 166 | 0.237 | 118 | 87 | 2 | 20 | 134 | 31 | 148 | Uncharacterized protein | Uncharacterized protein | | afdb-uniprot50 | AF-A0A192IJE2-F1-MODEL\_V4 | 1.0 | 0.0003393 | 166 | 0.255 | 133 | 97 | 2 | 3 | 135 | 22 | 152 | Uncharacterized protein | Uncharacterized protein | | afdb-uniprot50 | AF-A0A3B8QTC1-F1-MODEL\_V4 | 1.0 | 0.0001434 | 166 | 0.25 | 128 | 85 | 4 | 2 | 128 | 22 | 139 | Uncharacterized protein | Uncharacterized protein | | afdb-uniprot50 | AF-A0A7G8BY94-F1-MODEL\_V4 | 1.0 | 0.0002494 | 166 | 0.272 | 158 | 96 | 5 | 2 | 146 | 28 | 179 | Terminase small subunit | Terminase small subunit | | afdb-uniprot50 | AF-A0A7Z8R5N7-F1-MODEL\_V4 | 1.0 | 0.0003191 | 165 | 0.205 | 136 | 101 | 3 | 2 | 130 | 17 | 152 | Terminase small subunit | Terminase small subunit | | afdb-uniprot50 | AF-A0A1V5Z7S9-F1-MODEL\_V4 | 1.0 | 0.001314 | 165 | 0.259 | 108 | 78 | 1 | 22 | 129 | 63 | 168 | Phage DNA packaging protein Nu1 | Phage DNA packaging protein Nu1 | | afdb-uniprot50 | AF-A0A0C9PQA6-F1-MODEL\_V4 | 1.0 | 0.000195 | 164 | 0.234 | 149 | 101 | 5 | 2 | 144 | 45 | 186 | Uncharacterized protein | Uncharacterized protein | | afdb-uniprot50 | AF-A0A2D8RC70-F1-MODEL\_V4 | 1.0 | 0.0003838 | 162 | 0.222 | 148 | 109 | 4 | 5 | 147 | 18 | 164 | Uncharacterized protein | Uncharacterized protein | | afdb-uniprot50 | AF-A0A7X1W7E9-F1-MODEL\_V4 | 1.0 | 0.001398 | 161 | 0.287 | 132 | 88 | 4 | 4 | 135 | 25 | 150 | Terminase small subunit, Nu1 | Terminase small subunit, Nu1 | | afdb-uniprot50 | AF-A0A7C6RL71-F1-MODEL\_V4 | 1.0 | 8.238e-05 | 161 | 0.261 | 157 | 87 | 4 | 2 | 134 | 20 | 171 | Terminase small subunit | Terminase small subunit | | afdb-uniprot50 | AF-A0A7Z0KXQ8-F1-MODEL\_V4 | 1.0 | 0.000434 | 159 | 0.279 | 143 | 93 | 5 | 3 | 143 | 27 | 161 | Terminase small subunit, Nu1 | Terminase small subunit, Nu1 | | afdb-uniprot50 | AF-A0A7U9IVZ5-F1-MODEL\_V4 | 1.0 | 0.0003393 | 159 | 0.238 | 147 | 99 | 4 | 2 | 147 | 25 | 159 | Uncharacterized protein | Uncharacterized protein | | afdb-uniprot50 | AF-A0A6A4R6A4-F1-MODEL\_V4 | 1.0 | 0.0005552 | 158 | 0.27 | 137 | 92 | 1 | 2 | 130 | 24 | 160 | Uncharacterized protein | Uncharacterized protein | | afdb-uniprot50 | AF-A0A8A5HVC6-F1-MODEL\_V4 | 1.0 | 0.0004081 | 156 | 0.238 | 147 | 99 | 4 | 2 | 147 | 12 | 146 | Uncharacterized protein | Uncharacterized protein | | afdb-uniprot50 | AF-A0A7L6A869-F1-MODEL\_V4 | 1.0 | 0.001681 | 156 | 0.304 | 125 | 84 | 2 | 24 | 147 | 38 | 160 | Uncharacterized protein | Uncharacterized protein | | afdb-uniprot50 | AF-A0A7C5CB12-F1-MODEL\_V4 | 1.0 | 0.002586 | 156 | 0.214 | 126 | 93 | 2 | 24 | 147 | 46 | 167 | Terminase small subunit, Nu1 | Terminase small subunit, Nu1 | | afdb-uniprot50 | AF-A0A3B1BSM9-F1-MODEL\_V4 | 1.0 | 0.002022 | 155 | 0.248 | 137 | 89 | 3 | 11 | 147 | 3 | 125 | Uncharacterized protein | Uncharacterized protein | | afdb-uniprot50 | AF-A0A6N6NL26-F1-MODEL\_V4 | 1.0 | 0.0005221 | 155 | 0.211 | 151 | 104 | 5 | 2 | 147 | 54 | 194 | Terminase small subunit | Terminase small subunit | | afdb-uniprot50 | AF-A0A1I5MM69-F1-MODEL\_V4 | 1.0 | 0.004786 | 154 | 0.22 | 127 | 99 | 0 | 21 | 147 | 46 | 172 | Phage DNA packaging protein, Nu1 subunit of terminase | Phage DNA packaging protein, Nu1 subunit of terminase | | afdb-uniprot50 | AF-E8V6R1-F1-MODEL\_V4 | 1.0 | 0.0006678 | 153 | 0.266 | 150 | 87 | 4 | 2 | 134 | 13 | 156 | Uncharacterized protein | Uncharacterized protein | | afdb-uniprot50 | AF-A0A0S9LPH7-F1-MODEL\_V4 | 1.0 | 0.003111 | 151 | 0.347 | 118 | 73 | 2 | 24 | 140 | 44 | 158 | Uncharacterized protein | Uncharacterized protein | | afdb-uniprot50 | AF-A0A3M2C0J2-F1-MODEL\_V4 | 1.0 | 0.002586 | 150 | 0.233 | 133 | 94 | 3 | 3 | 132 | 13 | 140 | Uncharacterized protein | Uncharacterized protein | | afdb-uniprot50 | AF-A0A1H9NYA0-F1-MODEL\_V4 | 1.0 | 0.0005904 | 150 | 0.258 | 139 | 89 | 2 | 2 | 130 | 24 | 158 | Phage DNA packaging protein, Nu1 subunit of terminase | Phage DNA packaging protein, Nu1 subunit of terminase | | afdb-uniprot50 | AF-U7T5L9-F1-MODEL\_V4 | 1.0 | 0.001398 | 150 | 0.183 | 147 | 114 | 4 | 3 | 147 | 28 | 170 | Uncharacterized protein | Uncharacterized protein | | afdb-uniprot50 | AF-A0A2T5K719-F1-MODEL\_V4 | 1.0 | 0.0003838 | 150 | 0.186 | 166 | 115 | 4 | 2 | 147 | 44 | 209 | Phage terminase Nu1 subunit (DNA packaging protein) | Phage terminase Nu1 subunit (DNA packaging protein) | | afdb-uniprot50 | AF-A0A075KAS7-F1-MODEL\_V4 | 1.0 | 0.005413 | 149 | 0.223 | 121 | 86 | 2 | 21 | 139 | 42 | 156 | Uncharacterized protein | Uncharacterized protein | | afdb-uniprot50 | AF-A0A661I0F5-F1-MODEL\_V4 | 1.0 | 0.001027 | 148 | 0.218 | 133 | 102 | 1 | 2 | 132 | 53 | 185 | Uncharacterized protein | Uncharacterized protein | | afdb-uniprot50 | AF-A0A4Q2B780-F1-MODEL\_V4 | 1.0 | 0.002751 | 147 | 0.174 | 143 | 108 | 4 | 3 | 139 | 38 | 176 | Protoporphyrinogen oxidase | Protoporphyrinogen oxidase | | afdb-uniprot50 | AF-A0A8A5CXW5-F1-MODEL\_V4 | 1.0 | 0.003308 | 146 | 0.207 | 135 | 103 | 3 | 14 | 147 | 38 | 169 | Uncharacterized protein | Uncharacterized protein | | afdb-uniprot50 | AF-A0A2V8RWY8-F1-MODEL\_V4 | 1.0 | 0.003979 | 146 | 0.211 | 123 | 86 | 2 | 25 | 147 | 46 | 157 | Uncharacterized protein | Uncharacterized protein | | afdb-uniprot50 | AF-A0A2U1XZ16-F1-MODEL\_V4 | 1.0 | 0.0001192 | 146 | 0.3 | 150 | 99 | 4 | 2 | 147 | 24 | 171 | Uncharacterized protein | Uncharacterized protein | | afdb-uniprot50 | AF-A0A7G9WG98-F1-MODEL\_V4 | 1.0 | 0.002432 | 146 | 0.211 | 142 | 100 | 4 | 3 | 139 | 24 | 158 | Excisionase family DNA-binding protein | Excisionase family DNA-binding protein | | afdb-uniprot50 | AF-A0A1Q9PIU8-F1-MODEL\_V4 | 1.0 | 0.003518 | 146 | 0.216 | 143 | 95 | 2 | 16 | 147 | 41 | 177 | Uncharacterized protein | Uncharacterized protein | | afdb-uniprot50 | AF-A0A853K663-F1-MODEL\_V4 | 1.0 | 0.003742 | 145 | 0.321 | 112 | 74 | 1 | 36 | 147 | 2 | 111 | Terminase small subunit | Terminase small subunit | | afdb-uniprot50 | AF-A0A2I8QTZ5-F1-MODEL\_V4 | 1.0 | 0.002287 | 145 | 0.231 | 138 | 101 | 2 | 11 | 147 | 1 | 134 | Uncharacterized protein | Uncharacterized protein | | afdb-uniprot50 | AF-A0A1M6SLK9-F1-MODEL\_V4 | 1.0 | 0.004232 | 145 | 0.248 | 129 | 91 | 4 | 22 | 147 | 30 | 155 | Uncharacterized protein | Uncharacterized protein | | afdb-uniprot50 | AF-A0A752JIU9-F1-MODEL\_V4 | 1.0 | 0.001162 | 145 | 0.193 | 150 | 107 | 3 | 2 | 147 | 8 | 147 | Terminase small subunit | Terminase small subunit | | afdb-uniprot50 | AF-A0A5B9Y7V3-F1-MODEL\_V4 | 1.0 | 0.001901 | 145 | 0.234 | 141 | 98 | 5 | 3 | 138 | 36 | 171 | Uncharacterized protein | Uncharacterized protein | | afdb-uniprot50 | AF-A0A846KET6-F1-MODEL\_V4 | 1.0 | 0.00509 | 144 | 0.205 | 146 | 110 | 4 | 3 | 147 | 23 | 163 | DNA-packaging protein | DNA-packaging protein | | afdb-uniprot50 | AF-A0A098AXF9-F1-MODEL\_V4 | 1.0 | 0.002287 | 144 | 0.215 | 158 | 108 | 4 | 3 | 147 | 34 | 188 | Uncharacterized protein | Uncharacterized protein | | afdb-uniprot50 | AF-A0A854GZ82-F1-MODEL\_V4 | 1.0 | 0.002287 | 143 | 0.215 | 153 | 108 | 3 | 3 | 147 | 29 | 177 | Uncharacterized protein | Uncharacterized protein | | afdb-uniprot50 | AF-A0A7V2AKR6-F1-MODEL\_V4 | 1.0 | 0.003979 | 142 | 0.208 | 149 | 104 | 5 | 2 | 146 | 26 | 164 | Terminase small subunit, Nu1 | Terminase small subunit, Nu1 | | afdb-uniprot50 | AF-A0A3B9ZIR9-F1-MODEL\_V4 | 1.0 | 0.001901 | 142 | 0.231 | 147 | 100 | 6 | 2 | 147 | 73 | 207 | Uncharacterized protein | Uncharacterized protein | | afdb-uniprot50 | AF-A0A7C9M626-F1-MODEL\_V4 | 1.0 | 0.009419 | 141 | 0.204 | 127 | 97 | 1 | 16 | 138 | 44 | 170 | Uncharacterized protein | Uncharacterized protein | | afdb-uniprot50 | AF-A0A4R2YZM0-F1-MODEL\_V4 | 1.0 | 0.00215 | 140 | 0.177 | 152 | 114 | 5 | 2 | 147 | 29 | 175 | Uncharacterized protein | Uncharacterized protein | | afdb-uniprot50 | AF-A0A2S0MHN3-F1-MODEL\_V4 | 1.0 | 0.00509 | 139 | 0.284 | 109 | 75 | 2 | 23 | 130 | 39 | 145 | Uncharacterized protein | Uncharacterized protein | | afdb-uniprot50 | AF-A0A139GP13-F1-MODEL\_V4 | 1.0 | 0.0009084 | 139 | 0.212 | 146 | 104 | 4 | 2 | 147 | 21 | 155 | Uncharacterized protein | Uncharacterized protein | | afdb-uniprot50 | AF-A0A1V5QJC7-F1-MODEL\_V4 | 1.0 | 0.009419 | 139 | 0.215 | 116 | 84 | 2 | 21 | 130 | 50 | 164 | Uncharacterized protein | Uncharacterized protein | | afdb-uniprot50 | AF-A0A442XVU9-F1-MODEL\_V4 | 1.0 | 0.0045 | 139 | 0.244 | 147 | 103 | 4 | 4 | 147 | 27 | 168 | Terminase small subunit, Nu1 | Terminase small subunit, Nu1 | | afdb-uniprot50 | AF-A0A510KMJ0-F1-MODEL\_V4 | 1.0 | 0.005757 | 139 | 0.147 | 136 | 112 | 2 | 16 | 147 | 38 | 173 | Uncharacterized protein | Uncharacterized protein | | afdb-uniprot50 | AF-A0A2I8QQ64-F1-MODEL\_V4 | 1.0 | 0.002586 | 138 | 0.238 | 151 | 99 | 3 | 2 | 147 | 25 | 164 | Uncharacterized protein | Uncharacterized protein | | afdb-uniprot50 | AF-A0A516SJA0-F1-MODEL\_V4 | 1.0 | 0.007831 | 138 | 0.205 | 146 | 110 | 4 | 3 | 147 | 24 | 164 | Uncharacterized protein | Uncharacterized protein | | afdb-uniprot50 | AF-A0A2U3QE23-F1-MODEL\_V4 | 1.0 | 0.002432 | 137 | 0.261 | 134 | 87 | 4 | 4 | 133 | 27 | 152 | Uncharacterized protein | Uncharacterized protein | | afdb-uniprot50 | AF-A0A0B6D5F6-F1-MODEL\_V4 | 1.0 | 0.00215 | 137 | 0.21 | 114 | 86 | 3 | 19 | 130 | 55 | 166 | Uncharacterized protein | Uncharacterized protein | | afdb-uniprot50 | AF-A0A7R8B6S8-F1-MODEL\_V4 | 1.0 | 0.0005904 | 137 | 0.312 | 147 | 78 | 5 | 2 | 130 | 30 | 171 | Uncharacterized protein | Uncharacterized protein | | afdb-uniprot50 | AF-A0A7V8TMM0-F1-MODEL\_V4 | 1.0 | 0.001681 | 136 | 0.231 | 147 | 100 | 3 | 2 | 147 | 23 | 157 | Uncharacterized protein | Uncharacterized protein | | afdb-uniprot50 | AF-A0A2X2Q8M2-F1-MODEL\_V4 | 1.0 | 0.007831 | 136 | 0.246 | 146 | 98 | 6 | 4 | 147 | 30 | 165 | Uncharacterized protein | Uncharacterized protein | | afdb-uniprot50 | AF-A0A317PTI1-F1-MODEL\_V4 | 1.0 | 0.001901 | 136 | 0.24 | 129 | 89 | 3 | 22 | 147 | 32 | 154 | Uncharacterized protein | Uncharacterized protein | | afdb-uniprot50 | AF-A0A0H3ZUV8-F1-MODEL\_V4 | 1.0 | 0.0045 | 136 | 0.266 | 139 | 90 | 2 | 2 | 130 | 23 | 159 | Phage DNA packaging | Phage DNA packaging | | afdb-uniprot50 | AF-A0A843B8J5-F1-MODEL\_V4 | 1.0 | 0.005757 | 136 | 0.224 | 125 | 82 | 1 | 6 | 130 | 69 | 178 | Uncharacterized protein | Uncharacterized protein | | afdb-uniprot50 | AF-A0A1F9DZH8-F1-MODEL\_V4 | 1.0 | 0.0005221 | 136 | 0.264 | 136 | 87 | 4 | 2 | 130 | 24 | 153 | Uncharacterized protein | Uncharacterized protein | | afdb-uniprot50 | AF-A0A090UYZ9-F1-MODEL\_V4 | 1.0 | 0.001581 | 135 | 0.234 | 132 | 85 | 3 | 2 | 130 | 22 | 140 | Uncharacterized protein | Uncharacterized protein | | afdb-uniprot50 | AF-A0A371JBP9-F1-MODEL\_V4 | 1.0 | 0.004786 | 134 | 0.19 | 142 | 103 | 5 | 2 | 139 | 28 | 161 | Protoporphyrinogen oxidase | Protoporphyrinogen oxidase | | afdb-uniprot50 | AF-A0A2E9M7Y8-F1-MODEL\_V4 | 1.0 | 0.005413 | 133 | 0.2 | 120 | 89 | 3 | 11 | 130 | 30 | 142 | Uncharacterized protein | Uncharacterized protein | | afdb-uniprot50 | AF-A0A2E1P375-F1-MODEL\_V4 | 1.0 | 0.006924 | 133 | 0.198 | 131 | 96 | 3 | 3 | 129 | 22 | 147 | Uncharacterized protein | Uncharacterized protein | | afdb-uniprot50 | AF-A0A3P6JQE4-F1-MODEL\_V4 | 1.0 | 0.005757 | 133 | 0.22 | 154 | 107 | 5 | 3 | 146 | 37 | 187 | Phage DNA packaging protein, Nu1 subunit of terminase | Phage DNA packaging protein, Nu1 subunit of terminase | | afdb-uniprot50 | AF-A0A096AL02-F1-MODEL\_V4 | 1.0 | 0.0045 | 132 | 0.178 | 140 | 101 | 4 | 3 | 139 | 31 | 159 | Uncharacterized protein | Uncharacterized protein | | afdb-uniprot50 | AF-A0A0E4G066-F1-MODEL\_V4 | 1.0 | 0.001681 | 132 | 0.209 | 162 | 106 | 5 | 2 | 147 | 21 | 176 | Uncharacterized protein | Uncharacterized protein | | afdb-uniprot50 | AF-A0A554W5S1-F1-MODEL\_V4 | 1.0 | 0.002022 | 131 | 0.232 | 146 | 101 | 2 | 3 | 138 | 26 | 170 | Uncharacterized protein | Uncharacterized protein | | afdb-uniprot50 | AF-A0A1B1C3B3-F1-MODEL\_V4 | 1.0 | 0.0009084 | 131 | 0.253 | 150 | 91 | 5 | 2 | 144 | 45 | 180 | Uncharacterized protein | Uncharacterized protein | | afdb-uniprot50 | AF-A0A2A5ADY0-F1-MODEL\_V4 | 1.0 | 0.008857 | 129 | 0.198 | 131 | 95 | 4 | 3 | 129 | 22 | 146 | Uncharacterized protein | Uncharacterized protein | | afdb-uniprot50 | AF-A0A841I4T7-F1-MODEL\_V4 | 1.0 | 0.003518 | 129 | 0.228 | 118 | 86 | 2 | 15 | 129 | 36 | 151 | Uncharacterized protein | Uncharacterized protein | | afdb-uniprot50 | AF-A0A6I4RSJ3-F1-MODEL\_V4 | 1.0 | 0.003979 | 129 | 0.233 | 124 | 88 | 2 | 12 | 130 | 33 | 154 | Uncharacterized protein | Uncharacterized protein | | afdb-uniprot50 | AF-A0A1I0D922-F1-MODEL\_V4 | 1.0 | 0.006924 | 129 | 0.231 | 138 | 90 | 4 | 4 | 139 | 26 | 149 | Phage DNA packaging protein, Nu1 subunit of terminase | Phage DNA packaging protein, Nu1 subunit of terminase | | afdb-uniprot50 | AF-A0A160U3F2-F1-MODEL\_V4 | 1.0 | 0.003308 | 129 | 0.18 | 133 | 104 | 1 | 3 | 130 | 53 | 185 | Uncharacterized protein | Uncharacterized protein | | afdb-uniprot50 | AF-X1GDY6-F1-MODEL\_V4 | 1.0 | 0.006511 | 128 | 0.165 | 133 | 100 | 3 | 2 | 131 | 25 | 149 | Uncharacterized protein | Uncharacterized protein | | afdb-uniprot50 | AF-A0A316DDI3-F1-MODEL\_V4 | 1.0 | 0.00509 | 128 | 0.2 | 155 | 105 | 7 | 3 | 147 | 29 | 174 | Phage terminase Nu1 subunit (DNA packaging protein) | Phage terminase Nu1 subunit (DNA packaging protein) | | afdb-uniprot50 | AF-A0A7S7J7F6-F1-MODEL\_V4 | 1.0 | 0.003308 | 128 | 0.189 | 148 | 110 | 5 | 2 | 147 | 26 | 165 | Uncharacterized protein | Uncharacterized protein | | afdb-uniprot50 | AF-A4J3T3-F1-MODEL\_V4 | 1.0 | 0.004786 | 128 | 0.209 | 148 | 100 | 4 | 4 | 147 | 30 | 164 | Uncharacterized protein | Uncharacterized protein | | afdb-uniprot50 | AF-A0A358PNV0-F1-MODEL\_V4 | 1.0 | 0.005413 | 127 | 0.215 | 139 | 98 | 4 | 3 | 131 | 32 | 169 | Protoporphyrinogen oxidase | Protoporphyrinogen oxidase | | afdb-uniprot50 | AF-E7C7Y8-F1-MODEL\_V4 | 1.0 | 0.006122 | 126 | 0.289 | 152 | 91 | 5 | 3 | 147 | 25 | 166 | Uncharacterized protein | Uncharacterized protein | | afdb-uniprot50 | AF-A0A0U9HMP1-F1-MODEL\_V4 | 1.0 | 0.0045 | 126 | 0.262 | 141 | 90 | 5 | 3 | 139 | 33 | 163 | Phage DNA packaging protein, Nu1 subunit of terminase | Phage DNA packaging protein, Nu1 subunit of terminase | | afdb-uniprot50 | AF-A0A553SNI5-F1-MODEL\_V4 | 1.0 | 0.003979 | 126 | 0.232 | 159 | 104 | 6 | 2 | 147 | 51 | 204 | Uncharacterized protein | Uncharacterized protein | | afdb-uniprot50 | AF-A0A2S4QSD8-F1-MODEL\_V4 | 1.0 | 0.006122 | 125 | 0.185 | 135 | 105 | 2 | 14 | 147 | 31 | 161 | Uncharacterized protein | Uncharacterized protein | | afdb-uniprot50 | AF-A0A382N6L8-F1-MODEL\_V4 | 1.0 | 0.007831 | 124 | 0.191 | 136 | 94 | 3 | 20 | 147 | 34 | 161 | Uncharacterized protein | Uncharacterized protein | | afdb-uniprot50 | AF-A0A8B0KWW7-F1-MODEL\_V4 | 1.0 | 0.007364 | 124 | 0.221 | 149 | 100 | 5 | 2 | 147 | 21 | 156 | Uncharacterized protein | Uncharacterized protein | | afdb-uniprot50 | AF-A0A2N8HQG2-F1-MODEL\_V4 | 1.0 | 0.008328 | 124 | 0.207 | 140 | 101 | 4 | 2 | 139 | 42 | 173 | Uncharacterized protein | Uncharacterized protein | | afdb-uniprot50 | AF-G1WGD7-F1-MODEL\_V4 | 1.0 | 0.008857 | 123 | 0.209 | 143 | 99 | 5 | 3 | 139 | 25 | 159 | Uncharacterized protein | Uncharacterized protein | | afdb-uniprot50 | AF-A0A7I8DI47-F1-MODEL\_V4 | 1.0 | 0.009419 | 123 | 0.212 | 155 | 109 | 7 | 3 | 147 | 35 | 186 | Uncharacterized protein | Uncharacterized protein | | afdb-uniprot50 | AF-Q89JP3-F1-MODEL\_V4 | 1.0 | 0.003979 | 123 | 0.208 | 144 | 97 | 3 | 20 | 147 | 56 | 198 | Bll5240 protein | Bll5240 protein | | afdb-uniprot50 | AF-A0A7T5R3N3-F1-MODEL\_V4 | 1.0 | 0.008328 | 120 | 0.173 | 121 | 81 | 2 | 10 | 130 | 70 | 171 | Uncharacterized protein | Uncharacterized protein | | afdb-uniprot50 | AF-A0A839W5R0-F1-MODEL\_V4 | 1.0 | 0.003742 | 120 | 0.202 | 143 | 101 | 6 | 2 | 138 | 25 | 160 | Phage terminase Nu1 subunit (DNA packaging protein) | Phage terminase Nu1 subunit (DNA packaging protein) | | afdb-uniprot50 | AF-A0A1E7WZ77-F1-MODEL\_V4 | 1.0 | 0.006924 | 118 | 0.221 | 122 | 79 | 2 | 11 | 130 | 77 | 184 | Uncharacterized protein | Uncharacterized protein | | afdb-uniprot50 | AF-A0A1C6BMG4-F1-MODEL\_V4 | 1.0 | 0.00509 | 117 | 0.164 | 158 | 110 | 6 | 2 | 147 | 22 | 169 | Uncharacterized protein | Uncharacterized protein | | afdb-uniprot50 | AF-D7XY77-F1-MODEL\_V4 | 1.0 | 0.007364 | 117 | 0.181 | 154 | 112 | 6 | 2 | 147 | 26 | 173 | Uncharacterized protein | Uncharacterized protein | | afdb-uniprot50 | AF-A0A4D7QKX8-F1-MODEL\_V4 | 1.0 | 0.006924 | 117 | 0.207 | 130 | 89 | 3 | 3 | 132 | 82 | 197 | Uncharacterized protein | Uncharacterized protein | | afdb-uniprot50 | AF-A0A554WU06-F1-MODEL\_V4 | 1.0 | 0.006924 | 115 | 0.226 | 137 | 84 | 1 | 2 | 138 | 16 | 130 | Uncharacterized protein | Uncharacterized protein | | afdb-uniprot50 | AF-A0A7L6ENJ2-F1-MODEL\_V4 | 1.0 | 0.004232 | 115 | 0.207 | 159 | 100 | 5 | 2 | 147 | 22 | 167 | Uncharacterized protein | Uncharacterized protein | | afdb-uniprot50 | AF-A0A413EDA4-F1-MODEL\_V4 | 1.0 | 0.008328 | 115 | 0.191 | 157 | 100 | 6 | 3 | 147 | 31 | 172 | Uncharacterized protein | Uncharacterized protein | | afdb-uniprot50 | AF-A0A4Q8WZ92-F1-MODEL\_V4 | 1.0 | 0.007364 | 115 | 0.216 | 143 | 94 | 5 | 21 | 147 | 40 | 180 | Uncharacterized protein | Uncharacterized protein | | afdb-uniprot50 | AF-A0A496KCG1-F1-MODEL\_V4 | 1.0 | 0.006924 | 114 | 0.225 | 151 | 102 | 4 | 2 | 147 | 22 | 162 | DNA packaging protein | DNA packaging protein | | afdb-uniprot50 | AF-A0A2D5TTA5-F1-MODEL\_V4 | 1.0 | 0.001788 | 113 | 0.189 | 164 | 105 | 4 | 2 | 147 | 25 | 178 | Uncharacterized protein | Uncharacterized protein | | afdb-uniprot50 | AF-A0A081MYL8-F1-MODEL\_V4 | 1.0 | 0.004786 | 113 | 0.171 | 163 | 118 | 4 | 2 | 147 | 24 | 186 | Uncharacterized protein | Uncharacterized protein | | afdb-uniprot50 | AF-A0A844Q904-F1-MODEL\_V4 | 1.0 | 0.008328 | 113 | 0.205 | 151 | 91 | 5 | 2 | 139 | 33 | 167 | Uncharacterized protein | Uncharacterized protein | | afdb-uniprot50 | AF-A0A3D4L0I6-F1-MODEL\_V4 | 1.0 | 0.007364 | 113 | 0.216 | 148 | 98 | 6 | 2 | 139 | 171 | 310 | Uncharacterized protein | Uncharacterized protein | | afdb-uniprot50 | AF-A0A7U0PNM6-F1-MODEL\_V4 | 1.0 | 0.0045 | 111 | 0.198 | 151 | 106 | 4 | 2 | 147 | 25 | 165 | Uncharacterized protein | Uncharacterized protein | | afdb-uniprot50 | AF-A0A4Y8EEM3-F1-MODEL\_V4 | 1.0 | 0.008328 | 111 | 0.189 | 148 | 111 | 5 | 2 | 147 | 12 | 152 | Uncharacterized protein | Uncharacterized protein | |
| Top keywords  (threshold 1.00e-02 (evalue)) | **Terminase, Nu1, small, DNA, packaging, Phage, DNA\_packaging, Protoporphyrinogen, oxidase, homolog** |
| Output files | ../../similar\_structures/01\_FANPEZAQ\_CDS\_0001\_afdb-proteome\_foldseek.tsv ../../similar\_structures/01\_FANPEZAQ\_CDS\_0001\_afdb-uniprot50\_foldseek.tsv ../../similar\_structures/01\_FANPEZAQ\_CDS\_0001\_merged.svg ../../similar\_structures/01\_FANPEZAQ\_CDS\_0001\_pdb\_foldseek.tsv |

  
  
  

Return to summary | Go to next

  


---

**Sequence/structure alignments coloring**  
Each object in the alignment figures is colored according to its E-value following this color coding:

1e-100
10

**References:**  
1) Steinegger M, Meier M, Mirdita M, Vöhringer H, Haunsberger S J, and Söding J (2019) HH-suite3 for fast remote homology detection and deep protein annotation, BMC Bioinformatics, 473. doi: 10.1186/s12859-019-3019-7  
2) Jumper J, Evans R, Pritzel A, ..., Hassabis D (2021) Highly accurate protein structure prediction with AlphaFold, Nature, 596. doi: 10.1038/s41586-021-03819-2  
3) van Kempen M, Kim S, Tumescheit C, Mirdita M, Lee J, Gilchrist CLM, Söding J, and Steinegger M (2023) Fast and accurate protein structure search with Foldseek. Nature Biotechnology. doi: 10.1038/s41587-023-01773-0
