# Supplementary material for: Completing the BASEL phage collection to unlock hidden diversity for systematic exploration of phage–host interactions
Source: PLoS Biol. 2025 Apr 7;23(4):e3003063. doi: 10.1371/journal.pbio.3003063 (PMC11990801; doi:10.1371/journal.pbio.3003063)
Supplement: S2 Data — (ZIP) [file pbio.3003063.s009.zip › entries/10.html]

FANPEZAQ\_CDS\_0010


Return to summary | Go to previous | Go to next

|  |  |
| --- | --- |
| FANPEZAQ\_CDS\_0010 Page creation date: 02 Sep 2024, 12:00  Project folder: n/a  Input sequences file: Escherichia\_virus\_HeidiAbel.gb | hypothetical gp37 phage duf1834 tail prophage domain\_containing mu\_like minor u fragment putative duf3168 ankyrin phage\_related duf4128 associated gp37\_like duf806 atp\_binding |

### Sequence information

|  |  |
| --- | --- |
| Name | FANPEZAQ\_CDS\_0010  10\_FANPEZAQ\_CDS\_0010 (pipeline id) |
| Imported annotations | Escherichia\_virus\_HeidiAbel Bas97 |
| Protein sequence | MNADTHIDLEVLHNAIVADIKAAFPQLVTVEFYQDDPEARKTLPIPACLLTISELEADNE IDPGTEQLAVVATFEAHFIINSIRTPRAALAIRTLAAAFMAWLRKRRWTNPADPARKLPT GPANVVGGFPDDFTPELDKYEVWRVEWQQVIHLGESVWNDDGITPSEVYFSWSPEIGFGH EQDYEKVTP |
| Number of residues | 189 |
| Molecular weight (Da) | 21268.69 |
| Output files | ../../query\_sequences/10\_FANPEZAQ\_CDS\_0010.fasta |

### Putative domain architecture and protein family

#### Search results (HHblits)1

|  |  |
| --- | --- |
| Domain family databases searched | Pfam, Ncbi-cd, Cath, Phrogs |
| Results, scheme(s)  (Top layers only; threshold 1.00e-03 (evalue)) | xml version="1.0" encoding="utf-8" standalone="no"?       2024-09-02T21:08:13.914589 image/svg+xml   Matplotlib v3.7.2, https://matplotlib.org/ |
| Results, table  (E-value ≤ 1.00e-03 (evalue)) | | db | id | prob | evalue | pvalue | score | cols | query | query\_len | template | template\_len | name | description | | --- | --- | --- | --- | --- | --- | --- | --- | --- | --- | --- | --- | --- | | phrogs | 1072 | 100.0 | 1.7e-39 | 2.1e-43 | 254.3 | 166 | (9, 188) | 189 | (1, 177) | 183 | NA | NA; Category: unknown function; p140225 VI\_01698 | | phrogs | 490 | 99.9 | 5.2e-27 | 6.8e-31 | 183.3 | 138 | (9, 157) | 189 | (2, 145) | 183 | portal protein | portal protein; Category: head and packaging; p377723 VI\_06234 | | phrogs | 16426 | 99.0 | 4e-14 | 4.6e-18 | 106.3 | 144 | (8, 159) | 189 | (6, 161) | 188 | NA | NA; Category: unknown function; p190981 VI\_07687 | | phrogs | 6911 | 96.5 | 6.8e-06 | 8.4e-10 | 59.5 | 130 | (10, 153) | 189 | (2, 144) | 149 | NA | NA; Category: unknown function; p352299 VI\_01044 | | phrogs | 20428 | 95.3 | 0.0002 | 2.3e-08 | 51.0 | 161 | (11, 188) | 189 | (6, 171) | 175 | tail terminator | tail terminator; Category: connector; p329161 VI\_04355 | | phrogs | 209 | 95.3 | 0.00018 | 2.3e-08 | 50.2 | 94 | (12, 109) | 189 | (2, 106) | 131 | portal protein | portal protein; Category: head and packaging; p338775 VI\_10901 | |
| Top keywords  (threshold 1.00e-03 (evalue)) | **portal, head, and, packaging, p140225, VI\_01698, p377723, VI\_06234, p190981, VI\_07687** |
| Output files | ../../domain\_architecture/10\_FANPEZAQ\_CDS\_0010\_cath.hhr ../../domain\_architecture/10\_FANPEZAQ\_CDS\_0010\_merged.svg ../../domain\_architecture/10\_FANPEZAQ\_CDS\_0010\_ncbi-cd.hhr ../../domain\_architecture/10\_FANPEZAQ\_CDS\_0010\_pfam.hhr ../../domain\_architecture/10\_FANPEZAQ\_CDS\_0010\_phrogs.hhr |

### Identical protein sequences/structures

#### Search results

|  |  |
| --- | --- |
| Protein sequence databases searched | Pdb, Swissprot, Refseq |
| Identical proteins found | -- |
| Top keywords | -- |
| Output files | -- |

### Similar protein sequences/structures

#### Sequence similarity search results (HHblits)1

|  |  |
| --- | --- |
| Sequence databases searched | Uniclust, Pdb70 |
| Results, scheme(s)  (Top layers only, threshold 1.00e-03 (evalue)) | xml version="1.0" encoding="utf-8" standalone="no"?       2024-09-02T21:08:31.783454 image/svg+xml   Matplotlib v3.7.2, https://matplotlib.org/ |
| Results, table(s)  (threshold 1.00e-03 (evalue)) | | db | id | prob | evalue | pvalue | score | cols | query | query\_len | template | template\_len | name | description | | --- | --- | --- | --- | --- | --- | --- | --- | --- | --- | --- | --- | --- | | uniclust | UniRef100\_A0A024E9X0 | 100.0 | 1.8e-45 | 3.8e-51 | 283.3 | 173 | (2, 188) | 189 | (11, 183) | 201 | Phage protein | Phage protein | | uniclust | UniRef100\_A0A088FRT4 | 100.0 | 2.1e-45 | 4.5e-51 | 290.1 | 179 | (1, 189) | 189 | (17, 195) | 231 | Uncharacterized protein | Uncharacterized protein | | uniclust | UniRef100\_A0A142BA55 | 100.0 | 3.4e-42 | 7.5e-48 | 267.1 | 175 | (1, 189) | 189 | (3, 178) | 196 | Phage protein | Phage protein | | uniclust | UniRef100\_A0A024HA76 | 100.0 | 4.3e-40 | 9.1e-46 | 255.0 | 171 | (4, 188) | 189 | (29, 200) | 213 | Phage protein | Phage protein | | uniclust | UniRef100\_A0A081MYL3 | 100.0 | 5.7e-38 | 1.2e-43 | 238.1 | 176 | (3, 188) | 189 | (8, 184) | 191 | Phage protein | Phage protein | | uniclust | UniRef100\_A0A066T1B0 | 100.0 | 1.2e-36 | 2.5e-42 | 236.8 | 173 | (2, 189) | 189 | (5, 181) | 205 | Uncharacterized protein | Uncharacterized protein | | uniclust | UniRef100\_A0A167H3U2 | 100.0 | 1.3e-36 | 2.8e-42 | 242.1 | 175 | (2, 188) | 189 | (42, 222) | 243 | Uncharacterized protein | Uncharacterized protein | | uniclust | UniRef100\_A0A066RR18 | 100.0 | 3.8e-36 | 7.6e-42 | 224.6 | 174 | (3, 189) | 189 | (1, 174) | 181 | Phage protein | Phage protein | | uniclust | UniRef100\_A0A059IUK2 | 100.0 | 3.1e-34 | 6e-40 | 215.7 | 172 | (4, 188) | 189 | (42, 213) | 217 | Phage related protein | Phage related protein | | uniclust | UniRef100\_A0A0Q2U909 | 99.9 | 3.9e-32 | 8.2e-38 | 209.2 | 177 | (2, 189) | 189 | (12, 193) | 198 | Uncharacterized protein | Uncharacterized protein | | uniclust | UniRef100\_A0A031FSS6 | 99.9 | 8e-31 | 1.6e-36 | 198.2 | 172 | (1, 189) | 189 | (1, 173) | 188 | Putative phage-like protein | Putative phage-like protein | | uniclust | UniRef100\_A0A853IM63 | 99.9 | 1.3e-30 | 2.5e-36 | 192.8 | 174 | (3, 186) | 189 | (5, 182) | 185 | Uncharacterized protein (Fragment) | Uncharacterized protein (Fragment) | | uniclust | UniRef100\_A0A0J7J7S1 | 99.9 | 1.7e-30 | 3.6e-36 | 201.6 | 144 | (2, 156) | 189 | (3, 153) | 192 | Mu-like prophage protein gp37 | Mu-like prophage protein gp37 | | uniclust | UniRef100\_A0A104N6L4 | 99.9 | 3.5e-30 | 6.9e-36 | 185.8 | 123 | (55, 188) | 189 | (5, 127) | 139 | Phage tail protein (Fragment) | Phage tail protein (Fragment) | | uniclust | UniRef100\_A0A219Y8Y8 | 99.9 | 7.1e-30 | 1.4e-35 | 189.2 | 156 | (3, 170) | 189 | (1, 156) | 180 | Uncharacterized protein | Uncharacterized protein | | uniclust | UniRef100\_A0A1C2K2M7 | 99.9 | 5.2e-29 | 1.1e-34 | 190.5 | 176 | (1, 188) | 189 | (10, 186) | 191 | Uncharacterized protein | Uncharacterized protein | | uniclust | UniRef100\_A0A142BH88 | 99.9 | 3.1e-28 | 6.3e-34 | 198.9 | 165 | (7, 189) | 189 | (136, 300) | 305 | Uncharacterized protein | Uncharacterized protein | | uniclust | UniRef100\_A0A2J8GXZ0 | 99.9 | 8.3e-28 | 1.6e-33 | 179.9 | 171 | (5, 188) | 189 | (1, 175) | 180 | Phage tail protein | Phage tail protein | | uniclust | UniRef100\_A0A060H8T0 | 99.9 | 2e-27 | 3.7e-33 | 173.1 | 176 | (4, 189) | 189 | (5, 180) | 180 | Tail protein | Tail protein | | uniclust | UniRef100\_A0A0J5JHL4 | 99.9 | 2.4e-27 | 4.8e-33 | 177.7 | 134 | (8, 156) | 189 | (4, 139) | 167 | Gp37 protein | Gp37 protein | | uniclust | UniRef100\_A0A1A9VKF9 | 99.9 | 3.4e-26 | 6.5e-32 | 185.1 | 160 | (4, 176) | 189 | (52, 211) | 378 | Phage\_base\_V domain-containing protein | Phage\_base\_V domain-containing protein | | uniclust | UniRef100\_UPI0021CE98C7 | 99.9 | 7.7e-26 | 1.4e-31 | 165.2 | 175 | (4, 187) | 189 | (4, 184) | 185 | hypothetical protein | hypothetical protein | | uniclust | UniRef100\_A0A1T2CHW9 | 99.9 | 1.4e-25 | 2.8e-31 | 167.5 | 154 | (5, 174) | 189 | (1, 154) | 170 | Phage protein | Phage protein | | uniclust | UniRef100\_A0A9D8J6R2 | 99.8 | 1.2e-23 | 2.2e-29 | 147.6 | 126 | (6, 146) | 189 | (1, 126) | 126 | Uncharacterized protein (Fragment) | Uncharacterized protein (Fragment) | | uniclust | UniRef100\_Q9PFF2 | 99.8 | 7.2e-23 | 1.3e-28 | 143.6 | 137 | (45, 189) | 189 | (1, 137) | 137 | Phage-related protein | Phage-related protein | | uniclust | UniRef100\_A0A654AL97 | 99.8 | 2.2e-22 | 4.1e-28 | 148.1 | 138 | (7, 155) | 189 | (13, 150) | 189 | Phage protein | Phage protein | | uniclust | UniRef100\_A0A0W8JFJ1 | 99.8 | 3.2e-22 | 6.1e-28 | 150.8 | 171 | (6, 188) | 189 | (8, 178) | 185 | Phage protein | Phage protein | | uniclust | UniRef100\_A0A085AFN8 | 99.8 | 4.1e-22 | 7.9e-28 | 151.5 | 175 | (2, 189) | 189 | (1, 176) | 197 | Phage protein | Phage protein | | uniclust | UniRef100\_UPI002263F815 | 99.7 | 1.7e-21 | 3.2e-27 | 157.8 | 153 | (3, 168) | 189 | (2, 154) | 411 | DUF4113 domain-containing protein | DUF4113 domain-containing protein | | uniclust | UniRef100\_A0A380T1F7 | 99.7 | 2.1e-21 | 4e-27 | 127.5 | 79 | (101, 187) | 189 | (3, 81) | 85 | Uncharacterized protein | Uncharacterized protein | | uniclust | UniRef100\_UPI000B195A94 | 99.7 | 2.3e-21 | 4.5e-27 | 140.5 | 129 | (49, 188) | 189 | (2, 133) | 143 | hypothetical protein | hypothetical protein | | uniclust | UniRef100\_A0A5E4NQZ0 | 99.7 | 4.8e-21 | 8.7e-27 | 163.1 | 148 | (10, 171) | 189 | (282, 429) | 691 | Bacteriophage lambda, GpZ, minor tail,Ankyrin repeat-containing domain,Ankyrin repeat | Bacteriophage lambda, GpZ, minor tail,Ankyrin repeat-containing domain,Ankyrin repeat | | uniclust | UniRef100\_A0A1G8I0M9 | 99.7 | 6.6e-21 | 1.3e-26 | 144.3 | 171 | (3, 188) | 189 | (6, 181) | 192 | Gp37 protein | Gp37 protein | | uniclust | UniRef100\_A0A1H2FMJ7 | 99.7 | 7.6e-21 | 1.5e-26 | 145.5 | 157 | (2, 171) | 189 | (13, 176) | 192 | Phage protein Gp37/Gp68 | Phage protein Gp37/Gp68 | | uniclust | UniRef100\_A0A0H2M1V4 | 99.7 | 8.3e-21 | 1.7e-26 | 148.4 | 144 | (1, 154) | 189 | (1, 152) | 206 | DUF1834 family protein | DUF1834 family protein | | uniclust | UniRef100\_A0A1Z3U7W0 | 99.7 | 4.9e-20 | 9.3e-26 | 139.5 | 171 | (6, 186) | 189 | (1, 181) | 195 | Uncharacterized protein | Uncharacterized protein | | uniclust | UniRef100\_F2K221 | 99.7 | 4.9e-20 | 9.5e-26 | 139.6 | 173 | (2, 188) | 189 | (1, 177) | 184 | Gp37 protein | Gp37 protein | | uniclust | UniRef100\_A0A839IZ53 | 99.7 | 7.9e-20 | 1.5e-25 | 137.7 | 173 | (2, 188) | 189 | (5, 185) | 197 | Uncharacterized protein | Uncharacterized protein | | uniclust | UniRef100\_A0A853IIQ5 | 99.7 | 1e-19 | 1.9e-25 | 126.8 | 118 | (63, 187) | 189 | (5, 125) | 127 | Uncharacterized protein | Uncharacterized protein | | uniclust | UniRef100\_A0A1E7HY00 | 99.6 | 3.4e-19 | 6.5e-25 | 134.7 | 161 | (13, 188) | 189 | (6, 179) | 187 | Uncharacterized protein | Uncharacterized protein | | uniclust | UniRef100\_A0A059IW70 | 99.6 | 3.4e-19 | 6.5e-25 | 124.1 | 110 | (53, 172) | 189 | (2, 111) | 115 | Phage related protein | Phage related protein | | uniclust | UniRef100\_A0A1N6I1G9 | 99.6 | 3.7e-19 | 7.1e-25 | 135.0 | 170 | (7, 188) | 189 | (15, 190) | 195 | Uncharacterized protein | Uncharacterized protein | | uniclust | UniRef100\_A0A7J6YLT3 | 99.6 | 3.9e-19 | 7.2e-25 | 147.8 | 144 | (20, 176) | 189 | (2, 145) | 508 | Baseplate protein J-like domain-containing protein | Baseplate protein J-like domain-containing protein | | uniclust | UniRef100\_A0A090SU28 | 99.6 | 4.8e-19 | 8.9e-25 | 131.9 | 172 | (9, 188) | 189 | (3, 183) | 197 | Phage protein | Phage protein | | uniclust | UniRef100\_A0A071M5B9 | 99.6 | 6.4e-19 | 1.3e-24 | 141.3 | 154 | (12, 177) | 189 | (26, 194) | 234 | Uncharacterized protein | Uncharacterized protein | | uniclust | UniRef100\_UPI00037FEC58 | 99.6 | 1.4e-18 | 2.5e-24 | 127.7 | 169 | (6, 187) | 189 | (9, 177) | 177 | hypothetical protein | hypothetical protein | | uniclust | UniRef100\_A0A1I5KYE0 | 99.6 | 2.1e-18 | 3.9e-24 | 126.4 | 155 | (6, 174) | 189 | (2, 158) | 170 | Gp37 protein | Gp37 protein | | uniclust | UniRef100\_A0A068T9Q2 | 99.6 | 2e-18 | 4.1e-24 | 138.4 | 141 | (8, 159) | 189 | (20, 177) | 223 | Uncharacterized protein | Uncharacterized protein | | uniclust | UniRef100\_UPI001565E1E4 | 99.6 | 3.6e-18 | 6.6e-24 | 125.9 | 172 | (3, 188) | 189 | (1, 174) | 181 | hypothetical protein | hypothetical protein | | uniclust | UniRef100\_A0A8S4QQF2 | 99.5 | 1.1e-17 | 2.1e-23 | 138.2 | 131 | (28, 176) | 189 | (152, 282) | 409 | Jg15362 protein | Jg15362 protein | | uniclust | UniRef100\_A0A0P7IJV4 | 99.5 | 1.3e-17 | 2.6e-23 | 128.4 | 153 | (4, 168) | 189 | (1, 161) | 181 | Uncharacterized protein | Uncharacterized protein | | uniclust | UniRef100\_A0A1G5ACN2 | 99.5 | 3.7e-17 | 6.8e-23 | 120.9 | 169 | (7, 188) | 189 | (1, 174) | 177 | Uncharacterized protein | Uncharacterized protein | | uniclust | UniRef100\_UPI000F02A975 | 99.5 | 4.5e-17 | 8.5e-23 | 121.6 | 145 | (1, 157) | 189 | (1, 145) | 170 | hypothetical protein | hypothetical protein | | uniclust | UniRef100\_A0A0A1Z018 | 99.5 | 5.8e-17 | 1.1e-22 | 120.6 | 155 | (3, 171) | 189 | (1, 157) | 185 | Uncharacterized protein | Uncharacterized protein | | uniclust | UniRef100\_A0A1D2X525 | 99.5 | 9.8e-17 | 1.9e-22 | 118.9 | 140 | (5, 152) | 189 | (7, 153) | 156 | Uncharacterized protein | Uncharacterized protein | | uniclust | UniRef100\_A0A4P0TMC0 | 99.5 | 2.1e-16 | 3.9e-22 | 104.3 | 71 | (117, 187) | 189 | (8, 78) | 82 | Phage protein | Phage protein | | uniclust | UniRef100\_A0A4V2PRI1 | 99.4 | 3.2e-16 | 5.8e-22 | 115.9 | 165 | (7, 189) | 189 | (10, 174) | 179 | Gp37 protein | Gp37 protein | | uniclust | UniRef100\_A0A0C5VSK4 | 99.4 | 3.6e-16 | 7.3e-22 | 123.8 | 143 | (6, 157) | 189 | (1, 175) | 214 | Uncharacterized protein | Uncharacterized protein | | uniclust | UniRef100\_A0A4P9VGP3 | 99.4 | 4.4e-16 | 8.2e-22 | 116.4 | 133 | (41, 186) | 189 | (55, 187) | 193 | DUF1834 family protein | DUF1834 family protein | | uniclust | UniRef100\_A0A327JK07 | 99.4 | 4.8e-16 | 9.3e-22 | 118.7 | 158 | (1, 171) | 189 | (8, 172) | 184 | Uncharacterized protein | Uncharacterized protein | | uniclust | UniRef100\_UPI000B7E93C5 | 99.4 | 3e-15 | 5.6e-21 | 109.7 | 132 | (3, 149) | 189 | (1, 137) | 154 | hypothetical protein | hypothetical protein | | uniclust | UniRef100\_A0A0X8JJA6 | 99.2 | 5.3e-14 | 1e-19 | 107.6 | 138 | (7, 157) | 189 | (1, 146) | 185 | Osmotically inducible protein OsmC | Osmotically inducible protein OsmC | | uniclust | UniRef100\_A0A1F9BJG0 | 99.2 | 4.9e-14 | 1e-19 | 108.4 | 136 | (3, 152) | 189 | (7, 150) | 155 | DUF1834 domain-containing protein | DUF1834 domain-containing protein | | uniclust | UniRef100\_A0A450Z1H3 | 99.2 | 6e-14 | 1.1e-19 | 99.9 | 128 | (6, 145) | 189 | (1, 132) | 133 | Uncharacterized protein | Uncharacterized protein | | uniclust | UniRef100\_A0A5E4XFY4 | 99.2 | 1e-13 | 1.9e-19 | 105.4 | 143 | (7, 160) | 189 | (1, 151) | 188 | Uncharacterized protein | Uncharacterized protein | | uniclust | UniRef100\_UPI001E3A84FA | 99.1 | 2.6e-13 | 4.8e-19 | 85.4 | 64 | (126, 189) | 189 | (2, 65) | 65 | hypothetical protein | hypothetical protein | | uniclust | UniRef100\_UPI0004168F22 | 99.1 | 2.6e-13 | 4.9e-19 | 104.6 | 167 | (7, 188) | 189 | (8, 192) | 195 | hypothetical protein | hypothetical protein | | uniclust | UniRef100\_A0A450TJH1 | 99.1 | 2.7e-13 | 5e-19 | 89.3 | 61 | (128, 188) | 189 | (16, 76) | 79 | Uncharacterized protein | Uncharacterized protein | | uniclust | UniRef100\_A0A1E3G659 | 99.1 | 3.6e-13 | 6.6e-19 | 100.6 | 163 | (9, 187) | 189 | (2, 164) | 181 | Uncharacterized protein | Uncharacterized protein | | uniclust | UniRef100\_A0A0W1G855 | 99.1 | 3.7e-13 | 7.8e-19 | 111.8 | 144 | (2, 155) | 189 | (8, 171) | 256 | DUF1834 family protein | DUF1834 family protein | | uniclust | UniRef100\_A0A3N2E1B1 | 99.1 | 4.6e-13 | 8.5e-19 | 100.1 | 173 | (3, 188) | 189 | (1, 175) | 181 | Gp37 protein | Gp37 protein | | uniclust | UniRef100\_UPI0013D3F786 | 99.1 | 9.3e-13 | 1.7e-18 | 97.3 | 146 | (2, 156) | 189 | (1, 147) | 165 | hypothetical protein | hypothetical protein | | uniclust | UniRef100\_A0A5S9P2S6 | 99.1 | 9.9e-13 | 1.8e-18 | 99.9 | 172 | (9, 189) | 189 | (10, 198) | 201 | Uncharacterized protein | Uncharacterized protein | | uniclust | UniRef100\_A0A059ZVM0 | 99.0 | 1.3e-12 | 2.7e-18 | 106.4 | 166 | (1, 188) | 189 | (37, 216) | 234 | Uncharacterized protein | Uncharacterized protein | | uniclust | UniRef100\_A0A0E4BWM4 | 99.0 | 1.5e-12 | 3e-18 | 101.9 | 139 | (6, 156) | 189 | (1, 146) | 194 | Uncharacterized protein | Uncharacterized protein | | uniclust | UniRef100\_UPI001E56994E | 99.0 | 1.8e-12 | 3.3e-18 | 87.2 | 86 | (1, 87) | 189 | (1, 88) | 92 | hypothetical protein | hypothetical protein | | uniclust | UniRef100\_A0A8H8X087 | 99.0 | 2e-12 | 3.8e-18 | 100.5 | 160 | (5, 174) | 189 | (1, 179) | 208 | Uncharacterized protein | Uncharacterized protein | | uniclust | UniRef100\_A0A9E3PK95 | 99.0 | 3.1e-12 | 6e-18 | 99.5 | 146 | (10, 165) | 189 | (19, 180) | 204 | Uncharacterized protein | Uncharacterized protein | | uniclust | UniRef100\_A0A1C3EBP2 | 99.0 | 3.4e-12 | 6.2e-18 | 96.2 | 165 | (3, 177) | 189 | (1, 181) | 187 | Phage tail protein | Phage tail protein | | uniclust | UniRef100\_A0A2A4XUH6 | 99.0 | 3.5e-12 | 6.4e-18 | 94.7 | 140 | (6, 157) | 189 | (1, 147) | 169 | Uncharacterized protein | Uncharacterized protein | | uniclust | UniRef100\_A0A009YIC9 | 98.9 | 5.7e-12 | 1.2e-17 | 103.4 | 143 | (2, 155) | 189 | (17, 176) | 224 | DUF1834 family protein | DUF1834 family protein | | uniclust | UniRef100\_A0A650ENI9 | 98.9 | 1e-11 | 2.1e-17 | 97.4 | 135 | (3, 155) | 189 | (1, 146) | 184 | Uncharacterized protein | Uncharacterized protein | | uniclust | UniRef100\_A0A2K8U790 | 98.9 | 1.2e-11 | 2.2e-17 | 90.8 | 138 | (8, 153) | 189 | (7, 151) | 154 | Uncharacterized protein | Uncharacterized protein | | uniclust | UniRef100\_UPI002152489E | 98.8 | 2.4e-11 | 4.3e-17 | 82.6 | 85 | (93, 187) | 189 | (2, 87) | 95 | hypothetical protein | hypothetical protein | | uniclust | UniRef100\_A0A2D8QD62 | 98.8 | 2.8e-11 | 5.1e-17 | 92.8 | 177 | (7, 187) | 189 | (4, 194) | 207 | Uncharacterized protein | Uncharacterized protein | | uniclust | UniRef100\_A0A4P0TLN7 | 98.8 | 3.2e-11 | 5.9e-17 | 85.5 | 107 | (4, 129) | 189 | (3, 109) | 117 | DUF3168 domain-containing protein | DUF3168 domain-containing protein | | uniclust | UniRef100\_UPI001EDABC2D | 98.8 | 3.2e-11 | 5.9e-17 | 87.3 | 130 | (46, 186) | 189 | (1, 135) | 139 | hypothetical protein | hypothetical protein | | uniclust | UniRef100\_A0A4U9HEQ6 | 98.8 | 3.8e-11 | 7.1e-17 | 80.0 | 70 | (119, 188) | 189 | (11, 80) | 85 | Uncharacterized protein | Uncharacterized protein | | uniclust | UniRef100\_A0A8J2Z5Y1 | 98.8 | 3.6e-11 | 7.1e-17 | 92.7 | 161 | (2, 177) | 189 | (1, 161) | 175 | Uncharacterized protein | Uncharacterized protein | | uniclust | UniRef100\_A0A022PGL8 | 98.8 | 3.7e-11 | 7.8e-17 | 97.3 | 135 | (2, 153) | 189 | (14, 157) | 209 | Mu-like prophage protein gp37 | Mu-like prophage protein gp37 | | uniclust | UniRef100\_A0A0H3ZQZ5 | 98.8 | 5.2e-11 | 1.1e-16 | 93.9 | 158 | (2, 174) | 189 | (4, 161) | 183 | Uncharacterized protein | Uncharacterized protein | | uniclust | UniRef100\_A0A7X3ZHV1 | 98.8 | 6.6e-11 | 1.2e-16 | 91.8 | 154 | (24, 187) | 189 | (47, 216) | 223 | Uncharacterized protein | Uncharacterized protein | | uniclust | UniRef100\_A0A2N8GRJ2 | 98.7 | 9e-11 | 1.7e-16 | 78.4 | 77 | (40, 127) | 189 | (8, 84) | 85 | Uncharacterized protein (Fragment) | Uncharacterized protein (Fragment) | | uniclust | UniRef100\_A0A1H1G3G9 | 98.7 | 8.7e-11 | 1.7e-16 | 92.2 | 139 | (7, 155) | 189 | (11, 158) | 189 | Uncharacterized protein | Uncharacterized protein | | uniclust | UniRef100\_A0A846QLX3 | 98.7 | 9.4e-11 | 1.7e-16 | 79.7 | 86 | (92, 188) | 189 | (3, 88) | 94 | Uncharacterized protein | Uncharacterized protein | | uniclust | UniRef100\_UPI001E4FC916 | 98.7 | 9.6e-11 | 1.8e-16 | 84.6 | 98 | (6, 109) | 189 | (1, 98) | 135 | hypothetical protein | hypothetical protein | | uniclust | UniRef100\_A0A1S8YKZ2 | 98.7 | 1.4e-10 | 2.5e-16 | 87.7 | 166 | (9, 188) | 189 | (7, 176) | 182 | Phage tail protein | Phage tail protein | | uniclust | UniRef100\_UPI0018F1F2B8 | 98.7 | 1.5e-10 | 2.7e-16 | 87.8 | 168 | (5, 188) | 189 | (2, 170) | 175 | hypothetical protein | hypothetical protein | | uniclust | UniRef100\_B7RNM1 | 98.7 | 2.1e-10 | 3.9e-16 | 77.3 | 70 | (117, 186) | 189 | (15, 84) | 89 | Uncharacterized protein | Uncharacterized protein | | uniclust | UniRef100\_A0A9D9F620 | 98.6 | 2.7e-10 | 4.9e-16 | 87.2 | 161 | (7, 176) | 189 | (13, 183) | 197 | Uncharacterized protein | Uncharacterized protein | | uniclust | UniRef100\_A0A3N0V7T1 | 98.6 | 2.7e-10 | 5.2e-16 | 87.6 | 137 | (7, 156) | 189 | (1, 143) | 182 | DUF1834 family protein | DUF1834 family protein | | uniclust | UniRef100\_UPI001596FEA7 | 98.6 | 3.3e-10 | 6.1e-16 | 86.3 | 112 | (42, 161) | 189 | (41, 155) | 191 | hypothetical protein | hypothetical protein | | uniclust | UniRef100\_A0A962EV07 | 98.6 | 5e-10 | 9.2e-16 | 83.7 | 143 | (3, 157) | 189 | (1, 150) | 166 | Uncharacterized protein | Uncharacterized protein | | uniclust | UniRef100\_UPI001C25C53F | 98.6 | 6.1e-10 | 1.1e-15 | 75.8 | 70 | (5, 79) | 189 | (1, 70) | 93 | hypothetical protein | hypothetical protein | | uniclust | UniRef100\_UPI0004983960 | 98.6 | 7.1e-10 | 1.3e-15 | 85.9 | 136 | (7, 153) | 189 | (1, 148) | 213 | hypothetical protein | hypothetical protein | | uniclust | UniRef100\_A0A3R8IUE9 | 98.6 | 7.2e-10 | 1.3e-15 | 84.8 | 159 | (4, 176) | 189 | (6, 176) | 187 | DUF1834 family protein | DUF1834 family protein | | uniclust | UniRef100\_UPI001F146AA5 | 98.6 | 7.8e-10 | 1.4e-15 | 82.6 | 113 | (13, 145) | 189 | (3, 121) | 164 | hypothetical protein | hypothetical protein | | uniclust | UniRef100\_A0A2V3UBZ9 | 98.5 | 8.5e-10 | 1.6e-15 | 83.9 | 142 | (6, 155) | 189 | (1, 148) | 185 | Uncharacterized protein DUF1834 | Uncharacterized protein DUF1834 | | uniclust | UniRef100\_A0A1E3UZA7 | 98.5 | 8.8e-10 | 1.6e-15 | 84.1 | 153 | (15, 181) | 189 | (16, 183) | 191 | Uncharacterized protein | Uncharacterized protein | | uniclust | UniRef100\_A0A063BJE5 | 98.5 | 7.9e-10 | 1.7e-15 | 92.3 | 162 | (1, 173) | 189 | (8, 192) | 243 | Mu-like prophage protein gp37 | Mu-like prophage protein gp37 | | uniclust | UniRef100\_A0A0J7LV93 | 98.5 | 9.2e-10 | 1.9e-15 | 89.4 | 155 | (4, 176) | 189 | (8, 176) | 201 | Mu-like prophage protein gp37 | Mu-like prophage protein gp37 | | uniclust | UniRef100\_A0A136Q971 | 98.5 | 1.5e-09 | 2.7e-15 | 72.6 | 70 | (6, 80) | 189 | (13, 82) | 83 | DUF3168 domain-containing protein (Fragment) | DUF3168 domain-containing protein (Fragment) | | uniclust | UniRef100\_A0A5P9F0H4 | 98.5 | 1.8e-09 | 3.5e-15 | 83.4 | 179 | (6, 189) | 189 | (1, 186) | 190 | Uncharacterized protein | Uncharacterized protein | | uniclust | UniRef100\_A0A8T5Z499 | 98.4 | 2.3e-09 | 4.3e-15 | 73.6 | 83 | (99, 188) | 189 | (2, 85) | 96 | Phage tail protein | Phage tail protein | | uniclust | UniRef100\_UPI0020A2AFB5 | 98.4 | 3.3e-09 | 6.1e-15 | 88.5 | 152 | (13, 177) | 189 | (16, 182) | 379 | hypothetical protein | hypothetical protein | | uniclust | UniRef100\_A0A439U2R2 | 98.4 | 3.5e-09 | 6.7e-15 | 82.8 | 172 | (2, 188) | 189 | (1, 182) | 188 | Uncharacterized protein | Uncharacterized protein | | uniclust | UniRef100\_UPI000B8EB34D | 98.4 | 4.2e-09 | 7.8e-15 | 74.8 | 95 | (87, 188) | 189 | (13, 111) | 116 | hypothetical protein | hypothetical protein | | uniclust | UniRef100\_UPI00227F3D8E | 98.4 | 4.5e-09 | 8.2e-15 | 79.8 | 155 | (8, 175) | 189 | (7, 164) | 179 | hypothetical protein | hypothetical protein | | uniclust | UniRef100\_A0A8S5QI65 | 98.4 | 5.2e-09 | 1e-14 | 79.7 | 140 | (6, 155) | 189 | (1, 148) | 157 | Uncharacterized protein | Uncharacterized protein | | uniclust | UniRef100\_UPI0020C04467 | 98.4 | 5.6e-09 | 1e-14 | 72.0 | 95 | (5, 108) | 189 | (1, 95) | 97 | hypothetical protein | hypothetical protein | | uniclust | UniRef100\_A0A4U8YKI5 | 98.4 | 5.7e-09 | 1.1e-14 | 78.7 | 154 | (6, 170) | 189 | (1, 160) | 171 | Uncharacterized protein | Uncharacterized protein | | uniclust | UniRef100\_UPI001C99755B | 98.3 | 6.6e-09 | 1.2e-14 | 72.6 | 94 | (3, 98) | 189 | (1, 102) | 105 | DUF1834 family protein | DUF1834 family protein | | uniclust | UniRef100\_A0A0X8JK81 | 98.3 | 6.5e-09 | 1.3e-14 | 85.7 | 141 | (3, 153) | 189 | (1, 156) | 231 | Uncharacterized protein | Uncharacterized protein | | uniclust | UniRef100\_F2J640 | 98.3 | 9.5e-09 | 1.8e-14 | 88.3 | 136 | (6, 152) | 189 | (1, 142) | 489 | Uncharacterized protein | Uncharacterized protein | | uniclust | UniRef100\_UPI00234BD1FE | 98.3 | 1.1e-08 | 2.1e-14 | 72.7 | 78 | (5, 85) | 189 | (6, 83) | 115 | hypothetical protein | hypothetical protein | | uniclust | UniRef100\_UPI0020BFFED6 | 98.3 | 1.2e-08 | 2.1e-14 | 71.2 | 80 | (88, 174) | 189 | (9, 88) | 102 | hypothetical protein | hypothetical protein | | uniclust | UniRef100\_UPI001903E94F | 98.2 | 1.5e-08 | 2.7e-14 | 76.9 | 149 | (1, 156) | 189 | (1, 166) | 175 | hypothetical protein | hypothetical protein | | uniclust | UniRef100\_A0A1D2QS75 | 98.2 | 2.3e-08 | 4.3e-14 | 84.6 | 153 | (6, 162) | 189 | (1, 182) | 411 | Uncharacterized protein | Uncharacterized protein | | uniclust | UniRef100\_A0A1G8G2T2 | 98.2 | 3e-08 | 5.9e-14 | 79.8 | 141 | (4, 155) | 189 | (3, 162) | 206 | Mu-like prophage protein gp37 (Fragment) | Mu-like prophage protein gp37 (Fragment) | | uniclust | UniRef100\_UPI000AB590E9 | 98.2 | 3.2e-08 | 6e-14 | 71.5 | 104 | (64, 177) | 189 | (7, 112) | 126 | hypothetical protein | hypothetical protein | | uniclust | UniRef100\_A0A345DE57 | 98.1 | 3.5e-08 | 6.5e-14 | 75.6 | 140 | (7, 161) | 189 | (1, 141) | 184 | Uncharacterized protein | Uncharacterized protein | | uniclust | UniRef100\_A0A432QS63 | 98.1 | 3.9e-08 | 7.1e-14 | 75.5 | 144 | (9, 162) | 189 | (8, 157) | 186 | DUF1834 domain-containing protein | DUF1834 domain-containing protein | | uniclust | UniRef100\_UPI00217D2FA9 | 98.1 | 4.9e-08 | 9e-14 | 69.5 | 92 | (5, 102) | 189 | (4, 99) | 114 | hypothetical protein | hypothetical protein | | uniclust | UniRef100\_A0A142BHP0 | 98.1 | 5.7e-08 | 1e-13 | 68.3 | 84 | (64, 154) | 189 | (15, 101) | 105 | Phage protein | Phage protein | | uniclust | UniRef100\_Q4E9T1 | 98.0 | 7.8e-08 | 1.5e-13 | 61.2 | 52 | (119, 170) | 189 | (3, 54) | 58 | Uncharacterized protein | Uncharacterized protein | | uniclust | UniRef100\_UPI002271FB79 | 98.0 | 8.3e-08 | 1.5e-13 | 73.9 | 138 | (7, 155) | 189 | (1, 151) | 188 | hypothetical protein | hypothetical protein | | uniclust | UniRef100\_UPI001F062539 | 98.0 | 1e-07 | 1.9e-13 | 65.4 | 79 | (2, 85) | 189 | (3, 82) | 91 | hypothetical protein | hypothetical protein | | uniclust | UniRef100\_A0A0A1HZ01 | 98.0 | 1.2e-07 | 2.2e-13 | 64.9 | 78 | (103, 188) | 189 | (10, 88) | 89 | Uncharacterized protein | Uncharacterized protein | | uniclust | UniRef100\_A0A431IBE3 | 98.0 | 1.2e-07 | 2.2e-13 | 76.6 | 129 | (26, 171) | 189 | (110, 245) | 266 | DUF1834 family protein | DUF1834 family protein | | uniclust | UniRef100\_Q4EDG4 | 98.0 | 1.3e-07 | 2.5e-13 | 62.8 | 50 | (7, 59) | 189 | (1, 50) | 69 | Uncharacterized protein | Uncharacterized protein | | uniclust | UniRef100\_A0A7X6JH33 | 98.0 | 1.7e-07 | 3e-13 | 60.3 | 39 | (148, 186) | 189 | (19, 57) | 63 | Uncharacterized protein | Uncharacterized protein | | uniclust | UniRef100\_A0A0A2Y1K5 | 97.9 | 1.7e-07 | 3.5e-13 | 78.3 | 141 | (3, 151) | 189 | (20, 174) | 244 | Mu-like prophage protein gp37 | Mu-like prophage protein gp37 | | uniclust | UniRef100\_A0A059IWS3 | 97.9 | 2.3e-07 | 4.3e-13 | 61.1 | 59 | (8, 71) | 189 | (2, 60) | 67 | Uncharacterized protein | Uncharacterized protein | | uniclust | UniRef100\_A0A193QHJ5 | 97.8 | 3.3e-07 | 6.6e-13 | 75.2 | 139 | (4, 151) | 189 | (1, 153) | 219 | Uncharacterized protein | Uncharacterized protein | | uniclust | UniRef100\_UPI001931093B | 97.8 | 3.7e-07 | 6.9e-13 | 60.9 | 69 | (75, 152) | 189 | (1, 74) | 76 | hypothetical protein | hypothetical protein | | uniclust | UniRef100\_UPI001FC8A895 | 97.8 | 3.9e-07 | 7.2e-13 | 60.4 | 59 | (127, 187) | 189 | (9, 67) | 73 | hypothetical protein | hypothetical protein | | uniclust | UniRef100\_A0A8J3HUP9 | 97.8 | 4.5e-07 | 8.3e-13 | 57.9 | 56 | (83, 145) | 189 | (5, 60) | 60 | Uncharacterized protein | Uncharacterized protein | | uniclust | UniRef100\_UPI0013D70C2A | 97.8 | 4.6e-07 | 8.5e-13 | 62.8 | 89 | (46, 148) | 189 | (2, 92) | 94 | hypothetical protein | hypothetical protein | | uniclust | UniRef100\_A0A962YS06 | 97.8 | 5.2e-07 | 9.5e-13 | 67.6 | 137 | (6, 152) | 189 | (3, 147) | 150 | Uncharacterized protein | Uncharacterized protein | | uniclust | UniRef100\_A0A0Q8AN87 | 97.8 | 5.4e-07 | 1e-12 | 70.9 | 146 | (6, 159) | 189 | (1, 160) | 182 | Suppressor of fused-like domain-containing protein | Suppressor of fused-like domain-containing protein | | uniclust | UniRef100\_A0A962B4M7 | 97.7 | 7.8e-07 | 1.4e-12 | 69.5 | 151 | (2, 160) | 189 | (16, 172) | 198 | Uncharacterized protein | Uncharacterized protein | | uniclust | UniRef100\_A0A969Q5I0 | 97.7 | 8.7e-07 | 1.6e-12 | 58.4 | 44 | (142, 188) | 189 | (24, 67) | 70 | Uncharacterized protein | Uncharacterized protein | | uniclust | UniRef100\_A0A085NVH9 | 97.7 | 1.1e-06 | 2e-12 | 70.0 | 126 | (6, 148) | 189 | (1, 135) | 190 | DUF1834 family protein | DUF1834 family protein | | uniclust | UniRef100\_A0A1X4N607 | 97.7 | 1.1e-06 | 2.2e-12 | 71.4 | 163 | (7, 182) | 189 | (1, 181) | 198 | Uncharacterized protein | Uncharacterized protein | | uniclust | UniRef100\_A0A933SHU2 | 97.6 | 1.5e-06 | 2.9e-12 | 67.5 | 135 | (5, 153) | 189 | (1, 145) | 148 | DUF1834 family protein | DUF1834 family protein | | uniclust | UniRef100\_A0A800BWL1 | 97.6 | 2.3e-06 | 4.1e-12 | 58.4 | 75 | (6, 87) | 189 | (1, 76) | 83 | Uncharacterized protein (Fragment) | Uncharacterized protein (Fragment) | | uniclust | UniRef100\_Z9JKS2 | 97.6 | 2.4e-06 | 4.4e-12 | 67.9 | 76 | (4, 81) | 189 | (5, 80) | 217 | Uncharacterized protein | Uncharacterized protein | | uniclust | UniRef100\_A0A090KJ28 | 97.5 | 3.1e-06 | 6.3e-12 | 68.2 | 137 | (8, 157) | 189 | (15, 151) | 180 | Uncharacterized phage protein | Uncharacterized phage protein | | uniclust | UniRef100\_UPI00082BE2D3 | 97.5 | 3.9e-06 | 7.1e-12 | 64.0 | 137 | (7, 157) | 189 | (5, 146) | 162 | hypothetical protein | hypothetical protein | | uniclust | UniRef100\_UPI0004263E9E | 97.5 | 4.4e-06 | 8e-12 | 65.0 | 138 | (8, 157) | 189 | (4, 147) | 185 | hypothetical protein | hypothetical protein | | uniclust | UniRef100\_UPI001C04E967 | 97.4 | 5.6e-06 | 1e-11 | 61.0 | 98 | (5, 109) | 189 | (3, 104) | 129 | hypothetical protein | hypothetical protein | | uniclust | UniRef100\_A0A1G3LVL6 | 97.4 | 7.1e-06 | 1.4e-11 | 64.9 | 130 | (5, 149) | 189 | (1, 144) | 152 | Uncharacterized protein | Uncharacterized protein | | uniclust | UniRef100\_UPI001A9E6345 | 97.3 | 1.2e-05 | 2.1e-11 | 55.4 | 70 | (5, 81) | 189 | (4, 73) | 83 | hypothetical protein | hypothetical protein | | uniclust | UniRef100\_UPI001BAEFF64 | 97.3 | 1.2e-05 | 2.2e-11 | 67.1 | 146 | (7, 161) | 189 | (4, 184) | 307 | hypothetical protein | hypothetical protein | | uniclust | UniRef100\_A0A853I2M1 | 97.3 | 1.2e-05 | 2.2e-11 | 64.0 | 170 | (7, 187) | 189 | (1, 177) | 182 | Uncharacterized protein | Uncharacterized protein | | uniclust | UniRef100\_UPI001C98E712 | 97.3 | 1.2e-05 | 2.3e-11 | 57.6 | 67 | (83, 157) | 189 | (7, 75) | 107 | hypothetical protein | hypothetical protein | | uniclust | UniRef100\_A0A0Q0ZM37 | 97.3 | 1.2e-05 | 2.5e-11 | 66.8 | 148 | (8, 173) | 189 | (23, 173) | 206 | Uncharacterized protein | Uncharacterized protein | | uniclust | UniRef100\_A0A0F4VEU8 | 97.2 | 1.5e-05 | 2.9e-11 | 62.4 | 137 | (7, 154) | 189 | (1, 148) | 148 | Uncharacterized protein | Uncharacterized protein | | uniclust | UniRef100\_A0A6L8GFP2 | 97.2 | 2.3e-05 | 4.2e-11 | 63.4 | 148 | (28, 187) | 189 | (40, 227) | 234 | Uncharacterized protein | Uncharacterized protein | | uniclust | UniRef100\_A0A145VQF5 | 97.2 | 2.5e-05 | 4.6e-11 | 62.8 | 109 | (44, 163) | 189 | (23, 133) | 212 | Uncharacterized protein | Uncharacterized protein | | uniclust | UniRef100\_A0A376K4X6 | 97.1 | 2.6e-05 | 4.8e-11 | 56.6 | 86 | (83, 177) | 189 | (5, 93) | 113 | Uncharacterized protein | Uncharacterized protein | | uniclust | UniRef100\_A0A127MKR6 | 97.1 | 2.7e-05 | 5.1e-11 | 60.7 | 135 | (8, 151) | 189 | (5, 149) | 155 | DUF1834 domain-containing protein | DUF1834 domain-containing protein | | uniclust | UniRef100\_A0A956AIV7 | 97.1 | 3e-05 | 5.5e-11 | 60.8 | 137 | (8, 155) | 189 | (2, 149) | 186 | Uncharacterized protein | Uncharacterized protein | | uniclust | UniRef100\_A0A072T9J4 | 97.1 | 2.8e-05 | 6.1e-11 | 65.5 | 144 | (1, 156) | 189 | (26, 178) | 198 | DUF1834 family protein | DUF1834 family protein | | uniclust | UniRef100\_A0A839HEF9 | 97.1 | 3.6e-05 | 6.5e-11 | 59.7 | 147 | (1, 155) | 189 | (1, 166) | 171 | Uncharacterized protein | Uncharacterized protein | | uniclust | UniRef100\_A0A1E3G7H1 | 97.1 | 3.7e-05 | 6.9e-11 | 60.0 | 169 | (7, 187) | 189 | (3, 178) | 178 | Uncharacterized protein | Uncharacterized protein | | uniclust | UniRef100\_A0A0K0VKS5 | 97.0 | 5.1e-05 | 9.4e-11 | 57.1 | 76 | (4, 85) | 189 | (3, 79) | 139 | Uncharacterized protein | Uncharacterized protein | | uniclust | UniRef100\_UPI0010CB9643 | 97.0 | 5.1e-05 | 9.4e-11 | 49.4 | 46 | (143, 188) | 189 | (2, 48) | 59 | hypothetical protein | hypothetical protein | | uniclust | UniRef100\_A0A8D9PAR5 | 96.9 | 7.3e-05 | 1.3e-10 | 56.4 | 135 | (4, 153) | 189 | (2, 136) | 140 | Uncharacterized protein | Uncharacterized protein | | uniclust | UniRef100\_A0A066RQJ8 | 96.9 | 7.3e-05 | 1.5e-10 | 61.5 | 166 | (2, 188) | 189 | (4, 178) | 192 | Phage protein | Phage protein | | uniclust | UniRef100\_UPI001F4243D2 | 96.9 | 9.5e-05 | 1.7e-10 | 54.6 | 112 | (7, 133) | 189 | (5, 117) | 121 | hypothetical protein | hypothetical protein | | uniclust | UniRef100\_A0A1F4PEF5 | 96.8 | 0.00012 | 2.5e-10 | 58.8 | 122 | (3, 136) | 189 | (1, 135) | 154 | DUF1834 domain-containing protein | DUF1834 domain-containing protein | | uniclust | UniRef100\_A0A0G0CA56 | 96.8 | 0.00013 | 2.7e-10 | 59.7 | 125 | (5, 148) | 189 | (1, 129) | 167 | Gp37 protein | Gp37 protein | | uniclust | UniRef100\_A0A1A8TCK2 | 96.7 | 0.00017 | 3.4e-10 | 58.4 | 125 | (12, 151) | 189 | (5, 134) | 176 | Uncharacterized protein | Uncharacterized protein | | uniclust | UniRef100\_A0A0X6ZPF0 | 96.7 | 0.00021 | 4.5e-10 | 61.0 | 159 | (4, 176) | 189 | (9, 181) | 208 | Uncharacterized protein | Uncharacterized protein | | uniclust | UniRef100\_A0A8J3HUL9 | 96.7 | 0.00025 | 4.7e-10 | 43.1 | 40 | (25, 69) | 189 | (2, 41) | 42 | Uncharacterized protein | Uncharacterized protein | | uniclust | UniRef100\_A0A5P9CRF5 | 96.6 | 0.00029 | 5.5e-10 | 56.1 | 106 | (41, 157) | 189 | (43, 148) | 180 | Uncharacterized protein | Uncharacterized protein | | uniclust | UniRef100\_A0A0T7E1W9 | 96.5 | 0.00037 | 7.1e-10 | 55.9 | 128 | (15, 156) | 189 | (16, 144) | 173 | Uncharacterized protein | Uncharacterized protein | | uniclust | UniRef100\_A0A8T7D0H9 | 96.5 | 0.00041 | 7.5e-10 | 63.6 | 101 | (7, 109) | 189 | (8, 113) | 624 | Uncharacterized protein | Uncharacterized protein | | uniclust | UniRef100\_A0A522X2S5 | 96.5 | 0.00041 | 7.8e-10 | 57.5 | 135 | (7, 153) | 189 | (1, 159) | 210 | DUF1834 family protein | DUF1834 family protein | | uniclust | UniRef100\_A0A833L7J3 | 96.5 | 0.00044 | 8e-10 | 55.3 | 135 | (6, 151) | 189 | (1, 145) | 195 | DUF1834 family protein | DUF1834 family protein | | uniclust | UniRef100\_A0A3A5AI19 | 96.5 | 0.00041 | 8.4e-10 | 56.1 | 109 | (5, 124) | 189 | (1, 120) | 155 | DUF1834 family protein | DUF1834 family protein | | uniclust | UniRef100\_A0A074LMC7 | 96.5 | 0.0004 | 8.7e-10 | 57.7 | 98 | (6, 108) | 189 | (1, 98) | 172 | Uncharacterized protein | Uncharacterized protein | | uniclust | UniRef100\_A0A095ZCL4 | 96.5 | 0.00042 | 9.2e-10 | 57.7 | 135 | (5, 152) | 189 | (2, 149) | 172 | Uncharacterized protein | Uncharacterized protein | | uniclust | UniRef100\_A0A134CJH4 | 96.4 | 0.0007 | 1.4e-09 | 54.4 | 100 | (2, 108) | 189 | (4, 103) | 143 | Uncharacterized protein | Uncharacterized protein | | uniclust | UniRef100\_A0A0C5E1K8 | 96.3 | 0.00078 | 1.5e-09 | 55.8 | 111 | (8, 128) | 189 | (1, 128) | 189 | DUF1834 family protein | DUF1834 family protein | | uniclust | UniRef100\_A0A1V5IG09 | 96.3 | 0.00088 | 1.7e-09 | 53.2 | 136 | (4, 154) | 189 | (1, 141) | 146 | Uncharacterized protein | Uncharacterized protein | | uniclust | UniRef100\_A0A1B8Q520 | 96.3 | 0.00084 | 1.7e-09 | 55.1 | 141 | (1, 157) | 189 | (4, 146) | 165 | Gp37 protein | Gp37 protein | | uniclust | UniRef100\_A0A5M8P6C2 | 96.3 | 0.00092 | 1.8e-09 | 55.4 | 136 | (5, 152) | 189 | (20, 161) | 187 | DUF3168 domain-containing protein | DUF3168 domain-containing protein | |
| Top keywords  (threshold 1.00e-03 (evalue)) | **hypothetical, Phage, DUF1834, Gp37, domain\_containing, Fragment, tail, Mu\_like, prophage, DUF3168** |
| Output files | ../../similar\_sequences/10\_FANPEZAQ\_CDS\_0010\_merged.svg ../../similar\_sequences/10\_FANPEZAQ\_CDS\_0010\_pdb70.a3m ../../similar\_sequences/10\_FANPEZAQ\_CDS\_0010\_pdb70.hhr ../../similar\_sequences/10\_FANPEZAQ\_CDS\_0010\_uniclust.a3m ../../similar\_sequences/10\_FANPEZAQ\_CDS\_0010\_uniclust.hhr |

#### Structure prediction (AlphaFold)2

|  |  |
| --- | --- |
| Stats | xml version="1.0" encoding="utf-8" standalone="no"?       2024-09-02T21:09:09.106565 image/svg+xml   Matplotlib v3.7.2, https://matplotlib.org/ |
| Predicted structure | **NGL Viewer Controls:**  - Center: *Left-Click* - Rotate: *Left-Click + Drag* - Translate: *Right-Click + Drag* - Zoom: *Shift + Left-Click + Drag* |
| Output files | ../../predicted\_structures/10\_FANPEZAQ\_CDS\_0010/features.pkl ../../predicted\_structures/10\_FANPEZAQ\_CDS\_0010/ranked\_0.pdb ../../predicted\_structures/10\_FANPEZAQ\_CDS\_0010/ranked\_0\_plots.svg ../../predicted\_structures/10\_FANPEZAQ\_CDS\_0010/result\_model\_1\_ptm\_pred\_0.pkl |

#### Structure similarity search results (Foldseek)3

|  |  |
| --- | --- |
| Structure databases searched | Pdb, Afdb-proteome, Afdb-uniprot50 |
| Results, scheme(s)  (Top layers only, threshold 1.00e-02 (evalue)) | xml version="1.0" encoding="utf-8" standalone="no"?       2024-09-02T21:10:18.111435 image/svg+xml   Matplotlib v3.7.2, https://matplotlib.org/ |
| Results, table  (threshold 1.00e-02 (evalue)) | | db | id | prob | evalue | bits | fident | alnlen | mismatch | gapopen | qstart | qend | tstart | tend | name | description | | --- | --- | --- | --- | --- | --- | --- | --- | --- | --- | --- | --- | --- | --- | --- | | pdb | 6U5F\_A | 1.0 | 4.707e-09 | 294 | 0.279 | 179 | 110 | 4 | 6 | 184 | 2 | 161 | Tube PA0623 | Tube PA0623 | | pdb | 2GJV\_E | 1.0 | 0.001048 | 134 | 0.094 | 158 | 111 | 10 | 7 | 156 | 3 | 136 | putative cytoplasmic protein | putative cytoplasmic protein | | pdb | 3FZB\_A | 1.0 | 0.001768 | 112 | 0.102 | 156 | 106 | 9 | 6 | 153 | 1 | 130 | Minor tail protein U | Minor tail protein U | | pdb | 4ACV\_B | 1.0 | 0.00223 | 103 | 0.077 | 154 | 108 | 10 | 1 | 152 | 1 | 122 | PROPHAGE LAMBDALM01, ANTIGEN B | PROPHAGE LAMBDALM01, ANTIGEN B | | pdb | 3FZ2\_B | 1.0 | 0.004224 | 103 | 0.109 | 155 | 105 | 9 | 6 | 153 | 1 | 129 | Minor tail protein U | Minor tail protein U | | pdb | 3FZB\_F | 1.0 | 0.005028 | 103 | 0.111 | 153 | 108 | 8 | 6 | 153 | 1 | 130 | Minor tail protein U | Minor tail protein U | | pdb | 3FZB\_G | 1.0 | 0.008002 | 101 | 0.091 | 153 | 112 | 8 | 6 | 153 | 2 | 132 | Minor tail protein U | Minor tail protein U | | pdb | 3FZB\_B | 0.999 | 0.003348 | 99 | 0.108 | 147 | 105 | 7 | 13 | 153 | 4 | 130 | Minor tail protein U | Minor tail protein U | | pdb | 2L25\_A | 0.999 | 0.001247 | 99 | 0.125 | 168 | 101 | 13 | 7 | 156 | 2 | 141 | Uncharacterized protein | Uncharacterized protein | | pdb | 3FZB\_H | 0.999 | 0.004477 | 98 | 0.095 | 147 | 107 | 7 | 13 | 153 | 3 | 129 | Minor tail protein U | Minor tail protein U | | pdb | 3FZ2\_F | 0.999 | 0.004477 | 98 | 0.115 | 156 | 106 | 9 | 6 | 153 | 2 | 133 | Minor tail protein U | Minor tail protein U | | pdb | 3FZB\_I | 0.996 | 0.006722 | 90 | 0.108 | 148 | 102 | 9 | 13 | 153 | 3 | 127 | Minor tail protein U | Minor tail protein U | | pdb | 8GTF\_K | 0.978 | 0.006343 | 78 | 0.168 | 154 | 90 | 12 | 18 | 153 | 4 | 137 | Terminator protein | Terminator protein | | afdb-proteome | AF-G3XD82-F1-MODEL\_V4 | 1.0 | 8.093e-14 | 465 | 0.277 | 180 | 111 | 4 | 6 | 185 | 5 | 165 | Uncharacterized protein | Uncharacterized protein | | afdb-proteome | AF-P44231-F1-MODEL\_V4 | 1.0 | 1.18e-06 | 195 | 0.144 | 201 | 142 | 9 | 1 | 187 | 1 | 185 | Mu-like prophage FluMu protein gp37 | Mu-like prophage FluMu protein gp37 | | afdb-proteome | AF-Q8ZN03-F1-MODEL\_V4 | 1.0 | 0.0007022 | 140 | 0.096 | 145 | 112 | 7 | 10 | 152 | 5 | 132 | Gifsy-1 prophage protein | Gifsy-1 prophage protein | | afdb-proteome | AF-Q8ZKJ0-F1-MODEL\_V4 | 1.0 | 0.0005899 | 133 | 0.102 | 166 | 119 | 9 | 7 | 165 | 2 | 144 | Putative cytoplasmic protein | Putative cytoplasmic protein | | afdb-proteome | AF-Q8ZQG6-F1-MODEL\_V4 | 1.0 | 0.00133 | 129 | 0.104 | 144 | 108 | 8 | 12 | 152 | 7 | 132 | Fels-1 prophage protein | Fels-1 prophage protein | | afdb-proteome | AF-Q5F997-F1-MODEL\_V4 | 1.0 | 0.0005252 | 119 | 0.123 | 154 | 111 | 11 | 7 | 150 | 1 | 140 | Phage associated protein | Phage associated protein | | afdb-proteome | AF-Q8ZQ90-F1-MODEL\_V4 | 1.0 | 0.006022 | 112 | 0.124 | 145 | 106 | 8 | 12 | 153 | 7 | 133 | Gifsy-2 prophage probable minor tail protein | Gifsy-2 prophage probable minor tail protein | | afdb-proteome | AF-Q9I547-F1-MODEL\_V4 | 0.442 | 0.007597 | 49 | 0.152 | 190 | 93 | 13 | 13 | 166 | 10 | 167 | Uncharacterized protein | Uncharacterized protein | | afdb-uniprot50 | AF-A0A6M4YGW3-F1-MODEL\_V4 | 1.0 | 2.154e-23 | 889 | 0.56 | 189 | 69 | 3 | 1 | 178 | 1 | 186 | Uncharacterized protein | Uncharacterized protein | | afdb-uniprot50 | AF-A0A124PC40-F1-MODEL\_V4 | 1.0 | 3.371e-21 | 784 | 0.541 | 181 | 71 | 5 | 12 | 189 | 2 | 173 | Uncharacterized protein | Uncharacterized protein | | afdb-uniprot50 | AF-A0A430HF56-F1-MODEL\_V4 | 1.0 | 1.141e-20 | 780 | 0.492 | 189 | 85 | 4 | 1 | 189 | 1 | 178 | Uncharacterized protein | Uncharacterized protein | | afdb-uniprot50 | AF-A0A410UF72-F1-MODEL\_V4 | 1.0 | 1.1e-19 | 762 | 0.377 | 188 | 107 | 3 | 2 | 189 | 3 | 180 | Uncharacterized protein | Uncharacterized protein | | afdb-uniprot50 | AF-A0A291LZL3-F1-MODEL\_V4 | 1.0 | 6.519e-20 | 743 | 0.478 | 190 | 86 | 4 | 1 | 189 | 1 | 178 | Uncharacterized protein | Uncharacterized protein | | afdb-uniprot50 | AF-A0A315BPV6-F1-MODEL\_V4 | 1.0 | 6.655e-19 | 702 | 0.508 | 187 | 79 | 4 | 2 | 187 | 3 | 177 | Uncharacterized protein | Uncharacterized protein | | afdb-uniprot50 | AF-A0A0B4BHC6-F1-MODEL\_V4 | 1.0 | 2.683e-18 | 697 | 0.394 | 185 | 100 | 3 | 5 | 189 | 3 | 175 | Uncharacterized protein | Uncharacterized protein | | afdb-uniprot50 | AF-A0A143DEE7-F1-MODEL\_V4 | 1.0 | 1.59e-18 | 688 | 0.468 | 188 | 88 | 4 | 1 | 188 | 1 | 176 | Uncharacterized protein | Uncharacterized protein | | afdb-uniprot50 | AF-A0A844HSY5-F1-MODEL\_V4 | 1.0 | 3.076e-17 | 662 | 0.404 | 188 | 98 | 4 | 2 | 189 | 3 | 176 | Uncharacterized protein | Uncharacterized protein | | afdb-uniprot50 | AF-A0A2N1AP90-F1-MODEL\_V4 | 1.0 | 7.631e-18 | 661 | 0.423 | 189 | 97 | 3 | 1 | 189 | 1 | 177 | Uncharacterized protein | Uncharacterized protein | | afdb-uniprot50 | AF-A0A7V8FKG6-F1-MODEL\_V4 | 1.0 | 2.902e-17 | 660 | 0.38 | 189 | 105 | 4 | 1 | 188 | 1 | 178 | Uncharacterized protein | Uncharacterized protein | | afdb-uniprot50 | AF-A0A7J0BX95-F1-MODEL\_V4 | 1.0 | 2.301e-17 | 653 | 0.349 | 189 | 108 | 6 | 1 | 189 | 1 | 174 | Uncharacterized protein | Uncharacterized protein | | afdb-uniprot50 | AF-A0A1H9YBP8-F1-MODEL\_V4 | 1.0 | 4.895e-17 | 605 | 0.356 | 188 | 108 | 5 | 1 | 187 | 1 | 176 | Uncharacterized protein | Uncharacterized protein | | afdb-uniprot50 | AF-A0A238J9A3-F1-MODEL\_V4 | 1.0 | 8.428e-16 | 600 | 0.358 | 184 | 106 | 3 | 6 | 189 | 4 | 175 | Uncharacterized protein | Uncharacterized protein | | afdb-uniprot50 | AF-A0A846VUR4-F1-MODEL\_V4 | 1.0 | 2.963e-16 | 599 | 0.354 | 186 | 106 | 5 | 4 | 188 | 2 | 174 | Uncharacterized protein | Uncharacterized protein | | afdb-uniprot50 | AF-A0A1X7L1R0-F1-MODEL\_V4 | 1.0 | 4.449e-16 | 597 | 0.302 | 182 | 113 | 4 | 6 | 187 | 3 | 170 | Uncharacterized protein | Uncharacterized protein | | afdb-uniprot50 | AF-A0A158E8C2-F1-MODEL\_V4 | 1.0 | 2.091e-16 | 594 | 0.357 | 190 | 110 | 7 | 2 | 187 | 1 | 182 | Uncharacterized protein | Uncharacterized protein | | afdb-uniprot50 | AF-K5Z1V0-F1-MODEL\_V4 | 1.0 | 6.545e-17 | 585 | 0.321 | 190 | 113 | 4 | 2 | 189 | 3 | 178 | Uncharacterized protein | Uncharacterized protein | | afdb-uniprot50 | AF-A0A7W4WZS5-F1-MODEL\_V4 | 1.0 | 1.314e-16 | 583 | 0.338 | 201 | 110 | 8 | 2 | 189 | 1 | 191 | Uncharacterized protein | Uncharacterized protein | | afdb-uniprot50 | AF-A0A149SVH3-F1-MODEL\_V4 | 1.0 | 1.597e-15 | 577 | 0.366 | 191 | 104 | 6 | 2 | 188 | 4 | 181 | Uncharacterized protein | Uncharacterized protein | | afdb-uniprot50 | AF-A0A1X7CH01-F1-MODEL\_V4 | 1.0 | 1.597e-15 | 576 | 0.331 | 187 | 110 | 6 | 1 | 187 | 1 | 172 | Uncharacterized protein | Uncharacterized protein | | afdb-uniprot50 | AF-A0A3A8EIU2-F1-MODEL\_V4 | 1.0 | 4.449e-16 | 569 | 0.404 | 193 | 102 | 8 | 2 | 188 | 1 | 186 | Uncharacterized protein | Uncharacterized protein | | afdb-uniprot50 | AF-Q9PFF2-F1-MODEL\_V4 | 1.0 | 1.292e-14 | 546 | 0.4 | 145 | 79 | 2 | 45 | 189 | 1 | 137 | Uncharacterized protein | Uncharacterized protein | | afdb-uniprot50 | AF-A0A7H8NU11-F1-MODEL\_V4 | 1.0 | 7.504e-16 | 541 | 0.357 | 196 | 107 | 9 | 1 | 189 | 1 | 184 | Uncharacterized protein | Uncharacterized protein | | afdb-uniprot50 | AF-A0A2E3M0I4-F1-MODEL\_V4 | 1.0 | 2.693e-15 | 532 | 0.342 | 181 | 105 | 4 | 9 | 189 | 4 | 170 | Uncharacterized protein | Uncharacterized protein | | afdb-uniprot50 | AF-A0A7W2B841-F1-MODEL\_V4 | 1.0 | 3.206e-15 | 528 | 0.372 | 188 | 99 | 5 | 1 | 188 | 1 | 169 | Uncharacterized protein | Uncharacterized protein | | afdb-uniprot50 | AF-A0A4T2ACD2-F1-MODEL\_V4 | 1.0 | 5.73e-15 | 524 | 0.317 | 195 | 114 | 8 | 2 | 189 | 1 | 183 | Uncharacterized protein | Uncharacterized protein | | afdb-uniprot50 | AF-A0A2Z6AZ36-F1-MODEL\_V4 | 1.0 | 1.369e-14 | 518 | 0.281 | 181 | 116 | 3 | 8 | 188 | 1 | 167 | Uncharacterized protein | Uncharacterized protein | | afdb-uniprot50 | AF-A0A4U1KS61-F1-MODEL\_V4 | 1.0 | 3.468e-14 | 505 | 0.338 | 189 | 111 | 4 | 1 | 189 | 1 | 175 | Uncharacterized protein | Uncharacterized protein | | afdb-uniprot50 | AF-A0A081MYL3-F1-MODEL\_V4 | 1.0 | 7.821e-14 | 495 | 0.278 | 190 | 124 | 6 | 1 | 189 | 1 | 178 | Uncharacterized protein | Uncharacterized protein | | afdb-uniprot50 | AF-A0A446AZW9-F1-MODEL\_V4 | 1.0 | 2.807e-13 | 492 | 0.35 | 180 | 103 | 5 | 8 | 187 | 3 | 168 | Phage protein | Phage protein | | afdb-uniprot50 | AF-A0A1H6X2H3-F1-MODEL\_V4 | 1.0 | 4.734e-13 | 475 | 0.304 | 184 | 112 | 5 | 5 | 187 | 2 | 170 | Uncharacterized protein | Uncharacterized protein | | afdb-uniprot50 | AF-A0A3M3CQE2-F1-MODEL\_V4 | 1.0 | 5.317e-13 | 473 | 0.288 | 184 | 117 | 4 | 2 | 185 | 1 | 170 | Uncharacterized protein | Uncharacterized protein | | afdb-uniprot50 | AF-A0A450ZAR3-F1-MODEL\_V4 | 1.0 | 4.215e-13 | 473 | 0.245 | 187 | 125 | 7 | 7 | 189 | 2 | 176 | Uncharacterized protein | Uncharacterized protein | | afdb-uniprot50 | AF-A0A2S6H5L1-F1-MODEL\_V4 | 1.0 | 2.099e-13 | 472 | 0.312 | 189 | 117 | 5 | 5 | 189 | 2 | 181 | Uncharacterized protein | Uncharacterized protein | | afdb-uniprot50 | AF-S2EUJ0-F1-MODEL\_V4 | 1.0 | 8.461e-13 | 470 | 0.312 | 176 | 108 | 5 | 12 | 187 | 2 | 164 | Uncharacterized protein | Uncharacterized protein | | afdb-uniprot50 | AF-A6UZB9-F1-MODEL\_V4 | 1.0 | 2.099e-13 | 468 | 0.263 | 186 | 118 | 4 | 2 | 187 | 1 | 167 | Uncharacterized protein | Uncharacterized protein | | afdb-uniprot50 | AF-A0A061JGS2-F1-MODEL\_V4 | 1.0 | 1.57e-13 | 464 | 0.378 | 161 | 86 | 4 | 27 | 187 | 3 | 149 | Uncharacterized protein | Uncharacterized protein | | afdb-uniprot50 | AF-A0A0Q8D856-F1-MODEL\_V4 | 1.0 | 9.504e-13 | 464 | 0.301 | 186 | 116 | 4 | 2 | 187 | 1 | 172 | Uncharacterized protein | Uncharacterized protein | | afdb-uniprot50 | AF-A0A7S6S605-F1-MODEL\_V4 | 1.0 | 1.869e-13 | 463 | 0.296 | 182 | 114 | 4 | 8 | 189 | 4 | 171 | Uncharacterized protein | Uncharacterized protein | | afdb-uniprot50 | AF-A0A7V7NR68-F1-MODEL\_V4 | 1.0 | 7.533e-13 | 463 | 0.273 | 183 | 120 | 4 | 7 | 189 | 5 | 174 | Uncharacterized protein | Uncharacterized protein | | afdb-uniprot50 | AF-S2KFP5-F1-MODEL\_V4 | 1.0 | 6.707e-13 | 463 | 0.327 | 186 | 112 | 4 | 2 | 187 | 1 | 173 | Uncharacterized protein | Uncharacterized protein | | afdb-uniprot50 | AF-A0A7W5B8Q7-F1-MODEL\_V4 | 1.0 | 5.635e-13 | 461 | 0.263 | 186 | 123 | 4 | 4 | 189 | 2 | 173 | Uncharacterized protein | Uncharacterized protein | | afdb-uniprot50 | AF-Q73HC3-F1-MODEL\_V4 | 1.0 | 7.108e-13 | 460 | 0.325 | 172 | 102 | 5 | 7 | 178 | 1 | 158 | Uncharacterized protein | Uncharacterized protein | | afdb-uniprot50 | AF-A0A7C8M1D4-F1-MODEL\_V4 | 1.0 | 2.974e-13 | 456 | 0.319 | 191 | 114 | 5 | 2 | 188 | 5 | 183 | Uncharacterized protein | Uncharacterized protein | | afdb-uniprot50 | AF-A0A1Y3KWD3-F1-MODEL\_V4 | 1.0 | 1.271e-12 | 449 | 0.289 | 183 | 109 | 5 | 9 | 187 | 4 | 169 | Uncharacterized protein | Uncharacterized protein | | afdb-uniprot50 | AF-A0A142BA55-F1-MODEL\_V4 | 1.0 | 1.271e-12 | 445 | 0.268 | 186 | 119 | 9 | 7 | 188 | 3 | 175 | Uncharacterized protein | Uncharacterized protein | | afdb-uniprot50 | AF-H8L2J7-F1-MODEL\_V4 | 1.0 | 2.807e-13 | 441 | 0.297 | 195 | 115 | 6 | 1 | 187 | 1 | 181 | Uncharacterized protein | Uncharacterized protein | | afdb-uniprot50 | AF-A0A5E4NQZ0-F1-MODEL\_V4 | 1.0 | 1.427e-12 | 438 | 0.337 | 166 | 96 | 4 | 9 | 174 | 281 | 432 | Bacteriophage lambda, GpZ, minor tail,Ankyrin repeat-containing domain,Ankyrin repeat | Bacteriophage lambda, GpZ, minor tail,Ankyrin repeat-containing domain,Ankyrin repeat | | afdb-uniprot50 | AF-A0A7U3R1S8-F1-MODEL\_V4 | 1.0 | 1.603e-12 | 436 | 0.313 | 182 | 109 | 5 | 8 | 189 | 4 | 169 | Uncharacterized protein | Uncharacterized protein | | afdb-uniprot50 | AF-A0A258LKP1-F1-MODEL\_V4 | 1.0 | 6.847e-12 | 435 | 0.274 | 182 | 117 | 7 | 7 | 188 | 2 | 168 | Uncharacterized protein | Uncharacterized protein | | afdb-uniprot50 | AF-A0A240TSR2-F1-MODEL\_V4 | 1.0 | 1.427e-12 | 431 | 0.318 | 182 | 108 | 5 | 8 | 189 | 4 | 169 | Uncharacterized protein | Uncharacterized protein | | afdb-uniprot50 | AF-A0A1C3HH22-F1-MODEL\_V4 | 1.0 | 3.037e-12 | 430 | 0.234 | 188 | 122 | 6 | 6 | 188 | 4 | 174 | Uncharacterized protein | Uncharacterized protein | | afdb-uniprot50 | AF-A0A379YFI5-F1-MODEL\_V4 | 1.0 | 9.155e-12 | 423 | 0.229 | 192 | 127 | 11 | 1 | 188 | 1 | 175 | Uncharacterized protein | Uncharacterized protein | | afdb-uniprot50 | AF-A0A0D5Y351-F1-MODEL\_V4 | 1.0 | 1.375e-11 | 417 | 0.28 | 171 | 110 | 5 | 17 | 187 | 2 | 159 | Uncharacterized protein | Uncharacterized protein | | afdb-uniprot50 | AF-A0A059PCW0-F1-MODEL\_V4 | 1.0 | 1.457e-11 | 416 | 0.333 | 159 | 93 | 4 | 7 | 165 | 1 | 146 | Uncharacterized protein | Uncharacterized protein | | afdb-uniprot50 | AF-A0A1X7MHZ6-F1-MODEL\_V4 | 1.0 | 3.411e-12 | 415 | 0.274 | 182 | 110 | 6 | 8 | 189 | 3 | 162 | Uncharacterized protein | Uncharacterized protein | | afdb-uniprot50 | AF-A0A3N2E1B1-F1-MODEL\_V4 | 1.0 | 3.831e-12 | 414 | 0.209 | 186 | 132 | 7 | 4 | 187 | 2 | 174 | Uncharacterized protein | Uncharacterized protein | | afdb-uniprot50 | AF-A0A7Y3Z3N8-F1-MODEL\_V4 | 1.0 | 3.615e-12 | 414 | 0.216 | 189 | 130 | 6 | 6 | 189 | 4 | 179 | Uncharacterized protein | Uncharacterized protein | | afdb-uniprot50 | AF-A0A089PYZ4-F1-MODEL\_V4 | 1.0 | 6.096e-12 | 414 | 0.209 | 186 | 131 | 5 | 6 | 188 | 4 | 176 | Uncharacterized protein | Uncharacterized protein | | afdb-uniprot50 | AF-A0A3G6ZVQ7-F1-MODEL\_V4 | 1.0 | 6.461e-12 | 414 | 0.304 | 187 | 117 | 4 | 1 | 187 | 42 | 215 | Uncharacterized protein | Uncharacterized protein | | afdb-uniprot50 | AF-A0A7Z0MZG6-F1-MODEL\_V4 | 1.0 | 1.603e-12 | 413 | 0.287 | 188 | 109 | 7 | 7 | 188 | 2 | 170 | Uncharacterized protein | Uncharacterized protein | | afdb-uniprot50 | AF-A0A6L9FI07-F1-MODEL\_V4 | 1.0 | 1.09e-11 | 411 | 0.229 | 183 | 128 | 4 | 6 | 188 | 3 | 172 | Uncharacterized protein | Uncharacterized protein | | afdb-uniprot50 | AF-A0A509JCF5-F1-MODEL\_V4 | 1.0 | 9.702e-12 | 410 | 0.254 | 185 | 124 | 4 | 1 | 185 | 1 | 171 | Uncharacterized protein | Uncharacterized protein | | afdb-uniprot50 | AF-A0A1A9VKF9-F1-MODEL\_V4 | 1.0 | 3.285e-11 | 410 | 0.3 | 160 | 99 | 4 | 1 | 160 | 44 | 190 | Phage\_base\_V domain-containing protein | Phage\_base\_V domain-containing protein | | afdb-uniprot50 | AF-A0A1H1EGK5-F1-MODEL\_V4 | 1.0 | 5.228e-11 | 404 | 0.236 | 186 | 124 | 5 | 2 | 187 | 1 | 168 | Uncharacterized protein | Uncharacterized protein | | afdb-uniprot50 | AF-A0A450W6V5-F1-MODEL\_V4 | 1.0 | 1.838e-11 | 403 | 0.245 | 187 | 126 | 7 | 6 | 189 | 2 | 176 | Uncharacterized protein | Uncharacterized protein | | afdb-uniprot50 | AF-A0A7K0GNI0-F1-MODEL\_V4 | 1.0 | 5.121e-12 | 401 | 0.252 | 182 | 124 | 6 | 7 | 187 | 4 | 174 | Uncharacterized protein | Uncharacterized protein | | afdb-uniprot50 | AF-A0A4V2PRI1-F1-MODEL\_V4 | 1.0 | 1.224e-11 | 395 | 0.209 | 191 | 129 | 8 | 1 | 189 | 4 | 174 | Uncharacterized protein | Uncharacterized protein | | afdb-uniprot50 | AF-A0A2S9B8M0-F1-MODEL\_V4 | 1.0 | 2.064e-11 | 394 | 0.281 | 185 | 108 | 6 | 2 | 177 | 1 | 169 | Uncharacterized protein | Uncharacterized protein | | afdb-uniprot50 | AF-B8GS13-F1-MODEL\_V4 | 1.0 | 3.911e-11 | 393 | 0.267 | 191 | 120 | 9 | 7 | 189 | 1 | 179 | Uncharacterized protein | Uncharacterized protein | | afdb-uniprot50 | AF-A0A3M5CXK4-F1-MODEL\_V4 | 1.0 | 3.482e-11 | 390 | 0.322 | 152 | 92 | 3 | 36 | 187 | 1 | 141 | Uncharacterized protein | Uncharacterized protein | | afdb-uniprot50 | AF-A0A3M6E3B6-F1-MODEL\_V4 | 1.0 | 2.509e-10 | 390 | 0.313 | 166 | 98 | 5 | 14 | 178 | 2 | 152 | Uncharacterized protein | Uncharacterized protein | | afdb-uniprot50 | AF-A0A1I1UB35-F1-MODEL\_V4 | 1.0 | 1.324e-10 | 388 | 0.293 | 177 | 110 | 5 | 7 | 183 | 2 | 163 | Uncharacterized protein | Uncharacterized protein | | afdb-uniprot50 | AF-A0A826WKL5-F1-MODEL\_V4 | 1.0 | 2.925e-11 | 388 | 0.236 | 186 | 123 | 7 | 8 | 188 | 1 | 172 | Uncharacterized protein | Uncharacterized protein | | afdb-uniprot50 | AF-A0A2N0CX69-F1-MODEL\_V4 | 1.0 | 5.872e-11 | 387 | 0.234 | 188 | 125 | 7 | 6 | 189 | 3 | 175 | Uncharacterized protein | Uncharacterized protein | | afdb-uniprot50 | AF-A0A0H3ZM50-F1-MODEL\_V4 | 1.0 | 2.188e-11 | 384 | 0.286 | 157 | 102 | 3 | 33 | 189 | 1 | 147 | Uncharacterized protein | Uncharacterized protein | | afdb-uniprot50 | AF-A0A6S5X6Y7-F1-MODEL\_V4 | 1.0 | 7.408e-11 | 384 | 0.279 | 172 | 112 | 5 | 1 | 172 | 1 | 160 | Uncharacterized protein | Uncharacterized protein | | afdb-uniprot50 | AF-A0A6H2NT10-F1-MODEL\_V4 | 1.0 | 1.324e-10 | 382 | 0.368 | 144 | 78 | 4 | 30 | 173 | 2 | 132 | Uncharacterized protein | Uncharacterized protein | | afdb-uniprot50 | AF-A0A212KN54-F1-MODEL\_V4 | 1.0 | 6.461e-12 | 381 | 0.221 | 194 | 123 | 8 | 7 | 187 | 2 | 180 | Uncharacterized protein | Uncharacterized protein | | afdb-uniprot50 | AF-A0A7J6YLT3-F1-MODEL\_V4 | 1.0 | 1.576e-10 | 375 | 0.34 | 147 | 84 | 4 | 20 | 166 | 2 | 135 | Uncharacterized protein | Uncharacterized protein | | afdb-uniprot50 | AF-A0A3G2IKE0-F1-MODEL\_V4 | 1.0 | 2.064e-11 | 365 | 0.189 | 201 | 133 | 11 | 1 | 188 | 1 | 184 | Uncharacterized protein | Uncharacterized protein | | afdb-uniprot50 | AF-A0A059KU41-F1-MODEL\_V4 | 1.0 | 5.657e-10 | 364 | 0.226 | 181 | 122 | 8 | 8 | 187 | 3 | 166 | Uncharacterized protein | Uncharacterized protein | | afdb-uniprot50 | AF-A0A1V2DSI8-F1-MODEL\_V4 | 1.0 | 5.541e-11 | 363 | 0.305 | 170 | 102 | 7 | 9 | 178 | 4 | 157 | Uncharacterized protein | Uncharacterized protein | | afdb-uniprot50 | AF-A0A1E3G659-F1-MODEL\_V4 | 1.0 | 1.179e-10 | 361 | 0.187 | 181 | 127 | 7 | 8 | 186 | 1 | 163 | Uncharacterized protein | Uncharacterized protein | | afdb-uniprot50 | AF-A0A5E7KJV8-F1-MODEL\_V4 | 1.0 | 1.989e-10 | 359 | 0.267 | 198 | 117 | 6 | 1 | 189 | 4 | 182 | Uncharacterized protein | Uncharacterized protein | | afdb-uniprot50 | AF-Q31HU1-F1-MODEL\_V4 | 1.0 | 2.818e-10 | 357 | 0.232 | 172 | 117 | 6 | 8 | 178 | 3 | 160 | Uncharacterized protein | Uncharacterized protein | | afdb-uniprot50 | AF-A0A1E3LHB7-F1-MODEL\_V4 | 1.0 | 3.767e-10 | 352 | 0.319 | 166 | 95 | 5 | 8 | 173 | 4 | 151 | Uncharacterized protein | Uncharacterized protein | | afdb-uniprot50 | AF-A0A7X3ZHV1-F1-MODEL\_V4 | 1.0 | 2.76e-11 | 350 | 0.23 | 226 | 123 | 9 | 1 | 188 | 5 | 217 | Uncharacterized protein | Uncharacterized protein | | afdb-uniprot50 | AF-A0A4P9VGK5-F1-MODEL\_V4 | 1.0 | 3.767e-10 | 345 | 0.291 | 175 | 107 | 7 | 2 | 170 | 1 | 164 | Uncharacterized protein | Uncharacterized protein | | afdb-uniprot50 | AF-A0A266LMM3-F1-MODEL\_V4 | 1.0 | 2.561e-09 | 342 | 0.277 | 166 | 107 | 5 | 12 | 177 | 2 | 154 | Uncharacterized protein | Uncharacterized protein | | afdb-uniprot50 | AF-A0A6I2UNP3-F1-MODEL\_V4 | 1.0 | 5.995e-10 | 340 | 0.197 | 197 | 138 | 11 | 2 | 189 | 1 | 186 | Uncharacterized protein | Uncharacterized protein | | afdb-uniprot50 | AF-A0A1S8YKZ2-F1-MODEL\_V4 | 1.0 | 1.249e-10 | 339 | 0.207 | 183 | 129 | 6 | 9 | 188 | 7 | 176 | Uncharacterized protein | Uncharacterized protein | | afdb-uniprot50 | AF-A0A1W0CDL2-F1-MODEL\_V4 | 1.0 | 4.484e-10 | 337 | 0.279 | 172 | 103 | 8 | 8 | 179 | 1 | 151 | Uncharacterized protein | Uncharacterized protein | | afdb-uniprot50 | AF-A0A1T2CHW9-F1-MODEL\_V4 | 1.0 | 2.509e-10 | 337 | 0.227 | 185 | 118 | 9 | 2 | 181 | 1 | 165 | Uncharacterized protein | Uncharacterized protein | | afdb-uniprot50 | AF-A0A377QAJ9-F1-MODEL\_V4 | 1.0 | 5.657e-10 | 336 | 0.258 | 178 | 103 | 7 | 7 | 171 | 1 | 162 | Uncharacterized protein | Uncharacterized protein | | afdb-uniprot50 | AF-A0A1T4W4R6-F1-MODEL\_V4 | 1.0 | 6.354e-10 | 335 | 0.218 | 197 | 131 | 11 | 1 | 189 | 1 | 182 | Uncharacterized protein | Uncharacterized protein | | afdb-uniprot50 | AF-A0A654AL97-F1-MODEL\_V4 | 1.0 | 5.036e-10 | 334 | 0.248 | 177 | 113 | 6 | 1 | 168 | 1 | 166 | Uncharacterized protein | Uncharacterized protein | | afdb-uniprot50 | AF-F3LHZ3-F1-MODEL\_V4 | 1.0 | 3.554e-10 | 331 | 0.218 | 192 | 123 | 10 | 7 | 188 | 1 | 175 | Uncharacterized protein | Uncharacterized protein | | afdb-uniprot50 | AF-A0A090SU28-F1-MODEL\_V4 | 1.0 | 2.151e-09 | 325 | 0.235 | 191 | 129 | 5 | 7 | 188 | 1 | 183 | Uncharacterized protein | Uncharacterized protein | | afdb-uniprot50 | AF-A0A348HHI8-F1-MODEL\_V4 | 1.0 | 2.03e-09 | 324 | 0.216 | 189 | 134 | 9 | 1 | 188 | 1 | 176 | Aspartatecarbamoyltransferase, catalytic chain | Aspartatecarbamoyltransferase, catalytic chain | | afdb-uniprot50 | AF-A0A837E8U7-F1-MODEL\_V4 | 1.0 | 5.995e-10 | 323 | 0.197 | 187 | 129 | 8 | 9 | 188 | 8 | 180 | Uncharacterized protein | Uncharacterized protein | | afdb-uniprot50 | AF-A0A1G5ACN2-F1-MODEL\_V4 | 1.0 | 2.03e-09 | 319 | 0.235 | 195 | 119 | 9 | 7 | 189 | 1 | 177 | Uncharacterized protein | Uncharacterized protein | | afdb-uniprot50 | AF-A0A0Q2U909-F1-MODEL\_V4 | 1.0 | 2.416e-09 | 316 | 0.209 | 196 | 129 | 12 | 1 | 188 | 1 | 178 | Uncharacterized protein | Uncharacterized protein | | afdb-uniprot50 | AF-A0A853IEN6-F1-MODEL\_V4 | 1.0 | 3.231e-09 | 314 | 0.21 | 190 | 131 | 9 | 7 | 189 | 1 | 178 | Uncharacterized protein | Uncharacterized protein | | afdb-uniprot50 | AF-A0A1M6B7W3-F1-MODEL\_V4 | 1.0 | 4.076e-09 | 314 | 0.194 | 185 | 135 | 6 | 5 | 188 | 2 | 173 | Uncharacterized protein | Uncharacterized protein | | afdb-uniprot50 | AF-A0A145VQF5-F1-MODEL\_V4 | 1.0 | 5.895e-08 | 313 | 0.262 | 118 | 78 | 2 | 47 | 164 | 26 | 134 | Uncharacterized protein | Uncharacterized protein | | afdb-uniprot50 | AF-A0A7Z7VMF7-F1-MODEL\_V4 | 1.0 | 2.876e-09 | 308 | 0.226 | 199 | 121 | 11 | 5 | 178 | 2 | 192 | Uncharacterized protein | Uncharacterized protein | | afdb-uniprot50 | AF-A0A1I5W3T1-F1-MODEL\_V4 | 1.0 | 2.876e-09 | 298 | 0.203 | 187 | 129 | 11 | 9 | 188 | 7 | 180 | Uncharacterized protein | Uncharacterized protein | | afdb-uniprot50 | AF-A0A1S2TBK1-F1-MODEL\_V4 | 1.0 | 2.614e-08 | 297 | 0.248 | 165 | 104 | 5 | 25 | 189 | 2 | 146 | Uncharacterized protein | Uncharacterized protein | | afdb-uniprot50 | AF-A0A839IZ53-F1-MODEL\_V4 | 1.0 | 1.741e-08 | 292 | 0.184 | 201 | 135 | 13 | 1 | 188 | 1 | 185 | Uncharacterized protein | Uncharacterized protein | | afdb-uniprot50 | AF-F4BFQ7-F1-MODEL\_V4 | 1.0 | 2.328e-08 | 285 | 0.191 | 178 | 129 | 7 | 1 | 178 | 1 | 163 | Uncharacterized protein | Uncharacterized protein | | afdb-uniprot50 | AF-A0A1C3EBP2-F1-MODEL\_V4 | 1.0 | 8.672e-09 | 284 | 0.203 | 187 | 131 | 9 | 5 | 179 | 3 | 183 | Uncharacterized protein | Uncharacterized protein | | afdb-uniprot50 | AF-A0A2N0CKM3-F1-MODEL\_V4 | 1.0 | 1.054e-07 | 283 | 0.206 | 179 | 118 | 7 | 6 | 180 | 3 | 161 | Uncharacterized protein | Uncharacterized protein | | afdb-uniprot50 | AF-A0A447INZ1-F1-MODEL\_V4 | 1.0 | 1.094e-08 | 278 | 0.195 | 189 | 128 | 10 | 9 | 188 | 8 | 181 | Uncharacterized protein | Uncharacterized protein | | afdb-uniprot50 | AF-A0A4P9VGP3-F1-MODEL\_V4 | 1.0 | 9.19e-09 | 276 | 0.2 | 190 | 130 | 7 | 1 | 189 | 23 | 191 | Uncharacterized protein | Uncharacterized protein | | afdb-uniprot50 | AF-A0A345DE57-F1-MODEL\_V4 | 1.0 | 1.409e-07 | 271 | 0.243 | 164 | 102 | 8 | 7 | 166 | 1 | 146 | Uncharacterized protein | Uncharacterized protein | | afdb-uniprot50 | AF-A0A839HEF9-F1-MODEL\_V4 | 1.0 | 1.184e-07 | 269 | 0.166 | 168 | 125 | 7 | 1 | 155 | 1 | 166 | Uncharacterized protein | Uncharacterized protein | | afdb-uniprot50 | AF-A0A4U8YKI5-F1-MODEL\_V4 | 1.0 | 4.953e-08 | 265 | 0.215 | 181 | 117 | 8 | 7 | 179 | 2 | 165 | Uncharacterized protein | Uncharacterized protein | | afdb-uniprot50 | AF-A0A6A4R943-F1-MODEL\_V4 | 1.0 | 4.673e-08 | 262 | 0.182 | 197 | 140 | 10 | 1 | 189 | 1 | 184 | Uncharacterized protein | Uncharacterized protein | | afdb-uniprot50 | AF-A0A7D7X3U7-F1-MODEL\_V4 | 1.0 | 1.884e-07 | 260 | 0.151 | 192 | 139 | 11 | 1 | 185 | 1 | 175 | Uncharacterized protein | Uncharacterized protein | | afdb-uniprot50 | AF-A0A853IIQ5-F1-MODEL\_V4 | 1.0 | 7.592e-07 | 259 | 0.295 | 132 | 85 | 3 | 60 | 189 | 2 | 127 | Uncharacterized protein | Uncharacterized protein | | afdb-uniprot50 | AF-A7MWA0-F1-MODEL\_V4 | 1.0 | 1.677e-07 | 256 | 0.157 | 197 | 139 | 11 | 1 | 189 | 1 | 178 | Uncharacterized protein | Uncharacterized protein | | afdb-uniprot50 | AF-A0A379CNZ4-F1-MODEL\_V4 | 1.0 | 5.358e-07 | 255 | 0.165 | 193 | 137 | 11 | 2 | 188 | 1 | 175 | Uncharacterized protein | Uncharacterized protein | | afdb-uniprot50 | AF-A0A432QS63-F1-MODEL\_V4 | 1.0 | 1.184e-07 | 255 | 0.166 | 168 | 120 | 6 | 2 | 161 | 1 | 156 | Uncharacterized protein | Uncharacterized protein | | afdb-uniprot50 | AF-A0A7W6WLT8-F1-MODEL\_V4 | 1.0 | 3.177e-07 | 254 | 0.185 | 194 | 131 | 10 | 1 | 186 | 1 | 175 | Uncharacterized protein | Uncharacterized protein | | afdb-uniprot50 | AF-A0A4P9VH08-F1-MODEL\_V4 | 1.0 | 3.112e-08 | 254 | 0.198 | 186 | 133 | 4 | 2 | 187 | 120 | 289 | Uncharacterized protein | Uncharacterized protein | | afdb-uniprot50 | AF-A0A3S0H8F4-F1-MODEL\_V4 | 1.0 | 2.998e-07 | 253 | 0.164 | 189 | 133 | 10 | 7 | 189 | 2 | 171 | Uncharacterized protein | Uncharacterized protein | | afdb-uniprot50 | AF-A0A7T8NTX8-F1-MODEL\_V4 | 1.0 | 7.437e-08 | 250 | 0.186 | 188 | 132 | 10 | 8 | 185 | 3 | 179 | Uncharacterized protein | Uncharacterized protein | | afdb-uniprot50 | AF-A0A6I2KE43-F1-MODEL\_V4 | 1.0 | 2.669e-07 | 248 | 0.405 | 106 | 56 | 1 | 84 | 189 | 3 | 101 | Uncharacterized protein | Uncharacterized protein | | afdb-uniprot50 | AF-A0A292QWT2-F1-MODEL\_V4 | 1.0 | 2.426e-06 | 248 | 0.177 | 152 | 103 | 11 | 9 | 156 | 7 | 140 | Uncharacterized protein | Uncharacterized protein | | afdb-uniprot50 | AF-A0A7Y6Z4J4-F1-MODEL\_V4 | 1.0 | 6.018e-07 | 248 | 0.192 | 177 | 128 | 7 | 1 | 172 | 1 | 167 | Uncharacterized protein | Uncharacterized protein | | afdb-uniprot50 | AF-A0A085AFN8-F1-MODEL\_V4 | 1.0 | 6.248e-08 | 248 | 0.169 | 165 | 123 | 8 | 27 | 189 | 1 | 153 | Phage protein | Phage protein | | afdb-uniprot50 | AF-A0A5S9P2S6-F1-MODEL\_V4 | 1.0 | 6.248e-08 | 245 | 0.202 | 202 | 127 | 12 | 9 | 189 | 10 | 198 | Uncharacterized protein | Uncharacterized protein | | afdb-uniprot50 | AF-A0A5P9F0H4-F1-MODEL\_V4 | 1.0 | 1.777e-07 | 243 | 0.17 | 194 | 135 | 9 | 9 | 188 | 4 | 185 | Uncharacterized protein | Uncharacterized protein | | afdb-uniprot50 | AF-A0A522WEL2-F1-MODEL\_V4 | 1.0 | 2.998e-07 | 241 | 0.234 | 175 | 103 | 10 | 9 | 169 | 3 | 160 | Uncharacterized protein | Uncharacterized protein | | afdb-uniprot50 | AF-A0A1V5V144-F1-MODEL\_V4 | 1.0 | 1.076e-06 | 240 | 0.215 | 158 | 103 | 9 | 1 | 154 | 1 | 141 | Uncharacterized protein | Uncharacterized protein | | afdb-uniprot50 | AF-A0A849VN83-F1-MODEL\_V4 | 1.0 | 3.782e-07 | 239 | 0.17 | 193 | 133 | 13 | 7 | 189 | 1 | 176 | Uncharacterized protein | Uncharacterized protein | | afdb-uniprot50 | AF-A0A3B0MY09-F1-MODEL\_V4 | 1.0 | 7.592e-07 | 238 | 0.156 | 172 | 126 | 8 | 1 | 165 | 1 | 160 | Uncharacterized protein | Uncharacterized protein | | afdb-uniprot50 | AF-A0A853I879-F1-MODEL\_V4 | 1.0 | 4.008e-07 | 238 | 0.206 | 189 | 123 | 9 | 1 | 188 | 123 | 285 | Uncharacterized protein | Uncharacterized protein | | afdb-uniprot50 | AF-A0A515ERM5-F1-MODEL\_V4 | 1.0 | 1.281e-06 | 233 | 0.185 | 151 | 104 | 7 | 7 | 152 | 1 | 137 | Uncharacterized protein | Uncharacterized protein | | afdb-uniprot50 | AF-A0A2D8ACY1-F1-MODEL\_V4 | 1.0 | 2.998e-07 | 233 | 0.196 | 188 | 128 | 10 | 7 | 187 | 1 | 172 | Uncharacterized protein | Uncharacterized protein | | afdb-uniprot50 | AF-A0A7X4AMR2-F1-MODEL\_V4 | 1.0 | 1.615e-06 | 227 | 0.182 | 192 | 128 | 11 | 7 | 189 | 2 | 173 | Uncharacterized protein | Uncharacterized protein | | afdb-uniprot50 | AF-I3TTD7-F1-MODEL\_V4 | 1.0 | 4.502e-07 | 227 | 0.203 | 201 | 130 | 10 | 9 | 189 | 6 | 196 | Uncharacterized protein | Uncharacterized protein | | afdb-uniprot50 | AF-A0A365XR84-F1-MODEL\_V4 | 1.0 | 4.336e-06 | 226 | 0.128 | 171 | 127 | 6 | 9 | 173 | 5 | 159 | Uncharacterized protein | Uncharacterized protein | | afdb-uniprot50 | AF-A0A6S7D6J5-F1-MODEL\_V4 | 1.0 | 6.018e-07 | 225 | 0.17 | 200 | 137 | 10 | 1 | 183 | 1 | 188 | Uncharacterized protein | Uncharacterized protein | | afdb-uniprot50 | AF-A0A833IRE7-F1-MODEL\_V4 | 1.0 | 2.571e-06 | 224 | 0.174 | 189 | 138 | 7 | 7 | 189 | 1 | 177 | Uncharacterized protein | Uncharacterized protein | | afdb-uniprot50 | AF-A0A640W7I2-F1-MODEL\_V4 | 1.0 | 4.87e-06 | 223 | 0.153 | 156 | 109 | 7 | 1 | 154 | 1 | 135 | Uncharacterized protein | Uncharacterized protein | | afdb-uniprot50 | AF-A0A1G9UBD2-F1-MODEL\_V4 | 1.0 | 1.036e-05 | 223 | 0.182 | 148 | 100 | 5 | 13 | 152 | 3 | 137 | Uncharacterized protein | Uncharacterized protein | | afdb-uniprot50 | AF-A0A496VZB9-F1-MODEL\_V4 | 1.0 | 5.162e-06 | 223 | 0.158 | 164 | 112 | 7 | 1 | 159 | 1 | 143 | Uncharacterized protein | Uncharacterized protein | | afdb-uniprot50 | AF-A0A3D4RUC7-F1-MODEL\_V4 | 1.0 | 2.038e-06 | 223 | 0.126 | 221 | 135 | 10 | 7 | 189 | 2 | 202 | Uncharacterized protein | Uncharacterized protein | | afdb-uniprot50 | AF-A0A4S8HNS9-F1-MODEL\_V4 | 1.0 | 2.725e-06 | 222 | 0.136 | 176 | 128 | 7 | 9 | 177 | 6 | 164 | Uncharacterized protein | Uncharacterized protein | | afdb-uniprot50 | AF-A0A7X2NZB9-F1-MODEL\_V4 | 1.0 | 9.226e-06 | 221 | 0.43 | 93 | 45 | 1 | 96 | 188 | 2 | 86 | Uncharacterized protein | Uncharacterized protein | | afdb-uniprot50 | AF-A0A2N1TYS7-F1-MODEL\_V4 | 1.0 | 8.214e-06 | 221 | 0.152 | 151 | 110 | 7 | 7 | 154 | 2 | 137 | Uncharacterized protein | Uncharacterized protein | | afdb-uniprot50 | AF-A0A2I6S9D2-F1-MODEL\_V4 | 1.0 | 7.751e-06 | 220 | 0.177 | 158 | 113 | 7 | 2 | 155 | 1 | 145 | Uncharacterized protein | Uncharacterized protein | | afdb-uniprot50 | AF-A0A348FYH7-F1-MODEL\_V4 | 1.0 | 9.038e-07 | 220 | 0.178 | 174 | 118 | 11 | 1 | 164 | 1 | 159 | Uncharacterized protein | Uncharacterized protein | | afdb-uniprot50 | AF-A0A1X7NDV7-F1-MODEL\_V4 | 1.0 | 3.643e-06 | 219 | 0.165 | 175 | 126 | 8 | 1 | 165 | 1 | 165 | Uncharacterized protein | Uncharacterized protein | | afdb-uniprot50 | AF-A0A1M3PZ62-F1-MODEL\_V4 | 1.0 | 7.751e-06 | 215 | 0.177 | 152 | 112 | 5 | 10 | 159 | 7 | 147 | Uncharacterized protein | Uncharacterized protein | | afdb-uniprot50 | AF-A0A7C1ULK3-F1-MODEL\_V4 | 1.0 | 9.778e-06 | 214 | 0.125 | 152 | 103 | 8 | 13 | 152 | 2 | 135 | Uncharacterized protein | Uncharacterized protein | | afdb-uniprot50 | AF-A0A2P5N8U8-F1-MODEL\_V4 | 1.0 | 8.706e-06 | 214 | 0.104 | 153 | 119 | 7 | 12 | 159 | 5 | 144 | Uncharacterized protein | Uncharacterized protein | | afdb-uniprot50 | AF-A0A2W7JFE3-F1-MODEL\_V4 | 1.0 | 4.336e-06 | 214 | 0.148 | 155 | 117 | 5 | 15 | 166 | 5 | 147 | Uncharacterized protein | Uncharacterized protein | | afdb-uniprot50 | AF-A0A1F4PEF5-F1-MODEL\_V4 | 1.0 | 7.751e-06 | 213 | 0.117 | 162 | 112 | 9 | 6 | 152 | 2 | 147 | Uncharacterized protein | Uncharacterized protein | | afdb-uniprot50 | AF-A0A134B7B8-F1-MODEL\_V4 | 1.0 | 2.571e-06 | 212 | 0.128 | 179 | 127 | 7 | 10 | 172 | 2 | 167 | Uncharacterized protein | Uncharacterized protein | | afdb-uniprot50 | AF-A0A109C5L1-F1-MODEL\_V4 | 1.0 | 9.778e-06 | 209 | 0.165 | 163 | 113 | 9 | 7 | 165 | 2 | 145 | Uncharacterized protein | Uncharacterized protein | | afdb-uniprot50 | AF-A0A3S1EBT0-F1-MODEL\_V4 | 1.0 | 2.725e-06 | 209 | 0.197 | 192 | 128 | 12 | 1 | 178 | 1 | 180 | Uncharacterized protein | Uncharacterized protein | | afdb-uniprot50 | AF-A0A7U5AJB0-F1-MODEL\_V4 | 1.0 | 2.571e-06 | 209 | 0.208 | 182 | 126 | 9 | 4 | 177 | 2 | 173 | Uncharacterized protein | Uncharacterized protein | | afdb-uniprot50 | AF-A0A1Q3ZHY0-F1-MODEL\_V4 | 1.0 | 5.797e-06 | 208 | 0.127 | 172 | 126 | 11 | 9 | 173 | 4 | 158 | Uncharacterized protein | Uncharacterized protein | | afdb-uniprot50 | AF-A0A2W7QSY0-F1-MODEL\_V4 | 1.0 | 2.337e-05 | 207 | 0.196 | 183 | 126 | 11 | 1 | 174 | 1 | 171 | Uncharacterized protein | Uncharacterized protein | | afdb-uniprot50 | AF-A0A6L8MMN6-F1-MODEL\_V4 | 1.0 | 2.081e-05 | 205 | 0.141 | 148 | 105 | 5 | 13 | 152 | 2 | 135 | Uncharacterized protein | Uncharacterized protein | | afdb-uniprot50 | AF-A0A1H6CBN8-F1-MODEL\_V4 | 1.0 | 7.314e-06 | 205 | 0.133 | 157 | 112 | 7 | 7 | 156 | 5 | 144 | Uncharacterized protein | Uncharacterized protein | | afdb-uniprot50 | AF-R6VPC3-F1-MODEL\_V4 | 1.0 | 8.528e-07 | 205 | 0.179 | 178 | 114 | 9 | 10 | 172 | 2 | 162 | Uncharacterized protein | Uncharacterized protein | | afdb-uniprot50 | AF-T2G8C6-F1-MODEL\_V4 | 1.0 | 3.311e-05 | 204 | 0.186 | 161 | 116 | 9 | 1 | 155 | 1 | 152 | Uncharacterized protein | Uncharacterized protein | | afdb-uniprot50 | AF-A0A349PRD1-F1-MODEL\_V4 | 1.0 | 1.468e-05 | 204 | 0.123 | 170 | 126 | 12 | 8 | 168 | 1 | 156 | Uncharacterized protein | Uncharacterized protein | | afdb-uniprot50 | AF-A0A849MY44-F1-MODEL\_V4 | 1.0 | 1.712e-06 | 204 | 0.169 | 171 | 119 | 7 | 14 | 179 | 4 | 156 | Uncharacterized protein | Uncharacterized protein | | afdb-uniprot50 | AF-A0A844H9D9-F1-MODEL\_V4 | 1.0 | 3.243e-06 | 204 | 0.132 | 204 | 138 | 9 | 2 | 179 | 9 | 199 | Uncharacterized protein | Uncharacterized protein | | afdb-uniprot50 | AF-A0A7J5VW47-F1-MODEL\_V4 | 1.0 | 7.751e-06 | 202 | 0.131 | 183 | 129 | 9 | 8 | 178 | 1 | 165 | Uncharacterized protein | Uncharacterized protein | | afdb-uniprot50 | AF-A0A1G5ZJI4-F1-MODEL\_V4 | 1.0 | 1.357e-06 | 202 | 0.132 | 204 | 134 | 9 | 15 | 189 | 5 | 194 | Uncharacterized protein | Uncharacterized protein | | afdb-uniprot50 | AF-A0A446AZ98-F1-MODEL\_V4 | 1.0 | 7.164e-07 | 202 | 0.161 | 217 | 128 | 15 | 7 | 189 | 1 | 197 | Uncharacterized protein | Uncharacterized protein | | afdb-uniprot50 | AF-A0A450ZWN7-F1-MODEL\_V4 | 1.0 | 3.124e-05 | 201 | 0.151 | 158 | 107 | 7 | 1 | 153 | 1 | 136 | Uncharacterized protein | Uncharacterized protein | | afdb-uniprot50 | AF-I3TN74-F1-MODEL\_V4 | 1.0 | 1.556e-05 | 200 | 0.198 | 151 | 104 | 8 | 10 | 153 | 2 | 142 | Uncharacterized protein | Uncharacterized protein | | afdb-uniprot50 | AF-A0A1X3RM65-F1-MODEL\_V4 | 1.0 | 1.963e-05 | 200 | 0.177 | 169 | 123 | 6 | 7 | 173 | 1 | 155 | Uncharacterized protein | Uncharacterized protein | | afdb-uniprot50 | AF-A0A4R3UQ50-F1-MODEL\_V4 | 1.0 | 8.706e-06 | 200 | 0.169 | 159 | 113 | 7 | 14 | 165 | 4 | 150 | Uncharacterized protein | Uncharacterized protein | | afdb-uniprot50 | AF-W0HQ32-F1-MODEL\_V4 | 1.0 | 1.468e-05 | 200 | 0.164 | 164 | 117 | 9 | 8 | 165 | 1 | 150 | Uncharacterized protein | Uncharacterized protein | | afdb-uniprot50 | AF-A0A349MJN4-F1-MODEL\_V4 | 1.0 | 2.081e-05 | 199 | 0.147 | 149 | 106 | 7 | 12 | 153 | 5 | 139 | Uncharacterized protein | Uncharacterized protein | | afdb-uniprot50 | AF-A0A2A4XUH6-F1-MODEL\_V4 | 1.0 | 1.615e-06 | 199 | 0.21 | 176 | 111 | 10 | 8 | 168 | 3 | 165 | Uncharacterized protein | Uncharacterized protein | | afdb-uniprot50 | AF-A0A4Z0Q1Z6-F1-MODEL\_V4 | 1.0 | 4.092e-06 | 198 | 0.138 | 181 | 122 | 8 | 8 | 168 | 1 | 167 | Uncharacterized protein | Uncharacterized protein | | afdb-uniprot50 | AF-A0A3D5BS75-F1-MODEL\_V4 | 1.0 | 2.477e-05 | 196 | 0.112 | 169 | 117 | 10 | 8 | 166 | 1 | 146 | Uncharacterized protein | Uncharacterized protein | | afdb-uniprot50 | AF-A0A8B3TEH3-F1-MODEL\_V4 | 1.0 | 8.214e-06 | 196 | 0.112 | 187 | 142 | 9 | 9 | 187 | 4 | 174 | DUF1834 family protein | DUF1834 family protein | | afdb-uniprot50 | AF-A0A6A7YJM2-F1-MODEL\_V4 | 1.0 | 2.038e-06 | 196 | 0.158 | 189 | 124 | 10 | 14 | 182 | 4 | 177 | Uncharacterized protein | Uncharacterized protein | | afdb-uniprot50 | AF-A0A3N2E265-F1-MODEL\_V4 | 1.0 | 4.336e-06 | 196 | 0.141 | 191 | 120 | 9 | 7 | 165 | 2 | 180 | Uncharacterized protein | Uncharacterized protein | | afdb-uniprot50 | AF-A0A0Q4UF98-F1-MODEL\_V4 | 1.0 | 4.972e-05 | 195 | 0.18 | 150 | 101 | 8 | 7 | 153 | 2 | 132 | Uncharacterized protein | Uncharacterized protein | | afdb-uniprot50 | AF-A0A3M1MUF4-F1-MODEL\_V4 | 1.0 | 0.0001684 | 195 | 0.093 | 149 | 118 | 5 | 9 | 154 | 3 | 137 | Uncharacterized protein | Uncharacterized protein | | afdb-uniprot50 | AF-A0A840MMI4-F1-MODEL\_V4 | 1.0 | 5.269e-05 | 195 | 0.131 | 167 | 128 | 6 | 7 | 172 | 1 | 151 | Uncharacterized protein | Uncharacterized protein | | afdb-uniprot50 | AF-A0A450YW35-F1-MODEL\_V4 | 1.0 | 1.649e-05 | 195 | 0.174 | 172 | 123 | 7 | 1 | 171 | 1 | 154 | Gp37 protein | Gp37 protein | | afdb-uniprot50 | AF-V9HKZ2-F1-MODEL\_V4 | 1.0 | 2.948e-05 | 194 | 0.146 | 157 | 108 | 8 | 8 | 153 | 3 | 144 | Uncharacterized protein | Uncharacterized protein | | afdb-uniprot50 | AF-A0A6N7B6E2-F1-MODEL\_V4 | 1.0 | 2.337e-05 | 194 | 0.155 | 167 | 120 | 7 | 1 | 166 | 1 | 147 | Uncharacterized protein | Uncharacterized protein | | afdb-uniprot50 | AF-A0A450WGT7-F1-MODEL\_V4 | 1.0 | 2.625e-05 | 194 | 0.169 | 171 | 124 | 7 | 1 | 170 | 1 | 154 | Gp37 protein | Gp37 protein | | afdb-uniprot50 | AF-A0A5C7J5X6-F1-MODEL\_V4 | 1.0 | 6.144e-06 | 194 | 0.157 | 210 | 143 | 12 | 2 | 189 | 17 | 214 | DUF1834 family protein | DUF1834 family protein | | afdb-uniprot50 | AF-A0A1G8LDR9-F1-MODEL\_V4 | 1.0 | 3.311e-05 | 193 | 0.175 | 148 | 105 | 9 | 13 | 155 | 2 | 137 | Uncharacterized protein | Uncharacterized protein | | afdb-uniprot50 | AF-A0A177N1G7-F1-MODEL\_V4 | 1.0 | 9.419e-05 | 193 | 0.146 | 150 | 105 | 7 | 12 | 153 | 4 | 138 | Uncharacterized protein | Uncharacterized protein | | afdb-uniprot50 | AF-A0A5I6PT12-F1-MODEL\_V4 | 1.0 | 1.556e-05 | 193 | 0.142 | 175 | 120 | 9 | 9 | 166 | 3 | 164 | DUF1834 family protein | DUF1834 family protein | | afdb-uniprot50 | AF-V4NRC4-F1-MODEL\_V4 | 1.0 | 6.901e-06 | 193 | 0.152 | 203 | 130 | 13 | 12 | 186 | 5 | 193 | Uncharacterized protein | Uncharacterized protein | | afdb-uniprot50 | AF-A0A4R3LGT3-F1-MODEL\_V4 | 1.0 | 4.092e-06 | 192 | 0.19 | 184 | 129 | 8 | 10 | 189 | 5 | 172 | Uncharacterized protein | Uncharacterized protein | | afdb-uniprot50 | AF-A0A285P0D4-F1-MODEL\_V4 | 1.0 | 2.571e-06 | 192 | 0.136 | 205 | 132 | 11 | 15 | 188 | 5 | 195 | Uncharacterized protein | Uncharacterized protein | | afdb-uniprot50 | AF-A0A2C8YIV8-F1-MODEL\_V4 | 1.0 | 2.625e-05 | 191 | 0.184 | 163 | 111 | 8 | 1 | 156 | 9 | 156 | Uncharacterized protein | Uncharacterized protein | | afdb-uniprot50 | AF-G2H4N2-F1-MODEL\_V4 | 1.0 | 5.797e-06 | 191 | 0.154 | 207 | 140 | 13 | 8 | 189 | 1 | 197 | Uncharacterized protein | Uncharacterized protein | | afdb-uniprot50 | AF-A0A2S6N2V3-F1-MODEL\_V4 | 1.0 | 1.164e-05 | 191 | 0.134 | 178 | 124 | 9 | 8 | 166 | 1 | 167 | Uncharacterized protein | Uncharacterized protein | | afdb-uniprot50 | AF-A0A345DDG3-F1-MODEL\_V4 | 1.0 | 8.214e-06 | 191 | 0.152 | 217 | 125 | 13 | 12 | 180 | 4 | 209 | Uncharacterized protein | Uncharacterized protein | | afdb-uniprot50 | AF-A0A5C7TSP4-F1-MODEL\_V4 | 1.0 | 5.269e-05 | 190 | 0.15 | 166 | 114 | 7 | 1 | 163 | 1 | 142 | Uncharacterized protein | Uncharacterized protein | | afdb-uniprot50 | AF-A0A2S0MD53-F1-MODEL\_V4 | 1.0 | 4.427e-05 | 190 | 0.173 | 161 | 112 | 9 | 1 | 153 | 1 | 148 | Uncharacterized protein | Uncharacterized protein | | afdb-uniprot50 | AF-A0A6M3ZW34-F1-MODEL\_V4 | 1.0 | 3.06e-06 | 190 | 0.125 | 207 | 135 | 11 | 15 | 189 | 5 | 197 | Uncharacterized protein | Uncharacterized protein | | afdb-uniprot50 | AF-A0A0F9FP65-F1-MODEL\_V4 | 1.0 | 8.214e-06 | 190 | 0.145 | 179 | 121 | 8 | 10 | 166 | 6 | 174 | Uncharacterized protein | Uncharacterized protein | | afdb-uniprot50 | AF-A0A104N6L4-F1-MODEL\_V4 | 1.0 | 2.782e-05 | 189 | 0.286 | 115 | 71 | 3 | 71 | 185 | 3 | 106 | Uncharacterized protein | Uncharacterized protein | | afdb-uniprot50 | AF-A0A1G0I709-F1-MODEL\_V4 | 1.0 | 5.918e-05 | 189 | 0.18 | 150 | 106 | 6 | 7 | 152 | 1 | 137 | Uncharacterized protein | Uncharacterized protein | | afdb-uniprot50 | AF-I3YEI7-F1-MODEL\_V4 | 1.0 | 1.307e-05 | 189 | 0.204 | 176 | 107 | 11 | 1 | 166 | 1 | 153 | Uncharacterized protein | Uncharacterized protein | | afdb-uniprot50 | AF-A0A249STX7-F1-MODEL\_V4 | 1.0 | 1.556e-05 | 189 | 0.155 | 180 | 109 | 11 | 10 | 165 | 2 | 162 | Uncharacterized protein | Uncharacterized protein | | afdb-uniprot50 | AF-A0A2A2JXH5-F1-MODEL\_V4 | 1.0 | 3.719e-05 | 188 | 0.163 | 159 | 103 | 8 | 6 | 156 | 2 | 138 | Uncharacterized protein | Uncharacterized protein | | afdb-uniprot50 | AF-A0A194AGD2-F1-MODEL\_V4 | 1.0 | 0.0002528 | 188 | 0.13 | 153 | 117 | 6 | 7 | 155 | 2 | 142 | Uncharacterized protein | Uncharacterized protein | | afdb-uniprot50 | AF-W8F0Q4-F1-MODEL\_V4 | 1.0 | 1.098e-05 | 188 | 0.136 | 191 | 125 | 11 | 8 | 189 | 1 | 160 | Uncharacterized protein | Uncharacterized protein | | afdb-uniprot50 | AF-A0A379S6I8-F1-MODEL\_V4 | 1.0 | 3.861e-06 | 188 | 0.129 | 208 | 144 | 15 | 1 | 189 | 165 | 354 | Phage protein | Phage protein | | afdb-uniprot50 | AF-A0A059IW70-F1-MODEL\_V4 | 1.0 | 8.386e-05 | 187 | 0.315 | 95 | 57 | 2 | 71 | 165 | 3 | 89 | Uncharacterized protein | Uncharacterized protein | | afdb-uniprot50 | AF-A0A286GYR4-F1-MODEL\_V4 | 1.0 | 7.913e-05 | 187 | 0.168 | 148 | 107 | 6 | 13 | 155 | 4 | 140 | Uncharacterized protein | Uncharacterized protein | | afdb-uniprot50 | AF-A0A833GRL2-F1-MODEL\_V4 | 1.0 | 3.941e-05 | 187 | 0.158 | 164 | 113 | 9 | 8 | 164 | 1 | 146 | Uncharacterized protein | Uncharacterized protein | | afdb-uniprot50 | AF-B6IMF8-F1-MODEL\_V4 | 1.0 | 2.337e-05 | 187 | 0.177 | 169 | 121 | 6 | 7 | 173 | 1 | 153 | Uncharacterized protein | Uncharacterized protein | | afdb-uniprot50 | AF-A0A386HSH2-F1-MODEL\_V4 | 1.0 | 2.948e-05 | 187 | 0.134 | 186 | 134 | 7 | 1 | 166 | 7 | 185 | Uncharacterized protein | Uncharacterized protein | | afdb-uniprot50 | AF-A0A1M7B4C0-F1-MODEL\_V4 | 1.0 | 9.419e-05 | 185 | 0.142 | 161 | 104 | 7 | 1 | 156 | 2 | 133 | Uncharacterized protein | Uncharacterized protein | | afdb-uniprot50 | AF-A0A661DES6-F1-MODEL\_V4 | 1.0 | 1.556e-05 | 185 | 0.212 | 155 | 100 | 12 | 7 | 153 | 1 | 141 | Uncharacterized protein | Uncharacterized protein | | afdb-uniprot50 | AF-A0A7S8C7I5-F1-MODEL\_V4 | 1.0 | 0.0001499 | 185 | 0.169 | 165 | 117 | 9 | 7 | 165 | 2 | 152 | Uncharacterized protein | Uncharacterized protein | | afdb-uniprot50 | AF-A0A0Q0A4D7-F1-MODEL\_V4 | 1.0 | 6.901e-06 | 185 | 0.13 | 222 | 144 | 13 | 1 | 189 | 65 | 270 | Uncharacterized protein | Uncharacterized protein | | afdb-uniprot50 | AF-A0A2E1RHS9-F1-MODEL\_V4 | 1.0 | 9.982e-05 | 183 | 0.172 | 145 | 101 | 6 | 13 | 156 | 2 | 128 | Uncharacterized protein | Uncharacterized protein | | afdb-uniprot50 | AF-A0A3A0EP56-F1-MODEL\_V4 | 1.0 | 5.918e-05 | 183 | 0.153 | 150 | 108 | 6 | 7 | 153 | 1 | 134 | Uncharacterized protein | Uncharacterized protein | | afdb-uniprot50 | AF-A0A7C1A7F0-F1-MODEL\_V4 | 1.0 | 0.000284 | 183 | 0.131 | 152 | 114 | 7 | 8 | 154 | 1 | 139 | DUF1834 family protein | DUF1834 family protein | | afdb-uniprot50 | AF-A0A355TT27-F1-MODEL\_V4 | 1.0 | 0.0001335 | 183 | 0.135 | 162 | 119 | 7 | 7 | 165 | 1 | 144 | Uncharacterized protein | Uncharacterized protein | | afdb-uniprot50 | AF-A0A109BYV0-F1-MODEL\_V4 | 1.0 | 4.691e-05 | 183 | 0.137 | 153 | 118 | 9 | 7 | 153 | 2 | 146 | Uncharacterized protein | Uncharacterized protein | | afdb-uniprot50 | AF-A0A0F9PV67-F1-MODEL\_V4 | 1.0 | 2.081e-05 | 183 | 0.146 | 177 | 118 | 14 | 1 | 155 | 1 | 166 | Uncharacterized protein | Uncharacterized protein | | afdb-uniprot50 | AF-A0A2K4MRJ3-F1-MODEL\_V4 | 1.0 | 2.782e-05 | 183 | 0.128 | 179 | 126 | 11 | 2 | 174 | 34 | 188 | Uncharacterized protein | Uncharacterized protein | | afdb-uniprot50 | AF-A0A1B9LF31-F1-MODEL\_V4 | 1.0 | 5.584e-05 | 183 | 0.133 | 150 | 109 | 8 | 14 | 155 | 4 | 140 | Uncharacterized protein | Uncharacterized protein | | afdb-uniprot50 | AF-A0A3B9IJU4-F1-MODEL\_V4 | 1.0 | 7.913e-05 | 182 | 0.153 | 150 | 103 | 7 | 13 | 153 | 2 | 136 | Uncharacterized protein | Uncharacterized protein | | afdb-uniprot50 | AF-A0A853YIQ0-F1-MODEL\_V4 | 1.0 | 0.0001058 | 182 | 0.163 | 153 | 103 | 6 | 13 | 156 | 2 | 138 | Uncharacterized protein | Uncharacterized protein | | afdb-uniprot50 | AF-A0A6A8RIJ7-F1-MODEL\_V4 | 1.0 | 0.0002251 | 182 | 0.124 | 161 | 117 | 7 | 7 | 155 | 3 | 151 | Uncharacterized protein | Uncharacterized protein | | afdb-uniprot50 | AF-A0A4R1AH59-F1-MODEL\_V4 | 1.0 | 7.045e-05 | 182 | 0.114 | 166 | 118 | 11 | 7 | 159 | 1 | 150 | Uncharacterized protein | Uncharacterized protein | | afdb-uniprot50 | AF-A0A423PRY6-F1-MODEL\_V4 | 1.0 | 3.643e-06 | 182 | 0.159 | 194 | 120 | 11 | 1 | 188 | 1 | 157 | Uncharacterized protein | Uncharacterized protein | | afdb-uniprot50 | AF-V7HLN1-F1-MODEL\_V4 | 1.0 | 1.438e-06 | 182 | 0.179 | 201 | 131 | 11 | 1 | 188 | 1 | 180 | Uncharacterized protein | Uncharacterized protein | | afdb-uniprot50 | AF-A0A3N7BFR2-F1-MODEL\_V4 | 1.0 | 1.357e-06 | 182 | 0.176 | 210 | 120 | 17 | 15 | 189 | 5 | 196 | Uncharacterized protein | Uncharacterized protein | | afdb-uniprot50 | AF-A0A1B4FYB9-F1-MODEL\_V4 | 1.0 | 1.234e-05 | 182 | 0.138 | 216 | 134 | 14 | 6 | 179 | 2 | 207 | Uncharacterized protein | Uncharacterized protein | | afdb-uniprot50 | AF-A0A2P8EAQ1-F1-MODEL\_V4 | 1.0 | 3.124e-05 | 181 | 0.175 | 160 | 100 | 10 | 7 | 152 | 1 | 142 | Uncharacterized protein | Uncharacterized protein | | afdb-uniprot50 | AF-A0A2P5GLV5-F1-MODEL\_V4 | 1.0 | 5.584e-05 | 181 | 0.147 | 170 | 117 | 8 | 7 | 168 | 1 | 150 | Uncharacterized protein | Uncharacterized protein | | afdb-uniprot50 | AF-A0A7I0KP35-F1-MODEL\_V4 | 1.0 | 6.272e-05 | 181 | 0.155 | 167 | 127 | 5 | 7 | 173 | 1 | 153 | Uncharacterized protein | Uncharacterized protein | | afdb-uniprot50 | AF-A0A482IRV5-F1-MODEL\_V4 | 1.0 | 3.509e-05 | 181 | 0.173 | 173 | 125 | 6 | 8 | 179 | 1 | 156 | Uncharacterized protein | Uncharacterized protein | | afdb-uniprot50 | AF-A0A747D8U5-F1-MODEL\_V4 | 1.0 | 0.0001335 | 181 | 0.133 | 165 | 119 | 8 | 7 | 157 | 1 | 155 | DUF1834 family protein | DUF1834 family protein | | afdb-uniprot50 | AF-A0A0Q4UDE4-F1-MODEL\_V4 | 1.0 | 0.0001589 | 180 | 0.197 | 147 | 99 | 7 | 13 | 152 | 2 | 136 | Uncharacterized protein | Uncharacterized protein | | afdb-uniprot50 | AF-A0A844GC83-F1-MODEL\_V4 | 1.0 | 0.0003189 | 180 | 0.113 | 150 | 117 | 7 | 6 | 152 | 3 | 139 | Uncharacterized protein | Uncharacterized protein | | afdb-uniprot50 | AF-A0A418W4C9-F1-MODEL\_V4 | 1.0 | 5.584e-05 | 180 | 0.209 | 153 | 96 | 8 | 14 | 154 | 5 | 144 | Uncharacterized protein | Uncharacterized protein | | afdb-uniprot50 | AF-A0A0Q4TYV2-F1-MODEL\_V4 | 1.0 | 7.913e-05 | 180 | 0.182 | 148 | 101 | 9 | 9 | 150 | 5 | 138 | Uncharacterized protein | Uncharacterized protein | | afdb-uniprot50 | AF-A0A1B4SJZ4-F1-MODEL\_V4 | 1.0 | 4.691e-05 | 180 | 0.204 | 171 | 115 | 8 | 7 | 174 | 2 | 154 | Uncharacterized protein | Uncharacterized protein | | afdb-uniprot50 | AF-A0A450VPX4-F1-MODEL\_V4 | 1.0 | 2.205e-05 | 179 | 0.107 | 195 | 138 | 10 | 1 | 188 | 1 | 166 | Gp37 protein | Gp37 protein | | afdb-uniprot50 | AF-A0A4Y6VZN6-F1-MODEL\_V4 | 1.0 | 9.226e-06 | 179 | 0.19 | 194 | 125 | 14 | 10 | 183 | 7 | 188 | DUF1834 family protein | DUF1834 family protein | | afdb-uniprot50 | AF-A0A089YFT4-F1-MODEL\_V4 | 1.0 | 7.751e-06 | 178 | 0.128 | 203 | 138 | 11 | 7 | 187 | 1 | 186 | Uncharacterized protein | Uncharacterized protein | | afdb-uniprot50 | AF-A0A7W8DYF7-F1-MODEL\_V4 | 1.0 | 3.311e-05 | 178 | 0.175 | 194 | 118 | 12 | 1 | 168 | 1 | 178 | Uncharacterized protein | Uncharacterized protein | | afdb-uniprot50 | AF-A0A5M6I4T3-F1-MODEL\_V4 | 1.0 | 1.748e-05 | 178 | 0.212 | 188 | 117 | 10 | 13 | 187 | 6 | 175 | Uncharacterized protein | Uncharacterized protein | | afdb-uniprot50 | AF-A0A1E8PKI9-F1-MODEL\_V4 | 1.0 | 3.719e-05 | 178 | 0.156 | 211 | 135 | 11 | 8 | 189 | 1 | 197 | Uncharacterized protein | Uncharacterized protein | | afdb-uniprot50 | AF-A0A1T1AP14-F1-MODEL\_V4 | 1.0 | 5.584e-05 | 178 | 0.141 | 191 | 136 | 10 | 7 | 172 | 1 | 188 | Uncharacterized protein | Uncharacterized protein | | afdb-uniprot50 | AF-X6QBR1-F1-MODEL\_V4 | 1.0 | 8.887e-05 | 178 | 0.147 | 176 | 121 | 10 | 8 | 165 | 1 | 165 | PF08873 domain protein | PF08873 domain protein | | afdb-uniprot50 | AF-A0A4V2V2Q1-F1-MODEL\_V4 | 1.0 | 0.0001891 | 177 | 0.177 | 158 | 98 | 7 | 7 | 154 | 1 | 136 | Uncharacterized protein | Uncharacterized protein | | afdb-uniprot50 | AF-A0A1D2X525-F1-MODEL\_V4 | 1.0 | 8.386e-05 | 177 | 0.119 | 159 | 111 | 9 | 8 | 152 | 1 | 144 | Uncharacterized protein | Uncharacterized protein | | afdb-uniprot50 | AF-A0A7X7S173-F1-MODEL\_V4 | 1.0 | 5.269e-05 | 177 | 0.168 | 172 | 125 | 7 | 7 | 177 | 1 | 155 | Uncharacterized protein | Uncharacterized protein | | afdb-uniprot50 | AF-A0A847H596-F1-MODEL\_V4 | 1.0 | 0.0002528 | 176 | 0.113 | 150 | 110 | 6 | 7 | 152 | 1 | 131 | Uncharacterized protein | Uncharacterized protein | | afdb-uniprot50 | AF-A0A1C0SPE6-F1-MODEL\_V4 | 1.0 | 0.0001589 | 176 | 0.161 | 149 | 106 | 8 | 14 | 156 | 3 | 138 | Uncharacterized protein | Uncharacterized protein | | afdb-uniprot50 | AF-A0A2W6T3K4-F1-MODEL\_V4 | 1.0 | 0.0001188 | 176 | 0.131 | 160 | 112 | 10 | 14 | 166 | 9 | 148 | Uncharacterized protein | Uncharacterized protein | | afdb-uniprot50 | AF-A0A4V3WAW4-F1-MODEL\_V4 | 1.0 | 0.0001891 | 176 | 0.159 | 163 | 110 | 10 | 5 | 156 | 2 | 148 | Uncharacterized protein | Uncharacterized protein | | afdb-uniprot50 | AF-A0A1R1JMQ1-F1-MODEL\_V4 | 1.0 | 8.706e-06 | 176 | 0.118 | 203 | 134 | 10 | 13 | 185 | 3 | 190 | Uncharacterized protein | Uncharacterized protein | | afdb-uniprot50 | AF-A0A2M9WHN8-F1-MODEL\_V4 | 1.0 | 7.045e-05 | 176 | 0.137 | 175 | 119 | 10 | 12 | 166 | 5 | 167 | Uncharacterized protein | Uncharacterized protein | | afdb-uniprot50 | AF-A0A559Q8J5-F1-MODEL\_V4 | 1.0 | 9.419e-05 | 175 | 0.166 | 168 | 110 | 8 | 7 | 165 | 4 | 150 | Uncharacterized protein | Uncharacterized protein | | afdb-uniprot50 | AF-A0A4R1FMA6-F1-MODEL\_V4 | 1.0 | 0.0001589 | 175 | 0.113 | 168 | 130 | 6 | 7 | 172 | 2 | 152 | Gp37 protein | Gp37 protein | | afdb-uniprot50 | AF-A0A1Q3QXE1-F1-MODEL\_V4 | 1.0 | 3.509e-05 | 175 | 0.139 | 172 | 129 | 10 | 5 | 165 | 3 | 166 | Uncharacterized protein | Uncharacterized protein | | afdb-uniprot50 | AF-Q1MNR2-F1-MODEL\_V4 | 1.0 | 2.782e-05 | 175 | 0.174 | 189 | 125 | 11 | 8 | 177 | 1 | 177 | Uncharacterized protein | Uncharacterized protein | | afdb-uniprot50 | AF-A0A2V4BZS6-F1-MODEL\_V4 | 1.0 | 0.0001058 | 174 | 0.096 | 186 | 130 | 11 | 1 | 166 | 2 | 169 | Uncharacterized protein | Uncharacterized protein | | afdb-uniprot50 | AF-A0A0M4M4N7-F1-MODEL\_V4 | 1.0 | 4.427e-05 | 174 | 0.123 | 186 | 136 | 7 | 10 | 187 | 5 | 171 | Putative prophage protein | Putative prophage protein | | afdb-uniprot50 | AF-A0A376HZ26-F1-MODEL\_V4 | 1.0 | 3.124e-05 | 174 | 0.095 | 189 | 141 | 10 | 8 | 187 | 1 | 168 | Mu-like prophage protein gp37 | Mu-like prophage protein gp37 | | afdb-uniprot50 | AF-A0A6I1NPD5-F1-MODEL\_V4 | 1.0 | 7.314e-06 | 174 | 0.154 | 188 | 117 | 13 | 15 | 177 | 5 | 175 | Uncharacterized protein | Uncharacterized protein | | afdb-uniprot50 | AF-W0BVV9-F1-MODEL\_V4 | 1.0 | 5.918e-05 | 174 | 0.146 | 177 | 123 | 9 | 8 | 166 | 1 | 167 | Uncharacterized protein | Uncharacterized protein | | afdb-uniprot50 | AF-A0A1F4QWC5-F1-MODEL\_V4 | 1.0 | 0.0001784 | 173 | 0.196 | 153 | 102 | 7 | 7 | 155 | 1 | 136 | Uncharacterized protein | Uncharacterized protein | | afdb-uniprot50 | AF-A0A318KU15-F1-MODEL\_V4 | 1.0 | 8.887e-05 | 173 | 0.111 | 170 | 123 | 8 | 7 | 165 | 2 | 154 | Gp37 protein | Gp37 protein | | afdb-uniprot50 | AF-G4CJF9-F1-MODEL\_V4 | 1.0 | 0.0001891 | 173 | 0.111 | 170 | 130 | 7 | 8 | 174 | 3 | 154 | Phage Gp37 protein | Phage Gp37 protein | | afdb-uniprot50 | AF-A0A3G7TJT5-F1-MODEL\_V4 | 1.0 | 0.0001188 | 173 | 0.142 | 175 | 133 | 6 | 2 | 174 | 1 | 160 | Uncharacterized protein | Uncharacterized protein | | afdb-uniprot50 | AF-A0A6L8L6D7-F1-MODEL\_V4 | 1.0 | 4.177e-05 | 173 | 0.144 | 194 | 137 | 10 | 8 | 187 | 1 | 179 | DUF1834 family protein | DUF1834 family protein | | afdb-uniprot50 | AF-A0A143DC32-F1-MODEL\_V4 | 1.0 | 5.584e-05 | 173 | 0.14 | 178 | 120 | 9 | 13 | 172 | 2 | 164 | Uncharacterized protein | Uncharacterized protein | | afdb-uniprot50 | AF-A0A2Z6GCD5-F1-MODEL\_V4 | 1.0 | 0.0002004 | 172 | 0.141 | 148 | 108 | 8 | 7 | 152 | 1 | 131 | Uncharacterized protein | Uncharacterized protein | | afdb-uniprot50 | AF-A0A1G3JTJ3-F1-MODEL\_V4 | 1.0 | 0.0002386 | 172 | 0.16 | 150 | 106 | 7 | 7 | 152 | 2 | 135 | Uncharacterized protein | Uncharacterized protein | | afdb-uniprot50 | AF-A0A143XXV9-F1-MODEL\_V4 | 1.0 | 0.0001784 | 172 | 0.144 | 159 | 112 | 9 | 10 | 155 | 2 | 149 | Uncharacterized protein | Uncharacterized protein | | afdb-uniprot50 | AF-A0A1J5D4A8-F1-MODEL\_V4 | 1.0 | 0.0001121 | 172 | 0.118 | 169 | 122 | 9 | 6 | 163 | 2 | 154 | Uncharacterized protein | Uncharacterized protein | | afdb-uniprot50 | AF-A0A7L5ABA4-F1-MODEL\_V4 | 1.0 | 0.0001684 | 172 | 0.137 | 174 | 138 | 6 | 1 | 172 | 2 | 165 | Uncharacterized protein | Uncharacterized protein | | afdb-uniprot50 | AF-A0A5N0TG56-F1-MODEL\_V4 | 1.0 | 0.0002679 | 171 | 0.151 | 145 | 101 | 9 | 13 | 155 | 4 | 128 | Uncharacterized protein | Uncharacterized protein | | afdb-uniprot50 | AF-A0A5A8F1R4-F1-MODEL\_V4 | 1.0 | 0.0002251 | 171 | 0.142 | 154 | 105 | 8 | 8 | 153 | 1 | 135 | Uncharacterized protein | Uncharacterized protein | | afdb-uniprot50 | AF-A0A5E8D1X6-F1-MODEL\_V4 | 1.0 | 0.0002386 | 171 | 0.134 | 163 | 106 | 12 | 10 | 159 | 2 | 142 | Uncharacterized protein | Uncharacterized protein | | afdb-uniprot50 | AF-C1FA28-F1-MODEL\_V4 | 1.0 | 5.918e-05 | 171 | 0.168 | 178 | 112 | 11 | 1 | 162 | 1 | 158 | Uncharacterized protein | Uncharacterized protein | | afdb-uniprot50 | AF-A0A1B7KXZ2-F1-MODEL\_V4 | 1.0 | 7.314e-06 | 171 | 0.13 | 200 | 141 | 12 | 7 | 189 | 1 | 184 | Uncharacterized protein | Uncharacterized protein | | afdb-uniprot50 | AF-A0A3M3KWV5-F1-MODEL\_V4 | 1.0 | 9.226e-06 | 171 | 0.134 | 208 | 131 | 14 | 15 | 189 | 5 | 196 | Uncharacterized protein | Uncharacterized protein | | afdb-uniprot50 | AF-A0A3M5JCG4-F1-MODEL\_V4 | 1.0 | 9.226e-06 | 171 | 0.134 | 208 | 131 | 14 | 15 | 189 | 5 | 196 | Uncharacterized protein | Uncharacterized protein | | afdb-uniprot50 | AF-A0A1Z8PY23-F1-MODEL\_V4 | 1.0 | 9.778e-06 | 171 | 0.163 | 202 | 117 | 16 | 8 | 178 | 1 | 181 | Uncharacterized protein | Uncharacterized protein | | afdb-uniprot50 | AF-A0A2D0JLA8-F1-MODEL\_V4 | 1.0 | 6.272e-05 | 171 | 0.144 | 173 | 116 | 10 | 8 | 162 | 1 | 159 | Uncharacterized protein | Uncharacterized protein | | afdb-uniprot50 | AF-A0A2D9DW63-F1-MODEL\_V4 | 1.0 | 0.0001784 | 170 | 0.135 | 148 | 103 | 7 | 14 | 155 | 3 | 131 | Uncharacterized protein | Uncharacterized protein | | afdb-uniprot50 | AF-A0A560II09-F1-MODEL\_V4 | 1.0 | 0.0002004 | 170 | 0.164 | 152 | 103 | 10 | 11 | 152 | 2 | 139 | Uncharacterized protein | Uncharacterized protein | | afdb-uniprot50 | AF-A0A7V1ZNY4-F1-MODEL\_V4 | 1.0 | 0.000284 | 170 | 0.125 | 168 | 117 | 8 | 8 | 165 | 1 | 148 | DUF1834 family protein | DUF1834 family protein | | afdb-uniprot50 | AF-A0A329W903-F1-MODEL\_V4 | 1.0 | 0.0001259 | 170 | 0.173 | 167 | 124 | 4 | 7 | 173 | 1 | 153 | Uncharacterized protein | Uncharacterized protein | | afdb-uniprot50 | AF-A0A6M0FVW8-F1-MODEL\_V4 | 1.0 | 0.0001058 | 170 | 0.116 | 171 | 128 | 8 | 7 | 166 | 3 | 161 | Uncharacterized protein | Uncharacterized protein | | afdb-uniprot50 | AF-A0A6B2KN64-F1-MODEL\_V4 | 1.0 | 2.948e-05 | 170 | 0.123 | 203 | 134 | 12 | 15 | 189 | 5 | 191 | Uncharacterized protein | Uncharacterized protein | | afdb-uniprot50 | AF-A0A7T8TA87-F1-MODEL\_V4 | 1.0 | 1.234e-05 | 170 | 0.123 | 211 | 130 | 14 | 15 | 189 | 5 | 196 | Uncharacterized protein | Uncharacterized protein | | afdb-uniprot50 | AF-B9Z305-F1-MODEL\_V4 | 1.0 | 3.941e-05 | 170 | 0.155 | 174 | 120 | 10 | 9 | 166 | 7 | 169 | Uncharacterized protein | Uncharacterized protein | | afdb-uniprot50 | AF-A0A6M8SQE1-F1-MODEL\_V4 | 1.0 | 0.0005379 | 169 | 0.132 | 158 | 113 | 8 | 9 | 165 | 4 | 138 | Uncharacterized protein | Uncharacterized protein | | afdb-uniprot50 | AF-A0A376YHH1-F1-MODEL\_V4 | 1.0 | 4.972e-05 | 169 | 0.131 | 152 | 120 | 5 | 7 | 155 | 1 | 143 | Phage-related protein Gp37-like protein | Phage-related protein Gp37-like protein | | afdb-uniprot50 | AF-C1D844-F1-MODEL\_V4 | 1.0 | 0.0003797 | 169 | 0.124 | 161 | 117 | 9 | 8 | 164 | 1 | 141 | Gp37 domain containing protein | Gp37 domain containing protein | | afdb-uniprot50 | AF-A0A763CFX9-F1-MODEL\_V4 | 1.0 | 0.0001589 | 169 | 0.162 | 179 | 124 | 8 | 7 | 177 | 1 | 161 | Uncharacterized protein | Uncharacterized protein | | afdb-uniprot50 | AF-A0A653JWK4-F1-MODEL\_V4 | 1.0 | 5.584e-05 | 169 | 0.164 | 194 | 125 | 10 | 12 | 179 | 5 | 187 | Phage gp37-like protein | Phage gp37-like protein | | afdb-uniprot50 | AF-A0A812IPU7-F1-MODEL\_V4 | 1.0 | 0.0001188 | 169 | 0.127 | 157 | 117 | 7 | 2 | 156 | 569 | 707 | Hypothetical protein | Hypothetical protein | | afdb-uniprot50 | AF-A0A6B0W596-F1-MODEL\_V4 | 1.0 | 0.000338 | 168 | 0.118 | 152 | 124 | 5 | 7 | 156 | 1 | 144 | Uncharacterized protein | Uncharacterized protein | | afdb-uniprot50 | AF-A0A7C6JL55-F1-MODEL\_V4 | 1.0 | 0.0001684 | 168 | 0.16 | 156 | 101 | 8 | 10 | 152 | 2 | 140 | Uncharacterized protein | Uncharacterized protein | | afdb-uniprot50 | AF-A0A7C9K240-F1-MODEL\_V4 | 1.0 | 2.948e-05 | 168 | 0.105 | 208 | 148 | 13 | 1 | 186 | 4 | 195 | Uncharacterized protein | Uncharacterized protein | | afdb-uniprot50 | AF-A0A2X2DE00-F1-MODEL\_V4 | 1.0 | 0.0001259 | 168 | 0.125 | 184 | 131 | 10 | 8 | 176 | 1 | 169 | Mu-like prophage protein gp37 | Mu-like prophage protein gp37 | | afdb-uniprot50 | AF-A0A7T9FPJ0-F1-MODEL\_V4 | 1.0 | 3.311e-05 | 168 | 0.162 | 191 | 128 | 11 | 1 | 165 | 1 | 185 | DUF1834 family protein | DUF1834 family protein | | afdb-uniprot50 | AF-A0A221KCS7-F1-MODEL\_V4 | 1.0 | 0.0002528 | 167 | 0.166 | 156 | 104 | 12 | 8 | 153 | 13 | 152 | Uncharacterized protein | Uncharacterized protein | | afdb-uniprot50 | AF-A0A4R2GW33-F1-MODEL\_V4 | 1.0 | 0.0002251 | 167 | 0.109 | 165 | 128 | 8 | 2 | 163 | 16 | 164 | Gp37 protein | Gp37 protein | | afdb-uniprot50 | AF-Q126A0-F1-MODEL\_V4 | 1.0 | 5.269e-05 | 167 | 0.138 | 195 | 133 | 12 | 7 | 182 | 1 | 179 | Uncharacterized protein | Uncharacterized protein | | afdb-uniprot50 | AF-A0A2E2ISM1-F1-MODEL\_V4 | 1.0 | 3.941e-05 | 167 | 0.188 | 196 | 121 | 13 | 12 | 182 | 5 | 187 | Uncharacterized protein | Uncharacterized protein | | afdb-uniprot50 | AF-A0A085ELB4-F1-MODEL\_V4 | 1.0 | 0.0002004 | 166 | 0.128 | 171 | 116 | 12 | 7 | 163 | 3 | 154 | Uncharacterized protein | Uncharacterized protein | | afdb-uniprot50 | AF-A0A840LZ35-F1-MODEL\_V4 | 1.0 | 8.386e-05 | 166 | 0.138 | 180 | 122 | 9 | 1 | 165 | 1 | 162 | Uncharacterized protein | Uncharacterized protein | | afdb-uniprot50 | AF-A0A3S0EG19-F1-MODEL\_V4 | 1.0 | 5.269e-05 | 166 | 0.146 | 177 | 122 | 9 | 7 | 166 | 1 | 165 | Uncharacterized protein | Uncharacterized protein | | afdb-uniprot50 | AF-A0A0L0GQ01-F1-MODEL\_V4 | 1.0 | 9.419e-05 | 166 | 0.124 | 225 | 137 | 12 | 8 | 189 | 1 | 208 | Uncharacterized protein | Uncharacterized protein | | afdb-uniprot50 | AF-A0A327J9N9-F1-MODEL\_V4 | 1.0 | 0.0007192 | 165 | 0.089 | 146 | 113 | 7 | 9 | 152 | 5 | 132 | Uncharacterized protein | Uncharacterized protein | | afdb-uniprot50 | AF-A0A0C1G7G9-F1-MODEL\_V4 | 1.0 | 5.584e-05 | 165 | 0.144 | 180 | 121 | 12 | 2 | 173 | 3 | 157 | Uncharacterized protein | Uncharacterized protein | | afdb-uniprot50 | AF-A0A2D3T3I7-F1-MODEL\_V4 | 1.0 | 7.466e-05 | 165 | 0.122 | 171 | 120 | 6 | 15 | 166 | 5 | 164 | Uncharacterized protein | Uncharacterized protein | | afdb-uniprot50 | AF-A0A2A2H0E7-F1-MODEL\_V4 | 1.0 | 4.972e-05 | 165 | 0.106 | 187 | 141 | 11 | 15 | 189 | 5 | 177 | Uncharacterized protein | Uncharacterized protein | | afdb-uniprot50 | AF-A0A3R6C1V5-F1-MODEL\_V4 | 1.0 | 0.0005379 | 164 | 0.09 | 154 | 107 | 7 | 18 | 156 | 2 | 137 | Uncharacterized protein | Uncharacterized protein | | afdb-uniprot50 | AF-A0A0F5PGA2-F1-MODEL\_V4 | 1.0 | 0.0001589 | 164 | 0.173 | 150 | 94 | 10 | 17 | 153 | 3 | 135 | Uncharacterized protein | Uncharacterized protein | | afdb-uniprot50 | AF-A0A2N8NK38-F1-MODEL\_V4 | 1.0 | 0.0001684 | 164 | 0.12 | 166 | 111 | 10 | 1 | 152 | 1 | 145 | Uncharacterized protein | Uncharacterized protein | | afdb-uniprot50 | AF-A0A7U6QP29-F1-MODEL\_V4 | 1.0 | 0.0002679 | 164 | 0.117 | 170 | 123 | 8 | 1 | 165 | 1 | 148 | Uncharacterized protein | Uncharacterized protein | | afdb-uniprot50 | AF-F3PKG9-F1-MODEL\_V4 | 1.0 | 0.0002251 | 164 | 0.151 | 165 | 109 | 10 | 8 | 157 | 1 | 149 | Uncharacterized protein | Uncharacterized protein | | afdb-uniprot50 | AF-A0A1M7RI50-F1-MODEL\_V4 | 1.0 | 0.0001414 | 164 | 0.151 | 165 | 121 | 7 | 7 | 166 | 1 | 151 | Gp37 protein | Gp37 protein | | afdb-uniprot50 | AF-A0A225E4L8-F1-MODEL\_V4 | 1.0 | 5.584e-05 | 164 | 0.156 | 179 | 120 | 14 | 6 | 163 | 7 | 175 | Uncharacterized protein | Uncharacterized protein | | afdb-uniprot50 | AF-D2TJ30-F1-MODEL\_V4 | 1.0 | 7.913e-05 | 164 | 0.11 | 209 | 131 | 11 | 8 | 174 | 1 | 196 | Hypothetical prophage protein | Hypothetical prophage protein | | afdb-uniprot50 | AF-A0A0G0CA56-F1-MODEL\_V4 | 1.0 | 0.001213 | 163 | 0.117 | 153 | 116 | 7 | 7 | 157 | 1 | 136 | Uncharacterized protein | Uncharacterized protein | | afdb-uniprot50 | AF-A0A509KZR1-F1-MODEL\_V4 | 1.0 | 0.0003009 | 163 | 0.092 | 152 | 112 | 7 | 13 | 157 | 9 | 141 | Uncharacterized protein | Uncharacterized protein | | afdb-uniprot50 | AF-L1P217-F1-MODEL\_V4 | 1.0 | 0.0001414 | 163 | 0.12 | 182 | 134 | 8 | 1 | 179 | 1 | 159 | Gp37 protein | Gp37 protein | | afdb-uniprot50 | AF-A0A4Q3RNJ9-F1-MODEL\_V4 | 1.0 | 9.419e-05 | 163 | 0.127 | 181 | 130 | 9 | 7 | 174 | 2 | 167 | Uncharacterized protein | Uncharacterized protein | | afdb-uniprot50 | AF-A0A5C9D9K9-F1-MODEL\_V4 | 1.0 | 0.0001891 | 163 | 0.151 | 172 | 117 | 12 | 9 | 164 | 14 | 172 | Uncharacterized protein | Uncharacterized protein | | afdb-uniprot50 | AF-A0A6A2FMX2-F1-MODEL\_V4 | 1.0 | 8.386e-05 | 163 | 0.142 | 182 | 118 | 9 | 7 | 166 | 2 | 167 | Uncharacterized protein | Uncharacterized protein | | afdb-uniprot50 | AF-A0A837QHE5-F1-MODEL\_V4 | 1.0 | 3.124e-05 | 163 | 0.117 | 178 | 135 | 7 | 25 | 187 | 7 | 177 | Uncharacterized protein | Uncharacterized protein | | afdb-uniprot50 | AF-A0A2V3UBZ9-F1-MODEL\_V4 | 1.0 | 5.918e-05 | 163 | 0.164 | 182 | 125 | 12 | 8 | 179 | 3 | 167 | Uncharacterized protein DUF1834 | Uncharacterized protein DUF1834 | | afdb-uniprot50 | AF-A0A437T2J4-F1-MODEL\_V4 | 1.0 | 3.311e-05 | 163 | 0.105 | 200 | 136 | 9 | 15 | 189 | 5 | 186 | Uncharacterized protein | Uncharacterized protein | | afdb-uniprot50 | AF-A0A7C4DI82-F1-MODEL\_V4 | 1.0 | 0.001286 | 162 | 0.093 | 150 | 117 | 7 | 8 | 154 | 1 | 134 | DUF1834 family protein | DUF1834 family protein | | afdb-uniprot50 | AF-A0A2D5PCB8-F1-MODEL\_V4 | 1.0 | 0.0001891 | 162 | 0.132 | 158 | 111 | 11 | 7 | 159 | 2 | 138 | Uncharacterized protein | Uncharacterized protein | | afdb-uniprot50 | AF-A0A3C0TZ33-F1-MODEL\_V4 | 1.0 | 0.0003009 | 162 | 0.136 | 147 | 110 | 7 | 15 | 153 | 5 | 142 | Uncharacterized protein | Uncharacterized protein | | afdb-uniprot50 | AF-A0A5P9CRF5-F1-MODEL\_V4 | 1.0 | 0.0001684 | 162 | 0.162 | 160 | 118 | 7 | 9 | 166 | 11 | 156 | Uncharacterized protein | Uncharacterized protein | | afdb-uniprot50 | AF-A0A011NCS5-F1-MODEL\_V4 | 1.0 | 4.972e-05 | 162 | 0.152 | 184 | 127 | 11 | 1 | 177 | 1 | 162 | Uncharacterized protein | Uncharacterized protein | | afdb-uniprot50 | AF-B3QTJ8-F1-MODEL\_V4 | 1.0 | 0.0001058 | 162 | 0.102 | 176 | 131 | 8 | 9 | 168 | 8 | 172 | Uncharacterized protein | Uncharacterized protein | | afdb-uniprot50 | AF-A0A0F9GHP4-F1-MODEL\_V4 | 1.0 | 0.0001414 | 162 | 0.116 | 163 | 125 | 8 | 1 | 154 | 20 | 172 | Uncharacterized protein | Uncharacterized protein | | afdb-uniprot50 | AF-A0A4V3AUT9-F1-MODEL\_V4 | 1.0 | 0.0002386 | 162 | 0.122 | 172 | 137 | 4 | 7 | 178 | 1 | 158 | Uncharacterized protein | Uncharacterized protein | | afdb-uniprot50 | AF-A0A1Y1Q6H6-F1-MODEL\_V4 | 1.0 | 7.466e-05 | 162 | 0.102 | 176 | 125 | 10 | 12 | 176 | 3 | 156 | Uncharacterized protein | Uncharacterized protein | | afdb-uniprot50 | AF-A0A7C1A4S1-F1-MODEL\_V4 | 1.0 | 0.000284 | 161 | 0.12 | 166 | 118 | 9 | 6 | 155 | 3 | 156 | Uncharacterized protein | Uncharacterized protein | | afdb-uniprot50 | AF-A0A7I6PS33-F1-MODEL\_V4 | 1.0 | 8.887e-05 | 161 | 0.137 | 167 | 122 | 10 | 14 | 166 | 4 | 162 | Uncharacterized protein | Uncharacterized protein | | afdb-uniprot50 | AF-A0A7Y7NQN0-F1-MODEL\_V4 | 1.0 | 0.0001335 | 160 | 0.14 | 150 | 111 | 8 | 8 | 148 | 1 | 141 | Uncharacterized protein | Uncharacterized protein | | afdb-uniprot50 | AF-A0A381E3W2-F1-MODEL\_V4 | 1.0 | 0.0005379 | 160 | 0.12 | 158 | 123 | 10 | 7 | 156 | 2 | 151 | Uncharacterized protein | Uncharacterized protein | | afdb-uniprot50 | AF-R6T089-F1-MODEL\_V4 | 1.0 | 0.0001589 | 160 | 0.134 | 171 | 103 | 10 | 10 | 154 | 2 | 153 | Uncharacterized protein | Uncharacterized protein | | afdb-uniprot50 | AF-A0A1C3HHN1-F1-MODEL\_V4 | 1.0 | 0.0002124 | 160 | 0.156 | 172 | 129 | 6 | 7 | 177 | 1 | 157 | Gp37 protein | Gp37 protein | | afdb-uniprot50 | AF-A0A515EKE4-F1-MODEL\_V4 | 1.0 | 3.941e-05 | 160 | 0.137 | 204 | 132 | 11 | 10 | 178 | 3 | 197 | Uncharacterized protein | Uncharacterized protein | | afdb-uniprot50 | AF-A0A845BQV7-F1-MODEL\_V4 | 1.0 | 0.0001891 | 159 | 0.15 | 153 | 110 | 6 | 7 | 156 | 3 | 138 | Uncharacterized protein | Uncharacterized protein | | afdb-uniprot50 | AF-A0A249DY56-F1-MODEL\_V4 | 1.0 | 0.0001335 | 159 | 0.146 | 157 | 106 | 7 | 15 | 159 | 5 | 145 | Uncharacterized protein | Uncharacterized protein | | afdb-uniprot50 | AF-U6Q2Q7-F1-MODEL\_V4 | 1.0 | 0.00108 | 159 | 0.168 | 160 | 114 | 11 | 7 | 156 | 1 | 151 | Uncharacterized protein | Uncharacterized protein | | afdb-uniprot50 | AF-A0A1N6M5S0-F1-MODEL\_V4 | 1.0 | 3.509e-05 | 159 | 0.128 | 194 | 136 | 12 | 1 | 173 | 3 | 184 | Uncharacterized protein | Uncharacterized protein | | afdb-uniprot50 | AF-A0A0Q8STR6-F1-MODEL\_V4 | 1.0 | 0.0005701 | 158 | 0.132 | 159 | 106 | 8 | 7 | 156 | 1 | 136 | Uncharacterized protein | Uncharacterized protein | | afdb-uniprot50 | AF-A0A0T5PNX3-F1-MODEL\_V4 | 1.0 | 0.00108 | 158 | 0.131 | 152 | 118 | 8 | 10 | 156 | 6 | 148 | Uncharacterized protein | Uncharacterized protein | | afdb-uniprot50 | AF-G2IX39-F1-MODEL\_V4 | 1.0 | 0.0002386 | 158 | 0.127 | 172 | 125 | 9 | 8 | 174 | 1 | 152 | Gp37 protein | Gp37 protein | | afdb-uniprot50 | AF-A0A2A2GYG9-F1-MODEL\_V4 | 1.0 | 0.0007192 | 157 | 0.138 | 152 | 105 | 8 | 8 | 153 | 1 | 132 | Uncharacterized protein | Uncharacterized protein | | afdb-uniprot50 | AF-A0A1G0MRP2-F1-MODEL\_V4 | 1.0 | 0.0003797 | 157 | 0.082 | 157 | 128 | 5 | 8 | 154 | 1 | 151 | Uncharacterized protein | Uncharacterized protein | | afdb-uniprot50 | AF-A0A1J0WHQ0-F1-MODEL\_V4 | 1.0 | 0.0002124 | 157 | 0.159 | 169 | 104 | 12 | 1 | 153 | 1 | 147 | Uncharacterized protein | Uncharacterized protein | | afdb-uniprot50 | AF-A0A178HLQ9-F1-MODEL\_V4 | 1.0 | 0.0001784 | 157 | 0.123 | 178 | 127 | 8 | 8 | 179 | 1 | 155 | Uncharacterized protein | Uncharacterized protein | | afdb-uniprot50 | AF-A0A3M1IM86-F1-MODEL\_V4 | 1.0 | 0.0002251 | 157 | 0.143 | 174 | 113 | 9 | 10 | 172 | 2 | 150 | Uncharacterized protein | Uncharacterized protein | | afdb-uniprot50 | AF-A0A0J7L1A0-F1-MODEL\_V4 | 1.0 | 0.0002528 | 157 | 0.105 | 189 | 131 | 10 | 4 | 166 | 2 | 178 | Uncharacterized protein | Uncharacterized protein | | afdb-uniprot50 | AF-A0A1G6LS75-F1-MODEL\_V4 | 1.0 | 0.0002386 | 157 | 0.089 | 179 | 142 | 4 | 1 | 166 | 1 | 171 | Uncharacterized protein | Uncharacterized protein | | afdb-uniprot50 | AF-A0A7W4IJG5-F1-MODEL\_V4 | 1.0 | 0.0001121 | 157 | 0.129 | 193 | 141 | 11 | 1 | 179 | 1 | 180 | Uncharacterized protein | Uncharacterized protein | | afdb-uniprot50 | AF-A0A2D6E5V7-F1-MODEL\_V4 | 1.0 | 4.972e-05 | 157 | 0.104 | 172 | 128 | 11 | 7 | 165 | 5 | 163 | Uncharacterized protein | Uncharacterized protein | | afdb-uniprot50 | AF-A0A1V5PMC3-F1-MODEL\_V4 | 1.0 | 0.00108 | 156 | 0.104 | 153 | 115 | 8 | 7 | 155 | 1 | 135 | Gp37 protein | Gp37 protein | | afdb-uniprot50 | AF-A0A4Q3M822-F1-MODEL\_V4 | 1.0 | 0.0001684 | 156 | 0.15 | 159 | 102 | 9 | 8 | 152 | 1 | 140 | Uncharacterized protein | Uncharacterized protein | | afdb-uniprot50 | AF-X2HAW8-F1-MODEL\_V4 | 1.0 | 0.0002124 | 156 | 0.093 | 171 | 127 | 10 | 1 | 166 | 1 | 148 | Uncharacterized protein | Uncharacterized protein | | afdb-uniprot50 | AF-A0A4Y9VRF7-F1-MODEL\_V4 | 1.0 | 6.272e-05 | 156 | 0.133 | 203 | 133 | 13 | 13 | 189 | 2 | 187 | Uncharacterized protein | Uncharacterized protein | | afdb-uniprot50 | AF-A0A2S5TZJ7-F1-MODEL\_V4 | 1.0 | 0.0001589 | 156 | 0.132 | 204 | 143 | 13 | 1 | 189 | 1 | 185 | Uncharacterized protein | Uncharacterized protein | | afdb-uniprot50 | AF-A0A826W9J1-F1-MODEL\_V4 | 1.0 | 0.0002386 | 156 | 0.142 | 169 | 111 | 11 | 8 | 155 | 1 | 156 | DUF1834 family protein | DUF1834 family protein | | afdb-uniprot50 | AF-A0A545T5R9-F1-MODEL\_V4 | 1.0 | 0.0001589 | 156 | 0.122 | 221 | 141 | 9 | 1 | 182 | 1 | 207 | Uncharacterized protein | Uncharacterized protein | | afdb-uniprot50 | AF-A0A7V3JM37-F1-MODEL\_V4 | 1.0 | 0.0004519 | 155 | 0.12 | 158 | 114 | 10 | 8 | 155 | 1 | 143 | Uncharacterized protein | Uncharacterized protein | | afdb-uniprot50 | AF-A0A2W0B4M6-F1-MODEL\_V4 | 1.0 | 0.0009616 | 155 | 0.131 | 152 | 115 | 8 | 8 | 151 | 4 | 146 | Uncharacterized protein | Uncharacterized protein | | afdb-uniprot50 | AF-A0A411WIP5-F1-MODEL\_V4 | 1.0 | 0.0006786 | 155 | 0.118 | 160 | 123 | 6 | 7 | 165 | 4 | 146 | Uncharacterized protein | Uncharacterized protein | | afdb-uniprot50 | AF-A0A380MYP2-F1-MODEL\_V4 | 1.0 | 0.001444 | 155 | 0.1 | 160 | 126 | 9 | 8 | 156 | 3 | 155 | Uncharacterized protein | Uncharacterized protein | | afdb-uniprot50 | AF-A0A0Q3ETN6-F1-MODEL\_V4 | 1.0 | 9.982e-05 | 155 | 0.138 | 180 | 120 | 9 | 2 | 166 | 1 | 160 | Uncharacterized protein | Uncharacterized protein | | afdb-uniprot50 | AF-A0A7K1U6C2-F1-MODEL\_V4 | 1.0 | 0.000338 | 155 | 0.127 | 173 | 124 | 9 | 9 | 168 | 5 | 163 | Uncharacterized protein | Uncharacterized protein | | afdb-uniprot50 | AF-A0A2N1XYT5-F1-MODEL\_V4 | 1.0 | 9.982e-05 | 155 | 0.109 | 191 | 129 | 13 | 7 | 177 | 1 | 170 | Uncharacterized protein | Uncharacterized protein | | afdb-uniprot50 | AF-A0A3G9GEK2-F1-MODEL\_V4 | 1.0 | 9.982e-05 | 155 | 0.139 | 229 | 128 | 15 | 12 | 188 | 6 | 217 | Mu-like prophage FluMu protein gp37 | Mu-like prophage FluMu protein gp37 | | afdb-uniprot50 | AF-A0A2N9YI19-F1-MODEL\_V4 | 1.0 | 0.0008078 | 155 | 0.153 | 163 | 118 | 9 | 1 | 156 | 147 | 296 | Uncharacterized protein | Uncharacterized protein | | afdb-uniprot50 | AF-A0A661DU83-F1-MODEL\_V4 | 1.0 | 0.0004789 | 154 | 0.107 | 149 | 119 | 6 | 7 | 155 | 1 | 135 | Uncharacterized protein | Uncharacterized protein | | afdb-uniprot50 | AF-A0A2E4SSH5-F1-MODEL\_V4 | 1.0 | 0.0004789 | 154 | 0.101 | 157 | 119 | 7 | 6 | 154 | 2 | 144 | Uncharacterized protein | Uncharacterized protein | | afdb-uniprot50 | AF-A0A0R2LKL6-F1-MODEL\_V4 | 1.0 | 0.0005701 | 154 | 0.12 | 166 | 120 | 13 | 6 | 165 | 2 | 147 | Uncharacterized protein | Uncharacterized protein | | afdb-uniprot50 | AF-A0A2J9QIZ5-F1-MODEL\_V4 | 1.0 | 0.0003582 | 154 | 0.115 | 173 | 122 | 11 | 1 | 166 | 1 | 149 | Uncharacterized protein | Uncharacterized protein | | afdb-uniprot50 | AF-A5EUZ0-F1-MODEL\_V4 | 1.0 | 0.0002528 | 154 | 0.167 | 161 | 113 | 11 | 7 | 157 | 6 | 155 | Uncharacterized protein | Uncharacterized protein | | afdb-uniprot50 | AF-A0A4R6U5E4-F1-MODEL\_V4 | 1.0 | 6.647e-05 | 154 | 0.143 | 174 | 127 | 8 | 7 | 174 | 2 | 159 | Gp37 protein | Gp37 protein | | afdb-uniprot50 | AF-A0A4R8IH74-F1-MODEL\_V4 | 1.0 | 0.000284 | 154 | 0.089 | 190 | 133 | 9 | 7 | 178 | 3 | 170 | Uncharacterized protein | Uncharacterized protein | | afdb-uniprot50 | AF-A0A3B0JB18-F1-MODEL\_V4 | 1.0 | 7.466e-05 | 154 | 0.096 | 176 | 139 | 6 | 22 | 188 | 9 | 173 | Uncharacterized protein | Uncharacterized protein | | afdb-uniprot50 | AF-Q602Y7-F1-MODEL\_V4 | 1.0 | 0.0001414 | 154 | 0.189 | 174 | 117 | 9 | 1 | 170 | 1 | 154 | Uncharacterized protein | Uncharacterized protein | | afdb-uniprot50 | AF-A0A6M8HNB9-F1-MODEL\_V4 | 1.0 | 0.0001058 | 154 | 0.146 | 198 | 126 | 14 | 7 | 188 | 1 | 171 | Uncharacterized protein | Uncharacterized protein | | afdb-uniprot50 | AF-A0A1X0TIU2-F1-MODEL\_V4 | 1.0 | 0.0002251 | 154 | 0.081 | 172 | 134 | 9 | 1 | 166 | 31 | 184 | Uncharacterized protein | Uncharacterized protein | | afdb-uniprot50 | AF-A0A0A4ABV7-F1-MODEL\_V4 | 1.0 | 0.0002124 | 154 | 0.137 | 182 | 119 | 10 | 8 | 166 | 1 | 167 | Uncharacterized protein | Uncharacterized protein | | afdb-uniprot50 | AF-A0A518HBN8-F1-MODEL\_V4 | 1.0 | 0.0003009 | 154 | 0.141 | 177 | 135 | 10 | 7 | 178 | 3 | 167 | Uncharacterized protein | Uncharacterized protein | | afdb-uniprot50 | AF-A0A1E4AHP0-F1-MODEL\_V4 | 1.0 | 0.0001784 | 153 | 0.152 | 170 | 118 | 9 | 10 | 171 | 4 | 155 | Uncharacterized protein | Uncharacterized protein | | afdb-uniprot50 | AF-A0A166FAH8-F1-MODEL\_V4 | 1.0 | 0.0003797 | 153 | 0.117 | 170 | 123 | 7 | 9 | 166 | 9 | 163 | Uncharacterized protein | Uncharacterized protein | | afdb-uniprot50 | AF-E2CGF9-F1-MODEL\_V4 | 1.0 | 8.887e-05 | 153 | 0.109 | 183 | 134 | 10 | 3 | 177 | 14 | 175 | Uncharacterized protein | Uncharacterized protein | | afdb-uniprot50 | AF-A0A857FM52-F1-MODEL\_V4 | 1.0 | 7.045e-05 | 153 | 0.139 | 193 | 134 | 12 | 7 | 188 | 1 | 172 | Uncharacterized protein | Uncharacterized protein | | afdb-uniprot50 | AF-A0A420WGS9-F1-MODEL\_V4 | 1.0 | 0.0003582 | 152 | 0.111 | 153 | 118 | 6 | 7 | 157 | 1 | 137 | Uncharacterized protein DUF4128 | Uncharacterized protein DUF4128 | | afdb-uniprot50 | AF-A0A5C7P964-F1-MODEL\_V4 | 1.0 | 0.0008078 | 152 | 0.114 | 157 | 114 | 8 | 9 | 155 | 5 | 146 | Uncharacterized protein | Uncharacterized protein | | afdb-uniprot50 | AF-A0A1B1G143-F1-MODEL\_V4 | 1.0 | 0.0004789 | 152 | 0.109 | 173 | 111 | 11 | 10 | 165 | 2 | 148 | Uncharacterized protein | Uncharacterized protein | | afdb-uniprot50 | AF-A0A1C3FDG3-F1-MODEL\_V4 | 1.0 | 0.0006403 | 152 | 0.126 | 174 | 133 | 6 | 7 | 178 | 1 | 157 | Uncharacterized protein | Uncharacterized protein | | afdb-uniprot50 | AF-A0A1Q3Q8M2-F1-MODEL\_V4 | 1.0 | 0.0001121 | 152 | 0.17 | 199 | 121 | 8 | 1 | 185 | 1 | 169 | Uncharacterized protein | Uncharacterized protein | | afdb-uniprot50 | AF-A0A1G7SE70-F1-MODEL\_V4 | 1.0 | 6.272e-05 | 152 | 0.183 | 191 | 119 | 12 | 8 | 179 | 1 | 173 | Uncharacterized protein | Uncharacterized protein | | afdb-uniprot50 | AF-A0A2D3TE26-F1-MODEL\_V4 | 1.0 | 0.0001058 | 152 | 0.145 | 185 | 126 | 7 | 15 | 178 | 5 | 178 | Uncharacterized protein | Uncharacterized protein | | afdb-uniprot50 | AF-A0A0J6T1Z2-F1-MODEL\_V4 | 1.0 | 0.0008078 | 151 | 0.178 | 151 | 97 | 9 | 14 | 153 | 4 | 138 | Uncharacterized protein | Uncharacterized protein | | afdb-uniprot50 | AF-A0A5U3CNK9-F1-MODEL\_V4 | 1.0 | 0.0007622 | 151 | 0.142 | 161 | 117 | 9 | 7 | 163 | 1 | 144 | Uncharacterized protein | Uncharacterized protein | | afdb-uniprot50 | AF-A0A2J8HSC1-F1-MODEL\_V4 | 1.0 | 0.0004519 | 151 | 0.106 | 178 | 125 | 11 | 3 | 166 | 5 | 162 | Uncharacterized protein | Uncharacterized protein | | afdb-uniprot50 | AF-A0A2A2HG93-F1-MODEL\_V4 | 1.0 | 0.0004519 | 151 | 0.149 | 161 | 116 | 9 | 8 | 164 | 1 | 144 | Uncharacterized protein | Uncharacterized protein | | afdb-uniprot50 | AF-A0A444HEH6-F1-MODEL\_V4 | 1.0 | 0.0004519 | 151 | 0.13 | 184 | 127 | 9 | 9 | 178 | 4 | 168 | Uncharacterized protein | Uncharacterized protein | | afdb-uniprot50 | AF-A0A5B7ZK92-F1-MODEL\_V4 | 1.0 | 6.647e-05 | 151 | 0.135 | 199 | 138 | 10 | 7 | 188 | 1 | 182 | Uncharacterized protein | Uncharacterized protein | | afdb-uniprot50 | AF-A0A7Y1CQT2-F1-MODEL\_V4 | 1.0 | 5.584e-05 | 151 | 0.123 | 211 | 145 | 9 | 7 | 189 | 1 | 199 | Uncharacterized protein | Uncharacterized protein | | afdb-uniprot50 | AF-A0A1E3WMG0-F1-MODEL\_V4 | 1.0 | 8.386e-05 | 151 | 0.1 | 209 | 147 | 13 | 1 | 187 | 11 | 200 | Uncharacterized protein | Uncharacterized protein | | afdb-uniprot50 | AF-A0A1Q4T414-F1-MODEL\_V4 | 1.0 | 0.00153 | 150 | 0.125 | 159 | 114 | 10 | 1 | 156 | 3 | 139 | Uncharacterized protein | Uncharacterized protein | | afdb-uniprot50 | AF-A0A1G1N144-F1-MODEL\_V4 | 1.0 | 0.0004789 | 150 | 0.141 | 156 | 106 | 13 | 8 | 151 | 1 | 140 | Uncharacterized protein | Uncharacterized protein | | afdb-uniprot50 | AF-A0A3C2D818-F1-MODEL\_V4 | 1.0 | 0.000338 | 150 | 0.119 | 167 | 108 | 12 | 8 | 158 | 1 | 144 | Uncharacterized protein | Uncharacterized protein | | afdb-uniprot50 | AF-A0A4R1C4H1-F1-MODEL\_V4 | 1.0 | 0.001213 | 150 | 0.105 | 152 | 120 | 7 | 7 | 152 | 2 | 143 | Uncharacterized protein | Uncharacterized protein | | afdb-uniprot50 | AF-A0A075WUA5-F1-MODEL\_V4 | 1.0 | 0.00153 | 150 | 0.098 | 162 | 123 | 8 | 8 | 166 | 1 | 142 | Uncharacterized protein | Uncharacterized protein | | afdb-uniprot50 | AF-A0A806Z7M4-F1-MODEL\_V4 | 1.0 | 0.0001684 | 150 | 0.156 | 160 | 106 | 9 | 27 | 168 | 3 | 151 | Uncharacterized protein | Uncharacterized protein | | afdb-uniprot50 | AF-A0A2N7L8B1-F1-MODEL\_V4 | 1.0 | 0.0001335 | 150 | 0.128 | 194 | 139 | 12 | 1 | 174 | 1 | 184 | Uncharacterized protein | Uncharacterized protein | | afdb-uniprot50 | AF-A0A4P2QPQ9-F1-MODEL\_V4 | 1.0 | 0.0005701 | 150 | 0.148 | 148 | 104 | 9 | 27 | 166 | 124 | 257 | Uncharacterized protein | Uncharacterized protein | | afdb-uniprot50 | AF-A0A1Q3WT67-F1-MODEL\_V4 | 1.0 | 0.0006042 | 149 | 0.1 | 180 | 128 | 11 | 6 | 166 | 2 | 166 | Uncharacterized protein | Uncharacterized protein | | afdb-uniprot50 | AF-A0A0H3ZYQ3-F1-MODEL\_V4 | 1.0 | 0.0002528 | 149 | 0.094 | 190 | 139 | 11 | 8 | 187 | 6 | 172 | Uncharacterized protein | Uncharacterized protein | | afdb-uniprot50 | AF-B6WSR7-F1-MODEL\_V4 | 1.0 | 2.948e-05 | 149 | 0.164 | 201 | 133 | 11 | 10 | 189 | 5 | 191 | Uncharacterized protein | Uncharacterized protein | | afdb-uniprot50 | AF-A0A7U2KPY8-F1-MODEL\_V4 | 1.0 | 0.00153 | 148 | 0.102 | 156 | 111 | 7 | 7 | 154 | 1 | 135 | Uncharacterized protein | Uncharacterized protein | | afdb-uniprot50 | AF-A0A1G7TC54-F1-MODEL\_V4 | 1.0 | 0.0004024 | 148 | 0.095 | 157 | 101 | 11 | 18 | 156 | 9 | 142 | Uncharacterized protein | Uncharacterized protein | | afdb-uniprot50 | AF-A0A365QUT4-F1-MODEL\_V4 | 1.0 | 0.001444 | 148 | 0.185 | 156 | 106 | 12 | 7 | 152 | 3 | 147 | Uncharacterized protein | Uncharacterized protein | | afdb-uniprot50 | AF-A0A2D7VXQ5-F1-MODEL\_V4 | 1.0 | 0.003451 | 148 | 0.111 | 153 | 124 | 6 | 5 | 153 | 2 | 146 | Uncharacterized protein | Uncharacterized protein | | afdb-uniprot50 | AF-A0A5C7Q4P6-F1-MODEL\_V4 | 1.0 | 0.0006042 | 148 | 0.186 | 161 | 112 | 8 | 10 | 162 | 5 | 154 | Uncharacterized protein | Uncharacterized protein | | afdb-uniprot50 | AF-A0A7Y4DJI8-F1-MODEL\_V4 | 1.0 | 0.0003189 | 148 | 0.094 | 201 | 137 | 16 | 5 | 189 | 3 | 174 | Uncharacterized protein | Uncharacterized protein | | afdb-uniprot50 | AF-A0A377HPJ9-F1-MODEL\_V4 | 1.0 | 7.466e-05 | 148 | 0.135 | 206 | 143 | 12 | 1 | 187 | 1 | 190 | Uncharacterized protein | Uncharacterized protein | | afdb-uniprot50 | AF-A0A4V2W9H1-F1-MODEL\_V4 | 1.0 | 0.0009073 | 147 | 0.107 | 149 | 117 | 6 | 8 | 155 | 3 | 136 | Uncharacterized protein | Uncharacterized protein | | afdb-uniprot50 | AF-A0A5D9DCM1-F1-MODEL\_V4 | 1.0 | 0.0006403 | 147 | 0.137 | 153 | 114 | 6 | 7 | 156 | 2 | 139 | Uncharacterized protein | Uncharacterized protein | | afdb-uniprot50 | AF-A0A1G6A6F1-F1-MODEL\_V4 | 1.0 | 0.004353 | 147 | 0.067 | 148 | 122 | 5 | 10 | 153 | 5 | 140 | Gp37 protein | Gp37 protein | | afdb-uniprot50 | AF-T0YPM2-F1-MODEL\_V4 | 1.0 | 0.00153 | 147 | 0.114 | 157 | 120 | 9 | 6 | 154 | 3 | 148 | Phage associated protein | Phage associated protein | | afdb-uniprot50 | AF-A0A1U7GIF2-F1-MODEL\_V4 | 1.0 | 0.00153 | 147 | 0.122 | 163 | 120 | 10 | 9 | 165 | 6 | 151 | Uncharacterized protein | Uncharacterized protein | | afdb-uniprot50 | AF-A0A2G5KHB3-F1-MODEL\_V4 | 1.0 | 0.0005701 | 147 | 0.138 | 173 | 114 | 12 | 7 | 166 | 1 | 151 | Uncharacterized protein | Uncharacterized protein | | afdb-uniprot50 | AF-A0A2D6EYR9-F1-MODEL\_V4 | 1.0 | 0.0007192 | 147 | 0.13 | 168 | 124 | 6 | 5 | 154 | 3 | 166 | Uncharacterized protein | Uncharacterized protein | | afdb-uniprot50 | AF-A0A7W4PA56-F1-MODEL\_V4 | 1.0 | 0.0002528 | 147 | 0.122 | 179 | 134 | 10 | 13 | 179 | 8 | 175 | Uncharacterized protein | Uncharacterized protein | | afdb-uniprot50 | AF-A0A1J0EKE0-F1-MODEL\_V4 | 1.0 | 0.0001414 | 147 | 0.153 | 182 | 125 | 14 | 8 | 174 | 1 | 168 | Uncharacterized protein | Uncharacterized protein | | afdb-uniprot50 | AF-X5MMY5-F1-MODEL\_V4 | 1.0 | 0.001444 | 146 | 0.131 | 152 | 108 | 8 | 8 | 152 | 6 | 140 | Uncharacterized protein | Uncharacterized protein | | afdb-uniprot50 | AF-A0A1Y3QT12-F1-MODEL\_V4 | 1.0 | 0.0006042 | 146 | 0.124 | 161 | 119 | 8 | 7 | 155 | 5 | 155 | Uncharacterized protein | Uncharacterized protein | | afdb-uniprot50 | AF-A0A2R4VQW9-F1-MODEL\_V4 | 1.0 | 0.00153 | 146 | 0.136 | 176 | 122 | 10 | 1 | 165 | 1 | 157 | Uncharacterized protein | Uncharacterized protein | | afdb-uniprot50 | AF-A0A318NGV1-F1-MODEL\_V4 | 1.0 | 0.0006042 | 146 | 0.112 | 177 | 112 | 10 | 8 | 165 | 1 | 151 | Uncharacterized protein | Uncharacterized protein | | afdb-uniprot50 | AF-A0A4S2GWA7-F1-MODEL\_V4 | 1.0 | 0.002298 | 145 | 0.163 | 153 | 104 | 9 | 9 | 154 | 11 | 146 | DUF3168 domain-containing protein | DUF3168 domain-containing protein | | afdb-uniprot50 | AF-A0A840EF97-F1-MODEL\_V4 | 1.0 | 0.001213 | 145 | 0.096 | 176 | 120 | 11 | 1 | 166 | 1 | 147 | Uncharacterized protein | Uncharacterized protein | | afdb-uniprot50 | AF-A0A4P7L3J1-F1-MODEL\_V4 | 1.0 | 0.0001259 | 145 | 0.119 | 176 | 125 | 9 | 7 | 166 | 1 | 162 | Uncharacterized protein | Uncharacterized protein | | afdb-uniprot50 | AF-A0A1D9BCM1-F1-MODEL\_V4 | 1.0 | 0.0001058 | 145 | 0.129 | 193 | 129 | 12 | 15 | 184 | 5 | 181 | Uncharacterized protein | Uncharacterized protein | | afdb-uniprot50 | AF-A0A7C5WMU7-F1-MODEL\_V4 | 1.0 | 0.006168 | 144 | 0.097 | 154 | 120 | 7 | 7 | 155 | 2 | 141 | Uncharacterized protein | Uncharacterized protein | | afdb-uniprot50 | AF-A0A4R1PSF6-F1-MODEL\_V4 | 1.0 | 0.00153 | 144 | 0.132 | 159 | 107 | 10 | 7 | 152 | 1 | 141 | Uncharacterized protein | Uncharacterized protein | | afdb-uniprot50 | AF-A0A1T2L9Q7-F1-MODEL\_V4 | 1.0 | 0.001444 | 144 | 0.116 | 154 | 111 | 10 | 13 | 154 | 4 | 144 | Uncharacterized protein | Uncharacterized protein | | afdb-uniprot50 | AF-A0A5S9PHL1-F1-MODEL\_V4 | 1.0 | 0.001286 | 144 | 0.115 | 156 | 114 | 7 | 12 | 156 | 2 | 144 | Uncharacterized protein | Uncharacterized protein | | afdb-uniprot50 | AF-A0A7W6P0F7-F1-MODEL\_V4 | 1.0 | 0.0008078 | 144 | 0.116 | 163 | 133 | 6 | 7 | 164 | 1 | 157 | Uncharacterized protein | Uncharacterized protein | | afdb-uniprot50 | AF-L7U9W8-F1-MODEL\_V4 | 1.0 | 0.0008078 | 144 | 0.141 | 177 | 125 | 12 | 10 | 176 | 5 | 164 | Uncharacterized protein | Uncharacterized protein | | afdb-uniprot50 | AF-A0A2E3P790-F1-MODEL\_V4 | 1.0 | 0.0002386 | 144 | 0.162 | 203 | 129 | 14 | 8 | 185 | 1 | 187 | Uncharacterized protein | Uncharacterized protein | | afdb-uniprot50 | AF-A0A0D8CN10-F1-MODEL\_V4 | 1.0 | 0.0001589 | 144 | 0.136 | 205 | 143 | 11 | 6 | 188 | 2 | 194 | Uncharacterized protein | Uncharacterized protein | | afdb-uniprot50 | AF-G6XIS4-F1-MODEL\_V4 | 1.0 | 0.0001499 | 144 | 0.12 | 183 | 127 | 12 | 7 | 173 | 1 | 165 | Uncharacterized protein | Uncharacterized protein | | afdb-uniprot50 | AF-A0A5D0CT21-F1-MODEL\_V4 | 1.0 | 0.0006403 | 144 | 0.164 | 146 | 109 | 6 | 8 | 150 | 1 | 136 | Uncharacterized protein | Uncharacterized protein | | afdb-uniprot50 | AF-A0A6M3IP32-F1-MODEL\_V4 | 1.0 | 0.000338 | 143 | 0.131 | 190 | 143 | 11 | 1 | 179 | 1 | 179 | Uncharacterized protein | Uncharacterized protein | | afdb-uniprot50 | AF-D3A6M2-F1-MODEL\_V4 | 1.0 | 0.0006786 | 143 | 0.15 | 173 | 114 | 11 | 8 | 161 | 1 | 159 | Uncharacterized protein | Uncharacterized protein | | afdb-uniprot50 | AF-A0A2E9DC73-F1-MODEL\_V4 | 1.0 | 0.0009616 | 142 | 0.133 | 157 | 107 | 11 | 7 | 156 | 4 | 138 | Uncharacterized protein | Uncharacterized protein | | afdb-uniprot50 | AF-A0A644VLE7-F1-MODEL\_V4 | 1.0 | 0.001622 | 142 | 0.099 | 161 | 115 | 8 | 10 | 154 | 4 | 150 | Uncharacterized protein | Uncharacterized protein | | afdb-uniprot50 | AF-A0A096AIF8-F1-MODEL\_V4 | 1.0 | 0.001822 | 142 | 0.118 | 169 | 131 | 7 | 7 | 173 | 1 | 153 | Uncharacterized protein | Uncharacterized protein | | afdb-uniprot50 | AF-A0A223D2E0-F1-MODEL\_V4 | 1.0 | 0.0003189 | 142 | 0.112 | 187 | 133 | 14 | 5 | 166 | 2 | 180 | Uncharacterized protein | Uncharacterized protein | | afdb-uniprot50 | AF-A0A2S7SPC8-F1-MODEL\_V4 | 1.0 | 0.0001684 | 142 | 0.118 | 194 | 133 | 10 | 2 | 178 | 23 | 195 | Uncharacterized protein | Uncharacterized protein | | afdb-uniprot50 | AF-A0A7X6EJH3-F1-MODEL\_V4 | 1.0 | 6.647e-05 | 142 | 0.141 | 212 | 131 | 13 | 15 | 189 | 5 | 202 | Uncharacterized protein | Uncharacterized protein | | afdb-uniprot50 | AF-H0EA80-F1-MODEL\_V4 | 1.0 | 0.0001589 | 142 | 0.139 | 215 | 136 | 11 | 1 | 182 | 1 | 199 | Uncharacterized protein | Uncharacterized protein | | afdb-uniprot50 | AF-A0A5A9EMM4-F1-MODEL\_V4 | 1.0 | 0.0003797 | 142 | 0.147 | 190 | 136 | 11 | 1 | 178 | 1 | 176 | Uncharacterized protein | Uncharacterized protein | | afdb-uniprot50 | AF-A0A2D6AI45-F1-MODEL\_V4 | 1.0 | 0.004613 | 141 | 0.098 | 162 | 121 | 11 | 1 | 156 | 1 | 143 | Uncharacterized protein | Uncharacterized protein | | afdb-uniprot50 | AF-A0A521BNQ1-F1-MODEL\_V4 | 1.0 | 0.000338 | 141 | 0.131 | 167 | 99 | 12 | 9 | 157 | 6 | 144 | Uncharacterized protein | Uncharacterized protein | | afdb-uniprot50 | AF-C9MV92-F1-MODEL\_V4 | 1.0 | 0.0002679 | 140 | 0.125 | 167 | 114 | 11 | 10 | 158 | 2 | 154 | Uncharacterized protein | Uncharacterized protein | | afdb-uniprot50 | AF-A0A5C7QCW3-F1-MODEL\_V4 | 1.0 | 0.001719 | 140 | 0.134 | 171 | 121 | 10 | 9 | 164 | 5 | 163 | Uncharacterized protein | Uncharacterized protein | | afdb-uniprot50 | AF-A0A7D4FTT1-F1-MODEL\_V4 | 1.0 | 0.001286 | 139 | 0.089 | 179 | 117 | 11 | 10 | 166 | 2 | 156 | Uncharacterized protein | Uncharacterized protein | | afdb-uniprot50 | AF-A0A1D8UTD4-F1-MODEL\_V4 | 1.0 | 0.0002528 | 139 | 0.128 | 195 | 136 | 12 | 7 | 187 | 1 | 175 | Uncharacterized protein | Uncharacterized protein | | afdb-uniprot50 | AF-A0A1S1TJW6-F1-MODEL\_V4 | 1.0 | 5.584e-05 | 139 | 0.162 | 209 | 127 | 12 | 1 | 189 | 1 | 181 | Uncharacterized protein | Uncharacterized protein | | afdb-uniprot50 | AF-A0A644WAV0-F1-MODEL\_V4 | 1.0 | 0.0002124 | 139 | 0.149 | 201 | 122 | 13 | 8 | 189 | 1 | 171 | Uncharacterized protein | Uncharacterized protein | | afdb-uniprot50 | AF-A0A0N9Y5D3-F1-MODEL\_V4 | 1.0 | 0.002581 | 138 | 0.118 | 144 | 102 | 7 | 12 | 153 | 3 | 123 | Uncharacterized protein | Uncharacterized protein | | afdb-uniprot50 | AF-A0A3D5AF28-F1-MODEL\_V4 | 1.0 | 0.0008078 | 138 | 0.094 | 159 | 113 | 10 | 12 | 157 | 2 | 142 | Uncharacterized protein | Uncharacterized protein | | afdb-uniprot50 | AF-A0A1F8X037-F1-MODEL\_V4 | 1.0 | 0.003657 | 138 | 0.12 | 158 | 113 | 9 | 7 | 154 | 1 | 142 | Uncharacterized protein | Uncharacterized protein | | afdb-uniprot50 | AF-A0A7K3ZIE1-F1-MODEL\_V4 | 1.0 | 0.0008078 | 138 | 0.105 | 170 | 115 | 10 | 7 | 165 | 2 | 145 | Uncharacterized protein | Uncharacterized protein | | afdb-uniprot50 | AF-A0A1V4QIE0-F1-MODEL\_V4 | 1.0 | 0.0008561 | 138 | 0.129 | 154 | 116 | 4 | 7 | 153 | 1 | 143 | Uncharacterized protein | Uncharacterized protein | | afdb-uniprot50 | AF-A0A428MSH7-F1-MODEL\_V4 | 1.0 | 0.0004519 | 138 | 0.104 | 173 | 131 | 12 | 2 | 166 | 4 | 160 | Uncharacterized protein | Uncharacterized protein | | afdb-uniprot50 | AF-A0A1V6HT77-F1-MODEL\_V4 | 1.0 | 0.00193 | 138 | 0.104 | 173 | 127 | 12 | 2 | 153 | 1 | 166 | Uncharacterized protein | Uncharacterized protein | | afdb-uniprot50 | AF-A0A1Y0Y7R5-F1-MODEL\_V4 | 1.0 | 0.0002251 | 138 | 0.137 | 175 | 130 | 9 | 1 | 166 | 34 | 196 | Uncharacterized protein | Uncharacterized protein | | afdb-uniprot50 | AF-A0A317DUZ0-F1-MODEL\_V4 | 1.0 | 0.006537 | 138 | 0.201 | 154 | 106 | 10 | 9 | 156 | 170 | 312 | Uncharacterized protein | Uncharacterized protein | | afdb-uniprot50 | AF-A0A4R5VXB1-F1-MODEL\_V4 | 1.0 | 0.001286 | 137 | 0.138 | 152 | 106 | 12 | 7 | 152 | 1 | 133 | Uncharacterized protein | Uncharacterized protein | | afdb-uniprot50 | AF-A0A7G2T4T1-F1-MODEL\_V4 | 1.0 | 0.001213 | 137 | 0.136 | 169 | 106 | 12 | 10 | 162 | 2 | 146 | Uncharacterized protein | Uncharacterized protein | | afdb-uniprot50 | AF-A0A1L4D144-F1-MODEL\_V4 | 1.0 | 0.002168 | 137 | 0.121 | 165 | 125 | 6 | 9 | 168 | 5 | 154 | Uncharacterized protein | Uncharacterized protein | | afdb-uniprot50 | AF-B7RNM1-F1-MODEL\_V4 | 1.0 | 0.001213 | 136 | 0.442 | 70 | 39 | 0 | 120 | 189 | 18 | 87 | Uncharacterized protein | Uncharacterized protein | | afdb-uniprot50 | AF-A0A7W9S927-F1-MODEL\_V4 | 1.0 | 0.001286 | 136 | 0.111 | 152 | 110 | 7 | 7 | 152 | 2 | 134 | Uncharacterized protein | Uncharacterized protein | | afdb-uniprot50 | AF-A0A4Q7TEQ2-F1-MODEL\_V4 | 1.0 | 0.004353 | 136 | 0.107 | 149 | 111 | 6 | 10 | 153 | 4 | 135 | Uncharacterized protein | Uncharacterized protein | | afdb-uniprot50 | AF-A0A7J2K9N9-F1-MODEL\_V4 | 1.0 | 0.009262 | 136 | 0.12 | 149 | 117 | 7 | 7 | 152 | 1 | 138 | Uncharacterized protein | Uncharacterized protein | | afdb-uniprot50 | AF-A0A6M3IGG6-F1-MODEL\_V4 | 1.0 | 0.002168 | 136 | 0.134 | 163 | 116 | 10 | 9 | 162 | 6 | 152 | Uncharacterized protein | Uncharacterized protein | | afdb-uniprot50 | AF-R7JPZ3-F1-MODEL\_V4 | 1.0 | 0.0007622 | 136 | 0.109 | 173 | 130 | 7 | 1 | 168 | 3 | 156 | Phage-related protein Gp37-like protein | Phage-related protein Gp37-like protein | | afdb-uniprot50 | AF-A0A6C2YKQ7-F1-MODEL\_V4 | 1.0 | 0.001622 | 136 | 0.09 | 165 | 129 | 7 | 6 | 156 | 8 | 165 | Uncharacterized protein | Uncharacterized protein | | afdb-uniprot50 | AF-A0A6G9AQR9-F1-MODEL\_V4 | 1.0 | 0.0005701 | 136 | 0.166 | 174 | 118 | 12 | 7 | 162 | 1 | 165 | Uncharacterized protein | Uncharacterized protein | | afdb-uniprot50 | AF-A0A0R1RDJ7-F1-MODEL\_V4 | 1.0 | 0.002298 | 135 | 0.13 | 153 | 104 | 7 | 1 | 153 | 1 | 124 | Uncharacterized protein | Uncharacterized protein | | afdb-uniprot50 | AF-A0A3V2JIY3-F1-MODEL\_V4 | 1.0 | 0.003072 | 135 | 0.087 | 171 | 129 | 7 | 7 | 172 | 1 | 149 | Uncharacterized protein | Uncharacterized protein | | afdb-uniprot50 | AF-A0A6M0S828-F1-MODEL\_V4 | 1.0 | 0.001213 | 135 | 0.139 | 158 | 109 | 10 | 10 | 153 | 2 | 146 | Uncharacterized protein | Uncharacterized protein | | afdb-uniprot50 | AF-A0A4U0GUD8-F1-MODEL\_V4 | 1.0 | 0.001622 | 135 | 0.12 | 166 | 119 | 10 | 7 | 154 | 1 | 157 | Uncharacterized protein | Uncharacterized protein | | afdb-uniprot50 | AF-A0A7V7SWA7-F1-MODEL\_V4 | 1.0 | 0.000338 | 135 | 0.152 | 184 | 114 | 13 | 1 | 154 | 1 | 172 | Uncharacterized protein | Uncharacterized protein | | afdb-uniprot50 | AF-A0A3G9IET3-F1-MODEL\_V4 | 1.0 | 0.000284 | 135 | 0.1 | 180 | 129 | 8 | 8 | 166 | 7 | 174 | Uncharacterized protein | Uncharacterized protein | | afdb-uniprot50 | AF-A0A0F9TI92-F1-MODEL\_V4 | 1.0 | 0.0002124 | 135 | 0.111 | 161 | 125 | 8 | 9 | 159 | 53 | 205 | Uncharacterized protein | Uncharacterized protein | | afdb-uniprot50 | AF-A0A4Y9J0T5-F1-MODEL\_V4 | 1.0 | 0.003451 | 134 | 0.088 | 136 | 97 | 8 | 29 | 156 | 3 | 119 | Uncharacterized protein | Uncharacterized protein | | afdb-uniprot50 | AF-A0A0X8JIR5-F1-MODEL\_V4 | 1.0 | 0.00193 | 134 | 0.12 | 149 | 113 | 7 | 7 | 152 | 1 | 134 | Uncharacterized protein | Uncharacterized protein | | afdb-uniprot50 | AF-A0A3F3PDR2-F1-MODEL\_V4 | 1.0 | 0.003657 | 134 | 0.103 | 164 | 122 | 12 | 1 | 155 | 1 | 148 | Uncharacterized protein | Uncharacterized protein | | afdb-uniprot50 | AF-A0A3A4ZKA5-F1-MODEL\_V4 | 1.0 | 0.0009073 | 134 | 0.137 | 160 | 118 | 9 | 5 | 156 | 2 | 149 | Uncharacterized protein | Uncharacterized protein | | afdb-uniprot50 | AF-A0A838MQ51-F1-MODEL\_V4 | 1.0 | 0.001719 | 134 | 0.153 | 163 | 116 | 10 | 9 | 165 | 9 | 155 | Uncharacterized protein | Uncharacterized protein | | afdb-uniprot50 | AF-A0A1E4HY13-F1-MODEL\_V4 | 1.0 | 0.0006786 | 134 | 0.137 | 174 | 123 | 8 | 1 | 165 | 1 | 156 | Uncharacterized protein | Uncharacterized protein | | afdb-uniprot50 | AF-A0A5P8MWM5-F1-MODEL\_V4 | 1.0 | 0.0006042 | 134 | 0.158 | 196 | 128 | 18 | 9 | 188 | 14 | 188 | Uncharacterized protein | Uncharacterized protein | | afdb-uniprot50 | AF-A0A7W8JWA4-F1-MODEL\_V4 | 1.0 | 0.001822 | 133 | 0.111 | 161 | 129 | 5 | 7 | 165 | 1 | 149 | Uncharacterized protein | Uncharacterized protein | | afdb-uniprot50 | AF-A0A661KVG1-F1-MODEL\_V4 | 1.0 | 0.002581 | 133 | 0.105 | 161 | 121 | 10 | 9 | 159 | 13 | 160 | Uncharacterized protein | Uncharacterized protein | | afdb-uniprot50 | AF-A0A179CYM1-F1-MODEL\_V4 | 1.0 | 0.0001891 | 133 | 0.148 | 188 | 127 | 12 | 1 | 178 | 1 | 165 | Uncharacterized protein | Uncharacterized protein | | afdb-uniprot50 | AF-A0A524QZ15-F1-MODEL\_V4 | 1.0 | 0.001822 | 133 | 0.118 | 152 | 105 | 11 | 9 | 153 | 48 | 177 | Uncharacterized protein | Uncharacterized protein | | afdb-uniprot50 | AF-A0A850KD82-F1-MODEL\_V4 | 1.0 | 0.0008561 | 133 | 0.106 | 179 | 129 | 10 | 10 | 169 | 7 | 173 | DUF1834 family protein | DUF1834 family protein | | afdb-uniprot50 | AF-A0A2A2HEB9-F1-MODEL\_V4 | 1.0 | 0.000338 | 133 | 0.125 | 191 | 125 | 12 | 1 | 166 | 1 | 174 | Uncharacterized protein | Uncharacterized protein | | afdb-uniprot50 | AF-A0A4U9HEQ6-F1-MODEL\_V4 | 1.0 | 0.00108 | 132 | 0.333 | 69 | 46 | 0 | 120 | 188 | 12 | 80 | Uncharacterized protein | Uncharacterized protein | | afdb-uniprot50 | AF-A0A4Y6CYD7-F1-MODEL\_V4 | 1.0 | 0.005492 | 132 | 0.147 | 156 | 108 | 4 | 1 | 152 | 1 | 135 | Uncharacterized protein | Uncharacterized protein | | afdb-uniprot50 | AF-A0A7C1U118-F1-MODEL\_V4 | 1.0 | 0.006928 | 132 | 0.128 | 156 | 114 | 7 | 8 | 154 | 1 | 143 | Uncharacterized protein | Uncharacterized protein | | afdb-uniprot50 | AF-A0A7Z7NIL0-F1-MODEL\_V4 | 1.0 | 0.0007192 | 132 | 0.111 | 170 | 116 | 10 | 1 | 157 | 1 | 148 | Uncharacterized protein | Uncharacterized protein | | afdb-uniprot50 | AF-A8TYE4-F1-MODEL\_V4 | 1.0 | 0.0002004 | 132 | 0.165 | 163 | 107 | 9 | 33 | 187 | 2 | 143 | Uncharacterized protein | Uncharacterized protein | | afdb-uniprot50 | AF-A0A2A5A3B0-F1-MODEL\_V4 | 1.0 | 0.001362 | 132 | 0.136 | 176 | 129 | 12 | 9 | 173 | 5 | 168 | Uncharacterized protein | Uncharacterized protein | | afdb-uniprot50 | AF-A0A4R2USQ7-F1-MODEL\_V4 | 1.0 | 0.004108 | 131 | 0.121 | 156 | 107 | 11 | 5 | 154 | 2 | 133 | Uncharacterized protein | Uncharacterized protein | | afdb-uniprot50 | AF-F5R8A5-F1-MODEL\_V4 | 1.0 | 0.003657 | 131 | 0.164 | 146 | 106 | 8 | 10 | 153 | 6 | 137 | Uncharacterized protein | Uncharacterized protein | | afdb-uniprot50 | AF-A0A2N5XX45-F1-MODEL\_V4 | 1.0 | 0.001213 | 131 | 0.118 | 160 | 120 | 6 | 8 | 153 | 1 | 153 | Uncharacterized protein | Uncharacterized protein | | afdb-uniprot50 | AF-A0A7K1B1R7-F1-MODEL\_V4 | 1.0 | 0.002581 | 131 | 0.122 | 171 | 121 | 9 | 7 | 161 | 1 | 158 | Uncharacterized protein | Uncharacterized protein | | afdb-uniprot50 | AF-A0A327K8K4-F1-MODEL\_V4 | 1.0 | 0.0003189 | 131 | 0.172 | 191 | 129 | 11 | 1 | 177 | 1 | 176 | Uncharacterized protein | Uncharacterized protein | | afdb-uniprot50 | AF-A0A6P2CLK3-F1-MODEL\_V4 | 1.0 | 0.005492 | 130 | 0.093 | 150 | 109 | 5 | 7 | 155 | 1 | 124 | DUF806 family protein | DUF806 family protein | | afdb-uniprot50 | AF-A0A084XW78-F1-MODEL\_V4 | 1.0 | 0.001213 | 130 | 0.084 | 153 | 114 | 9 | 6 | 152 | 2 | 134 | Uncharacterized protein | Uncharacterized protein | | afdb-uniprot50 | AF-A0A543NXI2-F1-MODEL\_V4 | 1.0 | 0.002735 | 130 | 0.154 | 162 | 107 | 13 | 1 | 154 | 1 | 140 | Uncharacterized protein DUF3168 | Uncharacterized protein DUF3168 | | afdb-uniprot50 | AF-A0A1V4QT86-F1-MODEL\_V4 | 1.0 | 0.000338 | 130 | 0.15 | 146 | 106 | 8 | 15 | 154 | 8 | 141 | Uncharacterized protein | Uncharacterized protein | | afdb-uniprot50 | AF-A0A518CIC7-F1-MODEL\_V4 | 1.0 | 0.002168 | 130 | 0.108 | 157 | 122 | 10 | 7 | 154 | 1 | 148 | Uncharacterized protein | Uncharacterized protein | | afdb-uniprot50 | AF-A0A2E8E032-F1-MODEL\_V4 | 1.0 | 0.00582 | 130 | 0.097 | 154 | 121 | 8 | 9 | 155 | 5 | 147 | Uncharacterized protein | Uncharacterized protein | | afdb-uniprot50 | AF-A0A0G3XEA1-F1-MODEL\_V4 | 1.0 | 0.001622 | 130 | 0.11 | 163 | 121 | 9 | 9 | 163 | 9 | 155 | Uncharacterized protein | Uncharacterized protein | | afdb-uniprot50 | AF-D3DZY8-F1-MODEL\_V4 | 1.0 | 0.002046 | 130 | 0.122 | 171 | 126 | 9 | 9 | 163 | 7 | 169 | Uncharacterized protein | Uncharacterized protein | | afdb-uniprot50 | AF-A0A1Y2LFX2-F1-MODEL\_V4 | 1.0 | 0.0004024 | 130 | 0.144 | 201 | 133 | 13 | 8 | 187 | 1 | 183 | Uncharacterized protein | Uncharacterized protein | | afdb-uniprot50 | AF-A0A2G6EXL3-F1-MODEL\_V4 | 1.0 | 0.006168 | 129 | 0.127 | 110 | 86 | 4 | 46 | 152 | 5 | 107 | Uncharacterized protein | Uncharacterized protein | | afdb-uniprot50 | AF-A0A7T8RK97-F1-MODEL\_V4 | 1.0 | 0.006168 | 129 | 0.115 | 147 | 107 | 5 | 7 | 153 | 1 | 124 | DUF806 family protein | DUF806 family protein | | afdb-uniprot50 | AF-A0A6L7Y352-F1-MODEL\_V4 | 1.0 | 0.007342 | 129 | 0.129 | 155 | 113 | 7 | 7 | 156 | 1 | 138 | Uncharacterized protein | Uncharacterized protein | | afdb-uniprot50 | AF-A0A524GQA9-F1-MODEL\_V4 | 1.0 | 0.00193 | 129 | 0.13 | 168 | 122 | 13 | 1 | 155 | 1 | 157 | Uncharacterized protein | Uncharacterized protein | | afdb-uniprot50 | AF-A0A0T9QYM9-F1-MODEL\_V4 | 1.0 | 0.001362 | 129 | 0.107 | 158 | 112 | 9 | 27 | 166 | 2 | 148 | Mu-like prophage protein gp37 | Mu-like prophage protein gp37 | | afdb-uniprot50 | AF-A0A2W5QQ02-F1-MODEL\_V4 | 1.0 | 0.003451 | 128 | 0.136 | 147 | 100 | 10 | 14 | 153 | 2 | 128 | Uncharacterized protein | Uncharacterized protein | | afdb-uniprot50 | AF-A0A246S189-F1-MODEL\_V4 | 1.0 | 0.001719 | 128 | 0.124 | 153 | 106 | 9 | 7 | 152 | 3 | 134 | Uncharacterized protein | Uncharacterized protein | | afdb-uniprot50 | AF-A0A4Q4GUU5-F1-MODEL\_V4 | 1.0 | 0.001286 | 128 | 0.114 | 166 | 123 | 10 | 1 | 158 | 2 | 151 | Uncharacterized protein | Uncharacterized protein | | afdb-uniprot50 | AF-A0A2I0CTH9-F1-MODEL\_V4 | 1.0 | 0.004889 | 128 | 0.104 | 163 | 117 | 13 | 9 | 155 | 5 | 154 | Uncharacterized protein | Uncharacterized protein | | afdb-uniprot50 | AF-A0A016XM23-F1-MODEL\_V4 | 1.0 | 0.003072 | 128 | 0.109 | 183 | 132 | 9 | 9 | 179 | 4 | 167 | Uncharacterized protein | Uncharacterized protein | | afdb-uniprot50 | AF-A0A3N1J5Z6-F1-MODEL\_V4 | 1.0 | 0.001822 | 128 | 0.098 | 183 | 135 | 10 | 7 | 176 | 1 | 166 | Uncharacterized protein | Uncharacterized protein | | afdb-uniprot50 | AF-A0A1M3E549-F1-MODEL\_V4 | 1.0 | 0.001622 | 128 | 0.12 | 208 | 141 | 13 | 1 | 186 | 2 | 189 | Uncharacterized protein | Uncharacterized protein | | afdb-uniprot50 | AF-A0A179C349-F1-MODEL\_V4 | 1.0 | 0.003657 | 127 | 0.116 | 146 | 100 | 7 | 11 | 154 | 4 | 122 | Uncharacterized protein | Uncharacterized protein | | afdb-uniprot50 | AF-A0A0R2K2G6-F1-MODEL\_V4 | 1.0 | 0.002298 | 127 | 0.135 | 148 | 98 | 8 | 10 | 152 | 2 | 124 | Uncharacterized protein | Uncharacterized protein | | afdb-uniprot50 | AF-A0A1G3BXB4-F1-MODEL\_V4 | 1.0 | 0.003876 | 127 | 0.089 | 167 | 126 | 10 | 7 | 165 | 2 | 150 | Uncharacterized protein | Uncharacterized protein | | afdb-uniprot50 | AF-A0A7X9RUM6-F1-MODEL\_V4 | 1.0 | 0.002046 | 127 | 0.119 | 167 | 125 | 10 | 12 | 166 | 5 | 161 | Uncharacterized protein | Uncharacterized protein | | afdb-uniprot50 | AF-A0A6H1ZMY2-F1-MODEL\_V4 | 1.0 | 0.0004789 | 127 | 0.113 | 176 | 132 | 10 | 4 | 163 | 2 | 169 | Uncharacterized protein | Uncharacterized protein | | afdb-uniprot50 | AF-A0A841QIH0-F1-MODEL\_V4 | 1.0 | 0.0006786 | 127 | 0.126 | 182 | 121 | 13 | 1 | 165 | 4 | 164 | Uncharacterized protein | Uncharacterized protein | | afdb-uniprot50 | AF-A0A3A6MU72-F1-MODEL\_V4 | 1.0 | 0.0006042 | 127 | 0.101 | 167 | 125 | 8 | 7 | 166 | 1 | 149 | Uncharacterized protein | Uncharacterized protein | | afdb-uniprot50 | AF-A0A177PHJ4-F1-MODEL\_V4 | 1.0 | 0.003876 | 126 | 0.162 | 154 | 101 | 10 | 7 | 152 | 2 | 135 | Uncharacterized protein | Uncharacterized protein | | afdb-uniprot50 | AF-A0A2U2DWH8-F1-MODEL\_V4 | 1.0 | 0.002046 | 126 | 0.097 | 164 | 122 | 9 | 7 | 156 | 2 | 153 | Uncharacterized protein | Uncharacterized protein | | afdb-uniprot50 | AF-A0A3N5LHM6-F1-MODEL\_V4 | 1.0 | 0.001145 | 126 | 0.13 | 161 | 114 | 11 | 9 | 152 | 6 | 157 | Uncharacterized protein | Uncharacterized protein | | afdb-uniprot50 | AF-A0A089ZCH0-F1-MODEL\_V4 | 1.0 | 0.00108 | 126 | 0.124 | 161 | 117 | 9 | 9 | 153 | 9 | 161 | Phage-related protein | Phage-related protein | | afdb-uniprot50 | AF-X0VBA1-F1-MODEL\_V4 | 1.0 | 6.647e-05 | 126 | 0.141 | 184 | 125 | 10 | 30 | 187 | 14 | 190 | Uncharacterized protein | Uncharacterized protein | | afdb-uniprot50 | AF-A0A3N7CE51-F1-MODEL\_V4 | 1.0 | 0.001822 | 126 | 0.149 | 201 | 134 | 12 | 1 | 182 | 1 | 183 | Uncharacterized protein | Uncharacterized protein | | afdb-uniprot50 | AF-E6YRT6-F1-MODEL\_V4 | 1.0 | 0.001145 | 126 | 0.111 | 188 | 140 | 8 | 10 | 188 | 46 | 215 | Uncharacterized protein | Uncharacterized protein | | afdb-uniprot50 | AF-A0A2N6QWT3-F1-MODEL\_V4 | 1.0 | 0.004353 | 125 | 0.119 | 159 | 111 | 7 | 7 | 162 | 1 | 133 | Phage tail protein | Phage tail protein | | afdb-uniprot50 | AF-A0A0N1BU45-F1-MODEL\_V4 | 1.0 | 0.0006403 | 125 | 0.144 | 159 | 101 | 9 | 7 | 152 | 1 | 137 | Uncharacterized protein | Uncharacterized protein | | afdb-uniprot50 | AF-A0A255YQH8-F1-MODEL\_V4 | 1.0 | 0.002435 | 125 | 0.173 | 156 | 111 | 7 | 1 | 152 | 1 | 142 | Uncharacterized protein | Uncharacterized protein | | afdb-uniprot50 | AF-A0A1H3TG38-F1-MODEL\_V4 | 1.0 | 0.002298 | 125 | 0.156 | 172 | 109 | 11 | 9 | 165 | 6 | 156 | Uncharacterized protein | Uncharacterized protein | | afdb-uniprot50 | AF-A0A6I7MP73-F1-MODEL\_V4 | 1.0 | 0.002046 | 125 | 0.14 | 171 | 115 | 10 | 10 | 162 | 2 | 158 | Uncharacterized protein | Uncharacterized protein | | afdb-uniprot50 | AF-A0A1V6DHH5-F1-MODEL\_V4 | 1.0 | 0.001286 | 125 | 0.125 | 176 | 123 | 12 | 8 | 166 | 1 | 162 | Uncharacterized protein | Uncharacterized protein | | afdb-uniprot50 | AF-C1D6N8-F1-MODEL\_V4 | 1.0 | 0.001286 | 125 | 0.137 | 232 | 139 | 14 | 1 | 189 | 1 | 214 | DUF1834 domain containing protein | DUF1834 domain containing protein | | afdb-uniprot50 | AF-A0A844GBQ3-F1-MODEL\_V4 | 1.0 | 0.009262 | 124 | 0.1 | 129 | 94 | 10 | 29 | 152 | 5 | 116 | Uncharacterized protein | Uncharacterized protein | | afdb-uniprot50 | AF-A0A0R2BTK5-F1-MODEL\_V4 | 1.0 | 0.006928 | 124 | 0.099 | 151 | 108 | 7 | 7 | 155 | 2 | 126 | Uncharacterized protein | Uncharacterized protein | | afdb-uniprot50 | AF-A0A366FMK0-F1-MODEL\_V4 | 1.0 | 0.002168 | 124 | 0.201 | 154 | 103 | 11 | 7 | 151 | 1 | 143 | Uncharacterized protein | Uncharacterized protein | | afdb-uniprot50 | AF-W0RPE6-F1-MODEL\_V4 | 1.0 | 0.0009616 | 124 | 0.16 | 175 | 111 | 12 | 1 | 157 | 1 | 157 | Uncharacterized protein | Uncharacterized protein | | afdb-uniprot50 | AF-A0A126R8T4-F1-MODEL\_V4 | 1.0 | 0.0008078 | 124 | 0.174 | 166 | 111 | 10 | 9 | 165 | 9 | 157 | Uncharacterized protein | Uncharacterized protein | | afdb-uniprot50 | AF-A0A142XV75-F1-MODEL\_V4 | 1.0 | 0.00582 | 124 | 0.116 | 171 | 121 | 10 | 1 | 155 | 1 | 157 | Uncharacterized protein | Uncharacterized protein | | afdb-uniprot50 | AF-A0A7V3W3S9-F1-MODEL\_V4 | 1.0 | 0.001019 | 124 | 0.13 | 184 | 125 | 13 | 1 | 157 | 1 | 176 | Uncharacterized protein | Uncharacterized protein | | afdb-uniprot50 | AF-R7I5X1-F1-MODEL\_V4 | 1.0 | 0.001622 | 124 | 0.09 | 199 | 149 | 11 | 7 | 189 | 1 | 183 | Uncharacterized protein | Uncharacterized protein | | afdb-uniprot50 | AF-A0A502HRN3-F1-MODEL\_V4 | 1.0 | 0.0002124 | 124 | 0.135 | 192 | 118 | 13 | 30 | 189 | 108 | 283 | Uncharacterized protein | Uncharacterized protein | | afdb-uniprot50 | AF-A0A0F2IYW9-F1-MODEL\_V4 | 1.0 | 0.009262 | 123 | 0.134 | 119 | 90 | 5 | 40 | 157 | 2 | 108 | Uncharacterized protein | Uncharacterized protein | | afdb-uniprot50 | AF-A0A2E7W9M7-F1-MODEL\_V4 | 1.0 | 0.002735 | 123 | 0.129 | 154 | 110 | 7 | 7 | 154 | 1 | 136 | Uncharacterized protein | Uncharacterized protein | | afdb-uniprot50 | AF-A0A4R7BTN2-F1-MODEL\_V4 | 1.0 | 0.004108 | 123 | 0.129 | 154 | 108 | 9 | 7 | 153 | 1 | 135 | Uncharacterized protein DUF4128 | Uncharacterized protein DUF4128 | | afdb-uniprot50 | AF-A0A2D6XI96-F1-MODEL\_V4 | 1.0 | 0.006537 | 123 | 0.147 | 149 | 115 | 5 | 8 | 153 | 5 | 144 | Uncharacterized protein | Uncharacterized protein | | afdb-uniprot50 | AF-A0A7X9JN73-F1-MODEL\_V4 | 1.0 | 0.003072 | 123 | 0.111 | 152 | 107 | 8 | 9 | 152 | 19 | 150 | Uncharacterized protein | Uncharacterized protein | | afdb-uniprot50 | AF-A0A496QXT6-F1-MODEL\_V4 | 1.0 | 0.001286 | 123 | 0.118 | 152 | 117 | 9 | 9 | 154 | 12 | 152 | Uncharacterized protein | Uncharacterized protein | | afdb-uniprot50 | AF-A0A4Z0IPR0-F1-MODEL\_V4 | 1.0 | 0.006537 | 123 | 0.156 | 160 | 111 | 8 | 9 | 155 | 4 | 152 | Uncharacterized protein | Uncharacterized protein | | afdb-uniprot50 | AF-A0A2D6MCC7-F1-MODEL\_V4 | 1.0 | 0.002581 | 123 | 0.141 | 170 | 118 | 12 | 7 | 164 | 3 | 156 | Uncharacterized protein | Uncharacterized protein | | afdb-uniprot50 | AF-A0A4U6D146-F1-MODEL\_V4 | 1.0 | 0.002899 | 123 | 0.089 | 178 | 130 | 8 | 12 | 178 | 4 | 160 | Uncharacterized protein | Uncharacterized protein | | afdb-uniprot50 | AF-A0A521H788-F1-MODEL\_V4 | 1.0 | 0.003256 | 123 | 0.09 | 188 | 141 | 9 | 12 | 189 | 8 | 175 | Uncharacterized protein | Uncharacterized protein | | afdb-uniprot50 | AF-A0A4Z0SC48-F1-MODEL\_V4 | 1.0 | 0.006168 | 122 | 0.132 | 151 | 102 | 8 | 7 | 155 | 2 | 125 | Uncharacterized protein | Uncharacterized protein | | afdb-uniprot50 | AF-A0A069CSJ6-F1-MODEL\_V4 | 1.0 | 0.00582 | 122 | 0.111 | 152 | 107 | 7 | 7 | 153 | 1 | 129 | Phage tail protein | Phage tail protein | | afdb-uniprot50 | AF-A0A2P8FP87-F1-MODEL\_V4 | 1.0 | 0.004353 | 122 | 0.103 | 155 | 108 | 7 | 7 | 153 | 1 | 132 | Uncharacterized protein DUF4128 | Uncharacterized protein DUF4128 | | afdb-uniprot50 | AF-A0A661DGF6-F1-MODEL\_V4 | 1.0 | 0.001822 | 122 | 0.128 | 148 | 107 | 12 | 9 | 153 | 8 | 136 | Uncharacterized protein | Uncharacterized protein | | afdb-uniprot50 | AF-A0A2S5CGG2-F1-MODEL\_V4 | 1.0 | 0.00153 | 122 | 0.153 | 163 | 106 | 10 | 6 | 155 | 3 | 146 | Uncharacterized protein | Uncharacterized protein | | afdb-uniprot50 | AF-A0A1E7Q8E4-F1-MODEL\_V4 | 1.0 | 0.002435 | 122 | 0.115 | 164 | 109 | 12 | 9 | 154 | 4 | 149 | Uncharacterized protein | Uncharacterized protein | | afdb-uniprot50 | AF-K9RS62-F1-MODEL\_V4 | 1.0 | 0.005182 | 122 | 0.106 | 169 | 125 | 8 | 1 | 163 | 1 | 149 | Uncharacterized protein | Uncharacterized protein | | afdb-uniprot50 | AF-A0A1V5IG05-F1-MODEL\_V4 | 1.0 | 0.003072 | 122 | 0.122 | 171 | 117 | 9 | 1 | 155 | 1 | 154 | Uncharacterized protein | Uncharacterized protein | | afdb-uniprot50 | AF-A0A5C8UQH2-F1-MODEL\_V4 | 1.0 | 0.004353 | 122 | 0.119 | 167 | 125 | 9 | 10 | 166 | 6 | 160 | Uncharacterized protein | Uncharacterized protein | | afdb-uniprot50 | AF-A0A3D5AU90-F1-MODEL\_V4 | 1.0 | 0.006928 | 121 | 0.15 | 113 | 82 | 4 | 48 | 157 | 5 | 106 | Uncharacterized protein | Uncharacterized protein | | afdb-uniprot50 | AF-A0A2G2PNR6-F1-MODEL\_V4 | 1.0 | 0.003072 | 121 | 0.12 | 150 | 100 | 10 | 9 | 152 | 8 | 131 | Uncharacterized protein | Uncharacterized protein | | afdb-uniprot50 | AF-A0A175RB75-F1-MODEL\_V4 | 1.0 | 0.004613 | 121 | 0.136 | 147 | 103 | 10 | 16 | 155 | 9 | 138 | Uncharacterized protein | Uncharacterized protein | | afdb-uniprot50 | AF-A0A0F9QW21-F1-MODEL\_V4 | 1.0 | 0.003256 | 121 | 0.116 | 163 | 121 | 11 | 1 | 152 | 2 | 152 | Uncharacterized protein | Uncharacterized protein | | afdb-uniprot50 | AF-A0A830FZ37-F1-MODEL\_V4 | 1.0 | 0.001362 | 121 | 0.135 | 200 | 120 | 12 | 14 | 173 | 4 | 190 | Uncharacterized protein | Uncharacterized protein | | afdb-uniprot50 | AF-A0A1C0V8E1-F1-MODEL\_V4 | 1.0 | 0.001362 | 121 | 0.125 | 191 | 137 | 11 | 9 | 189 | 5 | 175 | Uncharacterized protein | Uncharacterized protein | | afdb-uniprot50 | AF-A0A645AWW2-F1-MODEL\_V4 | 1.0 | 0.006168 | 120 | 0.1 | 159 | 107 | 10 | 8 | 154 | 1 | 135 | Uncharacterized protein | Uncharacterized protein | | afdb-uniprot50 | AF-A0A848WCE3-F1-MODEL\_V4 | 1.0 | 0.007781 | 120 | 0.086 | 151 | 109 | 11 | 9 | 153 | 7 | 134 | Uncharacterized protein | Uncharacterized protein | | afdb-uniprot50 | AF-A0A0A8K5T5-F1-MODEL\_V4 | 1.0 | 0.004353 | 120 | 0.177 | 158 | 98 | 11 | 7 | 154 | 2 | 137 | Uncharacterized protein | Uncharacterized protein | | afdb-uniprot50 | AF-A0A1W9Z0J1-F1-MODEL\_V4 | 1.0 | 0.005182 | 120 | 0.103 | 165 | 116 | 13 | 1 | 156 | 2 | 143 | Uncharacterized protein | Uncharacterized protein | | afdb-uniprot50 | AF-A0A2S3W4U1-F1-MODEL\_V4 | 1.0 | 0.00153 | 120 | 0.109 | 155 | 111 | 8 | 43 | 188 | 1 | 137 | Uncharacterized protein | Uncharacterized protein | | afdb-uniprot50 | AF-A0A7W6K7J7-F1-MODEL\_V4 | 1.0 | 0.00193 | 120 | 0.12 | 166 | 109 | 8 | 1 | 152 | 13 | 155 | Uncharacterized protein | Uncharacterized protein | | afdb-uniprot50 | AF-A0A7C3USK7-F1-MODEL\_V4 | 1.0 | 0.001213 | 120 | 0.142 | 176 | 115 | 12 | 1 | 156 | 2 | 161 | Uncharacterized protein | Uncharacterized protein | | afdb-uniprot50 | AF-Q141R8-F1-MODEL\_V4 | 1.0 | 0.001145 | 120 | 0.169 | 165 | 121 | 7 | 10 | 172 | 8 | 158 | Uncharacterized protein | Uncharacterized protein | | afdb-uniprot50 | AF-A0A7C6E6J6-F1-MODEL\_V4 | 1.0 | 0.007342 | 119 | 0.102 | 136 | 96 | 7 | 30 | 154 | 9 | 129 | Uncharacterized protein | Uncharacterized protein | | afdb-uniprot50 | AF-Q5F997-F1-MODEL\_V4 | 1.0 | 0.00193 | 119 | 0.123 | 154 | 111 | 11 | 7 | 150 | 1 | 140 | Phage associated protein | Phage associated protein | | afdb-uniprot50 | AF-A0A1B7X3R8-F1-MODEL\_V4 | 1.0 | 0.002581 | 119 | 0.098 | 152 | 117 | 9 | 7 | 152 | 2 | 139 | Uncharacterized protein | Uncharacterized protein | | afdb-uniprot50 | AF-A0A7V0NNN3-F1-MODEL\_V4 | 1.0 | 0.008247 | 119 | 0.144 | 166 | 112 | 11 | 1 | 154 | 1 | 148 | Uncharacterized protein | Uncharacterized protein | | afdb-uniprot50 | AF-A0A6I1J9G2-F1-MODEL\_V4 | 1.0 | 0.002046 | 119 | 0.168 | 154 | 112 | 10 | 7 | 152 | 3 | 148 | Uncharacterized protein | Uncharacterized protein | | afdb-uniprot50 | AF-A0A661TVI5-F1-MODEL\_V4 | 1.0 | 0.005492 | 119 | 0.118 | 160 | 114 | 7 | 9 | 154 | 4 | 150 | Uncharacterized protein | Uncharacterized protein | | afdb-uniprot50 | AF-A0A402A527-F1-MODEL\_V4 | 1.0 | 0.002581 | 119 | 0.129 | 178 | 114 | 10 | 5 | 165 | 3 | 156 | Uncharacterized protein | Uncharacterized protein | | afdb-uniprot50 | AF-A0A0F9BJT1-F1-MODEL\_V4 | 1.0 | 0.006928 | 119 | 0.09 | 177 | 140 | 10 | 1 | 165 | 2 | 169 | Uncharacterized protein | Uncharacterized protein | | afdb-uniprot50 | AF-A0A2D6XLF9-F1-MODEL\_V4 | 1.0 | 0.00193 | 119 | 0.145 | 165 | 121 | 11 | 1 | 154 | 17 | 172 | Uncharacterized protein | Uncharacterized protein | | afdb-uniprot50 | AF-A0A1H9Q9R0-F1-MODEL\_V4 | 1.0 | 0.0004789 | 119 | 0.173 | 184 | 121 | 11 | 1 | 175 | 1 | 162 | Uncharacterized protein | Uncharacterized protein | | afdb-uniprot50 | AF-A0A1I6VDK5-F1-MODEL\_V4 | 1.0 | 0.0005701 | 119 | 0.13 | 207 | 142 | 15 | 1 | 187 | 1 | 189 | Uncharacterized protein | Uncharacterized protein | | afdb-uniprot50 | AF-A0A3N5UZL3-F1-MODEL\_V4 | 1.0 | 0.004108 | 118 | 0.111 | 162 | 113 | 11 | 5 | 155 | 2 | 143 | Uncharacterized protein | Uncharacterized protein | | afdb-uniprot50 | AF-A0A427UIF4-F1-MODEL\_V4 | 1.0 | 0.004889 | 118 | 0.132 | 166 | 117 | 11 | 1 | 160 | 2 | 146 | Uncharacterized protein | Uncharacterized protein | | afdb-uniprot50 | AF-A0A1E4HKH2-F1-MODEL\_V4 | 1.0 | 0.009262 | 118 | 0.143 | 153 | 109 | 11 | 9 | 152 | 10 | 149 | Uncharacterized protein | Uncharacterized protein | | afdb-uniprot50 | AF-A0A497AEL7-F1-MODEL\_V4 | 1.0 | 0.004613 | 118 | 0.086 | 162 | 126 | 11 | 6 | 152 | 2 | 156 | Uncharacterized protein | Uncharacterized protein | | afdb-uniprot50 | AF-A0A6M3LF59-F1-MODEL\_V4 | 1.0 | 0.003072 | 118 | 0.104 | 172 | 126 | 13 | 1 | 156 | 3 | 162 | Uncharacterized protein | Uncharacterized protein | | afdb-uniprot50 | AF-A0A843ACS1-F1-MODEL\_V4 | 1.0 | 0.0004789 | 118 | 0.104 | 182 | 126 | 12 | 1 | 164 | 1 | 163 | Uncharacterized protein | Uncharacterized protein | | afdb-uniprot50 | AF-A0A6M3IUD6-F1-MODEL\_V4 | 1.0 | 0.001822 | 118 | 0.136 | 169 | 121 | 7 | 7 | 168 | 1 | 151 | Uncharacterized protein | Uncharacterized protein | | afdb-uniprot50 | AF-A0A661HGP8-F1-MODEL\_V4 | 1.0 | 0.003876 | 118 | 0.094 | 180 | 142 | 10 | 11 | 181 | 6 | 173 | Uncharacterized protein | Uncharacterized protein | | afdb-uniprot50 | AF-A0A506TZ73-F1-MODEL\_V4 | 1.0 | 0.001145 | 117 | 0.165 | 163 | 105 | 13 | 1 | 152 | 3 | 145 | Uncharacterized protein | Uncharacterized protein | | afdb-uniprot50 | AF-A0A2I0FU84-F1-MODEL\_V4 | 1.0 | 0.002581 | 117 | 0.123 | 194 | 131 | 12 | 15 | 186 | 5 | 181 | Uncharacterized protein | Uncharacterized protein | | afdb-uniprot50 | AF-A0A1H8IRD0-F1-MODEL\_V4 | 1.0 | 0.006928 | 116 | 0.1 | 169 | 130 | 7 | 1 | 164 | 1 | 152 | Uncharacterized protein | Uncharacterized protein | | afdb-uniprot50 | AF-A0A5C5VW99-F1-MODEL\_V4 | 1.0 | 0.002046 | 116 | 0.107 | 167 | 116 | 14 | 14 | 165 | 4 | 152 | Uncharacterized protein | Uncharacterized protein | | afdb-uniprot50 | AF-D2U1L2-F1-MODEL\_V4 | 1.0 | 0.005492 | 116 | 0.094 | 137 | 99 | 5 | 43 | 165 | 16 | 141 | Conserved hypothetical phage protein | Conserved hypothetical phage protein | | afdb-uniprot50 | AF-A0A2D6F2E1-F1-MODEL\_V4 | 1.0 | 0.001622 | 116 | 0.165 | 181 | 112 | 14 | 7 | 166 | 2 | 164 | Uncharacterized protein | Uncharacterized protein | | afdb-uniprot50 | AF-A0A3D3SH89-F1-MODEL\_V4 | 1.0 | 0.002581 | 116 | 0.084 | 190 | 142 | 12 | 7 | 183 | 1 | 171 | Uncharacterized protein | Uncharacterized protein | | afdb-uniprot50 | AF-A0A1B7X0V1-F1-MODEL\_V4 | 1.0 | 0.000284 | 116 | 0.148 | 209 | 137 | 13 | 7 | 189 | 3 | 196 | Uncharacterized protein | Uncharacterized protein | | afdb-uniprot50 | AF-A0A841LE00-F1-MODEL\_V4 | 1.0 | 0.006537 | 116 | 0.11 | 227 | 140 | 15 | 1 | 189 | 2 | 204 | Uncharacterized protein | Uncharacterized protein | | afdb-uniprot50 | AF-A0A1I3HI47-F1-MODEL\_V4 | 1.0 | 0.008247 | 115 | 0.14 | 150 | 103 | 10 | 10 | 153 | 6 | 135 | Uncharacterized protein | Uncharacterized protein | | afdb-uniprot50 | AF-A0A1E4ZIU0-F1-MODEL\_V4 | 1.0 | 0.005182 | 115 | 0.093 | 161 | 117 | 12 | 9 | 164 | 6 | 142 | Uncharacterized protein | Uncharacterized protein | | afdb-uniprot50 | AF-A0A7W9VZR3-F1-MODEL\_V4 | 1.0 | 0.006537 | 115 | 0.136 | 161 | 108 | 12 | 7 | 156 | 1 | 141 | Uncharacterized protein | Uncharacterized protein | | afdb-uniprot50 | AF-A0A3S0BCC7-F1-MODEL\_V4 | 1.0 | 0.00874 | 115 | 0.124 | 169 | 115 | 12 | 9 | 159 | 4 | 157 | Uncharacterized protein | Uncharacterized protein | | afdb-uniprot50 | AF-A0A5C7MB17-F1-MODEL\_V4 | 1.0 | 0.004889 | 115 | 0.167 | 161 | 110 | 10 | 9 | 154 | 16 | 167 | Uncharacterized protein | Uncharacterized protein | | afdb-uniprot50 | AF-A0A523Z9A7-F1-MODEL\_V4 | 1.0 | 0.009262 | 115 | 0.133 | 165 | 125 | 8 | 6 | 156 | 7 | 167 | Uncharacterized protein | Uncharacterized protein | | afdb-uniprot50 | AF-A0A0U1K6V6-F1-MODEL\_V4 | 1.0 | 0.00153 | 115 | 0.117 | 205 | 133 | 14 | 1 | 187 | 26 | 200 | Putative ATP-binding protein | Putative ATP-binding protein | | afdb-uniprot50 | AF-A0A0Q5ZKT3-F1-MODEL\_V4 | 1.0 | 0.007342 | 114 | 0.113 | 150 | 113 | 9 | 7 | 152 | 1 | 134 | Uncharacterized protein | Uncharacterized protein | | afdb-uniprot50 | AF-A0A1H9QD02-F1-MODEL\_V4 | 1.0 | 0.002581 | 114 | 0.14 | 157 | 104 | 9 | 7 | 154 | 2 | 136 | Uncharacterized protein | Uncharacterized protein | | afdb-uniprot50 | AF-A0A257ZLI7-F1-MODEL\_V4 | 1.0 | 0.004353 | 114 | 0.104 | 162 | 113 | 7 | 7 | 152 | 2 | 147 | Uncharacterized protein | Uncharacterized protein | | afdb-uniprot50 | AF-A0A1H8A9M5-F1-MODEL\_V4 | 1.0 | 0.001719 | 114 | 0.131 | 182 | 127 | 9 | 7 | 165 | 2 | 175 | Uncharacterized protein | Uncharacterized protein | | afdb-uniprot50 | AF-I4E5S8-F1-MODEL\_V4 | 1.0 | 0.004108 | 114 | 0.133 | 203 | 119 | 11 | 27 | 187 | 2 | 189 | Uncharacterized protein | Uncharacterized protein | | afdb-uniprot50 | AF-A0A3A4VTU4-F1-MODEL\_V4 | 1.0 | 0.006928 | 113 | 0.118 | 152 | 110 | 10 | 12 | 154 | 3 | 139 | Uncharacterized protein | Uncharacterized protein | | afdb-uniprot50 | AF-A0A0F9N372-F1-MODEL\_V4 | 1.0 | 0.00874 | 113 | 0.115 | 165 | 123 | 12 | 9 | 165 | 5 | 154 | Uncharacterized protein | Uncharacterized protein | | afdb-uniprot50 | AF-A0A3D4ZXF2-F1-MODEL\_V4 | 1.0 | 0.004613 | 113 | 0.174 | 183 | 107 | 15 | 1 | 153 | 1 | 169 | Uncharacterized protein | Uncharacterized protein | | afdb-uniprot50 | AF-A0A249PA02-F1-MODEL\_V4 | 1.0 | 0.001213 | 112 | 0.118 | 161 | 112 | 10 | 1 | 154 | 1 | 138 | Uncharacterized protein | Uncharacterized protein | | afdb-uniprot50 | AF-A0A6B0W5K2-F1-MODEL\_V4 | 1.0 | 0.004613 | 112 | 0.144 | 159 | 109 | 10 | 11 | 156 | 8 | 152 | Uncharacterized protein | Uncharacterized protein | | afdb-uniprot50 | AF-A0A1S2VDH5-F1-MODEL\_V4 | 1.0 | 0.003072 | 112 | 0.141 | 184 | 107 | 14 | 13 | 165 | 3 | 166 | Uncharacterized protein | Uncharacterized protein | | afdb-uniprot50 | AF-A0A0F9X9T6-F1-MODEL\_V4 | 1.0 | 0.003256 | 112 | 0.111 | 179 | 116 | 12 | 1 | 153 | 1 | 162 | Uncharacterized protein | Uncharacterized protein | | afdb-uniprot50 | AF-A0A1B7LDB7-F1-MODEL\_V4 | 1.0 | 0.004108 | 112 | 0.142 | 169 | 115 | 9 | 9 | 166 | 10 | 159 | Uncharacterized protein | Uncharacterized protein | | afdb-uniprot50 | AF-A0A0F9UL68-F1-MODEL\_V4 | 1.0 | 0.001622 | 112 | 0.15 | 233 | 142 | 17 | 1 | 188 | 1 | 222 | Uncharacterized protein | Uncharacterized protein | | afdb-uniprot50 | AF-A0A2E8NYT8-F1-MODEL\_V4 | 1.0 | 0.001213 | 112 | 0.113 | 238 | 129 | 14 | 1 | 166 | 1 | 228 | Uncharacterized protein | Uncharacterized protein | | afdb-uniprot50 | AF-I9N4S3-F1-MODEL\_V4 | 1.0 | 0.005492 | 111 | 0.128 | 148 | 106 | 7 | 14 | 155 | 8 | 138 | Uncharacterized protein | Uncharacterized protein | | afdb-uniprot50 | AF-A0A1M4Y9T1-F1-MODEL\_V4 | 1.0 | 0.004613 | 111 | 0.138 | 152 | 106 | 9 | 14 | 152 | 4 | 143 | Uncharacterized protein | Uncharacterized protein | | afdb-uniprot50 | AF-A0A1V5WR40-F1-MODEL\_V4 | 1.0 | 0.006537 | 111 | 0.114 | 183 | 136 | 13 | 1 | 174 | 1 | 166 | Uncharacterized protein | Uncharacterized protein | | afdb-uniprot50 | AF-A0A5C7QH98-F1-MODEL\_V4 | 1.0 | 0.00193 | 111 | 0.142 | 190 | 120 | 15 | 10 | 168 | 22 | 199 | Uncharacterized protein | Uncharacterized protein | | afdb-uniprot50 | AF-A0A660MBK1-F1-MODEL\_V4 | 1.0 | 0.006928 | 110 | 0.169 | 159 | 108 | 9 | 12 | 156 | 5 | 153 | Uncharacterized protein | Uncharacterized protein | | afdb-uniprot50 | AF-A0A0F9BKL4-F1-MODEL\_V4 | 1.0 | 0.005182 | 110 | 0.118 | 186 | 136 | 10 | 7 | 179 | 1 | 171 | Uncharacterized protein | Uncharacterized protein | | afdb-uniprot50 | AF-A0A212KK97-F1-MODEL\_V4 | 1.0 | 0.005492 | 110 | 0.149 | 167 | 116 | 6 | 9 | 161 | 4 | 158 | Uncharacterized protein | Uncharacterized protein | | afdb-uniprot50 | AF-A0A1E3H1X1-F1-MODEL\_V4 | 1.0 | 0.007342 | 110 | 0.116 | 155 | 121 | 5 | 1 | 150 | 110 | 253 | Uncharacterized protein | Uncharacterized protein | | afdb-uniprot50 | AF-A0A2D7VXM3-F1-MODEL\_V4 | 1.0 | 0.004353 | 110 | 0.11 | 136 | 91 | 7 | 30 | 153 | 227 | 344 | Uncharacterized protein | Uncharacterized protein | | afdb-uniprot50 | AF-A0A7C1PIS6-F1-MODEL\_V4 | 1.0 | 0.003451 | 110 | 0.147 | 156 | 104 | 11 | 29 | 166 | 265 | 409 | Uncharacterized protein | Uncharacterized protein | | afdb-uniprot50 | AF-A0A3D9E5J7-F1-MODEL\_V4 | 1.0 | 0.007342 | 109 | 0.099 | 151 | 122 | 8 | 10 | 153 | 11 | 154 | Uncharacterized protein | Uncharacterized protein | | afdb-uniprot50 | AF-A0A371JLB5-F1-MODEL\_V4 | 1.0 | 0.002168 | 109 | 0.126 | 214 | 136 | 14 | 1 | 178 | 13 | 211 | Uncharacterized protein | Uncharacterized protein | | afdb-uniprot50 | AF-A0A523RTI9-F1-MODEL\_V4 | 1.0 | 0.003256 | 108 | 0.108 | 157 | 115 | 9 | 9 | 157 | 10 | 149 | Uncharacterized protein | Uncharacterized protein | | afdb-uniprot50 | AF-A0A1M7AHU1-F1-MODEL\_V4 | 1.0 | 0.003876 | 108 | 0.102 | 185 | 132 | 14 | 7 | 172 | 1 | 170 | Uncharacterized protein | Uncharacterized protein | | afdb-uniprot50 | AF-A0A559IZI9-F1-MODEL\_V4 | 1.0 | 0.006537 | 108 | 0.091 | 186 | 123 | 10 | 10 | 165 | 4 | 173 | Uncharacterized protein | Uncharacterized protein | | afdb-uniprot50 | AF-A0A2J9S7X7-F1-MODEL\_V4 | 1.0 | 0.004889 | 108 | 0.116 | 198 | 130 | 12 | 7 | 187 | 1 | 170 | ATP-binding protein | ATP-binding protein | | afdb-uniprot50 | AF-A0A2U8WAI8-F1-MODEL\_V4 | 1.0 | 0.003451 | 108 | 0.14 | 192 | 141 | 10 | 1 | 187 | 1 | 173 | Uncharacterized protein | Uncharacterized protein | | afdb-uniprot50 | AF-A0A2E7BJZ9-F1-MODEL\_V4 | 1.0 | 0.002899 | 107 | 0.13 | 168 | 121 | 9 | 9 | 164 | 5 | 159 | Uncharacterized protein | Uncharacterized protein | | afdb-uniprot50 | AF-A0A5C7JA81-F1-MODEL\_V4 | 1.0 | 0.003451 | 107 | 0.105 | 209 | 118 | 10 | 1 | 154 | 1 | 195 | Uncharacterized protein | Uncharacterized protein | | afdb-uniprot50 | AF-A0A1Z9SNZ4-F1-MODEL\_V4 | 1.0 | 0.004353 | 107 | 0.092 | 226 | 135 | 17 | 2 | 172 | 1 | 211 | Uncharacterized protein | Uncharacterized protein | | afdb-uniprot50 | AF-A0A7U7G943-F1-MODEL\_V4 | 1.0 | 0.006168 | 106 | 0.125 | 159 | 102 | 13 | 11 | 154 | 4 | 140 | Uncharacterized protein | Uncharacterized protein | | afdb-uniprot50 | AF-A0A2S5N3V3-F1-MODEL\_V4 | 1.0 | 0.00874 | 106 | 0.167 | 155 | 106 | 12 | 7 | 153 | 1 | 140 | Uncharacterized protein | Uncharacterized protein | | afdb-uniprot50 | AF-A0A1V5V8V5-F1-MODEL\_V4 | 1.0 | 0.005182 | 106 | 0.098 | 172 | 123 | 13 | 6 | 163 | 3 | 156 | Uncharacterized protein | Uncharacterized protein | | afdb-uniprot50 | AF-A0A5M9HA02-F1-MODEL\_V4 | 1.0 | 0.00874 | 106 | 0.128 | 187 | 117 | 13 | 7 | 165 | 1 | 169 | Uncharacterized protein | Uncharacterized protein | | afdb-uniprot50 | AF-A0A1A9RL24-F1-MODEL\_V4 | 1.0 | 0.002899 | 106 | 0.128 | 195 | 120 | 11 | 7 | 171 | 1 | 175 | Uncharacterized protein | Uncharacterized protein | | afdb-uniprot50 | AF-A0A0S8AR84-F1-MODEL\_V4 | 1.0 | 0.005492 | 106 | 0.097 | 184 | 123 | 12 | 7 | 178 | 1 | 153 | Uncharacterized protein | Uncharacterized protein | | afdb-uniprot50 | AF-A0A0R2MKJ7-F1-MODEL\_V4 | 1.0 | 0.003072 | 105 | 0.129 | 155 | 106 | 8 | 7 | 159 | 1 | 128 | Uncharacterized protein | Uncharacterized protein | | afdb-uniprot50 | AF-A0A7J2R9I8-F1-MODEL\_V4 | 1.0 | 0.00582 | 105 | 0.142 | 161 | 112 | 11 | 5 | 154 | 2 | 147 | Uncharacterized protein | Uncharacterized protein | | afdb-uniprot50 | AF-A0A348V8F1-F1-MODEL\_V4 | 1.0 | 0.009262 | 105 | 0.129 | 177 | 114 | 12 | 12 | 163 | 4 | 165 | Uncharacterized protein | Uncharacterized protein | | afdb-uniprot50 | AF-A0A239ABH2-F1-MODEL\_V4 | 1.0 | 0.004889 | 105 | 0.129 | 178 | 128 | 11 | 13 | 178 | 4 | 166 | Uncharacterized protein | Uncharacterized protein | | afdb-uniprot50 | AF-A0A7Y6T7P5-F1-MODEL\_V4 | 1.0 | 0.002168 | 105 | 0.107 | 196 | 148 | 11 | 1 | 187 | 1 | 178 | Uncharacterized protein | Uncharacterized protein | | afdb-uniprot50 | AF-N2AZG9-F1-MODEL\_V4 | 1.0 | 0.009262 | 105 | 0.109 | 183 | 127 | 15 | 7 | 166 | 1 | 170 | Uncharacterized protein | Uncharacterized protein | | afdb-uniprot50 | AF-A0A1Y4RYL6-F1-MODEL\_V4 | 1.0 | 0.006168 | 104 | 0.15 | 179 | 110 | 11 | 13 | 164 | 2 | 165 | Uncharacterized protein | Uncharacterized protein | | afdb-uniprot50 | AF-A0A2S2F818-F1-MODEL\_V4 | 1.0 | 0.001719 | 104 | 0.152 | 242 | 122 | 16 | 4 | 173 | 2 | 232 | Uncharacterized protein | Uncharacterized protein | | afdb-uniprot50 | AF-A0A661VEU5-F1-MODEL\_V4 | 1.0 | 0.004108 | 104 | 0.119 | 159 | 105 | 11 | 29 | 166 | 181 | 325 | Uncharacterized protein | Uncharacterized protein | | afdb-uniprot50 | AF-A0A833LAQ3-F1-MODEL\_V4 | 1.0 | 0.007342 | 103 | 0.131 | 145 | 96 | 7 | 15 | 153 | 6 | 126 | DUF806 family protein | DUF806 family protein | | afdb-uniprot50 | AF-A0A6M3IDV3-F1-MODEL\_V4 | 1.0 | 0.003256 | 103 | 0.1 | 169 | 128 | 10 | 1 | 156 | 3 | 160 | Uncharacterized protein | Uncharacterized protein | | afdb-uniprot50 | AF-A0A7R7T6R5-F1-MODEL\_V4 | 1.0 | 0.002435 | 103 | 0.123 | 194 | 145 | 13 | 2 | 187 | 1 | 177 | Uncharacterized protein | Uncharacterized protein | | afdb-uniprot50 | AF-A0A437MN64-F1-MODEL\_V4 | 1.0 | 0.008247 | 102 | 0.161 | 149 | 96 | 10 | 14 | 152 | 4 | 133 | Uncharacterized protein | Uncharacterized protein | | afdb-uniprot50 | AF-A0A7W8VCX7-F1-MODEL\_V4 | 1.0 | 0.002435 | 102 | 0.126 | 166 | 112 | 12 | 2 | 155 | 1 | 145 | Uncharacterized protein | Uncharacterized protein | | afdb-uniprot50 | AF-A0A0L7MPP8-F1-MODEL\_V4 | 1.0 | 0.00153 | 102 | 0.168 | 178 | 114 | 12 | 1 | 165 | 1 | 157 | Uncharacterized protein | Uncharacterized protein | | afdb-uniprot50 | AF-A0A7X6FP76-F1-MODEL\_V4 | 1.0 | 0.002899 | 102 | 0.14 | 164 | 118 | 5 | 30 | 189 | 21 | 165 | Uncharacterized protein | Uncharacterized protein | | afdb-uniprot50 | AF-A0A2D7IIR0-F1-MODEL\_V4 | 1.0 | 0.006928 | 102 | 0.11 | 200 | 110 | 10 | 28 | 166 | 40 | 232 | Uncharacterized protein | Uncharacterized protein | | afdb-uniprot50 | AF-A0A662EAC9-F1-MODEL\_V4 | 1.0 | 0.008247 | 101 | 0.13 | 153 | 104 | 11 | 6 | 152 | 2 | 131 | Uncharacterized protein | Uncharacterized protein | | afdb-uniprot50 | AF-A0A3N1M918-F1-MODEL\_V4 | 1.0 | 0.00582 | 101 | 0.133 | 150 | 101 | 11 | 9 | 152 | 7 | 133 | Uncharacterized protein DUF4128 | Uncharacterized protein DUF4128 | | afdb-uniprot50 | AF-A0A838TS93-F1-MODEL\_V4 | 1.0 | 0.007342 | 101 | 0.111 | 152 | 114 | 6 | 9 | 154 | 9 | 145 | Uncharacterized protein | Uncharacterized protein | | afdb-uniprot50 | AF-A0A7G3E4R6-F1-MODEL\_V4 | 1.0 | 0.009816 | 101 | 0.129 | 170 | 118 | 9 | 2 | 156 | 1 | 155 | Uncharacterized protein | Uncharacterized protein | | afdb-uniprot50 | AF-A0A1U7GKN0-F1-MODEL\_V4 | 1.0 | 0.002735 | 101 | 0.125 | 175 | 118 | 12 | 1 | 153 | 7 | 168 | Uncharacterized protein | Uncharacterized protein | | afdb-uniprot50 | AF-A0A1E4N746-F1-MODEL\_V4 | 1.0 | 0.001444 | 101 | 0.092 | 195 | 135 | 9 | 1 | 174 | 4 | 177 | Uncharacterized protein | Uncharacterized protein | | afdb-uniprot50 | AF-A0A1P9XTF9-F1-MODEL\_V4 | 1.0 | 0.00193 | 101 | 0.165 | 169 | 105 | 7 | 16 | 172 | 4 | 148 | Uncharacterized protein | Uncharacterized protein | | afdb-uniprot50 | AF-A0A0F9HRR7-F1-MODEL\_V4 | 1.0 | 0.002581 | 101 | 0.113 | 194 | 139 | 9 | 10 | 178 | 18 | 203 | Uncharacterized protein | Uncharacterized protein | | afdb-uniprot50 | AF-A0A0R2A4Y7-F1-MODEL\_V4 | 1.0 | 0.00874 | 100 | 0.098 | 152 | 110 | 7 | 7 | 157 | 1 | 126 | Phage tail protein | Phage tail protein | | afdb-uniprot50 | AF-A0A7Y8VDE0-F1-MODEL\_V4 | 1.0 | 0.007781 | 100 | 0.135 | 155 | 102 | 12 | 7 | 152 | 2 | 133 | Uncharacterized protein | Uncharacterized protein | | afdb-uniprot50 | AF-A0A1U7CX60-F1-MODEL\_V4 | 1.0 | 0.008247 | 100 | 0.094 | 158 | 121 | 7 | 1 | 152 | 13 | 154 | Uncharacterized protein | Uncharacterized protein | | afdb-uniprot50 | AF-A0A7C6KND4-F1-MODEL\_V4 | 1.0 | 0.005492 | 100 | 0.113 | 194 | 119 | 15 | 9 | 165 | 7 | 184 | Uncharacterized protein | Uncharacterized protein | | afdb-uniprot50 | AF-A0A0F9UER8-F1-MODEL\_V4 | 1.0 | 0.002581 | 100 | 0.112 | 186 | 123 | 12 | 29 | 187 | 187 | 357 | Uncharacterized protein | Uncharacterized protein | | afdb-uniprot50 | AF-A0A2E4IAJ2-F1-MODEL\_V4 | 1.0 | 0.009262 | 100 | 0.074 | 201 | 118 | 9 | 28 | 166 | 16 | 210 | Uncharacterized protein | Uncharacterized protein | | afdb-uniprot50 | AF-A0A380Q6K4-F1-MODEL\_V4 | 0.999 | 0.002735 | 99 | 0.095 | 189 | 136 | 11 | 1 | 172 | 12 | 182 | Putative ATP-binding protein | Putative ATP-binding protein | | afdb-uniprot50 | AF-A0A7Z0QH09-F1-MODEL\_V4 | 0.999 | 0.004108 | 99 | 0.121 | 198 | 144 | 14 | 1 | 186 | 1 | 180 | Uncharacterized protein | Uncharacterized protein | | afdb-uniprot50 | AF-A0A7Y6T8L2-F1-MODEL\_V4 | 0.999 | 0.004889 | 98 | 0.158 | 158 | 104 | 10 | 13 | 152 | 4 | 150 | Uncharacterized protein | Uncharacterized protein | | afdb-uniprot50 | AF-G0ER30-F1-MODEL\_V4 | 0.999 | 0.006168 | 98 | 0.135 | 185 | 124 | 11 | 1 | 171 | 1 | 163 | Uncharacterized protein | Uncharacterized protein | | afdb-uniprot50 | AF-A0A2D0AHM2-F1-MODEL\_V4 | 0.999 | 0.00874 | 98 | 0.098 | 173 | 118 | 15 | 9 | 166 | 11 | 160 | Uncharacterized protein | Uncharacterized protein | | afdb-uniprot50 | AF-A0A6H1ZM14-F1-MODEL\_V4 | 0.999 | 0.003657 | 97 | 0.126 | 214 | 127 | 15 | 2 | 189 | 1 | 180 | Uncharacterized protein | Uncharacterized protein | | afdb-uniprot50 | AF-A0A4R6IIY1-F1-MODEL\_V4 | 0.999 | 0.003451 | 96 | 0.151 | 165 | 112 | 11 | 1 | 154 | 1 | 148 | Uncharacterized protein | Uncharacterized protein | | afdb-uniprot50 | AF-A0A4R8FHZ6-F1-MODEL\_V4 | 0.999 | 0.004889 | 96 | 0.154 | 168 | 113 | 8 | 9 | 164 | 4 | 154 | Uncharacterized protein | Uncharacterized protein | | afdb-uniprot50 | AF-A0A3E0N2N0-F1-MODEL\_V4 | 0.998 | 0.006537 | 95 | 0.132 | 166 | 106 | 10 | 1 | 153 | 1 | 141 | Uncharacterized protein | Uncharacterized protein | | afdb-uniprot50 | AF-A0A2D6AID0-F1-MODEL\_V4 | 0.998 | 0.008247 | 95 | 0.09 | 166 | 107 | 13 | 7 | 153 | 2 | 142 | Uncharacterized protein | Uncharacterized protein | | afdb-uniprot50 | AF-A0A382XXG6-F1-MODEL\_V4 | 0.998 | 0.001362 | 95 | 0.117 | 179 | 124 | 9 | 9 | 166 | 19 | 184 | Uncharacterized protein | Uncharacterized protein | | afdb-uniprot50 | AF-A0A1H3W4X8-F1-MODEL\_V4 | 0.998 | 0.007342 | 94 | 0.12 | 166 | 119 | 9 | 1 | 156 | 2 | 150 | Uncharacterized protein | Uncharacterized protein | | afdb-uniprot50 | AF-A0A7X7Z267-F1-MODEL\_V4 | 0.998 | 0.005492 | 94 | 0.168 | 178 | 109 | 12 | 1 | 156 | 1 | 161 | Uncharacterized protein | Uncharacterized protein | | afdb-uniprot50 | AF-A0A564G526-F1-MODEL\_V4 | 0.998 | 0.004889 | 94 | 0.149 | 214 | 132 | 17 | 1 | 189 | 1 | 189 | Uncharacterized protein | Uncharacterized protein | | afdb-uniprot50 | AF-A0A0F9B3J1-F1-MODEL\_V4 | 0.998 | 0.005182 | 94 | 0.137 | 197 | 115 | 12 | 7 | 175 | 4 | 173 | Uncharacterized protein | Uncharacterized protein | | afdb-uniprot50 | AF-A0A2E2JTZ9-F1-MODEL\_V4 | 0.998 | 0.008247 | 93 | 0.18 | 166 | 96 | 14 | 7 | 155 | 2 | 144 | Uncharacterized protein | Uncharacterized protein | | afdb-uniprot50 | AF-A0A1C3WP40-F1-MODEL\_V4 | 0.998 | 0.001719 | 93 | 0.158 | 196 | 118 | 15 | 13 | 186 | 5 | 175 | Uncharacterized protein | Uncharacterized protein | | afdb-uniprot50 | AF-H1XTE3-F1-MODEL\_V4 | 0.997 | 0.007342 | 91 | 0.128 | 164 | 105 | 12 | 7 | 151 | 2 | 146 | Uncharacterized protein | Uncharacterized protein | | afdb-uniprot50 | AF-A0A5C7TWN4-F1-MODEL\_V4 | 0.996 | 0.004353 | 89 | 0.184 | 168 | 100 | 15 | 9 | 154 | 5 | 157 | Uncharacterized protein | Uncharacterized protein | | afdb-uniprot50 | AF-A0A1I5MX81-F1-MODEL\_V4 | 0.996 | 0.007342 | 89 | 0.137 | 174 | 113 | 11 | 8 | 168 | 1 | 150 | Uncharacterized protein | Uncharacterized protein | | afdb-uniprot50 | AF-A0A2D6AIL0-F1-MODEL\_V4 | 0.993 | 0.007781 | 86 | 0.143 | 146 | 99 | 10 | 21 | 154 | 10 | 141 | Uncharacterized protein | Uncharacterized protein | | afdb-uniprot50 | AF-A0A102DFT8-F1-MODEL\_V4 | 0.993 | 0.002735 | 86 | 0.111 | 206 | 129 | 14 | 3 | 179 | 11 | 191 | Uncharacterized protein | Uncharacterized protein | | afdb-uniprot50 | AF-A0A7C9TIT4-F1-MODEL\_V4 | 0.986 | 0.006928 | 81 | 0.134 | 208 | 121 | 13 | 4 | 166 | 23 | 216 | Uncharacterized protein | Uncharacterized protein | | afdb-uniprot50 | AF-A0A838DQ39-F1-MODEL\_V4 | 0.984 | 0.00582 | 80 | 0.137 | 197 | 113 | 13 | 1 | 165 | 1 | 172 | Uncharacterized protein | Uncharacterized protein | | afdb-uniprot50 | AF-A0A6J5FR96-F1-MODEL\_V4 | 0.984 | 0.006537 | 80 | 0.096 | 207 | 131 | 14 | 7 | 187 | 2 | 178 | Uncharacterized protein | Uncharacterized protein | | afdb-uniprot50 | AF-A0A7C1ZHV5-F1-MODEL\_V4 | 0.981 | 0.008247 | 79 | 0.12 | 183 | 107 | 13 | 11 | 152 | 10 | 179 | Uncharacterized protein | Uncharacterized protein | | afdb-uniprot50 | AF-A0A146H6I1-F1-MODEL\_V4 | 0.981 | 0.004108 | 79 | 0.134 | 193 | 130 | 10 | 12 | 185 | 408 | 582 | Uncharacterized protein | Uncharacterized protein | |
| Top keywords  (threshold 1.00e-02 (evalue)) | **Gp37, DUF1834, tail, Phage, prophage, Minor, U, Putative, Mu\_like, DUF4128** |
| Output files | ../../similar\_structures/10\_FANPEZAQ\_CDS\_0010\_afdb-proteome\_foldseek.tsv ../../similar\_structures/10\_FANPEZAQ\_CDS\_0010\_afdb-uniprot50\_foldseek.tsv ../../similar\_structures/10\_FANPEZAQ\_CDS\_0010\_merged.svg ../../similar\_structures/10\_FANPEZAQ\_CDS\_0010\_pdb\_foldseek.tsv |

  
  
  

Return to summary | Go to previous | Go to next

  


---

**Sequence/structure alignments coloring**  
Each object in the alignment figures is colored according to its E-value following this color coding:

1e-100
10

**References:**  
1) Steinegger M, Meier M, Mirdita M, Vöhringer H, Haunsberger S J, and Söding J (2019) HH-suite3 for fast remote homology detection and deep protein annotation, BMC Bioinformatics, 473. doi: 10.1186/s12859-019-3019-7  
2) Jumper J, Evans R, Pritzel A, ..., Hassabis D (2021) Highly accurate protein structure prediction with AlphaFold, Nature, 596. doi: 10.1038/s41586-021-03819-2  
3) van Kempen M, Kim S, Tumescheit C, Mirdita M, Lee J, Gilchrist CLM, Söding J, and Steinegger M (2023) Fast and accurate protein structure search with Foldseek. Nature Biotechnology. doi: 10.1038/s41587-023-01773-0
