# Supplementary material for: Completing the BASEL phage collection to unlock hidden diversity for systematic exploration of phage–host interactions
Source: PLoS Biol. 2025 Apr 7;23(4):e3003063. doi: 10.1371/journal.pbio.3003063 (PMC11990801; doi:10.1371/journal.pbio.3003063)
Supplement: S2 Data — (ZIP) [file pbio.3003063.s009.zip › entries/11.html]

FANPEZAQ\_CDS\_0011


Return to summary | Go to previous | Go to next

|  |  |
| --- | --- |
| FANPEZAQ\_CDS\_0011 Page creation date: 02 Sep 2024, 12:00  Project folder: n/a  Input sequences file: Escherichia\_virus\_HeidiAbel.gb | baseplate assembly phage v type secretion domain\_containing vi system vgr ob\_fold gp5 phage\_base\_v vgrg tip phage\_related fragment rhs gpv p2 putative element in beta\_helix spike bacteriophage vgrg1 n\_terminal conserved iv gp138 gp45 tail prophage gene bacteria mu secreted beta ob structural so4 yes fold d t4 and a engineered escherichia |

### Sequence information

|  |  |
| --- | --- |
| Name | FANPEZAQ\_CDS\_0011  11\_FANPEZAQ\_CDS\_0011 (pipeline id) |
| Imported annotations | Escherichia\_virus\_HeidiAbel Bas97 |
| Protein sequence | MSADVSELNRQLNNVVRIGTIKQLDLANARAKVSVAGCTTDWLPWGTNRAGKRRDWSPPV IGEQVVLFAPYGDLGQAVIGPSIFQEDHAAPAASADQETTVYPDGTTVDYNSASNTLTIT VAGAGNVIINCKQANVNTETATVTASTSVTLDTPDTFCTGNLTVAKSLTMGQEGGSATMK GAVSIEGPSLTHNGKNVGSDHSHTGVQPGGGNTGAPA |
| Number of residues | 217 |
| Molecular weight (Da) | 22267.36 |
| Output files | ../../query\_sequences/11\_FANPEZAQ\_CDS\_0011.fasta |

### Putative domain architecture and protein family

#### Search results (HHblits)1

|  |  |
| --- | --- |
| Domain family databases searched | Pfam, Ncbi-cd, Cath, Phrogs |
| Results, scheme(s)  (Top layers only; threshold 1.00e-03 (evalue)) | xml version="1.0" encoding="utf-8" standalone="no"?       2024-09-02T21:08:14.150912 image/svg+xml   Matplotlib v3.7.2, https://matplotlib.org/ |
| Results, table  (E-value ≤ 1.00e-03 (evalue)) | | db | id | prob | evalue | pvalue | score | cols | query | query\_len | template | template\_len | name | description | | --- | --- | --- | --- | --- | --- | --- | --- | --- | --- | --- | --- | --- | | pfam | PF04717 | 96.3 | 6.2e-06 | 1.6e-09 | 48.8 | 43 | (42, 84) | 217 | (32, 75) | 75 | Phage\_base\_V | Type VI secretion system/phage-baseplate injector OB domain | | pfam | PF18715 | 93.9 | 0.00069 | 1.6e-07 | 38.8 | 39 | (139, 182) | 217 | (3, 41) | 47 | Phage\_spike | Phage spike trimer | | cath | 3qr8A02 | 98.4 | 3.9e-11 | 6e-15 | 78.5 | 71 | (90, 162) | 217 | (2, 73) | 73 | Baseplate assembly protein v | CATHCODE: 6.20.150.10 NAME: Baseplate assembly protein v. Chain: a. Synonym: gpv. Engineered: yes SOURCE: Enterobacteria phage p2. Bacteriophage p2. Organism\_taxid: 10679. Gene: v. Expressed in: escherichia coli. Expression\_system\_taxid: 562 CLASS: Special, ARCH: Other non-globular, TOPOL: Chondroitinase Ac; Chain A, domain 3, HOMOL: Chondroitinase Ac; Chain A, domain 3 | | cath | 3qr8A01 | 98.2 | 1.8e-10 | 2.9e-14 | 76.2 | 87 | (1, 87) | 217 | (3, 89) | 90 | Baseplate assembly protein v | CATHCODE: 2.40.50.230 NAME: Baseplate assembly protein v. Chain: a. Synonym: gpv. Engineered: yes SOURCE: Enterobacteria phage p2. Bacteriophage p2. Organism\_taxid: 10679. Gene: v. Expressed in: escherichia coli. Expression\_system\_taxid: 562 CLASS: Mainly Beta, ARCH: Beta Barrel, TOPOL: OB fold (Dihydrolipoamide Acetyltransferase, E2P), HOMOL: Gp5 N-terminal domain | | cath | 1wthA01 | 97.6 | 2.1e-08 | 3.3e-12 | 72.1 | 81 | (14, 94) | 217 | (8, 111) | 128 | Tail-associated lysozyme | CATHCODE: 2.40.50.260 NAME: Tail-associated lysozyme. Chain: a. Synonym: protein gp5. Engineered: yes. Mutation: yes. Baseplate structural protein gp27. Chain: d. Synonym: hub protein 27. Engineered: yes SOURCE: Enterobacteria phage t4. Organism\_taxid: 10665. Gene: 5. Expressed in: escherichia coli bl21(de3). Expression\_system\_taxid: 469008. CLASS: Mainly Beta, ARCH: Beta Barrel, TOPOL: OB fold (Dihydrolipoamide Acetyltransferase, E2P), HOMOL: Nucleic acid-binding protein domain | | cath | 4uhvA04 | 96.2 | 1.3e-05 | 2.2e-09 | 50.2 | 74 | (14, 87) | 217 | (14, 102) | 103 | Vgrg1, valine-glycine repeat protein g1 | CATHCODE: 2.40.50.230 NAME: Vgrg1, valine-glycine repeat protein g1. Chain: a, b. Engineered: yes SOURCE: Pseudomonas aeruginosa. Organism\_taxid: 208964. Strain: pao1. Expressed in: escherichia coli. Expression\_system\_taxid: 469008. CLASS: Mainly Beta, ARCH: Beta Barrel, TOPOL: OB fold (Dihydrolipoamide Acetyltransferase, E2P), HOMOL: Gp5 N-terminal domain | | cath | 2p5zX04 | 96.2 | 1.5e-05 | 2.5e-09 | 48.4 | 55 | (29, 83) | 217 | (22, 85) | 85 | Type vi secretion system component | CATHCODE: 2.40.50.230 NAME: Type vi secretion system component. Chain: x. Fragment: n-terminal domain. Engineered: yes SOURCE: Escherichia coli o6. Organism\_taxid: 217992. Strain: o6:h1, cft073, upec. Atcc: 700928. Gene: c3393. Expressed in: escherichia coli bl21(de3). Expression\_system\_taxid: 469008. CLASS: Mainly Beta, ARCH: Beta Barrel, TOPOL: OB fold (Dihydrolipoamide Acetyltransferase, E2P), HOMOL: Gp5 N-terminal domain | | phrogs | 9992 | 99.8 | 2.8e-25 | 3.2e-29 | 164.5 | 127 | (1, 128) | 217 | (4, 136) | 223 | baseplate assembly protein | baseplate assembly protein; Category: tail; p200092 VI\_07688 | | phrogs | 11 | 99.7 | 1.8e-22 | 2.2e-26 | 160.8 | 180 | (7, 213) | 217 | (19, 255) | 257 | baseplate spike | baseplate spike; Category: tail; KY883640\_p56 | | phrogs | 6142 | 99.5 | 1.9e-18 | 2.2e-22 | 115.7 | 84 | (1, 85) | 217 | (3, 87) | 107 | baseplate assembly protein | baseplate assembly protein; Category: tail; p403853 VI\_11032 | |
| Top keywords  (threshold 1.00e-03 (evalue)) | **Beta, yes, Baseplate, a, Engineered, escherichia, coli, OB, Organism\_taxid, Expressed** |
| Output files | ../../domain\_architecture/11\_FANPEZAQ\_CDS\_0011\_cath.hhr ../../domain\_architecture/11\_FANPEZAQ\_CDS\_0011\_merged.svg ../../domain\_architecture/11\_FANPEZAQ\_CDS\_0011\_ncbi-cd.hhr ../../domain\_architecture/11\_FANPEZAQ\_CDS\_0011\_pfam.hhr ../../domain\_architecture/11\_FANPEZAQ\_CDS\_0011\_phrogs.hhr |

### Identical protein sequences/structures

#### Search results

|  |  |
| --- | --- |
| Protein sequence databases searched | Pdb, Swissprot, Refseq |
| Identical proteins found | -- |
| Top keywords | -- |
| Output files | -- |

### Similar protein sequences/structures

#### Sequence similarity search results (HHblits)1

|  |  |
| --- | --- |
| Sequence databases searched | Uniclust, Pdb70 |
| Results, scheme(s)  (Top layers only, threshold 1.00e-03 (evalue)) | xml version="1.0" encoding="utf-8" standalone="no"?       2024-09-02T21:08:32.411119 image/svg+xml   Matplotlib v3.7.2, https://matplotlib.org/ |
| Results, table(s)  (threshold 1.00e-03 (evalue)) | | db | id | prob | evalue | pvalue | score | cols | query | query\_len | template | template\_len | name | description | | --- | --- | --- | --- | --- | --- | --- | --- | --- | --- | --- | --- | --- | | uniclust | UniRef100\_A0A023Q1D6 | 100.0 | 1.1e-45 | 2.1e-51 | 295.6 | 214 | (1, 216) | 217 | (54, 274) | 306 | Gp5/Type VI secretion system Vgr protein OB-fold domain-containing protein | Gp5/Type VI secretion system Vgr protein OB-fold domain-containing protein | | uniclust | UniRef100\_A0A068Z7F4 | 100.0 | 6e-44 | 1.2e-49 | 283.6 | 209 | (1, 217) | 217 | (54, 264) | 282 | Phage baseplate assembly protein V | Phage baseplate assembly protein V | | uniclust | UniRef100\_A0A0L1KF84 | 100.0 | 4.3e-43 | 8.5e-49 | 262.2 | 184 | (1, 217) | 217 | (15, 198) | 209 | Phage baseplate assembly protein V | Phage baseplate assembly protein V | | uniclust | UniRef100\_A0A031GY36 | 100.0 | 4.9e-43 | 1e-48 | 280.1 | 201 | (1, 217) | 217 | (38, 246) | 291 | Phage P2 baseplate assembly gpV protein | Phage P2 baseplate assembly gpV protein | | uniclust | UniRef100\_A0A3A9HX61 | 100.0 | 5.9e-43 | 1.1e-48 | 256.5 | 204 | (1, 217) | 217 | (1, 207) | 208 | Phage baseplate assembly protein V | Phage baseplate assembly protein V | | uniclust | UniRef100\_A0A016XIR2 | 100.0 | 1e-42 | 2e-48 | 264.3 | 207 | (3, 217) | 217 | (6, 225) | 255 | Baseplate assembly protein | Baseplate assembly protein | | uniclust | UniRef100\_A0A011MGN7 | 100.0 | 1.8e-42 | 3.4e-48 | 259.7 | 197 | (1, 217) | 217 | (30, 229) | 232 | Baseplate assembly protein | Baseplate assembly protein | | uniclust | UniRef100\_A0A061JPC8 | 100.0 | 2.8e-42 | 5.6e-48 | 269.4 | 133 | (1, 133) | 217 | (30, 162) | 252 | Baseplate assembly protein | Baseplate assembly protein | | uniclust | UniRef100\_A0A0Y5J9M2 | 100.0 | 1.8e-40 | 3.5e-46 | 255.0 | 194 | (1, 217) | 217 | (42, 237) | 267 | Phage baseplate assembly protein V | Phage baseplate assembly protein V | | uniclust | UniRef100\_A0A0P6WI63 | 100.0 | 5.1e-40 | 9.9e-46 | 242.2 | 182 | (2, 217) | 217 | (12, 193) | 195 | Gp5/Type VI secretion system Vgr protein OB-fold domain-containing protein | Gp5/Type VI secretion system Vgr protein OB-fold domain-containing protein | | uniclust | UniRef100\_A0A077L0E8 | 100.0 | 7.3e-40 | 1.5e-45 | 247.5 | 190 | (1, 217) | 217 | (10, 199) | 207 | Gp5/Type VI secretion system Vgr protein OB-fold domain-containing protein | Gp5/Type VI secretion system Vgr protein OB-fold domain-containing protein | | uniclust | UniRef100\_A0A064AG27 | 100.0 | 2.1e-39 | 4.2e-45 | 246.4 | 205 | (1, 217) | 217 | (2, 217) | 220 | Baseplate assembly protein | Baseplate assembly protein | | uniclust | UniRef100\_A0A014N999 | 100.0 | 1.2e-38 | 2.2e-44 | 238.7 | 192 | (1, 217) | 217 | (54, 245) | 245 | Baseplate assembly protein | Baseplate assembly protein | | uniclust | UniRef100\_A0A0D8L226 | 100.0 | 2e-38 | 3.8e-44 | 234.6 | 205 | (3, 217) | 217 | (1, 207) | 210 | Baseplate assembly protein (Fragment) | Baseplate assembly protein (Fragment) | | uniclust | UniRef100\_A0A011VGA0 | 100.0 | 4.5e-37 | 9e-43 | 232.7 | 186 | (1, 217) | 217 | (17, 203) | 207 | Phage baseplate assembly protein V | Phage baseplate assembly protein V | | uniclust | UniRef100\_A0A1M7R7X5 | 100.0 | 5.3e-37 | 1.1e-42 | 237.6 | 212 | (2, 217) | 217 | (11, 247) | 249 | Phage baseplate assembly protein V | Phage baseplate assembly protein V | | uniclust | UniRef100\_A0A059IUA8 | 100.0 | 5.6e-37 | 1.1e-42 | 232.9 | 131 | (1, 133) | 217 | (23, 153) | 209 | Prophage LambdaW5, baseplate assembly protein V | Prophage LambdaW5, baseplate assembly protein V | | uniclust | UniRef100\_A0A149SVM5 | 100.0 | 1e-36 | 2.1e-42 | 234.8 | 182 | (2, 203) | 217 | (10, 197) | 223 | Gp5/Type VI secretion system Vgr protein OB-fold domain-containing protein | Gp5/Type VI secretion system Vgr protein OB-fold domain-containing protein | | uniclust | UniRef100\_A0A076PVW1 | 100.0 | 3.8e-36 | 7.2e-42 | 228.1 | 198 | (4, 217) | 217 | (42, 253) | 283 | Baseplate assembly protein | Baseplate assembly protein | | uniclust | UniRef100\_A0A239C9W6 | 100.0 | 4.8e-36 | 9.5e-42 | 228.2 | 194 | (1, 217) | 217 | (20, 219) | 227 | Phage baseplate assembly protein V | Phage baseplate assembly protein V | | uniclust | UniRef100\_A0A011LYC8 | 100.0 | 5.7e-36 | 1.1e-41 | 233.5 | 203 | (2, 216) | 217 | (24, 240) | 242 | Phage baseplate protein | Phage baseplate protein | | uniclust | UniRef100\_A0A1E3G706 | 100.0 | 7.2e-36 | 1.4e-41 | 221.3 | 124 | (1, 124) | 217 | (3, 126) | 198 | Gp5/Type VI secretion system Vgr protein OB-fold domain-containing protein | Gp5/Type VI secretion system Vgr protein OB-fold domain-containing protein | | uniclust | UniRef100\_A0A0L8A9N2 | 100.0 | 1.5e-35 | 2.9e-41 | 218.9 | 195 | (1, 216) | 217 | (1, 197) | 199 | Baseplate assembly protein | Baseplate assembly protein | | uniclust | UniRef100\_A0A5S9Q467 | 100.0 | 5.1e-35 | 9.7e-41 | 214.2 | 205 | (1, 217) | 217 | (3, 213) | 213 | Gp5/Type VI secretion system Vgr protein OB-fold domain-containing protein | Gp5/Type VI secretion system Vgr protein OB-fold domain-containing protein | | uniclust | UniRef100\_A0A136H344 | 100.0 | 6.9e-34 | 1.4e-39 | 213.8 | 184 | (1, 217) | 217 | (29, 212) | 214 | Gp5/Type VI secretion system Vgr protein OB-fold domain-containing protein | Gp5/Type VI secretion system Vgr protein OB-fold domain-containing protein | | uniclust | UniRef100\_A0A066RUV3 | 100.0 | 9.5e-34 | 1.9e-39 | 217.8 | 152 | (3, 161) | 217 | (37, 190) | 244 | Phage baseplate assembly protein V | Phage baseplate assembly protein V | | uniclust | UniRef100\_A0A0B4CLV9 | 100.0 | 8.6e-33 | 1.6e-38 | 199.0 | 182 | (1, 217) | 217 | (6, 189) | 189 | Baseplate assembly protein | Baseplate assembly protein | | uniclust | UniRef100\_A0A098AYV7 | 100.0 | 1e-32 | 2e-38 | 203.8 | 162 | (3, 217) | 217 | (12, 177) | 186 | Phage baseplate assembly protein V | Phage baseplate assembly protein V | | uniclust | UniRef100\_A0A009PIX5 | 100.0 | 1.9e-32 | 3.6e-38 | 195.2 | 183 | (9, 217) | 217 | (5, 187) | 188 | Phage baseplate assembly V family protein | Phage baseplate assembly V family protein | | uniclust | UniRef100\_A0A101WGP2 | 99.9 | 1.8e-31 | 3.5e-37 | 193.5 | 162 | (1, 217) | 217 | (4, 169) | 172 | Baseplate assembly protein | Baseplate assembly protein | | uniclust | UniRef100\_A0A4R2MLJ2 | 99.9 | 4.2e-31 | 8.1e-37 | 186.4 | 129 | (3, 133) | 217 | (7, 135) | 156 | Phage baseplate assembly protein V | Phage baseplate assembly protein V | | uniclust | UniRef100\_A0A085ASD7 | 99.9 | 4.9e-31 | 9.5e-37 | 199.4 | 133 | (1, 133) | 217 | (19, 153) | 231 | Phage-related baseplate protein | Phage-related baseplate protein | | uniclust | UniRef100\_A0A024E8J9 | 99.9 | 5e-31 | 9.6e-37 | 199.5 | 198 | (1, 210) | 217 | (31, 228) | 244 | Pyocin R2\_PP | Pyocin R2\_PP | | uniclust | UniRef100\_A0A0F7XZI7 | 99.9 | 2.4e-30 | 4.7e-36 | 184.7 | 124 | (1, 124) | 217 | (11, 134) | 157 | Phage baseplate assembly protein V | Phage baseplate assembly protein V | | uniclust | UniRef100\_A0A4V2RT09 | 99.9 | 4.2e-30 | 8e-36 | 181.9 | 118 | (4, 121) | 217 | (9, 126) | 150 | Phage baseplate assembly protein V | Phage baseplate assembly protein V | | uniclust | UniRef100\_V5ZC64 | 99.9 | 6.3e-30 | 1.2e-35 | 189.1 | 191 | (1, 216) | 217 | (84, 274) | 275 | Baseplate assembly protein V GpV | Baseplate assembly protein V GpV | | uniclust | UniRef100\_A0A0A1AFI6 | 99.9 | 1.4e-29 | 2.6e-35 | 192.5 | 194 | (16, 216) | 217 | (10, 243) | 257 | Phage baseplate assembly protein V | Phage baseplate assembly protein V | | uniclust | UniRef100\_A0A0M5KYZ3 | 99.9 | 2.8e-29 | 5.3e-35 | 185.3 | 214 | (3, 217) | 217 | (10, 232) | 232 | Phage-related baseplate assembly protein | Phage-related baseplate assembly protein | | uniclust | UniRef100\_A0A0U2B1K8 | 99.9 | 4.5e-29 | 8.5e-35 | 179.5 | 172 | (10, 216) | 217 | (6, 181) | 181 | Phage baseplate assembly protein V | Phage baseplate assembly protein V | | uniclust | UniRef100\_A0A1I3LN83 | 99.9 | 6.8e-29 | 1.3e-34 | 195.7 | 210 | (1, 217) | 217 | (1, 211) | 473 | Phage baseplate assembly protein V | Phage baseplate assembly protein V | | uniclust | UniRef100\_A0A156SSS7 | 99.9 | 1.5e-28 | 2.8e-34 | 173.6 | 130 | (1, 132) | 217 | (3, 132) | 159 | Phage baseplate assembly-like protein | Phage baseplate assembly-like protein | | uniclust | UniRef100\_A0A0M5MIR9 | 99.9 | 1.6e-28 | 3.1e-34 | 185.5 | 195 | (5, 207) | 217 | (3, 208) | 216 | Phage baseplate assembly protein V | Phage baseplate assembly protein V | | uniclust | UniRef100\_A0A1G5B4F2 | 99.9 | 5.7e-28 | 1.1e-33 | 181.0 | 129 | (3, 133) | 217 | (10, 141) | 206 | Phage baseplate assembly protein V | Phage baseplate assembly protein V | | uniclust | UniRef100\_A0A376DH93 | 99.9 | 1.1e-27 | 2.1e-33 | 186.6 | 191 | (4, 215) | 217 | (215, 405) | 418 | Putative phage tail completion protein S | Putative phage tail completion protein S | | uniclust | UniRef100\_A0A0G0BNF7 | 99.9 | 2.1e-27 | 4.1e-33 | 175.9 | 110 | (9, 119) | 217 | (6, 118) | 180 | Gp5/Type VI secretion system Vgr protein OB-fold domain-containing protein | Gp5/Type VI secretion system Vgr protein OB-fold domain-containing protein | | uniclust | UniRef100\_A0A0Q7A745 | 99.9 | 3.5e-27 | 6.9e-33 | 178.9 | 169 | (2, 217) | 217 | (22, 195) | 206 | Gp5/Type VI secretion system Vgr protein OB-fold domain-containing protein | Gp5/Type VI secretion system Vgr protein OB-fold domain-containing protein | | uniclust | UniRef100\_A0A2C9EHC3 | 99.9 | 3.7e-27 | 7e-33 | 178.3 | 193 | (3, 217) | 217 | (48, 241) | 243 | Baseplate assembly protein V | Baseplate assembly protein V | | uniclust | UniRef100\_A0A1C2K2I7 | 99.9 | 4.9e-27 | 9.4e-33 | 177.6 | 207 | (2, 216) | 217 | (24, 242) | 243 | Gp5/Type VI secretion system Vgr protein OB-fold domain-containing protein | Gp5/Type VI secretion system Vgr protein OB-fold domain-containing protein | | uniclust | UniRef100\_A0A074TD51 | 99.9 | 8.8e-27 | 1.7e-32 | 174.5 | 163 | (2, 217) | 217 | (36, 205) | 215 | Baseplate assembly protein | Baseplate assembly protein | | uniclust | UniRef100\_A0A0A6D4K9 | 99.9 | 9.1e-27 | 1.7e-32 | 170.8 | 186 | (10, 217) | 217 | (3, 195) | 195 | Baseplate assembly protein | Baseplate assembly protein | | uniclust | UniRef100\_A0A7X4AMQ9 | 99.9 | 1.1e-26 | 2e-32 | 174.9 | 131 | (3, 133) | 217 | (87, 219) | 308 | Phage baseplate assembly protein V | Phage baseplate assembly protein V | | uniclust | UniRef100\_A0A017HCC4 | 99.9 | 1.2e-26 | 2.3e-32 | 182.6 | 160 | (1, 217) | 217 | (73, 237) | 266 | Phage-related baseplate assembly protein V | Phage-related baseplate assembly protein V | | uniclust | UniRef100\_A0A074MGB6 | 99.9 | 2e-26 | 3.9e-32 | 174.3 | 117 | (3, 121) | 217 | (15, 134) | 190 | Phage baseplate assembly protein V | Phage baseplate assembly protein V | | uniclust | UniRef100\_A0A075KC29 | 99.9 | 2.8e-26 | 5.6e-32 | 172.2 | 161 | (1, 217) | 217 | (6, 173) | 181 | Phage baseplate assembly protein V | Phage baseplate assembly protein V | | uniclust | UniRef100\_A0A010S5G8 | 99.9 | 3.3e-26 | 6.6e-32 | 180.0 | 181 | (17, 217) | 217 | (53, 240) | 255 | Baseplate assembly protein | Baseplate assembly protein | | uniclust | UniRef100\_A0A177RV31 | 99.9 | 4.7e-26 | 8.8e-32 | 163.5 | 123 | (1, 123) | 217 | (21, 144) | 176 | Baseplate assembly protein (Fragment) | Baseplate assembly protein (Fragment) | | uniclust | UniRef100\_A0A377KA20 | 99.9 | 5.8e-26 | 1.1e-31 | 156.2 | 121 | (1, 121) | 217 | (3, 123) | 139 | Phage baseplate assembly protein V | Phage baseplate assembly protein V | | uniclust | UniRef100\_A0A378AZS7 | 99.9 | 7e-26 | 1.3e-31 | 155.4 | 120 | (3, 122) | 217 | (1, 120) | 142 | Baseplate assembly protein V | Baseplate assembly protein V | | uniclust | UniRef100\_A0A081MYL2 | 99.9 | 7.3e-26 | 1.4e-31 | 169.1 | 130 | (3, 133) | 217 | (11, 144) | 213 | Gp5/Type VI secretion system Vgr protein OB-fold domain-containing protein | Gp5/Type VI secretion system Vgr protein OB-fold domain-containing protein | | uniclust | UniRef100\_A0A1N7LRL3 | 99.9 | 7.4e-26 | 1.4e-31 | 169.5 | 119 | (3, 121) | 217 | (20, 138) | 215 | Phage baseplate assembly protein V | Phage baseplate assembly protein V | | uniclust | UniRef100\_A0A5M7Q9F8 | 99.9 | 7.9e-26 | 1.4e-31 | 166.9 | 193 | (1, 216) | 217 | (1, 261) | 262 | Phage baseplate assembly protein V | Phage baseplate assembly protein V | | uniclust | UniRef100\_A0A286C7H0 | 99.9 | 7.9e-26 | 1.5e-31 | 168.8 | 196 | (1, 216) | 217 | (6, 215) | 217 | Phage baseplate assembly protein V | Phage baseplate assembly protein V | | uniclust | UniRef100\_A0A1Y6CPE2 | 99.9 | 8.8e-26 | 1.7e-31 | 165.8 | 122 | (3, 124) | 217 | (5, 127) | 211 | Phage baseplate assembly protein V | Phage baseplate assembly protein V | | uniclust | UniRef100\_A0A2J4XY04 | 99.9 | 1.3e-25 | 2.4e-31 | 148.9 | 113 | (3, 115) | 217 | (1, 113) | 114 | Phage baseplate assembly protein V (Fragment) | Phage baseplate assembly protein V (Fragment) | | uniclust | UniRef100\_A0A0A1AKB5 | 99.9 | 1.5e-25 | 2.8e-31 | 162.3 | 185 | (4, 205) | 217 | (6, 200) | 212 | Phage baseplate assembly V family protein | Phage baseplate assembly V family protein | | uniclust | UniRef100\_A0A0A8H9Z0 | 99.9 | 2.2e-25 | 4.4e-31 | 169.5 | 146 | (1, 159) | 217 | (3, 148) | 187 | Phage baseplate assembly protein V | Phage baseplate assembly protein V | | uniclust | UniRef100\_A0A085AFN7 | 99.9 | 2.3e-25 | 4.5e-31 | 174.1 | 126 | (2, 131) | 217 | (37, 164) | 250 | Phage-related baseplate protein | Phage-related baseplate protein | | uniclust | UniRef100\_UPI001E4BCFD7 | 99.9 | 2.8e-25 | 5.2e-31 | 156.5 | 152 | (48, 217) | 217 | (3, 157) | 160 | phage baseplate assembly protein V | phage baseplate assembly protein V | | uniclust | UniRef100\_A0A381EC13 | 99.9 | 3.7e-25 | 7e-31 | 157.5 | 166 | (1, 217) | 217 | (1, 170) | 170 | Phage P2 baseplate assembly protein gpV | Phage P2 baseplate assembly protein gpV | | uniclust | UniRef100\_UPI0021A5E1A5 | 99.9 | 4.2e-25 | 7.7e-31 | 167.4 | 184 | (6, 217) | 217 | (110, 310) | 310 | phage baseplate assembly protein V | phage baseplate assembly protein V | | uniclust | UniRef100\_A0A167H3T4 | 99.9 | 4.2e-25 | 8e-31 | 163.6 | 122 | (1, 122) | 217 | (5, 126) | 191 | Gp5/Type VI secretion system Vgr protein OB-fold domain-containing protein | Gp5/Type VI secretion system Vgr protein OB-fold domain-containing protein | | uniclust | UniRef100\_A0A078MK26 | 99.9 | 4.1e-25 | 8.1e-31 | 162.1 | 129 | (3, 133) | 217 | (4, 135) | 175 | Phage-related baseplate assembly protein | Phage-related baseplate assembly protein | | uniclust | UniRef100\_A0A0B1R3F1 | 99.8 | 5.2e-25 | 1e-30 | 170.0 | 174 | (16, 216) | 217 | (25, 209) | 226 | Baseplate assembly protein | Baseplate assembly protein | | uniclust | UniRef100\_A0A013SVT5 | 99.8 | 6.9e-25 | 1.4e-30 | 178.8 | 175 | (17, 217) | 217 | (65, 278) | 304 | Putative transmembrane phage protein | Putative transmembrane phage protein | | uniclust | UniRef100\_A0A0M4DFV7 | 99.8 | 7.7e-25 | 1.5e-30 | 170.6 | 195 | (1, 217) | 217 | (13, 225) | 238 | Mu-like prophage protein gp45 | Mu-like prophage protein gp45 | | uniclust | UniRef100\_A0A0B2K158 | 99.8 | 9.4e-25 | 1.8e-30 | 155.5 | 117 | (7, 123) | 217 | (4, 123) | 150 | Gp5/Type VI secretion system Vgr protein OB-fold domain-containing protein | Gp5/Type VI secretion system Vgr protein OB-fold domain-containing protein | | uniclust | UniRef100\_A0A927DLG2 | 99.8 | 1.2e-24 | 2.3e-30 | 166.6 | 180 | (1, 205) | 217 | (1, 180) | 310 | Phage baseplate assembly protein V | Phage baseplate assembly protein V | | uniclust | UniRef100\_UPI001EFC9B91 | 99.8 | 3e-24 | 5.5e-30 | 161.2 | 162 | (1, 172) | 217 | (1, 162) | 294 | phage baseplate assembly protein V | phage baseplate assembly protein V | | uniclust | UniRef100\_A0A099PAQ9 | 99.8 | 2.9e-24 | 5.9e-30 | 161.7 | 141 | (14, 161) | 217 | (21, 174) | 187 | Gp5/Type VI secretion system Vgr protein OB-fold domain-containing protein | Gp5/Type VI secretion system Vgr protein OB-fold domain-containing protein | | uniclust | UniRef100\_A0A060GZS5 | 99.8 | 9.3e-24 | 1.7e-29 | 153.5 | 181 | (4, 208) | 217 | (33, 215) | 224 | Baseplate assembly protein | Baseplate assembly protein | | uniclust | UniRef100\_UPI0020131D42 | 99.8 | 1.3e-23 | 2.3e-29 | 163.7 | 148 | (1, 164) | 217 | (1, 148) | 396 | phage baseplate assembly protein V | phage baseplate assembly protein V | | uniclust | UniRef100\_A0A0J6K5M1 | 99.8 | 1.7e-23 | 3.2e-29 | 160.0 | 177 | (2, 216) | 217 | (15, 197) | 221 | Baseplate assembly protein | Baseplate assembly protein | | uniclust | UniRef100\_A0A066PI41 | 99.8 | 2e-23 | 4e-29 | 160.9 | 99 | (15, 113) | 217 | (26, 127) | 212 | Phage baseplate assembly protein V | Phage baseplate assembly protein V | | uniclust | UniRef100\_UPI0004DCBFF1 | 99.8 | 4.4e-23 | 8e-29 | 154.1 | 117 | (4, 122) | 217 | (8, 124) | 278 | phage baseplate assembly protein V | phage baseplate assembly protein V | | uniclust | UniRef100\_A0A1H4BQX8 | 99.8 | 5.5e-23 | 1e-28 | 150.6 | 166 | (49, 216) | 217 | (2, 192) | 194 | Phage baseplate assembly protein V | Phage baseplate assembly protein V | | uniclust | UniRef100\_A0A063BCW0 | 99.8 | 7.8e-23 | 1.6e-28 | 163.3 | 187 | (1, 205) | 217 | (36, 243) | 278 | Phage baseplate assembly protein V | Phage baseplate assembly protein V | | uniclust | UniRef100\_A0A0S4L9Z0 | 99.8 | 1.2e-22 | 2.4e-28 | 161.3 | 144 | (16, 159) | 217 | (25, 193) | 294 | Gp5/Type VI secretion system Vgr protein OB-fold domain-containing protein | Gp5/Type VI secretion system Vgr protein OB-fold domain-containing protein | | uniclust | UniRef100\_A0A0T7DUY3 | 99.8 | 1.4e-22 | 2.7e-28 | 155.3 | 118 | (4, 121) | 217 | (11, 134) | 265 | Putative Baseplate | Putative Baseplate | | uniclust | UniRef100\_A0A6L7TC77 | 99.8 | 2.1e-22 | 3.9e-28 | 158.5 | 127 | (3, 129) | 217 | (236, 368) | 428 | Phage baseplate assembly protein V | Phage baseplate assembly protein V | | uniclust | UniRef100\_A0A1M6LDL7 | 99.8 | 2.1e-22 | 4.2e-28 | 148.7 | 163 | (1, 217) | 217 | (6, 172) | 174 | Bacteriophage Mu Gp45 protein | Bacteriophage Mu Gp45 protein | | uniclust | UniRef100\_A0A1I5MP97 | 99.8 | 2.7e-22 | 5.2e-28 | 149.8 | 123 | (6, 129) | 217 | (7, 132) | 205 | Phage P2 baseplate assembly protein gpV | Phage P2 baseplate assembly protein gpV | | uniclust | UniRef100\_A0A0X1U7T7 | 99.8 | 3.3e-22 | 6.5e-28 | 154.0 | 97 | (12, 114) | 217 | (20, 122) | 227 | Phage-related baseplate assembly protein | Phage-related baseplate assembly protein | | uniclust | UniRef100\_A0A0Q2UFL2 | 99.8 | 3.9e-22 | 7.4e-28 | 144.8 | 157 | (8, 217) | 217 | (7, 166) | 172 | Gp5/Type VI secretion system Vgr protein OB-fold domain-containing protein | Gp5/Type VI secretion system Vgr protein OB-fold domain-containing protein | | uniclust | UniRef100\_A0A2G1CUF1 | 99.8 | 4.1e-22 | 7.8e-28 | 151.4 | 129 | (2, 132) | 217 | (1, 137) | 248 | Baseplate assembly protein | Baseplate assembly protein | | uniclust | UniRef100\_A0A022PBR6 | 99.8 | 4e-22 | 8.1e-28 | 156.8 | 175 | (1, 210) | 217 | (19, 197) | 238 | Phage baseplate assembly protein V | Phage baseplate assembly protein V | | uniclust | UniRef100\_A0A1J5E6Z0 | 99.8 | 5.4e-22 | 1.1e-27 | 155.4 | 143 | (18, 160) | 217 | (30, 188) | 253 | Gp5/Type VI secretion system Vgr protein OB-fold domain-containing protein | Gp5/Type VI secretion system Vgr protein OB-fold domain-containing protein | | uniclust | UniRef100\_A0A7W6WMQ5 | 99.8 | 6.8e-22 | 1.3e-27 | 141.7 | 128 | (4, 133) | 217 | (12, 139) | 203 | Phage baseplate assembly protein V | Phage baseplate assembly protein V | | uniclust | UniRef100\_A0A219Y8Y7 | 99.8 | 6.7e-22 | 1.3e-27 | 147.7 | 122 | (2, 124) | 217 | (4, 126) | 233 | Baseplate assembly protein V | Baseplate assembly protein V | | uniclust | UniRef100\_A0A1U9VIH8 | 99.8 | 6.9e-22 | 1.3e-27 | 147.1 | 180 | (2, 215) | 217 | (7, 197) | 202 | Phage baseplate protein | Phage baseplate protein | | uniclust | UniRef100\_A0A094ZPX1 | 99.8 | 6.9e-22 | 1.4e-27 | 150.4 | 178 | (1, 217) | 217 | (7, 191) | 200 | Phage baseplate assembly protein V | Phage baseplate assembly protein V | | uniclust | UniRef100\_A0A077QIF1 | 99.8 | 7.2e-22 | 1.4e-27 | 151.3 | 174 | (17, 217) | 217 | (29, 239) | 240 | Putative bacteriophage protein | Putative bacteriophage protein | | uniclust | UniRef100\_A0A0Q8B2S5 | 99.8 | 7.3e-22 | 1.4e-27 | 149.7 | 162 | (2, 217) | 217 | (26, 187) | 200 | Gp5/Type VI secretion system Vgr protein OB-fold domain-containing protein | Gp5/Type VI secretion system Vgr protein OB-fold domain-containing protein | | uniclust | UniRef100\_A0A062V419 | 99.8 | 8.8e-22 | 1.8e-27 | 158.9 | 116 | (16, 131) | 217 | (47, 179) | 284 | Gp5/Type VI secretion system Vgr protein OB-fold domain-containing protein | Gp5/Type VI secretion system Vgr protein OB-fold domain-containing protein | | uniclust | UniRef100\_A0A014LI06 | 99.8 | 1e-21 | 1.9e-27 | 147.5 | 143 | (18, 160) | 217 | (22, 180) | 234 | Type IV secretion protein Rhs | Type IV secretion protein Rhs | | uniclust | UniRef100\_A0A2G3L892 | 99.8 | 1.2e-21 | 2.3e-27 | 147.0 | 129 | (1, 132) | 217 | (1, 130) | 284 | Oxidoreductase | Oxidoreductase | | uniclust | UniRef100\_A0A376X398 | 99.7 | 1.5e-21 | 2.8e-27 | 141.5 | 149 | (52, 213) | 217 | (8, 164) | 168 | Phage baseplate assembly protein V | Phage baseplate assembly protein V | | uniclust | UniRef100\_A0A016XKF3 | 99.7 | 1.6e-21 | 3.2e-27 | 154.2 | 138 | (2, 162) | 217 | (22, 162) | 231 | Phage baseplate assembly protein V | Phage baseplate assembly protein V | | uniclust | UniRef100\_A0A238TH44 | 99.7 | 1.8e-21 | 3.3e-27 | 141.5 | 180 | (12, 213) | 217 | (17, 200) | 203 | Phage-related baseplate assembly protein | Phage-related baseplate assembly protein | | uniclust | UniRef100\_A0A0Q6VK61 | 99.7 | 2.1e-21 | 4.1e-27 | 154.0 | 145 | (17, 161) | 217 | (52, 217) | 281 | Gp5/Type VI secretion system Vgr protein OB-fold domain-containing protein | Gp5/Type VI secretion system Vgr protein OB-fold domain-containing protein | | uniclust | UniRef100\_A0A1T2XAL1 | 99.7 | 2.4e-21 | 4.7e-27 | 138.6 | 107 | (14, 123) | 217 | (3, 112) | 140 | Phage baseplate assembly protein V | Phage baseplate assembly protein V | | uniclust | UniRef100\_UPI000C8213C5 | 99.7 | 3.4e-21 | 6.2e-27 | 149.4 | 159 | (1, 168) | 217 | (1, 159) | 368 | phage baseplate assembly protein V | phage baseplate assembly protein V | | uniclust | UniRef100\_UPI001403B0E1 | 99.7 | 4e-21 | 7.4e-27 | 143.3 | 187 | (18, 215) | 217 | (79, 267) | 269 | phage baseplate assembly protein V | phage baseplate assembly protein V | | uniclust | UniRef100\_A0A7S8EHN4 | 99.7 | 4.4e-21 | 8.3e-27 | 141.7 | 88 | (4, 92) | 217 | (29, 116) | 198 | Phage baseplate assembly protein V | Phage baseplate assembly protein V | | uniclust | UniRef100\_A0A1Z5H6B0 | 99.7 | 4.6e-21 | 8.8e-27 | 135.7 | 133 | (47, 210) | 217 | (3, 135) | 145 | Gp5/Type VI secretion system Vgr protein OB-fold domain-containing protein | Gp5/Type VI secretion system Vgr protein OB-fold domain-containing protein | | uniclust | UniRef100\_A0A256CB18 | 99.7 | 5.4e-21 | 1e-26 | 143.6 | 206 | (2, 215) | 217 | (1, 218) | 220 | Uncharacterized protein | Uncharacterized protein | | uniclust | UniRef100\_A0A0A2WM62 | 99.7 | 5.4e-21 | 1.1e-26 | 146.8 | 121 | (12, 132) | 217 | (23, 158) | 221 | Rhs element Vgr protein | Rhs element Vgr protein | | uniclust | UniRef100\_A0A4Q6DAY4 | 99.7 | 6.8e-21 | 1.2e-26 | 136.6 | 123 | (1, 123) | 217 | (1, 123) | 195 | Phage baseplate assembly protein V (Fragment) | Phage baseplate assembly protein V (Fragment) | | uniclust | UniRef100\_A0A547PW88 | 99.7 | 7.2e-21 | 1.3e-26 | 136.4 | 149 | (1, 217) | 217 | (51, 199) | 200 | Phage baseplate assembly protein V | Phage baseplate assembly protein V | | uniclust | UniRef100\_UPI001558E326 | 99.7 | 8.8e-21 | 1.6e-26 | 131.3 | 126 | (6, 133) | 217 | (4, 129) | 159 | phage baseplate assembly protein V | phage baseplate assembly protein V | | uniclust | UniRef100\_A0A1Q7BLJ5 | 99.7 | 8.6e-21 | 1.7e-26 | 147.3 | 147 | (16, 166) | 217 | (25, 189) | 251 | Gp5/Type VI secretion system Vgr protein OB-fold domain-containing protein | Gp5/Type VI secretion system Vgr protein OB-fold domain-containing protein | | uniclust | UniRef100\_A0A074LQM8 | 99.7 | 1.2e-20 | 2.3e-26 | 141.4 | 152 | (17, 217) | 217 | (27, 181) | 182 | Gp5/Type VI secretion system Vgr protein OB-fold domain-containing protein | Gp5/Type VI secretion system Vgr protein OB-fold domain-containing protein | | uniclust | UniRef100\_A0A8X6Y3B4 | 99.7 | 1.5e-20 | 2.8e-26 | 127.9 | 118 | (3, 120) | 217 | (7, 124) | 135 | Spike protein | Spike protein | | uniclust | UniRef100\_A0A0H3WP77 | 99.7 | 1.5e-20 | 3e-26 | 144.8 | 150 | (3, 168) | 217 | (20, 175) | 219 | Baseplate assembly protein | Baseplate assembly protein | | uniclust | UniRef100\_A0A1X7MG63 | 99.7 | 2e-20 | 3.8e-26 | 122.0 | 86 | (3, 88) | 217 | (8, 95) | 98 | Phage-related baseplate assembly protein V (Fragment) | Phage-related baseplate assembly protein V (Fragment) | | uniclust | UniRef100\_A0A011NHQ0 | 99.7 | 2.9e-20 | 5.8e-26 | 148.1 | 116 | (16, 131) | 217 | (60, 191) | 271 | Rhs element Vgr protein | Rhs element Vgr protein | | uniclust | UniRef100\_UPI001BAEB46D | 99.7 | 5.7e-20 | 1.1e-25 | 118.4 | 92 | (3, 94) | 217 | (8, 99) | 102 | phage baseplate assembly protein V | phage baseplate assembly protein V | | uniclust | UniRef100\_A0A0Q4KYF8 | 99.7 | 6e-20 | 1.2e-25 | 138.8 | 141 | (15, 155) | 217 | (18, 172) | 213 | Gp5/Type VI secretion system Vgr protein OB-fold domain-containing protein | Gp5/Type VI secretion system Vgr protein OB-fold domain-containing protein | | uniclust | UniRef100\_A0A1Q7MAI9 | 99.7 | 6e-20 | 1.2e-25 | 141.1 | 145 | (16, 160) | 217 | (26, 188) | 237 | Gp5/Type VI secretion system Vgr protein OB-fold domain-containing protein | Gp5/Type VI secretion system Vgr protein OB-fold domain-containing protein | | uniclust | UniRef100\_UPI00156D5AAA | 99.7 | 8.6e-20 | 1.6e-25 | 132.1 | 117 | (4, 120) | 217 | (2, 119) | 212 | phage baseplate assembly protein V | phage baseplate assembly protein V | | uniclust | UniRef100\_G1UXN9 | 99.7 | 9.8e-20 | 1.8e-25 | 146.7 | 199 | (14, 216) | 217 | (22, 230) | 512 | Gp5/Type VI secretion system Vgr protein OB-fold domain-containing protein | Gp5/Type VI secretion system Vgr protein OB-fold domain-containing protein | | uniclust | UniRef100\_A0A376FH76 | 99.7 | 1e-19 | 1.9e-25 | 127.5 | 137 | (63, 207) | 217 | (1, 139) | 143 | Phage Baseplate Assembly protein V | Phage Baseplate Assembly protein V | | uniclust | UniRef100\_A0A1E3G5Z4 | 99.7 | 1.2e-19 | 2.2e-25 | 129.2 | 130 | (2, 133) | 217 | (20, 153) | 188 | Gp5/Type VI secretion system Vgr protein OB-fold domain-containing protein | Gp5/Type VI secretion system Vgr protein OB-fold domain-containing protein | | uniclust | UniRef100\_A0A165RMS4 | 99.7 | 1.3e-19 | 2.5e-25 | 133.7 | 168 | (3, 217) | 217 | (12, 179) | 180 | Phage-related baseplate assembly protein | Phage-related baseplate assembly protein | | uniclust | UniRef100\_A0A0C4WPL1 | 99.7 | 1.4e-19 | 2.7e-25 | 133.4 | 168 | (1, 217) | 217 | (3, 173) | 174 | Phage baseplate assembly V family protein | Phage baseplate assembly V family protein | | uniclust | UniRef100\_A0A1H2R423 | 99.7 | 1.5e-19 | 2.8e-25 | 126.7 | 84 | (2, 86) | 217 | (13, 97) | 140 | Phage baseplate assembly protein V | Phage baseplate assembly protein V | | uniclust | UniRef100\_UPI0008A6FAC2 | 99.7 | 1.7e-19 | 3.1e-25 | 128.9 | 152 | (4, 171) | 217 | (2, 154) | 193 | phage baseplate assembly protein V | phage baseplate assembly protein V | | uniclust | UniRef100\_UPI00135400ED | 99.7 | 1.8e-19 | 3.3e-25 | 126.3 | 130 | (3, 132) | 217 | (5, 136) | 150 | phage baseplate assembly protein V | phage baseplate assembly protein V | | uniclust | UniRef100\_A0A0A8WRA5 | 99.7 | 1.8e-19 | 3.4e-25 | 141.2 | 78 | (16, 93) | 217 | (20, 107) | 290 | Putative type VI secretion system protein VgrGA | Putative type VI secretion system protein VgrGA | | uniclust | UniRef100\_A0A087KRL2 | 99.7 | 2.1e-19 | 4.2e-25 | 139.4 | 167 | (1, 217) | 217 | (7, 181) | 211 | Prophage baseplate assembly protein V | Prophage baseplate assembly protein V | | uniclust | UniRef100\_A0A1D2QMX7 | 99.6 | 2.9e-19 | 5.4e-25 | 130.3 | 120 | (3, 122) | 217 | (11, 135) | 199 | Gp5/Type VI secretion system Vgr protein OB-fold domain-containing protein | Gp5/Type VI secretion system Vgr protein OB-fold domain-containing protein | | uniclust | UniRef100\_UPI0013D0D801 | 99.6 | 3e-19 | 5.6e-25 | 116.8 | 91 | (30, 120) | 217 | (2, 92) | 98 | phage baseplate assembly protein V | phage baseplate assembly protein V | | uniclust | UniRef100\_A0A061YJY3 | 99.6 | 3.6e-19 | 6.7e-25 | 137.8 | 127 | (3, 129) | 217 | (17, 144) | 316 | Phage baseplate assembly protein V | Phage baseplate assembly protein V | | uniclust | UniRef100\_A0A955WDS1 | 99.6 | 3.9e-19 | 7.6e-25 | 145.5 | 104 | (16, 119) | 217 | (23, 138) | 356 | Gp5/Type VI secretion system Vgr protein OB-fold domain-containing protein | Gp5/Type VI secretion system Vgr protein OB-fold domain-containing protein | | uniclust | UniRef100\_A0A021X9L8 | 99.6 | 4.9e-19 | 9.7e-25 | 139.4 | 103 | (2, 104) | 217 | (48, 155) | 235 | Phage P2 baseplate assembly protein GpV | Phage P2 baseplate assembly protein GpV | | uniclust | UniRef100\_A0A015YA37 | 99.6 | 5.2e-19 | 1e-24 | 158.1 | 119 | (15, 133) | 217 | (391, 522) | 651 | Phage late control D family protein | Phage late control D family protein | | uniclust | UniRef100\_A0A1I7E546 | 99.6 | 7.1e-19 | 1.3e-24 | 130.8 | 131 | (1, 133) | 217 | (1, 132) | 254 | Phage baseplate assembly protein V | Phage baseplate assembly protein V | | uniclust | UniRef100\_A0A2N2D1G0 | 99.6 | 8.1e-19 | 1.6e-24 | 136.1 | 108 | (16, 123) | 217 | (21, 142) | 248 | Phage tail protein | Phage tail protein | | uniclust | UniRef100\_UPI000DFC22FD | 99.6 | 9.1e-19 | 1.7e-24 | 123.0 | 137 | (52, 205) | 217 | (15, 158) | 170 | phage baseplate assembly protein V | phage baseplate assembly protein V | | uniclust | UniRef100\_A0A8S4QQF2 | 99.6 | 1e-18 | 1.9e-24 | 139.6 | 126 | (6, 133) | 217 | (264, 389) | 409 | Jg15362 protein | Jg15362 protein | | uniclust | UniRef100\_A0A080M624 | 99.6 | 9.4e-19 | 1.9e-24 | 143.8 | 117 | (17, 133) | 217 | (39, 170) | 304 | Phage-related baseplate assembly protein | Phage-related baseplate assembly protein | | uniclust | UniRef100\_A0A1A9VKF9 | 99.6 | 1.4e-18 | 2.6e-24 | 138.1 | 119 | (13, 133) | 217 | (200, 318) | 378 | Phage\_base\_V domain-containing protein | Phage\_base\_V domain-containing protein | | uniclust | UniRef100\_A0A085ZBA7 | 99.6 | 1.5e-18 | 3e-24 | 160.8 | 132 | (16, 153) | 217 | (475, 616) | 858 | Rhs element Vgr protein | Rhs element Vgr protein | | uniclust | UniRef100\_A0A7X2CFJ2 | 99.6 | 1.7e-18 | 3.2e-24 | 120.2 | 141 | (55, 217) | 217 | (2, 142) | 142 | Phage baseplate assembly protein V | Phage baseplate assembly protein V | | uniclust | UniRef100\_A0A077NCM3 | 99.6 | 1.8e-18 | 3.6e-24 | 138.3 | 169 | (17, 217) | 217 | (47, 249) | 257 | Putative Similar to bacteriophage protein | Putative Similar to bacteriophage protein | | uniclust | UniRef100\_A0A512ISE8 | 99.6 | 2e-18 | 3.9e-24 | 132.1 | 155 | (15, 171) | 217 | (25, 187) | 221 | Gp5/Type VI secretion system Vgr protein OB-fold domain-containing protein | Gp5/Type VI secretion system Vgr protein OB-fold domain-containing protein | | uniclust | UniRef100\_A0A285CU56 | 99.6 | 2.1e-18 | 4.1e-24 | 145.6 | 118 | (16, 133) | 217 | (236, 364) | 456 | Gp5/Type VI secretion system Vgr protein OB-fold domain-containing protein | Gp5/Type VI secretion system Vgr protein OB-fold domain-containing protein | | uniclust | UniRef100\_A0A1A1YQV4 | 99.6 | 2.3e-18 | 4.4e-24 | 136.0 | 117 | (16, 132) | 217 | (12, 139) | 275 | Gp5/Type VI secretion system Vgr protein OB-fold domain-containing protein | Gp5/Type VI secretion system Vgr protein OB-fold domain-containing protein | | uniclust | UniRef100\_A0A2N1PDD4 | 99.6 | 2.4e-18 | 4.8e-24 | 153.2 | 139 | (14, 152) | 217 | (412, 566) | 648 | Gp5/Type VI secretion system Vgr protein OB-fold domain-containing protein | Gp5/Type VI secretion system Vgr protein OB-fold domain-containing protein | | uniclust | UniRef100\_A0A014LZB8 | 99.6 | 2.5e-18 | 5e-24 | 155.3 | 118 | (16, 133) | 217 | (395, 524) | 640 | Type VI secretion protein VgrG | Type VI secretion protein VgrG | | uniclust | UniRef100\_A0A730JXJ6 | 99.6 | 3e-18 | 5.6e-24 | 110.2 | 90 | (22, 111) | 217 | (2, 91) | 91 | Phage baseplate assembly protein V (Fragment) | Phage baseplate assembly protein V (Fragment) | | uniclust | UniRef100\_A0A1M7YBP6 | 99.6 | 3.1e-18 | 5.9e-24 | 122.9 | 106 | (10, 121) | 217 | (4, 112) | 159 | Phage baseplate assembly protein V | Phage baseplate assembly protein V | | uniclust | UniRef100\_A0A0A1IUD8 | 99.6 | 3.3e-18 | 6.5e-24 | 135.0 | 187 | (17, 217) | 217 | (31, 257) | 259 | Phage protein Gp138 N-terminal domain-containing protein | Phage protein Gp138 N-terminal domain-containing protein | | uniclust | UniRef100\_A0A1M6PFI3 | 99.6 | 3.8e-18 | 7.2e-24 | 131.5 | 141 | (15, 155) | 217 | (20, 176) | 251 | Gp5/Type VI secretion system Vgr protein OB-fold domain-containing protein (Fragment) | Gp5/Type VI secretion system Vgr protein OB-fold domain-containing protein (Fragment) | | uniclust | UniRef100\_J1JE15 | 99.6 | 4.8e-18 | 8.8e-24 | 116.2 | 119 | (3, 122) | 217 | (7, 125) | 142 | Phage baseplate assembly protein V (Fragment) | Phage baseplate assembly protein V (Fragment) | | uniclust | UniRef100\_A0A090IQB5 | 99.6 | 4.8e-18 | 9e-24 | 130.3 | 198 | (3, 207) | 217 | (53, 257) | 268 | Phage baseplate assembly protein V | Phage baseplate assembly protein V | | uniclust | UniRef100\_A0A6I1HQM0 | 99.6 | 5.5e-18 | 1e-23 | 129.8 | 119 | (1, 121) | 217 | (4, 123) | 314 | Phage baseplate assembly protein V | Phage baseplate assembly protein V | | uniclust | UniRef100\_A0A190XCB5 | 99.6 | 5.5e-18 | 1.1e-23 | 132.6 | 160 | (16, 217) | 217 | (43, 217) | 222 | Putative baseplate assembly protein | Putative baseplate assembly protein | | uniclust | UniRef100\_A0A0H3ZTQ1 | 99.6 | 5.8e-18 | 1.1e-23 | 131.7 | 199 | (3, 216) | 217 | (35, 263) | 266 | Baseplate assembly protein V | Baseplate assembly protein V | | uniclust | UniRef100\_A0A0V0Q6U3 | 99.6 | 5.8e-18 | 1.1e-23 | 131.6 | 119 | (2, 120) | 217 | (17, 169) | 251 | Gp5/Type VI secretion system Vgr protein OB-fold domain-containing protein | Gp5/Type VI secretion system Vgr protein OB-fold domain-containing protein | | uniclust | UniRef100\_A0A516S9U8 | 99.6 | 6.7e-18 | 1.2e-23 | 122.5 | 130 | (3, 132) | 217 | (56, 186) | 209 | Phage baseplate assembly protein V | Phage baseplate assembly protein V | | uniclust | UniRef100\_A0A0C1ZK93 | 99.6 | 6.8e-18 | 1.3e-23 | 129.7 | 131 | (3, 133) | 217 | (5, 149) | 209 | VgrG protein | VgrG protein | | uniclust | UniRef100\_A0A0R9Q039 | 99.6 | 7.3e-18 | 1.3e-23 | 125.1 | 104 | (17, 121) | 217 | (7, 113) | 240 | Baseplate protein | Baseplate protein | | uniclust | UniRef100\_A0A139D6P1 | 99.6 | 7.7e-18 | 1.4e-23 | 121.9 | 120 | (5, 133) | 217 | (5, 127) | 184 | Phage baseplate assembly protein V | Phage baseplate assembly protein V | | uniclust | UniRef100\_A0A0B1XX70 | 99.6 | 7.4e-18 | 1.5e-23 | 149.1 | 119 | (15, 133) | 217 | (375, 504) | 589 | Type VI secretion protein VgrG | Type VI secretion protein VgrG | | uniclust | UniRef100\_A0A0X8C3M9 | 99.6 | 8.6e-18 | 1.7e-23 | 135.6 | 131 | (17, 153) | 217 | (72, 211) | 322 | Gp5/Type VI secretion system Vgr protein OB-fold domain-containing protein | Gp5/Type VI secretion system Vgr protein OB-fold domain-containing protein | | uniclust | UniRef100\_UPI000C036BF4 | 99.6 | 9.8e-18 | 1.8e-23 | 110.2 | 89 | (35, 123) | 217 | (3, 91) | 101 | phage baseplate assembly protein V | phage baseplate assembly protein V | | uniclust | UniRef100\_A0A016A3P0 | 99.6 | 9e-18 | 1.8e-23 | 152.7 | 134 | (16, 155) | 217 | (406, 553) | 721 | Phage late control D family protein | Phage late control D family protein | | uniclust | UniRef100\_A0A068R1Y7 | 99.6 | 9.2e-18 | 1.8e-23 | 132.9 | 178 | (17, 217) | 217 | (40, 237) | 243 | Phage protein Gp138 N-terminal domain-containing protein | Phage protein Gp138 N-terminal domain-containing protein | | uniclust | UniRef100\_A0A0J9EDT8 | 99.6 | 1e-17 | 2e-23 | 119.1 | 151 | (4, 217) | 217 | (8, 159) | 159 | Phage baseplate assembly protein V | Phage baseplate assembly protein V | | uniclust | UniRef100\_A0A927HQP9 | 99.5 | 1.4e-17 | 2.6e-23 | 122.7 | 171 | (10, 205) | 217 | (2, 172) | 232 | Phage baseplate assembly protein V | Phage baseplate assembly protein V | | uniclust | UniRef100\_A0A0K9NC70 | 99.5 | 1.3e-17 | 2.7e-23 | 135.8 | 138 | (17, 154) | 217 | (38, 208) | 279 | Gp5/Type VI secretion system Vgr protein OB-fold domain-containing protein | Gp5/Type VI secretion system Vgr protein OB-fold domain-containing protein | | uniclust | UniRef100\_A0A0M0SQ28 | 99.5 | 1.4e-17 | 2.7e-23 | 142.5 | 132 | (17, 153) | 217 | (377, 525) | 572 | Gp5/Type VI secretion system Vgr protein OB-fold domain-containing protein | Gp5/Type VI secretion system Vgr protein OB-fold domain-containing protein | | uniclust | UniRef100\_A0A4Q4AGX4 | 99.5 | 1.5e-17 | 2.8e-23 | 106.8 | 83 | (4, 86) | 217 | (10, 95) | 98 | Phage baseplate assembly protein V (Fragment) | Phage baseplate assembly protein V (Fragment) | | uniclust | UniRef100\_A0A069QGK5 | 99.5 | 1.5e-17 | 3e-23 | 135.1 | 117 | (17, 133) | 217 | (20, 150) | 280 | Gp5/Type VI secretion system Vgr protein OB-fold domain-containing protein | Gp5/Type VI secretion system Vgr protein OB-fold domain-containing protein | | uniclust | UniRef100\_A0A4P9VER7 | 99.5 | 1.7e-17 | 3.2e-23 | 119.3 | 130 | (3, 133) | 217 | (9, 142) | 191 | Phage baseplate assembly protein V | Phage baseplate assembly protein V | | uniclust | UniRef100\_A0A264Y5K0 | 99.5 | 1.7e-17 | 3.3e-23 | 132.2 | 118 | (16, 133) | 217 | (64, 192) | 285 | Gp5/Type VI secretion system Vgr protein OB-fold domain-containing protein | Gp5/Type VI secretion system Vgr protein OB-fold domain-containing protein | | uniclust | UniRef100\_UPI000488E995 | 99.5 | 2.1e-17 | 3.8e-23 | 122.3 | 128 | (3, 132) | 217 | (28, 155) | 239 | phage baseplate assembly protein V | phage baseplate assembly protein V | | uniclust | UniRef100\_A0A016QM46 | 99.5 | 2.2e-17 | 4.5e-23 | 153.1 | 117 | (15, 131) | 217 | (552, 686) | 899 | Rhs element Vgr protein | Rhs element Vgr protein | | uniclust | UniRef100\_A0A0D8L1Y3 | 99.5 | 2.5e-17 | 4.8e-23 | 114.4 | 117 | (80, 217) | 217 | (2, 118) | 119 | Baseplate assembly protein (Fragment) | Baseplate assembly protein (Fragment) | | uniclust | UniRef100\_A0A0D2JZP5 | 99.5 | 2.9e-17 | 5.4e-23 | 118.9 | 126 | (5, 132) | 217 | (12, 140) | 181 | Baseplate protein | Baseplate protein | | uniclust | UniRef100\_A0A353GTI7 | 99.5 | 3.1e-17 | 5.7e-23 | 120.5 | 177 | (11, 217) | 217 | (45, 225) | 225 | Phage baseplate assembly protein V | Phage baseplate assembly protein V | | uniclust | UniRef100\_A0A6I5RL13 | 99.5 | 3.1e-17 | 5.9e-23 | 108.3 | 85 | (1, 85) | 217 | (5, 89) | 91 | Phage baseplate assembly protein V (Fragment) | Phage baseplate assembly protein V (Fragment) | | uniclust | UniRef100\_F4QG87 | 99.5 | 3.1e-17 | 6e-23 | 122.7 | 186 | (1, 216) | 217 | (1, 189) | 199 | Phage baseplate assembly protein V family protein | Phage baseplate assembly protein V family protein | | uniclust | UniRef100\_A0A1A7KHP9 | 99.5 | 3.1e-17 | 6.1e-23 | 149.6 | 133 | (15, 153) | 217 | (146, 286) | 854 | Uncharacterized protein | Uncharacterized protein | | uniclust | UniRef100\_A0A0Q1FEE3 | 99.5 | 3.4e-17 | 6.5e-23 | 126.4 | 191 | (3, 217) | 217 | (9, 216) | 232 | Baseplate assembly protein | Baseplate assembly protein | | uniclust | UniRef100\_A0A023WVH6 | 99.5 | 3.6e-17 | 7.2e-23 | 126.3 | 115 | (1, 132) | 217 | (12, 129) | 205 | Phage baseplate assembly protein V | Phage baseplate assembly protein V | | uniclust | UniRef100\_A0A3N5MAF8 | 99.5 | 4.1e-17 | 8e-23 | 136.0 | 119 | (15, 133) | 217 | (187, 322) | 402 | Gp5/Type VI secretion system Vgr protein OB-fold domain-containing protein | Gp5/Type VI secretion system Vgr protein OB-fold domain-containing protein | | uniclust | UniRef100\_A0A840REX7 | 99.5 | 4.6e-17 | 8.4e-23 | 115.6 | 118 | (4, 121) | 217 | (3, 122) | 178 | Phage baseplate assembly protein V | Phage baseplate assembly protein V | | uniclust | UniRef100\_UPI001F2EC826 | 99.5 | 5.4e-17 | 1e-22 | 109.9 | 114 | (8, 121) | 217 | (2, 115) | 127 | phage baseplate assembly protein V | phage baseplate assembly protein V | | uniclust | UniRef100\_A0A257KYV8 | 99.5 | 5.4e-17 | 1e-22 | 124.2 | 118 | (16, 133) | 217 | (27, 155) | 232 | Type IV secretion protein Rhs (Fragment) | Type IV secretion protein Rhs (Fragment) | | uniclust | UniRef100\_A0A143XMY6 | 99.5 | 5.8e-17 | 1.1e-22 | 122.1 | 107 | (9, 121) | 217 | (11, 123) | 177 | Phage-related baseplate assembly protein | Phage-related baseplate assembly protein | | uniclust | UniRef100\_A0A0S8CC80 | 99.5 | 6.1e-17 | 1.2e-22 | 142.4 | 140 | (17, 156) | 217 | (503, 663) | 728 | Gp5/Type VI secretion system Vgr protein OB-fold domain-containing protein | Gp5/Type VI secretion system Vgr protein OB-fold domain-containing protein | | uniclust | UniRef100\_A0A4Q1SII8 | 99.5 | 6.1e-17 | 1.2e-22 | 140.0 | 116 | (9, 124) | 217 | (392, 523) | 596 | Type VI secretion system tip protein VgrG | Type VI secretion system tip protein VgrG | | uniclust | UniRef100\_A0A149TN28 | 99.5 | 6.2e-17 | 1.2e-22 | 121.1 | 178 | (1, 217) | 217 | (10, 192) | 193 | Baseplate assembly protein | Baseplate assembly protein | | uniclust | UniRef100\_A0A8S5LES5 | 99.5 | 6.6e-17 | 1.2e-22 | 118.2 | 165 | (15, 185) | 217 | (6, 174) | 208 | Baseplate assembly protein | Baseplate assembly protein | | uniclust | UniRef100\_A0A0G3A0D9 | 99.5 | 6.4e-17 | 1.3e-22 | 143.4 | 135 | (13, 154) | 217 | (379, 523) | 568 | Phage baseplate assembly protein V | Phage baseplate assembly protein V | | uniclust | UniRef100\_A0A4U0Z1D4 | 99.5 | 7.5e-17 | 1.4e-22 | 122.9 | 150 | (1, 217) | 217 | (146, 295) | 296 | Phage baseplate assembly protein V | Phage baseplate assembly protein V | | uniclust | UniRef100\_A0A085HGF5 | 99.5 | 7.9e-17 | 1.5e-22 | 122.8 | 181 | (1, 217) | 217 | (1, 224) | 225 | Putative phage protein | Putative phage protein | | uniclust | UniRef100\_A0A538PGR1 | 99.5 | 8.9e-17 | 1.7e-22 | 134.7 | 100 | (16, 115) | 217 | (22, 134) | 455 | OmpA-like domain-containing protein (Fragment) | OmpA-like domain-containing protein (Fragment) | | uniclust | UniRef100\_A0A248UJ04 | 99.5 | 1.4e-16 | 2.6e-22 | 114.3 | 94 | (1, 94) | 217 | (15, 113) | 149 | Phage-related baseplate assembly family protein | Phage-related baseplate assembly family protein | | uniclust | UniRef100\_A0A0J7KLX9 | 99.5 | 1.4e-16 | 2.7e-22 | 138.0 | 132 | (16, 153) | 217 | (366, 506) | 608 | Gp5/Type VI secretion system Vgr protein OB-fold domain-containing protein (Fragment) | Gp5/Type VI secretion system Vgr protein OB-fold domain-containing protein (Fragment) | | uniclust | UniRef100\_A0A1H4BCX2 | 99.5 | 1.5e-16 | 2.9e-22 | 139.3 | 117 | (16, 132) | 217 | (378, 505) | 583 | Rhs element Vgr protein | Rhs element Vgr protein | | uniclust | UniRef100\_UPI0005E17221 | 99.5 | 1.8e-16 | 3.5e-22 | 101.7 | 74 | (1, 74) | 217 | (2, 75) | 86 | phage baseplate assembly protein V | phage baseplate assembly protein V | | uniclust | UniRef100\_A0A0J7IFW6 | 99.5 | 1.8e-16 | 3.5e-22 | 123.5 | 119 | (29, 153) | 217 | (1, 126) | 227 | Gp5/Type VI secretion system Vgr protein OB-fold domain-containing protein | Gp5/Type VI secretion system Vgr protein OB-fold domain-containing protein | | uniclust | UniRef100\_A0A519RXB4 | 99.5 | 1.9e-16 | 3.6e-22 | 126.8 | 118 | (15, 132) | 217 | (100, 229) | 307 | Gp5/Type VI secretion system Vgr protein OB-fold domain-containing protein | Gp5/Type VI secretion system Vgr protein OB-fold domain-containing protein | | uniclust | UniRef100\_A0A0X3APK5 | 99.5 | 2.1e-16 | 4.2e-22 | 143.2 | 136 | (16, 156) | 217 | (412, 559) | 813 | Uncharacterized conserved protein, implicated in type VI secretion and phage assembly | Uncharacterized conserved protein, implicated in type VI secretion and phage assembly | | uniclust | UniRef100\_A0A1V5WGM0 | 99.5 | 2.2e-16 | 4.3e-22 | 120.3 | 145 | (15, 159) | 217 | (7, 176) | 214 | Phage-related baseplate assembly protein | Phage-related baseplate assembly protein | | uniclust | UniRef100\_A0A0M3AAX9 | 99.4 | 2.4e-16 | 4.6e-22 | 117.4 | 166 | (7, 217) | 217 | (8, 179) | 184 | Uncharacterized protein | Uncharacterized protein | | uniclust | UniRef100\_A0A077KP81 | 99.4 | 2.6e-16 | 5.2e-22 | 148.4 | 132 | (16, 153) | 217 | (439, 579) | 1149 | Gp5/Type VI secretion system Vgr protein OB-fold domain-containing protein | Gp5/Type VI secretion system Vgr protein OB-fold domain-containing protein | | uniclust | UniRef100\_UPI000D607F75 | 99.4 | 3.4e-16 | 6.3e-22 | 116.3 | 187 | (11, 217) | 217 | (5, 212) | 216 | phage baseplate assembly protein | phage baseplate assembly protein | | uniclust | UniRef100\_E8KKS1 | 99.4 | 3.5e-16 | 6.6e-22 | 107.0 | 110 | (11, 121) | 217 | (9, 121) | 125 | Gp5/Type VI secretion system Vgr protein OB-fold domain-containing protein (Fragment) | Gp5/Type VI secretion system Vgr protein OB-fold domain-containing protein (Fragment) | | uniclust | UniRef100\_A0A2V3UB47 | 99.4 | 3.8e-16 | 7.3e-22 | 113.3 | 93 | (2, 95) | 217 | (20, 112) | 158 | Phage baseplate assembly protein V | Phage baseplate assembly protein V | | uniclust | UniRef100\_A0A3D3TAS6 | 99.4 | 4e-16 | 7.6e-22 | 123.1 | 120 | (14, 133) | 217 | (19, 154) | 273 | DUF2345 domain-containing protein (Fragment) | DUF2345 domain-containing protein (Fragment) | | uniclust | UniRef100\_UPI002155723C | 99.4 | 4.3e-16 | 7.9e-22 | 120.3 | 148 | (15, 170) | 217 | (1, 150) | 324 | phage baseplate assembly protein V | phage baseplate assembly protein V | | uniclust | UniRef100\_A0A538CIT5 | 99.4 | 4.4e-16 | 8.1e-22 | 110.9 | 142 | (15, 161) | 217 | (16, 166) | 179 | Gp5/Type VI secretion system Vgr protein OB-fold domain-containing protein | Gp5/Type VI secretion system Vgr protein OB-fold domain-containing protein | | uniclust | UniRef100\_A0A958AIE9 | 99.4 | 4.5e-16 | 8.4e-22 | 112.5 | 114 | (17, 130) | 217 | (22, 151) | 176 | Gp5/Type VI secretion system Vgr protein OB-fold domain-containing protein | Gp5/Type VI secretion system Vgr protein OB-fold domain-containing protein | | uniclust | UniRef100\_A0A0A2ECB3 | 99.4 | 4.3e-16 | 8.5e-22 | 141.5 | 120 | (14, 133) | 217 | (463, 597) | 733 | Gp5/Type VI secretion system Vgr protein OB-fold domain-containing protein | Gp5/Type VI secretion system Vgr protein OB-fold domain-containing protein | | uniclust | UniRef100\_A0A1Q3KMT8 | 99.4 | 4.6e-16 | 8.7e-22 | 115.6 | 139 | (16, 160) | 217 | (19, 172) | 193 | Gp5/Type VI secretion system Vgr protein OB-fold domain-containing protein | Gp5/Type VI secretion system Vgr protein OB-fold domain-containing protein | | uniclust | UniRef100\_A0A239WJT3 | 99.4 | 4.6e-16 | 8.9e-22 | 131.6 | 117 | (17, 133) | 217 | (210, 340) | 473 | Uncharacterized protein conserved in bacteria | Uncharacterized protein conserved in bacteria | | uniclust | UniRef100\_A0A0D8FQF9 | 99.4 | 4.6e-16 | 9.2e-22 | 139.1 | 97 | (16, 112) | 217 | (420, 528) | 645 | Phage-like baseplate assembly protein | Phage-like baseplate assembly protein | | uniclust | UniRef100\_A0A016CPP4 | 99.4 | 5.4e-16 | 1.1e-21 | 139.1 | 118 | (16, 133) | 217 | (390, 521) | 745 | Phage-related baseplate assembly family protein | Phage-related baseplate assembly family protein | | uniclust | UniRef100\_A0A0X8C270 | 99.4 | 5.4e-16 | 1.1e-21 | 144.6 | 120 | (14, 133) | 217 | (401, 530) | 1104 | Gp5/Type VI secretion system Vgr protein OB-fold domain-containing protein | Gp5/Type VI secretion system Vgr protein OB-fold domain-containing protein | | uniclust | UniRef100\_F0IFV9 | 99.4 | 6e-16 | 1.1e-21 | 120.0 | 116 | (17, 133) | 217 | (60, 185) | 294 | Gp5/Type VI secretion system Vgr protein OB-fold domain-containing protein | Gp5/Type VI secretion system Vgr protein OB-fold domain-containing protein | | uniclust | UniRef100\_A0A174LDR4 | 99.4 | 6.3e-16 | 1.2e-21 | 121.9 | 144 | (15, 158) | 217 | (33, 205) | 269 | Uncharacterized protein conserved in bacteria | Uncharacterized protein conserved in bacteria | | uniclust | UniRef100\_A0A0C3MRL4 | 99.4 | 6.3e-16 | 1.2e-21 | 118.8 | 144 | (15, 160) | 217 | (17, 180) | 219 | Phage-related baseplate assembly family protein | Phage-related baseplate assembly family protein | | uniclust | UniRef100\_A0A1Z3U8S6 | 99.4 | 6.6e-16 | 1.2e-21 | 115.0 | 160 | (5, 164) | 217 | (26, 192) | 222 | Phage baseplate assembly protein V | Phage baseplate assembly protein V | | uniclust | UniRef100\_A0A1E7J785 | 99.4 | 6.3e-16 | 1.3e-21 | 125.4 | 107 | (15, 121) | 217 | (18, 143) | 279 | Gp5/Type VI secretion system Vgr protein OB-fold domain-containing protein | Gp5/Type VI secretion system Vgr protein OB-fold domain-containing protein | | uniclust | UniRef100\_A0A2A4XT07 | 99.4 | 6.8e-16 | 1.3e-21 | 112.8 | 143 | (6, 166) | 217 | (25, 167) | 190 | Gp5/Type VI secretion system Vgr protein OB-fold domain-containing protein | Gp5/Type VI secretion system Vgr protein OB-fold domain-containing protein | | uniclust | UniRef100\_UPI00217DEE8E | 99.4 | 6.9e-16 | 1.3e-21 | 112.6 | 130 | (3, 132) | 217 | (11, 141) | 211 | phage baseplate assembly protein V | phage baseplate assembly protein V | | uniclust | UniRef100\_A0A2V8SPQ8 | 99.4 | 7.2e-16 | 1.4e-21 | 112.5 | 99 | (17, 115) | 217 | (24, 133) | 164 | Gp5/Type VI secretion system Vgr protein OB-fold domain-containing protein (Fragment) | Gp5/Type VI secretion system Vgr protein OB-fold domain-containing protein (Fragment) | | uniclust | UniRef100\_A0A378VX02 | 99.4 | 8e-16 | 1.5e-21 | 104.9 | 132 | (48, 216) | 217 | (3, 134) | 135 | Putative phage associated protein | Putative phage associated protein | | uniclust | UniRef100\_A0A011QIW0 | 99.4 | 7.6e-16 | 1.5e-21 | 124.4 | 103 | (1, 106) | 217 | (4, 125) | 252 | Rhs element Vgr protein | Rhs element Vgr protein | | uniclust | UniRef100\_A0A2D9F2J4 | 99.4 | 8e-16 | 1.6e-21 | 118.6 | 132 | (2, 164) | 217 | (12, 146) | 198 | Phage baseplate assembly protein V | Phage baseplate assembly protein V | | uniclust | UniRef100\_A0A376KHX7 | 99.4 | 8.8e-16 | 1.6e-21 | 97.6 | 83 | (4, 86) | 217 | (1, 83) | 85 | Baseplate assembly protein V | Baseplate assembly protein V | | uniclust | UniRef100\_A0A261QLM5 | 99.4 | 8.8e-16 | 1.6e-21 | 103.9 | 104 | (17, 121) | 217 | (7, 113) | 118 | Baseplate protein (Fragment) | Baseplate protein (Fragment) | | uniclust | UniRef100\_A0A1G9EXB4 | 99.4 | 9.5e-16 | 1.8e-21 | 118.4 | 119 | (15, 133) | 217 | (22, 153) | 226 | Gp5/Type VI secretion system Vgr protein OB-fold domain-containing protein | Gp5/Type VI secretion system Vgr protein OB-fold domain-containing protein | | uniclust | UniRef100\_A0A1M6M230 | 99.4 | 1.2e-15 | 2.2e-21 | 124.0 | 139 | (15, 153) | 217 | (135, 285) | 378 | Gp5/Type VI secretion system Vgr protein OB-fold domain-containing protein (Fragment) | Gp5/Type VI secretion system Vgr protein OB-fold domain-containing protein (Fragment) | | uniclust | UniRef100\_A0A9E8D7C5 | 99.4 | 1.3e-15 | 2.4e-21 | 102.5 | 95 | (50, 152) | 217 | (9, 103) | 113 | Phage baseplate assembly protein V | Phage baseplate assembly protein V | | uniclust | UniRef100\_A0A090V6M7 | 99.4 | 1.3e-15 | 2.4e-21 | 117.2 | 100 | (16, 115) | 217 | (35, 171) | 223 | Phage protein Gp138 N-terminal domain-containing protein | Phage protein Gp138 N-terminal domain-containing protein | | uniclust | UniRef100\_A0A7Y6Z4G0 | 99.4 | 1.4e-15 | 2.5e-21 | 110.7 | 129 | (3, 133) | 217 | (9, 138) | 205 | Phage baseplate assembly protein V | Phage baseplate assembly protein V | | uniclust | UniRef100\_A0A090G3N9 | 99.4 | 1.4e-15 | 2.7e-21 | 112.4 | 110 | (2, 113) | 217 | (17, 132) | 184 | Gp5/Type VI secretion system Vgr protein OB-fold domain-containing protein | Gp5/Type VI secretion system Vgr protein OB-fold domain-containing protein | | uniclust | UniRef100\_A0A3D3M4P4 | 99.4 | 1.5e-15 | 2.8e-21 | 106.8 | 116 | (50, 172) | 217 | (2, 117) | 137 | Phage baseplate assembly protein V (Fragment) | Phage baseplate assembly protein V (Fragment) | | uniclust | UniRef100\_A0A0T9QI62 | 99.4 | 1.5e-15 | 2.8e-21 | 99.2 | 67 | (3, 69) | 217 | (17, 83) | 84 | Phage-related baseplate assembly protein | Phage-related baseplate assembly protein | | uniclust | UniRef100\_A0A031FUD1 | 99.4 | 1.5e-15 | 2.9e-21 | 113.3 | 122 | (1, 122) | 217 | (20, 143) | 203 | Phage baseplate assembly protein V | Phage baseplate assembly protein V | | uniclust | UniRef100\_A0A0E3V6K5 | 99.4 | 1.5e-15 | 3e-21 | 136.4 | 150 | (8, 159) | 217 | (361, 540) | 610 | Gp5/Type VI secretion system Vgr protein OB-fold domain-containing protein | Gp5/Type VI secretion system Vgr protein OB-fold domain-containing protein | | uniclust | UniRef100\_A0A538Q0J0 | 99.4 | 1.6e-15 | 3e-21 | 120.1 | 100 | (16, 115) | 217 | (22, 134) | 307 | Gp5/Type VI secretion system Vgr protein OB-fold domain-containing protein (Fragment) | Gp5/Type VI secretion system Vgr protein OB-fold domain-containing protein (Fragment) | | uniclust | UniRef100\_Q31HU0 | 99.4 | 2.1e-15 | 3.8e-21 | 111.0 | 191 | (15, 209) | 217 | (1, 215) | 221 | Phage-related baseplate assembly protein V | Phage-related baseplate assembly protein V | | uniclust | UniRef100\_A0A0E3KUE2 | 99.4 | 2.2e-15 | 4.1e-21 | 128.2 | 117 | (15, 131) | 217 | (376, 505) | 574 | VgrG protein | VgrG protein | | uniclust | UniRef100\_A0A1H2FMJ1 | 99.4 | 2.1e-15 | 4.2e-21 | 116.8 | 101 | (3, 104) | 217 | (23, 131) | 207 | Phage baseplate assembly protein V | Phage baseplate assembly protein V | | uniclust | UniRef100\_A0A0S2ZDV9 | 99.4 | 2.3e-15 | 4.4e-21 | 114.7 | 160 | (17, 209) | 217 | (21, 206) | 218 | Phage protein Gp138 N-terminal domain-containing protein | Phage protein Gp138 N-terminal domain-containing protein | | uniclust | UniRef100\_A0A134C1J7 | 99.4 | 2.4e-15 | 4.4e-21 | 102.7 | 111 | (4, 114) | 217 | (2, 115) | 120 | Uncharacterized protein (Fragment) | Uncharacterized protein (Fragment) | | uniclust | UniRef100\_A0A350LX97 | 99.4 | 2.9e-15 | 5.4e-21 | 117.0 | 84 | (5, 88) | 217 | (206, 289) | 349 | Gp5/Type VI secretion system Vgr protein OB-fold domain-containing protein | Gp5/Type VI secretion system Vgr protein OB-fold domain-containing protein | | uniclust | UniRef100\_A0A2D4SLA2 | 99.4 | 2.9e-15 | 5.7e-21 | 116.4 | 109 | (12, 120) | 217 | (8, 128) | 219 | Gp5/Type VI secretion system Vgr protein OB-fold domain-containing protein | Gp5/Type VI secretion system Vgr protein OB-fold domain-containing protein | | uniclust | UniRef100\_A0A1D8UTH1 | 99.3 | 3.5e-15 | 6.6e-21 | 109.4 | 159 | (1, 217) | 217 | (1, 164) | 166 | Baseplate assembly protein | Baseplate assembly protein | | uniclust | UniRef100\_A0A0N1CB70 | 99.3 | 3.4e-15 | 6.7e-21 | 117.3 | 103 | (2, 104) | 217 | (20, 129) | 231 | Gp5/Type VI secretion system Vgr protein OB-fold domain-containing protein | Gp5/Type VI secretion system Vgr protein OB-fold domain-containing protein | | uniclust | UniRef100\_A0A069QD16 | 99.3 | 4.4e-15 | 8.4e-21 | 121.0 | 139 | (17, 155) | 217 | (105, 262) | 354 | Gp5/Type VI secretion system Vgr protein OB-fold domain-containing protein (Fragment) | Gp5/Type VI secretion system Vgr protein OB-fold domain-containing protein (Fragment) | | uniclust | UniRef100\_A0A543G2B2 | 99.3 | 4.9e-15 | 9.3e-21 | 113.8 | 125 | (28, 153) | 217 | (10, 144) | 239 | Gp5/Type VI secretion system Vgr protein OB-fold domain-containing protein | Gp5/Type VI secretion system Vgr protein OB-fold domain-containing protein | | uniclust | UniRef100\_A0A096D7L8 | 99.3 | 4.6e-15 | 9.5e-21 | 131.3 | 143 | (7, 153) | 217 | (300, 452) | 493 | Gp5/Type VI secretion system Vgr protein OB-fold domain-containing protein | Gp5/Type VI secretion system Vgr protein OB-fold domain-containing protein | | uniclust | UniRef100\_A0A0T5NZY3 | 99.3 | 4.8e-15 | 9.5e-21 | 131.6 | 119 | (14, 132) | 217 | (380, 513) | 591 | Gp5/Type VI secretion system Vgr protein OB-fold domain-containing protein | Gp5/Type VI secretion system Vgr protein OB-fold domain-containing protein | | uniclust | UniRef100\_A0A3D9EV90 | 99.3 | 5.6e-15 | 1e-20 | 103.1 | 114 | (2, 115) | 217 | (1, 120) | 154 | Phage baseplate assembly protein V | Phage baseplate assembly protein V | | uniclust | UniRef100\_A0A381EFJ1 | 99.3 | 5.9e-15 | 1.1e-20 | 106.7 | 139 | (18, 217) | 217 | (22, 163) | 164 | Mu-like prophage protein gp45 | Mu-like prophage protein gp45 | | uniclust | UniRef100\_A0A023D2H7 | 99.3 | 5.7e-15 | 1.1e-20 | 115.8 | 76 | (17, 92) | 217 | (29, 106) | 228 | Phage related baseplate assembly protein | Phage related baseplate assembly protein | | uniclust | UniRef100\_A0A0Q8LLQ7 | 99.3 | 5.8e-15 | 1.1e-20 | 128.5 | 118 | (15, 132) | 217 | (376, 505) | 601 | Gp5/Type VI secretion system Vgr protein OB-fold domain-containing protein | Gp5/Type VI secretion system Vgr protein OB-fold domain-containing protein | | uniclust | UniRef100\_UPI0020135E1D | 99.3 | 6.1e-15 | 1.1e-20 | 104.7 | 134 | (69, 205) | 217 | (14, 158) | 172 | phage baseplate assembly protein V | phage baseplate assembly protein V | | uniclust | UniRef100\_A0A0D6TN34 | 99.3 | 6.2e-15 | 1.2e-20 | 116.0 | 124 | (29, 153) | 217 | (28, 161) | 254 | Uncharacterized protein | Uncharacterized protein | | uniclust | UniRef100\_A0A0C5AEP6 | 99.3 | 8.2e-15 | 1.5e-20 | 103.4 | 110 | (10, 120) | 217 | (20, 131) | 146 | Baseplate assembly protein | Baseplate assembly protein | | uniclust | UniRef100\_A0A2C7A8I5 | 99.3 | 8.5e-15 | 1.6e-20 | 106.6 | 185 | (3, 217) | 217 | (12, 202) | 202 | Gp5/Type VI secretion system Vgr protein OB-fold domain-containing protein | Gp5/Type VI secretion system Vgr protein OB-fold domain-containing protein | | uniclust | UniRef100\_A0A150S1K0 | 99.3 | 8.9e-15 | 1.7e-20 | 108.3 | 104 | (28, 131) | 217 | (21, 135) | 187 | Gp5/Type VI secretion system Vgr protein OB-fold domain-containing protein | Gp5/Type VI secretion system Vgr protein OB-fold domain-containing protein | | uniclust | UniRef100\_A0A1H4H7U1 | 99.3 | 1e-14 | 2e-20 | 129.9 | 133 | (16, 154) | 217 | (399, 550) | 643 | Uncharacterized conserved protein, implicated in type VI secretion and phage assembly | Uncharacterized conserved protein, implicated in type VI secretion and phage assembly | | uniclust | UniRef100\_A0A0D9NFY0 | 99.3 | 1e-14 | 2e-20 | 121.6 | 117 | (17, 133) | 217 | (152, 282) | 424 | Type IV secretion protein Rhs | Type IV secretion protein Rhs | | uniclust | UniRef100\_A0A2G6EWU3 | 99.3 | 1.1e-14 | 2.1e-20 | 102.3 | 108 | (5, 112) | 217 | (6, 123) | 160 | Gp5/Type VI secretion system Vgr protein OB-fold domain-containing protein | Gp5/Type VI secretion system Vgr protein OB-fold domain-containing protein | | uniclust | UniRef100\_A0A7J6YLT3 | 99.3 | 1.1e-14 | 2.1e-20 | 118.7 | 109 | (15, 123) | 217 | (136, 244) | 508 | Baseplate protein J-like domain-containing protein | Baseplate protein J-like domain-containing protein | | uniclust | UniRef100\_A0A345DE56 | 99.3 | 1.2e-14 | 2.2e-20 | 103.5 | 141 | (4, 162) | 217 | (7, 147) | 174 | Gp5/Type VI secretion system Vgr protein OB-fold domain-containing protein | Gp5/Type VI secretion system Vgr protein OB-fold domain-containing protein | | uniclust | UniRef100\_A0A1D2QM78 | 99.3 | 1.3e-14 | 2.5e-20 | 115.5 | 120 | (2, 121) | 217 | (20, 169) | 303 | Gp5/Type VI secretion system Vgr protein OB-fold domain-containing protein | Gp5/Type VI secretion system Vgr protein OB-fold domain-containing protein | | uniclust | UniRef100\_A0A3S4GGV8 | 99.3 | 1.3e-14 | 2.5e-20 | 94.5 | 70 | (54, 123) | 217 | (3, 72) | 93 | Phage baseplate assembly protein V | Phage baseplate assembly protein V | | uniclust | UniRef100\_A0A376DPX4 | 99.3 | 1.5e-14 | 2.9e-20 | 116.9 | 134 | (17, 156) | 217 | (81, 227) | 325 | Uncharacterized protein conserved in bacteria | Uncharacterized protein conserved in bacteria | | uniclust | UniRef100\_A0A1Y0Y2S8 | 99.3 | 1.6e-14 | 3.1e-20 | 110.5 | 189 | (3, 217) | 217 | (15, 210) | 210 | Baseplate assembly protein | Baseplate assembly protein | | uniclust | UniRef100\_A0A7W0QEQ5 | 99.3 | 1.7e-14 | 3.1e-20 | 118.6 | 116 | (18, 133) | 217 | (30, 156) | 454 | Gp5/Type VI secretion system Vgr protein OB-fold domain-containing protein | Gp5/Type VI secretion system Vgr protein OB-fold domain-containing protein | | uniclust | UniRef100\_A0A0E3ZX09 | 99.3 | 1.8e-14 | 3.5e-20 | 124.4 | 153 | (16, 168) | 217 | (351, 526) | 576 | Gp5/Type VI secretion system Vgr protein OB-fold domain-containing protein | Gp5/Type VI secretion system Vgr protein OB-fold domain-containing protein | | uniclust | UniRef100\_A0A2S8ACA2 | 99.3 | 2e-14 | 3.9e-20 | 117.1 | 140 | (14, 154) | 217 | (133, 287) | 372 | Type IV secretion protein Rhs (Fragment) | Type IV secretion protein Rhs (Fragment) | | uniclust | UniRef100\_A0A0W8FUT7 | 99.3 | 2.1e-14 | 4.1e-20 | 111.5 | 138 | (7, 168) | 217 | (34, 173) | 223 | Prophage baseplate assembly protein v | Prophage baseplate assembly protein v | | uniclust | UniRef100\_UPI000F060CC4 | 99.3 | 2.3e-14 | 4.2e-20 | 101.6 | 121 | (1, 121) | 217 | (21, 143) | 148 | phage baseplate assembly protein V | phage baseplate assembly protein V | | uniclust | UniRef100\_A0A349GR45 | 99.3 | 2.4e-14 | 4.5e-20 | 106.4 | 117 | (14, 133) | 217 | (4, 123) | 199 | Baseplate assembly protein | Baseplate assembly protein | | uniclust | UniRef100\_A0A077KAY0 | 99.3 | 2.3e-14 | 4.5e-20 | 117.7 | 172 | (15, 217) | 217 | (65, 260) | 313 | Putative baseplate protein | Putative baseplate protein | | uniclust | UniRef100\_A0A1J0MF38 | 99.3 | 2.4e-14 | 4.6e-20 | 113.4 | 195 | (13, 210) | 217 | (13, 252) | 263 | Baseplate central spike | Baseplate central spike | | uniclust | UniRef100\_A0A0Q0D424 | 99.3 | 2.7e-14 | 5e-20 | 96.5 | 110 | (2, 111) | 217 | (3, 113) | 113 | Baseplate assembly protein V | Baseplate assembly protein V | | uniclust | UniRef100\_A0A0F0H101 | 99.3 | 2.5e-14 | 5e-20 | 128.8 | 98 | (16, 113) | 217 | (424, 539) | 653 | Gp5/Type VI secretion system Vgr protein OB-fold domain-containing protein | Gp5/Type VI secretion system Vgr protein OB-fold domain-containing protein | | uniclust | UniRef100\_A0A450Z2A7 | 99.2 | 3e-14 | 5.6e-20 | 96.2 | 100 | (32, 133) | 217 | (2, 101) | 114 | Phage baseplate assembly protein V | Phage baseplate assembly protein V | | uniclust | UniRef100\_A0A075MMT0 | 99.2 | 3.1e-14 | 6e-20 | 108.3 | 117 | (17, 133) | 217 | (26, 153) | 185 | Gp5/Type VI secretion system Vgr protein OB-fold domain-containing protein | Gp5/Type VI secretion system Vgr protein OB-fold domain-containing protein | | uniclust | UniRef100\_A0A094QB96 | 99.2 | 3.4e-14 | 6.5e-20 | 121.2 | 97 | (14, 110) | 217 | (367, 476) | 596 | Gp5/Type VI secretion system Vgr protein OB-fold domain-containing protein | Gp5/Type VI secretion system Vgr protein OB-fold domain-containing protein | | uniclust | UniRef100\_A0A376U5W8 | 99.2 | 3.5e-14 | 6.7e-20 | 102.1 | 132 | (61, 217) | 217 | (15, 149) | 149 | Putative phage related protein | Putative phage related protein | | uniclust | UniRef100\_A0A0D1BTC3 | 99.2 | 3.4e-14 | 6.7e-20 | 112.7 | 90 | (17, 106) | 217 | (43, 168) | 232 | Phage protein Gp138 N-terminal domain-containing protein | Phage protein Gp138 N-terminal domain-containing protein | | uniclust | UniRef100\_A0A2M6UU70 | 99.2 | 3.6e-14 | 6.8e-20 | 105.5 | 173 | (1, 216) | 217 | (14, 196) | 203 | Baseplate assembly protein | Baseplate assembly protein | | uniclust | UniRef100\_A0A3G4V723 | 99.2 | 3.8e-14 | 7.2e-20 | 104.8 | 116 | (5, 121) | 217 | (23, 139) | 203 | Phage baseplate assembly protein V | Phage baseplate assembly protein V | | uniclust | UniRef100\_A0A1C6T0T6 | 99.2 | 4.1e-14 | 8.1e-20 | 110.7 | 117 | (17, 133) | 217 | (25, 161) | 220 | Phage-related baseplate assembly protein | Phage-related baseplate assembly protein | | uniclust | UniRef100\_UPI002355B909 | 99.2 | 4.8e-14 | 8.8e-20 | 104.8 | 128 | (4, 131) | 217 | (22, 151) | 230 | phage baseplate assembly protein V | phage baseplate assembly protein V | | uniclust | UniRef100\_A0A0C5V150 | 99.2 | 4.7e-14 | 8.8e-20 | 109.7 | 114 | (3, 116) | 217 | (24, 169) | 263 | Phage P2 baseplate assembly protein gpV | Phage P2 baseplate assembly protein gpV | | uniclust | UniRef100\_UPI001EE5592A | 99.2 | 4.9e-14 | 8.9e-20 | 100.6 | 91 | (6, 96) | 217 | (8, 101) | 174 | phage baseplate assembly protein V | phage baseplate assembly protein V | | uniclust | UniRef100\_A0A0Q4L4M4 | 99.2 | 4.6e-14 | 9e-20 | 113.5 | 129 | (1, 133) | 217 | (6, 147) | 276 | Gp5/Type VI secretion system Vgr protein OB-fold domain-containing protein | Gp5/Type VI secretion system Vgr protein OB-fold domain-containing protein | | uniclust | UniRef100\_A0A1G6Z7R3 | 99.2 | 4.9e-14 | 9.6e-20 | 125.5 | 104 | (17, 120) | 217 | (354, 466) | 627 | Uncharacterized conserved protein, implicated in type VI secretion and phage assembly | Uncharacterized conserved protein, implicated in type VI secretion and phage assembly | | uniclust | UniRef100\_A0A8S5QP55 | 99.2 | 5.1e-14 | 9.6e-20 | 111.3 | 166 | (41, 217) | 217 | (88, 287) | 287 | Baseplate protein | Baseplate protein | | uniclust | UniRef100\_A0A7C2HLH3 | 99.2 | 5.4e-14 | 1e-19 | 105.8 | 91 | (24, 114) | 217 | (96, 199) | 251 | Gp5/Type VI secretion system Vgr protein OB-fold domain-containing protein | Gp5/Type VI secretion system Vgr protein OB-fold domain-containing protein | | uniclust | UniRef100\_A0A1R1MK78 | 99.2 | 5.4e-14 | 1e-19 | 106.1 | 141 | (7, 153) | 217 | (4, 160) | 194 | Gp5/Type VI secretion system Vgr protein OB-fold domain-containing protein | Gp5/Type VI secretion system Vgr protein OB-fold domain-containing protein | | uniclust | UniRef100\_A0A0N0UY75 | 99.2 | 6e-14 | 1.1e-19 | 121.7 | 114 | (16, 129) | 217 | (376, 497) | 595 | Gp5/Type VI secretion system Vgr protein OB-fold domain-containing protein | Gp5/Type VI secretion system Vgr protein OB-fold domain-containing protein | | uniclust | UniRef100\_A0A7V8ABK9 | 99.2 | 6.3e-14 | 1.2e-19 | 96.6 | 119 | (9, 131) | 217 | (11, 129) | 139 | Baseplate assembly protein V | Baseplate assembly protein V | | uniclust | UniRef100\_A0A6L4A988 | 99.2 | 6.5e-14 | 1.2e-19 | 117.5 | 117 | (17, 133) | 217 | (255, 393) | 487 | VgrG-related protein (Fragment) | VgrG-related protein (Fragment) | | uniclust | UniRef100\_A0A2C9EHI6 | 99.2 | 7.1e-14 | 1.3e-19 | 105.2 | 161 | (11, 217) | 217 | (71, 234) | 237 | Prophage MuSo2, baseplate assembly protein V | Prophage MuSo2, baseplate assembly protein V | | uniclust | UniRef100\_A0A9D2KBF5 | 99.2 | 7.5e-14 | 1.4e-19 | 104.0 | 142 | (10, 154) | 217 | (4, 157) | 234 | Phage baseplate assembly protein V | Phage baseplate assembly protein V | | uniclust | UniRef100\_A0A014MQT2 | 99.2 | 7.2e-14 | 1.4e-19 | 110.8 | 107 | (17, 123) | 217 | (28, 144) | 220 | Baseplate assembly protein | Baseplate assembly protein | | uniclust | UniRef100\_A0A3M8AFK2 | 99.2 | 7.7e-14 | 1.5e-19 | 115.6 | 143 | (8, 154) | 217 | (103, 257) | 328 | Gp5/Type VI secretion system Vgr protein OB-fold domain-containing protein (Fragment) | Gp5/Type VI secretion system Vgr protein OB-fold domain-containing protein (Fragment) | | uniclust | UniRef100\_A0A066RIQ8 | 99.2 | 7.9e-14 | 1.6e-19 | 125.1 | 81 | (16, 96) | 217 | (366, 461) | 666 | Type IV secretion protein Rhs | Type IV secretion protein Rhs | | uniclust | UniRef100\_A0A812QV09 | 99.2 | 9.4e-14 | 1.7e-19 | 122.0 | 115 | (17, 133) | 217 | (944, 1058) | 1173 | XkdF protein | XkdF protein | | uniclust | UniRef100\_A0A2P8AQI4 | 99.2 | 9e-14 | 1.7e-19 | 110.8 | 138 | (10, 154) | 217 | (95, 242) | 283 | Gp5/Type VI secretion system Vgr protein OB-fold domain-containing protein | Gp5/Type VI secretion system Vgr protein OB-fold domain-containing protein | | uniclust | UniRef100\_A0A0B6S1Y0 | 99.2 | 9e-14 | 1.7e-19 | 108.5 | 169 | (16, 210) | 217 | (34, 222) | 233 | Phage baseplate assembly protein V | Phage baseplate assembly protein V | | uniclust | UniRef100\_A0A1E3WN49 | 99.2 | 1.1e-13 | 2.1e-19 | 104.2 | 182 | (2, 210) | 217 | (9, 193) | 205 | Baseplate puncturing device gp45 | Baseplate puncturing device gp45 | | uniclust | UniRef100\_A0A0A2ZWF3 | 99.2 | 1.2e-13 | 2.4e-19 | 111.0 | 71 | (1, 72) | 217 | (4, 98) | 231 | Phage baseplate protein | Phage baseplate protein | | uniclust | UniRef100\_A0A1Y0UW95 | 99.2 | 1.3e-13 | 2.4e-19 | 101.8 | 137 | (39, 217) | 217 | (16, 155) | 158 | Baseplate assembly protein | Baseplate assembly protein | | uniclust | UniRef100\_A0A075R0S9 | 99.2 | 1.3e-13 | 2.6e-19 | 126.9 | 112 | (9, 122) | 217 | (384, 504) | 753 | Putative phage protein | Putative phage protein | | uniclust | UniRef100\_A0A3D3TEQ1 | 99.2 | 1.4e-13 | 2.6e-19 | 119.4 | 130 | (17, 153) | 217 | (372, 516) | 620 | Gp5/Type VI secretion system Vgr protein OB-fold domain-containing protein | Gp5/Type VI secretion system Vgr protein OB-fold domain-containing protein | | uniclust | UniRef100\_A0A067Z827 | 99.2 | 1.3e-13 | 2.6e-19 | 114.5 | 175 | (17, 217) | 217 | (85, 279) | 299 | Phage protein Gp138 N-terminal domain-containing protein | Phage protein Gp138 N-terminal domain-containing protein | | uniclust | UniRef100\_A0A066ZM10 | 99.2 | 1.5e-13 | 2.9e-19 | 104.7 | 148 | (3, 172) | 217 | (9, 160) | 217 | Baseplate assembly protein | Baseplate assembly protein | | uniclust | UniRef100\_A0A376EWE4 | 99.2 | 1.7e-13 | 3.2e-19 | 113.1 | 142 | (17, 167) | 217 | (397, 541) | 550 | Gene D protein | Gene D protein | | uniclust | UniRef100\_A0A1N7HSV3 | 99.2 | 1.8e-13 | 3.4e-19 | 113.2 | 136 | (17, 158) | 217 | (84, 228) | 424 | Gp5/Type VI secretion system Vgr protein OB-fold domain-containing protein (Fragment) | Gp5/Type VI secretion system Vgr protein OB-fold domain-containing protein (Fragment) | | uniclust | UniRef100\_A0A6N6MEQ4 | 99.2 | 1.9e-13 | 3.5e-19 | 103.1 | 95 | (3, 99) | 217 | (16, 117) | 217 | Phage baseplate assembly protein V | Phage baseplate assembly protein V | | uniclust | UniRef100\_A0A3N5RDY0 | 99.2 | 1.9e-13 | 3.6e-19 | 108.6 | 105 | (17, 121) | 217 | (61, 181) | 284 | Phage tail protein | Phage tail protein | | uniclust | UniRef100\_A0A5C8AZF4 | 99.2 | 2e-13 | 3.7e-19 | 100.8 | 179 | (13, 205) | 217 | (4, 196) | 216 | Phage baseplate assembly protein V | Phage baseplate assembly protein V | | uniclust | UniRef100\_A0A0X3XDX9 | 99.2 | 1.9e-13 | 3.7e-19 | 119.0 | 75 | (16, 90) | 217 | (249, 329) | 505 | Gp5/Type VI secretion system Vgr protein OB-fold domain-containing protein | Gp5/Type VI secretion system Vgr protein OB-fold domain-containing protein | | uniclust | UniRef100\_A0A193QLB5 | 99.1 | 2e-13 | 3.8e-19 | 94.2 | 75 | (140, 217) | 217 | (30, 106) | 106 | Uncharacterized protein | Uncharacterized protein | | uniclust | UniRef100\_A0A0A8IL55 | 99.1 | 2.2e-13 | 4.2e-19 | 102.8 | 91 | (1, 96) | 217 | (12, 107) | 183 | Baseplate assembly protein | Baseplate assembly protein | | uniclust | UniRef100\_A0A158D125 | 99.1 | 2.8e-13 | 5.2e-19 | 99.1 | 134 | (43, 216) | 217 | (12, 145) | 168 | Bacteriophage Mu Gp45 protein | Bacteriophage Mu Gp45 protein | | uniclust | UniRef100\_A0A1H8YYE7 | 99.1 | 2.7e-13 | 5.3e-19 | 106.1 | 159 | (17, 216) | 217 | (34, 223) | 226 | Phage protein Gp138 N-terminal domain-containing protein | Phage protein Gp138 N-terminal domain-containing protein | | uniclust | UniRef100\_G8X7R7 | 99.1 | 3.1e-13 | 5.9e-19 | 101.6 | 119 | (29, 153) | 217 | (31, 156) | 209 | Rhs element Vgr protein | Rhs element Vgr protein | | uniclust | UniRef100\_A0A0D0H1Q9 | 99.1 | 3.3e-13 | 6.2e-19 | 100.3 | 185 | (6, 217) | 217 | (2, 194) | 198 | Bacteriophage Mu Gp45 family protein | Bacteriophage Mu Gp45 family protein | | uniclust | UniRef100\_UPI00201700A5 | 99.1 | 3.7e-13 | 6.7e-19 | 91.1 | 86 | (36, 121) | 217 | (4, 89) | 123 | phage baseplate assembly protein V | phage baseplate assembly protein V | | uniclust | UniRef100\_A0A0A2W1E5 | 99.1 | 3.9e-13 | 7.1e-19 | 121.8 | 160 | (16, 216) | 217 | (1052, 1248) | 1885 | Uncharacterized protein | Uncharacterized protein | | uniclust | UniRef100\_A0A088C4R9 | 99.1 | 4.4e-13 | 8.6e-19 | 108.2 | 162 | (17, 203) | 217 | (49, 231) | 264 | p2 gpV-like protein | p2 gpV-like protein | | uniclust | UniRef100\_A0A2E3N1P5 | 99.1 | 5.4e-13 | 9.9e-19 | 111.3 | 163 | (3, 169) | 217 | (18, 202) | 601 | Gp5/Type VI secretion system Vgr protein OB-fold domain-containing protein | Gp5/Type VI secretion system Vgr protein OB-fold domain-containing protein | | uniclust | UniRef100\_UPI00187624BB | 99.1 | 5.9e-13 | 1.1e-18 | 103.6 | 115 | (16, 131) | 217 | (18, 139) | 317 | phage baseplate assembly protein V | phage baseplate assembly protein V | | uniclust | UniRef100\_A0A155I046 | 99.1 | 6.4e-13 | 1.2e-18 | 100.9 | 173 | (2, 216) | 217 | (41, 216) | 227 | Phage baseplate assembly protein V | Phage baseplate assembly protein V | | uniclust | UniRef100\_A0A1E8PJ79 | 99.1 | 6.5e-13 | 1.2e-18 | 101.0 | 176 | (17, 217) | 217 | (33, 220) | 221 | Phage baseplate assembly protein V | Phage baseplate assembly protein V | | uniclust | UniRef100\_A0A369KV77 | 99.1 | 6.8e-13 | 1.3e-18 | 95.4 | 133 | (3, 158) | 217 | (9, 144) | 146 | Phage baseplate assembly protein V (Fragment) | Phage baseplate assembly protein V (Fragment) | | uniclust | UniRef100\_UPI0022B86340 | 99.1 | 7.2e-13 | 1.3e-18 | 104.8 | 150 | (41, 215) | 217 | (184, 361) | 363 | Gp138 family membrane-puncturing spike protein | Gp138 family membrane-puncturing spike protein | | uniclust | UniRef100\_A0A562GH31 | 99.1 | 7.6e-13 | 1.4e-18 | 92.0 | 100 | (12, 113) | 217 | (8, 111) | 132 | Phage baseplate assembly protein V | Phage baseplate assembly protein V | | uniclust | UniRef100\_A0A2V8QTC4 | 99.1 | 9e-13 | 1.7e-18 | 98.1 | 104 | (28, 131) | 217 | (7, 118) | 225 | Phage tail protein | Phage tail protein | | uniclust | UniRef100\_A0A126QN32 | 99.1 | 8.8e-13 | 1.7e-18 | 104.5 | 190 | (14, 216) | 217 | (37, 250) | 262 | Baseplate assembly protein | Baseplate assembly protein | | uniclust | UniRef100\_A0A9E3GUV8 | 99.1 | 9.1e-13 | 1.7e-18 | 99.2 | 186 | (6, 217) | 217 | (7, 195) | 201 | Uncharacterized protein | Uncharacterized protein | | uniclust | UniRef100\_UPI00222FD497 | 99.1 | 1e-12 | 1.8e-18 | 90.2 | 111 | (17, 127) | 217 | (2, 122) | 132 | phage baseplate assembly protein V | phage baseplate assembly protein V | | uniclust | UniRef100\_A0A318ELH1 | 99.1 | 9.8e-13 | 1.9e-18 | 102.6 | 141 | (13, 160) | 217 | (3, 150) | 236 | Tail fiber protein | Tail fiber protein | | uniclust | UniRef100\_A0A2Z5T171 | 99.1 | 1e-12 | 1.9e-18 | 101.8 | 171 | (41, 216) | 217 | (65, 272) | 276 | Baseplate assembly protein | Baseplate assembly protein | | uniclust | UniRef100\_UPI001CDCD071 | 99.1 | 1e-12 | 1.9e-18 | 91.1 | 109 | (15, 123) | 217 | (1, 112) | 141 | phage baseplate assembly protein V | phage baseplate assembly protein V | | uniclust | UniRef100\_A0A2I7R3I7 | 99.1 | 1e-12 | 1.9e-18 | 98.0 | 123 | (4, 132) | 217 | (9, 135) | 228 | Baseplate assembly protein V | Baseplate assembly protein V | | uniclust | UniRef100\_E0WUR2 | 99.1 | 1e-12 | 1.9e-18 | 88.8 | 104 | (5, 108) | 217 | (3, 107) | 121 | Putative phage baseplate protein | Putative phage baseplate protein | | uniclust | UniRef100\_A0A250KKR6 | 99.1 | 1e-12 | 1.9e-18 | 113.7 | 117 | (17, 133) | 217 | (359, 489) | 593 | Type IV secretion protein Rhs | Type IV secretion protein Rhs | | uniclust | UniRef100\_A0A285JXF0 | 99.1 | 1e-12 | 2e-18 | 109.3 | 153 | (4, 159) | 217 | (6, 195) | 494 | Gp5/Type VI secretion system Vgr protein OB-fold domain-containing protein | Gp5/Type VI secretion system Vgr protein OB-fold domain-containing protein | | uniclust | UniRef100\_A0A956GQI0 | 99.0 | 1.1e-12 | 2.1e-18 | 115.2 | 119 | (18, 136) | 217 | (28, 158) | 1114 | Gp5/Type VI secretion system Vgr protein OB-fold domain-containing protein | Gp5/Type VI secretion system Vgr protein OB-fold domain-containing protein | | uniclust | UniRef100\_A0A2D9B6H3 | 99.0 | 1.3e-12 | 2.5e-18 | 102.9 | 105 | (16, 120) | 217 | (28, 141) | 251 | Gp5/Type VI secretion system Vgr protein OB-fold domain-containing protein | Gp5/Type VI secretion system Vgr protein OB-fold domain-containing protein | | uniclust | UniRef100\_A0A061PST6 | 99.0 | 1.3e-12 | 2.6e-18 | 118.6 | 67 | (27, 93) | 217 | (399, 472) | 676 | VgrG protein | VgrG protein | | uniclust | UniRef100\_A0A0A7S6V5 | 99.0 | 1.4e-12 | 2.7e-18 | 104.3 | 70 | (2, 71) | 217 | (4, 92) | 266 | Phage protein Gp138 N-terminal domain-containing protein | Phage protein Gp138 N-terminal domain-containing protein | | uniclust | UniRef100\_A0A0C4YA02 | 99.0 | 1.5e-12 | 2.7e-18 | 95.4 | 95 | (16, 110) | 217 | (25, 122) | 167 | Phage-related baseplate assembly protein | Phage-related baseplate assembly protein | | uniclust | UniRef100\_UPI000A7B1761 | 99.0 | 1.8e-12 | 3.4e-18 | 91.2 | 102 | (29, 130) | 217 | (11, 124) | 154 | phage baseplate assembly protein V | phage baseplate assembly protein V | | uniclust | UniRef100\_A0A1A8ZH78 | 99.0 | 1.7e-12 | 3.4e-18 | 103.2 | 117 | (17, 133) | 217 | (29, 153) | 246 | Gp5/Type VI secretion system Vgr protein OB-fold domain-containing protein | Gp5/Type VI secretion system Vgr protein OB-fold domain-containing protein | | uniclust | UniRef100\_A0A5C4P976 | 99.0 | 2.4e-12 | 4.4e-18 | 90.3 | 89 | (2, 90) | 217 | (1, 89) | 150 | Phage baseplate assembly protein V | Phage baseplate assembly protein V | | uniclust | UniRef100\_A0A077EH50 | 99.0 | 2.3e-12 | 4.5e-18 | 117.2 | 117 | (17, 133) | 217 | (490, 619) | 842 | VgrG protein | VgrG protein | | uniclust | UniRef100\_A0A7V9PH58 | 99.0 | 2.3e-12 | 4.5e-18 | 112.6 | 105 | (17, 124) | 217 | (354, 466) | 627 | Gp5/Type VI secretion system Vgr protein OB-fold domain-containing protein | Gp5/Type VI secretion system Vgr protein OB-fold domain-containing protein | | uniclust | UniRef100\_A0A8I2CH63 | 99.0 | 2.5e-12 | 4.6e-18 | 90.7 | 85 | (2, 86) | 217 | (1, 85) | 155 | Phage baseplate assembly protein V | Phage baseplate assembly protein V | | uniclust | UniRef100\_A0A009YIF6 | 99.0 | 2.3e-12 | 4.6e-18 | 99.1 | 107 | (1, 122) | 217 | (14, 122) | 181 | Bacteriophage Mu Gp45 family protein | Bacteriophage Mu Gp45 family protein | | uniclust | UniRef100\_A0A348FYH6 | 99.0 | 2.7e-12 | 5e-18 | 96.2 | 93 | (2, 96) | 217 | (9, 101) | 200 | Gp5/Type VI secretion system Vgr protein OB-fold domain-containing protein | Gp5/Type VI secretion system Vgr protein OB-fold domain-containing protein | | uniclust | UniRef100\_A0A5C8XT18 | 99.0 | 2.9e-12 | 5.3e-18 | 84.3 | 88 | (58, 161) | 217 | (2, 89) | 102 | Phage baseplate assembly protein V (Fragment) | Phage baseplate assembly protein V (Fragment) | | uniclust | UniRef100\_A0A0H2Y5Z8 | 99.0 | 2.9e-12 | 5.3e-18 | 86.5 | 73 | (1, 73) | 217 | (36, 108) | 119 | Gp5/Type VI secretion system Vgr protein OB-fold domain-containing protein | Gp5/Type VI secretion system Vgr protein OB-fold domain-containing protein | | uniclust | UniRef100\_UPI00209A7807 | 99.0 | 3.1e-12 | 5.8e-18 | 91.0 | 78 | (1, 78) | 217 | (1, 78) | 164 | phage baseplate assembly protein V | phage baseplate assembly protein V | | uniclust | UniRef100\_A0A950GBI3 | 99.0 | 3.1e-12 | 5.8e-18 | 88.4 | 68 | (40, 107) | 217 | (2, 70) | 137 | Baseplate assembly protein | Baseplate assembly protein | | uniclust | UniRef100\_A0A3N9EW37 | 99.0 | 3.1e-12 | 5.9e-18 | 91.5 | 135 | (66, 216) | 217 | (3, 142) | 147 | Phage baseplate assembly protein V (Fragment) | Phage baseplate assembly protein V (Fragment) | | uniclust | UniRef100\_A0A072TF05 | 99.0 | 3.3e-12 | 6.1e-18 | 116.0 | 159 | (6, 173) | 217 | (470, 629) | 1809 | Bacteriophage regulatory protein, putative (Fragment) | Bacteriophage regulatory protein, putative (Fragment) | | uniclust | UniRef100\_A0A3D4Z671 | 99.0 | 3.3e-12 | 6.1e-18 | 92.9 | 136 | (3, 169) | 217 | (44, 182) | 190 | Phage baseplate assembly protein V | Phage baseplate assembly protein V | | uniclust | UniRef100\_A0A1Y4GV21 | 99.0 | 3.6e-12 | 6.9e-18 | 113.1 | 105 | (17, 121) | 217 | (500, 623) | 776 | Gp5/Type VI secretion system Vgr protein OB-fold domain-containing protein | Gp5/Type VI secretion system Vgr protein OB-fold domain-containing protein | | uniclust | UniRef100\_A0A8T7CZW0 | 99.0 | 3.8e-12 | 7e-18 | 99.0 | 111 | (4, 114) | 217 | (7, 151) | 308 | Phage baseplate assembly protein V | Phage baseplate assembly protein V | | uniclust | UniRef100\_UPI002021AE60 | 99.0 | 4e-12 | 7.4e-18 | 91.7 | 72 | (21, 92) | 217 | (2, 73) | 179 | phage baseplate assembly protein V | phage baseplate assembly protein V | | uniclust | UniRef100\_A0A193LS04 | 99.0 | 4.2e-12 | 7.8e-18 | 94.4 | 173 | (3, 216) | 217 | (11, 187) | 188 | Phage baseplate protein | Phage baseplate protein | | uniclust | UniRef100\_A0A0B1M335 | 99.0 | 4e-12 | 7.9e-18 | 103.9 | 192 | (14, 216) | 217 | (42, 245) | 291 | Phage baseplate protein | Phage baseplate protein | | uniclust | UniRef100\_A0A7Y5WP02 | 99.0 | 4.3e-12 | 8e-18 | 99.0 | 115 | (15, 130) | 217 | (26, 152) | 275 | Gp5/Type VI secretion system Vgr protein OB-fold domain-containing protein | Gp5/Type VI secretion system Vgr protein OB-fold domain-containing protein | | uniclust | UniRef100\_UPI00077A5E31 | 99.0 | 4.5e-12 | 8.4e-18 | 90.5 | 116 | (1, 133) | 217 | (1, 119) | 136 | phage baseplate assembly protein V | phage baseplate assembly protein V | | uniclust | UniRef100\_A0A344TT64 | 99.0 | 4.7e-12 | 8.7e-18 | 106.4 | 142 | (17, 159) | 217 | (242, 398) | 483 | Gp5/Type VI secretion system Vgr protein OB-fold domain-containing protein | Gp5/Type VI secretion system Vgr protein OB-fold domain-containing protein | | uniclust | UniRef100\_A0A0R3L044 | 99.0 | 4.9e-12 | 9e-18 | 94.8 | 100 | (4, 104) | 217 | (13, 118) | 233 | Gp5/Type VI secretion system Vgr protein OB-fold domain-containing protein | Gp5/Type VI secretion system Vgr protein OB-fold domain-containing protein | | uniclust | UniRef100\_A0A1Q6LL85 | 98.9 | 5.1e-12 | 9.9e-18 | 92.3 | 104 | (13, 122) | 217 | (2, 110) | 142 | Uncharacterized protein | Uncharacterized protein | | uniclust | UniRef100\_A0A0X8JVG9 | 98.9 | 5.4e-12 | 1e-17 | 97.5 | 163 | (14, 217) | 217 | (26, 207) | 208 | Phage protein Gp138 N-terminal domain-containing protein | Phage protein Gp138 N-terminal domain-containing protein | | uniclust | UniRef100\_A0A7H8YU12 | 98.9 | 5.7e-12 | 1.1e-17 | 113.2 | 137 | (16, 153) | 217 | (397, 546) | 1055 | Rhs element Vgr protein | Rhs element Vgr protein | | uniclust | UniRef100\_A0A827UUR8 | 98.9 | 5.6e-12 | 1.1e-17 | 90.0 | 107 | (2, 122) | 217 | (5, 114) | 137 | Baseplate assembly protein (Fragment) | Baseplate assembly protein (Fragment) | | uniclust | UniRef100\_UPI001BC86C81 | 98.9 | 6.3e-12 | 1.2e-17 | 99.8 | 115 | (17, 131) | 217 | (41, 165) | 296 | phage baseplate assembly protein V | phage baseplate assembly protein V | | uniclust | UniRef100\_UPI000C7FE3C4 | 98.9 | 6.7e-12 | 1.2e-17 | 89.7 | 58 | (48, 105) | 217 | (4, 61) | 167 | phage baseplate assembly protein V | phage baseplate assembly protein V | | uniclust | UniRef100\_A0A3S4S5U6 | 98.9 | 6.9e-12 | 1.3e-17 | 86.6 | 107 | (15, 123) | 217 | (1, 108) | 135 | Baseplate assembly protein V | Baseplate assembly protein V | | uniclust | UniRef100\_A0A1Q1PVM9 | 98.9 | 7.2e-12 | 1.4e-17 | 98.7 | 129 | (16, 152) | 217 | (24, 160) | 240 | D-like protein | D-like protein | | uniclust | UniRef100\_A0A653V686 | 98.9 | 7.8e-12 | 1.5e-17 | 108.2 | 118 | (16, 133) | 217 | (399, 526) | 636 | Gp5/Type VI secretion system Vgr protein OB-fold domain-containing protein | Gp5/Type VI secretion system Vgr protein OB-fold domain-containing protein | | uniclust | UniRef100\_C9MS63 | 98.9 | 8.2e-12 | 1.5e-17 | 113.3 | 117 | (17, 133) | 217 | (361, 491) | 1047 | Gp5/Type VI secretion system Vgr protein OB-fold domain-containing protein | Gp5/Type VI secretion system Vgr protein OB-fold domain-containing protein | | uniclust | UniRef100\_A0A022PDU6 | 98.9 | 7.9e-12 | 1.6e-17 | 112.7 | 107 | (16, 122) | 217 | (371, 491) | 575 | Gp5/Type VI secretion system Vgr protein OB-fold domain-containing protein | Gp5/Type VI secretion system Vgr protein OB-fold domain-containing protein | | uniclust | UniRef100\_A0A7C1CYC4 | 98.9 | 1e-11 | 1.9e-17 | 98.6 | 117 | (16, 132) | 217 | (141, 268) | 316 | Type IV secretion protein Rhs | Type IV secretion protein Rhs | | uniclust | UniRef100\_UPI000B280816 | 98.9 | 1e-11 | 2e-17 | 91.1 | 147 | (11, 169) | 217 | (3, 156) | 176 | phage baseplate assembly protein V | phage baseplate assembly protein V | | uniclust | UniRef100\_UPI001C8C52A2 | 98.9 | 1.1e-11 | 2e-17 | 95.5 | 80 | (16, 95) | 217 | (103, 189) | 283 | phage baseplate assembly protein V | phage baseplate assembly protein V | | uniclust | UniRef100\_A0A0D9NAU1 | 98.9 | 1.1e-11 | 2.2e-17 | 98.0 | 108 | (24, 131) | 217 | (5, 125) | 248 | Type IV secretion protein Rhs | Type IV secretion protein Rhs | | uniclust | UniRef100\_A0A951W2Z1 | 98.9 | 1.3e-11 | 2.3e-17 | 93.1 | 114 | (16, 132) | 217 | (2, 118) | 240 | Phage baseplate assembly protein V | Phage baseplate assembly protein V | | uniclust | UniRef100\_UPI00202674E5 | 98.9 | 1.3e-11 | 2.3e-17 | 104.0 | 133 | (27, 159) | 217 | (424, 571) | 626 | phage baseplate assembly protein V | phage baseplate assembly protein V | | uniclust | UniRef100\_A0A2D6I496 | 98.9 | 1.3e-11 | 2.5e-17 | 105.4 | 75 | (17, 91) | 217 | (347, 428) | 570 | Gp5/Type VI secretion system Vgr protein OB-fold domain-containing protein | Gp5/Type VI secretion system Vgr protein OB-fold domain-containing protein | | uniclust | UniRef100\_R9B5T6 | 98.9 | 1.4e-11 | 2.5e-17 | 85.5 | 98 | (6, 103) | 217 | (2, 99) | 137 | Gp5/Type VI secretion system Vgr protein OB-fold domain-containing protein | Gp5/Type VI secretion system Vgr protein OB-fold domain-containing protein | | uniclust | UniRef100\_A0A2V2GM48 | 98.9 | 1.4e-11 | 2.5e-17 | 95.7 | 97 | (12, 113) | 217 | (87, 189) | 300 | Gp5/Type VI secretion system Vgr protein OB-fold domain-containing protein | Gp5/Type VI secretion system Vgr protein OB-fold domain-containing protein | | uniclust | UniRef100\_A0A096CE48 | 98.9 | 1.6e-11 | 3.1e-17 | 107.6 | 97 | (12, 114) | 217 | (295, 400) | 482 | Gp5/Type VI secretion system Vgr protein OB-fold domain-containing protein | Gp5/Type VI secretion system Vgr protein OB-fold domain-containing protein | | uniclust | UniRef100\_A0A534P4W5 | 98.9 | 1.8e-11 | 3.2e-17 | 97.8 | 105 | (16, 120) | 217 | (26, 143) | 374 | Gp5/Type VI secretion system Vgr protein OB-fold domain-containing protein (Fragment) | Gp5/Type VI secretion system Vgr protein OB-fold domain-containing protein (Fragment) | | uniclust | UniRef100\_A0A2S6HRN7 | 98.9 | 1.7e-11 | 3.4e-17 | 105.8 | 110 | (11, 124) | 217 | (267, 384) | 452 | Late control gene D protein (GPD) | Late control gene D protein (GPD) | | uniclust | UniRef100\_A0A2S0MND7 | 98.9 | 1.9e-11 | 3.5e-17 | 88.1 | 77 | (5, 83) | 217 | (52, 129) | 175 | Gp5/Type VI secretion system Vgr protein OB-fold domain-containing protein | Gp5/Type VI secretion system Vgr protein OB-fold domain-containing protein | | uniclust | UniRef100\_A0A0B5KGS8 | 98.9 | 1.9e-11 | 3.6e-17 | 111.5 | 67 | (27, 93) | 217 | (594, 669) | 848 | ImpA family type VI secretion-associated protein | ImpA family type VI secretion-associated protein | | uniclust | UniRef100\_A0A1D2R0D3 | 98.9 | 1.9e-11 | 3.7e-17 | 100.2 | 94 | (15, 111) | 217 | (52, 156) | 314 | Gp5/Type VI secretion system Vgr protein OB-fold domain-containing protein | Gp5/Type VI secretion system Vgr protein OB-fold domain-containing protein | | uniclust | UniRef100\_A0A1H3JED8 | 98.9 | 2e-11 | 4e-17 | 108.5 | 113 | (16, 129) | 217 | (378, 503) | 567 | Rhs element Vgr protein | Rhs element Vgr protein | | uniclust | UniRef100\_A0A023XLV0 | 98.8 | 2.1e-11 | 4.2e-17 | 96.6 | 117 | (17, 133) | 217 | (39, 171) | 217 | Baseplate assembly protein V | Baseplate assembly protein V | | uniclust | UniRef100\_A0A1H9NJ96 | 98.8 | 2.2e-11 | 4.3e-17 | 92.9 | 69 | (14, 82) | 217 | (8, 82) | 180 | Baseplate assembly protein | Baseplate assembly protein | | uniclust | UniRef100\_UPI0011A2738F | 98.8 | 2.3e-11 | 4.4e-17 | 99.5 | 109 | (1, 116) | 217 | (1, 109) | 363 | contractile injection system protein, VgrG/Pvc8 family | contractile injection system protein, VgrG/Pvc8 family | | uniclust | UniRef100\_A0A0F9W262 | 98.8 | 2.3e-11 | 4.4e-17 | 95.4 | 143 | (16, 160) | 217 | (20, 185) | 228 | Gp5/Type VI secretion system Vgr protein OB-fold domain-containing protein (Fragment) | Gp5/Type VI secretion system Vgr protein OB-fold domain-containing protein (Fragment) | | uniclust | UniRef100\_A0A173SAA9 | 98.8 | 2.4e-11 | 4.6e-17 | 87.1 | 76 | (9, 84) | 217 | (9, 114) | 123 | Uncharacterized protein | Uncharacterized protein | | uniclust | UniRef100\_G0A9X5 | 98.8 | 2.7e-11 | 4.9e-17 | 92.0 | 76 | (2, 77) | 217 | (39, 114) | 249 | Site-specific recombinase | Site-specific recombinase | | uniclust | UniRef100\_A0A064AML4 | 98.8 | 2.6e-11 | 5.2e-17 | 93.1 | 86 | (17, 103) | 217 | (8, 96) | 183 | Phage baseplate protein | Phage baseplate protein | | uniclust | UniRef100\_A0A6P0Q5Z6 | 98.8 | 2.8e-11 | 5.2e-17 | 83.0 | 67 | (28, 94) | 217 | (37, 108) | 120 | Phage tail protein (Fragment) | Phage tail protein (Fragment) | | uniclust | UniRef100\_UPI001F3F1939 | 98.8 | 2.8e-11 | 5.2e-17 | 80.4 | 89 | (1, 91) | 217 | (1, 89) | 101 | phage baseplate assembly protein V | phage baseplate assembly protein V | | uniclust | UniRef100\_A0A1F8VKX0 | 98.8 | 2.9e-11 | 5.6e-17 | 92.1 | 166 | (13, 217) | 217 | (6, 173) | 187 | Baseplate assembly protein | Baseplate assembly protein | | uniclust | UniRef100\_A0A1P8ENB8 | 98.8 | 3.3e-11 | 6.3e-17 | 91.3 | 162 | (16, 215) | 217 | (8, 176) | 179 | Uncharacterized protein | Uncharacterized protein | | uniclust | UniRef100\_UPI001EE4E6FE | 98.8 | 3.6e-11 | 6.6e-17 | 80.5 | 73 | (16, 88) | 217 | (18, 97) | 110 | phage baseplate assembly protein V | phage baseplate assembly protein V | | uniclust | UniRef100\_A0A0T9PA02 | 98.8 | 3.8e-11 | 7.2e-17 | 94.3 | 184 | (17, 216) | 217 | (32, 245) | 254 | Phage protein Gp138 N-terminal domain-containing protein | Phage protein Gp138 N-terminal domain-containing protein | | uniclust | UniRef100\_UPI00201ED154 | 98.8 | 3.9e-11 | 7.2e-17 | 89.2 | 122 | (1, 122) | 217 | (1, 130) | 214 | hypothetical protein | hypothetical protein | | uniclust | UniRef100\_UPI001F403EA4 | 98.8 | 4.2e-11 | 7.8e-17 | 95.2 | 108 | (17, 124) | 217 | (174, 301) | 357 | phage baseplate assembly protein V | phage baseplate assembly protein V | | uniclust | UniRef100\_UPI00164A91CE | 98.8 | 4.3e-11 | 7.9e-17 | 79.5 | 76 | (52, 127) | 217 | (2, 77) | 105 | phage baseplate assembly protein V | phage baseplate assembly protein V | | uniclust | UniRef100\_A0A845R707 | 98.8 | 4.3e-11 | 7.9e-17 | 87.9 | 118 | (10, 131) | 217 | (60, 183) | 197 | Gp5/Type VI secretion system Vgr protein OB-fold domain-containing protein | Gp5/Type VI secretion system Vgr protein OB-fold domain-containing protein | | uniclust | UniRef100\_A0A0X3AMS8 | 98.8 | 4.4e-11 | 8.3e-17 | 100.5 | 117 | (17, 133) | 217 | (167, 293) | 414 | Phage-related baseplate assembly protein (Fragment) | Phage-related baseplate assembly protein (Fragment) | | uniclust | UniRef100\_A0A0C1ET35 | 98.8 | 4.6e-11 | 8.9e-17 | 93.2 | 99 | (53, 157) | 217 | (16, 115) | 222 | Uncharacterized protein | Uncharacterized protein | | uniclust | UniRef100\_A0A7S8C6X6 | 98.8 | 5.8e-11 | 1.1e-16 | 98.4 | 96 | (3, 99) | 217 | (330, 431) | 515 | Phage virion morphogenesis protein | Phage virion morphogenesis protein | | uniclust | UniRef100\_A0A377I2R7 | 98.8 | 5.9e-11 | 1.1e-16 | 92.0 | 169 | (18, 205) | 217 | (24, 208) | 289 | Phage baseplate assembly protein V | Phage baseplate assembly protein V | | uniclust | UniRef100\_A0A379VYI9 | 98.8 | 6.3e-11 | 1.2e-16 | 83.7 | 103 | (17, 120) | 217 | (7, 112) | 129 | Baseplate protein | Baseplate protein | | uniclust | UniRef100\_A0A919LS40 | 98.8 | 6.5e-11 | 1.2e-16 | 73.8 | 71 | (4, 74) | 217 | (1, 71) | 72 | Uncharacterized protein | Uncharacterized protein | | uniclust | UniRef100\_UPI0002DB02C0 | 98.8 | 6.9e-11 | 1.3e-16 | 87.2 | 133 | (17, 154) | 217 | (34, 182) | 201 | phage baseplate assembly protein V | phage baseplate assembly protein V | | uniclust | UniRef100\_UPI001A19B5C2 | 98.8 | 6.9e-11 | 1.3e-16 | 103.6 | 117 | (17, 133) | 217 | (137, 263) | 963 | phage baseplate assembly protein V | phage baseplate assembly protein V | | uniclust | UniRef100\_A0A088C368 | 98.8 | 6.5e-11 | 1.3e-16 | 97.1 | 192 | (17, 216) | 217 | (48, 281) | 283 | p2 gpV-like protein | p2 gpV-like protein | | uniclust | UniRef100\_UPI002115C689 | 98.8 | 7.1e-11 | 1.3e-16 | 74.5 | 69 | (23, 91) | 217 | (3, 71) | 79 | phage baseplate assembly protein V | phage baseplate assembly protein V | | uniclust | UniRef100\_UPI000371D434 | 98.8 | 7.2e-11 | 1.3e-16 | 87.2 | 102 | (3, 104) | 217 | (13, 119) | 181 | phage baseplate assembly protein V | phage baseplate assembly protein V | | uniclust | UniRef100\_A0A977FHW3 | 98.8 | 7.4e-11 | 1.4e-16 | 82.0 | 127 | (41, 216) | 217 | (10, 136) | 137 | Phage baseplate assembly protein | Phage baseplate assembly protein | | uniclust | UniRef100\_A0A928JDR0 | 98.7 | 8e-11 | 1.5e-16 | 90.3 | 74 | (17, 90) | 217 | (24, 128) | 265 | Phage protein Gp138 N-terminal domain-containing protein | Phage protein Gp138 N-terminal domain-containing protein | | uniclust | UniRef100\_A0A7V1ZNU7 | 98.7 | 9.6e-11 | 1.8e-16 | 85.2 | 90 | (5, 94) | 217 | (12, 104) | 181 | Gp5/Type VI secretion system Vgr protein OB-fold domain-containing protein | Gp5/Type VI secretion system Vgr protein OB-fold domain-containing protein | | uniclust | UniRef100\_A0A0Q7SRW7 | 98.7 | 9.4e-11 | 1.9e-16 | 111.1 | 140 | (11, 153) | 217 | (292, 450) | 1001 | Uncharacterized protein | Uncharacterized protein | | uniclust | UniRef100\_A0A161W0R2 | 98.7 | 1e-10 | 2e-16 | 79.1 | 71 | (7, 83) | 217 | (4, 77) | 84 | Uncharacterized protein | Uncharacterized protein | | uniclust | UniRef100\_A0A9E3FXI7 | 98.7 | 1.1e-10 | 2e-16 | 85.1 | 139 | (8, 158) | 217 | (19, 160) | 184 | Uncharacterized protein | Uncharacterized protein | | uniclust | UniRef100\_A0A0U5B8F1 | 98.7 | 1.1e-10 | 2.1e-16 | 101.0 | 141 | (11, 154) | 217 | (285, 438) | 476 | Gp5/Type VI secretion system Vgr protein OB-fold domain-containing protein | Gp5/Type VI secretion system Vgr protein OB-fold domain-containing protein | | uniclust | UniRef100\_UPI0004B6FA99 | 98.7 | 1.1e-10 | 2.1e-16 | 78.1 | 84 | (30, 113) | 217 | (15, 99) | 103 | phage baseplate assembly protein V | phage baseplate assembly protein V | | uniclust | UniRef100\_A0A1Y4DVJ7 | 98.7 | 1.2e-10 | 2.2e-16 | 104.3 | 99 | (17, 115) | 217 | (821, 940) | 1023 | Gp5/Type VI secretion system Vgr protein OB-fold domain-containing protein | Gp5/Type VI secretion system Vgr protein OB-fold domain-containing protein | | uniclust | UniRef100\_A0A2N5KH98 | 98.7 | 1.2e-10 | 2.3e-16 | 96.7 | 108 | (17, 124) | 217 | (194, 317) | 435 | Gp5/Type VI secretion system Vgr protein OB-fold domain-containing protein | Gp5/Type VI secretion system Vgr protein OB-fold domain-containing protein | | uniclust | UniRef100\_A0A162L1Y1 | 98.7 | 1.2e-10 | 2.3e-16 | 106.4 | 77 | (17, 93) | 217 | (387, 478) | 702 | Gp5/Type VI secretion system Vgr protein OB-fold domain-containing protein | Gp5/Type VI secretion system Vgr protein OB-fold domain-containing protein | | uniclust | UniRef100\_A0A2S0JRF5 | 98.7 | 1.3e-10 | 2.4e-16 | 83.2 | 115 | (15, 133) | 217 | (30, 147) | 163 | Uncharacterized protein | Uncharacterized protein | | uniclust | UniRef100\_A0A1H7UA08 | 98.7 | 1.3e-10 | 2.5e-16 | 89.8 | 88 | (16, 106) | 217 | (23, 117) | 219 | Gp5/Type VI secretion system Vgr protein OB-fold domain-containing protein | Gp5/Type VI secretion system Vgr protein OB-fold domain-containing protein | | uniclust | UniRef100\_A0A430BCY6 | 98.7 | 1.4e-10 | 2.6e-16 | 94.4 | 174 | (8, 216) | 217 | (175, 359) | 364 | Baseplate assembly protein | Baseplate assembly protein | | uniclust | UniRef100\_A0A1N6X9L1 | 98.7 | 1.5e-10 | 2.7e-16 | 80.6 | 82 | (12, 93) | 217 | (4, 92) | 137 | Type VI secretion system, phage-baseplate injector (Fragment) | Type VI secretion system, phage-baseplate injector (Fragment) | | uniclust | UniRef100\_A0A1A6FJR2 | 98.7 | 1.6e-10 | 2.9e-16 | 90.9 | 87 | (6, 94) | 217 | (22, 114) | 290 | Gp5/Type VI secretion system Vgr protein OB-fold domain-containing protein | Gp5/Type VI secretion system Vgr protein OB-fold domain-containing protein | | uniclust | UniRef100\_A0A023D7D4 | 98.7 | 1.5e-10 | 2.9e-16 | 105.2 | 67 | (27, 93) | 217 | (407, 482) | 675 | Secretion system Type VI Rhs element Vgr | Secretion system Type VI Rhs element Vgr | | uniclust | UniRef100\_A0A068CE12 | 98.7 | 1.5e-10 | 3e-16 | 87.8 | 98 | (42, 169) | 217 | (47, 144) | 170 | Baseplate assembly protein V | Baseplate assembly protein V | | uniclust | UniRef100\_A0A0D2GHS9 | 98.7 | 1.6e-10 | 3e-16 | 91.1 | 114 | (4, 133) | 217 | (13, 131) | 216 | Uncharacterized protein | Uncharacterized protein | | uniclust | UniRef100\_UPI001F5DA9EC | 98.7 | 1.9e-10 | 3.5e-16 | 79.5 | 93 | (39, 133) | 217 | (2, 99) | 131 | phage baseplate assembly protein V | phage baseplate assembly protein V | | uniclust | UniRef100\_A0A3M1ATA3 | 98.7 | 1.9e-10 | 3.5e-16 | 80.3 | 75 | (18, 92) | 217 | (24, 105) | 139 | Gp5/Type VI secretion system Vgr protein OB-fold domain-containing protein | Gp5/Type VI secretion system Vgr protein OB-fold domain-containing protein | | uniclust | UniRef100\_A0A968SXI1 | 98.7 | 1.9e-10 | 3.5e-16 | 97.1 | 110 | (6, 115) | 217 | (352, 468) | 605 | Type VI secretion system tip protein VgrG | Type VI secretion system tip protein VgrG | | uniclust | UniRef100\_UPI0004064B79 | 98.7 | 2e-10 | 3.6e-16 | 84.7 | 117 | (3, 121) | 217 | (40, 159) | 197 | phage baseplate assembly protein V | phage baseplate assembly protein V | | uniclust | UniRef100\_UPI002012D1F8 | 98.7 | 2e-10 | 3.6e-16 | 94.0 | 117 | (17, 133) | 217 | (24, 154) | 447 | phage baseplate assembly protein V | phage baseplate assembly protein V | | uniclust | UniRef100\_A0A327VQM2 | 98.7 | 2.1e-10 | 4e-16 | 98.6 | 115 | (17, 131) | 217 | (350, 474) | 583 | Uncharacterized protein involved in type VI secretion and phage assembly | Uncharacterized protein involved in type VI secretion and phage assembly | | uniclust | UniRef100\_A0A1C5P7Q4 | 98.7 | 2.3e-10 | 4.5e-16 | 98.4 | 117 | (14, 133) | 217 | (285, 412) | 474 | Phage protein D | Phage protein D | | uniclust | UniRef100\_UPI00068998DA | 98.7 | 2.5e-10 | 4.5e-16 | 92.3 | 116 | (17, 132) | 217 | (115, 248) | 368 | phage baseplate assembly protein V | phage baseplate assembly protein V | | uniclust | UniRef100\_A0A1I4TS69 | 98.6 | 2.4e-10 | 4.8e-16 | 106.1 | 77 | (17, 93) | 217 | (382, 473) | 713 | Type VI secretion system secreted protein VgrG | Type VI secretion system secreted protein VgrG | | uniclust | UniRef100\_A0A1M4ZE13 | 98.6 | 2.6e-10 | 4.9e-16 | 97.0 | 120 | (13, 132) | 217 | (364, 494) | 556 | Uncharacterized conserved protein, implicated in type VI secretion and phage assembly | Uncharacterized conserved protein, implicated in type VI secretion and phage assembly | | uniclust | UniRef100\_A0A377BG29 | 98.6 | 2.8e-10 | 5.2e-16 | 83.1 | 115 | (2, 133) | 217 | (17, 132) | 157 | Bacteriophage Mu Gp45 protein | Bacteriophage Mu Gp45 protein | | uniclust | UniRef100\_UPI0021AB61CB | 98.6 | 2.9e-10 | 5.4e-16 | 96.3 | 139 | (18, 161) | 217 | (78, 234) | 621 | phage baseplate assembly protein V | phage baseplate assembly protein V | | uniclust | UniRef100\_A0A917BFD9 | 98.6 | 2.9e-10 | 5.6e-16 | 84.1 | 97 | (15, 122) | 217 | (7, 108) | 162 | Gp5/Type VI secretion system Vgr protein OB-fold domain-containing protein | Gp5/Type VI secretion system Vgr protein OB-fold domain-containing protein | | uniclust | UniRef100\_A0A2W6ZDC9 | 98.6 | 3e-10 | 5.6e-16 | 86.7 | 83 | (3, 88) | 217 | (13, 100) | 220 | Gp5/Type VI secretion system Vgr protein OB-fold domain-containing protein | Gp5/Type VI secretion system Vgr protein OB-fold domain-containing protein | | uniclust | UniRef100\_A0A812U007 | 98.6 | 3.2e-10 | 5.8e-16 | 102.0 | 136 | (25, 160) | 217 | (1119, 1274) | 1306 | VgrG5 protein (Fragment) | VgrG5 protein (Fragment) | | uniclust | UniRef100\_A0A094RX34 | 98.6 | 3.2e-10 | 6e-16 | 88.2 | 57 | (17, 73) | 217 | (44, 112) | 225 | Phage protein Gp138 N-terminal domain-containing protein (Fragment) | Phage protein Gp138 N-terminal domain-containing protein (Fragment) | | uniclust | UniRef100\_A0A1W1Z4L7 | 98.6 | 3.2e-10 | 6.1e-16 | 83.8 | 105 | (3, 107) | 217 | (17, 128) | 164 | Type VI secretion system, phage-baseplate injector | Type VI secretion system, phage-baseplate injector | | uniclust | UniRef100\_A0A7Y1K0F9 | 98.6 | 3.3e-10 | 6.4e-16 | 98.0 | 76 | (17, 92) | 217 | (205, 295) | 514 | Type VI secretion system tip protein VgrG | Type VI secretion system tip protein VgrG | | uniclust | UniRef100\_A0A0S3U5G4 | 98.6 | 3.4e-10 | 6.6e-16 | 94.1 | 76 | (17, 92) | 217 | (36, 118) | 314 | Rhs element Vgr protein | Rhs element Vgr protein | | uniclust | UniRef100\_A0A075K3F1 | 98.6 | 3.4e-10 | 6.7e-16 | 106.6 | 79 | (17, 95) | 217 | (505, 598) | 918 | Type IV secretion protein Rhs | Type IV secretion protein Rhs | | uniclust | UniRef100\_A0A061Q576 | 98.6 | 3.6e-10 | 7e-16 | 105.1 | 78 | (16, 93) | 217 | (384, 476) | 848 | VgrG protein | VgrG protein | | uniclust | UniRef100\_UPI001569D794 | 98.6 | 3.9e-10 | 7.3e-16 | 77.1 | 104 | (81, 208) | 217 | (2, 106) | 115 | phage baseplate assembly protein V | phage baseplate assembly protein V | | uniclust | UniRef100\_A0A959CMX5 | 98.6 | 4.5e-10 | 8.3e-16 | 88.2 | 100 | (16, 115) | 217 | (29, 139) | 311 | Gp5/Type VI secretion system Vgr protein OB-fold domain-containing protein | Gp5/Type VI secretion system Vgr protein OB-fold domain-containing protein | | uniclust | UniRef100\_A0A371WU32 | 98.6 | 4.9e-10 | 8.9e-16 | 82.6 | 92 | (3, 95) | 217 | (20, 117) | 195 | Gp5/Type VI secretion system Vgr protein OB-fold domain-containing protein | Gp5/Type VI secretion system Vgr protein OB-fold domain-containing protein | | uniclust | UniRef100\_A0A6B8KG73 | 98.6 | 5e-10 | 9.2e-16 | 82.1 | 106 | (2, 111) | 217 | (14, 120) | 188 | Gp5/Type VI secretion system Vgr protein OB-fold domain-containing protein | Gp5/Type VI secretion system Vgr protein OB-fold domain-containing protein | | uniclust | UniRef100\_A0A1A3AFQ8 | 98.6 | 5.3e-10 | 9.8e-16 | 82.3 | 111 | (16, 133) | 217 | (14, 129) | 175 | Gp5/Type VI secretion system Vgr protein OB-fold domain-containing protein | Gp5/Type VI secretion system Vgr protein OB-fold domain-containing protein | | uniclust | UniRef100\_A0A023Q190 | 98.6 | 5e-10 | 1e-15 | 104.7 | 97 | (17, 113) | 217 | (439, 545) | 766 | Type IV secretion protein Rhs | Type IV secretion protein Rhs | | uniclust | UniRef100\_A0A8J6YCD9 | 98.6 | 5.5e-10 | 1e-15 | 73.2 | 86 | (44, 131) | 217 | (2, 88) | 95 | Phage baseplate assembly protein V | Phage baseplate assembly protein V | | uniclust | UniRef100\_UPI000E2E4993 | 98.6 | 5.7e-10 | 1.1e-15 | 68.1 | 62 | (1, 62) | 217 | (1, 62) | 62 | phage baseplate assembly protein V | phage baseplate assembly protein V | | uniclust | UniRef100\_A0A3C1JKA1 | 98.6 | 5.8e-10 | 1.1e-15 | 91.6 | 95 | (15, 109) | 217 | (225, 332) | 453 | Gp5/Type VI secretion system Vgr protein OB-fold domain-containing protein | Gp5/Type VI secretion system Vgr protein OB-fold domain-containing protein | | uniclust | UniRef100\_V5ZAQ6 | 98.6 | 5.9e-10 | 1.1e-15 | 74.0 | 72 | (1, 72) | 217 | (1, 72) | 102 | Baseplate assembly protein V GpV | Baseplate assembly protein V GpV | | uniclust | UniRef100\_A0A0P8YXD2 | 98.6 | 6.5e-10 | 1.2e-15 | 74.2 | 51 | (32, 82) | 217 | (7, 58) | 105 | Gp5/Type VI secretion system Vgr protein OB-fold domain-containing protein (Fragment) | Gp5/Type VI secretion system Vgr protein OB-fold domain-containing protein (Fragment) | | uniclust | UniRef100\_A0A925MG30 | 98.6 | 6.6e-10 | 1.2e-15 | 83.5 | 117 | (16, 133) | 217 | (18, 148) | 192 | Rhs element Vgr protein | Rhs element Vgr protein | | pdb70 | 3QR8\_A | 99.9 | 2.6e-33 | 2e-37 | 207.3 | 207 | (2, 217) | 217 | (4, 211) | 211 | Baseplate assembly protein V | 3QR8\_A Baseplate assembly protein V Beta-Helix, OB-Fold, Phage Baseplate, Iron-Binding | | pdb70 | 4S37\_K | 99.9 | 2.8e-32 | 2.1e-36 | 197.8 | 184 | (1, 203) | 217 | (1, 185) | 185 | Phage baseplate protein | 4S37\_K Phage baseplate protein Cell Puncturing Device, Trimer, beta-helix HET: SO4, PGE, CL, EDO | | pdb70 | 4S37\_L | 99.9 | 2.8e-32 | 2.1e-36 | 197.8 | 184 | (1, 203) | 217 | (1, 185) | 185 | Phage baseplate protein | 4S37\_L Phage baseplate protein Cell Puncturing Device, Trimer, beta-helix HET: PGE, CL, SO4, EDO | | pdb70 | 3PQI\_A | 99.7 | 1.3e-22 | 9.7e-27 | 152.7 | 176 | (13, 213) | 217 | (23, 239) | 247 | gene product 138 | 3PQI\_A gene product 138 Beta-Helix, OB-Fold, Phage Baseplate, Iron-Binding | | pdb70 | 4RU3\_A | 99.7 | 4.7e-21 | 3.5e-25 | 141.8 | 155 | (13, 216) | 217 | (32, 218) | 221 | puncturing protein gp41 | 4RU3\_A puncturing protein gp41 gp41, membrane translocation, phage baseplate | | pdb70 | 6J0M\_C | 99.4 | 1.3e-17 | 1e-21 | 137.7 | 140 | (13, 159) | 217 | (361, 514) | 538 | Pvc8 | 6J0M\_C Pvc8 assembly, Photorhabdus asymbiotica, PVC, contractile | | pdb70 | 6RBK\_C | 99.4 | 4.7e-17 | 3.6e-21 | 134.2 | 139 | (13, 158) | 217 | (352, 504) | 529 | Afp1, Afp2, Afp3, Afp16 | 6RBK\_C Afp1, Afp2, Afp3, Afp16 Anti-feeding prophage, secretion system, AFP | | pdb70 | 4MTK\_A | 99.2 | 1.5e-15 | 1.1e-19 | 128.2 | 109 | (13, 121) | 217 | (364, 500) | 643 | VgrG1 | 4MTK\_A VgrG1 Beta-barrel, OB-fold, Beta-helix, Type VI HET: SO4, TAM | | pdb70 | 4MTK\_B | 99.2 | 1.5e-15 | 1.1e-19 | 128.2 | 109 | (13, 121) | 217 | (364, 500) | 643 | VgrG1 | 4MTK\_B VgrG1 Beta-barrel, OB-fold, Beta-helix, Type VI HET: TAM, SO4 | | pdb70 | 4MTK\_C | 99.2 | 1.5e-15 | 1.1e-19 | 128.2 | 109 | (13, 121) | 217 | (364, 500) | 643 | VgrG1 | 4MTK\_C VgrG1 Beta-barrel, OB-fold, Beta-helix, Type VI HET: TAM, SO4 | | pdb70 | 4MTK\_D | 99.2 | 1.5e-15 | 1.1e-19 | 128.2 | 109 | (13, 121) | 217 | (364, 500) | 643 | VgrG1 | 4MTK\_D VgrG1 Beta-barrel, OB-fold, Beta-helix, Type VI HET: SO4, TAM | | pdb70 | 4MTK\_E | 99.2 | 1.5e-15 | 1.1e-19 | 128.2 | 109 | (13, 121) | 217 | (364, 500) | 643 | VgrG1 | 4MTK\_E VgrG1 Beta-barrel, OB-fold, Beta-helix, Type VI HET: SO4, TAM | | pdb70 | 4MTK\_F | 99.2 | 1.5e-15 | 1.1e-19 | 128.2 | 109 | (13, 121) | 217 | (364, 500) | 643 | VgrG1 | 4MTK\_F VgrG1 Beta-barrel, OB-fold, Beta-helix, Type VI HET: SO4, TAM | | pdb70 | 4UHV\_A | 99.2 | 1.5e-15 | 1.1e-19 | 128.3 | 109 | (13, 121) | 217 | (364, 500) | 651 | VGRG1, VALINE-GLYCINE REPEAT PROTEIN G1 | 4UHV\_A VGRG1, VALINE-GLYCINE REPEAT PROTEIN G1 STRUCTURAL PROTEIN, VGRG1, VIRULENCE, TOXIN HET: NA, MSE, CL | | pdb70 | 4UHV\_B | 99.2 | 1.5e-15 | 1.1e-19 | 128.3 | 109 | (13, 121) | 217 | (364, 500) | 651 | VGRG1, VALINE-GLYCINE REPEAT PROTEIN G1 | 4UHV\_B VGRG1, VALINE-GLYCINE REPEAT PROTEIN G1 STRUCTURAL PROTEIN, VGRG1, VIRULENCE, TOXIN HET: CL, NA, MSE | | pdb70 | 3AQJ\_A | 99.0 | 8.5e-14 | 6.3e-18 | 94.0 | 122 | (87, 217) | 217 | (12, 134) | 134 | Baseplate assembly protein V | 3AQJ\_A Baseplate assembly protein V bacteriophage, tail spike, iron binding | | pdb70 | 3AQJ\_Q | 99.0 | 8.5e-14 | 6.3e-18 | 94.0 | 122 | (87, 217) | 217 | (12, 134) | 134 | Baseplate assembly protein V | 3AQJ\_Q Baseplate assembly protein V bacteriophage, tail spike, iron binding | | pdb70 | 3QR7\_B | 98.9 | 4.4e-13 | 3.3e-17 | 88.2 | 112 | (97, 217) | 217 | (3, 115) | 115 | Baseplate assembly protein V | 3QR7\_B Baseplate assembly protein V Beta-Helix, OB-Fold, Phage Baseplate, Iron-Binding | | pdb70 | 2P5Z\_X | 98.8 | 1.3e-12 | 9.8e-17 | 106.5 | 81 | (14, 94) | 217 | (384, 481) | 491 | Type VI secretion system component | 2P5Z\_X Type VI secretion system component structural genomics, UNKNOWN FUNCTION, PSI-2 | | pdb70 | 2Z6B\_A | 98.7 | 4.5e-12 | 3.3e-16 | 105.0 | 81 | (12, 92) | 217 | (6, 109) | 584 | Tail-associated lysozyme (E.C.3.2.1.17), Baseplate structural | 2Z6B\_A Tail-associated lysozyme (E.C.3.2.1.17), Baseplate structural Protein containing metal complexes, Antimicrobial | | pdb70 | 3VTO\_B | 98.7 | 6.2e-12 | 4.6e-16 | 82.4 | 98 | (99, 211) | 217 | (6, 103) | 115 | Protein gp45 | 3VTO\_B Protein gp45 beta-helix, central spike, Mu phage | | pdb70 | 3VTO\_Q | 98.7 | 6.2e-12 | 4.6e-16 | 82.4 | 98 | (99, 211) | 217 | (6, 103) | 115 | Protein gp45 | 3VTO\_Q Protein gp45 beta-helix, central spike, Mu phage | | pdb70 | 3VTN\_A | 98.6 | 8.8e-12 | 6.6e-16 | 79.8 | 98 | (102, 216) | 217 | (3, 100) | 101 | Protein gp45 | 3VTN\_A Protein gp45 beta-helix, central spike, Mu phage | | pdb70 | 1K28\_A | 98.6 | 1.7e-11 | 1.3e-15 | 101.5 | 78 | (14, 91) | 217 | (8, 108) | 584 | TAIL-ASSOCIATED LYSOZYME, BASEPLATE STRUCTURAL PROTEIN | 1K28\_A TAIL-ASSOCIATED LYSOZYME, BASEPLATE STRUCTURAL PROTEIN Triple-stranded beta-helix, OB fold, pseudohexamer | | pdb70 | 5IV5\_YA | 98.1 | 1.1e-09 | 8e-14 | 90.6 | 75 | (15, 89) | 217 | (9, 106) | 575 | Baseplate wedge protein gp6, Baseplate | 5IV5\_YA Baseplate wedge protein gp6, Baseplate T4, baseplate-tail tube complex, pre-attachment | | pdb70 | 5IV5\_YB | 98.1 | 1.1e-09 | 8e-14 | 90.6 | 75 | (15, 89) | 217 | (9, 106) | 575 | Baseplate wedge protein gp6, Baseplate | 5IV5\_YB Baseplate wedge protein gp6, Baseplate T4, baseplate-tail tube complex, pre-attachment | | pdb70 | 5IV5\_YC | 98.1 | 1.1e-09 | 8e-14 | 90.6 | 75 | (15, 89) | 217 | (9, 106) | 575 | Baseplate wedge protein gp6, Baseplate | 5IV5\_YC Baseplate wedge protein gp6, Baseplate T4, baseplate-tail tube complex, pre-attachment | | pdb70 | 1WTH\_A | 98.1 | 1.3e-09 | 9.4e-14 | 90.2 | 73 | (14, 86) | 217 | (8, 103) | 584 | Tail-associated lysozyme (E.C.3.2.1.17)/Baseplate structural protein | 1WTH\_A Tail-associated lysozyme (E.C.3.2.1.17)/Baseplate structural protein Triple-stranded beta-helix, OB fold, pseudohexamer | | pdb70 | 4S36\_A | 98.1 | 1.4e-09 | 1e-13 | 67.7 | 88 | (98, 203) | 217 | (3, 90) | 90 | Phage baseplate protein | 4S36\_A Phage baseplate protein Cell Puncturing Device, Trimer, beta-helix | | pdb70 | 3PQH\_A | 97.4 | 2.1e-07 | 1.6e-11 | 60.9 | 75 | (101, 207) | 217 | (34, 111) | 127 | gene product 138 C-terminus | 3PQH\_A gene product 138 C-terminus Beta-Helix, OB-Fold, Phage Baseplate, Iron-Binding | | pdb70 | 5IV5\_YA | 97.3 | 3.4e-07 | 2.6e-11 | 75.3 | 82 | (40, 122) | 217 | (325, 417) | 575 | Baseplate wedge protein gp6, Baseplate | 5IV5\_YA Baseplate wedge protein gp6, Baseplate T4, baseplate-tail tube complex, pre-attachment | | pdb70 | 5IV5\_YB | 97.3 | 3.4e-07 | 2.6e-11 | 75.3 | 82 | (40, 122) | 217 | (325, 417) | 575 | Baseplate wedge protein gp6, Baseplate | 5IV5\_YB Baseplate wedge protein gp6, Baseplate T4, baseplate-tail tube complex, pre-attachment | | pdb70 | 5IV5\_YC | 97.3 | 3.4e-07 | 2.6e-11 | 75.3 | 82 | (40, 122) | 217 | (325, 417) | 575 | Baseplate wedge protein gp6, Baseplate | 5IV5\_YC Baseplate wedge protein gp6, Baseplate T4, baseplate-tail tube complex, pre-attachment | | pdb70 | 1K28\_A | 97.1 | 9e-07 | 6.7e-11 | 72.8 | 81 | (40, 121) | 217 | (314, 416) | 584 | TAIL-ASSOCIATED LYSOZYME, BASEPLATE STRUCTURAL PROTEIN | 1K28\_A TAIL-ASSOCIATED LYSOZYME, BASEPLATE STRUCTURAL PROTEIN Triple-stranded beta-helix, OB fold, pseudohexamer | |
| Top keywords  (threshold 1.00e-03 (evalue)) | **baseplate, assembly, Phage, V, Type, secretion, VI, domain\_containing, Vgr, system** |
| Output files | ../../similar\_sequences/11\_FANPEZAQ\_CDS\_0011\_merged.svg ../../similar\_sequences/11\_FANPEZAQ\_CDS\_0011\_pdb70.a3m ../../similar\_sequences/11\_FANPEZAQ\_CDS\_0011\_pdb70.hhr ../../similar\_sequences/11\_FANPEZAQ\_CDS\_0011\_uniclust.a3m ../../similar\_sequences/11\_FANPEZAQ\_CDS\_0011\_uniclust.hhr |

#### Structure prediction (AlphaFold)2

|  |  |
| --- | --- |
| Stats | xml version="1.0" encoding="utf-8" standalone="no"?       2024-09-02T21:09:09.981572 image/svg+xml   Matplotlib v3.7.2, https://matplotlib.org/ |
| Predicted structure | **NGL Viewer Controls:**  - Center: *Left-Click* - Rotate: *Left-Click + Drag* - Translate: *Right-Click + Drag* - Zoom: *Shift + Left-Click + Drag* |
| Output files | ../../predicted\_structures/11\_FANPEZAQ\_CDS\_0011/features.pkl ../../predicted\_structures/11\_FANPEZAQ\_CDS\_0011/ranked\_0.pdb ../../predicted\_structures/11\_FANPEZAQ\_CDS\_0011/ranked\_0\_plots.svg ../../predicted\_structures/11\_FANPEZAQ\_CDS\_0011/result\_model\_1\_ptm\_pred\_0.pkl |

#### Structure similarity search results (Foldseek)3

|  |  |
| --- | --- |
| Structure databases searched | Pdb, Afdb-proteome, Afdb-uniprot50 |
| Results, scheme(s)  (Top layers only, threshold 1.00e-02 (evalue)) | xml version="1.0" encoding="utf-8" standalone="no"?       2024-09-02T21:10:19.348302 image/svg+xml   Matplotlib v3.7.2, https://matplotlib.org/ |
| Results, table  (threshold 1.00e-02 (evalue)) | | db | id | prob | evalue | bits | fident | alnlen | mismatch | gapopen | qstart | qend | tstart | tend | name | description | | --- | --- | --- | --- | --- | --- | --- | --- | --- | --- | --- | --- | --- | --- | --- | | pdb | 3QR8\_A | 1.0 | 4.718e-16 | 601 | 0.382 | 157 | 96 | 1 | 6 | 162 | 2 | 157 | Baseplate assembly protein V | Baseplate assembly protein V | | pdb | 4S37\_G | 1.0 | 1.524e-13 | 488 | 0.274 | 186 | 123 | 3 | 2 | 186 | 1 | 175 | Phage baseplate protein | Phage baseplate protein | | pdb | 4S37\_Q | 1.0 | 8.936e-13 | 449 | 0.268 | 175 | 116 | 3 | 14 | 187 | 3 | 166 | Phage baseplate protein | Phage baseplate protein | | pdb | 3AQJ\_R | 1.0 | 4.55e-05 | 198 | 0.326 | 104 | 65 | 3 | 93 | 196 | 1 | 99 | Baseplate assembly protein V | Baseplate assembly protein V | | pdb | 7AEF\_Q | 1.0 | 2.54e-07 | 192 | 0.13 | 223 | 131 | 7 | 16 | 193 | 376 | 580 | Phosphoserine phosphatase SerB | Phosphoserine phosphatase SerB | | pdb | 8GRA\_G | 1.0 | 6.943e-08 | 191 | 0.195 | 245 | 146 | 12 | 15 | 216 | 363 | 599 | Type VI secretion system spike protein VgrG | Type VI secretion system spike protein VgrG | | pdb | 6H3N\_B | 1.0 | 5.448e-06 | 180 | 0.141 | 247 | 162 | 11 | 16 | 217 | 354 | 595 | VgrG1 | VgrG1 | | pdb | 6J0M\_A | 1.0 | 3.021e-06 | 176 | 0.102 | 176 | 124 | 9 | 16 | 159 | 362 | 535 | Pvc8 | Pvc8 | | pdb | 7B5H\_AJ | 1.0 | 2.54e-07 | 174 | 0.156 | 223 | 134 | 9 | 16 | 193 | 372 | 585 | All3320 protein | All3320 protein | | pdb | 4UHV\_A | 1.0 | 8.731e-06 | 162 | 0.151 | 244 | 159 | 14 | 16 | 215 | 367 | 606 | VGRG1, VALINE-GLYCINE REPEAT PROTEIN G1 | VGRG1, VALINE-GLYCINE REPEAT PROTEIN G1 | | pdb | 7Q5P\_B | 1.0 | 3.594e-05 | 159 | 0.135 | 244 | 167 | 11 | 16 | 217 | 354 | 595 | Type VI secretion protein VgrG | Type VI secretion protein VgrG | | pdb | 8EON\_P | 1.0 | 3.388e-05 | 158 | 0.146 | 177 | 108 | 10 | 4 | 144 | 20 | 189 | Baseplate spike gp43 | Baseplate spike gp43 | | pdb | 6RBK\_C | 1.0 | 4.55e-05 | 153 | 0.127 | 172 | 126 | 9 | 16 | 164 | 332 | 502 | Afp8 | Afp8 | | pdb | 4RU3\_A | 1.0 | 5.119e-05 | 126 | 0.141 | 233 | 107 | 12 | 4 | 197 | 6 | 184 | puncturing protein gp41 | puncturing protein gp41 | | pdb | 3I4O\_B | 1.0 | 0.005085 | 114 | 0.197 | 76 | 43 | 8 | 15 | 83 | 2 | 66 | Translation initiation factor IF-1 | Translation initiation factor IF-1 | | pdb | 2OQK\_A | 1.0 | 0.006069 | 108 | 0.146 | 75 | 56 | 6 | 15 | 88 | 11 | 78 | Putative translation initiation factor eIF-1A | Putative translation initiation factor eIF-1A | | pdb | 3PQI\_A | 1.0 | 0.006829 | 104 | 0.154 | 110 | 79 | 3 | 16 | 111 | 3 | 112 | gene product 138 | gene product 138 | | pdb | 7YFZ\_Y | 0.997 | 0.006829 | 92 | 0.115 | 156 | 96 | 7 | 1 | 117 | 11 | 163 | Pam3 spike gp20 | Pam3 spike gp20 | | pdb | 3FP9\_K | 0.992 | 0.004261 | 85 | 0.121 | 107 | 71 | 8 | 16 | 119 | 2 | 88 | Proteasome-associated ATPase | Proteasome-associated ATPase | | pdb | 3FP9\_G | 0.991 | 0.005085 | 84 | 0.121 | 107 | 71 | 8 | 16 | 119 | 2 | 88 | Proteasome-associated ATPase | Proteasome-associated ATPase | | pdb | 5KWA\_A | 0.986 | 0.008645 | 81 | 0.121 | 107 | 71 | 8 | 16 | 119 | 3 | 89 | Proteasome-associated ATPase | Proteasome-associated ATPase | | afdb-proteome | AF-A0A0H3GU25-F1-MODEL\_V4 | 1.0 | 2.964e-18 | 692 | 0.356 | 202 | 124 | 4 | 16 | 217 | 2 | 197 | Putative prophage baseplate assembly protein | Putative prophage baseplate assembly protein | | afdb-proteome | AF-Q8ZMU1-F1-MODEL\_V4 | 1.0 | 3.98e-18 | 558 | 0.269 | 223 | 126 | 3 | 1 | 217 | 1 | 192 | Fels-2 prophage protein | Fels-2 prophage protein | | afdb-proteome | AF-A0A0H3GM22-F1-MODEL\_V4 | 1.0 | 3.954e-16 | 517 | 0.265 | 196 | 120 | 3 | 1 | 196 | 1 | 172 | Baseplate assembly protein V | Baseplate assembly protein V | | afdb-proteome | AF-G3XCT9-F1-MODEL\_V4 | 1.0 | 5.954e-15 | 504 | 0.275 | 203 | 129 | 5 | 1 | 203 | 1 | 185 | Phage\_base\_V domain-containing protein | Phage\_base\_V domain-containing protein | | afdb-proteome | AF-Q9I0F3-F1-MODEL\_V4 | 1.0 | 5.137e-06 | 170 | 0.142 | 246 | 163 | 10 | 16 | 217 | 407 | 648 | Type VI secretion system spike protein VgrG1c | Type VI secretion system spike protein VgrG1c | | afdb-proteome | AF-Q9I1A6-F1-MODEL\_V4 | 1.0 | 1.772e-05 | 169 | 0.137 | 232 | 169 | 11 | 16 | 217 | 352 | 582 | Phage\_base\_V domain-containing protein | Phage\_base\_V domain-containing protein | | afdb-proteome | AF-Q9HTT3-F1-MODEL\_V4 | 1.0 | 4.057e-06 | 152 | 0.177 | 265 | 154 | 16 | 16 | 217 | 372 | 635 | Phage\_base\_V domain-containing protein | Phage\_base\_V domain-containing protein | | afdb-proteome | AF-Q9HU93-F1-MODEL\_V4 | 1.0 | 8.232e-06 | 147 | 0.162 | 264 | 158 | 14 | 16 | 217 | 377 | 639 | Type VI secretion system spike protein VgrG5 | Type VI secretion system spike protein VgrG5 | | afdb-proteome | AF-Q9I737-F1-MODEL\_V4 | 1.0 | 0.0004031 | 139 | 0.142 | 232 | 167 | 10 | 16 | 217 | 370 | 599 | Type VI secretion system spike protein VgrG1b | Type VI secretion system spike protein VgrG1b | | afdb-proteome | AF-Q9HYC3-F1-MODEL\_V4 | 1.0 | 3.012e-05 | 137 | 0.153 | 234 | 155 | 12 | 16 | 210 | 377 | 606 | Type VI secretion system spike protein VgrG4b | Type VI secretion system spike protein VgrG4b | | afdb-uniprot50 | AF-A0A8A6KD57-F1-MODEL\_V4 | 1.0 | 1.044e-22 | 799 | 0.471 | 214 | 104 | 3 | 4 | 217 | 5 | 209 | Phage baseplate assembly protein V | Phage baseplate assembly protein V | | afdb-uniprot50 | AF-A0A2Z3IDQ9-F1-MODEL\_V4 | 1.0 | 2.691e-23 | 782 | 0.536 | 218 | 93 | 2 | 1 | 217 | 1 | 211 | Phage baseplate assembly protein V | Phage baseplate assembly protein V | | afdb-uniprot50 | AF-A0A1I3LN83-F1-MODEL\_V4 | 1.0 | 3.577e-20 | 780 | 0.364 | 217 | 132 | 4 | 1 | 217 | 1 | 211 | Phage baseplate assembly protein V | Phage baseplate assembly protein V | | afdb-uniprot50 | AF-A0A2D8NW13-F1-MODEL\_V4 | 1.0 | 1.163e-19 | 774 | 0.359 | 217 | 135 | 4 | 1 | 217 | 1 | 213 | Baseplate assembly protein | Baseplate assembly protein | | afdb-uniprot50 | AF-A0A486S9U3-F1-MODEL\_V4 | 1.0 | 5.405e-20 | 769 | 0.376 | 215 | 128 | 5 | 3 | 217 | 39 | 247 | Baseplate assembly protein V | Baseplate assembly protein V | | afdb-uniprot50 | AF-A0A081RRN8-F1-MODEL\_V4 | 1.0 | 1.388e-19 | 764 | 0.367 | 215 | 128 | 5 | 4 | 217 | 32 | 239 | Phage baseplate assembly protein V | Phage baseplate assembly protein V | | afdb-uniprot50 | AF-A0A068QXN4-F1-MODEL\_V4 | 1.0 | 1.657e-19 | 759 | 0.331 | 214 | 137 | 4 | 4 | 217 | 29 | 236 | Baseplate assembly protein V | Baseplate assembly protein V | | afdb-uniprot50 | AF-A0A0W2DNN1-F1-MODEL\_V4 | 1.0 | 7.258e-20 | 758 | 0.388 | 211 | 123 | 4 | 3 | 213 | 5 | 209 | Baseplate assembly protein | Baseplate assembly protein | | afdb-uniprot50 | AF-A0A8B3QQT6-F1-MODEL\_V4 | 1.0 | 4.256e-19 | 753 | 0.36 | 194 | 119 | 3 | 3 | 196 | 5 | 193 | Phage baseplate assembly protein V | Phage baseplate assembly protein V | | afdb-uniprot50 | AF-A0A370T1W2-F1-MODEL\_V4 | 1.0 | 3.169e-19 | 749 | 0.358 | 215 | 132 | 4 | 3 | 217 | 21 | 229 | Phage baseplate assembly protein V | Phage baseplate assembly protein V | | afdb-uniprot50 | AF-E3G776-F1-MODEL\_V4 | 1.0 | 1.097e-19 | 741 | 0.398 | 221 | 119 | 8 | 1 | 217 | 1 | 211 | Phage baseplate assembly protein V | Phage baseplate assembly protein V | | afdb-uniprot50 | AF-A0A7Y1SGM7-F1-MODEL\_V4 | 1.0 | 1.752e-18 | 740 | 0.336 | 217 | 141 | 3 | 1 | 217 | 1 | 214 | Phage baseplate assembly protein V | Phage baseplate assembly protein V | | afdb-uniprot50 | AF-A0A7T9YBU7-F1-MODEL\_V4 | 1.0 | 5.387e-19 | 733 | 0.333 | 213 | 131 | 5 | 1 | 213 | 1 | 202 | Phage baseplate assembly protein V | Phage baseplate assembly protein V | | afdb-uniprot50 | AF-A0A0F3UMW4-F1-MODEL\_V4 | 1.0 | 1.159e-18 | 728 | 0.365 | 194 | 118 | 3 | 3 | 196 | 5 | 193 | Baseplate assembly protein | Baseplate assembly protein | | afdb-uniprot50 | AF-A0A376PNH2-F1-MODEL\_V4 | 1.0 | 1.159e-18 | 721 | 0.366 | 199 | 119 | 4 | 1 | 197 | 1 | 194 | Baseplate assembly protein V (GpV) | Baseplate assembly protein V (GpV) | | afdb-uniprot50 | AF-A0A6M8RDL6-F1-MODEL\_V4 | 1.0 | 1.971e-18 | 720 | 0.377 | 217 | 126 | 7 | 1 | 217 | 1 | 208 | Phage baseplate assembly protein V | Phage baseplate assembly protein V | | afdb-uniprot50 | AF-A0A0T9KKH2-F1-MODEL\_V4 | 1.0 | 4.514e-19 | 719 | 0.391 | 217 | 120 | 7 | 3 | 217 | 5 | 211 | Baseplate assembly protein V | Baseplate assembly protein V | | afdb-uniprot50 | AF-A0A2W5L0U8-F1-MODEL\_V4 | 1.0 | 1.769e-21 | 717 | 0.425 | 214 | 99 | 3 | 4 | 217 | 6 | 195 | Phage baseplate assembly protein V | Phage baseplate assembly protein V | | afdb-uniprot50 | AF-A0A2A7T2L7-F1-MODEL\_V4 | 1.0 | 6.43e-19 | 714 | 0.382 | 217 | 122 | 7 | 3 | 217 | 5 | 211 | Phage baseplate assembly protein V | Phage baseplate assembly protein V | | afdb-uniprot50 | AF-A0A7U3BDQ0-F1-MODEL\_V4 | 1.0 | 7.234e-19 | 706 | 0.364 | 217 | 126 | 6 | 3 | 217 | 5 | 211 | Baseplate assembly protein | Baseplate assembly protein | | afdb-uniprot50 | AF-A0A2S8HW35-F1-MODEL\_V4 | 1.0 | 7.234e-19 | 705 | 0.375 | 221 | 124 | 7 | 1 | 217 | 1 | 211 | Baseplate assembly protein | Baseplate assembly protein | | afdb-uniprot50 | AF-A0A377CCX9-F1-MODEL\_V4 | 1.0 | 8.14e-19 | 705 | 0.373 | 217 | 124 | 6 | 3 | 217 | 5 | 211 | Baseplate assembly protein V (GpV) | Baseplate assembly protein V (GpV) | | afdb-uniprot50 | AF-A0A8A7TZ35-F1-MODEL\_V4 | 1.0 | 9.158e-19 | 704 | 0.387 | 217 | 121 | 6 | 3 | 217 | 5 | 211 | Phage baseplate assembly protein V | Phage baseplate assembly protein V | | afdb-uniprot50 | AF-A0A810U4Z0-F1-MODEL\_V4 | 1.0 | 4.514e-19 | 704 | 0.373 | 217 | 124 | 6 | 3 | 217 | 17 | 223 | Phage baseplate assembly protein | Phage baseplate assembly protein | | afdb-uniprot50 | AF-A0A761QNR2-F1-MODEL\_V4 | 1.0 | 2.638e-17 | 702 | 0.371 | 164 | 102 | 1 | 3 | 166 | 5 | 167 | Phage baseplate assembly protein V | Phage baseplate assembly protein V | | afdb-uniprot50 | AF-A0A484X0R0-F1-MODEL\_V4 | 1.0 | 6.041e-18 | 700 | 0.369 | 195 | 116 | 4 | 1 | 193 | 1 | 190 | Baseplate assembly protein V (GpV) | Baseplate assembly protein V (GpV) | | afdb-uniprot50 | AF-F4N734-F1-MODEL\_V4 | 1.0 | 6.022e-17 | 691 | 0.38 | 176 | 106 | 2 | 1 | 176 | 1 | 173 | Baseplate assembly protein V | Baseplate assembly protein V | | afdb-uniprot50 | AF-A0A7Z1A3H5-F1-MODEL\_V4 | 1.0 | 1.379e-17 | 688 | 0.353 | 215 | 131 | 7 | 6 | 217 | 2 | 211 | Phage baseplate assembly protein V | Phage baseplate assembly protein V | | afdb-uniprot50 | AF-A0A376TE63-F1-MODEL\_V4 | 1.0 | 1.971e-18 | 683 | 0.364 | 217 | 126 | 6 | 3 | 217 | 28 | 234 | Baseplate assembly protein V (GpV) | Baseplate assembly protein V (GpV) | | afdb-uniprot50 | AF-A0A1T4W1B8-F1-MODEL\_V4 | 1.0 | 3.35e-18 | 680 | 0.36 | 222 | 135 | 3 | 1 | 217 | 1 | 220 | Phage baseplate assembly protein V | Phage baseplate assembly protein V | | afdb-uniprot50 | AF-A0A660NMC7-F1-MODEL\_V4 | 1.0 | 2.36e-19 | 678 | 0.486 | 189 | 83 | 5 | 1 | 187 | 1 | 177 | Phage baseplate assembly protein V | Phage baseplate assembly protein V | | afdb-uniprot50 | AF-A0A826YSP9-F1-MODEL\_V4 | 1.0 | 1.846e-16 | 670 | 0.375 | 149 | 92 | 1 | 3 | 151 | 5 | 152 | Phage baseplate assembly protein V | Phage baseplate assembly protein V | | afdb-uniprot50 | AF-A0A2P5N6T8-F1-MODEL\_V4 | 1.0 | 1.082e-15 | 664 | 0.344 | 145 | 95 | 0 | 1 | 145 | 1 | 145 | Baseplate assembly protein | Baseplate assembly protein | | afdb-uniprot50 | AF-A0A118DV18-F1-MODEL\_V4 | 1.0 | 1.037e-20 | 663 | 0.42 | 221 | 116 | 4 | 1 | 217 | 1 | 213 | Uncharacterized protein | Uncharacterized protein | | afdb-uniprot50 | AF-A0A3G9FLY0-F1-MODEL\_V4 | 1.0 | 7.674e-19 | 662 | 0.367 | 223 | 126 | 6 | 1 | 217 | 1 | 214 | Phage baseplate assembly protein | Phage baseplate assembly protein | | afdb-uniprot50 | AF-A0A212KMZ7-F1-MODEL\_V4 | 1.0 | 1.37e-15 | 660 | 0.411 | 136 | 79 | 1 | 4 | 138 | 7 | 142 | Phage baseplate assembly protein V (Modular protein) | Phage baseplate assembly protein V (Modular protein) | | afdb-uniprot50 | AF-A0A554X0S7-F1-MODEL\_V4 | 1.0 | 8.059e-16 | 658 | 0.379 | 137 | 80 | 1 | 1 | 132 | 1 | 137 | Phage baseplate assembly protein V | Phage baseplate assembly protein V | | afdb-uniprot50 | AF-A0A516WAE0-F1-MODEL\_V4 | 1.0 | 5.677e-17 | 657 | 0.292 | 219 | 147 | 6 | 1 | 217 | 1 | 213 | Phage baseplate assembly protein V | Phage baseplate assembly protein V | | afdb-uniprot50 | AF-A0A369RHM3-F1-MODEL\_V4 | 1.0 | 3.733e-15 | 656 | 0.423 | 118 | 68 | 0 | 4 | 121 | 7 | 124 | Phage P2 baseplate assembly protein gpV | Phage P2 baseplate assembly protein gpV | | afdb-uniprot50 | AF-A0A7V8FPR0-F1-MODEL\_V4 | 1.0 | 1.458e-16 | 655 | 0.317 | 227 | 138 | 8 | 3 | 216 | 5 | 227 | Phage\_base\_V domain-containing protein | Phage\_base\_V domain-containing protein | | afdb-uniprot50 | AF-A0A6N7JJ19-F1-MODEL\_V4 | 1.0 | 1.375e-16 | 654 | 0.316 | 202 | 129 | 6 | 4 | 203 | 7 | 201 | Phage baseplate assembly protein V | Phage baseplate assembly protein V | | afdb-uniprot50 | AF-A0A7X2BHN0-F1-MODEL\_V4 | 1.0 | 5.352e-17 | 654 | 0.298 | 218 | 146 | 6 | 1 | 215 | 1 | 214 | Phage baseplate assembly protein V | Phage baseplate assembly protein V | | afdb-uniprot50 | AF-N6USL5-F1-MODEL\_V4 | 1.0 | 5.045e-17 | 647 | 0.322 | 223 | 141 | 6 | 4 | 217 | 8 | 229 | Phage baseplate assembly protein V | Phage baseplate assembly protein V | | afdb-uniprot50 | AF-A0A430HF75-F1-MODEL\_V4 | 1.0 | 2.998e-20 | 645 | 0.492 | 207 | 89 | 3 | 5 | 197 | 2 | 206 | Phage baseplate assembly protein V | Phage baseplate assembly protein V | | afdb-uniprot50 | AF-A0A4R2MLJ2-F1-MODEL\_V4 | 1.0 | 5.982e-15 | 644 | 0.406 | 128 | 76 | 0 | 5 | 132 | 2 | 129 | Phage baseplate assembly protein V | Phage baseplate assembly protein V | | afdb-uniprot50 | AF-A0A3N9U432-F1-MODEL\_V4 | 1.0 | 2.503e-19 | 644 | 0.345 | 217 | 108 | 4 | 1 | 217 | 1 | 183 | Phage baseplate assembly protein V | Phage baseplate assembly protein V | | afdb-uniprot50 | AF-A0A7W8YF54-F1-MODEL\_V4 | 1.0 | 1.757e-19 | 641 | 0.385 | 218 | 110 | 7 | 1 | 217 | 1 | 195 | Phage baseplate assembly protein V | Phage baseplate assembly protein V | | afdb-uniprot50 | AF-A0A1M7KGY4-F1-MODEL\_V4 | 1.0 | 9.038e-15 | 637 | 0.391 | 120 | 73 | 0 | 2 | 121 | 1 | 120 | Phage baseplate assembly protein V | Phage baseplate assembly protein V | | afdb-uniprot50 | AF-A0A4Q6FFT3-F1-MODEL\_V4 | 1.0 | 3.959e-15 | 636 | 0.307 | 143 | 99 | 0 | 1 | 143 | 1 | 143 | Phage baseplate assembly protein V | Phage baseplate assembly protein V | | afdb-uniprot50 | AF-A0A418HAA9-F1-MODEL\_V4 | 1.0 | 2.337e-16 | 636 | 0.375 | 181 | 108 | 3 | 16 | 196 | 2 | 177 | Phage baseplate assembly protein V | Phage baseplate assembly protein V | | afdb-uniprot50 | AF-A0A258LJQ0-F1-MODEL\_V4 | 1.0 | 5.029e-16 | 635 | 0.335 | 194 | 118 | 6 | 1 | 194 | 1 | 183 | Phage\_base\_V domain-containing protein | Phage\_base\_V domain-containing protein | | afdb-uniprot50 | AF-A0A447N5E7-F1-MODEL\_V4 | 1.0 | 1.214e-14 | 634 | 0.344 | 125 | 82 | 0 | 1 | 125 | 1 | 125 | Phage baseplate assembly protein V | Phage baseplate assembly protein V | | afdb-uniprot50 | AF-A0A1Y2K0V2-F1-MODEL\_V4 | 1.0 | 2.188e-14 | 628 | 0.366 | 120 | 76 | 0 | 1 | 120 | 1 | 120 | Putative baseplate assembly protein V | Putative baseplate assembly protein V | | afdb-uniprot50 | AF-A1VSH0-F1-MODEL\_V4 | 1.0 | 4.241e-18 | 627 | 0.349 | 229 | 123 | 7 | 1 | 217 | 3 | 217 | Phage baseplate assembly protein V | Phage baseplate assembly protein V | | afdb-uniprot50 | AF-A0A376WR96-F1-MODEL\_V4 | 1.0 | 8.113e-18 | 627 | 0.301 | 219 | 147 | 4 | 1 | 217 | 1 | 215 | Baseplate assembly protein V (GpV) | Baseplate assembly protein V (GpV) | | afdb-uniprot50 | AF-A0A6C1U1Y1-F1-MODEL\_V4 | 1.0 | 5.639e-15 | 626 | 0.372 | 129 | 81 | 0 | 4 | 132 | 7 | 135 | Phage baseplate assembly protein V | Phage baseplate assembly protein V | | afdb-uniprot50 | AF-I3TTD6-F1-MODEL\_V4 | 1.0 | 2.196e-15 | 624 | 0.405 | 148 | 81 | 3 | 1 | 143 | 1 | 146 | Phage baseplate assembly protein V | Phage baseplate assembly protein V | | afdb-uniprot50 | AF-A0A2E3M0K1-F1-MODEL\_V4 | 1.0 | 1.079e-14 | 623 | 0.345 | 133 | 85 | 1 | 1 | 131 | 1 | 133 | Baseplate assembly protein V | Baseplate assembly protein V | | afdb-uniprot50 | AF-A0A1Y6CPE2-F1-MODEL\_V4 | 1.0 | 7.573e-15 | 623 | 0.315 | 133 | 88 | 1 | 4 | 136 | 6 | 135 | Phage baseplate assembly protein V | Phage baseplate assembly protein V | | afdb-uniprot50 | AF-A0A1C3EL99-F1-MODEL\_V4 | 1.0 | 2.62e-15 | 623 | 0.322 | 152 | 103 | 0 | 4 | 155 | 8 | 159 | Baseplate assembly protein | Baseplate assembly protein | | afdb-uniprot50 | AF-A0A376YK40-F1-MODEL\_V4 | 1.0 | 9.618e-16 | 622 | 0.368 | 171 | 103 | 3 | 16 | 186 | 2 | 167 | Baseplate assembly protein V (GpV) | Baseplate assembly protein V (GpV) | | afdb-uniprot50 | AF-A0A1N6UJB4-F1-MODEL\_V4 | 1.0 | 1.453e-15 | 619 | 0.229 | 218 | 156 | 6 | 1 | 217 | 1 | 207 | Phage baseplate assembly protein V | Phage baseplate assembly protein V | | afdb-uniprot50 | AF-A0A2E5BTY2-F1-MODEL\_V4 | 1.0 | 1.834e-14 | 618 | 0.372 | 129 | 81 | 0 | 4 | 132 | 15 | 143 | Baseplate assembly protein V | Baseplate assembly protein V | | afdb-uniprot50 | AF-A0A6P1S8D3-F1-MODEL\_V4 | 1.0 | 2.503e-19 | 616 | 0.368 | 217 | 114 | 7 | 1 | 217 | 1 | 194 | Phage baseplate assembly protein V | Phage baseplate assembly protein V | | afdb-uniprot50 | AF-A0A4P9VEW0-F1-MODEL\_V4 | 1.0 | 1.536e-14 | 615 | 0.341 | 129 | 85 | 0 | 9 | 137 | 2 | 130 | Phage baseplate assembly protein V | Phage baseplate assembly protein V | | afdb-uniprot50 | AF-A0A3B0LVV6-F1-MODEL\_V4 | 1.0 | 5.334e-16 | 613 | 0.294 | 217 | 143 | 6 | 1 | 217 | 33 | 239 | Uncharacterized protein | Uncharacterized protein | | afdb-uniprot50 | AF-A0A2J9GZ67-F1-MODEL\_V4 | 1.0 | 3.18e-20 | 612 | 0.36 | 233 | 133 | 3 | 1 | 217 | 7 | 239 | Phage baseplate assembly protein V | Phage baseplate assembly protein V | | afdb-uniprot50 | AF-A0A2S8HYS2-F1-MODEL\_V4 | 1.0 | 3.339e-17 | 611 | 0.292 | 226 | 144 | 4 | 2 | 213 | 1 | 224 | Phage baseplate assembly protein V | Phage baseplate assembly protein V | | afdb-uniprot50 | AF-A0A377KA20-F1-MODEL\_V4 | 1.0 | 2.462e-14 | 609 | 0.33 | 127 | 85 | 0 | 1 | 127 | 1 | 127 | Phage baseplate assembly protein V | Phage baseplate assembly protein V | | afdb-uniprot50 | AF-A0A4S3LTU7-F1-MODEL\_V4 | 1.0 | 2.817e-19 | 609 | 0.375 | 213 | 118 | 5 | 1 | 213 | 1 | 198 | Phage baseplate assembly protein V | Phage baseplate assembly protein V | | afdb-uniprot50 | AF-A0A143DE80-F1-MODEL\_V4 | 1.0 | 3.757e-17 | 608 | 0.367 | 215 | 123 | 6 | 1 | 205 | 1 | 212 | Uncharacterized protein | Uncharacterized protein | | afdb-uniprot50 | AF-A0A2U2DBJ1-F1-MODEL\_V4 | 1.0 | 2.638e-17 | 606 | 0.281 | 220 | 148 | 6 | 2 | 217 | 1 | 214 | Phage baseplate assembly protein V | Phage baseplate assembly protein V | | afdb-uniprot50 | AF-A0A4U8YLR0-F1-MODEL\_V4 | 1.0 | 6.73e-15 | 605 | 0.378 | 148 | 92 | 0 | 4 | 151 | 7 | 154 | Phage baseplate assembly protein v/gp45 | Phage baseplate assembly protein v/gp45 | | afdb-uniprot50 | AF-W1J949-F1-MODEL\_V4 | 1.0 | 9.587e-15 | 601 | 0.377 | 127 | 75 | 2 | 1 | 127 | 1 | 123 | Baseplate assembly protein V | Baseplate assembly protein V | | afdb-uniprot50 | AF-A0A2E3Q547-F1-MODEL\_V4 | 1.0 | 5.299e-14 | 601 | 0.31 | 132 | 91 | 0 | 1 | 132 | 1 | 132 | Phage baseplate protein | Phage baseplate protein | | afdb-uniprot50 | AF-C3X1Y6-F1-MODEL\_V4 | 1.0 | 1.468e-18 | 601 | 0.334 | 206 | 122 | 5 | 1 | 203 | 1 | 194 | Phage baseplate assembly protein V | Phage baseplate assembly protein V | | afdb-uniprot50 | AF-A0A771BAN4-F1-MODEL\_V4 | 1.0 | 2.62e-15 | 600 | 0.366 | 180 | 109 | 3 | 17 | 196 | 1 | 175 | Phage baseplate assembly protein V | Phage baseplate assembly protein V | | afdb-uniprot50 | AF-A0A741LDN8-F1-MODEL\_V4 | 1.0 | 2.47e-15 | 599 | 0.372 | 180 | 108 | 3 | 17 | 196 | 1 | 175 | Phage baseplate assembly protein V | Phage baseplate assembly protein V | | afdb-uniprot50 | AF-Q31Q99-F1-MODEL\_V4 | 1.0 | 1.079e-14 | 598 | 0.272 | 169 | 113 | 4 | 2 | 160 | 18 | 186 | Phage baseplate assembly protein V | Phage baseplate assembly protein V | | afdb-uniprot50 | AF-A0A5A9ENI4-F1-MODEL\_V4 | 1.0 | 3.117e-14 | 596 | 0.361 | 130 | 82 | 1 | 4 | 132 | 11 | 140 | Phage baseplate assembly protein V | Phage baseplate assembly protein V | | afdb-uniprot50 | AF-A0A7Z1R6V5-F1-MODEL\_V4 | 1.0 | 3.317e-15 | 595 | 0.25 | 204 | 146 | 4 | 1 | 203 | 1 | 198 | Phage baseplate protein | Phage baseplate protein | | afdb-uniprot50 | AF-B4EF38-F1-MODEL\_V4 | 1.0 | 4.227e-17 | 594 | 0.368 | 220 | 117 | 8 | 1 | 203 | 42 | 256 | Phage baseplate assembly protein | Phage baseplate assembly protein | | afdb-uniprot50 | AF-A0A5W3AAX1-F1-MODEL\_V4 | 1.0 | 2.07e-15 | 593 | 0.365 | 156 | 81 | 2 | 1 | 154 | 1 | 140 | Phage baseplate assembly protein V | Phage baseplate assembly protein V | | afdb-uniprot50 | AF-A0A4R7NQH3-F1-MODEL\_V4 | 1.0 | 9.747e-20 | 591 | 0.373 | 222 | 128 | 5 | 4 | 217 | 34 | 252 | Phage baseplate assembly protein V | Phage baseplate assembly protein V | | afdb-uniprot50 | AF-A0A7W8K684-F1-MODEL\_V4 | 1.0 | 2.07e-15 | 590 | 0.386 | 168 | 84 | 4 | 1 | 167 | 1 | 150 | Phage baseplate assembly protein V | Phage baseplate assembly protein V | | afdb-uniprot50 | AF-A0A396RYI9-F1-MODEL\_V4 | 1.0 | 6.022e-17 | 588 | 0.259 | 216 | 122 | 4 | 2 | 217 | 1 | 178 | Phage baseplate assembly protein V | Phage baseplate assembly protein V | | afdb-uniprot50 | AF-A0A7V8FKD6-F1-MODEL\_V4 | 1.0 | 3.159e-18 | 588 | 0.372 | 220 | 114 | 6 | 1 | 217 | 1 | 199 | Phage\_base\_V domain-containing protein | Phage\_base\_V domain-containing protein | | afdb-uniprot50 | AF-A0A142JGU2-F1-MODEL\_V4 | 1.0 | 1.226e-17 | 587 | 0.327 | 220 | 110 | 5 | 1 | 217 | 1 | 185 | Uncharacterized protein | Uncharacterized protein | | afdb-uniprot50 | AF-A0A3E0X1A7-F1-MODEL\_V4 | 1.0 | 6.002e-16 | 586 | 0.393 | 150 | 77 | 1 | 2 | 137 | 1 | 150 | Phage\_base\_V domain-containing protein | Phage\_base\_V domain-containing protein | | afdb-uniprot50 | AF-A0A741LDS5-F1-MODEL\_V4 | 1.0 | 1.84e-15 | 586 | 0.389 | 203 | 112 | 6 | 17 | 217 | 1 | 193 | Phage baseplate assembly protein V | Phage baseplate assembly protein V | | afdb-uniprot50 | AF-A0A6H2NSY1-F1-MODEL\_V4 | 1.0 | 1.828e-13 | 584 | 0.418 | 110 | 64 | 0 | 4 | 113 | 7 | 116 | Phage baseplate assembly protein V | Phage baseplate assembly protein V | | afdb-uniprot50 | AF-A0A1B8QD27-F1-MODEL\_V4 | 1.0 | 4.469e-16 | 581 | 0.289 | 214 | 129 | 5 | 1 | 214 | 1 | 191 | Phage\_base\_V domain-containing protein | Phage\_base\_V domain-containing protein | | afdb-uniprot50 | AF-A0A376DH93-F1-MODEL\_V4 | 1.0 | 4.012e-19 | 580 | 0.347 | 216 | 120 | 4 | 2 | 217 | 213 | 407 | Putative phage tail completion protein S | Putative phage tail completion protein S | | afdb-uniprot50 | AF-A0A5C8XUI7-F1-MODEL\_V4 | 1.0 | 3.53e-16 | 578 | 0.329 | 176 | 97 | 3 | 1 | 176 | 1 | 155 | Phage baseplate assembly protein V | Phage baseplate assembly protein V | | afdb-uniprot50 | AF-A0A235EWV9-F1-MODEL\_V4 | 1.0 | 4.788e-19 | 577 | 0.355 | 225 | 120 | 4 | 1 | 217 | 1 | 208 | Uncharacterized protein | Uncharacterized protein | | afdb-uniprot50 | AF-A0A6L2ZPM3-F1-MODEL\_V4 | 1.0 | 5.37e-18 | 576 | 0.351 | 216 | 119 | 4 | 2 | 217 | 3 | 197 | Baseplate assembly protein V (GpV) | Baseplate assembly protein V (GpV) | | afdb-uniprot50 | AF-A0A6N4PBL9-F1-MODEL\_V4 | 1.0 | 2.495e-18 | 575 | 0.333 | 213 | 121 | 4 | 5 | 217 | 2 | 193 | Phage baseplate assembly protein V | Phage baseplate assembly protein V | | afdb-uniprot50 | AF-A0A081B6D4-F1-MODEL\_V4 | 1.0 | 6.022e-17 | 575 | 0.311 | 212 | 139 | 3 | 4 | 215 | 7 | 211 | Phage baseplate assembly protein V | Phage baseplate assembly protein V | | afdb-uniprot50 | AF-A0A7X4JXC7-F1-MODEL\_V4 | 1.0 | 2.344e-17 | 574 | 0.3 | 216 | 113 | 2 | 2 | 217 | 1 | 178 | Phage baseplate assembly protein V | Phage baseplate assembly protein V | | afdb-uniprot50 | AF-A0A5E5AEH0-F1-MODEL\_V4 | 1.0 | 1.082e-15 | 574 | 0.299 | 224 | 136 | 5 | 1 | 217 | 1 | 210 | Baseplate assembly protein | Baseplate assembly protein | | afdb-uniprot50 | AF-A0A7H4NMM9-F1-MODEL\_V4 | 1.0 | 5.982e-15 | 571 | 0.307 | 140 | 93 | 1 | 1 | 136 | 1 | 140 | Baseplate assembly protein V | Baseplate assembly protein V | | afdb-uniprot50 | AF-A0A178GUC9-F1-MODEL\_V4 | 1.0 | 1.366e-14 | 571 | 0.331 | 148 | 85 | 1 | 4 | 137 | 7 | 154 | Baseplate assembly protein V | Baseplate assembly protein V | | afdb-uniprot50 | AF-A0A502CNP6-F1-MODEL\_V4 | 1.0 | 2.798e-17 | 571 | 0.296 | 216 | 114 | 4 | 2 | 217 | 1 | 178 | Phage baseplate assembly protein V | Phage baseplate assembly protein V | | afdb-uniprot50 | AF-A0A2U9LGE2-F1-MODEL\_V4 | 1.0 | 5.37e-18 | 571 | 0.342 | 213 | 119 | 4 | 5 | 217 | 2 | 193 | Phage baseplate assembly protein V | Phage baseplate assembly protein V | | afdb-uniprot50 | AF-A0A244BW49-F1-MODEL\_V4 | 1.0 | 5.696e-18 | 570 | 0.338 | 213 | 120 | 4 | 5 | 217 | 2 | 193 | Baseplate assembly protein | Baseplate assembly protein | | afdb-uniprot50 | AF-A0A379ZSB9-F1-MODEL\_V4 | 1.0 | 1.63e-14 | 569 | 0.304 | 138 | 90 | 1 | 1 | 132 | 1 | 138 | Phage baseplate assembly protein V | Phage baseplate assembly protein V | | afdb-uniprot50 | AF-A0A1V2N3K0-F1-MODEL\_V4 | 1.0 | 6.73e-15 | 568 | 0.351 | 148 | 82 | 2 | 4 | 137 | 7 | 154 | Phage baseplate assembly protein V family protein | Phage baseplate assembly protein V family protein | | afdb-uniprot50 | AF-A0A6N4T373-F1-MODEL\_V4 | 1.0 | 2.929e-13 | 568 | 0.365 | 126 | 79 | 1 | 1 | 125 | 1 | 126 | Phage\_base\_V domain-containing protein | Phage\_base\_V domain-containing protein | | afdb-uniprot50 | AF-A0A1C7W7X8-F1-MODEL\_V4 | 1.0 | 1.958e-16 | 567 | 0.292 | 222 | 144 | 5 | 2 | 217 | 1 | 215 | Phage\_base\_V domain-containing protein | Phage\_base\_V domain-containing protein | | afdb-uniprot50 | AF-A0A653KXX6-F1-MODEL\_V4 | 1.0 | 2.487e-17 | 566 | 0.337 | 237 | 87 | 6 | 1 | 217 | 1 | 187 | Contig\_41, whole genome shotgun sequence | Contig\_41, whole genome shotgun sequence | | afdb-uniprot50 | AF-A0A4Y9VQX4-F1-MODEL\_V4 | 1.0 | 2.091e-18 | 566 | 0.315 | 219 | 127 | 6 | 1 | 217 | 1 | 198 | Phage baseplate assembly protein V | Phage baseplate assembly protein V | | afdb-uniprot50 | AF-A0A2S6H5F0-F1-MODEL\_V4 | 1.0 | 1.531e-13 | 564 | 0.335 | 140 | 85 | 2 | 1 | 132 | 1 | 140 | Phage baseplate assembly protein V | Phage baseplate assembly protein V | | afdb-uniprot50 | AF-A0A1G6JDU4-F1-MODEL\_V4 | 1.0 | 3.35e-18 | 563 | 0.362 | 232 | 125 | 6 | 1 | 217 | 1 | 224 | Phage baseplate assembly protein V | Phage baseplate assembly protein V | | afdb-uniprot50 | AF-A0A853SGL8-F1-MODEL\_V4 | 1.0 | 2.612e-14 | 562 | 0.41 | 139 | 78 | 3 | 3 | 139 | 6 | 142 | Phage-related baseplate assembly protein | Phage-related baseplate assembly protein | | afdb-uniprot50 | AF-A0A2S8QRV5-F1-MODEL\_V4 | 1.0 | 8.059e-16 | 562 | 0.242 | 194 | 142 | 3 | 1 | 193 | 1 | 190 | Phage baseplate assembly protein V | Phage baseplate assembly protein V | | afdb-uniprot50 | AF-A0A3S5B410-F1-MODEL\_V4 | 1.0 | 7.648e-18 | 561 | 0.386 | 212 | 118 | 4 | 6 | 217 | 28 | 227 | Phage P2 baseplate assembly protein gpV | Phage P2 baseplate assembly protein gpV | | afdb-uniprot50 | AF-A0A7W6RFW2-F1-MODEL\_V4 | 1.0 | 5.352e-17 | 560 | 0.324 | 216 | 103 | 3 | 2 | 217 | 6 | 178 | Phage baseplate assembly protein V | Phage baseplate assembly protein V | | afdb-uniprot50 | AF-A0A774TGT6-F1-MODEL\_V4 | 1.0 | 5.696e-18 | 560 | 0.345 | 217 | 118 | 5 | 1 | 217 | 1 | 193 | Phage baseplate assembly protein V | Phage baseplate assembly protein V | | afdb-uniprot50 | AF-A0A6N8Q3A4-F1-MODEL\_V4 | 1.0 | 1.734e-15 | 559 | 0.287 | 181 | 118 | 3 | 1 | 181 | 1 | 170 | Phage baseplate assembly protein V | Phage baseplate assembly protein V | | afdb-uniprot50 | AF-A0A829E378-F1-MODEL\_V4 | 1.0 | 6.022e-17 | 559 | 0.313 | 217 | 115 | 6 | 1 | 217 | 1 | 183 | Uncharacterized protein | Uncharacterized protein | | afdb-uniprot50 | AF-A0A6N4T205-F1-MODEL\_V4 | 1.0 | 1.939e-13 | 558 | 0.359 | 128 | 81 | 1 | 1 | 127 | 1 | 128 | Phage\_base\_V domain-containing protein | Phage\_base\_V domain-containing protein | | afdb-uniprot50 | AF-Q8ZMU1-F1-MODEL\_V4 | 1.0 | 1.463e-17 | 558 | 0.269 | 223 | 126 | 3 | 1 | 217 | 1 | 192 | Fels-2 prophage protein | Fels-2 prophage protein | | afdb-uniprot50 | AF-A0A376HNC6-F1-MODEL\_V4 | 1.0 | 7.648e-18 | 558 | 0.341 | 217 | 119 | 5 | 1 | 217 | 1 | 193 | Baseplate assembly protein V | Baseplate assembly protein V | | afdb-uniprot50 | AF-A0A1H0NN53-F1-MODEL\_V4 | 1.0 | 2.647e-18 | 558 | 0.36 | 225 | 116 | 6 | 1 | 217 | 1 | 205 | Phage baseplate assembly protein V | Phage baseplate assembly protein V | | afdb-uniprot50 | AF-A0A1A9VKF9-F1-MODEL\_V4 | 1.0 | 2.063e-14 | 558 | 0.376 | 146 | 77 | 1 | 6 | 137 | 191 | 336 | Phage\_base\_V domain-containing protein | Phage\_base\_V domain-containing protein | | afdb-uniprot50 | AF-J1JE15-F1-MODEL\_V4 | 1.0 | 4.172e-13 | 557 | 0.333 | 135 | 88 | 2 | 4 | 137 | 8 | 141 | Phage baseplate assembly protein V | Phage baseplate assembly protein V | | afdb-uniprot50 | AF-A0A7Z8DPV0-F1-MODEL\_V4 | 1.0 | 7.21e-18 | 556 | 0.35 | 217 | 117 | 5 | 1 | 217 | 1 | 193 | Phage baseplate assembly protein V | Phage baseplate assembly protein V | | afdb-uniprot50 | AF-C0R4U2-F1-MODEL\_V4 | 1.0 | 1.63e-14 | 555 | 0.348 | 149 | 81 | 3 | 4 | 137 | 7 | 154 | Baseplate assembly protein V | Baseplate assembly protein V | | afdb-uniprot50 | AF-A0A3S5YL67-F1-MODEL\_V4 | 1.0 | 9.682e-18 | 555 | 0.341 | 217 | 119 | 5 | 1 | 217 | 1 | 193 | Baseplate assembly protein | Baseplate assembly protein | | afdb-uniprot50 | AF-A0A2X2ZC27-F1-MODEL\_V4 | 1.0 | 9.682e-18 | 555 | 0.341 | 217 | 119 | 5 | 1 | 217 | 1 | 193 | Baseplate assembly protein V | Baseplate assembly protein V | | afdb-uniprot50 | AF-A0A376SKQ2-F1-MODEL\_V4 | 1.0 | 7.163e-16 | 553 | 0.333 | 177 | 95 | 2 | 3 | 179 | 5 | 158 | Baseplate assembly protein V (GpV) | Baseplate assembly protein V (GpV) | | afdb-uniprot50 | AF-A0A6D0PZJ4-F1-MODEL\_V4 | 1.0 | 1.089e-17 | 553 | 0.341 | 217 | 119 | 5 | 1 | 217 | 1 | 193 | Phage baseplate assembly protein V | Phage baseplate assembly protein V | | afdb-uniprot50 | AF-C1DBK4-F1-MODEL\_V4 | 1.0 | 1.214e-14 | 552 | 0.342 | 143 | 86 | 2 | 1 | 137 | 1 | 141 | Phage baseplate assembly protein V | Phage baseplate assembly protein V | | afdb-uniprot50 | AF-A0A2Z6GCH3-F1-MODEL\_V4 | 1.0 | 4.484e-17 | 551 | 0.327 | 214 | 103 | 6 | 6 | 217 | 3 | 177 | Baseplate assembly protein | Baseplate assembly protein | | afdb-uniprot50 | AF-A0A1X7L1D4-F1-MODEL\_V4 | 1.0 | 5.299e-14 | 550 | 0.291 | 151 | 101 | 2 | 1 | 145 | 4 | 154 | Phage baseplate assembly protein V | Phage baseplate assembly protein V | | afdb-uniprot50 | AF-A0A2W5AAE8-F1-MODEL\_V4 | 1.0 | 2.077e-16 | 550 | 0.284 | 218 | 130 | 6 | 1 | 217 | 1 | 193 | Phage baseplate assembly protein V | Phage baseplate assembly protein V | | afdb-uniprot50 | AF-A0A166Z6Q3-F1-MODEL\_V4 | 1.0 | 1.024e-16 | 550 | 0.288 | 215 | 130 | 6 | 3 | 217 | 5 | 196 | Baseplate assembly protein | Baseplate assembly protein | | afdb-uniprot50 | AF-A0A0T9TB75-F1-MODEL\_V4 | 1.0 | 1.082e-15 | 549 | 0.278 | 219 | 99 | 4 | 1 | 217 | 1 | 162 | Baseplate assembly protein | Baseplate assembly protein | | afdb-uniprot50 | AF-A0A6S6Y3K5-F1-MODEL\_V4 | 1.0 | 1.746e-17 | 549 | 0.337 | 216 | 136 | 5 | 4 | 217 | 9 | 219 | Phage baseplate assembly protein V (Modular protein) | Phage baseplate assembly protein V (Modular protein) | | afdb-uniprot50 | AF-W0L460-F1-MODEL\_V4 | 1.0 | 2.337e-16 | 548 | 0.31 | 216 | 117 | 4 | 2 | 217 | 1 | 184 | Baseplate assembly protein | Baseplate assembly protein | | afdb-uniprot50 | AF-A0A482PH41-F1-MODEL\_V4 | 1.0 | 1.379e-17 | 548 | 0.331 | 217 | 121 | 5 | 1 | 217 | 1 | 193 | Phage baseplate assembly protein V | Phage baseplate assembly protein V | | afdb-uniprot50 | AF-A0A559QNI7-F1-MODEL\_V4 | 1.0 | 4.214e-16 | 548 | 0.331 | 193 | 111 | 2 | 2 | 194 | 1 | 175 | Phage baseplate assembly protein V | Phage baseplate assembly protein V | | afdb-uniprot50 | AF-A0A519FIN0-F1-MODEL\_V4 | 1.0 | 1.01e-12 | 546 | 0.31 | 132 | 89 | 2 | 1 | 131 | 1 | 131 | Phage baseplate assembly protein V | Phage baseplate assembly protein V | | afdb-uniprot50 | AF-A0A843NDI5-F1-MODEL\_V4 | 1.0 | 1.448e-14 | 545 | 0.331 | 160 | 91 | 1 | 5 | 164 | 2 | 145 | Phage baseplate assembly protein V | Phage baseplate assembly protein V | | afdb-uniprot50 | AF-A0A1E3G706-F1-MODEL\_V4 | 1.0 | 1.283e-13 | 545 | 0.331 | 145 | 95 | 1 | 1 | 143 | 1 | 145 | Phage\_base\_V domain-containing protein | Phage\_base\_V domain-containing protein | | afdb-uniprot50 | AF-A0A1X0W485-F1-MODEL\_V4 | 1.0 | 4.757e-17 | 545 | 0.269 | 223 | 118 | 4 | 1 | 217 | 1 | 184 | Baseplate assembly protein | Baseplate assembly protein | | afdb-uniprot50 | AF-A0A1E7PZX9-F1-MODEL\_V4 | 1.0 | 8.549e-16 | 545 | 0.336 | 193 | 91 | 4 | 1 | 193 | 1 | 156 | Phage\_base\_V domain-containing protein | Phage\_base\_V domain-containing protein | | afdb-uniprot50 | AF-A0A158E8E3-F1-MODEL\_V4 | 1.0 | 1.226e-17 | 545 | 0.288 | 225 | 136 | 4 | 1 | 208 | 1 | 218 | Phage baseplate assembly protein V | Phage baseplate assembly protein V | | afdb-uniprot50 | AF-W1HV66-F1-MODEL\_V4 | 1.0 | 1.932e-12 | 544 | 0.371 | 105 | 66 | 0 | 16 | 120 | 2 | 106 | Baseplate assembly protein V | Baseplate assembly protein V | | afdb-uniprot50 | AF-A0A149QJX7-F1-MODEL\_V4 | 1.0 | 3.542e-17 | 544 | 0.304 | 217 | 122 | 6 | 1 | 217 | 1 | 188 | Baseplate assembly protein | Baseplate assembly protein | | afdb-uniprot50 | AF-A0A0M5KYZ3-F1-MODEL\_V4 | 1.0 | 2.968e-17 | 544 | 0.308 | 217 | 136 | 4 | 4 | 217 | 8 | 213 | Phage-related baseplate assembly protein | Phage-related baseplate assembly protein | | afdb-uniprot50 | AF-A0A366IM94-F1-MODEL\_V4 | 1.0 | 1.027e-17 | 542 | 0.331 | 220 | 120 | 5 | 3 | 217 | 44 | 241 | Phage baseplate assembly protein V | Phage baseplate assembly protein V | | afdb-uniprot50 | AF-A0A1B3E8F5-F1-MODEL\_V4 | 1.0 | 5.029e-16 | 541 | 0.328 | 195 | 106 | 4 | 2 | 196 | 1 | 170 | Phage baseplate assembly protein V | Phage baseplate assembly protein V | | afdb-uniprot50 | AF-A0A7Z0MZS5-F1-MODEL\_V4 | 1.0 | 9.618e-16 | 540 | 0.331 | 223 | 124 | 8 | 4 | 217 | 10 | 216 | Phage baseplate assembly protein V | Phage baseplate assembly protein V | | afdb-uniprot50 | AF-A0A6D0J6U3-F1-MODEL\_V4 | 1.0 | 4.694e-13 | 539 | 0.346 | 130 | 84 | 1 | 16 | 145 | 2 | 130 | Phage baseplate assembly protein V | Phage baseplate assembly protein V | | afdb-uniprot50 | AF-A0A4V2RT09-F1-MODEL\_V4 | 1.0 | 3.496e-13 | 539 | 0.282 | 131 | 90 | 2 | 5 | 135 | 9 | 135 | Phage baseplate assembly protein V | Phage baseplate assembly protein V | | afdb-uniprot50 | AF-A0A5X8YW87-F1-MODEL\_V4 | 1.0 | 4.469e-16 | 538 | 0.322 | 192 | 107 | 4 | 1 | 192 | 1 | 169 | Phage baseplate assembly protein V | Phage baseplate assembly protein V | | afdb-uniprot50 | AF-A0A853I7W8-F1-MODEL\_V4 | 1.0 | 2.929e-13 | 538 | 0.307 | 140 | 97 | 0 | 16 | 155 | 2 | 141 | Phage baseplate assembly protein V | Phage baseplate assembly protein V | | afdb-uniprot50 | AF-A0A7W5K784-F1-MODEL\_V4 | 1.0 | 9.65e-17 | 538 | 0.304 | 217 | 129 | 7 | 1 | 217 | 1 | 195 | Phage baseplate assembly protein V | Phage baseplate assembly protein V | | afdb-uniprot50 | AF-A0A7W6WMQ5-F1-MODEL\_V4 | 1.0 | 7.187e-17 | 538 | 0.352 | 190 | 104 | 4 | 6 | 193 | 14 | 186 | Phage baseplate assembly protein V | Phage baseplate assembly protein V | | afdb-uniprot50 | AF-A0A7U9IYM4-F1-MODEL\_V4 | 1.0 | 3.757e-17 | 537 | 0.341 | 217 | 119 | 5 | 1 | 217 | 1 | 193 | Phage protein gp15 | Phage protein gp15 | | afdb-uniprot50 | AF-Q877J8-F1-MODEL\_V4 | 1.0 | 3.53e-16 | 537 | 0.319 | 219 | 123 | 6 | 1 | 217 | 1 | 195 | Phage-related baseplate assembly protein | Phage-related baseplate assembly protein | | afdb-uniprot50 | AF-A0A485AXI1-F1-MODEL\_V4 | 1.0 | 1.148e-15 | 537 | 0.273 | 194 | 117 | 3 | 3 | 196 | 22 | 191 | Phage P2 baseplate assembly protein gpV | Phage P2 baseplate assembly protein gpV | | afdb-uniprot50 | AF-A0A7W4KR34-F1-MODEL\_V4 | 1.0 | 1.822e-12 | 536 | 0.327 | 113 | 76 | 0 | 10 | 122 | 2 | 114 | Phage baseplate assembly protein V | Phage baseplate assembly protein V | | afdb-uniprot50 | AF-A0A4R3Y8K0-F1-MODEL\_V4 | 1.0 | 2.329e-15 | 536 | 0.289 | 214 | 110 | 5 | 4 | 217 | 6 | 177 | Phage baseplate assembly protein V | Phage baseplate assembly protein V | | afdb-uniprot50 | AF-A0A2U1XZ31-F1-MODEL\_V4 | 1.0 | 1.296e-16 | 536 | 0.304 | 230 | 136 | 8 | 4 | 217 | 18 | 239 | Phage\_base\_V domain-containing protein | Phage\_base\_V domain-containing protein | | afdb-uniprot50 | AF-A0A8B4LYP3-F1-MODEL\_V4 | 1.0 | 2.798e-17 | 534 | 0.36 | 219 | 110 | 7 | 1 | 217 | 1 | 191 | Baseplate assembly protein V | Baseplate assembly protein V | | afdb-uniprot50 | AF-A0A7X5KA07-F1-MODEL\_V4 | 1.0 | 6.041e-18 | 534 | 0.262 | 248 | 151 | 5 | 1 | 217 | 1 | 247 | Phage baseplate assembly protein V | Phage baseplate assembly protein V | | afdb-uniprot50 | AF-V5ZC64-F1-MODEL\_V4 | 1.0 | 5.677e-17 | 534 | 0.288 | 215 | 128 | 4 | 3 | 217 | 86 | 275 | Baseplate assembly protein V GpV | Baseplate assembly protein V GpV | | afdb-uniprot50 | AF-A0A061JPC8-F1-MODEL\_V4 | 1.0 | 4.725e-15 | 532 | 0.337 | 166 | 93 | 3 | 1 | 166 | 1 | 149 | Baseplate assembly protein | Baseplate assembly protein | | afdb-uniprot50 | AF-A0A1F2LPI2-F1-MODEL\_V4 | 1.0 | 9.068e-16 | 532 | 0.28 | 196 | 117 | 3 | 1 | 196 | 1 | 172 | Baseplate assembly protein | Baseplate assembly protein | | afdb-uniprot50 | AF-A0A3N8PDL7-F1-MODEL\_V4 | 1.0 | 3.127e-15 | 532 | 0.272 | 224 | 143 | 5 | 3 | 215 | 4 | 218 | Phage baseplate assembly protein V | Phage baseplate assembly protein V | | afdb-uniprot50 | AF-A0A369YJU0-F1-MODEL\_V4 | 1.0 | 3.985e-17 | 531 | 0.327 | 217 | 106 | 7 | 1 | 217 | 1 | 177 | Phage baseplate assembly protein V | Phage baseplate assembly protein V | | afdb-uniprot50 | AF-A0A2A5BPR3-F1-MODEL\_V4 | 1.0 | 1.292e-15 | 531 | 0.24 | 212 | 142 | 4 | 2 | 213 | 1 | 193 | Uncharacterized protein | Uncharacterized protein | | afdb-uniprot50 | AF-A0A4P9VFF4-F1-MODEL\_V4 | 1.0 | 8.059e-16 | 531 | 0.262 | 202 | 107 | 4 | 1 | 197 | 9 | 173 | Phage baseplate assembly protein V | Phage baseplate assembly protein V | | afdb-uniprot50 | AF-A0A2E4CSJ4-F1-MODEL\_V4 | 1.0 | 2.337e-16 | 531 | 0.299 | 204 | 127 | 5 | 14 | 217 | 22 | 209 | Baseplate assembly protein | Baseplate assembly protein | | afdb-uniprot50 | AF-A0A797M4Y5-F1-MODEL\_V4 | 1.0 | 1.723e-13 | 529 | 0.3 | 153 | 88 | 2 | 1 | 153 | 1 | 134 | Phage baseplate assembly protein V | Phage baseplate assembly protein V | | afdb-uniprot50 | AF-A0A420WVL0-F1-MODEL\_V4 | 1.0 | 3.328e-16 | 529 | 0.305 | 193 | 110 | 4 | 4 | 196 | 6 | 174 | Phage baseplate assembly protein V | Phage baseplate assembly protein V | | afdb-uniprot50 | AF-A0A2K4JEB7-F1-MODEL\_V4 | 1.0 | 5.029e-16 | 529 | 0.298 | 218 | 130 | 2 | 1 | 217 | 1 | 196 | Phage baseplate assembly protein V | Phage baseplate assembly protein V | | afdb-uniprot50 | AF-A0A833FRJ1-F1-MODEL\_V4 | 1.0 | 2.78e-15 | 528 | 0.273 | 219 | 117 | 4 | 4 | 217 | 8 | 189 | Phage baseplate assembly protein V | Phage baseplate assembly protein V | | afdb-uniprot50 | AF-A0A7X3ZHR6-F1-MODEL\_V4 | 1.0 | 1.137e-12 | 527 | 0.266 | 135 | 98 | 1 | 4 | 137 | 11 | 145 | Phage baseplate assembly protein V | Phage baseplate assembly protein V | | afdb-uniprot50 | AF-U2B529-F1-MODEL\_V4 | 1.0 | 4.425e-13 | 526 | 0.307 | 143 | 90 | 4 | 1 | 137 | 1 | 140 | Baseplate assembly protein | Baseplate assembly protein | | afdb-uniprot50 | AF-A0A516SJA1-F1-MODEL\_V4 | 1.0 | 1.137e-12 | 526 | 0.288 | 135 | 93 | 2 | 1 | 132 | 1 | 135 | Phage baseplate assembly protein V | Phage baseplate assembly protein V | | afdb-uniprot50 | AF-A0A4T2A4P9-F1-MODEL\_V4 | 1.0 | 2.62e-15 | 526 | 0.312 | 192 | 114 | 4 | 2 | 193 | 1 | 174 | Phage baseplate assembly protein V | Phage baseplate assembly protein V | | afdb-uniprot50 | AF-B0UTN4-F1-MODEL\_V4 | 1.0 | 1.296e-16 | 526 | 0.289 | 204 | 126 | 3 | 3 | 206 | 5 | 189 | Phage baseplate assembly protein V | Phage baseplate assembly protein V | | afdb-uniprot50 | AF-A0A318KM99-F1-MODEL\_V4 | 1.0 | 6.022e-17 | 526 | 0.331 | 229 | 137 | 7 | 1 | 217 | 1 | 225 | Phage baseplate assembly protein V | Phage baseplate assembly protein V | | afdb-uniprot50 | AF-A0A516SHS7-F1-MODEL\_V4 | 1.0 | 7.21e-18 | 526 | 0.328 | 225 | 113 | 4 | 4 | 217 | 41 | 238 | Phage baseplate assembly protein V | Phage baseplate assembly protein V | | afdb-uniprot50 | AF-A0A2G3L892-F1-MODEL\_V4 | 1.0 | 3.757e-17 | 526 | 0.32 | 243 | 121 | 9 | 1 | 217 | 1 | 225 | Oxidoreductase | Oxidoreductase | | afdb-uniprot50 | AF-A0A7Z7RJ47-F1-MODEL\_V4 | 1.0 | 3.554e-18 | 525 | 0.337 | 228 | 130 | 5 | 4 | 217 | 35 | 255 | Phage P2 baseplate assembly protein gpV | Phage P2 baseplate assembly protein gpV | | afdb-uniprot50 | AF-A0A7X0AGS3-F1-MODEL\_V4 | 1.0 | 4.214e-16 | 522 | 0.294 | 214 | 127 | 5 | 2 | 209 | 1 | 196 | Phage baseplate assembly protein V | Phage baseplate assembly protein V | | afdb-uniprot50 | AF-A0A2W5FLQ5-F1-MODEL\_V4 | 1.0 | 1.547e-16 | 522 | 0.255 | 239 | 120 | 6 | 2 | 217 | 3 | 206 | Phage baseplate assembly protein V | Phage baseplate assembly protein V | | afdb-uniprot50 | AF-A0A1V2N5A2-F1-MODEL\_V4 | 1.0 | 7.548e-14 | 521 | 0.352 | 139 | 76 | 2 | 13 | 137 | 2 | 140 | Phage baseplate assembly protein V family protein | Phage baseplate assembly protein V family protein | | afdb-uniprot50 | AF-A0A5J6LDB1-F1-MODEL\_V4 | 1.0 | 3.317e-15 | 521 | 0.271 | 217 | 110 | 4 | 1 | 217 | 1 | 169 | Phage baseplate assembly protein V | Phage baseplate assembly protein V | | afdb-uniprot50 | AF-A0A542V4K0-F1-MODEL\_V4 | 1.0 | 9.068e-16 | 521 | 0.282 | 195 | 121 | 4 | 2 | 196 | 1 | 176 | Phage baseplate assembly protein V | Phage baseplate assembly protein V | | afdb-uniprot50 | AF-A0A377A641-F1-MODEL\_V4 | 1.0 | 1.536e-14 | 520 | 0.335 | 170 | 94 | 2 | 1 | 170 | 1 | 151 | Baseplate assembly protein V | Baseplate assembly protein V | | afdb-uniprot50 | AF-E2CJU1-F1-MODEL\_V4 | 1.0 | 9.068e-16 | 520 | 0.276 | 217 | 132 | 3 | 3 | 217 | 9 | 202 | Phage-related baseplate assembly protein V | Phage-related baseplate assembly protein V | | afdb-uniprot50 | AF-A0A5S9Q467-F1-MODEL\_V4 | 1.0 | 3.733e-15 | 520 | 0.262 | 221 | 143 | 5 | 1 | 217 | 1 | 205 | Phage\_base\_V domain-containing protein | Phage\_base\_V domain-containing protein | | afdb-uniprot50 | AF-A0A375AA54-F1-MODEL\_V4 | 1.0 | 3.53e-16 | 520 | 0.357 | 210 | 95 | 3 | 7 | 216 | 3 | 172 | Baseplate assembly protein V | Baseplate assembly protein V | | afdb-uniprot50 | AF-A0A840REX7-F1-MODEL\_V4 | 1.0 | 1.723e-13 | 519 | 0.367 | 136 | 78 | 2 | 2 | 129 | 1 | 136 | Phage baseplate assembly protein V | Phage baseplate assembly protein V | | afdb-uniprot50 | AF-A0A430BCB8-F1-MODEL\_V4 | 1.0 | 4.214e-16 | 519 | 0.267 | 217 | 132 | 6 | 1 | 217 | 1 | 190 | Phage baseplate assembly protein V | Phage baseplate assembly protein V | | afdb-uniprot50 | AF-A0A516SJG1-F1-MODEL\_V4 | 1.0 | 3.92e-12 | 519 | 0.276 | 130 | 93 | 1 | 4 | 132 | 44 | 173 | Phage baseplate assembly protein V | Phage baseplate assembly protein V | | afdb-uniprot50 | AF-A0A212KBU0-F1-MODEL\_V4 | 1.0 | 4.455e-15 | 518 | 0.265 | 207 | 144 | 4 | 4 | 208 | 13 | 213 | Phage P2 baseplate assembly gpV-like protein (Modular protein) | Phage P2 baseplate assembly gpV-like protein (Modular protein) | | afdb-uniprot50 | AF-A0A410UF52-F1-MODEL\_V4 | 1.0 | 1.086e-16 | 518 | 0.279 | 215 | 135 | 5 | 4 | 217 | 20 | 215 | Phage baseplate assembly protein V | Phage baseplate assembly protein V | | afdb-uniprot50 | AF-A0A0H3GM22-F1-MODEL\_V4 | 1.0 | 1.453e-15 | 517 | 0.265 | 196 | 120 | 3 | 1 | 196 | 1 | 172 | Baseplate assembly protein V | Baseplate assembly protein V | | afdb-uniprot50 | AF-A0A377AJR1-F1-MODEL\_V4 | 1.0 | 4.996e-14 | 516 | 0.341 | 155 | 83 | 2 | 1 | 155 | 1 | 136 | Baseplate assembly protein V | Baseplate assembly protein V | | afdb-uniprot50 | AF-A0A5M6I3L2-F1-MODEL\_V4 | 1.0 | 3.097e-12 | 516 | 0.3 | 123 | 86 | 0 | 4 | 126 | 8 | 130 | Phage baseplate assembly protein V | Phage baseplate assembly protein V | | afdb-uniprot50 | AF-A0A1B3Z862-F1-MODEL\_V4 | 1.0 | 2.78e-15 | 516 | 0.262 | 217 | 126 | 6 | 1 | 217 | 1 | 183 | Baseplate assembly protein | Baseplate assembly protein | | afdb-uniprot50 | AF-A0A4Q6F5K6-F1-MODEL\_V4 | 1.0 | 8.949e-12 | 515 | 0.321 | 112 | 76 | 0 | 2 | 113 | 1 | 112 | Phage baseplate assembly protein V | Phage baseplate assembly protein V | | afdb-uniprot50 | AF-A0A653NAD7-F1-MODEL\_V4 | 1.0 | 2.063e-14 | 515 | 0.314 | 162 | 95 | 4 | 4 | 157 | 29 | 182 | Phage\_base\_V domain-containing protein | Phage\_base\_V domain-containing protein | | afdb-uniprot50 | AF-A0A5C7C8J3-F1-MODEL\_V4 | 1.0 | 2.203e-16 | 515 | 0.301 | 219 | 127 | 6 | 2 | 217 | 1 | 196 | Phage baseplate assembly protein V | Phage baseplate assembly protein V | | afdb-uniprot50 | AF-A0A177RV31-F1-MODEL\_V4 | 1.0 | 2.919e-12 | 514 | 0.385 | 114 | 67 | 2 | 1 | 113 | 1 | 112 | Baseplate assembly protein | Baseplate assembly protein | | afdb-uniprot50 | AF-A0A1M3PG72-F1-MODEL\_V4 | 1.0 | 3.328e-16 | 514 | 0.328 | 216 | 116 | 7 | 2 | 217 | 1 | 187 | Phage\_base\_V domain-containing protein | Phage\_base\_V domain-containing protein | | afdb-uniprot50 | AF-A0A1N7B0F6-F1-MODEL\_V4 | 1.0 | 9.618e-16 | 514 | 0.263 | 216 | 119 | 5 | 4 | 217 | 15 | 192 | Phage baseplate assembly protein V | Phage baseplate assembly protein V | | afdb-uniprot50 | AF-A0A1E1F5H0-F1-MODEL\_V4 | 1.0 | 1.292e-15 | 513 | 0.259 | 208 | 118 | 6 | 1 | 208 | 1 | 172 | Baseplate assembly protein | Baseplate assembly protein | | afdb-uniprot50 | AF-A0A5A7M6L4-F1-MODEL\_V4 | 1.0 | 1.024e-16 | 513 | 0.334 | 227 | 123 | 6 | 4 | 217 | 42 | 253 | Baseplate assembly protein | Baseplate assembly protein | | afdb-uniprot50 | AF-A0A250DVL7-F1-MODEL\_V4 | 1.0 | 5.029e-16 | 513 | 0.288 | 260 | 141 | 10 | 1 | 217 | 1 | 259 | Phage baseplate protein | Phage baseplate protein | | afdb-uniprot50 | AF-A0A515BE25-F1-MODEL\_V4 | 1.0 | 6.708e-14 | 511 | 0.244 | 217 | 117 | 4 | 1 | 217 | 1 | 170 | Phage baseplate assembly protein V | Phage baseplate assembly protein V | | afdb-uniprot50 | AF-G2HX98-F1-MODEL\_V4 | 1.0 | 1.541e-15 | 511 | 0.327 | 226 | 125 | 11 | 1 | 209 | 1 | 216 | Phage baseplate assembly protein | Phage baseplate assembly protein | | afdb-uniprot50 | AF-A0A3S8YQ07-F1-MODEL\_V4 | 1.0 | 5.299e-14 | 510 | 0.323 | 164 | 95 | 4 | 2 | 157 | 1 | 156 | Phage baseplate assembly protein V | Phage baseplate assembly protein V | | afdb-uniprot50 | AF-A0A2G3K3I8-F1-MODEL\_V4 | 1.0 | 2.798e-17 | 510 | 0.303 | 214 | 133 | 3 | 2 | 199 | 1 | 214 | Baseplate assembly protein | Baseplate assembly protein | | afdb-uniprot50 | AF-A0A6L6JFU3-F1-MODEL\_V4 | 1.0 | 7.548e-14 | 509 | 0.286 | 213 | 89 | 5 | 6 | 217 | 2 | 152 | Phage baseplate assembly protein V | Phage baseplate assembly protein V | | afdb-uniprot50 | AF-A0A8A8P9Z4-F1-MODEL\_V4 | 1.0 | 2.05e-12 | 508 | 0.336 | 125 | 81 | 1 | 1 | 123 | 1 | 125 | Phage baseplate assembly protein V | Phage baseplate assembly protein V | | afdb-uniprot50 | AF-A0A329VPT4-F1-MODEL\_V4 | 1.0 | 1.852e-17 | 508 | 0.266 | 259 | 147 | 6 | 1 | 217 | 1 | 258 | Phage baseplate protein | Phage baseplate protein | | afdb-uniprot50 | AF-A0A1V2DS20-F1-MODEL\_V4 | 1.0 | 3.519e-15 | 507 | 0.273 | 227 | 116 | 6 | 1 | 216 | 1 | 189 | Phage\_base\_V domain-containing protein | Phage\_base\_V domain-containing protein | | afdb-uniprot50 | AF-A0A2E9SIQ4-F1-MODEL\_V4 | 1.0 | 1.287e-14 | 507 | 0.231 | 229 | 148 | 9 | 1 | 217 | 1 | 213 | Baseplate assembly protein | Baseplate assembly protein | | afdb-uniprot50 | AF-A0A6B3LC16-F1-MODEL\_V4 | 1.0 | 1.082e-15 | 505 | 0.285 | 228 | 133 | 8 | 1 | 213 | 1 | 213 | Phage baseplate assembly protein V | Phage baseplate assembly protein V | | afdb-uniprot50 | AF-A0A378AZS7-F1-MODEL\_V4 | 1.0 | 3.72e-14 | 503 | 0.313 | 150 | 96 | 2 | 1 | 145 | 1 | 148 | Baseplate assembly protein V | Baseplate assembly protein V | | afdb-uniprot50 | AF-Q3RF60-F1-MODEL\_V4 | 1.0 | 4.741e-16 | 502 | 0.314 | 216 | 122 | 6 | 4 | 217 | 47 | 238 | Phage baseplate assembly protein V | Phage baseplate assembly protein V | | afdb-uniprot50 | AF-A0A516SJL7-F1-MODEL\_V4 | 1.0 | 1.816e-11 | 501 | 0.392 | 102 | 62 | 0 | 2 | 103 | 1 | 102 | Phage baseplate assembly protein V | Phage baseplate assembly protein V | | afdb-uniprot50 | AF-A0A1T0AWZ3-F1-MODEL\_V4 | 1.0 | 4.469e-16 | 500 | 0.336 | 196 | 105 | 6 | 1 | 196 | 1 | 171 | Baseplate assembly protein | Baseplate assembly protein | | afdb-uniprot50 | AF-A0A849VJT8-F1-MODEL\_V4 | 1.0 | 6.753e-16 | 500 | 0.275 | 218 | 150 | 5 | 1 | 217 | 1 | 211 | Phage baseplate assembly protein V | Phage baseplate assembly protein V | | afdb-uniprot50 | AF-A0A7U9AYN8-F1-MODEL\_V4 | 1.0 | 7.573e-15 | 499 | 0.344 | 174 | 94 | 3 | 1 | 174 | 1 | 154 | Baseplate assembly protein V | Baseplate assembly protein V | | afdb-uniprot50 | AF-A0A6I3XBP6-F1-MODEL\_V4 | 1.0 | 5.982e-15 | 499 | 0.252 | 202 | 123 | 5 | 1 | 198 | 1 | 178 | Phage baseplate assembly protein V | Phage baseplate assembly protein V | | afdb-uniprot50 | AF-A0A1W9H429-F1-MODEL\_V4 | 1.0 | 2.47e-15 | 498 | 0.271 | 217 | 117 | 6 | 1 | 217 | 1 | 176 | Phage\_base\_V domain-containing protein | Phage\_base\_V domain-containing protein | | afdb-uniprot50 | AF-A0A1X3DKD5-F1-MODEL\_V4 | 1.0 | 7.163e-16 | 498 | 0.326 | 233 | 116 | 6 | 5 | 217 | 2 | 213 | Uncharacterized protein | Uncharacterized protein | | afdb-uniprot50 | AF-W0ITY9-F1-MODEL\_V4 | 1.0 | 1.079e-14 | 497 | 0.276 | 224 | 112 | 7 | 1 | 217 | 1 | 181 | Baseplate assembly protein | Baseplate assembly protein | | afdb-uniprot50 | AF-A0A4Y6U991-F1-MODEL\_V4 | 1.0 | 2.62e-15 | 497 | 0.303 | 214 | 114 | 7 | 4 | 217 | 14 | 192 | Phage baseplate assembly protein V | Phage baseplate assembly protein V | | afdb-uniprot50 | AF-A0A418VVD7-F1-MODEL\_V4 | 1.0 | 9.587e-15 | 497 | 0.297 | 225 | 118 | 5 | 4 | 203 | 8 | 217 | Phage baseplate assembly protein V | Phage baseplate assembly protein V | | afdb-uniprot50 | AF-F3LHZ4-F1-MODEL\_V4 | 1.0 | 5.299e-14 | 496 | 0.254 | 212 | 115 | 4 | 4 | 213 | 8 | 178 | Phage\_base\_V domain-containing protein | Phage\_base\_V domain-containing protein | | afdb-uniprot50 | AF-A0A1I1UBL3-F1-MODEL\_V4 | 1.0 | 1.214e-14 | 495 | 0.261 | 191 | 123 | 2 | 4 | 194 | 6 | 178 | Phage baseplate assembly protein V | Phage baseplate assembly protein V | | afdb-uniprot50 | AF-A0A2D3WVI8-F1-MODEL\_V4 | 1.0 | 7.548e-14 | 494 | 0.3 | 180 | 109 | 6 | 1 | 177 | 1 | 166 | Phage\_base\_V domain-containing protein | Phage\_base\_V domain-containing protein | | afdb-uniprot50 | AF-A0A379C9Y4-F1-MODEL\_V4 | 1.0 | 8.549e-16 | 494 | 0.293 | 218 | 123 | 7 | 2 | 217 | 1 | 189 | Phage P2 baseplate assembly protein gpV | Phage P2 baseplate assembly protein gpV | | afdb-uniprot50 | AF-A0A7R7V081-F1-MODEL\_V4 | 1.0 | 1.375e-16 | 494 | 0.3 | 220 | 121 | 5 | 1 | 217 | 23 | 212 | Baseplate assembly protein | Baseplate assembly protein | | afdb-uniprot50 | AF-A0A2X2BK34-F1-MODEL\_V4 | 1.0 | 1.439e-12 | 493 | 0.371 | 194 | 116 | 5 | 24 | 217 | 1 | 188 | Phage baseplate assembly protein | Phage baseplate assembly protein | | afdb-uniprot50 | AF-A0A7G8VI14-F1-MODEL\_V4 | 1.0 | 5.352e-17 | 493 | 0.289 | 242 | 146 | 4 | 1 | 217 | 11 | 251 | Phage baseplate assembly protein V | Phage baseplate assembly protein V | | afdb-uniprot50 | AF-A0A1B8YNH2-F1-MODEL\_V4 | 1.0 | 9.65e-17 | 493 | 0.254 | 259 | 150 | 6 | 1 | 217 | 45 | 302 | Phage-related baseplate assembly protein | Phage-related baseplate assembly protein | | afdb-uniprot50 | AF-A0A016XIR2-F1-MODEL\_V4 | 1.0 | 1.02e-15 | 492 | 0.306 | 225 | 119 | 7 | 1 | 217 | 1 | 196 | Baseplate assembly protein | Baseplate assembly protein | | afdb-uniprot50 | AF-F3YY65-F1-MODEL\_V4 | 1.0 | 1.932e-12 | 492 | 0.237 | 164 | 119 | 1 | 4 | 161 | 8 | 171 | Phage baseplate assembly protein V | Phage baseplate assembly protein V | | afdb-uniprot50 | AF-A0A7X4GDK7-F1-MODEL\_V4 | 1.0 | 2.063e-14 | 491 | 0.227 | 207 | 119 | 5 | 12 | 217 | 11 | 177 | Phage baseplate assembly protein V | Phage baseplate assembly protein V | | afdb-uniprot50 | AF-A0A7W6REL9-F1-MODEL\_V4 | 1.0 | 1.635e-15 | 491 | 0.317 | 189 | 87 | 3 | 4 | 153 | 12 | 197 | Phage baseplate assembly protein V | Phage baseplate assembly protein V | | afdb-uniprot50 | AF-F1Z9C2-F1-MODEL\_V4 | 1.0 | 2.919e-12 | 489 | 0.294 | 136 | 89 | 5 | 13 | 146 | 12 | 142 | Phage P2 baseplate assembly gpV-like protein | Phage P2 baseplate assembly gpV-like protein | | afdb-uniprot50 | AF-A0A524RVZ0-F1-MODEL\_V4 | 1.0 | 5.264e-12 | 488 | 0.284 | 151 | 98 | 4 | 3 | 145 | 18 | 166 | Phage baseplate assembly protein V | Phage baseplate assembly protein V | | afdb-uniprot50 | AF-A0A1I7E546-F1-MODEL\_V4 | 1.0 | 3.117e-14 | 488 | 0.258 | 197 | 127 | 5 | 1 | 196 | 1 | 179 | Phage baseplate assembly protein V | Phage baseplate assembly protein V | | afdb-uniprot50 | AF-A0A0T9QWS2-F1-MODEL\_V4 | 1.0 | 5.299e-14 | 487 | 0.279 | 168 | 108 | 2 | 1 | 155 | 1 | 168 | Baseplate assembly protein V | Baseplate assembly protein V | | afdb-uniprot50 | AF-A0A6L8GG55-F1-MODEL\_V4 | 1.0 | 1.453e-15 | 487 | 0.277 | 227 | 131 | 9 | 4 | 217 | 9 | 215 | Phage baseplate assembly protein V | Phage baseplate assembly protein V | | afdb-uniprot50 | AF-A0A167H3T4-F1-MODEL\_V4 | 1.0 | 1.079e-14 | 486 | 0.283 | 194 | 105 | 3 | 1 | 194 | 1 | 160 | Phage\_base\_V domain-containing protein | Phage\_base\_V domain-containing protein | | afdb-uniprot50 | AF-U3U4P9-F1-MODEL\_V4 | 1.0 | 3.708e-13 | 486 | 0.308 | 198 | 121 | 5 | 16 | 209 | 6 | 191 | Uncharacterized protein | Uncharacterized protein | | afdb-uniprot50 | AF-A0A2C9EHC3-F1-MODEL\_V4 | 1.0 | 1.541e-15 | 486 | 0.304 | 223 | 122 | 6 | 1 | 217 | 46 | 241 | Baseplate assembly protein V | Baseplate assembly protein V | | afdb-uniprot50 | AF-A0A450W6W7-F1-MODEL\_V4 | 1.0 | 2.056e-13 | 485 | 0.306 | 150 | 98 | 1 | 1 | 144 | 1 | 150 | Phage baseplate assembly protein V | Phage baseplate assembly protein V | | afdb-uniprot50 | AF-A0A2U0SJV9-F1-MODEL\_V4 | 1.0 | 2.063e-14 | 485 | 0.308 | 201 | 106 | 5 | 17 | 217 | 2 | 169 | Phage baseplate assembly protein V | Phage baseplate assembly protein V | | afdb-uniprot50 | AF-D1P8A9-F1-MODEL\_V4 | 1.0 | 1.202e-11 | 484 | 0.31 | 145 | 98 | 2 | 17 | 161 | 1 | 143 | Phage-related baseplate assembly protein | Phage-related baseplate assembly protein | | afdb-uniprot50 | AF-A0A5E7YKZ5-F1-MODEL\_V4 | 1.0 | 9.618e-16 | 484 | 0.274 | 222 | 134 | 7 | 2 | 217 | 6 | 206 | Phage baseplate assembly protein V | Phage baseplate assembly protein V | | afdb-uniprot50 | AF-A0A521PR02-F1-MODEL\_V4 | 1.0 | 1.017e-14 | 483 | 0.281 | 213 | 113 | 7 | 12 | 217 | 5 | 184 | Phage baseplate assembly protein V | Phage baseplate assembly protein V | | afdb-uniprot50 | AF-A0A1G5ACL8-F1-MODEL\_V4 | 1.0 | 5.012e-15 | 480 | 0.284 | 204 | 103 | 4 | 4 | 206 | 8 | 169 | Phage baseplate assembly protein V | Phage baseplate assembly protein V | | afdb-uniprot50 | AF-A0A1M6B7Z3-F1-MODEL\_V4 | 1.0 | 8.033e-15 | 480 | 0.267 | 191 | 116 | 3 | 4 | 194 | 5 | 171 | Phage-related baseplate assembly protein | Phage-related baseplate assembly protein | | afdb-uniprot50 | AF-A0A4V0XDV9-F1-MODEL\_V4 | 1.0 | 3.117e-14 | 480 | 0.293 | 201 | 120 | 7 | 1 | 193 | 15 | 201 | Phage\_base\_V domain-containing protein | Phage\_base\_V domain-containing protein | | afdb-uniprot50 | AF-V4N894-F1-MODEL\_V4 | 1.0 | 5.658e-16 | 480 | 0.328 | 210 | 118 | 5 | 4 | 213 | 32 | 218 | Baseplate assembly protein | Baseplate assembly protein | | afdb-uniprot50 | AF-A0A381EC13-F1-MODEL\_V4 | 1.0 | 3.72e-14 | 479 | 0.271 | 221 | 106 | 3 | 1 | 217 | 1 | 170 | Phage P2 baseplate assembly protein gpV | Phage P2 baseplate assembly protein gpV | | afdb-uniprot50 | AF-A0A0P6WI63-F1-MODEL\_V4 | 1.0 | 3.306e-14 | 479 | 0.285 | 207 | 127 | 7 | 16 | 217 | 2 | 192 | Phage\_base\_V domain-containing protein | Phage\_base\_V domain-containing protein | | afdb-uniprot50 | AF-A0A7X4GVY2-F1-MODEL\_V4 | 1.0 | 1.74e-16 | 478 | 0.33 | 230 | 117 | 6 | 1 | 196 | 1 | 227 | Phage baseplate assembly protein V | Phage baseplate assembly protein V | | afdb-uniprot50 | AF-A0A1X7CH99-F1-MODEL\_V4 | 1.0 | 1.02e-15 | 478 | 0.284 | 253 | 143 | 7 | 1 | 216 | 1 | 252 | Phage baseplate assembly protein V | Phage baseplate assembly protein V | | afdb-uniprot50 | AF-A0A285JXF0-F1-MODEL\_V4 | 1.0 | 1.635e-15 | 477 | 0.237 | 236 | 135 | 3 | 1 | 192 | 3 | 237 | Phage\_base\_V domain-containing protein | Phage\_base\_V domain-containing protein | | afdb-uniprot50 | AF-A0A326LB63-F1-MODEL\_V4 | 1.0 | 1.287e-14 | 476 | 0.24 | 216 | 133 | 8 | 3 | 217 | 8 | 193 | Phage baseplate assembly protein V | Phage baseplate assembly protein V | | afdb-uniprot50 | AF-A0A5E4XG36-F1-MODEL\_V4 | 1.0 | 2.196e-15 | 476 | 0.294 | 224 | 142 | 4 | 1 | 217 | 1 | 215 | Baseplate protein | Baseplate protein | | afdb-uniprot50 | AF-A0A2N2S1D1-F1-MODEL\_V4 | 1.0 | 1.958e-16 | 476 | 0.305 | 236 | 127 | 7 | 1 | 217 | 1 | 218 | Phage baseplate assembly protein V | Phage baseplate assembly protein V | | afdb-uniprot50 | AF-A0A212J3Z5-F1-MODEL\_V4 | 1.0 | 3.117e-14 | 476 | 0.26 | 219 | 153 | 5 | 1 | 215 | 1 | 214 | Putative Phage baseplate assembly protein | Putative Phage baseplate assembly protein | | afdb-uniprot50 | AF-A0A1E3G5Z4-F1-MODEL\_V4 | 1.0 | 3.306e-14 | 474 | 0.276 | 195 | 106 | 6 | 4 | 192 | 15 | 180 | Phage\_base\_V domain-containing protein | Phage\_base\_V domain-containing protein | | afdb-uniprot50 | AF-A0A0M4TG07-F1-MODEL\_V4 | 1.0 | 5.317e-15 | 474 | 0.267 | 217 | 128 | 5 | 1 | 217 | 1 | 186 | Phage\_base\_V domain-containing protein | Phage\_base\_V domain-containing protein | | afdb-uniprot50 | AF-A0A7Y7HZL4-F1-MODEL\_V4 | 1.0 | 3.53e-16 | 474 | 0.323 | 210 | 124 | 5 | 1 | 198 | 1 | 204 | Phage baseplate assembly protein V | Phage baseplate assembly protein V | | afdb-uniprot50 | AF-A0A397NIY9-F1-MODEL\_V4 | 1.0 | 1.214e-14 | 472 | 0.354 | 189 | 99 | 7 | 16 | 197 | 2 | 174 | Phage baseplate assembly protein V | Phage baseplate assembly protein V | | afdb-uniprot50 | AF-A0A1G5B4F2-F1-MODEL\_V4 | 1.0 | 2.586e-11 | 471 | 0.231 | 138 | 103 | 2 | 4 | 141 | 7 | 141 | Phage baseplate assembly protein V | Phage baseplate assembly protein V | | afdb-uniprot50 | AF-K5XX39-F1-MODEL\_V4 | 1.0 | 1.828e-13 | 471 | 0.276 | 224 | 110 | 7 | 1 | 217 | 1 | 179 | Phage P2 baseplate assembly gpV-like protein | Phage P2 baseplate assembly gpV-like protein | | afdb-uniprot50 | AF-A4P0H7-F1-MODEL\_V4 | 1.0 | 5.012e-15 | 471 | 0.318 | 201 | 112 | 5 | 1 | 201 | 1 | 176 | Predicted phage P2-like baseplate assembly protein | Predicted phage P2-like baseplate assembly protein | | afdb-uniprot50 | AF-A0A2W4T7L3-F1-MODEL\_V4 | 1.0 | 1.712e-11 | 471 | 0.301 | 136 | 92 | 2 | 1 | 133 | 1 | 136 | Phage baseplate assembly protein V | Phage baseplate assembly protein V | | afdb-uniprot50 | AF-A0A3Q8U0Q9-F1-MODEL\_V4 | 1.0 | 5.299e-14 | 471 | 0.24 | 220 | 144 | 7 | 1 | 203 | 1 | 214 | Phage baseplate assembly protein V | Phage baseplate assembly protein V | | afdb-uniprot50 | AF-A0A0Q7ECU9-F1-MODEL\_V4 | 1.0 | 9.038e-15 | 469 | 0.266 | 210 | 115 | 6 | 1 | 203 | 1 | 178 | Phage\_base\_V domain-containing protein | Phage\_base\_V domain-containing protein | | afdb-uniprot50 | AF-A0A1H3ZWK9-F1-MODEL\_V4 | 1.0 | 1.84e-15 | 469 | 0.276 | 217 | 143 | 6 | 1 | 217 | 1 | 203 | Phage baseplate assembly protein V | Phage baseplate assembly protein V | | afdb-uniprot50 | AF-A0A345DE56-F1-MODEL\_V4 | 1.0 | 5.962e-14 | 468 | 0.225 | 200 | 123 | 6 | 1 | 196 | 1 | 172 | Phage\_base\_V domain-containing protein | Phage\_base\_V domain-containing protein | | afdb-uniprot50 | AF-G1UXN9-F1-MODEL\_V4 | 1.0 | 4.172e-13 | 468 | 0.25 | 236 | 150 | 10 | 2 | 217 | 3 | 231 | Uncharacterized protein | Uncharacterized protein | | afdb-uniprot50 | AF-S6GRC2-F1-MODEL\_V4 | 1.0 | 3.933e-13 | 467 | 0.272 | 143 | 96 | 2 | 1 | 137 | 1 | 141 | Phage\_base\_V domain-containing protein | Phage\_base\_V domain-containing protein | | afdb-uniprot50 | AF-A0A0J9EDT8-F1-MODEL\_V4 | 1.0 | 1.01e-12 | 467 | 0.237 | 219 | 102 | 7 | 1 | 217 | 1 | 156 | Phage baseplate assembly protein V | Phage baseplate assembly protein V | | afdb-uniprot50 | AF-H8L2J8-F1-MODEL\_V4 | 1.0 | 2.761e-13 | 467 | 0.244 | 209 | 136 | 3 | 1 | 203 | 1 | 193 | Phage baseplate assembly protein V | Phage baseplate assembly protein V | | afdb-uniprot50 | AF-A0A1E7HY32-F1-MODEL\_V4 | 1.0 | 4.214e-16 | 467 | 0.278 | 269 | 142 | 7 | 1 | 217 | 2 | 270 | Uncharacterized protein | Uncharacterized protein | | afdb-uniprot50 | AF-A0A7J6YLT3-F1-MODEL\_V4 | 1.0 | 4.425e-13 | 466 | 0.287 | 153 | 105 | 2 | 15 | 167 | 136 | 284 | Uncharacterized protein | Uncharacterized protein | | afdb-uniprot50 | AF-A0A367WWC3-F1-MODEL\_V4 | 1.0 | 1.932e-12 | 465 | 0.237 | 160 | 114 | 2 | 2 | 161 | 1 | 152 | Phage\_base\_V domain-containing protein | Phage\_base\_V domain-containing protein | | afdb-uniprot50 | AF-A0A6G8F2D8-F1-MODEL\_V4 | 1.0 | 5.317e-15 | 465 | 0.283 | 219 | 136 | 8 | 1 | 213 | 1 | 204 | Bacteriophage baseplate assembly protein V | Bacteriophage baseplate assembly protein V | | afdb-uniprot50 | AF-A0A4V0IE64-F1-MODEL\_V4 | 1.0 | 3.274e-11 | 463 | 0.457 | 107 | 53 | 2 | 1 | 107 | 1 | 102 | Phage P2 baseplate assembly protein gpV | Phage P2 baseplate assembly protein gpV | | afdb-uniprot50 | AF-A0A7W5BZS0-F1-MODEL\_V4 | 1.0 | 1.361e-13 | 463 | 0.288 | 201 | 106 | 5 | 1 | 193 | 1 | 172 | Phage baseplate assembly protein V | Phage baseplate assembly protein V | | afdb-uniprot50 | AF-A0A7U2VHG3-F1-MODEL\_V4 | 1.0 | 4.996e-14 | 462 | 0.287 | 209 | 112 | 5 | 9 | 217 | 3 | 174 | Phage baseplate assembly protein V | Phage baseplate assembly protein V | | afdb-uniprot50 | AF-A0A4R7Q4T9-F1-MODEL\_V4 | 1.0 | 5.658e-16 | 462 | 0.204 | 293 | 156 | 6 | 1 | 217 | 1 | 292 | Phage baseplate assembly protein V | Phage baseplate assembly protein V | | afdb-uniprot50 | AF-E1PLB0-F1-MODEL\_V4 | 1.0 | 2.9e-10 | 461 | 0.361 | 94 | 60 | 0 | 1 | 94 | 1 | 94 | Baseplate assembly protein V | Baseplate assembly protein V | | afdb-uniprot50 | AF-A0A4R1K4T2-F1-MODEL\_V4 | 1.0 | 3.296e-13 | 461 | 0.304 | 174 | 102 | 7 | 1 | 166 | 1 | 163 | Phage baseplate assembly protein V | Phage baseplate assembly protein V | | afdb-uniprot50 | AF-A0A623T421-F1-MODEL\_V4 | 1.0 | 1.075e-13 | 461 | 0.256 | 199 | 125 | 6 | 1 | 197 | 1 | 178 | Phage baseplate assembly protein V | Phage baseplate assembly protein V | | afdb-uniprot50 | AF-A0A373FPN0-F1-MODEL\_V4 | 1.0 | 5.012e-15 | 461 | 0.295 | 227 | 121 | 5 | 1 | 203 | 1 | 212 | Phage baseplate assembly protein V | Phage baseplate assembly protein V | | afdb-uniprot50 | AF-A0A1G8I115-F1-MODEL\_V4 | 1.0 | 1.945e-14 | 460 | 0.264 | 193 | 114 | 4 | 4 | 194 | 26 | 192 | Phage baseplate assembly protein V | Phage baseplate assembly protein V | | afdb-uniprot50 | AF-A0A692CV12-F1-MODEL\_V4 | 1.0 | 2.167e-11 | 456 | 0.302 | 142 | 93 | 4 | 16 | 156 | 2 | 138 | Phage baseplate assembly protein V | Phage baseplate assembly protein V | | afdb-uniprot50 | AF-A0A5Q0TIR4-F1-MODEL\_V4 | 1.0 | 2.752e-12 | 456 | 0.326 | 144 | 87 | 4 | 4 | 137 | 6 | 149 | Phage baseplate assembly protein V | Phage baseplate assembly protein V | | afdb-uniprot50 | AF-A0A812QV09-F1-MODEL\_V4 | 1.0 | 1.608e-10 | 456 | 0.322 | 124 | 82 | 1 | 15 | 138 | 942 | 1063 | XkdF protein | XkdF protein | | afdb-uniprot50 | AF-A0A7S7WDY0-F1-MODEL\_V4 | 1.0 | 3.959e-15 | 455 | 0.294 | 224 | 141 | 6 | 1 | 217 | 1 | 214 | Phage baseplate assembly protein V | Phage baseplate assembly protein V | | afdb-uniprot50 | AF-A0A1R1MK78-F1-MODEL\_V4 | 1.0 | 4.144e-11 | 452 | 0.258 | 143 | 97 | 6 | 15 | 151 | 4 | 143 | Phage\_base\_V domain-containing protein | Phage\_base\_V domain-containing protein | | afdb-uniprot50 | AF-A0A6G5QFQ9-F1-MODEL\_V4 | 1.0 | 8.978e-13 | 452 | 0.263 | 209 | 133 | 9 | 16 | 209 | 2 | 204 | Phage baseplate assembly protein V | Phage baseplate assembly protein V | | afdb-uniprot50 | AF-A7MWA1-F1-MODEL\_V4 | 1.0 | 2.603e-13 | 452 | 0.284 | 172 | 104 | 4 | 4 | 172 | 26 | 181 | Phage\_base\_V domain-containing protein | Phage\_base\_V domain-containing protein | | afdb-uniprot50 | AF-A0A5E7A8F9-F1-MODEL\_V4 | 1.0 | 2.036e-10 | 450 | 0.289 | 121 | 85 | 1 | 12 | 132 | 2 | 121 | Uncharacterized protein | Uncharacterized protein | | afdb-uniprot50 | AF-A0A2D3W2I8-F1-MODEL\_V4 | 1.0 | 1.939e-13 | 450 | 0.257 | 206 | 119 | 8 | 1 | 203 | 1 | 175 | Uncharacterized protein | Uncharacterized protein | | afdb-uniprot50 | AF-A0A2T5J1G2-F1-MODEL\_V4 | 1.0 | 8.492e-14 | 450 | 0.231 | 225 | 128 | 7 | 1 | 213 | 1 | 192 | Phage baseplate assembly protein V | Phage baseplate assembly protein V | | afdb-uniprot50 | AF-A0A2E2N1A4-F1-MODEL\_V4 | 1.0 | 5.903e-11 | 450 | 0.239 | 138 | 100 | 3 | 9 | 141 | 2 | 139 | Uncharacterized protein | Uncharacterized protein | | afdb-uniprot50 | AF-A0A837JGD3-F1-MODEL\_V4 | 1.0 | 1.357e-12 | 450 | 0.254 | 224 | 146 | 8 | 5 | 214 | 4 | 220 | Baseplate assembly protein | Baseplate assembly protein | | afdb-uniprot50 | AF-X1P9Y8-F1-MODEL\_V4 | 1.0 | 2.056e-13 | 449 | 0.247 | 194 | 133 | 6 | 1 | 185 | 1 | 190 | Phage\_base\_V domain-containing protein | Phage\_base\_V domain-containing protein | | afdb-uniprot50 | AF-A0A381C5Y2-F1-MODEL\_V4 | 1.0 | 1.723e-13 | 449 | 0.254 | 236 | 141 | 8 | 1 | 216 | 1 | 221 | Phage P2 baseplate assembly protein gpV | Phage P2 baseplate assembly protein gpV | | afdb-uniprot50 | AF-A0A5S3S1K6-F1-MODEL\_V4 | 1.0 | 5.621e-14 | 449 | 0.271 | 199 | 130 | 4 | 4 | 195 | 11 | 201 | Phage baseplate assembly protein V | Phage baseplate assembly protein V | | afdb-uniprot50 | AF-A0A2G6EI12-F1-MODEL\_V4 | 1.0 | 2.438e-11 | 448 | 0.236 | 161 | 116 | 5 | 1 | 158 | 1 | 157 | Phage\_base\_V domain-containing protein | Phage\_base\_V domain-containing protein | | afdb-uniprot50 | AF-A0A7U8ZG09-F1-MODEL\_V4 | 1.0 | 5.012e-15 | 448 | 0.287 | 216 | 116 | 6 | 3 | 217 | 36 | 214 | Uncharacterized protein | Uncharacterized protein | | afdb-uniprot50 | AF-A0A4Q0ZNI2-F1-MODEL\_V4 | 1.0 | 3.484e-12 | 447 | 0.303 | 158 | 102 | 5 | 1 | 155 | 1 | 153 | Uncharacterized protein | Uncharacterized protein | | afdb-uniprot50 | AF-A0A136H344-F1-MODEL\_V4 | 1.0 | 1.017e-14 | 447 | 0.301 | 202 | 118 | 3 | 3 | 197 | 8 | 193 | Uncharacterized protein | Uncharacterized protein | | afdb-uniprot50 | AF-A0A6L2ZS80-F1-MODEL\_V4 | 1.0 | 2.188e-14 | 446 | 0.307 | 218 | 121 | 7 | 1 | 215 | 1 | 191 | Putative phage baseplate protein | Putative phage baseplate protein | | afdb-uniprot50 | AF-A0A3B6Y4E8-F1-MODEL\_V4 | 1.0 | 7.116e-14 | 445 | 0.246 | 195 | 133 | 3 | 4 | 192 | 11 | 197 | Phage baseplate assembly protein V | Phage baseplate assembly protein V | | afdb-uniprot50 | AF-A0A6N3IEF8-F1-MODEL\_V4 | 1.0 | 3.285e-12 | 445 | 0.213 | 234 | 159 | 9 | 1 | 216 | 2 | 228 | Uncharacterized protein | Uncharacterized protein | | afdb-uniprot50 | AF-A0A423PRV3-F1-MODEL\_V4 | 1.0 | 2.948e-15 | 444 | 0.294 | 221 | 128 | 5 | 4 | 196 | 7 | 227 | Phage\_base\_V domain-containing protein | Phage\_base\_V domain-containing protein | | afdb-uniprot50 | AF-E4PPS8-F1-MODEL\_V4 | 1.0 | 3.972e-16 | 443 | 0.266 | 251 | 121 | 6 | 1 | 196 | 1 | 243 | Phage-related baseplate assembly protein V | Phage-related baseplate assembly protein V | | afdb-uniprot50 | AF-V4PX29-F1-MODEL\_V4 | 1.0 | 2.454e-13 | 442 | 0.239 | 217 | 150 | 4 | 1 | 217 | 1 | 202 | Uncharacterized protein | Uncharacterized protein | | afdb-uniprot50 | AF-A0A853IBN9-F1-MODEL\_V4 | 1.0 | 5.942e-13 | 440 | 0.283 | 201 | 102 | 6 | 1 | 190 | 1 | 170 | Phage baseplate assembly protein V | Phage baseplate assembly protein V | | afdb-uniprot50 | AF-A0A5C8XTJ4-F1-MODEL\_V4 | 1.0 | 9.43e-10 | 439 | 0.383 | 86 | 50 | 1 | 1 | 86 | 1 | 83 | Phage baseplate assembly protein V | Phage baseplate assembly protein V | | afdb-uniprot50 | AF-A0A6L9FGD0-F1-MODEL\_V4 | 1.0 | 8.006e-14 | 439 | 0.256 | 230 | 134 | 8 | 1 | 214 | 1 | 209 | Phage baseplate assembly protein V | Phage baseplate assembly protein V | | afdb-uniprot50 | AF-A0A2E6BPD3-F1-MODEL\_V4 | 1.0 | 1.828e-13 | 437 | 0.294 | 204 | 106 | 5 | 14 | 217 | 8 | 173 | Baseplate assembly protein | Baseplate assembly protein | | afdb-uniprot50 | AF-A0A258A0W3-F1-MODEL\_V4 | 1.0 | 8.006e-14 | 436 | 0.278 | 219 | 107 | 8 | 4 | 217 | 18 | 190 | Phage\_base\_V domain-containing protein | Phage\_base\_V domain-containing protein | | afdb-uniprot50 | AF-A0A5H2Y3Q4-F1-MODEL\_V4 | 1.0 | 7.573e-15 | 435 | 0.251 | 235 | 146 | 8 | 4 | 217 | 11 | 236 | Bacteriophage baseplate assembly protein V | Bacteriophage baseplate assembly protein V | | afdb-uniprot50 | AF-A0A1B7XDP0-F1-MODEL\_V4 | 1.0 | 1.287e-14 | 435 | 0.283 | 219 | 129 | 6 | 1 | 193 | 1 | 217 | Phage\_base\_V domain-containing protein | Phage\_base\_V domain-containing protein | | afdb-uniprot50 | AF-A0A8B2NVK9-F1-MODEL\_V4 | 1.0 | 1.516e-10 | 434 | 0.265 | 143 | 94 | 3 | 4 | 140 | 11 | 148 | Baseplate assembly protein | Baseplate assembly protein | | afdb-uniprot50 | AF-A0A1S6U8Y2-F1-MODEL\_V4 | 1.0 | 3.92e-12 | 434 | 0.267 | 202 | 123 | 7 | 16 | 217 | 4 | 180 | Phage baseplate assembly protein V | Phage baseplate assembly protein V | | afdb-uniprot50 | AF-A0A824EIG8-F1-MODEL\_V4 | 1.0 | 2.291e-10 | 433 | 0.318 | 113 | 74 | 2 | 16 | 127 | 2 | 112 | Phage baseplate assembly protein V | Phage baseplate assembly protein V | | afdb-uniprot50 | AF-A0A4R1J4B1-F1-MODEL\_V4 | 1.0 | 1.075e-13 | 433 | 0.268 | 227 | 154 | 6 | 1 | 217 | 1 | 225 | Phage baseplate assembly protein V | Phage baseplate assembly protein V | | afdb-uniprot50 | AF-A0A0U9HBQ1-F1-MODEL\_V4 | 1.0 | 6.642e-11 | 432 | 0.246 | 138 | 98 | 3 | 1 | 136 | 1 | 134 | Phage baseplate assembly protein V | Phage baseplate assembly protein V | | afdb-uniprot50 | AF-A0A4R0DU02-F1-MODEL\_V4 | 1.0 | 1.624e-13 | 432 | 0.221 | 217 | 122 | 6 | 1 | 217 | 1 | 170 | Phage baseplate assembly protein V | Phage baseplate assembly protein V | | afdb-uniprot50 | AF-F4QJB9-F1-MODEL\_V4 | 1.0 | 1.828e-13 | 432 | 0.267 | 202 | 125 | 7 | 16 | 217 | 2 | 180 | Baseplate assembly protein V | Baseplate assembly protein V | | afdb-uniprot50 | AF-A0A149SVM5-F1-MODEL\_V4 | 1.0 | 1.624e-13 | 432 | 0.253 | 201 | 118 | 7 | 1 | 194 | 1 | 176 | Phage\_base\_V domain-containing protein | Phage\_base\_V domain-containing protein | | afdb-uniprot50 | AF-N6VFT2-F1-MODEL\_V4 | 1.0 | 5.962e-14 | 432 | 0.263 | 224 | 134 | 7 | 4 | 208 | 8 | 219 | Phage baseplate assembly protein V | Phage baseplate assembly protein V | | afdb-uniprot50 | AF-A0A6S5X6Y8-F1-MODEL\_V4 | 1.0 | 1.129e-10 | 430 | 0.278 | 133 | 94 | 2 | 15 | 146 | 4 | 135 | Phage\_base\_V domain-containing protein | Phage\_base\_V domain-containing protein | | afdb-uniprot50 | AF-S5MPB7-F1-MODEL\_V4 | 1.0 | 1.511e-09 | 429 | 0.361 | 94 | 60 | 0 | 1 | 94 | 1 | 94 | Baseplate assembly protein V | Baseplate assembly protein V | | afdb-uniprot50 | AF-A0A3D9EV90-F1-MODEL\_V4 | 1.0 | 2.05e-12 | 429 | 0.272 | 154 | 102 | 3 | 2 | 145 | 1 | 154 | Phage baseplate assembly protein V | Phage baseplate assembly protein V | | afdb-uniprot50 | AF-A0A380AJE4-F1-MODEL\_V4 | 1.0 | 7.927e-11 | 428 | 0.193 | 145 | 108 | 3 | 2 | 137 | 1 | 145 | Phage P2 baseplate assembly protein gpV | Phage P2 baseplate assembly protein gpV | | afdb-uniprot50 | AF-A0A2D9W381-F1-MODEL\_V4 | 1.0 | 1.279e-12 | 426 | 0.236 | 233 | 112 | 4 | 2 | 217 | 1 | 184 | Baseplate assembly protein | Baseplate assembly protein | | afdb-uniprot50 | AF-A0A269PJ60-F1-MODEL\_V4 | 1.0 | 5.942e-13 | 424 | 0.248 | 221 | 149 | 4 | 4 | 217 | 11 | 221 | Phage\_base\_V domain-containing protein | Phage\_base\_V domain-containing protein | | afdb-uniprot50 | AF-A0A6G3I739-F1-MODEL\_V4 | 1.0 | 4.632e-09 | 423 | 0.349 | 83 | 54 | 0 | 1 | 83 | 1 | 83 | Phage baseplate assembly protein V | Phage baseplate assembly protein V | | afdb-uniprot50 | AF-A0A4Q0YIW9-F1-MODEL\_V4 | 1.0 | 2.603e-13 | 423 | 0.238 | 197 | 120 | 2 | 1 | 197 | 1 | 167 | Phage\_base\_V domain-containing protein | Phage\_base\_V domain-containing protein | | afdb-uniprot50 | AF-A0A7Z7KMS9-F1-MODEL\_V4 | 1.0 | 2.306e-12 | 422 | 0.242 | 206 | 132 | 6 | 1 | 203 | 1 | 185 | Phage P2 baseplate assembly protein gpV | Phage P2 baseplate assembly protein gpV | | afdb-uniprot50 | AF-A0A7W4RHJ5-F1-MODEL\_V4 | 1.0 | 1.075e-13 | 421 | 0.31 | 235 | 133 | 8 | 1 | 217 | 7 | 230 | Phage baseplate assembly protein V | Phage baseplate assembly protein V | | afdb-uniprot50 | AF-A0A024LSX6-F1-MODEL\_V4 | 1.0 | 2.89e-09 | 420 | 0.344 | 90 | 59 | 0 | 3 | 92 | 7 | 96 | Phage baseplate assembly protein V1 | Phage baseplate assembly protein V1 | | afdb-uniprot50 | AF-A0A2P7V3Q8-F1-MODEL\_V4 | 1.0 | 1.198e-10 | 420 | 0.189 | 148 | 117 | 1 | 2 | 146 | 1 | 148 | Baseplate assembly protein | Baseplate assembly protein | | afdb-uniprot50 | AF-A0A1N7LRL3-F1-MODEL\_V4 | 1.0 | 1.816e-11 | 418 | 0.285 | 147 | 93 | 5 | 1 | 138 | 1 | 144 | Phage baseplate assembly protein V | Phage baseplate assembly protein V | | afdb-uniprot50 | AF-A0A6I1J2X0-F1-MODEL\_V4 | 1.0 | 1.357e-12 | 418 | 0.228 | 201 | 130 | 7 | 1 | 197 | 1 | 180 | Phage baseplate assembly protein V | Phage baseplate assembly protein V | | afdb-uniprot50 | AF-A0A3S4JW88-F1-MODEL\_V4 | 1.0 | 6.241e-10 | 416 | 0.368 | 95 | 60 | 0 | 1 | 95 | 1 | 95 | Phage P2 baseplate assembly protein gpV | Phage P2 baseplate assembly protein gpV | | afdb-uniprot50 | AF-A0A081MYL2-F1-MODEL\_V4 | 1.0 | 6.686e-13 | 416 | 0.229 | 222 | 127 | 8 | 4 | 213 | 10 | 199 | Phage\_base\_V domain-containing protein | Phage\_base\_V domain-containing protein | | afdb-uniprot50 | AF-A0A7J5WFZ4-F1-MODEL\_V4 | 1.0 | 1.202e-11 | 416 | 0.217 | 221 | 162 | 5 | 4 | 213 | 10 | 230 | Phage baseplate assembly protein | Phage baseplate assembly protein | | afdb-uniprot50 | AF-A0A5T0IXE4-F1-MODEL\_V4 | 1.0 | 4.663e-11 | 415 | 0.312 | 144 | 86 | 5 | 16 | 151 | 2 | 140 | Phage baseplate assembly protein V | Phage baseplate assembly protein V | | afdb-uniprot50 | AF-A0A1H9YBJ7-F1-MODEL\_V4 | 1.0 | 1.007e-11 | 414 | 0.238 | 222 | 108 | 7 | 1 | 217 | 2 | 167 | Phage baseplate assembly protein V | Phage baseplate assembly protein V | | afdb-uniprot50 | AF-A0A2W5KZA5-F1-MODEL\_V4 | 1.0 | 7.98e-13 | 413 | 0.312 | 195 | 108 | 5 | 23 | 217 | 2 | 170 | Phage baseplate assembly protein V | Phage baseplate assembly protein V | | afdb-uniprot50 | AF-A0A839IVK7-F1-MODEL\_V4 | 1.0 | 4.382e-10 | 412 | 0.198 | 136 | 103 | 3 | 3 | 135 | 29 | 161 | Phage baseplate assembly protein V | Phage baseplate assembly protein V | | afdb-uniprot50 | AF-A0A6M8SWQ4-F1-MODEL\_V4 | 1.0 | 7.069e-12 | 412 | 0.227 | 207 | 141 | 8 | 16 | 217 | 8 | 200 | Phage baseplate assembly protein V | Phage baseplate assembly protein V | | afdb-uniprot50 | AF-A0A809B5H8-F1-MODEL\_V4 | 1.0 | 2.056e-13 | 412 | 0.242 | 243 | 156 | 5 | 1 | 215 | 1 | 243 | Phage baseplate assembly protein V | Phage baseplate assembly protein V | | afdb-uniprot50 | AF-A0A8B4NZR2-F1-MODEL\_V4 | 1.0 | 1.425e-09 | 411 | 0.389 | 95 | 55 | 1 | 1 | 95 | 1 | 92 | Baseplate assembly protein V | Baseplate assembly protein V | | afdb-uniprot50 | AF-A0A1G3UAP2-F1-MODEL\_V4 | 1.0 | 4.425e-13 | 411 | 0.234 | 209 | 123 | 7 | 1 | 208 | 1 | 173 | Uncharacterized protein | Uncharacterized protein | | afdb-uniprot50 | AF-A0A7J0BHU1-F1-MODEL\_V4 | 1.0 | 9.492e-12 | 411 | 0.202 | 222 | 163 | 7 | 4 | 217 | 8 | 223 | Baseplate protein | Baseplate protein | | afdb-uniprot50 | AF-J4UQU3-F1-MODEL\_V4 | 1.0 | 2.291e-10 | 408 | 0.228 | 140 | 98 | 3 | 1 | 137 | 1 | 133 | Phage-like baseplate assembly protein | Phage-like baseplate assembly protein | | afdb-uniprot50 | AF-A0A7V7G2T5-F1-MODEL\_V4 | 1.0 | 5.281e-13 | 407 | 0.257 | 241 | 99 | 6 | 1 | 217 | 1 | 185 | Phage baseplate assembly protein V | Phage baseplate assembly protein V | | afdb-uniprot50 | AF-G2ZT35-F1-MODEL\_V4 | 1.0 | 2.446e-12 | 403 | 0.22 | 227 | 159 | 9 | 4 | 217 | 7 | 228 | Putative Phage baseplate assembly protein V | Putative Phage baseplate assembly protein V | | afdb-uniprot50 | AF-A0A4V3M376-F1-MODEL\_V4 | 1.0 | 2.569e-09 | 402 | 0.393 | 89 | 54 | 0 | 8 | 96 | 5 | 93 | Phage baseplate assembly protein V | Phage baseplate assembly protein V | | afdb-uniprot50 | AF-A0A3G6WBA3-F1-MODEL\_V4 | 1.0 | 7.98e-13 | 402 | 0.231 | 233 | 90 | 6 | 1 | 214 | 1 | 163 | Phage baseplate assembly protein V | Phage baseplate assembly protein V | | afdb-uniprot50 | AF-A0A6L7TC77-F1-MODEL\_V4 | 1.0 | 2.603e-13 | 402 | 0.324 | 225 | 102 | 8 | 4 | 216 | 237 | 423 | Phage baseplate assembly protein V | Phage baseplate assembly protein V | | afdb-uniprot50 | AF-A0A3G4V723-F1-MODEL\_V4 | 1.0 | 1.926e-11 | 401 | 0.247 | 194 | 116 | 6 | 1 | 193 | 4 | 168 | Phage baseplate assembly protein V | Phage baseplate assembly protein V | | afdb-uniprot50 | AF-A0A2N1AP79-F1-MODEL\_V4 | 1.0 | 2.174e-12 | 401 | 0.288 | 177 | 92 | 2 | 18 | 194 | 2 | 144 | Phage baseplate assembly protein V | Phage baseplate assembly protein V | | afdb-uniprot50 | AF-D5EFA0-F1-MODEL\_V4 | 1.0 | 1.129e-10 | 400 | 0.168 | 160 | 114 | 3 | 4 | 160 | 7 | 150 | Phage baseplate assembly protein V | Phage baseplate assembly protein V | | afdb-uniprot50 | AF-E2CGG9-F1-MODEL\_V4 | 1.0 | 1.279e-12 | 400 | 0.278 | 205 | 104 | 6 | 16 | 217 | 14 | 177 | Phage baseplate component | Phage baseplate component | | afdb-uniprot50 | AF-A0A6A4R6B7-F1-MODEL\_V4 | 1.0 | 1.439e-12 | 399 | 0.229 | 231 | 112 | 3 | 4 | 217 | 26 | 207 | Phage baseplate assembly protein V | Phage baseplate assembly protein V | | afdb-uniprot50 | AF-E2CN36-F1-MODEL\_V4 | 1.0 | 1.614e-11 | 398 | 0.231 | 216 | 107 | 4 | 3 | 217 | 12 | 169 | Putative baseplate assembly protein V | Putative baseplate assembly protein V | | afdb-uniprot50 | AF-A0A1C3EE72-F1-MODEL\_V4 | 1.0 | 3.107e-13 | 397 | 0.239 | 205 | 123 | 3 | 4 | 208 | 31 | 202 | Phage\_base\_V domain-containing protein | Phage\_base\_V domain-containing protein | | afdb-uniprot50 | AF-A0A0K0VKK2-F1-MODEL\_V4 | 1.0 | 1.194e-09 | 396 | 0.171 | 134 | 107 | 3 | 3 | 134 | 22 | 153 | Uncharacterized protein | Uncharacterized protein | | afdb-uniprot50 | AF-A0A1Y1QXP2-F1-MODEL\_V4 | 1.0 | 2.743e-11 | 395 | 0.195 | 215 | 116 | 5 | 4 | 217 | 8 | 166 | Uncharacterized protein | Uncharacterized protein | | afdb-uniprot50 | AF-A0A827JR60-F1-MODEL\_V4 | 1.0 | 5.584e-12 | 394 | 0.279 | 197 | 117 | 4 | 21 | 217 | 2 | 173 | Phage baseplate assembly protein V | Phage baseplate assembly protein V | | afdb-uniprot50 | AF-A0A349GR45-F1-MODEL\_V4 | 1.0 | 4.648e-10 | 394 | 0.219 | 155 | 114 | 5 | 15 | 165 | 5 | 156 | Baseplate assembly protein | Baseplate assembly protein | | afdb-uniprot50 | AF-G2JAY8-F1-MODEL\_V4 | 1.0 | 7.069e-12 | 394 | 0.216 | 222 | 162 | 8 | 4 | 217 | 10 | 227 | Putative phage-related baseplate assembly protein V | Putative phage-related baseplate assembly protein V | | afdb-uniprot50 | AF-A0A6P1LVS1-F1-MODEL\_V4 | 1.0 | 7.069e-12 | 393 | 0.216 | 217 | 125 | 6 | 1 | 217 | 1 | 172 | Phage baseplate assembly protein V | Phage baseplate assembly protein V | | afdb-uniprot50 | AF-A0A828D047-F1-MODEL\_V4 | 1.0 | 2.595e-12 | 392 | 0.286 | 185 | 120 | 5 | 16 | 191 | 2 | 183 | Phage baseplate assembly protein V | Phage baseplate assembly protein V | | afdb-uniprot50 | AF-A0A806CKJ7-F1-MODEL\_V4 | 1.0 | 5.923e-12 | 391 | 0.23 | 200 | 121 | 6 | 16 | 215 | 2 | 168 | Phage baseplate assembly protein V | Phage baseplate assembly protein V | | afdb-uniprot50 | AF-A0A1I5RPU5-F1-MODEL\_V4 | 1.0 | 4.131e-10 | 390 | 0.24 | 137 | 90 | 6 | 1 | 129 | 1 | 131 | Phage P2 baseplate assembly protein gpV | Phage P2 baseplate assembly protein gpV | | afdb-uniprot50 | AF-A0A4R2GWQ4-F1-MODEL\_V4 | 1.0 | 1.275e-11 | 390 | 0.24 | 208 | 109 | 7 | 16 | 217 | 7 | 171 | Phage baseplate assembly protein V | Phage baseplate assembly protein V | | afdb-uniprot50 | AF-E1QHA2-F1-MODEL\_V4 | 1.0 | 1.27e-10 | 390 | 0.204 | 147 | 108 | 2 | 4 | 141 | 11 | 157 | Phage baseplate assembly protein V | Phage baseplate assembly protein V | | afdb-uniprot50 | AF-A0A2T3NIK5-F1-MODEL\_V4 | 1.0 | 2.422e-09 | 390 | 0.246 | 134 | 95 | 4 | 4 | 135 | 22 | 151 | Phage baseplate assembly protein V | Phage baseplate assembly protein V | | afdb-uniprot50 | AF-A0A708DHR8-F1-MODEL\_V4 | 1.0 | 1.907e-08 | 389 | 0.447 | 76 | 42 | 0 | 3 | 78 | 5 | 80 | Phage baseplate assembly protein V | Phage baseplate assembly protein V | | afdb-uniprot50 | AF-A0A259UCZ3-F1-MODEL\_V4 | 1.0 | 2.743e-11 | 389 | 0.204 | 220 | 113 | 5 | 1 | 217 | 1 | 161 | Phage-related baseplate assembly protein | Phage-related baseplate assembly protein | | afdb-uniprot50 | AF-A0A3N2E0Q5-F1-MODEL\_V4 | 1.0 | 5.903e-11 | 388 | 0.31 | 148 | 92 | 6 | 5 | 148 | 6 | 147 | Phage baseplate assembly protein V | Phage baseplate assembly protein V | | afdb-uniprot50 | AF-A0A1C3EBJ8-F1-MODEL\_V4 | 1.0 | 1.202e-11 | 387 | 0.223 | 224 | 148 | 10 | 4 | 213 | 7 | 218 | Phage\_base\_V domain-containing protein | Phage\_base\_V domain-containing protein | | afdb-uniprot50 | AF-A0A2J4XY04-F1-MODEL\_V4 | 1.0 | 1.506e-08 | 386 | 0.383 | 86 | 51 | 1 | 1 | 86 | 1 | 84 | Phage baseplate assembly protein V | Phage baseplate assembly protein V | | afdb-uniprot50 | AF-A0A2T5HH03-F1-MODEL\_V4 | 1.0 | 3.907e-11 | 386 | 0.29 | 141 | 83 | 5 | 1 | 130 | 1 | 135 | Phage baseplate assembly protein V | Phage baseplate assembly protein V | | afdb-uniprot50 | AF-A0A239C9W6-F1-MODEL\_V4 | 1.0 | 6.664e-12 | 386 | 0.222 | 220 | 153 | 4 | 4 | 217 | 8 | 215 | Phage baseplate assembly protein V | Phage baseplate assembly protein V | | afdb-uniprot50 | AF-A0A366D029-F1-MODEL\_V4 | 1.0 | 3.696e-12 | 386 | 0.217 | 193 | 123 | 5 | 4 | 194 | 26 | 192 | Phage baseplate assembly protein V | Phage baseplate assembly protein V | | afdb-uniprot50 | AF-A0A0A6D4K9-F1-MODEL\_V4 | 1.0 | 2.16e-10 | 385 | 0.235 | 178 | 120 | 7 | 16 | 187 | 5 | 172 | Baseplate assembly protein | Baseplate assembly protein | | afdb-uniprot50 | AF-A0A5S9P2X6-F1-MODEL\_V4 | 1.0 | 7.498e-12 | 384 | 0.23 | 230 | 128 | 13 | 1 | 217 | 1 | 194 | Phage\_base\_V domain-containing protein | Phage\_base\_V domain-containing protein | | afdb-uniprot50 | AF-A0A5Y3B0Q3-F1-MODEL\_V4 | 1.0 | 7.523e-13 | 384 | 0.217 | 234 | 155 | 8 | 1 | 215 | 1 | 225 | Phage baseplate assembly protein V | Phage baseplate assembly protein V | | afdb-uniprot50 | AF-A0A7M3MAY2-F1-MODEL\_V4 | 1.0 | 1.275e-11 | 383 | 0.198 | 237 | 163 | 9 | 4 | 217 | 8 | 240 | Uncharacterized protein | Uncharacterized protein | | afdb-uniprot50 | AF-A0A825DZB9-F1-MODEL\_V4 | 1.0 | 3.696e-12 | 382 | 0.293 | 191 | 118 | 7 | 16 | 195 | 2 | 186 | Phage baseplate assembly protein V | Phage baseplate assembly protein V | | afdb-uniprot50 | AF-A0A1L6I2B1-F1-MODEL\_V4 | 1.0 | 6.642e-11 | 381 | 0.256 | 218 | 146 | 9 | 4 | 217 | 8 | 213 | Uncharacterized protein | Uncharacterized protein | | afdb-uniprot50 | AF-A0A3Q0L1B6-F1-MODEL\_V4 | 1.0 | 2.446e-12 | 380 | 0.25 | 208 | 140 | 7 | 1 | 193 | 1 | 207 | Phage P2 baseplate assembly protein gpV | Phage P2 baseplate assembly protein gpV | | afdb-uniprot50 | AF-A0A0U2B1K8-F1-MODEL\_V4 | 1.0 | 1.822e-12 | 379 | 0.241 | 211 | 118 | 8 | 13 | 216 | 6 | 181 | Uncharacterized protein | Uncharacterized protein | | afdb-uniprot50 | AF-A0A6I1HQM0-F1-MODEL\_V4 | 1.0 | 1.624e-13 | 379 | 0.234 | 315 | 128 | 6 | 3 | 213 | 6 | 311 | Phage baseplate assembly protein V | Phage baseplate assembly protein V | | afdb-uniprot50 | AF-A0A6H0T7G9-F1-MODEL\_V4 | 1.0 | 2.752e-12 | 376 | 0.221 | 208 | 128 | 8 | 16 | 217 | 14 | 193 | Phage baseplate protein | Phage baseplate protein | | afdb-uniprot50 | AF-A0A1E4AHK4-F1-MODEL\_V4 | 1.0 | 1.348e-10 | 376 | 0.197 | 208 | 149 | 11 | 16 | 213 | 2 | 201 | Uncharacterized protein | Uncharacterized protein | | afdb-uniprot50 | AF-A0A0J9DZ44-F1-MODEL\_V4 | 1.0 | 5.923e-12 | 376 | 0.259 | 227 | 144 | 10 | 4 | 217 | 7 | 222 | Phage baseplate assembly protein V | Phage baseplate assembly protein V | | afdb-uniprot50 | AF-A0A0P0ADA7-F1-MODEL\_V4 | 1.0 | 2.036e-10 | 375 | 0.231 | 194 | 122 | 9 | 16 | 203 | 4 | 176 | Uncharacterized protein | Uncharacterized protein | | afdb-uniprot50 | AF-A0A2N0CXD6-F1-MODEL\_V4 | 1.0 | 5.923e-12 | 375 | 0.263 | 220 | 124 | 6 | 1 | 206 | 1 | 196 | Phage baseplate assembly protein V | Phage baseplate assembly protein V | | afdb-uniprot50 | AF-A0A602TUF8-F1-MODEL\_V4 | 1.0 | 5.565e-11 | 374 | 0.223 | 192 | 123 | 6 | 16 | 203 | 6 | 175 | Phage baseplate assembly protein V | Phage baseplate assembly protein V | | afdb-uniprot50 | AF-A0A2M7G5P7-F1-MODEL\_V4 | 1.0 | 3.671e-10 | 374 | 0.204 | 181 | 125 | 7 | 16 | 191 | 12 | 178 | Phage baseplate assembly protein V | Phage baseplate assembly protein V | | afdb-uniprot50 | AF-A0A2S2E577-F1-MODEL\_V4 | 1.0 | 1.434e-11 | 374 | 0.214 | 214 | 144 | 7 | 5 | 217 | 2 | 192 | Phage\_base\_V domain-containing protein | Phage\_base\_V domain-containing protein | | afdb-uniprot50 | AF-A0A6D2GFI2-F1-MODEL\_V4 | 1.0 | 6.241e-10 | 370 | 0.244 | 172 | 118 | 5 | 16 | 182 | 6 | 170 | Baseplate protein | Baseplate protein | | afdb-uniprot50 | AF-A0A376EWE4-F1-MODEL\_V4 | 1.0 | 6.642e-11 | 370 | 0.235 | 174 | 110 | 5 | 16 | 185 | 396 | 550 | Gene D protein | Gene D protein | | afdb-uniprot50 | AF-A0A6C1BQR0-F1-MODEL\_V4 | 1.0 | 8.381e-10 | 369 | 0.206 | 131 | 95 | 2 | 16 | 137 | 2 | 132 | Phage baseplate assembly protein V | Phage baseplate assembly protein V | | afdb-uniprot50 | AF-A0A7L6UL61-F1-MODEL\_V4 | 1.0 | 9.43e-10 | 369 | 0.323 | 167 | 105 | 5 | 32 | 196 | 16 | 176 | Phage baseplate assembly protein V | Phage baseplate assembly protein V | | afdb-uniprot50 | AF-A0A7L5ZYB0-F1-MODEL\_V4 | 1.0 | 5.529e-09 | 368 | 0.192 | 140 | 105 | 2 | 15 | 149 | 3 | 139 | Phage baseplate assembly protein V | Phage baseplate assembly protein V | | afdb-uniprot50 | AF-A0A1G0LH00-F1-MODEL\_V4 | 1.0 | 2.725e-09 | 368 | 0.254 | 153 | 104 | 6 | 17 | 163 | 2 | 150 | Phage\_base\_V domain-containing protein | Phage\_base\_V domain-containing protein | | afdb-uniprot50 | AF-A0A541BHN8-F1-MODEL\_V4 | 1.0 | 6.303e-13 | 367 | 0.227 | 264 | 150 | 9 | 4 | 217 | 11 | 270 | Phage baseplate assembly protein V | Phage baseplate assembly protein V | | afdb-uniprot50 | AF-R9B5T6-F1-MODEL\_V4 | 1.0 | 1.122e-08 | 366 | 0.27 | 111 | 76 | 2 | 1 | 111 | 1 | 106 | Phage\_base\_V domain-containing protein | Phage\_base\_V domain-containing protein | | afdb-uniprot50 | AF-A0A285NJ63-F1-MODEL\_V4 | 1.0 | 3.683e-11 | 366 | 0.18 | 222 | 125 | 4 | 4 | 211 | 11 | 189 | Phage baseplate assembly protein V | Phage baseplate assembly protein V | | afdb-uniprot50 | AF-A0A2T6F922-F1-MODEL\_V4 | 1.0 | 2.586e-11 | 366 | 0.212 | 226 | 155 | 9 | 1 | 217 | 1 | 212 | Phage baseplate assembly protein V | Phage baseplate assembly protein V | | afdb-uniprot50 | AF-A0A6B3ITL1-F1-MODEL\_V4 | 1.0 | 8.436e-12 | 365 | 0.223 | 233 | 124 | 7 | 1 | 216 | 1 | 193 | Phage baseplate assembly protein V | Phage baseplate assembly protein V | | afdb-uniprot50 | AF-A0A2N7G847-F1-MODEL\_V4 | 1.0 | 3.274e-11 | 365 | 0.231 | 207 | 151 | 6 | 1 | 203 | 1 | 203 | Uncharacterized protein | Uncharacterized protein | | afdb-uniprot50 | AF-A0A838Y7A3-F1-MODEL\_V4 | 1.0 | 7.927e-11 | 364 | 0.202 | 202 | 143 | 10 | 3 | 196 | 7 | 198 | Phage baseplate assembly protein V | Phage baseplate assembly protein V | | afdb-uniprot50 | AF-A0A4P6G5E4-F1-MODEL\_V4 | 1.0 | 4.963e-12 | 362 | 0.212 | 226 | 137 | 8 | 1 | 217 | 1 | 194 | Phage baseplate protein | Phage baseplate protein | | afdb-uniprot50 | AF-A0A602MX29-F1-MODEL\_V4 | 1.0 | 1.007e-11 | 362 | 0.248 | 217 | 133 | 7 | 1 | 207 | 1 | 197 | Phage baseplate assembly protein V | Phage baseplate assembly protein V | | afdb-uniprot50 | AF-A0A2K4IH05-F1-MODEL\_V4 | 1.0 | 1.926e-11 | 362 | 0.232 | 211 | 134 | 9 | 2 | 203 | 27 | 218 | Phage baseplate assembly protein V | Phage baseplate assembly protein V | | afdb-uniprot50 | AF-A0A4R5HEX7-F1-MODEL\_V4 | 1.0 | 5.903e-11 | 361 | 0.191 | 214 | 137 | 6 | 3 | 208 | 25 | 210 | Phage baseplate assembly protein V | Phage baseplate assembly protein V | | afdb-uniprot50 | AF-D1AFB2-F1-MODEL\_V4 | 1.0 | 2.9e-10 | 360 | 0.163 | 208 | 138 | 6 | 12 | 216 | 3 | 177 | Phage baseplate assembly protein V | Phage baseplate assembly protein V | | afdb-uniprot50 | AF-A0A327QB94-F1-MODEL\_V4 | 1.0 | 9.461e-11 | 360 | 0.231 | 207 | 128 | 11 | 16 | 217 | 3 | 183 | Phage baseplate assembly protein V | Phage baseplate assembly protein V | | afdb-uniprot50 | AF-A0A165THH2-F1-MODEL\_V4 | 1.0 | 3.086e-11 | 359 | 0.237 | 232 | 117 | 4 | 4 | 217 | 15 | 204 | Phage-related baseplate assembly protein | Phage-related baseplate assembly protein | | afdb-uniprot50 | AF-G8PUM2-F1-MODEL\_V4 | 1.0 | 2.043e-11 | 359 | 0.241 | 232 | 116 | 4 | 4 | 217 | 15 | 204 | Phage-related baseplate assembly protein V | Phage-related baseplate assembly protein V | | afdb-uniprot50 | AF-A0A2S7JRA1-F1-MODEL\_V4 | 1.0 | 5.903e-11 | 359 | 0.237 | 215 | 136 | 7 | 16 | 217 | 8 | 207 | Phage baseplate assembly protein V | Phage baseplate assembly protein V | | afdb-uniprot50 | AF-A0A411WI65-F1-MODEL\_V4 | 1.0 | 1.926e-11 | 358 | 0.219 | 205 | 135 | 8 | 16 | 217 | 3 | 185 | Phage baseplate assembly protein V | Phage baseplate assembly protein V | | afdb-uniprot50 | AF-A0A1N6M5J8-F1-MODEL\_V4 | 1.0 | 3.473e-11 | 358 | 0.219 | 223 | 141 | 9 | 1 | 216 | 4 | 200 | Phage-related baseplate assembly protein | Phage-related baseplate assembly protein | | afdb-uniprot50 | AF-A0A075KC29-F1-MODEL\_V4 | 1.0 | 2.029e-09 | 357 | 0.211 | 142 | 100 | 3 | 1 | 139 | 1 | 133 | Phage baseplate assembly protein V | Phage baseplate assembly protein V | | afdb-uniprot50 | AF-A0A0C1EUH5-F1-MODEL\_V4 | 1.0 | 1.129e-10 | 357 | 0.25 | 188 | 120 | 8 | 17 | 197 | 2 | 175 | Baseplate protein | Baseplate protein | | afdb-uniprot50 | AF-A0A2G6EWU3-F1-MODEL\_V4 | 1.0 | 7.901e-10 | 355 | 0.246 | 162 | 102 | 6 | 7 | 158 | 8 | 159 | Phage\_base\_V domain-containing protein | Phage\_base\_V domain-containing protein | | afdb-uniprot50 | AF-A0A660N066-F1-MODEL\_V4 | 1.0 | 4.158e-12 | 355 | 0.248 | 205 | 122 | 7 | 16 | 209 | 12 | 195 | Phage baseplate assembly protein V | Phage baseplate assembly protein V | | afdb-uniprot50 | AF-A0A4U7BE93-F1-MODEL\_V4 | 1.0 | 2.167e-11 | 354 | 0.222 | 189 | 125 | 7 | 16 | 203 | 2 | 169 | Phage baseplate assembly protein V | Phage baseplate assembly protein V | | afdb-uniprot50 | AF-A0A7D5LN82-F1-MODEL\_V4 | 1.0 | 7.901e-10 | 353 | 0.21 | 176 | 122 | 6 | 16 | 186 | 5 | 168 | Phage baseplate assembly protein V | Phage baseplate assembly protein V | | afdb-uniprot50 | AF-A0A5C4P976-F1-MODEL\_V4 | 1.0 | 1.064e-10 | 351 | 0.238 | 168 | 110 | 2 | 2 | 169 | 1 | 150 | Phage baseplate assembly protein V | Phage baseplate assembly protein V | | afdb-uniprot50 | AF-A0A1H1G342-F1-MODEL\_V4 | 1.0 | 2.276e-08 | 351 | 0.19 | 126 | 97 | 3 | 4 | 128 | 8 | 129 | Uncharacterized protein | Uncharacterized protein | | afdb-uniprot50 | AF-A0A840ML35-F1-MODEL\_V4 | 1.0 | 1.712e-11 | 351 | 0.28 | 200 | 116 | 8 | 16 | 197 | 12 | 201 | Phage baseplate assembly protein V | Phage baseplate assembly protein V | | afdb-uniprot50 | AF-A0A0D8L226-F1-MODEL\_V4 | 1.0 | 1.339e-08 | 350 | 0.329 | 170 | 106 | 5 | 49 | 217 | 2 | 164 | Baseplate assembly protein | Baseplate assembly protein | | afdb-uniprot50 | AF-A0A4R5W1B5-F1-MODEL\_V4 | 1.0 | 1.521e-11 | 348 | 0.218 | 224 | 128 | 11 | 1 | 216 | 1 | 185 | Phage baseplate assembly protein V | Phage baseplate assembly protein V | | afdb-uniprot50 | AF-A0A7C3GU98-F1-MODEL\_V4 | 1.0 | 4.144e-11 | 348 | 0.165 | 217 | 155 | 5 | 4 | 217 | 8 | 201 | Phage baseplate assembly protein V | Phage baseplate assembly protein V | | afdb-uniprot50 | AF-A0A5A8F6F2-F1-MODEL\_V4 | 1.0 | 6.642e-11 | 348 | 0.147 | 217 | 151 | 7 | 4 | 208 | 11 | 205 | Phage baseplate assembly protein V | Phage baseplate assembly protein V | | afdb-uniprot50 | AF-A0A257PQI7-F1-MODEL\_V4 | 1.0 | 2.036e-10 | 347 | 0.2 | 220 | 120 | 7 | 4 | 217 | 5 | 174 | Phage\_base\_V domain-containing protein | Phage\_base\_V domain-containing protein | | afdb-uniprot50 | AF-A0A6L8M2W9-F1-MODEL\_V4 | 1.0 | 2.43e-10 | 346 | 0.216 | 222 | 152 | 10 | 4 | 217 | 10 | 217 | Phage baseplate assembly protein V | Phage baseplate assembly protein V | | afdb-uniprot50 | AF-A0A5B9Y7V9-F1-MODEL\_V4 | 1.0 | 1.343e-09 | 345 | 0.207 | 183 | 122 | 4 | 12 | 191 | 9 | 171 | Phage baseplate assembly protein V | Phage baseplate assembly protein V | | afdb-uniprot50 | AF-A0A1D2QMX7-F1-MODEL\_V4 | 1.0 | 1.348e-10 | 345 | 0.244 | 209 | 122 | 6 | 4 | 208 | 12 | 188 | Phage\_base\_V domain-containing protein | Phage\_base\_V domain-containing protein | | afdb-uniprot50 | AF-A0A5C8BDY7-F1-MODEL\_V4 | 1.0 | 3.647e-08 | 344 | 0.238 | 126 | 91 | 3 | 14 | 137 | 5 | 127 | Phage baseplate assembly protein V | Phage baseplate assembly protein V | | afdb-uniprot50 | AF-A0A345J3I8-F1-MODEL\_V4 | 1.0 | 9.492e-12 | 344 | 0.237 | 219 | 145 | 8 | 1 | 211 | 2 | 206 | Phage baseplate assembly protein V | Phage baseplate assembly protein V | | afdb-uniprot50 | AF-A0A4Q8MEL0-F1-MODEL\_V4 | 1.0 | 3.907e-11 | 343 | 0.197 | 228 | 100 | 3 | 4 | 216 | 20 | 179 | Uncharacterized protein | Uncharacterized protein | | afdb-uniprot50 | AF-A0A3G2EA58-F1-MODEL\_V4 | 1.0 | 7.927e-11 | 342 | 0.186 | 220 | 143 | 8 | 3 | 217 | 4 | 192 | Phage\_base\_V domain-containing protein | Phage\_base\_V domain-containing protein | | afdb-uniprot50 | AF-A0A2S0JB32-F1-MODEL\_V4 | 1.0 | 2.43e-10 | 341 | 0.206 | 189 | 108 | 5 | 16 | 203 | 5 | 152 | Phage baseplate assembly protein V | Phage baseplate assembly protein V | | afdb-uniprot50 | AF-A0A1M7RIP4-F1-MODEL\_V4 | 1.0 | 1.198e-10 | 341 | 0.224 | 218 | 138 | 10 | 16 | 217 | 5 | 207 | Phage baseplate assembly protein V | Phage baseplate assembly protein V | | afdb-uniprot50 | AF-A0A2V2AA94-F1-MODEL\_V4 | 1.0 | 3.461e-10 | 341 | 0.216 | 254 | 127 | 8 | 1 | 217 | 1 | 219 | Phage baseplate assembly protein V | Phage baseplate assembly protein V | | afdb-uniprot50 | AF-A0A4Y7RWH3-F1-MODEL\_V4 | 1.0 | 4.396e-11 | 340 | 0.242 | 194 | 123 | 5 | 11 | 186 | 4 | 191 | Uncharacterized protein | Uncharacterized protein | | afdb-uniprot50 | AF-A0A7T5ENC6-F1-MODEL\_V4 | 1.0 | 1.348e-10 | 339 | 0.21 | 195 | 131 | 8 | 12 | 202 | 2 | 177 | Phage baseplate assembly protein V | Phage baseplate assembly protein V | | afdb-uniprot50 | AF-A0A1V4IV34-F1-MODEL\_V4 | 1.0 | 4.632e-09 | 336 | 0.228 | 149 | 99 | 4 | 1 | 145 | 1 | 137 | Phage-related baseplate assembly protein | Phage-related baseplate assembly protein | | afdb-uniprot50 | AF-A0A1W1Z4L7-F1-MODEL\_V4 | 1.0 | 3.881e-09 | 336 | 0.189 | 148 | 106 | 4 | 4 | 138 | 18 | 164 | Type VI secretion system, phage-baseplate injector | Type VI secretion system, phage-baseplate injector | | afdb-uniprot50 | AF-A0A6G5QN06-F1-MODEL\_V4 | 1.0 | 1.81e-10 | 335 | 0.259 | 189 | 103 | 7 | 16 | 203 | 2 | 154 | Phage baseplate assembly protein V | Phage baseplate assembly protein V | | afdb-uniprot50 | AF-A0A1H4QYN2-F1-MODEL\_V4 | 1.0 | 1.133e-11 | 335 | 0.216 | 226 | 143 | 9 | 1 | 217 | 1 | 201 | Phage\_base\_V domain-containing protein | Phage\_base\_V domain-containing protein | | afdb-uniprot50 | AF-A0A2X3AA95-F1-MODEL\_V4 | 1.0 | 5.903e-11 | 333 | 0.333 | 186 | 103 | 4 | 32 | 217 | 2 | 166 | Baseplate assembly protein V | Baseplate assembly protein V | | afdb-uniprot50 | AF-B3QTI6-F1-MODEL\_V4 | 1.0 | 7.022e-10 | 333 | 0.201 | 208 | 129 | 7 | 16 | 214 | 2 | 181 | Phage baseplate assembly protein V | Phage baseplate assembly protein V | | afdb-uniprot50 | AF-A0A2X0V6P6-F1-MODEL\_V4 | 1.0 | 1.27e-10 | 333 | 0.238 | 210 | 124 | 6 | 1 | 193 | 1 | 191 | Phage P2 baseplate assembly protein gpV | Phage P2 baseplate assembly protein gpV | | afdb-uniprot50 | AF-Q602Z7-F1-MODEL\_V4 | 1.0 | 7.901e-10 | 333 | 0.219 | 196 | 135 | 8 | 16 | 203 | 13 | 198 | Prophage MuMc02, baseplate assembly protein V | Prophage MuMc02, baseplate assembly protein V | | afdb-uniprot50 | AF-K1K036-F1-MODEL\_V4 | 1.0 | 5.565e-11 | 332 | 0.264 | 204 | 128 | 4 | 15 | 196 | 12 | 215 | Phage baseplate assembly protein V | Phage baseplate assembly protein V | | afdb-uniprot50 | AF-A0A2E2NAE7-F1-MODEL\_V4 | 1.0 | 1.19e-08 | 330 | 0.253 | 134 | 78 | 3 | 1 | 134 | 1 | 112 | Phage\_base\_V domain-containing protein | Phage\_base\_V domain-containing protein | | afdb-uniprot50 | AF-A0A827QZX9-F1-MODEL\_V4 | 1.0 | 7.449e-10 | 329 | 0.218 | 192 | 112 | 8 | 16 | 203 | 2 | 159 | Phage baseplate assembly protein V | Phage baseplate assembly protein V | | afdb-uniprot50 | AF-A0A375J5W5-F1-MODEL\_V4 | 1.0 | 4.396e-11 | 329 | 0.357 | 193 | 99 | 5 | 32 | 217 | 2 | 176 | Bacteriophage P2 Baseplate assembly protein GPV | Bacteriophage P2 Baseplate assembly protein GPV | | afdb-uniprot50 | AF-A0A4Q6DAY4-F1-MODEL\_V4 | 1.0 | 1.792e-07 | 328 | 0.289 | 107 | 76 | 0 | 40 | 146 | 1 | 107 | Phage baseplate assembly protein V | Phage baseplate assembly protein V | | afdb-uniprot50 | AF-A0A348AJ13-F1-MODEL\_V4 | 1.0 | 1.343e-09 | 327 | 0.205 | 195 | 111 | 6 | 12 | 203 | 4 | 157 | Phage-related baseplate assembly protein | Phage-related baseplate assembly protein | | afdb-uniprot50 | AF-G2IX47-F1-MODEL\_V4 | 1.0 | 3.076e-10 | 327 | 0.185 | 199 | 144 | 9 | 16 | 208 | 10 | 196 | Phage baseplate assembly protein V | Phage baseplate assembly protein V | | afdb-uniprot50 | AF-A0A7J4VI64-F1-MODEL\_V4 | 1.0 | 1.81e-10 | 326 | 0.207 | 227 | 120 | 6 | 1 | 217 | 1 | 177 | Phage\_base\_V domain-containing protein | Phage\_base\_V domain-containing protein | | afdb-uniprot50 | AF-A0A139D6P1-F1-MODEL\_V4 | 1.0 | 4.648e-10 | 326 | 0.203 | 211 | 133 | 7 | 1 | 203 | 1 | 184 | Phage baseplate assembly protein V | Phage baseplate assembly protein V | | afdb-uniprot50 | AF-A0A081NV30-F1-MODEL\_V4 | 1.0 | 3.252e-09 | 323 | 0.233 | 171 | 103 | 6 | 12 | 179 | 4 | 149 | Uncharacterized protein | Uncharacterized protein | | afdb-uniprot50 | AF-A0A5E5BJ14-F1-MODEL\_V4 | 1.0 | 2.881e-08 | 320 | 0.204 | 166 | 124 | 4 | 3 | 161 | 7 | 171 | Phage-related baseplate assembly protein | Phage-related baseplate assembly protein | | afdb-uniprot50 | AF-A0A096AIG4-F1-MODEL\_V4 | 1.0 | 7.022e-10 | 319 | 0.229 | 192 | 115 | 7 | 15 | 203 | 10 | 171 | Baseplate protein | Baseplate protein | | afdb-uniprot50 | AF-A0A0G3W8Q4-F1-MODEL\_V4 | 1.0 | 3.45e-09 | 317 | 0.198 | 166 | 117 | 4 | 1 | 157 | 3 | 161 | Phage baseplate assembly protein V | Phage baseplate assembly protein V | | afdb-uniprot50 | AF-R7JNY1-F1-MODEL\_V4 | 1.0 | 6.598e-09 | 317 | 0.24 | 162 | 106 | 6 | 16 | 172 | 2 | 151 | Putative bacteriophage baseplate protein | Putative bacteriophage baseplate protein | | afdb-uniprot50 | AF-A0A380Z445-F1-MODEL\_V4 | 1.0 | 2.036e-10 | 316 | 0.221 | 190 | 118 | 6 | 16 | 193 | 10 | 181 | Phage P2 baseplate assembly protein gpV | Phage P2 baseplate assembly protein gpV | | afdb-uniprot50 | AF-W4AM65-F1-MODEL\_V4 | 1.0 | 8.353e-09 | 313 | 0.196 | 183 | 113 | 3 | 11 | 190 | 3 | 154 | Phage baseplate assembly protein V | Phage baseplate assembly protein V | | afdb-uniprot50 | AF-A0A1B9NZR2-F1-MODEL\_V4 | 1.0 | 1.343e-09 | 312 | 0.168 | 196 | 136 | 5 | 4 | 197 | 22 | 192 | Uncharacterized protein | Uncharacterized protein | | afdb-uniprot50 | AF-A0A662Z9P3-F1-MODEL\_V4 | 1.0 | 2.569e-09 | 310 | 0.198 | 166 | 104 | 4 | 1 | 137 | 1 | 166 | Phage baseplate assembly protein V | Phage baseplate assembly protein V | | afdb-uniprot50 | AF-A0A285MD33-F1-MODEL\_V4 | 1.0 | 1.706e-10 | 310 | 0.207 | 236 | 112 | 5 | 4 | 217 | 13 | 195 | Phage baseplate assembly protein V | Phage baseplate assembly protein V | | afdb-uniprot50 | AF-A0A6S7B3J0-F1-MODEL\_V4 | 1.0 | 5.884e-10 | 308 | 0.202 | 207 | 121 | 7 | 1 | 191 | 1 | 179 | Phage\_base\_V domain-containing protein | Phage\_base\_V domain-containing protein | | afdb-uniprot50 | AF-A0A561IRQ8-F1-MODEL\_V4 | 1.0 | 7.045e-11 | 307 | 0.213 | 220 | 150 | 10 | 15 | 216 | 14 | 228 | Phage baseplate assembly protein V | Phage baseplate assembly protein V | | afdb-uniprot50 | AF-A0A209AE83-F1-MODEL\_V4 | 1.0 | 3.076e-10 | 306 | 0.223 | 179 | 83 | 6 | 1 | 125 | 1 | 177 | Phage\_base\_V domain-containing protein | Phage\_base\_V domain-containing protein | | afdb-uniprot50 | AF-Q31HU0-F1-MODEL\_V4 | 1.0 | 2.291e-10 | 306 | 0.244 | 225 | 142 | 10 | 16 | 217 | 2 | 221 | Phage-related baseplate assembly protein V | Phage-related baseplate assembly protein V | | afdb-uniprot50 | AF-A0A074MGB6-F1-MODEL\_V4 | 1.0 | 7.399e-08 | 303 | 0.15 | 160 | 117 | 3 | 12 | 168 | 3 | 146 | Uncharacterized protein | Uncharacterized protein | | afdb-uniprot50 | AF-A0A3C0RRX5-F1-MODEL\_V4 | 1.0 | 6.598e-09 | 303 | 0.18 | 161 | 115 | 6 | 4 | 158 | 8 | 157 | Uncharacterized protein | Uncharacterized protein | | afdb-uniprot50 | AF-D1Y2B5-F1-MODEL\_V4 | 1.0 | 1.122e-08 | 302 | 0.189 | 148 | 101 | 4 | 16 | 145 | 9 | 155 | Phage-related baseplate assembly protein | Phage-related baseplate assembly protein | | afdb-uniprot50 | AF-A0A561QKN0-F1-MODEL\_V4 | 1.0 | 1.689e-07 | 300 | 0.18 | 133 | 101 | 3 | 16 | 145 | 2 | 129 | Phage baseplate assembly protein V | Phage baseplate assembly protein V | | afdb-uniprot50 | AF-A0A1C3NRR4-F1-MODEL\_V4 | 1.0 | 1.061e-09 | 300 | 0.292 | 178 | 99 | 5 | 40 | 217 | 3 | 153 | Phage-related baseplate protein | Phage-related baseplate protein | | afdb-uniprot50 | AF-A0A149PP11-F1-MODEL\_V4 | 1.0 | 1.603e-09 | 299 | 0.368 | 179 | 93 | 6 | 40 | 217 | 1 | 160 | Baseplate assembly protein | Baseplate assembly protein | | afdb-uniprot50 | AF-A0A239EJ28-F1-MODEL\_V4 | 1.0 | 1.913e-09 | 297 | 0.301 | 146 | 77 | 5 | 4 | 149 | 5 | 125 | Phage baseplate assembly protein V | Phage baseplate assembly protein V | | afdb-uniprot50 | AF-A0A285M2J1-F1-MODEL\_V4 | 1.0 | 2.414e-08 | 297 | 0.182 | 159 | 109 | 7 | 1 | 145 | 1 | 152 | Uncharacterized protein | Uncharacterized protein | | afdb-uniprot50 | AF-A0A8A8M598-F1-MODEL\_V4 | 1.0 | 3.683e-11 | 293 | 0.238 | 252 | 143 | 11 | 1 | 217 | 1 | 238 | Uncharacterized protein | Uncharacterized protein | | afdb-uniprot50 | AF-A0A7Y3Z3N5-F1-MODEL\_V4 | 1.0 | 2.577e-10 | 293 | 0.199 | 241 | 158 | 9 | 4 | 213 | 11 | 247 | Phage baseplate assembly protein V | Phage baseplate assembly protein V | | afdb-uniprot50 | AF-G9PUJ1-F1-MODEL\_V4 | 1.0 | 6.576e-08 | 291 | 0.172 | 145 | 102 | 2 | 13 | 139 | 5 | 149 | Uncharacterized protein | Uncharacterized protein | | afdb-uniprot50 | AF-A0A843SF22-F1-MODEL\_V4 | 1.0 | 1.27e-10 | 291 | 0.205 | 238 | 145 | 10 | 4 | 217 | 7 | 224 | Phage baseplate assembly protein V | Phage baseplate assembly protein V | | afdb-uniprot50 | AF-A0A547PW88-F1-MODEL\_V4 | 1.0 | 6.62e-10 | 287 | 0.224 | 214 | 98 | 6 | 4 | 217 | 54 | 199 | Phage baseplate assembly protein V | Phage baseplate assembly protein V | | afdb-uniprot50 | AF-D8IV20-F1-MODEL\_V4 | 1.0 | 4.367e-09 | 284 | 0.252 | 206 | 133 | 10 | 16 | 217 | 8 | 196 | Bacteriophage baseplate assembly (GpV) protein | Bacteriophage baseplate assembly (GpV) protein | | afdb-uniprot50 | AF-A0A1I5W3T5-F1-MODEL\_V4 | 1.0 | 8.353e-09 | 283 | 0.201 | 218 | 103 | 8 | 1 | 217 | 1 | 148 | Phage baseplate assembly protein V | Phage baseplate assembly protein V | | afdb-uniprot50 | AF-A0A181WXG7-F1-MODEL\_V4 | 1.0 | 1.81e-10 | 280 | 0.259 | 231 | 125 | 8 | 16 | 203 | 6 | 233 | Baseplate assembly protein V | Baseplate assembly protein V | | afdb-uniprot50 | AF-A0A1I5MP97-F1-MODEL\_V4 | 1.0 | 1.334e-07 | 279 | 0.165 | 151 | 109 | 5 | 1 | 141 | 1 | 144 | Phage P2 baseplate assembly protein gpV | Phage P2 baseplate assembly protein gpV | | afdb-uniprot50 | AF-A0A377Z5C8-F1-MODEL\_V4 | 1.0 | 1.894e-06 | 273 | 0.346 | 130 | 80 | 3 | 67 | 196 | 2 | 126 | Putative prophage baseplate assembly protein | Putative prophage baseplate assembly protein | | afdb-uniprot50 | AF-A0A847G8C6-F1-MODEL\_V4 | 1.0 | 3.659e-09 | 271 | 0.204 | 244 | 128 | 9 | 16 | 198 | 2 | 240 | Phage baseplate assembly protein V | Phage baseplate assembly protein V | | afdb-uniprot50 | AF-A0A842J1J9-F1-MODEL\_V4 | 1.0 | 2.406e-07 | 270 | 0.15 | 180 | 137 | 7 | 16 | 192 | 7 | 173 | Uncharacterized protein | Uncharacterized protein | | afdb-uniprot50 | AF-A0A5E5P1X8-F1-MODEL\_V4 | 1.0 | 3.241e-08 | 270 | 0.174 | 223 | 156 | 10 | 3 | 209 | 7 | 217 | Baseplate assembly protein | Baseplate assembly protein | | afdb-uniprot50 | AF-A0A5C6Y6D1-F1-MODEL\_V4 | 1.0 | 4.632e-09 | 267 | 0.148 | 236 | 144 | 10 | 3 | 216 | 5 | 205 | Baseplate assembly protein | Baseplate assembly protein | | afdb-uniprot50 | AF-A0A447M7I0-F1-MODEL\_V4 | 1.0 | 3.231e-07 | 266 | 0.293 | 143 | 90 | 3 | 40 | 182 | 6 | 137 | Phage baseplate assembly protein V | Phage baseplate assembly protein V | | afdb-uniprot50 | AF-A0A1S2FCU4-F1-MODEL\_V4 | 1.0 | 5.195e-08 | 265 | 0.186 | 198 | 120 | 9 | 1 | 195 | 1 | 160 | Uncharacterized protein | Uncharacterized protein | | afdb-uniprot50 | AF-A0A6G7AI25-F1-MODEL\_V4 | 1.0 | 1.913e-09 | 264 | 0.245 | 224 | 112 | 7 | 14 | 217 | 11 | 197 | Phage baseplate assembly protein V | Phage baseplate assembly protein V | | afdb-uniprot50 | AF-A0A3A9CSA3-F1-MODEL\_V4 | 1.0 | 2.406e-07 | 262 | 0.177 | 192 | 136 | 8 | 14 | 202 | 3 | 175 | Uncharacterized protein | Uncharacterized protein | | afdb-uniprot50 | AF-A0A826UHE7-F1-MODEL\_V4 | 1.0 | 7.424e-09 | 261 | 0.337 | 169 | 91 | 4 | 49 | 217 | 1 | 148 | Phage baseplate assembly protein V | Phage baseplate assembly protein V | | afdb-uniprot50 | AF-U2F4C9-F1-MODEL\_V4 | 1.0 | 2.552e-07 | 260 | 0.258 | 186 | 116 | 8 | 40 | 209 | 3 | 182 | Baseplate assembly protein V, putative | Baseplate assembly protein V, putative | | afdb-uniprot50 | AF-A0A2J9HGD4-F1-MODEL\_V4 | 1.0 | 6.22e-09 | 258 | 0.32 | 184 | 109 | 3 | 48 | 215 | 7 | 190 | Uncharacterized protein | Uncharacterized protein | | afdb-uniprot50 | AF-A0A2A2HCZ4-F1-MODEL\_V4 | 1.0 | 5.178e-07 | 256 | 0.282 | 156 | 101 | 4 | 49 | 196 | 1 | 153 | Baseplate protein | Baseplate protein | | afdb-uniprot50 | AF-A0A353GTI7-F1-MODEL\_V4 | 1.0 | 1.262e-08 | 256 | 0.206 | 213 | 125 | 8 | 1 | 196 | 23 | 208 | Uncharacterized protein | Uncharacterized protein | | afdb-uniprot50 | AF-A0A1H4BQX8-F1-MODEL\_V4 | 1.0 | 5.884e-10 | 254 | 0.319 | 194 | 105 | 5 | 49 | 217 | 2 | 193 | Phage baseplate assembly protein V | Phage baseplate assembly protein V | | afdb-uniprot50 | AF-A0A133NE44-F1-MODEL\_V4 | 1.0 | 5.474e-06 | 251 | 0.163 | 183 | 136 | 8 | 16 | 195 | 7 | 175 | Uncharacterized protein | Uncharacterized protein | | afdb-uniprot50 | AF-A0A1M6IZQ5-F1-MODEL\_V4 | 1.0 | 5.492e-07 | 247 | 0.15 | 179 | 143 | 5 | 5 | 182 | 6 | 176 | Bacteriophage Mu Gp45 protein | Bacteriophage Mu Gp45 protein | | afdb-uniprot50 | AF-A0A367FZE3-F1-MODEL\_V4 | 1.0 | 1.415e-07 | 242 | 0.201 | 169 | 122 | 4 | 15 | 179 | 4 | 163 | Uncharacterized protein | Uncharacterized protein | | afdb-uniprot50 | AF-A0A6C8C1W7-F1-MODEL\_V4 | 1.0 | 1.786e-06 | 239 | 0.314 | 127 | 76 | 4 | 40 | 158 | 4 | 127 | Phage baseplate assembly protein V | Phage baseplate assembly protein V | | afdb-uniprot50 | AF-A0A198XEV1-F1-MODEL\_V4 | 1.0 | 2.707e-07 | 234 | 0.235 | 178 | 100 | 6 | 40 | 216 | 3 | 145 | Baseplate assembly protein V | Baseplate assembly protein V | | afdb-uniprot50 | AF-A0A2S9WYZ5-F1-MODEL\_V4 | 1.0 | 9.398e-09 | 233 | 0.191 | 224 | 140 | 9 | 16 | 217 | 3 | 207 | Phage\_base\_V domain-containing protein | Phage\_base\_V domain-containing protein | | afdb-uniprot50 | AF-R0KQF0-F1-MODEL\_V4 | 1.0 | 2.852e-05 | 231 | 0.32 | 128 | 82 | 3 | 67 | 194 | 2 | 124 | Baseplate assembly protein V | Baseplate assembly protein V | | afdb-uniprot50 | AF-A0A4P0TM83-F1-MODEL\_V4 | 1.0 | 2.871e-07 | 231 | 0.244 | 180 | 102 | 5 | 32 | 203 | 2 | 155 | Putative baseplate assembly protein V | Putative baseplate assembly protein V | | afdb-uniprot50 | AF-A0A064AML4-F1-MODEL\_V4 | 1.0 | 1.587e-06 | 224 | 0.189 | 179 | 117 | 7 | 19 | 190 | 2 | 159 | Phage baseplate protein | Phage baseplate protein | | afdb-uniprot50 | AF-A0A376FH76-F1-MODEL\_V4 | 1.0 | 4.572e-05 | 223 | 0.317 | 126 | 81 | 4 | 67 | 192 | 3 | 123 | Phage Baseplate Assembly protein V | Phage Baseplate Assembly protein V | | afdb-uniprot50 | AF-D0LVW0-F1-MODEL\_V4 | 1.0 | 1.901e-07 | 223 | 0.179 | 217 | 154 | 10 | 4 | 203 | 7 | 216 | Phage\_base\_V domain-containing protein | Phage\_base\_V domain-containing protein | | afdb-uniprot50 | AF-A0A3N2DJS8-F1-MODEL\_V4 | 1.0 | 4.338e-07 | 223 | 0.197 | 218 | 155 | 10 | 16 | 215 | 377 | 592 | Type VI secretion system secreted protein VgrG | Type VI secretion system secreted protein VgrG | | afdb-uniprot50 | AF-A0A210A7I4-F1-MODEL\_V4 | 1.0 | 2.268e-07 | 221 | 0.337 | 163 | 87 | 4 | 55 | 217 | 1 | 142 | Baseplate assembly protein | Baseplate assembly protein | | afdb-uniprot50 | AF-A0A2X3JT57-F1-MODEL\_V4 | 1.0 | 8.831e-08 | 220 | 0.323 | 170 | 94 | 4 | 48 | 217 | 20 | 168 | Baseplate assembly protein V | Baseplate assembly protein V | | afdb-uniprot50 | AF-A0A828EP35-F1-MODEL\_V4 | 1.0 | 2.016e-07 | 218 | 0.327 | 162 | 88 | 4 | 56 | 217 | 1 | 141 | Phage baseplate assembly protein V | Phage baseplate assembly protein V | | afdb-uniprot50 | AF-A0A1Z5H6B0-F1-MODEL\_V4 | 1.0 | 1.496e-06 | 218 | 0.217 | 175 | 94 | 4 | 48 | 216 | 4 | 141 | Phage\_base\_V domain-containing protein | Phage\_base\_V domain-containing protein | | afdb-uniprot50 | AF-A0A6F9JMD1-F1-MODEL\_V4 | 1.0 | 3.856e-07 | 214 | 0.26 | 173 | 112 | 6 | 45 | 203 | 2 | 172 | Phage baseplate assembly protein V | Phage baseplate assembly protein V | | afdb-uniprot50 | AF-A0A847TFE5-F1-MODEL\_V4 | 1.0 | 3.231e-07 | 213 | 0.176 | 238 | 160 | 8 | 16 | 217 | 365 | 602 | Uncharacterized protein | Uncharacterized protein | | afdb-uniprot50 | AF-A0A4U0Z1D4-F1-MODEL\_V4 | 1.0 | 3.046e-07 | 212 | 0.23 | 217 | 100 | 7 | 1 | 217 | 146 | 295 | Phage baseplate assembly protein V | Phage baseplate assembly protein V | | afdb-uniprot50 | AF-Q1H2H9-F1-MODEL\_V4 | 1.0 | 3.856e-07 | 212 | 0.148 | 222 | 126 | 8 | 15 | 193 | 344 | 545 | Rhs element Vgr protein | Rhs element Vgr protein | | afdb-uniprot50 | AF-A0A545SPM8-F1-MODEL\_V4 | 1.0 | 1.182e-06 | 212 | 0.156 | 243 | 164 | 12 | 16 | 217 | 374 | 616 | Type VI secretion system tip protein VgrG | Type VI secretion system tip protein VgrG | | afdb-uniprot50 | AF-A0A2Z5T171-F1-MODEL\_V4 | 1.0 | 3.869e-08 | 210 | 0.212 | 250 | 142 | 12 | 1 | 197 | 2 | 249 | Uncharacterized protein | Uncharacterized protein | | afdb-uniprot50 | AF-A0A350LX97-F1-MODEL\_V4 | 1.0 | 5.492e-07 | 208 | 0.212 | 216 | 98 | 6 | 2 | 217 | 203 | 346 | Phage\_base\_V domain-containing protein | Phage\_base\_V domain-containing protein | | afdb-uniprot50 | AF-A0A736MH22-F1-MODEL\_V4 | 1.0 | 4.338e-07 | 207 | 0.333 | 156 | 83 | 4 | 58 | 213 | 1 | 135 | Phage baseplate assembly protein V | Phage baseplate assembly protein V | | afdb-uniprot50 | AF-A0A2K9LLB3-F1-MODEL\_V4 | 1.0 | 1.33e-06 | 206 | 0.171 | 233 | 162 | 13 | 16 | 217 | 375 | 607 | Phage\_base\_V domain-containing protein | Phage\_base\_V domain-containing protein | | afdb-uniprot50 | AF-G6XKW3-F1-MODEL\_V4 | 1.0 | 1.411e-06 | 205 | 0.171 | 222 | 140 | 8 | 2 | 217 | 1 | 184 | Uncharacterized protein | Uncharacterized protein | | afdb-uniprot50 | AF-A0A358LCR7-F1-MODEL\_V4 | 1.0 | 1.689e-07 | 202 | 0.196 | 250 | 158 | 12 | 1 | 211 | 1 | 246 | Baseplate assembly protein | Baseplate assembly protein | | afdb-uniprot50 | AF-A5L163-F1-MODEL\_V4 | 1.0 | 3.036e-06 | 201 | 0.147 | 231 | 154 | 10 | 16 | 217 | 266 | 482 | Rhs element Vgr protein:Gp5-like protein | Rhs element Vgr protein:Gp5-like protein | | afdb-uniprot50 | AF-A0A376X398-F1-MODEL\_V4 | 1.0 | 9.306e-06 | 197 | 0.251 | 171 | 106 | 5 | 48 | 209 | 4 | 161 | Phage baseplate assembly protein V | Phage baseplate assembly protein V | | afdb-uniprot50 | AF-A0A6P1T758-F1-MODEL\_V4 | 1.0 | 2.268e-07 | 197 | 0.168 | 219 | 147 | 11 | 16 | 213 | 374 | 578 | Type VI secretion system tip protein VgrG | Type VI secretion system tip protein VgrG | | afdb-uniprot50 | AF-A0A1H7LDT2-F1-MODEL\_V4 | 1.0 | 5.178e-07 | 197 | 0.15 | 252 | 164 | 11 | 16 | 217 | 366 | 617 | Uncharacterized conserved protein, implicated in type VI secretion and phage assembly | Uncharacterized conserved protein, implicated in type VI secretion and phage assembly | | afdb-uniprot50 | AF-A0A847KZN7-F1-MODEL\_V4 | 1.0 | 7.351e-06 | 196 | 0.134 | 223 | 147 | 10 | 2 | 217 | 1 | 184 | Uncharacterized protein | Uncharacterized protein | | afdb-uniprot50 | AF-A0A150RNN9-F1-MODEL\_V4 | 1.0 | 1.406e-05 | 195 | 0.17 | 246 | 160 | 12 | 15 | 217 | 377 | 621 | Uncharacterized protein | Uncharacterized protein | | afdb-uniprot50 | AF-A0A1Y1RHY7-F1-MODEL\_V4 | 1.0 | 1.047e-05 | 194 | 0.132 | 227 | 170 | 9 | 14 | 217 | 372 | 594 | Uncharacterized protein | Uncharacterized protein | | afdb-uniprot50 | AF-A0A2R7QZ58-F1-MODEL\_V4 | 1.0 | 5.144e-05 | 193 | 0.24 | 154 | 95 | 6 | 51 | 203 | 6 | 138 | Phage baseplate assembly protein V | Phage baseplate assembly protein V | | afdb-uniprot50 | AF-A0A4Q7HXN1-F1-MODEL\_V4 | 1.0 | 1.491e-05 | 193 | 0.149 | 247 | 171 | 13 | 1 | 217 | 1 | 238 | Uncharacterized protein | Uncharacterized protein | | afdb-uniprot50 | AF-A0A2D5EKJ4-F1-MODEL\_V4 | 1.0 | 1.047e-05 | 193 | 0.147 | 258 | 163 | 12 | 16 | 217 | 24 | 280 | Type VI secretion system tip protein VgrG | Type VI secretion system tip protein VgrG | | afdb-uniprot50 | AF-A0A2L0ESL0-F1-MODEL\_V4 | 1.0 | 1.491e-05 | 192 | 0.16 | 237 | 163 | 12 | 15 | 216 | 422 | 657 | Uncharacterized protein | Uncharacterized protein | | afdb-uniprot50 | AF-A0A5M9RDN8-F1-MODEL\_V4 | 1.0 | 6.533e-06 | 191 | 0.146 | 218 | 158 | 6 | 14 | 207 | 352 | 565 | Type VI secretion system tip protein VgrG | Type VI secretion system tip protein VgrG | | afdb-uniprot50 | AF-A0A510UF59-F1-MODEL\_V4 | 1.0 | 3.209e-05 | 190 | 0.12 | 248 | 179 | 11 | 1 | 215 | 1 | 242 | Uncharacterized protein | Uncharacterized protein | | afdb-uniprot50 | AF-A0A1D7W984-F1-MODEL\_V4 | 1.0 | 1.111e-05 | 188 | 0.117 | 229 | 171 | 10 | 15 | 217 | 320 | 543 | Uncharacterized protein | Uncharacterized protein | | afdb-uniprot50 | AF-A0A3A6UBA1-F1-MODEL\_V4 | 1.0 | 1.684e-06 | 188 | 0.175 | 256 | 157 | 12 | 16 | 217 | 362 | 617 | Type VI secretion system tip protein VgrG | Type VI secretion system tip protein VgrG | | afdb-uniprot50 | AF-A0A1C3EDR2-F1-MODEL\_V4 | 1.0 | 8.773e-06 | 187 | 0.174 | 223 | 147 | 10 | 16 | 209 | 362 | 576 | Type IV secretion protein Rhs | Type IV secretion protein Rhs | | afdb-uniprot50 | AF-A0A7H0GLJ8-F1-MODEL\_V4 | 1.0 | 4.063e-05 | 186 | 0.176 | 238 | 160 | 11 | 16 | 217 | 426 | 663 | Type VI secretion system tip protein VgrG | Type VI secretion system tip protein VgrG | | afdb-uniprot50 | AF-A0A1Y1R6F0-F1-MODEL\_V4 | 1.0 | 1.25e-05 | 183 | 0.15 | 226 | 167 | 6 | 16 | 217 | 368 | 592 | Phage\_base\_V domain-containing protein | Phage\_base\_V domain-containing protein | | afdb-uniprot50 | AF-A0A607GYE7-F1-MODEL\_V4 | 1.0 | 9.871e-06 | 182 | 0.262 | 175 | 106 | 5 | 40 | 207 | 3 | 161 | Phage baseplate assembly protein V | Phage baseplate assembly protein V | | afdb-uniprot50 | AF-A0A5C6RYB4-F1-MODEL\_V4 | 1.0 | 2.852e-05 | 182 | 0.139 | 223 | 165 | 10 | 16 | 213 | 59 | 279 | Type VI secretion system tip protein VgrG | Type VI secretion system tip protein VgrG | | afdb-uniprot50 | AF-A0A7U7EJN7-F1-MODEL\_V4 | 1.0 | 1.178e-05 | 181 | 0.139 | 236 | 168 | 11 | 16 | 217 | 22 | 256 | Actin cross-linking toxin VgrG1 | Actin cross-linking toxin VgrG1 | | afdb-uniprot50 | AF-A0A3L8C1Y2-F1-MODEL\_V4 | 1.0 | 1.491e-05 | 180 | 0.114 | 228 | 175 | 10 | 16 | 217 | 339 | 565 | Type VI secretion system tip protein VgrG | Type VI secretion system tip protein VgrG | | afdb-uniprot50 | AF-A0A4P2QEK0-F1-MODEL\_V4 | 1.0 | 1.78e-05 | 180 | 0.142 | 232 | 165 | 10 | 15 | 216 | 373 | 600 | Uncharacterized protein | Uncharacterized protein | | afdb-uniprot50 | AF-A0A4U9UU72-F1-MODEL\_V4 | 1.0 | 3.83e-05 | 179 | 0.115 | 233 | 169 | 9 | 16 | 217 | 123 | 349 | Uncharacterized protein conserved in bacteria | Uncharacterized protein conserved in bacteria | | afdb-uniprot50 | AF-A0A448MIP6-F1-MODEL\_V4 | 1.0 | 4.063e-05 | 178 | 0.131 | 244 | 169 | 9 | 16 | 217 | 179 | 421 | Uncharacterized protein conserved in bacteria | Uncharacterized protein conserved in bacteria | | afdb-uniprot50 | AF-A0A509DVS8-F1-MODEL\_V4 | 1.0 | 4.063e-05 | 178 | 0.157 | 228 | 161 | 10 | 16 | 217 | 371 | 593 | Actin cross-linking toxin VgrG1 | Actin cross-linking toxin VgrG1 | | afdb-uniprot50 | AF-A0A3S4W3R1-F1-MODEL\_V4 | 1.0 | 3.026e-05 | 177 | 0.131 | 244 | 169 | 9 | 16 | 217 | 65 | 307 | Uncharacterized protein conserved in bacteria | Uncharacterized protein conserved in bacteria | | afdb-uniprot50 | AF-A0A7Y5KHH1-F1-MODEL\_V4 | 1.0 | 3.611e-05 | 177 | 0.156 | 230 | 166 | 9 | 16 | 217 | 97 | 326 | Uncharacterized protein | Uncharacterized protein | | afdb-uniprot50 | AF-A0A1N7GD70-F1-MODEL\_V4 | 1.0 | 3.83e-05 | 177 | 0.131 | 229 | 163 | 11 | 16 | 216 | 363 | 583 | Type VI secretion system secreted protein VgrG | Type VI secretion system secreted protein VgrG | | afdb-uniprot50 | AF-A0A3B0ZXQ5-F1-MODEL\_V4 | 1.0 | 2.689e-05 | 177 | 0.129 | 239 | 164 | 12 | 16 | 215 | 364 | 597 | VgrG protein | VgrG protein | | afdb-uniprot50 | AF-A0A368X9H0-F1-MODEL\_V4 | 1.0 | 5.144e-05 | 176 | 0.151 | 258 | 162 | 12 | 16 | 217 | 4 | 260 | Type VI secretion system secreted protein VgrG | Type VI secretion system secreted protein VgrG | | afdb-uniprot50 | AF-A0A2N1IN93-F1-MODEL\_V4 | 1.0 | 1.888e-05 | 176 | 0.144 | 236 | 167 | 10 | 16 | 217 | 77 | 311 | Type VI secretion system tip protein VgrG | Type VI secretion system tip protein VgrG | | afdb-uniprot50 | AF-A0A509MIG7-F1-MODEL\_V4 | 1.0 | 2.689e-05 | 175 | 0.147 | 223 | 159 | 10 | 16 | 210 | 167 | 386 | Type VI secretion system tip protein VgrG | Type VI secretion system tip protein VgrG | | afdb-uniprot50 | AF-A0A6M0CR85-F1-MODEL\_V4 | 1.0 | 4.849e-05 | 175 | 0.126 | 229 | 171 | 9 | 16 | 217 | 201 | 427 | Type VI secretion system tip protein VgrG | Type VI secretion system tip protein VgrG | | afdb-uniprot50 | AF-A0A3A0D0H8-F1-MODEL\_V4 | 1.0 | 6.139e-05 | 174 | 0.147 | 231 | 167 | 8 | 16 | 217 | 374 | 603 | Uncharacterized protein | Uncharacterized protein | | afdb-uniprot50 | AF-A0A1B8YCK0-F1-MODEL\_V4 | 1.0 | 2.253e-05 | 173 | 0.126 | 229 | 163 | 13 | 16 | 217 | 57 | 275 | Phage-related baseplate assembly protein | Phage-related baseplate assembly protein | | afdb-uniprot50 | AF-A0A7X4WP42-F1-MODEL\_V4 | 1.0 | 5.787e-05 | 173 | 0.142 | 231 | 165 | 10 | 16 | 217 | 61 | 287 | Type VI secretion system tip protein VgrG | Type VI secretion system tip protein VgrG | | afdb-uniprot50 | AF-A0A167ADJ3-F1-MODEL\_V4 | 1.0 | 2.535e-05 | 173 | 0.148 | 236 | 165 | 11 | 14 | 217 | 663 | 894 | Uncharacterized protein | Uncharacterized protein | | afdb-uniprot50 | AF-A0A1I6HDB9-F1-MODEL\_V4 | 1.0 | 3.026e-05 | 172 | 0.13 | 230 | 171 | 10 | 16 | 217 | 378 | 606 | Type VI secretion system secreted protein VgrG | Type VI secretion system secreted protein VgrG | | afdb-uniprot50 | AF-A0A0F7PK02-F1-MODEL\_V4 | 1.0 | 3.209e-05 | 171 | 0.155 | 232 | 165 | 12 | 16 | 217 | 12 | 242 | Rhs element Vgr protein | Rhs element Vgr protein | | afdb-uniprot50 | AF-A0A0F7LRQ6-F1-MODEL\_V4 | 1.0 | 1.582e-05 | 171 | 0.12 | 225 | 162 | 13 | 16 | 213 | 283 | 498 | Vrg family protein | Vrg family protein | | afdb-uniprot50 | AF-A0A178KKM7-F1-MODEL\_V4 | 1.0 | 8.773e-06 | 171 | 0.157 | 267 | 156 | 13 | 16 | 217 | 368 | 630 | Type IV secretion protein Rhs | Type IV secretion protein Rhs | | afdb-uniprot50 | AF-A0A1B1A454-F1-MODEL\_V4 | 1.0 | 5.144e-05 | 171 | 0.145 | 241 | 166 | 12 | 16 | 217 | 374 | 613 | Type IV secretion protein Rhs | Type IV secretion protein Rhs | | afdb-uniprot50 | AF-A0A6B2IKF0-F1-MODEL\_V4 | 1.0 | 0.0001044 | 171 | 0.144 | 236 | 167 | 12 | 16 | 217 | 376 | 610 | Type VI secretion system tip protein VgrG | Type VI secretion system tip protein VgrG | | afdb-uniprot50 | AF-A0A5P8P3V2-F1-MODEL\_V4 | 1.0 | 8.243e-05 | 171 | 0.12 | 233 | 176 | 8 | 14 | 217 | 379 | 611 | Type VI secretion system tip protein VgrG | Type VI secretion system tip protein VgrG | | afdb-uniprot50 | AF-A0A4U1H893-F1-MODEL\_V4 | 1.0 | 7.771e-05 | 171 | 0.152 | 236 | 156 | 13 | 16 | 216 | 381 | 607 | Type VI secretion system tip protein VgrG | Type VI secretion system tip protein VgrG | | afdb-uniprot50 | AF-A0A763CHS0-F1-MODEL\_V4 | 1.0 | 3.611e-05 | 170 | 0.148 | 250 | 163 | 13 | 1 | 217 | 93 | 325 | Uncharacterized protein | Uncharacterized protein | | afdb-uniprot50 | AF-A0A848EAM8-F1-MODEL\_V4 | 1.0 | 1.582e-05 | 170 | 0.164 | 225 | 161 | 11 | 16 | 217 | 388 | 608 | Type VI secretion system tip protein VgrG | Type VI secretion system tip protein VgrG | | afdb-uniprot50 | AF-A0A128EUM3-F1-MODEL\_V4 | 1.0 | 3.611e-05 | 169 | 0.157 | 222 | 156 | 7 | 16 | 210 | 362 | 579 | Phage-related baseplate assembly protein | Phage-related baseplate assembly protein | | afdb-uniprot50 | AF-A0A521TV16-F1-MODEL\_V4 | 1.0 | 9.306e-06 | 168 | 0.138 | 231 | 155 | 11 | 14 | 217 | 387 | 600 | Type VI secretion system tip protein VgrG | Type VI secretion system tip protein VgrG | | afdb-uniprot50 | AF-A0A561C8P1-F1-MODEL\_V4 | 1.0 | 6.512e-05 | 168 | 0.165 | 229 | 159 | 10 | 16 | 217 | 417 | 640 | Type VI secretion system secreted protein VgrG | Type VI secretion system secreted protein VgrG | | afdb-uniprot50 | AF-A0A4U1IUQ7-F1-MODEL\_V4 | 1.0 | 3.22e-06 | 168 | 0.165 | 260 | 157 | 11 | 15 | 217 | 467 | 723 | Type VI secretion system tip protein VgrG | Type VI secretion system tip protein VgrG | | afdb-uniprot50 | AF-A0A655SXW0-F1-MODEL\_V4 | 1.0 | 9.306e-06 | 167 | 0.126 | 246 | 171 | 12 | 1 | 208 | 10 | 249 | Uncharacterized protein | Uncharacterized protein | | afdb-uniprot50 | AF-U7NGG0-F1-MODEL\_V4 | 1.0 | 4.324e-06 | 167 | 0.178 | 230 | 142 | 12 | 16 | 217 | 52 | 262 | Phage\_base\_V domain-containing protein | Phage\_base\_V domain-containing protein | | afdb-uniprot50 | AF-A0A2S8Q0N4-F1-MODEL\_V4 | 1.0 | 4.849e-05 | 167 | 0.122 | 229 | 164 | 13 | 16 | 217 | 229 | 447 | Type VI secretion system tip protein VgrG | Type VI secretion system tip protein VgrG | | afdb-uniprot50 | AF-A0A017T821-F1-MODEL\_V4 | 1.0 | 4.849e-05 | 167 | 0.128 | 241 | 167 | 11 | 16 | 217 | 402 | 638 | VgrG protein | VgrG protein | | afdb-uniprot50 | AF-A0A2E4NX28-F1-MODEL\_V4 | 1.0 | 1.111e-05 | 166 | 0.154 | 226 | 153 | 12 | 16 | 213 | 5 | 220 | Type VI secretion system tip protein VgrG | Type VI secretion system tip protein VgrG | | afdb-uniprot50 | AF-A0A366AC90-F1-MODEL\_V4 | 1.0 | 3.404e-05 | 166 | 0.142 | 225 | 157 | 11 | 16 | 213 | 76 | 291 | Type VI secretion system tip protein VgrG | Type VI secretion system tip protein VgrG | | afdb-uniprot50 | AF-A0A0E4FZN6-F1-MODEL\_V4 | 1.0 | 6.953e-07 | 166 | 0.188 | 228 | 146 | 8 | 9 | 214 | 2 | 212 | Uncharacterized protein | Uncharacterized protein | | afdb-uniprot50 | AF-A0A291HKX6-F1-MODEL\_V4 | 1.0 | 8.744e-05 | 166 | 0.12 | 248 | 179 | 11 | 1 | 217 | 230 | 469 | Uncharacterized protein | Uncharacterized protein | | afdb-uniprot50 | AF-A0A2G6PY94-F1-MODEL\_V4 | 1.0 | 4.849e-05 | 166 | 0.122 | 220 | 166 | 9 | 16 | 213 | 368 | 582 | Phage\_base\_V domain-containing protein | Phage\_base\_V domain-containing protein | | afdb-uniprot50 | AF-A0A557PFK3-F1-MODEL\_V4 | 1.0 | 3.611e-05 | 166 | 0.15 | 232 | 155 | 12 | 16 | 217 | 375 | 594 | Type VI secretion system tip protein VgrG | Type VI secretion system tip protein VgrG | | afdb-uniprot50 | AF-A0A7Y5FUS3-F1-MODEL\_V4 | 1.0 | 0.0001486 | 165 | 0.116 | 231 | 174 | 8 | 16 | 217 | 246 | 475 | Type VI secretion system tip protein VgrG | Type VI secretion system tip protein VgrG | | afdb-uniprot50 | AF-A0A0N0IAF2-F1-MODEL\_V4 | 1.0 | 8.744e-05 | 165 | 0.139 | 223 | 161 | 9 | 16 | 210 | 357 | 576 | VgrG family protein | VgrG family protein | | afdb-uniprot50 | AF-A0A2X1VG03-F1-MODEL\_V4 | 1.0 | 9.275e-05 | 165 | 0.142 | 239 | 164 | 11 | 16 | 217 | 368 | 602 | Uncharacterized protein conserved in bacteria | Uncharacterized protein conserved in bacteria | | afdb-uniprot50 | AF-A0A844CPB5-F1-MODEL\_V4 | 1.0 | 5.787e-05 | 165 | 0.144 | 243 | 163 | 12 | 16 | 217 | 406 | 644 | Type VI secretion system tip protein VgrG | Type VI secretion system tip protein VgrG | | afdb-uniprot50 | AF-A0A2P1VB00-F1-MODEL\_V4 | 1.0 | 6.512e-05 | 165 | 0.171 | 228 | 159 | 10 | 16 | 217 | 416 | 639 | Type VI secretion system tip protein VgrG | Type VI secretion system tip protein VgrG | | afdb-uniprot50 | AF-U7HMH7-F1-MODEL\_V4 | 1.0 | 2.253e-05 | 164 | 0.147 | 258 | 163 | 9 | 16 | 217 | 55 | 311 | Phage\_base\_V domain-containing protein | Phage\_base\_V domain-containing protein | | afdb-uniprot50 | AF-A0A1C4BKP2-F1-MODEL\_V4 | 1.0 | 0.0001486 | 164 | 0.13 | 223 | 163 | 10 | 16 | 210 | 16 | 235 | Rhs element Vgr protein | Rhs element Vgr protein | | afdb-uniprot50 | AF-A0A6N7B6D2-F1-MODEL\_V4 | 1.0 | 6.93e-06 | 163 | 0.227 | 229 | 134 | 11 | 29 | 217 | 7 | 232 | Phage baseplate | Phage baseplate | | afdb-uniprot50 | AF-A0A349J5T5-F1-MODEL\_V4 | 1.0 | 1.178e-05 | 163 | 0.155 | 232 | 165 | 8 | 16 | 217 | 9 | 239 | Phage\_base\_V domain-containing protein | Phage\_base\_V domain-containing protein | | afdb-uniprot50 | AF-Q49TP6-F1-MODEL\_V4 | 1.0 | 2.852e-05 | 163 | 0.134 | 230 | 154 | 11 | 16 | 210 | 42 | 261 | VgrG | VgrG | | afdb-uniprot50 | AF-A0A2D5EBA9-F1-MODEL\_V4 | 1.0 | 1.111e-05 | 163 | 0.175 | 233 | 139 | 12 | 16 | 217 | 39 | 249 | Lysozyme | Lysozyme | | afdb-uniprot50 | AF-A0A7W1IND0-F1-MODEL\_V4 | 1.0 | 0.0001577 | 163 | 0.12 | 224 | 169 | 10 | 16 | 217 | 371 | 588 | Type VI secretion system tip protein VgrG | Type VI secretion system tip protein VgrG | | afdb-uniprot50 | AF-A0A7Y3ZDT9-F1-MODEL\_V4 | 1.0 | 6.512e-05 | 162 | 0.127 | 228 | 170 | 10 | 16 | 217 | 3 | 227 | Type VI secretion system tip protein VgrG | Type VI secretion system tip protein VgrG | | afdb-uniprot50 | AF-A0A2T1A7F3-F1-MODEL\_V4 | 1.0 | 1.25e-05 | 162 | 0.147 | 223 | 159 | 11 | 16 | 217 | 257 | 469 | Type VI secretion system secreted protein VgrG | Type VI secretion system secreted protein VgrG | | afdb-uniprot50 | AF-A0A2G6HUW3-F1-MODEL\_V4 | 1.0 | 0.000268 | 162 | 0.166 | 228 | 160 | 10 | 16 | 217 | 369 | 592 | Phage\_base\_V domain-containing protein | Phage\_base\_V domain-containing protein | | afdb-uniprot50 | AF-A0A3N9U6C8-F1-MODEL\_V4 | 1.0 | 7.326e-05 | 161 | 0.151 | 225 | 155 | 11 | 16 | 213 | 376 | 591 | Type VI secretion system tip protein VgrG | Type VI secretion system tip protein VgrG | | afdb-uniprot50 | AF-A0A4Q6DCT9-F1-MODEL\_V4 | 1.0 | 7.771e-05 | 160 | 0.181 | 231 | 156 | 10 | 16 | 217 | 64 | 290 | Type VI secretion system tip protein VgrG | Type VI secretion system tip protein VgrG | | afdb-uniprot50 | AF-A0A5C7CBJ7-F1-MODEL\_V4 | 1.0 | 0.0002843 | 160 | 0.116 | 250 | 184 | 12 | 1 | 217 | 237 | 482 | Uncharacterized protein | Uncharacterized protein | | afdb-uniprot50 | AF-A0A5E6WXA5-F1-MODEL\_V4 | 1.0 | 8.243e-05 | 160 | 0.139 | 237 | 168 | 10 | 16 | 217 | 266 | 501 | Actin cross-linking toxin VgrG1 | Actin cross-linking toxin VgrG1 | | afdb-uniprot50 | AF-A0A5J6LH20-F1-MODEL\_V4 | 1.0 | 2.689e-05 | 160 | 0.152 | 256 | 162 | 13 | 16 | 217 | 375 | 629 | Type VI secretion system tip protein VgrG | Type VI secretion system tip protein VgrG | | afdb-uniprot50 | AF-A0A2S5FHP1-F1-MODEL\_V4 | 1.0 | 1.78e-05 | 159 | 0.137 | 226 | 160 | 11 | 16 | 217 | 220 | 434 | Type VI secretion system tip protein VgrG | Type VI secretion system tip protein VgrG | | afdb-uniprot50 | AF-F8G2Q8-F1-MODEL\_V4 | 1.0 | 0.0003393 | 159 | 0.143 | 237 | 164 | 12 | 16 | 217 | 334 | 566 | Type VI secretion system Vgr family protein | Type VI secretion system Vgr family protein | | afdb-uniprot50 | AF-A0A1D2X8R5-F1-MODEL\_V4 | 1.0 | 0.0002382 | 159 | 0.152 | 242 | 158 | 14 | 16 | 217 | 372 | 606 | Phage\_base\_V domain-containing protein | Phage\_base\_V domain-containing protein | | afdb-uniprot50 | AF-A0A1I5SLG1-F1-MODEL\_V4 | 1.0 | 2.689e-05 | 158 | 0.14 | 228 | 161 | 11 | 16 | 217 | 110 | 328 | Rhs element Vgr protein | Rhs element Vgr protein | | afdb-uniprot50 | AF-A0A175VFY3-F1-MODEL\_V4 | 1.0 | 1.325e-05 | 158 | 0.146 | 226 | 158 | 11 | 16 | 217 | 156 | 370 | Type IV secretion protein Rhs | Type IV secretion protein Rhs | | afdb-uniprot50 | AF-A0A379DP27-F1-MODEL\_V4 | 1.0 | 1.582e-05 | 158 | 0.127 | 244 | 170 | 11 | 16 | 217 | 358 | 600 | Uncharacterized protein conserved in bacteria | Uncharacterized protein conserved in bacteria | | afdb-uniprot50 | AF-A0A5C6S553-F1-MODEL\_V4 | 1.0 | 2.535e-05 | 157 | 0.173 | 230 | 156 | 11 | 16 | 217 | 102 | 325 | Type VI secretion system tip protein VgrG | Type VI secretion system tip protein VgrG | | afdb-uniprot50 | AF-A0A6N7YBH3-F1-MODEL\_V4 | 1.0 | 9.871e-06 | 157 | 0.192 | 234 | 153 | 11 | 2 | 217 | 347 | 562 | VgrG-related protein | VgrG-related protein | | afdb-uniprot50 | AF-A0A5S9QB60-F1-MODEL\_V4 | 1.0 | 3.611e-05 | 157 | 0.162 | 227 | 155 | 9 | 16 | 217 | 367 | 583 | Actin cross-linking toxin VgrG1 | Actin cross-linking toxin VgrG1 | | afdb-uniprot50 | AF-U7QPZ3-F1-MODEL\_V4 | 1.0 | 0.0001577 | 156 | 0.1 | 218 | 170 | 8 | 16 | 209 | 39 | 254 | Phage\_base\_V domain-containing protein | Phage\_base\_V domain-containing protein | | afdb-uniprot50 | AF-A0A0X8XCU6-F1-MODEL\_V4 | 1.0 | 8.744e-05 | 156 | 0.151 | 231 | 161 | 12 | 16 | 217 | 403 | 627 | VgrG protein | VgrG protein | | afdb-uniprot50 | AF-R1INV5-F1-MODEL\_V4 | 1.0 | 2.852e-05 | 155 | 0.135 | 228 | 162 | 11 | 16 | 217 | 122 | 340 | VgrG protein | VgrG protein | | afdb-uniprot50 | AF-D0GK50-F1-MODEL\_V4 | 1.0 | 0.0001486 | 155 | 0.124 | 217 | 151 | 8 | 16 | 204 | 291 | 496 | Uncharacterized protein | Uncharacterized protein | | afdb-uniprot50 | AF-A0A379GKP7-F1-MODEL\_V4 | 1.0 | 2.253e-05 | 155 | 0.126 | 261 | 166 | 13 | 16 | 217 | 261 | 518 | Uncharacterized protein conserved in bacteria | Uncharacterized protein conserved in bacteria | | afdb-uniprot50 | AF-A0A447QXA9-F1-MODEL\_V4 | 1.0 | 2.689e-05 | 155 | 0.147 | 264 | 175 | 13 | 1 | 217 | 233 | 493 | Phage protein | Phage protein | | afdb-uniprot50 | AF-A0A1H5U3E3-F1-MODEL\_V4 | 1.0 | 5.144e-05 | 155 | 0.131 | 273 | 165 | 11 | 16 | 217 | 389 | 660 | Rhs element Vgr protein | Rhs element Vgr protein | | afdb-uniprot50 | AF-E6V5I3-F1-MODEL\_V4 | 1.0 | 5.787e-05 | 154 | 0.17 | 252 | 158 | 10 | 16 | 217 | 417 | 667 | Type VI secretion system Vgr family protein | Type VI secretion system Vgr family protein | | afdb-uniprot50 | AF-A0A8B3VQM5-F1-MODEL\_V4 | 1.0 | 0.0001245 | 153 | 0.24 | 166 | 87 | 6 | 53 | 217 | 1 | 128 | Putative phage associated protein | Putative phage associated protein | | afdb-uniprot50 | AF-A0A2S8PXI7-F1-MODEL\_V4 | 1.0 | 6.139e-05 | 153 | 0.114 | 227 | 161 | 11 | 16 | 213 | 286 | 501 | Type VI secretion system tip protein VgrG | Type VI secretion system tip protein VgrG | | afdb-uniprot50 | AF-A0A1E7PYR3-F1-MODEL\_V4 | 1.0 | 3.404e-05 | 152 | 0.153 | 235 | 154 | 13 | 16 | 217 | 129 | 351 | Phage\_base\_V domain-containing protein | Phage\_base\_V domain-containing protein | | afdb-uniprot50 | AF-A0A7H1J8Z0-F1-MODEL\_V4 | 1.0 | 4.572e-05 | 152 | 0.129 | 263 | 167 | 11 | 16 | 217 | 376 | 637 | Type VI secretion system tip protein VgrG | Type VI secretion system tip protein VgrG | | afdb-uniprot50 | AF-A0A1X4N450-F1-MODEL\_V4 | 1.0 | 0.0002843 | 152 | 0.154 | 233 | 165 | 10 | 16 | 217 | 362 | 593 | Phage\_base\_V domain-containing protein | Phage\_base\_V domain-containing protein | | afdb-uniprot50 | AF-M1FL72-F1-MODEL\_V4 | 1.0 | 2.689e-05 | 151 | 0.157 | 241 | 143 | 11 | 16 | 217 | 67 | 286 | Phage\_base\_V domain-containing protein | Phage\_base\_V domain-containing protein | | afdb-uniprot50 | AF-A0A2X1TPE1-F1-MODEL\_V4 | 1.0 | 3.209e-05 | 151 | 0.132 | 227 | 164 | 9 | 16 | 217 | 55 | 273 | Uncharacterized protein conserved in bacteria | Uncharacterized protein conserved in bacteria | | afdb-uniprot50 | AF-A6FCK4-F1-MODEL\_V4 | 1.0 | 5.787e-05 | 150 | 0.094 | 243 | 183 | 9 | 4 | 213 | 13 | 251 | Uncharacterized protein | Uncharacterized protein | | afdb-uniprot50 | AF-A0A624E833-F1-MODEL\_V4 | 1.0 | 0.0001401 | 150 | 0.104 | 248 | 182 | 12 | 1 | 213 | 43 | 285 | Uncharacterized protein | Uncharacterized protein | | afdb-uniprot50 | AF-A0A2C6DHW8-F1-MODEL\_V4 | 1.0 | 4.572e-05 | 150 | 0.107 | 241 | 178 | 10 | 4 | 211 | 13 | 249 | Uncharacterized protein | Uncharacterized protein | | afdb-uniprot50 | AF-A0A1G5PCP2-F1-MODEL\_V4 | 1.0 | 0.0002246 | 150 | 0.108 | 230 | 167 | 11 | 16 | 217 | 2 | 221 | Rhs element Vgr protein | Rhs element Vgr protein | | afdb-uniprot50 | AF-A0A1R4B2C8-F1-MODEL\_V4 | 1.0 | 0.0002117 | 150 | 0.138 | 224 | 159 | 10 | 16 | 213 | 375 | 590 | Phage-related baseplate assembly protein | Phage-related baseplate assembly protein | | afdb-uniprot50 | AF-A0A6L9FHW8-F1-MODEL\_V4 | 1.0 | 0.0001107 | 149 | 0.108 | 249 | 172 | 12 | 1 | 217 | 1 | 231 | Uncharacterized protein | Uncharacterized protein | | afdb-uniprot50 | AF-A0A855LDI3-F1-MODEL\_V4 | 1.0 | 0.0001882 | 149 | 0.129 | 231 | 168 | 9 | 16 | 217 | 13 | 239 | Type VI secretion system tip protein VgrG | Type VI secretion system tip protein VgrG | | afdb-uniprot50 | AF-A0A128FL67-F1-MODEL\_V4 | 1.0 | 6.139e-05 | 149 | 0.136 | 227 | 163 | 10 | 16 | 217 | 274 | 492 | Phage-related baseplate assembly protein | Phage-related baseplate assembly protein | | afdb-uniprot50 | AF-A0A246WKE1-F1-MODEL\_V4 | 1.0 | 9.838e-05 | 149 | 0.142 | 231 | 159 | 8 | 16 | 213 | 398 | 622 | Phage\_base\_V domain-containing protein | Phage\_base\_V domain-containing protein | | afdb-uniprot50 | AF-F3BJN5-F1-MODEL\_V4 | 1.0 | 3.209e-05 | 148 | 0.162 | 246 | 159 | 14 | 4 | 211 | 13 | 249 | Uncharacterized protein | Uncharacterized protein | | afdb-uniprot50 | AF-A0A840G0V0-F1-MODEL\_V4 | 1.0 | 0.0001774 | 147 | 0.165 | 248 | 160 | 12 | 16 | 217 | 416 | 662 | Type VI secretion system secreted protein VgrG | Type VI secretion system secreted protein VgrG | | afdb-uniprot50 | AF-A0A1Z2SEM0-F1-MODEL\_V4 | 1.0 | 4.063e-05 | 147 | 0.154 | 266 | 160 | 12 | 16 | 217 | 374 | 638 | Phage\_base\_V domain-containing protein | Phage\_base\_V domain-containing protein | | afdb-uniprot50 | AF-Q9KN42-F1-MODEL\_V4 | 1.0 | 0.0001774 | 147 | 0.15 | 266 | 158 | 14 | 16 | 217 | 375 | 636 | Type VI secretion system spike protein VgrG3 | Type VI secretion system spike protein VgrG3 | | afdb-uniprot50 | AF-B2PU61-F1-MODEL\_V4 | 1.0 | 0.0004556 | 146 | 0.13 | 238 | 167 | 11 | 16 | 216 | 227 | 461 | Rhs element Vgr protein | Rhs element Vgr protein | | afdb-uniprot50 | AF-A0A158C7T3-F1-MODEL\_V4 | 1.0 | 0.0004296 | 146 | 0.135 | 236 | 169 | 10 | 16 | 217 | 370 | 604 | Rhs element Vgr protein | Rhs element Vgr protein | | afdb-uniprot50 | AF-A0A3G5KE21-F1-MODEL\_V4 | 1.0 | 0.0002117 | 145 | 0.121 | 231 | 165 | 12 | 16 | 217 | 472 | 693 | Type VI secretion system tip protein VgrG | Type VI secretion system tip protein VgrG | | afdb-uniprot50 | AF-A0A161UY31-F1-MODEL\_V4 | 1.0 | 0.0004556 | 143 | 0.144 | 236 | 167 | 11 | 16 | 217 | 381 | 615 | Phage-related baseplate assembly protein | Phage-related baseplate assembly protein | | afdb-uniprot50 | AF-A0A271JCW4-F1-MODEL\_V4 | 1.0 | 0.0009244 | 143 | 0.165 | 236 | 161 | 11 | 16 | 217 | 375 | 608 | Phage\_base\_V domain-containing protein | Phage\_base\_V domain-containing protein | | afdb-uniprot50 | AF-A0A553JLK0-F1-MODEL\_V4 | 1.0 | 0.0001321 | 142 | 0.155 | 232 | 156 | 11 | 16 | 217 | 372 | 593 | Type VI secretion system tip protein VgrG | Type VI secretion system tip protein VgrG | | afdb-uniprot50 | AF-A0A833IVZ1-F1-MODEL\_V4 | 1.0 | 0.0001107 | 142 | 0.147 | 264 | 162 | 13 | 16 | 217 | 368 | 630 | Type VI secretion system tip protein VgrG | Type VI secretion system tip protein VgrG | | afdb-uniprot50 | AF-C5CK96-F1-MODEL\_V4 | 1.0 | 0.0001107 | 142 | 0.142 | 253 | 164 | 11 | 16 | 217 | 417 | 667 | Type VI secretion system Vgr family protein | Type VI secretion system Vgr family protein | | afdb-uniprot50 | AF-A0A7Y3Y1U2-F1-MODEL\_V4 | 1.0 | 0.0001486 | 142 | 0.145 | 268 | 161 | 11 | 14 | 217 | 374 | 637 | Type VI secretion system tip protein VgrG | Type VI secretion system tip protein VgrG | | afdb-uniprot50 | AF-A0A840FVA2-F1-MODEL\_V4 | 1.0 | 3.209e-05 | 141 | 0.292 | 140 | 80 | 3 | 81 | 201 | 9 | 148 | Phage baseplate assembly protein V | Phage baseplate assembly protein V | | afdb-uniprot50 | AF-A0A2W7GN04-F1-MODEL\_V4 | 1.0 | 6.139e-05 | 141 | 0.162 | 252 | 160 | 10 | 16 | 217 | 315 | 565 | Rhs element Vgr protein | Rhs element Vgr protein | | afdb-uniprot50 | AF-N6WVX3-F1-MODEL\_V4 | 1.0 | 0.0001774 | 140 | 0.14 | 263 | 164 | 12 | 16 | 217 | 369 | 630 | Type VI secretion system tip protein VgrG | Type VI secretion system tip protein VgrG | | afdb-uniprot50 | AF-A0A6L9PNP9-F1-MODEL\_V4 | 1.0 | 8.243e-05 | 140 | 0.15 | 252 | 163 | 10 | 16 | 217 | 417 | 667 | Type VI secretion system tip protein VgrG | Type VI secretion system tip protein VgrG | | afdb-uniprot50 | AF-A0A6B3LAR9-F1-MODEL\_V4 | 1.0 | 0.0001321 | 139 | 0.172 | 226 | 152 | 10 | 16 | 217 | 375 | 589 | Type VI secretion system tip protein VgrG | Type VI secretion system tip protein VgrG | | afdb-uniprot50 | AF-A0A0F5V8P1-F1-MODEL\_V4 | 1.0 | 0.0002117 | 139 | 0.143 | 230 | 159 | 11 | 16 | 213 | 380 | 603 | Type VI secretion protein VgrG | Type VI secretion protein VgrG | | afdb-uniprot50 | AF-A0A6G7LP93-F1-MODEL\_V4 | 1.0 | 0.0001882 | 138 | 0.147 | 244 | 164 | 9 | 16 | 216 | 376 | 618 | Type VI secretion system tip protein VgrG | Type VI secretion system tip protein VgrG | | afdb-uniprot50 | AF-A0A3A0E6C2-F1-MODEL\_V4 | 1.0 | 7.326e-05 | 137 | 0.093 | 266 | 176 | 8 | 16 | 217 | 374 | 638 | Phage\_base\_V domain-containing protein | Phage\_base\_V domain-containing protein | | afdb-uniprot50 | AF-D0XBY7-F1-MODEL\_V4 | 1.0 | 0.0001996 | 133 | 0.138 | 260 | 165 | 11 | 16 | 217 | 9 | 267 | Phage\_base\_V domain-containing protein | Phage\_base\_V domain-containing protein | | afdb-uniprot50 | AF-A0A0K2E151-F1-MODEL\_V4 | 1.0 | 0.0002246 | 133 | 0.144 | 221 | 157 | 10 | 16 | 208 | 372 | 588 | Rhs element Vgr family protein | Rhs element Vgr family protein | | afdb-uniprot50 | AF-D1AH50-F1-MODEL\_V4 | 1.0 | 0.0002246 | 132 | 0.143 | 209 | 136 | 10 | 16 | 192 | 308 | 505 | Uncharacterized protein | Uncharacterized protein | | afdb-uniprot50 | AF-A0A2D0J1V8-F1-MODEL\_V4 | 1.0 | 0.0001044 | 132 | 0.12 | 266 | 166 | 12 | 16 | 217 | 357 | 618 | Rhs element Vgr family protein | Rhs element Vgr family protein | | afdb-uniprot50 | AF-A0A2V5E8F6-F1-MODEL\_V4 | 1.0 | 0.0004556 | 131 | 0.156 | 236 | 164 | 10 | 16 | 217 | 392 | 626 | Type VI secretion system secreted protein VgrG | Type VI secretion system secreted protein VgrG | | afdb-uniprot50 | AF-A0A3M8FG17-F1-MODEL\_V4 | 1.0 | 0.0002843 | 129 | 0.134 | 215 | 165 | 9 | 16 | 217 | 227 | 433 | Type VI secretion system tip protein VgrG | Type VI secretion system tip protein VgrG | | afdb-uniprot50 | AF-A0A063Y972-F1-MODEL\_V4 | 1.0 | 0.0004556 | 129 | 0.139 | 244 | 164 | 13 | 16 | 217 | 354 | 593 | VgrG protein | VgrG protein | | afdb-uniprot50 | AF-A0A455UDC9-F1-MODEL\_V4 | 1.0 | 0.0001672 | 125 | 0.126 | 261 | 160 | 12 | 16 | 217 | 7 | 258 | Phage\_base\_V domain-containing protein | Phage\_base\_V domain-containing protein | | afdb-uniprot50 | AF-A0A660LA17-F1-MODEL\_V4 | 1.0 | 6.512e-05 | 123 | 0.151 | 218 | 145 | 10 | 16 | 214 | 372 | 568 | Uncharacterized protein involved in type VI secretion and phage assembly | Uncharacterized protein involved in type VI secretion and phage assembly | | afdb-uniprot50 | AF-A0A6J4RIX7-F1-MODEL\_V4 | 1.0 | 0.0001245 | 121 | 0.165 | 218 | 144 | 9 | 16 | 209 | 366 | 569 | Phage\_base\_V domain-containing protein | Phage\_base\_V domain-containing protein | | afdb-uniprot50 | AF-A0A7Y2C7P2-F1-MODEL\_V4 | 1.0 | 0.001768 | 121 | 0.134 | 246 | 168 | 9 | 16 | 217 | 325 | 569 | Type VI secretion system tip protein VgrG | Type VI secretion system tip protein VgrG | | afdb-uniprot50 | AF-A0A6L7D310-F1-MODEL\_V4 | 1.0 | 0.004281 | 119 | 0.13 | 192 | 151 | 6 | 39 | 217 | 547 | 735 | Phage\_base\_V domain-containing protein | Phage\_base\_V domain-containing protein | | afdb-uniprot50 | AF-A0A377B6W3-F1-MODEL\_V4 | 1.0 | 9.838e-05 | 117 | 0.284 | 183 | 88 | 8 | 61 | 203 | 1 | 180 | Putative phage baseplate component | Putative phage baseplate component | | afdb-uniprot50 | AF-A0A496WMG0-F1-MODEL\_V4 | 1.0 | 0.00117 | 115 | 0.117 | 263 | 164 | 10 | 16 | 217 | 407 | 662 | Uncharacterized protein | Uncharacterized protein | | afdb-uniprot50 | AF-A0A134A0Y4-F1-MODEL\_V4 | 1.0 | 0.008686 | 114 | 0.097 | 205 | 146 | 10 | 30 | 210 | 366 | 555 | Uncharacterized protein | Uncharacterized protein | | afdb-uniprot50 | AF-A0A3A9A3H4-F1-MODEL\_V4 | 1.0 | 6.512e-05 | 114 | 0.161 | 248 | 153 | 11 | 16 | 216 | 288 | 527 | Uncharacterized protein | Uncharacterized protein | | afdb-uniprot50 | AF-A0A177W637-F1-MODEL\_V4 | 1.0 | 0.002374 | 111 | 0.151 | 224 | 166 | 9 | 16 | 217 | 372 | 593 | Phage-related baseplate assembly protein | Phage-related baseplate assembly protein | | afdb-uniprot50 | AF-A0A376MVV2-F1-MODEL\_V4 | 1.0 | 0.0007302 | 107 | 0.272 | 154 | 89 | 6 | 65 | 217 | 3 | 134 | Baseplate assembly protein V | Baseplate assembly protein V | | afdb-uniprot50 | AF-A0A813EA03-F1-MODEL\_V4 | 0.872 | 0.009773 | 65 | 0.189 | 232 | 155 | 13 | 1 | 217 | 450 | 663 | Hypothetical protein | Hypothetical protein | |
| Top keywords  (threshold 1.00e-02 (evalue)) | **baseplate, assembly, V, Phage, Phage\_base\_V, domain\_containing, Type, secretion, VI, system** |
| Output files | ../../similar\_structures/11\_FANPEZAQ\_CDS\_0011\_afdb-proteome\_foldseek.tsv ../../similar\_structures/11\_FANPEZAQ\_CDS\_0011\_afdb-uniprot50\_foldseek.tsv ../../similar\_structures/11\_FANPEZAQ\_CDS\_0011\_merged.svg ../../similar\_structures/11\_FANPEZAQ\_CDS\_0011\_pdb\_foldseek.tsv |

  
  
  

Return to summary | Go to previous | Go to next

  


---

**Sequence/structure alignments coloring**  
Each object in the alignment figures is colored according to its E-value following this color coding:

1e-100
10

**References:**  
1) Steinegger M, Meier M, Mirdita M, Vöhringer H, Haunsberger S J, and Söding J (2019) HH-suite3 for fast remote homology detection and deep protein annotation, BMC Bioinformatics, 473. doi: 10.1186/s12859-019-3019-7  
2) Jumper J, Evans R, Pritzel A, ..., Hassabis D (2021) Highly accurate protein structure prediction with AlphaFold, Nature, 596. doi: 10.1038/s41586-021-03819-2  
3) van Kempen M, Kim S, Tumescheit C, Mirdita M, Lee J, Gilchrist CLM, Söding J, and Steinegger M (2023) Fast and accurate protein structure search with Foldseek. Nature Biotechnology. doi: 10.1038/s41587-023-01773-0
