# Supplementary material for: Completing the BASEL phage collection to unlock hidden diversity for systematic exploration of phage–host interactions
Source: PLoS Biol. 2025 Apr 7;23(4):e3003063. doi: 10.1371/journal.pbio.3003063 (PMC11990801; doi:10.1371/journal.pbio.3003063)
Supplement: S2 Data — (ZIP) [file pbio.3003063.s009.zip › entries/12.html]

FANPEZAQ\_CDS\_0012


Return to summary | Go to previous | Go to next

|  |  |
| --- | --- |
| FANPEZAQ\_CDS\_0012 Page creation date: 02 Sep 2024, 12:00  Project folder: n/a  Input sequences file: Escherichia\_virus\_HeidiAbel.gb | domain\_containing gpw\_gp25 baseplate gp25 gpw assembly irad gp25\_like phage lysozyme w putative 25\_like duf2634 gene wedge secretion type fragment vi system tail tsse hypothetical bacteriophage integrase lysozyme\_like phage\_like prophage a sheath t4 complex phage\_related element pbsx gp46 gp6 baseplate\_tail tube pre\_attachment xkds oxidoreductase |

### Sequence information

|  |  |
| --- | --- |
| Name | FANPEZAQ\_CDS\_0012  12\_FANPEZAQ\_CDS\_0012 (pipeline id) |
| Imported annotations | Escherichia\_virus\_HeidiAbel Bas97 |
| Protein sequence | MKGTNAATGAPLDGMDHLRQSIRDILTTPIGTRVMRRDYGSRLPYLVDAPMNRSTLLDLY AATAEALETWEPRISVQQVTATAAEPGRVELSISGEYLPDGKPITIDGIVVT |
| Number of residues | 112 |
| Molecular weight (Da) | 12140.70 |
| Output files | ../../query\_sequences/12\_FANPEZAQ\_CDS\_0012.fasta |

### Putative domain architecture and protein family

#### Search results (HHblits)1

|  |  |
| --- | --- |
| Domain family databases searched | Pfam, Ncbi-cd, Cath, Phrogs |
| Results, scheme(s)  (Top layers only; threshold 1.00e-03 (evalue)) | xml version="1.0" encoding="utf-8" standalone="no"?       2024-09-02T21:08:14.430277 image/svg+xml   Matplotlib v3.7.2, https://matplotlib.org/ |
| Results, table  (E-value ≤ 1.00e-03 (evalue)) | | db | id | prob | evalue | pvalue | score | cols | query | query\_len | template | template\_len | name | description | | --- | --- | --- | --- | --- | --- | --- | --- | --- | --- | --- | --- | --- | | pfam | PF04965 | 99.1 | 2.4e-15 | 4.5e-19 | 85.0 | 90 | (12, 101) | 112 | (1, 92) | 93 | GPW\_gp25 | Baseplate wedge protein gp25 | | pfam | PF10761 | 99.0 | 3.6e-14 | 6.4e-18 | 82.3 | 81 | (9, 102) | 112 | (17, 99) | 102 | DUF2590 | Protein of unknown function (DUF2590) | | pfam | PF10934 | 98.9 | 1.8e-13 | 3.3e-17 | 78.3 | 73 | (10, 83) | 112 | (19, 93) | 104 | DUF2634 | Protein of unknown function (DUF2634) | | pfam | PF07409 | 97.7 | 8e-09 | 1.5e-12 | 59.4 | 62 | (38, 100) | 112 | (44, 108) | 110 | GP46 | Phage protein GP46 | | cath | 2ia7A00 | 99.4 | 4.5e-18 | 6.1e-22 | 100.4 | 97 | (11, 107) | 112 | (27, 125) | 134 | Tail lysozyme, putative | CATHCODE: 3.10.450.40 NAME: Tail lysozyme, putative. Chain: a. Engineered: yes SOURCE: Geobacter sulfurreducens. Organism\_taxid: 35554. Gene: np\_952040.1. Expressed in: escherichia coli. Expression\_system\_taxid: 562. Expression\_system\_vector\_type: plasmid CLASS: Alpha Beta, ARCH: Roll, TOPOL: Nuclear Transport Factor 2; Chain: A, , HOMOL: Nuclear Transport Factor 2; Chain: A, | | cath | 4hrzB00 | 99.4 | 1.3e-17 | 1.8e-21 | 98.1 | 94 | (10, 103) | 112 | (26, 120) | 134 | Tail lysozyme | CATHCODE: 3.10.450.40 NAME: Tail lysozyme. Chain: a, b. Synonym: sheath polymerization initiator protein gp25, outer wedge of baseplate protein, protein gp25. Engineered: yes SOURCE: Enterobacteria phage t4. Organism\_taxid: 10665. Gene: 25. Expressed in: escherichia coli. Expression\_system\_taxid: 562. CLASS: Alpha Beta, ARCH: Roll, TOPOL: Nuclear Transport Factor 2; Chain: A, , HOMOL: Nuclear Transport Factor 2; Chain: A, | | phrogs | 261 | 99.9 | 3.2e-27 | 4e-31 | 152.7 | 99 | (5, 104) | 112 | (27, 127) | 139 | baseplate wedge subunit | baseplate wedge subunit; Category: tail; KU686208\_p77 | | phrogs | 43 | 99.8 | 2.8e-26 | 3.6e-30 | 146.3 | 107 | (1, 107) | 112 | (4, 111) | 127 | baseplate wedge subunit | baseplate wedge subunit; Category: tail; p108457 VI\_04298 | | phrogs | 975 | 99.5 | 2e-18 | 2.4e-22 | 118.2 | 96 | (10, 110) | 112 | (127, 223) | 236 | baseplate protein | baseplate protein; Category: tail; NC\_020871\_p93 | | phrogs | 120 | 99.2 | 1.2e-15 | 1.4e-19 | 95.1 | 70 | (10, 83) | 112 | (14, 84) | 118 | baseplate wedge subunit | baseplate wedge subunit; Category: tail; p345492 VI\_12445 | | phrogs | 37990 | 98.6 | 1.2e-11 | 1.3e-15 | 67.8 | 74 | (36, 110) | 112 | (1, 74) | 80 | baseplate wedge subunit | baseplate wedge subunit; Category: tail; NC\_028820\_p187 | | phrogs | 2669 | 98.0 | 2.1e-09 | 2.4e-13 | 66.1 | 59 | (10, 68) | 112 | (20, 78) | 138 | baseplate protein | baseplate protein; Category: tail; JN204348\_p134 | | phrogs | 1896 | 97.7 | 1.3e-08 | 1.4e-12 | 61.2 | 95 | (7, 101) | 112 | (15, 113) | 118 | NA | NA; Category: unknown function; p6628 VI\_08092 | | phrogs | 4233 | 97.3 | 1.7e-07 | 1.9e-11 | 61.1 | 80 | (12, 96) | 112 | (26, 107) | 208 | NA | NA; Category: unknown function; KX552041\_p157 | | phrogs | 15729 | 96.8 | 2.5e-06 | 2.8e-10 | 48.1 | 62 | (19, 84) | 112 | (10, 71) | 94 | baseplate protein | baseplate protein; Category: tail; p127195 VI\_01263 | | phrogs | 5026 | 96.4 | 9e-06 | 1e-09 | 47.8 | 74 | (16, 99) | 112 | (31, 106) | 117 | baseplate protein | baseplate protein; Category: tail; MF185718\_p38 | | phrogs | 33750 | 96.1 | 3.1e-05 | 3.5e-09 | 42.7 | 62 | (21, 84) | 112 | (16, 77) | 91 | NA | NA; Category: unknown function; p321351 VI\_06335 | | phrogs | 4033 | 96.0 | 3.3e-05 | 3.8e-09 | 52.5 | 87 | (10, 102) | 112 | (226, 313) | 320 | NA | NA; Category: unknown function; p55048 VI\_12418 | | phrogs | 180 | 94.6 | 0.00054 | 6.7e-08 | 40.8 | 75 | (28, 103) | 112 | (34, 108) | 120 | baseplate wedge subunit | baseplate wedge subunit; Category: tail; p189333 VI\_09191 | | phrogs | 33571 | 94.4 | 0.00076 | 8.5e-08 | 40.2 | 71 | (10, 83) | 112 | (59, 130) | 161 | NA | NA; Category: unknown function; NC\_008584\_p54 | |
| Top keywords  (threshold 1.00e-03 (evalue)) | **baseplate, tail, wedge, A, Nuclear, Transport, Factor, gp25, Phage, lysozyme** |
| Output files | ../../domain\_architecture/12\_FANPEZAQ\_CDS\_0012\_cath.hhr ../../domain\_architecture/12\_FANPEZAQ\_CDS\_0012\_merged.svg ../../domain\_architecture/12\_FANPEZAQ\_CDS\_0012\_ncbi-cd.hhr ../../domain\_architecture/12\_FANPEZAQ\_CDS\_0012\_pfam.hhr ../../domain\_architecture/12\_FANPEZAQ\_CDS\_0012\_phrogs.hhr |

### Identical protein sequences/structures

#### Search results

|  |  |
| --- | --- |
| Protein sequence databases searched | Pdb, Swissprot, Refseq |
| Identical proteins found | -- |
| Top keywords | -- |
| Output files | -- |

### Similar protein sequences/structures

#### Sequence similarity search results (HHblits)1

|  |  |
| --- | --- |
| Sequence databases searched | Uniclust, Pdb70 |
| Results, scheme(s)  (Top layers only, threshold 1.00e-03 (evalue)) | xml version="1.0" encoding="utf-8" standalone="no"?       2024-09-02T21:08:33.068464 image/svg+xml   Matplotlib v3.7.2, https://matplotlib.org/ |
| Results, table(s)  (threshold 1.00e-03 (evalue)) | | db | id | prob | evalue | pvalue | score | cols | query | query\_len | template | template\_len | name | description | | --- | --- | --- | --- | --- | --- | --- | --- | --- | --- | --- | --- | --- | | uniclust | UniRef100\_A0A011NBA4 | 99.9 | 2.1e-30 | 4.2e-36 | 164.1 | 110 | (1, 110) | 112 | (27, 136) | 154 | IraD/Gp25-like domain-containing protein | IraD/Gp25-like domain-containing protein | | uniclust | UniRef100\_A0A143DG48 | 99.9 | 4.2e-30 | 8.1e-36 | 158.6 | 110 | (1, 110) | 112 | (23, 132) | 140 | IraD/Gp25-like domain-containing protein | IraD/Gp25-like domain-containing protein | | uniclust | UniRef100\_A0A062INA0 | 99.9 | 2.5e-29 | 4.9e-35 | 157.1 | 111 | (1, 111) | 112 | (5, 115) | 144 | Lysozyme family protein | Lysozyme family protein | | uniclust | UniRef100\_A0A024E981 | 99.9 | 1.4e-28 | 2.8e-34 | 155.9 | 110 | (1, 111) | 112 | (31, 140) | 153 | Baseplate assembly protein W | Baseplate assembly protein W | | uniclust | UniRef100\_A0A016XHY4 | 99.9 | 1.7e-28 | 3.5e-34 | 155.6 | 111 | (1, 111) | 112 | (24, 135) | 151 | Baseplate assembly protein | Baseplate assembly protein | | uniclust | UniRef100\_A0A011NH43 | 99.9 | 2.6e-28 | 5.2e-34 | 164.3 | 106 | (4, 110) | 112 | (51, 160) | 227 | Baseplate wedge subunit | Baseplate wedge subunit | | uniclust | UniRef100\_A0A014PYG1 | 99.9 | 3.1e-28 | 6e-34 | 148.5 | 111 | (1, 111) | 112 | (3, 113) | 124 | Baseplate assembly protein (Fragment) | Baseplate assembly protein (Fragment) | | uniclust | UniRef100\_A0A069PL87 | 99.9 | 6.8e-28 | 1.3e-33 | 144.9 | 107 | (1, 107) | 112 | (7, 113) | 121 | IraD/Gp25-like domain-containing protein | IraD/Gp25-like domain-containing protein | | uniclust | UniRef100\_A0A062UZG7 | 99.9 | 9.7e-28 | 1.9e-33 | 148.6 | 100 | (11, 110) | 112 | (26, 127) | 137 | Phage baseplate assembly protein W | Phage baseplate assembly protein W | | uniclust | UniRef100\_A0A1H3KA32 | 99.9 | 1.3e-27 | 2.6e-33 | 149.2 | 101 | (11, 111) | 112 | (34, 135) | 144 | IraD/Gp25-like domain-containing protein | IraD/Gp25-like domain-containing protein | | uniclust | UniRef100\_A0A061KN01 | 99.9 | 5.7e-27 | 1.1e-32 | 146.9 | 110 | (1, 110) | 112 | (5, 114) | 144 | Baseplate assembly protein | Baseplate assembly protein | | uniclust | UniRef100\_A0A066RM20 | 99.9 | 6.5e-27 | 1.3e-32 | 143.9 | 107 | (1, 107) | 112 | (15, 121) | 129 | dTDP-glucose pyrophosphorylase | dTDP-glucose pyrophosphorylase | | uniclust | UniRef100\_A0A1M5Y4I2 | 99.9 | 7e-27 | 1.4e-32 | 146.3 | 103 | (8, 110) | 112 | (19, 125) | 149 | Gene 25-like lysozyme | Gene 25-like lysozyme | | uniclust | UniRef100\_A0A0Q4L1R6 | 99.9 | 1.5e-26 | 2.9e-32 | 146.0 | 108 | (3, 110) | 112 | (27, 138) | 145 | IraD/Gp25-like domain-containing protein | IraD/Gp25-like domain-containing protein | | uniclust | UniRef100\_A0A0Q8XJN5 | 99.9 | 2.1e-26 | 4.1e-32 | 145.0 | 100 | (11, 110) | 112 | (31, 132) | 150 | IraD/Gp25-like domain-containing protein | IraD/Gp25-like domain-containing protein | | uniclust | UniRef100\_A0A0C5AE75 | 99.9 | 2.4e-26 | 4.6e-32 | 141.7 | 107 | (4, 110) | 112 | (25, 135) | 139 | Baseplate wedge subunit | Baseplate wedge subunit | | uniclust | UniRef100\_A0A0E3EPQ9 | 99.9 | 2.5e-26 | 5.2e-32 | 146.3 | 108 | (3, 110) | 112 | (32, 142) | 148 | Base plate wedge subunit | Base plate wedge subunit | | uniclust | UniRef100\_A0A084SKL2 | 99.9 | 6.5e-26 | 1.3e-31 | 145.6 | 103 | (8, 110) | 112 | (37, 143) | 182 | IraD/Gp25-like domain-containing protein | IraD/Gp25-like domain-containing protein | | uniclust | UniRef100\_A0A0F9DR72 | 99.9 | 9.8e-26 | 1.9e-31 | 143.6 | 97 | (15, 111) | 112 | (38, 138) | 171 | IraD/Gp25-like domain-containing protein | IraD/Gp25-like domain-containing protein | | uniclust | UniRef100\_A0A1D7SKY7 | 99.9 | 2.1e-25 | 4.2e-31 | 144.2 | 109 | (2, 110) | 112 | (40, 151) | 167 | Baseplate wedge subunit | Baseplate wedge subunit | | uniclust | UniRef100\_A0A1I2FX92 | 99.9 | 3.5e-25 | 7e-31 | 135.6 | 100 | (12, 111) | 112 | (19, 118) | 123 | Gene 25-like lysozyme | Gene 25-like lysozyme | | uniclust | UniRef100\_A0A022PCH8 | 99.9 | 3.8e-25 | 7.6e-31 | 141.9 | 105 | (4, 108) | 112 | (40, 147) | 169 | Phage baseplate assembly protein W | Phage baseplate assembly protein W | | uniclust | UniRef100\_A0A060AMS0 | 99.9 | 4.1e-25 | 8.1e-31 | 139.2 | 109 | (2, 110) | 112 | (27, 139) | 147 | Putative baseplate wedge subunit | Putative baseplate wedge subunit | | uniclust | UniRef100\_A0A011NTL3 | 99.9 | 4.2e-25 | 8.2e-31 | 137.5 | 102 | (8, 109) | 112 | (29, 134) | 144 | Baseplate wedge subunit | Baseplate wedge subunit | | uniclust | UniRef100\_A0A067XRJ3 | 99.9 | 4.2e-25 | 8.4e-31 | 140.5 | 107 | (4, 110) | 112 | (37, 146) | 151 | IraD/Gp25-like domain-containing protein | IraD/Gp25-like domain-containing protein | | uniclust | UniRef100\_A0A103EKP3 | 99.8 | 8e-25 | 1.5e-30 | 127.6 | 84 | (1, 84) | 112 | (9, 92) | 101 | Phage baseplate protein (Fragment) | Phage baseplate protein (Fragment) | | uniclust | UniRef100\_A0A023Q0J9 | 99.8 | 1.3e-24 | 2.5e-30 | 142.0 | 100 | (11, 110) | 112 | (60, 161) | 179 | IraD/Gp25-like domain-containing protein | IraD/Gp25-like domain-containing protein | | uniclust | UniRef100\_A0A1H8VXY1 | 99.8 | 8.6e-24 | 1.7e-29 | 130.6 | 111 | (1, 111) | 112 | (17, 129) | 134 | IraD/Gp25-like domain-containing protein | IraD/Gp25-like domain-containing protein | | uniclust | UniRef100\_A0A348FYH4 | 99.8 | 1.8e-23 | 3.6e-29 | 125.8 | 103 | (1, 103) | 112 | (9, 112) | 119 | IraD/Gp25-like domain-containing protein | IraD/Gp25-like domain-containing protein | | uniclust | UniRef100\_A0A011UMM6 | 99.8 | 4.1e-23 | 8.2e-29 | 133.9 | 102 | (1, 102) | 112 | (34, 136) | 168 | IraD/Gp25-like domain-containing protein | IraD/Gp25-like domain-containing protein | | uniclust | UniRef100\_A0A4R2C4F1 | 99.8 | 7.3e-23 | 1.4e-28 | 128.0 | 103 | (8, 110) | 112 | (20, 126) | 169 | IraD/Gp25-like domain-containing protein | IraD/Gp25-like domain-containing protein | | uniclust | UniRef100\_A0A0Q7E412 | 99.8 | 1.3e-22 | 2.4e-28 | 126.0 | 103 | (8, 110) | 112 | (32, 140) | 148 | IraD/Gp25-like domain-containing protein | IraD/Gp25-like domain-containing protein | | uniclust | UniRef100\_A0A2E4ZEV5 | 99.8 | 2.9e-22 | 5.6e-28 | 122.4 | 100 | (11, 110) | 112 | (25, 125) | 130 | IraD/Gp25-like domain-containing protein | IraD/Gp25-like domain-containing protein | | uniclust | UniRef100\_A0A5S3XXE0 | 99.8 | 4.1e-22 | 7.9e-28 | 122.0 | 100 | (11, 110) | 112 | (20, 123) | 129 | IraD/Gp25-like domain-containing protein | IraD/Gp25-like domain-containing protein | | uniclust | UniRef100\_A0A0D0KR61 | 99.8 | 7.7e-22 | 1.5e-27 | 124.1 | 106 | (2, 107) | 112 | (5, 119) | 144 | Baseplate assembly protein W (GpW) | Baseplate assembly protein W (GpW) | | uniclust | UniRef100\_A0A291FP80 | 99.8 | 8.8e-22 | 1.6e-27 | 124.3 | 107 | (1, 107) | 112 | (73, 179) | 184 | Putative baseplate assembly protein W | Putative baseplate assembly protein W | | uniclust | UniRef100\_A0A1Z8UZN9 | 99.7 | 1.5e-21 | 2.8e-27 | 123.7 | 107 | (4, 110) | 112 | (44, 153) | 159 | IraD/Gp25-like domain-containing protein | IraD/Gp25-like domain-containing protein | | uniclust | UniRef100\_A0A0C1GGW4 | 99.7 | 1.5e-21 | 2.9e-27 | 123.7 | 94 | (8, 102) | 112 | (34, 127) | 149 | IraD/Gp25-like domain-containing protein | IraD/Gp25-like domain-containing protein | | uniclust | UniRef100\_A0A0E3G425 | 99.7 | 1.5e-21 | 3e-27 | 123.6 | 107 | (4, 110) | 112 | (32, 142) | 147 | Base plate wedge subunit | Base plate wedge subunit | | uniclust | UniRef100\_A0A376JCH0 | 99.7 | 1.7e-21 | 3.3e-27 | 114.8 | 81 | (2, 82) | 112 | (6, 86) | 98 | Phage baseplate protein | Phage baseplate protein | | uniclust | UniRef100\_D4HTV2 | 99.7 | 1.8e-21 | 3.3e-27 | 118.2 | 105 | (1, 105) | 112 | (43, 147) | 154 | Gp13 protein | Gp13 protein | | uniclust | UniRef100\_A0A059IUA3 | 99.7 | 2.4e-21 | 4.6e-27 | 115.1 | 107 | (1, 107) | 112 | (1, 107) | 119 | Putative prophage LambdaW5, baseplate assembly protein W,putative | Putative prophage LambdaW5, baseplate assembly protein W,putative | | uniclust | UniRef100\_A0A1R1MKK7 | 99.7 | 3.6e-21 | 7e-27 | 118.1 | 86 | (11, 98) | 112 | (20, 107) | 125 | IraD/Gp25-like domain-containing protein | IraD/Gp25-like domain-containing protein | | uniclust | UniRef100\_A0A410T6G4 | 99.7 | 6.3e-21 | 1.2e-26 | 114.7 | 91 | (11, 101) | 112 | (15, 106) | 120 | Baseplate wedge subunit | Baseplate wedge subunit | | uniclust | UniRef100\_A0A1V5RRZ4 | 99.7 | 7.2e-21 | 1.4e-26 | 116.7 | 100 | (11, 110) | 112 | (38, 138) | 139 | Gene 25-like lysozyme | Gene 25-like lysozyme | | uniclust | UniRef100\_A0A011P705 | 99.7 | 8e-21 | 1.6e-26 | 121.3 | 91 | (9, 101) | 112 | (35, 125) | 151 | Phage baseplate protein | Phage baseplate protein | | uniclust | UniRef100\_A0A7X5DY26 | 99.7 | 1.4e-20 | 2.5e-26 | 111.7 | 100 | (11, 110) | 112 | (19, 119) | 122 | Baseplate protein | Baseplate protein | | uniclust | UniRef100\_A0A0A0YS61 | 99.7 | 1.9e-20 | 3.7e-26 | 117.5 | 108 | (3, 110) | 112 | (25, 135) | 139 | Base plate wedge subunit | Base plate wedge subunit | | uniclust | UniRef100\_A0A8I0FE23 | 99.7 | 2.2e-20 | 4e-26 | 111.0 | 98 | (1, 98) | 112 | (12, 109) | 127 | GPW/gp25 family protein | GPW/gp25 family protein | | uniclust | UniRef100\_A0A023W5U8 | 99.7 | 2.2e-20 | 4.3e-26 | 114.7 | 92 | (11, 102) | 112 | (26, 118) | 137 | Baseplate wedge subunit | Baseplate wedge subunit | | uniclust | UniRef100\_A0A191W8N1 | 99.7 | 2.9e-20 | 5.6e-26 | 114.1 | 100 | (8, 109) | 112 | (14, 115) | 126 | IraD/Gp25-like domain-containing protein | IraD/Gp25-like domain-containing protein | | uniclust | UniRef100\_A0A0A8HAU6 | 99.7 | 3.4e-20 | 6.6e-26 | 109.5 | 82 | (16, 97) | 112 | (7, 88) | 100 | Phage baseplate assembly protein W | Phage baseplate assembly protein W | | uniclust | UniRef100\_A0A178HLN5 | 99.7 | 3.9e-20 | 7.6e-26 | 118.2 | 94 | (9, 103) | 112 | (47, 141) | 157 | IraD/Gp25-like domain-containing protein | IraD/Gp25-like domain-containing protein | | uniclust | UniRef100\_A0A031JP30 | 99.7 | 5.9e-20 | 1.2e-25 | 118.5 | 105 | (4, 110) | 112 | (36, 143) | 167 | GPW/gp25 family protein | GPW/gp25 family protein | | uniclust | UniRef100\_A0A1D2QY07 | 99.7 | 7.7e-20 | 1.5e-25 | 113.9 | 102 | (8, 110) | 112 | (29, 137) | 142 | IraD/Gp25-like domain-containing protein | IraD/Gp25-like domain-containing protein | | uniclust | UniRef100\_A0A6G3XJJ0 | 99.7 | 1.1e-19 | 2e-25 | 105.9 | 90 | (4, 93) | 112 | (16, 108) | 109 | GPW/gp25 family protein (Fragment) | GPW/gp25 family protein (Fragment) | | uniclust | UniRef100\_A0A3C1ALA6 | 99.7 | 1.6e-19 | 3e-25 | 104.1 | 92 | (12, 103) | 112 | (1, 92) | 97 | Phage baseplate protein (Fragment) | Phage baseplate protein (Fragment) | | uniclust | UniRef100\_A0A1Z9HXH3 | 99.6 | 2.3e-19 | 4.7e-25 | 114.3 | 98 | (12, 109) | 112 | (37, 136) | 144 | IraD/Gp25-like domain-containing protein | IraD/Gp25-like domain-containing protein | | uniclust | UniRef100\_A0A0F9S7G6 | 99.6 | 3.7e-19 | 7.4e-25 | 115.9 | 98 | (11, 110) | 112 | (43, 140) | 170 | IraD/Gp25-like domain-containing protein | IraD/Gp25-like domain-containing protein | | uniclust | UniRef100\_A0A091APV5 | 99.6 | 5e-19 | 9.8e-25 | 106.4 | 76 | (8, 83) | 112 | (26, 103) | 106 | IraD/Gp25-like domain-containing protein (Fragment) | IraD/Gp25-like domain-containing protein (Fragment) | | uniclust | UniRef100\_UPI001573BC7E | 99.6 | 6e-19 | 1.1e-24 | 108.3 | 101 | (11, 111) | 112 | (40, 142) | 150 | type VI secretion system baseplate subunit TssE | type VI secretion system baseplate subunit TssE | | uniclust | UniRef100\_A0A1M6M9N6 | 99.6 | 1e-18 | 2e-24 | 109.6 | 100 | (8, 108) | 112 | (34, 136) | 144 | Gene 25-like lysozyme | Gene 25-like lysozyme | | uniclust | UniRef100\_A0A0Q1ADI1 | 99.6 | 1.1e-18 | 2.2e-24 | 124.4 | 99 | (11, 110) | 112 | (33, 131) | 348 | IraD/Gp25-like domain-containing protein | IraD/Gp25-like domain-containing protein | | uniclust | UniRef100\_A0A927MQK2 | 99.6 | 1.4e-18 | 2.5e-24 | 109.2 | 103 | (8, 110) | 112 | (33, 139) | 189 | Phage baseplate assembly protein W | Phage baseplate assembly protein W | | uniclust | UniRef100\_A0A067ZJ13 | 99.6 | 1.6e-18 | 3.2e-24 | 108.9 | 88 | (13, 102) | 112 | (27, 116) | 125 | IraD/Gp25-like domain-containing protein | IraD/Gp25-like domain-containing protein | | uniclust | UniRef100\_D5SL83 | 99.6 | 2.2e-18 | 4e-24 | 105.2 | 108 | (3, 110) | 112 | (15, 125) | 149 | GPW/gp25 family protein | GPW/gp25 family protein | | uniclust | UniRef100\_A0A2E2FJP9 | 99.6 | 2.4e-18 | 4.5e-24 | 105.4 | 103 | (8, 110) | 112 | (21, 126) | 132 | Phage baseplate protein | Phage baseplate protein | | uniclust | UniRef100\_A8TYF6 | 99.6 | 2.6e-18 | 4.7e-24 | 104.8 | 95 | (4, 98) | 112 | (1, 95) | 153 | IraD/Gp25-like domain-containing protein | IraD/Gp25-like domain-containing protein | | uniclust | UniRef100\_A0A1V3IM03 | 99.6 | 3.1e-18 | 5.8e-24 | 100.6 | 97 | (13, 110) | 112 | (3, 99) | 105 | Baseplate assembly protein W (Fragment) | Baseplate assembly protein W (Fragment) | | uniclust | UniRef100\_A0A023XMK4 | 99.6 | 3e-18 | 6.1e-24 | 111.8 | 99 | (11, 110) | 112 | (42, 141) | 164 | IraD/Gp25-like domain-containing protein | IraD/Gp25-like domain-containing protein | | uniclust | UniRef100\_A0A0Q2YY05 | 99.6 | 3.1e-18 | 6.1e-24 | 108.9 | 96 | (4, 100) | 112 | (28, 123) | 147 | IraD/Gp25-like domain-containing protein | IraD/Gp25-like domain-containing protein | | uniclust | UniRef100\_A0A0F9FRJ8 | 99.6 | 3.8e-18 | 7.5e-24 | 106.9 | 97 | (11, 108) | 112 | (24, 121) | 133 | IraD/Gp25-like domain-containing protein | IraD/Gp25-like domain-containing protein | | uniclust | UniRef100\_A0A0S4XLR8 | 99.6 | 6.6e-18 | 1.3e-23 | 104.5 | 93 | (15, 107) | 112 | (36, 129) | 135 | Putative phage-related baseplate assembly protein (GPW-like) | Putative phage-related baseplate assembly protein (GPW-like) | | uniclust | UniRef100\_A0A011PP45 | 99.6 | 7.3e-18 | 1.5e-23 | 110.4 | 100 | (11, 111) | 112 | (57, 162) | 173 | Uncharacterized protein | Uncharacterized protein | | uniclust | UniRef100\_UPI001BAAC7D5 | 99.6 | 9.3e-18 | 1.7e-23 | 108.1 | 106 | (5, 110) | 112 | (108, 216) | 224 | GPW/gp25 family protein | GPW/gp25 family protein | | uniclust | UniRef100\_A0A1X7MCL8 | 99.6 | 9.6e-18 | 1.8e-23 | 97.8 | 91 | (20, 111) | 112 | (3, 93) | 98 | GPW/gp25 family protein | GPW/gp25 family protein | | uniclust | UniRef100\_A0A081C215 | 99.6 | 9.9e-18 | 2e-23 | 109.7 | 99 | (11, 110) | 112 | (57, 160) | 174 | Conserved domain protein | Conserved domain protein | | uniclust | UniRef100\_A0A0Q8LUA8 | 99.5 | 1.6e-17 | 3e-23 | 104.9 | 100 | (11, 110) | 112 | (40, 141) | 150 | IraD/Gp25-like domain-containing protein | IraD/Gp25-like domain-containing protein | | uniclust | UniRef100\_A0A1B1IUU0 | 99.5 | 1.7e-17 | 3.3e-23 | 106.2 | 106 | (5, 110) | 112 | (21, 130) | 156 | Baseplate protein | Baseplate protein | | uniclust | UniRef100\_A0A074LW06 | 99.5 | 2e-17 | 4e-23 | 102.6 | 86 | (13, 100) | 112 | (22, 108) | 119 | IraD/Gp25-like domain-containing protein | IraD/Gp25-like domain-containing protein | | uniclust | UniRef100\_A0A127AW49 | 99.5 | 2.3e-17 | 4.2e-23 | 102.6 | 107 | (4, 110) | 112 | (37, 147) | 156 | Sheath initiation protein | Sheath initiation protein | | uniclust | UniRef100\_UPI001F313682 | 99.5 | 2.5e-17 | 4.6e-23 | 100.6 | 105 | (1, 106) | 112 | (1, 107) | 152 | GPW/gp25 family protein | GPW/gp25 family protein | | uniclust | UniRef100\_A0A927DLG2 | 99.5 | 3.7e-17 | 6.9e-23 | 111.0 | 108 | (2, 110) | 112 | (195, 303) | 310 | Phage baseplate assembly protein V | Phage baseplate assembly protein V | | uniclust | UniRef100\_A0A014LIJ6 | 99.5 | 5.2e-17 | 1e-22 | 104.3 | 96 | (8, 104) | 112 | (55, 153) | 170 | IraD/Gp25-like domain-containing protein | IraD/Gp25-like domain-containing protein | | uniclust | UniRef100\_A0A061NG15 | 99.5 | 5.3e-17 | 1.1e-22 | 102.8 | 96 | (8, 108) | 112 | (18, 117) | 128 | DUF2634 domain-containing protein | DUF2634 domain-containing protein | | uniclust | UniRef100\_A0A2E3HQV9 | 99.5 | 5.8e-17 | 1.1e-22 | 99.7 | 100 | (5, 104) | 112 | (18, 120) | 131 | IraD/Gp25-like domain-containing protein | IraD/Gp25-like domain-containing protein | | uniclust | UniRef100\_A0A0N1C7H2 | 99.5 | 7.1e-17 | 1.4e-22 | 103.8 | 101 | (2, 102) | 112 | (21, 122) | 161 | Integrase | Integrase | | uniclust | UniRef100\_Q1QI83 | 99.5 | 7.5e-17 | 1.4e-22 | 99.8 | 100 | (2, 101) | 112 | (43, 143) | 165 | GPW/gp25 | GPW/gp25 | | uniclust | UniRef100\_A0A064AK59 | 99.5 | 7e-17 | 1.4e-22 | 102.1 | 87 | (12, 100) | 112 | (35, 122) | 132 | Isoleucyl-tRNA synthetase | Isoleucyl-tRNA synthetase | | uniclust | UniRef100\_A0A1T4WV80 | 99.5 | 8.2e-17 | 1.5e-22 | 101.8 | 82 | (2, 83) | 112 | (81, 162) | 192 | Phage baseplate assembly protein W | Phage baseplate assembly protein W | | uniclust | UniRef100\_A0A0F9T6N1 | 99.5 | 7.9e-17 | 1.5e-22 | 104.4 | 89 | (11, 99) | 112 | (38, 128) | 171 | IraD/Gp25-like domain-containing protein | IraD/Gp25-like domain-containing protein | | uniclust | UniRef100\_UPI000291EF94 | 99.5 | 8.6e-17 | 1.6e-22 | 94.4 | 108 | (2, 109) | 112 | (6, 113) | 116 | GPW/gp25 family protein | GPW/gp25 family protein | | uniclust | UniRef100\_A0A3D2C224 | 99.5 | 9e-17 | 1.7e-22 | 95.7 | 78 | (3, 80) | 112 | (46, 125) | 127 | IraD/Gp25-like domain-containing protein (Fragment) | IraD/Gp25-like domain-containing protein (Fragment) | | uniclust | UniRef100\_UPI0022B1D31B | 99.5 | 9.5e-17 | 1.7e-22 | 94.9 | 80 | (4, 83) | 112 | (1, 80) | 121 | GPW/gp25 family protein | GPW/gp25 family protein | | uniclust | UniRef100\_A0A086ML21 | 99.5 | 9.8e-17 | 1.9e-22 | 99.9 | 100 | (8, 108) | 112 | (20, 122) | 131 | IraD/Gp25-like domain-containing protein | IraD/Gp25-like domain-containing protein | | uniclust | UniRef100\_UPI0003640FF3 | 99.5 | 1.1e-16 | 2.1e-22 | 97.1 | 110 | (2, 111) | 112 | (14, 127) | 134 | GPW/gp25 family protein | GPW/gp25 family protein | | uniclust | UniRef100\_A0A0X1U7T5 | 99.5 | 1.8e-16 | 3.7e-22 | 99.7 | 87 | (12, 100) | 112 | (31, 117) | 126 | Protein 25-like lysozyme | Protein 25-like lysozyme | | uniclust | UniRef100\_A0A117MMK7 | 99.4 | 3.1e-16 | 6e-22 | 97.7 | 99 | (11, 110) | 112 | (28, 127) | 135 | IraD/Gp25-like domain-containing protein | IraD/Gp25-like domain-containing protein | | uniclust | UniRef100\_A0A2I7QP25 | 99.4 | 3.4e-16 | 6.7e-22 | 98.8 | 102 | (1, 102) | 112 | (19, 126) | 143 | Uncharacterized protein | Uncharacterized protein | | uniclust | UniRef100\_A0A1H1G2U4 | 99.4 | 4e-16 | 7.5e-22 | 94.4 | 108 | (1, 109) | 112 | (1, 120) | 122 | IraD/Gp25-like domain-containing protein | IraD/Gp25-like domain-containing protein | | uniclust | UniRef100\_A0A2D5K3P5 | 99.4 | 4.3e-16 | 8.1e-22 | 89.9 | 63 | (9, 71) | 112 | (23, 87) | 87 | IraD/Gp25-like domain-containing protein (Fragment) | IraD/Gp25-like domain-containing protein (Fragment) | | uniclust | UniRef100\_A0A069F3H4 | 99.4 | 4.9e-16 | 1e-21 | 98.3 | 93 | (11, 108) | 112 | (24, 118) | 125 | Uncharacterized protein | Uncharacterized protein | | uniclust | UniRef100\_A0A0E4BWU6 | 99.4 | 5.7e-16 | 1.1e-21 | 94.4 | 99 | (3, 101) | 112 | (12, 111) | 121 | IraD/Gp25-like domain-containing protein | IraD/Gp25-like domain-containing protein | | uniclust | UniRef100\_A0A031FQW9 | 99.4 | 5.6e-16 | 1.1e-21 | 96.8 | 107 | (1, 110) | 112 | (5, 114) | 124 | Phage baseplate assembly protein W | Phage baseplate assembly protein W | | uniclust | UniRef100\_A0A024B013 | 99.4 | 7.8e-16 | 1.6e-21 | 106.9 | 98 | (11, 110) | 112 | (129, 227) | 246 | Baseplate protein | Baseplate protein | | uniclust | UniRef100\_A0A0C2DUF9 | 99.4 | 8.4e-16 | 1.6e-21 | 99.5 | 104 | (6, 110) | 112 | (61, 173) | 182 | IraD/Gp25-like domain-containing protein | IraD/Gp25-like domain-containing protein | | uniclust | UniRef100\_A0A069QGW3 | 99.4 | 8.3e-16 | 1.7e-21 | 101.4 | 97 | (11, 107) | 112 | (48, 158) | 177 | Putative lysozyme | Putative lysozyme | | uniclust | UniRef100\_A0A0A7K9I0 | 99.4 | 9.4e-16 | 1.9e-21 | 101.5 | 98 | (11, 108) | 112 | (49, 161) | 175 | IraD/Gp25-like domain-containing protein | IraD/Gp25-like domain-containing protein | | uniclust | UniRef100\_A0A2E0GD41 | 99.4 | 1.1e-15 | 2.1e-21 | 97.7 | 98 | (11, 108) | 112 | (34, 136) | 156 | IraD/Gp25-like domain-containing protein | IraD/Gp25-like domain-containing protein | | uniclust | UniRef100\_A0A6J5L6U0 | 99.4 | 1.5e-15 | 2.9e-21 | 95.6 | 107 | (3, 110) | 112 | (17, 127) | 142 | GpW/Gp25/anti-adapter protein IraD | GpW/Gp25/anti-adapter protein IraD | | uniclust | UniRef100\_A0A370LRT3 | 99.4 | 1.7e-15 | 3.1e-21 | 93.9 | 73 | (11, 83) | 112 | (22, 94) | 143 | IraD/Gp25-like domain-containing protein | IraD/Gp25-like domain-containing protein | | uniclust | UniRef100\_A0A085ZBD9 | 99.4 | 1.8e-15 | 3.7e-21 | 99.2 | 98 | (11, 108) | 112 | (43, 153) | 163 | IraD/Gp25-like domain-containing protein | IraD/Gp25-like domain-containing protein | | uniclust | UniRef100\_A0A1F9LNZ8 | 99.4 | 2.6e-15 | 4.9e-21 | 94.0 | 96 | (4, 99) | 112 | (24, 123) | 150 | IraD/Gp25-like domain-containing protein (Fragment) | IraD/Gp25-like domain-containing protein (Fragment) | | uniclust | UniRef100\_A0A1Z9PTU6 | 99.4 | 2.8e-15 | 5.4e-21 | 95.6 | 94 | (11, 106) | 112 | (42, 135) | 156 | IraD/Gp25-like domain-containing protein | IraD/Gp25-like domain-containing protein | | uniclust | UniRef100\_A0A6P0Q329 | 99.3 | 3.3e-15 | 6.1e-21 | 84.7 | 73 | (4, 76) | 112 | (18, 92) | 92 | GPW/gp25 family protein (Fragment) | GPW/gp25 family protein (Fragment) | | uniclust | UniRef100\_A0A1V5LTW0 | 99.3 | 3.4e-15 | 6.3e-21 | 87.2 | 79 | (12, 91) | 112 | (29, 107) | 110 | Gene 25-like lysozyme | Gene 25-like lysozyme | | uniclust | UniRef100\_A0A068T9E9 | 99.3 | 3.5e-15 | 6.7e-21 | 92.6 | 102 | (2, 103) | 112 | (9, 111) | 137 | Putative phage gp25 protein | Putative phage gp25 protein | | uniclust | UniRef100\_A0A1J0EEV2 | 99.3 | 3.9e-15 | 7.4e-21 | 90.8 | 90 | (8, 99) | 112 | (22, 111) | 125 | Baseplate protein | Baseplate protein | | uniclust | UniRef100\_A0A090HXB8 | 99.3 | 4.1e-15 | 8.2e-21 | 94.1 | 97 | (8, 109) | 112 | (25, 125) | 130 | Conserved domain protein | Conserved domain protein | | uniclust | UniRef100\_A0A562GHU8 | 99.3 | 4.4e-15 | 8.3e-21 | 91.8 | 100 | (7, 108) | 112 | (31, 133) | 144 | Phage baseplate assembly protein W | Phage baseplate assembly protein W | | uniclust | UniRef100\_A0A081NYB0 | 99.3 | 5e-15 | 9.9e-21 | 94.2 | 88 | (8, 96) | 112 | (23, 114) | 142 | IraD/Gp25-like domain-containing protein | IraD/Gp25-like domain-containing protein | | uniclust | UniRef100\_A0A318L0U5 | 99.3 | 5.6e-15 | 1e-20 | 88.1 | 102 | (2, 103) | 112 | (9, 111) | 125 | IraD/Gp25-like domain-containing protein | IraD/Gp25-like domain-containing protein | | uniclust | UniRef100\_A0A3C1NZW4 | 99.3 | 6.5e-15 | 1.2e-20 | 77.2 | 57 | (20, 76) | 112 | (3, 59) | 59 | Baseplate protein (Fragment) | Baseplate protein (Fragment) | | uniclust | UniRef100\_A0A0B5FRN4 | 99.3 | 7.4e-15 | 1.4e-20 | 99.6 | 97 | (13, 109) | 112 | (31, 131) | 305 | IraD/Gp25-like domain-containing protein | IraD/Gp25-like domain-containing protein | | uniclust | UniRef100\_A0A381WMD8 | 99.3 | 7.5e-15 | 1.4e-20 | 84.9 | 73 | (4, 76) | 112 | (18, 92) | 94 | IraD/Gp25-like domain-containing protein | IraD/Gp25-like domain-containing protein | | uniclust | UniRef100\_A0A2T6NFS1 | 99.3 | 8.8e-15 | 1.6e-20 | 92.5 | 100 | (11, 110) | 112 | (77, 178) | 185 | IraD/Gp25-like domain-containing protein | IraD/Gp25-like domain-containing protein | | uniclust | UniRef100\_Q7MB49 | 99.3 | 9.1e-15 | 1.7e-20 | 95.7 | 96 | (6, 101) | 112 | (121, 219) | 239 | Photorhabdus luminescens subsp. laumondii TTO1 complete genome; segment 6/17 | Photorhabdus luminescens subsp. laumondii TTO1 complete genome; segment 6/17 | | uniclust | UniRef100\_A0A550H8T3 | 99.3 | 9.8e-15 | 1.8e-20 | 90.6 | 98 | (12, 109) | 112 | (39, 140) | 146 | IraD/Gp25-like domain-containing protein | IraD/Gp25-like domain-containing protein | | uniclust | UniRef100\_A0A6I7PSS5 | 99.3 | 1.9e-14 | 3.6e-20 | 86.2 | 98 | (13, 110) | 112 | (26, 125) | 127 | IraD/Gp25-like domain-containing protein | IraD/Gp25-like domain-containing protein | | uniclust | UniRef100\_A0A853IK11 | 99.3 | 2e-14 | 3.7e-20 | 77.5 | 52 | (1, 52) | 112 | (9, 60) | 63 | Baseplate assembly protein W (Fragment) | Baseplate assembly protein W (Fragment) | | uniclust | UniRef100\_A0A1T2XCA8 | 99.3 | 1.9e-14 | 3.7e-20 | 87.3 | 85 | (16, 103) | 112 | (17, 101) | 108 | Phage baseplate assembly protein W | Phage baseplate assembly protein W | | uniclust | UniRef100\_A0A023D1J8 | 99.3 | 1.9e-14 | 3.7e-20 | 91.2 | 100 | (8, 111) | 112 | (21, 125) | 130 | Phage tail protein | Phage tail protein | | uniclust | UniRef100\_A0A4U0FCL7 | 99.3 | 2.1e-14 | 3.9e-20 | 89.8 | 106 | (4, 110) | 112 | (24, 136) | 153 | DUF2634 domain-containing protein | DUF2634 domain-containing protein | | uniclust | UniRef100\_A0A080M8X4 | 99.3 | 2e-14 | 4e-20 | 91.1 | 98 | (11, 110) | 112 | (38, 140) | 145 | DUF2634 domain-containing protein | DUF2634 domain-containing protein | | uniclust | UniRef100\_UPI0022B9D4FA | 99.3 | 2.2e-14 | 4e-20 | 93.4 | 97 | (13, 109) | 112 | (31, 131) | 228 | GPW/gp25 family protein | GPW/gp25 family protein | | uniclust | UniRef100\_A0A2D6F2M5 | 99.3 | 2.1e-14 | 4.1e-20 | 92.3 | 87 | (13, 99) | 112 | (53, 140) | 161 | IraD/Gp25-like domain-containing protein | IraD/Gp25-like domain-containing protein | | uniclust | UniRef100\_A0A0X3VIR3 | 99.3 | 2.2e-14 | 4.4e-20 | 91.6 | 92 | (8, 99) | 112 | (18, 114) | 142 | IraD/Gp25-like domain-containing protein | IraD/Gp25-like domain-containing protein | | uniclust | UniRef100\_A0A1W9VM06 | 99.3 | 2.6e-14 | 4.8e-20 | 85.2 | 95 | (12, 107) | 112 | (15, 110) | 115 | IraD/Gp25-like domain-containing protein | IraD/Gp25-like domain-containing protein | | uniclust | UniRef100\_A0A090WBV0 | 99.2 | 2.8e-14 | 5.6e-20 | 93.3 | 96 | (11, 106) | 112 | (28, 137) | 154 | Gene 25-like lysozyme | Gene 25-like lysozyme | | uniclust | UniRef100\_A0A1J5PQ93 | 99.2 | 3.2e-14 | 5.8e-20 | 89.5 | 74 | (9, 83) | 112 | (19, 92) | 176 | Protein 25-like lysozyme | Protein 25-like lysozyme | | uniclust | UniRef100\_A0A4Q3KFV4 | 99.2 | 5.7e-14 | 1.1e-19 | 96.8 | 107 | (4, 110) | 112 | (1, 113) | 365 | Baseplate assembly protein | Baseplate assembly protein | | uniclust | UniRef100\_A0A0H4WV92 | 99.2 | 5.4e-14 | 1.1e-19 | 89.4 | 96 | (11, 108) | 112 | (16, 112) | 133 | IraD/Gp25-like domain-containing protein | IraD/Gp25-like domain-containing protein | | uniclust | UniRef100\_A0A936U591 | 99.2 | 6.1e-14 | 1.1e-19 | 90.1 | 72 | (12, 83) | 112 | (4, 75) | 203 | GPW/gp25 family protein | GPW/gp25 family protein | | uniclust | UniRef100\_A0A0J8DAG7 | 99.2 | 7.3e-14 | 1.4e-19 | 86.4 | 87 | (11, 99) | 112 | (19, 106) | 114 | Putative lysozyme | Putative lysozyme | | uniclust | UniRef100\_A0A0N7CEU8 | 99.2 | 8.2e-14 | 1.5e-19 | 85.5 | 106 | (2, 107) | 112 | (22, 130) | 137 | Putative baseplate wedge subunit | Putative baseplate wedge subunit | | uniclust | UniRef100\_A0A7X5D8Q1 | 99.2 | 8.7e-14 | 1.6e-19 | 86.6 | 101 | (3, 103) | 112 | (45, 149) | 162 | IraD/Gp25-like domain-containing protein | IraD/Gp25-like domain-containing protein | | uniclust | UniRef100\_A0A7L6A4Z7 | 99.2 | 1e-13 | 1.8e-19 | 89.1 | 103 | (8, 110) | 112 | (25, 130) | 202 | GPW/gp25 family protein | GPW/gp25 family protein | | uniclust | UniRef100\_A0A084SY44 | 99.2 | 9.6e-14 | 1.9e-19 | 89.1 | 90 | (11, 102) | 112 | (38, 129) | 150 | IraD/Gp25-like domain-containing protein | IraD/Gp25-like domain-containing protein | | uniclust | UniRef100\_A0A158DVL2 | 99.2 | 9.8e-14 | 1.9e-19 | 96.3 | 89 | (8, 98) | 112 | (109, 200) | 248 | Bacteriophage protein | Bacteriophage protein | | uniclust | UniRef100\_A0A074LKB3 | 99.2 | 1e-13 | 2e-19 | 87.4 | 93 | (8, 101) | 112 | (21, 116) | 133 | Phage baseplate protein | Phage baseplate protein | | uniclust | UniRef100\_A0A0E2HGZ0 | 99.2 | 1e-13 | 2e-19 | 89.2 | 103 | (7, 110) | 112 | (25, 130) | 142 | Phage protein | Phage protein | | uniclust | UniRef100\_A0A0F9Q0T0 | 99.2 | 1.2e-13 | 2.2e-19 | 88.7 | 93 | (11, 103) | 112 | (48, 151) | 164 | IraD/Gp25-like domain-containing protein | IraD/Gp25-like domain-containing protein | | uniclust | UniRef100\_UPI0011A0CDE0 | 99.2 | 1.3e-13 | 2.3e-19 | 95.3 | 99 | (1, 99) | 112 | (6, 104) | 368 | contractile injection system protein, VgrG/Pvc8 family | contractile injection system protein, VgrG/Pvc8 family | | uniclust | UniRef100\_A0A2N1PDC8 | 99.2 | 1.2e-13 | 2.4e-19 | 85.3 | 94 | (11, 104) | 112 | (17, 115) | 124 | IraD/Gp25-like domain-containing protein | IraD/Gp25-like domain-containing protein | | uniclust | UniRef100\_A0A017T4E9 | 99.2 | 1.3e-13 | 2.6e-19 | 92.6 | 88 | (13, 102) | 112 | (46, 136) | 178 | IraD/Gp25-like domain-containing protein | IraD/Gp25-like domain-containing protein | | uniclust | UniRef100\_A0A379WI79 | 99.2 | 1.4e-13 | 2.7e-19 | 81.1 | 74 | (9, 83) | 112 | (20, 93) | 101 | Baseplate protein | Baseplate protein | | uniclust | UniRef100\_A0A061NU27 | 99.2 | 1.4e-13 | 2.7e-19 | 84.2 | 85 | (13, 99) | 112 | (19, 104) | 107 | IraD/Gp25-like domain-containing protein | IraD/Gp25-like domain-containing protein | | uniclust | UniRef100\_A0A1E5XW96 | 99.2 | 1.4e-13 | 2.8e-19 | 89.8 | 99 | (11, 110) | 112 | (62, 165) | 170 | IraD/Gp25-like domain-containing protein | IraD/Gp25-like domain-containing protein | | uniclust | UniRef100\_A0A150Q962 | 99.2 | 1.7e-13 | 3.2e-19 | 86.9 | 99 | (8, 109) | 112 | (23, 123) | 141 | IraD/Gp25-like domain-containing protein | IraD/Gp25-like domain-containing protein | | uniclust | UniRef100\_A0A323U6E9 | 99.2 | 1.7e-13 | 3.4e-19 | 87.7 | 99 | (9, 110) | 112 | (21, 119) | 138 | IraD/Gp25-like domain-containing protein | IraD/Gp25-like domain-containing protein | | uniclust | UniRef100\_A0A812QV09 | 99.2 | 1.9e-13 | 3.5e-19 | 104.4 | 97 | (11, 107) | 112 | (1069, 1169) | 1173 | XkdF protein | XkdF protein | | uniclust | UniRef100\_A0A135ZED6 | 99.2 | 1.8e-13 | 3.6e-19 | 86.2 | 96 | (3, 101) | 112 | (16, 115) | 133 | DUF2634 domain-containing protein | DUF2634 domain-containing protein | | uniclust | UniRef100\_A0A1Y3QHP6 | 99.2 | 1.9e-13 | 3.6e-19 | 87.4 | 101 | (8, 109) | 112 | (34, 138) | 153 | Uncharacterized protein (Fragment) | Uncharacterized protein (Fragment) | | uniclust | UniRef100\_A0A174Z8M2 | 99.1 | 2e-13 | 4e-19 | 86.1 | 88 | (11, 100) | 112 | (36, 124) | 131 | Gene 25-like lysozyme | Gene 25-like lysozyme | | uniclust | UniRef100\_A0A381ZQG4 | 99.1 | 2.6e-13 | 4.9e-19 | 79.3 | 84 | (19, 102) | 112 | (5, 89) | 99 | IraD/Gp25-like domain-containing protein | IraD/Gp25-like domain-containing protein | | uniclust | UniRef100\_A0A662G9R2 | 99.1 | 3.7e-13 | 7e-19 | 82.8 | 97 | (8, 105) | 112 | (24, 127) | 137 | Uncharacterized protein (Fragment) | Uncharacterized protein (Fragment) | | uniclust | UniRef100\_A0A010SSS2 | 99.1 | 4e-13 | 7.7e-19 | 86.1 | 99 | (8, 111) | 112 | (25, 128) | 146 | Phage tail protein | Phage tail protein | | uniclust | UniRef100\_A0A1V5ZPA0 | 99.1 | 4.5e-13 | 8.4e-19 | 83.5 | 101 | (8, 108) | 112 | (30, 133) | 145 | Gene 25-like lysozyme | Gene 25-like lysozyme | | uniclust | UniRef100\_A0A2D5F9D6 | 99.1 | 5.1e-13 | 9.5e-19 | 83.8 | 96 | (5, 100) | 112 | (39, 136) | 147 | IraD/Gp25-like domain-containing protein | IraD/Gp25-like domain-containing protein | | uniclust | UniRef100\_A0A2D4SL70 | 99.1 | 5.8e-13 | 1.1e-18 | 85.9 | 72 | (11, 82) | 112 | (20, 91) | 151 | IraD/Gp25-like domain-containing protein | IraD/Gp25-like domain-containing protein | | uniclust | UniRef100\_A0A0C1PQ31 | 99.1 | 6.6e-13 | 1.3e-18 | 84.2 | 86 | (11, 99) | 112 | (22, 109) | 126 | DUF2634 domain-containing protein | DUF2634 domain-containing protein | | uniclust | UniRef100\_A0A060QLL0 | 99.1 | 6.8e-13 | 1.3e-18 | 84.0 | 100 | (8, 111) | 112 | (23, 127) | 130 | Putative phage tail protein | Putative phage tail protein | | uniclust | UniRef100\_A0A7Z0LWZ0 | 99.1 | 7.3e-13 | 1.4e-18 | 96.3 | 79 | (1, 79) | 112 | (1, 79) | 598 | GPW/gp25 family protein | GPW/gp25 family protein | | uniclust | UniRef100\_A0A069AW33 | 99.1 | 6.9e-13 | 1.4e-18 | 88.9 | 92 | (4, 98) | 112 | (45, 141) | 178 | Putative phage protein | Putative phage protein | | uniclust | UniRef100\_A0A4Y7RJW3 | 99.1 | 7.6e-13 | 1.5e-18 | 85.1 | 102 | (8, 110) | 112 | (25, 130) | 152 | IraD/Gp25-like domain-containing protein | IraD/Gp25-like domain-containing protein | | uniclust | UniRef100\_A0A3C2A343 | 99.1 | 8.4e-13 | 1.5e-18 | 83.5 | 99 | (11, 109) | 112 | (69, 169) | 176 | IraD/Gp25-like domain-containing protein | IraD/Gp25-like domain-containing protein | | uniclust | UniRef100\_A0A2G6EIV6 | 99.1 | 8.8e-13 | 1.6e-18 | 87.5 | 75 | (2, 76) | 112 | (11, 85) | 253 | IraD/Gp25-like domain-containing protein | IraD/Gp25-like domain-containing protein | | uniclust | UniRef100\_A0A6L3Z3G6 | 99.1 | 9e-13 | 1.6e-18 | 81.8 | 102 | (1, 102) | 112 | (43, 144) | 154 | IraD/Gp25-like domain-containing protein | IraD/Gp25-like domain-containing protein | | uniclust | UniRef100\_A0A2S6HUW9 | 99.1 | 8.9e-13 | 1.7e-18 | 81.6 | 84 | (13, 99) | 112 | (20, 103) | 123 | IraD/Gp25-like domain-containing protein | IraD/Gp25-like domain-containing protein | | uniclust | UniRef100\_A0A0H3ZTY4 | 99.1 | 8.9e-13 | 1.7e-18 | 81.1 | 100 | (1, 102) | 112 | (3, 105) | 112 | Phage protein | Phage protein | | uniclust | UniRef100\_A0A161YFA5 | 99.1 | 9.1e-13 | 1.7e-18 | 78.8 | 80 | (17, 98) | 112 | (16, 95) | 100 | Gene 25-like lysozyme | Gene 25-like lysozyme | | uniclust | UniRef100\_A0A0G3AVB6 | 99.1 | 9.6e-13 | 1.8e-18 | 81.6 | 96 | (13, 108) | 112 | (22, 119) | 124 | IraD/Gp25-like domain-containing protein | IraD/Gp25-like domain-containing protein | | uniclust | UniRef100\_UPI0011E4B62D | 99.1 | 1e-12 | 1.9e-18 | 72.5 | 71 | (11, 82) | 112 | (1, 71) | 75 | GPW/gp25 family protein | GPW/gp25 family protein | | uniclust | UniRef100\_A0A126QLP9 | 99.0 | 1.1e-12 | 2.2e-18 | 86.6 | 104 | (7, 111) | 112 | (51, 158) | 183 | Baseplate assembly protein | Baseplate assembly protein | | uniclust | UniRef100\_UPI001E5F099E | 99.0 | 1.4e-12 | 2.6e-18 | 76.0 | 70 | (15, 84) | 112 | (5, 74) | 103 | GPW/gp25 family protein | GPW/gp25 family protein | | uniclust | UniRef100\_A0A2V8VC79 | 99.0 | 1.3e-12 | 2.6e-18 | 85.1 | 88 | (13, 102) | 112 | (34, 124) | 146 | Type VI secretion system baseplate subunit TssE | Type VI secretion system baseplate subunit TssE | | uniclust | UniRef100\_A0A061Q550 | 99.0 | 1.3e-12 | 2.7e-18 | 85.2 | 94 | (13, 108) | 112 | (46, 142) | 157 | IraD/Gp25-like domain-containing protein | IraD/Gp25-like domain-containing protein | | uniclust | UniRef100\_A0A081NWR0 | 99.0 | 1.5e-12 | 2.8e-18 | 79.9 | 84 | (11, 98) | 112 | (21, 106) | 120 | Phage protein | Phage protein | | uniclust | UniRef100\_A0A7C2B4J0 | 99.0 | 1.5e-12 | 2.8e-18 | 79.1 | 86 | (11, 97) | 112 | (25, 110) | 134 | IraD/Gp25-like domain-containing protein | IraD/Gp25-like domain-containing protein | | uniclust | UniRef100\_A0A0R2JEB0 | 99.0 | 1.6e-12 | 3e-18 | 79.4 | 84 | (11, 98) | 112 | (17, 102) | 115 | IraD/Gp25-like domain-containing protein | IraD/Gp25-like domain-containing protein | | uniclust | UniRef100\_A0A5C7JCT6 | 99.0 | 1.7e-12 | 3.2e-18 | 83.1 | 79 | (5, 83) | 112 | (22, 104) | 159 | IraD/Gp25-like domain-containing protein | IraD/Gp25-like domain-containing protein | | uniclust | UniRef100\_A0A037YM48 | 99.0 | 1.6e-12 | 3.2e-18 | 86.1 | 96 | (12, 109) | 112 | (46, 145) | 172 | Type VI secretion protein | Type VI secretion protein | | uniclust | UniRef100\_A0A0F9TG53 | 99.0 | 1.8e-12 | 3.4e-18 | 80.8 | 98 | (11, 109) | 112 | (40, 139) | 159 | IraD/Gp25-like domain-containing protein | IraD/Gp25-like domain-containing protein | | uniclust | UniRef100\_A0A1C5ZER0 | 99.0 | 2e-12 | 3.8e-18 | 81.0 | 104 | (3, 110) | 112 | (10, 124) | 126 | Protein of uncharacterized function (DUF2634) | Protein of uncharacterized function (DUF2634) | | uniclust | UniRef100\_A0A1Q1PVL8 | 99.0 | 2.2e-12 | 4.3e-18 | 82.8 | 94 | (12, 106) | 112 | (37, 130) | 145 | Type IV secretion protein | Type IV secretion protein | | uniclust | UniRef100\_A0A061NKR2 | 99.0 | 2.2e-12 | 4.5e-18 | 86.6 | 93 | (3, 98) | 112 | (50, 147) | 177 | Phage-like element PBSX protein XkdS | Phage-like element PBSX protein XkdS | | uniclust | UniRef100\_A0A968VPK2 | 99.0 | 2.7e-12 | 5e-18 | 82.9 | 73 | (11, 83) | 112 | (27, 99) | 203 | GPW/gp25 family protein | GPW/gp25 family protein | | uniclust | UniRef100\_D6H7Q3 | 99.0 | 2.8e-12 | 5.2e-18 | 87.0 | 98 | (5, 102) | 112 | (195, 294) | 300 | IraD/Gp25-like domain-containing protein | IraD/Gp25-like domain-containing protein | | uniclust | UniRef100\_A0A4Q6D2R8 | 99.0 | 2.9e-12 | 5.4e-18 | 75.3 | 98 | (1, 99) | 112 | (1, 98) | 106 | Uncharacterized protein | Uncharacterized protein | | uniclust | UniRef100\_A0A973HYV5 | 99.0 | 3e-12 | 5.5e-18 | 76.5 | 104 | (5, 108) | 112 | (9, 116) | 119 | GPW/gp25 family protein | GPW/gp25 family protein | | uniclust | UniRef100\_B3QTI5 | 99.0 | 3.1e-12 | 5.7e-18 | 94.4 | 90 | (11, 102) | 112 | (22, 111) | 687 | GPW/gp25 family protein | GPW/gp25 family protein | | uniclust | UniRef100\_A0A023PTA0 | 99.0 | 3.1e-12 | 6.4e-18 | 85.3 | 89 | (11, 101) | 112 | (50, 141) | 166 | Type VI secretion system protein | Type VI secretion system protein | | uniclust | UniRef100\_A0A084JID6 | 99.0 | 3.6e-12 | 7.1e-18 | 81.6 | 89 | (7, 98) | 112 | (29, 122) | 138 | Phage protein | Phage protein | | uniclust | UniRef100\_UPI000B2BD334 | 99.0 | 4e-12 | 7.3e-18 | 78.3 | 74 | (9, 83) | 112 | (37, 110) | 144 | GPW/gp25 family protein | GPW/gp25 family protein | | uniclust | UniRef100\_A0A377AWY0 | 99.0 | 4.4e-12 | 8.2e-18 | 74.0 | 86 | (25, 110) | 112 | (1, 86) | 94 | Baseplate assembly protein W | Baseplate assembly protein W | | uniclust | UniRef100\_A0A6J5KRL7 | 99.0 | 4.4e-12 | 8.7e-18 | 81.9 | 86 | (13, 99) | 112 | (31, 117) | 143 | Baseplate wedge subunit | Baseplate wedge subunit | | uniclust | UniRef100\_A0A936LRJ0 | 99.0 | 4.8e-12 | 8.9e-18 | 78.2 | 95 | (14, 108) | 112 | (27, 124) | 135 | GPW/gp25 family protein | GPW/gp25 family protein | | uniclust | UniRef100\_A0A958S4Z7 | 99.0 | 5e-12 | 9.2e-18 | 75.8 | 100 | (11, 110) | 112 | (18, 119) | 121 | GPW/gp25 family protein | GPW/gp25 family protein | | uniclust | UniRef100\_A0A5J4RAP1 | 98.9 | 5.1e-12 | 9.8e-18 | 78.9 | 91 | (14, 104) | 112 | (29, 121) | 129 | IraD/Gp25-like domain-containing protein | IraD/Gp25-like domain-containing protein | | uniclust | UniRef100\_A0A3D4L1F4 | 98.9 | 5.5e-12 | 1e-17 | 75.3 | 85 | (13, 99) | 112 | (19, 103) | 107 | IraD/Gp25-like domain-containing protein | IraD/Gp25-like domain-containing protein | | uniclust | UniRef100\_A0A1Z9GYA1 | 98.9 | 5.6e-12 | 1e-17 | 81.4 | 98 | (11, 109) | 112 | (52, 150) | 179 | IraD/Gp25-like domain-containing protein | IraD/Gp25-like domain-containing protein | | uniclust | UniRef100\_E2CFL3 | 98.9 | 5.7e-12 | 1.1e-17 | 76.5 | 100 | (2, 102) | 112 | (3, 103) | 120 | GpW/gp25 family protein | GpW/gp25 family protein | | uniclust | UniRef100\_A0A645HFP9 | 98.9 | 5.9e-12 | 1.1e-17 | 78.1 | 100 | (11, 111) | 112 | (44, 148) | 151 | IraD/Gp25-like domain-containing protein | IraD/Gp25-like domain-containing protein | | uniclust | UniRef100\_A0A0M0SE81 | 98.9 | 6.6e-12 | 1.3e-17 | 78.2 | 93 | (3, 102) | 112 | (12, 108) | 117 | IraD/Gp25-like domain-containing protein | IraD/Gp25-like domain-containing protein | | uniclust | UniRef100\_UPI00106CED4F | 98.9 | 7.2e-12 | 1.3e-17 | 77.0 | 100 | (8, 107) | 112 | (23, 126) | 133 | GPW/gp25 family protein | GPW/gp25 family protein | | uniclust | UniRef100\_A0A2X1LMF2 | 98.9 | 8.7e-12 | 1.6e-17 | 66.5 | 51 | (2, 52) | 112 | (6, 56) | 58 | Baseplate assembly protein W | Baseplate assembly protein W | | uniclust | UniRef100\_A0A952LGA6 | 98.9 | 8.8e-12 | 1.7e-17 | 72.4 | 66 | (34, 99) | 112 | (2, 68) | 86 | GPW/gp25 family protein | GPW/gp25 family protein | | uniclust | UniRef100\_A0A090SWE0 | 98.9 | 8.9e-12 | 1.7e-17 | 76.3 | 108 | (2, 109) | 112 | (5, 115) | 124 | Uncharacterized protein | Uncharacterized protein | | uniclust | UniRef100\_A0A2U3KMN3 | 98.9 | 9.6e-12 | 1.8e-17 | 76.5 | 95 | (13, 108) | 112 | (26, 121) | 126 | IraD/Gp25-like domain-containing protein | IraD/Gp25-like domain-containing protein | | uniclust | UniRef100\_A0A8S5UG22 | 98.9 | 1.1e-11 | 2e-17 | 77.0 | 100 | (11, 110) | 112 | (24, 126) | 131 | Baseplate wedge protein | Baseplate wedge protein | | uniclust | UniRef100\_A0A015WIV6 | 98.9 | 1.2e-11 | 2.4e-17 | 79.5 | 96 | (12, 107) | 112 | (24, 133) | 144 | Lysozyme family protein | Lysozyme family protein | | uniclust | UniRef100\_A0A0W0VMK7 | 98.9 | 1.3e-11 | 2.5e-17 | 78.4 | 102 | (4, 108) | 112 | (26, 129) | 144 | IraD/Gp25-like domain-containing protein | IraD/Gp25-like domain-containing protein | | uniclust | UniRef100\_A0A1G8FCC5 | 98.9 | 1.3e-11 | 2.5e-17 | 78.0 | 80 | (11, 92) | 112 | (33, 113) | 138 | Phage baseplate assembly protein W | Phage baseplate assembly protein W | | uniclust | UniRef100\_A0A3A9EVM2 | 98.9 | 1.7e-11 | 3.1e-17 | 74.5 | 87 | (4, 93) | 112 | (40, 126) | 131 | IraD/Gp25-like domain-containing protein | IraD/Gp25-like domain-containing protein | | uniclust | UniRef100\_A0A096B5Z2 | 98.9 | 1.7e-11 | 3.4e-17 | 79.2 | 88 | (11, 101) | 112 | (21, 108) | 133 | Uncharacterized protein | Uncharacterized protein | | uniclust | UniRef100\_A0A0D0QWP6 | 98.9 | 1.8e-11 | 3.5e-17 | 79.5 | 93 | (11, 105) | 112 | (43, 138) | 146 | Phage-like element PBSX protein xkdS | Phage-like element PBSX protein xkdS | | uniclust | UniRef100\_A0A497PPN4 | 98.9 | 1.9e-11 | 3.5e-17 | 75.1 | 85 | (11, 96) | 112 | (30, 116) | 131 | IraD/Gp25-like domain-containing protein | IraD/Gp25-like domain-containing protein | | uniclust | UniRef100\_UPI0020131D42 | 98.9 | 1.9e-11 | 3.6e-17 | 85.8 | 75 | (1, 75) | 112 | (151, 225) | 396 | phage baseplate assembly protein V | phage baseplate assembly protein V | | uniclust | UniRef100\_A0A1V5IBU2 | 98.8 | 2.2e-11 | 4.1e-17 | 75.8 | 70 | (13, 82) | 112 | (26, 96) | 153 | Gene 25-like lysozyme | Gene 25-like lysozyme | | uniclust | UniRef100\_A0A0F7L855 | 98.8 | 2.6e-11 | 4.9e-17 | 76.3 | 91 | (12, 102) | 112 | (37, 131) | 144 | IraD/Gp25-like domain-containing protein | IraD/Gp25-like domain-containing protein | | uniclust | UniRef100\_A0A353BET6 | 98.8 | 2.8e-11 | 5.1e-17 | 76.0 | 86 | (13, 98) | 112 | (44, 137) | 161 | IraD/Gp25-like domain-containing protein | IraD/Gp25-like domain-containing protein | | uniclust | UniRef100\_A0A2K8LN18 | 98.8 | 3e-11 | 5.5e-17 | 77.2 | 88 | (15, 103) | 112 | (84, 172) | 182 | IraD/Gp25-like domain-containing protein | IraD/Gp25-like domain-containing protein | | uniclust | UniRef100\_A0A0B6CV24 | 98.8 | 3.4e-11 | 6.2e-17 | 71.5 | 96 | (3, 99) | 112 | (2, 98) | 112 | 25-like lysozyme family protein | 25-like lysozyme family protein | | uniclust | UniRef100\_A0A2D6XAB7 | 98.8 | 3.8e-11 | 7.2e-17 | 79.9 | 93 | (13, 105) | 112 | (91, 189) | 219 | IraD/Gp25-like domain-containing protein | IraD/Gp25-like domain-containing protein | | uniclust | UniRef100\_A0A023Q087 | 98.8 | 3.7e-11 | 7.4e-17 | 81.5 | 90 | (15, 106) | 112 | (47, 138) | 190 | Type VI secretion protein | Type VI secretion protein | | uniclust | UniRef100\_A0A7X6JWS6 | 98.8 | 4e-11 | 7.5e-17 | 71.9 | 74 | (33, 106) | 112 | (1, 74) | 109 | IraD/Gp25-like domain-containing protein | IraD/Gp25-like domain-containing protein | | uniclust | UniRef100\_A0A8S5MEP5 | 98.8 | 4.1e-11 | 7.6e-17 | 73.3 | 89 | (11, 99) | 112 | (25, 116) | 135 | Baseplate wedge protein | Baseplate wedge protein | | uniclust | UniRef100\_A0A1M7CV50 | 98.8 | 4.1e-11 | 7.7e-17 | 74.9 | 99 | (11, 109) | 112 | (23, 123) | 133 | Gene 25-like lysozyme | Gene 25-like lysozyme | | uniclust | UniRef100\_A0A0B0SDG5 | 98.8 | 3.9e-11 | 7.7e-17 | 81.9 | 99 | (11, 111) | 112 | (110, 208) | 214 | LysM domain-containing protein | LysM domain-containing protein | | uniclust | UniRef100\_A0A0R1NHW6 | 98.8 | 4.4e-11 | 8.3e-17 | 74.1 | 85 | (11, 100) | 112 | (23, 108) | 121 | Phage protein | Phage protein | | uniclust | UniRef100\_A0A1I1MPJ1 | 98.8 | 4.7e-11 | 8.9e-17 | 75.2 | 73 | (11, 83) | 112 | (23, 96) | 140 | Phage baseplate assembly protein W | Phage baseplate assembly protein W | | uniclust | UniRef100\_A0A2D3VZ43 | 98.8 | 5e-11 | 9.1e-17 | 69.9 | 87 | (18, 106) | 112 | (7, 93) | 103 | Baseplate assembly protein | Baseplate assembly protein | | uniclust | UniRef100\_A0A1Q6RI12 | 98.8 | 4.7e-11 | 9.2e-17 | 75.1 | 88 | (11, 100) | 112 | (21, 108) | 123 | IraD/Gp25-like domain-containing protein | IraD/Gp25-like domain-containing protein | | uniclust | UniRef100\_A0A1R4HML8 | 98.8 | 5.1e-11 | 9.6e-17 | 74.1 | 84 | (11, 99) | 112 | (19, 105) | 127 | Uncharacterized protein similar to VCA0109 | Uncharacterized protein similar to VCA0109 | | uniclust | UniRef100\_A0A268THM0 | 98.8 | 5.4e-11 | 1e-16 | 70.9 | 67 | (17, 84) | 112 | (5, 72) | 91 | IraD/Gp25-like domain-containing protein | IraD/Gp25-like domain-containing protein | | uniclust | UniRef100\_A0A0F7FGC4 | 98.8 | 6.1e-11 | 1.1e-16 | 71.2 | 97 | (11, 108) | 112 | (17, 113) | 119 | IraD/Gp25-like domain-containing protein | IraD/Gp25-like domain-containing protein | | uniclust | UniRef100\_A0A3S4FEP7 | 98.8 | 6e-11 | 1.1e-16 | 70.2 | 71 | (1, 71) | 112 | (4, 74) | 102 | Phage baseplate assembly protein W | Phage baseplate assembly protein W | | uniclust | UniRef100\_A0A482MLI0 | 98.8 | 6.1e-11 | 1.1e-16 | 76.2 | 99 | (10, 108) | 112 | (43, 146) | 156 | Baseplate assembly protein | Baseplate assembly protein | | uniclust | UniRef100\_X0WMB6 | 98.8 | 7.4e-11 | 1.4e-16 | 69.1 | 76 | (24, 99) | 112 | (2, 78) | 102 | IraD/Gp25-like domain-containing protein (Fragment) | IraD/Gp25-like domain-containing protein (Fragment) | | uniclust | UniRef100\_A0A0M1LJ08 | 98.8 | 7.3e-11 | 1.4e-16 | 71.9 | 71 | (12, 84) | 112 | (16, 86) | 104 | IraD/Gp25-like domain-containing protein | IraD/Gp25-like domain-containing protein | | uniclust | UniRef100\_A0A1Y6CPE4 | 98.7 | 8.3e-11 | 1.5e-16 | 69.4 | 97 | (1, 98) | 112 | (1, 97) | 106 | IraD/Gp25-like domain-containing protein | IraD/Gp25-like domain-containing protein | | uniclust | UniRef100\_A0A0A3Z8U7 | 98.7 | 7.9e-11 | 1.6e-16 | 74.5 | 105 | (4, 110) | 112 | (6, 119) | 122 | Phage GP46 family protein | Phage GP46 family protein | | uniclust | UniRef100\_A0A023WLJ8 | 98.7 | 8.1e-11 | 1.6e-16 | 78.4 | 92 | (13, 106) | 112 | (32, 126) | 173 | GPW/gp25 family protein | GPW/gp25 family protein | | uniclust | UniRef100\_A0A010SNZ9 | 98.7 | 8.1e-11 | 1.6e-16 | 77.4 | 92 | (14, 106) | 112 | (36, 134) | 154 | Type VI secretion protein | Type VI secretion protein | | uniclust | UniRef100\_A0A0A7GG36 | 98.7 | 8.6e-11 | 1.6e-16 | 72.1 | 86 | (11, 99) | 112 | (24, 110) | 119 | IraD/Gp25-like domain-containing protein | IraD/Gp25-like domain-containing protein | | uniclust | UniRef100\_A0A399IS01 | 98.7 | 8.8e-11 | 1.6e-16 | 71.4 | 97 | (8, 105) | 112 | (16, 115) | 123 | DUF2634 domain-containing protein | DUF2634 domain-containing protein | | uniclust | UniRef100\_A0A1B7X0G4 | 98.7 | 9e-11 | 1.8e-16 | 74.5 | 89 | (7, 102) | 112 | (22, 114) | 127 | Uncharacterized protein | Uncharacterized protein | | uniclust | UniRef100\_A0A126R1Q4 | 98.7 | 9.5e-11 | 1.8e-16 | 73.2 | 91 | (7, 99) | 112 | (20, 114) | 131 | Phage-related protein | Phage-related protein | | uniclust | UniRef100\_A0A0F4NK88 | 98.7 | 1e-10 | 1.8e-16 | 70.3 | 70 | (15, 84) | 112 | (35, 104) | 119 | IraD/Gp25-like domain-containing protein | IraD/Gp25-like domain-containing protein | | uniclust | UniRef100\_UPI00211A9560 | 98.7 | 1e-10 | 1.9e-16 | 72.5 | 70 | (13, 82) | 112 | (74, 143) | 145 | GPW/gp25 family protein | GPW/gp25 family protein | | uniclust | UniRef100\_A0A0F3RXN9 | 98.7 | 1e-10 | 1.9e-16 | 73.8 | 91 | (5, 99) | 112 | (22, 115) | 135 | IraD/Gp25-like domain-containing protein | IraD/Gp25-like domain-containing protein | | uniclust | UniRef100\_A0A833ALP0 | 98.7 | 1e-10 | 1.9e-16 | 65.3 | 46 | (8, 53) | 112 | (24, 71) | 71 | Phage tail protein (Fragment) | Phage tail protein (Fragment) | | uniclust | UniRef100\_UPI00114C9B31 | 98.7 | 1.3e-10 | 2.3e-16 | 69.1 | 79 | (1, 79) | 112 | (1, 79) | 103 | hypothetical protein | hypothetical protein | | uniclust | UniRef100\_A0A9D2RLV6 | 98.7 | 1.3e-10 | 2.5e-16 | 69.0 | 84 | (14, 99) | 112 | (10, 94) | 97 | Uncharacterized protein | Uncharacterized protein | | uniclust | UniRef100\_A0A2A4Y9P5 | 98.7 | 1.3e-10 | 2.6e-16 | 74.5 | 92 | (14, 105) | 112 | (26, 119) | 138 | Type VI secretion system baseplate subunit TssE | Type VI secretion system baseplate subunit TssE | | uniclust | UniRef100\_A0A1Q7YH61 | 98.7 | 1.4e-10 | 2.7e-16 | 73.3 | 98 | (11, 110) | 112 | (22, 131) | 144 | IraD/Gp25-like domain-containing protein | IraD/Gp25-like domain-containing protein | | uniclust | UniRef100\_A0A074VBM7 | 98.7 | 1.6e-10 | 2.9e-16 | 72.7 | 87 | (11, 99) | 112 | (57, 143) | 159 | Phage baseplate assembly protein W | Phage baseplate assembly protein W | | uniclust | UniRef100\_A0A3C0GL75 | 98.7 | 1.6e-10 | 3e-16 | 66.0 | 70 | (14, 83) | 112 | (3, 72) | 87 | IraD/Gp25-like domain-containing protein (Fragment) | IraD/Gp25-like domain-containing protein (Fragment) | | uniclust | UniRef100\_UPI001F535E75 | 98.7 | 1.8e-10 | 3.4e-16 | 73.7 | 72 | (2, 73) | 112 | (4, 75) | 179 | GPW/gp25 family protein | GPW/gp25 family protein | | uniclust | UniRef100\_A0A352A2I0 | 98.7 | 2e-10 | 4e-16 | 75.6 | 87 | (11, 99) | 112 | (46, 135) | 154 | DUF2634 domain-containing protein | DUF2634 domain-containing protein | | uniclust | UniRef100\_A0A075R4E3 | 98.7 | 2.2e-10 | 4.3e-16 | 73.2 | 103 | (8, 110) | 112 | (18, 128) | 134 | DUF2634 domain-containing protein | DUF2634 domain-containing protein | | uniclust | UniRef100\_A0A0C1PSI5 | 98.7 | 2.3e-10 | 4.6e-16 | 73.8 | 99 | (3, 106) | 112 | (14, 118) | 134 | DUF2634 domain-containing protein | DUF2634 domain-containing protein | | uniclust | UniRef100\_A0A843GHX7 | 98.7 | 2.5e-10 | 4.6e-16 | 70.0 | 104 | (4, 107) | 112 | (13, 123) | 134 | GPW/gp25 family protein | GPW/gp25 family protein | | uniclust | UniRef100\_A0A0W0SAI8 | 98.6 | 2.6e-10 | 4.8e-16 | 73.1 | 89 | (13, 102) | 112 | (39, 130) | 150 | IraD/Gp25-like domain-containing protein | IraD/Gp25-like domain-containing protein | | uniclust | UniRef100\_A0A8S5NX59 | 98.6 | 2.9e-10 | 5.2e-16 | 65.3 | 84 | (16, 100) | 112 | (2, 86) | 89 | Baseplate assembly protein | Baseplate assembly protein | | uniclust | UniRef100\_A0A350PF63 | 98.6 | 2.9e-10 | 5.4e-16 | 67.8 | 69 | (13, 82) | 112 | (26, 94) | 97 | IraD/Gp25-like domain-containing protein (Fragment) | IraD/Gp25-like domain-containing protein (Fragment) | | uniclust | UniRef100\_A0A014M2C2 | 98.6 | 2.9e-10 | 5.5e-16 | 60.8 | 49 | (2, 50) | 112 | (7, 55) | 55 | Baseplate assembly protein (Fragment) | Baseplate assembly protein (Fragment) | | uniclust | UniRef100\_A0A084J9B0 | 98.6 | 2.8e-10 | 5.6e-16 | 77.9 | 91 | (8, 100) | 112 | (52, 148) | 191 | DUF2634 domain-containing protein | DUF2634 domain-containing protein | | uniclust | UniRef100\_A0A1F5AKW5 | 98.6 | 3.2e-10 | 6.1e-16 | 73.9 | 85 | (15, 101) | 112 | (39, 126) | 156 | IraD/Gp25-like domain-containing protein | IraD/Gp25-like domain-containing protein | | uniclust | UniRef100\_A0A150QKC3 | 98.6 | 3.4e-10 | 6.8e-16 | 73.5 | 105 | (1, 109) | 112 | (9, 115) | 139 | IraD/Gp25-like domain-containing protein | IraD/Gp25-like domain-containing protein | | uniclust | UniRef100\_A0A7U3VTF8 | 98.6 | 4e-10 | 7.3e-16 | 67.2 | 106 | (1, 107) | 112 | (1, 106) | 111 | Baseplate assembly protein GpW | Baseplate assembly protein GpW | | uniclust | UniRef100\_A0A8S4QTC3 | 98.6 | 4e-10 | 7.4e-16 | 87.2 | 90 | (11, 100) | 112 | (641, 730) | 1131 | Jg18748 protein | Jg18748 protein | | uniclust | UniRef100\_A0A066TKD3 | 98.6 | 3.8e-10 | 7.4e-16 | 75.0 | 83 | (15, 98) | 112 | (43, 127) | 175 | IraD/Gp25-like domain-containing protein | IraD/Gp25-like domain-containing protein | | uniclust | UniRef100\_UPI00035FE005 | 98.6 | 3.9e-10 | 7.4e-16 | 79.3 | 97 | (8, 108) | 112 | (194, 295) | 304 | DUF2634 domain-containing protein | DUF2634 domain-containing protein | | uniclust | UniRef100\_UPI001F3F1559 | 98.6 | 4.2e-10 | 7.8e-16 | 70.6 | 94 | (11, 109) | 112 | (56, 151) | 155 | DUF2634 domain-containing protein | DUF2634 domain-containing protein | | uniclust | UniRef100\_UPI0020B36D29 | 98.6 | 4.7e-10 | 8.6e-16 | 62.1 | 62 | (1, 62) | 112 | (2, 63) | 72 | hypothetical protein | hypothetical protein | | uniclust | UniRef100\_A0A1F9MHG2 | 98.6 | 4.5e-10 | 8.7e-16 | 71.7 | 86 | (11, 97) | 112 | (25, 116) | 138 | IraD/Gp25-like domain-containing protein | IraD/Gp25-like domain-containing protein | | uniclust | UniRef100\_A0A1S6L331 | 98.6 | 4.7e-10 | 8.8e-16 | 71.1 | 99 | (10, 108) | 112 | (31, 135) | 141 | IraD/Gp25-like domain-containing protein | IraD/Gp25-like domain-containing protein | | uniclust | UniRef100\_UPI00211E9846 | 98.6 | 4.9e-10 | 9e-16 | 66.2 | 71 | (5, 75) | 112 | (19, 90) | 105 | GPW/gp25 family protein | GPW/gp25 family protein | | uniclust | UniRef100\_A0A014N481 | 98.6 | 4.8e-10 | 9.2e-16 | 76.3 | 85 | (16, 101) | 112 | (52, 137) | 204 | IraD/Gp25-like domain-containing protein | IraD/Gp25-like domain-containing protein | | uniclust | UniRef100\_S6ANJ8 | 98.6 | 5.2e-10 | 9.6e-16 | 70.3 | 84 | (11, 97) | 112 | (41, 126) | 156 | Uncharacterized protein | Uncharacterized protein | | uniclust | UniRef100\_A0A4P9VI48 | 98.6 | 5.3e-10 | 9.7e-16 | 64.6 | 78 | (2, 81) | 112 | (3, 80) | 92 | Baseplate assembly protein W | Baseplate assembly protein W | | uniclust | UniRef100\_A0A1F2QFX3 | 98.6 | 5.3e-10 | 1e-15 | 70.8 | 105 | (5, 110) | 112 | (15, 125) | 138 | IraD/Gp25-like domain-containing protein | IraD/Gp25-like domain-containing protein | | uniclust | UniRef100\_UPI001FEB107A | 98.6 | 5.5e-10 | 1e-15 | 63.8 | 81 | (27, 108) | 112 | (1, 82) | 86 | GPW/gp25 family protein | GPW/gp25 family protein | | uniclust | UniRef100\_A0A0B4ZXG6 | 98.6 | 5.7e-10 | 1e-15 | 63.0 | 66 | (37, 102) | 112 | (2, 68) | 80 | Baseplate wedge subunit | Baseplate wedge subunit | | uniclust | UniRef100\_A0A7U6KDW8 | 98.6 | 5.5e-10 | 1e-15 | 68.0 | 87 | (11, 99) | 112 | (15, 101) | 105 | IraD/Gp25-like domain-containing protein | IraD/Gp25-like domain-containing protein | | uniclust | UniRef100\_A0A317FGN1 | 98.6 | 5.8e-10 | 1.1e-15 | 75.2 | 95 | (11, 105) | 112 | (155, 251) | 262 | IraD/Gp25-like domain-containing protein | IraD/Gp25-like domain-containing protein | | uniclust | UniRef100\_A0A0N9ML22 | 98.6 | 6.2e-10 | 1.2e-15 | 70.1 | 83 | (11, 99) | 112 | (27, 111) | 123 | Uncharacterized protein | Uncharacterized protein | | uniclust | UniRef100\_A0A7V2KVL8 | 98.6 | 6.9e-10 | 1.3e-15 | 68.6 | 88 | (14, 101) | 112 | (15, 104) | 139 | GPW/gp25 family protein | GPW/gp25 family protein | | uniclust | UniRef100\_A0A9D7PFK3 | 98.6 | 7.7e-10 | 1.4e-15 | 63.6 | 74 | (26, 99) | 112 | (1, 75) | 89 | GPW/gp25 family protein | GPW/gp25 family protein | | uniclust | UniRef100\_A0A0M9DJQ4 | 98.6 | 7.5e-10 | 1.4e-15 | 68.2 | 84 | (11, 98) | 112 | (17, 102) | 118 | Phage portal protein | Phage portal protein | | uniclust | UniRef100\_A0A074M821 | 98.5 | 7.8e-10 | 1.5e-15 | 70.6 | 97 | (11, 109) | 112 | (36, 133) | 140 | Phage portal protein | Phage portal protein | | uniclust | UniRef100\_A0A8J3EAG9 | 98.5 | 9.2e-10 | 1.7e-15 | 60.0 | 52 | (1, 52) | 112 | (1, 52) | 66 | Uncharacterized protein | Uncharacterized protein | | uniclust | UniRef100\_A0A1C3NFC8 | 98.5 | 9.7e-10 | 1.8e-15 | 69.8 | 89 | (10, 98) | 112 | (58, 150) | 166 | IraD/Gp25-like domain-containing protein | IraD/Gp25-like domain-containing protein | | uniclust | UniRef100\_A0A350PFI1 | 98.5 | 9.8e-10 | 1.8e-15 | 66.6 | 90 | (11, 102) | 112 | (22, 111) | 122 | IraD/Gp25-like domain-containing protein | IraD/Gp25-like domain-containing protein | | uniclust | UniRef100\_A0A947D6A6 | 98.5 | 9.7e-10 | 1.8e-15 | 71.9 | 80 | (2, 81) | 112 | (36, 115) | 170 | Uncharacterized protein | Uncharacterized protein | | uniclust | UniRef100\_A0A1S1YVC0 | 98.5 | 1e-09 | 1.9e-15 | 66.4 | 85 | (13, 98) | 112 | (24, 108) | 120 | IraD/Gp25-like domain-containing protein | IraD/Gp25-like domain-containing protein | | uniclust | UniRef100\_A0A063BG98 | 98.5 | 1e-09 | 2e-15 | 72.2 | 107 | (3, 110) | 112 | (37, 146) | 164 | GP46 family protein | GP46 family protein | | uniclust | UniRef100\_F4BFR9 | 98.5 | 1.2e-09 | 2.3e-15 | 61.6 | 73 | (35, 107) | 112 | (1, 75) | 79 | IraD/Gp25-like domain-containing protein | IraD/Gp25-like domain-containing protein | | uniclust | UniRef100\_UPI000586A777 | 98.5 | 1.3e-09 | 2.3e-15 | 63.1 | 73 | (31, 103) | 112 | (1, 75) | 91 | GPW/gp25 family protein | GPW/gp25 family protein | | uniclust | UniRef100\_A0A0F6SF01 | 98.5 | 1.2e-09 | 2.3e-15 | 71.2 | 99 | (11, 110) | 112 | (45, 146) | 159 | IraD/Gp25-like domain-containing protein | IraD/Gp25-like domain-containing protein | | uniclust | UniRef100\_UPI001475626A | 98.5 | 1.3e-09 | 2.5e-15 | 57.2 | 45 | (14, 58) | 112 | (4, 48) | 54 | GPW/gp25 family protein | GPW/gp25 family protein | | uniclust | UniRef100\_A0A8S0HU87 | 98.5 | 1.4e-09 | 2.6e-15 | 59.5 | 57 | (27, 83) | 112 | (1, 57) | 67 | IraD/Gp25-like domain-containing protein | IraD/Gp25-like domain-containing protein | | uniclust | UniRef100\_A0A2V8SEE1 | 98.5 | 1.5e-09 | 2.8e-15 | 61.4 | 57 | (54, 110) | 112 | (5, 62) | 69 | Baseplate protein (Fragment) | Baseplate protein (Fragment) | | uniclust | UniRef100\_A0A1Z8WSX8 | 98.5 | 1.6e-09 | 3.1e-15 | 73.5 | 87 | (13, 99) | 112 | (67, 159) | 175 | IraD/Gp25-like domain-containing protein | IraD/Gp25-like domain-containing protein | | uniclust | UniRef100\_G8LQ32 | 98.5 | 1.8e-09 | 3.3e-15 | 64.7 | 65 | (2, 66) | 112 | (6, 70) | 112 | GPW/Gp25 Family Protein | GPW/Gp25 Family Protein | | uniclust | UniRef100\_UPI00058EE6FE | 98.5 | 1.8e-09 | 3.4e-15 | 63.3 | 81 | (13, 95) | 112 | (16, 96) | 99 | hypothetical protein | hypothetical protein | | uniclust | UniRef100\_A0A398BF99 | 98.5 | 1.8e-09 | 3.5e-15 | 69.8 | 102 | (5, 108) | 112 | (35, 142) | 147 | DUF2634 domain-containing protein | DUF2634 domain-containing protein | | uniclust | UniRef100\_UPI001D00D725 | 98.5 | 2.1e-09 | 3.8e-15 | 71.7 | 89 | (11, 99) | 112 | (127, 215) | 234 | GPW/gp25 family protein | GPW/gp25 family protein | | uniclust | UniRef100\_A0A0N1JQ98 | 98.5 | 2.1e-09 | 3.9e-15 | 69.5 | 68 | (15, 83) | 112 | (38, 105) | 145 | ImpF | ImpF | | uniclust | UniRef100\_A0A2D8ERB6 | 98.4 | 2.2e-09 | 4.1e-15 | 66.2 | 99 | (8, 106) | 112 | (17, 124) | 134 | IraD/Gp25-like domain-containing protein | IraD/Gp25-like domain-containing protein | | uniclust | UniRef100\_A0A174T7Q2 | 98.4 | 2.1e-09 | 4.2e-15 | 66.0 | 85 | (12, 98) | 112 | (7, 93) | 99 | Early E1A protein | Early E1A protein | | uniclust | UniRef100\_A0A7C4DJH8 | 98.4 | 2.5e-09 | 4.6e-15 | 69.2 | 65 | (11, 75) | 112 | (27, 91) | 186 | IraD/Gp25-like domain-containing protein | IraD/Gp25-like domain-containing protein | | uniclust | UniRef100\_A0A968I5W3 | 98.4 | 2.6e-09 | 4.7e-15 | 66.4 | 90 | (11, 100) | 112 | (20, 114) | 141 | GPW/gp25 family protein | GPW/gp25 family protein | | uniclust | UniRef100\_A0A0K1N0J6 | 98.4 | 2.6e-09 | 4.9e-15 | 67.9 | 83 | (13, 97) | 112 | (28, 113) | 138 | Type VI secretion system baseplate subunit TssE | Type VI secretion system baseplate subunit TssE | | uniclust | UniRef100\_A0A509B7Z6 | 98.4 | 3.2e-09 | 5.9e-15 | 64.9 | 86 | (14, 101) | 112 | (4, 93) | 113 | Type VI secretion system lysozyme-like protein | Type VI secretion system lysozyme-like protein | | uniclust | UniRef100\_A0A962Q7G8 | 98.4 | 3.6e-09 | 6.6e-15 | 64.6 | 87 | (11, 98) | 112 | (21, 108) | 125 | GPW/gp25 family protein | GPW/gp25 family protein | | uniclust | UniRef100\_UPI001EF54EB6 | 98.4 | 3.6e-09 | 6.6e-15 | 60.7 | 48 | (8, 55) | 112 | (30, 79) | 86 | GPW/gp25 family protein | GPW/gp25 family protein | | uniclust | UniRef100\_A0A327JB01 | 98.4 | 3.9e-09 | 7.2e-15 | 65.1 | 91 | (8, 100) | 112 | (33, 124) | 134 | IraD/Gp25-like domain-containing protein | IraD/Gp25-like domain-containing protein | | uniclust | UniRef100\_A0A7C3ETG1 | 98.4 | 4e-09 | 7.4e-15 | 66.2 | 84 | (15, 98) | 112 | (36, 127) | 141 | IraD/Gp25-like domain-containing protein | IraD/Gp25-like domain-containing protein | | uniclust | UniRef100\_A0A8J7EID1 | 98.4 | 4.8e-09 | 8.7e-15 | 62.6 | 71 | (13, 84) | 112 | (2, 75) | 108 | GPW/gp25 family protein | GPW/gp25 family protein | | uniclust | UniRef100\_A0A9D2GTM2 | 98.4 | 5e-09 | 9.1e-15 | 60.4 | 78 | (12, 92) | 112 | (6, 83) | 88 | GPW/gp25 family protein | GPW/gp25 family protein | | uniclust | UniRef100\_A0A248SJW8 | 98.4 | 5.1e-09 | 9.3e-15 | 63.9 | 98 | (11, 109) | 112 | (23, 121) | 124 | IraD/Gp25-like domain-containing protein | IraD/Gp25-like domain-containing protein | | uniclust | UniRef100\_A0A4R6LWW8 | 98.4 | 4.9e-09 | 9.4e-15 | 65.5 | 85 | (11, 99) | 112 | (22, 108) | 120 | Uncharacterized protein | Uncharacterized protein | | uniclust | UniRef100\_A0A662Q525 | 98.4 | 5.3e-09 | 9.8e-15 | 64.8 | 86 | (14, 99) | 112 | (38, 123) | 137 | IraD/Gp25-like domain-containing protein | IraD/Gp25-like domain-containing protein | | uniclust | UniRef100\_A0A509B7P4 | 98.4 | 5.5e-09 | 1e-14 | 58.7 | 42 | (11, 52) | 112 | (23, 64) | 71 | Putative bacteriophage baseplate protein | Putative bacteriophage baseplate protein | | uniclust | UniRef100\_UPI001CA5D6B8 | 98.3 | 6e-09 | 1.1e-14 | 63.2 | 93 | (12, 107) | 112 | (22, 115) | 119 | hypothetical protein | hypothetical protein | | uniclust | UniRef100\_A0A6J5RXQ6 | 98.3 | 6.2e-09 | 1.1e-14 | 63.4 | 100 | (11, 110) | 112 | (20, 119) | 123 | COG3628 Phage baseplate assembly protein W | COG3628 Phage baseplate assembly protein W | | uniclust | UniRef100\_U2F0H1 | 98.3 | 6.4e-09 | 1.2e-14 | 58.9 | 64 | (35, 98) | 112 | (1, 64) | 73 | Baseplate assembly protein W, putative | Baseplate assembly protein W, putative | | uniclust | UniRef100\_A0A1C5WXX2 | 98.3 | 6e-09 | 1.2e-14 | 67.5 | 82 | (11, 97) | 112 | (31, 112) | 131 | Uncharacterized protein | Uncharacterized protein | | uniclust | UniRef100\_A0A173R5E2 | 98.3 | 6.6e-09 | 1.3e-14 | 64.6 | 83 | (13, 98) | 112 | (7, 91) | 106 | Early E1A protein | Early E1A protein | | uniclust | UniRef100\_A0A0F6TGM0 | 98.3 | 7.2e-09 | 1.4e-14 | 65.0 | 100 | (5, 105) | 112 | (18, 120) | 129 | Putative base plate wedge subunit | Putative base plate wedge subunit | | uniclust | UniRef100\_A0A021X862 | 98.3 | 7.6e-09 | 1.5e-14 | 68.2 | 88 | (7, 98) | 112 | (36, 128) | 147 | Putative structural protein | Putative structural protein | | uniclust | UniRef100\_A0A1E3G6B2 | 98.3 | 8.5e-09 | 1.6e-14 | 63.1 | 104 | (2, 105) | 112 | (9, 120) | 125 | IraD/Gp25-like domain-containing protein | IraD/Gp25-like domain-containing protein | | uniclust | UniRef100\_A0A0C2UZ28 | 98.3 | 8.7e-09 | 1.6e-14 | 72.4 | 92 | (7, 100) | 112 | (184, 278) | 295 | LysM domain-containing protein | LysM domain-containing protein | | uniclust | UniRef100\_A0A069DI41 | 98.3 | 8.7e-09 | 1.7e-14 | 66.7 | 75 | (8, 83) | 112 | (26, 102) | 141 | Uncharacterized protein | Uncharacterized protein | | uniclust | UniRef100\_UPI0011682DE8 | 98.3 | 9.2e-09 | 1.7e-14 | 58.1 | 66 | (10, 77) | 112 | (12, 77) | 78 | GPW/gp25 family protein | GPW/gp25 family protein | | uniclust | UniRef100\_A0A9E7CRK2 | 98.3 | 9.7e-09 | 1.8e-14 | 64.7 | 85 | (11, 96) | 112 | (36, 121) | 142 | DUF2634 domain-containing protein | DUF2634 domain-containing protein | | uniclust | UniRef100\_R7UZ90 | 98.3 | 1e-08 | 1.9e-14 | 73.8 | 67 | (11, 77) | 112 | (366, 432) | 433 | Gp5/Type VI secretion system Vgr protein OB-fold domain-containing protein (Fragment) | Gp5/Type VI secretion system Vgr protein OB-fold domain-containing protein (Fragment) | | uniclust | UniRef100\_A0A1Y3AZE0 | 98.3 | 1e-08 | 1.9e-14 | 84.6 | 87 | (11, 97) | 112 | (3006, 3094) | 3230 | Chitinase-like protein | Chitinase-like protein | | uniclust | UniRef100\_A8V442 | 98.3 | 1.1e-08 | 2e-14 | 59.9 | 67 | (8, 75) | 112 | (19, 87) | 95 | Uncharacterized protein | Uncharacterized protein | | uniclust | UniRef100\_A0A017RUG6 | 98.3 | 9.6e-09 | 2e-14 | 69.1 | 87 | (11, 99) | 112 | (51, 140) | 156 | Phage protein | Phage protein | | uniclust | UniRef100\_A0A932TVE7 | 98.3 | 1.2e-08 | 2.2e-14 | 68.5 | 88 | (11, 99) | 112 | (131, 223) | 239 | DUF2634 domain-containing protein | DUF2634 domain-containing protein | | uniclust | UniRef100\_A0A933KI50 | 98.3 | 1.2e-08 | 2.2e-14 | 73.2 | 100 | (8, 109) | 112 | (317, 419) | 423 | DUF2634 domain-containing protein | DUF2634 domain-containing protein | | uniclust | UniRef100\_A0A3N7GF44 | 98.3 | 1.3e-08 | 2.4e-14 | 65.0 | 93 | (15, 108) | 112 | (69, 162) | 166 | IraD/Gp25-like domain-containing protein | IraD/Gp25-like domain-containing protein | | uniclust | UniRef100\_A0A920HWM8 | 98.3 | 1.4e-08 | 2.6e-14 | 60.2 | 78 | (5, 82) | 112 | (20, 99) | 103 | IraD/Gp25-like domain-containing protein | IraD/Gp25-like domain-containing protein | | uniclust | UniRef100\_A0A8I0H272 | 98.2 | 1.6e-08 | 2.9e-14 | 55.0 | 44 | (10, 53) | 112 | (6, 49) | 63 | Baseplate protein (Fragment) | Baseplate protein (Fragment) | | uniclust | UniRef100\_A0A1V3RQV3 | 98.2 | 1.8e-08 | 3.4e-14 | 63.6 | 72 | (11, 83) | 112 | (38, 109) | 153 | IraD/Gp25-like domain-containing protein | IraD/Gp25-like domain-containing protein | | uniclust | UniRef100\_UPI001C6791EA | 98.2 | 1.9e-08 | 3.4e-14 | 59.1 | 84 | (21, 105) | 112 | (4, 88) | 96 | GPW/gp25 family protein | GPW/gp25 family protein | | uniclust | UniRef100\_A0A166CB13 | 98.2 | 1.8e-08 | 3.5e-14 | 63.4 | 73 | (8, 81) | 112 | (17, 92) | 124 | DUF2634 domain-containing protein | DUF2634 domain-containing protein | | uniclust | UniRef100\_UPI001FCA211C | 98.2 | 2.1e-08 | 3.8e-14 | 61.0 | 52 | (11, 62) | 112 | (24, 75) | 119 | GPW/gp25 family protein | GPW/gp25 family protein | | uniclust | UniRef100\_UPI00132FC3C4 | 98.2 | 2.3e-08 | 4.2e-14 | 57.9 | 72 | (5, 81) | 112 | (8, 79) | 88 | type VI secretion system baseplate subunit TssE | type VI secretion system baseplate subunit TssE | | uniclust | UniRef100\_A0A1X1NFH7 | 98.2 | 2.4e-08 | 4.6e-14 | 72.8 | 92 | (8, 99) | 112 | (246, 341) | 366 | Baseplate protein J-like domain-containing protein | Baseplate protein J-like domain-containing protein | | uniclust | UniRef100\_A0A379FGB5 | 98.2 | 2.5e-08 | 4.8e-14 | 61.7 | 68 | (11, 80) | 112 | (33, 101) | 108 | TssC | TssC | | uniclust | UniRef100\_A0A061P919 | 98.2 | 2.6e-08 | 5.1e-14 | 66.9 | 92 | (4, 97) | 112 | (60, 159) | 178 | Phage-like element PBSX protein XkdS | Phage-like element PBSX protein XkdS | | uniclust | UniRef100\_A0A1C5PJ69 | 98.2 | 2.7e-08 | 5.4e-14 | 66.1 | 89 | (8, 98) | 112 | (48, 141) | 156 | Protein of uncharacterized function (DUF2634) | Protein of uncharacterized function (DUF2634) | | uniclust | UniRef100\_A0A521S4V9 | 98.2 | 2.9e-08 | 5.4e-14 | 70.2 | 87 | (14, 101) | 112 | (200, 287) | 300 | IraD/Gp25-like domain-containing protein | IraD/Gp25-like domain-containing protein | | uniclust | UniRef100\_A0A3S0EL96 | 98.2 | 3.2e-08 | 5.8e-14 | 64.5 | 87 | (15, 102) | 112 | (89, 176) | 187 | IraD/Gp25-like domain-containing protein | IraD/Gp25-like domain-containing protein | | uniclust | UniRef100\_UPI001F4C0C0A | 98.2 | 3.2e-08 | 5.9e-14 | 62.4 | 91 | (11, 102) | 112 | (44, 135) | 149 | GPW/gp25 family protein | GPW/gp25 family protein | | uniclust | UniRef100\_UPI001EF48225 | 98.1 | 3.5e-08 | 6.5e-14 | 63.1 | 87 | (13, 100) | 112 | (62, 149) | 164 | GPW/gp25 family protein | GPW/gp25 family protein | | uniclust | UniRef100\_A0A694VIP3 | 98.1 | 3.6e-08 | 6.7e-14 | 54.1 | 43 | (11, 53) | 112 | (15, 57) | 65 | Uncharacterized protein (Fragment) | Uncharacterized protein (Fragment) | | uniclust | UniRef100\_B1T4E9 | 98.1 | 3.7e-08 | 6.8e-14 | 57.2 | 73 | (35, 107) | 112 | (1, 75) | 89 | GPW/gp25 family protein | GPW/gp25 family protein | | uniclust | UniRef100\_A0A0S2ZE39 | 98.1 | 3.5e-08 | 6.9e-14 | 60.1 | 75 | (20, 97) | 112 | (15, 89) | 93 | Uncharacterized protein | Uncharacterized protein | | uniclust | UniRef100\_A0A961Y5S4 | 98.1 | 3.8e-08 | 7e-14 | 59.9 | 105 | (2, 108) | 112 | (4, 117) | 119 | Uncharacterized protein | Uncharacterized protein | | uniclust | UniRef100\_UPI0021D421C0 | 98.1 | 4e-08 | 7.3e-14 | 57.3 | 75 | (36, 110) | 112 | (9, 85) | 91 | GPW/gp25 family protein | GPW/gp25 family protein | | uniclust | UniRef100\_UPI000A5AA828 | 98.1 | 4e-08 | 7.4e-14 | 73.0 | 71 | (13, 83) | 112 | (34, 105) | 579 | GPW/gp25 family protein | GPW/gp25 family protein | | uniclust | UniRef100\_A0A084SH40 | 98.1 | 4e-08 | 7.7e-14 | 62.7 | 94 | (12, 108) | 112 | (20, 113) | 131 | IraD/Gp25-like domain-containing protein | IraD/Gp25-like domain-containing protein | | uniclust | UniRef100\_A0A4Z0GYW9 | 98.1 | 4.3e-08 | 8e-14 | 63.1 | 107 | (4, 111) | 112 | (57, 165) | 171 | IraD/Gp25-like domain-containing protein | IraD/Gp25-like domain-containing protein | | uniclust | UniRef100\_A0A017T1I7 | 98.1 | 4.2e-08 | 8.4e-14 | 68.1 | 96 | (14, 110) | 112 | (74, 179) | 197 | IraD/Gp25-like domain-containing protein | IraD/Gp25-like domain-containing protein | | uniclust | UniRef100\_UPI0008F12106 | 98.1 | 4.7e-08 | 8.6e-14 | 56.2 | 78 | (2, 110) | 112 | (6, 83) | 84 | baseplate assembly protein | baseplate assembly protein | | uniclust | UniRef100\_A0A661Z4X6 | 98.1 | 4.7e-08 | 8.6e-14 | 60.8 | 94 | (5, 98) | 112 | (12, 107) | 135 | IraD/Gp25-like domain-containing protein | IraD/Gp25-like domain-containing protein | | uniclust | UniRef100\_A0A428MS47 | 98.1 | 5e-08 | 9.3e-14 | 62.6 | 89 | (4, 95) | 112 | (33, 127) | 148 | DUF2634 domain-containing protein | DUF2634 domain-containing protein | | uniclust | UniRef100\_A0A0A1IWV2 | 98.1 | 4.8e-08 | 9.7e-14 | 64.5 | 88 | (8, 99) | 112 | (24, 116) | 140 | Uncharacterized protein | Uncharacterized protein | | uniclust | UniRef100\_A0A7C7FT58 | 98.1 | 5.3e-08 | 9.8e-14 | 57.9 | 81 | (18, 98) | 112 | (2, 88) | 102 | IraD/Gp25-like domain-containing protein | IraD/Gp25-like domain-containing protein | | uniclust | UniRef100\_UPI00053BFFF5 | 98.1 | 5.9e-08 | 1.1e-13 | 54.2 | 65 | (17, 83) | 112 | (5, 69) | 71 | GPW/gp25 family protein | GPW/gp25 family protein | | uniclust | UniRef100\_A0A959FP97 | 98.1 | 6e-08 | 1.1e-13 | 55.5 | 44 | (11, 54) | 112 | (30, 73) | 82 | GPW/gp25 family protein (Fragment) | GPW/gp25 family protein (Fragment) | | uniclust | UniRef100\_UPI0009E8DA5C | 98.1 | 6.4e-08 | 1.2e-13 | 61.7 | 66 | (16, 82) | 112 | (31, 96) | 149 | type VI secretion system baseplate subunit TssE | type VI secretion system baseplate subunit TssE | | uniclust | UniRef100\_A0A382IDM1 | 98.1 | 6.3e-08 | 1.2e-13 | 58.1 | 49 | (4, 52) | 112 | (43, 93) | 94 | Uncharacterized protein (Fragment) | Uncharacterized protein (Fragment) | | uniclust | UniRef100\_A0A3N7GKM9 | 98.1 | 6.6e-08 | 1.2e-13 | 54.3 | 39 | (4, 42) | 112 | (19, 59) | 73 | IraD/Gp25-like domain-containing protein (Fragment) | IraD/Gp25-like domain-containing protein (Fragment) | | uniclust | UniRef100\_A0A7K4MRV6 | 98.1 | 6.8e-08 | 1.3e-13 | 54.2 | 52 | (4, 55) | 112 | (17, 70) | 73 | Baseplate wedge subunit (Fragment) | Baseplate wedge subunit (Fragment) | | uniclust | UniRef100\_A0A143XKD4 | 98.1 | 6.8e-08 | 1.4e-13 | 60.8 | 86 | (12, 99) | 112 | (17, 102) | 106 | Gene 25-like lysozyme | Gene 25-like lysozyme | | uniclust | UniRef100\_A0A1S2W8T3 | 98.0 | 7.7e-08 | 1.4e-13 | 62.1 | 80 | (3, 83) | 112 | (35, 120) | 147 | DUF2634 domain-containing protein | DUF2634 domain-containing protein | | uniclust | UniRef100\_A0A6J5KJ56 | 98.0 | 8e-08 | 1.5e-13 | 60.6 | 80 | (18, 98) | 112 | (27, 107) | 132 | GpW/Gp25/anti-adapter protein IraD | GpW/Gp25/anti-adapter protein IraD | | uniclust | UniRef100\_A0A1I1H4X3 | 98.0 | 8e-08 | 1.5e-13 | 61.5 | 72 | (11, 82) | 112 | (14, 85) | 132 | Predicted component of the type VI protein secretion system | Predicted component of the type VI protein secretion system | | uniclust | UniRef100\_A0A1V5M2G2 | 98.0 | 8.8e-08 | 1.6e-13 | 55.5 | 40 | (13, 52) | 112 | (30, 69) | 87 | Gene 25-like lysozyme | Gene 25-like lysozyme | | uniclust | UniRef100\_A0A1W9S296 | 98.0 | 9.1e-08 | 1.7e-13 | 61.1 | 99 | (7, 109) | 112 | (25, 125) | 131 | IraD/Gp25-like domain-containing protein | IraD/Gp25-like domain-containing protein | | uniclust | UniRef100\_A0A0K0NKR9 | 98.0 | 8.7e-08 | 1.7e-13 | 62.0 | 87 | (4, 100) | 112 | (12, 102) | 125 | Putative lysozyme | Putative lysozyme | | uniclust | UniRef100\_D8NFC7 | 98.0 | 9.9e-08 | 1.8e-13 | 56.8 | 51 | (1, 51) | 112 | (1, 51) | 102 | Putative bacteriophage baseplate assembly-like protein | Putative bacteriophage baseplate assembly-like protein | | uniclust | UniRef100\_UPI0010AB85DE | 98.0 | 1e-07 | 1.8e-13 | 62.1 | 86 | (11, 97) | 112 | (75, 165) | 181 | GPW/gp25 family protein | GPW/gp25 family protein | | uniclust | UniRef100\_A0A076G5J9 | 98.0 | 9.7e-08 | 1.9e-13 | 62.1 | 84 | (11, 98) | 112 | (22, 108) | 128 | Baseplate component | Baseplate component | | uniclust | UniRef100\_A0A218MMN8 | 98.0 | 1.1e-07 | 2e-13 | 59.3 | 84 | (15, 100) | 112 | (40, 124) | 135 | Putative baseplate wedge protein | Putative baseplate wedge protein | | uniclust | UniRef100\_A0A164MCD3 | 98.0 | 1.1e-07 | 2.1e-13 | 62.7 | 95 | (11, 107) | 112 | (40, 140) | 147 | IraD/Gp25-like domain-containing protein | IraD/Gp25-like domain-containing protein | | uniclust | UniRef100\_A0A6N2TUW9 | 98.0 | 1.1e-07 | 2.1e-13 | 60.6 | 91 | (11, 103) | 112 | (20, 110) | 122 | Histidine kinase | Histidine kinase | | uniclust | UniRef100\_A0A450X8I7 | 98.0 | 1.2e-07 | 2.2e-13 | 55.0 | 40 | (11, 50) | 112 | (47, 86) | 87 | Uncharacterized protein (Fragment) | Uncharacterized protein (Fragment) | | uniclust | UniRef100\_A0A3B8IUD1 | 98.0 | 1.4e-07 | 2.5e-13 | 54.2 | 42 | (11, 52) | 112 | (32, 75) | 82 | Lysozyme (Fragment) | Lysozyme (Fragment) | | uniclust | UniRef100\_A0A0X8WQV4 | 98.0 | 1.5e-07 | 2.8e-13 | 56.3 | 93 | (13, 105) | 112 | (3, 96) | 105 | IraD/Gp25-like domain-containing protein | IraD/Gp25-like domain-containing protein | | uniclust | UniRef100\_A0A1F2Z7U0 | 98.0 | 1.5e-07 | 3e-13 | 63.4 | 94 | (15, 110) | 112 | (43, 147) | 167 | IraD/Gp25-like domain-containing protein | IraD/Gp25-like domain-containing protein | | uniclust | UniRef100\_A0A0P7GKB2 | 98.0 | 1.6e-07 | 3e-13 | 57.7 | 43 | (1, 43) | 112 | (79, 121) | 123 | Baseplate wedge subunit | Baseplate wedge subunit | | uniclust | UniRef100\_A0A1H4EGE0 | 98.0 | 1.6e-07 | 3e-13 | 62.1 | 94 | (14, 107) | 112 | (21, 144) | 152 | IraD/Gp25-like domain-containing protein | IraD/Gp25-like domain-containing protein | | uniclust | UniRef100\_UPI000BBDD989 | 98.0 | 1.7e-07 | 3.1e-13 | 56.4 | 68 | (15, 83) | 112 | (13, 80) | 107 | type VI secretion system baseplate subunit TssE | type VI secretion system baseplate subunit TssE | | uniclust | UniRef100\_A0A7C6EBT5 | 97.9 | 1.7e-07 | 3.1e-13 | 59.0 | 101 | (8, 110) | 112 | (21, 124) | 130 | IraD/Gp25-like domain-containing protein | IraD/Gp25-like domain-containing protein | | uniclust | UniRef100\_K8GN81 | 97.9 | 2e-07 | 3.7e-13 | 59.3 | 102 | (8, 110) | 112 | (35, 150) | 153 | Uncharacterized protein | Uncharacterized protein | | uniclust | UniRef100\_A0A509B4A1 | 97.9 | 2e-07 | 3.8e-13 | 54.0 | 65 | (36, 102) | 112 | (2, 66) | 77 | Putative bacteriophage baseplate protein | Putative bacteriophage baseplate protein | | uniclust | UniRef100\_A0A4S2ABK4 | 97.9 | 2e-07 | 3.8e-13 | 58.4 | 83 | (13, 97) | 112 | (18, 100) | 118 | Uncharacterized protein | Uncharacterized protein | | uniclust | UniRef100\_A0A8S5V9K4 | 97.9 | 2.1e-07 | 3.8e-13 | 57.1 | 87 | (11, 97) | 112 | (27, 113) | 120 | Baseplate wedge protein | Baseplate wedge protein | | uniclust | UniRef100\_UPI001D1663D5 | 97.9 | 2.1e-07 | 3.9e-13 | 52.5 | 69 | (4, 72) | 112 | (1, 70) | 74 | hypothetical protein | hypothetical protein | | uniclust | UniRef100\_A0A2E6PSE7 | 97.9 | 2.1e-07 | 3.9e-13 | 60.7 | 97 | (13, 110) | 112 | (57, 158) | 182 | IraD/Gp25-like domain-containing protein | IraD/Gp25-like domain-containing protein | | uniclust | UniRef100\_A0A1J0MHE5 | 97.9 | 2.2e-07 | 4.1e-13 | 52.2 | 42 | (13, 54) | 112 | (27, 68) | 72 | Baseplate wedge subunit | Baseplate wedge subunit | | uniclust | UniRef100\_A0A7C3UU77 | 97.9 | 2.3e-07 | 4.2e-13 | 57.6 | 85 | (15, 99) | 112 | (30, 116) | 130 | IraD/Gp25-like domain-containing protein | IraD/Gp25-like domain-containing protein | | uniclust | UniRef100\_A0A090QXN9 | 97.9 | 2e-07 | 4.2e-13 | 61.5 | 74 | (11, 84) | 112 | (23, 97) | 134 | Uncharacterized protein | Uncharacterized protein | | uniclust | UniRef100\_A0A0G9K5Z4 | 97.9 | 2.3e-07 | 4.2e-13 | 56.6 | 79 | (19, 97) | 112 | (8, 93) | 105 | Baseplate assembly protein | Baseplate assembly protein | | uniclust | UniRef100\_A0A068NSI2 | 97.9 | 2.2e-07 | 4.3e-13 | 63.8 | 87 | (11, 98) | 112 | (57, 154) | 190 | Bacteriophage protein | Bacteriophage protein | | uniclust | UniRef100\_A0A3C0GCP4 | 97.9 | 2.4e-07 | 4.4e-13 | 55.1 | 79 | (25, 103) | 112 | (2, 93) | 100 | IraD/Gp25-like domain-containing protein | IraD/Gp25-like domain-containing protein | | uniclust | UniRef100\_A0A060H4I9 | 97.9 | 2.6e-07 | 5.1e-13 | 61.4 | 86 | (11, 99) | 112 | (42, 128) | 147 | Phage-related protein | Phage-related protein | | uniclust | UniRef100\_A0A1C5REL3 | 97.9 | 2.7e-07 | 5.3e-13 | 62.7 | 97 | (11, 110) | 112 | (67, 167) | 173 | Protein of uncharacterized function (DUF2634) | Protein of uncharacterized function (DUF2634) | | uniclust | UniRef100\_A0A381SUY6 | 97.9 | 3.2e-07 | 5.8e-13 | 58.3 | 96 | (4, 99) | 112 | (37, 136) | 150 | IraD/Gp25-like domain-containing protein | IraD/Gp25-like domain-containing protein | | uniclust | UniRef100\_A0A2W4L8B5 | 97.9 | 3.4e-07 | 6.2e-13 | 57.6 | 95 | (12, 109) | 112 | (12, 107) | 128 | IraD/Gp25-like domain-containing protein | IraD/Gp25-like domain-containing protein | | uniclust | UniRef100\_A0A7C7EU41 | 97.9 | 3.2e-07 | 6.3e-13 | 58.0 | 83 | (11, 96) | 112 | (20, 103) | 113 | IraD/Gp25-like domain-containing protein | IraD/Gp25-like domain-containing protein | | uniclust | UniRef100\_A0A377AHZ9 | 97.9 | 3.4e-07 | 6.4e-13 | 55.3 | 67 | (16, 83) | 112 | (16, 82) | 98 | Type VI secretion system lysozyme-like protein | Type VI secretion system lysozyme-like protein | | uniclust | UniRef100\_A0A6J4HR49 | 97.8 | 3.7e-07 | 6.9e-13 | 59.1 | 72 | (11, 82) | 112 | (22, 102) | 145 | IraD/Gp25-like domain-containing protein | IraD/Gp25-like domain-containing protein | | uniclust | UniRef100\_UPI0013DCF42C | 97.8 | 3.8e-07 | 7.1e-13 | 53.3 | 51 | (8, 58) | 112 | (19, 71) | 83 | GPW/gp25 family protein | GPW/gp25 family protein | | uniclust | UniRef100\_A0A450Z097 | 97.8 | 4e-07 | 7.4e-13 | 53.0 | 42 | (1, 42) | 112 | (1, 42) | 87 | Uncharacterized protein | Uncharacterized protein | | uniclust | UniRef100\_A0A1T4W4S5 | 97.8 | 4.1e-07 | 7.5e-13 | 59.6 | 98 | (2, 99) | 112 | (78, 178) | 185 | Phage baseplate assembly protein W | Phage baseplate assembly protein W | | uniclust | UniRef100\_A0A843FHN1 | 97.8 | 4.2e-07 | 8e-13 | 58.4 | 87 | (11, 99) | 112 | (33, 121) | 134 | IraD/Gp25-like domain-containing protein | IraD/Gp25-like domain-containing protein | | uniclust | UniRef100\_A0A521U4A4 | 97.8 | 4.3e-07 | 8.1e-13 | 58.1 | 98 | (11, 110) | 112 | (25, 124) | 139 | IraD/Gp25-like domain-containing protein | IraD/Gp25-like domain-containing protein | | uniclust | UniRef100\_A0A1C6BMP3 | 97.8 | 4.3e-07 | 8.1e-13 | 54.8 | 79 | (13, 94) | 112 | (16, 94) | 95 | Uncharacterized protein | Uncharacterized protein | | uniclust | UniRef100\_A0A085W9S1 | 97.8 | 4.1e-07 | 8.2e-13 | 63.9 | 95 | (15, 110) | 112 | (71, 175) | 203 | IraD/Gp25-like domain-containing protein | IraD/Gp25-like domain-containing protein | | uniclust | UniRef100\_A0A2D6E551 | 97.8 | 4.7e-07 | 8.6e-13 | 58.5 | 93 | (6, 98) | 112 | (13, 111) | 166 | IraD/Gp25-like domain-containing protein | IraD/Gp25-like domain-containing protein | | uniclust | UniRef100\_A0A1V6N1Y4 | 97.8 | 5e-07 | 9.4e-13 | 57.5 | 103 | (4, 109) | 112 | (18, 125) | 131 | DUF2634 domain-containing protein | DUF2634 domain-containing protein | | uniclust | UniRef100\_A0A0B6RSR4 | 97.8 | 5.6e-07 | 1e-12 | 52.8 | 59 | (1, 59) | 112 | (1, 59) | 91 | Putative phage baseplate assembly protein W | Putative phage baseplate assembly protein W | | uniclust | UniRef100\_A0A0X8JJW6 | 97.8 | 5.9e-07 | 1.1e-12 | 56.7 | 69 | (11, 81) | 112 | (17, 86) | 129 | Type VI secretion protein | Type VI secretion protein | | uniclust | UniRef100\_A0A1Q6PX45 | 97.8 | 5.9e-07 | 1.1e-12 | 56.1 | 81 | (15, 98) | 112 | (21, 101) | 109 | IraD/Gp25-like domain-containing protein | IraD/Gp25-like domain-containing protein | | uniclust | UniRef100\_A0A090D3B0 | 97.8 | 5.9e-07 | 1.2e-12 | 60.2 | 82 | (11, 95) | 112 | (30, 113) | 147 | Phage protein | Phage protein | | uniclust | UniRef100\_A0A6J5N592 | 97.8 | 6.3e-07 | 1.2e-12 | 57.9 | 85 | (15, 99) | 112 | (22, 113) | 138 | Baseplate wedge subunit | Baseplate wedge subunit | | uniclust | UniRef100\_A0A1V3NED6 | 97.8 | 6.8e-07 | 1.3e-12 | 56.0 | 87 | (11, 98) | 112 | (26, 114) | 135 | IraD/Gp25-like domain-containing protein | IraD/Gp25-like domain-containing protein | | uniclust | UniRef100\_A0A7W2XXK3 | 97.8 | 7e-07 | 1.3e-12 | 56.3 | 81 | (15, 97) | 112 | (30, 114) | 141 | Type VI secretion system baseplate subunit TssE | Type VI secretion system baseplate subunit TssE | | uniclust | UniRef100\_A0A1C0B348 | 97.7 | 7.3e-07 | 1.4e-12 | 58.1 | 88 | (15, 103) | 112 | (29, 118) | 141 | 25-like lysozyme | 25-like lysozyme | | uniclust | UniRef100\_UPI001F083AAE | 97.7 | 8.5e-07 | 1.6e-12 | 58.1 | 100 | (8, 107) | 112 | (56, 171) | 181 | GPW/gp25 family protein | GPW/gp25 family protein | | uniclust | UniRef100\_A0A0G1YBV7 | 97.7 | 8.7e-07 | 1.6e-12 | 60.6 | 97 | (14, 111) | 112 | (151, 247) | 250 | Uncharacterized protein | Uncharacterized protein | | uniclust | UniRef100\_A0A2V2E4P3 | 97.7 | 8.8e-07 | 1.7e-12 | 56.1 | 97 | (8, 109) | 112 | (20, 119) | 122 | DUF2634 domain-containing protein | DUF2634 domain-containing protein | | uniclust | UniRef100\_A0A1I5RRA4 | 97.7 | 9.2e-07 | 1.7e-12 | 50.7 | 62 | (35, 97) | 112 | (1, 64) | 72 | Uncharacterized protein | Uncharacterized protein | | uniclust | UniRef100\_A0A430VBK1 | 97.7 | 1e-06 | 1.8e-12 | 55.4 | 89 | (14, 104) | 112 | (37, 126) | 137 | IraD/Gp25-like domain-containing protein | IraD/Gp25-like domain-containing protein | | uniclust | UniRef100\_E3HBK7 | 97.7 | 1e-06 | 1.9e-12 | 52.5 | 82 | (12, 96) | 112 | (14, 95) | 98 | GPW/gp25 family protein | GPW/gp25 family protein | | uniclust | UniRef100\_A0A344TT62 | 97.7 | 1e-06 | 1.9e-12 | 59.7 | 73 | (11, 83) | 112 | (90, 163) | 207 | IraD/Gp25-like domain-containing protein | IraD/Gp25-like domain-containing protein | | uniclust | UniRef100\_A0A0F4LD00 | 97.7 | 1.1e-06 | 2e-12 | 59.8 | 90 | (7, 98) | 112 | (62, 156) | 178 | DUF2634 domain-containing protein | DUF2634 domain-containing protein | | uniclust | UniRef100\_A0A8S1IKS8 | 97.7 | 1.1e-06 | 2.1e-12 | 68.0 | 92 | (8, 103) | 112 | (310, 405) | 822 | Baseplate protein J-like domain-containing protein | Baseplate protein J-like domain-containing protein | | uniclust | UniRef100\_A0A0P9KCS3 | 97.7 | 1.2e-06 | 2.2e-12 | 56.7 | 81 | (16, 98) | 112 | (16, 99) | 137 | Type VI secretion system lysozyme-related protein | Type VI secretion system lysozyme-related protein | | uniclust | UniRef100\_A0A8B3NLL6 | 97.7 | 1.2e-06 | 2.2e-12 | 63.7 | 80 | (2, 81) | 112 | (4, 83) | 419 | Baseplate protein J-like domain-containing protein | Baseplate protein J-like domain-containing protein | | uniclust | UniRef100\_A0A068ZX24 | 97.7 | 1.1e-06 | 2.3e-12 | 60.6 | 88 | (8, 97) | 112 | (55, 147) | 171 | DUF2634 domain-containing protein | DUF2634 domain-containing protein | | uniclust | UniRef100\_UPI001C89A3A3 | 97.7 | 1.2e-06 | 2.3e-12 | 53.5 | 58 | (13, 79) | 112 | (23, 80) | 103 | GPW/gp25 family protein | GPW/gp25 family protein | | uniclust | UniRef100\_A0A7C7BV91 | 97.7 | 1.3e-06 | 2.3e-12 | 52.3 | 49 | (11, 59) | 112 | (46, 94) | 100 | IraD/Gp25-like domain-containing protein | IraD/Gp25-like domain-containing protein | | uniclust | UniRef100\_A0A2D6MFM3 | 97.7 | 1.3e-06 | 2.4e-12 | 58.1 | 67 | (15, 81) | 112 | (91, 157) | 201 | IraD/Gp25-like domain-containing protein | IraD/Gp25-like domain-containing protein | | uniclust | UniRef100\_A0A3M2C415 | 97.6 | 1.5e-06 | 2.8e-12 | 52.8 | 85 | (13, 99) | 112 | (10, 94) | 109 | IraD/Gp25-like domain-containing protein (Fragment) | IraD/Gp25-like domain-containing protein (Fragment) | | uniclust | UniRef100\_A0A081P4G2 | 97.6 | 1.4e-06 | 2.8e-12 | 60.3 | 90 | (8, 99) | 112 | (51, 145) | 180 | DUF2634 domain-containing protein | DUF2634 domain-containing protein | | uniclust | UniRef100\_A0A9E3QY51 | 97.6 | 1.6e-06 | 3e-12 | 54.3 | 87 | (11, 99) | 112 | (35, 122) | 133 | Uncharacterized protein | Uncharacterized protein | | uniclust | UniRef100\_A0A021XFJ7 | 97.6 | 1.4e-06 | 3e-12 | 62.3 | 88 | (13, 102) | 112 | (82, 183) | 216 | Type VI secretion system lysozyme-like protein | Type VI secretion system lysozyme-like protein | | uniclust | UniRef100\_A0A0H5M096 | 97.6 | 1.6e-06 | 3e-12 | 57.6 | 85 | (16, 101) | 112 | (35, 120) | 178 | Type VI secretion system lysozyme-like protein | Type VI secretion system lysozyme-like protein | | uniclust | UniRef100\_A0A0F0HIV6 | 97.6 | 1.7e-06 | 3.2e-12 | 54.2 | 86 | (13, 99) | 112 | (21, 107) | 132 | IraD/Gp25-like domain-containing protein | IraD/Gp25-like domain-containing protein | | uniclust | UniRef100\_E1RFI1 | 97.6 | 1.7e-06 | 3.2e-12 | 54.3 | 90 | (11, 107) | 112 | (26, 116) | 119 | GPW/gp25 family protein | GPW/gp25 family protein | | uniclust | UniRef100\_UPI000BB729C8 | 97.6 | 1.8e-06 | 3.3e-12 | 56.4 | 89 | (5, 96) | 112 | (42, 135) | 154 | DUF2634 domain-containing protein | DUF2634 domain-containing protein | | uniclust | UniRef100\_UPI002020CE7C | 97.6 | 1.8e-06 | 3.3e-12 | 50.3 | 60 | (1, 60) | 112 | (1, 60) | 85 | hypothetical protein | hypothetical protein | | uniclust | UniRef100\_A0A2I7R3K5 | 97.6 | 1.8e-06 | 3.3e-12 | 52.7 | 95 | (2, 98) | 112 | (3, 100) | 112 | Lysozyme | Lysozyme | | uniclust | UniRef100\_A0A378EEK6 | 97.6 | 1.9e-06 | 3.5e-12 | 51.9 | 64 | (37, 101) | 112 | (2, 66) | 92 | Type VI secretion system lysozyme-like protein | Type VI secretion system lysozyme-like protein | | uniclust | UniRef100\_A0A1T1HD64 | 97.6 | 2e-06 | 3.7e-12 | 56.6 | 84 | (11, 94) | 112 | (19, 102) | 150 | IraD/Gp25-like domain-containing protein | IraD/Gp25-like domain-containing protein | | uniclust | UniRef100\_A0A1I1DKW3 | 97.6 | 2.1e-06 | 3.9e-12 | 52.9 | 91 | (11, 102) | 112 | (20, 111) | 119 | Phage baseplate assembly protein W | Phage baseplate assembly protein W | | uniclust | UniRef100\_UPI0014204378 | 97.6 | 2.2e-06 | 4e-12 | 50.3 | 69 | (4, 74) | 112 | (15, 87) | 88 | DUF2634 domain-containing protein | DUF2634 domain-containing protein | | uniclust | UniRef100\_UPI0001816813 | 97.6 | 2.3e-06 | 4.3e-12 | 47.3 | 52 | (4, 55) | 112 | (1, 52) | 64 | hypothetical protein | hypothetical protein | | uniclust | UniRef100\_A0A4R5N831 | 97.6 | 2.4e-06 | 4.4e-12 | 55.4 | 99 | (7, 107) | 112 | (51, 154) | 157 | DUF2634 domain-containing protein | DUF2634 domain-containing protein | | uniclust | UniRef100\_A0A023Y2J8 | 97.6 | 2.2e-06 | 4.5e-12 | 60.6 | 92 | (14, 107) | 112 | (55, 162) | 209 | Type VI secretion protein | Type VI secretion protein | | uniclust | UniRef100\_A0A075DXX6 | 97.6 | 2.4e-06 | 4.6e-12 | 55.8 | 84 | (13, 99) | 112 | (24, 108) | 131 | Putative baseplate component | Putative baseplate component | | uniclust | UniRef100\_A0A257Q2E2 | 97.5 | 2.7e-06 | 5e-12 | 59.5 | 99 | (8, 110) | 112 | (185, 288) | 289 | Gp5/Type VI secretion system Vgr protein OB-fold domain-containing protein | Gp5/Type VI secretion system Vgr protein OB-fold domain-containing protein | | uniclust | UniRef100\_H6WG16 | 97.5 | 2.8e-06 | 5.1e-12 | 55.7 | 85 | (11, 100) | 112 | (81, 165) | 177 | Virion structural protein | Virion structural protein | | uniclust | UniRef100\_UPI000A200276 | 97.5 | 2.9e-06 | 5.3e-12 | 50.9 | 66 | (35, 100) | 112 | (1, 67) | 100 | hypothetical protein | hypothetical protein | | uniclust | UniRef100\_A0A0F9MK12 | 97.5 | 2.8e-06 | 5.4e-12 | 54.9 | 74 | (11, 84) | 112 | (26, 100) | 131 | Uncharacterized protein (Fragment) | Uncharacterized protein (Fragment) | | uniclust | UniRef100\_A0A099VCP5 | 97.5 | 3.1e-06 | 6e-12 | 55.3 | 85 | (13, 99) | 112 | (23, 109) | 130 | Type VI secretion system baseplate subunit TssE | Type VI secretion system baseplate subunit TssE | | uniclust | UniRef100\_A0A0Q8PDY9 | 97.5 | 3e-06 | 6.1e-12 | 57.7 | 94 | (15, 110) | 112 | (35, 139) | 160 | Type VI secretion protein, lysozyme-like protein | Type VI secretion protein, lysozyme-like protein | | uniclust | UniRef100\_A0A0Q8ANH2 | 97.5 | 3.4e-06 | 6.3e-12 | 53.4 | 65 | (2, 66) | 112 | (5, 69) | 126 | IraD/Gp25-like domain-containing protein | IraD/Gp25-like domain-containing protein | | uniclust | UniRef100\_A0A1H8JI52 | 97.5 | 3.3e-06 | 6.3e-12 | 55.3 | 87 | (11, 99) | 112 | (40, 128) | 143 | DUF2634 domain-containing protein | DUF2634 domain-containing protein | | uniclust | UniRef100\_A0A009YPJ6 | 97.5 | 3.5e-06 | 6.7e-12 | 53.6 | 108 | (1, 109) | 112 | (1, 115) | 120 | Phage GP46 family protein | Phage GP46 family protein | | uniclust | UniRef100\_A0A1Q6LL95 | 97.5 | 3.6e-06 | 6.9e-12 | 51.1 | 77 | (14, 93) | 112 | (11, 87) | 93 | Uncharacterized protein | Uncharacterized protein | | uniclust | UniRef100\_A0A0K8MDE3 | 97.5 | 3.6e-06 | 7.1e-12 | 58.8 | 95 | (15, 110) | 112 | (65, 173) | 201 | Gene 25-like lysozyme | Gene 25-like lysozyme | | uniclust | UniRef100\_A0A497N9T7 | 97.5 | 3.9e-06 | 7.2e-12 | 52.8 | 89 | (11, 99) | 112 | (26, 116) | 133 | IraD/Gp25-like domain-containing protein | IraD/Gp25-like domain-containing protein | | uniclust | UniRef100\_A0A349NQ61 | 97.5 | 4e-06 | 7.3e-12 | 49.1 | 69 | (32, 100) | 112 | (1, 73) | 86 | IraD/Gp25-like domain-containing protein (Fragment) | IraD/Gp25-like domain-containing protein (Fragment) | | uniclust | UniRef100\_A0A022FQR5 | 97.5 | 3.7e-06 | 7.3e-12 | 58.7 | 95 | (15, 110) | 112 | (58, 162) | 186 | Type VI secretion system lysozyme | Type VI secretion system lysozyme | | uniclust | UniRef100\_A0A843GVE6 | 97.5 | 3.9e-06 | 7.4e-12 | 53.7 | 87 | (11, 97) | 112 | (19, 106) | 127 | IraD/Gp25-like domain-containing protein | IraD/Gp25-like domain-containing protein | | uniclust | UniRef100\_A0A352JSI9 | 97.5 | 4.1e-06 | 7.5e-12 | 53.6 | 97 | (11, 108) | 112 | (19, 123) | 139 | IraD/Gp25-like domain-containing protein | IraD/Gp25-like domain-containing protein | | uniclust | UniRef100\_A0A943Z1H4 | 97.5 | 4.2e-06 | 7.8e-12 | 50.4 | 81 | (13, 95) | 112 | (19, 99) | 102 | Uncharacterized protein | Uncharacterized protein | | uniclust | UniRef100\_A0A354D7Q5 | 97.5 | 4.1e-06 | 7.9e-12 | 68.9 | 99 | (8, 110) | 112 | (759, 860) | 863 | Baseplate protein J-like domain-containing protein | Baseplate protein J-like domain-containing protein | | uniclust | UniRef100\_X1ADF5 | 97.5 | 4.4e-06 | 8e-12 | 49.5 | 44 | (11, 54) | 112 | (34, 77) | 92 | Uncharacterized protein | Uncharacterized protein | | uniclust | UniRef100\_A0A1G3MBB2 | 97.5 | 4.4e-06 | 8.1e-12 | 53.1 | 70 | (14, 83) | 112 | (39, 108) | 143 | IraD/Gp25-like domain-containing protein | IraD/Gp25-like domain-containing protein | | pdb70 | 2IA7\_A | 99.6 | 5.4e-20 | 4.3e-24 | 111.2 | 101 | (8, 108) | 112 | (24, 126) | 134 | Crystal structure of putative tail | 2IA7\_A Crystal structure of putative tail NP\_952040.1, putative tail lysozyme, Structural | | pdb70 | 5IV5\_BD | 99.6 | 6.3e-20 | 5e-24 | 110.7 | 102 | (7, 108) | 112 | (21, 124) | 132 | Baseplate wedge protein gp6, Baseplate | 5IV5\_BD Baseplate wedge protein gp6, Baseplate T4, baseplate-tail tube complex, pre-attachment | | pdb70 | 5IV5\_DG | 99.6 | 6.3e-20 | 5e-24 | 110.7 | 102 | (7, 108) | 112 | (21, 124) | 132 | Baseplate wedge protein gp6, Baseplate | 5IV5\_DG Baseplate wedge protein gp6, Baseplate T4, baseplate-tail tube complex, pre-attachment | | pdb70 | 5IV5\_FJ | 99.6 | 6.3e-20 | 5e-24 | 110.7 | 102 | (7, 108) | 112 | (21, 124) | 132 | Baseplate wedge protein gp6, Baseplate | 5IV5\_FJ Baseplate wedge protein gp6, Baseplate T4, baseplate-tail tube complex, pre-attachment | | pdb70 | 5IV5\_IC | 99.6 | 6.3e-20 | 5e-24 | 110.7 | 102 | (7, 108) | 112 | (21, 124) | 132 | Baseplate wedge protein gp6, Baseplate | 5IV5\_IC Baseplate wedge protein gp6, Baseplate T4, baseplate-tail tube complex, pre-attachment | | pdb70 | 5IV5\_T | 99.6 | 6.3e-20 | 5e-24 | 110.7 | 102 | (7, 108) | 112 | (21, 124) | 132 | Baseplate wedge protein gp6, Baseplate | 5IV5\_T Baseplate wedge protein gp6, Baseplate T4, baseplate-tail tube complex, pre-attachment | | pdb70 | 5IW9\_A | 99.6 | 6.6e-20 | 5.3e-24 | 110.5 | 103 | (7, 109) | 112 | (20, 124) | 131 | Baseplate wedge protein gp25 | 5IW9\_A Baseplate wedge protein gp25 contractile sheath, baseplate, wedge, sheath HET: MSE | | pdb70 | 6OD1\_B | 99.6 | 1.5e-19 | 1.2e-23 | 106.9 | 97 | (11, 109) | 112 | (3, 100) | 115 | Regulator of RpoS, Anti-adapter protein | 6OD1\_B Regulator of RpoS, Anti-adapter protein response regulator, ClpXP adaptor, anti-adaptor | | pdb70 | 6RAO\_H | 99.6 | 1.8e-19 | 1.4e-23 | 109.7 | 102 | (7, 108) | 112 | (24, 129) | 140 | Afp1, Afp2, Afp3, Afp5, Afp9 | 6RAO\_H Afp1, Afp2, Afp3, Afp5, Afp9 Anti-feeding prophage, secretion system, AFP | | pdb70 | 6GJ1\_D | 99.6 | 2.3e-19 | 1.8e-23 | 109.9 | 100 | (8, 109) | 112 | (23, 123) | 143 | Putative type VI secretion protein | 6GJ1\_D Putative type VI secretion protein Secretion, baseplate, complex, STRUCTURAL PROTEIN | | pdb70 | 6J0N\_H | 99.5 | 3.5e-19 | 2.8e-23 | 108.5 | 103 | (7, 109) | 112 | (24, 130) | 140 | Pvc1, Pvc9, Pvc11, Pvc12, Pvc4 | 6J0N\_H Pvc1, Pvc9, Pvc11, Pvc12, Pvc4 assembly, Photorhabdus asymbiotica, PVC, contractile | |
| Top keywords  (threshold 1.00e-03 (evalue)) | **domain\_containing, IraD, Gp25\_like, Baseplate, GPW, gp25, assembly, Phage, Fragment, DUF2634** |
| Output files | ../../similar\_sequences/12\_FANPEZAQ\_CDS\_0012\_merged.svg ../../similar\_sequences/12\_FANPEZAQ\_CDS\_0012\_pdb70.a3m ../../similar\_sequences/12\_FANPEZAQ\_CDS\_0012\_pdb70.hhr ../../similar\_sequences/12\_FANPEZAQ\_CDS\_0012\_uniclust.a3m ../../similar\_sequences/12\_FANPEZAQ\_CDS\_0012\_uniclust.hhr |

#### Structure prediction (AlphaFold)2

|  |  |
| --- | --- |
| Stats | xml version="1.0" encoding="utf-8" standalone="no"?       2024-09-02T21:09:10.763989 image/svg+xml   Matplotlib v3.7.2, https://matplotlib.org/ |
| Predicted structure | **NGL Viewer Controls:**  - Center: *Left-Click* - Rotate: *Left-Click + Drag* - Translate: *Right-Click + Drag* - Zoom: *Shift + Left-Click + Drag* |
| Output files | ../../predicted\_structures/12\_FANPEZAQ\_CDS\_0012/features.pkl ../../predicted\_structures/12\_FANPEZAQ\_CDS\_0012/ranked\_0.pdb ../../predicted\_structures/12\_FANPEZAQ\_CDS\_0012/ranked\_0\_plots.svg ../../predicted\_structures/12\_FANPEZAQ\_CDS\_0012/result\_model\_1\_ptm\_pred\_0.pkl |

#### Structure similarity search results (Foldseek)3

|  |  |
| --- | --- |
| Structure databases searched | Pdb, Afdb-proteome, Afdb-uniprot50 |
| Results, scheme(s)  (Top layers only, threshold 1.00e-02 (evalue)) | xml version="1.0" encoding="utf-8" standalone="no"?       2024-09-02T21:10:23.559066 image/svg+xml   Matplotlib v3.7.2, https://matplotlib.org/ |
| Results, table  (threshold 1.00e-02 (evalue)) | | db | id | prob | evalue | bits | fident | alnlen | mismatch | gapopen | qstart | qend | tstart | tend | name | description | | --- | --- | --- | --- | --- | --- | --- | --- | --- | --- | --- | --- | --- | --- | --- | | pdb | 6U5B\_A | 1.0 | 2.212e-08 | 302 | 0.443 | 97 | 54 | 0 | 1 | 97 | 1 | 97 | Sheath Initiator PA0617 | Sheath Initiator PA0617 | | pdb | 6U5K\_B | 1.0 | 2.18e-07 | 255 | 0.443 | 97 | 54 | 0 | 1 | 97 | 1 | 97 | Sheath Initiator PA0617 | Sheath Initiator PA0617 | | pdb | 2IA7\_A | 1.0 | 5.181e-07 | 243 | 0.34 | 97 | 61 | 2 | 12 | 105 | 4 | 100 | Tail lysozyme, putative | Tail lysozyme, putative | | pdb | 5IW9\_B | 1.0 | 0.0001057 | 166 | 0.202 | 94 | 69 | 4 | 17 | 105 | 27 | 119 | Baseplate wedge protein gp25 | Baseplate wedge protein gp25 | | pdb | 6RAO\_H | 1.0 | 0.0009203 | 138 | 0.22 | 100 | 75 | 2 | 2 | 98 | 15 | 114 | Afp9 | Afp9 | | pdb | 7B5H\_AI | 1.0 | 9.934e-05 | 130 | 0.187 | 112 | 73 | 4 | 2 | 107 | 16 | 115 | All3318 protein | All3318 protein | | pdb | 7AEB\_S | 1.0 | 0.0002512 | 127 | 0.231 | 108 | 73 | 2 | 2 | 107 | 14 | 113 | Putative tail lysozyme | Putative tail lysozyme | | pdb | 6J0N\_D | 1.0 | 0.002056 | 126 | 0.163 | 98 | 79 | 2 | 2 | 96 | 17 | 114 | Pvc7 | Pvc7 | | afdb-proteome | AF-G3XD42-F1-MODEL\_V4 | 1.0 | 4.826e-12 | 463 | 0.416 | 108 | 61 | 1 | 1 | 108 | 1 | 106 | Probable bacteriophage protein | Probable bacteriophage protein | | afdb-proteome | AF-Q8ZMU2-F1-MODEL\_V4 | 1.0 | 3.714e-11 | 424 | 0.38 | 105 | 64 | 1 | 1 | 105 | 4 | 107 | Fels-2 prophage protein | Fels-2 prophage protein | | afdb-proteome | AF-A0A0H3H1Q9-F1-MODEL\_V4 | 1.0 | 3.085e-11 | 412 | 0.37 | 108 | 66 | 1 | 2 | 107 | 6 | 113 | Putative prophage baseplate protein | Putative prophage baseplate protein | | afdb-proteome | AF-A0A0H3GUQ6-F1-MODEL\_V4 | 1.0 | 1.063e-10 | 400 | 0.39 | 105 | 62 | 2 | 2 | 105 | 5 | 108 | Baseplate assembly protein W | Baseplate assembly protein W | | afdb-proteome | AF-A0A0H4IV38-F1-MODEL\_V4 | 1.0 | 5.228e-09 | 341 | 0.38 | 105 | 62 | 2 | 3 | 105 | 1 | 104 | Phage baseplate assembly protein | Phage baseplate assembly protein | | afdb-proteome | AF-Q8ZKK2-F1-MODEL\_V4 | 1.0 | 3.965e-07 | 271 | 0.25 | 108 | 77 | 4 | 1 | 107 | 11 | 115 | Putative phage baseplate protein | Putative phage baseplate protein | | afdb-proteome | AF-A0A0H3GZN2-F1-MODEL\_V4 | 1.0 | 0.0001922 | 145 | 0.189 | 95 | 68 | 5 | 15 | 105 | 30 | 119 | Type VI secretion system lysozyme-related protein | Type VI secretion system lysozyme-related protein | | afdb-proteome | AF-P44239-F1-MODEL\_V4 | 1.0 | 0.001674 | 123 | 0.221 | 131 | 80 | 10 | 1 | 112 | 5 | 132 | Mu-like prophage FluMu protein gp46 | Mu-like prophage FluMu protein gp46 | | afdb-proteome | AF-Q9I366-F1-MODEL\_V4 | 1.0 | 0.002745 | 118 | 0.244 | 98 | 64 | 6 | 16 | 106 | 26 | 120 | GPW\_gp25 domain-containing protein | GPW\_gp25 domain-containing protein | | afdb-proteome | AF-Q9I1B1-F1-MODEL\_V4 | 0.998 | 0.00479 | 95 | 0.178 | 123 | 81 | 6 | 1 | 105 | 1 | 121 | GPW\_gp25 domain-containing protein | GPW\_gp25 domain-containing protein | | afdb-uniprot50 | AF-A0A6M4YGB1-F1-MODEL\_V4 | 1.0 | 4.39e-18 | 740 | 0.675 | 111 | 36 | 0 | 1 | 111 | 1 | 111 | Phage baseplate protein | Phage baseplate protein | | afdb-uniprot50 | AF-A0A066RRA4-F1-MODEL\_V4 | 1.0 | 8.029e-17 | 695 | 0.598 | 112 | 45 | 0 | 1 | 112 | 1 | 112 | Phage baseplate protein | Phage baseplate protein | | afdb-uniprot50 | AF-A0A2S0MNS1-F1-MODEL\_V4 | 1.0 | 3.329e-16 | 655 | 0.589 | 112 | 46 | 0 | 1 | 112 | 1 | 112 | Phage baseplate protein | Phage baseplate protein | | afdb-uniprot50 | AF-I7DR34-F1-MODEL\_V4 | 1.0 | 4.825e-16 | 650 | 0.669 | 112 | 37 | 0 | 1 | 112 | 39 | 150 | Putative baseplate assembly protein W | Putative baseplate assembly protein W | | afdb-uniprot50 | AF-Q3RBV9-F1-MODEL\_V4 | 1.0 | 3.767e-16 | 649 | 0.598 | 112 | 45 | 0 | 1 | 112 | 1 | 112 | GPW/gp25 | GPW/gp25 | | afdb-uniprot50 | AF-A0A2U1XZ29-F1-MODEL\_V4 | 1.0 | 2.264e-15 | 633 | 0.594 | 111 | 45 | 0 | 1 | 111 | 1 | 111 | GPW\_gp25 domain-containing protein | GPW\_gp25 domain-containing protein | | afdb-uniprot50 | AF-A0A212KBR9-F1-MODEL\_V4 | 1.0 | 3.281e-15 | 631 | 0.473 | 112 | 59 | 0 | 1 | 112 | 1 | 112 | Putative Baseplate assembly protein W | Putative Baseplate assembly protein W | | afdb-uniprot50 | AF-A0A1D2QM84-F1-MODEL\_V4 | 1.0 | 6.478e-15 | 614 | 0.504 | 111 | 55 | 0 | 1 | 111 | 1 | 111 | GPW\_gp25 domain-containing protein | GPW\_gp25 domain-containing protein | | afdb-uniprot50 | AF-A0A7U9KG42-F1-MODEL\_V4 | 1.0 | 1.279e-14 | 602 | 0.5 | 112 | 56 | 0 | 1 | 112 | 1 | 112 | Putative lysozyme | Putative lysozyme | | afdb-uniprot50 | AF-A0A3N2E0S6-F1-MODEL\_V4 | 1.0 | 1.972e-14 | 585 | 0.526 | 112 | 53 | 0 | 1 | 112 | 1 | 112 | GPW\_gp25 domain-containing protein | GPW\_gp25 domain-containing protein | | afdb-uniprot50 | AF-A0A6G8F2Q6-F1-MODEL\_V4 | 1.0 | 6.384e-14 | 569 | 0.383 | 112 | 69 | 0 | 1 | 112 | 1 | 112 | Bacteriophage baseplate assembly protein W | Bacteriophage baseplate assembly protein W | | afdb-uniprot50 | AF-N6VCT6-F1-MODEL\_V4 | 1.0 | 3.187e-13 | 548 | 0.565 | 99 | 43 | 0 | 14 | 112 | 3 | 101 | Phage baseplate assembly protein GpW | Phage baseplate assembly protein GpW | | afdb-uniprot50 | AF-A0A3G2IL39-F1-MODEL\_V4 | 1.0 | 2.199e-13 | 545 | 0.446 | 112 | 62 | 0 | 1 | 112 | 1 | 112 | Phage baseplate protein | Phage baseplate protein | | afdb-uniprot50 | AF-A0A6L2ZTB2-F1-MODEL\_V4 | 1.0 | 6.384e-14 | 543 | 0.489 | 139 | 42 | 2 | 1 | 110 | 1 | 139 | Baseplate assembly protein | Baseplate assembly protein | | afdb-uniprot50 | AF-A0A0F4Q7B2-F1-MODEL\_V4 | 1.0 | 3.44e-14 | 542 | 0.518 | 110 | 53 | 0 | 1 | 110 | 1 | 110 | Baseplate assembly protein W | Baseplate assembly protein W | | afdb-uniprot50 | AF-A0A430DQS5-F1-MODEL\_V4 | 1.0 | 1.614e-13 | 541 | 0.513 | 115 | 51 | 3 | 1 | 111 | 1 | 114 | Oxidoreductase | Oxidoreductase | | afdb-uniprot50 | AF-A0A149SVH9-F1-MODEL\_V4 | 1.0 | 1.517e-13 | 535 | 0.517 | 112 | 53 | 1 | 1 | 112 | 1 | 111 | GPW\_gp25 domain-containing protein | GPW\_gp25 domain-containing protein | | afdb-uniprot50 | AF-A0A2Z4UFI3-F1-MODEL\_V4 | 1.0 | 2.647e-13 | 528 | 0.495 | 113 | 56 | 1 | 1 | 112 | 1 | 113 | Gene 25-like lysozyme | Gene 25-like lysozyme | | afdb-uniprot50 | AF-A0A3N2E0Q2-F1-MODEL\_V4 | 1.0 | 4.618e-13 | 527 | 0.414 | 111 | 65 | 0 | 1 | 111 | 1 | 111 | GPW\_gp25 domain-containing protein | GPW\_gp25 domain-containing protein | | afdb-uniprot50 | AF-A0A7X3ZHR5-F1-MODEL\_V4 | 1.0 | 2.816e-13 | 526 | 0.464 | 112 | 58 | 1 | 1 | 112 | 1 | 110 | Phage baseplate protein | Phage baseplate protein | | afdb-uniprot50 | AF-A0A432WB53-F1-MODEL\_V4 | 1.0 | 2.199e-13 | 526 | 0.526 | 112 | 53 | 0 | 1 | 112 | 3 | 114 | GPW\_gp25 domain-containing protein | GPW\_gp25 domain-containing protein | | afdb-uniprot50 | AF-A0A5P9F0H1-F1-MODEL\_V4 | 1.0 | 6.291e-13 | 521 | 0.427 | 110 | 63 | 0 | 1 | 110 | 3 | 112 | Gene 25-like lysozyme | Gene 25-like lysozyme | | afdb-uniprot50 | AF-A0A4U8YRG6-F1-MODEL\_V4 | 1.0 | 4.081e-13 | 519 | 0.509 | 110 | 53 | 1 | 1 | 110 | 2 | 110 | Gpw/gp25/anti-adapter protein irad | Gpw/gp25/anti-adapter protein irad | | afdb-uniprot50 | AF-A0A2D8QD63-F1-MODEL\_V4 | 1.0 | 5.914e-13 | 519 | 0.518 | 110 | 51 | 1 | 3 | 110 | 17 | 126 | Baseplate assembly protein | Baseplate assembly protein | | afdb-uniprot50 | AF-A0A192C892-F1-MODEL\_V4 | 1.0 | 1.242e-12 | 516 | 0.372 | 110 | 69 | 0 | 1 | 110 | 1 | 110 | Baseplate assembly protein W | Baseplate assembly protein W | | afdb-uniprot50 | AF-W8ZTA4-F1-MODEL\_V4 | 1.0 | 1.321e-12 | 514 | 0.372 | 110 | 69 | 0 | 1 | 110 | 17 | 126 | Putative phage baseplate assembly protein | Putative phage baseplate assembly protein | | afdb-uniprot50 | AF-A0A238UMY5-F1-MODEL\_V4 | 1.0 | 9.699e-13 | 503 | 0.513 | 111 | 49 | 2 | 1 | 111 | 1 | 106 | GPW\_gp25 domain-containing protein | GPW\_gp25 domain-containing protein | | afdb-uniprot50 | AF-A0A6B8QMM4-F1-MODEL\_V4 | 1.0 | 8.057e-13 | 498 | 0.527 | 108 | 49 | 1 | 1 | 106 | 1 | 108 | Baseplate assembly protein | Baseplate assembly protein | | afdb-uniprot50 | AF-A0A1I5W3Y1-F1-MODEL\_V4 | 1.0 | 5.15e-12 | 488 | 0.495 | 107 | 53 | 1 | 1 | 107 | 1 | 106 | GPW\_gp25 domain-containing protein | GPW\_gp25 domain-containing protein | | afdb-uniprot50 | AF-A0A7T8CNB6-F1-MODEL\_V4 | 1.0 | 2.775e-12 | 484 | 0.447 | 123 | 57 | 1 | 1 | 112 | 1 | 123 | Phage baseplate protein | Phage baseplate protein | | afdb-uniprot50 | AF-A0A2W4T7K0-F1-MODEL\_V4 | 1.0 | 2.775e-12 | 483 | 0.513 | 109 | 51 | 1 | 1 | 109 | 10 | 116 | Phage baseplate protein | Phage baseplate protein | | afdb-uniprot50 | AF-A0A7Y0FCZ2-F1-MODEL\_V4 | 1.0 | 6.2e-12 | 473 | 0.5 | 108 | 51 | 2 | 1 | 106 | 1 | 107 | GPW\_gp25 domain-containing protein | GPW\_gp25 domain-containing protein | | afdb-uniprot50 | AF-A0A0Q2Y5K4-F1-MODEL\_V4 | 1.0 | 4.841e-12 | 471 | 0.447 | 105 | 56 | 1 | 1 | 105 | 1 | 103 | GPW\_gp25 domain-containing protein | GPW\_gp25 domain-containing protein | | afdb-uniprot50 | AF-A0A2N7UDN8-F1-MODEL\_V4 | 1.0 | 2.167e-12 | 471 | 0.509 | 108 | 50 | 2 | 1 | 106 | 1 | 107 | Baseplate assembly protein | Baseplate assembly protein | | afdb-uniprot50 | AF-A0A6H2NTS6-F1-MODEL\_V4 | 1.0 | 7.94e-12 | 469 | 0.547 | 95 | 43 | 0 | 17 | 111 | 12 | 106 | Baseplate assembly protein W | Baseplate assembly protein W | | afdb-uniprot50 | AF-A0A2K4MJA3-F1-MODEL\_V4 | 1.0 | 7.94e-12 | 467 | 0.417 | 115 | 63 | 2 | 1 | 112 | 3 | 116 | Baseplate assembly protein | Baseplate assembly protein | | afdb-uniprot50 | AF-A0A369VRM6-F1-MODEL\_V4 | 1.0 | 3.341e-12 | 460 | 0.403 | 114 | 62 | 3 | 1 | 111 | 1 | 111 | Oxidoreductase | Oxidoreductase | | afdb-uniprot50 | AF-A0A2Z6GBZ8-F1-MODEL\_V4 | 1.0 | 1.474e-11 | 458 | 0.414 | 111 | 61 | 2 | 1 | 107 | 3 | 113 | Baseplate assembly protein | Baseplate assembly protein | | afdb-uniprot50 | AF-A0A1G5TK42-F1-MODEL\_V4 | 1.0 | 7.94e-12 | 458 | 0.4 | 115 | 62 | 3 | 1 | 111 | 1 | 112 | GPW\_gp25 domain-containing protein | GPW\_gp25 domain-containing protein | | afdb-uniprot50 | AF-B4F0J8-F1-MODEL\_V4 | 1.0 | 2.136e-11 | 457 | 0.401 | 112 | 65 | 2 | 1 | 112 | 3 | 112 | Putative phage baseplate assembly protein | Putative phage baseplate assembly protein | | afdb-uniprot50 | AF-A0A8A8MM88-F1-MODEL\_V4 | 1.0 | 1.474e-11 | 457 | 0.428 | 105 | 60 | 0 | 1 | 105 | 5 | 109 | GPW/gp25 family protein | GPW/gp25 family protein | | afdb-uniprot50 | AF-A0A4R7GGA6-F1-MODEL\_V4 | 1.0 | 1.668e-11 | 456 | 0.467 | 107 | 57 | 0 | 1 | 107 | 4 | 110 | GPW\_gp25 domain-containing protein | GPW\_gp25 domain-containing protein | | afdb-uniprot50 | AF-A0A1H9HC27-F1-MODEL\_V4 | 1.0 | 2.735e-11 | 454 | 0.428 | 112 | 63 | 1 | 1 | 112 | 1 | 111 | GPW\_gp25 domain-containing protein | GPW\_gp25 domain-containing protein | | afdb-uniprot50 | AF-A0A345RIW3-F1-MODEL\_V4 | 1.0 | 7.016e-12 | 454 | 0.482 | 112 | 56 | 2 | 1 | 111 | 1 | 111 | Baseplate assembly protein | Baseplate assembly protein | | afdb-uniprot50 | AF-A0A6I4T4J5-F1-MODEL\_V4 | 1.0 | 5.15e-12 | 453 | 0.459 | 111 | 55 | 2 | 1 | 106 | 4 | 114 | Oxidoreductase | Oxidoreductase | | afdb-uniprot50 | AF-A0A631DZ78-F1-MODEL\_V4 | 1.0 | 1.668e-11 | 452 | 0.367 | 106 | 67 | 0 | 1 | 106 | 5 | 110 | Baseplate assembly protein | Baseplate assembly protein | | afdb-uniprot50 | AF-C3X1Y5-F1-MODEL\_V4 | 1.0 | 2.136e-11 | 452 | 0.385 | 109 | 62 | 2 | 1 | 106 | 1 | 107 | GPW\_gp25 domain-containing protein | GPW\_gp25 domain-containing protein | | afdb-uniprot50 | AF-A0A7A6VT71-F1-MODEL\_V4 | 1.0 | 2.136e-11 | 447 | 0.358 | 106 | 68 | 0 | 1 | 106 | 5 | 110 | Baseplate assembly protein | Baseplate assembly protein | | afdb-uniprot50 | AF-A0A158S0X6-F1-MODEL\_V4 | 1.0 | 2.909e-11 | 447 | 0.459 | 111 | 55 | 3 | 1 | 107 | 6 | 115 | Baseplate assembly protein W | Baseplate assembly protein W | | afdb-uniprot50 | AF-A0A7G7WLN4-F1-MODEL\_V4 | 1.0 | 3.726e-11 | 446 | 0.387 | 111 | 66 | 2 | 2 | 112 | 4 | 112 | GPW/gp25 family protein | GPW/gp25 family protein | | afdb-uniprot50 | AF-A0A812QV09-F1-MODEL\_V4 | 1.0 | 5.479e-12 | 445 | 0.495 | 113 | 53 | 2 | 3 | 111 | 1061 | 1173 | XkdF protein | XkdF protein | | afdb-uniprot50 | AF-A0A4Z0W627-F1-MODEL\_V4 | 1.0 | 1.002e-10 | 444 | 0.37 | 108 | 68 | 0 | 1 | 108 | 1 | 108 | Baseplate assembly protein W | Baseplate assembly protein W | | afdb-uniprot50 | AF-A0A829QVE9-F1-MODEL\_V4 | 1.0 | 5.744e-11 | 444 | 0.351 | 108 | 70 | 0 | 1 | 108 | 3 | 110 | Putative lysozyme | Putative lysozyme | | afdb-uniprot50 | AF-A0A854BCP0-F1-MODEL\_V4 | 1.0 | 1.302e-11 | 442 | 0.428 | 112 | 62 | 2 | 1 | 111 | 1 | 111 | Baseplate assembly protein | Baseplate assembly protein | | afdb-uniprot50 | AF-A0A6H1ZRT2-F1-MODEL\_V4 | 1.0 | 8.324e-11 | 440 | 0.345 | 113 | 72 | 2 | 1 | 112 | 8 | 119 | Putative baseplate wedge subunit | Putative baseplate wedge subunit | | afdb-uniprot50 | AF-A0A1R3WEX9-F1-MODEL\_V4 | 1.0 | 2.909e-11 | 440 | 0.441 | 111 | 56 | 2 | 1 | 106 | 1 | 110 | GPW\_gp25 domain-containing protein | GPW\_gp25 domain-containing protein | | afdb-uniprot50 | AF-A0A2U3F139-F1-MODEL\_V4 | 1.0 | 3.292e-11 | 439 | 0.439 | 107 | 58 | 2 | 2 | 108 | 6 | 110 | Baseplate assembly protein | Baseplate assembly protein | | afdb-uniprot50 | AF-A0A3Y9JE76-F1-MODEL\_V4 | 1.0 | 3.963e-11 | 439 | 0.38 | 105 | 65 | 0 | 1 | 105 | 5 | 109 | Baseplate assembly protein | Baseplate assembly protein | | afdb-uniprot50 | AF-A0A142JGU1-F1-MODEL\_V4 | 1.0 | 1.302e-11 | 437 | 0.45 | 111 | 58 | 2 | 1 | 108 | 1 | 111 | GPW\_gp25 domain-containing protein | GPW\_gp25 domain-containing protein | | afdb-uniprot50 | AF-A0A8B2NXK0-F1-MODEL\_V4 | 1.0 | 1.887e-11 | 433 | 0.486 | 113 | 50 | 2 | 2 | 106 | 5 | 117 | Baseplate assembly protein | Baseplate assembly protein | | afdb-uniprot50 | AF-A0A1T4WV80-F1-MODEL\_V4 | 1.0 | 6.596e-12 | 433 | 0.477 | 111 | 53 | 1 | 1 | 106 | 80 | 190 | Phage baseplate assembly protein W | Phage baseplate assembly protein W | | afdb-uniprot50 | AF-A0A2L0ACC8-F1-MODEL\_V4 | 1.0 | 2.909e-11 | 432 | 0.444 | 108 | 57 | 2 | 1 | 105 | 1 | 108 | Oxidoreductase | Oxidoreductase | | afdb-uniprot50 | AF-A0A7V8SF12-F1-MODEL\_V4 | 1.0 | 4.771e-11 | 432 | 0.422 | 109 | 59 | 3 | 1 | 105 | 1 | 109 | GPW\_gp25 domain-containing protein | GPW\_gp25 domain-containing protein | | afdb-uniprot50 | AF-A0A6L2ZQK7-F1-MODEL\_V4 | 1.0 | 6.915e-11 | 431 | 0.413 | 104 | 61 | 0 | 2 | 105 | 4 | 107 | Phage baseplate assembly protein | Phage baseplate assembly protein | | afdb-uniprot50 | AF-A0A854E0F0-F1-MODEL\_V4 | 1.0 | 3.502e-11 | 431 | 0.443 | 106 | 58 | 1 | 1 | 105 | 1 | 106 | Baseplate assembly protein | Baseplate assembly protein | | afdb-uniprot50 | AF-A0A2V3UCJ0-F1-MODEL\_V4 | 1.0 | 9.559e-12 | 431 | 0.474 | 116 | 51 | 1 | 1 | 106 | 3 | 118 | GPW\_gp25 domain-containing protein | GPW\_gp25 domain-containing protein | | afdb-uniprot50 | AF-A0A326GHB1-F1-MODEL\_V4 | 1.0 | 2.909e-11 | 429 | 0.462 | 108 | 58 | 0 | 1 | 108 | 1 | 108 | Oxidoreductase | Oxidoreductase | | afdb-uniprot50 | AF-A0A2A4XT86-F1-MODEL\_V4 | 1.0 | 6.5e-11 | 428 | 0.419 | 105 | 61 | 0 | 1 | 105 | 1 | 105 | GPW\_gp25 domain-containing protein | GPW\_gp25 domain-containing protein | | afdb-uniprot50 | AF-A0A5E4SXB6-F1-MODEL\_V4 | 1.0 | 5.744e-11 | 428 | 0.364 | 118 | 68 | 3 | 1 | 112 | 3 | 119 | Phage baseplate protein | Phage baseplate protein | | afdb-uniprot50 | AF-A0A1V0LL55-F1-MODEL\_V4 | 1.0 | 6.5e-11 | 427 | 0.371 | 105 | 65 | 1 | 1 | 105 | 5 | 108 | Baseplate assembly protein | Baseplate assembly protein | | afdb-uniprot50 | AF-A0A328XFA0-F1-MODEL\_V4 | 1.0 | 8.855e-11 | 427 | 0.449 | 109 | 56 | 1 | 1 | 105 | 1 | 109 | GPW\_gp25 domain-containing protein | GPW\_gp25 domain-containing protein | | afdb-uniprot50 | AF-A0A367WUC5-F1-MODEL\_V4 | 1.0 | 3.502e-11 | 426 | 0.41 | 112 | 62 | 2 | 1 | 111 | 1 | 109 | GPW\_gp25 domain-containing protein | GPW\_gp25 domain-containing protein | | afdb-uniprot50 | AF-A0A7Z0LWZ0-F1-MODEL\_V4 | 1.0 | 2.735e-11 | 426 | 0.467 | 107 | 54 | 2 | 1 | 105 | 1 | 106 | GPW/gp25 family protein | GPW/gp25 family protein | | afdb-uniprot50 | AF-A0A828IES2-F1-MODEL\_V4 | 1.0 | 9.42e-11 | 425 | 0.419 | 105 | 60 | 1 | 2 | 105 | 7 | 111 | Baseplate assembly protein | Baseplate assembly protein | | afdb-uniprot50 | AF-A0A3R7GMZ6-F1-MODEL\_V4 | 1.0 | 7.825e-11 | 424 | 0.416 | 108 | 60 | 1 | 1 | 105 | 1 | 108 | Baseplate assembly protein | Baseplate assembly protein | | afdb-uniprot50 | AF-A0A6P1LA29-F1-MODEL\_V4 | 1.0 | 3.726e-11 | 423 | 0.467 | 109 | 54 | 2 | 1 | 105 | 1 | 109 | Baseplate assembly protein | Baseplate assembly protein | | afdb-uniprot50 | AF-A0A3E0X3W0-F1-MODEL\_V4 | 1.0 | 7.464e-12 | 423 | 0.465 | 116 | 54 | 2 | 1 | 108 | 1 | 116 | Baseplate assembly protein | Baseplate assembly protein | | afdb-uniprot50 | AF-Q1QI83-F1-MODEL\_V4 | 1.0 | 2.136e-11 | 422 | 0.426 | 122 | 56 | 3 | 1 | 108 | 42 | 163 | GPW/gp25 | GPW/gp25 | | afdb-uniprot50 | AF-A0A286B0L7-F1-MODEL\_V4 | 1.0 | 7.016e-12 | 420 | 0.5 | 112 | 50 | 2 | 1 | 106 | 1 | 112 | GPW\_gp25 domain-containing protein | GPW\_gp25 domain-containing protein | | afdb-uniprot50 | AF-Q9P9R9-F1-MODEL\_V4 | 1.0 | 2.417e-11 | 419 | 0.566 | 90 | 39 | 0 | 23 | 112 | 2 | 91 | Phage-related baseplate assembly protein | Phage-related baseplate assembly protein | | afdb-uniprot50 | AF-A0A2S4LWD4-F1-MODEL\_V4 | 1.0 | 7.356e-11 | 419 | 0.436 | 110 | 56 | 2 | 1 | 105 | 1 | 109 | GPW\_gp25 domain-containing protein | GPW\_gp25 domain-containing protein | | afdb-uniprot50 | AF-A0A1N7LRK4-F1-MODEL\_V4 | 1.0 | 1.86e-10 | 418 | 0.33 | 112 | 75 | 0 | 1 | 112 | 1 | 112 | GPW\_gp25 domain-containing protein | GPW\_gp25 domain-containing protein | | afdb-uniprot50 | AF-A0A6I3XA50-F1-MODEL\_V4 | 1.0 | 1.002e-10 | 418 | 0.398 | 113 | 63 | 2 | 1 | 111 | 1 | 110 | Baseplate assembly protein | Baseplate assembly protein | | afdb-uniprot50 | AF-A0A1Y1QY83-F1-MODEL\_V4 | 1.0 | 2.695e-10 | 417 | 0.504 | 105 | 50 | 2 | 4 | 107 | 1 | 104 | GPW\_gp25 domain-containing protein | GPW\_gp25 domain-containing protein | | afdb-uniprot50 | AF-A0A812RF50-F1-MODEL\_V4 | 1.0 | 2.037e-12 | 417 | 0.46 | 126 | 52 | 1 | 1 | 110 | 63 | 188 | W protein | W protein | | afdb-uniprot50 | AF-A0A1B4ETV1-F1-MODEL\_V4 | 1.0 | 4.155e-10 | 415 | 0.37 | 108 | 66 | 1 | 1 | 108 | 4 | 109 | Phage baseplate protein | Phage baseplate protein | | afdb-uniprot50 | AF-A0A806CDY2-F1-MODEL\_V4 | 1.0 | 1.206e-10 | 415 | 0.4 | 105 | 63 | 0 | 1 | 105 | 1 | 105 | GPW/gp25 family protein | GPW/gp25 family protein | | afdb-uniprot50 | AF-A8TYF6-F1-MODEL\_V4 | 1.0 | 2.534e-10 | 414 | 0.583 | 96 | 40 | 0 | 4 | 99 | 1 | 96 | GPW\_gp25 domain-containing protein | GPW\_gp25 domain-containing protein | | afdb-uniprot50 | AF-A0A4Q0Y939-F1-MODEL\_V4 | 1.0 | 4.155e-10 | 412 | 0.593 | 91 | 35 | 1 | 20 | 110 | 1 | 89 | Phage baseplate protein | Phage baseplate protein | | afdb-uniprot50 | AF-A0A0H3H1Q9-F1-MODEL\_V4 | 1.0 | 1.134e-10 | 412 | 0.37 | 108 | 66 | 1 | 2 | 107 | 6 | 113 | Putative prophage baseplate protein | Putative prophage baseplate protein | | afdb-uniprot50 | AF-A0A5M6I4S2-F1-MODEL\_V4 | 1.0 | 1.134e-10 | 412 | 0.373 | 107 | 66 | 1 | 3 | 108 | 4 | 110 | GPW\_gp25 domain-containing protein | GPW\_gp25 domain-containing protein | | afdb-uniprot50 | AF-A0A431QP25-F1-MODEL\_V4 | 1.0 | 5.399e-11 | 412 | 0.486 | 109 | 50 | 2 | 1 | 103 | 1 | 109 | Baseplate assembly protein | Baseplate assembly protein | | afdb-uniprot50 | AF-A0A829R2Z7-F1-MODEL\_V4 | 1.0 | 2.105e-10 | 411 | 0.375 | 104 | 65 | 0 | 2 | 105 | 4 | 107 | Putative lysozyme | Putative lysozyme | | afdb-uniprot50 | AF-A0A7Z1A3K0-F1-MODEL\_V4 | 1.0 | 2.867e-10 | 411 | 0.432 | 104 | 59 | 0 | 3 | 106 | 1 | 104 | Phage baseplate assembly protein | Phage baseplate assembly protein | | afdb-uniprot50 | AF-A0A1M4WDA9-F1-MODEL\_V4 | 1.0 | 1.643e-10 | 410 | 0.485 | 103 | 50 | 2 | 4 | 105 | 1 | 101 | GPW\_gp25 domain-containing protein | GPW\_gp25 domain-containing protein | | afdb-uniprot50 | AF-A0A250DSI4-F1-MODEL\_V4 | 1.0 | 2.105e-10 | 410 | 0.428 | 105 | 60 | 0 | 1 | 105 | 1 | 105 | GPW\_gp25 domain-containing protein | GPW\_gp25 domain-containing protein | | afdb-uniprot50 | AF-A0A2G6IRN8-F1-MODEL\_V4 | 1.0 | 3.906e-10 | 409 | 0.361 | 108 | 68 | 1 | 1 | 107 | 1 | 108 | GPW\_gp25 domain-containing protein | GPW\_gp25 domain-containing protein | | afdb-uniprot50 | AF-N9R592-F1-MODEL\_V4 | 1.0 | 2.239e-10 | 409 | 0.367 | 106 | 66 | 1 | 3 | 107 | 1 | 106 | GPW\_gp25 domain-containing protein | GPW\_gp25 domain-containing protein | | afdb-uniprot50 | AF-A0A6L9JNR0-F1-MODEL\_V4 | 1.0 | 7.825e-11 | 408 | 0.412 | 109 | 63 | 1 | 1 | 108 | 3 | 111 | Baseplate assembly protein | Baseplate assembly protein | | afdb-uniprot50 | AF-A0A1C3K3F0-F1-MODEL\_V4 | 1.0 | 1.002e-10 | 408 | 0.417 | 115 | 60 | 3 | 1 | 111 | 4 | 115 | Phage baseplate assembly protein | Phage baseplate assembly protein | | afdb-uniprot50 | AF-A0A2W6T5C9-F1-MODEL\_V4 | 1.0 | 2.909e-11 | 408 | 0.413 | 116 | 62 | 4 | 1 | 111 | 4 | 118 | Baseplate assembly protein | Baseplate assembly protein | | afdb-uniprot50 | AF-A0A7X2UKL5-F1-MODEL\_V4 | 1.0 | 3.05e-10 | 407 | 0.419 | 105 | 60 | 1 | 1 | 105 | 3 | 106 | Baseplate assembly protein | Baseplate assembly protein | | afdb-uniprot50 | AF-A0A4P7L9H6-F1-MODEL\_V4 | 1.0 | 2.867e-10 | 407 | 0.368 | 103 | 64 | 1 | 3 | 105 | 5 | 106 | Gene 25-like lysozyme | Gene 25-like lysozyme | | afdb-uniprot50 | AF-A0A7V8DBK9-F1-MODEL\_V4 | 1.0 | 3.05e-10 | 406 | 0.4 | 105 | 63 | 0 | 1 | 105 | 1 | 105 | GPW\_gp25 domain-containing protein | GPW\_gp25 domain-containing protein | | afdb-uniprot50 | AF-A0A8B4MIR0-F1-MODEL\_V4 | 1.0 | 3.906e-10 | 406 | 0.342 | 105 | 69 | 0 | 3 | 107 | 1 | 105 | Phage-related baseplate assembly protein (GPW-like) | Phage-related baseplate assembly protein (GPW-like) | | afdb-uniprot50 | AF-A0A1Z9Z2R2-F1-MODEL\_V4 | 1.0 | 4.485e-11 | 405 | 0.39 | 110 | 63 | 1 | 3 | 108 | 1 | 110 | Baseplate assembly protein | Baseplate assembly protein | | afdb-uniprot50 | AF-R9V0R8-F1-MODEL\_V4 | 1.0 | 2.382e-10 | 405 | 0.451 | 104 | 56 | 1 | 4 | 106 | 1 | 104 | Baseplate assembly protein | Baseplate assembly protein | | afdb-uniprot50 | AF-A0A0M1HPW6-F1-MODEL\_V4 | 1.0 | 5.002e-10 | 403 | 0.355 | 104 | 67 | 0 | 3 | 106 | 1 | 104 | Baseplate assembly protein | Baseplate assembly protein | | afdb-uniprot50 | AF-A0A1G7SDT3-F1-MODEL\_V4 | 1.0 | 3.672e-10 | 402 | 0.407 | 108 | 63 | 1 | 1 | 107 | 1 | 108 | GPW\_gp25 domain-containing protein | GPW\_gp25 domain-containing protein | | afdb-uniprot50 | AF-A0A5P2STB1-F1-MODEL\_V4 | 1.0 | 2.105e-10 | 402 | 0.359 | 103 | 65 | 1 | 4 | 105 | 1 | 103 | Baseplate assembly protein | Baseplate assembly protein | | afdb-uniprot50 | AF-A0A0E4BWU6-F1-MODEL\_V4 | 1.0 | 7.712e-10 | 402 | 0.292 | 106 | 74 | 1 | 1 | 105 | 8 | 113 | GPW\_gp25 domain-containing protein | GPW\_gp25 domain-containing protein | | afdb-uniprot50 | AF-A0A2S8VCL2-F1-MODEL\_V4 | 1.0 | 1.978e-10 | 401 | 0.464 | 112 | 55 | 3 | 4 | 111 | 1 | 111 | Baseplate assembly protein | Baseplate assembly protein | | afdb-uniprot50 | AF-A0A348HI76-F1-MODEL\_V4 | 1.0 | 4.155e-10 | 401 | 0.359 | 103 | 66 | 0 | 3 | 105 | 2 | 104 | Phage baseplate assemblyprotein W | Phage baseplate assemblyprotein W | | afdb-uniprot50 | AF-A0A1B9JGD1-F1-MODEL\_V4 | 1.0 | 6.406e-10 | 401 | 0.4 | 105 | 61 | 2 | 2 | 105 | 4 | 107 | GPW\_gp25 domain-containing protein | GPW\_gp25 domain-containing protein | | afdb-uniprot50 | AF-A0A0C4YEA8-F1-MODEL\_V4 | 1.0 | 5.744e-11 | 401 | 0.4 | 115 | 63 | 1 | 1 | 109 | 4 | 118 | Phage baseplate assembly protein | Phage baseplate assembly protein | | afdb-uniprot50 | AF-Q1QKS3-F1-MODEL\_V4 | 1.0 | 1.134e-10 | 401 | 0.396 | 121 | 60 | 3 | 1 | 108 | 3 | 123 | GPW/gp25 | GPW/gp25 | | afdb-uniprot50 | AF-A0A143I3T2-F1-MODEL\_V4 | 1.0 | 3.906e-10 | 399 | 0.413 | 104 | 60 | 1 | 4 | 106 | 1 | 104 | GPW\_gp25 domain-containing protein | GPW\_gp25 domain-containing protein | | afdb-uniprot50 | AF-A0A2V4DZL0-F1-MODEL\_V4 | 1.0 | 4.155e-10 | 399 | 0.378 | 111 | 64 | 3 | 2 | 111 | 4 | 110 | GPW\_gp25 domain-containing protein | GPW\_gp25 domain-containing protein | | afdb-uniprot50 | AF-A0A350LX95-F1-MODEL\_V4 | 1.0 | 1.283e-10 | 398 | 0.42 | 107 | 62 | 0 | 1 | 107 | 1 | 107 | Phage baseplate protein | Phage baseplate protein | | afdb-uniprot50 | AF-A0A376D4F9-F1-MODEL\_V4 | 1.0 | 9.284e-10 | 397 | 0.384 | 104 | 63 | 1 | 2 | 105 | 32 | 134 | GPW/gp25 family protein | GPW/gp25 family protein | | afdb-uniprot50 | AF-A0A5Y3W5M6-F1-MODEL\_V4 | 1.0 | 4.155e-10 | 396 | 0.409 | 110 | 63 | 2 | 1 | 109 | 3 | 111 | Baseplate assembly protein | Baseplate assembly protein | | afdb-uniprot50 | AF-G8PUM4-F1-MODEL\_V4 | 1.0 | 5.076e-11 | 396 | 0.396 | 121 | 60 | 2 | 1 | 108 | 3 | 123 | GPW/gp25 | GPW/gp25 | | afdb-uniprot50 | AF-E2CJU0-F1-MODEL\_V4 | 1.0 | 1.365e-10 | 396 | 0.378 | 119 | 61 | 3 | 1 | 107 | 3 | 120 | GPW/gp25 | GPW/gp25 | | afdb-uniprot50 | AF-A0A7X9X5F1-F1-MODEL\_V4 | 1.0 | 2.239e-10 | 394 | 0.417 | 115 | 60 | 1 | 1 | 108 | 1 | 115 | Baseplate assembly protein | Baseplate assembly protein | | afdb-uniprot50 | AF-A0A318L0U5-F1-MODEL\_V4 | 1.0 | 3.906e-10 | 394 | 0.371 | 105 | 65 | 1 | 2 | 105 | 9 | 113 | GPW\_gp25 domain-containing protein | GPW\_gp25 domain-containing protein | | afdb-uniprot50 | AF-A0A1I1UBH6-F1-MODEL\_V4 | 1.0 | 6.815e-10 | 393 | 0.419 | 105 | 59 | 2 | 3 | 106 | 2 | 105 | GPW\_gp25 domain-containing protein | GPW\_gp25 domain-containing protein | | afdb-uniprot50 | AF-V4PNX9-F1-MODEL\_V4 | 1.0 | 4.155e-10 | 393 | 0.439 | 107 | 58 | 1 | 1 | 105 | 1 | 107 | GPW\_gp25 domain-containing protein | GPW\_gp25 domain-containing protein | | afdb-uniprot50 | AF-A0A2E6BQ95-F1-MODEL\_V4 | 1.0 | 2.239e-10 | 393 | 0.44 | 109 | 55 | 2 | 4 | 108 | 1 | 107 | GPW\_gp25 domain-containing protein | GPW\_gp25 domain-containing protein | | afdb-uniprot50 | AF-A0A2N7P6U4-F1-MODEL\_V4 | 1.0 | 6.022e-10 | 393 | 0.327 | 113 | 73 | 1 | 1 | 110 | 2 | 114 | Uncharacterized protein | Uncharacterized protein | | afdb-uniprot50 | AF-A0A212J4Q2-F1-MODEL\_V4 | 1.0 | 7.249e-10 | 392 | 0.388 | 108 | 66 | 0 | 1 | 108 | 6 | 113 | Putative Baseplate assembly protein W | Putative Baseplate assembly protein W | | afdb-uniprot50 | AF-D4M9N2-F1-MODEL\_V4 | 1.0 | 2.534e-10 | 390 | 0.443 | 115 | 56 | 4 | 1 | 111 | 4 | 114 | Phage baseplate assembly protein W | Phage baseplate assembly protein W | | afdb-uniprot50 | AF-A0A2W5X821-F1-MODEL\_V4 | 1.0 | 3.906e-10 | 390 | 0.372 | 110 | 65 | 2 | 1 | 106 | 1 | 110 | Uncharacterized protein | Uncharacterized protein | | afdb-uniprot50 | AF-A0A5J6LCN8-F1-MODEL\_V4 | 1.0 | 2.382e-10 | 389 | 0.436 | 110 | 56 | 3 | 3 | 111 | 5 | 109 | GPW\_gp25 domain-containing protein | GPW\_gp25 domain-containing protein | | afdb-uniprot50 | AF-A0A556S966-F1-MODEL\_V4 | 1.0 | 3.05e-10 | 387 | 0.38 | 113 | 64 | 2 | 1 | 108 | 3 | 114 | Baseplate assembly protein | Baseplate assembly protein | | afdb-uniprot50 | AF-A0A6L3Y821-F1-MODEL\_V4 | 1.0 | 1.86e-10 | 387 | 0.404 | 121 | 58 | 2 | 1 | 107 | 3 | 123 | Baseplate assembly protein | Baseplate assembly protein | | afdb-uniprot50 | AF-A0A4Q3KFV4-F1-MODEL\_V4 | 1.0 | 5.321e-10 | 387 | 0.432 | 111 | 57 | 2 | 4 | 108 | 1 | 111 | Uncharacterized protein | Uncharacterized protein | | afdb-uniprot50 | AF-A0A1G8TJ30-F1-MODEL\_V4 | 1.0 | 2.867e-10 | 386 | 0.514 | 107 | 49 | 2 | 4 | 108 | 1 | 106 | GPW\_gp25 domain-containing protein | GPW\_gp25 domain-containing protein | | afdb-uniprot50 | AF-A0A856Y7R7-F1-MODEL\_V4 | 1.0 | 1.118e-09 | 386 | 0.5 | 98 | 48 | 1 | 1 | 97 | 1 | 98 | Baseplate assembly protein | Baseplate assembly protein | | afdb-uniprot50 | AF-A0A2S9RND6-F1-MODEL\_V4 | 1.0 | 9.284e-10 | 386 | 0.358 | 106 | 66 | 2 | 4 | 108 | 1 | 105 | Gene 25-like lysozyme | Gene 25-like lysozyme | | afdb-uniprot50 | AF-A0A348FYH4-F1-MODEL\_V4 | 1.0 | 3.906e-10 | 385 | 0.419 | 105 | 60 | 1 | 3 | 106 | 2 | 106 | GPW\_gp25 domain-containing protein | GPW\_gp25 domain-containing protein | | afdb-uniprot50 | AF-A0A2T5J1F8-F1-MODEL\_V4 | 1.0 | 2.695e-10 | 385 | 0.377 | 106 | 66 | 0 | 1 | 106 | 3 | 108 | GPW\_gp25 domain-containing protein | GPW\_gp25 domain-containing protein | | afdb-uniprot50 | AF-A0A2W7IMC6-F1-MODEL\_V4 | 1.0 | 4.42e-10 | 384 | 0.403 | 109 | 61 | 2 | 4 | 111 | 1 | 106 | GPW\_gp25 domain-containing protein | GPW\_gp25 domain-containing protein | | afdb-uniprot50 | AF-A0A5E4XG18-F1-MODEL\_V4 | 1.0 | 6.815e-10 | 384 | 0.382 | 115 | 64 | 2 | 1 | 111 | 1 | 112 | Phage baseplate protein | Phage baseplate protein | | afdb-uniprot50 | AF-Q31HT9-F1-MODEL\_V4 | 1.0 | 8.727e-10 | 383 | 0.299 | 117 | 74 | 4 | 1 | 112 | 1 | 114 | Phage baseplate assembly protein | Phage baseplate assembly protein | | afdb-uniprot50 | AF-A0A554XC26-F1-MODEL\_V4 | 1.0 | 8.727e-10 | 382 | 0.457 | 105 | 57 | 0 | 1 | 105 | 23 | 127 | Lysozyme | Lysozyme | | afdb-uniprot50 | AF-A0A5B9ACT0-F1-MODEL\_V4 | 1.0 | 6.022e-10 | 381 | 0.402 | 92 | 55 | 0 | 14 | 105 | 1 | 92 | Baseplate assembly protein | Baseplate assembly protein | | afdb-uniprot50 | AF-A0A256CAS9-F1-MODEL\_V4 | 1.0 | 2.206e-09 | 380 | 0.4 | 105 | 62 | 1 | 4 | 107 | 1 | 105 | GPW\_gp25 domain-containing protein | GPW\_gp25 domain-containing protein | | afdb-uniprot50 | AF-A0A0T9QIN0-F1-MODEL\_V4 | 1.0 | 9.876e-10 | 380 | 0.39 | 105 | 64 | 0 | 1 | 105 | 5 | 109 | Phage baseplate assembly protein W | Phage baseplate assembly protein W | | afdb-uniprot50 | AF-A0A542V4K3-F1-MODEL\_V4 | 1.0 | 1.523e-09 | 378 | 0.415 | 106 | 61 | 1 | 4 | 108 | 1 | 106 | GPW\_gp25 domain-containing protein | GPW\_gp25 domain-containing protein | | afdb-uniprot50 | AF-A0A2S2E6D5-F1-MODEL\_V4 | 1.0 | 8.727e-10 | 377 | 0.3 | 113 | 74 | 2 | 1 | 109 | 2 | 113 | Uncharacterized protein | Uncharacterized protein | | afdb-uniprot50 | AF-A0A423PQJ1-F1-MODEL\_V4 | 1.0 | 5.579e-09 | 375 | 0.417 | 103 | 60 | 0 | 4 | 106 | 1 | 103 | Baseplate assembly protein | Baseplate assembly protein | | afdb-uniprot50 | AF-A0A516W9Y9-F1-MODEL\_V4 | 1.0 | 6.022e-10 | 375 | 0.476 | 105 | 53 | 1 | 4 | 106 | 1 | 105 | Baseplate assembly protein | Baseplate assembly protein | | afdb-uniprot50 | AF-S6GUS5-F1-MODEL\_V4 | 1.0 | 1.189e-09 | 375 | 0.401 | 107 | 60 | 3 | 1 | 106 | 8 | 111 | W protein | W protein | | afdb-uniprot50 | AF-A0A1Y3CLD4-F1-MODEL\_V4 | 1.0 | 1.833e-09 | 375 | 0.38 | 105 | 63 | 2 | 3 | 105 | 1 | 105 | Baseplate assembly protein | Baseplate assembly protein | | afdb-uniprot50 | AF-A0A0U3ICT5-F1-MODEL\_V4 | 1.0 | 2.206e-09 | 374 | 0.388 | 103 | 63 | 0 | 3 | 105 | 1 | 103 | Baseplate assembly protein | Baseplate assembly protein | | afdb-uniprot50 | AF-A0A7T9Q7H3-F1-MODEL\_V4 | 1.0 | 2.074e-09 | 374 | 0.388 | 103 | 63 | 0 | 3 | 105 | 1 | 103 | GPW/gp25 family protein | GPW/gp25 family protein | | afdb-uniprot50 | AF-A0A166Z6R0-F1-MODEL\_V4 | 1.0 | 5.661e-10 | 373 | 0.412 | 109 | 60 | 2 | 1 | 108 | 3 | 108 | Baseplate assembly protein | Baseplate assembly protein | | afdb-uniprot50 | AF-A0A2N3KSL6-F1-MODEL\_V4 | 1.0 | 1.431e-09 | 373 | 0.364 | 107 | 66 | 1 | 1 | 105 | 1 | 107 | Baseplate assembly protein W | Baseplate assembly protein W | | afdb-uniprot50 | AF-A0A1M3HHS7-F1-MODEL\_V4 | 1.0 | 1.345e-09 | 372 | 0.416 | 108 | 60 | 2 | 1 | 105 | 1 | 108 | GPW\_gp25 domain-containing protein | GPW\_gp25 domain-containing protein | | afdb-uniprot50 | AF-A0A4R0EZX5-F1-MODEL\_V4 | 1.0 | 2.347e-09 | 372 | 0.378 | 103 | 64 | 0 | 3 | 105 | 1 | 103 | Baseplate assembly protein | Baseplate assembly protein | | afdb-uniprot50 | AF-A0A7S8HE74-F1-MODEL\_V4 | 1.0 | 5.321e-10 | 371 | 0.388 | 108 | 55 | 2 | 12 | 111 | 1 | 105 | GPW/gp25 family protein | GPW/gp25 family protein | | afdb-uniprot50 | AF-A0A8B0TIL9-F1-MODEL\_V4 | 1.0 | 2.656e-09 | 371 | 0.401 | 102 | 61 | 0 | 4 | 105 | 1 | 102 | GPW/gp25 family protein | GPW/gp25 family protein | | afdb-uniprot50 | AF-A0A4R0FND4-F1-MODEL\_V4 | 1.0 | 2.826e-09 | 369 | 0.368 | 103 | 65 | 0 | 3 | 105 | 1 | 103 | Baseplate assembly protein | Baseplate assembly protein | | afdb-uniprot50 | AF-C8N768-F1-MODEL\_V4 | 1.0 | 1.95e-09 | 369 | 0.361 | 108 | 67 | 2 | 2 | 108 | 19 | 125 | Putative lysozyme | Putative lysozyme | | afdb-uniprot50 | AF-A0A103EKP3-F1-MODEL\_V4 | 1.0 | 4.095e-09 | 368 | 0.675 | 80 | 26 | 0 | 1 | 80 | 1 | 80 | Phage baseplate protein | Phage baseplate protein | | afdb-uniprot50 | AF-A0A074TCW7-F1-MODEL\_V4 | 1.0 | 1.118e-09 | 368 | 0.371 | 113 | 61 | 1 | 3 | 105 | 2 | 114 | GPW\_gp25 domain-containing protein | GPW\_gp25 domain-containing protein | | afdb-uniprot50 | AF-A0A1I1UZQ7-F1-MODEL\_V4 | 1.0 | 1.95e-09 | 368 | 0.359 | 114 | 64 | 2 | 1 | 105 | 2 | 115 | GPW\_gp25 domain-containing protein | GPW\_gp25 domain-containing protein | | afdb-uniprot50 | AF-A0A7H2V930-F1-MODEL\_V4 | 1.0 | 3.198e-09 | 367 | 0.378 | 103 | 64 | 0 | 3 | 105 | 1 | 103 | GPW/gp25 family protein | GPW/gp25 family protein | | afdb-uniprot50 | AF-A0A239RWY0-F1-MODEL\_V4 | 1.0 | 3.619e-09 | 367 | 0.388 | 103 | 63 | 0 | 3 | 105 | 1 | 103 | Baseplate assembly protein | Baseplate assembly protein | | afdb-uniprot50 | AF-A0A258L738-F1-MODEL\_V4 | 1.0 | 1.265e-09 | 367 | 0.368 | 114 | 63 | 2 | 1 | 105 | 4 | 117 | GPW\_gp25 domain-containing protein | GPW\_gp25 domain-containing protein | | afdb-uniprot50 | AF-A0A5N4WTB4-F1-MODEL\_V4 | 1.0 | 3.198e-09 | 366 | 0.378 | 103 | 64 | 0 | 3 | 105 | 1 | 103 | Baseplate assembly protein | Baseplate assembly protein | | afdb-uniprot50 | AF-A0A2A2B5A6-F1-MODEL\_V4 | 1.0 | 9.876e-10 | 366 | 0.471 | 106 | 52 | 3 | 3 | 105 | 7 | 111 | Baseplate assembly protein | Baseplate assembly protein | | afdb-uniprot50 | AF-A0A1W1XJR9-F1-MODEL\_V4 | 1.0 | 1.345e-09 | 365 | 0.368 | 103 | 64 | 1 | 4 | 105 | 1 | 103 | GPW\_gp25 domain-containing protein | GPW\_gp25 domain-containing protein | | afdb-uniprot50 | AF-A0A846VGN2-F1-MODEL\_V4 | 1.0 | 4.356e-09 | 365 | 0.28 | 114 | 73 | 2 | 1 | 105 | 6 | 119 | Uncharacterized protein | Uncharacterized protein | | afdb-uniprot50 | AF-A0A345DE47-F1-MODEL\_V4 | 1.0 | 1.265e-09 | 364 | 0.467 | 107 | 54 | 2 | 4 | 107 | 1 | 107 | GPW\_gp25 domain-containing protein | GPW\_gp25 domain-containing protein | | afdb-uniprot50 | AF-A0A359KBY6-F1-MODEL\_V4 | 1.0 | 2.206e-09 | 362 | 0.373 | 107 | 64 | 2 | 1 | 105 | 1 | 106 | Uncharacterized protein | Uncharacterized protein | | afdb-uniprot50 | AF-A0A1B8QCY8-F1-MODEL\_V4 | 1.0 | 7.144e-09 | 361 | 0.352 | 105 | 68 | 0 | 4 | 108 | 1 | 105 | GPW\_gp25 domain-containing protein | GPW\_gp25 domain-containing protein | | afdb-uniprot50 | AF-A0A547PWD4-F1-MODEL\_V4 | 1.0 | 2.074e-09 | 361 | 0.495 | 103 | 49 | 1 | 1 | 103 | 1 | 100 | Phage baseplate protein | Phage baseplate protein | | afdb-uniprot50 | AF-A0A2G6E136-F1-MODEL\_V4 | 1.0 | 9.876e-10 | 360 | 0.419 | 112 | 54 | 2 | 4 | 106 | 1 | 110 | GPW\_gp25 domain-containing protein | GPW\_gp25 domain-containing protein | | afdb-uniprot50 | AF-A0A2G6EWW6-F1-MODEL\_V4 | 1.0 | 1.95e-09 | 359 | 0.446 | 103 | 57 | 0 | 3 | 105 | 10 | 112 | GPW\_gp25 domain-containing protein | GPW\_gp25 domain-containing protein | | afdb-uniprot50 | AF-A0A377AI41-F1-MODEL\_V4 | 1.0 | 6.313e-09 | 357 | 0.422 | 97 | 56 | 0 | 1 | 97 | 5 | 101 | Baseplate assembly protein W | Baseplate assembly protein W | | afdb-uniprot50 | AF-D6H7Q3-F1-MODEL\_V4 | 1.0 | 6.716e-09 | 357 | 0.377 | 106 | 63 | 2 | 2 | 105 | 192 | 296 | Uncharacterized protein | Uncharacterized protein | | afdb-uniprot50 | AF-C4GGH2-F1-MODEL\_V4 | 1.0 | 4.095e-09 | 355 | 0.386 | 106 | 60 | 3 | 3 | 105 | 1 | 104 | Putative lysozyme | Putative lysozyme | | afdb-uniprot50 | AF-A0A7U3VTF8-F1-MODEL\_V4 | 1.0 | 6.716e-09 | 355 | 0.294 | 112 | 78 | 1 | 1 | 112 | 1 | 111 | Baseplate assembly protein GpW | Baseplate assembly protein GpW | | afdb-uniprot50 | AF-C1DBK2-F1-MODEL\_V4 | 1.0 | 7.144e-09 | 354 | 0.411 | 107 | 59 | 3 | 4 | 106 | 1 | 107 | GpW/GP25 family protein | GpW/GP25 family protein | | afdb-uniprot50 | AF-A0A2M9VR53-F1-MODEL\_V4 | 1.0 | 4.095e-09 | 353 | 0.445 | 92 | 51 | 0 | 15 | 106 | 1 | 92 | Phage baseplate protein | Phage baseplate protein | | afdb-uniprot50 | AF-A0A0A2YRZ1-F1-MODEL\_V4 | 1.0 | 5.579e-09 | 351 | 0.388 | 103 | 61 | 2 | 4 | 105 | 1 | 102 | Baseplate wedge subunit | Baseplate wedge subunit | | afdb-uniprot50 | AF-A0A371WUQ6-F1-MODEL\_V4 | 1.0 | 3.619e-09 | 349 | 0.35 | 120 | 63 | 2 | 1 | 105 | 20 | 139 | Baseplate assembly protein | Baseplate assembly protein | | afdb-uniprot50 | AF-A0A241WJJ5-F1-MODEL\_V4 | 1.0 | 1.265e-09 | 348 | 0.408 | 120 | 59 | 4 | 1 | 108 | 1 | 120 | GPW\_gp25 domain-containing protein | GPW\_gp25 domain-containing protein | | afdb-uniprot50 | AF-A0A6B8KGJ0-F1-MODEL\_V4 | 1.0 | 2.074e-09 | 348 | 0.33 | 115 | 72 | 3 | 1 | 110 | 2 | 116 | Uncharacterized protein | Uncharacterized protein | | afdb-uniprot50 | AF-A0A7W6RF26-F1-MODEL\_V4 | 1.0 | 9.149e-09 | 347 | 0.352 | 125 | 64 | 4 | 1 | 108 | 1 | 125 | Uncharacterized protein | Uncharacterized protein | | afdb-uniprot50 | AF-A0A6D0J2V1-F1-MODEL\_V4 | 1.0 | 5.579e-09 | 346 | 0.382 | 89 | 55 | 0 | 17 | 105 | 6 | 94 | Baseplate assembly protein | Baseplate assembly protein | | afdb-uniprot50 | AF-A0A4R3Y632-F1-MODEL\_V4 | 1.0 | 4.634e-09 | 345 | 0.35 | 100 | 60 | 3 | 17 | 112 | 7 | 105 | GPW\_gp25 domain-containing protein | GPW\_gp25 domain-containing protein | | afdb-uniprot50 | AF-A0A326L1I8-F1-MODEL\_V4 | 1.0 | 5.579e-09 | 345 | 0.371 | 113 | 64 | 3 | 1 | 106 | 3 | 115 | GPW\_gp25 domain-containing protein | GPW\_gp25 domain-containing protein | | afdb-uniprot50 | AF-A0A809X5W8-F1-MODEL\_V4 | 1.0 | 1.41e-08 | 344 | 0.317 | 104 | 64 | 2 | 1 | 100 | 8 | 108 | Uncharacterized protein | Uncharacterized protein | | afdb-uniprot50 | AF-A0A516SJB4-F1-MODEL\_V4 | 1.0 | 3.198e-09 | 344 | 0.37 | 116 | 63 | 3 | 1 | 108 | 6 | 119 | Phage baseplate protein | Phage baseplate protein | | afdb-uniprot50 | AF-A0A535HZP7-F1-MODEL\_V4 | 1.0 | 6.716e-09 | 343 | 0.408 | 98 | 56 | 1 | 10 | 105 | 2 | 99 | GPW/gp25 family protein | GPW/gp25 family protein | | afdb-uniprot50 | AF-A0A7C6PU32-F1-MODEL\_V4 | 1.0 | 6.716e-09 | 343 | 0.279 | 111 | 73 | 2 | 1 | 105 | 7 | 116 | GPW/gp25 family protein | GPW/gp25 family protein | | afdb-uniprot50 | AF-A0A7W8YF20-F1-MODEL\_V4 | 1.0 | 1.806e-08 | 342 | 0.558 | 86 | 38 | 0 | 1 | 86 | 1 | 86 | GPW\_gp25 domain-containing protein | GPW\_gp25 domain-containing protein | | afdb-uniprot50 | AF-A0A037UUA6-F1-MODEL\_V4 | 1.0 | 5.244e-09 | 342 | 0.415 | 106 | 57 | 1 | 1 | 101 | 1 | 106 | GPW\_gp25 domain-containing protein | GPW\_gp25 domain-containing protein | | afdb-uniprot50 | AF-A0A0H4IV38-F1-MODEL\_V4 | 1.0 | 1.922e-08 | 341 | 0.38 | 105 | 62 | 2 | 3 | 105 | 1 | 104 | Phage baseplate assembly protein | Phage baseplate assembly protein | | afdb-uniprot50 | AF-A0A6L7EAJ2-F1-MODEL\_V4 | 1.0 | 1.172e-08 | 340 | 0.389 | 95 | 54 | 2 | 17 | 111 | 1 | 91 | Baseplate assembly protein | Baseplate assembly protein | | afdb-uniprot50 | AF-A0A3L7AJZ0-F1-MODEL\_V4 | 1.0 | 4.93e-09 | 340 | 0.312 | 112 | 68 | 2 | 1 | 103 | 1 | 112 | Baseplate assembly protein | Baseplate assembly protein | | afdb-uniprot50 | AF-A0A7W8AJL1-F1-MODEL\_V4 | 1.0 | 6.313e-09 | 340 | 0.324 | 114 | 68 | 3 | 1 | 105 | 8 | 121 | GPW\_gp25 domain-containing protein | GPW\_gp25 domain-containing protein | | afdb-uniprot50 | AF-A0A2A5BNQ3-F1-MODEL\_V4 | 1.0 | 1.118e-09 | 338 | 0.342 | 114 | 65 | 3 | 1 | 105 | 1 | 113 | GPW\_gp25 domain-containing protein | GPW\_gp25 domain-containing protein | | afdb-uniprot50 | AF-A0A839IXR3-F1-MODEL\_V4 | 1.0 | 8.601e-09 | 338 | 0.293 | 116 | 72 | 4 | 1 | 112 | 1 | 110 | Phage baseplate protein | Phage baseplate protein | | afdb-uniprot50 | AF-A0A1L6M2Z8-F1-MODEL\_V4 | 1.0 | 3.619e-09 | 338 | 0.324 | 117 | 67 | 2 | 1 | 105 | 9 | 125 | GPW\_gp25 domain-containing protein | GPW\_gp25 domain-containing protein | | afdb-uniprot50 | AF-B7RNM3-F1-MODEL\_V4 | 1.0 | 3.151e-08 | 337 | 0.576 | 78 | 33 | 0 | 35 | 112 | 1 | 78 | GPW/gp25 | GPW/gp25 | | afdb-uniprot50 | AF-A0A1Y6CPE4-F1-MODEL\_V4 | 1.0 | 1.596e-08 | 337 | 0.314 | 108 | 71 | 2 | 1 | 108 | 1 | 105 | Uncharacterized protein | Uncharacterized protein | | afdb-uniprot50 | AF-A0A5E8NU52-F1-MODEL\_V4 | 1.0 | 3.151e-08 | 334 | 0.387 | 93 | 57 | 0 | 1 | 93 | 5 | 97 | Baseplate assembly protein | Baseplate assembly protein | | afdb-uniprot50 | AF-E2CFL3-F1-MODEL\_V4 | 1.0 | 5.935e-09 | 334 | 0.275 | 116 | 79 | 3 | 1 | 112 | 2 | 116 | GpW/gp25 family protein | GpW/gp25 family protein | | afdb-uniprot50 | AF-Q4E960-F1-MODEL\_V4 | 1.0 | 3.352e-08 | 333 | 0.521 | 94 | 45 | 0 | 4 | 97 | 1 | 94 | GPW\_gp25 domain-containing protein | GPW\_gp25 domain-containing protein | | afdb-uniprot50 | AF-A0A7Y2L5A3-F1-MODEL\_V4 | 1.0 | 1.922e-08 | 330 | 0.5 | 84 | 41 | 1 | 23 | 105 | 2 | 85 | Baseplate assembly protein | Baseplate assembly protein | | afdb-uniprot50 | AF-A0A5C7PFA8-F1-MODEL\_V4 | 1.0 | 3.619e-09 | 329 | 0.363 | 121 | 64 | 3 | 1 | 108 | 6 | 126 | Baseplate assembly protein | Baseplate assembly protein | | afdb-uniprot50 | AF-A0A7W2BN75-F1-MODEL\_V4 | 1.0 | 1.172e-08 | 329 | 0.321 | 112 | 69 | 2 | 1 | 105 | 2 | 113 | Baseplate assembly protein | Baseplate assembly protein | | afdb-uniprot50 | AF-A0A6N7C1I5-F1-MODEL\_V4 | 1.0 | 1.596e-08 | 328 | 0.44 | 100 | 53 | 1 | 1 | 97 | 13 | 112 | Phage baseplate assembly protein | Phage baseplate assembly protein | | afdb-uniprot50 | AF-A0A1E3G6B2-F1-MODEL\_V4 | 1.0 | 2.044e-08 | 327 | 0.362 | 113 | 64 | 1 | 1 | 105 | 8 | 120 | Uncharacterized protein | Uncharacterized protein | | afdb-uniprot50 | AF-A0A6N8TE09-F1-MODEL\_V4 | 1.0 | 9.149e-09 | 327 | 0.313 | 115 | 72 | 2 | 1 | 108 | 8 | 122 | Integrase | Integrase | | afdb-uniprot50 | AF-A0A4V1LS71-F1-MODEL\_V4 | 1.0 | 2.461e-08 | 326 | 0.449 | 89 | 48 | 1 | 17 | 105 | 14 | 101 | GPW\_gp25 domain-containing protein | GPW\_gp25 domain-containing protein | | afdb-uniprot50 | AF-A0A6A4R9J3-F1-MODEL\_V4 | 1.0 | 2.175e-08 | 324 | 0.256 | 117 | 74 | 5 | 1 | 112 | 1 | 109 | Phage baseplate protein | Phage baseplate protein | | afdb-uniprot50 | AF-A0A258KUE2-F1-MODEL\_V4 | 1.0 | 8.085e-09 | 323 | 0.37 | 108 | 62 | 2 | 2 | 103 | 10 | 117 | GPW\_gp25 domain-containing protein | GPW\_gp25 domain-containing protein | | afdb-uniprot50 | AF-A0A484YKT9-F1-MODEL\_V4 | 1.0 | 3.151e-08 | 322 | 0.416 | 84 | 48 | 1 | 23 | 105 | 2 | 85 | Baseplate Assembly protein W | Baseplate Assembly protein W | | afdb-uniprot50 | AF-A0A379CBH3-F1-MODEL\_V4 | 1.0 | 3.352e-08 | 320 | 0.411 | 107 | 57 | 3 | 4 | 105 | 1 | 106 | Gene 25-like lysozyme | Gene 25-like lysozyme | | afdb-uniprot50 | AF-B7KB51-F1-MODEL\_V4 | 1.0 | 9.733e-09 | 320 | 0.339 | 109 | 68 | 3 | 2 | 107 | 19 | 126 | GPW/gp25 family protein | GPW/gp25 family protein | | afdb-uniprot50 | AF-A0A1M3AHU0-F1-MODEL\_V4 | 1.0 | 1.596e-08 | 320 | 0.351 | 108 | 64 | 2 | 2 | 103 | 7 | 114 | GPW\_gp25 domain-containing protein | GPW\_gp25 domain-containing protein | | afdb-uniprot50 | AF-A0A7W6N996-F1-MODEL\_V4 | 1.0 | 5.168e-08 | 319 | 0.373 | 123 | 61 | 3 | 1 | 107 | 1 | 123 | GPW\_gp25 domain-containing protein | GPW\_gp25 domain-containing protein | | afdb-uniprot50 | AF-A0A2H0PCR0-F1-MODEL\_V4 | 1.0 | 8.085e-09 | 319 | 0.296 | 108 | 71 | 3 | 2 | 105 | 13 | 119 | Baseplate protein | Baseplate protein | | afdb-uniprot50 | AF-A0A3G4W5G7-F1-MODEL\_V4 | 1.0 | 2.044e-08 | 318 | 0.327 | 107 | 71 | 1 | 2 | 107 | 14 | 120 | Gene 25-like lysozyme | Gene 25-like lysozyme | | afdb-uniprot50 | AF-A0A4Q8MC05-F1-MODEL\_V4 | 1.0 | 1.246e-08 | 318 | 0.361 | 119 | 62 | 3 | 1 | 105 | 3 | 121 | Uncharacterized protein | Uncharacterized protein | | afdb-uniprot50 | AF-A0A376ZFF8-F1-MODEL\_V4 | 1.0 | 1.155e-07 | 316 | 0.5 | 78 | 39 | 0 | 1 | 78 | 5 | 82 | Baseplate assembly protein W | Baseplate assembly protein W | | afdb-uniprot50 | AF-A0A0R3KWV8-F1-MODEL\_V4 | 1.0 | 3.352e-08 | 316 | 0.33 | 115 | 71 | 2 | 1 | 109 | 1 | 115 | GPW\_gp25 domain-containing protein | GPW\_gp25 domain-containing protein | | afdb-uniprot50 | AF-A0A836PEE7-F1-MODEL\_V4 | 1.0 | 7.49e-08 | 315 | 0.283 | 113 | 76 | 2 | 1 | 109 | 2 | 113 | dTDP-glucose pyrophosphorylase | dTDP-glucose pyrophosphorylase | | afdb-uniprot50 | AF-A0A2R4K2P8-F1-MODEL\_V4 | 1.0 | 1.806e-08 | 315 | 0.33 | 106 | 69 | 2 | 2 | 105 | 15 | 120 | Phage baseplate assembly protein W | Phage baseplate assembly protein W | | afdb-uniprot50 | AF-A0A367QB00-F1-MODEL\_V4 | 1.0 | 2.461e-08 | 315 | 0.348 | 109 | 67 | 3 | 2 | 107 | 18 | 125 | Baseplate protein | Baseplate protein | | afdb-uniprot50 | AF-A0A4Q6D2R8-F1-MODEL\_V4 | 1.0 | 3.794e-08 | 313 | 0.259 | 108 | 77 | 2 | 1 | 108 | 1 | 105 | Uncharacterized protein | Uncharacterized protein | | afdb-uniprot50 | AF-A0A327JMQ1-F1-MODEL\_V4 | 1.0 | 9.592e-08 | 312 | 0.341 | 123 | 70 | 5 | 1 | 112 | 3 | 125 | Baseplate assembly protein | Baseplate assembly protein | | afdb-uniprot50 | AF-A0A1Z4UHS5-F1-MODEL\_V4 | 1.0 | 2.785e-08 | 312 | 0.348 | 109 | 67 | 3 | 2 | 107 | 20 | 127 | GPW/gp25 family protein | GPW/gp25 family protein | | afdb-uniprot50 | AF-A0A2C9D702-F1-MODEL\_V4 | 1.0 | 5.498e-08 | 312 | 0.268 | 123 | 72 | 2 | 1 | 105 | 1 | 123 | Baseplate wedge subunit | Baseplate wedge subunit | | afdb-uniprot50 | AF-A0A3B8IJI9-F1-MODEL\_V4 | 1.0 | 1.035e-08 | 312 | 0.247 | 109 | 78 | 2 | 2 | 106 | 15 | 123 | GPW\_gp25 domain-containing protein | GPW\_gp25 domain-containing protein | | afdb-uniprot50 | AF-N2IFU2-F1-MODEL\_V4 | 1.0 | 6.222e-08 | 310 | 0.36 | 86 | 54 | 1 | 21 | 105 | 1 | 86 | GPW\_gp25 domain-containing protein | GPW\_gp25 domain-containing protein | | afdb-uniprot50 | AF-A0A7Y3X776-F1-MODEL\_V4 | 1.0 | 2.785e-08 | 310 | 0.348 | 109 | 67 | 3 | 2 | 107 | 13 | 120 | GPW/gp25 family protein | GPW/gp25 family protein | | afdb-uniprot50 | AF-E2CI67-F1-MODEL\_V4 | 1.0 | 7.041e-08 | 310 | 0.271 | 114 | 74 | 2 | 1 | 105 | 8 | 121 | Putative phage gp25 protein | Putative phage gp25 protein | | afdb-uniprot50 | AF-A0A845ZX24-F1-MODEL\_V4 | 1.0 | 3.352e-08 | 309 | 0.345 | 110 | 66 | 4 | 2 | 107 | 21 | 128 | GPW/gp25 family protein | GPW/gp25 family protein | | afdb-uniprot50 | AF-W5WMR1-F1-MODEL\_V4 | 1.0 | 3.151e-08 | 308 | 0.357 | 112 | 64 | 3 | 2 | 105 | 9 | 120 | GPW\_gp25 domain-containing protein | GPW\_gp25 domain-containing protein | | afdb-uniprot50 | AF-Q7NKR4-F1-MODEL\_V4 | 1.0 | 3.794e-08 | 308 | 0.357 | 109 | 66 | 3 | 2 | 107 | 24 | 131 | Gll1413 protein | Gll1413 protein | | afdb-uniprot50 | AF-A0A7X6FTX0-F1-MODEL\_V4 | 1.0 | 1.894e-07 | 307 | 0.33 | 100 | 66 | 1 | 1 | 99 | 8 | 107 | GPW\_gp25 domain-containing protein | GPW\_gp25 domain-containing protein | | afdb-uniprot50 | AF-A0A521CK88-F1-MODEL\_V4 | 1.0 | 1.228e-07 | 307 | 0.201 | 114 | 84 | 1 | 1 | 107 | 4 | 117 | Phage baseplate assembly protein W | Phage baseplate assembly protein W | | afdb-uniprot50 | AF-A0A6I5NLR7-F1-MODEL\_V4 | 1.0 | 2.313e-08 | 307 | 0.342 | 108 | 67 | 3 | 2 | 106 | 22 | 128 | GPW/gp25 family protein | GPW/gp25 family protein | | afdb-uniprot50 | AF-A0A0Q8ANH2-F1-MODEL\_V4 | 1.0 | 2.175e-08 | 306 | 0.318 | 113 | 70 | 2 | 2 | 107 | 5 | 117 | Uncharacterized protein | Uncharacterized protein | | afdb-uniprot50 | AF-A0A7C5ASQ0-F1-MODEL\_V4 | 1.0 | 2.785e-08 | 306 | 0.327 | 107 | 69 | 2 | 2 | 105 | 14 | 120 | Phage baseplate protein | Phage baseplate protein | | afdb-uniprot50 | AF-A0A3B8JYJ5-F1-MODEL\_V4 | 1.0 | 3.352e-08 | 306 | 0.345 | 110 | 66 | 4 | 2 | 107 | 21 | 128 | Baseplate protein | Baseplate protein | | afdb-uniprot50 | AF-A0A5S9QTJ7-F1-MODEL\_V4 | 1.0 | 5.498e-08 | 305 | 0.236 | 114 | 79 | 4 | 1 | 111 | 2 | 110 | Uncharacterized protein | Uncharacterized protein | | afdb-uniprot50 | AF-A0A1C3F4Y7-F1-MODEL\_V4 | 1.0 | 3.352e-08 | 305 | 0.268 | 108 | 75 | 2 | 2 | 105 | 14 | 121 | GPW/gp25 family protein | GPW/gp25 family protein | | afdb-uniprot50 | AF-A0A2E2NRP7-F1-MODEL\_V4 | 1.0 | 4.567e-08 | 305 | 0.273 | 106 | 75 | 1 | 2 | 105 | 16 | 121 | Baseplate protein | Baseplate protein | | afdb-uniprot50 | AF-A0A7X6CDB5-F1-MODEL\_V4 | 1.0 | 4.567e-08 | 305 | 0.357 | 109 | 66 | 3 | 2 | 107 | 22 | 129 | GPW/gp25 family protein | GPW/gp25 family protein | | afdb-uniprot50 | AF-A0A369RGC9-F1-MODEL\_V4 | 1.0 | 1.307e-07 | 304 | 0.519 | 77 | 37 | 0 | 35 | 111 | 1 | 77 | Baseplate assembly protein W | Baseplate assembly protein W | | afdb-uniprot50 | AF-A0A5M8P8S7-F1-MODEL\_V4 | 1.0 | 1.228e-07 | 304 | 0.299 | 117 | 70 | 3 | 1 | 105 | 2 | 118 | Baseplate assembly protein | Baseplate assembly protein | | afdb-uniprot50 | AF-A0A2T4UGD6-F1-MODEL\_V4 | 1.0 | 4.036e-08 | 304 | 0.289 | 107 | 73 | 2 | 2 | 105 | 13 | 119 | Baseplate protein | Baseplate protein | | afdb-uniprot50 | AF-A0A533SQR7-F1-MODEL\_V4 | 1.0 | 1.02e-07 | 304 | 0.278 | 115 | 74 | 3 | 1 | 106 | 11 | 125 | GPW/gp25 family protein | GPW/gp25 family protein | | afdb-uniprot50 | AF-A0A6I1JFW3-F1-MODEL\_V4 | 1.0 | 3.794e-08 | 304 | 0.315 | 114 | 68 | 2 | 1 | 104 | 7 | 120 | GPW\_gp25 domain-containing protein | GPW\_gp25 domain-containing protein | | afdb-uniprot50 | AF-A0A836SDZ9-F1-MODEL\_V4 | 1.0 | 3.566e-08 | 304 | 0.3 | 103 | 69 | 2 | 5 | 105 | 30 | 131 | Baseplate protein | Baseplate protein | | afdb-uniprot50 | AF-A0A4R5ICH3-F1-MODEL\_V4 | 1.0 | 2.313e-08 | 304 | 0.287 | 108 | 72 | 3 | 2 | 105 | 14 | 120 | Phage baseplate protein | Phage baseplate protein | | afdb-uniprot50 | AF-A0A6G3ZD83-F1-MODEL\_V4 | 1.0 | 4.036e-08 | 304 | 0.317 | 107 | 69 | 3 | 2 | 105 | 22 | 127 | GPW/gp25 family protein | GPW/gp25 family protein | | afdb-uniprot50 | AF-A0A4C2ENV8-F1-MODEL\_V4 | 1.0 | 5.849e-08 | 304 | 0.327 | 107 | 69 | 2 | 2 | 105 | 46 | 152 | Baseplate protein | Baseplate protein | | afdb-uniprot50 | AF-A0A2U0SVH0-F1-MODEL\_V4 | 1.0 | 4.501e-07 | 303 | 0.605 | 71 | 28 | 0 | 1 | 71 | 1 | 71 | Gene 25-like lysozyme | Gene 25-like lysozyme | | afdb-uniprot50 | AF-A0A1A9AYR2-F1-MODEL\_V4 | 1.0 | 1.573e-07 | 303 | 0.245 | 114 | 77 | 4 | 1 | 111 | 1 | 108 | Gene 25-like lysozyme | Gene 25-like lysozyme | | afdb-uniprot50 | AF-H8GWR0-F1-MODEL\_V4 | 1.0 | 1.02e-07 | 303 | 0.354 | 96 | 60 | 1 | 12 | 105 | 2 | 97 | GPW/gp25 family protein | GPW/gp25 family protein | | afdb-uniprot50 | AF-A0A090SWE0-F1-MODEL\_V4 | 1.0 | 1.228e-07 | 303 | 0.229 | 109 | 80 | 2 | 1 | 106 | 4 | 111 | Uncharacterized protein | Uncharacterized protein | | afdb-uniprot50 | AF-E2CHJ3-F1-MODEL\_V4 | 1.0 | 2.785e-08 | 303 | 0.376 | 117 | 60 | 3 | 1 | 104 | 3 | 119 | GPW/gp25 | GPW/gp25 | | afdb-uniprot50 | AF-A0A485AJM6-F1-MODEL\_V4 | 1.0 | 3.977e-07 | 302 | 0.445 | 83 | 46 | 0 | 1 | 83 | 5 | 87 | Gene 25-like lysozyme | Gene 25-like lysozyme | | afdb-uniprot50 | AF-A0A327JS28-F1-MODEL\_V4 | 1.0 | 1.307e-07 | 302 | 0.252 | 119 | 77 | 4 | 1 | 108 | 20 | 137 | Uncharacterized protein | Uncharacterized protein | | afdb-uniprot50 | AF-A0A484HJL0-F1-MODEL\_V4 | 1.0 | 2.313e-08 | 302 | 0.272 | 110 | 76 | 2 | 2 | 107 | 37 | 146 | Putative phage baseplate protein | Putative phage baseplate protein | | afdb-uniprot50 | AF-A0A286EJF8-F1-MODEL\_V4 | 1.0 | 5.849e-08 | 301 | 0.283 | 113 | 72 | 3 | 2 | 105 | 22 | 134 | GPW\_gp25 domain-containing protein | GPW\_gp25 domain-containing protein | | afdb-uniprot50 | AF-A0A0U3ESH1-F1-MODEL\_V4 | 1.0 | 4.036e-08 | 300 | 0.229 | 109 | 81 | 2 | 2 | 107 | 12 | 120 | GPW\_gp25 domain-containing protein | GPW\_gp25 domain-containing protein | | afdb-uniprot50 | AF-A0A0N1NB24-F1-MODEL\_V4 | 1.0 | 3.794e-08 | 300 | 0.345 | 113 | 66 | 3 | 1 | 105 | 28 | 140 | GPW/gp25 family protein | GPW/gp25 family protein | | afdb-uniprot50 | AF-A0A5P8MV09-F1-MODEL\_V4 | 1.0 | 9.017e-08 | 299 | 0.306 | 124 | 73 | 3 | 1 | 111 | 6 | 129 | Integrase | Integrase | | afdb-uniprot50 | AF-A0A521DJN8-F1-MODEL\_V4 | 1.0 | 5.849e-08 | 299 | 0.254 | 106 | 76 | 2 | 2 | 105 | 23 | 127 | GPW\_gp25 domain-containing protein | GPW\_gp25 domain-containing protein | | afdb-uniprot50 | AF-A0A6P0YQM2-F1-MODEL\_V4 | 1.0 | 3.794e-08 | 299 | 0.229 | 109 | 79 | 3 | 2 | 106 | 21 | 128 | GPW\_gp25 domain-containing protein | GPW\_gp25 domain-containing protein | | afdb-uniprot50 | AF-B9NUV2-F1-MODEL\_V4 | 1.0 | 7.041e-08 | 299 | 0.259 | 108 | 76 | 2 | 2 | 105 | 25 | 132 | Phage baseplate assembly protein W | Phage baseplate assembly protein W | | afdb-uniprot50 | AF-A0A528VJI5-F1-MODEL\_V4 | 1.0 | 1.78e-07 | 298 | 0.343 | 102 | 66 | 1 | 1 | 101 | 1 | 102 | GPW\_gp25 domain-containing protein | GPW\_gp25 domain-containing protein | | afdb-uniprot50 | AF-A0A1H2U8Y5-F1-MODEL\_V4 | 1.0 | 1.228e-07 | 298 | 0.305 | 108 | 72 | 2 | 2 | 107 | 14 | 120 | GPW\_gp25 domain-containing protein | GPW\_gp25 domain-containing protein | | afdb-uniprot50 | AF-A0A428YAU4-F1-MODEL\_V4 | 1.0 | 3.352e-08 | 297 | 0.311 | 106 | 70 | 2 | 2 | 105 | 14 | 118 | Phage baseplate protein | Phage baseplate protein | | afdb-uniprot50 | AF-A0A4R7QFY3-F1-MODEL\_V4 | 1.0 | 4.293e-08 | 297 | 0.259 | 108 | 77 | 2 | 2 | 106 | 21 | 128 | GPW\_gp25 domain-containing protein | GPW\_gp25 domain-containing protein | | afdb-uniprot50 | AF-A0A2D9B6I8-F1-MODEL\_V4 | 1.0 | 1.228e-07 | 296 | 0.245 | 114 | 79 | 2 | 1 | 107 | 8 | 121 | GPW\_gp25 domain-containing protein | GPW\_gp25 domain-containing protein | | afdb-uniprot50 | AF-A0A2G9WV35-F1-MODEL\_V4 | 1.0 | 8.476e-08 | 296 | 0.31 | 116 | 71 | 3 | 1 | 108 | 7 | 121 | Integrase | Integrase | | afdb-uniprot50 | AF-A0A428Z2I7-F1-MODEL\_V4 | 1.0 | 1.02e-07 | 296 | 0.268 | 108 | 77 | 2 | 2 | 107 | 18 | 125 | Baseplate protein | Baseplate protein | | afdb-uniprot50 | AF-A0A442I641-F1-MODEL\_V4 | 1.0 | 6.619e-08 | 296 | 0.305 | 118 | 73 | 3 | 1 | 109 | 27 | 144 | GPW\_gp25 domain-containing protein | GPW\_gp25 domain-containing protein | | afdb-uniprot50 | AF-A0A2A2HU34-F1-MODEL\_V4 | 1.0 | 9.592e-08 | 295 | 0.293 | 109 | 73 | 3 | 2 | 107 | 14 | 121 | Baseplate protein | Baseplate protein | | afdb-uniprot50 | AF-A0A1K2HLI9-F1-MODEL\_V4 | 1.0 | 1.155e-07 | 295 | 0.292 | 113 | 72 | 3 | 1 | 105 | 9 | 121 | GPW\_gp25 domain-containing protein | GPW\_gp25 domain-containing protein | | afdb-uniprot50 | AF-M0DEF1-F1-MODEL\_V4 | 1.0 | 1.155e-07 | 295 | 0.299 | 107 | 72 | 2 | 2 | 105 | 14 | 120 | GPW/gp25 family protein | GPW/gp25 family protein | | afdb-uniprot50 | AF-A0A6G3R8A8-F1-MODEL\_V4 | 1.0 | 7.968e-08 | 295 | 0.289 | 107 | 73 | 2 | 2 | 105 | 14 | 120 | GPW/gp25 family protein | GPW/gp25 family protein | | afdb-uniprot50 | AF-A0A7H9VBC7-F1-MODEL\_V4 | 1.0 | 1.573e-07 | 295 | 0.267 | 112 | 75 | 3 | 1 | 105 | 35 | 146 | Baseplate assembly protein | Baseplate assembly protein | | afdb-uniprot50 | AF-A0A3N5Q1L3-F1-MODEL\_V4 | 1.0 | 3.977e-07 | 294 | 0.315 | 95 | 63 | 1 | 14 | 106 | 1 | 95 | Phage baseplate protein | Phage baseplate protein | | afdb-uniprot50 | AF-R7JS48-F1-MODEL\_V4 | 1.0 | 4.788e-07 | 294 | 0.271 | 107 | 76 | 2 | 1 | 106 | 9 | 114 | Baseplate | Baseplate | | afdb-uniprot50 | AF-A0A661RQ76-F1-MODEL\_V4 | 1.0 | 9.017e-08 | 294 | 0.305 | 108 | 70 | 3 | 2 | 105 | 14 | 120 | Baseplate protein | Baseplate protein | | afdb-uniprot50 | AF-A0A3N0FWT9-F1-MODEL\_V4 | 1.0 | 4.036e-08 | 294 | 0.25 | 108 | 77 | 2 | 2 | 105 | 16 | 123 | GPW\_gp25 domain-containing protein | GPW\_gp25 domain-containing protein | | afdb-uniprot50 | AF-A0A840YD88-F1-MODEL\_V4 | 1.0 | 1.228e-07 | 293 | 0.291 | 96 | 66 | 1 | 12 | 105 | 2 | 97 | Uncharacterized protein | Uncharacterized protein | | afdb-uniprot50 | AF-A0A084T291-F1-MODEL\_V4 | 1.0 | 7.968e-08 | 293 | 0.293 | 109 | 73 | 2 | 2 | 106 | 14 | 122 | Baseplate protein | Baseplate protein | | afdb-uniprot50 | AF-A0A1W2F8A0-F1-MODEL\_V4 | 1.0 | 1.479e-07 | 293 | 0.333 | 105 | 66 | 1 | 1 | 101 | 1 | 105 | GPW\_gp25 domain-containing protein | GPW\_gp25 domain-containing protein | | afdb-uniprot50 | AF-A0A1P8FGT4-F1-MODEL\_V4 | 1.0 | 9.592e-08 | 293 | 0.307 | 114 | 70 | 3 | 1 | 105 | 11 | 124 | Baseplate assembly protein | Baseplate assembly protein | | afdb-uniprot50 | AF-A0A4S2RPJ9-F1-MODEL\_V4 | 1.0 | 1.894e-07 | 293 | 0.309 | 113 | 70 | 3 | 1 | 105 | 7 | 119 | Baseplate protein | Baseplate protein | | afdb-uniprot50 | AF-A0A561IRH5-F1-MODEL\_V4 | 1.0 | 1.673e-07 | 293 | 0.283 | 106 | 74 | 2 | 1 | 105 | 11 | 115 | Gene 25-like lysozyme | Gene 25-like lysozyme | | afdb-uniprot50 | AF-A0A2P2GC78-F1-MODEL\_V4 | 1.0 | 1.085e-07 | 292 | 0.289 | 107 | 73 | 2 | 2 | 105 | 14 | 120 | Baseplate protein | Baseplate protein | | afdb-uniprot50 | AF-A0A016QL71-F1-MODEL\_V4 | 1.0 | 6.222e-08 | 292 | 0.379 | 108 | 62 | 3 | 2 | 105 | 13 | 119 | GPW/gp25 family protein | GPW/gp25 family protein | | afdb-uniprot50 | AF-A0A3N5W7B2-F1-MODEL\_V4 | 1.0 | 4.858e-08 | 292 | 0.333 | 108 | 67 | 3 | 2 | 105 | 22 | 128 | Baseplate protein | Baseplate protein | | afdb-uniprot50 | AF-A0A2G6EIV6-F1-MODEL\_V4 | 1.0 | 3.977e-07 | 292 | 0.431 | 95 | 54 | 0 | 3 | 97 | 12 | 106 | GPW\_gp25 domain-containing protein | GPW\_gp25 domain-containing protein | | afdb-uniprot50 | AF-A0A5P9CRI3-F1-MODEL\_V4 | 1.0 | 4.501e-07 | 291 | 0.254 | 110 | 76 | 3 | 1 | 107 | 1 | 107 | Uncharacterized protein | Uncharacterized protein | | afdb-uniprot50 | AF-A0A7Y2SMU2-F1-MODEL\_V4 | 1.0 | 1.39e-07 | 291 | 0.261 | 111 | 75 | 4 | 2 | 107 | 13 | 121 | GPW/gp25 family protein | GPW/gp25 family protein | | afdb-uniprot50 | AF-A0A7X5QSP2-F1-MODEL\_V4 | 1.0 | 7.041e-08 | 291 | 0.203 | 108 | 82 | 2 | 2 | 105 | 13 | 120 | GPW/gp25 family protein | GPW/gp25 family protein | | afdb-uniprot50 | AF-A0A6C7EF79-F1-MODEL\_V4 | 1.0 | 1.02e-07 | 291 | 0.296 | 108 | 71 | 3 | 2 | 105 | 20 | 126 | GPW\_gp25 domain-containing protein | GPW\_gp25 domain-containing protein | | afdb-uniprot50 | AF-A0A833LM40-F1-MODEL\_V4 | 1.0 | 1.894e-07 | 290 | 0.312 | 112 | 70 | 3 | 1 | 105 | 8 | 119 | Baseplate assembly protein | Baseplate assembly protein | | afdb-uniprot50 | AF-A0A150P6E2-F1-MODEL\_V4 | 1.0 | 1.39e-07 | 290 | 0.28 | 107 | 74 | 2 | 2 | 105 | 17 | 123 | GPW\_gp25 domain-containing protein | GPW\_gp25 domain-containing protein | | afdb-uniprot50 | AF-A0A1T3NP84-F1-MODEL\_V4 | 1.0 | 1.894e-07 | 290 | 0.298 | 114 | 71 | 3 | 1 | 105 | 17 | 130 | GPW\_gp25 domain-containing protein | GPW\_gp25 domain-containing protein | | afdb-uniprot50 | AF-A0A842NAI3-F1-MODEL\_V4 | 1.0 | 5.418e-07 | 289 | 0.264 | 102 | 73 | 2 | 12 | 112 | 4 | 104 | GPW/gp25 family protein | GPW/gp25 family protein | | afdb-uniprot50 | AF-A0A212S7U4-F1-MODEL\_V4 | 1.0 | 1.479e-07 | 289 | 0.325 | 123 | 69 | 4 | 1 | 110 | 2 | 123 | GPW\_gp25 domain-containing protein | GPW\_gp25 domain-containing protein | | afdb-uniprot50 | AF-A0A7V9GIA2-F1-MODEL\_V4 | 1.0 | 9.592e-08 | 289 | 0.297 | 111 | 73 | 3 | 2 | 108 | 14 | 123 | GPW/gp25 family protein | GPW/gp25 family protein | | afdb-uniprot50 | AF-A0A4U7JDR4-F1-MODEL\_V4 | 1.0 | 1.78e-07 | 289 | 0.311 | 106 | 70 | 2 | 2 | 105 | 15 | 119 | GPW/gp25 family protein | GPW/gp25 family protein | | afdb-uniprot50 | AF-A0A4P8HN78-F1-MODEL\_V4 | 1.0 | 1.228e-07 | 289 | 0.272 | 110 | 75 | 3 | 2 | 107 | 16 | 124 | Uncharacterized protein | Uncharacterized protein | | afdb-uniprot50 | AF-A0A0N0JDW9-F1-MODEL\_V4 | 1.0 | 1.307e-07 | 289 | 0.283 | 113 | 73 | 3 | 2 | 106 | 14 | 126 | GPW\_gp25 domain-containing protein | GPW\_gp25 domain-containing protein | | afdb-uniprot50 | AF-A0A0R3MPT4-F1-MODEL\_V4 | 1.0 | 5.498e-08 | 289 | 0.35 | 120 | 63 | 4 | 1 | 105 | 1 | 120 | Phage baseplate protein | Phage baseplate protein | | afdb-uniprot50 | AF-B8IDQ3-F1-MODEL\_V4 | 1.0 | 9.592e-08 | 289 | 0.28 | 114 | 72 | 2 | 1 | 104 | 3 | 116 | GPW/gp25 family protein | GPW/gp25 family protein | | afdb-uniprot50 | AF-A0A2Z3Z422-F1-MODEL\_V4 | 1.0 | 3.106e-07 | 288 | 0.354 | 96 | 60 | 1 | 12 | 105 | 2 | 97 | Phage baseplate protein | Phage baseplate protein | | afdb-uniprot50 | AF-A0A3S0WS72-F1-MODEL\_V4 | 1.0 | 2.015e-07 | 288 | 0.22 | 109 | 79 | 3 | 1 | 106 | 1 | 106 | Uncharacterized protein | Uncharacterized protein | | afdb-uniprot50 | AF-A0A371K5T5-F1-MODEL\_V4 | 1.0 | 1.155e-07 | 288 | 0.259 | 104 | 76 | 1 | 2 | 105 | 11 | 113 | Baseplate assembly protein | Baseplate assembly protein | | afdb-uniprot50 | AF-A0A6N9YFS0-F1-MODEL\_V4 | 1.0 | 1.307e-07 | 288 | 0.292 | 106 | 72 | 2 | 3 | 105 | 2 | 107 | GPW/gp25 family protein | GPW/gp25 family protein | | afdb-uniprot50 | AF-A0A1I6IQR2-F1-MODEL\_V4 | 1.0 | 1.307e-07 | 288 | 0.308 | 107 | 71 | 2 | 2 | 105 | 14 | 120 | GPW\_gp25 domain-containing protein | GPW\_gp25 domain-containing protein | | afdb-uniprot50 | AF-A0A1F3S636-F1-MODEL\_V4 | 1.0 | 7.49e-08 | 288 | 0.259 | 108 | 76 | 2 | 2 | 105 | 15 | 122 | GPW\_gp25 domain-containing protein | GPW\_gp25 domain-containing protein | | afdb-uniprot50 | AF-A0A4Y9PT98-F1-MODEL\_V4 | 1.0 | 2.015e-07 | 288 | 0.289 | 114 | 72 | 3 | 1 | 105 | 7 | 120 | Baseplate protein | Baseplate protein | | afdb-uniprot50 | AF-A0A515KQR3-F1-MODEL\_V4 | 1.0 | 7.49e-08 | 288 | 0.218 | 110 | 82 | 2 | 2 | 107 | 17 | 126 | GPW/gp25 family protein | GPW/gp25 family protein | | afdb-uniprot50 | AF-A0A2M9J5R0-F1-MODEL\_V4 | 1.0 | 1.39e-07 | 288 | 0.299 | 107 | 72 | 2 | 2 | 105 | 14 | 120 | Baseplate protein | Baseplate protein | | afdb-uniprot50 | AF-A0A6P0NDR6-F1-MODEL\_V4 | 1.0 | 2.744e-07 | 287 | 0.276 | 112 | 74 | 2 | 1 | 105 | 7 | 118 | GPW/gp25 family protein | GPW/gp25 family protein | | afdb-uniprot50 | AF-A0A6V8KJ56-F1-MODEL\_V4 | 1.0 | 5.498e-08 | 287 | 0.297 | 111 | 75 | 2 | 1 | 108 | 9 | 119 | GPW\_gp25 domain-containing protein | GPW\_gp25 domain-containing protein | | afdb-uniprot50 | AF-A0A1H7YK45-F1-MODEL\_V4 | 1.0 | 9.017e-08 | 287 | 0.203 | 108 | 82 | 2 | 2 | 105 | 17 | 124 | GPW\_gp25 domain-containing protein | GPW\_gp25 domain-containing protein | | afdb-uniprot50 | AF-A0A7T7I7G9-F1-MODEL\_V4 | 1.0 | 2.58e-07 | 287 | 0.278 | 115 | 73 | 3 | 1 | 105 | 7 | 121 | GPW/gp25 family protein | GPW/gp25 family protein | | afdb-uniprot50 | AF-A0A1Z4QPU1-F1-MODEL\_V4 | 1.0 | 5.498e-08 | 287 | 0.35 | 114 | 64 | 4 | 1 | 105 | 42 | 154 | GPW / gp25 family protein | GPW / gp25 family protein | | afdb-uniprot50 | AF-A0A3C2A343-F1-MODEL\_V4 | 1.0 | 5.849e-08 | 287 | 0.222 | 108 | 80 | 2 | 2 | 105 | 58 | 165 | GPW\_gp25 domain-containing protein | GPW\_gp25 domain-containing protein | | afdb-uniprot50 | AF-A0A3M1SPN9-F1-MODEL\_V4 | 1.0 | 3.977e-07 | 286 | 0.351 | 91 | 57 | 1 | 17 | 105 | 7 | 97 | Baseplate protein | Baseplate protein | | afdb-uniprot50 | AF-A0A1N7S7K8-F1-MODEL\_V4 | 1.0 | 1.894e-07 | 286 | 0.311 | 106 | 71 | 1 | 2 | 105 | 14 | 119 | GPW/gp25 family protein | GPW/gp25 family protein | | afdb-uniprot50 | AF-A0A1V2QXE0-F1-MODEL\_V4 | 1.0 | 1.307e-07 | 285 | 0.273 | 106 | 74 | 2 | 2 | 105 | 11 | 115 | GPW\_gp25 domain-containing protein | GPW\_gp25 domain-containing protein | | afdb-uniprot50 | AF-A0A6M3K799-F1-MODEL\_V4 | 1.0 | 3.515e-07 | 285 | 0.173 | 115 | 87 | 3 | 1 | 107 | 7 | 121 | Putative baseplate wedge subunit | Putative baseplate wedge subunit | | afdb-uniprot50 | AF-E2SQJ7-F1-MODEL\_V4 | 1.0 | 7.968e-08 | 285 | 0.271 | 107 | 73 | 4 | 2 | 105 | 13 | 117 | Putative lysozyme | Putative lysozyme | | afdb-uniprot50 | AF-A0A3N5XQ15-F1-MODEL\_V4 | 1.0 | 7.968e-08 | 285 | 0.296 | 108 | 71 | 3 | 2 | 105 | 12 | 118 | Baseplate protein | Baseplate protein | | afdb-uniprot50 | AF-A0A1F8LUG1-F1-MODEL\_V4 | 1.0 | 1.78e-07 | 285 | 0.261 | 107 | 76 | 2 | 2 | 105 | 18 | 124 | Phage baseplate protein | Phage baseplate protein | | afdb-uniprot50 | AF-A0A371PUU5-F1-MODEL\_V4 | 1.0 | 1.307e-07 | 285 | 0.296 | 108 | 71 | 3 | 2 | 105 | 37 | 143 | Baseplate protein | Baseplate protein | | afdb-uniprot50 | AF-F5XJ49-F1-MODEL\_V4 | 1.0 | 1.39e-07 | 285 | 0.333 | 108 | 67 | 3 | 2 | 105 | 15 | 121 | GPW\_gp25 domain-containing protein | GPW\_gp25 domain-containing protein | | afdb-uniprot50 | AF-A0A7S8IJW2-F1-MODEL\_V4 | 1.0 | 1.479e-07 | 284 | 0.349 | 123 | 61 | 2 | 4 | 107 | 1 | 123 | Baseplate assembly protein | Baseplate assembly protein | | afdb-uniprot50 | AF-A0A6N6KES2-F1-MODEL\_V4 | 1.0 | 1.78e-07 | 284 | 0.252 | 107 | 78 | 1 | 2 | 106 | 20 | 126 | GPW\_gp25 domain-containing protein | GPW\_gp25 domain-containing protein | | afdb-uniprot50 | AF-A0A1V0Q088-F1-MODEL\_V4 | 1.0 | 3.515e-07 | 284 | 0.25 | 116 | 78 | 3 | 1 | 107 | 6 | 121 | GPW\_gp25 domain-containing protein | GPW\_gp25 domain-containing protein | | afdb-uniprot50 | AF-A0A6I2SHR9-F1-MODEL\_V4 | 1.0 | 1.573e-07 | 284 | 0.324 | 108 | 68 | 3 | 2 | 105 | 14 | 120 | Baseplate protein | Baseplate protein | | afdb-uniprot50 | AF-A0A437RHL5-F1-MODEL\_V4 | 1.0 | 1.78e-07 | 284 | 0.283 | 113 | 73 | 3 | 2 | 106 | 14 | 126 | Baseplate assembly protein | Baseplate assembly protein | | afdb-uniprot50 | AF-A0A356KNI6-F1-MODEL\_V4 | 1.0 | 1.673e-07 | 284 | 0.308 | 107 | 71 | 2 | 2 | 105 | 29 | 135 | Baseplate protein | Baseplate protein | | afdb-uniprot50 | AF-A0A7K0JP37-F1-MODEL\_V4 | 1.0 | 1.228e-07 | 284 | 0.318 | 110 | 68 | 4 | 2 | 105 | 28 | 136 | Baseplate protein | Baseplate protein | | afdb-uniprot50 | AF-A0A0U5IN79-F1-MODEL\_V4 | 1.0 | 3.566e-08 | 284 | 0.269 | 115 | 75 | 4 | 1 | 107 | 33 | 146 | GPW/gp25 family protein | GPW/gp25 family protein | | afdb-uniprot50 | AF-A0A828DTS9-F1-MODEL\_V4 | 1.0 | 1.457e-06 | 283 | 0.433 | 83 | 47 | 0 | 1 | 83 | 4 | 86 | Baseplate assembly protein | Baseplate assembly protein | | afdb-uniprot50 | AF-E8U3P0-F1-MODEL\_V4 | 1.0 | 1.228e-07 | 283 | 0.361 | 108 | 64 | 3 | 2 | 105 | 13 | 119 | GPW/gp25 family protein | GPW/gp25 family protein | | afdb-uniprot50 | AF-A0A239FMN3-F1-MODEL\_V4 | 1.0 | 1.085e-07 | 283 | 0.33 | 115 | 67 | 3 | 1 | 105 | 3 | 117 | GPW\_gp25 domain-containing protein | GPW\_gp25 domain-containing protein | | afdb-uniprot50 | AF-X5L3Y3-F1-MODEL\_V4 | 1.0 | 2.58e-07 | 283 | 0.263 | 114 | 75 | 3 | 1 | 105 | 7 | 120 | GPW/gp25 family protein | GPW/gp25 family protein | | afdb-uniprot50 | AF-A0A2M8MC75-F1-MODEL\_V4 | 1.0 | 1.673e-07 | 283 | 0.299 | 107 | 72 | 2 | 2 | 105 | 14 | 120 | Baseplate protein | Baseplate protein | | afdb-uniprot50 | AF-A0A1H6Z5N9-F1-MODEL\_V4 | 1.0 | 7.041e-08 | 283 | 0.339 | 109 | 69 | 2 | 1 | 106 | 18 | 126 | GPW\_gp25 domain-containing protein | GPW\_gp25 domain-containing protein | | afdb-uniprot50 | AF-A0A2U0SFJ6-F1-MODEL\_V4 | 1.0 | 1.155e-07 | 283 | 0.242 | 107 | 78 | 2 | 2 | 105 | 36 | 142 | GPW\_gp25 domain-containing protein | GPW\_gp25 domain-containing protein | | afdb-uniprot50 | AF-A0A2M8Q5J2-F1-MODEL\_V4 | 1.0 | 1.573e-07 | 282 | 0.305 | 108 | 70 | 3 | 2 | 105 | 13 | 119 | Baseplate protein | Baseplate protein | | afdb-uniprot50 | AF-A0A329LW89-F1-MODEL\_V4 | 1.0 | 1.39e-07 | 282 | 0.297 | 111 | 70 | 3 | 2 | 105 | 14 | 123 | Baseplate protein | Baseplate protein | | afdb-uniprot50 | AF-A0A2V8RLG8-F1-MODEL\_V4 | 1.0 | 1.573e-07 | 282 | 0.256 | 117 | 80 | 4 | 2 | 112 | 18 | 133 | Baseplate protein | Baseplate protein | | afdb-uniprot50 | AF-A0A561BKI6-F1-MODEL\_V4 | 1.0 | 1.479e-07 | 282 | 0.345 | 107 | 66 | 2 | 2 | 105 | 13 | 118 | GPW\_gp25 domain-containing protein | GPW\_gp25 domain-containing protein | | afdb-uniprot50 | AF-A0A2P2CID1-F1-MODEL\_V4 | 1.0 | 1.573e-07 | 282 | 0.287 | 108 | 72 | 3 | 2 | 105 | 23 | 129 | Putative GPW / gp25 family | Putative GPW / gp25 family | | afdb-uniprot50 | AF-A0A1B6AS33-F1-MODEL\_V4 | 1.0 | 1.39e-07 | 282 | 0.261 | 107 | 76 | 2 | 2 | 105 | 14 | 120 | GPW\_gp25 domain-containing protein | GPW\_gp25 domain-containing protein | | afdb-uniprot50 | AF-A0A7Y4FP63-F1-MODEL\_V4 | 1.0 | 9.453e-07 | 281 | 0.243 | 111 | 78 | 3 | 1 | 108 | 1 | 108 | Phage baseplate protein | Phage baseplate protein | | afdb-uniprot50 | AF-A0A2S5N393-F1-MODEL\_V4 | 1.0 | 8.886e-07 | 281 | 0.239 | 117 | 80 | 2 | 1 | 108 | 5 | 121 | Integrase | Integrase | | afdb-uniprot50 | AF-A0A412YNF3-F1-MODEL\_V4 | 1.0 | 1.894e-07 | 281 | 0.236 | 110 | 80 | 2 | 2 | 107 | 18 | 127 | GPW\_gp25 domain-containing protein | GPW\_gp25 domain-containing protein | | afdb-uniprot50 | AF-A0A497W433-F1-MODEL\_V4 | 1.0 | 9.017e-08 | 281 | 0.333 | 108 | 67 | 3 | 2 | 105 | 17 | 123 | GPW\_gp25 domain-containing protein | GPW\_gp25 domain-containing protein | | afdb-uniprot50 | AF-A0A1V5CGG0-F1-MODEL\_V4 | 1.0 | 1.39e-07 | 281 | 0.214 | 107 | 81 | 3 | 2 | 105 | 39 | 145 | Gene 25-like lysozyme | Gene 25-like lysozyme | | afdb-uniprot50 | AF-A0A376CQH6-F1-MODEL\_V4 | 1.0 | 1.866e-06 | 280 | 0.454 | 77 | 42 | 0 | 1 | 77 | 4 | 80 | GPW/gp25 family protein | GPW/gp25 family protein | | afdb-uniprot50 | AF-A0A3S6F5U6-F1-MODEL\_V4 | 1.0 | 9.453e-07 | 280 | 0.459 | 87 | 47 | 0 | 1 | 87 | 3 | 89 | GPW\_gp25 domain-containing protein | GPW\_gp25 domain-containing protein | | afdb-uniprot50 | AF-A0A0N8GF33-F1-MODEL\_V4 | 1.0 | 1.211e-06 | 280 | 0.238 | 113 | 77 | 2 | 4 | 107 | 1 | 113 | Uncharacterized protein | Uncharacterized protein | | afdb-uniprot50 | AF-A0A136LCY3-F1-MODEL\_V4 | 1.0 | 2.744e-07 | 280 | 0.28 | 107 | 73 | 3 | 2 | 105 | 12 | 117 | GPW/gp25 family protein | GPW/gp25 family protein | | afdb-uniprot50 | AF-A0A2D5YH56-F1-MODEL\_V4 | 1.0 | 2.425e-07 | 280 | 0.268 | 108 | 76 | 2 | 3 | 107 | 24 | 131 | GPW\_gp25 domain-containing protein | GPW\_gp25 domain-containing protein | | afdb-uniprot50 | AF-A0A2E5AW95-F1-MODEL\_V4 | 1.0 | 3.106e-07 | 280 | 0.212 | 108 | 82 | 2 | 3 | 107 | 25 | 132 | GPW\_gp25 domain-containing protein | GPW\_gp25 domain-containing protein | | afdb-uniprot50 | AF-A0A2D6MEG6-F1-MODEL\_V4 | 1.0 | 3.739e-07 | 280 | 0.203 | 108 | 83 | 2 | 3 | 107 | 26 | 133 | GPW\_gp25 domain-containing protein | GPW\_gp25 domain-containing protein | | afdb-uniprot50 | AF-A0A2T0T080-F1-MODEL\_V4 | 1.0 | 5.498e-08 | 279 | 0.307 | 114 | 72 | 3 | 2 | 108 | 14 | 127 | GPW\_gp25 domain-containing protein | GPW\_gp25 domain-containing protein | | afdb-uniprot50 | AF-A0A1R3VJW4-F1-MODEL\_V4 | 1.0 | 2.28e-07 | 279 | 0.238 | 109 | 81 | 1 | 2 | 108 | 17 | 125 | GPW\_gp25 domain-containing protein | GPW\_gp25 domain-containing protein | | afdb-uniprot50 | AF-A0A410CPI4-F1-MODEL\_V4 | 1.0 | 1.479e-07 | 279 | 0.24 | 108 | 78 | 2 | 2 | 105 | 16 | 123 | GPW\_gp25 domain-containing protein | GPW\_gp25 domain-containing protein | | afdb-uniprot50 | AF-A0A542Q250-F1-MODEL\_V4 | 1.0 | 3.515e-07 | 279 | 0.266 | 124 | 79 | 4 | 1 | 112 | 7 | 130 | GPW\_gp25 domain-containing protein | GPW\_gp25 domain-containing protein | | afdb-uniprot50 | AF-A0A5B8ASK0-F1-MODEL\_V4 | 1.0 | 2.015e-07 | 279 | 0.298 | 114 | 71 | 3 | 1 | 105 | 19 | 132 | GPW/gp25 family protein | GPW/gp25 family protein | | afdb-uniprot50 | AF-A0A4P8T4C7-F1-MODEL\_V4 | 1.0 | 3.106e-07 | 279 | 0.254 | 106 | 77 | 2 | 2 | 105 | 14 | 119 | Baseplate protein | Baseplate protein | | afdb-uniprot50 | AF-A0A376KHY4-F1-MODEL\_V4 | 1.0 | 1.754e-06 | 278 | 0.513 | 72 | 35 | 0 | 1 | 72 | 5 | 76 | Baseplate assembly protein W | Baseplate assembly protein W | | afdb-uniprot50 | AF-A0A1M4ZEM6-F1-MODEL\_V4 | 1.0 | 1.155e-07 | 278 | 0.229 | 109 | 80 | 2 | 2 | 106 | 16 | 124 | GPW\_gp25 domain-containing protein | GPW\_gp25 domain-containing protein | | afdb-uniprot50 | AF-A0A7W7H4Y6-F1-MODEL\_V4 | 1.0 | 2.919e-07 | 278 | 0.271 | 107 | 75 | 2 | 2 | 105 | 14 | 120 | Phage baseplate assembly protein W | Phage baseplate assembly protein W | | afdb-uniprot50 | AF-A0A3A0A4P4-F1-MODEL\_V4 | 1.0 | 3.106e-07 | 278 | 0.194 | 108 | 84 | 2 | 2 | 106 | 16 | 123 | GPW\_gp25 domain-containing protein | GPW\_gp25 domain-containing protein | | afdb-uniprot50 | AF-A0A1C5YWG8-F1-MODEL\_V4 | 1.0 | 3.515e-07 | 278 | 0.269 | 115 | 73 | 5 | 1 | 105 | 8 | 121 | Type VI secretion system lysozyme-like protein | Type VI secretion system lysozyme-like protein | | afdb-uniprot50 | AF-A0A3D1NSF4-F1-MODEL\_V4 | 1.0 | 5.093e-07 | 277 | 0.288 | 104 | 71 | 2 | 3 | 106 | 1 | 101 | Baseplate protein | Baseplate protein | | afdb-uniprot50 | AF-A0A1E5G2K2-F1-MODEL\_V4 | 1.0 | 1.155e-07 | 277 | 0.304 | 115 | 70 | 3 | 1 | 105 | 2 | 116 | GPW\_gp25 domain-containing protein | GPW\_gp25 domain-containing protein | | afdb-uniprot50 | AF-A0A3B8I8Y8-F1-MODEL\_V4 | 1.0 | 1.479e-07 | 277 | 0.222 | 108 | 80 | 2 | 2 | 105 | 15 | 122 | GPW\_gp25 domain-containing protein | GPW\_gp25 domain-containing protein | | afdb-uniprot50 | AF-A0A1Q9LK30-F1-MODEL\_V4 | 1.0 | 2.015e-07 | 277 | 0.261 | 107 | 76 | 2 | 2 | 105 | 14 | 120 | Baseplate protein | Baseplate protein | | afdb-uniprot50 | AF-A0A1C5CGJ3-F1-MODEL\_V4 | 1.0 | 2.744e-07 | 277 | 0.324 | 108 | 68 | 3 | 2 | 105 | 14 | 120 | GPW\_gp25 domain-containing protein | GPW\_gp25 domain-containing protein | | afdb-uniprot50 | AF-A0A7W3I8F7-F1-MODEL\_V4 | 1.0 | 2.247e-06 | 276 | 0.426 | 82 | 47 | 0 | 2 | 83 | 5 | 86 | GPW/gp25 family protein | GPW/gp25 family protein | | afdb-uniprot50 | AF-A0A7W7Z0T9-F1-MODEL\_V4 | 1.0 | 1.78e-07 | 276 | 0.304 | 115 | 62 | 4 | 1 | 106 | 1 | 106 | GPW\_gp25 domain-containing protein | GPW\_gp25 domain-containing protein | | afdb-uniprot50 | AF-A0A085AFN6-F1-MODEL\_V4 | 1.0 | 6.523e-07 | 276 | 0.23 | 117 | 77 | 5 | 1 | 112 | 1 | 109 | Phage baseplate assembly protein | Phage baseplate assembly protein | | afdb-uniprot50 | AF-A0A662AHS8-F1-MODEL\_V4 | 1.0 | 2.744e-07 | 276 | 0.216 | 111 | 81 | 3 | 1 | 106 | 1 | 110 | GPW\_gp25 domain-containing protein | GPW\_gp25 domain-containing protein | | afdb-uniprot50 | AF-A0A3A4NZZ6-F1-MODEL\_V4 | 1.0 | 1.02e-07 | 276 | 0.258 | 112 | 77 | 3 | 2 | 108 | 14 | 124 | Baseplate protein | Baseplate protein | | afdb-uniprot50 | AF-A0A2N7W3T6-F1-MODEL\_V4 | 1.0 | 5.764e-07 | 276 | 0.286 | 115 | 73 | 3 | 2 | 107 | 10 | 124 | Baseplate assembly protein | Baseplate assembly protein | | afdb-uniprot50 | AF-A0A1Q7WS59-F1-MODEL\_V4 | 1.0 | 2.28e-07 | 276 | 0.261 | 107 | 76 | 2 | 2 | 105 | 14 | 120 | Baseplate protein | Baseplate protein | | afdb-uniprot50 | AF-A0A7X0CIC5-F1-MODEL\_V4 | 1.0 | 2.919e-07 | 276 | 0.242 | 107 | 78 | 2 | 2 | 105 | 16 | 122 | GPW\_gp25 domain-containing protein | GPW\_gp25 domain-containing protein | | afdb-uniprot50 | AF-A0A2U0TQZ5-F1-MODEL\_V4 | 1.0 | 1.479e-07 | 276 | 0.24 | 108 | 78 | 2 | 2 | 105 | 15 | 122 | GPW\_gp25 domain-containing protein | GPW\_gp25 domain-containing protein | | afdb-uniprot50 | AF-A0A542J108-F1-MODEL\_V4 | 1.0 | 5.418e-07 | 276 | 0.289 | 114 | 72 | 3 | 1 | 105 | 7 | 120 | GPW\_gp25 domain-containing protein | GPW\_gp25 domain-containing protein | | afdb-uniprot50 | AF-A0A1J5PQ93-F1-MODEL\_V4 | 1.0 | 4.231e-07 | 276 | 0.247 | 109 | 78 | 3 | 1 | 106 | 10 | 117 | Protein 25-like lysozyme | Protein 25-like lysozyme | | afdb-uniprot50 | AF-A0A5E4XAT6-F1-MODEL\_V4 | 1.0 | 9.453e-07 | 275 | 0.263 | 91 | 65 | 1 | 17 | 105 | 7 | 97 | Baseplate protein | Baseplate protein | | afdb-uniprot50 | AF-A0A0F9DR72-F1-MODEL\_V4 | 1.0 | 3.106e-07 | 275 | 0.226 | 115 | 79 | 4 | 1 | 106 | 2 | 115 | GPW\_gp25 domain-containing protein | GPW\_gp25 domain-containing protein | | afdb-uniprot50 | AF-A0A2A3D7N8-F1-MODEL\_V4 | 1.0 | 3.977e-07 | 275 | 0.281 | 110 | 72 | 3 | 4 | 106 | 1 | 110 | Baseplate assembly protein | Baseplate assembly protein | | afdb-uniprot50 | AF-A0A6P0KR28-F1-MODEL\_V4 | 1.0 | 3.304e-07 | 275 | 0.266 | 109 | 77 | 2 | 2 | 107 | 14 | 122 | GPW/gp25 family protein | GPW/gp25 family protein | | afdb-uniprot50 | AF-A0A1Q8A383-F1-MODEL\_V4 | 1.0 | 3.515e-07 | 275 | 0.301 | 106 | 72 | 1 | 2 | 105 | 16 | 121 | Baseplate protein | Baseplate protein | | afdb-uniprot50 | AF-A0A1M7YV13-F1-MODEL\_V4 | 1.0 | 5.764e-07 | 275 | 0.247 | 117 | 78 | 3 | 1 | 107 | 7 | 123 | Gene 25-like lysozyme | Gene 25-like lysozyme | | afdb-uniprot50 | AF-I5B7H2-F1-MODEL\_V4 | 1.0 | 6.132e-07 | 275 | 0.263 | 114 | 75 | 3 | 1 | 105 | 8 | 121 | Phage baseplate assembly protein W | Phage baseplate assembly protein W | | afdb-uniprot50 | AF-A0A1I7EL93-F1-MODEL\_V4 | 1.0 | 2.785e-08 | 275 | 0.403 | 109 | 61 | 2 | 1 | 105 | 24 | 132 | GPW\_gp25 domain-containing protein | GPW\_gp25 domain-containing protein | | afdb-uniprot50 | AF-A0A386WG07-F1-MODEL\_V4 | 1.0 | 1.39e-07 | 275 | 0.333 | 108 | 69 | 2 | 1 | 105 | 18 | 125 | GPW\_gp25 domain-containing protein | GPW\_gp25 domain-containing protein | | afdb-uniprot50 | AF-A0A4Q5SKL8-F1-MODEL\_V4 | 1.0 | 2.425e-07 | 275 | 0.28 | 107 | 74 | 2 | 2 | 105 | 26 | 132 | GPW\_gp25 domain-containing protein | GPW\_gp25 domain-containing protein | | afdb-uniprot50 | AF-A0A4Z0H7I9-F1-MODEL\_V4 | 1.0 | 4.501e-07 | 275 | 0.289 | 114 | 72 | 3 | 1 | 105 | 5 | 118 | Baseplate protein | Baseplate protein | | afdb-uniprot50 | AF-A0A850GIX7-F1-MODEL\_V4 | 1.0 | 6.523e-07 | 274 | 0.204 | 98 | 76 | 1 | 12 | 107 | 2 | 99 | GPW/gp25 family protein | GPW/gp25 family protein | | afdb-uniprot50 | AF-A0A3M1B2Y6-F1-MODEL\_V4 | 1.0 | 1.307e-07 | 274 | 0.252 | 107 | 77 | 2 | 2 | 105 | 11 | 117 | GPW\_gp25 domain-containing protein | GPW\_gp25 domain-containing protein | | afdb-uniprot50 | AF-A0A382EFG4-F1-MODEL\_V4 | 1.0 | 3.739e-07 | 274 | 0.25 | 108 | 78 | 2 | 3 | 107 | 16 | 123 | GPW\_gp25 domain-containing protein | GPW\_gp25 domain-containing protein | | afdb-uniprot50 | AF-A0A6M0FMQ1-F1-MODEL\_V4 | 1.0 | 2.744e-07 | 274 | 0.22 | 109 | 82 | 2 | 2 | 107 | 16 | 124 | GPW/gp25 family protein | GPW/gp25 family protein | | afdb-uniprot50 | AF-A0A4Y3L060-F1-MODEL\_V4 | 1.0 | 7.381e-07 | 274 | 0.272 | 125 | 75 | 5 | 1 | 112 | 7 | 128 | GPW\_gp25 domain-containing protein | GPW\_gp25 domain-containing protein | | afdb-uniprot50 | AF-A0A6S6SM83-F1-MODEL\_V4 | 1.0 | 3.106e-07 | 274 | 0.275 | 109 | 76 | 2 | 2 | 107 | 23 | 131 | GPW\_gp25 domain-containing protein | GPW\_gp25 domain-containing protein | | afdb-uniprot50 | AF-A0A0F9TG53-F1-MODEL\_V4 | 1.0 | 2.919e-07 | 274 | 0.261 | 107 | 75 | 3 | 2 | 105 | 30 | 135 | GPW\_gp25 domain-containing protein | GPW\_gp25 domain-containing protein | | afdb-uniprot50 | AF-A0A1T4W4S5-F1-MODEL\_V4 | 1.0 | 1.07e-06 | 274 | 0.268 | 108 | 74 | 2 | 1 | 105 | 77 | 182 | Phage baseplate assembly protein W | Phage baseplate assembly protein W | | afdb-uniprot50 | AF-A0A810Z3I0-F1-MODEL\_V4 | 1.0 | 1.07e-06 | 273 | 0.228 | 92 | 68 | 2 | 17 | 106 | 7 | 97 | Baseplate protein | Baseplate protein | | afdb-uniprot50 | AF-A0A3B0VNW2-F1-MODEL\_V4 | 1.0 | 4.231e-07 | 273 | 0.273 | 106 | 74 | 2 | 3 | 105 | 16 | 121 | GPW\_gp25 domain-containing protein | GPW\_gp25 domain-containing protein | | afdb-uniprot50 | AF-A0A4Q9HLM9-F1-MODEL\_V4 | 1.0 | 4.501e-07 | 273 | 0.271 | 107 | 75 | 2 | 2 | 105 | 14 | 120 | Baseplate protein | Baseplate protein | | afdb-uniprot50 | AF-A0A0D8FVT8-F1-MODEL\_V4 | 1.0 | 3.739e-07 | 273 | 0.289 | 107 | 73 | 2 | 2 | 105 | 42 | 148 | 25-like lysozyme | 25-like lysozyme | | afdb-uniprot50 | AF-A0A2P8QYN7-F1-MODEL\_V4 | 1.0 | 2.744e-07 | 272 | 0.347 | 95 | 56 | 2 | 12 | 106 | 4 | 92 | Baseplate assembly protein | Baseplate assembly protein | | afdb-uniprot50 | AF-A0A4P8G0E4-F1-MODEL\_V4 | 1.0 | 4.231e-07 | 272 | 0.223 | 112 | 80 | 4 | 1 | 107 | 2 | 111 | Phage baseplate protein | Phage baseplate protein | | afdb-uniprot50 | AF-C1D831-F1-MODEL\_V4 | 1.0 | 3.106e-07 | 272 | 0.219 | 114 | 82 | 4 | 1 | 111 | 8 | 117 | Putative phage baseplate protein | Putative phage baseplate protein | | afdb-uniprot50 | AF-A0A1W1X795-F1-MODEL\_V4 | 1.0 | 5.093e-07 | 272 | 0.269 | 115 | 78 | 4 | 2 | 112 | 12 | 124 | GPW\_gp25 domain-containing protein | GPW\_gp25 domain-containing protein | | afdb-uniprot50 | AF-A0A7X5EZA1-F1-MODEL\_V4 | 1.0 | 7.852e-07 | 272 | 0.241 | 116 | 81 | 2 | 1 | 109 | 8 | 123 | Integrase | Integrase | | afdb-uniprot50 | AF-A0A1M7AAS8-F1-MODEL\_V4 | 1.0 | 2.143e-07 | 272 | 0.201 | 109 | 83 | 2 | 2 | 106 | 13 | 121 | GPW\_gp25 domain-containing protein | GPW\_gp25 domain-containing protein | | afdb-uniprot50 | AF-A0A2W6DBF0-F1-MODEL\_V4 | 1.0 | 6.939e-07 | 272 | 0.28 | 114 | 73 | 3 | 1 | 105 | 7 | 120 | Baseplate protein | Baseplate protein | | afdb-uniprot50 | AF-A0A3S2YJR9-F1-MODEL\_V4 | 1.0 | 7.381e-07 | 272 | 0.235 | 106 | 76 | 1 | 1 | 101 | 3 | 108 | Uncharacterized protein | Uncharacterized protein | | afdb-uniprot50 | AF-A0A6J4TRM4-F1-MODEL\_V4 | 1.0 | 3.304e-07 | 272 | 0.324 | 108 | 68 | 3 | 2 | 105 | 27 | 133 | GPW\_gp25 domain-containing protein | GPW\_gp25 domain-containing protein | | afdb-uniprot50 | AF-A0A1A9HJ71-F1-MODEL\_V4 | 1.0 | 3.106e-07 | 272 | 0.229 | 109 | 80 | 2 | 2 | 106 | 16 | 124 | GPW\_gp25 domain-containing protein | GPW\_gp25 domain-containing protein | | afdb-uniprot50 | AF-A0A1G3UAL2-F1-MODEL\_V4 | 1.0 | 9.017e-08 | 271 | 0.37 | 100 | 54 | 3 | 12 | 108 | 3 | 96 | Baseplate assembly protein | Baseplate assembly protein | | afdb-uniprot50 | AF-A0A285NMD3-F1-MODEL\_V4 | 1.0 | 3.061e-06 | 271 | 0.276 | 94 | 64 | 2 | 17 | 108 | 13 | 104 | GPW\_gp25 domain-containing protein | GPW\_gp25 domain-containing protein | | afdb-uniprot50 | AF-A0A0Q7TJ69-F1-MODEL\_V4 | 1.0 | 6.222e-08 | 271 | 0.321 | 109 | 69 | 3 | 1 | 105 | 13 | 120 | GPW\_gp25 domain-containing protein | GPW\_gp25 domain-containing protein | | afdb-uniprot50 | AF-A0A2S6I8X0-F1-MODEL\_V4 | 1.0 | 2.015e-07 | 271 | 0.275 | 109 | 75 | 2 | 2 | 106 | 14 | 122 | GPW\_gp25 domain-containing protein | GPW\_gp25 domain-containing protein | | afdb-uniprot50 | AF-A0A1Q4UKA2-F1-MODEL\_V4 | 1.0 | 2.58e-07 | 271 | 0.293 | 109 | 72 | 3 | 2 | 106 | 23 | 130 | GPW\_gp25 domain-containing protein | GPW\_gp25 domain-containing protein | | afdb-uniprot50 | AF-A0A7Y6XH67-F1-MODEL\_V4 | 1.0 | 2.919e-07 | 271 | 0.25 | 108 | 77 | 2 | 2 | 105 | 17 | 124 | GPW/gp25 family protein | GPW/gp25 family protein | | afdb-uniprot50 | AF-A0A2G1XB05-F1-MODEL\_V4 | 1.0 | 1.07e-06 | 271 | 0.263 | 114 | 75 | 3 | 1 | 105 | 14 | 127 | Baseplate protein | Baseplate protein | | afdb-uniprot50 | AF-A0A1N6M9S4-F1-MODEL\_V4 | 1.0 | 2.143e-07 | 271 | 0.203 | 108 | 82 | 2 | 2 | 105 | 15 | 122 | Baseplate wedge subunit | Baseplate wedge subunit | | afdb-uniprot50 | AF-A0A0M4DWU2-F1-MODEL\_V4 | 1.0 | 4.231e-07 | 271 | 0.342 | 108 | 66 | 3 | 2 | 105 | 14 | 120 | Baseplate protein | Baseplate protein | | afdb-uniprot50 | AF-A0A7Y7B5C7-F1-MODEL\_V4 | 1.0 | 4.231e-07 | 271 | 0.271 | 107 | 75 | 2 | 2 | 105 | 14 | 120 | GPW/gp25 family protein | GPW/gp25 family protein | | afdb-uniprot50 | AF-A0A376J3V4-F1-MODEL\_V4 | 1.0 | 3.256e-06 | 270 | 0.513 | 72 | 35 | 0 | 1 | 72 | 5 | 76 | Baseplate assembly protein W | Baseplate assembly protein W | | afdb-uniprot50 | AF-A0A535E2Y7-F1-MODEL\_V4 | 1.0 | 2.015e-07 | 270 | 0.292 | 106 | 69 | 3 | 12 | 112 | 2 | 106 | GPW/gp25 family protein | GPW/gp25 family protein | | afdb-uniprot50 | AF-A0A1D2QMW4-F1-MODEL\_V4 | 1.0 | 5.418e-07 | 270 | 0.225 | 111 | 75 | 5 | 1 | 105 | 2 | 107 | Uncharacterized protein | Uncharacterized protein | | afdb-uniprot50 | AF-A0A2D6WZT7-F1-MODEL\_V4 | 1.0 | 4.788e-07 | 270 | 0.24 | 108 | 79 | 2 | 3 | 107 | 16 | 123 | GPW\_gp25 domain-containing protein | GPW\_gp25 domain-containing protein | | afdb-uniprot50 | AF-A0A1S2N8L0-F1-MODEL\_V4 | 1.0 | 5.764e-07 | 270 | 0.207 | 111 | 83 | 3 | 2 | 108 | 19 | 128 | Putative tail lysozyme | Putative tail lysozyme | | afdb-uniprot50 | AF-A0A4R7ZWG3-F1-MODEL\_V4 | 1.0 | 9.453e-07 | 269 | 0.23 | 113 | 78 | 3 | 1 | 105 | 1 | 112 | GPW\_gp25 domain-containing protein | GPW\_gp25 domain-containing protein | | afdb-uniprot50 | AF-A0A0K1JCX4-F1-MODEL\_V4 | 1.0 | 5.764e-07 | 269 | 0.277 | 108 | 75 | 2 | 3 | 107 | 16 | 123 | GPW/gp25 family protein | GPW/gp25 family protein | | afdb-uniprot50 | AF-A0A841BZN7-F1-MODEL\_V4 | 1.0 | 3.106e-07 | 269 | 0.33 | 109 | 68 | 3 | 3 | 107 | 15 | 122 | Phage baseplate assembly protein W | Phage baseplate assembly protein W | | afdb-uniprot50 | AF-A0A3A9FX35-F1-MODEL\_V4 | 1.0 | 2.015e-07 | 269 | 0.275 | 116 | 72 | 4 | 1 | 106 | 10 | 123 | Baseplate protein | Baseplate protein | | afdb-uniprot50 | AF-A0A6I3M8T7-F1-MODEL\_V4 | 1.0 | 4.788e-07 | 269 | 0.287 | 108 | 72 | 3 | 2 | 105 | 14 | 120 | Baseplate protein | Baseplate protein | | afdb-uniprot50 | AF-A0A3N6FWS9-F1-MODEL\_V4 | 1.0 | 3.106e-07 | 269 | 0.293 | 109 | 74 | 2 | 2 | 107 | 14 | 122 | Gene 25-like lysozyme | Gene 25-like lysozyme | | afdb-uniprot50 | AF-A0A350PEH1-F1-MODEL\_V4 | 1.0 | 3.304e-07 | 268 | 0.198 | 111 | 83 | 3 | 3 | 107 | 19 | 129 | GPW\_gp25 domain-containing protein | GPW\_gp25 domain-containing protein | | afdb-uniprot50 | AF-A0A1Q4FZT8-F1-MODEL\_V4 | 1.0 | 2.919e-07 | 268 | 0.22 | 109 | 83 | 1 | 1 | 107 | 17 | 125 | GPW\_gp25 domain-containing protein | GPW\_gp25 domain-containing protein | | afdb-uniprot50 | AF-A0A2E0M339-F1-MODEL\_V4 | 1.0 | 7.852e-07 | 268 | 0.162 | 111 | 89 | 3 | 3 | 110 | 25 | 134 | GPW\_gp25 domain-containing protein | GPW\_gp25 domain-containing protein | | afdb-uniprot50 | AF-F9U9K7-F1-MODEL\_V4 | 1.0 | 2.744e-07 | 268 | 0.212 | 108 | 81 | 2 | 2 | 105 | 17 | 124 | GPW/gp25 family protein | GPW/gp25 family protein | | afdb-uniprot50 | AF-A0A518BPS1-F1-MODEL\_V4 | 1.0 | 6.619e-08 | 268 | 0.297 | 111 | 72 | 3 | 1 | 105 | 30 | 140 | Gene 25-like lysozyme | Gene 25-like lysozyme | | afdb-uniprot50 | AF-R5P778-F1-MODEL\_V4 | 1.0 | 1.288e-06 | 267 | 0.208 | 96 | 74 | 1 | 12 | 105 | 2 | 97 | GPW/gp25 family protein | GPW/gp25 family protein | | afdb-uniprot50 | AF-A0A6M0B625-F1-MODEL\_V4 | 1.0 | 5.418e-07 | 267 | 0.192 | 109 | 85 | 2 | 2 | 107 | 16 | 124 | GPW/gp25 family protein | GPW/gp25 family protein | | afdb-uniprot50 | AF-D8IV19-F1-MODEL\_V4 | 1.0 | 7.381e-07 | 267 | 0.3 | 103 | 69 | 2 | 5 | 105 | 31 | 132 | Bacteriophage baseplate assembly protein W | Bacteriophage baseplate assembly protein W | | afdb-uniprot50 | AF-A0A0M8SZZ8-F1-MODEL\_V4 | 1.0 | 4.501e-07 | 267 | 0.292 | 106 | 72 | 2 | 3 | 105 | 2 | 107 | Baseplate protein | Baseplate protein | | afdb-uniprot50 | AF-A0A7C2B4J0-F1-MODEL\_V4 | 1.0 | 3.977e-07 | 266 | 0.315 | 111 | 68 | 4 | 1 | 105 | 9 | 117 | GPW\_gp25 domain-containing protein | GPW\_gp25 domain-containing protein | | afdb-uniprot50 | AF-A0A1Z4U7A0-F1-MODEL\_V4 | 1.0 | 4.788e-07 | 266 | 0.183 | 109 | 85 | 2 | 2 | 106 | 17 | 125 | GPW\_gp25 domain-containing protein | GPW\_gp25 domain-containing protein | | afdb-uniprot50 | AF-A0A0S4XLR8-F1-MODEL\_V4 | 1.0 | 1.288e-06 | 265 | 0.309 | 97 | 63 | 2 | 12 | 107 | 4 | 97 | Putative phage-related baseplate assembly protein (GPW-like) | Putative phage-related baseplate assembly protein (GPW-like) | | afdb-uniprot50 | AF-A0A327JTI1-F1-MODEL\_V4 | 1.0 | 5.093e-07 | 265 | 0.252 | 115 | 70 | 4 | 1 | 107 | 1 | 107 | GPW\_gp25 domain-containing protein | GPW\_gp25 domain-containing protein | | afdb-uniprot50 | AF-A0A3D4RXG2-F1-MODEL\_V4 | 1.0 | 6.132e-07 | 265 | 0.25 | 108 | 76 | 3 | 2 | 105 | 16 | 122 | Phage tail protein | Phage tail protein | | afdb-uniprot50 | AF-A0A3S0SRP4-F1-MODEL\_V4 | 1.0 | 3.739e-07 | 265 | 0.268 | 108 | 75 | 3 | 1 | 106 | 16 | 121 | GPW\_gp25 domain-containing protein | GPW\_gp25 domain-containing protein | | afdb-uniprot50 | AF-A0A254S6U8-F1-MODEL\_V4 | 1.0 | 7.381e-07 | 265 | 0.168 | 107 | 88 | 1 | 2 | 107 | 15 | 121 | GPW\_gp25 domain-containing protein | GPW\_gp25 domain-containing protein | | afdb-uniprot50 | AF-A0A451AWS4-F1-MODEL\_V4 | 1.0 | 5.418e-07 | 265 | 0.209 | 110 | 83 | 2 | 2 | 107 | 16 | 125 | GPW\_gp25 domain-containing protein | GPW\_gp25 domain-containing protein | | afdb-uniprot50 | AF-A0A3S0JB46-F1-MODEL\_V4 | 1.0 | 4.231e-07 | 265 | 0.276 | 105 | 74 | 1 | 3 | 105 | 29 | 133 | GPW\_gp25 domain-containing protein | GPW\_gp25 domain-containing protein | | afdb-uniprot50 | AF-A0A2E5QDB5-F1-MODEL\_V4 | 1.0 | 6.132e-07 | 265 | 0.242 | 107 | 76 | 3 | 3 | 105 | 31 | 136 | GPW\_gp25 domain-containing protein | GPW\_gp25 domain-containing protein | | afdb-uniprot50 | AF-A0A1W9WDK2-F1-MODEL\_V4 | 1.0 | 6.132e-07 | 265 | 0.275 | 109 | 74 | 3 | 2 | 106 | 20 | 127 | GPW\_gp25 domain-containing protein | GPW\_gp25 domain-containing protein | | afdb-uniprot50 | AF-A0A2S2DR56-F1-MODEL\_V4 | 1.0 | 1.985e-06 | 264 | 0.234 | 94 | 70 | 1 | 17 | 108 | 7 | 100 | GPW\_gp25 domain-containing protein | GPW\_gp25 domain-containing protein | | afdb-uniprot50 | AF-A0A6P0Y0Z1-F1-MODEL\_V4 | 1.0 | 6.939e-07 | 264 | 0.237 | 97 | 70 | 2 | 17 | 109 | 16 | 112 | GPW/gp25 family protein | GPW/gp25 family protein | | afdb-uniprot50 | AF-A0A6M8STK6-F1-MODEL\_V4 | 1.0 | 1.55e-06 | 264 | 0.224 | 107 | 81 | 2 | 2 | 107 | 11 | 116 | GPW/gp25 family protein | GPW/gp25 family protein | | afdb-uniprot50 | AF-A0A6N8GMG1-F1-MODEL\_V4 | 1.0 | 6.939e-07 | 264 | 0.28 | 107 | 74 | 2 | 2 | 105 | 14 | 120 | Baseplate protein | Baseplate protein | | afdb-uniprot50 | AF-A0A6I7NUI7-F1-MODEL\_V4 | 1.0 | 5.093e-07 | 264 | 0.231 | 108 | 79 | 2 | 2 | 105 | 16 | 123 | GPW\_gp25 domain-containing protein | GPW\_gp25 domain-containing protein | | afdb-uniprot50 | AF-A0A6D1IH24-F1-MODEL\_V4 | 1.0 | 7.852e-07 | 264 | 0.311 | 109 | 68 | 4 | 2 | 105 | 14 | 120 | Baseplate protein | Baseplate protein | | afdb-uniprot50 | AF-A0A1S6FJH3-F1-MODEL\_V4 | 1.0 | 3.106e-07 | 264 | 0.296 | 108 | 72 | 2 | 2 | 105 | 17 | 124 | GPW\_gp25 domain-containing protein | GPW\_gp25 domain-containing protein | | afdb-uniprot50 | AF-A0A1I0ZTA3-F1-MODEL\_V4 | 1.0 | 4.501e-07 | 264 | 0.203 | 108 | 82 | 2 | 2 | 105 | 19 | 126 | GPW\_gp25 domain-containing protein | GPW\_gp25 domain-containing protein | | afdb-uniprot50 | AF-A0A2E0S336-F1-MODEL\_V4 | 1.0 | 6.939e-07 | 264 | 0.226 | 106 | 79 | 2 | 3 | 105 | 42 | 147 | GPW\_gp25 domain-containing protein | GPW\_gp25 domain-containing protein | | afdb-uniprot50 | AF-N1UP64-F1-MODEL\_V4 | 1.0 | 3.106e-07 | 264 | 0.324 | 114 | 73 | 3 | 1 | 111 | 24 | 136 | GPW\_gp25 domain-containing protein | GPW\_gp25 domain-containing protein | | afdb-uniprot50 | AF-A0A4R7JV64-F1-MODEL\_V4 | 1.0 | 3.304e-07 | 264 | 0.282 | 117 | 75 | 3 | 2 | 109 | 29 | 145 | GPW\_gp25 domain-containing protein | GPW\_gp25 domain-containing protein | | afdb-uniprot50 | AF-A0A3C0WX89-F1-MODEL\_V4 | 1.0 | 1.006e-06 | 263 | 0.238 | 109 | 80 | 2 | 2 | 107 | 12 | 120 | Baseplate protein | Baseplate protein | | afdb-uniprot50 | AF-A0A2M8BUW0-F1-MODEL\_V4 | 1.0 | 5.093e-07 | 263 | 0.275 | 109 | 72 | 5 | 2 | 105 | 13 | 119 | Baseplate protein | Baseplate protein | | afdb-uniprot50 | AF-A0A7W8JUU5-F1-MODEL\_V4 | 1.0 | 2.425e-07 | 263 | 0.231 | 108 | 80 | 2 | 1 | 105 | 26 | 133 | GPW\_gp25 domain-containing protein | GPW\_gp25 domain-containing protein | | afdb-uniprot50 | AF-A0A440VHY5-F1-MODEL\_V4 | 1.0 | 7.381e-07 | 262 | 0.289 | 107 | 69 | 3 | 6 | 105 | 1 | 107 | Baseplate assembly protein | Baseplate assembly protein | | afdb-uniprot50 | AF-A0A1Z8SB79-F1-MODEL\_V4 | 1.0 | 5.764e-07 | 262 | 0.226 | 106 | 79 | 2 | 3 | 105 | 18 | 123 | GPW\_gp25 domain-containing protein | GPW\_gp25 domain-containing protein | | afdb-uniprot50 | AF-R5L6F4-F1-MODEL\_V4 | 1.0 | 5.418e-07 | 262 | 0.25 | 108 | 76 | 4 | 2 | 105 | 16 | 122 | GPW\_gp25 domain-containing protein | GPW\_gp25 domain-containing protein | | afdb-uniprot50 | AF-A0A368JP81-F1-MODEL\_V4 | 1.0 | 6.132e-07 | 262 | 0.198 | 111 | 83 | 2 | 2 | 106 | 16 | 126 | GPW\_gp25 domain-containing protein | GPW\_gp25 domain-containing protein | | afdb-uniprot50 | AF-A0A0U3LKX5-F1-MODEL\_V4 | 1.0 | 4.788e-07 | 262 | 0.194 | 108 | 83 | 2 | 2 | 105 | 16 | 123 | GPW/gp25 family protein | GPW/gp25 family protein | | afdb-uniprot50 | AF-A0A2A4UTL9-F1-MODEL\_V4 | 1.0 | 3.061e-06 | 261 | 0.25 | 96 | 71 | 1 | 12 | 106 | 2 | 97 | GPW\_gp25 domain-containing protein | GPW\_gp25 domain-containing protein | | afdb-uniprot50 | AF-A0A416TLR2-F1-MODEL\_V4 | 1.0 | 5.764e-07 | 261 | 0.212 | 108 | 80 | 4 | 2 | 105 | 15 | 121 | Baseplate assembly protein | Baseplate assembly protein | | afdb-uniprot50 | AF-A0A1V5RRZ4-F1-MODEL\_V4 | 1.0 | 6.939e-07 | 261 | 0.279 | 111 | 78 | 2 | 3 | 112 | 30 | 139 | Gene 25-like lysozyme | Gene 25-like lysozyme | | afdb-uniprot50 | AF-A0A285QW58-F1-MODEL\_V4 | 1.0 | 3.106e-07 | 260 | 0.268 | 108 | 75 | 3 | 2 | 106 | 9 | 115 | GPW\_gp25 domain-containing protein | GPW\_gp25 domain-containing protein | | afdb-uniprot50 | AF-A0A4V2PNK1-F1-MODEL\_V4 | 1.0 | 9.453e-07 | 260 | 0.226 | 106 | 80 | 1 | 2 | 105 | 14 | 119 | GPW\_gp25 domain-containing protein | GPW\_gp25 domain-containing protein | | afdb-uniprot50 | AF-A0A0Q7WE07-F1-MODEL\_V4 | 1.0 | 2.143e-07 | 260 | 0.305 | 108 | 70 | 3 | 3 | 105 | 13 | 120 | GPW\_gp25 domain-containing protein | GPW\_gp25 domain-containing protein | | afdb-uniprot50 | AF-A0A0K3BU55-F1-MODEL\_V4 | 1.0 | 1.02e-07 | 260 | 0.28 | 114 | 73 | 2 | 1 | 105 | 5 | 118 | GPW\_gp25 domain-containing protein | GPW\_gp25 domain-containing protein | | afdb-uniprot50 | AF-W7IHM5-F1-MODEL\_V4 | 1.0 | 7.852e-07 | 260 | 0.194 | 108 | 82 | 3 | 2 | 105 | 15 | 121 | GPW\_gp25 domain-containing protein | GPW\_gp25 domain-containing protein | | afdb-uniprot50 | AF-A0A1G7A6G8-F1-MODEL\_V4 | 1.0 | 6.523e-07 | 260 | 0.194 | 108 | 84 | 2 | 2 | 106 | 17 | 124 | GPW\_gp25 domain-containing protein | GPW\_gp25 domain-containing protein | | afdb-uniprot50 | AF-A0A7W0L027-F1-MODEL\_V4 | 1.0 | 8.353e-07 | 259 | 0.287 | 108 | 74 | 2 | 2 | 106 | 15 | 122 | GPW/gp25 family protein | GPW/gp25 family protein | | afdb-uniprot50 | AF-A0A1T5EG95-F1-MODEL\_V4 | 1.0 | 7.852e-07 | 259 | 0.179 | 106 | 85 | 1 | 2 | 105 | 16 | 121 | GPW\_gp25 domain-containing protein | GPW\_gp25 domain-containing protein | | afdb-uniprot50 | AF-A0A5D0Q715-F1-MODEL\_V4 | 1.0 | 1.155e-07 | 259 | 0.357 | 112 | 66 | 3 | 3 | 108 | 19 | 130 | GPW/gp25 family protein | GPW/gp25 family protein | | afdb-uniprot50 | AF-A0A1Z9DJT3-F1-MODEL\_V4 | 1.0 | 1.754e-06 | 259 | 0.203 | 108 | 83 | 2 | 3 | 107 | 24 | 131 | GPW\_gp25 domain-containing protein | GPW\_gp25 domain-containing protein | | afdb-uniprot50 | AF-A0A2P7TGY9-F1-MODEL\_V4 | 1.0 | 1.006e-06 | 259 | 0.228 | 105 | 79 | 1 | 3 | 105 | 37 | 141 | GPW\_gp25 domain-containing protein | GPW\_gp25 domain-containing protein | | afdb-uniprot50 | AF-A0A081KAP0-F1-MODEL\_V4 | 1.0 | 6.523e-07 | 258 | 0.245 | 106 | 77 | 2 | 3 | 105 | 17 | 122 | GPW\_gp25 domain-containing protein | GPW\_gp25 domain-containing protein | | afdb-uniprot50 | AF-A0A2W4RIS3-F1-MODEL\_V4 | 1.0 | 1.138e-06 | 258 | 0.25 | 108 | 77 | 3 | 2 | 105 | 17 | 124 | GPW\_gp25 domain-containing protein | GPW\_gp25 domain-containing protein | | afdb-uniprot50 | AF-A0A3A8AUY7-F1-MODEL\_V4 | 1.0 | 1.649e-06 | 258 | 0.207 | 106 | 82 | 2 | 2 | 105 | 16 | 121 | Baseplate protein | Baseplate protein | | afdb-uniprot50 | AF-A0A2W2BBX1-F1-MODEL\_V4 | 1.0 | 7.852e-07 | 258 | 0.252 | 107 | 77 | 2 | 2 | 105 | 18 | 124 | GPW\_gp25 domain-containing protein | GPW\_gp25 domain-containing protein | | afdb-uniprot50 | AF-A0A7Y4WZF6-F1-MODEL\_V4 | 1.0 | 7.381e-07 | 258 | 0.207 | 111 | 82 | 2 | 2 | 106 | 22 | 132 | GPW/gp25 family protein | GPW/gp25 family protein | | afdb-uniprot50 | AF-A0A521BNH3-F1-MODEL\_V4 | 1.0 | 3.256e-06 | 257 | 0.186 | 102 | 81 | 1 | 8 | 107 | 3 | 104 | GPW\_gp25 domain-containing protein | GPW\_gp25 domain-containing protein | | afdb-uniprot50 | AF-A0A2Z3LGG4-F1-MODEL\_V4 | 1.0 | 5.418e-07 | 257 | 0.231 | 108 | 79 | 2 | 2 | 105 | 15 | 122 | GPW\_gp25 domain-containing protein | GPW\_gp25 domain-containing protein | | afdb-uniprot50 | AF-A0A1C6SZV0-F1-MODEL\_V4 | 1.0 | 9.453e-07 | 257 | 0.287 | 108 | 72 | 3 | 2 | 105 | 14 | 120 | GPW\_gp25 domain-containing protein | GPW\_gp25 domain-containing protein | | afdb-uniprot50 | AF-A0A4Y7B9B6-F1-MODEL\_V4 | 1.0 | 7.852e-07 | 257 | 0.241 | 116 | 78 | 2 | 1 | 106 | 30 | 145 | GPW\_gp25 domain-containing protein | GPW\_gp25 domain-containing protein | | afdb-uniprot50 | AF-A0A0A7KPC6-F1-MODEL\_V4 | 1.0 | 3.739e-07 | 256 | 0.271 | 107 | 74 | 2 | 3 | 105 | 12 | 118 | GPW\_gp25 domain-containing protein | GPW\_gp25 domain-containing protein | | afdb-uniprot50 | AF-A0A1Z9Q4U1-F1-MODEL\_V4 | 1.0 | 6.523e-07 | 256 | 0.232 | 112 | 78 | 4 | 3 | 107 | 15 | 125 | GPW\_gp25 domain-containing protein | GPW\_gp25 domain-containing protein | | afdb-uniprot50 | AF-A0A7W1N5U2-F1-MODEL\_V4 | 1.0 | 4.788e-07 | 256 | 0.214 | 107 | 81 | 2 | 2 | 105 | 13 | 119 | GPW/gp25 family protein | GPW/gp25 family protein | | afdb-uniprot50 | AF-A0A0S3UC95-F1-MODEL\_V4 | 1.0 | 1.006e-06 | 256 | 0.362 | 113 | 62 | 4 | 1 | 104 | 15 | 126 | GPW / gp25 family protein | GPW / gp25 family protein | | afdb-uniprot50 | AF-A0A6I5PFW1-F1-MODEL\_V4 | 1.0 | 1.288e-06 | 255 | 0.236 | 93 | 69 | 1 | 17 | 107 | 7 | 99 | GPW/gp25 family protein | GPW/gp25 family protein | | afdb-uniprot50 | AF-A0A0M8QQ48-F1-MODEL\_V4 | 1.0 | 1.138e-06 | 255 | 0.283 | 106 | 73 | 2 | 3 | 105 | 2 | 107 | Baseplate protein | Baseplate protein | | afdb-uniprot50 | AF-A0A7W7MKI6-F1-MODEL\_V4 | 1.0 | 8.886e-07 | 255 | 0.299 | 107 | 71 | 3 | 2 | 105 | 16 | 121 | Phage baseplate assembly protein W | Phage baseplate assembly protein W | | afdb-uniprot50 | AF-A0A1I3WUF2-F1-MODEL\_V4 | 1.0 | 4.231e-07 | 255 | 0.205 | 107 | 82 | 2 | 2 | 105 | 17 | 123 | GPW\_gp25 domain-containing protein | GPW\_gp25 domain-containing protein | | afdb-uniprot50 | AF-A0A3E5B1A9-F1-MODEL\_V4 | 1.0 | 2.247e-06 | 255 | 0.196 | 107 | 85 | 1 | 2 | 107 | 22 | 128 | GPW\_gp25 domain-containing protein | GPW\_gp25 domain-containing protein | | afdb-uniprot50 | AF-A0A2E4W5C2-F1-MODEL\_V4 | 1.0 | 1.649e-06 | 254 | 0.243 | 111 | 79 | 2 | 1 | 107 | 5 | 114 | GPW\_gp25 domain-containing protein | GPW\_gp25 domain-containing protein | | afdb-uniprot50 | AF-E7G9C5-F1-MODEL\_V4 | 1.0 | 5.418e-07 | 254 | 0.263 | 114 | 76 | 4 | 2 | 108 | 13 | 125 | GPW\_gp25 domain-containing protein | GPW\_gp25 domain-containing protein | | afdb-uniprot50 | AF-A0A177NGE0-F1-MODEL\_V4 | 1.0 | 9.453e-07 | 254 | 0.256 | 109 | 77 | 2 | 3 | 107 | 14 | 122 | GPW\_gp25 domain-containing protein | GPW\_gp25 domain-containing protein | | afdb-uniprot50 | AF-A0A3B1A623-F1-MODEL\_V4 | 1.0 | 9.453e-07 | 254 | 0.172 | 116 | 86 | 3 | 2 | 107 | 12 | 127 | GPW\_gp25 domain-containing protein | GPW\_gp25 domain-containing protein | | afdb-uniprot50 | AF-A0A1I0XDA2-F1-MODEL\_V4 | 1.0 | 1.211e-06 | 254 | 0.231 | 108 | 78 | 3 | 2 | 105 | 16 | 122 | GPW\_gp25 domain-containing protein | GPW\_gp25 domain-containing protein | | afdb-uniprot50 | AF-A0A424SVR1-F1-MODEL\_V4 | 1.0 | 1.754e-06 | 254 | 0.231 | 108 | 80 | 2 | 3 | 107 | 25 | 132 | GPW\_gp25 domain-containing protein | GPW\_gp25 domain-containing protein | | afdb-uniprot50 | AF-A0A291IN73-F1-MODEL\_V4 | 1.0 | 6.939e-07 | 254 | 0.27 | 111 | 78 | 2 | 1 | 108 | 25 | 135 | GPW\_gp25 domain-containing protein | GPW\_gp25 domain-containing protein | | afdb-uniprot50 | AF-A0A1E5BWS4-F1-MODEL\_V4 | 1.0 | 4.436e-06 | 253 | 0.254 | 118 | 78 | 5 | 1 | 110 | 1 | 116 | GPW\_gp25 domain-containing protein | GPW\_gp25 domain-containing protein | | afdb-uniprot50 | AF-X5QYV2-F1-MODEL\_V4 | 1.0 | 1.138e-06 | 253 | 0.234 | 111 | 78 | 2 | 2 | 105 | 16 | 126 | Baseplate protein | Baseplate protein | | afdb-uniprot50 | AF-A0A554VPK4-F1-MODEL\_V4 | 1.0 | 1.55e-06 | 253 | 0.165 | 109 | 87 | 2 | 2 | 106 | 18 | 126 | GPW/gp25 family protein | GPW/gp25 family protein | | afdb-uniprot50 | AF-A0A327QS96-F1-MODEL\_V4 | 1.0 | 8.886e-07 | 253 | 0.194 | 113 | 83 | 2 | 1 | 105 | 17 | 129 | GPW\_gp25 domain-containing protein | GPW\_gp25 domain-containing protein | | afdb-uniprot50 | AF-A0A5K7YHV8-F1-MODEL\_V4 | 1.0 | 1.07e-06 | 253 | 0.206 | 116 | 82 | 2 | 2 | 107 | 15 | 130 | GPW\_gp25 domain-containing protein | GPW\_gp25 domain-containing protein | | afdb-uniprot50 | AF-A0A701NJT1-F1-MODEL\_V4 | 1.0 | 3.061e-06 | 252 | 0.404 | 84 | 50 | 0 | 12 | 95 | 3 | 86 | Baseplate assembly protein | Baseplate assembly protein | | afdb-uniprot50 | AF-A0A1I2FX92-F1-MODEL\_V4 | 1.0 | 1.866e-06 | 252 | 0.311 | 106 | 70 | 2 | 2 | 105 | 12 | 116 | Gene 25-like lysozyme | Gene 25-like lysozyme | | afdb-uniprot50 | AF-A0A3P1UU22-F1-MODEL\_V4 | 1.0 | 7.381e-07 | 252 | 0.3 | 120 | 62 | 8 | 1 | 108 | 17 | 126 | Baseplate assembly protein | Baseplate assembly protein | | afdb-uniprot50 | AF-A0A2V7X7B0-F1-MODEL\_V4 | 1.0 | 6.838e-06 | 251 | 0.258 | 93 | 67 | 1 | 17 | 107 | 9 | 101 | Baseplate protein | Baseplate protein | | afdb-uniprot50 | AF-A0A2E6EKB3-F1-MODEL\_V4 | 1.0 | 1.866e-06 | 251 | 0.256 | 109 | 75 | 4 | 2 | 105 | 14 | 121 | Baseplate protein | Baseplate protein | | afdb-uniprot50 | AF-A0A4Q5Y1B4-F1-MODEL\_V4 | 1.0 | 1.37e-06 | 251 | 0.203 | 108 | 82 | 2 | 2 | 105 | 11 | 118 | GPW\_gp25 domain-containing protein | GPW\_gp25 domain-containing protein | | afdb-uniprot50 | AF-A0A7X7DI45-F1-MODEL\_V4 | 1.0 | 1.288e-06 | 251 | 0.266 | 109 | 77 | 2 | 2 | 107 | 22 | 130 | GPW/gp25 family protein | GPW/gp25 family protein | | afdb-uniprot50 | AF-A0A353GUZ2-F1-MODEL\_V4 | 1.0 | 1.37e-06 | 250 | 0.13 | 115 | 95 | 3 | 2 | 112 | 15 | 128 | GPW\_gp25 domain-containing protein | GPW\_gp25 domain-containing protein | | afdb-uniprot50 | AF-A0A165U8T9-F1-MODEL\_V4 | 1.0 | 4.719e-06 | 250 | 0.218 | 119 | 79 | 2 | 1 | 105 | 1 | 119 | Gene 25-like lysozyme | Gene 25-like lysozyme | | afdb-uniprot50 | AF-A9DX78-F1-MODEL\_V4 | 1.0 | 1.866e-06 | 250 | 0.183 | 109 | 85 | 2 | 2 | 106 | 17 | 125 | Tail lysozyme, putative | Tail lysozyme, putative | | afdb-uniprot50 | AF-A0A4Y8P3N7-F1-MODEL\_V4 | 1.0 | 1.985e-06 | 250 | 0.283 | 106 | 74 | 1 | 2 | 105 | 23 | 128 | Baseplate protein | Baseplate protein | | afdb-uniprot50 | AF-A0A0S2FBB8-F1-MODEL\_V4 | 1.0 | 5.093e-07 | 249 | 0.271 | 114 | 74 | 4 | 2 | 107 | 13 | 125 | Gene 25-like lysozyme family protein | Gene 25-like lysozyme family protein | | afdb-uniprot50 | AF-U2HWE8-F1-MODEL\_V4 | 1.0 | 1.985e-06 | 249 | 0.188 | 106 | 84 | 1 | 2 | 105 | 16 | 121 | GPW\_gp25 domain-containing protein | GPW\_gp25 domain-containing protein | | afdb-uniprot50 | AF-A0A1Z8QEK9-F1-MODEL\_V4 | 1.0 | 1.55e-06 | 249 | 0.185 | 108 | 84 | 3 | 3 | 106 | 18 | 125 | GPW\_gp25 domain-containing protein | GPW\_gp25 domain-containing protein | | afdb-uniprot50 | AF-A0A7Y6XND0-F1-MODEL\_V4 | 1.0 | 2.877e-06 | 249 | 0.181 | 116 | 85 | 3 | 1 | 106 | 11 | 126 | GPW/gp25 family protein | GPW/gp25 family protein | | afdb-uniprot50 | AF-K1RIV8-F1-MODEL\_V4 | 1.0 | 3.977e-07 | 249 | 0.218 | 110 | 79 | 4 | 1 | 105 | 22 | 129 | GPW/gp25 family protein | GPW/gp25 family protein | | afdb-uniprot50 | AF-A0A497CQ18-F1-MODEL\_V4 | 1.0 | 1.55e-06 | 249 | 0.206 | 116 | 82 | 3 | 2 | 107 | 11 | 126 | GPW\_gp25 domain-containing protein | GPW\_gp25 domain-containing protein | | afdb-uniprot50 | AF-A0A2T7L1T8-F1-MODEL\_V4 | 1.0 | 3.685e-06 | 249 | 0.263 | 114 | 75 | 3 | 1 | 105 | 19 | 132 | Baseplate protein | Baseplate protein | | afdb-uniprot50 | AF-A0A252DTP5-F1-MODEL\_V4 | 1.0 | 2.112e-06 | 249 | 0.201 | 109 | 83 | 2 | 2 | 106 | 26 | 134 | GPW\_gp25 domain-containing protein | GPW\_gp25 domain-containing protein | | afdb-uniprot50 | AF-A0A4U8UBX3-F1-MODEL\_V4 | 1.0 | 3.106e-07 | 248 | 0.285 | 105 | 62 | 2 | 1 | 105 | 1 | 92 | Baseplate assembly protein | Baseplate assembly protein | | afdb-uniprot50 | AF-A0A380Q839-F1-MODEL\_V4 | 1.0 | 3.061e-06 | 248 | 0.482 | 87 | 41 | 1 | 1 | 87 | 6 | 88 | Phage-like protein | Phage-like protein | | afdb-uniprot50 | AF-A0A6G3XXE1-F1-MODEL\_V4 | 1.0 | 2.39e-06 | 248 | 0.266 | 105 | 74 | 2 | 4 | 105 | 1 | 105 | GPW/gp25 family protein | GPW/gp25 family protein | | afdb-uniprot50 | AF-A0A2G2LH18-F1-MODEL\_V4 | 1.0 | 2.247e-06 | 248 | 0.196 | 107 | 83 | 2 | 3 | 106 | 21 | 127 | GPW\_gp25 domain-containing protein | GPW\_gp25 domain-containing protein | | afdb-uniprot50 | AF-A1ZC12-F1-MODEL\_V4 | 1.0 | 1.138e-06 | 248 | 0.201 | 109 | 81 | 3 | 2 | 105 | 17 | 124 | Phage baseplate assembly protein W | Phage baseplate assembly protein W | | afdb-uniprot50 | AF-C5BJK3-F1-MODEL\_V4 | 1.0 | 1.754e-06 | 248 | 0.179 | 117 | 85 | 3 | 2 | 107 | 18 | 134 | Phage GpW/Gp25 family protein | Phage GpW/Gp25 family protein | | afdb-uniprot50 | AF-A0A7C4AKA1-F1-MODEL\_V4 | 1.0 | 3.061e-06 | 247 | 0.247 | 109 | 75 | 4 | 3 | 108 | 6 | 110 | Baseplate protein | Baseplate protein | | afdb-uniprot50 | AF-A0A2D5ICS9-F1-MODEL\_V4 | 1.0 | 2.877e-06 | 247 | 0.234 | 111 | 81 | 3 | 3 | 110 | 18 | 127 | GPW\_gp25 domain-containing protein | GPW\_gp25 domain-containing protein | | afdb-uniprot50 | AF-A0A1H6RDH1-F1-MODEL\_V4 | 1.0 | 1.457e-06 | 247 | 0.224 | 107 | 80 | 2 | 2 | 105 | 17 | 123 | GPW\_gp25 domain-containing protein | GPW\_gp25 domain-containing protein | | afdb-uniprot50 | AF-A0A6S5Y5N2-F1-MODEL\_V4 | 1.0 | 1.07e-06 | 247 | 0.25 | 120 | 79 | 3 | 1 | 111 | 18 | 135 | Baseplate protein | Baseplate protein | | afdb-uniprot50 | AF-A0A1J4Z8F3-F1-MODEL\_V4 | 1.0 | 1.211e-06 | 247 | 0.188 | 106 | 84 | 1 | 2 | 105 | 20 | 125 | GPW\_gp25 domain-containing protein | GPW\_gp25 domain-containing protein | | afdb-uniprot50 | AF-A0A7W1U792-F1-MODEL\_V4 | 1.0 | 4.788e-07 | 247 | 0.212 | 108 | 81 | 2 | 2 | 105 | 22 | 129 | GPW/gp25 family protein | GPW/gp25 family protein | | afdb-uniprot50 | AF-U3AL81-F1-MODEL\_V4 | 1.0 | 8.757e-06 | 246 | 0.172 | 110 | 84 | 4 | 1 | 107 | 2 | 107 | Uncharacterized protein | Uncharacterized protein | | afdb-uniprot50 | AF-A0A8A3HHQ9-F1-MODEL\_V4 | 1.0 | 2.705e-06 | 246 | 0.265 | 113 | 74 | 4 | 1 | 105 | 1 | 112 | GPW/gp25 family protein | GPW/gp25 family protein | | afdb-uniprot50 | AF-A0A1C4VFP2-F1-MODEL\_V4 | 1.0 | 6.428e-06 | 246 | 0.201 | 114 | 82 | 3 | 1 | 106 | 1 | 113 | GPW\_gp25 domain-containing protein | GPW\_gp25 domain-containing protein | | afdb-uniprot50 | AF-A0A246RHT3-F1-MODEL\_V4 | 1.0 | 3.464e-06 | 246 | 0.205 | 107 | 82 | 3 | 2 | 106 | 8 | 113 | GPW\_gp25 domain-containing protein | GPW\_gp25 domain-containing protein | | afdb-uniprot50 | AF-A0A426VQD0-F1-MODEL\_V4 | 1.0 | 3.464e-06 | 246 | 0.162 | 111 | 86 | 4 | 1 | 105 | 4 | 113 | GPW\_gp25 domain-containing protein | GPW\_gp25 domain-containing protein | | afdb-uniprot50 | AF-A0A6I7PSS5-F1-MODEL\_V4 | 1.0 | 2.705e-06 | 246 | 0.279 | 93 | 61 | 2 | 17 | 105 | 30 | 120 | GPW\_gp25 domain-containing protein | GPW\_gp25 domain-containing protein | | afdb-uniprot50 | AF-A0A7G2RQA6-F1-MODEL\_V4 | 1.0 | 2.112e-06 | 246 | 0.149 | 107 | 88 | 2 | 3 | 106 | 19 | 125 | GPW\_gp25 domain-containing protein | GPW\_gp25 domain-containing protein | | afdb-uniprot50 | AF-A0A3D1U7G8-F1-MODEL\_V4 | 1.0 | 5.764e-07 | 246 | 0.179 | 117 | 82 | 5 | 1 | 105 | 5 | 119 | Baseplate assembly protein | Baseplate assembly protein | | afdb-uniprot50 | AF-A0A7K3ZDA3-F1-MODEL\_V4 | 1.0 | 2.247e-06 | 246 | 0.211 | 109 | 82 | 3 | 5 | 112 | 34 | 139 | GPW/gp25 family protein | GPW/gp25 family protein | | afdb-uniprot50 | AF-A0A0B6CV24-F1-MODEL\_V4 | 1.0 | 7.852e-07 | 245 | 0.272 | 110 | 76 | 3 | 3 | 109 | 2 | 110 | 25-like lysozyme family protein | 25-like lysozyme family protein | | afdb-uniprot50 | AF-A0A519PID9-F1-MODEL\_V4 | 1.0 | 1.649e-06 | 245 | 0.212 | 108 | 81 | 3 | 2 | 105 | 16 | 123 | GPW\_gp25 domain-containing protein | GPW\_gp25 domain-containing protein | | afdb-uniprot50 | AF-A0A0F9S7G6-F1-MODEL\_V4 | 1.0 | 1.37e-06 | 245 | 0.219 | 114 | 79 | 4 | 2 | 107 | 13 | 124 | GPW\_gp25 domain-containing protein | GPW\_gp25 domain-containing protein | | afdb-uniprot50 | AF-A0A1D9F2U1-F1-MODEL\_V4 | 1.0 | 9.453e-07 | 245 | 0.272 | 110 | 75 | 3 | 1 | 106 | 24 | 132 | GPW\_gp25 domain-containing protein | GPW\_gp25 domain-containing protein | | afdb-uniprot50 | AF-H0FAR8-F1-MODEL\_V4 | 1.0 | 2.112e-06 | 244 | 0.242 | 107 | 76 | 4 | 2 | 105 | 8 | 112 | GPW/gp25 family protein | GPW/gp25 family protein | | afdb-uniprot50 | AF-A0A0C5V444-F1-MODEL\_V4 | 1.0 | 3.464e-06 | 244 | 0.207 | 106 | 81 | 2 | 3 | 105 | 17 | 122 | Phage baseplate assembly protein W | Phage baseplate assembly protein W | | afdb-uniprot50 | AF-A0A4Q9KAQ7-F1-MODEL\_V4 | 1.0 | 1.07e-06 | 244 | 0.265 | 113 | 76 | 3 | 2 | 107 | 19 | 131 | Phage tail protein | Phage tail protein | | afdb-uniprot50 | AF-A0A6C0JT84-F1-MODEL\_V4 | 1.0 | 3.061e-06 | 244 | 0.165 | 109 | 86 | 3 | 3 | 107 | 21 | 128 | GPW\_gp25 domain-containing protein | GPW\_gp25 domain-containing protein | | afdb-uniprot50 | AF-A0A2T0HIB9-F1-MODEL\_V4 | 1.0 | 1.528e-05 | 243 | 0.519 | 77 | 37 | 0 | 7 | 83 | 2 | 78 | Baseplate assembly protein | Baseplate assembly protein | | afdb-uniprot50 | AF-A0A1H3JDF8-F1-MODEL\_V4 | 1.0 | 2.877e-06 | 243 | 0.244 | 98 | 69 | 2 | 17 | 109 | 7 | 104 | GPW\_gp25 domain-containing protein | GPW\_gp25 domain-containing protein | | afdb-uniprot50 | AF-A0A1W9VM06-F1-MODEL\_V4 | 1.0 | 2.247e-06 | 243 | 0.25 | 112 | 77 | 4 | 1 | 107 | 1 | 110 | GPW\_gp25 domain-containing protein | GPW\_gp25 domain-containing protein | | afdb-uniprot50 | AF-A0A1F4G5G2-F1-MODEL\_V4 | 1.0 | 7.381e-07 | 243 | 0.226 | 115 | 80 | 4 | 2 | 108 | 15 | 128 | Phage tail protein | Phage tail protein | | afdb-uniprot50 | AF-L7UKW4-F1-MODEL\_V4 | 1.0 | 7.739e-06 | 242 | 0.254 | 114 | 76 | 4 | 1 | 106 | 1 | 113 | GPW\_gp25 domain-containing protein | GPW\_gp25 domain-containing protein | | afdb-uniprot50 | AF-A0A172UEQ1-F1-MODEL\_V4 | 1.0 | 7.381e-07 | 242 | 0.262 | 118 | 81 | 3 | 1 | 112 | 8 | 125 | GPW\_gp25 domain-containing protein | GPW\_gp25 domain-containing protein | | afdb-uniprot50 | AF-A0A4Y4CYZ4-F1-MODEL\_V4 | 1.0 | 3.685e-06 | 242 | 0.273 | 106 | 75 | 1 | 4 | 107 | 15 | 120 | GPW\_gp25 domain-containing protein | GPW\_gp25 domain-containing protein | | afdb-uniprot50 | AF-A0A0Q6WZD8-F1-MODEL\_V4 | 1.0 | 2.705e-06 | 242 | 0.21 | 114 | 85 | 4 | 3 | 112 | 19 | 131 | GPW\_gp25 domain-containing protein | GPW\_gp25 domain-containing protein | | afdb-uniprot50 | AF-A0A2W5H172-F1-MODEL\_V4 | 1.0 | 4.17e-06 | 242 | 0.256 | 113 | 77 | 2 | 1 | 106 | 16 | 128 | Integrase | Integrase | | afdb-uniprot50 | AF-A0A1W0CS24-F1-MODEL\_V4 | 1.0 | 7.381e-07 | 242 | 0.238 | 109 | 79 | 2 | 1 | 105 | 18 | 126 | GPW\_gp25 domain-containing protein | GPW\_gp25 domain-containing protein | | afdb-uniprot50 | AF-G5IB78-F1-MODEL\_V4 | 1.0 | 1.211e-06 | 242 | 0.212 | 108 | 80 | 4 | 2 | 105 | 15 | 121 | GPW\_gp25 domain-containing protein | GPW\_gp25 domain-containing protein | | afdb-uniprot50 | AF-A0A3N9GJI3-F1-MODEL\_V4 | 1.0 | 6.939e-07 | 242 | 0.216 | 111 | 81 | 2 | 1 | 105 | 25 | 135 | Phage baseplate protein | Phage baseplate protein | | afdb-uniprot50 | AF-A0A349ZQF9-F1-MODEL\_V4 | 1.0 | 2.112e-06 | 242 | 0.2 | 110 | 83 | 4 | 2 | 107 | 16 | 124 | Baseplate protein | Baseplate protein | | afdb-uniprot50 | AF-A0A7V4G7W4-F1-MODEL\_V4 | 1.0 | 2.081e-05 | 241 | 0.266 | 90 | 64 | 2 | 17 | 105 | 2 | 90 | GPW\_gp25 domain-containing protein | GPW\_gp25 domain-containing protein | | afdb-uniprot50 | AF-A0A5B1BHT0-F1-MODEL\_V4 | 1.0 | 7.275e-06 | 241 | 0.244 | 98 | 71 | 2 | 12 | 106 | 1 | 98 | GPW/gp25 family protein | GPW/gp25 family protein | | afdb-uniprot50 | AF-A0A7K3XK95-F1-MODEL\_V4 | 1.0 | 9.91e-06 | 241 | 0.229 | 96 | 72 | 1 | 12 | 105 | 2 | 97 | GPW/gp25 family protein | GPW/gp25 family protein | | afdb-uniprot50 | AF-A0A511QWX5-F1-MODEL\_V4 | 1.0 | 5.34e-06 | 241 | 0.264 | 106 | 73 | 4 | 1 | 105 | 5 | 106 | Uncharacterized protein | Uncharacterized protein | | afdb-uniprot50 | AF-A0A2E6YZN4-F1-MODEL\_V4 | 1.0 | 5.02e-06 | 241 | 0.222 | 108 | 81 | 2 | 3 | 107 | 17 | 124 | GPW\_gp25 domain-containing protein | GPW\_gp25 domain-containing protein | | afdb-uniprot50 | AF-A0A318TME3-F1-MODEL\_V4 | 1.0 | 3.256e-06 | 241 | 0.309 | 110 | 66 | 2 | 4 | 103 | 1 | 110 | GPW\_gp25 domain-containing protein | GPW\_gp25 domain-containing protein | | afdb-uniprot50 | AF-A0A2R7JXZ8-F1-MODEL\_V4 | 1.0 | 9.453e-07 | 241 | 0.278 | 122 | 71 | 4 | 2 | 106 | 12 | 133 | Baseplate protein | Baseplate protein | | afdb-uniprot50 | AF-A0A2N8BBF2-F1-MODEL\_V4 | 1.0 | 6.939e-07 | 241 | 0.233 | 107 | 77 | 3 | 2 | 105 | 17 | 121 | Phage baseplate protein | Phage baseplate protein | | afdb-uniprot50 | AF-A0A1I5C9M8-F1-MODEL\_V4 | 1.0 | 9.453e-07 | 241 | 0.194 | 108 | 82 | 4 | 2 | 105 | 15 | 121 | GPW\_gp25 domain-containing protein | GPW\_gp25 domain-containing protein | | afdb-uniprot50 | AF-A0A2W4YAP4-F1-MODEL\_V4 | 1.0 | 4.501e-07 | 241 | 0.276 | 112 | 73 | 3 | 4 | 107 | 26 | 137 | Baseplate assembly protein | Baseplate assembly protein | | afdb-uniprot50 | AF-A0A5E4SAR3-F1-MODEL\_V4 | 1.0 | 2.112e-06 | 241 | 0.266 | 109 | 76 | 2 | 1 | 105 | 17 | 125 | Baseplate protein | Baseplate protein | | afdb-uniprot50 | AF-A0A652LXD1-F1-MODEL\_V4 | 1.0 | 5.68e-06 | 241 | 0.224 | 107 | 80 | 2 | 2 | 105 | 15 | 121 | Baseplate protein | Baseplate protein | | afdb-uniprot50 | AF-B9M7I0-F1-MODEL\_V4 | 1.0 | 1.649e-06 | 241 | 0.194 | 108 | 83 | 2 | 2 | 105 | 20 | 127 | Phage baseplate outer wedge protein (Acidic lysozyme), putative | Phage baseplate outer wedge protein (Acidic lysozyme), putative | | afdb-uniprot50 | AF-A0A2D3VZ43-F1-MODEL\_V4 | 1.0 | 1.866e-06 | 240 | 0.379 | 87 | 50 | 1 | 19 | 105 | 8 | 90 | Baseplate assembly protein | Baseplate assembly protein | | afdb-uniprot50 | AF-A0A7W7AUQ8-F1-MODEL\_V4 | 1.0 | 9.91e-06 | 240 | 0.252 | 91 | 65 | 3 | 17 | 105 | 7 | 96 | GPW\_gp25 domain-containing protein | GPW\_gp25 domain-containing protein | | afdb-uniprot50 | AF-A0A235IXZ5-F1-MODEL\_V4 | 1.0 | 4.436e-06 | 240 | 0.207 | 106 | 81 | 3 | 2 | 105 | 21 | 125 | GPW\_gp25 domain-containing protein | GPW\_gp25 domain-containing protein | | afdb-uniprot50 | AF-A0A2D5SX85-F1-MODEL\_V4 | 1.0 | 4.17e-06 | 240 | 0.179 | 106 | 85 | 1 | 3 | 106 | 29 | 134 | GPW\_gp25 domain-containing protein | GPW\_gp25 domain-containing protein | | afdb-uniprot50 | AF-A0A4R7CKT7-F1-MODEL\_V4 | 1.0 | 1.985e-06 | 240 | 0.203 | 108 | 83 | 2 | 2 | 106 | 24 | 131 | GPW\_gp25 domain-containing protein | GPW\_gp25 domain-containing protein | | afdb-uniprot50 | AF-A0A2E0HYC8-F1-MODEL\_V4 | 1.0 | 2.705e-06 | 240 | 0.174 | 109 | 86 | 3 | 3 | 107 | 31 | 139 | GPW\_gp25 domain-containing protein | GPW\_gp25 domain-containing protein | | afdb-uniprot50 | AF-A0A369UQV3-F1-MODEL\_V4 | 1.0 | 5.68e-06 | 239 | 0.216 | 106 | 80 | 3 | 2 | 105 | 9 | 113 | GPW\_gp25 domain-containing protein | GPW\_gp25 domain-containing protein | | afdb-uniprot50 | AF-A0A4Q3AGJ7-F1-MODEL\_V4 | 1.0 | 8.757e-06 | 239 | 0.2 | 110 | 81 | 3 | 1 | 105 | 7 | 114 | GPW\_gp25 domain-containing protein | GPW\_gp25 domain-containing protein | | afdb-uniprot50 | AF-A0A661I6D3-F1-MODEL\_V4 | 1.0 | 6.838e-06 | 239 | 0.269 | 104 | 69 | 2 | 1 | 97 | 8 | 111 | Baseplate protein | Baseplate protein | | afdb-uniprot50 | AF-A0A2S8QG71-F1-MODEL\_V4 | 1.0 | 1.985e-06 | 239 | 0.214 | 112 | 84 | 3 | 2 | 110 | 18 | 128 | Phage baseplate protein | Phage baseplate protein | | afdb-uniprot50 | AF-A0A7R7DSV2-F1-MODEL\_V4 | 1.0 | 8.886e-07 | 239 | 0.294 | 112 | 72 | 4 | 2 | 107 | 14 | 124 | GPW\_gp25 domain-containing protein | GPW\_gp25 domain-containing protein | | afdb-uniprot50 | AF-A0A1V5KBP1-F1-MODEL\_V4 | 1.0 | 2.39e-06 | 239 | 0.223 | 121 | 80 | 4 | 1 | 107 | 8 | 128 | Uncharacterized protein | Uncharacterized protein | | afdb-uniprot50 | AF-A0A4R8CYG7-F1-MODEL\_V4 | 1.0 | 4.17e-06 | 239 | 0.242 | 107 | 78 | 2 | 2 | 105 | 24 | 130 | GPW\_gp25 domain-containing protein | GPW\_gp25 domain-containing protein | | afdb-uniprot50 | AF-A0A1Z9KI13-F1-MODEL\_V4 | 1.0 | 6.043e-06 | 239 | 0.166 | 108 | 87 | 2 | 3 | 107 | 34 | 141 | GPW\_gp25 domain-containing protein | GPW\_gp25 domain-containing protein | | afdb-uniprot50 | AF-A0A136LBR9-F1-MODEL\_V4 | 1.0 | 2.112e-06 | 239 | 0.201 | 109 | 83 | 2 | 2 | 106 | 26 | 134 | Phage baseplate assembly protein W | Phage baseplate assembly protein W | | afdb-uniprot50 | AF-A0A4P2Q2Y6-F1-MODEL\_V4 | 1.0 | 7.739e-06 | 238 | 0.28 | 114 | 73 | 4 | 1 | 106 | 1 | 113 | GPW\_gp25 domain-containing protein | GPW\_gp25 domain-containing protein | | afdb-uniprot50 | AF-A0A1I2LH68-F1-MODEL\_V4 | 1.0 | 5.02e-06 | 238 | 0.216 | 120 | 82 | 5 | 1 | 109 | 1 | 119 | GPW\_gp25 domain-containing protein | GPW\_gp25 domain-containing protein | | afdb-uniprot50 | AF-A0A7Y5LM71-F1-MODEL\_V4 | 1.0 | 3.256e-06 | 238 | 0.302 | 109 | 69 | 5 | 2 | 105 | 14 | 120 | GPW/gp25 family protein | GPW/gp25 family protein | | afdb-uniprot50 | AF-A0A1X0D4T4-F1-MODEL\_V4 | 1.0 | 3.685e-06 | 238 | 0.241 | 112 | 81 | 4 | 2 | 111 | 20 | 129 | GPW\_gp25 domain-containing protein | GPW\_gp25 domain-containing protein | | afdb-uniprot50 | AF-W3ZZ38-F1-MODEL\_V4 | 1.0 | 1.07e-06 | 238 | 0.267 | 116 | 74 | 5 | 2 | 108 | 18 | 131 | GPW/gp25 family protein | GPW/gp25 family protein | | afdb-uniprot50 | AF-A0A1G5HAB2-F1-MODEL\_V4 | 1.0 | 1.457e-06 | 238 | 0.231 | 108 | 78 | 4 | 2 | 105 | 14 | 120 | GPW\_gp25 domain-containing protein | GPW\_gp25 domain-containing protein | | afdb-uniprot50 | AF-A0A2W6NE62-F1-MODEL\_V4 | 1.0 | 2.705e-06 | 237 | 0.301 | 93 | 59 | 2 | 12 | 104 | 4 | 90 | Baseplate assembly protein | Baseplate assembly protein | | afdb-uniprot50 | AF-A0A399IS01-F1-MODEL\_V4 | 1.0 | 3.061e-06 | 237 | 0.192 | 109 | 83 | 4 | 2 | 107 | 11 | 117 | DUF2634 domain-containing protein | DUF2634 domain-containing protein | | afdb-uniprot50 | AF-A0A2D8ESE3-F1-MODEL\_V4 | 1.0 | 6.428e-06 | 237 | 0.216 | 106 | 80 | 2 | 3 | 105 | 17 | 122 | GPW\_gp25 domain-containing protein | GPW\_gp25 domain-containing protein | | afdb-uniprot50 | AF-A0A1Q3WGE0-F1-MODEL\_V4 | 1.0 | 2.542e-06 | 237 | 0.212 | 108 | 81 | 3 | 2 | 105 | 16 | 123 | GPW\_gp25 domain-containing protein | GPW\_gp25 domain-containing protein | | afdb-uniprot50 | AF-A0A1H7PQT0-F1-MODEL\_V4 | 1.0 | 3.061e-06 | 237 | 0.194 | 108 | 83 | 2 | 2 | 105 | 16 | 123 | GPW\_gp25 domain-containing protein | GPW\_gp25 domain-containing protein | | afdb-uniprot50 | AF-A0A2L2XJP6-F1-MODEL\_V4 | 1.0 | 3.256e-06 | 236 | 0.212 | 108 | 80 | 4 | 2 | 106 | 16 | 121 | GPW\_gp25 domain-containing protein | GPW\_gp25 domain-containing protein | | afdb-uniprot50 | AF-C0BXW5-F1-MODEL\_V4 | 1.0 | 1.866e-06 | 236 | 0.268 | 108 | 74 | 4 | 2 | 105 | 15 | 121 | Putative lysozyme | Putative lysozyme | | afdb-uniprot50 | AF-A0A7W1PHH8-F1-MODEL\_V4 | 1.0 | 1.288e-06 | 236 | 0.219 | 114 | 80 | 4 | 2 | 107 | 15 | 127 | GPW/gp25 family protein | GPW/gp25 family protein | | afdb-uniprot50 | AF-A0A353G6I0-F1-MODEL\_V4 | 1.0 | 1.649e-06 | 236 | 0.25 | 120 | 77 | 4 | 1 | 111 | 16 | 131 | Baseplate | Baseplate | | afdb-uniprot50 | AF-A0A661CBH9-F1-MODEL\_V4 | 1.0 | 3.685e-06 | 236 | 0.225 | 111 | 81 | 2 | 4 | 109 | 27 | 137 | GPW\_gp25 domain-containing protein | GPW\_gp25 domain-containing protein | | afdb-uniprot50 | AF-A0A2E6C478-F1-MODEL\_V4 | 1.0 | 5.68e-06 | 236 | 0.242 | 107 | 78 | 2 | 3 | 106 | 27 | 133 | GPW\_gp25 domain-containing protein | GPW\_gp25 domain-containing protein | | afdb-uniprot50 | AF-A0A372IVD6-F1-MODEL\_V4 | 1.0 | 5.02e-06 | 236 | 0.323 | 99 | 64 | 2 | 2 | 97 | 14 | 112 | Baseplate protein | Baseplate protein | | afdb-uniprot50 | AF-A0A6L3Z3G6-F1-MODEL\_V4 | 1.0 | 2.247e-06 | 236 | 0.294 | 119 | 64 | 8 | 1 | 108 | 43 | 152 | GPW\_gp25 domain-containing protein | GPW\_gp25 domain-containing protein | | afdb-uniprot50 | AF-A0A2E8DY83-F1-MODEL\_V4 | 1.0 | 4.719e-06 | 236 | 0.18 | 105 | 84 | 2 | 3 | 105 | 40 | 144 | GPW\_gp25 domain-containing protein | GPW\_gp25 domain-containing protein | | afdb-uniprot50 | AF-A0A3S3QZP6-F1-MODEL\_V4 | 1.0 | 4.371e-05 | 235 | 0.27 | 85 | 62 | 0 | 1 | 85 | 3 | 87 | GPW\_gp25 domain-containing protein | GPW\_gp25 domain-containing protein | | afdb-uniprot50 | AF-A0A832Z8E4-F1-MODEL\_V4 | 1.0 | 4.17e-06 | 235 | 0.222 | 108 | 81 | 2 | 1 | 107 | 11 | 116 | Uncharacterized protein | Uncharacterized protein | | afdb-uniprot50 | AF-A0A7Y5NVN2-F1-MODEL\_V4 | 1.0 | 3.685e-06 | 235 | 0.191 | 120 | 87 | 5 | 1 | 111 | 10 | 128 | Uncharacterized protein | Uncharacterized protein | | afdb-uniprot50 | AF-A0A553JK30-F1-MODEL\_V4 | 1.0 | 2.705e-06 | 235 | 0.182 | 115 | 86 | 4 | 2 | 109 | 11 | 124 | GPW/gp25 family protein | GPW/gp25 family protein | | afdb-uniprot50 | AF-A0A6I2YZG1-F1-MODEL\_V4 | 1.0 | 8.232e-06 | 235 | 0.245 | 106 | 78 | 2 | 2 | 105 | 15 | 120 | Baseplate protein | Baseplate protein | | afdb-uniprot50 | AF-A0A7D7VPT3-F1-MODEL\_V4 | 1.0 | 3.464e-06 | 235 | 0.222 | 108 | 80 | 2 | 2 | 105 | 16 | 123 | GPW\_gp25 domain-containing protein | GPW\_gp25 domain-containing protein | | afdb-uniprot50 | AF-A0A661HHD5-F1-MODEL\_V4 | 1.0 | 1.985e-06 | 235 | 0.19 | 110 | 83 | 3 | 1 | 106 | 19 | 126 | GPW\_gp25 domain-containing protein | GPW\_gp25 domain-containing protein | | afdb-uniprot50 | AF-A0A832UUD0-F1-MODEL\_V4 | 1.0 | 5.68e-06 | 235 | 0.175 | 108 | 84 | 4 | 3 | 106 | 31 | 137 | Uncharacterized protein | Uncharacterized protein | | afdb-uniprot50 | AF-A0A2E9JHW6-F1-MODEL\_V4 | 1.0 | 5.02e-06 | 235 | 0.201 | 109 | 83 | 3 | 3 | 107 | 32 | 140 | GPW\_gp25 domain-containing protein | GPW\_gp25 domain-containing protein | | afdb-uniprot50 | AF-A0A4Y8PTA1-F1-MODEL\_V4 | 1.0 | 3.464e-06 | 235 | 0.33 | 106 | 67 | 2 | 4 | 105 | 32 | 137 | GPW\_gp25 domain-containing protein | GPW\_gp25 domain-containing protein | | afdb-uniprot50 | AF-Q7MB49-F1-MODEL\_V4 | 1.0 | 6.043e-06 | 235 | 0.214 | 98 | 75 | 1 | 10 | 105 | 126 | 223 | GPW\_gp25 domain-containing protein | GPW\_gp25 domain-containing protein | | afdb-uniprot50 | AF-M7XD14-F1-MODEL\_V4 | 1.0 | 8.757e-06 | 234 | 0.263 | 91 | 65 | 1 | 17 | 105 | 7 | 97 | GPW/gp25 family protein | GPW/gp25 family protein | | afdb-uniprot50 | AF-A0A419A4P0-F1-MODEL\_V4 | 1.0 | 2.247e-06 | 234 | 0.271 | 107 | 73 | 4 | 2 | 105 | 8 | 112 | GPW\_gp25 domain-containing protein | GPW\_gp25 domain-containing protein | | afdb-uniprot50 | AF-A0A1Y3QHP6-F1-MODEL\_V4 | 1.0 | 2.39e-06 | 234 | 0.207 | 106 | 80 | 3 | 3 | 105 | 2 | 106 | Uncharacterized protein | Uncharacterized protein | | afdb-uniprot50 | AF-A0A7T7L1C1-F1-MODEL\_V4 | 1.0 | 3.739e-07 | 234 | 0.263 | 110 | 77 | 2 | 2 | 107 | 15 | 124 | GPW/gp25 family protein | GPW/gp25 family protein | | afdb-uniprot50 | AF-A0A1J0VCH8-F1-MODEL\_V4 | 1.0 | 5.34e-06 | 234 | 0.228 | 114 | 78 | 4 | 1 | 105 | 8 | 120 | GPW\_gp25 domain-containing protein | GPW\_gp25 domain-containing protein | | afdb-uniprot50 | AF-A0A7Y9DPZ0-F1-MODEL\_V4 | 1.0 | 4.436e-06 | 234 | 0.24 | 108 | 77 | 4 | 2 | 106 | 22 | 127 | Uncharacterized protein | Uncharacterized protein | | afdb-uniprot50 | AF-A0A4V3QZC8-F1-MODEL\_V4 | 1.0 | 2.705e-06 | 234 | 0.256 | 109 | 77 | 3 | 1 | 106 | 23 | 130 | Baseplate assembly protein | Baseplate assembly protein | | afdb-uniprot50 | AF-A0A420YZC3-F1-MODEL\_V4 | 1.0 | 3.92e-06 | 234 | 0.16 | 112 | 86 | 3 | 2 | 105 | 11 | 122 | Uncharacterized protein | Uncharacterized protein | | afdb-uniprot50 | AF-A0A3C1ALA6-F1-MODEL\_V4 | 1.0 | 1.193e-05 | 233 | 0.363 | 77 | 47 | 1 | 31 | 107 | 1 | 75 | Phage baseplate protein | Phage baseplate protein | | afdb-uniprot50 | AF-A0A7C3GT18-F1-MODEL\_V4 | 1.0 | 6.838e-06 | 233 | 0.261 | 107 | 76 | 3 | 2 | 106 | 7 | 112 | GPW\_gp25 domain-containing protein | GPW\_gp25 domain-containing protein | | afdb-uniprot50 | AF-A0A3S4U5L3-F1-MODEL\_V4 | 1.0 | 4.501e-07 | 233 | 0.3 | 113 | 70 | 2 | 1 | 107 | 8 | 117 | Baseplate assembly protein W | Baseplate assembly protein W | | afdb-uniprot50 | AF-A0A1Z9B2C4-F1-MODEL\_V4 | 1.0 | 6.043e-06 | 233 | 0.188 | 101 | 81 | 1 | 5 | 105 | 12 | 111 | Uncharacterized protein | Uncharacterized protein | | afdb-uniprot50 | AF-A0A1B1G1R7-F1-MODEL\_V4 | 1.0 | 9.316e-06 | 233 | 0.162 | 111 | 88 | 2 | 2 | 107 | 11 | 121 | GPW/gp25 family protein | GPW/gp25 family protein | | afdb-uniprot50 | AF-A0A2S3UMZ3-F1-MODEL\_V4 | 1.0 | 1.193e-05 | 233 | 0.201 | 114 | 83 | 4 | 1 | 107 | 12 | 124 | Phage baseplate assembly protein W | Phage baseplate assembly protein W | | afdb-uniprot50 | AF-A0A2E5YV38-F1-MODEL\_V4 | 1.0 | 4.719e-06 | 233 | 0.205 | 112 | 79 | 4 | 3 | 107 | 18 | 126 | GPW\_gp25 domain-containing protein | GPW\_gp25 domain-containing protein | | afdb-uniprot50 | AF-A0A7Y6PTF8-F1-MODEL\_V4 | 1.0 | 8.886e-07 | 233 | 0.266 | 109 | 73 | 3 | 2 | 105 | 16 | 122 | GPW/gp25 family protein | GPW/gp25 family protein | | afdb-uniprot50 | AF-A0A383EU63-F1-MODEL\_V4 | 1.0 | 8.757e-06 | 233 | 0.175 | 108 | 86 | 2 | 3 | 107 | 23 | 130 | GPW\_gp25 domain-containing protein | GPW\_gp25 domain-containing protein | | afdb-uniprot50 | AF-A0A7X5H4C3-F1-MODEL\_V4 | 1.0 | 2.877e-06 | 233 | 0.186 | 107 | 84 | 2 | 2 | 105 | 18 | 124 | Phage baseplate protein | Phage baseplate protein | | afdb-uniprot50 | AF-A0A562GHU8-F1-MODEL\_V4 | 1.0 | 2.877e-06 | 233 | 0.252 | 107 | 74 | 5 | 2 | 105 | 27 | 130 | Phage baseplate assembly protein W | Phage baseplate assembly protein W | | afdb-uniprot50 | AF-A0A2D8I5K2-F1-MODEL\_V4 | 1.0 | 1.269e-05 | 233 | 0.173 | 115 | 87 | 2 | 1 | 107 | 24 | 138 | GPW\_gp25 domain-containing protein | GPW\_gp25 domain-containing protein | | afdb-uniprot50 | AF-A0A4P5VFE5-F1-MODEL\_V4 | 1.0 | 1.288e-06 | 233 | 0.245 | 114 | 79 | 3 | 1 | 107 | 34 | 147 | GPW\_gp25 domain-containing protein | GPW\_gp25 domain-containing protein | | afdb-uniprot50 | AF-A0A833DBQ1-F1-MODEL\_V4 | 1.0 | 5.68e-06 | 232 | 0.181 | 116 | 83 | 4 | 3 | 108 | 33 | 146 | Uncharacterized protein | Uncharacterized protein | | afdb-uniprot50 | AF-A0A2D7BZP7-F1-MODEL\_V4 | 1.0 | 3.685e-06 | 232 | 0.148 | 108 | 87 | 3 | 3 | 105 | 36 | 143 | GPW\_gp25 domain-containing protein | GPW\_gp25 domain-containing protein | | afdb-uniprot50 | AF-A0A2T6NFS1-F1-MODEL\_V4 | 1.0 | 9.316e-06 | 232 | 0.252 | 111 | 76 | 3 | 2 | 105 | 63 | 173 | GPW\_gp25 domain-containing protein | GPW\_gp25 domain-containing protein | | afdb-uniprot50 | AF-A0A7L6A4Z7-F1-MODEL\_V4 | 1.0 | 3.256e-06 | 232 | 0.198 | 106 | 83 | 1 | 2 | 105 | 20 | 125 | GPW/gp25 family protein | GPW/gp25 family protein | | afdb-uniprot50 | AF-A0A4R4TT05-F1-MODEL\_V4 | 1.0 | 7.275e-06 | 231 | 0.216 | 111 | 84 | 3 | 2 | 110 | 18 | 127 | GPW\_gp25 domain-containing protein | GPW\_gp25 domain-containing protein | | afdb-uniprot50 | AF-A0A7Y4XEU9-F1-MODEL\_V4 | 1.0 | 1.866e-06 | 231 | 0.22 | 118 | 75 | 4 | 5 | 107 | 14 | 129 | GPW\_gp25 domain-containing protein | GPW\_gp25 domain-containing protein | | afdb-uniprot50 | AF-A0A1Y4IQ66-F1-MODEL\_V4 | 1.0 | 6.428e-06 | 231 | 0.247 | 109 | 76 | 4 | 2 | 105 | 22 | 129 | GPW\_gp25 domain-containing protein | GPW\_gp25 domain-containing protein | | afdb-uniprot50 | AF-A6FZ69-F1-MODEL\_V4 | 1.0 | 5.34e-06 | 231 | 0.203 | 108 | 82 | 2 | 2 | 105 | 24 | 131 | GPW/gp25 | GPW/gp25 | | afdb-uniprot50 | AF-A0A1R3WBS2-F1-MODEL\_V4 | 1.0 | 1.866e-06 | 231 | 0.177 | 107 | 84 | 2 | 3 | 105 | 23 | 129 | GPW\_gp25 domain-containing protein | GPW\_gp25 domain-containing protein | | afdb-uniprot50 | AF-A0A3A8PSW3-F1-MODEL\_V4 | 1.0 | 9.316e-06 | 231 | 0.214 | 107 | 81 | 2 | 2 | 105 | 26 | 132 | GPW\_gp25 domain-containing protein | GPW\_gp25 domain-containing protein | | afdb-uniprot50 | AF-A0A3N7GF44-F1-MODEL\_V4 | 1.0 | 1.625e-05 | 231 | 0.226 | 119 | 81 | 5 | 1 | 110 | 47 | 163 | GPW\_gp25 domain-containing protein | GPW\_gp25 domain-containing protein | | afdb-uniprot50 | AF-A0A825MSY2-F1-MODEL\_V4 | 1.0 | 5.34e-06 | 230 | 0.272 | 99 | 63 | 3 | 17 | 107 | 21 | 118 | GPW/gp25 family protein | GPW/gp25 family protein | | afdb-uniprot50 | AF-A0A2D7G6P2-F1-MODEL\_V4 | 1.0 | 1.35e-05 | 230 | 0.181 | 110 | 87 | 2 | 5 | 112 | 25 | 133 | GPW\_gp25 domain-containing protein | GPW\_gp25 domain-containing protein | | afdb-uniprot50 | AF-A0A4P7CYP9-F1-MODEL\_V4 | 1.0 | 9.316e-06 | 230 | 0.261 | 107 | 74 | 4 | 2 | 105 | 27 | 131 | GPW\_gp25 domain-containing protein | GPW\_gp25 domain-containing protein | | afdb-uniprot50 | AF-A0A848G1D1-F1-MODEL\_V4 | 1.0 | 6.428e-06 | 230 | 0.16 | 106 | 87 | 1 | 2 | 105 | 16 | 121 | GPW/gp25 family protein | GPW/gp25 family protein | | afdb-uniprot50 | AF-A0A2D8EMQ8-F1-MODEL\_V4 | 1.0 | 2.705e-06 | 230 | 0.166 | 120 | 87 | 4 | 1 | 107 | 28 | 147 | GPW\_gp25 domain-containing protein | GPW\_gp25 domain-containing protein | | afdb-uniprot50 | AF-A0A6I6N486-F1-MODEL\_V4 | 1.0 | 6.043e-06 | 230 | 0.28 | 107 | 72 | 4 | 2 | 105 | 43 | 147 | GPW\_gp25 domain-containing protein | GPW\_gp25 domain-containing protein | | afdb-uniprot50 | AF-A0A2E6W7H5-F1-MODEL\_V4 | 1.0 | 7.275e-06 | 229 | 0.194 | 113 | 81 | 3 | 7 | 110 | 2 | 113 | GPW\_gp25 domain-containing protein | GPW\_gp25 domain-containing protein | | afdb-uniprot50 | AF-A4YW46-F1-MODEL\_V4 | 1.0 | 1.121e-05 | 229 | 0.214 | 107 | 81 | 3 | 2 | 106 | 8 | 113 | GPW\_gp25 domain-containing protein | GPW\_gp25 domain-containing protein | | afdb-uniprot50 | AF-A0A7X5DY26-F1-MODEL\_V4 | 1.0 | 3.061e-06 | 229 | 0.222 | 108 | 79 | 4 | 2 | 105 | 8 | 114 | Baseplate protein | Baseplate protein | | afdb-uniprot50 | AF-A0A7W3LYA6-F1-MODEL\_V4 | 1.0 | 3.92e-06 | 229 | 0.213 | 117 | 80 | 6 | 2 | 108 | 7 | 121 | GPW\_gp25 domain-containing protein | GPW\_gp25 domain-containing protein | | afdb-uniprot50 | AF-A0A535AQ84-F1-MODEL\_V4 | 1.0 | 5.68e-06 | 229 | 0.3 | 100 | 65 | 3 | 2 | 97 | 4 | 102 | GPW/gp25 family protein | GPW/gp25 family protein | | afdb-uniprot50 | AF-A0A524M6P3-F1-MODEL\_V4 | 1.0 | 7.275e-06 | 229 | 0.203 | 113 | 83 | 4 | 1 | 107 | 9 | 120 | Uncharacterized protein | Uncharacterized protein | | afdb-uniprot50 | AF-N1ZFS0-F1-MODEL\_V4 | 1.0 | 1.138e-06 | 229 | 0.218 | 110 | 78 | 3 | 2 | 105 | 11 | 118 | GPW\_gp25 domain-containing protein | GPW\_gp25 domain-containing protein | | afdb-uniprot50 | AF-A0A2N8ML17-F1-MODEL\_V4 | 1.0 | 1.138e-06 | 229 | 0.232 | 125 | 82 | 4 | 1 | 111 | 1 | 125 | GPW\_gp25 domain-containing protein | GPW\_gp25 domain-containing protein | | afdb-uniprot50 | AF-A0A0Q9UE51-F1-MODEL\_V4 | 1.0 | 1.457e-06 | 229 | 0.228 | 114 | 79 | 4 | 3 | 108 | 14 | 126 | GPW\_gp25 domain-containing protein | GPW\_gp25 domain-containing protein | | afdb-uniprot50 | AF-A0A2V7RA86-F1-MODEL\_V4 | 1.0 | 3.256e-06 | 229 | 0.266 | 109 | 75 | 4 | 1 | 105 | 16 | 123 | Baseplate assembly protein | Baseplate assembly protein | | afdb-uniprot50 | AF-A0A2D8UQ71-F1-MODEL\_V4 | 1.0 | 8.757e-06 | 229 | 0.24 | 108 | 79 | 2 | 2 | 106 | 24 | 131 | GPW\_gp25 domain-containing protein | GPW\_gp25 domain-containing protein | | afdb-uniprot50 | AF-A0A1H5RLH3-F1-MODEL\_V4 | 1.0 | 4.17e-06 | 229 | 0.185 | 108 | 83 | 4 | 2 | 105 | 16 | 122 | GPW\_gp25 domain-containing protein | GPW\_gp25 domain-containing protein | | afdb-uniprot50 | AF-A0A1G8B3G6-F1-MODEL\_V4 | 1.0 | 3.256e-06 | 229 | 0.2 | 110 | 81 | 6 | 1 | 107 | 97 | 202 | Phage baseplate assembly protein W | Phage baseplate assembly protein W | | afdb-uniprot50 | AF-A0A7H4NMM7-F1-MODEL\_V4 | 1.0 | 4.109e-05 | 228 | 0.478 | 69 | 36 | 0 | 3 | 71 | 6 | 74 | Baseplate assembly protein W | Baseplate assembly protein W | | afdb-uniprot50 | AF-A0A1M6UMM3-F1-MODEL\_V4 | 1.0 | 2.081e-05 | 228 | 0.211 | 90 | 70 | 1 | 17 | 105 | 7 | 96 | GPW\_gp25 domain-containing protein | GPW\_gp25 domain-containing protein | | afdb-uniprot50 | AF-A0A4Q7FYR9-F1-MODEL\_V4 | 1.0 | 1.649e-06 | 228 | 0.252 | 119 | 67 | 3 | 12 | 108 | 2 | 120 | Baseplate assembly protein | Baseplate assembly protein | | afdb-uniprot50 | AF-A0A497N9T7-F1-MODEL\_V4 | 1.0 | 1.193e-05 | 228 | 0.225 | 111 | 82 | 4 | 3 | 110 | 18 | 127 | Uncharacterized protein | Uncharacterized protein | | afdb-uniprot50 | AF-A0A2E2ZB32-F1-MODEL\_V4 | 1.0 | 1.436e-05 | 228 | 0.155 | 109 | 86 | 3 | 1 | 105 | 29 | 135 | GPW\_gp25 domain-containing protein | GPW\_gp25 domain-containing protein | | afdb-uniprot50 | AF-A0A7J5DNJ6-F1-MODEL\_V4 | 1.0 | 2.081e-05 | 227 | 0.282 | 85 | 59 | 2 | 22 | 105 | 2 | 85 | GPW/gp25 family protein | GPW/gp25 family protein | | afdb-uniprot50 | AF-A0A1I3ATS5-F1-MODEL\_V4 | 1.0 | 1.625e-05 | 227 | 0.254 | 114 | 75 | 5 | 1 | 105 | 1 | 113 | GPW\_gp25 domain-containing protein | GPW\_gp25 domain-containing protein | | afdb-uniprot50 | AF-A0A094QGT3-F1-MODEL\_V4 | 1.0 | 1.121e-05 | 227 | 0.275 | 98 | 69 | 2 | 2 | 97 | 14 | 111 | GPW/gp25 family protein | GPW/gp25 family protein | | afdb-uniprot50 | AF-A0A3A9G8M7-F1-MODEL\_V4 | 1.0 | 1.457e-06 | 227 | 0.247 | 109 | 75 | 3 | 2 | 105 | 15 | 121 | Baseplate protein | Baseplate protein | | afdb-uniprot50 | AF-A0A7X5D8Q1-F1-MODEL\_V4 | 1.0 | 1.649e-06 | 227 | 0.211 | 109 | 80 | 3 | 2 | 105 | 44 | 151 | GPW\_gp25 domain-containing protein | GPW\_gp25 domain-containing protein | | afdb-uniprot50 | AF-A0A5D4S1J9-F1-MODEL\_V4 | 1.0 | 3.92e-06 | 226 | 0.163 | 116 | 83 | 5 | 2 | 107 | 3 | 114 | DUF2634 domain-containing protein | DUF2634 domain-containing protein | | afdb-uniprot50 | AF-A0A1Q4ZX73-F1-MODEL\_V4 | 1.0 | 7.739e-06 | 226 | 0.242 | 107 | 76 | 4 | 2 | 105 | 9 | 113 | GPW\_gp25 domain-containing protein | GPW\_gp25 domain-containing protein | | afdb-uniprot50 | AF-A0A1V5V4U5-F1-MODEL\_V4 | 1.0 | 2.112e-06 | 226 | 0.23 | 117 | 79 | 4 | 2 | 108 | 12 | 127 | Gene 25-like lysozyme | Gene 25-like lysozyme | | afdb-uniprot50 | AF-A0A0F5JZG5-F1-MODEL\_V4 | 1.0 | 5.02e-06 | 226 | 0.203 | 108 | 83 | 2 | 2 | 106 | 19 | 126 | GPW\_gp25 domain-containing protein | GPW\_gp25 domain-containing protein | | afdb-uniprot50 | AF-A0A3R7S1T8-F1-MODEL\_V4 | 1.0 | 5.68e-06 | 226 | 0.203 | 108 | 82 | 3 | 3 | 107 | 20 | 126 | GPW\_gp25 domain-containing protein | GPW\_gp25 domain-containing protein | | afdb-uniprot50 | AF-A0A1Z9QID3-F1-MODEL\_V4 | 1.0 | 1.054e-05 | 225 | 0.18 | 105 | 82 | 3 | 5 | 107 | 12 | 114 | Uncharacterized protein | Uncharacterized protein | | afdb-uniprot50 | AF-A0A3B1EJD1-F1-MODEL\_V4 | 1.0 | 8.757e-06 | 225 | 0.263 | 110 | 76 | 4 | 2 | 107 | 12 | 120 | Mlr6561 protein | Mlr6561 protein | | afdb-uniprot50 | AF-A0A7J4UPF6-F1-MODEL\_V4 | 1.0 | 8.232e-06 | 225 | 0.169 | 106 | 85 | 2 | 3 | 105 | 14 | 119 | Uncharacterized protein | Uncharacterized protein | | afdb-uniprot50 | AF-A0A328FDP2-F1-MODEL\_V4 | 1.0 | 1.121e-05 | 225 | 0.252 | 107 | 78 | 1 | 1 | 105 | 23 | 129 | Baseplate assembly protein | Baseplate assembly protein | | afdb-uniprot50 | AF-A0A2S8QIP7-F1-MODEL\_V4 | 1.0 | 4.436e-06 | 225 | 0.214 | 107 | 81 | 2 | 2 | 105 | 18 | 124 | Phage baseplate protein | Phage baseplate protein | | afdb-uniprot50 | AF-A0A1Y3WKR7-F1-MODEL\_V4 | 1.0 | 6.428e-06 | 225 | 0.247 | 109 | 76 | 4 | 2 | 105 | 17 | 124 | GPW\_gp25 domain-containing protein | GPW\_gp25 domain-containing protein | | afdb-uniprot50 | AF-A0A1Y4HZE3-F1-MODEL\_V4 | 1.0 | 6.838e-06 | 225 | 0.222 | 108 | 79 | 4 | 2 | 105 | 38 | 144 | GPW\_gp25 domain-containing protein | GPW\_gp25 domain-containing protein | | afdb-uniprot50 | AF-F2K224-F1-MODEL\_V4 | 1.0 | 8.757e-06 | 224 | 0.247 | 109 | 76 | 3 | 3 | 108 | 2 | 107 | Uncharacterized protein | Uncharacterized protein | | afdb-uniprot50 | AF-A0A158EG26-F1-MODEL\_V4 | 1.0 | 1.729e-05 | 224 | 0.209 | 105 | 81 | 2 | 2 | 105 | 8 | 111 | Gene 25-like lysozyme | Gene 25-like lysozyme | | afdb-uniprot50 | AF-A0A4T0V9V5-F1-MODEL\_V4 | 1.0 | 5.68e-06 | 224 | 0.233 | 107 | 77 | 4 | 2 | 105 | 10 | 114 | GPW\_gp25 domain-containing protein | GPW\_gp25 domain-containing protein | | afdb-uniprot50 | AF-A0A2E6W7U8-F1-MODEL\_V4 | 1.0 | 2.355e-05 | 224 | 0.155 | 109 | 86 | 3 | 1 | 105 | 6 | 112 | GPW\_gp25 domain-containing protein | GPW\_gp25 domain-containing protein | | afdb-uniprot50 | AF-A0A2R4SX79-F1-MODEL\_V4 | 1.0 | 1.193e-05 | 224 | 0.235 | 106 | 78 | 3 | 2 | 105 | 15 | 119 | GPW\_gp25 domain-containing protein | GPW\_gp25 domain-containing protein | | afdb-uniprot50 | AF-A0A2D6E1F5-F1-MODEL\_V4 | 1.0 | 6.428e-06 | 224 | 0.212 | 113 | 82 | 2 | 2 | 107 | 10 | 122 | GPW\_gp25 domain-containing protein | GPW\_gp25 domain-containing protein | | afdb-uniprot50 | AF-A0A2D8ESJ7-F1-MODEL\_V4 | 1.0 | 1.436e-05 | 224 | 0.224 | 107 | 80 | 2 | 3 | 106 | 27 | 133 | GPW\_gp25 domain-containing protein | GPW\_gp25 domain-containing protein | | afdb-uniprot50 | AF-A0A2E8NRW1-F1-MODEL\_V4 | 1.0 | 1.121e-05 | 224 | 0.192 | 109 | 82 | 3 | 1 | 105 | 11 | 117 | GPW\_gp25 domain-containing protein | GPW\_gp25 domain-containing protein | | afdb-uniprot50 | AF-A0A1Q4RQU7-F1-MODEL\_V4 | 1.0 | 3.515e-07 | 224 | 0.342 | 108 | 63 | 2 | 2 | 108 | 21 | 121 | Baseplate protein | Baseplate protein | | afdb-uniprot50 | AF-A0A376ZFR1-F1-MODEL\_V4 | 1.0 | 1.839e-05 | 223 | 0.366 | 71 | 45 | 0 | 35 | 105 | 1 | 71 | Baseplate assembly protein W | Baseplate assembly protein W | | afdb-uniprot50 | AF-A0A1M7S3A9-F1-MODEL\_V4 | 1.0 | 1.625e-05 | 223 | 0.245 | 106 | 77 | 3 | 2 | 105 | 8 | 112 | GPW\_gp25 domain-containing protein | GPW\_gp25 domain-containing protein | | afdb-uniprot50 | AF-A0A2R6EXZ2-F1-MODEL\_V4 | 1.0 | 1.193e-05 | 223 | 0.33 | 100 | 62 | 3 | 2 | 97 | 14 | 112 | Baseplate protein | Baseplate protein | | afdb-uniprot50 | AF-A0A1M7TQV3-F1-MODEL\_V4 | 1.0 | 3.256e-06 | 223 | 0.234 | 115 | 77 | 4 | 1 | 105 | 11 | 124 | GPW\_gp25 domain-containing protein | GPW\_gp25 domain-containing protein | | afdb-uniprot50 | AF-A0A6B2YTY6-F1-MODEL\_V4 | 1.0 | 1.528e-05 | 223 | 0.273 | 106 | 68 | 3 | 1 | 97 | 8 | 113 | GPW/gp25 family protein | GPW/gp25 family protein | | afdb-uniprot50 | AF-A0A212K6S4-F1-MODEL\_V4 | 1.0 | 2.705e-06 | 223 | 0.205 | 117 | 79 | 5 | 1 | 105 | 13 | 127 | Uncharacterized protein | Uncharacterized protein | | afdb-uniprot50 | AF-A0A437M149-F1-MODEL\_V4 | 1.0 | 1.729e-05 | 223 | 0.173 | 121 | 85 | 5 | 1 | 111 | 94 | 209 | Uncharacterized protein | Uncharacterized protein | | afdb-uniprot50 | AF-A0A521S4V9-F1-MODEL\_V4 | 1.0 | 1.436e-05 | 223 | 0.245 | 106 | 77 | 3 | 2 | 105 | 188 | 292 | GPW\_gp25 domain-containing protein | GPW\_gp25 domain-containing protein | | afdb-uniprot50 | AF-A0A511N0N2-F1-MODEL\_V4 | 1.0 | 6.428e-06 | 222 | 0.223 | 112 | 81 | 2 | 2 | 107 | 14 | 125 | GPW\_gp25 domain-containing protein | GPW\_gp25 domain-containing protein | | afdb-uniprot50 | AF-A0A7Z9QJ22-F1-MODEL\_V4 | 1.0 | 1.436e-05 | 222 | 0.169 | 106 | 85 | 2 | 3 | 105 | 17 | 122 | GPW\_gp25 domain-containing protein | GPW\_gp25 domain-containing protein | | afdb-uniprot50 | AF-A0A2D8C9G0-F1-MODEL\_V4 | 1.0 | 1.839e-05 | 222 | 0.165 | 109 | 86 | 3 | 3 | 107 | 20 | 127 | GPW\_gp25 domain-containing protein | GPW\_gp25 domain-containing protein | | afdb-uniprot50 | AF-A0A7Y6UVS1-F1-MODEL\_V4 | 1.0 | 1.625e-05 | 222 | 0.306 | 98 | 66 | 1 | 2 | 97 | 20 | 117 | GPW/gp25 family protein | GPW/gp25 family protein | | afdb-uniprot50 | AF-A0A5S9NBS3-F1-MODEL\_V4 | 1.0 | 1.054e-05 | 222 | 0.227 | 110 | 79 | 3 | 1 | 105 | 13 | 121 | Uncharacterized protein | Uncharacterized protein | | afdb-uniprot50 | AF-A0A1Q4P0G2-F1-MODEL\_V4 | 1.0 | 9.316e-06 | 222 | 0.188 | 106 | 84 | 1 | 2 | 105 | 18 | 123 | GPW\_gp25 domain-containing protein | GPW\_gp25 domain-containing protein | | afdb-uniprot50 | AF-A0A4R0P0Z5-F1-MODEL\_V4 | 1.0 | 8.757e-06 | 222 | 0.189 | 111 | 85 | 2 | 1 | 106 | 17 | 127 | GPW\_gp25 domain-containing protein | GPW\_gp25 domain-containing protein | | afdb-uniprot50 | AF-A0A1W9Z4R0-F1-MODEL\_V4 | 1.0 | 3.016e-05 | 221 | 0.277 | 90 | 63 | 2 | 17 | 105 | 17 | 105 | GPW\_gp25 domain-containing protein | GPW\_gp25 domain-containing protein | | afdb-uniprot50 | AF-A0A525HH40-F1-MODEL\_V4 | 1.0 | 1.35e-05 | 221 | 0.259 | 108 | 75 | 4 | 2 | 106 | 9 | 114 | GPW\_gp25 domain-containing protein | GPW\_gp25 domain-containing protein | | afdb-uniprot50 | AF-A0A6J4Y8V7-F1-MODEL\_V4 | 1.0 | 1.436e-05 | 221 | 0.229 | 109 | 80 | 4 | 2 | 107 | 10 | 117 | GPW\_gp25 domain-containing protein | GPW\_gp25 domain-containing protein | | afdb-uniprot50 | AF-A0A2D5MRJ6-F1-MODEL\_V4 | 1.0 | 2.836e-05 | 221 | 0.166 | 108 | 85 | 3 | 1 | 105 | 27 | 132 | GPW\_gp25 domain-containing protein | GPW\_gp25 domain-containing protein | | afdb-uniprot50 | AF-A0A3C1NZC1-F1-MODEL\_V4 | 1.0 | 2.665e-05 | 221 | 0.219 | 105 | 74 | 3 | 1 | 97 | 8 | 112 | Baseplate protein | Baseplate protein | | afdb-uniprot50 | AF-A0A846DAZ9-F1-MODEL\_V4 | 1.0 | 1.957e-05 | 220 | 0.302 | 96 | 66 | 1 | 2 | 96 | 14 | 109 | GPW/gp25 family protein | GPW/gp25 family protein | | afdb-uniprot50 | AF-A0A800JQS2-F1-MODEL\_V4 | 1.0 | 4.719e-06 | 220 | 0.214 | 107 | 81 | 1 | 2 | 105 | 10 | 116 | GPW\_gp25 domain-containing protein | GPW\_gp25 domain-containing protein | | afdb-uniprot50 | AF-A0A2A5GV64-F1-MODEL\_V4 | 1.0 | 2.214e-05 | 220 | 0.168 | 113 | 90 | 3 | 3 | 112 | 14 | 125 | GPW\_gp25 domain-containing protein | GPW\_gp25 domain-containing protein | | afdb-uniprot50 | AF-A0A1V5QG84-F1-MODEL\_V4 | 1.0 | 8.232e-06 | 220 | 0.257 | 105 | 66 | 3 | 2 | 97 | 15 | 116 | Gene 25-like lysozyme | Gene 25-like lysozyme | | afdb-uniprot50 | AF-F6DTE8-F1-MODEL\_V4 | 1.0 | 4.719e-06 | 220 | 0.284 | 109 | 73 | 4 | 1 | 106 | 30 | 136 | GPW/gp25 family protein | GPW/gp25 family protein | | afdb-uniprot50 | AF-A0A1J5BI56-F1-MODEL\_V4 | 1.0 | 2.836e-05 | 219 | 0.268 | 93 | 63 | 3 | 16 | 105 | 9 | 99 | GPW\_gp25 domain-containing protein | GPW\_gp25 domain-containing protein | | afdb-uniprot50 | AF-A0A382YZB8-F1-MODEL\_V4 | 1.0 | 1.121e-05 | 219 | 0.186 | 107 | 83 | 3 | 3 | 105 | 4 | 110 | GPW\_gp25 domain-containing protein | GPW\_gp25 domain-containing protein | | afdb-uniprot50 | AF-A0A1I6DPB9-F1-MODEL\_V4 | 1.0 | 6.428e-06 | 219 | 0.212 | 113 | 82 | 4 | 2 | 108 | 12 | 123 | GPW\_gp25 domain-containing protein | GPW\_gp25 domain-containing protein | | afdb-uniprot50 | AF-A0A2D6MIZ8-F1-MODEL\_V4 | 1.0 | 8.757e-06 | 219 | 0.188 | 106 | 83 | 3 | 3 | 105 | 14 | 119 | GPW\_gp25 domain-containing protein | GPW\_gp25 domain-containing protein | | afdb-uniprot50 | AF-A0A2K3J5A4-F1-MODEL\_V4 | 1.0 | 2.214e-05 | 219 | 0.205 | 107 | 80 | 3 | 4 | 106 | 20 | 125 | GPW\_gp25 domain-containing protein | GPW\_gp25 domain-containing protein | | afdb-uniprot50 | AF-U1KRE2-F1-MODEL\_V4 | 1.0 | 2.112e-06 | 219 | 0.182 | 115 | 82 | 2 | 3 | 105 | 8 | 122 | GPW/gp25 family protein | GPW/gp25 family protein | | afdb-uniprot50 | AF-A0A2D4ZE95-F1-MODEL\_V4 | 1.0 | 1.957e-05 | 219 | 0.168 | 107 | 86 | 2 | 3 | 106 | 27 | 133 | GPW\_gp25 domain-containing protein | GPW\_gp25 domain-containing protein | | afdb-uniprot50 | AF-A0A5F1HX77-F1-MODEL\_V4 | 1.0 | 1.121e-05 | 219 | 0.185 | 108 | 85 | 2 | 2 | 106 | 17 | 124 | GPW/gp25 family protein | GPW/gp25 family protein | | afdb-uniprot50 | AF-A0A3R9V7X8-F1-MODEL\_V4 | 1.0 | 1.193e-05 | 219 | 0.31 | 100 | 64 | 3 | 2 | 97 | 32 | 130 | Baseplate protein | Baseplate protein | | afdb-uniprot50 | AF-A0A661PMX4-F1-MODEL\_V4 | 1.0 | 1.729e-05 | 218 | 0.189 | 116 | 87 | 4 | 1 | 112 | 5 | 117 | GPW\_gp25 domain-containing protein | GPW\_gp25 domain-containing protein | | afdb-uniprot50 | AF-A0A1V5BCJ8-F1-MODEL\_V4 | 1.0 | 2.081e-05 | 218 | 0.221 | 113 | 84 | 4 | 2 | 112 | 8 | 118 | Gene 25-like lysozyme | Gene 25-like lysozyme | | afdb-uniprot50 | AF-B0T4J0-F1-MODEL\_V4 | 1.0 | 1.436e-05 | 218 | 0.176 | 113 | 87 | 4 | 2 | 109 | 9 | 120 | GPW\_gp25 domain-containing protein | GPW\_gp25 domain-containing protein | | afdb-uniprot50 | AF-A0A117MMK7-F1-MODEL\_V4 | 1.0 | 2.355e-05 | 218 | 0.245 | 106 | 78 | 2 | 2 | 107 | 12 | 115 | GPW\_gp25 domain-containing protein | GPW\_gp25 domain-containing protein | | afdb-uniprot50 | AF-A0A0K9NA34-F1-MODEL\_V4 | 1.0 | 1.006e-06 | 218 | 0.275 | 109 | 72 | 3 | 2 | 105 | 15 | 121 | Putative tail lysozyme | Putative tail lysozyme | | afdb-uniprot50 | AF-A0A550H8T3-F1-MODEL\_V4 | 1.0 | 6.838e-06 | 218 | 0.206 | 116 | 80 | 4 | 4 | 107 | 16 | 131 | GPW\_gp25 domain-containing protein | GPW\_gp25 domain-containing protein | | afdb-uniprot50 | AF-A0A7W7CIY7-F1-MODEL\_V4 | 1.0 | 1.436e-05 | 218 | 0.261 | 107 | 74 | 4 | 2 | 105 | 28 | 132 | Phage baseplate assembly protein W | Phage baseplate assembly protein W | | afdb-uniprot50 | AF-A0A1Z8S7X6-F1-MODEL\_V4 | 1.0 | 6.838e-06 | 218 | 0.212 | 108 | 80 | 3 | 1 | 105 | 27 | 132 | GPW\_gp25 domain-containing protein | GPW\_gp25 domain-containing protein | | afdb-uniprot50 | AF-A0A4R1YZ54-F1-MODEL\_V4 | 1.0 | 1.121e-05 | 218 | 0.252 | 103 | 67 | 3 | 2 | 97 | 17 | 116 | GPW\_gp25 domain-containing protein | GPW\_gp25 domain-containing protein | | afdb-uniprot50 | AF-A0A1V5ZPA0-F1-MODEL\_V4 | 1.0 | 1.35e-05 | 218 | 0.214 | 112 | 80 | 4 | 1 | 106 | 20 | 129 | Gene 25-like lysozyme | Gene 25-like lysozyme | | afdb-uniprot50 | AF-A0A4D7BC41-F1-MODEL\_V4 | 1.0 | 2.355e-05 | 218 | 0.215 | 116 | 84 | 3 | 1 | 111 | 14 | 127 | Uncharacterized protein | Uncharacterized protein | | afdb-uniprot50 | AF-A0A381SJG5-F1-MODEL\_V4 | 1.0 | 1.729e-05 | 218 | 0.119 | 109 | 92 | 3 | 3 | 107 | 41 | 149 | GPW\_gp25 domain-containing protein | GPW\_gp25 domain-containing protein | | afdb-uniprot50 | AF-A0A3S0EL96-F1-MODEL\_V4 | 1.0 | 1.957e-05 | 218 | 0.245 | 118 | 79 | 5 | 1 | 110 | 68 | 183 | GPW\_gp25 domain-containing protein | GPW\_gp25 domain-containing protein | | afdb-uniprot50 | AF-A0A5M6ITN1-F1-MODEL\_V4 | 1.0 | 6.428e-06 | 218 | 0.272 | 110 | 75 | 5 | 1 | 108 | 104 | 210 | Uncharacterized protein | Uncharacterized protein | | afdb-uniprot50 | AF-A0A4Q3KH27-F1-MODEL\_V4 | 1.0 | 2.506e-05 | 217 | 0.189 | 116 | 83 | 4 | 1 | 107 | 6 | 119 | GPW\_gp25 domain-containing protein | GPW\_gp25 domain-containing protein | | afdb-uniprot50 | AF-A0A2S9XIG4-F1-MODEL\_V4 | 1.0 | 3.209e-05 | 217 | 0.172 | 110 | 86 | 3 | 2 | 107 | 12 | 120 | Gene 25-like lysozyme | Gene 25-like lysozyme | | afdb-uniprot50 | AF-A0A2N8PEC8-F1-MODEL\_V4 | 1.0 | 1.839e-05 | 217 | 0.242 | 107 | 76 | 4 | 2 | 105 | 17 | 121 | GPW\_gp25 domain-containing protein | GPW\_gp25 domain-containing protein | | afdb-uniprot50 | AF-A0A561S9T7-F1-MODEL\_V4 | 1.0 | 1.269e-05 | 217 | 0.16 | 143 | 83 | 7 | 1 | 111 | 1 | 138 | Uncharacterized protein | Uncharacterized protein | | afdb-uniprot50 | AF-A0A1R3VQV6-F1-MODEL\_V4 | 1.0 | 8.232e-06 | 217 | 0.203 | 113 | 82 | 3 | 2 | 106 | 19 | 131 | GPW\_gp25 domain-containing protein | GPW\_gp25 domain-containing protein | | afdb-uniprot50 | AF-A0A3D8NEX0-F1-MODEL\_V4 | 1.0 | 2.355e-05 | 217 | 0.283 | 106 | 67 | 3 | 1 | 97 | 19 | 124 | Baseplate protein | Baseplate protein | | afdb-uniprot50 | AF-A0A1Z9I0E6-F1-MODEL\_V4 | 1.0 | 1.528e-05 | 217 | 0.177 | 107 | 83 | 4 | 4 | 105 | 24 | 130 | GPW\_gp25 domain-containing protein | GPW\_gp25 domain-containing protein | | afdb-uniprot50 | AF-A0A1K1LB84-F1-MODEL\_V4 | 1.0 | 5.68e-06 | 217 | 0.181 | 116 | 83 | 4 | 1 | 105 | 34 | 148 | Baseplate assembly protein, putative | Baseplate assembly protein, putative | | afdb-uniprot50 | AF-A8ZRG2-F1-MODEL\_V4 | 1.0 | 5.68e-06 | 217 | 0.243 | 115 | 74 | 4 | 1 | 105 | 79 | 190 | GPW/gp25 family protein | GPW/gp25 family protein | | afdb-uniprot50 | AF-A0A844ZL85-F1-MODEL\_V4 | 1.0 | 1.269e-05 | 216 | 0.259 | 108 | 75 | 4 | 2 | 106 | 9 | 114 | Uncharacterized protein | Uncharacterized protein | | afdb-uniprot50 | AF-A0A285M3X1-F1-MODEL\_V4 | 1.0 | 2.081e-05 | 216 | 0.238 | 109 | 81 | 2 | 3 | 111 | 16 | 122 | Uncharacterized protein | Uncharacterized protein | | afdb-uniprot50 | AF-A0A0E3Z1I2-F1-MODEL\_V4 | 1.0 | 2.506e-05 | 216 | 0.224 | 107 | 80 | 3 | 2 | 106 | 11 | 116 | GPW\_gp25 domain-containing protein | GPW\_gp25 domain-containing protein | | afdb-uniprot50 | AF-A0A2P5KAU3-F1-MODEL\_V4 | 1.0 | 5.34e-06 | 216 | 0.166 | 114 | 89 | 3 | 1 | 108 | 18 | 131 | GPW\_gp25 domain-containing protein | GPW\_gp25 domain-containing protein | | afdb-uniprot50 | AF-A0A327Q7K0-F1-MODEL\_V4 | 1.0 | 1.729e-05 | 216 | 0.288 | 104 | 70 | 2 | 5 | 105 | 11 | 113 | Gene 25-like lysozyme | Gene 25-like lysozyme | | afdb-uniprot50 | AF-A0A4Q3TBW4-F1-MODEL\_V4 | 1.0 | 2.506e-05 | 215 | 0.24 | 104 | 73 | 3 | 3 | 105 | 2 | 100 | GPW\_gp25 domain-containing protein | GPW\_gp25 domain-containing protein | | afdb-uniprot50 | AF-A0A1M6K9Z9-F1-MODEL\_V4 | 1.0 | 3.209e-05 | 215 | 0.242 | 107 | 78 | 3 | 2 | 106 | 7 | 112 | GPW\_gp25 domain-containing protein | GPW\_gp25 domain-containing protein | | afdb-uniprot50 | AF-A0A5Q4DGZ4-F1-MODEL\_V4 | 1.0 | 1.121e-05 | 215 | 0.283 | 113 | 71 | 5 | 1 | 105 | 1 | 111 | GPW\_gp25 domain-containing protein | GPW\_gp25 domain-containing protein | | afdb-uniprot50 | AF-A0A238LBG6-F1-MODEL\_V4 | 1.0 | 2.355e-05 | 215 | 0.194 | 108 | 81 | 4 | 2 | 105 | 8 | 113 | Gene 25-like lysozyme | Gene 25-like lysozyme | | afdb-uniprot50 | AF-A0A2D5MUE6-F1-MODEL\_V4 | 1.0 | 3.631e-05 | 215 | 0.188 | 117 | 85 | 4 | 1 | 107 | 8 | 124 | GPW\_gp25 domain-containing protein | GPW\_gp25 domain-containing protein | | afdb-uniprot50 | AF-F2NYC6-F1-MODEL\_V4 | 1.0 | 1.957e-05 | 215 | 0.262 | 99 | 70 | 2 | 2 | 97 | 13 | 111 | GPW/gp25 family protein | GPW/gp25 family protein | | afdb-uniprot50 | AF-A0A369WU76-F1-MODEL\_V4 | 1.0 | 4.719e-06 | 215 | 0.212 | 108 | 80 | 2 | 3 | 105 | 15 | 122 | GPW\_gp25 domain-containing protein | GPW\_gp25 domain-containing protein | | afdb-uniprot50 | AF-A0A2K8LN18-F1-MODEL\_V4 | 1.0 | 2.665e-05 | 215 | 0.276 | 112 | 75 | 5 | 2 | 110 | 70 | 178 | GPW\_gp25 domain-containing protein | GPW\_gp25 domain-containing protein | | afdb-uniprot50 | AF-A0A4P9VI48-F1-MODEL\_V4 | 1.0 | 9.767e-05 | 214 | 0.402 | 67 | 40 | 0 | 1 | 67 | 2 | 68 | Baseplate assembly protein W | Baseplate assembly protein W | | afdb-uniprot50 | AF-H5SBU9-F1-MODEL\_V4 | 1.0 | 4.371e-05 | 214 | 0.322 | 93 | 62 | 1 | 13 | 105 | 8 | 99 | Prophage MuMc02, baseplate assembly protein W | Prophage MuMc02, baseplate assembly protein W | | afdb-uniprot50 | AF-A0A1I1DKW3-F1-MODEL\_V4 | 1.0 | 2.506e-05 | 214 | 0.179 | 117 | 81 | 5 | 1 | 105 | 1 | 114 | Uncharacterized protein | Uncharacterized protein | | afdb-uniprot50 | AF-A0A4R4UB04-F1-MODEL\_V4 | 1.0 | 1.193e-05 | 214 | 0.225 | 111 | 81 | 4 | 2 | 108 | 10 | 119 | GPW\_gp25 domain-containing protein | GPW\_gp25 domain-containing protein | | afdb-uniprot50 | AF-A0A1K1LCB6-F1-MODEL\_V4 | 1.0 | 1.269e-05 | 214 | 0.242 | 95 | 69 | 2 | 13 | 105 | 3 | 96 | Baseplate assembly protein, putative | Baseplate assembly protein, putative | | afdb-uniprot50 | AF-A0A075MN45-F1-MODEL\_V4 | 1.0 | 3.413e-05 | 214 | 0.198 | 106 | 82 | 3 | 2 | 105 | 17 | 121 | Phage baseplate assembly protein W | Phage baseplate assembly protein W | | afdb-uniprot50 | AF-A0A3M1VGJ9-F1-MODEL\_V4 | 1.0 | 2.665e-05 | 214 | 0.149 | 107 | 88 | 2 | 3 | 106 | 17 | 123 | GPW\_gp25 domain-containing protein | GPW\_gp25 domain-containing protein | | afdb-uniprot50 | AF-A0A2E8NZT2-F1-MODEL\_V4 | 1.0 | 4.109e-05 | 214 | 0.196 | 112 | 84 | 4 | 3 | 110 | 17 | 126 | GPW\_gp25 domain-containing protein | GPW\_gp25 domain-containing protein | | afdb-uniprot50 | AF-A0A0A6VNM1-F1-MODEL\_V4 | 1.0 | 2.214e-05 | 214 | 0.262 | 103 | 66 | 3 | 2 | 97 | 20 | 119 | Baseplate protein | Baseplate protein | | afdb-uniprot50 | AF-A0A3S0DBJ3-F1-MODEL\_V4 | 1.0 | 6.838e-06 | 214 | 0.204 | 127 | 81 | 5 | 2 | 109 | 12 | 137 | GPW\_gp25 domain-containing protein | GPW\_gp25 domain-containing protein | | afdb-uniprot50 | AF-A0A317I630-F1-MODEL\_V4 | 1.0 | 1.957e-05 | 214 | 0.233 | 124 | 75 | 4 | 2 | 106 | 17 | 139 | Baseplate protein | Baseplate protein | | afdb-uniprot50 | AF-A0A2D6RI59-F1-MODEL\_V4 | 1.0 | 6.428e-06 | 213 | 0.162 | 111 | 88 | 3 | 2 | 107 | 11 | 121 | GPW\_gp25 domain-containing protein | GPW\_gp25 domain-containing protein | | afdb-uniprot50 | AF-A0A2D5MQ93-F1-MODEL\_V4 | 1.0 | 2.355e-05 | 213 | 0.185 | 108 | 85 | 2 | 3 | 107 | 17 | 124 | GPW\_gp25 domain-containing protein | GPW\_gp25 domain-containing protein | | afdb-uniprot50 | AF-A0A0F9QCS5-F1-MODEL\_V4 | 1.0 | 2.836e-05 | 213 | 0.186 | 102 | 82 | 1 | 5 | 105 | 21 | 122 | Uncharacterized protein | Uncharacterized protein | | afdb-uniprot50 | AF-A0A5P2UWR7-F1-MODEL\_V4 | 1.0 | 3.016e-05 | 213 | 0.282 | 99 | 68 | 2 | 2 | 97 | 14 | 112 | Baseplate protein | Baseplate protein | | afdb-uniprot50 | AF-A0A6V8KGV1-F1-MODEL\_V4 | 1.0 | 2.081e-05 | 213 | 0.337 | 83 | 54 | 1 | 3 | 84 | 15 | 97 | Baseplate protein | Baseplate protein | | afdb-uniprot50 | AF-A0A7X6JAI8-F1-MODEL\_V4 | 1.0 | 4.65e-05 | 212 | 0.474 | 78 | 36 | 1 | 1 | 78 | 3 | 75 | Baseplate assembly protein | Baseplate assembly protein | | afdb-uniprot50 | AF-A0A399WMQ1-F1-MODEL\_V4 | 1.0 | 4.947e-05 | 212 | 0.196 | 107 | 83 | 3 | 2 | 106 | 11 | 116 | GPW\_gp25 domain-containing protein | GPW\_gp25 domain-containing protein | | afdb-uniprot50 | AF-A0A1I0F144-F1-MODEL\_V4 | 1.0 | 4.371e-05 | 212 | 0.205 | 107 | 80 | 4 | 2 | 105 | 12 | 116 | GPW\_gp25 domain-containing protein | GPW\_gp25 domain-containing protein | | afdb-uniprot50 | AF-A0A5C4QRE3-F1-MODEL\_V4 | 1.0 | 5.262e-05 | 212 | 0.168 | 113 | 86 | 4 | 1 | 106 | 4 | 115 | GPW/gp25 family protein | GPW/gp25 family protein | | afdb-uniprot50 | AF-A0A7C7GT66-F1-MODEL\_V4 | 1.0 | 1.957e-05 | 212 | 0.15 | 120 | 92 | 3 | 2 | 112 | 11 | 129 | GPW\_gp25 domain-containing protein | GPW\_gp25 domain-containing protein | | afdb-uniprot50 | AF-A0A535DVR5-F1-MODEL\_V4 | 1.0 | 1.957e-05 | 212 | 0.301 | 106 | 69 | 3 | 2 | 103 | 14 | 118 | GPW/gp25 family protein | GPW/gp25 family protein | | afdb-uniprot50 | AF-A0A6L9JRF5-F1-MODEL\_V4 | 1.0 | 9.316e-06 | 212 | 0.205 | 107 | 83 | 1 | 1 | 105 | 21 | 127 | Phage baseplate protein | Phage baseplate protein | | afdb-uniprot50 | AF-A0A2D0KA99-F1-MODEL\_V4 | 1.0 | 9.91e-06 | 212 | 0.171 | 111 | 86 | 2 | 1 | 105 | 19 | 129 | Phage baseplate protein | Phage baseplate protein | | afdb-uniprot50 | AF-A0A2M7WDH6-F1-MODEL\_V4 | 1.0 | 1.957e-05 | 212 | 0.201 | 109 | 81 | 5 | 3 | 108 | 94 | 199 | Uncharacterized protein | Uncharacterized protein | | afdb-uniprot50 | AF-A0A6B3IQI9-F1-MODEL\_V4 | 1.0 | 1.625e-05 | 211 | 0.282 | 99 | 68 | 2 | 2 | 97 | 15 | 113 | GPW/gp25 family protein | GPW/gp25 family protein | | afdb-uniprot50 | AF-A0A0F9FRJ8-F1-MODEL\_V4 | 1.0 | 9.91e-06 | 211 | 0.188 | 117 | 84 | 5 | 1 | 107 | 4 | 119 | GPW\_gp25 domain-containing protein | GPW\_gp25 domain-containing protein | | afdb-uniprot50 | AF-A0A382SJV7-F1-MODEL\_V4 | 1.0 | 2.506e-05 | 211 | 0.158 | 120 | 85 | 7 | 1 | 107 | 5 | 121 | GPW\_gp25 domain-containing protein | GPW\_gp25 domain-containing protein | | afdb-uniprot50 | AF-A0A842WRC3-F1-MODEL\_V4 | 1.0 | 3.209e-05 | 211 | 0.221 | 113 | 83 | 5 | 1 | 111 | 20 | 129 | DUF2634 domain-containing protein | DUF2634 domain-containing protein | | afdb-uniprot50 | AF-A0A5M8P5N3-F1-MODEL\_V4 | 1.0 | 2.355e-05 | 211 | 0.205 | 112 | 83 | 3 | 1 | 107 | 14 | 124 | Phage baseplate protein | Phage baseplate protein | | afdb-uniprot50 | AF-A0A3D5E673-F1-MODEL\_V4 | 1.0 | 2.506e-05 | 211 | 0.159 | 119 | 84 | 7 | 1 | 105 | 12 | 128 | GPW\_gp25 domain-containing protein | GPW\_gp25 domain-containing protein | | afdb-uniprot50 | AF-A0A7W5F3D6-F1-MODEL\_V4 | 1.0 | 1.193e-05 | 211 | 0.252 | 111 | 78 | 4 | 2 | 108 | 29 | 138 | GPW\_gp25 domain-containing protein | GPW\_gp25 domain-containing protein | | afdb-uniprot50 | AF-A0A2T6G6G7-F1-MODEL\_V4 | 1.0 | 1.528e-05 | 210 | 0.304 | 92 | 60 | 3 | 17 | 105 | 7 | 97 | Baseplate protein | Baseplate protein | | afdb-uniprot50 | AF-A0A7Z6PT91-F1-MODEL\_V4 | 1.0 | 2.836e-05 | 210 | 0.254 | 102 | 69 | 2 | 12 | 106 | 2 | 103 | Integrase | Integrase | | afdb-uniprot50 | AF-A0A5S4YIV1-F1-MODEL\_V4 | 1.0 | 4.109e-05 | 210 | 0.224 | 107 | 80 | 3 | 2 | 106 | 7 | 112 | GPW/gp25 family protein | GPW/gp25 family protein | | afdb-uniprot50 | AF-A0A838AGQ5-F1-MODEL\_V4 | 1.0 | 1.957e-05 | 210 | 0.148 | 108 | 87 | 3 | 1 | 105 | 4 | 109 | GPW/gp25 family protein | GPW/gp25 family protein | | afdb-uniprot50 | AF-A0A286DXR1-F1-MODEL\_V4 | 1.0 | 1.528e-05 | 210 | 0.214 | 107 | 79 | 4 | 2 | 105 | 15 | 119 | GPW\_gp25 domain-containing protein | GPW\_gp25 domain-containing protein | | afdb-uniprot50 | AF-A0A2E8FZA1-F1-MODEL\_V4 | 1.0 | 1.957e-05 | 210 | 0.201 | 109 | 81 | 3 | 4 | 106 | 28 | 136 | GPW\_gp25 domain-containing protein | GPW\_gp25 domain-containing protein | | afdb-uniprot50 | AF-A0A853ICS2-F1-MODEL\_V4 | 1.0 | 1.35e-05 | 210 | 0.254 | 106 | 70 | 3 | 1 | 97 | 20 | 125 | GPW/gp25 family protein | GPW/gp25 family protein | | afdb-uniprot50 | AF-A0A8B3NLL6-F1-MODEL\_V4 | 1.0 | 0.0002469 | 210 | 0.27 | 85 | 62 | 0 | 1 | 85 | 3 | 87 | Uncharacterized protein | Uncharacterized protein | | afdb-uniprot50 | AF-A0A4Q0ZJN2-F1-MODEL\_V4 | 1.0 | 1.436e-05 | 209 | 0.304 | 105 | 61 | 5 | 4 | 106 | 1 | 95 | GPW\_gp25 domain-containing protein | GPW\_gp25 domain-containing protein | | afdb-uniprot50 | AF-A0A1M6M9N6-F1-MODEL\_V4 | 1.0 | 2.081e-05 | 209 | 0.223 | 103 | 76 | 3 | 6 | 105 | 13 | 114 | Gene 25-like lysozyme | Gene 25-like lysozyme | | afdb-uniprot50 | AF-A0A0Q4KZB0-F1-MODEL\_V4 | 1.0 | 2.355e-05 | 209 | 0.276 | 105 | 74 | 1 | 2 | 104 | 18 | 122 | Baseplate assembly protein | Baseplate assembly protein | | afdb-uniprot50 | AF-A0A2T2YA09-F1-MODEL\_V4 | 1.0 | 1.35e-05 | 209 | 0.31 | 100 | 65 | 2 | 2 | 97 | 17 | 116 | GPW\_gp25 domain-containing protein | GPW\_gp25 domain-containing protein | | afdb-uniprot50 | AF-A0A7X5QIW4-F1-MODEL\_V4 | 1.0 | 5.955e-05 | 209 | 0.163 | 98 | 80 | 1 | 10 | 105 | 40 | 137 | Phage baseplate protein | Phage baseplate protein | | afdb-uniprot50 | AF-A0A238JEQ2-F1-MODEL\_V4 | 1.0 | 7.739e-06 | 209 | 0.241 | 116 | 76 | 3 | 1 | 105 | 31 | 145 | GPW\_gp25 domain-containing protein | GPW\_gp25 domain-containing protein | | afdb-uniprot50 | AF-A0A6I7P0B8-F1-MODEL\_V4 | 1.0 | 2.081e-05 | 209 | 0.28 | 100 | 68 | 2 | 2 | 97 | 38 | 137 | GPW\_gp25 domain-containing protein | GPW\_gp25 domain-containing protein | | afdb-uniprot50 | AF-A0A1Y6CTE9-F1-MODEL\_V4 | 1.0 | 2.355e-05 | 209 | 0.247 | 113 | 77 | 7 | 1 | 110 | 100 | 207 | Phage baseplate assembly protein W | Phage baseplate assembly protein W | | afdb-uniprot50 | AF-A0A6L4A963-F1-MODEL\_V4 | 1.0 | 3.209e-05 | 208 | 0.33 | 100 | 62 | 3 | 2 | 97 | 15 | 113 | GPW/gp25 family protein | GPW/gp25 family protein | | afdb-uniprot50 | AF-A0A523UWB7-F1-MODEL\_V4 | 1.0 | 4.109e-05 | 208 | 0.215 | 116 | 80 | 4 | 1 | 106 | 1 | 115 | GPW\_gp25 domain-containing protein | GPW\_gp25 domain-containing protein | | afdb-uniprot50 | AF-X5KSN9-F1-MODEL\_V4 | 1.0 | 5.598e-05 | 208 | 0.169 | 106 | 85 | 3 | 2 | 105 | 9 | 113 | GPW/gp25 family protein | GPW/gp25 family protein | | afdb-uniprot50 | AF-A0A1D2WBQ4-F1-MODEL\_V4 | 1.0 | 1.729e-05 | 208 | 0.258 | 112 | 74 | 6 | 3 | 111 | 14 | 119 | Uncharacterized protein | Uncharacterized protein | | afdb-uniprot50 | AF-A0A0X3VMP2-F1-MODEL\_V4 | 1.0 | 1.35e-05 | 208 | 0.258 | 112 | 76 | 5 | 2 | 108 | 17 | 126 | GPW\_gp25 domain-containing protein | GPW\_gp25 domain-containing protein | | afdb-uniprot50 | AF-A0A074M821-F1-MODEL\_V4 | 1.0 | 2.836e-05 | 208 | 0.194 | 108 | 83 | 4 | 1 | 106 | 22 | 127 | Uncharacterized protein | Uncharacterized protein | | afdb-uniprot50 | AF-A0A4R2GXL5-F1-MODEL\_V4 | 1.0 | 3.631e-05 | 208 | 0.191 | 115 | 85 | 3 | 1 | 108 | 18 | 131 | Uncharacterized protein | Uncharacterized protein | | afdb-uniprot50 | AF-A0A4U8YRK1-F1-MODEL\_V4 | 1.0 | 2.542e-06 | 208 | 0.172 | 133 | 84 | 5 | 1 | 108 | 8 | 139 | Uncharacterized protein | Uncharacterized protein | | afdb-uniprot50 | AF-A0A4Y3VBU6-F1-MODEL\_V4 | 1.0 | 1.625e-05 | 208 | 0.234 | 111 | 80 | 4 | 2 | 108 | 35 | 144 | GPW\_gp25 domain-containing protein | GPW\_gp25 domain-containing protein | | afdb-uniprot50 | AF-A0A1V5AF29-F1-MODEL\_V4 | 1.0 | 3.631e-05 | 207 | 0.261 | 107 | 74 | 4 | 2 | 105 | 34 | 138 | Gene 25-like lysozyme | Gene 25-like lysozyme | | afdb-uniprot50 | AF-A0A1G4U040-F1-MODEL\_V4 | 1.0 | 3.256e-06 | 206 | 0.275 | 116 | 73 | 7 | 2 | 112 | 15 | 124 | Uncharacterized protein | Uncharacterized protein | | afdb-uniprot50 | AF-A0A2T4TTD4-F1-MODEL\_V4 | 1.0 | 1.957e-05 | 206 | 0.174 | 109 | 85 | 2 | 2 | 105 | 13 | 121 | GPW\_gp25 domain-containing protein | GPW\_gp25 domain-containing protein | | afdb-uniprot50 | AF-A0A229UMK7-F1-MODEL\_V4 | 1.0 | 5.955e-05 | 206 | 0.132 | 113 | 91 | 3 | 2 | 107 | 14 | 126 | Uncharacterized protein | Uncharacterized protein | | afdb-uniprot50 | AF-A0A0Q1D4W1-F1-MODEL\_V4 | 1.0 | 2.214e-05 | 206 | 0.242 | 107 | 76 | 4 | 2 | 105 | 19 | 123 | GPW\_gp25 domain-containing protein | GPW\_gp25 domain-containing protein | | afdb-uniprot50 | AF-A8T9K2-F1-MODEL\_V4 | 1.0 | 2.506e-05 | 206 | 0.13 | 107 | 90 | 2 | 2 | 105 | 13 | 119 | GPW\_gp25 domain-containing protein | GPW\_gp25 domain-containing protein | | afdb-uniprot50 | AF-A0A1Z9ULM3-F1-MODEL\_V4 | 1.0 | 2.355e-05 | 205 | 0.2 | 100 | 73 | 3 | 14 | 107 | 36 | 134 | GPW\_gp25 domain-containing protein | GPW\_gp25 domain-containing protein | | afdb-uniprot50 | AF-A0A3N7HI14-F1-MODEL\_V4 | 1.0 | 8.757e-06 | 205 | 0.241 | 120 | 78 | 6 | 1 | 108 | 19 | 137 | Baseplate assembly protein | Baseplate assembly protein | | afdb-uniprot50 | AF-A0A843F7L3-F1-MODEL\_V4 | 1.0 | 3.863e-05 | 205 | 0.163 | 110 | 86 | 4 | 3 | 108 | 35 | 142 | Uncharacterized protein | Uncharacterized protein | | afdb-uniprot50 | AF-A0A381NWC8-F1-MODEL\_V4 | 1.0 | 4.65e-05 | 205 | 0.138 | 123 | 87 | 2 | 3 | 106 | 24 | 146 | GPW\_gp25 domain-containing protein | GPW\_gp25 domain-containing protein | | afdb-uniprot50 | AF-A0A381TRD1-F1-MODEL\_V4 | 1.0 | 2.355e-05 | 205 | 0.176 | 125 | 82 | 3 | 3 | 106 | 34 | 158 | GPW\_gp25 domain-containing protein | GPW\_gp25 domain-containing protein | | afdb-uniprot50 | AF-V4NPI9-F1-MODEL\_V4 | 1.0 | 0.0001928 | 204 | 0.476 | 63 | 33 | 0 | 1 | 63 | 6 | 68 | Baseplate assembly protein | Baseplate assembly protein | | afdb-uniprot50 | AF-G8LQ32-F1-MODEL\_V4 | 1.0 | 0.0001176 | 204 | 0.469 | 66 | 35 | 0 | 1 | 66 | 5 | 70 | GPW/Gp25 Family Protein | GPW/Gp25 Family Protein | | afdb-uniprot50 | AF-A0A2P5MMF8-F1-MODEL\_V4 | 1.0 | 4.371e-05 | 204 | 0.245 | 106 | 77 | 3 | 2 | 105 | 7 | 111 | GPW\_gp25 domain-containing protein | GPW\_gp25 domain-containing protein | | afdb-uniprot50 | AF-A0A7W5FEZ8-F1-MODEL\_V4 | 1.0 | 2.836e-05 | 204 | 0.252 | 107 | 75 | 4 | 2 | 105 | 9 | 113 | GPW\_gp25 domain-containing protein | GPW\_gp25 domain-containing protein | | afdb-uniprot50 | AF-A0A7W7HJ55-F1-MODEL\_V4 | 1.0 | 4.65e-05 | 204 | 0.242 | 107 | 76 | 4 | 2 | 105 | 9 | 113 | Phage baseplate assembly protein W | Phage baseplate assembly protein W | | afdb-uniprot50 | AF-A0A2E3DKA2-F1-MODEL\_V4 | 1.0 | 5.955e-05 | 204 | 0.196 | 122 | 78 | 6 | 1 | 107 | 7 | 123 | GPW\_gp25 domain-containing protein | GPW\_gp25 domain-containing protein | | afdb-uniprot50 | AF-D3CW99-F1-MODEL\_V4 | 1.0 | 4.371e-05 | 204 | 0.267 | 101 | 66 | 3 | 2 | 97 | 22 | 119 | GPW/gp25 family protein | GPW/gp25 family protein | | afdb-uniprot50 | AF-A0A7X6AE11-F1-MODEL\_V4 | 1.0 | 3.863e-05 | 204 | 0.372 | 86 | 51 | 2 | 2 | 85 | 14 | 98 | Baseplate protein | Baseplate protein | | afdb-uniprot50 | AF-A0A317FGN1-F1-MODEL\_V4 | 1.0 | 2.665e-05 | 204 | 0.194 | 118 | 84 | 3 | 1 | 107 | 140 | 257 | GPW\_gp25 domain-containing protein | GPW\_gp25 domain-containing protein | | afdb-uniprot50 | AF-F2K1R6-F1-MODEL\_V4 | 1.0 | 2.665e-05 | 203 | 0.302 | 96 | 63 | 2 | 3 | 97 | 10 | 102 | GPW/gp25 family protein | GPW/gp25 family protein | | afdb-uniprot50 | AF-A0A4Q5X4U8-F1-MODEL\_V4 | 1.0 | 5.262e-05 | 203 | 0.233 | 107 | 77 | 4 | 2 | 105 | 7 | 111 | GPW\_gp25 domain-containing protein | GPW\_gp25 domain-containing protein | | afdb-uniprot50 | AF-A0A4R5QHB6-F1-MODEL\_V4 | 1.0 | 3.631e-05 | 203 | 0.261 | 107 | 74 | 5 | 2 | 105 | 7 | 111 | Uncharacterized protein | Uncharacterized protein | | afdb-uniprot50 | AF-A0A1Z8QW37-F1-MODEL\_V4 | 1.0 | 1.528e-05 | 203 | 0.26 | 100 | 70 | 2 | 2 | 97 | 17 | 116 | GPW\_gp25 domain-containing protein | GPW\_gp25 domain-containing protein | | afdb-uniprot50 | AF-A0A432PWH9-F1-MODEL\_V4 | 1.0 | 2.836e-05 | 202 | 0.257 | 97 | 68 | 4 | 10 | 105 | 5 | 98 | GPW\_gp25 domain-containing protein | GPW\_gp25 domain-containing protein | | afdb-uniprot50 | AF-A0A0Q6XWC6-F1-MODEL\_V4 | 1.0 | 5.262e-05 | 202 | 0.23 | 113 | 80 | 5 | 2 | 110 | 7 | 116 | GPW\_gp25 domain-containing protein | GPW\_gp25 domain-containing protein | | afdb-uniprot50 | AF-A0A0H3ZLU4-F1-MODEL\_V4 | 1.0 | 1.193e-05 | 202 | 0.171 | 105 | 83 | 1 | 1 | 105 | 13 | 113 | Uncharacterized protein | Uncharacterized protein | | afdb-uniprot50 | AF-A0A2E4W7B1-F1-MODEL\_V4 | 1.0 | 1.625e-05 | 202 | 0.203 | 118 | 81 | 5 | 3 | 108 | 17 | 133 | GPW\_gp25 domain-containing protein | GPW\_gp25 domain-containing protein | | afdb-uniprot50 | AF-A0A1G3KNN2-F1-MODEL\_V4 | 1.0 | 2.506e-05 | 201 | 0.211 | 104 | 77 | 3 | 5 | 105 | 14 | 115 | Uncharacterized protein | Uncharacterized protein | | afdb-uniprot50 | AF-A0A2D6MIS0-F1-MODEL\_V4 | 1.0 | 1.193e-05 | 201 | 0.188 | 117 | 84 | 4 | 2 | 108 | 20 | 135 | GPW\_gp25 domain-containing protein | GPW\_gp25 domain-containing protein | | afdb-uniprot50 | AF-A0A2E6ZKV4-F1-MODEL\_V4 | 1.0 | 3.863e-05 | 201 | 0.13 | 107 | 88 | 3 | 3 | 105 | 36 | 141 | GPW\_gp25 domain-containing protein | GPW\_gp25 domain-containing protein | | afdb-uniprot50 | AF-A0A447N0I4-F1-MODEL\_V4 | 1.0 | 0.0001039 | 200 | 0.408 | 71 | 42 | 0 | 1 | 71 | 5 | 75 | Baseplate assembly protein | Baseplate assembly protein | | afdb-uniprot50 | AF-A0A3D2XG26-F1-MODEL\_V4 | 1.0 | 3.92e-06 | 200 | 0.207 | 106 | 70 | 2 | 2 | 105 | 17 | 110 | GPW\_gp25 domain-containing protein | GPW\_gp25 domain-containing protein | | afdb-uniprot50 | AF-A0A832CQ52-F1-MODEL\_V4 | 1.0 | 1.729e-05 | 200 | 0.25 | 108 | 74 | 5 | 1 | 105 | 13 | 116 | Uncharacterized protein | Uncharacterized protein | | afdb-uniprot50 | AF-A0A661Z4X6-F1-MODEL\_V4 | 1.0 | 2.836e-05 | 200 | 0.175 | 108 | 86 | 2 | 1 | 105 | 7 | 114 | GPW\_gp25 domain-containing protein | GPW\_gp25 domain-containing protein | | afdb-uniprot50 | AF-A0A662Q525-F1-MODEL\_V4 | 1.0 | 2.214e-05 | 200 | 0.161 | 124 | 87 | 4 | 1 | 107 | 12 | 135 | GPW\_gp25 domain-containing protein | GPW\_gp25 domain-containing protein | | afdb-uniprot50 | AF-A0A6N8MVK6-F1-MODEL\_V4 | 1.0 | 0.0002627 | 199 | 0.428 | 63 | 36 | 0 | 1 | 63 | 5 | 67 | GPW\_gp25 domain-containing protein | GPW\_gp25 domain-containing protein | | afdb-uniprot50 | AF-A0A843GVE6-F1-MODEL\_V4 | 1.0 | 0.0001415 | 199 | 0.141 | 113 | 88 | 3 | 1 | 105 | 1 | 112 | Uncharacterized protein | Uncharacterized protein | | afdb-uniprot50 | AF-A0A2D8EMY5-F1-MODEL\_V4 | 1.0 | 7.169e-05 | 199 | 0.168 | 107 | 86 | 2 | 3 | 106 | 12 | 118 | GPW\_gp25 domain-containing protein | GPW\_gp25 domain-containing protein | | afdb-uniprot50 | AF-A0A2E7WDX9-F1-MODEL\_V4 | 1.0 | 8.63e-05 | 199 | 0.137 | 109 | 89 | 3 | 3 | 107 | 15 | 122 | Uncharacterized protein | Uncharacterized protein | | afdb-uniprot50 | AF-A0A1G8FCC5-F1-MODEL\_V4 | 1.0 | 3.631e-05 | 199 | 0.14 | 107 | 88 | 3 | 1 | 106 | 23 | 126 | Phage baseplate assembly protein W | Phage baseplate assembly protein W | | afdb-uniprot50 | AF-A0A2E0GD41-F1-MODEL\_V4 | 1.0 | 3.413e-05 | 199 | 0.182 | 115 | 85 | 4 | 2 | 108 | 20 | 133 | GPW\_gp25 domain-containing protein | GPW\_gp25 domain-containing protein | | afdb-uniprot50 | AF-A0A2E5T0R5-F1-MODEL\_V4 | 1.0 | 0.0001602 | 199 | 0.139 | 136 | 88 | 5 | 4 | 112 | 21 | 154 | Uncharacterized protein | Uncharacterized protein | | afdb-uniprot50 | AF-A0A3C0G267-F1-MODEL\_V4 | 1.0 | 3.016e-05 | 199 | 0.186 | 107 | 80 | 2 | 6 | 105 | 83 | 189 | GPW\_gp25 domain-containing protein | GPW\_gp25 domain-containing protein | | afdb-uniprot50 | AF-A0A3G8C6Y1-F1-MODEL\_V4 | 1.0 | 0.0001704 | 198 | 0.362 | 69 | 42 | 1 | 39 | 107 | 3 | 69 | Phage baseplate assembly protein | Phage baseplate assembly protein | | afdb-uniprot50 | AF-A0A1H6APR4-F1-MODEL\_V4 | 1.0 | 3.631e-05 | 198 | 0.223 | 112 | 80 | 5 | 2 | 108 | 10 | 119 | GPW\_gp25 domain-containing protein | GPW\_gp25 domain-containing protein | | afdb-uniprot50 | AF-A0A3C0G5D2-F1-MODEL\_V4 | 1.0 | 3.631e-05 | 198 | 0.187 | 112 | 85 | 2 | 2 | 107 | 13 | 124 | GPW\_gp25 domain-containing protein | GPW\_gp25 domain-containing protein | | afdb-uniprot50 | AF-A0A101NH81-F1-MODEL\_V4 | 1.0 | 3.863e-05 | 198 | 0.225 | 111 | 81 | 4 | 2 | 108 | 13 | 122 | GPW\_gp25 domain-containing protein | GPW\_gp25 domain-containing protein | | afdb-uniprot50 | AF-A0A6M3ZUD3-F1-MODEL\_V4 | 1.0 | 4.371e-05 | 198 | 0.242 | 99 | 72 | 2 | 2 | 97 | 16 | 114 | GPW\_gp25 domain-containing protein | GPW\_gp25 domain-containing protein | | afdb-uniprot50 | AF-A0A2D7BZB0-F1-MODEL\_V4 | 1.0 | 8.113e-05 | 198 | 0.141 | 106 | 88 | 2 | 3 | 105 | 35 | 140 | GPW\_gp25 domain-containing protein | GPW\_gp25 domain-containing protein | | afdb-uniprot50 | AF-A0A5C7PYR2-F1-MODEL\_V4 | 1.0 | 4.371e-05 | 198 | 0.21 | 114 | 84 | 5 | 1 | 112 | 91 | 200 | Uncharacterized protein | Uncharacterized protein | | afdb-uniprot50 | AF-A0A009Q2W6-F1-MODEL\_V4 | 1.0 | 6.739e-05 | 197 | 0.366 | 71 | 45 | 0 | 35 | 105 | 1 | 71 | Lysozyme family protein | Lysozyme family protein | | afdb-uniprot50 | AF-A0A429AGL9-F1-MODEL\_V4 | 1.0 | 0.0001331 | 197 | 0.228 | 105 | 76 | 3 | 3 | 106 | 6 | 106 | GPW\_gp25 domain-containing protein | GPW\_gp25 domain-containing protein | | afdb-uniprot50 | AF-A0A1G3K418-F1-MODEL\_V4 | 1.0 | 8.113e-05 | 197 | 0.24 | 108 | 77 | 4 | 2 | 106 | 8 | 113 | GPW\_gp25 domain-containing protein | GPW\_gp25 domain-containing protein | | afdb-uniprot50 | AF-A0A1L3FAG4-F1-MODEL\_V4 | 1.0 | 3.631e-05 | 197 | 0.254 | 114 | 76 | 5 | 2 | 108 | 9 | 120 | GPW\_gp25 domain-containing protein | GPW\_gp25 domain-containing protein | | afdb-uniprot50 | AF-A0A1I6PRV0-F1-MODEL\_V4 | 1.0 | 0.0001039 | 197 | 0.224 | 107 | 78 | 4 | 2 | 105 | 12 | 116 | GPW\_gp25 domain-containing protein | GPW\_gp25 domain-containing protein | | afdb-uniprot50 | AF-A0A1Z9UJC8-F1-MODEL\_V4 | 1.0 | 7.626e-05 | 197 | 0.159 | 119 | 91 | 5 | 2 | 112 | 27 | 144 | GPW\_gp25 domain-containing protein | GPW\_gp25 domain-containing protein | | afdb-uniprot50 | AF-A0A4R2Q0H0-F1-MODEL\_V4 | 1.0 | 0.0001704 | 196 | 0.216 | 106 | 80 | 3 | 2 | 105 | 7 | 111 | GPW\_gp25 domain-containing protein | GPW\_gp25 domain-containing protein | | afdb-uniprot50 | AF-A0A831ZHY3-F1-MODEL\_V4 | 1.0 | 2.836e-05 | 196 | 0.191 | 115 | 84 | 5 | 2 | 108 | 12 | 125 | Uncharacterized protein | Uncharacterized protein | | afdb-uniprot50 | AF-A0A4Z0GYW9-F1-MODEL\_V4 | 1.0 | 2.665e-05 | 196 | 0.178 | 112 | 85 | 4 | 2 | 107 | 55 | 165 | GPW\_gp25 domain-containing protein | GPW\_gp25 domain-containing protein | | afdb-uniprot50 | AF-A0A3D2D4U8-F1-MODEL\_V4 | 1.0 | 0.0003807 | 195 | 0.359 | 64 | 41 | 0 | 1 | 64 | 2 | 65 | Baseplate assembly protein | Baseplate assembly protein | | afdb-uniprot50 | AF-A0A3T0L2L2-F1-MODEL\_V4 | 1.0 | 0.0001415 | 195 | 0.303 | 89 | 58 | 2 | 6 | 93 | 8 | 93 | Baseplate assembly protein | Baseplate assembly protein | | afdb-uniprot50 | AF-A0A1S1YVC0-F1-MODEL\_V4 | 1.0 | 0.0001176 | 195 | 0.198 | 106 | 82 | 3 | 3 | 107 | 14 | 117 | GPW\_gp25 domain-containing protein | GPW\_gp25 domain-containing protein | | afdb-uniprot50 | AF-A0A0G1YBV7-F1-MODEL\_V4 | 1.0 | 3.631e-05 | 195 | 0.182 | 126 | 84 | 3 | 1 | 111 | 123 | 244 | Uncharacterized protein | Uncharacterized protein | | afdb-uniprot50 | AF-A0A1G8I0U2-F1-MODEL\_V4 | 1.0 | 0.0005186 | 194 | 0.298 | 67 | 47 | 0 | 1 | 67 | 1 | 67 | Uncharacterized protein | Uncharacterized protein | | afdb-uniprot50 | AF-A0A0Q2Z3G8-F1-MODEL\_V4 | 1.0 | 5.955e-05 | 194 | 0.247 | 109 | 77 | 2 | 3 | 107 | 7 | 114 | GPW\_gp25 domain-containing protein | GPW\_gp25 domain-containing protein | | afdb-uniprot50 | AF-A0A7C6EBT5-F1-MODEL\_V4 | 1.0 | 5.262e-05 | 194 | 0.189 | 111 | 84 | 5 | 2 | 111 | 17 | 122 | Uncharacterized protein | Uncharacterized protein | | afdb-uniprot50 | AF-A0A2D8EIK4-F1-MODEL\_V4 | 1.0 | 0.0001331 | 194 | 0.205 | 107 | 82 | 2 | 3 | 106 | 27 | 133 | GPW\_gp25 domain-containing protein | GPW\_gp25 domain-containing protein | | afdb-uniprot50 | AF-A0A0B6RSR4-F1-MODEL\_V4 | 1.0 | 8.113e-05 | 193 | 0.435 | 78 | 41 | 1 | 1 | 78 | 1 | 75 | Putative phage baseplate assembly protein W | Putative phage baseplate assembly protein W | | afdb-uniprot50 | AF-A0A543PFP7-F1-MODEL\_V4 | 1.0 | 2.214e-05 | 193 | 0.205 | 112 | 82 | 5 | 2 | 108 | 7 | 116 | GPW\_gp25 domain-containing protein | GPW\_gp25 domain-containing protein | | afdb-uniprot50 | AF-A0A424JID7-F1-MODEL\_V4 | 1.0 | 1.625e-05 | 193 | 0.2 | 120 | 83 | 3 | 1 | 107 | 1 | 120 | Baseplate protein | Baseplate protein | | afdb-uniprot50 | AF-A0A0J1FSM7-F1-MODEL\_V4 | 1.0 | 3.016e-05 | 193 | 0.22 | 109 | 77 | 7 | 1 | 105 | 16 | 120 | 25-like lysozyme | 25-like lysozyme | | afdb-uniprot50 | AF-A0A350IEC1-F1-MODEL\_V4 | 1.0 | 0.0001105 | 193 | 0.132 | 113 | 90 | 3 | 1 | 105 | 27 | 139 | GPW\_gp25 domain-containing protein | GPW\_gp25 domain-containing protein | | afdb-uniprot50 | AF-A0A2D7XHE7-F1-MODEL\_V4 | 1.0 | 2.665e-05 | 193 | 0.203 | 113 | 82 | 4 | 1 | 105 | 33 | 145 | GPW\_gp25 domain-containing protein | GPW\_gp25 domain-containing protein | | afdb-uniprot50 | AF-A0A7C1ZK85-F1-MODEL\_V4 | 1.0 | 5.598e-05 | 192 | 0.26 | 119 | 73 | 5 | 3 | 108 | 6 | 122 | GPW\_gp25 domain-containing protein | GPW\_gp25 domain-containing protein | | afdb-uniprot50 | AF-A0A350PIZ7-F1-MODEL\_V4 | 1.0 | 2.355e-05 | 192 | 0.159 | 113 | 87 | 3 | 3 | 107 | 13 | 125 | GPW\_gp25 domain-containing protein | GPW\_gp25 domain-containing protein | | afdb-uniprot50 | AF-A0A1I3W1X3-F1-MODEL\_V4 | 1.0 | 7.626e-05 | 192 | 0.157 | 114 | 86 | 4 | 1 | 105 | 2 | 114 | GPW\_gp25 domain-containing protein | GPW\_gp25 domain-containing protein | | afdb-uniprot50 | AF-A0A7Y4R463-F1-MODEL\_V4 | 1.0 | 0.0001176 | 192 | 0.18 | 100 | 78 | 2 | 2 | 97 | 16 | 115 | GPW/gp25 family protein | GPW/gp25 family protein | | afdb-uniprot50 | AF-A0A2D9N2U0-F1-MODEL\_V4 | 1.0 | 5.262e-05 | 192 | 0.137 | 109 | 89 | 2 | 3 | 106 | 37 | 145 | GPW\_gp25 domain-containing protein | GPW\_gp25 domain-containing protein | | afdb-uniprot50 | AF-A0A353BET6-F1-MODEL\_V4 | 1.0 | 7.739e-06 | 192 | 0.2 | 135 | 77 | 4 | 3 | 106 | 27 | 161 | GPW\_gp25 domain-containing protein | GPW\_gp25 domain-containing protein | | afdb-uniprot50 | AF-A0A519DRW4-F1-MODEL\_V4 | 1.0 | 0.0002051 | 191 | 0.255 | 90 | 64 | 3 | 24 | 112 | 3 | 90 | GPW\_gp25 domain-containing protein | GPW\_gp25 domain-containing protein | | afdb-uniprot50 | AF-A0A7V8YDK1-F1-MODEL\_V4 | 1.0 | 4.65e-05 | 191 | 0.3 | 90 | 63 | 0 | 17 | 106 | 7 | 96 | GPW/gp25 family protein | GPW/gp25 family protein | | afdb-uniprot50 | AF-A0A7C7GCM5-F1-MODEL\_V4 | 1.0 | 7.626e-05 | 191 | 0.154 | 110 | 89 | 3 | 2 | 107 | 10 | 119 | GPW\_gp25 domain-containing protein | GPW\_gp25 domain-containing protein | | afdb-uniprot50 | AF-R5P4K2-F1-MODEL\_V4 | 1.0 | 4.371e-05 | 191 | 0.156 | 115 | 90 | 4 | 2 | 110 | 13 | 126 | GPW/gp25 family protein | GPW/gp25 family protein | | afdb-uniprot50 | AF-A0A0Q9UAP1-F1-MODEL\_V4 | 1.0 | 3.016e-05 | 191 | 0.3 | 113 | 70 | 3 | 1 | 104 | 20 | 132 | GPW\_gp25 domain-containing protein | GPW\_gp25 domain-containing protein | | afdb-uniprot50 | AF-A0A158DVL2-F1-MODEL\_V4 | 1.0 | 0.0001251 | 191 | 0.238 | 109 | 76 | 6 | 1 | 106 | 97 | 201 | Uncharacterized protein | Uncharacterized protein | | afdb-uniprot50 | AF-A0A7R7TBH0-F1-MODEL\_V4 | 1.0 | 8.63e-05 | 191 | 0.227 | 110 | 78 | 6 | 1 | 107 | 98 | 203 | Uncharacterized protein | Uncharacterized protein | | afdb-uniprot50 | AF-A0A328S0R0-F1-MODEL\_V4 | 1.0 | 0.0002051 | 191 | 0.198 | 106 | 80 | 5 | 3 | 105 | 16 | 119 | Uncharacterized protein | Uncharacterized protein | | afdb-uniprot50 | AF-A0A1E4AXV8-F1-MODEL\_V4 | 1.0 | 0.0002182 | 190 | 0.247 | 109 | 77 | 4 | 5 | 110 | 1 | 107 | GPW\_gp25 domain-containing protein | GPW\_gp25 domain-containing protein | | afdb-uniprot50 | AF-A0A0F9T6N1-F1-MODEL\_V4 | 1.0 | 8.63e-05 | 190 | 0.203 | 108 | 79 | 4 | 6 | 106 | 15 | 122 | GPW\_gp25 domain-containing protein | GPW\_gp25 domain-containing protein | | afdb-uniprot50 | AF-A0A3A8KT25-F1-MODEL\_V4 | 1.0 | 0.0002469 | 190 | 0.169 | 106 | 85 | 3 | 2 | 105 | 10 | 114 | GPW\_gp25 domain-containing protein | GPW\_gp25 domain-containing protein | | afdb-uniprot50 | AF-A0A0X3VIR3-F1-MODEL\_V4 | 1.0 | 3.92e-06 | 190 | 0.22 | 118 | 79 | 6 | 2 | 107 | 9 | 125 | GPW\_gp25 domain-containing protein | GPW\_gp25 domain-containing protein | | afdb-uniprot50 | AF-A0A1G3L9L2-F1-MODEL\_V4 | 1.0 | 3.863e-05 | 190 | 0.175 | 108 | 84 | 3 | 1 | 105 | 15 | 120 | Uncharacterized protein | Uncharacterized protein | | afdb-uniprot50 | AF-A0A1Z9M5W6-F1-MODEL\_V4 | 1.0 | 3.631e-05 | 190 | 0.165 | 115 | 86 | 4 | 4 | 108 | 22 | 136 | GPW\_gp25 domain-containing protein | GPW\_gp25 domain-containing protein | | afdb-uniprot50 | AF-A0A6F8YYN1-F1-MODEL\_V4 | 1.0 | 9.767e-05 | 190 | 0.29 | 100 | 66 | 3 | 2 | 97 | 26 | 124 | Baseplate protein | Baseplate protein | | afdb-uniprot50 | AF-A0A2S6WCH7-F1-MODEL\_V4 | 1.0 | 4.109e-05 | 189 | 0.241 | 116 | 77 | 5 | 2 | 108 | 13 | 126 | GPW\_gp25 domain-containing protein | GPW\_gp25 domain-containing protein | | afdb-uniprot50 | AF-G8R6Y2-F1-MODEL\_V4 | 1.0 | 8.63e-05 | 189 | 0.257 | 101 | 70 | 3 | 2 | 97 | 16 | 116 | Phage baseplate assembly protein W | Phage baseplate assembly protein W | | afdb-uniprot50 | AF-A0A6G3XJJ0-F1-MODEL\_V4 | 1.0 | 0.0003364 | 188 | 0.379 | 79 | 47 | 1 | 17 | 93 | 10 | 88 | GPW/gp25 family protein | GPW/gp25 family protein | | afdb-uniprot50 | AF-A0A1V5LTW0-F1-MODEL\_V4 | 1.0 | 6.335e-05 | 188 | 0.265 | 98 | 65 | 2 | 2 | 93 | 13 | 109 | Gene 25-like lysozyme | Gene 25-like lysozyme | | afdb-uniprot50 | AF-A0A1M7CV50-F1-MODEL\_V4 | 1.0 | 0.0002321 | 188 | 0.178 | 101 | 81 | 1 | 9 | 107 | 21 | 121 | Gene 25-like lysozyme | Gene 25-like lysozyme | | afdb-uniprot50 | AF-A0A2P2GFT5-F1-MODEL\_V4 | 1.0 | 4.65e-05 | 188 | 0.226 | 115 | 79 | 7 | 1 | 111 | 20 | 128 | Uncharacterized protein | Uncharacterized protein | | afdb-uniprot50 | AF-A0A656XVQ3-F1-MODEL\_V4 | 1.0 | 5.262e-05 | 188 | 0.263 | 110 | 75 | 2 | 1 | 104 | 18 | 127 | GPW\_gp25 domain-containing protein | GPW\_gp25 domain-containing protein | | afdb-uniprot50 | AF-A0A1Z8QIV7-F1-MODEL\_V4 | 1.0 | 1.193e-05 | 188 | 0.254 | 106 | 65 | 2 | 2 | 105 | 17 | 110 | GPW\_gp25 domain-containing protein | GPW\_gp25 domain-containing protein | | afdb-uniprot50 | AF-A0A258B3I1-F1-MODEL\_V4 | 1.0 | 9.767e-05 | 188 | 0.288 | 104 | 67 | 3 | 1 | 97 | 18 | 121 | GPW\_gp25 domain-containing protein | GPW\_gp25 domain-containing protein | | afdb-uniprot50 | AF-A0A5B0W7Y0-F1-MODEL\_V4 | 1.0 | 5.955e-05 | 188 | 0.163 | 110 | 85 | 3 | 3 | 105 | 43 | 152 | GPW/gp25 family protein | GPW/gp25 family protein | | afdb-uniprot50 | AF-A0A5C7Q1Z4-F1-MODEL\_V4 | 1.0 | 0.0001176 | 188 | 0.212 | 108 | 78 | 6 | 1 | 105 | 98 | 201 | Uncharacterized protein | Uncharacterized protein | | afdb-uniprot50 | AF-T0C511-F1-MODEL\_V4 | 1.0 | 3.016e-05 | 187 | 0.228 | 114 | 80 | 5 | 1 | 108 | 1 | 112 | Uncharacterized protein | Uncharacterized protein | | afdb-uniprot50 | AF-A0A2A5M1Q7-F1-MODEL\_V4 | 1.0 | 0.0001704 | 187 | 0.194 | 113 | 82 | 4 | 1 | 105 | 1 | 112 | GPW\_gp25 domain-containing protein | GPW\_gp25 domain-containing protein | | afdb-uniprot50 | AF-A0A2D7NVZ0-F1-MODEL\_V4 | 1.0 | 2.081e-05 | 187 | 0.241 | 112 | 71 | 3 | 2 | 109 | 17 | 118 | GPW\_gp25 domain-containing protein | GPW\_gp25 domain-containing protein | | afdb-uniprot50 | AF-A0A1P8YPY5-F1-MODEL\_V4 | 1.0 | 0.0005186 | 186 | 0.274 | 91 | 64 | 2 | 16 | 105 | 8 | 97 | Lysozyme family protein | Lysozyme family protein | | afdb-uniprot50 | AF-A0A2E7W0M6-F1-MODEL\_V4 | 1.0 | 0.0001176 | 186 | 0.14 | 114 | 90 | 3 | 2 | 107 | 10 | 123 | GPW\_gp25 domain-containing protein | GPW\_gp25 domain-containing protein | | afdb-uniprot50 | AF-R5PAT8-F1-MODEL\_V4 | 1.0 | 0.0001039 | 186 | 0.18 | 105 | 82 | 2 | 5 | 105 | 17 | 121 | GPW\_gp25 domain-containing protein | GPW\_gp25 domain-containing protein | | afdb-uniprot50 | AF-A0A511V854-F1-MODEL\_V4 | 1.0 | 4.371e-05 | 186 | 0.2 | 110 | 80 | 6 | 1 | 105 | 15 | 121 | Uncharacterized protein | Uncharacterized protein | | afdb-uniprot50 | AF-A0A5C1YDB3-F1-MODEL\_V4 | 1.0 | 1.528e-05 | 186 | 0.321 | 109 | 59 | 3 | 2 | 108 | 14 | 109 | GPW/gp25 family protein | GPW/gp25 family protein | | afdb-uniprot50 | AF-A0A1I0VPW8-F1-MODEL\_V4 | 1.0 | 0.0001039 | 185 | 0.146 | 116 | 87 | 4 | 1 | 105 | 1 | 115 | GPW\_gp25 domain-containing protein | GPW\_gp25 domain-containing protein | | afdb-uniprot50 | AF-A0A502HL99-F1-MODEL\_V4 | 1.0 | 0.0001331 | 185 | 0.18 | 105 | 78 | 5 | 3 | 106 | 18 | 115 | Uncharacterized protein | Uncharacterized protein | | afdb-uniprot50 | AF-A0A2E9V569-F1-MODEL\_V4 | 1.0 | 9.767e-05 | 185 | 0.163 | 110 | 88 | 3 | 2 | 107 | 10 | 119 | GPW\_gp25 domain-containing protein | GPW\_gp25 domain-containing protein | | afdb-uniprot50 | AF-A0A126QLP9-F1-MODEL\_V4 | 1.0 | 3.016e-05 | 185 | 0.165 | 133 | 83 | 6 | 1 | 107 | 8 | 138 | Baseplate assembly protein | Baseplate assembly protein | | afdb-uniprot50 | AF-A0A417HJ04-F1-MODEL\_V4 | 1.0 | 4.371e-05 | 184 | 0.189 | 111 | 81 | 4 | 2 | 105 | 11 | 119 | GPW\_gp25 domain-containing protein | GPW\_gp25 domain-containing protein | | afdb-uniprot50 | AF-A0A843GHX7-F1-MODEL\_V4 | 1.0 | 0.0001928 | 184 | 0.126 | 119 | 91 | 3 | 2 | 107 | 5 | 123 | GPW/gp25 family protein | GPW/gp25 family protein | | afdb-uniprot50 | AF-A0A2U1W193-F1-MODEL\_V4 | 1.0 | 0.0001105 | 184 | 0.185 | 108 | 83 | 5 | 1 | 106 | 96 | 200 | Uncharacterized protein | Uncharacterized protein | | afdb-uniprot50 | AF-A0A7Y5UCD9-F1-MODEL\_V4 | 1.0 | 0.0003162 | 183 | 0.325 | 83 | 54 | 1 | 25 | 105 | 2 | 84 | GPW/gp25 family protein | GPW/gp25 family protein | | afdb-uniprot50 | AF-A0A2T5J3U2-F1-MODEL\_V4 | 1.0 | 6.739e-05 | 183 | 0.215 | 116 | 80 | 3 | 1 | 106 | 1 | 115 | Phage gp46-like protein | Phage gp46-like protein | | afdb-uniprot50 | AF-A0A502IRR1-F1-MODEL\_V4 | 1.0 | 0.0001176 | 183 | 0.132 | 113 | 92 | 4 | 1 | 107 | 14 | 126 | DUF2634 domain-containing protein | DUF2634 domain-containing protein | | afdb-uniprot50 | AF-A0A1Y4WCG8-F1-MODEL\_V4 | 1.0 | 0.0003579 | 182 | 0.282 | 92 | 62 | 3 | 17 | 105 | 7 | 97 | GPW\_gp25 domain-containing protein | GPW\_gp25 domain-containing protein | | afdb-uniprot50 | AF-A0A1M7ZBF7-F1-MODEL\_V4 | 1.0 | 0.0002627 | 182 | 0.23 | 100 | 74 | 2 | 2 | 98 | 16 | 115 | GPW\_gp25 domain-containing protein | GPW\_gp25 domain-containing protein | | afdb-uniprot50 | AF-A0A2E5AVQ0-F1-MODEL\_V4 | 1.0 | 9.767e-05 | 182 | 0.207 | 111 | 80 | 4 | 3 | 107 | 22 | 130 | GPW\_gp25 domain-containing protein | GPW\_gp25 domain-containing protein | | afdb-uniprot50 | AF-E3PRX9-F1-MODEL\_V4 | 1.0 | 0.0001105 | 181 | 0.203 | 108 | 79 | 5 | 1 | 106 | 12 | 114 | Uncharacterized protein | Uncharacterized protein | | afdb-uniprot50 | AF-A0A327JB01-F1-MODEL\_V4 | 1.0 | 0.0005186 | 181 | 0.148 | 101 | 85 | 1 | 5 | 105 | 30 | 129 | Uncharacterized protein | Uncharacterized protein | | afdb-uniprot50 | AF-A0A2W6A5B1-F1-MODEL\_V4 | 1.0 | 4.371e-05 | 181 | 0.26 | 115 | 67 | 5 | 2 | 109 | 14 | 117 | Baseplate protein | Baseplate protein | | afdb-uniprot50 | AF-A0A0F7FGC4-F1-MODEL\_V4 | 1.0 | 0.0001251 | 180 | 0.186 | 107 | 80 | 4 | 5 | 107 | 9 | 112 | GPW\_gp25 domain-containing protein | GPW\_gp25 domain-containing protein | | afdb-uniprot50 | AF-A0A2D6AX80-F1-MODEL\_V4 | 1.0 | 0.0001039 | 180 | 0.15 | 113 | 88 | 4 | 2 | 107 | 28 | 139 | GPW\_gp25 domain-containing protein | GPW\_gp25 domain-containing protein | | afdb-uniprot50 | AF-A0A2E2ZDB2-F1-MODEL\_V4 | 1.0 | 9.767e-05 | 180 | 0.185 | 113 | 84 | 4 | 3 | 107 | 35 | 147 | GPW\_gp25 domain-containing protein | GPW\_gp25 domain-containing protein | | afdb-uniprot50 | AF-A0A1Z8W2C4-F1-MODEL\_V4 | 1.0 | 9.181e-05 | 180 | 0.193 | 119 | 83 | 4 | 2 | 107 | 27 | 145 | GPW\_gp25 domain-containing protein | GPW\_gp25 domain-containing protein | | afdb-uniprot50 | AF-A0A6P0S081-F1-MODEL\_V4 | 1.0 | 0.0001506 | 179 | 0.255 | 86 | 62 | 1 | 2 | 85 | 14 | 99 | GPW/gp25 family protein | GPW/gp25 family protein | | afdb-uniprot50 | AF-A0A7C3UU77-F1-MODEL\_V4 | 1.0 | 0.0001251 | 178 | 0.154 | 110 | 87 | 3 | 2 | 105 | 13 | 122 | GPW\_gp25 domain-containing protein | GPW\_gp25 domain-containing protein | | afdb-uniprot50 | AF-A0A223D2Z4-F1-MODEL\_V4 | 1.0 | 0.0001506 | 178 | 0.198 | 106 | 80 | 4 | 1 | 105 | 15 | 116 | Uncharacterized protein | Uncharacterized protein | | afdb-uniprot50 | AF-A0A1Z4H0Z3-F1-MODEL\_V4 | 1.0 | 0.0002469 | 178 | 0.24 | 100 | 72 | 2 | 2 | 97 | 17 | 116 | GPW\_gp25 domain-containing protein | GPW\_gp25 domain-containing protein | | afdb-uniprot50 | AF-A0A382CDC4-F1-MODEL\_V4 | 1.0 | 0.0004583 | 177 | 0.244 | 98 | 71 | 2 | 3 | 97 | 6 | 103 | GPW\_gp25 domain-containing protein | GPW\_gp25 domain-containing protein | | afdb-uniprot50 | AF-A0A6I5ZQ21-F1-MODEL\_V4 | 1.0 | 0.0001105 | 177 | 0.153 | 111 | 86 | 5 | 1 | 105 | 14 | 122 | Uncharacterized protein | Uncharacterized protein | | afdb-uniprot50 | AF-A0A2D6X0Z9-F1-MODEL\_V4 | 1.0 | 9.181e-05 | 177 | 0.118 | 118 | 92 | 5 | 2 | 107 | 27 | 144 | GPW\_gp25 domain-containing protein | GPW\_gp25 domain-containing protein | | afdb-uniprot50 | AF-A0A1V5IBU2-F1-MODEL\_V4 | 1.0 | 0.0003162 | 177 | 0.157 | 133 | 85 | 3 | 2 | 107 | 15 | 147 | Gene 25-like lysozyme | Gene 25-like lysozyme | | afdb-uniprot50 | AF-A0A7X6JWS6-F1-MODEL\_V4 | 1.0 | 0.0001039 | 176 | 0.415 | 77 | 40 | 1 | 33 | 104 | 1 | 77 | GPW\_gp25 domain-containing protein | GPW\_gp25 domain-containing protein | | afdb-uniprot50 | AF-A0A239C2Q9-F1-MODEL\_V4 | 1.0 | 7.169e-05 | 176 | 0.201 | 114 | 82 | 5 | 2 | 108 | 7 | 118 | GPW\_gp25 domain-containing protein | GPW\_gp25 domain-containing protein | | afdb-uniprot50 | AF-J1FA57-F1-MODEL\_V4 | 1.0 | 0.0002794 | 176 | 0.169 | 118 | 87 | 4 | 1 | 108 | 2 | 118 | Phage baseplate assembly protein W | Phage baseplate assembly protein W | | afdb-uniprot50 | AF-A0A2M9X3I2-F1-MODEL\_V4 | 1.0 | 0.0001602 | 176 | 0.176 | 102 | 76 | 3 | 10 | 105 | 24 | 123 | Baseplate wedge subunit | Baseplate wedge subunit | | afdb-uniprot50 | AF-A0A7C5ISD4-F1-MODEL\_V4 | 1.0 | 4.947e-05 | 176 | 0.188 | 106 | 71 | 2 | 2 | 105 | 17 | 109 | GPW\_gp25 domain-containing protein | GPW\_gp25 domain-containing protein | | afdb-uniprot50 | AF-A0A0P0QIR3-F1-MODEL\_V4 | 1.0 | 3.863e-05 | 176 | 0.203 | 113 | 79 | 4 | 1 | 105 | 21 | 130 | GPW\_gp25 domain-containing protein | GPW\_gp25 domain-containing protein | | afdb-uniprot50 | AF-A0A2E7N873-F1-MODEL\_V4 | 1.0 | 0.0001813 | 176 | 0.163 | 110 | 83 | 4 | 3 | 105 | 33 | 140 | GPW\_gp25 domain-containing protein | GPW\_gp25 domain-containing protein | | afdb-uniprot50 | AF-V4QXD0-F1-MODEL\_V4 | 1.0 | 0.0003364 | 175 | 0.309 | 71 | 49 | 0 | 35 | 105 | 2 | 72 | Baseplate assembly protein | Baseplate assembly protein | | afdb-uniprot50 | AF-A0A661CXS2-F1-MODEL\_V4 | 1.0 | 0.0002051 | 175 | 0.131 | 114 | 85 | 4 | 3 | 106 | 16 | 125 | GPW\_gp25 domain-containing protein | GPW\_gp25 domain-containing protein | | afdb-uniprot50 | AF-A0A3B8IBV9-F1-MODEL\_V4 | 1.0 | 0.0002794 | 175 | 0.242 | 99 | 73 | 1 | 1 | 97 | 17 | 115 | GPW\_gp25 domain-containing protein | GPW\_gp25 domain-containing protein | | afdb-uniprot50 | AF-A0A1Q4EDA0-F1-MODEL\_V4 | 1.0 | 0.0001415 | 175 | 0.176 | 113 | 84 | 5 | 2 | 107 | 9 | 119 | GPW\_gp25 domain-containing protein | GPW\_gp25 domain-containing protein | | afdb-uniprot50 | AF-A0A0F6SF01-F1-MODEL\_V4 | 1.0 | 0.0003364 | 175 | 0.18 | 105 | 79 | 6 | 6 | 105 | 40 | 142 | Uncharacterized protein | Uncharacterized protein | | afdb-uniprot50 | AF-A0A2D6E562-F1-MODEL\_V4 | 1.0 | 9.181e-05 | 175 | 0.195 | 123 | 83 | 5 | 2 | 111 | 48 | 167 | Uncharacterized protein | Uncharacterized protein | | afdb-uniprot50 | AF-E8WUR4-F1-MODEL\_V4 | 1.0 | 2.506e-05 | 175 | 0.221 | 113 | 72 | 4 | 2 | 108 | 14 | 116 | GPW/gp25 family protein | GPW/gp25 family protein | | afdb-uniprot50 | AF-A0A2A2B515-F1-MODEL\_V4 | 1.0 | 3.413e-05 | 174 | 0.203 | 113 | 82 | 4 | 3 | 108 | 10 | 121 | Uncharacterized protein | Uncharacterized protein | | afdb-uniprot50 | AF-A0A2E4W4D6-F1-MODEL\_V4 | 1.0 | 0.0001704 | 174 | 0.203 | 113 | 74 | 2 | 5 | 106 | 1 | 108 | Uncharacterized protein | Uncharacterized protein | | afdb-uniprot50 | AF-A0A1J0LUS2-F1-MODEL\_V4 | 1.0 | 0.0001176 | 174 | 0.228 | 114 | 76 | 7 | 1 | 107 | 5 | 113 | Uncharacterized protein | Uncharacterized protein | | afdb-uniprot50 | AF-A0A3P6JCI1-F1-MODEL\_V4 | 1.0 | 4.65e-05 | 174 | 0.236 | 114 | 75 | 3 | 3 | 105 | 10 | 122 | Phage baseplate assembly protein W | Phage baseplate assembly protein W | | afdb-uniprot50 | AF-A0A0C2UZ28-F1-MODEL\_V4 | 1.0 | 0.0001176 | 174 | 0.175 | 114 | 86 | 5 | 1 | 111 | 170 | 278 | Uncharacterized protein | Uncharacterized protein | | afdb-uniprot50 | AF-A0A7C7FT58-F1-MODEL\_V4 | 1.0 | 0.0002627 | 173 | 0.154 | 97 | 75 | 4 | 18 | 107 | 2 | 98 | GPW\_gp25 domain-containing protein | GPW\_gp25 domain-containing protein | | afdb-uniprot50 | AF-S4XZU3-F1-MODEL\_V4 | 1.0 | 0.0001415 | 173 | 0.194 | 113 | 86 | 5 | 2 | 111 | 12 | 122 | GPW\_gp25 domain-containing protein | GPW\_gp25 domain-containing protein | | afdb-uniprot50 | AF-A0A2E0MEK5-F1-MODEL\_V4 | 1.0 | 0.0003162 | 173 | 0.169 | 112 | 86 | 3 | 3 | 107 | 20 | 131 | GPW\_gp25 domain-containing protein | GPW\_gp25 domain-containing protein | | afdb-uniprot50 | AF-A0A3D1IZH3-F1-MODEL\_V4 | 1.0 | 0.0005186 | 172 | 0.163 | 110 | 87 | 5 | 1 | 108 | 76 | 182 | Uncharacterized protein | Uncharacterized protein | | afdb-uniprot50 | AF-A0A6I6E3I6-F1-MODEL\_V4 | 1.0 | 6.739e-05 | 171 | 0.256 | 113 | 75 | 3 | 1 | 105 | 1 | 112 | GPW\_gp25 domain-containing protein | GPW\_gp25 domain-containing protein | | afdb-uniprot50 | AF-A0A382V1Y7-F1-MODEL\_V4 | 1.0 | 7.169e-05 | 171 | 0.176 | 113 | 82 | 6 | 3 | 107 | 26 | 135 | GPW\_gp25 domain-containing protein | GPW\_gp25 domain-containing protein | | afdb-uniprot50 | AF-A0A1G3MBB2-F1-MODEL\_V4 | 1.0 | 0.0001813 | 171 | 0.223 | 112 | 82 | 3 | 5 | 112 | 28 | 138 | GPW\_gp25 domain-containing protein | GPW\_gp25 domain-containing protein | | afdb-uniprot50 | AF-A0A1Q7Z6P2-F1-MODEL\_V4 | 1.0 | 0.0006243 | 170 | 0.305 | 85 | 57 | 2 | 2 | 85 | 14 | 97 | GPW\_gp25 domain-containing protein | GPW\_gp25 domain-containing protein | | afdb-uniprot50 | AF-A0A2T5P176-F1-MODEL\_V4 | 1.0 | 0.0002469 | 170 | 0.25 | 108 | 75 | 5 | 2 | 105 | 3 | 108 | Uncharacterized protein | Uncharacterized protein | | afdb-uniprot50 | AF-A0A0F4NK88-F1-MODEL\_V4 | 1.0 | 0.0006642 | 170 | 0.342 | 76 | 49 | 1 | 8 | 82 | 27 | 102 | GPW\_gp25 domain-containing protein | GPW\_gp25 domain-containing protein | | afdb-uniprot50 | AF-A0A3D4UYF4-F1-MODEL\_V4 | 1.0 | 0.0001704 | 170 | 0.185 | 113 | 84 | 3 | 3 | 107 | 15 | 127 | Uncharacterized protein | Uncharacterized protein | | afdb-uniprot50 | AF-A0A7C3ETG1-F1-MODEL\_V4 | 1.0 | 0.0001928 | 170 | 0.155 | 116 | 86 | 4 | 2 | 105 | 10 | 125 | GPW\_gp25 domain-containing protein | GPW\_gp25 domain-containing protein | | afdb-uniprot50 | AF-A0A1Z4C3B0-F1-MODEL\_V4 | 1.0 | 0.0004308 | 170 | 0.165 | 115 | 89 | 6 | 1 | 112 | 85 | 195 | Uncharacterized protein | Uncharacterized protein | | afdb-uniprot50 | AF-A0A7M2RZX2-F1-MODEL\_V4 | 1.0 | 0.0002051 | 169 | 0.171 | 105 | 87 | 0 | 3 | 107 | 10 | 114 | Uncharacterized protein | Uncharacterized protein | | afdb-uniprot50 | AF-A0A350PIY9-F1-MODEL\_V4 | 1.0 | 0.0003364 | 169 | 0.119 | 109 | 91 | 2 | 2 | 105 | 18 | 126 | GPW\_gp25 domain-containing protein | GPW\_gp25 domain-containing protein | | afdb-uniprot50 | AF-A0A511N764-F1-MODEL\_V4 | 1.0 | 0.0006243 | 169 | 0.163 | 116 | 87 | 2 | 1 | 107 | 82 | 196 | Uncharacterized protein | Uncharacterized protein | | afdb-uniprot50 | AF-A0A5S9R4I1-F1-MODEL\_V4 | 1.0 | 0.0002973 | 168 | 0.257 | 97 | 60 | 2 | 23 | 107 | 2 | 98 | Uncharacterized protein | Uncharacterized protein | | afdb-uniprot50 | AF-A0A2D0N0Z6-F1-MODEL\_V4 | 1.0 | 9.181e-05 | 168 | 0.196 | 112 | 76 | 3 | 2 | 109 | 21 | 122 | GPW\_gp25 domain-containing protein | GPW\_gp25 domain-containing protein | | afdb-uniprot50 | AF-A0A209AE83-F1-MODEL\_V4 | 1.0 | 0.0001928 | 168 | 0.303 | 79 | 53 | 1 | 27 | 105 | 92 | 168 | Phage\_base\_V domain-containing protein | Phage\_base\_V domain-containing protein | | afdb-uniprot50 | AF-A0A3C0GIN9-F1-MODEL\_V4 | 1.0 | 0.0001251 | 167 | 0.162 | 123 | 82 | 6 | 2 | 106 | 10 | 129 | GPW\_gp25 domain-containing protein | GPW\_gp25 domain-containing protein | | afdb-uniprot50 | AF-A0A2K9P038-F1-MODEL\_V4 | 1.0 | 0.0002627 | 167 | 0.228 | 114 | 76 | 8 | 1 | 107 | 24 | 132 | Phage protein | Phage protein | | afdb-uniprot50 | AF-A0A8A6B8Z7-F1-MODEL\_V4 | 1.0 | 0.0002321 | 167 | 0.196 | 112 | 81 | 6 | 2 | 108 | 26 | 133 | DUF2634 domain-containing protein | DUF2634 domain-containing protein | | afdb-uniprot50 | AF-A0A7U9RXB1-F1-MODEL\_V4 | 1.0 | 0.0001176 | 167 | 0.247 | 113 | 78 | 5 | 1 | 108 | 36 | 146 | Uncharacterized protein | Uncharacterized protein | | afdb-uniprot50 | AF-A0A841VB05-F1-MODEL\_V4 | 1.0 | 0.0005869 | 166 | 0.325 | 86 | 54 | 2 | 2 | 86 | 14 | 96 | GPW/gp25 family protein | GPW/gp25 family protein | | afdb-uniprot50 | AF-A0A6M0RCZ2-F1-MODEL\_V4 | 1.0 | 0.0005186 | 166 | 0.166 | 126 | 87 | 6 | 1 | 112 | 1 | 122 | Uncharacterized protein | Uncharacterized protein | | afdb-uniprot50 | AF-A0A517VBS1-F1-MODEL\_V4 | 1.0 | 0.0005186 | 166 | 0.15 | 120 | 89 | 5 | 1 | 108 | 1 | 119 | Gene 25-like lysozyme | Gene 25-like lysozyme | | afdb-uniprot50 | AF-N1ZEV8-F1-MODEL\_V4 | 1.0 | 8.113e-05 | 166 | 0.196 | 122 | 82 | 5 | 1 | 107 | 1 | 121 | Uncharacterized protein | Uncharacterized protein | | afdb-uniprot50 | AF-V7ZNH0-F1-MODEL\_V4 | 1.0 | 0.0002321 | 166 | 0.187 | 112 | 84 | 5 | 2 | 108 | 16 | 125 | Uncharacterized protein | Uncharacterized protein | | afdb-uniprot50 | AF-A0A1W9S296-F1-MODEL\_V4 | 1.0 | 0.0004308 | 166 | 0.179 | 106 | 85 | 2 | 1 | 105 | 15 | 119 | Uncharacterized protein | Uncharacterized protein | | afdb-uniprot50 | AF-A0A1I0WHG9-F1-MODEL\_V4 | 1.0 | 0.0005186 | 166 | 0.221 | 113 | 77 | 6 | 2 | 107 | 23 | 131 | Uncharacterized protein | Uncharacterized protein | | afdb-uniprot50 | AF-A0A4R7BFE6-F1-MODEL\_V4 | 1.0 | 0.0001176 | 166 | 0.214 | 112 | 74 | 3 | 2 | 109 | 13 | 114 | GPW\_gp25 domain-containing protein | GPW\_gp25 domain-containing protein | | afdb-uniprot50 | AF-A0A1X1NFH7-F1-MODEL\_V4 | 1.0 | 0.0001251 | 166 | 0.201 | 124 | 79 | 4 | 2 | 105 | 247 | 370 | Uncharacterized protein | Uncharacterized protein | | afdb-uniprot50 | AF-A0A7W5K3U6-F1-MODEL\_V4 | 1.0 | 8.113e-05 | 165 | 0.209 | 110 | 77 | 4 | 2 | 105 | 14 | 119 | GPW\_gp25 domain-containing protein | GPW\_gp25 domain-containing protein | | afdb-uniprot50 | AF-A0A539E6I3-F1-MODEL\_V4 | 1.0 | 0.0007995 | 165 | 0.205 | 102 | 75 | 2 | 2 | 97 | 19 | 120 | GPW/gp25 | GPW/gp25 | | afdb-uniprot50 | AF-A0A2K8Z4R5-F1-MODEL\_V4 | 1.0 | 5.598e-05 | 165 | 0.203 | 108 | 72 | 2 | 2 | 107 | 14 | 109 | GPW\_gp25 domain-containing protein | GPW\_gp25 domain-containing protein | | afdb-uniprot50 | AF-A0A0L6JGL6-F1-MODEL\_V4 | 1.0 | 0.0005869 | 165 | 0.215 | 116 | 84 | 4 | 1 | 112 | 24 | 136 | Phage-like element PBSX protein, XkdS | Phage-like element PBSX protein, XkdS | | afdb-uniprot50 | AF-C6HYF2-F1-MODEL\_V4 | 1.0 | 0.0002051 | 164 | 0.276 | 94 | 62 | 5 | 17 | 106 | 32 | 123 | GPW/gp25 family protein | GPW/gp25 family protein | | afdb-uniprot50 | AF-A0A1H9YIU9-F1-MODEL\_V4 | 1.0 | 0.0004875 | 164 | 0.222 | 108 | 78 | 6 | 1 | 105 | 27 | 131 | Uncharacterized protein | Uncharacterized protein | | afdb-uniprot50 | AF-A0A2D6F2M5-F1-MODEL\_V4 | 1.0 | 0.0002469 | 164 | 0.229 | 109 | 74 | 8 | 3 | 105 | 54 | 158 | Uncharacterized protein | Uncharacterized protein | | afdb-uniprot50 | AF-A0A2A5HV32-F1-MODEL\_V4 | 1.0 | 0.0001039 | 164 | 0.226 | 106 | 68 | 2 | 2 | 105 | 49 | 142 | GPW\_gp25 domain-containing protein | GPW\_gp25 domain-containing protein | | afdb-uniprot50 | AF-A0A2D5F9D6-F1-MODEL\_V4 | 1.0 | 0.0009625 | 163 | 0.132 | 113 | 92 | 3 | 2 | 111 | 36 | 145 | GPW\_gp25 domain-containing protein | GPW\_gp25 domain-containing protein | | afdb-uniprot50 | AF-A0A252EC99-F1-MODEL\_V4 | 1.0 | 0.001024 | 163 | 0.205 | 107 | 81 | 2 | 2 | 104 | 33 | 139 | GPW\_gp25 domain-containing protein | GPW\_gp25 domain-containing protein | | afdb-uniprot50 | AF-A0A5J4RAP1-F1-MODEL\_V4 | 1.0 | 0.0007516 | 162 | 0.13 | 100 | 82 | 3 | 2 | 97 | 13 | 111 | GPW\_gp25 domain-containing protein | GPW\_gp25 domain-containing protein | | afdb-uniprot50 | AF-A0A7C7C1N4-F1-MODEL\_V4 | 1.0 | 0.0006642 | 162 | 0.155 | 109 | 88 | 3 | 2 | 106 | 10 | 118 | GPW\_gp25 domain-containing protein | GPW\_gp25 domain-containing protein | | afdb-uniprot50 | AF-A0A4Q7YHD4-F1-MODEL\_V4 | 1.0 | 0.0003364 | 162 | 0.245 | 106 | 74 | 4 | 2 | 102 | 9 | 113 | GPW\_gp25 domain-containing protein | GPW\_gp25 domain-containing protein | | afdb-uniprot50 | AF-A0A1M6M4V6-F1-MODEL\_V4 | 1.0 | 0.000405 | 162 | 0.176 | 113 | 82 | 7 | 1 | 107 | 36 | 143 | Uncharacterized protein | Uncharacterized protein | | afdb-uniprot50 | AF-C3X352-F1-MODEL\_V4 | 1.0 | 0.0006243 | 162 | 0.171 | 111 | 86 | 5 | 1 | 110 | 94 | 199 | Uncharacterized protein | Uncharacterized protein | | afdb-uniprot50 | AF-A0A5E4PHX7-F1-MODEL\_V4 | 1.0 | 0.0002973 | 161 | 0.184 | 114 | 81 | 5 | 1 | 107 | 10 | 118 | Uncharacterized protein | Uncharacterized protein | | afdb-uniprot50 | AF-A0A074LKB3-F1-MODEL\_V4 | 1.0 | 0.0001176 | 161 | 0.175 | 114 | 83 | 8 | 2 | 110 | 16 | 123 | Uncharacterized protein | Uncharacterized protein | | afdb-uniprot50 | AF-A0A842TDK5-F1-MODEL\_V4 | 1.0 | 0.0003364 | 161 | 0.224 | 107 | 79 | 3 | 3 | 106 | 17 | 122 | Uncharacterized protein | Uncharacterized protein | | afdb-uniprot50 | AF-L0R441-F1-MODEL\_V4 | 1.0 | 0.0007995 | 161 | 0.193 | 93 | 70 | 4 | 17 | 105 | 31 | 122 | Putative Type VI secretion system lysozyme-related protein | Putative Type VI secretion system lysozyme-related protein | | afdb-uniprot50 | AF-A0A2A5HX32-F1-MODEL\_V4 | 1.0 | 0.0001602 | 161 | 0.221 | 113 | 72 | 4 | 2 | 109 | 16 | 117 | GPW\_gp25 domain-containing protein | GPW\_gp25 domain-containing protein | | afdb-uniprot50 | AF-A0A3C0G474-F1-MODEL\_V4 | 1.0 | 0.0002469 | 161 | 0.121 | 123 | 87 | 6 | 1 | 105 | 22 | 141 | GPW\_gp25 domain-containing protein | GPW\_gp25 domain-containing protein | | afdb-uniprot50 | AF-A0A1J0LXL0-F1-MODEL\_V4 | 1.0 | 7.626e-05 | 160 | 0.192 | 109 | 77 | 6 | 1 | 107 | 11 | 110 | Uncharacterized protein | Uncharacterized protein | | afdb-uniprot50 | AF-A0A4Z0PFZ4-F1-MODEL\_V4 | 1.0 | 0.0001506 | 160 | 0.216 | 106 | 69 | 2 | 2 | 105 | 26 | 119 | GPW\_gp25 domain-containing protein | GPW\_gp25 domain-containing protein | | afdb-uniprot50 | AF-A0A090QXN9-F1-MODEL\_V4 | 1.0 | 0.0004875 | 159 | 0.213 | 103 | 77 | 2 | 3 | 105 | 1 | 99 | Uncharacterized protein | Uncharacterized protein | | afdb-uniprot50 | AF-A0A7J5EX88-F1-MODEL\_V4 | 1.0 | 0.0006243 | 159 | 0.18 | 105 | 80 | 3 | 2 | 106 | 9 | 107 | Uncharacterized protein | Uncharacterized protein | | afdb-uniprot50 | AF-C3X496-F1-MODEL\_V4 | 1.0 | 0.0001176 | 159 | 0.188 | 122 | 82 | 6 | 3 | 108 | 1 | 121 | GPW\_gp25 domain-containing protein | GPW\_gp25 domain-containing protein | | afdb-uniprot50 | AF-R5H3R4-F1-MODEL\_V4 | 1.0 | 0.0007516 | 159 | 0.177 | 118 | 85 | 8 | 1 | 112 | 20 | 131 | Uncharacterized protein | Uncharacterized protein | | afdb-uniprot50 | AF-A0A522CGL7-F1-MODEL\_V4 | 1.0 | 0.001395 | 159 | 0.16 | 112 | 84 | 6 | 1 | 105 | 10 | 118 | Uncharacterized protein | Uncharacterized protein | | afdb-uniprot50 | AF-A0A7M1PWR8-F1-MODEL\_V4 | 1.0 | 0.0008505 | 158 | 0.177 | 118 | 85 | 8 | 1 | 112 | 23 | 134 | DUF2634 domain-containing protein | DUF2634 domain-containing protein | | afdb-uniprot50 | AF-A0A1I3XYL5-F1-MODEL\_V4 | 1.0 | 0.0001039 | 158 | 0.208 | 115 | 73 | 5 | 2 | 109 | 16 | 119 | GPW\_gp25 domain-containing protein | GPW\_gp25 domain-containing protein | | afdb-uniprot50 | AF-A0A7C0UDU3-F1-MODEL\_V4 | 1.0 | 0.000405 | 157 | 0.18 | 111 | 79 | 6 | 2 | 105 | 13 | 118 | GPW\_gp25 domain-containing protein | GPW\_gp25 domain-containing protein | | afdb-uniprot50 | AF-A0A6N4GXB3-F1-MODEL\_V4 | 1.0 | 0.0002794 | 157 | 0.216 | 106 | 68 | 2 | 2 | 105 | 16 | 108 | GPW\_gp25 domain-containing protein | GPW\_gp25 domain-containing protein | | afdb-uniprot50 | AF-A0A2D0MZD6-F1-MODEL\_V4 | 1.0 | 0.0001506 | 157 | 0.216 | 106 | 69 | 2 | 2 | 105 | 16 | 109 | GPW\_gp25 domain-containing protein | GPW\_gp25 domain-containing protein | | afdb-uniprot50 | AF-A0A1C6BPB7-F1-MODEL\_V4 | 1.0 | 0.0002469 | 156 | 0.229 | 109 | 79 | 4 | 1 | 106 | 9 | 115 | Uncharacterized protein | Uncharacterized protein | | afdb-uniprot50 | AF-H6NDT0-F1-MODEL\_V4 | 1.0 | 0.0002627 | 156 | 0.196 | 117 | 79 | 8 | 1 | 108 | 11 | 121 | Uncharacterized protein | Uncharacterized protein | | afdb-uniprot50 | AF-A0A1F6G8V8-F1-MODEL\_V4 | 1.0 | 0.0001813 | 156 | 0.188 | 106 | 71 | 2 | 2 | 105 | 15 | 107 | GPW\_gp25 domain-containing protein | GPW\_gp25 domain-containing protein | | afdb-uniprot50 | AF-A0A2E3HR82-F1-MODEL\_V4 | 1.0 | 0.0007995 | 156 | 0.162 | 111 | 85 | 5 | 3 | 107 | 22 | 130 | GPW\_gp25 domain-containing protein | GPW\_gp25 domain-containing protein | | afdb-uniprot50 | AF-A0A2A6Z7N1-F1-MODEL\_V4 | 1.0 | 0.0005186 | 156 | 0.155 | 109 | 88 | 4 | 2 | 107 | 41 | 148 | Uncharacterized protein | Uncharacterized protein | | afdb-uniprot50 | AF-A0A2D6MFM3-F1-MODEL\_V4 | 1.0 | 0.0003579 | 156 | 0.131 | 122 | 88 | 5 | 4 | 107 | 76 | 197 | GPW\_gp25 domain-containing protein | GPW\_gp25 domain-containing protein | | afdb-uniprot50 | AF-A0A0R1ZJK1-F1-MODEL\_V4 | 1.0 | 0.0005186 | 155 | 0.169 | 118 | 84 | 8 | 2 | 112 | 15 | 125 | Uncharacterized protein | Uncharacterized protein | | afdb-uniprot50 | AF-A0A5C7JCT6-F1-MODEL\_V4 | 1.0 | 0.001484 | 155 | 0.11 | 109 | 92 | 4 | 2 | 105 | 13 | 121 | GPW\_gp25 domain-containing protein | GPW\_gp25 domain-containing protein | | afdb-uniprot50 | AF-A0A1I3LYC4-F1-MODEL\_V4 | 1.0 | 0.0005186 | 155 | 0.175 | 108 | 78 | 5 | 6 | 105 | 14 | 118 | Uncharacterized protein | Uncharacterized protein | | afdb-uniprot50 | AF-A0A822PRL6-F1-MODEL\_V4 | 1.0 | 0.001395 | 155 | 0.179 | 117 | 86 | 7 | 1 | 112 | 21 | 132 | Phage-like element PBSX protein | Phage-like element PBSX protein | | afdb-uniprot50 | AF-A0A1C6I381-F1-MODEL\_V4 | 1.0 | 0.0003579 | 155 | 0.154 | 123 | 90 | 7 | 1 | 112 | 16 | 135 | Uncharacterized protein | Uncharacterized protein | | afdb-uniprot50 | AF-A0A7X7JM68-F1-MODEL\_V4 | 1.0 | 0.0001813 | 154 | 0.174 | 109 | 79 | 6 | 3 | 106 | 16 | 118 | Type VI secretion system baseplate subunit TssE | Type VI secretion system baseplate subunit TssE | | afdb-uniprot50 | AF-A0A1V5B845-F1-MODEL\_V4 | 1.0 | 0.0002794 | 154 | 0.173 | 121 | 88 | 4 | 1 | 109 | 23 | 143 | Uncharacterized protein | Uncharacterized protein | | afdb-uniprot50 | AF-A0A7X9FIB8-F1-MODEL\_V4 | 1.0 | 0.0001602 | 152 | 0.184 | 125 | 79 | 6 | 1 | 108 | 1 | 119 | GPW/gp25 family protein | GPW/gp25 family protein | | afdb-uniprot50 | AF-X0PT30-F1-MODEL\_V4 | 1.0 | 0.001024 | 152 | 0.178 | 112 | 83 | 6 | 2 | 108 | 9 | 116 | Uncharacterized protein | Uncharacterized protein | | afdb-uniprot50 | AF-A0A7X8EVE5-F1-MODEL\_V4 | 1.0 | 0.000405 | 152 | 0.173 | 115 | 84 | 7 | 1 | 107 | 22 | 133 | DUF2634 domain-containing protein | DUF2634 domain-containing protein | | afdb-uniprot50 | AF-A0A1I4QKV8-F1-MODEL\_V4 | 1.0 | 0.0009625 | 152 | 0.196 | 117 | 84 | 7 | 1 | 112 | 23 | 134 | Uncharacterized protein | Uncharacterized protein | | afdb-uniprot50 | AF-A0A4R5N831-F1-MODEL\_V4 | 1.0 | 0.0005869 | 152 | 0.219 | 114 | 78 | 7 | 2 | 107 | 46 | 156 | Uncharacterized protein | Uncharacterized protein | | afdb-uniprot50 | AF-A0A3Q8US91-F1-MODEL\_V4 | 1.0 | 0.0002973 | 152 | 0.207 | 135 | 78 | 7 | 1 | 108 | 232 | 364 | GPW\_gp25 domain-containing protein | GPW\_gp25 domain-containing protein | | afdb-uniprot50 | AF-A0A431IHC8-F1-MODEL\_V4 | 1.0 | 0.0007065 | 151 | 0.137 | 116 | 88 | 4 | 3 | 107 | 10 | 124 | Uncharacterized protein | Uncharacterized protein | | afdb-uniprot50 | AF-A0A7X8C4E8-F1-MODEL\_V4 | 1.0 | 0.0009048 | 151 | 0.169 | 112 | 84 | 6 | 1 | 107 | 21 | 128 | DUF2634 domain-containing protein | DUF2634 domain-containing protein | | afdb-uniprot50 | AF-A0A2E1V253-F1-MODEL\_V4 | 1.0 | 0.0002182 | 151 | 0.198 | 106 | 71 | 2 | 2 | 105 | 17 | 110 | GPW\_gp25 domain-containing protein | GPW\_gp25 domain-containing protein | | afdb-uniprot50 | AF-A0A5C8AT77-F1-MODEL\_V4 | 1.0 | 0.001395 | 151 | 0.156 | 134 | 82 | 4 | 2 | 105 | 20 | 152 | GPW\_gp25 domain-containing protein | GPW\_gp25 domain-containing protein | | afdb-uniprot50 | AF-A0A2D5PHG3-F1-MODEL\_V4 | 1.0 | 0.0008505 | 150 | 0.169 | 106 | 82 | 2 | 2 | 105 | 12 | 113 | Uncharacterized protein | Uncharacterized protein | | afdb-uniprot50 | AF-V8I9F2-F1-MODEL\_V4 | 1.0 | 0.001786 | 150 | 0.207 | 111 | 79 | 6 | 2 | 107 | 15 | 121 | Uncharacterized protein | Uncharacterized protein | | afdb-uniprot50 | AF-G4Q4I2-F1-MODEL\_V4 | 1.0 | 0.002022 | 150 | 0.168 | 119 | 85 | 6 | 1 | 106 | 25 | 142 | Phage protein | Phage protein | | afdb-uniprot50 | AF-A0A4S2BQZ7-F1-MODEL\_V4 | 1.0 | 0.0002794 | 150 | 0.219 | 114 | 74 | 8 | 2 | 107 | 49 | 155 | DUF2634 domain-containing protein | DUF2634 domain-containing protein | | afdb-uniprot50 | AF-A0A4Q6BJX5-F1-MODEL\_V4 | 1.0 | 0.001395 | 149 | 0.178 | 112 | 81 | 6 | 1 | 105 | 6 | 113 | Uncharacterized protein | Uncharacterized protein | | afdb-uniprot50 | AF-A0A1H8JI52-F1-MODEL\_V4 | 1.0 | 0.002754 | 149 | 0.135 | 118 | 90 | 8 | 2 | 112 | 29 | 141 | Uncharacterized protein | Uncharacterized protein | | afdb-uniprot50 | AF-A0A7U4Y7W0-F1-MODEL\_V4 | 1.0 | 0.001089 | 149 | 0.139 | 93 | 74 | 3 | 17 | 105 | 41 | 131 | Type VI secretion protein | Type VI secretion protein | | afdb-uniprot50 | AF-A0A254RDD5-F1-MODEL\_V4 | 1.0 | 0.0008505 | 148 | 0.189 | 95 | 74 | 2 | 13 | 105 | 1 | 94 | Uncharacterized protein | Uncharacterized protein | | afdb-uniprot50 | AF-A0A559J3Y0-F1-MODEL\_V4 | 1.0 | 0.001484 | 148 | 0.149 | 107 | 85 | 5 | 3 | 107 | 1 | 103 | DUF2634 domain-containing protein | DUF2634 domain-containing protein | | afdb-uniprot50 | AF-A0A3F3H3F3-F1-MODEL\_V4 | 1.0 | 0.001311 | 148 | 0.154 | 110 | 84 | 6 | 2 | 106 | 14 | 119 | Uncharacterized protein | Uncharacterized protein | | afdb-uniprot50 | AF-A0A844ECA9-F1-MODEL\_V4 | 1.0 | 0.0003807 | 148 | 0.191 | 115 | 80 | 7 | 2 | 107 | 29 | 139 | DUF2634 domain-containing protein | DUF2634 domain-containing protein | | afdb-uniprot50 | AF-A0A1Y4UHE5-F1-MODEL\_V4 | 1.0 | 0.001089 | 147 | 0.194 | 113 | 84 | 5 | 1 | 108 | 20 | 130 | Uncharacterized protein | Uncharacterized protein | | afdb-uniprot50 | AF-A0A0S2W0M2-F1-MODEL\_V4 | 1.0 | 0.0019 | 147 | 0.215 | 116 | 81 | 7 | 1 | 111 | 17 | 127 | Phage-like element PBSX protein xkdS | Phage-like element PBSX protein xkdS | | afdb-uniprot50 | AF-J5GPF1-F1-MODEL\_V4 | 1.0 | 0.001395 | 147 | 0.188 | 117 | 85 | 7 | 1 | 112 | 23 | 134 | PF10934 family protein | PF10934 family protein | | afdb-uniprot50 | AF-A0A5B1BKF6-F1-MODEL\_V4 | 1.0 | 0.0003807 | 147 | 0.186 | 107 | 72 | 3 | 2 | 105 | 16 | 110 | GPW/gp25 family protein | GPW/gp25 family protein | | afdb-uniprot50 | AF-A0A1V6IG61-F1-MODEL\_V4 | 1.0 | 0.002754 | 146 | 0.106 | 113 | 92 | 4 | 1 | 105 | 2 | 113 | Uncharacterized protein | Uncharacterized protein | | afdb-uniprot50 | AF-A0A4U3FI15-F1-MODEL\_V4 | 1.0 | 0.001395 | 146 | 0.169 | 112 | 84 | 6 | 1 | 107 | 25 | 132 | DUF2634 domain-containing protein | DUF2634 domain-containing protein | | afdb-uniprot50 | AF-A0A1Y4U156-F1-MODEL\_V4 | 1.0 | 0.000405 | 146 | 0.225 | 120 | 78 | 8 | 2 | 112 | 22 | 135 | Uncharacterized protein | Uncharacterized protein | | afdb-uniprot50 | AF-A0A2T0WPJ3-F1-MODEL\_V4 | 1.0 | 0.0002794 | 146 | 0.245 | 106 | 66 | 2 | 2 | 105 | 16 | 109 | GPW\_gp25 domain-containing protein | GPW\_gp25 domain-containing protein | | afdb-uniprot50 | AF-A0A1P8Q4D0-F1-MODEL\_V4 | 1.0 | 0.002022 | 145 | 0.178 | 112 | 83 | 6 | 2 | 108 | 5 | 112 | Uncharacterized protein | Uncharacterized protein | | afdb-uniprot50 | AF-A0A2V2E0J3-F1-MODEL\_V4 | 1.0 | 0.001233 | 145 | 0.245 | 110 | 74 | 6 | 1 | 105 | 21 | 126 | DUF2634 domain-containing protein | DUF2634 domain-containing protein | | afdb-uniprot50 | AF-A0A396SP14-F1-MODEL\_V4 | 1.0 | 0.001159 | 144 | 0.177 | 118 | 83 | 8 | 2 | 112 | 22 | 132 | DUF2634 domain-containing protein | DUF2634 domain-containing protein | | afdb-uniprot50 | AF-A0A826VXZ4-F1-MODEL\_V4 | 1.0 | 0.001311 | 143 | 0.314 | 70 | 48 | 0 | 41 | 110 | 1 | 70 | Baseplate assembly protein | Baseplate assembly protein | | afdb-uniprot50 | AF-A0A3E3IVZ8-F1-MODEL\_V4 | 1.0 | 0.001311 | 143 | 0.155 | 116 | 86 | 6 | 1 | 107 | 11 | 123 | DUF2634 domain-containing protein | DUF2634 domain-containing protein | | afdb-uniprot50 | AF-A0A1T4UK52-F1-MODEL\_V4 | 1.0 | 0.001395 | 142 | 0.112 | 107 | 89 | 3 | 4 | 107 | 8 | 111 | Uncharacterized protein | Uncharacterized protein | | afdb-uniprot50 | AF-A0A7Y7HXH9-F1-MODEL\_V4 | 1.0 | 0.0007516 | 142 | 0.243 | 111 | 74 | 7 | 4 | 106 | 12 | 120 | Phage gp46-like protein | Phage gp46-like protein | | afdb-uniprot50 | AF-A8LSA1-F1-MODEL\_V4 | 1.0 | 0.000405 | 142 | 0.219 | 114 | 68 | 3 | 1 | 105 | 18 | 119 | GPW\_gp25 domain-containing protein | GPW\_gp25 domain-containing protein | | afdb-uniprot50 | AF-A0A3M0N0X0-F1-MODEL\_V4 | 1.0 | 0.001395 | 142 | 0.157 | 114 | 83 | 7 | 2 | 108 | 40 | 147 | DUF2634 domain-containing protein | DUF2634 domain-containing protein | | afdb-uniprot50 | AF-A0A2E9PUI9-F1-MODEL\_V4 | 1.0 | 0.001786 | 141 | 0.169 | 112 | 82 | 4 | 3 | 108 | 16 | 122 | GPW\_gp25 domain-containing protein | GPW\_gp25 domain-containing protein | | afdb-uniprot50 | AF-A0A3M0NK27-F1-MODEL\_V4 | 1.0 | 0.001311 | 141 | 0.157 | 114 | 83 | 7 | 2 | 108 | 55 | 162 | DUF2634 domain-containing protein | DUF2634 domain-containing protein | | afdb-uniprot50 | AF-E6X1N5-F1-MODEL\_V4 | 1.0 | 0.006153 | 139 | 0.228 | 92 | 65 | 1 | 2 | 93 | 8 | 93 | Uncharacterized protein | Uncharacterized protein | | afdb-uniprot50 | AF-R5SUR6-F1-MODEL\_V4 | 1.0 | 0.001311 | 139 | 0.194 | 118 | 87 | 5 | 1 | 112 | 24 | 139 | Phage protein | Phage protein | | afdb-uniprot50 | AF-A0A4R5NMZ8-F1-MODEL\_V4 | 1.0 | 0.0019 | 139 | 0.203 | 113 | 77 | 7 | 2 | 107 | 55 | 161 | Uncharacterized protein | Uncharacterized protein | | afdb-uniprot50 | AF-A0A1V5B2H8-F1-MODEL\_V4 | 1.0 | 0.00293 | 138 | 0.185 | 108 | 81 | 3 | 1 | 105 | 23 | 126 | Uncharacterized protein | Uncharacterized protein | | afdb-uniprot50 | AF-F4GSE7-F1-MODEL\_V4 | 1.0 | 0.001395 | 137 | 0.215 | 93 | 67 | 3 | 17 | 105 | 13 | 103 | GPW\_gp25 domain-containing protein | GPW\_gp25 domain-containing protein | | afdb-uniprot50 | AF-A0A1C6AKL0-F1-MODEL\_V4 | 1.0 | 0.00788 | 136 | 0.175 | 114 | 83 | 6 | 2 | 108 | 25 | 134 | Protein of uncharacterized function (DUF2634) | Protein of uncharacterized function (DUF2634) | | afdb-uniprot50 | AF-C4GJQ7-F1-MODEL\_V4 | 1.0 | 0.001089 | 136 | 0.241 | 116 | 75 | 5 | 1 | 107 | 40 | 151 | Uncharacterized protein | Uncharacterized protein | | afdb-uniprot50 | AF-A0A173S783-F1-MODEL\_V4 | 1.0 | 0.001395 | 135 | 0.17 | 123 | 82 | 8 | 1 | 107 | 25 | 143 | Protein of uncharacterized function (DUF2634) | Protein of uncharacterized function (DUF2634) | | afdb-uniprot50 | AF-A0A2D6XAB7-F1-MODEL\_V4 | 1.0 | 0.003991 | 134 | 0.174 | 126 | 79 | 5 | 5 | 108 | 52 | 174 | GPW\_gp25 domain-containing protein | GPW\_gp25 domain-containing protein | | afdb-uniprot50 | AF-A0A7C4DJH8-F1-MODEL\_V4 | 1.0 | 0.006153 | 133 | 0.25 | 84 | 57 | 2 | 5 | 86 | 21 | 100 | GPW\_gp25 domain-containing protein | GPW\_gp25 domain-containing protein | | afdb-uniprot50 | AF-A0A2E7CJE8-F1-MODEL\_V4 | 1.0 | 0.005111 | 132 | 0.159 | 88 | 66 | 2 | 3 | 90 | 8 | 87 | Uncharacterized protein | Uncharacterized protein | | afdb-uniprot50 | AF-A0A6H1Z7N3-F1-MODEL\_V4 | 1.0 | 0.006153 | 132 | 0.123 | 113 | 90 | 3 | 2 | 105 | 24 | 136 | Uncharacterized protein | Uncharacterized protein | | afdb-uniprot50 | AF-A0A2E5G1A5-F1-MODEL\_V4 | 1.0 | 0.003752 | 131 | 0.155 | 103 | 84 | 2 | 3 | 105 | 10 | 109 | Uncharacterized protein | Uncharacterized protein | | afdb-uniprot50 | AF-A0A1H8IY64-F1-MODEL\_V4 | 1.0 | 0.007407 | 131 | 0.159 | 113 | 86 | 7 | 2 | 108 | 19 | 128 | Uncharacterized protein | Uncharacterized protein | | afdb-uniprot50 | AF-A0A243EHU0-F1-MODEL\_V4 | 1.0 | 0.002288 | 131 | 0.25 | 104 | 70 | 5 | 3 | 101 | 133 | 233 | Uncharacterized protein | Uncharacterized protein | | afdb-uniprot50 | AF-A0A098CX13-F1-MODEL\_V4 | 1.0 | 0.004805 | 129 | 0.176 | 113 | 80 | 7 | 2 | 107 | 27 | 133 | Uncharacterized protein | Uncharacterized protein | | afdb-uniprot50 | AF-A0A3C0F5D4-F1-MODEL\_V4 | 1.0 | 0.008382 | 127 | 0.166 | 102 | 77 | 3 | 2 | 95 | 10 | 111 | GPW\_gp25 domain-containing protein | GPW\_gp25 domain-containing protein | | afdb-uniprot50 | AF-A0A431IP84-F1-MODEL\_V4 | 1.0 | 0.004246 | 127 | 0.178 | 112 | 85 | 5 | 3 | 110 | 16 | 124 | Uncharacterized protein | Uncharacterized protein | | afdb-uniprot50 | AF-A0A1Y4D1W7-F1-MODEL\_V4 | 1.0 | 0.001311 | 126 | 0.186 | 129 | 75 | 8 | 3 | 107 | 18 | 140 | GPW\_gp25 domain-containing protein | GPW\_gp25 domain-containing protein | | afdb-uniprot50 | AF-A0A3A9F4C1-F1-MODEL\_V4 | 1.0 | 0.00788 | 120 | 0.168 | 113 | 83 | 6 | 1 | 105 | 35 | 144 | DUF2634 domain-containing protein | DUF2634 domain-containing protein | | afdb-uniprot50 | AF-A0A1Y4GIV1-F1-MODEL\_V4 | 1.0 | 0.002754 | 116 | 0.209 | 124 | 78 | 8 | 5 | 111 | 16 | 136 | GPW\_gp25 domain-containing protein | GPW\_gp25 domain-containing protein | | afdb-uniprot50 | AF-F2Q843-F1-MODEL\_V4 | 1.0 | 0.006545 | 111 | 0.205 | 102 | 59 | 8 | 18 | 105 | 30 | 123 | GPW\_gp25 domain-containing protein | GPW\_gp25 domain-containing protein | |
| Top keywords  (threshold 1.00e-02 (evalue)) | **domain\_containing, GPW\_gp25, Baseplate, assembly, gp25, GPW, Phage, lysozyme, W, 25\_like** |
| Output files | ../../similar\_structures/12\_FANPEZAQ\_CDS\_0012\_afdb-proteome\_foldseek.tsv ../../similar\_structures/12\_FANPEZAQ\_CDS\_0012\_afdb-uniprot50\_foldseek.tsv ../../similar\_structures/12\_FANPEZAQ\_CDS\_0012\_merged.svg ../../similar\_structures/12\_FANPEZAQ\_CDS\_0012\_pdb\_foldseek.tsv |

  
  
  

Return to summary | Go to previous | Go to next

  


---

**Sequence/structure alignments coloring**  
Each object in the alignment figures is colored according to its E-value following this color coding:

1e-100
10

**References:**  
1) Steinegger M, Meier M, Mirdita M, Vöhringer H, Haunsberger S J, and Söding J (2019) HH-suite3 for fast remote homology detection and deep protein annotation, BMC Bioinformatics, 473. doi: 10.1186/s12859-019-3019-7  
2) Jumper J, Evans R, Pritzel A, ..., Hassabis D (2021) Highly accurate protein structure prediction with AlphaFold, Nature, 596. doi: 10.1038/s41586-021-03819-2  
3) van Kempen M, Kim S, Tumescheit C, Mirdita M, Lee J, Gilchrist CLM, Söding J, and Steinegger M (2023) Fast and accurate protein structure search with Foldseek. Nature Biotechnology. doi: 10.1038/s41587-023-01773-0
