# Supplementary material for: Completing the BASEL phage collection to unlock hidden diversity for systematic exploration of phage–host interactions
Source: PLoS Biol. 2025 Apr 7;23(4):e3003063. doi: 10.1371/journal.pbio.3003063 (PMC11990801; doi:10.1371/journal.pbio.3003063)
Supplement: S2 Data — (ZIP) [file pbio.3003063.s009.zip › entries/13.html]

FANPEZAQ\_CDS\_0013


Return to summary | Go to previous | Go to next

|  |  |
| --- | --- |
| FANPEZAQ\_CDS\_0013 Page creation date: 02 Sep 2024, 12:00  Project folder: n/a  Input sequences file: Escherichia\_virus\_HeidiAbel.gb | baseplate domain\_containing assembly baseplate\_j j\_like j gp47 phage putative phage\_related tail fragment jaye wedge mu bacteriophage homolog gp6 gpj tube t4 complex baseplate\_tail pre\_attachment structural prophage |

### Sequence information

|  |  |
| --- | --- |
| Name | FANPEZAQ\_CDS\_0013  13\_FANPEZAQ\_CDS\_0013 (pipeline id) |
| Imported annotations | Escherichia\_virus\_HeidiAbel Bas97 |
| Protein sequence | MAGSYTVIDLSQLPAPTIVEQLSFETILAEMIADLQARDSTFTALVESDPAYKILEVCAF RELLVRQRANEEAKAVMLAYASGTDLDQLGANVGVKRLVIRPADPNAVPPVPAVMESDNE FRARIQLAPEGYTTAGSEGSYAFHALGADADVKDVQPTSPTPGHVVVYVLSRTGDGAAPQ PLLDKVNATLTKEEVRPLTDNVTVQSAQIITYTISAELTLLPGPDSEVVRQAAFDAVAAY AEGQRRIGYDVTMSGLYRALHQPGVQNVKLTAPTVSLVLGDGQASYCTGITVTVAGETDV |
| Number of residues | 300 |
| Molecular weight (Da) | 31848.61 |
| Output files | ../../query\_sequences/13\_FANPEZAQ\_CDS\_0013.fasta |

### Putative domain architecture and protein family

#### Search results (HHblits)1

|  |  |
| --- | --- |
| Domain family databases searched | Pfam, Ncbi-cd, Cath, Phrogs |
| Results, scheme(s)  (Top layers only; threshold 1.00e-03 (evalue)) | xml version="1.0" encoding="utf-8" standalone="no"?       2024-09-02T21:08:14.706197 image/svg+xml   Matplotlib v3.7.2, https://matplotlib.org/ |
| Results, table  (E-value ≤ 1.00e-03 (evalue)) | | db | id | prob | evalue | pvalue | score | cols | query | query\_len | template | template\_len | name | description | | --- | --- | --- | --- | --- | --- | --- | --- | --- | --- | --- | --- | --- | | pfam | PF03434 | 99.4 | 3e-18 | 6.1e-22 | 152.2 | 155 | (115, 274) | 300 | (66, 247) | 289 | DUF276 | DUF276 | | pfam | PF04865 | 99.3 | 6.6e-17 | 1.4e-20 | 138.5 | 143 | (109, 259) | 300 | (104, 250) | 250 | Baseplate\_J | Baseplate J-like protein | | pfam | PF11041 | 98.6 | 3e-12 | 6.1e-16 | 104.4 | 103 | (69, 174) | 300 | (32, 156) | 181 | DUF2612 | Protein of unknown function (DUF2612) | | pfam | PF09684 | 94.2 | 0.00057 | 1.1e-07 | 50.0 | 68 | (75, 159) | 300 | (23, 90) | 138 | Tail\_P2\_I | Phage tail protein (Tail\_P2\_I) | | phrogs | 219 | 100.0 | 1.7e-36 | 2e-40 | 285.5 | 244 | (14, 262) | 300 | (1, 449) | 676 | baseplate wedge subunit | baseplate wedge subunit; Category: tail; KU686208\_p78 | | phrogs | 4226 | 99.8 | 3.9e-26 | 4.5e-30 | 182.8 | 230 | (70, 299) | 300 | (2, 231) | 231 | baseplate protein | baseplate protein; Category: tail; p428496 VI\_02892 | | phrogs | 6 | 99.8 | 8.9e-25 | 1.1e-28 | 199.5 | 283 | (8, 296) | 300 | (2, 471) | 472 | baseplate wedge subunit | baseplate wedge subunit; Category: tail; p248291 VI\_09499 | | phrogs | 1608 | 99.7 | 1.3e-21 | 1.5e-25 | 156.9 | 179 | (110, 292) | 300 | (34, 232) | 232 | tail protein | tail protein; Category: tail; p256756 VI\_05872 | | phrogs | 2103 | 99.2 | 1.3e-15 | 1.5e-19 | 119.2 | 157 | (14, 172) | 300 | (1, 234) | 245 | baseplate protein | baseplate protein; Category: tail; p226045 VI\_12003 | | phrogs | 1160 | 98.3 | 2.2e-10 | 2.6e-14 | 92.3 | 106 | (30, 157) | 300 | (4, 111) | 209 | baseplate protein | baseplate protein; Category: tail; p105495 VI\_00644 | | phrogs | 7378 | 98.2 | 3.3e-10 | 3.8e-14 | 96.8 | 98 | (47, 162) | 300 | (18, 117) | 462 | baseplate protein | baseplate protein; Category: tail; NC\_024792\_p167 | | phrogs | 4056 | 98.1 | 9.1e-10 | 1e-13 | 85.7 | 117 | (20, 159) | 300 | (10, 127) | 228 | baseplate protein | baseplate protein; Category: tail; NC\_024134\_p34 | | phrogs | 6277 | 98.0 | 2e-09 | 2.2e-13 | 87.1 | 105 | (49, 170) | 300 | (21, 125) | 283 | NA | NA; Category: unknown function; KY883640\_p59 | | phrogs | 4411 | 97.8 | 6.3e-09 | 7.1e-13 | 83.6 | 97 | (47, 161) | 300 | (18, 116) | 340 | virion structural protein | virion structural protein; Category: head and packaging; JN797798\_p181 | | phrogs | 2386 | 97.8 | 1.1e-08 | 1.2e-12 | 94.3 | 99 | (45, 161) | 300 | (19, 119) | 1309 | virion structural protein | virion structural protein; Category: head and packaging; NC\_020871\_p95 | | phrogs | 31189 | 97.8 | 1.1e-08 | 1.3e-12 | 85.3 | 143 | (110, 262) | 300 | (263, 406) | 662 | baseplate protein | baseplate protein; Category: tail; NC\_008584\_p150 | | phrogs | 22472 | 97.5 | 4.5e-08 | 5e-12 | 77.2 | 107 | (45, 168) | 300 | (20, 127) | 304 | NA | NA; Category: unknown function; MF370964\_p74 | | phrogs | 34452 | 97.3 | 2.3e-07 | 2.5e-11 | 65.7 | 86 | (12, 97) | 300 | (2, 87) | 175 | NA | NA; Category: unknown function; p43846 VI\_12438 | | phrogs | 13726 | 97.1 | 6e-07 | 6.8e-11 | 79.5 | 96 | (48, 161) | 300 | (22, 119) | 803 | NA | NA; Category: unknown function; NC\_027335\_p71 | | phrogs | 1369 | 96.9 | 1.7e-06 | 2e-10 | 75.3 | 128 | (110, 243) | 300 | (268, 410) | 477 | baseplate protein | baseplate protein; Category: tail; p133507 VI\_09775 | | phrogs | 23130 | 96.4 | 9.7e-06 | 1.1e-09 | 62.9 | 94 | (51, 161) | 300 | (19, 114) | 362 | tail protein | tail protein; Category: tail; NC\_022761\_p93 | | phrogs | 26379 | 96.2 | 1.9e-05 | 2.1e-09 | 58.9 | 105 | (24, 151) | 300 | (6, 114) | 220 | NA | NA; Category: unknown function; p152986 VI\_08595 | | phrogs | 521 | 96.2 | 1.8e-05 | 2.3e-09 | 61.1 | 107 | (47, 172) | 300 | (14, 123) | 180 | tail protein | tail protein; Category: tail; p222013 VI\_01195 | | phrogs | 5502 | 96.2 | 2e-05 | 2.3e-09 | 71.5 | 85 | (60, 161) | 300 | (31, 116) | 1585 | NA | NA; Category: unknown function; NC\_031037\_p116 | | phrogs | 10646 | 96.1 | 3e-05 | 3.5e-09 | 70.5 | 85 | (14, 98) | 300 | (23, 109) | 1154 | baseplate wedge subunit | baseplate wedge subunit; Category: tail; NC\_019526\_p71 | | phrogs | 14699 | 95.9 | 5.2e-05 | 5.8e-09 | 46.1 | 63 | (237, 299) | 300 | (4, 66) | 66 | NA | NA; Category: unknown function; p301057 VI\_10846 | | phrogs | 587 | 95.7 | 7.4e-05 | 9e-09 | 60.1 | 124 | (47, 171) | 300 | (35, 181) | 237 | structural protein | structural protein; Category: head and packaging; MF663761\_p45 | | phrogs | 21670 | 95.6 | 0.00011 | 1.3e-08 | 51.4 | 81 | (16, 98) | 300 | (5, 85) | 164 | NA | NA; Category: unknown function; p402686 VI\_09540 | | phrogs | 12386 | 95.3 | 0.00019 | 2.1e-08 | 42.9 | 62 | (20, 85) | 300 | (2, 63) | 65 | NA | NA; Category: unknown function; p419451 VI\_05447 | | phrogs | 30339 | 94.8 | 0.00044 | 4.8e-08 | 47.1 | 95 | (4, 98) | 300 | (6, 100) | 150 | NA | NA; Category: unknown function; p424932 VI\_08726 | | phrogs | 4017 | 94.8 | 0.00046 | 5.2e-08 | 53.0 | 109 | (48, 172) | 300 | (24, 135) | 285 | baseplate protein | baseplate protein; Category: tail; KY626162\_p27 | | phrogs | 5857 | 94.3 | 0.0009 | 1e-07 | 49.7 | 68 | (47, 129) | 300 | (26, 93) | 221 | baseplate wedge subunit | baseplate wedge subunit; Category: tail; p149245 VI\_12398 | |
| Top keywords  (threshold 1.00e-03 (evalue)) | **tail, baseplate, wedge, structural, head, and, packaging, virion, DUF276, J\_like** |
| Output files | ../../domain\_architecture/13\_FANPEZAQ\_CDS\_0013\_cath.hhr ../../domain\_architecture/13\_FANPEZAQ\_CDS\_0013\_merged.svg ../../domain\_architecture/13\_FANPEZAQ\_CDS\_0013\_ncbi-cd.hhr ../../domain\_architecture/13\_FANPEZAQ\_CDS\_0013\_pfam.hhr ../../domain\_architecture/13\_FANPEZAQ\_CDS\_0013\_phrogs.hhr |

### Identical protein sequences/structures

#### Search results

|  |  |
| --- | --- |
| Protein sequence databases searched | Pdb, Swissprot, Refseq |
| Identical proteins found | -- |
| Top keywords | -- |
| Output files | -- |

### Similar protein sequences/structures

#### Sequence similarity search results (HHblits)1

|  |  |
| --- | --- |
| Sequence databases searched | Uniclust, Pdb70 |
| Results, scheme(s)  (Top layers only, threshold 1.00e-03 (evalue)) | xml version="1.0" encoding="utf-8" standalone="no"?       2024-09-02T21:08:33.705521 image/svg+xml   Matplotlib v3.7.2, https://matplotlib.org/ |
| Results, table(s)  (threshold 1.00e-03 (evalue)) | | db | id | prob | evalue | pvalue | score | cols | query | query\_len | template | template\_len | name | description | | --- | --- | --- | --- | --- | --- | --- | --- | --- | --- | --- | --- | --- | | uniclust | UniRef100\_A0A016XHS1 | 100.0 | 3.9e-55 | 7.7e-61 | 373.6 | 298 | (1, 298) | 300 | (26, 325) | 337 | Baseplate assembly protein | Baseplate assembly protein | | uniclust | UniRef100\_A0A024E877 | 100.0 | 4.5e-54 | 9e-60 | 370.1 | 298 | (1, 298) | 300 | (32, 330) | 343 | Baseplate J-like protein | Baseplate J-like protein | | uniclust | UniRef100\_A0A009PKZ2 | 100.0 | 3.5e-51 | 7e-57 | 359.1 | 296 | (1, 297) | 300 | (57, 359) | 398 | Baseplate J-like family protein | Baseplate J-like family protein | | uniclust | UniRef100\_A0A0P9B5C1 | 100.0 | 4.2e-51 | 8e-57 | 332.2 | 298 | (1, 298) | 300 | (6, 310) | 312 | Baseplate protein J-like domain-containing protein (Fragment) | Baseplate protein J-like domain-containing protein (Fragment) | | uniclust | UniRef100\_A0A059IU93 | 100.0 | 1.4e-49 | 2.8e-55 | 329.6 | 282 | (2, 299) | 300 | (1, 282) | 301 | Putative prophage LambdaW1, baseplate assembly protein J,putative | Putative prophage LambdaW1, baseplate assembly protein J,putative | | uniclust | UniRef100\_A0A096AN02 | 100.0 | 1.9e-48 | 3.9e-54 | 343.3 | 288 | (2, 297) | 300 | (1, 369) | 376 | Baseplate protein J-like domain-containing protein | Baseplate protein J-like domain-containing protein | | uniclust | UniRef100\_A0A066RVH9 | 100.0 | 7.5e-48 | 1.5e-53 | 337.2 | 294 | (2, 299) | 300 | (35, 329) | 381 | Baseplate assembly protein | Baseplate assembly protein | | uniclust | UniRef100\_A0A011NCT2 | 100.0 | 1.8e-46 | 3.5e-52 | 339.1 | 293 | (1, 298) | 300 | (67, 442) | 523 | Phage baseplate protein | Phage baseplate protein | | uniclust | UniRef100\_A0A077Q3X4 | 100.0 | 8.2e-46 | 1.6e-51 | 303.5 | 295 | (5, 299) | 300 | (1, 302) | 307 | Baseplate assembly protein J (GpJ) | Baseplate assembly protein J (GpJ) | | uniclust | UniRef100\_A0A061P986 | 100.0 | 9.7e-46 | 2e-51 | 330.2 | 280 | (12, 297) | 300 | (38, 400) | 438 | Phage baseplate | Phage baseplate | | uniclust | UniRef100\_A0A096DBD2 | 100.0 | 1.3e-45 | 2.6e-51 | 326.0 | 269 | (21, 297) | 300 | (37, 389) | 400 | Baseplate protein J-like domain-containing protein | Baseplate protein J-like domain-containing protein | | uniclust | UniRef100\_A0A0A8HCY4 | 100.0 | 1.8e-44 | 3.6e-50 | 310.7 | 284 | (3, 296) | 300 | (12, 376) | 386 | Phage baseplate assembly protein J, putative | Phage baseplate assembly protein J, putative | | uniclust | UniRef100\_A0A068ZW73 | 100.0 | 2.3e-44 | 4.7e-50 | 320.5 | 279 | (10, 296) | 300 | (16, 386) | 407 | Baseplate protein J-like domain-containing protein | Baseplate protein J-like domain-containing protein | | uniclust | UniRef100\_A0A061NGS0 | 100.0 | 2.5e-44 | 4.8e-50 | 303.4 | 270 | (19, 296) | 300 | (7, 358) | 370 | Phage-like element PBSX protein XkdT | Phage-like element PBSX protein XkdT | | uniclust | UniRef100\_A0A081NYB1 | 100.0 | 3.5e-44 | 6.9e-50 | 315.2 | 271 | (19, 297) | 300 | (18, 394) | 418 | Baseplate protein J-like domain-containing protein | Baseplate protein J-like domain-containing protein | | uniclust | UniRef100\_A0A0B5QQX8 | 100.0 | 3.7e-43 | 7.2e-49 | 300.9 | 272 | (18, 296) | 300 | (12, 370) | 373 | Baseplate J protein | Baseplate J protein | | uniclust | UniRef100\_A0A1S9ZY08 | 100.0 | 1.3e-42 | 2.6e-48 | 282.4 | 273 | (6, 298) | 300 | (1, 274) | 278 | Baseplate protein J-like domain-containing protein | Baseplate protein J-like domain-containing protein | | uniclust | UniRef100\_A0A063BJF3 | 100.0 | 3e-42 | 6.2e-48 | 310.2 | 269 | (20, 296) | 300 | (64, 412) | 455 | Baseplate J family protein | Baseplate J family protein | | uniclust | UniRef100\_A0A090C6U6 | 100.0 | 1.5e-41 | 2.9e-47 | 289.4 | 269 | (18, 297) | 300 | (8, 356) | 360 | Putative baseplate-J protein | Putative baseplate-J protein | | uniclust | UniRef100\_A0A0B0S945 | 100.0 | 1.9e-41 | 3.7e-47 | 296.6 | 280 | (4, 296) | 300 | (7, 376) | 380 | Baseplate protein J-like domain-containing protein | Baseplate protein J-like domain-containing protein | | uniclust | UniRef100\_A0A0H3ZT21 | 100.0 | 5.8e-41 | 1.1e-46 | 275.4 | 275 | (2, 299) | 300 | (1, 282) | 294 | Phage-related baseplate assembly protein | Phage-related baseplate assembly protein | | uniclust | UniRef100\_A0A017RWE9 | 100.0 | 5.8e-41 | 1.2e-46 | 295.2 | 278 | (16, 299) | 300 | (26, 387) | 391 | Baseplate protein J-like domain-containing protein | Baseplate protein J-like domain-containing protein | | uniclust | UniRef100\_A0A072YE02 | 100.0 | 1.8e-40 | 3.5e-46 | 290.2 | 274 | (17, 296) | 300 | (19, 390) | 393 | Phage tail protein | Phage tail protein | | uniclust | UniRef100\_A0A016XIM7 | 100.0 | 2.9e-40 | 5.8e-46 | 297.4 | 270 | (20, 296) | 300 | (38, 387) | 451 | Baseplate J protein | Baseplate J protein | | uniclust | UniRef100\_UPI00082B4CAF | 100.0 | 3.5e-40 | 6.7e-46 | 275.4 | 280 | (5, 294) | 300 | (3, 362) | 363 | baseplate J/gp47 family protein | baseplate J/gp47 family protein | | uniclust | UniRef100\_A0A0B1YBX4 | 100.0 | 3.7e-40 | 7.4e-46 | 288.0 | 270 | (21, 296) | 300 | (14, 368) | 379 | Baseplate protein J-like domain-containing protein | Baseplate protein J-like domain-containing protein | | uniclust | UniRef100\_A0A2W1AVR7 | 100.0 | 4.2e-40 | 8.1e-46 | 277.4 | 266 | (21, 294) | 300 | (4, 355) | 357 | Baseplate protein J-like domain-containing protein | Baseplate protein J-like domain-containing protein | | uniclust | UniRef100\_A0A8T0C4P9 | 100.0 | 2.2e-39 | 4.3e-45 | 263.0 | 246 | (53, 298) | 300 | (3, 252) | 255 | Baseplate protein J-like domain-containing protein | Baseplate protein J-like domain-containing protein | | uniclust | UniRef100\_A0A2D9T280 | 100.0 | 5.1e-39 | 9.7e-45 | 266.8 | 266 | (20, 293) | 300 | (5, 346) | 346 | Baseplate protein J-like domain-containing protein | Baseplate protein J-like domain-containing protein | | uniclust | UniRef100\_A0A074LIS1 | 100.0 | 5.3e-39 | 1e-44 | 275.4 | 269 | (22, 295) | 300 | (7, 365) | 367 | Baseplate protein J-like domain-containing protein | Baseplate protein J-like domain-containing protein | | uniclust | UniRef100\_A0A059N0R3 | 100.0 | 8.9e-39 | 1.8e-44 | 282.4 | 279 | (2, 295) | 300 | (4, 387) | 393 | Baseplate protein J-like domain-containing protein | Baseplate protein J-like domain-containing protein | | uniclust | UniRef100\_A0A2P9HMM4 | 100.0 | 5.3e-38 | 1e-43 | 261.6 | 276 | (2, 281) | 300 | (1, 288) | 339 | Baseplate assembly protein J | Baseplate assembly protein J | | uniclust | UniRef100\_A0A0A1AFQ4 | 100.0 | 6.2e-38 | 1.2e-43 | 266.1 | 291 | (4, 299) | 300 | (1, 375) | 391 | Baseplate J/gp47 family protein | Baseplate J/gp47 family protein | | uniclust | UniRef100\_A0A021XBA6 | 100.0 | 1.9e-37 | 3.8e-43 | 260.0 | 295 | (1, 299) | 300 | (4, 306) | 307 | Baseplate J family protein | Baseplate J family protein | | uniclust | UniRef100\_A0A061N9X7 | 100.0 | 1.9e-37 | 3.9e-43 | 278.9 | 270 | (16, 295) | 300 | (26, 407) | 429 | Putative bacteriophage protein | Putative bacteriophage protein | | uniclust | UniRef100\_A0A0M0T6B2 | 100.0 | 2.8e-37 | 5.3e-43 | 257.0 | 267 | (22, 296) | 300 | (6, 333) | 339 | Tail protein (Fragment) | Tail protein (Fragment) | | uniclust | UniRef100\_A0A031FRM4 | 100.0 | 5.3e-37 | 1e-42 | 263.6 | 297 | (1, 297) | 300 | (1, 335) | 346 | Baseplate J-like protein | Baseplate J-like protein | | uniclust | UniRef100\_A0A5C0SDS7 | 100.0 | 9.9e-37 | 1.9e-42 | 252.9 | 267 | (21, 294) | 300 | (6, 360) | 361 | Baseplate J/gp47 family protein | Baseplate J/gp47 family protein | | uniclust | UniRef100\_A0A1I7ZUF6 | 100.0 | 1.4e-36 | 2.6e-42 | 242.0 | 220 | (2, 221) | 300 | (3, 223) | 229 | Baseplate\_J domain-containing protein | Baseplate\_J domain-containing protein | | uniclust | UniRef100\_A0A0C5VGB9 | 100.0 | 1.4e-36 | 2.7e-42 | 256.5 | 289 | (2, 292) | 300 | (1, 293) | 465 | Phage-related baseplate assembly protein | Phage-related baseplate assembly protein | | uniclust | UniRef100\_A0A161YLM2 | 100.0 | 2.8e-36 | 5.5e-42 | 248.6 | 278 | (4, 297) | 300 | (1, 284) | 286 | Baseplate protein J-like domain-containing protein | Baseplate protein J-like domain-containing protein | | uniclust | UniRef100\_A0A0C7NEI0 | 100.0 | 4.9e-36 | 9.4e-42 | 262.0 | 272 | (16, 293) | 300 | (8, 527) | 527 | Baseplate assembly protein J-like, predicted | Baseplate assembly protein J-like, predicted | | uniclust | UniRef100\_A0A068Z4R0 | 100.0 | 1.5e-35 | 2.9e-41 | 262.1 | 271 | (19, 295) | 300 | (55, 424) | 427 | Baseplate J/gp47 family protein | Baseplate J/gp47 family protein | | uniclust | UniRef100\_A0A0M0SY60 | 100.0 | 2e-35 | 3.7e-41 | 247.8 | 268 | (20, 293) | 300 | (14, 373) | 373 | Baseplate protein J-like domain-containing protein | Baseplate protein J-like domain-containing protein | | uniclust | UniRef100\_A0A0C1G7H8 | 100.0 | 5e-35 | 9.9e-41 | 254.9 | 283 | (9, 299) | 300 | (14, 379) | 382 | Baseplate protein J-like domain-containing protein | Baseplate protein J-like domain-containing protein | | uniclust | UniRef100\_A0A173RD27 | 100.0 | 5.2e-35 | 9.9e-41 | 247.7 | 285 | (6, 297) | 300 | (1, 381) | 389 | Uncharacterized homolog of phage Mu protein gp47 | Uncharacterized homolog of phage Mu protein gp47 | | uniclust | UniRef100\_A0A074LIV6 | 100.0 | 7.1e-35 | 1.3e-40 | 244.2 | 269 | (22, 296) | 300 | (4, 368) | 369 | Baseplate protein J-like domain-containing protein | Baseplate protein J-like domain-containing protein | | uniclust | UniRef100\_A0A0T9LR98 | 100.0 | 9.4e-35 | 1.8e-40 | 236.6 | 231 | (1, 231) | 300 | (19, 249) | 293 | Baseplate assembly protein | Baseplate assembly protein | | uniclust | UniRef100\_A0A0F4NJK8 | 100.0 | 2.1e-34 | 3.9e-40 | 230.2 | 268 | (6, 296) | 300 | (1, 268) | 274 | Baseplate protein J-like domain-containing protein | Baseplate protein J-like domain-containing protein | | uniclust | UniRef100\_A0A375ABY7 | 100.0 | 5.7e-34 | 1.1e-39 | 237.3 | 290 | (5, 294) | 300 | (1, 297) | 504 | Phage baseplate assembly protein J | Phage baseplate assembly protein J | | uniclust | UniRef100\_UPI000B7DA598 | 100.0 | 1.2e-33 | 2.2e-39 | 222.4 | 224 | (1, 224) | 300 | (1, 228) | 233 | baseplate J/gp47 family protein | baseplate J/gp47 family protein | | uniclust | UniRef100\_A0A085EXM5 | 100.0 | 1.3e-33 | 2.6e-39 | 242.4 | 269 | (19, 294) | 300 | (10, 376) | 379 | Putative bacteriophage protein | Putative bacteriophage protein | | uniclust | UniRef100\_A0A8J2Z5N4 | 100.0 | 1.6e-33 | 3e-39 | 225.1 | 277 | (4, 295) | 300 | (1, 281) | 283 | Bacteriophage protein | Bacteriophage protein | | uniclust | UniRef100\_A0A081MYF5 | 100.0 | 1.7e-33 | 3.3e-39 | 223.5 | 231 | (65, 297) | 300 | (2, 232) | 234 | Baseplate protein J-like domain-containing protein (Fragment) | Baseplate protein J-like domain-containing protein (Fragment) | | uniclust | UniRef100\_A0A0E2H3N7 | 100.0 | 5.8e-33 | 1.1e-38 | 239.5 | 272 | (21, 297) | 300 | (14, 377) | 385 | Baseplate protein J-like domain-containing protein | Baseplate protein J-like domain-containing protein | | uniclust | UniRef100\_A0A075RF08 | 100.0 | 6.9e-33 | 1.3e-38 | 227.5 | 265 | (3, 292) | 300 | (9, 283) | 286 | Baseplate assembly protein | Baseplate assembly protein | | uniclust | UniRef100\_A0A090BV61 | 100.0 | 1.2e-32 | 2.4e-38 | 253.9 | 277 | (14, 295) | 300 | (64, 553) | 580 | Baseplate protein J-like domain-containing protein | Baseplate protein J-like domain-containing protein | | uniclust | UniRef100\_A0A0F2QX06 | 100.0 | 1.5e-32 | 2.8e-38 | 221.7 | 250 | (21, 295) | 300 | (5, 259) | 260 | Baseplate protein J-like domain-containing protein | Baseplate protein J-like domain-containing protein | | uniclust | UniRef100\_A0A081BI31 | 100.0 | 1.7e-32 | 3.2e-38 | 237.8 | 271 | (16, 295) | 300 | (13, 389) | 400 | Phage protein | Phage protein | | uniclust | UniRef100\_A0A1H8P6M7 | 99.9 | 2.6e-32 | 5e-38 | 213.7 | 205 | (3, 207) | 300 | (6, 217) | 218 | Baseplate assembly protein | Baseplate assembly protein | | uniclust | UniRef100\_A0A1C5WAX4 | 99.9 | 6e-32 | 1.2e-37 | 229.2 | 270 | (18, 293) | 300 | (1, 352) | 354 | Uncharacterized homolog of phage Mu protein gp47 | Uncharacterized homolog of phage Mu protein gp47 | | uniclust | UniRef100\_A0A1I2BHZ6 | 99.9 | 8.7e-32 | 1.7e-37 | 225.3 | 246 | (22, 285) | 300 | (9, 332) | 341 | Uncharacterized phage protein gp47/JayE | Uncharacterized phage protein gp47/JayE | | uniclust | UniRef100\_A0A010RU55 | 99.9 | 3.4e-31 | 6.8e-37 | 232.9 | 270 | (21, 296) | 300 | (15, 387) | 415 | Baseplate protein | Baseplate protein | | uniclust | UniRef100\_A0A0T7DUX5 | 99.9 | 7.3e-31 | 1.4e-36 | 225.3 | 288 | (3, 294) | 300 | (33, 414) | 427 | Baseplate assembly protein J | Baseplate assembly protein J | | uniclust | UniRef100\_A0A926IEU8 | 99.9 | 8.8e-31 | 1.7e-36 | 210.6 | 193 | (17, 217) | 300 | (3, 264) | 264 | Baseplate J/gp47 family protein (Fragment) | Baseplate J/gp47 family protein (Fragment) | | uniclust | UniRef100\_A0A812QW15 | 99.9 | 9.1e-31 | 1.7e-36 | 233.4 | 290 | (2, 291) | 300 | (1, 297) | 1062 | J protein | J protein | | uniclust | UniRef100\_A0A064AIK0 | 99.9 | 1.4e-30 | 2.7e-36 | 222.6 | 271 | (22, 298) | 300 | (12, 366) | 372 | Baseplate assembly protein | Baseplate assembly protein | | uniclust | UniRef100\_A0A081J876 | 99.9 | 2e-30 | 4e-36 | 239.9 | 273 | (16, 297) | 300 | (52, 523) | 529 | Baseplate protein J-like domain-containing protein | Baseplate protein J-like domain-containing protein | | uniclust | UniRef100\_A0A174TPL6 | 99.9 | 3e-30 | 5.8e-36 | 218.7 | 279 | (16, 299) | 300 | (27, 397) | 398 | Uncharacterized homolog of phage Mu protein gp47 | Uncharacterized homolog of phage Mu protein gp47 | | uniclust | UniRef100\_A0A0F9B3X2 | 99.9 | 3.8e-30 | 7.5e-36 | 228.6 | 267 | (21, 293) | 300 | (6, 438) | 440 | Baseplate protein J-like domain-containing protein (Fragment) | Baseplate protein J-like domain-containing protein (Fragment) | | uniclust | UniRef100\_A0A166EHE8 | 99.9 | 1.3e-29 | 2.4e-35 | 215.6 | 269 | (21, 294) | 300 | (24, 393) | 395 | Baseplate J-like protein | Baseplate J-like protein | | uniclust | UniRef100\_A0A1I3REQ3 | 99.9 | 2.5e-29 | 4.9e-35 | 217.3 | 267 | (19, 295) | 300 | (12, 394) | 397 | Baseplate J-like protein | Baseplate J-like protein | | uniclust | UniRef100\_A0A1F1SBS6 | 99.9 | 2.5e-29 | 4.9e-35 | 210.5 | 193 | (20, 218) | 300 | (4, 273) | 280 | Baseplate protein J-like domain-containing protein (Fragment) | Baseplate protein J-like domain-containing protein (Fragment) | | uniclust | UniRef100\_A0A4Q7FYT9 | 99.9 | 2.8e-29 | 5.3e-35 | 205.0 | 284 | (1, 299) | 300 | (4, 302) | 310 | Baseplate protein J-like domain-containing protein | Baseplate protein J-like domain-containing protein | | uniclust | UniRef100\_A0A1D2QY08 | 99.9 | 3.7e-29 | 7.2e-35 | 222.6 | 242 | (42, 297) | 300 | (106, 526) | 531 | Baseplate protein J-like domain-containing protein | Baseplate protein J-like domain-containing protein | | uniclust | UniRef100\_A0A0F6YHW2 | 99.9 | 4.5e-29 | 8.9e-35 | 219.8 | 273 | (19, 296) | 300 | (11, 389) | 398 | Baseplate assembly protein, putative | Baseplate assembly protein, putative | | uniclust | UniRef100\_A0A0D1VV23 | 99.9 | 6e-29 | 1.1e-34 | 209.2 | 270 | (16, 294) | 300 | (8, 383) | 386 | Uncharacterized phage protein gp47/JayE | Uncharacterized phage protein gp47/JayE | | uniclust | UniRef100\_A0A0M1HGJ4 | 99.9 | 6.2e-29 | 1.2e-34 | 193.8 | 199 | (45, 243) | 300 | (15, 213) | 215 | Baseplate assembly protein (Fragment) | Baseplate assembly protein (Fragment) | | uniclust | UniRef100\_A0A2X1W9Z3 | 99.9 | 9.9e-29 | 1.8e-34 | 195.7 | 279 | (7, 287) | 300 | (3, 288) | 295 | Baseplate protein phage associated protein | Baseplate protein phage associated protein | | uniclust | UniRef100\_A0A0J1FTG2 | 99.9 | 3.9e-28 | 7.3e-34 | 193.0 | 231 | (17, 283) | 300 | (7, 239) | 248 | Baseplate J-like protein | Baseplate J-like protein | | uniclust | UniRef100\_A0A348Z5F5 | 99.9 | 4e-28 | 7.6e-34 | 194.4 | 180 | (111, 294) | 300 | (58, 243) | 244 | Baseplate protein J-like domain-containing protein (Fragment) | Baseplate protein J-like domain-containing protein (Fragment) | | uniclust | UniRef100\_A0A0C2VDI9 | 99.9 | 4e-28 | 8e-34 | 213.4 | 268 | (19, 298) | 300 | (4, 374) | 378 | Baseplate protein J-like domain-containing protein | Baseplate protein J-like domain-containing protein | | uniclust | UniRef100\_A0A135YQ64 | 99.9 | 4.4e-28 | 8.3e-34 | 202.1 | 262 | (21, 296) | 300 | (6, 345) | 347 | Baseplate J-like protein | Baseplate J-like protein | | uniclust | UniRef100\_A0A061NPP2 | 99.9 | 4.4e-28 | 8.6e-34 | 205.1 | 238 | (6, 298) | 300 | (8, 326) | 328 | Phage-related baseplate assembly protein | Phage-related baseplate assembly protein | | uniclust | UniRef100\_A0A1C6CKU7 | 99.9 | 7.6e-28 | 1.4e-33 | 189.6 | 247 | (30, 295) | 300 | (5, 258) | 260 | Uncharacterized homolog of phage Mu protein gp47 | Uncharacterized homolog of phage Mu protein gp47 | | uniclust | UniRef100\_A0A0B7GXK9 | 99.9 | 1.2e-27 | 2.4e-33 | 207.8 | 266 | (20, 298) | 300 | (8, 379) | 387 | Baseplate J/gp47 family protein | Baseplate J/gp47 family protein | | uniclust | UniRef100\_A0A193QL68 | 99.9 | 1.5e-27 | 2.7e-33 | 193.7 | 291 | (4, 298) | 300 | (79, 377) | 378 | Baseplate J-like protein | Baseplate J-like protein | | uniclust | UniRef100\_A0A258L4A6 | 99.9 | 1.8e-27 | 3.5e-33 | 186.1 | 193 | (1, 193) | 300 | (15, 209) | 213 | Baseplate protein J-like domain-containing protein | Baseplate protein J-like domain-containing protein | | uniclust | UniRef100\_A0A1S6TP77 | 99.9 | 2.1e-27 | 4.2e-33 | 203.8 | 266 | (16, 295) | 300 | (7, 343) | 358 | Phage baseplate assembly protein J, putative | Phage baseplate assembly protein J, putative | | uniclust | UniRef100\_A0A2I0CWU0 | 99.9 | 2.5e-27 | 4.6e-33 | 198.0 | 263 | (17, 294) | 300 | (9, 364) | 365 | Baseplate protein J-like domain-containing protein | Baseplate protein J-like domain-containing protein | | uniclust | UniRef100\_A0A7C6EBI1 | 99.9 | 3e-27 | 5.4e-33 | 191.2 | 266 | (24, 293) | 300 | (4, 354) | 364 | Baseplate protein J-like domain-containing protein | Baseplate protein J-like domain-containing protein | | uniclust | UniRef100\_A0A024AZH8 | 99.9 | 2.8e-27 | 5.5e-33 | 205.1 | 261 | (21, 297) | 300 | (7, 354) | 362 | Baseplate j protein | Baseplate j protein | | uniclust | UniRef100\_B6G011 | 99.9 | 3.7e-27 | 6.9e-33 | 192.8 | 267 | (20, 294) | 300 | (3, 350) | 354 | Baseplate J-like protein | Baseplate J-like protein | | uniclust | UniRef100\_A0A440V0E4 | 99.9 | 3.7e-27 | 7.1e-33 | 185.0 | 205 | (2, 223) | 300 | (1, 214) | 218 | Baseplate J protein | Baseplate J protein | | uniclust | UniRef100\_A9HP66 | 99.9 | 4.2e-27 | 8.1e-33 | 195.4 | 174 | (112, 293) | 300 | (109, 285) | 288 | Baseplate protein J-like domain-containing protein | Baseplate protein J-like domain-containing protein | | uniclust | UniRef100\_A0A6L2ZSF9 | 99.9 | 7.4e-27 | 1.4e-32 | 196.1 | 277 | (6, 282) | 300 | (6, 472) | 507 | Baseplate assembly protein | Baseplate assembly protein | | uniclust | UniRef100\_UPI0022DF30D1 | 99.9 | 8.2e-27 | 1.5e-32 | 193.2 | 240 | (19, 266) | 300 | (3, 318) | 446 | baseplate J/gp47 family protein | baseplate J/gp47 family protein | | uniclust | UniRef100\_A0A013SL59 | 99.9 | 1e-26 | 2.1e-32 | 209.9 | 273 | (14, 295) | 300 | (86, 467) | 490 | Baseplate J-like family protein | Baseplate J-like family protein | | uniclust | UniRef100\_A0A128FC77 | 99.9 | 1.2e-26 | 2.3e-32 | 199.4 | 274 | (19, 298) | 300 | (3, 363) | 365 | Baseplate J-like protein | Baseplate J-like protein | | uniclust | UniRef100\_A0A261QSM0 | 99.9 | 1.3e-26 | 2.4e-32 | 196.6 | 268 | (19, 292) | 300 | (6, 360) | 361 | Baseplate protein J-like domain-containing protein | Baseplate protein J-like domain-containing protein | | uniclust | UniRef100\_UPI00080E3582 | 99.9 | 1.3e-26 | 2.4e-32 | 191.4 | 284 | (4, 295) | 300 | (2, 380) | 382 | baseplate J/gp47 family protein | baseplate J/gp47 family protein | | uniclust | UniRef100\_A0A1F5C406 | 99.9 | 1.7e-26 | 3.3e-32 | 204.9 | 273 | (19, 294) | 300 | (4, 463) | 464 | Baseplate protein J-like domain-containing protein | Baseplate protein J-like domain-containing protein | | uniclust | UniRef100\_A0A376EX36 | 99.9 | 3.1e-26 | 5.9e-32 | 187.4 | 187 | (109, 298) | 300 | (85, 276) | 278 | Baseplate assembly protein J | Baseplate assembly protein J | | uniclust | UniRef100\_A0A8S4QUR0 | 99.9 | 4.7e-26 | 8.6e-32 | 197.4 | 249 | (14, 284) | 300 | (2, 252) | 611 | Jg15363 protein | Jg15363 protein | | uniclust | UniRef100\_A0A0W8FUR1 | 99.9 | 4.7e-26 | 8.8e-32 | 195.8 | 248 | (21, 293) | 300 | (53, 448) | 449 | Phage flumu protein gp47 | Phage flumu protein gp47 | | uniclust | UniRef100\_A0A6B8KJI2 | 99.9 | 4.9e-26 | 9.3e-32 | 186.6 | 280 | (1, 295) | 300 | (5, 312) | 315 | Baseplate protein J-like domain-containing protein | Baseplate protein J-like domain-containing protein | | uniclust | UniRef100\_A0A0F0HJ75 | 99.9 | 6.5e-26 | 1.2e-31 | 200.9 | 285 | (7, 296) | 300 | (2, 490) | 494 | Baseplate protein J-like domain-containing protein | Baseplate protein J-like domain-containing protein | | uniclust | UniRef100\_A0A8I1NZ44 | 99.9 | 6.7e-26 | 1.3e-31 | 187.0 | 287 | (7, 295) | 300 | (14, 336) | 338 | Baseplate J/gp47 family protein | Baseplate J/gp47 family protein | | uniclust | UniRef100\_A0A089K1A9 | 99.9 | 8.8e-26 | 1.7e-31 | 196.1 | 258 | (13, 295) | 300 | (16, 321) | 339 | Baseplate protein J-like domain-containing protein | Baseplate protein J-like domain-containing protein | | uniclust | UniRef100\_A0A0E2E9P5 | 99.9 | 9.7e-26 | 1.9e-31 | 198.0 | 274 | (21, 297) | 300 | (6, 394) | 396 | Baseplate protein J-like domain-containing protein | Baseplate protein J-like domain-containing protein | | uniclust | UniRef100\_A0A1U7N2A8 | 99.9 | 1.3e-25 | 2.4e-31 | 207.7 | 241 | (19, 268) | 300 | (3, 360) | 748 | Baseplate protein J-like domain-containing protein | Baseplate protein J-like domain-containing protein | | uniclust | UniRef100\_A0A3A9BP88 | 99.9 | 1.3e-25 | 2.5e-31 | 186.1 | 266 | (22, 295) | 300 | (3, 355) | 362 | Baseplate protein J-like domain-containing protein | Baseplate protein J-like domain-containing protein | | uniclust | UniRef100\_UPI0001AF5308 | 99.9 | 1.7e-25 | 3.1e-31 | 180.6 | 257 | (8, 267) | 300 | (4, 267) | 351 | baseplate J/gp47 family protein | baseplate J/gp47 family protein | | uniclust | UniRef100\_A0A3N6RV94 | 99.9 | 1.9e-25 | 3.5e-31 | 185.6 | 235 | (5, 239) | 300 | (1, 242) | 418 | Phage tail protein I | Phage tail protein I | | uniclust | UniRef100\_A0A2R3MZM3 | 99.9 | 2.3e-25 | 4.4e-31 | 188.8 | 266 | (18, 291) | 300 | (10, 380) | 388 | Baseplate protein J-like domain-containing protein | Baseplate protein J-like domain-containing protein | | uniclust | UniRef100\_A0A328CA52 | 99.9 | 2.3e-25 | 4.5e-31 | 191.2 | 271 | (19, 294) | 300 | (13, 387) | 390 | Baseplate protein J-like domain-containing protein | Baseplate protein J-like domain-containing protein | | uniclust | UniRef100\_A0A3A9EFF6 | 99.9 | 2.5e-25 | 4.8e-31 | 175.3 | 187 | (111, 299) | 300 | (17, 206) | 207 | Phage baseplate protein (Fragment) | Phage baseplate protein (Fragment) | | uniclust | UniRef100\_A0A080LY68 | 99.9 | 2.5e-25 | 4.9e-31 | 200.1 | 237 | (21, 269) | 300 | (14, 351) | 523 | Baseplate protein J-like domain-containing protein | Baseplate protein J-like domain-containing protein | | uniclust | UniRef100\_A0A0A2WKW8 | 99.9 | 2.7e-25 | 5.4e-31 | 210.1 | 181 | (112, 295) | 300 | (439, 648) | 679 | Baseplate J family protein | Baseplate J family protein | | uniclust | UniRef100\_A0A1M6UTJ7 | 99.8 | 5.7e-25 | 1.1e-30 | 186.2 | 234 | (17, 271) | 300 | (17, 275) | 311 | Baseplate J-like protein | Baseplate J-like protein | | uniclust | UniRef100\_A0A022PMN6 | 99.8 | 7.1e-25 | 1.4e-30 | 197.3 | 281 | (10, 297) | 300 | (92, 476) | 493 | Putative phage Mu protein gp47-like protein | Putative phage Mu protein gp47-like protein | | uniclust | UniRef100\_A0A0R3MV37 | 99.8 | 7.7e-25 | 1.4e-30 | 177.2 | 277 | (4, 297) | 300 | (4, 288) | 290 | Baseplate assembly protein | Baseplate assembly protein | | uniclust | UniRef100\_A0A0A7HBT3 | 99.8 | 7.6e-25 | 1.5e-30 | 191.9 | 276 | (8, 298) | 300 | (14, 389) | 391 | Baseplate J-like protein | Baseplate J-like protein | | uniclust | UniRef100\_UPI00188A944F | 99.8 | 1e-24 | 1.9e-30 | 175.7 | 222 | (74, 295) | 300 | (1, 249) | 251 | baseplate J/gp47 family protein | baseplate J/gp47 family protein | | uniclust | UniRef100\_A0A0F9SMT4 | 99.8 | 1.2e-24 | 2.3e-30 | 191.6 | 287 | (8, 296) | 300 | (36, 535) | 540 | Baseplate protein J-like domain-containing protein | Baseplate protein J-like domain-containing protein | | uniclust | UniRef100\_A0A068NXX0 | 99.8 | 1.4e-24 | 2.7e-30 | 195.3 | 264 | (16, 288) | 300 | (71, 484) | 498 | Baseplate assembly protein, putative | Baseplate assembly protein, putative | | uniclust | UniRef100\_A0A0A7G316 | 99.8 | 1.5e-24 | 2.9e-30 | 178.6 | 247 | (21, 292) | 300 | (6, 267) | 272 | Baseplate J-like family protein | Baseplate J-like family protein | | uniclust | UniRef100\_A0A164ZAU4 | 99.8 | 2.1e-24 | 3.9e-30 | 167.9 | 181 | (112, 294) | 300 | (8, 206) | 209 | Phage baseplate | Phage baseplate | | uniclust | UniRef100\_A0A062V567 | 99.8 | 2.4e-24 | 4.7e-30 | 181.8 | 261 | (13, 297) | 300 | (7, 311) | 324 | Baseplate J-like protein | Baseplate J-like protein | | uniclust | UniRef100\_A0A1I7E360 | 99.8 | 3e-24 | 5.6e-30 | 187.3 | 269 | (5, 273) | 300 | (1, 276) | 557 | Phage tail tape measure protein, TP901 family, core region | Phage tail tape measure protein, TP901 family, core region | | uniclust | UniRef100\_A0A0E3NKG6 | 99.8 | 2.9e-24 | 5.8e-30 | 206.6 | 183 | (112, 297) | 300 | (502, 716) | 756 | Baseplate protein J-like domain-containing protein | Baseplate protein J-like domain-containing protein | | uniclust | UniRef100\_A0A2A2HC74 | 99.8 | 3.3e-24 | 6.2e-30 | 167.0 | 185 | (111, 298) | 300 | (20, 205) | 209 | Baseplate protein J-like domain-containing protein | Baseplate protein J-like domain-containing protein | | uniclust | UniRef100\_A0A8I2FQ02 | 99.8 | 3.4e-24 | 6.5e-30 | 177.9 | 256 | (13, 295) | 300 | (3, 290) | 299 | Baseplate protein J-like domain-containing protein | Baseplate protein J-like domain-containing protein | | uniclust | UniRef100\_A0A258KUT3 | 99.8 | 3.8e-24 | 7.2e-30 | 165.6 | 190 | (109, 298) | 300 | (2, 191) | 193 | Baseplate protein J-like domain-containing protein | Baseplate protein J-like domain-containing protein | | uniclust | UniRef100\_UPI000469242B | 99.8 | 4e-24 | 7.4e-30 | 177.0 | 265 | (16, 292) | 300 | (14, 366) | 367 | baseplate J/gp47 family protein | baseplate J/gp47 family protein | | uniclust | UniRef100\_A0A0A8WTQ2 | 99.8 | 3.8e-24 | 7.5e-30 | 186.8 | 248 | (20, 289) | 300 | (48, 331) | 374 | Baseplate protein J-like domain-containing protein | Baseplate protein J-like domain-containing protein | | uniclust | UniRef100\_A0A3N9ESU6 | 99.8 | 4.6e-24 | 8.8e-30 | 163.7 | 170 | (115, 291) | 300 | (3, 176) | 183 | Phage tail protein (Fragment) | Phage tail protein (Fragment) | | uniclust | UniRef100\_A0A1H5ZLM5 | 99.8 | 5.4e-24 | 1e-29 | 184.2 | 268 | (19, 296) | 300 | (10, 409) | 413 | Baseplate J family protein | Baseplate J family protein | | uniclust | UniRef100\_A0A060H3Z0 | 99.8 | 6e-24 | 1.2e-29 | 187.1 | 276 | (9, 296) | 300 | (12, 391) | 423 | Baseplate protein J-like domain-containing protein | Baseplate protein J-like domain-containing protein | | uniclust | UniRef100\_A0A0A0GWF9 | 99.8 | 6.2e-24 | 1.2e-29 | 164.2 | 163 | (1, 163) | 300 | (7, 176) | 178 | Baseplate assembly protein J | Baseplate assembly protein J | | uniclust | UniRef100\_A0A7G5CAE3 | 99.8 | 7.8e-24 | 1.4e-29 | 159.4 | 193 | (14, 222) | 300 | (2, 194) | 206 | Baseplate assembly protein J | Baseplate assembly protein J | | uniclust | UniRef100\_A0A1E5NH39 | 99.8 | 8.5e-24 | 1.6e-29 | 180.8 | 268 | (21, 293) | 300 | (4, 366) | 367 | Baseplate protein J-like domain-containing protein | Baseplate protein J-like domain-containing protein | | uniclust | UniRef100\_A0A3D4RUD6 | 99.8 | 9.2e-24 | 1.7e-29 | 172.4 | 268 | (2, 285) | 300 | (1, 275) | 325 | Baseplate protein J-like domain-containing protein | Baseplate protein J-like domain-containing protein | | uniclust | UniRef100\_A0A8S5PDI9 | 99.8 | 9.5e-24 | 1.7e-29 | 171.0 | 261 | (18, 287) | 300 | (1, 339) | 354 | Baseplate J like protein | Baseplate J like protein | | uniclust | UniRef100\_A0A2S8P413 | 99.8 | 1.3e-23 | 2.5e-29 | 179.6 | 271 | (15, 292) | 300 | (4, 377) | 538 | Baseplate J family protein | Baseplate J family protein | | uniclust | UniRef100\_UPI000A8775AC | 99.8 | 1.7e-23 | 3.2e-29 | 169.7 | 211 | (9, 221) | 300 | (5, 300) | 303 | baseplate J/gp47 family protein | baseplate J/gp47 family protein | | uniclust | UniRef100\_A0A7V6NHE1 | 99.8 | 1.8e-23 | 3.5e-29 | 169.4 | 177 | (112, 295) | 300 | (88, 270) | 273 | Baseplate J protein | Baseplate J protein | | uniclust | UniRef100\_A0A212JYZ3 | 99.8 | 1.9e-23 | 3.6e-29 | 172.8 | 178 | (111, 295) | 300 | (100, 284) | 287 | Baseplate J family protein | Baseplate J family protein | | uniclust | UniRef100\_A0A3G2R557 | 99.8 | 2.1e-23 | 3.8e-29 | 174.5 | 268 | (22, 294) | 300 | (3, 466) | 467 | Baseplate protein J-like domain-containing protein | Baseplate protein J-like domain-containing protein | | uniclust | UniRef100\_A0A098MR91 | 99.8 | 2.2e-23 | 4.2e-29 | 178.6 | 272 | (15, 294) | 300 | (7, 391) | 395 | Baseplate protein J | Baseplate protein J | | uniclust | UniRef100\_A0A022MMA0 | 99.8 | 2.2e-23 | 4.4e-29 | 201.1 | 156 | (112, 269) | 300 | (564, 742) | 876 | Baseplate J family protein | Baseplate J family protein | | uniclust | UniRef100\_A0A0P9WA78 | 99.8 | 3e-23 | 5.7e-29 | 172.1 | 177 | (111, 294) | 300 | (97, 297) | 298 | Baseplate J-like protein | Baseplate J-like protein | | uniclust | UniRef100\_A0A2D6NHB6 | 99.8 | 3.2e-23 | 6.1e-29 | 177.6 | 260 | (21, 288) | 300 | (5, 368) | 381 | Baseplate protein J-like domain-containing protein | Baseplate protein J-like domain-containing protein | | uniclust | UniRef100\_A0A2N9MST4 | 99.8 | 3.3e-23 | 6.3e-29 | 172.8 | 238 | (13, 269) | 300 | (3, 265) | 327 | Baseplate protein J-like domain-containing protein | Baseplate protein J-like domain-containing protein | | uniclust | UniRef100\_A0A0R1YCE5 | 99.8 | 3.5e-23 | 6.7e-29 | 170.2 | 170 | (17, 192) | 300 | (8, 249) | 264 | Baseplate J-like protein | Baseplate J-like protein | | uniclust | UniRef100\_A0A4P0TK43 | 99.8 | 4.5e-23 | 8.3e-29 | 156.1 | 205 | (94, 298) | 300 | (2, 206) | 207 | Putative phage baseplate assembly protein | Putative phage baseplate assembly protein | | uniclust | UniRef100\_A0A084ZEL5 | 99.8 | 5.6e-23 | 1.1e-28 | 170.1 | 175 | (21, 201) | 300 | (6, 258) | 265 | Phage baseplate assembly protein J (Fragment) | Phage baseplate assembly protein J (Fragment) | | uniclust | UniRef100\_A0A3D4RUG0 | 99.8 | 6.2e-23 | 1.2e-28 | 175.3 | 270 | (8, 295) | 300 | (15, 305) | 416 | Baseplate protein J-like domain-containing protein | Baseplate protein J-like domain-containing protein | | uniclust | UniRef100\_A0A009ZC54 | 99.8 | 8e-23 | 1.5e-28 | 177.0 | 264 | (18, 294) | 300 | (89, 434) | 437 | Baseplate J-like family protein | Baseplate J-like family protein | | uniclust | UniRef100\_A0A0B6CQ96 | 99.8 | 1e-22 | 1.9e-28 | 159.6 | 256 | (19, 294) | 300 | (12, 267) | 270 | Baseplate J-like family protein | Baseplate J-like family protein | | uniclust | UniRef100\_A0A3B8INQ5 | 99.8 | 1.1e-22 | 2.2e-28 | 185.5 | 237 | (21, 268) | 300 | (5, 335) | 804 | Baseplate protein J-like domain-containing protein | Baseplate protein J-like domain-containing protein | | uniclust | UniRef100\_A0A074MBI6 | 99.8 | 1.1e-22 | 2.2e-28 | 192.6 | 181 | (112, 297) | 300 | (530, 746) | 751 | Baseplate protein J-like domain-containing protein | Baseplate protein J-like domain-containing protein | | uniclust | UniRef100\_A0A8S5MVE0 | 99.8 | 1.2e-22 | 2.2e-28 | 170.3 | 266 | (22, 293) | 300 | (107, 470) | 472 | Baseplate J like protein | Baseplate J like protein | | uniclust | UniRef100\_A0A2W4SAT6 | 99.8 | 1.2e-22 | 2.2e-28 | 169.2 | 173 | (16, 191) | 300 | (7, 264) | 296 | Baseplate protein J-like domain-containing protein (Fragment) | Baseplate protein J-like domain-containing protein (Fragment) | | uniclust | UniRef100\_A0A258DW01 | 99.8 | 1.6e-22 | 3.1e-28 | 169.7 | 269 | (21, 296) | 300 | (5, 363) | 367 | Baseplate protein J-like domain-containing protein | Baseplate protein J-like domain-containing protein | | uniclust | UniRef100\_A0A0B0SG84 | 99.8 | 1.6e-22 | 3.1e-28 | 173.2 | 256 | (21, 295) | 300 | (7, 354) | 356 | Baseplate protein J-like domain-containing protein | Baseplate protein J-like domain-containing protein | | uniclust | UniRef100\_A0A0C1PPW9 | 99.8 | 2e-22 | 3.8e-28 | 173.4 | 270 | (18, 295) | 300 | (10, 410) | 414 | Putative bacteriophage protein | Putative bacteriophage protein | | uniclust | UniRef100\_A0A0A0RK69 | 99.8 | 2.7e-22 | 5.1e-28 | 178.2 | 245 | (17, 270) | 300 | (9, 449) | 496 | Baseplate component | Baseplate component | | uniclust | UniRef100\_A0A8J4SQD0 | 99.8 | 2.8e-22 | 5.1e-28 | 179.1 | 267 | (21, 294) | 300 | (430, 779) | 935 | Baseplate protein J-like domain-containing protein | Baseplate protein J-like domain-containing protein | | uniclust | UniRef100\_A0A0P6XAW3 | 99.8 | 4.6e-22 | 9e-28 | 186.2 | 155 | (112, 268) | 300 | (490, 680) | 758 | Baseplate protein J-like domain-containing protein | Baseplate protein J-like domain-containing protein | | uniclust | UniRef100\_A0A2A2HFP1 | 99.8 | 4.9e-22 | 9.3e-28 | 174.2 | 256 | (7, 268) | 300 | (16, 385) | 481 | Baseplate J-like protein | Baseplate J-like protein | | uniclust | UniRef100\_A0A1Y3ZYD9 | 99.8 | 7.4e-22 | 1.4e-27 | 156.6 | 180 | (111, 294) | 300 | (26, 224) | 262 | Baseplate protein J-like domain-containing protein | Baseplate protein J-like domain-containing protein | | uniclust | UniRef100\_A0A662W1X1 | 99.8 | 8.3e-22 | 1.5e-27 | 159.1 | 218 | (21, 268) | 300 | (13, 232) | 337 | Baseplate protein J-like domain-containing protein | Baseplate protein J-like domain-containing protein | | uniclust | UniRef100\_A0A0K6HHF2 | 99.8 | 9e-22 | 1.7e-27 | 163.3 | 221 | (21, 245) | 300 | (5, 302) | 317 | Uncharacterized phage protein gp47/JayE (Fragment) | Uncharacterized phage protein gp47/JayE (Fragment) | | uniclust | UniRef100\_A0A0F9SSY1 | 99.8 | 9.3e-22 | 1.8e-27 | 170.6 | 240 | (43, 289) | 300 | (30, 362) | 385 | Baseplate protein J-like domain-containing protein | Baseplate protein J-like domain-containing protein | | uniclust | UniRef100\_A0A024QHR7 | 99.8 | 9.8e-22 | 1.9e-27 | 163.9 | 261 | (21, 291) | 300 | (6, 281) | 283 | Baseplate protein J-like domain-containing protein | Baseplate protein J-like domain-containing protein | | uniclust | UniRef100\_A0A1G6XUY3 | 99.8 | 1e-21 | 2e-27 | 183.7 | 268 | (21, 296) | 300 | (72, 492) | 608 | Uncharacterized phage protein gp47/JayE | Uncharacterized phage protein gp47/JayE | | uniclust | UniRef100\_A0A6J5KNL2 | 99.8 | 1.2e-21 | 2.4e-27 | 178.7 | 288 | (5, 295) | 300 | (1, 518) | 623 | Baseplate protein J-like | Baseplate protein J-like | | uniclust | UniRef100\_A0A1J5EX15 | 99.7 | 1.3e-21 | 2.6e-27 | 171.3 | 245 | (20, 270) | 300 | (14, 351) | 398 | Baseplate protein J-like domain-containing protein | Baseplate protein J-like domain-containing protein | | uniclust | UniRef100\_A0A068R2C5 | 99.7 | 1.4e-21 | 2.8e-27 | 176.4 | 269 | (17, 295) | 300 | (31, 415) | 421 | Phage protein | Phage protein | | uniclust | UniRef100\_A0A011MDS5 | 99.7 | 1.5e-21 | 3e-27 | 188.7 | 182 | (112, 296) | 300 | (512, 729) | 808 | Baseplate assembly protein | Baseplate assembly protein | | uniclust | UniRef100\_UPI00233ECD7D | 99.7 | 1.8e-21 | 3.2e-27 | 147.7 | 199 | (5, 203) | 300 | (1, 210) | 211 | baseplate J/gp47 family protein | baseplate J/gp47 family protein | | uniclust | UniRef100\_A0A220MKU4 | 99.7 | 1.8e-21 | 3.3e-27 | 153.1 | 182 | (111, 294) | 300 | (46, 239) | 240 | Baseplate protein J-like domain-containing protein | Baseplate protein J-like domain-containing protein | | uniclust | UniRef100\_A0A545T5R2 | 99.7 | 1.8e-21 | 3.3e-27 | 156.2 | 280 | (2, 289) | 300 | (1, 298) | 320 | Baseplate protein J-like domain-containing protein | Baseplate protein J-like domain-containing protein | | uniclust | UniRef100\_A0A068C8J3 | 99.7 | 1.8e-21 | 3.6e-27 | 178.7 | 272 | (17, 296) | 300 | (52, 440) | 510 | Putative baseplate J-like protein | Putative baseplate J-like protein | | uniclust | UniRef100\_D1P8B4 | 99.7 | 1.9e-21 | 3.6e-27 | 148.5 | 180 | (13, 192) | 300 | (2, 188) | 191 | Baseplate J-like protein (Fragment) | Baseplate J-like protein (Fragment) | | uniclust | UniRef100\_A0A133ZRX4 | 99.7 | 2.2e-21 | 4.3e-27 | 175.7 | 269 | (16, 293) | 300 | (22, 494) | 504 | Baseplate protein J-like domain-containing protein | Baseplate protein J-like domain-containing protein | | uniclust | UniRef100\_A0A0H5RM30 | 99.7 | 2.6e-21 | 5.1e-27 | 178.3 | 238 | (42, 288) | 300 | (181, 510) | 635 | Baseplate J-like protein | Baseplate J-like protein | | uniclust | UniRef100\_UPI0005E01794 | 99.7 | 3.3e-21 | 6.1e-27 | 153.0 | 199 | (5, 203) | 300 | (1, 206) | 292 | baseplate J/gp47 family protein | baseplate J/gp47 family protein | | uniclust | UniRef100\_A0A101WDK8 | 99.7 | 3.7e-21 | 7.2e-27 | 179.5 | 180 | (112, 298) | 300 | (488, 694) | 698 | Baseplate protein J-like domain-containing protein | Baseplate protein J-like domain-containing protein | | uniclust | UniRef100\_A0A3P8KWB0 | 99.7 | 4.2e-21 | 8e-27 | 146.3 | 147 | (154, 300) | 300 | (8, 154) | 154 | Uncharacterized homolog of phage Mu protein gp47 | Uncharacterized homolog of phage Mu protein gp47 | | uniclust | UniRef100\_A0A084SKL3 | 99.7 | 4.8e-21 | 9.5e-27 | 185.9 | 154 | (112, 268) | 300 | (303, 486) | 886 | Baseplate protein J-like domain-containing protein | Baseplate protein J-like domain-containing protein | | uniclust | UniRef100\_A0A0K1RXW7 | 99.7 | 5.2e-21 | 1e-26 | 168.8 | 231 | (12, 268) | 300 | (15, 271) | 457 | Baseplate protein J-like domain-containing protein | Baseplate protein J-like domain-containing protein | | uniclust | UniRef100\_A0A6P1Y489 | 99.7 | 5.4e-21 | 1e-26 | 158.5 | 264 | (16, 293) | 300 | (4, 348) | 352 | Baseplate protein J-like domain-containing protein | Baseplate protein J-like domain-containing protein | | uniclust | UniRef100\_A0A376CQY9 | 99.7 | 6.8e-21 | 1.3e-26 | 144.5 | 173 | (127, 299) | 300 | (10, 182) | 190 | Baseplate assembly protein GpJ | Baseplate assembly protein GpJ | | uniclust | UniRef100\_A0A835Z0B9 | 99.7 | 7.1e-21 | 1.3e-26 | 177.8 | 274 | (7, 296) | 300 | (5, 284) | 1777 | Baseplate protein J-like domain-containing protein | Baseplate protein J-like domain-containing protein | | uniclust | UniRef100\_A0A021X854 | 99.7 | 8.8e-21 | 1.7e-26 | 162.2 | 258 | (14, 295) | 300 | (9, 300) | 310 | Baseplate protein J-like domain-containing protein | Baseplate protein J-like domain-containing protein | | uniclust | UniRef100\_A0A101G5B9 | 99.7 | 9.8e-21 | 1.8e-26 | 151.8 | 183 | (9, 204) | 300 | (2, 273) | 275 | Baseplate J-like protein | Baseplate J-like protein | | uniclust | UniRef100\_A0A1B1IUR1 | 99.7 | 1.1e-20 | 2.1e-26 | 179.0 | 155 | (112, 268) | 300 | (399, 564) | 626 | Baseplate wedge subunit | Baseplate wedge subunit | | uniclust | UniRef100\_UPI000D012F51 | 99.7 | 1.3e-20 | 2.3e-26 | 158.5 | 245 | (18, 271) | 300 | (8, 353) | 390 | baseplate J/gp47 family protein | baseplate J/gp47 family protein | | uniclust | UniRef100\_A0A166CB08 | 99.7 | 1.2e-20 | 2.4e-26 | 162.8 | 273 | (8, 295) | 300 | (5, 368) | 374 | Baseplate J-like protein | Baseplate J-like protein | | uniclust | UniRef100\_A0A973IBV9 | 99.7 | 1.3e-20 | 2.5e-26 | 150.4 | 250 | (21, 292) | 300 | (5, 265) | 266 | Baseplate J/gp47 family protein | Baseplate J/gp47 family protein | | uniclust | UniRef100\_A0A134ARI0 | 99.7 | 1.5e-20 | 2.9e-26 | 160.5 | 246 | (43, 293) | 300 | (34, 366) | 369 | Baseplate protein J-like domain-containing protein | Baseplate protein J-like domain-containing protein | | uniclust | UniRef100\_A0A7J6YK41 | 99.7 | 1.7e-20 | 3.1e-26 | 152.7 | 192 | (12, 222) | 300 | (138, 329) | 353 | Baseplate protein J-like domain-containing protein | Baseplate protein J-like domain-containing protein | | uniclust | UniRef100\_A0A353REU8 | 99.7 | 1.8e-20 | 3.3e-26 | 151.6 | 179 | (111, 295) | 300 | (75, 256) | 258 | Baseplate protein J-like domain-containing protein | Baseplate protein J-like domain-containing protein | | uniclust | UniRef100\_UPI0020BFF1D4 | 99.7 | 1.8e-20 | 3.4e-26 | 157.8 | 202 | (43, 244) | 300 | (18, 223) | 475 | baseplate J/gp47 family protein | baseplate J/gp47 family protein | | uniclust | UniRef100\_A0A088C3H8 | 99.7 | 1.8e-20 | 3.5e-26 | 168.1 | 243 | (15, 266) | 300 | (6, 433) | 476 | p2 gpJ-like protein | p2 gpJ-like protein | | uniclust | UniRef100\_A0A1N7LRY2 | 99.7 | 1.9e-20 | 3.5e-26 | 150.2 | 274 | (14, 292) | 300 | (10, 289) | 299 | Phage-related baseplate assembly protein | Phage-related baseplate assembly protein | | uniclust | UniRef100\_A0A857ED91 | 99.7 | 2e-20 | 3.6e-26 | 146.2 | 185 | (111, 295) | 300 | (9, 199) | 257 | Baseplate protein J-like domain-containing protein | Baseplate protein J-like domain-containing protein | | uniclust | UniRef100\_A0A261QMI5 | 99.7 | 2.1e-20 | 3.9e-26 | 140.6 | 192 | (13, 211) | 300 | (5, 196) | 197 | Phage tail protein (Fragment) | Phage tail protein (Fragment) | | uniclust | UniRef100\_A0A7T7D8U4 | 99.7 | 2.1e-20 | 3.9e-26 | 161.1 | 263 | (10, 273) | 300 | (4, 354) | 596 | Phage tail protein I | Phage tail protein I | | uniclust | UniRef100\_A0A8D8XD97 | 99.7 | 2.3e-20 | 4.2e-26 | 138.9 | 168 | (27, 209) | 300 | (1, 170) | 177 | Baseplate protein J (Fragment) | Baseplate protein J (Fragment) | | uniclust | UniRef100\_UPI0012CD38E9 | 99.7 | 2.4e-20 | 4.4e-26 | 153.3 | 217 | (62, 293) | 300 | (159, 379) | 382 | prophage tail fiber N-terminal domain-containing protein | prophage tail fiber N-terminal domain-containing protein | | uniclust | UniRef100\_A0A1Q1PVP2 | 99.7 | 2.3e-20 | 4.5e-26 | 171.5 | 247 | (21, 271) | 300 | (12, 441) | 573 | Baseplate | Baseplate | | uniclust | UniRef100\_A0A1L8I3J9 | 99.7 | 2.7e-20 | 5e-26 | 150.4 | 155 | (16, 172) | 300 | (8, 247) | 257 | Baseplate protein J-like domain-containing protein | Baseplate protein J-like domain-containing protein | | uniclust | UniRef100\_A0A284VS02 | 99.7 | 2.9e-20 | 5.5e-26 | 158.2 | 249 | (13, 268) | 300 | (3, 274) | 313 | Baseplate protein J-like domain-containing protein | Baseplate protein J-like domain-containing protein | | uniclust | UniRef100\_A0A7G8VI16 | 99.7 | 3.3e-20 | 6e-26 | 138.5 | 144 | (6, 149) | 300 | (1, 144) | 187 | Baseplate J/gp47 family protein | Baseplate J/gp47 family protein | | uniclust | UniRef100\_UPI000327E1DD | 99.7 | 3.5e-20 | 6.3e-26 | 154.2 | 198 | (5, 202) | 300 | (1, 205) | 422 | contractile injection system protein, VgrG/Pvc8 family | contractile injection system protein, VgrG/Pvc8 family | | uniclust | UniRef100\_A0A2N2GRU7 | 99.7 | 3.8e-20 | 7.2e-26 | 152.2 | 178 | (112, 296) | 300 | (107, 295) | 299 | Baseplate assembly protein (Fragment) | Baseplate assembly protein (Fragment) | | uniclust | UniRef100\_A0A2D9F577 | 99.7 | 3.9e-20 | 7.3e-26 | 153.3 | 262 | (21, 289) | 300 | (5, 348) | 362 | Baseplate protein J-like domain-containing protein (Fragment) | Baseplate protein J-like domain-containing protein (Fragment) | | uniclust | UniRef100\_A0A8X6FB47 | 99.7 | 4.2e-20 | 7.8e-26 | 155.9 | 220 | (39, 280) | 300 | (9, 230) | 480 | Baseplate protein J | Baseplate protein J | | uniclust | UniRef100\_A0A8B3NLL6 | 99.7 | 4.5e-20 | 8.2e-26 | 153.4 | 276 | (7, 298) | 300 | (136, 418) | 419 | Baseplate protein J-like domain-containing protein | Baseplate protein J-like domain-containing protein | | uniclust | UniRef100\_E2CFL2 | 99.7 | 4.5e-20 | 8.3e-26 | 149.2 | 273 | (6, 295) | 300 | (1, 280) | 293 | Putative baseplate assembly protein J | Putative baseplate assembly protein J | | uniclust | UniRef100\_A0A644Z6C5 | 99.7 | 4.8e-20 | 8.9e-26 | 141.6 | 180 | (111, 296) | 300 | (19, 201) | 204 | Baseplate protein J-like domain-containing protein | Baseplate protein J-like domain-containing protein | | uniclust | UniRef100\_A0A011N873 | 99.7 | 5.6e-20 | 1.1e-25 | 170.9 | 215 | (42, 268) | 300 | (115, 484) | 644 | Baseplate protein J-like domain-containing protein | Baseplate protein J-like domain-containing protein | | uniclust | UniRef100\_A0A238HJ41 | 99.7 | 6.1e-20 | 1.1e-25 | 142.6 | 178 | (116, 296) | 300 | (17, 202) | 207 | Baseplate J-like protein | Baseplate J-like protein | | uniclust | UniRef100\_A0A094SKS8 | 99.7 | 6.3e-20 | 1.2e-25 | 164.0 | 271 | (16, 295) | 300 | (38, 433) | 471 | Phage baseplate protein (Fragment) | Phage baseplate protein (Fragment) | | uniclust | UniRef100\_A0A0M0SE30 | 99.7 | 6.6e-20 | 1.3e-25 | 160.9 | 271 | (13, 298) | 300 | (9, 376) | 396 | Baseplate protein J-like domain-containing protein | Baseplate protein J-like domain-containing protein | | uniclust | UniRef100\_A0A840NW02 | 99.7 | 7e-20 | 1.3e-25 | 141.2 | 204 | (78, 297) | 300 | (2, 206) | 208 | Phage-related baseplate assembly protein | Phage-related baseplate assembly protein | | uniclust | UniRef100\_A0A0N9N791 | 99.7 | 6.7e-20 | 1.3e-25 | 163.0 | 273 | (19, 294) | 300 | (28, 440) | 446 | Baseplate protein J-like domain-containing protein | Baseplate protein J-like domain-containing protein | | uniclust | UniRef100\_A0A7J4ETU4 | 99.7 | 8.2e-20 | 1.5e-25 | 152.5 | 220 | (37, 270) | 300 | (5, 391) | 433 | Baseplate protein J-like domain-containing protein | Baseplate protein J-like domain-containing protein | | uniclust | UniRef100\_A0A0Q7JQC0 | 99.7 | 7.7e-20 | 1.5e-25 | 174.1 | 183 | (112, 297) | 300 | (478, 694) | 748 | Baseplate protein J-like domain-containing protein | Baseplate protein J-like domain-containing protein | | uniclust | UniRef100\_UPI001F2080EB | 99.7 | 8.4e-20 | 1.5e-25 | 141.5 | 228 | (66, 293) | 300 | (3, 235) | 240 | baseplate J/gp47 family protein | baseplate J/gp47 family protein | | uniclust | UniRef100\_A0A2S6N2W1 | 99.7 | 1.1e-19 | 2.1e-25 | 150.6 | 277 | (4, 295) | 300 | (112, 403) | 407 | Baseplate protein J-like domain-containing protein | Baseplate protein J-like domain-containing protein | | uniclust | UniRef100\_A0A1C5PVS5 | 99.7 | 1.1e-19 | 2.1e-25 | 173.8 | 180 | (112, 296) | 300 | (503, 708) | 714 | Baseplate protein J-like domain-containing protein | Baseplate protein J-like domain-containing protein | | uniclust | UniRef100\_A0A3D8VNC0 | 99.7 | 1.2e-19 | 2.2e-25 | 144.8 | 151 | (11, 166) | 300 | (2, 234) | 235 | Baseplate J family protein (Fragment) | Baseplate J family protein (Fragment) | | uniclust | UniRef100\_A0A5C7QHU8 | 99.7 | 1.2e-19 | 2.3e-25 | 157.7 | 269 | (18, 294) | 300 | (11, 442) | 452 | Baseplate protein J-like domain-containing protein | Baseplate protein J-like domain-containing protein | | uniclust | UniRef100\_A0A423XTX1 | 99.7 | 1.6e-19 | 3e-25 | 152.0 | 240 | (53, 299) | 300 | (39, 359) | 467 | Phage baseplate protein | Phage baseplate protein | | uniclust | UniRef100\_A0A521CK67 | 99.7 | 1.6e-19 | 3e-25 | 151.3 | 271 | (21, 294) | 300 | (6, 446) | 447 | Baseplate J-like protein | Baseplate J-like protein | | uniclust | UniRef100\_A0A951P3G3 | 99.7 | 1.6e-19 | 3e-25 | 148.5 | 260 | (19, 287) | 300 | (3, 363) | 380 | Baseplate J/gp47 family protein | Baseplate J/gp47 family protein | | uniclust | UniRef100\_A0A6V7D981 | 99.7 | 1.7e-19 | 3.4e-25 | 142.1 | 130 | (2, 131) | 300 | (13, 142) | 169 | Baseplate assembly protein J | Baseplate assembly protein J | | uniclust | UniRef100\_UPI001AFE5812 | 99.7 | 1.9e-19 | 3.5e-25 | 155.4 | 269 | (18, 294) | 300 | (6, 382) | 593 | baseplate J/gp47 family protein | baseplate J/gp47 family protein | | uniclust | UniRef100\_A0A7J6YLT3 | 99.6 | 2.4e-19 | 4.5e-25 | 152.4 | 192 | (12, 222) | 300 | (293, 484) | 508 | Baseplate protein J-like domain-containing protein | Baseplate protein J-like domain-containing protein | | uniclust | UniRef100\_A0A327JML8 | 99.6 | 3.1e-19 | 5.8e-25 | 145.7 | 284 | (6, 297) | 300 | (7, 298) | 300 | Baseplate protein J-like domain-containing protein | Baseplate protein J-like domain-containing protein | | uniclust | UniRef100\_UPI0002DE2F33 | 99.6 | 3.2e-19 | 5.9e-25 | 150.6 | 221 | (19, 245) | 300 | (2, 293) | 474 | baseplate J/gp47 family protein | baseplate J/gp47 family protein | | uniclust | UniRef100\_A0A076G4G9 | 99.6 | 3.1e-19 | 6e-25 | 156.8 | 269 | (18, 295) | 300 | (12, 382) | 388 | Baseplate protein J-like domain-containing protein | Baseplate protein J-like domain-containing protein | | uniclust | UniRef100\_A0A0R3AT35 | 99.6 | 3.6e-19 | 6.8e-25 | 153.4 | 287 | (2, 295) | 300 | (1, 391) | 394 | Phage protein | Phage protein | | uniclust | UniRef100\_UPI000BB9B8A1 | 99.6 | 3.9e-19 | 7.3e-25 | 153.9 | 265 | (16, 287) | 300 | (7, 482) | 488 | baseplate J/gp47 family protein | baseplate J/gp47 family protein | | uniclust | UniRef100\_A0A8X6KGD0 | 99.6 | 4.1e-19 | 7.5e-25 | 153.0 | 218 | (43, 282) | 300 | (13, 232) | 573 | Baseplate protein J | Baseplate protein J | | uniclust | UniRef100\_J9PV89 | 99.6 | 4.2e-19 | 7.8e-25 | 150.0 | 272 | (21, 296) | 300 | (13, 470) | 476 | Putative baseplate J family protein | Putative baseplate J family protein | | uniclust | UniRef100\_A0A3S0DD06 | 99.6 | 4.4e-19 | 8.2e-25 | 156.7 | 178 | (112, 295) | 300 | (428, 616) | 621 | Baseplate protein J-like domain-containing protein | Baseplate protein J-like domain-containing protein | | uniclust | UniRef100\_A0A4P1QFN8 | 99.6 | 4.6e-19 | 8.6e-25 | 142.7 | 215 | (3, 221) | 300 | (1, 222) | 278 | Baseplate J-like family protein | Baseplate J-like family protein | | uniclust | UniRef100\_A0A081C216 | 99.6 | 4.8e-19 | 9.3e-25 | 161.2 | 220 | (42, 272) | 300 | (109, 415) | 588 | Baseplate protein J-like domain-containing protein | Baseplate protein J-like domain-containing protein | | uniclust | UniRef100\_A0A2M8G072 | 99.6 | 5.1e-19 | 9.3e-25 | 150.0 | 174 | (113, 293) | 300 | (301, 489) | 491 | Baseplate protein J-like domain-containing protein | Baseplate protein J-like domain-containing protein | | uniclust | UniRef100\_A0A8D9LH44 | 99.6 | 5.9e-19 | 1.1e-24 | 139.2 | 177 | (111, 294) | 300 | (84, 267) | 270 | Baseplate J-like protein | Baseplate J-like protein | | uniclust | UniRef100\_F5SA56 | 99.6 | 6.2e-19 | 1.2e-24 | 142.8 | 186 | (108, 296) | 300 | (95, 288) | 293 | Phage baseplate protein (Fragment) | Phage baseplate protein (Fragment) | | uniclust | UniRef100\_UPI001BAF66FD | 99.6 | 6.9e-19 | 1.3e-24 | 141.3 | 261 | (16, 291) | 300 | (14, 292) | 309 | baseplate J/gp47 family protein | baseplate J/gp47 family protein | | uniclust | UniRef100\_A0A7C2BDY4 | 99.6 | 7.2e-19 | 1.3e-24 | 143.7 | 258 | (21, 292) | 300 | (11, 351) | 354 | Baseplate protein J-like domain-containing protein | Baseplate protein J-like domain-containing protein | | uniclust | UniRef100\_A0A1V5Z4C1 | 99.6 | 9.3e-19 | 1.8e-24 | 149.1 | 157 | (111, 270) | 300 | (106, 278) | 322 | Baseplate J-like protein | Baseplate J-like protein | | uniclust | UniRef100\_A0A4Q3KFV4 | 99.6 | 1e-18 | 1.9e-24 | 143.3 | 237 | (45, 296) | 300 | (124, 363) | 365 | Baseplate assembly protein | Baseplate assembly protein | | uniclust | UniRef100\_A0A4V2AQW5 | 99.6 | 1e-18 | 2e-24 | 152.7 | 261 | (21, 294) | 300 | (20, 387) | 396 | Baseplate protein J-like domain-containing protein | Baseplate protein J-like domain-containing protein | | uniclust | UniRef100\_A0A0M0T5G9 | 99.6 | 1.3e-18 | 2.5e-24 | 132.4 | 147 | (141, 294) | 300 | (1, 155) | 157 | Tail protein (Fragment) | Tail protein (Fragment) | | uniclust | UniRef100\_A0A318KRB1 | 99.6 | 1.5e-18 | 2.7e-24 | 139.3 | 284 | (5, 292) | 300 | (1, 300) | 305 | Phage-related baseplate assembly protein | Phage-related baseplate assembly protein | | uniclust | UniRef100\_A0A2A5BN42 | 99.6 | 1.5e-18 | 2.7e-24 | 139.8 | 275 | (7, 296) | 300 | (11, 311) | 314 | Baseplate protein J-like domain-containing protein | Baseplate protein J-like domain-containing protein | | uniclust | UniRef100\_A0A060AG40 | 99.6 | 1.8e-18 | 3.6e-24 | 158.4 | 276 | (10, 295) | 300 | (21, 512) | 516 | Baseplate component | Baseplate component | | uniclust | UniRef100\_A0A6J5N751 | 99.6 | 2.1e-18 | 4e-24 | 152.2 | 269 | (21, 293) | 300 | (12, 478) | 488 | Baseplate wedge subunit | Baseplate wedge subunit | | uniclust | UniRef100\_A0A963HKG0 | 99.6 | 2.3e-18 | 4.4e-24 | 157.3 | 156 | (112, 269) | 300 | (304, 490) | 699 | Baseplate J/gp47 family protein (Fragment) | Baseplate J/gp47 family protein (Fragment) | | uniclust | UniRef100\_A0A0D8TUY8 | 99.6 | 2.8e-18 | 5.3e-24 | 139.3 | 154 | (19, 173) | 300 | (3, 244) | 264 | Phage baseplate J-like protein (Fragment) | Phage baseplate J-like protein (Fragment) | | uniclust | UniRef100\_A0A1A9WW65 | 99.6 | 3e-18 | 5.6e-24 | 132.9 | 170 | (126, 298) | 300 | (15, 189) | 191 | Baseplate\_J domain-containing protein | Baseplate\_J domain-containing protein | | uniclust | UniRef100\_H1D5E3 | 99.6 | 3.5e-18 | 6.6e-24 | 141.7 | 230 | (65, 296) | 300 | (9, 342) | 344 | Baseplate protein J-like domain-containing protein | Baseplate protein J-like domain-containing protein | | uniclust | UniRef100\_A0A016QLU4 | 99.6 | 3.6e-18 | 7e-24 | 146.6 | 183 | (111, 295) | 300 | (81, 300) | 308 | Baseplate protein J-like domain-containing protein | Baseplate protein J-like domain-containing protein | | uniclust | UniRef100\_A0A149VI46 | 99.6 | 3.6e-18 | 7.1e-24 | 147.8 | 169 | (17, 192) | 300 | (26, 278) | 326 | Baseplate protein J-like domain-containing protein (Fragment) | Baseplate protein J-like domain-containing protein (Fragment) | | uniclust | UniRef100\_A0A0A0YUY5 | 99.6 | 3.9e-18 | 7.6e-24 | 150.1 | 267 | (21, 292) | 300 | (5, 391) | 393 | Baseplate protein J-like domain-containing protein | Baseplate protein J-like domain-containing protein | | uniclust | UniRef100\_A0A2G2PNA8 | 99.6 | 5.4e-18 | 1.1e-23 | 153.8 | 231 | (18, 260) | 300 | (21, 360) | 486 | Baseplate protein J-like domain-containing protein | Baseplate protein J-like domain-containing protein | | uniclust | UniRef100\_A0A069PT37 | 99.6 | 5.7e-18 | 1.1e-23 | 150.2 | 232 | (19, 268) | 300 | (18, 286) | 390 | Baseplate protein J-like domain-containing protein | Baseplate protein J-like domain-containing protein | | uniclust | UniRef100\_A0A1C5KYZ3 | 99.6 | 6.1e-18 | 1.2e-23 | 156.7 | 179 | (112, 295) | 300 | (410, 618) | 619 | Uncharacterized homolog of phage Mu protein gp47 | Uncharacterized homolog of phage Mu protein gp47 | | uniclust | UniRef100\_UPI001C11CD17 | 99.6 | 7.4e-18 | 1.4e-23 | 131.7 | 171 | (19, 197) | 300 | (2, 247) | 247 | baseplate J/gp47 family protein | baseplate J/gp47 family protein | | uniclust | UniRef100\_A0A9E5G387 | 99.6 | 8.3e-18 | 1.6e-23 | 136.0 | 162 | (23, 189) | 300 | (2, 245) | 257 | Uncharacterized protein | Uncharacterized protein | | uniclust | UniRef100\_A0A075DXJ3 | 99.6 | 8.2e-18 | 1.6e-23 | 150.6 | 268 | (19, 295) | 300 | (10, 480) | 482 | Putative baseplate component | Putative baseplate component | | uniclust | UniRef100\_A0A383RGV8 | 99.6 | 8.8e-18 | 1.6e-23 | 137.3 | 179 | (112, 292) | 300 | (82, 278) | 283 | Baseplate J family protein | Baseplate J family protein | | uniclust | UniRef100\_UPI001EFEDC3E | 99.6 | 9.2e-18 | 1.7e-23 | 140.4 | 231 | (59, 296) | 300 | (2, 313) | 424 | baseplate J/gp47 family protein | baseplate J/gp47 family protein | | uniclust | UniRef100\_A0A8T3QP22 | 99.6 | 9.5e-18 | 1.8e-23 | 146.9 | 198 | (19, 221) | 300 | (3, 285) | 460 | Baseplate J/gp47 family protein | Baseplate J/gp47 family protein | | uniclust | UniRef100\_A0A929SH31 | 99.6 | 9.7e-18 | 1.8e-23 | 138.7 | 168 | (111, 287) | 300 | (102, 269) | 311 | Baseplate J/gp47 family protein | Baseplate J/gp47 family protein | | uniclust | UniRef100\_A0A5S9MGE0 | 99.5 | 1.1e-17 | 2.1e-23 | 134.8 | 180 | (112, 293) | 300 | (84, 274) | 278 | Baseplate protein J-like domain-containing protein | Baseplate protein J-like domain-containing protein | | uniclust | UniRef100\_A0A0A7NU29 | 99.5 | 1.5e-17 | 2.9e-23 | 147.4 | 235 | (21, 270) | 300 | (21, 341) | 505 | Baseplate J family protein | Baseplate J family protein | | uniclust | UniRef100\_A0A069PV97 | 99.5 | 1.5e-17 | 3e-23 | 168.7 | 180 | (111, 293) | 300 | (987, 1198) | 1219 | Baseplate protein J-like domain-containing protein | Baseplate protein J-like domain-containing protein | | uniclust | UniRef100\_A0A0S4I2J3 | 99.5 | 1.7e-17 | 3.2e-23 | 127.0 | 175 | (121, 296) | 300 | (1, 176) | 178 | Baseplate J-like protein | Baseplate J-like protein | | uniclust | UniRef100\_A0A072TF05 | 99.5 | 1.8e-17 | 3.4e-23 | 156.2 | 242 | (12, 258) | 300 | (652, 992) | 1809 | Bacteriophage regulatory protein, putative (Fragment) | Bacteriophage regulatory protein, putative (Fragment) | | uniclust | UniRef100\_A0A0H7L4M1 | 99.5 | 1.8e-17 | 3.4e-23 | 127.9 | 152 | (138, 296) | 300 | (2, 158) | 163 | Tail protein | Tail protein | | uniclust | UniRef100\_A0A1V5RRS7 | 99.5 | 1.9e-17 | 3.5e-23 | 149.9 | 244 | (21, 268) | 300 | (15, 442) | 787 | Baseplate protein J-like domain-containing protein | Baseplate protein J-like domain-containing protein | | uniclust | UniRef100\_B8IDQ2 | 99.5 | 2.5e-17 | 4.5e-23 | 130.0 | 231 | (4, 237) | 300 | (6, 257) | 263 | Baseplate J family protein | Baseplate J family protein | | uniclust | UniRef100\_A0A1L4D176 | 99.5 | 2.5e-17 | 4.7e-23 | 137.4 | 269 | (20, 295) | 300 | (4, 357) | 366 | Baseplate protein J-like domain-containing protein | Baseplate protein J-like domain-containing protein | | uniclust | UniRef100\_A0A080M2Y7 | 99.5 | 2.6e-17 | 5e-23 | 153.2 | 156 | (111, 268) | 300 | (356, 536) | 604 | Baseplate protein J-like domain-containing protein | Baseplate protein J-like domain-containing protein | | uniclust | UniRef100\_A0A0K9NAJ6 | 99.5 | 2.8e-17 | 5.5e-23 | 157.2 | 182 | (112, 297) | 300 | (497, 697) | 703 | Baseplate protein J-like domain-containing protein | Baseplate protein J-like domain-containing protein | | uniclust | UniRef100\_A0A0T9RK09 | 99.5 | 3.3e-17 | 6e-23 | 140.9 | 267 | (21, 291) | 300 | (166, 537) | 539 | Baseplate J family protein | Baseplate J family protein | | uniclust | UniRef100\_A0A812RFY7 | 99.5 | 4.3e-17 | 7.9e-23 | 145.0 | 221 | (44, 264) | 300 | (11, 259) | 772 | J protein | J protein | | uniclust | UniRef100\_A0A1E3G789 | 99.5 | 4.7e-17 | 8.7e-23 | 130.9 | 275 | (4, 293) | 300 | (1, 296) | 302 | Baseplate protein J-like domain-containing protein | Baseplate protein J-like domain-containing protein | | uniclust | UniRef100\_A0A430RXZ0 | 99.5 | 4.8e-17 | 8.9e-23 | 130.2 | 173 | (112, 293) | 300 | (98, 274) | 276 | Baseplate protein J-like domain-containing protein | Baseplate protein J-like domain-containing protein | | uniclust | UniRef100\_A0A0F9WMS5 | 99.5 | 5e-17 | 9.6e-23 | 145.7 | 284 | (9, 294) | 300 | (5, 513) | 521 | Baseplate protein J-like domain-containing protein | Baseplate protein J-like domain-containing protein | | uniclust | UniRef100\_UPI001EFE5B4D | 99.5 | 5.3e-17 | 9.8e-23 | 126.1 | 159 | (2, 160) | 300 | (1, 159) | 203 | hypothetical protein | hypothetical protein | | uniclust | UniRef100\_A0A353T659 | 99.5 | 5.2e-17 | 9.9e-23 | 142.2 | 273 | (16, 295) | 300 | (10, 410) | 413 | Baseplate protein J-like domain-containing protein | Baseplate protein J-like domain-containing protein | | uniclust | UniRef100\_A0A2G8MJ99 | 99.5 | 5.5e-17 | 1e-22 | 142.3 | 185 | (111, 297) | 300 | (178, 391) | 394 | Putative baseplate assembly protein (Fragment) | Putative baseplate assembly protein (Fragment) | | uniclust | UniRef100\_UPI00187D24B2 | 99.5 | 5.8e-17 | 1.1e-22 | 119.6 | 127 | (114, 244) | 300 | (2, 130) | 147 | baseplate J/gp47 family protein | baseplate J/gp47 family protein | | uniclust | UniRef100\_UPI001591BDFE | 99.5 | 6.4e-17 | 1.2e-22 | 123.1 | 179 | (54, 232) | 300 | (2, 180) | 202 | baseplate J/gp47 family protein | baseplate J/gp47 family protein | | uniclust | UniRef100\_A0A0R2FQN8 | 99.5 | 6.6e-17 | 1.3e-22 | 135.0 | 177 | (111, 295) | 300 | (78, 284) | 288 | Phage Mu gp47 related protein | Phage Mu gp47 related protein | | uniclust | UniRef100\_A0A2K9P040 | 99.5 | 7e-17 | 1.3e-22 | 141.1 | 242 | (21, 268) | 300 | (5, 336) | 574 | Baseplate J family protein | Baseplate J family protein | | uniclust | UniRef100\_A0A2N8K8B6 | 99.5 | 7e-17 | 1.3e-22 | 133.5 | 154 | (19, 173) | 300 | (3, 233) | 259 | Baseplate protein J-like domain-containing protein | Baseplate protein J-like domain-containing protein | | uniclust | UniRef100\_UPI001177B6DF | 99.5 | 7.5e-17 | 1.4e-22 | 133.6 | 182 | (110, 293) | 300 | (34, 381) | 381 | baseplate J/gp47 family protein | baseplate J/gp47 family protein | | uniclust | UniRef100\_A0A929JGZ9 | 99.5 | 7.6e-17 | 1.4e-22 | 136.2 | 173 | (111, 287) | 300 | (164, 350) | 373 | Baseplate J/gp47 family protein | Baseplate J/gp47 family protein | | uniclust | UniRef100\_A0A1H8RZN7 | 99.5 | 8.4e-17 | 1.6e-22 | 141.2 | 243 | (16, 268) | 300 | (6, 381) | 428 | Uncharacterized phage protein gp47/JayE | Uncharacterized phage protein gp47/JayE | | uniclust | UniRef100\_A0A1V6ET53 | 99.5 | 8.5e-17 | 1.6e-22 | 140.6 | 232 | (17, 260) | 300 | (8, 344) | 395 | Baseplate J-like protein | Baseplate J-like protein | | uniclust | UniRef100\_A0A6J5M9M0 | 99.5 | 1e-16 | 1.9e-22 | 133.1 | 242 | (21, 270) | 300 | (6, 348) | 389 | XkdT Uncharacterized homolog of phage Mu protein gp47 | XkdT Uncharacterized homolog of phage Mu protein gp47 | | uniclust | UniRef100\_A0A2E9U4S9 | 99.5 | 1e-16 | 1.9e-22 | 131.1 | 228 | (49, 285) | 300 | (28, 339) | 343 | Baseplate protein J-like domain-containing protein | Baseplate protein J-like domain-containing protein | | uniclust | UniRef100\_A0A1I0R9Q5 | 99.5 | 1e-16 | 2e-22 | 140.1 | 264 | (24, 289) | 300 | (9, 360) | 399 | Baseplate J-like protein | Baseplate J-like protein | | uniclust | UniRef100\_A0A943UN36 | 99.5 | 1.1e-16 | 2e-22 | 141.7 | 185 | (112, 298) | 300 | (525, 713) | 715 | Baseplate J/gp47 family protein | Baseplate J/gp47 family protein | | uniclust | UniRef100\_A0A090AGM4 | 99.5 | 1.1e-16 | 2e-22 | 140.1 | 156 | (112, 271) | 300 | (318, 475) | 638 | Baseplate protein J-like domain-containing protein | Baseplate protein J-like domain-containing protein | | uniclust | UniRef100\_A0A136KPX1 | 99.5 | 1.1e-16 | 2.2e-22 | 147.3 | 183 | (111, 295) | 300 | (268, 485) | 501 | Baseplate J-like protein | Baseplate J-like protein | | uniclust | UniRef100\_A0A511MW83 | 99.5 | 1.6e-16 | 2.9e-22 | 131.0 | 260 | (24, 293) | 300 | (13, 361) | 363 | Baseplate protein J-like domain-containing protein | Baseplate protein J-like domain-containing protein | | uniclust | UniRef100\_A0A098MIJ1 | 99.5 | 1.5e-16 | 2.9e-22 | 139.6 | 257 | (19, 288) | 300 | (35, 409) | 426 | Phage baseplate protein | Phage baseplate protein | | uniclust | UniRef100\_UPI00016A6C05 | 99.5 | 1.8e-16 | 3.4e-22 | 123.5 | 215 | (80, 298) | 300 | (1, 215) | 217 | baseplate J/gp47 family protein | baseplate J/gp47 family protein | | uniclust | UniRef100\_A0A0B0HE24 | 99.5 | 2e-16 | 3.8e-22 | 121.2 | 146 | (151, 297) | 300 | (3, 159) | 163 | Baseplate J-like protein | Baseplate J-like protein | | uniclust | UniRef100\_A0A1V0Q0D1 | 99.5 | 2.1e-16 | 4e-22 | 151.9 | 178 | (112, 292) | 300 | (574, 788) | 943 | Baseplate protein J-like domain-containing protein | Baseplate protein J-like domain-containing protein | | uniclust | UniRef100\_A0A094RWT6 | 99.5 | 2.1e-16 | 4.1e-22 | 135.0 | 209 | (3, 216) | 300 | (2, 302) | 303 | Phage protein (Fragment) | Phage protein (Fragment) | | uniclust | UniRef100\_UPI0009B77170 | 99.5 | 2.2e-16 | 4.1e-22 | 134.6 | 170 | (19, 192) | 300 | (2, 245) | 487 | baseplate J/gp47 family protein | baseplate J/gp47 family protein | | uniclust | UniRef100\_A0A5Y3ZZF9 | 99.5 | 2.3e-16 | 4.2e-22 | 138.3 | 234 | (55, 295) | 300 | (321, 634) | 639 | Uncharacterized protein | Uncharacterized protein | | uniclust | UniRef100\_A0A1S9JEE3 | 99.5 | 2.3e-16 | 4.3e-22 | 125.9 | 179 | (111, 296) | 300 | (54, 237) | 242 | Phage tail protein | Phage tail protein | | uniclust | UniRef100\_A0A0Q7YYN4 | 99.5 | 2.2e-16 | 4.3e-22 | 138.1 | 174 | (111, 288) | 300 | (147, 346) | 388 | Baseplate protein J-like domain-containing protein | Baseplate protein J-like domain-containing protein | | uniclust | UniRef100\_A0A0K4XJP0 | 99.5 | 2.4e-16 | 4.4e-22 | 130.9 | 234 | (53, 293) | 300 | (39, 352) | 352 | Putative phage baseplate protein | Putative phage baseplate protein | | uniclust | UniRef100\_A0A1G0RA06 | 99.4 | 2.5e-16 | 4.6e-22 | 130.7 | 269 | (22, 294) | 300 | (3, 366) | 383 | Baseplate protein J-like domain-containing protein | Baseplate protein J-like domain-containing protein | | uniclust | UniRef100\_A0A0S2ZD70 | 99.4 | 2.7e-16 | 5.2e-22 | 135.7 | 266 | (19, 294) | 300 | (9, 377) | 383 | Baseplate protein J-like domain-containing protein | Baseplate protein J-like domain-containing protein | | uniclust | UniRef100\_UPI001E545E66 | 99.4 | 3.3e-16 | 6.1e-22 | 135.4 | 234 | (20, 260) | 300 | (4, 314) | 556 | baseplate J/gp47 family protein | baseplate J/gp47 family protein | | uniclust | UniRef100\_A0A0F9SDC1 | 99.4 | 3.4e-16 | 6.4e-22 | 136.1 | 271 | (21, 295) | 300 | (4, 473) | 475 | Baseplate protein J-like domain-containing protein | Baseplate protein J-like domain-containing protein | | uniclust | UniRef100\_A0A165ZZT9 | 99.4 | 3.5e-16 | 6.6e-22 | 135.5 | 264 | (20, 293) | 300 | (11, 386) | 388 | Baseplate J-like protein | Baseplate J-like protein | | uniclust | UniRef100\_UPI001C0FE55C | 99.4 | 3.8e-16 | 7e-22 | 131.6 | 198 | (23, 243) | 300 | (2, 202) | 435 | baseplate J/gp47 family protein | baseplate J/gp47 family protein | | uniclust | UniRef100\_A0A067XR41 | 99.4 | 4.6e-16 | 9.2e-22 | 152.5 | 244 | (20, 267) | 300 | (13, 461) | 794 | Baseplate wedge | Baseplate wedge | | uniclust | UniRef100\_A0A0C2HQB0 | 99.4 | 5.2e-16 | 1e-21 | 141.0 | 215 | (42, 270) | 300 | (110, 419) | 533 | Baseplate protein J-like domain-containing protein | Baseplate protein J-like domain-containing protein | | uniclust | UniRef100\_A0A356K030 | 99.4 | 5.3e-16 | 1e-21 | 147.4 | 187 | (111, 299) | 300 | (624, 837) | 840 | Putative baseplate assembly protein (Fragment) | Putative baseplate assembly protein (Fragment) | | uniclust | UniRef100\_A0A109WD85 | 99.4 | 5.8e-16 | 1.1e-21 | 134.4 | 246 | (16, 270) | 300 | (19, 367) | 405 | Baseplate protein J-like domain-containing protein | Baseplate protein J-like domain-containing protein | | uniclust | UniRef100\_UPI00161E54F1 | 99.4 | 6.1e-16 | 1.1e-21 | 112.2 | 132 | (114, 252) | 300 | (3, 137) | 140 | baseplate J/gp47 family protein | baseplate J/gp47 family protein | | uniclust | UniRef100\_A0A0E3L2V3 | 99.4 | 6.6e-16 | 1.3e-21 | 152.2 | 183 | (111, 295) | 300 | (761, 971) | 979 | Baseplate protein J-like domain-containing protein | Baseplate protein J-like domain-containing protein | | uniclust | UniRef100\_A0A5C8CQS2 | 99.4 | 8.1e-16 | 1.5e-21 | 130.5 | 261 | (21, 292) | 300 | (5, 350) | 351 | Acetyltransferase | Acetyltransferase | | uniclust | UniRef100\_X1GT58 | 99.4 | 9e-16 | 1.7e-21 | 113.8 | 151 | (139, 293) | 300 | (1, 157) | 168 | Baseplate protein J-like domain-containing protein (Fragment) | Baseplate protein J-like domain-containing protein (Fragment) | | uniclust | UniRef100\_A0A830FUX9 | 99.4 | 1e-15 | 2e-21 | 130.7 | 259 | (20, 294) | 300 | (9, 389) | 390 | Putative phage protein gp47/JayE | Putative phage protein gp47/JayE | | uniclust | UniRef100\_A0A285J5J6 | 99.4 | 1.1e-15 | 2e-21 | 115.6 | 160 | (136, 296) | 300 | (1, 161) | 163 | Phage-related baseplate assembly protein (Fragment) | Phage-related baseplate assembly protein (Fragment) | | uniclust | UniRef100\_UPI00037164A3 | 99.4 | 1.2e-15 | 2.2e-21 | 122.7 | 190 | (23, 217) | 300 | (6, 286) | 293 | baseplate J/gp47 family protein | baseplate J/gp47 family protein | | uniclust | UniRef100\_A0A842IZ09 | 99.4 | 1.3e-15 | 2.3e-21 | 123.8 | 230 | (64, 297) | 300 | (5, 317) | 318 | Baseplate J/gp47 family protein | Baseplate J/gp47 family protein | | uniclust | UniRef100\_A0A3D4RY72 | 99.4 | 1.2e-15 | 2.4e-21 | 141.2 | 181 | (112, 295) | 300 | (494, 706) | 741 | Baseplate protein J-like domain-containing protein | Baseplate protein J-like domain-containing protein | | uniclust | UniRef100\_A0A086ML20 | 99.4 | 1.4e-15 | 2.7e-21 | 146.7 | 174 | (111, 288) | 300 | (636, 835) | 854 | Baseplate protein J-like domain-containing protein | Baseplate protein J-like domain-containing protein | | uniclust | UniRef100\_A0A098N215 | 99.4 | 1.4e-15 | 2.7e-21 | 132.7 | 263 | (22, 293) | 300 | (5, 371) | 379 | Phage baseplate protein | Phage baseplate protein | | uniclust | UniRef100\_A0A0C2QJG0 | 99.4 | 1.5e-15 | 2.8e-21 | 134.1 | 259 | (21, 295) | 300 | (9, 362) | 385 | Baseplate protein J-like domain-containing protein | Baseplate protein J-like domain-containing protein | | uniclust | UniRef100\_A0A7X6FRN3 | 99.4 | 1.6e-15 | 3e-21 | 116.3 | 153 | (5, 172) | 300 | (9, 161) | 208 | Baseplate protein J-like domain-containing protein | Baseplate protein J-like domain-containing protein | | uniclust | UniRef100\_A0A257P1Y5 | 99.4 | 1.6e-15 | 3e-21 | 126.4 | 179 | (111, 295) | 300 | (81, 270) | 272 | Baseplate protein J-like domain-containing protein | Baseplate protein J-like domain-containing protein | | uniclust | UniRef100\_UPI0015897FEC | 99.4 | 1.6e-15 | 3e-21 | 125.3 | 261 | (26, 294) | 300 | (14, 361) | 363 | baseplate J/gp47 family protein | baseplate J/gp47 family protein | | uniclust | UniRef100\_A0A077NCN1 | 99.4 | 1.6e-15 | 3e-21 | 130.5 | 274 | (10, 293) | 300 | (10, 394) | 405 | Bacteriophage protein | Bacteriophage protein | | uniclust | UniRef100\_A0A014N6D0 | 99.4 | 1.6e-15 | 3.3e-21 | 152.3 | 172 | (112, 287) | 300 | (761, 959) | 1002 | Baseplate protein J-like domain-containing protein | Baseplate protein J-like domain-containing protein | | uniclust | UniRef100\_UPI000A1870BF | 99.4 | 1.8e-15 | 3.5e-21 | 113.0 | 147 | (115, 264) | 300 | (4, 150) | 150 | baseplate J/gp47 family protein | baseplate J/gp47 family protein | | uniclust | UniRef100\_A0A242CHC8 | 99.4 | 2e-15 | 3.6e-21 | 120.2 | 250 | (21, 292) | 300 | (4, 268) | 269 | Baseplate protein J-like domain-containing protein | Baseplate protein J-like domain-containing protein | | uniclust | UniRef100\_A0A0S6WQZ6 | 99.4 | 1.9e-15 | 3.7e-21 | 139.2 | 175 | (111, 289) | 300 | (309, 509) | 528 | Baseplate protein J-like domain-containing protein | Baseplate protein J-like domain-containing protein | | uniclust | UniRef100\_A0A6M0A0U9 | 99.4 | 2.1e-15 | 3.9e-21 | 130.7 | 219 | (21, 270) | 300 | (10, 386) | 449 | Baseplate protein J-like domain-containing protein | Baseplate protein J-like domain-containing protein | | uniclust | UniRef100\_A0A3N5LBL1 | 99.4 | 2.2e-15 | 4e-21 | 128.8 | 245 | (23, 271) | 300 | (5, 348) | 487 | Baseplate protein J-like domain-containing protein | Baseplate protein J-like domain-containing protein | | uniclust | UniRef100\_A0A2W2DLG7 | 99.4 | 2.5e-15 | 4.8e-21 | 138.3 | 155 | (112, 270) | 300 | (421, 589) | 636 | Baseplate assembly protein | Baseplate assembly protein | | uniclust | UniRef100\_UPI001FAC64A8 | 99.4 | 2.7e-15 | 4.9e-21 | 123.6 | 223 | (70, 294) | 300 | (2, 348) | 350 | baseplate J/gp47 family protein | baseplate J/gp47 family protein | | uniclust | UniRef100\_A0A2E2FJ34 | 99.4 | 2.9e-15 | 5.5e-21 | 134.6 | 248 | (20, 271) | 300 | (12, 439) | 555 | Baseplate protein J-like domain-containing protein | Baseplate protein J-like domain-containing protein | | uniclust | UniRef100\_A0A3M4ZB42 | 99.4 | 3.1e-15 | 5.8e-21 | 108.9 | 128 | (117, 244) | 300 | (1, 128) | 131 | Baseplate assembly protein J (Fragment) | Baseplate assembly protein J (Fragment) | | uniclust | UniRef100\_A0A0U1DT04 | 99.3 | 3.3e-15 | 6.3e-21 | 131.8 | 194 | (42, 244) | 300 | (129, 406) | 420 | Baseplate J-like protein | Baseplate J-like protein | | uniclust | UniRef100\_K8GRQ2 | 99.3 | 3.5e-15 | 6.5e-21 | 123.3 | 267 | (20, 294) | 300 | (3, 358) | 361 | Putative phage Mu protein gp47-like protein | Putative phage Mu protein gp47-like protein | | uniclust | UniRef100\_A0A524PY89 | 99.3 | 3.7e-15 | 7.1e-21 | 136.2 | 187 | (111, 299) | 300 | (382, 592) | 594 | Putative baseplate assembly protein (Fragment) | Putative baseplate assembly protein (Fragment) | | uniclust | UniRef100\_A0A5C7PKD9 | 99.3 | 4.6e-15 | 8.5e-21 | 124.0 | 261 | (23, 287) | 300 | (16, 387) | 396 | Baseplate protein J-like domain-containing protein | Baseplate protein J-like domain-containing protein | | uniclust | UniRef100\_A0A1M6CQV3 | 99.3 | 5.5e-15 | 1e-20 | 104.9 | 107 | (134, 244) | 300 | (3, 111) | 113 | Baseplate J-like protein | Baseplate J-like protein | | uniclust | UniRef100\_A0A7W6DEC2 | 99.3 | 6.2e-15 | 1.1e-20 | 126.0 | 219 | (21, 245) | 300 | (5, 305) | 482 | Putative phage protein gp47/JayE | Putative phage protein gp47/JayE | | uniclust | UniRef100\_I8RM93 | 99.3 | 6.6e-15 | 1.2e-20 | 127.1 | 262 | (24, 292) | 300 | (15, 526) | 526 | Baseplate J family protein | Baseplate J family protein | | uniclust | UniRef100\_A0A8S5ST71 | 99.3 | 6.6e-15 | 1.2e-20 | 126.8 | 267 | (19, 293) | 300 | (12, 395) | 398 | Baseplate wedge protein | Baseplate wedge protein | | uniclust | UniRef100\_A0A1D9FVB1 | 99.3 | 7e-15 | 1.4e-20 | 135.3 | 174 | (112, 289) | 300 | (320, 519) | 537 | Baseplate assembly protein | Baseplate assembly protein | | uniclust | UniRef100\_A0A150SA95 | 99.3 | 7.6e-15 | 1.5e-20 | 132.9 | 199 | (20, 220) | 300 | (10, 291) | 498 | Baseplate protein J-like domain-containing protein | Baseplate protein J-like domain-containing protein | | uniclust | UniRef100\_A0A0Q6V809 | 99.3 | 7.6e-15 | 1.5e-20 | 147.3 | 184 | (111, 296) | 300 | (891, 1101) | 1139 | Baseplate protein J-like domain-containing protein | Baseplate protein J-like domain-containing protein | | uniclust | UniRef100\_A0A843G9W3 | 99.3 | 8.4e-15 | 1.6e-20 | 126.8 | 178 | (112, 295) | 300 | (263, 455) | 457 | Baseplate J/gp47 family protein | Baseplate J/gp47 family protein | | uniclust | UniRef100\_A0A1G6LTE0 | 99.3 | 8.5e-15 | 1.7e-20 | 130.2 | 253 | (22, 288) | 300 | (10, 369) | 380 | Baseplate J-like protein | Baseplate J-like protein | | uniclust | UniRef100\_A0A8S5UKQ2 | 99.3 | 9.2e-15 | 1.7e-20 | 124.8 | 280 | (12, 294) | 300 | (3, 473) | 474 | Baseplate wedge protein | Baseplate wedge protein | | uniclust | UniRef100\_A0A261E6U1 | 99.3 | 1.1e-14 | 2.1e-20 | 117.5 | 162 | (108, 270) | 300 | (27, 192) | 237 | Baseplate protein J-like domain-containing protein | Baseplate protein J-like domain-containing protein | | uniclust | UniRef100\_A0A2E5KBW0 | 99.3 | 1.2e-14 | 2.1e-20 | 124.9 | 267 | (17, 293) | 300 | (7, 492) | 496 | Baseplate protein J-like domain-containing protein | Baseplate protein J-like domain-containing protein | | uniclust | UniRef100\_A0A7X8LQY5 | 99.3 | 1.3e-14 | 2.4e-20 | 104.8 | 132 | (21, 170) | 300 | (3, 139) | 140 | Baseplate protein J-like domain-containing protein (Fragment) | Baseplate protein J-like domain-containing protein (Fragment) | | uniclust | UniRef100\_A0A3B8HH03 | 99.3 | 1.4e-14 | 2.6e-20 | 115.1 | 164 | (129, 295) | 300 | (9, 202) | 209 | Baseplate protein J-like domain-containing protein (Fragment) | Baseplate protein J-like domain-containing protein (Fragment) | | uniclust | UniRef100\_A0A5T0ECW8 | 99.3 | 1.6e-14 | 2.9e-20 | 119.3 | 218 | (42, 270) | 300 | (49, 344) | 353 | Baseplate assembly protein (Fragment) | Baseplate assembly protein (Fragment) | | uniclust | UniRef100\_A0A522CNZ0 | 99.3 | 1.6e-14 | 3e-20 | 120.3 | 247 | (42, 295) | 300 | (34, 374) | 379 | Baseplate protein J-like domain-containing protein | Baseplate protein J-like domain-containing protein | | uniclust | UniRef100\_A0A284VRY0 | 99.3 | 1.8e-14 | 3.4e-20 | 140.3 | 185 | (111, 295) | 300 | (793, 1004) | 1005 | Baseplate protein J-like domain-containing protein | Baseplate protein J-like domain-containing protein | | uniclust | UniRef100\_A0A2V8S3H9 | 99.3 | 1.7e-14 | 3.4e-20 | 138.4 | 181 | (112, 295) | 300 | (535, 774) | 777 | Putative baseplate assembly protein | Putative baseplate assembly protein | | uniclust | UniRef100\_A0A098LJS2 | 99.3 | 1.7e-14 | 3.4e-20 | 146.3 | 229 | (5, 269) | 300 | (14, 312) | 1032 | Uncharacterized protein | Uncharacterized protein | | uniclust | UniRef100\_UPI00038D138D | 99.3 | 1.9e-14 | 3.5e-20 | 114.5 | 158 | (21, 184) | 300 | (4, 238) | 243 | baseplate J/gp47 family protein | baseplate J/gp47 family protein | | uniclust | UniRef100\_A0A350Y2A6 | 99.3 | 2e-14 | 3.6e-20 | 123.2 | 146 | (19, 169) | 300 | (3, 238) | 482 | Baseplate protein J-like domain-containing protein | Baseplate protein J-like domain-containing protein | | uniclust | UniRef100\_UPI000F5FDBD6 | 99.3 | 2e-14 | 3.6e-20 | 124.7 | 176 | (112, 293) | 300 | (337, 540) | 541 | baseplate J/gp47 family protein | baseplate J/gp47 family protein | | uniclust | UniRef100\_A0A3L7X434 | 99.3 | 2.1e-14 | 3.9e-20 | 124.0 | 156 | (111, 268) | 300 | (124, 315) | 403 | Baseplate protein J-like domain-containing protein (Fragment) | Baseplate protein J-like domain-containing protein (Fragment) | | uniclust | UniRef100\_A0A136JVI8 | 99.3 | 2.2e-14 | 4.1e-20 | 136.4 | 175 | (111, 289) | 300 | (628, 828) | 846 | Baseplate protein J-like domain-containing protein | Baseplate protein J-like domain-containing protein | | uniclust | UniRef100\_A0A1F8MNF7 | 99.3 | 2.2e-14 | 4.2e-20 | 136.9 | 251 | (1, 287) | 300 | (1, 328) | 730 | Uncharacterized protein | Uncharacterized protein | | uniclust | UniRef100\_A0A5U6MH76 | 99.3 | 2.3e-14 | 4.2e-20 | 106.6 | 140 | (111, 256) | 300 | (22, 164) | 167 | Phage tail protein (Fragment) | Phage tail protein (Fragment) | | uniclust | UniRef100\_A0A1Q6LBP8 | 99.3 | 2.3e-14 | 4.2e-20 | 119.5 | 236 | (22, 265) | 300 | (3, 321) | 356 | Baseplate protein J-like domain-containing protein | Baseplate protein J-like domain-containing protein | | uniclust | UniRef100\_A0A842LGU8 | 99.3 | 2.5e-14 | 4.5e-20 | 119.5 | 235 | (52, 295) | 300 | (45, 381) | 383 | Baseplate J/gp47 family protein | Baseplate J/gp47 family protein | | uniclust | UniRef100\_A0A6V7F7S0 | 99.3 | 2.5e-14 | 4.6e-20 | 110.4 | 155 | (2, 158) | 300 | (1, 155) | 212 | Phage-related baseplate assembly protein | Phage-related baseplate assembly protein | | uniclust | UniRef100\_UPI0013748030 | 99.3 | 2.8e-14 | 5.2e-20 | 110.1 | 116 | (111, 230) | 300 | (82, 199) | 203 | baseplate J/gp47 family protein | baseplate J/gp47 family protein | | uniclust | UniRef100\_A0A4Q0YJP7 | 99.2 | 3.1e-14 | 5.7e-20 | 112.2 | 198 | (6, 203) | 300 | (5, 239) | 245 | Baseplate assembly protein | Baseplate assembly protein | | uniclust | UniRef100\_A0A1H4QY22 | 99.2 | 3.2e-14 | 5.9e-20 | 108.2 | 145 | (130, 280) | 300 | (2, 149) | 169 | Baseplate J-like protein | Baseplate J-like protein | | uniclust | UniRef100\_A0A1Y1QPN6 | 99.2 | 3.5e-14 | 6.4e-20 | 116.9 | 259 | (21, 290) | 300 | (8, 338) | 341 | Baseplate protein J-like domain-containing protein | Baseplate protein J-like domain-containing protein | | uniclust | UniRef100\_A0A058ZQ54 | 99.2 | 3.4e-14 | 6.7e-20 | 141.3 | 174 | (111, 288) | 300 | (838, 1037) | 1176 | Putative phage tail region protein | Putative phage tail region protein | | uniclust | UniRef100\_A0A3S0BRQ0 | 99.2 | 3.7e-14 | 6.9e-20 | 123.2 | 258 | (22, 287) | 300 | (13, 468) | 478 | Baseplate protein J-like domain-containing protein | Baseplate protein J-like domain-containing protein | | uniclust | UniRef100\_A0A2W4YN20 | 99.2 | 4.5e-14 | 8.5e-20 | 128.5 | 157 | (111, 269) | 300 | (322, 516) | 568 | Putative baseplate assembly protein | Putative baseplate assembly protein | | uniclust | UniRef100\_A0A3P1VZS5 | 99.2 | 4.7e-14 | 9.1e-20 | 124.6 | 226 | (19, 256) | 300 | (4, 326) | 374 | Baseplate protein J-like domain-containing protein | Baseplate protein J-like domain-containing protein | | uniclust | UniRef100\_A0A0E2HC00 | 99.2 | 5.1e-14 | 9.6e-20 | 113.5 | 219 | (22, 295) | 300 | (5, 236) | 237 | Baseplate protein J-like domain-containing protein | Baseplate protein J-like domain-containing protein | | uniclust | UniRef100\_A0A1Q7YH65 | 99.2 | 5.4e-14 | 1e-19 | 120.9 | 242 | (40, 294) | 300 | (60, 400) | 402 | Baseplate protein J-like domain-containing protein | Baseplate protein J-like domain-containing protein | | uniclust | UniRef100\_A0A074LTY1 | 99.2 | 5.2e-14 | 1e-19 | 140.1 | 156 | (111, 268) | 300 | (828, 1005) | 1071 | Baseplate protein J-like domain-containing protein | Baseplate protein J-like domain-containing protein | | uniclust | UniRef100\_A0A398CJB0 | 99.2 | 5.9e-14 | 1.1e-19 | 119.5 | 237 | (22, 269) | 300 | (8, 363) | 451 | Baseplate protein J-like domain-containing protein | Baseplate protein J-like domain-containing protein | | uniclust | UniRef100\_UPI0017498F18 | 99.2 | 6e-14 | 1.1e-19 | 108.9 | 105 | (112, 220) | 300 | (78, 184) | 186 | baseplate J/gp47 family protein | baseplate J/gp47 family protein | | uniclust | UniRef100\_A0A080LYP8 | 99.2 | 6e-14 | 1.2e-19 | 126.1 | 267 | (21, 290) | 300 | (40, 379) | 398 | Uncharacterized protein | Uncharacterized protein | | uniclust | UniRef100\_A0A1V5QEC7 | 99.2 | 6.4e-14 | 1.2e-19 | 108.3 | 181 | (111, 295) | 300 | (20, 208) | 213 | Baseplate J-like protein | Baseplate J-like protein | | uniclust | UniRef100\_A0A150RG01 | 99.2 | 6.4e-14 | 1.2e-19 | 131.2 | 155 | (112, 269) | 300 | (499, 690) | 756 | Baseplate protein J-like domain-containing protein | Baseplate protein J-like domain-containing protein | | uniclust | UniRef100\_A0A336NF78 | 99.2 | 6.5e-14 | 1.2e-19 | 100.3 | 111 | (2, 127) | 300 | (1, 116) | 118 | Phage related protein | Phage related protein | | uniclust | UniRef100\_A0A2E6X3G2 | 99.2 | 6.9e-14 | 1.3e-19 | 121.0 | 273 | (20, 300) | 300 | (6, 416) | 416 | Baseplate protein J-like domain-containing protein | Baseplate protein J-like domain-containing protein | | uniclust | UniRef100\_A0A6M0L2F8 | 99.2 | 7.2e-14 | 1.4e-19 | 126.9 | 152 | (112, 267) | 300 | (409, 577) | 625 | Baseplate protein J-like domain-containing protein | Baseplate protein J-like domain-containing protein | | uniclust | UniRef100\_A0A7C1ESV1 | 99.2 | 7.6e-14 | 1.4e-19 | 96.9 | 106 | (22, 143) | 300 | (5, 110) | 110 | Baseplate protein J-like domain-containing protein (Fragment) | Baseplate protein J-like domain-containing protein (Fragment) | | uniclust | UniRef100\_A0A1B8SLD9 | 99.2 | 7.8e-14 | 1.5e-19 | 127.6 | 269 | (21, 293) | 300 | (3, 576) | 588 | Baseplate protein J-like domain-containing protein | Baseplate protein J-like domain-containing protein | | uniclust | UniRef100\_A0A928YGD7 | 99.2 | 8.4e-14 | 1.5e-19 | 118.6 | 244 | (21, 270) | 300 | (13, 356) | 451 | Baseplate protein J-like domain-containing protein | Baseplate protein J-like domain-containing protein | | uniclust | UniRef100\_A0A0U1DD70 | 99.2 | 8.2e-14 | 1.6e-19 | 105.8 | 133 | (165, 298) | 300 | (7, 139) | 140 | Baseplate assembly protein GpJ | Baseplate assembly protein GpJ | | uniclust | UniRef100\_A0A3A8I9C4 | 99.2 | 8.1e-14 | 1.6e-19 | 123.9 | 245 | (21, 267) | 300 | (12, 332) | 405 | Baseplate protein J-like domain-containing protein | Baseplate protein J-like domain-containing protein | | uniclust | UniRef100\_A0A2A5EI74 | 99.2 | 8.6e-14 | 1.6e-19 | 121.2 | 150 | (21, 170) | 300 | (9, 237) | 552 | Baseplate protein J-like domain-containing protein | Baseplate protein J-like domain-containing protein | | uniclust | UniRef100\_A0A2X3LXX5 | 99.2 | 8.6e-14 | 1.6e-19 | 103.3 | 105 | (194, 298) | 300 | (14, 118) | 123 | Baseplate assembly protein J | Baseplate assembly protein J | | uniclust | UniRef100\_A0A9D0GYQ9 | 99.2 | 9.1e-14 | 1.7e-19 | 122.4 | 180 | (112, 294) | 300 | (404, 606) | 617 | Uncharacterized protein | Uncharacterized protein | | uniclust | UniRef100\_A0A521U4G1 | 99.2 | 9.5e-14 | 1.8e-19 | 119.4 | 241 | (21, 266) | 300 | (12, 356) | 396 | Baseplate protein J-like domain-containing protein | Baseplate protein J-like domain-containing protein | | uniclust | UniRef100\_UPI000374DC89 | 99.2 | 9.7e-14 | 1.8e-19 | 120.6 | 236 | (20, 265) | 300 | (10, 339) | 540 | baseplate J/gp47 family protein | baseplate J/gp47 family protein | | uniclust | UniRef100\_A0A645G9Q1 | 99.2 | 1e-13 | 1.9e-19 | 106.8 | 184 | (111, 296) | 300 | (15, 202) | 206 | Baseplate protein J-like domain-containing protein | Baseplate protein J-like domain-containing protein | | uniclust | UniRef100\_A0A953PSF3 | 99.2 | 1.1e-13 | 2e-19 | 115.3 | 224 | (20, 269) | 300 | (9, 335) | 369 | Baseplate J/gp47 family protein | Baseplate J/gp47 family protein | | uniclust | UniRef100\_A0A2X3CRG2 | 99.2 | 1.1e-13 | 2.1e-19 | 117.9 | 183 | (2, 191) | 300 | (2, 267) | 305 | Putative bacteriophage protein | Putative bacteriophage protein | | uniclust | UniRef100\_A0A7Z2GCV1 | 99.2 | 1.1e-13 | 2.1e-19 | 135.2 | 174 | (111, 288) | 300 | (880, 1079) | 1098 | Putative baseplate assembly protein | Putative baseplate assembly protein | | uniclust | UniRef100\_A0A398CKB0 | 99.2 | 1.3e-13 | 2.4e-19 | 122.9 | 182 | (112, 298) | 300 | (299, 516) | 518 | Putative baseplate assembly protein | Putative baseplate assembly protein | | uniclust | UniRef100\_A0A967LZV4 | 99.2 | 1.4e-13 | 2.6e-19 | 120.7 | 252 | (18, 277) | 300 | (9, 463) | 587 | Baseplate protein J-like domain-containing protein | Baseplate protein J-like domain-containing protein | | uniclust | UniRef100\_UPI0006C89834 | 99.2 | 1.7e-13 | 3.2e-19 | 107.7 | 183 | (113, 296) | 300 | (17, 233) | 236 | baseplate J/gp47 family protein | baseplate J/gp47 family protein | | uniclust | UniRef100\_A0A523TX46 | 99.2 | 1.8e-13 | 3.4e-19 | 109.7 | 163 | (131, 296) | 300 | (42, 237) | 242 | Baseplate protein J-like domain-containing protein | Baseplate protein J-like domain-containing protein | | uniclust | UniRef100\_A0A3P1VXU5 | 99.2 | 1.9e-13 | 3.6e-19 | 118.2 | 260 | (21, 289) | 300 | (19, 385) | 394 | Baseplate protein J-like domain-containing protein | Baseplate protein J-like domain-containing protein | | uniclust | UniRef100\_A0A524BHK3 | 99.1 | 2.2e-13 | 4e-19 | 114.0 | 271 | (19, 294) | 300 | (17, 376) | 381 | Baseplate protein J-like domain-containing protein (Fragment) | Baseplate protein J-like domain-containing protein (Fragment) | | uniclust | UniRef100\_A0A1H4BJM8 | 99.1 | 2.4e-13 | 4.8e-19 | 140.0 | 229 | (21, 288) | 300 | (20, 327) | 1375 | Baseplate J-like protein | Baseplate J-like protein | | uniclust | UniRef100\_A0A376TVP9 | 99.1 | 2.5e-13 | 5e-19 | 103.9 | 104 | (73, 176) | 300 | (16, 119) | 125 | Baseplate assembly protein GpJ | Baseplate assembly protein GpJ | | uniclust | UniRef100\_A0A8S5LX07 | 99.1 | 3.1e-13 | 5.8e-19 | 119.3 | 277 | (21, 299) | 300 | (14, 492) | 496 | Baseplate wedge protein | Baseplate wedge protein | | uniclust | UniRef100\_A0A0T9TB71 | 99.1 | 3.3e-13 | 6e-19 | 111.1 | 145 | (5, 149) | 300 | (1, 152) | 332 | Regulator of late gene expression | Regulator of late gene expression | | uniclust | UniRef100\_A0A6L6YM94 | 99.1 | 3.3e-13 | 6.1e-19 | 105.0 | 102 | (70, 172) | 300 | (2, 180) | 192 | Baseplate J protein (Fragment) | Baseplate J protein (Fragment) | | uniclust | UniRef100\_A0A7U6KQX5 | 99.1 | 3.4e-13 | 6.2e-19 | 99.0 | 129 | (30, 159) | 300 | (7, 137) | 150 | Baseplate protein J-like domain-containing protein | Baseplate protein J-like domain-containing protein | | uniclust | UniRef100\_A0A524PSP0 | 99.1 | 3.3e-13 | 6.3e-19 | 110.4 | 185 | (111, 296) | 300 | (52, 251) | 255 | Baseplate protein J-like domain-containing protein (Fragment) | Baseplate protein J-like domain-containing protein (Fragment) | | uniclust | UniRef100\_A0A0M0T7J9 | 99.1 | 3.4e-13 | 6.4e-19 | 109.7 | 123 | (20, 143) | 300 | (4, 200) | 219 | Tail protein (Fragment) | Tail protein (Fragment) | | uniclust | UniRef100\_A0A062UU35 | 99.1 | 3.6e-13 | 6.9e-19 | 134.1 | 158 | (111, 268) | 300 | (820, 998) | 1288 | Baseplate protein J-like domain-containing protein | Baseplate protein J-like domain-containing protein | | uniclust | UniRef100\_UPI0018D02FB2 | 99.1 | 3.8e-13 | 7.2e-19 | 106.9 | 176 | (111, 292) | 300 | (7, 215) | 217 | baseplate J/gp47 family protein | baseplate J/gp47 family protein | | uniclust | UniRef100\_A0A0A2VPK4 | 99.1 | 4e-13 | 7.4e-19 | 127.2 | 251 | (23, 279) | 300 | (958, 1317) | 1510 | Protein gp47 | Protein gp47 | | uniclust | UniRef100\_A0A072TEA8 | 99.1 | 4.3e-13 | 8e-19 | 131.1 | 245 | (27, 277) | 300 | (1133, 1491) | 2834 | Phage late control protein | Phage late control protein | | uniclust | UniRef100\_A0A1E4QYI6 | 99.1 | 4.7e-13 | 9e-19 | 113.1 | 229 | (19, 270) | 300 | (4, 235) | 270 | Baseplate protein J-like domain-containing protein | Baseplate protein J-like domain-containing protein | | uniclust | UniRef100\_A0A328TDR2 | 99.1 | 5.2e-13 | 9.7e-19 | 97.8 | 87 | (111, 202) | 300 | (32, 120) | 130 | Baseplate J-like family protein (Fragment) | Baseplate J-like family protein (Fragment) | | uniclust | UniRef100\_A0A931K983 | 99.1 | 5.6e-13 | 1e-18 | 113.4 | 251 | (31, 288) | 300 | (85, 426) | 433 | Baseplate protein J-like domain-containing protein | Baseplate protein J-like domain-containing protein | | uniclust | UniRef100\_A0A5B8AU34 | 99.1 | 5.8e-13 | 1.1e-18 | 109.8 | 264 | (20, 296) | 300 | (9, 326) | 332 | Baseplate protein J-like domain-containing protein | Baseplate protein J-like domain-containing protein | | uniclust | UniRef100\_A0A8S5M026 | 99.1 | 6e-13 | 1.1e-18 | 111.8 | 269 | (18, 293) | 300 | (7, 381) | 389 | Baseplate J like protein | Baseplate J like protein | | uniclust | UniRef100\_UPI00082E9BF2 | 99.1 | 6.2e-13 | 1.1e-18 | 106.8 | 166 | (23, 192) | 300 | (4, 248) | 272 | baseplate J/gp47 family protein | baseplate J/gp47 family protein | | uniclust | UniRef100\_J8V6S9 | 99.1 | 6.3e-13 | 1.2e-18 | 103.1 | 154 | (86, 239) | 300 | (1, 155) | 212 | Baseplate J family protein (Fragment) | Baseplate J family protein (Fragment) | | uniclust | UniRef100\_A0A0A6VQK1 | 99.1 | 5.9e-13 | 1.2e-18 | 129.2 | 182 | (112, 295) | 300 | (418, 627) | 643 | Baseplate protein J-like domain-containing protein | Baseplate protein J-like domain-containing protein | | uniclust | UniRef100\_A0A3D4L1P0 | 99.1 | 6.4e-13 | 1.2e-18 | 121.3 | 181 | (112, 296) | 300 | (488, 700) | 701 | Baseplate protein J-like domain-containing protein | Baseplate protein J-like domain-containing protein | | uniclust | UniRef100\_A0A6S6TBW8 | 99.1 | 6.8e-13 | 1.3e-18 | 132.1 | 248 | (2, 288) | 300 | (1, 304) | 937 | Uncharacterized protein | Uncharacterized protein | | uniclust | UniRef100\_A0A2X3LRF3 | 99.1 | 7.4e-13 | 1.4e-18 | 112.0 | 154 | (138, 293) | 300 | (5, 161) | 362 | Tail protein I | Tail protein I | | uniclust | UniRef100\_A0A3M2G4S3 | 99.1 | 7.1e-13 | 1.4e-18 | 121.5 | 180 | (111, 293) | 300 | (268, 481) | 515 | Putative baseplate assembly protein (Fragment) | Putative baseplate assembly protein (Fragment) | | uniclust | UniRef100\_M1PWF4 | 99.1 | 7.3e-13 | 1.4e-18 | 121.0 | 85 | (21, 105) | 300 | (10, 95) | 609 | Uncharacterized protein | Uncharacterized protein | | uniclust | UniRef100\_UPI0018CE8811 | 99.1 | 8e-13 | 1.5e-18 | 105.2 | 176 | (112, 292) | 300 | (60, 252) | 253 | baseplate J/gp47 family protein | baseplate J/gp47 family protein | | uniclust | UniRef100\_A0A951FB51 | 99.1 | 8.1e-13 | 1.5e-18 | 110.7 | 157 | (111, 270) | 300 | (98, 257) | 322 | Baseplate J/gp47 family protein | Baseplate J/gp47 family protein | | uniclust | UniRef100\_A9H6K2 | 99.1 | 8.5e-13 | 1.6e-18 | 107.7 | 152 | (19, 171) | 300 | (3, 232) | 267 | Putative bacteriophage related protein | Putative bacteriophage related protein | | uniclust | UniRef100\_A0A059V4B9 | 99.1 | 8.3e-13 | 1.6e-18 | 97.4 | 110 | (188, 297) | 300 | (3, 113) | 117 | Phage-related baseplate assembly protein | Phage-related baseplate assembly protein | | uniclust | UniRef100\_A0A6P0SM54 | 99.1 | 9.1e-13 | 1.7e-18 | 101.8 | 174 | (20, 220) | 300 | (9, 205) | 206 | Baseplate protein J-like domain-containing protein (Fragment) | Baseplate protein J-like domain-containing protein (Fragment) | | uniclust | UniRef100\_UPI0002EC6E08 | 99.1 | 9.3e-13 | 1.7e-18 | 97.3 | 138 | (150, 289) | 300 | (12, 149) | 154 | baseplate J/gp47 family protein | baseplate J/gp47 family protein | | uniclust | UniRef100\_UPI00167ACCF4 | 99.1 | 9.3e-13 | 1.7e-18 | 108.7 | 285 | (1, 297) | 300 | (1, 329) | 334 | baseplate J/gp47 family protein | baseplate J/gp47 family protein | | uniclust | UniRef100\_A0A661PQS9 | 99.1 | 1.1e-12 | 2e-18 | 115.1 | 240 | (42, 288) | 300 | (117, 449) | 570 | Baseplate protein J-like domain-containing protein | Baseplate protein J-like domain-containing protein | | uniclust | UniRef100\_A0A6L9YMA4 | 99.1 | 1.1e-12 | 2e-18 | 116.1 | 226 | (21, 287) | 300 | (18, 319) | 387 | Uncharacterized protein | Uncharacterized protein | | uniclust | UniRef100\_A0A1D8TTT2 | 99.1 | 1.1e-12 | 2e-18 | 123.1 | 156 | (112, 271) | 300 | (609, 778) | 826 | Baseplate assembly protein | Baseplate assembly protein | | uniclust | UniRef100\_UPI001FA8C027 | 99.0 | 1.1e-12 | 2.1e-18 | 99.5 | 132 | (112, 245) | 300 | (29, 179) | 183 | baseplate J/gp47 family protein | baseplate J/gp47 family protein | | uniclust | UniRef100\_A0A1V1PGV0 | 99.0 | 1.3e-12 | 2.3e-18 | 106.1 | 246 | (19, 285) | 300 | (7, 280) | 292 | Baseplate protein J-like domain-containing protein | Baseplate protein J-like domain-containing protein | | uniclust | UniRef100\_A0A081P6S8 | 99.0 | 1.2e-12 | 2.4e-18 | 134.6 | 157 | (111, 269) | 300 | (825, 1003) | 1055 | Baseplate protein J-like domain-containing protein | Baseplate protein J-like domain-containing protein | | uniclust | UniRef100\_A0A0J7K9G9 | 99.0 | 1.4e-12 | 2.5e-18 | 105.9 | 162 | (54, 218) | 300 | (4, 273) | 290 | Baseplate j family protein (Fragment) | Baseplate j family protein (Fragment) | | uniclust | UniRef100\_A0A084Z0M7 | 99.0 | 1.3e-12 | 2.5e-18 | 102.4 | 155 | (133, 293) | 300 | (8, 175) | 177 | Gp47 family phage protein | Gp47 family phage protein | | uniclust | UniRef100\_A0A0M2LYF3 | 99.0 | 1.3e-12 | 2.5e-18 | 108.4 | 135 | (12, 149) | 300 | (7, 223) | 240 | Baseplate protein (Fragment) | Baseplate protein (Fragment) | | uniclust | UniRef100\_A0A844GAV7 | 99.0 | 1.4e-12 | 2.6e-18 | 107.7 | 170 | (19, 191) | 300 | (3, 255) | 290 | Baseplate protein J-like domain-containing protein | Baseplate protein J-like domain-containing protein | | uniclust | UniRef100\_UPI002272A788 | 99.0 | 1.5e-12 | 2.7e-18 | 110.9 | 270 | (22, 295) | 300 | (24, 395) | 405 | baseplate J/gp47 family protein | baseplate J/gp47 family protein | | uniclust | UniRef100\_A0A0T9P9X2 | 99.0 | 1.5e-12 | 2.8e-18 | 116.2 | 268 | (21, 296) | 300 | (13, 472) | 478 | Uncharacterized homolog of phage Mu protein gp47 | Uncharacterized homolog of phage Mu protein gp47 | | uniclust | UniRef100\_A0A2G1CU44 | 99.0 | 1.7e-12 | 3.1e-18 | 108.5 | 267 | (10, 285) | 300 | (8, 353) | 367 | Baseplate protein J-like domain-containing protein | Baseplate protein J-like domain-containing protein | | uniclust | UniRef100\_A0A1X3ISQ9 | 99.0 | 1.7e-12 | 3.2e-18 | 94.4 | 124 | (175, 298) | 300 | (2, 125) | 126 | Phage baseplate assembly protein | Phage baseplate assembly protein | | uniclust | UniRef100\_A0A2G6CJI5 | 99.0 | 1.8e-12 | 3.5e-18 | 109.4 | 182 | (111, 296) | 300 | (96, 313) | 318 | T4-like baseplate wedge (Fragment) | T4-like baseplate wedge (Fragment) | | uniclust | UniRef100\_UPI000B20AC30 | 99.0 | 1.9e-12 | 3.5e-18 | 97.0 | 140 | (5, 160) | 300 | (1, 140) | 167 | baseplate J/gp47 family protein | baseplate J/gp47 family protein | | uniclust | UniRef100\_A0A0A0DRH3 | 99.0 | 1.8e-12 | 3.6e-18 | 131.5 | 155 | (112, 268) | 300 | (808, 985) | 1055 | Baseplate protein J-like domain-containing protein | Baseplate protein J-like domain-containing protein | | uniclust | UniRef100\_UPI0016896A8C | 99.0 | 2e-12 | 3.6e-18 | 107.2 | 230 | (43, 286) | 300 | (13, 328) | 340 | baseplate J/gp47 family protein | baseplate J/gp47 family protein | | uniclust | UniRef100\_A0A2Z7A2S1 | 99.0 | 2e-12 | 3.7e-18 | 125.5 | 227 | (56, 289) | 300 | (1585, 1929) | 2224 | Protein gp47 | Protein gp47 | | uniclust | UniRef100\_A0A8I2K3Q2 | 99.0 | 2e-12 | 3.7e-18 | 112.3 | 184 | (111, 296) | 300 | (312, 513) | 515 | Putative baseplate assembly protein | Putative baseplate assembly protein | | uniclust | UniRef100\_UPI001F4C956E | 99.0 | 2.1e-12 | 3.9e-18 | 96.6 | 136 | (8, 158) | 300 | (2, 142) | 166 | hypothetical protein | hypothetical protein | | uniclust | UniRef100\_A0A0B0EQK2 | 99.0 | 2.2e-12 | 4.4e-18 | 129.3 | 157 | (111, 271) | 300 | (623, 784) | 852 | Baseplate protein J-like domain-containing protein | Baseplate protein J-like domain-containing protein | | uniclust | UniRef100\_A0A963MCN6 | 99.0 | 2.6e-12 | 4.8e-18 | 118.6 | 158 | (111, 270) | 300 | (447, 633) | 675 | Baseplate assembly protein | Baseplate assembly protein | | uniclust | UniRef100\_A0A146H551 | 99.0 | 2.7e-12 | 5e-18 | 116.6 | 151 | (21, 172) | 300 | (5, 245) | 818 | Baseplate protein J-like domain-containing protein (Fragment) | Baseplate protein J-like domain-containing protein (Fragment) | | uniclust | UniRef100\_A0A7V7B0G8 | 99.0 | 3e-12 | 5.4e-18 | 103.3 | 217 | (21, 260) | 300 | (12, 233) | 276 | Baseplate protein J-like domain-containing protein | Baseplate protein J-like domain-containing protein | | uniclust | UniRef100\_A0A1W9S1W4 | 99.0 | 3.1e-12 | 5.8e-18 | 100.7 | 171 | (22, 210) | 300 | (6, 179) | 184 | Uncharacterized protein | Uncharacterized protein | | uniclust | UniRef100\_UPI0020C2AC94 | 99.0 | 3.3e-12 | 6e-18 | 96.1 | 107 | (112, 223) | 300 | (53, 162) | 171 | baseplate J/gp47 family protein | baseplate J/gp47 family protein | | uniclust | UniRef100\_UPI00064655BD | 99.0 | 3.3e-12 | 6.1e-18 | 100.0 | 176 | (113, 294) | 300 | (3, 198) | 200 | baseplate J/gp47 family protein | baseplate J/gp47 family protein | | uniclust | UniRef100\_A0A5E4HLP5 | 99.0 | 3.3e-12 | 6.1e-18 | 109.2 | 251 | (21, 276) | 300 | (5, 413) | 442 | Baseplate J-like protein | Baseplate J-like protein | | uniclust | UniRef100\_UPI0011BCFB36 | 99.0 | 3.4e-12 | 6.3e-18 | 98.1 | 85 | (111, 200) | 300 | (80, 166) | 167 | baseplate J/gp47 family protein | baseplate J/gp47 family protein | | uniclust | UniRef100\_A0A6G5YFG6 | 99.0 | 3.5e-12 | 6.5e-18 | 117.8 | 237 | (19, 271) | 300 | (7, 329) | 752 | Baseplate protein J-like domain-containing protein | Baseplate protein J-like domain-containing protein | | uniclust | UniRef100\_X5F9B2 | 99.0 | 3.6e-12 | 6.7e-18 | 90.6 | 110 | (130, 245) | 300 | (5, 117) | 121 | Phage-like element PBSX protein xkdT | Phage-like element PBSX protein xkdT | | pdb70 | 6RAO\_J | 99.9 | 1.4e-32 | 1.1e-36 | 279.9 | 219 | (16, 271) | 300 | (9, 320) | 963 | Afp1, Afp2, Afp3, Afp5, Afp9 | 6RAO\_J Afp1, Afp2, Afp3, Afp5, Afp9 Anti-feeding prophage, secretion system, AFP | | pdb70 | 6J0N\_S | 99.9 | 1.9e-32 | 1.6e-36 | 278.2 | 218 | (16, 267) | 300 | (9, 314) | 950 | Pvc1, Pvc9, Pvc11, Pvc12, Pvc4 | 6J0N\_S Pvc1, Pvc9, Pvc11, Pvc12, Pvc4 assembly, Photorhabdus asymbiotica, PVC, contractile | | pdb70 | 6J0N\_O | 99.7 | 2.4e-21 | 2.1e-25 | 194.3 | 185 | (107, 293) | 300 | (500, 718) | 728 | Pvc1, Pvc9, Pvc11, Pvc12, Pvc4 | 6J0N\_O Pvc1, Pvc9, Pvc11, Pvc12, Pvc4 assembly, Photorhabdus asymbiotica, PVC, contractile | | pdb70 | 6RAO\_I | 99.7 | 3.2e-21 | 2.8e-25 | 189.9 | 181 | (107, 289) | 300 | (382, 601) | 607 | Afp1, Afp2, Afp3, Afp5, Afp9 | 6RAO\_I Afp1, Afp2, Afp3, Afp5, Afp9 Anti-feeding prophage, secretion system, AFP | | pdb70 | 5IV5\_BH | 99.3 | 1.5e-16 | 1.3e-20 | 157.1 | 158 | (107, 267) | 300 | (311, 477) | 660 | Baseplate wedge protein gp6, Baseplate | 5IV5\_BH Baseplate wedge protein gp6, Baseplate T4, baseplate-tail tube complex, pre-attachment | | pdb70 | 5IV5\_BI | 99.3 | 1.5e-16 | 1.3e-20 | 157.1 | 158 | (107, 267) | 300 | (311, 477) | 660 | Baseplate wedge protein gp6, Baseplate | 5IV5\_BI Baseplate wedge protein gp6, Baseplate T4, baseplate-tail tube complex, pre-attachment | | pdb70 | 5IV5\_EA | 99.3 | 1.5e-16 | 1.3e-20 | 157.1 | 158 | (107, 267) | 300 | (311, 477) | 660 | Baseplate wedge protein gp6, Baseplate | 5IV5\_EA Baseplate wedge protein gp6, Baseplate T4, baseplate-tail tube complex, pre-attachment | | pdb70 | 5IV5\_EB | 99.3 | 1.5e-16 | 1.3e-20 | 157.1 | 158 | (107, 267) | 300 | (311, 477) | 660 | Baseplate wedge protein gp6, Baseplate | 5IV5\_EB Baseplate wedge protein gp6, Baseplate T4, baseplate-tail tube complex, pre-attachment | | pdb70 | 5IV5\_GD | 99.3 | 1.5e-16 | 1.3e-20 | 157.1 | 158 | (107, 267) | 300 | (311, 477) | 660 | Baseplate wedge protein gp6, Baseplate | 5IV5\_GD Baseplate wedge protein gp6, Baseplate T4, baseplate-tail tube complex, pre-attachment | | pdb70 | 5IV5\_GE | 99.3 | 1.5e-16 | 1.3e-20 | 157.1 | 158 | (107, 267) | 300 | (311, 477) | 660 | Baseplate wedge protein gp6, Baseplate | 5IV5\_GE Baseplate wedge protein gp6, Baseplate T4, baseplate-tail tube complex, pre-attachment | | pdb70 | 5HX2\_E | 99.3 | 1.5e-16 | 1.3e-20 | 157.1 | 158 | (107, 267) | 300 | (311, 477) | 660 | Baseplate wedge protein gp7, Baseplate | 5HX2\_E Baseplate wedge protein gp7, Baseplate T4, baseplate, complex, VIRAL PROTEIN | | pdb70 | 5IV5\_A | 99.3 | 1.5e-16 | 1.3e-20 | 157.1 | 158 | (107, 267) | 300 | (311, 477) | 660 | Baseplate wedge protein gp6, Baseplate | 5IV5\_A Baseplate wedge protein gp6, Baseplate T4, baseplate-tail tube complex, pre-attachment | | pdb70 | 5IV5\_B | 99.3 | 1.5e-16 | 1.3e-20 | 157.1 | 158 | (107, 267) | 300 | (311, 477) | 660 | Baseplate wedge protein gp6, Baseplate | 5IV5\_B Baseplate wedge protein gp6, Baseplate T4, baseplate-tail tube complex, pre-attachment | | pdb70 | 5IV5\_X | 99.3 | 1.5e-16 | 1.3e-20 | 157.1 | 158 | (107, 267) | 300 | (311, 477) | 660 | Baseplate wedge protein gp6, Baseplate | 5IV5\_X Baseplate wedge protein gp6, Baseplate T4, baseplate-tail tube complex, pre-attachment | | pdb70 | 5IV5\_Y | 99.3 | 1.5e-16 | 1.3e-20 | 157.1 | 158 | (107, 267) | 300 | (311, 477) | 660 | Baseplate wedge protein gp6, Baseplate | 5IV5\_Y Baseplate wedge protein gp6, Baseplate T4, baseplate-tail tube complex, pre-attachment | | pdb70 | 3H2T\_A | 99.3 | 1.7e-16 | 1.4e-20 | 144.5 | 132 | (131, 265) | 300 | (2, 141) | 335 | Baseplate structural protein Gp6 | 3H2T\_A Baseplate structural protein Gp6 viral protein, Virion | | pdb70 | 3H3Y\_I | 99.3 | 1.7e-16 | 1.4e-20 | 144.5 | 132 | (131, 265) | 300 | (2, 141) | 335 | Baseplate structural protein Gp6 | 3H3Y\_I Baseplate structural protein Gp6 viral structural protein, Virion, VIRAL | | pdb70 | 5CES\_A | 97.9 | 5.4e-09 | 4.8e-13 | 77.7 | 92 | (207, 298) | 300 | (2, 93) | 102 | PA0618 | 5CES\_A PA0618 gpJ, gp6, structural protein | |
| Top keywords  (threshold 1.00e-03 (evalue)) | **Baseplate, J\_like, domain\_containing, J, gp47, assembly, Phage, Fragment, Putative, wedge** |
| Output files | ../../similar\_sequences/13\_FANPEZAQ\_CDS\_0013\_merged.svg ../../similar\_sequences/13\_FANPEZAQ\_CDS\_0013\_pdb70.a3m ../../similar\_sequences/13\_FANPEZAQ\_CDS\_0013\_pdb70.hhr ../../similar\_sequences/13\_FANPEZAQ\_CDS\_0013\_uniclust.a3m ../../similar\_sequences/13\_FANPEZAQ\_CDS\_0013\_uniclust.hhr |

#### Structure prediction (AlphaFold)2

|  |  |
| --- | --- |
| Stats | xml version="1.0" encoding="utf-8" standalone="no"?       2024-09-02T21:09:11.794890 image/svg+xml   Matplotlib v3.7.2, https://matplotlib.org/ |
| Predicted structure | **NGL Viewer Controls:**  - Center: *Left-Click* - Rotate: *Left-Click + Drag* - Translate: *Right-Click + Drag* - Zoom: *Shift + Left-Click + Drag* |
| Output files | ../../predicted\_structures/13\_FANPEZAQ\_CDS\_0013/features.pkl ../../predicted\_structures/13\_FANPEZAQ\_CDS\_0013/ranked\_0.pdb ../../predicted\_structures/13\_FANPEZAQ\_CDS\_0013/ranked\_0\_plots.svg ../../predicted\_structures/13\_FANPEZAQ\_CDS\_0013/result\_model\_1\_ptm\_pred\_0.pkl |

#### Structure similarity search results (Foldseek)3

|  |  |
| --- | --- |
| Structure databases searched | Pdb, Afdb-proteome, Afdb-uniprot50 |
| Results, scheme(s)  (Top layers only, threshold 1.00e-02 (evalue)) | xml version="1.0" encoding="utf-8" standalone="no"?       2024-09-02T21:10:27.755946 image/svg+xml   Matplotlib v3.7.2, https://matplotlib.org/ |
| Results, table  (threshold 1.00e-02 (evalue)) | | db | id | prob | evalue | bits | fident | alnlen | mismatch | gapopen | qstart | qend | tstart | tend | name | description | | --- | --- | --- | --- | --- | --- | --- | --- | --- | --- | --- | --- | --- | --- | --- | | pdb | 6U5B\_M | 1.0 | 7.017e-27 | 787 | 0.482 | 294 | 143 | 5 | 8 | 296 | 2 | 291 | Sheath PA0622 | Sheath PA0622 | | pdb | 6U5B\_S | 1.0 | 5.705e-28 | 683 | 0.498 | 291 | 143 | 3 | 8 | 296 | 3 | 292 | Tube PA0623 | Tube PA0623 | | pdb | 5CES\_B | 1.0 | 3.943e-09 | 354 | 0.439 | 91 | 51 | 0 | 206 | 296 | 1 | 91 | PA0618 | PA0618 | | pdb | 7YFZ\_E | 1.0 | 5.489e-10 | 217 | 0.17 | 317 | 191 | 17 | 21 | 295 | 12 | 298 | Pam3 tube initiator gp17 | Pam3 tube initiator gp17 | | pdb | 5HX2\_D | 1.0 | 5.777e-06 | 159 | 0.12 | 183 | 140 | 10 | 102 | 270 | 292 | 467 | Baseplate wedge protein gp6 | Baseplate wedge protein gp6 | | pdb | 7AEB\_A | 1.0 | 7.136e-07 | 149 | 0.107 | 372 | 205 | 19 | 13 | 296 | 6 | 338 | Phospholipid/glycerol acyltransferase | Phospholipid/glycerol acyltransferase | | pdb | 3H2T\_A | 1.0 | 2.573e-05 | 144 | 0.115 | 130 | 102 | 7 | 151 | 271 | 17 | 142 | Baseplate structural protein Gp6 | Baseplate structural protein Gp6 | | pdb | 7B5H\_AH | 1.0 | 1.183e-05 | 137 | 0.147 | 231 | 141 | 14 | 114 | 294 | 974 | 1198 | All3317 protein | All3317 protein | | pdb | 5IV7\_B | 1.0 | 5.94e-05 | 129 | 0.127 | 181 | 139 | 9 | 104 | 271 | 296 | 470 | Baseplate wedge protein gp11 | Baseplate wedge protein gp11 | | pdb | 8EON\_Q | 1.0 | 2.573e-05 | 125 | 0.15 | 413 | 192 | 27 | 27 | 296 | 7 | 403 | Triplex gp44-b | Triplex gp44-b | | pdb | 6J0N\_J | 1.0 | 3.909e-05 | 121 | 0.16 | 218 | 136 | 15 | 110 | 289 | 393 | 601 | Pvc2 | Pvc2 | | pdb | 7AEB\_G | 1.0 | 0.0001292 | 113 | 0.131 | 221 | 144 | 12 | 110 | 284 | 789 | 1007 | Putative phage tail sheath protein FI | Putative phage tail sheath protein FI | | pdb | 7KH1\_A5 | 1.0 | 4.285e-06 | 111 | 0.128 | 411 | 202 | 25 | 20 | 294 | 11 | 401 | baseplate wedge protein, gp16 | baseplate wedge protein, gp16 | | pdb | 7B5H\_AD | 1.0 | 4.406e-05 | 103 | 0.134 | 401 | 172 | 21 | 15 | 295 | 13 | 358 | All3315 protein | All3315 protein | | pdb | 6RAO\_I | 0.999 | 0.0007755 | 98 | 0.169 | 206 | 140 | 12 | 110 | 293 | 246 | 442 | Afp11 | Afp11 | | pdb | 7YFZ\_G | 0.998 | 0.001046 | 93 | 0.128 | 225 | 130 | 13 | 21 | 204 | 5 | 204 | Pam3 baseplate wedge gp23 | Pam3 baseplate wedge gp23 | | pdb | 6J0N\_P | 0.996 | 0.0005752 | 89 | 0.129 | 387 | 191 | 22 | 17 | 296 | 5 | 352 | Pvc5 | Pvc5 | | pdb | 6RAO\_J | 0.967 | 0.000874 | 75 | 0.109 | 348 | 182 | 22 | 16 | 287 | 6 | 301 | Afp12 | Afp12 | | afdb-proteome | AF-G3XCX5-F1-MODEL\_V4 | 1.0 | 3.625e-42 | 1612 | 0.498 | 293 | 146 | 1 | 8 | 299 | 3 | 295 | Probable bacteriophage protein | Probable bacteriophage protein | | afdb-proteome | AF-A0A0H3GRZ1-F1-MODEL\_V4 | 1.0 | 2.604e-41 | 1554 | 0.485 | 301 | 148 | 1 | 6 | 299 | 2 | 302 | Putative prophage baseplate assembly protein | Putative prophage baseplate assembly protein | | afdb-proteome | AF-A0A0H3GKN7-F1-MODEL\_V4 | 1.0 | 7.192e-41 | 1524 | 0.471 | 301 | 152 | 1 | 6 | 299 | 2 | 302 | Baseplate assembly protein J | Baseplate assembly protein J | | afdb-proteome | AF-Q8ZMU3-F1-MODEL\_V4 | 1.0 | 2.593e-39 | 1471 | 0.485 | 301 | 148 | 2 | 6 | 299 | 2 | 302 | Fels-2 prophage protein | Fels-2 prophage protein | | afdb-proteome | AF-Q8ZKK3-F1-MODEL\_V4 | 1.0 | 2.758e-26 | 843 | 0.26 | 376 | 184 | 10 | 14 | 300 | 1 | 371 | Putative phage baseplate protein | Putative phage baseplate protein | | afdb-proteome | AF-Q5F8P1-F1-MODEL\_V4 | 1.0 | 1.197e-13 | 459 | 0.431 | 169 | 79 | 5 | 1 | 162 | 1 | 159 | Baseplate protein | Baseplate protein | | afdb-proteome | AF-P44240-F1-MODEL\_V4 | 1.0 | 6.587e-14 | 384 | 0.13 | 367 | 219 | 15 | 13 | 292 | 1 | 354 | Mu-like prophage FluMu protein gp47 | Mu-like prophage FluMu protein gp47 | | afdb-proteome | AF-P75981-F1-MODEL\_V4 | 1.0 | 5.795e-10 | 294 | 0.145 | 199 | 147 | 9 | 110 | 293 | 73 | 263 | Putative protein JayE | Putative protein JayE | | afdb-proteome | AF-A0A0H3GMA9-F1-MODEL\_V4 | 1.0 | 1.118e-09 | 242 | 0.107 | 390 | 223 | 18 | 20 | 295 | 20 | 398 | Putative bacteriophage protein | Putative bacteriophage protein | | afdb-proteome | AF-A0A0H3GKS8-F1-MODEL\_V4 | 1.0 | 7.128e-09 | 214 | 0.13 | 407 | 208 | 18 | 20 | 296 | 19 | 409 | Baseplate\_J domain-containing protein | Baseplate\_J domain-containing protein | | afdb-proteome | AF-A0A0H4ISQ6-F1-MODEL\_V4 | 1.0 | 0.002261 | 134 | 0.271 | 107 | 76 | 1 | 158 | 264 | 1 | 105 | Baseplate protein | Baseplate protein | | afdb-proteome | AF-Q32CZ0-F1-MODEL\_V4 | 1.0 | 0.0001838 | 107 | 0.131 | 198 | 131 | 12 | 40 | 206 | 6 | 193 | Hypothetical bacteriophage protein | Hypothetical bacteriophage protein | | afdb-proteome | AF-A0A0H3GT66-F1-MODEL\_V4 | 0.991 | 0.002548 | 84 | 0.093 | 203 | 137 | 12 | 38 | 206 | 4 | 193 | Uncharacterized protein | Uncharacterized protein | | afdb-proteome | AF-A0A0H3GSD8-F1-MODEL\_V4 | 0.941 | 0.004363 | 71 | 0.094 | 191 | 116 | 10 | 74 | 208 | 6 | 195 | Putative bacteriophage protein | Putative bacteriophage protein | | afdb-proteome | AF-A0A077ZHG9-F1-MODEL\_V4 | 0.941 | 0.005219 | 71 | 0.145 | 193 | 125 | 13 | 25 | 206 | 1966 | 2129 | DUF2184 and DUF2612 domain containing protein | DUF2184 and DUF2612 domain containing protein | | afdb-uniprot50 | AF-A0A7W8U3F8-F1-MODEL\_V4 | 1.0 | 2.582e-43 | 1697 | 0.579 | 295 | 124 | 0 | 5 | 299 | 3 | 297 | Phage-related baseplate assembly protein | Phage-related baseplate assembly protein | | afdb-uniprot50 | AF-A0A066RR24-F1-MODEL\_V4 | 1.0 | 2.501e-42 | 1654 | 0.518 | 291 | 140 | 0 | 9 | 299 | 5 | 295 | Baseplate assembly protein | Baseplate assembly protein | | afdb-uniprot50 | AF-A0A0V8T620-F1-MODEL\_V4 | 1.0 | 2.501e-42 | 1631 | 0.529 | 287 | 134 | 1 | 15 | 300 | 1 | 287 | Baseplate assembly protein | Baseplate assembly protein | | afdb-uniprot50 | AF-A0A2T6F1A4-F1-MODEL\_V4 | 1.0 | 4.282e-42 | 1629 | 0.568 | 292 | 124 | 1 | 8 | 299 | 6 | 295 | Baseplate assembly protein | Baseplate assembly protein | | afdb-uniprot50 | AF-G3XCX5-F1-MODEL\_V4 | 1.0 | 1.332e-41 | 1612 | 0.498 | 293 | 146 | 1 | 8 | 299 | 3 | 295 | Probable bacteriophage protein | Probable bacteriophage protein | | afdb-uniprot50 | AF-A0A149SVY4-F1-MODEL\_V4 | 1.0 | 2.729e-41 | 1587 | 0.513 | 300 | 146 | 0 | 1 | 300 | 2 | 301 | Baseplate\_J domain-containing protein | Baseplate\_J domain-containing protein | | afdb-uniprot50 | AF-A0A5N7XEQ3-F1-MODEL\_V4 | 1.0 | 2.729e-41 | 1585 | 0.518 | 305 | 139 | 2 | 1 | 298 | 1 | 304 | Baseplate assembly protein | Baseplate assembly protein | | afdb-uniprot50 | AF-A0A2S6H5I2-F1-MODEL\_V4 | 1.0 | 6.327e-43 | 1583 | 0.535 | 314 | 123 | 3 | 8 | 298 | 6 | 319 | Phage-related baseplate assembly protein | Phage-related baseplate assembly protein | | afdb-uniprot50 | AF-A0A410SH26-F1-MODEL\_V4 | 1.0 | 4.961e-41 | 1572 | 0.478 | 303 | 150 | 1 | 5 | 299 | 2 | 304 | Baseplate assembly protein | Baseplate assembly protein | | afdb-uniprot50 | AF-A0A0H3GRZ1-F1-MODEL\_V4 | 1.0 | 9.572e-41 | 1554 | 0.485 | 301 | 148 | 1 | 6 | 299 | 2 | 302 | Putative prophage baseplate assembly protein | Putative prophage baseplate assembly protein | | afdb-uniprot50 | AF-B5JY66-F1-MODEL\_V4 | 1.0 | 2.21e-40 | 1551 | 0.449 | 334 | 148 | 4 | 1 | 299 | 1 | 333 | Baseplate J-like protein | Baseplate J-like protein | | afdb-uniprot50 | AF-A0A0Q8D2W6-F1-MODEL\_V4 | 1.0 | 5.414e-40 | 1546 | 0.46 | 332 | 143 | 2 | 2 | 299 | 3 | 332 | Baseplate assembly protein | Baseplate assembly protein | | afdb-uniprot50 | AF-A0A812QW15-F1-MODEL\_V4 | 1.0 | 1.961e-40 | 1545 | 0.514 | 305 | 140 | 2 | 3 | 300 | 2 | 305 | J protein | J protein | | afdb-uniprot50 | AF-A0A291N2G8-F1-MODEL\_V4 | 1.0 | 2.081e-40 | 1544 | 0.49 | 300 | 149 | 3 | 1 | 299 | 1 | 297 | Baseplate assembly protein | Baseplate assembly protein | | afdb-uniprot50 | AF-A0A2T5J1G0-F1-MODEL\_V4 | 1.0 | 1.216e-40 | 1538 | 0.479 | 298 | 153 | 1 | 3 | 298 | 2 | 299 | Phage-related baseplate assembly protein | Phage-related baseplate assembly protein | | afdb-uniprot50 | AF-A0A4Z0E2N6-F1-MODEL\_V4 | 1.0 | 1.454e-40 | 1532 | 0.481 | 299 | 148 | 1 | 8 | 299 | 4 | 302 | Baseplate assembly protein | Baseplate assembly protein | | afdb-uniprot50 | AF-A0A0A1FE10-F1-MODEL\_V4 | 1.0 | 6.477e-40 | 1531 | 0.478 | 297 | 154 | 1 | 4 | 299 | 2 | 298 | Baseplate assembly protein J | Baseplate assembly protein J | | afdb-uniprot50 | AF-E1SFX6-F1-MODEL\_V4 | 1.0 | 1.74e-40 | 1528 | 0.5 | 306 | 142 | 2 | 1 | 299 | 1 | 302 | Baseplate assembly protein J (GpJ) | Baseplate assembly protein J (GpJ) | | afdb-uniprot50 | AF-A0A0H3GKN7-F1-MODEL\_V4 | 1.0 | 2.643e-40 | 1524 | 0.471 | 301 | 152 | 1 | 6 | 299 | 2 | 302 | Baseplate assembly protein J | Baseplate assembly protein J | | afdb-uniprot50 | AF-G0AIQ8-F1-MODEL\_V4 | 1.0 | 9.27e-40 | 1509 | 0.506 | 300 | 143 | 2 | 1 | 300 | 1 | 295 | Baseplate J-like protein | Baseplate J-like protein | | afdb-uniprot50 | AF-A0A3R8WZD4-F1-MODEL\_V4 | 1.0 | 1.495e-39 | 1506 | 0.46 | 302 | 156 | 1 | 5 | 299 | 2 | 303 | Baseplate assembly protein | Baseplate assembly protein | | afdb-uniprot50 | AF-A0A806D8P7-F1-MODEL\_V4 | 1.0 | 6.477e-40 | 1505 | 0.447 | 302 | 161 | 3 | 2 | 299 | 6 | 305 | Baseplate J family protein | Baseplate J family protein | | afdb-uniprot50 | AF-A0A397NE13-F1-MODEL\_V4 | 1.0 | 2.14e-39 | 1501 | 0.481 | 297 | 152 | 1 | 3 | 299 | 2 | 296 | Phage-related baseplate assembly protein | Phage-related baseplate assembly protein | | afdb-uniprot50 | AF-A0A094SSD9-F1-MODEL\_V4 | 1.0 | 8.226e-40 | 1500 | 0.473 | 306 | 151 | 2 | 1 | 299 | 1 | 303 | Baseplate assembly protein | Baseplate assembly protein | | afdb-uniprot50 | AF-A0A2Z6GCU5-F1-MODEL\_V4 | 1.0 | 2.016e-39 | 1494 | 0.458 | 301 | 157 | 3 | 1 | 298 | 1 | 298 | Baseplate Assembly protein | Baseplate Assembly protein | | afdb-uniprot50 | AF-A0A1S1NVP6-F1-MODEL\_V4 | 1.0 | 1.685e-39 | 1492 | 0.478 | 299 | 149 | 1 | 8 | 299 | 5 | 303 | Baseplate assembly protein | Baseplate assembly protein | | afdb-uniprot50 | AF-A4WEL5-F1-MODEL\_V4 | 1.0 | 3.251e-39 | 1489 | 0.478 | 299 | 149 | 1 | 8 | 299 | 4 | 302 | Baseplate J family protein | Baseplate J family protein | | afdb-uniprot50 | AF-A0A064DPN7-F1-MODEL\_V4 | 1.0 | 2.718e-39 | 1487 | 0.481 | 299 | 148 | 1 | 8 | 299 | 4 | 302 | Baseplate\_J domain-containing protein | Baseplate\_J domain-containing protein | | afdb-uniprot50 | AF-A0A3N1XDJ3-F1-MODEL\_V4 | 1.0 | 3.063e-39 | 1486 | 0.489 | 298 | 145 | 1 | 8 | 298 | 2 | 299 | Phage-related baseplate assembly protein | Phage-related baseplate assembly protein | | afdb-uniprot50 | AF-A0A1H9HC55-F1-MODEL\_V4 | 1.0 | 5.244e-39 | 1485 | 0.469 | 296 | 155 | 1 | 5 | 298 | 4 | 299 | Phage-related baseplate assembly protein | Phage-related baseplate assembly protein | | afdb-uniprot50 | AF-A0A561GUB6-F1-MODEL\_V4 | 1.0 | 4.129e-39 | 1484 | 0.508 | 299 | 141 | 2 | 1 | 299 | 1 | 293 | Phage-related baseplate assembly protein | Phage-related baseplate assembly protein | | afdb-uniprot50 | AF-A0A483GRP4-F1-MODEL\_V4 | 1.0 | 3.063e-39 | 1483 | 0.471 | 299 | 151 | 1 | 8 | 299 | 4 | 302 | Baseplate assembly protein | Baseplate assembly protein | | afdb-uniprot50 | AF-A0A1D2QS87-F1-MODEL\_V4 | 1.0 | 7.069e-39 | 1483 | 0.459 | 298 | 159 | 1 | 5 | 300 | 4 | 301 | Baseplate\_J domain-containing protein | Baseplate\_J domain-containing protein | | afdb-uniprot50 | AF-A0A375ABY7-F1-MODEL\_V4 | 1.0 | 3.664e-39 | 1482 | 0.511 | 297 | 138 | 2 | 6 | 295 | 2 | 298 | Phage baseplate assembly protein J | Phage baseplate assembly protein J | | afdb-uniprot50 | AF-A0A0Q4N0X4-F1-MODEL\_V4 | 1.0 | 2.412e-39 | 1472 | 0.498 | 301 | 144 | 1 | 6 | 299 | 2 | 302 | Baseplate assembly protein | Baseplate assembly protein | | afdb-uniprot50 | AF-C6BVX8-F1-MODEL\_V4 | 1.0 | 3.548e-38 | 1469 | 0.452 | 292 | 159 | 1 | 4 | 295 | 2 | 292 | Baseplate J family protein | Baseplate J family protein | | afdb-uniprot50 | AF-A0A1Q8DQX3-F1-MODEL\_V4 | 1.0 | 5.244e-39 | 1469 | 0.464 | 297 | 155 | 3 | 6 | 300 | 2 | 296 | Baseplate\_J domain-containing protein | Baseplate\_J domain-containing protein | | afdb-uniprot50 | AF-A0A4Q9EIG5-F1-MODEL\_V4 | 1.0 | 3.063e-39 | 1467 | 0.493 | 302 | 140 | 2 | 8 | 299 | 4 | 302 | Baseplate assembly protein | Baseplate assembly protein | | afdb-uniprot50 | AF-A0A845BNS7-F1-MODEL\_V4 | 1.0 | 1.14e-38 | 1461 | 0.464 | 299 | 153 | 2 | 8 | 299 | 4 | 302 | Baseplate assembly protein | Baseplate assembly protein | | afdb-uniprot50 | AF-A0A0D8CM68-F1-MODEL\_V4 | 1.0 | 2.072e-38 | 1460 | 0.438 | 308 | 160 | 2 | 4 | 298 | 3 | 310 | Baseplate J protein | Baseplate J protein | | afdb-uniprot50 | AF-A0A6B3J9L7-F1-MODEL\_V4 | 1.0 | 1.21e-38 | 1457 | 0.48 | 275 | 139 | 2 | 28 | 299 | 2 | 275 | Baseplate assembly protein | Baseplate assembly protein | | afdb-uniprot50 | AF-A0A447R011-F1-MODEL\_V4 | 1.0 | 1.012e-38 | 1456 | 0.465 | 288 | 147 | 1 | 19 | 299 | 2 | 289 | Baseplate assembly protein | Baseplate assembly protein | | afdb-uniprot50 | AF-A0A1I1UBY4-F1-MODEL\_V4 | 1.0 | 2.072e-38 | 1454 | 0.438 | 303 | 159 | 1 | 8 | 299 | 4 | 306 | Phage-related baseplate assembly protein | Phage-related baseplate assembly protein | | afdb-uniprot50 | AF-A0A269PHY2-F1-MODEL\_V4 | 1.0 | 4.506e-38 | 1442 | 0.47 | 291 | 151 | 2 | 5 | 295 | 2 | 289 | Baseplate assembly protein | Baseplate assembly protein | | afdb-uniprot50 | AF-A0A2N2S1G7-F1-MODEL\_V4 | 1.0 | 7.716e-38 | 1442 | 0.433 | 295 | 164 | 2 | 8 | 299 | 3 | 297 | Baseplate assembly protein | Baseplate assembly protein | | afdb-uniprot50 | AF-A0A2S9S4Q7-F1-MODEL\_V4 | 1.0 | 3.767e-38 | 1441 | 0.426 | 300 | 164 | 2 | 8 | 300 | 5 | 303 | Baseplate assembly protein | Baseplate assembly protein | | afdb-uniprot50 | AF-A0A636GAS7-F1-MODEL\_V4 | 1.0 | 3.548e-38 | 1440 | 0.448 | 299 | 158 | 1 | 8 | 299 | 4 | 302 | Baseplate assembly protein | Baseplate assembly protein | | afdb-uniprot50 | AF-A0A6I1IU11-F1-MODEL\_V4 | 1.0 | 4.245e-38 | 1438 | 0.45 | 284 | 152 | 1 | 21 | 300 | 1 | 284 | Baseplate assembly protein | Baseplate assembly protein | | afdb-uniprot50 | AF-A0A1E7RC48-F1-MODEL\_V4 | 1.0 | 5.567e-39 | 1437 | 0.488 | 293 | 142 | 2 | 8 | 296 | 4 | 292 | Baseplate J family protein | Baseplate J family protein | | afdb-uniprot50 | AF-A0A4Y9VR74-F1-MODEL\_V4 | 1.0 | 6.847e-38 | 1437 | 0.436 | 298 | 163 | 3 | 5 | 298 | 2 | 298 | Baseplate assembly protein | Baseplate assembly protein | | afdb-uniprot50 | AF-A0A1E7HY03-F1-MODEL\_V4 | 1.0 | 4.245e-38 | 1435 | 0.43 | 297 | 167 | 2 | 1 | 296 | 1 | 296 | Baseplate\_J domain-containing protein | Baseplate\_J domain-containing protein | | afdb-uniprot50 | AF-A0A7X6WY54-F1-MODEL\_V4 | 1.0 | 2.2e-38 | 1435 | 0.43 | 300 | 163 | 2 | 4 | 295 | 2 | 301 | Baseplate assembly protein | Baseplate assembly protein | | afdb-uniprot50 | AF-H8L2K1-F1-MODEL\_V4 | 1.0 | 6.847e-38 | 1430 | 0.427 | 295 | 166 | 2 | 6 | 299 | 2 | 294 | Phage-related baseplate assembly protein | Phage-related baseplate assembly protein | | afdb-uniprot50 | AF-A0A3F3J766-F1-MODEL\_V4 | 1.0 | 6.45e-38 | 1429 | 0.453 | 298 | 156 | 1 | 8 | 298 | 4 | 301 | Baseplate assembly protein | Baseplate assembly protein | | afdb-uniprot50 | AF-A0A541BB49-F1-MODEL\_V4 | 1.0 | 2.632e-38 | 1429 | 0.426 | 326 | 154 | 2 | 6 | 298 | 7 | 332 | Baseplate protein | Baseplate protein | | afdb-uniprot50 | AF-A0A162BXM4-F1-MODEL\_V4 | 1.0 | 2.966e-38 | 1427 | 0.416 | 324 | 156 | 2 | 8 | 298 | 9 | 332 | Baseplate assembly protein | Baseplate assembly protein | | afdb-uniprot50 | AF-Q31HT8-F1-MODEL\_V4 | 1.0 | 5.078e-38 | 1424 | 0.452 | 296 | 156 | 3 | 8 | 298 | 3 | 297 | Phage baseplate J-like protein | Phage baseplate J-like protein | | afdb-uniprot50 | AF-A0A454TSQ4-F1-MODEL\_V4 | 1.0 | 5.078e-38 | 1417 | 0.438 | 301 | 162 | 2 | 6 | 299 | 2 | 302 | Baseplate assembly protein | Baseplate assembly protein | | afdb-uniprot50 | AF-A0A4Z0WEF0-F1-MODEL\_V4 | 1.0 | 6.075e-38 | 1417 | 0.415 | 330 | 158 | 3 | 1 | 295 | 1 | 330 | Baseplate assembly protein | Baseplate assembly protein | | afdb-uniprot50 | AF-A0A376S4C0-F1-MODEL\_V4 | 1.0 | 1.104e-37 | 1414 | 0.489 | 282 | 137 | 1 | 25 | 299 | 2 | 283 | Baseplate assembly protein | Baseplate assembly protein | | afdb-uniprot50 | AF-A0A199YL26-F1-MODEL\_V4 | 1.0 | 6.847e-38 | 1410 | 0.449 | 309 | 159 | 2 | 1 | 298 | 1 | 309 | Baseplate\_J domain-containing protein | Baseplate\_J domain-containing protein | | afdb-uniprot50 | AF-A0A7W6RGM7-F1-MODEL\_V4 | 1.0 | 2.401e-37 | 1409 | 0.385 | 366 | 154 | 5 | 4 | 298 | 3 | 368 | Phage-related baseplate assembly protein | Phage-related baseplate assembly protein | | afdb-uniprot50 | AF-A0A482ISR2-F1-MODEL\_V4 | 1.0 | 2.131e-37 | 1404 | 0.44 | 300 | 160 | 2 | 8 | 299 | 5 | 304 | Baseplate assembly protein | Baseplate assembly protein | | afdb-uniprot50 | AF-A0A6L8GFW9-F1-MODEL\_V4 | 1.0 | 8.421e-37 | 1403 | 0.413 | 300 | 171 | 1 | 1 | 295 | 1 | 300 | Baseplate assembly protein | Baseplate assembly protein | | afdb-uniprot50 | AF-A0A4R1ZHR1-F1-MODEL\_V4 | 1.0 | 2.706e-37 | 1402 | 0.451 | 297 | 158 | 2 | 8 | 300 | 3 | 298 | Phage-related baseplate assembly protein | Phage-related baseplate assembly protein | | afdb-uniprot50 | AF-A0A7V7NR69-F1-MODEL\_V4 | 1.0 | 5.078e-38 | 1399 | 0.444 | 299 | 163 | 2 | 1 | 298 | 2 | 298 | Baseplate assembly protein | Baseplate assembly protein | | afdb-uniprot50 | AF-B8GS00-F1-MODEL\_V4 | 1.0 | 8.94e-37 | 1398 | 0.457 | 291 | 154 | 4 | 6 | 295 | 2 | 289 | Baseplate J-like protein | Baseplate J-like protein | | afdb-uniprot50 | AF-A0A2W6Y6Y1-F1-MODEL\_V4 | 1.0 | 7.039e-37 | 1395 | 0.433 | 300 | 164 | 3 | 1 | 298 | 1 | 296 | Baseplate assembly protein | Baseplate assembly protein | | afdb-uniprot50 | AF-A0A776QGR6-F1-MODEL\_V4 | 1.0 | 1.725e-36 | 1393 | 0.503 | 254 | 126 | 0 | 46 | 299 | 6 | 259 | Baseplate assembly protein | Baseplate assembly protein | | afdb-uniprot50 | AF-A0A4P9VSJ3-F1-MODEL\_V4 | 1.0 | 6.659e-39 | 1391 | 0.476 | 296 | 146 | 3 | 3 | 294 | 2 | 292 | Baseplate assembly protein | Baseplate assembly protein | | afdb-uniprot50 | AF-A0A2A9KD31-F1-MODEL\_V4 | 1.0 | 1.678e-37 | 1388 | 0.464 | 299 | 155 | 2 | 1 | 299 | 1 | 294 | Phage-related baseplate assembly protein | Phage-related baseplate assembly protein | | afdb-uniprot50 | AF-A0A1M4WDH6-F1-MODEL\_V4 | 1.0 | 8.421e-37 | 1386 | 0.445 | 292 | 158 | 2 | 5 | 295 | 2 | 290 | Phage-related baseplate assembly protein | Phage-related baseplate assembly protein | | afdb-uniprot50 | AF-A0A4P7J5J5-F1-MODEL\_V4 | 1.0 | 9.799e-38 | 1386 | 0.461 | 310 | 148 | 3 | 1 | 299 | 1 | 302 | Baseplate assembly protein | Baseplate assembly protein | | afdb-uniprot50 | AF-A0A7H8UVW2-F1-MODEL\_V4 | 1.0 | 4.364e-37 | 1386 | 0.404 | 304 | 167 | 2 | 8 | 300 | 5 | 305 | Baseplate J/gp47 family protein | Baseplate J/gp47 family protein | | afdb-uniprot50 | AF-A0A380TV19-F1-MODEL\_V4 | 1.0 | 4.918e-37 | 1384 | 0.416 | 300 | 167 | 3 | 8 | 300 | 5 | 303 | Uncharacterized homolog of phage Mu protein gp47 | Uncharacterized homolog of phage Mu protein gp47 | | afdb-uniprot50 | AF-S6HS98-F1-MODEL\_V4 | 1.0 | 3.135e-36 | 1383 | 0.392 | 303 | 179 | 3 | 1 | 299 | 1 | 302 | Baseplate\_J domain-containing protein | Baseplate\_J domain-containing protein | | afdb-uniprot50 | AF-A0A1G7S895-F1-MODEL\_V4 | 1.0 | 4.364e-37 | 1374 | 0.456 | 298 | 157 | 3 | 4 | 298 | 2 | 297 | Phage-related baseplate assembly protein | Phage-related baseplate assembly protein | | afdb-uniprot50 | AF-A0A285VRF8-F1-MODEL\_V4 | 1.0 | 6.049e-36 | 1370 | 0.429 | 298 | 162 | 3 | 5 | 294 | 2 | 299 | Phage-related baseplate assembly protein | Phage-related baseplate assembly protein | | afdb-uniprot50 | AF-A0A0T7PAJ5-F1-MODEL\_V4 | 1.0 | 6.422e-36 | 1369 | 0.527 | 254 | 120 | 0 | 46 | 299 | 7 | 260 | Baseplate assembly protein J | Baseplate assembly protein J | | afdb-uniprot50 | AF-A0A1I3U816-F1-MODEL\_V4 | 1.0 | 3.982e-36 | 1369 | 0.411 | 294 | 171 | 1 | 8 | 299 | 4 | 297 | Phage-related baseplate assembly protein | Phage-related baseplate assembly protein | | afdb-uniprot50 | AF-A0A423PQH8-F1-MODEL\_V4 | 1.0 | 1.725e-36 | 1364 | 0.426 | 326 | 153 | 4 | 8 | 299 | 4 | 329 | Baseplate assembly protein | Baseplate assembly protein | | afdb-uniprot50 | AF-A0A1B3E8E6-F1-MODEL\_V4 | 1.0 | 1.358e-36 | 1362 | 0.423 | 333 | 151 | 4 | 8 | 300 | 3 | 334 | Baseplate assembly protein | Baseplate assembly protein | | afdb-uniprot50 | AF-A0A0E3BVE1-F1-MODEL\_V4 | 1.0 | 8.658e-36 | 1360 | 0.427 | 295 | 152 | 3 | 8 | 300 | 3 | 282 | Baseplate assembly protein | Baseplate assembly protein | | afdb-uniprot50 | AF-A0A1H0SWN7-F1-MODEL\_V4 | 1.0 | 3.135e-36 | 1359 | 0.425 | 301 | 165 | 2 | 6 | 298 | 3 | 303 | Phage-related baseplate assembly protein | Phage-related baseplate assembly protein | | afdb-uniprot50 | AF-A0A853STM6-F1-MODEL\_V4 | 1.0 | 2.469e-36 | 1357 | 0.459 | 296 | 153 | 2 | 8 | 296 | 5 | 300 | Baseplate J-like protein | Baseplate J-like protein | | afdb-uniprot50 | AF-A0A4V6X7E1-F1-MODEL\_V4 | 1.0 | 2.621e-36 | 1350 | 0.475 | 303 | 136 | 4 | 5 | 300 | 2 | 288 | Baseplate assembly protein | Baseplate assembly protein | | afdb-uniprot50 | AF-A0A285V0V5-F1-MODEL\_V4 | 1.0 | 6.422e-36 | 1350 | 0.385 | 301 | 176 | 4 | 1 | 299 | 1 | 294 | Phage-related baseplate assembly protein | Phage-related baseplate assembly protein | | afdb-uniprot50 | AF-A0A5Y3B1N0-F1-MODEL\_V4 | 1.0 | 4.227e-36 | 1348 | 0.435 | 280 | 152 | 1 | 21 | 294 | 1 | 280 | Baseplate assembly protein | Baseplate assembly protein | | afdb-uniprot50 | AF-A0A7Y7D7U6-F1-MODEL\_V4 | 1.0 | 1.725e-36 | 1348 | 0.402 | 363 | 146 | 6 | 5 | 299 | 4 | 363 | Baseplate J/gp47 family protein | Baseplate J/gp47 family protein | | afdb-uniprot50 | AF-A0A2N1AP80-F1-MODEL\_V4 | 1.0 | 3.751e-36 | 1346 | 0.411 | 299 | 168 | 2 | 6 | 296 | 3 | 301 | Baseplate assembly protein | Baseplate assembly protein | | afdb-uniprot50 | AF-A0A3T0W3K4-F1-MODEL\_V4 | 1.0 | 2.191e-36 | 1344 | 0.417 | 304 | 164 | 2 | 5 | 295 | 2 | 305 | Baseplate J protein | Baseplate J protein | | afdb-uniprot50 | AF-A0A377LB58-F1-MODEL\_V4 | 1.0 | 3.223e-35 | 1342 | 0.46 | 256 | 138 | 0 | 44 | 299 | 5 | 260 | Baseplate assembly protein | Baseplate assembly protein | | afdb-uniprot50 | AF-A0A4U8YL31-F1-MODEL\_V4 | 1.0 | 1.531e-36 | 1339 | 0.424 | 332 | 149 | 4 | 6 | 295 | 2 | 333 | Baseplate protein j-like | Baseplate protein j-like | | afdb-uniprot50 | AF-A0A1E3G8B9-F1-MODEL\_V4 | 1.0 | 4.613e-35 | 1332 | 0.404 | 287 | 167 | 1 | 10 | 296 | 8 | 290 | Baseplate\_J domain-containing protein | Baseplate\_J domain-containing protein | | afdb-uniprot50 | AF-A0A1H2M4W7-F1-MODEL\_V4 | 1.0 | 1.036e-35 | 1329 | 0.453 | 300 | 155 | 4 | 4 | 295 | 3 | 301 | Phage-related baseplate assembly protein | Phage-related baseplate assembly protein | | afdb-uniprot50 | AF-A0A1D7ZGU3-F1-MODEL\_V4 | 1.0 | 4.897e-35 | 1326 | 0.356 | 297 | 188 | 2 | 6 | 300 | 2 | 297 | Baseplate J protein | Baseplate J protein | | afdb-uniprot50 | AF-A0A3N2E0Q3-F1-MODEL\_V4 | 1.0 | 9.757e-36 | 1325 | 0.389 | 298 | 175 | 2 | 5 | 295 | 4 | 301 | Phage-related baseplate assembly protein | Phage-related baseplate assembly protein | | afdb-uniprot50 | AF-A0A377U0P4-F1-MODEL\_V4 | 1.0 | 1.524e-34 | 1323 | 0.488 | 254 | 130 | 0 | 46 | 299 | 7 | 260 | Baseplate assembly protein J | Baseplate assembly protein J | | afdb-uniprot50 | AF-A0A3N2E200-F1-MODEL\_V4 | 1.0 | 2.782e-36 | 1323 | 0.447 | 286 | 156 | 1 | 14 | 299 | 3 | 286 | Phage-related baseplate assembly protein | Phage-related baseplate assembly protein | | afdb-uniprot50 | AF-A0A6C8X2I9-F1-MODEL\_V4 | 1.0 | 7.009e-35 | 1321 | 0.513 | 255 | 122 | 2 | 45 | 299 | 1 | 253 | Baseplate assembly protein | Baseplate assembly protein | | afdb-uniprot50 | AF-A0A212KBX8-F1-MODEL\_V4 | 1.0 | 1.773e-35 | 1321 | 0.389 | 298 | 172 | 2 | 8 | 295 | 7 | 304 | Baseplate assembly protein J (GPJ) | Baseplate assembly protein J (GPJ) | | afdb-uniprot50 | AF-A0A3S0YC89-F1-MODEL\_V4 | 1.0 | 1.315e-35 | 1316 | 0.466 | 296 | 148 | 4 | 8 | 296 | 5 | 297 | Baseplate assembly protein | Baseplate assembly protein | | afdb-uniprot50 | AF-A0A837E5S2-F1-MODEL\_V4 | 1.0 | 1.999e-35 | 1316 | 0.382 | 335 | 169 | 5 | 1 | 298 | 1 | 334 | Uncharacterized protein | Uncharacterized protein | | afdb-uniprot50 | AF-A0A7W6RE52-F1-MODEL\_V4 | 1.0 | 1.936e-34 | 1313 | 0.416 | 298 | 157 | 4 | 3 | 300 | 2 | 282 | Phage-related baseplate assembly protein | Phage-related baseplate assembly protein | | afdb-uniprot50 | AF-A0A5N9VV02-F1-MODEL\_V4 | 1.0 | 2.694e-35 | 1312 | 0.481 | 266 | 138 | 0 | 34 | 299 | 2 | 267 | Baseplate assembly protein | Baseplate assembly protein | | afdb-uniprot50 | AF-A0A0C2IH57-F1-MODEL\_V4 | 1.0 | 2.86e-35 | 1312 | 0.423 | 300 | 164 | 3 | 4 | 295 | 3 | 301 | Phage tail fiber protein | Phage tail fiber protein | | afdb-uniprot50 | AF-A0A2S9M0X9-F1-MODEL\_V4 | 1.0 | 4.743e-34 | 1311 | 0.387 | 297 | 179 | 2 | 6 | 300 | 2 | 297 | Baseplate J protein | Baseplate J protein | | afdb-uniprot50 | AF-A0A1F0H4T9-F1-MODEL\_V4 | 1.0 | 2.064e-36 | 1307 | 0.385 | 306 | 174 | 2 | 5 | 299 | 2 | 304 | Baseplate\_J domain-containing protein | Baseplate\_J domain-containing protein | | afdb-uniprot50 | AF-F4QJC2-F1-MODEL\_V4 | 1.0 | 8.658e-36 | 1302 | 0.447 | 306 | 146 | 5 | 1 | 299 | 1 | 290 | Baseplate J-like family protein | Baseplate J-like family protein | | afdb-uniprot50 | AF-A0A016XHS1-F1-MODEL\_V4 | 1.0 | 1.574e-35 | 1301 | 0.383 | 297 | 181 | 1 | 5 | 299 | 2 | 298 | Baseplate assembly protein | Baseplate assembly protein | | afdb-uniprot50 | AF-A0A2E5BTS9-F1-MODEL\_V4 | 1.0 | 7.933e-37 | 1300 | 0.423 | 321 | 156 | 2 | 8 | 299 | 7 | 327 | Baseplate assembly protein | Baseplate assembly protein | | afdb-uniprot50 | AF-A0A6P3BRR2-F1-MODEL\_V4 | 1.0 | 7.441e-35 | 1298 | 0.382 | 306 | 179 | 3 | 1 | 299 | 1 | 303 | Baseplate assembly protein | Baseplate assembly protein | | afdb-uniprot50 | AF-A0A514B9U7-F1-MODEL\_V4 | 1.0 | 4.094e-35 | 1297 | 0.434 | 304 | 155 | 4 | 4 | 295 | 3 | 301 | Baseplate assembly protein | Baseplate assembly protein | | afdb-uniprot50 | AF-A0A1E7WJG0-F1-MODEL\_V4 | 1.0 | 1.574e-35 | 1296 | 0.462 | 296 | 149 | 3 | 8 | 296 | 4 | 296 | Baseplate J-like protein | Baseplate J-like protein | | afdb-uniprot50 | AF-A0A1X7L2D2-F1-MODEL\_V4 | 1.0 | 1.352e-34 | 1295 | 0.447 | 293 | 146 | 3 | 8 | 300 | 2 | 278 | Phage-related baseplate assembly protein | Phage-related baseplate assembly protein | | afdb-uniprot50 | AF-A0A5E4XFI6-F1-MODEL\_V4 | 1.0 | 3.633e-35 | 1295 | 0.389 | 311 | 172 | 4 | 1 | 300 | 1 | 304 | Baseplate assembly protein | Baseplate assembly protein | | afdb-uniprot50 | AF-Q9KW05-F1-MODEL\_V4 | 1.0 | 2.527e-33 | 1291 | 0.495 | 230 | 116 | 0 | 70 | 299 | 3 | 232 | Baseplate/ tail fiber protein | Baseplate/ tail fiber protein | | afdb-uniprot50 | AF-A0A1C3EE52-F1-MODEL\_V4 | 1.0 | 1.065e-34 | 1285 | 0.371 | 326 | 168 | 4 | 10 | 298 | 9 | 334 | Baseplate\_J domain-containing protein | Baseplate\_J domain-containing protein | | afdb-uniprot50 | AF-A0A7Y1LY14-F1-MODEL\_V4 | 1.0 | 5.519e-35 | 1283 | 0.438 | 303 | 155 | 4 | 4 | 295 | 3 | 301 | Baseplate assembly protein | Baseplate assembly protein | | afdb-uniprot50 | AF-A0A1X4NS19-F1-MODEL\_V4 | 1.0 | 1.2e-34 | 1282 | 0.381 | 299 | 177 | 3 | 4 | 300 | 3 | 295 | Baseplate\_J domain-containing protein | Baseplate\_J domain-containing protein | | afdb-uniprot50 | AF-A0A8A8LZL8-F1-MODEL\_V4 | 1.0 | 3.422e-35 | 1277 | 0.417 | 302 | 165 | 3 | 4 | 298 | 2 | 299 | Uncharacterized protein | Uncharacterized protein | | afdb-uniprot50 | AF-A0A496L501-F1-MODEL\_V4 | 1.0 | 1.936e-34 | 1276 | 0.372 | 295 | 178 | 1 | 8 | 295 | 4 | 298 | Baseplate protein | Baseplate protein | | afdb-uniprot50 | AF-A0A1V0B6E1-F1-MODEL\_V4 | 1.0 | 1.352e-34 | 1275 | 0.438 | 301 | 152 | 4 | 8 | 296 | 6 | 301 | Baseplate assembly protein | Baseplate assembly protein | | afdb-uniprot50 | AF-A0A2J8GXT0-F1-MODEL\_V4 | 1.0 | 1.875e-33 | 1269 | 0.39 | 287 | 174 | 1 | 8 | 294 | 3 | 288 | Phage baseplate protein | Phage baseplate protein | | afdb-uniprot50 | AF-A0A6S4USR9-F1-MODEL\_V4 | 1.0 | 1.065e-34 | 1269 | 0.442 | 296 | 156 | 3 | 8 | 295 | 4 | 298 | Baseplate assembly protein | Baseplate assembly protein | | afdb-uniprot50 | AF-A0A6S4ZT36-F1-MODEL\_V4 | 1.0 | 5.035e-34 | 1258 | 0.428 | 292 | 157 | 3 | 8 | 292 | 4 | 292 | Baseplate assembly protein | Baseplate assembly protein | | afdb-uniprot50 | AF-A0A853I3D0-F1-MODEL\_V4 | 1.0 | 6.049e-36 | 1257 | 0.465 | 307 | 148 | 5 | 5 | 298 | 2 | 305 | Baseplate J/gp47 family protein | Baseplate J/gp47 family protein | | afdb-uniprot50 | AF-A0A345DE46-F1-MODEL\_V4 | 1.0 | 2.609e-34 | 1253 | 0.394 | 307 | 175 | 5 | 1 | 298 | 7 | 311 | Baseplate\_J domain-containing protein | Baseplate\_J domain-containing protein | | afdb-uniprot50 | AF-A0A348FYH3-F1-MODEL\_V4 | 1.0 | 5.035e-34 | 1250 | 0.417 | 297 | 165 | 4 | 1 | 296 | 1 | 290 | Baseplate assembly protein | Baseplate assembly protein | | afdb-uniprot50 | AF-A0A3L7AM87-F1-MODEL\_V4 | 1.0 | 8.121e-34 | 1250 | 0.381 | 299 | 170 | 2 | 9 | 296 | 7 | 301 | Baseplate assembly protein | Baseplate assembly protein | | afdb-uniprot50 | AF-A0A4R0DR47-F1-MODEL\_V4 | 1.0 | 3.518e-34 | 1250 | 0.347 | 322 | 179 | 3 | 6 | 296 | 2 | 323 | Baseplate\_J domain-containing protein | Baseplate\_J domain-containing protein | | afdb-uniprot50 | AF-A0A2C9D6M2-F1-MODEL\_V4 | 1.0 | 2.243e-33 | 1248 | 0.352 | 360 | 170 | 7 | 3 | 300 | 2 | 360 | Baseplate assembly protein | Baseplate assembly protein | | afdb-uniprot50 | AF-A0A8A8M399-F1-MODEL\_V4 | 1.0 | 1.618e-34 | 1247 | 0.42 | 300 | 163 | 3 | 8 | 300 | 6 | 301 | Uncharacterized protein | Uncharacterized protein | | afdb-uniprot50 | AF-A0A258L8U5-F1-MODEL\_V4 | 1.0 | 1.352e-34 | 1247 | 0.379 | 306 | 176 | 3 | 3 | 298 | 2 | 303 | Baseplate\_J domain-containing protein | Baseplate\_J domain-containing protein | | afdb-uniprot50 | AF-A0A3E0X3Z4-F1-MODEL\_V4 | 1.0 | 4.209e-34 | 1246 | 0.385 | 301 | 180 | 3 | 1 | 298 | 1 | 299 | Baseplate\_J domain-containing protein | Baseplate\_J domain-containing protein | | afdb-uniprot50 | AF-A0A1X7MCB0-F1-MODEL\_V4 | 1.0 | 5.013e-32 | 1244 | 0.511 | 223 | 109 | 0 | 77 | 299 | 1 | 223 | Phage baseplate assembly protein | Phage baseplate assembly protein | | afdb-uniprot50 | AF-A0A212J3W2-F1-MODEL\_V4 | 1.0 | 4.743e-34 | 1244 | 0.402 | 323 | 161 | 3 | 5 | 295 | 4 | 326 | Baseplate assembly protein J | Baseplate assembly protein J | | afdb-uniprot50 | AF-A0A2S2E5D8-F1-MODEL\_V4 | 1.0 | 8.121e-34 | 1240 | 0.409 | 288 | 154 | 3 | 8 | 295 | 3 | 274 | Baseplate protein J | Baseplate protein J | | afdb-uniprot50 | AF-A0A7Y3Z3Q6-F1-MODEL\_V4 | 1.0 | 2.046e-32 | 1236 | 0.324 | 290 | 195 | 1 | 6 | 295 | 2 | 290 | Phage baseplate protein | Phage baseplate protein | | afdb-uniprot50 | AF-A0A7V8DKV5-F1-MODEL\_V4 | 1.0 | 3.314e-34 | 1235 | 0.408 | 299 | 159 | 5 | 1 | 299 | 1 | 281 | Putative phage baseplate assembly protein | Putative phage baseplate assembly protein | | afdb-uniprot50 | AF-A0A3N8B0H1-F1-MODEL\_V4 | 1.0 | 2.941e-34 | 1232 | 0.421 | 304 | 165 | 3 | 4 | 300 | 2 | 301 | Baseplate assembly protein | Baseplate assembly protein | | afdb-uniprot50 | AF-A0A4Z1QXZ8-F1-MODEL\_V4 | 1.0 | 1.815e-32 | 1223 | 0.391 | 294 | 171 | 4 | 8 | 298 | 3 | 291 | Baseplate assembly protein | Baseplate assembly protein | | afdb-uniprot50 | AF-A0A0K6GTQ4-F1-MODEL\_V4 | 1.0 | 1.095e-33 | 1221 | 0.417 | 297 | 159 | 4 | 8 | 293 | 3 | 296 | Phage-related baseplate assembly protein | Phage-related baseplate assembly protein | | afdb-uniprot50 | AF-A0A3G9FXX2-F1-MODEL\_V4 | 1.0 | 3.84e-33 | 1220 | 0.361 | 304 | 178 | 4 | 1 | 296 | 1 | 296 | Baseplate assembly protein J | Baseplate assembly protein J | | afdb-uniprot50 | AF-A0A484X2Q2-F1-MODEL\_V4 | 1.0 | 3.314e-34 | 1220 | 0.469 | 296 | 142 | 4 | 8 | 294 | 4 | 293 | Baseplate assembly protein | Baseplate assembly protein | | afdb-uniprot50 | AF-A0A840X390-F1-MODEL\_V4 | 1.0 | 4.209e-34 | 1219 | 0.413 | 334 | 146 | 4 | 1 | 299 | 1 | 319 | Phage-related baseplate assembly protein | Phage-related baseplate assembly protein | | afdb-uniprot50 | AF-Q7NAB1-F1-MODEL\_V4 | 1.0 | 1.031e-33 | 1217 | 0.429 | 328 | 136 | 5 | 8 | 300 | 4 | 315 | Baseplate\_J domain-containing protein | Baseplate\_J domain-containing protein | | afdb-uniprot50 | AF-A0A318TPV5-F1-MODEL\_V4 | 1.0 | 1.823e-34 | 1216 | 0.379 | 316 | 174 | 6 | 1 | 296 | 3 | 316 | Phage-related baseplate assembly protein | Phage-related baseplate assembly protein | | afdb-uniprot50 | AF-A0A2K2G607-F1-MODEL\_V4 | 1.0 | 6.979e-33 | 1216 | 0.352 | 329 | 174 | 3 | 4 | 298 | 7 | 330 | Baseplate\_J domain-containing protein | Baseplate\_J domain-containing protein | | afdb-uniprot50 | AF-A0A258UWD9-F1-MODEL\_V4 | 1.0 | 1.162e-33 | 1213 | 0.408 | 301 | 159 | 4 | 1 | 298 | 1 | 285 | Baseplate\_J domain-containing protein | Baseplate\_J domain-containing protein | | afdb-uniprot50 | AF-A0A6I4T7M6-F1-MODEL\_V4 | 1.0 | 6.979e-33 | 1209 | 0.4 | 290 | 167 | 3 | 8 | 296 | 6 | 289 | Baseplate assembly protein | Baseplate assembly protein | | afdb-uniprot50 | AF-A0A7S8ACS8-F1-MODEL\_V4 | 1.0 | 3.617e-33 | 1207 | 0.365 | 328 | 166 | 5 | 8 | 295 | 2 | 327 | Phage tail protein | Phage tail protein | | afdb-uniprot50 | AF-A0A510U3L2-F1-MODEL\_V4 | 1.0 | 1.06e-32 | 1204 | 0.372 | 303 | 165 | 4 | 4 | 296 | 2 | 289 | Baseplate assembly protein | Baseplate assembly protein | | afdb-uniprot50 | AF-A0A5S9Q3R8-F1-MODEL\_V4 | 1.0 | 1.99e-33 | 1203 | 0.356 | 362 | 160 | 6 | 9 | 300 | 7 | 365 | Baseplate\_J domain-containing protein | Baseplate\_J domain-containing protein | | afdb-uniprot50 | AF-A0A256CB14-F1-MODEL\_V4 | 1.0 | 1.766e-33 | 1202 | 0.314 | 296 | 200 | 2 | 8 | 300 | 2 | 297 | Baseplate\_J domain-containing protein | Baseplate\_J domain-containing protein | | afdb-uniprot50 | AF-A0A1Q6U844-F1-MODEL\_V4 | 1.0 | 8.863e-33 | 1199 | 0.352 | 372 | 152 | 5 | 1 | 299 | 1 | 356 | Baseplate\_J domain-containing protein | Baseplate\_J domain-containing protein | | afdb-uniprot50 | AF-A0A2A4XTY4-F1-MODEL\_V4 | 1.0 | 3.407e-33 | 1198 | 0.37 | 294 | 178 | 3 | 5 | 296 | 4 | 292 | Baseplate assembly protein | Baseplate assembly protein | | afdb-uniprot50 | AF-A0A7X9WRR9-F1-MODEL\_V4 | 1.0 | 4.327e-33 | 1195 | 0.424 | 299 | 149 | 5 | 1 | 295 | 1 | 280 | Baseplate assembly protein | Baseplate assembly protein | | afdb-uniprot50 | AF-A0A193QL68-F1-MODEL\_V4 | 1.0 | 1.31e-33 | 1194 | 0.4 | 302 | 169 | 4 | 6 | 299 | 81 | 378 | Baseplate J-like protein | Baseplate J-like protein | | afdb-uniprot50 | AF-A0A495BKE9-F1-MODEL\_V4 | 1.0 | 1.567e-33 | 1192 | 0.379 | 324 | 162 | 4 | 8 | 295 | 2 | 322 | Phage-related baseplate assembly protein | Phage-related baseplate assembly protein | | afdb-uniprot50 | AF-A0A6L2ZSF9-F1-MODEL\_V4 | 1.0 | 6.367e-32 | 1192 | 0.289 | 491 | 152 | 3 | 1 | 294 | 1 | 491 | Baseplate assembly protein | Baseplate assembly protein | | afdb-uniprot50 | AF-A0A7H2V929-F1-MODEL\_V4 | 1.0 | 4.191e-32 | 1191 | 0.332 | 298 | 187 | 3 | 7 | 296 | 2 | 295 | Baseplate J/gp47 family protein | Baseplate J/gp47 family protein | | afdb-uniprot50 | AF-A0A1A6FKN6-F1-MODEL\_V4 | 1.0 | 1.567e-33 | 1191 | 0.395 | 301 | 169 | 3 | 8 | 299 | 9 | 305 | Baseplate\_J domain-containing protein | Baseplate\_J domain-containing protein | | afdb-uniprot50 | AF-A0A0N9WAW7-F1-MODEL\_V4 | 1.0 | 2.448e-32 | 1189 | 0.416 | 300 | 158 | 4 | 8 | 295 | 1 | 295 | Baseplate assembly protein | Baseplate assembly protein | | afdb-uniprot50 | AF-A0A2T5UR67-F1-MODEL\_V4 | 1.0 | 2.37e-31 | 1188 | 0.37 | 289 | 166 | 2 | 9 | 296 | 8 | 281 | Phage-related baseplate assembly protein | Phage-related baseplate assembly protein | | afdb-uniprot50 | AF-A0A8A8MKX7-F1-MODEL\_V4 | 1.0 | 8.863e-33 | 1185 | 0.399 | 313 | 166 | 6 | 1 | 299 | 1 | 305 | Baseplate J/gp47 family protein | Baseplate J/gp47 family protein | | afdb-uniprot50 | AF-A0A5P9F1N3-F1-MODEL\_V4 | 1.0 | 3.023e-33 | 1185 | 0.37 | 329 | 155 | 5 | 4 | 295 | 3 | 316 | Baseplate J-like protein | Baseplate J-like protein | | afdb-uniprot50 | AF-A0A1H2N8N7-F1-MODEL\_V4 | 1.0 | 8.584e-32 | 1182 | 0.351 | 293 | 174 | 3 | 8 | 299 | 3 | 280 | Phage-related baseplate assembly protein | Phage-related baseplate assembly protein | | afdb-uniprot50 | AF-G8PUM5-F1-MODEL\_V4 | 1.0 | 7.175e-32 | 1182 | 0.338 | 319 | 185 | 4 | 3 | 300 | 2 | 315 | Baseplate assembly protein J, predicted | Baseplate assembly protein J, predicted | | afdb-uniprot50 | AF-A0A0L0ETV6-F1-MODEL\_V4 | 1.0 | 4.191e-32 | 1180 | 0.347 | 305 | 174 | 4 | 5 | 299 | 4 | 293 | Baseplate\_J domain-containing protein | Baseplate\_J domain-containing protein | | afdb-uniprot50 | AF-A0A5S9HNH2-F1-MODEL\_V4 | 1.0 | 4.876e-33 | 1172 | 0.387 | 307 | 162 | 6 | 8 | 293 | 2 | 303 | Baseplate assembly protein | Baseplate assembly protein | | afdb-uniprot50 | AF-A0A2U1XZ04-F1-MODEL\_V4 | 1.0 | 1.611e-32 | 1167 | 0.41 | 292 | 156 | 3 | 7 | 298 | 2 | 277 | Baseplate\_J domain-containing protein | Baseplate\_J domain-containing protein | | afdb-uniprot50 | AF-A0A6N7JGM8-F1-MODEL\_V4 | 1.0 | 5.035e-34 | 1163 | 0.411 | 289 | 155 | 2 | 8 | 296 | 3 | 276 | Baseplate assembly protein | Baseplate assembly protein | | afdb-uniprot50 | AF-A0A2W5GCC1-F1-MODEL\_V4 | 1.0 | 5.65e-32 | 1163 | 0.371 | 304 | 172 | 4 | 8 | 296 | 9 | 308 | Baseplate assembly protein | Baseplate assembly protein | | afdb-uniprot50 | AF-A0A611ETK6-F1-MODEL\_V4 | 1.0 | 1.085e-29 | 1160 | 0.461 | 208 | 112 | 0 | 92 | 299 | 1 | 208 | Baseplate assembly protein | Baseplate assembly protein | | afdb-uniprot50 | AF-A0A3S0TV89-F1-MODEL\_V4 | 1.0 | 5.322e-32 | 1160 | 0.372 | 290 | 166 | 3 | 9 | 298 | 3 | 276 | Baseplate assembly protein J | Baseplate assembly protein J | | afdb-uniprot50 | AF-A0A3A9I1H5-F1-MODEL\_V4 | 1.0 | 7.175e-32 | 1160 | 0.36 | 372 | 155 | 5 | 8 | 296 | 2 | 373 | Baseplate\_J domain-containing protein | Baseplate\_J domain-containing protein | | afdb-uniprot50 | AF-A0A359KE54-F1-MODEL\_V4 | 1.0 | 3.823e-31 | 1153 | 0.376 | 292 | 164 | 4 | 7 | 296 | 4 | 279 | Baseplate assembly protein | Baseplate assembly protein | | afdb-uniprot50 | AF-A0A1T4WV84-F1-MODEL\_V4 | 1.0 | 8.584e-32 | 1153 | 0.369 | 330 | 170 | 5 | 1 | 296 | 2 | 327 | Phage-related baseplate assembly protein | Phage-related baseplate assembly protein | | afdb-uniprot50 | AF-A0A166WKK1-F1-MODEL\_V4 | 1.0 | 2.928e-32 | 1145 | 0.348 | 310 | 174 | 7 | 1 | 296 | 1 | 296 | Baseplate\_J domain-containing protein | Baseplate\_J domain-containing protein | | afdb-uniprot50 | AF-A0A0T9RLX7-F1-MODEL\_V4 | 1.0 | 1.031e-33 | 1144 | 0.468 | 301 | 142 | 6 | 6 | 295 | 2 | 295 | Bacteriophage P2-like protein | Bacteriophage P2-like protein | | afdb-uniprot50 | AF-A0A1S1U393-F1-MODEL\_V4 | 1.0 | 9.674e-32 | 1137 | 0.403 | 300 | 155 | 5 | 5 | 295 | 2 | 286 | Baseplate\_J domain-containing protein | Baseplate\_J domain-containing protein | | afdb-uniprot50 | AF-A0A1S1TKX1-F1-MODEL\_V4 | 1.0 | 1.384e-31 | 1137 | 0.339 | 327 | 172 | 5 | 1 | 298 | 5 | 316 | Baseplate\_J domain-containing protein | Baseplate\_J domain-containing protein | | afdb-uniprot50 | AF-A0A6N6MK70-F1-MODEL\_V4 | 1.0 | 1.758e-31 | 1137 | 0.325 | 320 | 186 | 6 | 5 | 299 | 4 | 318 | Baseplate J protein | Baseplate J protein | | afdb-uniprot50 | AF-I3TTD3-F1-MODEL\_V4 | 1.0 | 6.759e-32 | 1132 | 0.4 | 307 | 157 | 6 | 1 | 299 | 1 | 288 | Phage baseplate J-like protein | Phage baseplate J-like protein | | afdb-uniprot50 | AF-A0A2P9HMM4-F1-MODEL\_V4 | 1.0 | 9.674e-32 | 1127 | 0.368 | 301 | 173 | 3 | 7 | 295 | 2 | 297 | Baseplate assembly protein J | Baseplate assembly protein J | | afdb-uniprot50 | AF-A0A4P5VKK1-F1-MODEL\_V4 | 1.0 | 1.703e-30 | 1126 | 0.419 | 291 | 153 | 3 | 8 | 298 | 4 | 278 | Baseplate assembly protein | Baseplate assembly protein | | afdb-uniprot50 | AF-A0A7Z0MZG7-F1-MODEL\_V4 | 1.0 | 1.604e-30 | 1124 | 0.363 | 305 | 163 | 6 | 8 | 296 | 3 | 292 | Baseplate J/gp47 family protein | Baseplate J/gp47 family protein | | afdb-uniprot50 | AF-A0A4Q6CUH1-F1-MODEL\_V4 | 1.0 | 3.931e-30 | 1123 | 0.44 | 236 | 130 | 2 | 61 | 296 | 2 | 235 | Baseplate\_J domain-containing protein | Baseplate\_J domain-containing protein | | afdb-uniprot50 | AF-A0A833KQV3-F1-MODEL\_V4 | 1.0 | 2.824e-29 | 1122 | 0.418 | 232 | 131 | 1 | 68 | 299 | 2 | 229 | Baseplate assembly protein | Baseplate assembly protein | | afdb-uniprot50 | AF-A0A5S9P369-F1-MODEL\_V4 | 1.0 | 5.472e-31 | 1119 | 0.402 | 291 | 156 | 5 | 7 | 296 | 4 | 277 | Baseplate\_J domain-containing protein | Baseplate\_J domain-containing protein | | afdb-uniprot50 | AF-A0A1C3CVC7-F1-MODEL\_V4 | 1.0 | 4.059e-31 | 1117 | 0.303 | 303 | 200 | 5 | 8 | 299 | 4 | 306 | Phage baseplate protein | Phage baseplate protein | | afdb-uniprot50 | AF-A0A7W6S3E7-F1-MODEL\_V4 | 1.0 | 1.751e-29 | 1113 | 0.484 | 250 | 129 | 0 | 3 | 252 | 2 | 251 | Phage-related baseplate assembly protein | Phage-related baseplate assembly protein | | afdb-uniprot50 | AF-A0A5E8GZJ4-F1-MODEL\_V4 | 1.0 | 1.703e-30 | 1112 | 0.31 | 328 | 187 | 6 | 8 | 296 | 7 | 334 | Phage-related baseplate assembly protein | Phage-related baseplate assembly protein | | afdb-uniprot50 | AF-A0A1I1WB92-F1-MODEL\_V4 | 1.0 | 5.472e-31 | 1108 | 0.42 | 300 | 159 | 6 | 5 | 295 | 4 | 297 | Phage-related baseplate assembly protein | Phage-related baseplate assembly protein | | afdb-uniprot50 | AF-E2CN33-F1-MODEL\_V4 | 1.0 | 7.831e-31 | 1108 | 0.356 | 306 | 187 | 4 | 1 | 300 | 1 | 302 | Baseplate assembly protein J | Baseplate assembly protein J | | afdb-uniprot50 | AF-V0X2S6-F1-MODEL\_V4 | 1.0 | 3.272e-28 | 1105 | 0.485 | 202 | 104 | 0 | 98 | 299 | 2 | 203 | Baseplate J-like protein | Baseplate J-like protein | | afdb-uniprot50 | AF-A0A6I6R2A9-F1-MODEL\_V4 | 1.0 | 8.051e-30 | 1104 | 0.354 | 293 | 171 | 4 | 7 | 296 | 2 | 279 | Baseplate assembly protein | Baseplate assembly protein | | afdb-uniprot50 | AF-A0A5S3XLF5-F1-MODEL\_V4 | 1.0 | 1.19e-30 | 1097 | 0.304 | 309 | 194 | 4 | 4 | 299 | 3 | 303 | Baseplate J protein | Baseplate J protein | | afdb-uniprot50 | AF-F4BFR8-F1-MODEL\_V4 | 1.0 | 1.299e-29 | 1093 | 0.318 | 295 | 182 | 4 | 5 | 295 | 2 | 281 | Phage-related baseplate assembly protein | Phage-related baseplate assembly protein | | afdb-uniprot50 | AF-A0A4R7SRP0-F1-MODEL\_V4 | 1.0 | 4.173e-30 | 1092 | 0.31 | 296 | 202 | 2 | 1 | 296 | 1 | 294 | Phage-related baseplate assembly protein | Phage-related baseplate assembly protein | | afdb-uniprot50 | AF-A0A447M7C9-F1-MODEL\_V4 | 1.0 | 7.145e-30 | 1085 | 0.481 | 245 | 127 | 0 | 36 | 280 | 9 | 253 | Phage baseplate assembly protein J | Phage baseplate assembly protein J | | afdb-uniprot50 | AF-A0A1H1G315-F1-MODEL\_V4 | 1.0 | 2.36e-29 | 1083 | 0.29 | 300 | 206 | 4 | 1 | 300 | 1 | 293 | Phage-related baseplate assembly protein | Phage-related baseplate assembly protein | | afdb-uniprot50 | AF-A0A6S5X4A4-F1-MODEL\_V4 | 1.0 | 1.604e-30 | 1079 | 0.38 | 302 | 159 | 8 | 1 | 298 | 1 | 278 | Bacteriophage protein | Bacteriophage protein | | afdb-uniprot50 | AF-A0A0E4BVF6-F1-MODEL\_V4 | 1.0 | 3.378e-29 | 1075 | 0.309 | 304 | 188 | 6 | 2 | 300 | 3 | 289 | Baseplate\_J domain-containing protein | Baseplate\_J domain-containing protein | | afdb-uniprot50 | AF-A0A6L7TC79-F1-MODEL\_V4 | 1.0 | 5.626e-30 | 1072 | 0.475 | 282 | 116 | 4 | 31 | 295 | 1 | 267 | Baseplate assembly protein | Baseplate assembly protein | | afdb-uniprot50 | AF-A0A2N3KSP3-F1-MODEL\_V4 | 1.0 | 4.29e-29 | 1067 | 0.337 | 290 | 182 | 4 | 13 | 295 | 1 | 287 | Baseplate\_J domain-containing protein | Baseplate\_J domain-containing protein | | afdb-uniprot50 | AF-A0A7X3ZHS2-F1-MODEL\_V4 | 1.0 | 1.703e-30 | 1064 | 0.328 | 283 | 187 | 2 | 7 | 287 | 2 | 283 | Baseplate\_J domain-containing protein | Baseplate\_J domain-containing protein | | afdb-uniprot50 | AF-A0A285M4J8-F1-MODEL\_V4 | 1.0 | 2.094e-29 | 1062 | 0.341 | 293 | 182 | 5 | 8 | 300 | 8 | 289 | Phage-related baseplate assembly protein | Phage-related baseplate assembly protein | | afdb-uniprot50 | AF-A0A1D2QMV6-F1-MODEL\_V4 | 1.0 | 7.345e-29 | 1061 | 0.346 | 297 | 176 | 5 | 5 | 300 | 2 | 281 | Baseplate\_J domain-containing protein | Baseplate\_J domain-containing protein | | afdb-uniprot50 | AF-A0A0M3V9B1-F1-MODEL\_V4 | 1.0 | 1.649e-29 | 1061 | 0.316 | 313 | 193 | 5 | 5 | 299 | 5 | 314 | Baseplate assembly protein | Baseplate assembly protein | | afdb-uniprot50 | AF-A0A833LKM6-F1-MODEL\_V4 | 1.0 | 2.506e-29 | 1059 | 0.337 | 299 | 177 | 4 | 3 | 296 | 2 | 284 | Baseplate assembly protein | Baseplate assembly protein | | afdb-uniprot50 | AF-A0A1S1NBP5-F1-MODEL\_V4 | 1.0 | 3.807e-29 | 1053 | 0.305 | 304 | 184 | 4 | 5 | 296 | 4 | 292 | Baseplate\_J domain-containing protein | Baseplate\_J domain-containing protein | | afdb-uniprot50 | AF-A0A0P7JS55-F1-MODEL\_V4 | 1.0 | 2.998e-29 | 1051 | 0.303 | 300 | 188 | 6 | 3 | 298 | 2 | 284 | Baseplate\_J domain-containing protein | Baseplate\_J domain-containing protein | | afdb-uniprot50 | AF-A0A0R3L0F6-F1-MODEL\_V4 | 1.0 | 3.931e-30 | 1051 | 0.344 | 308 | 185 | 6 | 1 | 296 | 1 | 303 | Baseplate\_J domain-containing protein | Baseplate\_J domain-containing protein | | afdb-uniprot50 | AF-A0A1Y1QY37-F1-MODEL\_V4 | 1.0 | 8.313e-31 | 1051 | 0.334 | 359 | 153 | 7 | 8 | 298 | 5 | 345 | Baseplate\_J domain-containing protein | Baseplate\_J domain-containing protein | | afdb-uniprot50 | AF-A0A847G9J0-F1-MODEL\_V4 | 1.0 | 1.696e-28 | 1049 | 0.319 | 294 | 179 | 6 | 8 | 300 | 3 | 276 | Phage tail protein | Phage tail protein | | afdb-uniprot50 | AF-A0A0P6VRH8-F1-MODEL\_V4 | 1.0 | 8.825e-31 | 1042 | 0.322 | 301 | 189 | 7 | 8 | 300 | 9 | 302 | Baseplate\_J domain-containing protein | Baseplate\_J domain-containing protein | | afdb-uniprot50 | AF-A0A4U9HA10-F1-MODEL\_V4 | 1.0 | 2.36e-29 | 1036 | 0.483 | 271 | 130 | 3 | 6 | 267 | 2 | 271 | Uncharacterized homolog of phage Mu protein gp47 | Uncharacterized homolog of phage Mu protein gp47 | | afdb-uniprot50 | AF-A0A6M4Y9L2-F1-MODEL\_V4 | 1.0 | 2.286e-28 | 1034 | 0.592 | 228 | 93 | 0 | 1 | 228 | 1 | 228 | Baseplate assembly protein | Baseplate assembly protein | | afdb-uniprot50 | AF-A0A2A5BN42-F1-MODEL\_V4 | 1.0 | 3.807e-29 | 1031 | 0.294 | 323 | 187 | 5 | 3 | 299 | 7 | 314 | Baseplate\_J domain-containing protein | Baseplate\_J domain-containing protein | | afdb-uniprot50 | AF-A0A5S3R796-F1-MODEL\_V4 | 1.0 | 4.041e-29 | 1026 | 0.311 | 321 | 180 | 9 | 1 | 300 | 1 | 301 | Baseplate J protein | Baseplate J protein | | afdb-uniprot50 | AF-A0A2V4KD55-F1-MODEL\_V4 | 1.0 | 4.059e-31 | 1024 | 0.319 | 322 | 181 | 4 | 12 | 296 | 10 | 330 | Baseplate J protein | Baseplate J protein | | afdb-uniprot50 | AF-A0A4P9VEN9-F1-MODEL\_V4 | 1.0 | 1.185e-28 | 1016 | 0.363 | 297 | 164 | 7 | 5 | 299 | 4 | 277 | Baseplate\_J domain-containing protein | Baseplate\_J domain-containing protein | | afdb-uniprot50 | AF-A0A8B2NS25-F1-MODEL\_V4 | 1.0 | 5.784e-29 | 1016 | 0.321 | 302 | 177 | 5 | 8 | 296 | 8 | 294 | Baseplate assembly protein | Baseplate assembly protein | | afdb-uniprot50 | AF-A0A090SXL0-F1-MODEL\_V4 | 1.0 | 6.14e-29 | 1016 | 0.324 | 296 | 184 | 3 | 5 | 299 | 2 | 282 | Phage-related baseplate assembly protein | Phage-related baseplate assembly protein | | afdb-uniprot50 | AF-A0A4R6U4A6-F1-MODEL\_V4 | 1.0 | 2.085e-27 | 1014 | 0.332 | 289 | 173 | 5 | 13 | 300 | 1 | 270 | Phage-related baseplate assembly protein | Phage-related baseplate assembly protein | | afdb-uniprot50 | AF-A0A212LCZ5-F1-MODEL\_V4 | 1.0 | 4.29e-29 | 1014 | 0.311 | 305 | 187 | 7 | 8 | 296 | 3 | 300 | Putative phage-related baseplate assembly protein | Putative phage-related baseplate assembly protein | | afdb-uniprot50 | AF-A0A085ASA5-F1-MODEL\_V4 | 1.0 | 4.43e-30 | 1012 | 0.37 | 294 | 158 | 5 | 31 | 300 | 2 | 292 | Baseplate assembly protein J | Baseplate assembly protein J | | afdb-uniprot50 | AF-A0A838CFD0-F1-MODEL\_V4 | 1.0 | 2.416e-26 | 1008 | 0.382 | 235 | 143 | 1 | 68 | 300 | 2 | 236 | Baseplate J protein | Baseplate J protein | | afdb-uniprot50 | AF-A0A7U5UIH5-F1-MODEL\_V4 | 1.0 | 3.364e-27 | 1008 | 0.446 | 262 | 138 | 1 | 8 | 262 | 4 | 265 | Baseplate assembly protein | Baseplate assembly protein | | afdb-uniprot50 | AF-A0A1H3ZWS2-F1-MODEL\_V4 | 1.0 | 4.041e-29 | 1008 | 0.31 | 328 | 177 | 5 | 6 | 299 | 2 | 314 | Phage-related baseplate assembly protein | Phage-related baseplate assembly protein | | afdb-uniprot50 | AF-A0A2E3N1J6-F1-MODEL\_V4 | 1.0 | 1.696e-28 | 1007 | 0.33 | 342 | 163 | 4 | 7 | 298 | 2 | 327 | Baseplate\_J domain-containing protein | Baseplate\_J domain-containing protein | | afdb-uniprot50 | AF-A0A5A9EMT3-F1-MODEL\_V4 | 1.0 | 6.919e-29 | 1004 | 0.375 | 288 | 162 | 4 | 12 | 298 | 8 | 278 | Baseplate assembly protein J | Baseplate assembly protein J | | afdb-uniprot50 | AF-A0A142JGU0-F1-MODEL\_V4 | 1.0 | 1.973e-29 | 1004 | 0.346 | 323 | 164 | 6 | 8 | 298 | 4 | 311 | Baseplate\_J domain-containing protein | Baseplate\_J domain-containing protein | | afdb-uniprot50 | AF-A0A318KRB1-F1-MODEL\_V4 | 1.0 | 1.505e-28 | 1001 | 0.351 | 304 | 177 | 8 | 9 | 296 | 5 | 304 | Phage-related baseplate assembly protein | Phage-related baseplate assembly protein | | afdb-uniprot50 | AF-A0A554XFX2-F1-MODEL\_V4 | 1.0 | 6.14e-29 | 993 | 0.331 | 329 | 169 | 7 | 6 | 299 | 2 | 314 | Baseplate J-like protein | Baseplate J-like protein | | afdb-uniprot50 | AF-A0A2V3UAW4-F1-MODEL\_V4 | 1.0 | 7.345e-29 | 990 | 0.361 | 310 | 174 | 9 | 4 | 296 | 3 | 305 | Phage-related baseplate assembly protein | Phage-related baseplate assembly protein | | afdb-uniprot50 | AF-A0A1N7LRY2-F1-MODEL\_V4 | 1.0 | 1.218e-27 | 988 | 0.321 | 302 | 188 | 7 | 8 | 300 | 1 | 294 | Phage-related baseplate assembly protein | Phage-related baseplate assembly protein | | afdb-uniprot50 | AF-A0A1S0V677-F1-MODEL\_V4 | 1.0 | 6.087e-25 | 987 | 0.491 | 185 | 94 | 0 | 115 | 299 | 1 | 185 | Baseplate assembly protein | Baseplate assembly protein | | afdb-uniprot50 | AF-A0A7W8XVJ7-F1-MODEL\_V4 | 1.0 | 3.082e-28 | 986 | 0.313 | 300 | 189 | 8 | 6 | 298 | 7 | 296 | Phage-related baseplate assembly protein | Phage-related baseplate assembly protein | | afdb-uniprot50 | AF-A0A433LI32-F1-MODEL\_V4 | 1.0 | 1.051e-28 | 984 | 0.269 | 338 | 203 | 8 | 1 | 296 | 1 | 336 | Baseplate\_J domain-containing protein | Baseplate\_J domain-containing protein | | afdb-uniprot50 | AF-A0A4D7AUY0-F1-MODEL\_V4 | 1.0 | 1.417e-28 | 983 | 0.32 | 303 | 195 | 6 | 1 | 296 | 4 | 302 | Baseplate\_J domain-containing protein | Baseplate\_J domain-containing protein | | afdb-uniprot50 | AF-A0A0F4QK12-F1-MODEL\_V4 | 1.0 | 1.751e-29 | 981 | 0.37 | 294 | 160 | 7 | 5 | 296 | 2 | 272 | Baseplate protein | Baseplate protein | | afdb-uniprot50 | AF-A0A1Y6ITR8-F1-MODEL\_V4 | 1.0 | 7.552e-28 | 980 | 0.337 | 302 | 172 | 5 | 8 | 300 | 4 | 286 | Baseplate J-like protein | Baseplate J-like protein | | afdb-uniprot50 | AF-A0A317PF16-F1-MODEL\_V4 | 1.0 | 6.313e-28 | 979 | 0.28 | 314 | 202 | 8 | 1 | 298 | 1 | 306 | Phage-related baseplate assembly protein | Phage-related baseplate assembly protein | | afdb-uniprot50 | AF-A0A6N8K8T8-F1-MODEL\_V4 | 1.0 | 3.571e-27 | 978 | 0.465 | 247 | 128 | 1 | 1 | 243 | 1 | 247 | Baseplate assembly protein | Baseplate assembly protein | | afdb-uniprot50 | AF-A0A1C3EBK2-F1-MODEL\_V4 | 1.0 | 1.051e-28 | 976 | 0.341 | 302 | 168 | 9 | 8 | 295 | 4 | 288 | Baseplate\_J domain-containing protein | Baseplate\_J domain-containing protein | | afdb-uniprot50 | AF-A0A839IVB1-F1-MODEL\_V4 | 1.0 | 7.345e-29 | 976 | 0.304 | 322 | 186 | 4 | 12 | 296 | 7 | 327 | Baseplate J/gp47 family protein | Baseplate J/gp47 family protein | | afdb-uniprot50 | AF-A0A1N6M6H6-F1-MODEL\_V4 | 1.0 | 2.735e-28 | 976 | 0.257 | 326 | 203 | 4 | 12 | 298 | 7 | 332 | Baseplate J-like protein | Baseplate J-like protein | | afdb-uniprot50 | AF-A0A5P0JGH1-F1-MODEL\_V4 | 1.0 | 3.671e-26 | 973 | 0.522 | 222 | 99 | 1 | 8 | 222 | 4 | 225 | Baseplate assembly protein | Baseplate assembly protein | | afdb-uniprot50 | AF-A0A1W2EJW9-F1-MODEL\_V4 | 1.0 | 1.218e-27 | 972 | 0.247 | 315 | 212 | 9 | 1 | 298 | 1 | 307 | Phage-related baseplate assembly protein | Phage-related baseplate assembly protein | | afdb-uniprot50 | AF-A0A371WU65-F1-MODEL\_V4 | 1.0 | 1.121e-30 | 962 | 0.336 | 306 | 192 | 2 | 1 | 296 | 2 | 306 | Baseplate\_J domain-containing protein | Baseplate\_J domain-containing protein | | afdb-uniprot50 | AF-B0UK53-F1-MODEL\_V4 | 1.0 | 2.812e-27 | 961 | 0.349 | 335 | 165 | 12 | 5 | 296 | 4 | 328 | Baseplate J family protein | Baseplate J family protein | | afdb-uniprot50 | AF-A0A1R1MK94-F1-MODEL\_V4 | 1.0 | 1.147e-27 | 956 | 0.279 | 379 | 183 | 10 | 8 | 300 | 2 | 376 | Baseplate\_J domain-containing protein | Baseplate\_J domain-containing protein | | afdb-uniprot50 | AF-A0A7X3VHX5-F1-MODEL\_V4 | 1.0 | 5.734e-25 | 955 | 0.518 | 216 | 100 | 1 | 4 | 219 | 2 | 213 | Baseplate assembly protein | Baseplate assembly protein | | afdb-uniprot50 | AF-A0A2D2D5N1-F1-MODEL\_V4 | 1.0 | 1.792e-26 | 955 | 0.39 | 238 | 137 | 2 | 66 | 299 | 2 | 235 | Baseplate assembly protein | Baseplate assembly protein | | afdb-uniprot50 | AF-A0A450W779-F1-MODEL\_V4 | 1.0 | 2.649e-27 | 954 | 0.311 | 295 | 181 | 6 | 6 | 298 | 2 | 276 | Phage-related baseplate assembly protein | Phage-related baseplate assembly protein | | afdb-uniprot50 | AF-A0A7T8CNP9-F1-MODEL\_V4 | 1.0 | 2.812e-27 | 951 | 0.308 | 318 | 186 | 7 | 8 | 296 | 2 | 314 | Baseplate\_J domain-containing protein | Baseplate\_J domain-containing protein | | afdb-uniprot50 | AF-Q3RBT0-F1-MODEL\_V4 | 1.0 | 4.137e-26 | 943 | 0.517 | 224 | 106 | 1 | 1 | 224 | 1 | 222 | Baseplate J-like protein | Baseplate J-like protein | | afdb-uniprot50 | AF-A0A1G8HUM0-F1-MODEL\_V4 | 1.0 | 1.018e-27 | 942 | 0.251 | 330 | 204 | 6 | 10 | 296 | 7 | 336 | Phage-related baseplate assembly protein | Phage-related baseplate assembly protein | | afdb-uniprot50 | AF-A0A7K1PHL9-F1-MODEL\_V4 | 1.0 | 8.242e-27 | 937 | 0.276 | 300 | 193 | 4 | 8 | 299 | 2 | 285 | Baseplate J protein | Baseplate J protein | | afdb-uniprot50 | AF-A0A545T5R2-F1-MODEL\_V4 | 1.0 | 7.552e-28 | 937 | 0.317 | 318 | 183 | 6 | 4 | 295 | 3 | 312 | Baseplate\_J domain-containing protein | Baseplate\_J domain-containing protein | | afdb-uniprot50 | AF-A0A383SE97-F1-MODEL\_V4 | 1.0 | 8.996e-26 | 935 | 0.518 | 239 | 110 | 3 | 1 | 239 | 1 | 234 | Phage-related baseplate assembly protein | Phage-related baseplate assembly protein | | afdb-uniprot50 | AF-A0A2N7P721-F1-MODEL\_V4 | 1.0 | 1.018e-27 | 935 | 0.343 | 300 | 170 | 8 | 1 | 296 | 1 | 277 | Baseplate\_J domain-containing protein | Baseplate\_J domain-containing protein | | afdb-uniprot50 | AF-A0A6B8KJI2-F1-MODEL\_V4 | 1.0 | 1.965e-27 | 930 | 0.33 | 324 | 169 | 11 | 8 | 298 | 7 | 315 | Baseplate\_J domain-containing protein | Baseplate\_J domain-containing protein | | afdb-uniprot50 | AF-A0A2N6FYW7-F1-MODEL\_V4 | 1.0 | 1.965e-27 | 930 | 0.237 | 366 | 196 | 6 | 8 | 295 | 2 | 362 | Baseplate assembly protein | Baseplate assembly protein | | afdb-uniprot50 | AF-A0A7W2BN55-F1-MODEL\_V4 | 1.0 | 7.764e-27 | 929 | 0.309 | 320 | 189 | 7 | 4 | 296 | 3 | 317 | Baseplate J/gp47 family protein | Baseplate J/gp47 family protein | | afdb-uniprot50 | AF-A0A853IJD9-F1-MODEL\_V4 | 1.0 | 1.451e-25 | 927 | 0.403 | 208 | 120 | 2 | 92 | 295 | 20 | 227 | Baseplate J/gp47 family protein | Baseplate J/gp47 family protein | | afdb-uniprot50 | AF-A0A7U2YWJ9-F1-MODEL\_V4 | 1.0 | 1.252e-26 | 927 | 0.357 | 277 | 160 | 4 | 10 | 275 | 6 | 275 | Baseplate protein | Baseplate protein | | afdb-uniprot50 | AF-A0A1D2QRG1-F1-MODEL\_V4 | 1.0 | 1.142e-25 | 921 | 0.266 | 293 | 192 | 7 | 8 | 299 | 2 | 272 | Baseplate\_J domain-containing protein | Baseplate\_J domain-containing protein | | afdb-uniprot50 | AF-A0A651FVY8-F1-MODEL\_V4 | 1.0 | 1.903e-26 | 921 | 0.298 | 302 | 190 | 5 | 3 | 298 | 2 | 287 | Baseplate\_J domain-containing protein | Baseplate\_J domain-containing protein | | afdb-uniprot50 | AF-A0A379CNV1-F1-MODEL\_V4 | 1.0 | 2.812e-27 | 920 | 0.262 | 331 | 201 | 6 | 7 | 296 | 5 | 333 | Baseplate J-like protein | Baseplate J-like protein | | afdb-uniprot50 | AF-A0A2S6N2W1-F1-MODEL\_V4 | 1.0 | 5.759e-27 | 919 | 0.279 | 311 | 194 | 9 | 1 | 296 | 109 | 404 | Baseplate\_J domain-containing protein | Baseplate\_J domain-containing protein | | afdb-uniprot50 | AF-A0A524RVY7-F1-MODEL\_V4 | 1.0 | 1.293e-27 | 918 | 0.331 | 293 | 174 | 5 | 4 | 294 | 2 | 274 | Baseplate\_J domain-containing protein | Baseplate\_J domain-containing protein | | afdb-uniprot50 | AF-A0A0Q8AUB0-F1-MODEL\_V4 | 1.0 | 3.671e-26 | 916 | 0.31 | 296 | 181 | 6 | 8 | 296 | 3 | 282 | Baseplate\_J domain-containing protein | Baseplate\_J domain-containing protein | | afdb-uniprot50 | AF-A0A0N0KIE2-F1-MODEL\_V4 | 1.0 | 3.169e-27 | 916 | 0.273 | 318 | 201 | 9 | 1 | 296 | 1 | 310 | Baseplate\_J domain-containing protein | Baseplate\_J domain-containing protein | | afdb-uniprot50 | AF-A0A6L2YE65-F1-MODEL\_V4 | 1.0 | 2.723e-26 | 914 | 0.361 | 271 | 155 | 4 | 10 | 269 | 6 | 269 | Baseplate protein | Baseplate protein | | afdb-uniprot50 | AF-A0A0J9BHG4-F1-MODEL\_V4 | 1.0 | 1.445e-23 | 911 | 0.462 | 173 | 93 | 0 | 127 | 299 | 3 | 175 | Baseplate\_J domain-containing protein | Baseplate\_J domain-containing protein | | afdb-uniprot50 | AF-A0A5P9BN28-F1-MODEL\_V4 | 1.0 | 2.276e-26 | 911 | 0.292 | 297 | 188 | 6 | 8 | 298 | 2 | 282 | Baseplate J-like protein | Baseplate J-like protein | | afdb-uniprot50 | AF-A0A654BHD8-F1-MODEL\_V4 | 1.0 | 4.773e-23 | 910 | 0.472 | 165 | 87 | 0 | 135 | 299 | 2 | 166 | Phage-related baseplate assembly protein | Phage-related baseplate assembly protein | | afdb-uniprot50 | AF-A0A2W6Z6F0-F1-MODEL\_V4 | 1.0 | 2.565e-26 | 909 | 0.287 | 351 | 186 | 11 | 4 | 296 | 3 | 347 | Baseplate\_J domain-containing protein | Baseplate\_J domain-containing protein | | afdb-uniprot50 | AF-A0A1S9ZY08-F1-MODEL\_V4 | 1.0 | 5.402e-25 | 905 | 0.263 | 292 | 193 | 6 | 12 | 300 | 3 | 275 | Baseplate\_J domain-containing protein | Baseplate\_J domain-containing protein | | afdb-uniprot50 | AF-A0A381GJS6-F1-MODEL\_V4 | 1.0 | 1.785e-24 | 903 | 0.473 | 226 | 112 | 1 | 8 | 226 | 4 | 229 | Phage baseplate assembly protein J | Phage baseplate assembly protein J | | afdb-uniprot50 | AF-A0A377VBV5-F1-MODEL\_V4 | 1.0 | 2.34e-25 | 903 | 0.46 | 250 | 128 | 1 | 6 | 248 | 2 | 251 | Baseplate assembly protein J | Baseplate assembly protein J | | afdb-uniprot50 | AF-Q4EB01-F1-MODEL\_V4 | 1.0 | 1.635e-25 | 902 | 0.378 | 251 | 140 | 3 | 42 | 292 | 1 | 235 | Baseplate J-like protein | Baseplate J-like protein | | afdb-uniprot50 | AF-A0A3D1NTG9-F1-MODEL\_V4 | 1.0 | 1.047e-26 | 894 | 0.243 | 370 | 191 | 9 | 14 | 300 | 6 | 369 | Baseplate protein | Baseplate protein | | afdb-uniprot50 | AF-A0A645F3X1-F1-MODEL\_V4 | 1.0 | 8.957e-24 | 892 | 0.317 | 208 | 139 | 2 | 94 | 298 | 7 | 214 | Baseplate\_J domain-containing protein | Baseplate\_J domain-containing protein | | afdb-uniprot50 | AF-A0A327JML8-F1-MODEL\_V4 | 1.0 | 4.662e-26 | 892 | 0.262 | 308 | 211 | 8 | 1 | 300 | 1 | 300 | Baseplate\_J domain-containing protein | Baseplate\_J domain-containing protein | | afdb-uniprot50 | AF-A0A0S9F1A4-F1-MODEL\_V4 | 1.0 | 3.349e-25 | 891 | 0.322 | 298 | 179 | 5 | 6 | 298 | 2 | 281 | Baseplate J protein | Baseplate J protein | | afdb-uniprot50 | AF-F2J637-F1-MODEL\_V4 | 1.0 | 1.014e-25 | 889 | 0.304 | 292 | 181 | 5 | 13 | 298 | 6 | 281 | Phage protein gp26 | Phage protein gp26 | | afdb-uniprot50 | AF-A0A0M4LII5-F1-MODEL\_V4 | 1.0 | 8.474e-26 | 888 | 0.294 | 289 | 186 | 5 | 11 | 298 | 3 | 274 | Baseplate assembly protein J | Baseplate assembly protein J | | afdb-uniprot50 | AF-A0A0L6KDG2-F1-MODEL\_V4 | 1.0 | 1.956e-25 | 884 | 0.257 | 315 | 202 | 11 | 8 | 298 | 6 | 312 | Baseplate\_J domain-containing protein | Baseplate\_J domain-containing protein | | afdb-uniprot50 | AF-A0A1M7YYV2-F1-MODEL\_V4 | 1.0 | 1.498e-26 | 881 | 0.304 | 296 | 185 | 6 | 6 | 298 | 3 | 280 | Baseplate J-like protein | Baseplate J-like protein | | afdb-uniprot50 | AF-E2CI68-F1-MODEL\_V4 | 1.0 | 1.014e-25 | 881 | 0.309 | 297 | 183 | 5 | 8 | 298 | 2 | 282 | Baseplate assembly protein J | Baseplate assembly protein J | | afdb-uniprot50 | AF-A0A5M8P9P5-F1-MODEL\_V4 | 1.0 | 6.286e-26 | 880 | 0.279 | 293 | 189 | 5 | 12 | 298 | 4 | 280 | Baseplate assembly protein | Baseplate assembly protein | | afdb-uniprot50 | AF-A0A2W5VFF5-F1-MODEL\_V4 | 1.0 | 7.983e-26 | 880 | 0.273 | 326 | 187 | 7 | 5 | 296 | 3 | 312 | Uncharacterized protein | Uncharacterized protein | | afdb-uniprot50 | AF-G2HXA0-F1-MODEL\_V4 | 1.0 | 7.983e-26 | 879 | 0.205 | 370 | 209 | 8 | 8 | 296 | 2 | 367 | Phage baseplate assembly protein | Phage baseplate assembly protein | | afdb-uniprot50 | AF-A0A1I1V0V6-F1-MODEL\_V4 | 1.0 | 1.843e-25 | 877 | 0.25 | 316 | 204 | 9 | 1 | 296 | 1 | 303 | Phage-related baseplate assembly protein | Phage-related baseplate assembly protein | | afdb-uniprot50 | AF-A0A5B9DJ78-F1-MODEL\_V4 | 1.0 | 1.213e-25 | 874 | 0.299 | 297 | 186 | 6 | 8 | 298 | 5 | 285 | Baseplate\_J domain-containing protein | Baseplate\_J domain-containing protein | | afdb-uniprot50 | AF-A0A7X4WDI4-F1-MODEL\_V4 | 1.0 | 8.75e-27 | 870 | 0.25 | 328 | 198 | 8 | 13 | 296 | 1 | 324 | Baseplate J protein | Baseplate J protein | | afdb-uniprot50 | AF-A0A4R6M7P6-F1-MODEL\_V4 | 1.0 | 1.635e-25 | 869 | 0.29 | 296 | 183 | 6 | 1 | 296 | 1 | 269 | Phage-related baseplate assembly protein | Phage-related baseplate assembly protein | | afdb-uniprot50 | AF-A0A1G3UAG2-F1-MODEL\_V4 | 1.0 | 8.511e-28 | 868 | 0.264 | 367 | 184 | 9 | 8 | 296 | 1 | 359 | Baseplate\_J domain-containing protein | Baseplate\_J domain-containing protein | | afdb-uniprot50 | AF-A0A0F4NJK8-F1-MODEL\_V4 | 1.0 | 1.213e-25 | 867 | 0.283 | 293 | 188 | 5 | 8 | 300 | 2 | 272 | Baseplate\_J domain-containing protein | Baseplate\_J domain-containing protein | | afdb-uniprot50 | AF-A0A4Q7FYT9-F1-MODEL\_V4 | 1.0 | 4.392e-26 | 865 | 0.259 | 316 | 202 | 8 | 1 | 299 | 1 | 301 | Baseplate\_J domain-containing protein | Baseplate\_J domain-containing protein | | afdb-uniprot50 | AF-A0A1S7MRG2-F1-MODEL\_V4 | 1.0 | 8.207e-25 | 861 | 0.273 | 318 | 201 | 10 | 4 | 298 | 2 | 312 | Phage-related baseplate assembly protein | Phage-related baseplate assembly protein | | afdb-uniprot50 | AF-A0A376BTH5-F1-MODEL\_V4 | 1.0 | 2.8e-25 | 861 | 0.237 | 383 | 197 | 11 | 8 | 300 | 1 | 378 | Uncharacterized homolog of phage Mu protein gp47 | Uncharacterized homolog of phage Mu protein gp47 | | afdb-uniprot50 | AF-A0A136Q9J0-F1-MODEL\_V4 | 1.0 | 4.007e-25 | 857 | 0.392 | 285 | 137 | 5 | 8 | 257 | 2 | 285 | Baseplate assembly protein | Baseplate assembly protein | | afdb-uniprot50 | AF-A0A1I5RPD0-F1-MODEL\_V4 | 1.0 | 1.843e-25 | 856 | 0.248 | 370 | 188 | 11 | 8 | 295 | 2 | 363 | Baseplate J-like protein | Baseplate J-like protein | | afdb-uniprot50 | AF-A0A239CA17-F1-MODEL\_V4 | 1.0 | 4.007e-25 | 854 | 0.248 | 382 | 198 | 10 | 1 | 298 | 1 | 377 | Phage-related baseplate assembly protein | Phage-related baseplate assembly protein | | afdb-uniprot50 | AF-A0A833KP28-F1-MODEL\_V4 | 1.0 | 5.71e-23 | 853 | 0.438 | 203 | 110 | 1 | 68 | 270 | 2 | 200 | Baseplate assembly protein | Baseplate assembly protein | | afdb-uniprot50 | AF-U3A3E9-F1-MODEL\_V4 | 1.0 | 5.578e-26 | 851 | 0.238 | 331 | 202 | 7 | 12 | 296 | 3 | 329 | Putative phage baseplate assembly protein | Putative phage baseplate assembly protein | | afdb-uniprot50 | AF-A0A857ED91-F1-MODEL\_V4 | 1.0 | 3.54e-23 | 849 | 0.442 | 199 | 105 | 1 | 104 | 296 | 2 | 200 | Uncharacterized protein | Uncharacterized protein | | afdb-uniprot50 | AF-A0A2D3VSP7-F1-MODEL\_V4 | 1.0 | 3.671e-26 | 847 | 0.272 | 370 | 182 | 11 | 8 | 296 | 1 | 364 | Baseplate\_J domain-containing protein | Baseplate\_J domain-containing protein | | afdb-uniprot50 | AF-B4RLZ3-F1-MODEL\_V4 | 1.0 | 5.896e-24 | 845 | 0.369 | 271 | 157 | 5 | 1 | 264 | 1 | 264 | Putative baseplate protein, putative phage associated protein | Putative baseplate protein, putative phage associated protein | | afdb-uniprot50 | AF-C8N767-F1-MODEL\_V4 | 1.0 | 8.242e-27 | 845 | 0.314 | 302 | 177 | 6 | 4 | 298 | 2 | 280 | Baseplate J-like protein | Baseplate J-like protein | | afdb-uniprot50 | AF-A0A0F6A5M1-F1-MODEL\_V4 | 1.0 | 1.736e-25 | 844 | 0.309 | 297 | 182 | 7 | 1 | 294 | 1 | 277 | Baseplate\_J domain-containing protein | Baseplate\_J domain-containing protein | | afdb-uniprot50 | AF-A0A841GJI4-F1-MODEL\_V4 | 1.0 | 1.681e-24 | 842 | 0.286 | 342 | 188 | 13 | 5 | 300 | 4 | 335 | Phage-related baseplate assembly protein | Phage-related baseplate assembly protein | | afdb-uniprot50 | AF-A0A659JW46-F1-MODEL\_V4 | 1.0 | 3.897e-26 | 842 | 0.257 | 377 | 183 | 10 | 13 | 300 | 3 | 371 | Baseplate protein | Baseplate protein | | afdb-uniprot50 | AF-A0A7W8AJB6-F1-MODEL\_V4 | 1.0 | 2.34e-25 | 840 | 0.322 | 295 | 179 | 7 | 8 | 298 | 3 | 280 | Phage-related baseplate assembly protein | Phage-related baseplate assembly protein | | afdb-uniprot50 | AF-A0A061JGS7-F1-MODEL\_V4 | 1.0 | 3.335e-23 | 839 | 0.327 | 226 | 136 | 3 | 73 | 298 | 2 | 211 | Baseplate assembly protein J | Baseplate assembly protein J | | afdb-uniprot50 | AF-A0A411WHR4-F1-MODEL\_V4 | 1.0 | 7.487e-24 | 839 | 0.222 | 378 | 199 | 9 | 12 | 300 | 3 | 374 | Baseplate protein | Baseplate protein | | afdb-uniprot50 | AF-A0A6M8SXS4-F1-MODEL\_V4 | 1.0 | 1.175e-24 | 838 | 0.245 | 379 | 187 | 12 | 12 | 300 | 5 | 374 | Baseplate J/gp47 family protein | Baseplate J/gp47 family protein | | afdb-uniprot50 | AF-A0A1X3ITE5-F1-MODEL\_V4 | 1.0 | 3.758e-23 | 837 | 0.43 | 244 | 128 | 3 | 8 | 244 | 4 | 243 | Baseplate assembly protein J (GpJ) | Baseplate assembly protein J (GpJ) | | afdb-uniprot50 | AF-A0A6L8LZX8-F1-MODEL\_V4 | 1.0 | 5.088e-25 | 835 | 0.235 | 327 | 201 | 6 | 13 | 294 | 1 | 323 | Baseplate\_J domain-containing protein | Baseplate\_J domain-containing protein | | afdb-uniprot50 | AF-A0A837JBU7-F1-MODEL\_V4 | 1.0 | 2.554e-24 | 834 | 0.219 | 396 | 194 | 8 | 8 | 295 | 2 | 390 | Baseplate assembly protein | Baseplate assembly protein | | afdb-uniprot50 | AF-L0RFG6-F1-MODEL\_V4 | 1.0 | 1.202e-21 | 831 | 0.384 | 182 | 111 | 1 | 114 | 295 | 6 | 186 | Baseplate J family protein | Baseplate J family protein | | afdb-uniprot50 | AF-A0A7U6KQJ6-F1-MODEL\_V4 | 1.0 | 5.379e-23 | 829 | 0.336 | 247 | 146 | 4 | 55 | 300 | 2 | 231 | Baseplate assembly protein | Baseplate assembly protein | | afdb-uniprot50 | AF-A0A3S4DC92-F1-MODEL\_V4 | 1.0 | 4.354e-22 | 828 | 0.414 | 181 | 104 | 1 | 116 | 296 | 3 | 181 | Baseplate J-like protein | Baseplate J-like protein | | afdb-uniprot50 | AF-A0A6I5SY72-F1-MODEL\_V4 | 1.0 | 5.554e-24 | 827 | 0.382 | 259 | 139 | 5 | 18 | 274 | 3 | 242 | Baseplate assembly protein J | Baseplate assembly protein J | | afdb-uniprot50 | AF-A0A327Q7V2-F1-MODEL\_V4 | 1.0 | 1.405e-24 | 825 | 0.219 | 369 | 201 | 9 | 13 | 298 | 1 | 365 | Phage-related baseplate assembly protein | Phage-related baseplate assembly protein | | afdb-uniprot50 | AF-A0A7W5FTX4-F1-MODEL\_V4 | 1.0 | 1.681e-24 | 823 | 0.297 | 296 | 181 | 7 | 7 | 298 | 3 | 275 | Phage-related baseplate assembly protein | Phage-related baseplate assembly protein | | afdb-uniprot50 | AF-A0A8B3NLL6-F1-MODEL\_V4 | 1.0 | 3.897e-26 | 822 | 0.318 | 308 | 184 | 7 | 1 | 298 | 127 | 418 | Uncharacterized protein | Uncharacterized protein | | afdb-uniprot50 | AF-A0A7X7RZR2-F1-MODEL\_V4 | 1.0 | 2.406e-24 | 819 | 0.253 | 386 | 183 | 12 | 12 | 300 | 3 | 380 | Baseplate J/gp47 family protein | Baseplate J/gp47 family protein | | afdb-uniprot50 | AF-A0A0B6CQ96-F1-MODEL\_V4 | 1.0 | 6.86e-25 | 818 | 0.233 | 296 | 200 | 7 | 1 | 296 | 1 | 269 | Baseplate J-like family protein | Baseplate J-like family protein | | afdb-uniprot50 | AF-A0A3A9CZV9-F1-MODEL\_V4 | 1.0 | 3.443e-24 | 818 | 0.223 | 376 | 202 | 9 | 13 | 300 | 6 | 379 | Baseplate J/gp47 family protein | Baseplate J/gp47 family protein | | afdb-uniprot50 | AF-A0A849V9Q1-F1-MODEL\_V4 | 1.0 | 6.644e-24 | 814 | 0.232 | 382 | 195 | 12 | 8 | 300 | 1 | 373 | Baseplate J/gp47 family protein | Baseplate J/gp47 family protein | | afdb-uniprot50 | AF-A0A7S8C8N1-F1-MODEL\_V4 | 1.0 | 1.142e-25 | 813 | 0.336 | 291 | 172 | 7 | 12 | 298 | 3 | 276 | Baseplate J/gp47 family protein | Baseplate J/gp47 family protein | | afdb-uniprot50 | AF-A0A1M3AMW9-F1-MODEL\_V4 | 1.0 | 1.728e-23 | 811 | 0.257 | 318 | 197 | 7 | 13 | 296 | 3 | 315 | Baseplate\_J domain-containing protein | Baseplate\_J domain-containing protein | | afdb-uniprot50 | AF-A0A165U8U7-F1-MODEL\_V4 | 1.0 | 2.068e-23 | 809 | 0.25 | 304 | 199 | 6 | 1 | 298 | 1 | 281 | Baseplate J-like protein | Baseplate J-like protein | | afdb-uniprot50 | AF-A0A1C0V8C6-F1-MODEL\_V4 | 1.0 | 1.628e-23 | 807 | 0.229 | 379 | 191 | 10 | 13 | 295 | 1 | 374 | Baseplate\_J domain-containing protein | Baseplate\_J domain-containing protein | | afdb-uniprot50 | AF-A0A021XBA6-F1-MODEL\_V4 | 1.0 | 3.897e-26 | 806 | 0.298 | 312 | 184 | 9 | 1 | 294 | 1 | 295 | Baseplate J family protein | Baseplate J family protein | | afdb-uniprot50 | AF-A0A4U8YSZ3-F1-MODEL\_V4 | 1.0 | 1.138e-23 | 806 | 0.227 | 378 | 201 | 12 | 8 | 300 | 1 | 372 | Baseplate protein j-like | Baseplate protein j-like | | afdb-uniprot50 | AF-A0A659UR57-F1-MODEL\_V4 | 1.0 | 1.736e-25 | 805 | 0.333 | 300 | 174 | 7 | 9 | 298 | 3 | 286 | Baseplate J protein | Baseplate J protein | | afdb-uniprot50 | AF-A0A327KHD7-F1-MODEL\_V4 | 1.0 | 3.774e-25 | 805 | 0.293 | 300 | 184 | 7 | 8 | 295 | 4 | 287 | Baseplate\_J domain-containing protein | Baseplate\_J domain-containing protein | | afdb-uniprot50 | AF-A0A521DS17-F1-MODEL\_V4 | 1.0 | 6.259e-24 | 804 | 0.221 | 366 | 194 | 11 | 22 | 300 | 5 | 366 | Phage-related baseplate assembly protein | Phage-related baseplate assembly protein | | afdb-uniprot50 | AF-A0A3T0L2T8-F1-MODEL\_V4 | 1.0 | 3.774e-25 | 802 | 0.24 | 366 | 194 | 9 | 13 | 300 | 1 | 360 | Baseplate\_J domain-containing protein | Baseplate\_J domain-containing protein | | afdb-uniprot50 | AF-E6X1N4-F1-MODEL\_V4 | 1.0 | 1.009e-23 | 802 | 0.237 | 366 | 195 | 9 | 13 | 300 | 1 | 360 | Baseplate J family protein | Baseplate J family protein | | afdb-uniprot50 | AF-A0A1M7R7L6-F1-MODEL\_V4 | 1.0 | 7.283e-25 | 802 | 0.237 | 371 | 196 | 9 | 13 | 300 | 1 | 367 | Phage-related baseplate assembly protein | Phage-related baseplate assembly protein | | afdb-uniprot50 | AF-A0A0P9B5C1-F1-MODEL\_V4 | 1.0 | 6.035e-21 | 801 | 0.401 | 214 | 124 | 2 | 3 | 214 | 2 | 213 | Baseplate\_J domain-containing protein | Baseplate\_J domain-containing protein | | afdb-uniprot50 | AF-A0A2N9Y980-F1-MODEL\_V4 | 1.0 | 1.956e-25 | 801 | 0.308 | 305 | 186 | 8 | 1 | 298 | 1 | 287 | Baseplate assembly protein | Baseplate assembly protein | | afdb-uniprot50 | AF-Q2NRT7-F1-MODEL\_V4 | 1.0 | 8.675e-23 | 800 | 0.48 | 227 | 104 | 3 | 8 | 227 | 4 | 223 | Baseplate J-like protein | Baseplate J-like protein | | afdb-uniprot50 | AF-A0A5S9R4F2-F1-MODEL\_V4 | 1.0 | 8.957e-24 | 799 | 0.245 | 310 | 204 | 7 | 1 | 298 | 1 | 292 | Baseplate\_J domain-containing protein | Baseplate\_J domain-containing protein | | afdb-uniprot50 | AF-A0A5C7WA37-F1-MODEL\_V4 | 1.0 | 1.208e-23 | 795 | 0.245 | 379 | 185 | 11 | 13 | 300 | 4 | 372 | Baseplate protein | Baseplate protein | | afdb-uniprot50 | AF-A0A8A9QTJ7-F1-MODEL\_V4 | 1.0 | 7.698e-23 | 791 | 0.259 | 293 | 191 | 7 | 13 | 298 | 3 | 276 | Baseplate J/gp47 family protein | Baseplate J/gp47 family protein | | afdb-uniprot50 | AF-A0A7X6G3F7-F1-MODEL\_V4 | 1.0 | 1.57e-20 | 789 | 0.54 | 174 | 80 | 0 | 1 | 174 | 1 | 174 | Baseplate\_J domain-containing protein | Baseplate\_J domain-containing protein | | afdb-uniprot50 | AF-A0A5C1DKC4-F1-MODEL\_V4 | 1.0 | 1.242e-22 | 788 | 0.241 | 381 | 190 | 12 | 11 | 300 | 61 | 433 | Baseplate protein | Baseplate protein | | afdb-uniprot50 | AF-A0A1T4W4N2-F1-MODEL\_V4 | 1.0 | 7.487e-24 | 785 | 0.269 | 301 | 188 | 8 | 12 | 298 | 5 | 287 | Phage-related baseplate assembly protein | Phage-related baseplate assembly protein | | afdb-uniprot50 | AF-J6H0S6-F1-MODEL\_V4 | 1.0 | 1.102e-22 | 785 | 0.205 | 370 | 207 | 9 | 14 | 300 | 1 | 366 | Baseplate J-like protein | Baseplate J-like protein | | afdb-uniprot50 | AF-A0A5B9YAA6-F1-MODEL\_V4 | 1.0 | 1.102e-22 | 785 | 0.232 | 374 | 199 | 10 | 10 | 300 | 3 | 371 | Baseplate J/gp47 family protein | Baseplate J/gp47 family protein | | afdb-uniprot50 | AF-A0A2A7AQP1-F1-MODEL\_V4 | 1.0 | 2.474e-23 | 785 | 0.205 | 379 | 211 | 8 | 10 | 300 | 8 | 384 | Phage tail protein | Phage tail protein | | afdb-uniprot50 | AF-A0A485AH88-F1-MODEL\_V4 | 1.0 | 9.734e-21 | 783 | 0.472 | 197 | 97 | 1 | 8 | 197 | 4 | 200 | Baseplate J-like protein | Baseplate J-like protein | | afdb-uniprot50 | AF-A0A779QIS1-F1-MODEL\_V4 | 1.0 | 9.17e-21 | 782 | 0.449 | 207 | 103 | 3 | 8 | 207 | 4 | 206 | Baseplate assembly protein | Baseplate assembly protein | | afdb-uniprot50 | AF-A0A3L7JDR9-F1-MODEL\_V4 | 1.0 | 1.175e-24 | 782 | 0.299 | 307 | 183 | 9 | 8 | 298 | 7 | 297 | Baseplate\_J domain-containing protein | Baseplate\_J domain-containing protein | | afdb-uniprot50 | AF-A0A4V3CHL3-F1-MODEL\_V4 | 1.0 | 2.33e-23 | 782 | 0.236 | 377 | 196 | 10 | 12 | 300 | 4 | 376 | Phage-related baseplate assembly protein | Phage-related baseplate assembly protein | | afdb-uniprot50 | AF-A0A497UL74-F1-MODEL\_V4 | 1.0 | 3.897e-26 | 781 | 0.258 | 371 | 188 | 8 | 13 | 300 | 1 | 367 | Phage-related baseplate assembly protein | Phage-related baseplate assembly protein | | afdb-uniprot50 | AF-A0A380Z425-F1-MODEL\_V4 | 1.0 | 4.119e-24 | 781 | 0.239 | 364 | 186 | 10 | 20 | 300 | 9 | 364 | Uncharacterized homolog of phage Mu protein gp47 | Uncharacterized homolog of phage Mu protein gp47 | | afdb-uniprot50 | AF-A0A3D4RUG0-F1-MODEL\_V4 | 1.0 | 1.843e-25 | 779 | 0.299 | 321 | 180 | 10 | 1 | 294 | 1 | 303 | Baseplate\_J domain-containing protein | Baseplate\_J domain-containing protein | | afdb-uniprot50 | AF-A0A0K1NFZ3-F1-MODEL\_V4 | 1.0 | 5.209e-22 | 778 | 0.253 | 292 | 192 | 8 | 11 | 295 | 2 | 274 | Baseplate\_J domain-containing protein | Baseplate\_J domain-containing protein | | afdb-uniprot50 | AF-A0A075WT46-F1-MODEL\_V4 | 1.0 | 5.896e-24 | 777 | 0.218 | 362 | 195 | 9 | 23 | 300 | 8 | 365 | Baseplate\_J domain-containing protein | Baseplate\_J domain-containing protein | | afdb-uniprot50 | AF-M5JP61-F1-MODEL\_V4 | 1.0 | 1.009e-23 | 776 | 0.272 | 297 | 192 | 7 | 9 | 298 | 6 | 285 | Baseplate J family protein | Baseplate J family protein | | afdb-uniprot50 | AF-A0A098MGN3-F1-MODEL\_V4 | 1.0 | 1.242e-22 | 776 | 0.244 | 380 | 195 | 12 | 8 | 300 | 2 | 376 | Baseplate J protein | Baseplate J protein | | afdb-uniprot50 | AF-A0A450VII4-F1-MODEL\_V4 | 1.0 | 8.675e-23 | 771 | 0.3 | 260 | 162 | 5 | 39 | 298 | 3 | 242 | Phage-related baseplate assembly protein | Phage-related baseplate assembly protein | | afdb-uniprot50 | AF-D1P879-F1-MODEL\_V4 | 1.0 | 1.931e-19 | 766 | 0.424 | 153 | 88 | 0 | 147 | 299 | 3 | 155 | Baseplate J-like protein | Baseplate J-like protein | | afdb-uniprot50 | AF-A0A1E3G789-F1-MODEL\_V4 | 1.0 | 4.928e-24 | 765 | 0.247 | 319 | 199 | 10 | 1 | 296 | 1 | 301 | Baseplate\_J domain-containing protein | Baseplate\_J domain-containing protein | | afdb-uniprot50 | AF-A0A0D6ARM2-F1-MODEL\_V4 | 1.0 | 4.102e-22 | 760 | 0.173 | 421 | 211 | 8 | 12 | 300 | 3 | 418 | Phage-related baseplate assembly protein | Phage-related baseplate assembly protein | | afdb-uniprot50 | AF-E0LW08-F1-MODEL\_V4 | 1.0 | 2.385e-20 | 759 | 0.437 | 167 | 88 | 2 | 138 | 299 | 5 | 170 | Baseplate J family protein | Baseplate J family protein | | afdb-uniprot50 | AF-Q4EBT1-F1-MODEL\_V4 | 1.0 | 1.827e-21 | 759 | 0.396 | 227 | 122 | 2 | 13 | 239 | 1 | 212 | Baseplate J-like protein | Baseplate J-like protein | | afdb-uniprot50 | AF-A0A6G5QY03-F1-MODEL\_V4 | 1.0 | 1.282e-23 | 756 | 0.256 | 374 | 183 | 15 | 8 | 300 | 2 | 361 | Phage baseplate assembly protein J, putative | Phage baseplate assembly protein J, putative | | afdb-uniprot50 | AF-A0A512JPE9-F1-MODEL\_V4 | 1.0 | 9.468e-22 | 756 | 0.234 | 366 | 202 | 15 | 2 | 296 | 10 | 368 | Baseplate\_J domain-containing protein | Baseplate\_J domain-containing protein | | afdb-uniprot50 | AF-A0A367DB17-F1-MODEL\_V4 | 1.0 | 1.674e-22 | 754 | 0.193 | 373 | 212 | 12 | 12 | 300 | 7 | 374 | Baseplate J protein | Baseplate J protein | | afdb-uniprot50 | AF-A0A1V4GFY9-F1-MODEL\_V4 | 1.0 | 4.622e-22 | 746 | 0.227 | 378 | 198 | 13 | 12 | 300 | 6 | 378 | Baseplate\_J domain-containing protein | Baseplate\_J domain-containing protein | | afdb-uniprot50 | AF-A0A4D9IGF7-F1-MODEL\_V4 | 1.0 | 9.209e-23 | 745 | 0.271 | 331 | 155 | 6 | 53 | 300 | 2 | 329 | Baseplate protein | Baseplate protein | | afdb-uniprot50 | AF-D1AFB5-F1-MODEL\_V4 | 1.0 | 3.64e-22 | 744 | 0.191 | 365 | 204 | 11 | 20 | 298 | 9 | 368 | Baseplate J family protein | Baseplate J family protein | | afdb-uniprot50 | AF-A0A0R3MV37-F1-MODEL\_V4 | 1.0 | 6.232e-22 | 743 | 0.216 | 296 | 205 | 9 | 12 | 298 | 7 | 284 | Baseplate assembly protein | Baseplate assembly protein | | afdb-uniprot50 | AF-N6VUC8-F1-MODEL\_V4 | 1.0 | 1.728e-23 | 742 | 0.296 | 277 | 175 | 7 | 25 | 298 | 2 | 261 | Phage baseplate assembly protein GpJ | Phage baseplate assembly protein GpJ | | afdb-uniprot50 | AF-A0A1D9LNN0-F1-MODEL\_V4 | 1.0 | 6.061e-23 | 742 | 0.231 | 380 | 194 | 11 | 12 | 298 | 6 | 380 | Baseplate\_J domain-containing protein | Baseplate\_J domain-containing protein | | afdb-uniprot50 | AF-A0A823V5N8-F1-MODEL\_V4 | 1.0 | 2.959e-23 | 738 | 0.225 | 395 | 191 | 13 | 4 | 300 | 10 | 387 | Baseplate assembly protein | Baseplate assembly protein | | afdb-uniprot50 | AF-A0A4Y7RWI7-F1-MODEL\_V4 | 1.0 | 2.7e-22 | 736 | 0.219 | 369 | 201 | 10 | 12 | 298 | 5 | 368 | Baseplate\_J domain-containing protein | Baseplate\_J domain-containing protein | | afdb-uniprot50 | AF-A0A2N1QGA1-F1-MODEL\_V4 | 1.0 | 7.455e-22 | 736 | 0.191 | 381 | 217 | 11 | 8 | 300 | 3 | 380 | Baseplate\_J domain-containing protein | Baseplate\_J domain-containing protein | | afdb-uniprot50 | AF-A0A6I2URJ4-F1-MODEL\_V4 | 1.0 | 3.955e-19 | 735 | 0.494 | 186 | 92 | 1 | 1 | 184 | 1 | 186 | Baseplate assembly protein | Baseplate assembly protein | | afdb-uniprot50 | AF-A0A7X5KSN5-F1-MODEL\_V4 | 1.0 | 2.543e-22 | 735 | 0.162 | 382 | 227 | 10 | 8 | 300 | 2 | 379 | Phage tail protein | Phage tail protein | | afdb-uniprot50 | AF-A0A4Q8MDG2-F1-MODEL\_V4 | 1.0 | 2.406e-24 | 732 | 0.273 | 303 | 191 | 9 | 13 | 298 | 5 | 295 | Baseplate\_J domain-containing protein | Baseplate\_J domain-containing protein | | afdb-uniprot50 | AF-N9KZE1-F1-MODEL\_V4 | 1.0 | 4.732e-19 | 728 | 0.278 | 183 | 129 | 2 | 119 | 298 | 3 | 185 | Baseplate\_J domain-containing protein | Baseplate\_J domain-containing protein | | afdb-uniprot50 | AF-A0A4Q8MFD0-F1-MODEL\_V4 | 1.0 | 5.209e-22 | 728 | 0.223 | 331 | 208 | 12 | 1 | 300 | 1 | 313 | Baseplate\_J domain-containing protein | Baseplate\_J domain-containing protein | | afdb-uniprot50 | AF-A0A4Q6D773-F1-MODEL\_V4 | 1.0 | 1.318e-22 | 723 | 0.26 | 300 | 184 | 9 | 14 | 300 | 3 | 277 | Baseplate\_J domain-containing protein | Baseplate\_J domain-containing protein | | afdb-uniprot50 | AF-F3YY52-F1-MODEL\_V4 | 1.0 | 2.002e-22 | 722 | 0.222 | 382 | 200 | 12 | 8 | 300 | 1 | 374 | Baseplate J family protein | Baseplate J family protein | | afdb-uniprot50 | AF-A0A812RFY7-F1-MODEL\_V4 | 1.0 | 8.919e-22 | 720 | 0.437 | 258 | 116 | 2 | 36 | 264 | 2 | 259 | J protein | J protein | | afdb-uniprot50 | AF-A0A840NW02-F1-MODEL\_V4 | 1.0 | 5.506e-20 | 717 | 0.312 | 224 | 135 | 6 | 77 | 298 | 1 | 207 | Phage-related baseplate assembly protein | Phage-related baseplate assembly protein | | afdb-uniprot50 | AF-A0A2B7YKZ8-F1-MODEL\_V4 | 1.0 | 1.005e-21 | 717 | 0.199 | 376 | 206 | 11 | 13 | 300 | 2 | 370 | Bacteriocin | Bacteriocin | | afdb-uniprot50 | AF-A0A2H1SQX0-F1-MODEL\_V4 | 1.0 | 4.732e-19 | 716 | 0.531 | 173 | 79 | 1 | 2 | 174 | 9 | 179 | Baseplate assembly protein J | Baseplate assembly protein J | | afdb-uniprot50 | AF-A0A2G6EWY7-F1-MODEL\_V4 | 1.0 | 9.17e-21 | 714 | 0.26 | 292 | 186 | 6 | 13 | 298 | 1 | 268 | Baseplate\_J domain-containing protein | Baseplate\_J domain-containing protein | | afdb-uniprot50 | AF-E5Y5X0-F1-MODEL\_V4 | 1.0 | 1.485e-22 | 714 | 0.234 | 379 | 196 | 10 | 9 | 300 | 5 | 376 | Baseplate\_J domain-containing protein | Baseplate\_J domain-containing protein | | afdb-uniprot50 | AF-A0A7X4ZSW6-F1-MODEL\_V4 | 1.0 | 8.402e-22 | 714 | 0.208 | 378 | 207 | 10 | 10 | 298 | 6 | 380 | Baseplate J/gp47 family protein | Baseplate J/gp47 family protein | | afdb-uniprot50 | AF-R7JDJ8-F1-MODEL\_V4 | 1.0 | 1.242e-22 | 713 | 0.22 | 377 | 196 | 11 | 12 | 300 | 3 | 369 | Probable bacteriophage tail fiber protein | Probable bacteriophage tail fiber protein | | afdb-uniprot50 | AF-A0A4Q2XB46-F1-MODEL\_V4 | 1.0 | 2.237e-18 | 712 | 0.383 | 154 | 95 | 0 | 146 | 299 | 2 | 155 | Baseplate assembly protein | Baseplate assembly protein | | afdb-uniprot50 | AF-A0A0S4XLP9-F1-MODEL\_V4 | 1.0 | 1.527e-21 | 708 | 0.234 | 282 | 186 | 6 | 13 | 294 | 1 | 252 | Putative Baseplate J family protein | Putative Baseplate J family protein | | afdb-uniprot50 | AF-A0A6M3J3B0-F1-MODEL\_V4 | 1.0 | 2.565e-26 | 707 | 0.276 | 322 | 196 | 4 | 12 | 296 | 8 | 329 | Putative baseplate protein | Putative baseplate protein | | afdb-uniprot50 | AF-A0A4Q3KFV4-F1-MODEL\_V4 | 1.0 | 2.7e-22 | 704 | 0.302 | 271 | 163 | 6 | 31 | 298 | 118 | 365 | Uncharacterized protein | Uncharacterized protein | | afdb-uniprot50 | AF-A0A3Q9PYZ0-F1-MODEL\_V4 | 1.0 | 4.865e-18 | 700 | 0.465 | 146 | 78 | 0 | 154 | 299 | 2 | 147 | Baseplate assembly protein | Baseplate assembly protein | | afdb-uniprot50 | AF-A0A2S5F8Y6-F1-MODEL\_V4 | 1.0 | 1.621e-21 | 700 | 0.207 | 385 | 208 | 13 | 1 | 298 | 1 | 375 | Phage baseplate protein | Phage baseplate protein | | afdb-uniprot50 | AF-A0A836P245-F1-MODEL\_V4 | 1.0 | 6.38e-19 | 698 | 0.53 | 179 | 81 | 2 | 1 | 179 | 1 | 176 | Baseplate assembly protein | Baseplate assembly protein | | afdb-uniprot50 | AF-A0A0Q1F5V0-F1-MODEL\_V4 | 1.0 | 8.996e-26 | 696 | 0.275 | 370 | 182 | 8 | 13 | 300 | 3 | 368 | Baseplate\_J domain-containing protein | Baseplate\_J domain-containing protein | | afdb-uniprot50 | AF-E2CJT9-F1-MODEL\_V4 | 1.0 | 3.847e-20 | 694 | 0.216 | 291 | 205 | 7 | 13 | 296 | 1 | 275 | Baseplate J family protein | Baseplate J family protein | | afdb-uniprot50 | AF-A0A4U7JBE4-F1-MODEL\_V4 | 1.0 | 1.276e-21 | 694 | 0.217 | 373 | 200 | 10 | 14 | 299 | 1 | 368 | Baseplate J/gp47 family protein | Baseplate J/gp47 family protein | | afdb-uniprot50 | AF-K1JKP8-F1-MODEL\_V4 | 1.0 | 7.053e-24 | 694 | 0.235 | 374 | 197 | 10 | 12 | 300 | 9 | 378 | Baseplate\_J domain-containing protein | Baseplate\_J domain-containing protein | | afdb-uniprot50 | AF-Q0FZ02-F1-MODEL\_V4 | 1.0 | 3.23e-22 | 689 | 0.254 | 295 | 196 | 9 | 12 | 296 | 8 | 288 | Baseplate J-like protein | Baseplate J-like protein | | afdb-uniprot50 | AF-A8TTZ7-F1-MODEL\_V4 | 1.0 | 6.963e-18 | 688 | 0.448 | 145 | 80 | 0 | 152 | 296 | 2 | 146 | Baseplate J-like protein | Baseplate J-like protein | | afdb-uniprot50 | AF-A0A6G8CYG2-F1-MODEL\_V4 | 1.0 | 7.665e-21 | 686 | 0.307 | 270 | 158 | 6 | 32 | 298 | 8 | 251 | Baseplate J protein | Baseplate J protein | | afdb-uniprot50 | AF-A0A378XFF5-F1-MODEL\_V4 | 1.0 | 3.016e-18 | 683 | 0.339 | 174 | 111 | 3 | 128 | 300 | 28 | 198 | Baseplate J-like protein | Baseplate J-like protein | | afdb-uniprot50 | AF-A0A2J4P2N7-F1-MODEL\_V4 | 1.0 | 4.317e-18 | 682 | 0.506 | 164 | 81 | 0 | 77 | 240 | 3 | 166 | Baseplate assembly protein | Baseplate assembly protein | | afdb-uniprot50 | AF-A0A827ZIF5-F1-MODEL\_V4 | 1.0 | 8.601e-19 | 681 | 0.34 | 200 | 117 | 4 | 113 | 300 | 22 | 218 | Baseplate protein | Baseplate protein | | afdb-uniprot50 | AF-A0A1T4QDZ6-F1-MODEL\_V4 | 1.0 | 7.665e-21 | 680 | 0.177 | 360 | 208 | 9 | 23 | 298 | 10 | 365 | Phage-related baseplate assembly protein | Phage-related baseplate assembly protein | | afdb-uniprot50 | AF-A0A376EX36-F1-MODEL\_V4 | 1.0 | 4.732e-19 | 679 | 0.315 | 200 | 122 | 4 | 113 | 300 | 74 | 270 | Baseplate assembly protein J | Baseplate assembly protein J | | afdb-uniprot50 | AF-A0A1H1FF82-F1-MODEL\_V4 | 1.0 | 4.752e-21 | 679 | 0.244 | 295 | 192 | 11 | 13 | 296 | 9 | 283 | Phage-related baseplate assembly protein | Phage-related baseplate assembly protein | | afdb-uniprot50 | AF-A0A545T5S2-F1-MODEL\_V4 | 1.0 | 1.827e-21 | 678 | 0.262 | 373 | 173 | 12 | 7 | 300 | 8 | 357 | Uncharacterized protein | Uncharacterized protein | | afdb-uniprot50 | AF-A0A7D5QQY9-F1-MODEL\_V4 | 1.0 | 6.616e-22 | 676 | 0.237 | 316 | 152 | 6 | 72 | 300 | 6 | 319 | Baseplate J/gp47 family protein | Baseplate J/gp47 family protein | | afdb-uniprot50 | AF-A0A6M6IDN5-F1-MODEL\_V4 | 1.0 | 1.521e-19 | 674 | 0.383 | 237 | 128 | 3 | 6 | 239 | 3 | 224 | Baseplate assembly protein J | Baseplate assembly protein J | | afdb-uniprot50 | AF-D1P8B4-F1-MODEL\_V4 | 1.0 | 4.583e-18 | 673 | 0.477 | 180 | 87 | 1 | 13 | 185 | 2 | 181 | Baseplate J-like protein | Baseplate J-like protein | | afdb-uniprot50 | AF-A0A828HR86-F1-MODEL\_V4 | 1.0 | 7.847e-18 | 673 | 0.476 | 191 | 89 | 3 | 8 | 191 | 4 | 190 | Baseplate assembly protein | Baseplate assembly protein | | afdb-uniprot50 | AF-A0A238HJ41-F1-MODEL\_V4 | 1.0 | 5.661e-19 | 672 | 0.34 | 191 | 115 | 4 | 118 | 300 | 11 | 198 | Baseplate J-like protein | Baseplate J-like protein | | afdb-uniprot50 | AF-A0A064AIK0-F1-MODEL\_V4 | 1.0 | 1.393e-20 | 671 | 0.181 | 374 | 212 | 13 | 13 | 299 | 1 | 367 | Baseplate assembly protein | Baseplate assembly protein | | afdb-uniprot50 | AF-G2FT51-F1-MODEL\_V4 | 1.0 | 2.059e-21 | 671 | 0.237 | 396 | 183 | 15 | 14 | 298 | 1 | 388 | Baseplate J-like family protein | Baseplate J-like family protein | | afdb-uniprot50 | AF-A0A081MYF5-F1-MODEL\_V4 | 1.0 | 2.592e-17 | 670 | 0.41 | 156 | 89 | 2 | 144 | 299 | 2 | 154 | Baseplate\_J domain-containing protein | Baseplate\_J domain-containing protein | | afdb-uniprot50 | AF-A0A1V3RQM2-F1-MODEL\_V4 | 1.0 | 2.077e-25 | 670 | 0.246 | 378 | 188 | 9 | 13 | 300 | 8 | 378 | Phage tail protein | Phage tail protein | | afdb-uniprot50 | AF-D8IV11-F1-MODEL\_V4 | 1.0 | 8.207e-25 | 669 | 0.261 | 379 | 179 | 10 | 12 | 300 | 7 | 374 | Bacteriophage tail fiber protein | Bacteriophage tail fiber protein | | afdb-uniprot50 | AF-A0A2G6F072-F1-MODEL\_V4 | 1.0 | 1.312e-20 | 668 | 0.172 | 371 | 216 | 10 | 14 | 300 | 1 | 364 | Baseplate assembly protein | Baseplate assembly protein | | afdb-uniprot50 | AF-G2IX29-F1-MODEL\_V4 | 1.0 | 5.187e-20 | 668 | 0.218 | 370 | 194 | 11 | 13 | 294 | 1 | 363 | Phage baseplate J-like protein | Phage baseplate J-like protein | | afdb-uniprot50 | AF-A0A2X1N0C4-F1-MODEL\_V4 | 1.0 | 2.452e-19 | 667 | 0.396 | 212 | 121 | 2 | 31 | 239 | 1 | 208 | Baseplate assembly protein J | Baseplate assembly protein J | | afdb-uniprot50 | AF-J8V6S9-F1-MODEL\_V4 | 1.0 | 4.886e-20 | 666 | 0.34 | 220 | 124 | 5 | 87 | 295 | 2 | 211 | Baseplate J family protein | Baseplate J family protein | | afdb-uniprot50 | AF-A0A258KUT3-F1-MODEL\_V4 | 1.0 | 5.82e-18 | 658 | 0.352 | 170 | 110 | 0 | 131 | 300 | 5 | 174 | Baseplate\_J domain-containing protein | Baseplate\_J domain-containing protein | | afdb-uniprot50 | AF-F5SA56-F1-MODEL\_V4 | 1.0 | 1.762e-18 | 655 | 0.319 | 197 | 122 | 5 | 113 | 300 | 94 | 287 | Phage baseplate protein | Phage baseplate protein | | afdb-uniprot50 | AF-A0A1W0C8U1-F1-MODEL\_V4 | 1.0 | 4.928e-24 | 651 | 0.254 | 385 | 185 | 10 | 14 | 300 | 1 | 381 | Baseplate protein | Baseplate protein | | afdb-uniprot50 | AF-D8IV18-F1-MODEL\_V4 | 1.0 | 6.462e-25 | 651 | 0.251 | 386 | 186 | 10 | 13 | 300 | 8 | 388 | Bacteriophage tail fiber protein | Bacteriophage tail fiber protein | | afdb-uniprot50 | AF-A0A095Z753-F1-MODEL\_V4 | 1.0 | 1.894e-24 | 649 | 0.26 | 372 | 181 | 13 | 13 | 298 | 1 | 364 | Baseplate\_J domain-containing protein | Baseplate\_J domain-containing protein | | afdb-uniprot50 | AF-A0A2W5J717-F1-MODEL\_V4 | 1.0 | 9.091e-17 | 647 | 0.534 | 159 | 72 | 1 | 1 | 159 | 2 | 158 | Baseplate assembly protein | Baseplate assembly protein | | afdb-uniprot50 | AF-A0A1W1Z473-F1-MODEL\_V4 | 1.0 | 3.655e-24 | 647 | 0.283 | 293 | 186 | 6 | 11 | 296 | 12 | 287 | Phage-related baseplate assembly protein | Phage-related baseplate assembly protein | | afdb-uniprot50 | AF-E2CFL2-F1-MODEL\_V4 | 1.0 | 1.621e-21 | 645 | 0.278 | 294 | 186 | 10 | 10 | 295 | 5 | 280 | Putative baseplate assembly protein J | Putative baseplate assembly protein J | | afdb-uniprot50 | AF-A0A2M7G5N1-F1-MODEL\_V4 | 1.0 | 6.587e-20 | 645 | 0.19 | 373 | 206 | 11 | 13 | 300 | 1 | 362 | Baseplate protein | Baseplate protein | | afdb-uniprot50 | AF-A0A4Q0YJP7-F1-MODEL\_V4 | 1.0 | 6.38e-19 | 644 | 0.35 | 237 | 117 | 4 | 6 | 205 | 5 | 241 | Baseplate assembly protein | Baseplate assembly protein | | afdb-uniprot50 | AF-A0A832CMI4-F1-MODEL\_V4 | 1.0 | 2.934e-19 | 637 | 0.163 | 373 | 210 | 11 | 18 | 298 | 2 | 364 | Baseplate J/gp47 family protein | Baseplate J/gp47 family protein | | afdb-uniprot50 | AF-A0A1Y6CV72-F1-MODEL\_V4 | 1.0 | 5.187e-20 | 636 | 0.244 | 299 | 186 | 10 | 13 | 298 | 1 | 272 | Phage-related baseplate assembly protein | Phage-related baseplate assembly protein | | afdb-uniprot50 | AF-A0A827RX96-F1-MODEL\_V4 | 1.0 | 9.091e-17 | 632 | 0.459 | 159 | 82 | 1 | 68 | 226 | 2 | 156 | Baseplate assembly protein | Baseplate assembly protein | | afdb-uniprot50 | AF-A0A845A584-F1-MODEL\_V4 | 1.0 | 1.226e-16 | 629 | 0.515 | 165 | 78 | 1 | 1 | 165 | 4 | 166 | Uncharacterized protein | Uncharacterized protein | | afdb-uniprot50 | AF-A0A410X4X2-F1-MODEL\_V4 | 1.0 | 6.587e-20 | 628 | 0.24 | 329 | 159 | 9 | 61 | 300 | 4 | 330 | Baseplate J protein | Baseplate J protein | | afdb-uniprot50 | AF-A0A1M7RIH8-F1-MODEL\_V4 | 1.0 | 2.959e-23 | 628 | 0.218 | 371 | 201 | 9 | 13 | 300 | 5 | 369 | Phage-related baseplate assembly protein | Phage-related baseplate assembly protein | | afdb-uniprot50 | AF-A0A7Z1M1I0-F1-MODEL\_V4 | 1.0 | 1.282e-23 | 626 | 0.23 | 373 | 192 | 10 | 19 | 300 | 5 | 373 | Phage-related baseplate assembly protein | Phage-related baseplate assembly protein | | afdb-uniprot50 | AF-A0A482ISU8-F1-MODEL\_V4 | 1.0 | 2.788e-23 | 626 | 0.241 | 377 | 192 | 7 | 13 | 300 | 7 | 378 | Baseplate protein | Baseplate protein | | afdb-uniprot50 | AF-R7HY31-F1-MODEL\_V4 | 1.0 | 1.948e-23 | 625 | 0.244 | 380 | 198 | 10 | 8 | 300 | 3 | 380 | Baseplate J-like protein | Baseplate J-like protein | | afdb-uniprot50 | AF-A0A7J6YLT3-F1-MODEL\_V4 | 1.0 | 1.001e-19 | 625 | 0.373 | 249 | 132 | 7 | 1 | 249 | 283 | 507 | Uncharacterized protein | Uncharacterized protein | | afdb-uniprot50 | AF-A0A6A2F414-F1-MODEL\_V4 | 1.0 | 7.487e-24 | 624 | 0.237 | 370 | 197 | 10 | 12 | 300 | 5 | 370 | Baseplate J/gp47 family protein | Baseplate J/gp47 family protein | | afdb-uniprot50 | AF-A0A0X1U7S3-F1-MODEL\_V4 | 1.0 | 6.259e-24 | 624 | 0.23 | 378 | 202 | 9 | 10 | 300 | 7 | 382 | Baseplate J-like protein | Baseplate J-like protein | | afdb-uniprot50 | AF-A0A7I8DIE6-F1-MODEL\_V4 | 1.0 | 7.423e-20 | 622 | 0.189 | 364 | 205 | 11 | 20 | 298 | 5 | 363 | Baseplate\_J domain-containing protein | Baseplate\_J domain-containing protein | | afdb-uniprot50 | AF-A0A7U6QPG4-F1-MODEL\_V4 | 1.0 | 6.743e-17 | 621 | 0.421 | 147 | 83 | 2 | 155 | 300 | 6 | 151 | Baseplate\_J domain-containing protein | Baseplate\_J domain-containing protein | | afdb-uniprot50 | AF-A0A285J5J6-F1-MODEL\_V4 | 1.0 | 3.188e-16 | 621 | 0.377 | 159 | 97 | 2 | 138 | 295 | 3 | 160 | Phage-related baseplate assembly protein | Phage-related baseplate assembly protein | | afdb-uniprot50 | AF-A0A716MJC6-F1-MODEL\_V4 | 1.0 | 2.452e-19 | 619 | 0.257 | 345 | 167 | 7 | 14 | 273 | 1 | 341 | Baseplate J/gp47 family protein | Baseplate J/gp47 family protein | | afdb-uniprot50 | AF-A0A5S9NBD4-F1-MODEL\_V4 | 1.0 | 3.115e-19 | 616 | 0.179 | 356 | 201 | 10 | 23 | 294 | 26 | 374 | Baseplate\_J domain-containing protein | Baseplate\_J domain-containing protein | | afdb-uniprot50 | AF-A0A3A6CL84-F1-MODEL\_V4 | 1.0 | 5.165e-18 | 616 | 0.187 | 383 | 219 | 11 | 6 | 300 | 3 | 381 | Baseplate J/gp47 family protein | Baseplate J/gp47 family protein | | afdb-uniprot50 | AF-A0A078MEN5-F1-MODEL\_V4 | 1.0 | 6.435e-23 | 615 | 0.215 | 372 | 204 | 10 | 12 | 300 | 3 | 369 | Baseplate J-like protein | Baseplate J-like protein | | afdb-uniprot50 | AF-A0A0C1G7H8-F1-MODEL\_V4 | 1.0 | 2.108e-18 | 615 | 0.188 | 376 | 208 | 13 | 6 | 296 | 7 | 370 | Baseplate\_J domain-containing protein | Baseplate\_J domain-containing protein | | afdb-uniprot50 | AF-A0A4Q2QS73-F1-MODEL\_V4 | 1.0 | 2.365e-16 | 614 | 0.539 | 141 | 65 | 0 | 86 | 226 | 2 | 142 | Baseplate assembly protein | Baseplate assembly protein | | afdb-uniprot50 | AF-A0A2G2BGS6-F1-MODEL\_V4 | 1.0 | 3.51e-19 | 614 | 0.157 | 382 | 231 | 11 | 1 | 298 | 4 | 378 | Phage baseplate assembly protein | Phage baseplate assembly protein | | afdb-uniprot50 | AF-A0A327JA39-F1-MODEL\_V4 | 1.0 | 1.87e-18 | 613 | 0.16 | 418 | 211 | 11 | 13 | 296 | 1 | 412 | Baseplate\_J domain-containing protein | Baseplate\_J domain-containing protein | | afdb-uniprot50 | AF-A0A447T5K9-F1-MODEL\_V4 | 1.0 | 1.426e-17 | 612 | 0.364 | 195 | 104 | 4 | 116 | 292 | 18 | 210 | Uncharacterized homolog of phage Mu protein gp47 | Uncharacterized homolog of phage Mu protein gp47 | | afdb-uniprot50 | AF-A0A7C3HFY9-F1-MODEL\_V4 | 1.0 | 2.764e-19 | 612 | 0.196 | 367 | 202 | 13 | 13 | 296 | 1 | 357 | Baseplate J protein | Baseplate J protein | | afdb-uniprot50 | AF-A0A827JCJ0-F1-MODEL\_V4 | 1.0 | 4.844e-16 | 611 | 0.537 | 160 | 67 | 1 | 8 | 160 | 4 | 163 | Baseplate assembly protein | Baseplate assembly protein | | afdb-uniprot50 | AF-A0A0B0HE83-F1-MODEL\_V4 | 1.0 | 5.31e-17 | 611 | 0.281 | 192 | 133 | 4 | 112 | 300 | 88 | 277 | Baseplate J-like protein | Baseplate J-like protein | | afdb-uniprot50 | AF-A0A0T7DUX5-F1-MODEL\_V4 | 1.0 | 7.189e-19 | 611 | 0.178 | 381 | 210 | 15 | 12 | 296 | 7 | 380 | Baseplate assembly protein J | Baseplate assembly protein J | | afdb-uniprot50 | AF-A0A6M0STE5-F1-MODEL\_V4 | 1.0 | 1.001e-19 | 609 | 0.194 | 386 | 197 | 15 | 20 | 298 | 5 | 383 | Baseplate\_J domain-containing protein | Baseplate\_J domain-containing protein | | afdb-uniprot50 | AF-A0A3A9B522-F1-MODEL\_V4 | 1.0 | 2.615e-21 | 609 | 0.187 | 379 | 218 | 10 | 10 | 300 | 9 | 385 | Baseplate J/gp47 family protein | Baseplate J/gp47 family protein | | afdb-uniprot50 | AF-A0A450WH23-F1-MODEL\_V4 | 1.0 | 1.674e-22 | 608 | 0.246 | 377 | 189 | 12 | 12 | 300 | 3 | 372 | Phage-related baseplate assembly protein | Phage-related baseplate assembly protein | | afdb-uniprot50 | AF-A0A7U7FD77-F1-MODEL\_V4 | 1.0 | 5.53e-22 | 607 | 0.337 | 361 | 139 | 8 | 6 | 294 | 2 | 334 | Putative phage-related protein | Putative phage-related protein | | afdb-uniprot50 | AF-A0A5M8P6R0-F1-MODEL\_V4 | 1.0 | 6.772e-19 | 606 | 0.185 | 383 | 219 | 13 | 1 | 300 | 5 | 377 | Baseplate\_J domain-containing protein | Baseplate\_J domain-containing protein | | afdb-uniprot50 | AF-A0A377AJ05-F1-MODEL\_V4 | 1.0 | 6.743e-17 | 603 | 0.276 | 188 | 131 | 4 | 116 | 300 | 94 | 279 | Phage-related baseplate assembly protein J | Phage-related baseplate assembly protein J | | afdb-uniprot50 | AF-A0A2U2DG57-F1-MODEL\_V4 | 1.0 | 1.226e-16 | 602 | 0.44 | 186 | 95 | 2 | 8 | 188 | 8 | 189 | Baseplate assembly protein | Baseplate assembly protein | | afdb-uniprot50 | AF-A0A654C1I4-F1-MODEL\_V4 | 1.0 | 1.66e-18 | 602 | 0.379 | 232 | 136 | 4 | 1 | 231 | 3 | 227 | Uncharacterized protein | Uncharacterized protein | | afdb-uniprot50 | AF-A0A729S941-F1-MODEL\_V4 | 1.0 | 4.163e-15 | 601 | 0.503 | 129 | 64 | 0 | 87 | 215 | 1 | 129 | Baseplate assembly protein | Baseplate assembly protein | | afdb-uniprot50 | AF-A0A0T9KKG0-F1-MODEL\_V4 | 1.0 | 3.814e-16 | 598 | 0.471 | 142 | 75 | 0 | 158 | 299 | 2 | 143 | Baseplate assembly protein | Baseplate assembly protein | | afdb-uniprot50 | AF-A0A844Q921-F1-MODEL\_V4 | 1.0 | 7.698e-23 | 598 | 0.213 | 365 | 203 | 10 | 12 | 298 | 7 | 365 | Baseplate J protein | Baseplate J protein | | afdb-uniprot50 | AF-D6H7Q2-F1-MODEL\_V4 | 1.0 | 7.813e-16 | 597 | 0.414 | 181 | 96 | 4 | 10 | 183 | 6 | 183 | Baseplate\_J domain-containing protein | Baseplate\_J domain-containing protein | | afdb-uniprot50 | AF-A0A553SNF2-F1-MODEL\_V4 | 1.0 | 1.777e-22 | 595 | 0.18 | 372 | 218 | 10 | 12 | 300 | 5 | 372 | Baseplate\_J domain-containing protein | Baseplate\_J domain-containing protein | | afdb-uniprot50 | AF-A0A133NQP8-F1-MODEL\_V4 | 1.0 | 1.762e-18 | 592 | 0.163 | 361 | 211 | 14 | 23 | 298 | 10 | 364 | Baseplate J-like protein | Baseplate J-like protein | | afdb-uniprot50 | AF-R7Z8V1-F1-MODEL\_V4 | 1.0 | 8.402e-22 | 592 | 0.22 | 372 | 198 | 11 | 12 | 298 | 5 | 369 | Baseplate\_J domain-containing protein | Baseplate\_J domain-containing protein | | afdb-uniprot50 | AF-A0A285NE15-F1-MODEL\_V4 | 1.0 | 2.126e-22 | 591 | 0.258 | 364 | 178 | 12 | 19 | 295 | 2 | 360 | Phage-related baseplate assembly protein | Phage-related baseplate assembly protein | | afdb-uniprot50 | AF-A0A7G8VI16-F1-MODEL\_V4 | 1.0 | 2.665e-16 | 589 | 0.535 | 181 | 79 | 3 | 8 | 184 | 3 | 182 | Baseplate J/gp47 family protein | Baseplate J/gp47 family protein | | afdb-uniprot50 | AF-R6IMP8-F1-MODEL\_V4 | 1.0 | 2.7e-22 | 588 | 0.217 | 377 | 206 | 10 | 8 | 300 | 1 | 372 | Putative bacteriophage baseplate assembly protein | Putative bacteriophage baseplate assembly protein | | afdb-uniprot50 | AF-A0A7U9RBG4-F1-MODEL\_V4 | 1.0 | 6.743e-17 | 587 | 0.288 | 194 | 130 | 6 | 111 | 300 | 41 | 230 | Baseplate\_J domain-containing protein | Baseplate\_J domain-containing protein | | afdb-uniprot50 | AF-A0A098F587-F1-MODEL\_V4 | 1.0 | 2.126e-22 | 586 | 0.203 | 373 | 208 | 10 | 12 | 300 | 5 | 372 | Baseplate J-like protein | Baseplate J-like protein | | afdb-uniprot50 | AF-A0A143HC25-F1-MODEL\_V4 | 1.0 | 2.866e-22 | 580 | 0.192 | 369 | 215 | 9 | 12 | 300 | 5 | 370 | Baseplate\_J domain-containing protein | Baseplate\_J domain-containing protein | | afdb-uniprot50 | AF-A0A1A9G8H2-F1-MODEL\_V4 | 1.0 | 3.831e-18 | 580 | 0.158 | 378 | 228 | 11 | 6 | 300 | 3 | 373 | Phage baseplate assembly protein | Phage baseplate assembly protein | | afdb-uniprot50 | AF-A0A162QMQ9-F1-MODEL\_V4 | 1.0 | 3.864e-22 | 580 | 0.215 | 381 | 208 | 10 | 8 | 300 | 1 | 378 | Baseplate J-like protein | Baseplate J-like protein | | afdb-uniprot50 | AF-A0A511Z870-F1-MODEL\_V4 | 1.0 | 2.002e-22 | 578 | 0.233 | 381 | 200 | 12 | 8 | 300 | 1 | 377 | Baseplate\_J domain-containing protein | Baseplate\_J domain-containing protein | | afdb-uniprot50 | AF-A0A2S0JKS5-F1-MODEL\_V4 | 1.0 | 2.543e-22 | 577 | 0.218 | 376 | 200 | 14 | 12 | 300 | 4 | 372 | Baseplate J protein | Baseplate J protein | | afdb-uniprot50 | AF-G9PUJ5-F1-MODEL\_V4 | 1.0 | 1.577e-22 | 577 | 0.242 | 391 | 187 | 14 | 10 | 300 | 4 | 385 | Baseplate\_J domain-containing protein | Baseplate\_J domain-containing protein | | afdb-uniprot50 | AF-A0A2U2DFB6-F1-MODEL\_V4 | 1.0 | 9.923e-16 | 576 | 0.355 | 149 | 96 | 0 | 148 | 296 | 1 | 149 | Baseplate\_J domain-containing protein | Baseplate\_J domain-containing protein | | afdb-uniprot50 | AF-A0A6L9AAM5-F1-MODEL\_V4 | 1.0 | 9.052e-15 | 576 | 0.329 | 161 | 104 | 3 | 141 | 300 | 2 | 159 | Baseplate protein | Baseplate protein | | afdb-uniprot50 | AF-A0A830KCY6-F1-MODEL\_V4 | 1.0 | 2.603e-19 | 574 | 0.236 | 355 | 175 | 9 | 1 | 270 | 1 | 344 | Baseplate assembly protein | Baseplate assembly protein | | afdb-uniprot50 | AF-A0A7X2NKK3-F1-MODEL\_V4 | 1.0 | 7.455e-22 | 572 | 0.214 | 383 | 212 | 8 | 4 | 300 | 2 | 381 | Baseplate J/gp47 family protein | Baseplate J/gp47 family protein | | afdb-uniprot50 | AF-A0A645G9Q1-F1-MODEL\_V4 | 1.0 | 2.829e-16 | 571 | 0.291 | 192 | 130 | 5 | 113 | 300 | 17 | 206 | Baseplate\_J domain-containing protein | Baseplate\_J domain-containing protein | | afdb-uniprot50 | AF-A0A5Z4ZU36-F1-MODEL\_V4 | 1.0 | 7.813e-16 | 571 | 0.271 | 188 | 132 | 4 | 116 | 300 | 25 | 210 | Baseplate J protein | Baseplate J protein | | afdb-uniprot50 | AF-A0A7J6YK41-F1-MODEL\_V4 | 1.0 | 1.058e-17 | 571 | 0.379 | 237 | 124 | 6 | 13 | 249 | 139 | 352 | Baseplate\_J domain-containing protein | Baseplate\_J domain-containing protein | | afdb-uniprot50 | AF-A0A3A9EFF6-F1-MODEL\_V4 | 1.0 | 3.385e-16 | 568 | 0.291 | 192 | 128 | 4 | 116 | 300 | 22 | 212 | Phage baseplate protein | Phage baseplate protein | | afdb-uniprot50 | AF-A0A542AK95-F1-MODEL\_V4 | 1.0 | 7.023e-22 | 568 | 0.216 | 365 | 196 | 10 | 20 | 298 | 3 | 363 | Phage-related baseplate assembly protein | Phage-related baseplate assembly protein | | afdb-uniprot50 | AF-A0A7T2U7T2-F1-MODEL\_V4 | 1.0 | 1.854e-14 | 567 | 0.437 | 135 | 74 | 1 | 166 | 300 | 2 | 134 | Baseplate J/gp47 family protein | Baseplate J/gp47 family protein | | afdb-uniprot50 | AF-B8IDQ2-F1-MODEL\_V4 | 1.0 | 5.637e-17 | 566 | 0.353 | 269 | 138 | 11 | 1 | 241 | 1 | 261 | Baseplate J family protein | Baseplate J family protein | | afdb-uniprot50 | AF-A0A4U8UDU1-F1-MODEL\_V4 | 1.0 | 6.061e-23 | 565 | 0.225 | 368 | 193 | 11 | 15 | 300 | 1 | 358 | Baseplate\_J domain-containing protein | Baseplate\_J domain-containing protein | | afdb-uniprot50 | AF-A0A6L9HNT2-F1-MODEL\_V4 | 1.0 | 1.133e-21 | 564 | 0.212 | 382 | 210 | 9 | 8 | 300 | 3 | 382 | Baseplate J/gp47 family protein | Baseplate J/gp47 family protein | | afdb-uniprot50 | AF-A0A7J0BHX2-F1-MODEL\_V4 | 1.0 | 9.17e-21 | 563 | 0.228 | 372 | 197 | 10 | 13 | 300 | 1 | 366 | Baseplate protein | Baseplate protein | | afdb-uniprot50 | AF-Q4E9A8-F1-MODEL\_V4 | 1.0 | 1.155e-16 | 562 | 0.285 | 210 | 130 | 5 | 85 | 294 | 94 | 283 | Baseplate\_J domain-containing protein | Baseplate\_J domain-containing protein | | afdb-uniprot50 | AF-A0A496P7D6-F1-MODEL\_V4 | 1.0 | 3.23e-22 | 562 | 0.232 | 365 | 192 | 10 | 10 | 290 | 3 | 363 | Baseplate J/gp47 family protein | Baseplate J/gp47 family protein | | afdb-uniprot50 | AF-A0A7J5WEF0-F1-MODEL\_V4 | 1.0 | 1.202e-21 | 562 | 0.237 | 379 | 192 | 11 | 10 | 300 | 5 | 374 | Baseplate J family | Baseplate J family | | afdb-uniprot50 | AF-A0A1Y4WAU9-F1-MODEL\_V4 | 1.0 | 3.742e-21 | 562 | 0.221 | 383 | 207 | 10 | 7 | 300 | 5 | 385 | Phage tail protein | Phage tail protein | | afdb-uniprot50 | AF-A0A1M7LUN9-F1-MODEL\_V4 | 1.0 | 3.525e-21 | 561 | 0.195 | 378 | 215 | 8 | 10 | 300 | 8 | 383 | Phage-related baseplate assembly protein | Phage-related baseplate assembly protein | | afdb-uniprot50 | AF-V7ZEQ2-F1-MODEL\_V4 | 1.0 | 2.431e-15 | 560 | 0.436 | 174 | 94 | 3 | 1 | 174 | 1 | 170 | Phage-related baseplate assembly protein | Phage-related baseplate assembly protein | | afdb-uniprot50 | AF-A0A150KS38-F1-MODEL\_V4 | 1.0 | 3.128e-21 | 559 | 0.193 | 373 | 212 | 9 | 12 | 300 | 5 | 372 | Baseplate\_J domain-containing protein | Baseplate\_J domain-containing protein | | afdb-uniprot50 | AF-A0A6N7B8U0-F1-MODEL\_V4 | 1.0 | 1.621e-21 | 555 | 0.204 | 396 | 200 | 13 | 13 | 300 | 4 | 392 | Baseplate\_J domain-containing protein | Baseplate\_J domain-containing protein | | afdb-uniprot50 | AF-A0A1X7M9Q2-F1-MODEL\_V4 | 1.0 | 2.817e-14 | 553 | 0.454 | 121 | 66 | 0 | 179 | 299 | 1 | 121 | Baseplate assembly protein J | Baseplate assembly protein J | | afdb-uniprot50 | AF-A0A4U8S342-F1-MODEL\_V4 | 1.0 | 1.058e-17 | 550 | 0.16 | 356 | 211 | 12 | 13 | 294 | 1 | 342 | Baseplate\_J domain-containing protein | Baseplate\_J domain-containing protein | | afdb-uniprot50 | AF-A0A0M1HFG5-F1-MODEL\_V4 | 1.0 | 2.909e-15 | 549 | 0.381 | 173 | 98 | 3 | 55 | 226 | 2 | 166 | Baseplate assembly protein | Baseplate assembly protein | | afdb-uniprot50 | AF-A0A7X5CJX7-F1-MODEL\_V4 | 1.0 | 5.685e-21 | 547 | 0.214 | 383 | 209 | 10 | 7 | 300 | 5 | 384 | Baseplate J/gp47 family protein | Baseplate J/gp47 family protein | | afdb-uniprot50 | AF-F0CB87-F1-MODEL\_V4 | 1.0 | 1.854e-14 | 547 | 0.503 | 163 | 77 | 3 | 1 | 163 | 1 | 159 | Alpha-L-arabinofuranosidase | Alpha-L-arabinofuranosidase | | afdb-uniprot50 | AF-A0A714TMI2-F1-MODEL\_V4 | 1.0 | 7.128e-15 | 546 | 0.518 | 164 | 72 | 2 | 6 | 162 | 2 | 165 | Baseplate assembly protein | Baseplate assembly protein | | afdb-uniprot50 | AF-V7IAG1-F1-MODEL\_V4 | 1.0 | 3.694e-15 | 546 | 0.331 | 172 | 104 | 4 | 132 | 295 | 3 | 171 | Baseplate\_J domain-containing protein | Baseplate\_J domain-containing protein | | afdb-uniprot50 | AF-A0A744CQV0-F1-MODEL\_V4 | 1.0 | 7.392e-18 | 546 | 0.204 | 313 | 163 | 9 | 72 | 300 | 4 | 314 | Baseplate J/gp47 family protein | Baseplate J/gp47 family protein | | afdb-uniprot50 | AF-A0A5C8BGL4-F1-MODEL\_V4 | 1.0 | 4.084e-20 | 546 | 0.181 | 374 | 215 | 12 | 10 | 298 | 5 | 372 | Baseplate\_J domain-containing protein | Baseplate\_J domain-containing protein | | afdb-uniprot50 | AF-E5VKI5-F1-MODEL\_V4 | 1.0 | 9.734e-21 | 546 | 0.196 | 382 | 217 | 10 | 7 | 300 | 5 | 384 | Baseplate J-like protein | Baseplate J-like protein | | afdb-uniprot50 | AF-A0A0T9LR98-F1-MODEL\_V4 | 1.0 | 1.053e-15 | 545 | 0.378 | 193 | 109 | 3 | 6 | 190 | 2 | 191 | Baseplate assembly protein | Baseplate assembly protein | | afdb-uniprot50 | AF-F8KPM6-F1-MODEL\_V4 | 1.0 | 1.473e-18 | 544 | 0.193 | 361 | 201 | 14 | 14 | 298 | 1 | 347 | Baseplate assembly protein J, putative | Baseplate assembly protein J, putative | | afdb-uniprot50 | AF-A0A3R6RHW9-F1-MODEL\_V4 | 1.0 | 1.557e-16 | 544 | 0.143 | 382 | 234 | 11 | 10 | 300 | 8 | 387 | Baseplate\_J domain-containing protein | Baseplate\_J domain-containing protein | | afdb-uniprot50 | AF-A0A261QMI5-F1-MODEL\_V4 | 1.0 | 2.158e-15 | 543 | 0.336 | 196 | 117 | 6 | 12 | 204 | 4 | 189 | Phage tail protein | Phage tail protein | | afdb-uniprot50 | AF-A0A6N3FJC1-F1-MODEL\_V4 | 1.0 | 2.947e-21 | 543 | 0.205 | 390 | 211 | 12 | 4 | 300 | 11 | 394 | Baseplate J-like protein | Baseplate J-like protein | | afdb-uniprot50 | AF-A0A385Q5A3-F1-MODEL\_V4 | 1.0 | 4.217e-21 | 542 | 0.174 | 379 | 223 | 10 | 10 | 300 | 2 | 378 | Baseplate J/gp47 family protein | Baseplate J/gp47 family protein | | afdb-uniprot50 | AF-A0A416B7S2-F1-MODEL\_V4 | 1.0 | 2.032e-15 | 541 | 0.313 | 172 | 113 | 4 | 132 | 300 | 5 | 174 | Baseplate\_J domain-containing protein | Baseplate\_J domain-containing protein | | afdb-uniprot50 | AF-A0A377BF30-F1-MODEL\_V4 | 1.0 | 3.188e-16 | 541 | 0.265 | 211 | 137 | 6 | 106 | 300 | 39 | 247 | Phage-related baseplate assembly protein J | Phage-related baseplate assembly protein J | | afdb-uniprot50 | AF-A0A379YH24-F1-MODEL\_V4 | 1.0 | 2.218e-14 | 540 | 0.555 | 144 | 57 | 1 | 8 | 144 | 4 | 147 | Baseplate J-like protein | Baseplate J-like protein | | afdb-uniprot50 | AF-A0A369Y1S1-F1-MODEL\_V4 | 1.0 | 2.688e-20 | 538 | 0.19 | 363 | 208 | 8 | 20 | 300 | 7 | 365 | Baseplate\_J domain-containing protein | Baseplate\_J domain-containing protein | | afdb-uniprot50 | AF-A0A0Q3ETP3-F1-MODEL\_V4 | 1.0 | 3.71e-17 | 537 | 0.186 | 381 | 211 | 15 | 1 | 294 | 1 | 369 | Baseplate\_J domain-containing protein | Baseplate\_J domain-containing protein | | afdb-uniprot50 | AF-A0A285M2R0-F1-MODEL\_V4 | 1.0 | 9.387e-18 | 537 | 0.172 | 388 | 222 | 13 | 1 | 300 | 5 | 381 | Phage-related baseplate assembly protein | Phage-related baseplate assembly protein | | afdb-uniprot50 | AF-A0A2N3D694-F1-MODEL\_V4 | 1.0 | 1.46e-14 | 536 | 0.537 | 162 | 71 | 1 | 1 | 162 | 7 | 164 | Baseplate assembly protein | Baseplate assembly protein | | afdb-uniprot50 | AF-U2EPS9-F1-MODEL\_V4 | 1.0 | 4.98e-15 | 530 | 0.276 | 181 | 116 | 8 | 122 | 300 | 2 | 169 | Baseplate assembly protein J, putative | Baseplate assembly protein J, putative | | afdb-uniprot50 | AF-A0A810Q3S1-F1-MODEL\_V4 | 1.0 | 4.084e-20 | 530 | 0.212 | 381 | 207 | 11 | 10 | 300 | 7 | 384 | Uncharacterized protein | Uncharacterized protein | | afdb-uniprot50 | AF-B3QTJ3-F1-MODEL\_V4 | 1.0 | 2.776e-21 | 529 | 0.22 | 381 | 193 | 13 | 12 | 294 | 3 | 377 | Baseplate J family protein | Baseplate J family protein | | afdb-uniprot50 | AF-A0A6V7E4B2-F1-MODEL\_V4 | 1.0 | 3.694e-15 | 529 | 0.456 | 186 | 88 | 4 | 1 | 179 | 1 | 180 | Alpha-L-AF\_C domain-containing protein | Alpha-L-AF\_C domain-containing protein | | afdb-uniprot50 | AF-E3HBK6-F1-MODEL\_V4 | 1.0 | 4.199e-19 | 528 | 0.153 | 371 | 220 | 11 | 20 | 300 | 7 | 373 | Baseplate J family protein | Baseplate J family protein | | afdb-uniprot50 | AF-A0A2X3LRF3-F1-MODEL\_V4 | 1.0 | 7.567e-15 | 527 | 0.327 | 162 | 105 | 4 | 133 | 292 | 1 | 160 | Tail protein I | Tail protein I | | afdb-uniprot50 | AF-A0A2E3R244-F1-MODEL\_V4 | 1.0 | 6.587e-20 | 527 | 0.224 | 370 | 195 | 13 | 13 | 298 | 1 | 362 | Baseplate\_J domain-containing protein | Baseplate\_J domain-containing protein | | afdb-uniprot50 | AF-A0A1K1LB82-F1-MODEL\_V4 | 1.0 | 2.167e-17 | 527 | 0.176 | 391 | 208 | 20 | 15 | 299 | 1 | 383 | Baseplate assembly protein, putative | Baseplate assembly protein, putative | | afdb-uniprot50 | AF-A0A7U9S6B3-F1-MODEL\_V4 | 1.0 | 2.355e-14 | 526 | 0.269 | 171 | 118 | 5 | 133 | 298 | 5 | 173 | Baseplate\_J domain-containing protein | Baseplate\_J domain-containing protein | | afdb-uniprot50 | AF-A0A416HFK7-F1-MODEL\_V4 | 1.0 | 2.921e-17 | 526 | 0.137 | 378 | 223 | 12 | 17 | 300 | 9 | 377 | Baseplate\_J domain-containing protein | Baseplate\_J domain-containing protein | | afdb-uniprot50 | AF-A0A2X2UEX9-F1-MODEL\_V4 | 1.0 | 7.423e-20 | 526 | 0.195 | 378 | 213 | 10 | 12 | 300 | 4 | 379 | Baseplate J family protein | Baseplate J family protein | | afdb-uniprot50 | AF-A0A1G3LAY8-F1-MODEL\_V4 | 1.0 | 1.301e-16 | 525 | 0.159 | 377 | 216 | 14 | 18 | 300 | 2 | 371 | Baseplate\_J domain-containing protein | Baseplate\_J domain-containing protein | | afdb-uniprot50 | AF-A0A1W2CW87-F1-MODEL\_V4 | 1.0 | 4.865e-18 | 525 | 0.171 | 403 | 216 | 14 | 6 | 300 | 2 | 394 | Phage-related baseplate assembly protein | Phage-related baseplate assembly protein | | afdb-uniprot50 | AF-A0A1Q6JPW8-F1-MODEL\_V4 | 1.0 | 2.688e-20 | 524 | 0.218 | 366 | 197 | 8 | 22 | 300 | 1 | 364 | Baseplate\_J domain-containing protein | Baseplate\_J domain-containing protein | | afdb-uniprot50 | AF-A0A7U9RWZ8-F1-MODEL\_V4 | 1.0 | 2.375e-18 | 523 | 0.19 | 325 | 171 | 11 | 64 | 300 | 2 | 322 | Baseplate\_J domain-containing protein | Baseplate\_J domain-containing protein | | afdb-uniprot50 | AF-A0A1H8P6M7-F1-MODEL\_V4 | 1.0 | 1.692e-13 | 520 | 0.46 | 150 | 74 | 1 | 8 | 150 | 4 | 153 | Uncharacterized protein | Uncharacterized protein | | afdb-uniprot50 | AF-A0A4R2GWK6-F1-MODEL\_V4 | 1.0 | 9.651e-17 | 515 | 0.17 | 393 | 217 | 14 | 1 | 295 | 6 | 387 | Phage-related baseplate assembly protein | Phage-related baseplate assembly protein | | afdb-uniprot50 | AF-A0A4P0TK43-F1-MODEL\_V4 | 1.0 | 8.767e-14 | 514 | 0.452 | 146 | 80 | 0 | 116 | 261 | 6 | 151 | Putative phage baseplate assembly protein | Putative phage baseplate assembly protein | | afdb-uniprot50 | AF-A0A841I6H3-F1-MODEL\_V4 | 1.0 | 2.728e-13 | 512 | 0.5 | 148 | 67 | 1 | 8 | 148 | 4 | 151 | Phage-related baseplate assembly protein | Phage-related baseplate assembly protein | | afdb-uniprot50 | AF-A0A2V2GNU0-F1-MODEL\_V4 | 1.0 | 1.128e-19 | 510 | 0.195 | 378 | 212 | 8 | 12 | 300 | 9 | 383 | Phage tail protein | Phage tail protein | | afdb-uniprot50 | AF-A0A7C7ANJ6-F1-MODEL\_V4 | 1.0 | 1.301e-16 | 508 | 0.16 | 362 | 210 | 13 | 21 | 296 | 1 | 354 | Baseplate J/gp47 family protein | Baseplate J/gp47 family protein | | afdb-uniprot50 | AF-A0A6I5ZPJ9-F1-MODEL\_V4 | 1.0 | 8.564e-17 | 508 | 0.165 | 381 | 211 | 16 | 20 | 300 | 8 | 381 | Baseplate J-like protein | Baseplate J-like protein | | afdb-uniprot50 | AF-A0A3A9GRB5-F1-MODEL\_V4 | 1.0 | 1.479e-20 | 508 | 0.196 | 377 | 208 | 12 | 12 | 300 | 12 | 381 | Baseplate J/gp47 family protein | Baseplate J/gp47 family protein | | afdb-uniprot50 | AF-A0A1B1NTC2-F1-MODEL\_V4 | 1.0 | 1.57e-20 | 506 | 0.2 | 379 | 207 | 14 | 10 | 300 | 5 | 375 | Baseplate\_J domain-containing protein | Baseplate\_J domain-containing protein | | afdb-uniprot50 | AF-A0A7C9M632-F1-MODEL\_V4 | 1.0 | 1.667e-20 | 505 | 0.212 | 372 | 194 | 12 | 20 | 298 | 5 | 370 | Baseplate J/gp47 family protein | Baseplate J/gp47 family protein | | afdb-uniprot50 | AF-A0A3N6RV94-F1-MODEL\_V4 | 1.0 | 4.336e-20 | 505 | 0.302 | 367 | 154 | 12 | 6 | 300 | 2 | 338 | Phage tail protein I | Phage tail protein I | | afdb-uniprot50 | AF-D1P8F2-F1-MODEL\_V4 | 1.0 | 2.5e-14 | 501 | 0.481 | 158 | 79 | 1 | 25 | 179 | 2 | 159 | Baseplate J-like protein | Baseplate J-like protein | | afdb-uniprot50 | AF-A0A2V2FW36-F1-MODEL\_V4 | 1.0 | 2.041e-17 | 501 | 0.169 | 461 | 198 | 16 | 15 | 300 | 10 | 460 | Uncharacterized protein | Uncharacterized protein | | afdb-uniprot50 | AF-A0A3G2Q4N8-F1-MODEL\_V4 | 1.0 | 1.349e-19 | 499 | 0.188 | 381 | 213 | 13 | 12 | 300 | 8 | 384 | Phage baseplate protein | Phage baseplate protein | | afdb-uniprot50 | AF-A0A4V3WD86-F1-MODEL\_V4 | 1.0 | 1.923e-17 | 494 | 0.191 | 376 | 202 | 16 | 12 | 296 | 8 | 372 | Baseplate J protein | Baseplate J protein | | afdb-uniprot50 | AF-A0A2C6MEH4-F1-MODEL\_V4 | 1.0 | 1.652e-16 | 493 | 0.134 | 373 | 224 | 12 | 17 | 296 | 9 | 375 | Baseplate J protein | Baseplate J protein | | afdb-uniprot50 | AF-A0A2A7AEU6-F1-MODEL\_V4 | 1.0 | 1.652e-16 | 493 | 0.151 | 384 | 227 | 12 | 13 | 300 | 1 | 381 | Phage baseplate protein | Phage baseplate protein | | afdb-uniprot50 | AF-A0A323U4H9-F1-MODEL\_V4 | 1.0 | 1.26e-15 | 492 | 0.175 | 377 | 211 | 15 | 13 | 300 | 1 | 366 | Baseplate J protein | Baseplate J protein | | afdb-uniprot50 | AF-A0A373C6W2-F1-MODEL\_V4 | 1.0 | 5.023e-19 | 492 | 0.212 | 382 | 206 | 13 | 11 | 300 | 2 | 380 | Baseplate J/gp47 family protein | Baseplate J/gp47 family protein | | afdb-uniprot50 | AF-A0A2N8HQF9-F1-MODEL\_V4 | 1.0 | 5.023e-19 | 489 | 0.204 | 391 | 199 | 17 | 12 | 298 | 2 | 384 | Baseplate\_J domain-containing protein | Baseplate\_J domain-containing protein | | afdb-uniprot50 | AF-A0A413J6B9-F1-MODEL\_V4 | 1.0 | 9.131e-19 | 489 | 0.198 | 378 | 212 | 11 | 10 | 298 | 8 | 383 | Phage tail protein | Phage tail protein | | afdb-uniprot50 | AF-A0A0T9TB71-F1-MODEL\_V4 | 1.0 | 7.328e-14 | 488 | 0.464 | 168 | 75 | 2 | 6 | 158 | 2 | 169 | Regulator of late gene expression | Regulator of late gene expression | | afdb-uniprot50 | AF-A0A3D9PVP8-F1-MODEL\_V4 | 1.0 | 1.6e-15 | 488 | 0.162 | 364 | 211 | 11 | 13 | 292 | 1 | 354 | Putative phage protein gp47/JayE | Putative phage protein gp47/JayE | | afdb-uniprot50 | AF-A0A0K6A654-F1-MODEL\_V4 | 1.0 | 6.298e-13 | 487 | 0.395 | 134 | 81 | 0 | 166 | 299 | 2 | 135 | Baseplate assembly protein J | Baseplate assembly protein J | | afdb-uniprot50 | AF-A0A824DTY0-F1-MODEL\_V4 | 1.0 | 3.922e-15 | 487 | 0.246 | 203 | 119 | 5 | 116 | 300 | 16 | 202 | Baseplate assembly protein | Baseplate assembly protein | | afdb-uniprot50 | AF-A0A3S5XQX9-F1-MODEL\_V4 | 1.0 | 2.489e-12 | 484 | 0.554 | 128 | 50 | 1 | 6 | 126 | 3 | 130 | Baseplate J-like protein | Baseplate J-like protein | | afdb-uniprot50 | AF-A0A366IJ99-F1-MODEL\_V4 | 1.0 | 6.475e-12 | 482 | 0.484 | 99 | 51 | 0 | 201 | 299 | 1 | 99 | Uncharacterized protein | Uncharacterized protein | | afdb-uniprot50 | AF-A0A352X1L5-F1-MODEL\_V4 | 1.0 | 5.795e-16 | 481 | 0.163 | 380 | 214 | 14 | 1 | 295 | 1 | 361 | Baseplate\_J domain-containing protein | Baseplate\_J domain-containing protein | | afdb-uniprot50 | AF-A0A418GPV5-F1-MODEL\_V4 | 1.0 | 9.88e-14 | 480 | 0.348 | 175 | 100 | 3 | 113 | 275 | 68 | 240 | Baseplate J/gp47 family protein | Baseplate J/gp47 family protein | | afdb-uniprot50 | AF-A0A1H8DLC4-F1-MODEL\_V4 | 1.0 | 1.301e-16 | 478 | 0.176 | 368 | 204 | 15 | 21 | 300 | 1 | 357 | Uncharacterized phage protein gp47/JayE | Uncharacterized phage protein gp47/JayE | | afdb-uniprot50 | AF-A0A080HTY9-F1-MODEL\_V4 | 1.0 | 1.016e-12 | 477 | 0.355 | 135 | 87 | 0 | 161 | 295 | 1 | 135 | Baseplate J-like family protein | Baseplate J-like family protein | | afdb-uniprot50 | AF-A0A2G2D437-F1-MODEL\_V4 | 1.0 | 2.237e-18 | 477 | 0.164 | 383 | 231 | 11 | 1 | 300 | 4 | 380 | Phage baseplate assembly protein | Phage baseplate assembly protein | | afdb-uniprot50 | AF-W4RXX9-F1-MODEL\_V4 | 1.0 | 6.686e-13 | 475 | 0.532 | 139 | 62 | 2 | 1 | 139 | 1 | 136 | Phage-related baseplate assembly protein | Phage-related baseplate assembly protein | | afdb-uniprot50 | AF-A0A7I9SRD3-F1-MODEL\_V4 | 1.0 | 7.159e-17 | 474 | 0.205 | 331 | 149 | 12 | 67 | 300 | 2 | 315 | Baseplate assembly protein | Baseplate assembly protein | | afdb-uniprot50 | AF-A0A268TIH4-F1-MODEL\_V4 | 1.0 | 2.099e-16 | 474 | 0.171 | 356 | 200 | 14 | 16 | 294 | 7 | 344 | Baseplate\_J domain-containing protein | Baseplate\_J domain-containing protein | | afdb-uniprot50 | AF-A0A7V5GUY7-F1-MODEL\_V4 | 1.0 | 7.78e-14 | 473 | 0.254 | 185 | 128 | 6 | 116 | 296 | 4 | 182 | Baseplate\_J domain-containing protein | Baseplate\_J domain-containing protein | | afdb-uniprot50 | AF-A0A842IZ09-F1-MODEL\_V4 | 1.0 | 5.264e-13 | 472 | 0.225 | 186 | 138 | 5 | 115 | 298 | 137 | 318 | Baseplate J/gp47 family protein | Baseplate J/gp47 family protein | | afdb-uniprot50 | AF-W4RXF7-F1-MODEL\_V4 | 1.0 | 3.678e-13 | 471 | 0.484 | 163 | 78 | 3 | 1 | 163 | 1 | 157 | Phage-related baseplate assembly protein | Phage-related baseplate assembly protein | | afdb-uniprot50 | AF-W4SEG4-F1-MODEL\_V4 | 1.0 | 4.015e-12 | 470 | 0.514 | 138 | 64 | 2 | 1 | 138 | 1 | 135 | Uncharacterized protein | Uncharacterized protein | | afdb-uniprot50 | AF-A0A5U3E105-F1-MODEL\_V4 | 1.0 | 5.933e-13 | 469 | 0.51 | 145 | 64 | 1 | 8 | 145 | 1 | 145 | Baseplate assembly protein | Baseplate assembly protein | | afdb-uniprot50 | AF-A0A511NB39-F1-MODEL\_V4 | 1.0 | 6.715e-15 | 468 | 0.155 | 372 | 213 | 15 | 14 | 295 | 3 | 363 | Baseplate\_J domain-containing protein | Baseplate\_J domain-containing protein | | afdb-uniprot50 | AF-A0A767SN15-F1-MODEL\_V4 | 1.0 | 4.651e-11 | 466 | 0.44 | 93 | 52 | 0 | 207 | 299 | 1 | 93 | Baseplate assembly protein | Baseplate assembly protein | | afdb-uniprot50 | AF-A0A4D7AZ74-F1-MODEL\_V4 | 1.0 | 9.88e-14 | 464 | 0.214 | 196 | 140 | 7 | 110 | 296 | 100 | 290 | Baseplate\_J domain-containing protein | Baseplate\_J domain-containing protein | | afdb-uniprot50 | AF-A0A4S0JS99-F1-MODEL\_V4 | 1.0 | 3.356e-12 | 463 | 0.532 | 122 | 55 | 1 | 66 | 187 | 2 | 121 | Baseplate assembly protein | Baseplate assembly protein | | afdb-uniprot50 | AF-D8A2F7-F1-MODEL\_V4 | 1.0 | 1.699e-15 | 463 | 0.265 | 286 | 122 | 6 | 14 | 215 | 1 | 282 | Baseplate J-like protein | Baseplate J-like protein | | afdb-uniprot50 | AF-A0A3S4VYP8-F1-MODEL\_V4 | 1.0 | 5.795e-16 | 463 | 0.254 | 299 | 139 | 7 | 25 | 243 | 2 | 296 | Uncharacterized homolog of phage Mu protein gp47 | Uncharacterized homolog of phage Mu protein gp47 | | afdb-uniprot50 | AF-A0A1T5KWL5-F1-MODEL\_V4 | 1.0 | 1.26e-15 | 462 | 0.134 | 379 | 224 | 13 | 17 | 299 | 8 | 378 | Uncharacterized phage protein gp47/JayE | Uncharacterized phage protein gp47/JayE | | afdb-uniprot50 | AF-W4RY38-F1-MODEL\_V4 | 1.0 | 1.906e-13 | 461 | 0.469 | 162 | 81 | 3 | 1 | 162 | 1 | 157 | Phage-related baseplate assembly protein | Phage-related baseplate assembly protein | | afdb-uniprot50 | AF-Q5F8P1-F1-MODEL\_V4 | 1.0 | 4.401e-13 | 459 | 0.431 | 169 | 79 | 5 | 1 | 162 | 1 | 159 | Baseplate protein | Baseplate protein | | afdb-uniprot50 | AF-A0A842J386-F1-MODEL\_V4 | 1.0 | 1.369e-12 | 457 | 0.227 | 185 | 137 | 4 | 116 | 298 | 87 | 267 | Baseplate J/gp47 family protein | Baseplate J/gp47 family protein | | afdb-uniprot50 | AF-A0A846JQB4-F1-MODEL\_V4 | 1.0 | 7.813e-16 | 456 | 0.148 | 371 | 216 | 18 | 12 | 294 | 3 | 361 | Baseplate J/gp47 family protein | Baseplate J/gp47 family protein | | afdb-uniprot50 | AF-A0A2T4JNF5-F1-MODEL\_V4 | 1.0 | 1.326e-11 | 455 | 0.43 | 123 | 70 | 0 | 173 | 295 | 1 | 123 | Baseplate assembly protein | Baseplate assembly protein | | afdb-uniprot50 | AF-A0A3G2R4D9-F1-MODEL\_V4 | 1.0 | 1.426e-17 | 455 | 0.221 | 325 | 164 | 9 | 61 | 298 | 6 | 328 | Baseplate J/gp47 family protein | Baseplate J/gp47 family protein | | afdb-uniprot50 | AF-A0A1L5KT79-F1-MODEL\_V4 | 1.0 | 2.805e-12 | 454 | 0.309 | 152 | 97 | 4 | 154 | 300 | 1 | 149 | Baseplate J-like protein | Baseplate J-like protein | | afdb-uniprot50 | AF-A0A3G4W4D3-F1-MODEL\_V4 | 1.0 | 5.958e-15 | 454 | 0.185 | 431 | 187 | 16 | 22 | 296 | 13 | 435 | Baseplate J-like protein | Baseplate J-like protein | | afdb-uniprot50 | AF-A0A0J9B6B6-F1-MODEL\_V4 | 1.0 | 1.408e-11 | 453 | 0.534 | 131 | 50 | 2 | 7 | 128 | 3 | 131 | Uncharacterized protein | Uncharacterized protein | | afdb-uniprot50 | AF-A0A1M3AIG6-F1-MODEL\_V4 | 1.0 | 7.998e-13 | 453 | 0.216 | 194 | 136 | 7 | 115 | 296 | 1 | 190 | Uncharacterized protein | Uncharacterized protein | | afdb-uniprot50 | AF-A0A695HWH1-F1-MODEL\_V4 | 1.0 | 2.218e-14 | 452 | 0.241 | 203 | 120 | 5 | 116 | 300 | 68 | 254 | Baseplate assembly protein | Baseplate assembly protein | | afdb-uniprot50 | AF-E1SEF6-F1-MODEL\_V4 | 1.0 | 8.767e-14 | 447 | 0.493 | 166 | 70 | 4 | 6 | 163 | 2 | 161 | Baseplate assembly protein J (GpJ) | Baseplate assembly protein J (GpJ) | | afdb-uniprot50 | AF-A0A1M6M9S1-F1-MODEL\_V4 | 1.0 | 5.483e-18 | 447 | 0.173 | 357 | 202 | 12 | 21 | 292 | 4 | 352 | Uncharacterized phage protein gp47/JayE | Uncharacterized phage protein gp47/JayE | | afdb-uniprot50 | AF-A0A5C7PTQ6-F1-MODEL\_V4 | 1.0 | 3.922e-15 | 446 | 0.156 | 389 | 217 | 19 | 12 | 300 | 4 | 381 | Baseplate\_J domain-containing protein | Baseplate\_J domain-containing protein | | afdb-uniprot50 | AF-A0A3D3Y478-F1-MODEL\_V4 | 1.0 | 3.593e-16 | 445 | 0.163 | 361 | 205 | 13 | 22 | 295 | 1 | 351 | Baseplate protein J | Baseplate protein J | | afdb-uniprot50 | AF-A0A7Y7LP48-F1-MODEL\_V4 | 1.0 | 1.466e-16 | 445 | 0.175 | 364 | 203 | 14 | 13 | 292 | 1 | 351 | Putative phage protein gp47/JayE | Putative phage protein gp47/JayE | | afdb-uniprot50 | AF-A0A5D4RZE4-F1-MODEL\_V4 | 1.0 | 1.053e-15 | 445 | 0.132 | 376 | 213 | 16 | 20 | 295 | 4 | 366 | Baseplate\_J domain-containing protein | Baseplate\_J domain-containing protein | | afdb-uniprot50 | AF-A0A345J3J0-F1-MODEL\_V4 | 1.0 | 1.563e-18 | 444 | 0.201 | 377 | 209 | 15 | 4 | 300 | 2 | 366 | Phage baseplate assembly protein J | Phage baseplate assembly protein J | | afdb-uniprot50 | AF-A0A1E3UGI8-F1-MODEL\_V4 | 1.0 | 3.593e-16 | 444 | 0.163 | 372 | 212 | 11 | 22 | 299 | 14 | 380 | Baseplate J protein | Baseplate J protein | | afdb-uniprot50 | AF-A0A074LIS1-F1-MODEL\_V4 | 1.0 | 3.016e-18 | 442 | 0.187 | 357 | 196 | 14 | 22 | 293 | 3 | 350 | Baseplate\_J domain-containing protein | Baseplate\_J domain-containing protein | | afdb-uniprot50 | AF-A0A7U6KQX5-F1-MODEL\_V4 | 1.0 | 1.543e-12 | 441 | 0.445 | 157 | 77 | 3 | 25 | 179 | 2 | 150 | Baseplate\_J domain-containing protein | Baseplate\_J domain-containing protein | | afdb-uniprot50 | AF-A0A521CK67-F1-MODEL\_V4 | 1.0 | 2.654e-14 | 440 | 0.137 | 465 | 213 | 14 | 1 | 295 | 1 | 447 | Baseplate J-like protein | Baseplate J-like protein | | afdb-uniprot50 | AF-A0A7X6FRN3-F1-MODEL\_V4 | 1.0 | 2.281e-13 | 438 | 0.346 | 202 | 112 | 5 | 1 | 198 | 4 | 189 | Baseplate\_J domain-containing protein | Baseplate\_J domain-containing protein | | afdb-uniprot50 | AF-A0A376J4L9-F1-MODEL\_V4 | 1.0 | 2.642e-12 | 437 | 0.412 | 155 | 84 | 2 | 31 | 182 | 1 | 151 | Baseplate assembly protein J | Baseplate assembly protein J | | afdb-uniprot50 | AF-A0A316MUY0-F1-MODEL\_V4 | 1.0 | 4.438e-17 | 437 | 0.152 | 360 | 208 | 14 | 22 | 295 | 1 | 349 | Baseplate protein J | Baseplate protein J | | afdb-uniprot50 | AF-A0A7W6S2L4-F1-MODEL\_V4 | 1.0 | 5.099e-12 | 436 | 0.467 | 122 | 64 | 1 | 1 | 122 | 1 | 121 | Phage-related baseplate assembly protein | Phage-related baseplate assembly protein | | afdb-uniprot50 | AF-A0A379ZTU5-F1-MODEL\_V4 | 1.0 | 3.356e-12 | 436 | 0.514 | 138 | 60 | 1 | 7 | 137 | 3 | 140 | Phage baseplate assembly protein J | Phage baseplate assembly protein J | | afdb-uniprot50 | AF-E3PRX8-F1-MODEL\_V4 | 1.0 | 9.347e-16 | 434 | 0.129 | 364 | 222 | 11 | 19 | 294 | 3 | 359 | Baseplate\_J domain-containing protein | Baseplate\_J domain-containing protein | | afdb-uniprot50 | AF-A0A1G3KNM2-F1-MODEL\_V4 | 1.0 | 5.483e-18 | 434 | 0.172 | 376 | 207 | 13 | 18 | 300 | 2 | 366 | Baseplate\_J domain-containing protein | Baseplate\_J domain-containing protein | | afdb-uniprot50 | AF-R6BFZ4-F1-MODEL\_V4 | 1.0 | 2.581e-15 | 433 | 0.126 | 355 | 216 | 11 | 23 | 293 | 1 | 345 | Baseplate J-like protein | Baseplate J-like protein | | afdb-uniprot50 | AF-A0A357B384-F1-MODEL\_V4 | 1.0 | 9.387e-18 | 432 | 0.194 | 359 | 192 | 12 | 21 | 293 | 1 | 348 | Phage tail protein | Phage tail protein | | afdb-uniprot50 | AF-A0A7Z8LKE5-F1-MODEL\_V4 | 1.0 | 1.02e-14 | 432 | 0.166 | 378 | 204 | 16 | 20 | 295 | 3 | 371 | Putative phage protein gp47/JayE | Putative phage protein gp47/JayE | | afdb-uniprot50 | AF-A0A843AG30-F1-MODEL\_V4 | 1.0 | 9.651e-17 | 431 | 0.158 | 373 | 211 | 11 | 22 | 295 | 13 | 381 | Baseplate J/gp47 family protein | Baseplate J/gp47 family protein | | afdb-uniprot50 | AF-A0A1A9WW65-F1-MODEL\_V4 | 1.0 | 1.788e-11 | 427 | 0.323 | 142 | 92 | 3 | 158 | 298 | 1 | 139 | Baseplate\_J domain-containing protein | Baseplate\_J domain-containing protein | | afdb-uniprot50 | AF-A0A2A2K3F7-F1-MODEL\_V4 | 1.0 | 9.528e-11 | 427 | 0.504 | 123 | 60 | 1 | 8 | 129 | 4 | 126 | Uncharacterized protein | Uncharacterized protein | | afdb-uniprot50 | AF-A0A6H1Z9D6-F1-MODEL\_V4 | 1.0 | 9.88e-14 | 427 | 0.152 | 375 | 204 | 16 | 23 | 296 | 6 | 367 | Putative baseplate protein | Putative baseplate protein | | afdb-uniprot50 | AF-Q9K5D9-F1-MODEL\_V4 | 1.0 | 1.375e-14 | 425 | 0.24 | 325 | 154 | 7 | 1 | 239 | 1 | 318 | Putative base plate assembly protein J | Putative base plate assembly protein J | | afdb-uniprot50 | AF-J5WM03-F1-MODEL\_V4 | 1.0 | 2.281e-13 | 424 | 0.113 | 388 | 218 | 14 | 20 | 295 | 10 | 383 | Baseplate J-like protein | Baseplate J-like protein | | afdb-uniprot50 | AF-A0A7X6FPD5-F1-MODEL\_V4 | 1.0 | 1.495e-11 | 422 | 0.376 | 117 | 72 | 1 | 184 | 300 | 2 | 117 | Baseplate\_J domain-containing protein | Baseplate\_J domain-containing protein | | afdb-uniprot50 | AF-A0A0A0GWF9-F1-MODEL\_V4 | 1.0 | 1.074e-10 | 421 | 0.495 | 123 | 55 | 1 | 8 | 123 | 4 | 126 | Baseplate assembly protein J | Baseplate assembly protein J | | afdb-uniprot50 | AF-A6NQ14-F1-MODEL\_V4 | 1.0 | 9.651e-17 | 421 | 0.17 | 364 | 206 | 13 | 21 | 300 | 1 | 352 | Baseplate J-like protein | Baseplate J-like protein | | afdb-uniprot50 | AF-A0A1E8PKJ1-F1-MODEL\_V4 | 1.0 | 9.923e-16 | 421 | 0.164 | 364 | 209 | 14 | 12 | 293 | 2 | 352 | Baseplate\_J domain-containing protein | Baseplate\_J domain-containing protein | | afdb-uniprot50 | AF-A0A6L5X9Z4-F1-MODEL\_V4 | 1.0 | 2.167e-17 | 420 | 0.189 | 374 | 197 | 12 | 20 | 298 | 9 | 371 | Baseplate\_J domain-containing protein | Baseplate\_J domain-containing protein | | afdb-uniprot50 | AF-A0A3D4CFS7-F1-MODEL\_V4 | 1.0 | 2.909e-15 | 419 | 0.149 | 369 | 208 | 14 | 20 | 296 | 1 | 355 | Baseplate\_J domain-containing protein | Baseplate\_J domain-containing protein | | afdb-uniprot50 | AF-A0A376YPQ4-F1-MODEL\_V4 | 1.0 | 6.073e-10 | 418 | 0.468 | 94 | 50 | 0 | 206 | 299 | 3 | 96 | Baseplate assembly protein J | Baseplate assembly protein J | | afdb-uniprot50 | AF-A0A5T1QS83-F1-MODEL\_V4 | 1.0 | 9.61e-15 | 418 | 0.231 | 319 | 154 | 9 | 30 | 270 | 2 | 307 | Baseplate assembly protein | Baseplate assembly protein | | afdb-uniprot50 | AF-A0A268TWY5-F1-MODEL\_V4 | 1.0 | 7.813e-16 | 418 | 0.158 | 354 | 213 | 13 | 13 | 296 | 1 | 339 | Baseplate\_J domain-containing protein | Baseplate\_J domain-containing protein | | afdb-uniprot50 | AF-Q0F1S6-F1-MODEL\_V4 | 1.0 | 7.36e-16 | 418 | 0.151 | 364 | 215 | 15 | 13 | 293 | 1 | 353 | Tail protein, putative | Tail protein, putative | | afdb-uniprot50 | AF-A0A378VWY1-F1-MODEL\_V4 | 1.0 | 4.128e-11 | 416 | 0.444 | 144 | 69 | 3 | 1 | 137 | 1 | 140 | Baseplate protein, phage associated protein | Baseplate protein, phage associated protein | | afdb-uniprot50 | AF-A0A1Y4NG73-F1-MODEL\_V4 | 1.0 | 1.754e-16 | 416 | 0.157 | 363 | 209 | 13 | 20 | 296 | 1 | 352 | Baseplate\_J domain-containing protein | Baseplate\_J domain-containing protein | | afdb-uniprot50 | AF-A0A6B4SDA4-F1-MODEL\_V4 | 1.0 | 2.5e-14 | 415 | 0.146 | 361 | 209 | 16 | 21 | 294 | 3 | 351 | Baseplate J/gp47 family protein | Baseplate J/gp47 family protein | | afdb-uniprot50 | AF-A0A3D4I4D2-F1-MODEL\_V4 | 1.0 | 4.844e-16 | 414 | 0.16 | 361 | 208 | 12 | 21 | 296 | 1 | 351 | Baseplate protein J | Baseplate protein J | | afdb-uniprot50 | AF-A0A177XJU5-F1-MODEL\_V4 | 1.0 | 5.143e-16 | 413 | 0.182 | 291 | 209 | 9 | 12 | 294 | 5 | 274 | Baseplate\_J domain-containing protein | Baseplate\_J domain-containing protein | | afdb-uniprot50 | AF-A0A6N3FBS6-F1-MODEL\_V4 | 1.0 | 8.564e-17 | 412 | 0.213 | 384 | 196 | 18 | 14 | 300 | 1 | 375 | Baseplate J-like protein | Baseplate J-like protein | | afdb-uniprot50 | AF-A0A485AEW3-F1-MODEL\_V4 | 1.0 | 1.14e-10 | 411 | 0.46 | 100 | 54 | 0 | 196 | 295 | 3 | 102 | Bacteriophage P2-related tail formation protein | Bacteriophage P2-related tail formation protein | | afdb-uniprot50 | AF-A0A417N973-F1-MODEL\_V4 | 1.0 | 2.829e-16 | 411 | 0.189 | 358 | 199 | 12 | 21 | 295 | 1 | 350 | Phage tail protein | Phage tail protein | | afdb-uniprot50 | AF-A0A380C6G5-F1-MODEL\_V4 | 1.0 | 3.25e-11 | 410 | 0.504 | 105 | 52 | 0 | 191 | 295 | 11 | 115 | Baseplate J-like protein | Baseplate J-like protein | | afdb-uniprot50 | AF-A0A0L0W6R4-F1-MODEL\_V4 | 1.0 | 1.508e-15 | 409 | 0.166 | 354 | 204 | 16 | 23 | 294 | 6 | 350 | Baseplate\_J domain-containing protein | Baseplate\_J domain-containing protein | | afdb-uniprot50 | AF-A0A6V6Y4B3-F1-MODEL\_V4 | 1.0 | 1.22e-14 | 409 | 0.16 | 362 | 208 | 16 | 16 | 292 | 1 | 351 | Baseplate\_J domain-containing protein | Baseplate\_J domain-containing protein | | afdb-uniprot50 | AF-R6GTF5-F1-MODEL\_V4 | 1.0 | 3.814e-16 | 409 | 0.141 | 361 | 214 | 13 | 21 | 294 | 1 | 352 | Baseplate J-like protein | Baseplate J-like protein | | afdb-uniprot50 | AF-A0A502HRM3-F1-MODEL\_V4 | 1.0 | 5.121e-14 | 409 | 0.127 | 438 | 211 | 14 | 20 | 292 | 4 | 435 | Baseplate\_J domain-containing protein | Baseplate\_J domain-containing protein | | afdb-uniprot50 | AF-A0A5C0SDS7-F1-MODEL\_V4 | 1.0 | 5.31e-17 | 408 | 0.157 | 362 | 209 | 12 | 20 | 293 | 2 | 355 | Baseplate J/gp47 family protein | Baseplate J/gp47 family protein | | afdb-uniprot50 | AF-K8GRQ2-F1-MODEL\_V4 | 1.0 | 1.854e-14 | 408 | 0.144 | 368 | 216 | 12 | 19 | 296 | 2 | 360 | Putative phage Mu protein gp47-like protein | Putative phage Mu protein gp47-like protein | | afdb-uniprot50 | AF-A0A108C9J6-F1-MODEL\_V4 | 1.0 | 7.067e-11 | 407 | 0.343 | 137 | 87 | 2 | 150 | 284 | 5 | 140 | Baseplate\_J domain-containing protein | Baseplate\_J domain-containing protein | | afdb-uniprot50 | AF-A0A1G4VD53-F1-MODEL\_V4 | 1.0 | 4.049e-16 | 407 | 0.166 | 361 | 208 | 11 | 20 | 295 | 1 | 353 | Uncharacterized phage protein gp47/JayE | Uncharacterized phage protein gp47/JayE | | afdb-uniprot50 | AF-Q03RF8-F1-MODEL\_V4 | 1.0 | 4.824e-14 | 407 | 0.142 | 400 | 215 | 17 | 16 | 300 | 1 | 387 | Phage Mu gp47 related protein | Phage Mu gp47 related protein | | afdb-uniprot50 | AF-A0A1A9S102-F1-MODEL\_V4 | 1.0 | 6.933e-16 | 406 | 0.157 | 356 | 212 | 15 | 20 | 294 | 2 | 350 | Baseplate\_J domain-containing protein | Baseplate\_J domain-containing protein | | afdb-uniprot50 | AF-A0A848RMJ7-F1-MODEL\_V4 | 1.0 | 6.503e-14 | 406 | 0.138 | 361 | 215 | 13 | 20 | 295 | 2 | 351 | Baseplate J/gp47 family protein | Baseplate J/gp47 family protein | | afdb-uniprot50 | AF-A0A374UL92-F1-MODEL\_V4 | 1.0 | 9.347e-16 | 406 | 0.136 | 360 | 216 | 13 | 21 | 294 | 1 | 351 | Baseplate\_J domain-containing protein | Baseplate\_J domain-containing protein | | afdb-uniprot50 | AF-A0A0E1UFA6-F1-MODEL\_V4 | 1.0 | 2.13e-09 | 405 | 0.361 | 94 | 60 | 0 | 207 | 300 | 6 | 99 | Phage baseplate assembly protein | Phage baseplate assembly protein | | afdb-uniprot50 | AF-A0A8B4NGU9-F1-MODEL\_V4 | 1.0 | 1.587e-11 | 405 | 0.354 | 155 | 97 | 2 | 116 | 269 | 14 | 166 | Phage-related baseplate assembly protein J | Phage-related baseplate assembly protein J | | afdb-uniprot50 | AF-A0A3D0Z2Q3-F1-MODEL\_V4 | 1.0 | 8.805e-16 | 405 | 0.164 | 358 | 207 | 11 | 21 | 294 | 1 | 350 | Baseplate\_J domain-containing protein | Baseplate\_J domain-containing protein | | afdb-uniprot50 | AF-A0A644UZQ0-F1-MODEL\_V4 | 1.0 | 4.299e-16 | 405 | 0.147 | 380 | 215 | 14 | 18 | 294 | 8 | 381 | Baseplate\_J domain-containing protein | Baseplate\_J domain-containing protein | | afdb-uniprot50 | AF-G9YSH5-F1-MODEL\_V4 | 1.0 | 2.345e-12 | 404 | 0.252 | 182 | 127 | 6 | 116 | 294 | 24 | 199 | Baseplate J-like protein | Baseplate J-like protein | | afdb-uniprot50 | AF-A0A3D0NPG6-F1-MODEL\_V4 | 1.0 | 1.862e-16 | 404 | 0.146 | 362 | 214 | 15 | 20 | 295 | 1 | 353 | Baseplate\_J domain-containing protein | Baseplate\_J domain-containing protein | | afdb-uniprot50 | AF-A0A2S8I2A0-F1-MODEL\_V4 | 1.0 | 1.631e-10 | 403 | 0.431 | 139 | 69 | 3 | 1 | 132 | 1 | 136 | Baseplate assembly protein | Baseplate assembly protein | | afdb-uniprot50 | AF-R5AB96-F1-MODEL\_V4 | 1.0 | 2.581e-15 | 403 | 0.175 | 360 | 202 | 12 | 21 | 294 | 1 | 351 | Baseplate J-like protein | Baseplate J-like protein | | afdb-uniprot50 | AF-A0A5I0MME8-F1-MODEL\_V4 | 1.0 | 3.922e-15 | 402 | 0.141 | 368 | 219 | 16 | 13 | 296 | 1 | 355 | Baseplate J/gp47 family protein | Baseplate J/gp47 family protein | | afdb-uniprot50 | AF-A0A7M1Q5P2-F1-MODEL\_V4 | 1.0 | 1.55e-14 | 401 | 0.147 | 365 | 205 | 17 | 23 | 293 | 7 | 359 | Baseplate J/gp47 family protein | Baseplate J/gp47 family protein | | afdb-uniprot50 | AF-B6WSP9-F1-MODEL\_V4 | 1.0 | 4.419e-15 | 398 | 0.151 | 363 | 214 | 12 | 13 | 294 | 1 | 350 | Baseplate J-like protein | Baseplate J-like protein | | afdb-uniprot50 | AF-A0A3S0DD06-F1-MODEL\_V4 | 1.0 | 3.264e-13 | 398 | 0.127 | 519 | 217 | 19 | 1 | 296 | 1 | 506 | Uncharacterized protein | Uncharacterized protein | | afdb-uniprot50 | AF-A0A1C7FSD0-F1-MODEL\_V4 | 1.0 | 7.998e-13 | 397 | 0.243 | 193 | 132 | 7 | 110 | 294 | 100 | 286 | Baseplate J protein | Baseplate J protein | | afdb-uniprot50 | AF-C0DSG2-F1-MODEL\_V4 | 1.0 | 2.909e-15 | 397 | 0.156 | 358 | 210 | 14 | 20 | 294 | 2 | 350 | Baseplate J-like protein | Baseplate J-like protein | | afdb-uniprot50 | AF-A0A412AW35-F1-MODEL\_V4 | 1.0 | 9.091e-17 | 397 | 0.163 | 367 | 209 | 14 | 20 | 299 | 1 | 356 | Baseplate protein J | Baseplate protein J | | afdb-uniprot50 | AF-R6QTQ5-F1-MODEL\_V4 | 1.0 | 9.61e-15 | 396 | 0.142 | 364 | 213 | 13 | 20 | 295 | 1 | 353 | Baseplate J-like protein | Baseplate J-like protein | | afdb-uniprot50 | AF-A0A4Q3L8H7-F1-MODEL\_V4 | 1.0 | 3.578e-14 | 395 | 0.146 | 472 | 199 | 19 | 20 | 293 | 4 | 469 | Baseplate\_J domain-containing protein | Baseplate\_J domain-containing protein | | afdb-uniprot50 | AF-U7UJ67-F1-MODEL\_V4 | 1.0 | 1.747e-14 | 394 | 0.151 | 362 | 206 | 15 | 19 | 294 | 2 | 348 | Baseplate J-like protein | Baseplate J-like protein | | afdb-uniprot50 | AF-A0A4R2XGE2-F1-MODEL\_V4 | 1.0 | 2.896e-13 | 394 | 0.15 | 366 | 217 | 15 | 14 | 293 | 1 | 358 | Putative phage protein gp47/JayE | Putative phage protein gp47/JayE | | afdb-uniprot50 | AF-A0A1I1ENG9-F1-MODEL\_V4 | 1.0 | 3.814e-16 | 394 | 0.149 | 368 | 211 | 14 | 20 | 296 | 3 | 359 | Uncharacterized phage protein gp47/JayE | Uncharacterized phage protein gp47/JayE | | afdb-uniprot50 | AF-A0A7C3YSU9-F1-MODEL\_V4 | 1.0 | 2.024e-13 | 394 | 0.124 | 482 | 219 | 16 | 18 | 299 | 2 | 480 | Baseplate\_J domain-containing protein | Baseplate\_J domain-containing protein | | afdb-uniprot50 | AF-A0A826UG88-F1-MODEL\_V4 | 1.0 | 1.104e-09 | 393 | 0.435 | 108 | 57 | 1 | 98 | 205 | 1 | 104 | Baseplate assembly protein | Baseplate assembly protein | | afdb-uniprot50 | AF-X1XQF5-F1-MODEL\_V4 | 1.0 | 1.364e-10 | 392 | 0.426 | 122 | 70 | 0 | 179 | 300 | 1 | 122 | Baseplate\_J domain-containing protein | Baseplate\_J domain-containing protein | | afdb-uniprot50 | AF-A0A4Q9TJX4-F1-MODEL\_V4 | 1.0 | 2.29e-15 | 391 | 0.156 | 357 | 213 | 9 | 18 | 293 | 2 | 351 | Baseplate J/gp47 family protein | Baseplate J/gp47 family protein | | afdb-uniprot50 | AF-A0A2S6QXZ3-F1-MODEL\_V4 | 1.0 | 4.98e-15 | 391 | 0.129 | 362 | 225 | 10 | 13 | 293 | 1 | 353 | Baseplate\_J domain-containing protein | Baseplate\_J domain-containing protein | | afdb-uniprot50 | AF-A0A0F6LF31-F1-MODEL\_V4 | 1.0 | 4.782e-10 | 390 | 0.381 | 110 | 68 | 0 | 191 | 300 | 5 | 114 | Baseplate J-like family protein | Baseplate J-like family protein | | afdb-uniprot50 | AF-A0A1H0EKE3-F1-MODEL\_V4 | 1.0 | 2.355e-14 | 390 | 0.137 | 364 | 222 | 12 | 21 | 300 | 1 | 356 | Uncharacterized phage protein gp47/JayE | Uncharacterized phage protein gp47/JayE | | afdb-uniprot50 | AF-A0A7C6EBI1-F1-MODEL\_V4 | 1.0 | 1.053e-15 | 390 | 0.169 | 360 | 210 | 11 | 21 | 295 | 1 | 356 | Baseplate\_J domain-containing protein | Baseplate\_J domain-containing protein | | afdb-uniprot50 | AF-A0A090ZAN9-F1-MODEL\_V4 | 1.0 | 3.593e-16 | 389 | 0.188 | 376 | 198 | 13 | 14 | 295 | 3 | 365 | Baseplate J-like family protein | Baseplate J-like family protein | | afdb-uniprot50 | AF-B6WS15-F1-MODEL\_V4 | 1.0 | 1.26e-15 | 388 | 0.148 | 363 | 218 | 12 | 20 | 300 | 4 | 357 | Baseplate J-like protein | Baseplate J-like protein | | afdb-uniprot50 | AF-C0XGU3-F1-MODEL\_V4 | 1.0 | 2.421e-13 | 388 | 0.138 | 384 | 214 | 17 | 20 | 295 | 8 | 382 | Baseplate\_J domain-containing protein | Baseplate\_J domain-containing protein | | afdb-uniprot50 | AF-A0A1Q4ZD92-F1-MODEL\_V4 | 1.0 | 9.88e-14 | 388 | 0.185 | 437 | 187 | 19 | 22 | 296 | 13 | 442 | Baseplate\_J domain-containing protein | Baseplate\_J domain-containing protein | | afdb-uniprot50 | AF-A0A1F8WPI2-F1-MODEL\_V4 | 1.0 | 1.118e-15 | 387 | 0.149 | 354 | 214 | 10 | 20 | 293 | 4 | 350 | Baseplate\_J domain-containing protein | Baseplate\_J domain-containing protein | | afdb-uniprot50 | AF-A0A3D5NIG5-F1-MODEL\_V4 | 1.0 | 5.287e-15 | 387 | 0.146 | 363 | 209 | 14 | 21 | 294 | 1 | 351 | Baseplate\_J domain-containing protein | Baseplate\_J domain-containing protein | | afdb-uniprot50 | AF-A0A7G3F0Q5-F1-MODEL\_V4 | 1.0 | 8.692e-10 | 386 | 0.526 | 112 | 46 | 1 | 15 | 119 | 7 | 118 | Baseplate J-like protein | Baseplate J-like protein | | afdb-uniprot50 | AF-A0A2A7WUL9-F1-MODEL\_V4 | 1.0 | 1.55e-14 | 386 | 0.164 | 353 | 206 | 14 | 21 | 293 | 1 | 344 | Baseplate j family protein | Baseplate j family protein | | afdb-uniprot50 | AF-A0A223D2X1-F1-MODEL\_V4 | 1.0 | 7.36e-16 | 386 | 0.175 | 364 | 206 | 13 | 20 | 295 | 4 | 361 | Baseplate\_J domain-containing protein | Baseplate\_J domain-containing protein | | afdb-uniprot50 | AF-A0A6I6XZA3-F1-MODEL\_V4 | 1.0 | 8.033e-15 | 384 | 0.128 | 358 | 226 | 12 | 18 | 296 | 2 | 352 | Baseplate J/gp47 family protein | Baseplate J/gp47 family protein | | afdb-uniprot50 | AF-A0A6Y1Z4C1-F1-MODEL\_V4 | 1.0 | 7.93e-09 | 383 | 0.459 | 87 | 47 | 0 | 213 | 299 | 1 | 87 | Baseplate assembly protein | Baseplate assembly protein | | afdb-uniprot50 | AF-A0A844UU35-F1-MODEL\_V4 | 1.0 | 3.148e-10 | 383 | 0.482 | 116 | 60 | 0 | 1 | 116 | 1 | 116 | Baseplate assembly protein | Baseplate assembly protein | | afdb-uniprot50 | AF-A0A7X5DX78-F1-MODEL\_V4 | 1.0 | 1.684e-11 | 383 | 0.165 | 206 | 149 | 6 | 113 | 300 | 77 | 277 | Baseplate\_J domain-containing protein | Baseplate\_J domain-containing protein | | afdb-uniprot50 | AF-C6LG69-F1-MODEL\_V4 | 1.0 | 9.052e-15 | 383 | 0.143 | 384 | 223 | 17 | 8 | 295 | 2 | 375 | Baseplate J-like protein | Baseplate J-like protein | | afdb-uniprot50 | AF-U2QTS5-F1-MODEL\_V4 | 1.0 | 3.264e-13 | 383 | 0.16 | 392 | 198 | 17 | 20 | 298 | 9 | 382 | Baseplate\_J domain-containing protein | Baseplate\_J domain-containing protein | | afdb-uniprot50 | AF-S7V8X9-F1-MODEL\_V4 | 1.0 | 6.126e-14 | 383 | 0.151 | 377 | 210 | 13 | 23 | 295 | 22 | 392 | Baseplate J family protein | Baseplate J family protein | | afdb-uniprot50 | AF-Q727K4-F1-MODEL\_V4 | 1.0 | 3.48e-15 | 382 | 0.189 | 364 | 203 | 11 | 13 | 295 | 1 | 353 | Tail protein, putative | Tail protein, putative | | afdb-uniprot50 | AF-A0A3C1LVA0-F1-MODEL\_V4 | 1.0 | 2.431e-15 | 382 | 0.16 | 367 | 204 | 15 | 23 | 300 | 3 | 354 | Baseplate\_J domain-containing protein | Baseplate\_J domain-containing protein | | afdb-uniprot50 | AF-R6U9B8-F1-MODEL\_V4 | 1.0 | 2.355e-14 | 381 | 0.138 | 361 | 216 | 12 | 21 | 295 | 1 | 352 | Baseplate J-like protein | Baseplate J-like protein | | afdb-uniprot50 | AF-A0A2T5J3V8-F1-MODEL\_V4 | 1.0 | 4.98e-15 | 380 | 0.137 | 363 | 220 | 14 | 13 | 294 | 1 | 351 | Putative phage protein gp47/JayE | Putative phage protein gp47/JayE | | afdb-uniprot50 | AF-A0A176L6Z2-F1-MODEL\_V4 | 1.0 | 1.049e-13 | 380 | 0.153 | 442 | 206 | 15 | 14 | 298 | 1 | 431 | Baseplate\_J domain-containing protein | Baseplate\_J domain-containing protein | | afdb-uniprot50 | AF-A0A0D2GH44-F1-MODEL\_V4 | 1.0 | 1.915e-15 | 379 | 0.155 | 353 | 214 | 9 | 20 | 292 | 4 | 352 | Baseplate J protein | Baseplate J protein | | afdb-uniprot50 | AF-A0A7C6PX97-F1-MODEL\_V4 | 1.0 | 1.638e-12 | 379 | 0.12 | 481 | 215 | 16 | 13 | 295 | 3 | 473 | Baseplate\_J domain-containing protein | Baseplate\_J domain-containing protein | | afdb-uniprot50 | AF-S5MVA2-F1-MODEL\_V4 | 1.0 | 5.219e-09 | 378 | 0.411 | 90 | 53 | 0 | 206 | 295 | 3 | 92 | Baseplate assembly protein J | Baseplate assembly protein J | | afdb-uniprot50 | AF-A0A413G370-F1-MODEL\_V4 | 1.0 | 4.98e-15 | 378 | 0.162 | 363 | 212 | 14 | 20 | 300 | 1 | 353 | Baseplate\_J domain-containing protein | Baseplate\_J domain-containing protein | | afdb-uniprot50 | AF-A0A7V2IPE5-F1-MODEL\_V4 | 1.0 | 2.139e-11 | 378 | 0.186 | 198 | 145 | 9 | 110 | 296 | 247 | 439 | Baseplate J protein | Baseplate J protein | | afdb-uniprot50 | AF-A0A4P9VHM3-F1-MODEL\_V4 | 1.0 | 2.199e-10 | 377 | 0.336 | 116 | 77 | 0 | 180 | 295 | 4 | 119 | Baseplate assembly protein | Baseplate assembly protein | | afdb-uniprot50 | AF-W1L2P9-F1-MODEL\_V4 | 1.0 | 5.565e-11 | 377 | 0.323 | 167 | 95 | 5 | 1 | 154 | 2 | 163 | Uncharacterized protein | Uncharacterized protein | | afdb-uniprot50 | AF-A0A2H1SQY8-F1-MODEL\_V4 | 1.0 | 7.713e-10 | 375 | 0.432 | 104 | 59 | 0 | 196 | 299 | 2 | 105 | Baseplate assembly protein J | Baseplate assembly protein J | | afdb-uniprot50 | AF-A0A329KAH2-F1-MODEL\_V4 | 1.0 | 9.923e-16 | 375 | 0.176 | 363 | 200 | 13 | 19 | 295 | 2 | 351 | Baseplate j family protein | Baseplate j family protein | | afdb-uniprot50 | AF-R5KQQ2-F1-MODEL\_V4 | 1.0 | 1.02e-14 | 375 | 0.142 | 365 | 213 | 14 | 20 | 294 | 1 | 355 | Baseplate J-like protein | Baseplate J-like protein | | afdb-uniprot50 | AF-A0A328UJU0-F1-MODEL\_V4 | 1.0 | 1.747e-14 | 375 | 0.162 | 363 | 213 | 12 | 20 | 300 | 7 | 360 | Baseplate protein J | Baseplate protein J | | afdb-uniprot50 | AF-A0A371WT16-F1-MODEL\_V4 | 1.0 | 4.824e-14 | 375 | 0.153 | 379 | 208 | 13 | 20 | 293 | 5 | 375 | Baseplate protein | Baseplate protein | | afdb-uniprot50 | AF-A0A077Q3X4-F1-MODEL\_V4 | 1.0 | 8.692e-10 | 373 | 0.432 | 104 | 59 | 0 | 196 | 299 | 2 | 105 | Baseplate assembly protein J (GpJ) | Baseplate assembly protein J (GpJ) | | afdb-uniprot50 | AF-A0A243TCZ4-F1-MODEL\_V4 | 1.0 | 7.266e-10 | 373 | 0.388 | 103 | 60 | 1 | 198 | 300 | 119 | 218 | Uncharacterized protein | Uncharacterized protein | | afdb-uniprot50 | AF-A0A1T2XKG9-F1-MODEL\_V4 | 1.0 | 2.431e-15 | 373 | 0.158 | 360 | 205 | 14 | 20 | 293 | 3 | 350 | Baseplate\_J domain-containing protein | Baseplate\_J domain-containing protein | | afdb-uniprot50 | AF-A0A1Y0G5P4-F1-MODEL\_V4 | 1.0 | 4.544e-14 | 372 | 0.15 | 360 | 215 | 15 | 20 | 295 | 4 | 356 | Phage tail protein | Phage tail protein | | afdb-uniprot50 | AF-A0A1G8FC75-F1-MODEL\_V4 | 1.0 | 1.02e-14 | 372 | 0.155 | 372 | 211 | 14 | 20 | 295 | 4 | 368 | Uncharacterized phage protein gp47/JayE | Uncharacterized phage protein gp47/JayE | | afdb-uniprot50 | AF-A0A379YIU1-F1-MODEL\_V4 | 1.0 | 5.541e-09 | 371 | 0.344 | 93 | 61 | 0 | 207 | 299 | 17 | 109 | Baseplate J-like protein | Baseplate J-like protein | | afdb-uniprot50 | AF-A0A522WFF2-F1-MODEL\_V4 | 1.0 | 2.355e-14 | 370 | 0.147 | 360 | 219 | 13 | 20 | 296 | 4 | 358 | Baseplate J/gp47 family protein | Baseplate J/gp47 family protein | | afdb-uniprot50 | AF-I0GWS4-F1-MODEL\_V4 | 1.0 | 5.287e-15 | 367 | 0.16 | 361 | 207 | 14 | 19 | 294 | 3 | 352 | Putative baseplate protein | Putative baseplate protein | | afdb-uniprot50 | AF-A0A348Z8Z5-F1-MODEL\_V4 | 1.0 | 1.854e-14 | 367 | 0.136 | 359 | 214 | 13 | 22 | 293 | 4 | 353 | Baseplate\_J domain-containing protein | Baseplate\_J domain-containing protein | | afdb-uniprot50 | AF-A0A6N2T8J3-F1-MODEL\_V4 | 1.0 | 4.98e-15 | 366 | 0.181 | 364 | 196 | 18 | 22 | 295 | 1 | 352 | Baseplate J-like protein | Baseplate J-like protein | | afdb-uniprot50 | AF-A0A1S2WAC6-F1-MODEL\_V4 | 1.0 | 2.829e-16 | 366 | 0.193 | 373 | 198 | 15 | 12 | 293 | 2 | 362 | Baseplate\_J domain-containing protein | Baseplate\_J domain-containing protein | | afdb-uniprot50 | AF-A0A1V2H5A7-F1-MODEL\_V4 | 1.0 | 3.694e-15 | 366 | 0.185 | 389 | 196 | 17 | 11 | 293 | 2 | 375 | Baseplate\_J domain-containing protein | Baseplate\_J domain-containing protein | | afdb-uniprot50 | AF-A0A3M4Z394-F1-MODEL\_V4 | 1.0 | 1.321e-09 | 365 | 0.433 | 120 | 67 | 1 | 117 | 235 | 1 | 120 | Baseplate assembly protein J | Baseplate assembly protein J | | afdb-uniprot50 | AF-A0A2A7HD50-F1-MODEL\_V4 | 1.0 | 5.589e-13 | 364 | 0.129 | 354 | 214 | 17 | 21 | 290 | 1 | 344 | Baseplate J protein | Baseplate J protein | | afdb-uniprot50 | AF-A0A3D0XAJ1-F1-MODEL\_V4 | 1.0 | 1.249e-11 | 363 | 0.225 | 186 | 135 | 6 | 112 | 294 | 76 | 255 | Baseplate\_J domain-containing protein | Baseplate\_J domain-containing protein | | afdb-uniprot50 | AF-A0A845SWX3-F1-MODEL\_V4 | 1.0 | 8.527e-15 | 363 | 0.175 | 360 | 206 | 14 | 23 | 300 | 5 | 355 | Uncharacterized protein | Uncharacterized protein | | afdb-uniprot50 | AF-A0A081BI31-F1-MODEL\_V4 | 1.0 | 5.933e-13 | 363 | 0.152 | 381 | 202 | 13 | 22 | 294 | 16 | 383 | Phage protein | Phage protein | | afdb-uniprot50 | AF-A0A7Y4S5C2-F1-MODEL\_V4 | 1.0 | 1.182e-13 | 362 | 0.137 | 357 | 219 | 13 | 20 | 293 | 4 | 354 | Baseplate J/gp47 family protein | Baseplate J/gp47 family protein | | afdb-uniprot50 | AF-B9Z2Z3-F1-MODEL\_V4 | 1.0 | 1.255e-13 | 362 | 0.135 | 361 | 217 | 14 | 20 | 294 | 4 | 355 | Baseplate J family protein | Baseplate J family protein | | afdb-uniprot50 | AF-J1H457-F1-MODEL\_V4 | 1.0 | 3.175e-14 | 361 | 0.166 | 367 | 209 | 14 | 21 | 300 | 1 | 357 | Baseplate J-like protein | Baseplate J-like protein | | afdb-uniprot50 | AF-A0A1S8PIF2-F1-MODEL\_V4 | 1.0 | 1.332e-13 | 361 | 0.14 | 370 | 210 | 17 | 22 | 295 | 6 | 363 | Baseplate J-like protein | Baseplate J-like protein | | afdb-uniprot50 | AF-A0A662W1X1-F1-MODEL\_V4 | 1.0 | 9.307e-14 | 360 | 0.169 | 355 | 183 | 13 | 22 | 295 | 14 | 337 | Baseplate\_J domain-containing protein | Baseplate\_J domain-containing protein | | afdb-uniprot50 | AF-M3AAC0-F1-MODEL\_V4 | 1.0 | 8.767e-14 | 360 | 0.136 | 367 | 216 | 12 | 13 | 293 | 1 | 352 | Tail protein | Tail protein | | afdb-uniprot50 | AF-A0A1C6CHI6-F1-MODEL\_V4 | 1.0 | 5.436e-14 | 359 | 0.145 | 386 | 207 | 15 | 20 | 294 | 8 | 381 | Uncharacterized homolog of phage Mu protein gp47 | Uncharacterized homolog of phage Mu protein gp47 | | afdb-uniprot50 | AF-G9XBC3-F1-MODEL\_V4 | 1.0 | 9.052e-15 | 358 | 0.154 | 357 | 207 | 14 | 21 | 295 | 4 | 347 | Baseplate\_J domain-containing protein | Baseplate\_J domain-containing protein | | afdb-uniprot50 | AF-A0A358PSA7-F1-MODEL\_V4 | 1.0 | 3.088e-15 | 358 | 0.175 | 377 | 206 | 16 | 1 | 294 | 11 | 365 | Baseplate\_J domain-containing protein | Baseplate\_J domain-containing protein | | afdb-uniprot50 | AF-A0A1J4X5M8-F1-MODEL\_V4 | 1.0 | 1.215e-12 | 358 | 0.136 | 381 | 211 | 15 | 19 | 293 | 3 | 371 | Baseplate\_J domain-containing protein | Baseplate\_J domain-containing protein | | afdb-uniprot50 | AF-D7MYP8-F1-MODEL\_V4 | 1.0 | 7.502e-11 | 357 | 0.216 | 189 | 133 | 10 | 116 | 295 | 8 | 190 | Baseplate J-like protein | Baseplate J-like protein | | afdb-uniprot50 | AF-A0A3Q9S8Y2-F1-MODEL\_V4 | 1.0 | 4.28e-14 | 356 | 0.143 | 363 | 216 | 14 | 18 | 293 | 2 | 356 | Baseplate J/gp47 family protein | Baseplate J/gp47 family protein | | afdb-uniprot50 | AF-A0A502HGK3-F1-MODEL\_V4 | 1.0 | 2.158e-15 | 355 | 0.163 | 372 | 202 | 15 | 20 | 295 | 4 | 362 | Baseplate\_J domain-containing protein | Baseplate\_J domain-containing protein | | afdb-uniprot50 | AF-A0A7C3WLZ2-F1-MODEL\_V4 | 1.0 | 2.642e-12 | 354 | 0.117 | 468 | 216 | 16 | 20 | 294 | 1 | 464 | Baseplate\_J domain-containing protein | Baseplate\_J domain-containing protein | | afdb-uniprot50 | AF-A0A7G9WGX3-F1-MODEL\_V4 | 1.0 | 3.37e-14 | 353 | 0.169 | 360 | 203 | 15 | 21 | 293 | 1 | 351 | Baseplate J/gp47 family protein | Baseplate J/gp47 family protein | | afdb-uniprot50 | AF-S0FX69-F1-MODEL\_V4 | 1.0 | 1.645e-14 | 353 | 0.154 | 362 | 217 | 13 | 23 | 300 | 1 | 357 | Baseplate J-like protein | Baseplate J-like protein | | afdb-uniprot50 | AF-A0A5C8XTI4-F1-MODEL\_V4 | 1.0 | 1.89e-09 | 352 | 0.479 | 98 | 51 | 0 | 195 | 292 | 3 | 100 | Baseplate assembly protein | Baseplate assembly protein | | afdb-uniprot50 | AF-A0A1X3ISQ9-F1-MODEL\_V4 | 1.0 | 1.279e-08 | 352 | 0.354 | 93 | 60 | 0 | 207 | 299 | 17 | 109 | Phage baseplate assembly protein | Phage baseplate assembly protein | | afdb-uniprot50 | AF-A0A3D0E1W7-F1-MODEL\_V4 | 1.0 | 1.508e-15 | 351 | 0.155 | 372 | 215 | 15 | 13 | 293 | 6 | 369 | Baseplate\_J domain-containing protein | Baseplate\_J domain-containing protein | | afdb-uniprot50 | AF-A0A258L253-F1-MODEL\_V4 | 1.0 | 1.58e-09 | 350 | 0.411 | 107 | 63 | 0 | 190 | 296 | 12 | 118 | Baseplate\_J domain-containing protein | Baseplate\_J domain-containing protein | | afdb-uniprot50 | AF-X1GT58-F1-MODEL\_V4 | 1.0 | 5.077e-10 | 350 | 0.209 | 162 | 118 | 6 | 140 | 295 | 2 | 159 | Baseplate\_J domain-containing protein | Baseplate\_J domain-containing protein | | afdb-uniprot50 | AF-A0A418H6F8-F1-MODEL\_V4 | 1.0 | 3.048e-09 | 348 | 0.416 | 108 | 63 | 0 | 192 | 299 | 1 | 108 | Baseplate assembly protein | Baseplate assembly protein | | afdb-uniprot50 | AF-A0A1V5QEC7-F1-MODEL\_V4 | 1.0 | 8.692e-10 | 348 | 0.183 | 174 | 130 | 6 | 131 | 296 | 40 | 209 | Baseplate J-like protein | Baseplate J-like protein | | afdb-uniprot50 | AF-A0A1M5REH3-F1-MODEL\_V4 | 1.0 | 1.645e-14 | 348 | 0.139 | 386 | 220 | 18 | 20 | 300 | 4 | 382 | Uncharacterized phage protein gp47/JayE | Uncharacterized phage protein gp47/JayE | | afdb-uniprot50 | AF-A0A3D0KMD1-F1-MODEL\_V4 | 1.0 | 3.905e-13 | 347 | 0.139 | 351 | 209 | 11 | 31 | 296 | 1 | 343 | Baseplate\_J domain-containing protein | Baseplate\_J domain-containing protein | | afdb-uniprot50 | AF-A0A3N0V7H0-F1-MODEL\_V4 | 1.0 | 6.126e-14 | 347 | 0.161 | 354 | 210 | 10 | 20 | 293 | 4 | 350 | Baseplate J/gp47 family protein | Baseplate J/gp47 family protein | | afdb-uniprot50 | AF-A0A376TU70-F1-MODEL\_V4 | 1.0 | 1.04e-09 | 346 | 0.407 | 113 | 67 | 0 | 187 | 299 | 13 | 125 | Baseplate assembly protein GpJ | Baseplate assembly protein GpJ | | afdb-uniprot50 | AF-W4BPJ9-F1-MODEL\_V4 | 1.0 | 3.264e-13 | 346 | 0.158 | 303 | 213 | 13 | 7 | 295 | 2 | 276 | Baseplate\_J domain-containing protein | Baseplate\_J domain-containing protein | | afdb-uniprot50 | AF-A0A2G1Z9I9-F1-MODEL\_V4 | 1.0 | 7.328e-14 | 346 | 0.128 | 366 | 228 | 11 | 13 | 293 | 1 | 360 | Baseplate\_J domain-containing protein | Baseplate\_J domain-containing protein | | afdb-uniprot50 | AF-Q15XZ6-F1-MODEL\_V4 | 1.0 | 1.215e-12 | 346 | 0.132 | 400 | 227 | 19 | 1 | 294 | 1 | 386 | Uncharacterized phage Mu protein GP47-like protein | Uncharacterized phage Mu protein GP47-like protein | | afdb-uniprot50 | AF-A0A291RK52-F1-MODEL\_V4 | 1.0 | 6.903e-14 | 346 | 0.148 | 438 | 194 | 19 | 22 | 296 | 14 | 435 | Baseplate\_J domain-containing protein | Baseplate\_J domain-containing protein | | afdb-uniprot50 | AF-A0A644Z6C5-F1-MODEL\_V4 | 1.0 | 3.766e-10 | 345 | 0.217 | 170 | 120 | 7 | 129 | 295 | 9 | 168 | Baseplate\_J domain-containing protein | Baseplate\_J domain-containing protein | | afdb-uniprot50 | AF-A0A2W1CQX1-F1-MODEL\_V4 | 1.0 | 2.199e-10 | 345 | 0.209 | 191 | 139 | 6 | 112 | 293 | 92 | 279 | TehA, Tellurite resistance protein | TehA, Tellurite resistance protein | | afdb-uniprot50 | AF-A0A0Q4XSB4-F1-MODEL\_V4 | 1.0 | 8.767e-14 | 345 | 0.152 | 367 | 215 | 15 | 19 | 295 | 4 | 364 | Baseplate\_J domain-containing protein | Baseplate\_J domain-containing protein | | afdb-uniprot50 | AF-A0A2U1W182-F1-MODEL\_V4 | 1.0 | 1.96e-12 | 345 | 0.135 | 376 | 212 | 16 | 20 | 295 | 4 | 366 | Baseplate\_J domain-containing protein | Baseplate\_J domain-containing protein | | afdb-uniprot50 | AF-A0A2V2YWP1-F1-MODEL\_V4 | 1.0 | 9.838e-12 | 345 | 0.206 | 208 | 140 | 7 | 112 | 296 | 430 | 635 | Putative phage baseplate assembly protein | Putative phage baseplate assembly protein | | afdb-uniprot50 | AF-B1TCV6-F1-MODEL\_V4 | 1.0 | 4.782e-10 | 344 | 0.387 | 124 | 69 | 3 | 101 | 224 | 3 | 119 | Baseplate J family protein | Baseplate J family protein | | afdb-uniprot50 | AF-A0A3A9FCY9-F1-MODEL\_V4 | 1.0 | 9.307e-14 | 344 | 0.12 | 372 | 230 | 11 | 17 | 296 | 9 | 375 | Baseplate\_J domain-containing protein | Baseplate\_J domain-containing protein | | afdb-uniprot50 | AF-A0A4V2EGN7-F1-MODEL\_V4 | 1.0 | 1.369e-12 | 344 | 0.133 | 382 | 216 | 16 | 21 | 295 | 19 | 392 | Baseplate\_J domain-containing protein | Baseplate\_J domain-containing protein | | afdb-uniprot50 | AF-A0A3D1LC94-F1-MODEL\_V4 | 1.0 | 5.121e-14 | 343 | 0.196 | 311 | 156 | 13 | 74 | 300 | 2 | 302 | Phage tail protein | Phage tail protein | | afdb-uniprot50 | AF-A0A5S3YLY6-F1-MODEL\_V4 | 1.0 | 3.048e-09 | 342 | 0.413 | 116 | 60 | 3 | 74 | 189 | 2 | 109 | Baseplate J protein | Baseplate J protein | | afdb-uniprot50 | AF-A0A3S4J4W8-F1-MODEL\_V4 | 1.0 | 2.705e-09 | 342 | 0.538 | 117 | 47 | 1 | 8 | 117 | 4 | 120 | Baseplate assembly protein | Baseplate assembly protein | | afdb-uniprot50 | AF-A0A7C1VC68-F1-MODEL\_V4 | 1.0 | 9.88e-14 | 342 | 0.139 | 359 | 217 | 12 | 20 | 295 | 4 | 353 | Baseplate J/gp47 family protein | Baseplate J/gp47 family protein | | afdb-uniprot50 | AF-A0A4P8QBU6-F1-MODEL\_V4 | 1.0 | 2.148e-13 | 342 | 0.15 | 378 | 210 | 11 | 20 | 293 | 4 | 374 | Baseplate protein | Baseplate protein | | afdb-uniprot50 | AF-A0A4R3HUV0-F1-MODEL\_V4 | 1.0 | 1.113e-13 | 341 | 0.15 | 358 | 215 | 11 | 20 | 296 | 4 | 353 | Putative phage protein gp47/JayE | Putative phage protein gp47/JayE | | afdb-uniprot50 | AF-A0A5Z1E065-F1-MODEL\_V4 | 1.0 | 1.537e-10 | 340 | 0.282 | 163 | 101 | 4 | 113 | 270 | 28 | 179 | Baseplate assembly protein | Baseplate assembly protein | | afdb-uniprot50 | AF-A0A7J5WLS9-F1-MODEL\_V4 | 1.0 | 1.638e-12 | 339 | 0.159 | 364 | 204 | 16 | 20 | 294 | 3 | 353 | Baseplate J family | Baseplate J family | | afdb-uniprot50 | AF-A0A2E7U201-F1-MODEL\_V4 | 1.0 | 1.501e-13 | 339 | 0.139 | 459 | 210 | 21 | 14 | 293 | 1 | 453 | Baseplate\_J domain-containing protein | Baseplate\_J domain-containing protein | | afdb-uniprot50 | AF-A0A826JR68-F1-MODEL\_V4 | 1.0 | 3.766e-10 | 338 | 0.366 | 139 | 75 | 2 | 113 | 240 | 61 | 197 | Baseplate J/gp47 family protein | Baseplate J/gp47 family protein | | afdb-uniprot50 | AF-A0A1Y1S2G0-F1-MODEL\_V4 | 1.0 | 5.121e-14 | 338 | 0.133 | 368 | 223 | 12 | 15 | 293 | 1 | 361 | Baseplate\_J domain-containing protein | Baseplate\_J domain-containing protein | | afdb-uniprot50 | AF-A0A0C2VDI9-F1-MODEL\_V4 | 1.0 | 2.345e-12 | 338 | 0.156 | 389 | 202 | 19 | 13 | 293 | 1 | 371 | Baseplate\_J domain-containing protein | Baseplate\_J domain-containing protein | | afdb-uniprot50 | AF-A0A5C4QQ94-F1-MODEL\_V4 | 1.0 | 1.587e-11 | 338 | 0.124 | 500 | 220 | 15 | 11 | 298 | 2 | 495 | Baseplate\_J domain-containing protein | Baseplate\_J domain-containing protein | | afdb-uniprot50 | AF-A0A1Y4L6I3-F1-MODEL\_V4 | 1.0 | 9.88e-14 | 337 | 0.155 | 359 | 209 | 15 | 23 | 295 | 1 | 351 | Baseplate\_J domain-containing protein | Baseplate\_J domain-containing protein | | afdb-uniprot50 | AF-A0A1M6ZB03-F1-MODEL\_V4 | 1.0 | 2.896e-13 | 337 | 0.13 | 397 | 219 | 19 | 20 | 296 | 2 | 392 | Uncharacterized phage protein gp47/JayE | Uncharacterized phage protein gp47/JayE | | afdb-uniprot50 | AF-A0A2N1TNI5-F1-MODEL\_V4 | 1.0 | 5.121e-14 | 336 | 0.152 | 388 | 216 | 18 | 13 | 295 | 1 | 380 | Baseplate\_J domain-containing protein | Baseplate\_J domain-containing protein | | afdb-uniprot50 | AF-A0A3E2SY13-F1-MODEL\_V4 | 1.0 | 7.998e-13 | 335 | 0.148 | 349 | 205 | 13 | 34 | 300 | 4 | 342 | Baseplate protein J | Baseplate protein J | | afdb-uniprot50 | AF-C8NCB0-F1-MODEL\_V4 | 1.0 | 3.647e-09 | 333 | 0.289 | 121 | 85 | 1 | 178 | 298 | 1 | 120 | Uncharacterized protein | Uncharacterized protein | | afdb-uniprot50 | AF-A0A3A9HYN4-F1-MODEL\_V4 | 1.0 | 2.148e-13 | 333 | 0.172 | 377 | 210 | 17 | 12 | 293 | 3 | 372 | Baseplate J/gp47 family protein | Baseplate J/gp47 family protein | | afdb-uniprot50 | AF-R4YKQ9-F1-MODEL\_V4 | 1.0 | 3.161e-12 | 333 | 0.115 | 382 | 225 | 14 | 21 | 296 | 22 | 396 | Baseplate\_J domain-containing protein | Baseplate\_J domain-containing protein | | afdb-uniprot50 | AF-D1JAN0-F1-MODEL\_V4 | 1.0 | 9.267e-12 | 333 | 0.175 | 216 | 150 | 10 | 110 | 300 | 410 | 622 | Baseplate\_J domain-containing protein | Baseplate\_J domain-containing protein | | afdb-uniprot50 | AF-A0A379YC97-F1-MODEL\_V4 | 1.0 | 1.285e-10 | 332 | 0.438 | 130 | 73 | 0 | 170 | 299 | 1 | 130 | Baseplate J-like protein | Baseplate J-like protein | | afdb-uniprot50 | AF-A0A1B9ZS99-F1-MODEL\_V4 | 1.0 | 3.905e-13 | 332 | 0.211 | 298 | 141 | 9 | 77 | 285 | 1 | 293 | Baseplate\_J domain-containing protein | Baseplate\_J domain-containing protein | | afdb-uniprot50 | AF-A0A3Q9SAV9-F1-MODEL\_V4 | 1.0 | 3.798e-14 | 332 | 0.175 | 337 | 188 | 13 | 44 | 293 | 10 | 343 | Baseplate J/gp47 family protein | Baseplate J/gp47 family protein | | afdb-uniprot50 | AF-A0A3G2R557-F1-MODEL\_V4 | 1.0 | 8.491e-13 | 332 | 0.123 | 470 | 215 | 12 | 20 | 295 | 1 | 467 | Baseplate\_J domain-containing protein | Baseplate\_J domain-containing protein | | afdb-uniprot50 | AF-A0A4Q3L733-F1-MODEL\_V4 | 1.0 | 3.465e-13 | 332 | 0.138 | 476 | 209 | 19 | 13 | 293 | 1 | 470 | Baseplate\_J domain-containing protein | Baseplate\_J domain-containing protein | | afdb-uniprot50 | AF-A0A7C2U0W7-F1-MODEL\_V4 | 1.0 | 8.73e-12 | 331 | 0.125 | 446 | 215 | 15 | 14 | 296 | 1 | 434 | Baseplate\_J domain-containing protein | Baseplate\_J domain-containing protein | | afdb-uniprot50 | AF-A0A6G4XH46-F1-MODEL\_V4 | 1.0 | 3.45e-11 | 331 | 0.214 | 224 | 140 | 13 | 110 | 300 | 416 | 636 | Putative baseplate assembly protein | Putative baseplate assembly protein | | afdb-uniprot50 | AF-R6P8N6-F1-MODEL\_V4 | 1.0 | 2.631e-10 | 330 | 0.226 | 172 | 117 | 9 | 129 | 295 | 9 | 169 | Baseplate J-like protein | Baseplate J-like protein | | afdb-uniprot50 | AF-A0A0T9QWQ1-F1-MODEL\_V4 | 1.0 | 1.724e-08 | 329 | 0.532 | 109 | 51 | 0 | 42 | 150 | 2 | 110 | Baseplate assembly protein | Baseplate assembly protein | | afdb-uniprot50 | AF-Q1MNP3-F1-MODEL\_V4 | 1.0 | 1.732e-10 | 329 | 0.163 | 196 | 143 | 9 | 113 | 294 | 49 | 237 | Baseplate\_J domain-containing protein | Baseplate\_J domain-containing protein | | afdb-uniprot50 | AF-A0A1A7PWG3-F1-MODEL\_V4 | 1.0 | 5.242e-11 | 329 | 0.211 | 274 | 126 | 7 | 14 | 202 | 4 | 272 | Phage baseplate protein | Phage baseplate protein | | afdb-uniprot50 | AF-A0A2W7M1F1-F1-MODEL\_V4 | 1.0 | 2.642e-12 | 329 | 0.125 | 397 | 218 | 17 | 20 | 295 | 2 | 390 | Putative phage protein gp47/JayE | Putative phage protein gp47/JayE | | afdb-uniprot50 | AF-A0A2D5TJV2-F1-MODEL\_V4 | 1.0 | 3.161e-12 | 328 | 0.142 | 457 | 206 | 21 | 20 | 293 | 5 | 458 | Baseplate\_J domain-containing protein | Baseplate\_J domain-containing protein | | afdb-uniprot50 | AF-A0A7Y7LSY4-F1-MODEL\_V4 | 1.0 | 5.589e-13 | 327 | 0.12 | 358 | 225 | 10 | 20 | 293 | 4 | 355 | Putative phage protein gp47/JayE | Putative phage protein gp47/JayE | | afdb-uniprot50 | AF-E1RFI2-F1-MODEL\_V4 | 1.0 | 8.767e-14 | 327 | 0.154 | 370 | 203 | 15 | 20 | 295 | 12 | 365 | Baseplate J family protein | Baseplate J family protein | | afdb-uniprot50 | AF-A0A1Y6CTB1-F1-MODEL\_V4 | 1.0 | 2.642e-12 | 327 | 0.166 | 385 | 200 | 18 | 20 | 294 | 4 | 377 | Uncharacterized phage protein gp47/JayE | Uncharacterized phage protein gp47/JayE | | afdb-uniprot50 | AF-A0A524BHK3-F1-MODEL\_V4 | 1.0 | 2.654e-14 | 327 | 0.129 | 377 | 216 | 15 | 17 | 295 | 15 | 377 | Baseplate\_J domain-containing protein | Baseplate\_J domain-containing protein | | afdb-uniprot50 | AF-A0A2V4HQX5-F1-MODEL\_V4 | 1.0 | 4.524e-12 | 327 | 0.126 | 396 | 219 | 18 | 20 | 295 | 2 | 390 | Baseplate\_J domain-containing protein | Baseplate\_J domain-containing protein | | afdb-uniprot50 | AF-A0A2E2FJ34-F1-MODEL\_V4 | 1.0 | 1.402e-09 | 327 | 0.18 | 161 | 126 | 5 | 113 | 271 | 275 | 431 | Baseplate\_J domain-containing protein | Baseplate\_J domain-containing protein | | afdb-uniprot50 | AF-A0A017RWE9-F1-MODEL\_V4 | 1.0 | 1.078e-12 | 326 | 0.168 | 363 | 207 | 13 | 21 | 295 | 1 | 356 | Baseplate\_J domain-containing protein | Baseplate\_J domain-containing protein | | afdb-uniprot50 | AF-A0A510IHW7-F1-MODEL\_V4 | 1.0 | 1.684e-11 | 326 | 0.151 | 402 | 220 | 17 | 14 | 300 | 5 | 400 | Baseplate\_J domain-containing protein | Baseplate\_J domain-containing protein | | afdb-uniprot50 | AF-A0A2S3UN17-F1-MODEL\_V4 | 1.0 | 2.411e-11 | 326 | 0.111 | 510 | 220 | 19 | 12 | 300 | 5 | 502 | Putative phage baseplate assembly protein | Putative phage baseplate assembly protein | | afdb-uniprot50 | AF-A0A1Q6UET1-F1-MODEL\_V4 | 1.0 | 9.88e-14 | 325 | 0.171 | 362 | 199 | 15 | 13 | 292 | 4 | 346 | Baseplate\_J domain-containing protein | Baseplate\_J domain-containing protein | | afdb-uniprot50 | AF-A0A109QIW4-F1-MODEL\_V4 | 1.0 | 5.589e-13 | 325 | 0.142 | 372 | 215 | 15 | 20 | 294 | 3 | 367 | Baseplate\_J domain-containing protein | Baseplate\_J domain-containing protein | | afdb-uniprot50 | AF-A0A431JII9-F1-MODEL\_V4 | 1.0 | 9.569e-13 | 325 | 0.132 | 468 | 209 | 14 | 20 | 293 | 4 | 468 | Baseplate\_J domain-containing protein | Baseplate\_J domain-containing protein | | afdb-uniprot50 | AF-A0A522CGM2-F1-MODEL\_V4 | 1.0 | 4.128e-11 | 325 | 0.118 | 496 | 219 | 18 | 1 | 294 | 1 | 480 | Baseplate\_J domain-containing protein | Baseplate\_J domain-containing protein | | afdb-uniprot50 | AF-A0A7X6ADI4-F1-MODEL\_V4 | 1.0 | 7.067e-11 | 325 | 0.178 | 207 | 148 | 9 | 110 | 296 | 309 | 513 | Putative baseplate assembly protein | Putative baseplate assembly protein | | afdb-uniprot50 | AF-A0A255Y0J9-F1-MODEL\_V4 | 1.0 | 1.96e-12 | 324 | 0.167 | 347 | 203 | 11 | 29 | 293 | 2 | 344 | Baseplate\_J domain-containing protein | Baseplate\_J domain-containing protein | | afdb-uniprot50 | AF-Q5E379-F1-MODEL\_V4 | 1.0 | 1.332e-13 | 324 | 0.136 | 359 | 211 | 12 | 21 | 294 | 4 | 348 | Phage protein | Phage protein | | afdb-uniprot50 | AF-A0A7X6JCW7-F1-MODEL\_V4 | 1.0 | 3.134e-08 | 323 | 0.3 | 113 | 78 | 1 | 184 | 295 | 3 | 115 | Baseplate\_J domain-containing protein | Baseplate\_J domain-containing protein | | afdb-uniprot50 | AF-A0A412G6U6-F1-MODEL\_V4 | 1.0 | 2.281e-13 | 323 | 0.17 | 363 | 202 | 19 | 23 | 296 | 5 | 357 | Baseplate protein J | Baseplate protein J | | afdb-uniprot50 | AF-A0A3A3GJ63-F1-MODEL\_V4 | 1.0 | 2.57e-13 | 323 | 0.141 | 395 | 212 | 17 | 20 | 296 | 8 | 393 | Baseplate\_J domain-containing protein | Baseplate\_J domain-containing protein | | afdb-uniprot50 | AF-A0A1I0CD50-F1-MODEL\_V4 | 1.0 | 6.271e-11 | 321 | 0.117 | 390 | 209 | 17 | 20 | 294 | 10 | 379 | Uncharacterized phage protein gp47/JayE | Uncharacterized phage protein gp47/JayE | | afdb-uniprot50 | AF-A0A853IQN6-F1-MODEL\_V4 | 1.0 | 1.773e-07 | 320 | 0.285 | 91 | 64 | 1 | 209 | 299 | 8 | 97 | Uncharacterized protein | Uncharacterized protein | | afdb-uniprot50 | AF-A0A5B8AU34-F1-MODEL\_V4 | 1.0 | 7.535e-13 | 320 | 0.161 | 352 | 201 | 16 | 13 | 296 | 1 | 326 | Baseplate\_J domain-containing protein | Baseplate\_J domain-containing protein | | afdb-uniprot50 | AF-A0A510KG83-F1-MODEL\_V4 | 1.0 | 3.264e-13 | 320 | 0.128 | 359 | 221 | 14 | 22 | 294 | 1 | 353 | Baseplate J-like protein | Baseplate J-like protein | | afdb-uniprot50 | AF-A0A0F9LBF1-F1-MODEL\_V4 | 1.0 | 1.692e-13 | 320 | 0.161 | 389 | 209 | 18 | 13 | 295 | 1 | 378 | Baseplate\_J domain-containing protein | Baseplate\_J domain-containing protein | | afdb-uniprot50 | AF-A0A6I1HEM5-F1-MODEL\_V4 | 1.0 | 3.663e-11 | 319 | 0.405 | 185 | 90 | 4 | 8 | 180 | 5 | 181 | Uncharacterized protein | Uncharacterized protein | | afdb-uniprot50 | AF-A0A412CI32-F1-MODEL\_V4 | 1.0 | 1.182e-13 | 319 | 0.151 | 356 | 213 | 12 | 19 | 292 | 2 | 350 | Baseplate\_J domain-containing protein | Baseplate\_J domain-containing protein | | afdb-uniprot50 | AF-A0A258NW90-F1-MODEL\_V4 | 1.0 | 1.796e-13 | 319 | 0.157 | 369 | 214 | 15 | 11 | 295 | 2 | 357 | Baseplate\_J domain-containing protein | Baseplate\_J domain-containing protein | | afdb-uniprot50 | AF-A0A0F9F7E7-F1-MODEL\_V4 | 1.0 | 4.015e-12 | 319 | 0.137 | 372 | 210 | 16 | 20 | 295 | 1 | 357 | Baseplate\_J domain-containing protein | Baseplate\_J domain-containing protein | | afdb-uniprot50 | AF-A0A2G2PW46-F1-MODEL\_V4 | 1.0 | 9.569e-13 | 319 | 0.142 | 386 | 210 | 16 | 23 | 298 | 28 | 402 | Baseplate\_J domain-containing protein | Baseplate\_J domain-containing protein | | afdb-uniprot50 | AF-A0A653R9L3-F1-MODEL\_V4 | 1.0 | 4.672e-13 | 318 | 0.161 | 359 | 210 | 14 | 20 | 293 | 4 | 356 | Baseplate\_J domain-containing protein | Baseplate\_J domain-containing protein | | afdb-uniprot50 | AF-A0A1G9EXV1-F1-MODEL\_V4 | 1.0 | 1.011e-10 | 318 | 0.204 | 215 | 144 | 9 | 110 | 299 | 421 | 633 | Putative baseplate assembly protein | Putative baseplate assembly protein | | afdb-uniprot50 | AF-A0A450RV24-F1-MODEL\_V4 | 1.0 | 4.803e-12 | 317 | 0.143 | 397 | 216 | 20 | 14 | 295 | 6 | 393 | Uncharacterized phage protein gp47/JayE | Uncharacterized phage protein gp47/JayE | | afdb-uniprot50 | AF-A0A3N5XVA3-F1-MODEL\_V4 | 1.0 | 1.074e-10 | 317 | 0.185 | 216 | 143 | 10 | 111 | 296 | 813 | 1025 | Putative baseplate assembly protein | Putative baseplate assembly protein | | afdb-uniprot50 | AF-A0A6I2SNH4-F1-MODEL\_V4 | 1.0 | 1.255e-13 | 316 | 0.193 | 336 | 195 | 16 | 1 | 295 | 34 | 334 | Baseplate\_J domain-containing protein | Baseplate\_J domain-containing protein | | afdb-uniprot50 | AF-A0A2N2BUM8-F1-MODEL\_V4 | 1.0 | 5.907e-11 | 316 | 0.121 | 396 | 213 | 17 | 20 | 295 | 12 | 392 | Baseplate\_J domain-containing protein | Baseplate\_J domain-containing protein | | afdb-uniprot50 | AF-A0A4Q3NS83-F1-MODEL\_V4 | 1.0 | 1.998e-07 | 315 | 0.538 | 91 | 41 | 1 | 1 | 91 | 1 | 90 | Baseplate assembly protein | Baseplate assembly protein | | afdb-uniprot50 | AF-A0A166EHE8-F1-MODEL\_V4 | 1.0 | 2.57e-13 | 315 | 0.142 | 365 | 212 | 16 | 20 | 293 | 23 | 377 | Baseplate J-like protein | Baseplate J-like protein | | afdb-uniprot50 | AF-A0A7X9CZY0-F1-MODEL\_V4 | 1.0 | 4.959e-13 | 315 | 0.136 | 397 | 208 | 17 | 20 | 295 | 12 | 394 | Baseplate J/gp47 family protein | Baseplate J/gp47 family protein | | afdb-uniprot50 | AF-A0A154LLK7-F1-MODEL\_V4 | 1.0 | 1.638e-12 | 314 | 0.159 | 364 | 209 | 13 | 20 | 293 | 8 | 364 | Baseplate\_J domain-containing protein | Baseplate\_J domain-containing protein | | afdb-uniprot50 | AF-A0A3D1IYK7-F1-MODEL\_V4 | 1.0 | 1.408e-11 | 314 | 0.142 | 392 | 207 | 18 | 14 | 295 | 1 | 373 | Baseplate\_J domain-containing protein | Baseplate\_J domain-containing protein | | afdb-uniprot50 | AF-A0A1L6I8I3-F1-MODEL\_V4 | 1.0 | 4.401e-13 | 314 | 0.144 | 402 | 205 | 15 | 20 | 293 | 4 | 394 | Baseplate\_J domain-containing protein | Baseplate\_J domain-containing protein | | afdb-uniprot50 | AF-A0A1Y1QPN6-F1-MODEL\_V4 | 1.0 | 5.933e-13 | 312 | 0.142 | 357 | 212 | 12 | 12 | 292 | 2 | 340 | Baseplate\_J domain-containing protein | Baseplate\_J domain-containing protein | | afdb-uniprot50 | AF-R6PWP6-F1-MODEL\_V4 | 1.0 | 1.255e-13 | 312 | 0.169 | 359 | 200 | 14 | 23 | 293 | 5 | 353 | Baseplate J family protein | Baseplate J family protein | | afdb-uniprot50 | AF-A0A437M1E1-F1-MODEL\_V4 | 1.0 | 3.356e-12 | 312 | 0.148 | 390 | 206 | 17 | 14 | 293 | 1 | 374 | Baseplate\_J domain-containing protein | Baseplate\_J domain-containing protein | | afdb-uniprot50 | AF-A0A5A9X997-F1-MODEL\_V4 | 1.0 | 8.73e-12 | 312 | 0.153 | 385 | 206 | 18 | 20 | 295 | 4 | 377 | Baseplate\_J domain-containing protein | Baseplate\_J domain-containing protein | | afdb-uniprot50 | AF-A0A3D5BKD5-F1-MODEL\_V4 | 1.0 | 7.78e-14 | 312 | 0.152 | 399 | 219 | 15 | 14 | 299 | 3 | 395 | Baseplate\_J domain-containing protein | Baseplate\_J domain-containing protein | | afdb-uniprot50 | AF-A0A5C7QAD1-F1-MODEL\_V4 | 1.0 | 3.997e-10 | 312 | 0.148 | 209 | 150 | 10 | 112 | 295 | 310 | 515 | Uncharacterized protein | Uncharacterized protein | | afdb-uniprot50 | AF-A0A7Z7J4X3-F1-MODEL\_V4 | 1.0 | 2.62e-08 | 311 | 0.451 | 124 | 59 | 2 | 21 | 137 | 1 | 122 | Uncharacterized protein | Uncharacterized protein | | afdb-uniprot50 | AF-M5DNC9-F1-MODEL\_V4 | 1.0 | 7.998e-13 | 311 | 0.149 | 361 | 214 | 14 | 13 | 293 | 1 | 348 | Baseplate J-like protein | Baseplate J-like protein | | afdb-uniprot50 | AF-A0A381M382-F1-MODEL\_V4 | 1.0 | 2.859e-07 | 310 | 0.319 | 94 | 61 | 1 | 209 | 299 | 40 | 133 | Phage-related baseplate assembly protein | Phage-related baseplate assembly protein | | afdb-uniprot50 | AF-A0A2V7X4R7-F1-MODEL\_V4 | 1.0 | 1.04e-09 | 309 | 0.177 | 214 | 147 | 10 | 112 | 299 | 201 | 411 | Baseplate\_J domain-containing protein | Baseplate\_J domain-containing protein | | afdb-uniprot50 | AF-A0A174T558-F1-MODEL\_V4 | 1.0 | 1.952e-10 | 309 | 0.14 | 220 | 159 | 10 | 96 | 296 | 525 | 733 | Uncharacterized homolog of phage Mu protein gp47 | Uncharacterized homolog of phage Mu protein gp47 | | afdb-uniprot50 | AF-A0A7U6AEJ8-F1-MODEL\_V4 | 1.0 | 3.134e-08 | 308 | 0.454 | 110 | 60 | 0 | 135 | 244 | 2 | 111 | Baseplate\_J domain-containing protein | Baseplate\_J domain-containing protein | | afdb-uniprot50 | AF-A0A2H0IXG9-F1-MODEL\_V4 | 1.0 | 1.205e-08 | 308 | 0.203 | 182 | 119 | 8 | 133 | 293 | 3 | 179 | Uncharacterized protein | Uncharacterized protein | | afdb-uniprot50 | AF-A0A0A8WTQ2-F1-MODEL\_V4 | 1.0 | 8.491e-13 | 308 | 0.163 | 324 | 201 | 14 | 13 | 296 | 1 | 294 | Baseplate\_J domain-containing protein | Baseplate\_J domain-containing protein | | afdb-uniprot50 | AF-M3JG00-F1-MODEL\_V4 | 1.0 | 1.21e-10 | 308 | 0.13 | 391 | 213 | 16 | 13 | 296 | 2 | 372 | Baseplate\_J domain-containing protein | Baseplate\_J domain-containing protein | | afdb-uniprot50 | AF-A0A534UQ31-F1-MODEL\_V4 | 1.0 | 1.364e-10 | 308 | 0.179 | 217 | 145 | 10 | 110 | 296 | 778 | 991 | Putative baseplate assembly protein | Putative baseplate assembly protein | | afdb-uniprot50 | AF-A0A379TSV1-F1-MODEL\_V4 | 1.0 | 3.631e-07 | 307 | 0.322 | 90 | 59 | 2 | 210 | 298 | 1 | 89 | Putative bacteriophage baseplate assembly protein | Putative bacteriophage baseplate assembly protein | | afdb-uniprot50 | AF-A0A1G3LLV8-F1-MODEL\_V4 | 1.0 | 3.148e-10 | 307 | 0.119 | 495 | 217 | 19 | 12 | 295 | 7 | 493 | Baseplate\_J domain-containing protein | Baseplate\_J domain-containing protein | | afdb-uniprot50 | AF-A0A150TFN7-F1-MODEL\_V4 | 1.0 | 1.631e-10 | 307 | 0.147 | 223 | 153 | 10 | 110 | 300 | 454 | 671 | Baseplate\_J domain-containing protein | Baseplate\_J domain-containing protein | | afdb-uniprot50 | AF-A0A2I0CWU0-F1-MODEL\_V4 | 1.0 | 4.959e-13 | 306 | 0.147 | 366 | 204 | 13 | 22 | 294 | 14 | 364 | Baseplate\_J domain-containing protein | Baseplate\_J domain-containing protein | | afdb-uniprot50 | AF-A0A7C9QTR2-F1-MODEL\_V4 | 1.0 | 4.505e-10 | 306 | 0.175 | 211 | 148 | 11 | 110 | 296 | 791 | 999 | Uncharacterized protein | Uncharacterized protein | | afdb-uniprot50 | AF-A0A6N8PP11-F1-MODEL\_V4 | 1.0 | 3.222e-07 | 305 | 0.554 | 83 | 37 | 0 | 80 | 162 | 1 | 83 | Baseplate assembly protein | Baseplate assembly protein | | afdb-uniprot50 | AF-E6LJD1-F1-MODEL\_V4 | 1.0 | 6.874e-12 | 305 | 0.127 | 385 | 212 | 18 | 22 | 295 | 12 | 383 | Baseplate\_J domain-containing protein | Baseplate\_J domain-containing protein | | afdb-uniprot50 | AF-E3R381-F1-MODEL\_V4 | 1.0 | 4.803e-12 | 305 | 0.123 | 397 | 207 | 20 | 22 | 296 | 15 | 392 | Baseplate\_J domain-containing protein | Baseplate\_J domain-containing protein | | afdb-uniprot50 | AF-A0A355JCM0-F1-MODEL\_V4 | 1.0 | 2.884e-11 | 305 | 0.136 | 417 | 213 | 17 | 20 | 300 | 9 | 414 | Baseplate\_J domain-containing protein | Baseplate\_J domain-containing protein | | afdb-uniprot50 | AF-A0A2E1AI28-F1-MODEL\_V4 | 1.0 | 6.844e-10 | 305 | 0.189 | 216 | 146 | 10 | 111 | 300 | 431 | 643 | Putative baseplate assembly protein | Putative baseplate assembly protein | | afdb-uniprot50 | AF-A0A3S4LRQ5-F1-MODEL\_V4 | 1.0 | 2.468e-08 | 304 | 0.342 | 111 | 71 | 2 | 191 | 300 | 2 | 111 | Baseplate protein | Baseplate protein | | afdb-uniprot50 | AF-A0A317HNT2-F1-MODEL\_V4 | 1.0 | 1.501e-13 | 304 | 0.169 | 349 | 203 | 17 | 1 | 294 | 4 | 320 | Baseplate\_J domain-containing protein | Baseplate\_J domain-containing protein | | afdb-uniprot50 | AF-A0A0E3NKG6-F1-MODEL\_V4 | 1.0 | 7.502e-11 | 304 | 0.163 | 233 | 151 | 11 | 110 | 300 | 579 | 809 | Baseplate\_J domain-containing protein | Baseplate\_J domain-containing protein | | afdb-uniprot50 | AF-J9ZB16-F1-MODEL\_V4 | 1.0 | 3.356e-12 | 303 | 0.152 | 368 | 213 | 12 | 20 | 295 | 2 | 362 | Baseplate J family protein | Baseplate J family protein | | afdb-uniprot50 | AF-A0A3R7UR03-F1-MODEL\_V4 | 1.0 | 1.952e-10 | 303 | 0.148 | 222 | 147 | 10 | 113 | 294 | 380 | 599 | Baseplate\_J domain-containing protein | Baseplate\_J domain-containing protein | | afdb-uniprot50 | AF-A0A1H4QY22-F1-MODEL\_V4 | 1.0 | 3.435e-09 | 302 | 0.187 | 171 | 124 | 8 | 132 | 295 | 4 | 166 | Baseplate J-like protein | Baseplate J-like protein | | afdb-uniprot50 | AF-A0A3F3A315-F1-MODEL\_V4 | 1.0 | 1.29e-12 | 302 | 0.116 | 360 | 225 | 13 | 23 | 295 | 1 | 354 | Baseplate\_J domain-containing protein | Baseplate\_J domain-containing protein | | afdb-uniprot50 | AF-A0A7T8FM21-F1-MODEL\_V4 | 1.0 | 1.594e-13 | 302 | 0.141 | 374 | 216 | 15 | 12 | 292 | 3 | 364 | Baseplate J/gp47 family protein | Baseplate J/gp47 family protein | | afdb-uniprot50 | AF-A0A1S1HMN1-F1-MODEL\_V4 | 1.0 | 3.997e-10 | 302 | 0.095 | 378 | 225 | 16 | 20 | 294 | 10 | 373 | Baseplate\_J domain-containing protein | Baseplate\_J domain-containing protein | | afdb-uniprot50 | AF-A0A7Y6W8F8-F1-MODEL\_V4 | 1.0 | 1.846e-12 | 302 | 0.17 | 404 | 194 | 17 | 21 | 293 | 6 | 399 | Baseplate J/gp47 family protein | Baseplate J/gp47 family protein | | afdb-uniprot50 | AF-A0A1D2R572-F1-MODEL\_V4 | 1.0 | 2.793e-10 | 302 | 0.164 | 194 | 140 | 9 | 111 | 296 | 333 | 512 | Uncharacterized protein | Uncharacterized protein | | afdb-uniprot50 | AF-A0A431IBF5-F1-MODEL\_V4 | 1.0 | 2.345e-12 | 301 | 0.136 | 375 | 214 | 18 | 13 | 293 | 1 | 359 | Baseplate J protein | Baseplate J protein | | afdb-uniprot50 | AF-A0A433WZS4-F1-MODEL\_V4 | 1.0 | 4.524e-12 | 301 | 0.176 | 379 | 198 | 17 | 13 | 296 | 3 | 362 | Baseplate\_J domain-containing protein | Baseplate\_J domain-containing protein | | afdb-uniprot50 | AF-A0A838CGI4-F1-MODEL\_V4 | 1.0 | 1.435e-06 | 300 | 0.4 | 95 | 57 | 0 | 8 | 102 | 4 | 98 | Baseplate J protein | Baseplate J protein | | afdb-uniprot50 | AF-A0A257PB54-F1-MODEL\_V4 | 1.0 | 1.326e-11 | 299 | 0.131 | 366 | 217 | 15 | 20 | 293 | 4 | 360 | Baseplate\_J domain-containing protein | Baseplate\_J domain-containing protein | | afdb-uniprot50 | AF-A0A1Y3QLM5-F1-MODEL\_V4 | 1.0 | 5.933e-13 | 299 | 0.168 | 398 | 197 | 18 | 20 | 295 | 7 | 392 | Baseplate\_J domain-containing protein | Baseplate\_J domain-containing protein | | afdb-uniprot50 | AF-A0A0F9SDC1-F1-MODEL\_V4 | 1.0 | 1.326e-11 | 299 | 0.122 | 472 | 211 | 18 | 22 | 294 | 5 | 472 | Baseplate\_J domain-containing protein | Baseplate\_J domain-containing protein | | afdb-uniprot50 | AF-A0A0D5NAR8-F1-MODEL\_V4 | 1.0 | 1.364e-10 | 299 | 0.166 | 228 | 151 | 10 | 110 | 300 | 447 | 672 | Baseplate\_J domain-containing protein | Baseplate\_J domain-containing protein | | afdb-uniprot50 | AF-A0A0S4LB89-F1-MODEL\_V4 | 1.0 | 3.547e-10 | 298 | 0.202 | 227 | 143 | 9 | 110 | 300 | 434 | 658 | Baseplate\_J domain-containing protein | Baseplate\_J domain-containing protein | | afdb-uniprot50 | AF-A0A655DHR5-F1-MODEL\_V4 | 1.0 | 6.244e-09 | 297 | 0.323 | 133 | 87 | 3 | 170 | 300 | 3 | 134 | Phage baseplate protein | Phage baseplate protein | | afdb-uniprot50 | AF-A0A164ZAU4-F1-MODEL\_V4 | 1.0 | 2.261e-09 | 296 | 0.201 | 194 | 134 | 8 | 116 | 294 | 12 | 199 | Phage baseplate | Phage baseplate | | afdb-uniprot50 | AF-A0A824RZ05-F1-MODEL\_V4 | 1.0 | 1.78e-09 | 296 | 0.245 | 236 | 100 | 4 | 10 | 167 | 17 | 252 | Baseplate assembly protein | Baseplate assembly protein | | afdb-uniprot50 | AF-A0A7K4BK92-F1-MODEL\_V4 | 1.0 | 1.906e-13 | 296 | 0.145 | 393 | 196 | 20 | 20 | 295 | 11 | 380 | Baseplate\_J domain-containing protein | Baseplate\_J domain-containing protein | | afdb-uniprot50 | AF-A0A2T4N3C8-F1-MODEL\_V4 | 1.0 | 2.421e-13 | 294 | 0.138 | 398 | 223 | 14 | 11 | 294 | 2 | 393 | Baseplate\_J domain-containing protein | Baseplate\_J domain-containing protein | | afdb-uniprot50 | AF-L0L0C7-F1-MODEL\_V4 | 1.0 | 5.077e-10 | 294 | 0.164 | 225 | 150 | 9 | 111 | 299 | 997 | 1219 | Baseplate\_J domain-containing protein | Baseplate\_J domain-containing protein | | afdb-uniprot50 | AF-A0A1Z4C4M5-F1-MODEL\_V4 | 1.0 | 2.559e-11 | 293 | 0.152 | 380 | 200 | 17 | 20 | 293 | 3 | 366 | Baseplate\_J domain-containing protein | Baseplate\_J domain-containing protein | | afdb-uniprot50 | AF-A0A5C7Q1H5-F1-MODEL\_V4 | 1.0 | 6.271e-11 | 293 | 0.152 | 381 | 208 | 17 | 20 | 295 | 3 | 373 | Baseplate\_J domain-containing protein | Baseplate\_J domain-containing protein | | afdb-uniprot50 | AF-A0A5F1RNH5-F1-MODEL\_V4 | 1.0 | 3.075e-13 | 293 | 0.15 | 386 | 210 | 18 | 20 | 296 | 8 | 384 | Baseplate\_J domain-containing protein | Baseplate\_J domain-containing protein | | afdb-uniprot50 | AF-A0A059V4B9-F1-MODEL\_V4 | 1.0 | 2.859e-07 | 292 | 0.261 | 111 | 81 | 1 | 191 | 300 | 6 | 116 | Phage-related baseplate assembly protein | Phage-related baseplate assembly protein | | afdb-uniprot50 | AF-A0A1Z4HHP6-F1-MODEL\_V4 | 1.0 | 1.016e-12 | 292 | 0.185 | 345 | 197 | 22 | 13 | 299 | 3 | 321 | Baseplate\_J domain-containing protein | Baseplate\_J domain-containing protein | | afdb-uniprot50 | AF-A0A1R0WGM4-F1-MODEL\_V4 | 1.0 | 1.369e-12 | 291 | 0.157 | 355 | 202 | 16 | 28 | 296 | 2 | 345 | Baseplate\_J domain-containing protein | Baseplate\_J domain-containing protein | | afdb-uniprot50 | AF-A0A316PC92-F1-MODEL\_V4 | 1.0 | 6.686e-13 | 291 | 0.121 | 355 | 222 | 11 | 22 | 292 | 1 | 349 | Baseplate\_J domain-containing protein | Baseplate\_J domain-containing protein | | afdb-uniprot50 | AF-A0A1G8B3H6-F1-MODEL\_V4 | 1.0 | 7.502e-11 | 291 | 0.139 | 381 | 209 | 19 | 20 | 300 | 3 | 364 | Baseplate J-like protein | Baseplate J-like protein | | afdb-uniprot50 | AF-A0A662L870-F1-MODEL\_V4 | 1.0 | 4.505e-10 | 291 | 0.166 | 210 | 148 | 10 | 110 | 294 | 422 | 629 | Putative baseplate assembly protein | Putative baseplate assembly protein | | afdb-uniprot50 | AF-A0A7W7H569-F1-MODEL\_V4 | 1.0 | 1.321e-09 | 291 | 0.181 | 215 | 144 | 10 | 110 | 300 | 966 | 1172 | Baseplate\_J domain-containing protein | Baseplate\_J domain-containing protein | | afdb-uniprot50 | AF-A0A2X3LXX5-F1-MODEL\_V4 | 1.0 | 1.352e-06 | 290 | 0.345 | 84 | 55 | 0 | 216 | 299 | 2 | 85 | Baseplate assembly protein J | Baseplate assembly protein J | | afdb-uniprot50 | AF-D4DSI2-F1-MODEL\_V4 | 1.0 | 1.069e-08 | 290 | 0.233 | 154 | 103 | 7 | 151 | 294 | 2 | 150 | Baseplate\_J domain-containing protein | Baseplate\_J domain-containing protein | | afdb-uniprot50 | AF-A0A398BBK4-F1-MODEL\_V4 | 1.0 | 2.978e-12 | 290 | 0.138 | 383 | 213 | 17 | 20 | 295 | 8 | 380 | Baseplate J/gp47 family protein | Baseplate J/gp47 family protein | | afdb-uniprot50 | AF-A0A0F7FBD4-F1-MODEL\_V4 | 1.0 | 1.838e-10 | 290 | 0.127 | 391 | 214 | 19 | 20 | 295 | 4 | 382 | Baseplate\_J domain-containing protein | Baseplate\_J domain-containing protein | | afdb-uniprot50 | AF-A0A1G9EXR2-F1-MODEL\_V4 | 1.0 | 9.227e-10 | 290 | 0.221 | 212 | 134 | 13 | 110 | 296 | 870 | 1075 | Putative baseplate assembly protein | Putative baseplate assembly protein | | afdb-uniprot50 | AF-A0A7R7TB47-F1-MODEL\_V4 | 1.0 | 5.907e-11 | 289 | 0.132 | 384 | 210 | 17 | 14 | 294 | 1 | 364 | Uncharacterized protein | Uncharacterized protein | | afdb-uniprot50 | AF-H6NDS9-F1-MODEL\_V4 | 1.0 | 7.535e-13 | 288 | 0.157 | 394 | 204 | 20 | 20 | 295 | 8 | 391 | Baseplate protein | Baseplate protein | | afdb-uniprot50 | AF-A0A1I1TQL3-F1-MODEL\_V4 | 1.0 | 1.215e-12 | 288 | 0.146 | 396 | 203 | 17 | 20 | 296 | 14 | 393 | Uncharacterized phage protein gp47/JayE | Uncharacterized phage protein gp47/JayE | | afdb-uniprot50 | AF-Q2L2U7-F1-MODEL\_V4 | 1.0 | 1.677e-09 | 288 | 0.12 | 415 | 214 | 19 | 13 | 295 | 1 | 396 | Phage protein | Phage protein | | afdb-uniprot50 | AF-A0A810LC53-F1-MODEL\_V4 | 1.0 | 3.148e-10 | 288 | 0.214 | 224 | 137 | 14 | 110 | 300 | 405 | 622 | Putative baseplate assembly protein | Putative baseplate assembly protein | | afdb-uniprot50 | AF-A0A321LRK0-F1-MODEL\_V4 | 1.0 | 8.692e-10 | 288 | 0.158 | 214 | 147 | 11 | 111 | 295 | 557 | 766 | Putative baseplate assembly protein | Putative baseplate assembly protein | | afdb-uniprot50 | AF-V4IZ11-F1-MODEL\_V4 | 1.0 | 2.19e-08 | 287 | 0.271 | 151 | 85 | 3 | 168 | 295 | 2 | 150 | Baseplate\_J domain-containing protein | Baseplate\_J domain-containing protein | | afdb-uniprot50 | AF-A0A353REU8-F1-MODEL\_V4 | 1.0 | 1.244e-09 | 287 | 0.201 | 199 | 135 | 10 | 111 | 294 | 39 | 228 | Baseplate\_J domain-containing protein | Baseplate\_J domain-containing protein | | afdb-uniprot50 | AF-A0A2V1HBX8-F1-MODEL\_V4 | 1.0 | 1.78e-09 | 287 | 0.103 | 377 | 219 | 20 | 22 | 294 | 12 | 373 | Baseplate\_J domain-containing protein | Baseplate\_J domain-containing protein | | afdb-uniprot50 | AF-A0A1F5C406-F1-MODEL\_V4 | 1.0 | 1.898e-11 | 287 | 0.119 | 469 | 210 | 18 | 20 | 292 | 4 | 465 | Baseplate\_J domain-containing protein | Baseplate\_J domain-containing protein | | afdb-uniprot50 | AF-A0A7G9KUY0-F1-MODEL\_V4 | 1.0 | 2.39e-07 | 285 | 0.549 | 102 | 45 | 1 | 1 | 102 | 1 | 101 | Baseplate assembly protein J | Baseplate assembly protein J | | afdb-uniprot50 | AF-A0A432ULA7-F1-MODEL\_V4 | 1.0 | 1.587e-11 | 285 | 0.14 | 350 | 211 | 14 | 20 | 293 | 2 | 337 | Baseplate\_J domain-containing protein | Baseplate\_J domain-containing protein | | afdb-uniprot50 | AF-A0A3A0GCC9-F1-MODEL\_V4 | 1.0 | 8.692e-10 | 285 | 0.169 | 218 | 148 | 11 | 110 | 296 | 527 | 742 | Putative baseplate assembly protein | Putative baseplate assembly protein | | afdb-uniprot50 | AF-A0A239C2Z7-F1-MODEL\_V4 | 1.0 | 7.502e-11 | 285 | 0.191 | 245 | 136 | 13 | 110 | 296 | 507 | 747 | Putative baseplate assembly protein | Putative baseplate assembly protein | | afdb-uniprot50 | AF-A0A1Z4HHL8-F1-MODEL\_V4 | 1.0 | 1.104e-09 | 284 | 0.144 | 235 | 145 | 11 | 110 | 294 | 844 | 1072 | Baseplate\_J domain-containing protein | Baseplate\_J domain-containing protein | | afdb-uniprot50 | AF-A0A251ZY30-F1-MODEL\_V4 | 1.0 | 3.562e-12 | 283 | 0.132 | 376 | 215 | 15 | 16 | 292 | 1 | 364 | Baseplate\_J domain-containing protein | Baseplate\_J domain-containing protein | | afdb-uniprot50 | AF-A0A7T4UTX7-F1-MODEL\_V4 | 1.0 | 1.408e-11 | 283 | 0.124 | 386 | 218 | 19 | 21 | 296 | 23 | 398 | Uncharacterized protein | Uncharacterized protein | | afdb-uniprot50 | AF-G0ABR9-F1-MODEL\_V4 | 1.0 | 3.435e-09 | 283 | 0.153 | 209 | 153 | 10 | 110 | 296 | 824 | 1030 | Baseplate\_J domain-containing protein | Baseplate\_J domain-containing protein | | afdb-uniprot50 | AF-I5B7H1-F1-MODEL\_V4 | 1.0 | 7.266e-10 | 283 | 0.164 | 237 | 155 | 12 | 101 | 296 | 794 | 1028 | Baseplate J-like protein | Baseplate J-like protein | | afdb-uniprot50 | AF-A0A1Q6YE39-F1-MODEL\_V4 | 1.0 | 6.073e-10 | 282 | 0.165 | 218 | 148 | 10 | 110 | 295 | 442 | 657 | Putative baseplate assembly protein | Putative baseplate assembly protein | | afdb-uniprot50 | AF-A0A7C6P3L4-F1-MODEL\_V4 | 1.0 | 9.796e-10 | 282 | 0.15 | 212 | 153 | 10 | 110 | 298 | 496 | 703 | Baseplate\_J domain-containing protein | Baseplate\_J domain-containing protein | | afdb-uniprot50 | AF-A0A1S0V4H6-F1-MODEL\_V4 | 1.0 | 2.693e-07 | 281 | 0.504 | 105 | 52 | 0 | 1 | 105 | 1 | 105 | Uncharacterized protein | Uncharacterized protein | | afdb-uniprot50 | AF-A0A7W7HM91-F1-MODEL\_V4 | 1.0 | 1.402e-09 | 281 | 0.161 | 217 | 148 | 9 | 110 | 294 | 396 | 610 | Putative phage baseplate assembly protein | Putative phage baseplate assembly protein | | afdb-uniprot50 | AF-A0A3D4UXE9-F1-MODEL\_V4 | 1.0 | 4.917e-09 | 280 | 0.147 | 217 | 147 | 9 | 114 | 294 | 21 | 235 | Uncharacterized protein | Uncharacterized protein | | afdb-uniprot50 | AF-A0A1P8MVC1-F1-MODEL\_V4 | 1.0 | 8.188e-10 | 280 | 0.179 | 234 | 137 | 12 | 110 | 294 | 426 | 653 | Putative baseplate assembly protein | Putative baseplate assembly protein | | afdb-uniprot50 | AF-A0A7X8K102-F1-MODEL\_V4 | 1.0 | 1.321e-09 | 280 | 0.118 | 211 | 154 | 10 | 115 | 298 | 494 | 699 | Baseplate\_J domain-containing protein | Baseplate\_J domain-containing protein | | afdb-uniprot50 | AF-A0A4U2ZFI7-F1-MODEL\_V4 | 1.0 | 1.454e-12 | 278 | 0.138 | 382 | 224 | 14 | 8 | 296 | 2 | 371 | Baseplate J/gp47 family protein | Baseplate J/gp47 family protein | | afdb-uniprot50 | AF-A0A3M4S7G9-F1-MODEL\_V4 | 1.0 | 8.382e-07 | 277 | 0.438 | 98 | 55 | 0 | 101 | 198 | 2 | 99 | Phage baseplate assembly protein J | Phage baseplate assembly protein J | | afdb-uniprot50 | AF-A0A2E6KN58-F1-MODEL\_V4 | 1.0 | 6.657e-11 | 277 | 0.11 | 362 | 224 | 13 | 20 | 294 | 10 | 360 | Baseplate J protein | Baseplate J protein | | afdb-uniprot50 | AF-A0A6N4HYA5-F1-MODEL\_V4 | 1.0 | 9.569e-13 | 277 | 0.147 | 408 | 209 | 18 | 13 | 296 | 1 | 393 | Baseplate\_J domain-containing protein | Baseplate\_J domain-containing protein | | afdb-uniprot50 | AF-A0A550JHH0-F1-MODEL\_V4 | 1.0 | 5.099e-12 | 276 | 0.189 | 328 | 189 | 18 | 13 | 294 | 1 | 297 | Baseplate\_J domain-containing protein | Baseplate\_J domain-containing protein | | afdb-uniprot50 | AF-A0A150HQF5-F1-MODEL\_V4 | 1.0 | 1.177e-11 | 276 | 0.134 | 380 | 208 | 13 | 20 | 293 | 4 | 368 | Baseplate J-like protein | Baseplate J-like protein | | afdb-uniprot50 | AF-A0A2N0F3D7-F1-MODEL\_V4 | 1.0 | 1.177e-11 | 276 | 0.121 | 395 | 215 | 17 | 20 | 294 | 9 | 391 | Putative phage protein gp47/JayE | Putative phage protein gp47/JayE | | afdb-uniprot50 | AF-A0A535END5-F1-MODEL\_V4 | 1.0 | 1.78e-09 | 276 | 0.193 | 232 | 138 | 13 | 111 | 300 | 190 | 414 | Putative baseplate assembly protein | Putative baseplate assembly protein | | afdb-uniprot50 | AF-A0A7C4C5M9-F1-MODEL\_V4 | 1.0 | 2.139e-11 | 276 | 0.12 | 439 | 192 | 12 | 42 | 300 | 97 | 521 | Baseplate\_J domain-containing protein | Baseplate\_J domain-containing protein | | afdb-uniprot50 | AF-A0A6P2EU18-F1-MODEL\_V4 | 1.0 | 2.4e-09 | 276 | 0.172 | 214 | 144 | 10 | 110 | 294 | 501 | 710 | Baseplate J-like protein | Baseplate J-like protein | | afdb-uniprot50 | AF-A0A2K3JD84-F1-MODEL\_V4 | 1.0 | 2.717e-11 | 275 | 0.114 | 446 | 218 | 15 | 16 | 292 | 1 | 438 | Uncharacterized protein | Uncharacterized protein | | afdb-uniprot50 | AF-A0A4T3EY99-F1-MODEL\_V4 | 1.0 | 8.692e-10 | 275 | 0.162 | 228 | 150 | 11 | 110 | 300 | 442 | 665 | Putative baseplate assembly protein | Putative baseplate assembly protein | | afdb-uniprot50 | AF-A0A376ZP12-F1-MODEL\_V4 | 1.0 | 2.181e-06 | 274 | 0.298 | 87 | 59 | 1 | 210 | 296 | 1 | 85 | Baseplate assembly protein J | Baseplate assembly protein J | | afdb-uniprot50 | AF-A0A661L8M5-F1-MODEL\_V4 | 1.0 | 4.244e-10 | 274 | 0.164 | 207 | 137 | 10 | 116 | 295 | 18 | 215 | Baseplate\_J domain-containing protein | Baseplate\_J domain-containing protein | | afdb-uniprot50 | AF-A0A4Q3RYT5-F1-MODEL\_V4 | 1.0 | 6.844e-10 | 274 | 0.204 | 225 | 145 | 11 | 102 | 294 | 407 | 629 | Putative baseplate assembly protein | Putative baseplate assembly protein | | afdb-uniprot50 | AF-A0A4U9DA24-F1-MODEL\_V4 | 1.0 | 4.11e-09 | 273 | 0.189 | 206 | 136 | 11 | 113 | 293 | 25 | 224 | Uncharacterized homolog of phage Mu protein gp47 | Uncharacterized homolog of phage Mu protein gp47 | | afdb-uniprot50 | AF-A0A2R7QCT8-F1-MODEL\_V4 | 1.0 | 8.188e-10 | 273 | 0.186 | 204 | 138 | 11 | 110 | 294 | 115 | 309 | Baseplate\_J domain-containing protein | Baseplate\_J domain-containing protein | | afdb-uniprot50 | AF-A0A6H5RIG3-F1-MODEL\_V4 | 1.0 | 1.96e-12 | 273 | 0.142 | 387 | 210 | 18 | 20 | 296 | 8 | 382 | Baseplate J/gp47 family protein | Baseplate J/gp47 family protein | | afdb-uniprot50 | AF-A0A5D8QH11-F1-MODEL\_V4 | 1.0 | 1.205e-08 | 273 | 0.087 | 479 | 224 | 19 | 22 | 296 | 14 | 483 | Uncharacterized protein | Uncharacterized protein | | afdb-uniprot50 | AF-I3ZGQ5-F1-MODEL\_V4 | 1.0 | 5.721e-10 | 273 | 0.207 | 227 | 136 | 11 | 111 | 298 | 588 | 809 | Putative phage Mu protein gp47-like protein | Putative phage Mu protein gp47-like protein | | afdb-uniprot50 | AF-A0A1Z9HXQ8-F1-MODEL\_V4 | 1.0 | 4.363e-09 | 272 | 0.147 | 217 | 147 | 9 | 114 | 294 | 386 | 600 | Uncharacterized protein | Uncharacterized protein | | afdb-uniprot50 | AF-A0A7Y4U5C5-F1-MODEL\_V4 | 1.0 | 2.13e-09 | 272 | 0.186 | 225 | 146 | 10 | 110 | 300 | 438 | 659 | Putative baseplate assembly protein | Putative baseplate assembly protein | | afdb-uniprot50 | AF-A0A2I0F8R2-F1-MODEL\_V4 | 1.0 | 1.369e-12 | 271 | 0.125 | 398 | 223 | 15 | 16 | 294 | 1 | 392 | Baseplate\_J domain-containing protein | Baseplate\_J domain-containing protein | | afdb-uniprot50 | AF-A0A2V7W195-F1-MODEL\_V4 | 1.0 | 2.19e-08 | 271 | 0.189 | 216 | 144 | 11 | 111 | 299 | 429 | 640 | Putative baseplate assembly protein | Putative baseplate assembly protein | | afdb-uniprot50 | AF-A0A660N0R2-F1-MODEL\_V4 | 1.0 | 2.859e-07 | 270 | 0.306 | 111 | 75 | 2 | 190 | 299 | 1 | 110 | Baseplate assembly protein | Baseplate assembly protein | | afdb-uniprot50 | AF-A0A2K2TVT0-F1-MODEL\_V4 | 1.0 | 2.884e-11 | 270 | 0.137 | 394 | 212 | 17 | 18 | 295 | 4 | 385 | Baseplate\_J domain-containing protein | Baseplate\_J domain-containing protein | | afdb-uniprot50 | AF-A0A6I3MDQ0-F1-MODEL\_V4 | 1.0 | 2.072e-10 | 270 | 0.138 | 252 | 150 | 14 | 110 | 296 | 415 | 664 | Uncharacterized protein | Uncharacterized protein | | afdb-uniprot50 | AF-A0A0U1DD70-F1-MODEL\_V4 | 1.0 | 1.239e-07 | 269 | 0.26 | 115 | 81 | 2 | 180 | 294 | 3 | 113 | Baseplate assembly protein GpJ | Baseplate assembly protein GpJ | | afdb-uniprot50 | AF-A0A5T0H790-F1-MODEL\_V4 | 1.0 | 1.069e-08 | 269 | 0.23 | 243 | 107 | 6 | 1 | 163 | 3 | 245 | Baseplate assembly protein | Baseplate assembly protein | | afdb-uniprot50 | AF-A0A522CNZ0-F1-MODEL\_V4 | 1.0 | 2.489e-12 | 269 | 0.135 | 390 | 215 | 17 | 20 | 300 | 3 | 379 | Baseplate\_J domain-containing protein | Baseplate\_J domain-containing protein | | afdb-uniprot50 | AF-A0A7J3V0A5-F1-MODEL\_V4 | 1.0 | 1.21e-10 | 269 | 0.111 | 438 | 196 | 15 | 42 | 300 | 101 | 524 | DUF4815 domain-containing protein | DUF4815 domain-containing protein | | afdb-uniprot50 | AF-A0A849PJE9-F1-MODEL\_V4 | 1.0 | 2.548e-09 | 269 | 0.162 | 227 | 149 | 13 | 110 | 299 | 424 | 646 | Putative baseplate assembly protein | Putative baseplate assembly protein | | afdb-uniprot50 | AF-A0A133PS66-F1-MODEL\_V4 | 1.0 | 6.874e-12 | 268 | 0.109 | 394 | 219 | 16 | 20 | 296 | 22 | 400 | Baseplate\_J domain-containing protein | Baseplate\_J domain-containing protein | | afdb-uniprot50 | AF-A0A3N5LBL1-F1-MODEL\_V4 | 1.0 | 3.663e-11 | 268 | 0.157 | 368 | 198 | 13 | 20 | 280 | 2 | 364 | Baseplate\_J domain-containing protein | Baseplate\_J domain-containing protein | | afdb-uniprot50 | AF-A0A0D6KGS5-F1-MODEL\_V4 | 1.0 | 1.244e-09 | 268 | 0.141 | 248 | 152 | 10 | 110 | 300 | 951 | 1194 | Baseplate\_J domain-containing protein | Baseplate\_J domain-containing protein | | afdb-uniprot50 | AF-A0A2D6RI92-F1-MODEL\_V4 | 1.0 | 2.13e-09 | 267 | 0.154 | 226 | 148 | 11 | 110 | 294 | 371 | 594 | Uncharacterized protein | Uncharacterized protein | | afdb-uniprot50 | AF-A0A7Y3CTP7-F1-MODEL\_V4 | 1.0 | 6.447e-10 | 266 | 0.144 | 443 | 184 | 18 | 42 | 293 | 6 | 444 | Baseplate\_J domain-containing protein | Baseplate\_J domain-containing protein | | afdb-uniprot50 | AF-A0A854ZKD2-F1-MODEL\_V4 | 1.0 | 2.4e-09 | 266 | 0.159 | 220 | 148 | 11 | 111 | 295 | 422 | 639 | Putative baseplate assembly protein | Putative baseplate assembly protein | | afdb-uniprot50 | AF-A0A1V4VN91-F1-MODEL\_V4 | 1.0 | 1.677e-09 | 266 | 0.131 | 229 | 158 | 11 | 111 | 300 | 522 | 748 | Baseplate J-like protein | Baseplate J-like protein | | afdb-uniprot50 | AF-A0A3C0F0C1-F1-MODEL\_V4 | 1.0 | 1.104e-09 | 265 | 0.15 | 226 | 146 | 12 | 110 | 294 | 350 | 570 | Uncharacterized protein | Uncharacterized protein | | afdb-uniprot50 | AF-A0A7X2DY09-F1-MODEL\_V4 | 1.0 | 6.6e-07 | 264 | 0.505 | 87 | 43 | 0 | 143 | 229 | 2 | 88 | Baseplate assembly protein | Baseplate assembly protein | | afdb-uniprot50 | AF-A0A399W6F7-F1-MODEL\_V4 | 1.0 | 1.04e-09 | 264 | 0.146 | 252 | 146 | 13 | 110 | 296 | 439 | 686 | Putative baseplate assembly protein | Putative baseplate assembly protein | | afdb-uniprot50 | AF-A0A645HBP4-F1-MODEL\_V4 | 1.0 | 1.67e-07 | 263 | 0.21 | 138 | 103 | 5 | 158 | 294 | 4 | 136 | Baseplate\_J domain-containing protein | Baseplate\_J domain-containing protein | | afdb-uniprot50 | AF-A0A3D1R508-F1-MODEL\_V4 | 1.0 | 2.19e-08 | 263 | 0.185 | 210 | 127 | 11 | 127 | 295 | 85 | 291 | T4-like baseplate wedge | T4-like baseplate wedge | | afdb-uniprot50 | AF-A0A7T8EB75-F1-MODEL\_V4 | 1.0 | 9.267e-12 | 263 | 0.115 | 400 | 230 | 21 | 13 | 295 | 1 | 393 | Baseplate\_J domain-containing protein | Baseplate\_J domain-containing protein | | afdb-uniprot50 | AF-A0A4R8GX56-F1-MODEL\_V4 | 1.0 | 8.223e-12 | 263 | 0.119 | 401 | 212 | 18 | 20 | 296 | 14 | 397 | Putative phage protein gp47/JayE | Putative phage protein gp47/JayE | | afdb-uniprot50 | AF-A0A2W2DLG7-F1-MODEL\_V4 | 1.0 | 1.205e-08 | 263 | 0.215 | 213 | 137 | 11 | 111 | 299 | 406 | 612 | Baseplate\_J domain-containing protein | Baseplate\_J domain-containing protein | | afdb-uniprot50 | AF-A0A6P1MHD6-F1-MODEL\_V4 | 1.0 | 4.11e-09 | 263 | 0.153 | 209 | 143 | 10 | 116 | 296 | 483 | 685 | Baseplate\_J domain-containing protein | Baseplate\_J domain-containing protein | | afdb-uniprot50 | AF-A0A1T3NNP8-F1-MODEL\_V4 | 1.0 | 8.692e-10 | 263 | 0.161 | 248 | 144 | 14 | 111 | 296 | 486 | 731 | Baseplate\_J domain-containing protein | Baseplate\_J domain-containing protein | | afdb-uniprot50 | AF-A0A1Z4PZG8-F1-MODEL\_V4 | 1.0 | 4.11e-09 | 263 | 0.157 | 228 | 142 | 12 | 113 | 296 | 642 | 863 | Baseplate\_J domain-containing protein | Baseplate\_J domain-containing protein | | afdb-uniprot50 | AF-A0A783X0V2-F1-MODEL\_V4 | 1.0 | 1.14e-10 | 262 | 0.14 | 385 | 212 | 17 | 20 | 293 | 4 | 380 | Baseplate J/gp47 family protein | Baseplate J/gp47 family protein | | afdb-uniprot50 | AF-A0A336NB80-F1-MODEL\_V4 | 1.0 | 3.733e-06 | 261 | 0.236 | 93 | 68 | 3 | 207 | 298 | 18 | 108 | Uncharacterized protein | Uncharacterized protein | | afdb-uniprot50 | AF-A0A0R2IBD9-F1-MODEL\_V4 | 1.0 | 7.297e-12 | 261 | 0.118 | 396 | 218 | 20 | 20 | 296 | 8 | 391 | Phage Mu gp47 related protein | Phage Mu gp47 related protein | | afdb-uniprot50 | AF-A0A1C7W237-F1-MODEL\_V4 | 1.0 | 6.244e-09 | 261 | 0.107 | 411 | 222 | 20 | 13 | 296 | 1 | 393 | Baseplate\_J domain-containing protein | Baseplate\_J domain-containing protein | | afdb-uniprot50 | AF-A0A7C7CAA3-F1-MODEL\_V4 | 1.0 | 7.47e-09 | 261 | 0.152 | 216 | 143 | 11 | 116 | 294 | 389 | 601 | Uncharacterized protein | Uncharacterized protein | | afdb-uniprot50 | AF-A0A7X5W2W2-F1-MODEL\_V4 | 1.0 | 1.89e-09 | 261 | 0.162 | 227 | 146 | 10 | 110 | 294 | 452 | 676 | Putative baseplate assembly protein | Putative baseplate assembly protein | | afdb-uniprot50 | AF-A0A0E3WU79-F1-MODEL\_V4 | 1.0 | 1.58e-09 | 261 | 0.181 | 226 | 146 | 13 | 111 | 300 | 990 | 1212 | Baseplate\_J domain-containing protein | Baseplate\_J domain-containing protein | | afdb-uniprot50 | AF-A0A6M0BMD7-F1-MODEL\_V4 | 1.0 | 6.244e-09 | 260 | 0.184 | 211 | 127 | 11 | 125 | 296 | 21 | 225 | Baseplate protein J | Baseplate protein J | | afdb-uniprot50 | AF-A0A843FGQ1-F1-MODEL\_V4 | 1.0 | 3.161e-12 | 260 | 0.155 | 392 | 183 | 16 | 20 | 270 | 16 | 400 | Baseplate J/gp47 family protein | Baseplate J/gp47 family protein | | afdb-uniprot50 | AF-A0A4Y7S2C9-F1-MODEL\_V4 | 1.0 | 5.882e-09 | 259 | 0.177 | 214 | 145 | 13 | 110 | 300 | 417 | 622 | Baseplate J-like protein | Baseplate J-like protein | | afdb-uniprot50 | AF-A0A844MB01-F1-MODEL\_V4 | 1.0 | 1.89e-09 | 259 | 0.194 | 221 | 136 | 10 | 110 | 295 | 745 | 958 | Putative baseplate assembly protein | Putative baseplate assembly protein | | afdb-uniprot50 | AF-A0A518BPM4-F1-MODEL\_V4 | 1.0 | 8.418e-09 | 259 | 0.169 | 207 | 145 | 9 | 113 | 294 | 754 | 958 | Uncharacterized protein | Uncharacterized protein | | afdb-uniprot50 | AF-A1ZLI5-F1-MODEL\_V4 | 1.0 | 3.341e-10 | 258 | 0.136 | 316 | 182 | 13 | 20 | 300 | 1 | 260 | Uncharacterized protein | Uncharacterized protein | | afdb-uniprot50 | AF-A0A662VR35-F1-MODEL\_V4 | 1.0 | 5.541e-09 | 257 | 0.175 | 217 | 140 | 14 | 110 | 296 | 96 | 303 | Putative baseplate assembly protein | Putative baseplate assembly protein | | afdb-uniprot50 | AF-A0A1Z4TUW1-F1-MODEL\_V4 | 1.0 | 2.559e-11 | 257 | 0.145 | 351 | 206 | 16 | 13 | 296 | 3 | 326 | Baseplate\_J domain-containing protein | Baseplate\_J domain-containing protein | | afdb-uniprot50 | AF-A0A510PDS8-F1-MODEL\_V4 | 1.0 | 4.363e-09 | 257 | 0.177 | 231 | 147 | 9 | 110 | 299 | 940 | 1168 | Putative baseplate assembly protein | Putative baseplate assembly protein | | afdb-uniprot50 | AF-A0A418VPI8-F1-MODEL\_V4 | 1.0 | 3.236e-09 | 256 | 0.199 | 211 | 136 | 11 | 111 | 295 | 192 | 395 | Putative baseplate assembly protein | Putative baseplate assembly protein | | afdb-uniprot50 | AF-A0A0Q1A8V2-F1-MODEL\_V4 | 1.0 | 6.629e-09 | 256 | 0.132 | 226 | 155 | 9 | 110 | 296 | 805 | 1028 | Baseplate\_J domain-containing protein | Baseplate\_J domain-containing protein | | afdb-uniprot50 | AF-A0A2V2EBS4-F1-MODEL\_V4 | 1.0 | 2.015e-11 | 255 | 0.129 | 386 | 214 | 20 | 20 | 295 | 12 | 385 | Baseplate\_J domain-containing protein | Baseplate\_J domain-containing protein | | afdb-uniprot50 | AF-A0A6C2C7Q0-F1-MODEL\_V4 | 1.0 | 3.062e-11 | 255 | 0.137 | 393 | 209 | 17 | 20 | 293 | 8 | 389 | Baseplate J/gp47 family protein | Baseplate J/gp47 family protein | | afdb-uniprot50 | AF-A0A559TCY2-F1-MODEL\_V4 | 1.0 | 1.135e-08 | 255 | 0.116 | 405 | 218 | 18 | 12 | 294 | 5 | 391 | Baseplate J-like protein | Baseplate J-like protein | | afdb-uniprot50 | AF-A0A5Q0TCA5-F1-MODEL\_V4 | 1.0 | 9.838e-12 | 255 | 0.124 | 403 | 218 | 16 | 16 | 294 | 1 | 392 | Baseplate\_J domain-containing protein | Baseplate\_J domain-containing protein | | afdb-uniprot50 | AF-A0A2N9MSM6-F1-MODEL\_V4 | 1.0 | 2.325e-08 | 254 | 0.138 | 217 | 149 | 8 | 112 | 296 | 69 | 279 | Baseplate\_J domain-containing protein | Baseplate\_J domain-containing protein | | afdb-uniprot50 | AF-C4K4X2-F1-MODEL\_V4 | 1.0 | 8.937e-09 | 254 | 0.196 | 209 | 135 | 12 | 111 | 293 | 102 | 303 | Phage baseplate J-like protein | Phage baseplate J-like protein | | afdb-uniprot50 | AF-D1YVC1-F1-MODEL\_V4 | 1.0 | 9.528e-11 | 254 | 0.118 | 431 | 182 | 13 | 42 | 294 | 100 | 510 | Baseplate\_J domain-containing protein | Baseplate\_J domain-containing protein | | afdb-uniprot50 | AF-A0A315XDX8-F1-MODEL\_V4 | 1.0 | 2.325e-08 | 254 | 0.142 | 211 | 148 | 9 | 113 | 294 | 738 | 944 | Putative baseplate assembly protein | Putative baseplate assembly protein | | afdb-uniprot50 | AF-A0A318XA71-F1-MODEL\_V4 | 1.0 | 2.62e-08 | 253 | 0.172 | 237 | 144 | 12 | 110 | 299 | 443 | 674 | Putative phage baseplate assembly protein | Putative phage baseplate assembly protein | | afdb-uniprot50 | AF-A0A661IT72-F1-MODEL\_V4 | 1.0 | 7.037e-09 | 252 | 0.105 | 218 | 159 | 11 | 111 | 298 | 723 | 934 | Putative baseplate assembly protein | Putative baseplate assembly protein | | afdb-uniprot50 | AF-A0A069EWX3-F1-MODEL\_V4 | 1.0 | 2.139e-11 | 251 | 0.115 | 400 | 211 | 18 | 20 | 295 | 14 | 394 | Baseplate\_J domain-containing protein | Baseplate\_J domain-containing protein | | afdb-uniprot50 | AF-C2Z1K9-F1-MODEL\_V4 | 1.0 | 1.788e-11 | 251 | 0.124 | 411 | 217 | 23 | 13 | 296 | 1 | 395 | Baseplate\_J domain-containing protein | Baseplate\_J domain-containing protein | | afdb-uniprot50 | AF-A0A3M1QRQ3-F1-MODEL\_V4 | 1.0 | 2.063e-08 | 251 | 0.142 | 217 | 151 | 12 | 111 | 300 | 909 | 1117 | Putative baseplate assembly protein | Putative baseplate assembly protein | | afdb-uniprot50 | AF-A0A1H4G0X1-F1-MODEL\_V4 | 1.0 | 1.58e-09 | 250 | 0.159 | 244 | 144 | 12 | 110 | 294 | 489 | 730 | Putative baseplate assembly protein | Putative baseplate assembly protein | | afdb-uniprot50 | AF-A0A5B9EFW7-F1-MODEL\_V4 | 1.0 | 2.62e-08 | 250 | 0.172 | 220 | 138 | 11 | 112 | 296 | 773 | 983 | Putative baseplate assembly protein | Putative baseplate assembly protein | | afdb-uniprot50 | AF-A0A6M0AC30-F1-MODEL\_V4 | 1.0 | 1.89e-09 | 249 | 0.183 | 251 | 141 | 13 | 101 | 296 | 213 | 454 | Putative baseplate assembly protein | Putative baseplate assembly protein | | afdb-uniprot50 | AF-A0A3M3Y5F7-F1-MODEL\_V4 | 1.0 | 4.11e-09 | 248 | 0.14 | 228 | 153 | 11 | 102 | 294 | 14 | 233 | Baseplate\_J domain-containing protein | Baseplate\_J domain-containing protein | | afdb-uniprot50 | AF-A0A1I0C9X9-F1-MODEL\_V4 | 1.0 | 1.831e-08 | 248 | 0.16 | 212 | 140 | 12 | 115 | 296 | 495 | 698 | Putative baseplate assembly protein | Putative baseplate assembly protein | | afdb-uniprot50 | AF-A0A5D0UQC1-F1-MODEL\_V4 | 1.0 | 3.048e-09 | 248 | 0.216 | 226 | 134 | 14 | 110 | 298 | 616 | 835 | Uncharacterized protein | Uncharacterized protein | | afdb-uniprot50 | AF-A0A6P0LHK6-F1-MODEL\_V4 | 1.0 | 7.037e-09 | 248 | 0.168 | 232 | 139 | 14 | 110 | 296 | 791 | 1013 | Putative baseplate assembly protein | Putative baseplate assembly protein | | afdb-uniprot50 | AF-A0A1W1VPN7-F1-MODEL\_V4 | 1.0 | 8.455e-11 | 247 | 0.116 | 396 | 213 | 18 | 20 | 294 | 12 | 391 | Uncharacterized phage protein gp47/JayE | Uncharacterized phage protein gp47/JayE | | afdb-uniprot50 | AF-A0A521U5U0-F1-MODEL\_V4 | 1.0 | 3.663e-11 | 246 | 0.161 | 391 | 199 | 19 | 20 | 295 | 13 | 389 | Baseplate\_J domain-containing protein | Baseplate\_J domain-containing protein | | afdb-uniprot50 | AF-A0A6L9Z4K9-F1-MODEL\_V4 | 1.0 | 7.47e-09 | 246 | 0.177 | 237 | 139 | 13 | 110 | 296 | 345 | 575 | Putative baseplate assembly protein | Putative baseplate assembly protein | | afdb-uniprot50 | AF-A0A850I740-F1-MODEL\_V4 | 1.0 | 2.19e-08 | 245 | 0.122 | 221 | 161 | 12 | 110 | 299 | 414 | 632 | Uncharacterized protein | Uncharacterized protein | | afdb-uniprot50 | AF-A0A7Y5GDB4-F1-MODEL\_V4 | 1.0 | 1.677e-09 | 245 | 0.16 | 243 | 142 | 14 | 110 | 294 | 528 | 766 | Baseplate J/gp47 family protein | Baseplate J/gp47 family protein | | afdb-uniprot50 | AF-C3X351-F1-MODEL\_V4 | 1.0 | 2.13e-09 | 244 | 0.13 | 382 | 206 | 19 | 20 | 294 | 4 | 366 | Baseplate\_J domain-containing protein | Baseplate\_J domain-containing protein | | afdb-uniprot50 | AF-E3IXC3-F1-MODEL\_V4 | 1.0 | 2.705e-09 | 244 | 0.191 | 266 | 135 | 11 | 110 | 298 | 964 | 1226 | Baseplate J family protein | Baseplate J family protein | | afdb-uniprot50 | AF-A0A2D6E3S6-F1-MODEL\_V4 | 1.0 | 1.007e-08 | 243 | 0.151 | 224 | 148 | 12 | 110 | 294 | 379 | 599 | Uncharacterized protein | Uncharacterized protein | | afdb-uniprot50 | AF-A0A1W9V6S3-F1-MODEL\_V4 | 1.0 | 8.692e-10 | 242 | 0.102 | 480 | 211 | 18 | 22 | 294 | 13 | 479 | Baseplate\_J domain-containing protein | Baseplate\_J domain-containing protein | | afdb-uniprot50 | AF-A0A4Y6PV84-F1-MODEL\_V4 | 1.0 | 1.205e-08 | 242 | 0.157 | 260 | 136 | 10 | 115 | 294 | 457 | 713 | Putative baseplate assembly protein | Putative baseplate assembly protein | | afdb-uniprot50 | AF-A0A6P0ZY62-F1-MODEL\_V4 | 1.0 | 6.244e-09 | 242 | 0.185 | 237 | 136 | 14 | 110 | 296 | 936 | 1165 | Putative baseplate assembly protein | Putative baseplate assembly protein | | afdb-uniprot50 | AF-A0A527W6E1-F1-MODEL\_V4 | 1.0 | 1.099e-07 | 241 | 0.304 | 128 | 86 | 2 | 171 | 298 | 7 | 131 | Uncharacterized protein | Uncharacterized protein | | afdb-uniprot50 | AF-A0A061NPP2-F1-MODEL\_V4 | 1.0 | 4.762e-08 | 240 | 0.21 | 252 | 113 | 8 | 8 | 174 | 1 | 251 | Phage-related baseplate assembly protein | Phage-related baseplate assembly protein | | afdb-uniprot50 | AF-A0A0P8CHE4-F1-MODEL\_V4 | 1.0 | 4.486e-08 | 240 | 0.125 | 224 | 156 | 9 | 111 | 296 | 870 | 1091 | Baseplate J-like protein | Baseplate J-like protein | | afdb-uniprot50 | AF-A0A0F9A493-F1-MODEL\_V4 | 1.0 | 2.609e-06 | 239 | 0.19 | 110 | 87 | 2 | 187 | 295 | 2 | 110 | Uncharacterized protein | Uncharacterized protein | | afdb-uniprot50 | AF-A0A1V3IJ84-F1-MODEL\_V4 | 1.0 | 5.565e-11 | 239 | 0.135 | 383 | 210 | 16 | 20 | 294 | 10 | 379 | Baseplate\_J domain-containing protein | Baseplate\_J domain-containing protein | | afdb-uniprot50 | AF-A0A823TIV2-F1-MODEL\_V4 | 1.0 | 1.162e-05 | 238 | 0.355 | 107 | 65 | 3 | 1 | 104 | 1 | 106 | Baseplate assembly protein | Baseplate assembly protein | | afdb-uniprot50 | AF-A0A1I4DEF2-F1-MODEL\_V4 | 1.0 | 4.225e-08 | 238 | 0.129 | 208 | 155 | 9 | 110 | 295 | 421 | 624 | Uncharacterized protein | Uncharacterized protein | | afdb-uniprot50 | AF-A0A7C3XHE7-F1-MODEL\_V4 | 1.0 | 4.896e-07 | 237 | 0.202 | 138 | 96 | 6 | 152 | 278 | 18 | 152 | Baseplate\_J domain-containing protein | Baseplate\_J domain-containing protein | | afdb-uniprot50 | AF-A0A544QQX2-F1-MODEL\_V4 | 1.0 | 7.502e-11 | 237 | 0.137 | 423 | 209 | 23 | 13 | 295 | 1 | 407 | Baseplate J family protein | Baseplate J family protein | | afdb-uniprot50 | AF-A0A3A9ATA2-F1-MODEL\_V4 | 1.0 | 1.67e-07 | 237 | 0.08 | 483 | 230 | 16 | 20 | 296 | 12 | 486 | Uncharacterized protein | Uncharacterized protein | | afdb-uniprot50 | AF-A0A1V5BAG1-F1-MODEL\_V4 | 1.0 | 4.486e-08 | 237 | 0.168 | 232 | 147 | 13 | 110 | 300 | 904 | 1130 | Baseplate J-like protein | Baseplate J-like protein | | afdb-uniprot50 | AF-A0A825LZJ7-F1-MODEL\_V4 | 1.0 | 6.815e-08 | 235 | 0.24 | 225 | 85 | 7 | 77 | 222 | 1 | 218 | Baseplate assembly protein | Baseplate assembly protein | | afdb-uniprot50 | AF-A0A2S9SX29-F1-MODEL\_V4 | 1.0 | 2.063e-08 | 235 | 0.175 | 200 | 138 | 10 | 111 | 293 | 53 | 242 | Baseplate\_J domain-containing protein | Baseplate\_J domain-containing protein | | afdb-uniprot50 | AF-A0A2V2DR45-F1-MODEL\_V4 | 1.0 | 2.4e-09 | 235 | 0.134 | 327 | 191 | 13 | 52 | 293 | 2 | 321 | Baseplate J protein | Baseplate J protein | | afdb-uniprot50 | AF-A0A897N0F2-F1-MODEL\_V4 | 1.0 | 3.148e-10 | 235 | 0.122 | 433 | 210 | 20 | 13 | 296 | 1 | 412 | Putative phage protein gp47/JayE | Putative phage protein gp47/JayE | | afdb-uniprot50 | AF-A0A2P8VTN4-F1-MODEL\_V4 | 1.0 | 2.781e-08 | 235 | 0.167 | 227 | 137 | 16 | 110 | 296 | 647 | 861 | Uncharacterized protein | Uncharacterized protein | | afdb-uniprot50 | AF-A0A841VH02-F1-MODEL\_V4 | 1.0 | 1.624e-08 | 234 | 0.15 | 259 | 145 | 11 | 111 | 296 | 259 | 515 | Baseplate J/gp47 family protein | Baseplate J/gp47 family protein | | afdb-uniprot50 | AF-W2CWP2-F1-MODEL\_V4 | 1.0 | 9.149e-06 | 233 | 0.465 | 101 | 54 | 0 | 166 | 266 | 2 | 102 | Baseplate\_J domain-containing protein | Baseplate\_J domain-containing protein | | afdb-uniprot50 | AF-A0A086ML19-F1-MODEL\_V4 | 1.0 | 1.943e-08 | 233 | 0.211 | 222 | 128 | 14 | 110 | 294 | 701 | 912 | Baseplate\_J domain-containing protein | Baseplate\_J domain-containing protein | | afdb-uniprot50 | AF-A0A508B8W8-F1-MODEL\_V4 | 1.0 | 2.468e-08 | 233 | 0.135 | 243 | 145 | 13 | 111 | 298 | 812 | 1044 | Putative baseplate assembly protein | Putative baseplate assembly protein | | afdb-uniprot50 | AF-A0A1B6BCR6-F1-MODEL\_V4 | 1.0 | 8.937e-09 | 232 | 0.124 | 209 | 151 | 10 | 112 | 294 | 406 | 608 | Uncharacterized protein | Uncharacterized protein | | afdb-uniprot50 | AF-A0A4R2KD98-F1-MODEL\_V4 | 1.0 | 8.655e-08 | 232 | 0.163 | 239 | 142 | 14 | 110 | 300 | 720 | 948 | Putative phage baseplate assembly protein | Putative phage baseplate assembly protein | | afdb-uniprot50 | AF-A0A836SK26-F1-MODEL\_V4 | 1.0 | 7.713e-10 | 231 | 0.122 | 367 | 226 | 19 | 12 | 294 | 2 | 356 | Uncharacterized protein | Uncharacterized protein | | afdb-uniprot50 | AF-A0A610PH99-F1-MODEL\_V4 | 1.0 | 5.494e-05 | 230 | 0.385 | 96 | 57 | 2 | 10 | 103 | 17 | 112 | Baseplate assembly protein | Baseplate assembly protein | | afdb-uniprot50 | AF-A0A6M0AIH3-F1-MODEL\_V4 | 1.0 | 5.055e-08 | 230 | 0.18 | 199 | 113 | 12 | 142 | 296 | 2 | 194 | Baseplate\_J domain-containing protein | Baseplate\_J domain-containing protein | | afdb-uniprot50 | AF-A0A7U1HJI5-F1-MODEL\_V4 | 1.0 | 5.697e-08 | 230 | 0.187 | 240 | 138 | 14 | 110 | 300 | 652 | 883 | Uncharacterized protein | Uncharacterized protein | | afdb-uniprot50 | AF-A0A3M1B3R2-F1-MODEL\_V4 | 1.0 | 5.697e-08 | 229 | 0.159 | 207 | 141 | 10 | 110 | 294 | 425 | 620 | Uncharacterized protein | Uncharacterized protein | | afdb-uniprot50 | AF-A0A846BNC7-F1-MODEL\_V4 | 1.0 | 9.754e-08 | 229 | 0.115 | 242 | 155 | 11 | 111 | 296 | 966 | 1204 | Putative baseplate assembly protein | Putative baseplate assembly protein | | afdb-uniprot50 | AF-A0A846TRD8-F1-MODEL\_V4 | 1.0 | 6.42e-08 | 228 | 0.159 | 226 | 143 | 11 | 114 | 300 | 704 | 921 | Uncharacterized protein | Uncharacterized protein | | afdb-uniprot50 | AF-A0A3C0GH36-F1-MODEL\_V4 | 1.0 | 2.39e-07 | 227 | 0.159 | 176 | 113 | 8 | 152 | 294 | 17 | 190 | Uncharacterized protein | Uncharacterized protein | | afdb-uniprot50 | AF-A0A3N9V016-F1-MODEL\_V4 | 1.0 | 4.11e-09 | 223 | 0.106 | 500 | 227 | 22 | 13 | 296 | 1 | 496 | Putative baseplate assembly protein | Putative baseplate assembly protein | | afdb-uniprot50 | AF-A0A540WCV7-F1-MODEL\_V4 | 1.0 | 1.724e-08 | 223 | 0.198 | 247 | 140 | 14 | 101 | 296 | 406 | 645 | Putative baseplate assembly protein | Putative baseplate assembly protein | | afdb-uniprot50 | AF-A0A3G2IBU6-F1-MODEL\_V4 | 1.0 | 0.000161 | 222 | 0.432 | 74 | 42 | 0 | 8 | 81 | 6 | 79 | Uncharacterized protein | Uncharacterized protein | | afdb-uniprot50 | AF-A0A6L9YWI8-F1-MODEL\_V4 | 1.0 | 1.035e-07 | 222 | 0.171 | 233 | 134 | 12 | 116 | 296 | 26 | 251 | Baseplate\_J domain-containing protein | Baseplate\_J domain-containing protein | | afdb-uniprot50 | AF-A0A5S3XXB9-F1-MODEL\_V4 | 1.0 | 1.624e-08 | 222 | 0.127 | 211 | 144 | 14 | 112 | 295 | 270 | 467 | Uncharacterized protein | Uncharacterized protein | | afdb-uniprot50 | AF-G2G240-F1-MODEL\_V4 | 1.0 | 1.396e-07 | 222 | 0.183 | 212 | 138 | 10 | 111 | 294 | 433 | 637 | Baseplate\_J domain-containing protein | Baseplate\_J domain-containing protein | | afdb-uniprot50 | AF-A0A1F8S3X9-F1-MODEL\_V4 | 1.0 | 7.235e-08 | 222 | 0.126 | 253 | 144 | 11 | 115 | 299 | 719 | 962 | Uncharacterized protein | Uncharacterized protein | | afdb-uniprot50 | AF-A0A660E4D8-F1-MODEL\_V4 | 1.0 | 3.997e-10 | 221 | 0.125 | 397 | 215 | 16 | 20 | 294 | 11 | 397 | Baseplate\_J domain-containing protein | Baseplate\_J domain-containing protein | | afdb-uniprot50 | AF-A0A226RDN1-F1-MODEL\_V4 | 1.0 | 1.172e-09 | 220 | 0.108 | 404 | 220 | 17 | 20 | 293 | 8 | 401 | Baseplate\_J domain-containing protein | Baseplate\_J domain-containing protein | | afdb-uniprot50 | AF-A0A352AD62-F1-MODEL\_V4 | 1.0 | 4.896e-07 | 220 | 0.151 | 224 | 138 | 15 | 114 | 296 | 627 | 839 | Uncharacterized protein | Uncharacterized protein | | afdb-uniprot50 | AF-A0A1Z3HRW5-F1-MODEL\_V4 | 1.0 | 1.67e-07 | 219 | 0.203 | 231 | 136 | 13 | 107 | 296 | 548 | 771 | Uncharacterized protein | Uncharacterized protein | | afdb-uniprot50 | AF-I3IJH1-F1-MODEL\_V4 | 1.0 | 6.42e-08 | 219 | 0.162 | 240 | 142 | 14 | 110 | 300 | 635 | 864 | Uncharacterized protein | Uncharacterized protein | | afdb-uniprot50 | AF-A0A3B0YCG0-F1-MODEL\_V4 | 1.0 | 1.167e-07 | 219 | 0.152 | 236 | 139 | 15 | 115 | 300 | 665 | 889 | Uncharacterized protein | Uncharacterized protein | | afdb-uniprot50 | AF-A0A1Z4LU77-F1-MODEL\_V4 | 1.0 | 6.815e-08 | 219 | 0.126 | 253 | 149 | 11 | 111 | 294 | 817 | 1066 | Baseplate\_J domain-containing protein | Baseplate\_J domain-containing protein | | afdb-uniprot50 | AF-A0A7W7V276-F1-MODEL\_V4 | 1.0 | 6.42e-08 | 219 | 0.142 | 260 | 144 | 11 | 110 | 296 | 952 | 1205 | Putative phage baseplate assembly protein | Putative phage baseplate assembly protein | | afdb-uniprot50 | AF-A0A8A5XIZ9-F1-MODEL\_V4 | 1.0 | 2.682e-05 | 218 | 0.456 | 92 | 50 | 0 | 1 | 92 | 4 | 95 | Uncharacterized protein | Uncharacterized protein | | afdb-uniprot50 | AF-A0A5M7L0X9-F1-MODEL\_V4 | 1.0 | 3.997e-10 | 218 | 0.135 | 391 | 211 | 16 | 21 | 300 | 11 | 385 | Baseplate J/gp47 family protein | Baseplate J/gp47 family protein | | afdb-uniprot50 | AF-A0A5Q2NZU8-F1-MODEL\_V4 | 1.0 | 2.199e-10 | 218 | 0.135 | 412 | 206 | 17 | 20 | 295 | 8 | 405 | Baseplate\_J domain-containing protein | Baseplate\_J domain-containing protein | | afdb-uniprot50 | AF-A0A2V9KQL4-F1-MODEL\_V4 | 1.0 | 4.896e-07 | 218 | 0.174 | 218 | 140 | 13 | 115 | 300 | 655 | 864 | Uncharacterized protein | Uncharacterized protein | | afdb-uniprot50 | AF-A0A1W9S1W4-F1-MODEL\_V4 | 1.0 | 1.239e-07 | 217 | 0.123 | 194 | 148 | 5 | 15 | 206 | 1 | 174 | Uncharacterized protein | Uncharacterized protein | | afdb-uniprot50 | AF-A0A178LTM7-F1-MODEL\_V4 | 1.0 | 5.197e-07 | 217 | 0.207 | 222 | 127 | 12 | 115 | 296 | 659 | 871 | Baseplate\_J domain-containing protein | Baseplate\_J domain-containing protein | | afdb-uniprot50 | AF-A0A0D0H1J7-F1-MODEL\_V4 | 1.0 | 5.672e-06 | 216 | 0.375 | 104 | 65 | 0 | 123 | 226 | 6 | 109 | KY49.ctg7180000000025\_quiver, whole genome shotgun sequence | KY49.ctg7180000000025\_quiver, whole genome shotgun sequence | | afdb-uniprot50 | AF-A0A6P0RHI9-F1-MODEL\_V4 | 1.0 | 1.239e-07 | 216 | 0.143 | 258 | 143 | 11 | 111 | 294 | 899 | 1152 | Baseplate protein J | Baseplate protein J | | afdb-uniprot50 | AF-A0A354WSY4-F1-MODEL\_V4 | 1.0 | 1.011e-10 | 215 | 0.113 | 398 | 213 | 18 | 20 | 293 | 14 | 395 | Baseplate\_J domain-containing protein | Baseplate\_J domain-containing protein | | afdb-uniprot50 | AF-A0A832EBT2-F1-MODEL\_V4 | 1.0 | 1.274e-06 | 213 | 0.132 | 234 | 150 | 14 | 110 | 295 | 675 | 903 | Uncharacterized protein | Uncharacterized protein | | afdb-uniprot50 | AF-A0A0F9MQV1-F1-MODEL\_V4 | 1.0 | 5.366e-08 | 212 | 0.138 | 282 | 134 | 10 | 119 | 294 | 2 | 280 | Uncharacterized protein | Uncharacterized protein | | afdb-uniprot50 | AF-A0A352X2K1-F1-MODEL\_V4 | 1.0 | 1.035e-07 | 211 | 0.136 | 257 | 165 | 10 | 98 | 300 | 628 | 881 | Putative baseplate assembly protein | Putative baseplate assembly protein | | afdb-uniprot50 | AF-A0A497BHK9-F1-MODEL\_V4 | 1.0 | 2.793e-10 | 210 | 0.136 | 389 | 209 | 19 | 22 | 295 | 13 | 389 | Baseplate\_J domain-containing protein | Baseplate\_J domain-containing protein | | afdb-uniprot50 | AF-A0A661PHR5-F1-MODEL\_V4 | 1.0 | 4.782e-10 | 209 | 0.127 | 399 | 211 | 19 | 20 | 295 | 10 | 394 | Baseplate\_J domain-containing protein | Baseplate\_J domain-containing protein | | afdb-uniprot50 | AF-A0A1F1L5J3-F1-MODEL\_V4 | 1.0 | 4.11e-09 | 206 | 0.106 | 393 | 218 | 19 | 20 | 294 | 14 | 391 | Baseplate\_J domain-containing protein | Baseplate\_J domain-containing protein | | afdb-uniprot50 | AF-A0A1C5V4K5-F1-MODEL\_V4 | 1.0 | 8.346e-05 | 204 | 0.126 | 95 | 81 | 1 | 207 | 299 | 20 | 114 | Uncharacterized protein | Uncharacterized protein | | afdb-uniprot50 | AF-A0A367QYY9-F1-MODEL\_V4 | 1.0 | 7.68e-08 | 204 | 0.159 | 232 | 146 | 11 | 113 | 298 | 620 | 848 | Putative baseplate assembly protein | Putative baseplate assembly protein | | afdb-uniprot50 | AF-A0A7X5IBM0-F1-MODEL\_V4 | 1.0 | 2.4e-09 | 202 | 0.102 | 392 | 223 | 15 | 20 | 294 | 13 | 392 | Uncharacterized protein | Uncharacterized protein | | afdb-uniprot50 | AF-A0A7G2RX52-F1-MODEL\_V4 | 1.0 | 3.871e-09 | 200 | 0.118 | 397 | 214 | 19 | 20 | 296 | 11 | 391 | Baseplate\_J domain-containing protein | Baseplate\_J domain-containing protein | | afdb-uniprot50 | AF-A0A5E4HHS7-F1-MODEL\_V4 | 1.0 | 2.537e-07 | 200 | 0.173 | 236 | 134 | 18 | 110 | 299 | 850 | 1070 | PA14 domain protein | PA14 domain protein | | afdb-uniprot50 | AF-A0A7X8EVJ9-F1-MODEL\_V4 | 1.0 | 8.655e-08 | 199 | 0.099 | 221 | 157 | 12 | 111 | 295 | 495 | 709 | Uncharacterized protein | Uncharacterized protein | | afdb-uniprot50 | AF-A0A7X7LHT1-F1-MODEL\_V4 | 1.0 | 2.13e-09 | 197 | 0.121 | 305 | 194 | 18 | 20 | 295 | 4 | 263 | Baseplate\_J domain-containing protein | Baseplate\_J domain-containing protein | | afdb-uniprot50 | AF-A0A2U3KMN2-F1-MODEL\_V4 | 1.0 | 2.054e-06 | 197 | 0.121 | 238 | 144 | 14 | 110 | 296 | 640 | 863 | Uncharacterized protein | Uncharacterized protein | | afdb-uniprot50 | AF-A0A5Y6SBS8-F1-MODEL\_V4 | 1.0 | 0.000171 | 196 | 0.349 | 106 | 61 | 2 | 1 | 98 | 1 | 106 | Baseplate assembly protein | Baseplate assembly protein | | afdb-uniprot50 | AF-A0A806KJW5-F1-MODEL\_V4 | 1.0 | 1.358e-08 | 196 | 0.11 | 489 | 207 | 21 | 21 | 300 | 12 | 481 | Uncharacterized protein | Uncharacterized protein | | afdb-uniprot50 | AF-A1ZMF0-F1-MODEL\_V4 | 1.0 | 4.632e-09 | 195 | 0.132 | 309 | 194 | 17 | 20 | 294 | 1 | 269 | Baseplate\_J domain-containing protein | Baseplate\_J domain-containing protein | | afdb-uniprot50 | AF-N1WF81-F1-MODEL\_V4 | 1.0 | 6.629e-09 | 194 | 0.121 | 411 | 208 | 22 | 21 | 296 | 11 | 403 | Baseplate J-like protein | Baseplate J-like protein | | afdb-uniprot50 | AF-N1ZQP9-F1-MODEL\_V4 | 1.0 | 4.612e-07 | 194 | 0.106 | 207 | 157 | 10 | 116 | 298 | 500 | 702 | Baseplate\_J domain-containing protein | Baseplate\_J domain-containing protein | | afdb-uniprot50 | AF-A0A836ZSV8-F1-MODEL\_V4 | 1.0 | 0.0004189 | 193 | 0.338 | 71 | 47 | 0 | 210 | 280 | 2 | 72 | Baseplate assembly protein | Baseplate assembly protein | | afdb-uniprot50 | AF-A0A431I9K0-F1-MODEL\_V4 | 1.0 | 1.358e-08 | 190 | 0.09 | 475 | 220 | 15 | 21 | 293 | 12 | 476 | Baseplate\_J domain-containing protein | Baseplate\_J domain-containing protein | | afdb-uniprot50 | AF-A0A841JK07-F1-MODEL\_V4 | 1.0 | 1.943e-08 | 189 | 0.134 | 313 | 192 | 19 | 20 | 295 | 1 | 271 | Uncharacterized protein | Uncharacterized protein | | afdb-uniprot50 | AF-A0A6P0JNB2-F1-MODEL\_V4 | 1.0 | 6.022e-06 | 189 | 0.167 | 155 | 105 | 8 | 159 | 295 | 149 | 297 | Uncharacterized protein | Uncharacterized protein | | afdb-uniprot50 | AF-A0A1W6B8F3-F1-MODEL\_V4 | 1.0 | 3.532e-08 | 189 | 0.097 | 402 | 216 | 21 | 20 | 295 | 11 | 391 | Baseplate\_J domain-containing protein | Baseplate\_J domain-containing protein | | afdb-uniprot50 | AF-A0A7X5QI95-F1-MODEL\_V4 | 1.0 | 6.392e-06 | 184 | 0.177 | 163 | 103 | 6 | 155 | 293 | 2 | 157 | Phage baseplate protein | Phage baseplate protein | | afdb-uniprot50 | AF-F9D775-F1-MODEL\_V4 | 1.0 | 6.815e-08 | 179 | 0.134 | 313 | 197 | 17 | 21 | 296 | 1 | 276 | Uncharacterized protein | Uncharacterized protein | | afdb-uniprot50 | AF-A0A1I1E3U7-F1-MODEL\_V4 | 1.0 | 6.047e-08 | 178 | 0.171 | 309 | 180 | 17 | 20 | 295 | 1 | 266 | Uncharacterized protein | Uncharacterized protein | | afdb-uniprot50 | AF-A0A1B8SLD9-F1-MODEL\_V4 | 1.0 | 3.517e-06 | 178 | 0.086 | 591 | 219 | 20 | 20 | 296 | 1 | 584 | Baseplate\_J domain-containing protein | Baseplate\_J domain-containing protein | | afdb-uniprot50 | AF-A0A8B3TC69-F1-MODEL\_V4 | 1.0 | 2.325e-08 | 177 | 0.119 | 394 | 213 | 19 | 20 | 294 | 9 | 387 | Uncharacterized protein | Uncharacterized protein | | afdb-uniprot50 | AF-A0A0Q0ZIP1-F1-MODEL\_V4 | 1.0 | 9.188e-08 | 177 | 0.13 | 346 | 201 | 19 | 1 | 295 | 1 | 297 | Uncharacterized protein | Uncharacterized protein | | afdb-uniprot50 | AF-A0A520BF88-F1-MODEL\_V4 | 1.0 | 8.153e-08 | 176 | 0.146 | 362 | 188 | 21 | 13 | 295 | 4 | 323 | Uncharacterized protein | Uncharacterized protein | | afdb-uniprot50 | AF-A0A7V1ZEF9-F1-MODEL\_V4 | 1.0 | 4.896e-07 | 176 | 0.096 | 260 | 150 | 13 | 110 | 294 | 291 | 540 | Uncharacterized protein | Uncharacterized protein | | afdb-uniprot50 | AF-A0A661FKB4-F1-MODEL\_V4 | 1.0 | 1.065e-06 | 175 | 0.177 | 186 | 109 | 9 | 151 | 295 | 802 | 984 | Baseplate\_J domain-containing protein | Baseplate\_J domain-containing protein | | afdb-uniprot50 | AF-A0A1U7I513-F1-MODEL\_V4 | 1.0 | 4.466e-06 | 174 | 0.149 | 167 | 108 | 9 | 158 | 296 | 980 | 1140 | Baseplate\_J domain-containing protein | Baseplate\_J domain-containing protein | | afdb-uniprot50 | AF-A0A7C1Z9L5-F1-MODEL\_V4 | 1.0 | 7.204e-06 | 170 | 0.097 | 453 | 206 | 19 | 1 | 273 | 1 | 430 | Uncharacterized protein | Uncharacterized protein | | afdb-uniprot50 | AF-A0A257P8Z7-F1-MODEL\_V4 | 1.0 | 0.002232 | 169 | 0.261 | 84 | 62 | 0 | 20 | 103 | 4 | 87 | Baseplate protein | Baseplate protein | | afdb-uniprot50 | AF-A0A848JKX1-F1-MODEL\_V4 | 1.0 | 2.39e-07 | 165 | 0.156 | 339 | 187 | 17 | 23 | 300 | 9 | 309 | Uncharacterized protein | Uncharacterized protein | | afdb-uniprot50 | AF-K9PW54-F1-MODEL\_V4 | 1.0 | 3.517e-06 | 164 | 0.156 | 269 | 139 | 12 | 111 | 296 | 949 | 1212 | Baseplate\_J domain-containing protein | Baseplate\_J domain-containing protein | | afdb-uniprot50 | AF-A0A2E4BK49-F1-MODEL\_V4 | 1.0 | 1.476e-05 | 160 | 0.136 | 183 | 130 | 9 | 112 | 270 | 297 | 475 | Uncharacterized protein | Uncharacterized protein | | afdb-uniprot50 | AF-G6F3I2-F1-MODEL\_V4 | 1.0 | 4.486e-08 | 159 | 0.121 | 470 | 206 | 20 | 20 | 295 | 12 | 468 | Baseplate\_J domain-containing protein | Baseplate\_J domain-containing protein | | afdb-uniprot50 | AF-A0A7S9CLQ2-F1-MODEL\_V4 | 1.0 | 1.035e-07 | 158 | 0.121 | 370 | 192 | 17 | 20 | 270 | 17 | 372 | Baseplate J/gp47 family protein | Baseplate J/gp47 family protein | | afdb-uniprot50 | AF-A0A0F8Y9S2-F1-MODEL\_V4 | 1.0 | 0.006164 | 157 | 0.228 | 92 | 65 | 2 | 13 | 104 | 1 | 86 | Baseplate\_J domain-containing protein | Baseplate\_J domain-containing protein | | afdb-uniprot50 | AF-A0A6N8HUB6-F1-MODEL\_V4 | 1.0 | 1.566e-05 | 155 | 0.136 | 220 | 142 | 13 | 110 | 295 | 725 | 930 | Baseplate\_J domain-containing protein | Baseplate\_J domain-containing protein | | afdb-uniprot50 | AF-A0A1L8I3J9-F1-MODEL\_V4 | 1.0 | 0.0001517 | 151 | 0.172 | 214 | 100 | 5 | 20 | 158 | 12 | 223 | Baseplate\_J domain-containing protein | Baseplate\_J domain-containing protein | | afdb-uniprot50 | AF-A0A3P7PM03-F1-MODEL\_V4 | 1.0 | 1.352e-06 | 144 | 0.087 | 332 | 215 | 15 | 13 | 298 | 215 | 504 | Uncharacterized protein | Uncharacterized protein | | afdb-uniprot50 | AF-A0A3L7ZAM7-F1-MODEL\_V4 | 1.0 | 1.765e-05 | 121 | 0.096 | 343 | 191 | 21 | 20 | 294 | 1 | 292 | Uncharacterized protein | Uncharacterized protein | | afdb-uniprot50 | AF-A0A2W4SAT6-F1-MODEL\_V4 | 1.0 | 0.001981 | 119 | 0.146 | 212 | 104 | 5 | 22 | 158 | 13 | 222 | Baseplate\_J domain-containing protein | Baseplate\_J domain-containing protein | | afdb-uniprot50 | AF-A0A2G8MLJ1-F1-MODEL\_V4 | 1.0 | 0.0001517 | 118 | 0.115 | 295 | 154 | 10 | 20 | 227 | 17 | 291 | Baseplate\_J domain-containing protein | Baseplate\_J domain-containing protein | | afdb-uniprot50 | AF-A0A6A8LR27-F1-MODEL\_V4 | 1.0 | 0.007829 | 108 | 0.115 | 234 | 110 | 5 | 20 | 158 | 8 | 239 | Baseplate\_J domain-containing protein | Baseplate\_J domain-containing protein | | afdb-uniprot50 | AF-A0A3S4F3P3-F1-MODEL\_V4 | 1.0 | 0.000967 | 101 | 0.116 | 317 | 155 | 11 | 20 | 225 | 22 | 324 | Bacteriophage protein | Bacteriophage protein | | afdb-uniprot50 | AF-A0A5Y6EZV3-F1-MODEL\_V4 | 0.999 | 0.0002927 | 99 | 0.111 | 304 | 164 | 14 | 8 | 225 | 2 | 285 | Baseplate\_J domain-containing protein | Baseplate\_J domain-containing protein | | afdb-uniprot50 | AF-A0A849R7X1-F1-MODEL\_V4 | 0.995 | 0.005807 | 88 | 0.094 | 498 | 201 | 20 | 22 | 278 | 14 | 502 | Uncharacterized protein | Uncharacterized protein | | afdb-uniprot50 | AF-A0A828Z879-F1-MODEL\_V4 | 0.994 | 0.004057 | 87 | 0.111 | 322 | 170 | 10 | 22 | 242 | 12 | 318 | Baseplate J-like protein | Baseplate J-like protein | | afdb-uniprot50 | AF-A0A426VQ90-F1-MODEL\_V4 | 0.986 | 0.003195 | 81 | 0.098 | 384 | 158 | 11 | 20 | 226 | 9 | 381 | Uncharacterized protein | Uncharacterized protein | |
| Top keywords  (threshold 1.00e-02 (evalue)) | **Baseplate, assembly, domain\_containing, Baseplate\_J, J, gp47, Phage, J\_like, Phage\_related, Putative** |
| Output files | ../../similar\_structures/13\_FANPEZAQ\_CDS\_0013\_afdb-proteome\_foldseek.tsv ../../similar\_structures/13\_FANPEZAQ\_CDS\_0013\_afdb-uniprot50\_foldseek.tsv ../../similar\_structures/13\_FANPEZAQ\_CDS\_0013\_merged.svg ../../similar\_structures/13\_FANPEZAQ\_CDS\_0013\_pdb\_foldseek.tsv |

  
  
  

Return to summary | Go to previous | Go to next

  


---

**Sequence/structure alignments coloring**  
Each object in the alignment figures is colored according to its E-value following this color coding:

1e-100
10

**References:**  
1) Steinegger M, Meier M, Mirdita M, Vöhringer H, Haunsberger S J, and Söding J (2019) HH-suite3 for fast remote homology detection and deep protein annotation, BMC Bioinformatics, 473. doi: 10.1186/s12859-019-3019-7  
2) Jumper J, Evans R, Pritzel A, ..., Hassabis D (2021) Highly accurate protein structure prediction with AlphaFold, Nature, 596. doi: 10.1038/s41586-021-03819-2  
3) van Kempen M, Kim S, Tumescheit C, Mirdita M, Lee J, Gilchrist CLM, Söding J, and Steinegger M (2023) Fast and accurate protein structure search with Foldseek. Nature Biotechnology. doi: 10.1038/s41587-023-01773-0
