# Supplementary material for: Completing the BASEL phage collection to unlock hidden diversity for systematic exploration of phage–host interactions
Source: PLoS Biol. 2025 Apr 7;23(4):e3003063. doi: 10.1371/journal.pbio.3003063 (PMC11990801; doi:10.1371/journal.pbio.3003063)
Supplement: S2 Data — (ZIP) [file pbio.3003063.s009.zip › entries/14.html]

FANPEZAQ\_CDS\_0014


Return to summary | Go to previous | Go to next

|  |  |
| --- | --- |
| FANPEZAQ\_CDS\_0014 Page creation date: 02 Sep 2024, 12:00  Project folder: n/a  Input sequences file: Escherichia\_virus\_HeidiAbel.gb | tail phage i p2 bacteriophage formation p2\_related p2\_like putative fragment domain\_containing tail\_p2\_i gpi duf2313 baseplate fiber i\_like j prophage assembly phage\_related tail\_like |

### Sequence information

|  |  |
| --- | --- |
| Name | FANPEZAQ\_CDS\_0014  14\_FANPEZAQ\_CDS\_0014 (pipeline id) |
| Imported annotations | Escherichia\_virus\_HeidiAbel Bas97 |
| Protein sequence | MSDLLPPNATAQERALANTIARISDVPLAARKMWNPDTIPANLLPWMAWAFSVDDWNNNW TEQEKRNVIKNSLFVHKHKGTLAAIKSAVEPLGYIIRIVEWWEDTPQAEPYTFRLEVGLL DKGVDESIYDQFTRLIETNKNLRSHMKALTIKSEINGVAYFGAGMVSGITTTVYPYIAEN LESTGGVFTAAAEQSVDAVSVYPFT |
| Number of residues | 205 |
| Molecular weight (Da) | 22921.75 |
| Output files | ../../query\_sequences/14\_FANPEZAQ\_CDS\_0014.fasta |

### Putative domain architecture and protein family

#### Search results (HHblits)1

|  |  |
| --- | --- |
| Domain family databases searched | Pfam, Ncbi-cd, Cath, Phrogs |
| Results, scheme(s)  (Top layers only; threshold 1.00e-03 (evalue)) | xml version="1.0" encoding="utf-8" standalone="no"?       2024-09-02T21:08:14.957402 image/svg+xml   Matplotlib v3.7.2, https://matplotlib.org/ |
| Results, table  (E-value ≤ 1.00e-03 (evalue)) | | db | id | prob | evalue | pvalue | score | cols | query | query\_len | template | template\_len | name | description | | --- | --- | --- | --- | --- | --- | --- | --- | --- | --- | --- | --- | --- | | pfam | PF09684 | 99.5 | 2e-18 | 3.9e-22 | 125.7 | 130 | (7, 141) | 205 | (1, 138) | 138 | Tail\_P2\_I | Phage tail protein (Tail\_P2\_I) | | pfam | PF10076 | 99.2 | 3e-16 | 6e-20 | 118.6 | 138 | (9, 148) | 205 | (18, 185) | 188 | Phage\_Mu\_Gp48 | Bacteriophage Mu-like, Gp48 | | pfam | PF11041 | 99.2 | 1e-15 | 2e-19 | 116.0 | 129 | (8, 147) | 205 | (10, 180) | 181 | DUF2612 | Protein of unknown function (DUF2612) | | phrogs | 48 | 100.0 | 4.3e-47 | 5.7e-51 | 309.0 | 201 | (1, 204) | 205 | (7, 209) | 211 | tail protein | tail protein; Category: tail; p200917 VI\_08608 | | phrogs | 5726 | 99.9 | 4e-32 | 4.7e-36 | 196.5 | 114 | (2, 117) | 205 | (3, 122) | 128 | tail protein | tail protein; Category: tail; p214505 VI\_09983 | | phrogs | 9915 | 99.9 | 6.9e-31 | 8e-35 | 236.4 | 167 | (2, 169) | 205 | (7, 175) | 1008 | NA | NA; Category: unknown function; p204625 VI\_11278 | | phrogs | 14404 | 99.9 | 1.4e-27 | 1.6e-31 | 196.4 | 152 | (2, 157) | 205 | (12, 170) | 378 | baseplate protein | baseplate protein; Category: tail; p127197 VI\_01263 | | phrogs | 29945 | 99.7 | 5.3e-22 | 5.9e-26 | 158.2 | 131 | (25, 156) | 205 | (119, 256) | 369 | NA | NA; Category: unknown function; p52203 VI\_08944 | | phrogs | 1228 | 99.6 | 8.2e-20 | 9.7e-24 | 143.7 | 117 | (30, 153) | 205 | (43, 160) | 218 | tail protein | tail protein; Category: tail; p284533 VI\_03834 | | phrogs | 19272 | 99.5 | 4.5e-19 | 5.1e-23 | 141.9 | 148 | (2, 156) | 205 | (1, 149) | 301 | NA | NA; Category: unknown function; p52189 VI\_08944 | | phrogs | 34934 | 99.4 | 6.5e-18 | 7.3e-22 | 137.4 | 96 | (1, 96) | 205 | (2, 98) | 392 | tail protein | tail protein; Category: tail; p102007 VI\_04294 | | phrogs | 17612 | 99.4 | 4.3e-17 | 4.9e-21 | 133.3 | 140 | (3, 153) | 205 | (1, 141) | 386 | tail protein | tail protein; Category: tail; p217156 VI\_00068 | | phrogs | 8207 | 98.8 | 3.8e-13 | 4.4e-17 | 109.1 | 115 | (37, 158) | 205 | (20, 134) | 348 | NA | NA; Category: unknown function; p142933 VI\_00095 | | phrogs | 2871 | 95.9 | 5e-05 | 5.9e-09 | 69.2 | 88 | (24, 147) | 205 | (21, 108) | 1049 | tail protein | tail protein; Category: tail; p28620 VI\_01314 | | phrogs | 32946 | 94.9 | 0.00042 | 4.6e-08 | 49.3 | 71 | (28, 98) | 205 | (54, 127) | 191 | tail protein | tail protein; Category: tail; KU234533\_p33 | |
| Top keywords  (threshold 1.00e-03 (evalue)) | **tail, VI\_08944, Phage, Tail\_P2\_I, Bacteriophage, Mu\_like, Gp48, DUF2612, p200917, VI\_08608** |
| Output files | ../../domain\_architecture/14\_FANPEZAQ\_CDS\_0014\_cath.hhr ../../domain\_architecture/14\_FANPEZAQ\_CDS\_0014\_merged.svg ../../domain\_architecture/14\_FANPEZAQ\_CDS\_0014\_ncbi-cd.hhr ../../domain\_architecture/14\_FANPEZAQ\_CDS\_0014\_pfam.hhr ../../domain\_architecture/14\_FANPEZAQ\_CDS\_0014\_phrogs.hhr |

### Identical protein sequences/structures

#### Search results

|  |  |
| --- | --- |
| Protein sequence databases searched | Pdb, Swissprot, Refseq |
| Identical proteins found | -- |
| Top keywords | -- |
| Output files | -- |

### Similar protein sequences/structures

#### Sequence similarity search results (HHblits)1

|  |  |
| --- | --- |
| Sequence databases searched | Uniclust, Pdb70 |
| Results, scheme(s)  (Top layers only, threshold 1.00e-03 (evalue)) | xml version="1.0" encoding="utf-8" standalone="no"?       2024-09-02T21:08:34.315745 image/svg+xml   Matplotlib v3.7.2, https://matplotlib.org/ |
| Results, table(s)  (threshold 1.00e-03 (evalue)) | | db | id | prob | evalue | pvalue | score | cols | query | query\_len | template | template\_len | name | description | | --- | --- | --- | --- | --- | --- | --- | --- | --- | --- | --- | --- | --- | | uniclust | UniRef100\_A0A009QFG0 | 100.0 | 1.9e-54 | 4.2e-60 | 357.7 | 202 | (1, 204) | 205 | (47, 248) | 282 | Phage tail protein I | Phage tail protein I | | uniclust | UniRef100\_A0A024E9W6 | 100.0 | 2.1e-54 | 4.5e-60 | 354.2 | 204 | (1, 204) | 205 | (50, 253) | 294 | Tail protein I | Tail protein I | | uniclust | UniRef100\_A0A1G7SAK3 | 100.0 | 8.4e-52 | 1.8e-57 | 333.0 | 201 | (1, 204) | 205 | (4, 204) | 264 | Phage tail protein, P2 protein I family | Phage tail protein, P2 protein I family | | uniclust | UniRef100\_A0A077N9G9 | 100.0 | 2e-50 | 4e-56 | 318.1 | 201 | (2, 204) | 205 | (39, 239) | 267 | Tail protein I (GpI) | Tail protein I (GpI) | | uniclust | UniRef100\_A0A103EG21 | 100.0 | 4.7e-49 | 9.4e-55 | 309.7 | 176 | (1, 176) | 205 | (4, 179) | 249 | Phage tail protein | Phage tail protein | | uniclust | UniRef100\_A0A066T5L9 | 100.0 | 9.9e-49 | 2.1e-54 | 312.3 | 204 | (1, 204) | 205 | (30, 235) | 241 | Phage tail protein I | Phage tail protein I | | uniclust | UniRef100\_A0A066RUL7 | 100.0 | 1.7e-48 | 3.6e-54 | 320.2 | 187 | (1, 188) | 205 | (14, 202) | 278 | Tail protein | Tail protein | | uniclust | UniRef100\_A0A1C3EBR0 | 100.0 | 7.2e-47 | 1.5e-52 | 308.1 | 199 | (2, 203) | 205 | (14, 219) | 276 | Phage tail protein I | Phage tail protein I | | uniclust | UniRef100\_A0A0D7V3M6 | 100.0 | 1e-44 | 1.9e-50 | 278.5 | 202 | (2, 203) | 205 | (4, 206) | 238 | Phage tail protein I | Phage tail protein I | | uniclust | UniRef100\_A0A016XH76 | 100.0 | 1.3e-44 | 2.7e-50 | 291.4 | 169 | (1, 170) | 205 | (26, 197) | 256 | Tail protein | Tail protein | | uniclust | UniRef100\_A0A2U9LD38 | 100.0 | 2.8e-43 | 5.5e-49 | 279.0 | 175 | (2, 178) | 205 | (14, 188) | 283 | Phage tail protein I | Phage tail protein I | | uniclust | UniRef100\_A0A377XEC8 | 100.0 | 1.9e-42 | 3.8e-48 | 256.2 | 158 | (1, 160) | 205 | (3, 160) | 165 | Tail protein I | Tail protein I | | uniclust | UniRef100\_A0A0S1B435 | 100.0 | 1.9e-42 | 4.1e-48 | 282.8 | 159 | (2, 163) | 205 | (20, 185) | 280 | Phage P2 baseplate assembly gpI-like protein | Phage P2 baseplate assembly gpI-like protein | | uniclust | UniRef100\_A0A0J8DB16 | 100.0 | 2.1e-42 | 4.5e-48 | 278.8 | 202 | (2, 205) | 205 | (17, 224) | 259 | Phage tail protein, P2 protein I family | Phage tail protein, P2 protein I family | | uniclust | UniRef100\_A0A0A1AHN2 | 100.0 | 6e-42 | 1.2e-47 | 274.1 | 203 | (1, 205) | 205 | (10, 219) | 257 | Phage tail protein I | Phage tail protein I | | uniclust | UniRef100\_A0A0B6CYD1 | 100.0 | 1.9e-41 | 4.2e-47 | 283.8 | 164 | (1, 165) | 205 | (16, 187) | 299 | Phage tail protein I | Phage tail protein I | | uniclust | UniRef100\_A0A074LXC9 | 100.0 | 3.8e-41 | 7.8e-47 | 265.7 | 199 | (2, 204) | 205 | (19, 223) | 224 | Tail protein | Tail protein | | uniclust | UniRef100\_A0A1W9H3W1 | 100.0 | 6.4e-41 | 1.3e-46 | 264.3 | 153 | (2, 154) | 205 | (14, 169) | 245 | Phage tail protein I | Phage tail protein I | | uniclust | UniRef100\_A0A087NAA8 | 100.0 | 2e-40 | 4e-46 | 266.3 | 170 | (1, 171) | 205 | (46, 218) | 271 | Phage tail protein I | Phage tail protein I | | uniclust | UniRef100\_A0A1X7CGW5 | 100.0 | 5.3e-40 | 1.1e-45 | 258.1 | 164 | (1, 173) | 205 | (20, 184) | 247 | Phage tail protein, P2 protein I family | Phage tail protein, P2 protein I family | | uniclust | UniRef100\_A0A031FSG0 | 100.0 | 5.8e-40 | 1.2e-45 | 263.0 | 202 | (2, 205) | 205 | (11, 213) | 244 | Phage tail protein I | Phage tail protein I | | uniclust | UniRef100\_A0A017HBC1 | 100.0 | 7.9e-40 | 1.7e-45 | 263.2 | 164 | (1, 169) | 205 | (32, 211) | 234 | Phage tail fiber protein | Phage tail fiber protein | | uniclust | UniRef100\_A0A1G7HXV1 | 100.0 | 4.5e-39 | 8.2e-45 | 249.9 | 201 | (2, 204) | 205 | (3, 204) | 295 | Phage tail protein, P2 protein I family (Fragment) | Phage tail protein, P2 protein I family (Fragment) | | uniclust | UniRef100\_A0A1A9R6A9 | 100.0 | 2.3e-38 | 4.6e-44 | 260.7 | 155 | (1, 156) | 205 | (18, 178) | 333 | Phage tail protein I | Phage tail protein I | | uniclust | UniRef100\_A0A061NN14 | 100.0 | 2.9e-38 | 6.7e-44 | 259.9 | 199 | (2, 204) | 205 | (15, 219) | 249 | Putative phage tail protein | Putative phage tail protein | | uniclust | UniRef100\_A0A2X3LRF3 | 100.0 | 7.6e-38 | 1.4e-43 | 251.7 | 186 | (2, 189) | 205 | (169, 355) | 362 | Tail protein I | Tail protein I | | uniclust | UniRef100\_UPI0002D8351F | 100.0 | 9.8e-38 | 1.8e-43 | 243.5 | 203 | (2, 204) | 205 | (4, 207) | 267 | phage tail protein I | phage tail protein I | | uniclust | UniRef100\_A0A0F9Z9G5 | 100.0 | 1.2e-37 | 2.4e-43 | 251.7 | 203 | (1, 205) | 205 | (41, 247) | 262 | Phage tail protein | Phage tail protein | | uniclust | UniRef100\_A0A0N8R6G9 | 100.0 | 2.2e-37 | 4e-43 | 236.5 | 200 | (1, 202) | 205 | (56, 255) | 256 | Tail protein I | Tail protein I | | uniclust | UniRef100\_A0A239C7G7 | 100.0 | 2.4e-37 | 5.1e-43 | 246.2 | 201 | (2, 205) | 205 | (18, 224) | 229 | Phage tail protein, P2 protein I family | Phage tail protein, P2 protein I family | | uniclust | UniRef100\_A0A0A8H9H6 | 100.0 | 2.7e-37 | 5.9e-43 | 251.0 | 185 | (1, 191) | 205 | (8, 194) | 242 | Phage P2 family tail protein | Phage P2 family tail protein | | uniclust | UniRef100\_A0A1X3I4G6 | 100.0 | 3.4e-37 | 6.3e-43 | 235.2 | 181 | (2, 184) | 205 | (3, 183) | 254 | Putative phage tail protein | Putative phage tail protein | | uniclust | UniRef100\_A0A134C9I1 | 100.0 | 3.1e-37 | 6.5e-43 | 247.0 | 200 | (3, 204) | 205 | (11, 214) | 249 | Phage tail protein I-like protein | Phage tail protein I-like protein | | uniclust | UniRef100\_A0A375ABY7 | 100.0 | 9e-37 | 1.6e-42 | 251.3 | 197 | (2, 200) | 205 | (304, 500) | 504 | Phage baseplate assembly protein J | Phage baseplate assembly protein J | | uniclust | UniRef100\_A0A8T5Z8P8 | 100.0 | 1.9e-36 | 3.8e-42 | 220.9 | 139 | (2, 140) | 205 | (7, 146) | 148 | Phage tail protein I (Fragment) | Phage tail protein I (Fragment) | | uniclust | UniRef100\_A0A077KVE4 | 100.0 | 2e-36 | 4.1e-42 | 231.0 | 172 | (1, 174) | 205 | (16, 187) | 193 | Phage tail protein I | Phage tail protein I | | uniclust | UniRef100\_A0A1B9NST5 | 100.0 | 6.9e-36 | 1.4e-41 | 211.0 | 105 | (1, 105) | 205 | (6, 110) | 115 | Phage tail protein I | Phage tail protein I | | uniclust | UniRef100\_A0A1E7HY16 | 100.0 | 8.7e-36 | 1.7e-41 | 216.5 | 146 | (2, 147) | 205 | (3, 148) | 150 | Phage tail protein I (Fragment) | Phage tail protein I (Fragment) | | uniclust | UniRef100\_A0A7D6BR59 | 100.0 | 1.4e-35 | 2.6e-41 | 243.8 | 173 | (2, 175) | 205 | (6, 179) | 376 | Phage tail protein I | Phage tail protein I | | uniclust | UniRef100\_UPI001080C16A | 100.0 | 1.6e-35 | 3.1e-41 | 257.2 | 202 | (1, 203) | 205 | (5, 208) | 631 | phage tail protein I | phage tail protein I | | uniclust | UniRef100\_UPI0011A48CC0 | 100.0 | 1.7e-35 | 3.1e-41 | 230.9 | 185 | (17, 203) | 205 | (117, 301) | 301 | phage tail protein I | phage tail protein I | | uniclust | UniRef100\_A0A1J5B6D6 | 100.0 | 2e-35 | 4e-41 | 244.4 | 159 | (1, 159) | 205 | (7, 182) | 341 | Phage tail protein | Phage tail protein | | uniclust | UniRef100\_A0A0H3ZS74 | 100.0 | 4e-35 | 7.5e-41 | 226.5 | 183 | (2, 186) | 205 | (4, 187) | 237 | Tail protein I | Tail protein I | | uniclust | UniRef100\_A0A085ASA6 | 100.0 | 6.6e-35 | 1.3e-40 | 269.1 | 201 | (2, 203) | 205 | (168, 370) | 1175 | Tail protein I | Tail protein I | | uniclust | UniRef100\_A0A975LVB5 | 100.0 | 8.3e-35 | 1.6e-40 | 223.6 | 158 | (2, 160) | 205 | (5, 163) | 246 | Phage tail protein I | Phage tail protein I | | uniclust | UniRef100\_A0A0P7CQM9 | 100.0 | 1.3e-34 | 2.5e-40 | 228.4 | 162 | (1, 165) | 205 | (9, 176) | 250 | Phage tail protein | Phage tail protein | | uniclust | UniRef100\_A0A0C5VFB1 | 100.0 | 1.3e-34 | 2.7e-40 | 236.1 | 164 | (2, 166) | 205 | (121, 293) | 317 | Bacteriophage P2-related tail formation protein | Bacteriophage P2-related tail formation protein | | uniclust | UniRef100\_A0A285VTA9 | 100.0 | 2.2e-34 | 4.5e-40 | 244.7 | 146 | (1, 147) | 205 | (3, 181) | 399 | Phage tail protein, P2 protein I family | Phage tail protein, P2 protein I family | | uniclust | UniRef100\_A0A3N6RV94 | 100.0 | 3.1e-34 | 5.7e-40 | 234.1 | 189 | (13, 203) | 205 | (230, 418) | 418 | Phage tail protein I | Phage tail protein I | | uniclust | UniRef100\_A0A925L380 | 100.0 | 3.7e-34 | 7.1e-40 | 226.8 | 170 | (1, 171) | 205 | (6, 179) | 276 | Phage tail protein I | Phage tail protein I | | uniclust | UniRef100\_A0A5D8SJY0 | 100.0 | 1e-33 | 2e-39 | 208.0 | 152 | (23, 176) | 205 | (7, 158) | 159 | Phage tail protein I (Fragment) | Phage tail protein I (Fragment) | | uniclust | UniRef100\_A0A0E1S4D3 | 100.0 | 1.4e-33 | 2.7e-39 | 205.8 | 119 | (2, 120) | 205 | (7, 125) | 148 | Phage tail protein I | Phage tail protein I | | uniclust | UniRef100\_A0A1B3E8I5 | 100.0 | 1.5e-33 | 2.8e-39 | 222.1 | 160 | (1, 165) | 205 | (4, 170) | 281 | Phage tail protein I | Phage tail protein I | | uniclust | UniRef100\_A0A1W1UNV3 | 100.0 | 1.6e-33 | 3.2e-39 | 216.5 | 163 | (2, 169) | 205 | (8, 187) | 193 | Phage tail protein, P2 protein I family | Phage tail protein, P2 protein I family | | uniclust | UniRef100\_A0A840WZA1 | 100.0 | 1.8e-33 | 3.6e-39 | 222.1 | 169 | (2, 172) | 205 | (13, 188) | 222 | Phage tail P2-like protein | Phage tail P2-like protein | | uniclust | UniRef100\_A0A1S1MWK0 | 100.0 | 2.6e-33 | 5.1e-39 | 216.4 | 174 | (30, 204) | 205 | (36, 209) | 227 | Phage tail protein I | Phage tail protein I | | uniclust | UniRef100\_A0A329B4G1 | 100.0 | 2.7e-33 | 5.3e-39 | 199.6 | 117 | (1, 117) | 205 | (5, 121) | 124 | Phage tail P2-like protein (Fragment) | Phage tail P2-like protein (Fragment) | | uniclust | UniRef100\_A0A0T7DV65 | 100.0 | 4.2e-33 | 8.4e-39 | 220.2 | 197 | (4, 202) | 205 | (35, 238) | 244 | Phage tail protein I | Phage tail protein I | | uniclust | UniRef100\_A0A547PS50 | 100.0 | 5.3e-33 | 1.1e-38 | 218.9 | 180 | (2, 183) | 205 | (6, 190) | 230 | Phage tail protein I | Phage tail protein I | | uniclust | UniRef100\_A0A2X4TXF0 | 100.0 | 7.5e-33 | 1.4e-38 | 216.5 | 173 | (2, 176) | 205 | (3, 175) | 302 | Bacteriophage P2-related tail formation protein | Bacteriophage P2-related tail formation protein | | uniclust | UniRef100\_UPI000DFE0774 | 100.0 | 1.7e-32 | 3.1e-38 | 215.2 | 196 | (2, 199) | 205 | (3, 200) | 309 | phage tail protein I | phage tail protein I | | uniclust | UniRef100\_A0A085AFN4 | 100.0 | 1.7e-32 | 3.7e-38 | 220.7 | 190 | (2, 194) | 205 | (13, 206) | 224 | Tail protein I | Tail protein I | | uniclust | UniRef100\_A0A1Q6JPU8 | 100.0 | 1.9e-32 | 3.9e-38 | 227.3 | 199 | (3, 203) | 205 | (18, 222) | 310 | Phage tail protein I | Phage tail protein I | | uniclust | UniRef100\_A0A1C3FFM0 | 99.9 | 2.4e-32 | 5.1e-38 | 221.0 | 172 | (2, 178) | 205 | (20, 197) | 253 | Putative phage tail fiber protein | Putative phage tail fiber protein | | uniclust | UniRef100\_UPI000C7DCBEE | 99.9 | 5.9e-32 | 1.1e-37 | 222.0 | 170 | (2, 172) | 205 | (7, 178) | 412 | phage tail protein I | phage tail protein I | | uniclust | UniRef100\_A0A0C1QG10 | 99.9 | 7.4e-32 | 1.4e-37 | 216.0 | 172 | (2, 174) | 205 | (5, 198) | 305 | Phage tail protein I | Phage tail protein I | | uniclust | UniRef100\_A0A939NK51 | 99.9 | 9.3e-32 | 1.8e-37 | 191.4 | 122 | (2, 125) | 205 | (3, 124) | 128 | Phage tail protein I | Phage tail protein I | | uniclust | UniRef100\_A0A1C3ELC5 | 99.9 | 1e-31 | 1.9e-37 | 203.9 | 157 | (2, 159) | 205 | (3, 174) | 231 | Phage tail protein I | Phage tail protein I | | uniclust | UniRef100\_A0A7H4P5M4 | 99.9 | 1.9e-31 | 3.4e-37 | 204.4 | 170 | (2, 172) | 205 | (7, 178) | 253 | Putative prophage tail protein | Putative prophage tail protein | | uniclust | UniRef100\_A0A1H2N8K0 | 99.9 | 2.5e-31 | 5.2e-37 | 223.6 | 152 | (1, 152) | 205 | (6, 191) | 371 | Phage tail protein I | Phage tail protein I | | uniclust | UniRef100\_A0A145VQK9 | 99.9 | 1e-30 | 1.9e-36 | 198.2 | 153 | (2, 155) | 205 | (6, 159) | 232 | Phage tail protein I | Phage tail protein I | | uniclust | UniRef100\_UPI0008FEF499 | 99.9 | 1.3e-30 | 2.6e-36 | 228.6 | 170 | (2, 172) | 205 | (7, 178) | 713 | phage tail protein | phage tail protein | | uniclust | UniRef100\_A0A1C6DZY7 | 99.9 | 1.5e-30 | 3.2e-36 | 214.4 | 173 | (2, 176) | 205 | (37, 215) | 261 | Bacteriophage P2-related tail formation protein | Bacteriophage P2-related tail formation protein | | uniclust | UniRef100\_A0A074TKV9 | 99.9 | 2.1e-30 | 4.3e-36 | 207.1 | 172 | (2, 177) | 205 | (10, 195) | 257 | Tail protein | Tail protein | | uniclust | UniRef100\_UPI0009B30C98 | 99.9 | 3.2e-30 | 5.8e-36 | 199.4 | 191 | (4, 194) | 205 | (71, 262) | 269 | phage tail protein I | phage tail protein I | | uniclust | UniRef100\_UPI0006B4121B | 99.9 | 3.8e-30 | 6.9e-36 | 203.3 | 169 | (1, 169) | 205 | (1, 170) | 322 | phage tail protein I | phage tail protein I | | uniclust | UniRef100\_A0A1D2QS86 | 99.9 | 3.9e-30 | 7.8e-36 | 193.4 | 139 | (26, 165) | 205 | (4, 145) | 163 | Phage tail protein I | Phage tail protein I | | uniclust | UniRef100\_A0A1X7MNV1 | 99.9 | 7.5e-30 | 1.4e-35 | 183.2 | 118 | (2, 121) | 205 | (6, 123) | 140 | Phage tail protein I (Fragment) | Phage tail protein I (Fragment) | | uniclust | UniRef100\_A0A376RLD7 | 99.9 | 8.2e-30 | 1.5e-35 | 192.0 | 126 | (2, 129) | 205 | (86, 211) | 219 | Putative tail protein I (Gpi) | Putative tail protein I (Gpi) | | uniclust | UniRef100\_UPI000641FAAA | 99.9 | 9.7e-30 | 1.8e-35 | 181.4 | 138 | (27, 164) | 205 | (2, 139) | 142 | phage tail protein I | phage tail protein I | | uniclust | UniRef100\_A0A142BH84 | 99.9 | 1e-29 | 2e-35 | 207.5 | 120 | (1, 120) | 205 | (1, 120) | 352 | Phage tail protein | Phage tail protein | | uniclust | UniRef100\_A0A011MIC4 | 99.9 | 1.3e-29 | 2.6e-35 | 201.5 | 148 | (1, 153) | 205 | (21, 184) | 214 | Tail fiber protein | Tail fiber protein | | uniclust | UniRef100\_A0A162A844 | 99.9 | 1.5e-29 | 2.8e-35 | 204.4 | 163 | (2, 167) | 205 | (9, 178) | 305 | Tail protein | Tail protein | | uniclust | UniRef100\_UPI00073535E0 | 99.9 | 2.7e-29 | 5.2e-35 | 181.8 | 131 | (2, 135) | 205 | (6, 138) | 140 | phage tail protein I | phage tail protein I | | uniclust | UniRef100\_A0A2A2C599 | 99.9 | 2.9e-29 | 5.8e-35 | 198.5 | 196 | (2, 198) | 205 | (11, 215) | 228 | Phage tail protein I | Phage tail protein I | | uniclust | UniRef100\_D1Y2C1 | 99.9 | 3.9e-29 | 7.6e-35 | 195.7 | 196 | (3, 204) | 205 | (12, 215) | 240 | Phage tail protein | Phage tail protein | | uniclust | UniRef100\_A0A7G2U2N6 | 99.9 | 4.8e-29 | 8.8e-35 | 178.4 | 143 | (2, 146) | 205 | (5, 152) | 152 | Phage tail protein I | Phage tail protein I | | uniclust | UniRef100\_A0A7W6WC48 | 99.9 | 7.4e-29 | 1.4e-34 | 202.6 | 152 | (3, 154) | 205 | (4, 166) | 348 | Phage tail P2-like protein | Phage tail P2-like protein | | uniclust | UniRef100\_UPI00044AA162 | 99.9 | 8e-29 | 1.5e-34 | 183.3 | 148 | (44, 193) | 205 | (4, 154) | 161 | phage tail protein I | phage tail protein I | | uniclust | UniRef100\_UPI000DF97472 | 99.9 | 8.4e-29 | 1.5e-34 | 215.8 | 152 | (1, 153) | 205 | (2, 154) | 925 | phage tail protein I | phage tail protein I | | uniclust | UniRef100\_A0A1X7NDR7 | 99.9 | 1e-28 | 2.1e-34 | 196.3 | 149 | (1, 152) | 205 | (4, 196) | 238 | Phage tail protein, P2 protein I family | Phage tail protein, P2 protein I family | | uniclust | UniRef100\_A0A2G6HAI3 | 99.9 | 2.3e-28 | 4.2e-34 | 197.4 | 158 | (3, 163) | 205 | (6, 168) | 385 | Phage tail protein I (Fragment) | Phage tail protein I (Fragment) | | uniclust | UniRef100\_A0A8I1NYE7 | 99.9 | 2.9e-28 | 5.6e-34 | 188.0 | 203 | (2, 204) | 205 | (13, 220) | 221 | Phage tail protein I | Phage tail protein I | | uniclust | UniRef100\_A0A167GZS5 | 99.9 | 3.1e-28 | 5.8e-34 | 194.6 | 163 | (2, 167) | 205 | (3, 172) | 328 | Tail protein | Tail protein | | uniclust | UniRef100\_A0A149PPI8 | 99.9 | 3.5e-28 | 6.5e-34 | 189.6 | 151 | (2, 154) | 205 | (86, 239) | 263 | Tail protein | Tail protein | | uniclust | UniRef100\_A0A137SPR3 | 99.9 | 4.7e-28 | 9.9e-34 | 202.5 | 167 | (2, 171) | 205 | (20, 200) | 309 | Phage tail protein I-like protein | Phage tail protein I-like protein | | uniclust | UniRef100\_A0A078MHK2 | 99.9 | 5e-28 | 1.1e-33 | 202.9 | 154 | (2, 159) | 205 | (14, 173) | 293 | Phage tail protein (Tail\_P2\_I) | Phage tail protein (Tail\_P2\_I) | | uniclust | UniRef100\_A0A3N2E0S8 | 99.9 | 6.1e-28 | 1.2e-33 | 186.3 | 150 | (2, 152) | 205 | (13, 195) | 234 | Phage tail P2-like protein | Phage tail P2-like protein | | uniclust | UniRef100\_UPI0005DFBD90 | 99.9 | 6.8e-28 | 1.2e-33 | 196.4 | 149 | (29, 179) | 205 | (265, 413) | 417 | phage tail protein I | phage tail protein I | | uniclust | UniRef100\_A0A0F6A4X0 | 99.9 | 7.1e-28 | 1.4e-33 | 195.0 | 150 | (5, 157) | 205 | (11, 167) | 302 | Tail protein | Tail protein | | uniclust | UniRef100\_A0A316MKT1 | 99.9 | 7.6e-28 | 1.5e-33 | 193.8 | 164 | (37, 204) | 205 | (75, 241) | 278 | Phage tail protein I | Phage tail protein I | | uniclust | UniRef100\_UPI00210E7D19 | 99.9 | 9.4e-28 | 1.7e-33 | 191.4 | 196 | (2, 204) | 205 | (3, 199) | 342 | phage tail protein I | phage tail protein I | | uniclust | UniRef100\_A0A447N0G4 | 99.9 | 9.4e-28 | 1.8e-33 | 162.5 | 89 | (1, 89) | 205 | (3, 91) | 91 | Orf38 p2 I-like tail protein | Orf38 p2 I-like tail protein | | uniclust | UniRef100\_A0A968H8P4 | 99.9 | 1.2e-27 | 2.2e-33 | 183.7 | 199 | (4, 204) | 205 | (6, 210) | 217 | Phage tail protein I | Phage tail protein I | | uniclust | UniRef100\_A0A178HP38 | 99.9 | 1.1e-27 | 2.3e-33 | 201.6 | 153 | (3, 155) | 205 | (7, 180) | 343 | Phage tail protein I | Phage tail protein I | | uniclust | UniRef100\_A0A7W7N2Q5 | 99.9 | 1.5e-27 | 2.7e-33 | 189.7 | 163 | (2, 165) | 205 | (10, 175) | 332 | Phage tail P2-like protein | Phage tail P2-like protein | | uniclust | UniRef100\_A0A376J7K4 | 99.9 | 1.7e-27 | 3.2e-33 | 167.5 | 115 | (42, 158) | 205 | (2, 116) | 124 | Putative tail protein I (Gpi) | Putative tail protein I (Gpi) | | uniclust | UniRef100\_A0A1S1MZR1 | 99.9 | 1.8e-27 | 3.4e-33 | 192.2 | 149 | (2, 153) | 205 | (8, 163) | 313 | Phage tail protein I | Phage tail protein I | | uniclust | UniRef100\_UPI00235456C8 | 99.9 | 1.9e-27 | 3.6e-33 | 192.1 | 142 | (2, 150) | 205 | (6, 148) | 384 | phage tail protein I | phage tail protein I | | uniclust | UniRef100\_A0A0A8RA06 | 99.9 | 3.1e-27 | 5.9e-33 | 213.4 | 150 | (1, 150) | 205 | (1, 150) | 970 | Putative bacteriophage protein | Putative bacteriophage protein | | uniclust | UniRef100\_A0A269PJC4 | 99.9 | 3.8e-27 | 6.9e-33 | 174.0 | 149 | (29, 177) | 205 | (35, 183) | 186 | Phage tail protein I | Phage tail protein I | | uniclust | UniRef100\_A0A1D2QMU0 | 99.9 | 4e-27 | 8e-33 | 174.6 | 101 | (1, 101) | 205 | (3, 104) | 151 | Phage tail protein I | Phage tail protein I | | uniclust | UniRef100\_A0A0L8A9U9 | 99.9 | 5.9e-27 | 1.1e-32 | 194.0 | 151 | (2, 154) | 205 | (256, 409) | 433 | Tail protein | Tail protein | | uniclust | UniRef100\_UPI0020352A08 | 99.9 | 7.7e-27 | 1.4e-32 | 191.2 | 151 | (2, 153) | 205 | (7, 159) | 434 | phage tail protein I | phage tail protein I | | uniclust | UniRef100\_A0A059UZC6 | 99.9 | 7.6e-27 | 1.6e-32 | 198.8 | 149 | (3, 152) | 205 | (6, 195) | 385 | Tail protein I | Tail protein I | | uniclust | UniRef100\_UPI0022A77364 | 99.9 | 1.9e-26 | 3.4e-32 | 172.1 | 179 | (24, 203) | 205 | (4, 183) | 199 | phage tail protein I | phage tail protein I | | uniclust | UniRef100\_A0A5C7PF23 | 99.9 | 2.4e-26 | 5e-32 | 181.9 | 149 | (2, 153) | 205 | (12, 185) | 211 | Phage tail protein I | Phage tail protein I | | uniclust | UniRef100\_A0A1X7L2M2 | 99.9 | 2.8e-26 | 5.5e-32 | 196.5 | 146 | (1, 147) | 205 | (8, 189) | 496 | Phage tail protein, P2 protein I family | Phage tail protein, P2 protein I family | | uniclust | UniRef100\_A0A6L7TCB7 | 99.9 | 3e-26 | 5.5e-32 | 178.2 | 146 | (2, 153) | 205 | (101, 246) | 272 | Phage tail protein I | Phage tail protein I | | uniclust | UniRef100\_A0A239CJC5 | 99.9 | 3.2e-26 | 5.9e-32 | 182.3 | 152 | (2, 154) | 205 | (1, 154) | 332 | Phage tail protein, P2 protein I family | Phage tail protein, P2 protein I family | | uniclust | UniRef100\_A0A415HAB7 | 99.9 | 3.7e-26 | 7.5e-32 | 186.0 | 161 | (1, 166) | 205 | (14, 180) | 277 | Phage tail protein I | Phage tail protein I | | uniclust | UniRef100\_A0A090V3M0 | 99.9 | 3.7e-26 | 7.6e-32 | 214.4 | 156 | (2, 159) | 205 | (3, 158) | 1137 | Tail protein | Tail protein | | uniclust | UniRef100\_UPI0020300C06 | 99.9 | 5.6e-26 | 1e-31 | 163.5 | 119 | (2, 120) | 205 | (4, 122) | 154 | phage tail protein I | phage tail protein I | | uniclust | UniRef100\_A0A0Q0ZJE5 | 99.9 | 5.5e-26 | 1.1e-31 | 182.3 | 150 | (1, 154) | 205 | (11, 167) | 225 | Phage tail protein | Phage tail protein | | uniclust | UniRef100\_A0A0B7J8H6 | 99.9 | 5.9e-26 | 1.2e-31 | 185.2 | 163 | (2, 165) | 205 | (18, 181) | 258 | Phage tail protein I | Phage tail protein I | | uniclust | UniRef100\_UPI0009E5A335 | 99.9 | 6.8e-26 | 1.2e-31 | 173.2 | 151 | (2, 153) | 205 | (5, 162) | 237 | phage tail protein I | phage tail protein I | | uniclust | UniRef100\_A0A0F4QK22 | 99.9 | 7.4e-26 | 1.5e-31 | 190.0 | 124 | (28, 154) | 205 | (126, 260) | 376 | Phage tail protein I | Phage tail protein I | | uniclust | UniRef100\_UPI0012EB4EFA | 99.9 | 8.3e-26 | 1.5e-31 | 185.7 | 158 | (2, 159) | 205 | (4, 162) | 444 | phage tail protein I | phage tail protein I | | uniclust | UniRef100\_A0A1Y3DKT1 | 99.9 | 9.6e-26 | 1.8e-31 | 188.0 | 174 | (1, 176) | 205 | (1, 174) | 434 | Phage tail protein I | Phage tail protein I | | uniclust | UniRef100\_A0A8S0FTH7 | 99.9 | 1.3e-25 | 2.4e-31 | 175.7 | 144 | (2, 145) | 205 | (138, 282) | 285 | Baseplate protein J-like domain-containing protein | Baseplate protein J-like domain-containing protein | | uniclust | UniRef100\_A0A934JMB0 | 99.9 | 1.6e-25 | 3.1e-31 | 173.8 | 193 | (2, 195) | 205 | (13, 206) | 220 | Phage tail protein I | Phage tail protein I | | uniclust | UniRef100\_A0A0E4G100 | 99.9 | 1.6e-25 | 3.6e-31 | 199.1 | 95 | (1, 95) | 205 | (18, 112) | 426 | Phage tail protein I | Phage tail protein I | | uniclust | UniRef100\_UPI001B39611C | 99.9 | 2.2e-25 | 4e-31 | 178.3 | 163 | (2, 165) | 205 | (5, 174) | 342 | phage tail protein I | phage tail protein I | | uniclust | UniRef100\_A0A1G7SDN3 | 99.9 | 3.4e-25 | 6.2e-31 | 169.6 | 149 | (2, 153) | 205 | (54, 218) | 238 | Phage tail protein, P2 protein I family | Phage tail protein, P2 protein I family | | uniclust | UniRef100\_A0A348HHI4 | 99.9 | 4.5e-25 | 8.3e-31 | 171.3 | 201 | (2, 204) | 205 | (4, 206) | 266 | Bacteriophage P2-related tail formation protein | Bacteriophage P2-related tail formation protein | | uniclust | UniRef100\_UPI0013D775D4 | 99.9 | 4.5e-25 | 8.3e-31 | 167.9 | 148 | (2, 155) | 205 | (3, 151) | 227 | phage tail protein I | phage tail protein I | | uniclust | UniRef100\_A0A9E6RJL8 | 99.9 | 4.5e-25 | 8.4e-31 | 167.1 | 198 | (5, 202) | 205 | (6, 213) | 219 | Phage tail protein I | Phage tail protein I | | uniclust | UniRef100\_A0A8T7D0V5 | 99.8 | 6.4e-25 | 1.2e-30 | 175.1 | 159 | (2, 161) | 205 | (3, 174) | 332 | Phage tail protein I | Phage tail protein I | | uniclust | UniRef100\_UPI000F04D2C2 | 99.8 | 7.1e-25 | 1.3e-30 | 149.5 | 109 | (33, 141) | 205 | (1, 109) | 109 | phage tail protein I | phage tail protein I | | uniclust | UniRef100\_A0A022PNX6 | 99.8 | 6.9e-25 | 1.4e-30 | 175.0 | 160 | (4, 168) | 205 | (24, 203) | 219 | Phage tail protein, P2 protein I family | Phage tail protein, P2 protein I family | | uniclust | UniRef100\_UPI002264A9C4 | 99.8 | 9.1e-25 | 1.7e-30 | 152.0 | 114 | (2, 115) | 205 | (4, 117) | 123 | phage tail protein I | phage tail protein I | | uniclust | UniRef100\_UPI0013A68137 | 99.8 | 9.2e-25 | 1.7e-30 | 167.0 | 165 | (23, 188) | 205 | (23, 190) | 207 | phage tail protein I | phage tail protein I | | uniclust | UniRef100\_I3TTD2 | 99.8 | 1.2e-24 | 2.2e-30 | 170.3 | 164 | (2, 166) | 205 | (6, 175) | 282 | Gp15 protein | Gp15 protein | | uniclust | UniRef100\_A0A541BHK6 | 99.8 | 1.3e-24 | 2.5e-30 | 173.9 | 163 | (3, 166) | 205 | (23, 196) | 297 | Phage tail protein I | Phage tail protein I | | uniclust | UniRef100\_A0A3S4GL93 | 99.8 | 1.6e-24 | 3e-30 | 156.6 | 104 | (2, 105) | 205 | (16, 119) | 147 | Bacteriophage P2-related tail formation protein | Bacteriophage P2-related tail formation protein | | uniclust | UniRef100\_UPI00069850DB | 99.8 | 1.6e-24 | 3e-30 | 173.7 | 151 | (2, 153) | 205 | (16, 202) | 314 | phage tail protein I | phage tail protein I | | uniclust | UniRef100\_UPI000D57C2B6 | 99.8 | 1.7e-24 | 3e-30 | 157.4 | 158 | (44, 203) | 205 | (2, 163) | 163 | phage tail protein I | phage tail protein I | | uniclust | UniRef100\_A0A7U9H2I1 | 99.8 | 1.7e-24 | 3.2e-30 | 169.4 | 191 | (12, 204) | 205 | (21, 217) | 281 | Phage tail protein I | Phage tail protein I | | uniclust | UniRef100\_A0A0W8JFZ8 | 99.8 | 1.6e-24 | 3.2e-30 | 175.5 | 163 | (2, 165) | 205 | (29, 193) | 270 | Phage tail protein I | Phage tail protein I | | uniclust | UniRef100\_A0A266LLA0 | 99.8 | 1.8e-24 | 3.4e-30 | 147.7 | 90 | (2, 91) | 205 | (4, 93) | 95 | Phage tail protein I (Fragment) | Phage tail protein I (Fragment) | | uniclust | UniRef100\_A0A927WW89 | 99.8 | 2e-24 | 4e-30 | 187.9 | 174 | (3, 180) | 205 | (12, 195) | 583 | Phage tail protein I | Phage tail protein I | | uniclust | UniRef100\_A0A174T2Y7 | 99.8 | 2.3e-24 | 4.5e-30 | 170.1 | 170 | (2, 175) | 205 | (12, 187) | 226 | Bacteriophage P2-related tail formation protein | Bacteriophage P2-related tail formation protein | | uniclust | UniRef100\_A0A1N7LRG2 | 99.8 | 2.8e-24 | 5.3e-30 | 163.3 | 153 | (2, 154) | 205 | (6, 160) | 209 | Phage tail protein, P2 protein I family | Phage tail protein, P2 protein I family | | uniclust | UniRef100\_A0A173SBX4 | 99.8 | 3.2e-24 | 6.4e-30 | 185.6 | 149 | (2, 153) | 205 | (25, 179) | 457 | Bacteriophage P2-related tail formation protein | Bacteriophage P2-related tail formation protein | | uniclust | UniRef100\_A0A428VMX7 | 99.8 | 3.4e-24 | 6.5e-30 | 180.3 | 161 | (2, 169) | 205 | (288, 465) | 471 | Phage tail protein I | Phage tail protein I | | uniclust | UniRef100\_A0A2S7JR38 | 99.8 | 4e-24 | 7.8e-30 | 169.7 | 152 | (2, 154) | 205 | (7, 172) | 249 | Phage tail protein I | Phage tail protein I | | uniclust | UniRef100\_A0A2D1CSN0 | 99.8 | 4.1e-24 | 7.8e-30 | 188.2 | 142 | (3, 144) | 205 | (5, 146) | 733 | Putative tail fiber protein | Putative tail fiber protein | | uniclust | UniRef100\_A0A1E3G6D5 | 99.8 | 4.3e-24 | 7.9e-30 | 160.5 | 162 | (1, 164) | 205 | (1, 162) | 205 | Phage tail protein I | Phage tail protein I | | uniclust | UniRef100\_A0A318KN12 | 99.8 | 6.9e-24 | 1.3e-29 | 170.9 | 147 | (3, 150) | 205 | (5, 152) | 358 | Phage tail P2-like protein (Fragment) | Phage tail P2-like protein (Fragment) | | uniclust | UniRef100\_A0A067ZG50 | 99.8 | 9.5e-24 | 1.8e-29 | 159.8 | 172 | (2, 175) | 205 | (10, 191) | 192 | Tail protein I | Tail protein I | | uniclust | UniRef100\_A0A064AHQ3 | 99.8 | 2.3e-23 | 4.6e-29 | 165.0 | 158 | (3, 164) | 205 | (13, 178) | 218 | Phage tail protein | Phage tail protein | | uniclust | UniRef100\_UPI001F0C5BA4 | 99.8 | 2.6e-23 | 4.8e-29 | 147.8 | 104 | (2, 105) | 205 | (1, 104) | 139 | phage tail protein I | phage tail protein I | | uniclust | UniRef100\_A0A150MJA7 | 99.8 | 2.5e-23 | 4.8e-29 | 169.4 | 149 | (2, 154) | 205 | (10, 161) | 298 | Phage tail protein I | Phage tail protein I | | uniclust | UniRef100\_A0A2D3WQP3 | 99.8 | 2.7e-23 | 5.5e-29 | 162.5 | 150 | (1, 153) | 205 | (5, 177) | 201 | Phage tail protein I | Phage tail protein I | | uniclust | UniRef100\_UPI001D0F882A | 99.8 | 3.2e-23 | 5.8e-29 | 176.1 | 152 | (2, 154) | 205 | (5, 159) | 603 | phage tail protein I | phage tail protein I | | uniclust | UniRef100\_A0A1T4WXE7 | 99.8 | 3.4e-23 | 6.2e-29 | 160.3 | 154 | (2, 157) | 205 | (6, 168) | 253 | Phage tail protein, P2 protein I family | Phage tail protein, P2 protein I family | | uniclust | UniRef100\_A0A2B2B8I7 | 99.8 | 3.5e-23 | 6.4e-29 | 136.9 | 85 | (34, 120) | 205 | (1, 85) | 90 | Phage tail protein I (Fragment) | Phage tail protein I (Fragment) | | uniclust | UniRef100\_A0A812QW15 | 99.8 | 4e-23 | 7.4e-29 | 183.2 | 146 | (13, 159) | 205 | (289, 442) | 1062 | J protein | J protein | | uniclust | UniRef100\_UPI000AD33DF5 | 99.8 | 4.4e-23 | 8.2e-29 | 147.9 | 127 | (47, 174) | 205 | (1, 129) | 135 | phage tail protein | phage tail protein | | uniclust | UniRef100\_A0A016QLD8 | 99.8 | 4.2e-23 | 8.8e-29 | 180.6 | 118 | (29, 155) | 205 | (251, 370) | 429 | FHA domain-containing protein | FHA domain-containing protein | | uniclust | UniRef100\_A0A1W9USS5 | 99.8 | 6.2e-23 | 1.3e-28 | 178.1 | 124 | (30, 155) | 205 | (213, 362) | 397 | Phage tail protein I | Phage tail protein I | | uniclust | UniRef100\_A0A5Q2UG14 | 99.8 | 7.9e-23 | 1.5e-28 | 171.7 | 152 | (2, 154) | 205 | (7, 164) | 428 | Phage tail protein I | Phage tail protein I | | uniclust | UniRef100\_A0A3C1WTS9 | 99.8 | 8.8e-23 | 1.7e-28 | 160.2 | 187 | (3, 194) | 205 | (18, 208) | 247 | Phage tail protein I | Phage tail protein I | | uniclust | UniRef100\_A0A0A8JLX9 | 99.8 | 8.7e-23 | 1.7e-28 | 167.9 | 148 | (3, 153) | 205 | (11, 164) | 348 | Phage tail protein | Phage tail protein | | uniclust | UniRef100\_A0A1H8VRL6 | 99.8 | 1.2e-22 | 2.3e-28 | 163.9 | 147 | (2, 153) | 205 | (39, 191) | 315 | Phage tail protein, P2 protein I family | Phage tail protein, P2 protein I family | | uniclust | UniRef100\_A0A418VVG6 | 99.8 | 1.2e-22 | 2.3e-28 | 170.1 | 152 | (1, 152) | 205 | (1, 169) | 515 | Phage tail protein I | Phage tail protein I | | uniclust | UniRef100\_A0A812RFY7 | 99.8 | 1.3e-22 | 2.4e-28 | 176.0 | 150 | (1, 153) | 205 | (266, 431) | 772 | J protein | J protein | | uniclust | UniRef100\_A0A069Q1X1 | 99.8 | 1.5e-22 | 3.2e-28 | 170.0 | 122 | (29, 152) | 205 | (101, 240) | 300 | Uncharacterized protein | Uncharacterized protein | | uniclust | UniRef100\_UPI0018DE8610 | 99.8 | 1.8e-22 | 3.3e-28 | 142.3 | 130 | (43, 172) | 205 | (1, 130) | 131 | phage tail protein I | phage tail protein I | | uniclust | UniRef100\_UPI0005BBF981 | 99.8 | 2e-22 | 3.8e-28 | 140.7 | 88 | (33, 120) | 205 | (2, 89) | 111 | phage tail protein I | phage tail protein I | | uniclust | UniRef100\_A0A1E7J923 | 99.8 | 1.8e-22 | 3.8e-28 | 174.1 | 124 | (29, 154) | 205 | (145, 288) | 392 | Phage tail protein I | Phage tail protein I | | uniclust | UniRef100\_A0A966HEM8 | 99.8 | 2e-22 | 3.8e-28 | 164.4 | 147 | (3, 153) | 205 | (12, 165) | 336 | Phage tail protein I | Phage tail protein I | | uniclust | UniRef100\_A0A0L6JHZ7 | 99.8 | 1.8e-22 | 3.9e-28 | 178.6 | 172 | (12, 189) | 205 | (211, 401) | 442 | Phage tail protein | Phage tail protein | | uniclust | UniRef100\_A0A1Y1QXH2 | 99.8 | 2.3e-22 | 4.3e-28 | 154.5 | 149 | (2, 152) | 205 | (5, 155) | 237 | Phage tail protein I | Phage tail protein I | | uniclust | UniRef100\_A0A376MUF5 | 99.8 | 3e-22 | 5.4e-28 | 143.2 | 94 | (32, 127) | 205 | (43, 136) | 143 | Putative tail protein I (Gpi) | Putative tail protein I (Gpi) | | uniclust | UniRef100\_A0A2X2DI53 | 99.8 | 2.8e-22 | 5.5e-28 | 144.8 | 118 | (85, 204) | 205 | (1, 118) | 124 | Phage tail protein | Phage tail protein | | uniclust | UniRef100\_A0A521CHZ6 | 99.8 | 3.8e-22 | 7.3e-28 | 160.1 | 150 | (2, 153) | 205 | (4, 164) | 287 | Phage tail protein, P2 protein I family | Phage tail protein, P2 protein I family | | uniclust | UniRef100\_UPI002244F0CF | 99.8 | 5.9e-22 | 1.1e-27 | 132.0 | 87 | (35, 121) | 205 | (2, 88) | 93 | phage tail protein I | phage tail protein I | | uniclust | UniRef100\_UPI001CF0BE9A | 99.8 | 6.8e-22 | 1.2e-27 | 159.4 | 161 | (2, 169) | 205 | (167, 344) | 350 | phage tail protein I | phage tail protein I | | uniclust | UniRef100\_A0A845R5N3 | 99.8 | 9.5e-22 | 1.8e-27 | 157.0 | 197 | (2, 200) | 205 | (10, 213) | 250 | Phage tail protein I | Phage tail protein I | | uniclust | UniRef100\_A0A1C5WN41 | 99.8 | 9.4e-22 | 1.9e-27 | 164.9 | 171 | (3, 176) | 205 | (47, 222) | 345 | Bacteriophage P2-related tail formation protein | Bacteriophage P2-related tail formation protein | | uniclust | UniRef100\_UPI00210D76C9 | 99.8 | 1.2e-21 | 2.2e-27 | 163.0 | 152 | (2, 155) | 205 | (14, 184) | 471 | phage tail protein I | phage tail protein I | | uniclust | UniRef100\_A0A074LMF0 | 99.8 | 1.1e-21 | 2.4e-27 | 183.6 | 158 | (12, 175) | 205 | (496, 672) | 713 | Phage tail protein | Phage tail protein | | uniclust | UniRef100\_A0A2J4JSF3 | 99.7 | 2.1e-21 | 4e-27 | 155.4 | 160 | (3, 164) | 205 | (11, 178) | 255 | Phage tail protein I | Phage tail protein I | | uniclust | UniRef100\_UPI000DFB77B5 | 99.7 | 2.3e-21 | 4.3e-27 | 140.6 | 128 | (44, 172) | 205 | (10, 139) | 147 | phage tail protein I | phage tail protein I | | uniclust | UniRef100\_A0A0C1IR17 | 99.7 | 2.1e-21 | 4.3e-27 | 158.1 | 187 | (2, 189) | 205 | (11, 206) | 234 | Phage tail protein I | Phage tail protein I | | uniclust | UniRef100\_A0A1W2DZK8 | 99.7 | 2.9e-21 | 5.7e-27 | 154.3 | 201 | (3, 204) | 205 | (29, 238) | 243 | Phage tail protein, P2 protein I family | Phage tail protein, P2 protein I family | | uniclust | UniRef100\_A0A847XBJ9 | 99.7 | 3.1e-21 | 5.9e-27 | 144.5 | 132 | (39, 173) | 205 | (1, 132) | 173 | Phage tail protein I | Phage tail protein I | | uniclust | UniRef100\_A0A126QLJ0 | 99.7 | 2.9e-21 | 6e-27 | 158.9 | 191 | (13, 205) | 205 | (33, 224) | 257 | Uncharacterized protein | Uncharacterized protein | | uniclust | UniRef100\_UPI0007DC4380 | 99.7 | 3.4e-21 | 6.2e-27 | 141.4 | 148 | (3, 152) | 205 | (4, 157) | 168 | phage tail protein I | phage tail protein I | | uniclust | UniRef100\_A0A1W9VL85 | 99.7 | 3.1e-21 | 6.3e-27 | 165.2 | 124 | (30, 155) | 205 | (202, 351) | 384 | Phage tail protein I | Phage tail protein I | | uniclust | UniRef100\_A0A023PZC5 | 99.7 | 3.5e-21 | 6.7e-27 | 155.0 | 125 | (28, 153) | 205 | (124, 257) | 307 | Tail protein I | Tail protein I | | uniclust | UniRef100\_A0A080M1T9 | 99.7 | 3.2e-21 | 6.7e-27 | 158.2 | 123 | (29, 153) | 205 | (67, 198) | 249 | Phage tail protein domain protein | Phage tail protein domain protein | | uniclust | UniRef100\_A0A6G5QFK7 | 99.7 | 3.5e-21 | 7.3e-27 | 152.7 | 154 | (45, 202) | 205 | (42, 195) | 204 | Phage P2 family tail protein | Phage P2 family tail protein | | uniclust | UniRef100\_A0A6L3X1R5 | 99.7 | 4.9e-21 | 9.6e-27 | 132.8 | 85 | (2, 86) | 205 | (6, 92) | 96 | Phage tail protein I (Fragment) | Phage tail protein I (Fragment) | | uniclust | UniRef100\_A0A315X9K7 | 99.7 | 5.1e-21 | 1e-26 | 150.9 | 121 | (29, 153) | 205 | (73, 197) | 212 | Phage tail protein | Phage tail protein | | uniclust | UniRef100\_UPI000471CB6A | 99.7 | 5.5e-21 | 1e-26 | 163.7 | 150 | (2, 152) | 205 | (7, 162) | 530 | phage tail protein I | phage tail protein I | | uniclust | UniRef100\_A0A2P8QYP1 | 99.7 | 6.1e-21 | 1.1e-26 | 150.2 | 186 | (1, 192) | 205 | (1, 188) | 282 | Phage tail protein I | Phage tail protein I | | uniclust | UniRef100\_UPI0013D51FFC | 99.7 | 6.2e-21 | 1.1e-26 | 147.0 | 143 | (30, 176) | 205 | (46, 188) | 238 | phage tail protein I | phage tail protein I | | uniclust | UniRef100\_A0A973ALZ7 | 99.7 | 6.9e-21 | 1.3e-26 | 155.3 | 152 | (2, 153) | 205 | (3, 172) | 383 | Phage tail protein I | Phage tail protein I | | uniclust | UniRef100\_UPI001E610C52 | 99.7 | 7.1e-21 | 1.3e-26 | 154.2 | 145 | (3, 151) | 205 | (12, 162) | 360 | phage tail protein I | phage tail protein I | | uniclust | UniRef100\_A0A838RLH5 | 99.7 | 9.3e-21 | 1.8e-26 | 144.1 | 121 | (30, 154) | 205 | (21, 158) | 176 | Phage tail protein I | Phage tail protein I | | uniclust | UniRef100\_A0A089JZ90 | 99.7 | 9.1e-21 | 1.8e-26 | 156.5 | 122 | (29, 153) | 205 | (64, 207) | 289 | Tail protein | Tail protein | | uniclust | UniRef100\_UPI001F490D58 | 99.7 | 1.1e-20 | 2.1e-26 | 133.6 | 118 | (2, 119) | 205 | (6, 124) | 131 | phage tail protein I | phage tail protein I | | uniclust | UniRef100\_A0A1X3J0Q7 | 99.7 | 1.1e-20 | 2.1e-26 | 131.8 | 104 | (71, 176) | 205 | (2, 105) | 108 | Tail protein I (GpI) | Tail protein I (GpI) | | uniclust | UniRef100\_UPI000E3DDFBE | 99.7 | 1.9e-20 | 3.4e-26 | 123.7 | 82 | (30, 111) | 205 | (6, 87) | 87 | phage tail protein I | phage tail protein I | | uniclust | UniRef100\_A0A0H3ZYQ7 | 99.7 | 1.9e-20 | 3.5e-26 | 145.6 | 194 | (2, 196) | 205 | (9, 211) | 220 | Tail protein I | Tail protein I | | uniclust | UniRef100\_UPI000BA1955F | 99.7 | 2.1e-20 | 3.9e-26 | 129.3 | 97 | (9, 105) | 205 | (5, 101) | 106 | phage tail protein I | phage tail protein I | | uniclust | UniRef100\_A0A6N9P8X1 | 99.7 | 2.5e-20 | 4.7e-26 | 153.8 | 152 | (4, 158) | 205 | (16, 174) | 357 | Phage tail protein I | Phage tail protein I | | uniclust | UniRef100\_F4BFR7 | 99.7 | 4.3e-20 | 8e-26 | 140.8 | 165 | (1, 171) | 205 | (1, 167) | 195 | Phage tail protein | Phage tail protein | | uniclust | UniRef100\_A0A968XWL3 | 99.7 | 5e-20 | 9.2e-26 | 125.3 | 102 | (2, 103) | 205 | (1, 103) | 103 | Phage tail protein I | Phage tail protein I | | uniclust | UniRef100\_A0A3A6E7Q8 | 99.7 | 5.8e-20 | 1.1e-25 | 150.3 | 148 | (3, 153) | 205 | (14, 168) | 339 | Phage tail protein I | Phage tail protein I | | uniclust | UniRef100\_A0A1B9NSL6 | 99.7 | 6.3e-20 | 1.2e-25 | 133.3 | 128 | (75, 204) | 205 | (1, 128) | 155 | Phage tail protein I | Phage tail protein I | | uniclust | UniRef100\_A0A6N7JGU4 | 99.7 | 6.5e-20 | 1.2e-25 | 154.2 | 155 | (2, 156) | 205 | (3, 170) | 507 | Phage tail protein I | Phage tail protein I | | uniclust | UniRef100\_I7LG06 | 99.7 | 1.5e-19 | 2.7e-25 | 143.5 | 147 | (3, 153) | 205 | (11, 164) | 295 | Phage tail protein I | Phage tail protein I | | uniclust | UniRef100\_A0A2A5JYU3 | 99.7 | 1.5e-19 | 2.9e-25 | 130.0 | 130 | (42, 175) | 205 | (7, 136) | 138 | Phage tail protein I | Phage tail protein I | | uniclust | UniRef100\_UPI0009718AD0 | 99.7 | 1.7e-19 | 3.2e-25 | 129.0 | 103 | (3, 105) | 205 | (6, 109) | 139 | phage tail protein I | phage tail protein I | | uniclust | UniRef100\_UPI00197B9417 | 99.7 | 1.8e-19 | 3.3e-25 | 139.8 | 151 | (2, 153) | 205 | (7, 167) | 222 | phage tail protein I | phage tail protein I | | uniclust | UniRef100\_A0A1C6IIK7 | 99.7 | 1.8e-19 | 3.6e-25 | 151.1 | 142 | (10, 153) | 205 | (26, 173) | 331 | Bacteriophage P2-related tail formation protein | Bacteriophage P2-related tail formation protein | | uniclust | UniRef100\_A0A0E3SE24 | 99.7 | 2e-19 | 4.1e-25 | 164.1 | 158 | (10, 174) | 205 | (447, 648) | 709 | NHL repeat domain protein | NHL repeat domain protein | | uniclust | UniRef100\_A0A2S6N2X4 | 99.6 | 2.3e-19 | 4.5e-25 | 139.5 | 195 | (2, 204) | 205 | (9, 206) | 207 | Uncharacterized protein | Uncharacterized protein | | uniclust | UniRef100\_A0A3D5LSM9 | 99.6 | 2.4e-19 | 4.7e-25 | 148.0 | 158 | (2, 164) | 205 | (18, 181) | 352 | Phage tail protein I | Phage tail protein I | | uniclust | UniRef100\_A0A0C1MY76 | 99.6 | 2.3e-19 | 4.7e-25 | 158.6 | 136 | (13, 154) | 205 | (185, 349) | 520 | Phage tail protein | Phage tail protein | | uniclust | UniRef100\_A0A0X8JTG7 | 99.6 | 2.6e-19 | 5e-25 | 140.4 | 130 | (32, 165) | 205 | (45, 174) | 214 | Phage tail protein | Phage tail protein | | uniclust | UniRef100\_A0A0S2ZDH3 | 99.6 | 2.6e-19 | 5.3e-25 | 144.0 | 143 | (30, 178) | 205 | (49, 191) | 225 | Phage tail protein | Phage tail protein | | uniclust | UniRef100\_A0A1G3UAH8 | 99.6 | 2.8e-19 | 5.3e-25 | 140.2 | 145 | (3, 153) | 205 | (2, 150) | 245 | Phage tail protein I | Phage tail protein I | | uniclust | UniRef100\_A0A7X1ZFY9 | 99.6 | 3.1e-19 | 5.7e-25 | 134.7 | 131 | (73, 205) | 205 | (40, 170) | 199 | Phage tail protein I | Phage tail protein I | | uniclust | UniRef100\_A0A6I2UKH8 | 99.6 | 3.2e-19 | 5.8e-25 | 142.0 | 158 | (31, 192) | 205 | (44, 201) | 300 | Phage tail protein I | Phage tail protein I | | uniclust | UniRef100\_UPI001C7642B4 | 99.6 | 3.7e-19 | 6.8e-25 | 137.0 | 111 | (35, 150) | 205 | (48, 159) | 230 | phage tail protein I | phage tail protein I | | uniclust | UniRef100\_A0A6N9P2M1 | 99.6 | 4.1e-19 | 7.7e-25 | 146.2 | 140 | (30, 172) | 205 | (49, 188) | 344 | Phage tail protein I | Phage tail protein I | | uniclust | UniRef100\_A0A7U3VT62 | 99.6 | 4.4e-19 | 8.2e-25 | 149.5 | 150 | (1, 157) | 205 | (1, 162) | 513 | Fused protein containing phage-related tail protein GpI | Fused protein containing phage-related tail protein GpI | | uniclust | UniRef100\_A0A073CHG0 | 99.6 | 4.5e-19 | 9.1e-25 | 153.3 | 136 | (13, 154) | 205 | (218, 382) | 409 | Phage tail protein | Phage tail protein | | uniclust | UniRef100\_A0A268EA24 | 99.6 | 4.8e-19 | 9.3e-25 | 150.3 | 146 | (2, 153) | 205 | (16, 166) | 402 | Phage tail protein I | Phage tail protein I | | uniclust | UniRef100\_A0A2Z5STI6 | 99.6 | 4.9e-19 | 1e-24 | 162.1 | 167 | (12, 185) | 205 | (483, 703) | 733 | Phage tail protein | Phage tail protein | | uniclust | UniRef100\_A0A839IX64 | 99.6 | 5.7e-19 | 1.1e-24 | 139.6 | 158 | (2, 165) | 205 | (4, 162) | 284 | Phage tail protein I | Phage tail protein I | | uniclust | UniRef100\_A0A318KXU1 | 99.6 | 6.4e-19 | 1.2e-24 | 143.1 | 139 | (13, 154) | 205 | (15, 158) | 356 | Phage tail P2-like protein | Phage tail P2-like protein | | uniclust | UniRef100\_A0A928YGD5 | 99.6 | 6.8e-19 | 1.3e-24 | 136.7 | 157 | (3, 164) | 205 | (13, 175) | 217 | Phage tail protein I | Phage tail protein I | | uniclust | UniRef100\_A0A1U7IY23 | 99.6 | 8.3e-19 | 1.7e-24 | 148.8 | 119 | (29, 153) | 205 | (68, 194) | 360 | Phage tail protein I | Phage tail protein I | | uniclust | UniRef100\_A0A143XK95 | 99.6 | 1e-18 | 2e-24 | 145.1 | 166 | (2, 170) | 205 | (10, 182) | 331 | Phage tail protein (Tail\_P2\_I) | Phage tail protein (Tail\_P2\_I) | | uniclust | UniRef100\_A0A1C6EQC2 | 99.6 | 9.7e-19 | 2e-24 | 149.0 | 151 | (2, 154) | 205 | (10, 166) | 337 | Bacteriophage P2-related tail formation protein | Bacteriophage P2-related tail formation protein | | uniclust | UniRef100\_A0A022MS75 | 99.6 | 9.8e-19 | 2.1e-24 | 148.9 | 118 | (29, 152) | 205 | (124, 253) | 321 | Tail protein | Tail protein | | uniclust | UniRef100\_UPI0010454A05 | 99.6 | 1.3e-18 | 2.4e-24 | 137.8 | 148 | (3, 153) | 205 | (10, 170) | 286 | phage tail protein I | phage tail protein I | | uniclust | UniRef100\_UPI00209B7E43 | 99.6 | 1.4e-18 | 2.6e-24 | 146.1 | 146 | (4, 150) | 205 | (5, 190) | 491 | phage tail protein I | phage tail protein I | | uniclust | UniRef100\_A0A143HC79 | 99.6 | 1.5e-18 | 2.8e-24 | 135.4 | 147 | (3, 153) | 205 | (11, 160) | 219 | Phage tail protein I | Phage tail protein I | | uniclust | UniRef100\_A0A4S2HBU2 | 99.6 | 1.6e-18 | 2.9e-24 | 138.3 | 178 | (4, 183) | 205 | (12, 196) | 304 | Phage tail protein I | Phage tail protein I | | uniclust | UniRef100\_A0A3C2C7T1 | 99.6 | 1.7e-18 | 3.3e-24 | 144.5 | 156 | (12, 173) | 205 | (106, 293) | 333 | Phage tail protein (Fragment) | Phage tail protein (Fragment) | | uniclust | UniRef100\_UPI001CB6F6EA | 99.6 | 1.9e-18 | 3.4e-24 | 122.9 | 92 | (29, 120) | 205 | (34, 125) | 132 | phage tail protein I | phage tail protein I | | uniclust | UniRef100\_A0A1H2SW83 | 99.6 | 2e-18 | 3.6e-24 | 121.6 | 86 | (31, 118) | 205 | (29, 114) | 124 | Phage tail protein, P2 protein I family | Phage tail protein, P2 protein I family | | uniclust | UniRef100\_UPI0020A62330 | 99.6 | 2.3e-18 | 4.2e-24 | 125.9 | 153 | (47, 201) | 205 | (1, 154) | 158 | phage tail protein I | phage tail protein I | | uniclust | UniRef100\_A0A844Q9W2 | 99.6 | 2.9e-18 | 5.3e-24 | 133.3 | 195 | (3, 198) | 205 | (32, 237) | 245 | Phage tail protein I | Phage tail protein I | | uniclust | UniRef100\_A0A1C3HHS1 | 99.6 | 2.9e-18 | 5.5e-24 | 133.7 | 136 | (14, 153) | 205 | (31, 180) | 216 | Phage tail protein (Tail\_P2\_I) | Phage tail protein (Tail\_P2\_I) | | uniclust | UniRef100\_A0A0D6KHP6 | 99.6 | 2.8e-18 | 5.5e-24 | 151.9 | 119 | (29, 153) | 205 | (68, 194) | 535 | Putative bacteriophage tail protein | Putative bacteriophage tail protein | | uniclust | UniRef100\_A0A5A8F1G5 | 99.6 | 3.9e-18 | 7.2e-24 | 135.3 | 141 | (8, 153) | 205 | (15, 156) | 287 | Phage tail protein I | Phage tail protein I | | uniclust | UniRef100\_A0A136KPW8 | 99.6 | 3.6e-18 | 7.3e-24 | 146.0 | 123 | (28, 154) | 205 | (236, 375) | 381 | Phage tail protein | Phage tail protein | | uniclust | UniRef100\_UPI0018A3C604 | 99.6 | 3.9e-18 | 7.3e-24 | 133.1 | 170 | (3, 176) | 205 | (7, 179) | 236 | phage tail protein I | phage tail protein I | | uniclust | UniRef100\_UPI000887A604 | 99.6 | 4e-18 | 7.4e-24 | 127.1 | 90 | (1, 92) | 205 | (1, 90) | 180 | phage tail protein I | phage tail protein I | | uniclust | UniRef100\_A0A6G5Y431 | 99.6 | 4.2e-18 | 7.7e-24 | 128.1 | 121 | (31, 153) | 205 | (46, 169) | 191 | Phage tail protein I | Phage tail protein I | | uniclust | UniRef100\_A0A3A9DQT7 | 99.6 | 4.4e-18 | 8.2e-24 | 125.6 | 120 | (30, 151) | 205 | (46, 165) | 168 | Phage tail protein I (Fragment) | Phage tail protein I (Fragment) | | uniclust | UniRef100\_A0A2X0WX70 | 99.6 | 6.7e-18 | 1.2e-23 | 133.3 | 165 | (1, 166) | 205 | (10, 182) | 275 | Bacteriophage P2-related tail formation protein | Bacteriophage P2-related tail formation protein | | uniclust | UniRef100\_A0A174TLI5 | 99.6 | 9.8e-18 | 1.8e-23 | 138.1 | 148 | (14, 164) | 205 | (50, 201) | 366 | Bacteriophage P2-related tail formation protein | Bacteriophage P2-related tail formation protein | | uniclust | UniRef100\_A0A072YA28 | 99.6 | 8.8e-18 | 1.9e-23 | 138.7 | 159 | (33, 205) | 205 | (63, 225) | 236 | Phage protein | Phage protein | | uniclust | UniRef100\_A0A1V0Q0B5 | 99.5 | 1.3e-17 | 2.6e-23 | 153.1 | 164 | (12, 181) | 205 | (473, 657) | 690 | NHL repeat domain protein | NHL repeat domain protein | | uniclust | UniRef100\_UPI001BB07F4E | 99.5 | 1.4e-17 | 2.6e-23 | 143.3 | 148 | (3, 152) | 205 | (7, 164) | 622 | phage tail protein I | phage tail protein I | | uniclust | UniRef100\_A0A927WKT3 | 99.5 | 1.8e-17 | 3.3e-23 | 133.7 | 120 | (35, 157) | 205 | (47, 166) | 327 | Phage tail protein I | Phage tail protein I | | uniclust | UniRef100\_UPI00200D63BF | 99.5 | 2e-17 | 3.7e-23 | 114.6 | 108 | (53, 161) | 205 | (2, 109) | 111 | phage tail protein I | phage tail protein I | | uniclust | UniRef100\_A0A6A0HXA4 | 99.5 | 2e-17 | 4e-23 | 149.8 | 164 | (12, 181) | 205 | (448, 664) | 699 | Phage tail protein | Phage tail protein | | uniclust | UniRef100\_UPI001C0048D9 | 99.5 | 2.3e-17 | 4.2e-23 | 130.7 | 120 | (30, 153) | 205 | (66, 185) | 280 | phage tail protein I | phage tail protein I | | uniclust | UniRef100\_A0A345DE45 | 99.5 | 2.5e-17 | 4.6e-23 | 125.6 | 145 | (2, 153) | 205 | (16, 182) | 208 | Phage tail protein I | Phage tail protein I | | uniclust | UniRef100\_A0A1D9FX43 | 99.5 | 2.6e-17 | 5.1e-23 | 133.8 | 122 | (29, 153) | 205 | (56, 214) | 261 | Phage tail protein | Phage tail protein | | uniclust | UniRef100\_UPI001CC0D908 | 99.5 | 3.1e-17 | 5.8e-23 | 132.9 | 149 | (2, 152) | 205 | (8, 157) | 338 | phage tail protein I | phage tail protein I | | uniclust | UniRef100\_A0A0A8IL57 | 99.5 | 4.9e-17 | 9.9e-23 | 132.9 | 96 | (2, 101) | 205 | (37, 133) | 248 | Tail protein | Tail protein | | uniclust | UniRef100\_UPI002020F9DF | 99.5 | 6.2e-17 | 1.1e-22 | 106.7 | 75 | (2, 76) | 205 | (7, 83) | 83 | phage tail protein I | phage tail protein I | | uniclust | UniRef100\_UPI0018AC4BC9 | 99.5 | 6.5e-17 | 1.2e-22 | 118.7 | 125 | (73, 199) | 205 | (1, 126) | 159 | phage tail protein | phage tail protein | | uniclust | UniRef100\_A0A1C4SGP1 | 99.5 | 7.6e-17 | 1.5e-22 | 125.7 | 121 | (29, 153) | 205 | (55, 185) | 192 | Phage tail protein, P2 protein I family (Fragment) | Phage tail protein, P2 protein I family (Fragment) | | uniclust | UniRef100\_A0A173R5F4 | 99.5 | 8.3e-17 | 1.6e-22 | 131.1 | 147 | (2, 152) | 205 | (12, 165) | 301 | Bacteriophage P2-related tail formation protein | Bacteriophage P2-related tail formation protein | | uniclust | UniRef100\_A0A5M4DA93 | 99.5 | 9.6e-17 | 1.8e-22 | 129.3 | 121 | (29, 153) | 205 | (131, 268) | 274 | Phage tail protein | Phage tail protein | | uniclust | UniRef100\_A0A3N5LKH2 | 99.5 | 9.8e-17 | 1.9e-22 | 133.7 | 161 | (12, 179) | 205 | (59, 282) | 318 | Phage tail protein I (Fragment) | Phage tail protein I (Fragment) | | uniclust | UniRef100\_A0A1G5H9F7 | 99.5 | 9e-17 | 2e-22 | 141.0 | 145 | (29, 182) | 205 | (194, 351) | 374 | Phage tail protein domain-containing protein | Phage tail protein domain-containing protein | | uniclust | UniRef100\_A0A7M1L784 | 99.5 | 1.3e-16 | 2.4e-22 | 116.2 | 143 | (59, 204) | 205 | (6, 150) | 150 | Phage tail protein | Phage tail protein | | uniclust | UniRef100\_A0A3M2ARC6 | 99.5 | 1.3e-16 | 2.4e-22 | 125.3 | 115 | (30, 152) | 205 | (92, 207) | 210 | Phage tail protein | Phage tail protein | | uniclust | UniRef100\_A0A1N6X8I0 | 99.5 | 1.2e-16 | 2.4e-22 | 133.8 | 123 | (29, 153) | 205 | (106, 246) | 277 | Phage tail protein domain-containing protein | Phage tail protein domain-containing protein | | uniclust | UniRef100\_A0A4U0YVX5 | 99.5 | 1.5e-16 | 3e-22 | 101.9 | 58 | (1, 58) | 205 | (6, 63) | 64 | Phage tail protein I (Fragment) | Phage tail protein I (Fragment) | | uniclust | UniRef100\_A4J3V2 | 99.5 | 1.9e-16 | 3.6e-22 | 137.2 | 146 | (3, 152) | 205 | (12, 164) | 649 | Phage tail protein I | Phage tail protein I | | uniclust | UniRef100\_A0A062V414 | 99.5 | 2e-16 | 3.9e-22 | 131.9 | 122 | (30, 153) | 205 | (144, 282) | 309 | Phage tail protein | Phage tail protein | | uniclust | UniRef100\_A0A3M5GB96 | 99.5 | 2.2e-16 | 4.1e-22 | 112.5 | 81 | (13, 95) | 205 | (45, 125) | 130 | Tail protein I | Tail protein I | | uniclust | UniRef100\_A0A0F0HY11 | 99.4 | 3.1e-16 | 6.7e-22 | 147.4 | 156 | (12, 175) | 205 | (490, 691) | 736 | Uncharacterized protein | Uncharacterized protein | | uniclust | UniRef100\_A0A3A8YWF4 | 99.4 | 3.6e-16 | 6.9e-22 | 123.7 | 192 | (5, 201) | 205 | (14, 211) | 219 | Phage tail protein I | Phage tail protein I | | uniclust | UniRef100\_A0A8S5Q6A9 | 99.4 | 3.9e-16 | 7.3e-22 | 120.4 | 166 | (3, 172) | 205 | (14, 185) | 222 | Tail protein | Tail protein | | uniclust | UniRef100\_A0A0Q8XJJ0 | 99.4 | 3.3e-16 | 7.3e-22 | 147.9 | 159 | (12, 176) | 205 | (454, 696) | 748 | Phage tail protein | Phage tail protein | | uniclust | UniRef100\_A0A1M6N7M0 | 99.4 | 4e-16 | 7.3e-22 | 130.3 | 118 | (31, 152) | 205 | (41, 158) | 435 | Phage tail protein, P2 protein I family | Phage tail protein, P2 protein I family | | uniclust | UniRef100\_A0A0A8WVT7 | 99.4 | 4.2e-16 | 8.3e-22 | 130.1 | 88 | (13, 101) | 205 | (27, 116) | 316 | Phage tail protein | Phage tail protein | | uniclust | UniRef100\_A0A2E0L127 | 99.4 | 4.4e-16 | 8.8e-22 | 135.2 | 116 | (30, 154) | 205 | (234, 350) | 408 | FHA domain-containing protein | FHA domain-containing protein | | uniclust | UniRef100\_A0A285NFM9 | 99.4 | 4.8e-16 | 9.1e-22 | 121.6 | 175 | (28, 205) | 205 | (45, 220) | 221 | Phage tail protein (Tail\_P2\_I) | Phage tail protein (Tail\_P2\_I) | | uniclust | UniRef100\_A0A0N8IBW6 | 99.4 | 5.2e-16 | 9.5e-22 | 117.9 | 146 | (4, 152) | 205 | (7, 168) | 198 | Phage tail protein | Phage tail protein | | uniclust | UniRef100\_A0A3A9CZ71 | 99.4 | 5.3e-16 | 9.9e-22 | 130.3 | 145 | (3, 151) | 205 | (13, 164) | 410 | Phage tail protein | Phage tail protein | | uniclust | UniRef100\_A0A523SGB7 | 99.4 | 5.9e-16 | 1.1e-21 | 123.8 | 122 | (29, 154) | 205 | (146, 283) | 295 | OmpR/PhoB-type domain-containing protein | OmpR/PhoB-type domain-containing protein | | uniclust | UniRef100\_A0A014MDU0 | 99.4 | 5.8e-16 | 1.2e-21 | 141.3 | 172 | (2, 182) | 205 | (376, 609) | 649 | Tail protein | Tail protein | | uniclust | UniRef100\_A0A4Y7S1X8 | 99.4 | 6.2e-16 | 1.2e-21 | 136.9 | 154 | (12, 171) | 205 | (379, 550) | 593 | Phage tail protein | Phage tail protein | | uniclust | UniRef100\_A0A1Q6KUF2 | 99.4 | 6.3e-16 | 1.2e-21 | 126.1 | 159 | (3, 166) | 205 | (12, 175) | 275 | Phage tail protein I | Phage tail protein I | | uniclust | UniRef100\_A0A7X4ZQB7 | 99.4 | 7.2e-16 | 1.4e-21 | 123.1 | 171 | (2, 175) | 205 | (10, 187) | 252 | Phage tail protein I | Phage tail protein I | | uniclust | UniRef100\_A0A0Q0T5D2 | 99.4 | 7.4e-16 | 1.4e-21 | 108.9 | 110 | (65, 174) | 205 | (2, 111) | 117 | Phage tail protein | Phage tail protein | | uniclust | UniRef100\_A0A095WFB1 | 99.4 | 9e-16 | 1.8e-21 | 121.9 | 130 | (30, 165) | 205 | (45, 174) | 212 | Phage tail protein I | Phage tail protein I | | uniclust | UniRef100\_A0A2P9HMM9 | 99.4 | 9.3e-16 | 1.9e-21 | 134.9 | 92 | (2, 93) | 205 | (18, 111) | 440 | Phage tail protein I | Phage tail protein I | | uniclust | UniRef100\_A0A069AQR6 | 99.4 | 8.7e-16 | 1.9e-21 | 127.1 | 189 | (3, 205) | 205 | (14, 211) | 228 | DUF2313 domain-containing protein | DUF2313 domain-containing protein | | uniclust | UniRef100\_A0A6B9SWZ5 | 99.4 | 1e-15 | 2e-21 | 122.6 | 141 | (2, 151) | 205 | (4, 148) | 221 | Phage tail protein | Phage tail protein | | uniclust | UniRef100\_A0A1M7YBP4 | 99.4 | 1.2e-15 | 2.2e-21 | 126.9 | 145 | (3, 152) | 205 | (11, 162) | 339 | Phage tail protein (Tail\_P2\_I) | Phage tail protein (Tail\_P2\_I) | | uniclust | UniRef100\_UPI00189D0918 | 99.4 | 1.2e-15 | 2.3e-21 | 122.8 | 151 | (2, 153) | 205 | (5, 158) | 310 | phage tail protein I | phage tail protein I | | uniclust | UniRef100\_UPI001B3C6DE8 | 99.4 | 1.3e-15 | 2.4e-21 | 117.7 | 148 | (3, 154) | 205 | (11, 164) | 222 | phage tail protein | phage tail protein | | uniclust | UniRef100\_A0A1G9EY78 | 99.4 | 1.1e-15 | 2.4e-21 | 143.3 | 157 | (12, 174) | 205 | (465, 683) | 740 | Phage tail protein domain-containing protein | Phage tail protein domain-containing protein | | uniclust | UniRef100\_A0A099PD40 | 99.4 | 1.3e-15 | 2.7e-21 | 136.6 | 159 | (12, 175) | 205 | (221, 431) | 537 | Phage tail protein | Phage tail protein | | uniclust | UniRef100\_A0A2N6CT40 | 99.4 | 1.7e-15 | 3.2e-21 | 125.7 | 159 | (28, 194) | 205 | (110, 315) | 342 | Phage tail protein I | Phage tail protein I | | uniclust | UniRef100\_A0A6D0ZU35 | 99.4 | 2.4e-15 | 4.5e-21 | 110.6 | 121 | (63, 185) | 205 | (7, 127) | 152 | Phage tail protein I (Fragment) | Phage tail protein I (Fragment) | | uniclust | UniRef100\_A0A1C6BNG9 | 99.4 | 2.4e-15 | 4.6e-21 | 119.9 | 172 | (2, 175) | 205 | (10, 188) | 250 | Bacteriophage P2-related tail formation protein | Bacteriophage P2-related tail formation protein | | uniclust | UniRef100\_A0A3A9EQX9 | 99.4 | 2.9e-15 | 5.4e-21 | 122.8 | 128 | (35, 164) | 205 | (54, 182) | 346 | Phage tail protein I | Phage tail protein I | | uniclust | UniRef100\_UPI0009BF8F77 | 99.4 | 3.1e-15 | 5.7e-21 | 131.9 | 119 | (32, 151) | 205 | (34, 153) | 767 | phage tail protein I | phage tail protein I | | uniclust | UniRef100\_UPI00202CF454 | 99.4 | 3.1e-15 | 5.7e-21 | 99.7 | 74 | (47, 120) | 205 | (1, 74) | 86 | phage tail protein I | phage tail protein I | | uniclust | UniRef100\_A0A218Q5U8 | 99.4 | 3e-15 | 5.8e-21 | 125.4 | 119 | (29, 153) | 205 | (64, 190) | 365 | Phage tail protein | Phage tail protein | | uniclust | UniRef100\_A0A1Q6PWS5 | 99.3 | 3.2e-15 | 6.1e-21 | 126.0 | 136 | (12, 153) | 205 | (23, 159) | 369 | Phage tail protein I | Phage tail protein I | | uniclust | UniRef100\_UPI001F4B27F2 | 99.3 | 3.5e-15 | 6.4e-21 | 99.0 | 76 | (51, 126) | 205 | (2, 77) | 84 | phage tail protein I | phage tail protein I | | uniclust | UniRef100\_Q9KW54 | 99.3 | 3.7e-15 | 6.9e-21 | 111.9 | 128 | (14, 144) | 205 | (18, 163) | 164 | Tail protein | Tail protein | | uniclust | UniRef100\_A0A2W6SVJ0 | 99.3 | 3.8e-15 | 7.4e-21 | 128.0 | 94 | (1, 94) | 205 | (2, 96) | 415 | Phage tail protein I | Phage tail protein I | | uniclust | UniRef100\_A0A1T4W599 | 99.3 | 4.3e-15 | 8.5e-21 | 126.6 | 141 | (2, 150) | 205 | (12, 155) | 358 | Phage tail protein (Tail\_P2\_I) | Phage tail protein (Tail\_P2\_I) | | uniclust | UniRef100\_UPI001FEBD5CB | 99.3 | 4.7e-15 | 8.7e-21 | 107.4 | 112 | (92, 205) | 205 | (1, 112) | 141 | phage tail protein | phage tail protein | | uniclust | UniRef100\_A0A7T7D8U4 | 99.3 | 5.5e-15 | 1e-20 | 127.7 | 191 | (3, 196) | 205 | (390, 587) | 596 | Phage tail protein I | Phage tail protein I | | uniclust | UniRef100\_UPI001C997A2E | 99.3 | 6.1e-15 | 1.1e-20 | 99.7 | 85 | (3, 87) | 205 | (6, 92) | 93 | phage tail protein I | phage tail protein I | | uniclust | UniRef100\_A0A969G7N1 | 99.3 | 6.4e-15 | 1.2e-20 | 115.6 | 117 | (29, 153) | 205 | (18, 144) | 247 | Phage tail protein I | Phage tail protein I | | uniclust | UniRef100\_UPI0021795371 | 99.3 | 6.6e-15 | 1.3e-20 | 110.3 | 119 | (2, 120) | 205 | (10, 134) | 155 | phage tail protein I | phage tail protein I | | uniclust | UniRef100\_A0A2D3VXF3 | 99.3 | 7.5e-15 | 1.4e-20 | 110.0 | 146 | (2, 150) | 205 | (1, 147) | 176 | Phage tail protein I | Phage tail protein I | | uniclust | UniRef100\_A0A3N5MGM2 | 99.3 | 9.6e-15 | 1.8e-20 | 119.1 | 164 | (12, 182) | 205 | (91, 306) | 338 | Phage tail protein I (Fragment) | Phage tail protein I (Fragment) | | uniclust | UniRef100\_A0A158HNT1 | 99.3 | 9.6e-15 | 2e-20 | 135.4 | 157 | (12, 174) | 205 | (428, 667) | 710 | Serine/threonine-protein kinase PknD | Serine/threonine-protein kinase PknD | | uniclust | UniRef100\_D0LX69 | 99.3 | 1.3e-14 | 2.4e-20 | 122.1 | 123 | (30, 154) | 205 | (118, 260) | 448 | Phage tail protein | Phage tail protein | | uniclust | UniRef100\_A0A021X9K5 | 99.3 | 1.2e-14 | 2.5e-20 | 127.9 | 95 | (5, 101) | 205 | (11, 109) | 413 | Phage tail protein I | Phage tail protein I | | uniclust | UniRef100\_UPI000798D5FE | 99.3 | 1.3e-14 | 2.5e-20 | 100.0 | 95 | (80, 176) | 205 | (1, 95) | 96 | phage tail protein I | phage tail protein I | | uniclust | UniRef100\_UPI001126ED48 | 99.3 | 1.4e-14 | 2.6e-20 | 118.8 | 90 | (3, 92) | 205 | (15, 106) | 353 | phage tail protein I | phage tail protein I | | uniclust | UniRef100\_A0A160TAS9 | 99.3 | 1.3e-14 | 2.7e-20 | 135.7 | 158 | (12, 175) | 205 | (473, 695) | 748 | Phage tail protein | Phage tail protein | | uniclust | UniRef100\_A0A655CJ05 | 99.3 | 1.7e-14 | 3.4e-20 | 108.9 | 98 | (3, 100) | 205 | (11, 114) | 139 | Phage Tail protein I | Phage Tail protein I | | uniclust | UniRef100\_UPI0015626642 | 99.3 | 1.8e-14 | 3.4e-20 | 105.8 | 114 | (2, 121) | 205 | (10, 124) | 133 | phage tail protein | phage tail protein | | uniclust | UniRef100\_UPI001EEDE119 | 99.3 | 1.9e-14 | 3.4e-20 | 104.2 | 98 | (50, 147) | 205 | (1, 98) | 139 | phage tail protein I | phage tail protein I | | uniclust | UniRef100\_UPI001EEE1D57 | 99.3 | 1.9e-14 | 3.5e-20 | 94.7 | 67 | (23, 89) | 205 | (10, 77) | 79 | phage tail protein I | phage tail protein I | | uniclust | UniRef100\_A0A6I1I488 | 99.3 | 2e-14 | 3.7e-20 | 102.2 | 72 | (4, 75) | 205 | (7, 78) | 124 | Phage tail protein I | Phage tail protein I | | uniclust | UniRef100\_A0A535XYK6 | 99.3 | 2.1e-14 | 3.8e-20 | 110.3 | 110 | (30, 147) | 205 | (93, 203) | 208 | Phage tail protein I | Phage tail protein I | | uniclust | UniRef100\_A0A7Z7WAU5 | 99.3 | 2.1e-14 | 3.8e-20 | 98.5 | 90 | (75, 165) | 205 | (1, 96) | 100 | Bacteriophage P2-related tail formation protein | Bacteriophage P2-related tail formation protein | | uniclust | UniRef100\_A0A7X1ZG08 | 99.3 | 2.2e-14 | 4e-20 | 103.6 | 72 | (2, 73) | 205 | (60, 133) | 137 | Phage tail protein I | Phage tail protein I | | uniclust | UniRef100\_A0A2T4UGC8 | 99.3 | 2.1e-14 | 4.1e-20 | 117.2 | 121 | (29, 154) | 205 | (98, 227) | 253 | Phage tail protein | Phage tail protein | | uniclust | UniRef100\_A0A024LRF3 | 99.3 | 2.2e-14 | 4.6e-20 | 129.2 | 145 | (2, 153) | 205 | (49, 196) | 468 | Phage protein | Phage protein | | uniclust | UniRef100\_A0A2T4JG36 | 99.2 | 3.3e-14 | 6e-20 | 114.6 | 95 | (2, 96) | 205 | (4, 98) | 300 | Phage tail protein I | Phage tail protein I | | uniclust | UniRef100\_A0A238WY20 | 99.2 | 3.3e-14 | 6.4e-20 | 131.6 | 162 | (6, 171) | 205 | (663, 851) | 910 | Phage tail protein domain-containing protein | Phage tail protein domain-containing protein | | uniclust | UniRef100\_A0A084SKL4 | 99.2 | 3.8e-14 | 7.8e-20 | 120.5 | 115 | (28, 152) | 205 | (84, 208) | 318 | Tail protein | Tail protein | | uniclust | UniRef100\_A0A2T1ENV4 | 99.2 | 4.6e-14 | 8.6e-20 | 112.6 | 121 | (29, 153) | 205 | (57, 188) | 252 | Phage tail protein I | Phage tail protein I | | uniclust | UniRef100\_A0A011UNJ4 | 99.2 | 5.7e-14 | 1.1e-19 | 115.6 | 142 | (2, 151) | 205 | (8, 152) | 272 | Phage tail protein | Phage tail protein | | uniclust | UniRef100\_A0A607GXP1 | 99.2 | 5.9e-14 | 1.1e-19 | 100.8 | 95 | (69, 164) | 205 | (2, 99) | 114 | Phage tail protein | Phage tail protein | | uniclust | UniRef100\_A0A4R3LI10 | 99.2 | 6e-14 | 1.2e-19 | 125.9 | 140 | (2, 151) | 205 | (8, 151) | 532 | P2-related tail formation protein | P2-related tail formation protein | | uniclust | UniRef100\_A0A0P6W260 | 99.2 | 6.9e-14 | 1.3e-19 | 118.4 | 90 | (3, 92) | 205 | (15, 106) | 465 | Phage tail protein | Phage tail protein | | uniclust | UniRef100\_A0A4Q8MC19 | 99.2 | 7.7e-14 | 1.5e-19 | 119.2 | 94 | (2, 95) | 205 | (3, 97) | 422 | Phage tail protein I | Phage tail protein I | | uniclust | UniRef100\_A0A9D5W5Y6 | 99.2 | 7.8e-14 | 1.5e-19 | 109.0 | 148 | (2, 152) | 205 | (4, 188) | 206 | Phage tail protein | Phage tail protein | | uniclust | UniRef100\_UPI001E4BC782 | 99.2 | 8.2e-14 | 1.5e-19 | 97.1 | 71 | (3, 73) | 205 | (8, 79) | 104 | phage tail protein I | phage tail protein I | | uniclust | UniRef100\_A0A5M6I8Y5 | 99.2 | 9e-14 | 1.6e-19 | 103.1 | 117 | (85, 204) | 205 | (40, 156) | 160 | Phage tail protein I | Phage tail protein I | | uniclust | UniRef100\_UPI000DE39F06 | 99.2 | 1.2e-13 | 2.1e-19 | 93.5 | 78 | (2, 79) | 205 | (3, 82) | 91 | phage tail protein I | phage tail protein I | | uniclust | UniRef100\_A0A7X3ZHW1 | 99.2 | 1.6e-13 | 2.9e-19 | 113.5 | 144 | (3, 149) | 205 | (191, 340) | 366 | Phage tail protein I | Phage tail protein I | | uniclust | UniRef100\_A0A1G6NCN4 | 99.2 | 1.4e-13 | 3.1e-19 | 130.7 | 157 | (11, 175) | 205 | (496, 715) | 753 | Phage tail protein domain-containing protein | Phage tail protein domain-containing protein | | uniclust | UniRef100\_A0A381EC89 | 99.2 | 1.6e-13 | 3.2e-19 | 114.4 | 146 | (1, 153) | 205 | (2, 161) | 298 | Bacteriophage P2-related tail formation protein | Bacteriophage P2-related tail formation protein | | uniclust | UniRef100\_A0A1D9LHX5 | 99.2 | 1.7e-13 | 3.2e-19 | 118.5 | 145 | (1, 150) | 205 | (2, 150) | 444 | Phage tail protein | Phage tail protein | | uniclust | UniRef100\_A0A6B8KI89 | 99.2 | 2e-13 | 3.7e-19 | 110.1 | 190 | (2, 196) | 205 | (86, 278) | 295 | Phage tail protein I | Phage tail protein I | | uniclust | UniRef100\_A0A1H3ZWU0 | 99.1 | 1.9e-13 | 4e-19 | 121.7 | 96 | (2, 99) | 205 | (15, 111) | 413 | Phage tail protein, P2 protein I family | Phage tail protein, P2 protein I family | | uniclust | UniRef100\_A0A645H901 | 99.1 | 2.2e-13 | 4.1e-19 | 97.5 | 119 | (52, 174) | 205 | (4, 123) | 127 | Phage tail protein I | Phage tail protein I | | uniclust | UniRef100\_UPI0013771264 | 99.1 | 2.3e-13 | 4.3e-19 | 114.1 | 90 | (2, 93) | 205 | (6, 95) | 418 | phage tail protein I | phage tail protein I | | uniclust | UniRef100\_A0A072TF05 | 99.1 | 2.6e-13 | 4.8e-19 | 127.3 | 148 | (4, 153) | 205 | (1006, 1174) | 1809 | Bacteriophage regulatory protein, putative (Fragment) | Bacteriophage regulatory protein, putative (Fragment) | | uniclust | UniRef100\_UPI000F016F72 | 99.1 | 2.9e-13 | 5.3e-19 | 92.9 | 89 | (39, 128) | 205 | (3, 91) | 98 | phage tail protein I | phage tail protein I | | uniclust | UniRef100\_A0A7W6E8X0 | 99.1 | 2.8e-13 | 5.3e-19 | 107.2 | 97 | (3, 103) | 205 | (6, 102) | 214 | P2-related tail formation protein | P2-related tail formation protein | | uniclust | UniRef100\_A0A9D2DDK1 | 99.1 | 3.5e-13 | 6.6e-19 | 112.6 | 139 | (11, 151) | 205 | (15, 166) | 357 | Phage tail protein | Phage tail protein | | uniclust | UniRef100\_A0A448QV26 | 99.1 | 3.9e-13 | 7.2e-19 | 105.5 | 134 | (15, 153) | 205 | (67, 217) | 237 | Bacteriophage P2-related tail formation protein | Bacteriophage P2-related tail formation protein | | uniclust | UniRef100\_A0A031HNM3 | 99.1 | 3.5e-13 | 7.3e-19 | 117.7 | 135 | (2, 153) | 205 | (40, 178) | 350 | Phage tail protein | Phage tail protein | | uniclust | UniRef100\_A0A1T2XBJ5 | 99.1 | 4.3e-13 | 7.9e-19 | 99.4 | 135 | (59, 195) | 205 | (8, 142) | 158 | Phage P2 family tail protein | Phage P2 family tail protein | | uniclust | UniRef100\_A0A1C0V8J7 | 99.1 | 4.5e-13 | 8.3e-19 | 105.6 | 151 | (2, 153) | 205 | (2, 163) | 244 | Phage tail protein I | Phage tail protein I | | uniclust | UniRef100\_A0A064AK86 | 99.1 | 4.7e-13 | 8.6e-19 | 100.8 | 117 | (36, 155) | 205 | (6, 122) | 175 | Tail protein | Tail protein | | uniclust | UniRef100\_A0A2E2NRK6 | 99.1 | 4.4e-13 | 8.6e-19 | 117.3 | 122 | (30, 153) | 205 | (206, 366) | 421 | Uncharacterized protein | Uncharacterized protein | | uniclust | UniRef100\_A0A2W5WSP6 | 99.1 | 4.7e-13 | 8.9e-19 | 114.4 | 98 | (3, 100) | 205 | (5, 103) | 407 | Phage tail protein I | Phage tail protein I | | uniclust | UniRef100\_A0A1W1Z3V2 | 99.1 | 5.3e-13 | 1.1e-18 | 116.8 | 93 | (2, 95) | 205 | (6, 99) | 427 | Phage tail protein, P2 protein I family | Phage tail protein, P2 protein I family | | uniclust | UniRef100\_A0A3P6JJC9 | 99.1 | 5.9e-13 | 1.1e-18 | 90.7 | 77 | (75, 153) | 205 | (1, 77) | 94 | Phage tail protein | Phage tail protein | | uniclust | UniRef100\_A0A6I2UWE9 | 99.1 | 6.1e-13 | 1.1e-18 | 95.3 | 69 | (33, 101) | 205 | (1, 70) | 126 | Phage tail protein I (Fragment) | Phage tail protein I (Fragment) | | uniclust | UniRef100\_A0A1M6B964 | 99.1 | 6.1e-13 | 1.2e-18 | 115.9 | 142 | (1, 151) | 205 | (4, 148) | 437 | Phage tail protein (Tail\_P2\_I) | Phage tail protein (Tail\_P2\_I) | | uniclust | UniRef100\_A0A3A8UCJ4 | 99.1 | 6.4e-13 | 1.2e-18 | 115.2 | 143 | (5, 152) | 205 | (18, 167) | 518 | Phage tail protein I | Phage tail protein I | | uniclust | UniRef100\_UPI000B329C9F | 99.1 | 7.7e-13 | 1.5e-18 | 100.1 | 95 | (54, 151) | 205 | (11, 121) | 151 | phage tail protein | phage tail protein | | uniclust | UniRef100\_A0A968NW80 | 99.1 | 7.9e-13 | 1.5e-18 | 114.3 | 135 | (34, 174) | 205 | (240, 422) | 465 | Phage tail protein I | Phage tail protein I | | uniclust | UniRef100\_A0A1C5SWA5 | 99.1 | 7.2e-13 | 1.5e-18 | 116.4 | 158 | (12, 174) | 205 | (175, 352) | 373 | Phage tail protein domain | Phage tail protein domain | | uniclust | UniRef100\_A0A8H2LSC2 | 99.1 | 8.7e-13 | 1.6e-18 | 106.9 | 68 | (2, 69) | 205 | (6, 73) | 303 | Methyltransferase | Methyltransferase | | uniclust | UniRef100\_A0A957FSB1 | 99.1 | 1.1e-12 | 2.1e-18 | 111.5 | 114 | (30, 153) | 205 | (337, 451) | 464 | Phage tail protein I | Phage tail protein I | | uniclust | UniRef100\_A0A955Q574 | 99.0 | 1.3e-12 | 2.4e-18 | 102.0 | 117 | (35, 153) | 205 | (61, 196) | 225 | Uncharacterized protein | Uncharacterized protein | | uniclust | UniRef100\_A0A1G3M9I7 | 99.0 | 1.4e-12 | 2.7e-18 | 105.0 | 148 | (27, 177) | 205 | (41, 191) | 220 | Uncharacterized protein | Uncharacterized protein | | uniclust | UniRef100\_UPI000B5A0976 | 99.0 | 1.6e-12 | 2.9e-18 | 108.1 | 151 | (2, 153) | 205 | (1, 170) | 374 | phage tail protein | phage tail protein | | uniclust | UniRef100\_A0A7X6FP85 | 99.0 | 1.6e-12 | 3e-18 | 91.6 | 88 | (2, 90) | 205 | (5, 93) | 113 | Phage tail protein I | Phage tail protein I | | uniclust | UniRef100\_A0A0D8FVA9 | 99.0 | 1.7e-12 | 3.4e-18 | 107.2 | 120 | (29, 153) | 205 | (64, 188) | 244 | Phage tail protein | Phage tail protein | | uniclust | UniRef100\_I3D8X8 | 99.0 | 1.8e-12 | 3.6e-18 | 95.7 | 99 | (1, 101) | 205 | (1, 108) | 123 | Phage tail protein | Phage tail protein | | uniclust | UniRef100\_A0A0C1MU06 | 99.0 | 1.9e-12 | 3.9e-18 | 111.4 | 143 | (3, 153) | 205 | (18, 162) | 327 | Phage tail protein | Phage tail protein | | uniclust | UniRef100\_A0A508B349 | 99.0 | 2.1e-12 | 3.9e-18 | 107.8 | 120 | (30, 152) | 205 | (197, 320) | 387 | Uncharacterized protein | Uncharacterized protein | | uniclust | UniRef100\_A0A2W6YVX7 | 99.0 | 2.3e-12 | 4.4e-18 | 103.3 | 197 | (3, 204) | 205 | (23, 236) | 238 | Phage tail protein | Phage tail protein | | uniclust | UniRef100\_A0A352AM66 | 99.0 | 2.4e-12 | 4.5e-18 | 94.6 | 60 | (29, 89) | 205 | (70, 129) | 129 | Phage tail protein (Fragment) | Phage tail protein (Fragment) | | uniclust | UniRef100\_A0A0Q7UJR8 | 99.0 | 2.5e-12 | 4.8e-18 | 104.3 | 122 | (28, 153) | 205 | (43, 172) | 259 | Phage tail protein | Phage tail protein | | uniclust | UniRef100\_A0A0Q8B1P1 | 99.0 | 2.6e-12 | 4.8e-18 | 108.3 | 94 | (2, 95) | 205 | (13, 106) | 423 | Phage tail protein | Phage tail protein | | uniclust | UniRef100\_A0A1Q3SJJ0 | 99.0 | 2.8e-12 | 5.2e-18 | 105.0 | 121 | (28, 152) | 205 | (60, 199) | 325 | Phage tail protein I | Phage tail protein I | | uniclust | UniRef100\_A0A1X7Q586 | 99.0 | 3e-12 | 6e-18 | 111.4 | 142 | (1, 151) | 205 | (2, 145) | 401 | p2-related tail formation protein | p2-related tail formation protein | | uniclust | UniRef100\_A0A2N5DAD0 | 99.0 | 3.5e-12 | 7.1e-18 | 120.2 | 94 | (10, 104) | 205 | (561, 658) | 848 | Uncharacterized protein | Uncharacterized protein | | uniclust | UniRef100\_UPI001EDCD62C | 99.0 | 4.1e-12 | 7.6e-18 | 93.9 | 117 | (13, 142) | 205 | (13, 131) | 151 | phage tail protein | phage tail protein | | uniclust | UniRef100\_A0A1I1V086 | 99.0 | 4.8e-12 | 9.3e-18 | 109.3 | 93 | (2, 94) | 205 | (5, 98) | 404 | Phage tail protein, P2 protein I family | Phage tail protein, P2 protein I family | | uniclust | UniRef100\_A0A3D1NS20 | 98.9 | 5.3e-12 | 9.8e-18 | 97.4 | 139 | (11, 151) | 205 | (15, 166) | 203 | Phage tail protein | Phage tail protein | | uniclust | UniRef100\_A0A3A4NQI1 | 98.9 | 6e-12 | 1.1e-17 | 98.2 | 119 | (28, 148) | 205 | (45, 182) | 206 | Phage tail protein I | Phage tail protein I | | uniclust | UniRef100\_A0A4Y6PWX7 | 98.9 | 7e-12 | 1.3e-17 | 110.7 | 126 | (20, 147) | 205 | (432, 572) | 670 | Phage tail protein I | Phage tail protein I | | uniclust | UniRef100\_UPI0021D519AB | 98.9 | 7.7e-12 | 1.4e-17 | 91.2 | 132 | (73, 204) | 205 | (5, 137) | 138 | phage tail protein | phage tail protein | | uniclust | UniRef100\_UPI001C49953D | 98.9 | 1e-11 | 1.9e-17 | 95.5 | 142 | (2, 147) | 205 | (5, 168) | 172 | phage tail protein | phage tail protein | | uniclust | UniRef100\_A0A929K3N6 | 98.9 | 1.1e-11 | 2e-17 | 92.3 | 92 | (12, 104) | 205 | (24, 119) | 157 | Phage tail protein I (Fragment) | Phage tail protein I (Fragment) | | uniclust | UniRef100\_A0A7C4AJB7 | 98.9 | 1.1e-11 | 2e-17 | 106.5 | 94 | (2, 95) | 205 | (4, 98) | 397 | Phage tail protein I (Fragment) | Phage tail protein I (Fragment) | | uniclust | UniRef100\_A0A3A9B594 | 98.9 | 1.1e-11 | 2e-17 | 100.3 | 148 | (3, 153) | 205 | (16, 174) | 288 | Phage tail protein I (Fragment) | Phage tail protein I (Fragment) | | uniclust | UniRef100\_A0A7C3GTL1 | 98.9 | 1.2e-11 | 2.2e-17 | 109.8 | 140 | (12, 153) | 205 | (464, 637) | 707 | Phage tail protein I | Phage tail protein I | | uniclust | UniRef100\_UPI0021C5C126 | 98.9 | 1.2e-11 | 2.2e-17 | 81.9 | 64 | (38, 101) | 205 | (4, 70) | 78 | phage tail protein I | phage tail protein I | | uniclust | UniRef100\_A0A411CW10 | 98.9 | 1.2e-11 | 2.4e-17 | 99.6 | 116 | (36, 153) | 205 | (58, 175) | 205 | Tail protein | Tail protein | | uniclust | UniRef100\_A0A3P8M2T9 | 98.9 | 1.4e-11 | 2.6e-17 | 85.7 | 66 | (2, 67) | 205 | (3, 68) | 103 | Bacteriophage P2-related tail formation protein | Bacteriophage P2-related tail formation protein | | uniclust | UniRef100\_UPI000417C6A3 | 98.9 | 1.5e-11 | 2.7e-17 | 103.8 | 121 | (33, 153) | 205 | (41, 180) | 414 | phage tail protein | phage tail protein | | uniclust | UniRef100\_UPI00201AC909 | 98.9 | 1.6e-11 | 2.9e-17 | 77.7 | 46 | (30, 75) | 205 | (10, 55) | 61 | phage tail protein I | phage tail protein I | | uniclust | UniRef100\_A0A969IZC1 | 98.9 | 1.7e-11 | 3.2e-17 | 98.0 | 116 | (29, 151) | 205 | (52, 175) | 261 | Phage tail protein I | Phage tail protein I | | uniclust | UniRef100\_A0A087KKF1 | 98.9 | 1.7e-11 | 3.2e-17 | 87.8 | 81 | (5, 85) | 205 | (7, 91) | 108 | Tail fiber protein (Fragment) | Tail fiber protein (Fragment) | | uniclust | UniRef100\_A0A496RFZ9 | 98.9 | 1.7e-11 | 3.3e-17 | 109.3 | 159 | (12, 176) | 205 | (381, 549) | 587 | Phage tail protein (Fragment) | Phage tail protein (Fragment) | | uniclust | UniRef100\_A0A3D2IPZ0 | 98.9 | 1.8e-11 | 3.4e-17 | 97.2 | 133 | (30, 168) | 205 | (49, 181) | 247 | Phage tail protein I | Phage tail protein I | | uniclust | UniRef100\_UPI000C6CA109 | 98.9 | 1.9e-11 | 3.4e-17 | 104.4 | 141 | (29, 175) | 205 | (254, 418) | 460 | phage tail protein | phage tail protein | | uniclust | UniRef100\_A0A929PG22 | 98.9 | 1.9e-11 | 3.5e-17 | 99.5 | 75 | (29, 104) | 205 | (31, 106) | 299 | Uncharacterized protein | Uncharacterized protein | | uniclust | UniRef100\_UPI00209C94E4 | 98.9 | 1.9e-11 | 3.6e-17 | 96.7 | 135 | (30, 171) | 205 | (54, 204) | 240 | phage tail protein | phage tail protein | | uniclust | UniRef100\_UPI000E352F0F | 98.9 | 1.9e-11 | 3.7e-17 | 91.1 | 84 | (91, 176) | 205 | (5, 88) | 137 | phage tail protein I | phage tail protein I | | uniclust | UniRef100\_UPI0006865A37 | 98.9 | 2.1e-11 | 3.9e-17 | 105.2 | 141 | (3, 145) | 205 | (12, 193) | 511 | phage tail protein | phage tail protein | | uniclust | UniRef100\_A0A090SU32 | 98.8 | 2.4e-11 | 4.5e-17 | 78.9 | 65 | (19, 83) | 205 | (4, 69) | 70 | Uncharacterized protein | Uncharacterized protein | | uniclust | UniRef100\_A0A6S6YHP9 | 98.8 | 2.4e-11 | 4.5e-17 | 99.4 | 140 | (2, 151) | 205 | (4, 147) | 291 | Phage tail protein | Phage tail protein | | uniclust | UniRef100\_A0A928VPI1 | 98.8 | 2.8e-11 | 5.2e-17 | 102.8 | 121 | (30, 153) | 205 | (253, 412) | 435 | Uncharacterized protein | Uncharacterized protein | | uniclust | UniRef100\_A0A1J0LXM1 | 98.8 | 2.6e-11 | 5.2e-17 | 103.2 | 116 | (35, 151) | 205 | (53, 183) | 308 | Phage tail protein (Tail\_P2\_I) | Phage tail protein (Tail\_P2\_I) | | uniclust | UniRef100\_A0A0Q6MMI2 | 98.8 | 2.7e-11 | 5.8e-17 | 117.9 | 161 | (12, 178) | 205 | (635, 867) | 968 | Phage tail protein | Phage tail protein | | uniclust | UniRef100\_A0A9E6AVE8 | 98.8 | 3.2e-11 | 5.8e-17 | 96.0 | 165 | (3, 168) | 205 | (14, 186) | 249 | Phage tail protein I | Phage tail protein I | | uniclust | UniRef100\_A0A1S2E3G0 | 98.8 | 3.1e-11 | 5.8e-17 | 99.8 | 139 | (13, 152) | 205 | (19, 166) | 286 | Phage tail protein I | Phage tail protein I | | uniclust | UniRef100\_UPI001CF59578 | 98.8 | 3.2e-11 | 5.9e-17 | 101.4 | 114 | (29, 147) | 205 | (202, 333) | 396 | phage tail protein | phage tail protein | | uniclust | UniRef100\_A0A2V8RW14 | 98.8 | 3.2e-11 | 6.3e-17 | 105.2 | 136 | (30, 171) | 205 | (195, 383) | 431 | Phage tail protein | Phage tail protein | | uniclust | UniRef100\_UPI0003FB0752 | 98.8 | 3.5e-11 | 6.4e-17 | 96.1 | 171 | (30, 203) | 205 | (68, 240) | 256 | phage tail protein | phage tail protein | | uniclust | UniRef100\_U1KSX1 | 98.8 | 3.7e-11 | 6.7e-17 | 92.0 | 98 | (53, 153) | 205 | (1, 105) | 187 | Phage tail protein I | Phage tail protein I | | uniclust | UniRef100\_A0A2P8W8C8 | 98.8 | 3.6e-11 | 6.9e-17 | 110.7 | 134 | (12, 150) | 205 | (467, 644) | 709 | Phage tail protein (Fragment) | Phage tail protein (Fragment) | | uniclust | UniRef100\_UPI0018E0E16F | 98.8 | 3.9e-11 | 7.1e-17 | 102.2 | 111 | (36, 147) | 205 | (50, 196) | 446 | phage tail protein I | phage tail protein I | | uniclust | UniRef100\_UPI000DE5AB00 | 98.8 | 4e-11 | 7.4e-17 | 86.9 | 110 | (1, 110) | 205 | (1, 116) | 130 | phage tail protein I | phage tail protein I | | uniclust | UniRef100\_UPI000B064F58 | 98.8 | 4.1e-11 | 7.6e-17 | 85.2 | 97 | (13, 115) | 205 | (13, 111) | 112 | phage tail protein | phage tail protein | | uniclust | UniRef100\_A0A1H0YPQ4 | 98.8 | 4.7e-11 | 8.8e-17 | 96.0 | 202 | (3, 204) | 205 | (9, 248) | 250 | Phage tail protein, P2 protein I family | Phage tail protein, P2 protein I family | | uniclust | UniRef100\_A0A3A8IIH2 | 98.8 | 5.1e-11 | 1e-16 | 100.7 | 122 | (42, 168) | 205 | (134, 281) | 298 | Phage tail protein | Phage tail protein | | uniclust | UniRef100\_UPI0016870817 | 98.8 | 5.6e-11 | 1e-16 | 107.9 | 133 | (12, 147) | 205 | (646, 832) | 898 | phage tail protein | phage tail protein | | uniclust | UniRef100\_A0A0M2RHZ0 | 98.8 | 5.6e-11 | 1.1e-16 | 97.8 | 115 | (29, 152) | 205 | (52, 175) | 266 | Phage tail protein | Phage tail protein | | uniclust | UniRef100\_UPI0021F725AA | 98.8 | 5.8e-11 | 1.1e-16 | 76.4 | 56 | (30, 85) | 205 | (10, 65) | 66 | phage tail protein I | phage tail protein I | | uniclust | UniRef100\_A0A238TE88 | 98.8 | 5.7e-11 | 1.1e-16 | 87.5 | 86 | (65, 153) | 205 | (2, 103) | 123 | Uncharacterized protein | Uncharacterized protein | | uniclust | UniRef100\_UPI0021AC41D2 | 98.8 | 6.8e-11 | 1.2e-16 | 95.6 | 147 | (4, 153) | 205 | (5, 164) | 278 | phage tail protein | phage tail protein | | uniclust | UniRef100\_A0A4V2ZG90 | 98.8 | 6.9e-11 | 1.3e-16 | 106.1 | 142 | (30, 177) | 205 | (509, 676) | 704 | Phage tail protein | Phage tail protein | | uniclust | UniRef100\_A0A0P8A262 | 98.8 | 7e-11 | 1.3e-16 | 93.6 | 120 | (45, 171) | 205 | (2, 171) | 214 | Uncharacterized protein | Uncharacterized protein | | uniclust | UniRef100\_A0A968LQ35 | 98.7 | 8.3e-11 | 1.5e-16 | 92.6 | 116 | (30, 153) | 205 | (100, 215) | 226 | Phage tail protein I | Phage tail protein I | | uniclust | UniRef100\_A0A059PCS6 | 98.7 | 8.6e-11 | 1.6e-16 | 101.5 | 137 | (1, 149) | 205 | (15, 153) | 417 | Tail I | Tail I | | uniclust | UniRef100\_A0A938E8C9 | 98.7 | 9.1e-11 | 1.7e-16 | 89.0 | 70 | (30, 100) | 205 | (67, 140) | 173 | Uncharacterized protein (Fragment) | Uncharacterized protein (Fragment) | | uniclust | UniRef100\_A0A0B5FHF2 | 98.7 | 8.5e-11 | 1.7e-16 | 106.0 | 101 | (3, 104) | 205 | (11, 120) | 529 | Tail protein | Tail protein | | uniclust | UniRef100\_A0A450WSG3 | 98.7 | 9.9e-11 | 1.8e-16 | 96.1 | 145 | (1, 150) | 205 | (1, 149) | 315 | P2-related tail formation protein | P2-related tail formation protein | | uniclust | UniRef100\_A0A533MJS2 | 98.7 | 1e-10 | 1.9e-16 | 102.5 | 89 | (12, 101) | 205 | (333, 425) | 582 | Phage tail protein I | Phage tail protein I | | uniclust | UniRef100\_A0A9E8EP50 | 98.7 | 1e-10 | 1.9e-16 | 94.2 | 109 | (39, 153) | 205 | (38, 164) | 270 | Phage tail protein | Phage tail protein | | uniclust | UniRef100\_A0A124IN49 | 98.7 | 9.6e-11 | 2e-16 | 103.4 | 152 | (30, 188) | 205 | (196, 363) | 381 | Phage tail protein | Phage tail protein | | uniclust | UniRef100\_UPI001FC81059 | 98.7 | 1.1e-10 | 2.1e-16 | 96.0 | 119 | (29, 153) | 205 | (153, 283) | 321 | phage tail protein | phage tail protein | | uniclust | UniRef100\_UPI001FDF4D5B | 98.7 | 1.2e-10 | 2.2e-16 | 86.9 | 69 | (30, 98) | 205 | (33, 101) | 155 | phage tail protein I | phage tail protein I | | uniclust | UniRef100\_A0A969BUE2 | 98.7 | 1.3e-10 | 2.3e-16 | 99.8 | 117 | (30, 151) | 205 | (240, 397) | 466 | Uncharacterized protein | Uncharacterized protein | | uniclust | UniRef100\_A0A8J7KWD7 | 98.7 | 1.2e-10 | 2.3e-16 | 88.5 | 110 | (30, 151) | 205 | (42, 156) | 158 | Phage tail-like protein | Phage tail-like protein | | uniclust | UniRef100\_A0A1J7BCP7 | 98.7 | 1.4e-10 | 2.6e-16 | 94.1 | 120 | (30, 153) | 205 | (60, 189) | 252 | Phage tail protein | Phage tail protein | | uniclust | UniRef100\_A0A2W5C641 | 98.7 | 1.6e-10 | 3.1e-16 | 95.6 | 139 | (2, 147) | 205 | (5, 146) | 266 | Phage tail protein (Fragment) | Phage tail protein (Fragment) | | uniclust | UniRef100\_A0A8S5UB99 | 98.7 | 1.9e-10 | 3.4e-16 | 93.9 | 108 | (38, 151) | 205 | (75, 182) | 295 | Tail protein | Tail protein | | uniclust | UniRef100\_U2F716 | 98.7 | 1.9e-10 | 3.6e-16 | 87.0 | 118 | (43, 167) | 205 | (1, 133) | 143 | Tail fiber | Tail fiber | | uniclust | UniRef100\_A0A7C6KQ05 | 98.7 | 2e-10 | 3.8e-16 | 87.7 | 133 | (14, 150) | 205 | (21, 158) | 180 | Phage tail protein | Phage tail protein | | uniclust | UniRef100\_A0A7J9Y123 | 98.7 | 2.2e-10 | 4e-16 | 92.2 | 117 | (30, 153) | 205 | (64, 181) | 262 | Phage tail protein | Phage tail protein | | uniclust | UniRef100\_A0A0K2RLR3 | 98.7 | 2.3e-10 | 4.3e-16 | 91.4 | 113 | (30, 153) | 205 | (69, 194) | 209 | Phage tail protein | Phage tail protein | | uniclust | UniRef100\_A0A7C4ELX4 | 98.7 | 2.4e-10 | 4.4e-16 | 88.6 | 94 | (2, 95) | 205 | (5, 102) | 199 | Phage tail protein (Fragment) | Phage tail protein (Fragment) | | uniclust | UniRef100\_UPI000DEEDDBB | 98.7 | 2.4e-10 | 4.4e-16 | 72.9 | 61 | (1, 61) | 205 | (1, 61) | 62 | phage tail protein I | phage tail protein I | | uniclust | UniRef100\_A0A938CLU1 | 98.7 | 2.4e-10 | 4.5e-16 | 71.6 | 56 | (27, 82) | 205 | (2, 57) | 57 | Phage tail protein I | Phage tail protein I | | uniclust | UniRef100\_A0A059IUT0 | 98.7 | 2.5e-10 | 4.7e-16 | 93.7 | 137 | (1, 149) | 205 | (15, 153) | 269 | Phage tail protein | Phage tail protein | | uniclust | UniRef100\_A0A327JSB1 | 98.6 | 2.9e-10 | 5.4e-16 | 82.7 | 89 | (2, 91) | 205 | (6, 98) | 130 | Phage tail protein I (Fragment) | Phage tail protein I (Fragment) | | uniclust | UniRef100\_A0A371J258 | 98.6 | 3e-10 | 5.6e-16 | 82.4 | 108 | (2, 111) | 205 | (10, 123) | 123 | Phage tail protein (Fragment) | Phage tail protein (Fragment) | | uniclust | UniRef100\_A0A239INL8 | 98.6 | 3.2e-10 | 6.3e-16 | 94.7 | 119 | (29, 152) | 205 | (64, 187) | 273 | Phage tail protein domain-containing protein | Phage tail protein domain-containing protein | | uniclust | UniRef100\_A0A212KXG2 | 98.6 | 3.3e-10 | 6.3e-16 | 100.3 | 105 | (40, 150) | 205 | (77, 181) | 469 | Phage tail protein | Phage tail protein | | uniclust | UniRef100\_A0A4R7I2Z4 | 98.6 | 3.6e-10 | 6.7e-16 | 92.3 | 121 | (30, 154) | 205 | (51, 181) | 271 | Phage tail-like protein | Phage tail-like protein | | uniclust | UniRef100\_A0A4R2JPC5 | 98.6 | 4.2e-10 | 7.8e-16 | 85.3 | 116 | (28, 151) | 205 | (41, 157) | 158 | Phage tail-like protein | Phage tail-like protein | | uniclust | UniRef100\_UPI0022473D3F | 98.6 | 4.4e-10 | 8.2e-16 | 86.5 | 110 | (37, 147) | 205 | (27, 161) | 188 | phage tail protein | phage tail protein | | uniclust | UniRef100\_A0A925IPT4 | 98.6 | 5.1e-10 | 9.4e-16 | 96.2 | 115 | (34, 153) | 205 | (207, 355) | 463 | Phage tail protein | Phage tail protein | | uniclust | UniRef100\_UPI002356C388 | 98.6 | 5.2e-10 | 9.5e-16 | 85.0 | 136 | (3, 147) | 205 | (5, 144) | 171 | phage tail protein | phage tail protein | | uniclust | UniRef100\_UPI001916A594 | 98.6 | 5.2e-10 | 9.6e-16 | 84.3 | 139 | (3, 153) | 205 | (6, 145) | 162 | phage tail protein | phage tail protein | | uniclust | UniRef100\_G0FVT8 | 98.6 | 5.1e-10 | 9.6e-16 | 103.2 | 120 | (30, 151) | 205 | (711, 844) | 847 | Baseplate protein J-like domain-containing protein | Baseplate protein J-like domain-containing protein | | uniclust | UniRef100\_UPI001EF24CD4 | 98.6 | 5.5e-10 | 1e-15 | 94.3 | 132 | (30, 163) | 205 | (180, 312) | 391 | phage tail protein | phage tail protein | | uniclust | UniRef100\_A0A7W0VDI4 | 98.6 | 5.6e-10 | 1e-15 | 86.4 | 70 | (31, 101) | 205 | (118, 188) | 194 | PASTA domain-containing protein (Fragment) | PASTA domain-containing protein (Fragment) | | uniclust | UniRef100\_A0A1G9PB45 | 98.6 | 5.2e-10 | 1.1e-15 | 103.7 | 63 | (30, 92) | 205 | (74, 140) | 561 | Phage tail protein domain-containing protein | Phage tail protein domain-containing protein | | uniclust | UniRef100\_A0A962F9E9 | 98.6 | 5.9e-10 | 1.1e-15 | 87.2 | 148 | (2, 153) | 205 | (8, 158) | 209 | Phage tail protein I | Phage tail protein I | | uniclust | UniRef100\_UPI001E6408FD | 98.6 | 6e-10 | 1.1e-15 | 82.3 | 134 | (59, 204) | 205 | (6, 142) | 142 | DUF2313 domain-containing protein | DUF2313 domain-containing protein | |
| Top keywords  (threshold 1.00e-03 (evalue)) | **tail, Phage, I, Fragment, P2, P2\_related, formation, Bacteriophage, domain\_containing, Putative** |
| Output files | ../../similar\_sequences/14\_FANPEZAQ\_CDS\_0014\_merged.svg ../../similar\_sequences/14\_FANPEZAQ\_CDS\_0014\_pdb70.a3m ../../similar\_sequences/14\_FANPEZAQ\_CDS\_0014\_pdb70.hhr ../../similar\_sequences/14\_FANPEZAQ\_CDS\_0014\_uniclust.a3m ../../similar\_sequences/14\_FANPEZAQ\_CDS\_0014\_uniclust.hhr |

#### Structure prediction (AlphaFold)2

|  |  |
| --- | --- |
| Stats | xml version="1.0" encoding="utf-8" standalone="no"?       2024-09-02T21:09:12.909538 image/svg+xml   Matplotlib v3.7.2, https://matplotlib.org/ |
| Predicted structure | **NGL Viewer Controls:**  - Center: *Left-Click* - Rotate: *Left-Click + Drag* - Translate: *Right-Click + Drag* - Zoom: *Shift + Left-Click + Drag* |
| Output files | ../../predicted\_structures/14\_FANPEZAQ\_CDS\_0014/features.pkl ../../predicted\_structures/14\_FANPEZAQ\_CDS\_0014/ranked\_0.pdb ../../predicted\_structures/14\_FANPEZAQ\_CDS\_0014/ranked\_0\_plots.svg ../../predicted\_structures/14\_FANPEZAQ\_CDS\_0014/result\_model\_1\_ptm\_pred\_0.pkl |

#### Structure similarity search results (Foldseek)3

|  |  |
| --- | --- |
| Structure databases searched | Pdb, Afdb-proteome, Afdb-uniprot50 |
| Results, scheme(s)  (Top layers only, threshold 1.00e-02 (evalue)) | xml version="1.0" encoding="utf-8" standalone="no"?       2024-09-02T21:10:29.376195 image/svg+xml   Matplotlib v3.7.2, https://matplotlib.org/ |
| Results, table  (threshold 1.00e-02 (evalue)) | | db | id | prob | evalue | bits | fident | alnlen | mismatch | gapopen | qstart | qend | tstart | tend | name | description | | --- | --- | --- | --- | --- | --- | --- | --- | --- | --- | --- | --- | --- | --- | --- | | pdb | 6U5B\_0 | 1.0 | 1.456e-10 | 303 | 0.348 | 152 | 97 | 1 | 1 | 152 | 1 | 150 | Tri2 PA0619 | Tri2 PA0619 | | afdb-proteome | AF-Q8ZMU4-F1-MODEL\_V4 | 1.0 | 7.453e-23 | 701 | 0.418 | 203 | 116 | 1 | 1 | 203 | 1 | 201 | Fels-2 prophage protein | Fels-2 prophage protein | | afdb-proteome | AF-A0A0H3GQR7-F1-MODEL\_V4 | 1.0 | 1.573e-20 | 600 | 0.37 | 200 | 121 | 2 | 1 | 197 | 2 | 199 | Tail protein I | Tail protein I | | afdb-proteome | AF-A0A0H3GWZ9-F1-MODEL\_V4 | 1.0 | 6.461e-19 | 513 | 0.359 | 181 | 114 | 1 | 5 | 185 | 1 | 179 | Tail protein I | Tail protein I | | afdb-proteome | AF-Q8ZKK4-F1-MODEL\_V4 | 1.0 | 6.181e-16 | 454 | 0.317 | 211 | 134 | 6 | 1 | 203 | 1 | 209 | Putative phage tail protein | Putative phage tail protein | | afdb-proteome | AF-G3XD92-F1-MODEL\_V4 | 1.0 | 9.347e-17 | 443 | 0.324 | 179 | 118 | 2 | 1 | 178 | 1 | 177 | Probable bacteriophage protein | Probable bacteriophage protein | | afdb-proteome | AF-Q6NXT4-F1-MODEL\_V4 | 0.817 | 0.008268 | 62 | 0.128 | 234 | 147 | 13 | 8 | 203 | 169 | 383 | Zinc transporter 6 | Zinc transporter 6 | | afdb-uniprot50 | AF-A0A2Z3I623-F1-MODEL\_V4 | 1.0 | 4.101e-28 | 866 | 0.645 | 203 | 72 | 0 | 2 | 204 | 20 | 222 | Phage tail protein I | Phage tail protein I | | afdb-uniprot50 | AF-C3X1Y3-F1-MODEL\_V4 | 1.0 | 1.568e-24 | 765 | 0.453 | 205 | 110 | 1 | 1 | 205 | 2 | 204 | Phage tail protein I | Phage tail protein I | | afdb-uniprot50 | AF-A0A3S8ZPY7-F1-MODEL\_V4 | 1.0 | 8.51e-22 | 730 | 0.405 | 180 | 107 | 0 | 1 | 180 | 2 | 181 | Phage tail protein I | Phage tail protein I | | afdb-uniprot50 | AF-A0A5C9AIB0-F1-MODEL\_V4 | 1.0 | 6.211e-22 | 713 | 0.435 | 186 | 103 | 1 | 1 | 186 | 1 | 184 | Phage tail protein I | Phage tail protein I | | afdb-uniprot50 | AF-E4PPS5-F1-MODEL\_V4 | 1.0 | 9.652e-22 | 712 | 0.392 | 191 | 116 | 0 | 1 | 191 | 1 | 191 | Phage tail protein I | Phage tail protein I | | afdb-uniprot50 | AF-A0A1N6I1G1-F1-MODEL\_V4 | 1.0 | 7.99e-22 | 708 | 0.388 | 193 | 117 | 1 | 4 | 196 | 3 | 194 | Phage tail protein, P2 protein I family | Phage tail protein, P2 protein I family | | afdb-uniprot50 | AF-A0A4Y5WA04-F1-MODEL\_V4 | 1.0 | 2.188e-21 | 705 | 0.451 | 175 | 96 | 0 | 1 | 175 | 1 | 175 | Phage tail protein I | Phage tail protein I | | afdb-uniprot50 | AF-A0A846QRB9-F1-MODEL\_V4 | 1.0 | 2.666e-23 | 703 | 0.443 | 203 | 113 | 0 | 1 | 203 | 2 | 204 | Phage tail P2-like protein | Phage tail P2-like protein | | afdb-uniprot50 | AF-A0A0D7V3M6-F1-MODEL\_V4 | 1.0 | 1.065e-22 | 703 | 0.386 | 207 | 124 | 2 | 1 | 204 | 1 | 207 | Uncharacterized protein | Uncharacterized protein | | afdb-uniprot50 | AF-A0A430HF69-F1-MODEL\_V4 | 1.0 | 1.541e-20 | 703 | 0.415 | 178 | 102 | 2 | 1 | 176 | 1 | 178 | Phage tail protein I | Phage tail protein I | | afdb-uniprot50 | AF-Q8ZMU4-F1-MODEL\_V4 | 1.0 | 2.74e-22 | 701 | 0.418 | 203 | 116 | 1 | 1 | 203 | 1 | 201 | Fels-2 prophage protein | Fels-2 prophage protein | | afdb-uniprot50 | AF-A0A5Y2X4S5-F1-MODEL\_V4 | 1.0 | 1.929e-21 | 695 | 0.46 | 176 | 91 | 2 | 1 | 174 | 1 | 174 | Phage tail protein I | Phage tail protein I | | afdb-uniprot50 | AF-A0A7W5P3V3-F1-MODEL\_V4 | 1.0 | 6.437e-23 | 689 | 0.438 | 203 | 112 | 1 | 1 | 203 | 2 | 202 | Phage tail P2-like protein | Phage tail P2-like protein | | afdb-uniprot50 | AF-A0A250DSF2-F1-MODEL\_V4 | 1.0 | 5.43e-20 | 689 | 0.382 | 178 | 107 | 1 | 1 | 175 | 1 | 178 | Phage tail protein I | Phage tail protein I | | afdb-uniprot50 | AF-A0A328X6D0-F1-MODEL\_V4 | 1.0 | 2.331e-21 | 685 | 0.418 | 184 | 103 | 2 | 1 | 181 | 1 | 183 | Phage tail P2-like protein | Phage tail P2-like protein | | afdb-uniprot50 | AF-A0A5Y8G7Y1-F1-MODEL\_V4 | 1.0 | 9.063e-22 | 684 | 0.448 | 185 | 98 | 2 | 1 | 183 | 1 | 183 | Phage tail protein I | Phage tail protein I | | afdb-uniprot50 | AF-A0A7U7FJK2-F1-MODEL\_V4 | 1.0 | 2.13e-22 | 684 | 0.428 | 203 | 114 | 1 | 1 | 203 | 29 | 229 | Putative bacteriophage protein | Putative bacteriophage protein | | afdb-uniprot50 | AF-A0A1Y3P6W7-F1-MODEL\_V4 | 1.0 | 1.701e-21 | 683 | 0.41 | 185 | 106 | 2 | 1 | 185 | 1 | 182 | Phage tail protein | Phage tail protein | | afdb-uniprot50 | AF-A0A1I5KZ34-F1-MODEL\_V4 | 1.0 | 4.828e-22 | 680 | 0.414 | 205 | 118 | 1 | 1 | 203 | 1 | 205 | Phage tail protein, P2 protein I family | Phage tail protein, P2 protein I family | | afdb-uniprot50 | AF-A0A137YJL7-F1-MODEL\_V4 | 1.0 | 1.276e-20 | 678 | 0.375 | 176 | 109 | 1 | 1 | 175 | 1 | 176 | Phage tail protein | Phage tail protein | | afdb-uniprot50 | AF-A0A144SRX6-F1-MODEL\_V4 | 1.0 | 1.396e-19 | 677 | 0.474 | 156 | 78 | 2 | 1 | 154 | 1 | 154 | Phage Tail protein I | Phage Tail protein I | | afdb-uniprot50 | AF-A0A1G7HXV1-F1-MODEL\_V4 | 1.0 | 5.832e-22 | 677 | 0.429 | 205 | 114 | 2 | 2 | 205 | 3 | 205 | Phage tail protein, P2 protein I family | Phage tail protein, P2 protein I family | | afdb-uniprot50 | AF-A0A061JKL1-F1-MODEL\_V4 | 1.0 | 1.641e-20 | 676 | 0.357 | 182 | 116 | 1 | 1 | 182 | 1 | 181 | Uncharacterized protein | Uncharacterized protein | | afdb-uniprot50 | AF-A0A1N6Q2Z7-F1-MODEL\_V4 | 1.0 | 2.716e-20 | 675 | 0.396 | 179 | 107 | 1 | 1 | 178 | 1 | 179 | Phage tail protein, P2 protein I family | Phage tail protein, P2 protein I family | | afdb-uniprot50 | AF-A0A0N8R6G9-F1-MODEL\_V4 | 1.0 | 3.107e-22 | 673 | 0.413 | 203 | 117 | 1 | 1 | 203 | 56 | 256 | Tail protein I | Tail protein I | | afdb-uniprot50 | AF-A0A0Q0CU47-F1-MODEL\_V4 | 1.0 | 1.701e-21 | 670 | 0.41 | 190 | 110 | 1 | 1 | 190 | 1 | 188 | Tail protein I | Tail protein I | | afdb-uniprot50 | AF-A0A4Y6UC71-F1-MODEL\_V4 | 1.0 | 8.438e-20 | 670 | 0.346 | 173 | 112 | 1 | 4 | 175 | 18 | 190 | Phage tail protein I | Phage tail protein I | | afdb-uniprot50 | AF-A0A1H0NP46-F1-MODEL\_V4 | 1.0 | 2.112e-20 | 670 | 0.388 | 188 | 115 | 0 | 5 | 192 | 1 | 188 | Phage tail protein, P2 protein I family | Phage tail protein, P2 protein I family | | afdb-uniprot50 | AF-A0A6I3XGM5-F1-MODEL\_V4 | 1.0 | 2.461e-19 | 666 | 0.41 | 178 | 103 | 2 | 1 | 178 | 1 | 176 | Phage tail protein I | Phage tail protein I | | afdb-uniprot50 | AF-A0A254PRB8-F1-MODEL\_V4 | 1.0 | 1.584e-19 | 661 | 0.359 | 178 | 112 | 1 | 1 | 178 | 6 | 181 | Phage tail protein I | Phage tail protein I | | afdb-uniprot50 | AF-A0A395R2T8-F1-MODEL\_V4 | 1.0 | 4.787e-20 | 659 | 0.386 | 181 | 109 | 1 | 1 | 179 | 1 | 181 | Uncharacterized protein | Uncharacterized protein | | afdb-uniprot50 | AF-A0A0S1B435-F1-MODEL\_V4 | 1.0 | 2.188e-21 | 658 | 0.371 | 202 | 127 | 0 | 4 | 205 | 3 | 204 | Phage P2 baseplate assembly gpI-like protein | Phage P2 baseplate assembly gpI-like protein | | afdb-uniprot50 | AF-A0A485AEW3-F1-MODEL\_V4 | 1.0 | 3.622e-21 | 657 | 0.448 | 194 | 103 | 2 | 1 | 192 | 105 | 296 | Bacteriophage P2-related tail formation protein | Bacteriophage P2-related tail formation protein | | afdb-uniprot50 | AF-A0A2N7UDJ2-F1-MODEL\_V4 | 1.0 | 1.701e-21 | 654 | 0.423 | 203 | 114 | 3 | 2 | 203 | 6 | 206 | Phage tail protein I | Phage tail protein I | | afdb-uniprot50 | AF-A0A6B8QMK9-F1-MODEL\_V4 | 1.0 | 2.188e-21 | 653 | 0.366 | 202 | 128 | 0 | 4 | 205 | 9 | 210 | Phage tail protein I | Phage tail protein I | | afdb-uniprot50 | AF-A0A1F1QBB0-F1-MODEL\_V4 | 1.0 | 9.063e-22 | 652 | 0.381 | 202 | 123 | 1 | 1 | 202 | 1 | 200 | Phage tail protein | Phage tail protein | | afdb-uniprot50 | AF-A0A0D0TMA8-F1-MODEL\_V4 | 1.0 | 9.918e-21 | 649 | 0.432 | 185 | 103 | 1 | 1 | 185 | 1 | 183 | Phage tail protein | Phage tail protein | | afdb-uniprot50 | AF-A0A3N6RV94-F1-MODEL\_V4 | 1.0 | 3.193e-21 | 648 | 0.428 | 203 | 114 | 1 | 1 | 203 | 182 | 382 | Phage tail protein I | Phage tail protein I | | afdb-uniprot50 | AF-A0A2T5J1G3-F1-MODEL\_V4 | 1.0 | 1.276e-20 | 644 | 0.429 | 205 | 114 | 3 | 1 | 205 | 1 | 202 | Phage tail P2-like protein | Phage tail P2-like protein | | afdb-uniprot50 | AF-A0A1X3ISP6-F1-MODEL\_V4 | 1.0 | 1.474e-17 | 643 | 0.496 | 133 | 65 | 1 | 22 | 154 | 8 | 138 | Phage tail protein I | Phage tail protein I | | afdb-uniprot50 | AF-A0A7W6RFJ4-F1-MODEL\_V4 | 1.0 | 1.701e-21 | 643 | 0.404 | 205 | 119 | 2 | 2 | 205 | 5 | 207 | Phage tail P2-like protein | Phage tail P2-like protein | | afdb-uniprot50 | AF-C1DS15-F1-MODEL\_V4 | 1.0 | 2.893e-20 | 643 | 0.383 | 206 | 123 | 2 | 1 | 203 | 1 | 205 | Phage P2 baseplate assembly gpI-like protein | Phage P2 baseplate assembly gpI-like protein | | afdb-uniprot50 | AF-A0A7Z3GTI5-F1-MODEL\_V4 | 1.0 | 2.249e-20 | 638 | 0.424 | 179 | 100 | 2 | 1 | 178 | 1 | 177 | Phage tail protein I | Phage tail protein I | | afdb-uniprot50 | AF-A0A5E9SCQ9-F1-MODEL\_V4 | 1.0 | 6.383e-21 | 638 | 0.421 | 190 | 108 | 1 | 14 | 203 | 2 | 189 | Phage tail protein I | Phage tail protein I | | afdb-uniprot50 | AF-A0A143DE04-F1-MODEL\_V4 | 1.0 | 5.627e-21 | 638 | 0.371 | 207 | 127 | 3 | 1 | 205 | 2 | 207 | Uncharacterized protein | Uncharacterized protein | | afdb-uniprot50 | AF-A0A1Y2SIU0-F1-MODEL\_V4 | 1.0 | 9.063e-22 | 637 | 0.436 | 204 | 112 | 2 | 1 | 203 | 1 | 202 | Phage tail protein I | Phage tail protein I | | afdb-uniprot50 | AF-A0A2X4TXF0-F1-MODEL\_V4 | 1.0 | 6.159e-20 | 637 | 0.431 | 176 | 97 | 2 | 1 | 175 | 1 | 174 | Bacteriophage P2-related tail formation protein | Bacteriophage P2-related tail formation protein | | afdb-uniprot50 | AF-A0A7W2HYN2-F1-MODEL\_V4 | 1.0 | 4.962e-21 | 636 | 0.376 | 207 | 125 | 3 | 1 | 205 | 1 | 205 | Phage tail protein I | Phage tail protein I | | afdb-uniprot50 | AF-A0A2X3LRF3-F1-MODEL\_V4 | 1.0 | 7.71e-21 | 633 | 0.394 | 195 | 115 | 2 | 1 | 194 | 168 | 360 | Tail protein I | Tail protein I | | afdb-uniprot50 | AF-A0A7C8HXE3-F1-MODEL\_V4 | 1.0 | 1.231e-19 | 632 | 0.369 | 184 | 112 | 3 | 1 | 180 | 1 | 184 | Uncharacterized protein | Uncharacterized protein | | afdb-uniprot50 | AF-A0A5N9VY60-F1-MODEL\_V4 | 1.0 | 5.384e-18 | 631 | 0.42 | 150 | 85 | 1 | 24 | 173 | 2 | 149 | Phage tail protein I | Phage tail protein I | | afdb-uniprot50 | AF-A0A6L2ZSC6-F1-MODEL\_V4 | 1.0 | 8.745e-21 | 630 | 0.442 | 208 | 112 | 3 | 1 | 205 | 6 | 212 | Phage tail protein I | Phage tail protein I | | afdb-uniprot50 | AF-A0A2E3KNC1-F1-MODEL\_V4 | 1.0 | 1.276e-20 | 626 | 0.385 | 192 | 108 | 3 | 1 | 188 | 1 | 186 | Phage tail protein I | Phage tail protein I | | afdb-uniprot50 | AF-A0A0H3ZIR2-F1-MODEL\_V4 | 1.0 | 2.112e-20 | 621 | 0.359 | 203 | 130 | 0 | 1 | 203 | 3 | 205 | Phage tail fiber protein | Phage tail fiber protein | | afdb-uniprot50 | AF-A0A4U8YMN4-F1-MODEL\_V4 | 1.0 | 1.796e-19 | 620 | 0.393 | 188 | 109 | 5 | 1 | 184 | 1 | 187 | Tail p2 i: phage tail protein i | Tail p2 i: phage tail protein i | | afdb-uniprot50 | AF-A0A2E9SIS4-F1-MODEL\_V4 | 1.0 | 1.796e-19 | 620 | 0.352 | 193 | 125 | 0 | 1 | 193 | 26 | 218 | Phage tail protein I | Phage tail protein I | | afdb-uniprot50 | AF-A0A5D3J470-F1-MODEL\_V4 | 1.0 | 5.099e-20 | 618 | 0.379 | 187 | 111 | 2 | 1 | 187 | 2 | 183 | Phage tail protein I | Phage tail protein I | | afdb-uniprot50 | AF-A0A7X4KDS0-F1-MODEL\_V4 | 1.0 | 4.92e-19 | 617 | 0.393 | 178 | 107 | 1 | 1 | 178 | 2 | 178 | Phage tail protein I | Phage tail protein I | | afdb-uniprot50 | AF-A0A447P481-F1-MODEL\_V4 | 1.0 | 3.93e-18 | 611 | 0.428 | 168 | 92 | 2 | 33 | 200 | 1 | 164 | Putative phage tail protein | Putative phage tail protein | | afdb-uniprot50 | AF-R6J7K3-F1-MODEL\_V4 | 1.0 | 9.234e-19 | 610 | 0.366 | 183 | 112 | 3 | 1 | 181 | 5 | 185 | Phage tail protein I | Phage tail protein I | | afdb-uniprot50 | AF-G9Y651-F1-MODEL\_V4 | 1.0 | 3.824e-19 | 609 | 0.36 | 183 | 113 | 3 | 1 | 180 | 2 | 183 | Phage tail protein I | Phage tail protein I | | afdb-uniprot50 | AF-A0A8A9XHG1-F1-MODEL\_V4 | 1.0 | 1.447e-20 | 609 | 0.365 | 208 | 127 | 3 | 1 | 205 | 1 | 206 | Phage tail protein I | Phage tail protein I | | afdb-uniprot50 | AF-A0A7S9AD83-F1-MODEL\_V4 | 1.0 | 2.037e-19 | 605 | 0.351 | 188 | 115 | 1 | 1 | 188 | 1 | 181 | Phage tail protein I | Phage tail protein I | | afdb-uniprot50 | AF-A0A0Q8Y302-F1-MODEL\_V4 | 1.0 | 3.193e-21 | 603 | 0.398 | 206 | 121 | 1 | 1 | 203 | 1 | 206 | Phage tail protein | Phage tail protein | | afdb-uniprot50 | AF-K4Y7C4-F1-MODEL\_V4 | 1.0 | 8.597e-17 | 600 | 0.481 | 133 | 67 | 1 | 43 | 175 | 2 | 132 | Phage tail protein | Phage tail protein | | afdb-uniprot50 | AF-A0A828QYR1-F1-MODEL\_V4 | 1.0 | 4.92e-19 | 598 | 0.42 | 176 | 100 | 2 | 28 | 203 | 1 | 174 | Phage tail protein I | Phage tail protein I | | afdb-uniprot50 | AF-A0A809HAG4-F1-MODEL\_V4 | 1.0 | 7.645e-19 | 595 | 0.372 | 177 | 109 | 2 | 1 | 176 | 5 | 180 | Phage tail protein I | Phage tail protein I | | afdb-uniprot50 | AF-A0A1Y3DKT1-F1-MODEL\_V4 | 1.0 | 2.461e-19 | 594 | 0.381 | 181 | 108 | 3 | 1 | 179 | 1 | 179 | Phage tail protein I | Phage tail protein I | | afdb-uniprot50 | AF-A0A378Q2Y3-F1-MODEL\_V4 | 1.0 | 7.376e-18 | 593 | 0.373 | 174 | 107 | 2 | 2 | 175 | 19 | 190 | Bacteriophage P2-related tail formation protein | Bacteriophage P2-related tail formation protein | | afdb-uniprot50 | AF-A0A2N0CX35-F1-MODEL\_V4 | 1.0 | 2.868e-18 | 593 | 0.397 | 176 | 104 | 2 | 1 | 175 | 5 | 179 | Phage tail protein I | Phage tail protein I | | afdb-uniprot50 | AF-A0A8B5FYA0-F1-MODEL\_V4 | 1.0 | 1.672e-17 | 591 | 0.47 | 153 | 77 | 2 | 1 | 151 | 1 | 151 | Phage tail protein I | Phage tail protein I | | afdb-uniprot50 | AF-A0A1F0H4V7-F1-MODEL\_V4 | 1.0 | 4.787e-20 | 591 | 0.331 | 208 | 131 | 3 | 1 | 205 | 4 | 206 | Uncharacterized protein | Uncharacterized protein | | afdb-uniprot50 | AF-A0A4R2Z4J2-F1-MODEL\_V4 | 1.0 | 8.671e-19 | 588 | 0.448 | 176 | 94 | 2 | 1 | 175 | 1 | 174 | Phage tail P2-like protein | Phage tail P2-like protein | | afdb-uniprot50 | AF-A0A6G8D863-F1-MODEL\_V4 | 1.0 | 2.791e-19 | 587 | 0.269 | 208 | 149 | 2 | 1 | 205 | 1 | 208 | Phage tail protein I | Phage tail protein I | | afdb-uniprot50 | AF-A0A4P9VQK1-F1-MODEL\_V4 | 1.0 | 2.868e-18 | 585 | 0.411 | 180 | 101 | 4 | 1 | 179 | 1 | 176 | Phage tail protein I | Phage tail protein I | | afdb-uniprot50 | AF-A0A5M7L3Q9-F1-MODEL\_V4 | 1.0 | 4.619e-19 | 583 | 0.395 | 172 | 102 | 1 | 4 | 175 | 9 | 178 | Phage tail protein I | Phage tail protein I | | afdb-uniprot50 | AF-A0A7D5XQF7-F1-MODEL\_V4 | 1.0 | 7.239e-21 | 581 | 0.359 | 203 | 128 | 1 | 1 | 203 | 1 | 201 | Phage tail protein I | Phage tail protein I | | afdb-uniprot50 | AF-A0A376J222-F1-MODEL\_V4 | 1.0 | 1.672e-17 | 577 | 0.48 | 150 | 76 | 1 | 26 | 175 | 7 | 154 | Tail protein I (GpI) | Tail protein I (GpI) | | afdb-uniprot50 | AF-N8XJQ6-F1-MODEL\_V4 | 1.0 | 4.337e-19 | 576 | 0.296 | 206 | 140 | 3 | 1 | 205 | 1 | 202 | Phage tail protein I | Phage tail protein I | | afdb-uniprot50 | AF-A0A2T5HH08-F1-MODEL\_V4 | 1.0 | 1.311e-19 | 572 | 0.336 | 211 | 127 | 4 | 1 | 205 | 2 | 205 | Phage tail P2-like protein | Phage tail P2-like protein | | afdb-uniprot50 | AF-A0A103EG21-F1-MODEL\_V4 | 1.0 | 8.986e-20 | 571 | 0.376 | 202 | 125 | 1 | 1 | 201 | 1 | 202 | Uncharacterized protein | Uncharacterized protein | | afdb-uniprot50 | AF-A0A085ASA6-F1-MODEL\_V4 | 1.0 | 7.58e-17 | 570 | 0.385 | 174 | 100 | 4 | 1 | 168 | 1 | 173 | Tail protein I | Tail protein I | | afdb-uniprot50 | AF-A0A2Y0P998-F1-MODEL\_V4 | 1.0 | 2.354e-16 | 567 | 0.372 | 153 | 94 | 1 | 1 | 153 | 13 | 163 | Putative tail protein I (Gpi) | Putative tail protein I (Gpi) | | afdb-uniprot50 | AF-A0A2U9LD38-F1-MODEL\_V4 | 1.0 | 3.372e-19 | 567 | 0.365 | 205 | 128 | 1 | 1 | 205 | 3 | 205 | Phage tail protein I | Phage tail protein I | | afdb-uniprot50 | AF-A0A853IMJ7-F1-MODEL\_V4 | 1.0 | 1.897e-17 | 566 | 0.321 | 171 | 113 | 3 | 1 | 169 | 4 | 173 | Phage tail protein I | Phage tail protein I | | afdb-uniprot50 | AF-A0A1Q5W016-F1-MODEL\_V4 | 1.0 | 4.457e-18 | 564 | 0.431 | 176 | 97 | 2 | 1 | 175 | 1 | 174 | Phage tail protein I | Phage tail protein I | | afdb-uniprot50 | AF-A0A853IED5-F1-MODEL\_V4 | 1.0 | 1.83e-16 | 563 | 0.335 | 155 | 101 | 2 | 26 | 179 | 3 | 156 | Phage tail protein I | Phage tail protein I | | afdb-uniprot50 | AF-V5ZB74-F1-MODEL\_V4 | 1.0 | 1.913e-19 | 562 | 0.36 | 186 | 116 | 2 | 1 | 185 | 1 | 184 | Tail protein I GpI | Tail protein I GpI | | afdb-uniprot50 | AF-A0A4T2AB33-F1-MODEL\_V4 | 1.0 | 3.93e-18 | 562 | 0.321 | 205 | 133 | 4 | 3 | 205 | 4 | 204 | Phage tail protein I | Phage tail protein I | | afdb-uniprot50 | AF-A0A7U1PSN1-F1-MODEL\_V4 | 1.0 | 4.073e-19 | 561 | 0.328 | 201 | 130 | 3 | 1 | 198 | 1 | 199 | Phage tail protein I | Phage tail protein I | | afdb-uniprot50 | AF-A0A0M4U7R5-F1-MODEL\_V4 | 1.0 | 1.178e-16 | 560 | 0.32 | 175 | 117 | 2 | 1 | 175 | 3 | 175 | Phage tail protein | Phage tail protein | | afdb-uniprot50 | AF-A0A829DXU4-F1-MODEL\_V4 | 1.0 | 2.599e-17 | 559 | 0.318 | 179 | 121 | 1 | 1 | 179 | 1 | 178 | Phage tail protein I | Phage tail protein I | | afdb-uniprot50 | AF-A0A743CBY9-F1-MODEL\_V4 | 1.0 | 2.037e-19 | 559 | 0.311 | 202 | 138 | 1 | 1 | 201 | 3 | 204 | Phage tail protein I | Phage tail protein I | | afdb-uniprot50 | AF-A0A0D8TVW9-F1-MODEL\_V4 | 1.0 | 1.076e-17 | 557 | 0.328 | 189 | 122 | 2 | 1 | 189 | 2 | 185 | Tail protein | Tail protein | | afdb-uniprot50 | AF-A0A5E6YIM8-F1-MODEL\_V4 | 1.0 | 1.897e-17 | 557 | 0.379 | 179 | 103 | 4 | 1 | 175 | 3 | 177 | Uncharacterized protein | Uncharacterized protein | | afdb-uniprot50 | AF-A0A2U3F145-F1-MODEL\_V4 | 1.0 | 2.094e-18 | 555 | 0.405 | 180 | 105 | 1 | 24 | 203 | 5 | 182 | Phage tail protein I | Phage tail protein I | | afdb-uniprot50 | AF-A0A1E7RBV3-F1-MODEL\_V4 | 1.0 | 4.073e-19 | 552 | 0.357 | 190 | 111 | 2 | 1 | 188 | 2 | 182 | Phage tail protein I | Phage tail protein I | | afdb-uniprot50 | AF-A0A145VQK9-F1-MODEL\_V4 | 1.0 | 9.489e-18 | 551 | 0.331 | 202 | 132 | 3 | 1 | 201 | 5 | 204 | Uncharacterized protein | Uncharacterized protein | | afdb-uniprot50 | AF-A0A362XRN1-F1-MODEL\_V4 | 1.0 | 2.791e-19 | 547 | 0.412 | 177 | 104 | 0 | 28 | 204 | 1 | 177 | Phage tail P2-like protein | Phage tail P2-like protein | | afdb-uniprot50 | AF-A0A828I9B9-F1-MODEL\_V4 | 1.0 | 2.058e-14 | 547 | 0.359 | 128 | 80 | 1 | 26 | 153 | 1 | 126 | Phage tail protein I | Phage tail protein I | | afdb-uniprot50 | AF-A0A246KFX4-F1-MODEL\_V4 | 1.0 | 3.435e-16 | 545 | 0.322 | 183 | 117 | 4 | 1 | 178 | 8 | 188 | Bacteriophage P2-related tail formation protein | Bacteriophage P2-related tail formation protein | | afdb-uniprot50 | AF-A0A7C1LB13-F1-MODEL\_V4 | 1.0 | 6.448e-16 | 544 | 0.344 | 151 | 95 | 1 | 38 | 188 | 1 | 147 | Phage tail protein I | Phage tail protein I | | afdb-uniprot50 | AF-A0A4U8YL85-F1-MODEL\_V4 | 1.0 | 4.58e-17 | 544 | 0.32 | 206 | 137 | 3 | 1 | 205 | 3 | 206 | Tail p2 i: phage tail protein i | Tail p2 i: phage tail protein i | | afdb-uniprot50 | AF-W0ITV5-F1-MODEL\_V4 | 1.0 | 3.93e-18 | 543 | 0.383 | 201 | 118 | 3 | 4 | 203 | 11 | 206 | Tail protein | Tail protein | | afdb-uniprot50 | AF-L3CBD8-F1-MODEL\_V4 | 1.0 | 8.141e-19 | 543 | 0.349 | 206 | 129 | 3 | 1 | 203 | 1 | 204 | Phage tail protein I | Phage tail protein I | | afdb-uniprot50 | AF-A0A2I8XW61-F1-MODEL\_V4 | 1.0 | 3.435e-16 | 543 | 0.369 | 173 | 106 | 3 | 2 | 172 | 7 | 178 | Phage tail protein I | Phage tail protein I | | afdb-uniprot50 | AF-A0A6N3DZD4-F1-MODEL\_V4 | 1.0 | 4.419e-16 | 540 | 0.374 | 171 | 104 | 3 | 4 | 172 | 9 | 178 | Phage tail protein (Tail\_P2\_I) | Phage tail protein (Tail\_P2\_I) | | afdb-uniprot50 | AF-A0A5D3JC96-F1-MODEL\_V4 | 1.0 | 1.188e-18 | 539 | 0.331 | 187 | 120 | 2 | 1 | 187 | 2 | 183 | Phage tail protein I | Phage tail protein I | | afdb-uniprot50 | AF-A0A5E4XFG2-F1-MODEL\_V4 | 1.0 | 1.178e-16 | 538 | 0.326 | 187 | 117 | 4 | 1 | 185 | 1 | 180 | Phage tail protein I | Phage tail protein I | | afdb-uniprot50 | AF-A0A7H4P5M4-F1-MODEL\_V4 | 1.0 | 7.58e-17 | 538 | 0.367 | 177 | 107 | 4 | 1 | 175 | 6 | 179 | Putative prophage tail protein | Putative prophage tail protein | | afdb-uniprot50 | AF-A0A7V8DKV8-F1-MODEL\_V4 | 1.0 | 2.599e-17 | 535 | 0.351 | 188 | 114 | 4 | 1 | 186 | 1 | 182 | Phage tail protein P2 protein I family | Phage tail protein P2 protein I family | | afdb-uniprot50 | AF-A0A7Y3N1Q0-F1-MODEL\_V4 | 1.0 | 8.366e-18 | 535 | 0.27 | 207 | 149 | 2 | 1 | 205 | 5 | 211 | Phage tail protein I | Phage tail protein I | | afdb-uniprot50 | AF-A0A849VNG3-F1-MODEL\_V4 | 1.0 | 1.136e-15 | 534 | 0.323 | 164 | 101 | 3 | 1 | 157 | 2 | 162 | Phage tail protein I | Phage tail protein I | | afdb-uniprot50 | AF-R9ARZ6-F1-MODEL\_V4 | 1.0 | 3.792e-17 | 533 | 0.318 | 176 | 120 | 0 | 1 | 176 | 1 | 176 | Phage tail protein I | Phage tail protein I | | afdb-uniprot50 | AF-A0A5M8FWX9-F1-MODEL\_V4 | 1.0 | 3.343e-17 | 533 | 0.276 | 210 | 145 | 4 | 1 | 205 | 1 | 208 | Phage tail protein I | Phage tail protein I | | afdb-uniprot50 | AF-A0A348HHI4-F1-MODEL\_V4 | 1.0 | 1.614e-16 | 533 | 0.296 | 209 | 141 | 5 | 1 | 205 | 1 | 207 | Bacteriophage P2-related tail formation protein | Bacteriophage P2-related tail formation protein | | afdb-uniprot50 | AF-A0A066RUL7-F1-MODEL\_V4 | 1.0 | 3.792e-17 | 532 | 0.286 | 192 | 133 | 2 | 13 | 203 | 2 | 190 | Tail protein | Tail protein | | afdb-uniprot50 | AF-A0A6D0PQW3-F1-MODEL\_V4 | 1.0 | 1.178e-16 | 531 | 0.355 | 177 | 106 | 5 | 1 | 171 | 1 | 175 | Phage tail protein I | Phage tail protein I | | afdb-uniprot50 | AF-F4V3B6-F1-MODEL\_V4 | 1.0 | 2.44e-17 | 530 | 0.324 | 197 | 126 | 4 | 1 | 191 | 1 | 196 | Tail protein I (GpI) | Tail protein I (GpI) | | afdb-uniprot50 | AF-G4CPH7-F1-MODEL\_V4 | 1.0 | 1.584e-19 | 528 | 0.385 | 205 | 123 | 2 | 1 | 205 | 3 | 204 | Phage tail protein I | Phage tail protein I | | afdb-uniprot50 | AF-A0A1I1QCJ1-F1-MODEL\_V4 | 1.0 | 9.489e-18 | 528 | 0.294 | 214 | 139 | 5 | 1 | 205 | 1 | 211 | Phage tail protein, P2 protein I family | Phage tail protein, P2 protein I family | | afdb-uniprot50 | AF-A0A547EQ25-F1-MODEL\_V4 | 1.0 | 4.337e-19 | 526 | 0.334 | 206 | 136 | 1 | 1 | 205 | 4 | 209 | Phage tail protein I | Phage tail protein I | | afdb-uniprot50 | AF-A0A827H7B9-F1-MODEL\_V4 | 1.0 | 8.597e-17 | 525 | 0.352 | 176 | 111 | 2 | 2 | 175 | 6 | 180 | Phage tail protein I | Phage tail protein I | | afdb-uniprot50 | AF-A0A702LDY9-F1-MODEL\_V4 | 1.0 | 1.254e-16 | 523 | 0.369 | 176 | 103 | 4 | 1 | 170 | 1 | 174 | Phage tail protein I | Phage tail protein I | | afdb-uniprot50 | AF-A0A853IAZ5-F1-MODEL\_V4 | 1.0 | 9.489e-18 | 522 | 0.378 | 198 | 112 | 4 | 1 | 191 | 2 | 195 | Phage tail protein I | Phage tail protein I | | afdb-uniprot50 | AF-A0A7Y7D7P6-F1-MODEL\_V4 | 1.0 | 1.423e-16 | 521 | 0.296 | 182 | 122 | 3 | 1 | 180 | 1 | 178 | Phage tail protein I | Phage tail protein I | | afdb-uniprot50 | AF-A0A7X5J840-F1-MODEL\_V4 | 1.0 | 5.532e-17 | 521 | 0.269 | 208 | 147 | 3 | 1 | 205 | 2 | 207 | Phage tail protein I | Phage tail protein I | | afdb-uniprot50 | AF-A1VSH3-F1-MODEL\_V4 | 1.0 | 8.671e-19 | 520 | 0.348 | 172 | 112 | 0 | 2 | 173 | 5 | 176 | Phage tail protein I | Phage tail protein I | | afdb-uniprot50 | AF-A0A7V7NR87-F1-MODEL\_V4 | 1.0 | 2.076e-16 | 520 | 0.301 | 196 | 134 | 2 | 1 | 195 | 3 | 196 | Phage tail protein I | Phage tail protein I | | afdb-uniprot50 | AF-A0A1X3I4G6-F1-MODEL\_V4 | 1.0 | 1.047e-18 | 520 | 0.342 | 207 | 125 | 5 | 1 | 200 | 2 | 204 | Putative phage tail protein | Putative phage tail protein | | afdb-uniprot50 | AF-A0A4Q3CYC6-F1-MODEL\_V4 | 1.0 | 1.336e-16 | 519 | 0.38 | 155 | 96 | 0 | 48 | 202 | 1 | 155 | Phage tail protein I | Phage tail protein I | | afdb-uniprot50 | AF-L0R6A3-F1-MODEL\_V4 | 1.0 | 6.503e-18 | 519 | 0.301 | 209 | 137 | 3 | 1 | 205 | 2 | 205 | Uncharacterized protein | Uncharacterized protein | | afdb-uniprot50 | AF-A0A7H9K2Z1-F1-MODEL\_V4 | 1.0 | 4.878e-17 | 519 | 0.366 | 180 | 106 | 4 | 1 | 174 | 1 | 178 | Phage tail protein I | Phage tail protein I | | afdb-uniprot50 | AF-A0A5S3R576-F1-MODEL\_V4 | 1.0 | 1.781e-17 | 518 | 0.331 | 211 | 133 | 4 | 1 | 205 | 1 | 209 | Phage tail protein I | Phage tail protein I | | afdb-uniprot50 | AF-A0A726YCV2-F1-MODEL\_V4 | 1.0 | 4.495e-20 | 518 | 0.366 | 224 | 121 | 4 | 1 | 205 | 2 | 223 | Phage tail protein I | Phage tail protein I | | afdb-uniprot50 | AF-A0A1N7LRG2-F1-MODEL\_V4 | 1.0 | 1.614e-16 | 517 | 0.251 | 207 | 150 | 4 | 1 | 205 | 5 | 208 | Phage tail protein, P2 protein I family | Phage tail protein, P2 protein I family | | afdb-uniprot50 | AF-A0A2J5PKJ6-F1-MODEL\_V4 | 1.0 | 1.766e-15 | 516 | 0.383 | 159 | 95 | 2 | 4 | 160 | 9 | 166 | Phage tail protein I | Phage tail protein I | | afdb-uniprot50 | AF-A0A2A3WJP6-F1-MODEL\_V4 | 1.0 | 7.58e-17 | 516 | 0.388 | 180 | 100 | 5 | 1 | 170 | 1 | 180 | Phage tail protein I | Phage tail protein I | | afdb-uniprot50 | AF-A0A3E0X4D1-F1-MODEL\_V4 | 1.0 | 3.055e-18 | 515 | 0.34 | 179 | 115 | 2 | 1 | 178 | 2 | 178 | Phage tail protein I | Phage tail protein I | | afdb-uniprot50 | AF-A0A7X9X6E3-F1-MODEL\_V4 | 1.0 | 1.336e-16 | 515 | 0.331 | 184 | 114 | 4 | 1 | 180 | 1 | 179 | Phage tail protein I | Phage tail protein I | | afdb-uniprot50 | AF-A0A1W1XKA3-F1-MODEL\_V4 | 1.0 | 6.106e-18 | 514 | 0.382 | 178 | 106 | 4 | 1 | 176 | 1 | 176 | Phage tail protein, P2 protein I family | Phage tail protein, P2 protein I family | | afdb-uniprot50 | AF-A0A6L9MLM2-F1-MODEL\_V4 | 1.0 | 4.878e-17 | 514 | 0.291 | 206 | 141 | 3 | 4 | 205 | 17 | 221 | Phage tail protein I | Phage tail protein I | | afdb-uniprot50 | AF-A0A7K0GMX4-F1-MODEL\_V4 | 1.0 | 4.004e-15 | 514 | 0.339 | 165 | 100 | 3 | 1 | 157 | 1 | 164 | Phage tail protein I | Phage tail protein I | | afdb-uniprot50 | AF-A0A2N3KX23-F1-MODEL\_V4 | 1.0 | 4.301e-17 | 513 | 0.338 | 201 | 130 | 3 | 4 | 202 | 3 | 202 | Phage tail protein I | Phage tail protein I | | afdb-uniprot50 | AF-A0A4V3E318-F1-MODEL\_V4 | 1.0 | 1.038e-16 | 513 | 0.297 | 195 | 124 | 5 | 1 | 191 | 1 | 186 | Phage tail P2-like protein | Phage tail P2-like protein | | afdb-uniprot50 | AF-A0A3Q8FAV9-F1-MODEL\_V4 | 1.0 | 4.073e-19 | 513 | 0.385 | 184 | 108 | 1 | 1 | 184 | 1 | 179 | Phage tail protein I | Phage tail protein I | | afdb-uniprot50 | AF-A0A495BJ44-F1-MODEL\_V4 | 1.0 | 2.768e-17 | 511 | 0.314 | 207 | 129 | 3 | 4 | 205 | 5 | 203 | Phage tail P2-like protein | Phage tail P2-like protein | | afdb-uniprot50 | AF-A0A5S3XWD0-F1-MODEL\_V4 | 1.0 | 5.151e-15 | 510 | 0.27 | 174 | 117 | 3 | 1 | 167 | 8 | 178 | Phage tail protein I | Phage tail protein I | | afdb-uniprot50 | AF-A0A377XEC8-F1-MODEL\_V4 | 1.0 | 1.83e-16 | 509 | 0.414 | 157 | 86 | 3 | 1 | 156 | 1 | 152 | Tail protein I | Tail protein I | | afdb-uniprot50 | AF-A0A2S6H5I0-F1-MODEL\_V4 | 1.0 | 1.21e-15 | 508 | 0.395 | 167 | 83 | 4 | 1 | 151 | 1 | 165 | Phage tail P2-like protein | Phage tail P2-like protein | | afdb-uniprot50 | AF-A0A853SGW9-F1-MODEL\_V4 | 1.0 | 3.166e-19 | 508 | 0.364 | 206 | 130 | 1 | 1 | 205 | 3 | 208 | Phage tail protein (Tail\_P2\_I) | Phage tail protein (Tail\_P2\_I) | | afdb-uniprot50 | AF-A0A1Y2K0Y0-F1-MODEL\_V4 | 1.0 | 7.117e-17 | 508 | 0.33 | 218 | 129 | 4 | 1 | 205 | 2 | 215 | Putative phage tail protein I | Putative phage tail protein I | | afdb-uniprot50 | AF-A0A5P9F0G1-F1-MODEL\_V4 | 1.0 | 5.012e-16 | 507 | 0.306 | 186 | 119 | 5 | 1 | 181 | 1 | 181 | Phage tail protein | Phage tail protein | | afdb-uniprot50 | AF-A0A4P0YQA9-F1-MODEL\_V4 | 1.0 | 1.474e-17 | 506 | 0.345 | 200 | 125 | 5 | 1 | 197 | 1 | 197 | Tail protein I (GpI) from prophage | Tail protein I (GpI) from prophage | | afdb-uniprot50 | AF-A0A5J6LD05-F1-MODEL\_V4 | 1.0 | 6.683e-17 | 506 | 0.309 | 207 | 136 | 4 | 1 | 205 | 1 | 202 | Phage tail protein I | Phage tail protein I | | afdb-uniprot50 | AF-A0A2G2L0X1-F1-MODEL\_V4 | 1.0 | 1.396e-19 | 506 | 0.368 | 209 | 123 | 6 | 1 | 205 | 2 | 205 | Phage tail protein I | Phage tail protein I | | afdb-uniprot50 | AF-A0A166Z6T7-F1-MODEL\_V4 | 1.0 | 1.672e-17 | 506 | 0.31 | 222 | 135 | 5 | 1 | 205 | 4 | 224 | Tail protein | Tail protein | | afdb-uniprot50 | AF-A0A0B6CYD1-F1-MODEL\_V4 | 1.0 | 1.038e-16 | 505 | 0.336 | 205 | 125 | 4 | 1 | 205 | 1 | 194 | Phage tail protein I | Phage tail protein I | | afdb-uniprot50 | AF-A0A2A3MGU4-F1-MODEL\_V4 | 1.0 | 3.253e-18 | 505 | 0.375 | 173 | 106 | 1 | 4 | 174 | 42 | 214 | Phage tail protein I | Phage tail protein I | | afdb-uniprot50 | AF-A0A4R0DV06-F1-MODEL\_V4 | 1.0 | 1.719e-16 | 504 | 0.352 | 176 | 113 | 1 | 1 | 176 | 1 | 175 | Phage tail protein I | Phage tail protein I | | afdb-uniprot50 | AF-A0A376SCH0-F1-MODEL\_V4 | 1.0 | 2.507e-16 | 504 | 0.397 | 176 | 104 | 1 | 1 | 176 | 3 | 176 | Tail protein | Tail protein | | afdb-uniprot50 | AF-A0A1M3PG28-F1-MODEL\_V4 | 1.0 | 4.419e-16 | 503 | 0.346 | 182 | 108 | 5 | 4 | 181 | 3 | 177 | Phage tail protein I | Phage tail protein I | | afdb-uniprot50 | AF-A0A1M7YYQ0-F1-MODEL\_V4 | 1.0 | 3.76e-15 | 503 | 0.354 | 161 | 95 | 3 | 1 | 152 | 1 | 161 | Phage tail protein (Tail\_P2\_I) | Phage tail protein (Tail\_P2\_I) | | afdb-uniprot50 | AF-A0A291LZR2-F1-MODEL\_V4 | 1.0 | 7.856e-18 | 501 | 0.306 | 183 | 125 | 1 | 1 | 183 | 1 | 181 | Phage tail protein I | Phage tail protein I | | afdb-uniprot50 | AF-A0A2A4XSX7-F1-MODEL\_V4 | 1.0 | 1.881e-15 | 501 | 0.337 | 166 | 101 | 3 | 1 | 159 | 1 | 164 | Phage tail protein I | Phage tail protein I | | afdb-uniprot50 | AF-A0A1A9KGZ0-F1-MODEL\_V4 | 1.0 | 1.557e-15 | 501 | 0.378 | 156 | 97 | 0 | 48 | 203 | 2 | 157 | Phage tail protein I | Phage tail protein I | | afdb-uniprot50 | AF-A0A380C5Q3-F1-MODEL\_V4 | 1.0 | 7.58e-17 | 500 | 0.31 | 206 | 136 | 2 | 1 | 201 | 2 | 206 | Bacteriophage P2-related tail formation protein | Bacteriophage P2-related tail formation protein | | afdb-uniprot50 | AF-A0A2G4U387-F1-MODEL\_V4 | 1.0 | 9.57e-20 | 500 | 0.377 | 204 | 124 | 2 | 1 | 203 | 17 | 218 | Phage tail protein I | Phage tail protein I | | afdb-uniprot50 | AF-A0A5Q2KBP8-F1-MODEL\_V4 | 1.0 | 6.926e-18 | 499 | 0.395 | 172 | 102 | 1 | 4 | 175 | 37 | 206 | Phage tail protein I | Phage tail protein I | | afdb-uniprot50 | AF-A0A853HRN2-F1-MODEL\_V4 | 1.0 | 6.683e-17 | 498 | 0.328 | 198 | 109 | 5 | 2 | 179 | 4 | 197 | Phage tail protein I | Phage tail protein I | | afdb-uniprot50 | AF-A0A857EA04-F1-MODEL\_V4 | 1.0 | 4.747e-18 | 496 | 0.364 | 181 | 112 | 2 | 1 | 180 | 3 | 181 | Phage tail protein I | Phage tail protein I | | afdb-uniprot50 | AF-A0A7J0BYT0-F1-MODEL\_V4 | 1.0 | 1.515e-16 | 496 | 0.352 | 207 | 129 | 3 | 1 | 205 | 1 | 204 | Uncharacterized protein | Uncharacterized protein | | afdb-uniprot50 | AF-A0A6S5X5R7-F1-MODEL\_V4 | 1.0 | 5.532e-17 | 496 | 0.292 | 226 | 138 | 4 | 1 | 205 | 2 | 226 | Uncharacterized protein | Uncharacterized protein | | afdb-uniprot50 | AF-A0A1V3IL14-F1-MODEL\_V4 | 1.0 | 5.055e-18 | 495 | 0.392 | 176 | 107 | 0 | 1 | 176 | 3 | 178 | Phage tail protein I | Phage tail protein I | | afdb-uniprot50 | AF-A0A4S3LTA7-F1-MODEL\_V4 | 1.0 | 1.3e-17 | 494 | 0.397 | 176 | 103 | 2 | 1 | 175 | 1 | 174 | Phage tail protein I | Phage tail protein I | | afdb-uniprot50 | AF-A0A0D8DEP2-F1-MODEL\_V4 | 1.0 | 2.211e-16 | 494 | 0.297 | 212 | 141 | 2 | 2 | 205 | 7 | 218 | Tail protein | Tail protein | | afdb-uniprot50 | AF-A0A125AY37-F1-MODEL\_V4 | 1.0 | 4.707e-16 | 493 | 0.304 | 184 | 120 | 3 | 1 | 180 | 1 | 180 | Phage tail protein | Phage tail protein | | afdb-uniprot50 | AF-A0A212KBJ6-F1-MODEL\_V4 | 1.0 | 9.751e-17 | 493 | 0.296 | 233 | 132 | 5 | 4 | 205 | 3 | 234 | Uncharacterized protein | Uncharacterized protein | | afdb-uniprot50 | AF-A0A0C5VFB1-F1-MODEL\_V4 | 1.0 | 1.178e-16 | 492 | 0.31 | 203 | 129 | 7 | 1 | 194 | 125 | 325 | Bacteriophage P2-related tail formation protein | Bacteriophage P2-related tail formation protein | | afdb-uniprot50 | AF-A0A6N6MG39-F1-MODEL\_V4 | 1.0 | 6.867e-16 | 491 | 0.322 | 208 | 135 | 5 | 3 | 205 | 11 | 217 | Phage tail protein I | Phage tail protein I | | afdb-uniprot50 | AF-A0A269PIH6-F1-MODEL\_V4 | 1.0 | 1.178e-16 | 491 | 0.318 | 226 | 131 | 6 | 1 | 205 | 5 | 228 | Phage tail protein I | Phage tail protein I | | afdb-uniprot50 | AF-A0A0H3ZYQ7-F1-MODEL\_V4 | 1.0 | 9.751e-17 | 489 | 0.242 | 202 | 146 | 4 | 4 | 201 | 11 | 209 | Tail protein I | Tail protein I | | afdb-uniprot50 | AF-A0A0U3HNX6-F1-MODEL\_V4 | 1.0 | 9.751e-17 | 489 | 0.281 | 217 | 138 | 5 | 2 | 201 | 5 | 220 | Uncharacterized protein | Uncharacterized protein | | afdb-uniprot50 | AF-A0A826V4P5-F1-MODEL\_V4 | 1.0 | 5.151e-15 | 488 | 0.489 | 143 | 69 | 2 | 1 | 141 | 1 | 141 | Phage tail protein I | Phage tail protein I | | afdb-uniprot50 | AF-A0A545T778-F1-MODEL\_V4 | 1.0 | 4.878e-17 | 488 | 0.313 | 214 | 130 | 4 | 1 | 202 | 2 | 210 | Phage tail protein I | Phage tail protein I | | afdb-uniprot50 | AF-A0A2G2D6J3-F1-MODEL\_V4 | 1.0 | 2.211e-16 | 488 | 0.309 | 207 | 133 | 6 | 5 | 205 | 26 | 228 | Phage tail protein I | Phage tail protein I | | afdb-uniprot50 | AF-A0A432WB46-F1-MODEL\_V4 | 1.0 | 3.792e-17 | 488 | 0.312 | 221 | 136 | 5 | 1 | 205 | 2 | 222 | Phage tail protein I | Phage tail protein I | | afdb-uniprot50 | AF-A0A2N3KSK0-F1-MODEL\_V4 | 1.0 | 3.315e-15 | 487 | 0.314 | 191 | 118 | 6 | 1 | 179 | 1 | 190 | Phage tail protein I | Phage tail protein I | | afdb-uniprot50 | AF-A0A0E3UDX0-F1-MODEL\_V4 | 1.0 | 5.293e-14 | 486 | 0.35 | 154 | 96 | 2 | 13 | 163 | 2 | 154 | Tail protein | Tail protein | | afdb-uniprot50 | AF-A0A1Z3UCW5-F1-MODEL\_V4 | 1.0 | 3.435e-16 | 486 | 0.308 | 191 | 119 | 5 | 1 | 187 | 2 | 183 | Phage tail protein I | Phage tail protein I | | afdb-uniprot50 | AF-A0A7W5G584-F1-MODEL\_V4 | 1.0 | 6.626e-15 | 486 | 0.377 | 167 | 86 | 5 | 1 | 156 | 1 | 160 | Phage tail P2-like protein | Phage tail P2-like protein | | afdb-uniprot50 | AF-Q1GH43-F1-MODEL\_V4 | 1.0 | 4.301e-17 | 485 | 0.344 | 177 | 115 | 1 | 1 | 176 | 5 | 181 | Phage tail protein I | Phage tail protein I | | afdb-uniprot50 | AF-A0A6M3J2X1-F1-MODEL\_V4 | 1.0 | 2.923e-15 | 485 | 0.221 | 203 | 153 | 4 | 1 | 202 | 3 | 201 | Putative tail protein | Putative tail protein | | afdb-uniprot50 | AF-A0A5N8ADV9-F1-MODEL\_V4 | 1.0 | 5.012e-16 | 485 | 0.324 | 197 | 117 | 4 | 1 | 192 | 1 | 186 | Phage tail protein I | Phage tail protein I | | afdb-uniprot50 | AF-G2HXA1-F1-MODEL\_V4 | 1.0 | 1.766e-15 | 483 | 0.251 | 187 | 129 | 3 | 1 | 183 | 3 | 182 | Phage tail protein | Phage tail protein | | afdb-uniprot50 | AF-A0A290WU73-F1-MODEL\_V4 | 1.0 | 5.734e-18 | 483 | 0.337 | 231 | 125 | 2 | 3 | 205 | 12 | 242 | Phage tail protein I | Phage tail protein I | | afdb-uniprot50 | AF-E6L5Z8-F1-MODEL\_V4 | 1.0 | 4.15e-16 | 482 | 0.241 | 211 | 152 | 3 | 1 | 205 | 1 | 209 | Phage tail protein | Phage tail protein | | afdb-uniprot50 | AF-A0A1C3EBR0-F1-MODEL\_V4 | 1.0 | 4.419e-16 | 481 | 0.269 | 197 | 131 | 5 | 4 | 187 | 3 | 199 | Phage tail protein I | Phage tail protein I | | afdb-uniprot50 | AF-A0A2S2E575-F1-MODEL\_V4 | 1.0 | 4.419e-16 | 481 | 0.33 | 206 | 122 | 5 | 1 | 194 | 1 | 202 | Baseplate protein | Baseplate protein | | afdb-uniprot50 | AF-A0A2G6IRQ3-F1-MODEL\_V4 | 1.0 | 8.295e-16 | 481 | 0.292 | 229 | 133 | 6 | 4 | 205 | 10 | 236 | Phage tail protein I | Phage tail protein I | | afdb-uniprot50 | AF-A0A7W7N2Q5-F1-MODEL\_V4 | 1.0 | 2.133e-15 | 481 | 0.31 | 200 | 133 | 4 | 2 | 197 | 10 | 208 | Phage tail P2-like protein | Phage tail P2-like protein | | afdb-uniprot50 | AF-A0A7W0BQ66-F1-MODEL\_V4 | 1.0 | 7.789e-16 | 480 | 0.301 | 186 | 123 | 3 | 1 | 184 | 3 | 183 | Phage tail P2-like protein | Phage tail P2-like protein | | afdb-uniprot50 | AF-A0A3A9HV48-F1-MODEL\_V4 | 1.0 | 1.83e-16 | 480 | 0.348 | 204 | 124 | 6 | 4 | 205 | 5 | 201 | Phage tail protein I | Phage tail protein I | | afdb-uniprot50 | AF-A0A516SLX1-F1-MODEL\_V4 | 1.0 | 2.577e-15 | 479 | 0.342 | 207 | 124 | 5 | 4 | 205 | 17 | 216 | Phage tail protein I | Phage tail protein I | | afdb-uniprot50 | AF-A0A1N6M5D6-F1-MODEL\_V4 | 1.0 | 4.15e-16 | 479 | 0.241 | 224 | 150 | 4 | 1 | 205 | 4 | 226 | Phage tail protein (Tail\_P2\_I) | Phage tail protein (Tail\_P2\_I) | | afdb-uniprot50 | AF-A0A4R5W2H2-F1-MODEL\_V4 | 1.0 | 3.112e-15 | 478 | 0.314 | 178 | 115 | 4 | 4 | 176 | 10 | 185 | Phage tail protein I | Phage tail protein I | | afdb-uniprot50 | AF-A0A354XLH7-F1-MODEL\_V4 | 1.0 | 2.076e-16 | 478 | 0.352 | 213 | 126 | 6 | 1 | 205 | 1 | 209 | Phage tail protein I | Phage tail protein I | | afdb-uniprot50 | AF-F4HB19-F1-MODEL\_V4 | 1.0 | 1.614e-16 | 477 | 0.331 | 178 | 115 | 3 | 1 | 176 | 4 | 179 | Phage tail protein | Phage tail protein | | afdb-uniprot50 | AF-A0A828HQ92-F1-MODEL\_V4 | 1.0 | 1.146e-17 | 477 | 0.323 | 207 | 136 | 2 | 1 | 205 | 2 | 206 | Phage tail protein I | Phage tail protein I | | afdb-uniprot50 | AF-A0A285Z1J9-F1-MODEL\_V4 | 1.0 | 3.69e-18 | 477 | 0.318 | 207 | 132 | 2 | 1 | 198 | 4 | 210 | Phage tail protein, P2 protein I family | Phage tail protein, P2 protein I family | | afdb-uniprot50 | AF-A0A1E3G6D5-F1-MODEL\_V4 | 1.0 | 2.419e-15 | 476 | 0.263 | 205 | 149 | 2 | 1 | 205 | 1 | 203 | Phage tail protein I | Phage tail protein I | | afdb-uniprot50 | AF-A0A080K8W0-F1-MODEL\_V4 | 1.0 | 8.366e-18 | 476 | 0.362 | 204 | 126 | 2 | 2 | 205 | 4 | 203 | Bacteriophage P2-related tail formation protein | Bacteriophage P2-related tail formation protein | | afdb-uniprot50 | AF-A0A3N2E0S8-F1-MODEL\_V4 | 1.0 | 3.003e-14 | 475 | 0.339 | 162 | 101 | 3 | 2 | 163 | 10 | 165 | Phage tail P2-like protein | Phage tail P2-like protein | | afdb-uniprot50 | AF-A0A166WJW3-F1-MODEL\_V4 | 1.0 | 9.408e-16 | 475 | 0.273 | 227 | 142 | 5 | 1 | 205 | 3 | 228 | Uncharacterized protein | Uncharacterized protein | | afdb-uniprot50 | AF-A0A4R5HGG6-F1-MODEL\_V4 | 1.0 | 1.168e-14 | 474 | 0.201 | 213 | 161 | 4 | 1 | 205 | 24 | 235 | Phage tail protein I | Phage tail protein I | | afdb-uniprot50 | AF-A0A7G8SH16-F1-MODEL\_V4 | 1.0 | 3.69e-18 | 473 | 0.386 | 176 | 106 | 2 | 1 | 175 | 4 | 178 | Phage tail protein I | Phage tail protein I | | afdb-uniprot50 | AF-A0A0E3BUJ7-F1-MODEL\_V4 | 1.0 | 1.221e-17 | 471 | 0.347 | 213 | 119 | 7 | 1 | 203 | 1 | 203 | Tail protein | Tail protein | | afdb-uniprot50 | AF-A0A1I1QA98-F1-MODEL\_V4 | 1.0 | 3.435e-16 | 471 | 0.259 | 239 | 140 | 5 | 2 | 205 | 20 | 256 | Phage tail protein, P2 protein I family | Phage tail protein, P2 protein I family | | afdb-uniprot50 | AF-A0A367M848-F1-MODEL\_V4 | 1.0 | 1.168e-14 | 470 | 0.339 | 153 | 91 | 3 | 4 | 152 | 5 | 151 | Phage tail protein I | Phage tail protein I | | afdb-uniprot50 | AF-A0A369VQL1-F1-MODEL\_V4 | 1.0 | 1.719e-16 | 470 | 0.267 | 183 | 129 | 3 | 1 | 179 | 4 | 185 | Phage tail protein I | Phage tail protein I | | afdb-uniprot50 | AF-A0A0L8A9U9-F1-MODEL\_V4 | 1.0 | 5.338e-16 | 469 | 0.336 | 184 | 113 | 4 | 2 | 182 | 256 | 433 | Tail protein | Tail protein | | afdb-uniprot50 | AF-A0A5S3XJT3-F1-MODEL\_V4 | 1.0 | 2.82e-14 | 467 | 0.345 | 162 | 92 | 6 | 1 | 152 | 7 | 164 | Phage tail protein I | Phage tail protein I | | afdb-uniprot50 | AF-A0A7T8NTY3-F1-MODEL\_V4 | 1.0 | 2.923e-15 | 465 | 0.275 | 185 | 119 | 5 | 4 | 178 | 7 | 186 | Phage tail protein I | Phage tail protein I | | afdb-uniprot50 | AF-A0A1E3G7K1-F1-MODEL\_V4 | 1.0 | 5.012e-16 | 464 | 0.267 | 213 | 144 | 4 | 1 | 201 | 1 | 213 | Phage tail protein I | Phage tail protein I | | afdb-uniprot50 | AF-A0A2A4ZMU3-F1-MODEL\_V4 | 1.0 | 2.003e-15 | 464 | 0.32 | 215 | 133 | 6 | 1 | 205 | 1 | 212 | Phage tail protein I | Phage tail protein I | | afdb-uniprot50 | AF-A0A5S9Q3G5-F1-MODEL\_V4 | 1.0 | 5.293e-14 | 463 | 0.371 | 159 | 85 | 4 | 1 | 151 | 1 | 152 | Uncharacterized protein | Uncharacterized protein | | afdb-uniprot50 | AF-A0A1X7MJV0-F1-MODEL\_V4 | 1.0 | 1.002e-15 | 462 | 0.275 | 229 | 139 | 8 | 1 | 205 | 5 | 230 | Phage tail protein I | Phage tail protein I | | afdb-uniprot50 | AF-A0A0F9Z9G5-F1-MODEL\_V4 | 1.0 | 2.192e-14 | 460 | 0.281 | 181 | 125 | 2 | 1 | 178 | 2 | 180 | Uncharacterized protein | Uncharacterized protein | | afdb-uniprot50 | AF-A0A269PJC4-F1-MODEL\_V4 | 1.0 | 3.896e-16 | 459 | 0.272 | 169 | 123 | 0 | 12 | 180 | 18 | 186 | Phage tail protein I | Phage tail protein I | | afdb-uniprot50 | AF-B5JY67-F1-MODEL\_V4 | 1.0 | 1.528e-18 | 459 | 0.323 | 198 | 127 | 3 | 1 | 196 | 5 | 197 | Phage tail protein I | Phage tail protein I | | afdb-uniprot50 | AF-A0A849VB51-F1-MODEL\_V4 | 1.0 | 1.278e-13 | 455 | 0.31 | 164 | 101 | 5 | 3 | 155 | 5 | 167 | Phage tail protein I | Phage tail protein I | | afdb-uniprot50 | AF-A0A2D3VXF3-F1-MODEL\_V4 | 1.0 | 1.933e-14 | 454 | 0.215 | 181 | 134 | 5 | 1 | 178 | 1 | 176 | Phage tail protein I | Phage tail protein I | | afdb-uniprot50 | AF-Q8ZKK4-F1-MODEL\_V4 | 1.0 | 2.272e-15 | 454 | 0.317 | 211 | 134 | 6 | 1 | 203 | 1 | 209 | Putative phage tail protein | Putative phage tail protein | | afdb-uniprot50 | AF-A0A6M1CS17-F1-MODEL\_V4 | 1.0 | 3.97e-13 | 453 | 0.374 | 131 | 77 | 2 | 30 | 158 | 6 | 133 | Phage tail protein I | Phage tail protein I | | afdb-uniprot50 | AF-A0A2A7UXW9-F1-MODEL\_V4 | 1.0 | 1.002e-15 | 452 | 0.312 | 208 | 135 | 5 | 1 | 205 | 41 | 243 | Phage tail protein I | Phage tail protein I | | afdb-uniprot50 | AF-A0A6C8GFU6-F1-MODEL\_V4 | 1.0 | 2.82e-14 | 451 | 0.308 | 172 | 113 | 3 | 36 | 203 | 6 | 175 | Phage tail protein | Phage tail protein | | afdb-uniprot50 | AF-A0A806LU75-F1-MODEL\_V4 | 1.0 | 1.002e-15 | 451 | 0.273 | 194 | 132 | 2 | 12 | 205 | 29 | 213 | Tail protein | Tail protein | | afdb-uniprot50 | AF-A0A6L3Y824-F1-MODEL\_V4 | 1.0 | 1.658e-15 | 451 | 0.287 | 205 | 139 | 5 | 5 | 205 | 18 | 219 | Phage tail protein I | Phage tail protein I | | afdb-uniprot50 | AF-A0A6L8GK81-F1-MODEL\_V4 | 1.0 | 2.898e-13 | 450 | 0.288 | 156 | 103 | 5 | 1 | 154 | 1 | 150 | Phage tail protein I | Phage tail protein I | | afdb-uniprot50 | AF-A0A482INU7-F1-MODEL\_V4 | 1.0 | 4.541e-15 | 450 | 0.275 | 214 | 144 | 4 | 1 | 205 | 1 | 212 | Phage tail protein I | Phage tail protein I | | afdb-uniprot50 | AF-A0A326GL50-F1-MODEL\_V4 | 1.0 | 8.295e-16 | 450 | 0.295 | 186 | 126 | 3 | 1 | 182 | 4 | 188 | Phage tail protein I | Phage tail protein I | | afdb-uniprot50 | AF-B7WUI3-F1-MODEL\_V4 | 1.0 | 1.2e-13 | 449 | 0.263 | 163 | 110 | 4 | 24 | 182 | 6 | 162 | Phage tail protein I | Phage tail protein I | | afdb-uniprot50 | AF-A0A3R9Y2T4-F1-MODEL\_V4 | 1.0 | 3.93e-18 | 449 | 0.333 | 204 | 132 | 2 | 3 | 205 | 2 | 202 | Phage tail protein I | Phage tail protein I | | afdb-uniprot50 | AF-A0A5S3USV2-F1-MODEL\_V4 | 1.0 | 9.078e-15 | 449 | 0.25 | 240 | 143 | 7 | 1 | 205 | 2 | 239 | Phage tail protein I | Phage tail protein I | | afdb-uniprot50 | AF-A0A3Q8E075-F1-MODEL\_V4 | 1.0 | 1.313e-12 | 448 | 0.314 | 143 | 93 | 3 | 20 | 161 | 3 | 141 | Phage tail protein I | Phage tail protein I | | afdb-uniprot50 | AF-A0A510U3R5-F1-MODEL\_V4 | 1.0 | 2.133e-15 | 448 | 0.265 | 211 | 142 | 6 | 4 | 202 | 9 | 218 | Uncharacterized protein | Uncharacterized protein | | afdb-uniprot50 | AF-A0A1G5B4I5-F1-MODEL\_V4 | 1.0 | 4.419e-16 | 447 | 0.306 | 212 | 129 | 6 | 3 | 203 | 12 | 216 | Phage tail protein, P2 protein I family | Phage tail protein, P2 protein I family | | afdb-uniprot50 | AF-A0A375ABY7-F1-MODEL\_V4 | 1.0 | 4.073e-19 | 447 | 0.41 | 200 | 116 | 1 | 4 | 203 | 306 | 503 | Phage baseplate assembly protein J | Phage baseplate assembly protein J | | afdb-uniprot50 | AF-A0A326L3P6-F1-MODEL\_V4 | 1.0 | 3.029e-16 | 446 | 0.352 | 187 | 112 | 4 | 4 | 186 | 21 | 202 | Phage tail protein I | Phage tail protein I | | afdb-uniprot50 | AF-A0A0C6FTI9-F1-MODEL\_V4 | 1.0 | 5.486e-15 | 446 | 0.296 | 216 | 135 | 7 | 1 | 201 | 7 | 220 | Phage tail protein I | Phage tail protein I | | afdb-uniprot50 | AF-A0A1U9RGY6-F1-MODEL\_V4 | 1.0 | 3.659e-16 | 446 | 0.283 | 233 | 131 | 7 | 1 | 203 | 2 | 228 | Phage tail protein I | Phage tail protein I | | afdb-uniprot50 | AF-A0A4P9VF49-F1-MODEL\_V4 | 1.0 | 1.106e-16 | 446 | 0.33 | 209 | 134 | 5 | 1 | 205 | 1 | 207 | Phage tail protein I | Phage tail protein I | | afdb-uniprot50 | AF-A0A3S7DS03-F1-MODEL\_V4 | 1.0 | 7.723e-14 | 445 | 0.295 | 169 | 109 | 7 | 4 | 164 | 2 | 168 | Phage tail protein I | Phage tail protein I | | afdb-uniprot50 | AF-A0A2N1APA2-F1-MODEL\_V4 | 1.0 | 8.225e-14 | 445 | 0.266 | 195 | 112 | 3 | 1 | 164 | 1 | 195 | Phage tail protein I | Phage tail protein I | | afdb-uniprot50 | AF-A0A1W9H3W1-F1-MODEL\_V4 | 1.0 | 1.361e-13 | 444 | 0.306 | 163 | 105 | 6 | 1 | 157 | 1 | 161 | Uncharacterized protein | Uncharacterized protein | | afdb-uniprot50 | AF-R5H8L2-F1-MODEL\_V4 | 1.0 | 9.329e-14 | 444 | 0.278 | 176 | 121 | 3 | 1 | 173 | 42 | 214 | Putative phage tail protein | Putative phage tail protein | | afdb-uniprot50 | AF-E2CN32-F1-MODEL\_V4 | 1.0 | 5.486e-15 | 444 | 0.29 | 210 | 127 | 5 | 4 | 193 | 10 | 217 | Putative phage tail protein | Putative phage tail protein | | afdb-uniprot50 | AF-A0A350DIF7-F1-MODEL\_V4 | 1.0 | 2.67e-16 | 444 | 0.315 | 219 | 130 | 5 | 1 | 203 | 2 | 216 | Phage tail protein I | Phage tail protein I | | afdb-uniprot50 | AF-G3XD92-F1-MODEL\_V4 | 1.0 | 3.435e-16 | 443 | 0.324 | 179 | 118 | 2 | 1 | 178 | 1 | 177 | Probable bacteriophage protein | Probable bacteriophage protein | | afdb-uniprot50 | AF-A0A2R3PXS6-F1-MODEL\_V4 | 1.0 | 1.881e-15 | 443 | 0.329 | 185 | 113 | 5 | 2 | 182 | 82 | 259 | Phage tail protein I | Phage tail protein I | | afdb-uniprot50 | AF-A0A3S8YQ57-F1-MODEL\_V4 | 1.0 | 2.272e-15 | 443 | 0.35 | 197 | 113 | 5 | 3 | 192 | 4 | 192 | Phage tail protein I | Phage tail protein I | | afdb-uniprot50 | AF-A0A516SJT0-F1-MODEL\_V4 | 1.0 | 8.524e-15 | 442 | 0.33 | 209 | 127 | 6 | 2 | 205 | 3 | 203 | Phage tail protein I | Phage tail protein I | | afdb-uniprot50 | AF-A0A1T4WXE7-F1-MODEL\_V4 | 1.0 | 1.557e-15 | 442 | 0.262 | 221 | 145 | 7 | 1 | 203 | 1 | 221 | Phage tail protein, P2 protein I family | Phage tail protein, P2 protein I family | | afdb-uniprot50 | AF-A0A853HRM2-F1-MODEL\_V4 | 1.0 | 1.115e-18 | 441 | 0.402 | 206 | 119 | 3 | 1 | 203 | 2 | 206 | Phage tail protein I | Phage tail protein I | | afdb-uniprot50 | AF-A0A1M7U2A5-F1-MODEL\_V4 | 1.0 | 5.151e-15 | 441 | 0.283 | 208 | 139 | 6 | 3 | 203 | 11 | 215 | Phage tail protein, P2 protein I family | Phage tail protein, P2 protein I family | | afdb-uniprot50 | AF-A0A2C9ZYU5-F1-MODEL\_V4 | 1.0 | 8.225e-14 | 441 | 0.342 | 152 | 94 | 3 | 1 | 148 | 81 | 230 | Phage tail protein I | Phage tail protein I | | afdb-uniprot50 | AF-A0A2C9D5L0-F1-MODEL\_V4 | 1.0 | 1.933e-14 | 440 | 0.274 | 211 | 141 | 6 | 4 | 203 | 20 | 229 | Phage tail protein I | Phage tail protein I | | afdb-uniprot50 | AF-A0A1G7SAK3-F1-MODEL\_V4 | 1.0 | 1.373e-15 | 440 | 0.336 | 217 | 127 | 5 | 1 | 205 | 1 | 212 | Phage tail protein, P2 protein I family | Phage tail protein, P2 protein I family | | afdb-uniprot50 | AF-A0A541BB46-F1-MODEL\_V4 | 1.0 | 2.67e-16 | 440 | 0.326 | 202 | 123 | 5 | 1 | 192 | 9 | 207 | Phage tail protein I | Phage tail protein I | | afdb-uniprot50 | AF-A0A1C3EE42-F1-MODEL\_V4 | 1.0 | 1.411e-14 | 439 | 0.292 | 198 | 114 | 6 | 4 | 176 | 5 | 201 | Phage tail protein I | Phage tail protein I | | afdb-uniprot50 | AF-A0A8A6BIR6-F1-MODEL\_V4 | 1.0 | 6.222e-15 | 439 | 0.256 | 222 | 136 | 8 | 4 | 203 | 9 | 223 | Phage tail protein I | Phage tail protein I | | afdb-uniprot50 | AF-A0A2T4JDB8-F1-MODEL\_V4 | 1.0 | 1.751e-13 | 438 | 0.321 | 168 | 95 | 4 | 1 | 152 | 1 | 165 | Phage tail protein I | Phage tail protein I | | afdb-uniprot50 | AF-A0A7Y8ADW1-F1-MODEL\_V4 | 1.0 | 2.335e-14 | 438 | 0.225 | 213 | 156 | 4 | 1 | 205 | 5 | 216 | Phage tail protein I | Phage tail protein I | | afdb-uniprot50 | AF-A0A350LX93-F1-MODEL\_V4 | 1.0 | 5.195e-17 | 437 | 0.363 | 212 | 119 | 5 | 1 | 202 | 1 | 206 | Phage tail protein I | Phage tail protein I | | afdb-uniprot50 | AF-A0A1W0C8U7-F1-MODEL\_V4 | 1.0 | 1.6e-14 | 437 | 0.253 | 217 | 149 | 6 | 1 | 205 | 1 | 216 | Phage tail protein I | Phage tail protein I | | afdb-uniprot50 | AF-A0A0M9GJD3-F1-MODEL\_V4 | 1.0 | 1.704e-14 | 436 | 0.229 | 209 | 150 | 6 | 4 | 203 | 10 | 216 | Phage tail protein, P2 protein I family | Phage tail protein, P2 protein I family | | afdb-uniprot50 | AF-A0A1M3AMT4-F1-MODEL\_V4 | 1.0 | 1.002e-15 | 436 | 0.269 | 208 | 146 | 6 | 4 | 205 | 15 | 222 | Phage tail protein I | Phage tail protein I | | afdb-uniprot50 | AF-A0A109RVS0-F1-MODEL\_V4 | 1.0 | 3.628e-14 | 435 | 0.283 | 212 | 141 | 7 | 1 | 205 | 1 | 208 | Uncharacterized protein | Uncharacterized protein | | afdb-uniprot50 | AF-A0A6S5P7S3-F1-MODEL\_V4 | 1.0 | 1.03e-14 | 435 | 0.229 | 244 | 144 | 8 | 1 | 200 | 1 | 244 | Uncharacterized protein | Uncharacterized protein | | afdb-uniprot50 | AF-A0A8A6KEH0-F1-MODEL\_V4 | 1.0 | 2.399e-13 | 434 | 0.321 | 165 | 93 | 4 | 1 | 152 | 2 | 160 | Phage tail protein I | Phage tail protein I | | afdb-uniprot50 | AF-A0A191WAB0-F1-MODEL\_V4 | 1.0 | 1.69e-12 | 434 | 0.201 | 174 | 131 | 3 | 1 | 173 | 7 | 173 | Phage tail protein | Phage tail protein | | afdb-uniprot50 | AF-A0A7X8PBJ4-F1-MODEL\_V4 | 1.0 | 5.338e-16 | 434 | 0.351 | 185 | 110 | 5 | 1 | 179 | 1 | 181 | Phage tail protein I | Phage tail protein I | | afdb-uniprot50 | AF-A0A2S7V2R9-F1-MODEL\_V4 | 1.0 | 7.723e-14 | 434 | 0.153 | 196 | 163 | 3 | 1 | 195 | 8 | 201 | Phage tail protein I | Phage tail protein I | | afdb-uniprot50 | AF-A0A844Q9W2-F1-MODEL\_V4 | 1.0 | 1.168e-14 | 434 | 0.254 | 208 | 142 | 6 | 5 | 202 | 37 | 241 | Phage tail protein I | Phage tail protein I | | afdb-uniprot50 | AF-G8PUM6-F1-MODEL\_V4 | 1.0 | 7.252e-14 | 434 | 0.271 | 210 | 144 | 5 | 4 | 205 | 10 | 218 | Phage tail protein I | Phage tail protein I | | afdb-uniprot50 | AF-A0A6P2IUC4-F1-MODEL\_V4 | 1.0 | 4.228e-13 | 433 | 0.295 | 149 | 99 | 2 | 35 | 180 | 2 | 147 | Phage tail protein I | Phage tail protein I | | afdb-uniprot50 | AF-A0A7G2U2N6-F1-MODEL\_V4 | 1.0 | 6.169e-13 | 433 | 0.326 | 153 | 93 | 3 | 1 | 145 | 1 | 151 | Phage tail protein I | Phage tail protein I | | afdb-uniprot50 | AF-A0A541BHK6-F1-MODEL\_V4 | 1.0 | 5.151e-15 | 433 | 0.276 | 224 | 136 | 8 | 4 | 205 | 6 | 225 | Phage tail protein I | Phage tail protein I | | afdb-uniprot50 | AF-A0A6N7BYW6-F1-MODEL\_V4 | 1.0 | 7.515e-15 | 432 | 0.262 | 179 | 127 | 3 | 1 | 178 | 2 | 176 | Uncharacterized protein | Uncharacterized protein | | afdb-uniprot50 | AF-A0A7X4JXH6-F1-MODEL\_V4 | 1.0 | 2.948e-17 | 432 | 0.37 | 205 | 120 | 6 | 1 | 203 | 4 | 201 | Phage tail protein I | Phage tail protein I | | afdb-uniprot50 | AF-A0A5S3YYC2-F1-MODEL\_V4 | 1.0 | 9.078e-15 | 432 | 0.223 | 233 | 150 | 7 | 1 | 205 | 2 | 231 | Phage tail protein I | Phage tail protein I | | afdb-uniprot50 | AF-A0A5P9HBR8-F1-MODEL\_V4 | 1.0 | 9.078e-15 | 432 | 0.26 | 200 | 125 | 6 | 2 | 184 | 5 | 198 | Phage tail protein | Phage tail protein | | afdb-uniprot50 | AF-A0A2A5BNC1-F1-MODEL\_V4 | 1.0 | 1.127e-13 | 431 | 0.325 | 163 | 93 | 6 | 1 | 151 | 1 | 158 | Phage tail protein I | Phage tail protein I | | afdb-uniprot50 | AF-A0A285NMK5-F1-MODEL\_V4 | 1.0 | 2.192e-14 | 431 | 0.265 | 196 | 135 | 3 | 12 | 205 | 23 | 211 | Phage tail protein, P2 protein I family | Phage tail protein, P2 protein I family | | afdb-uniprot50 | AF-A0A510U7K2-F1-MODEL\_V4 | 1.0 | 4.836e-15 | 431 | 0.268 | 227 | 140 | 7 | 4 | 205 | 7 | 232 | Uncharacterized protein | Uncharacterized protein | | afdb-uniprot50 | AF-C5A8Q4-F1-MODEL\_V4 | 1.0 | 1.544e-13 | 430 | 0.31 | 161 | 103 | 4 | 1 | 157 | 1 | 157 | Phage-related tail protein | Phage-related tail protein | | afdb-uniprot50 | AF-A0A7W4PUW2-F1-MODEL\_V4 | 1.0 | 4.878e-17 | 430 | 0.339 | 212 | 131 | 3 | 1 | 205 | 1 | 210 | Phage tail protein I | Phage tail protein I | | afdb-uniprot50 | AF-A0A4R2P790-F1-MODEL\_V4 | 1.0 | 5.637e-14 | 430 | 0.288 | 208 | 138 | 7 | 1 | 205 | 1 | 201 | Phage tail P2-like protein | Phage tail P2-like protein | | afdb-uniprot50 | AF-B7RNM5-F1-MODEL\_V4 | 1.0 | 1.325e-14 | 429 | 0.253 | 213 | 148 | 4 | 1 | 205 | 6 | 215 | Putative phage tail protein | Putative phage tail protein | | afdb-uniprot50 | AF-A0A8B1NMW4-F1-MODEL\_V4 | 1.0 | 3.863e-14 | 428 | 0.372 | 153 | 91 | 2 | 42 | 194 | 2 | 149 | Uncharacterized protein | Uncharacterized protein | | afdb-uniprot50 | AF-A0A847KR13-F1-MODEL\_V4 | 1.0 | 3.287e-13 | 428 | 0.293 | 167 | 111 | 5 | 4 | 165 | 17 | 181 | Phage tail protein I | Phage tail protein I | | afdb-uniprot50 | AF-A0A5Z0QYI5-F1-MODEL\_V4 | 1.0 | 1.897e-17 | 428 | 0.356 | 202 | 125 | 4 | 1 | 200 | 1 | 199 | Phage tail protein I | Phage tail protein I | | afdb-uniprot50 | AF-A0A8B2TV07-F1-MODEL\_V4 | 1.0 | 5.486e-15 | 428 | 0.22 | 209 | 151 | 4 | 1 | 205 | 1 | 201 | Phage tail protein I | Phage tail protein I | | afdb-uniprot50 | AF-A0A1M3HHZ6-F1-MODEL\_V4 | 1.0 | 2.419e-15 | 428 | 0.306 | 222 | 128 | 7 | 1 | 204 | 1 | 214 | Phage tail protein I | Phage tail protein I | | afdb-uniprot50 | AF-A0A3A3D8P6-F1-MODEL\_V4 | 1.0 | 8.524e-15 | 428 | 0.27 | 174 | 117 | 3 | 1 | 167 | 8 | 178 | Phage tail protein I | Phage tail protein I | | afdb-uniprot50 | AF-A0A233HGS3-F1-MODEL\_V4 | 1.0 | 8.225e-14 | 427 | 0.206 | 203 | 152 | 4 | 1 | 202 | 7 | 201 | Phage tail protein | Phage tail protein | | afdb-uniprot50 | AF-A0A238JAQ9-F1-MODEL\_V4 | 1.0 | 1.127e-13 | 425 | 0.3 | 180 | 106 | 5 | 1 | 165 | 1 | 175 | Phage tail protein (Tail\_P2\_I) | Phage tail protein (Tail\_P2\_I) | | afdb-uniprot50 | AF-A0A3S4IC27-F1-MODEL\_V4 | 1.0 | 3.198e-14 | 425 | 0.274 | 211 | 145 | 4 | 1 | 205 | 1 | 209 | Bacteriophage P2-related tail formation protein | Bacteriophage P2-related tail formation protein | | afdb-uniprot50 | AF-A0A7Y1X1Y1-F1-MODEL\_V4 | 1.0 | 1.57e-17 | 425 | 0.334 | 209 | 133 | 3 | 1 | 205 | 1 | 207 | Phage tail protein I | Phage tail protein I | | afdb-uniprot50 | AF-A0A7X6WY32-F1-MODEL\_V4 | 1.0 | 1.127e-13 | 425 | 0.243 | 201 | 113 | 4 | 1 | 162 | 2 | 202 | Phage tail protein I | Phage tail protein I | | afdb-uniprot50 | AF-D0IJ10-F1-MODEL\_V4 | 1.0 | 6.57e-13 | 424 | 0.27 | 159 | 111 | 3 | 19 | 176 | 2 | 156 | Tail protein I | Tail protein I | | afdb-uniprot50 | AF-A0A1B9NZR7-F1-MODEL\_V4 | 1.0 | 1.799e-12 | 424 | 0.158 | 177 | 141 | 3 | 1 | 176 | 7 | 176 | Uncharacterized protein | Uncharacterized protein | | afdb-uniprot50 | AF-A0A1W0CDK0-F1-MODEL\_V4 | 1.0 | 5.151e-15 | 424 | 0.269 | 215 | 133 | 8 | 4 | 205 | 5 | 208 | Phage tail protein I | Phage tail protein I | | afdb-uniprot50 | AF-A0A2S5F905-F1-MODEL\_V4 | 1.0 | 2.399e-13 | 423 | 0.294 | 170 | 105 | 7 | 4 | 163 | 2 | 166 | Phage tail protein I | Phage tail protein I | | afdb-uniprot50 | AF-A0A840WZA1-F1-MODEL\_V4 | 1.0 | 8.759e-14 | 423 | 0.28 | 200 | 130 | 5 | 2 | 192 | 7 | 201 | Phage tail P2-like protein | Phage tail P2-like protein | | afdb-uniprot50 | AF-A0A1H1G374-F1-MODEL\_V4 | 1.0 | 2.335e-14 | 423 | 0.25 | 212 | 148 | 4 | 4 | 205 | 7 | 217 | Phage tail protein, P2 protein I family | Phage tail protein, P2 protein I family | | afdb-uniprot50 | AF-A0A5S9QVU4-F1-MODEL\_V4 | 1.0 | 4.503e-13 | 422 | 0.303 | 178 | 111 | 5 | 1 | 167 | 3 | 178 | Uncharacterized protein | Uncharacterized protein | | afdb-uniprot50 | AF-A0A6N4CKX4-F1-MODEL\_V4 | 1.0 | 2.507e-16 | 422 | 0.354 | 192 | 114 | 4 | 4 | 194 | 3 | 185 | Uncharacterized protein | Uncharacterized protein | | afdb-uniprot50 | AF-A0A4U8UH95-F1-MODEL\_V4 | 1.0 | 2.335e-14 | 422 | 0.242 | 202 | 146 | 5 | 1 | 200 | 2 | 198 | Phage tail protein I | Phage tail protein I | | afdb-uniprot50 | AF-A0A1M3I801-F1-MODEL\_V4 | 1.0 | 5.151e-15 | 422 | 0.313 | 188 | 113 | 6 | 2 | 181 | 5 | 184 | Phage tail protein I | Phage tail protein I | | afdb-uniprot50 | AF-A0A840LX24-F1-MODEL\_V4 | 1.0 | 1.45e-13 | 422 | 0.223 | 215 | 155 | 7 | 1 | 205 | 7 | 219 | Phage tail P2-like protein | Phage tail P2-like protein | | afdb-uniprot50 | AF-A0A1N6M6A1-F1-MODEL\_V4 | 1.0 | 1.127e-13 | 422 | 0.208 | 201 | 155 | 4 | 4 | 202 | 15 | 213 | Phage tail protein (Tail\_P2\_I) | Phage tail protein (Tail\_P2\_I) | | afdb-uniprot50 | AF-A0A423PQM5-F1-MODEL\_V4 | 1.0 | 9.329e-14 | 421 | 0.287 | 188 | 117 | 7 | 2 | 183 | 8 | 184 | Tail protein | Tail protein | | afdb-uniprot50 | AF-K5Z1Z7-F1-MODEL\_V4 | 1.0 | 2.399e-13 | 421 | 0.333 | 171 | 95 | 4 | 1 | 155 | 5 | 172 | Phage tail protein I | Phage tail protein I | | afdb-uniprot50 | AF-A0A5C5NW28-F1-MODEL\_V4 | 1.0 | 1.865e-13 | 421 | 0.255 | 184 | 122 | 5 | 1 | 170 | 1 | 183 | Phage tail protein I | Phage tail protein I | | afdb-uniprot50 | AF-A0A0C1G372-F1-MODEL\_V4 | 1.0 | 3.086e-13 | 420 | 0.349 | 169 | 90 | 5 | 1 | 152 | 1 | 166 | Tail protein | Tail protein | | afdb-uniprot50 | AF-A0A261E3S3-F1-MODEL\_V4 | 1.0 | 8.004e-15 | 420 | 0.256 | 207 | 142 | 6 | 4 | 199 | 2 | 207 | Phage tail protein I | Phage tail protein I | | afdb-uniprot50 | AF-A0A2T5NQ88-F1-MODEL\_V4 | 1.0 | 9.329e-14 | 420 | 0.27 | 200 | 140 | 3 | 10 | 205 | 5 | 202 | Phage tail protein I | Phage tail protein I | | afdb-uniprot50 | AF-A0A2T6LZT5-F1-MODEL\_V4 | 1.0 | 1.6e-14 | 419 | 0.327 | 168 | 99 | 5 | 1 | 158 | 3 | 166 | Phage tail P2-like protein | Phage tail P2-like protein | | afdb-uniprot50 | AF-A0A0L9YAM1-F1-MODEL\_V4 | 1.0 | 8.225e-14 | 418 | 0.237 | 198 | 144 | 6 | 11 | 205 | 24 | 217 | Phage tail protein I | Phage tail protein I | | afdb-uniprot50 | AF-A0A413FZE3-F1-MODEL\_V4 | 1.0 | 2.721e-13 | 417 | 0.239 | 171 | 123 | 3 | 11 | 180 | 29 | 193 | Phage tail protein I | Phage tail protein I | | afdb-uniprot50 | AF-A0A6L9FPZ5-F1-MODEL\_V4 | 1.0 | 1.865e-13 | 417 | 0.275 | 185 | 123 | 5 | 4 | 183 | 17 | 195 | Phage tail protein I | Phage tail protein I | | afdb-uniprot50 | AF-A0A1R1MK88-F1-MODEL\_V4 | 1.0 | 1.986e-13 | 417 | 0.254 | 181 | 121 | 4 | 4 | 172 | 7 | 185 | Phage tail protein I | Phage tail protein I | | afdb-uniprot50 | AF-A0A806DA82-F1-MODEL\_V4 | 1.0 | 5.637e-14 | 416 | 0.276 | 188 | 124 | 5 | 1 | 182 | 2 | 183 | Phage tail protein I | Phage tail protein I | | afdb-uniprot50 | AF-E6X1N3-F1-MODEL\_V4 | 1.0 | 1.058e-13 | 416 | 0.247 | 194 | 130 | 4 | 1 | 180 | 2 | 193 | Uncharacterized protein | Uncharacterized protein | | afdb-uniprot50 | AF-A0A1I5MPV2-F1-MODEL\_V4 | 1.0 | 1.097e-14 | 416 | 0.247 | 214 | 146 | 6 | 3 | 205 | 11 | 220 | Phage tail protein, P2 protein I family | Phage tail protein, P2 protein I family | | afdb-uniprot50 | AF-A0A5C1DKB3-F1-MODEL\_V4 | 1.0 | 1.2e-13 | 416 | 0.276 | 195 | 133 | 4 | 1 | 189 | 1 | 193 | Phage tail protein I | Phage tail protein I | | afdb-uniprot50 | AF-A0A4Y8REX3-F1-MODEL\_V4 | 1.0 | 4.264e-15 | 414 | 0.317 | 214 | 128 | 8 | 4 | 203 | 20 | 229 | Phage tail protein I | Phage tail protein I | | afdb-uniprot50 | AF-A0A377AZI4-F1-MODEL\_V4 | 1.0 | 2.898e-13 | 413 | 0.401 | 137 | 80 | 1 | 56 | 192 | 4 | 138 | Tail protein I | Tail protein I | | afdb-uniprot50 | AF-F3YY39-F1-MODEL\_V4 | 1.0 | 3.003e-14 | 412 | 0.313 | 214 | 131 | 6 | 4 | 205 | 13 | 222 | Phage tail protein I | Phage tail protein I | | afdb-uniprot50 | AF-U3AMK5-F1-MODEL\_V4 | 1.0 | 7.723e-14 | 412 | 0.251 | 211 | 144 | 5 | 4 | 203 | 20 | 227 | Putative phage tail protein | Putative phage tail protein | | afdb-uniprot50 | AF-A0A2P8QYP1-F1-MODEL\_V4 | 1.0 | 3.863e-14 | 412 | 0.242 | 206 | 150 | 4 | 1 | 205 | 1 | 201 | Phage tail protein I | Phage tail protein I | | afdb-uniprot50 | AF-A0A827ZHP7-F1-MODEL\_V4 | 1.0 | 5.439e-13 | 411 | 0.504 | 125 | 60 | 1 | 1 | 125 | 2 | 124 | Phage tail protein I | Phage tail protein I | | afdb-uniprot50 | AF-A0A7Y3Z485-F1-MODEL\_V4 | 1.0 | 4.503e-13 | 411 | 0.237 | 177 | 124 | 4 | 2 | 176 | 8 | 175 | Uncharacterized protein | Uncharacterized protein | | afdb-uniprot50 | AF-A0A016XH76-F1-MODEL\_V4 | 1.0 | 2.507e-16 | 411 | 0.327 | 186 | 121 | 3 | 4 | 186 | 14 | 198 | Tail protein | Tail protein | | afdb-uniprot50 | AF-A0A7X4WDI7-F1-MODEL\_V4 | 1.0 | 2.898e-13 | 411 | 0.196 | 209 | 157 | 4 | 1 | 205 | 4 | 205 | Phage tail protein | Phage tail protein | | afdb-uniprot50 | AF-R6P8B0-F1-MODEL\_V4 | 1.0 | 7.252e-14 | 411 | 0.276 | 188 | 128 | 4 | 1 | 183 | 1 | 185 | Putative phage tail protein | Putative phage tail protein | | afdb-uniprot50 | AF-A0A5S3YYF1-F1-MODEL\_V4 | 1.0 | 5.293e-14 | 411 | 0.276 | 217 | 133 | 6 | 12 | 205 | 18 | 233 | Phage tail protein I | Phage tail protein I | | afdb-uniprot50 | AF-A0A1I7EL73-F1-MODEL\_V4 | 1.0 | 2.076e-16 | 409 | 0.325 | 203 | 125 | 4 | 1 | 200 | 1 | 194 | Phage tail protein, P2 protein I family | Phage tail protein, P2 protein I family | | afdb-uniprot50 | AF-A0A359KDK8-F1-MODEL\_V4 | 1.0 | 3.406e-14 | 409 | 0.226 | 212 | 146 | 7 | 1 | 197 | 5 | 213 | Phage tail protein I | Phage tail protein I | | afdb-uniprot50 | AF-A0A418VVG6-F1-MODEL\_V4 | 1.0 | 1.097e-14 | 409 | 0.283 | 243 | 134 | 5 | 1 | 205 | 1 | 241 | Phage tail protein I | Phage tail protein I | | afdb-uniprot50 | AF-A0A399MBD2-F1-MODEL\_V4 | 1.0 | 1.127e-13 | 408 | 0.264 | 208 | 141 | 7 | 1 | 200 | 1 | 204 | Phage tail protein I | Phage tail protein I | | afdb-uniprot50 | AF-A0A2E3Q532-F1-MODEL\_V4 | 1.0 | 1.69e-12 | 408 | 0.26 | 196 | 112 | 4 | 1 | 163 | 1 | 196 | Phage tail protein I | Phage tail protein I | | afdb-uniprot50 | AF-R8AR41-F1-MODEL\_V4 | 1.0 | 1.865e-13 | 407 | 0.237 | 206 | 147 | 4 | 1 | 203 | 1 | 199 | Phage tail protein I | Phage tail protein I | | afdb-uniprot50 | AF-A0A812QW15-F1-MODEL\_V4 | 1.0 | 3.003e-14 | 407 | 0.331 | 172 | 97 | 4 | 26 | 180 | 303 | 473 | J protein | J protein | | afdb-uniprot50 | AF-A0A660NKJ4-F1-MODEL\_V4 | 1.0 | 7.452e-13 | 406 | 0.319 | 169 | 95 | 5 | 1 | 152 | 1 | 166 | Phage tail protein I | Phage tail protein I | | afdb-uniprot50 | AF-I7E1B5-F1-MODEL\_V4 | 1.0 | 2.507e-16 | 406 | 0.358 | 209 | 115 | 10 | 1 | 200 | 1 | 199 | Phage tail protein I | Phage tail protein I | | afdb-uniprot50 | AF-A0A1T4UVP0-F1-MODEL\_V4 | 1.0 | 5.486e-15 | 406 | 0.279 | 215 | 134 | 8 | 4 | 205 | 1 | 207 | Phage tail protein, P2 protein I family | Phage tail protein, P2 protein I family | | afdb-uniprot50 | AF-A0A2G3J6I4-F1-MODEL\_V4 | 1.0 | 3.728e-13 | 406 | 0.231 | 212 | 153 | 5 | 1 | 205 | 1 | 209 | Phage tail protein | Phage tail protein | | afdb-uniprot50 | AF-A0A840Q0S0-F1-MODEL\_V4 | 1.0 | 4.382e-14 | 406 | 0.266 | 195 | 135 | 5 | 12 | 205 | 28 | 215 | Phage tail P2-like protein | Phage tail P2-like protein | | afdb-uniprot50 | AF-A0A7I9KDL4-F1-MODEL\_V4 | 1.0 | 6.003e-14 | 405 | 0.23 | 200 | 146 | 4 | 1 | 198 | 1 | 194 | Phage tail protein I | Phage tail protein I | | afdb-uniprot50 | AF-A0A7M3MBM5-F1-MODEL\_V4 | 1.0 | 2.486e-14 | 405 | 0.266 | 221 | 137 | 7 | 4 | 205 | 12 | 226 | Phage tail protein I | Phage tail protein I | | afdb-uniprot50 | AF-F2K226-F1-MODEL\_V4 | 1.0 | 3.287e-13 | 405 | 0.218 | 220 | 152 | 6 | 4 | 205 | 15 | 232 | Phage tail protein I | Phage tail protein I | | afdb-uniprot50 | AF-A0A512JPD4-F1-MODEL\_V4 | 1.0 | 1.815e-14 | 404 | 0.24 | 250 | 142 | 9 | 1 | 205 | 1 | 247 | Uncharacterized protein | Uncharacterized protein | | afdb-uniprot50 | AF-A0A855ISH0-F1-MODEL\_V4 | 1.0 | 5.107e-13 | 403 | 0.224 | 169 | 121 | 3 | 2 | 166 | 10 | 172 | Phage tail protein I | Phage tail protein I | | afdb-uniprot50 | AF-A0A547PS50-F1-MODEL\_V4 | 1.0 | 9.935e-14 | 403 | 0.279 | 215 | 130 | 8 | 4 | 200 | 8 | 215 | Phage tail protein I | Phage tail protein I | | afdb-uniprot50 | AF-W4M090-F1-MODEL\_V4 | 1.0 | 8.524e-15 | 403 | 0.31 | 229 | 131 | 8 | 1 | 205 | 1 | 226 | Uncharacterized protein | Uncharacterized protein | | afdb-uniprot50 | AF-A0A162A844-F1-MODEL\_V4 | 1.0 | 6.394e-14 | 403 | 0.271 | 173 | 115 | 4 | 1 | 165 | 1 | 170 | Tail protein | Tail protein | | afdb-uniprot50 | AF-A0A285M2N0-F1-MODEL\_V4 | 1.0 | 9.586e-13 | 402 | 0.281 | 167 | 110 | 4 | 2 | 158 | 5 | 171 | Phage tail protein, P2 protein I family | Phage tail protein, P2 protein I family | | afdb-uniprot50 | AF-A0A839IX64-F1-MODEL\_V4 | 1.0 | 2.097e-11 | 402 | 0.254 | 157 | 108 | 5 | 1 | 154 | 1 | 151 | Phage tail protein I | Phage tail protein I | | afdb-uniprot50 | AF-A0A1H7YJ96-F1-MODEL\_V4 | 1.0 | 2.115e-13 | 401 | 0.251 | 195 | 132 | 5 | 12 | 205 | 33 | 214 | Phage tail protein, P2 protein I family | Phage tail protein, P2 protein I family | | afdb-uniprot50 | AF-A0A3S2V1V0-F1-MODEL\_V4 | 1.0 | 2.82e-14 | 401 | 0.234 | 264 | 141 | 6 | 1 | 205 | 7 | 268 | Phage tail protein I | Phage tail protein I | | afdb-uniprot50 | AF-A0A239CJC5-F1-MODEL\_V4 | 1.0 | 2.844e-16 | 401 | 0.31 | 203 | 126 | 5 | 1 | 200 | 1 | 192 | Phage tail protein, P2 protein I family | Phage tail protein, P2 protein I family | | afdb-uniprot50 | AF-A0A4R6M881-F1-MODEL\_V4 | 1.0 | 1.021e-12 | 400 | 0.331 | 184 | 111 | 6 | 3 | 178 | 11 | 190 | Phage tail P2-like protein | Phage tail P2-like protein | | afdb-uniprot50 | AF-A0A1G8HUV2-F1-MODEL\_V4 | 1.0 | 1.69e-12 | 400 | 0.181 | 209 | 165 | 6 | 1 | 205 | 12 | 218 | Phage tail protein, P2 protein I family | Phage tail protein, P2 protein I family | | afdb-uniprot50 | AF-A0A7L5Y1L5-F1-MODEL\_V4 | 1.0 | 2.335e-14 | 399 | 0.282 | 184 | 127 | 4 | 2 | 182 | 6 | 187 | Phage tail protein I | Phage tail protein I | | afdb-uniprot50 | AF-A0A345J3J1-F1-MODEL\_V4 | 1.0 | 1.644e-13 | 399 | 0.243 | 205 | 149 | 4 | 1 | 203 | 4 | 204 | Phage tail protein, P2 family | Phage tail protein, P2 family | | afdb-uniprot50 | AF-A0A855N9J0-F1-MODEL\_V4 | 1.0 | 4.503e-13 | 399 | 0.233 | 206 | 152 | 4 | 1 | 205 | 1 | 201 | Phage tail protein I | Phage tail protein I | | afdb-uniprot50 | AF-F8FW00-F1-MODEL\_V4 | 1.0 | 1.45e-13 | 398 | 0.283 | 219 | 139 | 8 | 1 | 205 | 1 | 215 | Pyocin R2\_PP, tail formation protein | Pyocin R2\_PP, tail formation protein | | afdb-uniprot50 | AF-A0A1C3FFM0-F1-MODEL\_V4 | 1.0 | 3.377e-12 | 397 | 0.289 | 159 | 106 | 4 | 1 | 157 | 3 | 156 | Putative phage tail fiber protein | Putative phage tail fiber protein | | afdb-uniprot50 | AF-A0A0W8JFZ8-F1-MODEL\_V4 | 1.0 | 5.439e-13 | 396 | 0.2 | 200 | 149 | 3 | 16 | 205 | 2 | 200 | Uncharacterized protein | Uncharacterized protein | | afdb-uniprot50 | AF-A0A2M7MVB7-F1-MODEL\_V4 | 1.0 | 8.225e-14 | 396 | 0.257 | 229 | 121 | 7 | 1 | 181 | 1 | 228 | Phage tail protein I | Phage tail protein I | | afdb-uniprot50 | AF-A0A7U2UZK0-F1-MODEL\_V4 | 1.0 | 1.751e-13 | 396 | 0.246 | 215 | 148 | 8 | 4 | 205 | 10 | 223 | Phage tail protein I | Phage tail protein I | | afdb-uniprot50 | AF-R7JD91-F1-MODEL\_V4 | 1.0 | 1.986e-13 | 396 | 0.278 | 183 | 118 | 7 | 2 | 176 | 43 | 219 | Putative phage tail protein | Putative phage tail protein | | afdb-uniprot50 | AF-A0A4S2HBU2-F1-MODEL\_V4 | 1.0 | 5.248e-12 | 396 | 0.248 | 161 | 111 | 6 | 5 | 158 | 13 | 170 | Phage tail protein I | Phage tail protein I | | afdb-uniprot50 | AF-A0A7J0BHY1-F1-MODEL\_V4 | 1.0 | 1.158e-12 | 395 | 0.257 | 194 | 138 | 5 | 13 | 204 | 28 | 217 | Phage tail protein I | Phage tail protein I | | afdb-uniprot50 | AF-A0A855ITT2-F1-MODEL\_V4 | 1.0 | 8.452e-13 | 395 | 0.151 | 205 | 167 | 4 | 1 | 200 | 8 | 210 | Phage tail protein I | Phage tail protein I | | afdb-uniprot50 | AF-A0A7U9H2I1-F1-MODEL\_V4 | 1.0 | 2.721e-13 | 395 | 0.252 | 198 | 138 | 5 | 11 | 203 | 24 | 216 | Phage tail protein I | Phage tail protein I | | afdb-uniprot50 | AF-A0A1X3J174-F1-MODEL\_V4 | 1.0 | 3.471e-11 | 394 | 0.438 | 105 | 57 | 1 | 71 | 175 | 2 | 104 | Tail protein I (GpI) | Tail protein I (GpI) | | afdb-uniprot50 | AF-I3TTD2-F1-MODEL\_V4 | 1.0 | 2.253e-13 | 394 | 0.285 | 221 | 141 | 5 | 1 | 205 | 1 | 220 | Gp15 protein | Gp15 protein | | afdb-uniprot50 | AF-A0A7W6REA6-F1-MODEL\_V4 | 1.0 | 1.03e-14 | 393 | 0.264 | 227 | 145 | 6 | 1 | 205 | 9 | 235 | Phage tail P2-like protein | Phage tail P2-like protein | | afdb-uniprot50 | AF-A0A290TRG8-F1-MODEL\_V4 | 1.0 | 1.69e-12 | 393 | 0.245 | 183 | 123 | 6 | 14 | 186 | 108 | 285 | Uncharacterized protein | Uncharacterized protein | | afdb-uniprot50 | AF-A0A5S3WQZ3-F1-MODEL\_V4 | 1.0 | 1.69e-12 | 393 | 0.311 | 170 | 100 | 5 | 11 | 165 | 104 | 271 | Phage tail protein I | Phage tail protein I | | afdb-uniprot50 | AF-F3LHZ7-F1-MODEL\_V4 | 1.0 | 1.158e-12 | 392 | 0.287 | 174 | 102 | 6 | 1 | 156 | 1 | 170 | Tail protein I | Tail protein I | | afdb-uniprot50 | AF-A0A7X6GUL0-F1-MODEL\_V4 | 1.0 | 3.171e-12 | 392 | 0.263 | 171 | 110 | 6 | 4 | 162 | 8 | 174 | Phage tail protein I | Phage tail protein I | | afdb-uniprot50 | AF-U1JMH5-F1-MODEL\_V4 | 1.0 | 1.704e-14 | 392 | 0.29 | 210 | 138 | 5 | 1 | 202 | 1 | 207 | Putative prophage tail protein | Putative prophage tail protein | | afdb-uniprot50 | AF-R9KAQ1-F1-MODEL\_V4 | 1.0 | 8.759e-14 | 392 | 0.255 | 219 | 141 | 6 | 3 | 205 | 21 | 233 | Phage tail protein I | Phage tail protein I | | afdb-uniprot50 | AF-A0A891ZWB9-F1-MODEL\_V4 | 1.0 | 1.916e-12 | 391 | 0.228 | 175 | 125 | 5 | 11 | 181 | 3 | 171 | Bacteriophage P2-related tail formation protein | Bacteriophage P2-related tail formation protein | | afdb-uniprot50 | AF-A0A522IBS6-F1-MODEL\_V4 | 1.0 | 3.287e-13 | 391 | 0.271 | 225 | 140 | 7 | 1 | 203 | 1 | 223 | Phage tail protein I | Phage tail protein I | | afdb-uniprot50 | AF-A0A239EKX6-F1-MODEL\_V4 | 1.0 | 1.373e-15 | 391 | 0.272 | 213 | 138 | 4 | 1 | 196 | 4 | 216 | Phage tail protein, P2 protein I family | Phage tail protein, P2 protein I family | | afdb-uniprot50 | AF-A0A318KN12-F1-MODEL\_V4 | 1.0 | 1.19e-11 | 391 | 0.335 | 161 | 105 | 2 | 4 | 163 | 6 | 165 | Phage tail P2-like protein | Phage tail P2-like protein | | afdb-uniprot50 | AF-A0A5T0QSQ9-F1-MODEL\_V4 | 1.0 | 1.544e-13 | 390 | 0.234 | 196 | 140 | 6 | 1 | 193 | 1 | 189 | Phage tail protein I | Phage tail protein I | | afdb-uniprot50 | AF-A0A376YFL4-F1-MODEL\_V4 | 1.0 | 5.685e-16 | 390 | 0.372 | 196 | 119 | 2 | 1 | 194 | 1 | 194 | Tail protein | Tail protein | | afdb-uniprot50 | AF-A0A432PXG2-F1-MODEL\_V4 | 1.0 | 1.058e-13 | 390 | 0.258 | 213 | 142 | 7 | 4 | 205 | 6 | 213 | Phage tail protein I | Phage tail protein I | | afdb-uniprot50 | AF-A0A6N6XVM0-F1-MODEL\_V4 | 1.0 | 3.97e-13 | 389 | 0.238 | 189 | 128 | 7 | 3 | 181 | 11 | 193 | Phage tail protein I | Phage tail protein I | | afdb-uniprot50 | AF-A0A090IT51-F1-MODEL\_V4 | 1.0 | 9.586e-13 | 388 | 0.194 | 206 | 158 | 3 | 1 | 205 | 8 | 206 | Phage tail protein I | Phage tail protein I | | afdb-uniprot50 | AF-A0A8B2QT57-F1-MODEL\_V4 | 1.0 | 1.127e-13 | 387 | 0.293 | 201 | 131 | 7 | 4 | 202 | 3 | 194 | Phage tail protein I | Phage tail protein I | | afdb-uniprot50 | AF-A0A1E7HY16-F1-MODEL\_V4 | 1.0 | 7.19e-12 | 386 | 0.366 | 139 | 83 | 2 | 1 | 139 | 2 | 135 | Phage tail protein I | Phage tail protein I | | afdb-uniprot50 | AF-B8GRZ9-F1-MODEL\_V4 | 1.0 | 2.192e-14 | 386 | 0.337 | 157 | 98 | 3 | 1 | 153 | 1 | 155 | Bacteriophage P2-related tail formation protein-like protein | Bacteriophage P2-related tail formation protein-like protein | | afdb-uniprot50 | AF-A0A3S5I5C6-F1-MODEL\_V4 | 1.0 | 9.851e-12 | 386 | 0.307 | 169 | 100 | 6 | 11 | 164 | 104 | 270 | Phage tail protein I | Phage tail protein I | | afdb-uniprot50 | AF-B9NP80-F1-MODEL\_V4 | 1.0 | 3.97e-13 | 385 | 0.265 | 211 | 145 | 5 | 1 | 203 | 4 | 212 | Pyocin R2\_PP, tail formation protein GpI | Pyocin R2\_PP, tail formation protein GpI | | afdb-uniprot50 | AF-A0A7X7NDE5-F1-MODEL\_V4 | 1.0 | 2.041e-12 | 385 | 0.236 | 190 | 130 | 6 | 1 | 184 | 1 | 181 | Phage tail protein I | Phage tail protein I | | afdb-uniprot50 | AF-A0A1Y4W9W6-F1-MODEL\_V4 | 1.0 | 9.001e-13 | 385 | 0.264 | 204 | 138 | 7 | 4 | 198 | 20 | 220 | Phage tail protein I | Phage tail protein I | | afdb-uniprot50 | AF-A0A259C7Y6-F1-MODEL\_V4 | 1.0 | 3.97e-13 | 384 | 0.287 | 160 | 105 | 5 | 26 | 178 | 5 | 162 | Phage tail protein I | Phage tail protein I | | afdb-uniprot50 | AF-A0A8A7F3K5-F1-MODEL\_V4 | 1.0 | 1.6e-14 | 384 | 0.262 | 229 | 143 | 8 | 1 | 205 | 1 | 227 | Phage tail protein I | Phage tail protein I | | afdb-uniprot50 | AF-L0RFK5-F1-MODEL\_V4 | 1.0 | 1.361e-13 | 383 | 0.269 | 267 | 130 | 9 | 1 | 205 | 1 | 264 | Uncharacterized protein | Uncharacterized protein | | afdb-uniprot50 | AF-A0A134C9I1-F1-MODEL\_V4 | 1.0 | 1.799e-12 | 382 | 0.245 | 204 | 141 | 9 | 5 | 202 | 13 | 209 | Phage tail protein I-like protein | Phage tail protein I-like protein | | afdb-uniprot50 | AF-A0A1B3E8I5-F1-MODEL\_V4 | 1.0 | 1.087e-12 | 382 | 0.267 | 258 | 131 | 7 | 1 | 205 | 1 | 253 | Uncharacterized protein | Uncharacterized protein | | afdb-uniprot50 | AF-A0A849VC39-F1-MODEL\_V4 | 1.0 | 1.865e-13 | 381 | 0.299 | 167 | 107 | 3 | 3 | 162 | 9 | 172 | Phage tail protein I | Phage tail protein I | | afdb-uniprot50 | AF-A0A4R0F5F4-F1-MODEL\_V4 | 1.0 | 2.721e-13 | 380 | 0.267 | 176 | 127 | 2 | 1 | 176 | 1 | 174 | Phage tail protein I | Phage tail protein I | | afdb-uniprot50 | AF-A0A2A3MPV6-F1-MODEL\_V4 | 1.0 | 2.555e-13 | 380 | 0.35 | 180 | 106 | 6 | 1 | 173 | 46 | 221 | Phage tail protein I | Phage tail protein I | | afdb-uniprot50 | AF-A0A6N8I028-F1-MODEL\_V4 | 1.0 | 8.759e-14 | 379 | 0.278 | 194 | 124 | 5 | 14 | 203 | 35 | 216 | Phage tail protein | Phage tail protein | | afdb-uniprot50 | AF-A0A2G2D3E6-F1-MODEL\_V4 | 1.0 | 2.625e-12 | 379 | 0.216 | 194 | 140 | 5 | 4 | 187 | 14 | 205 | Phage tail protein I | Phage tail protein I | | afdb-uniprot50 | AF-A0A0A8JLX9-F1-MODEL\_V4 | 1.0 | 2.533e-11 | 379 | 0.246 | 158 | 110 | 5 | 4 | 155 | 12 | 166 | Phage tail protein | Phage tail protein | | afdb-uniprot50 | AF-A0A353G6I2-F1-MODEL\_V4 | 1.0 | 4.192e-11 | 377 | 0.233 | 167 | 112 | 8 | 1 | 152 | 1 | 166 | Phage tail protein I | Phage tail protein I | | afdb-uniprot50 | AF-A0A0Q2UZB0-F1-MODEL\_V4 | 1.0 | 1.021e-12 | 377 | 0.261 | 214 | 142 | 7 | 1 | 203 | 14 | 222 | Uncharacterized protein | Uncharacterized protein | | afdb-uniprot50 | AF-A0A165W2J4-F1-MODEL\_V4 | 1.0 | 2.465e-12 | 377 | 0.286 | 206 | 139 | 5 | 4 | 205 | 10 | 211 | Uncharacterized protein | Uncharacterized protein | | afdb-uniprot50 | AF-A0A2G6HAI3-F1-MODEL\_V4 | 1.0 | 6.626e-15 | 377 | 0.324 | 234 | 125 | 9 | 1 | 205 | 1 | 230 | Phage tail protein I | Phage tail protein I | | afdb-uniprot50 | AF-A0A1C6D4J3-F1-MODEL\_V4 | 1.0 | 5.589e-12 | 376 | 0.28 | 178 | 113 | 8 | 4 | 171 | 15 | 187 | Bacteriophage P2-related tail formation protein | Bacteriophage P2-related tail formation protein | | afdb-uniprot50 | AF-D5EF95-F1-MODEL\_V4 | 1.0 | 2.253e-13 | 376 | 0.285 | 217 | 134 | 8 | 3 | 204 | 8 | 218 | Phage tail protein I | Phage tail protein I | | afdb-uniprot50 | AF-A0A1G7SDN3-F1-MODEL\_V4 | 1.0 | 9.25e-12 | 376 | 0.301 | 169 | 99 | 4 | 4 | 156 | 56 | 221 | Phage tail protein, P2 protein I family | Phage tail protein, P2 protein I family | | afdb-uniprot50 | AF-G9PUJ6-F1-MODEL\_V4 | 1.0 | 1.45e-13 | 375 | 0.233 | 210 | 141 | 6 | 5 | 203 | 14 | 214 | Phage tail protein I | Phage tail protein I | | afdb-uniprot50 | AF-A0A7U3VT62-F1-MODEL\_V4 | 1.0 | 7.657e-12 | 375 | 0.337 | 169 | 93 | 5 | 1 | 157 | 1 | 162 | Fused protein containing phage-related tail protein GpI | Fused protein containing phage-related tail protein GpI | | afdb-uniprot50 | AF-A0A1C3E9B0-F1-MODEL\_V4 | 1.0 | 4.928e-12 | 374 | 0.179 | 201 | 155 | 7 | 1 | 196 | 7 | 202 | Phage tail protein I | Phage tail protein I | | afdb-uniprot50 | AF-A0A087NAA8-F1-MODEL\_V4 | 1.0 | 6.169e-13 | 374 | 0.325 | 181 | 111 | 5 | 1 | 174 | 3 | 179 | Uncharacterized protein | Uncharacterized protein | | afdb-uniprot50 | AF-E5Y5X1-F1-MODEL\_V4 | 1.0 | 6.394e-14 | 374 | 0.257 | 233 | 141 | 8 | 4 | 205 | 14 | 245 | Phage tail protein I | Phage tail protein I | | afdb-uniprot50 | AF-A0A545T5T7-F1-MODEL\_V4 | 1.0 | 3.97e-13 | 373 | 0.308 | 224 | 106 | 9 | 3 | 186 | 14 | 228 | Phage tail protein I | Phage tail protein I | | afdb-uniprot50 | AF-A0A1Q6PTY4-F1-MODEL\_V4 | 1.0 | 6.57e-13 | 373 | 0.246 | 219 | 144 | 7 | 4 | 205 | 14 | 228 | Phage tail protein I | Phage tail protein I | | afdb-uniprot50 | AF-A0A1W1UNV3-F1-MODEL\_V4 | 1.0 | 3.377e-12 | 371 | 0.296 | 172 | 101 | 4 | 1 | 155 | 1 | 169 | Phage tail protein, P2 protein I family | Phage tail protein, P2 protein I family | | afdb-uniprot50 | AF-A0A1Q6JPU8-F1-MODEL\_V4 | 1.0 | 2.379e-11 | 371 | 0.279 | 154 | 103 | 4 | 11 | 163 | 30 | 176 | Phage tail protein I | Phage tail protein I | | afdb-uniprot50 | AF-A0A7X5B8H9-F1-MODEL\_V4 | 1.0 | 4.08e-12 | 370 | 0.222 | 207 | 154 | 5 | 1 | 201 | 7 | 212 | Phage tail protein | Phage tail protein | | afdb-uniprot50 | AF-A0A2X0WX70-F1-MODEL\_V4 | 1.0 | 9.586e-13 | 370 | 0.208 | 211 | 156 | 5 | 4 | 205 | 13 | 221 | Bacteriophage P2-related tail formation protein | Bacteriophage P2-related tail formation protein | | afdb-uniprot50 | AF-A0A0F6A4X0-F1-MODEL\_V4 | 1.0 | 3.728e-13 | 370 | 0.266 | 165 | 110 | 5 | 1 | 157 | 1 | 162 | Tail protein | Tail protein | | afdb-uniprot50 | AF-A0A497UKP7-F1-MODEL\_V4 | 1.0 | 3.597e-12 | 369 | 0.247 | 194 | 125 | 7 | 1 | 188 | 1 | 179 | P2-related tail formation protein | P2-related tail formation protein | | afdb-uniprot50 | AF-F9S7B6-F1-MODEL\_V4 | 1.0 | 4.345e-12 | 369 | 0.227 | 180 | 130 | 6 | 4 | 176 | 28 | 205 | Bacteriophage tail protein I | Bacteriophage tail protein I | | afdb-uniprot50 | AF-A0A0J9EGJ6-F1-MODEL\_V4 | 1.0 | 2.721e-13 | 368 | 0.244 | 229 | 128 | 7 | 14 | 200 | 2 | 227 | Tail protein I | Tail protein I | | afdb-uniprot50 | AF-A0A0H5CXM9-F1-MODEL\_V4 | 1.0 | 2.379e-11 | 367 | 0.264 | 178 | 112 | 6 | 1 | 165 | 5 | 176 | Phage tail protein I | Phage tail protein I | | afdb-uniprot50 | AF-A0A1A9G374-F1-MODEL\_V4 | 1.0 | 1.19e-11 | 367 | 0.152 | 243 | 166 | 7 | 1 | 205 | 3 | 243 | Uncharacterized protein | Uncharacterized protein | | afdb-uniprot50 | AF-A0A5Q2UG14-F1-MODEL\_V4 | 1.0 | 7.452e-13 | 367 | 0.273 | 183 | 119 | 7 | 5 | 177 | 1 | 179 | Phage tail protein I | Phage tail protein I | | afdb-uniprot50 | AF-A0A2K2G515-F1-MODEL\_V4 | 1.0 | 2.898e-13 | 366 | 0.264 | 189 | 124 | 6 | 1 | 180 | 3 | 185 | Phage tail protein I | Phage tail protein I | | afdb-uniprot50 | AF-A0A1I5W407-F1-MODEL\_V4 | 1.0 | 1.097e-14 | 364 | 0.295 | 220 | 128 | 8 | 1 | 200 | 1 | 213 | Phage tail protein, P2 protein I family | Phage tail protein, P2 protein I family | | afdb-uniprot50 | AF-A0A1Q6JQM0-F1-MODEL\_V4 | 1.0 | 5.793e-13 | 364 | 0.273 | 212 | 136 | 6 | 5 | 205 | 15 | 219 | Phage tail protein I | Phage tail protein I | | afdb-uniprot50 | AF-A0A3G6WST2-F1-MODEL\_V4 | 1.0 | 1.544e-13 | 364 | 0.378 | 169 | 86 | 4 | 1 | 153 | 34 | 199 | Phage tail protein I | Phage tail protein I | | afdb-uniprot50 | AF-A0A1X7MNV1-F1-MODEL\_V4 | 1.0 | 7.869e-11 | 363 | 0.446 | 121 | 61 | 2 | 1 | 117 | 1 | 119 | Phage tail protein I | Phage tail protein I | | afdb-uniprot50 | AF-A0A1B1NTF0-F1-MODEL\_V4 | 1.0 | 4.345e-12 | 363 | 0.234 | 183 | 129 | 7 | 3 | 176 | 19 | 199 | Baseplate protein | Baseplate protein | | afdb-uniprot50 | AF-D8IV10-F1-MODEL\_V4 | 1.0 | 1.799e-12 | 363 | 0.206 | 232 | 156 | 7 | 2 | 205 | 6 | 237 | Tail-related protein | Tail-related protein | | afdb-uniprot50 | AF-A0A6L7TCB7-F1-MODEL\_V4 | 1.0 | 1.865e-13 | 363 | 0.345 | 171 | 100 | 5 | 1 | 167 | 100 | 262 | Phage tail protein I | Phage tail protein I | | afdb-uniprot50 | AF-A0A6C9G6K6-F1-MODEL\_V4 | 1.0 | 7.869e-11 | 362 | 0.514 | 103 | 50 | 0 | 1 | 103 | 1 | 103 | Phage tail protein I | Phage tail protein I | | afdb-uniprot50 | AF-A0A290TLP4-F1-MODEL\_V4 | 1.0 | 6.222e-15 | 362 | 0.298 | 181 | 122 | 3 | 2 | 179 | 5 | 183 | Uncharacterized protein | Uncharacterized protein | | afdb-uniprot50 | AF-A0A142BH84-F1-MODEL\_V4 | 1.0 | 2.041e-12 | 362 | 0.5 | 116 | 58 | 0 | 1 | 116 | 1 | 116 | Phage tail protein | Phage tail protein | | afdb-uniprot50 | AF-A0A645F841-F1-MODEL\_V4 | 1.0 | 3.287e-13 | 361 | 0.278 | 208 | 132 | 10 | 3 | 200 | 11 | 210 | Uncharacterized protein | Uncharacterized protein | | afdb-uniprot50 | AF-F4QJC1-F1-MODEL\_V4 | 1.0 | 3.003e-14 | 360 | 0.312 | 189 | 119 | 6 | 1 | 185 | 1 | 182 | Phage tail protein I | Phage tail protein I | | afdb-uniprot50 | AF-A0A655UT13-F1-MODEL\_V4 | 1.0 | 2.233e-11 | 360 | 0.251 | 171 | 107 | 5 | 1 | 155 | 2 | 167 | Bacteriophage P2-related tail formation protein | Bacteriophage P2-related tail formation protein | | afdb-uniprot50 | AF-A0A3D4MXN2-F1-MODEL\_V4 | 1.0 | 6.339e-12 | 360 | 0.279 | 186 | 114 | 8 | 4 | 174 | 39 | 219 | Phage tail protein I | Phage tail protein I | | afdb-uniprot50 | AF-A0A6L7FZ02-F1-MODEL\_V4 | 1.0 | 2.978e-12 | 360 | 0.228 | 223 | 150 | 8 | 1 | 205 | 1 | 219 | Phage tail protein I | Phage tail protein I | | afdb-uniprot50 | AF-F3YY51-F1-MODEL\_V4 | 1.0 | 1.233e-12 | 357 | 0.266 | 206 | 140 | 6 | 4 | 203 | 13 | 213 | Phage tail protein I | Phage tail protein I | | afdb-uniprot50 | AF-A0A3S2Y7C0-F1-MODEL\_V4 | 1.0 | 1.986e-13 | 357 | 0.288 | 184 | 117 | 4 | 4 | 184 | 16 | 188 | Phage tail protein I | Phage tail protein I | | afdb-uniprot50 | AF-A0A6L6VKC1-F1-MODEL\_V4 | 1.0 | 7.19e-12 | 357 | 0.191 | 225 | 162 | 8 | 1 | 205 | 1 | 225 | Phage tail protein I | Phage tail protein I | | afdb-uniprot50 | AF-A0A161XW60-F1-MODEL\_V4 | 1.0 | 7.869e-11 | 356 | 0.277 | 166 | 107 | 5 | 3 | 157 | 98 | 261 | Tail protein | Tail protein | | afdb-uniprot50 | AF-A0A3B6Y3G2-F1-MODEL\_V4 | 1.0 | 1.03e-14 | 354 | 0.275 | 185 | 123 | 3 | 4 | 179 | 1 | 183 | Phage tail protein I | Phage tail protein I | | afdb-uniprot50 | AF-A0A1Y4W8I7-F1-MODEL\_V4 | 1.0 | 1.916e-12 | 354 | 0.258 | 213 | 135 | 9 | 3 | 199 | 11 | 216 | Phage tail protein I | Phage tail protein I | | afdb-uniprot50 | AF-A0A3A9ENS4-F1-MODEL\_V4 | 1.0 | 1.158e-12 | 354 | 0.241 | 240 | 137 | 10 | 4 | 203 | 15 | 249 | Phage tail protein I | Phage tail protein I | | afdb-uniprot50 | AF-A0A1Y3WRA3-F1-MODEL\_V4 | 1.0 | 1.849e-11 | 353 | 0.219 | 187 | 135 | 7 | 5 | 183 | 13 | 196 | Phage tail protein I | Phage tail protein I | | afdb-uniprot50 | AF-A0A1G3UAH8-F1-MODEL\_V4 | 1.0 | 9.586e-13 | 353 | 0.242 | 227 | 138 | 10 | 4 | 205 | 3 | 220 | Phage tail protein I | Phage tail protein I | | afdb-uniprot50 | AF-A0A7C9LKP3-F1-MODEL\_V4 | 1.0 | 9.505e-11 | 351 | 0.189 | 179 | 135 | 6 | 4 | 177 | 18 | 191 | Phage tail protein I | Phage tail protein I | | afdb-uniprot50 | AF-A0A1B8QCU6-F1-MODEL\_V4 | 1.0 | 2.335e-14 | 351 | 0.284 | 179 | 123 | 3 | 4 | 182 | 19 | 192 | Phage tail protein I | Phage tail protein I | | afdb-uniprot50 | AF-F7L031-F1-MODEL\_V4 | 1.0 | 7.657e-12 | 351 | 0.248 | 189 | 122 | 5 | 3 | 175 | 11 | 195 | Putative phage tail protein | Putative phage tail protein | | afdb-uniprot50 | AF-A0A327Q855-F1-MODEL\_V4 | 1.0 | 4.627e-12 | 351 | 0.254 | 220 | 142 | 9 | 1 | 205 | 1 | 213 | Phage tail P2-like protein | Phage tail P2-like protein | | afdb-uniprot50 | AF-A0A239C7G7-F1-MODEL\_V4 | 1.0 | 4.928e-12 | 351 | 0.263 | 205 | 134 | 5 | 4 | 197 | 12 | 210 | Phage tail protein, P2 protein I family | Phage tail protein, P2 protein I family | | afdb-uniprot50 | AF-A0A135IPT9-F1-MODEL\_V4 | 1.0 | 6.626e-15 | 351 | 0.343 | 224 | 122 | 9 | 1 | 205 | 5 | 222 | Uncharacterized protein | Uncharacterized protein | | afdb-uniprot50 | AF-A0A085AFN4-F1-MODEL\_V4 | 1.0 | 8.155e-12 | 350 | 0.256 | 191 | 123 | 6 | 1 | 182 | 2 | 182 | Tail protein I | Tail protein I | | afdb-uniprot50 | AF-A0A1M7R7F1-F1-MODEL\_V4 | 1.0 | 7.19e-12 | 350 | 0.223 | 201 | 134 | 8 | 1 | 192 | 1 | 188 | P2-related tail formation protein | P2-related tail formation protein | | afdb-uniprot50 | AF-A0A0A2SKX6-F1-MODEL\_V4 | 1.0 | 2.796e-12 | 349 | 0.254 | 185 | 120 | 8 | 3 | 177 | 11 | 187 | Uncharacterized protein | Uncharacterized protein | | afdb-uniprot50 | AF-B6IMH1-F1-MODEL\_V4 | 1.0 | 1.531e-11 | 349 | 0.236 | 224 | 152 | 6 | 1 | 205 | 1 | 224 | Uncharacterized protein | Uncharacterized protein | | afdb-uniprot50 | AF-A0A0J7Y5C0-F1-MODEL\_V4 | 1.0 | 1.087e-12 | 348 | 0.266 | 229 | 138 | 4 | 1 | 200 | 3 | 230 | Tail protein | Tail protein | | afdb-uniprot50 | AF-A0A5S3YZW9-F1-MODEL\_V4 | 1.0 | 3.471e-11 | 348 | 0.255 | 192 | 125 | 7 | 5 | 180 | 97 | 286 | Phage tail protein I | Phage tail protein I | | afdb-uniprot50 | AF-A0A2S9GTF6-F1-MODEL\_V4 | 1.0 | 3.377e-12 | 347 | 0.346 | 150 | 90 | 4 | 1 | 149 | 3 | 145 | Phage tail protein I | Phage tail protein I | | afdb-uniprot50 | AF-A0A7T1BKU5-F1-MODEL\_V4 | 1.0 | 8.685e-12 | 347 | 0.278 | 183 | 119 | 7 | 5 | 177 | 11 | 190 | Phage tail protein I | Phage tail protein I | | afdb-uniprot50 | AF-A0A3A9EMV3-F1-MODEL\_V4 | 1.0 | 5.393e-11 | 346 | 0.258 | 147 | 97 | 5 | 11 | 154 | 31 | 168 | Phage tail protein I | Phage tail protein I | | afdb-uniprot50 | AF-A0A4P6B4N0-F1-MODEL\_V4 | 1.0 | 1.969e-11 | 346 | 0.268 | 186 | 116 | 8 | 3 | 177 | 11 | 187 | Phage tail protein | Phage tail protein | | afdb-uniprot50 | AF-A0A1H9HC51-F1-MODEL\_V4 | 1.0 | 1.644e-13 | 346 | 0.289 | 214 | 135 | 6 | 1 | 203 | 3 | 210 | Phage tail protein, P2 protein I family | Phage tail protein, P2 protein I family | | afdb-uniprot50 | AF-A0A6N9P645-F1-MODEL\_V4 | 1.0 | 1.302e-10 | 346 | 0.251 | 163 | 110 | 5 | 6 | 163 | 21 | 176 | Phage tail protein I | Phage tail protein I | | afdb-uniprot50 | AF-A0A249DWE7-F1-MODEL\_V4 | 1.0 | 2.173e-12 | 345 | 0.315 | 165 | 98 | 4 | 1 | 156 | 2 | 160 | Phage tail protein I | Phage tail protein I | | afdb-uniprot50 | AF-A0A1H0YPQ4-F1-MODEL\_V4 | 1.0 | 3.471e-11 | 345 | 0.211 | 222 | 156 | 9 | 1 | 205 | 1 | 220 | Phage tail protein, P2 protein I family | Phage tail protein, P2 protein I family | | afdb-uniprot50 | AF-B6WRR5-F1-MODEL\_V4 | 1.0 | 3.171e-12 | 345 | 0.228 | 206 | 144 | 5 | 1 | 205 | 54 | 245 | Phage tail protein I-like protein | Phage tail protein I-like protein | | afdb-uniprot50 | AF-A0A5C7PF23-F1-MODEL\_V4 | 1.0 | 1.399e-12 | 344 | 0.272 | 209 | 115 | 6 | 1 | 175 | 2 | 207 | Phage tail protein I | Phage tail protein I | | afdb-uniprot50 | AF-A0A1Y1QXH2-F1-MODEL\_V4 | 1.0 | 1.9e-10 | 344 | 0.264 | 151 | 106 | 4 | 1 | 148 | 4 | 152 | Phage tail protein I | Phage tail protein I | | afdb-uniprot50 | AF-A0A023PZC5-F1-MODEL\_V4 | 1.0 | 7.869e-11 | 343 | 0.269 | 178 | 118 | 6 | 1 | 166 | 97 | 274 | Tail protein I | Tail protein I | | afdb-uniprot50 | AF-A0A2P8EI45-F1-MODEL\_V4 | 1.0 | 5.064e-11 | 341 | 0.231 | 177 | 124 | 7 | 4 | 172 | 13 | 185 | Phage tail P2-like protein | Phage tail P2-like protein | | afdb-uniprot50 | AF-A0A351B539-F1-MODEL\_V4 | 1.0 | 9.586e-13 | 341 | 0.235 | 289 | 133 | 8 | 1 | 205 | 8 | 292 | Phage tail protein I | Phage tail protein I | | afdb-uniprot50 | AF-A0A2J4RC72-F1-MODEL\_V4 | 1.0 | 2.024e-10 | 340 | 0.441 | 120 | 63 | 2 | 1 | 117 | 6 | 124 | Phage tail protein I | Phage tail protein I | | afdb-uniprot50 | AF-A0A1E4MW51-F1-MODEL\_V4 | 1.0 | 4.465e-11 | 340 | 0.283 | 141 | 93 | 3 | 22 | 157 | 3 | 140 | Phage tail protein I | Phage tail protein I | | afdb-uniprot50 | AF-A0A3T0N1K0-F1-MODEL\_V4 | 1.0 | 3.406e-14 | 340 | 0.275 | 243 | 133 | 7 | 1 | 205 | 4 | 241 | Phage tail protein I | Phage tail protein I | | afdb-uniprot50 | AF-A0A5U3DU51-F1-MODEL\_V4 | 1.0 | 1.387e-10 | 339 | 0.391 | 120 | 71 | 1 | 75 | 194 | 2 | 119 | Phage tail protein I | Phage tail protein I | | afdb-uniprot50 | AF-A0A5C8XTJ9-F1-MODEL\_V4 | 1.0 | 5.952e-12 | 339 | 0.361 | 155 | 95 | 3 | 53 | 205 | 3 | 155 | Phage tail protein I | Phage tail protein I | | afdb-uniprot50 | AF-A0A6I2UKH8-F1-MODEL\_V4 | 1.0 | 7.19e-12 | 339 | 0.19 | 210 | 155 | 8 | 4 | 205 | 12 | 214 | Phage tail protein I | Phage tail protein I | | afdb-uniprot50 | AF-A0A285M2Z8-F1-MODEL\_V4 | 1.0 | 4.588e-10 | 338 | 0.213 | 164 | 118 | 5 | 4 | 157 | 14 | 176 | Phage tail protein, P2 protein I family | Phage tail protein, P2 protein I family | | afdb-uniprot50 | AF-A0A124INN1-F1-MODEL\_V4 | 1.0 | 5.248e-12 | 337 | 0.258 | 186 | 118 | 7 | 4 | 178 | 12 | 188 | Uncharacterized protein | Uncharacterized protein | | afdb-uniprot50 | AF-A0A081B6D0-F1-MODEL\_V4 | 1.0 | 3.06e-11 | 337 | 0.284 | 165 | 99 | 4 | 1 | 149 | 1 | 162 | Phage tail protein I | Phage tail protein I | | afdb-uniprot50 | AF-A0A413ED79-F1-MODEL\_V4 | 1.0 | 6.751e-12 | 337 | 0.221 | 208 | 149 | 7 | 3 | 201 | 11 | 214 | Phage tail protein I | Phage tail protein I | | afdb-uniprot50 | AF-R9M1L6-F1-MODEL\_V4 | 1.0 | 5.064e-11 | 337 | 0.274 | 171 | 104 | 7 | 5 | 163 | 15 | 177 | Phage tail protein I | Phage tail protein I | | afdb-uniprot50 | AF-A0A4R7SS34-F1-MODEL\_V4 | 1.0 | 1.573e-10 | 336 | 0.234 | 162 | 109 | 5 | 1 | 151 | 3 | 160 | Phage tail P2-like protein | Phage tail P2-like protein | | afdb-uniprot50 | AF-A0A101K5W9-F1-MODEL\_V4 | 1.0 | 7.657e-12 | 336 | 0.235 | 212 | 137 | 8 | 3 | 198 | 15 | 217 | Phage tail protein | Phage tail protein | | afdb-uniprot50 | AF-D8IV17-F1-MODEL\_V4 | 1.0 | 3.259e-11 | 336 | 0.209 | 220 | 154 | 6 | 4 | 205 | 9 | 226 | Phage-related tail protein | Phage-related tail protein | | afdb-uniprot50 | AF-A0A318KXU1-F1-MODEL\_V4 | 1.0 | 6.117e-11 | 335 | 0.301 | 166 | 98 | 6 | 1 | 156 | 1 | 158 | Phage tail P2-like protein | Phage tail P2-like protein | | afdb-uniprot50 | AF-E0NXI4-F1-MODEL\_V4 | 1.0 | 1.63e-11 | 331 | 0.23 | 213 | 143 | 8 | 3 | 203 | 12 | 215 | Phage tail protein | Phage tail protein | | afdb-uniprot50 | AF-A0A3T0L2N1-F1-MODEL\_V4 | 1.0 | 3.728e-13 | 331 | 0.235 | 212 | 144 | 7 | 1 | 205 | 3 | 203 | Uncharacterized protein | Uncharacterized protein | | afdb-uniprot50 | AF-A0A259U4Q0-F1-MODEL\_V4 | 1.0 | 2.041e-12 | 330 | 0.231 | 207 | 146 | 7 | 3 | 201 | 12 | 213 | Phage tail protein | Phage tail protein | | afdb-uniprot50 | AF-A0A1M6SJT3-F1-MODEL\_V4 | 1.0 | 1.9e-10 | 329 | 0.231 | 199 | 141 | 8 | 5 | 197 | 12 | 204 | Phage tail protein, P2 protein I family | Phage tail protein, P2 protein I family | | afdb-uniprot50 | AF-A0A376VMZ8-F1-MODEL\_V4 | 1.0 | 4.045e-10 | 328 | 0.552 | 105 | 45 | 1 | 1 | 103 | 1 | 105 | Tail protein I (GpI) | Tail protein I (GpI) | | afdb-uniprot50 | AF-A0A2V2FTF7-F1-MODEL\_V4 | 1.0 | 2.233e-11 | 328 | 0.241 | 170 | 113 | 4 | 12 | 179 | 37 | 192 | Phage tail protein I | Phage tail protein I | | afdb-uniprot50 | AF-E2CGH4-F1-MODEL\_V4 | 1.0 | 2.533e-11 | 328 | 0.211 | 213 | 144 | 7 | 4 | 194 | 15 | 225 | Phage tail protein I | Phage tail protein I | | afdb-uniprot50 | AF-A0A2S0PE89-F1-MODEL\_V4 | 1.0 | 7.19e-12 | 328 | 0.215 | 237 | 130 | 8 | 1 | 190 | 1 | 228 | Phage tail protein I | Phage tail protein I | | afdb-uniprot50 | AF-A0A1B1A2A5-F1-MODEL\_V4 | 1.0 | 5.637e-14 | 327 | 0.302 | 205 | 132 | 5 | 9 | 205 | 4 | 205 | Uncharacterized protein | Uncharacterized protein | | afdb-uniprot50 | AF-A0A845R5N3-F1-MODEL\_V4 | 1.0 | 7.869e-11 | 327 | 0.175 | 233 | 157 | 9 | 4 | 204 | 1 | 230 | Phage tail protein I | Phage tail protein I | | afdb-uniprot50 | AF-K1KKI1-F1-MODEL\_V4 | 1.0 | 4.08e-12 | 326 | 0.244 | 213 | 143 | 9 | 3 | 200 | 12 | 221 | Phage tail protein I | Phage tail protein I | | afdb-uniprot50 | AF-A0A827WDG4-F1-MODEL\_V4 | 1.0 | 4.886e-10 | 325 | 0.472 | 110 | 56 | 1 | 1 | 110 | 2 | 109 | Phage tail protein I | Phage tail protein I | | afdb-uniprot50 | AF-A0A0X8JTG7-F1-MODEL\_V4 | 1.0 | 3.06e-11 | 324 | 0.197 | 192 | 139 | 8 | 14 | 197 | 28 | 212 | Phage tail protein | Phage tail protein | | afdb-uniprot50 | AF-D4M9N0-F1-MODEL\_V4 | 1.0 | 9.001e-13 | 324 | 0.282 | 237 | 130 | 11 | 1 | 205 | 1 | 229 | Phage tail protein, P2 protein I family | Phage tail protein, P2 protein I family | | afdb-uniprot50 | AF-A0A433LI31-F1-MODEL\_V4 | 1.0 | 6.938e-11 | 323 | 0.221 | 190 | 132 | 6 | 1 | 184 | 1 | 180 | Phage tail protein I | Phage tail protein I | | afdb-uniprot50 | AF-A0A5M8P5X2-F1-MODEL\_V4 | 1.0 | 1.531e-11 | 323 | 0.215 | 209 | 151 | 7 | 5 | 202 | 31 | 237 | Phage tail protein I | Phage tail protein I | | afdb-uniprot50 | AF-A0A0J2JHP4-F1-MODEL\_V4 | 1.0 | 1.784e-10 | 322 | 0.318 | 138 | 89 | 3 | 64 | 198 | 3 | 138 | Phage tail protein I | Phage tail protein I | | afdb-uniprot50 | AF-A0A511Z872-F1-MODEL\_V4 | 1.0 | 2.097e-11 | 322 | 0.218 | 215 | 143 | 7 | 4 | 205 | 12 | 214 | Uncharacterized protein | Uncharacterized protein | | afdb-uniprot50 | AF-A0A7C8GR62-F1-MODEL\_V4 | 1.0 | 2.698e-11 | 322 | 0.231 | 212 | 140 | 9 | 4 | 205 | 12 | 210 | Phage tail protein I | Phage tail protein I | | afdb-uniprot50 | AF-A0A2S0JR95-F1-MODEL\_V4 | 1.0 | 1.849e-11 | 321 | 0.2 | 224 | 149 | 8 | 4 | 202 | 10 | 228 | Phage tail protein I | Phage tail protein I | | afdb-uniprot50 | AF-A0A4P9VH54-F1-MODEL\_V4 | 1.0 | 1.302e-10 | 320 | 0.325 | 120 | 81 | 0 | 26 | 145 | 1 | 120 | Phage tail protein I | Phage tail protein I | | afdb-uniprot50 | AF-A0A379ZTT6-F1-MODEL\_V4 | 1.0 | 2.772e-10 | 320 | 0.37 | 135 | 83 | 1 | 69 | 203 | 7 | 139 | Putative phage tail protein | Putative phage tail protein | | afdb-uniprot50 | AF-A0A376WQ79-F1-MODEL\_V4 | 1.0 | 5.695e-09 | 320 | 0.525 | 97 | 46 | 0 | 1 | 97 | 3 | 99 | Tail protein | Tail protein | | afdb-uniprot50 | AF-V4JM45-F1-MODEL\_V4 | 1.0 | 1.049e-11 | 320 | 0.323 | 195 | 90 | 9 | 2 | 159 | 14 | 203 | Tail protein | Tail protein | | afdb-uniprot50 | AF-A0A777HJF3-F1-MODEL\_V4 | 1.0 | 3.144e-10 | 319 | 0.392 | 125 | 74 | 1 | 79 | 203 | 1 | 123 | Phage tail protein I | Phage tail protein I | | afdb-uniprot50 | AF-A0A7W4RI07-F1-MODEL\_V4 | 1.0 | 1.799e-12 | 319 | 0.309 | 171 | 112 | 4 | 38 | 205 | 1 | 168 | Phage tail P2-like protein | Phage tail P2-like protein | | afdb-uniprot50 | AF-J4UR00-F1-MODEL\_V4 | 1.0 | 5.064e-11 | 319 | 0.225 | 182 | 136 | 4 | 23 | 203 | 4 | 181 | Phage tail protein I-like protein | Phage tail protein I-like protein | | afdb-uniprot50 | AF-A0A0X1U7S8-F1-MODEL\_V4 | 1.0 | 2.024e-10 | 319 | 0.201 | 174 | 128 | 6 | 10 | 178 | 20 | 187 | Phage tail protein | Phage tail protein | | afdb-uniprot50 | AF-A0A6N7W593-F1-MODEL\_V4 | 1.0 | 5.064e-11 | 319 | 0.219 | 214 | 144 | 8 | 4 | 205 | 13 | 215 | Phage tail protein I | Phage tail protein I | | afdb-uniprot50 | AF-A0A6B8KI89-F1-MODEL\_V4 | 1.0 | 1.35e-11 | 319 | 0.228 | 206 | 151 | 7 | 1 | 203 | 85 | 285 | Phage tail protein I | Phage tail protein I | | afdb-uniprot50 | AF-A0A6N7JGU4-F1-MODEL\_V4 | 1.0 | 1.45e-13 | 319 | 0.253 | 249 | 142 | 5 | 1 | 205 | 2 | 250 | Phage tail protein I | Phage tail protein I | | afdb-uniprot50 | AF-A0A143HC79-F1-MODEL\_V4 | 1.0 | 1.616e-09 | 318 | 0.23 | 156 | 107 | 7 | 9 | 159 | 19 | 166 | Uncharacterized protein | Uncharacterized protein | | afdb-uniprot50 | AF-E3H9A4-F1-MODEL\_V4 | 1.0 | 3.259e-11 | 318 | 0.179 | 184 | 134 | 6 | 4 | 179 | 12 | 186 | Phage tail protein | Phage tail protein | | afdb-uniprot50 | AF-A0A7C3CG54-F1-MODEL\_V4 | 1.0 | 8.612e-10 | 318 | 0.262 | 164 | 103 | 5 | 4 | 155 | 10 | 167 | Phage tail protein I | Phage tail protein I | | afdb-uniprot50 | AF-A0A3A6E7Q8-F1-MODEL\_V4 | 1.0 | 4.192e-11 | 318 | 0.242 | 239 | 143 | 9 | 1 | 205 | 10 | 244 | Phage tail protein I | Phage tail protein I | | afdb-uniprot50 | AF-A0A0B0HIN7-F1-MODEL\_V4 | 1.0 | 2.603e-10 | 318 | 0.227 | 167 | 112 | 8 | 4 | 160 | 12 | 171 | Phage tail protein (Tail\_P2\_I) | Phage tail protein (Tail\_P2\_I) | | afdb-uniprot50 | AF-B6VLW9-F1-MODEL\_V4 | 1.0 | 5.021e-09 | 317 | 0.49 | 100 | 51 | 0 | 1 | 100 | 2 | 101 | Putative bacteriophage protein | Putative bacteriophage protein | | afdb-uniprot50 | AF-A0A1M7LUF7-F1-MODEL\_V4 | 1.0 | 2.097e-11 | 317 | 0.223 | 188 | 126 | 6 | 1 | 178 | 9 | 186 | Phage tail protein, P2 protein I family | Phage tail protein, P2 protein I family | | afdb-uniprot50 | AF-A0A1H0P8S4-F1-MODEL\_V4 | 1.0 | 6.117e-11 | 317 | 0.206 | 208 | 153 | 9 | 5 | 205 | 12 | 214 | Phage tail protein, P2 protein I family | Phage tail protein, P2 protein I family | | afdb-uniprot50 | AF-A0A376RLD7-F1-MODEL\_V4 | 1.0 | 8.925e-11 | 316 | 0.515 | 130 | 60 | 2 | 1 | 129 | 84 | 211 | Putative tail protein I (Gpi) | Putative tail protein I (Gpi) | | afdb-uniprot50 | AF-A0A0P8W181-F1-MODEL\_V4 | 1.0 | 5.393e-11 | 316 | 0.219 | 205 | 150 | 5 | 6 | 205 | 19 | 218 | Phage tail protein | Phage tail protein | | afdb-uniprot50 | AF-A0A1M6GFJ1-F1-MODEL\_V4 | 1.0 | 3.259e-11 | 316 | 0.251 | 215 | 144 | 7 | 3 | 205 | 12 | 221 | Phage tail protein, P2 protein I family | Phage tail protein, P2 protein I family | | afdb-uniprot50 | AF-A0A0U9HK81-F1-MODEL\_V4 | 1.0 | 2.873e-11 | 315 | 0.239 | 217 | 139 | 8 | 3 | 205 | 11 | 215 | Phage tail protein, P2 protein I family | Phage tail protein, P2 protein I family | | afdb-uniprot50 | AF-A0A5B9Y8K3-F1-MODEL\_V4 | 1.0 | 2.155e-10 | 314 | 0.219 | 182 | 128 | 7 | 3 | 176 | 11 | 186 | Phage tail protein I | Phage tail protein I | | afdb-uniprot50 | AF-A0A2E2N191-F1-MODEL\_V4 | 1.0 | 5.107e-13 | 314 | 0.261 | 199 | 133 | 5 | 10 | 202 | 1 | 191 | Phage tail protein I | Phage tail protein I | | afdb-uniprot50 | AF-H1PQH3-F1-MODEL\_V4 | 1.0 | 5.902e-10 | 314 | 0.235 | 174 | 118 | 4 | 12 | 180 | 29 | 192 | Phage tail protein I | Phage tail protein I | | afdb-uniprot50 | AF-A0A7U9X3N6-F1-MODEL\_V4 | 1.0 | 3.798e-10 | 313 | 0.257 | 167 | 111 | 5 | 3 | 162 | 11 | 171 | Uncharacterized protein | Uncharacterized protein | | afdb-uniprot50 | AF-A0A162QMS0-F1-MODEL\_V4 | 1.0 | 3.471e-11 | 313 | 0.229 | 218 | 141 | 9 | 3 | 203 | 11 | 218 | Phage tail protein | Phage tail protein | | afdb-uniprot50 | AF-A0A1C6EQC2-F1-MODEL\_V4 | 1.0 | 5.204e-10 | 313 | 0.184 | 179 | 134 | 7 | 5 | 175 | 13 | 187 | Bacteriophage P2-related tail formation protein | Bacteriophage P2-related tail formation protein | | afdb-uniprot50 | AF-G4KQ65-F1-MODEL\_V4 | 1.0 | 4.465e-11 | 312 | 0.239 | 184 | 116 | 9 | 4 | 174 | 11 | 183 | Uncharacterized protein | Uncharacterized protein | | afdb-uniprot50 | AF-A0A369BHI8-F1-MODEL\_V4 | 1.0 | 2.295e-10 | 312 | 0.231 | 177 | 122 | 8 | 6 | 177 | 20 | 187 | Phage tail P2-like protein | Phage tail P2-like protein | | afdb-uniprot50 | AF-A0A844HQG5-F1-MODEL\_V4 | 1.0 | 2.873e-11 | 312 | 0.281 | 188 | 114 | 3 | 1 | 170 | 1 | 185 | Phage tail protein I | Phage tail protein I | | afdb-uniprot50 | AF-A0A7X5KSS8-F1-MODEL\_V4 | 1.0 | 4.465e-11 | 312 | 0.207 | 202 | 151 | 5 | 3 | 198 | 11 | 209 | Uncharacterized protein | Uncharacterized protein | | afdb-uniprot50 | AF-A0A1M7RI70-F1-MODEL\_V4 | 1.0 | 1.387e-10 | 311 | 0.199 | 206 | 147 | 7 | 1 | 197 | 1 | 197 | Phage tail protein (Tail\_P2\_I) | Phage tail protein (Tail\_P2\_I) | | afdb-uniprot50 | AF-A0A3S5D9L0-F1-MODEL\_V4 | 1.0 | 1.661e-08 | 310 | 0.471 | 89 | 45 | 1 | 35 | 123 | 2 | 88 | Phage tail protein I | Phage tail protein I | | afdb-uniprot50 | AF-A0A2X2UK87-F1-MODEL\_V4 | 1.0 | 2.533e-11 | 310 | 0.227 | 211 | 134 | 7 | 12 | 202 | 30 | 231 | Phage tail protein | Phage tail protein | | afdb-uniprot50 | AF-A0A173SBX4-F1-MODEL\_V4 | 1.0 | 2.097e-11 | 310 | 0.211 | 208 | 141 | 6 | 12 | 205 | 31 | 229 | Bacteriophage P2-related tail formation protein | Bacteriophage P2-related tail formation protein | | afdb-uniprot50 | AF-A0A3S4HEN1-F1-MODEL\_V4 | 1.0 | 1.387e-10 | 309 | 0.189 | 232 | 156 | 10 | 1 | 203 | 2 | 230 | Bacteriophage P2-related tail formation protein | Bacteriophage P2-related tail formation protein | | afdb-uniprot50 | AF-A0A7X5ICE8-F1-MODEL\_V4 | 1.0 | 1.338e-09 | 309 | 0.191 | 167 | 123 | 7 | 5 | 163 | 1 | 163 | Uncharacterized protein | Uncharacterized protein | | afdb-uniprot50 | AF-A0A0C1IR17-F1-MODEL\_V4 | 1.0 | 6.938e-11 | 307 | 0.189 | 211 | 149 | 6 | 1 | 190 | 1 | 210 | Uncharacterized protein | Uncharacterized protein | | afdb-uniprot50 | AF-A0A7Z0N0J2-F1-MODEL\_V4 | 1.0 | 1.19e-11 | 306 | 0.3 | 190 | 95 | 6 | 1 | 157 | 1 | 185 | Phage tail protein I | Phage tail protein I | | afdb-uniprot50 | AF-A0A385Q1K2-F1-MODEL\_V4 | 1.0 | 1.387e-10 | 305 | 0.211 | 180 | 128 | 8 | 3 | 175 | 16 | 188 | Phage tail protein I | Phage tail protein I | | afdb-uniprot50 | AF-A0A8A8BWX0-F1-MODEL\_V4 | 1.0 | 2.097e-11 | 305 | 0.289 | 166 | 98 | 4 | 1 | 149 | 2 | 164 | Phage tail protein I | Phage tail protein I | | afdb-uniprot50 | AF-A0A0C9LZZ8-F1-MODEL\_V4 | 1.0 | 1.19e-11 | 305 | 0.266 | 184 | 110 | 6 | 5 | 179 | 1 | 168 | DNA, contig: SP610 | DNA, contig: SP610 | | afdb-uniprot50 | AF-A0A3P6KXK0-F1-MODEL\_V4 | 1.0 | 7.869e-11 | 305 | 0.234 | 213 | 141 | 9 | 1 | 202 | 9 | 210 | Phage tail protein (Tail\_P2\_I) | Phage tail protein (Tail\_P2\_I) | | afdb-uniprot50 | AF-A0A5A8F1G5-F1-MODEL\_V4 | 1.0 | 6.459e-09 | 305 | 0.301 | 146 | 94 | 4 | 11 | 156 | 22 | 159 | Phage tail protein I | Phage tail protein I | | afdb-uniprot50 | AF-R5GZX5-F1-MODEL\_V4 | 1.0 | 5.393e-11 | 304 | 0.238 | 172 | 110 | 6 | 11 | 176 | 32 | 188 | Uncharacterized protein | Uncharacterized protein | | afdb-uniprot50 | AF-A0A2V2GDM7-F1-MODEL\_V4 | 1.0 | 7.869e-11 | 304 | 0.198 | 186 | 128 | 8 | 5 | 178 | 18 | 194 | Phage tail protein I | Phage tail protein I | | afdb-uniprot50 | AF-A0A3A9G247-F1-MODEL\_V4 | 1.0 | 2.379e-11 | 304 | 0.224 | 249 | 135 | 10 | 5 | 205 | 14 | 252 | Phage tail protein I | Phage tail protein I | | afdb-uniprot50 | AF-A0A2W5JKU7-F1-MODEL\_V4 | 1.0 | 1.18e-09 | 303 | 0.304 | 138 | 88 | 3 | 48 | 182 | 2 | 134 | Phage tail protein I | Phage tail protein I | | afdb-uniprot50 | AF-A0A7X1ZFY9-F1-MODEL\_V4 | 1.0 | 5.393e-11 | 303 | 0.342 | 149 | 90 | 2 | 55 | 203 | 28 | 168 | Phage tail protein I | Phage tail protein I | | afdb-uniprot50 | AF-A0A167C5L9-F1-MODEL\_V4 | 1.0 | 2.079e-09 | 302 | 0.245 | 163 | 117 | 5 | 12 | 173 | 25 | 182 | Phage tail protein | Phage tail protein | | afdb-uniprot50 | AF-A0A2Z6GC43-F1-MODEL\_V4 | 1.0 | 2.295e-10 | 302 | 0.294 | 173 | 101 | 5 | 3 | 159 | 2 | 169 | Tail protein | Tail protein | | afdb-uniprot50 | AF-A0A4Q2KVQ7-F1-MODEL\_V4 | 1.0 | 9.851e-12 | 302 | 0.234 | 179 | 125 | 5 | 5 | 175 | 13 | 187 | Phage tail protein I | Phage tail protein I | | afdb-uniprot50 | AF-A0A7G9GXJ3-F1-MODEL\_V4 | 1.0 | 2.233e-11 | 302 | 0.208 | 211 | 146 | 12 | 3 | 205 | 2 | 199 | Phage tail protein I | Phage tail protein I | | afdb-uniprot50 | AF-A0A174UHU3-F1-MODEL\_V4 | 1.0 | 1.148e-10 | 302 | 0.211 | 227 | 141 | 9 | 5 | 202 | 15 | 232 | Bacteriophage P2-related tail formation protein | Bacteriophage P2-related tail formation protein | | afdb-uniprot50 | AF-A0A825BCX2-F1-MODEL\_V4 | 1.0 | 2.079e-09 | 301 | 0.243 | 144 | 103 | 3 | 52 | 193 | 2 | 141 | Phage tail protein I | Phage tail protein I | | afdb-uniprot50 | AF-A0A212J3U9-F1-MODEL\_V4 | 1.0 | 1.675e-10 | 301 | 0.267 | 168 | 104 | 5 | 1 | 152 | 2 | 166 | Phage tail protein I | Phage tail protein I | | afdb-uniprot50 | AF-A0A1W2CVH4-F1-MODEL\_V4 | 1.0 | 2.952e-10 | 301 | 0.137 | 232 | 171 | 7 | 2 | 205 | 6 | 236 | Uncharacterized protein | Uncharacterized protein | | afdb-uniprot50 | AF-A0A2T4JNB1-F1-MODEL\_V4 | 1.0 | 1.19e-11 | 300 | 0.32 | 184 | 105 | 5 | 1 | 167 | 2 | 182 | Phage tail protein I | Phage tail protein I | | afdb-uniprot50 | AF-A0A414Q2J0-F1-MODEL\_V4 | 1.0 | 5.542e-10 | 300 | 0.21 | 166 | 110 | 7 | 5 | 158 | 15 | 171 | Phage tail protein I | Phage tail protein I | | afdb-uniprot50 | AF-A0A1M4WE79-F1-MODEL\_V4 | 1.0 | 5.952e-12 | 299 | 0.296 | 172 | 102 | 5 | 1 | 156 | 1 | 169 | Phage tail protein, P2 protein I family | Phage tail protein, P2 protein I family | | afdb-uniprot50 | AF-A0A4Z0R3W7-F1-MODEL\_V4 | 1.0 | 3.349e-10 | 299 | 0.215 | 172 | 123 | 5 | 12 | 176 | 38 | 204 | Phage tail protein I | Phage tail protein I | | afdb-uniprot50 | AF-A0A1X7NDR7-F1-MODEL\_V4 | 1.0 | 5.393e-11 | 299 | 0.246 | 219 | 108 | 8 | 1 | 165 | 4 | 219 | Phage tail protein, P2 protein I family | Phage tail protein, P2 protein I family | | afdb-uniprot50 | AF-A0A3C1WTS9-F1-MODEL\_V4 | 1.0 | 2.444e-10 | 299 | 0.235 | 187 | 130 | 6 | 11 | 194 | 28 | 204 | Phage tail protein I | Phage tail protein I | | afdb-uniprot50 | AF-A0A853IIL6-F1-MODEL\_V4 | 1.0 | 2.581e-08 | 298 | 0.519 | 104 | 49 | 1 | 1 | 104 | 1 | 103 | Phage tail protein I | Phage tail protein I | | afdb-uniprot50 | AF-A0A510KR89-F1-MODEL\_V4 | 1.0 | 4.588e-10 | 298 | 0.238 | 180 | 117 | 6 | 12 | 184 | 33 | 199 | Uncharacterized protein | Uncharacterized protein | | afdb-uniprot50 | AF-C0QSD8-F1-MODEL\_V4 | 1.0 | 2.155e-10 | 298 | 0.153 | 196 | 159 | 6 | 12 | 205 | 29 | 219 | Uncharacterized protein | Uncharacterized protein | | afdb-uniprot50 | AF-A0A266LJW6-F1-MODEL\_V4 | 1.0 | 5.347e-09 | 297 | 0.366 | 112 | 69 | 2 | 65 | 175 | 2 | 112 | Phage tail protein I | Phage tail protein I | | afdb-uniprot50 | AF-A0A0N8IBW6-F1-MODEL\_V4 | 1.0 | 6.065e-09 | 297 | 0.225 | 164 | 108 | 4 | 4 | 151 | 7 | 167 | Uncharacterized protein | Uncharacterized protein | | afdb-uniprot50 | AF-A0A2G6EZC5-F1-MODEL\_V4 | 1.0 | 1.518e-09 | 297 | 0.194 | 190 | 138 | 6 | 3 | 182 | 15 | 199 | Phage tail protein | Phage tail protein | | afdb-uniprot50 | AF-A0A2A2IJZ7-F1-MODEL\_V4 | 1.0 | 5.064e-11 | 297 | 0.234 | 205 | 146 | 7 | 5 | 202 | 13 | 213 | Phage tail protein I | Phage tail protein I | | afdb-uniprot50 | AF-A0A139D6J5-F1-MODEL\_V4 | 1.0 | 1.477e-10 | 297 | 0.207 | 212 | 149 | 6 | 5 | 205 | 14 | 217 | Uncharacterized protein | Uncharacterized protein | | afdb-uniprot50 | AF-L8MFE9-F1-MODEL\_V4 | 1.0 | 5.743e-11 | 296 | 0.289 | 166 | 94 | 5 | 1 | 147 | 1 | 161 | Putative TAIL-RELATED PROTEIN (TAIL FORMATION-LIKE PROTEIN) | Putative TAIL-RELATED PROTEIN (TAIL FORMATION-LIKE PROTEIN) | | afdb-uniprot50 | AF-D1ALM0-F1-MODEL\_V4 | 1.0 | 4.755e-11 | 296 | 0.241 | 215 | 135 | 8 | 3 | 202 | 12 | 213 | Uncharacterized protein | Uncharacterized protein | | afdb-uniprot50 | AF-A0A4Q3YY71-F1-MODEL\_V4 | 1.0 | 1.969e-11 | 295 | 0.252 | 198 | 120 | 8 | 2 | 178 | 6 | 196 | Phage tail protein I | Phage tail protein I | | afdb-uniprot50 | AF-A0A4U7J706-F1-MODEL\_V4 | 1.0 | 2.024e-10 | 294 | 0.22 | 186 | 121 | 11 | 5 | 177 | 13 | 187 | Phage tail protein I | Phage tail protein I | | afdb-uniprot50 | AF-A0A2N2S1K8-F1-MODEL\_V4 | 1.0 | 2.379e-11 | 294 | 0.309 | 165 | 94 | 6 | 2 | 149 | 4 | 165 | Phage tail protein I | Phage tail protein I | | afdb-uniprot50 | AF-A0A447IP21-F1-MODEL\_V4 | 1.0 | 1.078e-10 | 294 | 0.241 | 232 | 112 | 6 | 1 | 171 | 1 | 229 | Phage tail protein (Tail\_P2\_I) | Phage tail protein (Tail\_P2\_I) | | afdb-uniprot50 | AF-A0A1Y4NK51-F1-MODEL\_V4 | 1.0 | 1.675e-10 | 294 | 0.208 | 216 | 148 | 7 | 3 | 205 | 26 | 231 | Phage tail protein I | Phage tail protein I | | afdb-uniprot50 | AF-A0A6L3Y496-F1-MODEL\_V4 | 1.0 | 2.927e-08 | 293 | 0.33 | 100 | 64 | 2 | 78 | 175 | 1 | 99 | Phage tail protein I | Phage tail protein I | | afdb-uniprot50 | AF-A0A064AK86-F1-MODEL\_V4 | 1.0 | 1.518e-09 | 293 | 0.177 | 175 | 133 | 5 | 35 | 202 | 5 | 175 | Tail protein | Tail protein | | afdb-uniprot50 | AF-A0A2S0JKT1-F1-MODEL\_V4 | 1.0 | 4.886e-10 | 293 | 0.191 | 225 | 150 | 9 | 5 | 202 | 13 | 232 | Phage tail protein I | Phage tail protein I | | afdb-uniprot50 | AF-A0A165PIY6-F1-MODEL\_V4 | 1.0 | 7.593e-10 | 293 | 0.199 | 226 | 159 | 12 | 1 | 205 | 1 | 225 | Phage tail protein | Phage tail protein | | afdb-uniprot50 | AF-U2PI12-F1-MODEL\_V4 | 1.0 | 1.833e-09 | 292 | 0.196 | 168 | 115 | 4 | 12 | 176 | 37 | 187 | Phage tail protein | Phage tail protein | | afdb-uniprot50 | AF-D9R641-F1-MODEL\_V4 | 1.0 | 1.425e-09 | 290 | 0.165 | 175 | 125 | 7 | 1 | 163 | 9 | 174 | Uncharacterized protein | Uncharacterized protein | | afdb-uniprot50 | AF-A0A1T4QDN6-F1-MODEL\_V4 | 1.0 | 7.389e-11 | 289 | 0.217 | 184 | 127 | 8 | 4 | 178 | 9 | 184 | Phage tail protein, P2 protein I family | Phage tail protein, P2 protein I family | | afdb-uniprot50 | AF-A0A2D3WQP3-F1-MODEL\_V4 | 1.0 | 7.129e-10 | 289 | 0.194 | 190 | 112 | 5 | 1 | 152 | 5 | 191 | Phage tail protein I | Phage tail protein I | | afdb-uniprot50 | AF-A0A7T5EN83-F1-MODEL\_V4 | 1.0 | 1.148e-10 | 288 | 0.231 | 186 | 123 | 7 | 3 | 177 | 11 | 187 | Phage tail protein I | Phage tail protein I | | afdb-uniprot50 | AF-A0A6C1BSQ9-F1-MODEL\_V4 | 1.0 | 1.387e-10 | 288 | 0.235 | 217 | 139 | 8 | 4 | 205 | 5 | 209 | Phage tail protein I | Phage tail protein I | | afdb-uniprot50 | AF-A0A329TXY5-F1-MODEL\_V4 | 1.0 | 5.021e-09 | 288 | 0.217 | 161 | 114 | 7 | 5 | 157 | 13 | 169 | Phage tail protein I | Phage tail protein I | | afdb-uniprot50 | AF-A0A316Q9A4-F1-MODEL\_V4 | 1.0 | 4.755e-11 | 287 | 0.235 | 212 | 142 | 9 | 5 | 205 | 12 | 214 | Phage tail protein I | Phage tail protein I | | afdb-uniprot50 | AF-A0A7C5ZID1-F1-MODEL\_V4 | 1.0 | 3.665e-09 | 287 | 0.203 | 157 | 113 | 7 | 12 | 164 | 175 | 323 | Phage tail protein I | Phage tail protein I | | afdb-uniprot50 | AF-A0A318EJ45-F1-MODEL\_V4 | 1.0 | 2.952e-10 | 286 | 0.191 | 188 | 134 | 8 | 3 | 180 | 11 | 190 | Tail protein P2 I | Tail protein P2 I | | afdb-uniprot50 | AF-A0A2S6N2X4-F1-MODEL\_V4 | 1.0 | 6.938e-11 | 286 | 0.247 | 210 | 144 | 10 | 1 | 205 | 7 | 207 | Uncharacterized protein | Uncharacterized protein | | afdb-uniprot50 | AF-A0A416B7R3-F1-MODEL\_V4 | 1.0 | 5.204e-10 | 286 | 0.215 | 204 | 147 | 6 | 5 | 201 | 13 | 210 | Uncharacterized protein | Uncharacterized protein | | afdb-uniprot50 | AF-A0A7M1L784-F1-MODEL\_V4 | 1.0 | 2.512e-09 | 285 | 0.272 | 154 | 101 | 6 | 56 | 203 | 1 | 149 | Uncharacterized protein | Uncharacterized protein | | afdb-uniprot50 | AF-A0A7U9RV01-F1-MODEL\_V4 | 1.0 | 1.784e-10 | 285 | 0.193 | 207 | 146 | 9 | 4 | 199 | 13 | 209 | Uncharacterized protein | Uncharacterized protein | | afdb-uniprot50 | AF-A8SB35-F1-MODEL\_V4 | 1.0 | 1.338e-09 | 284 | 0.201 | 208 | 145 | 9 | 3 | 197 | 16 | 215 | Uncharacterized protein | Uncharacterized protein | | afdb-uniprot50 | AF-A0A7X4ZQB7-F1-MODEL\_V4 | 1.0 | 4.886e-10 | 284 | 0.206 | 218 | 152 | 7 | 3 | 202 | 11 | 225 | Uncharacterized protein | Uncharacterized protein | | afdb-uniprot50 | AF-A0A1M6LS85-F1-MODEL\_V4 | 1.0 | 1.148e-10 | 284 | 0.213 | 229 | 141 | 9 | 5 | 203 | 14 | 233 | Phage tail protein, P2 protein I family | Phage tail protein, P2 protein I family | | afdb-uniprot50 | AF-A0A836NZX7-F1-MODEL\_V4 | 1.0 | 1.936e-07 | 283 | 0.465 | 101 | 54 | 0 | 1 | 101 | 2 | 102 | Tail protein | Tail protein | | afdb-uniprot50 | AF-A0A1V4GEU5-F1-MODEL\_V4 | 1.0 | 4.045e-10 | 283 | 0.218 | 183 | 125 | 8 | 4 | 175 | 13 | 188 | Uncharacterized protein | Uncharacterized protein | | afdb-uniprot50 | AF-A0A0Q2XP81-F1-MODEL\_V4 | 1.0 | 2.512e-09 | 283 | 0.196 | 168 | 113 | 6 | 4 | 157 | 13 | 172 | Uncharacterized protein | Uncharacterized protein | | afdb-uniprot50 | AF-A0A373LAF9-F1-MODEL\_V4 | 1.0 | 9.767e-10 | 283 | 0.226 | 212 | 142 | 8 | 4 | 202 | 14 | 216 | Phage tail protein I | Phage tail protein I | | afdb-uniprot50 | AF-A0A316MKT1-F1-MODEL\_V4 | 1.0 | 1.256e-09 | 282 | 0.207 | 231 | 152 | 6 | 4 | 205 | 13 | 241 | Phage tail protein I | Phage tail protein I | | afdb-uniprot50 | AF-A0A3A6JUC8-F1-MODEL\_V4 | 1.0 | 1.108e-09 | 282 | 0.158 | 196 | 159 | 3 | 11 | 205 | 28 | 218 | Phage tail protein I | Phage tail protein I | | afdb-uniprot50 | AF-A0A7X3ZHW1-F1-MODEL\_V4 | 1.0 | 3.903e-09 | 281 | 0.267 | 161 | 105 | 5 | 2 | 155 | 190 | 344 | Phage tail protein I | Phage tail protein I | | afdb-uniprot50 | AF-A0A329MGA2-F1-MODEL\_V4 | 1.0 | 1.108e-09 | 280 | 0.215 | 246 | 144 | 10 | 3 | 205 | 40 | 279 | Uncharacterized protein | Uncharacterized protein | | afdb-uniprot50 | AF-A0A1Y4BCC7-F1-MODEL\_V4 | 1.0 | 2.772e-10 | 279 | 0.239 | 192 | 116 | 11 | 4 | 180 | 11 | 187 | Phage tail protein I | Phage tail protein I | | afdb-uniprot50 | AF-A0A377AJR9-F1-MODEL\_V4 | 1.0 | 2.137e-08 | 278 | 0.495 | 103 | 52 | 0 | 1 | 103 | 2 | 104 | Putative tail protein I (Gpi) | Putative tail protein I (Gpi) | | afdb-uniprot50 | AF-A0A3D2IPZ0-F1-MODEL\_V4 | 1.0 | 2.849e-09 | 278 | 0.199 | 201 | 141 | 8 | 5 | 198 | 18 | 205 | Uncharacterized protein | Uncharacterized protein | | afdb-uniprot50 | AF-A0A7U5GZ11-F1-MODEL\_V4 | 1.0 | 1.223e-10 | 278 | 0.236 | 211 | 147 | 7 | 4 | 205 | 12 | 217 | Phage tail protein I | Phage tail protein I | | afdb-uniprot50 | AF-A0A842J0C5-F1-MODEL\_V4 | 1.0 | 3.903e-09 | 276 | 0.245 | 159 | 106 | 7 | 12 | 167 | 28 | 175 | Uncharacterized protein | Uncharacterized protein | | afdb-uniprot50 | AF-C3WBC4-F1-MODEL\_V4 | 1.0 | 7.593e-10 | 276 | 0.267 | 157 | 102 | 5 | 5 | 153 | 15 | 166 | Phage tail protein | Phage tail protein | | afdb-uniprot50 | AF-A0A8B3RDD9-F1-MODEL\_V4 | 1.0 | 2.215e-09 | 276 | 0.223 | 179 | 108 | 6 | 4 | 157 | 6 | 178 | Phage tail protein I | Phage tail protein I | | afdb-uniprot50 | AF-A0A3A9CSI0-F1-MODEL\_V4 | 1.0 | 6.065e-09 | 276 | 0.182 | 175 | 124 | 6 | 3 | 167 | 18 | 183 | Uncharacterized protein | Uncharacterized protein | | afdb-uniprot50 | AF-B0PEZ8-F1-MODEL\_V4 | 1.0 | 9.767e-10 | 276 | 0.204 | 205 | 151 | 6 | 4 | 201 | 11 | 210 | Phage tail protein | Phage tail protein | | afdb-uniprot50 | AF-A0A7U9MZ97-F1-MODEL\_V4 | 1.0 | 1.18e-09 | 276 | 0.213 | 211 | 150 | 8 | 4 | 205 | 11 | 214 | Uncharacterized protein | Uncharacterized protein | | afdb-uniprot50 | AF-A0A348AJ17-F1-MODEL\_V4 | 1.0 | 7.869e-11 | 276 | 0.274 | 226 | 132 | 10 | 3 | 205 | 12 | 228 | Phage tail protein | Phage tail protein | | afdb-uniprot50 | AF-A0A3S4GL93-F1-MODEL\_V4 | 1.0 | 2.424e-08 | 275 | 0.504 | 101 | 50 | 0 | 4 | 104 | 18 | 118 | Bacteriophage P2-related tail formation protein | Bacteriophage P2-related tail formation protein | | afdb-uniprot50 | AF-A0A3A8WB53-F1-MODEL\_V4 | 1.0 | 7.593e-10 | 275 | 0.21 | 219 | 148 | 10 | 5 | 205 | 13 | 224 | Phage tail protein I | Phage tail protein I | | afdb-uniprot50 | AF-A0A285NFM9-F1-MODEL\_V4 | 1.0 | 2.772e-10 | 274 | 0.227 | 211 | 146 | 8 | 6 | 205 | 16 | 220 | Phage tail protein (Tail\_P2\_I) | Phage tail protein (Tail\_P2\_I) | | afdb-uniprot50 | AF-A0A1Q6KUF2-F1-MODEL\_V4 | 1.0 | 1.338e-09 | 274 | 0.183 | 223 | 152 | 11 | 4 | 205 | 13 | 226 | Uncharacterized protein | Uncharacterized protein | | afdb-uniprot50 | AF-A0A5S4VLN5-F1-MODEL\_V4 | 1.0 | 1.338e-09 | 273 | 0.216 | 217 | 151 | 8 | 3 | 205 | 11 | 222 | Uncharacterized protein | Uncharacterized protein | | afdb-uniprot50 | AF-R5BTZ8-F1-MODEL\_V4 | 1.0 | 1.138e-08 | 272 | 0.245 | 171 | 117 | 8 | 3 | 165 | 11 | 177 | Uncharacterized protein | Uncharacterized protein | | afdb-uniprot50 | AF-A0A078MHK2-F1-MODEL\_V4 | 1.0 | 2.079e-09 | 272 | 0.176 | 232 | 150 | 8 | 3 | 198 | 11 | 237 | Phage tail protein (Tail\_P2\_I) | Phage tail protein (Tail\_P2\_I) | | afdb-uniprot50 | AF-A0A3E2TEK9-F1-MODEL\_V4 | 1.0 | 2.952e-10 | 272 | 0.196 | 204 | 139 | 6 | 12 | 205 | 36 | 224 | Uncharacterized protein | Uncharacterized protein | | afdb-uniprot50 | AF-A0A3D5LSM9-F1-MODEL\_V4 | 1.0 | 7.593e-10 | 271 | 0.206 | 233 | 140 | 8 | 3 | 199 | 11 | 234 | Uncharacterized protein | Uncharacterized protein | | afdb-uniprot50 | AF-A0A416UB11-F1-MODEL\_V4 | 1.0 | 1.721e-09 | 271 | 0.153 | 247 | 157 | 10 | 1 | 202 | 9 | 248 | Phage tail protein | Phage tail protein | | afdb-uniprot50 | AF-A0A7T7D8U4-F1-MODEL\_V4 | 1.0 | 2.849e-09 | 271 | 0.257 | 214 | 136 | 10 | 3 | 200 | 390 | 596 | Phage tail protein I | Phage tail protein I | | afdb-uniprot50 | AF-A0A346QYG0-F1-MODEL\_V4 | 1.0 | 4.845e-08 | 270 | 0.45 | 100 | 54 | 1 | 2 | 100 | 19 | 118 | Phage tail protein I | Phage tail protein I | | afdb-uniprot50 | AF-A0A6L9HFG1-F1-MODEL\_V4 | 1.0 | 8.612e-10 | 269 | 0.175 | 234 | 153 | 10 | 3 | 205 | 21 | 245 | Phage tail protein I | Phage tail protein I | | afdb-uniprot50 | AF-A0A7V8HY86-F1-MODEL\_V4 | 1.0 | 3.231e-09 | 268 | 0.434 | 115 | 65 | 0 | 1 | 115 | 5 | 119 | Phage tail protein I | Phage tail protein I | | afdb-uniprot50 | AF-D4K5G8-F1-MODEL\_V4 | 1.0 | 5.204e-10 | 268 | 0.22 | 181 | 122 | 8 | 3 | 171 | 11 | 184 | Phage tail protein (Tail\_P2\_I) | Phage tail protein (Tail\_P2\_I) | | afdb-uniprot50 | AF-A0A1G5JKM4-F1-MODEL\_V4 | 1.0 | 3.441e-09 | 266 | 0.164 | 164 | 127 | 6 | 12 | 173 | 30 | 185 | Phage tail protein (Tail\_P2\_I) | Phage tail protein (Tail\_P2\_I) | | afdb-uniprot50 | AF-A0A1H8VRL6-F1-MODEL\_V4 | 1.0 | 1.04e-09 | 266 | 0.18 | 238 | 149 | 11 | 3 | 205 | 11 | 237 | Phage tail protein, P2 protein I family | Phage tail protein, P2 protein I family | | afdb-uniprot50 | AF-A0A7X4Z3Q6-F1-MODEL\_V4 | 1.0 | 7.326e-09 | 266 | 0.224 | 187 | 118 | 10 | 5 | 169 | 18 | 199 | Uncharacterized protein | Uncharacterized protein | | afdb-uniprot50 | AF-A0A812RFY7-F1-MODEL\_V4 | 1.0 | 1.477e-10 | 266 | 0.317 | 164 | 97 | 4 | 1 | 150 | 266 | 428 | J protein | J protein | | afdb-uniprot50 | AF-A0A8B4XHD1-F1-MODEL\_V4 | 1.0 | 6.404e-07 | 265 | 0.44 | 93 | 52 | 0 | 1 | 93 | 1 | 93 | Phage tail P2-like protein | Phage tail P2-like protein | | afdb-uniprot50 | AF-A0A6N3FBW1-F1-MODEL\_V4 | 1.0 | 3.441e-09 | 265 | 0.197 | 192 | 128 | 10 | 4 | 184 | 14 | 190 | Phage tail protein (Tail\_P2\_I) | Phage tail protein (Tail\_P2\_I) | | afdb-uniprot50 | AF-A0A6N9P2M1-F1-MODEL\_V4 | 1.0 | 2.358e-09 | 264 | 0.201 | 238 | 151 | 9 | 1 | 205 | 18 | 249 | Uncharacterized protein | Uncharacterized protein | | afdb-uniprot50 | AF-A0A1C6IIK7-F1-MODEL\_V4 | 1.0 | 2.952e-10 | 264 | 0.191 | 225 | 150 | 10 | 5 | 205 | 14 | 230 | Bacteriophage P2-related tail formation protein | Bacteriophage P2-related tail formation protein | | afdb-uniprot50 | AF-A0A2E4F7R5-F1-MODEL\_V4 | 1.0 | 1.518e-09 | 264 | 0.152 | 249 | 163 | 10 | 3 | 205 | 79 | 325 | PASTA domain-containing protein | PASTA domain-containing protein | | afdb-uniprot50 | AF-A0A7S7QIG1-F1-MODEL\_V4 | 1.0 | 6.232e-08 | 264 | 0.376 | 101 | 62 | 1 | 1 | 100 | 1 | 101 | Phage tail protein I | Phage tail protein I | | afdb-uniprot50 | AF-A0A1Y3S126-F1-MODEL\_V4 | 1.0 | 4.588e-10 | 263 | 0.161 | 254 | 152 | 10 | 3 | 205 | 17 | 260 | Phage tail protein I | Phage tail protein I | | afdb-uniprot50 | AF-A0A238JEV2-F1-MODEL\_V4 | 1.0 | 6.637e-08 | 262 | 0.242 | 128 | 92 | 4 | 1 | 124 | 7 | 133 | Phage tail protein (Tail\_P2\_I) | Phage tail protein (Tail\_P2\_I) | | afdb-uniprot50 | AF-A0A3A8UCJ4-F1-MODEL\_V4 | 1.0 | 3.231e-09 | 261 | 0.221 | 212 | 144 | 9 | 5 | 200 | 13 | 219 | Uncharacterized protein | Uncharacterized protein | | afdb-uniprot50 | AF-A0A2N8HQL6-F1-MODEL\_V4 | 1.0 | 5.695e-09 | 260 | 0.178 | 213 | 156 | 7 | 4 | 205 | 11 | 215 | Phage tail protein I | Phage tail protein I | | afdb-uniprot50 | AF-A0A7C8LUT2-F1-MODEL\_V4 | 1.0 | 3.231e-09 | 259 | 0.155 | 199 | 163 | 4 | 4 | 200 | 19 | 214 | Uncharacterized protein | Uncharacterized protein | | afdb-uniprot50 | AF-A0A238WY20-F1-MODEL\_V4 | 1.0 | 4.427e-09 | 259 | 0.155 | 244 | 162 | 7 | 2 | 205 | 659 | 898 | Phage tail protein domain-containing protein | Phage tail protein domain-containing protein | | afdb-uniprot50 | AF-A0A4P9VKB1-F1-MODEL\_V4 | 1.0 | 1.327e-07 | 258 | 0.408 | 93 | 55 | 0 | 1 | 93 | 2 | 94 | Phage tail protein I | Phage tail protein I | | afdb-uniprot50 | AF-A0A1W9UFI2-F1-MODEL\_V4 | 1.0 | 1.108e-09 | 258 | 0.195 | 230 | 143 | 10 | 12 | 205 | 28 | 251 | Uncharacterized protein | Uncharacterized protein | | afdb-uniprot50 | AF-A0A1H0BY95-F1-MODEL\_V4 | 1.0 | 1.464e-08 | 258 | 0.181 | 243 | 145 | 7 | 1 | 197 | 1 | 235 | Phage tail protein (Tail\_P2\_I) | Phage tail protein (Tail\_P2\_I) | | afdb-uniprot50 | AF-A0A1S1TLC3-F1-MODEL\_V4 | 1.0 | 1.603e-07 | 258 | 0.333 | 102 | 66 | 2 | 1 | 100 | 4 | 105 | Phage tail protein I | Phage tail protein I | | afdb-uniprot50 | AF-A8GLQ8-F1-MODEL\_V4 | 1.0 | 8.612e-10 | 255 | 0.28 | 171 | 103 | 5 | 16 | 182 | 4 | 158 | Phage tail protein I | Phage tail protein I | | afdb-uniprot50 | AF-A0A833LKH9-F1-MODEL\_V4 | 1.0 | 2.652e-07 | 254 | 0.32 | 100 | 67 | 1 | 1 | 99 | 2 | 101 | Phage tail protein I | Phage tail protein I | | afdb-uniprot50 | AF-A0A3L7AJI5-F1-MODEL\_V4 | 1.0 | 4.389e-07 | 254 | 0.303 | 99 | 67 | 2 | 4 | 100 | 6 | 104 | Phage tail protein I | Phage tail protein I | | afdb-uniprot50 | AF-A0A826R5C0-F1-MODEL\_V4 | 1.0 | 1.098e-07 | 252 | 0.417 | 103 | 58 | 1 | 1 | 101 | 1 | 103 | Phage tail protein I | Phage tail protein I | | afdb-uniprot50 | AF-A0A7X2NLM7-F1-MODEL\_V4 | 1.0 | 1.769e-08 | 252 | 0.153 | 248 | 161 | 12 | 1 | 202 | 9 | 253 | Phage tail protein | Phage tail protein | | afdb-uniprot50 | AF-A0A4S5JBM7-F1-MODEL\_V4 | 1.0 | 1.413e-07 | 252 | 0.346 | 101 | 65 | 1 | 1 | 100 | 3 | 103 | Phage tail protein I | Phage tail protein I | | afdb-uniprot50 | AF-A0A417IXT6-F1-MODEL\_V4 | 1.0 | 3.766e-08 | 251 | 0.202 | 158 | 110 | 4 | 4 | 152 | 13 | 163 | Uncharacterized protein | Uncharacterized protein | | afdb-uniprot50 | AF-A0A2G6EHZ3-F1-MODEL\_V4 | 1.0 | 2.952e-10 | 251 | 0.228 | 171 | 109 | 4 | 4 | 155 | 28 | 194 | Phage tail protein I | Phage tail protein I | | afdb-uniprot50 | AF-A0A6N2Z557-F1-MODEL\_V4 | 1.0 | 6.694e-10 | 251 | 0.251 | 207 | 137 | 9 | 3 | 200 | 11 | 208 | Phage tail protein (Tail\_P2\_I) | Phage tail protein (Tail\_P2\_I) | | afdb-uniprot50 | AF-A7MW91-F1-MODEL\_V4 | 1.0 | 8.539e-08 | 250 | 0.193 | 160 | 120 | 7 | 53 | 205 | 2 | 159 | Uncharacterized protein | Uncharacterized protein | | afdb-uniprot50 | AF-A0A6D0ZU35-F1-MODEL\_V4 | 1.0 | 3.118e-08 | 249 | 0.253 | 146 | 95 | 5 | 66 | 201 | 3 | 144 | Phage tail protein I | Phage tail protein I | | afdb-uniprot50 | AF-A0A2P7V3T2-F1-MODEL\_V4 | 1.0 | 3.903e-09 | 249 | 0.243 | 218 | 129 | 13 | 3 | 199 | 11 | 213 | Phage tail protein I | Phage tail protein I | | afdb-uniprot50 | AF-A0A7G9WG79-F1-MODEL\_V4 | 1.0 | 1.56e-08 | 249 | 0.183 | 202 | 151 | 6 | 4 | 196 | 14 | 210 | Phage tail protein I | Phage tail protein I | | afdb-uniprot50 | AF-A0A367FXP5-F1-MODEL\_V4 | 1.0 | 1.212e-08 | 249 | 0.188 | 260 | 149 | 9 | 3 | 205 | 11 | 265 | Uncharacterized protein | Uncharacterized protein | | afdb-uniprot50 | AF-I0GRI5-F1-MODEL\_V4 | 1.0 | 2.603e-10 | 248 | 0.22 | 213 | 145 | 7 | 3 | 203 | 11 | 214 | Uncharacterized protein | Uncharacterized protein | | afdb-uniprot50 | AF-A0A1Y4BD08-F1-MODEL\_V4 | 1.0 | 3.665e-09 | 248 | 0.2 | 229 | 145 | 10 | 4 | 203 | 14 | 233 | Phage tail protein I | Phage tail protein I | | afdb-uniprot50 | AF-A0A2U9T741-F1-MODEL\_V4 | 1.0 | 2.079e-09 | 247 | 0.19 | 210 | 136 | 11 | 16 | 205 | 61 | 256 | Phage tail protein | Phage tail protein | | afdb-uniprot50 | AF-A0A4Z1QZ30-F1-MODEL\_V4 | 1.0 | 7.736e-07 | 246 | 0.306 | 101 | 68 | 2 | 1 | 99 | 17 | 117 | Phage tail protein I | Phage tail protein I | | afdb-uniprot50 | AF-A0A5C8BFD6-F1-MODEL\_V4 | 1.0 | 1.769e-08 | 244 | 0.184 | 211 | 155 | 7 | 4 | 202 | 12 | 217 | Uncharacterized protein | Uncharacterized protein | | afdb-uniprot50 | AF-R9KX12-F1-MODEL\_V4 | 1.0 | 3.903e-09 | 244 | 0.158 | 221 | 144 | 9 | 11 | 204 | 34 | 239 | Phage tail protein I | Phage tail protein I | | afdb-uniprot50 | AF-A0A1C6DZY7-F1-MODEL\_V4 | 1.0 | 7.528e-08 | 241 | 0.191 | 178 | 124 | 7 | 5 | 168 | 14 | 185 | Bacteriophage P2-related tail formation protein | Bacteriophage P2-related tail formation protein | | afdb-uniprot50 | AF-A0A1G6EJP1-F1-MODEL\_V4 | 1.0 | 1.505e-07 | 241 | 0.366 | 112 | 65 | 2 | 1 | 111 | 3 | 109 | Phage tail protein, P2 protein I family | Phage tail protein, P2 protein I family | | afdb-uniprot50 | AF-Q72D30-F1-MODEL\_V4 | 1.0 | 3.118e-08 | 238 | 0.15 | 206 | 163 | 7 | 5 | 205 | 19 | 217 | Uncharacterized protein | Uncharacterized protein | | afdb-uniprot50 | AF-A0A8B2NSS2-F1-MODEL\_V4 | 1.0 | 6.404e-07 | 237 | 0.373 | 99 | 61 | 1 | 3 | 100 | 2 | 100 | Phage tail protein I | Phage tail protein I | | afdb-uniprot50 | AF-A0A7C3GTL1-F1-MODEL\_V4 | 1.0 | 1.661e-08 | 237 | 0.15 | 233 | 153 | 9 | 12 | 205 | 471 | 697 | Uncharacterized protein | Uncharacterized protein | | afdb-uniprot50 | AF-G4KPI3-F1-MODEL\_V4 | 1.0 | 3.903e-09 | 236 | 0.206 | 184 | 134 | 8 | 3 | 179 | 16 | 194 | Uncharacterized protein | Uncharacterized protein | | afdb-uniprot50 | AF-A0A2P1S8D0-F1-MODEL\_V4 | 1.0 | 6.694e-10 | 236 | 0.227 | 211 | 139 | 9 | 3 | 198 | 11 | 212 | Phage tail protein I | Phage tail protein I | | afdb-uniprot50 | AF-A0A1Q6PXA8-F1-MODEL\_V4 | 1.0 | 2.358e-09 | 236 | 0.184 | 211 | 155 | 8 | 5 | 202 | 13 | 219 | Phage tail protein I | Phage tail protein I | | afdb-uniprot50 | AF-A0A0N0N4N5-F1-MODEL\_V4 | 1.0 | 1.661e-08 | 236 | 0.175 | 200 | 152 | 7 | 15 | 205 | 34 | 229 | Phage tail protein | Phage tail protein | | afdb-uniprot50 | AF-R9LUW9-F1-MODEL\_V4 | 1.0 | 1.291e-08 | 236 | 0.202 | 212 | 141 | 9 | 12 | 205 | 29 | 230 | Uncharacterized protein | Uncharacterized protein | | afdb-uniprot50 | AF-A0A5C8S6W3-F1-MODEL\_V4 | 1.0 | 4.121e-07 | 235 | 0.362 | 102 | 63 | 2 | 1 | 100 | 8 | 109 | Phage tail protein I | Phage tail protein I | | afdb-uniprot50 | AF-A0A2K8MAJ7-F1-MODEL\_V4 | 1.0 | 9.685e-08 | 234 | 0.154 | 240 | 165 | 8 | 2 | 205 | 658 | 895 | Uncharacterized protein | Uncharacterized protein | | afdb-uniprot50 | AF-R6TSD6-F1-MODEL\_V4 | 1.0 | 2.581e-08 | 233 | 0.181 | 231 | 150 | 11 | 4 | 205 | 14 | 234 | Uncharacterized protein | Uncharacterized protein | | afdb-uniprot50 | AF-H1CIQ0-F1-MODEL\_V4 | 1.0 | 9.424e-09 | 232 | 0.179 | 229 | 149 | 9 | 3 | 200 | 12 | 232 | Phage tail protein I | Phage tail protein I | | afdb-uniprot50 | AF-A0A2T4JG36-F1-MODEL\_V4 | 1.0 | 1.989e-06 | 232 | 0.43 | 93 | 53 | 0 | 1 | 93 | 3 | 95 | Phage tail protein I | Phage tail protein I | | afdb-uniprot50 | AF-A0A6F9X0W8-F1-MODEL\_V4 | 1.0 | 3.118e-08 | 226 | 0.177 | 192 | 143 | 9 | 5 | 190 | 19 | 201 | Uncharacterized protein | Uncharacterized protein | | afdb-uniprot50 | AF-A0A502CNF8-F1-MODEL\_V4 | 1.0 | 1.546e-06 | 226 | 0.353 | 99 | 62 | 2 | 4 | 100 | 3 | 101 | Phage tail protein I | Phage tail protein I | | afdb-uniprot50 | AF-A0A497FKG3-F1-MODEL\_V4 | 1.0 | 3.87e-07 | 221 | 0.193 | 150 | 108 | 4 | 44 | 182 | 3 | 150 | Phage tail protein I | Phage tail protein I | | afdb-uniprot50 | AF-Q7NKR6-F1-MODEL\_V4 | 1.0 | 4.011e-08 | 221 | 0.157 | 228 | 147 | 11 | 12 | 205 | 183 | 399 | Gll1411 protein | Gll1411 protein | | afdb-uniprot50 | AF-A0A7J9Y123-F1-MODEL\_V4 | 1.0 | 1.17e-07 | 220 | 0.137 | 218 | 152 | 8 | 11 | 203 | 49 | 255 | Phage tail protein | Phage tail protein | | afdb-uniprot50 | AF-A0A854ZEC9-F1-MODEL\_V4 | 1.0 | 1.818e-07 | 220 | 0.172 | 185 | 138 | 8 | 12 | 189 | 493 | 669 | Uncharacterized protein | Uncharacterized protein | | afdb-uniprot50 | AF-A0A1D2QMU0-F1-MODEL\_V4 | 1.0 | 1.246e-07 | 219 | 0.284 | 151 | 85 | 6 | 4 | 136 | 6 | 151 | Phage tail protein I | Phage tail protein I | | afdb-uniprot50 | AF-A0A4U8S340-F1-MODEL\_V4 | 1.0 | 4.549e-08 | 218 | 0.175 | 205 | 151 | 8 | 4 | 205 | 3 | 192 | Uncharacterized protein | Uncharacterized protein | | afdb-uniprot50 | AF-A0A6G5QFK7-F1-MODEL\_V4 | 1.0 | 4.157e-09 | 218 | 0.192 | 208 | 155 | 7 | 1 | 205 | 1 | 198 | Phage P2 family tail protein | Phage P2 family tail protein | | afdb-uniprot50 | AF-A0A5W6Y441-F1-MODEL\_V4 | 1.0 | 2.825e-07 | 215 | 0.277 | 126 | 86 | 3 | 80 | 203 | 1 | 123 | Phage tail protein I | Phage tail protein I | | afdb-uniprot50 | AF-S5MV89-F1-MODEL\_V4 | 1.0 | 7.528e-08 | 215 | 0.319 | 122 | 81 | 1 | 55 | 176 | 17 | 136 | Phage tail fiber | Phage tail fiber | | afdb-uniprot50 | AF-A0A4D7B3A9-F1-MODEL\_V4 | 1.0 | 5.852e-08 | 215 | 0.371 | 132 | 69 | 3 | 4 | 134 | 11 | 129 | Phage tail protein I | Phage tail protein I | | afdb-uniprot50 | AF-A0A6P0Y575-F1-MODEL\_V4 | 1.0 | 2.581e-08 | 214 | 0.152 | 197 | 137 | 9 | 1 | 184 | 52 | 231 | Phage tail protein | Phage tail protein | | afdb-uniprot50 | AF-A0A6N8BB33-F1-MODEL\_V4 | 1.0 | 4.675e-07 | 214 | 0.127 | 212 | 155 | 4 | 12 | 194 | 29 | 239 | Uncharacterized protein | Uncharacterized protein | | afdb-uniprot50 | AF-A0A4Q6CU04-F1-MODEL\_V4 | 1.0 | 3.008e-07 | 213 | 0.285 | 133 | 94 | 1 | 1 | 133 | 6 | 137 | Phage tail protein I | Phage tail protein I | | afdb-uniprot50 | AF-A0A1I1V086-F1-MODEL\_V4 | 1.0 | 4.804e-06 | 213 | 0.297 | 101 | 69 | 2 | 2 | 100 | 4 | 104 | Phage tail protein, P2 protein I family | Phage tail protein, P2 protein I family | | afdb-uniprot50 | AF-A0A2W5WSP6-F1-MODEL\_V4 | 1.0 | 5.116e-06 | 211 | 0.326 | 104 | 66 | 3 | 1 | 100 | 1 | 104 | Phage tail protein I | Phage tail protein I | | afdb-uniprot50 | AF-A0A4R7I2Z4-F1-MODEL\_V4 | 1.0 | 1.413e-07 | 207 | 0.173 | 219 | 157 | 10 | 5 | 205 | 20 | 232 | Phage tail-like protein | Phage tail-like protein | | afdb-uniprot50 | AF-A0A1X3J0Q7-F1-MODEL\_V4 | 1.0 | 1.754e-06 | 206 | 0.454 | 88 | 46 | 1 | 88 | 175 | 4 | 89 | Tail protein I (GpI) | Tail protein I (GpI) | | afdb-uniprot50 | AF-A0A1T2XBJ5-F1-MODEL\_V4 | 1.0 | 6.014e-07 | 206 | 0.217 | 147 | 111 | 3 | 54 | 199 | 3 | 146 | Phage P2 family tail protein | Phage P2 family tail protein | | afdb-uniprot50 | AF-A0A827B048-F1-MODEL\_V4 | 1.0 | 1.28e-06 | 205 | 0.454 | 88 | 46 | 1 | 88 | 175 | 2 | 87 | Phage tail protein I | Phage tail protein I | | afdb-uniprot50 | AF-U5QMV0-F1-MODEL\_V4 | 1.0 | 1.413e-07 | 205 | 0.156 | 230 | 151 | 9 | 12 | 205 | 180 | 402 | Phage tail protein | Phage tail protein | | afdb-uniprot50 | AF-A0A1W1Z3V2-F1-MODEL\_V4 | 1.0 | 4.235e-06 | 203 | 0.318 | 110 | 73 | 2 | 4 | 111 | 8 | 117 | Phage tail protein, P2 protein I family | Phage tail protein, P2 protein I family | | afdb-uniprot50 | AF-A0A1M4V0G4-F1-MODEL\_V4 | 1.0 | 1.603e-07 | 201 | 0.168 | 214 | 153 | 10 | 3 | 205 | 7 | 206 | Uncharacterized protein | Uncharacterized protein | | afdb-uniprot50 | AF-A0A3A9FB62-F1-MODEL\_V4 | 1.0 | 1.868e-06 | 199 | 0.187 | 165 | 107 | 9 | 4 | 149 | 12 | 168 | Uncharacterized protein | Uncharacterized protein | | afdb-uniprot50 | AF-A0A1C5HA19-F1-MODEL\_V4 | 1.0 | 6.232e-08 | 198 | 0.15 | 233 | 156 | 11 | 4 | 205 | 9 | 230 | Phage tail protein domain-containing protein | Phage tail protein domain-containing protein | | afdb-uniprot50 | AF-A0A3L7JDQ4-F1-MODEL\_V4 | 1.0 | 7.009e-06 | 198 | 0.298 | 104 | 68 | 2 | 1 | 99 | 2 | 105 | Phage tail protein I | Phage tail protein I | | afdb-uniprot50 | AF-A0A239INL8-F1-MODEL\_V4 | 1.0 | 9.685e-08 | 196 | 0.143 | 202 | 156 | 6 | 12 | 202 | 37 | 232 | Phage tail protein domain-containing protein | Phage tail protein domain-containing protein | | afdb-uniprot50 | AF-A0A0E4G100-F1-MODEL\_V4 | 1.0 | 3.634e-07 | 194 | 0.264 | 136 | 82 | 4 | 4 | 137 | 9 | 128 | Uncharacterized protein | Uncharacterized protein | | afdb-uniprot50 | AF-A0A0Q8B1P1-F1-MODEL\_V4 | 1.0 | 1.28e-06 | 194 | 0.294 | 129 | 77 | 3 | 4 | 131 | 15 | 130 | Uncharacterized protein | Uncharacterized protein | | afdb-uniprot50 | AF-A0A2G2MJ30-F1-MODEL\_V4 | 1.0 | 8.239e-07 | 192 | 0.123 | 227 | 168 | 12 | 4 | 205 | 12 | 232 | Uncharacterized protein | Uncharacterized protein | | afdb-uniprot50 | AF-A0A5F1S592-F1-MODEL\_V4 | 1.0 | 1.17e-07 | 191 | 0.251 | 175 | 106 | 7 | 2 | 168 | 6 | 163 | Phage tail protein I | Phage tail protein I | | afdb-uniprot50 | AF-A0A1H4FZQ0-F1-MODEL\_V4 | 1.0 | 9.344e-07 | 190 | 0.103 | 261 | 156 | 8 | 14 | 198 | 466 | 724 | Phage tail protein domain-containing protein | Phage tail protein domain-containing protein | | afdb-uniprot50 | AF-A0A439EP98-F1-MODEL\_V4 | 1.0 | 1.129e-06 | 189 | 0.265 | 113 | 73 | 4 | 75 | 183 | 2 | 108 | Phage tail protein I | Phage tail protein I | | afdb-uniprot50 | AF-A0A4Q8MC19-F1-MODEL\_V4 | 1.0 | 0.000144 | 189 | 0.255 | 90 | 65 | 2 | 13 | 100 | 2 | 91 | Phage tail protein I | Phage tail protein I | | afdb-uniprot50 | AF-A0A5M6I8Y5-F1-MODEL\_V4 | 1.0 | 2.903e-06 | 187 | 0.365 | 115 | 70 | 1 | 89 | 203 | 44 | 155 | Phage tail protein I | Phage tail protein I | | afdb-uniprot50 | AF-A0A221SYK6-F1-MODEL\_V4 | 1.0 | 1.28e-06 | 187 | 0.157 | 216 | 154 | 10 | 5 | 201 | 150 | 356 | Phage tail protein | Phage tail protein | | afdb-uniprot50 | AF-A0A1A6FKQ9-F1-MODEL\_V4 | 1.0 | 9.602e-06 | 187 | 0.298 | 104 | 67 | 3 | 1 | 100 | 1 | 102 | Phage tail protein I | Phage tail protein I | | afdb-uniprot50 | AF-A0A0F4R541-F1-MODEL\_V4 | 1.0 | 3.634e-07 | 186 | 0.134 | 215 | 157 | 11 | 5 | 202 | 7 | 209 | Uncharacterized protein | Uncharacterized protein | | afdb-uniprot50 | AF-A0A4P6YWR5-F1-MODEL\_V4 | 1.0 | 1.06e-06 | 186 | 0.116 | 214 | 170 | 9 | 1 | 200 | 9 | 217 | Uncharacterized protein | Uncharacterized protein | | afdb-uniprot50 | AF-W1HTC4-F1-MODEL\_V4 | 1.0 | 4.51e-06 | 184 | 0.363 | 99 | 59 | 2 | 93 | 189 | 2 | 98 | Tail protein I | Tail protein I | | afdb-uniprot50 | AF-A0A542PWC5-F1-MODEL\_V4 | 1.0 | 5.802e-06 | 180 | 0.113 | 283 | 158 | 9 | 5 | 200 | 18 | 294 | Uncharacterized protein | Uncharacterized protein | | afdb-uniprot50 | AF-A0A5Z1E0Y7-F1-MODEL\_V4 | 1.0 | 1.316e-05 | 179 | 0.223 | 121 | 88 | 3 | 84 | 202 | 1 | 117 | Phage tail protein I | Phage tail protein I | | afdb-uniprot50 | AF-A0A0F5VGY1-F1-MODEL\_V4 | 1.0 | 1.202e-06 | 179 | 0.164 | 195 | 144 | 7 | 12 | 201 | 27 | 207 | Phage protein | Phage protein | | afdb-uniprot50 | AF-A0A021X9K5-F1-MODEL\_V4 | 1.0 | 1.754e-06 | 176 | 0.255 | 141 | 81 | 5 | 1 | 134 | 2 | 125 | Phage tail protein I | Phage tail protein I | | afdb-uniprot50 | AF-A0A7T7VP87-F1-MODEL\_V4 | 1.0 | 4.549e-08 | 175 | 0.244 | 213 | 121 | 7 | 3 | 200 | 10 | 197 | Phage tail protein I | Phage tail protein I | | afdb-uniprot50 | AF-A0A2U2DFB4-F1-MODEL\_V4 | 1.0 | 5.116e-06 | 173 | 0.276 | 134 | 80 | 5 | 1 | 130 | 5 | 125 | Phage tail protein I | Phage tail protein I | | afdb-uniprot50 | AF-A0A3M0SWH5-F1-MODEL\_V4 | 1.0 | 9.344e-07 | 170 | 0.157 | 203 | 143 | 13 | 12 | 205 | 24 | 207 | DUF2313 domain-containing protein | DUF2313 domain-containing protein | | afdb-uniprot50 | AF-A0A849PJG0-F1-MODEL\_V4 | 1.0 | 2.403e-06 | 170 | 0.14 | 264 | 151 | 10 | 5 | 197 | 140 | 398 | Uncharacterized protein | Uncharacterized protein | | afdb-uniprot50 | AF-A0A2W6SVJ0-F1-MODEL\_V4 | 1.0 | 1.692e-05 | 170 | 0.251 | 139 | 86 | 5 | 1 | 137 | 1 | 123 | Phage tail protein I | Phage tail protein I | | afdb-uniprot50 | AF-A0A1B9NSL6-F1-MODEL\_V4 | 1.0 | 7.464e-06 | 169 | 0.306 | 111 | 75 | 1 | 95 | 205 | 1 | 109 | Phage tail protein I | Phage tail protein I | | afdb-uniprot50 | AF-A0A744CNX5-F1-MODEL\_V4 | 1.0 | 7.009e-06 | 168 | 0.268 | 138 | 91 | 5 | 3 | 132 | 11 | 146 | Phage tail protein I | Phage tail protein I | | afdb-uniprot50 | AF-A0A6V6Y3Y7-F1-MODEL\_V4 | 1.0 | 5.802e-06 | 168 | 0.139 | 208 | 150 | 10 | 4 | 197 | 17 | 209 | Uncharacterized protein | Uncharacterized protein | | afdb-uniprot50 | AF-A0A1S8T002-F1-MODEL\_V4 | 1.0 | 2.338e-07 | 163 | 0.19 | 199 | 139 | 12 | 11 | 205 | 48 | 228 | Uncharacterized protein | Uncharacterized protein | | afdb-uniprot50 | AF-A0A450WI71-F1-MODEL\_V4 | 1.0 | 1.492e-05 | 160 | 0.139 | 215 | 167 | 9 | 1 | 205 | 3 | 209 | Concanavalin A-like lectin/glucanases superfamily protein | Concanavalin A-like lectin/glucanases superfamily protein | | afdb-uniprot50 | AF-A0A2M9VEP7-F1-MODEL\_V4 | 1.0 | 1.246e-07 | 159 | 0.246 | 203 | 97 | 2 | 1 | 203 | 1 | 147 | Phage tail protein I | Phage tail protein I | | afdb-uniprot50 | AF-U5MT65-F1-MODEL\_V4 | 1.0 | 1.098e-07 | 159 | 0.227 | 211 | 138 | 14 | 5 | 205 | 6 | 201 | Uncharacterized protein | Uncharacterized protein | | afdb-uniprot50 | AF-A5ZQ01-F1-MODEL\_V4 | 1.0 | 3.292e-06 | 158 | 0.156 | 198 | 143 | 10 | 12 | 204 | 35 | 213 | Uncharacterized protein | Uncharacterized protein | | afdb-uniprot50 | AF-A0A541BDT5-F1-MODEL\_V4 | 1.0 | 2.177e-05 | 155 | 0.138 | 224 | 160 | 10 | 1 | 205 | 3 | 212 | Uncharacterized protein | Uncharacterized protein | | afdb-uniprot50 | AF-G7MCE0-F1-MODEL\_V4 | 1.0 | 2.49e-07 | 151 | 0.215 | 200 | 134 | 12 | 11 | 205 | 27 | 208 | Uncharacterized protein | Uncharacterized protein | | afdb-uniprot50 | AF-A0A2P8EL31-F1-MODEL\_V4 | 1.0 | 5.116e-06 | 149 | 0.14 | 206 | 160 | 7 | 11 | 205 | 31 | 230 | Uncharacterized protein DUF2313 | Uncharacterized protein DUF2313 | | afdb-uniprot50 | AF-A0A4R3NVK9-F1-MODEL\_V4 | 1.0 | 3.506e-06 | 144 | 0.206 | 232 | 139 | 9 | 1 | 205 | 2 | 215 | Phage tail P2-like protein | Phage tail P2-like protein | | afdb-uniprot50 | AF-A0A7Z0TG82-F1-MODEL\_V4 | 1.0 | 7.95e-06 | 143 | 0.186 | 215 | 142 | 15 | 3 | 205 | 9 | 202 | YmfQ family protein | YmfQ family protein | | afdb-uniprot50 | AF-W4SMV0-F1-MODEL\_V4 | 1.0 | 0.0001192 | 142 | 0.243 | 119 | 78 | 4 | 69 | 182 | 3 | 114 | Phage-related tail protein | Phage-related tail protein | | afdb-uniprot50 | AF-A0A7X5DCQ4-F1-MODEL\_V4 | 1.0 | 3.177e-05 | 142 | 0.123 | 210 | 162 | 10 | 5 | 203 | 14 | 212 | DUF2313 domain-containing protein | DUF2313 domain-containing protein | | afdb-uniprot50 | AF-A0A1W7LUH0-F1-MODEL\_V4 | 1.0 | 6.581e-06 | 140 | 0.181 | 198 | 139 | 13 | 12 | 205 | 24 | 202 | Putative phage protein XkdU | Putative phage protein XkdU | | afdb-uniprot50 | AF-A0A412NHA8-F1-MODEL\_V4 | 1.0 | 6.763e-05 | 140 | 0.136 | 198 | 150 | 9 | 14 | 203 | 34 | 218 | DUF2313 domain-containing protein | DUF2313 domain-containing protein | | afdb-uniprot50 | AF-R9KLV1-F1-MODEL\_V4 | 1.0 | 9.602e-06 | 140 | 0.12 | 208 | 153 | 12 | 12 | 205 | 42 | 233 | Uncharacterized protein | Uncharacterized protein | | afdb-uniprot50 | AF-A0A2I9CLJ2-F1-MODEL\_V4 | 1.0 | 3.837e-05 | 137 | 0.172 | 215 | 153 | 9 | 2 | 202 | 5 | 208 | Uncharacterized protein | Uncharacterized protein | | afdb-uniprot50 | AF-A0A828HRI0-F1-MODEL\_V4 | 1.0 | 5.257e-05 | 135 | 0.253 | 126 | 79 | 3 | 93 | 205 | 6 | 129 | Phage tail protein I | Phage tail protein I | | afdb-uniprot50 | AF-A0A704VV40-F1-MODEL\_V4 | 1.0 | 0.0001192 | 132 | 0.347 | 95 | 62 | 0 | 109 | 203 | 4 | 98 | Phage tail protein I | Phage tail protein I | | afdb-uniprot50 | AF-A0A2A6Z7V1-F1-MODEL\_V4 | 1.0 | 0.0001192 | 131 | 0.113 | 229 | 169 | 11 | 4 | 205 | 19 | 240 | Uncharacterized protein | Uncharacterized protein | | afdb-uniprot50 | AF-A0A494X1U6-F1-MODEL\_V4 | 1.0 | 1.692e-05 | 131 | 0.129 | 232 | 150 | 12 | 12 | 202 | 125 | 345 | Uncharacterized protein | Uncharacterized protein | | afdb-uniprot50 | AF-A0A265Q495-F1-MODEL\_V4 | 1.0 | 6.18e-06 | 128 | 0.2 | 220 | 143 | 12 | 1 | 205 | 1 | 202 | Uncharacterized protein | Uncharacterized protein | | afdb-uniprot50 | AF-A0A417ZEM8-F1-MODEL\_V4 | 1.0 | 0.000144 | 125 | 0.09 | 210 | 174 | 6 | 3 | 205 | 5 | 204 | Uncharacterized protein | Uncharacterized protein | | afdb-uniprot50 | AF-A0A7U1GLI6-F1-MODEL\_V4 | 1.0 | 2.319e-05 | 124 | 0.153 | 215 | 157 | 11 | 5 | 205 | 8 | 211 | DUF2313 domain-containing protein | DUF2313 domain-containing protein | | afdb-uniprot50 | AF-A0A0C1PS25-F1-MODEL\_V4 | 1.0 | 8.169e-05 | 123 | 0.141 | 219 | 156 | 9 | 3 | 202 | 19 | 224 | Uncharacterized protein | Uncharacterized protein | | afdb-uniprot50 | AF-A0A7X5CZ78-F1-MODEL\_V4 | 1.0 | 0.0003702 | 119 | 0.127 | 212 | 153 | 9 | 14 | 205 | 34 | 233 | DUF2313 domain-containing protein | DUF2313 domain-containing protein | | afdb-uniprot50 | AF-A0A7X5L3R8-F1-MODEL\_V4 | 1.0 | 6.35e-05 | 114 | 0.156 | 211 | 154 | 11 | 4 | 203 | 10 | 207 | DUF2313 domain-containing protein | DUF2313 domain-containing protein | | afdb-uniprot50 | AF-A0A1V5NBE0-F1-MODEL\_V4 | 1.0 | 0.0002101 | 112 | 0.135 | 199 | 154 | 9 | 10 | 205 | 1 | 184 | Uncharacterized protein | Uncharacterized protein | | afdb-uniprot50 | AF-A0A415TAE8-F1-MODEL\_V4 | 1.0 | 0.0002878 | 102 | 0.086 | 209 | 161 | 11 | 5 | 197 | 11 | 205 | DUF2313 domain-containing protein | DUF2313 domain-containing protein | | afdb-uniprot50 | AF-A0A0P7H682-F1-MODEL\_V4 | 1.0 | 0.0001533 | 100 | 0.21 | 219 | 145 | 7 | 1 | 205 | 1 | 205 | Phage tail protein I | Phage tail protein I | | afdb-uniprot50 | AF-A0A2G6AV33-F1-MODEL\_V4 | 0.998 | 0.0003065 | 95 | 0.172 | 209 | 146 | 12 | 6 | 205 | 27 | 217 | Uncharacterized protein | Uncharacterized protein | | afdb-uniprot50 | AF-A0A6N9PSR9-F1-MODEL\_V4 | 0.998 | 0.000144 | 93 | 0.149 | 208 | 152 | 9 | 5 | 201 | 9 | 202 | DUF2313 domain-containing protein | DUF2313 domain-containing protein | | afdb-uniprot50 | AF-A0A7X6N9Z3-F1-MODEL\_V4 | 0.986 | 0.003805 | 81 | 0.141 | 212 | 129 | 11 | 6 | 203 | 203 | 375 | Flagellar hook-length control protein FliK | Flagellar hook-length control protein FliK | | afdb-uniprot50 | AF-A0A1I6IPJ1-F1-MODEL\_V4 | 0.912 | 0.005212 | 68 | 0.097 | 194 | 155 | 7 | 12 | 200 | 303 | 481 | TIGR00341 family protein | TIGR00341 family protein | | afdb-uniprot50 | AF-A0A2E0A3F5-F1-MODEL\_V4 | 0.663 | 0.009784 | 56 | 0.125 | 223 | 148 | 11 | 1 | 205 | 267 | 460 | Flg\_hook domain-containing protein | Flg\_hook domain-containing protein | |
| Top keywords  (threshold 1.00e-02 (evalue)) | **tail, Phage, I, P2, P2\_like, Bacteriophage, formation, Putative, P2\_related, Tail\_P2\_I** |
| Output files | ../../similar\_structures/14\_FANPEZAQ\_CDS\_0014\_afdb-proteome\_foldseek.tsv ../../similar\_structures/14\_FANPEZAQ\_CDS\_0014\_afdb-uniprot50\_foldseek.tsv ../../similar\_structures/14\_FANPEZAQ\_CDS\_0014\_merged.svg ../../similar\_structures/14\_FANPEZAQ\_CDS\_0014\_pdb\_foldseek.tsv |

  
  
  

Return to summary | Go to previous | Go to next

  


---

**Sequence/structure alignments coloring**  
Each object in the alignment figures is colored according to its E-value following this color coding:

1e-100
10

**References:**  
1) Steinegger M, Meier M, Mirdita M, Vöhringer H, Haunsberger S J, and Söding J (2019) HH-suite3 for fast remote homology detection and deep protein annotation, BMC Bioinformatics, 473. doi: 10.1186/s12859-019-3019-7  
2) Jumper J, Evans R, Pritzel A, ..., Hassabis D (2021) Highly accurate protein structure prediction with AlphaFold, Nature, 596. doi: 10.1038/s41586-021-03819-2  
3) van Kempen M, Kim S, Tumescheit C, Mirdita M, Lee J, Gilchrist CLM, Söding J, and Steinegger M (2023) Fast and accurate protein structure search with Foldseek. Nature Biotechnology. doi: 10.1038/s41587-023-01773-0
