# Supplementary material for: Completing the BASEL phage collection to unlock hidden diversity for systematic exploration of phage–host interactions
Source: PLoS Biol. 2025 Apr 7;23(4):e3003063. doi: 10.1371/journal.pbio.3003063 (PMC11990801; doi:10.1371/journal.pbio.3003063)
Supplement: S2 Data — (ZIP) [file pbio.3003063.s009.zip › entries/15.html]

FANPEZAQ\_CDS\_0015


Return to summary | Go to previous | Go to next

|  |  |
| --- | --- |
| FANPEZAQ\_CDS\_0015 Page creation date: 02 Sep 2024, 12:00  Project folder: n/a  Input sequences file: Escherichia\_virus\_HeidiAbel.gb | tail phage fiber fibre tail\_collar domain\_containing collar putative phage\_related repeat h fragment protein\_like variable bacteriophage gph repeat\_containing probable product fiber\_like duf3751 prophage dna inversion peptidase s74 duf2793 side adhesin pyocin short\_chain fatty acid transporter |

### Sequence information

|  |  |
| --- | --- |
| Name | FANPEZAQ\_CDS\_0015  15\_FANPEZAQ\_CDS\_0015 (pipeline id) |
| Imported annotations | Escherichia\_virus\_HeidiAbel Bas97 |
| Protein sequence | MATYYTLLTKIGQARIANAVALNQMVNLTYMAVGDGNGNPTTPNENQTALVREKYRATIN QLTVDPENPNYLVAEMIVPTTVGGWSIYEVGVFDDQNQLIAVANFPATYKPELVEGSGRD LIVRIIIQVSNTSVVTLKIDPAITLASQAWVAANYVKKITMAGGTTGQVLAKKSNANEDF QWVDPTAAVNVIVDSQTEEQTLASGQTIINLAKLTTQATAIYVEGVRLYPVTHYTINSDT RLTLKTPYPNGSKILAVQNDPTSTISLASTTQKGFTILSSATNSDDETKAATPKAVNDAV AALASTLGSASGRTVGNGANQIPDMNFFTSGTNWFKQPNGFIVQRGVSVAGAIGSPTFIT FPVPFTDYANVQIVVSYDNAEPYVTSIPTFAATPVNTGSGFSLMGSQVPGSNTTYARWIA IGK |
| Number of residues | 423 |
| Molecular weight (Da) | 45003.17 |
| Output files | ../../query\_sequences/15\_FANPEZAQ\_CDS\_0015.fasta |

### Putative domain architecture and protein family

#### Search results (HHblits)1

|  |  |
| --- | --- |
| Domain family databases searched | Pfam, Ncbi-cd, Cath, Phrogs |
| Results, scheme(s)  (Top layers only; threshold 1.00e-03 (evalue)) | xml version="1.0" encoding="utf-8" standalone="no"?       2024-09-02T21:08:15.225722 image/svg+xml   Matplotlib v3.7.2, https://matplotlib.org/ |
| Results, table  (E-value ≤ 1.00e-03 (evalue)) | | db | id | prob | evalue | pvalue | score | cols | query | query\_len | template | template\_len | name | description | | --- | --- | --- | --- | --- | --- | --- | --- | --- | --- | --- | --- | --- | | phrogs | 6162 | 100.0 | 7.4e-53 | 9.1e-57 | 406.7 | 313 | (2, 423) | 423 | (3, 326) | 326 | tail fiber protein | tail fiber protein; Category: tail; p300453 VI\_07590 | | phrogs | 381 | 100.0 | 2.4e-51 | 2.9e-55 | 425.5 | 203 | (1, 304) | 423 | (2, 205) | 748 | tail fiber protein | tail fiber protein; Category: tail; p250465 VI\_02803 | | phrogs | 6202 | 100.0 | 9.1e-50 | 1.1e-53 | 394.5 | 202 | (2, 304) | 423 | (6, 208) | 588 | tail fiber protein | tail fiber protein; Category: tail; p390163 VI\_08186 | | phrogs | 327 | 100.0 | 2.2e-44 | 2.6e-48 | 361.4 | 156 | (1, 156) | 423 | (2, 157) | 544 | tail protein | tail protein; Category: tail; p384764 VI\_09705 | | phrogs | 988 | 100.0 | 8.5e-43 | 1e-46 | 350.9 | 203 | (1, 304) | 423 | (5, 208) | 520 | tail protein | tail protein; Category: tail; p13596 VI\_07483 | | phrogs | 4618 | 100.0 | 9.5e-37 | 1.1e-40 | 289.3 | 204 | (2, 308) | 423 | (1, 206) | 365 | tail collar fiber protein | tail collar fiber protein; Category: tail; p204627 VI\_11278 | | phrogs | 1780 | 99.9 | 4.7e-34 | 5.9e-38 | 259.7 | 157 | (1, 160) | 423 | (3, 159) | 204 | tail collar fiber protein | tail collar fiber protein; Category: tail; p306437 VI\_06250 | | phrogs | 11518 | 99.9 | 8.5e-32 | 9.9e-36 | 269.0 | 153 | (1, 153) | 423 | (1, 154) | 648 | tail fiber protein | tail fiber protein; Category: tail; p203938 VI\_11941 | | phrogs | 5181 | 99.9 | 6.7e-31 | 7.9e-35 | 263.6 | 155 | (1, 155) | 423 | (1, 156) | 707 | NA | NA; Category: unknown function; p394343 VI\_08938 | | phrogs | 2137 | 99.9 | 1e-30 | 1.2e-34 | 237.6 | 154 | (2, 155) | 423 | (4, 157) | 281 | tail fiber protein | tail fiber protein; Category: tail; p58188 VI\_06099 | | phrogs | 1162 | 99.9 | 3.1e-29 | 3.7e-33 | 236.4 | 151 | (2, 155) | 423 | (4, 154) | 339 | tail fiber protein | tail fiber protein; Category: tail; p177110 VI\_06260 | | phrogs | 14570 | 99.9 | 1.6e-27 | 1.8e-31 | 223.6 | 154 | (2, 155) | 423 | (6, 159) | 540 | NA | NA; Category: unknown function; p191826 VI\_07392 | | phrogs | 2557 | 99.8 | 3.2e-24 | 4.2e-28 | 182.9 | 115 | (304, 423) | 423 | (12, 129) | 129 | virion structural protein | virion structural protein; Category: head and packaging; p173779 VI\_03738 | | phrogs | 10153 | 99.7 | 2.1e-22 | 2.3e-26 | 180.1 | 154 | (2, 155) | 423 | (3, 156) | 327 | tail fiber protein | tail fiber protein; Category: tail; p319252 VI\_06087 | | phrogs | 27782 | 99.6 | 1.2e-20 | 1.3e-24 | 182.7 | 155 | (2, 156) | 423 | (3, 158) | 641 | NA | NA; Category: unknown function; p342043 VI\_01070 | | phrogs | 3737 | 99.6 | 1.3e-20 | 1.6e-24 | 167.4 | 137 | (1, 143) | 423 | (3, 139) | 184 | tail fiber protein | tail fiber protein; Category: tail; p259608 VI\_12019 | | phrogs | 30680 | 99.6 | 1.9e-20 | 2.1e-24 | 168.4 | 154 | (2, 155) | 423 | (1, 154) | 338 | NA | NA; Category: unknown function; p169858 VI\_01362 | | phrogs | 295 | 99.6 | 3.5e-20 | 4.1e-24 | 187.4 | 194 | (2, 304) | 423 | (171, 392) | 813 | tail fiber protein | tail fiber protein; Category: tail; p109395 VI\_03810 | | phrogs | 4890 | 99.6 | 6.5e-20 | 7.3e-24 | 149.2 | 119 | (304, 423) | 423 | (29, 153) | 153 | NA | NA; Category: unknown function; p375303 VI\_02828 | | phrogs | 14531 | 99.5 | 2.2e-19 | 2.5e-23 | 162.9 | 173 | (3, 183) | 423 | (5, 177) | 359 | NA | NA; Category: unknown function; p181208 VI\_06278 | | phrogs | 1396 | 99.5 | 2.3e-19 | 3e-23 | 168.5 | 122 | (294, 423) | 423 | (119, 261) | 261 | tail fiber protein | tail fiber protein; Category: tail; p37946 VI\_07185 | | phrogs | 9532 | 99.4 | 2.3e-17 | 2.6e-21 | 158.7 | 154 | (2, 155) | 423 | (3, 156) | 759 | tail fiber protein | tail fiber protein; Category: tail; p329901 VI\_11396 | | phrogs | 5075 | 99.3 | 2.2e-16 | 2.5e-20 | 144.8 | 149 | (2, 155) | 423 | (4, 153) | 394 | NA | NA; Category: unknown function; p127048 VI\_01582 | | phrogs | 7037 | 99.2 | 1.3e-15 | 1.6e-19 | 135.2 | 146 | (3, 152) | 423 | (4, 157) | 185 | tail fiber protein | tail fiber protein; Category: tail; p172398 VI\_05814 | | phrogs | 17585 | 99.2 | 1.9e-15 | 2.2e-19 | 145.5 | 159 | (4, 163) | 423 | (5, 163) | 650 | NA | NA; Category: unknown function; p387047 VI\_09109 | | phrogs | 23632 | 99.0 | 7.9e-14 | 9.1e-18 | 125.7 | 152 | (1, 156) | 423 | (1, 160) | 290 | NA | NA; Category: unknown function; p130591 VI\_06329 | | phrogs | 7489 | 99.0 | 8.2e-14 | 9.5e-18 | 137.0 | 140 | (4, 145) | 423 | (1, 147) | 666 | tail fiber protein | tail fiber protein; Category: tail; p80663 VI\_09756 | | phrogs | 25305 | 98.9 | 1.2e-13 | 1.3e-17 | 136.8 | 159 | (4, 163) | 423 | (5, 163) | 870 | tail fiber protein | tail fiber protein; Category: tail; p176118 VI\_11367 | | phrogs | 4209 | 98.9 | 1.9e-13 | 2.3e-17 | 130.1 | 88 | (329, 423) | 423 | (253, 341) | 341 | tail fiber protein | tail fiber protein; Category: tail; p184403 VI\_05508 | | phrogs | 8239 | 98.8 | 5.4e-13 | 6.2e-17 | 129.4 | 140 | (4, 145) | 423 | (2, 148) | 654 | tail fiber protein | tail fiber protein; Category: tail; p255681 VI\_08125 | | phrogs | 13714 | 98.8 | 8.6e-13 | 1e-16 | 113.6 | 147 | (3, 154) | 423 | (4, 156) | 191 | tail fiber protein | tail fiber protein; Category: tail; p143339 VI\_09468 | | phrogs | 4545 | 98.7 | 1.3e-12 | 1.6e-16 | 126.2 | 138 | (3, 145) | 423 | (2, 144) | 512 | NA | NA; Category: unknown function; p341124 VI\_09299 | | phrogs | 13388 | 98.7 | 1.6e-12 | 1.9e-16 | 105.2 | 112 | (4, 117) | 423 | (2, 118) | 127 | tail collar fiber protein | tail collar fiber protein; Category: tail; p255876 VI\_08161 | | phrogs | 15547 | 98.1 | 6.1e-10 | 7e-14 | 100.9 | 103 | (1, 111) | 423 | (1, 104) | 323 | NA | NA; Category: unknown function; p391937 VI\_02991 | | phrogs | 38337 | 98.1 | 9.4e-10 | 1.1e-13 | 103.7 | 145 | (4, 152) | 423 | (9, 160) | 569 | NA | NA; Category: unknown function; p170432 VI\_01289 | | phrogs | 19961 | 98.1 | 1e-09 | 1.1e-13 | 101.9 | 150 | (1, 155) | 423 | (1, 159) | 439 | tail fiber protein | tail fiber protein; Category: tail; p321354 VI\_06335 | | phrogs | 19921 | 98.0 | 2.1e-09 | 2.4e-13 | 104.6 | 99 | (7, 105) | 423 | (24, 133) | 946 | tail spike protein | tail spike protein; Category: tail; NC\_027381\_p75 | | phrogs | 8443 | 97.6 | 3e-08 | 3.5e-12 | 95.8 | 106 | (1, 114) | 423 | (1, 107) | 530 | tail fiber protein | tail fiber protein; Category: tail; p142958 VI\_03022 | | phrogs | 6683 | 97.5 | 5e-08 | 5.8e-12 | 88.9 | 99 | (317, 423) | 423 | (257, 359) | 359 | tail fiber protein | tail fiber protein; Category: tail; NC\_010463\_p10 | | phrogs | 4849 | 97.5 | 5.7e-08 | 6.5e-12 | 95.1 | 105 | (1, 111) | 423 | (1, 106) | 790 | tail protein | tail protein; Category: tail; JX681814\_p23 | | phrogs | 33377 | 97.5 | 6.1e-08 | 6.8e-12 | 88.2 | 50 | (330, 380) | 423 | (349, 409) | 456 | NA | NA; Category: unknown function; p217392 VI\_05721 | | phrogs | 25563 | 97.5 | 7e-08 | 7.9e-12 | 94.6 | 102 | (2, 109) | 423 | (1, 103) | 829 | NA | NA; Category: unknown function; p252637 VI\_04196 | | phrogs | 30004 | 97.2 | 3.6e-07 | 4.1e-11 | 81.2 | 147 | (5, 152) | 423 | (3, 155) | 328 | NA | NA; Category: unknown function; p288306 VI\_01174 | | phrogs | 10372 | 97.2 | 3.8e-07 | 4.3e-11 | 76.7 | 43 | (262, 304) | 423 | (62, 104) | 200 | NA | NA; Category: unknown function; p28886 VI\_10606 | | phrogs | 2631 | 97.1 | 6e-07 | 6.9e-11 | 84.6 | 41 | (264, 304) | 423 | (2, 42) | 482 | tail fiber protein | tail fiber protein; Category: tail; p130317 VI\_07372 | | phrogs | 6043 | 97.1 | 7e-07 | 7.7e-11 | 84.9 | 139 | (5, 145) | 423 | (2, 147) | 814 | NA | NA; Category: unknown function; p336028 VI\_09513 | | phrogs | 17472 | 97.0 | 7.5e-07 | 8.7e-11 | 88.2 | 126 | (4, 138) | 423 | (11, 138) | 868 | tail protein | tail protein; Category: tail; KY290947\_p27 | | phrogs | 36614 | 96.8 | 2.1e-06 | 2.3e-10 | 66.3 | 40 | (265, 304) | 423 | (3, 42) | 131 | NA | NA; Category: unknown function; p237616 VI\_06905 | | phrogs | 24573 | 96.6 | 4.3e-06 | 4.8e-10 | 79.0 | 137 | (6, 144) | 423 | (3, 146) | 780 | NA | NA; Category: unknown function; p244508 VI\_06663 | | phrogs | 8713 | 94.8 | 0.00048 | 5.4e-08 | 63.6 | 40 | (264, 303) | 423 | (352, 391) | 541 | tail fiber protein | tail fiber protein; Category: tail; p399266 VI\_10066 | | phrogs | 17623 | 94.3 | 0.00091 | 1e-07 | 65.8 | 96 | (5, 106) | 423 | (7, 103) | 1121 | tail collar fiber protein | tail collar fiber protein; Category: tail; NC\_021343\_p35 | |
| Top keywords  (threshold 1.00e-03 (evalue)) | **tail, fiber, collar, p300453, VI\_07590, p250465, VI\_02803, p390163, VI\_08186, p384764** |
| Output files | ../../domain\_architecture/15\_FANPEZAQ\_CDS\_0015\_cath.hhr ../../domain\_architecture/15\_FANPEZAQ\_CDS\_0015\_merged.svg ../../domain\_architecture/15\_FANPEZAQ\_CDS\_0015\_ncbi-cd.hhr ../../domain\_architecture/15\_FANPEZAQ\_CDS\_0015\_pfam.hhr ../../domain\_architecture/15\_FANPEZAQ\_CDS\_0015\_phrogs.hhr |

### Identical protein sequences/structures

#### Search results

|  |  |
| --- | --- |
| Protein sequence databases searched | Pdb, Swissprot, Refseq |
| Identical proteins found | -- |
| Top keywords | -- |
| Output files | -- |

### Similar protein sequences/structures

#### Sequence similarity search results (HHblits)1

|  |  |
| --- | --- |
| Sequence databases searched | Uniclust, Pdb70 |
| Results, scheme(s)  (Top layers only, threshold 1.00e-03 (evalue)) | xml version="1.0" encoding="utf-8" standalone="no"?       2024-09-02T21:08:34.936126 image/svg+xml   Matplotlib v3.7.2, https://matplotlib.org/ |
| Results, table(s)  (threshold 1.00e-03 (evalue)) | | db | id | prob | evalue | pvalue | score | cols | query | query\_len | template | template\_len | name | description | | --- | --- | --- | --- | --- | --- | --- | --- | --- | --- | --- | --- | --- | | uniclust | UniRef100\_A0A381GJP7 | 100.0 | 1.2e-52 | 2.3e-58 | 364.7 | 284 | (1, 375) | 423 | (4, 351) | 397 | Phage tail fiber protein | Phage tail fiber protein | | uniclust | UniRef100\_A0A1G7VLT1 | 100.0 | 7.7e-52 | 1.5e-57 | 363.3 | 160 | (1, 160) | 423 | (1, 160) | 369 | Phage tail-collar fibre protein | Phage tail-collar fibre protein | | uniclust | UniRef100\_A0A0G2PMU5 | 100.0 | 4.6e-51 | 8.7e-57 | 374.3 | 165 | (1, 165) | 423 | (2, 166) | 528 | Tail fiber protein | Tail fiber protein | | uniclust | UniRef100\_A0A261E4L1 | 100.0 | 1.3e-49 | 2.5e-55 | 346.0 | 162 | (1, 162) | 423 | (2, 163) | 361 | DNA inversion product | DNA inversion product | | uniclust | UniRef100\_A0A088FQW7 | 100.0 | 5.2e-49 | 1e-54 | 364.8 | 280 | (1, 281) | 423 | (15, 299) | 515 | Tail protein | Tail protein | | uniclust | UniRef100\_A0A080I6W6 | 100.0 | 3.8e-48 | 7.1e-54 | 335.9 | 341 | (2, 375) | 423 | (13, 358) | 405 | Phage tail-collar fiber family protein | Phage tail-collar fiber family protein | | uniclust | UniRef100\_A0A061JKN2 | 100.0 | 9.7e-48 | 1.8e-53 | 352.0 | 276 | (1, 277) | 423 | (26, 304) | 521 | Tail protein | Tail protein | | uniclust | UniRef100\_A0A024HA82 | 100.0 | 2.4e-47 | 4.7e-53 | 333.8 | 155 | (2, 156) | 423 | (6, 160) | 358 | Uncharacterized protein | Uncharacterized protein | | uniclust | UniRef100\_A0A0E1LY00 | 100.0 | 5.6e-47 | 1e-52 | 343.6 | 160 | (1, 160) | 423 | (5, 165) | 585 | Putative phage tail fiber protein | Putative phage tail fiber protein | | uniclust | UniRef100\_A0A080KKZ3 | 100.0 | 2.1e-46 | 4e-52 | 331.9 | 158 | (2, 159) | 423 | (3, 160) | 343 | Tail fiber protein | Tail fiber protein | | uniclust | UniRef100\_A0A0J8YRQ6 | 100.0 | 3e-46 | 5.7e-52 | 329.5 | 171 | (2, 172) | 423 | (3, 173) | 409 | Phage tail protein | Phage tail protein | | uniclust | UniRef100\_A0A827WUC6 | 100.0 | 3.2e-46 | 5.8e-52 | 304.5 | 276 | (2, 369) | 423 | (3, 278) | 290 | Phage tail protein (Fragment) | Phage tail protein (Fragment) | | uniclust | UniRef100\_A0A1B8TDC2 | 100.0 | 4.1e-46 | 7.7e-52 | 341.0 | 154 | (3, 156) | 423 | (4, 157) | 566 | Phage tail protein | Phage tail protein | | uniclust | UniRef100\_A0A2N7UDN2 | 100.0 | 5.2e-46 | 1e-51 | 348.6 | 160 | (1, 160) | 423 | (15, 174) | 523 | Phage tail protein | Phage tail protein | | uniclust | UniRef100\_A0A0M2WUJ2 | 100.0 | 1.1e-45 | 2.1e-51 | 345.0 | 160 | (1, 160) | 423 | (5, 164) | 528 | Uncharacterized protein | Uncharacterized protein | | uniclust | UniRef100\_A0A022PCI5 | 100.0 | 1.4e-45 | 2.6e-51 | 328.1 | 205 | (2, 297) | 423 | (3, 207) | 438 | Phage-related tail fiber protein | Phage-related tail fiber protein | | uniclust | UniRef100\_A0A1C3HKM5 | 100.0 | 2.9e-45 | 5.5e-51 | 357.2 | 229 | (1, 330) | 423 | (16, 262) | 912 | Phage tail fiber repeat protein | Phage tail fiber repeat protein | | uniclust | UniRef100\_UPI0005E23890 | 100.0 | 3.8e-45 | 7e-51 | 311.9 | 283 | (2, 371) | 423 | (3, 337) | 384 | phage tail protein | phage tail protein | | uniclust | UniRef100\_A0A144P4D2 | 100.0 | 9.8e-45 | 1.9e-50 | 353.5 | 158 | (2, 159) | 423 | (5, 163) | 707 | Tail Fiber protein | Tail Fiber protein | | uniclust | UniRef100\_A0A1B8YGC4 | 100.0 | 3.9e-44 | 7.6e-50 | 340.4 | 159 | (2, 160) | 423 | (3, 161) | 616 | Phage tail fiber repeat protein | Phage tail fiber repeat protein | | uniclust | UniRef100\_A0A7D7ENG5 | 100.0 | 5.7e-44 | 1.1e-49 | 321.1 | 280 | (2, 281) | 423 | (3, 282) | 551 | Tail fiber | Tail fiber | | uniclust | UniRef100\_A0A0C2EE47 | 100.0 | 1.1e-43 | 2.1e-49 | 335.7 | 157 | (2, 158) | 423 | (10, 166) | 603 | Phage tail fiber protein | Phage tail fiber protein | | uniclust | UniRef100\_A0A1G5RGJ6 | 100.0 | 1.2e-43 | 2.3e-49 | 304.5 | 158 | (2, 159) | 423 | (3, 160) | 348 | Phage tail fibre repeat-containing protein (Fragment) | Phage tail fibre repeat-containing protein (Fragment) | | uniclust | UniRef100\_UPI0005C6F866 | 100.0 | 3.6e-43 | 6.8e-49 | 308.4 | 158 | (3, 160) | 423 | (4, 161) | 427 | phage tail protein | phage tail protein | | uniclust | UniRef100\_A0A077P0S8 | 100.0 | 4.4e-43 | 8.3e-49 | 318.9 | 202 | (1, 303) | 423 | (2, 203) | 500 | Uncharacterized protein | Uncharacterized protein | | uniclust | UniRef100\_A0A1Q5TUM5 | 100.0 | 1.2e-42 | 2.2e-48 | 322.0 | 159 | (2, 160) | 423 | (3, 161) | 573 | Phage tail protein | Phage tail protein | | uniclust | UniRef100\_A0A3L0W1T3 | 100.0 | 1.6e-42 | 3e-48 | 302.5 | 160 | (1, 160) | 423 | (2, 161) | 368 | Phage tail protein | Phage tail protein | | uniclust | UniRef100\_A0A0K0I9Z1 | 100.0 | 2.1e-42 | 4.1e-48 | 337.0 | 203 | (1, 304) | 423 | (2, 205) | 748 | Phage tail protein | Phage tail protein | | uniclust | UniRef100\_C6D9F9 | 100.0 | 4.1e-42 | 7.8e-48 | 317.8 | 158 | (2, 159) | 423 | (3, 160) | 597 | Tail fiber repeat 2 protein | Tail fiber repeat 2 protein | | uniclust | UniRef100\_A0A2X7EVU3 | 100.0 | 6.3e-42 | 1.2e-47 | 310.3 | 159 | (2, 160) | 423 | (3, 161) | 519 | Phage tail collar domain-containing protein | Phage tail collar domain-containing protein | | uniclust | UniRef100\_A0A0U4W4I1 | 100.0 | 9.8e-42 | 1.8e-47 | 308.5 | 279 | (3, 281) | 423 | (5, 290) | 577 | Ig-like domain-containing protein | Ig-like domain-containing protein | | uniclust | UniRef100\_A0A0B6X5X6 | 100.0 | 2.2e-41 | 4.1e-47 | 292.7 | 158 | (2, 159) | 423 | (3, 160) | 383 | E14 prophage putative tail fiber protein (Modular protein) | E14 prophage putative tail fiber protein (Modular protein) | | uniclust | UniRef100\_A0A081RRN4 | 100.0 | 2.9e-41 | 5.5e-47 | 310.1 | 159 | (2, 160) | 423 | (3, 161) | 684 | Phage-related tail fiber protein | Phage-related tail fiber protein | | uniclust | UniRef100\_A0A077NGB1 | 100.0 | 3.1e-41 | 5.9e-47 | 314.1 | 159 | (2, 160) | 423 | (3, 161) | 765 | Uncharacterized protein | Uncharacterized protein | | uniclust | UniRef100\_A0A0M0TBG4 | 100.0 | 3.7e-41 | 6.9e-47 | 281.2 | 204 | (2, 301) | 423 | (3, 206) | 274 | Tail protein (Fragment) | Tail protein (Fragment) | | uniclust | UniRef100\_A0A0D5Y3B7 | 100.0 | 3.6e-41 | 7e-47 | 321.4 | 157 | (2, 158) | 423 | (22, 178) | 498 | Phage tail protein | Phage tail protein | | uniclust | UniRef100\_A0A080KJ35 | 100.0 | 3.9e-41 | 7.4e-47 | 310.4 | 203 | (3, 302) | 423 | (16, 218) | 530 | Phage-related tail fiber protein | Phage-related tail fiber protein | | uniclust | UniRef100\_A0A2G1CTX9 | 100.0 | 4.3e-41 | 8.1e-47 | 299.6 | 173 | (1, 173) | 423 | (5, 177) | 391 | Phage tail protein | Phage tail protein | | uniclust | UniRef100\_A0A068QVC8 | 100.0 | 6.1e-41 | 1.2e-46 | 316.5 | 158 | (2, 159) | 423 | (3, 160) | 696 | Uncharacterized protein | Uncharacterized protein | | uniclust | UniRef100\_A0A3D1C6R7 | 100.0 | 7.1e-41 | 1.3e-46 | 304.0 | 171 | (2, 172) | 423 | (10, 180) | 511 | Phage tail protein | Phage tail protein | | uniclust | UniRef100\_A0A0A1ABU4 | 100.0 | 9.6e-41 | 1.8e-46 | 304.1 | 200 | (3, 303) | 423 | (5, 205) | 539 | Phage tail protein | Phage tail protein | | uniclust | UniRef100\_A0A2X5BQE6 | 100.0 | 1.1e-40 | 2.2e-46 | 308.8 | 160 | (1, 160) | 423 | (4, 163) | 540 | Phage tail fibre repeat | Phage tail fibre repeat | | uniclust | UniRef100\_A0A1D7Z5A8 | 100.0 | 2e-40 | 3.8e-46 | 299.2 | 159 | (2, 160) | 423 | (3, 161) | 555 | Phage tail protein | Phage tail protein | | uniclust | UniRef100\_A0A0P7LYC5 | 100.0 | 3.9e-40 | 7.4e-46 | 303.1 | 225 | (2, 232) | 423 | (10, 236) | 609 | Phage tail protein | Phage tail protein | | uniclust | UniRef100\_UPI000A487BB5 | 100.0 | 4.1e-40 | 7.6e-46 | 298.0 | 214 | (2, 305) | 423 | (3, 216) | 578 | phage tail protein | phage tail protein | | uniclust | UniRef100\_UPI0004A05D1E | 100.0 | 6.7e-40 | 1.2e-45 | 295.7 | 209 | (2, 216) | 423 | (3, 211) | 589 | phage tail protein | phage tail protein | | uniclust | UniRef100\_A0A1B1KLD7 | 100.0 | 1e-39 | 1.9e-45 | 305.5 | 158 | (2, 159) | 423 | (3, 160) | 679 | Uncharacterized protein | Uncharacterized protein | | uniclust | UniRef100\_A0A376D432 | 100.0 | 1.1e-39 | 2.1e-45 | 282.2 | 170 | (2, 171) | 423 | (3, 172) | 464 | Putative variable tail fiber protein | Putative variable tail fiber protein | | uniclust | UniRef100\_A0A0K9TAC2 | 100.0 | 1.2e-39 | 2.2e-45 | 279.0 | 157 | (3, 159) | 423 | (7, 164) | 341 | Phage tail fiber | Phage tail fiber | | uniclust | UniRef100\_A0A068R2V7 | 100.0 | 1.3e-39 | 2.4e-45 | 283.6 | 247 | (2, 349) | 423 | (3, 278) | 430 | Uncharacterized protein | Uncharacterized protein | | uniclust | UniRef100\_A0A1H5DGF8 | 100.0 | 1.4e-39 | 2.7e-45 | 311.9 | 156 | (2, 157) | 423 | (6, 161) | 545 | Phage tail-collar fibre protein | Phage tail-collar fibre protein | | uniclust | UniRef100\_A0A5M7L9F8 | 100.0 | 2.1e-39 | 3.9e-45 | 281.1 | 273 | (3, 374) | 423 | (13, 346) | 388 | Phage tail protein | Phage tail protein | | uniclust | UniRef100\_A0A1C4G344 | 100.0 | 2.3e-39 | 4.3e-45 | 305.5 | 159 | (2, 160) | 423 | (3, 162) | 733 | Phage tail fibre repeat-containing protein | Phage tail fibre repeat-containing protein | | uniclust | UniRef100\_A0A078LPZ8 | 100.0 | 2.9e-39 | 5.7e-45 | 305.2 | 169 | (2, 170) | 423 | (10, 178) | 566 | Phage tail fiber protein | Phage tail fiber protein | | uniclust | UniRef100\_UPI00042486BE | 100.0 | 3.2e-39 | 5.9e-45 | 296.7 | 292 | (2, 303) | 423 | (3, 315) | 695 | tail fiber protein | tail fiber protein | | uniclust | UniRef100\_A0A0R9N0A5 | 100.0 | 3.2e-39 | 6e-45 | 295.1 | 158 | (3, 160) | 423 | (6, 163) | 584 | Phage tail protein | Phage tail protein | | uniclust | UniRef100\_A0A2A2F373 | 100.0 | 7.4e-39 | 1.4e-44 | 266.5 | 170 | (1, 170) | 423 | (4, 173) | 250 | Phage tail protein | Phage tail protein | | uniclust | UniRef100\_A0A014MD74 | 100.0 | 1.1e-38 | 2.1e-44 | 307.2 | 178 | (2, 180) | 423 | (95, 272) | 647 | Variable tail fiber protein | Variable tail fiber protein | | uniclust | UniRef100\_A0A097R5S7 | 100.0 | 1.1e-38 | 2.2e-44 | 291.9 | 171 | (2, 172) | 423 | (17, 187) | 428 | Phage tail protein | Phage tail protein | | uniclust | UniRef100\_D1NYY3 | 100.0 | 1.6e-38 | 3.1e-44 | 296.7 | 155 | (2, 156) | 423 | (1, 155) | 501 | Phage tail fiber repeat protein | Phage tail fiber repeat protein | | uniclust | UniRef100\_A0A2S4QEE2 | 100.0 | 1.9e-38 | 3.6e-44 | 306.1 | 299 | (1, 304) | 423 | (2, 340) | 691 | Phage tail protein | Phage tail protein | | uniclust | UniRef100\_A0A2I5TL70 | 100.0 | 2.6e-38 | 5e-44 | 287.2 | 160 | (2, 161) | 423 | (25, 184) | 445 | Phage tail protein | Phage tail protein | | uniclust | UniRef100\_A0A0D8RJM6 | 100.0 | 2.7e-38 | 5.1e-44 | 257.9 | 201 | (2, 303) | 423 | (6, 207) | 240 | Phage tail protein | Phage tail protein | | uniclust | UniRef100\_A0A0X4EUS0 | 100.0 | 3.5e-38 | 6.8e-44 | 310.2 | 159 | (2, 160) | 423 | (98, 257) | 761 | Phage tail protein | Phage tail protein | | uniclust | UniRef100\_A0A1I6E807 | 100.0 | 3.9e-38 | 7.5e-44 | 306.7 | 299 | (4, 307) | 423 | (33, 370) | 825 | Phage tail fibre repeat-containing protein | Phage tail fibre repeat-containing protein | | uniclust | UniRef100\_A0A080I1Q9 | 100.0 | 4.3e-38 | 8.4e-44 | 299.3 | 172 | (2, 173) | 423 | (6, 177) | 599 | Phage tail-collar fiber family protein | Phage tail-collar fiber family protein | | uniclust | UniRef100\_A0A376PRJ2 | 100.0 | 4.9e-38 | 9e-44 | 253.0 | 235 | (2, 341) | 423 | (3, 237) | 241 | Phage tail collar protein | Phage tail collar protein | | uniclust | UniRef100\_A0A6H2GG96 | 100.0 | 6.7e-38 | 1.2e-43 | 285.0 | 158 | (2, 159) | 423 | (3, 160) | 694 | Phage tail protein | Phage tail protein | | uniclust | UniRef100\_A0A2D0ITF1 | 100.0 | 7e-38 | 1.3e-43 | 293.9 | 158 | (3, 160) | 423 | (4, 161) | 857 | Phage tail fiber protein | Phage tail fiber protein | | uniclust | UniRef100\_A0A077PGX7 | 100.0 | 7.2e-38 | 1.3e-43 | 277.9 | 154 | (3, 156) | 423 | (4, 157) | 512 | Uncharacterized protein | Uncharacterized protein | | uniclust | UniRef100\_A0A379VLX8 | 100.0 | 1.5e-37 | 2.8e-43 | 259.9 | 171 | (2, 172) | 423 | (3, 173) | 335 | Phage tail fiber protein | Phage tail fiber protein | | uniclust | UniRef100\_A0A080HIT0 | 100.0 | 1.7e-37 | 3.1e-43 | 279.4 | 170 | (2, 171) | 423 | (3, 172) | 499 | Phage tail-collar fiber family protein | Phage tail-collar fiber family protein | | uniclust | UniRef100\_A0A2R8CQR2 | 100.0 | 1.8e-37 | 3.3e-43 | 263.6 | 160 | (1, 160) | 423 | (1, 160) | 364 | Uncharacterized protein | Uncharacterized protein | | uniclust | UniRef100\_A0A0T9KM71 | 100.0 | 2e-37 | 3.7e-43 | 270.8 | 152 | (3, 154) | 423 | (39, 190) | 413 | Variable tail fiber protein | Variable tail fiber protein | | uniclust | UniRef100\_A0A1E7PZY5 | 100.0 | 2.4e-37 | 4.7e-43 | 262.0 | 160 | (1, 160) | 423 | (2, 161) | 271 | Phage tail protein | Phage tail protein | | uniclust | UniRef100\_A0A806X791 | 100.0 | 3e-37 | 5.7e-43 | 272.2 | 210 | (1, 300) | 423 | (5, 214) | 390 | Phage tail collar domain-containing protein | Phage tail collar domain-containing protein | | uniclust | UniRef100\_A0A2H4JF09 | 100.0 | 7.3e-37 | 1.4e-42 | 267.8 | 157 | (2, 158) | 423 | (6, 162) | 416 | Putative tail collar domain protein | Putative tail collar domain protein | | uniclust | UniRef100\_A0A7V8UDY7 | 100.0 | 1.1e-36 | 2.1e-42 | 276.8 | 155 | (2, 156) | 423 | (6, 160) | 572 | Phage tail protein | Phage tail protein | | uniclust | UniRef100\_A0A0A0GX64 | 100.0 | 1.3e-36 | 2.4e-42 | 299.1 | 203 | (1, 304) | 423 | (5, 208) | 791 | Putative tail fiber protein | Putative tail fiber protein | | uniclust | UniRef100\_A0A1G9EQI8 | 100.0 | 1.9e-36 | 3.5e-42 | 272.8 | 158 | (1, 158) | 423 | (5, 162) | 420 | Phage-related tail fibre protein | Phage-related tail fibre protein | | uniclust | UniRef100\_UPI001F4EC7D7 | 100.0 | 2e-36 | 3.6e-42 | 261.3 | 157 | (2, 158) | 423 | (3, 159) | 428 | phage tail protein | phage tail protein | | uniclust | UniRef100\_A0A3S4G527 | 100.0 | 2.2e-36 | 4.1e-42 | 265.2 | 244 | (2, 302) | 423 | (3, 247) | 426 | Phage variable tail-fiber protein | Phage variable tail-fiber protein | | uniclust | UniRef100\_A0A085GA02 | 100.0 | 2.3e-36 | 4.4e-42 | 285.9 | 160 | (1, 160) | 423 | (5, 165) | 622 | Putative tail fiber protein | Putative tail fiber protein | | uniclust | UniRef100\_A0A085JCX9 | 100.0 | 3e-36 | 5.9e-42 | 296.7 | 213 | (1, 303) | 423 | (96, 308) | 688 | Tail fiber protein | Tail fiber protein | | uniclust | UniRef100\_A0A3M2VPF6 | 100.0 | 3.7e-36 | 7e-42 | 262.9 | 151 | (2, 152) | 423 | (6, 156) | 316 | Putative Tail fiber protein H | Putative Tail fiber protein H | | uniclust | UniRef100\_A0A0Q0EQE1 | 100.0 | 3.8e-36 | 7.2e-42 | 270.2 | 156 | (1, 156) | 423 | (5, 160) | 434 | Phage tail fiber protein h | Phage tail fiber protein h | | uniclust | UniRef100\_A0A2K8QMP4 | 100.0 | 4.9e-36 | 9.1e-42 | 257.8 | 170 | (2, 171) | 423 | (3, 172) | 383 | Phage tail protein | Phage tail protein | | uniclust | UniRef100\_UPI0010D6282B | 100.0 | 5.7e-36 | 1.1e-41 | 228.6 | 153 | (1, 153) | 423 | (2, 154) | 169 | phage tail protein | phage tail protein | | uniclust | UniRef100\_A0A264VRT5 | 100.0 | 5.9e-36 | 1.1e-41 | 284.9 | 156 | (2, 157) | 423 | (3, 158) | 568 | Phage tail collar domain-containing protein | Phage tail collar domain-containing protein | | uniclust | UniRef100\_A0A0D6BKK2 | 100.0 | 6e-36 | 1.2e-41 | 286.7 | 157 | (1, 157) | 423 | (5, 161) | 605 | Phage-related tail fibre protein-like protein | Phage-related tail fibre protein-like protein | | uniclust | UniRef100\_A0A1H0SWR0 | 100.0 | 6.4e-36 | 1.2e-41 | 269.6 | 154 | (4, 157) | 423 | (5, 158) | 549 | Phage tail-collar fibre protein | Phage tail-collar fibre protein | | uniclust | UniRef100\_A0A0E1ST60 | 100.0 | 6.5e-36 | 1.3e-41 | 295.6 | 292 | (3, 301) | 423 | (6, 336) | 808 | GpH | GpH | | uniclust | UniRef100\_A0A068Z6W7 | 100.0 | 9.4e-36 | 1.8e-41 | 257.4 | 169 | (2, 170) | 423 | (3, 171) | 410 | Phage tail protein | Phage tail protein | | uniclust | UniRef100\_UPI0009E43696 | 100.0 | 9.6e-36 | 1.8e-41 | 269.7 | 169 | (2, 171) | 423 | (3, 171) | 583 | phage tail protein | phage tail protein | | uniclust | UniRef100\_A0A0P9YJT9 | 100.0 | 9.2e-36 | 1.8e-41 | 293.7 | 159 | (1, 159) | 423 | (5, 163) | 710 | Putative Tail fiber protein H | Putative Tail fiber protein H | | uniclust | UniRef100\_A0A2K8VZI6 | 100.0 | 1.1e-35 | 2e-41 | 268.0 | 153 | (3, 155) | 423 | (30, 182) | 542 | Phage tail fiber protein | Phage tail fiber protein | | uniclust | UniRef100\_V5YST5 | 100.0 | 1.1e-35 | 2.1e-41 | 259.9 | 279 | (1, 280) | 423 | (1, 279) | 550 | Putative tail-collar fibre protein | Putative tail-collar fibre protein | | uniclust | UniRef100\_A0A1X7LWW5 | 100.0 | 1.2e-35 | 2.3e-41 | 269.1 | 159 | (3, 161) | 423 | (4, 162) | 512 | Phage tail fibre repeat-containing protein | Phage tail fibre repeat-containing protein | | uniclust | UniRef100\_A0A024EJX5 | 100.0 | 1.2e-35 | 2.4e-41 | 293.9 | 157 | (1, 157) | 423 | (5, 161) | 754 | Tail fiber protein H, putative | Tail fiber protein H, putative | | uniclust | UniRef100\_A0A3B7L361 | 100.0 | 1.3e-35 | 2.4e-41 | 273.2 | 153 | (1, 153) | 423 | (3, 155) | 491 | Phage tail protein | Phage tail protein | | uniclust | UniRef100\_A0A747R6J3 | 100.0 | 1.4e-35 | 2.7e-41 | 255.4 | 157 | (2, 158) | 423 | (3, 159) | 359 | Phage tail protein | Phage tail protein | | uniclust | UniRef100\_A0A0N9MHE6 | 100.0 | 1.5e-35 | 3e-41 | 290.9 | 156 | (2, 157) | 423 | (6, 161) | 695 | Phage tail fiber protein | Phage tail fiber protein | | uniclust | UniRef100\_A0A379QSS5 | 100.0 | 2.2e-35 | 4.1e-41 | 262.4 | 295 | (3, 306) | 423 | (4, 313) | 540 | Tail protein | Tail protein | | uniclust | UniRef100\_A0A1B9KN89 | 100.0 | 2.5e-35 | 4.7e-41 | 269.5 | 156 | (1, 156) | 423 | (2, 157) | 619 | Phage tail protein | Phage tail protein | | uniclust | UniRef100\_A0A0Q8Y205 | 100.0 | 4.2e-35 | 7.9e-41 | 267.8 | 158 | (1, 158) | 423 | (2, 159) | 505 | Phage tail protein | Phage tail protein | | uniclust | UniRef100\_A0A0H3F5Q0 | 100.0 | 6.9e-35 | 1.3e-40 | 277.6 | 160 | (1, 160) | 423 | (1, 160) | 960 | Tail fiber repeat 2 protein | Tail fiber repeat 2 protein | | uniclust | UniRef100\_UPI00210164A2 | 100.0 | 9.3e-35 | 1.7e-40 | 239.9 | 252 | (2, 253) | 423 | (3, 261) | 333 | phage tail protein | phage tail protein | | uniclust | UniRef100\_A0A248LIJ6 | 100.0 | 9.2e-35 | 1.7e-40 | 263.3 | 154 | (2, 155) | 423 | (3, 156) | 521 | Tail fiber protein 2 | Tail fiber protein 2 | | uniclust | UniRef100\_A0A5J2VSE0 | 100.0 | 9.5e-35 | 1.8e-40 | 266.5 | 286 | (2, 304) | 423 | (3, 293) | 581 | Phage tail protein | Phage tail protein | | uniclust | UniRef100\_A0A0T9TRH4 | 100.0 | 9.2e-35 | 1.8e-40 | 278.7 | 163 | (2, 164) | 423 | (57, 219) | 551 | Tail fiber protein | Tail fiber protein | | uniclust | UniRef100\_A0A1A0FJZ1 | 100.0 | 9.5e-35 | 1.8e-40 | 267.6 | 158 | (3, 160) | 423 | (7, 164) | 418 | Phage tail protein | Phage tail protein | | uniclust | UniRef100\_A0A1G3JRW0 | 100.0 | 1.2e-34 | 2.2e-40 | 274.6 | 240 | (3, 246) | 423 | (6, 263) | 629 | Phage tail protein | Phage tail protein | | uniclust | UniRef100\_A0A0W0MDX6 | 100.0 | 1.8e-34 | 3.4e-40 | 242.6 | 160 | (1, 160) | 423 | (1, 161) | 312 | Phage tail protein | Phage tail protein | | uniclust | UniRef100\_A0A0H3NZ57 | 100.0 | 1.9e-34 | 3.5e-40 | 254.3 | 157 | (2, 158) | 423 | (3, 159) | 514 | Phage tail fiber protein | Phage tail fiber protein | | uniclust | UniRef100\_A0A0T9KX86 | 100.0 | 2.4e-34 | 4.4e-40 | 268.1 | 299 | (2, 304) | 423 | (1, 338) | 658 | Tail fiber repeat 2-containing protein | Tail fiber repeat 2-containing protein | | uniclust | UniRef100\_A0A1M4Y377 | 100.0 | 2.4e-34 | 4.6e-40 | 261.3 | 160 | (1, 160) | 423 | (1, 160) | 542 | Phage tail-collar fibre protein | Phage tail-collar fibre protein | | uniclust | UniRef100\_A0A1Y2SIM3 | 100.0 | 3.1e-34 | 5.6e-40 | 258.9 | 158 | (3, 160) | 423 | (4, 161) | 675 | Tail protein | Tail protein | | uniclust | UniRef100\_A0A0D1M6C4 | 100.0 | 4.5e-34 | 8.6e-40 | 272.2 | 155 | (2, 156) | 423 | (6, 160) | 756 | Phage tail collar domain-containing protein | Phage tail collar domain-containing protein | | uniclust | UniRef100\_UPI000AC960B9 | 100.0 | 1.1e-33 | 2.1e-39 | 224.9 | 156 | (2, 157) | 423 | (3, 158) | 244 | phage tail protein | phage tail protein | | uniclust | UniRef100\_A0A1C3H901 | 100.0 | 1.3e-33 | 2.4e-39 | 256.4 | 161 | (2, 162) | 423 | (3, 163) | 655 | Phage tail fiber repeat protein | Phage tail fiber repeat protein | | uniclust | UniRef100\_A0A2C9P0H8 | 100.0 | 2.1e-33 | 3.9e-39 | 253.8 | 157 | (2, 158) | 423 | (5, 162) | 581 | Phage tail protein | Phage tail protein | | uniclust | UniRef100\_A0A376DLB6 | 100.0 | 2.4e-33 | 4.5e-39 | 249.0 | 156 | (2, 157) | 423 | (3, 161) | 430 | Phage tail fibre repeat | Phage tail fibre repeat | | uniclust | UniRef100\_A0A0H3NQY6 | 100.0 | 2.5e-33 | 4.7e-39 | 210.0 | 131 | (3, 133) | 423 | (7, 137) | 139 | Phage tail fiber protein | Phage tail fiber protein | | uniclust | UniRef100\_A0A1I3XGI0 | 100.0 | 2.7e-33 | 4.9e-39 | 234.7 | 152 | (3, 154) | 423 | (4, 155) | 372 | Phage tail fibre repeat-containing protein (Fragment) | Phage tail fibre repeat-containing protein (Fragment) | | uniclust | UniRef100\_A0A2K9QFQ7 | 100.0 | 2.7e-33 | 5.1e-39 | 247.6 | 168 | (3, 170) | 423 | (4, 171) | 474 | Phage tail protein | Phage tail protein | | uniclust | UniRef100\_A0A4P7L2V6 | 100.0 | 3.1e-33 | 5.8e-39 | 249.9 | 154 | (2, 155) | 423 | (1, 154) | 508 | Phage tail-collar fiber protein | Phage tail-collar fiber protein | | uniclust | UniRef100\_A0A4R0FQS3 | 100.0 | 3.1e-33 | 5.8e-39 | 244.3 | 157 | (2, 160) | 423 | (3, 159) | 399 | Phage tail protein | Phage tail protein | | uniclust | UniRef100\_A0A1H0YPL5 | 100.0 | 3.3e-33 | 6.3e-39 | 248.1 | 165 | (1, 165) | 423 | (3, 167) | 405 | Phage tail-collar fibre protein | Phage tail-collar fibre protein | | uniclust | UniRef100\_A0A0A3AYU0 | 100.0 | 3.7e-33 | 6.9e-39 | 247.2 | 150 | (3, 152) | 423 | (4, 153) | 466 | Phage tail fiber protein | Phage tail fiber protein | | uniclust | UniRef100\_A0A0A1HYZ6 | 100.0 | 3.9e-33 | 7.2e-39 | 251.2 | 155 | (1, 155) | 423 | (1, 156) | 513 | Prophage long tail fiber protein | Prophage long tail fiber protein | | uniclust | UniRef100\_A0A143DGR5 | 100.0 | 3.8e-33 | 7.4e-39 | 257.5 | 154 | (1, 154) | 423 | (18, 171) | 425 | Phage tail collar domain-containing protein | Phage tail collar domain-containing protein | | uniclust | UniRef100\_A0A0F7Y0J9 | 100.0 | 4.6e-33 | 8.7e-39 | 269.9 | 155 | (2, 156) | 423 | (6, 160) | 1016 | Phage-related tail fiber protein-like protein | Phage-related tail fiber protein-like protein | | uniclust | UniRef100\_A0A6M0RCD0 | 100.0 | 4.9e-33 | 9.4e-39 | 234.5 | 152 | (2, 153) | 423 | (5, 156) | 261 | Phage tail protein (Fragment) | Phage tail protein (Fragment) | | uniclust | UniRef100\_A0A6D0UUI3 | 100.0 | 5.2e-33 | 9.6e-39 | 245.7 | 157 | (2, 158) | 423 | (3, 159) | 576 | Phage tail protein | Phage tail protein | | uniclust | UniRef100\_UPI0002FB3713 | 100.0 | 6.3e-33 | 1.2e-38 | 224.9 | 156 | (2, 157) | 423 | (6, 161) | 243 | phage tail protein | phage tail protein | | uniclust | UniRef100\_A0A376TTQ8 | 100.0 | 6.9e-33 | 1.3e-38 | 209.4 | 155 | (2, 156) | 423 | (3, 157) | 168 | Variable tail fiber protein | Variable tail fiber protein | | uniclust | UniRef100\_A0A077NSD4 | 100.0 | 7.5e-33 | 1.4e-38 | 257.0 | 158 | (3, 160) | 423 | (4, 161) | 922 | Phage tail collar domain-containing protein | Phage tail collar domain-containing protein | | uniclust | UniRef100\_A0A8T6A9G5 | 100.0 | 7.6e-33 | 1.4e-38 | 241.7 | 158 | (3, 160) | 423 | (7, 165) | 478 | Phage tail protein (Fragment) | Phage tail protein (Fragment) | | uniclust | UniRef100\_A0A077K818 | 100.0 | 7.6e-33 | 1.4e-38 | 255.3 | 155 | (3, 157) | 423 | (5, 159) | 555 | Phage tail collar domain-containing protein | Phage tail collar domain-containing protein | | uniclust | UniRef100\_A0A1D3JVK3 | 100.0 | 8.6e-33 | 1.6e-38 | 251.0 | 156 | (2, 157) | 423 | (6, 161) | 474 | Phage tail protein | Phage tail protein | | uniclust | UniRef100\_A0A2D0JYZ8 | 100.0 | 1e-32 | 1.8e-38 | 231.7 | 204 | (1, 301) | 423 | (1, 204) | 376 | Phage tail fiber protein | Phage tail fiber protein | | uniclust | UniRef100\_A0A1N6MS83 | 100.0 | 1.2e-32 | 2.3e-38 | 241.3 | 155 | (1, 155) | 423 | (1, 155) | 550 | Uncharacterized protein | Uncharacterized protein | | uniclust | UniRef100\_A0A4Y9K5F2 | 100.0 | 1.4e-32 | 2.6e-38 | 264.2 | 152 | (1, 152) | 423 | (3, 154) | 667 | Phage tail protein | Phage tail protein | | uniclust | UniRef100\_UPI000A46B6C7 | 100.0 | 1.8e-32 | 3.3e-38 | 249.4 | 159 | (2, 160) | 423 | (6, 165) | 653 | phage tail protein | phage tail protein | | uniclust | UniRef100\_A0A7S4LVA4 | 100.0 | 2.1e-32 | 3.9e-38 | 245.1 | 160 | (1, 160) | 423 | (1, 160) | 587 | Tail fiber protein | Tail fiber protein | | uniclust | UniRef100\_A0A1B9JGB5 | 100.0 | 2.3e-32 | 4.3e-38 | 247.9 | 254 | (2, 300) | 423 | (3, 262) | 665 | Phage tail collar domain-containing protein | Phage tail collar domain-containing protein | | uniclust | UniRef100\_UPI000445DE18 | 100.0 | 2.4e-32 | 4.5e-38 | 244.4 | 159 | (3, 161) | 423 | (7, 166) | 482 | tail fiber protein | tail fiber protein | | uniclust | UniRef100\_A0A0A1AI95 | 100.0 | 2.5e-32 | 4.7e-38 | 257.0 | 159 | (1, 159) | 423 | (2, 161) | 889 | Phage tail protein | Phage tail protein | | uniclust | UniRef100\_A0A0A7NV63 | 99.9 | 2.7e-32 | 5.1e-38 | 268.4 | 159 | (2, 160) | 423 | (295, 454) | 1033 | Tail fiber protein | Tail fiber protein | | uniclust | UniRef100\_A0A101LTG1 | 99.9 | 3.3e-32 | 6.2e-38 | 250.0 | 157 | (3, 159) | 423 | (5, 162) | 633 | Phage tail protein | Phage tail protein | | uniclust | UniRef100\_A0A0F3TBN3 | 99.9 | 3.3e-32 | 6.2e-38 | 264.0 | 296 | (4, 304) | 423 | (2, 336) | 726 | Phage tail protein | Phage tail protein | | uniclust | UniRef100\_A0A0N9MHW1 | 99.9 | 4.1e-32 | 7.7e-38 | 271.0 | 156 | (2, 157) | 423 | (6, 161) | 1312 | Phage tail fiber protein | Phage tail fiber protein | | uniclust | UniRef100\_A0A776QF13 | 99.9 | 5e-32 | 9.3e-38 | 231.5 | 198 | (2, 300) | 423 | (3, 201) | 389 | Phage tail protein (Fragment) | Phage tail protein (Fragment) | | uniclust | UniRef100\_A0A1N6I148 | 99.9 | 5.1e-32 | 9.6e-38 | 249.9 | 164 | (1, 164) | 423 | (2, 165) | 519 | Phage tail-collar fibre protein | Phage tail-collar fibre protein | | uniclust | UniRef100\_A0A2U8I2Y4 | 99.9 | 5.5e-32 | 1e-37 | 235.4 | 208 | (3, 300) | 423 | (4, 211) | 509 | Phage tail protein | Phage tail protein | | uniclust | UniRef100\_A0A0M1LJ12 | 99.9 | 5.5e-32 | 1e-37 | 229.7 | 152 | (3, 154) | 423 | (8, 159) | 299 | Phage tail protein | Phage tail protein | | uniclust | UniRef100\_A0A858NPD6 | 99.9 | 6.1e-32 | 1.1e-37 | 248.3 | 262 | (1, 263) | 423 | (1, 262) | 775 | Tail fiber protein | Tail fiber protein | | uniclust | UniRef100\_A0A398UJ84 | 99.9 | 6.5e-32 | 1.2e-37 | 242.4 | 161 | (1, 161) | 423 | (5, 166) | 575 | Phage tail protein (Fragment) | Phage tail protein (Fragment) | | uniclust | UniRef100\_UPI001BC85BAF | 99.9 | 8.4e-32 | 1.6e-37 | 246.4 | 160 | (1, 160) | 423 | (1, 160) | 715 | phage tail protein | phage tail protein | | uniclust | UniRef100\_A0A3F3IWG0 | 99.9 | 1.1e-31 | 2.1e-37 | 245.0 | 159 | (2, 160) | 423 | (5, 164) | 575 | Bacteriophage T7 Gp17 C-terminal domain-containing protein | Bacteriophage T7 Gp17 C-terminal domain-containing protein | | uniclust | UniRef100\_UPI001140E494 | 99.9 | 1.7e-31 | 3.2e-37 | 235.7 | 160 | (1, 160) | 423 | (1, 160) | 550 | phage tail protein | phage tail protein | | uniclust | UniRef100\_G9Z5T4 | 99.9 | 1.9e-31 | 3.6e-37 | 259.1 | 295 | (4, 303) | 423 | (2, 335) | 753 | Phage tail collar domain protein | Phage tail collar domain protein | | uniclust | UniRef100\_A0A0C1RAC0 | 99.9 | 2e-31 | 3.9e-37 | 247.4 | 153 | (2, 154) | 423 | (7, 159) | 477 | Phage tail-collar fiber family protein | Phage tail-collar fiber family protein | | uniclust | UniRef100\_A0A1W1XL57 | 99.9 | 2.2e-31 | 4e-37 | 216.1 | 157 | (1, 157) | 423 | (2, 158) | 234 | Phage tail-collar fibre protein | Phage tail-collar fibre protein | | uniclust | UniRef100\_A0A0H3FAF6 | 99.9 | 2.5e-31 | 4.6e-37 | 249.8 | 160 | (1, 160) | 423 | (1, 160) | 795 | Tail fiber repeat 2 protein | Tail fiber repeat 2 protein | | uniclust | UniRef100\_A0A430HF82 | 99.9 | 2.5e-31 | 4.6e-37 | 214.7 | 258 | (2, 263) | 423 | (1, 258) | 276 | Phage tail protein | Phage tail protein | | uniclust | UniRef100\_A0A068QZ58 | 99.9 | 2.8e-31 | 5.3e-37 | 256.5 | 301 | (2, 307) | 423 | (17, 356) | 709 | Bacteriophage T7 Gp17 C-terminal domain-containing protein | Bacteriophage T7 Gp17 C-terminal domain-containing protein | | uniclust | UniRef100\_A0A5B8HYX9 | 99.9 | 2.9e-31 | 5.3e-37 | 236.3 | 158 | (3, 160) | 423 | (24, 181) | 488 | Phage tail protein | Phage tail protein | | uniclust | UniRef100\_A0A077NM26 | 99.9 | 3e-31 | 5.6e-37 | 236.4 | 154 | (3, 156) | 423 | (4, 157) | 641 | Uncharacterized protein | Uncharacterized protein | | uniclust | UniRef100\_UPI00098C1720 | 99.9 | 3.2e-31 | 5.8e-37 | 251.7 | 159 | (2, 160) | 423 | (3, 161) | 1423 | tail fiber protein | tail fiber protein | | uniclust | UniRef100\_A0A1H5GVL6 | 99.9 | 3.1e-31 | 5.8e-37 | 238.0 | 155 | (2, 156) | 423 | (6, 160) | 476 | Phage-related tail fibre protein | Phage-related tail fibre protein | | uniclust | UniRef100\_A0A2X1N770 | 99.9 | 3.3e-31 | 6.1e-37 | 245.6 | 160 | (1, 160) | 423 | (5, 165) | 710 | Tail fiber protein | Tail fiber protein | | uniclust | UniRef100\_A0A0D4ZXS4 | 99.9 | 3.7e-31 | 7e-37 | 236.1 | 163 | (1, 163) | 423 | (17, 179) | 374 | Tail fiber protein | Tail fiber protein | | uniclust | UniRef100\_UPI000AE7EBAD | 99.9 | 3.8e-31 | 7.1e-37 | 233.3 | 155 | (2, 156) | 423 | (6, 160) | 434 | phage tail protein | phage tail protein | | uniclust | UniRef100\_A0A1H4Y3H3 | 99.9 | 3.8e-31 | 7.1e-37 | 253.1 | 243 | (1, 247) | 423 | (5, 264) | 787 | Phage Tail Collar Domain | Phage Tail Collar Domain | | uniclust | UniRef100\_A0A433ZYK3 | 99.9 | 3.9e-31 | 7.2e-37 | 213.2 | 156 | (2, 157) | 423 | (3, 158) | 249 | Phage tail protein (Fragment) | Phage tail protein (Fragment) | | uniclust | UniRef100\_A0A1S6KZZ8 | 99.9 | 3.8e-31 | 7.3e-37 | 253.0 | 169 | (2, 170) | 423 | (51, 219) | 738 | Tail fiber protein | Tail fiber protein | | uniclust | UniRef100\_UPI0005E045F7 | 99.9 | 4.3e-31 | 7.8e-37 | 222.5 | 162 | (2, 163) | 423 | (3, 164) | 364 | phage tail protein | phage tail protein | | uniclust | UniRef100\_UPI001FC9489E | 99.9 | 5.4e-31 | 9.9e-37 | 212.1 | 203 | (1, 304) | 423 | (1, 203) | 270 | phage tail protein | phage tail protein | | uniclust | UniRef100\_C5BEP6 | 99.9 | 5.8e-31 | 1.1e-36 | 224.7 | 158 | (2, 159) | 423 | (3, 160) | 426 | Phage tail fiber domain protein | Phage tail fiber domain protein | | uniclust | UniRef100\_A0A024E8J4 | 99.9 | 6.1e-31 | 1.2e-36 | 245.4 | 156 | (2, 157) | 423 | (6, 161) | 640 | Tail fiber protein H, putative | Tail fiber protein H, putative | | uniclust | UniRef100\_UPI000F950B8D | 99.9 | 6.5e-31 | 1.2e-36 | 228.0 | 159 | (1, 159) | 423 | (2, 160) | 423 | phage tail protein | phage tail protein | | uniclust | UniRef100\_A0A484ZG34 | 99.9 | 1e-30 | 1.8e-36 | 215.3 | 219 | (2, 310) | 423 | (3, 221) | 317 | Phage tail fibre repeat | Phage tail fibre repeat | | uniclust | UniRef100\_UPI000B30225B | 99.9 | 1e-30 | 1.9e-36 | 230.7 | 158 | (3, 160) | 423 | (4, 162) | 575 | tail fiber protein | tail fiber protein | | uniclust | UniRef100\_A0A0Q8DAL0 | 99.9 | 1.1e-30 | 2.1e-36 | 239.3 | 256 | (2, 262) | 423 | (7, 279) | 629 | Phage tail protein | Phage tail protein | | uniclust | UniRef100\_A0A836YZF5 | 99.9 | 1.2e-30 | 2.2e-36 | 229.1 | 150 | (3, 152) | 423 | (4, 153) | 394 | Tail fiber protein (Fragment) | Tail fiber protein (Fragment) | | uniclust | UniRef100\_A0A447PAU2 | 99.9 | 1.4e-30 | 2.5e-36 | 226.1 | 170 | (2, 171) | 423 | (3, 172) | 446 | Phage tail-like protein | Phage tail-like protein | | uniclust | UniRef100\_A0A495BJ48 | 99.9 | 1.4e-30 | 2.6e-36 | 223.2 | 155 | (1, 155) | 423 | (2, 156) | 380 | Tail-collar fiber protein | Tail-collar fiber protein | | uniclust | UniRef100\_A0A2X2BLJ3 | 99.9 | 1.6e-30 | 3e-36 | 212.0 | 155 | (2, 156) | 423 | (3, 157) | 277 | Phage variable tail fiber protein | Phage variable tail fiber protein | | uniclust | UniRef100\_UPI001CE1390D | 99.9 | 2e-30 | 3.6e-36 | 241.8 | 170 | (2, 171) | 423 | (3, 172) | 852 | phage tail protein | phage tail protein | | uniclust | UniRef100\_A0A3Z6GBS8 | 99.9 | 1.9e-30 | 3.6e-36 | 233.7 | 159 | (2, 160) | 423 | (5, 164) | 563 | Phage tail protein | Phage tail protein | | uniclust | UniRef100\_UPI001FFE7AE7 | 99.9 | 2.3e-30 | 4.2e-36 | 216.7 | 156 | (3, 158) | 423 | (4, 159) | 359 | phage tail protein | phage tail protein | | uniclust | UniRef100\_A0A1B9JGD4 | 99.9 | 2.4e-30 | 4.5e-36 | 221.5 | 156 | (3, 158) | 423 | (4, 159) | 381 | Phage tail collar domain-containing protein | Phage tail collar domain-containing protein | | uniclust | UniRef100\_UPI0018A29913 | 99.9 | 2.8e-30 | 5.2e-36 | 223.8 | 172 | (2, 173) | 423 | (3, 174) | 430 | phage tail protein | phage tail protein | | uniclust | UniRef100\_A0A072Y2M2 | 99.9 | 3e-30 | 5.7e-36 | 233.2 | 153 | (1, 153) | 423 | (3, 155) | 441 | Putative phage tail fiber protein | Putative phage tail fiber protein | | uniclust | UniRef100\_A0A0D6BCT9 | 99.9 | 3.9e-30 | 7.3e-36 | 248.3 | 243 | (1, 247) | 423 | (5, 265) | 1040 | Phage-related tail fibre protein-like protein | Phage-related tail fibre protein-like protein | | uniclust | UniRef100\_A0A2V1HJ68 | 99.9 | 3.9e-30 | 7.3e-36 | 229.5 | 368 | (4, 375) | 423 | (2, 484) | 526 | Phage tail protein | Phage tail protein | | uniclust | UniRef100\_UPI00224B8CEA | 99.9 | 4.3e-30 | 7.8e-36 | 219.1 | 160 | (1, 160) | 423 | (1, 160) | 419 | phage tail protein | phage tail protein | | uniclust | UniRef100\_A0A827QGW3 | 99.9 | 5.7e-30 | 1.1e-35 | 223.8 | 158 | (3, 160) | 423 | (4, 161) | 364 | Phage tail protein (Fragment) | Phage tail protein (Fragment) | | uniclust | UniRef100\_A0A0F4TBD1 | 99.9 | 6.1e-30 | 1.1e-35 | 235.5 | 156 | (2, 157) | 423 | (6, 161) | 585 | Phage tail protein | Phage tail protein | | uniclust | UniRef100\_A0A8E1QKA4 | 99.9 | 6.6e-30 | 1.2e-35 | 210.9 | 152 | (2, 153) | 423 | (3, 154) | 255 | Tail fiber protein (Fragment) | Tail fiber protein (Fragment) | | uniclust | UniRef100\_A0A853HVL0 | 99.9 | 7.1e-30 | 1.3e-35 | 214.8 | 163 | (3, 165) | 423 | (4, 169) | 332 | Phage tail protein | Phage tail protein | | uniclust | UniRef100\_UPI001AD852BE | 99.9 | 7.6e-30 | 1.4e-35 | 220.7 | 160 | (1, 160) | 423 | (1, 160) | 474 | phage tail protein | phage tail protein | | uniclust | UniRef100\_A0A5U3F244 | 99.9 | 7.8e-30 | 1.5e-35 | 227.4 | 319 | (4, 333) | 423 | (2, 359) | 431 | Phage tail protein (Fragment) | Phage tail protein (Fragment) | | uniclust | UniRef100\_A0A5T5IEY5 | 99.9 | 8.3e-30 | 1.6e-35 | 223.5 | 157 | (2, 158) | 423 | (5, 162) | 401 | Phage tail protein (Fragment) | Phage tail protein (Fragment) | | uniclust | UniRef100\_A0A0S4I2P4 | 99.9 | 9e-30 | 1.7e-35 | 207.2 | 157 | (2, 158) | 423 | (6, 162) | 255 | Uncharacterized protein | Uncharacterized protein | | uniclust | UniRef100\_UPI00041B59CC | 99.9 | 1e-29 | 1.9e-35 | 222.7 | 170 | (2, 171) | 423 | (3, 172) | 474 | phage tail protein | phage tail protein | | uniclust | UniRef100\_A0A7X1Y572 | 99.9 | 1e-29 | 1.9e-35 | 231.6 | 156 | (2, 157) | 423 | (6, 161) | 592 | Phage tail protein | Phage tail protein | | uniclust | UniRef100\_A0A2M8SLT6 | 99.9 | 1.3e-29 | 2.3e-35 | 220.3 | 152 | (2, 153) | 423 | (6, 157) | 409 | Phage tail protein | Phage tail protein | | uniclust | UniRef100\_A0A0F4VR40 | 99.9 | 1.3e-29 | 2.5e-35 | 236.2 | 221 | (2, 230) | 423 | (7, 231) | 497 | Phage tail fiber protein | Phage tail fiber protein | | uniclust | UniRef100\_A0A3S5ENC7 | 99.9 | 1.5e-29 | 2.7e-35 | 216.1 | 158 | (3, 160) | 423 | (4, 161) | 383 | Putative bacteriophage tail fiber protein | Putative bacteriophage tail fiber protein | | uniclust | UniRef100\_UPI001E2832F3 | 99.9 | 1.6e-29 | 2.9e-35 | 216.0 | 155 | (2, 156) | 423 | (1, 155) | 423 | phage tail protein | phage tail protein | | uniclust | UniRef100\_A0A1C6YVI6 | 99.9 | 1.9e-29 | 3.5e-35 | 236.7 | 156 | (1, 160) | 423 | (32, 190) | 595 | Phage-related tail fibre protein | Phage-related tail fibre protein | | uniclust | UniRef100\_A0A084ZPT2 | 99.9 | 2.1e-29 | 3.8e-35 | 214.6 | 155 | (1, 155) | 423 | (2, 156) | 330 | Phage tail fiber protein | Phage tail fiber protein | | uniclust | UniRef100\_UPI001EE069FF | 99.9 | 2.4e-29 | 4.4e-35 | 215.9 | 158 | (3, 160) | 423 | (4, 161) | 441 | phage tail protein | phage tail protein | | uniclust | UniRef100\_UPI001D141119 | 99.9 | 2.8e-29 | 5.2e-35 | 211.6 | 158 | (2, 159) | 423 | (3, 160) | 330 | phage tail protein | phage tail protein | | uniclust | UniRef100\_A0A4Q7BEF2 | 99.9 | 3.1e-29 | 5.8e-35 | 195.5 | 158 | (1, 158) | 423 | (2, 159) | 186 | Phage tail protein (Fragment) | Phage tail protein (Fragment) | | uniclust | UniRef100\_A0A1G4ZBJ6 | 99.9 | 3.1e-29 | 6e-35 | 244.6 | 250 | (1, 304) | 423 | (2, 253) | 728 | Phage T4 tail fibre | Phage T4 tail fibre | | uniclust | UniRef100\_UPI001C266F20 | 99.9 | 3.4e-29 | 6.2e-35 | 216.5 | 160 | (1, 160) | 423 | (1, 160) | 467 | phage tail protein | phage tail protein | | uniclust | UniRef100\_A0A1H0NP67 | 99.9 | 3.7e-29 | 6.9e-35 | 216.6 | 154 | (4, 157) | 423 | (5, 158) | 388 | Phage tail-collar fibre protein | Phage tail-collar fibre protein | | uniclust | UniRef100\_UPI0009B80218 | 99.9 | 4.1e-29 | 7.5e-35 | 211.0 | 167 | (2, 168) | 423 | (3, 169) | 382 | phage tail protein | phage tail protein | | uniclust | UniRef100\_A0A7V8F402 | 99.9 | 4.2e-29 | 7.8e-35 | 219.1 | 155 | (3, 157) | 423 | (4, 158) | 479 | Phage tail protein | Phage tail protein | | uniclust | UniRef100\_A0A069A3R7 | 99.9 | 4.6e-29 | 8.7e-35 | 212.6 | 152 | (3, 154) | 423 | (8, 159) | 301 | Putative phage tail fiber protein | Putative phage tail fiber protein | | uniclust | UniRef100\_UPI001476555E | 99.9 | 5e-29 | 9.3e-35 | 208.5 | 157 | (2, 158) | 423 | (6, 162) | 297 | phage tail protein | phage tail protein | | uniclust | UniRef100\_A0A381C7I1 | 99.9 | 5.2e-29 | 1e-34 | 233.8 | 149 | (1, 149) | 423 | (2, 150) | 504 | Phage tail fibre repeat | Phage tail fibre repeat | | uniclust | UniRef100\_A0A3M3BF19 | 99.9 | 5.5e-29 | 1e-34 | 181.2 | 116 | (2, 117) | 423 | (6, 121) | 122 | Tail fiber protein H (Fragment) | Tail fiber protein H (Fragment) | | uniclust | UniRef100\_UPI00201F34D1 | 99.9 | 5.8e-29 | 1.1e-34 | 221.7 | 159 | (2, 160) | 423 | (3, 162) | 624 | phage tail protein | phage tail protein | | uniclust | UniRef100\_UPI000A9C88D5 | 99.9 | 6e-29 | 1.1e-34 | 223.6 | 159 | (2, 160) | 423 | (3, 161) | 629 | phage tail protein | phage tail protein | | uniclust | UniRef100\_A0A1H7YIZ1 | 99.9 | 6.4e-29 | 1.2e-34 | 222.4 | 157 | (1, 157) | 423 | (2, 158) | 404 | Phage tail-collar fibre protein | Phage tail-collar fibre protein | | uniclust | UniRef100\_A0A381I8S9 | 99.9 | 7.5e-29 | 1.4e-34 | 205.9 | 154 | (3, 156) | 423 | (8, 161) | 266 | Phage tail fiber protein | Phage tail fiber protein | | uniclust | UniRef100\_UPI0009F41D80 | 99.9 | 7.5e-29 | 1.4e-34 | 231.1 | 156 | (2, 157) | 423 | (6, 161) | 607 | phage tail protein | phage tail protein | | uniclust | UniRef100\_UPI000516E280 | 99.9 | 8.9e-29 | 1.6e-34 | 205.0 | 155 | (2, 156) | 423 | (6, 160) | 308 | phage tail protein | phage tail protein | | uniclust | UniRef100\_A0A1I1UBJ2 | 99.9 | 9.3e-29 | 1.7e-34 | 212.8 | 154 | (1, 154) | 423 | (1, 154) | 353 | Phage tail-collar fibre protein | Phage tail-collar fibre protein | | uniclust | UniRef100\_A0A0B5QHJ2 | 99.9 | 1e-28 | 1.9e-34 | 205.3 | 150 | (3, 152) | 423 | (14, 163) | 280 | Phage tail protein | Phage tail protein | | uniclust | UniRef100\_UPI000DFE3857 | 99.9 | 1.2e-28 | 2.1e-34 | 183.7 | 129 | (2, 130) | 423 | (3, 131) | 153 | phage tail protein | phage tail protein | | uniclust | UniRef100\_A0A2M8YKL8 | 99.9 | 1.2e-28 | 2.3e-34 | 201.6 | 154 | (1, 154) | 423 | (1, 154) | 298 | Tail-collar fiber protein | Tail-collar fiber protein | | uniclust | UniRef100\_A0A080K6T3 | 99.9 | 1.3e-28 | 2.3e-34 | 180.6 | 123 | (2, 124) | 423 | (3, 125) | 127 | Tail fiber protein (Fragment) | Tail fiber protein (Fragment) | | uniclust | UniRef100\_UPI0009B7EA04 | 99.9 | 1.3e-28 | 2.4e-34 | 227.4 | 163 | (2, 164) | 423 | (3, 165) | 813 | phage tail protein | phage tail protein | | uniclust | UniRef100\_UPI000B7CBA84 | 99.9 | 1.7e-28 | 3e-34 | 221.3 | 157 | (3, 159) | 423 | (7, 164) | 700 | tail fiber protein | tail fiber protein | | uniclust | UniRef100\_UPI0012EBC142 | 99.9 | 1.9e-28 | 3.4e-34 | 213.8 | 259 | (4, 263) | 423 | (5, 263) | 503 | phage tail protein | phage tail protein | | uniclust | UniRef100\_A0A0N0E1T2 | 99.9 | 1.8e-28 | 3.4e-34 | 241.3 | 155 | (1, 155) | 423 | (9, 163) | 809 | Phage-related tail fiber protein | Phage-related tail fiber protein | | uniclust | UniRef100\_A0A0L8IPP7 | 99.9 | 2.6e-28 | 4.8e-34 | 223.9 | 157 | (1, 157) | 423 | (5, 161) | 688 | Tail fiber protein H | Tail fiber protein H | | uniclust | UniRef100\_UPI0002E0FE29 | 99.9 | 2.8e-28 | 5.1e-34 | 207.1 | 157 | (2, 158) | 423 | (6, 162) | 353 | phage tail protein | phage tail protein | | uniclust | UniRef100\_UPI0019693137 | 99.9 | 3e-28 | 5.5e-34 | 213.9 | 165 | (3, 167) | 423 | (4, 168) | 535 | phage tail protein | phage tail protein | | uniclust | UniRef100\_UPI0005DFBD90 | 99.9 | 3.3e-28 | 6.1e-34 | 207.8 | 154 | (3, 156) | 423 | (4, 157) | 417 | phage tail protein I | phage tail protein I | | uniclust | UniRef100\_UPI0002725035 | 99.9 | 3.3e-28 | 6.1e-34 | 206.7 | 157 | (2, 158) | 423 | (6, 162) | 365 | phage tail protein | phage tail protein | | uniclust | UniRef100\_UPI001EFE032C | 99.9 | 3.3e-28 | 6.2e-34 | 216.5 | 155 | (2, 156) | 423 | (3, 157) | 520 | phage tail protein | phage tail protein | | uniclust | UniRef100\_UPI001EFD6104 | 99.9 | 3.4e-28 | 6.2e-34 | 197.3 | 155 | (2, 156) | 423 | (3, 157) | 278 | phage tail protein | phage tail protein | | uniclust | UniRef100\_UPI000C9DD6CC | 99.9 | 3.4e-28 | 6.3e-34 | 215.4 | 184 | (3, 186) | 423 | (4, 187) | 582 | phage tail protein | phage tail protein | | uniclust | UniRef100\_A0A077QIY4 | 99.9 | 3.8e-28 | 7.3e-34 | 229.8 | 295 | (3, 303) | 423 | (1, 333) | 574 | Tail fiber protein | Tail fiber protein | | uniclust | UniRef100\_A0A7Y8UX39 | 99.9 | 4.2e-28 | 7.9e-34 | 222.0 | 155 | (2, 156) | 423 | (6, 160) | 619 | Phage tail protein | Phage tail protein | | uniclust | UniRef100\_A0A516S9W3 | 99.9 | 4.6e-28 | 8.5e-34 | 189.7 | 183 | (3, 185) | 423 | (6, 189) | 204 | Phage tail protein | Phage tail protein | | uniclust | UniRef100\_UPI0022643D07 | 99.9 | 4.7e-28 | 8.6e-34 | 199.2 | 205 | (2, 307) | 423 | (5, 210) | 308 | phage tail protein | phage tail protein | | uniclust | UniRef100\_A0A1N6Q2W5 | 99.9 | 4.6e-28 | 8.6e-34 | 190.8 | 184 | (2, 185) | 423 | (1, 185) | 205 | Phage tail-collar fibre protein | Phage tail-collar fibre protein | | uniclust | UniRef100\_A0A3M5GCU5 | 99.9 | 5.7e-28 | 1.1e-33 | 188.2 | 155 | (2, 156) | 423 | (6, 160) | 196 | Phage-related tail fiber protein-like protein | Phage-related tail fiber protein-like protein | | uniclust | UniRef100\_C3X1Y2 | 99.9 | 6.2e-28 | 1.1e-33 | 209.5 | 183 | (3, 185) | 423 | (4, 186) | 480 | Phage tail collar domain-containing protein | Phage tail collar domain-containing protein | | uniclust | UniRef100\_A0A7X2MJW4 | 99.9 | 6.4e-28 | 1.2e-33 | 220.6 | 299 | (4, 307) | 423 | (2, 339) | 635 | Phage tail protein (Fragment) | Phage tail protein (Fragment) | | uniclust | UniRef100\_C6DCN9 | 99.9 | 6.5e-28 | 1.2e-33 | 217.5 | 164 | (2, 165) | 423 | (3, 166) | 697 | Phage-related tail fibre protein-like protein | Phage-related tail fibre protein-like protein | | uniclust | UniRef100\_UPI001FF61CC4 | 99.9 | 7.7e-28 | 1.4e-33 | 211.2 | 160 | (1, 160) | 423 | (1, 160) | 530 | phage tail protein | phage tail protein | | uniclust | UniRef100\_UPI0016768C5C | 99.9 | 7.8e-28 | 1.4e-33 | 212.1 | 161 | (1, 161) | 423 | (2, 164) | 465 | phage tail protein | phage tail protein | | uniclust | UniRef100\_A0A1M7U2L6 | 99.9 | 8.5e-28 | 1.6e-33 | 205.3 | 157 | (3, 159) | 423 | (4, 160) | 360 | Phage tail-collar fibre protein | Phage tail-collar fibre protein | | uniclust | UniRef100\_UPI0009BDDDCE | 99.9 | 1e-27 | 1.9e-33 | 215.6 | 156 | (2, 157) | 423 | (6, 161) | 522 | phage tail protein | phage tail protein | | uniclust | UniRef100\_A0A077NG27 | 99.9 | 1e-27 | 2e-33 | 222.1 | 140 | (1, 145) | 423 | (1, 140) | 457 | Putative phage tail fiber protein | Putative phage tail fiber protein | | uniclust | UniRef100\_UPI002017ADC9 | 99.9 | 1.2e-27 | 2.2e-33 | 219.4 | 170 | (2, 171) | 423 | (3, 172) | 792 | phage tail protein | phage tail protein | | uniclust | UniRef100\_A0A0B6XER1 | 99.9 | 1.2e-27 | 2.3e-33 | 223.5 | 153 | (3, 160) | 423 | (4, 156) | 738 | Putative phage variable tail fiber protein | Putative phage variable tail fiber protein | | uniclust | UniRef100\_A0A8S5N9Y2 | 99.9 | 1.3e-27 | 2.3e-33 | 226.3 | 202 | (1, 303) | 423 | (2, 209) | 990 | Baseplate wedge protein | Baseplate wedge protein | | uniclust | UniRef100\_A0A4D6XN95 | 99.9 | 1.6e-27 | 2.9e-33 | 204.4 | 154 | (2, 155) | 423 | (6, 165) | 428 | Phage tail protein | Phage tail protein | | uniclust | UniRef100\_A0A137SPV5 | 99.9 | 1.7e-27 | 3.1e-33 | 205.0 | 164 | (2, 169) | 423 | (7, 170) | 294 | Tail fiber protein | Tail fiber protein | | uniclust | UniRef100\_A0A0N0FU17 | 99.9 | 1.8e-27 | 3.3e-33 | 211.8 | 154 | (2, 155) | 423 | (6, 159) | 540 | Tail fiber protein H | Tail fiber protein H | | uniclust | UniRef100\_UPI000942066E | 99.9 | 1.8e-27 | 3.3e-33 | 209.8 | 160 | (2, 161) | 423 | (3, 162) | 551 | phage tail protein | phage tail protein | | uniclust | UniRef100\_UPI0003EF618D | 99.9 | 1.8e-27 | 3.5e-33 | 205.0 | 112 | (60, 171) | 423 | (22, 133) | 284 | phage tail protein | phage tail protein | | uniclust | UniRef100\_A0A077N2I9 | 99.9 | 1.9e-27 | 3.6e-33 | 234.0 | 153 | (3, 160) | 423 | (4, 156) | 1303 | Tail protein | Tail protein | | uniclust | UniRef100\_UPI0003474E6B | 99.9 | 2.1e-27 | 3.9e-33 | 204.4 | 158 | (1, 158) | 423 | (1, 158) | 441 | phage tail protein | phage tail protein | | uniclust | UniRef100\_A0A0A7RTP0 | 99.9 | 2.1e-27 | 3.9e-33 | 217.5 | 152 | (2, 153) | 423 | (3, 154) | 603 | Tail fiber protein | Tail fiber protein | | uniclust | UniRef100\_A0A133PS36 | 99.9 | 2.2e-27 | 4e-33 | 204.5 | 156 | (1, 156) | 423 | (2, 157) | 330 | Uncharacterized protein | Uncharacterized protein | | uniclust | UniRef100\_M1MEX9 | 99.9 | 2.2e-27 | 4.2e-33 | 216.4 | 215 | (2, 230) | 423 | (3, 217) | 536 | Putative phage tail fiber protein | Putative phage tail fiber protein | | uniclust | UniRef100\_A0A1I0EJH2 | 99.9 | 2.4e-27 | 4.4e-33 | 190.3 | 158 | (2, 159) | 423 | (4, 161) | 214 | Phage tail-collar fibre protein | Phage tail-collar fibre protein | | uniclust | UniRef100\_G7LSU4 | 99.9 | 2.5e-27 | 4.5e-33 | 213.2 | 157 | (1, 157) | 423 | (1, 157) | 673 | Uncharacterized protein | Uncharacterized protein | | uniclust | UniRef100\_UPI001F5BB0E8 | 99.9 | 2.7e-27 | 5e-33 | 193.5 | 155 | (3, 157) | 423 | (4, 158) | 292 | phage tail protein | phage tail protein | | uniclust | UniRef100\_A0A066RQJ3 | 99.9 | 2.7e-27 | 5.1e-33 | 218.3 | 159 | (1, 159) | 423 | (8, 166) | 592 | Tail fiber protein | Tail fiber protein | | uniclust | UniRef100\_A0A351NNZ8 | 99.9 | 3.1e-27 | 5.6e-33 | 215.4 | 169 | (2, 170) | 423 | (3, 171) | 775 | Phage tail protein | Phage tail protein | | uniclust | UniRef100\_UPI000F0649BD | 99.9 | 3.4e-27 | 6.3e-33 | 208.3 | 157 | (2, 158) | 423 | (6, 162) | 481 | phage tail protein | phage tail protein | | uniclust | UniRef100\_A0A941I8B6 | 99.9 | 4e-27 | 7.4e-33 | 222.1 | 153 | (3, 155) | 423 | (4, 156) | 914 | Phage tail protein | Phage tail protein | | uniclust | UniRef100\_A0A485CDL0 | 99.9 | 4.2e-27 | 7.8e-33 | 198.1 | 157 | (2, 158) | 423 | (4, 160) | 282 | Phage tail fibre repeat | Phage tail fibre repeat | | uniclust | UniRef100\_UPI002280C8C9 | 99.9 | 4.5e-27 | 8.2e-33 | 204.1 | 155 | (3, 157) | 423 | (4, 158) | 475 | phage tail protein | phage tail protein | | uniclust | UniRef100\_A0A1A9KIH8 | 99.9 | 4.5e-27 | 8.3e-33 | 217.4 | 154 | (1, 154) | 423 | (1, 154) | 804 | SGNH hydrolase-type esterase domain-containing protein | SGNH hydrolase-type esterase domain-containing protein | | uniclust | UniRef100\_UPI000A85364E | 99.9 | 6.2e-27 | 1.1e-32 | 212.2 | 260 | (3, 263) | 423 | (4, 263) | 725 | phage tail protein | phage tail protein | | uniclust | UniRef100\_A0A2T5HH07 | 99.9 | 6.6e-27 | 1.2e-32 | 194.5 | 159 | (2, 160) | 423 | (3, 163) | 253 | Tail-collar fiber protein | Tail-collar fiber protein | | uniclust | UniRef100\_A0A1I6E7W8 | 99.9 | 6.7e-27 | 1.2e-32 | 202.8 | 157 | (2, 158) | 423 | (3, 159) | 469 | Phage-related tail fibre protein | Phage-related tail fibre protein | | uniclust | UniRef100\_UPI000D348A11 | 99.9 | 6.7e-27 | 1.2e-32 | 201.2 | 156 | (1, 156) | 423 | (1, 156) | 346 | phage tail protein | phage tail protein | | uniclust | UniRef100\_UPI000BE5AFC7 | 99.9 | 6.5e-27 | 1.2e-32 | 207.5 | 247 | (92, 374) | 423 | (17, 264) | 310 | phage tail protein | phage tail protein | | uniclust | UniRef100\_A0A3M4NA57 | 99.9 | 8.2e-27 | 1.5e-32 | 203.0 | 157 | (2, 158) | 423 | (6, 162) | 433 | Tail fiber protein H (Fragment) | Tail fiber protein H (Fragment) | | uniclust | UniRef100\_UPI002162F542 | 99.9 | 8.6e-27 | 1.6e-32 | 211.9 | 156 | (1, 156) | 423 | (1, 156) | 748 | phage tail protein | phage tail protein | | uniclust | UniRef100\_A0A0S4SND2 | 99.9 | 8.5e-27 | 1.6e-32 | 202.1 | 154 | (1, 157) | 423 | (2, 155) | 309 | Tail fiber protein H | Tail fiber protein H | | uniclust | UniRef100\_A0A1S7SGS6 | 99.9 | 9.3e-27 | 1.8e-32 | 224.0 | 153 | (3, 155) | 423 | (19, 171) | 585 | DUF2793 domain-containing protein | DUF2793 domain-containing protein | | uniclust | UniRef100\_UPI000DF33EE5 | 99.9 | 1.3e-26 | 2.3e-32 | 204.2 | 160 | (1, 160) | 423 | (2, 164) | 539 | phage tail protein | phage tail protein | | uniclust | UniRef100\_A0A0C2RHK4 | 99.9 | 1.3e-26 | 2.5e-32 | 225.5 | 157 | (1, 157) | 423 | (2, 159) | 926 | Tail protein | Tail protein | | uniclust | UniRef100\_UPI00168161AD | 99.9 | 1.5e-26 | 2.7e-32 | 183.3 | 154 | (3, 156) | 423 | (4, 157) | 222 | phage tail protein | phage tail protein | | uniclust | UniRef100\_A0A0S1B3R8 | 99.9 | 1.5e-26 | 2.9e-32 | 177.9 | 157 | (1, 157) | 423 | (1, 158) | 180 | Side tail phage protein | Side tail phage protein | | uniclust | UniRef100\_UPI0013B467C1 | 99.9 | 1.6e-26 | 2.9e-32 | 200.0 | 155 | (2, 156) | 423 | (3, 158) | 434 | phage tail protein | phage tail protein | | uniclust | UniRef100\_A0A0E1PUY8 | 99.9 | 1.8e-26 | 3.4e-32 | 233.2 | 159 | (1, 161) | 423 | (2, 160) | 966 | Phage tail fiber protein | Phage tail fiber protein | | uniclust | UniRef100\_A0A1H9AFH7 | 99.9 | 1.9e-26 | 3.4e-32 | 206.6 | 156 | (2, 157) | 423 | (6, 161) | 544 | Phage-related tail fibre protein | Phage-related tail fibre protein | | uniclust | UniRef100\_UPI0009AC2AD7 | 99.9 | 1.9e-26 | 3.5e-32 | 215.1 | 144 | (1, 148) | 423 | (2, 145) | 583 | phage tail protein | phage tail protein | | uniclust | UniRef100\_A0A318T0X1 | 99.9 | 1.9e-26 | 3.7e-32 | 220.5 | 154 | (2, 155) | 423 | (7, 160) | 635 | Uncharacterized protein DUF2793 | Uncharacterized protein DUF2793 | | uniclust | UniRef100\_A0A2T5NR62 | 99.9 | 2e-26 | 3.8e-32 | 208.5 | 156 | (3, 158) | 423 | (4, 160) | 515 | Phage tail protein | Phage tail protein | | uniclust | UniRef100\_UPI00025CA96A | 99.9 | 2.1e-26 | 4e-32 | 214.8 | 157 | (3, 159) | 423 | (7, 164) | 713 | tail fiber protein | tail fiber protein | | uniclust | UniRef100\_A0A285Z1M8 | 99.9 | 2.5e-26 | 4.6e-32 | 202.4 | 156 | (1, 156) | 423 | (1, 156) | 417 | Phage-related tail fibre protein | Phage-related tail fibre protein | | uniclust | UniRef100\_A0A348HI80 | 99.9 | 2.5e-26 | 4.6e-32 | 194.8 | 155 | (1, 155) | 423 | (1, 155) | 385 | Phage-related tail fibre | Phage-related tail fibre | | uniclust | UniRef100\_UPI001CC4633C | 99.9 | 2.6e-26 | 4.8e-32 | 210.4 | 157 | (3, 159) | 423 | (4, 160) | 814 | phage tail protein | phage tail protein | | uniclust | UniRef100\_M8EFI1 | 99.9 | 2.6e-26 | 4.8e-32 | 202.5 | 152 | (3, 154) | 423 | (4, 155) | 454 | Phage-related tail fiber protein-like protein | Phage-related tail fiber protein-like protein | | uniclust | UniRef100\_UPI00068A8B5B | 99.9 | 2.6e-26 | 4.8e-32 | 188.6 | 152 | (3, 154) | 423 | (4, 155) | 266 | phage tail protein | phage tail protein | | uniclust | UniRef100\_A0A411WIN2 | 99.9 | 2.6e-26 | 4.9e-32 | 201.8 | 145 | (3, 153) | 423 | (4, 152) | 396 | Phage tail protein | Phage tail protein | | uniclust | UniRef100\_A0A7V7KJT5 | 99.9 | 3.6e-26 | 6.5e-32 | 202.0 | 154 | (1, 154) | 423 | (1, 154) | 553 | Sialidase domain-containing protein | Sialidase domain-containing protein | | uniclust | UniRef100\_UPI0005F91E19 | 99.9 | 3.6e-26 | 6.6e-32 | 201.5 | 142 | (2, 147) | 423 | (3, 144) | 431 | phage tail protein | phage tail protein | | uniclust | UniRef100\_UPI001864AFE8 | 99.9 | 3.7e-26 | 6.9e-32 | 208.3 | 152 | (1, 152) | 423 | (2, 153) | 564 | phage tail protein | phage tail protein | | uniclust | UniRef100\_A0A076LIZ9 | 99.9 | 3.9e-26 | 7.3e-32 | 214.7 | 291 | (3, 299) | 423 | (1, 328) | 582 | Phage Tail Collar domain protein | Phage Tail Collar domain protein | | uniclust | UniRef100\_A0A1I9KF43 | 99.9 | 4.3e-26 | 7.9e-32 | 209.6 | 154 | (3, 156) | 423 | (4, 157) | 839 | Tail-collar fiber protein | Tail-collar fiber protein | | uniclust | UniRef100\_D5EF94 | 99.9 | 4.4e-26 | 8.1e-32 | 201.7 | 158 | (3, 160) | 423 | (4, 161) | 561 | Phage-related tail fibre protein-like protein | Phage-related tail fibre protein-like protein | | uniclust | UniRef100\_UPI001F14809F | 99.9 | 4.4e-26 | 8.2e-32 | 189.3 | 159 | (1, 159) | 423 | (1, 159) | 325 | phage tail protein | phage tail protein | | uniclust | UniRef100\_A0A845BZU2 | 99.9 | 4.9e-26 | 9e-32 | 205.9 | 156 | (3, 158) | 423 | (4, 159) | 701 | Phage tail protein | Phage tail protein | | uniclust | UniRef100\_A0A1S6TPA5 | 99.9 | 4.8e-26 | 9.1e-32 | 187.1 | 151 | (1, 154) | 423 | (3, 153) | 227 | Phage tail-collar fiber protein (DUF3751 domain) | Phage tail-collar fiber protein (DUF3751 domain) | | uniclust | UniRef100\_A0A2C9EHA1 | 99.9 | 4.9e-26 | 9.1e-32 | 195.7 | 156 | (2, 157) | 423 | (6, 161) | 408 | Putative tail fiber protein | Putative tail fiber protein | | uniclust | UniRef100\_A0A2Z6AZ11 | 99.9 | 6.2e-26 | 1.2e-31 | 197.1 | 148 | (1, 148) | 423 | (2, 149) | 348 | Tail Collar domain protein | Tail Collar domain protein | | uniclust | UniRef100\_UPI00068C6394 | 99.9 | 7.3e-26 | 1.3e-31 | 193.4 | 154 | (3, 156) | 423 | (7, 160) | 407 | phage tail protein | phage tail protein | | uniclust | UniRef100\_UPI001F2CA588 | 99.9 | 8.6e-26 | 1.6e-31 | 189.7 | 163 | (2, 164) | 423 | (6, 168) | 354 | phage tail protein | phage tail protein | | uniclust | UniRef100\_A0A0T9UVJ2 | 99.9 | 9e-26 | 1.6e-31 | 169.3 | 129 | (3, 131) | 423 | (4, 132) | 163 | Variable tail fiber protein | Variable tail fiber protein | | uniclust | UniRef100\_A0A4U3FAV2 | 99.9 | 8.9e-26 | 1.7e-31 | 197.4 | 156 | (2, 157) | 423 | (3, 158) | 354 | Phage tail protein | Phage tail protein | | uniclust | UniRef100\_UPI0017462346 | 99.9 | 9.1e-26 | 1.7e-31 | 184.4 | 161 | (1, 161) | 423 | (2, 166) | 237 | phage tail protein | phage tail protein | | uniclust | UniRef100\_UPI001295318C | 99.9 | 9.3e-26 | 1.7e-31 | 208.8 | 242 | (2, 247) | 423 | (6, 264) | 723 | phage tail protein | phage tail protein | | uniclust | UniRef100\_UPI0022E93E45 | 99.9 | 9.8e-26 | 1.8e-31 | 205.7 | 153 | (3, 155) | 423 | (4, 156) | 767 | phage tail protein | phage tail protein | | uniclust | UniRef100\_A0A2W5JZT7 | 99.9 | 9.7e-26 | 1.8e-31 | 198.9 | 156 | (1, 159) | 423 | (3, 158) | 370 | Phage tail protein | Phage tail protein | | uniclust | UniRef100\_A0A315WVX7 | 99.9 | 1e-25 | 1.9e-31 | 202.7 | 155 | (3, 157) | 423 | (7, 166) | 561 | Phage tail protein | Phage tail protein | | uniclust | UniRef100\_UPI0013F605E0 | 99.9 | 1.1e-25 | 2e-31 | 196.4 | 156 | (3, 159) | 423 | (4, 160) | 488 | phage tail protein | phage tail protein | | uniclust | UniRef100\_A0A5Q2KDB2 | 99.9 | 1.1e-25 | 2e-31 | 199.8 | 200 | (3, 303) | 423 | (4, 203) | 575 | Phage tail protein | Phage tail protein | | uniclust | UniRef100\_A0A0G9KCC4 | 99.9 | 1.3e-25 | 2.5e-31 | 201.7 | 155 | (1, 158) | 423 | (1, 155) | 405 | Phage tail protein | Phage tail protein | | uniclust | UniRef100\_A0A2N6J7T9 | 99.9 | 1.6e-25 | 2.9e-31 | 202.0 | 156 | (1, 156) | 423 | (1, 156) | 628 | Phage-related tail fiber protein-like protein | Phage-related tail fiber protein-like protein | | uniclust | UniRef100\_UPI0022CCF947 | 99.9 | 1.8e-25 | 3.3e-31 | 206.9 | 203 | (1, 304) | 423 | (2, 207) | 902 | phage tail protein | phage tail protein | | uniclust | UniRef100\_A0A0N9MHY1 | 99.9 | 1.9e-25 | 3.7e-31 | 217.4 | 155 | (2, 156) | 423 | (6, 160) | 843 | Phage tail collar domain-containing protein | Phage tail collar domain-containing protein | | uniclust | UniRef100\_A0A0H3B1B9 | 99.9 | 1.9e-25 | 3.7e-31 | 213.1 | 293 | (3, 301) | 423 | (1, 334) | 558 | Tail fiber repeat 2 protein | Tail fiber repeat 2 protein | | uniclust | UniRef100\_UPI001E3AD0D6 | 99.9 | 2e-25 | 3.7e-31 | 180.7 | 155 | (3, 157) | 423 | (4, 158) | 268 | phage tail protein | phage tail protein | | uniclust | UniRef100\_A0A853I101 | 99.9 | 2.1e-25 | 4e-31 | 198.6 | 155 | (3, 157) | 423 | (4, 161) | 435 | Phage tail protein | Phage tail protein | | uniclust | UniRef100\_UPI001AE086F0 | 99.9 | 2.3e-25 | 4.2e-31 | 204.3 | 159 | (1, 159) | 423 | (1, 159) | 808 | phage tail protein | phage tail protein | | uniclust | UniRef100\_A0A0J8DFT2 | 99.9 | 2.8e-25 | 5.2e-31 | 185.3 | 151 | (3, 153) | 423 | (4, 154) | 299 | Phage tail-collar fiber protein | Phage tail-collar fiber protein | | uniclust | UniRef100\_A0A377IMK2 | 99.9 | 4.2e-25 | 7.7e-31 | 206.7 | 149 | (3, 151) | 423 | (4, 152) | 833 | Putative phage-like tail fiber protein | Putative phage-like tail fiber protein | | uniclust | UniRef100\_A0A265Q5Q8 | 99.9 | 4.3e-25 | 7.9e-31 | 197.9 | 151 | (2, 152) | 423 | (3, 153) | 524 | Phage tail protein | Phage tail protein | | uniclust | UniRef100\_A0A380C811 | 99.9 | 4.5e-25 | 8.4e-31 | 184.9 | 156 | (1, 156) | 423 | (3, 164) | 257 | Phage tail protein | Phage tail protein | | uniclust | UniRef100\_UPI000A4D95A8 | 99.9 | 4.6e-25 | 8.4e-31 | 202.1 | 255 | (3, 314) | 423 | (4, 262) | 743 | phage tail protein | phage tail protein | | uniclust | UniRef100\_A0A921AXE4 | 99.9 | 4.7e-25 | 8.7e-31 | 181.2 | 153 | (3, 156) | 423 | (5, 157) | 256 | Phage tail protein | Phage tail protein | | uniclust | UniRef100\_A0A1Q6KPQ8 | 99.8 | 5.7e-25 | 1.1e-30 | 185.8 | 159 | (3, 161) | 423 | (39, 197) | 278 | Phage tail protein | Phage tail protein | | uniclust | UniRef100\_A0A6D2GE62 | 99.8 | 6.2e-25 | 1.1e-30 | 162.3 | 129 | (31, 159) | 423 | (1, 129) | 148 | Tail protein | Tail protein | | uniclust | UniRef100\_A0A069B2A9 | 99.8 | 6.6e-25 | 1.2e-30 | 191.0 | 154 | (3, 156) | 423 | (25, 178) | 371 | Putative tail fiber protein | Putative tail fiber protein | | uniclust | UniRef100\_A0A7Z7YC39 | 99.8 | 7e-25 | 1.3e-30 | 188.6 | 153 | (2, 154) | 423 | (3, 155) | 426 | Phage tail protein | Phage tail protein | | uniclust | UniRef100\_A0A1K1QNR1 | 99.8 | 6.9e-25 | 1.3e-30 | 207.1 | 153 | (1, 153) | 423 | (1, 154) | 682 | Phage tail-collar fibre protein | Phage tail-collar fibre protein | | uniclust | UniRef100\_UPI001FAEEA20 | 99.8 | 8e-25 | 1.5e-30 | 193.4 | 155 | (2, 156) | 423 | (6, 160) | 544 | phage tail protein | phage tail protein | | uniclust | UniRef100\_A0A3M5NCX5 | 99.8 | 9.1e-25 | 1.7e-30 | 181.2 | 156 | (2, 157) | 423 | (6, 161) | 318 | Phage tail protein | Phage tail protein | | uniclust | UniRef100\_A0A376FFE3 | 99.8 | 9.7e-25 | 1.8e-30 | 155.7 | 112 | (31, 142) | 423 | (1, 112) | 121 | Phage tail collar domain-containing protein | Phage tail collar domain-containing protein | | uniclust | UniRef100\_A0A828ZHJ0 | 99.8 | 9.9e-25 | 1.8e-30 | 200.7 | 159 | (1, 159) | 423 | (1, 159) | 592 | Tail fiber protein (GpH) | Tail fiber protein (GpH) | | uniclust | UniRef100\_UPI00209EEE42 | 99.8 | 1e-24 | 1.9e-30 | 182.3 | 160 | (4, 163) | 423 | (5, 164) | 338 | phage tail protein | phage tail protein | | uniclust | UniRef100\_A0A6I1HR03 | 99.8 | 1e-24 | 1.9e-30 | 201.0 | 153 | (3, 155) | 423 | (7, 159) | 753 | SGNH hydrolase-type esterase domain-containing protein | SGNH hydrolase-type esterase domain-containing protein | | uniclust | UniRef100\_UPI001C450459 | 99.8 | 1.1e-24 | 1.9e-30 | 193.2 | 158 | (3, 160) | 423 | (25, 182) | 558 | phage tail protein | phage tail protein | | uniclust | UniRef100\_A0A611ES10 | 99.8 | 1.2e-24 | 2.2e-30 | 166.6 | 149 | (59, 308) | 423 | (4, 152) | 169 | Phage tail protein (Fragment) | Phage tail protein (Fragment) | | uniclust | UniRef100\_UPI001FCEFC3F | 99.8 | 1.2e-24 | 2.2e-30 | 188.9 | 193 | (8, 301) | 423 | (1, 193) | 462 | phage tail protein | phage tail protein | | uniclust | UniRef100\_B8DLJ2 | 99.8 | 1.4e-24 | 2.7e-30 | 197.4 | 149 | (4, 153) | 423 | (5, 154) | 511 | Tail fiber protein, putative | Tail fiber protein, putative | | uniclust | UniRef100\_UPI00215BC1F7 | 99.8 | 1.5e-24 | 2.7e-30 | 195.8 | 155 | (2, 156) | 423 | (3, 157) | 669 | phage tail protein | phage tail protein | | uniclust | UniRef100\_A0A4P2WWZ3 | 99.8 | 1.5e-24 | 2.8e-30 | 174.7 | 157 | (3, 160) | 423 | (5, 161) | 225 | Uncharacterized protein | Uncharacterized protein | | uniclust | UniRef100\_A0A0A7RTQ0 | 99.8 | 1.5e-24 | 2.8e-30 | 191.9 | 153 | (1, 153) | 423 | (1, 153) | 436 | Tail-collar fiber family protein | Tail-collar fiber family protein | | uniclust | UniRef100\_A0A8S5MN89 | 99.8 | 1.6e-24 | 2.9e-30 | 168.4 | 155 | (3, 157) | 423 | (4, 158) | 201 | Tail collar fiber protein | Tail collar fiber protein | | uniclust | UniRef100\_A0A495AKT5 | 99.8 | 1.6e-24 | 2.9e-30 | 205.0 | 282 | (3, 304) | 423 | (129, 418) | 907 | Phage tail protein | Phage tail protein | | uniclust | UniRef100\_A0A974X0L5 | 99.8 | 1.7e-24 | 3.1e-30 | 188.1 | 153 | (2, 154) | 423 | (1, 153) | 463 | Phage tail protein | Phage tail protein | | uniclust | UniRef100\_UPI00158FB9FD | 99.8 | 1.7e-24 | 3.1e-30 | 159.5 | 129 | (3, 131) | 423 | (4, 132) | 133 | phage tail protein | phage tail protein | | uniclust | UniRef100\_A0A078LT99 | 99.8 | 1.8e-24 | 3.4e-30 | 183.2 | 163 | (3, 165) | 423 | (7, 169) | 339 | Tail fiber protein H | Tail fiber protein H | | uniclust | UniRef100\_UPI0018E08366 | 99.8 | 1.9e-24 | 3.6e-30 | 184.0 | 157 | (3, 159) | 423 | (5, 162) | 391 | phage tail protein | phage tail protein | | uniclust | UniRef100\_A0A2H4J194 | 99.8 | 2e-24 | 3.6e-30 | 178.1 | 157 | (1, 157) | 423 | (1, 157) | 303 | Putative tail-collar fiber protein | Putative tail-collar fiber protein | | uniclust | UniRef100\_A0A0H0GM37 | 99.8 | 2.3e-24 | 4.3e-30 | 205.5 | 153 | (3, 156) | 423 | (7, 160) | 687 | Phage tail protein | Phage tail protein | | uniclust | UniRef100\_A0A2X1IXU3 | 99.8 | 2.4e-24 | 4.5e-30 | 163.2 | 124 | (41, 164) | 423 | (7, 130) | 166 | Variable tail fiber protein | Variable tail fiber protein | | uniclust | UniRef100\_UPI001F1039DB | 99.8 | 2.4e-24 | 4.5e-30 | 198.7 | 154 | (3, 156) | 423 | (4, 157) | 853 | phage tail protein | phage tail protein | | uniclust | UniRef100\_UPI00065A80D1 | 99.8 | 2.8e-24 | 5.1e-30 | 182.8 | 155 | (2, 156) | 423 | (86, 240) | 384 | phage tail protein I | phage tail protein I | | uniclust | UniRef100\_A0A9E0KF93 | 99.8 | 2.9e-24 | 5.3e-30 | 182.2 | 163 | (1, 164) | 423 | (1, 163) | 377 | Phage tail protein | Phage tail protein | | uniclust | UniRef100\_A0A1E3GML3 | 99.8 | 2.9e-24 | 5.3e-30 | 203.7 | 156 | (2, 157) | 423 | (4, 160) | 768 | Phage tail protein | Phage tail protein | | uniclust | UniRef100\_A0A0D8DFZ0 | 99.8 | 3e-24 | 5.5e-30 | 173.1 | 153 | (2, 159) | 423 | (27, 179) | 204 | Tail fiber protein | Tail fiber protein | | uniclust | UniRef100\_UPI0018A82FE3 | 99.8 | 3.1e-24 | 5.8e-30 | 154.9 | 118 | (1, 118) | 423 | (2, 119) | 120 | phage tail protein | phage tail protein | | uniclust | UniRef100\_UPI001C5D3A71 | 99.8 | 3.5e-24 | 6.5e-30 | 170.4 | 114 | (47, 160) | 423 | (1, 114) | 224 | tail fiber protein | tail fiber protein | | uniclust | UniRef100\_UPI001C474985 | 99.8 | 3.6e-24 | 6.7e-30 | 193.0 | 158 | (3, 160) | 423 | (25, 182) | 655 | phage tail protein | phage tail protein | | uniclust | UniRef100\_A0A367LYA0 | 99.8 | 3.8e-24 | 7e-30 | 169.0 | 155 | (3, 157) | 423 | (7, 166) | 198 | Phage tail protein (Fragment) | Phage tail protein (Fragment) | | uniclust | UniRef100\_UPI001C48C03A | 99.8 | 4.2e-24 | 7.7e-30 | 155.0 | 131 | (14, 144) | 423 | (2, 132) | 132 | phage tail protein | phage tail protein | | uniclust | UniRef100\_A0A2D1IR12 | 99.8 | 4.7e-24 | 8.8e-30 | 200.7 | 157 | (1, 159) | 423 | (2, 158) | 604 | Phage tail protein | Phage tail protein | | uniclust | UniRef100\_A0A1Y5MYB7 | 99.8 | 4.7e-24 | 9e-30 | 187.8 | 153 | (2, 157) | 423 | (4, 157) | 316 | Phage tail protein | Phage tail protein | | uniclust | UniRef100\_A0A3E1QH42 | 99.8 | 5.1e-24 | 9.5e-30 | 195.1 | 150 | (3, 152) | 423 | (4, 153) | 636 | Phage tail protein (Fragment) | Phage tail protein (Fragment) | | uniclust | UniRef100\_UPI001CA43773 | 99.8 | 5.5e-24 | 1e-29 | 192.6 | 155 | (2, 156) | 423 | (1, 156) | 684 | phage tail protein | phage tail protein | | uniclust | UniRef100\_N8R6Q7 | 99.8 | 5.6e-24 | 1e-29 | 165.1 | 153 | (2, 154) | 423 | (1, 154) | 198 | Uncharacterized protein | Uncharacterized protein | | uniclust | UniRef100\_A0A1B1KPJ7 | 99.8 | 5.6e-24 | 1e-29 | 185.2 | 159 | (3, 161) | 423 | (5, 163) | 358 | Phage tail protein | Phage tail protein | | uniclust | UniRef100\_UPI000F76A94D | 99.8 | 5.8e-24 | 1.1e-29 | 170.7 | 155 | (1, 156) | 423 | (1, 159) | 248 | phage tail protein | phage tail protein | | uniclust | UniRef100\_A0A3F3A8F4 | 99.8 | 5.7e-24 | 1.1e-29 | 191.7 | 153 | (1, 153) | 423 | (2, 154) | 556 | Putative phage tail fiber protein | Putative phage tail fiber protein | | uniclust | UniRef100\_L1M5X1 | 99.8 | 7.2e-24 | 1.3e-29 | 193.5 | 155 | (2, 156) | 423 | (6, 160) | 748 | Tail fiber protein H | Tail fiber protein H | | uniclust | UniRef100\_UPI001F2A79DD | 99.8 | 7.3e-24 | 1.3e-29 | 191.6 | 153 | (2, 154) | 423 | (6, 158) | 673 | phage tail protein | phage tail protein | | uniclust | UniRef100\_A0A077NHN3 | 99.8 | 7.5e-24 | 1.4e-29 | 207.2 | 152 | (3, 159) | 423 | (4, 155) | 1195 | Tail protein | Tail protein | | uniclust | UniRef100\_A0A2N2KCW4 | 99.8 | 8.5e-24 | 1.6e-29 | 168.0 | 153 | (1, 153) | 423 | (1, 153) | 231 | Phage tail protein (Fragment) | Phage tail protein (Fragment) | | uniclust | UniRef100\_UPI00190502AF | 99.8 | 8.6e-24 | 1.6e-29 | 192.6 | 155 | (1, 155) | 423 | (1, 159) | 729 | phage tail protein | phage tail protein | | uniclust | UniRef100\_A0A290WU51 | 99.8 | 9e-24 | 1.6e-29 | 191.4 | 150 | (3, 152) | 423 | (4, 153) | 686 | SGNH hydrolase-type esterase domain-containing protein | SGNH hydrolase-type esterase domain-containing protein | | uniclust | UniRef100\_UPI0018D16FB5 | 99.8 | 9.6e-24 | 1.8e-29 | 197.1 | 153 | (3, 155) | 423 | (5, 157) | 668 | phage tail protein | phage tail protein | | uniclust | UniRef100\_UPI000240E034 | 99.8 | 1e-23 | 1.9e-29 | 178.8 | 164 | (2, 165) | 423 | (1, 164) | 341 | phage tail protein | phage tail protein | | uniclust | UniRef100\_A0A1T4W1B3 | 99.8 | 1.1e-23 | 2.1e-29 | 180.9 | 152 | (3, 154) | 423 | (4, 155) | 416 | Phage tail-collar fibre protein | Phage tail-collar fibre protein | | uniclust | UniRef100\_A0A5T3EMD8 | 99.8 | 1.1e-23 | 2.1e-29 | 151.6 | 98 | (2, 99) | 423 | (3, 101) | 103 | Phage tail protein (Fragment) | Phage tail protein (Fragment) | | uniclust | UniRef100\_A0A6G9RP95 | 99.8 | 1.1e-23 | 2.1e-29 | 196.5 | 140 | (1, 144) | 423 | (3, 142) | 466 | Phage tail protein | Phage tail protein | | uniclust | UniRef100\_UPI001F07DAE0 | 99.8 | 1.1e-23 | 2.1e-29 | 178.8 | 148 | (2, 153) | 423 | (6, 153) | 328 | phage tail protein | phage tail protein | | uniclust | UniRef100\_UPI001FEFA6B3 | 99.8 | 1.2e-23 | 2.3e-29 | 171.2 | 154 | (2, 155) | 423 | (3, 156) | 274 | phage tail protein | phage tail protein | | uniclust | UniRef100\_A0A1X3I4D9 | 99.8 | 1.3e-23 | 2.5e-29 | 195.3 | 147 | (1, 154) | 423 | (8, 154) | 442 | Putative tail fiber protein (GpH) | Putative tail fiber protein (GpH) | | uniclust | UniRef100\_UPI001191EDC2 | 99.8 | 1.4e-23 | 2.6e-29 | 151.1 | 116 | (22, 137) | 423 | (10, 125) | 126 | phage tail protein | phage tail protein | | uniclust | UniRef100\_A0A483Z142 | 99.8 | 1.4e-23 | 2.6e-29 | 169.5 | 159 | (1, 160) | 423 | (2, 160) | 220 | Phage tail protein (Fragment) | Phage tail protein (Fragment) | | uniclust | UniRef100\_A0A077N5A2 | 99.8 | 1.5e-23 | 2.8e-29 | 195.6 | 275 | (3, 281) | 423 | (1, 314) | 657 | Bacteriophage T7 Gp17 C-terminal domain-containing protein | Bacteriophage T7 Gp17 C-terminal domain-containing protein | | uniclust | UniRef100\_UPI0015E27F6A | 99.8 | 1.6e-23 | 3e-29 | 185.6 | 154 | (3, 156) | 423 | (4, 157) | 551 | phage tail protein | phage tail protein | | uniclust | UniRef100\_UPI000D6467B0 | 99.8 | 1.7e-23 | 3.1e-29 | 173.7 | 157 | (1, 157) | 423 | (1, 161) | 278 | phage tail protein | phage tail protein | | uniclust | UniRef100\_A0A0J1NB15 | 99.8 | 1.7e-23 | 3.1e-29 | 196.2 | 153 | (2, 154) | 423 | (3, 155) | 589 | Phage tail protein | Phage tail protein | | uniclust | UniRef100\_A0A0A8H9Y5 | 99.8 | 1.7e-23 | 3.2e-29 | 184.4 | 154 | (1, 157) | 423 | (3, 156) | 348 | Phage tail-collar fiber protein (DUF3751 domain) | Phage tail-collar fiber protein (DUF3751 domain) | | uniclust | UniRef100\_A0A239C905 | 99.8 | 1.9e-23 | 3.5e-29 | 185.6 | 153 | (2, 154) | 423 | (1, 153) | 563 | Phage tail-collar fibre protein | Phage tail-collar fibre protein | | uniclust | UniRef100\_A0A8I1WMJ8 | 99.8 | 2e-23 | 3.8e-29 | 172.0 | 160 | (1, 160) | 423 | (1, 164) | 298 | Phage tail protein | Phage tail protein | | uniclust | UniRef100\_UPI001F29E04B | 99.8 | 2.5e-23 | 4.6e-29 | 182.6 | 149 | (3, 151) | 423 | (4, 152) | 500 | phage tail protein | phage tail protein | | uniclust | UniRef100\_A0A7Z2A8B6 | 99.8 | 2.5e-23 | 4.8e-29 | 187.0 | 158 | (1, 160) | 423 | (1, 158) | 420 | Phage tail protein | Phage tail protein | | uniclust | UniRef100\_UPI001C106791 | 99.8 | 3e-23 | 5.5e-29 | 187.7 | 151 | (2, 152) | 423 | (3, 153) | 669 | phage tail protein | phage tail protein | | uniclust | UniRef100\_A0A7Y8JX08 | 99.8 | 3.1e-23 | 5.7e-29 | 167.2 | 156 | (1, 156) | 423 | (1, 157) | 244 | Phage tail protein (Fragment) | Phage tail protein (Fragment) | | uniclust | UniRef100\_UPI0020A40C1E | 99.8 | 3.2e-23 | 5.8e-29 | 179.1 | 157 | (2, 160) | 423 | (3, 159) | 434 | phage tail protein | phage tail protein | | uniclust | UniRef100\_UPI001C998269 | 99.8 | 3.2e-23 | 5.9e-29 | 189.9 | 159 | (1, 159) | 423 | (1, 163) | 769 | phage tail protein | phage tail protein | | uniclust | UniRef100\_A0A077NV23 | 99.8 | 3.2e-23 | 6.3e-29 | 205.9 | 244 | (3, 251) | 423 | (1, 282) | 689 | Phage tail collar domain-containing protein | Phage tail collar domain-containing protein | | uniclust | UniRef100\_A0A345J3J2 | 99.8 | 3.4e-23 | 6.3e-29 | 179.7 | 156 | (2, 157) | 423 | (1, 156) | 450 | Phage-related tail fiber protein | Phage-related tail fiber protein | | uniclust | UniRef100\_A0A9D1QZY4 | 99.8 | 3.5e-23 | 6.4e-29 | 179.6 | 161 | (3, 163) | 423 | (4, 164) | 417 | Phage tail protein (Fragment) | Phage tail protein (Fragment) | | uniclust | UniRef100\_UPI00206B758F | 99.8 | 3.8e-23 | 7e-29 | 171.3 | 152 | (2, 153) | 423 | (4, 155) | 310 | phage tail protein | phage tail protein | | uniclust | UniRef100\_A0A0J1LWH4 | 99.8 | 4.1e-23 | 7.7e-29 | 197.5 | 212 | (1, 308) | 423 | (96, 307) | 716 | Phage tail collar domain-containing protein | Phage tail collar domain-containing protein | | uniclust | UniRef100\_A0A943L988 | 99.8 | 5e-23 | 9.2e-29 | 151.3 | 136 | (3, 138) | 423 | (5, 140) | 141 | Phage tail protein (Fragment) | Phage tail protein (Fragment) | | uniclust | UniRef100\_A0A846TIU6 | 99.8 | 5.4e-23 | 1e-28 | 167.9 | 153 | (3, 155) | 423 | (4, 156) | 243 | DUF2793 domain-containing protein | DUF2793 domain-containing protein | | uniclust | UniRef100\_A0A022PLF6 | 99.8 | 5.4e-23 | 1e-28 | 199.6 | 244 | (3, 251) | 423 | (16, 297) | 629 | Phage tail-collar fiber protein | Phage tail-collar fiber protein | | uniclust | UniRef100\_J6HCZ5 | 99.8 | 6e-23 | 1.1e-28 | 174.9 | 152 | (3, 154) | 423 | (4, 155) | 384 | Tail fiber protein | Tail fiber protein | | uniclust | UniRef100\_UPI000B4A1C81 | 99.8 | 6.1e-23 | 1.1e-28 | 185.1 | 152 | (3, 154) | 423 | (4, 155) | 647 | phage tail protein | phage tail protein | | uniclust | UniRef100\_A0A2D1CSN0 | 99.8 | 6.1e-23 | 1.1e-28 | 192.4 | 154 | (3, 156) | 423 | (135, 288) | 733 | Putative tail fiber protein | Putative tail fiber protein | | uniclust | UniRef100\_UPI000AFA6AC9 | 99.8 | 6.5e-23 | 1.2e-28 | 190.2 | 149 | (3, 151) | 423 | (4, 152) | 885 | phage tail protein | phage tail protein | | uniclust | UniRef100\_A0A0F9Z9H8 | 99.8 | 6.5e-23 | 1.2e-28 | 167.5 | 152 | (1, 159) | 423 | (7, 158) | 234 | Tail fiber-like protein | Tail fiber-like protein | | uniclust | UniRef100\_A0A3G9G2L6 | 99.8 | 7.4e-23 | 1.4e-28 | 194.9 | 153 | (3, 156) | 423 | (5, 157) | 735 | Phage tail fiber protein | Phage tail fiber protein | | uniclust | UniRef100\_UPI0021CF5D59 | 99.8 | 7.6e-23 | 1.4e-28 | 165.6 | 123 | (36, 158) | 423 | (2, 124) | 260 | phage tail protein | phage tail protein | | uniclust | UniRef100\_A0A1G8FGR6 | 99.8 | 7.5e-23 | 1.4e-28 | 190.5 | 158 | (1, 158) | 423 | (2, 159) | 657 | Phage Tail Collar Domain | Phage Tail Collar Domain | | uniclust | UniRef100\_A0A6B8QPL4 | 99.8 | 8.6e-23 | 1.6e-28 | 168.0 | 153 | (3, 155) | 423 | (46, 198) | 292 | Phage tail protein | Phage tail protein | | uniclust | UniRef100\_UPI0006945938 | 99.8 | 8.7e-23 | 1.6e-28 | 178.7 | 156 | (1, 156) | 423 | (2, 159) | 407 | phage tail protein | phage tail protein | | uniclust | UniRef100\_A0A1I5MQ45 | 99.8 | 9.3e-23 | 1.7e-28 | 183.1 | 154 | (1, 154) | 423 | (1, 154) | 617 | Chaperone of endosialidase | Chaperone of endosialidase | | uniclust | UniRef100\_UPI000690FF63 | 99.8 | 9.3e-23 | 1.7e-28 | 177.2 | 154 | (3, 156) | 423 | (4, 157) | 406 | phage tail protein | phage tail protein | | uniclust | UniRef100\_UPI00208DA219 | 99.8 | 9.4e-23 | 1.7e-28 | 159.5 | 93 | (71, 163) | 423 | (2, 94) | 206 | phage tail protein | phage tail protein | | uniclust | UniRef100\_UPI0004CFD2CA | 99.8 | 9.7e-23 | 1.8e-28 | 171.2 | 154 | (2, 155) | 423 | (3, 156) | 343 | phage tail protein | phage tail protein | | uniclust | UniRef100\_UPI001356777F | 99.8 | 1.1e-22 | 2.1e-28 | 180.2 | 271 | (4, 277) | 423 | (5, 276) | 542 | phage tail protein | phage tail protein | | uniclust | UniRef100\_A0A2D3VW29 | 99.8 | 1.2e-22 | 2.2e-28 | 159.5 | 153 | (2, 154) | 423 | (1, 153) | 211 | Phage tail protein | Phage tail protein | | uniclust | UniRef100\_UPI000693D28B | 99.8 | 1.3e-22 | 2.4e-28 | 173.0 | 156 | (1, 156) | 423 | (1, 156) | 386 | phage tail protein | phage tail protein | | uniclust | UniRef100\_UPI000DFAA696 | 99.8 | 1.4e-22 | 2.6e-28 | 182.3 | 154 | (3, 156) | 423 | (7, 165) | 510 | phage tail protein | phage tail protein | | uniclust | UniRef100\_G4CPH8 | 99.8 | 1.4e-22 | 2.6e-28 | 186.0 | 154 | (2, 155) | 423 | (3, 156) | 779 | Uncharacterized protein | Uncharacterized protein | | uniclust | UniRef100\_A0A965NEJ7 | 99.8 | 1.5e-22 | 2.7e-28 | 147.8 | 131 | (2, 133) | 423 | (3, 133) | 135 | Uncharacterized protein (Fragment) | Uncharacterized protein (Fragment) | | uniclust | UniRef100\_A0A2G2D6M1 | 99.8 | 1.6e-22 | 2.9e-28 | 157.7 | 154 | (3, 157) | 423 | (22, 176) | 187 | Phage tail protein | Phage tail protein | | uniclust | UniRef100\_A0A7C8HT98 | 99.8 | 1.6e-22 | 2.9e-28 | 184.3 | 155 | (1, 155) | 423 | (2, 156) | 476 | Phage tail collar domain-containing protein | Phage tail collar domain-containing protein | | uniclust | UniRef100\_A0A1N6M5X0 | 99.8 | 1.5e-22 | 3e-28 | 186.4 | 161 | (2, 163) | 423 | (8, 168) | 363 | Phage Tail Collar Domain protein | Phage Tail Collar Domain protein | | uniclust | UniRef100\_UPI001E52CB3C | 99.8 | 1.7e-22 | 3.2e-28 | 166.5 | 204 | (72, 277) | 423 | (5, 210) | 296 | phage tail protein | phage tail protein | | uniclust | UniRef100\_UPI001967A92B | 99.8 | 2.3e-22 | 4.2e-28 | 175.4 | 150 | (3, 152) | 423 | (4, 153) | 466 | phage tail protein | phage tail protein | | uniclust | UniRef100\_UPI0015D48CC2 | 99.8 | 2.4e-22 | 4.4e-28 | 139.5 | 91 | (23, 113) | 423 | (2, 92) | 93 | phage tail protein | phage tail protein | | uniclust | UniRef100\_UPI000986ADFB | 99.8 | 2.8e-22 | 5.1e-28 | 169.2 | 151 | (2, 152) | 423 | (3, 153) | 353 | phage tail protein | phage tail protein | | uniclust | UniRef100\_UPI0023545723 | 99.8 | 2.9e-22 | 5.4e-28 | 181.4 | 154 | (1, 154) | 423 | (1, 154) | 667 | phage tail protein | phage tail protein | | uniclust | UniRef100\_A0A0A8JLH1 | 99.8 | 3.1e-22 | 5.7e-28 | 163.8 | 157 | (1, 157) | 423 | (1, 157) | 280 | Tail fiber protein | Tail fiber protein | | uniclust | UniRef100\_UPI000E267FDC | 99.8 | 3.2e-22 | 6e-28 | 169.5 | 136 | (2, 144) | 423 | (1, 137) | 294 | phage tail protein | phage tail protein | | uniclust | UniRef100\_UPI00069E8323 | 99.8 | 3.2e-22 | 6e-28 | 190.3 | 151 | (3, 154) | 423 | (5, 155) | 779 | phage tail protein | phage tail protein | | uniclust | UniRef100\_UPI00068D786B | 99.8 | 3.4e-22 | 6.2e-28 | 148.9 | 127 | (2, 133) | 423 | (3, 129) | 151 | phage tail protein | phage tail protein | | uniclust | UniRef100\_UPI0015AF74D6 | 99.8 | 3.4e-22 | 6.2e-28 | 158.5 | 154 | (1, 154) | 423 | (1, 154) | 224 | phage tail protein | phage tail protein | | uniclust | UniRef100\_A0A4R4E5X6 | 99.8 | 3.8e-22 | 6.9e-28 | 181.0 | 157 | (1, 157) | 423 | (1, 157) | 568 | Trimeric autotransporter adhesin YadA-like head domain-containing protein | Trimeric autotransporter adhesin YadA-like head domain-containing protein | | uniclust | UniRef100\_UPI00038C8FDF | 99.8 | 3.8e-22 | 7e-28 | 164.2 | 151 | (3, 153) | 423 | (5, 155) | 268 | phage tail protein | phage tail protein | | uniclust | UniRef100\_A0A4Y9GWV8 | 99.8 | 3.9e-22 | 7.3e-28 | 183.5 | 152 | (1, 152) | 423 | (2, 153) | 558 | Phage tail protein (Fragment) | Phage tail protein (Fragment) | | uniclust | UniRef100\_A0A0A8TLD1 | 99.8 | 4.3e-22 | 8.1e-28 | 196.3 | 157 | (1, 159) | 423 | (2, 158) | 998 | Phage tail fiber protein | Phage tail fiber protein | | uniclust | UniRef100\_UPI0014839BDF | 99.8 | 4.5e-22 | 8.3e-28 | 181.0 | 155 | (2, 156) | 423 | (6, 160) | 698 | phage tail protein | phage tail protein | | uniclust | UniRef100\_A0A376RL24 | 99.8 | 4.7e-22 | 8.8e-28 | 157.9 | 131 | (73, 304) | 423 | (2, 132) | 182 | Bcv gene product | Bcv gene product | | uniclust | UniRef100\_C6E6P3 | 99.8 | 4.9e-22 | 9e-28 | 160.3 | 156 | (2, 157) | 423 | (1, 157) | 251 | Phage-related tail fibre protein-like protein | Phage-related tail fibre protein-like protein | | uniclust | UniRef100\_A0A2N2GS05 | 99.8 | 5.2e-22 | 9.6e-28 | 174.5 | 153 | (1, 153) | 423 | (1, 153) | 497 | Phage tail protein | Phage tail protein | | uniclust | UniRef100\_UPI0008C2DC0F | 99.8 | 5.4e-22 | 1e-27 | 177.8 | 157 | (1, 157) | 423 | (1, 158) | 537 | phage tail protein | phage tail protein | | uniclust | UniRef100\_A0A4P9VIJ6 | 99.8 | 5.5e-22 | 1e-27 | 156.2 | 156 | (3, 158) | 423 | (4, 162) | 213 | Phage tail protein | Phage tail protein | | uniclust | UniRef100\_A0A7X2D3Y0 | 99.8 | 5.7e-22 | 1e-27 | 172.6 | 152 | (4, 155) | 423 | (5, 156) | 454 | Phage tail protein | Phage tail protein | | uniclust | UniRef100\_A0A6M0SQX7 | 99.8 | 5.7e-22 | 1.1e-27 | 180.1 | 153 | (1, 154) | 423 | (2, 154) | 574 | Phage tail protein | Phage tail protein | | uniclust | UniRef100\_A0A829RXG4 | 99.8 | 5.7e-22 | 1.1e-27 | 194.7 | 156 | (4, 160) | 423 | (6, 161) | 802 | Phage tail-collar fiber family protein | Phage tail-collar fiber family protein | | uniclust | UniRef100\_A0A853IIN2 | 99.8 | 6.4e-22 | 1.2e-27 | 169.2 | 161 | (3, 163) | 423 | (6, 169) | 349 | Phage tail protein | Phage tail protein | | uniclust | UniRef100\_A0A0M5MEM2 | 99.8 | 6.3e-22 | 1.2e-27 | 184.1 | 153 | (2, 157) | 423 | (1, 154) | 480 | Phage tail-collar fiber protein (DUF3751 domain) | Phage tail-collar fiber protein (DUF3751 domain) | | uniclust | UniRef100\_A0A2X2TYC3 | 99.8 | 6.7e-22 | 1.2e-27 | 170.7 | 138 | (1, 142) | 423 | (1, 138) | 356 | Phage tail fiber protein | Phage tail fiber protein | | uniclust | UniRef100\_A0A7X7NDN0 | 99.8 | 7.4e-22 | 1.4e-27 | 147.8 | 131 | (2, 133) | 423 | (4, 134) | 138 | Phage tail protein | Phage tail protein | | uniclust | UniRef100\_A0A1Y3CBA8 | 99.8 | 7.3e-22 | 1.4e-27 | 190.8 | 159 | (1, 161) | 423 | (2, 160) | 838 | Phage tail collar domain-containing protein | Phage tail collar domain-containing protein | | uniclust | UniRef100\_UPI0004685D26 | 99.8 | 7.7e-22 | 1.4e-27 | 175.3 | 156 | (3, 158) | 423 | (5, 160) | 517 | phage tail protein | phage tail protein | | uniclust | UniRef100\_A0A4P9VV53 | 99.8 | 7.6e-22 | 1.4e-27 | 175.8 | 158 | (3, 160) | 423 | (4, 164) | 431 | Phage tail protein | Phage tail protein | | uniclust | UniRef100\_A0A949CHK2 | 99.8 | 7.8e-22 | 1.5e-27 | 182.1 | 135 | (3, 137) | 423 | (4, 139) | 563 | Phage tail protein | Phage tail protein | | uniclust | UniRef100\_UPI0015E62003 | 99.8 | 8.3e-22 | 1.5e-27 | 132.8 | 84 | (74, 157) | 423 | (3, 86) | 87 | phage tail protein | phage tail protein | | uniclust | UniRef100\_UPI0003D2F6E8 | 99.8 | 9.8e-22 | 1.8e-27 | 158.3 | 139 | (67, 306) | 423 | (3, 141) | 228 | phage tail protein | phage tail protein | | uniclust | UniRef100\_A0A482IPK9 | 99.8 | 1.1e-21 | 2e-27 | 158.7 | 156 | (3, 158) | 423 | (4, 159) | 255 | Phage tail protein | Phage tail protein | | uniclust | UniRef100\_A0A5M6I3L7 | 99.8 | 1.1e-21 | 2.1e-27 | 167.9 | 156 | (1, 156) | 423 | (3, 158) | 312 | Phage tail protein | Phage tail protein | | uniclust | UniRef100\_A0A109RTW5 | 99.8 | 1.2e-21 | 2.1e-27 | 180.0 | 158 | (3, 161) | 423 | (4, 161) | 685 | Phage tail collar domain-containing protein | Phage tail collar domain-containing protein | | uniclust | UniRef100\_E5Y5X2 | 99.8 | 1.2e-21 | 2.1e-27 | 182.3 | 159 | (3, 161) | 423 | (4, 162) | 682 | Phage tail collar domain-containing protein | Phage tail collar domain-containing protein | | uniclust | UniRef100\_UPI001CF60DE5 | 99.8 | 1.2e-21 | 2.2e-27 | 182.3 | 151 | (3, 153) | 423 | (4, 154) | 823 | phage tail protein | phage tail protein | | uniclust | UniRef100\_A0A8S0FS69 | 99.8 | 1.2e-21 | 2.2e-27 | 153.8 | 137 | (2, 142) | 423 | (4, 140) | 165 | Phage tail protein | Phage tail protein | | uniclust | UniRef100\_A0A3D9UVK1 | 99.8 | 1.3e-21 | 2.5e-27 | 196.1 | 153 | (2, 159) | 423 | (3, 155) | 2092 | Tail fiber-like repeat protein | Tail fiber-like repeat protein | | uniclust | UniRef100\_UPI001CA5DA19 | 99.7 | 1.5e-21 | 2.7e-27 | 180.8 | 296 | (5, 305) | 423 | (3, 337) | 837 | phage tail protein | phage tail protein | | uniclust | UniRef100\_A0A4V1INX2 | 99.7 | 1.4e-21 | 2.7e-27 | 171.3 | 155 | (3, 157) | 423 | (4, 163) | 391 | Phage tail collar domain-containing protein | Phage tail collar domain-containing protein | | uniclust | UniRef100\_UPI001B39AF5A | 99.7 | 1.5e-21 | 2.7e-27 | 168.2 | 152 | (3, 154) | 423 | (5, 156) | 414 | phage tail protein | phage tail protein | | uniclust | UniRef100\_A0A380AJ63 | 99.7 | 1.6e-21 | 3e-27 | 186.5 | 145 | (1, 145) | 423 | (1, 145) | 670 | Caudovirales tail fibre assembly protein | Caudovirales tail fibre assembly protein | | uniclust | UniRef100\_UPI001F48AD60 | 99.7 | 1.7e-21 | 3.2e-27 | 150.8 | 154 | (3, 156) | 423 | (4, 161) | 190 | phage tail protein | phage tail protein | | uniclust | UniRef100\_UPI0021505079 | 99.7 | 1.9e-21 | 3.4e-27 | 180.3 | 154 | (3, 156) | 423 | (4, 157) | 849 | phage tail protein | phage tail protein | | uniclust | UniRef100\_UPI0002CDE47E | 99.7 | 1.9e-21 | 3.5e-27 | 178.8 | 153 | (3, 156) | 423 | (5, 157) | 606 | phage tail protein | phage tail protein | | pdb70 | 6CU2\_A | 98.8 | 1e-12 | 1.1e-16 | 120.4 | 105 | (309, 423) | 423 | (169, 273) | 273 | Probable bacteriophage protein | 6CU2\_A Probable bacteriophage protein adhesin, tail fiber, fiber, phage | | pdb70 | 6CT8\_A | 98.8 | 1e-12 | 1.1e-16 | 120.4 | 105 | (309, 423) | 423 | (169, 273) | 273 | Probable bacteriophage protein | 6CT8\_A Probable bacteriophage protein adhesin, tail fiber, fiber, phage HET: MSE | | pdb70 | 6CL6\_F | 98.7 | 3e-12 | 3e-16 | 123.9 | 105 | (309, 423) | 423 | (268, 372) | 372 | Tail fiber protein | 6CL6\_F Tail fiber protein R-type pyocin, Pseudomonas aeruginosa, contractile HET: EDO | | pdb70 | 6CXB\_A | 98.6 | 9.4e-12 | 1e-15 | 105.5 | 100 | (322, 423) | 423 | (41, 146) | 146 | Probable bacteriophage protein | 6CXB\_A Probable bacteriophage protein adhesin, tail fiber, fiber, phage | | pdb70 | 4MTM\_A | 98.4 | 6.1e-11 | 6.7e-15 | 101.7 | 97 | (321, 423) | 423 | (61, 164) | 164 | Putative tail fiber protein | 4MTM\_A Putative tail fiber protein Lectin fold, Tail fiber, receptor HET: EDO, BR, GOL | | pdb70 | 6CL5\_D | 97.8 | 8.2e-09 | 8.5e-13 | 99.5 | 99 | (323, 423) | 423 | (280, 384) | 384 | Phage tail protein | 6CL5\_D Phage tail protein R-type pyocin, Pseudomonas aeruginosa, contractile | |
| Top keywords  (threshold 1.00e-03 (evalue)) | **tail, Phage, fiber, fibre, tail\_collar, Fragment, domain\_containing, collar, Putative, Phage\_related** |
| Output files | ../../similar\_sequences/15\_FANPEZAQ\_CDS\_0015\_merged.svg ../../similar\_sequences/15\_FANPEZAQ\_CDS\_0015\_pdb70.a3m ../../similar\_sequences/15\_FANPEZAQ\_CDS\_0015\_pdb70.hhr ../../similar\_sequences/15\_FANPEZAQ\_CDS\_0015\_uniclust.a3m ../../similar\_sequences/15\_FANPEZAQ\_CDS\_0015\_uniclust.hhr |

#### Structure prediction (AlphaFold)2

|  |  |
| --- | --- |
| Stats | xml version="1.0" encoding="utf-8" standalone="no"?       2024-09-02T21:09:14.212967 image/svg+xml   Matplotlib v3.7.2, https://matplotlib.org/ |
| Predicted structure | **NGL Viewer Controls:**  - Center: *Left-Click* - Rotate: *Left-Click + Drag* - Translate: *Right-Click + Drag* - Zoom: *Shift + Left-Click + Drag* |
| Output files | ../../predicted\_structures/15\_FANPEZAQ\_CDS\_0015/features.pkl ../../predicted\_structures/15\_FANPEZAQ\_CDS\_0015/ranked\_0.pdb ../../predicted\_structures/15\_FANPEZAQ\_CDS\_0015/ranked\_0\_plots.svg ../../predicted\_structures/15\_FANPEZAQ\_CDS\_0015/result\_model\_1\_ptm\_pred\_0.pkl |

#### Structure similarity search results (Foldseek)3

|  |  |
| --- | --- |
| Structure databases searched | Pdb, Afdb-proteome, Afdb-uniprot50 |
| Results, scheme(s)  (Top layers only, threshold 1.00e-02 (evalue)) | xml version="1.0" encoding="utf-8" standalone="no"?       2024-09-02T21:10:33.642177 image/svg+xml   Matplotlib v3.7.2, https://matplotlib.org/ |
| Results, table  (threshold 1.00e-02 (evalue)) | | db | id | prob | evalue | bits | fident | alnlen | mismatch | gapopen | qstart | qend | tstart | tend | name | description | | --- | --- | --- | --- | --- | --- | --- | --- | --- | --- | --- | --- | --- | --- | --- | | pdb | 4MTM\_A | 1.0 | 5.97e-05 | 171 | 0.185 | 167 | 98 | 11 | 265 | 423 | 1 | 137 | Putative tail fiber protein | Putative tail fiber protein | | pdb | 6CU2\_A | 1.0 | 0.0007118 | 145 | 0.191 | 94 | 62 | 8 | 334 | 423 | 166 | 249 | R2-type pyocin | R2-type pyocin | | pdb | 6CL5\_F | 1.0 | 0.0006391 | 136 | 0.27 | 100 | 58 | 9 | 334 | 423 | 280 | 374 | Tail fiber protein | Tail fiber protein | | pdb | 7RFV\_A | 1.0 | 0.003584 | 128 | 0.187 | 80 | 59 | 4 | 179 | 258 | 166 | 239 | Tailspike protein | Tailspike protein | | pdb | 6CXB\_A | 1.0 | 0.002458 | 121 | 0.261 | 107 | 63 | 10 | 328 | 423 | 21 | 122 | R1-type pyocin tail fiber protein | R1-type pyocin tail fiber protein | | afdb-proteome | AF-Q8ZKK5-F1-MODEL\_V4 | 1.0 | 2.829e-22 | 488 | 0.217 | 551 | 249 | 24 | 1 | 412 | 1 | 508 | Putative phage tail fiber protein H | Putative phage tail fiber protein H | | afdb-proteome | AF-A0A0H3H1R2-F1-MODEL\_V4 | 1.0 | 1.45e-16 | 414 | 0.227 | 334 | 169 | 14 | 1 | 258 | 1 | 321 | Uncharacterized protein | Uncharacterized protein | | afdb-proteome | AF-G3XD71-F1-MODEL\_V4 | 1.0 | 1.12e-19 | 397 | 0.151 | 708 | 295 | 32 | 1 | 423 | 5 | 691 | Probable bacteriophage protein | Probable bacteriophage protein | | afdb-proteome | AF-A0A0H3GKQ5-F1-MODEL\_V4 | 1.0 | 3.083e-16 | 393 | 0.243 | 341 | 157 | 14 | 1 | 258 | 1 | 323 | Uncharacterized protein | Uncharacterized protein | | afdb-proteome | AF-Q8ZMU5-F1-MODEL\_V4 | 1.0 | 5.949e-19 | 378 | 0.207 | 583 | 224 | 23 | 1 | 421 | 2 | 508 | Fels-2 prophage protein | Fels-2 prophage protein | | afdb-proteome | AF-P76508-F1-MODEL\_V4 | 1.0 | 1.03e-06 | 245 | 0.287 | 132 | 82 | 6 | 296 | 423 | 49 | 172 | Putative uncharacterized protein YfdL | Putative uncharacterized protein YfdL | | afdb-proteome | AF-P45581-F1-MODEL\_V4 | 1.0 | 8.547e-05 | 206 | 0.295 | 88 | 48 | 6 | 341 | 423 | 131 | 209 | Uncharacterized protein StfP | Uncharacterized protein StfP | | afdb-proteome | AF-Q57793-F1-MODEL\_V4 | 1.0 | 0.0005634 | 122 | 0.156 | 134 | 88 | 8 | 307 | 423 | 26 | 151 | Uncharacterized protein MJ0347 | Uncharacterized protein MJ0347 | | afdb-proteome | AF-Q9I5S0-F1-MODEL\_V4 | 0.923 | 0.004607 | 69 | 0.114 | 368 | 169 | 21 | 193 | 423 | 1 | 348 | Uncharacterized protein | Uncharacterized protein | | afdb-proteome | AF-Q9I5S3-F1-MODEL\_V4 | 0.772 | 0.006032 | 60 | 0.125 | 389 | 148 | 24 | 198 | 423 | 2 | 361 | Uncharacterized protein | Uncharacterized protein | | afdb-uniprot50 | AF-A0A430HF82-F1-MODEL\_V4 | 1.0 | 1.815e-31 | 1105 | 0.473 | 262 | 126 | 4 | 4 | 261 | 3 | 256 | Uncharacterized protein | Uncharacterized protein | | afdb-uniprot50 | AF-A0A2A3MGV4-F1-MODEL\_V4 | 1.0 | 7.366e-31 | 1016 | 0.456 | 267 | 138 | 3 | 1 | 261 | 1 | 266 | Phage tail protein | Phage tail protein | | afdb-uniprot50 | AF-A0A250DS87-F1-MODEL\_V4 | 1.0 | 4.298e-31 | 990 | 0.43 | 265 | 146 | 4 | 1 | 263 | 1 | 262 | Phage tail protein | Phage tail protein | | afdb-uniprot50 | AF-A0A0F9VFY7-F1-MODEL\_V4 | 1.0 | 1.505e-29 | 962 | 0.415 | 265 | 151 | 3 | 1 | 263 | 1 | 263 | Collar domain-containing protein | Collar domain-containing protein | | afdb-uniprot50 | AF-A0A5E7KHM1-F1-MODEL\_V4 | 1.0 | 7.473e-30 | 929 | 0.436 | 266 | 143 | 4 | 1 | 261 | 1 | 264 | Collar domain-containing protein | Collar domain-containing protein | | afdb-uniprot50 | AF-A0A432RGY3-F1-MODEL\_V4 | 1.0 | 2.674e-19 | 857 | 0.531 | 158 | 71 | 1 | 1 | 155 | 1 | 158 | Uncharacterized protein | Uncharacterized protein | | afdb-uniprot50 | AF-A0A7V7KJT5-F1-MODEL\_V4 | 1.0 | 6.683e-19 | 853 | 0.503 | 153 | 76 | 0 | 1 | 153 | 1 | 153 | Sialidase domain-containing protein | Sialidase domain-containing protein | | afdb-uniprot50 | AF-G4CPH8-F1-MODEL\_V4 | 1.0 | 1.5e-18 | 848 | 0.525 | 154 | 72 | 1 | 1 | 153 | 1 | 154 | Uncharacterized protein | Uncharacterized protein | | afdb-uniprot50 | AF-A0A3N9U476-F1-MODEL\_V4 | 1.0 | 3.27e-20 | 844 | 0.465 | 176 | 90 | 1 | 1 | 176 | 1 | 172 | Collar domain-containing protein | Collar domain-containing protein | | afdb-uniprot50 | AF-A5N994-F1-MODEL\_V4 | 1.0 | 3.899e-19 | 843 | 0.47 | 153 | 81 | 0 | 1 | 153 | 2 | 154 | Uncharacterized protein | Uncharacterized protein | | afdb-uniprot50 | AF-A0A724JF68-F1-MODEL\_V4 | 1.0 | 7.855e-19 | 842 | 0.503 | 157 | 78 | 0 | 2 | 158 | 3 | 159 | Phage tail protein | Phage tail protein | | afdb-uniprot50 | AF-A0A396RYI5-F1-MODEL\_V4 | 1.0 | 2.57e-18 | 834 | 0.458 | 155 | 84 | 0 | 2 | 156 | 6 | 160 | Uncharacterized protein | Uncharacterized protein | | afdb-uniprot50 | AF-A0A4R1K4B3-F1-MODEL\_V4 | 1.0 | 9.233e-19 | 832 | 0.475 | 162 | 85 | 0 | 1 | 162 | 1 | 162 | Phage-related tail fiber protein | Phage-related tail fiber protein | | afdb-uniprot50 | AF-A0A3Z6GCN4-F1-MODEL\_V4 | 1.0 | 6e-19 | 830 | 0.493 | 158 | 80 | 0 | 1 | 158 | 2 | 159 | Phage tail protein | Phage tail protein | | afdb-uniprot50 | AF-A0A345LUV5-F1-MODEL\_V4 | 1.0 | 2.072e-18 | 830 | 0.519 | 152 | 73 | 0 | 4 | 155 | 3 | 154 | Collar domain-containing protein | Collar domain-containing protein | | afdb-uniprot50 | AF-A0A5T7A7I4-F1-MODEL\_V4 | 1.0 | 3.748e-18 | 828 | 0.481 | 158 | 82 | 0 | 1 | 158 | 2 | 159 | Phage tail protein | Phage tail protein | | afdb-uniprot50 | AF-A0A2P8VLU5-F1-MODEL\_V4 | 1.0 | 3.748e-18 | 827 | 0.487 | 158 | 81 | 0 | 1 | 158 | 2 | 159 | Phage tail protein | Phage tail protein | | afdb-uniprot50 | AF-A0A6M0RCD0-F1-MODEL\_V4 | 1.0 | 1.258e-19 | 825 | 0.403 | 166 | 98 | 1 | 1 | 166 | 1 | 165 | Phage tail protein | Phage tail protein | | afdb-uniprot50 | AF-A0A1I1WBP0-F1-MODEL\_V4 | 1.0 | 1.085e-18 | 824 | 0.478 | 163 | 83 | 1 | 1 | 163 | 1 | 161 | Phage tail-collar fibre protein | Phage tail-collar fibre protein | | afdb-uniprot50 | AF-A0A080IG54-F1-MODEL\_V4 | 1.0 | 5.767e-18 | 823 | 0.481 | 158 | 82 | 0 | 1 | 158 | 2 | 159 | Phage tail-collar fiber family protein | Phage tail-collar fiber family protein | | afdb-uniprot50 | AF-A0A5I1KPW3-F1-MODEL\_V4 | 1.0 | 1.763e-18 | 820 | 0.496 | 157 | 79 | 0 | 2 | 158 | 3 | 159 | Phage tail protein | Phage tail protein | | afdb-uniprot50 | AF-A0A4R1TM91-F1-MODEL\_V4 | 1.0 | 1.145e-18 | 819 | 0.452 | 157 | 82 | 2 | 1 | 153 | 1 | 157 | Tail-collar fiber protein | Tail-collar fiber protein | | afdb-uniprot50 | AF-G7LSU4-F1-MODEL\_V4 | 1.0 | 2.187e-18 | 819 | 0.512 | 164 | 79 | 1 | 1 | 163 | 1 | 164 | Uncharacterized protein | Uncharacterized protein | | afdb-uniprot50 | AF-X6QCI0-F1-MODEL\_V4 | 1.0 | 7.155e-18 | 816 | 0.513 | 150 | 73 | 0 | 4 | 153 | 3 | 152 | Tail fiber protein | Tail fiber protein | | afdb-uniprot50 | AF-D6IGH8-F1-MODEL\_V4 | 1.0 | 7.155e-18 | 816 | 0.481 | 158 | 82 | 0 | 1 | 158 | 2 | 159 | Phage tail fiber protein | Phage tail fiber protein | | afdb-uniprot50 | AF-A0A7U3LRL7-F1-MODEL\_V4 | 1.0 | 8.749e-19 | 814 | 0.506 | 160 | 79 | 0 | 1 | 160 | 2 | 161 | Uncharacterized protein | Uncharacterized protein | | afdb-uniprot50 | AF-A0A7T3MYV0-F1-MODEL\_V4 | 1.0 | 1.276e-18 | 813 | 0.437 | 153 | 86 | 0 | 1 | 153 | 2 | 154 | Phage tail protein | Phage tail protein | | afdb-uniprot50 | AF-A0A5W6HQH5-F1-MODEL\_V4 | 1.0 | 9.885e-18 | 813 | 0.487 | 158 | 81 | 0 | 1 | 158 | 2 | 159 | Phage tail protein | Phage tail protein | | afdb-uniprot50 | AF-A0A604P221-F1-MODEL\_V4 | 1.0 | 4.405e-18 | 813 | 0.496 | 157 | 79 | 0 | 2 | 158 | 3 | 159 | Phage tail protein | Phage tail protein | | afdb-uniprot50 | AF-A0A6B3YWL7-F1-MODEL\_V4 | 1.0 | 1.963e-18 | 811 | 0.424 | 153 | 88 | 0 | 1 | 153 | 2 | 154 | Phage tail protein | Phage tail protein | | afdb-uniprot50 | AF-A0A2S8QLP1-F1-MODEL\_V4 | 1.0 | 3.188e-18 | 806 | 0.453 | 161 | 88 | 0 | 1 | 161 | 2 | 162 | Uncharacterized protein | Uncharacterized protein | | afdb-uniprot50 | AF-A0A5U9RX76-F1-MODEL\_V4 | 1.0 | 2.072e-18 | 806 | 0.493 | 158 | 80 | 0 | 1 | 158 | 2 | 159 | Phage tail protein | Phage tail protein | | afdb-uniprot50 | AF-A0A292AIP5-F1-MODEL\_V4 | 1.0 | 1.043e-17 | 806 | 0.496 | 155 | 76 | 1 | 1 | 155 | 2 | 154 | Uncharacterized protein | Uncharacterized protein | | afdb-uniprot50 | AF-A0A0U3LJE0-F1-MODEL\_V4 | 1.0 | 4.837e-19 | 805 | 0.448 | 176 | 97 | 0 | 1 | 176 | 1 | 176 | Bacteriophage variable tail fiber protein | Bacteriophage variable tail fiber protein | | afdb-uniprot50 | AF-F3YY38-F1-MODEL\_V4 | 1.0 | 3.551e-18 | 804 | 0.519 | 154 | 73 | 1 | 1 | 153 | 2 | 155 | Uncharacterized protein | Uncharacterized protein | | afdb-uniprot50 | AF-A0A5T6DFY3-F1-MODEL\_V4 | 1.0 | 1.67e-18 | 803 | 0.496 | 157 | 79 | 0 | 2 | 158 | 3 | 159 | Phage tail protein | Phage tail protein | | afdb-uniprot50 | AF-A0A376D432-F1-MODEL\_V4 | 1.0 | 1.145e-18 | 803 | 0.481 | 158 | 82 | 0 | 1 | 158 | 2 | 159 | Putative variable tail fiber protein | Putative variable tail fiber protein | | afdb-uniprot50 | AF-A0A447PAU2-F1-MODEL\_V4 | 1.0 | 1.5e-18 | 803 | 0.487 | 158 | 81 | 0 | 1 | 158 | 2 | 159 | Phage tail-like protein | Phage tail-like protein | | afdb-uniprot50 | AF-A0A3N9UIV0-F1-MODEL\_V4 | 1.0 | 1.763e-18 | 801 | 0.458 | 155 | 84 | 0 | 1 | 155 | 1 | 155 | Uncharacterized protein | Uncharacterized protein | | afdb-uniprot50 | AF-A0A348HHI2-F1-MODEL\_V4 | 1.0 | 2.218e-17 | 798 | 0.419 | 155 | 90 | 0 | 1 | 155 | 1 | 155 | Phage-related tail fibre protein | Phage-related tail fibre protein | | afdb-uniprot50 | AF-A0A7L9R9Q1-F1-MODEL\_V4 | 1.0 | 2.072e-18 | 798 | 0.487 | 162 | 83 | 0 | 1 | 162 | 2 | 163 | Phage tail protein | Phage tail protein | | afdb-uniprot50 | AF-A0A7D6C2F1-F1-MODEL\_V4 | 1.0 | 1.366e-17 | 798 | 0.432 | 155 | 88 | 0 | 1 | 155 | 2 | 156 | Phage tail protein | Phage tail protein | | afdb-uniprot50 | AF-A0A5T2YBP8-F1-MODEL\_V4 | 1.0 | 2.471e-17 | 797 | 0.481 | 158 | 82 | 0 | 1 | 158 | 2 | 159 | Phage tail protein | Phage tail protein | | afdb-uniprot50 | AF-A0A2Z4WEE0-F1-MODEL\_V4 | 1.0 | 3.188e-18 | 796 | 0.4 | 155 | 93 | 0 | 1 | 155 | 1 | 155 | Uncharacterized protein | Uncharacterized protein | | afdb-uniprot50 | AF-A0A6L5EC52-F1-MODEL\_V4 | 1.0 | 1.86e-18 | 795 | 0.515 | 159 | 76 | 1 | 1 | 158 | 1 | 159 | Phage tail protein | Phage tail protein | | afdb-uniprot50 | AF-A0A2G8C8T9-F1-MODEL\_V4 | 1.0 | 1.421e-18 | 790 | 0.496 | 159 | 79 | 1 | 1 | 158 | 1 | 159 | Phage tail protein | Phage tail protein | | afdb-uniprot50 | AF-A0A0P9SFR0-F1-MODEL\_V4 | 1.0 | 2.102e-17 | 790 | 0.441 | 154 | 86 | 0 | 2 | 155 | 6 | 159 | Phage-related tail fiber protein-like protein | Phage-related tail fiber protein-like protein | | afdb-uniprot50 | AF-A0A084ZPT2-F1-MODEL\_V4 | 1.0 | 7.155e-18 | 788 | 0.506 | 156 | 76 | 1 | 1 | 155 | 1 | 156 | Phage tail fiber protein | Phage tail fiber protein | | afdb-uniprot50 | AF-A0A2R8CQR2-F1-MODEL\_V4 | 1.0 | 1.411e-26 | 786 | 0.235 | 476 | 203 | 16 | 1 | 423 | 1 | 368 | Uncharacterized protein | Uncharacterized protein | | afdb-uniprot50 | AF-J6HCZ5-F1-MODEL\_V4 | 1.0 | 7.551e-18 | 785 | 0.434 | 161 | 91 | 0 | 1 | 161 | 2 | 162 | Tail fiber protein | Tail fiber protein | | afdb-uniprot50 | AF-A0A5Y2E3R8-F1-MODEL\_V4 | 1.0 | 5.685e-19 | 784 | 0.429 | 177 | 96 | 2 | 1 | 172 | 1 | 177 | Phage tail protein | Phage tail protein | | afdb-uniprot50 | AF-A0A447P489-F1-MODEL\_V4 | 1.0 | 5.851e-17 | 783 | 0.507 | 136 | 67 | 0 | 1 | 136 | 2 | 137 | Phage tail fiber protein | Phage tail fiber protein | | afdb-uniprot50 | AF-A0A7U6FCV4-F1-MODEL\_V4 | 1.0 | 3.414e-17 | 783 | 0.424 | 153 | 88 | 0 | 1 | 153 | 5 | 157 | Uncharacterized protein | Uncharacterized protein | | afdb-uniprot50 | AF-A0A714EVS6-F1-MODEL\_V4 | 1.0 | 4.649e-18 | 783 | 0.503 | 157 | 78 | 0 | 2 | 158 | 3 | 159 | Phage tail protein | Phage tail protein | | afdb-uniprot50 | AF-A0A377Q9P4-F1-MODEL\_V4 | 1.0 | 1.366e-17 | 783 | 0.487 | 154 | 76 | 1 | 3 | 153 | 2 | 155 | Uncharacterized protein | Uncharacterized protein | | afdb-uniprot50 | AF-C2BG12-F1-MODEL\_V4 | 1.0 | 4.235e-17 | 782 | 0.346 | 153 | 100 | 0 | 1 | 153 | 1 | 153 | Uncharacterized protein | Uncharacterized protein | | afdb-uniprot50 | AF-A0A827L2X9-F1-MODEL\_V4 | 1.0 | 2.435e-18 | 781 | 0.49 | 159 | 80 | 1 | 1 | 158 | 1 | 159 | Phage tail protein | Phage tail protein | | afdb-uniprot50 | AF-A0A3M6E5R0-F1-MODEL\_V4 | 1.0 | 3.234e-17 | 780 | 0.432 | 155 | 88 | 0 | 1 | 155 | 5 | 159 | Tail fiber protein H | Tail fiber protein H | | afdb-uniprot50 | AF-A0A433ZYK3-F1-MODEL\_V4 | 1.0 | 4.174e-18 | 779 | 0.465 | 161 | 86 | 0 | 1 | 161 | 2 | 162 | Uncharacterized protein | Uncharacterized protein | | afdb-uniprot50 | AF-D1P881-F1-MODEL\_V4 | 1.0 | 2.713e-18 | 778 | 0.453 | 163 | 87 | 1 | 4 | 164 | 3 | 165 | Uncharacterized protein | Uncharacterized protein | | afdb-uniprot50 | AF-A0A506QSU0-F1-MODEL\_V4 | 1.0 | 5.253e-17 | 777 | 0.474 | 158 | 83 | 0 | 1 | 158 | 2 | 159 | Phage tail protein | Phage tail protein | | afdb-uniprot50 | AF-A0A4P9VIJ6-F1-MODEL\_V4 | 1.0 | 4.649e-18 | 777 | 0.397 | 166 | 95 | 3 | 1 | 161 | 1 | 166 | Uncharacterized protein | Uncharacterized protein | | afdb-uniprot50 | AF-A0A6G2EFZ9-F1-MODEL\_V4 | 1.0 | 1.86e-18 | 775 | 0.445 | 166 | 88 | 1 | 1 | 162 | 1 | 166 | Phage tail protein | Phage tail protein | | afdb-uniprot50 | AF-A0A659NRL8-F1-MODEL\_V4 | 1.0 | 4.39e-22 | 774 | 0.321 | 330 | 111 | 5 | 2 | 331 | 3 | 219 | Phage tail protein | Phage tail protein | | afdb-uniprot50 | AF-A0A5W0Y2X8-F1-MODEL\_V4 | 1.0 | 5.465e-18 | 772 | 0.481 | 158 | 82 | 0 | 1 | 158 | 2 | 159 | Phage tail protein | Phage tail protein | | afdb-uniprot50 | AF-A0A080HXC3-F1-MODEL\_V4 | 1.0 | 1.192e-19 | 771 | 0.419 | 193 | 101 | 2 | 1 | 184 | 2 | 192 | Phage tail-collar fiber family protein | Phage tail-collar fiber family protein | | afdb-uniprot50 | AF-F4VH24-F1-MODEL\_V4 | 1.0 | 1.294e-17 | 771 | 0.481 | 158 | 82 | 0 | 1 | 158 | 2 | 159 | Putative bacteriophage variable tail fiber protein H | Putative bacteriophage variable tail fiber protein H | | afdb-uniprot50 | AF-A0A3N0FYV1-F1-MODEL\_V4 | 1.0 | 5.178e-18 | 771 | 0.484 | 163 | 83 | 1 | 1 | 162 | 1 | 163 | Phage tail protein | Phage tail protein | | afdb-uniprot50 | AF-A0A0T9KM71-F1-MODEL\_V4 | 1.0 | 2.471e-17 | 771 | 0.465 | 161 | 86 | 0 | 1 | 161 | 2 | 162 | Variable tail fiber protein | Variable tail fiber protein | | afdb-uniprot50 | AF-D4GNL8-F1-MODEL\_V4 | 1.0 | 8.531e-17 | 771 | 0.481 | 158 | 82 | 0 | 1 | 158 | 2 | 159 | H | H | | afdb-uniprot50 | AF-E0SFL4-F1-MODEL\_V4 | 1.0 | 2.072e-18 | 769 | 0.5 | 164 | 79 | 2 | 1 | 163 | 93 | 254 | Tail fiber protein | Tail fiber protein | | afdb-uniprot50 | AF-A0A5F1ELN5-F1-MODEL\_V4 | 1.0 | 9.233e-19 | 768 | 0.484 | 159 | 81 | 1 | 1 | 158 | 1 | 159 | Phage tail protein | Phage tail protein | | afdb-uniprot50 | AF-A0A3M5N4H6-F1-MODEL\_V4 | 1.0 | 9.003e-17 | 768 | 0.441 | 154 | 86 | 0 | 2 | 155 | 6 | 159 | Phage-related tail fiber protein-like protein | Phage-related tail fiber protein-like protein | | afdb-uniprot50 | AF-A0A0T9TRH4-F1-MODEL\_V4 | 1.0 | 2.874e-29 | 766 | 0.299 | 468 | 184 | 15 | 1 | 423 | 2 | 370 | Tail fiber protein | Tail fiber protein | | afdb-uniprot50 | AF-A0A1M7FCH9-F1-MODEL\_V4 | 1.0 | 2.471e-17 | 766 | 0.477 | 155 | 81 | 0 | 1 | 155 | 2 | 156 | Phage tail-collar fibre protein | Phage tail-collar fibre protein | | afdb-uniprot50 | AF-A0A385XY40-F1-MODEL\_V4 | 1.0 | 8.41e-18 | 764 | 0.49 | 159 | 80 | 1 | 1 | 158 | 1 | 159 | Collar domain-containing protein | Collar domain-containing protein | | afdb-uniprot50 | AF-A0A741RNT9-F1-MODEL\_V4 | 1.0 | 1.605e-17 | 763 | 0.481 | 158 | 82 | 0 | 1 | 158 | 2 | 159 | Phage tail protein | Phage tail protein | | afdb-uniprot50 | AF-A0A2T5HH07-F1-MODEL\_V4 | 1.0 | 3.414e-17 | 761 | 0.439 | 157 | 84 | 2 | 1 | 153 | 1 | 157 | Tail-collar fiber protein | Tail-collar fiber protein | | afdb-uniprot50 | AF-A0A482IPK9-F1-MODEL\_V4 | 1.0 | 2.218e-17 | 760 | 0.359 | 153 | 98 | 0 | 1 | 153 | 2 | 154 | Uncharacterized protein | Uncharacterized protein | | afdb-uniprot50 | AF-A0A2P7NQL6-F1-MODEL\_V4 | 1.0 | 6.516e-17 | 758 | 0.393 | 155 | 94 | 0 | 1 | 155 | 3 | 157 | Uncharacterized protein | Uncharacterized protein | | afdb-uniprot50 | AF-W2C9W5-F1-MODEL\_V4 | 1.0 | 1.441e-17 | 757 | 0.509 | 151 | 74 | 0 | 1 | 151 | 2 | 152 | Phage tail protein | Phage tail protein | | afdb-uniprot50 | AF-A0A741LDP6-F1-MODEL\_V4 | 1.0 | 8.084e-17 | 757 | 0.503 | 151 | 75 | 0 | 1 | 151 | 2 | 152 | Phage tail protein | Phage tail protein | | afdb-uniprot50 | AF-A0A1S6TPA5-F1-MODEL\_V4 | 1.0 | 2.752e-17 | 757 | 0.352 | 156 | 98 | 1 | 3 | 158 | 2 | 154 | Phage tail-collar fiber protein (DUF3751 domain) | Phage tail-collar fiber protein (DUF3751 domain) | | afdb-uniprot50 | AF-A0A377LB56-F1-MODEL\_V4 | 1.0 | 1.162e-17 | 757 | 0.469 | 162 | 86 | 0 | 1 | 162 | 2 | 163 | Phage tail collar protein | Phage tail collar protein | | afdb-uniprot50 | AF-A0A329VCK4-F1-MODEL\_V4 | 1.0 | 4.453e-21 | 755 | 0.343 | 306 | 100 | 3 | 4 | 309 | 3 | 207 | Phage tail protein | Phage tail protein | | afdb-uniprot50 | AF-A0A2D5S2E6-F1-MODEL\_V4 | 1.0 | 6.877e-17 | 753 | 0.337 | 151 | 100 | 0 | 3 | 153 | 2 | 152 | Phage tail protein | Phage tail protein | | afdb-uniprot50 | AF-A0A0S8ZMG3-F1-MODEL\_V4 | 1.0 | 1.179e-16 | 751 | 0.424 | 153 | 88 | 0 | 1 | 153 | 5 | 157 | Collar domain-containing protein | Collar domain-containing protein | | afdb-uniprot50 | AF-V0XNW2-F1-MODEL\_V4 | 1.0 | 4.235e-17 | 750 | 0.448 | 154 | 84 | 1 | 1 | 153 | 2 | 155 | Uncharacterized protein | Uncharacterized protein | | afdb-uniprot50 | AF-A0A1Y0FPI9-F1-MODEL\_V4 | 1.0 | 1.521e-17 | 750 | 0.468 | 160 | 83 | 2 | 1 | 158 | 1 | 160 | 6-phosphogluconolactonase | 6-phosphogluconolactonase | | afdb-uniprot50 | AF-A0A5X2Z7L4-F1-MODEL\_V4 | 1.0 | 7.337e-20 | 749 | 0.322 | 307 | 107 | 3 | 1 | 307 | 2 | 207 | Phage tail protein | Phage tail protein | | afdb-uniprot50 | AF-A0A839C2U0-F1-MODEL\_V4 | 1.0 | 4.716e-17 | 749 | 0.493 | 158 | 80 | 0 | 1 | 158 | 1 | 158 | Phage tail protein | Phage tail protein | | afdb-uniprot50 | AF-A0A7W3F3N4-F1-MODEL\_V4 | 1.0 | 2.471e-17 | 747 | 0.487 | 158 | 81 | 0 | 1 | 158 | 2 | 159 | Phage tail protein | Phage tail protein | | afdb-uniprot50 | AF-A0A522IBP7-F1-MODEL\_V4 | 1.0 | 2.471e-17 | 746 | 0.435 | 163 | 89 | 2 | 1 | 162 | 2 | 162 | Uncharacterized protein | Uncharacterized protein | | afdb-uniprot50 | AF-A0A5S9NC05-F1-MODEL\_V4 | 1.0 | 7.258e-17 | 746 | 0.376 | 154 | 95 | 1 | 1 | 153 | 1 | 154 | Uncharacterized protein | Uncharacterized protein | | afdb-uniprot50 | AF-A0A1I0EJH2-F1-MODEL\_V4 | 1.0 | 1.719e-16 | 745 | 0.418 | 153 | 88 | 1 | 1 | 153 | 1 | 152 | Phage tail-collar fibre protein | Phage tail-collar fibre protein | | afdb-uniprot50 | AF-D7XVP6-F1-MODEL\_V4 | 1.0 | 1.516e-21 | 745 | 0.327 | 324 | 113 | 5 | 1 | 324 | 2 | 220 | Phage tail fiber repeat protein | Phage tail fiber repeat protein | | afdb-uniprot50 | AF-A0A1G5RGJ6-F1-MODEL\_V4 | 1.0 | 2.742e-21 | 744 | 0.349 | 323 | 103 | 5 | 4 | 326 | 3 | 218 | Phage tail fibre repeat-containing protein | Phage tail fibre repeat-containing protein | | afdb-uniprot50 | AF-A0A351NNZ8-F1-MODEL\_V4 | 1.0 | 2.102e-17 | 744 | 0.484 | 161 | 83 | 0 | 2 | 162 | 3 | 163 | Uncharacterized protein | Uncharacterized protein | | afdb-uniprot50 | AF-A0A7A6RGS2-F1-MODEL\_V4 | 1.0 | 3.414e-17 | 743 | 0.453 | 172 | 94 | 0 | 1 | 172 | 2 | 173 | Phage tail protein | Phage tail protein | | afdb-uniprot50 | AF-A0A417HJB2-F1-MODEL\_V4 | 1.0 | 2.904e-17 | 743 | 0.358 | 156 | 100 | 0 | 3 | 158 | 5 | 160 | Uncharacterized protein | Uncharacterized protein | | afdb-uniprot50 | AF-A0A5U6FPA7-F1-MODEL\_V4 | 1.0 | 1.462e-16 | 742 | 0.503 | 151 | 75 | 0 | 1 | 151 | 2 | 152 | Phage tail protein | Phage tail protein | | afdb-uniprot50 | AF-A0A1M5TXK3-F1-MODEL\_V4 | 1.0 | 1.653e-30 | 742 | 0.298 | 469 | 205 | 18 | 1 | 423 | 2 | 392 | Phage tail-collar fibre protein | Phage tail-collar fibre protein | | afdb-uniprot50 | AF-D8J073-F1-MODEL\_V4 | 1.0 | 1.196e-30 | 741 | 0.28 | 449 | 249 | 14 | 1 | 418 | 3 | 408 | Bacteriophage variable tail fibre protein | Bacteriophage variable tail fibre protein | | afdb-uniprot50 | AF-A0A1H5GVL6-F1-MODEL\_V4 | 1.0 | 6.516e-17 | 741 | 0.442 | 156 | 86 | 1 | 1 | 155 | 1 | 156 | Phage-related tail fibre protein | Phage-related tail fibre protein | | afdb-uniprot50 | AF-A0A7X5IDT3-F1-MODEL\_V4 | 1.0 | 5.544e-17 | 740 | 0.31 | 158 | 109 | 0 | 1 | 158 | 1 | 158 | Peptidase S74 domain-containing protein | Peptidase S74 domain-containing protein | | afdb-uniprot50 | AF-A0A6B4D2K2-F1-MODEL\_V4 | 1.0 | 6e-19 | 738 | 0.4 | 195 | 110 | 2 | 1 | 188 | 2 | 196 | Phage tail protein | Phage tail protein | | afdb-uniprot50 | AF-A0A6G4CRK8-F1-MODEL\_V4 | 1.0 | 8.875e-18 | 738 | 0.465 | 161 | 86 | 0 | 1 | 161 | 2 | 162 | Phage tail protein | Phage tail protein | | afdb-uniprot50 | AF-A0A2W5TDM3-F1-MODEL\_V4 | 1.0 | 9.003e-17 | 738 | 0.445 | 157 | 86 | 1 | 3 | 158 | 2 | 158 | Peptidase S74 domain-containing protein | Peptidase S74 domain-containing protein | | afdb-uniprot50 | AF-A0A6A1RN19-F1-MODEL\_V4 | 1.0 | 1.058e-16 | 738 | 0.381 | 160 | 98 | 1 | 3 | 162 | 5 | 163 | Uncharacterized protein | Uncharacterized protein | | afdb-uniprot50 | AF-A0A1H9EZ18-F1-MODEL\_V4 | 1.0 | 1.162e-17 | 737 | 0.493 | 164 | 81 | 1 | 1 | 162 | 2 | 165 | Phage Tail Collar Domain | Phage Tail Collar Domain | | afdb-uniprot50 | AF-A0A8B5VHZ4-F1-MODEL\_V4 | 1.0 | 4.469e-17 | 736 | 0.444 | 153 | 85 | 0 | 1 | 153 | 2 | 154 | Phage tail protein | Phage tail protein | | afdb-uniprot50 | AF-A0A806LIS8-F1-MODEL\_V4 | 1.0 | 2.471e-17 | 736 | 0.434 | 161 | 90 | 1 | 1 | 161 | 2 | 161 | Uncharacterized protein | Uncharacterized protein | | afdb-uniprot50 | AF-A0A1W1XL57-F1-MODEL\_V4 | 1.0 | 3.802e-17 | 734 | 0.37 | 162 | 101 | 1 | 1 | 161 | 1 | 162 | Phage tail-collar fibre protein | Phage tail-collar fibre protein | | afdb-uniprot50 | AF-N1ZHX9-F1-MODEL\_V4 | 1.0 | 1.629e-16 | 734 | 0.322 | 158 | 107 | 0 | 1 | 158 | 3 | 160 | Peptidase S74 domain-containing protein | Peptidase S74 domain-containing protein | | afdb-uniprot50 | AF-A0A7W6RGC7-F1-MODEL\_V4 | 1.0 | 7.633e-21 | 731 | 0.423 | 229 | 110 | 6 | 1 | 219 | 1 | 217 | Phage-related tail fiber protein | Phage-related tail fiber protein | | afdb-uniprot50 | AF-A0A418H7E0-F1-MODEL\_V4 | 1.0 | 1.244e-16 | 728 | 0.427 | 159 | 90 | 1 | 1 | 158 | 2 | 160 | Phage tail protein | Phage tail protein | | afdb-uniprot50 | AF-A0A3D9EDX5-F1-MODEL\_V4 | 1.0 | 7.77e-16 | 728 | 0.5 | 154 | 76 | 1 | 1 | 153 | 1 | 154 | Tail-collar fiber protein | Tail-collar fiber protein | | afdb-uniprot50 | AF-A0A1H4Y3H3-F1-MODEL\_V4 | 1.0 | 8.084e-17 | 728 | 0.44 | 150 | 84 | 0 | 4 | 153 | 8 | 157 | Phage Tail Collar Domain | Phage Tail Collar Domain | | afdb-uniprot50 | AF-A0A5C9ABQ6-F1-MODEL\_V4 | 1.0 | 5.049e-16 | 726 | 0.507 | 130 | 64 | 0 | 1 | 130 | 2 | 131 | Phage tail protein | Phage tail protein | | afdb-uniprot50 | AF-A0A0N9MHW1-F1-MODEL\_V4 | 1.0 | 1.386e-16 | 726 | 0.428 | 154 | 88 | 0 | 2 | 155 | 6 | 159 | Phage tail fiber protein | Phage tail fiber protein | | afdb-uniprot50 | AF-A0A3M2VPF6-F1-MODEL\_V4 | 1.0 | 4.012e-17 | 725 | 0.403 | 166 | 99 | 0 | 1 | 166 | 5 | 170 | Putative Tail fiber protein H | Putative Tail fiber protein H | | afdb-uniprot50 | AF-A0A3D4FGS6-F1-MODEL\_V4 | 1.0 | 2.072e-18 | 724 | 0.443 | 178 | 99 | 0 | 1 | 178 | 2 | 179 | Uncharacterized protein | Uncharacterized protein | | afdb-uniprot50 | AF-A0A1I7J4R3-F1-MODEL\_V4 | 1.0 | 1.411e-26 | 723 | 0.252 | 432 | 221 | 17 | 1 | 423 | 1 | 339 | Phage tail-collar fibre protein | Phage tail-collar fibre protein | | afdb-uniprot50 | AF-A0A3S6F0P7-F1-MODEL\_V4 | 1.0 | 1.105e-28 | 723 | 0.279 | 473 | 198 | 11 | 1 | 423 | 2 | 381 | Uncharacterized protein | Uncharacterized protein | | afdb-uniprot50 | AF-A0A290WU51-F1-MODEL\_V4 | 1.0 | 1.101e-17 | 722 | 0.375 | 181 | 110 | 1 | 1 | 178 | 2 | 182 | Uncharacterized protein | Uncharacterized protein | | afdb-uniprot50 | AF-A0A248XR00-F1-MODEL\_V4 | 1.0 | 2.645e-16 | 722 | 0.444 | 153 | 85 | 0 | 1 | 153 | 2 | 154 | Phage tail protein | Phage tail protein | | afdb-uniprot50 | AF-D1NYY3-F1-MODEL\_V4 | 1.0 | 8.875e-18 | 721 | 0.472 | 163 | 84 | 1 | 4 | 164 | 3 | 165 | Uncharacterized protein | Uncharacterized protein | | afdb-uniprot50 | AF-A0A7I0L7S4-F1-MODEL\_V4 | 1.0 | 8.2e-16 | 721 | 0.437 | 151 | 84 | 1 | 4 | 153 | 3 | 153 | Peptidase S74 domain-containing protein | Peptidase S74 domain-containing protein | | afdb-uniprot50 | AF-A0A6N6MLW5-F1-MODEL\_V4 | 1.0 | 4.296e-16 | 720 | 0.364 | 151 | 96 | 0 | 1 | 151 | 1 | 151 | DUF2793 domain-containing protein | DUF2793 domain-containing protein | | afdb-uniprot50 | AF-A0A6B8QPL4-F1-MODEL\_V4 | 1.0 | 2.218e-17 | 720 | 0.453 | 161 | 87 | 1 | 2 | 162 | 45 | 204 | Uncharacterized protein | Uncharacterized protein | | afdb-uniprot50 | AF-A0A1I7C9J8-F1-MODEL\_V4 | 1.0 | 8.689e-27 | 719 | 0.263 | 440 | 203 | 21 | 1 | 423 | 1 | 336 | Phage tail-collar fibre protein | Phage tail-collar fibre protein | | afdb-uniprot50 | AF-A0A7T4ZYC8-F1-MODEL\_V4 | 1.0 | 4.617e-26 | 718 | 0.275 | 439 | 186 | 11 | 1 | 423 | 2 | 324 | Phage tail protein | Phage tail protein | | afdb-uniprot50 | AF-A0A839BSH3-F1-MODEL\_V4 | 1.0 | 7.004e-27 | 718 | 0.293 | 447 | 177 | 13 | 1 | 423 | 1 | 332 | Phage tail protein | Phage tail protein | | afdb-uniprot50 | AF-A0A829HHD3-F1-MODEL\_V4 | 1.0 | 2.946e-16 | 717 | 0.345 | 162 | 105 | 1 | 1 | 162 | 2 | 162 | Uncharacterized protein | Uncharacterized protein | | afdb-uniprot50 | AF-A0A418H6B6-F1-MODEL\_V4 | 1.0 | 6.264e-16 | 715 | 0.507 | 132 | 65 | 0 | 1 | 132 | 2 | 133 | Phage tail protein | Phage tail protein | | afdb-uniprot50 | AF-A0A3S4I322-F1-MODEL\_V4 | 1.0 | 6.516e-17 | 715 | 0.456 | 160 | 85 | 2 | 1 | 158 | 1 | 160 | Uncharacterized protein | Uncharacterized protein | | afdb-uniprot50 | AF-A0A411WMD6-F1-MODEL\_V4 | 1.0 | 2.516e-27 | 715 | 0.262 | 530 | 182 | 14 | 1 | 423 | 1 | 428 | Phage tail protein | Phage tail protein | | afdb-uniprot50 | AF-A0A2N2GS05-F1-MODEL\_V4 | 1.0 | 3.109e-16 | 715 | 0.407 | 162 | 96 | 0 | 1 | 162 | 1 | 162 | Uncharacterized protein | Uncharacterized protein | | afdb-uniprot50 | AF-A0A3S0LIM0-F1-MODEL\_V4 | 1.0 | 3.857e-16 | 715 | 0.327 | 162 | 108 | 1 | 1 | 162 | 2 | 162 | Alkaline phosphatase | Alkaline phosphatase | | afdb-uniprot50 | AF-A0A776X733-F1-MODEL\_V4 | 1.0 | 2.187e-18 | 713 | 0.291 | 298 | 109 | 4 | 1 | 297 | 2 | 198 | Phage tail protein | Phage tail protein | | afdb-uniprot50 | AF-A0A0N1HPL4-F1-MODEL\_V4 | 1.0 | 7.053e-19 | 713 | 0.405 | 185 | 103 | 2 | 1 | 179 | 2 | 185 | Tail protein | Tail protein | | afdb-uniprot50 | AF-A0A375A5G5-F1-MODEL\_V4 | 1.0 | 4.649e-18 | 712 | 0.437 | 185 | 93 | 2 | 1 | 174 | 2 | 186 | Phage tail fiber protein | Phage tail fiber protein | | afdb-uniprot50 | AF-A0A2N2KCW4-F1-MODEL\_V4 | 1.0 | 7.258e-17 | 712 | 0.398 | 163 | 98 | 0 | 1 | 163 | 1 | 163 | Uncharacterized protein | Uncharacterized protein | | afdb-uniprot50 | AF-A0A560QZS3-F1-MODEL\_V4 | 1.0 | 1.914e-16 | 712 | 0.409 | 154 | 90 | 1 | 1 | 153 | 1 | 154 | Tail collar domain | Tail collar domain | | afdb-uniprot50 | AF-A0A8B5S4N3-F1-MODEL\_V4 | 1.0 | 1.196e-15 | 711 | 0.346 | 153 | 99 | 1 | 1 | 153 | 2 | 153 | Uncharacterized protein | Uncharacterized protein | | afdb-uniprot50 | AF-A0A506K1B3-F1-MODEL\_V4 | 1.0 | 5.935e-16 | 711 | 0.356 | 160 | 100 | 2 | 1 | 158 | 2 | 160 | Peptidase S74 domain-containing protein | Peptidase S74 domain-containing protein | | afdb-uniprot50 | AF-A0A398PZR3-F1-MODEL\_V4 | 1.0 | 1.055e-20 | 709 | 0.301 | 318 | 142 | 9 | 1 | 306 | 2 | 251 | Phage tail protein | Phage tail protein | | afdb-uniprot50 | AF-A0A7D6WRG1-F1-MODEL\_V4 | 1.0 | 1.505e-29 | 709 | 0.26 | 507 | 184 | 13 | 1 | 423 | 2 | 401 | Phage tail protein | Phage tail protein | | afdb-uniprot50 | AF-A0A258E4J6-F1-MODEL\_V4 | 1.0 | 1.719e-16 | 709 | 0.402 | 154 | 91 | 1 | 1 | 153 | 1 | 154 | Uncharacterized protein | Uncharacterized protein | | afdb-uniprot50 | AF-A0A774MST8-F1-MODEL\_V4 | 1.0 | 3.365e-18 | 708 | 0.28 | 300 | 112 | 3 | 1 | 298 | 2 | 199 | Phage tail protein | Phage tail protein | | afdb-uniprot50 | AF-A0A0S4I2P4-F1-MODEL\_V4 | 1.0 | 8.41e-18 | 708 | 0.39 | 182 | 111 | 0 | 2 | 183 | 6 | 187 | Uncharacterized protein | Uncharacterized protein | | afdb-uniprot50 | AF-A0A375G4X8-F1-MODEL\_V4 | 1.0 | 1.226e-17 | 708 | 0.365 | 186 | 114 | 4 | 1 | 182 | 1 | 186 | Peptidase S74 domain-containing protein | Peptidase S74 domain-containing protein | | afdb-uniprot50 | AF-A0A6G6NFC5-F1-MODEL\_V4 | 1.0 | 3.234e-17 | 707 | 0.473 | 173 | 87 | 2 | 1 | 173 | 1 | 169 | Phage tail protein | Phage tail protein | | afdb-uniprot50 | AF-A0A2D9EKL2-F1-MODEL\_V4 | 1.0 | 5.178e-18 | 705 | 0.407 | 184 | 105 | 3 | 1 | 180 | 1 | 184 | Uncharacterized protein | Uncharacterized protein | | afdb-uniprot50 | AF-A0A7X1KJH1-F1-MODEL\_V4 | 1.0 | 5.465e-18 | 704 | 0.406 | 187 | 103 | 3 | 2 | 180 | 6 | 192 | Phage tail protein | Phage tail protein | | afdb-uniprot50 | AF-A0A265Q5Q8-F1-MODEL\_V4 | 1.0 | 3.802e-17 | 704 | 0.45 | 160 | 86 | 1 | 1 | 158 | 2 | 161 | Uncharacterized protein | Uncharacterized protein | | afdb-uniprot50 | AF-A0A2K4ISX8-F1-MODEL\_V4 | 1.0 | 2.471e-17 | 704 | 0.377 | 172 | 106 | 1 | 2 | 172 | 6 | 177 | Phage tail protein | Phage tail protein | | afdb-uniprot50 | AF-A0A376ZKW5-F1-MODEL\_V4 | 1.0 | 1.629e-16 | 703 | 0.467 | 154 | 76 | 1 | 1 | 148 | 2 | 155 | Phage tail collar protein | Phage tail collar protein | | afdb-uniprot50 | AF-A0A2G8D691-F1-MODEL\_V4 | 1.0 | 3.843e-20 | 703 | 0.27 | 329 | 134 | 3 | 1 | 324 | 2 | 229 | Phage tail protein | Phage tail protein | | afdb-uniprot50 | AF-F3G6R6-F1-MODEL\_V4 | 1.0 | 2.904e-17 | 702 | 0.385 | 187 | 105 | 3 | 2 | 181 | 6 | 189 | Phage-related tail fiber protein-like protein | Phage-related tail fiber protein-like protein | | afdb-uniprot50 | AF-A0A4S3LPH8-F1-MODEL\_V4 | 1.0 | 8.352e-26 | 702 | 0.315 | 428 | 180 | 12 | 1 | 423 | 2 | 321 | Phage tail protein | Phage tail protein | | afdb-uniprot50 | AF-A0A7Y1JSW6-F1-MODEL\_V4 | 1.0 | 6.61e-16 | 702 | 0.393 | 155 | 94 | 0 | 1 | 155 | 5 | 159 | Phage tail protein | Phage tail protein | | afdb-uniprot50 | AF-A0A1B9LPY6-F1-MODEL\_V4 | 1.0 | 2.733e-25 | 700 | 0.281 | 434 | 183 | 16 | 1 | 423 | 2 | 317 | Uncharacterized protein | Uncharacterized protein | | afdb-uniprot50 | AF-A0A376J6K3-F1-MODEL\_V4 | 1.0 | 1.84e-15 | 699 | 0.412 | 148 | 86 | 1 | 1 | 147 | 5 | 152 | Phage protein gpH | Phage protein gpH | | afdb-uniprot50 | AF-A0A4R7GU78-F1-MODEL\_V4 | 1.0 | 7.208e-25 | 698 | 0.279 | 429 | 179 | 10 | 1 | 423 | 2 | 306 | Tail-collar fiber protein | Tail-collar fiber protein | | afdb-uniprot50 | AF-A0A377K993-F1-MODEL\_V4 | 1.0 | 7.053e-19 | 698 | 0.416 | 209 | 100 | 3 | 1 | 187 | 2 | 210 | Phage-related tail fiber protein-like protein | Phage-related tail fiber protein-like protein | | afdb-uniprot50 | AF-A0A4R0HWF8-F1-MODEL\_V4 | 1.0 | 1.6e-21 | 697 | 0.312 | 346 | 115 | 5 | 1 | 324 | 1 | 245 | Phage tail protein | Phage tail protein | | afdb-uniprot50 | AF-A0A1C7WI46-F1-MODEL\_V4 | 1.0 | 2.132e-16 | 696 | 0.372 | 161 | 98 | 2 | 1 | 158 | 1 | 161 | Uncharacterized protein | Uncharacterized protein | | afdb-uniprot50 | AF-A0A2U1TNF9-F1-MODEL\_V4 | 1.0 | 2.57e-18 | 695 | 0.435 | 193 | 92 | 1 | 1 | 176 | 2 | 194 | Phage tail protein | Phage tail protein | | afdb-uniprot50 | AF-F9MRF3-F1-MODEL\_V4 | 1.0 | 3.857e-16 | 694 | 0.366 | 153 | 97 | 0 | 1 | 153 | 2 | 154 | Uncharacterized protein | Uncharacterized protein | | afdb-uniprot50 | AF-A0A4Y5WA69-F1-MODEL\_V4 | 1.0 | 6.045e-26 | 694 | 0.268 | 439 | 197 | 19 | 1 | 423 | 2 | 332 | Uncharacterized protein | Uncharacterized protein | | afdb-uniprot50 | AF-A0A1A9KIH8-F1-MODEL\_V4 | 1.0 | 4.07e-16 | 694 | 0.421 | 166 | 96 | 0 | 1 | 166 | 1 | 166 | SGNH\_hydro domain-containing protein | SGNH\_hydro domain-containing protein | | afdb-uniprot50 | AF-A0A3R8V706-F1-MODEL\_V4 | 1.0 | 1.929e-23 | 693 | 0.226 | 442 | 181 | 13 | 2 | 423 | 6 | 306 | Uncharacterized protein | Uncharacterized protein | | afdb-uniprot50 | AF-A0A2D0LDE2-F1-MODEL\_V4 | 1.0 | 1.047e-28 | 693 | 0.252 | 523 | 187 | 13 | 1 | 423 | 2 | 420 | Phage tail protein | Phage tail protein | | afdb-uniprot50 | AF-A0A161X4B1-F1-MODEL\_V4 | 1.0 | 4.533e-16 | 693 | 0.496 | 153 | 77 | 0 | 1 | 153 | 2 | 154 | SGNH\_hydro domain-containing protein | SGNH\_hydro domain-containing protein | | afdb-uniprot50 | AF-A0A069A3R7-F1-MODEL\_V4 | 1.0 | 1.003e-16 | 691 | 0.397 | 161 | 97 | 0 | 1 | 161 | 6 | 166 | Putative phage tail fiber protein | Putative phage tail fiber protein | | afdb-uniprot50 | AF-A0A3E0X1N8-F1-MODEL\_V4 | 1.0 | 2.409e-15 | 691 | 0.359 | 153 | 98 | 0 | 1 | 153 | 10 | 162 | Collar domain-containing protein | Collar domain-containing protein | | afdb-uniprot50 | AF-A0A5E6S0H5-F1-MODEL\_V4 | 1.0 | 1.694e-17 | 690 | 0.373 | 174 | 106 | 1 | 1 | 171 | 5 | 178 | Uncharacterized protein | Uncharacterized protein | | afdb-uniprot50 | AF-A0A3S4HKY0-F1-MODEL\_V4 | 1.0 | 8.381e-22 | 689 | 0.322 | 350 | 128 | 6 | 1 | 331 | 1 | 260 | Phage tail fibre repeat | Phage tail fibre repeat | | afdb-uniprot50 | AF-A0A4U8UED2-F1-MODEL\_V4 | 1.0 | 1.483e-15 | 689 | 0.36 | 158 | 98 | 1 | 4 | 161 | 3 | 157 | Phage tail protein | Phage tail protein | | afdb-uniprot50 | AF-A0A771BD76-F1-MODEL\_V4 | 1.0 | 4.358e-15 | 689 | 0.437 | 151 | 84 | 1 | 4 | 153 | 3 | 153 | Uncharacterized protein | Uncharacterized protein | | afdb-uniprot50 | AF-A0A2N0DUE2-F1-MODEL\_V4 | 1.0 | 4.296e-16 | 687 | 0.402 | 159 | 94 | 1 | 1 | 158 | 1 | 159 | Tail collar domain | Tail collar domain | | afdb-uniprot50 | AF-A0A625JTE0-F1-MODEL\_V4 | 1.0 | 5.329e-16 | 686 | 0.36 | 158 | 98 | 1 | 1 | 158 | 3 | 157 | Phage tail protein | Phage tail protein | | afdb-uniprot50 | AF-A0A7X4KF01-F1-MODEL\_V4 | 1.0 | 5.049e-16 | 684 | 0.339 | 165 | 104 | 3 | 1 | 163 | 2 | 163 | Uncharacterized protein | Uncharacterized protein | | afdb-uniprot50 | AF-J2PVQ7-F1-MODEL\_V4 | 1.0 | 5.049e-16 | 684 | 0.402 | 159 | 94 | 1 | 1 | 158 | 1 | 159 | Phage-related tail fiber protein | Phage-related tail fiber protein | | afdb-uniprot50 | AF-A0A1G5MGW4-F1-MODEL\_V4 | 1.0 | 5.049e-16 | 684 | 0.396 | 159 | 95 | 1 | 1 | 158 | 1 | 159 | Phage-related tail fibre protein | Phage-related tail fibre protein | | afdb-uniprot50 | AF-A0A2A2F373-F1-MODEL\_V4 | 1.0 | 1.565e-15 | 683 | 0.408 | 159 | 88 | 2 | 1 | 153 | 4 | 162 | Uncharacterized protein | Uncharacterized protein | | afdb-uniprot50 | AF-A0A6L7E7U0-F1-MODEL\_V4 | 1.0 | 6.683e-19 | 682 | 0.401 | 204 | 96 | 2 | 1 | 178 | 2 | 205 | Phage tail protein | Phage tail protein | | afdb-uniprot50 | AF-A0A3A6RIP6-F1-MODEL\_V4 | 1.0 | 3.602e-17 | 681 | 0.393 | 188 | 104 | 2 | 1 | 178 | 2 | 189 | Phage tail protein | Phage tail protein | | afdb-uniprot50 | AF-A0A7W4RHL5-F1-MODEL\_V4 | 1.0 | 2.283e-15 | 681 | 0.41 | 156 | 91 | 1 | 1 | 155 | 2 | 157 | Uncharacterized protein | Uncharacterized protein | | afdb-uniprot50 | AF-A0A516SJM3-F1-MODEL\_V4 | 1.0 | 1.346e-18 | 680 | 0.415 | 195 | 110 | 2 | 1 | 191 | 1 | 195 | Uncharacterized protein | Uncharacterized protein | | afdb-uniprot50 | AF-A0A1Q6KPQ8-F1-MODEL\_V4 | 1.0 | 3.463e-16 | 680 | 0.339 | 165 | 106 | 2 | 1 | 164 | 1 | 163 | Uncharacterized protein | Uncharacterized protein | | afdb-uniprot50 | AF-A0A2U2UFI2-F1-MODEL\_V4 | 1.0 | 3.899e-19 | 679 | 0.284 | 330 | 124 | 5 | 1 | 329 | 3 | 221 | Phage tail protein | Phage tail protein | | afdb-uniprot50 | AF-A0A419AS73-F1-MODEL\_V4 | 1.0 | 2.498e-20 | 679 | 0.313 | 325 | 119 | 5 | 1 | 324 | 2 | 223 | Phage tail protein | Phage tail protein | | afdb-uniprot50 | AF-C6E6P3-F1-MODEL\_V4 | 1.0 | 1.117e-16 | 678 | 0.39 | 169 | 97 | 2 | 4 | 166 | 3 | 171 | Phage-related tail fibre protein-like protein | Phage-related tail fibre protein-like protein | | afdb-uniprot50 | AF-A0A7Z2QM66-F1-MODEL\_V4 | 1.0 | 8.2e-16 | 678 | 0.396 | 159 | 95 | 1 | 1 | 158 | 1 | 159 | Phage tail protein | Phage tail protein | | afdb-uniprot50 | AF-A0A845RKE3-F1-MODEL\_V4 | 1.0 | 6.264e-16 | 676 | 0.326 | 156 | 105 | 0 | 1 | 156 | 29 | 184 | Uncharacterized protein | Uncharacterized protein | | afdb-uniprot50 | AF-A0A1M7U2L6-F1-MODEL\_V4 | 1.0 | 1.043e-17 | 676 | 0.439 | 189 | 92 | 2 | 1 | 175 | 1 | 189 | Phage tail-collar fibre protein | Phage tail-collar fibre protein | | afdb-uniprot50 | AF-A0A1Z1SPG3-F1-MODEL\_V4 | 1.0 | 7.66e-17 | 676 | 0.433 | 173 | 91 | 1 | 1 | 166 | 2 | 174 | Phage tail protein | Phage tail protein | | afdb-uniprot50 | AF-A0A774SHB9-F1-MODEL\_V4 | 1.0 | 3.955e-18 | 675 | 0.298 | 305 | 110 | 4 | 1 | 302 | 1 | 204 | Phage tail protein | Phage tail protein | | afdb-uniprot50 | AF-A0A1X3RYJ6-F1-MODEL\_V4 | 1.0 | 3.39e-25 | 675 | 0.266 | 428 | 200 | 15 | 1 | 423 | 1 | 319 | Phage tail protein | Phage tail protein | | afdb-uniprot50 | AF-A0A828EEN1-F1-MODEL\_V4 | 1.0 | 2.674e-19 | 675 | 0.411 | 209 | 101 | 3 | 1 | 187 | 2 | 210 | Phage tail protein | Phage tail protein | | afdb-uniprot50 | AF-A0A4Y8GSR2-F1-MODEL\_V4 | 1.0 | 4.567e-23 | 675 | 0.335 | 331 | 147 | 8 | 1 | 323 | 2 | 267 | Short-chain fatty acid transporter | Short-chain fatty acid transporter | | afdb-uniprot50 | AF-C6DCN9-F1-MODEL\_V4 | 1.0 | 4.649e-18 | 674 | 0.441 | 204 | 88 | 3 | 1 | 178 | 2 | 205 | Phage-related tail fibre protein-like protein | Phage-related tail fibre protein-like protein | | afdb-uniprot50 | AF-A0A542AK78-F1-MODEL\_V4 | 1.0 | 2.409e-15 | 673 | 0.353 | 150 | 96 | 1 | 4 | 153 | 3 | 151 | Tail-collar fiber protein | Tail-collar fiber protein | | afdb-uniprot50 | AF-A0A824XYH8-F1-MODEL\_V4 | 1.0 | 8.2e-16 | 673 | 0.379 | 158 | 95 | 1 | 1 | 158 | 3 | 157 | Phage tail protein | Phage tail protein | | afdb-uniprot50 | AF-A0A2C9EHA1-F1-MODEL\_V4 | 1.0 | 1.441e-17 | 673 | 0.363 | 187 | 111 | 3 | 4 | 186 | 8 | 190 | Putative tail fiber protein | Putative tail fiber protein | | afdb-uniprot50 | AF-A0A072Y7K9-F1-MODEL\_V4 | 1.0 | 5.767e-18 | 673 | 0.338 | 195 | 120 | 2 | 1 | 187 | 2 | 195 | Tail fiber protein H | Tail fiber protein H | | afdb-uniprot50 | AF-N8RAV5-F1-MODEL\_V4 | 1.0 | 2.132e-16 | 673 | 0.394 | 175 | 101 | 2 | 3 | 172 | 2 | 176 | Uncharacterized protein | Uncharacterized protein | | afdb-uniprot50 | AF-A0A745FXY9-F1-MODEL\_V4 | 1.0 | 1.719e-16 | 672 | 0.368 | 187 | 105 | 3 | 1 | 178 | 2 | 184 | Phage tail protein | Phage tail protein | | afdb-uniprot50 | AF-A0A380C811-F1-MODEL\_V4 | 1.0 | 1.992e-17 | 672 | 0.353 | 181 | 107 | 3 | 1 | 176 | 1 | 176 | Uncharacterized protein | Uncharacterized protein | | afdb-uniprot50 | AF-A0A261E4L1-F1-MODEL\_V4 | 1.0 | 2.203e-25 | 672 | 0.278 | 434 | 207 | 17 | 1 | 423 | 2 | 340 | DNA inversion product | DNA inversion product | | afdb-uniprot50 | AF-A0A556RUG3-F1-MODEL\_V4 | 1.0 | 3.054e-21 | 672 | 0.33 | 306 | 108 | 1 | 1 | 306 | 2 | 210 | Phage tail protein | Phage tail protein | | afdb-uniprot50 | AF-A0A1E7RCE5-F1-MODEL\_V4 | 1.0 | 1.231e-28 | 672 | 0.282 | 496 | 230 | 19 | 4 | 417 | 3 | 454 | Phage tail protein | Phage tail protein | | afdb-uniprot50 | AF-L1M5X1-F1-MODEL\_V4 | 1.0 | 4.235e-17 | 672 | 0.381 | 186 | 113 | 2 | 2 | 186 | 6 | 190 | Tail fiber protein H | Tail fiber protein H | | afdb-uniprot50 | AF-A0A6H7NTP2-F1-MODEL\_V4 | 1.0 | 8.2e-16 | 671 | 0.392 | 158 | 93 | 1 | 1 | 158 | 3 | 157 | Phage tail protein | Phage tail protein | | afdb-uniprot50 | AF-A0A624F4N8-F1-MODEL\_V4 | 1.0 | 1.133e-15 | 671 | 0.386 | 158 | 94 | 1 | 1 | 158 | 3 | 157 | Phage tail protein | Phage tail protein | | afdb-uniprot50 | AF-A0A4Q0UTE8-F1-MODEL\_V4 | 1.0 | 2.791e-16 | 671 | 0.367 | 177 | 107 | 3 | 1 | 176 | 3 | 175 | Uncharacterized protein | Uncharacterized protein | | afdb-uniprot50 | AF-A0A024HA82-F1-MODEL\_V4 | 1.0 | 5.142e-26 | 671 | 0.271 | 445 | 237 | 20 | 1 | 423 | 2 | 381 | Uncharacterized protein | Uncharacterized protein | | afdb-uniprot50 | AF-A0A2Z6AZ11-F1-MODEL\_V4 | 1.0 | 3.451e-20 | 670 | 0.351 | 236 | 112 | 6 | 1 | 219 | 1 | 212 | Tail Collar domain protein | Tail Collar domain protein | | afdb-uniprot50 | AF-A0A690UYS0-F1-MODEL\_V4 | 1.0 | 9.639e-16 | 669 | 0.386 | 158 | 94 | 1 | 1 | 158 | 3 | 157 | Phage tail protein | Phage tail protein | | afdb-uniprot50 | AF-A0A0M0TBG4-F1-MODEL\_V4 | 1.0 | 2.242e-20 | 668 | 0.319 | 307 | 111 | 4 | 1 | 306 | 1 | 210 | Tail protein | Tail protein | | afdb-uniprot50 | AF-A0A826S3K6-F1-MODEL\_V4 | 1.0 | 1.235e-24 | 668 | 0.276 | 438 | 184 | 11 | 1 | 423 | 2 | 321 | Phage tail protein | Phage tail protein | | afdb-uniprot50 | AF-A0A2N0DX16-F1-MODEL\_V4 | 1.0 | 1.84e-15 | 668 | 0.409 | 154 | 91 | 0 | 2 | 155 | 6 | 159 | Phage tail protein | Phage tail protein | | afdb-uniprot50 | AF-A0A1I3I6J5-F1-MODEL\_V4 | 1.0 | 6.132e-25 | 667 | 0.26 | 438 | 187 | 17 | 1 | 423 | 1 | 316 | Phage tail-collar fibre protein | Phage tail-collar fibre protein | | afdb-uniprot50 | AF-A0A1B9JGD4-F1-MODEL\_V4 | 1.0 | 4.649e-18 | 667 | 0.394 | 195 | 95 | 2 | 1 | 172 | 2 | 196 | Collar domain-containing protein | Collar domain-containing protein | | afdb-uniprot50 | AF-A0A4T2A2H5-F1-MODEL\_V4 | 1.0 | 3.439e-24 | 667 | 0.212 | 451 | 199 | 15 | 1 | 423 | 2 | 324 | DNA inversion product | DNA inversion product | | afdb-uniprot50 | AF-A0A5C1A538-F1-MODEL\_V4 | 1.0 | 1.093e-25 | 667 | 0.225 | 497 | 210 | 16 | 1 | 423 | 1 | 396 | DNA inversion product | DNA inversion product | | afdb-uniprot50 | AF-A0A0F4TBD1-F1-MODEL\_V4 | 1.0 | 2.542e-15 | 667 | 0.396 | 154 | 93 | 0 | 2 | 155 | 6 | 159 | Phage tail protein | Phage tail protein | | afdb-uniprot50 | AF-A0A379UXV3-F1-MODEL\_V4 | 1.0 | 5.104e-19 | 666 | 0.411 | 209 | 101 | 3 | 1 | 187 | 2 | 210 | Phage tail fiber protein | Phage tail fiber protein | | afdb-uniprot50 | AF-A0A447XE38-F1-MODEL\_V4 | 1.0 | 1.07e-19 | 665 | 0.308 | 324 | 123 | 2 | 1 | 324 | 2 | 224 | Tail fiber protein | Tail fiber protein | | afdb-uniprot50 | AF-A0A721ZMC9-F1-MODEL\_V4 | 1.0 | 2.179e-22 | 665 | 0.304 | 335 | 162 | 7 | 2 | 324 | 3 | 278 | Phage tail protein | Phage tail protein | | afdb-uniprot50 | AF-A0A221ZMW8-F1-MODEL\_V4 | 1.0 | 4.115e-19 | 664 | 0.414 | 210 | 100 | 4 | 1 | 187 | 1 | 210 | Phage tail fiber protein | Phage tail fiber protein | | afdb-uniprot50 | AF-A0A7G5NY25-F1-MODEL\_V4 | 1.0 | 2.163e-15 | 664 | 0.375 | 157 | 98 | 0 | 2 | 158 | 7 | 163 | Phage tail protein | Phage tail protein | | afdb-uniprot50 | AF-A0A3Q8FR82-F1-MODEL\_V4 | 1.0 | 2.48e-28 | 664 | 0.277 | 429 | 217 | 20 | 1 | 407 | 1 | 358 | Phage tail protein | Phage tail protein | | afdb-uniprot50 | AF-A0A5P3ME88-F1-MODEL\_V4 | 1.0 | 4.716e-17 | 664 | 0.357 | 190 | 103 | 4 | 1 | 172 | 2 | 190 | Phage tail-collar fiber family protein | Phage tail-collar fiber family protein | | afdb-uniprot50 | AF-A0A2N1CWE2-F1-MODEL\_V4 | 1.0 | 3.629e-24 | 662 | 0.242 | 442 | 180 | 13 | 2 | 423 | 6 | 312 | DNA inversion product | DNA inversion product | | afdb-uniprot50 | AF-A0A6G5QMQ8-F1-MODEL\_V4 | 1.0 | 4.296e-16 | 660 | 0.365 | 164 | 98 | 2 | 3 | 163 | 2 | 162 | Phage tail-collar fiber protein (DUF3751 domain) | Phage tail-collar fiber protein (DUF3751 domain) | | afdb-uniprot50 | AF-A0A717EDS8-F1-MODEL\_V4 | 1.0 | 3.281e-16 | 659 | 0.347 | 187 | 109 | 3 | 1 | 178 | 5 | 187 | Phage tail protein | Phage tail protein | | afdb-uniprot50 | AF-A0A1E7PZY5-F1-MODEL\_V4 | 1.0 | 2.155e-19 | 659 | 0.297 | 329 | 132 | 8 | 1 | 324 | 1 | 235 | Uncharacterized protein | Uncharacterized protein | | afdb-uniprot50 | AF-A0A1B9LET1-F1-MODEL\_V4 | 1.0 | 8.352e-26 | 659 | 0.238 | 473 | 205 | 13 | 1 | 423 | 1 | 368 | Uncharacterized protein | Uncharacterized protein | | afdb-uniprot50 | AF-A0A5B8HDY3-F1-MODEL\_V4 | 1.0 | 3.669e-27 | 659 | 0.265 | 485 | 201 | 15 | 1 | 423 | 1 | 392 | Phage tail protein | Phage tail protein | | afdb-uniprot50 | AF-J2UA87-F1-MODEL\_V4 | 1.0 | 2.988e-15 | 659 | 0.389 | 159 | 96 | 1 | 1 | 158 | 1 | 159 | Phage-related tail fiber protein | Phage-related tail fiber protein | | afdb-uniprot50 | AF-A0A4R7YKL0-F1-MODEL\_V4 | 1.0 | 1.782e-21 | 658 | 0.296 | 320 | 146 | 11 | 1 | 306 | 1 | 255 | Tail-collar fiber protein | Tail-collar fiber protein | | afdb-uniprot50 | AF-A0A4R0GNU2-F1-MODEL\_V4 | 1.0 | 1.145e-18 | 657 | 0.423 | 210 | 98 | 4 | 1 | 187 | 1 | 210 | Phage tail protein | Phage tail protein | | afdb-uniprot50 | AF-A0A6N8PLL2-F1-MODEL\_V4 | 1.0 | 7.443e-19 | 655 | 0.312 | 333 | 120 | 7 | 1 | 330 | 2 | 228 | Phage tail protein | Phage tail protein | | afdb-uniprot50 | AF-A0A8B6JH22-F1-MODEL\_V4 | 1.0 | 5.935e-16 | 655 | 0.365 | 164 | 101 | 1 | 1 | 161 | 1 | 164 | Tail fiber protein | Tail fiber protein | | afdb-uniprot50 | AF-A0A8A8PF66-F1-MODEL\_V4 | 1.0 | 5.31e-20 | 655 | 0.364 | 236 | 130 | 5 | 1 | 223 | 1 | 229 | Phage tail protein | Phage tail protein | | afdb-uniprot50 | AF-A0A1I7HNR6-F1-MODEL\_V4 | 1.0 | 3.342e-26 | 654 | 0.239 | 484 | 199 | 17 | 1 | 423 | 2 | 377 | Phage tail fibre repeat-containing protein | Phage tail fibre repeat-containing protein | | afdb-uniprot50 | AF-A0A1E3GML3-F1-MODEL\_V4 | 1.0 | 1.003e-16 | 654 | 0.39 | 182 | 99 | 3 | 3 | 172 | 2 | 183 | Uncharacterized protein | Uncharacterized protein | | afdb-uniprot50 | AF-A0A5E8H5R3-F1-MODEL\_V4 | 1.0 | 4.854e-15 | 653 | 0.409 | 154 | 89 | 2 | 5 | 157 | 2 | 154 | Phage-related tail fiber protein | Phage-related tail fiber protein | | afdb-uniprot50 | AF-A0A743JKX6-F1-MODEL\_V4 | 1.0 | 1.854e-22 | 653 | 0.312 | 336 | 154 | 8 | 1 | 324 | 1 | 271 | Phage tail protein | Phage tail protein | | afdb-uniprot50 | AF-A0A7Z3MBJ3-F1-MODEL\_V4 | 1.0 | 1.222e-21 | 653 | 0.316 | 325 | 120 | 3 | 1 | 324 | 1 | 224 | Phage tail protein | Phage tail protein | | afdb-uniprot50 | AF-A0A6V8GZ93-F1-MODEL\_V4 | 1.0 | 3.329e-15 | 652 | 0.373 | 158 | 96 | 1 | 1 | 158 | 3 | 157 | Phage tail protein | Phage tail protein | | afdb-uniprot50 | AF-D6JBE2-F1-MODEL\_V4 | 1.0 | 1.834e-19 | 652 | 0.289 | 307 | 138 | 8 | 2 | 296 | 6 | 244 | GpH | GpH | | afdb-uniprot50 | AF-A0A843NZ40-F1-MODEL\_V4 | 1.0 | 4.296e-16 | 651 | 0.402 | 164 | 92 | 2 | 1 | 163 | 2 | 160 | Phage tail protein | Phage tail protein | | afdb-uniprot50 | AF-U6ZRT4-F1-MODEL\_V4 | 1.0 | 1.117e-16 | 651 | 0.412 | 172 | 98 | 1 | 4 | 172 | 8 | 179 | Collar domain-containing protein | Collar domain-containing protein | | afdb-uniprot50 | AF-A0A7U5TL09-F1-MODEL\_V4 | 1.0 | 6.379e-26 | 650 | 0.283 | 483 | 188 | 15 | 1 | 423 | 2 | 386 | Phage tail protein | Phage tail protein | | afdb-uniprot50 | AF-A0A5C7E1E3-F1-MODEL\_V4 | 1.0 | 5.122e-15 | 649 | 0.354 | 158 | 99 | 1 | 1 | 158 | 3 | 157 | Phage tail protein | Phage tail protein | | afdb-uniprot50 | AF-A0A1Y4J5U1-F1-MODEL\_V4 | 1.0 | 7.882e-15 | 649 | 0.299 | 157 | 110 | 0 | 4 | 160 | 40 | 196 | Uncharacterized protein | Uncharacterized protein | | afdb-uniprot50 | AF-A0A5C4YDH8-F1-MODEL\_V4 | 1.0 | 2.049e-15 | 648 | 0.392 | 158 | 93 | 1 | 1 | 158 | 3 | 157 | Phage tail protein | Phage tail protein | | afdb-uniprot50 | AF-A0A7Y7YIA9-F1-MODEL\_V4 | 1.0 | 4.599e-15 | 648 | 0.422 | 154 | 89 | 0 | 2 | 155 | 6 | 159 | Phage tail protein | Phage tail protein | | afdb-uniprot50 | AF-A0A381GJP7-F1-MODEL\_V4 | 1.0 | 4.343e-19 | 647 | 0.305 | 314 | 116 | 4 | 1 | 308 | 2 | 219 | Phage tail fiber protein | Phage tail fiber protein | | afdb-uniprot50 | AF-A0A3Z0G4I0-F1-MODEL\_V4 | 1.0 | 4.803e-27 | 647 | 0.267 | 493 | 196 | 14 | 1 | 423 | 2 | 399 | Phage tail protein | Phage tail protein | | afdb-uniprot50 | AF-A0A2D3VW29-F1-MODEL\_V4 | 1.0 | 3.513e-15 | 646 | 0.377 | 151 | 94 | 0 | 3 | 153 | 2 | 152 | Uncharacterized protein | Uncharacterized protein | | afdb-uniprot50 | AF-A0A2N7P6T1-F1-MODEL\_V4 | 1.0 | 1.901e-24 | 646 | 0.214 | 438 | 226 | 18 | 3 | 423 | 2 | 338 | Uncharacterized protein | Uncharacterized protein | | afdb-uniprot50 | AF-C1DBJ9-F1-MODEL\_V4 | 1.0 | 3.439e-24 | 646 | 0.252 | 456 | 197 | 18 | 2 | 422 | 3 | 349 | Probable phage tail fiber protein | Probable phage tail fiber protein | | afdb-uniprot50 | AF-A0A542V4K6-F1-MODEL\_V4 | 1.0 | 2.516e-27 | 646 | 0.245 | 505 | 260 | 24 | 1 | 423 | 2 | 467 | Tail-collar fiber protein | Tail-collar fiber protein | | afdb-uniprot50 | AF-A0A1S1HUY3-F1-MODEL\_V4 | 1.0 | 9.778e-15 | 646 | 0.36 | 161 | 101 | 2 | 1 | 159 | 1 | 161 | Uncharacterized protein | Uncharacterized protein | | afdb-uniprot50 | AF-A0A0B5QHJ2-F1-MODEL\_V4 | 1.0 | 2.02e-16 | 645 | 0.342 | 187 | 115 | 2 | 1 | 179 | 1 | 187 | Phage tail protein | Phage tail protein | | afdb-uniprot50 | AF-A0A376DLB6-F1-MODEL\_V4 | 1.0 | 2.978e-19 | 644 | 0.341 | 249 | 107 | 5 | 1 | 221 | 2 | 221 | Uncharacterized protein | Uncharacterized protein | | afdb-uniprot50 | AF-A0A379QPW4-F1-MODEL\_V4 | 1.0 | 6.904e-28 | 644 | 0.256 | 506 | 244 | 21 | 1 | 423 | 1 | 457 | Tail protein | Tail protein | | afdb-uniprot50 | AF-A0A318T0X1-F1-MODEL\_V4 | 1.0 | 3.109e-16 | 644 | 0.345 | 162 | 106 | 0 | 1 | 162 | 2 | 163 | Uncharacterized protein DUF2793 | Uncharacterized protein DUF2793 | | afdb-uniprot50 | AF-A0A1N6Q2W5-F1-MODEL\_V4 | 1.0 | 4.174e-18 | 642 | 0.36 | 200 | 113 | 5 | 3 | 194 | 2 | 194 | Phage tail-collar fibre protein | Phage tail-collar fibre protein | | afdb-uniprot50 | AF-A0A5M0YJG9-F1-MODEL\_V4 | 1.0 | 3.513e-15 | 641 | 0.375 | 152 | 92 | 1 | 2 | 153 | 3 | 151 | Uncharacterized protein | Uncharacterized protein | | afdb-uniprot50 | AF-A0A6C8Y4A3-F1-MODEL\_V4 | 1.0 | 4.518e-20 | 641 | 0.326 | 315 | 108 | 4 | 1 | 308 | 2 | 219 | Phage tail protein | Phage tail protein | | afdb-uniprot50 | AF-A0A081RRN4-F1-MODEL\_V4 | 1.0 | 8.845e-22 | 641 | 0.331 | 314 | 132 | 6 | 4 | 306 | 3 | 249 | Phage-related tail fiber protein | Phage-related tail fiber protein | | afdb-uniprot50 | AF-A0A0N1IDW0-F1-MODEL\_V4 | 1.0 | 9.639e-16 | 640 | 0.353 | 164 | 103 | 1 | 1 | 161 | 1 | 164 | Tail protein | Tail protein | | afdb-uniprot50 | AF-A0A2X3H8W1-F1-MODEL\_V4 | 1.0 | 3.329e-15 | 640 | 0.401 | 167 | 92 | 2 | 1 | 159 | 1 | 167 | Tail fiber protein | Tail fiber protein | | afdb-uniprot50 | AF-A0A1T4WZ85-F1-MODEL\_V4 | 1.0 | 2.636e-20 | 640 | 0.336 | 244 | 125 | 10 | 1 | 223 | 1 | 228 | Phage tail-collar fibre protein | Phage tail-collar fibre protein | | afdb-uniprot50 | AF-A0A0S1B3R8-F1-MODEL\_V4 | 1.0 | 6.354e-15 | 639 | 0.378 | 156 | 96 | 1 | 1 | 155 | 1 | 156 | Side tail phage protein | Side tail phage protein | | afdb-uniprot50 | AF-A0A4P5QDV9-F1-MODEL\_V4 | 1.0 | 1.213e-14 | 639 | 0.285 | 161 | 115 | 0 | 1 | 161 | 1 | 161 | Phage tail protein | Phage tail protein | | afdb-uniprot50 | AF-A0A3D9DW60-F1-MODEL\_V4 | 1.0 | 1.431e-25 | 639 | 0.232 | 490 | 215 | 19 | 1 | 423 | 1 | 396 | Tail-collar fiber protein | Tail-collar fiber protein | | afdb-uniprot50 | AF-A0A379X3P6-F1-MODEL\_V4 | 1.0 | 4.16e-22 | 638 | 0.298 | 342 | 155 | 8 | 1 | 324 | 1 | 275 | Gp19 | Gp19 | | afdb-uniprot50 | AF-A0A4P2WWZ3-F1-MODEL\_V4 | 1.0 | 3.414e-17 | 637 | 0.31 | 203 | 119 | 5 | 1 | 183 | 2 | 203 | Uncharacterized protein | Uncharacterized protein | | afdb-uniprot50 | AF-A0A485CDL0-F1-MODEL\_V4 | 1.0 | 2.542e-15 | 637 | 0.38 | 168 | 98 | 1 | 1 | 162 | 2 | 169 | Uncharacterized protein | Uncharacterized protein | | afdb-uniprot50 | AF-A0A2S0JB19-F1-MODEL\_V4 | 1.0 | 6.706e-15 | 637 | 0.358 | 162 | 98 | 3 | 1 | 162 | 1 | 156 | Uncharacterized protein | Uncharacterized protein | | afdb-uniprot50 | AF-V4NM72-F1-MODEL\_V4 | 1.0 | 6.588e-20 | 637 | 0.287 | 344 | 126 | 3 | 1 | 326 | 1 | 243 | Uncharacterized protein | Uncharacterized protein | | afdb-uniprot50 | AF-A0A1V0LL73-F1-MODEL\_V4 | 1.0 | 2.227e-28 | 637 | 0.271 | 515 | 228 | 17 | 1 | 423 | 2 | 461 | Phage tail protein | Phage tail protein | | afdb-uniprot50 | AF-A0A2S4SSM1-F1-MODEL\_V4 | 1.0 | 6.087e-18 | 637 | 0.393 | 211 | 99 | 3 | 1 | 182 | 2 | 212 | Phage tail protein | Phage tail protein | | afdb-uniprot50 | AF-A0A7U6ITD4-F1-MODEL\_V4 | 1.0 | 3.513e-15 | 637 | 0.343 | 157 | 98 | 2 | 1 | 156 | 2 | 154 | Uncharacterized protein | Uncharacterized protein | | afdb-uniprot50 | AF-A0A380TT23-F1-MODEL\_V4 | 1.0 | 7.77e-16 | 637 | 0.409 | 166 | 93 | 2 | 3 | 163 | 2 | 167 | Plasmin and fibronectin-binding protein A | Plasmin and fibronectin-binding protein A | | afdb-uniprot50 | AF-A0A369YED8-F1-MODEL\_V4 | 1.0 | 9.134e-16 | 636 | 0.375 | 168 | 100 | 1 | 1 | 163 | 1 | 168 | Uncharacterized protein | Uncharacterized protein | | afdb-uniprot50 | AF-A5MYJ9-F1-MODEL\_V4 | 1.0 | 4.977e-17 | 636 | 0.369 | 195 | 114 | 2 | 1 | 187 | 2 | 195 | Predicted phage tail fibre protein | Predicted phage tail fibre protein | | afdb-uniprot50 | AF-A0A0S4SND2-F1-MODEL\_V4 | 1.0 | 8.531e-17 | 635 | 0.397 | 186 | 85 | 2 | 1 | 162 | 1 | 183 | Tail fiber protein H | Tail fiber protein H | | afdb-uniprot50 | AF-A0A6S4UU02-F1-MODEL\_V4 | 1.0 | 9.134e-16 | 635 | 0.31 | 190 | 112 | 3 | 1 | 172 | 2 | 190 | Mannuronan 5-epimerase | Mannuronan 5-epimerase | | afdb-uniprot50 | AF-A0A6D0LVZ5-F1-MODEL\_V4 | 1.0 | 3.489e-23 | 634 | 0.29 | 420 | 127 | 6 | 2 | 346 | 3 | 326 | Phage tail protein | Phage tail protein | | afdb-uniprot50 | AF-A0A1Q6AW37-F1-MODEL\_V4 | 1.0 | 2.172e-26 | 634 | 0.255 | 505 | 193 | 19 | 1 | 423 | 2 | 405 | Phage tail protein | Phage tail protein | | afdb-uniprot50 | AF-C6D9F9-F1-MODEL\_V4 | 1.0 | 4.518e-20 | 634 | 0.332 | 307 | 103 | 5 | 1 | 306 | 1 | 206 | Tail fiber repeat 2 protein | Tail fiber repeat 2 protein | | afdb-uniprot50 | AF-A0A2P8QYP4-F1-MODEL\_V4 | 1.0 | 3.065e-17 | 633 | 0.366 | 199 | 118 | 2 | 1 | 194 | 1 | 196 | Uncharacterized protein | Uncharacterized protein | | afdb-uniprot50 | AF-A0A777TPN4-F1-MODEL\_V4 | 1.0 | 2.782e-20 | 632 | 0.277 | 321 | 159 | 7 | 1 | 307 | 2 | 263 | Short-chain fatty acid transporter | Short-chain fatty acid transporter | | afdb-uniprot50 | AF-A0A7U4KUX0-F1-MODEL\_V4 | 1.0 | 3.212e-25 | 632 | 0.276 | 485 | 198 | 14 | 1 | 423 | 2 | 395 | Tail protein | Tail protein | | afdb-uniprot50 | AF-A0A5T2TRA4-F1-MODEL\_V4 | 1.0 | 3.402e-21 | 632 | 0.326 | 334 | 144 | 9 | 1 | 323 | 2 | 265 | Uncharacterized protein | Uncharacterized protein | | afdb-uniprot50 | AF-A0A8A5HZ74-F1-MODEL\_V4 | 1.0 | 1.549e-27 | 630 | 0.278 | 532 | 210 | 20 | 1 | 423 | 1 | 467 | Phage tail protein | Phage tail protein | | afdb-uniprot50 | AF-A0A2R3MZM8-F1-MODEL\_V4 | 1.0 | 4.07e-16 | 629 | 0.312 | 179 | 115 | 4 | 1 | 179 | 2 | 172 | Uncharacterized protein | Uncharacterized protein | | afdb-uniprot50 | AF-A0A823Q4U5-F1-MODEL\_V4 | 1.0 | 1.149e-14 | 628 | 0.375 | 144 | 85 | 3 | 1 | 143 | 3 | 142 | Phage tail protein | Phage tail protein | | afdb-uniprot50 | AF-A0A3U9HGD2-F1-MODEL\_V4 | 1.0 | 6.379e-26 | 628 | 0.264 | 496 | 189 | 18 | 1 | 423 | 2 | 394 | Phage tail protein | Phage tail protein | | afdb-uniprot50 | AF-A0A3E0X2Y9-F1-MODEL\_V4 | 1.0 | 8.2e-16 | 627 | 0.376 | 178 | 108 | 2 | 1 | 177 | 1 | 176 | Uncharacterized protein | Uncharacterized protein | | afdb-uniprot50 | AF-A0A080HQP8-F1-MODEL\_V4 | 1.0 | 5.604e-20 | 627 | 0.322 | 257 | 130 | 7 | 1 | 223 | 2 | 248 | Phage tail-collar fiber family protein | Phage tail-collar fiber family protein | | afdb-uniprot50 | AF-A0A447XW04-F1-MODEL\_V4 | 1.0 | 7.233e-21 | 627 | 0.326 | 334 | 144 | 8 | 1 | 323 | 2 | 265 | Tail fiber protein | Tail fiber protein | | afdb-uniprot50 | AF-A0A6L6ITL6-F1-MODEL\_V4 | 1.0 | 1.538e-20 | 626 | 0.29 | 351 | 126 | 6 | 1 | 324 | 2 | 256 | Phage tail protein | Phage tail protein | | afdb-uniprot50 | AF-A0A1S6U821-F1-MODEL\_V4 | 1.0 | 1.814e-16 | 626 | 0.326 | 184 | 116 | 2 | 3 | 181 | 1 | 181 | Phage tail collar fiber protein | Phage tail collar fiber protein | | afdb-uniprot50 | AF-A0A7H9S4N1-F1-MODEL\_V4 | 1.0 | 1.361e-21 | 625 | 0.335 | 334 | 141 | 9 | 1 | 323 | 2 | 265 | Phage tail protein | Phage tail protein | | afdb-uniprot50 | AF-A0A2A3VZP6-F1-MODEL\_V4 | 1.0 | 6.242e-20 | 624 | 0.29 | 330 | 150 | 9 | 1 | 314 | 1 | 262 | Phage tail protein | Phage tail protein | | afdb-uniprot50 | AF-A0A2G2D6M1-F1-MODEL\_V4 | 1.0 | 3.329e-15 | 623 | 0.375 | 165 | 101 | 2 | 2 | 165 | 21 | 184 | Uncharacterized protein | Uncharacterized protein | | afdb-uniprot50 | AF-A0A426VL03-F1-MODEL\_V4 | 1.0 | 1.017e-15 | 623 | 0.303 | 181 | 113 | 4 | 1 | 178 | 26 | 196 | Uncharacterized protein | Uncharacterized protein | | afdb-uniprot50 | AF-W7PLX3-F1-MODEL\_V4 | 1.0 | 3.212e-25 | 623 | 0.202 | 666 | 178 | 16 | 1 | 423 | 1 | 556 | Side tail fiber protein | Side tail fiber protein | | afdb-uniprot50 | AF-A0A2T9VBX3-F1-MODEL\_V4 | 1.0 | 3e-26 | 622 | 0.252 | 495 | 197 | 18 | 1 | 423 | 2 | 395 | Phage tail protein | Phage tail protein | | afdb-uniprot50 | AF-A0A1T2X6C6-F1-MODEL\_V4 | 1.0 | 5.329e-16 | 621 | 0.329 | 182 | 106 | 3 | 6 | 178 | 4 | 178 | Phage tail-collar fiber protein (DUF3751 domain) | Phage tail-collar fiber protein (DUF3751 domain) | | afdb-uniprot50 | AF-A0A827GY52-F1-MODEL\_V4 | 1.0 | 4.205e-25 | 621 | 0.263 | 501 | 188 | 21 | 1 | 423 | 2 | 399 | Phage tail protein | Phage tail protein | | afdb-uniprot50 | AF-A0A1S8PIF5-F1-MODEL\_V4 | 1.0 | 6.706e-15 | 621 | 0.414 | 164 | 93 | 2 | 1 | 161 | 1 | 164 | Glycosyl hydrolases family 43 | Glycosyl hydrolases family 43 | | afdb-uniprot50 | AF-A0A1H0YPL5-F1-MODEL\_V4 | 1.0 | 6.516e-17 | 620 | 0.32 | 200 | 118 | 3 | 3 | 194 | 5 | 194 | Phage tail-collar fibre protein | Phage tail-collar fibre protein | | afdb-uniprot50 | AF-A0A4P8YDH1-F1-MODEL\_V4 | 1.0 | 1.646e-19 | 619 | 0.321 | 252 | 116 | 5 | 1 | 221 | 2 | 229 | Uncharacterized protein | Uncharacterized protein | | afdb-uniprot50 | AF-A0A7X5J7V1-F1-MODEL\_V4 | 1.0 | 1.426e-14 | 618 | 0.373 | 150 | 92 | 2 | 5 | 153 | 2 | 150 | Uncharacterized protein | Uncharacterized protein | | afdb-uniprot50 | AF-A0A6B2FQ21-F1-MODEL\_V4 | 1.0 | 9.993e-21 | 618 | 0.236 | 432 | 164 | 15 | 1 | 423 | 1 | 275 | Phage tail protein | Phage tail protein | | afdb-uniprot50 | AF-A0A0R9N0A5-F1-MODEL\_V4 | 1.0 | 1.04e-21 | 618 | 0.316 | 332 | 154 | 9 | 1 | 322 | 1 | 269 | Phage tail protein | Phage tail protein | | afdb-uniprot50 | AF-A0A4S2HC06-F1-MODEL\_V4 | 1.0 | 2.079e-14 | 617 | 0.32 | 153 | 104 | 0 | 1 | 153 | 2 | 154 | Uncharacterized protein | Uncharacterized protein | | afdb-uniprot50 | AF-A0A421HAJ6-F1-MODEL\_V4 | 1.0 | 3.513e-15 | 617 | 0.395 | 167 | 93 | 2 | 1 | 159 | 1 | 167 | Phage tail protein | Phage tail protein | | afdb-uniprot50 | AF-A0A0F6RH94-F1-MODEL\_V4 | 1.0 | 5.427e-26 | 617 | 0.239 | 496 | 207 | 14 | 1 | 423 | 2 | 400 | Tail protein | Tail protein | | afdb-uniprot50 | AF-A0A4Q6YW23-F1-MODEL\_V4 | 1.0 | 7.363e-16 | 615 | 0.309 | 181 | 112 | 4 | 1 | 178 | 3 | 173 | Uncharacterized protein | Uncharacterized protein | | afdb-uniprot50 | AF-A0A0A8FI71-F1-MODEL\_V4 | 1.0 | 6.401e-22 | 615 | 0.24 | 433 | 189 | 17 | 1 | 422 | 2 | 305 | Tail protein | Tail protein | | afdb-uniprot50 | AF-A0A6L9JTE5-F1-MODEL\_V4 | 1.0 | 2.095e-21 | 615 | 0.35 | 331 | 126 | 7 | 4 | 322 | 3 | 256 | Phage tail protein | Phage tail protein | | afdb-uniprot50 | AF-A0A653EAR8-F1-MODEL\_V4 | 1.0 | 2.358e-24 | 615 | 0.249 | 453 | 196 | 19 | 1 | 423 | 2 | 340 | Phage tail-collar fibre protein | Phage tail-collar fibre protein | | afdb-uniprot50 | AF-A0A0U1KJN9-F1-MODEL\_V4 | 1.0 | 2.884e-25 | 614 | 0.249 | 489 | 207 | 16 | 1 | 423 | 2 | 396 | Tail fiber protein | Tail fiber protein | | afdb-uniprot50 | AF-A0A849JYK8-F1-MODEL\_V4 | 1.0 | 4.145e-26 | 614 | 0.249 | 497 | 215 | 16 | 1 | 423 | 2 | 414 | Phage tail protein | Phage tail protein | | afdb-uniprot50 | AF-C3X1Y2-F1-MODEL\_V4 | 1.0 | 1.121e-27 | 614 | 0.313 | 501 | 222 | 19 | 1 | 412 | 2 | 469 | Collar domain-containing protein | Collar domain-containing protein | | afdb-uniprot50 | AF-A0A828AFE6-F1-MODEL\_V4 | 1.0 | 1.782e-21 | 614 | 0.273 | 336 | 167 | 9 | 1 | 322 | 2 | 274 | Phage tail protein | Phage tail protein | | afdb-uniprot50 | AF-A0A4R5W1D0-F1-MODEL\_V4 | 1.0 | 1.101e-17 | 614 | 0.388 | 216 | 109 | 6 | 1 | 194 | 1 | 215 | Uncharacterized protein | Uncharacterized protein | | afdb-uniprot50 | AF-A0A1R7Q9M1-F1-MODEL\_V4 | 1.0 | 1.426e-14 | 614 | 0.341 | 158 | 102 | 2 | 1 | 158 | 1 | 156 | Uncharacterized protein | Uncharacterized protein | | afdb-uniprot50 | AF-A0A853IMF0-F1-MODEL\_V4 | 1.0 | 4.421e-14 | 613 | 0.368 | 133 | 81 | 1 | 1 | 130 | 1 | 133 | Phage tail protein | Phage tail protein | | afdb-uniprot50 | AF-A0A826DDX5-F1-MODEL\_V4 | 1.0 | 1.213e-14 | 613 | 0.373 | 158 | 96 | 1 | 1 | 158 | 3 | 157 | Phage tail protein | Phage tail protein | | afdb-uniprot50 | AF-H3R957-F1-MODEL\_V4 | 1.0 | 8.261e-23 | 613 | 0.259 | 447 | 186 | 16 | 1 | 423 | 1 | 326 | Uncharacterized protein | Uncharacterized protein | | afdb-uniprot50 | AF-A0A6I3JS90-F1-MODEL\_V4 | 1.0 | 1.327e-19 | 613 | 0.323 | 303 | 107 | 3 | 4 | 306 | 3 | 207 | Collar domain-containing protein | Collar domain-containing protein | | afdb-uniprot50 | AF-A0A811XII5-F1-MODEL\_V4 | 1.0 | 2.203e-25 | 612 | 0.223 | 488 | 213 | 14 | 1 | 422 | 2 | 389 | Phage tail fiber protein | Phage tail fiber protein | | afdb-uniprot50 | AF-A0A423D1H4-F1-MODEL\_V4 | 1.0 | 8.814e-26 | 612 | 0.222 | 490 | 234 | 19 | 1 | 423 | 2 | 411 | Uncharacterized protein | Uncharacterized protein | | afdb-uniprot50 | AF-A0A747Y1U2-F1-MODEL\_V4 | 1.0 | 5.31e-20 | 611 | 0.25 | 351 | 137 | 7 | 1 | 326 | 4 | 253 | Phage tail protein | Phage tail protein | | afdb-uniprot50 | AF-A0A726YJW6-F1-MODEL\_V4 | 1.0 | 1.171e-24 | 610 | 0.238 | 483 | 197 | 12 | 4 | 423 | 14 | 388 | Uncharacterized protein | Uncharacterized protein | | afdb-uniprot50 | AF-A0A2G0Q045-F1-MODEL\_V4 | 1.0 | 2.674e-19 | 610 | 0.316 | 313 | 113 | 3 | 1 | 313 | 2 | 213 | Tail protein | Tail protein | | afdb-uniprot50 | AF-A0A447N0H8-F1-MODEL\_V4 | 1.0 | 2.367e-20 | 609 | 0.292 | 338 | 157 | 10 | 1 | 323 | 1 | 271 | Gp19 | Gp19 | | afdb-uniprot50 | AF-A0A7I9NTD9-F1-MODEL\_V4 | 1.0 | 1.676e-14 | 609 | 0.36 | 158 | 98 | 1 | 1 | 158 | 3 | 157 | Phage tail protein | Phage tail protein | | afdb-uniprot50 | AF-A0A4P0YQ56-F1-MODEL\_V4 | 1.0 | 4.281e-20 | 609 | 0.261 | 318 | 156 | 10 | 1 | 306 | 1 | 251 | Putative phage tail fiber protein | Putative phage tail fiber protein | | afdb-uniprot50 | AF-A0A857F214-F1-MODEL\_V4 | 1.0 | 3.451e-20 | 609 | 0.33 | 306 | 115 | 4 | 1 | 306 | 2 | 217 | Phage tail protein | Phage tail protein | | afdb-uniprot50 | AF-A0A5X4GC92-F1-MODEL\_V4 | 1.0 | 6.401e-22 | 609 | 0.303 | 349 | 163 | 9 | 1 | 328 | 1 | 290 | Short-chain fatty acid transporter | Short-chain fatty acid transporter | | afdb-uniprot50 | AF-A0A5Z0R0R1-F1-MODEL\_V4 | 1.0 | 8.319e-15 | 609 | 0.375 | 157 | 96 | 2 | 3 | 159 | 12 | 166 | Collar domain-containing protein | Collar domain-containing protein | | afdb-uniprot50 | AF-A0A830Q9A3-F1-MODEL\_V4 | 1.0 | 6.221e-24 | 608 | 0.264 | 457 | 191 | 14 | 1 | 423 | 2 | 347 | Putative phage tail collar domain-containing protein | Putative phage tail collar domain-containing protein | | afdb-uniprot50 | AF-C5BEP6-F1-MODEL\_V4 | 1.0 | 2.772e-24 | 608 | 0.223 | 475 | 206 | 15 | 1 | 422 | 2 | 366 | Phage tail fiber domain protein | Phage tail fiber domain protein | | afdb-uniprot50 | AF-A0A6I2IHE3-F1-MODEL\_V4 | 1.0 | 3.476e-27 | 608 | 0.257 | 509 | 221 | 20 | 1 | 423 | 2 | 439 | Phage tail protein | Phage tail protein | | afdb-uniprot50 | AF-A0A5X9L6P6-F1-MODEL\_V4 | 1.0 | 3.59e-21 | 608 | 0.314 | 331 | 153 | 9 | 2 | 322 | 3 | 269 | Phage tail protein | Phage tail protein | | afdb-uniprot50 | AF-A0A5D3JF06-F1-MODEL\_V4 | 1.0 | 3.761e-14 | 606 | 0.417 | 151 | 87 | 1 | 1 | 151 | 2 | 151 | Phage tail protein | Phage tail protein | | afdb-uniprot50 | AF-A0A826YSE8-F1-MODEL\_V4 | 1.0 | 4.22e-21 | 606 | 0.326 | 334 | 144 | 8 | 1 | 323 | 2 | 265 | Phage tail protein | Phage tail protein | | afdb-uniprot50 | AF-A0A2X1PYX0-F1-MODEL\_V4 | 1.0 | 5.032e-20 | 606 | 0.293 | 327 | 150 | 10 | 1 | 314 | 1 | 259 | Bacteriophage tail protein | Bacteriophage tail protein | | afdb-uniprot50 | AF-A0A024E8J4-F1-MODEL\_V4 | 1.0 | 2.579e-14 | 606 | 0.42 | 145 | 84 | 0 | 14 | 158 | 2 | 146 | Tail fiber protein H, putative | Tail fiber protein H, putative | | afdb-uniprot50 | AF-A0A5H6KLG3-F1-MODEL\_V4 | 1.0 | 1.209e-18 | 605 | 0.267 | 307 | 123 | 5 | 1 | 306 | 4 | 209 | Phage tail protein | Phage tail protein | | afdb-uniprot50 | AF-A0A2Y0PD72-F1-MODEL\_V4 | 1.0 | 8.595e-24 | 605 | 0.219 | 483 | 203 | 12 | 1 | 423 | 2 | 370 | Tail fiber domain-containing protein | Tail fiber domain-containing protein | | afdb-uniprot50 | AF-A0A7W0B3B2-F1-MODEL\_V4 | 1.0 | 1.093e-25 | 605 | 0.236 | 512 | 248 | 25 | 1 | 423 | 1 | 458 | Phage-related tail fiber protein | Phage-related tail fiber protein | | afdb-uniprot50 | AF-N1NKC6-F1-MODEL\_V4 | 1.0 | 1.381e-20 | 605 | 0.306 | 330 | 122 | 5 | 1 | 324 | 2 | 230 | Uncharacterized protein | Uncharacterized protein | | afdb-uniprot50 | AF-A0A5U3G5W3-F1-MODEL\_V4 | 1.0 | 1.028e-18 | 604 | 0.244 | 347 | 138 | 6 | 1 | 324 | 1 | 246 | Uncharacterized protein | Uncharacterized protein | | afdb-uniprot50 | AF-A0A4D7AWU2-F1-MODEL\_V4 | 1.0 | 6.108e-14 | 604 | 0.324 | 154 | 101 | 3 | 1 | 153 | 1 | 152 | DUF2793 domain-containing protein | DUF2793 domain-containing protein | | afdb-uniprot50 | AF-A0A0U1HL65-F1-MODEL\_V4 | 1.0 | 1.141e-22 | 604 | 0.252 | 491 | 190 | 20 | 1 | 423 | 2 | 383 | Tail fiber protein | Tail fiber protein | | afdb-uniprot50 | AF-D2BZE6-F1-MODEL\_V4 | 1.0 | 4.343e-19 | 604 | 0.31 | 264 | 138 | 7 | 1 | 223 | 2 | 262 | Phage-related tail fibre protein-like protein | Phage-related tail fibre protein-like protein | | afdb-uniprot50 | AF-A0A433NXC7-F1-MODEL\_V4 | 1.0 | 9.469e-21 | 602 | 0.335 | 331 | 113 | 6 | 2 | 332 | 3 | 226 | Tail fiber protein | Tail fiber protein | | afdb-uniprot50 | AF-N9QTJ7-F1-MODEL\_V4 | 1.0 | 6.264e-16 | 602 | 0.333 | 207 | 118 | 5 | 3 | 190 | 5 | 210 | Uncharacterized protein | Uncharacterized protein | | afdb-uniprot50 | AF-A0A7Y1SG14-F1-MODEL\_V4 | 1.0 | 7.77e-16 | 601 | 0.351 | 202 | 111 | 6 | 1 | 183 | 1 | 201 | Phage tail protein | Phage tail protein | | afdb-uniprot50 | AF-A0A7H4NYM5-F1-MODEL\_V4 | 1.0 | 7.855e-19 | 601 | 0.266 | 334 | 138 | 7 | 1 | 324 | 2 | 238 | Putative prophage tail fiber protein | Putative prophage tail fiber protein | | afdb-uniprot50 | AF-A0A5M6I3L7-F1-MODEL\_V4 | 1.0 | 8.749e-19 | 601 | 0.287 | 316 | 135 | 9 | 1 | 306 | 1 | 236 | Uncharacterized protein | Uncharacterized protein | | afdb-uniprot50 | AF-A0A827RXE4-F1-MODEL\_V4 | 1.0 | 1.516e-21 | 600 | 0.344 | 334 | 138 | 10 | 1 | 324 | 2 | 264 | Phage tail protein | Phage tail protein | | afdb-uniprot50 | AF-A0A1G5QGK4-F1-MODEL\_V4 | 1.0 | 1.436e-21 | 600 | 0.338 | 340 | 118 | 9 | 4 | 322 | 3 | 256 | Phage tail fibre repeat-containing protein | Phage tail fibre repeat-containing protein | | afdb-uniprot50 | AF-A0A077P0S8-F1-MODEL\_V4 | 1.0 | 2.926e-24 | 600 | 0.215 | 622 | 187 | 15 | 1 | 423 | 2 | 521 | Uncharacterized protein | Uncharacterized protein | | afdb-uniprot50 | AF-A0A853HVL0-F1-MODEL\_V4 | 1.0 | 2.525e-23 | 599 | 0.217 | 450 | 207 | 21 | 1 | 423 | 1 | 332 | Phage tail protein | Phage tail protein | | afdb-uniprot50 | AF-I6DBN4-F1-MODEL\_V4 | 1.0 | 8.502e-21 | 598 | 0.286 | 342 | 165 | 12 | 1 | 308 | 1 | 297 | Phage tail fiber repeat family protein | Phage tail fiber repeat family protein | | afdb-uniprot50 | AF-A0A2H9EM17-F1-MODEL\_V4 | 1.0 | 3.342e-26 | 598 | 0.265 | 535 | 211 | 22 | 1 | 423 | 2 | 466 | Phage tail protein | Phage tail protein | | afdb-uniprot50 | AF-J2V596-F1-MODEL\_V4 | 1.0 | 3.463e-16 | 597 | 0.356 | 199 | 119 | 5 | 2 | 193 | 6 | 202 | Uncharacterized protein | Uncharacterized protein | | afdb-uniprot50 | AF-A0A7A4NFG1-F1-MODEL\_V4 | 1.0 | 2.367e-20 | 596 | 0.267 | 344 | 170 | 9 | 1 | 332 | 5 | 278 | Phage tail protein | Phage tail protein | | afdb-uniprot50 | AF-F7SVK4-F1-MODEL\_V4 | 1.0 | 1.478e-19 | 596 | 0.337 | 264 | 134 | 9 | 1 | 244 | 1 | 243 | Uncharacterized protein | Uncharacterized protein | | afdb-uniprot50 | AF-A0A1I3S1C5-F1-MODEL\_V4 | 1.0 | 6.588e-20 | 595 | 0.303 | 330 | 123 | 6 | 1 | 324 | 2 | 230 | Phage tail fibre repeat-containing protein | Phage tail fibre repeat-containing protein | | afdb-uniprot50 | AF-A0A853I7M0-F1-MODEL\_V4 | 1.0 | 4.438e-25 | 595 | 0.211 | 467 | 248 | 23 | 1 | 423 | 1 | 391 | Phage tail protein | Phage tail protein | | afdb-uniprot50 | AF-A0A066RYY3-F1-MODEL\_V4 | 1.0 | 2.636e-20 | 595 | 0.295 | 305 | 144 | 8 | 1 | 259 | 1 | 280 | Uncharacterized protein | Uncharacterized protein | | afdb-uniprot50 | AF-A0A1C6Z3Q6-F1-MODEL\_V4 | 1.0 | 3.789e-21 | 594 | 0.313 | 332 | 155 | 7 | 1 | 324 | 2 | 268 | Phage tail fibre repeat-containing protein | Phage tail fibre repeat-containing protein | | afdb-uniprot50 | AF-A0A1B9JGB9-F1-MODEL\_V4 | 1.0 | 3.999e-21 | 593 | 0.276 | 355 | 130 | 3 | 1 | 343 | 2 | 241 | Collar domain-containing protein | Collar domain-containing protein | | afdb-uniprot50 | AF-A0A0T9UVJ2-F1-MODEL\_V4 | 1.0 | 6.803e-14 | 592 | 0.47 | 134 | 70 | 1 | 1 | 133 | 1 | 134 | Variable tail fiber protein | Variable tail fiber protein | | afdb-uniprot50 | AF-A0A0B6FER7-F1-MODEL\_V4 | 1.0 | 8.261e-23 | 592 | 0.254 | 436 | 211 | 21 | 1 | 423 | 2 | 336 | Phage tail-collar fiber family protein | Phage tail-collar fiber family protein | | afdb-uniprot50 | AF-A0A3S5DM66-F1-MODEL\_V4 | 1.0 | 4.101e-23 | 592 | 0.258 | 449 | 197 | 17 | 1 | 408 | 1 | 354 | Phage tail fiber protein H | Phage tail fiber protein H | | afdb-uniprot50 | AF-A0A077PK87-F1-MODEL\_V4 | 1.0 | 4.056e-20 | 592 | 0.308 | 331 | 121 | 6 | 1 | 324 | 1 | 230 | E14 prophage putative tail fiber protein (Modular protein) | E14 prophage putative tail fiber protein (Modular protein) | | afdb-uniprot50 | AF-A0A833UC36-F1-MODEL\_V4 | 1.0 | 9.134e-16 | 592 | 0.368 | 179 | 103 | 4 | 1 | 178 | 1 | 170 | Uncharacterized protein | Uncharacterized protein | | afdb-uniprot50 | AF-A0A831GBG9-F1-MODEL\_V4 | 1.0 | 7.882e-15 | 591 | 0.396 | 169 | 88 | 5 | 1 | 159 | 1 | 165 | Phage tail protein | Phage tail protein | | afdb-uniprot50 | AF-A0A4T5FQZ6-F1-MODEL\_V4 | 1.0 | 1.113e-20 | 591 | 0.278 | 359 | 180 | 12 | 1 | 324 | 5 | 319 | Phage tail protein | Phage tail protein | | afdb-uniprot50 | AF-A0A2X5PQJ0-F1-MODEL\_V4 | 1.0 | 3.928e-26 | 591 | 0.271 | 537 | 207 | 24 | 1 | 423 | 1 | 467 | Phage tail collar protein | Phage tail collar protein | | afdb-uniprot50 | AF-A0A5M6IAF3-F1-MODEL\_V4 | 1.0 | 1.814e-16 | 590 | 0.418 | 184 | 104 | 2 | 1 | 183 | 1 | 182 | Uncharacterized protein | Uncharacterized protein | | afdb-uniprot50 | AF-A0A1V2UTE0-F1-MODEL\_V4 | 1.0 | 4.784e-16 | 590 | 0.341 | 208 | 114 | 5 | 1 | 206 | 1 | 187 | Uncharacterized protein | Uncharacterized protein | | afdb-uniprot50 | AF-A0A1A0FJZ1-F1-MODEL\_V4 | 1.0 | 3.83e-24 | 589 | 0.218 | 481 | 208 | 17 | 2 | 423 | 6 | 377 | Uncharacterized protein | Uncharacterized protein | | afdb-uniprot50 | AF-R8WJW4-F1-MODEL\_V4 | 1.0 | 1.629e-16 | 589 | 0.425 | 209 | 98 | 2 | 1 | 187 | 2 | 210 | Uncharacterized protein | Uncharacterized protein | | afdb-uniprot50 | AF-A0A7M3FHK4-F1-MODEL\_V4 | 1.0 | 2.683e-15 | 589 | 0.319 | 197 | 115 | 4 | 1 | 179 | 2 | 197 | Phage tail protein | Phage tail protein | | afdb-uniprot50 | AF-A0A1G9YF31-F1-MODEL\_V4 | 1.0 | 5.728e-26 | 588 | 0.226 | 512 | 260 | 30 | 2 | 423 | 6 | 471 | Phage tail-collar fibre protein | Phage tail-collar fibre protein | | afdb-uniprot50 | AF-A0A2X2BLJ3-F1-MODEL\_V4 | 1.0 | 2.752e-17 | 587 | 0.363 | 223 | 113 | 3 | 1 | 194 | 2 | 224 | Phage variable tail fiber protein | Phage variable tail fiber protein | | afdb-uniprot50 | AF-A0A7Y8JX08-F1-MODEL\_V4 | 1.0 | 2.946e-16 | 587 | 0.341 | 214 | 112 | 5 | 1 | 198 | 1 | 201 | Phage tail protein | Phage tail protein | | afdb-uniprot50 | AF-A0A3N6UMU5-F1-MODEL\_V4 | 1.0 | 7.233e-21 | 587 | 0.231 | 453 | 166 | 15 | 1 | 423 | 1 | 301 | Phage tail protein | Phage tail protein | | afdb-uniprot50 | AF-A0A2V4E3U5-F1-MODEL\_V4 | 1.0 | 1.641e-23 | 587 | 0.271 | 457 | 188 | 17 | 1 | 423 | 2 | 347 | DNA inversion product | DNA inversion product | | afdb-uniprot50 | AF-A0A6Y2VPA8-F1-MODEL\_V4 | 1.0 | 3.098e-20 | 587 | 0.27 | 344 | 169 | 9 | 1 | 332 | 5 | 278 | Phage tail protein | Phage tail protein | | afdb-uniprot50 | AF-A0A6H8PL82-F1-MODEL\_V4 | 1.0 | 3.564e-14 | 586 | 0.381 | 160 | 94 | 2 | 1 | 158 | 1 | 157 | Phage tail protein | Phage tail protein | | afdb-uniprot50 | AF-A0A5Y7WJU3-F1-MODEL\_V4 | 1.0 | 3.088e-24 | 586 | 0.209 | 620 | 187 | 17 | 1 | 423 | 2 | 515 | Phage tail protein | Phage tail protein | | afdb-uniprot50 | AF-A0A1C4DJ79-F1-MODEL\_V4 | 1.0 | 3.223e-21 | 585 | 0.247 | 437 | 184 | 14 | 1 | 423 | 2 | 307 | Phage tail-collar fibre protein | Phage tail-collar fibre protein | | afdb-uniprot50 | AF-A0A3X7LX61-F1-MODEL\_V4 | 1.0 | 1.908e-20 | 585 | 0.251 | 346 | 136 | 5 | 1 | 324 | 4 | 248 | Phage tail protein | Phage tail protein | | afdb-uniprot50 | AF-A0A8A7FP84-F1-MODEL\_V4 | 1.0 | 6.588e-20 | 585 | 0.303 | 330 | 123 | 6 | 1 | 324 | 2 | 230 | Phage tail protein | Phage tail protein | | afdb-uniprot50 | AF-A0A367LYA0-F1-MODEL\_V4 | 1.0 | 7.577e-14 | 584 | 0.371 | 148 | 88 | 1 | 6 | 148 | 3 | 150 | Phage tail protein | Phage tail protein | | afdb-uniprot50 | AF-A0A827KPR0-F1-MODEL\_V4 | 1.0 | 1.694e-17 | 584 | 0.237 | 312 | 133 | 6 | 1 | 308 | 1 | 211 | Phage tail protein | Phage tail protein | | afdb-uniprot50 | AF-A0A1H0FNR9-F1-MODEL\_V4 | 1.0 | 1.361e-21 | 584 | 0.22 | 440 | 198 | 15 | 1 | 423 | 1 | 312 | Phage tail-collar fibre protein | Phage tail-collar fibre protein | | afdb-uniprot50 | AF-A0A443WD25-F1-MODEL\_V4 | 1.0 | 5.014e-24 | 584 | 0.277 | 440 | 200 | 21 | 2 | 423 | 3 | 342 | DNA inversion product | DNA inversion product | | afdb-uniprot50 | AF-A0A140NE20-F1-MODEL\_V4 | 1.0 | 7.105e-26 | 584 | 0.243 | 580 | 241 | 16 | 1 | 423 | 2 | 540 | Phage-related tail fibre protein-like protein | Phage-related tail fibre protein-like protein | | afdb-uniprot50 | AF-A0A2G2KYA8-F1-MODEL\_V4 | 1.0 | 3.899e-19 | 584 | 0.303 | 264 | 134 | 9 | 1 | 257 | 2 | 222 | Uncharacterized protein | Uncharacterized protein | | afdb-uniprot50 | AF-A0A068QVC8-F1-MODEL\_V4 | 1.0 | 1.07e-19 | 583 | 0.306 | 330 | 122 | 6 | 1 | 324 | 2 | 230 | Uncharacterized protein | Uncharacterized protein | | afdb-uniprot50 | AF-A0A7W4PXC5-F1-MODEL\_V4 | 1.0 | 4.174e-18 | 583 | 0.308 | 253 | 132 | 9 | 1 | 247 | 1 | 216 | Phage tail protein | Phage tail protein | | afdb-uniprot50 | AF-A0A1B9JGB5-F1-MODEL\_V4 | 1.0 | 1.29e-21 | 582 | 0.295 | 359 | 154 | 11 | 1 | 324 | 1 | 295 | Collar domain-containing protein | Collar domain-containing protein | | afdb-uniprot50 | AF-A0A1Q5TLV3-F1-MODEL\_V4 | 1.0 | 4.056e-20 | 582 | 0.298 | 335 | 125 | 8 | 1 | 326 | 1 | 234 | Tail protein | Tail protein | | afdb-uniprot50 | AF-A0A2Z2JIM9-F1-MODEL\_V4 | 1.0 | 1.834e-19 | 582 | 0.271 | 335 | 162 | 8 | 1 | 323 | 5 | 269 | Phage tail fiber repeat protein | Phage tail fiber repeat protein | | afdb-uniprot50 | AF-A0A7W3G1H3-F1-MODEL\_V4 | 1.0 | 1.105e-13 | 581 | 0.351 | 148 | 92 | 1 | 1 | 148 | 2 | 145 | Phage tail protein | Phage tail protein | | afdb-uniprot50 | AF-A0A143DG88-F1-MODEL\_V4 | 1.0 | 1.07e-19 | 581 | 0.281 | 316 | 143 | 9 | 1 | 299 | 3 | 251 | Collar domain-containing protein | Collar domain-containing protein | | afdb-uniprot50 | AF-A0A6I6IHZ7-F1-MODEL\_V4 | 1.0 | 2.627e-24 | 581 | 0.256 | 491 | 209 | 15 | 1 | 423 | 2 | 404 | Phage tail protein | Phage tail protein | | afdb-uniprot50 | AF-A0A7A2HGT8-F1-MODEL\_V4 | 1.0 | 3.642e-20 | 580 | 0.274 | 339 | 160 | 9 | 1 | 323 | 1 | 269 | Phage tail protein | Phage tail protein | | afdb-uniprot50 | AF-A0A3L2NND0-F1-MODEL\_V4 | 1.0 | 2.782e-20 | 580 | 0.299 | 341 | 159 | 12 | 1 | 324 | 1 | 278 | Short-chain fatty acid transporter | Short-chain fatty acid transporter | | afdb-uniprot50 | AF-A0A1B9JGC2-F1-MODEL\_V4 | 1.0 | 1.985e-21 | 580 | 0.277 | 367 | 157 | 10 | 1 | 322 | 1 | 304 | Uncharacterized protein | Uncharacterized protein | | afdb-uniprot50 | AF-A0A3E3I3G7-F1-MODEL\_V4 | 1.0 | 7.179e-14 | 579 | 0.341 | 158 | 96 | 3 | 1 | 153 | 1 | 155 | Uncharacterized protein | Uncharacterized protein | | afdb-uniprot50 | AF-A0A2K1QCR8-F1-MODEL\_V4 | 1.0 | 2.333e-21 | 579 | 0.241 | 439 | 189 | 13 | 1 | 422 | 2 | 313 | Phage tail protein | Phage tail protein | | afdb-uniprot50 | AF-A0A3N6UVH7-F1-MODEL\_V4 | 1.0 | 2.853e-22 | 578 | 0.26 | 450 | 196 | 15 | 1 | 423 | 2 | 341 | Phage tail protein | Phage tail protein | | afdb-uniprot50 | AF-A0A7U7FJJ3-F1-MODEL\_V4 | 1.0 | 6.132e-25 | 578 | 0.238 | 546 | 197 | 16 | 1 | 423 | 2 | 451 | Hypothetical phage tail fiber protein | Hypothetical phage tail fiber protein | | afdb-uniprot50 | AF-A0A377H523-F1-MODEL\_V4 | 1.0 | 6.83e-25 | 578 | 0.201 | 572 | 205 | 16 | 1 | 423 | 2 | 470 | Phage tail fibre repeat | Phage tail fibre repeat | | afdb-uniprot50 | AF-A0A5C8XUF6-F1-MODEL\_V4 | 1.0 | 7.337e-20 | 577 | 0.254 | 318 | 158 | 10 | 1 | 306 | 1 | 251 | Uncharacterized protein | Uncharacterized protein | | afdb-uniprot50 | AF-A0A7W5P3C6-F1-MODEL\_V4 | 1.0 | 1.533e-24 | 577 | 0.262 | 506 | 202 | 16 | 1 | 423 | 2 | 419 | Phage-related tail fiber protein | Phage-related tail fiber protein | | afdb-uniprot50 | AF-A0A3G2IM13-F1-MODEL\_V4 | 1.0 | 1.56e-19 | 577 | 0.267 | 352 | 129 | 2 | 1 | 324 | 2 | 252 | Uncharacterized protein | Uncharacterized protein | | afdb-uniprot50 | AF-A0A846QFW6-F1-MODEL\_V4 | 1.0 | 6.588e-20 | 576 | 0.332 | 286 | 142 | 9 | 1 | 249 | 1 | 274 | Phage-related tail fiber protein | Phage-related tail fiber protein | | afdb-uniprot50 | AF-A0A1B8YGC4-F1-MODEL\_V4 | 1.0 | 2.095e-21 | 576 | 0.34 | 344 | 120 | 6 | 4 | 326 | 3 | 260 | Phage tail fiber repeat protein | Phage tail fiber repeat protein | | afdb-uniprot50 | AF-A0A5T2CS27-F1-MODEL\_V4 | 1.0 | 2.616e-13 | 575 | 0.385 | 127 | 75 | 1 | 1 | 127 | 3 | 126 | Phage tail protein | Phage tail protein | | afdb-uniprot50 | AF-A0A5W3T5U2-F1-MODEL\_V4 | 1.0 | 1.308e-20 | 575 | 0.288 | 343 | 156 | 12 | 1 | 323 | 1 | 275 | Phage tail protein | Phage tail protein | | afdb-uniprot50 | AF-A0A827K3J7-F1-MODEL\_V4 | 1.0 | 7.053e-19 | 575 | 0.294 | 309 | 116 | 4 | 1 | 308 | 2 | 209 | Phage tail protein | Phage tail protein | | afdb-uniprot50 | AF-A0A4R2GW55-F1-MODEL\_V4 | 1.0 | 5.871e-13 | 575 | 0.353 | 147 | 93 | 2 | 1 | 146 | 1 | 146 | Phage-related tail fiber protein | Phage-related tail fiber protein | | afdb-uniprot50 | AF-A0A1Q8DQY0-F1-MODEL\_V4 | 1.0 | 7.996e-14 | 574 | 0.325 | 160 | 103 | 3 | 1 | 158 | 1 | 157 | Uncharacterized protein | Uncharacterized protein | | afdb-uniprot50 | AF-A0A2V4EMJ8-F1-MODEL\_V4 | 1.0 | 2.401e-19 | 574 | 0.33 | 284 | 130 | 9 | 1 | 237 | 2 | 272 | Uncharacterized protein | Uncharacterized protein | | afdb-uniprot50 | AF-A0A731FN07-F1-MODEL\_V4 | 1.0 | 5.747e-22 | 574 | 0.295 | 366 | 183 | 9 | 1 | 321 | 1 | 336 | Phage tail protein | Phage tail protein | | afdb-uniprot50 | AF-A0A077QF37-F1-MODEL\_V4 | 1.0 | 2.674e-19 | 574 | 0.306 | 330 | 122 | 5 | 1 | 324 | 2 | 230 | Uncharacterized protein | Uncharacterized protein | | afdb-uniprot50 | AF-A0A4R1NAG8-F1-MODEL\_V4 | 1.0 | 8.624e-20 | 573 | 0.304 | 315 | 113 | 5 | 1 | 305 | 1 | 219 | Tail fiber-like repeat protein | Tail fiber-like repeat protein | | afdb-uniprot50 | AF-D4F114-F1-MODEL\_V4 | 1.0 | 8.624e-20 | 573 | 0.324 | 330 | 131 | 5 | 2 | 331 | 3 | 240 | Phage tail fiber repeat protein | Phage tail fiber repeat protein | | afdb-uniprot50 | AF-A0A5W1P1H8-F1-MODEL\_V4 | 1.0 | 7.743e-20 | 573 | 0.282 | 343 | 158 | 10 | 1 | 323 | 1 | 275 | Phage tail protein | Phage tail protein | | afdb-uniprot50 | AF-A0A3L5HA13-F1-MODEL\_V4 | 1.0 | 2.936e-20 | 572 | 0.283 | 342 | 158 | 9 | 1 | 323 | 2 | 275 | Phage tail protein | Phage tail protein | | afdb-uniprot50 | AF-A0A3E2BGR8-F1-MODEL\_V4 | 1.0 | 7.443e-19 | 572 | 0.299 | 307 | 113 | 5 | 4 | 306 | 3 | 211 | Phage tail protein | Phage tail protein | | afdb-uniprot50 | AF-A0A5U6Z5U0-F1-MODEL\_V4 | 1.0 | 2.087e-25 | 572 | 0.231 | 562 | 224 | 17 | 1 | 423 | 2 | 494 | Phage tail protein | Phage tail protein | | afdb-uniprot50 | AF-A0A3G9G2L6-F1-MODEL\_V4 | 1.0 | 3.707e-15 | 572 | 0.32 | 200 | 117 | 4 | 1 | 182 | 3 | 201 | Phage tail fiber protein | Phage tail fiber protein | | afdb-uniprot50 | AF-D3V0E0-F1-MODEL\_V4 | 1.0 | 2.534e-19 | 572 | 0.303 | 330 | 123 | 5 | 1 | 324 | 2 | 230 | E14 prophage putative tail fiber protein (Modular protein) | E14 prophage putative tail fiber protein (Modular protein) | | afdb-uniprot50 | AF-A0A5E6YEG9-F1-MODEL\_V4 | 1.0 | 2.392e-23 | 571 | 0.209 | 454 | 234 | 20 | 4 | 423 | 8 | 370 | Uncharacterized protein | Uncharacterized protein | | afdb-uniprot50 | AF-F5MFA9-F1-MODEL\_V4 | 1.0 | 2.013e-20 | 571 | 0.28 | 342 | 159 | 10 | 1 | 323 | 2 | 275 | Phage tail fibre repeat family protein | Phage tail fibre repeat family protein | | afdb-uniprot50 | AF-A0A8B2FET2-F1-MODEL\_V4 | 1.0 | 5.104e-19 | 571 | 0.306 | 303 | 112 | 4 | 4 | 306 | 3 | 207 | Phage tail protein | Phage tail protein | | afdb-uniprot50 | AF-A0A2D0JSC8-F1-MODEL\_V4 | 1.0 | 1.646e-19 | 571 | 0.31 | 335 | 121 | 8 | 1 | 326 | 1 | 234 | Tail protein | Tail protein | | afdb-uniprot50 | AF-A0A242PF12-F1-MODEL\_V4 | 1.0 | 6.153e-21 | 571 | 0.315 | 345 | 130 | 5 | 1 | 344 | 1 | 240 | Collar domain-containing protein | Collar domain-containing protein | | afdb-uniprot50 | AF-R9B4A5-F1-MODEL\_V4 | 1.0 | 1.047e-13 | 570 | 0.254 | 165 | 113 | 5 | 1 | 161 | 2 | 160 | Collar domain-containing protein | Collar domain-containing protein | | afdb-uniprot50 | AF-A0A376MUF9-F1-MODEL\_V4 | 1.0 | 1.381e-20 | 570 | 0.293 | 341 | 158 | 9 | 1 | 322 | 1 | 277 | Putative tail fiber protein (GpH) | Putative tail fiber protein (GpH) | | afdb-uniprot50 | AF-D3QUP0-F1-MODEL\_V4 | 1.0 | 9.851e-22 | 570 | 0.317 | 343 | 153 | 7 | 1 | 332 | 2 | 274 | DNA inversion product | DNA inversion product | | afdb-uniprot50 | AF-A0A379F8Y3-F1-MODEL\_V4 | 1.0 | 8.531e-17 | 570 | 0.364 | 222 | 116 | 4 | 2 | 207 | 3 | 215 | Phage variable tail fiber protein | Phage variable tail fiber protein | | afdb-uniprot50 | AF-A0A1C3K388-F1-MODEL\_V4 | 1.0 | 4.042e-24 | 569 | 0.252 | 451 | 229 | 25 | 3 | 423 | 2 | 374 | Phage tail fiber protein | Phage tail fiber protein | | afdb-uniprot50 | AF-A0A389MCH5-F1-MODEL\_V4 | 1.0 | 6.683e-19 | 569 | 0.286 | 304 | 116 | 3 | 1 | 304 | 1 | 203 | Uncharacterized protein | Uncharacterized protein | | afdb-uniprot50 | AF-A0A2N7UDN2-F1-MODEL\_V4 | 1.0 | 6.732e-26 | 569 | 0.215 | 595 | 268 | 27 | 1 | 423 | 1 | 568 | Uncharacterized protein | Uncharacterized protein | | afdb-uniprot50 | AF-A0A3G2IDY2-F1-MODEL\_V4 | 1.0 | 1.56e-19 | 569 | 0.269 | 352 | 128 | 4 | 1 | 324 | 2 | 252 | Uncharacterized protein | Uncharacterized protein | | afdb-uniprot50 | AF-A0A3M8C902-F1-MODEL\_V4 | 1.0 | 4.837e-19 | 568 | 0.333 | 285 | 133 | 12 | 1 | 252 | 2 | 262 | Phage tail protein | Phage tail protein | | afdb-uniprot50 | AF-A0A2D0JYZ8-F1-MODEL\_V4 | 1.0 | 1.688e-21 | 568 | 0.288 | 367 | 141 | 8 | 1 | 344 | 1 | 270 | Phage tail fiber protein | Phage tail fiber protein | | afdb-uniprot50 | AF-A0A5U3Y0T7-F1-MODEL\_V4 | 1.0 | 2.936e-20 | 568 | 0.251 | 346 | 136 | 5 | 1 | 324 | 4 | 248 | Phage tail protein | Phage tail protein | | afdb-uniprot50 | AF-A0A7U0C064-F1-MODEL\_V4 | 1.0 | 3.642e-20 | 568 | 0.305 | 344 | 149 | 9 | 1 | 324 | 2 | 275 | Tail fiber protein | Tail fiber protein | | afdb-uniprot50 | AF-A0A2D0IW09-F1-MODEL\_V4 | 1.0 | 5.032e-20 | 568 | 0.31 | 329 | 120 | 5 | 1 | 324 | 2 | 228 | Tail protein | Tail protein | | afdb-uniprot50 | AF-A0A3D9ULH6-F1-MODEL\_V4 | 1.0 | 5.685e-19 | 568 | 0.309 | 323 | 121 | 5 | 1 | 322 | 2 | 223 | Tail fiber-like repeat protein | Tail fiber-like repeat protein | | afdb-uniprot50 | AF-A0A7X7NDN0-F1-MODEL\_V4 | 1.0 | 5.871e-13 | 567 | 0.375 | 128 | 79 | 1 | 4 | 131 | 3 | 129 | Phage tail protein | Phage tail protein | | afdb-uniprot50 | AF-A0A702QMM0-F1-MODEL\_V4 | 1.0 | 1.366e-17 | 566 | 0.23 | 312 | 134 | 5 | 1 | 307 | 1 | 211 | Phage tail protein | Phage tail protein | | afdb-uniprot50 | AF-A0A2A7UY14-F1-MODEL\_V4 | 1.0 | 2.275e-19 | 566 | 0.216 | 434 | 165 | 12 | 1 | 405 | 1 | 288 | Uncharacterized protein | Uncharacterized protein | | afdb-uniprot50 | AF-A0A391N963-F1-MODEL\_V4 | 1.0 | 1.125e-23 | 566 | 0.232 | 465 | 206 | 21 | 1 | 423 | 1 | 356 | Uncharacterized protein | Uncharacterized protein | | afdb-uniprot50 | AF-A0A348HHI3-F1-MODEL\_V4 | 1.0 | 8.875e-18 | 566 | 0.301 | 249 | 129 | 7 | 1 | 219 | 1 | 234 | Phage-related tail fibre protein | Phage-related tail fibre protein | | afdb-uniprot50 | AF-A6UZC4-F1-MODEL\_V4 | 1.0 | 2.392e-23 | 566 | 0.23 | 473 | 239 | 19 | 1 | 423 | 2 | 399 | GpH | GpH | | afdb-uniprot50 | AF-L3C905-F1-MODEL\_V4 | 1.0 | 5.604e-20 | 566 | 0.264 | 318 | 155 | 11 | 1 | 306 | 1 | 251 | Uncharacterized protein | Uncharacterized protein | | afdb-uniprot50 | AF-A0A2D0KDJ9-F1-MODEL\_V4 | 1.0 | 4.056e-20 | 566 | 0.299 | 334 | 125 | 7 | 1 | 326 | 2 | 234 | Tail protein | Tail protein | | afdb-uniprot50 | AF-A0A0F7J9Z5-F1-MODEL\_V4 | 1.0 | 1.623e-20 | 566 | 0.338 | 334 | 140 | 10 | 1 | 323 | 2 | 265 | Variable tail fiber protein | Variable tail fiber protein | | afdb-uniprot50 | AF-A0A829RXG4-F1-MODEL\_V4 | 1.0 | 6.61e-16 | 565 | 0.325 | 212 | 130 | 4 | 1 | 212 | 3 | 201 | Phage tail-collar fiber family protein | Phage tail-collar fiber family protein | | afdb-uniprot50 | AF-A0A1V9IMJ2-F1-MODEL\_V4 | 1.0 | 1.258e-19 | 564 | 0.272 | 305 | 149 | 12 | 1 | 258 | 2 | 280 | Phage tail fiber protein | Phage tail fiber protein | | afdb-uniprot50 | AF-A0A484ZG34-F1-MODEL\_V4 | 1.0 | 1.381e-20 | 564 | 0.253 | 430 | 188 | 12 | 1 | 411 | 2 | 317 | Phage tail fibre repeat | Phage tail fibre repeat | | afdb-uniprot50 | AF-A0A078LT99-F1-MODEL\_V4 | 1.0 | 4.89e-22 | 564 | 0.213 | 450 | 206 | 17 | 4 | 423 | 8 | 339 | Tail fiber protein H | Tail fiber protein H | | afdb-uniprot50 | AF-A0A2I8Q7M1-F1-MODEL\_V4 | 1.0 | 1.494e-22 | 564 | 0.241 | 476 | 216 | 20 | 1 | 423 | 2 | 385 | Phage tail protein | Phage tail protein | | afdb-uniprot50 | AF-A0A7W4RI03-F1-MODEL\_V4 | 1.0 | 8.472e-25 | 564 | 0.245 | 537 | 259 | 27 | 1 | 423 | 1 | 505 | Uncharacterized protein | Uncharacterized protein | | afdb-uniprot50 | AF-A0A840PS60-F1-MODEL\_V4 | 1.0 | 1.935e-19 | 563 | 0.271 | 280 | 160 | 9 | 1 | 248 | 2 | 269 | Phage-related tail fiber protein | Phage-related tail fiber protein | | afdb-uniprot50 | AF-A0A5V1PKK2-F1-MODEL\_V4 | 1.0 | 6e-19 | 563 | 0.282 | 308 | 119 | 4 | 1 | 307 | 4 | 210 | Uncharacterized protein | Uncharacterized protein | | afdb-uniprot50 | AF-A0A2X3E9B4-F1-MODEL\_V4 | 1.0 | 2.187e-18 | 563 | 0.333 | 249 | 126 | 5 | 1 | 223 | 1 | 235 | Uncharacterized protein | Uncharacterized protein | | afdb-uniprot50 | AF-A0A2R8CKT4-F1-MODEL\_V4 | 1.0 | 1.109e-24 | 563 | 0.2 | 545 | 270 | 22 | 1 | 423 | 1 | 501 | Uncharacterized protein | Uncharacterized protein | | afdb-uniprot50 | AF-A0A3D8XED5-F1-MODEL\_V4 | 1.0 | 4.281e-20 | 562 | 0.277 | 342 | 160 | 9 | 1 | 323 | 5 | 278 | Phage tail protein | Phage tail protein | | afdb-uniprot50 | AF-A0A827CPD5-F1-MODEL\_V4 | 1.0 | 8.172e-20 | 561 | 0.277 | 335 | 160 | 10 | 1 | 323 | 5 | 269 | Phage tail protein | Phage tail protein | | afdb-uniprot50 | AF-A0A502FV80-F1-MODEL\_V4 | 1.0 | 1.763e-18 | 559 | 0.338 | 278 | 109 | 7 | 1 | 221 | 2 | 261 | Phage tail protein | Phage tail protein | | afdb-uniprot50 | AF-A0A7W9XSU5-F1-MODEL\_V4 | 1.0 | 7.208e-25 | 559 | 0.232 | 537 | 261 | 21 | 1 | 423 | 3 | 502 | Uncharacterized protein | Uncharacterized protein | | afdb-uniprot50 | AF-A0A4R0E5Y1-F1-MODEL\_V4 | 1.0 | 9.399e-14 | 558 | 0.324 | 157 | 100 | 3 | 1 | 155 | 2 | 154 | Uncharacterized protein | Uncharacterized protein | | afdb-uniprot50 | AF-U1ZYL0-F1-MODEL\_V4 | 1.0 | 1.158e-21 | 558 | 0.229 | 448 | 201 | 17 | 1 | 423 | 1 | 329 | Uncharacterized protein | Uncharacterized protein | | afdb-uniprot50 | AF-W7PY95-F1-MODEL\_V4 | 1.0 | 3.054e-21 | 558 | 0.271 | 364 | 149 | 16 | 1 | 259 | 1 | 353 | Phage tail protein | Phage tail protein | | afdb-uniprot50 | AF-W1J986-F1-MODEL\_V4 | 1.0 | 1.401e-19 | 558 | 0.306 | 330 | 122 | 6 | 1 | 324 | 2 | 230 | Collar domain-containing protein | Collar domain-containing protein | | afdb-uniprot50 | AF-A0A7C8HVY4-F1-MODEL\_V4 | 1.0 | 1.346e-18 | 557 | 0.223 | 371 | 147 | 6 | 1 | 342 | 1 | 259 | Uncharacterized protein | Uncharacterized protein | | afdb-uniprot50 | AF-A0A2I5TL70-F1-MODEL\_V4 | 1.0 | 7.969e-18 | 556 | 0.342 | 248 | 126 | 4 | 1 | 221 | 1 | 238 | Phage tail protein | Phage tail protein | | afdb-uniprot50 | AF-A0A3G7TJE0-F1-MODEL\_V4 | 1.0 | 1.356e-25 | 556 | 0.23 | 508 | 281 | 25 | 2 | 423 | 6 | 489 | Phage tail fiber protein | Phage tail fiber protein | | afdb-uniprot50 | AF-A0A8B4SMY3-F1-MODEL\_V4 | 1.0 | 3.776e-25 | 556 | 0.233 | 562 | 223 | 18 | 1 | 423 | 2 | 494 | Tail fiber protein | Tail fiber protein | | afdb-uniprot50 | AF-A0A828ZHJ0-F1-MODEL\_V4 | 1.0 | 9.102e-20 | 556 | 0.243 | 320 | 153 | 9 | 1 | 257 | 1 | 294 | Tail fiber protein (GpH) | Tail fiber protein (GpH) | | afdb-uniprot50 | AF-A0A4Q7BEF2-F1-MODEL\_V4 | 1.0 | 1.867e-14 | 555 | 0.327 | 171 | 107 | 4 | 1 | 166 | 1 | 168 | Uncharacterized protein | Uncharacterized protein | | afdb-uniprot50 | AF-C1DS14-F1-MODEL\_V4 | 1.0 | 1.032e-14 | 555 | 0.314 | 197 | 116 | 5 | 1 | 178 | 1 | 197 | Phage P2 tail fiber gpH-like protein | Phage P2 tail fiber gpH-like protein | | afdb-uniprot50 | AF-A0A7T0RCA3-F1-MODEL\_V4 | 1.0 | 1.226e-17 | 555 | 0.322 | 242 | 132 | 7 | 2 | 223 | 6 | 235 | Phage tail protein | Phage tail protein | | afdb-uniprot50 | AF-A0A7R7ZA19-F1-MODEL\_V4 | 1.0 | 3.776e-25 | 555 | 0.251 | 489 | 238 | 22 | 2 | 423 | 3 | 430 | Uncharacterized protein | Uncharacterized protein | | afdb-uniprot50 | AF-A0A5U4CIE5-F1-MODEL\_V4 | 1.0 | 5.31e-20 | 555 | 0.251 | 346 | 136 | 5 | 1 | 324 | 4 | 248 | Phage tail protein | Phage tail protein | | afdb-uniprot50 | AF-A5I4A1-F1-MODEL\_V4 | 1.0 | 5.851e-17 | 555 | 0.323 | 229 | 133 | 4 | 1 | 216 | 2 | 221 | Putative tail fiber protein | Putative tail fiber protein | | afdb-uniprot50 | AF-A0A0A8JLH1-F1-MODEL\_V4 | 1.0 | 1.738e-19 | 554 | 0.253 | 300 | 161 | 11 | 1 | 260 | 1 | 277 | Tail fiber protein | Tail fiber protein | | afdb-uniprot50 | AF-M8EFI1-F1-MODEL\_V4 | 1.0 | 3.629e-24 | 554 | 0.245 | 460 | 232 | 21 | 1 | 407 | 2 | 399 | Phage-related tail fiber protein-like protein | Phage-related tail fiber protein-like protein | | afdb-uniprot50 | AF-A0A348D9H5-F1-MODEL\_V4 | 1.0 | 9.367e-18 | 553 | 0.27 | 310 | 123 | 6 | 1 | 308 | 1 | 209 | Uncharacterized protein | Uncharacterized protein | | afdb-uniprot50 | AF-V4NRK3-F1-MODEL\_V4 | 1.0 | 1.634e-12 | 552 | 0.487 | 119 | 61 | 0 | 1 | 119 | 1 | 119 | Uncharacterized protein | Uncharacterized protein | | afdb-uniprot50 | AF-A0A827S3F2-F1-MODEL\_V4 | 1.0 | 2.155e-19 | 552 | 0.248 | 410 | 139 | 10 | 1 | 345 | 2 | 307 | Phage tail protein | Phage tail protein | | afdb-uniprot50 | AF-A0A810U842-F1-MODEL\_V4 | 1.0 | 1.431e-25 | 552 | 0.225 | 598 | 265 | 27 | 1 | 423 | 2 | 576 | Uncharacterized protein | Uncharacterized protein | | afdb-uniprot50 | AF-A0A4S3LTE6-F1-MODEL\_V4 | 1.0 | 1.763e-18 | 551 | 0.28 | 307 | 120 | 3 | 1 | 307 | 2 | 207 | Uncharacterized protein | Uncharacterized protein | | afdb-uniprot50 | AF-A0A7B5Z917-F1-MODEL\_V4 | 1.0 | 4.837e-19 | 551 | 0.274 | 339 | 133 | 7 | 1 | 327 | 1 | 238 | Phage tail protein | Phage tail protein | | afdb-uniprot50 | AF-A0A7X1Y572-F1-MODEL\_V4 | 1.0 | 2.863e-18 | 550 | 0.276 | 318 | 149 | 10 | 2 | 306 | 6 | 255 | Uncharacterized protein | Uncharacterized protein | | afdb-uniprot50 | AF-A0A077NGB1-F1-MODEL\_V4 | 1.0 | 1.085e-18 | 550 | 0.309 | 330 | 121 | 6 | 1 | 324 | 2 | 230 | Uncharacterized protein | Uncharacterized protein | | afdb-uniprot50 | AF-A0A376TTQ8-F1-MODEL\_V4 | 1.0 | 3.075e-13 | 549 | 0.521 | 119 | 57 | 0 | 1 | 119 | 2 | 120 | Variable tail fiber protein | Variable tail fiber protein | | afdb-uniprot50 | AF-A0A5C8XYZ1-F1-MODEL\_V4 | 1.0 | 2.179e-22 | 549 | 0.255 | 474 | 206 | 22 | 1 | 423 | 1 | 378 | Phage tail protein | Phage tail protein | | afdb-uniprot50 | AF-A0A8A7FKW4-F1-MODEL\_V4 | 1.0 | 3.132e-23 | 549 | 0.203 | 581 | 197 | 12 | 1 | 423 | 1 | 473 | Phage tail protein | Phage tail protein | | afdb-uniprot50 | AF-A0A4R6E2J7-F1-MODEL\_V4 | 1.0 | 1.617e-24 | 549 | 0.24 | 557 | 240 | 21 | 1 | 423 | 1 | 508 | Tail fiber-like repeat protein | Tail fiber-like repeat protein | | afdb-uniprot50 | AF-A0A427Q396-F1-MODEL\_V4 | 1.0 | 2.489e-24 | 549 | 0.188 | 573 | 271 | 25 | 2 | 423 | 6 | 535 | Uncharacterized protein | Uncharacterized protein | | afdb-uniprot50 | AF-A0A854BC76-F1-MODEL\_V4 | 1.0 | 2.203e-25 | 548 | 0.229 | 496 | 284 | 22 | 4 | 423 | 8 | 481 | Phage tail protein | Phage tail protein | | afdb-uniprot50 | AF-A0A747EHS2-F1-MODEL\_V4 | 1.0 | 8.719e-23 | 548 | 0.177 | 751 | 175 | 15 | 1 | 423 | 1 | 636 | Phage tail protein | Phage tail protein | | afdb-uniprot50 | AF-A0A6C8RYR2-F1-MODEL\_V4 | 1.0 | 2.57e-18 | 547 | 0.274 | 393 | 127 | 10 | 1 | 329 | 2 | 300 | Phage tail protein | Phage tail protein | | afdb-uniprot50 | AF-A0A2D0LF95-F1-MODEL\_V4 | 1.0 | 1.956e-22 | 547 | 0.197 | 604 | 196 | 13 | 1 | 423 | 2 | 497 | Tail protein | Tail protein | | afdb-uniprot50 | AF-A0A1V2GLB1-F1-MODEL\_V4 | 1.0 | 1.86e-18 | 547 | 0.276 | 325 | 130 | 6 | 1 | 323 | 1 | 222 | Phage tail protein | Phage tail protein | | afdb-uniprot50 | AF-A0A2J9EBQ6-F1-MODEL\_V4 | 1.0 | 5.292e-24 | 546 | 0.243 | 509 | 222 | 15 | 1 | 421 | 2 | 435 | Phage tail protein | Phage tail protein | | afdb-uniprot50 | AF-A0A752D550-F1-MODEL\_V4 | 1.0 | 2.148e-23 | 546 | 0.225 | 595 | 240 | 24 | 1 | 423 | 1 | 546 | Phage tail protein | Phage tail protein | | afdb-uniprot50 | AF-A0A2X3JIY7-F1-MODEL\_V4 | 1.0 | 1.921e-12 | 545 | 0.376 | 130 | 76 | 2 | 3 | 131 | 7 | 132 | Phage protein gpH | Phage protein gpH | | afdb-uniprot50 | AF-A0A377AV74-F1-MODEL\_V4 | 1.0 | 4.502e-24 | 545 | 0.251 | 525 | 192 | 22 | 1 | 423 | 1 | 426 | Phage tail collar domain-containing protein | Phage tail collar domain-containing protein | | afdb-uniprot50 | AF-A0A659PJA5-F1-MODEL\_V4 | 1.0 | 4.82e-23 | 545 | 0.215 | 543 | 215 | 22 | 1 | 423 | 1 | 452 | Phage tail protein | Phage tail protein | | afdb-uniprot50 | AF-A0A6A4RC06-F1-MODEL\_V4 | 1.0 | 2.627e-24 | 545 | 0.198 | 573 | 269 | 27 | 1 | 423 | 1 | 532 | Uncharacterized protein | Uncharacterized protein | | afdb-uniprot50 | AF-A0A2V1Y908-F1-MODEL\_V4 | 1.0 | 6.31e-23 | 543 | 0.22 | 477 | 229 | 17 | 4 | 423 | 8 | 398 | Phage tail-collar fibre protein | Phage tail-collar fibre protein | | afdb-uniprot50 | AF-A0A7W5DMQ9-F1-MODEL\_V4 | 1.0 | 1.956e-22 | 543 | 0.235 | 502 | 211 | 25 | 1 | 423 | 1 | 408 | Uncharacterized protein | Uncharacterized protein | | afdb-uniprot50 | AF-A0A4U8YH51-F1-MODEL\_V4 | 1.0 | 2.926e-24 | 542 | 0.207 | 468 | 246 | 17 | 1 | 423 | 1 | 388 | Phage tail fibre protein | Phage tail fibre protein | | afdb-uniprot50 | AF-A0A6N3HNW3-F1-MODEL\_V4 | 1.0 | 3.054e-21 | 542 | 0.173 | 761 | 213 | 22 | 1 | 423 | 1 | 683 | Phage tail fibre repeat protein | Phage tail fibre repeat protein | | afdb-uniprot50 | AF-A0A3T2UQY7-F1-MODEL\_V4 | 1.0 | 1.623e-20 | 542 | 0.28 | 364 | 179 | 16 | 1 | 322 | 1 | 323 | Phage tail protein | Phage tail protein | | afdb-uniprot50 | AF-A0A328TYP4-F1-MODEL\_V4 | 1.0 | 1.664e-22 | 541 | 0.235 | 472 | 220 | 17 | 1 | 421 | 2 | 383 | Phage tail-collar fiber family protein | Phage tail-collar fiber family protein | | afdb-uniprot50 | AF-A0A4R0FQS3-F1-MODEL\_V4 | 1.0 | 4.042e-24 | 541 | 0.22 | 485 | 259 | 25 | 1 | 423 | 2 | 429 | Uncharacterized protein | Uncharacterized protein | | afdb-uniprot50 | AF-A0A1I4ZMZ1-F1-MODEL\_V4 | 1.0 | 4.684e-25 | 541 | 0.231 | 510 | 246 | 22 | 4 | 423 | 8 | 461 | Phage tail-collar fibre protein | Phage tail-collar fibre protein | | afdb-uniprot50 | AF-A0A0H3MMX0-F1-MODEL\_V4 | 1.0 | 4.96e-21 | 541 | 0.288 | 357 | 200 | 12 | 1 | 324 | 1 | 336 | Putative side tail phage protein | Putative side tail phage protein | | afdb-uniprot50 | AF-A0A8B3UK72-F1-MODEL\_V4 | 1.0 | 2.155e-19 | 541 | 0.286 | 335 | 162 | 8 | 1 | 323 | 2 | 271 | Tail Fiber protein | Tail Fiber protein | | afdb-uniprot50 | AF-B1LMZ0-F1-MODEL\_V4 | 1.0 | 2.435e-18 | 541 | 0.276 | 325 | 130 | 6 | 1 | 323 | 1 | 222 | Putative phage tail fiber protein | Putative phage tail fiber protein | | afdb-uniprot50 | AF-A0A7Z8BNV6-F1-MODEL\_V4 | 1.0 | 3.551e-18 | 541 | 0.274 | 324 | 131 | 5 | 1 | 323 | 2 | 222 | Phage tail protein | Phage tail protein | | afdb-uniprot50 | AF-A0A5K1MSE4-F1-MODEL\_V4 | 1.0 | 3.615e-13 | 541 | 0.352 | 156 | 98 | 1 | 1 | 156 | 2 | 154 | Uncharacterized protein | Uncharacterized protein | | afdb-uniprot50 | AF-A0A5P2SMC4-F1-MODEL\_V4 | 1.0 | 4.751e-24 | 540 | 0.278 | 478 | 251 | 24 | 1 | 423 | 1 | 439 | Phage tail protein | Phage tail protein | | afdb-uniprot50 | AF-A0A742CYR3-F1-MODEL\_V4 | 1.0 | 6.565e-24 | 540 | 0.239 | 509 | 227 | 24 | 1 | 419 | 1 | 439 | Phage tail protein | Phage tail protein | | afdb-uniprot50 | AF-A0A5D4YFX8-F1-MODEL\_V4 | 1.0 | 5.368e-23 | 540 | 0.2 | 629 | 240 | 22 | 1 | 423 | 1 | 572 | Phage tail protein | Phage tail protein | | afdb-uniprot50 | AF-A0A0L6FWL7-F1-MODEL\_V4 | 1.0 | 2.627e-24 | 540 | 0.181 | 715 | 280 | 27 | 1 | 423 | 5 | 705 | Collar domain-containing protein | Collar domain-containing protein | | afdb-uniprot50 | AF-A0A398UJ84-F1-MODEL\_V4 | 1.0 | 2.713e-18 | 539 | 0.262 | 324 | 135 | 5 | 1 | 323 | 5 | 225 | Phage tail protein | Phage tail protein | | afdb-uniprot50 | AF-A0A5W0I1C1-F1-MODEL\_V4 | 1.0 | 1.881e-21 | 539 | 0.281 | 387 | 183 | 12 | 1 | 333 | 1 | 346 | Phage tail protein | Phage tail protein | | afdb-uniprot50 | AF-A0A6F9WXH3-F1-MODEL\_V4 | 1.0 | 2.479e-13 | 539 | 0.326 | 156 | 105 | 0 | 1 | 156 | 2 | 157 | Uncharacterized protein | Uncharacterized protein | | afdb-uniprot50 | AF-A0A399Q0B5-F1-MODEL\_V4 | 1.0 | 3.489e-23 | 539 | 0.178 | 548 | 278 | 29 | 4 | 423 | 8 | 511 | Collar domain-containing protein | Collar domain-containing protein | | afdb-uniprot50 | AF-A0A2S4QLD3-F1-MODEL\_V4 | 1.0 | 1.827e-23 | 539 | 0.233 | 607 | 258 | 32 | 1 | 423 | 1 | 583 | Uncharacterized protein | Uncharacterized protein | | afdb-uniprot50 | AF-A0A080K6T3-F1-MODEL\_V4 | 1.0 | 2.654e-12 | 538 | 0.491 | 116 | 59 | 0 | 1 | 116 | 2 | 117 | Tail fiber protein | Tail fiber protein | | afdb-uniprot50 | AF-A0A7R7HR43-F1-MODEL\_V4 | 1.0 | 2.148e-23 | 538 | 0.216 | 577 | 273 | 24 | 2 | 423 | 6 | 558 | Uncharacterized protein | Uncharacterized protein | | afdb-uniprot50 | AF-D5EF94-F1-MODEL\_V4 | 1.0 | 6.242e-20 | 538 | 0.297 | 306 | 166 | 10 | 1 | 258 | 2 | 306 | Phage-related tail fibre protein-like protein | Phage-related tail fibre protein-like protein | | afdb-uniprot50 | AF-A0A1E7WJE2-F1-MODEL\_V4 | 1.0 | 7.882e-15 | 538 | 0.301 | 212 | 126 | 2 | 1 | 190 | 4 | 215 | SGNH\_hydro domain-containing protein | SGNH\_hydro domain-containing protein | | afdb-uniprot50 | AF-A0A376VKB4-F1-MODEL\_V4 | 1.0 | 5.032e-20 | 537 | 0.286 | 359 | 176 | 13 | 1 | 324 | 1 | 314 | Putative tail fiber protein (GpH) | Putative tail fiber protein (GpH) | | afdb-uniprot50 | AF-A0A837JCH1-F1-MODEL\_V4 | 1.0 | 5.329e-16 | 537 | 0.267 | 232 | 151 | 5 | 1 | 223 | 1 | 222 | Uncharacterized protein | Uncharacterized protein | | afdb-uniprot50 | AF-A0A701UXD7-F1-MODEL\_V4 | 1.0 | 2.561e-22 | 537 | 0.254 | 463 | 223 | 21 | 1 | 423 | 1 | 381 | Phage tail protein | Phage tail protein | | afdb-uniprot50 | AF-A0A6M0YIJ7-F1-MODEL\_V4 | 1.0 | 1.6e-21 | 537 | 0.209 | 491 | 211 | 16 | 2 | 422 | 3 | 386 | Phage tail protein | Phage tail protein | | afdb-uniprot50 | AF-A0A2V4LES5-F1-MODEL\_V4 | 1.0 | 1.732e-23 | 537 | 0.226 | 494 | 259 | 28 | 1 | 423 | 3 | 444 | Uncharacterized protein | Uncharacterized protein | | afdb-uniprot50 | AF-A0A8A2FEP4-F1-MODEL\_V4 | 1.0 | 3.59e-21 | 537 | 0.244 | 413 | 190 | 17 | 1 | 361 | 1 | 343 | Phage tail protein | Phage tail protein | | afdb-uniprot50 | AF-A0A827VE52-F1-MODEL\_V4 | 1.0 | 2.782e-20 | 537 | 0.27 | 366 | 183 | 13 | 1 | 333 | 5 | 319 | Phage tail protein | Phage tail protein | | afdb-uniprot50 | AF-A0A806Z174-F1-MODEL\_V4 | 1.0 | 3.629e-24 | 537 | 0.195 | 695 | 241 | 23 | 1 | 423 | 1 | 649 | Uncharacterized protein | Uncharacterized protein | | afdb-uniprot50 | AF-R9K2R2-F1-MODEL\_V4 | 1.0 | 2.283e-15 | 536 | 0.242 | 219 | 129 | 5 | 1 | 186 | 1 | 215 | Uncharacterized protein | Uncharacterized protein | | afdb-uniprot50 | AF-A0A5Y3PXB4-F1-MODEL\_V4 | 1.0 | 2.978e-19 | 536 | 0.25 | 340 | 134 | 7 | 1 | 334 | 4 | 228 | Phage tail protein | Phage tail protein | | afdb-uniprot50 | AF-A0A7B6C8V7-F1-MODEL\_V4 | 1.0 | 4.056e-20 | 536 | 0.271 | 364 | 187 | 10 | 1 | 332 | 5 | 322 | Phage tail protein | Phage tail protein | | afdb-uniprot50 | AF-A0A6C8Y9W3-F1-MODEL\_V4 | 1.0 | 1.025e-22 | 535 | 0.245 | 469 | 211 | 18 | 1 | 423 | 1 | 372 | Uncharacterized protein | Uncharacterized protein | | afdb-uniprot50 | AF-A0A1E3XL37-F1-MODEL\_V4 | 1.0 | 5.604e-20 | 535 | 0.253 | 332 | 146 | 7 | 1 | 322 | 1 | 240 | Uncharacterized protein | Uncharacterized protein | | afdb-uniprot50 | AF-A0A349XXC0-F1-MODEL\_V4 | 1.0 | 1.565e-15 | 534 | 0.292 | 219 | 116 | 4 | 1 | 183 | 1 | 216 | Phage tail protein | Phage tail protein | | afdb-uniprot50 | AF-G9Z0C3-F1-MODEL\_V4 | 1.0 | 3.212e-25 | 534 | 0.255 | 563 | 257 | 24 | 1 | 423 | 2 | 542 | Uncharacterized protein | Uncharacterized protein | | afdb-uniprot50 | AF-A0A375ABE7-F1-MODEL\_V4 | 1.0 | 3.317e-19 | 533 | 0.281 | 316 | 145 | 9 | 1 | 251 | 2 | 300 | Phage tail fiber protein | Phage tail fiber protein | | afdb-uniprot50 | AF-A0A444RCG1-F1-MODEL\_V4 | 1.0 | 3.748e-18 | 532 | 0.266 | 394 | 131 | 9 | 1 | 330 | 2 | 301 | Phage tail protein | Phage tail protein | | afdb-uniprot50 | AF-A0A3S4G527-F1-MODEL\_V4 | 1.0 | 2.636e-20 | 532 | 0.292 | 359 | 172 | 14 | 1 | 324 | 1 | 312 | Phage variable tail-fiber protein | Phage variable tail-fiber protein | | afdb-uniprot50 | AF-A0A5U3EXM5-F1-MODEL\_V4 | 1.0 | 9.573e-24 | 531 | 0.232 | 494 | 231 | 23 | 1 | 423 | 2 | 418 | Uncharacterized protein | Uncharacterized protein | | afdb-uniprot50 | AF-A0A2C6DJ56-F1-MODEL\_V4 | 1.0 | 1.538e-20 | 531 | 0.266 | 394 | 155 | 12 | 1 | 377 | 2 | 278 | Uncharacterized protein | Uncharacterized protein | | afdb-uniprot50 | AF-A0A370S8A4-F1-MODEL\_V4 | 1.0 | 1.361e-21 | 531 | 0.245 | 464 | 213 | 18 | 1 | 423 | 1 | 368 | Tail fiber-like repeat protein | Tail fiber-like repeat protein | | afdb-uniprot50 | AF-W2CTP2-F1-MODEL\_V4 | 1.0 | 6.539e-13 | 530 | 0.52 | 125 | 60 | 0 | 27 | 151 | 2 | 126 | Phage tail protein | Phage tail protein | | afdb-uniprot50 | AF-A0A064DL20-F1-MODEL\_V4 | 1.0 | 6.565e-24 | 530 | 0.269 | 501 | 218 | 22 | 1 | 423 | 2 | 432 | Uncharacterized protein | Uncharacterized protein | | afdb-uniprot50 | AF-A0A5S4U4U6-F1-MODEL\_V4 | 1.0 | 2.926e-24 | 529 | 0.258 | 495 | 218 | 24 | 1 | 423 | 2 | 419 | Phage tail protein | Phage tail protein | | afdb-uniprot50 | AF-A0A1V2G5C9-F1-MODEL\_V4 | 1.0 | 5.292e-24 | 529 | 0.244 | 528 | 218 | 24 | 1 | 423 | 1 | 452 | Phage tail protein | Phage tail protein | | afdb-uniprot50 | AF-A0A7Y9B7N8-F1-MODEL\_V4 | 1.0 | 1.376e-24 | 529 | 0.245 | 600 | 239 | 25 | 1 | 423 | 2 | 564 | Phage tail protein | Phage tail protein | | afdb-uniprot50 | AF-A0A626YJZ8-F1-MODEL\_V4 | 1.0 | 2.401e-19 | 529 | 0.245 | 346 | 138 | 7 | 1 | 324 | 4 | 248 | Phage tail protein | Phage tail protein | | afdb-uniprot50 | AF-A0A7U1Z2W3-F1-MODEL\_V4 | 1.0 | 2.187e-18 | 529 | 0.265 | 339 | 131 | 6 | 1 | 326 | 1 | 234 | Phage tail protein | Phage tail protein | | afdb-uniprot50 | AF-I3DCB6-F1-MODEL\_V4 | 1.0 | 1.623e-20 | 528 | 0.245 | 359 | 172 | 12 | 1 | 322 | 2 | 298 | Phage tail fiber repeat protein | Phage tail fiber repeat protein | | afdb-uniprot50 | AF-A0A0G9KCC4-F1-MODEL\_V4 | 1.0 | 8.531e-17 | 528 | 0.3 | 236 | 140 | 5 | 1 | 223 | 1 | 224 | Uncharacterized protein | Uncharacterized protein | | afdb-uniprot50 | AF-A0A5Y2KP10-F1-MODEL\_V4 | 1.0 | 9.711e-23 | 528 | 0.201 | 581 | 233 | 21 | 1 | 423 | 1 | 508 | Phage tail protein | Phage tail protein | | afdb-uniprot50 | AF-A0A1Q9QWT4-F1-MODEL\_V4 | 1.0 | 1.641e-23 | 528 | 0.184 | 574 | 278 | 22 | 4 | 423 | 8 | 545 | Uncharacterized protein | Uncharacterized protein | | afdb-uniprot50 | AF-A0A4D6XN95-F1-MODEL\_V4 | 1.0 | 4.82e-23 | 527 | 0.216 | 495 | 245 | 23 | 1 | 423 | 5 | 428 | Uncharacterized protein | Uncharacterized protein | | afdb-uniprot50 | AF-A0A4Y9GYI2-F1-MODEL\_V4 | 1.0 | 5.104e-19 | 527 | 0.232 | 331 | 153 | 7 | 1 | 322 | 2 | 240 | Uncharacterized protein | Uncharacterized protein | | afdb-uniprot50 | AF-A0A2G1CTX9-F1-MODEL\_V4 | 1.0 | 9.573e-24 | 526 | 0.237 | 479 | 229 | 20 | 4 | 423 | 3 | 404 | Uncharacterized protein | Uncharacterized protein | | afdb-uniprot50 | AF-A0A6G4BMA1-F1-MODEL\_V4 | 1.0 | 4.82e-23 | 526 | 0.232 | 517 | 229 | 22 | 1 | 423 | 2 | 444 | Phage tail protein | Phage tail protein | | afdb-uniprot50 | AF-A0A062L6U3-F1-MODEL\_V4 | 1.0 | 5.271e-13 | 526 | 0.354 | 161 | 103 | 1 | 2 | 162 | 3 | 162 | Phage tail-collar fiber family protein | Phage tail-collar fiber family protein | | afdb-uniprot50 | AF-D1Y2C2-F1-MODEL\_V4 | 1.0 | 2.148e-23 | 525 | 0.207 | 521 | 265 | 18 | 1 | 423 | 1 | 471 | Uncharacterized protein | Uncharacterized protein | | afdb-uniprot50 | AF-A0A827W3S7-F1-MODEL\_V4 | 1.0 | 4.281e-20 | 524 | 0.281 | 370 | 175 | 12 | 1 | 335 | 5 | 318 | Uncharacterized protein | Uncharacterized protein | | afdb-uniprot50 | AF-G5QJV8-F1-MODEL\_V4 | 1.0 | 4.16e-22 | 524 | 0.214 | 481 | 212 | 15 | 1 | 423 | 4 | 376 | Phage tail fiber protein | Phage tail fiber protein | | afdb-uniprot50 | AF-A0A7H9FY19-F1-MODEL\_V4 | 1.0 | 3.489e-23 | 524 | 0.263 | 485 | 232 | 25 | 1 | 422 | 1 | 423 | Phage tail protein | Phage tail protein | | afdb-uniprot50 | AF-A0A1I4WBK0-F1-MODEL\_V4 | 1.0 | 3.885e-23 | 524 | 0.198 | 646 | 256 | 23 | 2 | 423 | 6 | 613 | Phage tail-collar fibre protein | Phage tail-collar fibre protein | | afdb-uniprot50 | AF-A0A807Z4S1-F1-MODEL\_V4 | 1.0 | 1.317e-12 | 523 | 0.314 | 159 | 101 | 3 | 1 | 152 | 3 | 160 | Uncharacterized protein | Uncharacterized protein | | afdb-uniprot50 | AF-R7ZJ78-F1-MODEL\_V4 | 1.0 | 6.332e-19 | 523 | 0.276 | 322 | 148 | 12 | 1 | 258 | 1 | 301 | Phage-related tail fiber protein-like protein | Phage-related tail fiber protein-like protein | | afdb-uniprot50 | AF-A0A3M5NCX5-F1-MODEL\_V4 | 1.0 | 1.346e-18 | 523 | 0.275 | 312 | 155 | 11 | 2 | 247 | 6 | 312 | Uncharacterized protein | Uncharacterized protein | | afdb-uniprot50 | AF-A0A5E1A2X0-F1-MODEL\_V4 | 1.0 | 1.003e-16 | 523 | 0.316 | 250 | 130 | 12 | 1 | 223 | 2 | 237 | Phage tail collar domain-containing protein | Phage tail collar domain-containing protein | | afdb-uniprot50 | AF-A0A4Z0B2H6-F1-MODEL\_V4 | 1.0 | 4.82e-23 | 523 | 0.213 | 530 | 261 | 24 | 2 | 423 | 6 | 487 | Uncharacterized protein | Uncharacterized protein | | afdb-uniprot50 | AF-A4P0H3-F1-MODEL\_V4 | 1.0 | 3.143e-19 | 523 | 0.234 | 332 | 152 | 5 | 1 | 322 | 2 | 241 | Probable tail fiber protein | Probable tail fiber protein | | afdb-uniprot50 | AF-A0A1B7IQU1-F1-MODEL\_V4 | 1.0 | 1.294e-17 | 523 | 0.281 | 320 | 124 | 7 | 1 | 316 | 1 | 218 | Phage tail fiber protein | Phage tail fiber protein | | afdb-uniprot50 | AF-A0A1G5B425-F1-MODEL\_V4 | 1.0 | 2.102e-17 | 522 | 0.281 | 270 | 144 | 12 | 1 | 244 | 1 | 246 | Phage Tail Collar Domain | Phage Tail Collar Domain | | afdb-uniprot50 | AF-A0A7S4LVA4-F1-MODEL\_V4 | 1.0 | 1.583e-18 | 522 | 0.267 | 344 | 133 | 3 | 1 | 326 | 1 | 243 | Uncharacterized protein | Uncharacterized protein | | afdb-uniprot50 | AF-G5S7G0-F1-MODEL\_V4 | 1.0 | 7.053e-19 | 522 | 0.236 | 346 | 141 | 7 | 1 | 324 | 4 | 248 | Phage tail fiber protein | Phage tail fiber protein | | afdb-uniprot50 | AF-A0A1B8TDC2-F1-MODEL\_V4 | 1.0 | 1.025e-22 | 521 | 0.241 | 564 | 239 | 29 | 1 | 423 | 1 | 516 | Phage tail protein | Phage tail protein | | afdb-uniprot50 | AF-A0A7I9ALZ6-F1-MODEL\_V4 | 1.0 | 2.148e-23 | 520 | 0.246 | 523 | 243 | 23 | 1 | 421 | 1 | 474 | Putative tail fiber protein (GpH),Phage tail fibre repeat | Putative tail fiber protein (GpH),Phage tail fibre repeat | | afdb-uniprot50 | AF-A0A7H8UZA3-F1-MODEL\_V4 | 1.0 | 2.042e-19 | 520 | 0.239 | 334 | 147 | 9 | 1 | 322 | 3 | 241 | Phage tail protein | Phage tail protein | | afdb-uniprot50 | AF-A0A837CRU8-F1-MODEL\_V4 | 1.0 | 7.388e-12 | 519 | 0.504 | 113 | 55 | 1 | 1 | 112 | 1 | 113 | Phage tail fiber protein | Phage tail fiber protein | | afdb-uniprot50 | AF-A4N472-F1-MODEL\_V4 | 1.0 | 2.978e-19 | 519 | 0.24 | 332 | 150 | 8 | 1 | 322 | 2 | 241 | Probable tail fiber protein | Probable tail fiber protein | | afdb-uniprot50 | AF-A0A6X8RGM9-F1-MODEL\_V4 | 1.0 | 2.333e-21 | 519 | 0.179 | 715 | 214 | 20 | 1 | 423 | 1 | 634 | Phage tail protein | Phage tail protein | | afdb-uniprot50 | AF-A0A5S3V1C2-F1-MODEL\_V4 | 1.0 | 5.705e-15 | 519 | 0.259 | 212 | 139 | 5 | 2 | 203 | 4 | 207 | Uncharacterized protein | Uncharacterized protein | | afdb-uniprot50 | AF-A0A2G4UEQ4-F1-MODEL\_V4 | 1.0 | 6.683e-19 | 518 | 0.228 | 438 | 184 | 12 | 1 | 413 | 2 | 310 | Phage tail protein | Phage tail protein | | afdb-uniprot50 | AF-A0A4S3Y564-F1-MODEL\_V4 | 1.0 | 1.01e-23 | 518 | 0.212 | 570 | 260 | 29 | 2 | 423 | 6 | 534 | Phage tail protein | Phage tail protein | | afdb-uniprot50 | AF-A0A3D1C6R7-F1-MODEL\_V4 | 1.0 | 2.358e-24 | 518 | 0.246 | 572 | 248 | 23 | 1 | 423 | 2 | 539 | Phage tail protein | Phage tail protein | | afdb-uniprot50 | AF-A0A1I5L005-F1-MODEL\_V4 | 1.0 | 1.226e-17 | 517 | 0.285 | 291 | 145 | 10 | 1 | 246 | 1 | 273 | Phage tail-collar fibre protein | Phage tail-collar fibre protein | | afdb-uniprot50 | AF-A0A3R9RZV6-F1-MODEL\_V4 | 1.0 | 1.406e-15 | 517 | 0.271 | 236 | 128 | 6 | 1 | 194 | 1 | 234 | Uncharacterized protein | Uncharacterized protein | | afdb-uniprot50 | AF-A0A5S4U6Y9-F1-MODEL\_V4 | 1.0 | 4.837e-19 | 516 | 0.246 | 328 | 167 | 9 | 1 | 255 | 2 | 322 | Phage tail protein | Phage tail protein | | afdb-uniprot50 | AF-A0A0J8YRQ6-F1-MODEL\_V4 | 1.0 | 1.082e-22 | 516 | 0.248 | 500 | 200 | 22 | 1 | 423 | 2 | 402 | Uncharacterized protein | Uncharacterized protein | | afdb-uniprot50 | AF-A0A5E4SRD1-F1-MODEL\_V4 | 1.0 | 6.087e-18 | 516 | 0.245 | 318 | 155 | 10 | 1 | 253 | 2 | 299 | Uncharacterized protein | Uncharacterized protein | | afdb-uniprot50 | AF-A0A5Z2TND1-F1-MODEL\_V4 | 1.0 | 1.025e-22 | 516 | 0.232 | 534 | 247 | 25 | 1 | 422 | 1 | 483 | Phage tail protein | Phage tail protein | | afdb-uniprot50 | AF-A0A7Y8F9X6-F1-MODEL\_V4 | 1.0 | 2.148e-23 | 516 | 0.173 | 653 | 293 | 27 | 2 | 423 | 6 | 642 | Phage tail protein | Phage tail protein | | afdb-uniprot50 | AF-A0A4S2QLK6-F1-MODEL\_V4 | 1.0 | 7.053e-19 | 516 | 0.265 | 331 | 142 | 7 | 1 | 322 | 2 | 240 | Uncharacterized protein | Uncharacterized protein | | afdb-uniprot50 | AF-A0A4R8JQJ0-F1-MODEL\_V4 | 1.0 | 2.782e-20 | 515 | 0.246 | 438 | 217 | 14 | 1 | 417 | 2 | 347 | Tail fiber-like repeat protein | Tail fiber-like repeat protein | | afdb-uniprot50 | AF-A0A6N4NZG6-F1-MODEL\_V4 | 1.0 | 2.607e-17 | 515 | 0.276 | 325 | 127 | 8 | 1 | 323 | 1 | 219 | Phage tail protein | Phage tail protein | | afdb-uniprot50 | AF-A0A7Y1CCL3-F1-MODEL\_V4 | 1.0 | 3.365e-18 | 514 | 0.263 | 296 | 147 | 8 | 2 | 235 | 6 | 292 | Phage tail protein | Phage tail protein | | afdb-uniprot50 | AF-A0A5M9IW52-F1-MODEL\_V4 | 1.0 | 4.82e-23 | 514 | 0.187 | 539 | 279 | 25 | 1 | 423 | 1 | 496 | Collar domain-containing protein | Collar domain-containing protein | | afdb-uniprot50 | AF-A0A336NWA0-F1-MODEL\_V4 | 1.0 | 7.053e-19 | 514 | 0.29 | 351 | 126 | 5 | 1 | 324 | 2 | 256 | Phage tail fiber protein | Phage tail fiber protein | | afdb-uniprot50 | AF-A0A403NCR6-F1-MODEL\_V4 | 1.0 | 1.162e-17 | 514 | 0.242 | 346 | 139 | 5 | 1 | 324 | 2 | 246 | Phage tail protein | Phage tail protein | | afdb-uniprot50 | AF-A0A625UNB3-F1-MODEL\_V4 | 1.0 | 1.24e-20 | 513 | 0.241 | 455 | 221 | 16 | 1 | 411 | 2 | 376 | Phage tail protein | Phage tail protein | | afdb-uniprot50 | AF-A0A2V4KQ48-F1-MODEL\_V4 | 1.0 | 6.242e-20 | 513 | 0.238 | 377 | 174 | 11 | 1 | 272 | 5 | 373 | Uncharacterized protein | Uncharacterized protein | | afdb-uniprot50 | AF-A0A701ZGL9-F1-MODEL\_V4 | 1.0 | 1.158e-21 | 512 | 0.24 | 475 | 208 | 19 | 1 | 413 | 1 | 384 | Phage tail protein | Phage tail protein | | afdb-uniprot50 | AF-A0A1X3I4D9-F1-MODEL\_V4 | 1.0 | 1.985e-21 | 511 | 0.254 | 500 | 181 | 18 | 1 | 417 | 1 | 391 | Putative tail fiber protein (GpH) | Putative tail fiber protein (GpH) | | afdb-uniprot50 | AF-A0A2U0W405-F1-MODEL\_V4 | 1.0 | 2.148e-23 | 511 | 0.218 | 513 | 270 | 24 | 1 | 423 | 1 | 472 | Tail collar domain | Tail collar domain | | afdb-uniprot50 | AF-F7U608-F1-MODEL\_V4 | 1.0 | 2.832e-15 | 511 | 0.289 | 221 | 118 | 4 | 1 | 185 | 1 | 218 | Phage-related tail fibre protein-like protein | Phage-related tail fibre protein-like protein | | afdb-uniprot50 | AF-A0A829QY27-F1-MODEL\_V4 | 1.0 | 7.417e-23 | 511 | 0.211 | 620 | 254 | 29 | 4 | 423 | 3 | 587 | Tail fiber protein | Tail fiber protein | | afdb-uniprot50 | AF-A0A4Q2B3R1-F1-MODEL\_V4 | 1.0 | 3.377e-14 | 510 | 0.314 | 197 | 116 | 5 | 2 | 194 | 11 | 192 | Uncharacterized protein | Uncharacterized protein | | afdb-uniprot50 | AF-A0A826UY05-F1-MODEL\_V4 | 1.0 | 2.065e-22 | 510 | 0.224 | 494 | 245 | 24 | 1 | 421 | 2 | 430 | Phage tail protein | Phage tail protein | | afdb-uniprot50 | AF-A0A1N6MS83-F1-MODEL\_V4 | 1.0 | 7.941e-22 | 510 | 0.247 | 452 | 179 | 15 | 1 | 412 | 1 | 331 | Uncharacterized protein | Uncharacterized protein | | afdb-uniprot50 | AF-A0A1G4ZBJ6-F1-MODEL\_V4 | 1.0 | 7.312e-24 | 510 | 0.189 | 627 | 256 | 29 | 1 | 423 | 2 | 580 | Phage T4 tail fibre | Phage T4 tail fibre | | afdb-uniprot50 | AF-A0A722H4T9-F1-MODEL\_V4 | 1.0 | 1.014e-19 | 510 | 0.255 | 418 | 183 | 17 | 1 | 362 | 2 | 347 | Phage tail protein | Phage tail protein | | afdb-uniprot50 | AF-B3Y0S1-F1-MODEL\_V4 | 1.0 | 6.424e-18 | 509 | 0.271 | 324 | 132 | 7 | 1 | 323 | 5 | 225 | Putative phage tail fiber protein | Putative phage tail fiber protein | | afdb-uniprot50 | AF-A0A5C7CK81-F1-MODEL\_V4 | 1.0 | 4.16e-22 | 509 | 0.265 | 449 | 200 | 19 | 1 | 405 | 2 | 364 | Uncharacterized protein | Uncharacterized protein | | afdb-uniprot50 | AF-A0A1I3XGI0-F1-MODEL\_V4 | 1.0 | 3.27e-20 | 508 | 0.234 | 473 | 168 | 14 | 1 | 417 | 2 | 336 | Phage tail fibre repeat-containing protein | Phage tail fibre repeat-containing protein | | afdb-uniprot50 | AF-A0A285Z1M8-F1-MODEL\_V4 | 1.0 | 4.328e-23 | 508 | 0.217 | 478 | 239 | 19 | 1 | 423 | 1 | 398 | Phage-related tail fibre protein | Phage-related tail fibre protein | | afdb-uniprot50 | AF-A0A1J0EGB1-F1-MODEL\_V4 | 1.0 | 1.416e-22 | 508 | 0.221 | 560 | 254 | 27 | 1 | 423 | 3 | 517 | Phage tail protein | Phage tail protein | | afdb-uniprot50 | AF-A0A778CR53-F1-MODEL\_V4 | 1.0 | 9.851e-22 | 508 | 0.237 | 485 | 219 | 20 | 1 | 417 | 2 | 403 | Phage tail protein | Phage tail protein | | afdb-uniprot50 | AF-A0A2X2TYC3-F1-MODEL\_V4 | 1.0 | 1.605e-17 | 507 | 0.241 | 307 | 137 | 5 | 1 | 307 | 1 | 211 | Phage tail fiber protein | Phage tail fiber protein | | afdb-uniprot50 | AF-A0A6G8F290-F1-MODEL\_V4 | 1.0 | 3.188e-18 | 507 | 0.237 | 291 | 153 | 8 | 1 | 246 | 7 | 273 | Tail protein | Tail protein | | afdb-uniprot50 | AF-A0A7T9YBY6-F1-MODEL\_V4 | 1.0 | 3.353e-22 | 507 | 0.27 | 458 | 170 | 14 | 1 | 417 | 1 | 335 | Phage tail protein | Phage tail protein | | afdb-uniprot50 | AF-W1J3T6-F1-MODEL\_V4 | 1.0 | 1.07e-19 | 506 | 0.282 | 390 | 154 | 8 | 1 | 377 | 2 | 278 | Uncharacterized protein | Uncharacterized protein | | afdb-uniprot50 | AF-A0A2V5BHI2-F1-MODEL\_V4 | 1.0 | 1.757e-22 | 506 | 0.222 | 557 | 256 | 24 | 2 | 423 | 6 | 520 | Phage-related tail fiber protein | Phage-related tail fiber protein | | afdb-uniprot50 | AF-A0A316MM73-F1-MODEL\_V4 | 1.0 | 6.779e-18 | 505 | 0.219 | 342 | 198 | 12 | 1 | 323 | 4 | 295 | Uncharacterized protein | Uncharacterized protein | | afdb-uniprot50 | AF-A0A836YZF5-F1-MODEL\_V4 | 1.0 | 1.421e-18 | 505 | 0.258 | 337 | 145 | 7 | 1 | 324 | 2 | 246 | Tail fiber protein | Tail fiber protein | | afdb-uniprot50 | AF-A0A376LG26-F1-MODEL\_V4 | 1.0 | 1.516e-21 | 505 | 0.23 | 460 | 243 | 19 | 1 | 408 | 2 | 402 | Putative side tail phage protein | Putative side tail phage protein | | afdb-uniprot50 | AF-A0A5T5I9M4-F1-MODEL\_V4 | 1.0 | 1.222e-21 | 505 | 0.199 | 512 | 237 | 17 | 1 | 421 | 2 | 431 | Phage tail protein | Phage tail protein | | afdb-uniprot50 | AF-A0A2M8YKL8-F1-MODEL\_V4 | 1.0 | 1.583e-18 | 504 | 0.318 | 298 | 143 | 8 | 1 | 246 | 1 | 290 | Tail-collar fiber protein | Tail-collar fiber protein | | afdb-uniprot50 | AF-A0A853I4D4-F1-MODEL\_V4 | 1.0 | 1.5e-18 | 504 | 0.268 | 317 | 155 | 12 | 1 | 258 | 3 | 301 | Phage tail protein | Phage tail protein | | afdb-uniprot50 | AF-A0A753WFH7-F1-MODEL\_V4 | 1.0 | 5.747e-22 | 504 | 0.207 | 579 | 216 | 23 | 1 | 421 | 1 | 494 | Phage tail protein | Phage tail protein | | afdb-uniprot50 | AF-A0A1M4Y377-F1-MODEL\_V4 | 1.0 | 3.353e-22 | 504 | 0.193 | 574 | 263 | 33 | 1 | 421 | 1 | 527 | Phage tail-collar fibre protein | Phage tail-collar fibre protein | | afdb-uniprot50 | AF-A0A7X5D8F7-F1-MODEL\_V4 | 1.0 | 6.087e-18 | 502 | 0.206 | 348 | 188 | 13 | 1 | 318 | 4 | 293 | Uncharacterized protein | Uncharacterized protein | | afdb-uniprot50 | AF-A0A1B9JGD0-F1-MODEL\_V4 | 1.0 | 6.242e-20 | 502 | 0.274 | 372 | 139 | 7 | 1 | 349 | 1 | 264 | Collar domain-containing protein | Collar domain-containing protein | | afdb-uniprot50 | AF-A0A172YYW4-F1-MODEL\_V4 | 1.0 | 1.757e-22 | 502 | 0.221 | 546 | 263 | 26 | 2 | 423 | 6 | 513 | Putative phage tail fiber protein | Putative phage tail fiber protein | | afdb-uniprot50 | AF-A0A776LTW8-F1-MODEL\_V4 | 1.0 | 3.885e-23 | 502 | 0.273 | 439 | 173 | 17 | 1 | 377 | 1 | 355 | Phage tail protein | Phage tail protein | | afdb-uniprot50 | AF-A0A0W0MX65-F1-MODEL\_V4 | 1.0 | 3.177e-22 | 502 | 0.224 | 569 | 245 | 29 | 2 | 422 | 6 | 525 | Uncharacterized protein | Uncharacterized protein | | afdb-uniprot50 | AF-A0A243TCZ2-F1-MODEL\_V4 | 1.0 | 8.875e-18 | 502 | 0.253 | 308 | 134 | 5 | 1 | 308 | 1 | 212 | Phage tail protein | Phage tail protein | | afdb-uniprot50 | AF-A0A1W7LPS7-F1-MODEL\_V4 | 1.0 | 6.175e-17 | 502 | 0.274 | 266 | 146 | 9 | 1 | 255 | 1 | 230 | Uncharacterized protein | Uncharacterized protein | | afdb-uniprot50 | AF-A0A1D3JVK3-F1-MODEL\_V4 | 1.0 | 3.011e-22 | 501 | 0.179 | 518 | 275 | 21 | 2 | 423 | 6 | 469 | Uncharacterized protein | Uncharacterized protein | | afdb-uniprot50 | AF-A0A1B7HT63-F1-MODEL\_V4 | 1.0 | 1.29e-21 | 501 | 0.248 | 462 | 224 | 17 | 1 | 403 | 2 | 399 | Phage tail fiber protein | Phage tail fiber protein | | afdb-uniprot50 | AF-A0A4P9VLW7-F1-MODEL\_V4 | 1.0 | 4.837e-19 | 500 | 0.227 | 427 | 180 | 15 | 2 | 419 | 3 | 288 | Uncharacterized protein | Uncharacterized protein | | afdb-uniprot50 | AF-M1RN38-F1-MODEL\_V4 | 1.0 | 9.102e-20 | 500 | 0.259 | 359 | 161 | 11 | 1 | 261 | 2 | 353 | Uncharacterized protein | Uncharacterized protein | | afdb-uniprot50 | AF-A0A3F3I8L4-F1-MODEL\_V4 | 1.0 | 2.095e-21 | 500 | 0.192 | 567 | 203 | 16 | 1 | 423 | 4 | 459 | Uncharacterized protein | Uncharacterized protein | | afdb-uniprot50 | AF-A0A1H0SWR0-F1-MODEL\_V4 | 1.0 | 9.851e-22 | 500 | 0.188 | 615 | 279 | 18 | 1 | 423 | 1 | 587 | Phage tail-collar fibre protein | Phage tail-collar fibre protein | | afdb-uniprot50 | AF-A0A1Q5VBN3-F1-MODEL\_V4 | 1.0 | 1.881e-21 | 500 | 0.262 | 438 | 179 | 10 | 1 | 414 | 1 | 318 | Uncharacterized protein | Uncharacterized protein | | afdb-uniprot50 | AF-A0A0M7E7A2-F1-MODEL\_V4 | 1.0 | 6.31e-23 | 499 | 0.241 | 522 | 214 | 20 | 1 | 423 | 2 | 440 | Variable tail fiber protein | Variable tail fiber protein | | afdb-uniprot50 | AF-A0A7T9UBZ9-F1-MODEL\_V4 | 1.0 | 1.117e-16 | 499 | 0.267 | 258 | 168 | 8 | 1 | 238 | 1 | 257 | Phage tail protein | Phage tail protein | | afdb-uniprot50 | AF-N9L859-F1-MODEL\_V4 | 1.0 | 5.049e-16 | 499 | 0.3 | 256 | 134 | 8 | 3 | 246 | 2 | 224 | Uncharacterized protein | Uncharacterized protein | | afdb-uniprot50 | AF-A0A1I1UBJ2-F1-MODEL\_V4 | 1.0 | 6.952e-20 | 498 | 0.261 | 382 | 187 | 19 | 1 | 322 | 1 | 347 | Phage tail-collar fibre protein | Phage tail-collar fibre protein | | afdb-uniprot50 | AF-A0A285M2J9-F1-MODEL\_V4 | 1.0 | 3.27e-20 | 498 | 0.21 | 455 | 237 | 22 | 1 | 423 | 1 | 365 | Phage tail-collar fibre protein | Phage tail-collar fibre protein | | afdb-uniprot50 | AF-A0A556S900-F1-MODEL\_V4 | 1.0 | 5.747e-22 | 498 | 0.26 | 488 | 171 | 13 | 1 | 409 | 2 | 378 | Phage tail protein | Phage tail protein | | afdb-uniprot50 | AF-A0A2S5C7R9-F1-MODEL\_V4 | 1.0 | 2.936e-20 | 498 | 0.175 | 719 | 227 | 21 | 1 | 423 | 1 | 649 | Phage tail protein | Phage tail protein | | afdb-uniprot50 | AF-A0A5T8G1R8-F1-MODEL\_V4 | 1.0 | 7.525e-22 | 498 | 0.205 | 491 | 204 | 15 | 1 | 423 | 4 | 376 | Phage tail protein | Phage tail protein | | afdb-uniprot50 | AF-A0A4U3FAV2-F1-MODEL\_V4 | 1.0 | 4.056e-20 | 497 | 0.25 | 440 | 212 | 17 | 1 | 413 | 2 | 350 | Uncharacterized protein | Uncharacterized protein | | afdb-uniprot50 | AF-A0A7D6BYB5-F1-MODEL\_V4 | 1.0 | 2.853e-22 | 497 | 0.217 | 528 | 198 | 14 | 1 | 406 | 2 | 436 | Phage tail protein | Phage tail protein | | afdb-uniprot50 | AF-A0A829CQA7-F1-MODEL\_V4 | 1.0 | 8.845e-22 | 497 | 0.167 | 680 | 236 | 21 | 1 | 423 | 1 | 607 | Phage tail fiber repeat family protein | Phage tail fiber repeat family protein | | afdb-uniprot50 | AF-A0A1V0BI47-F1-MODEL\_V4 | 1.0 | 1.141e-22 | 497 | 0.161 | 779 | 268 | 30 | 1 | 423 | 2 | 750 | Uncharacterized protein | Uncharacterized protein | | afdb-uniprot50 | AF-A0A1C3HKM5-F1-MODEL\_V4 | 1.0 | 1.24e-20 | 497 | 0.248 | 451 | 174 | 13 | 1 | 417 | 1 | 320 | Phage tail fiber repeat protein | Phage tail fiber repeat protein | | afdb-uniprot50 | AF-A0A1Y2S899-F1-MODEL\_V4 | 1.0 | 3.098e-20 | 496 | 0.239 | 501 | 172 | 17 | 1 | 416 | 2 | 378 | Tail protein | Tail protein | | afdb-uniprot50 | AF-A0A3A9I3B0-F1-MODEL\_V4 | 1.0 | 5.329e-16 | 496 | 0.265 | 252 | 152 | 6 | 1 | 221 | 1 | 250 | Collar domain-containing protein | Collar domain-containing protein | | afdb-uniprot50 | AF-A0A855M4U4-F1-MODEL\_V4 | 1.0 | 1.04e-21 | 496 | 0.242 | 541 | 179 | 16 | 1 | 423 | 1 | 428 | Phage tail protein | Phage tail protein | | afdb-uniprot50 | AF-A0A2K9M2M1-F1-MODEL\_V4 | 1.0 | 1.097e-21 | 496 | 0.179 | 662 | 281 | 26 | 2 | 423 | 6 | 644 | Phage tail protein | Phage tail protein | | afdb-uniprot50 | AF-A0A7U9G0P5-F1-MODEL\_V4 | 1.0 | 1.077e-11 | 495 | 0.496 | 127 | 64 | 0 | 32 | 158 | 2 | 128 | Putative tail fiber protein (GpH) | Putative tail fiber protein (GpH) | | afdb-uniprot50 | AF-A0A483Z189-F1-MODEL\_V4 | 1.0 | 1.82e-12 | 494 | 0.416 | 137 | 77 | 3 | 23 | 158 | 4 | 138 | Phage tail protein | Phage tail protein | | afdb-uniprot50 | AF-A0A2S8JIV1-F1-MODEL\_V4 | 1.0 | 7.577e-14 | 494 | 0.308 | 198 | 111 | 5 | 1 | 176 | 1 | 194 | Phage tail protein | Phage tail protein | | afdb-uniprot50 | AF-A0A5U6G5Z8-F1-MODEL\_V4 | 1.0 | 7.633e-21 | 494 | 0.23 | 481 | 206 | 18 | 1 | 405 | 1 | 393 | Collar domain-containing protein | Collar domain-containing protein | | afdb-uniprot50 | AF-A0A2U3F153-F1-MODEL\_V4 | 1.0 | 2.211e-21 | 494 | 0.252 | 467 | 164 | 13 | 1 | 409 | 2 | 341 | Uncharacterized protein | Uncharacterized protein | | afdb-uniprot50 | AF-L0MEM1-F1-MODEL\_V4 | 1.0 | 1.688e-21 | 493 | 0.171 | 695 | 192 | 15 | 1 | 423 | 1 | 583 | Phage-related tail fiber protein | Phage-related tail fiber protein | | afdb-uniprot50 | AF-A0A1W5DTY4-F1-MODEL\_V4 | 1.0 | 1.175e-20 | 492 | 0.267 | 452 | 167 | 10 | 1 | 417 | 2 | 324 | Phage tail fiber repeat protein | Phage tail fiber repeat protein | | afdb-uniprot50 | AF-A0A6I1H834-F1-MODEL\_V4 | 1.0 | 8.719e-23 | 492 | 0.227 | 563 | 240 | 28 | 1 | 423 | 1 | 508 | Phage tail protein | Phage tail protein | | afdb-uniprot50 | AF-A0A5P2SLL5-F1-MODEL\_V4 | 1.0 | 5.387e-19 | 491 | 0.297 | 373 | 156 | 17 | 1 | 298 | 2 | 343 | Phage tail protein | Phage tail protein | | afdb-uniprot50 | AF-A0A1G3JRW0-F1-MODEL\_V4 | 1.0 | 5.16e-22 | 491 | 0.228 | 543 | 259 | 22 | 1 | 423 | 4 | 506 | Uncharacterized protein | Uncharacterized protein | | afdb-uniprot50 | AF-A0A2U0SUN8-F1-MODEL\_V4 | 1.0 | 1.577e-22 | 491 | 0.206 | 590 | 272 | 24 | 1 | 423 | 1 | 561 | Tail-collar fiber protein | Tail-collar fiber protein | | afdb-uniprot50 | AF-A0A5U6SMK2-F1-MODEL\_V4 | 1.0 | 1.782e-21 | 491 | 0.205 | 481 | 215 | 16 | 1 | 423 | 4 | 375 | Phage tail protein | Phage tail protein | | afdb-uniprot50 | AF-A0A2U8I2Y4-F1-MODEL\_V4 | 1.0 | 2.462e-21 | 490 | 0.24 | 529 | 192 | 18 | 1 | 421 | 1 | 427 | Uncharacterized protein | Uncharacterized protein | | afdb-uniprot50 | AF-A0A7L7BSE0-F1-MODEL\_V4 | 1.0 | 1.401e-19 | 490 | 0.239 | 451 | 209 | 17 | 1 | 405 | 2 | 364 | Phage tail protein | Phage tail protein | | afdb-uniprot50 | AF-A0A1X3K047-F1-MODEL\_V4 | 1.0 | 3.735e-22 | 489 | 0.205 | 556 | 243 | 25 | 1 | 423 | 2 | 491 | Putative phage tail fiber protein H | Putative phage tail fiber protein H | | afdb-uniprot50 | AF-A0A854J8Q1-F1-MODEL\_V4 | 1.0 | 3.098e-20 | 489 | 0.247 | 453 | 171 | 13 | 1 | 417 | 1 | 319 | Uncharacterized protein | Uncharacterized protein | | afdb-uniprot50 | AF-D2MWT7-F1-MODEL\_V4 | 1.0 | 1.914e-16 | 488 | 0.244 | 298 | 131 | 8 | 1 | 248 | 3 | 256 | Tail fiber protein H, putative | Tail fiber protein H, putative | | afdb-uniprot50 | AF-A0A125V1R9-F1-MODEL\_V4 | 1.0 | 3.501e-19 | 488 | 0.22 | 359 | 190 | 8 | 1 | 306 | 1 | 322 | Phage tail fiber protein | Phage tail fiber protein | | afdb-uniprot50 | AF-A0A447QPL2-F1-MODEL\_V4 | 1.0 | 1.24e-20 | 488 | 0.236 | 456 | 174 | 13 | 1 | 409 | 1 | 329 | Phage tail fibre repeat | Phage tail fibre repeat | | afdb-uniprot50 | AF-Q8ZKK5-F1-MODEL\_V4 | 1.0 | 1.04e-21 | 488 | 0.217 | 551 | 249 | 24 | 1 | 412 | 1 | 508 | Putative phage tail fiber protein H | Putative phage tail fiber protein H | | afdb-uniprot50 | AF-A0A8B3U7S3-F1-MODEL\_V4 | 1.0 | 1.175e-20 | 488 | 0.184 | 725 | 236 | 25 | 1 | 423 | 1 | 671 | Tail Fiber protein | Tail Fiber protein | | afdb-uniprot50 | AF-A0A4U9D1H6-F1-MODEL\_V4 | 1.0 | 2.914e-13 | 487 | 0.349 | 186 | 109 | 3 | 1 | 176 | 1 | 184 | Uncharacterized protein | Uncharacterized protein | | afdb-uniprot50 | AF-G1UYT0-F1-MODEL\_V4 | 1.0 | 5.387e-19 | 487 | 0.277 | 335 | 179 | 13 | 1 | 296 | 1 | 311 | Uncharacterized protein | Uncharacterized protein | | afdb-uniprot50 | AF-A0A0T9T666-F1-MODEL\_V4 | 1.0 | 5.767e-18 | 487 | 0.29 | 331 | 145 | 9 | 1 | 309 | 2 | 264 | Variable tail fiber protein | Variable tail fiber protein | | afdb-uniprot50 | AF-A0A031FSS2-F1-MODEL\_V4 | 1.0 | 7.525e-22 | 487 | 0.216 | 555 | 260 | 25 | 1 | 423 | 1 | 512 | Prophage long tail fiber protein H | Prophage long tail fiber protein H | | afdb-uniprot50 | AF-A0A831HRR1-F1-MODEL\_V4 | 1.0 | 1.436e-21 | 487 | 0.222 | 517 | 264 | 25 | 1 | 421 | 1 | 475 | Phage tail protein | Phage tail protein | | afdb-uniprot50 | AF-A0A2N7RX48-F1-MODEL\_V4 | 1.0 | 4.16e-22 | 486 | 0.221 | 491 | 240 | 23 | 1 | 423 | 1 | 417 | Uncharacterized protein | Uncharacterized protein | | afdb-uniprot50 | AF-A0A454TSP7-F1-MODEL\_V4 | 1.0 | 7.258e-17 | 486 | 0.293 | 296 | 152 | 12 | 1 | 247 | 1 | 288 | Phage tail protein | Phage tail protein | | afdb-uniprot50 | AF-A0A380Q8N6-F1-MODEL\_V4 | 1.0 | 3.353e-22 | 486 | 0.239 | 510 | 269 | 21 | 1 | 423 | 2 | 479 | Tail fiber protein | Tail fiber protein | | afdb-uniprot50 | AF-A0A376U5P4-F1-MODEL\_V4 | 1.0 | 1.058e-16 | 485 | 0.299 | 344 | 151 | 16 | 1 | 324 | 2 | 275 | Tail fiber protein | Tail fiber protein | | afdb-uniprot50 | AF-H8NR92-F1-MODEL\_V4 | 1.0 | 2.863e-18 | 485 | 0.287 | 320 | 156 | 8 | 1 | 254 | 2 | 315 | Tail Collar domain-containing protein | Tail Collar domain-containing protein | | afdb-uniprot50 | AF-A0A5Y2LQP0-F1-MODEL\_V4 | 1.0 | 5.235e-21 | 485 | 0.252 | 464 | 183 | 13 | 1 | 405 | 1 | 359 | Collar domain-containing protein | Collar domain-containing protein | | afdb-uniprot50 | AF-A0A6N8PUZ8-F1-MODEL\_V4 | 1.0 | 8.056e-21 | 485 | 0.244 | 474 | 221 | 20 | 1 | 413 | 1 | 398 | Phage tail protein | Phage tail protein | | afdb-uniprot50 | AF-A0A7Z0UXS0-F1-MODEL\_V4 | 1.0 | 1.421e-18 | 485 | 0.249 | 349 | 188 | 12 | 1 | 324 | 1 | 300 | Phage tail fiber protein | Phage tail fiber protein | | afdb-uniprot50 | AF-A0A7V8F402-F1-MODEL\_V4 | 1.0 | 2.713e-18 | 484 | 0.302 | 350 | 170 | 15 | 1 | 309 | 1 | 317 | Uncharacterized protein | Uncharacterized protein | | afdb-uniprot50 | AF-A0A144L7H0-F1-MODEL\_V4 | 1.0 | 8.845e-22 | 484 | 0.219 | 607 | 186 | 23 | 1 | 423 | 1 | 503 | Phage tail fiber protein | Phage tail fiber protein | | afdb-uniprot50 | AF-A0A1Y3L4U4-F1-MODEL\_V4 | 1.0 | 3.539e-22 | 484 | 0.188 | 674 | 254 | 34 | 2 | 423 | 6 | 638 | Phage tail protein | Phage tail protein | | afdb-uniprot50 | AF-A0A7U5ZFC7-F1-MODEL\_V4 | 1.0 | 2.211e-21 | 484 | 0.258 | 460 | 229 | 18 | 1 | 413 | 2 | 396 | Phage tail protein | Phage tail protein | | afdb-uniprot50 | AF-A0A844Q9H3-F1-MODEL\_V4 | 1.0 | 1.308e-20 | 483 | 0.215 | 505 | 214 | 24 | 1 | 422 | 3 | 408 | Uncharacterized protein | Uncharacterized protein | | afdb-uniprot50 | AF-A0A7M2QP04-F1-MODEL\_V4 | 1.0 | 1.555e-23 | 483 | 0.181 | 591 | 261 | 24 | 4 | 423 | 3 | 541 | Tail fiber protein | Tail fiber protein | | afdb-uniprot50 | AF-A0A2J9H2E6-F1-MODEL\_V4 | 1.0 | 5.915e-20 | 483 | 0.221 | 487 | 183 | 12 | 1 | 409 | 1 | 369 | Phage tail protein | Phage tail protein | | afdb-uniprot50 | AF-A0A143HDH4-F1-MODEL\_V4 | 1.0 | 5.196e-14 | 482 | 0.31 | 193 | 109 | 5 | 1 | 177 | 1 | 185 | Uncharacterized protein | Uncharacterized protein | | afdb-uniprot50 | AF-A0A1N6I148-F1-MODEL\_V4 | 1.0 | 2.179e-22 | 482 | 0.198 | 548 | 273 | 27 | 1 | 423 | 2 | 508 | Phage tail-collar fibre protein | Phage tail-collar fibre protein | | afdb-uniprot50 | AF-A0A1Q5TR71-F1-MODEL\_V4 | 1.0 | 1.538e-20 | 482 | 0.261 | 448 | 173 | 16 | 1 | 408 | 2 | 331 | Tail protein | Tail protein | | afdb-uniprot50 | AF-A0A822NDL8-F1-MODEL\_V4 | 1.0 | 8.972e-21 | 482 | 0.258 | 434 | 185 | 9 | 1 | 400 | 2 | 332 | Uncharacterized protein | Uncharacterized protein | | afdb-uniprot50 | AF-A0A2C9NUX8-F1-MODEL\_V4 | 1.0 | 4.012e-17 | 482 | 0.244 | 307 | 136 | 5 | 1 | 307 | 1 | 211 | Phage tail protein | Phage tail protein | | afdb-uniprot50 | AF-K7ZZ34-F1-MODEL\_V4 | 1.0 | 7.797e-12 | 481 | 0.504 | 121 | 58 | 1 | 1 | 119 | 2 | 122 | Phage tail fiber protein | Phage tail fiber protein | | afdb-uniprot50 | AF-N8R6Q7-F1-MODEL\_V4 | 1.0 | 5.705e-15 | 481 | 0.365 | 197 | 121 | 3 | 4 | 198 | 3 | 197 | Uncharacterized protein | Uncharacterized protein | | afdb-uniprot50 | AF-A0A1Y5MYB7-F1-MODEL\_V4 | 1.0 | 2.607e-17 | 481 | 0.206 | 315 | 181 | 15 | 3 | 259 | 2 | 305 | Uncharacterized protein | Uncharacterized protein | | afdb-uniprot50 | AF-A0A6B3ZPL8-F1-MODEL\_V4 | 1.0 | 1.129e-19 | 481 | 0.205 | 369 | 181 | 12 | 1 | 257 | 2 | 370 | Phage tail protein | Phage tail protein | | afdb-uniprot50 | AF-A0A7U5SYK9-F1-MODEL\_V4 | 1.0 | 5.425e-11 | 481 | 0.248 | 153 | 108 | 3 | 6 | 155 | 10 | 158 | Phage tail protein | Phage tail protein | | afdb-uniprot50 | AF-A0A4Y6UDH3-F1-MODEL\_V4 | 1.0 | 3.353e-22 | 481 | 0.206 | 518 | 271 | 28 | 1 | 423 | 18 | 490 | Uncharacterized protein | Uncharacterized protein | | afdb-uniprot50 | AF-A0A377LGA7-F1-MODEL\_V4 | 1.0 | 6.066e-22 | 481 | 0.254 | 492 | 266 | 26 | 1 | 423 | 5 | 464 | Tail fiber protein | Tail fiber protein | | afdb-uniprot50 | AF-A0A825RJE7-F1-MODEL\_V4 | 1.0 | 5.465e-18 | 480 | 0.237 | 320 | 169 | 11 | 1 | 259 | 3 | 308 | Phage tail protein | Phage tail protein | | afdb-uniprot50 | AF-A0A4Y9K5F2-F1-MODEL\_V4 | 1.0 | 4.907e-18 | 480 | 0.259 | 332 | 144 | 8 | 1 | 322 | 2 | 241 | Uncharacterized protein | Uncharacterized protein | | afdb-uniprot50 | AF-A0A6H9S1K4-F1-MODEL\_V4 | 1.0 | 4.87e-11 | 479 | 0.428 | 112 | 64 | 0 | 1 | 112 | 5 | 116 | Phage tail protein | Phage tail protein | | afdb-uniprot50 | AF-A0A1G7D1W6-F1-MODEL\_V4 | 1.0 | 1.014e-19 | 479 | 0.253 | 410 | 145 | 11 | 1 | 405 | 2 | 255 | Phage tail-collar fibre protein | Phage tail-collar fibre protein | | afdb-uniprot50 | AF-A0A379CXC0-F1-MODEL\_V4 | 1.0 | 1.29e-21 | 479 | 0.21 | 599 | 260 | 32 | 1 | 423 | 2 | 563 | Phage tail fibre repeat | Phage tail fibre repeat | | afdb-uniprot50 | AF-A0A2D1CSN0-F1-MODEL\_V4 | 1.0 | 5.525e-21 | 479 | 0.186 | 629 | 276 | 28 | 2 | 423 | 6 | 605 | Putative tail fiber protein | Putative tail fiber protein | | afdb-uniprot50 | AF-A0A1Y2SIM3-F1-MODEL\_V4 | 1.0 | 1.24e-20 | 479 | 0.254 | 452 | 178 | 14 | 1 | 412 | 1 | 333 | Tail protein | Tail protein | | afdb-uniprot50 | AF-A0A4U6GR68-F1-MODEL\_V4 | 1.0 | 3.021e-18 | 478 | 0.276 | 405 | 147 | 14 | 1 | 331 | 2 | 334 | Phage tail protein | Phage tail protein | | afdb-uniprot50 | AF-A0A5N7JNT7-F1-MODEL\_V4 | 1.0 | 6.494e-21 | 478 | 0.205 | 530 | 257 | 24 | 1 | 423 | 3 | 475 | Phage tail protein | Phage tail protein | | afdb-uniprot50 | AF-A0A766JGR3-F1-MODEL\_V4 | 1.0 | 1.478e-19 | 478 | 0.211 | 486 | 210 | 17 | 1 | 417 | 5 | 386 | Phage tail protein | Phage tail protein | | afdb-uniprot50 | AF-A0A077NM26-F1-MODEL\_V4 | 1.0 | 4.768e-20 | 478 | 0.257 | 431 | 177 | 12 | 1 | 412 | 1 | 307 | Uncharacterized protein | Uncharacterized protein | | afdb-uniprot50 | AF-A0A754E741-F1-MODEL\_V4 | 1.0 | 2.936e-20 | 477 | 0.152 | 713 | 201 | 17 | 1 | 423 | 4 | 602 | Uncharacterized protein | Uncharacterized protein | | afdb-uniprot50 | AF-A0A2G8N573-F1-MODEL\_V4 | 1.0 | 6.066e-22 | 477 | 0.207 | 666 | 260 | 24 | 2 | 423 | 6 | 647 | Phage tail protein | Phage tail protein | | afdb-uniprot50 | AF-A4WEL3-F1-MODEL\_V4 | 1.0 | 3.365e-18 | 476 | 0.27 | 340 | 158 | 12 | 1 | 255 | 2 | 336 | Phage Tail Collar domain protein | Phage Tail Collar domain protein | | afdb-uniprot50 | AF-A0A1H6YID8-F1-MODEL\_V4 | 1.0 | 1.24e-20 | 476 | 0.208 | 479 | 243 | 21 | 2 | 423 | 6 | 405 | Phage tail-collar fibre protein | Phage tail-collar fibre protein | | afdb-uniprot50 | AF-A0A1B9KN89-F1-MODEL\_V4 | 1.0 | 1.757e-22 | 476 | 0.213 | 619 | 250 | 27 | 1 | 423 | 1 | 578 | Uncharacterized protein | Uncharacterized protein | | afdb-uniprot50 | AF-A0A1G7S9W8-F1-MODEL\_V4 | 1.0 | 1.175e-20 | 476 | 0.205 | 448 | 233 | 20 | 1 | 423 | 1 | 350 | Uncharacterized protein | Uncharacterized protein | | afdb-uniprot50 | AF-A0A4U9HA58-F1-MODEL\_V4 | 1.0 | 3.27e-20 | 475 | 0.253 | 415 | 157 | 12 | 1 | 363 | 1 | 314 | Phage tail fibre repeat | Phage tail fibre repeat | | afdb-uniprot50 | AF-A0A0T9UMM8-F1-MODEL\_V4 | 1.0 | 1.381e-20 | 475 | 0.244 | 470 | 175 | 14 | 1 | 405 | 1 | 355 | Variable tail fiber protein | Variable tail fiber protein | | afdb-uniprot50 | AF-A0A1N6MV94-F1-MODEL\_V4 | 1.0 | 1.129e-19 | 475 | 0.232 | 474 | 176 | 11 | 1 | 417 | 2 | 344 | Uncharacterized protein | Uncharacterized protein | | afdb-uniprot50 | AF-Q1I687-F1-MODEL\_V4 | 1.0 | 2.333e-21 | 475 | 0.144 | 908 | 278 | 29 | 1 | 423 | 5 | 898 | Putative phage variable tail fibre protein | Putative phage variable tail fibre protein | | afdb-uniprot50 | AF-A0A5T5IEY5-F1-MODEL\_V4 | 1.0 | 5.915e-20 | 474 | 0.214 | 509 | 187 | 18 | 1 | 419 | 4 | 389 | Phage tail protein | Phage tail protein | | afdb-uniprot50 | AF-A0A068R2V7-F1-MODEL\_V4 | 1.0 | 1.457e-20 | 474 | 0.209 | 540 | 192 | 16 | 1 | 420 | 2 | 426 | Uncharacterized protein | Uncharacterized protein | | afdb-uniprot50 | AF-A0A7D5LM12-F1-MODEL\_V4 | 1.0 | 1.436e-21 | 474 | 0.225 | 537 | 238 | 26 | 4 | 423 | 8 | 483 | Phage tail protein | Phage tail protein | | afdb-uniprot50 | AF-A0A6D0PJP5-F1-MODEL\_V4 | 1.0 | 4.7e-21 | 474 | 0.19 | 600 | 243 | 26 | 1 | 421 | 2 | 537 | Phage tail protein | Phage tail protein | | afdb-uniprot50 | AF-A0A236MFK9-F1-MODEL\_V4 | 1.0 | 1.07e-19 | 474 | 0.225 | 444 | 179 | 15 | 1 | 392 | 5 | 335 | Phage tail protein | Phage tail protein | | afdb-uniprot50 | AF-A0A1B2FA13-F1-MODEL\_V4 | 1.0 | 1.985e-21 | 474 | 0.206 | 615 | 243 | 31 | 2 | 423 | 6 | 568 | Uncharacterized protein | Uncharacterized protein | | afdb-uniprot50 | AF-A0A7I7E0P7-F1-MODEL\_V4 | 1.0 | 2.013e-20 | 474 | 0.261 | 401 | 209 | 14 | 1 | 361 | 2 | 355 | Uncharacterized protein | Uncharacterized protein | | afdb-uniprot50 | AF-A0A7Y0N7P7-F1-MODEL\_V4 | 1.0 | 8.624e-20 | 473 | 0.226 | 371 | 206 | 12 | 1 | 324 | 1 | 337 | Phage tail protein | Phage tail protein | | afdb-uniprot50 | AF-W1J3V9-F1-MODEL\_V4 | 1.0 | 1.713e-20 | 473 | 0.199 | 586 | 200 | 16 | 1 | 422 | 1 | 481 | Uncharacterized protein | Uncharacterized protein | | afdb-uniprot50 | AF-A0A1H8TEQ6-F1-MODEL\_V4 | 1.0 | 3.789e-21 | 473 | 0.219 | 566 | 249 | 29 | 1 | 423 | 1 | 516 | Phage tail-collar fibre protein | Phage tail-collar fibre protein | | afdb-uniprot50 | AF-A0A2D1IR12-F1-MODEL\_V4 | 1.0 | 4.633e-22 | 473 | 0.194 | 577 | 263 | 25 | 1 | 423 | 1 | 529 | Uncharacterized protein | Uncharacterized protein | | afdb-uniprot50 | AF-A0A5A9YWL3-F1-MODEL\_V4 | 1.0 | 9.535e-13 | 472 | 0.295 | 169 | 105 | 3 | 24 | 183 | 6 | 169 | Phage tail protein | Phage tail protein | | afdb-uniprot50 | AF-A0A495BJ48-F1-MODEL\_V4 | 1.0 | 3.843e-20 | 472 | 0.22 | 498 | 202 | 20 | 1 | 423 | 1 | 387 | Tail-collar fiber protein | Tail-collar fiber protein | | afdb-uniprot50 | AF-A0A482IM57-F1-MODEL\_V4 | 1.0 | 8.749e-19 | 472 | 0.214 | 368 | 174 | 15 | 1 | 260 | 1 | 361 | Uncharacterized protein | Uncharacterized protein | | afdb-uniprot50 | AF-A0A4R7GFV2-F1-MODEL\_V4 | 1.0 | 3.843e-20 | 472 | 0.225 | 511 | 223 | 21 | 1 | 421 | 2 | 429 | Tail-collar fiber protein | Tail-collar fiber protein | | afdb-uniprot50 | AF-A0A3F3P2W1-F1-MODEL\_V4 | 1.0 | 1.516e-21 | 472 | 0.222 | 518 | 269 | 22 | 1 | 421 | 1 | 481 | Phage tail protein | Phage tail protein | | afdb-uniprot50 | AF-A0A198GSC6-F1-MODEL\_V4 | 1.0 | 6.31e-23 | 472 | 0.216 | 573 | 263 | 24 | 1 | 423 | 2 | 538 | Phage tail fiber protein | Phage tail fiber protein | | afdb-uniprot50 | AF-A0A376M6X6-F1-MODEL\_V4 | 1.0 | 9.993e-21 | 472 | 0.234 | 504 | 235 | 25 | 1 | 392 | 5 | 469 | Tail fiber protein (Gph) | Tail fiber protein (Gph) | | afdb-uniprot50 | AF-A0A7A6VQX8-F1-MODEL\_V4 | 1.0 | 1.623e-20 | 472 | 0.245 | 480 | 215 | 23 | 1 | 392 | 2 | 422 | Phage tail protein | Phage tail protein | | afdb-uniprot50 | AF-N9CZ59-F1-MODEL\_V4 | 1.0 | 1.313e-16 | 472 | 0.215 | 329 | 173 | 11 | 1 | 278 | 2 | 296 | Uncharacterized protein | Uncharacterized protein | | afdb-uniprot50 | AF-A0A853IIN2-F1-MODEL\_V4 | 1.0 | 4.405e-18 | 471 | 0.265 | 339 | 149 | 19 | 1 | 258 | 1 | 320 | Phage tail protein | Phage tail protein | | afdb-uniprot50 | AF-A0A315WVX7-F1-MODEL\_V4 | 1.0 | 3.999e-21 | 471 | 0.218 | 567 | 264 | 28 | 1 | 423 | 1 | 532 | Phage tail protein | Phage tail protein | | afdb-uniprot50 | AF-A0A4P6GED1-F1-MODEL\_V4 | 1.0 | 5.747e-22 | 471 | 0.178 | 644 | 283 | 34 | 1 | 423 | 1 | 619 | Phage tail protein | Phage tail protein | | afdb-uniprot50 | AF-A0A7V8EWA4-F1-MODEL\_V4 | 1.0 | 2.211e-21 | 471 | 0.155 | 655 | 285 | 26 | 2 | 423 | 6 | 625 | Phage tail protein | Phage tail protein | | afdb-uniprot50 | AF-A0A1Y3CBA8-F1-MODEL\_V4 | 1.0 | 1.588e-14 | 471 | 0.256 | 238 | 153 | 6 | 1 | 223 | 1 | 229 | Collar domain-containing protein | Collar domain-containing protein | | afdb-uniprot50 | AF-A0A380PQB1-F1-MODEL\_V4 | 1.0 | 2.401e-19 | 470 | 0.254 | 464 | 182 | 15 | 1 | 417 | 2 | 348 | Tail fiber protein | Tail fiber protein | | afdb-uniprot50 | AF-A0A5T8J817-F1-MODEL\_V4 | 1.0 | 1.24e-20 | 470 | 0.211 | 483 | 211 | 16 | 1 | 423 | 4 | 376 | Phage tail protein | Phage tail protein | | afdb-uniprot50 | AF-A0A7I7ESP2-F1-MODEL\_V4 | 1.0 | 3.843e-20 | 469 | 0.266 | 461 | 220 | 21 | 1 | 418 | 2 | 387 | gp37\_C domain-containing protein | gp37\_C domain-containing protein | | afdb-uniprot50 | AF-R5ITN8-F1-MODEL\_V4 | 1.0 | 2.194e-14 | 468 | 0.247 | 234 | 137 | 7 | 2 | 198 | 6 | 237 | Possible tail fiber protein | Possible tail fiber protein | | afdb-uniprot50 | AF-A0A0M9GBQ6-F1-MODEL\_V4 | 1.0 | 3.054e-21 | 468 | 0.235 | 514 | 254 | 31 | 2 | 423 | 6 | 472 | Phage-related tail fiber protein | Phage-related tail fiber protein | | afdb-uniprot50 | AF-A0A3S6EZT4-F1-MODEL\_V4 | 1.0 | 1.808e-20 | 467 | 0.216 | 508 | 207 | 21 | 1 | 421 | 2 | 405 | Uncharacterized protein | Uncharacterized protein | | afdb-uniprot50 | AF-B8DLJ2-F1-MODEL\_V4 | 1.0 | 5.446e-22 | 467 | 0.196 | 533 | 290 | 20 | 1 | 423 | 2 | 505 | Tail fiber protein, putative | Tail fiber protein, putative | | afdb-uniprot50 | AF-A0A6I1L6W4-F1-MODEL\_V4 | 1.0 | 1.782e-21 | 467 | 0.197 | 576 | 275 | 27 | 1 | 423 | 1 | 541 | Uncharacterized protein | Uncharacterized protein | | afdb-uniprot50 | AF-A0A4U3LF57-F1-MODEL\_V4 | 1.0 | 2.498e-20 | 467 | 0.23 | 446 | 209 | 17 | 1 | 405 | 2 | 354 | Phage tail protein | Phage tail protein | | afdb-uniprot50 | AF-H8NWL9-F1-MODEL\_V4 | 1.0 | 8.624e-20 | 467 | 0.249 | 441 | 188 | 12 | 1 | 412 | 1 | 327 | Tail fiber repeat 2 protein | Tail fiber repeat 2 protein | | afdb-uniprot50 | AF-A0A825XI72-F1-MODEL\_V4 | 1.0 | 4.907e-18 | 466 | 0.24 | 328 | 167 | 12 | 1 | 258 | 1 | 316 | Phage tail protein | Phage tail protein | | afdb-uniprot50 | AF-A0A2X4TXE4-F1-MODEL\_V4 | 1.0 | 4.056e-20 | 466 | 0.231 | 453 | 179 | 13 | 1 | 417 | 2 | 321 | Phage tail fibre repeat | Phage tail fibre repeat | | afdb-uniprot50 | AF-A0A737EIK7-F1-MODEL\_V4 | 1.0 | 4.22e-21 | 465 | 0.228 | 495 | 228 | 21 | 1 | 423 | 1 | 413 | Phage tail protein | Phage tail protein | | afdb-uniprot50 | AF-A0A362XHI5-F1-MODEL\_V4 | 1.0 | 5.685e-19 | 465 | 0.225 | 425 | 171 | 12 | 1 | 380 | 1 | 312 | Tail-collar fiber protein | Tail-collar fiber protein | | afdb-uniprot50 | AF-A0A756ZEH1-F1-MODEL\_V4 | 1.0 | 9.334e-22 | 465 | 0.227 | 584 | 242 | 32 | 2 | 423 | 3 | 539 | Phage tail protein | Phage tail protein | | afdb-uniprot50 | AF-A0A7X1BUI6-F1-MODEL\_V4 | 1.0 | 8.502e-21 | 465 | 0.225 | 505 | 219 | 16 | 1 | 411 | 2 | 428 | Phage tail protein | Phage tail protein | | afdb-uniprot50 | AF-A0A383RYZ5-F1-MODEL\_V4 | 1.0 | 5.235e-21 | 465 | 0.191 | 759 | 252 | 28 | 2 | 423 | 6 | 739 | Putative tail fiber protein | Putative tail fiber protein | | afdb-uniprot50 | AF-A0A7W3F2A9-F1-MODEL\_V4 | 1.0 | 1.457e-20 | 464 | 0.243 | 477 | 222 | 22 | 1 | 417 | 2 | 399 | Phage tail protein | Phage tail protein | | afdb-uniprot50 | AF-A0A263JA61-F1-MODEL\_V4 | 1.0 | 1.113e-20 | 464 | 0.251 | 421 | 196 | 14 | 1 | 409 | 1 | 314 | Uncharacterized protein | Uncharacterized protein | | afdb-uniprot50 | AF-A0A3A9I672-F1-MODEL\_V4 | 1.0 | 4.469e-17 | 463 | 0.181 | 292 | 173 | 10 | 3 | 258 | 16 | 277 | Uncharacterized protein | Uncharacterized protein | | afdb-uniprot50 | AF-A0A6X8FNY2-F1-MODEL\_V4 | 1.0 | 3.143e-19 | 463 | 0.239 | 468 | 202 | 19 | 1 | 412 | 1 | 370 | Phage tail protein | Phage tail protein | | afdb-uniprot50 | AF-A0A6G5QFT1-F1-MODEL\_V4 | 1.0 | 1.04e-21 | 463 | 0.193 | 542 | 268 | 22 | 4 | 423 | 3 | 497 | Phage tail-collar fiber protein (DUF3751 domain) | Phage tail-collar fiber protein (DUF3751 domain) | | afdb-uniprot50 | AF-A0A7Y8XK01-F1-MODEL\_V4 | 1.0 | 1.623e-20 | 463 | 0.252 | 491 | 183 | 16 | 1 | 418 | 2 | 381 | Phage tail protein | Phage tail protein | | afdb-uniprot50 | AF-A0A656VM29-F1-MODEL\_V4 | 1.0 | 8.624e-20 | 463 | 0.254 | 448 | 175 | 13 | 1 | 417 | 1 | 320 | Uncharacterized protein | Uncharacterized protein | | afdb-uniprot50 | AF-A0A3S4IZL9-F1-MODEL\_V4 | 1.0 | 7.337e-20 | 461 | 0.258 | 468 | 175 | 18 | 1 | 417 | 2 | 348 | Tail fiber protein | Tail fiber protein | | afdb-uniprot50 | AF-A0A7Z7L7Z7-F1-MODEL\_V4 | 1.0 | 2.242e-20 | 461 | 0.218 | 494 | 252 | 21 | 1 | 421 | 5 | 437 | Putative tail fiber protein | Putative tail fiber protein | | afdb-uniprot50 | AF-A0A364GR27-F1-MODEL\_V4 | 1.0 | 2.013e-20 | 460 | 0.208 | 642 | 221 | 23 | 1 | 423 | 1 | 574 | Tail-collar fiber protein | Tail-collar fiber protein | | afdb-uniprot50 | AF-A0A8B5NF75-F1-MODEL\_V4 | 1.0 | 7.743e-20 | 460 | 0.211 | 530 | 202 | 17 | 1 | 423 | 1 | 421 | Phage tail protein | Phage tail protein | | afdb-uniprot50 | AF-A0A828HMS4-F1-MODEL\_V4 | 1.0 | 2.742e-21 | 459 | 0.252 | 491 | 234 | 25 | 2 | 408 | 4 | 445 | Uncharacterized protein | Uncharacterized protein | | afdb-uniprot50 | AF-A0A855N7K5-F1-MODEL\_V4 | 1.0 | 5.329e-16 | 459 | 0.22 | 268 | 178 | 9 | 3 | 247 | 2 | 261 | Uncharacterized protein | Uncharacterized protein | | afdb-uniprot50 | AF-A0A5R8ZE78-F1-MODEL\_V4 | 1.0 | 3.955e-18 | 459 | 0.229 | 375 | 180 | 11 | 1 | 272 | 5 | 373 | Collar domain-containing protein | Collar domain-containing protein | | afdb-uniprot50 | AF-A0A7A3AF76-F1-MODEL\_V4 | 1.0 | 3.899e-19 | 459 | 0.238 | 457 | 170 | 17 | 1 | 408 | 1 | 328 | Phage tail protein | Phage tail protein | | afdb-uniprot50 | AF-A0A852Q0N8-F1-MODEL\_V4 | 1.0 | 1.192e-19 | 459 | 0.25 | 443 | 177 | 12 | 1 | 418 | 1 | 313 | Phage tail protein | Phage tail protein | | afdb-uniprot50 | AF-A0A747DAY4-F1-MODEL\_V4 | 1.0 | 9.502e-17 | 458 | 0.217 | 304 | 164 | 8 | 1 | 258 | 32 | 307 | Collar domain-containing protein | Collar domain-containing protein | | afdb-uniprot50 | AF-A0A2X5DKB2-F1-MODEL\_V4 | 1.0 | 7.053e-19 | 458 | 0.227 | 435 | 180 | 13 | 1 | 405 | 2 | 310 | Side tail phage protein | Side tail phage protein | | afdb-uniprot50 | AF-A0A7X5IEB0-F1-MODEL\_V4 | 1.0 | 5.705e-15 | 458 | 0.218 | 247 | 177 | 3 | 1 | 240 | 3 | 240 | Peptidase S74 domain-containing protein | Peptidase S74 domain-containing protein | | afdb-uniprot50 | AF-A0A4R7GCL9-F1-MODEL\_V4 | 1.0 | 1.258e-19 | 458 | 0.211 | 516 | 194 | 13 | 1 | 414 | 1 | 405 | Tail fiber protein gp37 | Tail fiber protein gp37 | | afdb-uniprot50 | AF-A0A2J9QNN2-F1-MODEL\_V4 | 1.0 | 2.125e-20 | 458 | 0.216 | 438 | 188 | 15 | 1 | 392 | 1 | 329 | Phage tail protein | Phage tail protein | | afdb-uniprot50 | AF-A0A370T1V6-F1-MODEL\_V4 | 1.0 | 1.381e-20 | 458 | 0.253 | 450 | 175 | 14 | 1 | 417 | 1 | 322 | Phage-related tail fiber protein | Phage-related tail fiber protein | | afdb-uniprot50 | AF-A0A827ZET3-F1-MODEL\_V4 | 1.0 | 5.685e-19 | 457 | 0.231 | 458 | 174 | 17 | 1 | 409 | 1 | 329 | Phage tail protein | Phage tail protein | | afdb-uniprot50 | AF-A0A1I7H4F9-F1-MODEL\_V4 | 1.0 | 4.518e-20 | 457 | 0.145 | 699 | 282 | 30 | 1 | 423 | 1 | 659 | Phage Tail Collar Domain | Phage Tail Collar Domain | | afdb-uniprot50 | AF-A0A1B8PH65-F1-MODEL\_V4 | 1.0 | 2.367e-20 | 457 | 0.153 | 718 | 256 | 24 | 1 | 423 | 2 | 662 | Collar domain-containing protein | Collar domain-containing protein | | afdb-uniprot50 | AF-A0A0C1UK48-F1-MODEL\_V4 | 1.0 | 8.348e-11 | 456 | 0.435 | 108 | 61 | 0 | 1 | 108 | 2 | 109 | Phage tail-collar fiber family protein | Phage tail-collar fiber family protein | | afdb-uniprot50 | AF-A0A564M9T5-F1-MODEL\_V4 | 1.0 | 1.327e-19 | 456 | 0.22 | 468 | 213 | 17 | 1 | 422 | 2 | 363 | Uncharacterized protein | Uncharacterized protein | | afdb-uniprot50 | AF-A0A7W7KQY3-F1-MODEL\_V4 | 1.0 | 1.538e-20 | 456 | 0.186 | 704 | 264 | 28 | 1 | 421 | 1 | 678 | Uncharacterized protein | Uncharacterized protein | | afdb-uniprot50 | AF-A0A558FD57-F1-MODEL\_V4 | 1.0 | 1.262e-15 | 455 | 0.237 | 290 | 162 | 10 | 1 | 248 | 1 | 273 | Uncharacterized protein | Uncharacterized protein | | afdb-uniprot50 | AF-A0A4R2YQ67-F1-MODEL\_V4 | 1.0 | 1.834e-19 | 455 | 0.235 | 437 | 193 | 13 | 1 | 415 | 1 | 318 | Tail fiber-like repeat protein | Tail fiber-like repeat protein | | afdb-uniprot50 | AF-A0A2C8EXU4-F1-MODEL\_V4 | 1.0 | 4.7e-21 | 454 | 0.173 | 657 | 255 | 20 | 4 | 423 | 8 | 613 | Putative Phage-related tail fiber protein | Putative Phage-related tail fiber protein | | afdb-uniprot50 | AF-A0A1I7E346-F1-MODEL\_V4 | 1.0 | 4.281e-20 | 454 | 0.218 | 499 | 254 | 24 | 1 | 420 | 1 | 442 | Phage tail fibre repeat-containing protein | Phage tail fibre repeat-containing protein | | afdb-uniprot50 | AF-A0A7Z7YC39-F1-MODEL\_V4 | 1.0 | 4.583e-19 | 453 | 0.227 | 395 | 158 | 15 | 1 | 255 | 2 | 389 | Uncharacterized protein | Uncharacterized protein | | afdb-uniprot50 | AF-A0A377PB01-F1-MODEL\_V4 | 1.0 | 1.158e-21 | 453 | 0.211 | 590 | 252 | 24 | 2 | 423 | 5 | 548 | Phage tail fibre repeat | Phage tail fibre repeat | | afdb-uniprot50 | AF-N1ZEN3-F1-MODEL\_V4 | 1.0 | 1.196e-15 | 453 | 0.22 | 281 | 172 | 9 | 1 | 250 | 1 | 265 | Peptidase S74 domain-containing protein | Peptidase S74 domain-containing protein | | afdb-uniprot50 | AF-A0A031GY26-F1-MODEL\_V4 | 1.0 | 9.993e-21 | 453 | 0.178 | 711 | 281 | 30 | 1 | 423 | 1 | 696 | Phage-related tail fiber protein-like protein | Phage-related tail fiber protein-like protein | | afdb-uniprot50 | AF-A0A560Z585-F1-MODEL\_V4 | 1.0 | 2.013e-20 | 452 | 0.204 | 533 | 246 | 26 | 2 | 422 | 6 | 472 | Tail-collar fiber protein | Tail-collar fiber protein | | afdb-uniprot50 | AF-A0A8B2ZR20-F1-MODEL\_V4 | 1.0 | 1.244e-16 | 452 | 0.266 | 319 | 153 | 11 | 1 | 306 | 1 | 251 | Uncharacterized protein | Uncharacterized protein | | afdb-uniprot50 | AF-A0A7Y0ZFL0-F1-MODEL\_V4 | 1.0 | 2.367e-20 | 452 | 0.184 | 651 | 287 | 28 | 2 | 423 | 6 | 641 | Uncharacterized protein | Uncharacterized protein | | afdb-uniprot50 | AF-A0A1M4V0T2-F1-MODEL\_V4 | 1.0 | 4.281e-20 | 451 | 0.225 | 483 | 224 | 23 | 1 | 423 | 2 | 394 | Phage tail-collar fibre protein | Phage tail-collar fibre protein | | afdb-uniprot50 | AF-A0A2A2PHW8-F1-MODEL\_V4 | 1.0 | 2.242e-20 | 451 | 0.191 | 679 | 265 | 27 | 2 | 423 | 6 | 657 | Uncharacterized protein | Uncharacterized protein | | afdb-uniprot50 | AF-A0A845KEW0-F1-MODEL\_V4 | 1.0 | 1.014e-19 | 451 | 0.26 | 449 | 195 | 18 | 1 | 406 | 1 | 355 | Phage tail protein | Phage tail protein | | afdb-uniprot50 | AF-A0A4S1DI76-F1-MODEL\_V4 | 1.0 | 2.57e-18 | 451 | 0.209 | 396 | 174 | 16 | 1 | 259 | 1 | 394 | Uncharacterized protein | Uncharacterized protein | | afdb-uniprot50 | AF-A0A0H3ZQZ8-F1-MODEL\_V4 | 1.0 | 3.211e-10 | 450 | 0.211 | 151 | 112 | 3 | 6 | 153 | 10 | 156 | Phage tail fiber protein | Phage tail fiber protein | | afdb-uniprot50 | AF-A0A7X2D3Y0-F1-MODEL\_V4 | 1.0 | 4.281e-20 | 450 | 0.201 | 506 | 256 | 27 | 1 | 421 | 1 | 443 | Uncharacterized protein | Uncharacterized protein | | afdb-uniprot50 | AF-H2IRJ0-F1-MODEL\_V4 | 1.0 | 1.538e-20 | 450 | 0.188 | 620 | 264 | 27 | 1 | 423 | 1 | 578 | Phage-related tail fiber protein | Phage-related tail fiber protein | | afdb-uniprot50 | AF-A0A822X4Z2-F1-MODEL\_V4 | 1.0 | 5.465e-18 | 450 | 0.25 | 355 | 164 | 12 | 1 | 326 | 2 | 283 | Tail Fiber protein | Tail Fiber protein | | afdb-uniprot50 | AF-A0A1S7SGS6-F1-MODEL\_V4 | 1.0 | 3.955e-18 | 449 | 0.215 | 389 | 167 | 14 | 1 | 258 | 1 | 382 | Uncharacterized protein | Uncharacterized protein | | afdb-uniprot50 | AF-A0A853I101-F1-MODEL\_V4 | 1.0 | 5.387e-19 | 449 | 0.204 | 513 | 244 | 26 | 1 | 422 | 1 | 440 | Phage tail protein | Phage tail protein | | afdb-uniprot50 | AF-A0A7U7FC33-F1-MODEL\_V4 | 1.0 | 7.337e-20 | 449 | 0.244 | 507 | 177 | 16 | 1 | 413 | 2 | 396 | Putative phage-related protein | Putative phage-related protein | | afdb-uniprot50 | AF-A0A850F4G9-F1-MODEL\_V4 | 1.0 | 5.83e-21 | 449 | 0.182 | 549 | 271 | 21 | 1 | 423 | 2 | 498 | Phage tail protein | Phage tail protein | | afdb-uniprot50 | AF-A0A826V725-F1-MODEL\_V4 | 1.0 | 1.175e-20 | 449 | 0.211 | 653 | 248 | 31 | 1 | 421 | 1 | 618 | Phage tail protein | Phage tail protein | | afdb-uniprot50 | AF-A0A2V5B0I4-F1-MODEL\_V4 | 1.0 | 7.633e-21 | 449 | 0.185 | 669 | 287 | 33 | 1 | 423 | 1 | 657 | Tail-collar fiber protein | Tail-collar fiber protein | | afdb-uniprot50 | AF-A0A7M1NUJ9-F1-MODEL\_V4 | 1.0 | 9.606e-20 | 448 | 0.176 | 647 | 227 | 28 | 1 | 422 | 2 | 567 | Phage tail protein | Phage tail protein | | afdb-uniprot50 | AF-A0A5R9QTW1-F1-MODEL\_V4 | 1.0 | 2.598e-21 | 448 | 0.227 | 598 | 265 | 24 | 1 | 423 | 1 | 576 | Phage tail protein | Phage tail protein | | afdb-uniprot50 | AF-A0A826TQX2-F1-MODEL\_V4 | 1.0 | 1.738e-19 | 448 | 0.242 | 487 | 192 | 17 | 1 | 417 | 2 | 381 | Phage tail protein | Phage tail protein | | afdb-uniprot50 | AF-A0A1T0AVV7-F1-MODEL\_V4 | 1.0 | 5.31e-20 | 448 | 0.235 | 459 | 195 | 16 | 1 | 418 | 1 | 344 | Collar domain-containing protein | Collar domain-containing protein | | afdb-uniprot50 | AF-A0A0C1RAC0-F1-MODEL\_V4 | 1.0 | 2.978e-19 | 447 | 0.222 | 413 | 164 | 13 | 1 | 258 | 1 | 411 | Phage tail-collar fiber family protein | Phage tail-collar fiber family protein | | afdb-uniprot50 | AF-A0A3G6ZV45-F1-MODEL\_V4 | 1.0 | 2.636e-20 | 447 | 0.179 | 630 | 285 | 29 | 2 | 423 | 6 | 611 | Phage tail fiber protein | Phage tail fiber protein | | afdb-uniprot50 | AF-G9PUJ7-F1-MODEL\_V4 | 1.0 | 2.02e-16 | 446 | 0.248 | 322 | 155 | 13 | 5 | 260 | 3 | 303 | Uncharacterized protein | Uncharacterized protein | | afdb-uniprot50 | AF-A0A379Z2I6-F1-MODEL\_V4 | 1.0 | 3.843e-20 | 446 | 0.232 | 456 | 175 | 14 | 1 | 417 | 2 | 321 | Phage tail fibre repeat | Phage tail fibre repeat | | afdb-uniprot50 | AF-A0A4R2YZB4-F1-MODEL\_V4 | 1.0 | 3.098e-20 | 446 | 0.192 | 625 | 267 | 26 | 1 | 420 | 1 | 592 | Tail fiber-like repeat protein | Tail fiber-like repeat protein | | afdb-uniprot50 | AF-A0A5U3EWV3-F1-MODEL\_V4 | 1.0 | 3.843e-20 | 445 | 0.227 | 480 | 212 | 14 | 1 | 412 | 2 | 390 | Phage tail protein | Phage tail protein | | afdb-uniprot50 | AF-A0A0E3CGK1-F1-MODEL\_V4 | 1.0 | 1.129e-19 | 445 | 0.221 | 491 | 215 | 24 | 1 | 377 | 1 | 438 | Uncharacterized protein | Uncharacterized protein | | afdb-uniprot50 | AF-A0A0B6XER1-F1-MODEL\_V4 | 1.0 | 2.02e-16 | 445 | 0.247 | 311 | 117 | 8 | 1 | 306 | 2 | 200 | Putative phage variable tail fiber protein | Putative phage variable tail fiber protein | | afdb-uniprot50 | AF-A0A2D0KQU5-F1-MODEL\_V4 | 1.0 | 2.367e-20 | 445 | 0.208 | 676 | 262 | 32 | 1 | 423 | 2 | 657 | Phage tail fiber protein | Phage tail fiber protein | | afdb-uniprot50 | AF-A0A152BV35-F1-MODEL\_V4 | 1.0 | 1.028e-18 | 445 | 0.207 | 486 | 212 | 16 | 1 | 417 | 5 | 386 | Phage tail protein | Phage tail protein | | afdb-uniprot50 | AF-A7GFS8-F1-MODEL\_V4 | 1.0 | 2.308e-18 | 444 | 0.227 | 395 | 165 | 10 | 1 | 258 | 2 | 393 | Tail fiber protein H | Tail fiber protein H | | afdb-uniprot50 | AF-A0A5K4Y4X7-F1-MODEL\_V4 | 1.0 | 5.915e-20 | 444 | 0.216 | 540 | 211 | 27 | 1 | 421 | 1 | 447 | Uncharacterized protein | Uncharacterized protein | | afdb-uniprot50 | AF-B1JB17-F1-MODEL\_V4 | 1.0 | 7.743e-20 | 444 | 0.176 | 737 | 266 | 26 | 1 | 423 | 5 | 714 | Phage-related tail fibre protein-like protein | Phage-related tail fibre protein-like protein | | afdb-uniprot50 | AF-A0A0A1HV98-F1-MODEL\_V4 | 1.0 | 2.636e-20 | 444 | 0.16 | 743 | 274 | 25 | 2 | 423 | 6 | 719 | Phage tail fiber protein | Phage tail fiber protein | | afdb-uniprot50 | AF-A0A7Y8TQ40-F1-MODEL\_V4 | 1.0 | 4.977e-17 | 443 | 0.224 | 321 | 171 | 14 | 1 | 259 | 1 | 305 | Phage tail protein | Phage tail protein | | afdb-uniprot50 | AF-A0A2W0FKV2-F1-MODEL\_V4 | 1.0 | 4.453e-21 | 443 | 0.216 | 535 | 252 | 27 | 2 | 423 | 6 | 486 | Phage tail protein | Phage tail protein | | afdb-uniprot50 | AF-A0A1B1KLD7-F1-MODEL\_V4 | 1.0 | 4.056e-20 | 443 | 0.236 | 448 | 183 | 15 | 1 | 417 | 2 | 321 | Uncharacterized protein | Uncharacterized protein | | afdb-uniprot50 | AF-A0A3I8FWP0-F1-MODEL\_V4 | 1.0 | 3.451e-20 | 442 | 0.185 | 562 | 244 | 27 | 1 | 420 | 4 | 493 | Uncharacterized protein | Uncharacterized protein | | afdb-uniprot50 | AF-A0A3G7JDV4-F1-MODEL\_V4 | 1.0 | 1.56e-19 | 442 | 0.203 | 526 | 258 | 21 | 2 | 410 | 6 | 487 | Phage tail fiber protein | Phage tail fiber protein | | afdb-uniprot50 | AF-A0A7G8SK23-F1-MODEL\_V4 | 1.0 | 1.276e-18 | 442 | 0.227 | 449 | 193 | 11 | 1 | 412 | 1 | 332 | Phage tail protein | Phage tail protein | | afdb-uniprot50 | AF-A0A3V2YUH3-F1-MODEL\_V4 | 1.0 | 2.367e-20 | 442 | 0.201 | 644 | 252 | 31 | 1 | 423 | 1 | 602 | Uncharacterized protein | Uncharacterized protein | | afdb-uniprot50 | AF-A0A1H8UEW5-F1-MODEL\_V4 | 1.0 | 1.209e-18 | 442 | 0.25 | 455 | 224 | 15 | 1 | 405 | 2 | 389 | Phage-related tail fibre protein | Phage-related tail fibre protein | | afdb-uniprot50 | AF-A0A822N451-F1-MODEL\_V4 | 1.0 | 2.072e-18 | 442 | 0.233 | 480 | 168 | 14 | 1 | 417 | 2 | 344 | Tail fiber protein XnpH1 of the Xenorhabdicin, a R-type bacteriocin | Tail fiber protein XnpH1 of the Xenorhabdicin, a R-type bacteriocin | | afdb-uniprot50 | AF-A0A2L0TU26-F1-MODEL\_V4 | 1.0 | 2.367e-20 | 441 | 0.233 | 506 | 241 | 18 | 2 | 416 | 3 | 452 | Phage tail protein | Phage tail protein | | afdb-uniprot50 | AF-H8NT04-F1-MODEL\_V4 | 1.0 | 5.387e-19 | 441 | 0.229 | 445 | 192 | 12 | 1 | 412 | 1 | 327 | Tail fiber repeat 2 protein | Tail fiber repeat 2 protein | | afdb-uniprot50 | AF-A0A5S3XN15-F1-MODEL\_V4 | 1.0 | 2.873e-14 | 441 | 0.218 | 266 | 174 | 9 | 1 | 249 | 3 | 251 | Uncharacterized protein | Uncharacterized protein | | afdb-uniprot50 | AF-A0A840UDD6-F1-MODEL\_V4 | 1.0 | 1.478e-19 | 440 | 0.22 | 480 | 222 | 23 | 2 | 417 | 3 | 394 | Phage-related tail fiber protein | Phage-related tail fiber protein | | afdb-uniprot50 | AF-A0A2A2MBN2-F1-MODEL\_V4 | 1.0 | 8.502e-21 | 439 | 0.254 | 492 | 204 | 19 | 1 | 409 | 1 | 412 | Uncharacterized protein | Uncharacterized protein | | afdb-uniprot50 | AF-J2Y2U3-F1-MODEL\_V4 | 1.0 | 2.042e-19 | 439 | 0.167 | 667 | 284 | 30 | 1 | 423 | 1 | 639 | Putative phage tail protein | Putative phage tail protein | | afdb-uniprot50 | AF-A0A7B5VCM3-F1-MODEL\_V4 | 1.0 | 2.791e-16 | 438 | 0.241 | 298 | 159 | 15 | 1 | 256 | 1 | 273 | Tail fiber | Tail fiber | | afdb-uniprot50 | AF-A0A6G4HNB6-F1-MODEL\_V4 | 1.0 | 8.875e-18 | 438 | 0.221 | 357 | 180 | 10 | 1 | 260 | 1 | 356 | Phage tail protein | Phage tail protein | | afdb-uniprot50 | AF-A0A1I1KYG8-F1-MODEL\_V4 | 1.0 | 3.021e-18 | 438 | 0.209 | 488 | 233 | 21 | 1 | 423 | 1 | 400 | Phage tail-collar fibre protein | Phage tail-collar fibre protein | | afdb-uniprot50 | AF-A0A2W7IK50-F1-MODEL\_V4 | 1.0 | 5.604e-20 | 438 | 0.216 | 512 | 220 | 31 | 1 | 423 | 1 | 420 | Tail-collar fiber protein | Tail-collar fiber protein | | afdb-uniprot50 | AF-A0A5B0C2P7-F1-MODEL\_V4 | 1.0 | 1.175e-20 | 438 | 0.18 | 709 | 281 | 30 | 2 | 423 | 6 | 701 | Phage tail protein | Phage tail protein | | afdb-uniprot50 | AF-A0A827LBC4-F1-MODEL\_V4 | 1.0 | 2.822e-19 | 438 | 0.219 | 470 | 204 | 19 | 1 | 423 | 5 | 358 | Phage tail protein | Phage tail protein | | afdb-uniprot50 | AF-A0A5V0QDA0-F1-MODEL\_V4 | 1.0 | 2.791e-16 | 437 | 0.228 | 306 | 164 | 12 | 1 | 260 | 1 | 280 | Collar domain-containing protein | Collar domain-containing protein | | afdb-uniprot50 | AF-A0A7G9WG78-F1-MODEL\_V4 | 1.0 | 5.14e-11 | 436 | 0.404 | 121 | 72 | 0 | 28 | 148 | 1 | 121 | Phage tail protein | Phage tail protein | | afdb-uniprot50 | AF-A0A2I4NEU5-F1-MODEL\_V4 | 1.0 | 1.808e-20 | 436 | 0.21 | 500 | 263 | 25 | 1 | 422 | 2 | 447 | Phage tail-collar fiber family protein | Phage tail-collar fiber family protein | | afdb-uniprot50 | AF-A0A429K136-F1-MODEL\_V4 | 1.0 | 1.196e-15 | 436 | 0.2 | 339 | 177 | 12 | 1 | 259 | 1 | 325 | Uncharacterized protein | Uncharacterized protein | | afdb-uniprot50 | AF-A0A6D0S3Z5-F1-MODEL\_V4 | 1.0 | 1.963e-18 | 436 | 0.213 | 534 | 195 | 18 | 1 | 423 | 2 | 421 | Phage tail protein | Phage tail protein | | afdb-uniprot50 | AF-A0A8A6J8G1-F1-MODEL\_V4 | 1.0 | 2.435e-18 | 436 | 0.236 | 453 | 177 | 10 | 1 | 417 | 2 | 321 | Phage tail protein | Phage tail protein | | afdb-uniprot50 | AF-R6P5W1-F1-MODEL\_V4 | 1.0 | 6e-19 | 435 | 0.168 | 451 | 222 | 18 | 4 | 423 | 3 | 331 | Putative tail fiber-related protein | Putative tail fiber-related protein | | afdb-uniprot50 | AF-A0A3V5LF01-F1-MODEL\_V4 | 1.0 | 4.583e-19 | 435 | 0.224 | 494 | 225 | 19 | 1 | 403 | 1 | 427 | Phage tail protein | Phage tail protein | | afdb-uniprot50 | AF-A0A6B4FV55-F1-MODEL\_V4 | 1.0 | 2.978e-19 | 434 | 0.201 | 507 | 222 | 24 | 1 | 420 | 2 | 412 | Phage tail protein | Phage tail protein | | afdb-uniprot50 | AF-A0A4V1INX2-F1-MODEL\_V4 | 1.0 | 8.172e-20 | 434 | 0.209 | 510 | 214 | 22 | 1 | 416 | 1 | 415 | Uncharacterized protein | Uncharacterized protein | | afdb-uniprot50 | AF-A0A7T7R8V5-F1-MODEL\_V4 | 1.0 | 6.779e-18 | 433 | 0.197 | 451 | 230 | 21 | 1 | 423 | 1 | 347 | Phage tail protein | Phage tail protein | | afdb-uniprot50 | AF-A0A1X7LWW5-F1-MODEL\_V4 | 1.0 | 7.053e-19 | 433 | 0.192 | 567 | 199 | 20 | 1 | 419 | 1 | 456 | Phage tail fibre repeat-containing protein | Phage tail fibre repeat-containing protein | | afdb-uniprot50 | AF-A0A2K4JEE0-F1-MODEL\_V4 | 1.0 | 3.098e-20 | 433 | 0.19 | 572 | 252 | 26 | 2 | 421 | 6 | 518 | Phage tail protein | Phage tail protein | | afdb-uniprot50 | AF-A0A2N6J7T9-F1-MODEL\_V4 | 1.0 | 2.978e-19 | 433 | 0.224 | 521 | 221 | 25 | 1 | 422 | 1 | 437 | Phage-related tail fiber protein-like protein | Phage-related tail fiber protein-like protein | | afdb-uniprot50 | AF-A0A857ER02-F1-MODEL\_V4 | 1.0 | 1.457e-20 | 433 | 0.167 | 663 | 286 | 24 | 1 | 423 | 1 | 637 | Uncharacterized protein | Uncharacterized protein | | afdb-uniprot50 | AF-A0A8B6AAK0-F1-MODEL\_V4 | 1.0 | 1.478e-19 | 433 | 0.174 | 746 | 274 | 27 | 1 | 423 | 2 | 728 | Phage-related tail fiber protein-like protein | Phage-related tail fiber protein-like protein | | afdb-uniprot50 | AF-A0A4U3C4N5-F1-MODEL\_V4 | 1.0 | 1.209e-18 | 433 | 0.189 | 438 | 235 | 17 | 1 | 421 | 91 | 425 | DUF1566 domain-containing protein | DUF1566 domain-containing protein | | afdb-uniprot50 | AF-A0A269PIY6-F1-MODEL\_V4 | 1.0 | 2.02e-16 | 433 | 0.174 | 338 | 179 | 13 | 4 | 258 | 3 | 323 | Uncharacterized protein | Uncharacterized protein | | afdb-uniprot50 | AF-A0A261REI1-F1-MODEL\_V4 | 1.0 | 2.341e-17 | 432 | 0.219 | 456 | 209 | 21 | 1 | 412 | 1 | 353 | Uncharacterized protein | Uncharacterized protein | | afdb-uniprot50 | AF-A0A1I6E7W8-F1-MODEL\_V4 | 1.0 | 1.145e-18 | 432 | 0.195 | 569 | 210 | 26 | 1 | 423 | 2 | 468 | Phage-related tail fibre protein | Phage-related tail fibre protein | | afdb-uniprot50 | AF-A0A1D7Z5A8-F1-MODEL\_V4 | 1.0 | 6e-19 | 432 | 0.211 | 642 | 177 | 20 | 1 | 423 | 2 | 532 | Phage tail protein | Phage tail protein | | afdb-uniprot50 | AF-A0A853I581-F1-MODEL\_V4 | 1.0 | 6.952e-20 | 431 | 0.177 | 512 | 237 | 25 | 2 | 423 | 3 | 420 | Phage tail protein | Phage tail protein | | afdb-uniprot50 | AF-A0A7J0BJF8-F1-MODEL\_V4 | 1.0 | 9.993e-21 | 431 | 0.189 | 558 | 264 | 27 | 1 | 423 | 1 | 504 | Tail fiber protein | Tail fiber protein | | afdb-uniprot50 | AF-A0A2T5NWN9-F1-MODEL\_V4 | 1.0 | 3.098e-20 | 430 | 0.19 | 561 | 266 | 31 | 1 | 420 | 1 | 514 | Uncharacterized protein | Uncharacterized protein | | afdb-uniprot50 | AF-A0A7M1L6P4-F1-MODEL\_V4 | 1.0 | 8.172e-20 | 429 | 0.194 | 560 | 266 | 25 | 1 | 418 | 1 | 517 | Phage tail protein | Phage tail protein | | afdb-uniprot50 | AF-A0A329CFM1-F1-MODEL\_V4 | 1.0 | 2.341e-17 | 428 | 0.263 | 372 | 183 | 16 | 1 | 323 | 5 | 334 | Tail-collar fiber protein | Tail-collar fiber protein | | afdb-uniprot50 | AF-A0A376LF19-F1-MODEL\_V4 | 1.0 | 3.694e-19 | 428 | 0.203 | 535 | 200 | 18 | 1 | 423 | 1 | 421 | Phage tail fiber protein H (GpH) | Phage tail fiber protein H (GpH) | | afdb-uniprot50 | AF-A0A4R6PI57-F1-MODEL\_V4 | 1.0 | 2.978e-19 | 426 | 0.188 | 663 | 280 | 31 | 1 | 423 | 1 | 645 | Phage-related tail fiber protein | Phage-related tail fiber protein | | afdb-uniprot50 | AF-A0A3B0C125-F1-MODEL\_V4 | 1.0 | 2.349e-13 | 426 | 0.258 | 213 | 133 | 7 | 1 | 194 | 1 | 207 | Uncharacterized protein | Uncharacterized protein | | afdb-uniprot50 | AF-A0A376FFE3-F1-MODEL\_V4 | 1.0 | 2.086e-10 | 425 | 0.517 | 116 | 55 | 1 | 32 | 147 | 2 | 116 | Phage tail collar domain-containing protein | Phage tail collar domain-containing protein | | afdb-uniprot50 | AF-A0A285M654-F1-MODEL\_V4 | 1.0 | 2.308e-18 | 425 | 0.191 | 418 | 175 | 13 | 1 | 258 | 1 | 415 | Phage tail-collar fibre protein | Phage tail-collar fibre protein | | afdb-uniprot50 | AF-A0A0N9MHE6-F1-MODEL\_V4 | 1.0 | 2.102e-17 | 425 | 0.286 | 363 | 183 | 19 | 2 | 324 | 6 | 332 | Phage tail fiber protein | Phage tail fiber protein | | afdb-uniprot50 | AF-A0A1X1A8V7-F1-MODEL\_V4 | 1.0 | 2.752e-17 | 425 | 0.267 | 396 | 178 | 16 | 1 | 311 | 5 | 373 | Uncharacterized protein | Uncharacterized protein | | afdb-uniprot50 | AF-A0A1W7LRA1-F1-MODEL\_V4 | 1.0 | 9.606e-20 | 424 | 0.177 | 513 | 276 | 27 | 1 | 421 | 1 | 459 | Putative phage tail fiber protein | Putative phage tail fiber protein | | afdb-uniprot50 | AF-A0A1H2M4C6-F1-MODEL\_V4 | 1.0 | 3.694e-19 | 424 | 0.258 | 461 | 224 | 20 | 2 | 405 | 6 | 405 | Phage tail-collar fibre protein | Phage tail-collar fibre protein | | afdb-uniprot50 | AF-A0A5E1AHB0-F1-MODEL\_V4 | 1.0 | 7.443e-19 | 424 | 0.231 | 475 | 252 | 17 | 1 | 405 | 2 | 433 | Tail Fiber protein | Tail Fiber protein | | afdb-uniprot50 | AF-A0A5N9IEZ9-F1-MODEL\_V4 | 1.0 | 9.233e-19 | 424 | 0.154 | 813 | 269 | 29 | 2 | 423 | 6 | 790 | Phage tail protein | Phage tail protein | | afdb-uniprot50 | AF-A0A2X5NM48-F1-MODEL\_V4 | 1.0 | 5.032e-20 | 423 | 0.159 | 519 | 261 | 22 | 3 | 423 | 2 | 443 | Uncharacterized protein | Uncharacterized protein | | afdb-uniprot50 | AF-A0A315T3S6-F1-MODEL\_V4 | 1.0 | 1.014e-19 | 423 | 0.157 | 817 | 278 | 35 | 1 | 423 | 5 | 804 | Tail-collar fiber protein | Tail-collar fiber protein | | afdb-uniprot50 | AF-A0A377IMK2-F1-MODEL\_V4 | 1.0 | 8.624e-20 | 423 | 0.222 | 494 | 242 | 27 | 1 | 422 | 1 | 424 | Putative phage-like tail fiber protein | Putative phage-like tail fiber protein | | afdb-uniprot50 | AF-N9JQ34-F1-MODEL\_V4 | 1.0 | 6.087e-18 | 422 | 0.207 | 434 | 245 | 16 | 1 | 423 | 1 | 346 | Uncharacterized protein | Uncharacterized protein | | afdb-uniprot50 | AF-A0A348HI80-F1-MODEL\_V4 | 1.0 | 1.583e-18 | 422 | 0.226 | 495 | 190 | 21 | 1 | 417 | 1 | 380 | Phage-related tail fibre | Phage-related tail fibre | | afdb-uniprot50 | AF-A0A3E0FEW6-F1-MODEL\_V4 | 1.0 | 3.899e-19 | 421 | 0.212 | 531 | 249 | 22 | 4 | 411 | 8 | 492 | Tail-collar fiber protein | Tail-collar fiber protein | | afdb-uniprot50 | AF-A0A1X0VZ24-F1-MODEL\_V4 | 1.0 | 1.629e-16 | 420 | 0.232 | 322 | 173 | 13 | 1 | 252 | 1 | 318 | Phage tail protein | Phage tail protein | | afdb-uniprot50 | AF-A0A1B5ER81-F1-MODEL\_V4 | 1.0 | 6.242e-20 | 420 | 0.175 | 702 | 280 | 33 | 2 | 423 | 6 | 688 | Uncharacterized protein | Uncharacterized protein | | afdb-uniprot50 | AF-A0A446AZN6-F1-MODEL\_V4 | 1.0 | 5.387e-19 | 420 | 0.16 | 775 | 286 | 31 | 2 | 423 | 6 | 768 | Uncharacterized protein | Uncharacterized protein | | afdb-uniprot50 | AF-A0A1Q6JPI1-F1-MODEL\_V4 | 1.0 | 4.296e-16 | 419 | 0.197 | 349 | 192 | 13 | 4 | 313 | 15 | 314 | Uncharacterized protein | Uncharacterized protein | | afdb-uniprot50 | AF-A0A2G8BXY9-F1-MODEL\_V4 | 1.0 | 3.551e-18 | 419 | 0.213 | 472 | 208 | 21 | 1 | 423 | 1 | 358 | Phage tail protein | Phage tail protein | | afdb-uniprot50 | AF-A0A0U4W4I1-F1-MODEL\_V4 | 1.0 | 5.104e-19 | 419 | 0.218 | 572 | 252 | 29 | 1 | 423 | 2 | 527 | Ig-like domain-containing protein | Ig-like domain-containing protein | | afdb-uniprot50 | AF-A0A246GVB8-F1-MODEL\_V4 | 1.0 | 4.649e-18 | 419 | 0.229 | 419 | 180 | 15 | 1 | 311 | 5 | 388 | Collar domain-containing protein | Collar domain-containing protein | | afdb-uniprot50 | AF-A0A3S5ENC7-F1-MODEL\_V4 | 1.0 | 1.788e-17 | 418 | 0.24 | 486 | 187 | 17 | 2 | 412 | 3 | 381 | Putative bacteriophage tail fiber protein | Putative bacteriophage tail fiber protein | | afdb-uniprot50 | AF-A0A1H9AFH7-F1-MODEL\_V4 | 1.0 | 1.313e-16 | 418 | 0.274 | 361 | 179 | 16 | 2 | 311 | 6 | 334 | Phage-related tail fibre protein | Phage-related tail fibre protein | | afdb-uniprot50 | AF-A0A077P9Q6-F1-MODEL\_V4 | 1.0 | 4.07e-16 | 418 | 0.24 | 333 | 134 | 9 | 1 | 326 | 2 | 222 | Uncharacterized protein | Uncharacterized protein | | afdb-uniprot50 | AF-J2MLE3-F1-MODEL\_V4 | 1.0 | 7.855e-19 | 417 | 0.253 | 450 | 236 | 13 | 2 | 417 | 6 | 389 | Putative phage protein | Putative phage protein | | afdb-uniprot50 | AF-V8RA43-F1-MODEL\_V4 | 1.0 | 8.749e-19 | 417 | 0.14 | 842 | 267 | 35 | 1 | 423 | 1 | 804 | Tail protein | Tail protein | | afdb-uniprot50 | AF-A0A0E1PUY8-F1-MODEL\_V4 | 1.0 | 6.588e-20 | 417 | 0.193 | 485 | 267 | 23 | 1 | 423 | 1 | 423 | Uncharacterized protein | Uncharacterized protein | | afdb-uniprot50 | AF-A0A5C8BE21-F1-MODEL\_V4 | 1.0 | 6.446e-14 | 416 | 0.245 | 248 | 140 | 5 | 3 | 217 | 2 | 235 | Uncharacterized protein | Uncharacterized protein | | afdb-uniprot50 | AF-A0A806X791-F1-MODEL\_V4 | 1.0 | 7.969e-18 | 416 | 0.213 | 436 | 242 | 10 | 1 | 416 | 2 | 356 | Uncharacterized protein | Uncharacterized protein | | afdb-uniprot50 | AF-A0A828NYP4-F1-MODEL\_V4 | 1.0 | 4.649e-18 | 416 | 0.189 | 464 | 201 | 16 | 1 | 409 | 4 | 347 | Phage tail protein | Phage tail protein | | afdb-uniprot50 | AF-A0A1K2AJW9-F1-MODEL\_V4 | 1.0 | 1.9e-09 | 416 | 0.401 | 127 | 76 | 0 | 32 | 158 | 2 | 128 | Phage tail-collar fibre protein | Phage tail-collar fibre protein | | afdb-uniprot50 | AF-A0A0N9MHY1-F1-MODEL\_V4 | 1.0 | 2.904e-17 | 416 | 0.269 | 378 | 181 | 16 | 2 | 306 | 6 | 361 | Collar domain-containing protein | Collar domain-containing protein | | afdb-uniprot50 | AF-A0A853I1G7-F1-MODEL\_V4 | 1.0 | 9.233e-19 | 415 | 0.174 | 544 | 213 | 23 | 2 | 422 | 3 | 433 | Phage tail protein | Phage tail protein | | afdb-uniprot50 | AF-A0A1S8TWQ6-F1-MODEL\_V4 | 1.0 | 1.56e-19 | 415 | 0.201 | 531 | 260 | 30 | 1 | 423 | 2 | 476 | Uncharacterized protein | Uncharacterized protein | | afdb-uniprot50 | AF-A0A8B5G8Z2-F1-MODEL\_V4 | 1.0 | 2.234e-09 | 414 | 0.48 | 102 | 53 | 0 | 1 | 102 | 2 | 103 | Phage tail protein | Phage tail protein | | afdb-uniprot50 | AF-A0A2T5NR62-F1-MODEL\_V4 | 1.0 | 1.56e-19 | 414 | 0.193 | 544 | 217 | 28 | 1 | 422 | 1 | 444 | Uncharacterized protein | Uncharacterized protein | | afdb-uniprot50 | AF-A0A3M2YA40-F1-MODEL\_V4 | 1.0 | 1.788e-17 | 414 | 0.243 | 399 | 199 | 19 | 1 | 350 | 5 | 349 | Uncharacterized protein | Uncharacterized protein | | afdb-uniprot50 | AF-A0A3G6Z2B6-F1-MODEL\_V4 | 1.0 | 2.636e-20 | 413 | 0.176 | 572 | 255 | 25 | 1 | 423 | 1 | 505 | Uncharacterized protein | Uncharacterized protein | | afdb-uniprot50 | AF-A0A1I5MQ45-F1-MODEL\_V4 | 1.0 | 9.745e-19 | 413 | 0.202 | 513 | 244 | 22 | 1 | 412 | 1 | 449 | Chaperone of endosialidase | Chaperone of endosialidase | | afdb-uniprot50 | AF-A0A4V3CGG4-F1-MODEL\_V4 | 1.0 | 2.674e-19 | 412 | 0.189 | 534 | 246 | 29 | 1 | 405 | 1 | 476 | Tail-collar fiber protein | Tail-collar fiber protein | | afdb-uniprot50 | AF-A0A285M2V3-F1-MODEL\_V4 | 1.0 | 7.443e-19 | 410 | 0.202 | 500 | 204 | 19 | 2 | 422 | 4 | 387 | Phage tail-collar fibre protein | Phage tail-collar fibre protein | | afdb-uniprot50 | AF-A0A484QR79-F1-MODEL\_V4 | 1.0 | 4.837e-19 | 410 | 0.202 | 548 | 237 | 30 | 3 | 423 | 2 | 476 | Phage tail fiber protein | Phage tail fiber protein | | afdb-uniprot50 | AF-A0A3E1QH42-F1-MODEL\_V4 | 1.0 | 7.443e-19 | 410 | 0.213 | 441 | 216 | 20 | 1 | 415 | 2 | 337 | Phage tail protein | Phage tail protein | | afdb-uniprot50 | AF-A0A1W9UG34-F1-MODEL\_V4 | 1.0 | 1.346e-18 | 409 | 0.191 | 495 | 212 | 23 | 1 | 423 | 1 | 379 | Uncharacterized protein | Uncharacterized protein | | afdb-uniprot50 | AF-A0A1H8JBA9-F1-MODEL\_V4 | 1.0 | 9.035e-13 | 409 | 0.318 | 207 | 121 | 6 | 18 | 211 | 2 | 201 | Phage tail-collar fibre protein | Phage tail-collar fibre protein | | afdb-uniprot50 | AF-S4YR67-F1-MODEL\_V4 | 1.0 | 4.405e-18 | 408 | 0.228 | 420 | 197 | 10 | 1 | 412 | 2 | 302 | Uncharacterized protein | Uncharacterized protein | | afdb-uniprot50 | AF-A0A5Q2KDB2-F1-MODEL\_V4 | 1.0 | 7.443e-19 | 408 | 0.229 | 461 | 201 | 18 | 1 | 413 | 1 | 355 | Uncharacterized protein | Uncharacterized protein | | afdb-uniprot50 | AF-A0A5U3F095-F1-MODEL\_V4 | 1.0 | 1.192e-19 | 408 | 0.169 | 719 | 264 | 33 | 1 | 423 | 4 | 685 | Phage tail protein | Phage tail protein | | afdb-uniprot50 | AF-A0A3Q8TZG5-F1-MODEL\_V4 | 1.0 | 1.213e-14 | 408 | 0.265 | 290 | 165 | 11 | 1 | 255 | 5 | 281 | Collar domain-containing protein | Collar domain-containing protein | | afdb-uniprot50 | AF-A0A380T4V8-F1-MODEL\_V4 | 1.0 | 7.443e-19 | 408 | 0.174 | 786 | 276 | 32 | 2 | 423 | 6 | 782 | Putative tail fiber protein | Putative tail fiber protein | | afdb-uniprot50 | AF-A0A5E4UH46-F1-MODEL\_V4 | 1.0 | 3.021e-18 | 407 | 0.228 | 434 | 218 | 21 | 1 | 392 | 2 | 360 | Uncharacterized protein | Uncharacterized protein | | afdb-uniprot50 | AF-A0A1N6I1A6-F1-MODEL\_V4 | 1.0 | 8.2e-16 | 406 | 0.239 | 351 | 154 | 12 | 1 | 255 | 1 | 334 | Phage tail-collar fibre protein | Phage tail-collar fibre protein | | afdb-uniprot50 | AF-A0A4U8YRJ4-F1-MODEL\_V4 | 1.0 | 3.857e-16 | 406 | 0.236 | 342 | 170 | 11 | 1 | 257 | 1 | 336 | Phage tail fibre protein | Phage tail fibre protein | | afdb-uniprot50 | AF-Q1QXR9-F1-MODEL\_V4 | 1.0 | 1.763e-18 | 406 | 0.215 | 551 | 255 | 26 | 1 | 422 | 1 | 502 | Phage-related tail fibre protein-like protein | Phage-related tail fibre protein-like protein | | afdb-uniprot50 | AF-E1W2D8-F1-MODEL\_V4 | 1.0 | 2.275e-19 | 406 | 0.206 | 509 | 209 | 16 | 1 | 413 | 2 | 411 | Probable tail fiber protein | Probable tail fiber protein | | afdb-uniprot50 | AF-A0A1H1IHT2-F1-MODEL\_V4 | 1.0 | 1.145e-18 | 406 | 0.165 | 696 | 288 | 27 | 2 | 423 | 6 | 682 | Phage tail-collar fibre protein | Phage tail-collar fibre protein | | afdb-uniprot50 | AF-A0A616NKZ7-F1-MODEL\_V4 | 1.0 | 1.085e-18 | 406 | 0.244 | 494 | 212 | 23 | 2 | 423 | 3 | 407 | Phage tail protein | Phage tail protein | | afdb-uniprot50 | AF-A0A6I1HR03-F1-MODEL\_V4 | 1.0 | 6.424e-18 | 406 | 0.142 | 771 | 285 | 27 | 1 | 422 | 5 | 747 | Uncharacterized protein | Uncharacterized protein | | afdb-uniprot50 | AF-A0A2D0LG90-F1-MODEL\_V4 | 1.0 | 9.265e-15 | 406 | 0.227 | 316 | 117 | 8 | 1 | 306 | 2 | 200 | Tail protein | Tail protein | | afdb-uniprot50 | AF-A0A5Y3B2B3-F1-MODEL\_V4 | 1.0 | 3.414e-17 | 405 | 0.187 | 453 | 247 | 18 | 1 | 420 | 1 | 365 | Uncharacterized protein | Uncharacterized protein | | afdb-uniprot50 | AF-A0A3M0SWC3-F1-MODEL\_V4 | 1.0 | 3.143e-19 | 405 | 0.187 | 591 | 249 | 30 | 1 | 422 | 1 | 529 | Uncharacterized protein | Uncharacterized protein | | afdb-uniprot50 | AF-A0A2W5T9N3-F1-MODEL\_V4 | 1.0 | 7.363e-16 | 404 | 0.238 | 310 | 170 | 13 | 1 | 258 | 2 | 297 | Uncharacterized protein | Uncharacterized protein | | afdb-uniprot50 | AF-A0A2X4TX84-F1-MODEL\_V4 | 1.0 | 2.187e-18 | 404 | 0.226 | 500 | 197 | 21 | 4 | 419 | 3 | 396 | Uncharacterized protein | Uncharacterized protein | | afdb-uniprot50 | AF-N2IMU0-F1-MODEL\_V4 | 1.0 | 8.749e-19 | 404 | 0.23 | 600 | 226 | 34 | 1 | 422 | 1 | 542 | Uncharacterized protein | Uncharacterized protein | | afdb-uniprot50 | AF-E5Y5X2-F1-MODEL\_V4 | 1.0 | 1.276e-18 | 404 | 0.209 | 478 | 246 | 21 | 1 | 415 | 2 | 410 | Uncharacterized protein | Uncharacterized protein | | afdb-uniprot50 | AF-A0A7Z2TBJ7-F1-MODEL\_V4 | 1.0 | 2.863e-18 | 404 | 0.156 | 781 | 283 | 37 | 1 | 423 | 1 | 763 | Phage tail protein | Phage tail protein | | afdb-uniprot50 | AF-A0A8A7WRM1-F1-MODEL\_V4 | 1.0 | 1.014e-19 | 403 | 0.251 | 466 | 230 | 24 | 1 | 417 | 1 | 396 | Phage tail protein | Phage tail protein | | afdb-uniprot50 | AF-A0A3M4NA57-F1-MODEL\_V4 | 1.0 | 3.802e-17 | 401 | 0.244 | 405 | 201 | 18 | 1 | 355 | 5 | 354 | Tail fiber protein H | Tail fiber protein H | | afdb-uniprot50 | AF-A0A1G9EQI8-F1-MODEL\_V4 | 1.0 | 4.343e-19 | 401 | 0.197 | 581 | 267 | 29 | 2 | 416 | 6 | 552 | Phage-related tail fibre protein | Phage-related tail fibre protein | | afdb-uniprot50 | AF-A0A221FN03-F1-MODEL\_V4 | 1.0 | 2.506e-16 | 400 | 0.197 | 364 | 181 | 12 | 1 | 258 | 1 | 359 | Phage tail protein | Phage tail protein | | afdb-uniprot50 | AF-A0A2D0ITF1-F1-MODEL\_V4 | 1.0 | 9.885e-18 | 400 | 0.234 | 473 | 175 | 10 | 1 | 417 | 2 | 343 | Phage tail fiber protein | Phage tail fiber protein | | afdb-uniprot50 | AF-A0A7T1Y6B1-F1-MODEL\_V4 | 1.0 | 6.087e-18 | 399 | 0.228 | 477 | 214 | 23 | 2 | 416 | 6 | 390 | Phage tail protein | Phage tail protein | | afdb-uniprot50 | AF-A0A855K828-F1-MODEL\_V4 | 1.0 | 1.763e-18 | 399 | 0.196 | 515 | 243 | 21 | 1 | 421 | 1 | 438 | Uncharacterized protein | Uncharacterized protein | | afdb-uniprot50 | AF-A0A371YQG3-F1-MODEL\_V4 | 1.0 | 3.281e-16 | 399 | 0.194 | 390 | 181 | 15 | 1 | 260 | 2 | 388 | Uncharacterized protein | Uncharacterized protein | | afdb-uniprot50 | AF-A0A3L0W1T3-F1-MODEL\_V4 | 1.0 | 3.551e-18 | 398 | 0.231 | 466 | 219 | 20 | 1 | 416 | 2 | 378 | Phage tail protein | Phage tail protein | | afdb-uniprot50 | AF-A0A2J9GZ53-F1-MODEL\_V4 | 1.0 | 2.042e-19 | 398 | 0.221 | 505 | 261 | 17 | 1 | 414 | 4 | 467 | gp37\_C domain-containing protein | gp37\_C domain-containing protein | | afdb-uniprot50 | AF-A0A0E3BR31-F1-MODEL\_V4 | 1.0 | 1.028e-18 | 398 | 0.236 | 477 | 232 | 23 | 1 | 412 | 1 | 410 | Tail protein | Tail protein | | afdb-uniprot50 | AF-N9SPH7-F1-MODEL\_V4 | 1.0 | 6.087e-18 | 398 | 0.169 | 577 | 236 | 25 | 2 | 421 | 4 | 494 | Uncharacterized protein | Uncharacterized protein | | afdb-uniprot50 | AF-A0A2K9QFQ7-F1-MODEL\_V4 | 1.0 | 9.233e-19 | 397 | 0.186 | 567 | 217 | 23 | 1 | 421 | 2 | 469 | Phage tail protein | Phage tail protein | | afdb-uniprot50 | AF-A0A5C6YJN9-F1-MODEL\_V4 | 1.0 | 1.117e-16 | 397 | 0.237 | 391 | 206 | 18 | 1 | 349 | 10 | 350 | Uncharacterized protein | Uncharacterized protein | | afdb-uniprot50 | AF-G3XD71-F1-MODEL\_V4 | 1.0 | 4.115e-19 | 397 | 0.151 | 708 | 295 | 32 | 1 | 423 | 5 | 691 | Probable bacteriophage protein | Probable bacteriophage protein | | afdb-uniprot50 | AF-A0A3A8ECP1-F1-MODEL\_V4 | 1.0 | 1.196e-15 | 396 | 0.16 | 437 | 215 | 20 | 1 | 421 | 2 | 302 | Uncharacterized protein | Uncharacterized protein | | afdb-uniprot50 | AF-A0A0M5MEM2-F1-MODEL\_V4 | 1.0 | 7.443e-19 | 396 | 0.18 | 544 | 265 | 30 | 3 | 417 | 2 | 493 | Phage tail-collar fiber protein (DUF3751 domain) | Phage tail-collar fiber protein (DUF3751 domain) | | afdb-uniprot50 | AF-A0A4P8SH52-F1-MODEL\_V4 | 1.0 | 6.683e-19 | 396 | 0.205 | 593 | 241 | 27 | 1 | 421 | 2 | 536 | Uncharacterized protein | Uncharacterized protein | | afdb-uniprot50 | AF-A0A7L7MRD4-F1-MODEL\_V4 | 1.0 | 2.116e-09 | 395 | 0.376 | 109 | 66 | 2 | 1 | 108 | 1 | 108 | Uncharacterized protein | Uncharacterized protein | | afdb-uniprot50 | AF-A0A2I1RIW9-F1-MODEL\_V4 | 1.0 | 1.047e-13 | 395 | 0.234 | 269 | 174 | 10 | 1 | 261 | 1 | 245 | Uncharacterized protein | Uncharacterized protein | | afdb-uniprot50 | AF-A0A7Z2A8B6-F1-MODEL\_V4 | 1.0 | 1.67e-18 | 395 | 0.195 | 471 | 245 | 24 | 1 | 422 | 1 | 386 | Uncharacterized protein | Uncharacterized protein | | afdb-uniprot50 | AF-A0A2K8VZI6-F1-MODEL\_V4 | 1.0 | 1.276e-18 | 395 | 0.2 | 524 | 213 | 30 | 1 | 422 | 2 | 421 | Phage tail fiber protein | Phage tail fiber protein | | afdb-uniprot50 | AF-A0A345J3J2-F1-MODEL\_V4 | 1.0 | 2.042e-19 | 395 | 0.195 | 502 | 238 | 26 | 4 | 412 | 3 | 431 | Phage-related tail fiber protein | Phage-related tail fiber protein | | afdb-uniprot50 | AF-A0A3P6JSJ9-F1-MODEL\_V4 | 1.0 | 9.233e-19 | 395 | 0.205 | 541 | 237 | 25 | 1 | 421 | 2 | 469 | Putative phage tail fiber protein | Putative phage tail fiber protein | | afdb-uniprot50 | AF-A0A3F3A8F4-F1-MODEL\_V4 | 1.0 | 3.501e-19 | 395 | 0.219 | 492 | 252 | 25 | 1 | 413 | 1 | 439 | Putative phage tail fiber protein | Putative phage tail fiber protein | | afdb-uniprot50 | AF-A0A5B8I5F0-F1-MODEL\_V4 | 1.0 | 3.021e-18 | 395 | 0.227 | 496 | 218 | 19 | 1 | 385 | 1 | 442 | Phage tail protein | Phage tail protein | | afdb-uniprot50 | AF-A0A840MLL2-F1-MODEL\_V4 | 1.0 | 1.5e-18 | 394 | 0.209 | 520 | 252 | 26 | 1 | 423 | 1 | 458 | Phage-related tail fiber protein | Phage-related tail fiber protein | | afdb-uniprot50 | AF-A0A0M4TW35-F1-MODEL\_V4 | 1.0 | 1.676e-14 | 393 | 0.238 | 285 | 181 | 12 | 3 | 275 | 2 | 262 | Uncharacterized protein | Uncharacterized protein | | afdb-uniprot50 | AF-A0A0H3GKQ5-F1-MODEL\_V4 | 1.0 | 1.133e-15 | 393 | 0.243 | 341 | 157 | 14 | 1 | 258 | 1 | 323 | Uncharacterized protein | Uncharacterized protein | | afdb-uniprot50 | AF-A0A072Y2M2-F1-MODEL\_V4 | 1.0 | 2.187e-18 | 393 | 0.204 | 522 | 235 | 26 | 1 | 423 | 2 | 441 | Putative phage tail fiber protein | Putative phage tail fiber protein | | afdb-uniprot50 | AF-A0A248LIJ6-F1-MODEL\_V4 | 1.0 | 2.155e-19 | 393 | 0.204 | 612 | 226 | 30 | 2 | 420 | 3 | 546 | Tail fiber protein 2 | Tail fiber protein 2 | | afdb-uniprot50 | AF-A0A826U4X0-F1-MODEL\_V4 | 1.0 | 1.101e-17 | 393 | 0.216 | 484 | 231 | 21 | 1 | 422 | 1 | 398 | Phage tail protein | Phage tail protein | | afdb-uniprot50 | AF-A0A6H3LMB7-F1-MODEL\_V4 | 1.0 | 2.771e-09 | 392 | 0.372 | 129 | 81 | 0 | 33 | 161 | 1 | 129 | Phage tail protein | Phage tail protein | | afdb-uniprot50 | AF-A0A6S7CIQ6-F1-MODEL\_V4 | 1.0 | 5.178e-18 | 392 | 0.231 | 462 | 234 | 25 | 1 | 404 | 1 | 399 | Uncharacterized protein | Uncharacterized protein | | afdb-uniprot50 | AF-A0A2K9CH40-F1-MODEL\_V4 | 1.0 | 4.405e-18 | 392 | 0.148 | 849 | 257 | 32 | 1 | 423 | 5 | 813 | Uncharacterized protein | Uncharacterized protein | | afdb-uniprot50 | AF-A0A1B2QZE5-F1-MODEL\_V4 | 1.0 | 3.188e-18 | 391 | 0.229 | 506 | 223 | 29 | 1 | 413 | 1 | 432 | Uncharacterized protein | Uncharacterized protein | | afdb-uniprot50 | AF-A0A7Y8ZMJ1-F1-MODEL\_V4 | 1.0 | 7.053e-19 | 391 | 0.199 | 511 | 272 | 27 | 1 | 422 | 1 | 463 | Phage-related tail fiber protein | Phage-related tail fiber protein | | afdb-uniprot50 | AF-A0A1S8T0S9-F1-MODEL\_V4 | 1.0 | 2.401e-19 | 391 | 0.193 | 549 | 267 | 28 | 1 | 423 | 2 | 500 | Uncharacterized protein | Uncharacterized protein | | afdb-uniprot50 | AF-A0A0D6BCT9-F1-MODEL\_V4 | 1.0 | 1.583e-18 | 391 | 0.151 | 814 | 283 | 36 | 2 | 423 | 6 | 803 | Phage-related tail fibre protein-like protein | Phage-related tail fibre protein-like protein | | afdb-uniprot50 | AF-A0A0A8FH85-F1-MODEL\_V4 | 1.0 | 4.405e-18 | 390 | 0.21 | 518 | 257 | 20 | 1 | 416 | 1 | 468 | Collar domain-containing protein | Collar domain-containing protein | | afdb-uniprot50 | AF-A0A240TRE0-F1-MODEL\_V4 | 1.0 | 1.763e-18 | 389 | 0.222 | 499 | 214 | 24 | 1 | 416 | 1 | 408 | Uncharacterized protein | Uncharacterized protein | | afdb-uniprot50 | AF-A0A1N6MZM9-F1-MODEL\_V4 | 1.0 | 6.706e-15 | 389 | 0.233 | 334 | 121 | 11 | 1 | 315 | 2 | 219 | Uncharacterized protein | Uncharacterized protein | | afdb-uniprot50 | AF-B2TMU2-F1-MODEL\_V4 | 1.0 | 7.155e-18 | 388 | 0.197 | 521 | 261 | 27 | 1 | 423 | 2 | 463 | Putative tail fiber protein | Putative tail fiber protein | | afdb-uniprot50 | AF-A0A239C905-F1-MODEL\_V4 | 1.0 | 6.683e-19 | 388 | 0.171 | 612 | 250 | 28 | 4 | 423 | 3 | 549 | Phage tail-collar fibre protein | Phage tail-collar fibre protein | | afdb-uniprot50 | AF-A0A7Y3ILG7-F1-MODEL\_V4 | 1.0 | 1.846e-11 | 387 | 0.212 | 221 | 134 | 9 | 6 | 204 | 10 | 212 | Uncharacterized protein | Uncharacterized protein | | afdb-uniprot50 | AF-A7ZNA3-F1-MODEL\_V4 | 1.0 | 6.706e-15 | 387 | 0.217 | 322 | 168 | 15 | 1 | 258 | 3 | 304 | Uncharacterized protein | Uncharacterized protein | | afdb-uniprot50 | AF-A0A3M5P7X5-F1-MODEL\_V4 | 1.0 | 2.187e-18 | 387 | 0.182 | 538 | 213 | 18 | 2 | 423 | 6 | 432 | Phage tail fiber protein h | Phage tail fiber protein h | | afdb-uniprot50 | AF-A0A157QPJ3-F1-MODEL\_V4 | 1.0 | 1.441e-17 | 387 | 0.232 | 404 | 211 | 15 | 1 | 351 | 1 | 358 | Tail fiber protein | Tail fiber protein | | afdb-uniprot50 | AF-A0A0E3M8H3-F1-MODEL\_V4 | 1.0 | 3.748e-18 | 386 | 0.207 | 454 | 262 | 16 | 1 | 406 | 2 | 405 | Putative phage tail fiber protein | Putative phage tail fiber protein | | afdb-uniprot50 | AF-M1MEX9-F1-MODEL\_V4 | 1.0 | 4.174e-18 | 386 | 0.182 | 538 | 290 | 28 | 1 | 423 | 1 | 503 | Putative phage tail fiber protein | Putative phage tail fiber protein | | afdb-uniprot50 | AF-Q4QK27-F1-MODEL\_V4 | 1.0 | 1.043e-17 | 386 | 0.214 | 439 | 218 | 21 | 1 | 415 | 2 | 337 | Probable tail fiber protein | Probable tail fiber protein | | afdb-uniprot50 | AF-N9KFA7-F1-MODEL\_V4 | 1.0 | 8.779e-15 | 385 | 0.183 | 337 | 168 | 16 | 2 | 258 | 3 | 312 | Uncharacterized protein | Uncharacterized protein | | afdb-uniprot50 | AF-A0A0P9N6Y3-F1-MODEL\_V4 | 1.0 | 3.602e-17 | 385 | 0.214 | 550 | 255 | 26 | 2 | 423 | 6 | 506 | Tail fiber protein H | Tail fiber protein H | | afdb-uniprot50 | AF-A0A1C4DMT0-F1-MODEL\_V4 | 1.0 | 7.855e-19 | 384 | 0.203 | 579 | 216 | 28 | 1 | 421 | 2 | 493 | Phage tail-collar fibre protein | Phage tail-collar fibre protein | | afdb-uniprot50 | AF-A0A509JDS2-F1-MODEL\_V4 | 1.0 | 3.365e-18 | 384 | 0.163 | 717 | 282 | 31 | 1 | 423 | 1 | 693 | Phage tail protein | Phage tail protein | | afdb-uniprot50 | AF-A0A0H3ZXX7-F1-MODEL\_V4 | 1.0 | 3.365e-18 | 384 | 0.217 | 520 | 242 | 28 | 1 | 417 | 1 | 458 | Tail fiber protein | Tail fiber protein | | afdb-uniprot50 | AF-A0A0P9YJT9-F1-MODEL\_V4 | 1.0 | 4.907e-18 | 383 | 0.208 | 570 | 252 | 28 | 1 | 407 | 5 | 538 | Putative Tail fiber protein H | Putative Tail fiber protein H | | afdb-uniprot50 | AF-A0A725RYD2-F1-MODEL\_V4 | 1.0 | 1.028e-18 | 383 | 0.181 | 705 | 261 | 33 | 1 | 421 | 1 | 673 | Phage tail protein | Phage tail protein | | afdb-uniprot50 | AF-A0A2N8B960-F1-MODEL\_V4 | 1.0 | 1.887e-17 | 382 | 0.191 | 537 | 226 | 27 | 2 | 421 | 6 | 451 | Phage tail protein | Phage tail protein | | afdb-uniprot50 | AF-A0A2K4GCZ3-F1-MODEL\_V4 | 1.0 | 2.102e-17 | 382 | 0.207 | 487 | 261 | 20 | 2 | 411 | 6 | 444 | Uncharacterized protein | Uncharacterized protein | | afdb-uniprot50 | AF-A0A6D2GE62-F1-MODEL\_V4 | 1.0 | 1.153e-10 | 381 | 0.434 | 145 | 77 | 1 | 32 | 176 | 2 | 141 | Tail protein | Tail protein | | afdb-uniprot50 | AF-A0A127MM56-F1-MODEL\_V4 | 1.0 | 1.366e-17 | 380 | 0.259 | 490 | 217 | 28 | 1 | 417 | 1 | 417 | Uncharacterized protein | Uncharacterized protein | | afdb-uniprot50 | AF-A0A423IG29-F1-MODEL\_V4 | 1.0 | 2.471e-17 | 380 | 0.202 | 450 | 247 | 20 | 1 | 410 | 5 | 382 | Uncharacterized protein | Uncharacterized protein | | afdb-uniprot50 | AF-B5JYG6-F1-MODEL\_V4 | 1.0 | 5.122e-15 | 379 | 0.436 | 188 | 101 | 4 | 83 | 268 | 2 | 186 | Phage Tail Collar Domain family | Phage Tail Collar Domain family | | afdb-uniprot50 | AF-A0A6S5JPB2-F1-MODEL\_V4 | 1.0 | 4.649e-18 | 379 | 0.228 | 529 | 210 | 25 | 1 | 412 | 2 | 449 | Uncharacterized protein | Uncharacterized protein | | afdb-uniprot50 | AF-A0A1G6JDL2-F1-MODEL\_V4 | 1.0 | 1.244e-16 | 379 | 0.147 | 575 | 230 | 22 | 1 | 421 | 3 | 471 | Phage tail-collar fibre protein | Phage tail-collar fibre protein | | afdb-uniprot50 | AF-A0A855HWY9-F1-MODEL\_V4 | 1.0 | 6.779e-18 | 379 | 0.191 | 518 | 261 | 18 | 1 | 412 | 2 | 467 | Uncharacterized protein | Uncharacterized protein | | afdb-uniprot50 | AF-A0A8B6KUE2-F1-MODEL\_V4 | 1.0 | 6.087e-18 | 378 | 0.218 | 508 | 255 | 22 | 1 | 418 | 1 | 456 | Variable tail fiber protein | Variable tail fiber protein | | afdb-uniprot50 | AF-Q8ZMU5-F1-MODEL\_V4 | 1.0 | 2.187e-18 | 378 | 0.207 | 583 | 224 | 23 | 1 | 421 | 2 | 508 | Fels-2 prophage protein | Fels-2 prophage protein | | afdb-uniprot50 | AF-A0A5F0JKE5-F1-MODEL\_V4 | 1.0 | 2.341e-17 | 378 | 0.159 | 683 | 252 | 35 | 2 | 423 | 6 | 627 | Uncharacterized protein | Uncharacterized protein | | afdb-uniprot50 | AF-A0A7Y8Z4M8-F1-MODEL\_V4 | 1.0 | 9.885e-18 | 378 | 0.163 | 569 | 251 | 30 | 1 | 422 | 1 | 491 | Uncharacterized protein | Uncharacterized protein | | afdb-uniprot50 | AF-A0A1I5D5R7-F1-MODEL\_V4 | 1.0 | 6.354e-15 | 378 | 0.23 | 325 | 138 | 9 | 1 | 323 | 2 | 216 | Phage tail fibre repeat-containing protein | Phage tail fibre repeat-containing protein | | afdb-uniprot50 | AF-A0A7X1KID4-F1-MODEL\_V4 | 1.0 | 3.021e-18 | 377 | 0.19 | 551 | 234 | 24 | 4 | 423 | 8 | 477 | Phage tail protein | Phage tail protein | | afdb-uniprot50 | AF-A0A6I5RT98-F1-MODEL\_V4 | 1.0 | 1.652e-15 | 377 | 0.259 | 378 | 193 | 13 | 2 | 310 | 6 | 365 | Uncharacterized protein | Uncharacterized protein | | afdb-uniprot50 | AF-A0A353G7W2-F1-MODEL\_V4 | 1.0 | 4.129e-15 | 376 | 0.224 | 347 | 165 | 11 | 1 | 306 | 2 | 285 | Uncharacterized protein | Uncharacterized protein | | afdb-uniprot50 | AF-A0A1H8SZT3-F1-MODEL\_V4 | 1.0 | 4.649e-18 | 375 | 0.197 | 568 | 274 | 27 | 1 | 421 | 1 | 533 | Phage-related tail fibre protein | Phage-related tail fibre protein | | afdb-uniprot50 | AF-A0A3G2IKC5-F1-MODEL\_V4 | 1.0 | 1.887e-17 | 375 | 0.171 | 566 | 245 | 25 | 1 | 412 | 1 | 496 | Uncharacterized protein | Uncharacterized protein | | afdb-uniprot50 | AF-A0A379YEC2-F1-MODEL\_V4 | 1.0 | 2.506e-16 | 373 | 0.154 | 485 | 247 | 25 | 1 | 420 | 1 | 387 | Uncharacterized protein | Uncharacterized protein | | afdb-uniprot50 | AF-A0A4P7L2V6-F1-MODEL\_V4 | 1.0 | 2.57e-18 | 373 | 0.231 | 513 | 237 | 23 | 4 | 416 | 3 | 458 | Phage tail-collar fiber protein | Phage tail-collar fiber protein | | afdb-uniprot50 | AF-A0A2T5NRB6-F1-MODEL\_V4 | 1.0 | 6.877e-17 | 373 | 0.218 | 498 | 239 | 26 | 1 | 423 | 2 | 424 | Uncharacterized protein | Uncharacterized protein | | afdb-uniprot50 | AF-A0A7Y1MB50-F1-MODEL\_V4 | 1.0 | 1.605e-17 | 373 | 0.187 | 544 | 268 | 25 | 2 | 412 | 6 | 508 | Phage tail protein | Phage tail protein | | afdb-uniprot50 | AF-F9GSW5-F1-MODEL\_V4 | 1.0 | 7.443e-19 | 373 | 0.126 | 746 | 289 | 27 | 1 | 423 | 2 | 707 | Phage tail collar domain-containing protein | Phage tail collar domain-containing protein | | afdb-uniprot50 | AF-A0A855ISI7-F1-MODEL\_V4 | 1.0 | 4.682e-10 | 372 | 0.185 | 199 | 126 | 5 | 6 | 172 | 10 | 204 | Uncharacterized protein | Uncharacterized protein | | afdb-uniprot50 | AF-A0A0A8TLD1-F1-MODEL\_V4 | 1.0 | 9.885e-18 | 372 | 0.187 | 559 | 246 | 31 | 1 | 412 | 1 | 498 | Phage tail fiber protein | Phage tail fiber protein | | afdb-uniprot50 | AF-A0A6G9RP95-F1-MODEL\_V4 | 1.0 | 3.109e-16 | 371 | 0.166 | 480 | 215 | 20 | 1 | 421 | 3 | 356 | Phage tail protein | Phage tail protein | | afdb-uniprot50 | AF-A0A6M0YIR0-F1-MODEL\_V4 | 1.0 | 1.313e-16 | 371 | 0.179 | 452 | 246 | 20 | 1 | 416 | 2 | 364 | Uncharacterized protein | Uncharacterized protein | | afdb-uniprot50 | AF-A0A7C8HXB6-F1-MODEL\_V4 | 1.0 | 1.605e-17 | 371 | 0.217 | 533 | 231 | 23 | 1 | 414 | 1 | 466 | Uncharacterized protein | Uncharacterized protein | | afdb-uniprot50 | AF-A0A708DGG9-F1-MODEL\_V4 | 1.0 | 4.405e-18 | 371 | 0.196 | 576 | 222 | 28 | 1 | 421 | 1 | 490 | Uncharacterized protein | Uncharacterized protein | | afdb-uniprot50 | AF-A0A1N7LRE7-F1-MODEL\_V4 | 1.0 | 1.39e-12 | 371 | 0.19 | 315 | 147 | 8 | 1 | 310 | 1 | 212 | Phage tail-collar fibre protein | Phage tail-collar fibre protein | | afdb-uniprot50 | AF-A0A2H5E1S9-F1-MODEL\_V4 | 1.0 | 5.253e-17 | 371 | 0.212 | 457 | 235 | 20 | 2 | 418 | 3 | 374 | Uncharacterized protein | Uncharacterized protein | | afdb-uniprot50 | AF-A0A379CAC0-F1-MODEL\_V4 | 1.0 | 2.375e-16 | 371 | 0.178 | 414 | 177 | 10 | 1 | 412 | 2 | 254 | Phage tail fibre repeat | Phage tail fibre repeat | | afdb-uniprot50 | AF-M1MI28-F1-MODEL\_V4 | 1.0 | 6.087e-18 | 371 | 0.159 | 582 | 247 | 25 | 1 | 422 | 2 | 501 | Putative tail fiber protein | Putative tail fiber protein | | afdb-uniprot50 | AF-A0A7W4RSH8-F1-MODEL\_V4 | 1.0 | 3.463e-16 | 370 | 0.202 | 459 | 231 | 16 | 1 | 416 | 1 | 367 | Phage-related tail fiber protein | Phage-related tail fiber protein | | afdb-uniprot50 | AF-B1B8U4-F1-MODEL\_V4 | 1.0 | 1.226e-17 | 370 | 0.178 | 559 | 241 | 21 | 1 | 421 | 1 | 479 | Putative phage tail fiber protein | Putative phage tail fiber protein | | afdb-uniprot50 | AF-A0A4P7LY25-F1-MODEL\_V4 | 1.0 | 7.155e-18 | 370 | 0.184 | 705 | 259 | 37 | 1 | 421 | 1 | 673 | Phage tail protein | Phage tail protein | | afdb-uniprot50 | AF-A0A368TXZ3-F1-MODEL\_V4 | 1.0 | 7.66e-17 | 369 | 0.167 | 536 | 218 | 19 | 2 | 416 | 6 | 434 | Collar domain-containing protein | Collar domain-containing protein | | afdb-uniprot50 | AF-A0A840M5X8-F1-MODEL\_V4 | 1.0 | 3.748e-18 | 369 | 0.161 | 601 | 277 | 28 | 1 | 421 | 2 | 555 | Uncharacterized protein | Uncharacterized protein | | afdb-uniprot50 | AF-A0A488A766-F1-MODEL\_V4 | 1.0 | 7.308e-09 | 368 | 0.385 | 114 | 63 | 2 | 1 | 111 | 1 | 110 | Tail fiber protein | Tail fiber protein | | afdb-uniprot50 | AF-A0A379FY21-F1-MODEL\_V4 | 1.0 | 1.226e-17 | 367 | 0.194 | 612 | 248 | 31 | 4 | 421 | 3 | 563 | Uncharacterized protein | Uncharacterized protein | | afdb-uniprot50 | AF-A0A8B4MIL4-F1-MODEL\_V4 | 1.0 | 6.087e-18 | 367 | 0.192 | 536 | 272 | 24 | 1 | 412 | 1 | 499 | Bifunctional phage-like tail fiber protein/heme utilization protein | Bifunctional phage-like tail fiber protein/heme utilization protein | | afdb-uniprot50 | AF-B0P727-F1-MODEL\_V4 | 1.0 | 2.791e-16 | 366 | 0.146 | 497 | 242 | 23 | 3 | 420 | 2 | 395 | Uncharacterized protein | Uncharacterized protein | | afdb-uniprot50 | AF-A0A5T0CRZ1-F1-MODEL\_V4 | 1.0 | 6.264e-16 | 366 | 0.186 | 402 | 175 | 14 | 2 | 258 | 4 | 398 | Phage tail protein | Phage tail protein | | afdb-uniprot50 | AF-A0A7Z1GL46-F1-MODEL\_V4 | 1.0 | 3.065e-17 | 365 | 0.196 | 581 | 267 | 32 | 2 | 422 | 6 | 546 | Tail collar domain | Tail collar domain | | afdb-uniprot50 | AF-A0A6P2SH75-F1-MODEL\_V4 | 1.0 | 7.258e-17 | 364 | 0.216 | 555 | 257 | 26 | 1 | 420 | 2 | 513 | Uncharacterized protein | Uncharacterized protein | | afdb-uniprot50 | AF-A0A380AJ63-F1-MODEL\_V4 | 1.0 | 2.791e-16 | 364 | 0.16 | 516 | 259 | 24 | 1 | 414 | 1 | 444 | Caudovirales tail fibre assembly protein | Caudovirales tail fibre assembly protein | | afdb-uniprot50 | AF-A0A845BZU2-F1-MODEL\_V4 | 1.0 | 1.366e-17 | 364 | 0.204 | 573 | 264 | 29 | 1 | 421 | 1 | 533 | Uncharacterized protein | Uncharacterized protein | | afdb-uniprot50 | AF-A0A3M3YCB8-F1-MODEL\_V4 | 1.0 | 7.258e-17 | 364 | 0.214 | 521 | 263 | 27 | 1 | 412 | 1 | 483 | Uncharacterized protein | Uncharacterized protein | | afdb-uniprot50 | AF-N8XQE4-F1-MODEL\_V4 | 1.0 | 2.713e-18 | 362 | 0.171 | 612 | 267 | 28 | 1 | 421 | 4 | 566 | Pectate\_lyase\_3 domain-containing protein | Pectate\_lyase\_3 domain-containing protein | | afdb-uniprot50 | AF-A0A378NBM0-F1-MODEL\_V4 | 1.0 | 1.992e-17 | 362 | 0.19 | 578 | 223 | 27 | 1 | 422 | 2 | 490 | Catalytic beta propeller domain of bacteriophage endosialidase | Catalytic beta propeller domain of bacteriophage endosialidase | | afdb-uniprot50 | AF-E6WD86-F1-MODEL\_V4 | 1.0 | 1.605e-17 | 361 | 0.174 | 597 | 216 | 25 | 1 | 421 | 1 | 496 | Phage-related tail fibre protein-like protein | Phage-related tail fibre protein-like protein | | afdb-uniprot50 | AF-A0A2S9X9N0-F1-MODEL\_V4 | 1.0 | 1.462e-16 | 361 | 0.153 | 708 | 249 | 31 | 1 | 423 | 1 | 642 | Uncharacterized protein | Uncharacterized protein | | afdb-uniprot50 | AF-A0A6C2DDF4-F1-MODEL\_V4 | 1.0 | 3.365e-18 | 361 | 0.165 | 683 | 290 | 36 | 1 | 423 | 2 | 664 | gp37\_C domain-containing protein | gp37\_C domain-containing protein | | afdb-uniprot50 | AF-A0A2T5KPN3-F1-MODEL\_V4 | 1.0 | 1.543e-16 | 361 | 0.21 | 494 | 253 | 24 | 1 | 411 | 5 | 444 | Tail collar domain | Tail collar domain | | afdb-uniprot50 | AF-A0A530M9E5-F1-MODEL\_V4 | 1.0 | 1.162e-17 | 360 | 0.199 | 506 | 230 | 23 | 1 | 413 | 2 | 425 | Phage tail protein | Phage tail protein | | afdb-uniprot50 | AF-A0A7Y8FLP5-F1-MODEL\_V4 | 1.0 | 6.516e-17 | 360 | 0.205 | 512 | 268 | 26 | 1 | 412 | 1 | 473 | Phage tail protein | Phage tail protein | | afdb-uniprot50 | AF-A0A7U9D8U8-F1-MODEL\_V4 | 1.0 | 2.102e-17 | 358 | 0.183 | 517 | 266 | 29 | 1 | 418 | 2 | 461 | Uncharacterized protein | Uncharacterized protein | | afdb-uniprot50 | AF-A0A483W7T5-F1-MODEL\_V4 | 1.0 | 1.992e-17 | 358 | 0.203 | 635 | 228 | 31 | 1 | 422 | 1 | 570 | Phage tail protein | Phage tail protein | | afdb-uniprot50 | AF-A0A383RR92-F1-MODEL\_V4 | 1.0 | 6.877e-17 | 358 | 0.218 | 567 | 239 | 29 | 2 | 409 | 6 | 527 | Putative tail fiber protein | Putative tail fiber protein | | afdb-uniprot50 | AF-A0A5E4YCM3-F1-MODEL\_V4 | 1.0 | 4.469e-17 | 357 | 0.171 | 611 | 241 | 30 | 1 | 422 | 2 | 535 | Uncharacterized protein | Uncharacterized protein | | afdb-uniprot50 | AF-W7S9K3-F1-MODEL\_V4 | 1.0 | 1.366e-17 | 356 | 0.207 | 458 | 251 | 22 | 1 | 405 | 2 | 400 | Tail fiber protein | Tail fiber protein | | afdb-uniprot50 | AF-A0A857E7Z2-F1-MODEL\_V4 | 1.0 | 1.543e-16 | 356 | 0.169 | 454 | 245 | 17 | 1 | 421 | 1 | 355 | Uncharacterized protein | Uncharacterized protein | | afdb-uniprot50 | AF-A0A378YNC7-F1-MODEL\_V4 | 1.0 | 1.117e-16 | 355 | 0.205 | 517 | 206 | 26 | 1 | 417 | 2 | 413 | Uncharacterized protein | Uncharacterized protein | | afdb-uniprot50 | AF-A0A5E5AEQ9-F1-MODEL\_V4 | 1.0 | 6.516e-17 | 354 | 0.2 | 550 | 222 | 27 | 1 | 422 | 2 | 461 | Tail fiber protein | Tail fiber protein | | afdb-uniprot50 | AF-A0A5U0PXD3-F1-MODEL\_V4 | 1.0 | 2.752e-17 | 354 | 0.173 | 537 | 240 | 24 | 2 | 421 | 9 | 458 | Uncharacterized protein | Uncharacterized protein | | afdb-uniprot50 | AF-A0A4V1IMV1-F1-MODEL\_V4 | 1.0 | 1.992e-17 | 353 | 0.181 | 555 | 290 | 22 | 1 | 420 | 1 | 525 | Uncharacterized protein | Uncharacterized protein | | afdb-uniprot50 | AF-A0A239SWC2-F1-MODEL\_V4 | 1.0 | 2.946e-16 | 353 | 0.214 | 457 | 233 | 26 | 1 | 392 | 2 | 397 | Uncharacterized protein | Uncharacterized protein | | afdb-uniprot50 | AF-A0A1C7C2V4-F1-MODEL\_V4 | 1.0 | 4.469e-17 | 352 | 0.147 | 798 | 286 | 34 | 1 | 423 | 1 | 778 | Uncharacterized protein | Uncharacterized protein | | afdb-uniprot50 | AF-A0A5E7VM25-F1-MODEL\_V4 | 1.0 | 2.02e-16 | 351 | 0.2 | 529 | 282 | 21 | 2 | 422 | 6 | 501 | Uncharacterized protein | Uncharacterized protein | | afdb-uniprot50 | AF-A0A6G8D7U0-F1-MODEL\_V4 | 1.0 | 2.163e-15 | 349 | 0.185 | 480 | 253 | 20 | 1 | 423 | 2 | 400 | Phage tail protein | Phage tail protein | | afdb-uniprot50 | AF-A0A1Y3CH38-F1-MODEL\_V4 | 1.0 | 1.179e-16 | 348 | 0.18 | 487 | 277 | 25 | 1 | 413 | 1 | 439 | Collar domain-containing protein | Collar domain-containing protein | | afdb-uniprot50 | AF-A0A6N7BXN9-F1-MODEL\_V4 | 1.0 | 1.231e-13 | 347 | 0.203 | 325 | 185 | 11 | 1 | 256 | 1 | 320 | Uncharacterized protein | Uncharacterized protein | | afdb-uniprot50 | AF-A0A5E4XZ01-F1-MODEL\_V4 | 1.0 | 5.329e-16 | 343 | 0.196 | 499 | 245 | 28 | 1 | 413 | 2 | 430 | Uncharacterized protein | Uncharacterized protein | | afdb-uniprot50 | AF-A0A177RPE9-F1-MODEL\_V4 | 1.0 | 6.175e-17 | 342 | 0.207 | 486 | 252 | 23 | 1 | 412 | 1 | 427 | Tail protein | Tail protein | | afdb-uniprot50 | AF-A0A241WFG7-F1-MODEL\_V4 | 1.0 | 6.108e-14 | 341 | 0.209 | 334 | 169 | 16 | 2 | 255 | 3 | 321 | Uncharacterized protein | Uncharacterized protein | | afdb-uniprot50 | AF-A0A1W2DYX6-F1-MODEL\_V4 | 1.0 | 1.914e-16 | 340 | 0.185 | 554 | 251 | 30 | 1 | 422 | 1 | 485 | Uncharacterized protein | Uncharacterized protein | | afdb-uniprot50 | AF-X6QAX6-F1-MODEL\_V4 | 1.0 | 1.942e-15 | 338 | 0.186 | 462 | 232 | 23 | 1 | 419 | 1 | 361 | Tail fiber protein | Tail fiber protein | | afdb-uniprot50 | AF-A0A1H5GLX5-F1-MODEL\_V4 | 1.0 | 4.296e-16 | 338 | 0.19 | 536 | 244 | 27 | 1 | 423 | 1 | 459 | Phage Tail Collar Domain | Phage Tail Collar Domain | | afdb-uniprot50 | AF-A0A6M0CVM5-F1-MODEL\_V4 | 1.0 | 3.463e-16 | 338 | 0.208 | 480 | 227 | 26 | 2 | 378 | 6 | 435 | Phage tail protein | Phage tail protein | | afdb-uniprot50 | AF-C3KCU2-F1-MODEL\_V4 | 1.0 | 6.976e-16 | 337 | 0.157 | 678 | 288 | 31 | 1 | 421 | 1 | 651 | Putative phage tail fiber-related protein | Putative phage tail fiber-related protein | | afdb-uniprot50 | AF-A0A367MED4-F1-MODEL\_V4 | 1.0 | 2.163e-15 | 336 | 0.194 | 478 | 232 | 25 | 1 | 415 | 1 | 388 | Uncharacterized protein | Uncharacterized protein | | afdb-uniprot50 | AF-A0A2T5J1H4-F1-MODEL\_V4 | 1.0 | 9.134e-16 | 331 | 0.178 | 465 | 261 | 20 | 1 | 421 | 1 | 388 | Tail-collar fiber protein | Tail-collar fiber protein | | afdb-uniprot50 | AF-V8RCP1-F1-MODEL\_V4 | 1.0 | 1.942e-15 | 331 | 0.192 | 494 | 252 | 24 | 2 | 423 | 6 | 424 | Phage tail protein | Phage tail protein | | afdb-uniprot50 | AF-A0A1B8QCU5-F1-MODEL\_V4 | 1.0 | 4.599e-15 | 331 | 0.187 | 431 | 208 | 20 | 1 | 407 | 1 | 313 | Uncharacterized protein | Uncharacterized protein | | afdb-uniprot50 | AF-A0A2T0YWF7-F1-MODEL\_V4 | 1.0 | 1.133e-15 | 330 | 0.213 | 446 | 221 | 23 | 32 | 423 | 2 | 371 | Tail-collar fiber protein | Tail-collar fiber protein | | afdb-uniprot50 | AF-A0A6B3J8L4-F1-MODEL\_V4 | 1.0 | 9.639e-16 | 330 | 0.169 | 525 | 258 | 25 | 1 | 423 | 1 | 449 | Uncharacterized protein | Uncharacterized protein | | afdb-uniprot50 | AF-A0A1N6UIC0-F1-MODEL\_V4 | 1.0 | 5.049e-16 | 329 | 0.189 | 527 | 233 | 25 | 2 | 421 | 6 | 445 | Phage tail-collar fibre protein | Phage tail-collar fibre protein | | afdb-uniprot50 | AF-A0A376P7J4-F1-MODEL\_V4 | 1.0 | 6.633e-12 | 329 | 0.256 | 292 | 114 | 4 | 32 | 323 | 2 | 190 | Putative phage tail fiber protein | Putative phage tail fiber protein | | afdb-uniprot50 | AF-A0A330EWK9-F1-MODEL\_V4 | 1.0 | 1.074e-15 | 327 | 0.162 | 510 | 252 | 20 | 1 | 421 | 2 | 425 | Phage tail protein | Phage tail protein | | afdb-uniprot50 | AF-A0A6M0SQX7-F1-MODEL\_V4 | 1.0 | 6.976e-16 | 327 | 0.162 | 626 | 267 | 26 | 1 | 423 | 1 | 571 | Uncharacterized protein | Uncharacterized protein | | afdb-uniprot50 | AF-A0A1G5ACS0-F1-MODEL\_V4 | 1.0 | 5.544e-17 | 323 | 0.167 | 555 | 294 | 28 | 1 | 423 | 1 | 519 | Phage tail-collar fibre protein | Phage tail-collar fibre protein | | afdb-uniprot50 | AF-A0A1C3CVN3-F1-MODEL\_V4 | 1.0 | 1.744e-15 | 320 | 0.169 | 556 | 280 | 31 | 1 | 421 | 1 | 509 | Uncharacterized protein | Uncharacterized protein | | afdb-uniprot50 | AF-A0A368V9P8-F1-MODEL\_V4 | 1.0 | 1.386e-16 | 315 | 0.186 | 516 | 256 | 24 | 17 | 421 | 2 | 464 | Tail-collar fiber protein | Tail-collar fiber protein | | afdb-uniprot50 | AF-A0A2W6YCW4-F1-MODEL\_V4 | 1.0 | 9.778e-15 | 314 | 0.165 | 495 | 269 | 21 | 1 | 421 | 1 | 425 | Uncharacterized protein | Uncharacterized protein | | afdb-uniprot50 | AF-A0A150HM63-F1-MODEL\_V4 | 1.0 | 1.406e-15 | 314 | 0.17 | 552 | 268 | 27 | 1 | 402 | 1 | 512 | Uncharacterized protein | Uncharacterized protein | | afdb-uniprot50 | AF-U1JBI7-F1-MODEL\_V4 | 1.0 | 1.089e-14 | 312 | 0.179 | 467 | 273 | 27 | 1 | 414 | 3 | 412 | Putative side tail phage protein | Putative side tail phage protein | | afdb-uniprot50 | AF-A0A7Y3T9N6-F1-MODEL\_V4 | 1.0 | 3.154e-15 | 311 | 0.153 | 535 | 271 | 23 | 1 | 423 | 1 | 465 | DUF2793 domain-containing protein | DUF2793 domain-containing protein | | afdb-uniprot50 | AF-U5MTV4-F1-MODEL\_V4 | 1.0 | 3.377e-14 | 310 | 0.125 | 783 | 261 | 24 | 1 | 422 | 2 | 721 | H: tail fiber protein | H: tail fiber protein | | afdb-uniprot50 | AF-A0A6P1LU34-F1-MODEL\_V4 | 1.0 | 2.914e-13 | 309 | 0.148 | 459 | 225 | 21 | 1 | 421 | 2 | 332 | Uncharacterized protein | Uncharacterized protein | | afdb-uniprot50 | AF-A0A2U8DMY3-F1-MODEL\_V4 | 1.0 | 6.706e-15 | 306 | 0.193 | 512 | 265 | 23 | 1 | 418 | 2 | 459 | Uncharacterized protein | Uncharacterized protein | | afdb-uniprot50 | AF-A0A241V3C4-F1-MODEL\_V4 | 1.0 | 1.942e-15 | 306 | 0.2 | 518 | 261 | 20 | 1 | 412 | 2 | 472 | Uncharacterized protein | Uncharacterized protein | | afdb-uniprot50 | AF-A0A1C3CUP3-F1-MODEL\_V4 | 1.0 | 6.021e-15 | 305 | 0.187 | 475 | 265 | 25 | 1 | 423 | 1 | 406 | Uncharacterized protein | Uncharacterized protein | | afdb-uniprot50 | AF-A0A7Z2B2X5-F1-MODEL\_V4 | 1.0 | 3.513e-15 | 303 | 0.16 | 581 | 247 | 25 | 1 | 421 | 2 | 501 | Uncharacterized protein | Uncharacterized protein | | afdb-uniprot50 | AF-A0A376H5A1-F1-MODEL\_V4 | 1.0 | 1.426e-14 | 301 | 0.161 | 527 | 227 | 25 | 1 | 406 | 2 | 434 | Phage tail protein | Phage tail protein | | afdb-uniprot50 | AF-A0A5M8P6A1-F1-MODEL\_V4 | 1.0 | 1.28e-14 | 288 | 0.214 | 452 | 257 | 25 | 2 | 413 | 5 | 398 | Uncharacterized protein | Uncharacterized protein | | afdb-uniprot50 | AF-S7JLI8-F1-MODEL\_V4 | 1.0 | 5.067e-12 | 283 | 0.161 | 365 | 205 | 14 | 6 | 348 | 10 | 295 | Tail fiber protein | Tail fiber protein | | afdb-uniprot50 | AF-S4YWB1-F1-MODEL\_V4 | 1.0 | 1.105e-13 | 279 | 0.174 | 447 | 240 | 19 | 3 | 422 | 2 | 346 | Uncharacterized protein | Uncharacterized protein | | afdb-uniprot50 | AF-G2HXA2-F1-MODEL\_V4 | 1.0 | 4.421e-14 | 277 | 0.149 | 574 | 271 | 32 | 1 | 421 | 1 | 510 | Phage tail fiber protein | Phage tail fiber protein | | afdb-uniprot50 | AF-A0A3B0BXV7-F1-MODEL\_V4 | 1.0 | 1.371e-13 | 271 | 0.202 | 410 | 234 | 20 | 5 | 404 | 3 | 329 | Uncharacterized protein | Uncharacterized protein | | afdb-uniprot50 | AF-A0A329MQ29-F1-MODEL\_V4 | 1.0 | 2.663e-08 | 266 | 0.216 | 162 | 119 | 4 | 6 | 166 | 5 | 159 | Uncharacterized protein | Uncharacterized protein | | afdb-uniprot50 | AF-A0A849B068-F1-MODEL\_V4 | 1.0 | 2.722e-14 | 255 | 0.171 | 495 | 272 | 24 | 1 | 420 | 1 | 432 | Uncharacterized protein | Uncharacterized protein | | afdb-uniprot50 | AF-A0A1K1T5P9-F1-MODEL\_V4 | 1.0 | 2.551e-11 | 220 | 0.235 | 438 | 212 | 21 | 32 | 411 | 2 | 374 | Phage tail-collar fibre protein | Phage tail-collar fibre protein | | afdb-uniprot50 | AF-A0A378E622-F1-MODEL\_V4 | 1.0 | 8.14e-09 | 217 | 0.211 | 303 | 138 | 12 | 32 | 258 | 2 | 279 | Tail fiber protein | Tail fiber protein | |
| Top keywords  (threshold 1.00e-02 (evalue)) | **tail, Phage, fiber, fibre, tail\_collar, domain\_containing, Collar, Putative, Phage\_related, repeat** |
| Output files | ../../similar\_structures/15\_FANPEZAQ\_CDS\_0015\_afdb-proteome\_foldseek.tsv ../../similar\_structures/15\_FANPEZAQ\_CDS\_0015\_afdb-uniprot50\_foldseek.tsv ../../similar\_structures/15\_FANPEZAQ\_CDS\_0015\_merged.svg ../../similar\_structures/15\_FANPEZAQ\_CDS\_0015\_pdb\_foldseek.tsv |

  
  
  

Return to summary | Go to previous | Go to next

  


---

**Sequence/structure alignments coloring**  
Each object in the alignment figures is colored according to its E-value following this color coding:

1e-100
10

**References:**  
1) Steinegger M, Meier M, Mirdita M, Vöhringer H, Haunsberger S J, and Söding J (2019) HH-suite3 for fast remote homology detection and deep protein annotation, BMC Bioinformatics, 473. doi: 10.1186/s12859-019-3019-7  
2) Jumper J, Evans R, Pritzel A, ..., Hassabis D (2021) Highly accurate protein structure prediction with AlphaFold, Nature, 596. doi: 10.1038/s41586-021-03819-2  
3) van Kempen M, Kim S, Tumescheit C, Mirdita M, Lee J, Gilchrist CLM, Söding J, and Steinegger M (2023) Fast and accurate protein structure search with Foldseek. Nature Biotechnology. doi: 10.1038/s41587-023-01773-0
