# Supplementary material for: Completing the BASEL phage collection to unlock hidden diversity for systematic exploration of phage–host interactions
Source: PLoS Biol. 2025 Apr 7;23(4):e3003063. doi: 10.1371/journal.pbio.3003063 (PMC11990801; doi:10.1371/journal.pbio.3003063)
Supplement: S2 Data — (ZIP) [file pbio.3003063.s009.zip › entries/16.html]

FANPEZAQ\_CDS\_0016


Return to summary | Go to previous | Go to next

|  |  |
| --- | --- |
| FANPEZAQ\_CDS\_0016 Page creation date: 02 Sep 2024, 12:00  Project folder: n/a  Input sequences file: Escherichia\_virus\_HeidiAbel.gb | tail assembly fiber phage chaperone caudovirales putative fibre lambda gpk prophage virus gp38 domain\_containing fragment hypothetical duf4376 bacteriophage cps\_53 kple1 g homolog from lambdoid |

### Sequence information

|  |  |
| --- | --- |
| Name | FANPEZAQ\_CDS\_0016  16\_FANPEZAQ\_CDS\_0016 (pipeline id) |
| Imported annotations | Escherichia\_virus\_HeidiAbel Bas97 |
| Protein sequence | MSKYMFSPSTNAFYPVALQSIYEEANMWPNDGVGCEEDVFSEFTGVPPEGKRRGSDKDGN PAWVDVPPLTKEQQIANAKSMQQYLIDQANDFMNSKQWPGKAAIGRLKDAELNQYKIWLD YLDALYAVDTSTAPEITWPEQPQ |
| Number of residues | 143 |
| Molecular weight (Da) | 16173.84 |
| Output files | ../../query\_sequences/16\_FANPEZAQ\_CDS\_0016.fasta |

### Putative domain architecture and protein family

#### Search results (HHblits)1

|  |  |
| --- | --- |
| Domain family databases searched | Pfam, Ncbi-cd, Cath, Phrogs |
| Results, scheme(s)  (Top layers only; threshold 1.00e-03 (evalue)) | xml version="1.0" encoding="utf-8" standalone="no"?       2024-09-02T21:08:15.476921 image/svg+xml   Matplotlib v3.7.2, https://matplotlib.org/ |
| Results, table  (E-value ≤ 1.00e-03 (evalue)) | | db | id | prob | evalue | pvalue | score | cols | query | query\_len | template | template\_len | name | description | | --- | --- | --- | --- | --- | --- | --- | --- | --- | --- | --- | --- | --- | | pfam | PF02413 | 98.9 | 1.1e-13 | 2.3e-17 | 88.7 | 94 | (44, 143) | 143 | (27, 120) | 120 | Caudo\_TAP | Caudovirales tail fibre assembly protein, lambda gpK | | pfam | PF16778 | 97.4 | 4.9e-08 | 9.9e-12 | 53.6 | 56 | (77, 143) | 143 | (3, 60) | 60 | Phage\_tail\_APC | Phage tail assembly chaperone protein | | cath | 2kz6A01 | 99.1 | 4.5e-15 | 7.7e-19 | 95.0 | 73 | (28, 126) | 143 | (11, 83) | 83 | Uncharacterized protein | CATHCODE: 6.10.140.1310 NAME: Uncharacterized protein. Chain: a. Engineered: yes SOURCE: Chromobacterium violaceum. Organism\_taxid: 536. Gene: cv\_0426. Expressed in: escherichia coli. Expression\_system\_taxid: 562. Expression\_system\_vector: p15tv lic CLASS: Special, ARCH: Helix non-globular, TOPOL: Helix Hairpins, HOMOL: Helix Hairpins | | phrogs | 188 | 100.0 | 6.4e-39 | 8.4e-43 | 225.6 | 136 | (1, 143) | 143 | (4, 140) | 140 | tail fiber assembly protein | tail fiber assembly protein; Category: tail; p268800 VI\_09964 | | phrogs | 5572 | 99.7 | 8.3e-22 | 9.7e-26 | 123.0 | 75 | (67, 143) | 143 | (4, 78) | 80 | NA | NA; Category: unknown function; p62117 VI\_01572 | | phrogs | 105 | 99.7 | 2.5e-21 | 3.1e-25 | 141.8 | 80 | (62, 143) | 143 | (124, 203) | 203 | tail fiber assembly | tail fiber assembly; Category: tail; p319838 VI\_03642 | | phrogs | 3792 | 99.6 | 5.7e-21 | 6.6e-25 | 120.8 | 80 | (56, 143) | 143 | (9, 88) | 88 | tail assembly chaperone | tail assembly chaperone; Category: tail; p125307 VI\_08164 | | phrogs | 136 | 99.5 | 1e-18 | 1.3e-22 | 125.3 | 81 | (55, 143) | 143 | (95, 175) | 175 | tail fiber assembly protein | tail fiber assembly protein; Category: tail; p240703 VI\_01691 | | phrogs | 5844 | 99.1 | 1.2e-14 | 1.3e-18 | 101.5 | 111 | (30, 142) | 143 | (81, 193) | 199 | NA | NA; Category: unknown function; p304883 VI\_09788 | | phrogs | 19350 | 98.3 | 1.1e-10 | 1.3e-14 | 67.4 | 65 | (1, 65) | 143 | (1, 66) | 69 | NA | NA; Category: unknown function; p37945 VI\_07185 | | phrogs | 5400 | 98.3 | 1.2e-10 | 1.4e-14 | 69.5 | 59 | (3, 68) | 143 | (1, 59) | 74 | NA | NA; Category: unknown function; p80665 VI\_09756 | | phrogs | 1795 | 98.1 | 1.2e-09 | 1.4e-13 | 73.9 | 61 | (78, 143) | 143 | (70, 133) | 136 | tail fiber protein | tail fiber protein; Category: tail; KX660669\_p44 | | phrogs | 8746 | 98.0 | 1.3e-09 | 1.6e-13 | 74.1 | 61 | (73, 143) | 143 | (92, 154) | 154 | tail fiber assembly protein | tail fiber assembly protein; Category: tail; KP343639\_p7 | | phrogs | 4628 | 98.0 | 2.5e-09 | 2.8e-13 | 70.0 | 64 | (70, 143) | 143 | (58, 123) | 124 | tail fiber assembly | tail fiber assembly; Category: tail; NC\_026587\_p82 | | phrogs | 24907 | 97.6 | 3.4e-08 | 3.9e-12 | 68.1 | 66 | (76, 143) | 143 | (130, 197) | 206 | NA | NA; Category: unknown function; p115387 VI\_09903 | | phrogs | 8716 | 97.5 | 5.7e-08 | 6.7e-12 | 63.2 | 58 | (71, 143) | 143 | (56, 115) | 115 | tail fiber protein | tail fiber protein; Category: tail; NC\_020838\_p5 | | phrogs | 37801 | 97.4 | 1.2e-07 | 1.3e-11 | 54.4 | 36 | (2, 37) | 143 | (1, 36) | 73 | NA | NA; Category: unknown function; p165538 VI\_11050 | |
| Top keywords  (threshold 1.00e-03 (evalue)) | **tail, assembly, fiber, Helix, chaperone, Hairpins, Caudovirales, fibre, lambda, gpK** |
| Output files | ../../domain\_architecture/16\_FANPEZAQ\_CDS\_0016\_cath.hhr ../../domain\_architecture/16\_FANPEZAQ\_CDS\_0016\_merged.svg ../../domain\_architecture/16\_FANPEZAQ\_CDS\_0016\_ncbi-cd.hhr ../../domain\_architecture/16\_FANPEZAQ\_CDS\_0016\_pfam.hhr ../../domain\_architecture/16\_FANPEZAQ\_CDS\_0016\_phrogs.hhr |

### Identical protein sequences/structures

#### Search results

|  |  |
| --- | --- |
| Protein sequence databases searched | Pdb, Swissprot, Refseq |
| Identical proteins found | -- |
| Top keywords | -- |
| Output files | -- |

### Similar protein sequences/structures

#### Sequence similarity search results (HHblits)1

|  |  |
| --- | --- |
| Sequence databases searched | Uniclust, Pdb70 |
| Results, scheme(s)  (Top layers only, threshold 1.00e-03 (evalue)) | xml version="1.0" encoding="utf-8" standalone="no"?       2024-09-02T21:08:35.691326 image/svg+xml   Matplotlib v3.7.2, https://matplotlib.org/ |
| Results, table(s)  (threshold 1.00e-03 (evalue)) | | db | id | prob | evalue | pvalue | score | cols | query | query\_len | template | template\_len | name | description | | --- | --- | --- | --- | --- | --- | --- | --- | --- | --- | --- | --- | --- | | uniclust | UniRef100\_A0A071M0C5 | 100.0 | 1.3e-50 | 2.5e-56 | 261.9 | 141 | (1, 143) | 143 | (16, 156) | 172 | Tail fiber assembly protein | Tail fiber assembly protein | | uniclust | UniRef100\_A0A345CNR7 | 100.0 | 3.3e-47 | 6.2e-53 | 234.6 | 135 | (1, 143) | 143 | (3, 137) | 143 | Tail fiber assembly protein | Tail fiber assembly protein | | uniclust | UniRef100\_A0A022PLD3 | 100.0 | 1e-46 | 1.9e-52 | 251.1 | 141 | (1, 143) | 143 | (51, 191) | 214 | Caudovirales tail fiber assembly protein | Caudovirales tail fiber assembly protein | | uniclust | UniRef100\_A0A0M7GTK9 | 100.0 | 5.2e-45 | 9.9e-51 | 229.7 | 136 | (1, 143) | 143 | (5, 142) | 151 | Bacteriophage tail assembly protein | Bacteriophage tail assembly protein | | uniclust | UniRef100\_A0A077Q6L9 | 100.0 | 6e-45 | 1.1e-50 | 234.1 | 143 | (1, 143) | 143 | (17, 159) | 177 | Putative tail fiber assembly protein | Putative tail fiber assembly protein | | uniclust | UniRef100\_A0A080IB21 | 100.0 | 2.7e-43 | 5.3e-49 | 235.4 | 141 | (1, 143) | 143 | (26, 166) | 193 | Caudovirales tail fiber assembly family protein | Caudovirales tail fiber assembly family protein | | uniclust | UniRef100\_A0A022PIU1 | 100.0 | 9.4e-43 | 1.8e-48 | 237.7 | 141 | (1, 143) | 143 | (55, 195) | 224 | Caudovirales tail fiber assembly protein | Caudovirales tail fiber assembly protein | | uniclust | UniRef100\_A0A2X1RV26 | 100.0 | 1.4e-42 | 2.6e-48 | 216.5 | 141 | (1, 143) | 143 | (1, 142) | 146 | Caudovirales tail fibre assembly protein | Caudovirales tail fibre assembly protein | | uniclust | UniRef100\_A0A376YI45 | 100.0 | 3.6e-42 | 6.7e-48 | 214.1 | 143 | (1, 143) | 143 | (11, 153) | 153 | CPS-53 (KpLE1) prophage protein | CPS-53 (KpLE1) prophage protein | | uniclust | UniRef100\_A0A0E8XEB7 | 100.0 | 9.8e-41 | 1.9e-46 | 217.2 | 141 | (1, 143) | 143 | (16, 157) | 172 | Tail assembly chaperone gp38 | Tail assembly chaperone gp38 | | uniclust | UniRef100\_A0A0H2VSV7 | 100.0 | 6.5e-40 | 1.2e-45 | 206.1 | 136 | (1, 143) | 143 | (37, 172) | 188 | Putative phage tail fibre protein | Putative phage tail fibre protein | | uniclust | UniRef100\_A0A0V9JY68 | 100.0 | 7e-40 | 1.3e-45 | 207.7 | 140 | (1, 143) | 143 | (19, 158) | 159 | Phage tail protein | Phage tail protein | | uniclust | UniRef100\_A0A071M4A9 | 100.0 | 9.5e-40 | 1.8e-45 | 212.7 | 135 | (1, 143) | 143 | (24, 158) | 194 | Tail fiber assembly protein | Tail fiber assembly protein | | uniclust | UniRef100\_A0A071LRY7 | 100.0 | 4.7e-39 | 8.9e-45 | 199.8 | 136 | (1, 143) | 143 | (1, 136) | 140 | Tail fiber assembly protein | Tail fiber assembly protein | | uniclust | UniRef100\_A0A8R2D774 | 100.0 | 4.5e-38 | 8.5e-44 | 195.6 | 140 | (2, 143) | 143 | (1, 143) | 144 | Tail fiber assembly protein | Tail fiber assembly protein | | uniclust | UniRef100\_A0A3F3IA78 | 100.0 | 5.7e-35 | 1.1e-40 | 183.7 | 134 | (1, 143) | 143 | (4, 137) | 155 | Tail fiber assembly protein | Tail fiber assembly protein | | uniclust | UniRef100\_A0A0D0PTQ5 | 100.0 | 1.1e-33 | 2.2e-39 | 177.9 | 133 | (1, 143) | 143 | (1, 135) | 140 | Tail fiber assembly protein | Tail fiber assembly protein | | uniclust | UniRef100\_A0A4D7IX74 | 100.0 | 3.7e-33 | 6.8e-39 | 176.6 | 138 | (3, 142) | 143 | (59, 197) | 197 | Phage tail protein | Phage tail protein | | uniclust | UniRef100\_A0A080KGQ0 | 100.0 | 9.8e-33 | 1.9e-38 | 176.0 | 133 | (1, 143) | 143 | (16, 148) | 153 | Caudovirales tail fiber assembly protein | Caudovirales tail fiber assembly protein | | uniclust | UniRef100\_A0A031FRK0 | 100.0 | 1.4e-32 | 2.7e-38 | 176.8 | 135 | (1, 143) | 143 | (10, 146) | 164 | Prophage tail fimber assembly protein | Prophage tail fimber assembly protein | | uniclust | UniRef100\_A0A080HNE4 | 100.0 | 2.2e-32 | 4.3e-38 | 181.2 | 134 | (1, 143) | 143 | (23, 159) | 172 | Caudovirales tail fiber assembly family protein | Caudovirales tail fiber assembly family protein | | uniclust | UniRef100\_A0A127QUQ2 | 99.9 | 3.5e-32 | 6.7e-38 | 173.1 | 137 | (1, 143) | 143 | (4, 149) | 154 | Caudovirales tail fiber assembly family protein | Caudovirales tail fiber assembly family protein | | uniclust | UniRef100\_A0A024EI51 | 99.9 | 3.6e-31 | 7e-37 | 179.1 | 135 | (1, 143) | 143 | (34, 170) | 203 | Phage tail protein | Phage tail protein | | uniclust | UniRef100\_A0A068QUZ6 | 99.9 | 3.6e-31 | 7e-37 | 169.8 | 135 | (1, 143) | 143 | (2, 136) | 148 | Putative tail fiber assembly protein | Putative tail fiber assembly protein | | uniclust | UniRef100\_A0A0A0YSY3 | 99.9 | 4.1e-31 | 7.7e-37 | 171.4 | 135 | (3, 143) | 143 | (68, 202) | 220 | Tail fiber assembly protein | Tail fiber assembly protein | | uniclust | UniRef100\_A0A1Q6AW63 | 99.9 | 6.1e-31 | 1.1e-36 | 160.6 | 137 | (4, 143) | 143 | (6, 143) | 147 | Phage tail protein | Phage tail protein | | uniclust | UniRef100\_UPI0002D7ACE8 | 99.9 | 1.3e-30 | 2.5e-36 | 162.6 | 136 | (1, 143) | 143 | (2, 139) | 142 | tail assembly chaperone | tail assembly chaperone | | uniclust | UniRef100\_A0A0A6DH01 | 99.9 | 1.8e-30 | 3.4e-36 | 165.6 | 134 | (1, 142) | 143 | (5, 140) | 156 | Phage tail protein | Phage tail protein | | uniclust | UniRef100\_A0A2L1UQL2 | 99.9 | 2e-30 | 3.7e-36 | 159.7 | 132 | (2, 143) | 143 | (1, 132) | 134 | Phage tail protein | Phage tail protein | | uniclust | UniRef100\_A0A2D0IKG5 | 99.9 | 5.2e-30 | 9.5e-36 | 155.2 | 135 | (3, 143) | 143 | (15, 149) | 149 | Tail assembly chaperone | Tail assembly chaperone | | uniclust | UniRef100\_A0A0N0XL62 | 99.9 | 2e-29 | 3.9e-35 | 162.3 | 128 | (1, 143) | 143 | (5, 134) | 146 | Caudovirales tail fiber assembly protein | Caudovirales tail fiber assembly protein | | uniclust | UniRef100\_A0A1W0D5R5 | 99.9 | 2.2e-29 | 4e-35 | 153.8 | 135 | (3, 143) | 143 | (1, 137) | 143 | Phage tail protein | Phage tail protein | | uniclust | UniRef100\_A0A1M3PGE6 | 99.9 | 2.9e-29 | 5.5e-35 | 155.3 | 126 | (1, 143) | 143 | (1, 128) | 133 | Phage tail protein | Phage tail protein | | uniclust | UniRef100\_A0A0N9WEK9 | 99.9 | 6.4e-29 | 1.2e-34 | 156.0 | 135 | (1, 143) | 143 | (1, 137) | 151 | Phage tail protein | Phage tail protein | | uniclust | UniRef100\_A0A2S5GQX3 | 99.9 | 8.9e-29 | 1.7e-34 | 159.4 | 135 | (3, 143) | 143 | (62, 198) | 201 | Tail fiber assembly protein | Tail fiber assembly protein | | uniclust | UniRef100\_A0A031IRP6 | 99.9 | 1.5e-28 | 2.8e-34 | 165.1 | 141 | (1, 143) | 143 | (3, 198) | 210 | Prophage PSPPH06 tail fiber protein | Prophage PSPPH06 tail fiber protein | | uniclust | UniRef100\_A0A2S7HKK0 | 99.9 | 3e-28 | 5.6e-34 | 146.4 | 138 | (3, 143) | 143 | (1, 138) | 142 | Phage tail protein | Phage tail protein | | uniclust | UniRef100\_A0A1L4BM55 | 99.9 | 1.2e-27 | 2.3e-33 | 144.9 | 121 | (22, 143) | 143 | (3, 123) | 127 | Caudovirales tail fiber assembly protein | Caudovirales tail fiber assembly protein | | uniclust | UniRef100\_A0A1Q5TJA7 | 99.9 | 1.8e-27 | 3.3e-33 | 152.7 | 76 | (66, 143) | 143 | (64, 139) | 162 | Tail fiber assembly protein | Tail fiber assembly protein | | uniclust | UniRef100\_A0A501S8D9 | 99.9 | 3.8e-27 | 7.2e-33 | 146.8 | 140 | (1, 143) | 143 | (1, 140) | 142 | Tail fiber assembly protein | Tail fiber assembly protein | | uniclust | UniRef100\_UPI0015857BD7 | 99.9 | 4.1e-27 | 7.5e-33 | 149.7 | 142 | (2, 143) | 143 | (70, 211) | 211 | tail fiber assembly protein | tail fiber assembly protein | | uniclust | UniRef100\_A0A104MR71 | 99.9 | 4.5e-27 | 8.4e-33 | 147.8 | 133 | (1, 143) | 143 | (1, 139) | 149 | Tail fiber assembly protein | Tail fiber assembly protein | | uniclust | UniRef100\_A0A0K8QR29 | 99.9 | 4.8e-27 | 9.1e-33 | 152.2 | 127 | (1, 143) | 143 | (16, 144) | 160 | Tail fiber assembly protein | Tail fiber assembly protein | | uniclust | UniRef100\_A0A077P958 | 99.9 | 6.9e-27 | 1.3e-32 | 140.4 | 91 | (50, 143) | 143 | (17, 107) | 114 | Putative tail fiber chaperone Rac prophage | Putative tail fiber chaperone Rac prophage | | uniclust | UniRef100\_A0A1G9YD25 | 99.9 | 1.2e-26 | 2.3e-32 | 145.2 | 112 | (30, 143) | 143 | (20, 132) | 142 | Virus tail fibre assembly protein, lambda gpK | Virus tail fibre assembly protein, lambda gpK | | uniclust | UniRef100\_A0A0B1YWB2 | 99.9 | 1.2e-26 | 2.3e-32 | 151.4 | 136 | (1, 143) | 143 | (7, 144) | 171 | Phage tail protein | Phage tail protein | | uniclust | UniRef100\_A0A022PH83 | 99.9 | 2e-26 | 3.8e-32 | 137.6 | 95 | (46, 143) | 143 | (4, 98) | 110 | Caudovirales tail fiber assembly protein | Caudovirales tail fiber assembly protein | | uniclust | UniRef100\_A0A0E1SIM2 | 99.9 | 5.7e-26 | 1.1e-31 | 150.2 | 114 | (28, 143) | 143 | (45, 161) | 184 | Tail assembly chaperone | Tail assembly chaperone | | uniclust | UniRef100\_A0A2D0KV06 | 99.9 | 6.6e-26 | 1.2e-31 | 161.5 | 138 | (2, 143) | 143 | (421, 561) | 561 | Tail assembly chaperone | Tail assembly chaperone | | uniclust | UniRef100\_A0A3A3ZKX8 | 99.9 | 1.6e-25 | 3.1e-31 | 133.0 | 91 | (1, 91) | 143 | (2, 93) | 98 | Tail fiber assembly protein | Tail fiber assembly protein | | uniclust | UniRef100\_A0A014N9D4 | 99.9 | 2.4e-25 | 4.4e-31 | 140.8 | 139 | (1, 143) | 143 | (35, 175) | 176 | Tail fiber assembly protein | Tail fiber assembly protein | | uniclust | UniRef100\_A0A2K8QS96 | 99.9 | 2.4e-25 | 4.5e-31 | 133.8 | 123 | (2, 143) | 143 | (1, 126) | 128 | Phage tail protein | Phage tail protein | | uniclust | UniRef100\_A0A158S497 | 99.9 | 4.2e-25 | 8.1e-31 | 139.5 | 126 | (2, 143) | 143 | (1, 128) | 129 | Tail fiber assembly protein | Tail fiber assembly protein | | uniclust | UniRef100\_A0A369F960 | 99.9 | 5.1e-25 | 9.7e-31 | 140.5 | 131 | (1, 143) | 143 | (6, 137) | 151 | Tail fiber assembly protein | Tail fiber assembly protein | | uniclust | UniRef100\_A0A208ZVY5 | 99.8 | 6.1e-25 | 1.1e-30 | 128.9 | 76 | (66, 143) | 143 | (15, 90) | 94 | Phage tail protein | Phage tail protein | | uniclust | UniRef100\_A0A1H1NDT3 | 99.8 | 7.5e-25 | 1.4e-30 | 139.7 | 126 | (4, 143) | 143 | (1, 130) | 143 | Virus tail fibre assembly protein, lambda gpK | Virus tail fibre assembly protein, lambda gpK | | uniclust | UniRef100\_A0A3V9FI80 | 99.8 | 9.1e-25 | 1.7e-30 | 137.4 | 136 | (2, 143) | 143 | (10, 145) | 149 | Phage tail protein | Phage tail protein | | uniclust | UniRef100\_A0A0T9UQ83 | 99.8 | 9.5e-25 | 1.8e-30 | 130.7 | 94 | (48, 143) | 143 | (13, 106) | 110 | Tail assembly chaperone gp38 | Tail assembly chaperone gp38 | | uniclust | UniRef100\_A0A1G5QUE3 | 99.8 | 1.2e-24 | 2.2e-30 | 130.6 | 100 | (1, 100) | 143 | (6, 105) | 109 | Virus tail fibre assembly protein, lambda gpK | Virus tail fibre assembly protein, lambda gpK | | uniclust | UniRef100\_UPI000792F1CB | 99.8 | 1.2e-24 | 2.2e-30 | 131.9 | 140 | (2, 143) | 143 | (1, 144) | 144 | tail fiber assembly protein | tail fiber assembly protein | | uniclust | UniRef100\_A0A2S1BAI7 | 99.8 | 1.3e-24 | 2.5e-30 | 136.0 | 104 | (37, 143) | 143 | (36, 140) | 144 | Phage tail protein | Phage tail protein | | uniclust | UniRef100\_A0A0U3TL14 | 99.8 | 1.4e-24 | 2.7e-30 | 138.1 | 131 | (1, 143) | 143 | (19, 149) | 157 | Tail fiber assembly protein | Tail fiber assembly protein | | uniclust | UniRef100\_A0A0W1G7X1 | 99.8 | 1.5e-24 | 2.9e-30 | 140.4 | 130 | (1, 143) | 143 | (3, 154) | 163 | Phage tail protein | Phage tail protein | | uniclust | UniRef100\_A0A2X4UV66 | 99.8 | 1.7e-24 | 3.3e-30 | 132.5 | 94 | (1, 95) | 143 | (3, 96) | 116 | Uncharacterized protein | Uncharacterized protein | | uniclust | UniRef100\_A0A2J4V2S3 | 99.8 | 2.7e-24 | 5.1e-30 | 133.7 | 81 | (61, 143) | 143 | (46, 126) | 132 | Tail fiber assembly protein (Fragment) | Tail fiber assembly protein (Fragment) | | uniclust | UniRef100\_A0A0H5HS46 | 99.8 | 3.1e-24 | 5.9e-30 | 142.2 | 137 | (1, 143) | 143 | (31, 170) | 212 | Putative tail fiber assembly protein | Putative tail fiber assembly protein | | uniclust | UniRef100\_A0A4R4JRM7 | 99.8 | 8.3e-24 | 1.6e-29 | 127.4 | 74 | (68, 143) | 143 | (37, 110) | 117 | Tail fiber assembly protein | Tail fiber assembly protein | | uniclust | UniRef100\_A0A1S2TBU1 | 99.8 | 8.5e-24 | 1.6e-29 | 134.6 | 137 | (3, 142) | 143 | (27, 166) | 174 | Phage tail protein | Phage tail protein | | uniclust | UniRef100\_A0A411WM04 | 99.8 | 1.9e-23 | 3.7e-29 | 128.3 | 110 | (27, 143) | 143 | (11, 121) | 124 | Tail fiber assembly protein | Tail fiber assembly protein | | uniclust | UniRef100\_A0A2D0J0R2 | 99.8 | 2.3e-23 | 4.3e-29 | 141.1 | 76 | (66, 143) | 143 | (154, 229) | 230 | Phage tail fiber assembly | Phage tail fiber assembly | | uniclust | UniRef100\_A0A1X0VZT8 | 99.8 | 2.8e-23 | 5.3e-29 | 132.8 | 134 | (4, 143) | 143 | (1, 154) | 155 | Phage tail protein | Phage tail protein | | uniclust | UniRef100\_A0A7U5JH60 | 99.8 | 3.3e-23 | 6.2e-29 | 134.7 | 135 | (1, 140) | 143 | (21, 157) | 161 | Tail fiber assembly protein | Tail fiber assembly protein | | uniclust | UniRef100\_A0A2L1I8I3 | 99.8 | 4e-23 | 7.6e-29 | 131.1 | 77 | (65, 143) | 143 | (56, 134) | 141 | Phage tail protein | Phage tail protein | | uniclust | UniRef100\_A0A229A5L0 | 99.8 | 5.8e-23 | 1.1e-28 | 126.1 | 142 | (2, 143) | 143 | (7, 148) | 152 | Tail fiber assembly protein | Tail fiber assembly protein | | uniclust | UniRef100\_A0A2U8I8G2 | 99.8 | 7.4e-23 | 1.4e-28 | 120.1 | 84 | (58, 143) | 143 | (5, 89) | 94 | Phage tail protein | Phage tail protein | | uniclust | UniRef100\_A0A2X1MUF5 | 99.8 | 1.7e-22 | 3.2e-28 | 128.8 | 85 | (56, 143) | 143 | (63, 147) | 157 | Tail fiber assembly protein (Gpg) | Tail fiber assembly protein (Gpg) | | uniclust | UniRef100\_A0A7D6TWW6 | 99.8 | 2.3e-22 | 4.3e-28 | 126.3 | 120 | (1, 123) | 143 | (1, 120) | 184 | Tail fiber assembly protein | Tail fiber assembly protein | | uniclust | UniRef100\_A0A336QKS4 | 99.8 | 2.3e-22 | 4.4e-28 | 122.8 | 74 | (67, 143) | 143 | (41, 114) | 116 | Phage tail fiber assembly protein | Phage tail fiber assembly protein | | uniclust | UniRef100\_A0A068Z7G0 | 99.8 | 2.6e-22 | 5e-28 | 130.7 | 72 | (70, 143) | 143 | (80, 151) | 157 | Tail fiber assembly protein | Tail fiber assembly protein | | uniclust | UniRef100\_A0A076LFJ0 | 99.8 | 3.2e-22 | 6e-28 | 117.9 | 86 | (58, 143) | 143 | (2, 87) | 96 | Phage tail fiber assembly protein | Phage tail fiber assembly protein | | uniclust | UniRef100\_UPI000983374A | 99.8 | 3.6e-22 | 6.8e-28 | 120.2 | 105 | (34, 143) | 143 | (11, 116) | 117 | tail fiber assembly protein | tail fiber assembly protein | | uniclust | UniRef100\_A0A0K1QN38 | 99.8 | 4.1e-22 | 7.7e-28 | 126.4 | 76 | (66, 143) | 143 | (49, 126) | 143 | Phage tail protein | Phage tail protein | | uniclust | UniRef100\_A0A423XTX7 | 99.8 | 9.9e-22 | 1.8e-27 | 120.5 | 137 | (1, 143) | 143 | (1, 143) | 146 | Phage tail protein | Phage tail protein | | uniclust | UniRef100\_A0A0H3AZX4 | 99.8 | 9.7e-22 | 1.8e-27 | 130.4 | 73 | (69, 143) | 143 | (111, 183) | 197 | Tail assembly chaperone gp38 | Tail assembly chaperone gp38 | | uniclust | UniRef100\_A0A2C5TSB9 | 99.8 | 1.1e-21 | 2e-27 | 113.5 | 94 | (48, 143) | 143 | (3, 96) | 96 | Phage tail protein | Phage tail protein | | uniclust | UniRef100\_UPI000A9D9905 | 99.7 | 1.7e-21 | 3.2e-27 | 117.8 | 121 | (2, 143) | 143 | (1, 123) | 123 | phage tail assembly chaperone | phage tail assembly chaperone | | uniclust | UniRef100\_A0A548QSH7 | 99.7 | 2.2e-21 | 4e-27 | 117.7 | 131 | (1, 141) | 143 | (3, 133) | 137 | Tail fiber assembly protein (Fragment) | Tail fiber assembly protein (Fragment) | | uniclust | UniRef100\_A0A090V754 | 99.7 | 3.6e-21 | 6.7e-27 | 113.3 | 83 | (59, 143) | 143 | (6, 90) | 94 | Predicted tail fiber assembly protein | Predicted tail fiber assembly protein | | uniclust | UniRef100\_A0A5C5DE35 | 99.7 | 3.7e-21 | 6.9e-27 | 120.5 | 138 | (1, 143) | 143 | (1, 141) | 145 | Tail fiber assembly protein | Tail fiber assembly protein | | uniclust | UniRef100\_A0A2T8WWY8 | 99.7 | 4.1e-21 | 7.7e-27 | 126.8 | 68 | (74, 143) | 143 | (112, 179) | 194 | Tail fiber assembly protein (Fragment) | Tail fiber assembly protein (Fragment) | | uniclust | UniRef100\_A0A0N9ZQ35 | 99.7 | 4.4e-21 | 8.2e-27 | 121.5 | 134 | (2, 143) | 143 | (10, 145) | 156 | Phage tail protein | Phage tail protein | | uniclust | UniRef100\_E9CQ84 | 99.7 | 6.3e-21 | 1.2e-26 | 110.4 | 80 | (62, 143) | 143 | (5, 84) | 87 | Putative phage tail assembly protein (Fragment) | Putative phage tail assembly protein (Fragment) | | uniclust | UniRef100\_A0A0J1NAY6 | 99.7 | 6.3e-21 | 1.2e-26 | 128.8 | 70 | (72, 143) | 143 | (113, 182) | 190 | Tail fiber assembly protein | Tail fiber assembly protein | | uniclust | UniRef100\_A0A1C7W7E1 | 99.7 | 6.8e-21 | 1.3e-26 | 110.4 | 72 | (1, 72) | 143 | (1, 72) | 80 | Phage tail protein | Phage tail protein | | uniclust | UniRef100\_A0A077NP04 | 99.7 | 8.5e-21 | 1.6e-26 | 126.9 | 80 | (62, 143) | 143 | (118, 197) | 201 | Tail fiber assembly protein | Tail fiber assembly protein | | uniclust | UniRef100\_A0A2T8TCL9 | 99.7 | 1.2e-20 | 2.3e-26 | 122.4 | 132 | (2, 143) | 143 | (1, 169) | 180 | Tail assembly chaperone | Tail assembly chaperone | | uniclust | UniRef100\_UPI00094363AD | 99.7 | 1.7e-20 | 3.2e-26 | 116.7 | 106 | (2, 112) | 143 | (12, 117) | 121 | tail fiber assembly protein | tail fiber assembly protein | | uniclust | UniRef100\_A0A3N2RWI5 | 99.7 | 2.1e-20 | 3.9e-26 | 121.0 | 131 | (3, 143) | 143 | (33, 184) | 188 | Tail fiber assembly protein | Tail fiber assembly protein | | uniclust | UniRef100\_A0A2E3R781 | 99.7 | 2.7e-20 | 4.9e-26 | 114.3 | 126 | (1, 143) | 143 | (7, 135) | 138 | Phage tail protein | Phage tail protein | | uniclust | UniRef100\_A0A1D8UTE2 | 99.7 | 2.8e-20 | 5.3e-26 | 109.5 | 70 | (1, 70) | 143 | (6, 75) | 83 | Uncharacterized protein | Uncharacterized protein | | uniclust | UniRef100\_UPI001D07D678 | 99.7 | 3.3e-20 | 6.2e-26 | 115.0 | 139 | (2, 142) | 143 | (1, 159) | 159 | tail fiber assembly protein | tail fiber assembly protein | | uniclust | UniRef100\_A0A2T6LTZ2 | 99.7 | 3.5e-20 | 6.5e-26 | 115.2 | 127 | (3, 143) | 143 | (1, 127) | 134 | Virus tail fiber assembly protein lambda gpK | Virus tail fiber assembly protein lambda gpK | | uniclust | UniRef100\_A0A0T9T931 | 99.7 | 3.8e-20 | 7.2e-26 | 118.7 | 110 | (27, 143) | 143 | (44, 157) | 160 | Tail fiber assembly protein G | Tail fiber assembly protein G | | uniclust | UniRef100\_UPI001322C4D5 | 99.7 | 4.2e-20 | 7.8e-26 | 106.3 | 74 | (68, 143) | 143 | (13, 86) | 89 | tail fiber assembly protein | tail fiber assembly protein | | uniclust | UniRef100\_A0A0T9T1K4 | 99.7 | 4.5e-20 | 8.5e-26 | 123.2 | 85 | (57, 143) | 143 | (124, 208) | 225 | Tail fiber assembly protein G | Tail fiber assembly protein G | | uniclust | UniRef100\_A0A022PI45 | 99.7 | 5e-20 | 9.8e-26 | 128.7 | 80 | (62, 143) | 143 | (134, 213) | 230 | Caudovirales tail fiber assembly protein | Caudovirales tail fiber assembly protein | | uniclust | UniRef100\_A0A1Z1STM3 | 99.7 | 6e-20 | 1.1e-25 | 113.7 | 133 | (3, 143) | 143 | (1, 134) | 134 | Tail fiber assembly protein | Tail fiber assembly protein | | uniclust | UniRef100\_A0A022PBR1 | 99.7 | 6e-20 | 1.2e-25 | 128.5 | 80 | (62, 143) | 143 | (155, 234) | 245 | Bacteriophage tail assembly protein | Bacteriophage tail assembly protein | | uniclust | UniRef100\_A0A0T9R7L9 | 99.7 | 7.9e-20 | 1.5e-25 | 123.0 | 128 | (1, 143) | 143 | (35, 174) | 202 | Tail fiber assembly protein G | Tail fiber assembly protein G | | uniclust | UniRef100\_A0A1Q6B6N7 | 99.7 | 8.5e-20 | 1.6e-25 | 107.6 | 84 | (58, 143) | 143 | (29, 112) | 116 | Phage tail protein | Phage tail protein | | uniclust | UniRef100\_A0A127Q904 | 99.7 | 8.8e-20 | 1.7e-25 | 123.8 | 104 | (38, 143) | 143 | (86, 196) | 213 | Caudovirales tail fiber assembly family protein | Caudovirales tail fiber assembly family protein | | uniclust | UniRef100\_A0A0A1IVW2 | 99.7 | 1e-19 | 1.9e-25 | 119.7 | 75 | (67, 143) | 143 | (76, 150) | 161 | Tail fiber component | Tail fiber component | | uniclust | UniRef100\_A0A068R0K4 | 99.7 | 1.2e-19 | 2.3e-25 | 121.8 | 72 | (67, 143) | 143 | (110, 181) | 205 | Tail fiber assembly protein | Tail fiber assembly protein | | uniclust | UniRef100\_A0A0A0CIE4 | 99.7 | 1.2e-19 | 2.3e-25 | 115.5 | 86 | (56, 143) | 143 | (46, 131) | 143 | Tail assembly protein (Fragment) | Tail assembly protein (Fragment) | | uniclust | UniRef100\_A0A024L1Y3 | 99.7 | 1.4e-19 | 2.8e-25 | 129.0 | 81 | (61, 143) | 143 | (149, 229) | 266 | Tail fiber assembly protein | Tail fiber assembly protein | | uniclust | UniRef100\_UPI00131B923D | 99.7 | 1.8e-19 | 3.3e-25 | 109.5 | 126 | (4, 142) | 143 | (1, 127) | 130 | tail fiber assembly protein | tail fiber assembly protein | | uniclust | UniRef100\_UPI001FC7C713 | 99.7 | 1.9e-19 | 3.5e-25 | 110.1 | 114 | (26, 143) | 143 | (34, 148) | 149 | tail fiber assembly protein | tail fiber assembly protein | | uniclust | UniRef100\_UPI000664EF55 | 99.6 | 2.4e-19 | 4.4e-25 | 101.0 | 80 | (4, 88) | 143 | (1, 80) | 82 | tail fiber assembly protein | tail fiber assembly protein | | uniclust | UniRef100\_A0A0H3I5Y1 | 99.6 | 2.5e-19 | 4.7e-25 | 113.9 | 81 | (61, 143) | 143 | (46, 128) | 145 | Tail fiber chaperone, Qin prophage | Tail fiber chaperone, Qin prophage | | uniclust | UniRef100\_A0A0T9RSX1 | 99.6 | 2.8e-19 | 5.2e-25 | 101.8 | 75 | (67, 143) | 143 | (6, 80) | 84 | Tail fiber assembly protein p37 | Tail fiber assembly protein p37 | | uniclust | UniRef100\_A0A2S8PUH6 | 99.6 | 3.1e-19 | 5.7e-25 | 96.6 | 59 | (82, 142) | 143 | (2, 60) | 64 | Phage tail protein (Fragment) | Phage tail protein (Fragment) | | uniclust | UniRef100\_A0A0D1LLW8 | 99.6 | 3.3e-19 | 6.3e-25 | 109.5 | 72 | (70, 143) | 143 | (33, 106) | 117 | Tail fiber protein | Tail fiber protein | | uniclust | UniRef100\_A0A1X8WD02 | 99.6 | 4.1e-19 | 7.7e-25 | 102.6 | 79 | (60, 143) | 143 | (11, 89) | 91 | Caudovirales tail fiber assembly protein | Caudovirales tail fiber assembly protein | | uniclust | UniRef100\_A0A2R7PZL5 | 99.6 | 4.2e-19 | 7.9e-25 | 109.4 | 66 | (76, 143) | 143 | (52, 119) | 121 | Phage tail protein (Fragment) | Phage tail protein (Fragment) | | uniclust | UniRef100\_A0A851FVU6 | 99.6 | 5.2e-19 | 9.7e-25 | 109.8 | 134 | (2, 143) | 143 | (1, 135) | 142 | Tail fiber assembly protein | Tail fiber assembly protein | | uniclust | UniRef100\_UPI000C786B2F | 99.6 | 6.9e-19 | 1.3e-24 | 105.0 | 113 | (29, 143) | 143 | (6, 122) | 123 | tail fiber assembly protein | tail fiber assembly protein | | uniclust | UniRef100\_A0A418GGH7 | 99.6 | 7.2e-19 | 1.3e-24 | 109.5 | 80 | (61, 142) | 143 | (63, 142) | 144 | Phage tail protein (Fragment) | Phage tail protein (Fragment) | | uniclust | UniRef100\_A0A2E0R6L6 | 99.6 | 7e-19 | 1.3e-24 | 113.7 | 122 | (2, 143) | 143 | (4, 127) | 148 | Phage tail protein | Phage tail protein | | uniclust | UniRef100\_A0A0P9WFS5 | 99.6 | 7.1e-19 | 1.4e-24 | 113.9 | 81 | (61, 143) | 143 | (68, 150) | 157 | Tail fiber assembly protein | Tail fiber assembly protein | | uniclust | UniRef100\_UPI000560D39E | 99.6 | 7.4e-19 | 1.4e-24 | 111.1 | 101 | (30, 143) | 143 | (45, 147) | 147 | tail fiber assembly protein | tail fiber assembly protein | | uniclust | UniRef100\_A0A0N1JDF2 | 99.6 | 7.6e-19 | 1.4e-24 | 112.3 | 111 | (30, 142) | 143 | (29, 141) | 154 | Prophage PSPPH06 | Prophage PSPPH06 | | uniclust | UniRef100\_A0A060AGS0 | 99.6 | 8.4e-19 | 1.7e-24 | 123.5 | 74 | (68, 143) | 143 | (127, 200) | 232 | Putative tail fiber assembly protein | Putative tail fiber assembly protein | | uniclust | UniRef100\_A0A485I3W6 | 99.6 | 1.2e-18 | 2.2e-24 | 109.6 | 134 | (2, 142) | 143 | (6, 143) | 167 | Tail fiber assembly protein | Tail fiber assembly protein | | uniclust | UniRef100\_A0A0H3KZW0 | 99.6 | 1.4e-18 | 2.7e-24 | 117.3 | 82 | (60, 143) | 143 | (123, 204) | 208 | Tail fiber assembly protein | Tail fiber assembly protein | | uniclust | UniRef100\_A0A158FMJ5 | 99.6 | 1.5e-18 | 2.7e-24 | 111.0 | 74 | (67, 143) | 143 | (70, 143) | 156 | Caudovirales tail fiber assembly protein | Caudovirales tail fiber assembly protein | | uniclust | UniRef100\_UPI000695DC3B | 99.6 | 1.8e-18 | 3.3e-24 | 103.1 | 75 | (67, 143) | 143 | (46, 120) | 120 | tail fiber assembly protein | tail fiber assembly protein | | uniclust | UniRef100\_A0A376EWH3 | 99.6 | 2.3e-18 | 4.3e-24 | 103.5 | 86 | (56, 143) | 143 | (12, 103) | 105 | Phage tail assembly chaperone gp38 | Phage tail assembly chaperone gp38 | | uniclust | UniRef100\_UPI00068E9C67 | 99.6 | 2.7e-18 | 4.9e-24 | 102.7 | 80 | (62, 143) | 143 | (44, 123) | 124 | tail fiber assembly protein | tail fiber assembly protein | | uniclust | UniRef100\_A0A2A9KIG4 | 99.6 | 3.1e-18 | 5.9e-24 | 110.2 | 84 | (58, 143) | 143 | (59, 148) | 153 | Virus tail fiber assembly protein lambda gpK | Virus tail fiber assembly protein lambda gpK | | uniclust | UniRef100\_UPI00210832D5 | 99.6 | 3.8e-18 | 7e-24 | 108.7 | 76 | (66, 143) | 143 | (118, 193) | 193 | tail fiber assembly protein | tail fiber assembly protein | | uniclust | UniRef100\_A0A1G7SHF9 | 99.6 | 4.3e-18 | 8.4e-24 | 117.0 | 90 | (1, 90) | 143 | (9, 99) | 207 | Uncharacterized protein (Fragment) | Uncharacterized protein (Fragment) | | uniclust | UniRef100\_A0A336P4J4 | 99.6 | 5.1e-18 | 9.6e-24 | 100.2 | 91 | (49, 143) | 143 | (7, 98) | 102 | Phage tail fiber assembly protein | Phage tail fiber assembly protein | | uniclust | UniRef100\_A0A2J9ED04 | 99.6 | 5.1e-18 | 9.8e-24 | 110.7 | 101 | (30, 143) | 143 | (51, 153) | 158 | Tail fiber assembly protein | Tail fiber assembly protein | | uniclust | UniRef100\_UPI00200582ED | 99.6 | 5.8e-18 | 1.1e-23 | 105.5 | 93 | (2, 94) | 143 | (1, 93) | 165 | tail fiber assembly protein | tail fiber assembly protein | | uniclust | UniRef100\_A0A1F4JLP3 | 99.6 | 6.7e-18 | 1.3e-23 | 107.1 | 124 | (2, 142) | 143 | (15, 140) | 146 | Phage tail protein | Phage tail protein | | uniclust | UniRef100\_UPI0004B0C64D | 99.5 | 1.3e-17 | 2.3e-23 | 97.3 | 92 | (46, 143) | 143 | (2, 93) | 96 | tail fiber assembly protein | tail fiber assembly protein | | uniclust | UniRef100\_A0A0T9RKV0 | 99.5 | 1.4e-17 | 2.6e-23 | 106.2 | 87 | (50, 143) | 143 | (53, 140) | 151 | Putative phage tail fiber assembly protein | Putative phage tail fiber assembly protein | | uniclust | UniRef100\_UPI0007359171 | 99.5 | 1.6e-17 | 3e-23 | 103.1 | 139 | (2, 143) | 143 | (1, 141) | 141 | hypothetical protein | hypothetical protein | | uniclust | UniRef100\_A0A1C7WD91 | 99.5 | 1.6e-17 | 3e-23 | 116.8 | 83 | (58, 143) | 143 | (213, 295) | 296 | Phage tail protein | Phage tail protein | | uniclust | UniRef100\_A0A5T9PXD7 | 99.5 | 1.6e-17 | 3.1e-23 | 105.2 | 84 | (57, 143) | 143 | (46, 129) | 134 | Tail fiber assembly protein | Tail fiber assembly protein | | uniclust | UniRef100\_A0A2A2PEQ9 | 99.5 | 2.2e-17 | 4.1e-23 | 92.6 | 60 | (82, 143) | 143 | (9, 68) | 71 | Phage tail protein | Phage tail protein | | uniclust | UniRef100\_A0A022PAN9 | 99.5 | 2.3e-17 | 4.3e-23 | 115.8 | 72 | (70, 143) | 143 | (138, 209) | 252 | Caudovirales tail fiber assembly protein | Caudovirales tail fiber assembly protein | | uniclust | UniRef100\_A0A081RRP7 | 99.5 | 2.4e-17 | 4.5e-23 | 106.5 | 74 | (68, 143) | 143 | (97, 170) | 171 | Caudovirales tail fiber assembly protein | Caudovirales tail fiber assembly protein | | uniclust | UniRef100\_A0A4R4K582 | 99.5 | 2.5e-17 | 4.6e-23 | 97.2 | 73 | (69, 143) | 143 | (14, 86) | 95 | Phage tail protein | Phage tail protein | | uniclust | UniRef100\_A0A081RR89 | 99.5 | 4.9e-17 | 9.1e-23 | 99.5 | 63 | (78, 143) | 143 | (54, 116) | 124 | Caudovirales tail fiber assembly protein | Caudovirales tail fiber assembly protein | | uniclust | UniRef100\_A0A1H8RBN5 | 99.5 | 4.7e-17 | 9.1e-23 | 108.5 | 113 | (2, 121) | 143 | (5, 124) | 168 | Phage tail assembly chaperone protein | Phage tail assembly chaperone protein | | uniclust | UniRef100\_A0A5K4YG87 | 99.5 | 6.4e-17 | 1.2e-22 | 91.7 | 75 | (1, 75) | 143 | (1, 76) | 79 | Uncharacterized protein | Uncharacterized protein | | uniclust | UniRef100\_A0A2Z3I402 | 99.5 | 6.5e-17 | 1.2e-22 | 105.1 | 81 | (61, 143) | 143 | (98, 180) | 181 | Phage tail protein | Phage tail protein | | uniclust | UniRef100\_UPI001D06A5C9 | 99.5 | 7.5e-17 | 1.4e-22 | 88.9 | 71 | (60, 132) | 143 | (4, 74) | 78 | tail fiber assembly protein | tail fiber assembly protein | | uniclust | UniRef100\_A0A0F0FPS3 | 99.5 | 8.9e-17 | 1.7e-22 | 97.6 | 81 | (61, 143) | 143 | (43, 125) | 125 | Tail fiber assembly protein | Tail fiber assembly protein | | uniclust | UniRef100\_A0A0F7Y2W8 | 99.5 | 9.6e-17 | 1.8e-22 | 105.8 | 68 | (74, 143) | 143 | (116, 185) | 186 | Phage tail protein | Phage tail protein | | uniclust | UniRef100\_A0A0A1FEP8 | 99.5 | 1e-16 | 2e-22 | 113.8 | 71 | (71, 143) | 143 | (173, 245) | 261 | Phage tail fiber assembly protein | Phage tail fiber assembly protein | | uniclust | UniRef100\_UPI0021BD8A01 | 99.5 | 1.5e-16 | 2.7e-22 | 94.4 | 67 | (75, 143) | 143 | (42, 108) | 108 | tail fiber assembly protein | tail fiber assembly protein | | uniclust | UniRef100\_A0A5N3CRX4 | 99.5 | 1.5e-16 | 2.9e-22 | 93.7 | 70 | (1, 70) | 143 | (9, 79) | 86 | Phage tail protein | Phage tail protein | | uniclust | UniRef100\_A0A0C5DSP9 | 99.5 | 1.7e-16 | 3.3e-22 | 108.2 | 72 | (70, 143) | 143 | (116, 189) | 208 | Tail fiber assembly protein | Tail fiber assembly protein | | uniclust | UniRef100\_A0A2X2RKD0 | 99.5 | 1.9e-16 | 3.4e-22 | 94.9 | 85 | (50, 143) | 143 | (26, 110) | 114 | Phage tail fiber assembly protein | Phage tail fiber assembly protein | | uniclust | UniRef100\_A0A0K4WW72 | 99.4 | 2.5e-16 | 4.7e-22 | 92.5 | 85 | (1, 85) | 143 | (1, 85) | 111 | Tail fiber assembly protein | Tail fiber assembly protein | | uniclust | UniRef100\_A0A2N1H596 | 99.4 | 3.3e-16 | 6.2e-22 | 100.1 | 133 | (2, 143) | 143 | (1, 137) | 147 | Phage tail protein | Phage tail protein | | uniclust | UniRef100\_A0A743TIT6 | 99.4 | 3.4e-16 | 6.2e-22 | 91.5 | 92 | (46, 143) | 143 | (6, 97) | 99 | Tail fiber assembly protein (Fragment) | Tail fiber assembly protein (Fragment) | | uniclust | UniRef100\_A0A5V7Y4W7 | 99.4 | 3.5e-16 | 6.4e-22 | 91.8 | 81 | (58, 143) | 143 | (18, 98) | 101 | Tail fiber assembly protein | Tail fiber assembly protein | | uniclust | UniRef100\_UPI001E56960D | 99.4 | 3.7e-16 | 6.7e-22 | 92.3 | 97 | (44, 143) | 143 | (3, 99) | 110 | tail fiber assembly protein | tail fiber assembly protein | | uniclust | UniRef100\_A0A2I8Q8G5 | 99.4 | 3.9e-16 | 7.1e-22 | 93.8 | 69 | (73, 143) | 143 | (57, 125) | 127 | Phage tail protein | Phage tail protein | | uniclust | UniRef100\_UPI00224466F1 | 99.4 | 4e-16 | 7.4e-22 | 94.7 | 128 | (2, 142) | 143 | (1, 130) | 135 | phage tail assembly chaperone | phage tail assembly chaperone | | uniclust | UniRef100\_C4K6I6 | 99.4 | 4.2e-16 | 7.8e-22 | 82.2 | 55 | (87, 143) | 143 | (2, 56) | 57 | Putative tail fiber assembly protein | Putative tail fiber assembly protein | | uniclust | UniRef100\_A0A225SMD4 | 99.4 | 4.3e-16 | 8e-22 | 102.4 | 72 | (70, 143) | 143 | (86, 159) | 184 | Phage tail protein | Phage tail protein | | uniclust | UniRef100\_A0A380AA26 | 99.4 | 5.1e-16 | 9.6e-22 | 89.0 | 68 | (1, 68) | 143 | (1, 69) | 80 | Tail fiber assembly protein | Tail fiber assembly protein | | uniclust | UniRef100\_A0A1Q8SPJ2 | 99.4 | 5.9e-16 | 1.1e-21 | 103.0 | 88 | (2, 89) | 143 | (8, 96) | 213 | DUF4376 domain-containing protein | DUF4376 domain-containing protein | | uniclust | UniRef100\_UPI0003FF973E | 99.4 | 6.2e-16 | 1.2e-21 | 88.1 | 67 | (2, 68) | 143 | (3, 69) | 79 | hypothetical protein | hypothetical protein | | uniclust | UniRef100\_UPI001F296025 | 99.4 | 6.9e-16 | 1.3e-21 | 94.2 | 79 | (65, 143) | 143 | (49, 127) | 129 | tail fiber assembly protein | tail fiber assembly protein | | uniclust | UniRef100\_UPI00067AAE6F | 99.4 | 7.5e-16 | 1.4e-21 | 91.6 | 100 | (3, 114) | 143 | (1, 101) | 112 | tail fiber assembly protein | tail fiber assembly protein | | uniclust | UniRef100\_A0A1G7PVA6 | 99.4 | 7.5e-16 | 1.5e-21 | 102.2 | 64 | (2, 70) | 143 | (1, 64) | 160 | Uncharacterized protein | Uncharacterized protein | | uniclust | UniRef100\_A0A5T6NIG6 | 99.4 | 8e-16 | 1.5e-21 | 86.8 | 70 | (69, 143) | 143 | (6, 75) | 78 | Tail fiber assembly protein | Tail fiber assembly protein | | uniclust | UniRef100\_A0A6I3WIV8 | 99.4 | 8.2e-16 | 1.5e-21 | 98.7 | 67 | (75, 143) | 143 | (85, 153) | 157 | Tail fiber assembly protein | Tail fiber assembly protein | | uniclust | UniRef100\_UPI001883862A | 99.4 | 8.5e-16 | 1.6e-21 | 97.8 | 137 | (3, 142) | 143 | (35, 179) | 183 | tail fiber assembly protein | tail fiber assembly protein | | uniclust | UniRef100\_A0A0W0MTR4 | 99.4 | 8.9e-16 | 1.7e-21 | 96.0 | 70 | (72, 143) | 143 | (69, 140) | 140 | Phage tail protein (Fragment) | Phage tail protein (Fragment) | | uniclust | UniRef100\_A0A080KAQ9 | 99.4 | 9e-16 | 1.7e-21 | 94.7 | 65 | (77, 143) | 143 | (50, 114) | 124 | Caudovirales tail fiber assembly protein (Fragment) | Caudovirales tail fiber assembly protein (Fragment) | | uniclust | UniRef100\_A0A432UNX0 | 99.4 | 1.2e-15 | 2.2e-21 | 100.2 | 73 | (2, 74) | 143 | (1, 74) | 194 | Tail fiber assembly protein | Tail fiber assembly protein | | uniclust | UniRef100\_A0A0N9WN70 | 99.4 | 1.4e-15 | 2.5e-21 | 100.5 | 136 | (4, 143) | 143 | (1, 236) | 239 | Tail fiber assembly protein | Tail fiber assembly protein | | uniclust | UniRef100\_A0A2R8CKR9 | 99.4 | 1.4e-15 | 2.5e-21 | 101.3 | 67 | (2, 68) | 143 | (3, 69) | 202 | Uncharacterized protein | Uncharacterized protein | | uniclust | UniRef100\_A0A0A0CJA3 | 99.4 | 1.4e-15 | 2.5e-21 | 80.6 | 44 | (100, 143) | 143 | (9, 52) | 55 | Tail assembly protein (Fragment) | Tail assembly protein (Fragment) | | uniclust | UniRef100\_A0A1H3DNJ2 | 99.4 | 1.5e-15 | 2.8e-21 | 90.5 | 66 | (75, 142) | 143 | (38, 105) | 107 | Virus tail fibre assembly protein, lambda gpK | Virus tail fibre assembly protein, lambda gpK | | uniclust | UniRef100\_A0A1D9LI34 | 99.4 | 1.6e-15 | 3.1e-21 | 96.9 | 68 | (2, 74) | 143 | (1, 68) | 149 | Tail fiber assembly protein | Tail fiber assembly protein | | uniclust | UniRef100\_UPI0009EA3590 | 99.4 | 1.7e-15 | 3.2e-21 | 97.2 | 68 | (74, 143) | 143 | (95, 164) | 164 | tail fiber assembly protein | tail fiber assembly protein | | uniclust | UniRef100\_UPI0002198843 | 99.4 | 1.9e-15 | 3.6e-21 | 99.7 | 63 | (2, 69) | 143 | (1, 63) | 183 | DUF4376 domain-containing protein | DUF4376 domain-containing protein | | uniclust | UniRef100\_UPI000DDB98E6 | 99.4 | 3e-15 | 5.5e-21 | 89.3 | 99 | (1, 102) | 143 | (3, 103) | 107 | tail fiber assembly protein | tail fiber assembly protein | | uniclust | UniRef100\_A0A1C4G4F0 | 99.3 | 3.3e-15 | 6.1e-21 | 100.2 | 66 | (73, 143) | 143 | (138, 203) | 216 | Virus tail fibre assembly protein, lambda gpK | Virus tail fibre assembly protein, lambda gpK | | uniclust | UniRef100\_A0A0A7S0J4 | 99.3 | 3.6e-15 | 6.8e-21 | 92.2 | 74 | (67, 143) | 143 | (42, 115) | 117 | Caudovirales tail fiber assembly protein | Caudovirales tail fiber assembly protein | | uniclust | UniRef100\_UPI00208F983D | 99.3 | 3.9e-15 | 7.1e-21 | 91.4 | 81 | (60, 142) | 143 | (62, 142) | 143 | tail fiber assembly protein | tail fiber assembly protein | | uniclust | UniRef100\_D4I2C0 | 99.3 | 3.9e-15 | 7.2e-21 | 85.7 | 56 | (86, 143) | 143 | (2, 57) | 97 | Tail fiber assembly protein homolog from lambdoid prophage DLP12 | Tail fiber assembly protein homolog from lambdoid prophage DLP12 | | uniclust | UniRef100\_A0A0F4T3H0 | 99.3 | 4e-15 | 7.6e-21 | 97.7 | 74 | (66, 143) | 143 | (70, 144) | 150 | Tail fiber assembly protein | Tail fiber assembly protein | | uniclust | UniRef100\_A0A087NAA6 | 99.3 | 4e-15 | 7.6e-21 | 99.0 | 71 | (1, 77) | 143 | (10, 80) | 170 | Uncharacterized protein | Uncharacterized protein | | uniclust | UniRef100\_UPI001EE2F813 | 99.3 | 4.1e-15 | 7.6e-21 | 104.2 | 73 | (69, 143) | 143 | (288, 360) | 360 | tail fiber assembly protein | tail fiber assembly protein | | uniclust | UniRef100\_A0A2N8AP07 | 99.3 | 4.8e-15 | 8.8e-21 | 98.1 | 134 | (2, 142) | 143 | (96, 231) | 240 | Phage tail protein | Phage tail protein | | uniclust | UniRef100\_A0A8S5V3M4 | 99.3 | 4.8e-15 | 8.9e-21 | 94.5 | 112 | (10, 143) | 143 | (23, 137) | 156 | Tail assembly chaperone protein | Tail assembly chaperone protein | | uniclust | UniRef100\_A0A225SMJ4 | 99.3 | 5.1e-15 | 9.4e-21 | 90.8 | 66 | (2, 72) | 143 | (1, 66) | 129 | Phage tail protein | Phage tail protein | | uniclust | UniRef100\_A0A031FRM8 | 99.3 | 5.4e-15 | 1e-20 | 99.0 | 79 | (62, 143) | 143 | (86, 168) | 171 | Caudovirales tail fiber assembly protein | Caudovirales tail fiber assembly protein | | uniclust | UniRef100\_A0A077F610 | 99.3 | 6.5e-15 | 1.2e-20 | 102.3 | 70 | (72, 143) | 143 | (158, 229) | 246 | Phage tail fiber assembly protein | Phage tail fiber assembly protein | | uniclust | UniRef100\_G9E597 | 99.3 | 7e-15 | 1.3e-20 | 92.0 | 127 | (1, 143) | 143 | (19, 147) | 151 | Tail fiber assembly protein | Tail fiber assembly protein | | uniclust | UniRef100\_A0A5C7CFK8 | 99.3 | 7.1e-15 | 1.3e-20 | 94.3 | 76 | (66, 143) | 143 | (100, 175) | 191 | Tail fiber assembly protein | Tail fiber assembly protein | | uniclust | UniRef100\_UPI000680C4D0 | 99.3 | 7.6e-15 | 1.4e-20 | 98.3 | 84 | (58, 143) | 143 | (177, 260) | 260 | tail fiber assembly protein | tail fiber assembly protein | | uniclust | UniRef100\_A0A024E8T8 | 99.3 | 8.7e-15 | 1.6e-20 | 97.9 | 70 | (72, 143) | 143 | (116, 187) | 200 | Tail fiber assembly protein | Tail fiber assembly protein | | uniclust | UniRef100\_UPI00068D9C5E | 99.3 | 9.1e-15 | 1.7e-20 | 91.8 | 65 | (76, 143) | 143 | (66, 130) | 131 | tail fiber assembly protein | tail fiber assembly protein | | uniclust | UniRef100\_A0A022PFM9 | 99.3 | 9.8e-15 | 1.8e-20 | 108.2 | 84 | (58, 143) | 143 | (450, 533) | 534 | Phage-related tail fiber protein | Phage-related tail fiber protein | | uniclust | UniRef100\_A0A192A522 | 99.3 | 2e-14 | 3.8e-20 | 100.7 | 76 | (2, 77) | 143 | (4, 105) | 240 | DUF4376 domain-containing protein | DUF4376 domain-containing protein | | uniclust | UniRef100\_A0A7Y0Z1A9 | 99.3 | 2.1e-14 | 3.9e-20 | 85.6 | 67 | (75, 143) | 143 | (37, 105) | 105 | Tail fiber assembly protein | Tail fiber assembly protein | | uniclust | UniRef100\_UPI0002EF02CE | 99.3 | 2.2e-14 | 4e-20 | 81.4 | 80 | (2, 82) | 143 | (1, 80) | 81 | hypothetical protein | hypothetical protein | | uniclust | UniRef100\_A0A246GTR2 | 99.3 | 2.4e-14 | 4.6e-20 | 93.9 | 128 | (2, 143) | 143 | (1, 133) | 145 | Phage tail protein | Phage tail protein | | uniclust | UniRef100\_A0A0S4UUF1 | 99.3 | 2.7e-14 | 5.1e-20 | 98.4 | 69 | (72, 143) | 143 | (152, 220) | 228 | Integrase/recombinase | Integrase/recombinase | | uniclust | UniRef100\_A0A0H3PBT9 | 99.2 | 2.8e-14 | 5.4e-20 | 102.2 | 79 | (63, 143) | 143 | (129, 207) | 256 | Uncharacterized protein (Fragment) | Uncharacterized protein (Fragment) | | uniclust | UniRef100\_UPI00077BD77D | 99.2 | 3.2e-14 | 5.8e-20 | 91.0 | 94 | (2, 95) | 143 | (1, 94) | 184 | tail fiber assembly protein | tail fiber assembly protein | | uniclust | UniRef100\_A0A077NN52 | 99.2 | 3.3e-14 | 6.1e-20 | 88.8 | 81 | (61, 143) | 143 | (72, 152) | 152 | Tail fiber assembly protein | Tail fiber assembly protein | | uniclust | UniRef100\_A0A2A5RDH4 | 99.2 | 3.3e-14 | 6.3e-20 | 92.5 | 74 | (68, 143) | 143 | (69, 146) | 146 | Phage tail protein | Phage tail protein | | uniclust | UniRef100\_UPI00190065B7 | 99.2 | 3.5e-14 | 6.5e-20 | 87.6 | 130 | (1, 130) | 143 | (1, 132) | 145 | hypothetical protein | hypothetical protein | | uniclust | UniRef100\_A0A1A9VZ06 | 99.2 | 3.6e-14 | 6.7e-20 | 102.2 | 73 | (69, 143) | 143 | (403, 475) | 479 | Bacteriophage P22 tailspike N-terminal domain-containing protein | Bacteriophage P22 tailspike N-terminal domain-containing protein | | uniclust | UniRef100\_A0A344SLS6 | 99.2 | 3.7e-14 | 6.9e-20 | 81.0 | 70 | (69, 143) | 143 | (5, 74) | 80 | Tail fiber assembly protein | Tail fiber assembly protein | | uniclust | UniRef100\_A0A6L7C6M0 | 99.2 | 4.3e-14 | 8e-20 | 86.4 | 64 | (78, 143) | 143 | (68, 131) | 131 | Tail fiber assembly protein | Tail fiber assembly protein | | uniclust | UniRef100\_UPI001883B016 | 99.2 | 4.4e-14 | 8e-20 | 101.8 | 137 | (3, 142) | 143 | (333, 477) | 481 | tail fiber assembly protein | tail fiber assembly protein | | uniclust | UniRef100\_A0A7L6CMI7 | 99.2 | 4.5e-14 | 8.4e-20 | 86.3 | 64 | (76, 143) | 143 | (48, 112) | 113 | Tail fiber assembly protein | Tail fiber assembly protein | | uniclust | UniRef100\_A0A023WVR1 | 99.2 | 4.5e-14 | 8.6e-20 | 91.6 | 61 | (2, 68) | 143 | (1, 61) | 137 | Phage tail protein | Phage tail protein | | uniclust | UniRef100\_A0A0Q8NI86 | 99.2 | 4.9e-14 | 9.4e-20 | 91.8 | 107 | (11, 143) | 143 | (26, 134) | 150 | Phage tail protein | Phage tail protein | | uniclust | UniRef100\_A0A1C1W012 | 99.2 | 6.1e-14 | 1.1e-19 | 92.9 | 110 | (31, 142) | 143 | (84, 195) | 203 | Phage tail protein | Phage tail protein | | uniclust | UniRef100\_A0A2L0M0L4 | 99.2 | 6.2e-14 | 1.1e-19 | 82.3 | 73 | (69, 143) | 143 | (26, 100) | 106 | Phage tail protein | Phage tail protein | | uniclust | UniRef100\_A0A2V1HJ93 | 99.2 | 6.9e-14 | 1.3e-19 | 85.8 | 74 | (67, 142) | 143 | (46, 121) | 122 | Phage tail protein | Phage tail protein | | uniclust | UniRef100\_A0A0L1KFJ7 | 99.2 | 6.9e-14 | 1.3e-19 | 91.8 | 59 | (1, 64) | 143 | (5, 64) | 156 | Putative tail fiber assembly protein | Putative tail fiber assembly protein | | uniclust | UniRef100\_A0A0K1QML5 | 99.2 | 7e-14 | 1.3e-19 | 97.7 | 79 | (63, 143) | 143 | (150, 230) | 255 | Tail fiber assembly protein | Tail fiber assembly protein | | uniclust | UniRef100\_A0A077NTU9 | 99.2 | 7.2e-14 | 1.3e-19 | 75.5 | 47 | (92, 143) | 143 | (7, 53) | 59 | Tail fiber assembly protein | Tail fiber assembly protein | | uniclust | UniRef100\_A0A261SNT8 | 99.2 | 1e-13 | 1.9e-19 | 91.5 | 133 | (2, 143) | 143 | (4, 151) | 154 | Phage tail protein | Phage tail protein | | uniclust | UniRef100\_UPI000190DFD3 | 99.2 | 1.1e-13 | 2e-19 | 79.4 | 72 | (70, 143) | 143 | (7, 79) | 83 | tail fiber assembly protein | tail fiber assembly protein | | uniclust | UniRef100\_UPI0022FDBD1A | 99.2 | 1.1e-13 | 2.1e-19 | 81.4 | 72 | (70, 143) | 143 | (35, 107) | 107 | tail fiber assembly protein | tail fiber assembly protein | | uniclust | UniRef100\_UPI001101862B | 99.2 | 1.3e-13 | 2.4e-19 | 76.1 | 62 | (77, 143) | 143 | (3, 64) | 65 | tail fiber assembly protein | tail fiber assembly protein | | uniclust | UniRef100\_UPI001124FB43 | 99.2 | 1.3e-13 | 2.5e-19 | 79.3 | 84 | (58, 143) | 143 | (3, 87) | 89 | tail fiber assembly protein | tail fiber assembly protein | | uniclust | UniRef100\_A0A0A8VHP2 | 99.2 | 1.3e-13 | 2.5e-19 | 90.3 | 133 | (1, 142) | 143 | (1, 139) | 169 | Putative tail fiber assembly protein | Putative tail fiber assembly protein | | uniclust | UniRef100\_UPI001256250B | 99.2 | 1.5e-13 | 2.7e-19 | 82.1 | 82 | (1, 90) | 143 | (1, 82) | 109 | tail fiber assembly protein | tail fiber assembly protein | | uniclust | UniRef100\_A0A0J7J5L4 | 99.2 | 1.4e-13 | 2.7e-19 | 95.2 | 63 | (1, 68) | 143 | (6, 68) | 202 | Uncharacterized protein | Uncharacterized protein | | uniclust | UniRef100\_A0A1Z9Q5B5 | 99.2 | 1.5e-13 | 2.9e-19 | 88.2 | 118 | (4, 141) | 143 | (1, 122) | 137 | Uncharacterized protein | Uncharacterized protein | | uniclust | UniRef100\_A0A1B9KI83 | 99.2 | 1.6e-13 | 3e-19 | 79.8 | 72 | (69, 143) | 143 | (12, 83) | 85 | Tail fiber assembly protein | Tail fiber assembly protein | | uniclust | UniRef100\_UPI001CCF0462 | 99.2 | 1.8e-13 | 3.3e-19 | 73.6 | 59 | (83, 143) | 143 | (3, 63) | 63 | tail fiber assembly protein | tail fiber assembly protein | | uniclust | UniRef100\_A0A031GY32 | 99.2 | 1.9e-13 | 3.7e-19 | 92.1 | 87 | (1, 88) | 143 | (8, 98) | 183 | Uncharacterized protein | Uncharacterized protein | | uniclust | UniRef100\_A0A1C7WAJ7 | 99.1 | 2.2e-13 | 4.1e-19 | 77.0 | 64 | (78, 143) | 143 | (5, 68) | 75 | Phage tail protein | Phage tail protein | | uniclust | UniRef100\_A0A2N8B7K5 | 99.1 | 2.5e-13 | 4.7e-19 | 85.4 | 69 | (72, 142) | 143 | (71, 141) | 147 | Phage tail protein | Phage tail protein | | uniclust | UniRef100\_UPI00164D51E1 | 99.1 | 2.9e-13 | 5.2e-19 | 85.1 | 110 | (30, 141) | 143 | (47, 158) | 160 | phage tail assembly chaperone | phage tail assembly chaperone | | uniclust | UniRef100\_A0A3S0YDP2 | 99.1 | 2.8e-13 | 5.3e-19 | 92.9 | 88 | (1, 89) | 143 | (1, 133) | 240 | DUF4376 domain-containing protein | DUF4376 domain-containing protein | | uniclust | UniRef100\_A0A7W4JZ22 | 99.1 | 3.1e-13 | 5.8e-19 | 85.7 | 125 | (3, 142) | 143 | (7, 133) | 139 | Tail fiber assembly protein | Tail fiber assembly protein | | uniclust | UniRef100\_UPI00080DBA8F | 99.1 | 3.2e-13 | 5.8e-19 | 87.3 | 128 | (4, 142) | 143 | (58, 185) | 193 | tail fiber assembly protein | tail fiber assembly protein | | uniclust | UniRef100\_A0A6N9S5D4 | 99.1 | 3.2e-13 | 6e-19 | 88.2 | 67 | (75, 143) | 143 | (141, 207) | 209 | Chromosome partitioning protein ParB | Chromosome partitioning protein ParB | | uniclust | UniRef100\_UPI0023423F92 | 99.1 | 3.9e-13 | 7.1e-19 | 84.8 | 56 | (85, 142) | 143 | (105, 160) | 163 | tail fiber assembly protein | tail fiber assembly protein | | uniclust | UniRef100\_A0A024E960 | 99.1 | 3.9e-13 | 7.5e-19 | 91.2 | 72 | (70, 143) | 143 | (79, 152) | 171 | Phage tail protein | Phage tail protein | | uniclust | UniRef100\_A0A7J0BMD4 | 99.1 | 4.3e-13 | 8.1e-19 | 82.9 | 60 | (2, 68) | 143 | (1, 60) | 129 | Uncharacterized protein | Uncharacterized protein | | uniclust | UniRef100\_A0A1X3J0U4 | 99.1 | 4.8e-13 | 8.9e-19 | 64.4 | 33 | (111, 143) | 143 | (2, 34) | 35 | Tail fiber assembly protein-like protein | Tail fiber assembly protein-like protein | | uniclust | UniRef100\_UPI001C47AEF2 | 99.1 | 4.9e-13 | 9.1e-19 | 81.9 | 124 | (2, 143) | 143 | (1, 130) | 135 | phage tail assembly chaperone | phage tail assembly chaperone | | uniclust | UniRef100\_UPI001FD41782 | 99.1 | 5.3e-13 | 9.7e-19 | 75.7 | 58 | (83, 143) | 143 | (29, 86) | 86 | tail fiber assembly protein | tail fiber assembly protein | | uniclust | UniRef100\_UPI0011B1F365 | 99.1 | 5.7e-13 | 1.1e-18 | 80.8 | 73 | (69, 143) | 143 | (24, 101) | 112 | tail fiber assembly protein | tail fiber assembly protein | | uniclust | UniRef100\_A0A5V6PU83 | 99.1 | 5.8e-13 | 1.1e-18 | 85.6 | 110 | (1, 118) | 143 | (1, 111) | 184 | Tail fiber assembly protein | Tail fiber assembly protein | | uniclust | UniRef100\_A0A376D3X0 | 99.1 | 5.9e-13 | 1.1e-18 | 77.8 | 93 | (2, 101) | 143 | (1, 93) | 101 | Tail assembly chaperone gp38 | Tail assembly chaperone gp38 | | uniclust | UniRef100\_A0A2J7VZP8 | 99.1 | 6.2e-13 | 1.1e-18 | 85.2 | 65 | (77, 143) | 143 | (114, 180) | 180 | Uncharacterized protein | Uncharacterized protein | | uniclust | UniRef100\_A0A192CNA1 | 99.1 | 6.1e-13 | 1.1e-18 | 84.1 | 70 | (69, 143) | 143 | (69, 138) | 140 | Tail fiber assembly protein | Tail fiber assembly protein | | uniclust | UniRef100\_UPI001F210AA1 | 99.1 | 6.3e-13 | 1.2e-18 | 82.0 | 67 | (74, 142) | 143 | (72, 140) | 141 | tail fiber assembly protein | tail fiber assembly protein | | uniclust | UniRef100\_A0A351RD40 | 99.1 | 7.4e-13 | 1.4e-18 | 83.2 | 83 | (58, 142) | 143 | (47, 130) | 130 | Tail fiber assembly protein | Tail fiber assembly protein | | uniclust | UniRef100\_A0A1C4BVV1 | 99.1 | 7.8e-13 | 1.4e-18 | 71.7 | 56 | (2, 63) | 143 | (1, 56) | 60 | Phage protein | Phage protein | | uniclust | UniRef100\_A0A0H8H6L6 | 99.1 | 8.2e-13 | 1.5e-18 | 87.0 | 69 | (73, 143) | 143 | (104, 178) | 183 | Tail fiber assembly protein | Tail fiber assembly protein | | uniclust | UniRef100\_A0A379CM87 | 99.1 | 8.7e-13 | 1.6e-18 | 90.0 | 80 | (62, 143) | 143 | (127, 206) | 210 | Caudovirales tail fibre assembly protein | Caudovirales tail fibre assembly protein | | uniclust | UniRef100\_A0A1H1V7V6 | 99.1 | 8.7e-13 | 1.7e-18 | 83.3 | 81 | (60, 143) | 143 | (30, 112) | 122 | Virus tail fibre assembly protein, lambda gpK | Virus tail fibre assembly protein, lambda gpK | | uniclust | UniRef100\_A0A1A9RM66 | 99.1 | 8.9e-13 | 1.7e-18 | 90.5 | 67 | (2, 74) | 143 | (18, 84) | 228 | Uncharacterized protein (Fragment) | Uncharacterized protein (Fragment) | | uniclust | UniRef100\_A0A0T9RR95 | 99.1 | 1.1e-12 | 2e-18 | 67.7 | 44 | (100, 143) | 143 | (4, 47) | 48 | Tail assembly chaperone gp38 | Tail assembly chaperone gp38 | | uniclust | UniRef100\_A0A0K4CN17 | 99.0 | 1.1e-12 | 2.1e-18 | 87.1 | 84 | (58, 143) | 143 | (115, 199) | 203 | Tail fiber assembly protein | Tail fiber assembly protein | | uniclust | UniRef100\_UPI001F1FB5E9 | 99.0 | 1.2e-12 | 2.2e-18 | 81.6 | 131 | (2, 143) | 143 | (1, 149) | 149 | phage tail assembly chaperone | phage tail assembly chaperone | | uniclust | UniRef100\_A0A1Q5TR83 | 99.0 | 1.3e-12 | 2.4e-18 | 88.8 | 77 | (65, 143) | 143 | (106, 182) | 196 | Tail assembly chaperone | Tail assembly chaperone | | uniclust | UniRef100\_A0A3J2DEH3 | 99.0 | 1.3e-12 | 2.4e-18 | 77.4 | 65 | (77, 143) | 143 | (8, 78) | 89 | Phage tail protein (Fragment) | Phage tail protein (Fragment) | | uniclust | UniRef100\_A0A2X1PXX8 | 99.0 | 1.3e-12 | 2.5e-18 | 80.1 | 81 | (61, 143) | 143 | (27, 108) | 116 | Tail fiber assembly protein from lambdoid prophage | Tail fiber assembly protein from lambdoid prophage | | uniclust | UniRef100\_E0M480 | 99.0 | 1.4e-12 | 2.6e-18 | 86.7 | 76 | (66, 143) | 143 | (119, 194) | 196 | Tail assembly chaperone gp38 | Tail assembly chaperone gp38 | | uniclust | UniRef100\_A0A5D8M825 | 99.0 | 1.4e-12 | 2.6e-18 | 64.3 | 34 | (110, 143) | 143 | (2, 35) | 38 | Tail fiber assembly protein | Tail fiber assembly protein | | uniclust | UniRef100\_UPI000B7D225B | 99.0 | 1.5e-12 | 2.7e-18 | 80.7 | 124 | (9, 141) | 143 | (9, 132) | 133 | tail fiber assembly protein | tail fiber assembly protein | | uniclust | UniRef100\_UPI00138F4819 | 99.0 | 1.6e-12 | 2.9e-18 | 76.7 | 69 | (70, 143) | 143 | (6, 74) | 106 | tail fiber assembly protein | tail fiber assembly protein | | uniclust | UniRef100\_UPI001B8D2E1C | 99.0 | 1.7e-12 | 3.1e-18 | 83.3 | 105 | (36, 143) | 143 | (68, 180) | 180 | tail fiber assembly protein | tail fiber assembly protein | | uniclust | UniRef100\_A0A090NC98 | 99.0 | 1.7e-12 | 3.2e-18 | 64.7 | 31 | (113, 143) | 143 | (8, 38) | 40 | Terminase large subunit | Terminase large subunit | | uniclust | UniRef100\_UPI001E48B2CF | 99.0 | 2e-12 | 3.6e-18 | 75.3 | 70 | (72, 143) | 143 | (27, 96) | 98 | tail fiber assembly protein | tail fiber assembly protein | | uniclust | UniRef100\_UPI0018F0CC46 | 99.0 | 2.2e-12 | 4e-18 | 78.8 | 67 | (74, 143) | 143 | (65, 131) | 131 | tail fiber assembly protein | tail fiber assembly protein | | uniclust | UniRef100\_A0A8G2C8I7 | 99.0 | 2.6e-12 | 4.8e-18 | 80.9 | 117 | (2, 127) | 143 | (1, 121) | 159 | Caudovirales tail fibre assembly protein | Caudovirales tail fibre assembly protein | | uniclust | UniRef100\_A0A315BKG8 | 99.0 | 3e-12 | 5.6e-18 | 76.9 | 75 | (3, 82) | 143 | (1, 75) | 118 | Phage tail protein | Phage tail protein | | uniclust | UniRef100\_UPI002097513D | 99.0 | 3.4e-12 | 6.2e-18 | 80.1 | 77 | (64, 143) | 143 | (63, 139) | 150 | tail fiber assembly protein | tail fiber assembly protein | | uniclust | UniRef100\_A0A0F4VEV3 | 99.0 | 3.4e-12 | 6.4e-18 | 82.1 | 123 | (2, 142) | 143 | (4, 127) | 131 | Phage tail protein | Phage tail protein | | uniclust | UniRef100\_A0A066SRH2 | 99.0 | 4e-12 | 7.3e-18 | 75.4 | 56 | (85, 142) | 143 | (31, 86) | 101 | Phage tail protein | Phage tail protein | | uniclust | UniRef100\_A0A376YM78 | 99.0 | 4.8e-12 | 8.9e-18 | 66.4 | 46 | (93, 143) | 143 | (2, 47) | 51 | Tail fiber assembly protein | Tail fiber assembly protein | | uniclust | UniRef100\_UPI0013C49323 | 99.0 | 5e-12 | 9.2e-18 | 75.2 | 75 | (67, 143) | 143 | (36, 110) | 110 | tail fiber assembly protein | tail fiber assembly protein | | uniclust | UniRef100\_A0A0T9UB25 | 99.0 | 5.1e-12 | 9.5e-18 | 73.2 | 66 | (76, 143) | 143 | (3, 73) | 85 | Putative phage tail fiber assembly protein | Putative phage tail fiber assembly protein | | uniclust | UniRef100\_A0A221FYR6 | 99.0 | 5.2e-12 | 9.5e-18 | 67.2 | 58 | (82, 143) | 143 | (2, 59) | 60 | Tail fiber assembly protein | Tail fiber assembly protein | | uniclust | UniRef100\_J8UT63 | 99.0 | 5.2e-12 | 9.6e-18 | 71.8 | 63 | (79, 143) | 143 | (15, 79) | 85 | Tail fiber assembly protein | Tail fiber assembly protein | | uniclust | UniRef100\_A0A242NTF1 | 98.9 | 5.3e-12 | 9.8e-18 | 76.1 | 104 | (35, 142) | 143 | (3, 109) | 110 | Tail fiber assembly protein | Tail fiber assembly protein | | uniclust | UniRef100\_UPI001C7D3E88 | 98.9 | 5.4e-12 | 1e-17 | 78.6 | 135 | (1, 142) | 143 | (1, 147) | 147 | tail fiber assembly protein | tail fiber assembly protein | | uniclust | UniRef100\_UPI001FD7E4A7 | 98.9 | 5.6e-12 | 1e-17 | 72.1 | 73 | (67, 141) | 143 | (12, 84) | 88 | tail fiber assembly protein | tail fiber assembly protein | | uniclust | UniRef100\_A0A1H4Y2W8 | 98.9 | 5.9e-12 | 1.1e-17 | 85.5 | 70 | (1, 75) | 143 | (3, 74) | 201 | Phage tail protein | Phage tail protein | | uniclust | UniRef100\_A0A370S0C2 | 98.9 | 6e-12 | 1.1e-17 | 79.2 | 66 | (76, 143) | 143 | (56, 123) | 132 | Virus tail fiber assembly protein lambda gpK | Virus tail fiber assembly protein lambda gpK | | uniclust | UniRef100\_A0A076G4D7 | 98.9 | 6.1e-12 | 1.2e-17 | 88.0 | 66 | (76, 143) | 143 | (113, 187) | 213 | Uncharacterized protein | Uncharacterized protein | | uniclust | UniRef100\_E0SM49 | 98.9 | 6.6e-12 | 1.2e-17 | 74.6 | 59 | (73, 133) | 143 | (25, 83) | 105 | Tail fiber assembly protein | Tail fiber assembly protein | | uniclust | UniRef100\_A0A1A9WW69 | 98.9 | 7.1e-12 | 1.3e-17 | 92.0 | 72 | (69, 142) | 143 | (98, 171) | 529 | Tail fiber assembly protein | Tail fiber assembly protein | | uniclust | UniRef100\_UPI0015624E47 | 98.9 | 7.4e-12 | 1.4e-17 | 76.6 | 64 | (78, 143) | 143 | (67, 130) | 130 | tail fiber assembly protein | tail fiber assembly protein | | uniclust | UniRef100\_A0A7X5ZI37 | 98.9 | 7.7e-12 | 1.4e-17 | 75.9 | 74 | (67, 143) | 143 | (47, 120) | 124 | Tail fiber assembly protein | Tail fiber assembly protein | | uniclust | UniRef100\_A0A8I0PZM9 | 98.9 | 8.9e-12 | 1.7e-17 | 69.6 | 62 | (80, 143) | 143 | (5, 66) | 70 | Phage tail protein | Phage tail protein | | uniclust | UniRef100\_UPI001BA53F6E | 98.9 | 9.1e-12 | 1.7e-17 | 68.9 | 67 | (73, 141) | 143 | (6, 72) | 73 | tail fiber assembly protein | tail fiber assembly protein | | uniclust | UniRef100\_A0A3L2VMZ7 | 98.9 | 9.8e-12 | 1.8e-17 | 63.9 | 39 | (105, 143) | 143 | (2, 40) | 50 | Tail fiber assembly protein | Tail fiber assembly protein | | uniclust | UniRef100\_A0A478GUA7 | 98.9 | 9.8e-12 | 1.8e-17 | 75.3 | 69 | (73, 143) | 143 | (19, 93) | 100 | Phage tail fiber assembly protein | Phage tail fiber assembly protein | | uniclust | UniRef100\_UPI00039E0AEB | 98.9 | 1e-11 | 1.9e-17 | 83.5 | 66 | (2, 68) | 143 | (3, 68) | 206 | hypothetical protein | hypothetical protein | | uniclust | UniRef100\_UPI000BCBA4DC | 98.9 | 1.1e-11 | 1.9e-17 | 70.0 | 61 | (77, 139) | 143 | (21, 81) | 81 | tail fiber assembly protein | tail fiber assembly protein | | uniclust | UniRef100\_UPI001CDACA06 | 98.9 | 1.2e-11 | 2.3e-17 | 71.1 | 80 | (31, 110) | 143 | (8, 88) | 90 | tail fiber assembly protein | tail fiber assembly protein | | uniclust | UniRef100\_A0A235HBB2 | 98.9 | 1.4e-11 | 2.5e-17 | 83.3 | 83 | (2, 89) | 143 | (1, 87) | 193 | DUF4376 domain-containing protein | DUF4376 domain-containing protein | | uniclust | UniRef100\_A0A2V4F8Z2 | 98.9 | 1.5e-11 | 2.7e-17 | 70.2 | 60 | (2, 66) | 143 | (3, 63) | 70 | Uncharacterized protein | Uncharacterized protein | | uniclust | UniRef100\_A0A176NIZ0 | 98.9 | 1.6e-11 | 3e-17 | 81.3 | 80 | (62, 143) | 143 | (58, 139) | 147 | Phage tail assembly chaperone protein | Phage tail assembly chaperone protein | | uniclust | UniRef100\_A0A066T5Y8 | 98.9 | 1.6e-11 | 3e-17 | 81.1 | 66 | (75, 142) | 143 | (129, 194) | 197 | Phage tail protein | Phage tail protein | | uniclust | UniRef100\_UPI0021E99FC3 | 98.9 | 1.7e-11 | 3.2e-17 | 75.4 | 65 | (77, 143) | 143 | (68, 134) | 134 | tail fiber assembly protein | tail fiber assembly protein | | uniclust | UniRef100\_A0A953LSW1 | 98.9 | 1.8e-11 | 3.2e-17 | 76.6 | 70 | (4, 78) | 143 | (1, 70) | 148 | Uncharacterized protein (Fragment) | Uncharacterized protein (Fragment) | | uniclust | UniRef100\_A0A141RRG9 | 98.9 | 1.8e-11 | 3.3e-17 | 68.5 | 65 | (70, 136) | 143 | (12, 76) | 77 | Caudovirales tail fibre assembly protein | Caudovirales tail fibre assembly protein | | uniclust | UniRef100\_A0A366F728 | 98.9 | 1.9e-11 | 3.5e-17 | 76.0 | 99 | (32, 143) | 143 | (17, 117) | 120 | Phage tail assembly chaperone | Phage tail assembly chaperone | | uniclust | UniRef100\_A0A388SKY9 | 98.8 | 2.4e-11 | 4.5e-17 | 77.9 | 68 | (2, 75) | 143 | (1, 68) | 152 | Putative tail fiber assembly protein p37 | Putative tail fiber assembly protein p37 | | uniclust | UniRef100\_UPI0020A40FBA | 98.8 | 2.5e-11 | 4.5e-17 | 69.4 | 64 | (76, 142) | 143 | (23, 86) | 86 | tail fiber assembly protein | tail fiber assembly protein | | uniclust | UniRef100\_A0A2G0Y6Y2 | 98.8 | 2.5e-11 | 4.6e-17 | 81.5 | 64 | (77, 142) | 143 | (155, 220) | 228 | Phage tail protein | Phage tail protein | | uniclust | UniRef100\_A0A078LUM4 | 98.8 | 2.5e-11 | 4.7e-17 | 84.7 | 63 | (1, 69) | 143 | (2, 64) | 203 | Putative tail fiber assembly-like protein | Putative tail fiber assembly-like protein | | uniclust | UniRef100\_Q9B026 | 98.8 | 2.7e-11 | 4.9e-17 | 75.8 | 72 | (70, 143) | 143 | (73, 144) | 147 | Probable tail fiber assembly protein | Probable tail fiber assembly protein | | uniclust | UniRef100\_UPI001C256683 | 98.8 | 3.1e-11 | 5.7e-17 | 74.4 | 103 | (30, 143) | 143 | (23, 133) | 134 | tail fiber assembly protein | tail fiber assembly protein | | uniclust | UniRef100\_UPI002157772D | 98.8 | 3.5e-11 | 6.4e-17 | 63.6 | 51 | (47, 97) | 143 | (5, 55) | 57 | tail fiber assembly protein | tail fiber assembly protein | | uniclust | UniRef100\_A0A3R0JSW1 | 98.8 | 3.6e-11 | 6.6e-17 | 66.6 | 64 | (1, 64) | 143 | (4, 67) | 70 | GST N-terminal domain-containing protein | GST N-terminal domain-containing protein | | uniclust | UniRef100\_A0A1G9YTU7 | 98.8 | 3.6e-11 | 6.6e-17 | 76.3 | 98 | (30, 143) | 143 | (46, 144) | 144 | Phage tail assembly chaperone protein | Phage tail assembly chaperone protein | | uniclust | UniRef100\_A0A5D4YEH1 | 98.8 | 3.9e-11 | 7.2e-17 | 73.2 | 73 | (66, 143) | 143 | (38, 110) | 114 | Tail fiber assembly protein | Tail fiber assembly protein | | uniclust | UniRef100\_A0A0D9API2 | 98.8 | 4.4e-11 | 8.2e-17 | 81.5 | 69 | (1, 75) | 143 | (11, 81) | 213 | Phage tail protein | Phage tail protein | | uniclust | UniRef100\_A0A2E3R7C7 | 98.8 | 5.5e-11 | 1e-16 | 71.4 | 89 | (50, 143) | 143 | (7, 100) | 102 | Tail fiber assembly protein | Tail fiber assembly protein | | uniclust | UniRef100\_A0A0F6B2I6 | 98.8 | 6.3e-11 | 1.2e-16 | 84.3 | 77 | (62, 143) | 143 | (299, 375) | 378 | Phage side tail fiber assembly protein | Phage side tail fiber assembly protein | | uniclust | UniRef100\_UPI0012642E8B | 98.8 | 6.3e-11 | 1.2e-16 | 67.2 | 55 | (87, 143) | 143 | (25, 80) | 82 | tail fiber assembly protein | tail fiber assembly protein | | uniclust | UniRef100\_A0A2D5R456 | 98.8 | 6.7e-11 | 1.3e-16 | 75.8 | 98 | (27, 143) | 143 | (36, 135) | 136 | Phage tail protein | Phage tail protein | | uniclust | UniRef100\_A0A7X0SZA7 | 98.7 | 7.9e-11 | 1.5e-16 | 65.4 | 44 | (100, 143) | 143 | (7, 50) | 69 | Tail fiber assembly protein | Tail fiber assembly protein | | uniclust | UniRef100\_A0A0W8JAN2 | 98.7 | 7.9e-11 | 1.5e-16 | 76.3 | 122 | (1, 131) | 143 | (2, 126) | 147 | Phage tail protein | Phage tail protein | | uniclust | UniRef100\_UPI000CF05EB2 | 98.7 | 8.2e-11 | 1.5e-16 | 79.3 | 60 | (80, 141) | 143 | (184, 243) | 244 | IS5 family transposase | IS5 family transposase | | uniclust | UniRef100\_A0A3A6R813 | 98.7 | 9e-11 | 1.7e-16 | 76.1 | 115 | (29, 143) | 143 | (38, 171) | 185 | Tail fiber assembly protein | Tail fiber assembly protein | | uniclust | UniRef100\_A0A399PZJ5 | 98.7 | 9.4e-11 | 1.7e-16 | 74.3 | 60 | (3, 70) | 143 | (1, 60) | 133 | Phage tail protein | Phage tail protein | | uniclust | UniRef100\_A0A0P1FIZ6 | 98.7 | 9.3e-11 | 1.8e-16 | 78.2 | 65 | (68, 143) | 143 | (76, 142) | 148 | Caudovirales tail fibre assembly protein | Caudovirales tail fibre assembly protein | | uniclust | UniRef100\_A0A1H4V2G1 | 98.7 | 9.8e-11 | 1.8e-16 | 74.9 | 117 | (2, 128) | 143 | (1, 123) | 133 | Uncharacterized protein | Uncharacterized protein | | uniclust | UniRef100\_A0A8I1G1G7 | 98.7 | 1e-10 | 1.9e-16 | 63.4 | 55 | (3, 63) | 143 | (1, 56) | 61 | Uncharacterized protein (Fragment) | Uncharacterized protein (Fragment) | | uniclust | UniRef100\_A0A0H3NQF5 | 98.7 | 1.2e-10 | 2.2e-16 | 67.9 | 68 | (73, 143) | 143 | (22, 89) | 89 | Tail fiber assembly protein | Tail fiber assembly protein | | uniclust | UniRef100\_B4RM01 | 98.7 | 1.2e-10 | 2.3e-16 | 75.5 | 58 | (2, 68) | 143 | (1, 58) | 157 | Phage associated protein | Phage associated protein | | uniclust | UniRef100\_UPI001FCA6AA2 | 98.7 | 1.3e-10 | 2.3e-16 | 75.2 | 50 | (91, 142) | 143 | (131, 180) | 180 | DUF1983 domain-containing protein | DUF1983 domain-containing protein | | uniclust | UniRef100\_UPI000272327A | 98.7 | 1.5e-10 | 2.8e-16 | 67.7 | 56 | (4, 65) | 143 | (1, 56) | 97 | hypothetical protein | hypothetical protein | | uniclust | UniRef100\_UPI0020C96B49 | 98.7 | 1.6e-10 | 2.9e-16 | 66.4 | 67 | (72, 142) | 143 | (21, 87) | 87 | tail fiber assembly protein | tail fiber assembly protein | | uniclust | UniRef100\_A0A2S8YX24 | 98.7 | 1.6e-10 | 3e-16 | 73.2 | 60 | (82, 143) | 143 | (32, 93) | 157 | Phage tail protein | Phage tail protein | | uniclust | UniRef100\_UPI0006877F13 | 98.7 | 1.7e-10 | 3.1e-16 | 64.7 | 64 | (1, 69) | 143 | (1, 64) | 76 | hypothetical protein | hypothetical protein | | uniclust | UniRef100\_A0A2D0YIG7 | 98.7 | 1.7e-10 | 3.2e-16 | 75.2 | 115 | (1, 130) | 143 | (15, 131) | 150 | Tail fibers protein | Tail fibers protein | | uniclust | UniRef100\_UPI001F153A13 | 98.7 | 1.8e-10 | 3.4e-16 | 70.7 | 60 | (81, 143) | 143 | (68, 127) | 128 | tail fiber assembly protein | tail fiber assembly protein | | uniclust | UniRef100\_A0A078LGE4 | 98.7 | 2e-10 | 3.7e-16 | 68.6 | 56 | (74, 143) | 143 | (20, 77) | 86 | Uncharacterized protein | Uncharacterized protein | | uniclust | UniRef100\_A0A0J6KHP8 | 98.7 | 2e-10 | 3.8e-16 | 73.8 | 55 | (78, 143) | 143 | (60, 116) | 123 | Phage tail protein | Phage tail protein | | uniclust | UniRef100\_UPI001E61745F | 98.7 | 2.1e-10 | 3.9e-16 | 73.4 | 72 | (1, 77) | 143 | (1, 72) | 167 | hypothetical protein | hypothetical protein | | uniclust | UniRef100\_A0A080IVY2 | 98.7 | 2.2e-10 | 4.1e-16 | 79.2 | 69 | (73, 143) | 143 | (116, 190) | 216 | Caudovirales tail fiber assembly family protein | Caudovirales tail fiber assembly family protein | | uniclust | UniRef100\_UPI00227A8565 | 98.7 | 2.5e-10 | 4.5e-16 | 71.4 | 68 | (70, 142) | 143 | (76, 143) | 143 | tail fiber assembly protein | tail fiber assembly protein | | uniclust | UniRef100\_A0A447L1E5 | 98.6 | 2.8e-10 | 5.1e-16 | 76.3 | 73 | (69, 143) | 143 | (156, 228) | 229 | Caudovirales tail fibre assembly protein | Caudovirales tail fibre assembly protein | | uniclust | UniRef100\_A0A0T9KYK9 | 98.6 | 2.9e-10 | 5.3e-16 | 73.9 | 71 | (71, 143) | 143 | (88, 158) | 165 | Tail fiber assembly protein G | Tail fiber assembly protein G | | uniclust | UniRef100\_UPI0009BA29CF | 98.6 | 3e-10 | 5.5e-16 | 80.8 | 64 | (78, 143) | 143 | (304, 369) | 369 | tail fiber assembly protein | tail fiber assembly protein | | uniclust | UniRef100\_A0A2C6DTU1 | 98.6 | 3e-10 | 5.6e-16 | 55.4 | 29 | (115, 143) | 143 | (2, 30) | 36 | Tail fiber assembly protein | Tail fiber assembly protein | | uniclust | UniRef100\_A0A0T9P2Y6 | 98.6 | 3.1e-10 | 5.7e-16 | 73.7 | 71 | (71, 143) | 143 | (92, 162) | 164 | Tail fiber assembly protein G | Tail fiber assembly protein G | | uniclust | UniRef100\_UPI001061FCA3 | 98.6 | 3.3e-10 | 6e-16 | 71.0 | 55 | (86, 142) | 143 | (88, 142) | 144 | tail fiber assembly protein | tail fiber assembly protein | | uniclust | UniRef100\_UPI00046AFBFA | 98.6 | 3.3e-10 | 6.1e-16 | 72.8 | 62 | (80, 143) | 143 | (99, 162) | 162 | tail fiber assembly protein | tail fiber assembly protein | | uniclust | UniRef100\_A0A3D1IY78 | 98.6 | 3.5e-10 | 6.4e-16 | 66.1 | 87 | (2, 91) | 143 | (1, 94) | 95 | Uncharacterized protein (Fragment) | Uncharacterized protein (Fragment) | | uniclust | UniRef100\_Q126B3 | 98.6 | 3.7e-10 | 6.8e-16 | 75.4 | 82 | (4, 85) | 143 | (1, 112) | 223 | Phage tail protein | Phage tail protein | | uniclust | UniRef100\_A0A0J6LG96 | 98.6 | 3.7e-10 | 7e-16 | 69.3 | 73 | (69, 143) | 143 | (19, 93) | 102 | Phage-like protein | Phage-like protein | | uniclust | UniRef100\_UPI001BD46F23 | 98.6 | 3.9e-10 | 7.2e-16 | 74.1 | 71 | (70, 142) | 143 | (106, 178) | 180 | tail fiber assembly protein | tail fiber assembly protein | | uniclust | UniRef100\_J3CFF5 | 98.6 | 4.4e-10 | 8.1e-16 | 64.0 | 82 | (60, 143) | 143 | (1, 82) | 82 | Caudovirales tail fiber assembly protein | Caudovirales tail fiber assembly protein | | uniclust | UniRef100\_UPI00192B3CC5 | 98.6 | 4.5e-10 | 8.3e-16 | 86.9 | 69 | (72, 142) | 143 | (796, 864) | 867 | phage tail protein | phage tail protein | | uniclust | UniRef100\_A0A2A2T4I5 | 98.6 | 4.3e-10 | 8.4e-16 | 75.8 | 66 | (67, 143) | 143 | (78, 144) | 156 | Phage tail protein | Phage tail protein | | uniclust | UniRef100\_UPI001BE0504F | 98.6 | 5e-10 | 9.2e-16 | 64.9 | 57 | (85, 143) | 143 | (30, 86) | 90 | tail fiber assembly protein | tail fiber assembly protein | | uniclust | UniRef100\_A0A5J6WBQ9 | 98.6 | 5.3e-10 | 9.8e-16 | 71.5 | 85 | (57, 143) | 143 | (55, 145) | 146 | Tail fiber assembly protein | Tail fiber assembly protein | | uniclust | UniRef100\_UPI001F5BCB89 | 98.6 | 5.8e-10 | 1.1e-15 | 63.7 | 68 | (63, 132) | 143 | (4, 71) | 83 | tail fiber assembly protein | tail fiber assembly protein | | uniclust | UniRef100\_A0A732CWE2 | 98.6 | 6.6e-10 | 1.2e-15 | 55.9 | 38 | (100, 137) | 143 | (4, 41) | 41 | Tail fiber assembly protein | Tail fiber assembly protein | | uniclust | UniRef100\_A0A1W0CCP9 | 98.6 | 6.7e-10 | 1.3e-15 | 63.8 | 63 | (79, 143) | 143 | (2, 66) | 72 | Phage tail protein | Phage tail protein | | uniclust | UniRef100\_UPI001F44B6A4 | 98.6 | 7e-10 | 1.3e-15 | 73.2 | 60 | (82, 143) | 143 | (143, 202) | 202 | tail fiber assembly protein | tail fiber assembly protein | | uniclust | UniRef100\_UPI00117AE413 | 98.6 | 7.1e-10 | 1.3e-15 | 65.1 | 69 | (73, 143) | 143 | (27, 95) | 97 | tail fiber assembly protein | tail fiber assembly protein | | uniclust | UniRef100\_A0A8S5R0T0 | 98.6 | 7.1e-10 | 1.3e-15 | 76.9 | 68 | (65, 143) | 143 | (122, 195) | 203 | Tail assembly chaperone protein | Tail assembly chaperone protein | | uniclust | UniRef100\_UPI0020A688E0 | 98.6 | 7.6e-10 | 1.4e-15 | 58.2 | 52 | (88, 142) | 143 | (3, 54) | 54 | tail fiber assembly protein | tail fiber assembly protein | | uniclust | UniRef100\_A0A077LR29 | 98.5 | 7.6e-10 | 1.4e-15 | 76.0 | 67 | (75, 143) | 143 | (115, 183) | 199 | Tail fiber assembly protein | Tail fiber assembly protein | | uniclust | UniRef100\_UPI000DEF41CE | 98.5 | 8.2e-10 | 1.5e-15 | 72.2 | 112 | (24, 143) | 143 | (54, 170) | 170 | phage tail assembly chaperone | phage tail assembly chaperone | | uniclust | UniRef100\_A0A0H5AKI0 | 98.5 | 8e-10 | 1.5e-15 | 66.1 | 70 | (72, 143) | 143 | (4, 75) | 85 | Phage-like protein | Phage-like protein | | uniclust | UniRef100\_A0A3W0FGG3 | 98.5 | 8.2e-10 | 1.5e-15 | 57.6 | 41 | (100, 143) | 143 | (4, 44) | 47 | Tail fiber assembly protein | Tail fiber assembly protein | | uniclust | UniRef100\_A0A377HWU3 | 98.5 | 9.4e-10 | 1.7e-15 | 66.3 | 58 | (2, 68) | 143 | (1, 58) | 104 | Putative tail fiber assembly protein | Putative tail fiber assembly protein | | uniclust | UniRef100\_A0A381C907 | 98.5 | 1.1e-09 | 2e-15 | 63.7 | 62 | (24, 86) | 143 | (30, 91) | 91 | Caudovirales tail fibre assembly protein | Caudovirales tail fibre assembly protein | | uniclust | UniRef100\_A0A1H2HWK3 | 98.5 | 1.1e-09 | 2.1e-15 | 72.5 | 74 | (67, 142) | 143 | (69, 144) | 158 | Phage tail assembly chaperone protein | Phage tail assembly chaperone protein | | uniclust | UniRef100\_A0A090K647 | 98.5 | 1.1e-09 | 2.1e-15 | 66.9 | 58 | (74, 143) | 143 | (23, 82) | 89 | Uncharacterized phage protein | Uncharacterized phage protein | | uniclust | UniRef100\_A0A2A2GZS3 | 98.5 | 1.2e-09 | 2.2e-15 | 61.6 | 61 | (1, 66) | 143 | (5, 65) | 70 | Uncharacterized protein | Uncharacterized protein | | uniclust | UniRef100\_A0A977GFE5 | 98.5 | 1.2e-09 | 2.2e-15 | 61.7 | 56 | (86, 143) | 143 | (14, 78) | 78 | Tail fiber assembly protein | Tail fiber assembly protein | | uniclust | UniRef100\_UPI00191DD07F | 98.5 | 1.5e-09 | 2.7e-15 | 62.4 | 69 | (73, 143) | 143 | (13, 82) | 85 | tail fiber assembly protein | tail fiber assembly protein | | uniclust | UniRef100\_UPI001E5F398A | 98.5 | 1.5e-09 | 2.7e-15 | 68.2 | 80 | (62, 143) | 143 | (64, 143) | 143 | tail fiber assembly protein | tail fiber assembly protein | | uniclust | UniRef100\_UPI000FAB35A7 | 98.5 | 1.6e-09 | 2.9e-15 | 59.1 | 54 | (69, 124) | 143 | (9, 62) | 64 | tail fiber assembly protein | tail fiber assembly protein | | uniclust | UniRef100\_A0A2S9RN83 | 98.5 | 1.6e-09 | 3e-15 | 65.5 | 58 | (2, 68) | 143 | (4, 61) | 106 | Phage tail protein | Phage tail protein | | uniclust | UniRef100\_A0A0A1I628 | 98.5 | 1.6e-09 | 3.1e-15 | 74.3 | 58 | (76, 143) | 143 | (87, 146) | 166 | Tail fiber assembly protein | Tail fiber assembly protein | | uniclust | UniRef100\_A0A382DWT1 | 98.5 | 1.7e-09 | 3.1e-15 | 68.7 | 58 | (75, 143) | 143 | (64, 123) | 126 | Phage tail protein (Fragment) | Phage tail protein (Fragment) | | uniclust | UniRef100\_UPI00111B018F | 98.5 | 1.7e-09 | 3.1e-15 | 64.0 | 97 | (1, 98) | 143 | (1, 98) | 100 | tail fiber assembly protein | tail fiber assembly protein | | uniclust | UniRef100\_A0A0B7JBQ8 | 98.5 | 1.6e-09 | 3.1e-15 | 70.1 | 61 | (72, 143) | 143 | (49, 111) | 118 | Phage tail protein | Phage tail protein | | uniclust | UniRef100\_A0A5J6B771 | 98.5 | 1.7e-09 | 3.1e-15 | 68.8 | 60 | (4, 68) | 143 | (1, 60) | 156 | Tail fibers protein | Tail fibers protein | | uniclust | UniRef100\_A0A3P3QNW6 | 98.5 | 1.7e-09 | 3.2e-15 | 64.5 | 70 | (69, 143) | 143 | (16, 87) | 88 | Phage tail protein | Phage tail protein | | uniclust | UniRef100\_UPI0009416878 | 98.5 | 2e-09 | 3.6e-15 | 65.1 | 62 | (77, 143) | 143 | (39, 100) | 105 | tail fiber assembly protein | tail fiber assembly protein | | uniclust | UniRef100\_UPI0022FED9A9 | 98.5 | 2e-09 | 3.7e-15 | 55.9 | 49 | (92, 143) | 143 | (2, 50) | 50 | tail fiber assembly protein | tail fiber assembly protein | | uniclust | UniRef100\_A0A2D4ZXJ1 | 98.5 | 2e-09 | 3.9e-15 | 73.1 | 60 | (73, 143) | 143 | (96, 157) | 158 | Tail fiber assembly protein | Tail fiber assembly protein | | uniclust | UniRef100\_A0A023Q0D1 | 98.5 | 2.1e-09 | 4e-15 | 65.9 | 58 | (75, 143) | 143 | (24, 83) | 90 | Phage tail protein | Phage tail protein | | uniclust | UniRef100\_A0A553ZT71 | 98.5 | 2.2e-09 | 4e-15 | 53.0 | 34 | (110, 143) | 143 | (1, 34) | 38 | Tail fiber assembly protein (Fragment) | Tail fiber assembly protein (Fragment) | | uniclust | UniRef100\_A0A6J5MT50 | 98.4 | 2.2e-09 | 4.1e-15 | 70.6 | 57 | (74, 143) | 143 | (81, 139) | 141 | Phage tail assembly chaperone protein | Phage tail assembly chaperone protein | | uniclust | UniRef100\_A0A5E7AJE0 | 98.4 | 2.4e-09 | 4.5e-15 | 71.2 | 62 | (1, 68) | 143 | (1, 62) | 187 | DUF4376 domain-containing protein | DUF4376 domain-containing protein | | uniclust | UniRef100\_U1ZF67 | 98.4 | 2.6e-09 | 4.7e-15 | 60.8 | 69 | (2, 76) | 143 | (1, 71) | 80 | Uncharacterized protein (Fragment) | Uncharacterized protein (Fragment) | | uniclust | UniRef100\_A0A1Z8T124 | 98.4 | 2.5e-09 | 4.7e-15 | 65.9 | 58 | (74, 143) | 143 | (39, 97) | 99 | Phage tail protein | Phage tail protein | | uniclust | UniRef100\_A0A826QMU1 | 98.4 | 2.6e-09 | 4.8e-15 | 61.1 | 64 | (78, 143) | 143 | (3, 72) | 77 | Tail fiber assembly protein | Tail fiber assembly protein | | uniclust | UniRef100\_A0A0H0K3J8 | 98.4 | 3e-09 | 5.5e-15 | 60.8 | 66 | (1, 66) | 143 | (1, 67) | 74 | CPS-53 (KpLE1) prophage protein | CPS-53 (KpLE1) prophage protein | | uniclust | UniRef100\_A0A096GRC5 | 98.4 | 2.9e-09 | 5.5e-15 | 70.7 | 58 | (75, 143) | 143 | (91, 149) | 153 | Tail fiber assembly protein | Tail fiber assembly protein | | uniclust | UniRef100\_A0A418X1B6 | 98.4 | 3.1e-09 | 5.7e-15 | 72.0 | 75 | (3, 77) | 143 | (1, 126) | 214 | Uncharacterized protein | Uncharacterized protein | | uniclust | UniRef100\_A0A380X5D8 | 98.4 | 3.1e-09 | 5.7e-15 | 63.1 | 58 | (3, 67) | 143 | (1, 58) | 101 | Tail fiber assembly protein | Tail fiber assembly protein | | uniclust | UniRef100\_A0A2G8MZ68 | 98.4 | 3.1e-09 | 5.9e-15 | 74.3 | 60 | (2, 68) | 143 | (1, 60) | 203 | Phage tail protein | Phage tail protein | | uniclust | UniRef100\_A0A7Y8YCH4 | 98.4 | 3.4e-09 | 6.3e-15 | 55.7 | 45 | (95, 141) | 143 | (6, 50) | 53 | Tail fiber assembly protein | Tail fiber assembly protein | | uniclust | UniRef100\_UPI000288F1B1 | 98.4 | 3.4e-09 | 6.4e-15 | 67.2 | 109 | (27, 143) | 143 | (15, 128) | 128 | hypothetical protein | hypothetical protein | | uniclust | UniRef100\_A0A286QHW2 | 98.4 | 3.4e-09 | 6.4e-15 | 70.5 | 68 | (74, 143) | 143 | (113, 180) | 180 | Putative tail fiber assembly protein | Putative tail fiber assembly protein | | uniclust | UniRef100\_A0A1R0GHS9 | 98.4 | 3.5e-09 | 6.6e-15 | 73.6 | 79 | (63, 143) | 143 | (112, 192) | 200 | Phage tail protein | Phage tail protein | | uniclust | UniRef100\_A0A855K755 | 98.4 | 3.8e-09 | 7.1e-15 | 65.9 | 75 | (66, 143) | 143 | (50, 124) | 125 | Tail fiber assembly protein | Tail fiber assembly protein | | uniclust | UniRef100\_A0A763STN1 | 98.4 | 3.9e-09 | 7.1e-15 | 54.0 | 41 | (93, 135) | 143 | (1, 41) | 46 | Tail fiber assembly protein | Tail fiber assembly protein | | uniclust | UniRef100\_UPI002023B97A | 98.4 | 4.2e-09 | 7.7e-15 | 58.1 | 49 | (77, 127) | 143 | (15, 63) | 66 | tail fiber assembly protein | tail fiber assembly protein | | uniclust | UniRef100\_A0A167MRB5 | 98.4 | 4e-09 | 7.7e-15 | 68.6 | 62 | (71, 143) | 143 | (59, 122) | 125 | Tail fiber assembly protein | Tail fiber assembly protein | | uniclust | UniRef100\_A0A6M4XJ55 | 98.4 | 4.2e-09 | 7.7e-15 | 58.4 | 60 | (3, 67) | 143 | (1, 60) | 64 | Uncharacterized protein | Uncharacterized protein | | uniclust | UniRef100\_UPI001F0AB459 | 98.4 | 4.3e-09 | 7.9e-15 | 57.5 | 63 | (63, 125) | 143 | (2, 64) | 64 | tail fiber assembly protein | tail fiber assembly protein | | uniclust | UniRef100\_UPI001CC0D473 | 98.4 | 4.5e-09 | 8.3e-15 | 57.4 | 57 | (2, 64) | 143 | (1, 57) | 64 | hypothetical protein | hypothetical protein | | uniclust | UniRef100\_UPI001BD5EA33 | 98.4 | 4.5e-09 | 8.3e-15 | 58.4 | 65 | (77, 143) | 143 | (6, 70) | 70 | tail fiber assembly protein | tail fiber assembly protein | | uniclust | UniRef100\_A0A2S9X7U2 | 98.4 | 4.5e-09 | 8.3e-15 | 75.9 | 69 | (64, 143) | 143 | (219, 289) | 295 | Phage tail protein | Phage tail protein | | uniclust | UniRef100\_A0A0C5EHZ3 | 98.4 | 4.8e-09 | 9e-15 | 64.3 | 61 | (72, 143) | 143 | (29, 91) | 95 | Phage tail assembly chaperone protein | Phage tail assembly chaperone protein | | uniclust | UniRef100\_A0A0H4VYX9 | 98.4 | 4.9e-09 | 9e-15 | 69.4 | 88 | (3, 95) | 143 | (42, 131) | 201 | Tail assembly chaperone | Tail assembly chaperone | | uniclust | UniRef100\_UPI001F55841F | 98.4 | 4.9e-09 | 9.1e-15 | 57.9 | 56 | (77, 143) | 143 | (4, 61) | 63 | tail fiber assembly protein | tail fiber assembly protein | | uniclust | UniRef100\_A0A6S6RXB7 | 98.4 | 5e-09 | 9.3e-15 | 56.5 | 54 | (57, 112) | 143 | (6, 59) | 60 | Uncharacterized protein | Uncharacterized protein | | uniclust | UniRef100\_A0A2D4QWA0 | 98.4 | 4.9e-09 | 9.3e-15 | 64.5 | 53 | (77, 143) | 143 | (37, 91) | 98 | Phage tail protein | Phage tail protein | | uniclust | UniRef100\_UPI002301F898 | 98.4 | 5.3e-09 | 9.7e-15 | 65.6 | 66 | (1, 66) | 143 | (1, 66) | 140 | hypothetical protein | hypothetical protein | | uniclust | UniRef100\_A0A411GJZ7 | 98.4 | 5.6e-09 | 1e-14 | 64.4 | 79 | (60, 143) | 143 | (40, 118) | 122 | Uncharacterized protein | Uncharacterized protein | | uniclust | UniRef100\_A0A126QKP2 | 98.3 | 6e-09 | 1.1e-14 | 70.5 | 62 | (71, 143) | 143 | (88, 151) | 163 | Phage tail protein | Phage tail protein | | uniclust | UniRef100\_A0A1B7JW24 | 98.3 | 6.4e-09 | 1.2e-14 | 84.7 | 80 | (62, 143) | 143 | (672, 753) | 754 | Phage minor tail protein | Phage minor tail protein | | uniclust | UniRef100\_B0VK51 | 98.3 | 6.7e-09 | 1.2e-14 | 70.1 | 58 | (4, 66) | 143 | (1, 58) | 228 | Uncharacterized protein 51 | Uncharacterized protein 51 | | uniclust | UniRef100\_Q7N1H8 | 98.3 | 6.8e-09 | 1.2e-14 | 71.2 | 58 | (75, 134) | 143 | (140, 197) | 258 | Photorhabdus luminescens subsp. laumondii TTO1 complete genome; segment 12/17 | Photorhabdus luminescens subsp. laumondii TTO1 complete genome; segment 12/17 | | uniclust | UniRef100\_A0A022PK72 | 98.3 | 6.8e-09 | 1.3e-14 | 79.1 | 71 | (71, 143) | 143 | (394, 464) | 569 | Phage tail protein | Phage tail protein | | uniclust | UniRef100\_UPI0009339C22 | 98.3 | 6.9e-09 | 1.3e-14 | 60.9 | 75 | (10, 84) | 143 | (8, 82) | 90 | tail fiber assembly protein | tail fiber assembly protein | | uniclust | UniRef100\_A0A0J6K2V7 | 98.3 | 8.2e-09 | 1.6e-14 | 63.7 | 61 | (72, 143) | 143 | (29, 91) | 94 | Phage tail protein | Phage tail protein | | uniclust | UniRef100\_UPI000984B09A | 98.3 | 8.7e-09 | 1.6e-14 | 66.4 | 68 | (74, 143) | 143 | (64, 133) | 134 | phage tail assembly chaperone | phage tail assembly chaperone | | uniclust | UniRef100\_A0A1A8TEZ4 | 98.3 | 8.8e-09 | 1.6e-14 | 69.0 | 63 | (69, 143) | 143 | (99, 163) | 168 | Caudovirales tail fibre assembly protein | Caudovirales tail fibre assembly protein | | uniclust | UniRef100\_A0A2S3X1I2 | 98.3 | 9.2e-09 | 1.7e-14 | 80.1 | 63 | (79, 143) | 143 | (783, 847) | 847 | Phage tail protein | Phage tail protein | | uniclust | UniRef100\_UPI0018F81469 | 98.3 | 9.4e-09 | 1.7e-14 | 56.7 | 63 | (2, 69) | 143 | (1, 63) | 67 | hypothetical protein | hypothetical protein | | uniclust | UniRef100\_A0A1L5C071 | 98.3 | 9.1e-09 | 1.7e-14 | 69.9 | 65 | (70, 143) | 143 | (74, 141) | 161 | Putative tail fiber assembly protein | Putative tail fiber assembly protein | | uniclust | UniRef100\_A0A6N6K3R1 | 98.3 | 9.5e-09 | 1.7e-14 | 59.3 | 84 | (4, 93) | 143 | (1, 84) | 85 | Phage tail protein | Phage tail protein | | uniclust | UniRef100\_A0A7G2IV99 | 98.3 | 9.4e-09 | 1.8e-14 | 56.9 | 47 | (75, 123) | 143 | (12, 58) | 60 | Tail fiber assembly protein | Tail fiber assembly protein | | uniclust | UniRef100\_A0A3N6V447 | 98.3 | 9.6e-09 | 1.8e-14 | 61.8 | 68 | (74, 143) | 143 | (33, 106) | 108 | Tail fiber assembly protein | Tail fiber assembly protein | | uniclust | UniRef100\_A0A1N6I101 | 98.3 | 9.6e-09 | 1.8e-14 | 65.7 | 117 | (2, 127) | 143 | (1, 126) | 140 | Uncharacterized protein | Uncharacterized protein | | uniclust | UniRef100\_A0A2S3X8M0 | 98.3 | 1e-08 | 1.9e-14 | 78.2 | 63 | (79, 143) | 143 | (603, 667) | 667 | Phage tail protein | Phage tail protein | | uniclust | UniRef100\_A0A0U5EW02 | 98.3 | 1.1e-08 | 2.1e-14 | 69.7 | 53 | (2, 61) | 143 | (20, 72) | 184 | Uncharacterized protein | Uncharacterized protein | | uniclust | UniRef100\_UPI000484336E | 98.3 | 1.2e-08 | 2.2e-14 | 62.7 | 59 | (83, 143) | 143 | (61, 121) | 121 | tail fiber assembly protein | tail fiber assembly protein | | uniclust | UniRef100\_UPI0003353EC4 | 98.3 | 1.2e-08 | 2.2e-14 | 64.6 | 110 | (32, 142) | 143 | (14, 131) | 132 | tail fiber assembly protein | tail fiber assembly protein | | uniclust | UniRef100\_A0A1A9VZ50 | 98.3 | 1.2e-08 | 2.2e-14 | 73.1 | 68 | (74, 143) | 143 | (293, 360) | 363 | Tail fiber assembly protein | Tail fiber assembly protein | | uniclust | UniRef100\_UPI001C97D201 | 98.3 | 1.2e-08 | 2.2e-14 | 57.6 | 44 | (3, 46) | 143 | (1, 44) | 75 | hypothetical protein | hypothetical protein | | uniclust | UniRef100\_UPI0015A437FB | 98.3 | 1.2e-08 | 2.2e-14 | 56.2 | 60 | (2, 67) | 143 | (1, 60) | 66 | hypothetical protein | hypothetical protein | | uniclust | UniRef100\_UPI001F4303E0 | 98.3 | 1.2e-08 | 2.2e-14 | 54.6 | 53 | (89, 143) | 143 | (4, 56) | 57 | tail fiber assembly protein | tail fiber assembly protein | | uniclust | UniRef100\_A0A443IQF6 | 98.3 | 1.2e-08 | 2.2e-14 | 68.4 | 61 | (72, 143) | 143 | (84, 146) | 153 | Tail fiber assembly protein | Tail fiber assembly protein | | uniclust | UniRef100\_A0A1I1B6J1 | 98.3 | 1.3e-08 | 2.4e-14 | 68.5 | 62 | (71, 143) | 143 | (84, 146) | 156 | Phage tail assembly chaperone protein | Phage tail assembly chaperone protein | | uniclust | UniRef100\_A0A345CT33 | 98.3 | 1.3e-08 | 2.4e-14 | 56.7 | 59 | (2, 67) | 143 | (1, 59) | 66 | Uncharacterized protein | Uncharacterized protein | | uniclust | UniRef100\_UPI001F45C5CD | 98.3 | 1.4e-08 | 2.5e-14 | 58.3 | 65 | (66, 132) | 143 | (9, 74) | 82 | tail fiber assembly protein | tail fiber assembly protein | | uniclust | UniRef100\_UPI0003061D81 | 98.3 | 1.4e-08 | 2.6e-14 | 53.1 | 33 | (110, 142) | 143 | (19, 51) | 51 | tail fiber assembly protein | tail fiber assembly protein | | uniclust | UniRef100\_A0A0U1HXL6 | 98.2 | 1.5e-08 | 2.7e-14 | 63.5 | 71 | (71, 143) | 143 | (26, 101) | 122 | Putative phage tail fiber assembly protein | Putative phage tail fiber assembly protein | | uniclust | UniRef100\_A0A0U1ZGY3 | 98.2 | 1.4e-08 | 2.7e-14 | 69.0 | 59 | (77, 143) | 143 | (92, 152) | 167 | Uncharacterized protein | Uncharacterized protein | | uniclust | UniRef100\_A0A076GCL9 | 98.2 | 1.5e-08 | 2.9e-14 | 70.0 | 69 | (73, 143) | 143 | (112, 180) | 186 | Putative tail fiber assembly protein | Putative tail fiber assembly protein | | uniclust | UniRef100\_A0A0H0G3F6 | 98.2 | 1.6e-08 | 2.9e-14 | 58.7 | 46 | (85, 132) | 143 | (12, 57) | 82 | Tail fiber assembly protein | Tail fiber assembly protein | | uniclust | UniRef100\_UPI00143FE79E | 98.2 | 1.6e-08 | 2.9e-14 | 70.5 | 66 | (1, 67) | 143 | (84, 149) | 289 | tail fiber assembly protein | tail fiber assembly protein | | uniclust | UniRef100\_A0A0T9V566 | 98.2 | 1.7e-08 | 3.2e-14 | 67.1 | 132 | (1, 142) | 143 | (2, 137) | 143 | Tail fiber assembly protein | Tail fiber assembly protein | | uniclust | UniRef100\_A0A0A0HKV0 | 98.2 | 1.8e-08 | 3.3e-14 | 59.9 | 62 | (71, 143) | 143 | (14, 77) | 80 | Caudovirales tail fiber assembly protein | Caudovirales tail fiber assembly protein | | uniclust | UniRef100\_A0A2X2RPY1 | 98.2 | 1.8e-08 | 3.3e-14 | 65.9 | 64 | (76, 141) | 143 | (114, 177) | 180 | Caudovirales tail fibre assembly protein | Caudovirales tail fibre assembly protein | | uniclust | UniRef100\_A0A376J3U5 | 98.2 | 1.9e-08 | 3.6e-14 | 64.8 | 72 | (69, 143) | 143 | (73, 144) | 144 | Tail fiber assembly protein (Gpg) | Tail fiber assembly protein (Gpg) | | uniclust | UniRef100\_A0A7L6U9J7 | 98.2 | 2e-08 | 3.8e-14 | 54.9 | 49 | (92, 142) | 143 | (14, 62) | 63 | Tail fiber assembly protein | Tail fiber assembly protein | | uniclust | UniRef100\_UPI00214F3669 | 98.2 | 2.1e-08 | 3.8e-14 | 58.4 | 52 | (87, 143) | 143 | (5, 56) | 88 | tail fiber assembly protein | tail fiber assembly protein | | uniclust | UniRef100\_A0A8T6AMS3 | 98.2 | 2.1e-08 | 3.9e-14 | 54.6 | 30 | (113, 142) | 143 | (33, 62) | 62 | Tail fiber assembly protein | Tail fiber assembly protein | | uniclust | UniRef100\_A0A444RIF9 | 98.2 | 2.1e-08 | 3.9e-14 | 62.9 | 73 | (69, 143) | 143 | (47, 126) | 126 | Tail fiber assembly protein | Tail fiber assembly protein | | uniclust | UniRef100\_A0A0D5Y4U0 | 98.2 | 2.3e-08 | 4.2e-14 | 59.5 | 57 | (85, 143) | 143 | (32, 97) | 98 | Phage tail protein | Phage tail protein | | uniclust | UniRef100\_UPI001ED8DAE3 | 98.2 | 2.3e-08 | 4.3e-14 | 77.3 | 68 | (74, 143) | 143 | (593, 660) | 660 | tail fiber assembly protein | tail fiber assembly protein | | uniclust | UniRef100\_A0A2D6P997 | 98.2 | 2.3e-08 | 4.4e-14 | 63.7 | 58 | (75, 143) | 143 | (63, 121) | 122 | Tail fiber assembly protein | Tail fiber assembly protein | | uniclust | UniRef100\_A0A2N7P764 | 98.2 | 2.6e-08 | 4.9e-14 | 63.2 | 93 | (4, 96) | 143 | (1, 94) | 147 | Uncharacterized protein | Uncharacterized protein | | uniclust | UniRef100\_A0A2X1PJL4 | 98.2 | 2.7e-08 | 5e-14 | 60.0 | 65 | (27, 91) | 143 | (41, 105) | 108 | Putative phage tail assembly chaperone | Putative phage tail assembly chaperone | | uniclust | UniRef100\_UPI001B82413B | 98.2 | 2.8e-08 | 5.1e-14 | 56.9 | 56 | (3, 64) | 143 | (1, 56) | 80 | hypothetical protein | hypothetical protein | | uniclust | UniRef100\_UPI0004661D35 | 98.2 | 2.8e-08 | 5.1e-14 | 59.8 | 70 | (72, 143) | 143 | (16, 86) | 93 | phage tail assembly chaperone | phage tail assembly chaperone | | uniclust | UniRef100\_UPI0021155A40 | 98.2 | 2.9e-08 | 5.4e-14 | 56.2 | 64 | (77, 142) | 143 | (4, 71) | 72 | tail fiber assembly protein | tail fiber assembly protein | | uniclust | UniRef100\_C1D954 | 98.2 | 3.1e-08 | 5.7e-14 | 69.0 | 65 | (4, 74) | 143 | (1, 65) | 283 | HsdM | HsdM | | uniclust | UniRef100\_A0A4R4J6I0 | 98.2 | 3.2e-08 | 5.9e-14 | 48.5 | 28 | (115, 142) | 143 | (9, 36) | 37 | Tail fiber assembly protein | Tail fiber assembly protein | | uniclust | UniRef100\_A0A1A9WSI7 | 98.2 | 3.4e-08 | 6.2e-14 | 74.9 | 78 | (5, 82) | 143 | (509, 587) | 604 | Uncharacterized protein | Uncharacterized protein | | uniclust | UniRef100\_A0A0F7Y2W3 | 98.2 | 3.3e-08 | 6.3e-14 | 77.3 | 62 | (80, 143) | 143 | (430, 493) | 493 | Tail fiber protein | Tail fiber protein | | uniclust | UniRef100\_A0A484QPN5 | 98.1 | 3.4e-08 | 6.4e-14 | 63.2 | 79 | (63, 143) | 143 | (51, 131) | 139 | Tail fiber assembly protein | Tail fiber assembly protein | | uniclust | UniRef100\_A0A2M7HGM4 | 98.1 | 3.5e-08 | 6.4e-14 | 57.5 | 81 | (47, 143) | 143 | (4, 84) | 88 | Phage tail protein | Phage tail protein | | uniclust | UniRef100\_UPI001F25180A | 98.1 | 3.6e-08 | 6.6e-14 | 57.2 | 72 | (70, 143) | 143 | (8, 86) | 86 | tail fiber assembly protein | tail fiber assembly protein | | uniclust | UniRef100\_A0A8T5ZT04 | 98.1 | 3.8e-08 | 7e-14 | 47.1 | 30 | (114, 143) | 143 | (3, 32) | 33 | Tail fiber assembly protein | Tail fiber assembly protein | | uniclust | UniRef100\_UPI000734FD4D | 98.1 | 4.1e-08 | 7.6e-14 | 70.6 | 81 | (60, 142) | 143 | (134, 216) | 294 | tail fiber assembly protein | tail fiber assembly protein | | uniclust | UniRef100\_S2KT03 | 98.1 | 4.3e-08 | 7.8e-14 | 64.1 | 65 | (76, 142) | 143 | (104, 176) | 176 | Phage tail fiber assembly protein | Phage tail fiber assembly protein | | uniclust | UniRef100\_A0A5A9DBI3 | 98.1 | 4.3e-08 | 8e-14 | 48.9 | 33 | (109, 141) | 143 | (5, 37) | 40 | Tail fiber assembly protein | Tail fiber assembly protein | | uniclust | UniRef100\_UPI0021CFB339 | 98.1 | 4.3e-08 | 8e-14 | 58.6 | 64 | (75, 143) | 143 | (38, 101) | 102 | tail fiber assembly protein | tail fiber assembly protein | | uniclust | UniRef100\_A0A411H8C5 | 98.1 | 4.4e-08 | 8.2e-14 | 61.8 | 64 | (77, 143) | 143 | (69, 132) | 134 | Tail fiber assembly protein | Tail fiber assembly protein | | pdb70 | 5YVQ\_B | 99.2 | 2.3e-15 | 2.3e-19 | 112.6 | 75 | (64, 143) | 143 | (101, 175) | 175 | Gene product J | 5YVQ\_B Gene product J bacteriophage, VIRAL PROTEIN HET: GOL | | pdb70 | 2KZ6\_A | 98.5 | 2.6e-11 | 2.5e-15 | 84.2 | 63 | (70, 143) | 143 | (38, 102) | 102 | Uncharacterized protein | 2KZ6\_A Uncharacterized protein protein of unknown function, Structural | |
| Top keywords  (threshold 1.00e-03 (evalue)) | **tail, assembly, fiber, Phage, chaperone, Caudovirales, Fragment, Putative, fibre, hypothetical** |
| Output files | ../../similar\_sequences/16\_FANPEZAQ\_CDS\_0016\_merged.svg ../../similar\_sequences/16\_FANPEZAQ\_CDS\_0016\_pdb70.a3m ../../similar\_sequences/16\_FANPEZAQ\_CDS\_0016\_pdb70.hhr ../../similar\_sequences/16\_FANPEZAQ\_CDS\_0016\_uniclust.a3m ../../similar\_sequences/16\_FANPEZAQ\_CDS\_0016\_uniclust.hhr |

#### Structure prediction (AlphaFold)2

|  |  |
| --- | --- |
| Stats | xml version="1.0" encoding="utf-8" standalone="no"?       2024-09-02T21:09:15.217395 image/svg+xml   Matplotlib v3.7.2, https://matplotlib.org/ |
| Predicted structure | **NGL Viewer Controls:**  - Center: *Left-Click* - Rotate: *Left-Click + Drag* - Translate: *Right-Click + Drag* - Zoom: *Shift + Left-Click + Drag* |
| Output files | ../../predicted\_structures/16\_FANPEZAQ\_CDS\_0016/features.pkl ../../predicted\_structures/16\_FANPEZAQ\_CDS\_0016/ranked\_0.pdb ../../predicted\_structures/16\_FANPEZAQ\_CDS\_0016/ranked\_0\_plots.svg ../../predicted\_structures/16\_FANPEZAQ\_CDS\_0016/result\_model\_1\_ptm\_pred\_0.pkl |

#### Structure similarity search results (Foldseek)3

|  |  |
| --- | --- |
| Structure databases searched | Pdb, Afdb-proteome, Afdb-uniprot50 |
| Results, scheme(s)  (Top layers only, threshold 1.00e-02 (evalue)) | xml version="1.0" encoding="utf-8" standalone="no"?       2024-09-02T21:10:34.592546 image/svg+xml   Matplotlib v3.7.2, https://matplotlib.org/ |
| Results, table  (threshold 1.00e-02 (evalue)) | | db | id | prob | evalue | bits | fident | alnlen | mismatch | gapopen | qstart | qend | tstart | tend | name | description | | --- | --- | --- | --- | --- | --- | --- | --- | --- | --- | --- | --- | --- | --- | --- | | afdb-proteome | AF-P77656-F1-MODEL\_V4 | 1.0 | 9.056e-18 | 683 | 0.564 | 140 | 61 | 0 | 4 | 143 | 3 | 142 | Uncharacterized protein YfdK | Uncharacterized protein YfdK | | afdb-proteome | AF-Q8ZNJ2-F1-MODEL\_V4 | 1.0 | 6.388e-13 | 448 | 0.429 | 142 | 79 | 2 | 1 | 141 | 1 | 141 | Putative cytoplasmic protein | Putative cytoplasmic protein | | afdb-proteome | AF-P09154-F1-MODEL\_V4 | 1.0 | 1.137e-09 | 313 | 0.368 | 141 | 78 | 4 | 3 | 142 | 2 | 132 | Uncharacterized protein YmfS | Uncharacterized protein YmfS | | afdb-proteome | AF-G3XD08-F1-MODEL\_V4 | 1.0 | 8.406e-08 | 260 | 0.276 | 123 | 87 | 2 | 4 | 126 | 5 | 125 | Uncharacterized protein | Uncharacterized protein | | afdb-proteome | AF-Q8ZMU7-F1-MODEL\_V4 | 1.0 | 4.528e-07 | 202 | 0.212 | 146 | 97 | 6 | 1 | 142 | 1 | 132 | Fels-2 prophage protein | Fels-2 prophage protein | | afdb-proteome | AF-P09153-F1-MODEL\_V4 | 1.0 | 4.276e-06 | 176 | 0.256 | 148 | 77 | 8 | 2 | 143 | 79 | 199 | Prophage tail fiber assembly protein homolog TfaE | Prophage tail fiber assembly protein homolog TfaE | | afdb-proteome | AF-P77326-F1-MODEL\_V4 | 1.0 | 6.248e-05 | 171 | 0.34 | 100 | 57 | 5 | 46 | 143 | 21 | 113 | Putative protein TfaS | Putative protein TfaS | | afdb-proteome | AF-P77699-F1-MODEL\_V4 | 1.0 | 3.794e-05 | 161 | 0.208 | 144 | 84 | 5 | 3 | 142 | 67 | 184 | Protein TfaD | Protein TfaD | | afdb-proteome | AF-P76155-F1-MODEL\_V4 | 1.0 | 0.0001406 | 140 | 0.205 | 146 | 82 | 5 | 3 | 142 | 74 | 191 | Prophage tail fiber assembly protein homolog TfaQ | Prophage tail fiber assembly protein homolog TfaQ | | afdb-proteome | AF-Q8ZQ80-F1-MODEL\_V4 | 1.0 | 0.0001804 | 140 | 0.164 | 146 | 91 | 6 | 2 | 143 | 73 | 191 | Gifsy-2 prophage tail fiber assembly like-protein | Gifsy-2 prophage tail fiber assembly like-protein | | afdb-proteome | AF-P40784-F1-MODEL\_V4 | 1.0 | 0.000192 | 138 | 0.178 | 146 | 89 | 7 | 2 | 143 | 73 | 191 | Tail fiber assembly protein homolog from lambdoid prophage Fels-1 | Tail fiber assembly protein homolog from lambdoid prophage Fels-1 | | afdb-proteome | AF-P77163-F1-MODEL\_V4 | 1.0 | 0.0002315 | 136 | 0.205 | 146 | 82 | 5 | 3 | 142 | 74 | 191 | Prophage tail fiber assembly protein homolog TfaR | Prophage tail fiber assembly protein homolog TfaR | | afdb-proteome | AF-Q8ZN15-F1-MODEL\_V4 | 1.0 | 0.0006279 | 127 | 0.157 | 146 | 90 | 6 | 2 | 143 | 73 | 189 | Gifsy-1 prophage protein | Gifsy-1 prophage protein | | afdb-proteome | AF-Q8ZNJ1-F1-MODEL\_V4 | 1.0 | 0.0002971 | 122 | 0.155 | 161 | 100 | 7 | 5 | 143 | 28 | 174 | Putative tail fiber assembly protein | Putative tail fiber assembly protein | | afdb-proteome | AF-H9L459-F1-MODEL\_V4 | 1.0 | 0.001703 | 112 | 0.136 | 147 | 92 | 7 | 3 | 143 | 173 | 290 | Phage-tail assembly protein-like protein | Phage-tail assembly protein-like protein | | afdb-uniprot50 | AF-A0A2Y9TWM4-F1-MODEL\_V4 | 1.0 | 4.014e-17 | 676 | 0.46 | 141 | 76 | 0 | 3 | 143 | 2 | 142 | Tail fiber assembly protein | Tail fiber assembly protein | | afdb-uniprot50 | AF-A0A5Z5REI7-F1-MODEL\_V4 | 1.0 | 3.752e-18 | 672 | 0.566 | 143 | 61 | 1 | 1 | 143 | 1 | 142 | Tail assembly chaperone | Tail assembly chaperone | | afdb-uniprot50 | AF-A0A3P5HII9-F1-MODEL\_V4 | 1.0 | 7.93e-18 | 664 | 0.566 | 143 | 61 | 1 | 1 | 143 | 1 | 142 | Prophage tail fiber assembly protein TfaE | Prophage tail fiber assembly protein TfaE | | afdb-uniprot50 | AF-A0A1X3I4W0-F1-MODEL\_V4 | 1.0 | 1.306e-17 | 661 | 0.573 | 143 | 60 | 1 | 1 | 143 | 24 | 165 | Caudovirales tail fiber assembly protein | Caudovirales tail fiber assembly protein | | afdb-uniprot50 | AF-A0A639BZQ5-F1-MODEL\_V4 | 1.0 | 6.61e-17 | 658 | 0.552 | 143 | 63 | 1 | 1 | 143 | 1 | 142 | Tail fiber assembly protein | Tail fiber assembly protein | | afdb-uniprot50 | AF-A0A828GQS5-F1-MODEL\_V4 | 1.0 | 5.151e-17 | 653 | 0.51 | 143 | 70 | 0 | 1 | 143 | 1 | 143 | Tail fiber assembly protein | Tail fiber assembly protein | | afdb-uniprot50 | AF-A0A3A6RJK8-F1-MODEL\_V4 | 1.0 | 1.023e-16 | 651 | 0.51 | 143 | 70 | 0 | 1 | 143 | 1 | 143 | Tail fiber assembly protein | Tail fiber assembly protein | | afdb-uniprot50 | AF-A0A6I6DJ31-F1-MODEL\_V4 | 1.0 | 6.211e-17 | 645 | 0.564 | 140 | 60 | 1 | 4 | 143 | 4 | 142 | Tail fiber assembly protein | Tail fiber assembly protein | | afdb-uniprot50 | AF-A0A8A5IWD8-F1-MODEL\_V4 | 1.0 | 1.583e-16 | 637 | 0.517 | 143 | 69 | 0 | 1 | 143 | 1 | 143 | Tail fiber assembly protein | Tail fiber assembly protein | | afdb-uniprot50 | AF-A0A0Q4MYZ8-F1-MODEL\_V4 | 1.0 | 1.028e-15 | 620 | 0.442 | 140 | 76 | 1 | 4 | 143 | 2 | 139 | Uncharacterized protein | Uncharacterized protein | | afdb-uniprot50 | AF-A0A411WHE0-F1-MODEL\_V4 | 1.0 | 1.028e-15 | 618 | 0.439 | 141 | 79 | 0 | 3 | 143 | 11 | 151 | Tail fiber assembly protein | Tail fiber assembly protein | | afdb-uniprot50 | AF-A0A0H2V5L4-F1-MODEL\_V4 | 1.0 | 3.579e-15 | 610 | 0.423 | 144 | 82 | 1 | 1 | 143 | 11 | 154 | Uncharacterized protein | Uncharacterized protein | | afdb-uniprot50 | AF-A0A7U2JL75-F1-MODEL\_V4 | 1.0 | 1.404e-15 | 607 | 0.454 | 143 | 78 | 0 | 1 | 143 | 1 | 143 | Tail fiber assembly protein | Tail fiber assembly protein | | afdb-uniprot50 | AF-A0A433ZQC5-F1-MODEL\_V4 | 1.0 | 9.075e-16 | 604 | 0.489 | 141 | 69 | 2 | 3 | 143 | 2 | 139 | Uncharacterized protein | Uncharacterized protein | | afdb-uniprot50 | AF-A0A0Q4MZH0-F1-MODEL\_V4 | 1.0 | 1.033e-14 | 584 | 0.417 | 139 | 79 | 1 | 4 | 142 | 2 | 138 | Uncharacterized protein | Uncharacterized protein | | afdb-uniprot50 | AF-A0A827CVF2-F1-MODEL\_V4 | 1.0 | 2.313e-15 | 569 | 0.556 | 142 | 59 | 3 | 4 | 143 | 3 | 142 | Tail fiber assembly protein | Tail fiber assembly protein | | afdb-uniprot50 | AF-A0A826JA78-F1-MODEL\_V4 | 1.0 | 9.12e-15 | 568 | 0.482 | 141 | 72 | 1 | 4 | 143 | 3 | 143 | Tail assembly chaperone | Tail assembly chaperone | | afdb-uniprot50 | AF-A0A6N8NTV2-F1-MODEL\_V4 | 1.0 | 6.242e-16 | 564 | 0.526 | 133 | 63 | 0 | 1 | 133 | 1 | 133 | Tail fiber assembly protein | Tail fiber assembly protein | | afdb-uniprot50 | AF-A0A6N4ED12-F1-MODEL\_V4 | 1.0 | 5.894e-15 | 564 | 0.363 | 143 | 89 | 1 | 1 | 143 | 11 | 151 | Phage tail protein | Phage tail protein | | afdb-uniprot50 | AF-A0A377ALH1-F1-MODEL\_V4 | 1.0 | 6.677e-15 | 561 | 0.592 | 125 | 51 | 0 | 19 | 143 | 2 | 126 | CPS-53 (KpLE1) prophage protein | CPS-53 (KpLE1) prophage protein | | afdb-uniprot50 | AF-A0A6G6JM87-F1-MODEL\_V4 | 1.0 | 1.1e-14 | 560 | 0.442 | 140 | 75 | 2 | 4 | 143 | 2 | 138 | Tail fiber assembly protein | Tail fiber assembly protein | | afdb-uniprot50 | AF-A0A6D0JBZ2-F1-MODEL\_V4 | 1.0 | 7.107e-15 | 558 | 0.531 | 141 | 59 | 2 | 4 | 143 | 5 | 139 | Tail fiber assembly protein | Tail fiber assembly protein | | afdb-uniprot50 | AF-A0A2U3F206-F1-MODEL\_V4 | 1.0 | 2.324e-14 | 556 | 0.394 | 142 | 84 | 1 | 1 | 142 | 1 | 140 | Tail fiber assembly protein | Tail fiber assembly protein | | afdb-uniprot50 | AF-A0A6I2IQI1-F1-MODEL\_V4 | 1.0 | 2.803e-14 | 552 | 0.364 | 140 | 89 | 0 | 4 | 143 | 3 | 142 | Tail fiber assembly protein | Tail fiber assembly protein | | afdb-uniprot50 | AF-A0A6B2FQM4-F1-MODEL\_V4 | 1.0 | 1.591e-15 | 550 | 0.398 | 143 | 84 | 1 | 1 | 143 | 7 | 147 | Tail fiber assembly protein | Tail fiber assembly protein | | afdb-uniprot50 | AF-A0A732IA80-F1-MODEL\_V4 | 1.0 | 4.913e-14 | 549 | 0.446 | 141 | 76 | 2 | 4 | 143 | 5 | 144 | Tail fiber assembly protein | Tail fiber assembly protein | | afdb-uniprot50 | AF-A0A411WM32-F1-MODEL\_V4 | 1.0 | 1.033e-14 | 547 | 0.321 | 143 | 95 | 1 | 1 | 143 | 14 | 154 | Tail fiber assembly protein | Tail fiber assembly protein | | afdb-uniprot50 | AF-A0A4R4JDX7-F1-MODEL\_V4 | 1.0 | 1.502e-14 | 546 | 0.394 | 142 | 84 | 1 | 1 | 142 | 3 | 142 | Phage tail protein | Phage tail protein | | afdb-uniprot50 | AF-A0A7Z3MDV7-F1-MODEL\_V4 | 1.0 | 1.502e-14 | 545 | 0.365 | 145 | 88 | 2 | 1 | 143 | 1 | 143 | Phage tail assembly chaperone gp38 | Phage tail assembly chaperone gp38 | | afdb-uniprot50 | AF-A0A064DHX9-F1-MODEL\_V4 | 1.0 | 2.474e-14 | 542 | 0.391 | 143 | 83 | 3 | 2 | 143 | 22 | 161 | Uncharacterized protein | Uncharacterized protein | | afdb-uniprot50 | AF-A0A411WII7-F1-MODEL\_V4 | 1.0 | 7.143e-14 | 534 | 0.375 | 144 | 87 | 2 | 1 | 143 | 1 | 142 | Tail fiber assembly protein | Tail fiber assembly protein | | afdb-uniprot50 | AF-A0A7Y7YPN7-F1-MODEL\_V4 | 1.0 | 4.913e-14 | 532 | 0.378 | 140 | 83 | 2 | 4 | 143 | 2 | 137 | Tail fiber assembly protein | Tail fiber assembly protein | | afdb-uniprot50 | AF-A0A4V2X8Y2-F1-MODEL\_V4 | 1.0 | 6.305e-14 | 532 | 0.382 | 141 | 84 | 2 | 4 | 143 | 2 | 140 | Phage tail protein | Phage tail protein | | afdb-uniprot50 | AF-A0A077QBZ2-F1-MODEL\_V4 | 1.0 | 7.143e-14 | 530 | 0.378 | 140 | 84 | 2 | 4 | 143 | 2 | 138 | Uncharacterized protein | Uncharacterized protein | | afdb-uniprot50 | AF-A0A077NV63-F1-MODEL\_V4 | 1.0 | 9.756e-14 | 524 | 0.371 | 140 | 85 | 2 | 4 | 143 | 2 | 138 | Uncharacterized protein | Uncharacterized protein | | afdb-uniprot50 | AF-A0A3D1C8C5-F1-MODEL\_V4 | 1.0 | 1.71e-13 | 522 | 0.386 | 145 | 85 | 3 | 1 | 143 | 1 | 143 | Tail fiber assembly protein | Tail fiber assembly protein | | afdb-uniprot50 | AF-A0A7L6U240-F1-MODEL\_V4 | 1.0 | 3.175e-14 | 521 | 0.425 | 141 | 78 | 2 | 4 | 143 | 2 | 140 | Tail assembly chaperone | Tail assembly chaperone | | afdb-uniprot50 | AF-A0A0H0RZ18-F1-MODEL\_V4 | 1.0 | 3.597e-14 | 519 | 0.397 | 141 | 84 | 1 | 4 | 143 | 3 | 143 | Phage tail protein | Phage tail protein | | afdb-uniprot50 | AF-A0A376ST16-F1-MODEL\_V4 | 1.0 | 2.062e-13 | 519 | 0.507 | 126 | 62 | 0 | 1 | 126 | 1 | 126 | Tail fiber assembly protein | Tail fiber assembly protein | | afdb-uniprot50 | AF-A0A1I3WQ77-F1-MODEL\_V4 | 1.0 | 3.175e-14 | 518 | 0.335 | 143 | 93 | 1 | 1 | 143 | 3 | 143 | Virus tail fibre assembly protein, lambda gpK | Virus tail fibre assembly protein, lambda gpK | | afdb-uniprot50 | AF-A0A1V2BDV7-F1-MODEL\_V4 | 1.0 | 2.336e-13 | 514 | 0.378 | 140 | 85 | 1 | 4 | 143 | 2 | 139 | Uncharacterized protein | Uncharacterized protein | | afdb-uniprot50 | AF-A0A2X1RV26-F1-MODEL\_V4 | 1.0 | 6.745e-13 | 511 | 0.342 | 143 | 92 | 1 | 1 | 143 | 1 | 141 | Caudovirales tail fibre assembly protein | Caudovirales tail fibre assembly protein | | afdb-uniprot50 | AF-A0A7X5FY77-F1-MODEL\_V4 | 1.0 | 1.333e-13 | 510 | 0.393 | 145 | 84 | 3 | 1 | 143 | 1 | 143 | Tail fiber assembly protein | Tail fiber assembly protein | | afdb-uniprot50 | AF-A0A0P0QDS8-F1-MODEL\_V4 | 1.0 | 2.998e-13 | 507 | 0.335 | 140 | 91 | 1 | 4 | 143 | 3 | 140 | Tail fiber assembly protein | Tail fiber assembly protein | | afdb-uniprot50 | AF-A0A2X4V2H3-F1-MODEL\_V4 | 1.0 | 9.756e-14 | 505 | 0.356 | 143 | 89 | 2 | 1 | 143 | 1 | 140 | Caudovirales tail fibre assembly protein | Caudovirales tail fibre assembly protein | | afdb-uniprot50 | AF-A0A351NIR9-F1-MODEL\_V4 | 1.0 | 3.396e-13 | 504 | 0.35 | 140 | 87 | 3 | 4 | 143 | 3 | 138 | Phage tail protein | Phage tail protein | | afdb-uniprot50 | AF-A0A377AI92-F1-MODEL\_V4 | 1.0 | 7.143e-14 | 504 | 0.57 | 128 | 54 | 1 | 16 | 143 | 6 | 132 | CPS-53 (KpLE1) prophage protein | CPS-53 (KpLE1) prophage protein | | afdb-uniprot50 | AF-A0A254PQ93-F1-MODEL\_V4 | 1.0 | 1.607e-13 | 503 | 0.349 | 143 | 89 | 3 | 1 | 142 | 2 | 141 | Phage tail protein | Phage tail protein | | afdb-uniprot50 | AF-A0A3D1C5Q5-F1-MODEL\_V4 | 1.0 | 3.847e-13 | 499 | 0.333 | 144 | 93 | 2 | 1 | 143 | 1 | 142 | Phage tail protein | Phage tail protein | | afdb-uniprot50 | AF-A0A2S8PTN0-F1-MODEL\_V4 | 1.0 | 5.954e-13 | 497 | 0.335 | 140 | 90 | 2 | 4 | 142 | 2 | 139 | Phage tail protein | Phage tail protein | | afdb-uniprot50 | AF-A0A482PI23-F1-MODEL\_V4 | 1.0 | 5.566e-14 | 497 | 0.423 | 144 | 75 | 2 | 1 | 143 | 1 | 137 | Tail fiber assembly protein | Tail fiber assembly protein | | afdb-uniprot50 | AF-A0A794RD75-F1-MODEL\_V4 | 1.0 | 2.195e-13 | 496 | 0.576 | 125 | 51 | 2 | 21 | 143 | 2 | 126 | Tail fiber assembly protein | Tail fiber assembly protein | | afdb-uniprot50 | AF-A0A0H3FAT1-F1-MODEL\_V4 | 1.0 | 2.998e-13 | 491 | 0.354 | 141 | 88 | 2 | 4 | 143 | 3 | 141 | Tail assembly chaperone gp38 | Tail assembly chaperone gp38 | | afdb-uniprot50 | AF-A0A370S9Q0-F1-MODEL\_V4 | 1.0 | 6.745e-13 | 490 | 0.288 | 142 | 99 | 1 | 2 | 143 | 20 | 159 | Virus tail fiber assembly protein lambda gpK | Virus tail fiber assembly protein lambda gpK | | afdb-uniprot50 | AF-A0A1C4G0U3-F1-MODEL\_V4 | 1.0 | 7.564e-15 | 487 | 0.45 | 140 | 74 | 1 | 4 | 143 | 2 | 138 | Virus tail fibre assembly protein, lambda gpK | Virus tail fibre assembly protein, lambda gpK | | afdb-uniprot50 | AF-A0A857DVJ6-F1-MODEL\_V4 | 1.0 | 7.641e-13 | 487 | 0.392 | 140 | 82 | 2 | 4 | 143 | 2 | 138 | Tail fiber assembly protein | Tail fiber assembly protein | | afdb-uniprot50 | AF-A0A7U5UFF6-F1-MODEL\_V4 | 1.0 | 1.111e-12 | 483 | 0.349 | 143 | 90 | 2 | 2 | 143 | 17 | 157 | Tail assembly chaperone | Tail assembly chaperone | | afdb-uniprot50 | AF-A4W7Q4-F1-MODEL\_V4 | 1.0 | 4.963e-12 | 481 | 0.335 | 140 | 91 | 1 | 4 | 143 | 3 | 140 | Phage tail assembly chaperone gp38 | Phage tail assembly chaperone gp38 | | afdb-uniprot50 | AF-A0A831I510-F1-MODEL\_V4 | 1.0 | 1.044e-12 | 478 | 0.433 | 143 | 73 | 1 | 1 | 143 | 1 | 135 | Tail fiber assembly protein | Tail fiber assembly protein | | afdb-uniprot50 | AF-W0HMV2-F1-MODEL\_V4 | 1.0 | 3.396e-13 | 478 | 0.321 | 143 | 95 | 1 | 1 | 143 | 1 | 141 | Uncharacterized protein | Uncharacterized protein | | afdb-uniprot50 | AF-A0A4D7IX74-F1-MODEL\_V4 | 1.0 | 1.044e-12 | 478 | 0.412 | 143 | 80 | 3 | 2 | 142 | 57 | 197 | Phage tail protein | Phage tail protein | | afdb-uniprot50 | AF-A0A087KXL6-F1-MODEL\_V4 | 1.0 | 6.337e-13 | 477 | 0.34 | 144 | 89 | 4 | 1 | 143 | 1 | 139 | Uncharacterized protein | Uncharacterized protein | | afdb-uniprot50 | AF-A0A7U5TH15-F1-MODEL\_V4 | 1.0 | 9.213e-13 | 476 | 0.358 | 145 | 88 | 4 | 1 | 143 | 1 | 142 | Uncharacterized protein | Uncharacterized protein | | afdb-uniprot50 | AF-A0A2A2HLN3-F1-MODEL\_V4 | 1.0 | 1.719e-12 | 476 | 0.34 | 141 | 90 | 2 | 4 | 143 | 2 | 140 | Phage tail protein | Phage tail protein | | afdb-uniprot50 | AF-A0A6L9JIU7-F1-MODEL\_V4 | 1.0 | 1.719e-12 | 474 | 0.326 | 141 | 92 | 2 | 4 | 143 | 2 | 140 | Tail fiber assembly protein | Tail fiber assembly protein | | afdb-uniprot50 | AF-A0A4R6PIE0-F1-MODEL\_V4 | 1.0 | 1.339e-12 | 474 | 0.364 | 140 | 87 | 1 | 4 | 143 | 3 | 140 | Virus tail fiber assembly protein lambda gpK | Virus tail fiber assembly protein lambda gpK | | afdb-uniprot50 | AF-A0A5U1N744-F1-MODEL\_V4 | 1.0 | 6.337e-13 | 474 | 0.453 | 141 | 74 | 3 | 4 | 142 | 3 | 142 | Tail fiber assembly protein | Tail fiber assembly protein | | afdb-uniprot50 | AF-A0A0A8VIE2-F1-MODEL\_V4 | 1.0 | 8.132e-13 | 473 | 0.335 | 143 | 92 | 2 | 1 | 143 | 1 | 140 | Tail fiber assembly protein p37 | Tail fiber assembly protein p37 | | afdb-uniprot50 | AF-A0A2V2BI76-F1-MODEL\_V4 | 1.0 | 1.24e-15 | 472 | 0.461 | 143 | 77 | 0 | 1 | 143 | 1 | 143 | Virus tail fiber assembly protein lambda gpK | Virus tail fiber assembly protein lambda gpK | | afdb-uniprot50 | AF-U3U6G6-F1-MODEL\_V4 | 1.0 | 1.719e-12 | 472 | 0.355 | 138 | 86 | 2 | 4 | 140 | 2 | 137 | Uncharacterized protein | Uncharacterized protein | | afdb-uniprot50 | AF-A0A826NIC5-F1-MODEL\_V4 | 1.0 | 2.195e-13 | 471 | 0.374 | 139 | 85 | 1 | 2 | 140 | 3 | 139 | Tail fiber assembly protein | Tail fiber assembly protein | | afdb-uniprot50 | AF-A0A3S5G566-F1-MODEL\_V4 | 1.0 | 2.66e-12 | 469 | 0.345 | 142 | 89 | 3 | 1 | 140 | 1 | 140 | Tail fiber assembly protein | Tail fiber assembly protein | | afdb-uniprot50 | AF-A0A811D365-F1-MODEL\_V4 | 1.0 | 3.013e-12 | 465 | 0.35 | 140 | 86 | 2 | 4 | 143 | 2 | 136 | Uncharacterized protein | Uncharacterized protein | | afdb-uniprot50 | AF-A0A765X9T6-F1-MODEL\_V4 | 1.0 | 5.594e-13 | 465 | 0.46 | 141 | 69 | 1 | 3 | 143 | 2 | 135 | Tail fiber assembly protein | Tail fiber assembly protein | | afdb-uniprot50 | AF-A0A7U5G183-F1-MODEL\_V4 | 1.0 | 7.215e-12 | 461 | 0.333 | 144 | 90 | 4 | 1 | 143 | 1 | 139 | Uncharacterized protein | Uncharacterized protein | | afdb-uniprot50 | AF-H2IRJ1-F1-MODEL\_V4 | 1.0 | 3.867e-12 | 461 | 0.358 | 145 | 89 | 3 | 1 | 143 | 1 | 143 | Caudovirales tail fiber assembly protein | Caudovirales tail fiber assembly protein | | afdb-uniprot50 | AF-A0A5B8I5R9-F1-MODEL\_V4 | 1.0 | 5.594e-13 | 459 | 0.356 | 146 | 88 | 4 | 1 | 143 | 1 | 143 | Tail assembly chaperone | Tail assembly chaperone | | afdb-uniprot50 | AF-A0A6B0TPS5-F1-MODEL\_V4 | 1.0 | 6.369e-12 | 457 | 0.335 | 143 | 93 | 1 | 1 | 143 | 9 | 149 | Tail fiber assembly protein | Tail fiber assembly protein | | afdb-uniprot50 | AF-A0A3N6SH38-F1-MODEL\_V4 | 1.0 | 7.679e-12 | 456 | 0.324 | 145 | 92 | 5 | 1 | 143 | 1 | 141 | Phage tail protein | Phage tail protein | | afdb-uniprot50 | AF-A0A6I6IFF1-F1-MODEL\_V4 | 1.0 | 7.179e-13 | 455 | 0.375 | 149 | 83 | 3 | 3 | 143 | 2 | 148 | Tail fiber assembly protein | Tail fiber assembly protein | | afdb-uniprot50 | AF-A0A3Q8DJE8-F1-MODEL\_V4 | 1.0 | 9.855e-12 | 452 | 0.392 | 140 | 79 | 3 | 4 | 143 | 4 | 137 | Phage tail protein | Phage tail protein | | afdb-uniprot50 | AF-A0A2I8Q7J9-F1-MODEL\_V4 | 1.0 | 1.829e-12 | 452 | 0.349 | 143 | 89 | 3 | 1 | 143 | 11 | 149 | Phage tail protein | Phage tail protein | | afdb-uniprot50 | AF-A0A7I6TII6-F1-MODEL\_V4 | 1.0 | 3.413e-12 | 451 | 0.298 | 144 | 98 | 2 | 1 | 143 | 1 | 142 | Tail assembly chaperone | Tail assembly chaperone | | afdb-uniprot50 | AF-R8ANC1-F1-MODEL\_V4 | 1.0 | 2.817e-13 | 451 | 0.384 | 143 | 86 | 2 | 1 | 143 | 2 | 142 | Putative tail fiber assembly protein | Putative tail fiber assembly protein | | afdb-uniprot50 | AF-A0A857EZE8-F1-MODEL\_V4 | 1.0 | 1.615e-12 | 449 | 0.357 | 140 | 87 | 2 | 4 | 143 | 3 | 139 | Tail fiber assembly protein | Tail fiber assembly protein | | afdb-uniprot50 | AF-A0A3G2I9A2-F1-MODEL\_V4 | 1.0 | 8.173e-12 | 448 | 0.342 | 140 | 87 | 2 | 4 | 143 | 2 | 136 | Tail fiber assembly protein | Tail fiber assembly protein | | afdb-uniprot50 | AF-Q8ZNJ2-F1-MODEL\_V4 | 1.0 | 2.348e-12 | 448 | 0.429 | 142 | 79 | 2 | 1 | 141 | 1 | 141 | Putative cytoplasmic protein | Putative cytoplasmic protein | | afdb-uniprot50 | AF-A0A4P7KSD4-F1-MODEL\_V4 | 1.0 | 2.499e-12 | 442 | 0.312 | 141 | 94 | 2 | 4 | 143 | 9 | 147 | Caudovirales tail fiber assembly protein, lambda gpK | Caudovirales tail fiber assembly protein, lambda gpK | | afdb-uniprot50 | AF-A0A7X1F2W4-F1-MODEL\_V4 | 1.0 | 2.083e-11 | 439 | 0.328 | 140 | 91 | 2 | 4 | 143 | 2 | 138 | Tail fiber assembly protein | Tail fiber assembly protein | | afdb-uniprot50 | AF-A0A859TBI1-F1-MODEL\_V4 | 1.0 | 6.779e-12 | 435 | 0.333 | 141 | 91 | 2 | 3 | 143 | 2 | 139 | Tail fiber assembly protein | Tail fiber assembly protein | | afdb-uniprot50 | AF-A0A0A3ZBR4-F1-MODEL\_V4 | 1.0 | 8.699e-12 | 433 | 0.342 | 143 | 85 | 3 | 4 | 143 | 3 | 139 | Uncharacterized protein | Uncharacterized protein | | afdb-uniprot50 | AF-A0A564UCV1-F1-MODEL\_V4 | 1.0 | 6.014e-11 | 431 | 0.546 | 108 | 48 | 1 | 37 | 143 | 3 | 110 | Caudovirales tail fibre assembly protein | Caudovirales tail fibre assembly protein | | afdb-uniprot50 | AF-A0A7D7ACY9-F1-MODEL\_V4 | 1.0 | 3.223e-11 | 429 | 0.347 | 141 | 88 | 3 | 4 | 143 | 3 | 140 | Tail fiber assembly protein | Tail fiber assembly protein | | afdb-uniprot50 | AF-A0A212I9D1-F1-MODEL\_V4 | 1.0 | 1.525e-11 | 428 | 0.338 | 142 | 89 | 3 | 4 | 143 | 3 | 141 | Uncharacterized protein | Uncharacterized protein | | afdb-uniprot50 | AF-A0A7I6VNU6-F1-MODEL\_V4 | 1.0 | 4.663e-12 | 423 | 0.335 | 143 | 91 | 3 | 1 | 143 | 1 | 139 | Tail assembly chaperone | Tail assembly chaperone | | afdb-uniprot50 | AF-A0A7W5JQR9-F1-MODEL\_V4 | 1.0 | 2.217e-11 | 420 | 0.278 | 140 | 99 | 1 | 4 | 143 | 3 | 140 | Uncharacterized protein | Uncharacterized protein | | afdb-uniprot50 | AF-A0A344S5G9-F1-MODEL\_V4 | 1.0 | 3.431e-11 | 420 | 0.345 | 139 | 87 | 3 | 3 | 140 | 2 | 137 | Phage tail protein | Phage tail protein | | afdb-uniprot50 | AF-A0A6M5D0K4-F1-MODEL\_V4 | 1.0 | 2.36e-11 | 419 | 0.304 | 148 | 91 | 3 | 4 | 143 | 3 | 146 | Phage tail protein | Phage tail protein | | afdb-uniprot50 | AF-A0A826S2B1-F1-MODEL\_V4 | 1.0 | 1.957e-11 | 417 | 0.34 | 141 | 84 | 3 | 4 | 143 | 22 | 154 | Tail fiber assembly protein | Tail fiber assembly protein | | afdb-uniprot50 | AF-A0A7A3AEP4-F1-MODEL\_V4 | 1.0 | 6.369e-12 | 416 | 0.404 | 141 | 77 | 2 | 4 | 143 | 5 | 139 | Tail fiber assembly protein | Tail fiber assembly protein | | afdb-uniprot50 | AF-Q2NQF0-F1-MODEL\_V4 | 1.0 | 6.014e-11 | 415 | 0.378 | 132 | 79 | 2 | 4 | 135 | 12 | 140 | Caudovirales tail fibre assembly protein | Caudovirales tail fibre assembly protein | | afdb-uniprot50 | AF-A0A0J1N7J0-F1-MODEL\_V4 | 1.0 | 8.743e-11 | 414 | 0.29 | 141 | 98 | 1 | 3 | 143 | 2 | 140 | Uncharacterized protein | Uncharacterized protein | | afdb-uniprot50 | AF-A0A810TM02-F1-MODEL\_V4 | 1.0 | 4.403e-11 | 414 | 0.305 | 144 | 94 | 3 | 1 | 143 | 1 | 139 | Phage tail fiber assembly protein | Phage tail fiber assembly protein | | afdb-uniprot50 | AF-A0A1R0Q9D4-F1-MODEL\_V4 | 1.0 | 4.136e-11 | 412 | 0.34 | 138 | 84 | 3 | 4 | 141 | 2 | 132 | Phage tail protein | Phage tail protein | | afdb-uniprot50 | AF-A0A5D4YFI5-F1-MODEL\_V4 | 1.0 | 5.309e-11 | 410 | 0.307 | 140 | 95 | 1 | 4 | 143 | 3 | 140 | Tail fiber assembly protein | Tail fiber assembly protein | | afdb-uniprot50 | AF-A0A377F879-F1-MODEL\_V4 | 1.0 | 1.353e-10 | 409 | 0.491 | 118 | 60 | 0 | 26 | 143 | 23 | 140 | Putative phage tail assembly chaperone | Putative phage tail assembly chaperone | | afdb-uniprot50 | AF-D2TYN1-F1-MODEL\_V4 | 1.0 | 4.136e-11 | 406 | 0.297 | 141 | 95 | 3 | 4 | 143 | 2 | 139 | Caudovirales tail fiber assembly protein, lambda gpK | Caudovirales tail fiber assembly protein, lambda gpK | | afdb-uniprot50 | AF-A0A2D0LEZ5-F1-MODEL\_V4 | 1.0 | 2.983e-14 | 405 | 0.342 | 146 | 91 | 2 | 1 | 143 | 2 | 145 | Phage tail fiber assembly | Phage tail fiber assembly | | afdb-uniprot50 | AF-A0A3N0FQC3-F1-MODEL\_V4 | 1.0 | 1.44e-10 | 401 | 0.335 | 140 | 85 | 2 | 4 | 143 | 5 | 136 | Phage tail protein | Phage tail protein | | afdb-uniprot50 | AF-A0A3M4RIN5-F1-MODEL\_V4 | 1.0 | 2.083e-11 | 400 | 0.286 | 143 | 98 | 3 | 1 | 142 | 1 | 140 | Phage tail fiber assembly | Phage tail fiber assembly | | afdb-uniprot50 | AF-A0A830Q7F2-F1-MODEL\_V4 | 1.0 | 2.512e-11 | 400 | 0.351 | 145 | 89 | 4 | 1 | 143 | 1 | 142 | Putative phage tail fiber assembly protein | Putative phage tail fiber assembly protein | | afdb-uniprot50 | AF-A0A1H8UDQ4-F1-MODEL\_V4 | 1.0 | 2.093e-10 | 398 | 0.298 | 144 | 91 | 3 | 4 | 143 | 3 | 140 | Virus tail fibre assembly protein, lambda gpK | Virus tail fibre assembly protein, lambda gpK | | afdb-uniprot50 | AF-A0A1Q6B0D0-F1-MODEL\_V4 | 1.0 | 7.251e-11 | 398 | 0.359 | 142 | 87 | 3 | 3 | 143 | 5 | 143 | Phage tail protein | Phage tail protein | | afdb-uniprot50 | AF-A0A7L6YDG5-F1-MODEL\_V4 | 1.0 | 6.401e-11 | 397 | 0.292 | 147 | 93 | 4 | 1 | 143 | 4 | 143 | Tail fiber assembly protein | Tail fiber assembly protein | | afdb-uniprot50 | AF-A0A7L6CIZ9-F1-MODEL\_V4 | 1.0 | 1.054e-10 | 395 | 0.342 | 143 | 88 | 5 | 4 | 143 | 4 | 143 | Tail fiber assembly protein | Tail fiber assembly protein | | afdb-uniprot50 | AF-A0A4V3F6N1-F1-MODEL\_V4 | 1.0 | 1.533e-10 | 394 | 0.32 | 125 | 82 | 2 | 4 | 127 | 10 | 132 | Virus tail fiber assembly protein lambda gpK | Virus tail fiber assembly protein lambda gpK | | afdb-uniprot50 | AF-A0A5F1T5X9-F1-MODEL\_V4 | 1.0 | 7.288e-10 | 394 | 0.33 | 142 | 89 | 3 | 2 | 143 | 23 | 158 | Tail fiber assembly protein | Tail fiber assembly protein | | afdb-uniprot50 | AF-A0A2D0KV06-F1-MODEL\_V4 | 1.0 | 2.831e-12 | 393 | 0.356 | 143 | 85 | 3 | 4 | 143 | 423 | 561 | Tail assembly chaperone | Tail assembly chaperone | | afdb-uniprot50 | AF-A0A6N8NR58-F1-MODEL\_V4 | 1.0 | 3.256e-09 | 389 | 0.623 | 93 | 34 | 1 | 51 | 143 | 1 | 92 | Tail fiber assembly protein | Tail fiber assembly protein | | afdb-uniprot50 | AF-T0QGQ9-F1-MODEL\_V4 | 1.0 | 3.044e-10 | 386 | 0.366 | 120 | 74 | 1 | 1 | 120 | 3 | 120 | Putative tail fiber protein | Putative tail fiber protein | | afdb-uniprot50 | AF-A0A1Q5V7C8-F1-MODEL\_V4 | 1.0 | 4.686e-11 | 385 | 0.323 | 142 | 90 | 4 | 4 | 143 | 2 | 139 | Uncharacterized protein | Uncharacterized protein | | afdb-uniprot50 | AF-A0A4T4IVD4-F1-MODEL\_V4 | 1.0 | 4.136e-11 | 385 | 0.291 | 144 | 97 | 3 | 1 | 143 | 1 | 140 | Tail assembly chaperone | Tail assembly chaperone | | afdb-uniprot50 | AF-A0A209ALQ9-F1-MODEL\_V4 | 1.0 | 2.073e-12 | 385 | 0.388 | 144 | 81 | 4 | 4 | 143 | 13 | 153 | Tail fiber assembly protein | Tail fiber assembly protein | | afdb-uniprot50 | AF-A0A744A7C6-F1-MODEL\_V4 | 1.0 | 2.384e-09 | 383 | 0.541 | 96 | 43 | 1 | 47 | 142 | 1 | 95 | Tail fiber assembly protein | Tail fiber assembly protein | | afdb-uniprot50 | AF-A0A654DRC8-F1-MODEL\_V4 | 1.0 | 2.998e-13 | 378 | 0.335 | 140 | 88 | 3 | 4 | 143 | 2 | 136 | Uncharacterized protein | Uncharacterized protein | | afdb-uniprot50 | AF-A0A3S6EWT1-F1-MODEL\_V4 | 1.0 | 3.906e-10 | 377 | 0.302 | 142 | 94 | 4 | 4 | 143 | 2 | 140 | Uncharacterized protein | Uncharacterized protein | | afdb-uniprot50 | AF-A0A1M5U130-F1-MODEL\_V4 | 1.0 | 5.594e-13 | 377 | 0.371 | 140 | 85 | 2 | 4 | 143 | 3 | 139 | Virus tail fibre assembly protein, lambda gpK | Virus tail fibre assembly protein, lambda gpK | | afdb-uniprot50 | AF-A0A085JCX8-F1-MODEL\_V4 | 1.0 | 6.847e-10 | 376 | 0.292 | 140 | 96 | 2 | 4 | 143 | 2 | 138 | Tail fiber assembly protein | Tail fiber assembly protein | | afdb-uniprot50 | AF-A0A068Z8F6-F1-MODEL\_V4 | 1.0 | 2.817e-13 | 374 | 0.349 | 143 | 88 | 2 | 4 | 143 | 3 | 143 | Tail fiber assembly protein | Tail fiber assembly protein | | afdb-uniprot50 | AF-A0A5U3DTV8-F1-MODEL\_V4 | 1.0 | 1.122e-10 | 373 | 0.321 | 140 | 90 | 2 | 4 | 143 | 3 | 137 | Tail fiber assembly protein | Tail fiber assembly protein | | afdb-uniprot50 | AF-A0A495AL45-F1-MODEL\_V4 | 1.0 | 1.194e-10 | 372 | 0.316 | 142 | 91 | 3 | 4 | 143 | 2 | 139 | Tail fiber assembly protein | Tail fiber assembly protein | | afdb-uniprot50 | AF-A0A7U4I2C5-F1-MODEL\_V4 | 1.0 | 8.743e-11 | 372 | 0.3 | 140 | 96 | 1 | 4 | 143 | 3 | 140 | Uncharacterized protein | Uncharacterized protein | | afdb-uniprot50 | AF-A0A806YUH0-F1-MODEL\_V4 | 1.0 | 2.073e-12 | 372 | 0.349 | 143 | 88 | 3 | 4 | 143 | 3 | 143 | Uncharacterized protein | Uncharacterized protein | | afdb-uniprot50 | AF-A0A5A9D4L2-F1-MODEL\_V4 | 1.0 | 2.687e-10 | 372 | 0.351 | 145 | 83 | 5 | 1 | 140 | 41 | 179 | Tail fiber assembly protein | Tail fiber assembly protein | | afdb-uniprot50 | AF-A0A5B0K5E4-F1-MODEL\_V4 | 1.0 | 2.348e-12 | 370 | 0.286 | 143 | 97 | 3 | 4 | 143 | 9 | 149 | Tail fiber assembly protein | Tail fiber assembly protein | | afdb-uniprot50 | AF-A0A3D3HLN8-F1-MODEL\_V4 | 1.0 | 1.977e-09 | 368 | 0.441 | 111 | 59 | 2 | 33 | 143 | 5 | 112 | Phage tail protein | Phage tail protein | | afdb-uniprot50 | AF-A0A5V3MS23-F1-MODEL\_V4 | 1.0 | 1.36e-09 | 368 | 0.264 | 140 | 101 | 1 | 4 | 143 | 3 | 140 | Tail fiber assembly protein | Tail fiber assembly protein | | afdb-uniprot50 | AF-A0A8B3UIH1-F1-MODEL\_V4 | 1.0 | 2.524e-10 | 368 | 0.316 | 142 | 86 | 4 | 4 | 140 | 3 | 138 | Tail assembly chaperone gp38 | Tail assembly chaperone gp38 | | afdb-uniprot50 | AF-A0A7L6DU13-F1-MODEL\_V4 | 1.0 | 6.044e-10 | 368 | 0.321 | 146 | 93 | 5 | 1 | 143 | 1 | 143 | Tail fiber assembly protein | Tail fiber assembly protein | | afdb-uniprot50 | AF-A0A376U5W9-F1-MODEL\_V4 | 1.0 | 6.498e-08 | 367 | 0.612 | 80 | 31 | 0 | 64 | 143 | 2 | 81 | Putative phage tail assembly chaperone | Putative phage tail assembly chaperone | | afdb-uniprot50 | AF-A0A701UP36-F1-MODEL\_V4 | 1.0 | 1.54e-09 | 366 | 0.312 | 144 | 92 | 5 | 4 | 143 | 3 | 143 | Tail assembly chaperone | Tail assembly chaperone | | afdb-uniprot50 | AF-A0A0P8I676-F1-MODEL\_V4 | 1.0 | 1.36e-09 | 365 | 0.364 | 118 | 72 | 2 | 27 | 143 | 1 | 116 | Phage tail protein | Phage tail protein | | afdb-uniprot50 | AF-A0A2N5DU56-F1-MODEL\_V4 | 1.0 | 3.413e-12 | 362 | 0.354 | 144 | 90 | 2 | 1 | 143 | 9 | 150 | Phage tail protein | Phage tail protein | | afdb-uniprot50 | AF-A0A069J7E8-F1-MODEL\_V4 | 1.0 | 1.54e-09 | 361 | 0.51 | 98 | 48 | 0 | 4 | 101 | 3 | 100 | Uncharacterized protein | Uncharacterized protein | | afdb-uniprot50 | AF-A0A0T9UK09-F1-MODEL\_V4 | 1.0 | 2.831e-12 | 361 | 0.378 | 148 | 79 | 4 | 3 | 143 | 2 | 143 | Tail assembly chaperone gp38 | Tail assembly chaperone gp38 | | afdb-uniprot50 | AF-A0A501S8D9-F1-MODEL\_V4 | 1.0 | 8.743e-11 | 360 | 0.3 | 143 | 91 | 3 | 4 | 143 | 3 | 139 | Tail fiber assembly protein | Tail fiber assembly protein | | afdb-uniprot50 | AF-A0A376WPX8-F1-MODEL\_V4 | 1.0 | 1.006e-07 | 359 | 0.637 | 80 | 29 | 0 | 64 | 143 | 7 | 86 | CPS-53 (KpLE1) prophage protein | CPS-53 (KpLE1) prophage protein | | afdb-uniprot50 | AF-A0A0A2V4M0-F1-MODEL\_V4 | 1.0 | 2.36e-11 | 359 | 0.285 | 147 | 96 | 4 | 1 | 143 | 1 | 142 | Uncharacterized protein yfdK | Uncharacterized protein yfdK | | afdb-uniprot50 | AF-A0A849JYU2-F1-MODEL\_V4 | 1.0 | 1.271e-10 | 358 | 0.307 | 143 | 97 | 1 | 1 | 143 | 2 | 142 | Tail fiber assembly protein | Tail fiber assembly protein | | afdb-uniprot50 | AF-A0A2V1HEP7-F1-MODEL\_V4 | 1.0 | 1.271e-10 | 354 | 0.316 | 142 | 91 | 3 | 3 | 143 | 5 | 141 | Phage tail protein | Phage tail protein | | afdb-uniprot50 | AF-A0A3W4K3C3-F1-MODEL\_V4 | 1.0 | 3.707e-08 | 353 | 0.584 | 89 | 37 | 0 | 55 | 143 | 1 | 89 | Phage tail protein | Phage tail protein | | afdb-uniprot50 | AF-A0A827UX27-F1-MODEL\_V4 | 1.0 | 6.847e-10 | 353 | 0.257 | 140 | 102 | 1 | 4 | 143 | 3 | 140 | Tail fiber assembly protein | Tail fiber assembly protein | | afdb-uniprot50 | AF-A0A7Y6NBC3-F1-MODEL\_V4 | 1.0 | 4.447e-09 | 352 | 0.302 | 139 | 90 | 2 | 4 | 142 | 3 | 134 | Tail fiber assembly protein | Tail fiber assembly protein | | afdb-uniprot50 | AF-A0A6N3HR67-F1-MODEL\_V4 | 1.0 | 3.926e-09 | 351 | 0.291 | 144 | 86 | 4 | 4 | 143 | 3 | 134 | Caudovirales tail fibre assembly protein | Caudovirales tail fibre assembly protein | | afdb-uniprot50 | AF-A0A424WAM4-F1-MODEL\_V4 | 1.0 | 1.745e-09 | 348 | 0.286 | 143 | 97 | 3 | 4 | 143 | 29 | 169 | Tail fiber assembly protein | Tail fiber assembly protein | | afdb-uniprot50 | AF-A0A7D7HTP8-F1-MODEL\_V4 | 1.0 | 3.906e-10 | 346 | 0.244 | 143 | 103 | 2 | 1 | 143 | 4 | 141 | Tail assembly chaperone | Tail assembly chaperone | | afdb-uniprot50 | AF-A0A837LIM0-F1-MODEL\_V4 | 1.0 | 3.028e-11 | 346 | 0.364 | 159 | 71 | 5 | 4 | 143 | 3 | 150 | Uncharacterized protein | Uncharacterized protein | | afdb-uniprot50 | AF-A0A5C5DE35-F1-MODEL\_V4 | 1.0 | 1.06e-09 | 344 | 0.234 | 145 | 105 | 4 | 1 | 142 | 1 | 142 | Tail fiber assembly protein | Tail fiber assembly protein | | afdb-uniprot50 | AF-A0A8A6CV85-F1-MODEL\_V4 | 1.0 | 5.736e-08 | 343 | 0.511 | 88 | 43 | 0 | 56 | 143 | 5 | 92 | Tail fiber assembly protein | Tail fiber assembly protein | | afdb-uniprot50 | AF-A0A410XDP5-F1-MODEL\_V4 | 1.0 | 2.36e-11 | 343 | 0.276 | 141 | 99 | 2 | 4 | 143 | 2 | 140 | Phage tail protein | Phage tail protein | | afdb-uniprot50 | AF-A0A376FB91-F1-MODEL\_V4 | 1.0 | 7.361e-08 | 342 | 0.515 | 66 | 32 | 0 | 3 | 68 | 2 | 67 | Phage tail assembly chaperone gp38 | Phage tail assembly chaperone gp38 | | afdb-uniprot50 | AF-A0A5P0JJ43-F1-MODEL\_V4 | 1.0 | 4.178e-09 | 340 | 0.344 | 119 | 74 | 2 | 4 | 122 | 2 | 116 | Tail fiber assembly protein | Tail fiber assembly protein | | afdb-uniprot50 | AF-Q2NWF3-F1-MODEL\_V4 | 1.0 | 1.548e-08 | 339 | 0.333 | 114 | 74 | 1 | 1 | 114 | 19 | 130 | Uncharacterized protein | Uncharacterized protein | | afdb-uniprot50 | AF-A0A7U5JH60-F1-MODEL\_V4 | 1.0 | 9.954e-10 | 339 | 0.314 | 124 | 82 | 2 | 4 | 127 | 3 | 123 | Uncharacterized protein | Uncharacterized protein | | afdb-uniprot50 | AF-W1J7J0-F1-MODEL\_V4 | 1.0 | 5.013e-10 | 338 | 0.301 | 146 | 91 | 4 | 1 | 143 | 3 | 140 | Uncharacterized protein | Uncharacterized protein | | afdb-uniprot50 | AF-A0A2Y0RH22-F1-MODEL\_V4 | 1.0 | 1.128e-09 | 338 | 0.298 | 144 | 86 | 3 | 4 | 143 | 3 | 135 | Tail assembly chaperone gp38 | Tail assembly chaperone gp38 | | afdb-uniprot50 | AF-A0A1I6E7B8-F1-MODEL\_V4 | 1.0 | 9.353e-10 | 337 | 0.28 | 139 | 96 | 3 | 4 | 140 | 3 | 139 | Virus tail fibre assembly protein, lambda gpK | Virus tail fibre assembly protein, lambda gpK | | afdb-uniprot50 | AF-A0A5X3P5F8-F1-MODEL\_V4 | 1.0 | 4.71e-10 | 336 | 0.5 | 138 | 50 | 3 | 3 | 140 | 2 | 120 | Tail fiber assembly protein | Tail fiber assembly protein | | afdb-uniprot50 | AF-D2U4A6-F1-MODEL\_V4 | 1.0 | 4.447e-09 | 336 | 0.283 | 127 | 88 | 2 | 4 | 129 | 12 | 136 | Phage tail assembly protein | Phage tail assembly protein | | afdb-uniprot50 | AF-A0A0U1HYV0-F1-MODEL\_V4 | 1.0 | 4.447e-09 | 336 | 0.308 | 146 | 89 | 5 | 3 | 143 | 2 | 140 | Phage tail protein | Phage tail protein | | afdb-uniprot50 | AF-A0A7X5FY66-F1-MODEL\_V4 | 1.0 | 1.2e-09 | 335 | 0.263 | 144 | 91 | 4 | 4 | 143 | 3 | 135 | Tail fiber assembly protein | Tail fiber assembly protein | | afdb-uniprot50 | AF-A0A2D0IKG5-F1-MODEL\_V4 | 1.0 | 2.384e-09 | 331 | 0.282 | 145 | 90 | 4 | 3 | 143 | 15 | 149 | Tail assembly chaperone | Tail assembly chaperone | | afdb-uniprot50 | AF-A0A777TWY1-F1-MODEL\_V4 | 1.0 | 7.398e-07 | 329 | 0.657 | 70 | 24 | 0 | 74 | 143 | 2 | 71 | Tail fiber assembly protein | Tail fiber assembly protein | | afdb-uniprot50 | AF-A0A5D4YD83-F1-MODEL\_V4 | 1.0 | 6.847e-10 | 329 | 0.321 | 143 | 86 | 5 | 4 | 143 | 2 | 136 | Tail fiber assembly protein | Tail fiber assembly protein | | afdb-uniprot50 | AF-W0BPG2-F1-MODEL\_V4 | 1.0 | 1.857e-09 | 329 | 0.279 | 143 | 98 | 4 | 3 | 143 | 2 | 141 | Uncharacterized protein | Uncharacterized protein | | afdb-uniprot50 | AF-A0A376YI45-F1-MODEL\_V4 | 1.0 | 5.038e-09 | 327 | 0.44 | 100 | 56 | 0 | 4 | 103 | 3 | 102 | CPS-53 (KpLE1) prophage protein | CPS-53 (KpLE1) prophage protein | | afdb-uniprot50 | AF-A0A2D0LAF6-F1-MODEL\_V4 | 1.0 | 6.075e-09 | 327 | 0.279 | 143 | 91 | 4 | 1 | 143 | 1 | 131 | Tail assembly chaperone | Tail assembly chaperone | | afdb-uniprot50 | AF-A0A5R8TTA1-F1-MODEL\_V4 | 1.0 | 1.145e-06 | 325 | 0.657 | 70 | 24 | 0 | 74 | 143 | 2 | 71 | Tail fiber assembly protein | Tail fiber assembly protein | | afdb-uniprot50 | AF-A0A7Y8YCI4-F1-MODEL\_V4 | 1.0 | 1.284e-08 | 324 | 0.271 | 140 | 94 | 2 | 4 | 143 | 2 | 133 | Tail fiber assembly protein | Tail fiber assembly protein | | afdb-uniprot50 | AF-A0A411WM65-F1-MODEL\_V4 | 1.0 | 1.867e-08 | 324 | 0.292 | 140 | 90 | 3 | 4 | 143 | 3 | 133 | Tail fiber assembly protein | Tail fiber assembly protein | | afdb-uniprot50 | AF-A0A7M3J414-F1-MODEL\_V4 | 1.0 | 2.384e-09 | 323 | 0.555 | 108 | 47 | 1 | 1 | 108 | 1 | 107 | Tail fiber assembly protein | Tail fiber assembly protein | | afdb-uniprot50 | AF-A0A6N9SA34-F1-MODEL\_V4 | 1.0 | 2.7e-09 | 323 | 0.451 | 124 | 61 | 1 | 3 | 126 | 2 | 118 | Tail fiber assembly protein | Tail fiber assembly protein | | afdb-uniprot50 | AF-A0A857F028-F1-MODEL\_V4 | 1.0 | 3.688e-09 | 323 | 0.346 | 124 | 78 | 2 | 20 | 143 | 2 | 122 | Tail fiber assembly protein | Tail fiber assembly protein | | afdb-uniprot50 | AF-A0A847KVC2-F1-MODEL\_V4 | 1.0 | 3.965e-07 | 319 | 0.447 | 67 | 36 | 1 | 1 | 66 | 5 | 71 | Uncharacterized protein | Uncharacterized protein | | afdb-uniprot50 | AF-A0A7U4HR21-F1-MODEL\_V4 | 1.0 | 2.251e-08 | 319 | 0.248 | 145 | 93 | 4 | 3 | 143 | 4 | 136 | Uncharacterized protein | Uncharacterized protein | | afdb-uniprot50 | AF-A0A4C6ZPY9-F1-MODEL\_V4 | 1.0 | 9.4e-09 | 319 | 0.273 | 139 | 94 | 2 | 4 | 142 | 19 | 150 | Phage tail fiber assembly protein | Phage tail fiber assembly protein | | afdb-uniprot50 | AF-A0A807LDM0-F1-MODEL\_V4 | 1.0 | 6.075e-09 | 317 | 0.266 | 150 | 90 | 6 | 4 | 143 | 3 | 142 | Uncharacterized protein | Uncharacterized protein | | afdb-uniprot50 | AF-A0A7D6TWW6-F1-MODEL\_V4 | 1.0 | 6.044e-10 | 317 | 0.377 | 127 | 76 | 2 | 1 | 127 | 1 | 124 | Tail fiber assembly protein | Tail fiber assembly protein | | afdb-uniprot50 | AF-A0A7L6EL48-F1-MODEL\_V4 | 1.0 | 1.987e-08 | 314 | 0.255 | 145 | 94 | 5 | 4 | 143 | 3 | 138 | Tail fiber assembly protein | Tail fiber assembly protein | | afdb-uniprot50 | AF-A0A7Z2EIW0-F1-MODEL\_V4 | 1.0 | 6.136e-07 | 313 | 0.512 | 82 | 38 | 1 | 64 | 143 | 1 | 82 | Tail fiber assembly protein | Tail fiber assembly protein | | afdb-uniprot50 | AF-A0A5E8P1V0-F1-MODEL\_V4 | 1.0 | 1.754e-08 | 313 | 0.322 | 124 | 81 | 2 | 20 | 143 | 5 | 125 | Phage tail protein | Phage tail protein | | afdb-uniprot50 | AF-P09154-F1-MODEL\_V4 | 1.0 | 4.178e-09 | 313 | 0.368 | 141 | 78 | 4 | 3 | 142 | 2 | 132 | Uncharacterized protein YmfS | Uncharacterized protein YmfS | | afdb-uniprot50 | AF-A0A829ITR5-F1-MODEL\_V4 | 1.0 | 2.575e-06 | 312 | 0.589 | 73 | 30 | 0 | 71 | 143 | 16 | 88 | Uncharacterized protein | Uncharacterized protein | | afdb-uniprot50 | AF-A0A7U6IRQ5-F1-MODEL\_V4 | 1.0 | 5.65e-11 | 312 | 0.393 | 145 | 81 | 5 | 3 | 143 | 2 | 143 | Tail assembly chaperone | Tail assembly chaperone | | afdb-uniprot50 | AF-A0A7W3BQ09-F1-MODEL\_V4 | 1.0 | 6.531e-07 | 309 | 0.409 | 66 | 39 | 0 | 1 | 66 | 1 | 66 | Uncharacterized protein | Uncharacterized protein | | afdb-uniprot50 | AF-A0A243UU47-F1-MODEL\_V4 | 1.0 | 1.997e-07 | 308 | 0.435 | 78 | 44 | 0 | 4 | 81 | 3 | 80 | Phage tail fiber assembly protein | Phage tail fiber assembly protein | | afdb-uniprot50 | AF-A0A5B9ATE9-F1-MODEL\_V4 | 1.0 | 4.757e-08 | 308 | 0.266 | 139 | 95 | 2 | 5 | 143 | 2 | 133 | Tail fiber assembly protein | Tail fiber assembly protein | | afdb-uniprot50 | AF-A0A1Q5VEU1-F1-MODEL\_V4 | 1.0 | 8.787e-10 | 308 | 0.267 | 146 | 95 | 6 | 1 | 142 | 3 | 140 | Uncharacterized protein | Uncharacterized protein | | afdb-uniprot50 | AF-A0A847KQA9-F1-MODEL\_V4 | 1.0 | 5.089e-07 | 307 | 0.458 | 72 | 37 | 1 | 1 | 70 | 6 | 77 | Tail fiber assembly protein | Tail fiber assembly protein | | afdb-uniprot50 | AF-A0A1Z1STM3-F1-MODEL\_V4 | 1.0 | 5.389e-08 | 307 | 0.256 | 117 | 83 | 3 | 28 | 143 | 4 | 117 | Uncharacterized protein | Uncharacterized protein | | afdb-uniprot50 | AF-A0A336NXC7-F1-MODEL\_V4 | 1.0 | 1.278e-09 | 306 | 0.358 | 145 | 66 | 3 | 1 | 143 | 3 | 122 | Phage tail fiber assembly protein | Phage tail fiber assembly protein | | afdb-uniprot50 | AF-A0A5K6DP22-F1-MODEL\_V4 | 1.0 | 5.707e-09 | 306 | 0.241 | 141 | 104 | 2 | 3 | 143 | 9 | 146 | Uncharacterized protein | Uncharacterized protein | | afdb-uniprot50 | AF-A0A5A9CAD4-F1-MODEL\_V4 | 1.0 | 1.284e-08 | 305 | 0.415 | 113 | 52 | 1 | 45 | 143 | 1 | 113 | Tail fiber assembly protein | Tail fiber assembly protein | | afdb-uniprot50 | AF-A0A837F9A4-F1-MODEL\_V4 | 1.0 | 7.796e-09 | 305 | 0.326 | 141 | 84 | 4 | 4 | 143 | 3 | 133 | Uncharacterized protein | Uncharacterized protein | | afdb-uniprot50 | AF-A0A376WQ73-F1-MODEL\_V4 | 1.0 | 6.136e-07 | 304 | 0.417 | 67 | 39 | 0 | 4 | 70 | 3 | 69 | CPS-53 (KpLE1) prophage protein | CPS-53 (KpLE1) prophage protein | | afdb-uniprot50 | AF-A0A1N6MWN0-F1-MODEL\_V4 | 1.0 | 6.105e-08 | 304 | 0.312 | 112 | 74 | 2 | 3 | 114 | 2 | 110 | Uncharacterized protein | Uncharacterized protein | | afdb-uniprot50 | AF-A0A741M1S9-F1-MODEL\_V4 | 1.0 | 8.831e-09 | 301 | 0.253 | 138 | 97 | 2 | 4 | 141 | 2 | 133 | Tail fiber assembly protein | Tail fiber assembly protein | | afdb-uniprot50 | AF-A0A5M7L6C3-F1-MODEL\_V4 | 1.0 | 1.367e-08 | 301 | 0.276 | 152 | 92 | 7 | 1 | 142 | 1 | 144 | Tail fiber assembly protein | Tail fiber assembly protein | | afdb-uniprot50 | AF-A0A7W3H786-F1-MODEL\_V4 | 1.0 | 1.011e-06 | 300 | 0.393 | 66 | 40 | 0 | 1 | 66 | 1 | 66 | Uncharacterized protein | Uncharacterized protein | | afdb-uniprot50 | AF-A0A6C6ZQ08-F1-MODEL\_V4 | 1.0 | 2.115e-08 | 300 | 0.482 | 114 | 57 | 2 | 27 | 139 | 1 | 113 | Tail fiber assembly protein | Tail fiber assembly protein | | afdb-uniprot50 | AF-A0A1V8NRE6-F1-MODEL\_V4 | 1.0 | 4.157e-10 | 300 | 0.361 | 163 | 74 | 6 | 4 | 143 | 2 | 157 | Uncharacterized protein | Uncharacterized protein | | afdb-uniprot50 | AF-A0A2L0TKF4-F1-MODEL\_V4 | 1.0 | 1.867e-08 | 300 | 0.244 | 143 | 102 | 3 | 1 | 143 | 27 | 163 | Phage tail protein | Phage tail protein | | afdb-uniprot50 | AF-A0A1C7W7E1-F1-MODEL\_V4 | 1.0 | 6.951e-07 | 297 | 0.465 | 73 | 38 | 1 | 1 | 73 | 1 | 72 | Uncharacterized protein | Uncharacterized protein | | afdb-uniprot50 | AF-W1IUZ4-F1-MODEL\_V4 | 1.0 | 1.373e-07 | 295 | 0.368 | 103 | 65 | 0 | 41 | 143 | 2 | 104 | Putative tail fiber assembly protein | Putative tail fiber assembly protein | | afdb-uniprot50 | AF-A0A5H5WYN2-F1-MODEL\_V4 | 1.0 | 4.47e-08 | 295 | 0.49 | 104 | 52 | 1 | 37 | 140 | 3 | 105 | Tail fiber assembly protein | Tail fiber assembly protein | | afdb-uniprot50 | AF-A0A763SVG3-F1-MODEL\_V4 | 1.0 | 6.564e-06 | 294 | 0.671 | 67 | 22 | 0 | 77 | 143 | 2 | 68 | Tail fiber assembly protein | Tail fiber assembly protein | | afdb-uniprot50 | AF-A0A4U6GPM8-F1-MODEL\_V4 | 1.0 | 1.2e-09 | 293 | 0.316 | 142 | 74 | 3 | 1 | 141 | 1 | 120 | Tail fiber assembly protein | Tail fiber assembly protein | | afdb-uniprot50 | AF-A0A7M2VBL5-F1-MODEL\_V4 | 1.0 | 8.256e-10 | 291 | 0.304 | 141 | 87 | 4 | 4 | 143 | 3 | 133 | Tail fiber assembly protein | Tail fiber assembly protein | | afdb-uniprot50 | AF-A0A0M0T7T7-F1-MODEL\_V4 | 1.0 | 3.67e-10 | 291 | 0.319 | 147 | 88 | 4 | 4 | 142 | 7 | 149 | Uncharacterized protein | Uncharacterized protein | | afdb-uniprot50 | AF-A0A402TSN0-F1-MODEL\_V4 | 1.0 | 2.007e-06 | 290 | 0.402 | 67 | 39 | 1 | 4 | 69 | 3 | 69 | Tail fiber assembly protein | Tail fiber assembly protein | | afdb-uniprot50 | AF-A0A7I6QQY2-F1-MODEL\_V4 | 1.0 | 2.42e-06 | 290 | 0.402 | 67 | 40 | 0 | 4 | 70 | 3 | 69 | Uncharacterized protein | Uncharacterized protein | | afdb-uniprot50 | AF-A0A7I8Z101-F1-MODEL\_V4 | 1.0 | 1.664e-06 | 289 | 0.347 | 69 | 44 | 1 | 1 | 69 | 1 | 68 | Hypothetical protein | Hypothetical protein | | afdb-uniprot50 | AF-A0A3S6EV28-F1-MODEL\_V4 | 1.0 | 3.272e-08 | 289 | 0.27 | 144 | 100 | 4 | 1 | 143 | 1 | 140 | Uncharacterized protein | Uncharacterized protein | | afdb-uniprot50 | AF-A0A7U6IVZ9-F1-MODEL\_V4 | 1.0 | 3.483e-08 | 287 | 0.441 | 120 | 58 | 2 | 24 | 143 | 14 | 124 | Phage tail protein | Phage tail protein | | afdb-uniprot50 | AF-A0A6G3I5P6-F1-MODEL\_V4 | 1.0 | 4.492e-07 | 283 | 0.435 | 78 | 44 | 0 | 4 | 81 | 3 | 80 | Tail fiber assembly protein | Tail fiber assembly protein | | afdb-uniprot50 | AF-A0A0V9JY68-F1-MODEL\_V4 | 1.0 | 9.447e-08 | 282 | 0.235 | 140 | 101 | 3 | 4 | 142 | 3 | 137 | Uncharacterized protein | Uncharacterized protein | | afdb-uniprot50 | AF-A0A5Q2VF37-F1-MODEL\_V4 | 1.0 | 2.575e-06 | 281 | 0.402 | 67 | 40 | 0 | 4 | 70 | 3 | 69 | Uncharacterized protein | Uncharacterized protein | | afdb-uniprot50 | AF-A0A5N9VV86-F1-MODEL\_V4 | 1.0 | 2.903e-07 | 281 | 0.414 | 99 | 56 | 1 | 4 | 102 | 2 | 98 | Tail fiber assembly protein | Tail fiber assembly protein | | afdb-uniprot50 | AF-A0A1C0U180-F1-MODEL\_V4 | 1.0 | 8.381e-07 | 280 | 0.377 | 98 | 59 | 1 | 46 | 143 | 1 | 96 | Caudovirales tail fiber assembly protein | Caudovirales tail fiber assembly protein | | afdb-uniprot50 | AF-A0A198GQI9-F1-MODEL\_V4 | 1.0 | 1.07e-07 | 280 | 0.244 | 139 | 98 | 3 | 5 | 143 | 2 | 133 | Tail fiber assembly protein | Tail fiber assembly protein | | afdb-uniprot50 | AF-A0A4P9D4P7-F1-MODEL\_V4 | 1.0 | 3.707e-08 | 280 | 0.504 | 119 | 47 | 4 | 31 | 143 | 19 | 131 | Phage tail protein | Phage tail protein | | afdb-uniprot50 | AF-A0A2N0VZQ8-F1-MODEL\_V4 | 1.0 | 3.726e-07 | 279 | 0.251 | 139 | 94 | 3 | 5 | 143 | 2 | 130 | Phage tail protein | Phage tail protein | | afdb-uniprot50 | AF-A0A0H5HS46-F1-MODEL\_V4 | 1.0 | 1.54e-09 | 279 | 0.299 | 147 | 86 | 7 | 4 | 143 | 2 | 138 | Putative tail fiber assembly protein | Putative tail fiber assembly protein | | afdb-uniprot50 | AF-A0A827R0P6-F1-MODEL\_V4 | 1.0 | 3.09e-07 | 278 | 0.489 | 92 | 46 | 1 | 1 | 92 | 1 | 91 | Tail fiber assembly protein | Tail fiber assembly protein | | afdb-uniprot50 | AF-A0A5M7KZH6-F1-MODEL\_V4 | 1.0 | 5.736e-08 | 276 | 0.258 | 147 | 96 | 5 | 4 | 143 | 5 | 145 | Tail fiber assembly protein | Tail fiber assembly protein | | afdb-uniprot50 | AF-A0A1D8UTE2-F1-MODEL\_V4 | 1.0 | 6.986e-06 | 273 | 0.38 | 71 | 43 | 1 | 1 | 70 | 2 | 72 | Uncharacterized protein | Uncharacterized protein | | afdb-uniprot50 | AF-J2U9X8-F1-MODEL\_V4 | 1.0 | 8.297e-09 | 271 | 0.268 | 145 | 89 | 6 | 3 | 143 | 2 | 133 | Caudovirales tail fiber assembly protein | Caudovirales tail fiber assembly protein | | afdb-uniprot50 | AF-A0A5V6PU83-F1-MODEL\_V4 | 1.0 | 9.954e-10 | 270 | 0.3 | 143 | 87 | 5 | 1 | 142 | 1 | 131 | Uncharacterized protein | Uncharacterized protein | | afdb-uniprot50 | AF-A0A7I6ZLB1-F1-MODEL\_V4 | 1.0 | 8.423e-06 | 268 | 0.363 | 66 | 42 | 0 | 3 | 68 | 2 | 67 | Uncharacterized protein | Uncharacterized protein | | afdb-uniprot50 | AF-A0A2S9ASE0-F1-MODEL\_V4 | 1.0 | 2.408e-07 | 268 | 0.238 | 147 | 93 | 5 | 4 | 143 | 39 | 173 | Phage tail protein | Phage tail protein | | afdb-uniprot50 | AF-A0A6M5DB00-F1-MODEL\_V4 | 1.0 | 1.133e-08 | 268 | 0.315 | 146 | 72 | 6 | 2 | 143 | 77 | 198 | Phage tail protein | Phage tail protein | | afdb-uniprot50 | AF-A0A1G5QUE3-F1-MODEL\_V4 | 1.0 | 1.218e-06 | 267 | 0.433 | 83 | 46 | 1 | 4 | 85 | 2 | 84 | Virus tail fibre assembly protein, lambda gpK | Virus tail fibre assembly protein, lambda gpK | | afdb-uniprot50 | AF-E0LV13-F1-MODEL\_V4 | 1.0 | 3.926e-09 | 267 | 0.312 | 141 | 89 | 5 | 4 | 143 | 3 | 136 | Tail assembly chaperone gp38 | Tail assembly chaperone gp38 | | afdb-uniprot50 | AF-A0A7W4P415-F1-MODEL\_V4 | 1.0 | 2.874e-09 | 266 | 0.297 | 148 | 81 | 8 | 1 | 143 | 2 | 131 | Uncharacterized protein | Uncharacterized protein | | afdb-uniprot50 | AF-A0A7V7JCF9-F1-MODEL\_V4 | 1.0 | 9.954e-10 | 265 | 0.344 | 148 | 80 | 7 | 3 | 143 | 2 | 139 | Phage tail protein | Phage tail protein | | afdb-uniprot50 | AF-A0A7D7KF73-F1-MODEL\_V4 | 1.0 | 1.139e-07 | 264 | 0.286 | 143 | 83 | 5 | 5 | 143 | 2 | 129 | Tail fiber assembly protein | Tail fiber assembly protein | | afdb-uniprot50 | AF-A0A2C5TSB9-F1-MODEL\_V4 | 1.0 | 1.477e-05 | 263 | 0.412 | 80 | 45 | 1 | 64 | 143 | 9 | 86 | Phage tail protein | Phage tail protein | | afdb-uniprot50 | AF-A0A2I0FUR0-F1-MODEL\_V4 | 1.0 | 1.133e-08 | 263 | 0.255 | 141 | 102 | 2 | 3 | 142 | 6 | 144 | Phage tail protein | Phage tail protein | | afdb-uniprot50 | AF-A0A5F1BTK6-F1-MODEL\_V4 | 1.0 | 2.903e-07 | 262 | 0.284 | 123 | 86 | 2 | 4 | 126 | 5 | 125 | Phage tail protein | Phage tail protein | | afdb-uniprot50 | AF-A0A6B1U976-F1-MODEL\_V4 | 1.0 | 6.951e-07 | 260 | 0.242 | 140 | 95 | 3 | 4 | 143 | 2 | 130 | Tail fiber assembly protein | Tail fiber assembly protein | | afdb-uniprot50 | AF-G3XD08-F1-MODEL\_V4 | 1.0 | 3.09e-07 | 260 | 0.276 | 123 | 87 | 2 | 4 | 126 | 5 | 125 | Uncharacterized protein | Uncharacterized protein | | afdb-uniprot50 | AF-A0A379QMG1-F1-MODEL\_V4 | 1.0 | 2.115e-08 | 259 | 0.289 | 128 | 74 | 4 | 1 | 123 | 1 | 116 | Caudovirales tail fibre assembly protein | Caudovirales tail fibre assembly protein | | afdb-uniprot50 | AF-A0A2T4Y153-F1-MODEL\_V4 | 1.0 | 1.867e-08 | 259 | 0.3 | 130 | 82 | 4 | 1 | 126 | 1 | 125 | Uncharacterized protein | Uncharacterized protein | | afdb-uniprot50 | AF-A0A7X3M9Z9-F1-MODEL\_V4 | 1.0 | 6.465e-09 | 259 | 0.288 | 142 | 91 | 6 | 3 | 143 | 21 | 153 | Tail fiber assembly protein | Tail fiber assembly protein | | afdb-uniprot50 | AF-A0A5N3CRX4-F1-MODEL\_V4 | 1.0 | 1.387e-05 | 258 | 0.414 | 70 | 40 | 1 | 1 | 69 | 1 | 70 | Phage tail protein | Phage tail protein | | afdb-uniprot50 | AF-A0A6B1U9P0-F1-MODEL\_V4 | 1.0 | 5.794e-06 | 258 | 0.394 | 71 | 42 | 1 | 1 | 70 | 1 | 71 | Phage tail protein | Phage tail protein | | afdb-uniprot50 | AF-A0A345CNV0-F1-MODEL\_V4 | 1.0 | 1.997e-07 | 258 | 0.221 | 149 | 99 | 6 | 4 | 143 | 3 | 143 | Phage tail protein | Phage tail protein | | afdb-uniprot50 | AF-A0A380SAU4-F1-MODEL\_V4 | 1.0 | 1.225e-05 | 256 | 0.376 | 69 | 42 | 1 | 1 | 69 | 1 | 68 | Uncharacterized protein | Uncharacterized protein | | afdb-uniprot50 | AF-A0A4R7NR00-F1-MODEL\_V4 | 1.0 | 1.772e-06 | 255 | 0.302 | 86 | 59 | 1 | 5 | 89 | 2 | 87 | Uncharacterized protein DUF4376 | Uncharacterized protein DUF4376 | | afdb-uniprot50 | AF-A0A6S5KC07-F1-MODEL\_V4 | 1.0 | 1.876e-07 | 254 | 0.289 | 145 | 87 | 6 | 1 | 143 | 1 | 131 | Uncharacterized protein | Uncharacterized protein | | afdb-uniprot50 | AF-A0A2K4PA33-F1-MODEL\_V4 | 1.0 | 1.07e-07 | 253 | 0.35 | 120 | 75 | 2 | 1 | 120 | 4 | 120 | Phage tail fiber assembly protein | Phage tail fiber assembly protein | | afdb-uniprot50 | AF-A0A3V3S8C9-F1-MODEL\_V4 | 1.0 | 2.741e-06 | 253 | 0.285 | 119 | 79 | 4 | 28 | 143 | 10 | 125 | Tail assembly chaperone | Tail assembly chaperone | | afdb-uniprot50 | AF-A0A6H1FWY0-F1-MODEL\_V4 | 1.0 | 1.455e-08 | 253 | 0.273 | 128 | 85 | 3 | 20 | 143 | 6 | 129 | Tail fiber assembly protein | Tail fiber assembly protein | | afdb-uniprot50 | AF-A0A8B3T6D4-F1-MODEL\_V4 | 1.0 | 4.199e-08 | 252 | 0.279 | 118 | 80 | 3 | 4 | 120 | 3 | 116 | Uncharacterized protein | Uncharacterized protein | | afdb-uniprot50 | AF-A0A855M959-F1-MODEL\_V4 | 1.0 | 4.47e-08 | 251 | 0.27 | 144 | 89 | 4 | 4 | 143 | 2 | 133 | Uncharacterized protein | Uncharacterized protein | | afdb-uniprot50 | AF-A0A4Q1B5G0-F1-MODEL\_V4 | 1.0 | 6.564e-06 | 249 | 0.388 | 72 | 43 | 1 | 4 | 74 | 9 | 80 | Uncharacterized protein | Uncharacterized protein | | afdb-uniprot50 | AF-A0A1U9Q763-F1-MODEL\_V4 | 1.0 | 6.531e-07 | 249 | 0.255 | 141 | 96 | 6 | 5 | 139 | 2 | 139 | Uncharacterized protein | Uncharacterized protein | | afdb-uniprot50 | AF-A0A5B0C0W6-F1-MODEL\_V4 | 1.0 | 1.011e-06 | 249 | 0.241 | 124 | 90 | 4 | 4 | 126 | 28 | 148 | Tail fiber assembly protein | Tail fiber assembly protein | | afdb-uniprot50 | AF-A0A352MQ92-F1-MODEL\_V4 | 1.0 | 6.951e-07 | 247 | 0.343 | 102 | 63 | 3 | 4 | 105 | 3 | 100 | Phage tail protein | Phage tail protein | | afdb-uniprot50 | AF-U2MIZ1-F1-MODEL\_V4 | 1.0 | 6.167e-06 | 246 | 0.318 | 91 | 60 | 1 | 53 | 143 | 2 | 90 | Tail fiber assembly like-protein | Tail fiber assembly like-protein | | afdb-uniprot50 | AF-A0A5Y3W528-F1-MODEL\_V4 | 1.0 | 8.921e-07 | 246 | 0.369 | 92 | 53 | 2 | 4 | 94 | 2 | 89 | Phage tail protein | Phage tail protein | | afdb-uniprot50 | AF-A0A731EUV7-F1-MODEL\_V4 | 1.0 | 4.781e-07 | 246 | 0.376 | 109 | 66 | 2 | 1 | 108 | 1 | 108 | Tail fiber assembly protein | Tail fiber assembly protein | | afdb-uniprot50 | AF-A0A2K9QKP4-F1-MODEL\_V4 | 1.0 | 2.888e-08 | 245 | 0.262 | 145 | 95 | 4 | 4 | 143 | 2 | 139 | Uncharacterized protein | Uncharacterized protein | | afdb-uniprot50 | AF-A0A4D0B479-F1-MODEL\_V4 | 1.0 | 8.381e-07 | 244 | 0.282 | 117 | 81 | 2 | 28 | 143 | 11 | 125 | Phage tail fiber assembly protein | Phage tail fiber assembly protein | | afdb-uniprot50 | AF-A0A7Y9CEQ5-F1-MODEL\_V4 | 1.0 | 1.455e-08 | 244 | 0.285 | 140 | 97 | 2 | 4 | 143 | 3 | 139 | Tail fiber assembly protein | Tail fiber assembly protein | | afdb-uniprot50 | AF-A0A5X9FP93-F1-MODEL\_V4 | 1.0 | 9.011e-05 | 243 | 0.716 | 60 | 17 | 0 | 84 | 143 | 2 | 61 | Tail fiber assembly protein | Tail fiber assembly protein | | afdb-uniprot50 | AF-A0A0H3AZJ6-F1-MODEL\_V4 | 1.0 | 7.796e-09 | 243 | 0.295 | 149 | 90 | 7 | 4 | 143 | 3 | 145 | Uncharacterized protein | Uncharacterized protein | | afdb-uniprot50 | AF-A0A743U4J6-F1-MODEL\_V4 | 1.0 | 3.289e-07 | 242 | 0.244 | 127 | 92 | 2 | 1 | 127 | 1 | 123 | Tail assembly chaperone | Tail assembly chaperone | | afdb-uniprot50 | AF-A0A6N3R610-F1-MODEL\_V4 | 1.0 | 7.473e-05 | 240 | 0.701 | 57 | 17 | 0 | 86 | 142 | 2 | 58 | Uncharacterized protein | Uncharacterized protein | | afdb-uniprot50 | AF-W1F7R7-F1-MODEL\_V4 | 1.0 | 1.477e-05 | 240 | 0.323 | 99 | 64 | 2 | 45 | 143 | 1 | 96 | Phage tail fiber assembly protein | Phage tail fiber assembly protein | | afdb-uniprot50 | AF-A0A7T5A242-F1-MODEL\_V4 | 1.0 | 1.469e-06 | 238 | 0.29 | 117 | 76 | 3 | 28 | 143 | 1 | 111 | Tail fiber assembly protein | Tail fiber assembly protein | | afdb-uniprot50 | AF-A0A411WM04-F1-MODEL\_V4 | 1.0 | 7.361e-08 | 238 | 0.28 | 125 | 84 | 3 | 19 | 143 | 3 | 121 | Tail fiber assembly protein | Tail fiber assembly protein | | afdb-uniprot50 | AF-A0A2U3F338-F1-MODEL\_V4 | 1.0 | 1.772e-06 | 237 | 0.212 | 141 | 100 | 4 | 4 | 143 | 5 | 135 | Uncharacterized protein | Uncharacterized protein | | afdb-uniprot50 | AF-A0A433ZYI6-F1-MODEL\_V4 | 1.0 | 0.0001394 | 236 | 0.44 | 75 | 40 | 1 | 69 | 143 | 1 | 73 | Uncharacterized protein | Uncharacterized protein | | afdb-uniprot50 | AF-A0A428L7S5-F1-MODEL\_V4 | 1.0 | 1.145e-06 | 236 | 0.241 | 149 | 97 | 5 | 1 | 143 | 1 | 139 | Tail assembly chaperone | Tail assembly chaperone | | afdb-uniprot50 | AF-A0A6S7DGX0-F1-MODEL\_V4 | 1.0 | 3.105e-06 | 236 | 0.214 | 149 | 101 | 5 | 1 | 143 | 60 | 198 | Uncharacterized protein | Uncharacterized protein | | afdb-uniprot50 | AF-A0A7Y0ZWM6-F1-MODEL\_V4 | 1.0 | 5.765e-07 | 235 | 0.27 | 144 | 95 | 5 | 4 | 142 | 2 | 140 | Tail fiber assembly protein | Tail fiber assembly protein | | afdb-uniprot50 | AF-A0A828KRF7-F1-MODEL\_V4 | 1.0 | 3.105e-06 | 234 | 0.466 | 90 | 44 | 3 | 4 | 91 | 3 | 90 | Tail fiber assembly protein | Tail fiber assembly protein | | afdb-uniprot50 | AF-A0A0A8FI31-F1-MODEL\_V4 | 1.0 | 1.876e-07 | 233 | 0.28 | 139 | 87 | 2 | 5 | 143 | 2 | 127 | Tail fiber protein | Tail fiber protein | | afdb-uniprot50 | AF-A0A793IKX6-F1-MODEL\_V4 | 1.0 | 5.471e-05 | 232 | 0.369 | 65 | 41 | 0 | 1 | 65 | 1 | 65 | Tail fiber assembly protein | Tail fiber assembly protein | | afdb-uniprot50 | AF-A0A2H5DVM4-F1-MODEL\_V4 | 1.0 | 3.985e-06 | 231 | 0.217 | 147 | 100 | 4 | 1 | 143 | 3 | 138 | Uncharacterized protein | Uncharacterized protein | | afdb-uniprot50 | AF-A0A7Z2J1J3-F1-MODEL\_V4 | 1.0 | 1.139e-07 | 231 | 0.275 | 145 | 98 | 4 | 4 | 143 | 3 | 145 | Tail fiber assembly protein | Tail fiber assembly protein | | afdb-uniprot50 | AF-A0A0F1B154-F1-MODEL\_V4 | 1.0 | 5.794e-06 | 230 | 0.214 | 140 | 97 | 3 | 4 | 143 | 2 | 128 | Uncharacterized protein | Uncharacterized protein | | afdb-uniprot50 | AF-A0A1G7SVK8-F1-MODEL\_V4 | 1.0 | 2.125e-07 | 229 | 0.235 | 157 | 86 | 5 | 4 | 126 | 3 | 159 | Uncharacterized protein | Uncharacterized protein | | afdb-uniprot50 | AF-A0A763CKN9-F1-MODEL\_V4 | 1.0 | 1.656e-07 | 229 | 0.181 | 193 | 100 | 5 | 4 | 141 | 2 | 191 | DUF4376 domain-containing protein | DUF4376 domain-containing protein | | afdb-uniprot50 | AF-A0A6M5HRU9-F1-MODEL\_V4 | 1.0 | 0.0001394 | 228 | 0.375 | 64 | 39 | 1 | 3 | 66 | 2 | 64 | Uncharacterized protein | Uncharacterized protein | | afdb-uniprot50 | AF-A0A0M7GTK9-F1-MODEL\_V4 | 1.0 | 1.469e-06 | 228 | 0.243 | 152 | 92 | 6 | 1 | 142 | 1 | 139 | Bacteriophage tail assembly protein | Bacteriophage tail assembly protein | | afdb-uniprot50 | AF-A0A2N7P764-F1-MODEL\_V4 | 1.0 | 8.381e-07 | 228 | 0.231 | 147 | 98 | 5 | 5 | 143 | 2 | 141 | Uncharacterized protein | Uncharacterized protein | | afdb-uniprot50 | AF-A0A0N0XL62-F1-MODEL\_V4 | 1.0 | 6.564e-06 | 227 | 0.213 | 145 | 100 | 5 | 1 | 142 | 1 | 134 | Caudovirales tail fiber assembly protein | Caudovirales tail fiber assembly protein | | afdb-uniprot50 | AF-A0A1H0BDJ1-F1-MODEL\_V4 | 1.0 | 9.543e-06 | 226 | 0.182 | 148 | 106 | 6 | 1 | 143 | 1 | 138 | Virus tail fibre assembly protein, lambda gpK | Virus tail fibre assembly protein, lambda gpK | | afdb-uniprot50 | AF-A0A5E8P460-F1-MODEL\_V4 | 1.0 | 7.914e-06 | 225 | 0.333 | 90 | 60 | 0 | 1 | 90 | 1 | 90 | Phage tail protein | Phage tail protein | | afdb-uniprot50 | AF-A0A844U1B6-F1-MODEL\_V4 | 1.0 | 3.744e-06 | 225 | 0.231 | 147 | 101 | 4 | 1 | 143 | 1 | 139 | Phage tail protein | Phage tail protein | | afdb-uniprot50 | AF-E3G2V8-F1-MODEL\_V4 | 1.0 | 1.772e-06 | 224 | 0.25 | 148 | 86 | 8 | 1 | 143 | 1 | 128 | Tail assembly chaperone gp38 | Tail assembly chaperone gp38 | | afdb-uniprot50 | AF-A0A077Q6L9-F1-MODEL\_V4 | 1.0 | 3.985e-06 | 223 | 0.371 | 97 | 56 | 2 | 50 | 143 | 2 | 96 | Putative tail fiber assembly protein | Putative tail fiber assembly protein | | afdb-uniprot50 | AF-A0A077P958-F1-MODEL\_V4 | 1.0 | 0.0001681 | 222 | 0.333 | 78 | 50 | 1 | 66 | 143 | 2 | 77 | Putative tail fiber chaperone Rac prophage | Putative tail fiber chaperone Rac prophage | | afdb-uniprot50 | AF-A0A8B2Y1X9-F1-MODEL\_V4 | 1.0 | 5.089e-07 | 222 | 0.246 | 146 | 101 | 6 | 1 | 143 | 1 | 140 | Phage tail protein | Phage tail protein | | afdb-uniprot50 | AF-A0A643FMC7-F1-MODEL\_V4 | 1.0 | 7.874e-07 | 221 | 0.258 | 143 | 91 | 4 | 3 | 143 | 2 | 131 | Phage tail assembly chaperone | Phage tail assembly chaperone | | afdb-uniprot50 | AF-W1EMA3-F1-MODEL\_V4 | 1.0 | 7.914e-06 | 221 | 0.229 | 144 | 93 | 7 | 4 | 143 | 3 | 132 | Uncharacterized protein | Uncharacterized protein | | afdb-uniprot50 | AF-A0A2N8KM43-F1-MODEL\_V4 | 1.0 | 2.918e-06 | 221 | 0.256 | 144 | 88 | 6 | 4 | 143 | 3 | 131 | Tail assembly chaperone | Tail assembly chaperone | | afdb-uniprot50 | AF-A0A377LDC8-F1-MODEL\_V4 | 1.0 | 1.218e-06 | 220 | 0.541 | 85 | 39 | 0 | 20 | 104 | 3 | 87 | CPS-53 (KpLE1) prophage protein | CPS-53 (KpLE1) prophage protein | | afdb-uniprot50 | AF-A0A1I7J4J1-F1-MODEL\_V4 | 1.0 | 3.483e-08 | 220 | 0.238 | 176 | 80 | 7 | 5 | 126 | 2 | 177 | DUF4376 domain-containing protein | DUF4376 domain-containing protein | | afdb-uniprot50 | AF-A0A2Z6EW35-F1-MODEL\_V4 | 1.0 | 2.741e-06 | 218 | 0.239 | 146 | 94 | 6 | 4 | 143 | 3 | 137 | Uncharacterized protein | Uncharacterized protein | | afdb-uniprot50 | AF-A0A1H9W380-F1-MODEL\_V4 | 1.0 | 5.736e-08 | 218 | 0.238 | 193 | 87 | 7 | 4 | 141 | 3 | 190 | DUF4376 domain-containing protein | DUF4376 domain-containing protein | | afdb-uniprot50 | AF-A0A2W5F2S4-F1-MODEL\_V4 | 1.0 | 5.794e-06 | 217 | 0.246 | 142 | 94 | 5 | 5 | 142 | 2 | 134 | Phage tail protein | Phage tail protein | | afdb-uniprot50 | AF-A0A076LFJ0-F1-MODEL\_V4 | 1.0 | 0.0004285 | 216 | 0.373 | 67 | 40 | 1 | 77 | 143 | 2 | 66 | Phage tail fiber assembly protein | Phage tail fiber assembly protein | | afdb-uniprot50 | AF-H3RFA6-F1-MODEL\_V4 | 1.0 | 0.0005853 | 216 | 0.342 | 73 | 46 | 1 | 71 | 143 | 8 | 78 | Uncharacterized protein | Uncharacterized protein | | afdb-uniprot50 | AF-A0A849JQA2-F1-MODEL\_V4 | 1.0 | 1.076e-06 | 216 | 0.273 | 146 | 78 | 7 | 4 | 143 | 3 | 126 | Tail assembly chaperone | Tail assembly chaperone | | afdb-uniprot50 | AF-A0A0D8L6B2-F1-MODEL\_V4 | 1.0 | 1.297e-06 | 216 | 0.218 | 165 | 95 | 8 | 3 | 143 | 21 | 175 | Uncharacterized protein | Uncharacterized protein | | afdb-uniprot50 | AF-A0A447UEL9-F1-MODEL\_V4 | 1.0 | 6.167e-06 | 215 | 0.192 | 140 | 102 | 3 | 4 | 143 | 2 | 130 | Phage tail fiber assembly protein | Phage tail fiber assembly protein | | afdb-uniprot50 | AF-A0A2I8DIP5-F1-MODEL\_V4 | 1.0 | 6.564e-06 | 215 | 0.244 | 147 | 95 | 6 | 4 | 143 | 24 | 161 | Phage tail protein | Phage tail protein | | afdb-uniprot50 | AF-X1XFW3-F1-MODEL\_V4 | 1.0 | 2.136e-06 | 215 | 0.248 | 141 | 85 | 5 | 17 | 142 | 577 | 711 | DNA\_circ\_N domain-containing protein | DNA\_circ\_N domain-containing protein | | afdb-uniprot50 | AF-A0A7X2R448-F1-MODEL\_V4 | 1.0 | 0.0002027 | 213 | 0.376 | 77 | 46 | 1 | 66 | 142 | 11 | 85 | Tail fiber assembly protein | Tail fiber assembly protein | | afdb-uniprot50 | AF-A0A6G2GGE2-F1-MODEL\_V4 | 1.0 | 2.932e-05 | 212 | 0.317 | 85 | 57 | 1 | 4 | 88 | 2 | 85 | Tail fiber assembly protein | Tail fiber assembly protein | | afdb-uniprot50 | AF-A0A4D8QSV8-F1-MODEL\_V4 | 1.0 | 1.016e-05 | 210 | 0.215 | 144 | 95 | 5 | 4 | 142 | 3 | 133 | Phage tail protein | Phage tail protein | | afdb-uniprot50 | AF-A0A0D0PTQ5-F1-MODEL\_V4 | 1.0 | 2.285e-05 | 210 | 0.204 | 142 | 100 | 4 | 4 | 143 | 3 | 133 | Contig\_41, whole genome shotgun sequence | Contig\_41, whole genome shotgun sequence | | afdb-uniprot50 | AF-A0A1J0EK84-F1-MODEL\_V4 | 1.0 | 8.381e-07 | 210 | 0.251 | 147 | 96 | 4 | 3 | 143 | 2 | 140 | Uncharacterized protein | Uncharacterized protein | | afdb-uniprot50 | AF-A0A0M7JWH2-F1-MODEL\_V4 | 1.0 | 4.515e-06 | 208 | 0.282 | 131 | 83 | 5 | 1 | 126 | 1 | 125 | Caudovirales tail fibre assembly protein | Caudovirales tail fibre assembly protein | | afdb-uniprot50 | AF-A0A0H2VSV7-F1-MODEL\_V4 | 1.0 | 7.914e-06 | 208 | 0.233 | 124 | 88 | 2 | 4 | 127 | 35 | 151 | Putative phage tail fibre protein | Putative phage tail fibre protein | | afdb-uniprot50 | AF-A0A6H1S397-F1-MODEL\_V4 | 1.0 | 1.212e-07 | 208 | 0.214 | 168 | 93 | 7 | 1 | 133 | 84 | 247 | Uncharacterized protein | Uncharacterized protein | | afdb-uniprot50 | AF-A0A7H8I663-F1-MODEL\_V4 | 1.0 | 0.0004854 | 206 | 0.3 | 80 | 52 | 2 | 66 | 143 | 1 | 78 | Tail fiber assembly protein | Tail fiber assembly protein | | afdb-uniprot50 | AF-A0A775TK41-F1-MODEL\_V4 | 1.0 | 0.0003553 | 205 | 0.345 | 81 | 50 | 2 | 64 | 143 | 9 | 87 | Phage tail protein | Phage tail protein | | afdb-uniprot50 | AF-A0A1I4ZNN3-F1-MODEL\_V4 | 1.0 | 3.744e-06 | 205 | 0.238 | 151 | 94 | 6 | 4 | 143 | 2 | 142 | Uncharacterized protein | Uncharacterized protein | | afdb-uniprot50 | AF-A0A854BW95-F1-MODEL\_V4 | 1.0 | 1.297e-06 | 204 | 0.237 | 143 | 96 | 5 | 4 | 142 | 2 | 135 | Phage tail protein | Phage tail protein | | afdb-uniprot50 | AF-A0A0T9RNX7-F1-MODEL\_V4 | 1.0 | 2.262e-07 | 204 | 0.258 | 151 | 95 | 6 | 1 | 143 | 2 | 143 | Putative tail fiber assembly protein | Putative tail fiber assembly protein | | afdb-uniprot50 | AF-A0A6M5CYI8-F1-MODEL\_V4 | 1.0 | 0.001588 | 203 | 0.342 | 70 | 46 | 0 | 74 | 143 | 2 | 71 | Uncharacterized protein | Uncharacterized protein | | afdb-uniprot50 | AF-A0A2C5TPI7-F1-MODEL\_V4 | 1.0 | 8.423e-06 | 203 | 0.26 | 138 | 77 | 3 | 4 | 141 | 2 | 114 | Phage tail protein | Phage tail protein | | afdb-uniprot50 | AF-W1J6H1-F1-MODEL\_V4 | 1.0 | 5.444e-06 | 203 | 0.223 | 143 | 80 | 4 | 4 | 143 | 2 | 116 | Putaive tail fiber assembly protein | Putaive tail fiber assembly protein | | afdb-uniprot50 | AF-A0A1S8CP93-F1-MODEL\_V4 | 1.0 | 1.772e-06 | 203 | 0.205 | 141 | 102 | 5 | 4 | 143 | 3 | 134 | Uncharacterized protein | Uncharacterized protein | | afdb-uniprot50 | AF-G5QY84-F1-MODEL\_V4 | 1.0 | 0.0005499 | 202 | 0.343 | 64 | 42 | 0 | 4 | 67 | 2 | 65 | Uncharacterized protein | Uncharacterized protein | | afdb-uniprot50 | AF-C5AKX8-F1-MODEL\_V4 | 1.0 | 2.432e-05 | 202 | 0.201 | 144 | 99 | 5 | 4 | 143 | 6 | 137 | Uncharacterized protein | Uncharacterized protein | | afdb-uniprot50 | AF-A0A1B8HT06-F1-MODEL\_V4 | 1.0 | 2.273e-06 | 202 | 0.275 | 145 | 75 | 5 | 2 | 142 | 61 | 179 | Uncharacterized protein | Uncharacterized protein | | afdb-uniprot50 | AF-A0A855M0Y7-F1-MODEL\_V4 | 1.0 | 4.242e-06 | 202 | 0.236 | 148 | 79 | 7 | 2 | 143 | 77 | 196 | Tail assembly chaperone | Tail assembly chaperone | | afdb-uniprot50 | AF-A0A0M0T628-F1-MODEL\_V4 | 1.0 | 3.5e-07 | 201 | 0.279 | 129 | 87 | 3 | 1 | 126 | 1 | 126 | Uncharacterized protein | Uncharacterized protein | | afdb-uniprot50 | AF-R8WJ06-F1-MODEL\_V4 | 1.0 | 1.664e-06 | 201 | 0.263 | 144 | 89 | 4 | 3 | 142 | 2 | 132 | Uncharacterized protein | Uncharacterized protein | | afdb-uniprot50 | AF-A0A379QQG2-F1-MODEL\_V4 | 1.0 | 2.563e-07 | 201 | 0.223 | 161 | 89 | 4 | 1 | 127 | 1 | 159 | Phage tail fiber assembly | Phage tail fiber assembly | | afdb-uniprot50 | AF-A0A826XDH3-F1-MODEL\_V4 | 1.0 | 0.000158 | 200 | 0.276 | 94 | 61 | 4 | 52 | 143 | 45 | 133 | Tail fiber assembly protein | Tail fiber assembly protein | | afdb-uniprot50 | AF-A0A1B8HTT9-F1-MODEL\_V4 | 1.0 | 5.444e-06 | 199 | 0.277 | 148 | 66 | 5 | 4 | 143 | 2 | 116 | Uncharacterized protein | Uncharacterized protein | | afdb-uniprot50 | AF-A0A432UNX0-F1-MODEL\_V4 | 1.0 | 2.563e-07 | 199 | 0.218 | 192 | 95 | 8 | 4 | 140 | 3 | 194 | Uncharacterized protein | Uncharacterized protein | | afdb-uniprot50 | AF-A0A8A5H0Q5-F1-MODEL\_V4 | 1.0 | 1.673e-05 | 198 | 0.264 | 117 | 73 | 4 | 28 | 143 | 24 | 128 | Tail fiber assembly protein | Tail fiber assembly protein | | afdb-uniprot50 | AF-A0A1A9WSI7-F1-MODEL\_V4 | 1.0 | 0.0001394 | 198 | 0.282 | 85 | 60 | 1 | 2 | 85 | 506 | 590 | Uncharacterized protein | Uncharacterized protein | | afdb-uniprot50 | AF-A0A763SYU9-F1-MODEL\_V4 | 1.0 | 0.0004026 | 197 | 0.369 | 65 | 40 | 1 | 4 | 68 | 3 | 66 | Tail assembly chaperone | Tail assembly chaperone | | afdb-uniprot50 | AF-A0A177SRB6-F1-MODEL\_V4 | 1.0 | 9.543e-06 | 197 | 0.229 | 144 | 97 | 6 | 3 | 142 | 2 | 135 | Phage tail protein | Phage tail protein | | afdb-uniprot50 | AF-E0LW01-F1-MODEL\_V4 | 1.0 | 9.543e-06 | 196 | 0.183 | 147 | 88 | 7 | 2 | 143 | 12 | 131 | Tail assembly chaperone gp38 | Tail assembly chaperone gp38 | | afdb-uniprot50 | AF-A0A7W7ING5-F1-MODEL\_V4 | 1.0 | 1.673e-05 | 196 | 0.236 | 152 | 85 | 8 | 4 | 142 | 3 | 136 | Uncharacterized protein | Uncharacterized protein | | afdb-uniprot50 | AF-A0A1Q8EUF2-F1-MODEL\_V4 | 1.0 | 4.537e-05 | 195 | 0.227 | 123 | 87 | 2 | 4 | 126 | 3 | 117 | Uncharacterized protein | Uncharacterized protein | | afdb-uniprot50 | AF-D8MKJ6-F1-MODEL\_V4 | 1.0 | 6.986e-06 | 195 | 0.205 | 146 | 86 | 6 | 2 | 143 | 77 | 196 | Phage tail assembly chaperone gp38 | Phage tail assembly chaperone gp38 | | afdb-uniprot50 | AF-A0A1J7C8B7-F1-MODEL\_V4 | 1.0 | 4.242e-06 | 195 | 0.219 | 146 | 82 | 6 | 2 | 142 | 73 | 191 | Phage tail protein | Phage tail protein | | afdb-uniprot50 | AF-A0A6L9LZN9-F1-MODEL\_V4 | 1.0 | 1.78e-05 | 193 | 0.248 | 145 | 90 | 7 | 4 | 142 | 3 | 134 | Phage tail protein | Phage tail protein | | afdb-uniprot50 | AF-A0A2V1Y818-F1-MODEL\_V4 | 1.0 | 5.14e-05 | 193 | 0.171 | 146 | 102 | 5 | 4 | 143 | 2 | 134 | Virus tail fiber assembly protein lambda gpK | Virus tail fiber assembly protein lambda gpK | | afdb-uniprot50 | AF-A0A1G9YD25-F1-MODEL\_V4 | 1.0 | 0.0001086 | 191 | 0.242 | 140 | 93 | 3 | 4 | 143 | 3 | 129 | Virus tail fibre assembly protein, lambda gpK | Virus tail fibre assembly protein, lambda gpK | | afdb-uniprot50 | AF-A0A4Q9ESD9-F1-MODEL\_V4 | 1.0 | 2.42e-06 | 191 | 0.231 | 164 | 87 | 6 | 4 | 142 | 2 | 151 | Tail fiber assembly protein | Tail fiber assembly protein | | afdb-uniprot50 | AF-A0A2U3F166-F1-MODEL\_V4 | 1.0 | 3.518e-06 | 191 | 0.226 | 146 | 91 | 8 | 4 | 142 | 5 | 135 | Phage tail protein | Phage tail protein | | afdb-uniprot50 | AF-A0A209A5U3-F1-MODEL\_V4 | 1.0 | 2.147e-05 | 190 | 0.232 | 146 | 91 | 7 | 1 | 142 | 1 | 129 | Uncharacterized protein | Uncharacterized protein | | afdb-uniprot50 | AF-A0A379FUE8-F1-MODEL\_V4 | 1.0 | 0.001026 | 189 | 0.383 | 73 | 43 | 1 | 70 | 142 | 4 | 74 | Bacteriophage tail assembly protein | Bacteriophage tail assembly protein | | afdb-uniprot50 | AF-A0A3D1C7H0-F1-MODEL\_V4 | 1.0 | 0.0004026 | 189 | 0.28 | 89 | 62 | 1 | 55 | 143 | 4 | 90 | Phage tail protein | Phage tail protein | | afdb-uniprot50 | AF-A0A2J9HPR0-F1-MODEL\_V4 | 1.0 | 0.0001156 | 189 | 0.226 | 115 | 78 | 4 | 33 | 143 | 4 | 111 | Tail assembly chaperone | Tail assembly chaperone | | afdb-uniprot50 | AF-A0A7W8U3D6-F1-MODEL\_V4 | 1.0 | 3.518e-06 | 189 | 0.254 | 157 | 77 | 7 | 4 | 126 | 2 | 152 | Peptidoglycan/xylan/chitin deacetylase (PgdA/CDA1 family) | Peptidoglycan/xylan/chitin deacetylase (PgdA/CDA1 family) | | afdb-uniprot50 | AF-A0A3L1P348-F1-MODEL\_V4 | 1.0 | 0.000456 | 188 | 0.344 | 90 | 57 | 1 | 54 | 143 | 1 | 88 | Tail fiber assembly protein | Tail fiber assembly protein | | afdb-uniprot50 | AF-A0A411SXP7-F1-MODEL\_V4 | 1.0 | 1.477e-05 | 188 | 0.208 | 144 | 96 | 6 | 2 | 143 | 6 | 133 | Uncharacterized protein | Uncharacterized protein | | afdb-uniprot50 | AF-A0A7H9BG12-F1-MODEL\_V4 | 1.0 | 2.432e-05 | 188 | 0.221 | 131 | 86 | 2 | 5 | 126 | 2 | 125 | Uncharacterized protein | Uncharacterized protein | | afdb-uniprot50 | AF-A0A7X5TMC5-F1-MODEL\_V4 | 1.0 | 0.0002158 | 187 | 0.282 | 99 | 69 | 1 | 45 | 143 | 8 | 104 | Phage tail protein | Phage tail protein | | afdb-uniprot50 | AF-D0ZBI3-F1-MODEL\_V4 | 1.0 | 1.572e-05 | 187 | 0.204 | 147 | 85 | 7 | 2 | 143 | 73 | 192 | Phage tail assembly chaperone gp38 | Phage tail assembly chaperone gp38 | | afdb-uniprot50 | AF-W1ITK8-F1-MODEL\_V4 | 1.0 | 0.001588 | 186 | 0.315 | 76 | 50 | 1 | 68 | 143 | 13 | 86 | Putaive tail fiber assembly protein | Putaive tail fiber assembly protein | | afdb-uniprot50 | AF-A0A0J7J7Q7-F1-MODEL\_V4 | 1.0 | 7.398e-07 | 186 | 0.217 | 156 | 88 | 4 | 5 | 126 | 2 | 157 | Uncharacterized protein | Uncharacterized protein | | afdb-uniprot50 | AF-A0A618PKJ7-F1-MODEL\_V4 | 1.0 | 3.322e-05 | 185 | 0.238 | 105 | 80 | 0 | 1 | 105 | 2 | 106 | Tail fiber assembly protein | Tail fiber assembly protein | | afdb-uniprot50 | AF-A0A376DH18-F1-MODEL\_V4 | 1.0 | 0.0001484 | 185 | 0.278 | 104 | 66 | 4 | 42 | 143 | 21 | 117 | Caudovirales tail fibre assembly protein | Caudovirales tail fibre assembly protein | | afdb-uniprot50 | AF-A0A0U1HLA4-F1-MODEL\_V4 | 1.0 | 0.001914 | 184 | 0.276 | 76 | 53 | 1 | 68 | 143 | 6 | 79 | Tail assembly chaperone gp38 | Tail assembly chaperone gp38 | | afdb-uniprot50 | AF-A0A3A3ZKX8-F1-MODEL\_V4 | 1.0 | 0.0002444 | 184 | 0.32 | 78 | 53 | 0 | 4 | 81 | 3 | 80 | Uncharacterized protein | Uncharacterized protein | | afdb-uniprot50 | AF-A0A838YFR4-F1-MODEL\_V4 | 1.0 | 9.591e-05 | 184 | 0.229 | 144 | 91 | 6 | 3 | 143 | 2 | 128 | Uncharacterized protein | Uncharacterized protein | | afdb-uniprot50 | AF-A0A1M3PGE6-F1-MODEL\_V4 | 1.0 | 2.147e-05 | 184 | 0.248 | 149 | 86 | 6 | 1 | 143 | 1 | 129 | Uncharacterized protein | Uncharacterized protein | | afdb-uniprot50 | AF-A0A5A9DGY0-F1-MODEL\_V4 | 1.0 | 0.0001394 | 183 | 0.352 | 102 | 59 | 6 | 45 | 143 | 52 | 149 | Tail fiber assembly protein | Tail fiber assembly protein | | afdb-uniprot50 | AF-A0A420WVJ7-F1-MODEL\_V4 | 1.0 | 7.874e-07 | 183 | 0.229 | 161 | 85 | 6 | 5 | 126 | 2 | 162 | Uncharacterized protein | Uncharacterized protein | | afdb-uniprot50 | AF-A0A7I6QR37-F1-MODEL\_V4 | 1.0 | 0.0004026 | 182 | 0.232 | 99 | 68 | 4 | 46 | 143 | 16 | 107 | Uncharacterized protein | Uncharacterized protein | | afdb-uniprot50 | AF-A0A5Y9TWT7-F1-MODEL\_V4 | 1.0 | 4.829e-05 | 182 | 0.231 | 147 | 83 | 6 | 2 | 143 | 79 | 200 | Tail fiber assembly protein | Tail fiber assembly protein | | afdb-uniprot50 | AF-A0A855SIT2-F1-MODEL\_V4 | 1.0 | 0.001492 | 181 | 0.392 | 56 | 34 | 0 | 1 | 56 | 1 | 56 | Tail fiber assembly protein | Tail fiber assembly protein | | afdb-uniprot50 | AF-A0A080I832-F1-MODEL\_V4 | 1.0 | 0.0001789 | 181 | 0.295 | 88 | 59 | 2 | 1 | 88 | 1 | 85 | Tail assembly chaperone gp38 domain protein | Tail assembly chaperone gp38 domain protein | | afdb-uniprot50 | AF-A0A8A5IKF3-F1-MODEL\_V4 | 1.0 | 0.0002947 | 181 | 0.245 | 102 | 69 | 4 | 45 | 143 | 6 | 102 | Tail fiber assembly protein | Tail fiber assembly protein | | afdb-uniprot50 | AF-A0A0T9KM84-F1-MODEL\_V4 | 1.0 | 0.0007057 | 179 | 0.329 | 82 | 52 | 2 | 62 | 143 | 11 | 89 | Putative phage tail fiber assembly protein | Putative phage tail fiber assembly protein | | afdb-uniprot50 | AF-J2SUJ2-F1-MODEL\_V4 | 1.0 | 2.147e-05 | 179 | 0.21 | 147 | 96 | 6 | 3 | 142 | 2 | 135 | Caudovirales tail fiber assembly protein | Caudovirales tail fiber assembly protein | | afdb-uniprot50 | AF-A0A0H3I5Y1-F1-MODEL\_V4 | 1.0 | 5.471e-05 | 179 | 0.251 | 147 | 86 | 8 | 1 | 143 | 1 | 127 | Tail fiber chaperone, Qin prophage | Tail fiber chaperone, Qin prophage | | afdb-uniprot50 | AF-A0A2W7L5R6-F1-MODEL\_V4 | 1.0 | 0.0001789 | 179 | 0.233 | 154 | 99 | 7 | 4 | 142 | 3 | 152 | Phage tail assembly chaperone | Phage tail assembly chaperone | | afdb-uniprot50 | AF-A0A4U2U962-F1-MODEL\_V4 | 1.0 | 2.588e-05 | 179 | 0.183 | 147 | 88 | 7 | 2 | 143 | 60 | 179 | Tail fiber assembly protein | Tail fiber assembly protein | | afdb-uniprot50 | AF-A0A2R8CKR9-F1-MODEL\_V4 | 1.0 | 6.136e-07 | 179 | 0.302 | 139 | 80 | 5 | 3 | 140 | 2 | 124 | Uncharacterized protein | Uncharacterized protein | | afdb-uniprot50 | AF-A0A2K9MAQ1-F1-MODEL\_V4 | 1.0 | 0.000158 | 179 | 0.182 | 148 | 102 | 5 | 2 | 143 | 72 | 206 | Uncharacterized protein | Uncharacterized protein | | afdb-uniprot50 | AF-A0A7Y9WJF3-F1-MODEL\_V4 | 1.0 | 4.537e-05 | 178 | 0.226 | 146 | 93 | 8 | 5 | 143 | 2 | 134 | Uncharacterized protein | Uncharacterized protein | | afdb-uniprot50 | AF-U3TVP9-F1-MODEL\_V4 | 1.0 | 0.0007057 | 178 | 0.242 | 99 | 67 | 4 | 45 | 143 | 86 | 176 | Uncharacterized protein | Uncharacterized protein | | afdb-uniprot50 | AF-A0A4P7L7D3-F1-MODEL\_V4 | 1.0 | 2.432e-05 | 178 | 0.231 | 147 | 81 | 7 | 2 | 143 | 87 | 206 | Uncharacterized protein | Uncharacterized protein | | afdb-uniprot50 | AF-D2U2G7-F1-MODEL\_V4 | 1.0 | 2.932e-05 | 177 | 0.238 | 147 | 80 | 7 | 2 | 143 | 58 | 177 | Phage tail fiber assembly protein | Phage tail fiber assembly protein | | afdb-uniprot50 | AF-A0A5C4RHG7-F1-MODEL\_V4 | 1.0 | 2.588e-05 | 177 | 0.226 | 146 | 83 | 7 | 2 | 143 | 75 | 194 | Tail fiber assembly protein | Tail fiber assembly protein | | afdb-uniprot50 | AF-A0A484QG78-F1-MODEL\_V4 | 1.0 | 0.000131 | 176 | 0.195 | 148 | 99 | 7 | 4 | 143 | 3 | 138 | Tail fiber assembly protein | Tail fiber assembly protein | | afdb-uniprot50 | AF-A0A7X5QHH8-F1-MODEL\_V4 | 1.0 | 3.322e-05 | 176 | 0.226 | 146 | 83 | 6 | 2 | 143 | 753 | 872 | Phage-tail\_3 domain-containing protein | Phage-tail\_3 domain-containing protein | | afdb-uniprot50 | AF-R9VMU8-F1-MODEL\_V4 | 1.0 | 0.0005853 | 175 | 0.273 | 95 | 63 | 2 | 53 | 143 | 50 | 142 | Uncharacterized protein | Uncharacterized protein | | afdb-uniprot50 | AF-A0A5C7CFK8-F1-MODEL\_V4 | 1.0 | 0.002962 | 175 | 0.278 | 79 | 54 | 2 | 66 | 143 | 99 | 175 | Tail fiber assembly protein | Tail fiber assembly protein | | afdb-uniprot50 | AF-A0A7H8I670-F1-MODEL\_V4 | 1.0 | 6.198e-05 | 175 | 0.191 | 146 | 88 | 7 | 2 | 143 | 79 | 198 | Tail fiber assembly protein | Tail fiber assembly protein | | afdb-uniprot50 | AF-A0A1G5QW41-F1-MODEL\_V4 | 1.0 | 2.932e-05 | 175 | 0.238 | 147 | 80 | 6 | 2 | 143 | 410 | 529 | Phage Tail Collar Domain | Phage Tail Collar Domain | | afdb-uniprot50 | AF-A0A833NNS7-F1-MODEL\_V4 | 1.0 | 0.0003553 | 174 | 0.265 | 98 | 67 | 2 | 46 | 143 | 22 | 114 | Tail fiber assembly protein | Tail fiber assembly protein | | afdb-uniprot50 | AF-A0A223W0F0-F1-MODEL\_V4 | 1.0 | 0.0004026 | 174 | 0.266 | 109 | 73 | 3 | 37 | 143 | 26 | 129 | Uncharacterized protein | Uncharacterized protein | | afdb-uniprot50 | AF-A0A7H8UFS0-F1-MODEL\_V4 | 1.0 | 7.954e-05 | 174 | 0.314 | 108 | 57 | 5 | 30 | 133 | 26 | 120 | Tail fiber assembly protein | Tail fiber assembly protein | | afdb-uniprot50 | AF-A0A2G0WBJ8-F1-MODEL\_V4 | 1.0 | 0.0001021 | 173 | 0.253 | 146 | 89 | 7 | 4 | 142 | 2 | 134 | Uncharacterized protein | Uncharacterized protein | | afdb-uniprot50 | AF-E9TCL4-F1-MODEL\_V4 | 1.0 | 0.0004026 | 172 | 0.285 | 98 | 65 | 3 | 46 | 143 | 24 | 116 | Caudovirales tail fiber assembly protein | Caudovirales tail fiber assembly protein | | afdb-uniprot50 | AF-A0A857BPZ5-F1-MODEL\_V4 | 1.0 | 7.021e-05 | 172 | 0.217 | 147 | 83 | 6 | 2 | 143 | 79 | 198 | Tail fiber assembly protein | Tail fiber assembly protein | | afdb-uniprot50 | AF-T0QEZ4-F1-MODEL\_V4 | 1.0 | 0.001092 | 171 | 0.242 | 99 | 68 | 4 | 45 | 143 | 1 | 92 | Uncharacterized protein | Uncharacterized protein | | afdb-uniprot50 | AF-A0A5N7JNQ3-F1-MODEL\_V4 | 1.0 | 9.591e-05 | 171 | 0.237 | 143 | 95 | 5 | 4 | 142 | 37 | 169 | Phage tail protein | Phage tail protein | | afdb-uniprot50 | AF-A0A6I4LQ79-F1-MODEL\_V4 | 1.0 | 0.000663 | 170 | 0.265 | 98 | 67 | 2 | 46 | 143 | 1 | 93 | Tail fiber assembly protein | Tail fiber assembly protein | | afdb-uniprot50 | AF-A0A403LVI5-F1-MODEL\_V4 | 1.0 | 0.000456 | 170 | 0.285 | 98 | 65 | 3 | 46 | 143 | 9 | 101 | Tail fiber assembly protein | Tail fiber assembly protein | | afdb-uniprot50 | AF-A0A2I8Q8G5-F1-MODEL\_V4 | 1.0 | 0.0009639 | 170 | 0.305 | 85 | 55 | 3 | 60 | 143 | 44 | 125 | Uncharacterized protein | Uncharacterized protein | | afdb-uniprot50 | AF-A0A7X5TM28-F1-MODEL\_V4 | 1.0 | 5.14e-05 | 170 | 0.217 | 147 | 83 | 7 | 2 | 143 | 89 | 208 | Phage tail protein | Phage tail protein | | afdb-uniprot50 | AF-A0A6N8PP82-F1-MODEL\_V4 | 1.0 | 0.006663 | 169 | 0.454 | 44 | 24 | 0 | 1 | 44 | 1 | 44 | Tail fiber assembly protein | Tail fiber assembly protein | | afdb-uniprot50 | AF-A0A208ZVY5-F1-MODEL\_V4 | 1.0 | 0.005526 | 169 | 0.26 | 73 | 52 | 1 | 71 | 143 | 4 | 74 | Phage tail protein | Phage tail protein | | afdb-uniprot50 | AF-A0A411H8C5-F1-MODEL\_V4 | 1.0 | 0.0006229 | 169 | 0.265 | 98 | 58 | 3 | 46 | 143 | 26 | 109 | Uncharacterized protein | Uncharacterized protein | | afdb-uniprot50 | AF-A0A1G5QVS6-F1-MODEL\_V4 | 1.0 | 0.0001789 | 169 | 0.226 | 146 | 83 | 6 | 2 | 143 | 6 | 125 | Virus tail fibre assembly protein, lambda gpK | Virus tail fibre assembly protein, lambda gpK | | afdb-uniprot50 | AF-A0A3L2NWY4-F1-MODEL\_V4 | 1.0 | 0.0005166 | 169 | 0.265 | 98 | 67 | 2 | 46 | 143 | 35 | 127 | Tail fiber assembly protein | Tail fiber assembly protein | | afdb-uniprot50 | AF-A0A345EKD5-F1-MODEL\_V4 | 1.0 | 9.591e-05 | 169 | 0.252 | 119 | 76 | 6 | 28 | 143 | 27 | 135 | Phage tail protein | Phage tail protein | | afdb-uniprot50 | AF-F3YY47-F1-MODEL\_V4 | 1.0 | 0.0002947 | 169 | 0.195 | 148 | 102 | 4 | 4 | 143 | 3 | 141 | Prophage PSPPH06, putative tail fiber protein | Prophage PSPPH06, putative tail fiber protein | | afdb-uniprot50 | AF-A0A855V8H1-F1-MODEL\_V4 | 1.0 | 0.0001156 | 169 | 0.208 | 144 | 105 | 4 | 4 | 142 | 3 | 142 | Phage tail protein | Phage tail protein | | afdb-uniprot50 | AF-A0A736RL65-F1-MODEL\_V4 | 1.0 | 4.537e-05 | 169 | 0.214 | 154 | 88 | 9 | 1 | 143 | 44 | 175 | Tail fiber assembly protein | Tail fiber assembly protein | | afdb-uniprot50 | AF-A0A849JE86-F1-MODEL\_V4 | 1.0 | 4.005e-05 | 169 | 0.2 | 145 | 86 | 7 | 2 | 142 | 82 | 200 | Phage tail protein | Phage tail protein | | afdb-uniprot50 | AF-A0A1Q6B6N7-F1-MODEL\_V4 | 1.0 | 0.0009639 | 168 | 0.271 | 92 | 60 | 3 | 53 | 142 | 3 | 89 | Phage tail protein | Phage tail protein | | afdb-uniprot50 | AF-A0A6F8VBJ7-F1-MODEL\_V4 | 1.0 | 0.0002296 | 168 | 0.222 | 144 | 88 | 5 | 5 | 143 | 2 | 126 | Uncharacterized protein | Uncharacterized protein | | afdb-uniprot50 | AF-A0A7Z7PAS9-F1-MODEL\_V4 | 1.0 | 1.895e-05 | 168 | 0.22 | 154 | 90 | 9 | 1 | 143 | 41 | 175 | Bacteriophage tail assembly protein | Bacteriophage tail assembly protein | | afdb-uniprot50 | AF-A0A2D0IJS5-F1-MODEL\_V4 | 1.0 | 4.005e-05 | 168 | 0.239 | 146 | 81 | 7 | 2 | 143 | 78 | 197 | Tail assembly protein | Tail assembly protein | | afdb-uniprot50 | AF-A0A5C4RJQ8-F1-MODEL\_V4 | 1.0 | 6.597e-05 | 168 | 0.227 | 145 | 84 | 6 | 2 | 143 | 79 | 198 | Tail fiber assembly protein | Tail fiber assembly protein | | afdb-uniprot50 | AF-A0A7X0T437-F1-MODEL\_V4 | 1.0 | 9.591e-05 | 168 | 0.191 | 146 | 88 | 7 | 2 | 143 | 81 | 200 | Tail fiber assembly protein | Tail fiber assembly protein | | afdb-uniprot50 | AF-E7CGJ6-F1-MODEL\_V4 | 1.0 | 9.011e-05 | 167 | 0.201 | 144 | 85 | 5 | 3 | 142 | 114 | 231 | Phage-related tail fiber assembly protein G | Phage-related tail fiber assembly protein G | | afdb-uniprot50 | AF-A0A250BJZ7-F1-MODEL\_V4 | 1.0 | 4.005e-05 | 166 | 0.227 | 145 | 81 | 6 | 2 | 142 | 176 | 293 | Phage tail protein | Phage tail protein | | afdb-uniprot50 | AF-A0A6B1U8Y3-F1-MODEL\_V4 | 1.0 | 4.005e-05 | 165 | 0.208 | 139 | 93 | 4 | 4 | 142 | 3 | 124 | Tail fiber assembly protein | Tail fiber assembly protein | | afdb-uniprot50 | AF-A0A4P8SN34-F1-MODEL\_V4 | 1.0 | 4.263e-05 | 165 | 0.206 | 155 | 87 | 10 | 1 | 141 | 61 | 193 | Tail assembly chaperone | Tail assembly chaperone | | afdb-uniprot50 | AF-A0A443ZGX7-F1-MODEL\_V4 | 1.0 | 0.0002769 | 164 | 0.202 | 143 | 101 | 6 | 4 | 142 | 3 | 136 | Phage tail protein | Phage tail protein | | afdb-uniprot50 | AF-A0A430BB89-F1-MODEL\_V4 | 1.0 | 3.105e-06 | 164 | 0.217 | 161 | 96 | 4 | 4 | 141 | 3 | 156 | Uncharacterized protein | Uncharacterized protein | | afdb-uniprot50 | AF-A0A097QXF3-F1-MODEL\_V4 | 1.0 | 6.198e-05 | 164 | 0.207 | 154 | 89 | 9 | 1 | 143 | 44 | 175 | Uncharacterized protein | Uncharacterized protein | | afdb-uniprot50 | AF-A0A422ZK35-F1-MODEL\_V4 | 1.0 | 0.0001394 | 164 | 0.213 | 145 | 86 | 6 | 2 | 143 | 73 | 192 | Tail assembly chaperone | Tail assembly chaperone | | afdb-uniprot50 | AF-Q7N2W3-F1-MODEL\_V4 | 1.0 | 0.0009639 | 164 | 0.27 | 100 | 60 | 3 | 55 | 143 | 374 | 471 | Uncharacterized protein | Uncharacterized protein | | afdb-uniprot50 | AF-A0A376EYU4-F1-MODEL\_V4 | 1.0 | 0.007092 | 163 | 0.416 | 48 | 28 | 0 | 4 | 51 | 3 | 50 | Phage tail assembly chaperone gp38 | Phage tail assembly chaperone gp38 | | afdb-uniprot50 | AF-A0A2U3TGW6-F1-MODEL\_V4 | 1.0 | 0.0002027 | 163 | 0.202 | 148 | 92 | 8 | 1 | 142 | 1 | 128 | Uncharacterized protein | Uncharacterized protein | | afdb-uniprot50 | AF-A0A851GEG3-F1-MODEL\_V4 | 1.0 | 4.829e-05 | 163 | 0.208 | 149 | 88 | 8 | 4 | 143 | 50 | 177 | Tail fiber assembly protein | Tail fiber assembly protein | | afdb-uniprot50 | AF-A0A4R7P8A5-F1-MODEL\_V4 | 1.0 | 5.823e-05 | 163 | 0.219 | 146 | 82 | 7 | 2 | 142 | 88 | 206 | Virus tail fiber assembly protein lambda gpK | Virus tail fiber assembly protein lambda gpK | | afdb-uniprot50 | AF-A0A2A2GZI5-F1-MODEL\_V4 | 1.0 | 0.0001231 | 163 | 0.204 | 147 | 85 | 7 | 2 | 143 | 87 | 206 | Uncharacterized protein | Uncharacterized protein | | afdb-uniprot50 | AF-A0A7U4N670-F1-MODEL\_V4 | 1.0 | 6.597e-05 | 162 | 0.206 | 155 | 90 | 9 | 1 | 143 | 40 | 173 | Uncharacterized protein | Uncharacterized protein | | afdb-uniprot50 | AF-D3V998-F1-MODEL\_V4 | 1.0 | 0.000456 | 161 | 0.267 | 101 | 69 | 3 | 43 | 143 | 5 | 100 | Uncharacterized protein | Uncharacterized protein | | afdb-uniprot50 | AF-A0A0T9UVT2-F1-MODEL\_V4 | 1.0 | 9.591e-05 | 161 | 0.294 | 95 | 62 | 4 | 4 | 95 | 2 | 94 | Uncharacterized protein | Uncharacterized protein | | afdb-uniprot50 | AF-A0A077P8M4-F1-MODEL\_V4 | 1.0 | 0.0001021 | 161 | 0.179 | 156 | 91 | 10 | 1 | 143 | 45 | 176 | Putative tail fiber assembly protein | Putative tail fiber assembly protein | | afdb-uniprot50 | AF-A0A3D9UGT7-F1-MODEL\_V4 | 1.0 | 9.011e-05 | 161 | 0.236 | 144 | 85 | 6 | 1 | 143 | 58 | 177 | Virus tail fiber assembly protein lambda gpK | Virus tail fiber assembly protein lambda gpK | | afdb-uniprot50 | AF-P77699-F1-MODEL\_V4 | 1.0 | 0.0001394 | 161 | 0.208 | 144 | 84 | 5 | 3 | 142 | 67 | 184 | Protein TfaD | Protein TfaD | | afdb-uniprot50 | AF-A0A2G8BY00-F1-MODEL\_V4 | 1.0 | 0.000158 | 161 | 0.171 | 146 | 91 | 5 | 2 | 143 | 76 | 195 | Uncharacterized protein | Uncharacterized protein | | afdb-uniprot50 | AF-W7NUC6-F1-MODEL\_V4 | 1.0 | 0.0001681 | 161 | 0.172 | 145 | 92 | 7 | 2 | 143 | 79 | 198 | Tail fiber assembly protein | Tail fiber assembly protein | | afdb-uniprot50 | AF-A0A7Z3MKM2-F1-MODEL\_V4 | 1.0 | 9.591e-05 | 160 | 0.185 | 156 | 92 | 10 | 1 | 143 | 2 | 135 | Tail fiber assembly protein | Tail fiber assembly protein | | afdb-uniprot50 | AF-A0A1X1A8Y4-F1-MODEL\_V4 | 1.0 | 0.0007511 | 160 | 0.178 | 123 | 93 | 2 | 4 | 126 | 5 | 119 | Phage tail protein | Phage tail protein | | afdb-uniprot50 | AF-A0A0M0T4V1-F1-MODEL\_V4 | 1.0 | 0.0001086 | 160 | 0.227 | 145 | 84 | 7 | 2 | 143 | 78 | 197 | Tail assembly protein | Tail assembly protein | | afdb-uniprot50 | AF-A0A7D6NVZ1-F1-MODEL\_V4 | 1.0 | 0.0001789 | 159 | 0.259 | 127 | 67 | 3 | 17 | 143 | 54 | 153 | Tail fiber assembly protein | Tail fiber assembly protein | | afdb-uniprot50 | AF-A0A6N8QLY1-F1-MODEL\_V4 | 1.0 | 0.000131 | 159 | 0.188 | 143 | 86 | 5 | 3 | 141 | 69 | 185 | Tail fiber assembly protein | Tail fiber assembly protein | | afdb-uniprot50 | AF-A0A455W0B7-F1-MODEL\_V4 | 1.0 | 0.0005166 | 158 | 0.225 | 120 | 77 | 5 | 25 | 143 | 22 | 126 | Uncharacterized protein | Uncharacterized protein | | afdb-uniprot50 | AF-A0A4P6GKN4-F1-MODEL\_V4 | 1.0 | 0.0003782 | 158 | 0.182 | 137 | 94 | 6 | 3 | 135 | 2 | 124 | Phage tail protein | Phage tail protein | | afdb-uniprot50 | AF-A0A455VVP5-F1-MODEL\_V4 | 1.0 | 0.0001789 | 158 | 0.179 | 145 | 91 | 7 | 2 | 143 | 33 | 152 | Prophage tail fiber assembly protein homolog TfaE | Prophage tail fiber assembly protein homolog TfaE | | afdb-uniprot50 | AF-A0A2K4IBZ4-F1-MODEL\_V4 | 1.0 | 5.471e-05 | 158 | 0.206 | 145 | 100 | 6 | 3 | 143 | 2 | 135 | Phage tail protein | Phage tail protein | | afdb-uniprot50 | AF-A0A1B9L6I4-F1-MODEL\_V4 | 1.0 | 0.0001484 | 158 | 0.217 | 147 | 83 | 6 | 2 | 143 | 82 | 201 | Uncharacterized protein | Uncharacterized protein | | afdb-uniprot50 | AF-A0A7X2R1K4-F1-MODEL\_V4 | 1.0 | 0.0002027 | 157 | 0.261 | 126 | 66 | 3 | 17 | 142 | 37 | 135 | Tail fiber assembly protein | Tail fiber assembly protein | | afdb-uniprot50 | AF-A0A0L1C3B6-F1-MODEL\_V4 | 1.0 | 0.0004854 | 156 | 0.285 | 119 | 72 | 7 | 28 | 143 | 27 | 135 | Tail fiber assembly protein | Tail fiber assembly protein | | afdb-uniprot50 | AF-A0A4S3LMC6-F1-MODEL\_V4 | 1.0 | 8.466e-05 | 156 | 0.206 | 155 | 90 | 9 | 1 | 143 | 38 | 171 | Tail fiber assembly protein | Tail fiber assembly protein | | afdb-uniprot50 | AF-A0A1Y0KR81-F1-MODEL\_V4 | 1.0 | 1.225e-05 | 156 | 0.181 | 154 | 93 | 5 | 4 | 126 | 2 | 153 | Uncharacterized protein | Uncharacterized protein | | afdb-uniprot50 | AF-A0A2D0J0R2-F1-MODEL\_V4 | 1.0 | 0.0002601 | 156 | 0.232 | 129 | 72 | 3 | 15 | 143 | 112 | 213 | Phage tail fiber assembly | Phage tail fiber assembly | | afdb-uniprot50 | AF-A0A4P7L774-F1-MODEL\_V4 | 1.0 | 0.0001394 | 156 | 0.21 | 147 | 84 | 7 | 2 | 143 | 97 | 216 | Uncharacterized protein | Uncharacterized protein | | afdb-uniprot50 | AF-L4J2A0-F1-MODEL\_V4 | 1.0 | 0.0001905 | 156 | 0.229 | 148 | 81 | 8 | 2 | 143 | 79 | 199 | Phage tail fiber assembly protein | Phage tail fiber assembly protein | | afdb-uniprot50 | AF-A0A2Z4RQA3-F1-MODEL\_V4 | 1.0 | 0.0002947 | 155 | 0.254 | 122 | 84 | 4 | 5 | 126 | 2 | 116 | Phage tail protein | Phage tail protein | | afdb-uniprot50 | AF-A0A3B0MF92-F1-MODEL\_V4 | 1.0 | 0.0002296 | 155 | 0.197 | 152 | 92 | 9 | 1 | 143 | 39 | 169 | Uncharacterized protein | Uncharacterized protein | | afdb-uniprot50 | AF-A7MLN9-F1-MODEL\_V4 | 1.0 | 0.0002601 | 155 | 0.166 | 144 | 92 | 6 | 2 | 142 | 75 | 193 | Uncharacterized protein | Uncharacterized protein | | afdb-uniprot50 | AF-A0A2D0IXB9-F1-MODEL\_V4 | 1.0 | 0.0002027 | 155 | 0.19 | 147 | 87 | 7 | 2 | 143 | 87 | 206 | Tail fiber protein of a prophage | Tail fiber protein of a prophage | | afdb-uniprot50 | AF-A0A1H9EZ77-F1-MODEL\_V4 | 1.0 | 7.954e-05 | 155 | 0.16 | 199 | 89 | 7 | 4 | 143 | 26 | 205 | Virus tail fibre assembly protein, lambda gpK | Virus tail fibre assembly protein, lambda gpK | | afdb-uniprot50 | AF-A0A2P5IM29-F1-MODEL\_V4 | 1.0 | 0.0001681 | 154 | 0.278 | 115 | 69 | 6 | 28 | 140 | 27 | 129 | Phage tail protein | Phage tail protein | | afdb-uniprot50 | AF-A0A5U6ST04-F1-MODEL\_V4 | 1.0 | 0.0001086 | 154 | 0.19 | 147 | 95 | 7 | 1 | 143 | 11 | 137 | Tail fiber assembly protein | Tail fiber assembly protein | | afdb-uniprot50 | AF-F2K8I0-F1-MODEL\_V4 | 1.0 | 6.597e-05 | 154 | 0.203 | 123 | 91 | 4 | 4 | 126 | 3 | 118 | Conserved hypothetical phage protein Putative tail fiber assembly protein | Conserved hypothetical phage protein Putative tail fiber assembly protein | | afdb-uniprot50 | AF-A0A2S8QNI7-F1-MODEL\_V4 | 1.0 | 0.0002947 | 154 | 0.209 | 148 | 83 | 7 | 2 | 143 | 25 | 144 | Phage tail protein | Phage tail protein | | afdb-uniprot50 | AF-A0A3N8BA59-F1-MODEL\_V4 | 1.0 | 0.0002296 | 154 | 0.221 | 158 | 89 | 11 | 1 | 142 | 1 | 140 | Phage tail protein | Phage tail protein | | afdb-uniprot50 | AF-A0A080KEX6-F1-MODEL\_V4 | 1.0 | 0.0002027 | 154 | 0.225 | 142 | 88 | 5 | 2 | 143 | 31 | 150 | Caudovirales tail fiber assembly protein | Caudovirales tail fiber assembly protein | | afdb-uniprot50 | AF-A0A6S7EC07-F1-MODEL\_V4 | 1.0 | 8.466e-05 | 154 | 0.251 | 139 | 87 | 6 | 1 | 127 | 1 | 134 | Uncharacterized protein | Uncharacterized protein | | afdb-uniprot50 | AF-A0A5F1I731-F1-MODEL\_V4 | 1.0 | 0.0001484 | 154 | 0.211 | 151 | 91 | 8 | 1 | 143 | 39 | 169 | Tail assembly chaperone | Tail assembly chaperone | | afdb-uniprot50 | AF-A0A379FLW3-F1-MODEL\_V4 | 1.0 | 0.0003137 | 154 | 0.236 | 127 | 74 | 2 | 17 | 143 | 119 | 222 | Bacteriophage tail assembly protein | Bacteriophage tail assembly protein | | afdb-uniprot50 | AF-A0A346B7G2-F1-MODEL\_V4 | 1.0 | 0.0003339 | 153 | 0.194 | 149 | 104 | 7 | 1 | 142 | 1 | 140 | Phage tail protein | Phage tail protein | | afdb-uniprot50 | AF-A0A6P1Q4F9-F1-MODEL\_V4 | 1.0 | 0.000131 | 153 | 0.197 | 162 | 96 | 8 | 5 | 143 | 25 | 175 | Uncharacterized protein | Uncharacterized protein | | afdb-uniprot50 | AF-A0A2L2B7F2-F1-MODEL\_V4 | 1.0 | 0.0002947 | 153 | 0.247 | 89 | 58 | 3 | 4 | 89 | 3 | 85 | DUF4376 domain-containing protein | DUF4376 domain-containing protein | | afdb-uniprot50 | AF-A0A7X5TFD8-F1-MODEL\_V4 | 1.0 | 0.0002027 | 153 | 0.21 | 147 | 84 | 7 | 2 | 143 | 78 | 197 | Phage tail protein | Phage tail protein | | afdb-uniprot50 | AF-A0A2T4HXL0-F1-MODEL\_V4 | 1.0 | 0.0002027 | 153 | 0.205 | 146 | 84 | 7 | 2 | 142 | 87 | 205 | Phage tail protein | Phage tail protein | | afdb-uniprot50 | AF-A0A3A6RRJ9-F1-MODEL\_V4 | 1.0 | 0.007092 | 152 | 0.293 | 75 | 51 | 1 | 66 | 140 | 9 | 81 | Uncharacterized protein | Uncharacterized protein | | afdb-uniprot50 | AF-A0A1U6INB7-F1-MODEL\_V4 | 1.0 | 0.001317 | 152 | 0.179 | 145 | 94 | 7 | 4 | 143 | 3 | 127 | Phage tail assembly chaperone protein | Phage tail assembly chaperone protein | | afdb-uniprot50 | AF-A0A837WAY8-F1-MODEL\_V4 | 1.0 | 0.0007057 | 152 | 0.197 | 142 | 100 | 6 | 4 | 143 | 3 | 132 | Phage tail protein | Phage tail protein | | afdb-uniprot50 | AF-A0A2D0IW15-F1-MODEL\_V4 | 1.0 | 0.0002444 | 152 | 0.2 | 155 | 91 | 10 | 1 | 143 | 38 | 171 | Tail assembly chaperone | Tail assembly chaperone | | afdb-uniprot50 | AF-A0A2S8Q9I9-F1-MODEL\_V4 | 1.0 | 0.0002769 | 152 | 0.219 | 146 | 84 | 7 | 2 | 143 | 61 | 180 | Phage tail protein | Phage tail protein | | afdb-uniprot50 | AF-A0A2S5K611-F1-MODEL\_V4 | 1.0 | 0.0003553 | 152 | 0.142 | 147 | 94 | 7 | 2 | 143 | 78 | 197 | Phage tail protein | Phage tail protein | | afdb-uniprot50 | AF-A0A447L1E5-F1-MODEL\_V4 | 1.0 | 0.0005499 | 152 | 0.169 | 124 | 90 | 6 | 25 | 143 | 113 | 228 | Caudovirales tail fibre assembly protein | Caudovirales tail fibre assembly protein | | afdb-uniprot50 | AF-A0A2P1VLZ0-F1-MODEL\_V4 | 1.0 | 0.0002296 | 151 | 0.205 | 146 | 86 | 5 | 2 | 143 | 80 | 199 | Phage tail protein | Phage tail protein | | afdb-uniprot50 | AF-A0A375AA84-F1-MODEL\_V4 | 1.0 | 0.0003553 | 151 | 0.162 | 148 | 90 | 7 | 2 | 143 | 83 | 202 | Tail fiber assembly protein | Tail fiber assembly protein | | afdb-uniprot50 | AF-V0AZV2-F1-MODEL\_V4 | 1.0 | 0.0002296 | 151 | 0.222 | 144 | 80 | 7 | 2 | 140 | 82 | 198 | Caudovirales tail fiber assembly protein | Caudovirales tail fiber assembly protein | | afdb-uniprot50 | AF-A0A336QKS4-F1-MODEL\_V4 | 1.0 | 0.000663 | 150 | 0.209 | 143 | 81 | 5 | 4 | 143 | 3 | 116 | Phage tail fiber assembly protein | Phage tail fiber assembly protein | | afdb-uniprot50 | AF-A0A119AU52-F1-MODEL\_V4 | 1.0 | 0.000456 | 150 | 0.18 | 144 | 102 | 6 | 4 | 140 | 5 | 139 | Uncharacterized protein | Uncharacterized protein | | afdb-uniprot50 | AF-A0A4Z0TBQ0-F1-MODEL\_V4 | 1.0 | 0.0005166 | 150 | 0.194 | 144 | 86 | 5 | 3 | 142 | 27 | 144 | Phage tail protein | Phage tail protein | | afdb-uniprot50 | AF-A0A7V8IHT1-F1-MODEL\_V4 | 1.0 | 0.0002444 | 150 | 0.191 | 146 | 86 | 6 | 2 | 142 | 86 | 204 | Tail assembly protein | Tail assembly protein | | afdb-uniprot50 | AF-A0A376D3X0-F1-MODEL\_V4 | 1.0 | 0.0002027 | 149 | 0.363 | 99 | 56 | 1 | 3 | 101 | 2 | 93 | Tail assembly chaperone gp38 | Tail assembly chaperone gp38 | | afdb-uniprot50 | AF-A0A2P9EJZ1-F1-MODEL\_V4 | 1.0 | 0.002783 | 149 | 0.302 | 86 | 57 | 2 | 55 | 140 | 29 | 111 | Tail fiber assembly protein homolog from lambdoid prophage DLP12 | Tail fiber assembly protein homolog from lambdoid prophage DLP12 | | afdb-uniprot50 | AF-A0A330EV64-F1-MODEL\_V4 | 1.0 | 0.0006229 | 149 | 0.21 | 157 | 85 | 8 | 1 | 143 | 1 | 132 | Uncharacterized protein | Uncharacterized protein | | afdb-uniprot50 | AF-A0A4R5L703-F1-MODEL\_V4 | 1.0 | 0.001401 | 149 | 0.21 | 152 | 98 | 7 | 1 | 142 | 1 | 140 | Phage tail protein | Phage tail protein | | afdb-uniprot50 | AF-A0A315WUN1-F1-MODEL\_V4 | 1.0 | 0.001401 | 149 | 0.186 | 123 | 92 | 3 | 4 | 126 | 7 | 121 | Phage tail protein | Phage tail protein | | afdb-uniprot50 | AF-A0A2A7U735-F1-MODEL\_V4 | 1.0 | 0.0003782 | 149 | 0.207 | 154 | 89 | 8 | 1 | 143 | 39 | 170 | Tail assembly chaperone | Tail assembly chaperone | | afdb-uniprot50 | AF-A0A379GMK5-F1-MODEL\_V4 | 1.0 | 0.0002947 | 149 | 0.198 | 146 | 87 | 7 | 1 | 142 | 86 | 205 | Bacteriophage tail assembly protein | Bacteriophage tail assembly protein | | afdb-uniprot50 | AF-Q7N5D1-F1-MODEL\_V4 | 1.0 | 0.0003339 | 149 | 0.19 | 147 | 87 | 7 | 2 | 143 | 374 | 493 | Collar domain-containing protein | Collar domain-containing protein | | afdb-uniprot50 | AF-A0A7D6IG48-F1-MODEL\_V4 | 1.0 | 0.0005499 | 148 | 0.209 | 143 | 99 | 4 | 5 | 143 | 2 | 134 | Tail fiber assembly protein | Tail fiber assembly protein | | afdb-uniprot50 | AF-A0A261SNT8-F1-MODEL\_V4 | 1.0 | 0.0004285 | 148 | 0.218 | 119 | 81 | 5 | 29 | 143 | 33 | 143 | Uncharacterized protein | Uncharacterized protein | | afdb-uniprot50 | AF-A0A077NP04-F1-MODEL\_V4 | 1.0 | 0.0004026 | 148 | 0.163 | 147 | 91 | 7 | 2 | 143 | 62 | 181 | Uncharacterized protein | Uncharacterized protein | | afdb-uniprot50 | AF-A0A379DRV6-F1-MODEL\_V4 | 1.0 | 0.000456 | 148 | 0.189 | 148 | 86 | 7 | 2 | 143 | 77 | 196 | Bacteriophage tail assembly protein | Bacteriophage tail assembly protein | | afdb-uniprot50 | AF-A0A1B9MVS0-F1-MODEL\_V4 | 1.0 | 0.0003782 | 148 | 0.197 | 147 | 86 | 7 | 2 | 143 | 83 | 202 | Uncharacterized protein | Uncharacterized protein | | afdb-uniprot50 | AF-A0A077NJ56-F1-MODEL\_V4 | 1.0 | 0.0003137 | 147 | 0.243 | 148 | 75 | 10 | 4 | 143 | 5 | 123 | Uncharacterized protein | Uncharacterized protein | | afdb-uniprot50 | AF-W1IQA1-F1-MODEL\_V4 | 1.0 | 0.001401 | 147 | 0.195 | 123 | 82 | 5 | 25 | 143 | 28 | 137 | Putative tail fiber assembly protein | Putative tail fiber assembly protein | | afdb-uniprot50 | AF-A0A068Z1I4-F1-MODEL\_V4 | 1.0 | 0.0002947 | 147 | 0.212 | 141 | 87 | 6 | 2 | 141 | 70 | 187 | Phage tail assembly protein | Phage tail assembly protein | | afdb-uniprot50 | AF-A0A7X1BIN5-F1-MODEL\_V4 | 1.0 | 0.0004285 | 147 | 0.182 | 148 | 87 | 6 | 2 | 143 | 77 | 196 | Tail fiber assembly protein | Tail fiber assembly protein | | afdb-uniprot50 | AF-A0A1A9VZ50-F1-MODEL\_V4 | 1.0 | 0.000456 | 147 | 0.178 | 146 | 90 | 6 | 2 | 143 | 241 | 360 | Uncharacterized protein | Uncharacterized protein | | afdb-uniprot50 | AF-A0A7Y0VF10-F1-MODEL\_V4 | 1.0 | 0.001317 | 146 | 0.204 | 142 | 99 | 4 | 3 | 142 | 2 | 131 | Phage tail protein | Phage tail protein | | afdb-uniprot50 | AF-A0A7M2QM89-F1-MODEL\_V4 | 1.0 | 0.0004285 | 146 | 0.187 | 144 | 85 | 7 | 2 | 140 | 82 | 198 | Uncharacterized protein | Uncharacterized protein | | afdb-uniprot50 | AF-A1JND9-F1-MODEL\_V4 | 1.0 | 0.0003137 | 146 | 0.19 | 147 | 86 | 7 | 2 | 143 | 86 | 204 | Putative phage tail fiber assembly protein | Putative phage tail fiber assembly protein | | afdb-uniprot50 | AF-A0A3R8W675-F1-MODEL\_V4 | 1.0 | 0.0004026 | 146 | 0.191 | 146 | 88 | 7 | 2 | 143 | 87 | 206 | Tail fiber assembly protein | Tail fiber assembly protein | | afdb-uniprot50 | AF-A0A2N4VAI3-F1-MODEL\_V4 | 1.0 | 1.303e-05 | 145 | 0.221 | 140 | 89 | 5 | 1 | 127 | 1 | 133 | Uncharacterized protein | Uncharacterized protein | | afdb-uniprot50 | AF-A0A410UEB7-F1-MODEL\_V4 | 1.0 | 0.003153 | 145 | 0.197 | 86 | 65 | 2 | 4 | 89 | 3 | 84 | Uncharacterized protein | Uncharacterized protein | | afdb-uniprot50 | AF-A0A6L6FGL7-F1-MODEL\_V4 | 1.0 | 0.0002947 | 145 | 0.206 | 145 | 85 | 7 | 2 | 142 | 65 | 183 | Tail fiber assembly protein | Tail fiber assembly protein | | afdb-uniprot50 | AF-A0A2N5EJ48-F1-MODEL\_V4 | 1.0 | 0.0004026 | 145 | 0.21 | 147 | 84 | 7 | 2 | 143 | 96 | 215 | Phage tail protein | Phage tail protein | | afdb-uniprot50 | AF-A0A658A1F9-F1-MODEL\_V4 | 1.0 | 0.005192 | 144 | 0.257 | 66 | 47 | 2 | 2 | 66 | 19 | 83 | Uncharacterized protein | Uncharacterized protein | | afdb-uniprot50 | AF-A0A423NVP6-F1-MODEL\_V4 | 1.0 | 0.0002947 | 144 | 0.174 | 143 | 103 | 6 | 4 | 142 | 3 | 134 | Uncharacterized protein | Uncharacterized protein | | afdb-uniprot50 | AF-A0A554PRY0-F1-MODEL\_V4 | 1.0 | 0.001026 | 144 | 0.167 | 143 | 105 | 4 | 4 | 142 | 2 | 134 | Phage tail protein | Phage tail protein | | afdb-uniprot50 | AF-A0A344UII6-F1-MODEL\_V4 | 1.0 | 1.78e-05 | 144 | 0.248 | 149 | 92 | 7 | 4 | 137 | 3 | 146 | Uncharacterized protein | Uncharacterized protein | | afdb-uniprot50 | AF-B8DLJ3-F1-MODEL\_V4 | 1.0 | 0.0006229 | 144 | 0.169 | 159 | 101 | 5 | 4 | 140 | 3 | 152 | Uncharacterized protein | Uncharacterized protein | | afdb-uniprot50 | AF-A0A379Z175-F1-MODEL\_V4 | 1.0 | 0.0002769 | 144 | 0.162 | 154 | 96 | 9 | 1 | 142 | 40 | 172 | Caudovirales tail fibre assembly protein | Caudovirales tail fibre assembly protein | | afdb-uniprot50 | AF-A0A1C0U9B5-F1-MODEL\_V4 | 1.0 | 0.0007994 | 144 | 0.193 | 155 | 94 | 9 | 1 | 143 | 42 | 177 | Caudovirales tail fiber assembly protein | Caudovirales tail fiber assembly protein | | afdb-uniprot50 | AF-A0A2A7T5Q6-F1-MODEL\_V4 | 1.0 | 0.0004026 | 144 | 0.198 | 146 | 85 | 7 | 3 | 143 | 74 | 192 | Tail assembly chaperone | Tail assembly chaperone | | afdb-uniprot50 | AF-U7QV10-F1-MODEL\_V4 | 1.0 | 0.0007057 | 143 | 0.169 | 153 | 88 | 8 | 2 | 143 | 59 | 183 | Uncharacterized protein | Uncharacterized protein | | afdb-uniprot50 | AF-A0A4U9HJ43-F1-MODEL\_V4 | 1.0 | 0.0004854 | 141 | 0.208 | 149 | 81 | 8 | 2 | 143 | 71 | 189 | Caudovirales tail fibre assembly protein | Caudovirales tail fibre assembly protein | | afdb-uniprot50 | AF-A0A556RUH4-F1-MODEL\_V4 | 1.0 | 0.007092 | 140 | 0.204 | 98 | 71 | 3 | 46 | 143 | 1 | 91 | Tail fiber assembly protein | Tail fiber assembly protein | | afdb-uniprot50 | AF-A0A1B3Z882-F1-MODEL\_V4 | 1.0 | 0.000131 | 139 | 0.19 | 131 | 94 | 4 | 4 | 127 | 3 | 128 | Uncharacterized protein | Uncharacterized protein | | afdb-uniprot50 | AF-A0A7V8TLT1-F1-MODEL\_V4 | 1.0 | 0.0005166 | 139 | 0.219 | 155 | 86 | 10 | 1 | 143 | 38 | 169 | Tail fiber assembly protein | Tail fiber assembly protein | | afdb-uniprot50 | AF-A0A4V2W777-F1-MODEL\_V4 | 1.0 | 0.001237 | 139 | 0.156 | 147 | 92 | 7 | 1 | 142 | 175 | 294 | Virus tail fiber assembly protein lambda gpK | Virus tail fiber assembly protein lambda gpK | | afdb-uniprot50 | AF-A0A315BKG8-F1-MODEL\_V4 | 1.0 | 0.0005853 | 138 | 0.24 | 137 | 84 | 6 | 4 | 140 | 2 | 118 | Uncharacterized protein | Uncharacterized protein | | afdb-uniprot50 | AF-A0A3G5LCQ7-F1-MODEL\_V4 | 1.0 | 0.0005499 | 138 | 0.216 | 125 | 67 | 6 | 45 | 143 | 3 | 122 | Phage tail protein | Phage tail protein | | afdb-uniprot50 | AF-A0A2N8AP07-F1-MODEL\_V4 | 1.0 | 0.002615 | 138 | 0.146 | 143 | 109 | 6 | 4 | 142 | 98 | 231 | Uncharacterized protein | Uncharacterized protein | | afdb-uniprot50 | AF-A0A485A861-F1-MODEL\_V4 | 1.0 | 0.0004026 | 138 | 0.193 | 145 | 92 | 9 | 4 | 143 | 618 | 742 | Caudovirales tail fibre assembly protein | Caudovirales tail fibre assembly protein | | afdb-uniprot50 | AF-U1TQF0-F1-MODEL\_V4 | 1.0 | 0.0008509 | 137 | 0.179 | 139 | 105 | 4 | 5 | 143 | 2 | 131 | Uncharacterized protein | Uncharacterized protein | | afdb-uniprot50 | AF-A0A3D9YP94-F1-MODEL\_V4 | 1.0 | 0.001237 | 137 | 0.214 | 149 | 88 | 8 | 5 | 142 | 2 | 132 | Uncharacterized protein | Uncharacterized protein | | afdb-uniprot50 | AF-A0A1S8CIT8-F1-MODEL\_V4 | 1.0 | 0.001588 | 137 | 0.171 | 146 | 91 | 7 | 2 | 143 | 77 | 196 | Uncharacterized protein | Uncharacterized protein | | afdb-uniprot50 | AF-S1IG16-F1-MODEL\_V4 | 1.0 | 0.0003137 | 136 | 0.206 | 145 | 88 | 9 | 4 | 140 | 6 | 131 | Uncharacterized protein | Uncharacterized protein | | afdb-uniprot50 | AF-A0A1N6MS03-F1-MODEL\_V4 | 1.0 | 0.0009639 | 136 | 0.165 | 163 | 97 | 8 | 5 | 143 | 22 | 169 | Uncharacterized protein | Uncharacterized protein | | afdb-uniprot50 | AF-A0A7T9HE50-F1-MODEL\_V4 | 1.0 | 0.0006229 | 135 | 0.209 | 124 | 80 | 4 | 4 | 126 | 3 | 109 | Tail fiber assembly protein | Tail fiber assembly protein | | afdb-uniprot50 | AF-A0A3S9K111-F1-MODEL\_V4 | 1.0 | 0.0008509 | 135 | 0.185 | 140 | 97 | 5 | 4 | 141 | 3 | 127 | Phage tail protein | Phage tail protein | | afdb-uniprot50 | AF-A0A2V4KJM6-F1-MODEL\_V4 | 1.0 | 0.001026 | 135 | 0.167 | 137 | 102 | 6 | 4 | 140 | 3 | 127 | Uncharacterized protein | Uncharacterized protein | | afdb-uniprot50 | AF-D4BTZ7-F1-MODEL\_V4 | 1.0 | 0.0007057 | 135 | 0.18 | 150 | 97 | 8 | 1 | 143 | 39 | 169 | Caudovirales tail fiber assembly protein | Caudovirales tail fiber assembly protein | | afdb-uniprot50 | AF-A0A2J9H2E1-F1-MODEL\_V4 | 1.0 | 0.001092 | 135 | 0.171 | 146 | 91 | 7 | 2 | 143 | 74 | 193 | Phage tail protein | Phage tail protein | | afdb-uniprot50 | AF-U1ZF67-F1-MODEL\_V4 | 1.0 | 0.002783 | 133 | 0.262 | 80 | 51 | 2 | 4 | 81 | 3 | 76 | Uncharacterized protein | Uncharacterized protein | | afdb-uniprot50 | AF-A0A1F8VMN7-F1-MODEL\_V4 | 1.0 | 0.001026 | 132 | 0.184 | 130 | 91 | 5 | 5 | 130 | 2 | 120 | Uncharacterized protein | Uncharacterized protein | | afdb-uniprot50 | AF-A0A085H8Z3-F1-MODEL\_V4 | 1.0 | 0.001492 | 132 | 0.193 | 145 | 87 | 6 | 3 | 143 | 78 | 196 | Tail fiber assembly protein | Tail fiber assembly protein | | afdb-uniprot50 | AF-A0A225SMJ4-F1-MODEL\_V4 | 1.0 | 0.0006229 | 131 | 0.217 | 124 | 78 | 5 | 5 | 127 | 2 | 107 | Uncharacterized protein | Uncharacterized protein | | afdb-uniprot50 | AF-A0A3S0J0M3-F1-MODEL\_V4 | 1.0 | 0.003571 | 131 | 0.136 | 146 | 104 | 7 | 4 | 142 | 3 | 133 | Phage tail protein | Phage tail protein | | afdb-uniprot50 | AF-A0A345CRH0-F1-MODEL\_V4 | 1.0 | 2.432e-05 | 131 | 0.219 | 132 | 87 | 7 | 4 | 127 | 3 | 126 | Uncharacterized protein | Uncharacterized protein | | afdb-uniprot50 | AF-A0A1G6QQ09-F1-MODEL\_V4 | 1.0 | 0.003801 | 131 | 0.167 | 155 | 88 | 5 | 4 | 126 | 3 | 148 | Uncharacterized protein | Uncharacterized protein | | afdb-uniprot50 | AF-A0A2A2PTQ0-F1-MODEL\_V4 | 1.0 | 0.003801 | 130 | 0.165 | 109 | 86 | 3 | 28 | 135 | 1 | 105 | Uncharacterized protein | Uncharacterized protein | | afdb-uniprot50 | AF-A0A7D6E693-F1-MODEL\_V4 | 1.0 | 0.001162 | 130 | 0.186 | 123 | 93 | 3 | 4 | 126 | 2 | 117 | Phage tail protein | Phage tail protein | | afdb-uniprot50 | AF-A0A1B4FRS3-F1-MODEL\_V4 | 1.0 | 0.001798 | 130 | 0.192 | 135 | 94 | 6 | 12 | 142 | 16 | 139 | Phage tail protein | Phage tail protein | | afdb-uniprot50 | AF-V0ATP8-F1-MODEL\_V4 | 1.0 | 0.0003137 | 130 | 0.218 | 151 | 87 | 9 | 1 | 143 | 40 | 167 | Caudovirales tail fiber assembly protein | Caudovirales tail fiber assembly protein | | afdb-uniprot50 | AF-A0A2C8EYE8-F1-MODEL\_V4 | 1.0 | 0.004583 | 130 | 0.186 | 123 | 90 | 5 | 27 | 143 | 78 | 196 | Uncharacterized protein | Uncharacterized protein | | afdb-uniprot50 | AF-A0A7W3CXQ1-F1-MODEL\_V4 | 1.0 | 0.0007057 | 129 | 0.186 | 134 | 91 | 4 | 4 | 133 | 3 | 122 | Uncharacterized protein | Uncharacterized protein | | afdb-uniprot50 | AF-A0A2K4MJ83-F1-MODEL\_V4 | 1.0 | 0.0001156 | 129 | 0.197 | 147 | 95 | 5 | 4 | 142 | 2 | 133 | Uncharacterized protein | Uncharacterized protein | | afdb-uniprot50 | AF-A0A291MWI8-F1-MODEL\_V4 | 1.0 | 3.536e-05 | 129 | 0.2 | 165 | 94 | 6 | 4 | 141 | 3 | 156 | Uncharacterized protein | Uncharacterized protein | | afdb-uniprot50 | AF-A0A0U1KG08-F1-MODEL\_V4 | 1.0 | 0.001237 | 128 | 0.227 | 145 | 90 | 8 | 4 | 143 | 3 | 130 | Putative phage tail fiber assembly protein | Putative phage tail fiber assembly protein | | afdb-uniprot50 | AF-A0A5P1DHX9-F1-MODEL\_V4 | 1.0 | 0.005526 | 128 | 0.171 | 99 | 80 | 2 | 28 | 126 | 49 | 145 | Phage tail protein | Phage tail protein | | afdb-uniprot50 | AF-A0A6L6H6J7-F1-MODEL\_V4 | 1.0 | 0.001092 | 128 | 0.155 | 167 | 98 | 6 | 14 | 143 | 13 | 173 | Tail fiber assembly protein | Tail fiber assembly protein | | afdb-uniprot50 | AF-A0A3M4M4E6-F1-MODEL\_V4 | 1.0 | 0.0002601 | 128 | 0.2 | 110 | 69 | 3 | 4 | 99 | 3 | 107 | Uncharacterized protein | Uncharacterized protein | | afdb-uniprot50 | AF-A0A5N7JQ80-F1-MODEL\_V4 | 1.0 | 0.003801 | 127 | 0.191 | 120 | 88 | 3 | 3 | 122 | 2 | 112 | Phage tail protein | Phage tail protein | | afdb-uniprot50 | AF-A0A7W2PYW0-F1-MODEL\_V4 | 1.0 | 0.006261 | 127 | 0.144 | 125 | 95 | 3 | 4 | 126 | 5 | 119 | Phage tail protein | Phage tail protein | | afdb-uniprot50 | AF-A0A0C1W5C6-F1-MODEL\_V4 | 1.0 | 0.003355 | 126 | 0.237 | 118 | 82 | 4 | 28 | 140 | 15 | 129 | Uncharacterized protein | Uncharacterized protein | | afdb-uniprot50 | AF-A0A7D4DZS1-F1-MODEL\_V4 | 1.0 | 0.005882 | 125 | 0.133 | 142 | 94 | 6 | 4 | 143 | 3 | 117 | Uncharacterized protein | Uncharacterized protein | | afdb-uniprot50 | AF-A0A1S1V1A2-F1-MODEL\_V4 | 1.0 | 0.005526 | 125 | 0.222 | 108 | 80 | 3 | 28 | 135 | 1 | 104 | Uncharacterized protein | Uncharacterized protein | | afdb-uniprot50 | AF-A0A2U8TMA5-F1-MODEL\_V4 | 1.0 | 0.005526 | 125 | 0.165 | 127 | 92 | 4 | 3 | 126 | 2 | 117 | Phage tail protein | Phage tail protein | | afdb-uniprot50 | AF-F3YW31-F1-MODEL\_V4 | 1.0 | 0.002168 | 125 | 0.226 | 128 | 80 | 3 | 1 | 127 | 1 | 110 | Uncharacterized protein | Uncharacterized protein | | afdb-uniprot50 | AF-A0A6N0JI93-F1-MODEL\_V4 | 1.0 | 0.001401 | 124 | 0.164 | 152 | 88 | 6 | 4 | 142 | 3 | 128 | Uncharacterized protein | Uncharacterized protein | | afdb-uniprot50 | AF-A0A109L8T1-F1-MODEL\_V4 | 1.0 | 0.00169 | 124 | 0.164 | 146 | 101 | 9 | 4 | 142 | 3 | 134 | Uncharacterized protein | Uncharacterized protein | | afdb-uniprot50 | AF-A0A418WP23-F1-MODEL\_V4 | 1.0 | 0.0002769 | 124 | 0.172 | 151 | 106 | 4 | 4 | 143 | 3 | 145 | Uncharacterized protein | Uncharacterized protein | | afdb-uniprot50 | AF-A0A1D9BC70-F1-MODEL\_V4 | 1.0 | 0.0003553 | 124 | 0.142 | 183 | 102 | 5 | 4 | 140 | 2 | 175 | DUF4376 domain-containing protein | DUF4376 domain-containing protein | | afdb-uniprot50 | AF-A0A5E7Q4J6-F1-MODEL\_V4 | 1.0 | 0.0005166 | 123 | 0.208 | 139 | 93 | 6 | 5 | 135 | 2 | 131 | Uncharacterized protein | Uncharacterized protein | | afdb-uniprot50 | AF-A0A5S9HN81-F1-MODEL\_V4 | 1.0 | 0.0007511 | 123 | 0.198 | 141 | 92 | 5 | 4 | 129 | 3 | 137 | Uncharacterized protein | Uncharacterized protein | | afdb-uniprot50 | AF-A0A377N9X0-F1-MODEL\_V4 | 1.0 | 0.002615 | 123 | 0.174 | 149 | 87 | 7 | 2 | 143 | 70 | 189 | Caudovirales tail fibre assembly protein | Caudovirales tail fibre assembly protein | | afdb-uniprot50 | AF-A0A4R6YXJ0-F1-MODEL\_V4 | 1.0 | 0.0007994 | 123 | 0.183 | 158 | 97 | 5 | 1 | 126 | 1 | 158 | Uncharacterized protein | Uncharacterized protein | | afdb-uniprot50 | AF-A0A250KYB2-F1-MODEL\_V4 | 1.0 | 0.001492 | 121 | 0.16 | 193 | 94 | 5 | 4 | 138 | 2 | 184 | DUF4376 domain-containing protein | DUF4376 domain-containing protein | | afdb-uniprot50 | AF-Q1I688-F1-MODEL\_V4 | 1.0 | 0.002037 | 120 | 0.181 | 143 | 102 | 6 | 4 | 142 | 2 | 133 | Putative phage protein | Putative phage protein | | afdb-uniprot50 | AF-A0A2A7T6M2-F1-MODEL\_V4 | 1.0 | 0.004878 | 120 | 0.177 | 118 | 77 | 6 | 46 | 143 | 29 | 146 | Phage tail protein | Phage tail protein | | afdb-uniprot50 | AF-A0A806X527-F1-MODEL\_V4 | 1.0 | 0.002308 | 120 | 0.193 | 145 | 69 | 4 | 45 | 143 | 53 | 195 | Phage tail protein | Phage tail protein | | afdb-uniprot50 | AF-A0A7V8RB58-F1-MODEL\_V4 | 1.0 | 0.0002947 | 119 | 0.223 | 152 | 98 | 6 | 4 | 143 | 3 | 146 | Uncharacterized protein | Uncharacterized protein | | afdb-uniprot50 | AF-A0A0T9T410-F1-MODEL\_V4 | 1.0 | 0.004583 | 119 | 0.164 | 146 | 103 | 7 | 4 | 143 | 32 | 164 | Tail fiber assembly protein G | Tail fiber assembly protein G | | afdb-uniprot50 | AF-A0A2N1H596-F1-MODEL\_V4 | 1.0 | 0.003571 | 118 | 0.217 | 124 | 80 | 6 | 28 | 143 | 1 | 115 | Phage tail protein | Phage tail protein | | afdb-uniprot50 | AF-A0A261SHH5-F1-MODEL\_V4 | 1.0 | 0.0004285 | 118 | 0.18 | 144 | 94 | 6 | 4 | 143 | 3 | 126 | Uncharacterized protein | Uncharacterized protein | | afdb-uniprot50 | AF-A0A537MFJ5-F1-MODEL\_V4 | 1.0 | 0.0005166 | 118 | 0.195 | 138 | 89 | 7 | 1 | 126 | 1 | 128 | Uncharacterized protein | Uncharacterized protein | | afdb-uniprot50 | AF-A0A2V3Y7F0-F1-MODEL\_V4 | 1.0 | 0.002037 | 118 | 0.2 | 140 | 87 | 8 | 4 | 135 | 3 | 125 | Phage tail assembly chaperone | Phage tail assembly chaperone | | afdb-uniprot50 | AF-A0A5E6WPB4-F1-MODEL\_V4 | 1.0 | 0.004583 | 118 | 0.255 | 86 | 58 | 1 | 4 | 89 | 2 | 81 | Uncharacterized protein | Uncharacterized protein | | afdb-uniprot50 | AF-A0A5P0J6I2-F1-MODEL\_V4 | 1.0 | 0.0001484 | 116 | 0.234 | 132 | 80 | 5 | 3 | 127 | 2 | 119 | Uncharacterized protein | Uncharacterized protein | | afdb-uniprot50 | AF-A0A5C7Q2A1-F1-MODEL\_V4 | 1.0 | 0.002168 | 116 | 0.195 | 169 | 95 | 8 | 1 | 140 | 1 | 157 | Uncharacterized protein | Uncharacterized protein | | afdb-uniprot50 | AF-A0A1C7WD91-F1-MODEL\_V4 | 1.0 | 0.001401 | 116 | 0.154 | 201 | 92 | 7 | 2 | 142 | 71 | 253 | Uncharacterized protein | Uncharacterized protein | | afdb-uniprot50 | AF-A0A083ZV50-F1-MODEL\_V4 | 1.0 | 0.004046 | 115 | 0.159 | 163 | 80 | 4 | 28 | 142 | 48 | 201 | Caudovirales tail fiber assembly protein | Caudovirales tail fiber assembly protein | | afdb-uniprot50 | AF-A0A0E8XEB7-F1-MODEL\_V4 | 1.0 | 0.0004854 | 114 | 0.242 | 161 | 82 | 8 | 2 | 143 | 18 | 157 | Tail assembly chaperone gp38 | Tail assembly chaperone gp38 | | afdb-uniprot50 | AF-A0A1V5QIZ0-F1-MODEL\_V4 | 1.0 | 0.002457 | 112 | 0.16 | 187 | 95 | 6 | 5 | 138 | 2 | 179 | DUF4376 domain-containing protein | DUF4376 domain-containing protein | | afdb-uniprot50 | AF-H9BMI8-F1-MODEL\_V4 | 1.0 | 0.006663 | 111 | 0.194 | 159 | 93 | 10 | 4 | 143 | 6 | 148 | Uncharacterized protein | Uncharacterized protein | | afdb-uniprot50 | AF-A0A1A9VZ06-F1-MODEL\_V4 | 1.0 | 0.007092 | 110 | 0.188 | 159 | 87 | 9 | 5 | 142 | 337 | 474 | Head\_binding domain-containing protein | Head\_binding domain-containing protein | | afdb-uniprot50 | AF-A0A4R3J9P7-F1-MODEL\_V4 | 1.0 | 0.006261 | 108 | 0.146 | 198 | 101 | 6 | 4 | 139 | 3 | 194 | Uncharacterized protein | Uncharacterized protein | | afdb-uniprot50 | AF-A0A6G6ZV78-F1-MODEL\_V4 | 1.0 | 0.004306 | 105 | 0.142 | 169 | 103 | 7 | 4 | 143 | 3 | 158 | Phage tail protein | Phage tail protein | | afdb-uniprot50 | AF-A0A2T6LN68-F1-MODEL\_V4 | 1.0 | 0.0002158 | 105 | 0.197 | 142 | 89 | 5 | 1 | 127 | 1 | 132 | Uncharacterized protein | Uncharacterized protein | | afdb-uniprot50 | AF-A0A2V2AD22-F1-MODEL\_V4 | 1.0 | 0.008034 | 105 | 0.245 | 102 | 70 | 4 | 27 | 126 | 81 | 177 | Uncharacterized protein | Uncharacterized protein | | afdb-uniprot50 | AF-A0A1I2QMV9-F1-MODEL\_V4 | 1.0 | 0.0006229 | 104 | 0.181 | 138 | 91 | 5 | 4 | 134 | 2 | 124 | Uncharacterized protein | Uncharacterized protein | | afdb-uniprot50 | AF-A0A377QEM3-F1-MODEL\_V4 | 1.0 | 0.002783 | 104 | 0.171 | 134 | 89 | 5 | 1 | 126 | 1 | 120 | Uncharacterized protein | Uncharacterized protein | | afdb-uniprot50 | AF-A0A427DLX8-F1-MODEL\_V4 | 1.0 | 0.0002947 | 102 | 0.209 | 148 | 92 | 8 | 4 | 142 | 2 | 133 | Uncharacterized protein | Uncharacterized protein | | afdb-uniprot50 | AF-A0A7T7KK36-F1-MODEL\_V4 | 1.0 | 0.005526 | 101 | 0.159 | 188 | 102 | 8 | 4 | 140 | 2 | 184 | Uncharacterized protein | Uncharacterized protein | | afdb-uniprot50 | AF-A0A354UB04-F1-MODEL\_V4 | 1.0 | 0.009688 | 100 | 0.205 | 146 | 96 | 5 | 3 | 138 | 2 | 137 | Uncharacterized protein | Uncharacterized protein | | afdb-uniprot50 | AF-A0A2S9AZ00-F1-MODEL\_V4 | 1.0 | 0.003801 | 100 | 0.201 | 124 | 74 | 4 | 3 | 109 | 2 | 117 | Uncharacterized protein | Uncharacterized protein | | afdb-uniprot50 | AF-A0A7Z8DSQ9-F1-MODEL\_V4 | 1.0 | 0.0008509 | 100 | 0.163 | 196 | 90 | 6 | 1 | 141 | 10 | 186 | DUF4376 domain-containing protein | DUF4376 domain-containing protein | | afdb-uniprot50 | AF-A0A845BIX0-F1-MODEL\_V4 | 0.999 | 0.005526 | 99 | 0.125 | 262 | 105 | 8 | 2 | 141 | 21 | 280 | DUF4376 domain-containing protein | DUF4376 domain-containing protein | | afdb-uniprot50 | AF-A0A557QX70-F1-MODEL\_V4 | 0.999 | 0.006261 | 98 | 0.163 | 184 | 96 | 6 | 4 | 139 | 3 | 176 | Uncharacterized protein | Uncharacterized protein | | afdb-uniprot50 | AF-A0A142BH82-F1-MODEL\_V4 | 0.999 | 0.0009056 | 97 | 0.17 | 176 | 88 | 8 | 4 | 130 | 3 | 169 | Uncharacterized protein | Uncharacterized protein | | afdb-uniprot50 | AF-A0A4R2MGV0-F1-MODEL\_V4 | 0.999 | 0.002962 | 96 | 0.205 | 136 | 80 | 6 | 4 | 127 | 3 | 122 | Uncharacterized protein | Uncharacterized protein | | afdb-uniprot50 | AF-A0A7I8EBT6-F1-MODEL\_V4 | 0.999 | 0.002308 | 96 | 0.16 | 187 | 98 | 6 | 12 | 141 | 2 | 186 | Uncharacterized protein | Uncharacterized protein | | afdb-uniprot50 | AF-W9BTD0-F1-MODEL\_V4 | 0.997 | 0.001588 | 92 | 0.175 | 165 | 101 | 10 | 2 | 143 | 11 | 163 | Uncharacterized protein | Uncharacterized protein | | afdb-uniprot50 | AF-A0A1Y0N4F4-F1-MODEL\_V4 | 0.996 | 0.007092 | 90 | 0.183 | 131 | 80 | 6 | 5 | 127 | 2 | 113 | Uncharacterized protein | Uncharacterized protein | | afdb-uniprot50 | AF-A0A4D8RIN6-F1-MODEL\_V4 | 0.996 | 0.008034 | 90 | 0.161 | 198 | 98 | 8 | 2 | 140 | 329 | 517 | DUF4376 domain-containing protein | DUF4376 domain-containing protein | | afdb-uniprot50 | AF-A0A2W0FDX5-F1-MODEL\_V4 | 0.961 | 0.003153 | 74 | 0.232 | 116 | 66 | 6 | 28 | 134 | 5 | 106 | Phage tail protein | Phage tail protein | |
| Top keywords  (threshold 1.00e-02 (evalue)) | **tail, assembly, fiber, Phage, chaperone, Caudovirales, fibre, Putative, prophage, lambda** |
| Output files | ../../similar\_structures/16\_FANPEZAQ\_CDS\_0016\_afdb-proteome\_foldseek.tsv ../../similar\_structures/16\_FANPEZAQ\_CDS\_0016\_afdb-uniprot50\_foldseek.tsv ../../similar\_structures/16\_FANPEZAQ\_CDS\_0016\_merged.svg ../../similar\_structures/16\_FANPEZAQ\_CDS\_0016\_pdb\_foldseek.tsv |

  
  
  

Return to summary | Go to previous | Go to next

  


---

**Sequence/structure alignments coloring**  
Each object in the alignment figures is colored according to its E-value following this color coding:

1e-100
10

**References:**  
1) Steinegger M, Meier M, Mirdita M, Vöhringer H, Haunsberger S J, and Söding J (2019) HH-suite3 for fast remote homology detection and deep protein annotation, BMC Bioinformatics, 473. doi: 10.1186/s12859-019-3019-7  
2) Jumper J, Evans R, Pritzel A, ..., Hassabis D (2021) Highly accurate protein structure prediction with AlphaFold, Nature, 596. doi: 10.1038/s41586-021-03819-2  
3) van Kempen M, Kim S, Tumescheit C, Mirdita M, Lee J, Gilchrist CLM, Söding J, and Steinegger M (2023) Fast and accurate protein structure search with Foldseek. Nature Biotechnology. doi: 10.1038/s41587-023-01773-0
