# Supplementary material for: Completing the BASEL phage collection to unlock hidden diversity for systematic exploration of phage–host interactions
Source: PLoS Biol. 2025 Apr 7;23(4):e3003063. doi: 10.1371/journal.pbio.3003063 (PMC11990801; doi:10.1371/journal.pbio.3003063)
Supplement: S2 Data — (ZIP) [file pbio.3003063.s009.zip › entries/17.html]

FANPEZAQ\_CDS\_0017


Return to summary | Go to previous | Go to next

|  |  |
| --- | --- |
| FANPEZAQ\_CDS\_0017 Page creation date: 02 Sep 2024, 12:00  Project folder: n/a  Input sequences file: Escherichia\_virus\_HeidiAbel.gb | tail collar domain\_containing phage fiber microcystin\_dependent hypothetical baseplate t4 wedge gp6 complex tube putative baseplate\_tail pre\_attachment fragment phage\_related side microcystin dependent prophage fibre short protein\_like repeat acid transporter structural fatty short\_chain containing n\_terminal bacteriophage long |

### Sequence information

|  |  |
| --- | --- |
| Name | FANPEZAQ\_CDS\_0017  17\_FANPEZAQ\_CDS\_0017 (pipeline id) |
| Imported annotations | Escherichia\_virus\_HeidiAbel Bas97 |
| Protein sequence | MSQILEFWKGLSGAVLPFAGANAPTGWLMCDGSAVSRTTFAELFASIGTTYGTGDGSTTF NLPDLRGRVAAGKDNMGGTPANRLTAAGAGITGTALGATGGSQTHTMTTGQMPAHSHGVN DPTHAHSVYDPTHTHAVYDPGHVHSYGRVTTGNGQGSDIGTANNHVTANTGAATTGISLY ASATGIGIYGAATGISIQNQGGGAAHNITQPTIVLNHIIKI |
| Number of residues | 221 |
| Molecular weight (Da) | 22212.26 |
| Output files | ../../query\_sequences/17\_FANPEZAQ\_CDS\_0017.fasta |

### Putative domain architecture and protein family

#### Search results (HHblits)1

|  |  |
| --- | --- |
| Domain family databases searched | Pfam, Ncbi-cd, Cath, Phrogs |
| Results, scheme(s)  (Top layers only; threshold 1.00e-03 (evalue)) | xml version="1.0" encoding="utf-8" standalone="no"?       2024-09-02T21:08:15.827230 image/svg+xml   Matplotlib v3.7.2, https://matplotlib.org/ |
| Results, table  (E-value ≤ 1.00e-03 (evalue)) | | db | id | prob | evalue | pvalue | score | cols | query | query\_len | template | template\_len | name | description | | --- | --- | --- | --- | --- | --- | --- | --- | --- | --- | --- | --- | --- | | phrogs | 8763 | 99.9 | 4.4e-34 | 5.4e-38 | 233.1 | 170 | (28, 221) | 221 | (1, 170) | 170 | tail protein | tail protein; Category: tail; NC\_023006\_p17 | | phrogs | 8469 | 99.9 | 9.4e-32 | 1.1e-35 | 230.8 | 168 | (9, 221) | 221 | (114, 290) | 292 | tail fiber protein | tail fiber protein; Category: tail; NC\_019917\_p61 | | phrogs | 523 | 99.9 | 1.3e-28 | 1.6e-32 | 204.1 | 89 | (6, 123) | 221 | (40, 128) | 183 | tail collar fiber protein | tail collar fiber protein; Category: tail; p284593 VI\_03272 | | phrogs | 1800 | 99.8 | 1.3e-26 | 1.6e-30 | 193.7 | 155 | (8, 221) | 221 | (70, 224) | 224 | tail collar fiber protein | tail collar fiber protein; Category: tail; KJ019100\_p265 | | phrogs | 1858 | 99.8 | 9.8e-24 | 1.3e-27 | 182.0 | 86 | (8, 122) | 221 | (103, 188) | 256 | tail fiber protein | tail fiber protein; Category: tail; p362065 VI\_01943 | | phrogs | 9116 | 99.7 | 4.7e-23 | 5.4e-27 | 182.6 | 100 | (9, 121) | 221 | (58, 157) | 443 | tail protein | tail protein; Category: tail; KU160641\_p20 | | phrogs | 2097 | 99.7 | 7.3e-23 | 8.8e-27 | 185.7 | 117 | (8, 145) | 221 | (255, 371) | 454 | tail fiber protein | tail fiber protein; Category: tail; p203462 VI\_04227 | | phrogs | 31611 | 99.7 | 3.4e-22 | 3.8e-26 | 171.9 | 198 | (7, 221) | 221 | (156, 384) | 384 | tail collar protein | tail collar protein; Category: tail; KU234532\_p33 | | phrogs | 14307 | 99.7 | 6.7e-22 | 7.7e-26 | 141.2 | 81 | (6, 115) | 221 | (3, 83) | 83 | NA | NA; Category: unknown function; p28392 VI\_07752 | | phrogs | 20472 | 99.5 | 1.7e-18 | 1.9e-22 | 147.4 | 86 | (7, 121) | 221 | (186, 271) | 331 | NA | NA; Category: unknown function; p276897 VI\_03881 | | phrogs | 25747 | 99.4 | 7.2e-18 | 8e-22 | 153.9 | 95 | (11, 124) | 221 | (679, 773) | 901 | tail fiber protein | tail fiber protein; Category: tail; KR052480\_p88 | | phrogs | 295 | 99.4 | 1.6e-17 | 1.9e-21 | 156.2 | 108 | (5, 146) | 221 | (624, 746) | 813 | tail fiber protein | tail fiber protein; Category: tail; p109395 VI\_03810 | | phrogs | 4849 | 99.4 | 2.5e-17 | 2.9e-21 | 152.3 | 96 | (7, 121) | 221 | (550, 662) | 790 | tail protein | tail protein; Category: tail; JX681814\_p23 | | phrogs | 3100 | 99.3 | 9.5e-17 | 1.1e-20 | 143.0 | 100 | (7, 140) | 221 | (249, 363) | 436 | tail fiber protein | tail fiber protein; Category: tail; p172841 VI\_09914 | | phrogs | 5795 | 99.3 | 9.4e-17 | 1.2e-20 | 134.3 | 89 | (7, 122) | 221 | (62, 152) | 215 | tail protein | tail protein; Category: tail; JN254801\_p12 | | phrogs | 327 | 99.3 | 1.7e-16 | 2.1e-20 | 146.2 | 87 | (5, 120) | 221 | (381, 467) | 544 | tail protein | tail protein; Category: tail; p384764 VI\_09705 | | phrogs | 6256 | 99.1 | 6.9e-15 | 8.2e-19 | 134.5 | 108 | (4, 147) | 221 | (251, 379) | 579 | tail fiber protein | tail fiber protein; Category: tail; KY883648\_p41 | | phrogs | 6459 | 99.1 | 1.1e-14 | 1.2e-18 | 110.4 | 66 | (27, 121) | 221 | (3, 68) | 121 | tail collar protein | tail collar protein; Category: tail; p30245 VI\_11136 | | phrogs | 19618 | 99.0 | 6.4e-14 | 7.2e-18 | 121.0 | 90 | (7, 120) | 221 | (133, 222) | 387 | tail collar protein | tail collar protein; Category: tail; p51395 VI\_03026 | | phrogs | 12846 | 98.9 | 2.7e-13 | 3e-17 | 119.8 | 91 | (5, 121) | 221 | (150, 240) | 544 | tail fiber protein; Ig-domain containing | tail fiber protein; Ig-domain containing; Category: tail; MF979563\_p29 | | phrogs | 4711 | 98.7 | 3.9e-12 | 4.5e-16 | 111.4 | 101 | (9, 121) | 221 | (223, 400) | 461 | NA | NA; Category: unknown function; p26028 VI\_12353 | | phrogs | 7765 | 98.6 | 1.1e-11 | 1.3e-15 | 110.7 | 102 | (8, 121) | 221 | (340, 517) | 578 | NA | NA; Category: unknown function; p66770 VI\_12458 | | phrogs | 38759 | 98.1 | 9.8e-10 | 1.1e-13 | 100.0 | 78 | (37, 139) | 221 | (648, 727) | 787 | NA | NA; Category: unknown function; p217390 VI\_05721 | | phrogs | 9370 | 98.0 | 1.4e-09 | 1.5e-13 | 94.2 | 100 | (10, 121) | 221 | (226, 401) | 458 | tail fiber protein | tail fiber protein; Category: tail; p122631 VI\_12395 | | phrogs | 26622 | 97.9 | 4.8e-09 | 5.4e-13 | 94.5 | 84 | (9, 119) | 221 | (174, 257) | 691 | tail fiber protein | tail fiber protein; Category: tail; p171543 VI\_11794 | | phrogs | 2631 | 97.6 | 3.9e-08 | 4.6e-12 | 86.8 | 85 | (7, 125) | 221 | (337, 421) | 482 | tail fiber protein | tail fiber protein; Category: tail; p130317 VI\_07372 | | phrogs | 8239 | 97.5 | 5.3e-08 | 6.1e-12 | 87.8 | 84 | (6, 124) | 221 | (442, 525) | 654 | tail fiber protein | tail fiber protein; Category: tail; p255681 VI\_08125 | | phrogs | 20242 | 97.4 | 8e-08 | 9.2e-12 | 89.5 | 75 | (14, 118) | 221 | (913, 987) | 1001 | tail fiber protein | tail fiber protein; Category: tail; NC\_014660\_p244 | | phrogs | 6921 | 97.3 | 2.2e-07 | 2.6e-11 | 86.7 | 67 | (8, 74) | 221 | (234, 326) | 767 | tail fiber protein | tail fiber protein; Category: tail; KM979355\_p107 | | phrogs | 4591 | 97.2 | 3.7e-07 | 4.3e-11 | 81.5 | 53 | (2, 54) | 221 | (5, 59) | 567 | short tail fiber protein | short tail fiber protein; Category: tail; KX349292\_p35 | | phrogs | 7489 | 97.0 | 9.6e-07 | 1.1e-10 | 80.2 | 42 | (8, 49) | 221 | (497, 539) | 666 | tail fiber protein | tail fiber protein; Category: tail; p80663 VI\_09756 | | phrogs | 24267 | 96.7 | 3.1e-06 | 3.5e-10 | 68.7 | 29 | (93, 121) | 221 | (185, 213) | 266 | NA | NA; Category: unknown function; p146200 VI\_04950 | | phrogs | 4924 | 96.6 | 4.7e-06 | 5.6e-10 | 78.4 | 67 | (6, 74) | 221 | (537, 617) | 981 | short tail fiber protein | short tail fiber protein; Category: tail; NC\_031900\_p33 | | phrogs | 38064 | 96.6 | 5.5e-06 | 6.2e-10 | 67.4 | 62 | (3, 64) | 221 | (213, 276) | 295 | tail collar protein | tail collar protein; Category: tail; NC\_029002\_p19 | | phrogs | 31602 | 96.2 | 1.9e-05 | 2.2e-09 | 67.7 | 67 | (7, 73) | 221 | (11, 97) | 428 | virion structural protein | virion structural protein; Category: head and packaging; NC\_013085\_p212 | | phrogs | 2631 | 95.7 | 8.8e-05 | 1e-08 | 64.9 | 33 | (4, 36) | 221 | (179, 225) | 482 | tail fiber protein | tail fiber protein; Category: tail; p130317 VI\_07372 | | phrogs | 13173 | 94.7 | 0.0005 | 5.8e-08 | 63.3 | 95 | (12, 122) | 221 | (445, 557) | 920 | tail fiber protein | tail fiber protein; Category: tail; MG201401\_p40 | |
| Top keywords  (threshold 1.00e-03 (evalue)) | **tail, fiber, collar, p130317, VI\_07372, short, NC\_023006\_p17, NC\_019917\_p61, p284593, VI\_03272** |
| Output files | ../../domain\_architecture/17\_FANPEZAQ\_CDS\_0017\_cath.hhr ../../domain\_architecture/17\_FANPEZAQ\_CDS\_0017\_merged.svg ../../domain\_architecture/17\_FANPEZAQ\_CDS\_0017\_ncbi-cd.hhr ../../domain\_architecture/17\_FANPEZAQ\_CDS\_0017\_pfam.hhr ../../domain\_architecture/17\_FANPEZAQ\_CDS\_0017\_phrogs.hhr |

### Identical protein sequences/structures

#### Search results

|  |  |
| --- | --- |
| Protein sequence databases searched | Pdb, Swissprot, Refseq |
| Identical proteins found | -- |
| Top keywords | -- |
| Output files | -- |

### Similar protein sequences/structures

#### Sequence similarity search results (HHblits)1

|  |  |
| --- | --- |
| Sequence databases searched | Uniclust, Pdb70 |
| Results, scheme(s)  (Top layers only, threshold 1.00e-03 (evalue)) | xml version="1.0" encoding="utf-8" standalone="no"?       2024-09-02T21:08:36.325454 image/svg+xml   Matplotlib v3.7.2, https://matplotlib.org/ |
| Results, table(s)  (threshold 1.00e-03 (evalue)) | | db | id | prob | evalue | pvalue | score | cols | query | query\_len | template | template\_len | name | description | | --- | --- | --- | --- | --- | --- | --- | --- | --- | --- | --- | --- | --- | | uniclust | UniRef100\_A0A0P0EA18 | 100.0 | 8e-41 | 1.5e-46 | 242.0 | 180 | (6, 221) | 221 | (63, 242) | 247 | Phage tail collar domain-containing protein | Phage tail collar domain-containing protein | | uniclust | UniRef100\_A0A0M4T0P4 | 100.0 | 8.7e-38 | 1.6e-43 | 241.3 | 200 | (8, 221) | 221 | (157, 367) | 368 | Phage tail collar domain-containing protein | Phage tail collar domain-containing protein | | uniclust | UniRef100\_A0A0M5IIJ7 | 100.0 | 1.6e-37 | 2.9e-43 | 234.2 | 171 | (9, 221) | 221 | (127, 297) | 302 | Microcystin-dependent protein-like protein | Microcystin-dependent protein-like protein | | uniclust | UniRef100\_A0A813X408 | 100.0 | 1.4e-36 | 2.7e-42 | 222.5 | 195 | (8, 221) | 221 | (59, 256) | 270 | Phage tail collar domain-containing protein | Phage tail collar domain-containing protein | | uniclust | UniRef100\_A0A1C2DSX6 | 100.0 | 6.8e-36 | 1.3e-41 | 214.0 | 113 | (6, 118) | 221 | (30, 142) | 218 | Phage tail collar domain-containing protein | Phage tail collar domain-containing protein | | uniclust | UniRef100\_A0A0B3RIK5 | 100.0 | 3.2e-35 | 6e-41 | 217.5 | 186 | (7, 221) | 221 | (78, 263) | 265 | Phage Tail Collar | Phage Tail Collar | | uniclust | UniRef100\_A0A1V1P4C0 | 100.0 | 8.1e-34 | 1.5e-39 | 219.8 | 175 | (10, 221) | 221 | (208, 386) | 386 | Microcystin-dependent protein-like protein | Microcystin-dependent protein-like protein | | uniclust | UniRef100\_A0A1F3ABU4 | 100.0 | 8e-34 | 1.5e-39 | 199.8 | 100 | (8, 119) | 221 | (19, 118) | 176 | Phage tail collar domain-containing protein | Phage tail collar domain-containing protein | | uniclust | UniRef100\_A0A813P3R8 | 100.0 | 9.5e-34 | 1.8e-39 | 199.8 | 174 | (9, 221) | 221 | (38, 211) | 220 | Phage tail collar domain-containing protein | Phage tail collar domain-containing protein | | uniclust | UniRef100\_A0A1V4VVI6 | 100.0 | 3.4e-33 | 6.4e-39 | 213.1 | 178 | (7, 221) | 221 | (205, 382) | 386 | Phage Tail Collar Domain protein | Phage Tail Collar Domain protein | | uniclust | UniRef100\_A0A3D0M2I4 | 100.0 | 4.4e-33 | 8.2e-39 | 199.0 | 99 | (8, 119) | 221 | (71, 169) | 230 | Phage tail collar domain-containing protein | Phage tail collar domain-containing protein | | uniclust | UniRef100\_A0A6J5M5U3 | 100.0 | 1.6e-32 | 3e-38 | 197.3 | 94 | (8, 119) | 221 | (72, 165) | 238 | MdpB Microcystin-dependent protein | MdpB Microcystin-dependent protein | | uniclust | UniRef100\_A0A074J4T7 | 100.0 | 2.6e-32 | 4.8e-38 | 188.4 | 100 | (8, 119) | 221 | (48, 147) | 194 | Phage tail collar domain-containing protein | Phage tail collar domain-containing protein | | uniclust | UniRef100\_A0A2E7V2E5 | 99.9 | 3.8e-32 | 7e-38 | 203.8 | 108 | (7, 117) | 221 | (152, 259) | 325 | Phage tail collar domain-containing protein | Phage tail collar domain-containing protein | | uniclust | UniRef100\_A0A0H4IKC1 | 99.9 | 4.2e-32 | 7.8e-38 | 198.2 | 181 | (8, 221) | 221 | (82, 262) | 264 | Putative tail collar protein | Putative tail collar protein | | uniclust | UniRef100\_A0A0D7Q2R9 | 99.9 | 7.7e-32 | 1.4e-37 | 207.0 | 116 | (9, 126) | 221 | (133, 248) | 335 | Phage tail collar domain-containing protein | Phage tail collar domain-containing protein | | uniclust | UniRef100\_A0A813VP22 | 99.9 | 1.2e-31 | 2.1e-37 | 200.4 | 196 | (7, 221) | 221 | (136, 339) | 346 | Phage tail collar domain-containing protein | Phage tail collar domain-containing protein | | uniclust | UniRef100\_A0A177Q1Y6 | 99.9 | 5.3e-31 | 9.9e-37 | 195.1 | 108 | (8, 119) | 221 | (132, 239) | 301 | Phage tail collar domain-containing protein | Phage tail collar domain-containing protein | | uniclust | UniRef100\_A0A814BNA3 | 99.9 | 7.4e-31 | 1.4e-36 | 190.3 | 176 | (7, 221) | 221 | (104, 279) | 280 | Phage tail collar domain-containing protein | Phage tail collar domain-containing protein | | uniclust | UniRef100\_A0A814NW00 | 99.9 | 8.6e-31 | 1.6e-36 | 216.5 | 198 | (10, 221) | 221 | (585, 787) | 792 | Phage tail collar domain-containing protein | Phage tail collar domain-containing protein | | uniclust | UniRef100\_A0A6N4T611 | 99.9 | 1.1e-30 | 2e-36 | 178.0 | 90 | (8, 119) | 221 | (21, 110) | 168 | Phage tail collar domain-containing protein | Phage tail collar domain-containing protein | | uniclust | UniRef100\_A0A2E4T1Y0 | 99.9 | 1.4e-30 | 2.6e-36 | 189.3 | 178 | (10, 221) | 221 | (108, 286) | 286 | Phage tail collar domain-containing protein | Phage tail collar domain-containing protein | | uniclust | UniRef100\_A0A6C0EEV3 | 99.9 | 2e-30 | 3.7e-36 | 191.4 | 173 | (8, 221) | 221 | (176, 348) | 349 | Phage tail collar domain-containing protein | Phage tail collar domain-containing protein | | uniclust | UniRef100\_A0A060DEV3 | 99.9 | 4.2e-30 | 7.9e-36 | 177.4 | 158 | (7, 221) | 221 | (28, 186) | 186 | Phage tail collar domain-containing protein | Phage tail collar domain-containing protein | | uniclust | UniRef100\_A0A1G0C5V8 | 99.9 | 8e-30 | 1.5e-35 | 193.7 | 157 | (10, 221) | 221 | (116, 276) | 280 | Phage tail collar domain-containing protein | Phage tail collar domain-containing protein | | uniclust | UniRef100\_A0A0Q6Z9B8 | 99.9 | 9.4e-30 | 1.7e-35 | 196.1 | 111 | (9, 120) | 221 | (202, 313) | 387 | Phage tail collar domain-containing protein | Phage tail collar domain-containing protein | | uniclust | UniRef100\_A0A074J2N7 | 99.9 | 1.3e-29 | 2.4e-35 | 178.8 | 97 | (10, 120) | 221 | (6, 102) | 188 | Phage tail collar domain-containing protein | Phage tail collar domain-containing protein | | uniclust | UniRef100\_A0A814FG22 | 99.9 | 1.6e-29 | 3e-35 | 183.5 | 197 | (8, 221) | 221 | (41, 245) | 255 | Phage tail collar domain-containing protein | Phage tail collar domain-containing protein | | uniclust | UniRef100\_A0A1F3PPS7 | 99.9 | 1.6e-29 | 3.1e-35 | 196.5 | 159 | (9, 221) | 221 | (156, 317) | 319 | Phage tail collar domain-containing protein | Phage tail collar domain-containing protein | | uniclust | UniRef100\_A0A813X8J9 | 99.9 | 2e-29 | 3.8e-35 | 198.7 | 196 | (9, 221) | 221 | (262, 465) | 466 | Phage tail collar domain-containing protein | Phage tail collar domain-containing protein | | uniclust | UniRef100\_A0A0B4EK47 | 99.9 | 2.9e-29 | 5.4e-35 | 184.8 | 99 | (9, 120) | 221 | (59, 157) | 237 | Phage tail collar domain-containing protein | Phage tail collar domain-containing protein | | uniclust | UniRef100\_A0A0L0ENS5 | 99.9 | 5.2e-29 | 9.8e-35 | 187.1 | 98 | (9, 119) | 221 | (50, 147) | 249 | Phage tail collar domain-containing protein | Phage tail collar domain-containing protein | | uniclust | UniRef100\_A0A063KYT2 | 99.9 | 5.5e-29 | 1e-34 | 184.2 | 99 | (9, 120) | 221 | (49, 147) | 233 | Phage tail protein | Phage tail protein | | uniclust | UniRef100\_A0A0Q0XB69 | 99.9 | 7.2e-29 | 1.3e-34 | 178.6 | 97 | (10, 119) | 221 | (45, 141) | 219 | Phage tail collar domain-containing protein | Phage tail collar domain-containing protein | | uniclust | UniRef100\_A0A010YCA6 | 99.9 | 8e-29 | 1.5e-34 | 183.5 | 99 | (9, 120) | 221 | (30, 128) | 228 | Microcystin-dependent protein | Microcystin-dependent protein | | uniclust | UniRef100\_A0A0C3ENX2 | 99.9 | 1.1e-28 | 2.1e-34 | 180.4 | 97 | (9, 118) | 221 | (32, 128) | 213 | Tail protein | Tail protein | | uniclust | UniRef100\_A0A077L9W7 | 99.9 | 1.4e-28 | 2.7e-34 | 180.8 | 100 | (8, 120) | 221 | (26, 125) | 249 | Tail Collar domain protein | Tail Collar domain protein | | uniclust | UniRef100\_A0A0D6AS89 | 99.9 | 1.5e-28 | 2.8e-34 | 180.6 | 92 | (7, 120) | 221 | (52, 145) | 216 | Microcystin dependent protein | Microcystin dependent protein | | uniclust | UniRef100\_A0A0U4JQ24 | 99.9 | 4.3e-28 | 7.9e-34 | 186.0 | 97 | (10, 119) | 221 | (59, 155) | 455 | Tail protein | Tail protein | | uniclust | UniRef100\_A0A0A1DL90 | 99.9 | 4.5e-28 | 8.3e-34 | 166.9 | 99 | (9, 120) | 221 | (12, 110) | 180 | Microcystin dependent protein | Microcystin dependent protein | | uniclust | UniRef100\_A0A5C7MBK6 | 99.9 | 6.6e-28 | 1.2e-33 | 182.3 | 182 | (9, 221) | 221 | (204, 385) | 387 | Tail fiber protein | Tail fiber protein | | uniclust | UniRef100\_A0A157ZM93 | 99.9 | 8.8e-28 | 1.6e-33 | 175.4 | 170 | (9, 221) | 221 | (83, 266) | 269 | Trimeric autotransporter adhesin YadA-like stalk domain-containing protein | Trimeric autotransporter adhesin YadA-like stalk domain-containing protein | | uniclust | UniRef100\_A0A023Y4H2 | 99.9 | 9e-28 | 1.7e-33 | 179.6 | 98 | (9, 119) | 221 | (51, 148) | 242 | Phage tail collar domain-containing protein | Phage tail collar domain-containing protein | | uniclust | UniRef100\_A0A160TGR6 | 99.9 | 9.5e-28 | 1.8e-33 | 178.0 | 99 | (9, 120) | 221 | (34, 132) | 243 | Microcystin dependent protein | Microcystin dependent protein | | uniclust | UniRef100\_A0A814Z5L0 | 99.9 | 1e-27 | 1.9e-33 | 177.1 | 189 | (8, 221) | 221 | (117, 320) | 322 | Phage tail collar domain-containing protein | Phage tail collar domain-containing protein | | uniclust | UniRef100\_A0A0Q6S010 | 99.9 | 1.1e-27 | 2e-33 | 179.1 | 97 | (10, 119) | 221 | (41, 137) | 247 | Phage tail collar domain-containing protein | Phage tail collar domain-containing protein | | uniclust | UniRef100\_A0A073AGH6 | 99.9 | 1.1e-27 | 2e-33 | 176.6 | 94 | (9, 120) | 221 | (48, 141) | 227 | Phage tail protein | Phage tail protein | | uniclust | UniRef100\_UPI000693AA4D | 99.9 | 1.1e-27 | 2.1e-33 | 184.8 | 117 | (9, 138) | 221 | (377, 501) | 614 | tail fiber protein | tail fiber protein | | uniclust | UniRef100\_A0A814K3I7 | 99.9 | 1.4e-27 | 2.5e-33 | 186.3 | 189 | (11, 221) | 221 | (286, 484) | 489 | Phage tail collar domain-containing protein | Phage tail collar domain-containing protein | | uniclust | UniRef100\_A0A072T796 | 99.9 | 1.5e-27 | 2.7e-33 | 180.1 | 98 | (9, 119) | 221 | (56, 153) | 267 | Tail collar protein | Tail collar protein | | uniclust | UniRef100\_A0A010ZGK4 | 99.9 | 1.5e-27 | 2.9e-33 | 180.6 | 98 | (9, 119) | 221 | (70, 167) | 255 | Microcystin-dependent protein | Microcystin-dependent protein | | uniclust | UniRef100\_A0A0A8WR50 | 99.9 | 1.8e-27 | 3.5e-33 | 184.4 | 145 | (9, 221) | 221 | (143, 290) | 291 | Phage Tail Collar Domain protein | Phage Tail Collar Domain protein | | uniclust | UniRef100\_A0A8S5MVZ6 | 99.9 | 2.1e-27 | 3.9e-33 | 170.3 | 92 | (8, 120) | 221 | (88, 179) | 242 | Baseplate structural protein | Baseplate structural protein | | uniclust | UniRef100\_A0A011A738 | 99.9 | 2.1e-27 | 3.9e-33 | 182.1 | 98 | (9, 119) | 221 | (67, 164) | 292 | Microcystin-dependent protein | Microcystin-dependent protein | | uniclust | UniRef100\_A0A813NFR6 | 99.9 | 2.3e-27 | 4.2e-33 | 195.9 | 195 | (9, 221) | 221 | (499, 702) | 707 | Phage tail collar domain-containing protein | Phage tail collar domain-containing protein | | uniclust | UniRef100\_A0A1E4LGS2 | 99.9 | 2.5e-27 | 4.6e-33 | 186.8 | 124 | (9, 139) | 221 | (224, 347) | 432 | Phage tail collar domain-containing protein | Phage tail collar domain-containing protein | | uniclust | UniRef100\_UPI0018C60E32 | 99.9 | 2.7e-27 | 5e-33 | 174.5 | 175 | (7, 221) | 221 | (204, 378) | 378 | tail fiber protein | tail fiber protein | | uniclust | UniRef100\_A0A1G8XFZ8 | 99.9 | 3.1e-27 | 5.7e-33 | 179.8 | 172 | (7, 221) | 221 | (148, 321) | 323 | Microcystin-dependent protein | Microcystin-dependent protein | | uniclust | UniRef100\_A0A1G6EJM0 | 99.9 | 3.5e-27 | 6.4e-33 | 170.4 | 192 | (7, 220) | 221 | (117, 313) | 316 | Microcystin-dependent protein | Microcystin-dependent protein | | uniclust | UniRef100\_A0A2I7SF00 | 99.9 | 4.3e-27 | 8.1e-33 | 178.3 | 144 | (9, 221) | 221 | (146, 291) | 293 | Phage tail collar domain-containing protein | Phage tail collar domain-containing protein | | uniclust | UniRef100\_A0A0F9MAR7 | 99.9 | 5e-27 | 9.4e-33 | 167.6 | 70 | (7, 119) | 221 | (27, 98) | 178 | Phage tail collar domain-containing protein | Phage tail collar domain-containing protein | | uniclust | UniRef100\_A0A150TEA7 | 99.9 | 5.1e-27 | 9.4e-33 | 190.2 | 164 | (8, 221) | 221 | (499, 664) | 664 | Phage tail collar domain-containing protein | Phage tail collar domain-containing protein | | uniclust | UniRef100\_A0A0P9DVX3 | 99.9 | 7.3e-27 | 1.4e-32 | 183.5 | 97 | (10, 119) | 221 | (215, 311) | 389 | Phage tail collar domain-containing protein | Phage tail collar domain-containing protein | | uniclust | UniRef100\_A0A163AZI2 | 99.9 | 9.7e-27 | 1.8e-32 | 180.0 | 153 | (9, 221) | 221 | (232, 384) | 387 | Phage tail collar domain-containing protein | Phage tail collar domain-containing protein | | uniclust | UniRef100\_A0A2D6U0S1 | 99.9 | 1.9e-26 | 3.5e-32 | 172.4 | 109 | (11, 119) | 221 | (77, 191) | 271 | Phage tail collar domain-containing protein | Phage tail collar domain-containing protein | | uniclust | UniRef100\_A0A9E8ELC9 | 99.9 | 2e-26 | 3.7e-32 | 162.4 | 97 | (10, 119) | 221 | (108, 204) | 273 | Tail fiber protein | Tail fiber protein | | uniclust | UniRef100\_A0A522ETL0 | 99.9 | 2.8e-26 | 5.2e-32 | 165.1 | 106 | (9, 119) | 221 | (83, 188) | 243 | Tail fiber protein | Tail fiber protein | | uniclust | UniRef100\_A0A1H3XG57 | 99.9 | 2.9e-26 | 5.5e-32 | 161.2 | 97 | (11, 120) | 221 | (10, 106) | 182 | Microcystin-dependent protein | Microcystin-dependent protein | | uniclust | UniRef100\_A0A815GMY2 | 99.9 | 3.3e-26 | 6.2e-32 | 149.1 | 121 | (7, 144) | 221 | (14, 134) | 135 | Phage tail collar domain-containing protein | Phage tail collar domain-containing protein | | uniclust | UniRef100\_A0A0R3DQ44 | 99.9 | 3.3e-26 | 6.2e-32 | 179.2 | 112 | (8, 119) | 221 | (196, 308) | 383 | Phage tail collar domain-containing protein | Phage tail collar domain-containing protein | | uniclust | UniRef100\_A0A7C5Z090 | 99.9 | 5.8e-26 | 1.1e-31 | 163.2 | 102 | (6, 120) | 221 | (165, 266) | 315 | Phage tail collar domain-containing protein | Phage tail collar domain-containing protein | | uniclust | UniRef100\_A0A173LYL4 | 99.9 | 5.9e-26 | 1.1e-31 | 182.7 | 104 | (9, 127) | 221 | (542, 645) | 704 | Collagen triple helix repeat protein | Collagen triple helix repeat protein | | uniclust | UniRef100\_A0A1Z8ZQ15 | 99.9 | 5.8e-26 | 1.1e-31 | 175.1 | 139 | (8, 221) | 221 | (212, 352) | 353 | Phage tail collar domain-containing protein | Phage tail collar domain-containing protein | | uniclust | UniRef100\_A0A2W6Y1Z1 | 99.9 | 6.1e-26 | 1.1e-31 | 172.7 | 168 | (11, 221) | 221 | (124, 302) | 312 | Phage tail protein | Phage tail protein | | uniclust | UniRef100\_A0A813ML39 | 99.9 | 1.1e-25 | 2e-31 | 181.2 | 194 | (10, 220) | 221 | (548, 751) | 753 | Phage tail collar domain-containing protein | Phage tail collar domain-containing protein | | uniclust | UniRef100\_A0A940VND6 | 99.9 | 1.1e-25 | 2.1e-31 | 168.9 | 95 | (9, 119) | 221 | (270, 364) | 416 | Phage tail collar domain-containing protein | Phage tail collar domain-containing protein | | uniclust | UniRef100\_A0A0Q5Z2P4 | 99.9 | 1.4e-25 | 2.7e-31 | 175.5 | 104 | (10, 119) | 221 | (148, 251) | 421 | Phage tail collar domain-containing protein | Phage tail collar domain-containing protein | | uniclust | UniRef100\_A0A0C2D114 | 99.9 | 1.5e-25 | 2.8e-31 | 164.2 | 143 | (8, 221) | 221 | (70, 214) | 216 | Phage tail fiber protein | Phage tail fiber protein | | uniclust | UniRef100\_A0A0P9DVX3 | 99.9 | 1.6e-25 | 3.1e-31 | 176.1 | 92 | (10, 119) | 221 | (47, 138) | 389 | Phage tail collar domain-containing protein | Phage tail collar domain-containing protein | | uniclust | UniRef100\_A0A8S5RTE1 | 99.9 | 1.7e-25 | 3.1e-31 | 159.9 | 98 | (7, 121) | 221 | (86, 183) | 249 | Tail collar domain | Tail collar domain | | uniclust | UniRef100\_A0A369RCN8 | 99.9 | 2.6e-25 | 4.9e-31 | 158.9 | 98 | (9, 119) | 221 | (115, 212) | 275 | Phage tail collar domain-containing protein | Phage tail collar domain-containing protein | | uniclust | UniRef100\_A0A031GQD8 | 99.9 | 3.3e-25 | 6.1e-31 | 154.6 | 102 | (9, 119) | 221 | (3, 104) | 177 | Microcystin dependent protein | Microcystin dependent protein | | uniclust | UniRef100\_A0A060DHI4 | 99.9 | 4.1e-25 | 7.6e-31 | 166.4 | 99 | (10, 121) | 221 | (47, 145) | 267 | Phage tail collar domain-containing protein | Phage tail collar domain-containing protein | | uniclust | UniRef100\_A0A8T3YWH3 | 99.9 | 4.3e-25 | 7.9e-31 | 158.5 | 94 | (9, 126) | 221 | (88, 181) | 241 | Tail fiber protein | Tail fiber protein | | uniclust | UniRef100\_A0A0A1FCT1 | 99.9 | 4.2e-25 | 7.9e-31 | 155.3 | 98 | (9, 119) | 221 | (8, 105) | 177 | Microcystin dependent protein | Microcystin dependent protein | | uniclust | UniRef100\_UPI0020119B83 | 99.8 | 5.8e-25 | 1.1e-30 | 157.1 | 99 | (9, 119) | 221 | (155, 253) | 299 | tail fiber protein | tail fiber protein | | uniclust | UniRef100\_A0A813PLM5 | 99.8 | 5.9e-25 | 1.1e-30 | 154.4 | 193 | (12, 221) | 221 | (19, 219) | 220 | Uncharacterized protein | Uncharacterized protein | | uniclust | UniRef100\_A0A0Q5ZY68 | 99.8 | 6.1e-25 | 1.1e-30 | 167.9 | 112 | (6, 118) | 221 | (136, 247) | 316 | Phage tail collar domain-containing protein | Phage tail collar domain-containing protein | | uniclust | UniRef100\_A0A0A1PS49 | 99.8 | 1.3e-24 | 2.5e-30 | 163.9 | 185 | (7, 221) | 221 | (194, 379) | 379 | Phage Tail Collar Domain protein | Phage Tail Collar Domain protein | | uniclust | UniRef100\_A0A4Y6CGU0 | 99.8 | 1.4e-24 | 2.6e-30 | 180.5 | 151 | (8, 221) | 221 | (546, 698) | 718 | Phage tail collar domain-containing protein | Phage tail collar domain-containing protein | | uniclust | UniRef100\_UPI0018C7C22C | 99.8 | 1.4e-24 | 2.6e-30 | 158.7 | 177 | (8, 221) | 221 | (181, 357) | 357 | tail fiber protein | tail fiber protein | | uniclust | UniRef100\_A0A6L5FV64 | 99.8 | 1.5e-24 | 2.9e-30 | 155.0 | 98 | (9, 120) | 221 | (111, 208) | 268 | Phage tail collar domain-containing protein | Phage tail collar domain-containing protein | | uniclust | UniRef100\_A0A481Z0P9 | 99.8 | 1.6e-24 | 3.1e-30 | 158.2 | 74 | (9, 120) | 221 | (96, 169) | 231 | Tail collar domain protein | Tail collar domain protein | | uniclust | UniRef100\_A0A3N6N9Q9 | 99.8 | 1.8e-24 | 3.3e-30 | 163.8 | 97 | (10, 119) | 221 | (139, 235) | 316 | Phage tail collar domain-containing protein | Phage tail collar domain-containing protein | | uniclust | UniRef100\_A0A059FRJ9 | 99.8 | 1.9e-24 | 3.5e-30 | 166.6 | 98 | (9, 119) | 221 | (60, 157) | 272 | Phage tail collar domain-containing protein | Phage tail collar domain-containing protein | | uniclust | UniRef100\_A0A943I3K4 | 99.8 | 3e-24 | 5.5e-30 | 143.2 | 88 | (11, 115) | 221 | (4, 91) | 165 | Tail fiber protein | Tail fiber protein | | uniclust | UniRef100\_A0A3M1ZWN2 | 99.8 | 4e-24 | 7.3e-30 | 153.6 | 97 | (10, 119) | 221 | (34, 131) | 269 | Phage tail collar domain-containing protein | Phage tail collar domain-containing protein | | uniclust | UniRef100\_A0A1X7I1H1 | 99.8 | 4.3e-24 | 8e-30 | 166.0 | 93 | (9, 119) | 221 | (181, 273) | 346 | Microcystin-dependent protein | Microcystin-dependent protein | | uniclust | UniRef100\_A0A5K0UBR7 | 99.8 | 4.6e-24 | 8.6e-30 | 158.2 | 72 | (8, 119) | 221 | (94, 165) | 264 | Uncharacterized protein | Uncharacterized protein | | uniclust | UniRef100\_A0A0F8YBQ7 | 99.8 | 5.8e-24 | 1.1e-29 | 154.4 | 93 | (9, 120) | 221 | (74, 166) | 227 | Phage tail collar domain-containing protein (Fragment) | Phage tail collar domain-containing protein (Fragment) | | uniclust | UniRef100\_A0A1Y4GY33 | 99.8 | 6.1e-24 | 1.1e-29 | 157.7 | 97 | (8, 121) | 221 | (184, 280) | 339 | Phage tail collar domain-containing protein | Phage tail collar domain-containing protein | | uniclust | UniRef100\_A0A7V2Y2J7 | 99.8 | 6.6e-24 | 1.2e-29 | 160.9 | 112 | (7, 120) | 221 | (150, 261) | 330 | Phage tail collar domain-containing protein | Phage tail collar domain-containing protein | | uniclust | UniRef100\_A0A516KT37 | 99.8 | 6.7e-24 | 1.2e-29 | 160.8 | 99 | (9, 120) | 221 | (263, 363) | 430 | Minor tail protein | Minor tail protein | | uniclust | UniRef100\_A0A6C0HZC1 | 99.8 | 6.9e-24 | 1.3e-29 | 151.5 | 191 | (8, 221) | 221 | (42, 256) | 256 | Phage tail collar domain-containing protein | Phage tail collar domain-containing protein | | uniclust | UniRef100\_A0A1X7I1H1 | 99.8 | 6.9e-24 | 1.3e-29 | 164.9 | 98 | (9, 119) | 221 | (7, 104) | 346 | Microcystin-dependent protein | Microcystin-dependent protein | | uniclust | UniRef100\_A0A1F3IL82 | 99.8 | 7.1e-24 | 1.3e-29 | 160.8 | 86 | (9, 118) | 221 | (141, 228) | 319 | Phage tail collar domain-containing protein | Phage tail collar domain-containing protein | | uniclust | UniRef100\_A0A937XD69 | 99.8 | 7.5e-24 | 1.4e-29 | 144.5 | 146 | (7, 221) | 221 | (65, 210) | 211 | Tail fiber protein | Tail fiber protein | | uniclust | UniRef100\_UPI000A039BE3 | 99.8 | 8.3e-24 | 1.5e-29 | 157.5 | 102 | (9, 122) | 221 | (239, 340) | 412 | tail fiber protein | tail fiber protein | | uniclust | UniRef100\_A0A7G9A427 | 99.8 | 9.3e-24 | 1.7e-29 | 157.7 | 197 | (8, 221) | 221 | (222, 423) | 424 | Phage tail collar domain-containing protein | Phage tail collar domain-containing protein | | uniclust | UniRef100\_A0A7K0RNX1 | 99.8 | 1.1e-23 | 2e-29 | 162.1 | 105 | (10, 120) | 221 | (344, 448) | 562 | Phage tail collar domain-containing protein | Phage tail collar domain-containing protein | | uniclust | UniRef100\_A0A386HRX7 | 99.8 | 1.2e-23 | 2.2e-29 | 158.7 | 69 | (9, 120) | 221 | (158, 228) | 295 | Tail fiber protein | Tail fiber protein | | uniclust | UniRef100\_A0A2M7SZQ3 | 99.8 | 1.3e-23 | 2.5e-29 | 165.3 | 106 | (8, 119) | 221 | (249, 357) | 438 | Phage tail collar domain-containing protein | Phage tail collar domain-containing protein | | uniclust | UniRef100\_A0A2S5DRN7 | 99.8 | 1.8e-23 | 3.3e-29 | 150.1 | 170 | (9, 221) | 221 | (104, 281) | 282 | Phage tail protein | Phage tail protein | | uniclust | UniRef100\_A0A150WBY6 | 99.8 | 1.8e-23 | 3.5e-29 | 157.1 | 110 | (6, 120) | 221 | (86, 195) | 257 | Phage tail collar domain-containing protein | Phage tail collar domain-containing protein | | uniclust | UniRef100\_A0A3A8TU84 | 99.8 | 2.1e-23 | 4e-29 | 142.0 | 97 | (10, 119) | 221 | (4, 100) | 144 | Phage tail protein (Fragment) | Phage tail protein (Fragment) | | uniclust | UniRef100\_A0A1A6CD73 | 99.8 | 2.4e-23 | 4.7e-29 | 164.1 | 142 | (11, 221) | 221 | (144, 287) | 291 | Phage tail collar domain-containing protein | Phage tail collar domain-containing protein | | uniclust | UniRef100\_A0A8J7Q9N7 | 99.8 | 2.6e-23 | 4.7e-29 | 151.2 | 104 | (8, 124) | 221 | (120, 224) | 289 | Tail fiber protein | Tail fiber protein | | uniclust | UniRef100\_A0A974Z143 | 99.8 | 2.6e-23 | 4.8e-29 | 160.3 | 99 | (8, 119) | 221 | (387, 485) | 538 | Tail fiber protein | Tail fiber protein | | uniclust | UniRef100\_A0A2L1HSI4 | 99.8 | 2.6e-23 | 4.9e-29 | 157.0 | 172 | (8, 221) | 221 | (178, 349) | 349 | Phage tail collar domain-containing protein | Phage tail collar domain-containing protein | | uniclust | UniRef100\_A0A969SMF4 | 99.8 | 3.9e-23 | 7.1e-29 | 155.7 | 195 | (10, 220) | 221 | (246, 450) | 453 | Phage tail collar domain-containing protein | Phage tail collar domain-containing protein | | uniclust | UniRef100\_A0A3D1PJI0 | 99.8 | 4.1e-23 | 7.6e-29 | 150.2 | 99 | (8, 119) | 221 | (44, 142) | 337 | Phage tail collar domain-containing protein | Phage tail collar domain-containing protein | | uniclust | UniRef100\_A0A661AU03 | 99.8 | 4.1e-23 | 7.6e-29 | 162.9 | 171 | (9, 221) | 221 | (552, 722) | 722 | Phage tail collar domain-containing protein (Fragment) | Phage tail collar domain-containing protein (Fragment) | | uniclust | UniRef100\_A0A1D9LMZ6 | 99.8 | 5.4e-23 | 1e-28 | 152.3 | 94 | (8, 120) | 221 | (99, 192) | 252 | Phage tail collar domain-containing protein | Phage tail collar domain-containing protein | | uniclust | UniRef100\_E3T4R8 | 99.8 | 6.5e-23 | 1.2e-28 | 142.9 | 99 | (9, 119) | 221 | (77, 175) | 242 | Phage tail collar domain-containing protein | Phage tail collar domain-containing protein | | uniclust | UniRef100\_A0A1Z9FAS1 | 99.8 | 6.8e-23 | 1.3e-28 | 169.4 | 107 | (9, 119) | 221 | (493, 601) | 679 | Phage tail collar domain-containing protein | Phage tail collar domain-containing protein | | uniclust | UniRef100\_A0A1Z7ZRI5 | 99.8 | 6.9e-23 | 1.3e-28 | 159.7 | 107 | (8, 120) | 221 | (125, 231) | 382 | Phage tail collar domain-containing protein | Phage tail collar domain-containing protein | | uniclust | UniRef100\_A0A098S053 | 99.8 | 1e-22 | 1.9e-28 | 147.3 | 92 | (9, 119) | 221 | (65, 156) | 219 | Phage tail collar domain-containing protein | Phage tail collar domain-containing protein | | uniclust | UniRef100\_D3F201 | 99.8 | 1.2e-22 | 2.1e-28 | 147.4 | 184 | (18, 221) | 221 | (73, 260) | 262 | Tail Collar domain protein | Tail Collar domain protein | | uniclust | UniRef100\_A0A7Y7P107 | 99.8 | 1.2e-22 | 2.3e-28 | 161.4 | 108 | (8, 120) | 221 | (213, 321) | 380 | Tail fiber protein | Tail fiber protein | | uniclust | UniRef100\_A0A2E1UKG0 | 99.8 | 1.3e-22 | 2.3e-28 | 159.8 | 169 | (8, 221) | 221 | (305, 490) | 491 | Major tropism determinant N-terminal domain-containing protein (Fragment) | Major tropism determinant N-terminal domain-containing protein (Fragment) | | uniclust | UniRef100\_A0A844YWZ8 | 99.8 | 1.3e-22 | 2.3e-28 | 146.4 | 93 | (8, 119) | 221 | (115, 207) | 254 | Phage tail protein | Phage tail protein | | uniclust | UniRef100\_A0A6P1ZJ46 | 99.8 | 1.3e-22 | 2.4e-28 | 161.9 | 185 | (5, 221) | 221 | (248, 438) | 439 | Phage tail collar domain-containing protein | Phage tail collar domain-containing protein | | uniclust | UniRef100\_A0A1V5QDU0 | 99.8 | 1.4e-22 | 2.6e-28 | 171.9 | 109 | (8, 120) | 221 | (1889, 1997) | 2045 | Phage Tail Collar Domain protein | Phage Tail Collar Domain protein | | uniclust | UniRef100\_A0A1Q8TCI9 | 99.8 | 1.4e-22 | 2.7e-28 | 152.6 | 88 | (13, 119) | 221 | (108, 195) | 290 | Phage tail collar domain-containing protein | Phage tail collar domain-containing protein | | uniclust | UniRef100\_A0A2A3DSC8 | 99.8 | 1.7e-22 | 3.1e-28 | 153.3 | 90 | (10, 118) | 221 | (131, 220) | 282 | Phage tail collar domain-containing protein | Phage tail collar domain-containing protein | | uniclust | UniRef100\_A0A143DGR5 | 99.8 | 1.7e-22 | 3.1e-28 | 162.2 | 93 | (7, 118) | 221 | (266, 358) | 425 | Phage tail collar domain-containing protein | Phage tail collar domain-containing protein | | uniclust | UniRef100\_A0A074JN26 | 99.8 | 2.1e-22 | 3.9e-28 | 146.9 | 98 | (10, 120) | 221 | (54, 151) | 231 | Phage tail collar domain-containing protein | Phage tail collar domain-containing protein | | uniclust | UniRef100\_A0A158D1B4 | 99.8 | 2.2e-22 | 4e-28 | 151.3 | 193 | (7, 221) | 221 | (172, 373) | 373 | Phage tail collar domain-containing protein | Phage tail collar domain-containing protein | | uniclust | UniRef100\_D0LMW0 | 99.8 | 3e-22 | 5.5e-28 | 141.4 | 95 | (13, 119) | 221 | (113, 207) | 264 | Tail Collar domain protein | Tail Collar domain protein | | uniclust | UniRef100\_A0A820P0S5 | 99.8 | 3.1e-22 | 5.7e-28 | 153.4 | 194 | (13, 220) | 221 | (250, 447) | 449 | Phage tail collar domain-containing protein | Phage tail collar domain-containing protein | | uniclust | UniRef100\_A0A8S5PNQ0 | 99.8 | 3.8e-22 | 6.9e-28 | 140.7 | 105 | (7, 126) | 221 | (63, 167) | 231 | Baseplate wedge protein | Baseplate wedge protein | | uniclust | UniRef100\_UPI000CE3D78C | 99.8 | 3.8e-22 | 7e-28 | 149.2 | 100 | (8, 120) | 221 | (269, 368) | 419 | tail fiber protein | tail fiber protein | | uniclust | UniRef100\_J0MTG7 | 99.8 | 4.4e-22 | 8.2e-28 | 145.0 | 87 | (9, 119) | 221 | (160, 246) | 310 | Phage tail collar domain protein | Phage tail collar domain protein | | uniclust | UniRef100\_A0A945NP07 | 99.8 | 5.6e-22 | 1e-27 | 151.9 | 99 | (9, 122) | 221 | (309, 407) | 520 | Phage tail collar domain-containing protein | Phage tail collar domain-containing protein | | uniclust | UniRef100\_A0A358QWR4 | 99.8 | 5.9e-22 | 1.1e-27 | 140.5 | 120 | (8, 221) | 221 | (123, 244) | 244 | Phage tail collar domain-containing protein | Phage tail collar domain-containing protein | | uniclust | UniRef100\_A0A813U148 | 99.8 | 6.4e-22 | 1.2e-27 | 154.6 | 194 | (9, 221) | 221 | (432, 633) | 633 | Phage tail collar domain-containing protein (Fragment) | Phage tail collar domain-containing protein (Fragment) | | uniclust | UniRef100\_A0A3B0MDZ4 | 99.8 | 7e-22 | 1.3e-27 | 147.2 | 77 | (9, 85) | 221 | (149, 225) | 315 | Phage tail collar domain-containing protein | Phage tail collar domain-containing protein | | uniclust | UniRef100\_A0A2W4QXT5 | 99.8 | 7.1e-22 | 1.3e-27 | 138.3 | 149 | (8, 221) | 221 | (99, 247) | 247 | Uncharacterized protein | Uncharacterized protein | | uniclust | UniRef100\_A0A024LSG2 | 99.8 | 8.4e-22 | 1.6e-27 | 153.1 | 92 | (9, 119) | 221 | (178, 269) | 345 | Putative phage tail fiber protein | Putative phage tail fiber protein | | uniclust | UniRef100\_A0A0S4U5F8 | 99.8 | 8.6e-22 | 1.6e-27 | 145.2 | 91 | (10, 119) | 221 | (73, 163) | 236 | Putative phage tail collar protein | Putative phage tail collar protein | | uniclust | UniRef100\_A0A1G7NQ56 | 99.8 | 9.2e-22 | 1.7e-27 | 146.0 | 67 | (8, 76) | 221 | (77, 144) | 250 | Phage Tail Collar Domain | Phage Tail Collar Domain | | uniclust | UniRef100\_A0A0G1QR78 | 99.8 | 1e-21 | 1.9e-27 | 139.6 | 99 | (9, 119) | 221 | (131, 229) | 276 | Tail Collar domain protein | Tail Collar domain protein | | uniclust | UniRef100\_A0A1G7VYT4 | 99.8 | 1.1e-21 | 2e-27 | 156.7 | 92 | (10, 119) | 221 | (4, 95) | 518 | Microcystin-dependent protein | Microcystin-dependent protein | | uniclust | UniRef100\_A0A291LA10 | 99.8 | 1.2e-21 | 2.3e-27 | 132.5 | 92 | (9, 119) | 221 | (35, 126) | 176 | Putative tail-collar fiber protein | Putative tail-collar fiber protein | | uniclust | UniRef100\_A0A516M2K7 | 99.8 | 1.3e-21 | 2.5e-27 | 150.6 | 167 | (6, 221) | 221 | (283, 449) | 449 | Putative tail fiber protein | Putative tail fiber protein | | uniclust | UniRef100\_A0A0A0DZA9 | 99.7 | 1.8e-21 | 3.3e-27 | 149.7 | 97 | (11, 120) | 221 | (59, 156) | 297 | Phage tail collar domain-containing protein | Phage tail collar domain-containing protein | | uniclust | UniRef100\_A0A6G6K382 | 99.7 | 1.8e-21 | 3.3e-27 | 150.1 | 111 | (9, 119) | 221 | (252, 365) | 419 | Phage tail collar domain-containing protein | Phage tail collar domain-containing protein | | uniclust | UniRef100\_A0A351KZF5 | 99.7 | 1.9e-21 | 3.4e-27 | 153.0 | 164 | (8, 221) | 221 | (510, 677) | 677 | Phage tail collar domain-containing protein | Phage tail collar domain-containing protein | | uniclust | UniRef100\_A0A1G6T0X4 | 99.7 | 1.9e-21 | 3.5e-27 | 140.9 | 97 | (10, 119) | 221 | (21, 117) | 224 | Microcystin-dependent protein | Microcystin-dependent protein | | uniclust | UniRef100\_UPI0010939542 | 99.7 | 2.1e-21 | 3.8e-27 | 138.7 | 96 | (8, 103) | 221 | (106, 201) | 284 | tail fiber protein | tail fiber protein | | uniclust | UniRef100\_A0A5J4Y931 | 99.7 | 2.5e-21 | 4.5e-27 | 140.6 | 93 | (21, 126) | 221 | (162, 254) | 323 | Phage tail collar domain-containing protein | Phage tail collar domain-containing protein | | uniclust | UniRef100\_A0A1F7BR55 | 99.7 | 2.6e-21 | 4.8e-27 | 148.5 | 110 | (10, 119) | 221 | (365, 474) | 523 | Phage tail collar domain-containing protein | Phage tail collar domain-containing protein | | uniclust | UniRef100\_E4T5W3 | 99.7 | 2.7e-21 | 5e-27 | 155.9 | 96 | (11, 119) | 221 | (739, 838) | 898 | Tail Collar domain protein | Tail Collar domain protein | | uniclust | UniRef100\_A0A936WGZ5 | 99.7 | 2.8e-21 | 5.2e-27 | 148.0 | 98 | (9, 119) | 221 | (288, 388) | 449 | Tail fiber protein | Tail fiber protein | | uniclust | UniRef100\_A0A1G3BQZ8 | 99.7 | 2.8e-21 | 5.2e-27 | 143.7 | 107 | (8, 119) | 221 | (39, 151) | 227 | Phage tail collar domain-containing protein | Phage tail collar domain-containing protein | | uniclust | UniRef100\_A0A9E0UQ04 | 99.7 | 2.9e-21 | 5.4e-27 | 137.2 | 97 | (11, 120) | 221 | (75, 171) | 242 | Phage tail protein | Phage tail protein | | uniclust | UniRef100\_A0A1G7VYT4 | 99.7 | 3e-21 | 5.5e-27 | 154.4 | 96 | (10, 119) | 221 | (173, 268) | 518 | Microcystin-dependent protein | Microcystin-dependent protein | | uniclust | UniRef100\_A0A958X9M4 | 99.7 | 3.2e-21 | 5.8e-27 | 155.6 | 157 | (8, 221) | 221 | (581, 737) | 737 | Uncharacterized protein | Uncharacterized protein | | uniclust | UniRef100\_A0A0N9MHY1 | 99.7 | 3.2e-21 | 5.9e-27 | 162.5 | 193 | (9, 221) | 221 | (651, 843) | 843 | Phage tail collar domain-containing protein | Phage tail collar domain-containing protein | | uniclust | UniRef100\_A0A1G0CK00 | 99.7 | 3.4e-21 | 6.4e-27 | 133.5 | 98 | (9, 119) | 221 | (6, 103) | 185 | Phage tail protein | Phage tail protein | | uniclust | UniRef100\_A0A5C7LV16 | 99.7 | 3.5e-21 | 6.4e-27 | 154.7 | 174 | (8, 221) | 221 | (97, 270) | 856 | Phage tail collar domain-containing protein | Phage tail collar domain-containing protein | | uniclust | UniRef100\_A0A0D0N756 | 99.7 | 3.4e-21 | 6.4e-27 | 139.2 | 102 | (9, 119) | 221 | (29, 138) | 202 | Phage tail collar domain-containing protein | Phage tail collar domain-containing protein | | uniclust | UniRef100\_A0A815YNV7 | 99.7 | 4e-21 | 7.3e-27 | 134.7 | 124 | (7, 147) | 221 | (118, 241) | 248 | Phage tail collar domain-containing protein | Phage tail collar domain-containing protein | | uniclust | UniRef100\_A0A2W7PA10 | 99.7 | 4e-21 | 7.6e-27 | 141.1 | 92 | (9, 119) | 221 | (77, 168) | 224 | Tail collar domain | Tail collar domain | | uniclust | UniRef100\_A0A2K9MAN8 | 99.7 | 4.7e-21 | 8.6e-27 | 132.2 | 103 | (10, 119) | 221 | (13, 115) | 221 | Phage tail collar domain-containing protein | Phage tail collar domain-containing protein | | uniclust | UniRef100\_A0A845XUZ4 | 99.7 | 4.7e-21 | 8.7e-27 | 146.6 | 103 | (8, 127) | 221 | (339, 441) | 507 | Phage tail collar domain-containing protein | Phage tail collar domain-containing protein | | uniclust | UniRef100\_A0A1Q4DT89 | 99.7 | 4.7e-21 | 8.7e-27 | 138.3 | 185 | (8, 221) | 221 | (33, 227) | 227 | Phage tail collar domain-containing protein | Phage tail collar domain-containing protein | | uniclust | UniRef100\_A0A966D4Q4 | 99.7 | 5.2e-21 | 9.7e-27 | 139.7 | 77 | (9, 85) | 221 | (40, 116) | 262 | Phage tail collar domain-containing protein | Phage tail collar domain-containing protein | | uniclust | UniRef100\_A0A0Q6ZRG1 | 99.7 | 5.3e-21 | 1e-26 | 147.9 | 94 | (10, 122) | 221 | (166, 259) | 316 | Phage tail collar domain-containing protein | Phage tail collar domain-containing protein | | uniclust | UniRef100\_A0A835XN54 | 99.7 | 5.7e-21 | 1.1e-26 | 152.1 | 99 | (9, 119) | 221 | (447, 545) | 631 | Phage tail collar domain-containing protein | Phage tail collar domain-containing protein | | uniclust | UniRef100\_A0A0P7BJN4 | 99.7 | 6e-21 | 1.1e-26 | 148.6 | 91 | (9, 119) | 221 | (458, 548) | 599 | Phage tail collar domain-containing protein | Phage tail collar domain-containing protein | | uniclust | UniRef100\_A0A815F2F0 | 99.7 | 6.2e-21 | 1.2e-26 | 144.6 | 128 | (8, 148) | 221 | (172, 300) | 355 | Phage tail collar domain-containing protein | Phage tail collar domain-containing protein | | uniclust | UniRef100\_A0A3M9M907 | 99.7 | 6.6e-21 | 1.2e-26 | 140.5 | 177 | (7, 221) | 221 | (137, 317) | 317 | Phage tail collar domain-containing protein | Phage tail collar domain-containing protein | | uniclust | UniRef100\_A0A6B3UTZ7 | 99.7 | 6.6e-21 | 1.2e-26 | 142.6 | 79 | (7, 85) | 221 | (159, 237) | 312 | Phage tail protein | Phage tail protein | | uniclust | UniRef100\_UPI001F3D999F | 99.7 | 8.5e-21 | 1.6e-26 | 139.8 | 141 | (8, 221) | 221 | (218, 360) | 360 | hypothetical protein | hypothetical protein | | uniclust | UniRef100\_A0A1X7F9Q3 | 99.7 | 9.3e-21 | 1.7e-26 | 139.0 | 75 | (9, 83) | 221 | (77, 151) | 219 | Phage Tail Collar Domain | Phage Tail Collar Domain | | uniclust | UniRef100\_A0A899ISM6 | 99.7 | 9.7e-21 | 1.8e-26 | 134.7 | 76 | (8, 83) | 221 | (29, 104) | 207 | Tail protein | Tail protein | | uniclust | UniRef100\_A0A0B9H1Q2 | 99.7 | 1e-20 | 1.9e-26 | 139.9 | 98 | (9, 119) | 221 | (51, 148) | 252 | Phage tail collar domain-containing protein | Phage tail collar domain-containing protein | | uniclust | UniRef100\_A0A927Y330 | 99.7 | 1e-20 | 1.9e-26 | 152.8 | 108 | (8, 128) | 221 | (648, 755) | 901 | Fibronectin type-III domain-containing protein | Fibronectin type-III domain-containing protein | | uniclust | UniRef100\_A0A0M2NH56 | 99.7 | 1e-20 | 1.9e-26 | 133.3 | 99 | (6, 119) | 221 | (107, 207) | 256 | Microcystin dependent protein | Microcystin dependent protein | | uniclust | UniRef100\_A0A120FHB0 | 99.7 | 1e-20 | 1.9e-26 | 143.9 | 176 | (8, 221) | 221 | (209, 384) | 384 | Phage tail collar domain-containing protein | Phage tail collar domain-containing protein | | uniclust | UniRef100\_A0A814HPR8 | 99.7 | 1e-20 | 1.9e-26 | 134.8 | 161 | (8, 221) | 221 | (41, 215) | 219 | Phage tail collar domain-containing protein | Phage tail collar domain-containing protein | | uniclust | UniRef100\_A0A225DUJ5 | 99.7 | 1.2e-20 | 2.1e-26 | 140.0 | 125 | (7, 145) | 221 | (105, 242) | 326 | Phage tail fiber | Phage tail fiber | | uniclust | UniRef100\_UPI0009F51E47 | 99.7 | 1.2e-20 | 2.2e-26 | 142.4 | 95 | (10, 119) | 221 | (296, 390) | 444 | tail fiber protein | tail fiber protein | | uniclust | UniRef100\_A0A350MM43 | 99.7 | 1.2e-20 | 2.3e-26 | 134.2 | 69 | (8, 119) | 221 | (93, 163) | 215 | Tail fiber protein (Fragment) | Tail fiber protein (Fragment) | | uniclust | UniRef100\_A0A815DS73 | 99.7 | 1.3e-20 | 2.4e-26 | 120.8 | 108 | (7, 126) | 221 | (25, 132) | 137 | Phage tail collar domain-containing protein | Phage tail collar domain-containing protein | | uniclust | UniRef100\_A0A815JLE8 | 99.7 | 1.3e-20 | 2.4e-26 | 145.5 | 194 | (10, 221) | 221 | (271, 473) | 474 | Phage tail collar domain-containing protein | Phage tail collar domain-containing protein | | uniclust | UniRef100\_A0A162G8C3 | 99.7 | 1.4e-20 | 2.7e-26 | 138.4 | 69 | (8, 77) | 221 | (54, 122) | 224 | Phage tail collar domain-containing protein | Phage tail collar domain-containing protein | | uniclust | UniRef100\_A0A0Q2UK27 | 99.7 | 1.6e-20 | 2.9e-26 | 129.7 | 94 | (7, 119) | 221 | (23, 116) | 166 | Phage tail collar domain-containing protein | Phage tail collar domain-containing protein | | uniclust | UniRef100\_A0A0F9B578 | 99.7 | 1.6e-20 | 3e-26 | 136.7 | 98 | (11, 119) | 221 | (110, 210) | 262 | Phage tail collar domain-containing protein | Phage tail collar domain-containing protein | | uniclust | UniRef100\_A0A1H0FQQ1 | 99.7 | 1.6e-20 | 3e-26 | 127.8 | 97 | (10, 119) | 221 | (4, 100) | 176 | Microcystin-dependent protein | Microcystin-dependent protein | | uniclust | UniRef100\_A0A0H2MCN6 | 99.7 | 1.7e-20 | 3.2e-26 | 139.4 | 197 | (2, 221) | 221 | (63, 280) | 280 | Minor tail protein | Minor tail protein | | uniclust | UniRef100\_A0A816HQP8 | 99.7 | 1.8e-20 | 3.3e-26 | 118.9 | 112 | (23, 147) | 221 | (3, 114) | 129 | Phage tail collar domain-containing protein (Fragment) | Phage tail collar domain-containing protein (Fragment) | | uniclust | UniRef100\_A0A354T924 | 99.7 | 1.8e-20 | 3.4e-26 | 139.0 | 110 | (7, 121) | 221 | (118, 228) | 279 | Phage tail collar domain-containing protein | Phage tail collar domain-containing protein | | uniclust | UniRef100\_A0A814J2Q1 | 99.7 | 2.1e-20 | 3.8e-26 | 156.9 | 190 | (9, 221) | 221 | (1103, 1304) | 1304 | Elongation of very long chain fatty acids protein | Elongation of very long chain fatty acids protein | | uniclust | UniRef100\_A0A7W8G0N9 | 99.7 | 2.2e-20 | 4.1e-26 | 125.7 | 90 | (12, 119) | 221 | (6, 95) | 168 | Microcystin-dependent protein | Microcystin-dependent protein | | uniclust | UniRef100\_A0A819QFI6 | 99.7 | 2.3e-20 | 4.2e-26 | 121.1 | 120 | (11, 149) | 221 | (2, 126) | 147 | Phage tail collar domain-containing protein (Fragment) | Phage tail collar domain-containing protein (Fragment) | | uniclust | UniRef100\_A0A964YWP9 | 99.7 | 2.5e-20 | 4.6e-26 | 137.7 | 184 | (10, 215) | 221 | (173, 358) | 367 | Phage tail collar domain-containing protein | Phage tail collar domain-containing protein | | uniclust | UniRef100\_A0A1M4TNW1 | 99.7 | 2.5e-20 | 4.7e-26 | 151.7 | 84 | (9, 119) | 221 | (566, 649) | 728 | Microcystin-dependent protein | Microcystin-dependent protein | | uniclust | UniRef100\_A0A9E9KT91 | 99.7 | 2.6e-20 | 4.8e-26 | 139.5 | 76 | (10, 85) | 221 | (138, 213) | 352 | Phage tail protein | Phage tail protein | | uniclust | UniRef100\_A0A968JEA1 | 99.7 | 2.9e-20 | 5.4e-26 | 134.0 | 65 | (9, 73) | 221 | (72, 136) | 300 | Tail fiber protein | Tail fiber protein | | uniclust | UniRef100\_A0A9E3L5V0 | 99.7 | 3.3e-20 | 6e-26 | 136.4 | 107 | (9, 119) | 221 | (147, 255) | 329 | Tail fiber protein | Tail fiber protein | | uniclust | UniRef100\_A0A0J5GKU1 | 99.7 | 3.3e-20 | 6.2e-26 | 129.2 | 93 | (8, 119) | 221 | (22, 114) | 172 | Phage tail collar domain-containing protein | Phage tail collar domain-containing protein | | uniclust | UniRef100\_A0A1I3Z6T6 | 99.7 | 3.4e-20 | 6.3e-26 | 122.3 | 98 | (9, 119) | 221 | (17, 114) | 135 | Phage Tail Collar Domain (Fragment) | Phage Tail Collar Domain (Fragment) | | uniclust | UniRef100\_A0A090N7J5 | 99.7 | 3.4e-20 | 6.4e-26 | 148.9 | 189 | (7, 221) | 221 | (205, 417) | 456 | Microcystin-dependent protein | Microcystin-dependent protein | | uniclust | UniRef100\_A0A1G0LDM4 | 99.7 | 3.4e-20 | 6.4e-26 | 138.1 | 74 | (8, 120) | 221 | (159, 234) | 306 | Phage tail collar domain-containing protein | Phage tail collar domain-containing protein | | uniclust | UniRef100\_A0A480A6W0 | 99.7 | 3.5e-20 | 6.5e-26 | 148.6 | 145 | (11, 221) | 221 | (548, 695) | 696 | Phage tail collar domain-containing protein | Phage tail collar domain-containing protein | | uniclust | UniRef100\_A0A1V5X7T8 | 99.7 | 3.6e-20 | 6.6e-26 | 129.3 | 151 | (8, 221) | 221 | (84, 237) | 237 | Phage Tail Collar Domain protein | Phage Tail Collar Domain protein | | uniclust | UniRef100\_A0A7C8AIK9 | 99.7 | 3.6e-20 | 6.6e-26 | 139.5 | 107 | (9, 120) | 221 | (176, 284) | 338 | Tail fiber protein | Tail fiber protein | | uniclust | UniRef100\_A0A1F3PPY1 | 99.7 | 4e-20 | 7.4e-26 | 140.5 | 89 | (8, 120) | 221 | (212, 300) | 378 | Uncharacterized protein | Uncharacterized protein | | uniclust | UniRef100\_A0A947MTH0 | 99.7 | 4.4e-20 | 8.1e-26 | 141.2 | 101 | (7, 119) | 221 | (229, 329) | 497 | Tail fiber protein | Tail fiber protein | | uniclust | UniRef100\_UPI00158EA18A | 99.7 | 4.8e-20 | 8.7e-26 | 122.1 | 151 | (24, 221) | 221 | (10, 165) | 166 | hypothetical protein | hypothetical protein | | uniclust | UniRef100\_A0A1G4PCJ5 | 99.7 | 5.1e-20 | 9.5e-26 | 137.2 | 70 | (8, 120) | 221 | (126, 197) | 250 | Microcystin-dependent protein | Microcystin-dependent protein | | uniclust | UniRef100\_A0A2E4EDR2 | 99.7 | 5.5e-20 | 1e-25 | 119.1 | 98 | (9, 119) | 221 | (24, 121) | 124 | Phage tail collar domain-containing protein | Phage tail collar domain-containing protein | | uniclust | UniRef100\_UPI001D053F05 | 99.7 | 5.6e-20 | 1e-25 | 144.5 | 99 | (9, 120) | 221 | (471, 569) | 651 | uncharacterized protein | uncharacterized protein | | uniclust | UniRef100\_A0A846FV65 | 99.7 | 5.8e-20 | 1.1e-25 | 136.5 | 92 | (9, 119) | 221 | (225, 316) | 381 | Phage tail collar domain-containing protein | Phage tail collar domain-containing protein | | uniclust | UniRef100\_A0A0Q4T3S1 | 99.7 | 6.2e-20 | 1.1e-25 | 140.2 | 107 | (13, 119) | 221 | (148, 255) | 423 | Phage tail collar domain-containing protein | Phage tail collar domain-containing protein | | uniclust | UniRef100\_A0A1H8IGX2 | 99.7 | 6.3e-20 | 1.2e-25 | 150.2 | 75 | (10, 84) | 221 | (285, 359) | 462 | Phage Tail Collar Domain | Phage Tail Collar Domain | | uniclust | UniRef100\_A0A941A575 | 99.7 | 7.2e-20 | 1.3e-25 | 128.3 | 94 | (10, 119) | 221 | (48, 156) | 215 | Phage tail collar domain-containing protein | Phage tail collar domain-containing protein | | uniclust | UniRef100\_A0A212J771 | 99.7 | 8.2e-20 | 1.5e-25 | 135.3 | 96 | (9, 119) | 221 | (132, 229) | 301 | Phage tail collar domain-containing protein | Phage tail collar domain-containing protein | | uniclust | UniRef100\_A9AVC5 | 99.7 | 8.7e-20 | 1.6e-25 | 151.1 | 152 | (7, 221) | 221 | (782, 935) | 935 | Tail Collar domain protein | Tail Collar domain protein | | uniclust | UniRef100\_A0A820I8T0 | 99.7 | 8.7e-20 | 1.6e-25 | 122.4 | 125 | (10, 147) | 221 | (11, 136) | 180 | Phage tail collar domain-containing protein (Fragment) | Phage tail collar domain-containing protein (Fragment) | | uniclust | UniRef100\_A0A822BDI4 | 99.7 | 9.2e-20 | 1.7e-25 | 121.7 | 120 | (9, 146) | 221 | (26, 146) | 174 | Phage tail collar domain-containing protein (Fragment) | Phage tail collar domain-containing protein (Fragment) | | uniclust | UniRef100\_A0A323UBG8 | 99.7 | 9.8e-20 | 1.8e-25 | 132.6 | 97 | (4, 119) | 221 | (103, 201) | 322 | Phage tail collar domain-containing protein | Phage tail collar domain-containing protein | | uniclust | UniRef100\_A0A0C1EKM6 | 99.7 | 9.9e-20 | 1.8e-25 | 140.6 | 92 | (9, 119) | 221 | (204, 295) | 358 | Phage tail collar domain-containing protein | Phage tail collar domain-containing protein | | uniclust | UniRef100\_A0A163Z5D0 | 99.7 | 1e-19 | 1.9e-25 | 139.9 | 65 | (8, 74) | 221 | (114, 178) | 308 | Phage tail collar domain-containing protein | Phage tail collar domain-containing protein | | uniclust | UniRef100\_A0A819MQL5 | 99.7 | 1.1e-19 | 2e-25 | 129.6 | 126 | (11, 149) | 221 | (49, 175) | 228 | Phage tail collar domain-containing protein | Phage tail collar domain-containing protein | | uniclust | UniRef100\_A0A1D7Y2A0 | 99.7 | 1.2e-19 | 2.3e-25 | 139.0 | 159 | (9, 221) | 221 | (132, 303) | 309 | Phage tail collar domain-containing protein | Phage tail collar domain-containing protein | | uniclust | UniRef100\_A0A2N2CG59 | 99.7 | 1.3e-19 | 2.4e-25 | 124.1 | 144 | (8, 221) | 221 | (61, 206) | 206 | Phage tail collar domain-containing protein | Phage tail collar domain-containing protein | | uniclust | UniRef100\_A0A4Y5NZ21 | 99.7 | 1.4e-19 | 2.6e-25 | 127.7 | 96 | (10, 119) | 221 | (74, 170) | 225 | Tail fiber | Tail fiber | | uniclust | UniRef100\_A0A3S9QK74 | 99.7 | 1.4e-19 | 2.6e-25 | 131.4 | 98 | (8, 118) | 221 | (133, 232) | 303 | Phage tail collar domain-containing protein | Phage tail collar domain-containing protein | | uniclust | UniRef100\_A0A349QFL3 | 99.7 | 1.4e-19 | 2.6e-25 | 127.0 | 93 | (8, 119) | 221 | (25, 135) | 186 | Phage tail collar domain-containing protein | Phage tail collar domain-containing protein | | uniclust | UniRef100\_UPI001FB54CC0 | 99.7 | 1.5e-19 | 2.8e-25 | 133.3 | 100 | (7, 120) | 221 | (195, 294) | 356 | tail fiber protein | tail fiber protein | | uniclust | UniRef100\_A0A4V0I998 | 99.7 | 1.5e-19 | 2.8e-25 | 147.6 | 100 | (8, 119) | 221 | (505, 604) | 1012 | Phage tail collar domain-containing protein | Phage tail collar domain-containing protein | | uniclust | UniRef100\_A0A1V5WWW4 | 99.7 | 1.9e-19 | 3.4e-25 | 130.6 | 98 | (9, 119) | 221 | (158, 256) | 311 | Phage Tail Collar Domain protein | Phage Tail Collar Domain protein | | uniclust | UniRef100\_UPI001EF32E70 | 99.7 | 2e-19 | 3.7e-25 | 123.7 | 166 | (12, 221) | 221 | (47, 212) | 212 | tail fiber protein | tail fiber protein | | uniclust | UniRef100\_A0A077PNS6 | 99.7 | 2e-19 | 3.7e-25 | 131.9 | 90 | (4, 119) | 221 | (10, 107) | 190 | Phage tail collar domain-containing protein | Phage tail collar domain-containing protein | | uniclust | UniRef100\_A0A0G4FSR3 | 99.7 | 2.1e-19 | 3.9e-25 | 140.9 | 98 | (10, 119) | 221 | (479, 576) | 625 | Phage tail collar domain-containing protein | Phage tail collar domain-containing protein | | uniclust | UniRef100\_A0A2A2TNN2 | 99.7 | 2.1e-19 | 4e-25 | 152.8 | 158 | (8, 221) | 221 | (762, 924) | 924 | LamG-like jellyroll fold domain-containing protein | LamG-like jellyroll fold domain-containing protein | | uniclust | UniRef100\_I0KE34 | 99.7 | 2.3e-19 | 4.3e-25 | 124.4 | 97 | (11, 120) | 221 | (53, 149) | 224 | Tail collar domain protein | Tail collar domain protein | | uniclust | UniRef100\_A0A813P951 | 99.6 | 2.4e-19 | 4.5e-25 | 140.1 | 108 | (9, 128) | 221 | (397, 504) | 579 | LamG-like jellyroll fold domain-containing protein | LamG-like jellyroll fold domain-containing protein | | uniclust | UniRef100\_A0A1H8SWG1 | 99.6 | 2.5e-19 | 4.5e-25 | 127.3 | 85 | (7, 119) | 221 | (125, 211) | 265 | Microcystin-dependent protein | Microcystin-dependent protein | | uniclust | UniRef100\_A0A923UU29 | 99.6 | 2.7e-19 | 5e-25 | 128.4 | 80 | (6, 85) | 221 | (39, 118) | 231 | Tail fiber protein | Tail fiber protein | | uniclust | UniRef100\_A0A937CVC5 | 99.6 | 3.3e-19 | 6.1e-25 | 131.8 | 119 | (8, 137) | 221 | (175, 293) | 363 | Tail fiber protein | Tail fiber protein | | uniclust | UniRef100\_A0A388TAF9 | 99.6 | 3.3e-19 | 6.2e-25 | 125.7 | 145 | (9, 221) | 221 | (25, 176) | 177 | Phage tail fiber protein (Fragment) | Phage tail fiber protein (Fragment) | | uniclust | UniRef100\_A0A959MGB6 | 99.6 | 3.4e-19 | 6.3e-25 | 130.7 | 100 | (11, 120) | 221 | (177, 276) | 340 | Tail fiber protein | Tail fiber protein | | uniclust | UniRef100\_A0A8J7LAJ2 | 99.6 | 3.5e-19 | 6.5e-25 | 128.3 | 97 | (8, 119) | 221 | (140, 236) | 294 | Tail fiber protein | Tail fiber protein | | uniclust | UniRef100\_A0A062GTY4 | 99.6 | 3.5e-19 | 6.5e-25 | 129.1 | 77 | (7, 84) | 221 | (70, 146) | 218 | Phage Tail Collar domain protein | Phage Tail Collar domain protein | | uniclust | UniRef100\_A0A923NCB6 | 99.6 | 3.7e-19 | 6.9e-25 | 142.7 | 99 | (9, 120) | 221 | (617, 716) | 796 | Tail fiber protein | Tail fiber protein | | uniclust | UniRef100\_A0A235AB52 | 99.6 | 3.8e-19 | 7.1e-25 | 150.0 | 97 | (8, 117) | 221 | (479, 575) | 677 | Phage tail collar domain-containing protein | Phage tail collar domain-containing protein | | uniclust | UniRef100\_A0A074JA67 | 99.6 | 4.1e-19 | 7.6e-25 | 126.0 | 91 | (10, 119) | 221 | (52, 142) | 207 | Phage tail collar domain-containing protein | Phage tail collar domain-containing protein | | uniclust | UniRef100\_A0A1V4BWF5 | 99.6 | 4.3e-19 | 7.9e-25 | 127.5 | 70 | (8, 120) | 221 | (89, 160) | 258 | Uncharacterized protein | Uncharacterized protein | | uniclust | UniRef100\_A0A1G1PZ25 | 99.6 | 5.1e-19 | 9.5e-25 | 131.1 | 80 | (7, 119) | 221 | (145, 226) | 278 | Prepilin-type N-terminal cleavage/methylation domain-containing protein | Prepilin-type N-terminal cleavage/methylation domain-containing protein | | uniclust | UniRef100\_A0A1W9JSL6 | 99.6 | 5.2e-19 | 9.6e-25 | 131.5 | 74 | (12, 85) | 221 | (136, 209) | 312 | Phage tail collar domain-containing protein | Phage tail collar domain-containing protein | | uniclust | UniRef100\_A0A0A1FH46 | 99.6 | 5.8e-19 | 1.1e-24 | 133.4 | 80 | (6, 85) | 221 | (150, 229) | 320 | Phage tail fiber protein | Phage tail fiber protein | | uniclust | UniRef100\_A0A832HQN6 | 99.6 | 5.9e-19 | 1.1e-24 | 122.0 | 76 | (8, 122) | 221 | (51, 126) | 191 | Phage tail collar domain-containing protein | Phage tail collar domain-containing protein | | uniclust | UniRef100\_A0A120IHK0 | 99.6 | 6.2e-19 | 1.1e-24 | 125.1 | 90 | (11, 119) | 221 | (38, 127) | 187 | Phage tail collar domain-containing protein | Phage tail collar domain-containing protein | | uniclust | UniRef100\_UPI000C7DD9BD | 99.6 | 6.7e-19 | 1.2e-24 | 133.5 | 95 | (8, 119) | 221 | (298, 392) | 447 | tail fiber protein | tail fiber protein | | uniclust | UniRef100\_A0A1Q6UCI5 | 99.6 | 6.7e-19 | 1.3e-24 | 126.7 | 77 | (8, 84) | 221 | (36, 112) | 195 | Phage tail collar domain-containing protein | Phage tail collar domain-containing protein | | uniclust | UniRef100\_A0A2E5T0N1 | 99.6 | 7.7e-19 | 1.4e-24 | 137.4 | 92 | (9, 122) | 221 | (291, 382) | 426 | Phage tail collar domain-containing protein | Phage tail collar domain-containing protein | | uniclust | UniRef100\_A0A3M0CFX7 | 99.6 | 8.2e-19 | 1.5e-24 | 124.5 | 99 | (10, 120) | 221 | (7, 105) | 209 | Microcystin-dependent protein | Microcystin-dependent protein | | uniclust | UniRef100\_A0A1H9HCA5 | 99.6 | 8.3e-19 | 1.5e-24 | 133.1 | 94 | (7, 119) | 221 | (199, 292) | 345 | Microcystin-dependent protein | Microcystin-dependent protein | | uniclust | UniRef100\_A0A433WUA5 | 99.6 | 8.6e-19 | 1.6e-24 | 131.3 | 53 | (7, 75) | 221 | (60, 114) | 261 | Phage tail collar domain-containing protein | Phage tail collar domain-containing protein | | uniclust | UniRef100\_A0A095S9W8 | 99.6 | 8.8e-19 | 1.6e-24 | 125.0 | 66 | (9, 75) | 221 | (29, 94) | 170 | Phage tail Collar | Phage tail Collar | | uniclust | UniRef100\_A0A6C0DW18 | 99.6 | 9.8e-19 | 1.8e-24 | 118.4 | 94 | (13, 119) | 221 | (37, 130) | 188 | Phage tail collar domain-containing protein | Phage tail collar domain-containing protein | | uniclust | UniRef100\_A0A2N2SSE1 | 99.6 | 9.8e-19 | 1.8e-24 | 124.7 | 95 | (8, 108) | 221 | (75, 169) | 271 | Phage tail protein | Phage tail protein | | uniclust | UniRef100\_A0A083UIH7 | 99.6 | 1e-18 | 1.9e-24 | 146.2 | 76 | (10, 85) | 221 | (353, 428) | 636 | Tail fiber protein | Tail fiber protein | | uniclust | UniRef100\_A0A6M5Z7F0 | 99.6 | 1.1e-18 | 2e-24 | 135.2 | 102 | (7, 119) | 221 | (391, 492) | 544 | Phage tail collar domain-containing protein | Phage tail collar domain-containing protein | | uniclust | UniRef100\_A0A2N2HV87 | 99.6 | 1.1e-18 | 2.1e-24 | 128.8 | 109 | (11, 119) | 221 | (174, 287) | 354 | Phage tail collar domain-containing protein | Phage tail collar domain-containing protein | | uniclust | UniRef100\_UPI000E5A2982 | 99.6 | 1.2e-18 | 2.2e-24 | 127.9 | 94 | (22, 119) | 221 | (174, 267) | 337 | tail fiber protein | tail fiber protein | | uniclust | UniRef100\_A0A1Y1Q2G4 | 99.6 | 1.2e-18 | 2.3e-24 | 132.3 | 91 | (10, 119) | 221 | (71, 161) | 326 | Phage tail collar domain-containing protein | Phage tail collar domain-containing protein | | uniclust | UniRef100\_A0A6C0JGK4 | 99.6 | 1.2e-18 | 2.3e-24 | 121.6 | 97 | (9, 119) | 221 | (76, 172) | 232 | Phage tail collar domain-containing protein | Phage tail collar domain-containing protein | | uniclust | UniRef100\_A0A926SHI8 | 99.6 | 1.3e-18 | 2.3e-24 | 124.3 | 73 | (10, 119) | 221 | (140, 214) | 272 | Tail fiber protein | Tail fiber protein | | uniclust | UniRef100\_A0A969MIB8 | 99.6 | 1.3e-18 | 2.4e-24 | 139.3 | 98 | (10, 119) | 221 | (514, 611) | 767 | Uncharacterized protein | Uncharacterized protein | | uniclust | UniRef100\_A0A0Q0Y1I2 | 99.6 | 1.3e-18 | 2.4e-24 | 133.9 | 102 | (8, 119) | 221 | (54, 174) | 268 | Putative rhizosphere induced protein RhiB | Putative rhizosphere induced protein RhiB | | uniclust | UniRef100\_A0A088FQW7 | 99.6 | 1.3e-18 | 2.4e-24 | 143.0 | 93 | (8, 119) | 221 | (316, 408) | 515 | Tail protein | Tail protein | | uniclust | UniRef100\_UPI0015862BE5 | 99.6 | 1.4e-18 | 2.5e-24 | 121.2 | 71 | (8, 78) | 221 | (11, 81) | 182 | tail fiber protein | tail fiber protein | | uniclust | UniRef100\_A0A821B254 | 99.6 | 1.4e-18 | 2.5e-24 | 126.6 | 121 | (11, 147) | 221 | (106, 228) | 318 | Phage tail collar domain-containing protein | Phage tail collar domain-containing protein | | uniclust | UniRef100\_A0A075X7J1 | 99.6 | 1.6e-18 | 2.9e-24 | 128.5 | 149 | (7, 221) | 221 | (160, 313) | 313 | Phage tail collar domain-containing protein | Phage tail collar domain-containing protein | | uniclust | UniRef100\_A0A2S9GTJ9 | 99.6 | 1.6e-18 | 3e-24 | 123.2 | 90 | (8, 119) | 221 | (17, 108) | 205 | Phage Tail Collar Domain | Phage Tail Collar Domain | | uniclust | UniRef100\_A0A930BHL5 | 99.6 | 1.6e-18 | 3e-24 | 113.3 | 56 | (21, 119) | 221 | (7, 62) | 128 | Uncharacterized protein | Uncharacterized protein | | uniclust | UniRef100\_A0A369KVY1 | 99.6 | 1.6e-18 | 3e-24 | 131.8 | 104 | (8, 119) | 221 | (80, 186) | 314 | Phage tail collar domain-containing protein | Phage tail collar domain-containing protein | | uniclust | UniRef100\_A0A7M2CD16 | 99.6 | 1.6e-18 | 3e-24 | 124.0 | 67 | (9, 119) | 221 | (54, 120) | 188 | Phage tail collar domain-containing protein | Phage tail collar domain-containing protein | | uniclust | UniRef100\_UPI001591FAB7 | 99.6 | 1.7e-18 | 3.1e-24 | 125.2 | 192 | (8, 221) | 221 | (89, 284) | 284 | tail fiber protein | tail fiber protein | | uniclust | UniRef100\_A0A942VA29 | 99.6 | 1.7e-18 | 3.1e-24 | 127.1 | 99 | (9, 119) | 221 | (104, 202) | 293 | Tail fiber protein | Tail fiber protein | | uniclust | UniRef100\_A0A3M1QYC2 | 99.6 | 1.7e-18 | 3.2e-24 | 127.3 | 105 | (9, 119) | 221 | (132, 238) | 306 | Phage tail collar domain-containing protein (Fragment) | Phage tail collar domain-containing protein (Fragment) | | uniclust | UniRef100\_A0A136Q4S0 | 99.6 | 1.9e-18 | 3.5e-24 | 136.7 | 95 | (10, 119) | 221 | (144, 240) | 567 | Phage Tail Collar domain protein | Phage Tail Collar domain protein | | uniclust | UniRef100\_X0Z504 | 99.6 | 2e-18 | 3.7e-24 | 120.4 | 98 | (9, 119) | 221 | (76, 173) | 229 | Phage tail collar domain-containing protein (Fragment) | Phage tail collar domain-containing protein (Fragment) | | uniclust | UniRef100\_A0A212BGA2 | 99.6 | 2e-18 | 3.7e-24 | 121.8 | 74 | (12, 85) | 221 | (67, 140) | 249 | Phage tail collar domain-containing protein | Phage tail collar domain-containing protein | | uniclust | UniRef100\_A0A085FNZ1 | 99.6 | 2.1e-18 | 4e-24 | 141.8 | 95 | (20, 114) | 221 | (233, 393) | 486 | Uncharacterized protein | Uncharacterized protein | | uniclust | UniRef100\_A0A4P6FA25 | 99.6 | 2.4e-18 | 4.5e-24 | 116.7 | 86 | (22, 120) | 221 | (7, 92) | 190 | Phage tail collar domain-containing protein | Phage tail collar domain-containing protein | | uniclust | UniRef100\_A0A0J5P3M7 | 99.6 | 2.4e-18 | 4.6e-24 | 122.4 | 65 | (7, 84) | 221 | (16, 80) | 160 | Phage tail collar domain-containing protein | Phage tail collar domain-containing protein | | uniclust | UniRef100\_A0A1W6NZ62 | 99.6 | 2.5e-18 | 4.6e-24 | 130.8 | 101 | (9, 119) | 221 | (278, 378) | 456 | Phage tail collar domain-containing protein | Phage tail collar domain-containing protein | | uniclust | UniRef100\_A0A934ZLY9 | 99.6 | 2.6e-18 | 4.8e-24 | 125.6 | 114 | (8, 126) | 221 | (145, 258) | 315 | Tail fiber protein | Tail fiber protein | | uniclust | UniRef100\_UPI00168913C5 | 99.6 | 2.7e-18 | 4.9e-24 | 137.3 | 97 | (9, 119) | 221 | (556, 659) | 748 | tail fiber protein | tail fiber protein | | uniclust | UniRef100\_A0A926VM20 | 99.6 | 2.7e-18 | 5e-24 | 128.5 | 85 | (8, 119) | 221 | (252, 336) | 394 | Tail fiber protein | Tail fiber protein | | uniclust | UniRef100\_A0A072T5I3 | 99.6 | 2.7e-18 | 5e-24 | 115.8 | 94 | (10, 119) | 221 | (3, 96) | 175 | Phage tail protein | Phage tail protein | | uniclust | UniRef100\_A0A820RLX9 | 99.6 | 2.9e-18 | 5.3e-24 | 111.0 | 98 | (8, 119) | 221 | (25, 122) | 141 | Phage tail collar domain-containing protein | Phage tail collar domain-containing protein | | uniclust | UniRef100\_A0A0E9MQR3 | 99.6 | 3.1e-18 | 5.8e-24 | 143.8 | 94 | (11, 119) | 221 | (359, 452) | 693 | PEP-CTERM protein-sorting domain-containing protein | PEP-CTERM protein-sorting domain-containing protein | | uniclust | UniRef100\_A0A977UYB6 | 99.6 | 3.6e-18 | 6.7e-24 | 140.1 | 76 | (8, 83) | 221 | (829, 904) | 1018 | Phage tail protein | Phage tail protein | | uniclust | UniRef100\_A0A6L4A8E9 | 99.6 | 3.7e-18 | 6.8e-24 | 122.5 | 100 | (8, 119) | 221 | (47, 146) | 281 | Phage tail collar domain-containing protein | Phage tail collar domain-containing protein | | uniclust | UniRef100\_A0A1G7VYT4 | 99.6 | 4.7e-18 | 8.8e-24 | 136.9 | 98 | (10, 119) | 221 | (345, 442) | 518 | Microcystin-dependent protein | Microcystin-dependent protein | | uniclust | UniRef100\_A0A0F1B0R1 | 99.6 | 4.9e-18 | 9.2e-24 | 123.7 | 72 | (4, 85) | 221 | (38, 109) | 194 | Phage tail collar domain-containing protein | Phage tail collar domain-containing protein | | uniclust | UniRef100\_A0A2D9HKR9 | 99.6 | 5.4e-18 | 1e-23 | 116.5 | 94 | (7, 103) | 221 | (74, 167) | 206 | Phage tail collar domain-containing protein | Phage tail collar domain-containing protein | | uniclust | UniRef100\_A0A2A5VXT6 | 99.6 | 5.4e-18 | 1e-23 | 135.5 | 103 | (8, 118) | 221 | (268, 376) | 435 | Phage tail collar domain-containing protein | Phage tail collar domain-containing protein | | uniclust | UniRef100\_A0A6C0JIE1 | 99.6 | 6.3e-18 | 1.2e-23 | 118.9 | 95 | (9, 119) | 221 | (12, 118) | 196 | Phage tail collar domain-containing protein | Phage tail collar domain-containing protein | | uniclust | UniRef100\_A0A7C7C9X1 | 99.6 | 6.5e-18 | 1.2e-23 | 118.8 | 94 | (13, 127) | 221 | (87, 182) | 242 | Tail fiber protein | Tail fiber protein | | uniclust | UniRef100\_A0A257UMQ9 | 99.6 | 6.6e-18 | 1.2e-23 | 118.5 | 96 | (12, 119) | 221 | (69, 165) | 238 | Phage tail collar domain-containing protein | Phage tail collar domain-containing protein | | uniclust | UniRef100\_A0A1V5WG08 | 99.6 | 7.1e-18 | 1.3e-23 | 118.2 | 79 | (8, 121) | 221 | (90, 168) | 235 | Phage tail collar domain-containing protein | Phage tail collar domain-containing protein | | uniclust | UniRef100\_A0A2D5S2C0 | 99.6 | 7e-18 | 1.3e-23 | 119.6 | 78 | (8, 85) | 221 | (16, 93) | 177 | Phage tail protein (Fragment) | Phage tail protein (Fragment) | | uniclust | UniRef100\_A0A845WN63 | 99.6 | 7.2e-18 | 1.3e-23 | 121.2 | 88 | (9, 119) | 221 | (136, 224) | 282 | Phage tail collar domain-containing protein | Phage tail collar domain-containing protein | | uniclust | UniRef100\_A0A553JK32 | 99.6 | 7.2e-18 | 1.3e-23 | 131.7 | 75 | (9, 126) | 221 | (324, 398) | 483 | Phage tail collar domain-containing protein | Phage tail collar domain-containing protein | | uniclust | UniRef100\_K7XWX8 | 99.6 | 7.5e-18 | 1.4e-23 | 125.3 | 77 | (9, 85) | 221 | (199, 275) | 370 | Phage tail fiber protein | Phage tail fiber protein | | uniclust | UniRef100\_A0A800M671 | 99.6 | 7.6e-18 | 1.4e-23 | 120.9 | 96 | (11, 119) | 221 | (114, 209) | 279 | Phage tail protein | Phage tail protein | | uniclust | UniRef100\_D1BW55 | 99.6 | 7.7e-18 | 1.4e-23 | 127.9 | 85 | (22, 119) | 221 | (236, 320) | 443 | Tail Collar domain protein | Tail Collar domain protein | | uniclust | UniRef100\_A0A967KK39 | 99.6 | 7.9e-18 | 1.4e-23 | 126.3 | 96 | (11, 119) | 221 | (6, 101) | 399 | Phage tail protein | Phage tail protein | | uniclust | UniRef100\_A0A0E9MQR3 | 99.6 | 7.8e-18 | 1.5e-23 | 141.6 | 96 | (11, 120) | 221 | (501, 596) | 693 | PEP-CTERM protein-sorting domain-containing protein | PEP-CTERM protein-sorting domain-containing protein | | uniclust | UniRef100\_A0A2V9WHC2 | 99.6 | 8.1e-18 | 1.5e-23 | 122.6 | 99 | (7, 126) | 221 | (130, 230) | 315 | Phage tail collar domain-containing protein | Phage tail collar domain-containing protein | | uniclust | UniRef100\_A0A969GKG4 | 99.6 | 8.8e-18 | 1.6e-23 | 123.9 | 84 | (9, 119) | 221 | (205, 288) | 346 | Phage tail collar domain-containing protein | Phage tail collar domain-containing protein | | uniclust | UniRef100\_A0A8T6N729 | 99.6 | 9e-18 | 1.6e-23 | 109.7 | 67 | (9, 75) | 221 | (27, 93) | 148 | Phage tail collar domain-containing protein (Fragment) | Phage tail collar domain-containing protein (Fragment) | | uniclust | UniRef100\_A0A3B8Q1P4 | 99.6 | 9.9e-18 | 1.8e-23 | 124.7 | 68 | (9, 119) | 221 | (252, 319) | 371 | Uncharacterized protein | Uncharacterized protein | | uniclust | UniRef100\_A0A8S0HYQ4 | 99.6 | 1.1e-17 | 1.9e-23 | 118.6 | 127 | (8, 221) | 221 | (115, 243) | 243 | Phage tail collar domain-containing protein | Phage tail collar domain-containing protein | | uniclust | UniRef100\_A0A5B8XJ48 | 99.6 | 1.1e-17 | 2e-23 | 130.8 | 96 | (12, 119) | 221 | (434, 529) | 580 | Phage tail collar domain protein | Phage tail collar domain protein | | uniclust | UniRef100\_A0A965JC12 | 99.6 | 1.1e-17 | 2e-23 | 122.2 | 76 | (7, 82) | 221 | (89, 164) | 281 | Phage tail collar domain-containing protein | Phage tail collar domain-containing protein | | uniclust | UniRef100\_A0A959IFW3 | 99.6 | 1.1e-17 | 2e-23 | 133.1 | 84 | (8, 119) | 221 | (561, 644) | 701 | Tail fiber protein | Tail fiber protein | | uniclust | UniRef100\_A0A7C5IR70 | 99.6 | 1.1e-17 | 2.1e-23 | 130.0 | 146 | (11, 221) | 221 | (405, 550) | 550 | Phage tail collar domain-containing protein | Phage tail collar domain-containing protein | | uniclust | UniRef100\_UPI0018DC42F4 | 99.5 | 1.2e-17 | 2.1e-23 | 129.1 | 93 | (22, 128) | 221 | (361, 453) | 519 | tail fiber protein | tail fiber protein | | uniclust | UniRef100\_A0A1F9IXH9 | 99.5 | 1.2e-17 | 2.2e-23 | 115.8 | 105 | (10, 118) | 221 | (65, 170) | 217 | Phage tail collar domain-containing protein | Phage tail collar domain-containing protein | | uniclust | UniRef100\_A0A955C5E6 | 99.5 | 1.2e-17 | 2.2e-23 | 126.5 | 101 | (11, 119) | 221 | (265, 368) | 431 | Tail fiber protein | Tail fiber protein | | uniclust | UniRef100\_A0A022PFL8 | 99.5 | 1.2e-17 | 2.2e-23 | 119.1 | 84 | (7, 119) | 221 | (50, 133) | 206 | Phage-related tail fiber protein | Phage-related tail fiber protein | | uniclust | UniRef100\_A0A2E1T177 | 99.5 | 1.3e-17 | 2.4e-23 | 128.2 | 89 | (8, 139) | 221 | (108, 198) | 278 | Uncharacterized protein | Uncharacterized protein | | uniclust | UniRef100\_A0A3S0C1T6 | 99.5 | 1.3e-17 | 2.4e-23 | 125.8 | 67 | (9, 75) | 221 | (83, 149) | 354 | Tail fiber protein | Tail fiber protein | | uniclust | UniRef100\_A0A9E0PA08 | 99.5 | 1.4e-17 | 2.6e-23 | 122.8 | 111 | (8, 119) | 221 | (182, 295) | 344 | Tail fiber protein | Tail fiber protein | | uniclust | UniRef100\_A0A0D6GNQ8 | 99.5 | 1.4e-17 | 2.6e-23 | 126.1 | 72 | (4, 85) | 221 | (115, 186) | 281 | Tail fiber protein | Tail fiber protein | | uniclust | UniRef100\_A0A2X1ULZ5 | 99.5 | 1.5e-17 | 2.7e-23 | 123.8 | 98 | (9, 145) | 221 | (75, 174) | 258 | Phage tail fibre repeat | Phage tail fibre repeat | | uniclust | UniRef100\_A0A6C0JYK5 | 99.5 | 1.5e-17 | 2.8e-23 | 140.5 | 149 | (8, 221) | 221 | (1367, 1519) | 1521 | Trimeric autotransporter adhesin YadA-like head domain-containing protein | Trimeric autotransporter adhesin YadA-like head domain-containing protein | | uniclust | UniRef100\_UPI001E32DA79 | 99.5 | 1.5e-17 | 2.8e-23 | 115.5 | 98 | (9, 119) | 221 | (121, 218) | 221 | tail fiber protein | tail fiber protein | | uniclust | UniRef100\_UPI001FC84116 | 99.5 | 1.6e-17 | 2.9e-23 | 131.5 | 92 | (8, 117) | 221 | (462, 553) | 624 | tail fiber protein | tail fiber protein | | uniclust | UniRef100\_A0A0E3P079 | 99.5 | 1.6e-17 | 3e-23 | 130.8 | 98 | (9, 119) | 221 | (331, 428) | 431 | Microcystin dependent protein | Microcystin dependent protein | | uniclust | UniRef100\_UPI0004242599 | 99.5 | 1.7e-17 | 3.1e-23 | 119.8 | 77 | (8, 84) | 221 | (111, 187) | 291 | tail fiber protein | tail fiber protein | | uniclust | UniRef100\_A0A2E4L524 | 99.5 | 1.8e-17 | 3.3e-23 | 135.2 | 87 | (9, 118) | 221 | (789, 875) | 927 | Phage tail collar domain-containing protein | Phage tail collar domain-containing protein | | uniclust | UniRef100\_A0A938GHZ7 | 99.5 | 1.9e-17 | 3.5e-23 | 131.6 | 72 | (8, 120) | 221 | (372, 445) | 514 | Uncharacterized protein | Uncharacterized protein | | uniclust | UniRef100\_A0A1H1FGW5 | 99.5 | 1.9e-17 | 3.5e-23 | 126.0 | 112 | (8, 119) | 221 | (199, 375) | 449 | Phage Tail Collar Domain | Phage Tail Collar Domain | | uniclust | UniRef100\_A0A1K0IP40 | 99.5 | 2.1e-17 | 3.9e-23 | 136.7 | 91 | (10, 119) | 221 | (406, 496) | 548 | Phage tail collar domain-containing protein | Phage tail collar domain-containing protein | | uniclust | UniRef100\_A0A7C3YVS4 | 99.5 | 2.3e-17 | 4.2e-23 | 112.4 | 90 | (11, 119) | 221 | (46, 135) | 192 | Phage tail collar domain-containing protein | Phage tail collar domain-containing protein | | uniclust | UniRef100\_A0A835XXH8 | 99.5 | 2.3e-17 | 4.3e-23 | 128.3 | 109 | (7, 127) | 221 | (356, 464) | 550 | Phage tail collar domain-containing protein | Phage tail collar domain-containing protein | | uniclust | UniRef100\_A0A375EED5 | 99.5 | 2.4e-17 | 4.4e-23 | 125.2 | 94 | (11, 121) | 221 | (4, 97) | 440 | Phage tail collar domain-containing protein | Phage tail collar domain-containing protein | | uniclust | UniRef100\_A0A418RLT6 | 99.5 | 2.4e-17 | 4.5e-23 | 122.0 | 64 | (8, 119) | 221 | (105, 170) | 247 | Tail fiber protein | Tail fiber protein | | uniclust | UniRef100\_A0A1X3I976 | 99.5 | 2.6e-17 | 4.8e-23 | 113.5 | 72 | (4, 85) | 221 | (16, 87) | 166 | Side tail fiber protein-like protein (Fragment) | Side tail fiber protein-like protein (Fragment) | | uniclust | UniRef100\_A0A024L5N7 | 99.5 | 2.6e-17 | 4.9e-23 | 126.8 | 87 | (4, 119) | 221 | (112, 198) | 289 | Phage tail protein | Phage tail protein | | uniclust | UniRef100\_A0A2G1DGN7 | 99.5 | 2.7e-17 | 5.1e-23 | 122.9 | 77 | (8, 84) | 221 | (165, 241) | 317 | Phage tail collar domain-containing protein | Phage tail collar domain-containing protein | | uniclust | UniRef100\_A0A1G7NGA5 | 99.5 | 2.7e-17 | 5.1e-23 | 125.5 | 76 | (9, 84) | 221 | (113, 202) | 333 | Phage Tail Collar Domain | Phage Tail Collar Domain | | uniclust | UniRef100\_A0A1I5LHR7 | 99.5 | 2.9e-17 | 5.3e-23 | 109.5 | 67 | (10, 119) | 221 | (40, 108) | 167 | Microcystin-dependent protein | Microcystin-dependent protein | | uniclust | UniRef100\_A0A816UR79 | 99.5 | 2.9e-17 | 5.3e-23 | 109.6 | 145 | (24, 221) | 221 | (21, 166) | 168 | Phage tail collar domain-containing protein | Phage tail collar domain-containing protein | | uniclust | UniRef100\_A0A370CFE4 | 99.5 | 2.9e-17 | 5.4e-23 | 126.6 | 100 | (9, 119) | 221 | (231, 331) | 382 | Phage tail collar domain-containing protein | Phage tail collar domain-containing protein | | uniclust | UniRef100\_K8GNE3 | 99.5 | 3e-17 | 5.4e-23 | 130.9 | 196 | (8, 221) | 221 | (480, 703) | 703 | Phage tail collar family protein | Phage tail collar family protein | | uniclust | UniRef100\_A0A7R9WFI0 | 99.5 | 3e-17 | 5.5e-23 | 118.5 | 97 | (11, 120) | 221 | (48, 144) | 257 | Phage tail collar domain-containing protein | Phage tail collar domain-containing protein | | uniclust | UniRef100\_A0A346HW44 | 99.5 | 3.2e-17 | 6e-23 | 133.8 | 110 | (9, 137) | 221 | (275, 384) | 574 | Putative tail-fiber protein | Putative tail-fiber protein | | uniclust | UniRef100\_A0A9D1L441 | 99.5 | 3.3e-17 | 6.1e-23 | 127.0 | 98 | (9, 119) | 221 | (239, 336) | 527 | Tail fiber protein | Tail fiber protein | | uniclust | UniRef100\_A0A819C810 | 99.5 | 3.5e-17 | 6.3e-23 | 137.1 | 198 | (10, 220) | 221 | (352, 562) | 1314 | RNA helicase | RNA helicase | | uniclust | UniRef100\_A0A6C0HLH9 | 99.5 | 3.5e-17 | 6.4e-23 | 119.4 | 88 | (8, 119) | 221 | (145, 233) | 312 | Phage tail collar domain-containing protein | Phage tail collar domain-containing protein | | uniclust | UniRef100\_A0A022PDV2 | 99.5 | 3.6e-17 | 6.8e-23 | 128.9 | 87 | (6, 118) | 221 | (173, 267) | 353 | Phage tail collar family protein | Phage tail collar family protein | | uniclust | UniRef100\_A0A7X5TNA6 | 99.5 | 3.8e-17 | 7.1e-23 | 115.2 | 83 | (8, 119) | 221 | (52, 134) | 188 | Phage tail collar domain-containing protein | Phage tail collar domain-containing protein | | uniclust | UniRef100\_A0A1J5P6P6 | 99.5 | 3.9e-17 | 7.1e-23 | 117.9 | 100 | (16, 119) | 221 | (111, 210) | 288 | Phage terminase large subunit | Phage terminase large subunit | | uniclust | UniRef100\_A0A6C0KWH9 | 99.5 | 4.1e-17 | 7.5e-23 | 132.0 | 164 | (10, 221) | 221 | (667, 831) | 831 | Phage tail collar domain-containing protein | Phage tail collar domain-containing protein | | uniclust | UniRef100\_UPI000E5A64BF | 99.5 | 4.3e-17 | 7.9e-23 | 122.4 | 105 | (9, 119) | 221 | (236, 341) | 394 | tail fiber protein | tail fiber protein | | uniclust | UniRef100\_A0A098UFE1 | 99.5 | 5.1e-17 | 9.6e-23 | 127.5 | 98 | (9, 119) | 221 | (74, 171) | 305 | Phage tail collar domain-containing protein | Phage tail collar domain-containing protein | | uniclust | UniRef100\_A0A024FDZ3 | 99.5 | 5.3e-17 | 1e-22 | 128.1 | 99 | (6, 136) | 221 | (161, 260) | 331 | Phage tail fibers | Phage tail fibers | | uniclust | UniRef100\_UPI001AFA07E9 | 99.5 | 5.5e-17 | 1e-22 | 126.6 | 162 | (8, 221) | 221 | (395, 561) | 561 | hypothetical protein | hypothetical protein | | uniclust | UniRef100\_A0A972W8A9 | 99.5 | 5.9e-17 | 1.1e-22 | 120.7 | 107 | (8, 119) | 221 | (211, 321) | 368 | Tail fiber protein | Tail fiber protein | | uniclust | UniRef100\_A0A1Y5K9L9 | 99.5 | 6.1e-17 | 1.1e-22 | 116.9 | 101 | (10, 123) | 221 | (5, 105) | 233 | Phage tail collar domain-containing protein | Phage tail collar domain-containing protein | | uniclust | UniRef100\_F2Y385 | 99.5 | 6.2e-17 | 1.1e-22 | 117.7 | 150 | (8, 221) | 221 | (154, 303) | 303 | Putative phage tail collar domain family protein | Putative phage tail collar domain family protein | | uniclust | UniRef100\_A0A5A9GP38 | 99.5 | 6.3e-17 | 1.2e-22 | 118.8 | 109 | (8, 124) | 221 | (89, 204) | 256 | Tail fiber protein | Tail fiber protein | | uniclust | UniRef100\_A0A5C5PXD1 | 99.5 | 6.4e-17 | 1.2e-22 | 122.9 | 90 | (10, 118) | 221 | (185, 274) | 335 | Phage tail collar domain-containing protein | Phage tail collar domain-containing protein | | uniclust | UniRef100\_A0A0F9I8V3 | 99.5 | 6.4e-17 | 1.2e-22 | 115.4 | 69 | (7, 118) | 221 | (26, 100) | 184 | Phage tail collar domain-containing protein | Phage tail collar domain-containing protein | | uniclust | UniRef100\_A0A937FTQ8 | 99.5 | 6.6e-17 | 1.2e-22 | 122.0 | 178 | (10, 221) | 221 | (206, 409) | 410 | Uncharacterized protein | Uncharacterized protein | | uniclust | UniRef100\_A0A835XV31 | 99.5 | 6.6e-17 | 1.2e-22 | 137.9 | 108 | (8, 127) | 221 | (1556, 1663) | 1750 | EF-hand domain-containing protein | EF-hand domain-containing protein | | uniclust | UniRef100\_A0A815B3Y9 | 99.5 | 6.6e-17 | 1.2e-22 | 129.3 | 196 | (9, 221) | 221 | (380, 583) | 585 | Phage tail collar domain-containing protein | Phage tail collar domain-containing protein | | uniclust | UniRef100\_A0A2Z6B2C1 | 99.5 | 6.6e-17 | 1.2e-22 | 117.3 | 74 | (8, 83) | 221 | (119, 192) | 257 | Putative phage tail fiber-related protein | Putative phage tail fiber-related protein | | uniclust | UniRef100\_A0A077PK15 | 99.5 | 7.6e-17 | 1.4e-22 | 124.4 | 69 | (7, 85) | 221 | (160, 228) | 318 | Putative E14 prophage tail fiber protein (Modular protein) | Putative E14 prophage tail fiber protein (Modular protein) | | uniclust | UniRef100\_UPI0020139BF7 | 99.5 | 7.7e-17 | 1.4e-22 | 122.6 | 131 | (9, 146) | 221 | (204, 334) | 440 | tail fiber protein | tail fiber protein | | uniclust | UniRef100\_A0A6J5LZ45 | 99.5 | 7.8e-17 | 1.4e-22 | 116.2 | 108 | (12, 119) | 221 | (124, 232) | 285 | MdpB Microcystin-dependent protein | MdpB Microcystin-dependent protein | | uniclust | UniRef100\_A0A0N9MHW1 | 99.5 | 7.8e-17 | 1.5e-22 | 142.1 | 169 | (10, 221) | 221 | (1144, 1312) | 1312 | Phage tail fiber protein | Phage tail fiber protein | | uniclust | UniRef100\_U3A9G3 | 99.5 | 8.1e-17 | 1.5e-22 | 128.2 | 157 | (9, 221) | 221 | (531, 687) | 687 | Phage tail collar domain-containing protein | Phage tail collar domain-containing protein | | uniclust | UniRef100\_Q55EP2 | 99.5 | 8.1e-17 | 1.5e-22 | 107.4 | 90 | (10, 124) | 221 | (25, 114) | 166 | Phage tail collar domain-containing protein | Phage tail collar domain-containing protein | | uniclust | UniRef100\_A0A5Q2W2K9 | 99.5 | 8.2e-17 | 1.5e-22 | 127.2 | 146 | (8, 221) | 221 | (405, 634) | 634 | Tail collar | Tail collar | | uniclust | UniRef100\_A0A845XBF3 | 99.5 | 8.4e-17 | 1.6e-22 | 132.6 | 76 | (8, 121) | 221 | (456, 541) | 610 | Tail fiber protein | Tail fiber protein | | uniclust | UniRef100\_A0A6J5S6B7 | 99.5 | 8.6e-17 | 1.6e-22 | 123.9 | 112 | (8, 119) | 221 | (299, 415) | 494 | Phage tail collar domain containing protein | Phage tail collar domain containing protein | | uniclust | UniRef100\_A0A1Y1IPX0 | 99.5 | 9.8e-17 | 1.8e-22 | 111.9 | 65 | (38, 120) | 221 | (29, 93) | 164 | Phage tail collar domain-containing protein | Phage tail collar domain-containing protein | | uniclust | UniRef100\_A0A1E4J0W6 | 99.5 | 1e-16 | 1.9e-22 | 119.4 | 116 | (11, 145) | 221 | (121, 236) | 337 | Phage tail collar domain-containing protein | Phage tail collar domain-containing protein | | uniclust | UniRef100\_A0A0H5Q3U7 | 99.5 | 1e-16 | 1.9e-22 | 116.3 | 89 | (11, 120) | 221 | (71, 159) | 213 | Phage tail collar domain-containing protein | Phage tail collar domain-containing protein | | uniclust | UniRef100\_A0A1G3JKE1 | 99.5 | 1e-16 | 1.9e-22 | 121.9 | 78 | (8, 85) | 221 | (137, 214) | 286 | Peptidase S74 domain-containing protein | Peptidase S74 domain-containing protein | | uniclust | UniRef100\_A0A814Z794 | 99.5 | 1e-16 | 1.9e-22 | 116.6 | 122 | (9, 145) | 221 | (119, 241) | 252 | Phage tail collar domain-containing protein | Phage tail collar domain-containing protein | | uniclust | UniRef100\_A0A835XQ12 | 99.5 | 1.1e-16 | 2e-22 | 120.8 | 98 | (10, 119) | 221 | (235, 335) | 409 | Phage tail collar domain-containing protein | Phage tail collar domain-containing protein | | uniclust | UniRef100\_A0A2I7SAV6 | 99.5 | 1.1e-16 | 2e-22 | 118.2 | 87 | (8, 119) | 221 | (121, 207) | 268 | Short tail fiber protein | Short tail fiber protein | | uniclust | UniRef100\_A0A3M1AQT8 | 99.5 | 1.1e-16 | 2e-22 | 132.3 | 195 | (9, 221) | 221 | (853, 1066) | 1066 | Phage tail collar domain-containing protein | Phage tail collar domain-containing protein | | uniclust | UniRef100\_A0A814VVY1 | 99.5 | 1.1e-16 | 2e-22 | 124.1 | 92 | (10, 119) | 221 | (330, 421) | 423 | Phage tail collar domain-containing protein | Phage tail collar domain-containing protein | | uniclust | UniRef100\_A0A1M6Z489 | 99.5 | 1.1e-16 | 2.1e-22 | 114.9 | 104 | (9, 118) | 221 | (114, 217) | 264 | Phage Tail Collar Domain | Phage Tail Collar Domain | | uniclust | UniRef100\_A0A2S4LWD3 | 99.5 | 1.1e-16 | 2.1e-22 | 141.0 | 190 | (10, 221) | 221 | (844, 1066) | 1067 | Microcystin-dependent protein | Microcystin-dependent protein | | uniclust | UniRef100\_A0A522BAB4 | 99.5 | 1.2e-16 | 2.1e-22 | 113.3 | 97 | (11, 120) | 221 | (98, 194) | 249 | Phage tail collar domain-containing protein | Phage tail collar domain-containing protein | | uniclust | UniRef100\_A0A133XLD8 | 99.5 | 1.2e-16 | 2.2e-22 | 116.7 | 78 | (8, 85) | 221 | (124, 201) | 268 | Phage tail collar domain-containing protein | Phage tail collar domain-containing protein | | uniclust | UniRef100\_A0A376BTN0 | 99.5 | 1.2e-16 | 2.3e-22 | 132.3 | 94 | (7, 119) | 221 | (507, 600) | 669 | Phage Tail Collar Domain | Phage Tail Collar Domain | | uniclust | UniRef100\_A0A6J5QPP2 | 99.5 | 1.3e-16 | 2.4e-22 | 118.2 | 93 | (10, 121) | 221 | (137, 229) | 348 | Phage tail collar domain containing protein | Phage tail collar domain containing protein | | uniclust | UniRef100\_A0A150PN60 | 99.5 | 1.4e-16 | 2.5e-22 | 120.1 | 69 | (8, 119) | 221 | (273, 343) | 404 | Phage tail collar domain-containing protein | Phage tail collar domain-containing protein | | uniclust | UniRef100\_A0A966QWS9 | 99.5 | 1.4e-16 | 2.5e-22 | 118.8 | 69 | (8, 76) | 221 | (208, 277) | 368 | Phage tail collar domain-containing protein | Phage tail collar domain-containing protein | | uniclust | UniRef100\_A0A2E6GQF0 | 99.5 | 1.4e-16 | 2.6e-22 | 118.0 | 98 | (10, 119) | 221 | (199, 297) | 351 | Phage tail collar domain-containing protein | Phage tail collar domain-containing protein | | uniclust | UniRef100\_A0A821TQI6 | 99.5 | 1.4e-16 | 2.6e-22 | 115.8 | 106 | (25, 149) | 221 | (14, 119) | 279 | Tesmin/TSO1-like CXC domain-containing protein (Fragment) | Tesmin/TSO1-like CXC domain-containing protein (Fragment) | | uniclust | UniRef100\_A0A1G3HMJ8 | 99.5 | 1.4e-16 | 2.7e-22 | 125.7 | 75 | (10, 84) | 221 | (174, 248) | 324 | Phage tail collar domain-containing protein | Phage tail collar domain-containing protein | | uniclust | UniRef100\_A0A158DQ00 | 99.5 | 1.4e-16 | 2.7e-22 | 123.6 | 72 | (9, 119) | 221 | (79, 152) | 278 | Phage-related tail fiber protein | Phage-related tail fiber protein | | uniclust | UniRef100\_A0A822B9W3 | 99.5 | 1.5e-16 | 2.8e-22 | 103.2 | 96 | (8, 119) | 221 | (32, 132) | 140 | Phage tail collar domain-containing protein | Phage tail collar domain-containing protein | | uniclust | UniRef100\_A0A084EG14 | 99.5 | 1.6e-16 | 2.9e-22 | 110.7 | 97 | (10, 119) | 221 | (14, 111) | 195 | Tail collar domain-containing protein | Tail collar domain-containing protein | | uniclust | UniRef100\_UPI001C0EAFD0 | 99.5 | 1.6e-16 | 3e-22 | 125.9 | 151 | (8, 221) | 221 | (495, 647) | 647 | hypothetical protein | hypothetical protein | | uniclust | UniRef100\_A0A2R4H0T7 | 99.5 | 1.6e-16 | 3e-22 | 122.7 | 97 | (10, 119) | 221 | (265, 445) | 501 | Phage tail collar domain-containing protein | Phage tail collar domain-containing protein | | uniclust | UniRef100\_A0A1Y4GXK4 | 99.5 | 1.7e-16 | 3e-22 | 113.0 | 94 | (9, 119) | 221 | (94, 187) | 245 | Phage tail collar domain-containing protein | Phage tail collar domain-containing protein | | uniclust | UniRef100\_UPI001D104AA5 | 99.5 | 1.7e-16 | 3.1e-22 | 113.5 | 100 | (9, 119) | 221 | (77, 176) | 265 | tail fiber protein | tail fiber protein | | uniclust | UniRef100\_A0A1C3YQ05 | 99.5 | 1.7e-16 | 3.1e-22 | 121.9 | 78 | (8, 85) | 221 | (151, 228) | 314 | Phage tail fibre repeat-containing protein | Phage tail fibre repeat-containing protein | | uniclust | UniRef100\_A0A0G3CP38 | 99.5 | 1.8e-16 | 3.4e-22 | 111.7 | 73 | (4, 86) | 221 | (15, 87) | 179 | Phage tail collar domain-containing protein | Phage tail collar domain-containing protein | | uniclust | UniRef100\_A0A074VFM7 | 99.5 | 2.1e-16 | 3.9e-22 | 127.9 | 78 | (8, 85) | 221 | (226, 303) | 411 | Phage Tail Collar Domain | Phage Tail Collar Domain | | uniclust | UniRef100\_UPI000532B154 | 99.5 | 2.2e-16 | 4e-22 | 112.9 | 83 | (8, 119) | 221 | (72, 154) | 218 | tail fiber protein | tail fiber protein | | uniclust | UniRef100\_A0A7W4IK36 | 99.5 | 2.2e-16 | 4e-22 | 125.4 | 107 | (20, 133) | 221 | (3, 109) | 659 | Phage tail collar domain-containing protein | Phage tail collar domain-containing protein | | uniclust | UniRef100\_A0A4R1FMB3 | 99.5 | 2.2e-16 | 4.1e-22 | 130.3 | 92 | (9, 119) | 221 | (668, 759) | 817 | Tail collar domain | Tail collar domain | | uniclust | UniRef100\_A0A817Z300 | 99.4 | 2.5e-16 | 4.6e-22 | 120.2 | 198 | (10, 221) | 221 | (243, 448) | 449 | Phage tail collar domain-containing protein | Phage tail collar domain-containing protein | | uniclust | UniRef100\_A0A8S2DDE4 | 99.4 | 2.6e-16 | 4.7e-22 | 126.2 | 177 | (15, 220) | 221 | (2, 189) | 728 | Amino acid permease/ SLC12A domain-containing protein | Amino acid permease/ SLC12A domain-containing protein | | uniclust | UniRef100\_A0A2D8EVB5 | 99.4 | 2.6e-16 | 4.8e-22 | 124.9 | 75 | (9, 126) | 221 | (508, 586) | 652 | Major tropism determinant N-terminal domain-containing protein | Major tropism determinant N-terminal domain-containing protein | | uniclust | UniRef100\_A0A1X0TJA2 | 99.4 | 2.6e-16 | 4.8e-22 | 114.2 | 76 | (10, 85) | 221 | (66, 141) | 224 | Phage tail collar domain-containing protein | Phage tail collar domain-containing protein | | uniclust | UniRef100\_A0A7L4ZU76 | 99.4 | 2.7e-16 | 4.9e-22 | 116.9 | 95 | (11, 119) | 221 | (4, 98) | 356 | Phage tail collar domain-containing protein | Phage tail collar domain-containing protein | | uniclust | UniRef100\_A0A8S5VPB3 | 99.4 | 2.9e-16 | 5.4e-22 | 116.1 | 40 | (9, 74) | 221 | (34, 75) | 262 | Tail fiber protein | Tail fiber protein | | uniclust | UniRef100\_A0A6J5PAS1 | 99.4 | 3e-16 | 5.4e-22 | 113.2 | 93 | (7, 119) | 221 | (140, 232) | 281 | Phage tail collar domain containing protein | Phage tail collar domain containing protein | | uniclust | UniRef100\_A0A534A2U7 | 99.4 | 3.1e-16 | 5.6e-22 | 105.1 | 86 | (23, 119) | 221 | (36, 121) | 170 | Phage tail collar domain-containing protein | Phage tail collar domain-containing protein | | uniclust | UniRef100\_A0A3M2BJC7 | 99.4 | 3.1e-16 | 5.8e-22 | 106.3 | 76 | (9, 84) | 221 | (92, 167) | 183 | Tail fiber protein (Fragment) | Tail fiber protein (Fragment) | | uniclust | UniRef100\_A0A957SMD6 | 99.4 | 3.2e-16 | 5.9e-22 | 125.9 | 67 | (10, 119) | 221 | (617, 685) | 742 | Uncharacterized protein | Uncharacterized protein | | uniclust | UniRef100\_A0A212LXT2 | 99.4 | 3.3e-16 | 6e-22 | 114.8 | 74 | (12, 119) | 221 | (140, 218) | 273 | Phage Tail Collar domain protein | Phage Tail Collar domain protein | | uniclust | UniRef100\_A0A2Z6AZ11 | 99.4 | 3.6e-16 | 6.7e-22 | 119.8 | 74 | (10, 83) | 221 | (187, 260) | 348 | Tail Collar domain protein | Tail Collar domain protein | | uniclust | UniRef100\_A0A3D4BUX3 | 99.4 | 3.7e-16 | 6.9e-22 | 92.9 | 64 | (10, 74) | 221 | (5, 68) | 72 | Phage tail protein (Fragment) | Phage tail protein (Fragment) | | uniclust | UniRef100\_A0A963Z1H4 | 99.4 | 3.9e-16 | 7.1e-22 | 111.0 | 60 | (17, 76) | 221 | (27, 86) | 252 | Tail fiber protein | Tail fiber protein | | uniclust | UniRef100\_A0A1B8YGC4 | 99.4 | 4.2e-16 | 7.8e-22 | 131.8 | 68 | (8, 85) | 221 | (470, 537) | 616 | Phage tail fiber repeat protein | Phage tail fiber repeat protein | | uniclust | UniRef100\_UPI001FFB714D | 99.4 | 4.3e-16 | 7.8e-22 | 112.2 | 91 | (10, 119) | 221 | (108, 198) | 277 | phage tail protein | phage tail protein | | uniclust | UniRef100\_A0A0K4CUX8 | 99.4 | 4.2e-16 | 7.9e-22 | 107.1 | 64 | (12, 85) | 221 | (8, 71) | 148 | Alternative C-terminus of phage tail fiber protein, fused to tail fiber protein by inversion | Alternative C-terminus of phage tail fiber protein, fused to tail fiber protein by inversion | | uniclust | UniRef100\_A0A0T9PN75 | 99.4 | 4.5e-16 | 8.4e-22 | 127.5 | 87 | (7, 119) | 221 | (270, 364) | 447 | Phage Tail Collar Domain | Phage Tail Collar Domain | | uniclust | UniRef100\_A0A0A2S0D5 | 99.4 | 4.6e-16 | 8.5e-22 | 110.2 | 86 | (4, 118) | 221 | (34, 119) | 186 | Phage tail protein | Phage tail protein | | uniclust | UniRef100\_A0A6J7VL93 | 99.4 | 4.6e-16 | 8.5e-22 | 116.0 | 98 | (22, 119) | 221 | (207, 307) | 363 | Phage tail collar domain containing protein | Phage tail collar domain containing protein | | uniclust | UniRef100\_A0A5E7XPA5 | 99.4 | 4.7e-16 | 8.6e-22 | 111.9 | 80 | (10, 128) | 221 | (110, 191) | 275 | Phage tail collar domain-containing protein | Phage tail collar domain-containing protein | | uniclust | UniRef100\_A0A071MNQ9 | 99.4 | 4.8e-16 | 9e-22 | 129.9 | 191 | (9, 221) | 221 | (430, 652) | 653 | Tail protein | Tail protein | | uniclust | UniRef100\_UPI0006A999EC | 99.4 | 5e-16 | 9.2e-22 | 115.3 | 73 | (8, 119) | 221 | (191, 265) | 352 | tail fiber protein | tail fiber protein | | uniclust | UniRef100\_A0A0F8WDG3 | 99.4 | 5.1e-16 | 9.7e-22 | 107.2 | 55 | (9, 106) | 221 | (19, 75) | 135 | Phage tail collar domain-containing protein | Phage tail collar domain-containing protein | | uniclust | UniRef100\_UPI001FC8B715 | 99.4 | 5.3e-16 | 9.7e-22 | 99.3 | 67 | (9, 75) | 221 | (47, 113) | 128 | phage tail protein | phage tail protein | | uniclust | UniRef100\_C6ABW9 | 99.4 | 5.3e-16 | 9.7e-22 | 117.5 | 75 | (9, 83) | 221 | (220, 294) | 370 | Phage tail collar protein | Phage tail collar protein | | uniclust | UniRef100\_UPI000736F1C9 | 99.4 | 5.6e-16 | 1e-21 | 119.7 | 73 | (13, 85) | 221 | (335, 407) | 496 | tail fiber protein | tail fiber protein | | uniclust | UniRef100\_A0A821Z358 | 99.4 | 5.7e-16 | 1e-21 | 102.3 | 93 | (11, 119) | 221 | (2, 99) | 154 | Phage tail collar domain-containing protein | Phage tail collar domain-containing protein | | uniclust | UniRef100\_A0A954EH12 | 99.4 | 6e-16 | 1.1e-21 | 116.6 | 100 | (10, 121) | 221 | (114, 213) | 395 | Tail fiber protein (Fragment) | Tail fiber protein (Fragment) | | uniclust | UniRef100\_A0A061JKN2 | 99.4 | 6e-16 | 1.1e-21 | 126.9 | 76 | (10, 85) | 221 | (327, 402) | 521 | Tail protein | Tail protein | | uniclust | UniRef100\_A0A0K8JHT0 | 99.4 | 6.3e-16 | 1.2e-21 | 118.7 | 92 | (9, 119) | 221 | (193, 285) | 469 | Phage tail collar domain-containing protein | Phage tail collar domain-containing protein | | uniclust | UniRef100\_A0A022PG07 | 99.4 | 6.3e-16 | 1.2e-21 | 124.6 | 88 | (6, 119) | 221 | (256, 351) | 434 | Phage tail collar family protein | Phage tail collar family protein | | uniclust | UniRef100\_A0A6M0S0R8 | 99.4 | 6.5e-16 | 1.2e-21 | 99.9 | 62 | (15, 119) | 221 | (2, 65) | 125 | Tail fiber protein | Tail fiber protein | | uniclust | UniRef100\_A0A5E4KZA2 | 99.4 | 6.7e-16 | 1.2e-21 | 111.2 | 67 | (10, 119) | 221 | (117, 185) | 277 | Phage Tail Collar Domain protein | Phage Tail Collar Domain protein | | uniclust | UniRef100\_A0A081RQS4 | 99.4 | 6.9e-16 | 1.3e-21 | 123.6 | 83 | (8, 119) | 221 | (331, 413) | 474 | Phage-related tail fiber protein (Fragment) | Phage-related tail fiber protein (Fragment) | | uniclust | UniRef100\_A0A6J5QSS9 | 99.4 | 7.2e-16 | 1.3e-21 | 113.0 | 102 | (7, 119) | 221 | (109, 210) | 317 | MdpB Microcystin-dependent protein | MdpB Microcystin-dependent protein | | uniclust | UniRef100\_A0A1S8LFT6 | 99.4 | 7.1e-16 | 1.3e-21 | 112.1 | 66 | (10, 75) | 221 | (23, 88) | 178 | Phage tail collar domain protein | Phage tail collar domain protein | | uniclust | UniRef100\_A0A7T8IWR9 | 99.4 | 7.4e-16 | 1.4e-21 | 120.8 | 82 | (9, 119) | 221 | (423, 506) | 568 | Tail fiber protein | Tail fiber protein | | uniclust | UniRef100\_A0A2E0ZP65 | 99.4 | 7.8e-16 | 1.4e-21 | 100.8 | 75 | (9, 83) | 221 | (20, 94) | 146 | Phage tail collar domain-containing protein | Phage tail collar domain-containing protein | | uniclust | UniRef100\_A0A4U6CQW2 | 99.4 | 7.8e-16 | 1.4e-21 | 117.5 | 98 | (9, 119) | 221 | (238, 335) | 342 | Phage tail collar domain-containing protein | Phage tail collar domain-containing protein | | uniclust | UniRef100\_A0A6P2HBE9 | 99.4 | 7.8e-16 | 1.5e-21 | 112.9 | 171 | (9, 218) | 221 | (54, 228) | 233 | Tail fiber protein | Tail fiber protein | | uniclust | UniRef100\_A0A957NRE5 | 99.4 | 8.1e-16 | 1.5e-21 | 115.6 | 67 | (10, 119) | 221 | (265, 333) | 388 | Uncharacterized protein (Fragment) | Uncharacterized protein (Fragment) | | uniclust | UniRef100\_A0A228IJ11 | 99.4 | 8.3e-16 | 1.5e-21 | 122.6 | 174 | (5, 221) | 221 | (305, 527) | 531 | Tail fiber protein | Tail fiber protein | | uniclust | UniRef100\_A0A973FDY3 | 99.4 | 8.9e-16 | 1.6e-21 | 109.1 | 182 | (10, 221) | 221 | (70, 251) | 251 | Tail fiber protein | Tail fiber protein | | uniclust | UniRef100\_A0A1X3DKU1 | 99.4 | 8.9e-16 | 1.7e-21 | 112.8 | 76 | (8, 83) | 221 | (80, 155) | 256 | Phage tail collar domain-containing protein | Phage tail collar domain-containing protein | | uniclust | UniRef100\_A0A835ZHE3 | 99.4 | 9.7e-16 | 1.8e-21 | 111.3 | 79 | (24, 119) | 221 | (16, 94) | 294 | Phage tail collar domain-containing protein | Phage tail collar domain-containing protein | | uniclust | UniRef100\_A0A1G5MZE4 | 99.4 | 1.2e-15 | 2.2e-21 | 126.1 | 74 | (12, 85) | 221 | (662, 735) | 825 | Phage Tail Collar Domain | Phage Tail Collar Domain | | uniclust | UniRef100\_A0A1V5WU45 | 99.4 | 1.2e-15 | 2.2e-21 | 108.1 | 125 | (11, 143) | 221 | (8, 138) | 210 | Phage Tail Collar Domain protein | Phage Tail Collar Domain protein | | uniclust | UniRef100\_A0A2N9Y859 | 99.4 | 1.2e-15 | 2.2e-21 | 120.4 | 78 | (8, 85) | 221 | (157, 234) | 390 | Phage tail protein | Phage tail protein | | uniclust | UniRef100\_A0A090ZDZ3 | 99.4 | 1.3e-15 | 2.4e-21 | 102.9 | 91 | (10, 119) | 221 | (38, 128) | 130 | Phage Tail Collar domain protein | Phage Tail Collar domain protein | | uniclust | UniRef100\_A0A2Z2P0U1 | 99.4 | 1.3e-15 | 2.5e-21 | 112.1 | 67 | (9, 117) | 221 | (67, 133) | 193 | Phage tail collar domain-containing protein | Phage tail collar domain-containing protein | | uniclust | UniRef100\_A0A849R972 | 99.4 | 1.4e-15 | 2.6e-21 | 109.8 | 67 | (8, 119) | 221 | (164, 232) | 280 | Tail fiber protein | Tail fiber protein | | uniclust | UniRef100\_A0A1I2LDU5 | 99.4 | 1.4e-15 | 2.6e-21 | 119.0 | 94 | (7, 119) | 221 | (305, 398) | 450 | Microcystin-dependent protein | Microcystin-dependent protein | | uniclust | UniRef100\_UPI001F3862FB | 99.4 | 1.4e-15 | 2.6e-21 | 112.5 | 92 | (9, 119) | 221 | (175, 266) | 338 | phage tail protein | phage tail protein | | uniclust | UniRef100\_A0A815V480 | 99.4 | 1.5e-15 | 2.7e-21 | 123.1 | 195 | (10, 221) | 221 | (500, 702) | 803 | Phage tail collar domain-containing protein | Phage tail collar domain-containing protein | | uniclust | UniRef100\_A0A069PMJ9 | 99.4 | 1.6e-15 | 3e-21 | 124.4 | 76 | (10, 85) | 221 | (294, 385) | 521 | Tail protein | Tail protein | | uniclust | UniRef100\_A0A956Q788 | 99.4 | 1.7e-15 | 3.1e-21 | 108.9 | 112 | (8, 119) | 221 | (83, 221) | 270 | Uncharacterized protein | Uncharacterized protein | | uniclust | UniRef100\_A0A2I2L5J8 | 99.4 | 1.7e-15 | 3.1e-21 | 125.6 | 168 | (8, 221) | 221 | (890, 1059) | 1059 | Collagen-like protein with Phage Tail Collar Domain | Collagen-like protein with Phage Tail Collar Domain | | uniclust | UniRef100\_A0A0A0CRJ5 | 99.4 | 1.8e-15 | 3.3e-21 | 108.0 | 67 | (7, 83) | 221 | (52, 118) | 195 | Phage tail collar domain-containing protein | Phage tail collar domain-containing protein | | uniclust | UniRef100\_A0A0H3GJI2 | 99.4 | 1.8e-15 | 3.3e-21 | 117.8 | 75 | (11, 85) | 221 | (233, 307) | 385 | Phage tail collar domain-containing protein | Phage tail collar domain-containing protein | | uniclust | UniRef100\_UPI00210D7C8A | 99.4 | 1.8e-15 | 3.4e-21 | 91.8 | 83 | (24, 119) | 221 | (1, 92) | 94 | tail fiber protein | tail fiber protein | | uniclust | UniRef100\_A0A928V6C5 | 99.4 | 2.2e-15 | 3.9e-21 | 117.8 | 70 | (10, 119) | 221 | (401, 472) | 544 | FHA domain-containing protein | FHA domain-containing protein | | uniclust | UniRef100\_A0A516LLH4 | 99.4 | 2.3e-15 | 4.2e-21 | 97.2 | 80 | (8, 87) | 221 | (15, 94) | 133 | Putative tail fiber protein | Putative tail fiber protein | | uniclust | UniRef100\_UPI0004113DCB | 99.4 | 2.3e-15 | 4.3e-21 | 119.3 | 147 | (9, 221) | 221 | (476, 627) | 627 | hypothetical protein | hypothetical protein | | uniclust | UniRef100\_A0A1G8CBW8 | 99.4 | 2.4e-15 | 4.4e-21 | 104.9 | 77 | (9, 85) | 221 | (23, 99) | 181 | Phage Tail Collar Domain | Phage Tail Collar Domain | | uniclust | UniRef100\_A0A6V8M1A9 | 99.4 | 2.4e-15 | 4.5e-21 | 110.4 | 101 | (7, 119) | 221 | (76, 178) | 243 | Phage tail collar domain-containing protein | Phage tail collar domain-containing protein | | uniclust | UniRef100\_A0A1I3YTE4 | 99.4 | 2.5e-15 | 4.6e-21 | 116.6 | 83 | (8, 119) | 221 | (227, 309) | 358 | Phage Tail Collar Domain (Fragment) | Phage Tail Collar Domain (Fragment) | | uniclust | UniRef100\_UPI001F13FAFE | 99.4 | 2.5e-15 | 4.6e-21 | 104.6 | 79 | (7, 85) | 221 | (72, 150) | 215 | phage tail protein | phage tail protein | | uniclust | UniRef100\_A0A1H0KC46 | 99.4 | 2.6e-15 | 4.8e-21 | 111.5 | 105 | (7, 119) | 221 | (36, 151) | 215 | Microcystin-dependent protein | Microcystin-dependent protein | | pdb70 | 1PDI\_F | 99.4 | 3.5e-17 | 3.3e-21 | 142.9 | 112 | (8, 123) | 221 | (90, 204) | 278 | Short tail fiber protein | 1PDI\_F Short tail fiber protein STRUCTURAL PROTEIN | | pdb70 | 5IV5\_AI | 99.3 | 4.4e-16 | 4.1e-20 | 146.1 | 112 | (8, 123) | 221 | (339, 453) | 527 | Baseplate wedge protein gp6, Baseplate | 5IV5\_AI Baseplate wedge protein gp6, Baseplate T4, baseplate-tail tube complex, pre-attachment | | pdb70 | 5IV5\_AJ | 99.3 | 4.4e-16 | 4.1e-20 | 146.1 | 112 | (8, 123) | 221 | (339, 453) | 527 | Baseplate wedge protein gp6, Baseplate | 5IV5\_AJ Baseplate wedge protein gp6, Baseplate T4, baseplate-tail tube complex, pre-attachment | | pdb70 | 5IV5\_BA | 99.3 | 4.4e-16 | 4.1e-20 | 146.1 | 112 | (8, 123) | 221 | (339, 453) | 527 | Baseplate wedge protein gp6, Baseplate | 5IV5\_BA Baseplate wedge protein gp6, Baseplate T4, baseplate-tail tube complex, pre-attachment | | pdb70 | 5IV5\_DB | 99.3 | 4.4e-16 | 4.1e-20 | 146.1 | 112 | (8, 123) | 221 | (339, 453) | 527 | Baseplate wedge protein gp6, Baseplate | 5IV5\_DB Baseplate wedge protein gp6, Baseplate T4, baseplate-tail tube complex, pre-attachment | | pdb70 | 5IV5\_DC | 99.3 | 4.4e-16 | 4.1e-20 | 146.1 | 112 | (8, 123) | 221 | (339, 453) | 527 | Baseplate wedge protein gp6, Baseplate | 5IV5\_DC Baseplate wedge protein gp6, Baseplate T4, baseplate-tail tube complex, pre-attachment | | pdb70 | 5IV5\_DD | 99.3 | 4.4e-16 | 4.1e-20 | 146.1 | 112 | (8, 123) | 221 | (339, 453) | 527 | Baseplate wedge protein gp6, Baseplate | 5IV5\_DD Baseplate wedge protein gp6, Baseplate T4, baseplate-tail tube complex, pre-attachment | | pdb70 | 5IV5\_FE | 99.3 | 4.4e-16 | 4.1e-20 | 146.1 | 112 | (8, 123) | 221 | (339, 453) | 527 | Baseplate wedge protein gp6, Baseplate | 5IV5\_FE Baseplate wedge protein gp6, Baseplate T4, baseplate-tail tube complex, pre-attachment | | pdb70 | 5IV5\_FF | 99.3 | 4.4e-16 | 4.1e-20 | 146.1 | 112 | (8, 123) | 221 | (339, 453) | 527 | Baseplate wedge protein gp6, Baseplate | 5IV5\_FF Baseplate wedge protein gp6, Baseplate T4, baseplate-tail tube complex, pre-attachment | | pdb70 | 5IV5\_FG | 99.3 | 4.4e-16 | 4.1e-20 | 146.1 | 112 | (8, 123) | 221 | (339, 453) | 527 | Baseplate wedge protein gp6, Baseplate | 5IV5\_FG Baseplate wedge protein gp6, Baseplate T4, baseplate-tail tube complex, pre-attachment | | pdb70 | 5IV5\_HH | 99.3 | 4.4e-16 | 4.1e-20 | 146.1 | 112 | (8, 123) | 221 | (339, 453) | 527 | Baseplate wedge protein gp6, Baseplate | 5IV5\_HH Baseplate wedge protein gp6, Baseplate T4, baseplate-tail tube complex, pre-attachment | | pdb70 | 5IV5\_HJ | 99.3 | 4.4e-16 | 4.1e-20 | 146.1 | 112 | (8, 123) | 221 | (339, 453) | 527 | Baseplate wedge protein gp6, Baseplate | 5IV5\_HJ Baseplate wedge protein gp6, Baseplate T4, baseplate-tail tube complex, pre-attachment | | pdb70 | 5IV5\_O | 99.3 | 4.4e-16 | 4.1e-20 | 146.1 | 112 | (8, 123) | 221 | (339, 453) | 527 | Baseplate wedge protein gp6, Baseplate | 5IV5\_O Baseplate wedge protein gp6, Baseplate T4, baseplate-tail tube complex, pre-attachment | | pdb70 | 5IV5\_P | 99.3 | 4.4e-16 | 4.1e-20 | 146.1 | 112 | (8, 123) | 221 | (339, 453) | 527 | Baseplate wedge protein gp6, Baseplate | 5IV5\_P Baseplate wedge protein gp6, Baseplate T4, baseplate-tail tube complex, pre-attachment | | pdb70 | 5IV5\_Q | 99.3 | 4.4e-16 | 4.1e-20 | 146.1 | 112 | (8, 123) | 221 | (339, 453) | 527 | Baseplate wedge protein gp6, Baseplate | 5IV5\_Q Baseplate wedge protein gp6, Baseplate T4, baseplate-tail tube complex, pre-attachment | | pdb70 | 5IV5\_n | 99.3 | 4.4e-16 | 4.1e-20 | 146.1 | 112 | (8, 123) | 221 | (339, 453) | 527 | Baseplate wedge protein gp6, Baseplate | 5IV5\_n Baseplate wedge protein gp6, Baseplate T4, baseplate-tail tube complex, pre-attachment | | pdb70 | 1OCY\_A | 99.2 | 6.1e-16 | 6.1e-20 | 127.3 | 111 | (7, 121) | 221 | (9, 122) | 198 | BACTERIOPHAGE T4 SHORT TAIL FIBRE | 1OCY\_A BACTERIOPHAGE T4 SHORT TAIL FIBRE STRUCTURAL PROTEIN, FIBROUS PROTEIN, LIPO-POLYSACCHARIDE HET: CIT, SO4 | | pdb70 | 2XGF\_A | 99.1 | 1.7e-14 | 1.6e-18 | 122.8 | 80 | (7, 121) | 221 | (26, 105) | 242 | LONG TAIL FIBER PROTEIN P37 | 2XGF\_A LONG TAIL FIBER PROTEIN P37 VIRAL PROTEIN, FIBER PROTEIN | | pdb70 | 5LYE\_A | 98.4 | 5.4e-11 | 5e-15 | 104.9 | 65 | (7, 73) | 221 | (254, 319) | 322 | Gp12 | 5LYE\_A Gp12 STRUCTURAL PROTEIN, GENE PRODUCT 12 | | pdb70 | 2FKK\_A | 98.0 | 2.8e-09 | 2.7e-13 | 88.1 | 55 | (66, 121) | 221 | (87, 145) | 206 | Baseplate structural protein Gp10 | 2FKK\_A Baseplate structural protein Gp10 Bacteriophage T4 Baseplate HET: EDO | | pdb70 | 5IV5\_HI | 97.3 | 1.7e-07 | 1.6e-11 | 86.7 | 112 | (8, 123) | 221 | (339, 453) | 527 | Baseplate wedge protein gp6, Baseplate | 5IV5\_HI Baseplate wedge protein gp6, Baseplate T4, baseplate-tail tube complex, pre-attachment | | pdb70 | 5HX2\_H | 97.0 | 1.2e-06 | 1e-10 | 82.2 | 55 | (66, 121) | 221 | (483, 541) | 602 | Baseplate wedge protein gp7, Baseplate | 5HX2\_H Baseplate wedge protein gp7, Baseplate T4, baseplate, complex, VIRAL PROTEIN | | pdb70 | 5IV5\_AC | 97.0 | 1.2e-06 | 1e-10 | 82.2 | 55 | (66, 121) | 221 | (483, 541) | 602 | Baseplate wedge protein gp6, Baseplate | 5IV5\_AC Baseplate wedge protein gp6, Baseplate T4, baseplate-tail tube complex, pre-attachment | | pdb70 | 5IV5\_AD | 97.0 | 1.2e-06 | 1e-10 | 82.2 | 55 | (66, 121) | 221 | (483, 541) | 602 | Baseplate wedge protein gp6, Baseplate | 5IV5\_AD Baseplate wedge protein gp6, Baseplate T4, baseplate-tail tube complex, pre-attachment | | pdb70 | 5IV5\_AE | 97.0 | 1.2e-06 | 1e-10 | 82.2 | 55 | (66, 121) | 221 | (483, 541) | 602 | Baseplate wedge protein gp6, Baseplate | 5IV5\_AE Baseplate wedge protein gp6, Baseplate T4, baseplate-tail tube complex, pre-attachment | | pdb70 | 5IV5\_CF | 97.0 | 1.2e-06 | 1e-10 | 82.2 | 55 | (66, 121) | 221 | (483, 541) | 602 | Baseplate wedge protein gp6, Baseplate | 5IV5\_CF Baseplate wedge protein gp6, Baseplate T4, baseplate-tail tube complex, pre-attachment | | pdb70 | 5IV5\_CG | 97.0 | 1.2e-06 | 1e-10 | 82.2 | 55 | (66, 121) | 221 | (483, 541) | 602 | Baseplate wedge protein gp6, Baseplate | 5IV5\_CG Baseplate wedge protein gp6, Baseplate T4, baseplate-tail tube complex, pre-attachment | | pdb70 | 5IV5\_CH | 97.0 | 1.2e-06 | 1e-10 | 82.2 | 55 | (66, 121) | 221 | (483, 541) | 602 | Baseplate wedge protein gp6, Baseplate | 5IV5\_CH Baseplate wedge protein gp6, Baseplate T4, baseplate-tail tube complex, pre-attachment | | pdb70 | 5IV5\_EI | 97.0 | 1.2e-06 | 1e-10 | 82.2 | 55 | (66, 121) | 221 | (483, 541) | 602 | Baseplate wedge protein gp6, Baseplate | 5IV5\_EI Baseplate wedge protein gp6, Baseplate T4, baseplate-tail tube complex, pre-attachment | | pdb70 | 5IV5\_EJ | 97.0 | 1.2e-06 | 1e-10 | 82.2 | 55 | (66, 121) | 221 | (483, 541) | 602 | Baseplate wedge protein gp6, Baseplate | 5IV5\_EJ Baseplate wedge protein gp6, Baseplate T4, baseplate-tail tube complex, pre-attachment | | pdb70 | 5IV5\_FA | 97.0 | 1.2e-06 | 1e-10 | 82.2 | 55 | (66, 121) | 221 | (483, 541) | 602 | Baseplate wedge protein gp6, Baseplate | 5IV5\_FA Baseplate wedge protein gp6, Baseplate T4, baseplate-tail tube complex, pre-attachment | | pdb70 | 5IV5\_HB | 97.0 | 1.2e-06 | 1e-10 | 82.2 | 55 | (66, 121) | 221 | (483, 541) | 602 | Baseplate wedge protein gp6, Baseplate | 5IV5\_HB Baseplate wedge protein gp6, Baseplate T4, baseplate-tail tube complex, pre-attachment | | pdb70 | 5IV5\_HC | 97.0 | 1.2e-06 | 1e-10 | 82.2 | 55 | (66, 121) | 221 | (483, 541) | 602 | Baseplate wedge protein gp6, Baseplate | 5IV5\_HC Baseplate wedge protein gp6, Baseplate T4, baseplate-tail tube complex, pre-attachment | | pdb70 | 5IV5\_HD | 97.0 | 1.2e-06 | 1e-10 | 82.2 | 55 | (66, 121) | 221 | (483, 541) | 602 | Baseplate wedge protein gp6, Baseplate | 5IV5\_HD Baseplate wedge protein gp6, Baseplate T4, baseplate-tail tube complex, pre-attachment | | pdb70 | 5IV5\_I | 97.0 | 1.2e-06 | 1e-10 | 82.2 | 55 | (66, 121) | 221 | (483, 541) | 602 | Baseplate wedge protein gp6, Baseplate | 5IV5\_I Baseplate wedge protein gp6, Baseplate T4, baseplate-tail tube complex, pre-attachment | | pdb70 | 5IV5\_J | 97.0 | 1.2e-06 | 1e-10 | 82.2 | 55 | (66, 121) | 221 | (483, 541) | 602 | Baseplate wedge protein gp6, Baseplate | 5IV5\_J Baseplate wedge protein gp6, Baseplate T4, baseplate-tail tube complex, pre-attachment | | pdb70 | 5IV5\_K | 97.0 | 1.2e-06 | 1e-10 | 82.2 | 55 | (66, 121) | 221 | (483, 541) | 602 | Baseplate wedge protein gp6, Baseplate | 5IV5\_K Baseplate wedge protein gp6, Baseplate T4, baseplate-tail tube complex, pre-attachment | | pdb70 | 5IV7\_FC | 97.0 | 1.2e-06 | 1e-10 | 82.2 | 55 | (66, 121) | 221 | (483, 541) | 602 | Baseplate wedge protein gp6, Baseplate | 5IV7\_FC Baseplate wedge protein gp6, Baseplate T4, baseplate, post-attachment, bacteriophage, bacterial | |
| Top keywords  (threshold 1.00e-03 (evalue)) | **tail, Phage, collar, domain\_containing, fiber, Baseplate, T4, wedge, gp6, complex** |
| Output files | ../../similar\_sequences/17\_FANPEZAQ\_CDS\_0017\_merged.svg ../../similar\_sequences/17\_FANPEZAQ\_CDS\_0017\_pdb70.a3m ../../similar\_sequences/17\_FANPEZAQ\_CDS\_0017\_pdb70.hhr ../../similar\_sequences/17\_FANPEZAQ\_CDS\_0017\_uniclust.a3m ../../similar\_sequences/17\_FANPEZAQ\_CDS\_0017\_uniclust.hhr |

#### Structure prediction (AlphaFold)2

|  |  |
| --- | --- |
| Stats | xml version="1.0" encoding="utf-8" standalone="no"?       2024-09-02T21:09:16.168346 image/svg+xml   Matplotlib v3.7.2, https://matplotlib.org/ |
| Predicted structure | **NGL Viewer Controls:**  - Center: *Left-Click* - Rotate: *Left-Click + Drag* - Translate: *Right-Click + Drag* - Zoom: *Shift + Left-Click + Drag* |
| Output files | ../../predicted\_structures/17\_FANPEZAQ\_CDS\_0017/features.pkl ../../predicted\_structures/17\_FANPEZAQ\_CDS\_0017/ranked\_0.pdb ../../predicted\_structures/17\_FANPEZAQ\_CDS\_0017/ranked\_0\_plots.svg ../../predicted\_structures/17\_FANPEZAQ\_CDS\_0017/result\_model\_1\_ptm\_pred\_0.pkl |

#### Structure similarity search results (Foldseek)3

|  |  |
| --- | --- |
| Structure databases searched | Pdb, Afdb-proteome, Afdb-uniprot50 |
| Results, scheme(s)  (Top layers only, threshold 1.00e-02 (evalue)) | xml version="1.0" encoding="utf-8" standalone="no"?       2024-09-02T21:10:37.577455 image/svg+xml   Matplotlib v3.7.2, https://matplotlib.org/ |
| Results, table  (threshold 1.00e-02 (evalue)) | | db | id | prob | evalue | bits | fident | alnlen | mismatch | gapopen | qstart | qend | tstart | tend | name | description | | --- | --- | --- | --- | --- | --- | --- | --- | --- | --- | --- | --- | --- | --- | --- | | pdb | 1OCY\_A | 1.0 | 0.0001505 | 113 | 0.18 | 238 | 110 | 10 | 12 | 220 | 14 | 195 | BACTERIOPHAGE T4 SHORT TAIL FIBRE | BACTERIOPHAGE T4 SHORT TAIL FIBRE | | pdb | 1PDI\_A | 1.0 | 0.0001695 | 111 | 0.236 | 237 | 120 | 16 | 1 | 220 | 83 | 275 | Short tail fiber protein | Short tail fiber protein | | pdb | 5IV5\_O | 1.0 | 0.0001337 | 110 | 0.207 | 231 | 127 | 12 | 5 | 220 | 334 | 523 | Tail tube protein gp19 | Tail tube protein gp19 | | pdb | 2XGF\_B | 1.0 | 8.824e-05 | 106 | 0.191 | 246 | 128 | 11 | 11 | 220 | 4 | 214 | LONG TAIL FIBER PROTEIN P37 | LONG TAIL FIBER PROTEIN P37 | | pdb | 6OV6\_C | 0.986 | 0.001717 | 81 | 0.135 | 237 | 112 | 16 | 1 | 221 | 84 | 243 | C24 PROTEIN | C24 PROTEIN | | pdb | 6OV6\_A | 0.967 | 0.003108 | 75 | 0.14 | 235 | 113 | 15 | 1 | 221 | 95 | 254 | C24 PROTEIN | C24 PROTEIN | | pdb | 6OV6\_B | 0.956 | 0.004438 | 73 | 0.16 | 231 | 113 | 16 | 1 | 221 | 80 | 239 | C24 PROTEIN | C24 PROTEIN | | afdb-proteome | AF-A0A0H3GJI2-F1-MODEL\_V4 | 1.0 | 1.608e-08 | 198 | 0.251 | 223 | 77 | 9 | 10 | 221 | 231 | 374 | Collar domain-containing protein | Collar domain-containing protein | | afdb-proteome | AF-Q8ZQF5-F1-MODEL\_V4 | 1.0 | 7.814e-05 | 115 | 0.174 | 212 | 142 | 8 | 11 | 220 | 608 | 788 | Putative Fels-1 prophage minor tail protein | Putative Fels-1 prophage minor tail protein | | afdb-proteome | AF-Q8ZQ81-F1-MODEL\_V4 | 1.0 | 0.0002562 | 115 | 0.155 | 212 | 146 | 9 | 11 | 220 | 630 | 810 | Gifsy-2 prophage probable tail fiber protein | Gifsy-2 prophage probable tail fiber protein | | afdb-proteome | AF-P77515-F1-MODEL\_V4 | 0.988 | 0.0005881 | 82 | 0.202 | 242 | 144 | 10 | 12 | 220 | 93 | 318 | Prophage side tail fiber protein homolog StfQ | Prophage side tail fiber protein homolog StfQ | | afdb-uniprot50 | AF-A0A7W4VIA3-F1-MODEL\_V4 | 1.0 | 3.91e-22 | 772 | 0.489 | 231 | 107 | 5 | 1 | 221 | 1 | 230 | Microcystin-dependent protein | Microcystin-dependent protein | | afdb-uniprot50 | AF-A0A512NCR2-F1-MODEL\_V4 | 1.0 | 1.571e-19 | 622 | 0.479 | 223 | 75 | 9 | 3 | 221 | 22 | 207 | Tail protein | Tail protein | | afdb-uniprot50 | AF-A0A5C7Q4X8-F1-MODEL\_V4 | 1.0 | 4.111e-18 | 585 | 0.433 | 212 | 80 | 6 | 12 | 221 | 20 | 193 | Phage tail protein | Phage tail protein | | afdb-uniprot50 | AF-A0A522VPY7-F1-MODEL\_V4 | 1.0 | 3.441e-18 | 583 | 0.459 | 211 | 63 | 3 | 11 | 221 | 61 | 220 | Tail fiber protein | Tail fiber protein | | afdb-uniprot50 | AF-A0A6A7M412-F1-MODEL\_V4 | 1.0 | 4.852e-19 | 556 | 0.466 | 223 | 75 | 10 | 2 | 221 | 65 | 246 | Collar domain-containing protein | Collar domain-containing protein | | afdb-uniprot50 | AF-A0A5C7MBC1-F1-MODEL\_V4 | 1.0 | 6.23e-18 | 553 | 0.459 | 211 | 59 | 5 | 11 | 221 | 108 | 263 | Phage tail protein | Phage tail protein | | afdb-uniprot50 | AF-A0A523UNM2-F1-MODEL\_V4 | 1.0 | 5.871e-18 | 552 | 0.364 | 211 | 104 | 7 | 11 | 221 | 35 | 215 | Collar domain-containing protein | Collar domain-containing protein | | afdb-uniprot50 | AF-A0A7L7MLE0-F1-MODEL\_V4 | 1.0 | 1.61e-17 | 546 | 0.377 | 220 | 111 | 6 | 1 | 220 | 54 | 247 | Tail fiber protein | Tail fiber protein | | afdb-uniprot50 | AF-A0A5C7LV16-F1-MODEL\_V4 | 1.0 | 2.271e-18 | 543 | 0.466 | 210 | 72 | 4 | 11 | 220 | 100 | 269 | Collar domain-containing protein | Collar domain-containing protein | | afdb-uniprot50 | AF-A0A516H786-F1-MODEL\_V4 | 1.0 | 3.484e-17 | 536 | 0.438 | 212 | 67 | 5 | 11 | 221 | 66 | 226 | Collar domain-containing protein | Collar domain-containing protein | | afdb-uniprot50 | AF-A0A844QL60-F1-MODEL\_V4 | 1.0 | 2.3e-17 | 534 | 0.416 | 221 | 90 | 7 | 2 | 220 | 81 | 264 | Uncharacterized protein | Uncharacterized protein | | afdb-uniprot50 | AF-A0A5C7PH71-F1-MODEL\_V4 | 1.0 | 2.194e-16 | 526 | 0.382 | 212 | 109 | 4 | 11 | 221 | 40 | 230 | Tail fiber protein | Tail fiber protein | | afdb-uniprot50 | AF-H0HNH5-F1-MODEL\_V4 | 1.0 | 1.076e-16 | 520 | 0.403 | 213 | 93 | 5 | 11 | 221 | 206 | 386 | Microcystin-dependent protein-like protein | Microcystin-dependent protein-like protein | | afdb-uniprot50 | AF-A0A6G6K382-F1-MODEL\_V4 | 1.0 | 1.076e-16 | 520 | 0.381 | 223 | 83 | 4 | 3 | 221 | 251 | 422 | Collar domain-containing protein | Collar domain-containing protein | | afdb-uniprot50 | AF-A0A1Y2J6J7-F1-MODEL\_V4 | 1.0 | 2.622e-16 | 517 | 0.402 | 216 | 88 | 8 | 12 | 221 | 25 | 205 | Collar domain-containing protein | Collar domain-containing protein | | afdb-uniprot50 | AF-A0A060DEV3-F1-MODEL\_V4 | 1.0 | 2.358e-15 | 514 | 0.355 | 211 | 79 | 8 | 12 | 221 | 13 | 167 | Collar domain-containing protein | Collar domain-containing protein | | afdb-uniprot50 | AF-D5BQR2-F1-MODEL\_V4 | 1.0 | 7.103e-17 | 511 | 0.386 | 212 | 87 | 6 | 11 | 221 | 119 | 288 | Microcystin-dependent protein-like protein | Microcystin-dependent protein-like protein | | afdb-uniprot50 | AF-A0A1M3FXB5-F1-MODEL\_V4 | 1.0 | 5.945e-17 | 501 | 0.413 | 225 | 106 | 7 | 11 | 221 | 93 | 305 | Collar domain-containing protein | Collar domain-containing protein | | afdb-uniprot50 | AF-A0A1F7BR55-F1-MODEL\_V4 | 1.0 | 6.02e-16 | 501 | 0.386 | 220 | 81 | 3 | 2 | 221 | 358 | 523 | Collar domain-containing protein | Collar domain-containing protein | | afdb-uniprot50 | AF-A0A516IWE1-F1-MODEL\_V4 | 1.0 | 2.471e-16 | 499 | 0.398 | 216 | 89 | 8 | 12 | 221 | 149 | 329 | Phage tail protein | Phage tail protein | | afdb-uniprot50 | AF-B5ZFN5-F1-MODEL\_V4 | 1.0 | 1.002e-17 | 496 | 0.454 | 211 | 94 | 6 | 12 | 220 | 86 | 277 | Tail Collar domain protein | Tail Collar domain protein | | afdb-uniprot50 | AF-A0A6H1ZGL2-F1-MODEL\_V4 | 1.0 | 1.731e-16 | 493 | 0.426 | 211 | 96 | 5 | 11 | 221 | 152 | 337 | Putative tail collar protein | Putative tail collar protein | | afdb-uniprot50 | AF-A0A1G6EJM0-F1-MODEL\_V4 | 1.0 | 3.973e-16 | 486 | 0.409 | 210 | 105 | 4 | 12 | 220 | 122 | 313 | Microcystin-dependent protein | Microcystin-dependent protein | | afdb-uniprot50 | AF-A0A840HY65-F1-MODEL\_V4 | 1.0 | 4.474e-16 | 484 | 0.444 | 209 | 80 | 5 | 12 | 220 | 53 | 225 | Microcystin-dependent protein | Microcystin-dependent protein | | afdb-uniprot50 | AF-A0A1X3FVC4-F1-MODEL\_V4 | 1.0 | 4.474e-16 | 476 | 0.443 | 212 | 81 | 6 | 12 | 220 | 150 | 327 | Phage tail protein | Phage tail protein | | afdb-uniprot50 | AF-A0A4Q7TER5-F1-MODEL\_V4 | 1.0 | 4.474e-16 | 475 | 0.4 | 215 | 95 | 8 | 10 | 220 | 200 | 384 | Microcystin-dependent protein | Microcystin-dependent protein | | afdb-uniprot50 | AF-A0A836CG45-F1-MODEL\_V4 | 1.0 | 4.377e-13 | 464 | 0.386 | 212 | 70 | 4 | 11 | 221 | 2 | 154 | Uncharacterized protein | Uncharacterized protein | | afdb-uniprot50 | AF-A0A225DUJ5-F1-MODEL\_V4 | 1.0 | 3.744e-16 | 463 | 0.421 | 211 | 92 | 5 | 11 | 221 | 130 | 310 | Phage tail fiber | Phage tail fiber | | afdb-uniprot50 | AF-A0A1E4LGS2-F1-MODEL\_V4 | 1.0 | 5.346e-16 | 458 | 0.409 | 210 | 101 | 4 | 11 | 220 | 178 | 364 | Collar domain-containing protein | Collar domain-containing protein | | afdb-uniprot50 | AF-A0A7J4F737-F1-MODEL\_V4 | 1.0 | 4.023e-15 | 457 | 0.386 | 212 | 75 | 6 | 11 | 221 | 100 | 257 | Collar domain-containing protein | Collar domain-containing protein | | afdb-uniprot50 | AF-A0A2E7V2E5-F1-MODEL\_V4 | 1.0 | 7.729e-15 | 450 | 0.345 | 211 | 100 | 7 | 11 | 221 | 135 | 307 | Collar domain-containing protein | Collar domain-containing protein | | afdb-uniprot50 | AF-A0A7C1IMI0-F1-MODEL\_V4 | 1.0 | 7.284e-15 | 447 | 0.374 | 211 | 76 | 5 | 11 | 221 | 137 | 291 | Collar domain-containing protein | Collar domain-containing protein | | afdb-uniprot50 | AF-A0A4Q1VB42-F1-MODEL\_V4 | 1.0 | 1.399e-14 | 446 | 0.331 | 214 | 109 | 6 | 12 | 221 | 152 | 335 | Phage tail protein | Phage tail protein | | afdb-uniprot50 | AF-A0A814Y0G9-F1-MODEL\_V4 | 1.0 | 1.319e-14 | 445 | 0.361 | 213 | 93 | 5 | 11 | 221 | 108 | 279 | Hypothetical protein | Hypothetical protein | | afdb-uniprot50 | AF-A0A3D0M2I4-F1-MODEL\_V4 | 1.0 | 1.774e-14 | 442 | 0.336 | 220 | 86 | 4 | 2 | 221 | 57 | 216 | Uncharacterized protein | Uncharacterized protein | | afdb-uniprot50 | AF-A0A815SI59-F1-MODEL\_V4 | 1.0 | 1.672e-14 | 440 | 0.376 | 210 | 92 | 4 | 11 | 220 | 399 | 569 | Hypothetical protein | Hypothetical protein | | afdb-uniprot50 | AF-A0A815N1T3-F1-MODEL\_V4 | 1.0 | 2.418e-13 | 439 | 0.35 | 211 | 94 | 4 | 11 | 221 | 2 | 169 | Hypothetical protein | Hypothetical protein | | afdb-uniprot50 | AF-A0A7C1UDS7-F1-MODEL\_V4 | 1.0 | 2.388e-14 | 437 | 0.35 | 214 | 81 | 5 | 8 | 221 | 134 | 289 | Collar domain-containing protein | Collar domain-containing protein | | afdb-uniprot50 | AF-A0A819R841-F1-MODEL\_V4 | 1.0 | 7.284e-15 | 436 | 0.333 | 219 | 120 | 8 | 11 | 220 | 308 | 509 | Hypothetical protein | Hypothetical protein | | afdb-uniprot50 | AF-G9RT16-F1-MODEL\_V4 | 1.0 | 1.693e-13 | 433 | 0.369 | 211 | 67 | 6 | 11 | 221 | 11 | 155 | Collar domain-containing protein | Collar domain-containing protein | | afdb-uniprot50 | AF-W9GZ80-F1-MODEL\_V4 | 1.0 | 1.883e-14 | 433 | 0.369 | 219 | 87 | 8 | 4 | 220 | 95 | 264 | Collar domain-containing protein | Collar domain-containing protein | | afdb-uniprot50 | AF-A0A817QHN6-F1-MODEL\_V4 | 1.0 | 2.094e-15 | 433 | 0.36 | 219 | 114 | 8 | 11 | 220 | 373 | 574 | Hypothetical protein | Hypothetical protein | | afdb-uniprot50 | AF-A0A821J8J7-F1-MODEL\_V4 | 1.0 | 9.801e-15 | 432 | 0.334 | 218 | 120 | 7 | 11 | 220 | 191 | 391 | Hypothetical protein | Hypothetical protein | | afdb-uniprot50 | AF-A0A7T7YU12-F1-MODEL\_V4 | 1.0 | 6.55e-14 | 430 | 0.393 | 216 | 88 | 5 | 12 | 220 | 109 | 288 | Tail fiber protein | Tail fiber protein | | afdb-uniprot50 | AF-A0A820NDG9-F1-MODEL\_V4 | 1.0 | 2.818e-15 | 429 | 0.361 | 221 | 111 | 11 | 11 | 220 | 104 | 305 | Hypothetical protein | Hypothetical protein | | afdb-uniprot50 | AF-A0A1V1P4C0-F1-MODEL\_V4 | 1.0 | 1.774e-14 | 429 | 0.369 | 211 | 97 | 6 | 11 | 221 | 189 | 363 | Microcystin-dependent protein-like protein | Microcystin-dependent protein-like protein | | afdb-uniprot50 | AF-A0A0D6AS89-F1-MODEL\_V4 | 1.0 | 6.779e-16 | 428 | 0.341 | 240 | 105 | 8 | 3 | 221 | 37 | 244 | Microcystin dependent protein | Microcystin dependent protein | | afdb-uniprot50 | AF-A0A177Q1Y6-F1-MODEL\_V4 | 1.0 | 2.534e-14 | 428 | 0.342 | 228 | 88 | 8 | 1 | 221 | 81 | 253 | Collar domain-containing protein | Collar domain-containing protein | | afdb-uniprot50 | AF-A0A846V389-F1-MODEL\_V4 | 1.0 | 2.25e-14 | 428 | 0.303 | 224 | 120 | 8 | 1 | 220 | 140 | 331 | Microcystin-dependent protein | Microcystin-dependent protein | | afdb-uniprot50 | AF-A0A6B2LL29-F1-MODEL\_V4 | 1.0 | 1.352e-12 | 427 | 0.317 | 211 | 83 | 6 | 11 | 221 | 3 | 152 | Collar domain-containing protein | Collar domain-containing protein | | afdb-uniprot50 | AF-A0A815B3Y9-F1-MODEL\_V4 | 1.0 | 4.53e-15 | 427 | 0.343 | 233 | 119 | 13 | 3 | 220 | 369 | 582 | Hypothetical protein | Hypothetical protein | | afdb-uniprot50 | AF-A0A815K0V8-F1-MODEL\_V4 | 1.0 | 6.468e-15 | 423 | 0.32 | 225 | 120 | 11 | 11 | 220 | 184 | 390 | Hypothetical protein | Hypothetical protein | | afdb-uniprot50 | AF-A0A813P3R8-F1-MODEL\_V4 | 1.0 | 1.053e-13 | 422 | 0.368 | 225 | 93 | 7 | 3 | 220 | 17 | 199 | Hypothetical protein | Hypothetical protein | | afdb-uniprot50 | AF-A0A816E9L0-F1-MODEL\_V4 | 1.0 | 3.791e-15 | 422 | 0.336 | 220 | 117 | 9 | 11 | 220 | 481 | 681 | Hypothetical protein | Hypothetical protein | | afdb-uniprot50 | AF-A0A6C0ALD8-F1-MODEL\_V4 | 1.0 | 9.353e-14 | 420 | 0.345 | 211 | 107 | 7 | 12 | 221 | 49 | 229 | Collar domain-containing protein | Collar domain-containing protein | | afdb-uniprot50 | AF-A0A1I7KTY4-F1-MODEL\_V4 | 1.0 | 1.485e-14 | 420 | 0.448 | 212 | 76 | 9 | 11 | 220 | 199 | 371 | Microcystin-dependent protein | Microcystin-dependent protein | | afdb-uniprot50 | AF-A0A818BYP4-F1-MODEL\_V4 | 1.0 | 6.864e-15 | 420 | 0.327 | 220 | 119 | 9 | 11 | 220 | 407 | 607 | Hypothetical protein | Hypothetical protein | | afdb-uniprot50 | AF-A0A7W4IKD8-F1-MODEL\_V4 | 1.0 | 5.101e-15 | 418 | 0.409 | 220 | 102 | 7 | 8 | 220 | 140 | 338 | Tail fiber protein | Tail fiber protein | | afdb-uniprot50 | AF-A0A816GY78-F1-MODEL\_V4 | 1.0 | 5.744e-15 | 417 | 0.337 | 222 | 113 | 9 | 11 | 220 | 57 | 256 | Hypothetical protein | Hypothetical protein | | afdb-uniprot50 | AF-A0A2M7SZQ3-F1-MODEL\_V4 | 1.0 | 1.973e-15 | 416 | 0.386 | 220 | 91 | 9 | 3 | 221 | 234 | 410 | Collar domain-containing protein | Collar domain-containing protein | | afdb-uniprot50 | AF-A0A4Y6CGU0-F1-MODEL\_V4 | 1.0 | 1.417e-13 | 413 | 0.355 | 211 | 87 | 4 | 11 | 221 | 522 | 683 | Collar domain-containing protein | Collar domain-containing protein | | afdb-uniprot50 | AF-A0A820E4P5-F1-MODEL\_V4 | 1.0 | 2.723e-13 | 411 | 0.364 | 211 | 95 | 7 | 11 | 221 | 25 | 196 | Hypothetical protein | Hypothetical protein | | afdb-uniprot50 | AF-A0A1J5P6P6-F1-MODEL\_V4 | 1.0 | 9.924e-14 | 411 | 0.427 | 213 | 88 | 7 | 12 | 221 | 106 | 287 | Phage terminase large subunit | Phage terminase large subunit | | afdb-uniprot50 | AF-A0A822CIC7-F1-MODEL\_V4 | 1.0 | 9.236e-15 | 411 | 0.337 | 219 | 119 | 8 | 11 | 220 | 132 | 333 | Hypothetical protein | Hypothetical protein | | afdb-uniprot50 | AF-A0A821HM98-F1-MODEL\_V4 | 1.0 | 8.704e-15 | 411 | 0.319 | 222 | 118 | 9 | 11 | 219 | 231 | 432 | Hypothetical protein | Hypothetical protein | | afdb-uniprot50 | AF-A0A0P0EA18-F1-MODEL\_V4 | 1.0 | 1.693e-13 | 407 | 0.317 | 230 | 91 | 6 | 1 | 221 | 18 | 190 | Collar domain-containing protein | Collar domain-containing protein | | afdb-uniprot50 | AF-A0A5J4Y931-F1-MODEL\_V4 | 1.0 | 1.907e-13 | 404 | 0.334 | 212 | 98 | 5 | 12 | 221 | 153 | 323 | Collar domain-containing protein | Collar domain-containing protein | | afdb-uniprot50 | AF-A0A2E3QAG3-F1-MODEL\_V4 | 1.0 | 2.049e-12 | 402 | 0.291 | 223 | 99 | 7 | 1 | 221 | 10 | 175 | Phage tail protein | Phage tail protein | | afdb-uniprot50 | AF-A0A3S0TER0-F1-MODEL\_V4 | 1.0 | 7.827e-14 | 400 | 0.377 | 209 | 83 | 6 | 12 | 220 | 488 | 649 | Collar domain-containing protein | Collar domain-containing protein | | afdb-uniprot50 | AF-A0A4Q3WNL6-F1-MODEL\_V4 | 1.0 | 3.618e-14 | 399 | 0.33 | 227 | 95 | 6 | 3 | 221 | 239 | 416 | Tail fiber protein | Tail fiber protein | | afdb-uniprot50 | AF-A0A820D1C7-F1-MODEL\_V4 | 1.0 | 2.689e-14 | 397 | 0.328 | 222 | 116 | 11 | 12 | 220 | 171 | 372 | Hypothetical protein | Hypothetical protein | | afdb-uniprot50 | AF-A0A817IDX0-F1-MODEL\_V4 | 1.0 | 8.813e-14 | 397 | 0.348 | 218 | 117 | 8 | 11 | 220 | 399 | 599 | Hypothetical protein | Hypothetical protein | | afdb-uniprot50 | AF-A0A821YM97-F1-MODEL\_V4 | 1.0 | 7.376e-14 | 396 | 0.283 | 219 | 131 | 7 | 11 | 220 | 31 | 232 | Hypothetical protein | Hypothetical protein | | afdb-uniprot50 | AF-A0A6C0HZC1-F1-MODEL\_V4 | 1.0 | 4.868e-14 | 396 | 0.302 | 228 | 124 | 6 | 12 | 221 | 46 | 256 | Collar domain-containing protein | Collar domain-containing protein | | afdb-uniprot50 | AF-A0A816FNH1-F1-MODEL\_V4 | 1.0 | 5.482e-14 | 395 | 0.321 | 224 | 115 | 10 | 11 | 219 | 431 | 632 | Hypothetical protein | Hypothetical protein | | afdb-uniprot50 | AF-A0A7V3QID4-F1-MODEL\_V4 | 1.0 | 2.023e-13 | 392 | 0.341 | 211 | 78 | 5 | 11 | 221 | 207 | 356 | Collar domain-containing protein | Collar domain-containing protein | | afdb-uniprot50 | AF-A0A821GMF9-F1-MODEL\_V4 | 1.0 | 7.827e-14 | 391 | 0.324 | 219 | 113 | 9 | 11 | 220 | 15 | 207 | Hypothetical protein | Hypothetical protein | | afdb-uniprot50 | AF-A0A818P2B2-F1-MODEL\_V4 | 1.0 | 6.173e-14 | 390 | 0.339 | 233 | 103 | 7 | 1 | 221 | 46 | 239 | Hypothetical protein | Hypothetical protein | | afdb-uniprot50 | AF-A0A814FG22-F1-MODEL\_V4 | 1.0 | 1.319e-14 | 390 | 0.336 | 220 | 116 | 10 | 11 | 220 | 49 | 248 | Hypothetical protein | Hypothetical protein | | afdb-uniprot50 | AF-A0A6N6JSU8-F1-MODEL\_V4 | 1.0 | 4.645e-13 | 388 | 0.354 | 217 | 101 | 9 | 11 | 221 | 25 | 208 | Collar domain-containing protein | Collar domain-containing protein | | afdb-uniprot50 | AF-A0A815V480-F1-MODEL\_V4 | 1.0 | 2.023e-13 | 388 | 0.322 | 217 | 122 | 9 | 12 | 220 | 502 | 701 | Hypothetical protein | Hypothetical protein | | afdb-uniprot50 | AF-A0A4Q8ARL3-F1-MODEL\_V4 | 1.0 | 1.274e-12 | 387 | 0.31 | 229 | 89 | 6 | 3 | 221 | 26 | 195 | Microcystin-dependent protein | Microcystin-dependent protein | | afdb-uniprot50 | AF-A0A3M1NVN9-F1-MODEL\_V4 | 1.0 | 2.723e-13 | 387 | 0.364 | 269 | 112 | 11 | 2 | 220 | 59 | 318 | Collar domain-containing protein | Collar domain-containing protein | | afdb-uniprot50 | AF-A0A158D1B4-F1-MODEL\_V4 | 1.0 | 1.09e-15 | 385 | 0.414 | 229 | 104 | 6 | 11 | 221 | 176 | 392 | Phage tail collar domain-containing protein | Phage tail collar domain-containing protein | | afdb-uniprot50 | AF-A0A4Q0AIU5-F1-MODEL\_V4 | 1.0 | 2.336e-11 | 384 | 0.293 | 211 | 88 | 8 | 11 | 221 | 2 | 151 | Collar domain-containing protein | Collar domain-containing protein | | afdb-uniprot50 | AF-A0A822DWL8-F1-MODEL\_V4 | 1.0 | 1.907e-13 | 384 | 0.322 | 220 | 120 | 8 | 11 | 220 | 12 | 212 | Hypothetical protein | Hypothetical protein | | afdb-uniprot50 | AF-A0A818VB53-F1-MODEL\_V4 | 1.0 | 1.053e-13 | 384 | 0.309 | 233 | 126 | 9 | 2 | 220 | 11 | 222 | Hypothetical protein | Hypothetical protein | | afdb-uniprot50 | AF-A0A818SKZ2-F1-MODEL\_V4 | 1.0 | 1.053e-13 | 384 | 0.327 | 220 | 112 | 9 | 12 | 220 | 33 | 227 | Hypothetical protein | Hypothetical protein | | afdb-uniprot50 | AF-A0A815JLE8-F1-MODEL\_V4 | 1.0 | 8.813e-14 | 384 | 0.353 | 218 | 115 | 9 | 11 | 220 | 272 | 471 | Hypothetical protein | Hypothetical protein | | afdb-uniprot50 | AF-A0A1Y4GYF8-F1-MODEL\_V4 | 1.0 | 2.336e-11 | 381 | 0.308 | 211 | 85 | 4 | 11 | 221 | 9 | 158 | Collar domain-containing protein | Collar domain-containing protein | | afdb-uniprot50 | AF-A0A819LSZ1-F1-MODEL\_V4 | 1.0 | 7.469e-13 | 379 | 0.343 | 230 | 95 | 8 | 3 | 221 | 30 | 214 | Hypothetical protein | Hypothetical protein | | afdb-uniprot50 | AF-A0A369RCN8-F1-MODEL\_V4 | 1.0 | 1.066e-12 | 379 | 0.319 | 210 | 81 | 4 | 12 | 221 | 73 | 220 | Collar domain-containing protein | Collar domain-containing protein | | afdb-uniprot50 | AF-A0A1C3XJM3-F1-MODEL\_V4 | 1.0 | 6.193e-09 | 379 | 0.579 | 88 | 36 | 1 | 16 | 102 | 128 | 215 | Phage Tail Collar Domain | Phage Tail Collar Domain | | afdb-uniprot50 | AF-A0A559TE11-F1-MODEL\_V4 | 1.0 | 9.924e-14 | 378 | 0.384 | 229 | 109 | 9 | 2 | 221 | 183 | 388 | Microcystin-dependent protein | Microcystin-dependent protein | | afdb-uniprot50 | AF-A0A815U1H6-F1-MODEL\_V4 | 1.0 | 3.452e-13 | 378 | 0.31 | 219 | 125 | 8 | 11 | 220 | 514 | 715 | Hypothetical protein | Hypothetical protein | | afdb-uniprot50 | AF-A0A2N2HV87-F1-MODEL\_V4 | 1.0 | 5.482e-14 | 375 | 0.346 | 222 | 93 | 8 | 11 | 221 | 174 | 354 | Uncharacterized protein | Uncharacterized protein | | afdb-uniprot50 | AF-A0A7C5Z090-F1-MODEL\_V4 | 1.0 | 4.125e-13 | 374 | 0.371 | 221 | 74 | 7 | 1 | 221 | 160 | 315 | Collar domain-containing protein | Collar domain-containing protein | | afdb-uniprot50 | AF-A0A840BX98-F1-MODEL\_V4 | 1.0 | 7.926e-13 | 374 | 0.333 | 225 | 113 | 9 | 2 | 220 | 296 | 489 | Microcystin-dependent protein | Microcystin-dependent protein | | afdb-uniprot50 | AF-A0A816MB52-F1-MODEL\_V4 | 1.0 | 2.023e-13 | 374 | 0.323 | 229 | 115 | 12 | 11 | 220 | 505 | 712 | Hypothetical protein | Hypothetical protein | | afdb-uniprot50 | AF-A0A816Z751-F1-MODEL\_V4 | 1.0 | 1.186e-13 | 373 | 0.314 | 229 | 110 | 10 | 11 | 221 | 2 | 201 | Hypothetical protein | Hypothetical protein | | afdb-uniprot50 | AF-A0A835XXH8-F1-MODEL\_V4 | 1.0 | 1.132e-12 | 373 | 0.313 | 230 | 115 | 8 | 2 | 220 | 350 | 547 | Uncharacterized protein | Uncharacterized protein | | afdb-uniprot50 | AF-A0A521K5D5-F1-MODEL\_V4 | 1.0 | 1.335e-13 | 372 | 0.361 | 216 | 83 | 6 | 12 | 221 | 49 | 215 | Collar domain-containing protein | Collar domain-containing protein | | afdb-uniprot50 | AF-A0A820QPV6-F1-MODEL\_V4 | 1.0 | 4.929e-13 | 372 | 0.313 | 217 | 127 | 8 | 11 | 220 | 92 | 293 | Hypothetical protein | Hypothetical protein | | afdb-uniprot50 | AF-A0A814LGM5-F1-MODEL\_V4 | 1.0 | 6.633e-13 | 371 | 0.35 | 211 | 96 | 8 | 12 | 221 | 48 | 218 | Hypothetical protein | Hypothetical protein | | afdb-uniprot50 | AF-A0A1F3B1K2-F1-MODEL\_V4 | 1.0 | 1.931e-12 | 370 | 0.316 | 212 | 79 | 5 | 11 | 221 | 374 | 520 | Collar domain-containing protein | Collar domain-containing protein | | afdb-uniprot50 | AF-A0A815QNH7-F1-MODEL\_V4 | 1.0 | 3.663e-13 | 369 | 0.327 | 217 | 122 | 7 | 11 | 220 | 297 | 496 | Hypothetical protein | Hypothetical protein | | afdb-uniprot50 | AF-A0A814Z5Y2-F1-MODEL\_V4 | 1.0 | 7.376e-14 | 368 | 0.313 | 220 | 121 | 11 | 11 | 220 | 124 | 323 | Hypothetical protein | Hypothetical protein | | afdb-uniprot50 | AF-A0A4D7B3R7-F1-MODEL\_V4 | 1.0 | 1.931e-12 | 366 | 0.35 | 214 | 114 | 9 | 11 | 221 | 125 | 316 | Tail fiber protein | Tail fiber protein | | afdb-uniprot50 | AF-A0A3N7JCP5-F1-MODEL\_V4 | 1.0 | 3.496e-12 | 364 | 0.271 | 214 | 128 | 6 | 10 | 220 | 3 | 191 | Phage tail protein | Phage tail protein | | afdb-uniprot50 | AF-A0A2D9HKR9-F1-MODEL\_V4 | 1.0 | 2.049e-12 | 364 | 0.355 | 211 | 54 | 4 | 11 | 221 | 78 | 206 | Collar domain-containing protein | Collar domain-containing protein | | afdb-uniprot50 | AF-A0A3D1PJI0-F1-MODEL\_V4 | 1.0 | 2.174e-12 | 364 | 0.325 | 209 | 78 | 5 | 12 | 220 | 48 | 193 | Collar domain-containing protein | Collar domain-containing protein | | afdb-uniprot50 | AF-A0A813ML39-F1-MODEL\_V4 | 1.0 | 1.258e-13 | 363 | 0.319 | 219 | 124 | 9 | 11 | 220 | 515 | 717 | Hypothetical protein | Hypothetical protein | | afdb-uniprot50 | AF-A0A821Z962-F1-MODEL\_V4 | 1.0 | 2.889e-13 | 362 | 0.297 | 225 | 122 | 9 | 11 | 220 | 220 | 423 | Hypothetical protein | Hypothetical protein | | afdb-uniprot50 | AF-A0A819Y0C9-F1-MODEL\_V4 | 1.0 | 1.258e-13 | 360 | 0.299 | 227 | 115 | 9 | 11 | 221 | 50 | 248 | Hypothetical protein | Hypothetical protein | | afdb-uniprot50 | AF-A0A817VC28-F1-MODEL\_V4 | 1.0 | 1.005e-12 | 359 | 0.319 | 216 | 119 | 8 | 12 | 220 | 29 | 223 | Hypothetical protein | Hypothetical protein | | afdb-uniprot50 | AF-A0A1Y4GY33-F1-MODEL\_V4 | 1.0 | 8.41e-13 | 359 | 0.321 | 224 | 88 | 6 | 2 | 221 | 174 | 337 | Collar domain-containing protein | Collar domain-containing protein | | afdb-uniprot50 | AF-A0A6A7MGA2-F1-MODEL\_V4 | 1.0 | 7.127e-12 | 359 | 0.312 | 211 | 107 | 4 | 11 | 221 | 171 | 343 | Collar domain-containing protein | Collar domain-containing protein | | afdb-uniprot50 | AF-A0A7W2NTN7-F1-MODEL\_V4 | 1.0 | 8.516e-12 | 357 | 0.281 | 217 | 103 | 8 | 10 | 220 | 3 | 172 | Phage tail protein | Phage tail protein | | afdb-uniprot50 | AF-A0A5S3SAM7-F1-MODEL\_V4 | 1.0 | 8.026e-12 | 355 | 0.41 | 200 | 80 | 9 | 24 | 220 | 1 | 165 | Phage tail protein | Phage tail protein | | afdb-uniprot50 | AF-A0A817QZ18-F1-MODEL\_V4 | 1.0 | 9.151e-11 | 355 | 0.311 | 209 | 103 | 5 | 14 | 221 | 4 | 172 | Hypothetical protein | Hypothetical protein | | afdb-uniprot50 | AF-A0A6N3F5J0-F1-MODEL\_V4 | 1.0 | 1.274e-12 | 355 | 0.323 | 213 | 74 | 9 | 11 | 221 | 49 | 193 | Phage Tail Collar Domain protein | Phage Tail Collar Domain protein | | afdb-uniprot50 | AF-A0A1V5DL01-F1-MODEL\_V4 | 1.0 | 2.418e-13 | 355 | 0.374 | 219 | 87 | 10 | 11 | 221 | 160 | 336 | Phage Tail Collar Domain protein | Phage Tail Collar Domain protein | | afdb-uniprot50 | AF-A0A7V8IVS3-F1-MODEL\_V4 | 1.0 | 8.925e-13 | 355 | 0.331 | 211 | 80 | 8 | 12 | 221 | 256 | 406 | Collar domain-containing protein | Collar domain-containing protein | | afdb-uniprot50 | AF-E4T5W3-F1-MODEL\_V4 | 1.0 | 2.448e-12 | 354 | 0.362 | 215 | 78 | 7 | 11 | 221 | 739 | 898 | Tail Collar domain protein | Tail Collar domain protein | | afdb-uniprot50 | AF-A0A512NCS0-F1-MODEL\_V4 | 1.0 | 3.452e-13 | 353 | 0.338 | 213 | 97 | 7 | 12 | 221 | 145 | 316 | Collar domain-containing protein | Collar domain-containing protein | | afdb-uniprot50 | AF-A0A1F3ABU4-F1-MODEL\_V4 | 1.0 | 2.366e-10 | 352 | 0.314 | 207 | 79 | 6 | 16 | 221 | 1 | 145 | Collar domain-containing protein | Collar domain-containing protein | | afdb-uniprot50 | AF-A0A2I0N9H0-F1-MODEL\_V4 | 1.0 | 2.598e-12 | 352 | 0.285 | 224 | 111 | 11 | 1 | 221 | 168 | 345 | Collar domain-containing protein | Collar domain-containing protein | | afdb-uniprot50 | AF-A0A6N4RF73-F1-MODEL\_V4 | 1.0 | 1.258e-13 | 351 | 0.344 | 215 | 109 | 7 | 11 | 221 | 39 | 225 | Tail fiber protein | Tail fiber protein | | afdb-uniprot50 | AF-A0A257UMQ9-F1-MODEL\_V4 | 1.0 | 2.174e-12 | 351 | 0.378 | 214 | 87 | 8 | 11 | 221 | 68 | 238 | Collar domain-containing protein | Collar domain-containing protein | | afdb-uniprot50 | AF-A0A2A6FNG7-F1-MODEL\_V4 | 1.0 | 1.453e-11 | 350 | 0.311 | 225 | 101 | 6 | 3 | 221 | 11 | 187 | Collar domain-containing protein | Collar domain-containing protein | | afdb-uniprot50 | AF-A0A817WT08-F1-MODEL\_V4 | 1.0 | 5.231e-13 | 348 | 0.308 | 227 | 113 | 7 | 12 | 221 | 55 | 254 | Hypothetical protein | Hypothetical protein | | afdb-uniprot50 | AF-A0A7V2Y2J7-F1-MODEL\_V4 | 1.0 | 1.435e-12 | 348 | 0.297 | 215 | 86 | 10 | 11 | 221 | 161 | 314 | Collar domain-containing protein | Collar domain-containing protein | | afdb-uniprot50 | AF-A0A0G1QRC3-F1-MODEL\_V4 | 1.0 | 8.516e-12 | 347 | 0.331 | 211 | 74 | 7 | 11 | 221 | 133 | 276 | Tail Collar domain protein | Tail Collar domain protein | | afdb-uniprot50 | AF-A0A1V4VVI6-F1-MODEL\_V4 | 1.0 | 3.936e-12 | 346 | 0.304 | 223 | 92 | 10 | 2 | 221 | 219 | 381 | Phage Tail Collar Domain protein | Phage Tail Collar Domain protein | | afdb-uniprot50 | AF-A0A661AU03-F1-MODEL\_V4 | 1.0 | 6.716e-12 | 345 | 0.341 | 211 | 97 | 7 | 11 | 221 | 554 | 722 | Collar domain-containing protein | Collar domain-containing protein | | afdb-uniprot50 | AF-A0A816AZ21-F1-MODEL\_V4 | 1.0 | 7.469e-13 | 344 | 0.286 | 220 | 125 | 8 | 11 | 220 | 262 | 459 | Hypothetical protein | Hypothetical protein | | afdb-uniprot50 | AF-A0A813U148-F1-MODEL\_V4 | 1.0 | 1.186e-13 | 343 | 0.298 | 241 | 124 | 10 | 2 | 221 | 300 | 516 | Hypothetical protein | Hypothetical protein | | afdb-uniprot50 | AF-A0A818QJV6-F1-MODEL\_V4 | 1.0 | 1.005e-12 | 342 | 0.266 | 221 | 128 | 9 | 11 | 220 | 450 | 647 | Hypothetical protein | Hypothetical protein | | afdb-uniprot50 | AF-A0A816H4T3-F1-MODEL\_V4 | 1.0 | 3.104e-12 | 341 | 0.297 | 222 | 124 | 8 | 11 | 220 | 352 | 553 | Hypothetical protein | Hypothetical protein | | afdb-uniprot50 | AF-A0A0Q4T3S1-F1-MODEL\_V4 | 1.0 | 3.71e-12 | 340 | 0.334 | 284 | 108 | 11 | 12 | 221 | 125 | 401 | Collar domain-containing protein | Collar domain-containing protein | | afdb-uniprot50 | AF-A0A431IBK6-F1-MODEL\_V4 | 1.0 | 1.955e-11 | 339 | 0.299 | 224 | 82 | 7 | 2 | 221 | 29 | 181 | Collar domain-containing protein | Collar domain-containing protein | | afdb-uniprot50 | AF-A0A086D2K0-F1-MODEL\_V4 | 1.0 | 3.756e-11 | 339 | 0.276 | 224 | 105 | 8 | 11 | 220 | 6 | 186 | Tail protein | Tail protein | | afdb-uniprot50 | AF-A0A6M5Z7F0-F1-MODEL\_V4 | 1.0 | 1.216e-11 | 339 | 0.364 | 211 | 73 | 8 | 11 | 221 | 395 | 544 | Collar domain-containing protein | Collar domain-containing protein | | afdb-uniprot50 | AF-A0A228K8W8-F1-MODEL\_V4 | 1.0 | 1.736e-11 | 338 | 0.269 | 234 | 115 | 8 | 1 | 221 | 94 | 284 | Uncharacterized protein | Uncharacterized protein | | afdb-uniprot50 | AF-A0A656QC36-F1-MODEL\_V4 | 1.0 | 1.08e-11 | 337 | 0.359 | 220 | 93 | 7 | 9 | 220 | 104 | 283 | Uncharacterized protein | Uncharacterized protein | | afdb-uniprot50 | AF-A0A7S6S8F8-F1-MODEL\_V4 | 1.0 | 3.144e-11 | 336 | 0.294 | 221 | 123 | 8 | 11 | 220 | 4 | 202 | Phage tail protein | Phage tail protein | | afdb-uniprot50 | AF-A0A813XM12-F1-MODEL\_V4 | 1.0 | 4.587e-14 | 336 | 0.333 | 240 | 117 | 12 | 2 | 221 | 73 | 289 | Hypothetical protein | Hypothetical protein | | afdb-uniprot50 | AF-A0A815S9R3-F1-MODEL\_V4 | 1.0 | 1.931e-12 | 335 | 0.283 | 236 | 127 | 9 | 11 | 220 | 337 | 556 | Hypothetical protein | Hypothetical protein | | afdb-uniprot50 | AF-A0A2V9WHC2-F1-MODEL\_V4 | 1.0 | 1.82e-12 | 334 | 0.372 | 215 | 93 | 7 | 12 | 220 | 135 | 313 | Collar domain-containing protein | Collar domain-containing protein | | afdb-uniprot50 | AF-D0LMW0-F1-MODEL\_V4 | 1.0 | 1.842e-11 | 333 | 0.291 | 223 | 96 | 7 | 2 | 220 | 98 | 262 | Tail Collar domain protein | Tail Collar domain protein | | afdb-uniprot50 | AF-A0A0F0DN26-F1-MODEL\_V4 | 1.0 | 9.151e-11 | 332 | 0.278 | 223 | 106 | 7 | 11 | 220 | 6 | 186 | Tail protein | Tail protein | | afdb-uniprot50 | AF-A0A819AK34-F1-MODEL\_V4 | 1.0 | 1.132e-12 | 332 | 0.313 | 230 | 127 | 10 | 2 | 221 | 98 | 306 | Hypothetical protein | Hypothetical protein | | afdb-uniprot50 | AF-A0A2L2NDQ4-F1-MODEL\_V4 | 1.0 | 2.889e-13 | 332 | 0.328 | 256 | 112 | 7 | 11 | 221 | 160 | 400 | Phage tail collar domain protein | Phage tail collar domain protein | | afdb-uniprot50 | AF-A0A367Q7H7-F1-MODEL\_V4 | 1.0 | 3.54e-11 | 331 | 0.305 | 219 | 86 | 6 | 4 | 220 | 312 | 466 | Collar domain-containing protein | Collar domain-containing protein | | afdb-uniprot50 | AF-A0A561GTC3-F1-MODEL\_V4 | 1.0 | 3.54e-11 | 330 | 0.294 | 221 | 116 | 11 | 10 | 220 | 3 | 193 | Microcystin-dependent protein | Microcystin-dependent protein | | afdb-uniprot50 | AF-A0A1Y2LD10-F1-MODEL\_V4 | 1.0 | 8.127e-11 | 328 | 0.298 | 211 | 77 | 5 | 12 | 221 | 52 | 192 | Collar domain-containing protein | Collar domain-containing protein | | afdb-uniprot50 | AF-D9SMM5-F1-MODEL\_V4 | 1.0 | 1.842e-11 | 328 | 0.275 | 225 | 110 | 7 | 11 | 220 | 4 | 190 | Tail Collar domain protein | Tail Collar domain protein | | afdb-uniprot50 | AF-A0A199XMG2-F1-MODEL\_V4 | 1.0 | 6.409e-11 | 327 | 0.304 | 217 | 93 | 9 | 12 | 220 | 5 | 171 | Phage tail collar domain protein | Phage tail collar domain protein | | afdb-uniprot50 | AF-A0A818C432-F1-MODEL\_V4 | 1.0 | 4.992e-12 | 327 | 0.295 | 237 | 121 | 8 | 3 | 220 | 14 | 223 | Hypothetical protein | Hypothetical protein | | afdb-uniprot50 | AF-A0A6L4A8E9-F1-MODEL\_V4 | 1.0 | 7.563e-12 | 327 | 0.319 | 210 | 89 | 7 | 12 | 220 | 51 | 207 | Collar domain-containing protein | Collar domain-containing protein | | afdb-uniprot50 | AF-A0A3B9AMF4-F1-MODEL\_V4 | 1.0 | 2.827e-10 | 325 | 0.291 | 216 | 99 | 6 | 10 | 220 | 3 | 169 | Phage tail protein | Phage tail protein | | afdb-uniprot50 | AF-X0Z504-F1-MODEL\_V4 | 1.0 | 2.962e-11 | 325 | 0.317 | 211 | 85 | 6 | 11 | 221 | 78 | 229 | Collar domain-containing protein | Collar domain-containing protein | | afdb-uniprot50 | AF-A0A4Y8ADI2-F1-MODEL\_V4 | 1.0 | 1.955e-11 | 323 | 0.295 | 223 | 104 | 9 | 11 | 220 | 5 | 187 | Phage tail protein | Phage tail protein | | afdb-uniprot50 | AF-A0A6J5DWF4-F1-MODEL\_V4 | 1.0 | 1.657e-10 | 322 | 0.282 | 230 | 111 | 8 | 3 | 221 | 79 | 265 | Uncharacterized protein | Uncharacterized protein | | afdb-uniprot50 | AF-A0A820P0S5-F1-MODEL\_V4 | 1.0 | 2.598e-12 | 322 | 0.355 | 211 | 107 | 8 | 20 | 220 | 224 | 415 | Hypothetical protein | Hypothetical protein | | afdb-uniprot50 | AF-A0A845XUZ4-F1-MODEL\_V4 | 1.0 | 1.542e-11 | 322 | 0.342 | 210 | 93 | 6 | 12 | 221 | 343 | 507 | Uncharacterized protein | Uncharacterized protein | | afdb-uniprot50 | AF-A0A832E9G7-F1-MODEL\_V4 | 1.0 | 5.118e-10 | 321 | 0.278 | 212 | 96 | 7 | 10 | 220 | 3 | 158 | Phage tail protein | Phage tail protein | | afdb-uniprot50 | AF-A0A2U8WK05-F1-MODEL\_V4 | 1.0 | 5.692e-11 | 321 | 0.227 | 237 | 131 | 9 | 11 | 220 | 4 | 215 | Collar domain-containing protein | Collar domain-containing protein | | afdb-uniprot50 | AF-A0A816ETV2-F1-MODEL\_V4 | 1.0 | 5.551e-13 | 321 | 0.314 | 232 | 124 | 10 | 1 | 221 | 66 | 273 | Hypothetical protein | Hypothetical protein | | afdb-uniprot50 | AF-A0A1J5PBH2-F1-MODEL\_V4 | 1.0 | 6.04e-11 | 321 | 0.288 | 215 | 118 | 10 | 12 | 221 | 138 | 322 | Phage tail collar domain protein | Phage tail collar domain protein | | afdb-uniprot50 | AF-A0A1Q3W4R7-F1-MODEL\_V4 | 1.0 | 2.229e-10 | 320 | 0.202 | 276 | 129 | 6 | 11 | 220 | 218 | 468 | Uncharacterized protein | Uncharacterized protein | | afdb-uniprot50 | AF-A0A1W2GA44-F1-MODEL\_V4 | 1.0 | 1.387e-10 | 319 | 0.227 | 224 | 135 | 8 | 11 | 220 | 4 | 203 | Microcystin-dependent protein | Microcystin-dependent protein | | afdb-uniprot50 | AF-A0A815G9G8-F1-MODEL\_V4 | 1.0 | 7.038e-13 | 319 | 0.311 | 225 | 114 | 10 | 12 | 221 | 100 | 298 | Hypothetical protein | Hypothetical protein | | afdb-uniprot50 | AF-D1BW55-F1-MODEL\_V4 | 1.0 | 2.075e-11 | 319 | 0.302 | 245 | 124 | 9 | 1 | 220 | 215 | 437 | Tail Collar domain protein | Tail Collar domain protein | | afdb-uniprot50 | AF-A0A3A4PGH4-F1-MODEL\_V4 | 1.0 | 8.733e-10 | 318 | 0.266 | 210 | 103 | 5 | 11 | 220 | 5 | 163 | Phage tail protein | Phage tail protein | | afdb-uniprot50 | AF-A9H7F1-F1-MODEL\_V4 | 1.0 | 5.231e-13 | 318 | 0.468 | 177 | 72 | 5 | 28 | 198 | 1 | 161 | Putative membrane protein | Putative membrane protein | | afdb-uniprot50 | AF-A0A3E0DWJ8-F1-MODEL\_V4 | 1.0 | 3.183e-10 | 318 | 0.276 | 213 | 111 | 7 | 10 | 220 | 3 | 174 | Microcystin-dependent protein | Microcystin-dependent protein | | afdb-uniprot50 | AF-A0A1X7NXK8-F1-MODEL\_V4 | 1.0 | 5.054e-11 | 317 | 0.216 | 236 | 137 | 9 | 11 | 220 | 4 | 217 | Microcystin-dependent protein | Microcystin-dependent protein | | afdb-uniprot50 | AF-A0A0D7Q2R9-F1-MODEL\_V4 | 1.0 | 4.489e-11 | 317 | 0.251 | 219 | 116 | 9 | 12 | 220 | 120 | 300 | Collar domain-containing protein | Collar domain-containing protein | | afdb-uniprot50 | AF-A0A0G4FSR3-F1-MODEL\_V4 | 1.0 | 1.636e-11 | 317 | 0.261 | 226 | 95 | 11 | 2 | 221 | 466 | 625 | Collar domain-containing protein | Collar domain-containing protein | | afdb-uniprot50 | AF-A0A4R2H649-F1-MODEL\_V4 | 1.0 | 1.758e-10 | 316 | 0.303 | 221 | 89 | 9 | 11 | 220 | 4 | 170 | Microcystin-dependent protein | Microcystin-dependent protein | | afdb-uniprot50 | AF-A0A4V0I5Q5-F1-MODEL\_V4 | 1.0 | 5.364e-11 | 314 | 0.336 | 211 | 75 | 7 | 11 | 220 | 287 | 433 | Uncharacterized protein | Uncharacterized protein | | afdb-uniprot50 | AF-A0A2D8BVW2-F1-MODEL\_V4 | 1.0 | 1.842e-11 | 313 | 0.273 | 223 | 89 | 7 | 12 | 221 | 24 | 186 | Collar domain-containing protein | Collar domain-containing protein | | afdb-uniprot50 | AF-A0A316AC07-F1-MODEL\_V4 | 1.0 | 5.692e-11 | 313 | 0.313 | 223 | 101 | 8 | 2 | 220 | 21 | 195 | Microcystin-dependent protein | Microcystin-dependent protein | | afdb-uniprot50 | AF-A0A2N2SSE1-F1-MODEL\_V4 | 1.0 | 3.496e-12 | 313 | 0.378 | 219 | 103 | 8 | 11 | 221 | 78 | 271 | Phage tail protein | Phage tail protein | | afdb-uniprot50 | AF-A0A1W6NZ62-F1-MODEL\_V4 | 1.0 | 2.336e-11 | 313 | 0.293 | 225 | 116 | 7 | 4 | 220 | 265 | 454 | Collar domain-containing protein | Collar domain-containing protein | | afdb-uniprot50 | AF-A0A154VNW1-F1-MODEL\_V4 | 1.0 | 9.711e-11 | 312 | 0.208 | 235 | 149 | 6 | 11 | 220 | 38 | 260 | Collar domain-containing protein | Collar domain-containing protein | | afdb-uniprot50 | AF-A0A820N1U4-F1-MODEL\_V4 | 1.0 | 4.704e-12 | 312 | 0.283 | 236 | 126 | 11 | 2 | 220 | 79 | 288 | Hypothetical protein | Hypothetical protein | | afdb-uniprot50 | AF-A0A4R6F7Q8-F1-MODEL\_V4 | 1.0 | 7.658e-11 | 312 | 0.308 | 246 | 101 | 8 | 1 | 220 | 102 | 304 | Uncharacterized protein | Uncharacterized protein | | afdb-uniprot50 | AF-A0A835XN54-F1-MODEL\_V4 | 1.0 | 1.842e-11 | 312 | 0.316 | 224 | 110 | 8 | 3 | 221 | 436 | 621 | Uncharacterized protein | Uncharacterized protein | | afdb-uniprot50 | AF-A0A819RFW5-F1-MODEL\_V4 | 1.0 | 6.716e-12 | 311 | 0.301 | 222 | 114 | 7 | 12 | 220 | 37 | 230 | Hypothetical protein | Hypothetical protein | | afdb-uniprot50 | AF-A0A821GTZ3-F1-MODEL\_V4 | 1.0 | 1.523e-12 | 311 | 0.28 | 225 | 120 | 10 | 11 | 220 | 165 | 362 | Hypothetical protein | Hypothetical protein | | afdb-uniprot50 | AF-A0A1J5HLH8-F1-MODEL\_V4 | 1.0 | 1.018e-11 | 311 | 0.339 | 212 | 104 | 8 | 12 | 221 | 439 | 616 | Collar domain-containing protein | Collar domain-containing protein | | afdb-uniprot50 | AF-A0A328LF96-F1-MODEL\_V4 | 1.0 | 5.118e-10 | 310 | 0.246 | 211 | 110 | 7 | 11 | 220 | 5 | 167 | Microcystin-dependent protein | Microcystin-dependent protein | | afdb-uniprot50 | AF-A0A199XM71-F1-MODEL\_V4 | 1.0 | 2.664e-10 | 309 | 0.295 | 213 | 105 | 6 | 10 | 220 | 3 | 172 | Phage tail collar domain protein | Phage tail collar domain protein | | afdb-uniprot50 | AF-A0A814GRD0-F1-MODEL\_V4 | 1.0 | 3.336e-11 | 309 | 0.313 | 217 | 111 | 8 | 14 | 220 | 1 | 189 | Hypothetical protein | Hypothetical protein | | afdb-uniprot50 | AF-A0A503X4M5-F1-MODEL\_V4 | 1.0 | 1.758e-10 | 309 | 0.243 | 234 | 123 | 9 | 12 | 221 | 112 | 315 | Uncharacterized protein | Uncharacterized protein | | afdb-uniprot50 | AF-A0A7Z0J594-F1-MODEL\_V4 | 1.0 | 7.658e-11 | 309 | 0.309 | 210 | 83 | 7 | 12 | 221 | 409 | 556 | Microcystin-dependent protein | Microcystin-dependent protein | | afdb-uniprot50 | AF-A0A173LYL4-F1-MODEL\_V4 | 1.0 | 3.104e-12 | 309 | 0.378 | 219 | 106 | 11 | 12 | 221 | 578 | 775 | Collagen triple helix repeat protein | Collagen triple helix repeat protein | | afdb-uniprot50 | AF-A0A0Q4QY73-F1-MODEL\_V4 | 1.0 | 1.107e-09 | 308 | 0.289 | 211 | 99 | 6 | 11 | 220 | 4 | 164 | Collar domain-containing protein | Collar domain-containing protein | | afdb-uniprot50 | AF-A0A1E3L3M0-F1-MODEL\_V4 | 1.0 | 1.175e-09 | 308 | 0.248 | 213 | 113 | 6 | 11 | 220 | 5 | 173 | Collar domain-containing protein | Collar domain-containing protein | | afdb-uniprot50 | AF-A0A4S8NSY4-F1-MODEL\_V4 | 1.0 | 4.763e-11 | 308 | 0.232 | 237 | 139 | 9 | 11 | 220 | 47 | 267 | Collar domain-containing protein | Collar domain-containing protein | | afdb-uniprot50 | AF-D3F201-F1-MODEL\_V4 | 1.0 | 2.075e-11 | 307 | 0.359 | 220 | 112 | 10 | 12 | 221 | 61 | 261 | Tail Collar domain protein | Tail Collar domain protein | | afdb-uniprot50 | AF-A0A816HBL7-F1-MODEL\_V4 | 1.0 | 2.926e-12 | 307 | 0.32 | 225 | 100 | 10 | 11 | 220 | 194 | 380 | Hypothetical protein | Hypothetical protein | | afdb-uniprot50 | AF-A0A4V2W2R3-F1-MODEL\_V4 | 1.0 | 1.107e-09 | 306 | 0.275 | 214 | 92 | 7 | 11 | 220 | 4 | 158 | Microcystin-dependent protein | Microcystin-dependent protein | | afdb-uniprot50 | AF-A0A1Y6CS75-F1-MODEL\_V4 | 1.0 | 4.545e-10 | 306 | 0.226 | 225 | 114 | 9 | 11 | 220 | 6 | 185 | Microcystin-dependent protein | Microcystin-dependent protein | | afdb-uniprot50 | AF-A0A3D6BMR4-F1-MODEL\_V4 | 1.0 | 3.54e-11 | 306 | 0.29 | 231 | 136 | 7 | 1 | 221 | 4 | 216 | Collar domain-containing protein | Collar domain-containing protein | | afdb-uniprot50 | AF-A0A1Y4GXK4-F1-MODEL\_V4 | 1.0 | 7.658e-11 | 306 | 0.289 | 211 | 89 | 6 | 11 | 221 | 83 | 232 | Collar domain-containing protein | Collar domain-containing protein | | afdb-uniprot50 | AF-A0A5C1ACN0-F1-MODEL\_V4 | 1.0 | 8.229e-10 | 306 | 0.261 | 252 | 102 | 6 | 2 | 221 | 337 | 536 | Uncharacterized protein | Uncharacterized protein | | afdb-uniprot50 | AF-A0A371WXZ3-F1-MODEL\_V4 | 1.0 | 4.489e-11 | 305 | 0.276 | 213 | 124 | 8 | 11 | 220 | 4 | 189 | Phage tail protein | Phage tail protein | | afdb-uniprot50 | AF-A0A816TS03-F1-MODEL\_V4 | 1.0 | 1.369e-11 | 305 | 0.289 | 228 | 118 | 10 | 11 | 220 | 427 | 628 | Hypothetical protein | Hypothetical protein | | afdb-uniprot50 | AF-A0A818AVD1-F1-MODEL\_V4 | 1.0 | 1.715e-12 | 305 | 0.277 | 220 | 127 | 9 | 11 | 220 | 494 | 691 | Hypothetical protein | Hypothetical protein | | afdb-uniprot50 | AF-A0A3S0R516-F1-MODEL\_V4 | 1.0 | 1.657e-10 | 304 | 0.269 | 223 | 127 | 7 | 11 | 220 | 5 | 204 | Phage tail protein | Phage tail protein | | afdb-uniprot50 | AF-A0A835XPQ2-F1-MODEL\_V4 | 1.0 | 2.336e-11 | 304 | 0.318 | 220 | 116 | 7 | 3 | 220 | 440 | 627 | Uncharacterized protein | Uncharacterized protein | | afdb-uniprot50 | AF-A0A397M176-F1-MODEL\_V4 | 1.0 | 4.545e-10 | 303 | 0.265 | 211 | 111 | 6 | 11 | 220 | 5 | 172 | Microcystin-dependent protein | Microcystin-dependent protein | | afdb-uniprot50 | AF-A0A7W0BUS8-F1-MODEL\_V4 | 1.0 | 3.038e-09 | 302 | 0.247 | 210 | 109 | 5 | 11 | 220 | 5 | 165 | Microcystin-dependent protein | Microcystin-dependent protein | | afdb-uniprot50 | AF-G8THK5-F1-MODEL\_V4 | 1.0 | 8.733e-10 | 302 | 0.281 | 213 | 101 | 7 | 12 | 220 | 5 | 169 | Tail Collar domain protein | Tail Collar domain protein | | afdb-uniprot50 | AF-A0A5N1IZX8-F1-MODEL\_V4 | 1.0 | 1.404e-09 | 301 | 0.278 | 212 | 108 | 5 | 11 | 220 | 4 | 172 | Phage tail protein | Phage tail protein | | afdb-uniprot50 | AF-A0A4Q3L744-F1-MODEL\_V4 | 1.0 | 1.323e-09 | 301 | 0.294 | 214 | 115 | 9 | 11 | 220 | 4 | 185 | Phage tail protein | Phage tail protein | | afdb-uniprot50 | AF-A0A3B8I8V5-F1-MODEL\_V4 | 1.0 | 7.308e-10 | 301 | 0.27 | 218 | 121 | 8 | 10 | 220 | 3 | 189 | Phage tail protein | Phage tail protein | | afdb-uniprot50 | AF-A0A7K1Y201-F1-MODEL\_V4 | 1.0 | 6.116e-10 | 301 | 0.262 | 217 | 125 | 9 | 11 | 220 | 4 | 192 | Phage tail protein | Phage tail protein | | afdb-uniprot50 | AF-G3ISC7-F1-MODEL\_V4 | 1.0 | 9.711e-11 | 301 | 0.296 | 229 | 123 | 9 | 11 | 220 | 5 | 214 | Tail Collar domain protein | Tail Collar domain protein | | afdb-uniprot50 | AF-A0A6A8A4D1-F1-MODEL\_V4 | 1.0 | 2.101e-10 | 301 | 0.244 | 237 | 136 | 11 | 11 | 220 | 70 | 290 | Collar domain-containing protein | Collar domain-containing protein | | afdb-uniprot50 | AF-A0A2E0J1D8-F1-MODEL\_V4 | 1.0 | 1.758e-10 | 301 | 0.253 | 221 | 87 | 5 | 11 | 221 | 215 | 367 | Collar domain-containing protein | Collar domain-containing protein | | afdb-uniprot50 | AF-A0A4R6FF43-F1-MODEL\_V4 | 1.0 | 2.127e-09 | 300 | 0.275 | 214 | 100 | 7 | 10 | 220 | 3 | 164 | Microcystin-dependent protein | Microcystin-dependent protein | | afdb-uniprot50 | AF-A0A6L5E183-F1-MODEL\_V4 | 1.0 | 1.247e-09 | 300 | 0.274 | 215 | 130 | 6 | 12 | 221 | 5 | 198 | Phage tail protein | Phage tail protein | | afdb-uniprot50 | AF-A0A2D8SKJ2-F1-MODEL\_V4 | 1.0 | 1.561e-10 | 300 | 0.281 | 227 | 116 | 9 | 12 | 220 | 5 | 202 | Phage tail protein | Phage tail protein | | afdb-uniprot50 | AF-A0A7Z3TTQ5-F1-MODEL\_V4 | 1.0 | 1.561e-10 | 300 | 0.202 | 237 | 148 | 8 | 11 | 220 | 29 | 251 | Tail fiber protein | Tail fiber protein | | afdb-uniprot50 | AF-A0A2P8G260-F1-MODEL\_V4 | 1.0 | 1.49e-09 | 299 | 0.271 | 210 | 100 | 5 | 11 | 220 | 4 | 160 | Microcystin-dependent protein | Microcystin-dependent protein | | afdb-uniprot50 | AF-A0A3B1BHP0-F1-MODEL\_V4 | 1.0 | 2.229e-10 | 299 | 0.298 | 211 | 117 | 8 | 11 | 220 | 5 | 185 | Microcystin dependent protein | Microcystin dependent protein | | afdb-uniprot50 | AF-A0A0U3HN04-F1-MODEL\_V4 | 1.0 | 5.763e-10 | 299 | 0.226 | 212 | 142 | 6 | 11 | 221 | 6 | 196 | Collar domain-containing protein | Collar domain-containing protein | | afdb-uniprot50 | AF-A0A522BAB4-F1-MODEL\_V4 | 1.0 | 8.229e-10 | 299 | 0.363 | 212 | 72 | 7 | 12 | 221 | 99 | 249 | Collar domain-containing protein | Collar domain-containing protein | | afdb-uniprot50 | AF-A0A157ZM93-F1-MODEL\_V4 | 1.0 | 6.409e-11 | 299 | 0.282 | 237 | 110 | 8 | 1 | 221 | 76 | 268 | YadA\_stalk domain-containing protein | YadA\_stalk domain-containing protein | | afdb-uniprot50 | AF-A0A817VYB8-F1-MODEL\_V4 | 1.0 | 1.471e-10 | 299 | 0.266 | 221 | 124 | 9 | 1 | 220 | 252 | 435 | Hypothetical protein | Hypothetical protein | | afdb-uniprot50 | AF-A0A815HLR0-F1-MODEL\_V4 | 1.0 | 1.616e-12 | 299 | 0.303 | 237 | 128 | 9 | 2 | 221 | 417 | 633 | Hypothetical protein | Hypothetical protein | | afdb-uniprot50 | AF-A0A286G5D7-F1-MODEL\_V4 | 1.0 | 6.49e-10 | 298 | 0.266 | 210 | 108 | 7 | 11 | 220 | 4 | 167 | Microcystin-dependent protein | Microcystin-dependent protein | | afdb-uniprot50 | AF-A0A6N8HFD9-F1-MODEL\_V4 | 1.0 | 1.581e-09 | 298 | 0.254 | 212 | 113 | 7 | 10 | 220 | 4 | 171 | Phage tail protein | Phage tail protein | | afdb-uniprot50 | AF-A0A2P8D2J1-F1-MODEL\_V4 | 1.0 | 1.49e-09 | 298 | 0.292 | 212 | 108 | 5 | 10 | 220 | 3 | 173 | Microcystin-dependent protein | Microcystin-dependent protein | | afdb-uniprot50 | AF-A0A173M9U9-F1-MODEL\_V4 | 1.0 | 1.231e-10 | 298 | 0.278 | 219 | 117 | 10 | 12 | 220 | 5 | 192 | Microcystin-dependent protein | Microcystin-dependent protein | | afdb-uniprot50 | AF-A0A1B9VN74-F1-MODEL\_V4 | 1.0 | 9.151e-11 | 298 | 0.229 | 235 | 137 | 9 | 11 | 220 | 33 | 248 | Tail Collar domain protein | Tail Collar domain protein | | afdb-uniprot50 | AF-A0A2E0M2L0-F1-MODEL\_V4 | 1.0 | 1.758e-10 | 298 | 0.282 | 251 | 124 | 8 | 9 | 220 | 18 | 251 | Collar domain-containing protein | Collar domain-containing protein | | afdb-uniprot50 | AF-A0A1V9FKC3-F1-MODEL\_V4 | 1.0 | 6.887e-10 | 297 | 0.251 | 211 | 113 | 7 | 12 | 220 | 6 | 173 | Collar domain-containing protein | Collar domain-containing protein | | afdb-uniprot50 | AF-A0A4Z0NDY8-F1-MODEL\_V4 | 1.0 | 2.229e-10 | 297 | 0.283 | 289 | 116 | 9 | 11 | 221 | 143 | 418 | Collar domain-containing protein | Collar domain-containing protein | | afdb-uniprot50 | AF-A0A7W4IK36-F1-MODEL\_V4 | 1.0 | 1.453e-11 | 296 | 0.424 | 205 | 98 | 6 | 18 | 220 | 1 | 187 | Collar domain-containing protein | Collar domain-containing protein | | afdb-uniprot50 | AF-A0A2P8D2H1-F1-MODEL\_V4 | 1.0 | 2.127e-09 | 295 | 0.267 | 217 | 105 | 7 | 10 | 220 | 3 | 171 | Microcystin-dependent protein | Microcystin-dependent protein | | afdb-uniprot50 | AF-A0A3N7CVV8-F1-MODEL\_V4 | 1.0 | 1.581e-09 | 295 | 0.255 | 219 | 110 | 8 | 11 | 220 | 5 | 179 | Phage tail protein | Phage tail protein | | afdb-uniprot50 | AF-A0A1M6T5E4-F1-MODEL\_V4 | 1.0 | 7.658e-11 | 295 | 0.288 | 225 | 130 | 6 | 11 | 220 | 4 | 213 | Microcystin-dependent protein | Microcystin-dependent protein | | afdb-uniprot50 | AF-A0A6L5FV64-F1-MODEL\_V4 | 1.0 | 1.842e-11 | 295 | 0.327 | 235 | 113 | 11 | 3 | 221 | 45 | 250 | Collar domain-containing protein | Collar domain-containing protein | | afdb-uniprot50 | AF-A0A6L8KW90-F1-MODEL\_V4 | 1.0 | 1.107e-09 | 295 | 0.234 | 247 | 138 | 10 | 11 | 220 | 34 | 266 | Phage tail protein | Phage tail protein | | afdb-uniprot50 | AF-A0A835XQ12-F1-MODEL\_V4 | 1.0 | 2.962e-11 | 295 | 0.273 | 227 | 117 | 11 | 3 | 221 | 223 | 409 | Uncharacterized protein | Uncharacterized protein | | afdb-uniprot50 | AF-A0A5R8ZI20-F1-MODEL\_V4 | 1.0 | 2.698e-09 | 294 | 0.261 | 214 | 102 | 7 | 11 | 220 | 4 | 165 | Phage tail protein | Phage tail protein | | afdb-uniprot50 | AF-A0A4Q0RK75-F1-MODEL\_V4 | 1.0 | 1.889e-09 | 294 | 0.256 | 218 | 102 | 9 | 11 | 220 | 4 | 169 | Phage tail protein | Phage tail protein | | afdb-uniprot50 | AF-A0A1I1NQ92-F1-MODEL\_V4 | 1.0 | 3.804e-10 | 294 | 0.282 | 216 | 117 | 10 | 11 | 220 | 4 | 187 | Microcystin-dependent protein | Microcystin-dependent protein | | afdb-uniprot50 | AF-A0A521AUE6-F1-MODEL\_V4 | 1.0 | 1.107e-09 | 294 | 0.273 | 216 | 136 | 8 | 10 | 220 | 3 | 202 | Microcystin-dependent protein | Microcystin-dependent protein | | afdb-uniprot50 | AF-A0A1G8MR82-F1-MODEL\_V4 | 1.0 | 8.624e-11 | 294 | 0.282 | 258 | 129 | 9 | 3 | 221 | 28 | 268 | Phage Tail Collar Domain | Phage Tail Collar Domain | | afdb-uniprot50 | AF-E5APE0-F1-MODEL\_V4 | 1.0 | 6.49e-10 | 294 | 0.318 | 226 | 102 | 7 | 2 | 220 | 161 | 341 | PHAGE TAIL PROTEIN | PHAGE TAIL PROTEIN | | afdb-uniprot50 | AF-A0A1I7LHC6-F1-MODEL\_V4 | 1.0 | 2.51e-10 | 294 | 0.293 | 279 | 121 | 12 | 11 | 221 | 143 | 413 | Microcystin-dependent protein | Microcystin-dependent protein | | afdb-uniprot50 | AF-A0A819YKS0-F1-MODEL\_V4 | 1.0 | 2.792e-11 | 293 | 0.253 | 225 | 126 | 9 | 11 | 220 | 6 | 203 | Hypothetical protein | Hypothetical protein | | afdb-uniprot50 | AF-A0A2E0ZP65-F1-MODEL\_V4 | 1.0 | 2.732e-08 | 292 | 0.566 | 90 | 33 | 1 | 2 | 85 | 7 | 96 | Collar domain-containing protein | Collar domain-containing protein | | afdb-uniprot50 | AF-A0A7W7Z7K7-F1-MODEL\_V4 | 1.0 | 1.889e-09 | 292 | 0.251 | 211 | 108 | 7 | 11 | 220 | 5 | 166 | Microcystin-dependent protein | Microcystin-dependent protein | | afdb-uniprot50 | AF-A0A1V9FKH6-F1-MODEL\_V4 | 1.0 | 5.183e-09 | 292 | 0.243 | 218 | 110 | 8 | 10 | 221 | 3 | 171 | Collar domain-containing protein | Collar domain-containing protein | | afdb-uniprot50 | AF-A0A6C0DW18-F1-MODEL\_V4 | 1.0 | 5.763e-10 | 292 | 0.28 | 214 | 88 | 7 | 11 | 220 | 35 | 186 | Collar domain-containing protein | Collar domain-containing protein | | afdb-uniprot50 | AF-A0A7C3YVS4-F1-MODEL\_V4 | 1.0 | 1.231e-10 | 292 | 0.276 | 210 | 88 | 9 | 12 | 221 | 47 | 192 | Collar domain-containing protein | Collar domain-containing protein | | afdb-uniprot50 | AF-A0A7H9DPU9-F1-MODEL\_V4 | 1.0 | 1.49e-09 | 292 | 0.278 | 219 | 111 | 7 | 11 | 220 | 5 | 185 | Phage tail protein | Phage tail protein | | afdb-uniprot50 | AF-A0A1Q3GJM7-F1-MODEL\_V4 | 1.0 | 6.49e-10 | 292 | 0.274 | 226 | 110 | 10 | 10 | 220 | 3 | 189 | Collar domain-containing protein | Collar domain-containing protein | | afdb-uniprot50 | AF-A0A3T0W1B7-F1-MODEL\_V4 | 1.0 | 6.887e-10 | 292 | 0.235 | 217 | 133 | 8 | 11 | 220 | 6 | 196 | Phage tail protein | Phage tail protein | | afdb-uniprot50 | AF-A0A521AUD9-F1-MODEL\_V4 | 1.0 | 4.036e-10 | 292 | 0.281 | 224 | 127 | 9 | 10 | 220 | 4 | 206 | Microcystin-dependent protein | Microcystin-dependent protein | | afdb-uniprot50 | AF-A0A850KVP1-F1-MODEL\_V4 | 1.0 | 2.366e-10 | 292 | 0.246 | 215 | 117 | 9 | 11 | 220 | 38 | 212 | Tail fiber protein | Tail fiber protein | | afdb-uniprot50 | AF-A0A821ML04-F1-MODEL\_V4 | 1.0 | 3.804e-10 | 292 | 0.254 | 212 | 110 | 10 | 11 | 220 | 195 | 360 | Hypothetical protein | Hypothetical protein | | afdb-uniprot50 | AF-A0A1V2H5U5-F1-MODEL\_V4 | 1.0 | 1.561e-10 | 292 | 0.319 | 213 | 118 | 9 | 12 | 220 | 213 | 402 | Collar domain-containing protein | Collar domain-containing protein | | afdb-uniprot50 | AF-A0A1E4J0W6-F1-MODEL\_V4 | 1.0 | 1.352e-12 | 291 | 0.344 | 247 | 121 | 9 | 1 | 221 | 106 | 337 | Collar domain-containing protein | Collar domain-containing protein | | afdb-uniprot50 | AF-A0A2E5YUW9-F1-MODEL\_V4 | 1.0 | 1.369e-11 | 291 | 0.265 | 241 | 124 | 5 | 11 | 221 | 148 | 365 | Collar domain-containing protein | Collar domain-containing protein | | afdb-uniprot50 | AF-A0A258K539-F1-MODEL\_V4 | 1.0 | 2.396e-09 | 290 | 0.25 | 220 | 103 | 7 | 11 | 221 | 4 | 170 | Collar domain-containing protein | Collar domain-containing protein | | afdb-uniprot50 | AF-A0A1T4L5J9-F1-MODEL\_V4 | 1.0 | 2.258e-09 | 290 | 0.279 | 215 | 102 | 7 | 11 | 220 | 5 | 171 | Microcystin-dependent protein | Microcystin-dependent protein | | afdb-uniprot50 | AF-A0A6L9IQ28-F1-MODEL\_V4 | 1.0 | 2.366e-10 | 290 | 0.276 | 213 | 117 | 9 | 11 | 220 | 21 | 199 | Phage tail protein | Phage tail protein | | afdb-uniprot50 | AF-A0A3N6N9Q9-F1-MODEL\_V4 | 1.0 | 3e-10 | 290 | 0.259 | 216 | 105 | 7 | 9 | 220 | 133 | 297 | Collar domain-containing protein | Collar domain-containing protein | | afdb-uniprot50 | AF-A6EAC0-F1-MODEL\_V4 | 1.0 | 1.247e-09 | 289 | 0.252 | 214 | 115 | 6 | 11 | 220 | 4 | 176 | Microcystin-dependent protein | Microcystin-dependent protein | | afdb-uniprot50 | AF-A0A7U0PKD0-F1-MODEL\_V4 | 1.0 | 6.801e-11 | 289 | 0.272 | 224 | 127 | 11 | 11 | 220 | 5 | 206 | Phage tail protein | Phage tail protein | | afdb-uniprot50 | AF-A0A5C7MBK6-F1-MODEL\_V4 | 1.0 | 2.51e-10 | 289 | 0.279 | 236 | 99 | 9 | 12 | 221 | 276 | 466 | Uncharacterized protein | Uncharacterized protein | | afdb-uniprot50 | AF-A0A1H7BRU9-F1-MODEL\_V4 | 1.0 | 1.043e-09 | 288 | 0.271 | 217 | 102 | 9 | 11 | 220 | 4 | 171 | Microcystin-dependent protein | Microcystin-dependent protein | | afdb-uniprot50 | AF-A0A1G4QLN4-F1-MODEL\_V4 | 1.0 | 1.107e-09 | 288 | 0.276 | 210 | 116 | 7 | 11 | 220 | 4 | 177 | Microcystin-dependent protein | Microcystin-dependent protein | | afdb-uniprot50 | AF-A0A7W9ZEX5-F1-MODEL\_V4 | 1.0 | 1.49e-09 | 288 | 0.269 | 215 | 111 | 10 | 11 | 220 | 4 | 177 | Microcystin-dependent protein | Microcystin-dependent protein | | afdb-uniprot50 | AF-F0SE37-F1-MODEL\_V4 | 1.0 | 1.107e-09 | 288 | 0.263 | 224 | 118 | 8 | 11 | 221 | 4 | 193 | Tail Collar domain protein | Tail Collar domain protein | | afdb-uniprot50 | AF-A0A1G6Y0D3-F1-MODEL\_V4 | 1.0 | 1.758e-10 | 288 | 0.207 | 212 | 130 | 7 | 11 | 220 | 40 | 215 | Microcystin-dependent protein | Microcystin-dependent protein | | afdb-uniprot50 | AF-A0A1Y4GLN6-F1-MODEL\_V4 | 1.0 | 1.458e-06 | 287 | 0.538 | 65 | 30 | 0 | 12 | 76 | 25 | 89 | Collar domain-containing protein | Collar domain-containing protein | | afdb-uniprot50 | AF-D8GPZ1-F1-MODEL\_V4 | 1.0 | 7.755e-10 | 287 | 0.274 | 222 | 137 | 6 | 11 | 220 | 5 | 214 | Collar domain-containing protein | Collar domain-containing protein | | afdb-uniprot50 | AF-K8GNE3-F1-MODEL\_V4 | 1.0 | 8.516e-12 | 287 | 0.334 | 254 | 118 | 8 | 1 | 221 | 468 | 703 | Phage tail collar family protein | Phage tail collar family protein | | afdb-uniprot50 | AF-A0A2C6C321-F1-MODEL\_V4 | 1.0 | 1.175e-09 | 286 | 0.24 | 212 | 121 | 7 | 10 | 220 | 3 | 175 | Phage tail protein | Phage tail protein | | afdb-uniprot50 | AF-A0A444WG53-F1-MODEL\_V4 | 1.0 | 4.087e-09 | 286 | 0.291 | 213 | 107 | 5 | 11 | 220 | 5 | 176 | Tail Collar domain protein | Tail Collar domain protein | | afdb-uniprot50 | AF-A0A7T8SK15-F1-MODEL\_V4 | 1.0 | 8.229e-10 | 286 | 0.273 | 216 | 107 | 9 | 12 | 220 | 9 | 181 | Tail fiber protein | Tail fiber protein | | afdb-uniprot50 | AF-A0A7Y4RAK2-F1-MODEL\_V4 | 1.0 | 9.711e-11 | 286 | 0.292 | 239 | 141 | 8 | 2 | 221 | 64 | 293 | Tail fiber protein | Tail fiber protein | | afdb-uniprot50 | AF-A0A222EY63-F1-MODEL\_V4 | 1.0 | 1.889e-09 | 285 | 0.289 | 211 | 79 | 6 | 11 | 221 | 21 | 160 | Collar domain-containing protein | Collar domain-containing protein | | afdb-uniprot50 | AF-A0A817V4J7-F1-MODEL\_V4 | 1.0 | 1.107e-09 | 285 | 0.305 | 219 | 104 | 6 | 11 | 221 | 17 | 195 | Hypothetical protein | Hypothetical protein | | afdb-uniprot50 | AF-A0A7L4ZNP4-F1-MODEL\_V4 | 1.0 | 5.118e-10 | 285 | 0.254 | 224 | 112 | 9 | 2 | 220 | 21 | 194 | Collar domain-containing protein | Collar domain-containing protein | | afdb-uniprot50 | AF-A0A1F9IXH9-F1-MODEL\_V4 | 1.0 | 5.054e-11 | 285 | 0.33 | 230 | 84 | 6 | 2 | 221 | 48 | 217 | Collar domain-containing protein | Collar domain-containing protein | | afdb-uniprot50 | AF-A0A222FDY7-F1-MODEL\_V4 | 1.0 | 2.542e-09 | 285 | 0.223 | 224 | 136 | 8 | 11 | 220 | 29 | 228 | Phage tail protein | Phage tail protein | | afdb-uniprot50 | AF-A0A833JCH6-F1-MODEL\_V4 | 1.0 | 2.51e-10 | 285 | 0.235 | 259 | 146 | 12 | 2 | 221 | 68 | 313 | Uncharacterized protein | Uncharacterized protein | | afdb-uniprot50 | AF-A0A2U2BCA2-F1-MODEL\_V4 | 1.0 | 3.585e-10 | 284 | 0.278 | 233 | 118 | 7 | 2 | 220 | 24 | 220 | Phage tail protein | Phage tail protein | | afdb-uniprot50 | AF-A0A1H8W0U3-F1-MODEL\_V4 | 1.0 | 6.193e-09 | 283 | 0.235 | 212 | 111 | 6 | 10 | 220 | 3 | 164 | Microcystin-dependent protein | Microcystin-dependent protein | | afdb-uniprot50 | AF-A0A519S7T0-F1-MODEL\_V4 | 1.0 | 7.4e-09 | 283 | 0.257 | 214 | 105 | 10 | 11 | 220 | 4 | 167 | Phage tail protein | Phage tail protein | | afdb-uniprot50 | AF-A0A3S1AVP9-F1-MODEL\_V4 | 1.0 | 2.127e-09 | 283 | 0.265 | 222 | 115 | 6 | 11 | 220 | 5 | 190 | Phage tail protein | Phage tail protein | | afdb-uniprot50 | AF-A0A3S9QK74-F1-MODEL\_V4 | 1.0 | 1.03e-10 | 283 | 0.252 | 214 | 111 | 10 | 11 | 221 | 136 | 303 | Collar domain-containing protein | Collar domain-containing protein | | afdb-uniprot50 | AF-A0A1I5JPD8-F1-MODEL\_V4 | 1.0 | 2.258e-09 | 283 | 0.28 | 214 | 123 | 7 | 12 | 220 | 171 | 358 | Uncharacterized protein | Uncharacterized protein | | afdb-uniprot50 | AF-A0A2N9X676-F1-MODEL\_V4 | 1.0 | 1.657e-10 | 283 | 0.281 | 213 | 119 | 8 | 11 | 221 | 245 | 425 | Collar domain-containing protein | Collar domain-containing protein | | afdb-uniprot50 | AF-A0A848FSQ8-F1-MODEL\_V4 | 1.0 | 5.5e-09 | 282 | 0.24 | 212 | 114 | 5 | 12 | 220 | 5 | 172 | Phage tail protein | Phage tail protein | | afdb-uniprot50 | AF-A0A4Q7N378-F1-MODEL\_V4 | 1.0 | 3.63e-09 | 282 | 0.267 | 217 | 106 | 7 | 10 | 220 | 3 | 172 | Microcystin-dependent protein | Microcystin-dependent protein | | afdb-uniprot50 | AF-A0A2E3FMJ4-F1-MODEL\_V4 | 1.0 | 3.378e-10 | 282 | 0.328 | 210 | 102 | 8 | 11 | 220 | 504 | 674 | Collar domain-containing protein | Collar domain-containing protein | | afdb-uniprot50 | AF-A0A7S7U734-F1-MODEL\_V4 | 1.0 | 8.843e-09 | 281 | 0.255 | 215 | 102 | 9 | 11 | 220 | 5 | 166 | Phage tail protein | Phage tail protein | | afdb-uniprot50 | AF-A0A521AU55-F1-MODEL\_V4 | 1.0 | 3.183e-10 | 281 | 0.252 | 222 | 131 | 10 | 11 | 220 | 13 | 211 | Microcystin-dependent protein | Microcystin-dependent protein | | afdb-uniprot50 | AF-A0A348AH31-F1-MODEL\_V4 | 1.0 | 7.4e-09 | 280 | 0.284 | 211 | 103 | 7 | 11 | 220 | 4 | 167 | Phage Tail Collar Domain protein | Phage Tail Collar Domain protein | | afdb-uniprot50 | AF-A0A1C2GP00-F1-MODEL\_V4 | 1.0 | 9.834e-10 | 280 | 0.248 | 213 | 120 | 9 | 11 | 220 | 5 | 180 | Collar domain-containing protein | Collar domain-containing protein | | afdb-uniprot50 | AF-F0SE36-F1-MODEL\_V4 | 1.0 | 6.887e-10 | 280 | 0.297 | 222 | 110 | 9 | 11 | 220 | 5 | 192 | Tail Collar domain protein | Tail Collar domain protein | | afdb-uniprot50 | AF-F8GEA0-F1-MODEL\_V4 | 1.0 | 4.087e-09 | 280 | 0.227 | 229 | 130 | 7 | 11 | 220 | 69 | 269 | Tail Collar domain protein | Tail Collar domain protein | | afdb-uniprot50 | AF-A0A2T9K1M7-F1-MODEL\_V4 | 1.0 | 1.107e-09 | 279 | 0.281 | 224 | 116 | 8 | 11 | 220 | 4 | 196 | Phage tail protein | Phage tail protein | | afdb-uniprot50 | AF-A0A3G8WSK6-F1-MODEL\_V4 | 1.0 | 3.421e-09 | 279 | 0.266 | 225 | 120 | 10 | 10 | 220 | 3 | 196 | Phage tail protein | Phage tail protein | | afdb-uniprot50 | AF-A0A2E4T1Y0-F1-MODEL\_V4 | 1.0 | 8.516e-12 | 279 | 0.295 | 230 | 115 | 9 | 11 | 221 | 118 | 319 | Collar domain-containing protein | Collar domain-containing protein | | afdb-uniprot50 | AF-A0A818V6Z8-F1-MODEL\_V4 | 1.0 | 4.823e-10 | 279 | 0.269 | 223 | 128 | 10 | 11 | 220 | 389 | 589 | Hypothetical protein | Hypothetical protein | | afdb-uniprot50 | AF-A0A1J0WLQ5-F1-MODEL\_V4 | 1.0 | 2.698e-09 | 278 | 0.248 | 217 | 108 | 9 | 12 | 220 | 14 | 183 | Collar domain-containing protein | Collar domain-containing protein | | afdb-uniprot50 | AF-A0A818YA82-F1-MODEL\_V4 | 1.0 | 5.118e-10 | 277 | 0.235 | 225 | 135 | 9 | 11 | 220 | 98 | 300 | Hypothetical protein | Hypothetical protein | | afdb-uniprot50 | AF-A0A1F3K302-F1-MODEL\_V4 | 1.0 | 5.763e-10 | 276 | 0.279 | 222 | 124 | 8 | 10 | 220 | 3 | 199 | Collar domain-containing protein | Collar domain-containing protein | | afdb-uniprot50 | AF-W6RSW9-F1-MODEL\_V4 | 1.0 | 3.144e-11 | 276 | 0.282 | 244 | 130 | 11 | 5 | 221 | 36 | 261 | Putative tail fiber protein | Putative tail fiber protein | | afdb-uniprot50 | AF-A0A7H1JCB2-F1-MODEL\_V4 | 1.0 | 1.98e-10 | 276 | 0.252 | 242 | 124 | 9 | 11 | 220 | 41 | 257 | Tail fiber protein | Tail fiber protein | | afdb-uniprot50 | AF-A0A553JK32-F1-MODEL\_V4 | 1.0 | 1.247e-09 | 276 | 0.316 | 224 | 108 | 6 | 2 | 220 | 297 | 480 | Collar domain-containing protein | Collar domain-containing protein | | afdb-uniprot50 | AF-A0A7C8AIS3-F1-MODEL\_V4 | 1.0 | 1.404e-09 | 275 | 0.242 | 231 | 113 | 8 | 1 | 221 | 164 | 342 | Collar domain-containing protein | Collar domain-containing protein | | afdb-uniprot50 | AF-A0A849EVQ5-F1-MODEL\_V4 | 1.0 | 3.421e-09 | 274 | 0.271 | 221 | 111 | 9 | 11 | 220 | 6 | 187 | Phage tail protein | Phage tail protein | | afdb-uniprot50 | AF-A0A7W6LK96-F1-MODEL\_V4 | 1.0 | 6.887e-10 | 274 | 0.2 | 235 | 148 | 8 | 11 | 220 | 41 | 260 | Microcystin-dependent protein | Microcystin-dependent protein | | afdb-uniprot50 | AF-A0A423L7N4-F1-MODEL\_V4 | 1.0 | 9.037e-12 | 274 | 0.318 | 226 | 119 | 9 | 12 | 221 | 176 | 382 | Collar domain-containing protein | Collar domain-containing protein | | afdb-uniprot50 | AF-A0A5B8XJ48-F1-MODEL\_V4 | 1.0 | 1.889e-09 | 274 | 0.301 | 212 | 79 | 5 | 11 | 220 | 433 | 577 | Phage tail collar domain protein | Phage tail collar domain protein | | afdb-uniprot50 | AF-A0A395JR72-F1-MODEL\_V4 | 1.0 | 2.863e-09 | 273 | 0.282 | 223 | 108 | 9 | 11 | 221 | 5 | 187 | Phage tail protein | Phage tail protein | | afdb-uniprot50 | AF-A0A3B7MPY4-F1-MODEL\_V4 | 1.0 | 4.884e-09 | 272 | 0.287 | 216 | 99 | 7 | 12 | 220 | 5 | 172 | Phage tail protein | Phage tail protein | | afdb-uniprot50 | AF-A0A2S5DKY5-F1-MODEL\_V4 | 1.0 | 2.315e-07 | 272 | 0.45 | 100 | 40 | 3 | 9 | 106 | 23 | 109 | Phage tail protein | Phage tail protein | | afdb-uniprot50 | AF-A0A359L8X4-F1-MODEL\_V4 | 1.0 | 2.698e-09 | 272 | 0.259 | 231 | 115 | 11 | 11 | 220 | 4 | 199 | Phage tail protein | Phage tail protein | | afdb-uniprot50 | AF-A0A0Q6ZRG1-F1-MODEL\_V4 | 1.0 | 4.823e-10 | 272 | 0.273 | 223 | 112 | 10 | 1 | 221 | 156 | 330 | Collar domain-containing protein | Collar domain-containing protein | | afdb-uniprot50 | AF-A0A6N1X655-F1-MODEL\_V4 | 1.0 | 1.657e-10 | 272 | 0.293 | 215 | 120 | 9 | 11 | 221 | 201 | 387 | Tail fiber protein | Tail fiber protein | | afdb-uniprot50 | AF-A0A2W5K0J5-F1-MODEL\_V4 | 1.0 | 1.453e-11 | 272 | 0.325 | 227 | 116 | 6 | 9 | 221 | 228 | 431 | Collar domain-containing protein | Collar domain-containing protein | | afdb-uniprot50 | AF-A0A152A3A6-F1-MODEL\_V4 | 1.0 | 3.852e-09 | 272 | 0.247 | 214 | 120 | 6 | 10 | 221 | 442 | 616 | Collar domain-containing protein | Collar domain-containing protein | | afdb-uniprot50 | AF-A0A1H2GA33-F1-MODEL\_V4 | 1.0 | 4.036e-10 | 271 | 0.261 | 237 | 110 | 9 | 11 | 220 | 34 | 232 | Microcystin-dependent protein | Microcystin-dependent protein | | afdb-uniprot50 | AF-A0A1W2F866-F1-MODEL\_V4 | 1.0 | 2.664e-10 | 271 | 0.339 | 224 | 87 | 8 | 1 | 221 | 123 | 288 | Phage Tail Collar Domain | Phage Tail Collar Domain | | afdb-uniprot50 | AF-L0H2G4-F1-MODEL\_V4 | 1.0 | 8.843e-09 | 270 | 0.245 | 216 | 111 | 8 | 11 | 220 | 6 | 175 | Microcystin-dependent protein | Microcystin-dependent protein | | afdb-uniprot50 | AF-A0A2D3RDN9-F1-MODEL\_V4 | 1.0 | 3.038e-09 | 270 | 0.284 | 211 | 102 | 8 | 11 | 220 | 32 | 194 | Collar domain-containing protein | Collar domain-containing protein | | afdb-uniprot50 | AF-A0A0M9DM99-F1-MODEL\_V4 | 1.0 | 7.755e-10 | 270 | 0.261 | 241 | 124 | 9 | 11 | 220 | 4 | 221 | Phage tail collar protein | Phage tail collar protein | | afdb-uniprot50 | AF-A0A178MM09-F1-MODEL\_V4 | 1.0 | 5.763e-10 | 270 | 0.224 | 218 | 136 | 7 | 11 | 220 | 31 | 223 | Collar domain-containing protein | Collar domain-containing protein | | afdb-uniprot50 | AF-A0A839IKX9-F1-MODEL\_V4 | 1.0 | 7.4e-09 | 269 | 0.213 | 220 | 125 | 5 | 11 | 220 | 4 | 185 | Tail fiber protein | Tail fiber protein | | afdb-uniprot50 | AF-A0A1K1QMP3-F1-MODEL\_V4 | 1.0 | 1.34e-08 | 269 | 0.276 | 224 | 106 | 10 | 11 | 220 | 4 | 185 | Microcystin-dependent protein | Microcystin-dependent protein | | afdb-uniprot50 | AF-A0A4Z1BHZ3-F1-MODEL\_V4 | 1.0 | 6.572e-09 | 269 | 0.235 | 234 | 104 | 7 | 11 | 220 | 4 | 186 | Phage tail protein | Phage tail protein | | afdb-uniprot50 | AF-A0A2N3KBQ2-F1-MODEL\_V4 | 1.0 | 1.889e-09 | 269 | 0.261 | 218 | 128 | 7 | 10 | 220 | 3 | 194 | Collar domain-containing protein | Collar domain-containing protein | | afdb-uniprot50 | AF-A0A1H8GZ50-F1-MODEL\_V4 | 1.0 | 2.479e-11 | 269 | 0.268 | 235 | 123 | 11 | 11 | 220 | 4 | 214 | Microcystin-dependent protein | Microcystin-dependent protein | | afdb-uniprot50 | AF-A0A4R7BEN1-F1-MODEL\_V4 | 1.0 | 1.98e-10 | 269 | 0.327 | 214 | 102 | 9 | 12 | 221 | 241 | 416 | Microcystin-dependent protein | Microcystin-dependent protein | | afdb-uniprot50 | AF-A0A2N3T4D1-F1-MODEL\_V4 | 1.0 | 6.193e-09 | 268 | 0.262 | 213 | 117 | 8 | 11 | 220 | 4 | 179 | Microcystin-dependent protein | Microcystin-dependent protein | | afdb-uniprot50 | AF-A0A162JQP9-F1-MODEL\_V4 | 1.0 | 1.404e-09 | 268 | 0.24 | 225 | 115 | 9 | 11 | 220 | 5 | 188 | Collar domain-containing protein | Collar domain-containing protein | | afdb-uniprot50 | AF-A0A4Q5WXF2-F1-MODEL\_V4 | 1.0 | 4.545e-10 | 267 | 0.282 | 230 | 119 | 9 | 11 | 220 | 5 | 208 | Phage tail protein | Phage tail protein | | afdb-uniprot50 | AF-A0A212BGA2-F1-MODEL\_V4 | 1.0 | 2.366e-10 | 267 | 0.336 | 217 | 107 | 11 | 10 | 221 | 65 | 249 | Collar domain-containing protein | Collar domain-containing protein | | afdb-uniprot50 | AF-A0A4D8R397-F1-MODEL\_V4 | 1.0 | 1.175e-09 | 267 | 0.242 | 239 | 131 | 11 | 11 | 220 | 31 | 248 | Collar domain-containing protein | Collar domain-containing protein | | afdb-uniprot50 | AF-A0A800M671-F1-MODEL\_V4 | 1.0 | 1.78e-09 | 267 | 0.279 | 211 | 97 | 8 | 11 | 220 | 114 | 270 | Phage tail protein | Phage tail protein | | afdb-uniprot50 | AF-A0NQ95-F1-MODEL\_V4 | 1.0 | 1.387e-10 | 267 | 0.27 | 229 | 128 | 9 | 2 | 221 | 131 | 329 | Putative tail fiber-related protein | Putative tail fiber-related protein | | afdb-uniprot50 | AF-A0A844A272-F1-MODEL\_V4 | 1.0 | 1.889e-09 | 267 | 0.3 | 233 | 128 | 8 | 1 | 221 | 245 | 454 | Tail fiber protein | Tail fiber protein | | afdb-uniprot50 | AF-A0A4S2GXX0-F1-MODEL\_V4 | 1.0 | 5.836e-09 | 266 | 0.251 | 211 | 108 | 7 | 11 | 220 | 29 | 190 | Phage tail protein | Phage tail protein | | afdb-uniprot50 | AF-A0A177MPX3-F1-MODEL\_V4 | 1.0 | 5.836e-09 | 266 | 0.251 | 215 | 121 | 6 | 11 | 220 | 19 | 198 | Collar domain-containing protein | Collar domain-containing protein | | afdb-uniprot50 | AF-A0A2H0IRE9-F1-MODEL\_V4 | 1.0 | 4.087e-09 | 266 | 0.256 | 230 | 118 | 7 | 11 | 220 | 4 | 200 | Phage tail protein | Phage tail protein | | afdb-uniprot50 | AF-A0A845L736-F1-MODEL\_V4 | 1.0 | 6.887e-10 | 266 | 0.305 | 229 | 113 | 11 | 11 | 220 | 4 | 205 | Uncharacterized protein | Uncharacterized protein | | afdb-uniprot50 | AF-F7P1I1-F1-MODEL\_V4 | 1.0 | 1.509e-08 | 265 | 0.216 | 217 | 116 | 6 | 11 | 220 | 4 | 173 | Microcystin-dependent protein | Microcystin-dependent protein | | afdb-uniprot50 | AF-A0A2E4PG81-F1-MODEL\_V4 | 1.0 | 9.958e-09 | 265 | 0.229 | 218 | 112 | 8 | 11 | 220 | 6 | 175 | Phage tail protein | Phage tail protein | | afdb-uniprot50 | AF-A0A653P8Z9-F1-MODEL\_V4 | 1.0 | 9.834e-10 | 265 | 0.242 | 231 | 124 | 10 | 11 | 220 | 33 | 233 | Collar domain-containing protein | Collar domain-containing protein | | afdb-uniprot50 | AF-A0A2S1SFX4-F1-MODEL\_V4 | 1.0 | 7.853e-09 | 264 | 0.257 | 214 | 110 | 9 | 11 | 220 | 6 | 174 | Phage tail protein | Phage tail protein | | afdb-uniprot50 | AF-A0A4Q6AGP9-F1-MODEL\_V4 | 1.0 | 7.4e-09 | 264 | 0.255 | 215 | 112 | 6 | 10 | 220 | 3 | 173 | Phage tail protein | Phage tail protein | | afdb-uniprot50 | AF-A0A7H9DR54-F1-MODEL\_V4 | 1.0 | 2.005e-09 | 264 | 0.278 | 219 | 113 | 11 | 11 | 220 | 4 | 186 | Phage tail protein | Phage tail protein | | afdb-uniprot50 | AF-A0A1Y4GJI2-F1-MODEL\_V4 | 1.0 | 1.164e-05 | 263 | 0.516 | 60 | 29 | 0 | 12 | 71 | 23 | 82 | Collar domain-containing protein | Collar domain-containing protein | | afdb-uniprot50 | AF-A0A383RWZ2-F1-MODEL\_V4 | 1.0 | 3.223e-09 | 263 | 0.263 | 216 | 110 | 7 | 11 | 220 | 4 | 176 | Collar domain-containing protein | Collar domain-containing protein | | afdb-uniprot50 | AF-A0A291QWR7-F1-MODEL\_V4 | 1.0 | 4.603e-09 | 263 | 0.263 | 216 | 122 | 8 | 11 | 220 | 4 | 188 | Phage tail protein | Phage tail protein | | afdb-uniprot50 | AF-A0A0G4ICD0-F1-MODEL\_V4 | 1.0 | 2.396e-09 | 263 | 0.27 | 211 | 103 | 7 | 11 | 220 | 42 | 202 | Collar domain-containing protein | Collar domain-containing protein | | afdb-uniprot50 | AF-A0A7Y7EEW4-F1-MODEL\_V4 | 1.0 | 4.603e-09 | 263 | 0.217 | 234 | 140 | 13 | 11 | 220 | 7 | 221 | Tail fiber protein | Tail fiber protein | | afdb-uniprot50 | AF-A0A2Z4PXV1-F1-MODEL\_V4 | 1.0 | 1.78e-09 | 263 | 0.222 | 238 | 138 | 10 | 11 | 220 | 13 | 231 | Collar domain-containing protein | Collar domain-containing protein | | afdb-uniprot50 | AF-U1ID74-F1-MODEL\_V4 | 1.0 | 6.116e-10 | 263 | 0.287 | 216 | 126 | 7 | 12 | 220 | 98 | 292 | NitT/TauT family transport system permease | NitT/TauT family transport system permease | | afdb-uniprot50 | AF-A0A7W6ICU7-F1-MODEL\_V4 | 1.0 | 1.758e-10 | 263 | 0.266 | 233 | 118 | 9 | 11 | 220 | 358 | 560 | Microcystin-dependent protein | Microcystin-dependent protein | | afdb-uniprot50 | AF-A0A2K9MAN8-F1-MODEL\_V4 | 1.0 | 1.955e-11 | 262 | 0.321 | 233 | 131 | 7 | 1 | 221 | 4 | 221 | Collar domain-containing protein | Collar domain-containing protein | | afdb-uniprot50 | AF-A0A3D9L8W2-F1-MODEL\_V4 | 1.0 | 2.542e-09 | 262 | 0.246 | 227 | 135 | 11 | 11 | 220 | 5 | 212 | Microcystin-dependent protein | Microcystin-dependent protein | | afdb-uniprot50 | AF-A0A2L1HSI4-F1-MODEL\_V4 | 1.0 | 1.678e-09 | 262 | 0.256 | 234 | 125 | 11 | 1 | 221 | 151 | 348 | Collar domain-containing protein | Collar domain-containing protein | | afdb-uniprot50 | AF-A0A5B8UCR2-F1-MODEL\_V4 | 1.0 | 1.175e-09 | 261 | 0.311 | 231 | 89 | 11 | 3 | 220 | 35 | 208 | Tail fiber protein | Tail fiber protein | | afdb-uniprot50 | AF-A0A7V1GDM0-F1-MODEL\_V4 | 1.0 | 3.038e-09 | 261 | 0.267 | 232 | 134 | 10 | 11 | 220 | 6 | 223 | Phage tail protein | Phage tail protein | | afdb-uniprot50 | AF-A0A433BEE5-F1-MODEL\_V4 | 1.0 | 3.63e-09 | 260 | 0.233 | 214 | 109 | 8 | 11 | 220 | 13 | 175 | Collar domain-containing protein | Collar domain-containing protein | | afdb-uniprot50 | AF-A0A7T4YH49-F1-MODEL\_V4 | 1.0 | 1.175e-09 | 260 | 0.29 | 217 | 91 | 11 | 11 | 221 | 42 | 201 | Tail fiber protein | Tail fiber protein | | afdb-uniprot50 | AF-A0A0F9W1V5-F1-MODEL\_V4 | 1.0 | 2.396e-09 | 259 | 0.29 | 217 | 80 | 7 | 12 | 220 | 22 | 172 | Collar domain-containing protein | Collar domain-containing protein | | afdb-uniprot50 | AF-A0A6J4FWR0-F1-MODEL\_V4 | 1.0 | 4.337e-09 | 259 | 0.283 | 215 | 114 | 9 | 11 | 220 | 5 | 184 | Putative Microcystin dependent protein | Putative Microcystin dependent protein | | afdb-uniprot50 | AF-A0A1V4X328-F1-MODEL\_V4 | 1.0 | 2.51e-10 | 259 | 0.342 | 213 | 98 | 8 | 12 | 221 | 228 | 401 | Phage Tail Collar Domain protein | Phage Tail Collar Domain protein | | afdb-uniprot50 | AF-A0A6N1WZL5-F1-MODEL\_V4 | 1.0 | 4.823e-10 | 259 | 0.297 | 215 | 119 | 6 | 11 | 221 | 824 | 1010 | Tail fiber protein | Tail fiber protein | | afdb-uniprot50 | AF-A1AK23-F1-MODEL\_V4 | 1.0 | 7.4e-09 | 258 | 0.25 | 224 | 127 | 8 | 11 | 220 | 4 | 200 | Phage Tail Collar domain protein | Phage Tail Collar domain protein | | afdb-uniprot50 | AF-A0A848FK93-F1-MODEL\_V4 | 1.0 | 3.038e-09 | 258 | 0.235 | 212 | 123 | 7 | 11 | 220 | 29 | 203 | Phage tail protein | Phage tail protein | | afdb-uniprot50 | AF-A0A251WMF7-F1-MODEL\_V4 | 1.0 | 3.464e-08 | 257 | 0.242 | 214 | 88 | 8 | 11 | 220 | 5 | 148 | Collar domain-containing protein | Collar domain-containing protein | | afdb-uniprot50 | AF-L0H3J2-F1-MODEL\_V4 | 1.0 | 2.574e-08 | 257 | 0.267 | 213 | 108 | 12 | 11 | 220 | 5 | 172 | Microcystin-dependent protein | Microcystin-dependent protein | | afdb-uniprot50 | AF-A0A1V9FC14-F1-MODEL\_V4 | 1.0 | 6.193e-09 | 257 | 0.23 | 217 | 116 | 8 | 10 | 220 | 4 | 175 | Collar domain-containing protein | Collar domain-containing protein | | afdb-uniprot50 | AF-A0A351HL10-F1-MODEL\_V4 | 1.0 | 4.603e-09 | 257 | 0.266 | 218 | 133 | 8 | 10 | 220 | 3 | 200 | Phage tail protein | Phage tail protein | | afdb-uniprot50 | AF-A0A2D8AUD5-F1-MODEL\_V4 | 1.0 | 1.231e-10 | 257 | 0.263 | 224 | 121 | 8 | 12 | 221 | 24 | 217 | Collar domain-containing protein | Collar domain-containing protein | | afdb-uniprot50 | AF-A0A2E3CYM6-F1-MODEL\_V4 | 1.0 | 5.5e-09 | 257 | 0.265 | 211 | 129 | 6 | 11 | 220 | 34 | 219 | Phage tail protein | Phage tail protein | | afdb-uniprot50 | AF-A0A538RHD6-F1-MODEL\_V4 | 1.0 | 1.323e-09 | 257 | 0.296 | 219 | 114 | 10 | 11 | 221 | 56 | 242 | Collar domain-containing protein | Collar domain-containing protein | | afdb-uniprot50 | AF-A0A3E0WQ94-F1-MODEL\_V4 | 1.0 | 3.378e-10 | 257 | 0.274 | 237 | 116 | 12 | 1 | 221 | 279 | 475 | Collar domain-containing protein | Collar domain-containing protein | | afdb-uniprot50 | AF-A0A3M0CW52-F1-MODEL\_V4 | 1.0 | 1.34e-08 | 256 | 0.231 | 220 | 123 | 8 | 11 | 221 | 7 | 189 | Microcystin-dependent protein | Microcystin-dependent protein | | afdb-uniprot50 | AF-A0A090VAI2-F1-MODEL\_V4 | 1.0 | 1.121e-08 | 256 | 0.278 | 223 | 106 | 8 | 11 | 220 | 28 | 208 | Microcystin dependent protein | Microcystin dependent protein | | afdb-uniprot50 | AF-A0A1M3LS74-F1-MODEL\_V4 | 1.0 | 1.803e-08 | 255 | 0.26 | 211 | 115 | 9 | 11 | 220 | 5 | 175 | Collar domain-containing protein | Collar domain-containing protein | | afdb-uniprot50 | AF-A8HTM6-F1-MODEL\_V4 | 1.0 | 2.574e-08 | 255 | 0.225 | 235 | 125 | 10 | 12 | 221 | 148 | 350 | Microcystin-dependent protein | Microcystin-dependent protein | | afdb-uniprot50 | AF-A0A7Z6VNA8-F1-MODEL\_V4 | 1.0 | 5.183e-09 | 255 | 0.283 | 226 | 129 | 10 | 2 | 221 | 456 | 654 | Phage tail protein | Phage tail protein | | afdb-uniprot50 | AF-A0A2E1HKS8-F1-MODEL\_V4 | 1.0 | 3.464e-08 | 254 | 0.24 | 212 | 115 | 6 | 11 | 220 | 6 | 173 | Phage tail protein | Phage tail protein | | afdb-uniprot50 | AF-A0A7Z0I2P9-F1-MODEL\_V4 | 1.0 | 8.333e-09 | 254 | 0.234 | 213 | 122 | 6 | 11 | 220 | 40 | 214 | Tail fiber protein | Tail fiber protein | | afdb-uniprot50 | AF-A0A1J0EHY7-F1-MODEL\_V4 | 1.0 | 2.792e-11 | 254 | 0.33 | 227 | 115 | 9 | 12 | 221 | 131 | 337 | Collar domain-containing protein | Collar domain-containing protein | | afdb-uniprot50 | AF-A0A354WRB2-F1-MODEL\_V4 | 1.0 | 1.263e-08 | 253 | 0.285 | 210 | 87 | 6 | 12 | 221 | 22 | 168 | Collar domain-containing protein | Collar domain-containing protein | | afdb-uniprot50 | AF-A0A1P8X333-F1-MODEL\_V4 | 1.0 | 3.076e-08 | 253 | 0.248 | 217 | 108 | 8 | 11 | 220 | 6 | 174 | Collar domain-containing protein | Collar domain-containing protein | | afdb-uniprot50 | AF-A0A3N5E5J5-F1-MODEL\_V4 | 1.0 | 2.154e-08 | 253 | 0.254 | 216 | 117 | 9 | 11 | 220 | 5 | 182 | Phage tail protein | Phage tail protein | | afdb-uniprot50 | AF-A0A233RHW0-F1-MODEL\_V4 | 1.0 | 1.121e-08 | 253 | 0.229 | 222 | 128 | 10 | 11 | 220 | 5 | 195 | Collar domain-containing protein | Collar domain-containing protein | | afdb-uniprot50 | AF-A0A0U3F2K0-F1-MODEL\_V4 | 1.0 | 5.836e-09 | 253 | 0.269 | 215 | 98 | 9 | 11 | 220 | 43 | 203 | Collar domain-containing protein | Collar domain-containing protein | | afdb-uniprot50 | AF-A0A6N6VYM1-F1-MODEL\_V4 | 1.0 | 5.118e-10 | 253 | 0.277 | 245 | 144 | 8 | 2 | 221 | 45 | 281 | Collar domain-containing protein | Collar domain-containing protein | | afdb-uniprot50 | AF-B5JYG6-F1-MODEL\_V4 | 1.0 | 3.421e-09 | 253 | 0.26 | 234 | 124 | 10 | 1 | 220 | 200 | 398 | Phage Tail Collar Domain family | Phage Tail Collar Domain family | | afdb-uniprot50 | AF-A0A366FYJ6-F1-MODEL\_V4 | 1.0 | 1.247e-09 | 253 | 0.323 | 213 | 89 | 8 | 11 | 221 | 646 | 805 | Tail collar domain | Tail collar domain | | afdb-uniprot50 | AF-A0A1Y0FCL5-F1-MODEL\_V4 | 1.0 | 6.974e-09 | 252 | 0.251 | 223 | 110 | 7 | 3 | 221 | 24 | 193 | Collar domain-containing protein | Collar domain-containing protein | | afdb-uniprot50 | AF-I3TXM0-F1-MODEL\_V4 | 1.0 | 2.396e-09 | 252 | 0.256 | 222 | 115 | 10 | 11 | 220 | 39 | 222 | Tail Collar domain protein | Tail Collar domain protein | | afdb-uniprot50 | AF-A0A7C1MNT2-F1-MODEL\_V4 | 1.0 | 4.036e-10 | 252 | 0.302 | 225 | 119 | 11 | 11 | 220 | 68 | 269 | Tail fiber protein | Tail fiber protein | | afdb-uniprot50 | AF-A0A2H5E1S5-F1-MODEL\_V4 | 1.0 | 3.378e-10 | 252 | 0.301 | 219 | 126 | 6 | 5 | 221 | 218 | 411 | Collar domain-containing protein | Collar domain-containing protein | | afdb-uniprot50 | AF-A0A382GIC3-F1-MODEL\_V4 | 1.0 | 1.323e-09 | 251 | 0.278 | 223 | 93 | 9 | 8 | 221 | 29 | 192 | Collar domain-containing protein | Collar domain-containing protein | | afdb-uniprot50 | AF-A0A4R1A3G5-F1-MODEL\_V4 | 1.0 | 2.542e-09 | 251 | 0.244 | 237 | 121 | 8 | 2 | 220 | 19 | 215 | Phage tail protein | Phage tail protein | | afdb-uniprot50 | AF-N9TWJ4-F1-MODEL\_V4 | 1.0 | 3.223e-09 | 251 | 0.269 | 223 | 124 | 10 | 11 | 220 | 29 | 225 | Tail collar domain-containing protein | Tail collar domain-containing protein | | afdb-uniprot50 | AF-A0A419H9E0-F1-MODEL\_V4 | 1.0 | 1.404e-09 | 251 | 0.229 | 253 | 132 | 10 | 11 | 220 | 24 | 256 | Collar domain-containing protein | Collar domain-containing protein | | afdb-uniprot50 | AF-A0A0Q5F3R8-F1-MODEL\_V4 | 1.0 | 2.863e-09 | 251 | 0.201 | 248 | 135 | 12 | 11 | 220 | 40 | 262 | Collar domain-containing protein | Collar domain-containing protein | | afdb-uniprot50 | AF-A0A1I4FJV1-F1-MODEL\_V4 | 1.0 | 5.183e-09 | 250 | 0.277 | 220 | 106 | 8 | 11 | 220 | 29 | 205 | Microcystin-dependent protein | Microcystin-dependent protein | | afdb-uniprot50 | AF-A0A1S8LIP9-F1-MODEL\_V4 | 1.0 | 6.974e-09 | 250 | 0.227 | 237 | 131 | 10 | 11 | 220 | 4 | 215 | Phage tail collar domain protein | Phage tail collar domain protein | | afdb-uniprot50 | AF-A0A819NXH0-F1-MODEL\_V4 | 1.0 | 1.094e-10 | 250 | 0.262 | 248 | 123 | 14 | 2 | 220 | 13 | 229 | Hypothetical protein | Hypothetical protein | | afdb-uniprot50 | AF-A0A2S1XFQ2-F1-MODEL\_V4 | 1.0 | 7.755e-10 | 250 | 0.215 | 237 | 140 | 8 | 11 | 220 | 31 | 248 | Collar domain-containing protein | Collar domain-containing protein | | afdb-uniprot50 | AF-F4KQ07-F1-MODEL\_V4 | 1.0 | 2.127e-09 | 250 | 0.243 | 234 | 116 | 13 | 2 | 221 | 129 | 315 | Tail Collar domain protein | Tail Collar domain protein | | afdb-uniprot50 | AF-A0A257ULB7-F1-MODEL\_V4 | 1.0 | 1.561e-10 | 250 | 0.368 | 244 | 116 | 10 | 12 | 220 | 133 | 373 | Collar domain-containing protein | Collar domain-containing protein | | afdb-uniprot50 | AF-A0A1G5E937-F1-MODEL\_V4 | 1.0 | 1.699e-08 | 249 | 0.231 | 229 | 115 | 8 | 11 | 220 | 4 | 190 | Microcystin-dependent protein | Microcystin-dependent protein | | afdb-uniprot50 | AF-A0A7U3BZ46-F1-MODEL\_V4 | 1.0 | 1.581e-09 | 249 | 0.303 | 224 | 93 | 8 | 1 | 220 | 142 | 306 | Collar domain-containing protein | Collar domain-containing protein | | afdb-uniprot50 | AF-A0A814Z5L0-F1-MODEL\_V4 | 1.0 | 3.183e-10 | 249 | 0.291 | 230 | 116 | 10 | 12 | 220 | 111 | 314 | Hypothetical protein | Hypothetical protein | | afdb-uniprot50 | AF-A0A2T4JN94-F1-MODEL\_V4 | 1.0 | 1.404e-09 | 249 | 0.255 | 219 | 100 | 8 | 6 | 221 | 209 | 367 | Collar domain-containing protein | Collar domain-containing protein | | afdb-uniprot50 | AF-A0A2K8QRG9-F1-MODEL\_V4 | 1.0 | 3.076e-08 | 248 | 0.209 | 267 | 138 | 10 | 11 | 220 | 37 | 287 | Collar domain-containing protein | Collar domain-containing protein | | afdb-uniprot50 | AF-A0A8A7KLE0-F1-MODEL\_V4 | 1.0 | 9.958e-09 | 247 | 0.278 | 223 | 93 | 8 | 3 | 221 | 38 | 196 | Phage tail protein | Phage tail protein | | afdb-uniprot50 | AF-A0A835ZKI7-F1-MODEL\_V4 | 1.0 | 2.698e-09 | 247 | 0.293 | 225 | 120 | 10 | 12 | 220 | 25 | 226 | Uncharacterized protein | Uncharacterized protein | | afdb-uniprot50 | AF-B8FJJ3-F1-MODEL\_V4 | 1.0 | 4.884e-09 | 247 | 0.259 | 216 | 86 | 8 | 12 | 221 | 117 | 264 | Tail Collar domain protein | Tail Collar domain protein | | afdb-uniprot50 | AF-A0A1X0T8R2-F1-MODEL\_V4 | 1.0 | 4.392e-08 | 246 | 0.251 | 215 | 109 | 8 | 11 | 220 | 19 | 186 | Phage tail collar domain-containing protein | Phage tail collar domain-containing protein | | afdb-uniprot50 | AF-A0A1G0MZ06-F1-MODEL\_V4 | 1.0 | 6.655e-08 | 246 | 0.236 | 224 | 130 | 9 | 11 | 220 | 4 | 200 | Collar domain-containing protein | Collar domain-containing protein | | afdb-uniprot50 | AF-A0A0B3RIK5-F1-MODEL\_V4 | 1.0 | 3.421e-09 | 246 | 0.293 | 276 | 121 | 9 | 3 | 221 | 27 | 285 | Phage Tail Collar | Phage Tail Collar | | afdb-uniprot50 | AF-A0A7Y7P107-F1-MODEL\_V4 | 1.0 | 2.005e-09 | 246 | 0.215 | 237 | 107 | 10 | 1 | 221 | 122 | 295 | Tail fiber protein | Tail fiber protein | | afdb-uniprot50 | AF-A0A7C1Q6C6-F1-MODEL\_V4 | 1.0 | 1.49e-09 | 245 | 0.276 | 235 | 101 | 13 | 1 | 221 | 50 | 229 | Collar domain-containing protein | Collar domain-containing protein | | afdb-uniprot50 | AF-A0A7Z2VX81-F1-MODEL\_V4 | 1.0 | 2.827e-10 | 245 | 0.218 | 252 | 135 | 12 | 11 | 220 | 33 | 264 | Phage tail protein | Phage tail protein | | afdb-uniprot50 | AF-A0A1R3EDN7-F1-MODEL\_V4 | 1.0 | 1.509e-08 | 244 | 0.238 | 222 | 137 | 10 | 13 | 220 | 2 | 205 | Collar domain-containing protein | Collar domain-containing protein | | afdb-uniprot50 | AF-A0A120IHK0-F1-MODEL\_V4 | 1.0 | 3.676e-08 | 243 | 0.23 | 213 | 101 | 8 | 11 | 221 | 24 | 175 | Collar domain-containing protein | Collar domain-containing protein | | afdb-uniprot50 | AF-A0A1J4ST39-F1-MODEL\_V4 | 1.0 | 1.422e-08 | 243 | 0.254 | 216 | 110 | 9 | 11 | 220 | 5 | 175 | Collar domain-containing protein | Collar domain-containing protein | | afdb-uniprot50 | AF-C6DJW4-F1-MODEL\_V4 | 1.0 | 2.03e-08 | 243 | 0.192 | 229 | 139 | 6 | 11 | 220 | 35 | 236 | Tail Collar domain protein | Tail Collar domain protein | | afdb-uniprot50 | AF-A0A1B9VN93-F1-MODEL\_V4 | 1.0 | 8.333e-09 | 243 | 0.173 | 242 | 143 | 11 | 11 | 220 | 35 | 251 | Tail Collar domain protein | Tail Collar domain protein | | afdb-uniprot50 | AF-A0A661ZRY0-F1-MODEL\_V4 | 1.0 | 2.005e-09 | 243 | 0.272 | 220 | 133 | 6 | 11 | 221 | 94 | 295 | Collar domain-containing protein | Collar domain-containing protein | | afdb-uniprot50 | AF-A0A3S9H6F8-F1-MODEL\_V4 | 1.0 | 7.853e-09 | 243 | 0.264 | 219 | 95 | 5 | 3 | 221 | 143 | 295 | Collar domain-containing protein | Collar domain-containing protein | | afdb-uniprot50 | AF-A0A3S9SJT2-F1-MODEL\_V4 | 1.0 | 2.542e-09 | 243 | 0.319 | 219 | 72 | 9 | 5 | 221 | 183 | 326 | Phage tail protein | Phage tail protein | | afdb-uniprot50 | AF-A0A3B0MDZ4-F1-MODEL\_V4 | 1.0 | 7.217e-11 | 243 | 0.283 | 236 | 128 | 8 | 3 | 221 | 155 | 366 | Collar domain-containing protein | Collar domain-containing protein | | afdb-uniprot50 | AF-A0A7W3S4S1-F1-MODEL\_V4 | 1.0 | 1.19e-08 | 243 | 0.302 | 281 | 120 | 11 | 11 | 221 | 145 | 419 | Microcystin-dependent protein | Microcystin-dependent protein | | afdb-uniprot50 | AF-A0A411MQE7-F1-MODEL\_V4 | 1.0 | 1.121e-08 | 243 | 0.267 | 217 | 127 | 7 | 11 | 221 | 256 | 446 | Collar domain-containing protein | Collar domain-containing protein | | afdb-uniprot50 | AF-L8MKX1-F1-MODEL\_V4 | 1.0 | 2.664e-10 | 243 | 0.317 | 217 | 111 | 9 | 10 | 221 | 342 | 526 | Phage tail fiber protein | Phage tail fiber protein | | afdb-uniprot50 | AF-A0A819H8M6-F1-MODEL\_V4 | 1.0 | 5.431e-10 | 243 | 0.338 | 201 | 104 | 9 | 11 | 198 | 481 | 665 | Hypothetical protein | Hypothetical protein | | afdb-uniprot50 | AF-A0A7V8INF4-F1-MODEL\_V4 | 1.0 | 8.954e-08 | 242 | 0.252 | 210 | 99 | 6 | 11 | 220 | 1 | 152 | Collar domain-containing protein | Collar domain-containing protein | | afdb-uniprot50 | AF-A0A8A6KKJ5-F1-MODEL\_V4 | 1.0 | 4.504e-06 | 242 | 0.437 | 80 | 43 | 1 | 2 | 79 | 72 | 151 | Tail fiber protein | Tail fiber protein | | afdb-uniprot50 | AF-A0A4D8PL44-F1-MODEL\_V4 | 1.0 | 2.005e-09 | 242 | 0.235 | 242 | 129 | 11 | 11 | 220 | 31 | 248 | Collar domain-containing protein | Collar domain-containing protein | | afdb-uniprot50 | AF-A0A0G9JNT3-F1-MODEL\_V4 | 1.0 | 2.698e-09 | 242 | 0.284 | 225 | 104 | 12 | 3 | 221 | 158 | 331 | Collar domain-containing protein | Collar domain-containing protein | | afdb-uniprot50 | AF-A0A125Q5U9-F1-MODEL\_V4 | 1.0 | 1.803e-08 | 242 | 0.253 | 233 | 131 | 11 | 7 | 221 | 139 | 346 | Collar domain-containing protein | Collar domain-containing protein | | afdb-uniprot50 | AF-A0A7V9P271-F1-MODEL\_V4 | 1.0 | 2.005e-09 | 242 | 0.274 | 211 | 107 | 5 | 11 | 221 | 256 | 420 | Tail fiber protein | Tail fiber protein | | afdb-uniprot50 | AF-A0A6L3AU25-F1-MODEL\_V4 | 1.0 | 9.384e-09 | 241 | 0.176 | 238 | 148 | 11 | 11 | 220 | 31 | 248 | Phage tail protein | Phage tail protein | | afdb-uniprot50 | AF-A0A3N5NQW9-F1-MODEL\_V4 | 1.0 | 8.333e-09 | 241 | 0.268 | 212 | 65 | 6 | 12 | 221 | 266 | 389 | Tail fiber protein | Tail fiber protein | | afdb-uniprot50 | AF-A0A150J402-F1-MODEL\_V4 | 1.0 | 1.057e-08 | 241 | 0.215 | 218 | 95 | 5 | 12 | 221 | 267 | 416 | Phage Tail Collar Domain protein | Phage Tail Collar Domain protein | | afdb-uniprot50 | AF-A0A1S8LYQ0-F1-MODEL\_V4 | 1.0 | 2.732e-08 | 240 | 0.259 | 212 | 93 | 6 | 9 | 220 | 30 | 177 | Phage tail collar domain protein | Phage tail collar domain protein | | afdb-uniprot50 | AF-A0A3S0V0B8-F1-MODEL\_V4 | 1.0 | 1.057e-08 | 240 | 0.227 | 246 | 131 | 10 | 18 | 220 | 2 | 231 | Collar domain-containing protein | Collar domain-containing protein | | afdb-uniprot50 | AF-A0A1M6BZM6-F1-MODEL\_V4 | 1.0 | 9.502e-08 | 239 | 0.22 | 213 | 118 | 6 | 11 | 220 | 4 | 171 | Phage Tail Collar Domain protein | Phage Tail Collar Domain protein | | afdb-uniprot50 | AF-A0A7W9FLI3-F1-MODEL\_V4 | 1.0 | 6.572e-09 | 239 | 0.261 | 218 | 124 | 10 | 11 | 220 | 8 | 196 | Microcystin-dependent protein | Microcystin-dependent protein | | afdb-uniprot50 | AF-A0A2U2HDJ5-F1-MODEL\_V4 | 1.0 | 6.193e-09 | 239 | 0.263 | 239 | 110 | 12 | 11 | 220 | 4 | 205 | Phage tail protein | Phage tail protein | | afdb-uniprot50 | AF-A0A4Y7XFR7-F1-MODEL\_V4 | 1.0 | 1.581e-09 | 239 | 0.268 | 227 | 116 | 9 | 2 | 221 | 49 | 232 | Collar domain-containing protein | Collar domain-containing protein | | afdb-uniprot50 | AF-A0A2E5YV74-F1-MODEL\_V4 | 1.0 | 9.267e-10 | 239 | 0.237 | 269 | 123 | 9 | 11 | 221 | 147 | 391 | Collar domain-containing protein | Collar domain-containing protein | | afdb-uniprot50 | AF-A0A833JB53-F1-MODEL\_V4 | 1.0 | 2.863e-09 | 238 | 0.216 | 249 | 151 | 11 | 3 | 220 | 38 | 273 | Uncharacterized protein | Uncharacterized protein | | afdb-uniprot50 | AF-A0A446AXF9-F1-MODEL\_V4 | 1.0 | 4.087e-09 | 238 | 0.269 | 219 | 126 | 8 | 11 | 221 | 116 | 308 | Collar domain-containing protein | Collar domain-containing protein | | afdb-uniprot50 | AF-A0A366D7H9-F1-MODEL\_V4 | 1.0 | 1.121e-08 | 238 | 0.253 | 221 | 88 | 7 | 2 | 221 | 356 | 500 | Microcystin-dependent protein | Microcystin-dependent protein | | afdb-uniprot50 | AF-A0A1H2FLZ8-F1-MODEL\_V4 | 1.0 | 3.223e-09 | 238 | 0.276 | 210 | 94 | 6 | 11 | 220 | 268 | 419 | Microcystin-dependent protein | Microcystin-dependent protein | | afdb-uniprot50 | AF-A0A820I8T0-F1-MODEL\_V4 | 1.0 | 2.542e-09 | 237 | 0.391 | 184 | 85 | 8 | 11 | 182 | 12 | 180 | Hypothetical protein | Hypothetical protein | | afdb-uniprot50 | AF-A0A495JVM4-F1-MODEL\_V4 | 1.0 | 1.422e-08 | 237 | 0.236 | 228 | 115 | 10 | 9 | 220 | 11 | 195 | Tail collar domain | Tail collar domain | | afdb-uniprot50 | AF-A0A560CBU6-F1-MODEL\_V4 | 1.0 | 3.852e-09 | 237 | 0.217 | 235 | 142 | 9 | 11 | 220 | 31 | 248 | Microcystin-dependent protein | Microcystin-dependent protein | | afdb-uniprot50 | AF-A0A2A5VXT6-F1-MODEL\_V4 | 1.0 | 1.509e-08 | 237 | 0.235 | 229 | 112 | 7 | 1 | 221 | 246 | 419 | Collar domain-containing protein | Collar domain-containing protein | | afdb-uniprot50 | AF-A0A098S053-F1-MODEL\_V4 | 1.0 | 5.248e-08 | 236 | 0.244 | 225 | 97 | 10 | 11 | 221 | 14 | 179 | Collar domain-containing protein | Collar domain-containing protein | | afdb-uniprot50 | AF-A0A5C7LB37-F1-MODEL\_V4 | 1.0 | 5.183e-09 | 236 | 0.207 | 222 | 125 | 12 | 3 | 221 | 51 | 224 | Tail fiber protein | Tail fiber protein | | afdb-uniprot50 | AF-A0A2R7UAN8-F1-MODEL\_V4 | 1.0 | 2.127e-09 | 236 | 0.3 | 220 | 113 | 9 | 12 | 221 | 46 | 234 | Collar domain-containing protein | Collar domain-containing protein | | afdb-uniprot50 | AF-A0A1X3DKU1-F1-MODEL\_V4 | 1.0 | 3.038e-09 | 236 | 0.3 | 220 | 116 | 8 | 3 | 221 | 61 | 243 | Collar domain-containing protein | Collar domain-containing protein | | afdb-uniprot50 | AF-A0A448VHU0-F1-MODEL\_V4 | 1.0 | 4.337e-09 | 236 | 0.246 | 231 | 111 | 11 | 1 | 221 | 255 | 432 | Putative phage tail fiber protein | Putative phage tail fiber protein | | afdb-uniprot50 | AF-A0A480ATV8-F1-MODEL\_V4 | 1.0 | 7.4e-09 | 235 | 0.286 | 220 | 100 | 8 | 4 | 221 | 64 | 228 | Tail protein | Tail protein | | afdb-uniprot50 | AF-A0A533TV88-F1-MODEL\_V4 | 1.0 | 9.958e-09 | 235 | 0.225 | 262 | 131 | 13 | 11 | 220 | 39 | 280 | Tail fiber protein | Tail fiber protein | | afdb-uniprot50 | AF-A0A4R7MXL7-F1-MODEL\_V4 | 1.0 | 8.843e-09 | 235 | 0.291 | 223 | 108 | 10 | 1 | 221 | 593 | 767 | Tail collar domain | Tail collar domain | | afdb-uniprot50 | AF-A0A1X0ZXQ4-F1-MODEL\_V4 | 1.0 | 7.4e-09 | 235 | 0.274 | 222 | 110 | 10 | 1 | 221 | 701 | 872 | Collar domain-containing protein | Collar domain-containing protein | | afdb-uniprot50 | AF-A0A367V8J5-F1-MODEL\_V4 | 1.0 | 2.574e-08 | 234 | 0.25 | 220 | 105 | 9 | 1 | 220 | 14 | 173 | Collar domain-containing protein | Collar domain-containing protein | | afdb-uniprot50 | AF-A0A821SA43-F1-MODEL\_V4 | 1.0 | 5.836e-09 | 234 | 0.286 | 206 | 111 | 9 | 23 | 219 | 14 | 192 | Hypothetical protein | Hypothetical protein | | afdb-uniprot50 | AF-A0A7X4H185-F1-MODEL\_V4 | 1.0 | 7.308e-10 | 234 | 0.23 | 243 | 130 | 12 | 11 | 220 | 4 | 222 | Collar domain-containing protein | Collar domain-containing protein | | afdb-uniprot50 | AF-A0A2R6VDR8-F1-MODEL\_V4 | 1.0 | 9.958e-09 | 234 | 0.224 | 227 | 138 | 9 | 11 | 220 | 34 | 239 | Collar domain-containing protein | Collar domain-containing protein | | afdb-uniprot50 | AF-A0A323UBG8-F1-MODEL\_V4 | 1.0 | 8.333e-09 | 234 | 0.257 | 245 | 130 | 10 | 3 | 220 | 101 | 320 | Collar domain-containing protein | Collar domain-containing protein | | afdb-uniprot50 | AF-A0A8A3LGF1-F1-MODEL\_V4 | 1.0 | 1.175e-09 | 234 | 0.316 | 212 | 94 | 10 | 11 | 221 | 181 | 342 | Tail fiber protein | Tail fiber protein | | afdb-uniprot50 | AF-A0A378UDT9-F1-MODEL\_V4 | 1.0 | 1.057e-08 | 234 | 0.265 | 211 | 95 | 8 | 11 | 221 | 429 | 579 | Putative phage tail fiber protein | Putative phage tail fiber protein | | afdb-uniprot50 | AF-A0A1E4UZN2-F1-MODEL\_V4 | 1.0 | 1.34e-08 | 233 | 0.25 | 216 | 121 | 10 | 11 | 220 | 34 | 214 | Collar domain-containing protein | Collar domain-containing protein | | afdb-uniprot50 | AF-A0A222FGG6-F1-MODEL\_V4 | 1.0 | 4.946e-08 | 233 | 0.233 | 227 | 125 | 9 | 11 | 220 | 54 | 248 | Phage tail protein | Phage tail protein | | afdb-uniprot50 | AF-I8TWE2-F1-MODEL\_V4 | 1.0 | 2.154e-08 | 233 | 0.244 | 229 | 103 | 6 | 3 | 221 | 114 | 282 | Tail Collar domain protein | Tail Collar domain protein | | afdb-uniprot50 | AF-A0A5P3MW99-F1-MODEL\_V4 | 1.0 | 1.78e-09 | 233 | 0.28 | 232 | 92 | 9 | 2 | 221 | 147 | 315 | Tail fiber protein | Tail fiber protein | | afdb-uniprot50 | AF-A0A031IW68-F1-MODEL\_V4 | 1.0 | 9.384e-09 | 233 | 0.297 | 222 | 110 | 12 | 1 | 221 | 600 | 776 | Tail fiber protein H | Tail fiber protein H | | afdb-uniprot50 | AF-A0A7H9BG73-F1-MODEL\_V4 | 1.0 | 1.121e-08 | 232 | 0.285 | 214 | 91 | 11 | 11 | 221 | 25 | 179 | Tail fiber protein | Tail fiber protein | | afdb-uniprot50 | AF-A6FCS9-F1-MODEL\_V4 | 1.0 | 2.899e-08 | 232 | 0.217 | 239 | 120 | 11 | 11 | 220 | 4 | 204 | Collar domain-containing protein | Collar domain-containing protein | | afdb-uniprot50 | AF-A0A5C6V133-F1-MODEL\_V4 | 1.0 | 1.121e-08 | 232 | 0.288 | 215 | 108 | 9 | 11 | 221 | 128 | 301 | Tail fiber protein | Tail fiber protein | | afdb-uniprot50 | AF-A0A498REH0-F1-MODEL\_V4 | 1.0 | 1.34e-08 | 232 | 0.279 | 211 | 91 | 6 | 11 | 221 | 253 | 402 | Collar domain-containing protein | Collar domain-containing protein | | afdb-uniprot50 | AF-A0A359D4J8-F1-MODEL\_V4 | 1.0 | 2.426e-08 | 232 | 0.229 | 218 | 93 | 7 | 12 | 221 | 379 | 529 | Collar domain-containing protein | Collar domain-containing protein | | afdb-uniprot50 | AF-A0A1M7JT24-F1-MODEL\_V4 | 1.0 | 6.655e-08 | 231 | 0.233 | 227 | 116 | 11 | 11 | 220 | 4 | 189 | Microcystin-dependent protein | Microcystin-dependent protein | | afdb-uniprot50 | AF-A0A821FDS7-F1-MODEL\_V4 | 1.0 | 1.422e-08 | 231 | 0.41 | 173 | 81 | 8 | 11 | 172 | 49 | 211 | Hypothetical protein | Hypothetical protein | | afdb-uniprot50 | AF-A0A0Q2UK27-F1-MODEL\_V4 | 1.0 | 2.456e-07 | 230 | 0.222 | 220 | 96 | 9 | 3 | 220 | 17 | 163 | Collar domain-containing protein | Collar domain-containing protein | | afdb-uniprot50 | AF-A0A2U1XUJ9-F1-MODEL\_V4 | 1.0 | 1.205e-07 | 230 | 0.251 | 231 | 89 | 7 | 2 | 221 | 18 | 175 | Collar domain-containing protein | Collar domain-containing protein | | afdb-uniprot50 | AF-A0A1D9LMZ6-F1-MODEL\_V4 | 1.0 | 8.333e-09 | 230 | 0.283 | 229 | 84 | 10 | 2 | 221 | 39 | 196 | Collar domain-containing protein | Collar domain-containing protein | | afdb-uniprot50 | AF-A0A661CQ93-F1-MODEL\_V4 | 1.0 | 2.005e-09 | 230 | 0.235 | 221 | 119 | 7 | 11 | 220 | 13 | 194 | Collar domain-containing protein | Collar domain-containing protein | | afdb-uniprot50 | AF-A0A1X7F9Q3-F1-MODEL\_V4 | 1.0 | 9.384e-09 | 230 | 0.283 | 222 | 83 | 8 | 4 | 221 | 73 | 222 | Phage Tail Collar Domain | Phage Tail Collar Domain | | afdb-uniprot50 | AF-A0A7W3FCC0-F1-MODEL\_V4 | 1.0 | 1.601e-08 | 230 | 0.272 | 224 | 110 | 13 | 11 | 220 | 30 | 214 | Tail fiber protein | Tail fiber protein | | afdb-uniprot50 | AF-A0A2R4VPM6-F1-MODEL\_V4 | 1.0 | 3.676e-08 | 230 | 0.241 | 215 | 129 | 9 | 11 | 220 | 35 | 220 | Collar domain-containing protein | Collar domain-containing protein | | afdb-uniprot50 | AF-A0A318V634-F1-MODEL\_V4 | 1.0 | 5.91e-08 | 230 | 0.213 | 230 | 146 | 8 | 11 | 220 | 38 | 252 | Microcystin-dependent protein | Microcystin-dependent protein | | afdb-uniprot50 | AF-A0A3C2AA29-F1-MODEL\_V4 | 1.0 | 8.333e-09 | 230 | 0.251 | 219 | 94 | 6 | 3 | 221 | 136 | 284 | Collar domain-containing protein | Collar domain-containing protein | | afdb-uniprot50 | AF-A0A651FYR1-F1-MODEL\_V4 | 1.0 | 1.008e-07 | 230 | 0.217 | 234 | 124 | 9 | 12 | 221 | 114 | 312 | Uncharacterized protein | Uncharacterized protein | | afdb-uniprot50 | AF-A0A7X4KFU6-F1-MODEL\_V4 | 1.0 | 8.733e-10 | 229 | 0.247 | 238 | 133 | 11 | 11 | 220 | 4 | 223 | Collar domain-containing protein | Collar domain-containing protein | | afdb-uniprot50 | AF-A0A522ETL0-F1-MODEL\_V4 | 1.0 | 9.384e-09 | 229 | 0.252 | 214 | 86 | 6 | 12 | 221 | 110 | 253 | Tail fiber protein | Tail fiber protein | | afdb-uniprot50 | AF-A0A1U9MIU8-F1-MODEL\_V4 | 1.0 | 8.333e-09 | 229 | 0.241 | 211 | 108 | 9 | 12 | 221 | 154 | 313 | Phage Tail Collar Domain | Phage Tail Collar Domain | | afdb-uniprot50 | AF-A0A0M0H0I1-F1-MODEL\_V4 | 1.0 | 7.952e-08 | 228 | 0.245 | 212 | 89 | 7 | 11 | 221 | 14 | 155 | Phage Tail Collar Domain | Phage Tail Collar Domain | | afdb-uniprot50 | AF-A0A0J5P3M7-F1-MODEL\_V4 | 1.0 | 2.574e-08 | 228 | 0.279 | 215 | 99 | 10 | 11 | 221 | 14 | 176 | Collar domain-containing protein | Collar domain-containing protein | | afdb-uniprot50 | AF-A0A2Z6AZX8-F1-MODEL\_V4 | 1.0 | 1.913e-08 | 228 | 0.244 | 225 | 112 | 11 | 1 | 221 | 37 | 207 | Phage Tail Collar Domain protein | Phage Tail Collar Domain protein | | afdb-uniprot50 | AF-A0A239KLT7-F1-MODEL\_V4 | 1.0 | 1.678e-09 | 228 | 0.232 | 254 | 138 | 12 | 11 | 220 | 4 | 244 | Microcystin-dependent protein | Microcystin-dependent protein | | afdb-uniprot50 | AF-A0A2S5KL32-F1-MODEL\_V4 | 1.0 | 7.952e-08 | 228 | 0.23 | 243 | 132 | 11 | 11 | 220 | 51 | 271 | Phage tail protein | Phage tail protein | | afdb-uniprot50 | AF-A0A1V2R607-F1-MODEL\_V4 | 1.0 | 3.676e-08 | 227 | 0.189 | 248 | 139 | 11 | 11 | 220 | 35 | 258 | Phage tail protein | Phage tail protein | | afdb-uniprot50 | AF-A0A2A6HIY1-F1-MODEL\_V4 | 1.0 | 6.193e-09 | 227 | 0.303 | 227 | 99 | 10 | 1 | 221 | 183 | 356 | Collar domain-containing protein | Collar domain-containing protein | | afdb-uniprot50 | AF-A0A7G8ZSD1-F1-MODEL\_V4 | 1.0 | 1.19e-08 | 227 | 0.265 | 222 | 99 | 10 | 12 | 221 | 246 | 415 | Tail fiber protein | Tail fiber protein | | afdb-uniprot50 | AF-A0A833JEF0-F1-MODEL\_V4 | 1.0 | 7.952e-08 | 226 | 0.22 | 222 | 104 | 10 | 2 | 221 | 47 | 201 | Uncharacterized protein | Uncharacterized protein | | afdb-uniprot50 | AF-A0A560GUX4-F1-MODEL\_V4 | 1.0 | 1.889e-09 | 226 | 0.223 | 269 | 134 | 12 | 11 | 220 | 5 | 257 | Microcystin-dependent protein | Microcystin-dependent protein | | afdb-uniprot50 | AF-A0A0U1KXP1-F1-MODEL\_V4 | 1.0 | 9.958e-09 | 226 | 0.295 | 210 | 90 | 7 | 12 | 221 | 172 | 323 | Phage tail fibers | Phage tail fibers | | afdb-uniprot50 | AF-A0A846QFW6-F1-MODEL\_V4 | 1.0 | 3.676e-08 | 226 | 0.27 | 211 | 80 | 6 | 11 | 221 | 317 | 453 | Phage-related tail fiber protein | Phage-related tail fiber protein | | afdb-uniprot50 | AF-A0A662BD73-F1-MODEL\_V4 | 1.0 | 6.271e-08 | 225 | 0.276 | 210 | 75 | 7 | 12 | 221 | 39 | 171 | Phage tail protein | Phage tail protein | | afdb-uniprot50 | AF-A0A2N3PZP5-F1-MODEL\_V4 | 1.0 | 7.4e-09 | 225 | 0.242 | 256 | 129 | 10 | 11 | 220 | 4 | 240 | Collar domain-containing protein | Collar domain-containing protein | | afdb-uniprot50 | AF-A0A7S7WJF1-F1-MODEL\_V4 | 1.0 | 2.286e-08 | 225 | 0.266 | 214 | 104 | 7 | 9 | 221 | 143 | 304 | Collar domain-containing protein | Collar domain-containing protein | | afdb-uniprot50 | AF-A0A7Y8AZA7-F1-MODEL\_V4 | 1.0 | 1.422e-08 | 225 | 0.274 | 211 | 89 | 7 | 12 | 221 | 190 | 337 | Tail fiber protein | Tail fiber protein | | afdb-uniprot50 | AF-A0A7Z0F930-F1-MODEL\_V4 | 1.0 | 4.337e-09 | 225 | 0.247 | 218 | 131 | 8 | 9 | 221 | 161 | 350 | Phage-related tail fiber protein | Phage-related tail fiber protein | | afdb-uniprot50 | AF-C8PDQ5-F1-MODEL\_V4 | 1.0 | 1.34e-08 | 225 | 0.237 | 227 | 108 | 11 | 1 | 221 | 224 | 391 | Phage Tail Collar Domain protein | Phage Tail Collar Domain protein | | afdb-uniprot50 | AF-A0A0F9S6G2-F1-MODEL\_V4 | 1.0 | 2.732e-08 | 225 | 0.28 | 214 | 84 | 6 | 12 | 220 | 313 | 461 | Collar domain-containing protein | Collar domain-containing protein | | afdb-uniprot50 | AF-A0A378TUW3-F1-MODEL\_V4 | 1.0 | 2.426e-08 | 225 | 0.285 | 221 | 105 | 11 | 3 | 221 | 343 | 512 | Putative phage tail fiber protein | Putative phage tail fiber protein | | afdb-uniprot50 | AF-A0A2E9QLA8-F1-MODEL\_V4 | 1.0 | 3.076e-08 | 224 | 0.236 | 241 | 128 | 13 | 3 | 221 | 106 | 312 | Collar domain-containing protein | Collar domain-containing protein | | afdb-uniprot50 | AF-A0A250DS87-F1-MODEL\_V4 | 1.0 | 7.853e-09 | 224 | 0.271 | 232 | 124 | 11 | 1 | 220 | 290 | 488 | Phage tail protein | Phage tail protein | | afdb-uniprot50 | AF-A0A1H6MNN9-F1-MODEL\_V4 | 1.0 | 1.263e-08 | 224 | 0.294 | 221 | 105 | 9 | 2 | 221 | 455 | 625 | Microcystin-dependent protein | Microcystin-dependent protein | | afdb-uniprot50 | AF-A0A0L6FYH4-F1-MODEL\_V4 | 1.0 | 9.267e-10 | 224 | 0.285 | 210 | 130 | 6 | 12 | 221 | 493 | 682 | Phage tail protein | Phage tail protein | | afdb-uniprot50 | AF-A0A1G3BQZ8-F1-MODEL\_V4 | 1.0 | 6.193e-09 | 223 | 0.285 | 221 | 107 | 7 | 3 | 221 | 32 | 203 | Collar domain-containing protein | Collar domain-containing protein | | afdb-uniprot50 | AF-A0A559ZVU9-F1-MODEL\_V4 | 1.0 | 2.286e-08 | 223 | 0.255 | 223 | 108 | 11 | 11 | 220 | 19 | 196 | Tail collar domain | Tail collar domain | | afdb-uniprot50 | AF-A0A5S9PGT0-F1-MODEL\_V4 | 1.0 | 1.34e-08 | 223 | 0.235 | 225 | 121 | 10 | 11 | 220 | 37 | 225 | Collar domain-containing protein | Collar domain-containing protein | | afdb-uniprot50 | AF-E2PCZ5-F1-MODEL\_V4 | 1.0 | 2.574e-08 | 223 | 0.295 | 220 | 101 | 9 | 5 | 221 | 141 | 309 | Phage Tail Collar Domain protein | Phage Tail Collar Domain protein | | afdb-uniprot50 | AF-A0A075KIT9-F1-MODEL\_V4 | 1.0 | 6.193e-09 | 223 | 0.287 | 219 | 99 | 6 | 3 | 221 | 156 | 317 | Tail Collar domain protein | Tail Collar domain protein | | afdb-uniprot50 | AF-A0A0Q4WR10-F1-MODEL\_V4 | 1.0 | 4.884e-09 | 223 | 0.381 | 215 | 118 | 6 | 12 | 216 | 108 | 317 | Collar domain-containing protein | Collar domain-containing protein | | afdb-uniprot50 | AF-A0A4R6DCB1-F1-MODEL\_V4 | 1.0 | 2.426e-08 | 223 | 0.245 | 220 | 117 | 9 | 2 | 221 | 235 | 405 | Phage tail fiber protein | Phage tail fiber protein | | afdb-uniprot50 | AF-A0A6L5BI96-F1-MODEL\_V4 | 1.0 | 2.863e-09 | 223 | 0.28 | 214 | 86 | 7 | 10 | 221 | 295 | 442 | Collar domain-containing protein | Collar domain-containing protein | | afdb-uniprot50 | AF-A0A2N8HQI7-F1-MODEL\_V4 | 1.0 | 1.121e-08 | 223 | 0.274 | 215 | 95 | 8 | 12 | 221 | 321 | 479 | Collar domain-containing protein | Collar domain-containing protein | | afdb-uniprot50 | AF-A0A1H5GLX5-F1-MODEL\_V4 | 1.0 | 3.264e-08 | 223 | 0.262 | 221 | 114 | 10 | 2 | 221 | 315 | 487 | Phage Tail Collar Domain | Phage Tail Collar Domain | | afdb-uniprot50 | AF-A0A7W2L2C3-F1-MODEL\_V4 | 1.0 | 7.494e-08 | 223 | 0.245 | 220 | 122 | 9 | 3 | 221 | 384 | 560 | Tail fiber protein | Tail fiber protein | | afdb-uniprot50 | AF-A0A498DEB1-F1-MODEL\_V4 | 1.0 | 1.07e-07 | 222 | 0.247 | 222 | 97 | 8 | 12 | 220 | 23 | 187 | Collar domain-containing protein | Collar domain-containing protein | | afdb-uniprot50 | AF-A0A509KW42-F1-MODEL\_V4 | 1.0 | 2.574e-08 | 222 | 0.231 | 220 | 95 | 5 | 1 | 220 | 51 | 196 | Collar domain-containing protein | Collar domain-containing protein | | afdb-uniprot50 | AF-A0A381TMQ4-F1-MODEL\_V4 | 1.0 | 8.843e-09 | 222 | 0.252 | 214 | 82 | 9 | 11 | 221 | 69 | 207 | Uncharacterized protein | Uncharacterized protein | | afdb-uniprot50 | AF-A0A344UNR3-F1-MODEL\_V4 | 1.0 | 5.248e-08 | 222 | 0.264 | 223 | 102 | 7 | 1 | 221 | 162 | 324 | Collar domain-containing protein | Collar domain-containing protein | | afdb-uniprot50 | AF-A0A3S0C1T6-F1-MODEL\_V4 | 1.0 | 9.384e-09 | 222 | 0.276 | 300 | 117 | 12 | 3 | 221 | 73 | 353 | Tail fiber protein | Tail fiber protein | | afdb-uniprot50 | AF-A0A1K0IP40-F1-MODEL\_V4 | 1.0 | 2.732e-08 | 222 | 0.27 | 218 | 90 | 9 | 6 | 221 | 320 | 470 | Collar domain-containing protein | Collar domain-containing protein | | afdb-uniprot50 | AF-A0A154VGC7-F1-MODEL\_V4 | 1.0 | 6.271e-08 | 221 | 0.25 | 220 | 107 | 10 | 1 | 220 | 14 | 175 | Collar domain-containing protein | Collar domain-containing protein | | afdb-uniprot50 | AF-A0A7Z0FDF9-F1-MODEL\_V4 | 1.0 | 4.139e-08 | 221 | 0.236 | 224 | 128 | 10 | 11 | 220 | 6 | 200 | Microcystin-dependent protein | Microcystin-dependent protein | | afdb-uniprot50 | AF-A0A844YWZ8-F1-MODEL\_V4 | 1.0 | 1.057e-08 | 221 | 0.279 | 211 | 76 | 6 | 11 | 221 | 96 | 230 | Phage tail protein | Phage tail protein | | afdb-uniprot50 | AF-E2CN30-F1-MODEL\_V4 | 1.0 | 1.34e-08 | 221 | 0.257 | 225 | 86 | 9 | 3 | 221 | 116 | 265 | Tail fiber protein gpH | Tail fiber protein gpH | | afdb-uniprot50 | AF-A0A1Z7ZRI5-F1-MODEL\_V4 | 1.0 | 1.107e-09 | 221 | 0.293 | 259 | 131 | 10 | 11 | 221 | 95 | 349 | Collar domain-containing protein | Collar domain-containing protein | | afdb-uniprot50 | AF-A0A348HHI3-F1-MODEL\_V4 | 1.0 | 7.853e-09 | 221 | 0.275 | 225 | 103 | 8 | 2 | 221 | 204 | 373 | Phage-related tail fibre protein | Phage-related tail fibre protein | | afdb-uniprot50 | AF-A0A376BTN0-F1-MODEL\_V4 | 1.0 | 2.574e-08 | 221 | 0.312 | 221 | 82 | 9 | 1 | 221 | 479 | 629 | Phage Tail Collar Domain | Phage Tail Collar Domain | | afdb-uniprot50 | AF-A0A845XCJ3-F1-MODEL\_V4 | 1.0 | 5.248e-08 | 220 | 0.254 | 212 | 103 | 8 | 11 | 221 | 25 | 182 | Uncharacterized protein | Uncharacterized protein | | afdb-uniprot50 | AF-A0A7H1CRS4-F1-MODEL\_V4 | 1.0 | 1.357e-07 | 220 | 0.257 | 210 | 124 | 5 | 11 | 220 | 5 | 182 | Collar domain-containing protein | Collar domain-containing protein | | afdb-uniprot50 | AF-N9NNT1-F1-MODEL\_V4 | 1.0 | 3.676e-08 | 220 | 0.285 | 221 | 81 | 8 | 2 | 221 | 61 | 205 | Collar domain-containing protein | Collar domain-containing protein | | afdb-uniprot50 | AF-A0A6I1ND50-F1-MODEL\_V4 | 1.0 | 8.843e-09 | 220 | 0.246 | 211 | 85 | 6 | 11 | 221 | 212 | 348 | Collar domain-containing protein | Collar domain-containing protein | | afdb-uniprot50 | AF-A0A509LEA5-F1-MODEL\_V4 | 1.0 | 3.852e-09 | 220 | 0.288 | 222 | 86 | 11 | 12 | 220 | 195 | 357 | Collar domain-containing protein | Collar domain-containing protein | | afdb-uniprot50 | AF-A0A0F8Z7I6-F1-MODEL\_V4 | 1.0 | 3.95e-07 | 219 | 0.212 | 212 | 90 | 6 | 12 | 221 | 21 | 157 | Collar domain-containing protein | Collar domain-containing protein | | afdb-uniprot50 | AF-A0A5J6QYJ5-F1-MODEL\_V4 | 1.0 | 3.223e-09 | 219 | 0.311 | 231 | 131 | 8 | 1 | 221 | 37 | 249 | Tail fiber protein | Tail fiber protein | | afdb-uniprot50 | AF-A0A430HC49-F1-MODEL\_V4 | 1.0 | 1.34e-08 | 219 | 0.227 | 251 | 119 | 14 | 11 | 220 | 55 | 271 | Phage tail protein | Phage tail protein | | afdb-uniprot50 | AF-A0A263JBT7-F1-MODEL\_V4 | 1.0 | 1.509e-08 | 219 | 0.269 | 219 | 95 | 10 | 12 | 221 | 131 | 293 | Collar domain-containing protein | Collar domain-containing protein | | afdb-uniprot50 | AF-C3X8Y3-F1-MODEL\_V4 | 1.0 | 2.574e-08 | 218 | 0.257 | 225 | 93 | 6 | 1 | 221 | 116 | 270 | Phage Tail Collar Domain protein | Phage Tail Collar Domain protein | | afdb-uniprot50 | AF-A0A2E2N138-F1-MODEL\_V4 | 1.0 | 5.984e-07 | 217 | 0.241 | 211 | 87 | 8 | 11 | 221 | 1 | 138 | Collar domain-containing protein | Collar domain-containing protein | | afdb-uniprot50 | AF-G9PUJ8-F1-MODEL\_V4 | 1.0 | 1.528e-07 | 217 | 0.211 | 217 | 100 | 9 | 11 | 221 | 16 | 167 | Collar domain-containing protein | Collar domain-containing protein | | afdb-uniprot50 | AF-A0A498RAD1-F1-MODEL\_V4 | 1.0 | 5.91e-08 | 217 | 0.25 | 212 | 88 | 6 | 11 | 221 | 46 | 187 | Collar domain-containing protein | Collar domain-containing protein | | afdb-uniprot50 | AF-A0A4D8QIM4-F1-MODEL\_V4 | 1.0 | 2.426e-08 | 217 | 0.25 | 251 | 112 | 12 | 11 | 220 | 4 | 219 | Phage tail protein | Phage tail protein | | afdb-uniprot50 | AF-A0A351LDF1-F1-MODEL\_V4 | 1.0 | 2.03e-08 | 217 | 0.267 | 247 | 137 | 10 | 3 | 221 | 61 | 291 | Collar domain-containing protein | Collar domain-containing protein | | afdb-uniprot50 | AF-A0A258ZJM9-F1-MODEL\_V4 | 1.0 | 3.076e-08 | 217 | 0.287 | 212 | 82 | 8 | 11 | 221 | 228 | 371 | Collar domain-containing protein | Collar domain-containing protein | | afdb-uniprot50 | AF-A0A7U2B2P4-F1-MODEL\_V4 | 1.0 | 1.357e-07 | 216 | 0.24 | 237 | 88 | 11 | 1 | 221 | 24 | 184 | Tail fiber protein | Tail fiber protein | | afdb-uniprot50 | AF-C4GFX3-F1-MODEL\_V4 | 1.0 | 1.803e-08 | 216 | 0.242 | 227 | 110 | 9 | 2 | 221 | 139 | 310 | Phage Tail Collar Domain protein | Phage Tail Collar Domain protein | | afdb-uniprot50 | AF-A0A7X3H8V8-F1-MODEL\_V4 | 1.0 | 4.946e-08 | 216 | 0.261 | 222 | 97 | 8 | 1 | 221 | 175 | 330 | Collar domain-containing protein | Collar domain-containing protein | | afdb-uniprot50 | AF-A0A4R1FMB3-F1-MODEL\_V4 | 1.0 | 3.464e-08 | 216 | 0.239 | 221 | 95 | 6 | 1 | 221 | 585 | 732 | Tail collar domain | Tail collar domain | | afdb-uniprot50 | AF-I3CVE0-F1-MODEL\_V4 | 1.0 | 5.569e-08 | 215 | 0.26 | 223 | 100 | 9 | 2 | 221 | 28 | 188 | Collar domain-containing protein | Collar domain-containing protein | | afdb-uniprot50 | AF-A0A1E4LLZ9-F1-MODEL\_V4 | 1.0 | 1.057e-08 | 215 | 0.228 | 219 | 135 | 10 | 11 | 220 | 11 | 204 | Collar domain-containing protein | Collar domain-containing protein | | afdb-uniprot50 | AF-A0A2D5I712-F1-MODEL\_V4 | 1.0 | 2.366e-10 | 215 | 0.311 | 225 | 127 | 7 | 12 | 221 | 17 | 228 | Collar domain-containing protein | Collar domain-containing protein | | afdb-uniprot50 | AF-A0A1H9M0F5-F1-MODEL\_V4 | 1.0 | 2.181e-07 | 215 | 0.208 | 230 | 123 | 8 | 10 | 220 | 38 | 227 | Microcystin-dependent protein | Microcystin-dependent protein | | afdb-uniprot50 | AF-A0A2V2BG48-F1-MODEL\_V4 | 1.0 | 4.392e-08 | 215 | 0.19 | 262 | 142 | 13 | 11 | 220 | 35 | 278 | Microcystin-dependent protein | Microcystin-dependent protein | | afdb-uniprot50 | AF-A0A0B6CXA9-F1-MODEL\_V4 | 1.0 | 4.946e-08 | 215 | 0.227 | 220 | 89 | 7 | 3 | 221 | 186 | 325 | Phage Tail Collar domain protein | Phage Tail Collar domain protein | | afdb-uniprot50 | AF-A0A2N9X5V1-F1-MODEL\_V4 | 1.0 | 2.574e-08 | 215 | 0.261 | 218 | 90 | 10 | 11 | 221 | 182 | 335 | Collar domain-containing protein | Collar domain-containing protein | | afdb-uniprot50 | AF-A0A1V5JDC5-F1-MODEL\_V4 | 1.0 | 5.248e-08 | 215 | 0.29 | 217 | 80 | 9 | 11 | 220 | 228 | 377 | Phage Tail Collar Domain protein | Phage Tail Collar Domain protein | | afdb-uniprot50 | AF-A0A7Z1M4M9-F1-MODEL\_V4 | 1.0 | 1.34e-08 | 215 | 0.298 | 221 | 106 | 7 | 1 | 221 | 217 | 388 | Short tail fiber-like protein | Short tail fiber-like protein | | afdb-uniprot50 | AF-A0A6M1M2X3-F1-MODEL\_V4 | 1.0 | 7.588e-07 | 214 | 0.195 | 220 | 102 | 9 | 12 | 221 | 4 | 158 | Tail fiber protein | Tail fiber protein | | afdb-uniprot50 | AF-A0A661CMF7-F1-MODEL\_V4 | 1.0 | 4.661e-08 | 214 | 0.224 | 245 | 120 | 11 | 1 | 221 | 1 | 199 | Collar domain-containing protein | Collar domain-containing protein | | afdb-uniprot50 | AF-Q8XYR6-F1-MODEL\_V4 | 1.0 | 8.843e-09 | 214 | 0.272 | 220 | 109 | 8 | 3 | 221 | 56 | 225 | Probable phage tail collar protein | Probable phage tail collar protein | | afdb-uniprot50 | AF-A0A841R3L5-F1-MODEL\_V4 | 1.0 | 9.958e-09 | 214 | 0.237 | 215 | 134 | 7 | 12 | 221 | 53 | 242 | Microcystin-dependent protein | Microcystin-dependent protein | | afdb-uniprot50 | AF-A0A4Q0ZFJ9-F1-MODEL\_V4 | 1.0 | 5.91e-08 | 214 | 0.224 | 214 | 97 | 5 | 12 | 221 | 169 | 317 | Collar domain-containing protein | Collar domain-containing protein | | afdb-uniprot50 | AF-A0A348HHI2-F1-MODEL\_V4 | 1.0 | 3.264e-08 | 213 | 0.28 | 210 | 82 | 9 | 12 | 221 | 277 | 417 | Phage-related tail fibre protein | Phage-related tail fibre protein | | afdb-uniprot50 | AF-A0A3E0X1N8-F1-MODEL\_V4 | 1.0 | 2.396e-09 | 213 | 0.257 | 225 | 110 | 8 | 9 | 221 | 250 | 429 | Collar domain-containing protein | Collar domain-containing protein | | afdb-uniprot50 | AF-A0A840RBH8-F1-MODEL\_V4 | 1.0 | 8.052e-07 | 212 | 0.186 | 214 | 105 | 6 | 11 | 221 | 26 | 173 | Microcystin-dependent protein | Microcystin-dependent protein | | afdb-uniprot50 | AF-A4TXJ3-F1-MODEL\_V4 | 1.0 | 2.766e-07 | 212 | 0.2 | 229 | 129 | 9 | 11 | 220 | 34 | 227 | Phage Tail Collar | Phage Tail Collar | | afdb-uniprot50 | AF-A0A0A1FH46-F1-MODEL\_V4 | 1.0 | 5.248e-08 | 212 | 0.253 | 221 | 95 | 10 | 2 | 221 | 98 | 249 | Phage tail fiber protein | Phage tail fiber protein | | afdb-uniprot50 | AF-A0A7D7SIN8-F1-MODEL\_V4 | 1.0 | 3.9e-08 | 212 | 0.275 | 225 | 102 | 12 | 5 | 221 | 210 | 381 | Tail fiber protein | Tail fiber protein | | afdb-uniprot50 | AF-A0A522WRR5-F1-MODEL\_V4 | 1.0 | 6.271e-08 | 211 | 0.257 | 214 | 128 | 9 | 11 | 220 | 5 | 191 | Collar domain-containing protein | Collar domain-containing protein | | afdb-uniprot50 | AF-A0A1B6BGB3-F1-MODEL\_V4 | 1.0 | 7.062e-08 | 211 | 0.222 | 225 | 132 | 11 | 11 | 220 | 4 | 200 | Microcystin dependent protein | Microcystin dependent protein | | afdb-uniprot50 | AF-A0A2K1FR86-F1-MODEL\_V4 | 1.0 | 3.676e-08 | 211 | 0.26 | 223 | 91 | 8 | 2 | 221 | 63 | 214 | Collar domain-containing protein | Collar domain-containing protein | | afdb-uniprot50 | AF-A0A424HEY5-F1-MODEL\_V4 | 1.0 | 6.193e-09 | 211 | 0.247 | 222 | 113 | 7 | 7 | 221 | 102 | 276 | Tail fiber protein | Tail fiber protein | | afdb-uniprot50 | AF-A0A5C7LV11-F1-MODEL\_V4 | 1.0 | 2.426e-08 | 211 | 0.273 | 219 | 93 | 6 | 3 | 221 | 153 | 305 | Collar domain-containing protein | Collar domain-containing protein | | afdb-uniprot50 | AF-X2H9P4-F1-MODEL\_V4 | 1.0 | 3.264e-08 | 211 | 0.283 | 212 | 91 | 9 | 12 | 221 | 204 | 356 | Phage tail fiber protein | Phage tail fiber protein | | afdb-uniprot50 | AF-A0A251WMU1-F1-MODEL\_V4 | 1.0 | 1.008e-07 | 210 | 0.27 | 211 | 100 | 8 | 11 | 220 | 5 | 162 | Collar domain-containing protein | Collar domain-containing protein | | afdb-uniprot50 | AF-A0A4Q0HQ98-F1-MODEL\_V4 | 1.0 | 2.154e-08 | 210 | 0.252 | 234 | 142 | 9 | 3 | 221 | 2 | 217 | Collar domain-containing protein | Collar domain-containing protein | | afdb-uniprot50 | AF-A5ENH3-F1-MODEL\_V4 | 1.0 | 2.426e-08 | 210 | 0.404 | 205 | 86 | 8 | 16 | 213 | 116 | 291 | Putative phage tail Collar domain protein | Putative phage tail Collar domain protein | | afdb-uniprot50 | AF-A0A816MWF3-F1-MODEL\_V4 | 1.0 | 8.545e-07 | 210 | 0.395 | 149 | 66 | 5 | 11 | 152 | 186 | 317 | Hypothetical protein | Hypothetical protein | | afdb-uniprot50 | AF-F3YY59-F1-MODEL\_V4 | 1.0 | 1.803e-08 | 210 | 0.252 | 218 | 91 | 9 | 5 | 221 | 281 | 427 | Tail Collar domain protein | Tail Collar domain protein | | afdb-uniprot50 | AF-A0A1H9AFH7-F1-MODEL\_V4 | 1.0 | 2.732e-08 | 210 | 0.256 | 226 | 115 | 9 | 1 | 221 | 367 | 544 | Phage-related tail fibre protein | Phage-related tail fibre protein | | afdb-uniprot50 | AF-A0A4R1SCQ5-F1-MODEL\_V4 | 1.0 | 1.601e-08 | 210 | 0.308 | 211 | 78 | 8 | 11 | 221 | 428 | 570 | Phage-related tail fiber protein | Phage-related tail fiber protein | | afdb-uniprot50 | AF-A0A4R1G1E3-F1-MODEL\_V4 | 1.0 | 4.946e-08 | 210 | 0.262 | 221 | 90 | 7 | 1 | 221 | 554 | 701 | Tail collar domain | Tail collar domain | | afdb-uniprot50 | AF-A0A4P7BDA5-F1-MODEL\_V4 | 1.0 | 2.154e-08 | 209 | 0.225 | 239 | 122 | 9 | 11 | 220 | 4 | 208 | Phage tail protein | Phage tail protein | | afdb-uniprot50 | AF-A0A6C0BHW3-F1-MODEL\_V4 | 1.0 | 3.076e-08 | 209 | 0.236 | 241 | 142 | 10 | 1 | 220 | 8 | 227 | Collar domain-containing protein | Collar domain-containing protein | | afdb-uniprot50 | AF-A0A1W0CQA8-F1-MODEL\_V4 | 1.0 | 1.19e-08 | 209 | 0.339 | 221 | 79 | 9 | 1 | 221 | 171 | 324 | Collar domain-containing protein | Collar domain-containing protein | | afdb-uniprot50 | AF-A0A0N9MHW1-F1-MODEL\_V4 | 1.0 | 2.286e-08 | 209 | 0.25 | 220 | 117 | 11 | 3 | 221 | 978 | 1150 | Phage tail fiber protein | Phage tail fiber protein | | afdb-uniprot50 | AF-A0A133XLD8-F1-MODEL\_V4 | 1.0 | 1.528e-07 | 208 | 0.242 | 210 | 94 | 6 | 11 | 220 | 77 | 221 | Collar domain-containing protein | Collar domain-containing protein | | afdb-uniprot50 | AF-A0A7Y2P2Q3-F1-MODEL\_V4 | 1.0 | 4.087e-09 | 208 | 0.223 | 251 | 115 | 13 | 11 | 220 | 4 | 215 | Collar domain-containing protein | Collar domain-containing protein | | afdb-uniprot50 | AF-A0A3G9GF41-F1-MODEL\_V4 | 1.0 | 2.732e-08 | 208 | 0.246 | 211 | 88 | 7 | 11 | 221 | 187 | 326 | Phage tail fiber protein | Phage tail fiber protein | | afdb-uniprot50 | AF-A0A4P7LA05-F1-MODEL\_V4 | 1.0 | 1.621e-07 | 208 | 0.233 | 227 | 116 | 9 | 1 | 221 | 192 | 366 | Phage Tail Collar Domain protein | Phage Tail Collar Domain protein | | afdb-uniprot50 | AF-A0A1S1CJX7-F1-MODEL\_V4 | 1.0 | 2.181e-07 | 208 | 0.265 | 211 | 80 | 5 | 11 | 221 | 373 | 508 | Collar domain-containing protein | Collar domain-containing protein | | afdb-uniprot50 | AF-A0A2N2HSX5-F1-MODEL\_V4 | 1.0 | 5.91e-08 | 207 | 0.273 | 212 | 81 | 6 | 11 | 221 | 58 | 197 | Collar domain-containing protein | Collar domain-containing protein | | afdb-uniprot50 | AF-A0A2W7PA10-F1-MODEL\_V4 | 1.0 | 3.464e-08 | 207 | 0.274 | 211 | 80 | 6 | 11 | 221 | 79 | 216 | Tail collar domain | Tail collar domain | | afdb-uniprot50 | AF-C3X8U2-F1-MODEL\_V4 | 1.0 | 5.248e-08 | 207 | 0.224 | 223 | 117 | 10 | 3 | 221 | 96 | 266 | Phage Tail Collar Domain protein | Phage Tail Collar Domain protein | | afdb-uniprot50 | AF-A0A1C3YPF7-F1-MODEL\_V4 | 1.0 | 7.494e-08 | 207 | 0.245 | 212 | 101 | 8 | 11 | 221 | 132 | 285 | Phage Tail Collar Domain | Phage Tail Collar Domain | | afdb-uniprot50 | AF-A0A1X3GF36-F1-MODEL\_V4 | 1.0 | 8.052e-07 | 207 | 0.213 | 243 | 129 | 9 | 12 | 221 | 104 | 317 | Uncharacterized protein | Uncharacterized protein | | afdb-uniprot50 | AF-A0A4Y9GKA5-F1-MODEL\_V4 | 1.0 | 8.954e-08 | 207 | 0.254 | 232 | 100 | 11 | 2 | 221 | 177 | 347 | Tail fiber protein | Tail fiber protein | | afdb-uniprot50 | AF-A0A1Q6U9D9-F1-MODEL\_V4 | 1.0 | 2.154e-08 | 207 | 0.233 | 227 | 84 | 11 | 12 | 221 | 208 | 361 | Collar domain-containing protein | Collar domain-containing protein | | afdb-uniprot50 | AF-A0A1E7RCE5-F1-MODEL\_V4 | 1.0 | 7.952e-08 | 207 | 0.232 | 215 | 100 | 9 | 11 | 221 | 303 | 456 | Phage tail protein | Phage tail protein | | afdb-uniprot50 | AF-U0ZCR8-F1-MODEL\_V4 | 1.0 | 4.946e-08 | 207 | 0.265 | 222 | 99 | 7 | 1 | 221 | 307 | 465 | Collar domain-containing protein | Collar domain-containing protein | | afdb-uniprot50 | AF-A0A3A9HSP0-F1-MODEL\_V4 | 1.0 | 3.305e-07 | 206 | 0.263 | 209 | 95 | 7 | 12 | 220 | 24 | 173 | Tail fiber protein | Tail fiber protein | | afdb-uniprot50 | AF-A0A367VXU5-F1-MODEL\_V4 | 1.0 | 4.72e-07 | 206 | 0.244 | 213 | 94 | 9 | 12 | 220 | 25 | 174 | Collar domain-containing protein | Collar domain-containing protein | | afdb-uniprot50 | AF-A0A822CRA9-F1-MODEL\_V4 | 1.0 | 5.984e-07 | 206 | 0.365 | 164 | 82 | 5 | 11 | 166 | 222 | 371 | Hypothetical protein | Hypothetical protein | | afdb-uniprot50 | AF-A0A7Y8AQ99-F1-MODEL\_V4 | 1.0 | 1.44e-07 | 206 | 0.257 | 210 | 95 | 8 | 12 | 221 | 300 | 448 | Tail fiber protein | Tail fiber protein | | afdb-uniprot50 | AF-A0A2T5UPX7-F1-MODEL\_V4 | 1.0 | 1.205e-07 | 206 | 0.251 | 215 | 91 | 9 | 11 | 220 | 184 | 333 | Microcystin-dependent protein | Microcystin-dependent protein | | afdb-uniprot50 | AF-H8FV07-F1-MODEL\_V4 | 1.0 | 2.732e-08 | 206 | 0.285 | 210 | 73 | 7 | 12 | 220 | 484 | 617 | Collar domain-containing protein | Collar domain-containing protein | | afdb-uniprot50 | AF-A0A7X7EMF1-F1-MODEL\_V4 | 1.0 | 1.205e-07 | 205 | 0.253 | 221 | 92 | 9 | 3 | 221 | 90 | 239 | Tail fiber protein | Tail fiber protein | | afdb-uniprot50 | AF-A0A4R4G488-F1-MODEL\_V4 | 1.0 | 6.655e-08 | 205 | 0.244 | 221 | 92 | 9 | 2 | 221 | 150 | 296 | Collar domain-containing protein | Collar domain-containing protein | | afdb-uniprot50 | AF-A0A4R7WSX9-F1-MODEL\_V4 | 1.0 | 6.655e-08 | 205 | 0.245 | 212 | 111 | 7 | 11 | 221 | 153 | 316 | Tail collar domain | Tail collar domain | | afdb-uniprot50 | AF-A0A2K2G606-F1-MODEL\_V4 | 1.0 | 2.181e-07 | 205 | 0.245 | 220 | 107 | 9 | 3 | 221 | 194 | 355 | Collar domain-containing protein | Collar domain-containing protein | | afdb-uniprot50 | AF-C6ABW9-F1-MODEL\_V4 | 1.0 | 9.502e-08 | 205 | 0.207 | 226 | 106 | 9 | 2 | 221 | 212 | 370 | Phage tail collar protein | Phage tail collar protein | | afdb-uniprot50 | AF-A0A818RWQ3-F1-MODEL\_V4 | 1.0 | 2.154e-08 | 205 | 0.281 | 206 | 130 | 7 | 11 | 214 | 31 | 220 | Hypothetical protein | Hypothetical protein | | afdb-uniprot50 | AF-A0A291LYY5-F1-MODEL\_V4 | 1.0 | 5.569e-08 | 205 | 0.247 | 222 | 93 | 7 | 1 | 221 | 296 | 444 | Collar domain-containing protein | Collar domain-containing protein | | afdb-uniprot50 | AF-A0A2N6FYL6-F1-MODEL\_V4 | 1.0 | 2.315e-07 | 204 | 0.208 | 221 | 118 | 7 | 2 | 221 | 47 | 211 | Collar domain-containing protein | Collar domain-containing protein | | afdb-uniprot50 | AF-C4GGG9-F1-MODEL\_V4 | 1.0 | 7.062e-08 | 204 | 0.242 | 227 | 110 | 10 | 2 | 221 | 98 | 269 | Phage Tail Collar Domain protein | Phage Tail Collar Domain protein | | afdb-uniprot50 | AF-A0A286B0I3-F1-MODEL\_V4 | 1.0 | 5.91e-08 | 204 | 0.208 | 216 | 126 | 7 | 11 | 221 | 301 | 476 | Phage Tail Collar Domain | Phage Tail Collar Domain | | afdb-uniprot50 | AF-U1BY63-F1-MODEL\_V4 | 1.0 | 5.248e-08 | 204 | 0.258 | 213 | 91 | 6 | 9 | 221 | 424 | 569 | Collar domain-containing protein | Collar domain-containing protein | | afdb-uniprot50 | AF-A0A446AZ89-F1-MODEL\_V4 | 1.0 | 2.732e-08 | 204 | 0.307 | 221 | 115 | 8 | 11 | 221 | 562 | 754 | Collar domain-containing protein | Collar domain-containing protein | | afdb-uniprot50 | AF-A0A3M5GHQ5-F1-MODEL\_V4 | 1.0 | 2.181e-07 | 203 | 0.222 | 225 | 127 | 9 | 12 | 221 | 5 | 196 | Phage tail Collar | Phage tail Collar | | afdb-uniprot50 | AF-A0A1V5LAC3-F1-MODEL\_V4 | 1.0 | 1.72e-07 | 203 | 0.2 | 224 | 110 | 10 | 3 | 221 | 50 | 209 | Phage Tail Collar Domain protein | Phage Tail Collar Domain protein | | afdb-uniprot50 | AF-A0A7X8LQL7-F1-MODEL\_V4 | 1.0 | 3.464e-08 | 203 | 0.253 | 217 | 88 | 6 | 11 | 221 | 70 | 218 | Phage tail protein | Phage tail protein | | afdb-uniprot50 | AF-A0A5C5PXD1-F1-MODEL\_V4 | 1.0 | 4.139e-08 | 203 | 0.24 | 216 | 93 | 7 | 11 | 221 | 92 | 241 | Collar domain-containing protein | Collar domain-containing protein | | afdb-uniprot50 | AF-A0A5E6WIQ1-F1-MODEL\_V4 | 1.0 | 3.464e-08 | 203 | 0.27 | 229 | 106 | 11 | 1 | 221 | 107 | 282 | Collar domain-containing protein | Collar domain-containing protein | | afdb-uniprot50 | AF-A0A7H1M8U3-F1-MODEL\_V4 | 1.0 | 4.661e-08 | 203 | 0.28 | 228 | 112 | 9 | 2 | 221 | 103 | 286 | Phage Tail Collar domain protein | Phage Tail Collar domain protein | | afdb-uniprot50 | AF-A0A2N9XQU6-F1-MODEL\_V4 | 1.0 | 1.601e-08 | 203 | 0.251 | 219 | 96 | 12 | 11 | 221 | 140 | 298 | Collar domain-containing protein | Collar domain-containing protein | | afdb-uniprot50 | AF-A0A3M9M907-F1-MODEL\_V4 | 1.0 | 1.205e-07 | 203 | 0.273 | 238 | 97 | 13 | 2 | 221 | 131 | 310 | Collar domain-containing protein | Collar domain-containing protein | | afdb-uniprot50 | AF-A0A162GDK8-F1-MODEL\_V4 | 1.0 | 1.135e-07 | 203 | 0.273 | 212 | 93 | 7 | 12 | 220 | 461 | 614 | Collar domain-containing protein | Collar domain-containing protein | | afdb-uniprot50 | AF-A0A5A7M8N1-F1-MODEL\_V4 | 1.0 | 5.91e-08 | 203 | 0.224 | 223 | 112 | 10 | 4 | 220 | 1057 | 1224 | Collar domain-containing protein | Collar domain-containing protein | | afdb-uniprot50 | AF-A0A367WHK4-F1-MODEL\_V4 | 1.0 | 7.062e-08 | 202 | 0.263 | 209 | 96 | 7 | 12 | 220 | 51 | 201 | Collar domain-containing protein | Collar domain-containing protein | | afdb-uniprot50 | AF-A0A2L2BGH7-F1-MODEL\_V4 | 1.0 | 5.569e-08 | 202 | 0.267 | 213 | 89 | 7 | 9 | 221 | 109 | 254 | Collar domain-containing protein | Collar domain-containing protein | | afdb-uniprot50 | AF-A0A5M6CCN1-F1-MODEL\_V4 | 1.0 | 8.333e-09 | 202 | 0.252 | 250 | 133 | 11 | 3 | 220 | 34 | 261 | Collar domain-containing protein | Collar domain-containing protein | | afdb-uniprot50 | AF-A0A1V5RYX1-F1-MODEL\_V4 | 1.0 | 1.803e-08 | 202 | 0.3 | 210 | 111 | 9 | 11 | 220 | 6 | 179 | Phage Tail Collar Domain protein | Phage Tail Collar Domain protein | | afdb-uniprot50 | AF-A0A1W9JSL6-F1-MODEL\_V4 | 1.0 | 2.766e-07 | 202 | 0.267 | 217 | 120 | 10 | 9 | 221 | 133 | 314 | Collar domain-containing protein | Collar domain-containing protein | | afdb-uniprot50 | AF-A0A817X4U4-F1-MODEL\_V4 | 1.0 | 2.766e-07 | 202 | 0.237 | 223 | 94 | 8 | 2 | 221 | 191 | 340 | Hypothetical protein | Hypothetical protein | | afdb-uniprot50 | AF-A0A7U3LRL7-F1-MODEL\_V4 | 1.0 | 2.315e-07 | 202 | 0.233 | 227 | 98 | 11 | 4 | 221 | 200 | 359 | Uncharacterized protein | Uncharacterized protein | | afdb-uniprot50 | AF-A0A6I6QS22-F1-MODEL\_V4 | 1.0 | 5.569e-08 | 202 | 0.271 | 210 | 83 | 7 | 12 | 221 | 418 | 557 | Collar domain-containing protein | Collar domain-containing protein | | afdb-uniprot50 | AF-A0A0N9MHY1-F1-MODEL\_V4 | 1.0 | 2.03e-08 | 202 | 0.283 | 219 | 124 | 9 | 11 | 221 | 555 | 748 | Collar domain-containing protein | Collar domain-containing protein | | afdb-uniprot50 | AF-A0A512NQJ7-F1-MODEL\_V4 | 1.0 | 2.972e-06 | 201 | 0.386 | 132 | 70 | 3 | 28 | 159 | 1 | 121 | Collar domain-containing protein | Collar domain-containing protein | | afdb-uniprot50 | AF-A0A7W6MM04-F1-MODEL\_V4 | 1.0 | 3.347e-06 | 201 | 0.195 | 220 | 100 | 10 | 12 | 220 | 4 | 157 | Collar domain-containing protein | Collar domain-containing protein | | afdb-uniprot50 | AF-A0A1Y4GG17-F1-MODEL\_V4 | 1.0 | 2.181e-07 | 201 | 0.227 | 211 | 114 | 9 | 11 | 220 | 25 | 187 | Collar domain-containing protein | Collar domain-containing protein | | afdb-uniprot50 | AF-A0A077L9W7-F1-MODEL\_V4 | 1.0 | 1.621e-07 | 201 | 0.199 | 221 | 153 | 5 | 11 | 220 | 5 | 212 | Tail Collar domain protein | Tail Collar domain protein | | afdb-uniprot50 | AF-A0A486SU05-F1-MODEL\_V4 | 1.0 | 5.569e-08 | 201 | 0.234 | 230 | 98 | 10 | 1 | 221 | 119 | 279 | Phage Tail Collar Domain | Phage Tail Collar Domain | | afdb-uniprot50 | AF-A0A1H9HCA5-F1-MODEL\_V4 | 1.0 | 2.607e-07 | 201 | 0.224 | 218 | 96 | 10 | 3 | 220 | 167 | 311 | Microcystin-dependent protein | Microcystin-dependent protein | | afdb-uniprot50 | AF-A0A7D5QQI0-F1-MODEL\_V4 | 1.0 | 1.07e-07 | 201 | 0.312 | 211 | 70 | 7 | 11 | 221 | 323 | 458 | Tail fiber protein | Tail fiber protein | | afdb-uniprot50 | AF-A0A202B4T0-F1-MODEL\_V4 | 1.0 | 7.062e-08 | 200 | 0.234 | 230 | 95 | 7 | 9 | 221 | 66 | 231 | Collar domain-containing protein | Collar domain-containing protein | | afdb-uniprot50 | AF-A0A1W9LKS6-F1-MODEL\_V4 | 1.0 | 1.826e-07 | 200 | 0.269 | 226 | 127 | 7 | 9 | 221 | 122 | 322 | Collar domain-containing protein | Collar domain-containing protein | | afdb-uniprot50 | AF-A0A7W6RGC7-F1-MODEL\_V4 | 1.0 | 3.076e-08 | 200 | 0.253 | 229 | 96 | 9 | 3 | 221 | 192 | 355 | Phage-related tail fiber protein | Phage-related tail fiber protein | | afdb-uniprot50 | AF-A0A7Y1MB50-F1-MODEL\_V4 | 1.0 | 1.279e-07 | 200 | 0.255 | 211 | 84 | 5 | 11 | 221 | 515 | 652 | Phage tail protein | Phage tail protein | | afdb-uniprot50 | AF-A0A8A9YBB3-F1-MODEL\_V4 | 1.0 | 4.661e-08 | 200 | 0.245 | 220 | 123 | 9 | 12 | 221 | 646 | 832 | Phage tail protein | Phage tail protein | | afdb-uniprot50 | AF-A0A6N4RFI0-F1-MODEL\_V4 | 1.0 | 1.528e-07 | 199 | 0.341 | 208 | 110 | 6 | 16 | 214 | 3 | 192 | Tail fiber protein | Tail fiber protein | | afdb-uniprot50 | AF-A0A6C0KMM6-F1-MODEL\_V4 | 1.0 | 6.271e-08 | 199 | 0.24 | 212 | 116 | 10 | 11 | 220 | 46 | 214 | Collar domain-containing protein | Collar domain-containing protein | | afdb-uniprot50 | AF-A0A5S9HP15-F1-MODEL\_V4 | 1.0 | 2.607e-07 | 199 | 0.254 | 224 | 95 | 8 | 5 | 221 | 121 | 279 | Collar domain-containing protein | Collar domain-containing protein | | afdb-uniprot50 | AF-A0A353PD69-F1-MODEL\_V4 | 1.0 | 1.135e-07 | 199 | 0.228 | 232 | 127 | 11 | 11 | 221 | 89 | 289 | Collar domain-containing protein | Collar domain-containing protein | | afdb-uniprot50 | AF-A0A515EKF3-F1-MODEL\_V4 | 1.0 | 3.9e-08 | 199 | 0.298 | 211 | 84 | 8 | 11 | 221 | 179 | 325 | Collar domain-containing protein | Collar domain-containing protein | | afdb-uniprot50 | AF-A0A454TSP7-F1-MODEL\_V4 | 1.0 | 3.9e-08 | 199 | 0.283 | 212 | 95 | 8 | 11 | 221 | 291 | 446 | Phage tail protein | Phage tail protein | | afdb-uniprot50 | AF-A0A0F9VFY7-F1-MODEL\_V4 | 1.0 | 2.315e-07 | 199 | 0.256 | 218 | 98 | 10 | 5 | 221 | 294 | 448 | Collar domain-containing protein | Collar domain-containing protein | | afdb-uniprot50 | AF-A0A076PYE4-F1-MODEL\_V4 | 1.0 | 3.9e-08 | 199 | 0.269 | 226 | 107 | 8 | 1 | 220 | 908 | 1081 | Collar domain-containing protein | Collar domain-containing protein | | afdb-uniprot50 | AF-A0A7C5BRR0-F1-MODEL\_V4 | 1.0 | 1.44e-07 | 198 | 0.242 | 210 | 90 | 6 | 12 | 221 | 54 | 194 | Uncharacterized protein | Uncharacterized protein | | afdb-uniprot50 | AF-C3X3R4-F1-MODEL\_V4 | 1.0 | 7.062e-08 | 198 | 0.228 | 236 | 89 | 11 | 2 | 220 | 70 | 229 | Collar domain-containing protein | Collar domain-containing protein | | afdb-uniprot50 | AF-A0A1Q6U9C4-F1-MODEL\_V4 | 1.0 | 5.639e-07 | 198 | 0.243 | 222 | 98 | 8 | 2 | 220 | 91 | 245 | Collar domain-containing protein | Collar domain-containing protein | | afdb-uniprot50 | AF-A0A2E3Q9T6-F1-MODEL\_V4 | 1.0 | 5.91e-08 | 198 | 0.268 | 212 | 90 | 9 | 11 | 221 | 105 | 252 | Collar domain-containing protein | Collar domain-containing protein | | afdb-uniprot50 | AF-A0A0H3GJI2-F1-MODEL\_V4 | 1.0 | 5.91e-08 | 198 | 0.251 | 223 | 77 | 9 | 10 | 221 | 231 | 374 | Collar domain-containing protein | Collar domain-containing protein | | afdb-uniprot50 | AF-A0A212J3U4-F1-MODEL\_V4 | 1.0 | 1.07e-07 | 198 | 0.246 | 215 | 91 | 8 | 11 | 221 | 267 | 414 | Collar domain-containing protein | Collar domain-containing protein | | afdb-uniprot50 | AF-A0A840MKP4-F1-MODEL\_V4 | 1.0 | 6.271e-08 | 198 | 0.274 | 211 | 94 | 6 | 11 | 221 | 285 | 436 | Phage-related tail fiber protein | Phage-related tail fiber protein | | afdb-uniprot50 | AF-A0A2S8IPM3-F1-MODEL\_V4 | 1.0 | 6.655e-08 | 198 | 0.242 | 256 | 136 | 13 | 1 | 220 | 421 | 654 | Uncharacterized protein | Uncharacterized protein | | afdb-uniprot50 | AF-A0A7W8S7J7-F1-MODEL\_V4 | 1.0 | 3.507e-07 | 198 | 0.198 | 257 | 147 | 12 | 1 | 220 | 487 | 721 | Uncharacterized protein | Uncharacterized protein | | afdb-uniprot50 | AF-A0A077LFW5-F1-MODEL\_V4 | 1.0 | 7.952e-08 | 197 | 0.242 | 235 | 118 | 9 | 11 | 220 | 5 | 204 | Tail collar domain-containing protein | Tail collar domain-containing protein | | afdb-uniprot50 | AF-A0A143DC03-F1-MODEL\_V4 | 1.0 | 2.056e-07 | 197 | 0.248 | 221 | 97 | 9 | 1 | 220 | 116 | 268 | Collar domain-containing protein | Collar domain-containing protein | | afdb-uniprot50 | AF-A0A2I1XBM6-F1-MODEL\_V4 | 1.0 | 6.655e-08 | 197 | 0.259 | 216 | 94 | 10 | 12 | 221 | 186 | 341 | Phage tail protein | Phage tail protein | | afdb-uniprot50 | AF-A0A0S9N328-F1-MODEL\_V4 | 1.0 | 6.271e-08 | 197 | 0.257 | 237 | 142 | 12 | 1 | 221 | 226 | 444 | Collar domain-containing protein | Collar domain-containing protein | | afdb-uniprot50 | AF-A0A381F1Q4-F1-MODEL\_V4 | 1.0 | 3.464e-08 | 197 | 0.272 | 213 | 116 | 8 | 11 | 221 | 271 | 446 | Phage Tail Collar Domain | Phage Tail Collar Domain | | afdb-uniprot50 | AF-A0A1H8IGX2-F1-MODEL\_V4 | 1.0 | 1.528e-07 | 197 | 0.231 | 220 | 97 | 6 | 2 | 221 | 304 | 451 | Phage Tail Collar Domain | Phage Tail Collar Domain | | afdb-uniprot50 | AF-A0A7W6RFU8-F1-MODEL\_V4 | 1.0 | 7.588e-07 | 196 | 0.222 | 211 | 121 | 9 | 11 | 220 | 41 | 209 | Microcystin-dependent protein | Microcystin-dependent protein | | afdb-uniprot50 | AF-A0A6A7Y806-F1-MODEL\_V4 | 1.0 | 7.494e-08 | 196 | 0.228 | 214 | 140 | 8 | 10 | 220 | 20 | 211 | Collar domain-containing protein | Collar domain-containing protein | | afdb-uniprot50 | AF-A0A177NMW9-F1-MODEL\_V4 | 1.0 | 6.655e-08 | 196 | 0.259 | 239 | 119 | 11 | 11 | 220 | 40 | 249 | Collar domain-containing protein | Collar domain-containing protein | | afdb-uniprot50 | AF-A0A3Q9BAD5-F1-MODEL\_V4 | 1.0 | 2.181e-07 | 196 | 0.216 | 226 | 110 | 8 | 3 | 221 | 163 | 328 | Collar domain-containing protein | Collar domain-containing protein | | afdb-uniprot50 | AF-A0A496KQQ3-F1-MODEL\_V4 | 1.0 | 3.95e-07 | 196 | 0.274 | 211 | 78 | 9 | 11 | 221 | 226 | 361 | Collar domain-containing protein | Collar domain-containing protein | | afdb-uniprot50 | AF-A0A530YYK5-F1-MODEL\_V4 | 1.0 | 5.569e-08 | 195 | 0.251 | 227 | 113 | 6 | 3 | 221 | 70 | 247 | Collar domain-containing protein | Collar domain-containing protein | | afdb-uniprot50 | AF-A0A6L5HA01-F1-MODEL\_V4 | 1.0 | 6.739e-07 | 195 | 0.247 | 222 | 93 | 6 | 1 | 221 | 110 | 258 | Collar domain-containing protein | Collar domain-containing protein | | afdb-uniprot50 | AF-A0A509LD13-F1-MODEL\_V4 | 1.0 | 5.008e-07 | 195 | 0.252 | 214 | 85 | 6 | 12 | 220 | 46 | 189 | Collar domain-containing protein | Collar domain-containing protein | | afdb-uniprot50 | AF-A0A2A3MGV4-F1-MODEL\_V4 | 1.0 | 1.621e-07 | 195 | 0.233 | 210 | 88 | 6 | 12 | 221 | 307 | 443 | Phage tail protein | Phage tail protein | | afdb-uniprot50 | AF-F4KS41-F1-MODEL\_V4 | 1.0 | 5.984e-07 | 195 | 0.243 | 218 | 104 | 11 | 11 | 221 | 312 | 475 | Tail Collar domain protein | Tail Collar domain protein | | afdb-uniprot50 | AF-A0A840MLL2-F1-MODEL\_V4 | 1.0 | 1.44e-07 | 195 | 0.225 | 235 | 124 | 7 | 1 | 221 | 469 | 659 | Phage-related tail fiber protein | Phage-related tail fiber protein | | afdb-uniprot50 | AF-A0A378UFL6-F1-MODEL\_V4 | 1.0 | 6.655e-08 | 195 | 0.262 | 221 | 112 | 8 | 2 | 221 | 532 | 702 | Putative phage tail fiber protein | Putative phage tail fiber protein | | afdb-uniprot50 | AF-A0A496KG49-F1-MODEL\_V4 | 1.0 | 2.154e-08 | 194 | 0.279 | 222 | 101 | 10 | 11 | 221 | 106 | 279 | Collar domain-containing protein | Collar domain-containing protein | | afdb-uniprot50 | AF-A0A7Z7RIG7-F1-MODEL\_V4 | 1.0 | 1.44e-07 | 194 | 0.216 | 222 | 112 | 9 | 1 | 221 | 144 | 304 | Phage Tail Collar Domain | Phage Tail Collar Domain | | afdb-uniprot50 | AF-A0A6P1ZJ46-F1-MODEL\_V4 | 1.0 | 1.44e-07 | 194 | 0.287 | 216 | 112 | 9 | 12 | 221 | 204 | 383 | Collar domain-containing protein | Collar domain-containing protein | | afdb-uniprot50 | AF-A0A378UGB4-F1-MODEL\_V4 | 1.0 | 1.135e-07 | 194 | 0.225 | 222 | 110 | 8 | 11 | 220 | 471 | 642 | Putative phage tail fiber protein | Putative phage tail fiber protein | | afdb-uniprot50 | AF-A0A7Y1CQT0-F1-MODEL\_V4 | 1.0 | 1.528e-07 | 194 | 0.256 | 218 | 87 | 6 | 11 | 221 | 503 | 652 | Collar domain-containing protein | Collar domain-containing protein | | afdb-uniprot50 | AF-R7I616-F1-MODEL\_V4 | 1.0 | 6.655e-08 | 193 | 0.217 | 230 | 97 | 9 | 2 | 221 | 94 | 250 | Collar domain-containing protein | Collar domain-containing protein | | afdb-uniprot50 | AF-A0A2H0ZA63-F1-MODEL\_V4 | 1.0 | 5.314e-07 | 193 | 0.235 | 212 | 92 | 8 | 11 | 221 | 129 | 271 | Collar domain-containing protein | Collar domain-containing protein | | afdb-uniprot50 | AF-A0A817YYG9-F1-MODEL\_V4 | 1.0 | 1.19e-08 | 193 | 0.32 | 206 | 97 | 6 | 20 | 216 | 395 | 566 | Hypothetical protein | Hypothetical protein | | afdb-uniprot50 | AF-G9PUK2-F1-MODEL\_V4 | 1.0 | 2.082e-06 | 192 | 0.238 | 214 | 96 | 10 | 12 | 220 | 24 | 175 | Collar domain-containing protein | Collar domain-containing protein | | afdb-uniprot50 | AF-A0A285UDY9-F1-MODEL\_V4 | 1.0 | 3.464e-08 | 192 | 0.303 | 231 | 121 | 11 | 11 | 221 | 156 | 366 | Tail collar domain | Tail collar domain | | afdb-uniprot50 | AF-C3X912-F1-MODEL\_V4 | 1.0 | 2.456e-07 | 192 | 0.224 | 218 | 95 | 10 | 11 | 221 | 286 | 436 | Phage Tail Collar Domain protein | Phage Tail Collar Domain protein | | afdb-uniprot50 | AF-C5B185-F1-MODEL\_V4 | 1.0 | 3.305e-07 | 192 | 0.235 | 212 | 91 | 6 | 11 | 221 | 308 | 449 | Collar domain-containing protein | Collar domain-containing protein | | afdb-uniprot50 | AF-A0A5E7KHM1-F1-MODEL\_V4 | 1.0 | 4.946e-08 | 192 | 0.302 | 251 | 120 | 9 | 12 | 221 | 305 | 541 | Collar domain-containing protein | Collar domain-containing protein | | afdb-uniprot50 | AF-A0A1G9EQI8-F1-MODEL\_V4 | 1.0 | 3.305e-07 | 192 | 0.2 | 215 | 108 | 10 | 11 | 221 | 401 | 555 | Phage-related tail fibre protein | Phage-related tail fibre protein | | afdb-uniprot50 | AF-A0A7T9LJD7-F1-MODEL\_V4 | 1.0 | 2.181e-07 | 191 | 0.251 | 223 | 98 | 11 | 3 | 221 | 13 | 170 | Tail fiber protein | Tail fiber protein | | afdb-uniprot50 | AF-A0A815S8I3-F1-MODEL\_V4 | 1.0 | 1.295e-06 | 191 | 0.355 | 169 | 91 | 5 | 11 | 171 | 16 | 174 | Hypothetical protein | Hypothetical protein | | afdb-uniprot50 | AF-A0A0P9CHF4-F1-MODEL\_V4 | 1.0 | 8.545e-07 | 191 | 0.218 | 220 | 115 | 9 | 4 | 221 | 70 | 234 | Collar domain-containing protein | Collar domain-containing protein | | afdb-uniprot50 | AF-A0A6B3UTZ7-F1-MODEL\_V4 | 1.0 | 1.135e-07 | 191 | 0.283 | 212 | 79 | 7 | 11 | 221 | 106 | 245 | Phage tail protein | Phage tail protein | | afdb-uniprot50 | AF-A0A662DPJ2-F1-MODEL\_V4 | 1.0 | 1.44e-07 | 191 | 0.264 | 246 | 130 | 13 | 2 | 221 | 50 | 270 | Collar domain-containing protein | Collar domain-containing protein | | afdb-uniprot50 | AF-A0A2S9XA95-F1-MODEL\_V4 | 1.0 | 2.456e-07 | 191 | 0.246 | 223 | 104 | 9 | 1 | 221 | 158 | 318 | Collar domain-containing protein | Collar domain-containing protein | | afdb-uniprot50 | AF-A0A2K9CDC4-F1-MODEL\_V4 | 1.0 | 2.315e-07 | 191 | 0.241 | 232 | 108 | 8 | 1 | 221 | 145 | 319 | Collar domain-containing protein | Collar domain-containing protein | | afdb-uniprot50 | AF-A0A2N9Y0U7-F1-MODEL\_V4 | 1.0 | 3.115e-07 | 191 | 0.243 | 222 | 93 | 9 | 11 | 221 | 172 | 329 | Collar domain-containing protein | Collar domain-containing protein | | afdb-uniprot50 | AF-A0A2W0GCJ0-F1-MODEL\_V4 | 1.0 | 8.954e-08 | 191 | 0.284 | 211 | 89 | 7 | 11 | 221 | 233 | 381 | Phage tail protein | Phage tail protein | | afdb-uniprot50 | AF-A0A3B8Q380-F1-MODEL\_V4 | 1.0 | 3.95e-07 | 191 | 0.215 | 232 | 119 | 10 | 1 | 221 | 220 | 399 | Collar domain-containing protein | Collar domain-containing protein | | afdb-uniprot50 | AF-V9H7W8-F1-MODEL\_V4 | 1.0 | 3.95e-07 | 191 | 0.25 | 228 | 108 | 11 | 2 | 221 | 249 | 421 | Collar domain-containing protein | Collar domain-containing protein | | afdb-uniprot50 | AF-A0A2K4ISX8-F1-MODEL\_V4 | 1.0 | 5.314e-07 | 191 | 0.284 | 211 | 90 | 9 | 11 | 221 | 508 | 657 | Phage tail protein | Phage tail protein | | afdb-uniprot50 | AF-A0A3S0SEE9-F1-MODEL\_V4 | 1.0 | 8.954e-08 | 190 | 0.277 | 227 | 101 | 9 | 4 | 221 | 91 | 263 | Collar domain-containing protein | Collar domain-containing protein | | afdb-uniprot50 | AF-A0A835ZHE3-F1-MODEL\_V4 | 1.0 | 5.984e-07 | 190 | 0.284 | 225 | 120 | 10 | 12 | 220 | 4 | 203 | Uncharacterized protein | Uncharacterized protein | | afdb-uniprot50 | AF-A0A3C1RWZ2-F1-MODEL\_V4 | 1.0 | 7.062e-08 | 190 | 0.261 | 268 | 147 | 9 | 3 | 221 | 49 | 314 | Collar domain-containing protein | Collar domain-containing protein | | afdb-uniprot50 | AF-A0A5M8P654-F1-MODEL\_V4 | 1.0 | 5.569e-08 | 190 | 0.254 | 216 | 110 | 11 | 11 | 221 | 171 | 340 | Phage tail protein | Phage tail protein | | afdb-uniprot50 | AF-A0A3C0UIR2-F1-MODEL\_V4 | 1.0 | 9.502e-08 | 190 | 0.271 | 210 | 87 | 9 | 12 | 220 | 168 | 312 | Collar domain-containing protein | Collar domain-containing protein | | afdb-uniprot50 | AF-A0A143DG88-F1-MODEL\_V4 | 1.0 | 6.739e-07 | 190 | 0.291 | 158 | 83 | 7 | 11 | 162 | 255 | 389 | Collar domain-containing protein | Collar domain-containing protein | | afdb-uniprot50 | AF-A0A822BJ94-F1-MODEL\_V4 | 1.0 | 2.209e-06 | 190 | 0.368 | 163 | 83 | 5 | 11 | 166 | 407 | 556 | Hypothetical protein | Hypothetical protein | | afdb-uniprot50 | AF-A0A1S8LA53-F1-MODEL\_V4 | 1.0 | 1.937e-07 | 190 | 0.247 | 210 | 91 | 7 | 12 | 221 | 669 | 811 | Phage tail collar domain protein | Phage tail collar domain protein | | afdb-uniprot50 | AF-A0A1Y4GGY2-F1-MODEL\_V4 | 1.0 | 1.07e-07 | 189 | 0.256 | 222 | 104 | 10 | 12 | 220 | 24 | 197 | Collar domain-containing protein | Collar domain-containing protein | | afdb-uniprot50 | AF-A0A292RR50-F1-MODEL\_V4 | 1.0 | 2.181e-07 | 188 | 0.168 | 237 | 99 | 11 | 3 | 221 | 49 | 205 | Collar domain-containing protein | Collar domain-containing protein | | afdb-uniprot50 | AF-A0A222FFN0-F1-MODEL\_V4 | 1.0 | 4.448e-07 | 188 | 0.19 | 226 | 140 | 10 | 11 | 220 | 30 | 228 | Collar domain-containing protein | Collar domain-containing protein | | afdb-uniprot50 | AF-C3X3V5-F1-MODEL\_V4 | 1.0 | 3.676e-08 | 188 | 0.226 | 225 | 114 | 8 | 3 | 220 | 87 | 258 | Collar domain-containing protein | Collar domain-containing protein | | afdb-uniprot50 | AF-K9F0F3-F1-MODEL\_V4 | 1.0 | 1.357e-07 | 188 | 0.27 | 248 | 139 | 11 | 3 | 220 | 31 | 266 | Microcystin-dependent protein | Microcystin-dependent protein | | afdb-uniprot50 | AF-A0A1Y6D0G3-F1-MODEL\_V4 | 1.0 | 3.722e-07 | 188 | 0.246 | 211 | 98 | 7 | 12 | 221 | 166 | 316 | Phage Tail Collar Domain | Phage Tail Collar Domain | | afdb-uniprot50 | AF-A0A5B7ZM63-F1-MODEL\_V4 | 1.0 | 9.502e-08 | 188 | 0.232 | 237 | 89 | 11 | 3 | 221 | 179 | 340 | Tail fiber protein | Tail fiber protein | | afdb-uniprot50 | AF-A0A7U2FZZ3-F1-MODEL\_V4 | 1.0 | 2.181e-07 | 188 | 0.226 | 230 | 101 | 10 | 1 | 221 | 254 | 415 | Tail fiber protein | Tail fiber protein | | afdb-uniprot50 | AF-A0A7S7WIR9-F1-MODEL\_V4 | 1.0 | 1.135e-07 | 187 | 0.272 | 224 | 91 | 11 | 10 | 221 | 80 | 243 | Collar domain-containing protein | Collar domain-containing protein | | afdb-uniprot50 | AF-A0A135WED4-F1-MODEL\_V4 | 1.0 | 1.205e-07 | 187 | 0.254 | 232 | 92 | 13 | 11 | 221 | 147 | 318 | Collar domain-containing protein | Collar domain-containing protein | | afdb-uniprot50 | AF-U1AK99-F1-MODEL\_V4 | 1.0 | 3.305e-07 | 187 | 0.263 | 228 | 91 | 10 | 2 | 221 | 263 | 421 | Collar domain-containing protein | Collar domain-containing protein | | afdb-uniprot50 | AF-A0A0K8JHT0-F1-MODEL\_V4 | 1.0 | 1.07e-07 | 187 | 0.264 | 212 | 90 | 9 | 12 | 220 | 196 | 344 | Collar domain-containing protein | Collar domain-containing protein | | afdb-uniprot50 | AF-A7INV5-F1-MODEL\_V4 | 1.0 | 4.448e-07 | 187 | 0.203 | 211 | 94 | 7 | 11 | 221 | 356 | 492 | Tail Collar domain protein | Tail Collar domain protein | | afdb-uniprot50 | AF-A0A0D9LFX9-F1-MODEL\_V4 | 1.0 | 1.72e-07 | 186 | 0.303 | 211 | 70 | 9 | 12 | 221 | 206 | 340 | Phage Tail Collar domain protein | Phage Tail Collar domain protein | | afdb-uniprot50 | AF-A0A381EVQ5-F1-MODEL\_V4 | 1.0 | 6.271e-08 | 186 | 0.265 | 230 | 96 | 9 | 11 | 221 | 190 | 365 | Phage Tail Collar Domain | Phage Tail Collar Domain | | afdb-uniprot50 | AF-C3X3K6-F1-MODEL\_V4 | 1.0 | 3.507e-07 | 186 | 0.227 | 237 | 104 | 11 | 2 | 220 | 198 | 373 | Collar domain-containing protein | Collar domain-containing protein | | afdb-uniprot50 | AF-A0A1Q3ZE37-F1-MODEL\_V4 | 1.0 | 1.458e-06 | 186 | 0.202 | 217 | 102 | 8 | 12 | 220 | 456 | 609 | Collar domain-containing protein | Collar domain-containing protein | | afdb-uniprot50 | AF-A0A817T034-F1-MODEL\_V4 | 1.0 | 2.574e-08 | 186 | 0.312 | 211 | 106 | 10 | 16 | 216 | 420 | 601 | Hypothetical protein | Hypothetical protein | | afdb-uniprot50 | AF-A0A353WK11-F1-MODEL\_V4 | 1.0 | 2.766e-07 | 185 | 0.258 | 217 | 85 | 7 | 6 | 221 | 85 | 226 | Collar domain-containing protein | Collar domain-containing protein | | afdb-uniprot50 | AF-C3X3V2-F1-MODEL\_V4 | 1.0 | 1.72e-07 | 185 | 0.227 | 233 | 92 | 10 | 3 | 220 | 129 | 288 | Collar domain-containing protein | Collar domain-containing protein | | afdb-uniprot50 | AF-C3X8V2-F1-MODEL\_V4 | 1.0 | 2.607e-07 | 185 | 0.243 | 226 | 96 | 9 | 3 | 220 | 70 | 228 | Phage Tail Collar Domain protein | Phage Tail Collar Domain protein | | afdb-uniprot50 | AF-A0A352DBM0-F1-MODEL\_V4 | 1.0 | 3.722e-07 | 185 | 0.219 | 214 | 103 | 7 | 9 | 221 | 351 | 501 | Collar domain-containing protein | Collar domain-containing protein | | afdb-uniprot50 | AF-A0A3M2DZ18-F1-MODEL\_V4 | 1.0 | 1.07e-07 | 184 | 0.276 | 235 | 109 | 8 | 1 | 220 | 223 | 411 | Tail fiber protein | Tail fiber protein | | afdb-uniprot50 | AF-Q7P176-F1-MODEL\_V4 | 1.0 | 5.008e-07 | 184 | 0.206 | 223 | 115 | 10 | 1 | 221 | 273 | 435 | Probable bacteriophge tail fiber protein | Probable bacteriophge tail fiber protein | | afdb-uniprot50 | AF-A0A7R7UYC3-F1-MODEL\_V4 | 1.0 | 4.448e-07 | 184 | 0.279 | 211 | 84 | 7 | 11 | 221 | 453 | 595 | Collar domain-containing protein | Collar domain-containing protein | | afdb-uniprot50 | AF-A0A2N3LS20-F1-MODEL\_V4 | 1.0 | 1.083e-06 | 183 | 0.203 | 211 | 91 | 8 | 13 | 221 | 176 | 311 | Collar domain-containing protein | Collar domain-containing protein | | afdb-uniprot50 | AF-A0A822BDI4-F1-MODEL\_V4 | 1.0 | 9.067e-07 | 182 | 0.349 | 166 | 71 | 7 | 12 | 162 | 29 | 172 | Hypothetical protein | Hypothetical protein | | afdb-uniprot50 | AF-A0A821UTN8-F1-MODEL\_V4 | 1.0 | 1.295e-06 | 182 | 0.261 | 172 | 78 | 6 | 11 | 179 | 348 | 473 | Hypothetical protein | Hypothetical protein | | afdb-uniprot50 | AF-A0A3N9U476-F1-MODEL\_V4 | 1.0 | 1.742e-06 | 182 | 0.218 | 211 | 101 | 8 | 11 | 221 | 362 | 508 | Collar domain-containing protein | Collar domain-containing protein | | afdb-uniprot50 | AF-A0A7W9ZHL4-F1-MODEL\_V4 | 1.0 | 1.295e-06 | 181 | 0.24 | 212 | 122 | 7 | 11 | 220 | 5 | 179 | Microcystin-dependent protein | Microcystin-dependent protein | | afdb-uniprot50 | AF-A0A661CNJ0-F1-MODEL\_V4 | 1.0 | 3.115e-07 | 181 | 0.229 | 222 | 122 | 8 | 12 | 220 | 13 | 198 | Collar domain-containing protein | Collar domain-containing protein | | afdb-uniprot50 | AF-A0A6C0KKB8-F1-MODEL\_V4 | 1.0 | 2.456e-07 | 181 | 0.266 | 214 | 113 | 7 | 11 | 221 | 31 | 203 | Collar domain-containing protein | Collar domain-containing protein | | afdb-uniprot50 | AF-A0A7W9FLH6-F1-MODEL\_V4 | 1.0 | 1.15e-06 | 181 | 0.204 | 220 | 139 | 8 | 10 | 220 | 14 | 206 | Microcystin-dependent protein | Microcystin-dependent protein | | afdb-uniprot50 | AF-C3X192-F1-MODEL\_V4 | 1.0 | 3.95e-07 | 181 | 0.245 | 228 | 100 | 9 | 1 | 220 | 67 | 230 | Collar domain-containing protein | Collar domain-containing protein | | afdb-uniprot50 | AF-A0A2S5DKP9-F1-MODEL\_V4 | 1.0 | 2.607e-07 | 181 | 0.231 | 225 | 107 | 9 | 2 | 221 | 218 | 381 | Collar domain-containing protein | Collar domain-containing protein | | afdb-uniprot50 | AF-A0A2M9R257-F1-MODEL\_V4 | 1.0 | 8.843e-09 | 181 | 0.286 | 269 | 125 | 14 | 11 | 221 | 149 | 408 | Collar domain-containing protein | Collar domain-containing protein | | afdb-uniprot50 | AF-C3X7Z8-F1-MODEL\_V4 | 1.0 | 4.448e-07 | 181 | 0.244 | 209 | 94 | 7 | 12 | 220 | 201 | 345 | Phage Tail Collar Domain protein | Phage Tail Collar Domain protein | | afdb-uniprot50 | AF-A0A7Y6S8C6-F1-MODEL\_V4 | 1.0 | 2.209e-06 | 180 | 0.29 | 200 | 90 | 8 | 22 | 221 | 2 | 149 | Tail fiber protein | Tail fiber protein | | afdb-uniprot50 | AF-A0A177M7V7-F1-MODEL\_V4 | 1.0 | 5.984e-07 | 180 | 0.221 | 239 | 128 | 13 | 11 | 220 | 5 | 214 | Collar domain-containing protein | Collar domain-containing protein | | afdb-uniprot50 | AF-A0A816UWW5-F1-MODEL\_V4 | 1.0 | 4.779e-06 | 180 | 0.228 | 171 | 89 | 4 | 11 | 179 | 180 | 309 | Hypothetical protein | Hypothetical protein | | afdb-uniprot50 | AF-C3X8I7-F1-MODEL\_V4 | 1.0 | 6.35e-07 | 180 | 0.23 | 226 | 97 | 9 | 2 | 220 | 96 | 251 | Phage Tail Collar Domain protein | Phage Tail Collar Domain protein | | afdb-uniprot50 | AF-A0A143DGR5-F1-MODEL\_V4 | 1.0 | 4.191e-07 | 180 | 0.218 | 224 | 92 | 8 | 11 | 220 | 329 | 483 | Collar domain-containing protein | Collar domain-containing protein | | afdb-uniprot50 | AF-A0A820VF90-F1-MODEL\_V4 | 1.0 | 1.458e-06 | 179 | 0.256 | 222 | 115 | 8 | 11 | 220 | 1 | 184 | Hypothetical protein | Hypothetical protein | | afdb-uniprot50 | AF-A0A074JSZ8-F1-MODEL\_V4 | 1.0 | 2.766e-07 | 179 | 0.235 | 221 | 91 | 7 | 12 | 220 | 57 | 211 | Collar domain-containing protein | Collar domain-containing protein | | afdb-uniprot50 | AF-A0A1G8DPL3-F1-MODEL\_V4 | 1.0 | 2.286e-08 | 179 | 0.245 | 253 | 130 | 15 | 11 | 220 | 4 | 238 | Microcystin-dependent protein | Microcystin-dependent protein | | afdb-uniprot50 | AF-A0A356FYY9-F1-MODEL\_V4 | 1.0 | 1.826e-07 | 178 | 0.198 | 237 | 108 | 11 | 2 | 221 | 87 | 258 | Collar domain-containing protein | Collar domain-containing protein | | afdb-uniprot50 | AF-A0A6P0WRL1-F1-MODEL\_V4 | 1.0 | 3.115e-07 | 178 | 0.24 | 254 | 150 | 5 | 6 | 220 | 67 | 316 | Collar domain-containing protein | Collar domain-containing protein | | afdb-uniprot50 | AF-A0A336NEY6-F1-MODEL\_V4 | 1.0 | 5.008e-07 | 178 | 0.211 | 218 | 120 | 7 | 12 | 221 | 160 | 333 | Phage Tail Collar Domain | Phage Tail Collar Domain | | afdb-uniprot50 | AF-C3X3R8-F1-MODEL\_V4 | 1.0 | 6.35e-07 | 178 | 0.218 | 233 | 95 | 9 | 3 | 220 | 78 | 238 | Collar domain-containing protein | Collar domain-containing protein | | afdb-uniprot50 | AF-A0A8B2QXZ8-F1-MODEL\_V4 | 1.0 | 2.315e-07 | 178 | 0.231 | 212 | 95 | 8 | 11 | 221 | 571 | 715 | Tail collar domain | Tail collar domain | | afdb-uniprot50 | AF-C3XAA4-F1-MODEL\_V4 | 1.0 | 1.083e-06 | 177 | 0.207 | 217 | 81 | 8 | 11 | 220 | 15 | 147 | Phage Tail Collar Domain protein | Phage Tail Collar Domain protein | | afdb-uniprot50 | AF-A0A7Z7RHC2-F1-MODEL\_V4 | 1.0 | 1.021e-06 | 177 | 0.224 | 218 | 103 | 8 | 11 | 221 | 586 | 744 | Phage Tail Collar Domain | Phage Tail Collar Domain | | afdb-uniprot50 | AF-A0A521ZHZ2-F1-MODEL\_V4 | 1.0 | 1.642e-06 | 176 | 0.242 | 227 | 99 | 13 | 2 | 220 | 211 | 372 | Tail fiber protein | Tail fiber protein | | afdb-uniprot50 | AF-C3X8V7-F1-MODEL\_V4 | 1.0 | 1.22e-06 | 175 | 0.236 | 224 | 99 | 8 | 3 | 220 | 181 | 338 | Phage Tail Collar Domain protein | Phage Tail Collar Domain protein | | afdb-uniprot50 | AF-A0A7Z0JL73-F1-MODEL\_V4 | 1.0 | 1.021e-06 | 175 | 0.208 | 254 | 142 | 15 | 3 | 220 | 693 | 923 | Microcystin-dependent protein | Microcystin-dependent protein | | afdb-uniprot50 | AF-A0A6P3BUP6-F1-MODEL\_V4 | 1.0 | 9.622e-07 | 174 | 0.237 | 240 | 150 | 8 | 3 | 220 | 93 | 321 | Putative phage tail protein | Putative phage tail protein | | afdb-uniprot50 | AF-A0A3R8V3H5-F1-MODEL\_V4 | 1.0 | 7.952e-08 | 174 | 0.284 | 271 | 122 | 11 | 11 | 221 | 68 | 326 | Tail fiber protein | Tail fiber protein | | afdb-uniprot50 | AF-J1JT46-F1-MODEL\_V4 | 1.0 | 3.722e-07 | 174 | 0.195 | 256 | 141 | 9 | 1 | 221 | 149 | 374 | Collar domain-containing protein | Collar domain-containing protein | | afdb-uniprot50 | AF-C3X1Y2-F1-MODEL\_V4 | 1.0 | 1.374e-06 | 174 | 0.255 | 211 | 92 | 7 | 12 | 220 | 191 | 338 | Collar domain-containing protein | Collar domain-containing protein | | afdb-uniprot50 | AF-A0A2W5JYE1-F1-MODEL\_V4 | 1.0 | 5.314e-07 | 173 | 0.236 | 220 | 94 | 7 | 1 | 220 | 154 | 299 | Collar domain-containing protein | Collar domain-containing protein | | afdb-uniprot50 | AF-A0A661P6R3-F1-MODEL\_V4 | 1.0 | 1.008e-07 | 173 | 0.303 | 231 | 113 | 10 | 3 | 214 | 63 | 264 | Collar domain-containing protein | Collar domain-containing protein | | afdb-uniprot50 | AF-A0A0C4Y828-F1-MODEL\_V4 | 1.0 | 4.448e-07 | 173 | 0.195 | 246 | 162 | 8 | 1 | 220 | 286 | 521 | Phage-related tail fiber protein | Phage-related tail fiber protein | | afdb-uniprot50 | AF-A0A251X838-F1-MODEL\_V4 | 1.0 | 2.801e-06 | 172 | 0.184 | 244 | 136 | 14 | 3 | 221 | 149 | 354 | Collar domain-containing protein | Collar domain-containing protein | | afdb-uniprot50 | AF-A0A7W8WHR4-F1-MODEL\_V4 | 1.0 | 5.984e-07 | 172 | 0.189 | 259 | 149 | 11 | 2 | 220 | 457 | 694 | Microcystin-dependent protein | Microcystin-dependent protein | | afdb-uniprot50 | AF-A0A1H9M0F1-F1-MODEL\_V4 | 1.0 | 3.154e-06 | 171 | 0.193 | 227 | 129 | 10 | 8 | 220 | 31 | 217 | Microcystin-dependent protein | Microcystin-dependent protein | | afdb-uniprot50 | AF-A0A3B9Q8G5-F1-MODEL\_V4 | 1.0 | 7.151e-07 | 171 | 0.247 | 226 | 80 | 8 | 11 | 221 | 152 | 302 | Collar domain-containing protein | Collar domain-containing protein | | afdb-uniprot50 | AF-A0A819J9H2-F1-MODEL\_V4 | 1.0 | 4.191e-07 | 171 | 0.217 | 221 | 91 | 9 | 11 | 220 | 183 | 332 | Hypothetical protein | Hypothetical protein | | afdb-uniprot50 | AF-A0A7X3HHB3-F1-MODEL\_V4 | 1.0 | 3.722e-07 | 170 | 0.204 | 250 | 155 | 13 | 1 | 220 | 207 | 442 | Uncharacterized protein | Uncharacterized protein | | afdb-uniprot50 | AF-A0A660N0Q8-F1-MODEL\_V4 | 1.0 | 5.382e-06 | 169 | 0.254 | 220 | 101 | 8 | 16 | 221 | 2 | 172 | Collar domain-containing protein | Collar domain-containing protein | | afdb-uniprot50 | AF-A0A3D5BA67-F1-MODEL\_V4 | 1.0 | 8.052e-07 | 169 | 0.217 | 221 | 97 | 10 | 3 | 220 | 92 | 239 | Collar domain-containing protein | Collar domain-containing protein | | afdb-uniprot50 | AF-A0A6P0WKN5-F1-MODEL\_V4 | 1.0 | 5.314e-07 | 169 | 0.252 | 222 | 121 | 7 | 3 | 214 | 51 | 237 | Tail fiber protein | Tail fiber protein | | afdb-uniprot50 | AF-A0A2E1KV02-F1-MODEL\_V4 | 1.0 | 5.248e-08 | 169 | 0.336 | 229 | 110 | 8 | 11 | 214 | 49 | 260 | Uncharacterized protein | Uncharacterized protein | | afdb-uniprot50 | AF-A0A2N8HS50-F1-MODEL\_V4 | 1.0 | 1.458e-06 | 168 | 0.21 | 214 | 104 | 8 | 11 | 221 | 95 | 246 | Collar domain-containing protein | Collar domain-containing protein | | afdb-uniprot50 | AF-A0A820D142-F1-MODEL\_V4 | 1.0 | 5.984e-07 | 168 | 0.317 | 192 | 106 | 6 | 19 | 210 | 28 | 194 | Hypothetical protein | Hypothetical protein | | afdb-uniprot50 | AF-C3X910-F1-MODEL\_V4 | 1.0 | 3.722e-07 | 168 | 0.264 | 212 | 86 | 9 | 12 | 220 | 265 | 409 | Phage Tail Collar Domain protein | Phage Tail Collar Domain protein | | afdb-uniprot50 | AF-A0A381EST0-F1-MODEL\_V4 | 1.0 | 3.95e-07 | 167 | 0.233 | 240 | 91 | 10 | 11 | 221 | 235 | 410 | Phage Tail Collar Domain | Phage Tail Collar Domain | | afdb-uniprot50 | AF-A0A2S9X7Q4-F1-MODEL\_V4 | 1.0 | 1.44e-07 | 167 | 0.284 | 211 | 103 | 11 | 11 | 221 | 392 | 554 | Collar domain-containing protein | Collar domain-containing protein | | afdb-uniprot50 | AF-A0A5S9PHC1-F1-MODEL\_V4 | 1.0 | 7.241e-06 | 166 | 0.192 | 223 | 132 | 10 | 9 | 220 | 27 | 212 | Collar domain-containing protein | Collar domain-containing protein | | afdb-uniprot50 | AF-A0A7Y8KXK1-F1-MODEL\_V4 | 1.0 | 6.739e-07 | 166 | 0.216 | 226 | 120 | 12 | 2 | 220 | 129 | 304 | Tail fiber protein | Tail fiber protein | | afdb-uniprot50 | AF-A0A1X0TJA2-F1-MODEL\_V4 | 1.0 | 1.567e-05 | 165 | 0.364 | 118 | 60 | 4 | 20 | 136 | 1 | 104 | Collar domain-containing protein | Collar domain-containing protein | | afdb-uniprot50 | AF-A0A6L5JUV3-F1-MODEL\_V4 | 1.0 | 1.235e-05 | 165 | 0.228 | 206 | 83 | 6 | 15 | 220 | 35 | 164 | Collar domain-containing protein | Collar domain-containing protein | | afdb-uniprot50 | AF-A0A1Y1Q392-F1-MODEL\_V4 | 1.0 | 2.519e-05 | 165 | 0.325 | 166 | 87 | 6 | 2 | 162 | 54 | 199 | Collar domain-containing protein | Collar domain-containing protein | | afdb-uniprot50 | AF-A0A7V8JNI0-F1-MODEL\_V4 | 1.0 | 3.676e-08 | 165 | 0.266 | 270 | 138 | 8 | 1 | 221 | 172 | 430 | Collar domain-containing protein | Collar domain-containing protein | | afdb-uniprot50 | AF-A0A816C073-F1-MODEL\_V4 | 1.0 | 1.295e-06 | 165 | 0.307 | 195 | 110 | 6 | 20 | 214 | 42 | 211 | Hypothetical protein | Hypothetical protein | | afdb-uniprot50 | AF-A0A1Y4GG09-F1-MODEL\_V4 | 1.0 | 1.22e-06 | 164 | 0.205 | 238 | 93 | 10 | 12 | 220 | 24 | 194 | Collar domain-containing protein | Collar domain-containing protein | | afdb-uniprot50 | AF-A0A522VZY9-F1-MODEL\_V4 | 1.0 | 1.15e-06 | 164 | 0.219 | 232 | 138 | 9 | 12 | 220 | 2 | 213 | Tail fiber protein | Tail fiber protein | | afdb-uniprot50 | AF-A0A2D5I6Z8-F1-MODEL\_V4 | 1.0 | 1.826e-07 | 164 | 0.268 | 227 | 137 | 9 | 11 | 220 | 25 | 239 | Collar domain-containing protein | Collar domain-containing protein | | afdb-uniprot50 | AF-A0A7Y5MLB7-F1-MODEL\_V4 | 1.0 | 9.622e-07 | 163 | 0.259 | 227 | 117 | 12 | 6 | 221 | 175 | 361 | Tail fiber protein | Tail fiber protein | | afdb-uniprot50 | AF-A0A3A9HNI7-F1-MODEL\_V4 | 1.0 | 2.64e-06 | 162 | 0.252 | 218 | 92 | 9 | 11 | 220 | 20 | 174 | Tail fiber protein | Tail fiber protein | | afdb-uniprot50 | AF-A0A7H2RJ25-F1-MODEL\_V4 | 1.0 | 5.382e-06 | 162 | 0.165 | 218 | 116 | 7 | 3 | 220 | 64 | 215 | Tail fiber protein | Tail fiber protein | | afdb-uniprot50 | AF-A0A6I2KGM5-F1-MODEL\_V4 | 1.0 | 2.607e-07 | 162 | 0.214 | 270 | 132 | 12 | 11 | 220 | 79 | 328 | Tail fiber protein | Tail fiber protein | | afdb-uniprot50 | AF-A0A7T1Y6B1-F1-MODEL\_V4 | 1.0 | 4.779e-06 | 162 | 0.203 | 221 | 106 | 9 | 11 | 221 | 233 | 393 | Phage tail protein | Phage tail protein | | afdb-uniprot50 | AF-A0A448V8J3-F1-MODEL\_V4 | 1.0 | 6.739e-07 | 162 | 0.208 | 292 | 135 | 11 | 12 | 221 | 159 | 436 | Phage Tail Collar Domain | Phage Tail Collar Domain | | afdb-uniprot50 | AF-A0A1Z7Z823-F1-MODEL\_V4 | 1.0 | 6.35e-07 | 162 | 0.281 | 213 | 81 | 6 | 9 | 221 | 418 | 558 | Collar domain-containing protein | Collar domain-containing protein | | afdb-uniprot50 | AF-A0A077LCE0-F1-MODEL\_V4 | 1.0 | 7.588e-07 | 161 | 0.238 | 235 | 119 | 11 | 11 | 220 | 5 | 204 | Phage Tail Collar domain protein | Phage Tail Collar domain protein | | afdb-uniprot50 | AF-A0A6L6YMH3-F1-MODEL\_V4 | 1.0 | 2.64e-06 | 160 | 0.198 | 227 | 106 | 8 | 3 | 221 | 114 | 272 | Collar domain-containing protein | Collar domain-containing protein | | afdb-uniprot50 | AF-A9ITY1-F1-MODEL\_V4 | 1.0 | 1.872e-05 | 160 | 0.168 | 244 | 119 | 5 | 2 | 221 | 116 | 299 | Phage related protein | Phage related protein | | afdb-uniprot50 | AF-Q0BEK5-F1-MODEL\_V4 | 1.0 | 2.209e-06 | 159 | 0.243 | 246 | 142 | 9 | 1 | 220 | 506 | 733 | Phage Tail Collar domain protein | Phage Tail Collar domain protein | | afdb-uniprot50 | AF-A0A7C4AHI4-F1-MODEL\_V4 | 1.0 | 2.972e-06 | 155 | 0.221 | 212 | 93 | 8 | 11 | 220 | 79 | 220 | Collar domain-containing protein | Collar domain-containing protein | | afdb-uniprot50 | AF-A0A246KV99-F1-MODEL\_V4 | 1.0 | 3.507e-07 | 155 | 0.29 | 248 | 135 | 10 | 11 | 221 | 246 | 489 | Collar domain-containing protein | Collar domain-containing protein | | afdb-uniprot50 | AF-A0A1Y4GRC1-F1-MODEL\_V4 | 1.0 | 1.235e-05 | 154 | 0.22 | 222 | 94 | 11 | 11 | 221 | 23 | 176 | Collar domain-containing protein | Collar domain-containing protein | | afdb-uniprot50 | AF-A0A368TXZ3-F1-MODEL\_V4 | 1.0 | 4.244e-06 | 154 | 0.216 | 217 | 112 | 9 | 11 | 221 | 273 | 437 | Collar domain-containing protein | Collar domain-containing protein | | afdb-uniprot50 | AF-A0A1Y5MHL2-F1-MODEL\_V4 | 1.0 | 1.742e-06 | 154 | 0.21 | 223 | 97 | 10 | 11 | 221 | 507 | 662 | Collar domain-containing protein | Collar domain-containing protein | | afdb-uniprot50 | AF-A0A817QMQ8-F1-MODEL\_V4 | 1.0 | 5.639e-07 | 152 | 0.293 | 218 | 126 | 10 | 11 | 221 | 20 | 216 | Hypothetical protein | Hypothetical protein | | afdb-uniprot50 | AF-C3X9T8-F1-MODEL\_V4 | 1.0 | 7.241e-06 | 151 | 0.23 | 217 | 83 | 11 | 12 | 220 | 196 | 336 | Phage Tail Collar Domain protein | Phage Tail Collar Domain protein | | afdb-uniprot50 | AF-A0A1G9MSY8-F1-MODEL\_V4 | 1.0 | 1.476e-05 | 150 | 0.187 | 315 | 134 | 16 | 2 | 221 | 103 | 390 | Uncharacterized protein | Uncharacterized protein | | afdb-uniprot50 | AF-A0A1A9RLT3-F1-MODEL\_V4 | 1.0 | 1.097e-05 | 150 | 0.218 | 211 | 87 | 8 | 12 | 220 | 155 | 289 | Uncharacterized protein | Uncharacterized protein | | afdb-uniprot50 | AF-C1D6M7-F1-MODEL\_V4 | 1.0 | 3.154e-06 | 149 | 0.239 | 209 | 101 | 10 | 12 | 220 | 105 | 255 | Phage-related protein | Phage-related protein | | afdb-uniprot50 | AF-A0A662BH77-F1-MODEL\_V4 | 1.0 | 1.391e-05 | 149 | 0.211 | 227 | 130 | 10 | 3 | 220 | 354 | 540 | Uncharacterized protein | Uncharacterized protein | | afdb-uniprot50 | AF-A0A437M1B9-F1-MODEL\_V4 | 1.0 | 2.108e-05 | 149 | 0.229 | 309 | 135 | 14 | 1 | 221 | 337 | 630 | Uncharacterized protein | Uncharacterized protein | | afdb-uniprot50 | AF-A0A560C055-F1-MODEL\_V4 | 1.0 | 6.824e-06 | 147 | 0.209 | 229 | 77 | 5 | 3 | 221 | 728 | 862 | Tail collar domain | Tail collar domain | | afdb-uniprot50 | AF-I4FLZ9-F1-MODEL\_V4 | 1.0 | 4.05e-05 | 147 | 0.244 | 213 | 113 | 6 | 11 | 221 | 782 | 948 | Uncharacterized protein | Uncharacterized protein | | afdb-uniprot50 | AF-A0A349P466-F1-MODEL\_V4 | 1.0 | 1.567e-05 | 146 | 0.206 | 218 | 131 | 9 | 12 | 220 | 94 | 278 | Uncharacterized protein | Uncharacterized protein | | afdb-uniprot50 | AF-A0A3S4IBW8-F1-MODEL\_V4 | 1.0 | 2.972e-06 | 146 | 0.272 | 224 | 120 | 9 | 9 | 214 | 323 | 521 | Phage Tail Collar Domain | Phage Tail Collar Domain | | afdb-uniprot50 | AF-A0A4Q3MBS5-F1-MODEL\_V4 | 1.0 | 8.545e-07 | 145 | 0.225 | 239 | 129 | 9 | 12 | 220 | 5 | 217 | Collar domain-containing protein | Collar domain-containing protein | | afdb-uniprot50 | AF-A0A818P2E2-F1-MODEL\_V4 | 1.0 | 3.194e-05 | 142 | 0.267 | 183 | 94 | 7 | 2 | 161 | 25 | 190 | Hypothetical protein | Hypothetical protein | | afdb-uniprot50 | AF-A0A813X408-F1-MODEL\_V4 | 1.0 | 3.552e-06 | 142 | 0.37 | 170 | 76 | 5 | 16 | 174 | 67 | 216 | Hypothetical protein | Hypothetical protein | | afdb-uniprot50 | AF-A0A1H1GAM2-F1-MODEL\_V4 | 1.0 | 0.0001409 | 142 | 0.2 | 335 | 141 | 14 | 12 | 221 | 107 | 439 | Uncharacterized protein | Uncharacterized protein | | afdb-uniprot50 | AF-A0A292S0E4-F1-MODEL\_V4 | 1.0 | 2.519e-05 | 141 | 0.203 | 226 | 98 | 8 | 3 | 220 | 78 | 229 | Collar domain-containing protein | Collar domain-containing protein | | afdb-uniprot50 | AF-A0A3C0GBI9-F1-MODEL\_V4 | 1.0 | 1.135e-07 | 141 | 0.323 | 229 | 123 | 10 | 14 | 214 | 1 | 225 | Collar domain-containing protein | Collar domain-containing protein | | afdb-uniprot50 | AF-A0A2S9X536-F1-MODEL\_V4 | 1.0 | 8.154e-06 | 141 | 0.234 | 235 | 127 | 12 | 3 | 220 | 283 | 481 | Collar domain-containing protein | Collar domain-containing protein | | afdb-uniprot50 | AF-A0A821TQI6-F1-MODEL\_V4 | 1.0 | 1.642e-06 | 139 | 0.34 | 191 | 95 | 8 | 25 | 214 | 13 | 173 | Hypothetical protein | Hypothetical protein | | afdb-uniprot50 | AF-A0A1I1D6G6-F1-MODEL\_V4 | 1.0 | 2.082e-06 | 139 | 0.221 | 221 | 125 | 11 | 11 | 221 | 93 | 276 | Phage Tail Collar Domain | Phage Tail Collar Domain | | afdb-uniprot50 | AF-U1BY59-F1-MODEL\_V4 | 1.0 | 4.779e-06 | 136 | 0.275 | 189 | 96 | 6 | 11 | 188 | 389 | 547 | Collar domain-containing protein | Collar domain-containing protein | | afdb-uniprot50 | AF-A0A5V1PKQ6-F1-MODEL\_V4 | 1.0 | 9.866e-05 | 135 | 0.176 | 227 | 147 | 10 | 3 | 220 | 86 | 281 | Tail fiber protein | Tail fiber protein | | afdb-uniprot50 | AF-A0A2D6P7B0-F1-MODEL\_V4 | 1.0 | 1.391e-05 | 133 | 0.309 | 213 | 110 | 8 | 12 | 214 | 116 | 301 | Collar domain-containing protein | Collar domain-containing protein | | afdb-uniprot50 | AF-A0A806X791-F1-MODEL\_V4 | 1.0 | 5.783e-05 | 133 | 0.2 | 234 | 143 | 9 | 2 | 220 | 154 | 358 | Uncharacterized protein | Uncharacterized protein | | afdb-uniprot50 | AF-A0A5V0QBY5-F1-MODEL\_V4 | 1.0 | 9.866e-05 | 131 | 0.171 | 228 | 147 | 11 | 3 | 220 | 115 | 310 | Phage tail protein | Phage tail protein | | afdb-uniprot50 | AF-A0A6C8YBT0-F1-MODEL\_V4 | 1.0 | 0.0001111 | 130 | 0.18 | 211 | 142 | 7 | 11 | 220 | 166 | 346 | Tail fiber protein | Tail fiber protein | | afdb-uniprot50 | AF-A0A060HJ12-F1-MODEL\_V4 | 1.0 | 1.849e-06 | 129 | 0.273 | 205 | 129 | 5 | 11 | 214 | 137 | 322 | Tail protein | Tail protein | | afdb-uniprot50 | AF-A0A5V0QBB4-F1-MODEL\_V4 | 1.0 | 0.0002551 | 127 | 0.171 | 222 | 150 | 9 | 3 | 220 | 115 | 306 | Phage tail protein | Phage tail protein | | afdb-uniprot50 | AF-A0A5U0QPU6-F1-MODEL\_V4 | 1.0 | 9.298e-05 | 127 | 0.183 | 224 | 138 | 10 | 5 | 220 | 197 | 383 | Tail fiber protein | Tail fiber protein | | afdb-uniprot50 | AF-A0A410UF18-F1-MODEL\_V4 | 1.0 | 1.458e-06 | 127 | 0.295 | 227 | 122 | 8 | 11 | 214 | 183 | 394 | Collar domain-containing protein | Collar domain-containing protein | | afdb-uniprot50 | AF-A0A1E7YUN4-F1-MODEL\_V4 | 1.0 | 7.333e-05 | 126 | 0.2 | 239 | 136 | 11 | 2 | 220 | 9 | 212 | Long tail fiber protein p37 | Long tail fiber protein p37 | | afdb-uniprot50 | AF-A0A821RQV6-F1-MODEL\_V4 | 1.0 | 2.519e-05 | 126 | 0.275 | 200 | 107 | 5 | 19 | 212 | 51 | 218 | Hypothetical protein | Hypothetical protein | | afdb-uniprot50 | AF-A0A610C2R2-F1-MODEL\_V4 | 1.0 | 0.0001586 | 126 | 0.185 | 221 | 147 | 8 | 3 | 220 | 290 | 480 | Tail fiber protein | Tail fiber protein | | afdb-uniprot50 | AF-A0A1C7W6K6-F1-MODEL\_V4 | 1.0 | 0.0002707 | 125 | 0.219 | 214 | 130 | 7 | 12 | 220 | 85 | 266 | Collar domain-containing protein | Collar domain-containing protein | | afdb-uniprot50 | AF-A0A6L4BC09-F1-MODEL\_V4 | 1.0 | 9.298e-05 | 125 | 0.316 | 155 | 93 | 5 | 16 | 167 | 75 | 219 | Tail fiber protein | Tail fiber protein | | afdb-uniprot50 | AF-A0A821JPY2-F1-MODEL\_V4 | 1.0 | 3.01e-05 | 124 | 0.266 | 206 | 109 | 8 | 16 | 214 | 139 | 309 | Hypothetical protein | Hypothetical protein | | afdb-uniprot50 | AF-A0A376IY78-F1-MODEL\_V4 | 1.0 | 0.0001896 | 124 | 0.169 | 230 | 141 | 10 | 3 | 220 | 217 | 408 | Phage tail fibre repeat | Phage tail fibre repeat | | afdb-uniprot50 | AF-A0A719G1Y9-F1-MODEL\_V4 | 1.0 | 0.0006214 | 123 | 0.146 | 211 | 149 | 8 | 11 | 220 | 27 | 207 | Tail fiber protein | Tail fiber protein | | afdb-uniprot50 | AF-A0A639YPN2-F1-MODEL\_V4 | 1.0 | 0.0001586 | 122 | 0.201 | 228 | 138 | 10 | 2 | 220 | 141 | 333 | Tail fiber protein | Tail fiber protein | | afdb-uniprot50 | AF-A0A1J7C8B9-F1-MODEL\_V4 | 1.0 | 0.0002135 | 121 | 0.192 | 228 | 140 | 12 | 2 | 220 | 77 | 269 | Collar domain-containing protein | Collar domain-containing protein | | afdb-uniprot50 | AF-H3R9T3-F1-MODEL\_V4 | 1.0 | 8.762e-05 | 120 | 0.211 | 227 | 133 | 11 | 1 | 220 | 155 | 342 | Phage tail collar protein | Phage tail collar protein | | afdb-uniprot50 | AF-A0A379QX05-F1-MODEL\_V4 | 1.0 | 9.298e-05 | 120 | 0.213 | 220 | 129 | 11 | 11 | 220 | 234 | 419 | DNA recombinase-like protein | DNA recombinase-like protein | | afdb-uniprot50 | AF-A0A741N024-F1-MODEL\_V4 | 1.0 | 0.0003865 | 120 | 0.18 | 211 | 142 | 7 | 11 | 220 | 518 | 698 | Collar domain-containing protein | Collar domain-containing protein | | afdb-uniprot50 | AF-A0A833J123-F1-MODEL\_V4 | 1.0 | 0.0002404 | 119 | 0.164 | 231 | 146 | 11 | 2 | 220 | 155 | 350 | Phage tail protein | Phage tail protein | | afdb-uniprot50 | AF-A0A5U0QU90-F1-MODEL\_V4 | 1.0 | 0.0008872 | 119 | 0.162 | 221 | 152 | 9 | 3 | 220 | 177 | 367 | Tail fiber protein | Tail fiber protein | | afdb-uniprot50 | AF-A0A707P6F2-F1-MODEL\_V4 | 1.0 | 0.0008872 | 118 | 0.151 | 211 | 148 | 7 | 11 | 220 | 14 | 194 | Tail fiber protein | Tail fiber protein | | afdb-uniprot50 | AF-V7IMA5-F1-MODEL\_V4 | 1.0 | 0.0002135 | 118 | 0.2 | 215 | 133 | 8 | 11 | 220 | 290 | 470 | Phage Tail Collar domain protein | Phage Tail Collar domain protein | | afdb-uniprot50 | AF-A0A439EPD8-F1-MODEL\_V4 | 1.0 | 6.512e-05 | 117 | 0.28 | 214 | 102 | 10 | 11 | 207 | 17 | 195 | Phage tail protein | Phage tail protein | | afdb-uniprot50 | AF-A0A759ZPD9-F1-MODEL\_V4 | 1.0 | 0.0009415 | 117 | 0.155 | 212 | 146 | 9 | 11 | 220 | 179 | 359 | Collar domain-containing protein | Collar domain-containing protein | | afdb-uniprot50 | AF-A0A602CNX1-F1-MODEL\_V4 | 1.0 | 0.0004101 | 117 | 0.169 | 212 | 143 | 8 | 11 | 220 | 463 | 643 | Phage tail protein | Phage tail protein | | afdb-uniprot50 | AF-A0A3U5FGR7-F1-MODEL\_V4 | 1.0 | 0.0007425 | 117 | 0.169 | 213 | 142 | 10 | 11 | 220 | 630 | 810 | Phage tail protein | Phage tail protein | | afdb-uniprot50 | AF-A0A2E1UKG0-F1-MODEL\_V4 | 1.0 | 0.0003048 | 116 | 0.247 | 226 | 112 | 10 | 11 | 221 | 315 | 497 | Mtd\_N domain-containing protein | Mtd\_N domain-containing protein | | afdb-uniprot50 | AF-A0A706NAW6-F1-MODEL\_V4 | 1.0 | 0.0005518 | 116 | 0.169 | 212 | 143 | 8 | 11 | 220 | 360 | 540 | Phage tail protein | Phage tail protein | | afdb-uniprot50 | AF-A0A762B2E8-F1-MODEL\_V4 | 1.0 | 0.00106 | 116 | 0.16 | 212 | 145 | 9 | 11 | 220 | 510 | 690 | Phage tail protein | Phage tail protein | | afdb-uniprot50 | AF-A0A085HGE9-F1-MODEL\_V4 | 1.0 | 0.0002551 | 115 | 0.212 | 226 | 138 | 9 | 3 | 220 | 92 | 285 | Phage tail fiber protein | Phage tail fiber protein | | afdb-uniprot50 | AF-A0A778CR53-F1-MODEL\_V4 | 1.0 | 6.136e-05 | 115 | 0.194 | 236 | 138 | 12 | 11 | 220 | 416 | 625 | Phage tail protein | Phage tail protein | | afdb-uniprot50 | AF-Q8ZQ81-F1-MODEL\_V4 | 1.0 | 0.0009415 | 115 | 0.155 | 212 | 146 | 9 | 11 | 220 | 630 | 810 | Gifsy-2 prophage probable tail fiber protein | Gifsy-2 prophage probable tail fiber protein | | afdb-uniprot50 | AF-H3RF68-F1-MODEL\_V4 | 1.0 | 0.0002404 | 114 | 0.187 | 224 | 142 | 10 | 2 | 220 | 106 | 294 | Putative tail fiber protein | Putative tail fiber protein | | afdb-uniprot50 | AF-A0A701J2Z3-F1-MODEL\_V4 | 1.0 | 0.001125 | 114 | 0.156 | 211 | 147 | 7 | 11 | 220 | 160 | 340 | Phage tail protein | Phage tail protein | | afdb-uniprot50 | AF-A0A619AEI5-F1-MODEL\_V4 | 1.0 | 0.0002265 | 114 | 0.195 | 215 | 134 | 8 | 11 | 220 | 237 | 417 | Tail fiber protein | Tail fiber protein | | afdb-uniprot50 | AF-A0A6H2Y0W8-F1-MODEL\_V4 | 1.0 | 0.0005518 | 114 | 0.204 | 215 | 131 | 9 | 11 | 220 | 267 | 446 | Shikimate transporter | Shikimate transporter | | afdb-uniprot50 | AF-A0A757ZPK5-F1-MODEL\_V4 | 1.0 | 0.0002707 | 114 | 0.204 | 215 | 132 | 8 | 11 | 220 | 509 | 689 | Phage tail protein | Phage tail protein | | afdb-uniprot50 | AF-A0A738DCD2-F1-MODEL\_V4 | 1.0 | 0.0007879 | 113 | 0.165 | 212 | 144 | 8 | 11 | 220 | 165 | 345 | Tail fiber protein | Tail fiber protein | | afdb-uniprot50 | AF-A0A757D915-F1-MODEL\_V4 | 1.0 | 0.0005518 | 113 | 0.165 | 212 | 144 | 8 | 11 | 220 | 267 | 447 | Phage tail protein | Phage tail protein | | afdb-uniprot50 | AF-A0A5U6SMK2-F1-MODEL\_V4 | 1.0 | 0.0005856 | 113 | 0.174 | 212 | 140 | 8 | 12 | 220 | 429 | 608 | Phage tail protein | Phage tail protein | | afdb-uniprot50 | AF-A0A3V2NRU9-F1-MODEL\_V4 | 1.0 | 0.001514 | 112 | 0.161 | 211 | 146 | 8 | 11 | 220 | 220 | 400 | Shikimate transporter | Shikimate transporter | | afdb-uniprot50 | AF-A0A707VIZ3-F1-MODEL\_V4 | 1.0 | 0.0008872 | 112 | 0.165 | 212 | 144 | 8 | 11 | 220 | 339 | 519 | Phage tail protein | Phage tail protein | | afdb-uniprot50 | AF-A0A604P221-F1-MODEL\_V4 | 1.0 | 0.0002265 | 112 | 0.195 | 220 | 128 | 10 | 11 | 220 | 419 | 599 | Phage tail protein | Phage tail protein | | afdb-uniprot50 | AF-A0A2X4T8S9-F1-MODEL\_V4 | 1.0 | 0.0003642 | 111 | 0.19 | 221 | 133 | 12 | 11 | 220 | 102 | 287 | DNA recombinase-like protein | DNA recombinase-like protein | | afdb-uniprot50 | AF-A0A820G8G8-F1-MODEL\_V4 | 1.0 | 0.0001786 | 111 | 0.232 | 181 | 95 | 6 | 11 | 162 | 179 | 344 | Hypothetical protein | Hypothetical protein | | afdb-uniprot50 | AF-A0A7Z0Y4I4-F1-MODEL\_V4 | 1.0 | 0.0003642 | 111 | 0.195 | 215 | 134 | 8 | 11 | 220 | 198 | 378 | Phage tail protein | Phage tail protein | | afdb-uniprot50 | AF-A0A5W2GRF4-F1-MODEL\_V4 | 1.0 | 0.0009415 | 110 | 0.156 | 211 | 147 | 7 | 11 | 220 | 284 | 464 | Phage tail protein | Phage tail protein | | afdb-uniprot50 | AF-A0A5Z0R0R1-F1-MODEL\_V4 | 1.0 | 0.0002872 | 110 | 0.213 | 220 | 129 | 12 | 11 | 220 | 318 | 503 | Collar domain-containing protein | Collar domain-containing protein | | afdb-uniprot50 | AF-A0A731WFF8-F1-MODEL\_V4 | 1.0 | 0.0001251 | 110 | 0.213 | 215 | 130 | 11 | 11 | 220 | 341 | 521 | Collar domain-containing protein | Collar domain-containing protein | | afdb-uniprot50 | AF-G5RWL7-F1-MODEL\_V4 | 1.0 | 0.0002265 | 109 | 0.185 | 221 | 129 | 9 | 11 | 220 | 83 | 263 | Tail fiber assembly like-protein | Tail fiber assembly like-protein | | afdb-uniprot50 | AF-A0A600RD36-F1-MODEL\_V4 | 1.0 | 0.0003865 | 109 | 0.209 | 220 | 125 | 11 | 11 | 220 | 95 | 275 | Tail fiber protein | Tail fiber protein | | afdb-uniprot50 | AF-A0A5Y2ZE76-F1-MODEL\_V4 | 1.0 | 0.001194 | 109 | 0.16 | 212 | 145 | 8 | 11 | 220 | 163 | 343 | Tail fiber protein | Tail fiber protein | | afdb-uniprot50 | AF-A0A708RGM0-F1-MODEL\_V4 | 1.0 | 0.00106 | 109 | 0.165 | 212 | 144 | 8 | 11 | 220 | 339 | 519 | Phage tail protein | Phage tail protein | | afdb-uniprot50 | AF-A0A6C0BYV0-F1-MODEL\_V4 | 1.0 | 0.001514 | 108 | 0.216 | 240 | 120 | 10 | 12 | 221 | 85 | 286 | Uncharacterized protein | Uncharacterized protein | | afdb-uniprot50 | AF-A0A7Y5M3U8-F1-MODEL\_V4 | 1.0 | 0.001194 | 108 | 0.158 | 214 | 144 | 8 | 11 | 220 | 206 | 387 | Tail fiber protein | Tail fiber protein | | afdb-uniprot50 | AF-A0A7H8WZS4-F1-MODEL\_V4 | 1.0 | 0.0006214 | 108 | 0.177 | 225 | 126 | 11 | 11 | 220 | 512 | 692 | Phage tail protein | Phage tail protein | | afdb-uniprot50 | AF-A0A827KJI0-F1-MODEL\_V4 | 1.0 | 0.001344 | 107 | 0.176 | 221 | 143 | 9 | 7 | 220 | 6 | 194 | Tail fiber protein | Tail fiber protein | | afdb-uniprot50 | AF-A0A855SIZ9-F1-MODEL\_V4 | 1.0 | 0.0007425 | 107 | 0.181 | 232 | 136 | 11 | 11 | 220 | 17 | 216 | Phage tail protein | Phage tail protein | | afdb-uniprot50 | AF-A0A736VG58-F1-MODEL\_V4 | 1.0 | 0.0004101 | 107 | 0.198 | 217 | 128 | 11 | 12 | 220 | 297 | 475 | Tail fiber protein | Tail fiber protein | | afdb-uniprot50 | AF-A0A5Y2LQP0-F1-MODEL\_V4 | 1.0 | 0.0004101 | 107 | 0.209 | 215 | 131 | 9 | 11 | 220 | 364 | 544 | Collar domain-containing protein | Collar domain-containing protein | | afdb-uniprot50 | AF-A0A2E9W8F9-F1-MODEL\_V4 | 1.0 | 0.0005518 | 106 | 0.22 | 204 | 139 | 6 | 13 | 214 | 8 | 193 | Collar domain-containing protein | Collar domain-containing protein | | afdb-uniprot50 | AF-A0A5T3NHC4-F1-MODEL\_V4 | 1.0 | 0.003475 | 106 | 0.137 | 211 | 151 | 7 | 11 | 220 | 525 | 705 | Collar domain-containing protein | Collar domain-containing protein | | afdb-uniprot50 | AF-A0A0W7WFQ0-F1-MODEL\_V4 | 1.0 | 0.0004352 | 105 | 0.191 | 235 | 143 | 7 | 11 | 214 | 30 | 248 | Collar domain-containing protein | Collar domain-containing protein | | afdb-uniprot50 | AF-A0A5A9C9Z6-F1-MODEL\_V4 | 1.0 | 0.0008361 | 105 | 0.188 | 233 | 136 | 10 | 11 | 220 | 671 | 873 | Tail fiber protein | Tail fiber protein | | afdb-uniprot50 | AF-A0A714KQT2-F1-MODEL\_V4 | 1.0 | 0.0009415 | 104 | 0.171 | 221 | 132 | 10 | 11 | 220 | 27 | 207 | Tail fiber protein | Tail fiber protein | | afdb-uniprot50 | AF-A0A707NFK7-F1-MODEL\_V4 | 1.0 | 0.0008361 | 104 | 0.175 | 216 | 137 | 11 | 11 | 220 | 280 | 460 | Phage tail protein | Phage tail protein | | afdb-uniprot50 | AF-A0A6J7EVX5-F1-MODEL\_V4 | 1.0 | 4.504e-06 | 104 | 0.405 | 227 | 106 | 4 | 13 | 221 | 347 | 562 | Unannotated protein | Unannotated protein | | afdb-uniprot50 | AF-A0A5E8KNR2-F1-MODEL\_V4 | 1.0 | 0.0001683 | 103 | 0.263 | 201 | 95 | 7 | 16 | 216 | 2 | 149 | Collar domain-containing protein | Collar domain-containing protein | | afdb-uniprot50 | AF-L3Q3I4-F1-MODEL\_V4 | 1.0 | 0.001427 | 103 | 0.197 | 228 | 130 | 13 | 8 | 220 | 243 | 432 | Collar domain-containing protein | Collar domain-containing protein | | afdb-uniprot50 | AF-A0A2I5TL70-F1-MODEL\_V4 | 1.0 | 0.0009415 | 102 | 0.177 | 236 | 155 | 9 | 1 | 220 | 208 | 420 | Phage tail protein | Phage tail protein | | afdb-uniprot50 | AF-A0A4Y8GSR2-F1-MODEL\_V4 | 1.0 | 0.0006594 | 101 | 0.188 | 234 | 152 | 8 | 3 | 220 | 290 | 501 | Short-chain fatty acid transporter | Short-chain fatty acid transporter | | afdb-uniprot50 | AF-A0A717LCK5-F1-MODEL\_V4 | 1.0 | 0.00106 | 100 | 0.183 | 224 | 126 | 12 | 11 | 220 | 29 | 209 | Tail fiber protein | Tail fiber protein | | afdb-uniprot50 | AF-A0A479JBS8-F1-MODEL\_V4 | 1.0 | 0.00052 | 100 | 0.222 | 247 | 148 | 11 | 1 | 220 | 134 | 363 | Phage tail fiber protein | Phage tail fiber protein | | afdb-uniprot50 | AF-A0A815QGL9-F1-MODEL\_V4 | 1.0 | 0.0001683 | 100 | 0.183 | 223 | 136 | 7 | 2 | 214 | 646 | 832 | Hypothetical protein | Hypothetical protein | | afdb-uniprot50 | AF-A0A485DQI3-F1-MODEL\_V4 | 0.999 | 0.0004101 | 99 | 0.205 | 214 | 136 | 8 | 11 | 220 | 260 | 443 | Phage lambda-related protein | Phage lambda-related protein | | afdb-uniprot50 | AF-I6CNP4-F1-MODEL\_V4 | 0.999 | 0.001809 | 98 | 0.188 | 239 | 136 | 11 | 11 | 220 | 42 | 251 | Long tail fiber protein p37 | Long tail fiber protein p37 | | afdb-uniprot50 | AF-A0A328TN35-F1-MODEL\_V4 | 0.999 | 0.001809 | 98 | 0.207 | 255 | 143 | 13 | 2 | 221 | 34 | 264 | Phage tail fiber repeat family protein | Phage tail fiber repeat family protein | | afdb-uniprot50 | AF-D2TUQ3-F1-MODEL\_V4 | 0.999 | 0.0002404 | 98 | 0.166 | 246 | 143 | 14 | 3 | 220 | 69 | 280 | Putative phage tail fibre protein | Putative phage tail fibre protein | | afdb-uniprot50 | AF-A0A8A7UB48-F1-MODEL\_V4 | 0.999 | 0.001606 | 98 | 0.184 | 239 | 137 | 10 | 11 | 220 | 96 | 305 | Tail fiber protein | Tail fiber protein | | afdb-uniprot50 | AF-A0A828NYP4-F1-MODEL\_V4 | 0.999 | 0.003086 | 96 | 0.189 | 222 | 137 | 12 | 8 | 220 | 362 | 549 | Phage tail protein | Phage tail protein | | afdb-uniprot50 | AF-A0A0T9T368-F1-MODEL\_V4 | 0.998 | 0.00106 | 95 | 0.199 | 236 | 136 | 11 | 1 | 220 | 86 | 284 | Phage tail protein | Phage tail protein | | afdb-uniprot50 | AF-A0A8B4KTQ3-F1-MODEL\_V4 | 0.998 | 0.00593 | 95 | 0.171 | 256 | 135 | 10 | 12 | 220 | 452 | 677 | PPE-repeat protein | PPE-repeat protein | | afdb-uniprot50 | AF-A0A830SMV6-F1-MODEL\_V4 | 0.998 | 0.002583 | 94 | 0.184 | 239 | 137 | 10 | 11 | 220 | 205 | 414 | Short-chain fatty acid transporter | Short-chain fatty acid transporter | | afdb-uniprot50 | AF-A0A243TCZ2-F1-MODEL\_V4 | 0.998 | 0.0009415 | 94 | 0.193 | 238 | 129 | 12 | 11 | 220 | 386 | 588 | Phage tail protein | Phage tail protein | | afdb-uniprot50 | AF-A0A1F3CD68-F1-MODEL\_V4 | 0.997 | 0.002908 | 92 | 0.22 | 240 | 114 | 11 | 12 | 221 | 388 | 584 | Uncharacterized protein | Uncharacterized protein | | afdb-uniprot50 | AF-A0A8B4K950-F1-MODEL\_V4 | 0.997 | 0.0004618 | 92 | 0.239 | 242 | 136 | 11 | 11 | 220 | 924 | 1149 | Putative tail fiber protein | Putative tail fiber protein | | afdb-uniprot50 | AF-A0A828H8Q0-F1-MODEL\_V4 | 0.997 | 0.0003234 | 92 | 0.237 | 240 | 139 | 11 | 11 | 220 | 949 | 1174 | Short-chain fatty acid transporter | Short-chain fatty acid transporter | | afdb-uniprot50 | AF-W0BPN1-F1-MODEL\_V4 | 0.997 | 0.0007425 | 91 | 0.2 | 220 | 143 | 8 | 12 | 220 | 176 | 373 | Collar domain-containing protein | Collar domain-containing protein | | afdb-uniprot50 | AF-A0A4U3LF57-F1-MODEL\_V4 | 0.997 | 0.001194 | 91 | 0.232 | 219 | 141 | 10 | 11 | 220 | 359 | 559 | Phage tail protein | Phage tail protein | | afdb-uniprot50 | AF-A0A7A3AF76-F1-MODEL\_V4 | 0.997 | 0.001919 | 91 | 0.2 | 220 | 146 | 8 | 11 | 220 | 668 | 867 | Phage tail protein | Phage tail protein | | afdb-uniprot50 | AF-A0A827K9D2-F1-MODEL\_V4 | 0.996 | 0.001344 | 90 | 0.175 | 228 | 143 | 8 | 11 | 220 | 87 | 287 | Tail fiber protein | Tail fiber protein | | afdb-uniprot50 | AF-A0A1E7Z390-F1-MODEL\_V4 | 0.996 | 0.002161 | 90 | 0.208 | 249 | 150 | 11 | 2 | 221 | 76 | 306 | Phage tail protein | Phage tail protein | | afdb-uniprot50 | AF-A0A7Z3MBJ3-F1-MODEL\_V4 | 0.996 | 0.001514 | 90 | 0.215 | 246 | 151 | 9 | 1 | 220 | 200 | 429 | Phage tail protein | Phage tail protein | | afdb-uniprot50 | AF-A0A2J9H2E6-F1-MODEL\_V4 | 0.996 | 0.002294 | 90 | 0.194 | 231 | 136 | 10 | 11 | 220 | 468 | 669 | Phage tail protein | Phage tail protein | | afdb-uniprot50 | AF-A0A7T7XQ37-F1-MODEL\_V4 | 0.996 | 0.003086 | 90 | 0.214 | 257 | 116 | 13 | 1 | 220 | 540 | 747 | Uncharacterized protein | Uncharacterized protein | | afdb-uniprot50 | AF-A0A2J0PYI7-F1-MODEL\_V4 | 0.996 | 0.001267 | 90 | 0.196 | 254 | 141 | 10 | 11 | 220 | 572 | 806 | Collar domain-containing protein | Collar domain-containing protein | | afdb-uniprot50 | AF-K4VSX7-F1-MODEL\_V4 | 0.996 | 0.001427 | 89 | 0.2 | 224 | 141 | 7 | 11 | 220 | 101 | 300 | Major tail fiber protein | Major tail fiber protein | | afdb-uniprot50 | AF-A0A090R0D4-F1-MODEL\_V4 | 0.996 | 0.001919 | 89 | 0.157 | 242 | 134 | 10 | 1 | 220 | 4 | 197 | Phage tail fibers | Phage tail fibers | | afdb-uniprot50 | AF-A0A5F0PX95-F1-MODEL\_V4 | 0.996 | 0.002037 | 89 | 0.176 | 226 | 145 | 8 | 11 | 220 | 189 | 389 | Tail fiber protein | Tail fiber protein | | afdb-uniprot50 | AF-A0A4C8VIP7-F1-MODEL\_V4 | 0.996 | 0.002583 | 89 | 0.175 | 211 | 150 | 6 | 11 | 220 | 650 | 837 | Phage side tail fiber protein | Phage side tail fiber protein | | afdb-uniprot50 | AF-A0A517N136-F1-MODEL\_V4 | 0.995 | 0.003475 | 88 | 0.262 | 217 | 120 | 7 | 17 | 214 | 958 | 1153 | Phage Tail Collar Domain protein | Phage Tail Collar Domain protein | | afdb-uniprot50 | AF-A0A2P5GM50-F1-MODEL\_V4 | 0.994 | 0.00106 | 87 | 0.206 | 228 | 136 | 9 | 12 | 220 | 225 | 426 | Collar domain-containing protein | Collar domain-containing protein | | afdb-uniprot50 | AF-A0A236MFK9-F1-MODEL\_V4 | 0.994 | 0.003688 | 87 | 0.187 | 262 | 129 | 12 | 11 | 220 | 317 | 546 | Phage tail protein | Phage tail protein | | afdb-uniprot50 | AF-A0A7W4KMJ0-F1-MODEL\_V4 | 0.994 | 0.001514 | 87 | 0.182 | 225 | 145 | 7 | 11 | 220 | 453 | 653 | Tail fiber protein | Tail fiber protein | | afdb-uniprot50 | AF-A0A1Q4PEJ7-F1-MODEL\_V4 | 0.994 | 0.001705 | 87 | 0.21 | 214 | 137 | 10 | 11 | 220 | 501 | 686 | Phage tail protein | Phage tail protein | | afdb-uniprot50 | AF-A0A8B5NF75-F1-MODEL\_V4 | 0.994 | 0.002161 | 87 | 0.174 | 223 | 149 | 7 | 11 | 220 | 670 | 870 | Phage tail protein | Phage tail protein | | afdb-uniprot50 | AF-A0A827FX06-F1-MODEL\_V4 | 0.994 | 0.002583 | 87 | 0.195 | 220 | 147 | 7 | 11 | 220 | 942 | 1141 | Phage tail protein | Phage tail protein | | afdb-uniprot50 | AF-A0A5P0PY25-F1-MODEL\_V4 | 0.993 | 0.0007879 | 86 | 0.212 | 235 | 133 | 10 | 12 | 220 | 2 | 210 | Tail fiber protein | Tail fiber protein | | afdb-uniprot50 | AF-A0A7A6N7A4-F1-MODEL\_V4 | 0.993 | 0.002741 | 86 | 0.179 | 228 | 142 | 8 | 11 | 220 | 251 | 451 | Tail fiber protein | Tail fiber protein | | afdb-uniprot50 | AF-A0A2X5DKB2-F1-MODEL\_V4 | 0.993 | 0.002037 | 86 | 0.232 | 219 | 141 | 10 | 11 | 220 | 315 | 515 | Side tail phage protein | Side tail phage protein | | afdb-uniprot50 | AF-D6I5M1-F1-MODEL\_V4 | 0.993 | 0.0005856 | 86 | 0.229 | 231 | 135 | 10 | 12 | 220 | 342 | 551 | Collar domain-containing protein | Collar domain-containing protein | | afdb-uniprot50 | AF-A0A1X3JXT7-F1-MODEL\_V4 | 0.993 | 0.002908 | 86 | 0.214 | 242 | 140 | 11 | 12 | 220 | 419 | 643 | Side tail fiber protein-like protein | Side tail fiber protein-like protein | | afdb-uniprot50 | AF-A0A478KQU1-F1-MODEL\_V4 | 0.993 | 0.0007879 | 86 | 0.232 | 241 | 137 | 10 | 12 | 220 | 611 | 835 | Phage side tail fiber protein | Phage side tail fiber protein | | afdb-uniprot50 | AF-A0A2Z2JLN6-F1-MODEL\_V4 | 0.993 | 0.002294 | 86 | 0.2 | 219 | 147 | 6 | 11 | 220 | 748 | 947 | Putative membrane protein | Putative membrane protein | | afdb-uniprot50 | AF-A0A827JSA7-F1-MODEL\_V4 | 0.992 | 0.001344 | 85 | 0.185 | 248 | 145 | 10 | 11 | 220 | 23 | 251 | Tail fiber protein | Tail fiber protein | | afdb-uniprot50 | AF-A0A7D6J6I4-F1-MODEL\_V4 | 0.992 | 0.004407 | 85 | 0.217 | 239 | 143 | 10 | 12 | 220 | 539 | 763 | Tail fiber protein | Tail fiber protein | | afdb-uniprot50 | AF-A0A827LBC4-F1-MODEL\_V4 | 0.992 | 0.007519 | 85 | 0.204 | 235 | 127 | 13 | 11 | 220 | 616 | 815 | Phage tail protein | Phage tail protein | | afdb-uniprot50 | AF-A0A1R0G2E0-F1-MODEL\_V4 | 0.992 | 0.00106 | 85 | 0.181 | 243 | 139 | 10 | 11 | 220 | 698 | 913 | Prophage tail fiber N-terminal domain-containing protein | Prophage tail fiber N-terminal domain-containing protein | | afdb-uniprot50 | AF-A0A3P5DNK9-F1-MODEL\_V4 | 0.992 | 0.001705 | 85 | 0.208 | 249 | 146 | 11 | 5 | 220 | 722 | 952 | Uncharacterized protein | Uncharacterized protein | | afdb-uniprot50 | AF-A0A2X8FBW3-F1-MODEL\_V4 | 0.992 | 0.003913 | 85 | 0.231 | 238 | 141 | 10 | 12 | 220 | 760 | 984 | Putative tail fiber protein | Putative tail fiber protein | | afdb-uniprot50 | AF-A0A0H3MMX0-F1-MODEL\_V4 | 0.991 | 0.004407 | 84 | 0.16 | 237 | 158 | 9 | 1 | 220 | 312 | 524 | Putative side tail phage protein | Putative side tail phage protein | | afdb-uniprot50 | AF-A0A6N6XUS5-F1-MODEL\_V4 | 0.991 | 0.001514 | 84 | 0.215 | 241 | 143 | 11 | 11 | 220 | 585 | 810 | Short-chain fatty acid transporter | Short-chain fatty acid transporter | | afdb-uniprot50 | AF-A0A377NAA6-F1-MODEL\_V4 | 0.991 | 0.003086 | 84 | 0.17 | 229 | 149 | 9 | 11 | 220 | 650 | 856 | Tail fiber protein | Tail fiber protein | | afdb-uniprot50 | AF-A0A828UHZ0-F1-MODEL\_V4 | 0.99 | 0.003475 | 83 | 0.187 | 224 | 144 | 7 | 11 | 220 | 403 | 602 | Phage tail collar domain protein | Phage tail collar domain protein | | afdb-uniprot50 | AF-A0A8B4JKQ6-F1-MODEL\_V4 | 0.99 | 0.001344 | 83 | 0.198 | 252 | 139 | 12 | 11 | 220 | 384 | 614 | Tail fiber protein | Tail fiber protein | | afdb-uniprot50 | AF-A0A379R3M2-F1-MODEL\_V4 | 0.99 | 0.002294 | 83 | 0.204 | 220 | 146 | 8 | 11 | 220 | 524 | 724 | Gifsy-2 prophage tail fiber protein | Gifsy-2 prophage tail fiber protein | | afdb-uniprot50 | AF-A0A6C8XTX3-F1-MODEL\_V4 | 0.99 | 0.003688 | 83 | 0.163 | 232 | 142 | 8 | 11 | 220 | 608 | 809 | Phage tail protein | Phage tail protein | | afdb-uniprot50 | AF-W0BR44-F1-MODEL\_V4 | 0.988 | 0.001919 | 82 | 0.208 | 221 | 141 | 8 | 12 | 220 | 364 | 562 | Collar domain-containing protein | Collar domain-containing protein | | afdb-uniprot50 | AF-A0A793GNT0-F1-MODEL\_V4 | 0.986 | 0.001606 | 81 | 0.235 | 234 | 130 | 12 | 12 | 220 | 33 | 242 | Tail fiber protein | Tail fiber protein | | afdb-uniprot50 | AF-F4V1R4-F1-MODEL\_V4 | 0.986 | 0.001606 | 81 | 0.226 | 234 | 132 | 11 | 12 | 220 | 143 | 352 | Side tail fiber protein lambdoid prophage Qin-like protein | Side tail fiber protein lambdoid prophage Qin-like protein | | afdb-uniprot50 | AF-A0A8B4L7W3-F1-MODEL\_V4 | 0.986 | 0.001705 | 81 | 0.229 | 231 | 135 | 10 | 12 | 220 | 183 | 392 | Phage tail domain-containing protein | Phage tail domain-containing protein | | afdb-uniprot50 | AF-A0A1X3M1P4-F1-MODEL\_V4 | 0.986 | 0.004962 | 81 | 0.207 | 227 | 134 | 10 | 12 | 220 | 250 | 448 | Side tail fiber protein-like protein | Side tail fiber protein-like protein | | afdb-uniprot50 | AF-A0A7U0GZW4-F1-MODEL\_V4 | 0.986 | 0.003475 | 81 | 0.205 | 239 | 147 | 9 | 12 | 220 | 241 | 466 | Phage tail tape measure protein | Phage tail tape measure protein | | afdb-uniprot50 | AF-A0A826TQX2-F1-MODEL\_V4 | 0.986 | 0.002583 | 81 | 0.194 | 221 | 144 | 9 | 12 | 220 | 505 | 703 | Phage tail protein | Phage tail protein | | afdb-uniprot50 | AF-A0A7B5PWV2-F1-MODEL\_V4 | 0.986 | 0.004407 | 81 | 0.207 | 222 | 140 | 10 | 12 | 220 | 544 | 742 | Collar domain-containing protein | Collar domain-containing protein | | afdb-uniprot50 | AF-B2TVZ0-F1-MODEL\_V4 | 0.984 | 0.002294 | 80 | 0.198 | 252 | 139 | 11 | 11 | 220 | 209 | 439 | Side tail fiber protein | Side tail fiber protein | | afdb-uniprot50 | AF-A0A376VIM7-F1-MODEL\_V4 | 0.984 | 0.002037 | 80 | 0.194 | 252 | 140 | 12 | 11 | 220 | 273 | 503 | Side tail fiber protein from bacteriophage origin | Side tail fiber protein from bacteriophage origin | | afdb-uniprot50 | AF-A0A6L9FK66-F1-MODEL\_V4 | 0.984 | 0.006677 | 80 | 0.188 | 234 | 152 | 9 | 1 | 220 | 309 | 518 | Uncharacterized protein | Uncharacterized protein | | afdb-uniprot50 | AF-A0A646JEL0-F1-MODEL\_V4 | 0.984 | 0.001809 | 80 | 0.183 | 245 | 145 | 10 | 12 | 220 | 626 | 851 | Phage tail protein | Phage tail protein | | afdb-uniprot50 | AF-A0A4S4IN19-F1-MODEL\_V4 | 0.984 | 0.006677 | 80 | 0.227 | 246 | 137 | 12 | 12 | 220 | 804 | 1033 | Qin prophage, putative side tail fiber assembly protein | Qin prophage, putative side tail fiber assembly protein | | afdb-uniprot50 | AF-A0A376SP24-F1-MODEL\_V4 | 0.981 | 0.002294 | 79 | 0.235 | 234 | 130 | 12 | 12 | 220 | 108 | 317 | Prophage tail fiber domain-containing protein | Prophage tail fiber domain-containing protein | | afdb-uniprot50 | AF-A0A2X6JPY3-F1-MODEL\_V4 | 0.981 | 0.002294 | 79 | 0.225 | 226 | 142 | 9 | 12 | 220 | 461 | 670 | Prophage tail fiber N-terminal domain-containing protein | Prophage tail fiber N-terminal domain-containing protein | | afdb-uniprot50 | AF-A0A2X2HTT9-F1-MODEL\_V4 | 0.981 | 0.006677 | 79 | 0.219 | 228 | 142 | 9 | 11 | 220 | 712 | 921 | Side tail fiber protein | Side tail fiber protein | | afdb-uniprot50 | AF-A0A775EW34-F1-MODEL\_V4 | 0.978 | 0.002908 | 78 | 0.234 | 235 | 131 | 12 | 11 | 220 | 54 | 264 | Tail fiber protein | Tail fiber protein | | afdb-uniprot50 | AF-A0A6C0LBP0-F1-MODEL\_V4 | 0.978 | 0.001606 | 78 | 0.197 | 269 | 141 | 13 | 12 | 221 | 15 | 267 | Collar domain-containing protein | Collar domain-containing protein | | afdb-uniprot50 | AF-A0A5X4GC92-F1-MODEL\_V4 | 0.978 | 0.007519 | 78 | 0.221 | 248 | 147 | 10 | 3 | 220 | 253 | 484 | Short-chain fatty acid transporter | Short-chain fatty acid transporter | | afdb-uniprot50 | AF-A0A810TS17-F1-MODEL\_V4 | 0.978 | 0.002741 | 78 | 0.221 | 235 | 134 | 11 | 11 | 220 | 297 | 507 | Short-chain dehydrogenase | Short-chain dehydrogenase | | afdb-uniprot50 | AF-A0A4B9BPF7-F1-MODEL\_V4 | 0.978 | 0.002434 | 78 | 0.222 | 229 | 139 | 9 | 12 | 220 | 305 | 514 | Phage tail protein | Phage tail protein | | afdb-uniprot50 | AF-A0A6D0EXL5-F1-MODEL\_V4 | 0.978 | 0.002161 | 78 | 0.216 | 236 | 133 | 11 | 11 | 220 | 449 | 658 | Tail fiber protein | Tail fiber protein | | afdb-uniprot50 | AF-A0A152BV35-F1-MODEL\_V4 | 0.978 | 0.004676 | 78 | 0.195 | 225 | 139 | 8 | 12 | 220 | 510 | 708 | Phage tail protein | Phage tail protein | | afdb-uniprot50 | AF-A0A836ZDU5-F1-MODEL\_V4 | 0.975 | 0.003475 | 77 | 0.227 | 237 | 129 | 12 | 11 | 220 | 319 | 528 | Phage tail fiber repeat family protein | Phage tail fiber repeat family protein | | afdb-uniprot50 | AF-A0A222QUV6-F1-MODEL\_V4 | 0.971 | 0.007979 | 76 | 0.209 | 229 | 131 | 11 | 12 | 220 | 338 | 536 | Putative tail fiber protein | Putative tail fiber protein | | afdb-uniprot50 | AF-A0A377F1I4-F1-MODEL\_V4 | 0.971 | 0.005266 | 76 | 0.209 | 248 | 139 | 11 | 12 | 220 | 349 | 578 | Phage tail protein | Phage tail protein | | afdb-uniprot50 | AF-Q7BTW5-F1-MODEL\_V4 | 0.971 | 0.004962 | 76 | 0.217 | 258 | 135 | 10 | 11 | 220 | 620 | 858 | Tail fiber protein homolog | Tail fiber protein homolog | | afdb-uniprot50 | AF-A0A6N8NGX5-F1-MODEL\_V4 | 0.967 | 0.003913 | 75 | 0.235 | 234 | 130 | 12 | 12 | 220 | 365 | 574 | Collar domain-containing protein | Collar domain-containing protein | | afdb-uniprot50 | AF-A0A854RAR2-F1-MODEL\_V4 | 0.961 | 0.007979 | 74 | 0.198 | 252 | 139 | 13 | 11 | 220 | 36 | 266 | Phage tail protein | Phage tail protein | | afdb-uniprot50 | AF-A0A7U8W607-F1-MODEL\_V4 | 0.956 | 0.007979 | 73 | 0.183 | 251 | 144 | 10 | 11 | 220 | 213 | 443 | Tail protein 2 | Tail protein 2 | | afdb-uniprot50 | AF-A0A7B3JM82-F1-MODEL\_V4 | 0.956 | 0.006292 | 73 | 0.19 | 252 | 141 | 9 | 11 | 220 | 678 | 908 | Uncharacterized protein | Uncharacterized protein | |
| Top keywords  (threshold 1.00e-02 (evalue)) | **Collar, tail, domain\_containing, Phage, fiber, Hypothetical, Microcystin\_dependent, Putative, Side, Phage\_related** |
| Output files | ../../similar\_structures/17\_FANPEZAQ\_CDS\_0017\_afdb-proteome\_foldseek.tsv ../../similar\_structures/17\_FANPEZAQ\_CDS\_0017\_afdb-uniprot50\_foldseek.tsv ../../similar\_structures/17\_FANPEZAQ\_CDS\_0017\_merged.svg ../../similar\_structures/17\_FANPEZAQ\_CDS\_0017\_pdb\_foldseek.tsv |

  
  
  

Return to summary | Go to previous | Go to next

  


---

**Sequence/structure alignments coloring**  
Each object in the alignment figures is colored according to its E-value following this color coding:

1e-100
10

**References:**  
1) Steinegger M, Meier M, Mirdita M, Vöhringer H, Haunsberger S J, and Söding J (2019) HH-suite3 for fast remote homology detection and deep protein annotation, BMC Bioinformatics, 473. doi: 10.1186/s12859-019-3019-7  
2) Jumper J, Evans R, Pritzel A, ..., Hassabis D (2021) Highly accurate protein structure prediction with AlphaFold, Nature, 596. doi: 10.1038/s41586-021-03819-2  
3) van Kempen M, Kim S, Tumescheit C, Mirdita M, Lee J, Gilchrist CLM, Söding J, and Steinegger M (2023) Fast and accurate protein structure search with Foldseek. Nature Biotechnology. doi: 10.1038/s41587-023-01773-0
