# Supplementary material for: Completing the BASEL phage collection to unlock hidden diversity for systematic exploration of phage–host interactions
Source: PLoS Biol. 2025 Apr 7;23(4):e3003063. doi: 10.1371/journal.pbio.3003063 (PMC11990801; doi:10.1371/journal.pbio.3003063)
Supplement: S2 Data — (ZIP) [file pbio.3003063.s009.zip › entries/18.html]

FANPEZAQ\_CDS\_0018


Return to summary | Go to previous | Go to next

|  |  |
| --- | --- |
| FANPEZAQ\_CDS\_0018 Page creation date: 02 Sep 2024, 12:00  Project folder: n/a  Input sequences file: Escherichia\_virus\_HeidiAbel.gb | tail phage sheath domain\_containing fragment subtilisin\_like fi c\_terminal phage\_sheath\_1c major phage\_sheath\_1 putative prophage monomer outer membrane secretion hypothetical contractile ragb susd nutrient uptake structural system bacteriophage afp3 type vi afp2 phage\_related afp1 anti\_feeding afp |

### Sequence information

|  |  |
| --- | --- |
| Name | FANPEZAQ\_CDS\_0018  18\_FANPEZAQ\_CDS\_0018 (pipeline id) |
| Imported annotations | Escherichia\_virus\_HeidiAbel Bas97 |
| Protein sequence | MAGTDFLHGVEVLEIDSGSRVIQTVKSSVIGIVGTAPNADPDAFPLNTPTLIAGSQREAA KLDMLGTGEGTLPKALDAILDQIGAVVIVVRVEEGESDTATLANIMGGVNASTGKYEGVH ALLGAESVVGYSPRILCVPGFTHTRVQDGVTAISIGNGGSGYTEAPTITLAGGGGGSGAT AQATVVGGVITKITVTNAGMGYTEAPTVTITGGNGTGATATASVGIAANAVVSELLGIAN RLRAVIIADGPNTNDADAIAYRKDFGSKRVYVVDPKVLVLDEDGTTITDWASPRVAGLIA RIDNERGFWWSPSNQEIYGIQGTARTIDFTLGDTSSRANLLNENEVTTIIRQDGFRLWGN RTTSSDPKWAFLCVVRTADIINDSLLRAHLWAVDRGITKQYVNDVIEGVNAYLRNLVSLG AILGGTCWADPDLNTADQIADGKVYFDFDFTPVYPAEHIIFRSQLTNDYISEVFE |
| Number of residues | 475 |
| Molecular weight (Da) | 49868.35 |
| Output files | ../../query\_sequences/18\_FANPEZAQ\_CDS\_0018.fasta |

### Putative domain architecture and protein family

#### Search results (HHblits)1

|  |  |
| --- | --- |
| Domain family databases searched | Pfam, Ncbi-cd, Cath, Phrogs |
| Results, scheme(s)  (Top layers only; threshold 1.00e-03 (evalue)) | xml version="1.0" encoding="utf-8" standalone="no"?       2024-09-02T21:08:16.093537 image/svg+xml   Matplotlib v3.7.2, https://matplotlib.org/ |
| Results, table  (E-value ≤ 1.00e-03 (evalue)) | | db | id | prob | evalue | pvalue | score | cols | query | query\_len | template | template\_len | name | description | | --- | --- | --- | --- | --- | --- | --- | --- | --- | --- | --- | --- | --- | | pfam | PF17541 | 99.8 | 3.7e-26 | 8.1e-30 | 231.7 | 231 | (229, 472) | 475 | (173, 422) | 430 | TssC | Type VI secretion system, TssC, VipB | | pfam | PF10758 | 99.5 | 8.8e-19 | 1.8e-22 | 178.3 | 175 | (287, 469) | 475 | (244, 435) | 436 | DUF2586 | Protein of unknown function (DUF2586) | | pfam | PF17482 | 98.0 | 9.7e-10 | 2.2e-13 | 83.6 | 97 | (366, 465) | 475 | (3, 99) | 100 | Phage\_sheath\_1C | Phage tail sheath C-terminal domain | | pfam | PF05943 | 97.8 | 3.7e-09 | 7.6e-13 | 100.2 | 130 | (229, 363) | 475 | (107, 301) | 302 | VipB | EvpB/VC\_A0108, tail sheath N-terminal domain | | pfam | PF18945 | 96.6 | 2.5e-06 | 5.2e-10 | 68.2 | 85 | (371, 467) | 475 | (15, 110) | 112 | VipB\_2 | EvpB/VC\_A0108, tail sheath gpW/gp25-like domain | | cath | 3foaB01 | 99.9 | 2e-27 | 3.9e-31 | 207.7 | 199 | (26, 327) | 475 | (2, 230) | 231 | Tail sheath protein gp18 | CATHCODE: 3.40.50.11780 NAME: Tail sheath protein gp18. Chain: a, b, c, d. Fragment: deletion mutantgp18m: unp residues 1-510. Engineered: yes. Mutation: yes SOURCE: Enterobacteria phage t4. Bacteriophage t4. Organism\_taxid: 10665. Gene: 18, gb aaa32541. Expressed in: escherichia coli. Expression\_system\_taxid: 562. CLASS: Alpha Beta, ARCH: 3-Layer(aba) Sandwich, TOPOL: Rossmann fold, HOMOL: Rossmann fold | | phrogs | 23 | 100.0 | 2.6e-57 | 3.2e-61 | 471.2 | 439 | (3, 474) | 475 | (5, 523) | 530 | tail sheath | tail sheath; Category: tail; NC\_017972\_p61 | | phrogs | 279 | 100.0 | 9.5e-45 | 1.2e-48 | 368.3 | 417 | (4, 465) | 475 | (10, 472) | 473 | tail sheath | tail sheath; Category: tail; p47709 VI\_05997 | | phrogs | 13288 | 99.9 | 7.5e-29 | 8.5e-33 | 207.2 | 195 | (280, 474) | 475 | (4, 204) | 208 | NA | NA; Category: unknown function; p370787 VI\_12002 | | phrogs | 1036 | 99.9 | 2.6e-27 | 3.1e-31 | 231.4 | 421 | (9, 471) | 475 | (15, 518) | 529 | tail sheath | tail sheath; Category: tail; p43549 VI\_12111 | | phrogs | 17610 | 99.8 | 2.4e-24 | 2.7e-28 | 185.3 | 227 | (245, 472) | 475 | (2, 250) | 257 | tail sheath | tail sheath; Category: tail; p306105 VI\_06232 | | phrogs | 10581 | 99.7 | 3e-22 | 3.4e-26 | 196.7 | 204 | (265, 468) | 475 | (673, 887) | 888 | tail sheath | tail sheath; Category: tail; NC\_019526\_p41 | | phrogs | 907 | 99.6 | 6.2e-21 | 7.3e-25 | 181.1 | 184 | (287, 472) | 475 | (171, 368) | 370 | tail sheath | tail sheath; Category: tail; NC\_009542\_p28 | | phrogs | 10244 | 99.6 | 4.6e-20 | 5.3e-24 | 166.9 | 323 | (9, 467) | 475 | (11, 346) | 348 | tail sheath | tail sheath; Category: tail; p54311 VI\_12213 | | phrogs | 15814 | 99.1 | 3.8e-15 | 4.2e-19 | 138.1 | 294 | (162, 471) | 475 | (411, 730) | 738 | NA | NA; Category: unknown function; p401786 VI\_06225 | | phrogs | 16736 | 99.1 | 6.5e-15 | 7.3e-19 | 108.5 | 100 | (374, 473) | 475 | (2, 101) | 108 | NA | NA; Category: unknown function; p404698 VI\_10663 | | phrogs | 30392 | 99.0 | 2.8e-14 | 3.1e-18 | 131.6 | 279 | (162, 469) | 475 | (478, 769) | 774 | tail sheath | tail sheath; Category: tail; NC\_029002\_p92 | | phrogs | 16991 | 98.9 | 1.2e-13 | 1.4e-17 | 132.6 | 168 | (285, 465) | 475 | (545, 715) | 717 | tail sheath | tail sheath; Category: tail; NC\_009760\_p40 | | phrogs | 11242 | 98.9 | 2.8e-13 | 3.2e-17 | 101.6 | 94 | (2, 100) | 475 | (1, 95) | 112 | NA | NA; Category: unknown function; p435652 VI\_11847 | | phrogs | 1480 | 98.8 | 9e-13 | 1.1e-16 | 122.7 | 313 | (9, 464) | 475 | (5, 354) | 355 | tail sheath | tail sheath; Category: tail; NC\_029116\_p9 | | phrogs | 20240 | 98.7 | 1.8e-12 | 2e-16 | 108.3 | 228 | (2, 263) | 475 | (1, 249) | 272 | NA | NA; Category: unknown function; p370788 VI\_12002 | | phrogs | 34521 | 98.7 | 3.3e-12 | 3.7e-16 | 122.0 | 204 | (268, 473) | 475 | (734, 940) | 944 | tail sheath | tail sheath; Category: tail; LC168164\_p135 | | phrogs | 19666 | 98.6 | 7e-12 | 7.8e-16 | 120.1 | 233 | (227, 465) | 475 | (743, 985) | 987 | tail sheath | tail sheath; Category: tail; MF360957\_p13 | | phrogs | 165 | 98.0 | 1.3e-09 | 1.6e-13 | 106.5 | 364 | (9, 427) | 475 | (19, 433) | 473 | tail sheath | tail sheath; Category: tail; p220370 VI\_01324 | | phrogs | 12477 | 97.0 | 1e-06 | 1.2e-10 | 54.0 | 48 | (3, 50) | 475 | (1, 48) | 48 | NA | NA; Category: unknown function; p311722 VI\_10874 | | phrogs | 38392 | 96.7 | 3.8e-06 | 4.3e-10 | 70.7 | 92 | (2, 101) | 475 | (1, 94) | 329 | tail sheath | tail sheath; Category: tail; MF001355\_p127 | | phrogs | 20365 | 95.6 | 0.0001 | 1.1e-08 | 45.7 | 50 | (424, 473) | 475 | (2, 51) | 58 | NA | NA; Category: unknown function; p352521 VI\_10849 | |
| Top keywords  (threshold 1.00e-03 (evalue)) | **tail, sheath, Phage, EvpB, VC\_A0108, yes, t4, Rossmann, fold, VI\_12002** |
| Output files | ../../domain\_architecture/18\_FANPEZAQ\_CDS\_0018\_cath.hhr ../../domain\_architecture/18\_FANPEZAQ\_CDS\_0018\_merged.svg ../../domain\_architecture/18\_FANPEZAQ\_CDS\_0018\_ncbi-cd.hhr ../../domain\_architecture/18\_FANPEZAQ\_CDS\_0018\_pfam.hhr ../../domain\_architecture/18\_FANPEZAQ\_CDS\_0018\_phrogs.hhr |

### Identical protein sequences/structures

#### Search results

|  |  |
| --- | --- |
| Protein sequence databases searched | Pdb, Swissprot, Refseq |
| Identical proteins found | -- |
| Top keywords | -- |
| Output files | -- |

### Similar protein sequences/structures

#### Sequence similarity search results (HHblits)1

|  |  |
| --- | --- |
| Sequence databases searched | Uniclust, Pdb70 |
| Results, scheme(s)  (Top layers only, threshold 1.00e-03 (evalue)) | xml version="1.0" encoding="utf-8" standalone="no"?       2024-09-02T21:08:37.015043 image/svg+xml   Matplotlib v3.7.2, https://matplotlib.org/ |
| Results, table(s)  (threshold 1.00e-03 (evalue)) | | db | id | prob | evalue | pvalue | score | cols | query | query\_len | template | template\_len | name | description | | --- | --- | --- | --- | --- | --- | --- | --- | --- | --- | --- | --- | --- | | uniclust | UniRef100\_A0A088FVH5 | 100.0 | 3.3e-79 | 6.5e-85 | 563.7 | 392 | (1, 475) | 475 | (21, 412) | 424 | Tail protein | Tail protein | | uniclust | UniRef100\_A0A009Q2W1 | 100.0 | 2.3e-74 | 4.5e-80 | 555.2 | 385 | (1, 475) | 475 | (82, 468) | 525 | Phage tail sheath family protein | Phage tail sheath family protein | | uniclust | UniRef100\_A0A095ULV3 | 100.0 | 1.4e-72 | 2.7e-78 | 527.7 | 364 | (1, 468) | 475 | (52, 437) | 446 | Phage tail sheath protein | Phage tail sheath protein | | uniclust | UniRef100\_A0A2D7W1J0 | 100.0 | 6.9e-72 | 1.3e-77 | 520.6 | 441 | (1, 468) | 475 | (1, 507) | 513 | Phage tail protein | Phage tail protein | | uniclust | UniRef100\_A0A024E974 | 100.0 | 8.4e-71 | 1.7e-76 | 523.6 | 385 | (1, 475) | 475 | (72, 456) | 475 | Major tail sheath protein | Major tail sheath protein | | uniclust | UniRef100\_A0A068TBL7 | 100.0 | 1.9e-70 | 3.7e-76 | 519.2 | 383 | (6, 475) | 475 | (29, 415) | 440 | Phage tail sheath protein | Phage tail sheath protein | | uniclust | UniRef100\_A0A094QDP2 | 100.0 | 2e-70 | 3.9e-76 | 517.9 | 233 | (229, 467) | 475 | (282, 530) | 544 | Putative phage tail sheath protein | Putative phage tail sheath protein | | uniclust | UniRef100\_A0A061YI42 | 100.0 | 2.1e-70 | 4.1e-76 | 522.2 | 381 | (1, 475) | 475 | (1, 495) | 507 | Phage tail protein | Phage tail protein | | uniclust | UniRef100\_A0A062UZH4 | 100.0 | 2.7e-69 | 5.3e-75 | 525.6 | 356 | (1, 469) | 475 | (125, 501) | 536 | Phage tail sheath protein FI | Phage tail sheath protein FI | | uniclust | UniRef100\_A0A011NBA1 | 100.0 | 3.7e-67 | 7.2e-73 | 482.8 | 384 | (1, 474) | 475 | (2, 387) | 400 | Tail sheath protein | Tail sheath protein | | uniclust | UniRef100\_A0A0P6X2M2 | 100.0 | 3.9e-67 | 7.4e-73 | 489.6 | 235 | (229, 469) | 475 | (290, 538) | 552 | Tail protein | Tail protein | | uniclust | UniRef100\_A0A016QTR2 | 100.0 | 1.1e-66 | 2.2e-72 | 523.5 | 447 | (7, 470) | 475 | (74, 587) | 629 | Phage tail sheath protein | Phage tail sheath protein | | uniclust | UniRef100\_A0A2X2RMN3 | 100.0 | 3.5e-66 | 6.7e-72 | 460.8 | 375 | (2, 474) | 475 | (1, 375) | 383 | Phage tail sheath protein | Phage tail sheath protein | | uniclust | UniRef100\_A0A0K1RXU5 | 100.0 | 2.4e-65 | 4.6e-71 | 470.3 | 361 | (2, 467) | 475 | (1, 418) | 432 | Phage tail sheath protein FI | Phage tail sheath protein FI | | uniclust | UniRef100\_A0A0B2YHC3 | 100.0 | 4.4e-65 | 8.5e-71 | 476.9 | 360 | (7, 467) | 475 | (31, 418) | 458 | Tail protein | Tail protein | | uniclust | UniRef100\_A0A011NH28 | 100.0 | 2.6e-64 | 5.2e-70 | 501.9 | 233 | (230, 468) | 475 | (380, 623) | 643 | Major tail sheath protein | Major tail sheath protein | | uniclust | UniRef100\_A0A059VPR9 | 100.0 | 3e-64 | 5.8e-70 | 459.2 | 364 | (7, 467) | 475 | (21, 422) | 432 | Putative phage tail sheath protein | Putative phage tail sheath protein | | uniclust | UniRef100\_A0A011MIB3 | 100.0 | 5e-64 | 9.7e-70 | 482.3 | 380 | (1, 474) | 475 | (53, 525) | 538 | Phage tail protein | Phage tail protein | | uniclust | UniRef100\_A0A1G6NJX4 | 100.0 | 8.8e-64 | 1.7e-69 | 479.3 | 357 | (5, 469) | 475 | (112, 486) | 502 | Phage tail sheath protein FI | Phage tail sheath protein FI | | uniclust | UniRef100\_A0A0D0HFB9 | 100.0 | 1.3e-62 | 2.5e-68 | 443.6 | 384 | (1, 474) | 475 | (1, 386) | 393 | Phage tail sheath family protein | Phage tail sheath family protein | | uniclust | UniRef100\_A0A1Q7VC09 | 100.0 | 4.2e-62 | 8.2e-68 | 477.1 | 238 | (225, 468) | 475 | (422, 673) | 680 | Phage tail protein | Phage tail protein | | uniclust | UniRef100\_A0A0R3MVK4 | 100.0 | 5.3e-62 | 9.8e-68 | 433.5 | 468 | (1, 475) | 475 | (1, 469) | 469 | Phage tail protein | Phage tail protein | | uniclust | UniRef100\_A0A1J5DEX6 | 100.0 | 7.4e-62 | 1.4e-67 | 462.9 | 233 | (229, 468) | 475 | (337, 579) | 591 | Tail sheath protein C-terminal domain-containing protein | Tail sheath protein C-terminal domain-containing protein | | uniclust | UniRef100\_A0A099PD65 | 100.0 | 1.3e-60 | 2.7e-66 | 482.7 | 237 | (229, 471) | 475 | (461, 712) | 745 | Tail sheath protein C-terminal domain-containing protein | Tail sheath protein C-terminal domain-containing protein | | uniclust | UniRef100\_A0A1E7Q659 | 100.0 | 4.2e-59 | 7.9e-65 | 412.6 | 358 | (2, 467) | 475 | (1, 375) | 377 | Phage tail protein | Phage tail protein | | uniclust | UniRef100\_A0A090AMB4 | 100.0 | 9e-59 | 1.8e-64 | 467.2 | 237 | (227, 469) | 475 | (417, 673) | 689 | Putative phage tail sheath protein | Putative phage tail sheath protein | | uniclust | UniRef100\_A0A014LUF2 | 100.0 | 5.2e-58 | 1e-63 | 457.6 | 445 | (2, 468) | 475 | (155, 677) | 748 | Tail protein | Tail protein | | uniclust | UniRef100\_A0A0C1QXU3 | 100.0 | 1.1e-57 | 2.3e-63 | 466.5 | 235 | (229, 469) | 475 | (558, 804) | 810 | Tail protein | Tail protein | | uniclust | UniRef100\_A0A059WKP9 | 100.0 | 1.2e-57 | 2.5e-63 | 445.8 | 429 | (1, 475) | 475 | (3, 488) | 501 | Tail sheath protein | Tail sheath protein | | uniclust | UniRef100\_A0A060H1U5 | 100.0 | 3.1e-56 | 5.6e-62 | 384.5 | 392 | (2, 474) | 475 | (5, 396) | 396 | Tail protein | Tail protein | | uniclust | UniRef100\_A0A011MDR6 | 100.0 | 3.7e-56 | 7.5e-62 | 453.7 | 232 | (231, 468) | 475 | (486, 730) | 737 | Major tail sheath protein | Major tail sheath protein | | uniclust | UniRef100\_A0A022PIB5 | 100.0 | 7e-56 | 1.3e-61 | 394.9 | 366 | (19, 474) | 475 | (18, 388) | 394 | Phage tail sheath protein FI | Phage tail sheath protein FI | | uniclust | UniRef100\_A0A1A7PR96 | 100.0 | 7.2e-56 | 1.4e-61 | 406.5 | 429 | (2, 473) | 475 | (1, 473) | 478 | Phage tail protein (Fragment) | Phage tail protein (Fragment) | | uniclust | UniRef100\_A0A0F0HJ39 | 100.0 | 9.2e-56 | 1.7e-61 | 403.6 | 369 | (1, 468) | 475 | (1, 495) | 501 | Phage tail protein (Fragment) | Phage tail protein (Fragment) | | uniclust | UniRef100\_A0A069PUW5 | 100.0 | 1.5e-55 | 2.9e-61 | 430.5 | 234 | (229, 468) | 475 | (427, 689) | 701 | Tail protein | Tail protein | | uniclust | UniRef100\_A0A812WA44 | 100.0 | 2.9e-55 | 5.2e-61 | 404.1 | 442 | (2, 467) | 475 | (1, 513) | 780 | 18 protein | 18 protein | | uniclust | UniRef100\_A0A085ART8 | 100.0 | 1e-54 | 2e-60 | 404.6 | 368 | (4, 471) | 475 | (51, 532) | 546 | Phage tail sheath monomer | Phage tail sheath monomer | | uniclust | UniRef100\_A0A022PCH2 | 100.0 | 1.1e-54 | 2.1e-60 | 415.3 | 348 | (1, 469) | 475 | (60, 425) | 449 | Phage tail sheath protein FI | Phage tail sheath protein FI | | uniclust | UniRef100\_A0A011PSV2 | 100.0 | 3.1e-54 | 6.1e-60 | 437.5 | 181 | (287, 468) | 475 | (412, 602) | 768 | Tail sheath protein | Tail sheath protein | | uniclust | UniRef100\_A0A5A9EN65 | 100.0 | 6.6e-54 | 1.2e-59 | 394.8 | 462 | (2, 475) | 475 | (1, 617) | 617 | Phage tail protein | Phage tail protein | | uniclust | UniRef100\_A0A1A9VKH7 | 100.0 | 9.6e-54 | 1.8e-59 | 380.4 | 375 | (4, 462) | 475 | (2, 383) | 535 | Phage tail protein | Phage tail protein | | uniclust | UniRef100\_A0A1G3FX38 | 100.0 | 9.9e-54 | 1.9e-59 | 404.7 | 358 | (7, 468) | 475 | (77, 459) | 472 | Phage tail protein (Fragment) | Phage tail protein (Fragment) | | uniclust | UniRef100\_A0A1D2QMV4 | 100.0 | 1.7e-53 | 3.2e-59 | 386.2 | 379 | (1, 475) | 475 | (15, 395) | 399 | Phage tail protein | Phage tail protein | | uniclust | UniRef100\_A0A2M9PCU5 | 100.0 | 3.1e-53 | 5.8e-59 | 371.2 | 246 | (230, 475) | 475 | (55, 301) | 315 | Phage tail protein (Fragment) | Phage tail protein (Fragment) | | uniclust | UniRef100\_A0A0P9N092 | 100.0 | 4.4e-53 | 8.2e-59 | 373.0 | 385 | (1, 474) | 475 | (29, 418) | 421 | Major tail sheath protein | Major tail sheath protein | | uniclust | UniRef100\_A0A080LVP7 | 100.0 | 7.4e-53 | 1.4e-58 | 380.9 | 233 | (231, 470) | 475 | (114, 359) | 372 | Tail sheath protein | Tail sheath protein | | uniclust | UniRef100\_A0A0A8WRE5 | 100.0 | 9.7e-53 | 1.8e-58 | 387.6 | 353 | (7, 470) | 475 | (11, 381) | 521 | Phage tail sheath protein | Phage tail sheath protein | | uniclust | UniRef100\_A0A031JIL8 | 100.0 | 1.1e-52 | 2.3e-58 | 433.0 | 235 | (230, 470) | 475 | (520, 775) | 782 | Phage tail sheath protein | Phage tail sheath protein | | uniclust | UniRef100\_A0A812QV43 | 100.0 | 1.7e-52 | 3.1e-58 | 396.7 | 386 | (2, 474) | 475 | (1, 390) | 1104 | GpFI protein | GpFI protein | | uniclust | UniRef100\_A0A1F3S987 | 100.0 | 2e-52 | 4e-58 | 395.4 | 355 | (2, 467) | 475 | (1, 443) | 447 | Tail sheath protein C-terminal domain-containing protein | Tail sheath protein C-terminal domain-containing protein | | uniclust | UniRef100\_A0A7R9HD16 | 100.0 | 2.4e-52 | 4.4e-58 | 367.9 | 378 | (2, 466) | 475 | (100, 484) | 487 | Phage tail sheath protein | Phage tail sheath protein | | uniclust | UniRef100\_A0A011TTH8 | 100.0 | 9.1e-52 | 1.8e-57 | 395.0 | 458 | (2, 475) | 475 | (1, 476) | 492 | Tail sheath protein | Tail sheath protein | | uniclust | UniRef100\_A0A2U3LUJ3 | 100.0 | 1.4e-51 | 2.6e-57 | 380.6 | 234 | (229, 468) | 475 | (206, 451) | 477 | Phage tail sheath protein | Phage tail sheath protein | | uniclust | UniRef100\_A0A022PDU0 | 100.0 | 1.4e-51 | 2.8e-57 | 401.1 | 177 | (286, 468) | 475 | (272, 458) | 520 | Phage tail sheath protein FI | Phage tail sheath protein FI | | uniclust | UniRef100\_A0A356K0G5 | 100.0 | 2.3e-51 | 4.3e-57 | 360.3 | 296 | (1, 396) | 475 | (1, 325) | 325 | Phage tail protein (Fragment) | Phage tail protein (Fragment) | | uniclust | UniRef100\_A0A1H3QMK3 | 100.0 | 3.3e-51 | 6.2e-57 | 392.6 | 232 | (230, 467) | 475 | (550, 805) | 809 | Uncharacterized protein | Uncharacterized protein | | uniclust | UniRef100\_A0A0Q8LR77 | 100.0 | 3.4e-51 | 6.5e-57 | 401.5 | 232 | (230, 467) | 475 | (621, 867) | 870 | Phage tail protein | Phage tail protein | | uniclust | UniRef100\_A0A1Q3SJK3 | 100.0 | 3.6e-51 | 6.7e-57 | 374.8 | 233 | (229, 467) | 475 | (364, 611) | 615 | Phage tail protein | Phage tail protein | | uniclust | UniRef100\_A0A7R9HA11 | 100.0 | 6.3e-51 | 1.2e-56 | 392.6 | 364 | (15, 467) | 475 | (627, 991) | 1133 | Uncharacterized protein | Uncharacterized protein | | uniclust | UniRef100\_A0A0F3IT60 | 100.0 | 2e-50 | 3.9e-56 | 380.9 | 362 | (5, 467) | 475 | (5, 500) | 504 | Phage tail protein (Fragment) | Phage tail protein (Fragment) | | uniclust | UniRef100\_A0A2V9GXL0 | 100.0 | 2.4e-50 | 4.6e-56 | 388.0 | 234 | (229, 468) | 475 | (530, 782) | 786 | Phage tail protein | Phage tail protein | | uniclust | UniRef100\_A0A1A1YQW6 | 100.0 | 1.7e-49 | 3.3e-55 | 402.4 | 234 | (229, 468) | 475 | (859, 1114) | 1121 | Phage tail protein | Phage tail protein | | uniclust | UniRef100\_A0A5Q4D9T1 | 100.0 | 1.8e-49 | 3.4e-55 | 360.3 | 359 | (7, 468) | 475 | (14, 401) | 436 | Phage tail sheath family protein | Phage tail sheath family protein | | uniclust | UniRef100\_A0A061JGM3 | 100.0 | 3.9e-49 | 7.3e-55 | 341.2 | 304 | (2, 397) | 475 | (1, 305) | 306 | Major tail sheath protein | Major tail sheath protein | | uniclust | UniRef100\_A0A2P8VUG9 | 100.0 | 7.1e-49 | 1.3e-54 | 364.5 | 233 | (229, 467) | 475 | (293, 536) | 541 | Phage tail protein | Phage tail protein | | uniclust | UniRef100\_A0A519FU64 | 100.0 | 1.8e-48 | 3.4e-54 | 343.4 | 305 | (86, 475) | 475 | (13, 318) | 324 | Phage tail sheath family protein (Fragment) | Phage tail sheath family protein (Fragment) | | uniclust | UniRef100\_A0A0B5FRP8 | 100.0 | 2.9e-48 | 5.4e-54 | 362.1 | 423 | (2, 468) | 475 | (1, 501) | 513 | Tail protein | Tail protein | | uniclust | UniRef100\_A0A1Q7MAI6 | 100.0 | 3.9e-48 | 7.2e-54 | 343.8 | 369 | (3, 464) | 475 | (2, 381) | 515 | Phage tail protein | Phage tail protein | | uniclust | UniRef100\_A0A4Q5SQU1 | 100.0 | 3.8e-48 | 7.4e-54 | 344.1 | 234 | (230, 469) | 475 | (47, 304) | 307 | Phage tail sheath family protein (Fragment) | Phage tail sheath family protein (Fragment) | | uniclust | UniRef100\_A0A6J5N355 | 100.0 | 3.9e-48 | 7.5e-54 | 367.6 | 433 | (2, 469) | 475 | (1, 503) | 511 | Tail sheath protein | Tail sheath protein | | uniclust | UniRef100\_A0A7J6YK69 | 100.0 | 5.1e-48 | 9.4e-54 | 383.6 | 384 | (2, 468) | 475 | (1278, 1668) | 2523 | Uncharacterized protein | Uncharacterized protein | | uniclust | UniRef100\_A0A2W5Z8T4 | 100.0 | 1.4e-47 | 2.5e-53 | 343.7 | 347 | (6, 468) | 475 | (6, 376) | 565 | AAA+ ATPase domain-containing protein | AAA+ ATPase domain-containing protein | | uniclust | UniRef100\_A0A367ZMD7 | 100.0 | 1.4e-47 | 2.7e-53 | 358.7 | 235 | (228, 468) | 475 | (356, 607) | 621 | Phage tail sheath protein FI | Phage tail sheath protein FI | | uniclust | UniRef100\_A0A2X3JUE5 | 100.0 | 2.2e-47 | 4.3e-53 | 355.1 | 343 | (2, 435) | 475 | (1, 349) | 394 | Major tail sheath protein FI | Major tail sheath protein FI | | uniclust | UniRef100\_A0A0D6L3S9 | 100.0 | 6.8e-47 | 1.3e-52 | 351.5 | 368 | (2, 466) | 475 | (3, 466) | 470 | Uncharacterized protein (Fragment) | Uncharacterized protein (Fragment) | | uniclust | UniRef100\_A0A0W8I564 | 100.0 | 1.3e-46 | 2.5e-52 | 354.8 | 334 | (18, 469) | 475 | (25, 377) | 508 | Tail sheath protein subtilisin-like domain-containing protein | Tail sheath protein subtilisin-like domain-containing protein | | uniclust | UniRef100\_A0A2E2NTG5 | 100.0 | 1.6e-46 | 2.9e-52 | 352.6 | 229 | (230, 468) | 475 | (329, 593) | 600 | Phage tail protein | Phage tail protein | | uniclust | UniRef100\_A0A956NBW1 | 100.0 | 3.3e-46 | 6.1e-52 | 340.0 | 336 | (18, 468) | 475 | (53, 405) | 521 | Phage tail sheath subtilisin-like domain-containing protein | Phage tail sheath subtilisin-like domain-containing protein | | uniclust | UniRef100\_A0A074M4V8 | 100.0 | 7e-46 | 1.4e-51 | 356.3 | 409 | (7, 468) | 475 | (12, 470) | 481 | Phage tail protein | Phage tail protein | | uniclust | UniRef100\_A0A1D8TT95 | 100.0 | 1.1e-45 | 2e-51 | 352.3 | 232 | (229, 466) | 475 | (536, 788) | 792 | Phage tail protein | Phage tail protein | | uniclust | UniRef100\_A0A0J0Y2J6 | 100.0 | 1.3e-45 | 2.5e-51 | 358.4 | 179 | (287, 466) | 475 | (516, 704) | 708 | Phage tail sheath protein | Phage tail sheath protein | | uniclust | UniRef100\_A0A0Q8XJ68 | 100.0 | 3.2e-45 | 6.2e-51 | 360.8 | 234 | (229, 471) | 475 | (515, 767) | 783 | Tail sheath protein C-terminal domain-containing protein | Tail sheath protein C-terminal domain-containing protein | | uniclust | UniRef100\_A0A0P0QJ01 | 100.0 | 5.3e-45 | 1e-50 | 342.5 | 376 | (7, 469) | 475 | (16, 416) | 433 | Phage tail sheath family protein | Phage tail sheath family protein | | uniclust | UniRef100\_A0A350QJ90 | 100.0 | 1.3e-44 | 2.5e-50 | 327.4 | 233 | (230, 468) | 475 | (119, 369) | 409 | Phage tail sheath family protein (Fragment) | Phage tail sheath family protein (Fragment) | | uniclust | UniRef100\_A0A014LI00 | 100.0 | 2.3e-44 | 4.5e-50 | 342.4 | 420 | (7, 472) | 475 | (31, 524) | 528 | Tail protein | Tail protein | | uniclust | UniRef100\_A0A1U7N9U3 | 100.0 | 5.5e-44 | 1e-49 | 326.7 | 378 | (1, 466) | 475 | (1, 476) | 480 | Phage tail protein | Phage tail protein | | uniclust | UniRef100\_A0A410G7I7 | 100.0 | 5.7e-44 | 1.1e-49 | 345.3 | 231 | (231, 468) | 475 | (533, 785) | 789 | Phage tail sheath family protein | Phage tail sheath family protein | | uniclust | UniRef100\_A0A2V9K9Q7 | 100.0 | 8.7e-44 | 1.7e-49 | 335.5 | 235 | (229, 470) | 475 | (242, 498) | 500 | Phage tail protein | Phage tail protein | | uniclust | UniRef100\_A0A0T9RBT0 | 100.0 | 2.5e-43 | 4.7e-49 | 301.3 | 236 | (240, 475) | 475 | (3, 240) | 247 | Phage tail sheath monomer | Phage tail sheath monomer | | uniclust | UniRef100\_A0A0N0JDZ8 | 100.0 | 1e-42 | 2e-48 | 349.9 | 236 | (229, 470) | 475 | (420, 667) | 729 | Tail sheath protein C-terminal domain-containing protein | Tail sheath protein C-terminal domain-containing protein | | uniclust | UniRef100\_A0A2N5D506 | 100.0 | 1.4e-42 | 2.6e-48 | 309.1 | 346 | (1, 466) | 475 | (1, 367) | 371 | Phage tail sheath family protein | Phage tail sheath family protein | | uniclust | UniRef100\_A0A1A9VZ70 | 100.0 | 2.3e-42 | 4.3e-48 | 310.2 | 289 | (101, 474) | 475 | (52, 354) | 359 | Tail sheath protein subtilisin-like domain-containing protein | Tail sheath protein subtilisin-like domain-containing protein | | uniclust | UniRef100\_A0A0L9YBE2 | 100.0 | 2.3e-42 | 4.5e-48 | 333.7 | 377 | (1, 475) | 475 | (1, 490) | 497 | Phage tail sheath protein | Phage tail sheath protein | | uniclust | UniRef100\_A0A952H001 | 100.0 | 3.3e-42 | 6.1e-48 | 307.8 | 233 | (229, 467) | 475 | (218, 461) | 466 | Phage tail sheath family protein | Phage tail sheath family protein | | uniclust | UniRef100\_A0A031FQW7 | 100.0 | 4.4e-42 | 8.5e-48 | 322.4 | 366 | (7, 475) | 475 | (14, 390) | 414 | Phage tail sheath monomer | Phage tail sheath monomer | | uniclust | UniRef100\_A0A0S7Z2I8 | 100.0 | 7.2e-42 | 1.4e-47 | 340.4 | 437 | (6, 468) | 475 | (80, 582) | 655 | Tail sheath protein subtilisin-like domain-containing protein | Tail sheath protein subtilisin-like domain-containing protein | | uniclust | UniRef100\_A0A5B7V907 | 100.0 | 7.8e-42 | 1.4e-47 | 302.7 | 232 | (231, 468) | 475 | (231, 483) | 488 | Phage tail sheath protein | Phage tail sheath protein | | uniclust | UniRef100\_A0A0Q6AB86 | 100.0 | 1.5e-41 | 2.9e-47 | 328.5 | 180 | (286, 466) | 475 | (451, 640) | 644 | VWFA domain-containing protein | VWFA domain-containing protein | | uniclust | UniRef100\_A0A2E2NTG3 | 100.0 | 1.9e-41 | 3.6e-47 | 311.5 | 228 | (230, 468) | 475 | (149, 395) | 401 | Phage tail sheath family protein | Phage tail sheath family protein | | uniclust | UniRef100\_A0A143G8E1 | 100.0 | 3.7e-41 | 7.1e-47 | 294.1 | 274 | (1, 364) | 475 | (3, 283) | 285 | Phage tail protein | Phage tail protein | | uniclust | UniRef100\_A0A1B7IQW9 | 100.0 | 6.3e-41 | 1.2e-46 | 308.6 | 248 | (227, 474) | 475 | (395, 644) | 649 | Phage tail sheath monomer | Phage tail sheath monomer | | uniclust | UniRef100\_A0A1B1NT66 | 100.0 | 2.2e-40 | 4.1e-46 | 300.9 | 376 | (4, 474) | 475 | (1, 426) | 434 | Putative prophage major tail sheath protein | Putative prophage major tail sheath protein | | uniclust | UniRef100\_A0A090GUB3 | 100.0 | 4.6e-40 | 8.8e-46 | 301.7 | 395 | (2, 474) | 475 | (1, 420) | 435 | Phage tail protein | Phage tail protein | | uniclust | UniRef100\_A0A010SSQ8 | 100.0 | 8e-40 | 1.6e-45 | 319.5 | 430 | (7, 466) | 475 | (71, 553) | 609 | Phage tail protein | Phage tail protein | | uniclust | UniRef100\_A0A4V1UFD5 | 100.0 | 1.1e-39 | 2.1e-45 | 298.7 | 236 | (229, 470) | 475 | (204, 461) | 464 | Phage tail sheath family protein | Phage tail sheath family protein | | uniclust | UniRef100\_A0A1C4YCD9 | 100.0 | 1.3e-39 | 2.4e-45 | 284.2 | 198 | (268, 471) | 475 | (16, 233) | 270 | Phage tail sheath protein | Phage tail sheath protein | | uniclust | UniRef100\_A0A3M2D088 | 100.0 | 1.4e-39 | 2.7e-45 | 266.8 | 176 | (289, 470) | 475 | (1, 184) | 193 | Phage tail sheath family protein (Fragment) | Phage tail sheath family protein (Fragment) | | uniclust | UniRef100\_A0A2A7RYU0 | 100.0 | 1.5e-39 | 2.8e-45 | 281.3 | 310 | (3, 402) | 475 | (1, 320) | 339 | Tail sheath protein subtilisin-like domain-containing protein | Tail sheath protein subtilisin-like domain-containing protein | | uniclust | UniRef100\_A0A077KVI5 | 100.0 | 2.3e-39 | 4.3e-45 | 278.3 | 239 | (230, 473) | 475 | (4, 244) | 262 | Tail sheath protein subtilisin-like domain-containing protein | Tail sheath protein subtilisin-like domain-containing protein | | uniclust | UniRef100\_A0A1C3NFI6 | 100.0 | 2.4e-39 | 4.8e-45 | 312.4 | 238 | (225, 468) | 475 | (265, 515) | 530 | Phage tail sheath protein FI | Phage tail sheath protein FI | | uniclust | UniRef100\_A0A0Q8AN78 | 100.0 | 2.6e-39 | 4.9e-45 | 289.8 | 388 | (1, 474) | 475 | (1, 400) | 413 | Phage tail protein | Phage tail protein | | uniclust | UniRef100\_A0A1A7R3U7 | 100.0 | 3.4e-39 | 6.4e-45 | 303.9 | 372 | (2, 468) | 475 | (1, 463) | 727 | Phage tail sheath protein FI | Phage tail sheath protein FI | | uniclust | UniRef100\_A0A1V5CVP8 | 100.0 | 6.5e-39 | 1.2e-44 | 292.7 | 234 | (228, 467) | 475 | (249, 502) | 507 | Phage tail sheath protein | Phage tail sheath protein | | uniclust | UniRef100\_A0A963BNV3 | 100.0 | 7e-39 | 1.3e-44 | 282.7 | 233 | (229, 467) | 475 | (218, 461) | 464 | Phage tail sheath family protein | Phage tail sheath family protein | | uniclust | UniRef100\_A0A0A8H9Y0 | 100.0 | 7.6e-39 | 1.5e-44 | 294.6 | 366 | (5, 474) | 475 | (3, 377) | 385 | Major tail sheath protein | Major tail sheath protein | | uniclust | UniRef100\_A0A1Q7BHJ3 | 100.0 | 8.2e-39 | 1.5e-44 | 285.6 | 334 | (30, 464) | 475 | (2, 353) | 516 | Phage tail protein | Phage tail protein | | uniclust | UniRef100\_A0A136QAK0 | 100.0 | 1.5e-38 | 2.8e-44 | 262.3 | 243 | (233, 475) | 475 | (3, 246) | 249 | Tail protein (Fragment) | Tail protein (Fragment) | | uniclust | UniRef100\_A0A2D5XZB8 | 100.0 | 1.6e-38 | 3.1e-44 | 304.8 | 229 | (235, 469) | 475 | (416, 670) | 677 | Tail sheath protein C-terminal domain-containing protein | Tail sheath protein C-terminal domain-containing protein | | uniclust | UniRef100\_A0A150S1N9 | 100.0 | 1.7e-38 | 3.2e-44 | 292.8 | 399 | (7, 471) | 475 | (11, 452) | 462 | Tail sheath protein subtilisin-like domain-containing protein | Tail sheath protein subtilisin-like domain-containing protein | | uniclust | UniRef100\_A0A8S5LXM1 | 100.0 | 1.8e-38 | 3.4e-44 | 289.1 | 431 | (2, 467) | 475 | (1, 496) | 500 | Tail sheath protein | Tail sheath protein | | uniclust | UniRef100\_A0A7V9BVW5 | 100.0 | 1.9e-38 | 3.6e-44 | 291.3 | 227 | (232, 468) | 475 | (326, 587) | 593 | Phage tail sheath family protein | Phage tail sheath family protein | | uniclust | UniRef100\_A0A0M3V6G8 | 100.0 | 1.9e-38 | 3.7e-44 | 299.2 | 414 | (7, 466) | 475 | (17, 476) | 509 | Tail sheath protein subtilisin-like domain-containing protein | Tail sheath protein subtilisin-like domain-containing protein | | uniclust | UniRef100\_A0A433VYQ0 | 100.0 | 2.9e-38 | 5.3e-44 | 303.0 | 231 | (231, 467) | 475 | (893, 1149) | 1173 | Inter-alpha-trypsin inhibitor domain-containing protein | Inter-alpha-trypsin inhibitor domain-containing protein | | uniclust | UniRef100\_A0A134CAP7 | 100.0 | 5.6e-38 | 1.1e-43 | 295.0 | 281 | (101, 475) | 475 | (126, 425) | 430 | Phage tail protein (Fragment) | Phage tail protein (Fragment) | | uniclust | UniRef100\_A0A1X7M8N6 | 100.0 | 7.8e-38 | 1.5e-43 | 268.6 | 227 | (1, 315) | 475 | (1, 228) | 231 | Phage major tail sheath protein | Phage major tail sheath protein | | uniclust | UniRef100\_A0A7C3H8R7 | 100.0 | 9.4e-38 | 1.7e-43 | 281.0 | 233 | (229, 467) | 475 | (247, 499) | 504 | Phage tail sheath family protein (Fragment) | Phage tail sheath family protein (Fragment) | | uniclust | UniRef100\_A0A0M2S0L1 | 100.0 | 9.6e-38 | 1.9e-43 | 311.0 | 237 | (228, 469) | 475 | (523, 779) | 786 | Tail sheath protein subtilisin-like domain-containing protein | Tail sheath protein subtilisin-like domain-containing protein | | uniclust | UniRef100\_A0A0Q4LKW2 | 100.0 | 1.7e-37 | 3.2e-43 | 260.7 | 182 | (286, 468) | 475 | (15, 206) | 213 | Tail sheath protein C-terminal domain-containing protein | Tail sheath protein C-terminal domain-containing protein | | uniclust | UniRef100\_A0A5D0URY7 | 100.0 | 4.3e-37 | 8e-43 | 290.0 | 225 | (235, 467) | 475 | (556, 800) | 804 | Phage tail sheath family protein | Phage tail sheath family protein | | uniclust | UniRef100\_A0A7Y6Z423 | 100.0 | 5e-37 | 9.3e-43 | 283.1 | 233 | (229, 467) | 475 | (295, 553) | 557 | Phage tail sheath family protein | Phage tail sheath family protein | | uniclust | UniRef100\_A0A3N5KUP2 | 100.0 | 7.6e-37 | 1.4e-42 | 253.9 | 190 | (269, 465) | 475 | (4, 201) | 214 | Phage tail protein (Fragment) | Phage tail protein (Fragment) | | uniclust | UniRef100\_A0A285NJC0 | 100.0 | 9.1e-37 | 1.7e-42 | 268.1 | 374 | (3, 474) | 475 | (1, 391) | 399 | Tail sheath protein subtilisin-like domain-containing protein | Tail sheath protein subtilisin-like domain-containing protein | | uniclust | UniRef100\_A0A0A7HAU9 | 100.0 | 9.5e-37 | 1.9e-42 | 293.1 | 426 | (1, 466) | 475 | (1, 482) | 490 | Tail sheath protein | Tail sheath protein | | uniclust | UniRef100\_A0A2S9WYU3 | 100.0 | 1e-36 | 2e-42 | 250.1 | 157 | (306, 468) | 475 | (1, 165) | 184 | Phage tail protein (Fragment) | Phage tail protein (Fragment) | | uniclust | UniRef100\_A0A6N7C2F0 | 100.0 | 2e-36 | 3.7e-42 | 263.4 | 233 | (231, 469) | 475 | (25, 269) | 286 | Uncharacterized protein | Uncharacterized protein | | uniclust | UniRef100\_A0A2S7K9H5 | 100.0 | 3.6e-36 | 6.7e-42 | 266.5 | 345 | (7, 465) | 475 | (106, 469) | 473 | Phage tail protein | Phage tail protein | | uniclust | UniRef100\_A0A2G6CJB9 | 100.0 | 4.4e-36 | 8.3e-42 | 277.4 | 244 | (226, 473) | 475 | (203, 474) | 481 | Phage tail protein | Phage tail protein | | uniclust | UniRef100\_A0A6J7X5P4 | 100.0 | 4.9e-36 | 9e-42 | 272.9 | 232 | (231, 468) | 475 | (345, 604) | 610 | Phage tail sheath C-terminal domain containing protein | Phage tail sheath C-terminal domain containing protein | | uniclust | UniRef100\_A0A0F9ST52 | 100.0 | 1.1e-35 | 2.1e-41 | 295.7 | 242 | (229, 474) | 475 | (517, 786) | 796 | Tail sheath protein C-terminal domain-containing protein | Tail sheath protein C-terminal domain-containing protein | | uniclust | UniRef100\_A0A1S6HVK6 | 100.0 | 1.3e-35 | 2.5e-41 | 261.9 | 180 | (286, 467) | 475 | (77, 266) | 279 | Phage tail sheath protein | Phage tail sheath protein | | uniclust | UniRef100\_A0A6G9ZDK1 | 100.0 | 1.5e-35 | 2.8e-41 | 273.1 | 230 | (231, 466) | 475 | (447, 691) | 695 | Phage tail protein | Phage tail protein | | uniclust | UniRef100\_A0A1C3FCE3 | 100.0 | 1.5e-35 | 2.8e-41 | 250.7 | 209 | (266, 474) | 475 | (11, 225) | 232 | Phage tail sheath monomer | Phage tail sheath monomer | | uniclust | UniRef100\_A0A6V8KE03 | 100.0 | 2e-35 | 3.8e-41 | 260.4 | 270 | (2, 388) | 475 | (9, 343) | 345 | Tail sheath protein subtilisin-like domain-containing protein | Tail sheath protein subtilisin-like domain-containing protein | | uniclust | UniRef100\_A0A3A0B079 | 100.0 | 2e-35 | 3.8e-41 | 270.2 | 236 | (229, 470) | 475 | (257, 510) | 523 | Phage tail sheath family protein (Fragment) | Phage tail sheath family protein (Fragment) | | uniclust | UniRef100\_UPI001E51F530 | 100.0 | 3.6e-35 | 6.6e-41 | 271.0 | 343 | (16, 469) | 475 | (238, 609) | 646 | phage tail sheath subtilisin-like domain-containing protein | phage tail sheath subtilisin-like domain-containing protein | | uniclust | UniRef100\_A0A1Z4RAC9 | 100.0 | 5.1e-35 | 9.8e-41 | 268.8 | 360 | (3, 466) | 475 | (8, 384) | 398 | Phage tail sheath protein fi-like protein | Phage tail sheath protein fi-like protein | | uniclust | UniRef100\_A0A1Q7YH55 | 100.0 | 5.3e-35 | 9.9e-41 | 266.9 | 433 | (7, 464) | 475 | (10, 494) | 508 | Tail sheath protein subtilisin-like domain-containing protein | Tail sheath protein subtilisin-like domain-containing protein | | uniclust | UniRef100\_A0A2K8ZCA4 | 100.0 | 5.9e-35 | 1.2e-40 | 281.9 | 197 | (270, 467) | 475 | (301, 509) | 514 | Phage tail protein | Phage tail protein | | uniclust | UniRef100\_A0A518BPN9 | 100.0 | 8.9e-35 | 1.6e-40 | 270.0 | 232 | (229, 466) | 475 | (489, 744) | 750 | Phage tail sheath protein | Phage tail sheath protein | | uniclust | UniRef100\_A0A0A0YVK8 | 100.0 | 1.3e-34 | 2.5e-40 | 279.9 | 235 | (229, 469) | 475 | (307, 559) | 575 | Tail sheath protein | Tail sheath protein | | uniclust | UniRef100\_A0A7Z2GDE3 | 100.0 | 1.4e-34 | 2.7e-40 | 282.0 | 232 | (230, 467) | 475 | (717, 979) | 983 | BIG2 domain-containing protein | BIG2 domain-containing protein | | uniclust | UniRef100\_A0A8X6MUK6 | 100.0 | 2.6e-34 | 4.7e-40 | 256.5 | 212 | (230, 445) | 475 | (45, 256) | 498 | Putative prophage major tail sheath protein | Putative prophage major tail sheath protein | | uniclust | UniRef100\_A0A0T7DUW1 | 100.0 | 3.1e-34 | 5.8e-40 | 272.8 | 232 | (242, 474) | 475 | (352, 604) | 612 | Putative bacteriophage tail sheath protein | Putative bacteriophage tail sheath protein | | uniclust | UniRef100\_A0A2E7CJ71 | 100.0 | 3.8e-34 | 7.1e-40 | 271.9 | 195 | (267, 467) | 475 | (426, 635) | 643 | Phage tail sheath family protein | Phage tail sheath family protein | | uniclust | UniRef100\_A0A1W0CRX8 | 100.0 | 6.1e-34 | 1.2e-39 | 267.1 | 176 | (287, 468) | 475 | (375, 560) | 562 | Phage tail protein | Phage tail protein | | uniclust | UniRef100\_A0A5N8UQV4 | 100.0 | 8.5e-34 | 1.6e-39 | 247.6 | 230 | (231, 468) | 475 | (110, 366) | 367 | Phage tail sheath family protein | Phage tail sheath family protein | | uniclust | UniRef100\_A0A2S2DPT2 | 100.0 | 8.6e-34 | 1.6e-39 | 265.7 | 362 | (7, 466) | 475 | (296, 819) | 823 | Tail sheath protein C-terminal domain-containing protein | Tail sheath protein C-terminal domain-containing protein | | uniclust | UniRef100\_A0A2A8B6F9 | 100.0 | 1.6e-33 | 3.1e-39 | 226.4 | 168 | (295, 468) | 475 | (1, 177) | 181 | Phage tail sheath protein | Phage tail sheath protein | | uniclust | UniRef100\_UPI00035D90AA | 100.0 | 3.5e-33 | 6.6e-39 | 271.1 | 231 | (231, 467) | 475 | (157, 427) | 837 | phage tail sheath C-terminal domain-containing protein | phage tail sheath C-terminal domain-containing protein | | uniclust | UniRef100\_A0A126V0R7 | 100.0 | 4.8e-33 | 9.1e-39 | 257.6 | 225 | (231, 465) | 475 | (264, 505) | 511 | Phage tail protein | Phage tail protein | | uniclust | UniRef100\_A0A3S4DC63 | 100.0 | 8.4e-33 | 1.6e-38 | 228.1 | 196 | (268, 468) | 475 | (7, 213) | 228 | Phage tail sheath protein | Phage tail sheath protein | | uniclust | UniRef100\_A0A838FVS3 | 100.0 | 1e-32 | 1.9e-38 | 253.3 | 333 | (26, 466) | 475 | (32, 382) | 465 | Uncharacterized protein | Uncharacterized protein | | uniclust | UniRef100\_A0A1M3D377 | 100.0 | 1.2e-32 | 2.3e-38 | 246.9 | 354 | (5, 461) | 475 | (30, 428) | 430 | Tail sheath protein C-terminal domain-containing protein | Tail sheath protein C-terminal domain-containing protein | | uniclust | UniRef100\_A0A6J5S5A9 | 100.0 | 1.3e-32 | 2.5e-38 | 256.9 | 231 | (230, 466) | 475 | (532, 780) | 783 | Tail sheath protein | Tail sheath protein | | uniclust | UniRef100\_A0A3N5DYH3 | 100.0 | 1.4e-32 | 2.6e-38 | 258.8 | 180 | (286, 466) | 475 | (325, 514) | 518 | Phage tail sheath family protein | Phage tail sheath family protein | | uniclust | UniRef100\_A0A4Q6CEC2 | 100.0 | 1.6e-32 | 3.1e-38 | 233.4 | 237 | (230, 474) | 475 | (7, 245) | 249 | Phage tail protein (Fragment) | Phage tail protein (Fragment) | | uniclust | UniRef100\_A0A385ISI4 | 100.0 | 1.9e-32 | 3.7e-38 | 270.9 | 236 | (229, 470) | 475 | (359, 610) | 622 | Putative tail sheath protein | Putative tail sheath protein | | uniclust | UniRef100\_A0A7K2KKT6 | 99.9 | 3.3e-32 | 6e-38 | 229.6 | 167 | (231, 403) | 475 | (131, 305) | 306 | Phage tail sheath family protein (Fragment) | Phage tail sheath family protein (Fragment) | | uniclust | UniRef100\_A0A2D6SUT0 | 99.9 | 7.2e-32 | 1.3e-37 | 247.8 | 224 | (230, 459) | 475 | (226, 470) | 553 | Phage tail protein | Phage tail protein | | uniclust | UniRef100\_A0A353GA80 | 99.9 | 8.2e-32 | 1.5e-37 | 233.0 | 292 | (2, 381) | 475 | (1, 323) | 325 | Phage tail protein (Fragment) | Phage tail protein (Fragment) | | uniclust | UniRef100\_A0A925M9A4 | 99.9 | 9.8e-32 | 1.8e-37 | 229.7 | 183 | (286, 469) | 475 | (72, 264) | 273 | Phage tail sheath family protein | Phage tail sheath family protein | | uniclust | UniRef100\_A0A0N7C9H8 | 99.9 | 1.2e-31 | 2.3e-37 | 251.4 | 233 | (231, 469) | 475 | (161, 411) | 427 | Tail sheath protein | Tail sheath protein | | uniclust | UniRef100\_A0A2X2BJR7 | 99.9 | 2.5e-31 | 4.8e-37 | 223.4 | 221 | (115, 421) | 475 | (18, 240) | 241 | Major tail sheath protein | Major tail sheath protein | | uniclust | UniRef100\_A0A954RU13 | 99.9 | 2.6e-31 | 4.8e-37 | 243.4 | 235 | (232, 467) | 475 | (360, 624) | 627 | Phage tail sheath family protein | Phage tail sheath family protein | | uniclust | UniRef100\_A0A662Q4X3 | 99.9 | 6.4e-31 | 1.2e-36 | 236.7 | 190 | (267, 468) | 475 | (312, 511) | 526 | Uncharacterized protein | Uncharacterized protein | | uniclust | UniRef100\_A0A960XNF8 | 99.9 | 7.4e-31 | 1.4e-36 | 213.3 | 194 | (268, 467) | 475 | (14, 229) | 232 | Phage tail sheath family protein | Phage tail sheath family protein | | uniclust | UniRef100\_A0A2W4QEG8 | 99.9 | 2.2e-30 | 4.2e-36 | 243.7 | 175 | (286, 466) | 475 | (356, 545) | 549 | Tail sheath protein C-terminal domain-containing protein | Tail sheath protein C-terminal domain-containing protein | | uniclust | UniRef100\_A0A958D3A0 | 99.9 | 2.6e-30 | 4.7e-36 | 208.6 | 186 | (231, 422) | 475 | (19, 220) | 220 | Phage tail sheath family protein (Fragment) | Phage tail sheath family protein (Fragment) | | uniclust | UniRef100\_A0A953FW81 | 99.9 | 2.8e-30 | 5.2e-36 | 232.7 | 345 | (7, 466) | 475 | (155, 523) | 526 | Tail sheath protein C-terminal domain-containing protein | Tail sheath protein C-terminal domain-containing protein | | uniclust | UniRef100\_A0A7V9U445 | 99.9 | 3.3e-30 | 6.2e-36 | 220.8 | 200 | (264, 469) | 475 | (42, 251) | 259 | Phage tail sheath family protein | Phage tail sheath family protein | | uniclust | UniRef100\_A0A955QN58 | 99.9 | 3.7e-30 | 6.8e-36 | 212.2 | 225 | (237, 467) | 475 | (7, 251) | 255 | Phage tail sheath family protein | Phage tail sheath family protein | | uniclust | UniRef100\_A0A951KAP4 | 99.9 | 4.3e-30 | 7.9e-36 | 240.1 | 235 | (229, 469) | 475 | (499, 759) | 765 | Phage tail sheath family protein | Phage tail sheath family protein | | uniclust | UniRef100\_A0A957BWX3 | 99.9 | 4.4e-30 | 8e-36 | 233.4 | 366 | (2, 465) | 475 | (1, 409) | 569 | TIR domain-containing protein | TIR domain-containing protein | | uniclust | UniRef100\_A0A0M7QCF0 | 99.9 | 6.8e-30 | 1.3e-35 | 230.1 | 233 | (231, 469) | 475 | (33, 283) | 295 | Putative tail sheath monomer [Shigella phage Shfl2] | Putative tail sheath monomer [Shigella phage Shfl2] | | uniclust | UniRef100\_UPI001ABB0727 | 99.9 | 1.4e-29 | 2.6e-35 | 237.8 | 174 | (287, 466) | 475 | (498, 685) | 689 | phage tail sheath C-terminal domain-containing protein | phage tail sheath C-terminal domain-containing protein | | uniclust | UniRef100\_A0A7X8L470 | 99.9 | 1.5e-29 | 2.8e-35 | 229.6 | 227 | (229, 464) | 475 | (322, 557) | 559 | Phage tail sheath family protein | Phage tail sheath family protein | | uniclust | UniRef100\_A0A353BET0 | 99.9 | 1.7e-29 | 3e-35 | 235.4 | 232 | (229, 466) | 475 | (478, 729) | 734 | Tail sheath protein C-terminal domain-containing protein | Tail sheath protein C-terminal domain-containing protein | | uniclust | UniRef100\_A0A7X5ZX35 | 99.9 | 3.4e-29 | 6.3e-35 | 226.4 | 227 | (235, 467) | 475 | (282, 531) | 536 | Phage tail sheath family protein | Phage tail sheath family protein | | uniclust | UniRef100\_A0A2W1BAD9 | 99.9 | 4.3e-29 | 8e-35 | 228.0 | 425 | (3, 466) | 475 | (1, 450) | 460 | Tail sheath protein C-terminal domain-containing protein | Tail sheath protein C-terminal domain-containing protein | | uniclust | UniRef100\_A0A2D7XCW1 | 99.9 | 4.3e-29 | 8.4e-35 | 234.4 | 235 | (229, 469) | 475 | (155, 421) | 426 | Phage tail protein | Phage tail protein | | uniclust | UniRef100\_A0A023W640 | 99.9 | 4.4e-29 | 8.8e-35 | 257.0 | 233 | (231, 469) | 475 | (410, 661) | 699 | Tail sheath protein | Tail sheath protein | | uniclust | UniRef100\_A0A1I4LMX5 | 99.9 | 5.1e-29 | 1e-34 | 235.6 | 293 | (1, 352) | 475 | (1, 383) | 400 | Phage tail protein | Phage tail protein | | uniclust | UniRef100\_UPI000A44B44F | 99.9 | 7.1e-29 | 1.3e-34 | 203.4 | 181 | (231, 421) | 475 | (33, 243) | 243 | phage tail sheath subtilisin-like domain-containing protein | phage tail sheath subtilisin-like domain-containing protein | | uniclust | UniRef100\_A0A011NTQ5 | 99.9 | 1.3e-28 | 2.5e-34 | 262.3 | 236 | (229, 470) | 475 | (671, 936) | 946 | Phage tail sheath protein | Phage tail sheath protein | | uniclust | UniRef100\_A0A849TMA6 | 99.9 | 1.4e-28 | 2.7e-34 | 234.8 | 432 | (7, 467) | 475 | (16, 501) | 635 | Phage tail protein | Phage tail protein | | uniclust | UniRef100\_A0A9E1IB57 | 99.9 | 1.5e-28 | 2.7e-34 | 218.2 | 194 | (268, 467) | 475 | (235, 443) | 451 | Phage tail sheath family protein | Phage tail sheath family protein | | uniclust | UniRef100\_A0A2D5F6D1 | 99.9 | 2.2e-28 | 4.1e-34 | 233.2 | 238 | (227, 468) | 475 | (425, 679) | 686 | Tail sheath protein C-terminal domain-containing protein | Tail sheath protein C-terminal domain-containing protein | | uniclust | UniRef100\_A0A011NTT3 | 99.9 | 3.2e-28 | 6.1e-34 | 238.9 | 219 | (229, 464) | 475 | (330, 558) | 560 | Phage tail sheath protein | Phage tail sheath protein | | uniclust | UniRef100\_A0A0Q8V0M7 | 99.9 | 3.7e-28 | 7.2e-34 | 242.1 | 239 | (229, 473) | 475 | (449, 709) | 713 | Tail sheath protein C-terminal domain-containing protein | Tail sheath protein C-terminal domain-containing protein | | uniclust | UniRef100\_A0A429I985 | 99.9 | 4e-28 | 7.5e-34 | 223.7 | 427 | (2, 468) | 475 | (18, 506) | 514 | Uncharacterized protein (Fragment) | Uncharacterized protein (Fragment) | | uniclust | UniRef100\_UPI0020BD5A97 | 99.9 | 4.5e-28 | 8.3e-34 | 197.4 | 188 | (106, 377) | 475 | (43, 231) | 231 | phage tail sheath subtilisin-like domain-containing protein | phage tail sheath subtilisin-like domain-containing protein | | uniclust | UniRef100\_A0A1Y3AZE0 | 99.9 | 4.6e-28 | 8.4e-34 | 246.9 | 322 | (6, 454) | 475 | (933, 1270) | 3230 | Chitinase-like protein | Chitinase-like protein | | uniclust | UniRef100\_UPI0022869F9D | 99.9 | 5.2e-28 | 9.6e-34 | 206.0 | 177 | (286, 466) | 475 | (127, 315) | 319 | phage tail sheath subtilisin-like domain-containing protein | phage tail sheath subtilisin-like domain-containing protein | | uniclust | UniRef100\_A0A0B0SCR7 | 99.9 | 5.6e-28 | 1.1e-33 | 236.9 | 428 | (2, 472) | 475 | (1, 470) | 487 | DUF2586 family protein | DUF2586 family protein | | uniclust | UniRef100\_UPI0009DD7DD7 | 99.9 | 6.9e-28 | 1.3e-33 | 219.6 | 222 | (234, 466) | 475 | (332, 568) | 572 | phage tail sheath C-terminal domain-containing protein | phage tail sheath C-terminal domain-containing protein | | uniclust | UniRef100\_A0A952SK04 | 99.9 | 8.5e-28 | 1.6e-33 | 219.9 | 235 | (231, 469) | 475 | (325, 588) | 593 | Phage tail sheath family protein | Phage tail sheath family protein | | uniclust | UniRef100\_A0A1Z4PN29 | 99.9 | 9.1e-28 | 1.7e-33 | 216.0 | 355 | (6, 466) | 475 | (3, 369) | 376 | Putative prophage major tail sheath protein | Putative prophage major tail sheath protein | | uniclust | UniRef100\_A0A447T5K7 | 99.9 | 9.5e-28 | 1.8e-33 | 211.8 | 320 | (2, 363) | 475 | (1, 364) | 377 | Phage tail sheath protein | Phage tail sheath protein | | uniclust | UniRef100\_A0A2D5FCB7 | 99.9 | 1.1e-27 | 2e-33 | 212.4 | 233 | (231, 469) | 475 | (86, 337) | 341 | Phage tail protein (Fragment) | Phage tail protein (Fragment) | | uniclust | UniRef100\_A0A0Q6MQW8 | 99.9 | 1.1e-27 | 2.1e-33 | 239.1 | 202 | (266, 468) | 475 | (351, 563) | 580 | Phage tail protein | Phage tail protein | | uniclust | UniRef100\_A0A800F6T4 | 99.9 | 1.4e-27 | 2.7e-33 | 203.9 | 177 | (287, 466) | 475 | (96, 283) | 291 | Phage tail sheath family protein | Phage tail sheath family protein | | uniclust | UniRef100\_UPI0013159F4D | 99.9 | 1.5e-27 | 2.7e-33 | 196.0 | 191 | (266, 467) | 475 | (38, 241) | 244 | phage tail sheath C-terminal domain-containing protein | phage tail sheath C-terminal domain-containing protein | | uniclust | UniRef100\_E2CFK5 | 99.9 | 1.7e-27 | 3.1e-33 | 211.8 | 378 | (5, 474) | 475 | (6, 387) | 398 | Putative tail sheath protein | Putative tail sheath protein | | uniclust | UniRef100\_A0A081C205 | 99.9 | 1.9e-27 | 3.6e-33 | 216.5 | 339 | (5, 465) | 475 | (10, 360) | 361 | Phage tail sheath protein | Phage tail sheath protein | | uniclust | UniRef100\_A0A1F7S379 | 99.9 | 2.2e-27 | 4.1e-33 | 211.6 | 240 | (229, 468) | 475 | (185, 448) | 464 | Phage tail protein (Fragment) | Phage tail protein (Fragment) | | uniclust | UniRef100\_A0A064A4E1 | 99.9 | 2.6e-27 | 4.9e-33 | 200.9 | 222 | (243, 475) | 475 | (12, 250) | 251 | Phage tail protein (Fragment) | Phage tail protein (Fragment) | | uniclust | UniRef100\_UPI0005EF3930 | 99.9 | 3e-27 | 5.5e-33 | 195.4 | 247 | (42, 388) | 475 | (6, 253) | 254 | phage tail sheath subtilisin-like domain-containing protein | phage tail sheath subtilisin-like domain-containing protein | | uniclust | UniRef100\_A0A403T4T3 | 99.9 | 3e-27 | 5.6e-33 | 221.2 | 284 | (1, 374) | 475 | (1, 290) | 751 | Tail sheath protein subtilisin-like domain-containing protein | Tail sheath protein subtilisin-like domain-containing protein | | uniclust | UniRef100\_A0A6P0I039 | 99.9 | 5.6e-27 | 1e-32 | 205.3 | 175 | (286, 466) | 475 | (148, 336) | 340 | Phage tail sheath family protein | Phage tail sheath family protein | | uniclust | UniRef100\_A0A0Q5TAS8 | 99.9 | 6.9e-27 | 1.3e-32 | 207.7 | 179 | (287, 466) | 475 | (183, 371) | 375 | Tail sheath protein C-terminal domain-containing protein | Tail sheath protein C-terminal domain-containing protein | | uniclust | UniRef100\_UPI00211EA2BE | 99.9 | 8.6e-27 | 1.6e-32 | 180.3 | 136 | (296, 437) | 475 | (3, 148) | 164 | phage tail sheath subtilisin-like domain-containing protein | phage tail sheath subtilisin-like domain-containing protein | | uniclust | UniRef100\_A0A2T4JE69 | 99.9 | 8.6e-27 | 1.6e-32 | 193.4 | 206 | (114, 397) | 475 | (14, 220) | 226 | Phage tail protein (Fragment) | Phage tail protein (Fragment) | | uniclust | UniRef100\_A0A849EW92 | 99.9 | 9.2e-27 | 1.7e-32 | 218.3 | 230 | (231, 467) | 475 | (507, 758) | 761 | Phage tail sheath family protein | Phage tail sheath family protein | | uniclust | UniRef100\_A0A813CV22 | 99.9 | 9.4e-27 | 1.7e-32 | 242.1 | 226 | (233, 464) | 475 | (292, 546) | 7673 | FtsH protein | FtsH protein | | uniclust | UniRef100\_A0A955ZJD4 | 99.9 | 9.4e-27 | 1.8e-32 | 217.2 | 234 | (229, 468) | 475 | (324, 586) | 597 | Phage tail sheath subtilisin-like domain-containing protein | Phage tail sheath subtilisin-like domain-containing protein | | uniclust | UniRef100\_A0A959ZZ97 | 99.9 | 9.9e-27 | 1.9e-32 | 218.7 | 429 | (2, 467) | 475 | (1, 474) | 479 | Uncharacterized protein | Uncharacterized protein | | uniclust | UniRef100\_A0A813BIE1 | 99.9 | 1.1e-26 | 2.1e-32 | 232.3 | 220 | (232, 457) | 475 | (291, 539) | 1968 | FtsH protein | FtsH protein | | uniclust | UniRef100\_A0A1Q7Z022 | 99.9 | 1.4e-26 | 2.6e-32 | 203.1 | 231 | (232, 467) | 475 | (146, 391) | 399 | Uncharacterized protein | Uncharacterized protein | | uniclust | UniRef100\_UPI000D34BEAD | 99.9 | 2.3e-26 | 4.3e-32 | 197.4 | 204 | (257, 467) | 475 | (112, 324) | 334 | phage tail sheath C-terminal domain-containing protein | phage tail sheath C-terminal domain-containing protein | | uniclust | UniRef100\_A0A1Y3AZE0 | 99.9 | 2.7e-26 | 4.9e-32 | 234.6 | 161 | (286, 452) | 475 | (1543, 1712) | 3230 | Chitinase-like protein | Chitinase-like protein | | uniclust | UniRef100\_A0A3M1ZRW3 | 99.9 | 2.8e-26 | 5.2e-32 | 213.6 | 235 | (231, 469) | 475 | (445, 699) | 702 | Phage tail sheath family protein (Fragment) | Phage tail sheath family protein (Fragment) | | uniclust | UniRef100\_A0A6J5KV31 | 99.9 | 4.2e-26 | 8.1e-32 | 228.4 | 235 | (229, 469) | 475 | (544, 788) | 794 | Tail sheath protein | Tail sheath protein | | uniclust | UniRef100\_A0A0A8J9Q3 | 99.9 | 4.1e-26 | 8.1e-32 | 240.0 | 237 | (229, 469) | 475 | (752, 1020) | 1043 | Tail sheath protein | Tail sheath protein | | uniclust | UniRef100\_A0A376TUN6 | 99.9 | 4.7e-26 | 9.2e-32 | 194.5 | 172 | (229, 400) | 475 | (17, 190) | 202 | Tail sheath protein | Tail sheath protein | | uniclust | UniRef100\_A0A1Y3TJF4 | 99.9 | 6.1e-26 | 1.1e-31 | 205.0 | 379 | (4, 474) | 475 | (1, 484) | 490 | Phage tail protein | Phage tail protein | | uniclust | UniRef100\_A0A1G6LSA3 | 99.9 | 6e-26 | 1.1e-31 | 208.4 | 351 | (4, 466) | 475 | (1, 366) | 373 | Phage tail sheath protein | Phage tail sheath protein | | uniclust | UniRef100\_A0A0B0HE64 | 99.9 | 8.3e-26 | 1.6e-31 | 204.7 | 274 | (4, 374) | 475 | (1, 371) | 383 | Phage tail sheath protein | Phage tail sheath protein | | uniclust | UniRef100\_A0A956GJ63 | 99.9 | 9.1e-26 | 1.7e-31 | 195.3 | 232 | (233, 470) | 475 | (43, 305) | 311 | Phage tail sheath family protein | Phage tail sheath family protein | | uniclust | UniRef100\_A0A0F0HL05 | 99.9 | 9.7e-26 | 1.8e-31 | 193.1 | 234 | (231, 470) | 475 | (14, 278) | 289 | Tail protein (Fragment) | Tail protein (Fragment) | | uniclust | UniRef100\_A0A257MWQ1 | 99.9 | 1.4e-25 | 2.7e-31 | 179.8 | 126 | (343, 468) | 475 | (13, 148) | 152 | Putative phage tail sheath protein FI (Fragment) | Putative phage tail sheath protein FI (Fragment) | | uniclust | UniRef100\_UPI001F56A12B | 99.9 | 1.4e-25 | 2.7e-31 | 210.9 | 243 | (230, 472) | 475 | (24, 267) | 772 | phage tail tape measure protein | phage tail tape measure protein | | uniclust | UniRef100\_A0A0S8A5K3 | 99.9 | 1.5e-25 | 2.8e-31 | 168.2 | 122 | (273, 400) | 475 | (1, 125) | 126 | Tail sheath protein subtilisin-like domain-containing protein | Tail sheath protein subtilisin-like domain-containing protein | | uniclust | UniRef100\_A0A090ZAL6 | 99.9 | 1.6e-25 | 3.1e-31 | 219.7 | 408 | (7, 467) | 475 | (15, 465) | 475 | Phage tail sheath family protein | Phage tail sheath family protein | | uniclust | UniRef100\_A0A8X6HJI7 | 99.9 | 2e-25 | 3.7e-31 | 197.8 | 217 | (22, 325) | 475 | (175, 392) | 428 | Phage tail protein | Phage tail protein | | uniclust | UniRef100\_A0A1Z9H610 | 99.9 | 2e-25 | 3.9e-31 | 236.3 | 236 | (228, 469) | 475 | (709, 977) | 982 | Tail sheath protein C-terminal domain-containing protein | Tail sheath protein C-terminal domain-containing protein | | uniclust | UniRef100\_UPI000FEEF31E | 99.9 | 2.1e-25 | 4e-31 | 186.3 | 221 | (33, 342) | 475 | (2, 224) | 224 | phage tail sheath subtilisin-like domain-containing protein | phage tail sheath subtilisin-like domain-containing protein | | uniclust | UniRef100\_A0A523DTF3 | 99.9 | 2.2e-25 | 4.1e-31 | 169.3 | 131 | (288, 424) | 475 | (2, 136) | 146 | Phage tail sheath family protein | Phage tail sheath family protein | | uniclust | UniRef100\_UPI00138F8D04 | 99.9 | 2.3e-25 | 4.1e-31 | 198.9 | 233 | (230, 468) | 475 | (199, 439) | 456 | phage tail sheath subtilisin-like domain-containing protein | phage tail sheath subtilisin-like domain-containing protein | | uniclust | UniRef100\_A0A941J0D5 | 99.9 | 2.4e-25 | 4.5e-31 | 185.4 | 229 | (229, 466) | 475 | (13, 258) | 264 | Phage tail sheath family protein | Phage tail sheath family protein | | uniclust | UniRef100\_A0A370LRA8 | 99.9 | 3.1e-25 | 5.7e-31 | 214.2 | 237 | (229, 469) | 475 | (647, 902) | 911 | Tail sheath protein C-terminal domain-containing protein | Tail sheath protein C-terminal domain-containing protein | | uniclust | UniRef100\_A0A4Q3AFM2 | 99.9 | 3.2e-25 | 6.2e-31 | 211.6 | 260 | (5, 363) | 475 | (9, 405) | 453 | Phage tail protein (Fragment) | Phage tail protein (Fragment) | | uniclust | UniRef100\_A0A0F9FWT7 | 99.9 | 3.7e-25 | 7.2e-31 | 219.8 | 232 | (229, 466) | 475 | (400, 641) | 655 | Tail sheath protein subtilisin-like domain-containing protein (Fragment) | Tail sheath protein subtilisin-like domain-containing protein (Fragment) | | uniclust | UniRef100\_A0A2Z4FJ67 | 99.9 | 4.2e-25 | 7.7e-31 | 199.0 | 333 | (19, 467) | 475 | (126, 487) | 495 | Tail sheath protein subtilisin-like domain-containing protein | Tail sheath protein subtilisin-like domain-containing protein | | uniclust | UniRef100\_A0A7C4ZXL6 | 99.9 | 4.2e-25 | 7.8e-31 | 197.7 | 338 | (1, 464) | 475 | (1, 358) | 360 | Phage tail protein | Phage tail protein | | uniclust | UniRef100\_UPI000AE5212C | 99.9 | 4.8e-25 | 8.9e-31 | 181.2 | 199 | (87, 371) | 475 | (5, 211) | 213 | phage tail sheath subtilisin-like domain-containing protein | phage tail sheath subtilisin-like domain-containing protein | | uniclust | UniRef100\_A0A950FF97 | 99.8 | 6e-25 | 1.1e-30 | 205.7 | 244 | (230, 473) | 475 | (441, 716) | 725 | Phage tail sheath subtilisin-like domain-containing protein | Phage tail sheath subtilisin-like domain-containing protein | | uniclust | UniRef100\_A0A3C1Q7E4 | 99.8 | 6.1e-25 | 1.1e-30 | 188.8 | 205 | (261, 475) | 475 | (64, 279) | 280 | Phage tail protein | Phage tail protein | | uniclust | UniRef100\_A0A2W6P9S6 | 99.8 | 8.1e-25 | 1.5e-30 | 176.1 | 184 | (290, 474) | 475 | (2, 185) | 191 | Phage tail sheath family protein (Fragment) | Phage tail sheath family protein (Fragment) | | uniclust | UniRef100\_A0A448JE63 | 99.8 | 8.6e-25 | 1.6e-30 | 195.3 | 238 | (231, 474) | 475 | (66, 305) | 317 | Major tail sheath protein | Major tail sheath protein | | uniclust | UniRef100\_A0A1F3RZR2 | 99.8 | 1.1e-24 | 1.9e-30 | 193.6 | 180 | (286, 466) | 475 | (243, 431) | 434 | Uncharacterized protein | Uncharacterized protein | | uniclust | UniRef100\_A0A6M0F244 | 99.8 | 1.3e-24 | 2.4e-30 | 187.8 | 180 | (286, 466) | 475 | (151, 340) | 344 | Tail sheath protein C-terminal domain-containing protein | Tail sheath protein C-terminal domain-containing protein | | uniclust | UniRef100\_A0A965TRY7 | 99.8 | 1.3e-24 | 2.4e-30 | 199.1 | 205 | (258, 466) | 475 | (359, 573) | 576 | Phage tail sheath family protein | Phage tail sheath family protein | | uniclust | UniRef100\_A0A929F6P8 | 99.8 | 1.5e-24 | 2.7e-30 | 184.5 | 163 | (231, 399) | 475 | (126, 305) | 305 | Phage tail sheath family protein (Fragment) | Phage tail sheath family protein (Fragment) | | uniclust | UniRef100\_A0A080LR57 | 99.8 | 1.6e-24 | 2.9e-30 | 168.2 | 117 | (353, 469) | 475 | (7, 133) | 136 | Tail sheath protein | Tail sheath protein | | uniclust | UniRef100\_A0A327J8X6 | 99.8 | 2e-24 | 3.6e-30 | 193.9 | 427 | (6, 469) | 475 | (1, 468) | 475 | Tail sheath protein subtilisin-like domain-containing protein | Tail sheath protein subtilisin-like domain-containing protein | | uniclust | UniRef100\_A0A350EYQ4 | 99.8 | 2e-24 | 3.6e-30 | 172.6 | 145 | (317, 467) | 475 | (6, 161) | 197 | Tail sheath protein C-terminal domain-containing protein (Fragment) | Tail sheath protein C-terminal domain-containing protein (Fragment) | | uniclust | UniRef100\_A0A370E172 | 99.8 | 2e-24 | 3.6e-30 | 189.3 | 180 | (286, 466) | 475 | (193, 382) | 386 | Phage tail protein | Phage tail protein | | uniclust | UniRef100\_A0A1Y3AZE0 | 99.8 | 2e-24 | 3.6e-30 | 221.4 | 371 | (3, 466) | 475 | (1726, 2118) | 3230 | Chitinase-like protein | Chitinase-like protein | | uniclust | UniRef100\_UPI001EDA5694 | 99.8 | 2.2e-24 | 4e-30 | 186.3 | 326 | (8, 468) | 475 | (2, 340) | 343 | hypothetical protein | hypothetical protein | | uniclust | UniRef100\_A0A0F3RPG1 | 99.8 | 2.2e-24 | 4e-30 | 173.7 | 191 | (2, 275) | 475 | (1, 192) | 192 | Phage tail sheath family protein | Phage tail sheath family protein | | uniclust | UniRef100\_A0A085K2M7 | 99.8 | 2.7e-24 | 5.2e-30 | 183.6 | 188 | (1, 278) | 475 | (11, 199) | 201 | Phage tail protein | Phage tail protein | | uniclust | UniRef100\_A0A1J6QRE2 | 99.8 | 3.3e-24 | 6.2e-30 | 190.1 | 372 | (7, 474) | 475 | (4, 390) | 397 | Phage tail protein | Phage tail protein | | uniclust | UniRef100\_A0A8S0FXS2 | 99.8 | 3.7e-24 | 7.2e-30 | 189.4 | 161 | (231, 391) | 475 | (57, 231) | 232 | Tail sheath protein subtilisin-like domain-containing protein | Tail sheath protein subtilisin-like domain-containing protein | | uniclust | UniRef100\_A0A2E6EJU7 | 99.8 | 4.1e-24 | 7.6e-30 | 192.5 | 193 | (266, 468) | 475 | (281, 484) | 487 | Phage tail protein | Phage tail protein | | uniclust | UniRef100\_A0A1Q8A397 | 99.8 | 4.4e-24 | 8.3e-30 | 164.5 | 105 | (364, 468) | 475 | (2, 112) | 133 | Tail sheath protein C-terminal domain-containing protein | Tail sheath protein C-terminal domain-containing protein | | uniclust | UniRef100\_A0A925P9Z3 | 99.8 | 4.8e-24 | 9.2e-30 | 177.6 | 159 | (306, 470) | 475 | (10, 175) | 182 | Phage tail sheath protein | Phage tail sheath protein | | uniclust | UniRef100\_A0A355DNH3 | 99.8 | 5.3e-24 | 9.9e-30 | 194.1 | 128 | (230, 363) | 475 | (301, 455) | 464 | Phage tail protein (Fragment) | Phage tail protein (Fragment) | | uniclust | UniRef100\_A0A833AQN6 | 99.8 | 5.6e-24 | 1.1e-29 | 217.7 | 236 | (229, 470) | 475 | (691, 967) | 974 | Phage tail sheath family protein | Phage tail sheath family protein | | uniclust | UniRef100\_UPI0015C33B2A | 99.8 | 6.9e-24 | 1.3e-29 | 161.0 | 129 | (285, 419) | 475 | (9, 141) | 143 | phage tail sheath subtilisin-like domain-containing protein | phage tail sheath subtilisin-like domain-containing protein | | uniclust | UniRef100\_A0A376YN89 | 99.8 | 7.9e-24 | 1.4e-29 | 169.7 | 171 | (260, 430) | 475 | (3, 174) | 200 | Major tail sheath protein FI | Major tail sheath protein FI | | uniclust | UniRef100\_A0A1C0V8T7 | 99.8 | 1.1e-23 | 2e-29 | 196.7 | 244 | (229, 473) | 475 | (428, 686) | 688 | Tail sheath protein subtilisin-like domain-containing protein | Tail sheath protein subtilisin-like domain-containing protein | | uniclust | UniRef100\_UPI00116C6DAE | 99.8 | 1.1e-23 | 2.1e-29 | 164.5 | 147 | (291, 437) | 475 | (8, 154) | 158 | phage tail sheath subtilisin-like domain-containing protein | phage tail sheath subtilisin-like domain-containing protein | | uniclust | UniRef100\_A0A7W1Q988 | 99.8 | 1.2e-23 | 2.3e-29 | 180.9 | 239 | (3, 363) | 475 | (2, 249) | 329 | Phage tail sheath family protein | Phage tail sheath family protein | | uniclust | UniRef100\_A0A292GIW4 | 99.8 | 1.3e-23 | 2.6e-29 | 204.2 | 241 | (229, 469) | 475 | (374, 636) | 649 | Tail sheath protein | Tail sheath protein | | uniclust | UniRef100\_A0A2D6PBJ4 | 99.8 | 1.6e-23 | 3e-29 | 197.6 | 237 | (229, 469) | 475 | (394, 647) | 652 | Tail sheath protein C-terminal domain-containing protein | Tail sheath protein C-terminal domain-containing protein | | uniclust | UniRef100\_UPI0022347FD7 | 99.8 | 1.6e-23 | 3e-29 | 163.6 | 141 | (231, 371) | 475 | (28, 169) | 170 | phage tail sheath subtilisin-like domain-containing protein | phage tail sheath subtilisin-like domain-containing protein | | uniclust | UniRef100\_A0A266LLW5 | 99.8 | 1.8e-23 | 3.3e-29 | 161.5 | 143 | (231, 373) | 475 | (4, 147) | 149 | Phage tail protein (Fragment) | Phage tail protein (Fragment) | | uniclust | UniRef100\_A0A6S6PL19 | 99.8 | 1.9e-23 | 3.5e-29 | 192.8 | 429 | (7, 466) | 475 | (17, 487) | 497 | Phage tail protein | Phage tail protein | | uniclust | UniRef100\_A0A412P9T8 | 99.8 | 1.9e-23 | 3.6e-29 | 187.2 | 114 | (287, 403) | 475 | (265, 382) | 384 | Phage tail sheath family protein | Phage tail sheath family protein | | uniclust | UniRef100\_A0A3A8PQG2 | 99.8 | 1.9e-23 | 3.6e-29 | 194.5 | 232 | (230, 467) | 475 | (202, 472) | 481 | Phage tail sheath family protein (Fragment) | Phage tail sheath family protein (Fragment) | | uniclust | UniRef100\_A0A813AAM4 | 99.8 | 2e-23 | 3.7e-29 | 204.7 | 234 | (229, 468) | 475 | (1012, 1266) | 1276 | 18 protein | 18 protein | | uniclust | UniRef100\_A0A0C5ADZ8 | 99.8 | 2e-23 | 3.8e-29 | 208.8 | 232 | (232, 469) | 475 | (494, 744) | 763 | Tail sheath monomer | Tail sheath monomer | | uniclust | UniRef100\_A0A3A8JLS6 | 99.8 | 2.1e-23 | 3.9e-29 | 182.1 | 241 | (230, 474) | 475 | (74, 342) | 347 | Phage tail sheath family protein | Phage tail sheath family protein | | uniclust | UniRef100\_A0A382AQG4 | 99.8 | 3.2e-23 | 5.9e-29 | 192.6 | 234 | (229, 468) | 475 | (251, 494) | 500 | Tail sheath protein C-terminal domain-containing protein (Fragment) | Tail sheath protein C-terminal domain-containing protein (Fragment) | | uniclust | UniRef100\_A0A268THL2 | 99.8 | 4e-23 | 7.6e-29 | 189.0 | 357 | (5, 474) | 475 | (3, 362) | 374 | Phage tail protein | Phage tail protein | | uniclust | UniRef100\_A0A5F1HTD7 | 99.8 | 5.6e-23 | 1e-28 | 179.1 | 174 | (287, 466) | 475 | (168, 354) | 361 | Uncharacterized protein | Uncharacterized protein | | uniclust | UniRef100\_A0A1H9D327 | 99.8 | 5.4e-23 | 1e-28 | 204.7 | 236 | (229, 470) | 475 | (621, 887) | 894 | IPT/TIG domain-containing protein | IPT/TIG domain-containing protein | | uniclust | UniRef100\_A0A1Z8RYF4 | 99.8 | 6e-23 | 1.2e-28 | 218.7 | 234 | (231, 470) | 475 | (746, 1004) | 1008 | Tail sheath protein C-terminal domain-containing protein | Tail sheath protein C-terminal domain-containing protein | | uniclust | UniRef100\_A0A059WTY0 | 99.8 | 6.9e-23 | 1.3e-28 | 163.4 | 136 | (231, 372) | 475 | (51, 192) | 192 | Phage tail sheath protein (Fragment) | Phage tail sheath protein (Fragment) | | uniclust | UniRef100\_A0A3E1Y3Z4 | 99.8 | 7.1e-23 | 1.3e-28 | 188.4 | 179 | (287, 466) | 475 | (394, 582) | 586 | Phage tail sheath family protein | Phage tail sheath family protein | | uniclust | UniRef100\_A0A7C6A7S9 | 99.8 | 7.4e-23 | 1.4e-28 | 184.7 | 431 | (2, 469) | 475 | (1, 479) | 487 | Tail sheath protein C-terminal domain-containing protein | Tail sheath protein C-terminal domain-containing protein | | uniclust | UniRef100\_A0A0N8TDW0 | 99.8 | 9.2e-23 | 1.7e-28 | 165.1 | 128 | (340, 467) | 475 | (24, 161) | 165 | Phage tail sheath protein FI | Phage tail sheath protein FI | | uniclust | UniRef100\_UPI0009B437D5 | 99.8 | 1.2e-22 | 2.1e-28 | 190.4 | 233 | (229, 468) | 475 | (425, 685) | 702 | phage tail sheath family protein | phage tail sheath family protein | | uniclust | UniRef100\_A0A5Z1S5A9 | 99.8 | 1.2e-22 | 2.3e-28 | 171.8 | 215 | (257, 474) | 475 | (10, 226) | 233 | Phage tail sheath family protein (Fragment) | Phage tail sheath family protein (Fragment) | | uniclust | UniRef100\_A0A0G9K7V9 | 99.8 | 1.3e-22 | 2.4e-28 | 187.1 | 237 | (231, 472) | 475 | (140, 379) | 388 | Tail protein | Tail protein | | uniclust | UniRef100\_A0A5C7JAB2 | 99.8 | 1.4e-22 | 2.6e-28 | 192.6 | 237 | (229, 469) | 475 | (310, 575) | 580 | Tail sheath protein C-terminal domain-containing protein | Tail sheath protein C-terminal domain-containing protein | | uniclust | UniRef100\_A0A3N5G9W8 | 99.8 | 1.4e-22 | 2.7e-28 | 197.4 | 218 | (229, 464) | 475 | (323, 551) | 553 | Phage tail sheath protein (Fragment) | Phage tail sheath protein (Fragment) | | uniclust | UniRef100\_UPI00143D32B4 | 99.8 | 1.8e-22 | 3.3e-28 | 174.8 | 180 | (287, 468) | 475 | (151, 340) | 342 | phage tail sheath family protein | phage tail sheath family protein | | uniclust | UniRef100\_A0A2V8N5I3 | 99.8 | 2.6e-22 | 4.7e-28 | 170.3 | 197 | (266, 468) | 475 | (78, 283) | 291 | Tail sheath protein C-terminal domain-containing protein | Tail sheath protein C-terminal domain-containing protein | | uniclust | UniRef100\_A0A075E197 | 99.8 | 2.6e-22 | 5e-28 | 198.3 | 233 | (232, 467) | 475 | (395, 642) | 660 | Tail sheath family protein | Tail sheath family protein | | uniclust | UniRef100\_A0A085EKS0 | 99.8 | 2.6e-22 | 5.2e-28 | 193.9 | 363 | (2, 469) | 475 | (14, 411) | 419 | Tail sheath protein | Tail sheath protein | | uniclust | UniRef100\_A0A4R3Z7M7 | 99.8 | 3.1e-22 | 5.6e-28 | 154.7 | 140 | (231, 378) | 475 | (12, 156) | 157 | Tail sheath protein | Tail sheath protein | | uniclust | UniRef100\_A0A7I8MZ51 | 99.8 | 3.1e-22 | 5.8e-28 | 182.1 | 230 | (233, 468) | 475 | (271, 511) | 520 | Tail sheath protein C-terminal domain-containing protein | Tail sheath protein C-terminal domain-containing protein | | uniclust | UniRef100\_A0A0H4WYB2 | 99.8 | 4e-22 | 7.8e-28 | 194.1 | 400 | (7, 465) | 475 | (36, 490) | 493 | Phage tail sheath protein | Phage tail sheath protein | | uniclust | UniRef100\_A0A081NYA3 | 99.8 | 6.6e-22 | 1.3e-27 | 196.2 | 427 | (5, 470) | 475 | (3, 468) | 481 | Tail sheath protein subtilisin-like domain-containing protein | Tail sheath protein subtilisin-like domain-containing protein | | uniclust | UniRef100\_A0A6A1TYH7 | 99.8 | 8.7e-22 | 1.6e-27 | 169.9 | 243 | (225, 474) | 475 | (69, 313) | 329 | Phage tail protein | Phage tail protein | | uniclust | UniRef100\_UPI0006204962 | 99.8 | 9.9e-22 | 1.8e-27 | 168.8 | 258 | (5, 362) | 475 | (3, 271) | 271 | phage tail protein | phage tail protein | | uniclust | UniRef100\_A0A015U7W7 | 99.8 | 1.1e-21 | 2.3e-27 | 195.7 | 368 | (1, 471) | 475 | (20, 418) | 427 | DUF2586 family protein | DUF2586 family protein | | uniclust | UniRef100\_A0A061JGT4 | 99.7 | 1.4e-21 | 2.6e-27 | 153.9 | 115 | (360, 474) | 475 | (8, 122) | 138 | Major tail sheath protein | Major tail sheath protein | | uniclust | UniRef100\_J2WTG1 | 99.7 | 1.6e-21 | 2.9e-27 | 179.4 | 228 | (231, 466) | 475 | (323, 559) | 570 | Phage tail sheath protein FI (Fragment) | Phage tail sheath protein FI (Fragment) | | uniclust | UniRef100\_A0A4Q3UNL2 | 99.7 | 1.6e-21 | 2.9e-27 | 171.4 | 149 | (227, 381) | 475 | (227, 380) | 380 | Phage tail sheath family protein (Fragment) | Phage tail sheath family protein (Fragment) | | uniclust | UniRef100\_A0A842WMT0 | 99.7 | 2e-21 | 3.6e-27 | 187.3 | 233 | (230, 465) | 475 | (680, 929) | 949 | Tail sheath protein subtilisin-like domain-containing protein | Tail sheath protein subtilisin-like domain-containing protein | | uniclust | UniRef100\_A0A2E3R271 | 99.7 | 2.1e-21 | 3.9e-27 | 172.9 | 377 | (7, 474) | 475 | (3, 423) | 424 | Tail sheath protein subtilisin-like domain-containing protein | Tail sheath protein subtilisin-like domain-containing protein | | uniclust | UniRef100\_A0A2D6AJR1 | 99.7 | 2.2e-21 | 4.2e-27 | 185.1 | 176 | (287, 468) | 475 | (231, 412) | 452 | Phage tail protein | Phage tail protein | | uniclust | UniRef100\_A0A956ILR2 | 99.7 | 3.1e-21 | 5.7e-27 | 169.2 | 231 | (231, 467) | 475 | (100, 359) | 371 | Phage tail sheath family protein | Phage tail sheath family protein | | uniclust | UniRef100\_UPI001B8D85E7 | 99.7 | 3.2e-21 | 5.8e-27 | 184.7 | 131 | (287, 423) | 475 | (678, 812) | 876 | phage tail sheath family protein | phage tail sheath family protein | | uniclust | UniRef100\_A0A6A4R6J7 | 99.7 | 3.5e-21 | 6.5e-27 | 170.2 | 286 | (7, 394) | 475 | (12, 307) | 311 | Phage tail protein (Fragment) | Phage tail protein (Fragment) | | uniclust | UniRef100\_A0A6M0FV63 | 99.7 | 3.6e-21 | 6.8e-27 | 173.2 | 344 | (7, 466) | 475 | (4, 362) | 367 | Tail sheath protein subtilisin-like domain-containing protein | Tail sheath protein subtilisin-like domain-containing protein | | uniclust | UniRef100\_A0A976JLS4 | 99.7 | 3.7e-21 | 6.8e-27 | 171.0 | 233 | (230, 465) | 475 | (152, 408) | 415 | Phage tail sheath family protein | Phage tail sheath family protein | | uniclust | UniRef100\_A0A4S2S3L3 | 99.7 | 3.9e-21 | 7.2e-27 | 184.7 | 233 | (232, 470) | 475 | (668, 912) | 916 | Phage tail sheath family protein | Phage tail sheath family protein | | uniclust | UniRef100\_A0A7K1RD86 | 99.7 | 4.7e-21 | 8.7e-27 | 181.2 | 342 | (43, 470) | 475 | (384, 743) | 756 | Phage tail protein | Phage tail protein | | uniclust | UniRef100\_A0A2W6WG49 | 99.7 | 4.8e-21 | 8.9e-27 | 161.6 | 243 | (3, 474) | 475 | (1, 243) | 249 | Tail sheath protein C-terminal domain-containing protein | Tail sheath protein C-terminal domain-containing protein | | uniclust | UniRef100\_A0A0F8XJ22 | 99.7 | 5e-21 | 9.5e-27 | 157.4 | 155 | (309, 469) | 475 | (3, 163) | 176 | Tail sheath protein C-terminal domain-containing protein (Fragment) | Tail sheath protein C-terminal domain-containing protein (Fragment) | | uniclust | UniRef100\_A0A957QB84 | 99.7 | 5.4e-21 | 9.8e-27 | 172.3 | 235 | (229, 469) | 475 | (179, 458) | 466 | Phage tail sheath family protein | Phage tail sheath family protein | | uniclust | UniRef100\_A0A292SAA6 | 99.7 | 1.2e-20 | 2.2e-26 | 173.0 | 201 | (266, 469) | 475 | (239, 443) | 451 | Tail sheath protein C-terminal domain-containing protein | Tail sheath protein C-terminal domain-containing protein | | uniclust | UniRef100\_A0A924Q0Y1 | 99.7 | 1.5e-20 | 2.8e-26 | 170.1 | 208 | (265, 473) | 475 | (107, 327) | 340 | Phage tail protein | Phage tail protein | | uniclust | UniRef100\_E3BEQ6 | 99.7 | 1.8e-20 | 3.2e-26 | 163.8 | 215 | (240, 466) | 475 | (127, 353) | 356 | Uncharacterized protein | Uncharacterized protein | | uniclust | UniRef100\_A0A096CJ44 | 99.7 | 1.8e-20 | 3.5e-26 | 180.1 | 322 | (7, 466) | 475 | (56, 394) | 395 | Tail sheath protein C-terminal domain-containing protein | Tail sheath protein C-terminal domain-containing protein | | uniclust | UniRef100\_A0A3N7HIZ4 | 99.7 | 2.1e-20 | 4.2e-26 | 188.0 | 198 | (265, 467) | 475 | (406, 611) | 622 | Phage tail sheath protein | Phage tail sheath protein | | uniclust | UniRef100\_A0A2S5F8Z6 | 99.7 | 2.4e-20 | 4.4e-26 | 168.4 | 240 | (234, 474) | 475 | (204, 462) | 469 | Phage tail protein | Phage tail protein | | uniclust | UniRef100\_A0A011PBZ6 | 99.7 | 2.5e-20 | 4.7e-26 | 177.0 | 204 | (266, 471) | 475 | (298, 509) | 519 | Phage tail sheath protein | Phage tail sheath protein | | uniclust | UniRef100\_A0A482MLF8 | 99.7 | 3.6e-20 | 6.8e-26 | 179.2 | 234 | (229, 468) | 475 | (397, 642) | 657 | Tail sheath protein | Tail sheath protein | | uniclust | UniRef100\_A0A257GTL1 | 99.7 | 3.5e-20 | 6.8e-26 | 171.0 | 188 | (1, 280) | 475 | (1, 222) | 303 | Phage tail protein (Fragment) | Phage tail protein (Fragment) | | uniclust | UniRef100\_A0A923X4B7 | 99.7 | 3.7e-20 | 6.9e-26 | 139.2 | 102 | (366, 467) | 475 | (4, 111) | 120 | Phage tail sheath family protein | Phage tail sheath family protein | | uniclust | UniRef100\_A0A1X7MDT6 | 99.7 | 3.9e-20 | 7.2e-26 | 140.4 | 123 | (300, 422) | 475 | (1, 123) | 138 | Phage tail sheath protein (Fragment) | Phage tail sheath protein (Fragment) | | uniclust | UniRef100\_A0A7J4G8F5 | 99.7 | 4e-20 | 7.5e-26 | 171.5 | 233 | (231, 469) | 475 | (241, 493) | 502 | Tail sheath protein subtilisin-like domain-containing protein (Fragment) | Tail sheath protein subtilisin-like domain-containing protein (Fragment) | | uniclust | UniRef100\_A0A1Q7C2B6 | 99.7 | 4.1e-20 | 7.8e-26 | 144.8 | 115 | (353, 467) | 475 | (3, 123) | 129 | Uncharacterized protein | Uncharacterized protein | | uniclust | UniRef100\_A0A972PJ70 | 99.7 | 5e-20 | 9.2e-26 | 144.1 | 140 | (336, 475) | 475 | (20, 164) | 165 | Phage tail sheath family protein | Phage tail sheath family protein | | uniclust | UniRef100\_A0A2E6W8A2 | 99.7 | 5e-20 | 9.4e-26 | 179.2 | 239 | (229, 469) | 475 | (392, 660) | 668 | Tail sheath protein C-terminal domain-containing protein (Fragment) | Tail sheath protein C-terminal domain-containing protein (Fragment) | | uniclust | UniRef100\_UPI000470A430 | 99.7 | 5.1e-20 | 9.5e-26 | 155.9 | 221 | (2, 311) | 475 | (1, 259) | 259 | phage tail sheath family protein | phage tail sheath family protein | | uniclust | UniRef100\_A0A193QGV7 | 99.7 | 5.5e-20 | 1e-25 | 148.9 | 198 | (260, 473) | 475 | (3, 201) | 205 | Phage tail sheath protein | Phage tail sheath protein | | uniclust | UniRef100\_A0A3C1KVB2 | 99.7 | 5.9e-20 | 1.1e-25 | 169.4 | 119 | (229, 354) | 475 | (340, 460) | 461 | Tail sheath protein subtilisin-like domain-containing protein (Fragment) | Tail sheath protein subtilisin-like domain-containing protein (Fragment) | | uniclust | UniRef100\_UPI0020B107FC | 99.7 | 8.1e-20 | 1.5e-25 | 174.4 | 428 | (7, 465) | 475 | (15, 497) | 818 | hypothetical protein | hypothetical protein | | uniclust | UniRef100\_A0A1I5H9Z7 | 99.7 | 1e-19 | 1.9e-25 | 168.5 | 367 | (16, 471) | 475 | (15, 409) | 424 | Phage tail sheath protein FI | Phage tail sheath protein FI | | uniclust | UniRef100\_A0A6P0LXV0 | 99.7 | 1e-19 | 1.9e-25 | 145.9 | 130 | (1, 144) | 475 | (1, 136) | 171 | Phage tail sheath family protein (Fragment) | Phage tail sheath family protein (Fragment) | | uniclust | UniRef100\_A0A350EYQ5 | 99.7 | 1e-19 | 1.9e-25 | 148.6 | 201 | (4, 316) | 475 | (3, 213) | 216 | Phage tail sheath family protein (Fragment) | Phage tail sheath family protein (Fragment) | | uniclust | UniRef100\_A0A1G7 | 99.7 | 1e-19 | 1.9e-25 | 163.4 | 232 | (231, 468) | 475 | (79, 341) | 353 | Tail tube glycoprotein (Fragment) | Tail tube glycoprotein (Fragment) | | uniclust | UniRef100\_A0A1V5KBQ7 | 99.7 | 1.6e-19 | 2.8e-25 | 173.9 | 234 | (229, 466) | 475 | (625, 883) | 894 | Phage tail sheath protein | Phage tail sheath protein | | uniclust | UniRef100\_A0A926CBS6 | 99.7 | 1.6e-19 | 2.9e-25 | 148.8 | 195 | (267, 468) | 475 | (19, 220) | 228 | Phage tail sheath family protein | Phage tail sheath family protein | | uniclust | UniRef100\_UPI000A73B8BF | 99.7 | 1.6e-19 | 3e-25 | 147.4 | 193 | (2, 282) | 475 | (1, 215) | 215 | phage tail sheath subtilisin-like domain-containing protein | phage tail sheath subtilisin-like domain-containing protein | | uniclust | UniRef100\_A0A2A4T3R4 | 99.7 | 1.6e-19 | 3.1e-25 | 187.7 | 232 | (231, 469) | 475 | (784, 1035) | 1041 | Tail sheath protein C-terminal domain-containing protein | Tail sheath protein C-terminal domain-containing protein | | uniclust | UniRef100\_A0A950BPE5 | 99.7 | 2e-19 | 3.8e-25 | 158.2 | 311 | (2, 328) | 475 | (1, 363) | 370 | Phage tail sheath family protein (Fragment) | Phage tail sheath family protein (Fragment) | | uniclust | UniRef100\_A0A497CTY6 | 99.6 | 2.5e-19 | 4.7e-25 | 164.5 | 127 | (229, 361) | 475 | (357, 499) | 499 | Peptidase C30 domain-containing protein (Fragment) | Peptidase C30 domain-containing protein (Fragment) | | uniclust | UniRef100\_A0A2D6E608 | 99.6 | 2.7e-19 | 5.1e-25 | 176.5 | 240 | (229, 474) | 475 | (529, 800) | 801 | Tail sheath protein C-terminal domain-containing protein | Tail sheath protein C-terminal domain-containing protein | | uniclust | UniRef100\_A0A1L6I8K2 | 99.6 | 3.2e-19 | 5.9e-25 | 164.7 | 280 | (168, 466) | 475 | (97, 391) | 405 | Phage tail protein | Phage tail protein | | uniclust | UniRef100\_A0A661H7Y5 | 99.6 | 5.7e-19 | 1.1e-24 | 141.5 | 129 | (337, 467) | 475 | (20, 158) | 164 | Tail sheath protein C-terminal domain-containing protein | Tail sheath protein C-terminal domain-containing protein | | uniclust | UniRef100\_A0A261KJC2 | 99.6 | 5.8e-19 | 1.1e-24 | 165.9 | 91 | (1, 94) | 475 | (1, 94) | 327 | Tail protein (Fragment) | Tail protein (Fragment) | | uniclust | UniRef100\_A0A150TG83 | 99.6 | 6.2e-19 | 1.2e-24 | 169.0 | 197 | (268, 470) | 475 | (477, 685) | 699 | Tail sheath protein C-terminal domain-containing protein | Tail sheath protein C-terminal domain-containing protein | | uniclust | UniRef100\_A0A0H2YXP9 | 99.6 | 6.6e-19 | 1.2e-24 | 144.6 | 137 | (3, 144) | 475 | (1, 143) | 185 | GpF1 | GpF1 | | uniclust | UniRef100\_A0A068ZW53 | 99.6 | 6.4e-19 | 1.3e-24 | 173.8 | 414 | (6, 463) | 475 | (34, 502) | 508 | Phage tail sheath protein | Phage tail sheath protein | | uniclust | UniRef100\_A0A7X6FQF0 | 99.6 | 7e-19 | 1.3e-24 | 148.4 | 238 | (2, 251) | 475 | (1, 247) | 266 | Phage tail protein | Phage tail protein | | uniclust | UniRef100\_A0A951GZA7 | 99.6 | 7.3e-19 | 1.3e-24 | 157.6 | 163 | (231, 399) | 475 | (232, 427) | 428 | Phage tail sheath family protein (Fragment) | Phage tail sheath family protein (Fragment) | | uniclust | UniRef100\_J2VU54 | 99.6 | 7.2e-19 | 1.4e-24 | 157.2 | 73 | (287, 365) | 475 | (239, 313) | 318 | Uncharacterized protein (Fragment) | Uncharacterized protein (Fragment) | | uniclust | UniRef100\_A0A8J7Q6J0 | 99.6 | 8.9e-19 | 1.6e-24 | 148.1 | 116 | (353, 468) | 475 | (8, 129) | 270 | Phage tail sheath family protein | Phage tail sheath family protein | | uniclust | UniRef100\_A0A5D8RP33 | 99.6 | 9e-19 | 1.7e-24 | 135.2 | 117 | (357, 473) | 475 | (1, 120) | 129 | Phage tail sheath family protein (Fragment) | Phage tail sheath family protein (Fragment) | | uniclust | UniRef100\_A0A6V8KHY3 | 99.6 | 8.9e-19 | 1.7e-24 | 133.3 | 95 | (374, 468) | 475 | (1, 101) | 110 | Tail sheath protein C-terminal domain-containing protein | Tail sheath protein C-terminal domain-containing protein | | uniclust | UniRef100\_A0A6P0JV64 | 99.6 | 9.9e-19 | 1.9e-24 | 155.3 | 220 | (2, 323) | 475 | (1, 304) | 310 | Phage tail sheath family protein (Fragment) | Phage tail sheath family protein (Fragment) | | uniclust | UniRef100\_UPI000615A201 | 99.6 | 1.1e-18 | 2e-24 | 162.3 | 202 | (1, 303) | 475 | (1, 316) | 338 | phage tail sheath C-terminal domain-containing protein | phage tail sheath C-terminal domain-containing protein | | uniclust | UniRef100\_UPI00156E4C90 | 99.6 | 1.3e-18 | 2.4e-24 | 134.0 | 126 | (269, 394) | 475 | (3, 129) | 130 | phage tail sheath subtilisin-like domain-containing protein | phage tail sheath subtilisin-like domain-containing protein | | uniclust | UniRef100\_A0A4P9VIU9 | 99.6 | 1.3e-18 | 2.5e-24 | 137.7 | 130 | (2, 137) | 475 | (1, 130) | 141 | Phage tail protein | Phage tail protein | | uniclust | UniRef100\_A0A7L5Y128 | 99.6 | 1.8e-18 | 3.2e-24 | 163.7 | 337 | (43, 466) | 475 | (331, 687) | 713 | Right-handed parallel beta-helix repeat-containing protein | Right-handed parallel beta-helix repeat-containing protein | | uniclust | UniRef100\_A0A4U9D4B3 | 99.6 | 1.8e-18 | 3.3e-24 | 147.4 | 194 | (32, 315) | 475 | (1, 199) | 285 | Phage tail sheath protein | Phage tail sheath protein | | uniclust | UniRef100\_A0A3B8PUP4 | 99.6 | 2.1e-18 | 3.9e-24 | 156.9 | 140 | (229, 374) | 475 | (333, 485) | 485 | Phage tail protein (Fragment) | Phage tail protein (Fragment) | | uniclust | UniRef100\_A0A955R619 | 99.6 | 3.3e-18 | 6e-24 | 145.6 | 232 | (229, 467) | 475 | (12, 272) | 281 | Phage tail sheath family protein | Phage tail sheath family protein | | uniclust | UniRef100\_UPI000D149F10 | 99.6 | 3.3e-18 | 6.1e-24 | 140.3 | 150 | (267, 426) | 475 | (58, 210) | 217 | phage tail sheath C-terminal domain-containing protein | phage tail sheath C-terminal domain-containing protein | | uniclust | UniRef100\_A0A099P8J3 | 99.6 | 3.2e-18 | 6.1e-24 | 130.7 | 87 | (1, 95) | 475 | (1, 90) | 106 | Uncharacterized protein (Fragment) | Uncharacterized protein (Fragment) | | uniclust | UniRef100\_A0A379QW15 | 99.6 | 3.6e-18 | 6.8e-24 | 133.0 | 128 | (346, 474) | 475 | (2, 134) | 140 | Tail protein | Tail protein | | uniclust | UniRef100\_A0A7S5T3D9 | 99.6 | 3.7e-18 | 6.9e-24 | 165.7 | 229 | (229, 463) | 475 | (572, 817) | 833 | Tail sheath protein subtilisin-like domain-containing protein | Tail sheath protein subtilisin-like domain-containing protein | | uniclust | UniRef100\_A0A660SQZ6 | 99.6 | 4e-18 | 7.4e-24 | 149.2 | 234 | (231, 468) | 475 | (91, 341) | 347 | Tail sheath protein C-terminal domain-containing protein (Fragment) | Tail sheath protein C-terminal domain-containing protein (Fragment) | | uniclust | UniRef100\_UPI000A41458E | 99.6 | 4.9e-18 | 9e-24 | 133.6 | 135 | (266, 400) | 475 | (21, 161) | 163 | phage tail sheath subtilisin-like domain-containing protein | phage tail sheath subtilisin-like domain-containing protein | | uniclust | UniRef100\_A0A9C8Q3C5 | 99.6 | 5.4e-18 | 9.9e-24 | 134.7 | 137 | (337, 473) | 475 | (31, 168) | 177 | Phage tail sheath family protein | Phage tail sheath family protein | | uniclust | UniRef100\_UPI00158EB90D | 99.6 | 5.9e-18 | 1.1e-23 | 150.2 | 225 | (231, 464) | 475 | (120, 365) | 387 | phage tail sheath family protein | phage tail sheath family protein | | uniclust | UniRef100\_A0A963ER11 | 99.6 | 7.9e-18 | 1.5e-23 | 144.6 | 224 | (237, 470) | 475 | (7, 243) | 259 | Uncharacterized protein | Uncharacterized protein | | uniclust | UniRef100\_A0A0A0BPC6 | 99.6 | 1e-17 | 1.9e-23 | 147.8 | 208 | (259, 475) | 475 | (43, 265) | 269 | Tail protein | Tail protein | | uniclust | UniRef100\_A0A0F8VRZ6 | 99.6 | 1.1e-17 | 2e-23 | 131.1 | 127 | (343, 469) | 475 | (3, 132) | 139 | Tail sheath protein C-terminal domain-containing protein (Fragment) | Tail sheath protein C-terminal domain-containing protein (Fragment) | | uniclust | UniRef100\_A0A1Z4K7L4 | 99.5 | 1.1e-17 | 2.1e-23 | 150.5 | 224 | (230, 463) | 475 | (127, 367) | 394 | Phage tail sheath protein fi-like protein | Phage tail sheath protein fi-like protein | | uniclust | UniRef100\_A0A6G5QYL8 | 99.5 | 1.4e-17 | 2.6e-23 | 156.6 | 240 | (231, 474) | 475 | (411, 653) | 658 | Phage tail sheath protein FI (Integrase domain) | Phage tail sheath protein FI (Integrase domain) | | uniclust | UniRef100\_UPI00223C8DA3 | 99.5 | 2.1e-17 | 3.8e-23 | 145.1 | 200 | (266, 470) | 475 | (137, 344) | 350 | hypothetical protein | hypothetical protein | | uniclust | UniRef100\_A0A8S5MEQ3 | 99.5 | 2.1e-17 | 3.8e-23 | 157.8 | 235 | (229, 469) | 475 | (514, 764) | 768 | Tail sheath protein | Tail sheath protein | | uniclust | UniRef100\_UPI001C5FF8DD | 99.5 | 2.1e-17 | 3.9e-23 | 115.9 | 85 | (289, 373) | 475 | (3, 87) | 87 | phage tail sheath subtilisin-like domain-containing protein | phage tail sheath subtilisin-like domain-containing protein | | uniclust | UniRef100\_A0A0F9PP94 | 99.5 | 2.1e-17 | 4e-23 | 166.9 | 231 | (229, 465) | 475 | (504, 752) | 761 | Tail sheath protein C-terminal domain-containing protein | Tail sheath protein C-terminal domain-containing protein | | uniclust | UniRef100\_A0A7Y0GV13 | 99.5 | 3.3e-17 | 6.2e-23 | 152.8 | 90 | (229, 318) | 475 | (388, 483) | 487 | Phage tail sheath family protein (Fragment) | Phage tail sheath family protein (Fragment) | | uniclust | UniRef100\_A0A0C2VDI1 | 99.5 | 3.6e-17 | 7.1e-23 | 166.0 | 229 | (228, 467) | 475 | (322, 569) | 591 | Tail sheath protein subtilisin-like domain-containing protein | Tail sheath protein subtilisin-like domain-containing protein | | uniclust | UniRef100\_A0A2H0PCS0 | 99.5 | 4e-17 | 7.3e-23 | 155.2 | 244 | (229, 474) | 475 | (453, 724) | 728 | Tail sheath protein C-terminal domain-containing protein | Tail sheath protein C-terminal domain-containing protein | | uniclust | UniRef100\_A0A0F9KEX1 | 99.5 | 4e-17 | 7.4e-23 | 158.4 | 226 | (243, 468) | 475 | (681, 922) | 926 | Tail sheath protein C-terminal domain-containing protein (Fragment) | Tail sheath protein C-terminal domain-containing protein (Fragment) | | uniclust | UniRef100\_A0A6V8LKN3 | 99.5 | 4.4e-17 | 8.2e-23 | 125.4 | 117 | (353, 469) | 475 | (15, 138) | 144 | Tail sheath protein C-terminal domain-containing protein | Tail sheath protein C-terminal domain-containing protein | | uniclust | UniRef100\_A0A4Q5HCU8 | 99.5 | 4.8e-17 | 8.7e-23 | 122.9 | 113 | (287, 402) | 475 | (10, 126) | 129 | Phage tail sheath family protein | Phage tail sheath family protein | | uniclust | UniRef100\_A0A1F3SEE9 | 99.5 | 6e-17 | 1.1e-22 | 143.3 | 322 | (22, 463) | 475 | (23, 365) | 370 | Tail sheath protein C-terminal domain-containing protein | Tail sheath protein C-terminal domain-containing protein | | uniclust | UniRef100\_A0A434RRN7 | 99.5 | 6.7e-17 | 1.2e-22 | 139.6 | 241 | (230, 471) | 475 | (40, 293) | 307 | Phage tail protein (Fragment) | Phage tail protein (Fragment) | | uniclust | UniRef100\_A0A7W0HPR7 | 99.5 | 8.2e-17 | 1.5e-22 | 134.3 | 202 | (29, 360) | 475 | (4, 211) | 238 | Tail sheath protein subtilisin-like domain-containing protein | Tail sheath protein subtilisin-like domain-containing protein | | uniclust | UniRef100\_A0A1H2UW00 | 99.5 | 8.1e-17 | 1.5e-22 | 123.1 | 112 | (363, 474) | 475 | (2, 114) | 122 | Phage tail sheath protein | Phage tail sheath protein | | uniclust | UniRef100\_A0A2D7XGG3 | 99.5 | 9.7e-17 | 1.8e-22 | 155.1 | 196 | (266, 467) | 475 | (582, 784) | 793 | Tail sheath protein C-terminal domain-containing protein | Tail sheath protein C-terminal domain-containing protein | | uniclust | UniRef100\_UPI0013CE83EC | 99.5 | 1e-16 | 1.8e-22 | 117.8 | 104 | (259, 362) | 475 | (2, 106) | 106 | phage tail sheath subtilisin-like domain-containing protein | phage tail sheath subtilisin-like domain-containing protein | | uniclust | UniRef100\_A0A024AZG0 | 99.5 | 1.2e-16 | 2.4e-22 | 163.5 | 227 | (229, 471) | 475 | (337, 581) | 615 | Tail sheath protein | Tail sheath protein | | uniclust | UniRef100\_UPI001D12A7C3 | 99.5 | 1.3e-16 | 2.4e-22 | 143.0 | 305 | (7, 430) | 475 | (44, 368) | 410 | phage tail sheath C-terminal domain-containing protein | phage tail sheath C-terminal domain-containing protein | | uniclust | UniRef100\_A0A1Z8RYI1 | 99.5 | 1.4e-16 | 2.7e-22 | 156.9 | 200 | (265, 470) | 475 | (514, 720) | 723 | Tail sheath protein subtilisin-like domain-containing protein (Fragment) | Tail sheath protein subtilisin-like domain-containing protein (Fragment) | | uniclust | UniRef100\_A0A0F6W355 | 99.5 | 1.6e-16 | 3e-22 | 156.4 | 422 | (5, 472) | 475 | (1, 481) | 489 | Tail sheath protein subtilisin-like domain-containing protein | Tail sheath protein subtilisin-like domain-containing protein | | uniclust | UniRef100\_A0A0Q0D1V1 | 99.5 | 1.7e-16 | 3.1e-22 | 121.1 | 118 | (357, 474) | 475 | (1, 118) | 122 | Major tail sheath protein | Major tail sheath protein | | uniclust | UniRef100\_A0A7S5RC19 | 99.5 | 2.1e-16 | 3.8e-22 | 151.4 | 233 | (231, 469) | 475 | (516, 761) | 773 | Tail sheath protein | Tail sheath protein | | uniclust | UniRef100\_A0A2Z3EDS5 | 99.4 | 2.5e-16 | 4.6e-22 | 150.0 | 237 | (229, 468) | 475 | (459, 712) | 727 | Tail sheath protein | Tail sheath protein | | uniclust | UniRef100\_A0A4V6JGZ6 | 99.4 | 2.8e-16 | 5.1e-22 | 138.8 | 259 | (41, 390) | 475 | (75, 351) | 359 | Phage tail sheath protein | Phage tail sheath protein | | uniclust | UniRef100\_UPI00096A297B | 99.4 | 3e-16 | 5.5e-22 | 132.8 | 191 | (18, 321) | 475 | (9, 206) | 261 | phage tail sheath family protein | phage tail sheath family protein | | uniclust | UniRef100\_UPI001922CB15 | 99.4 | 3.7e-16 | 6.7e-22 | 127.6 | 184 | (41, 324) | 475 | (13, 197) | 204 | phage tail sheath subtilisin-like domain-containing protein | phage tail sheath subtilisin-like domain-containing protein | | uniclust | UniRef100\_UPI00197F3314 | 99.4 | 3.9e-16 | 7.2e-22 | 116.7 | 116 | (359, 474) | 475 | (2, 118) | 120 | phage tail sheath C-terminal domain-containing protein | phage tail sheath C-terminal domain-containing protein | | uniclust | UniRef100\_A0A1M5WCL1 | 99.4 | 4.1e-16 | 7.8e-22 | 124.8 | 86 | (2, 95) | 475 | (1, 92) | 136 | Phage tail sheath protein | Phage tail sheath protein | | uniclust | UniRef100\_A0A0T9RBS8 | 99.4 | 4.9e-16 | 9.2e-22 | 119.0 | 90 | (2, 95) | 475 | (1, 90) | 118 | Phage tail sheath monomer | Phage tail sheath monomer | | uniclust | UniRef100\_UPI00234B027B | 99.4 | 6.6e-16 | 1.2e-21 | 133.5 | 139 | (2, 145) | 475 | (1, 139) | 302 | ATP-binding protein | ATP-binding protein | | uniclust | UniRef100\_A0A257LFR8 | 99.4 | 6.3e-16 | 1.2e-21 | 142.4 | 85 | (5, 97) | 475 | (8, 94) | 272 | Phage tail protein (Fragment) | Phage tail protein (Fragment) | | uniclust | UniRef100\_A0A3D0UP20 | 99.4 | 6.7e-16 | 1.2e-21 | 115.3 | 88 | (310, 403) | 475 | (1, 90) | 119 | Tail sheath protein subtilisin-like domain-containing protein (Fragment) | Tail sheath protein subtilisin-like domain-containing protein (Fragment) | | uniclust | UniRef100\_A0A1C4JDU7 | 99.4 | 6.7e-16 | 1.3e-21 | 135.9 | 87 | (2, 96) | 475 | (1, 89) | 233 | Phage tail sheath protein | Phage tail sheath protein | | uniclust | UniRef100\_UPI0018EC0130 | 99.4 | 7.4e-16 | 1.4e-21 | 110.4 | 92 | (289, 381) | 475 | (1, 95) | 95 | phage tail sheath subtilisin-like domain-containing protein | phage tail sheath subtilisin-like domain-containing protein | | uniclust | UniRef100\_A0A3B9JCS2 | 99.4 | 8.1e-16 | 1.5e-21 | 121.1 | 91 | (1, 94) | 475 | (1, 103) | 125 | Phage tail sheath family protein (Fragment) | Phage tail sheath family protein (Fragment) | | uniclust | UniRef100\_A0A1M6F1L4 | 99.4 | 8.6e-16 | 1.6e-21 | 136.5 | 122 | (2, 142) | 475 | (1, 159) | 268 | Phage tail sheath protein | Phage tail sheath protein | | uniclust | UniRef100\_A0A0B0HIM0 | 99.4 | 1e-15 | 1.9e-21 | 117.3 | 109 | (367, 475) | 475 | (4, 112) | 114 | Phage tail sheath protein | Phage tail sheath protein | | uniclust | UniRef100\_A0A2I7QP51 | 99.4 | 1.3e-15 | 2.3e-21 | 155.2 | 231 | (229, 464) | 475 | (1030, 1276) | 1283 | Tail sheath protein | Tail sheath protein | | uniclust | UniRef100\_A0A484YNN0 | 99.4 | 1.4e-15 | 2.5e-21 | 122.0 | 134 | (233, 366) | 475 | (3, 138) | 180 | Tail Sheath protein | Tail Sheath protein | | uniclust | UniRef100\_A0A2E2FJ22 | 99.4 | 1.9e-15 | 3.5e-21 | 149.0 | 239 | (229, 469) | 475 | (360, 637) | 649 | Tail sheath protein subtilisin-like domain-containing protein | Tail sheath protein subtilisin-like domain-containing protein | | uniclust | UniRef100\_A0A0P7JRX0 | 99.4 | 2.1e-15 | 3.9e-21 | 139.1 | 404 | (2, 466) | 475 | (1, 428) | 434 | Tail sheath protein subtilisin-like domain-containing protein | Tail sheath protein subtilisin-like domain-containing protein | | uniclust | UniRef100\_A0A2X3JRA6 | 99.4 | 2.2e-15 | 4e-21 | 117.7 | 141 | (238, 379) | 475 | (8, 150) | 153 | Major tail sheath protein FI | Major tail sheath protein FI | | uniclust | UniRef100\_A0A1H5ZKW0 | 99.4 | 2.2e-15 | 4.1e-21 | 144.5 | 384 | (7, 466) | 475 | (10, 426) | 440 | Phage tail sheath protein | Phage tail sheath protein | | uniclust | UniRef100\_A0A1H8RZB9 | 99.4 | 2.3e-15 | 4.4e-21 | 146.1 | 411 | (1, 468) | 475 | (1, 441) | 451 | Phage tail sheath protein | Phage tail sheath protein | | uniclust | UniRef100\_X1DII4 | 99.4 | 2.8e-15 | 5.2e-21 | 113.6 | 85 | (343, 427) | 475 | (17, 104) | 128 | Tail sheath protein C-terminal domain-containing protein (Fragment) | Tail sheath protein C-terminal domain-containing protein (Fragment) | | uniclust | UniRef100\_A0A7V9TCY7 | 99.4 | 3.1e-15 | 5.7e-21 | 113.1 | 83 | (4, 94) | 475 | (4, 88) | 117 | Phage tail sheath family protein (Fragment) | Phage tail sheath family protein (Fragment) | | uniclust | UniRef100\_UPI0015CF41A0 | 99.3 | 3.8e-15 | 7.1e-21 | 108.5 | 83 | (287, 376) | 475 | (9, 94) | 94 | phage tail sheath subtilisin-like domain-containing protein | phage tail sheath subtilisin-like domain-containing protein | | uniclust | UniRef100\_A0A076YKJ9 | 99.3 | 4.6e-15 | 8.6e-21 | 149.0 | 236 | (232, 470) | 475 | (582, 831) | 839 | Putative tail sheath protein | Putative tail sheath protein | | uniclust | UniRef100\_A0A6B3GW63 | 99.3 | 5.3e-15 | 9.8e-21 | 119.5 | 98 | (231, 328) | 475 | (80, 183) | 187 | Phage tail sheath family protein (Fragment) | Phage tail sheath family protein (Fragment) | | uniclust | UniRef100\_UPI0006CC31C6 | 99.3 | 5.6e-15 | 1e-20 | 112.1 | 110 | (337, 446) | 475 | (12, 126) | 128 | hypothetical protein | hypothetical protein | | uniclust | UniRef100\_A0A946FFJ9 | 99.3 | 5.8e-15 | 1.1e-20 | 135.0 | 87 | (2, 96) | 475 | (1, 89) | 370 | Uncharacterized protein (Fragment) | Uncharacterized protein (Fragment) | | uniclust | UniRef100\_A0A7C3UJZ4 | 99.3 | 5.9e-15 | 1.1e-20 | 147.5 | 201 | (263, 470) | 475 | (566, 770) | 776 | Tail sheath protein C-terminal domain-containing protein | Tail sheath protein C-terminal domain-containing protein | | uniclust | UniRef100\_UPI001D0F62D8 | 99.3 | 6.2e-15 | 1.1e-20 | 126.2 | 180 | (231, 416) | 475 | (61, 244) | 275 | phage tail sheath subtilisin-like domain-containing protein | phage tail sheath subtilisin-like domain-containing protein | | uniclust | UniRef100\_A0A5C7M3L5 | 99.3 | 7.6e-15 | 1.4e-20 | 132.7 | 132 | (229, 363) | 475 | (273, 418) | 420 | Tail sheath protein subtilisin-like domain-containing protein (Fragment) | Tail sheath protein subtilisin-like domain-containing protein (Fragment) | | uniclust | UniRef100\_A0A644XLY2 | 99.3 | 8.2e-15 | 1.5e-20 | 113.4 | 123 | (353, 475) | 475 | (3, 129) | 132 | Prophage major tail sheath protein | Prophage major tail sheath protein | | uniclust | UniRef100\_W4MDK3 | 99.3 | 8.9e-15 | 1.6e-20 | 121.1 | 186 | (2, 285) | 475 | (1, 193) | 218 | Tail sheath protein subtilisin-like domain-containing protein | Tail sheath protein subtilisin-like domain-containing protein | | uniclust | UniRef100\_A0A0U2YXC1 | 99.3 | 9.8e-15 | 1.9e-20 | 143.6 | 90 | (1, 98) | 475 | (1, 92) | 476 | Phage tail sheath protein (Fragment) | Phage tail sheath protein (Fragment) | | uniclust | UniRef100\_A0A1Z8WSY0 | 99.3 | 1e-14 | 1.9e-20 | 152.1 | 199 | (265, 469) | 475 | (1466, 1672) | 1675 | Tail sheath protein C-terminal domain-containing protein | Tail sheath protein C-terminal domain-containing protein | | uniclust | UniRef100\_A0A8S0FU88 | 99.3 | 1.2e-14 | 2.3e-20 | 109.2 | 105 | (286, 390) | 475 | (16, 120) | 121 | Tail sheath protein subtilisin-like domain-containing protein | Tail sheath protein subtilisin-like domain-containing protein | | uniclust | UniRef100\_A0A1Y3CXG9 | 99.3 | 1.3e-14 | 2.4e-20 | 132.4 | 187 | (3, 279) | 475 | (1, 194) | 343 | FHA domain-containing protein | FHA domain-containing protein | | uniclust | UniRef100\_A0A967K953 | 99.3 | 1.4e-14 | 2.5e-20 | 96.0 | 63 | (355, 417) | 475 | (1, 63) | 63 | Phage tail sheath family protein (Fragment) | Phage tail sheath family protein (Fragment) | | uniclust | UniRef100\_A0A327KJ47 | 99.3 | 1.5e-14 | 2.8e-20 | 126.7 | 314 | (2, 326) | 475 | (2, 325) | 326 | Phage tail protein (Fragment) | Phage tail protein (Fragment) | | uniclust | UniRef100\_A0A3C2AP21 | 99.3 | 1.6e-14 | 3e-20 | 145.2 | 203 | (265, 468) | 475 | (851, 1064) | 1069 | Uncharacterized protein (Fragment) | Uncharacterized protein (Fragment) | | uniclust | UniRef100\_A0A3B8IT26 | 99.3 | 2e-14 | 3.7e-20 | 133.5 | 121 | (287, 408) | 475 | (404, 527) | 528 | Tail sheath protein subtilisin-like domain-containing protein (Fragment) | Tail sheath protein subtilisin-like domain-containing protein (Fragment) | | uniclust | UniRef100\_H6WFT7 | 99.3 | 2.7e-14 | 4.9e-20 | 142.1 | 191 | (268, 468) | 475 | (909, 1111) | 1137 | Tail sheath protein | Tail sheath protein | | uniclust | UniRef100\_A0A068NXW1 | 99.3 | 2.7e-14 | 5.3e-20 | 138.9 | 331 | (7, 467) | 475 | (23, 394) | 398 | Tail sheath protein subtilisin-like domain-containing protein | Tail sheath protein subtilisin-like domain-containing protein | | uniclust | UniRef100\_A0A524QBZ3 | 99.2 | 2.9e-14 | 5.4e-20 | 135.4 | 87 | (2, 95) | 475 | (44, 137) | 550 | Phage tail sheath family protein (Fragment) | Phage tail sheath family protein (Fragment) | | uniclust | UniRef100\_A0A926AT07 | 99.2 | 2.9e-14 | 5.5e-20 | 132.3 | 85 | (4, 96) | 475 | (4, 90) | 308 | Phage tail sheath family protein (Fragment) | Phage tail sheath family protein (Fragment) | | uniclust | UniRef100\_A0A327JRX8 | 99.2 | 3e-14 | 5.5e-20 | 114.5 | 156 | (307, 464) | 475 | (5, 160) | 178 | Tail sheath protein C-terminal domain-containing protein (Fragment) | Tail sheath protein C-terminal domain-containing protein (Fragment) | | uniclust | UniRef100\_A0A356X1N2 | 99.2 | 3e-14 | 5.6e-20 | 117.0 | 173 | (295, 469) | 475 | (2, 182) | 187 | Phage tail protein (Fragment) | Phage tail protein (Fragment) | | uniclust | UniRef100\_A0A0C3J1K3 | 99.2 | 3.1e-14 | 6e-20 | 142.8 | 186 | (267, 470) | 475 | (301, 507) | 523 | Phage tail sheath family protein | Phage tail sheath family protein | | uniclust | UniRef100\_A0A6V6ZU17 | 99.2 | 3.3e-14 | 6.1e-20 | 134.9 | 317 | (1, 330) | 475 | (1, 332) | 649 | Uncharacterized protein | Uncharacterized protein | | uniclust | UniRef100\_A0A075BS62 | 99.2 | 3.5e-14 | 6.5e-20 | 140.1 | 232 | (229, 468) | 475 | (522, 768) | 776 | Putative tail sheath protein | Putative tail sheath protein | | uniclust | UniRef100\_A0A239K6F1 | 99.2 | 3.9e-14 | 7.4e-20 | 118.6 | 88 | (1, 96) | 475 | (1, 90) | 187 | Phage tail sheath protein | Phage tail sheath protein | | uniclust | UniRef100\_A0A370E0M7 | 99.2 | 3.9e-14 | 7.4e-20 | 118.1 | 123 | (2, 144) | 475 | (3, 149) | 160 | Uncharacterized protein | Uncharacterized protein | | uniclust | UniRef100\_A0A931KX23 | 99.2 | 4.2e-14 | 7.8e-20 | 132.4 | 396 | (19, 467) | 475 | (15, 457) | 462 | Uncharacterized protein | Uncharacterized protein | | uniclust | UniRef100\_A0A956AK97 | 99.2 | 4.8e-14 | 8.9e-20 | 115.9 | 176 | (245, 424) | 475 | (2, 196) | 205 | Phage tail protein (Fragment) | Phage tail protein (Fragment) | | uniclust | UniRef100\_A0A4Y8ZK08 | 99.2 | 4.7e-14 | 8.9e-20 | 116.8 | 83 | (6, 96) | 475 | (6, 90) | 175 | Phage tail sheath family protein (Fragment) | Phage tail sheath family protein (Fragment) | | uniclust | UniRef100\_A0A7K0P9W3 | 99.2 | 4.9e-14 | 9.1e-20 | 133.1 | 232 | (230, 466) | 475 | (227, 480) | 486 | Tail sheath protein subtilisin-like domain-containing protein | Tail sheath protein subtilisin-like domain-containing protein | | uniclust | UniRef100\_A0A1I1S0H2 | 99.2 | 4.9e-14 | 9.2e-20 | 105.6 | 87 | (381, 467) | 475 | (2, 95) | 99 | Phage tail sheath protein | Phage tail sheath protein | | uniclust | UniRef100\_A0A6J5RUV9 | 99.2 | 5.1e-14 | 9.3e-20 | 125.8 | 336 | (8, 464) | 475 | (3, 362) | 373 | Uncharacterized protein | Uncharacterized protein | | uniclust | UniRef100\_A0A368A7G8 | 99.2 | 5.1e-14 | 9.4e-20 | 115.4 | 74 | (260, 334) | 475 | (110, 183) | 201 | Phage tail sheath protein FI | Phage tail sheath protein FI | | uniclust | UniRef100\_A0A839HMA6 | 99.2 | 5.2e-14 | 9.5e-20 | 128.8 | 180 | (287, 467) | 475 | (259, 454) | 457 | Phage tail sheath family protein | Phage tail sheath family protein | | uniclust | UniRef100\_A0A935W4P1 | 99.2 | 5.4e-14 | 1e-19 | 110.7 | 105 | (364, 468) | 475 | (23, 133) | 140 | Uncharacterized protein | Uncharacterized protein | | uniclust | UniRef100\_A0A7C5GVL1 | 99.2 | 6e-14 | 1.1e-19 | 127.8 | 126 | (1, 144) | 475 | (1, 159) | 325 | Phage tail protein (Fragment) | Phage tail protein (Fragment) | | uniclust | UniRef100\_A0A2E7WB84 | 99.2 | 7e-14 | 1.3e-19 | 109.8 | 112 | (358, 469) | 475 | (2, 114) | 124 | Tail sheath protein C-terminal domain-containing protein (Fragment) | Tail sheath protein C-terminal domain-containing protein (Fragment) | | uniclust | UniRef100\_A0A450ZA88 | 99.2 | 7.7e-14 | 1.4e-19 | 117.3 | 122 | (230, 351) | 475 | (27, 150) | 208 | Tail sheath protein subtilisin-like domain-containing protein | Tail sheath protein subtilisin-like domain-containing protein | | uniclust | UniRef100\_A0A3A8EJ92 | 99.2 | 7.7e-14 | 1.5e-19 | 113.8 | 136 | (2, 142) | 475 | (1, 139) | 150 | Phage tail protein (Fragment) | Phage tail protein (Fragment) | | uniclust | UniRef100\_A0A0G3M576 | 99.2 | 8.3e-14 | 1.7e-19 | 138.8 | 368 | (4, 468) | 475 | (2, 412) | 415 | DUF2586 family protein | DUF2586 family protein | | uniclust | UniRef100\_UPI001872E667 | 99.2 | 9.2e-14 | 1.7e-19 | 115.8 | 122 | (353, 474) | 475 | (97, 218) | 223 | P2 family phage major capsid protein | P2 family phage major capsid protein | | uniclust | UniRef100\_A0A0F9KVW3 | 99.2 | 9.2e-14 | 1.7e-19 | 138.0 | 223 | (244, 467) | 475 | (829, 1073) | 1079 | Tail sheath protein C-terminal domain-containing protein (Fragment) | Tail sheath protein C-terminal domain-containing protein (Fragment) | | uniclust | UniRef100\_UPI00193ADC5D | 99.2 | 9.6e-14 | 1.8e-19 | 110.3 | 145 | (326, 474) | 475 | (8, 159) | 164 | hypothetical protein | hypothetical protein | | uniclust | UniRef100\_UPI00047DF1B9 | 99.2 | 1.3e-13 | 2.4e-19 | 127.1 | 233 | (229, 467) | 475 | (220, 474) | 482 | hypothetical protein | hypothetical protein | | uniclust | UniRef100\_UPI001FEE2E79 | 99.2 | 1.7e-13 | 3.1e-19 | 113.2 | 133 | (340, 473) | 475 | (58, 193) | 208 | hypothetical protein | hypothetical protein | | uniclust | UniRef100\_A0A523V8M4 | 99.2 | 1.8e-13 | 3.3e-19 | 125.8 | 236 | (225, 466) | 475 | (206, 460) | 467 | Tail sheath protein subtilisin-like domain-containing protein (Fragment) | Tail sheath protein subtilisin-like domain-containing protein (Fragment) | | uniclust | UniRef100\_UPI001F1A55FA | 99.2 | 1.9e-13 | 3.5e-19 | 108.3 | 102 | (230, 331) | 475 | (10, 112) | 160 | phage tail sheath subtilisin-like domain-containing protein | phage tail sheath subtilisin-like domain-containing protein | | uniclust | UniRef100\_UPI001CC11497 | 99.1 | 2.2e-13 | 4.1e-19 | 103.6 | 94 | (372, 465) | 475 | (19, 118) | 126 | phage tail sheath C-terminal domain-containing protein | phage tail sheath C-terminal domain-containing protein | | uniclust | UniRef100\_UPI001EEA4F30 | 99.1 | 2.3e-13 | 4.2e-19 | 98.4 | 81 | (308, 388) | 475 | (1, 81) | 96 | phage tail sheath subtilisin-like domain-containing protein | phage tail sheath subtilisin-like domain-containing protein | | uniclust | UniRef100\_K8GN70 | 99.1 | 2.4e-13 | 4.4e-19 | 133.8 | 214 | (244, 467) | 475 | (704, 927) | 939 | Phage tail sheath protein FI | Phage tail sheath protein FI | | uniclust | UniRef100\_A0A950PI68 | 99.1 | 2.8e-13 | 5.2e-19 | 107.8 | 100 | (365, 464) | 475 | (57, 161) | 164 | Phage tail sheath family protein | Phage tail sheath family protein | | uniclust | UniRef100\_A0A3Q9LGI5 | 99.1 | 2.8e-13 | 5.2e-19 | 112.3 | 167 | (62, 315) | 475 | (6, 180) | 192 | Tail sheath protein subtilisin-like domain-containing protein | Tail sheath protein subtilisin-like domain-containing protein | | uniclust | UniRef100\_A0A3B0XDA8 | 99.1 | 3.3e-13 | 6.2e-19 | 113.8 | 87 | (1, 95) | 475 | (1, 89) | 196 | Uncharacterized protein (Fragment) | Uncharacterized protein (Fragment) | | uniclust | UniRef100\_UPI001FCE0DAA | 99.1 | 3.9e-13 | 7.2e-19 | 111.9 | 122 | (225, 346) | 475 | (94, 217) | 218 | phage tail sheath subtilisin-like domain-containing protein | phage tail sheath subtilisin-like domain-containing protein | | pdb70 | 6RBN\_D | 100.0 | 1.6e-43 | 1.2e-47 | 348.8 | 351 | (1, 468) | 475 | (1, 450) | 451 | Afp1, Afp2, Afp3, Afp16 | 6RBN\_D Afp1, Afp2, Afp3, Afp16 Anti-feeding prophage, secretion system, AFP | | pdb70 | 6RAO\_D | 100.0 | 2.1e-43 | 1.6e-47 | 347.9 | 351 | (1, 468) | 475 | (1, 450) | 451 | Afp1, Afp2, Afp3, Afp5, Afp9 | 6RAO\_D Afp1, Afp2, Afp3, Afp5, Afp9 Anti-feeding prophage, secretion system, AFP | | pdb70 | 3J9Q\_B | 100.0 | 2.9e-42 | 2.2e-46 | 336.4 | 382 | (5, 475) | 475 | (2, 384) | 386 | sheath, tube | 3J9Q\_B sheath, tube pyocin, bacteriocin, sheath, tube, STRUCTURAL | | pdb70 | 6J0B\_J | 100.0 | 9.1e-40 | 7e-44 | 314.9 | 337 | (1, 468) | 475 | (1, 353) | 355 | Pvc2, Conserved hypothetical phage tail | 6J0B\_J Pvc2, Conserved hypothetical phage tail assembly, Photorhabdus asymbiotica, PVC, contractile | | pdb70 | 6J0C\_c | 100.0 | 9.1e-40 | 7e-44 | 314.9 | 337 | (1, 468) | 475 | (1, 353) | 355 | Pvc2 | 6J0C\_c Pvc2 assembly, Photorhabdus asymbiotica, PVC, contractile | | pdb70 | 6J0N\_u | 100.0 | 2.7e-39 | 2e-43 | 318.8 | 351 | (1, 468) | 475 | (1, 439) | 440 | Pvc1, Pvc9, Pvc11, Pvc12, Pvc4 | 6J0N\_u Pvc1, Pvc9, Pvc11, Pvc12, Pvc4 assembly, Photorhabdus asymbiotica, PVC, contractile | | pdb70 | 6RAO\_C | 100.0 | 5.2e-39 | 4e-43 | 309.4 | 336 | (1, 468) | 475 | (1, 353) | 354 | Afp1, Afp2, Afp3, Afp5, Afp9 | 6RAO\_C Afp1, Afp2, Afp3, Afp5, Afp9 Anti-feeding prophage, secretion system, AFP | | pdb70 | 6RBN\_C | 100.0 | 5.2e-39 | 4e-43 | 309.4 | 336 | (1, 468) | 475 | (1, 353) | 354 | Afp1, Afp2, Afp3, Afp16 | 6RBN\_C Afp1, Afp2, Afp3, Afp16 Anti-feeding prophage, secretion system, AFP | | pdb70 | 6RAO\_E | 100.0 | 1.9e-37 | 1.5e-41 | 305.2 | 376 | (4, 468) | 475 | (2, 405) | 417 | Afp1, Afp2, Afp3, Afp5, Afp9 | 6RAO\_E Afp1, Afp2, Afp3, Afp5, Afp9 Anti-feeding prophage, secretion system, AFP | | pdb70 | 6J0N\_V | 100.0 | 3.4e-36 | 2.6e-40 | 295.5 | 369 | (4, 468) | 475 | (2, 405) | 410 | Pvc1, Pvc9, Pvc11, Pvc12, Pvc4 | 6J0N\_V Pvc1, Pvc9, Pvc11, Pvc12, Pvc4 assembly, Photorhabdus asymbiotica, PVC, contractile | | pdb70 | 3HXL\_A | 99.9 | 1e-33 | 7.8e-38 | 280.9 | 394 | (4, 469) | 475 | (10, 441) | 446 | uncharacterized protein DSY3957 | 3HXL\_A uncharacterized protein DSY3957 alpha-beta three-domained protein., Structural Genomics | | pdb70 | 3LML\_A | 99.9 | 6.9e-33 | 5.3e-37 | 275.8 | 396 | (3, 469) | 475 | (11, 455) | 460 | Lin1278 protein | 3LML\_A Lin1278 protein Structural Genomics, PSI-2, Protein Structure | | pdb70 | 3LML\_B | 99.9 | 6.9e-33 | 5.3e-37 | 275.8 | 396 | (3, 469) | 475 | (11, 455) | 460 | Lin1278 protein | 3LML\_B Lin1278 protein Structural Genomics, PSI-2, Protein Structure HET: MSE | | pdb70 | 3J2M\_V | 99.9 | 8e-33 | 6e-37 | 281.0 | 239 | (230, 474) | 475 | (388, 655) | 659 | Tail connector protein Gp15, Tail | 3J2M\_V Tail connector protein Gp15, Tail bacteriophage T4, phage tail terminator | | pdb70 | 5LI4\_B | 99.9 | 3.9e-27 | 3e-31 | 240.3 | 248 | (201, 470) | 475 | (319, 585) | 587 | Uncharacterized protein | 5LI4\_B Uncharacterized protein polyvalent staphylococcal bactoriophage, Myoviridae, tail | | pdb70 | 6GKW\_A | 99.9 | 6.5e-27 | 5e-31 | 224.8 | 316 | (3, 464) | 475 | (3, 355) | 356 | Putative phage XkdK-like protein | 6GKW\_A Putative phage XkdK-like protein Diffocin Sheath, structural protein HET: MSE | | pdb70 | 3FOA\_C | 98.8 | 6.7e-13 | 5e-17 | 129.6 | 84 | (3, 95) | 475 | (1, 86) | 510 | Tail sheath protein Gp18 | 3FOA\_C Tail sheath protein Gp18 alpha-beta, viral structural protein, bacteriophage | | pdb70 | 3FOI\_B | 98.8 | 6.7e-13 | 5e-17 | 129.6 | 84 | (3, 95) | 475 | (1, 86) | 510 | Tail sheath protein Gp18 | 3FOI\_B Tail sheath protein Gp18 alpha-beta, viral structural protein, bacteriophage | |
| Top keywords  (threshold 1.00e-03 (evalue)) | **tail, sheath, Phage, domain\_containing, Fragment, subtilisin\_like, C\_terminal, Major, FI, Putative** |
| Output files | ../../similar\_sequences/18\_FANPEZAQ\_CDS\_0018\_merged.svg ../../similar\_sequences/18\_FANPEZAQ\_CDS\_0018\_pdb70.a3m ../../similar\_sequences/18\_FANPEZAQ\_CDS\_0018\_pdb70.hhr ../../similar\_sequences/18\_FANPEZAQ\_CDS\_0018\_uniclust.a3m ../../similar\_sequences/18\_FANPEZAQ\_CDS\_0018\_uniclust.hhr |

#### Structure prediction (AlphaFold)2

|  |  |
| --- | --- |
| Stats | xml version="1.0" encoding="utf-8" standalone="no"?       2024-09-02T21:09:17.817824 image/svg+xml   Matplotlib v3.7.2, https://matplotlib.org/ |
| Predicted structure | **NGL Viewer Controls:**  - Center: *Left-Click* - Rotate: *Left-Click + Drag* - Translate: *Right-Click + Drag* - Zoom: *Shift + Left-Click + Drag* |
| Output files | ../../predicted\_structures/18\_FANPEZAQ\_CDS\_0018/features.pkl ../../predicted\_structures/18\_FANPEZAQ\_CDS\_0018/ranked\_0.pdb ../../predicted\_structures/18\_FANPEZAQ\_CDS\_0018/ranked\_0\_plots.svg ../../predicted\_structures/18\_FANPEZAQ\_CDS\_0018/result\_model\_1\_ptm\_pred\_0.pkl |

#### Structure similarity search results (Foldseek)3

|  |  |
| --- | --- |
| Structure databases searched | Pdb, Afdb-proteome, Afdb-uniprot50 |
| Results, scheme(s)  (Top layers only, threshold 1.00e-02 (evalue)) | xml version="1.0" encoding="utf-8" standalone="no"?       2024-09-02T21:10:41.882959 image/svg+xml   Matplotlib v3.7.2, https://matplotlib.org/ |
| Results, table  (threshold 1.00e-02 (evalue)) | | db | id | prob | evalue | bits | fident | alnlen | mismatch | gapopen | qstart | qend | tstart | tend | name | description | | --- | --- | --- | --- | --- | --- | --- | --- | --- | --- | --- | --- | --- | --- | --- | | pdb | 6U5B\_N | 1.0 | 2.288e-35 | 1224 | 0.372 | 473 | 201 | 8 | 5 | 473 | 1 | 381 | Sheath PA0622 | Sheath PA0622 | | pdb | 8BKY\_A | 1.0 | 9.482e-19 | 574 | 0.228 | 490 | 229 | 19 | 3 | 466 | 1 | 367 | Phage tail sheath protein | Phage tail sheath protein | | pdb | 6RAO\_D | 1.0 | 7.019e-18 | 536 | 0.161 | 484 | 256 | 17 | 5 | 466 | 2 | 357 | Afp3 | Afp3 | | pdb | 6RC8\_B | 1.0 | 4.835e-16 | 477 | 0.163 | 477 | 263 | 20 | 5 | 468 | 2 | 355 | Afp3 | Afp3 | | pdb | 6J0N\_H | 1.0 | 1.908e-15 | 452 | 0.137 | 493 | 256 | 21 | 3 | 468 | 2 | 352 | Pvc2 | Pvc2 | | pdb | 6J0N\_V | 1.0 | 8.089e-16 | 449 | 0.134 | 514 | 269 | 22 | 1 | 468 | 5 | 388 | Pvc3 | Pvc3 | | pdb | 8BL4\_D | 1.0 | 5.341e-15 | 437 | 0.199 | 457 | 246 | 23 | 12 | 448 | 8 | 364 | Phage tail protein | Phage tail protein | | pdb | 6RGL\_A | 1.0 | 2.166e-12 | 384 | 0.141 | 481 | 256 | 20 | 7 | 466 | 4 | 348 | Afp2 | Afp2 | | pdb | 6J0N\_T | 1.0 | 2.363e-14 | 374 | 0.142 | 526 | 254 | 20 | 9 | 468 | 5 | 399 | Pvc3 | Pvc3 | | pdb | 7AEK\_3A | 1.0 | 3.373e-11 | 365 | 0.229 | 192 | 136 | 5 | 285 | 466 | 465 | 654 | Putative phage tail sheath protein FI | Putative phage tail sheath protein FI | | pdb | 7AE0\_3A | 1.0 | 2.649e-14 | 363 | 0.142 | 611 | 274 | 25 | 4 | 466 | 1 | 509 | Phage tail protein | Phage tail protein | | pdb | 7B5H\_AO | 1.0 | 5.572e-14 | 360 | 0.144 | 581 | 270 | 25 | 3 | 466 | 1 | 471 | All3325 protein | All3325 protein | | pdb | 7B5H\_AP | 1.0 | 1.295e-12 | 317 | 0.147 | 585 | 271 | 29 | 3 | 466 | 1 | 478 | All3325 protein | All3325 protein | | pdb | 6RGL\_D | 1.0 | 3.945e-10 | 283 | 0.156 | 505 | 264 | 25 | 1 | 468 | 4 | 383 | Afp4 | Afp4 | | pdb | 8JAN\_N | 1.0 | 6.8e-12 | 251 | 0.131 | 578 | 322 | 38 | 7 | 466 | 12 | 527 | Gp22 | Gp22 | | pdb | 6GKW\_A | 1.0 | 2.492e-06 | 177 | 0.117 | 468 | 220 | 27 | 28 | 453 | 23 | 339 | Putative phage XkdK-like protein | Putative phage XkdK-like protein | | pdb | 8AU1\_A | 1.0 | 3.718e-06 | 157 | 0.106 | 282 | 193 | 14 | 231 | 472 | 425 | 687 | Putative tail sheath protein | Putative tail sheath protein | | pdb | 5N8N\_B | 1.0 | 4.674e-06 | 150 | 0.11 | 327 | 187 | 22 | 231 | 467 | 141 | 453 | EvpB family type VI secretion protein | EvpB family type VI secretion protein | | pdb | 5URX\_1B | 1.0 | 6.222e-06 | 140 | 0.116 | 334 | 180 | 21 | 231 | 468 | 114 | 428 | TssC | TssC | | pdb | 7KH1\_A6 | 1.0 | 2.099e-06 | 130 | 0.107 | 603 | 294 | 44 | 1 | 465 | 1 | 497 | tail sheath protein, gp6 | tail sheath protein, gp6 | | pdb | 3J9O\_D | 1.0 | 0.0001026 | 128 | 0.07 | 340 | 201 | 18 | 231 | 474 | 103 | 423 | Intracellular growth locus protein B | Intracellular growth locus protein B | | pdb | 3J9G\_B | 1.0 | 4.605e-05 | 127 | 0.083 | 334 | 191 | 20 | 231 | 468 | 114 | 428 | VipB | VipB | | pdb | 8HDR\_M | 1.0 | 9.148e-05 | 125 | 0.111 | 509 | 268 | 31 | 8 | 466 | 8 | 381 | Pam3 sheath protein | Pam3 sheath protein | | pdb | 5MXN\_A | 1.0 | 0.0001924 | 113 | 0.088 | 328 | 196 | 18 | 231 | 468 | 158 | 472 | Type VI secretion protein | Type VI secretion protein | | pdb | 5OJQ\_D | 1.0 | 0.0001924 | 113 | 0.088 | 328 | 196 | 18 | 231 | 468 | 158 | 472 | Type VI secretion protein | Type VI secretion protein | | pdb | 8FVG\_A | 1.0 | 4.949e-06 | 108 | 0.087 | 586 | 321 | 36 | 1 | 466 | 1 | 492 | Sheath protein gp31 | Sheath protein gp31 | | pdb | 3J2M\_U | 0.999 | 0.0009014 | 98 | 0.098 | 649 | 248 | 30 | 26 | 422 | 1 | 564 | Tail sheath protein Gp18 | Tail sheath protein Gp18 | | pdb | 8ENV\_E | 0.997 | 2.914e-05 | 91 | 0.096 | 593 | 304 | 39 | 6 | 467 | 10 | 501 | Structural protein gp45 | Structural protein gp45 | | afdb-proteome | AF-G3XD39-F1-MODEL\_V4 | 1.0 | 1.989e-44 | 1677 | 0.372 | 473 | 201 | 8 | 5 | 473 | 2 | 382 | Probable bacteriophage protein | Probable bacteriophage protein | | afdb-proteome | AF-A0A0H3GRZ6-F1-MODEL\_V4 | 1.0 | 2.361e-44 | 1663 | 0.339 | 477 | 220 | 8 | 3 | 474 | 1 | 387 | Putative prophage tail sheath | Putative prophage tail sheath | | afdb-proteome | AF-A0A0H3GQS2-F1-MODEL\_V4 | 1.0 | 8.541e-42 | 1591 | 0.323 | 476 | 229 | 6 | 1 | 474 | 1 | 385 | Phage tail sheath protein | Phage tail sheath protein | | afdb-proteome | AF-Q8ZMV0-F1-MODEL\_V4 | 1.0 | 2.133e-41 | 1578 | 0.327 | 474 | 227 | 5 | 3 | 474 | 2 | 385 | Fels-2 prophage protein | Fels-2 prophage protein | | afdb-proteome | AF-Q8ZKJ2-F1-MODEL\_V4 | 1.0 | 5.16e-35 | 1011 | 0.252 | 547 | 261 | 21 | 1 | 474 | 1 | 472 | Putative phage tail sheath protein | Putative phage tail sheath protein | | afdb-proteome | AF-P44233-F1-MODEL\_V4 | 1.0 | 3.131e-09 | 194 | 0.103 | 579 | 302 | 35 | 2 | 466 | 11 | 486 | Mu-like prophage FluMu tail sheath protein | Mu-like prophage FluMu tail sheath protein | | afdb-proteome | AF-A0A0H3GRW7-F1-MODEL\_V4 | 1.0 | 2.382e-06 | 181 | 0.104 | 517 | 270 | 27 | 6 | 468 | 1 | 378 | Putative bacteriophage protein | Putative bacteriophage protein | | afdb-proteome | AF-Q9I748-F1-MODEL\_V4 | 1.0 | 5.086e-07 | 168 | 0.104 | 335 | 196 | 21 | 231 | 475 | 178 | 498 | Type VI secretion system sheath protein TssC1 | Type VI secretion system sheath protein TssC1 | | afdb-proteome | AF-Q8ZRL7-F1-MODEL\_V4 | 1.0 | 6.77e-07 | 166 | 0.119 | 343 | 185 | 23 | 231 | 475 | 179 | 502 | Putative cytoplasmic protein | Putative cytoplasmic protein | | afdb-proteome | AF-Q9I1B3-F1-MODEL\_V4 | 1.0 | 1.01e-06 | 156 | 0.119 | 334 | 177 | 19 | 231 | 468 | 175 | 487 | Uricase PuuD | Uricase PuuD | | afdb-proteome | AF-A0A0H3GSE9-F1-MODEL\_V4 | 1.0 | 5.306e-06 | 152 | 0.091 | 328 | 194 | 21 | 230 | 468 | 196 | 508 | EvpB/family type VI secretion protein | EvpB/family type VI secretion protein | | afdb-proteome | AF-Q9I367-F1-MODEL\_V4 | 1.0 | 5.618e-06 | 142 | 0.093 | 331 | 191 | 19 | 231 | 468 | 173 | 487 | Uncharacterized protein | Uncharacterized protein | | afdb-uniprot50 | AF-A0A1T3BL82-F1-MODEL\_V4 | 1.0 | 1.03e-70 | 2600 | 0.585 | 473 | 185 | 7 | 1 | 473 | 1 | 462 | Uncharacterized protein | Uncharacterized protein | | afdb-uniprot50 | AF-A0A2G6HAE9-F1-MODEL\_V4 | 1.0 | 1.716e-62 | 2511 | 0.573 | 471 | 122 | 3 | 5 | 475 | 2 | 393 | Phage tail protein | Phage tail protein | | afdb-uniprot50 | AF-V7HPP7-F1-MODEL\_V4 | 1.0 | 1.745e-67 | 2446 | 0.606 | 478 | 168 | 10 | 1 | 473 | 1 | 463 | Tail sheath protein | Tail sheath protein | | afdb-uniprot50 | AF-A0A522WEK9-F1-MODEL\_V4 | 1.0 | 1.982e-64 | 2352 | 0.522 | 475 | 212 | 9 | 3 | 474 | 2 | 464 | Uncharacterized protein | Uncharacterized protein | | afdb-uniprot50 | AF-A0A7S8HDT1-F1-MODEL\_V4 | 1.0 | 1.982e-64 | 2330 | 0.488 | 473 | 232 | 5 | 3 | 474 | 2 | 465 | Phage tail sheath subtilisin-like domain-containing protein | Phage tail sheath subtilisin-like domain-containing protein | | afdb-uniprot50 | AF-A0A212KBR8-F1-MODEL\_V4 | 1.0 | 5.548e-64 | 2330 | 0.511 | 475 | 217 | 9 | 3 | 474 | 2 | 464 | Putative phage tail protein | Putative phage tail protein | | afdb-uniprot50 | AF-A0A3R8TA92-F1-MODEL\_V4 | 1.0 | 1.287e-58 | 2302 | 0.539 | 471 | 132 | 7 | 3 | 473 | 1 | 386 | Phage tail protein | Phage tail protein | | afdb-uniprot50 | AF-A0A524RVX4-F1-MODEL\_V4 | 1.0 | 9.543e-62 | 2282 | 0.466 | 476 | 238 | 7 | 5 | 473 | 4 | 470 | Phage tail protein | Phage tail protein | | afdb-uniprot50 | AF-A0A5M6IF21-F1-MODEL\_V4 | 1.0 | 2.523e-61 | 2272 | 0.564 | 473 | 127 | 4 | 1 | 473 | 52 | 445 | Phage tail protein | Phage tail protein | | afdb-uniprot50 | AF-A0A2V3UCK1-F1-MODEL\_V4 | 1.0 | 1.978e-60 | 2261 | 0.465 | 481 | 238 | 9 | 3 | 474 | 2 | 472 | Uncharacterized protein | Uncharacterized protein | | afdb-uniprot50 | AF-A0A5A9EN65-F1-MODEL\_V4 | 1.0 | 2.125e-61 | 2242 | 0.433 | 625 | 193 | 10 | 3 | 475 | 2 | 617 | Phage tail protein | Phage tail protein | | afdb-uniprot50 | AF-K5XWL1-F1-MODEL\_V4 | 1.0 | 9.956e-61 | 2238 | 0.508 | 482 | 217 | 7 | 3 | 474 | 2 | 473 | Phage\_sheath\_1 domain-containing protein | Phage\_sheath\_1 domain-containing protein | | afdb-uniprot50 | AF-A0A812QV43-F1-MODEL\_V4 | 1.0 | 7.37e-60 | 2168 | 0.554 | 474 | 125 | 3 | 1 | 473 | 1 | 389 | GpFI protein | GpFI protein | | afdb-uniprot50 | AF-Q31HT2-F1-MODEL\_V4 | 1.0 | 8.352e-53 | 2082 | 0.431 | 473 | 179 | 4 | 3 | 473 | 2 | 386 | Phage tail sheath protein | Phage tail sheath protein | | afdb-uniprot50 | AF-A0A1M5PX14-F1-MODEL\_V4 | 1.0 | 1.362e-58 | 2076 | 0.429 | 477 | 259 | 8 | 1 | 474 | 1 | 467 | Uncharacterized protein | Uncharacterized protein | | afdb-uniprot50 | AF-A0A7W6RFE6-F1-MODEL\_V4 | 1.0 | 7.788e-56 | 2065 | 0.425 | 482 | 252 | 12 | 5 | 474 | 3 | 471 | Phage\_sheath\_1 domain-containing protein | Phage\_sheath\_1 domain-containing protein | | afdb-uniprot50 | AF-A0A1A9VKH7-F1-MODEL\_V4 | 1.0 | 1.249e-56 | 2030 | 0.505 | 465 | 141 | 5 | 5 | 463 | 3 | 384 | Uncharacterized protein | Uncharacterized protein | | afdb-uniprot50 | AF-E2CJT2-F1-MODEL\_V4 | 1.0 | 1.731e-51 | 2025 | 0.405 | 471 | 190 | 5 | 3 | 473 | 2 | 382 | Phage tail sheath protein | Phage tail sheath protein | | afdb-uniprot50 | AF-A0A7V7KJN4-F1-MODEL\_V4 | 1.0 | 2.09e-56 | 2014 | 0.448 | 587 | 128 | 5 | 3 | 475 | 1 | 505 | Phage tail protein | Phage tail protein | | afdb-uniprot50 | AF-A0A8A8PHF1-F1-MODEL\_V4 | 1.0 | 4.151e-56 | 2007 | 0.453 | 587 | 125 | 5 | 3 | 475 | 1 | 505 | Phage tail protein | Phage tail protein | | afdb-uniprot50 | AF-A0A149SWC0-F1-MODEL\_V4 | 1.0 | 5.296e-57 | 1990 | 0.441 | 491 | 247 | 13 | 3 | 474 | 2 | 484 | Uncharacterized protein | Uncharacterized protein | | afdb-uniprot50 | AF-A0A2W5KVX7-F1-MODEL\_V4 | 1.0 | 1.734e-55 | 1983 | 0.429 | 584 | 136 | 5 | 5 | 474 | 2 | 502 | Phage tail protein | Phage tail protein | | afdb-uniprot50 | AF-F4BFQ2-F1-MODEL\_V4 | 1.0 | 2.695e-50 | 1977 | 0.421 | 474 | 181 | 8 | 5 | 475 | 2 | 385 | Uncharacterized protein | Uncharacterized protein | | afdb-uniprot50 | AF-A0A7R9HD16-F1-MODEL\_V4 | 1.0 | 2.741e-55 | 1974 | 0.504 | 472 | 142 | 5 | 2 | 467 | 100 | 485 | Hypothetical protein | Hypothetical protein | | afdb-uniprot50 | AF-A0A167FZT2-F1-MODEL\_V4 | 1.0 | 2.241e-53 | 1955 | 0.476 | 510 | 147 | 10 | 3 | 473 | 1 | 429 | Tail protein | Tail protein | | afdb-uniprot50 | AF-A0A6N7JIY2-F1-MODEL\_V4 | 1.0 | 1.262e-49 | 1932 | 0.416 | 471 | 188 | 6 | 5 | 474 | 2 | 386 | Phage tail protein | Phage tail protein | | afdb-uniprot50 | AF-A0A7R9HA11-F1-MODEL\_V4 | 1.0 | 6.104e-55 | 1920 | 0.507 | 467 | 136 | 7 | 13 | 474 | 422 | 799 | Hypothetical protein | Hypothetical protein | | afdb-uniprot50 | AF-A0A432QUT0-F1-MODEL\_V4 | 1.0 | 5.444e-55 | 1918 | 0.4 | 475 | 271 | 8 | 1 | 474 | 1 | 462 | Uncharacterized protein | Uncharacterized protein | | afdb-uniprot50 | AF-A0A345DE55-F1-MODEL\_V4 | 1.0 | 1.109e-48 | 1915 | 0.388 | 479 | 201 | 5 | 1 | 475 | 1 | 391 | Prophage major tail sheath protein | Prophage major tail sheath protein | | afdb-uniprot50 | AF-A0A239EII1-F1-MODEL\_V4 | 1.0 | 9.915e-53 | 1902 | 0.412 | 587 | 146 | 4 | 3 | 474 | 2 | 504 | Uncharacterized protein | Uncharacterized protein | | afdb-uniprot50 | AF-A0A410UFB0-F1-MODEL\_V4 | 1.0 | 1.705e-50 | 1900 | 0.449 | 478 | 180 | 7 | 1 | 473 | 1 | 400 | Phage tail protein | Phage tail protein | | afdb-uniprot50 | AF-A0A423PRQ5-F1-MODEL\_V4 | 1.0 | 1.779e-49 | 1898 | 0.401 | 478 | 207 | 8 | 1 | 474 | 1 | 403 | Tail protein | Tail protein | | afdb-uniprot50 | AF-A0A6M6IDP4-F1-MODEL\_V4 | 1.0 | 3.534e-49 | 1882 | 0.536 | 416 | 119 | 6 | 3 | 418 | 2 | 343 | Phage tail protein | Phage tail protein | | afdb-uniprot50 | AF-A0A0B6D4V4-F1-MODEL\_V4 | 1.0 | 1.455e-47 | 1852 | 0.365 | 473 | 209 | 5 | 3 | 474 | 1 | 383 | Phage tail sheath family protein | Phage tail sheath family protein | | afdb-uniprot50 | AF-A0A2N1ANM3-F1-MODEL\_V4 | 1.0 | 1.521e-50 | 1849 | 0.507 | 475 | 140 | 5 | 1 | 475 | 1 | 381 | Phage tail protein | Phage tail protein | | afdb-uniprot50 | AF-A0A3N2E0U0-F1-MODEL\_V4 | 1.0 | 1.607e-46 | 1825 | 0.372 | 470 | 206 | 4 | 5 | 474 | 2 | 382 | Uncharacterized protein | Uncharacterized protein | | afdb-uniprot50 | AF-A0A1G7PXY9-F1-MODEL\_V4 | 1.0 | 5.121e-47 | 1797 | 0.398 | 474 | 191 | 7 | 5 | 474 | 2 | 385 | Uncharacterized protein | Uncharacterized protein | | afdb-uniprot50 | AF-A0A5P9F317-F1-MODEL\_V4 | 1.0 | 3.798e-50 | 1787 | 0.354 | 589 | 179 | 6 | 1 | 475 | 1 | 502 | Phage tail sheath protein | Phage tail sheath protein | | afdb-uniprot50 | AF-A0A6L3Y4M8-F1-MODEL\_V4 | 1.0 | 4.918e-52 | 1782 | 0.412 | 480 | 258 | 14 | 1 | 474 | 1 | 462 | Phage tail protein | Phage tail protein | | afdb-uniprot50 | AF-A0A1Y1QXK5-F1-MODEL\_V4 | 1.0 | 1.496e-45 | 1777 | 0.348 | 471 | 214 | 6 | 5 | 474 | 2 | 380 | Phage\_sheath\_1 domain-containing protein | Phage\_sheath\_1 domain-containing protein | | afdb-uniprot50 | AF-A0A2E3N1I9-F1-MODEL\_V4 | 1.0 | 2.977e-49 | 1774 | 0.435 | 482 | 178 | 11 | 1 | 475 | 1 | 395 | Phage tail protein | Phage tail protein | | afdb-uniprot50 | AF-A0A258L5K8-F1-MODEL\_V4 | 1.0 | 1.377e-51 | 1774 | 0.379 | 496 | 269 | 14 | 1 | 474 | 1 | 479 | Uncharacterized protein | Uncharacterized protein | | afdb-uniprot50 | AF-A0A8B2NUE5-F1-MODEL\_V4 | 1.0 | 3.247e-51 | 1760 | 0.345 | 492 | 291 | 12 | 1 | 474 | 1 | 479 | Phage tail protein | Phage tail protein | | afdb-uniprot50 | AF-A0A3G4V9H2-F1-MODEL\_V4 | 1.0 | 2.363e-45 | 1754 | 0.336 | 475 | 225 | 5 | 3 | 475 | 2 | 388 | Phage tail protein | Phage tail protein | | afdb-uniprot50 | AF-A0A2N3KSI6-F1-MODEL\_V4 | 1.0 | 8.807e-45 | 1742 | 0.334 | 478 | 227 | 4 | 1 | 475 | 3 | 392 | Phage tail protein | Phage tail protein | | afdb-uniprot50 | AF-A0A847KPL0-F1-MODEL\_V4 | 1.0 | 7.232e-51 | 1737 | 0.374 | 475 | 271 | 14 | 5 | 474 | 2 | 455 | Phage tail protein | Phage tail protein | | afdb-uniprot50 | AF-A0A2S6N2V9-F1-MODEL\_V4 | 1.0 | 6.249e-45 | 1689 | 0.343 | 477 | 224 | 9 | 4 | 474 | 3 | 396 | Uncharacterized protein | Uncharacterized protein | | afdb-uniprot50 | AF-A0A258CZQ8-F1-MODEL\_V4 | 1.0 | 3.199e-50 | 1686 | 0.321 | 489 | 300 | 14 | 1 | 474 | 1 | 472 | Phage tail protein | Phage tail protein | | afdb-uniprot50 | AF-A0A1D2QS78-F1-MODEL\_V4 | 1.0 | 4.134e-48 | 1672 | 0.403 | 552 | 156 | 7 | 5 | 475 | 2 | 460 | Uncharacterized protein | Uncharacterized protein | | afdb-uniprot50 | AF-A0A2W5K1C0-F1-MODEL\_V4 | 1.0 | 5.186e-44 | 1670 | 0.523 | 403 | 117 | 4 | 72 | 474 | 3 | 330 | Phage tail protein | Phage tail protein | | afdb-uniprot50 | AF-A0A7T8NTW5-F1-MODEL\_V4 | 1.0 | 3.68e-44 | 1669 | 0.337 | 480 | 228 | 8 | 1 | 474 | 1 | 396 | Phage tail sheath subtilisin-like domain-containing protein | Phage tail sheath subtilisin-like domain-containing protein | | afdb-uniprot50 | AF-A0A6L7FX52-F1-MODEL\_V4 | 1.0 | 5.492e-44 | 1644 | 0.352 | 476 | 215 | 9 | 1 | 474 | 1 | 385 | Phage tail protein | Phage tail protein | | afdb-uniprot50 | AF-A0A774N4X1-F1-MODEL\_V4 | 1.0 | 2.394e-42 | 1634 | 0.32 | 474 | 230 | 5 | 3 | 474 | 2 | 385 | Phage tail protein | Phage tail protein | | afdb-uniprot50 | AF-A0A4R1K4A7-F1-MODEL\_V4 | 1.0 | 8.568e-47 | 1617 | 0.359 | 587 | 163 | 8 | 3 | 475 | 1 | 488 | Uncharacterized protein | Uncharacterized protein | | afdb-uniprot50 | AF-A0A285ZDJ9-F1-MODEL\_V4 | 1.0 | 6.329e-42 | 1601 | 0.348 | 477 | 214 | 7 | 3 | 475 | 1 | 384 | Uncharacterized protein | Uncharacterized protein | | afdb-uniprot50 | AF-A0A482NKP0-F1-MODEL\_V4 | 1.0 | 2.104e-41 | 1596 | 0.316 | 474 | 232 | 5 | 3 | 474 | 1 | 384 | Phage tail sheath protein | Phage tail sheath protein | | afdb-uniprot50 | AF-A0A7G7RNU6-F1-MODEL\_V4 | 1.0 | 3.783e-42 | 1583 | 0.321 | 479 | 227 | 7 | 3 | 473 | 2 | 390 | Phage tail sheath protein | Phage tail sheath protein | | afdb-uniprot50 | AF-A0A367WTL7-F1-MODEL\_V4 | 1.0 | 3.52e-41 | 1581 | 0.312 | 474 | 233 | 6 | 3 | 474 | 2 | 384 | Tail sheath protein | Tail sheath protein | | afdb-uniprot50 | AF-G8LQ42-F1-MODEL\_V4 | 1.0 | 1.933e-43 | 1580 | 0.329 | 480 | 222 | 6 | 5 | 474 | 39 | 428 | Tail Sheath Protein | Tail Sheath Protein | | afdb-uniprot50 | AF-Q8ZMV0-F1-MODEL\_V4 | 1.0 | 7.839e-41 | 1578 | 0.327 | 474 | 227 | 5 | 3 | 474 | 2 | 385 | Fels-2 prophage protein | Fels-2 prophage protein | | afdb-uniprot50 | AF-A0A2W6YVE1-F1-MODEL\_V4 | 1.0 | 1.077e-46 | 1578 | 0.349 | 498 | 286 | 16 | 3 | 474 | 1 | 486 | Phage tail protein | Phage tail protein | | afdb-uniprot50 | AF-Q5D5G3-F1-MODEL\_V4 | 1.0 | 3.093e-40 | 1570 | 0.464 | 377 | 115 | 5 | 19 | 395 | 2 | 291 | Phage tail sheath protein | Phage tail sheath protein | | afdb-uniprot50 | AF-A0A1N7LRF5-F1-MODEL\_V4 | 1.0 | 9.854e-41 | 1562 | 0.314 | 477 | 233 | 8 | 1 | 474 | 3 | 388 | Uncharacterized protein | Uncharacterized protein | | afdb-uniprot50 | AF-A0A350LX90-F1-MODEL\_V4 | 1.0 | 1.059e-41 | 1555 | 0.305 | 472 | 240 | 8 | 4 | 474 | 3 | 387 | Phage tail protein | Phage tail protein | | afdb-uniprot50 | AF-A0A836RQY2-F1-MODEL\_V4 | 1.0 | 2.606e-40 | 1538 | 0.397 | 428 | 168 | 5 | 48 | 473 | 1 | 340 | Phage tail protein | Phage tail protein | | afdb-uniprot50 | AF-A0A3R1AVG7-F1-MODEL\_V4 | 1.0 | 2.073e-40 | 1536 | 0.314 | 477 | 234 | 5 | 1 | 474 | 1 | 387 | Phage tail sheath protein | Phage tail sheath protein | | afdb-uniprot50 | AF-A0A806D7P7-F1-MODEL\_V4 | 1.0 | 6.144e-40 | 1534 | 0.284 | 471 | 244 | 5 | 5 | 474 | 2 | 380 | Tail sheath protein | Tail sheath protein | | afdb-uniprot50 | AF-A0A7W4X2I7-F1-MODEL\_V4 | 1.0 | 2.135e-42 | 1528 | 0.508 | 362 | 101 | 5 | 3 | 364 | 2 | 286 | Phage tail sheath protein FI | Phage tail sheath protein FI | | afdb-uniprot50 | AF-A0A3M4LWQ9-F1-MODEL\_V4 | 1.0 | 1.933e-43 | 1519 | 0.366 | 477 | 206 | 7 | 2 | 474 | 73 | 457 | Major tail sheath protein | Major tail sheath protein | | afdb-uniprot50 | AF-A0A1B2R8T9-F1-MODEL\_V4 | 1.0 | 7.295e-40 | 1501 | 0.292 | 476 | 243 | 7 | 3 | 474 | 2 | 387 | Phage tail protein | Phage tail protein | | afdb-uniprot50 | AF-A0A376QG66-F1-MODEL\_V4 | 1.0 | 2.016e-42 | 1500 | 0.333 | 480 | 222 | 8 | 3 | 474 | 1 | 390 | Major tail sheath protein FI | Major tail sheath protein FI | | afdb-uniprot50 | AF-A0A369Z1S6-F1-MODEL\_V4 | 1.0 | 1.449e-39 | 1498 | 0.28 | 475 | 249 | 6 | 3 | 474 | 2 | 386 | Phage tail protein | Phage tail protein | | afdb-uniprot50 | AF-A0A2G3K3C7-F1-MODEL\_V4 | 1.0 | 2.227e-41 | 1496 | 0.294 | 510 | 232 | 8 | 3 | 474 | 2 | 421 | Phage tail protein | Phage tail protein | | afdb-uniprot50 | AF-A0A0C2IBA1-F1-MODEL\_V4 | 1.0 | 1.698e-42 | 1493 | 0.327 | 473 | 226 | 5 | 4 | 474 | 84 | 466 | Phage tail sheath monomer | Phage tail sheath monomer | | afdb-uniprot50 | AF-A0A0K1NER8-F1-MODEL\_V4 | 1.0 | 1.849e-40 | 1492 | 0.291 | 481 | 241 | 7 | 3 | 474 | 1 | 390 | Uncharacterized protein | Uncharacterized protein | | afdb-uniprot50 | AF-A0A0Q8XV39-F1-MODEL\_V4 | 1.0 | 2.718e-39 | 1491 | 0.3 | 479 | 239 | 8 | 1 | 474 | 1 | 388 | Phage tail protein | Phage tail protein | | afdb-uniprot50 | AF-A0A348HI83-F1-MODEL\_V4 | 1.0 | 1.428e-38 | 1483 | 0.3 | 476 | 240 | 6 | 1 | 474 | 1 | 385 | Phage tail sheath protein FI | Phage tail sheath protein FI | | afdb-uniprot50 | AF-A0A4P9VGN2-F1-MODEL\_V4 | 1.0 | 4.816e-39 | 1478 | 0.305 | 478 | 230 | 9 | 3 | 474 | 2 | 383 | Phage tail protein | Phage tail protein | | afdb-uniprot50 | AF-A0A2M8RTB4-F1-MODEL\_V4 | 1.0 | 2.718e-39 | 1476 | 0.271 | 475 | 253 | 7 | 3 | 474 | 2 | 386 | Phage tail protein | Phage tail protein | | afdb-uniprot50 | AF-A0A6B8KJH9-F1-MODEL\_V4 | 1.0 | 1.043e-40 | 1475 | 0.319 | 485 | 237 | 12 | 1 | 474 | 1 | 403 | Phage tail protein | Phage tail protein | | afdb-uniprot50 | AF-A0A1A9RDH2-F1-MODEL\_V4 | 1.0 | 1.449e-39 | 1472 | 0.297 | 474 | 240 | 5 | 3 | 474 | 2 | 384 | Phage tail protein | Phage tail protein | | afdb-uniprot50 | AF-B1H6B6-F1-MODEL\_V4 | 1.0 | 2.389e-38 | 1470 | 0.305 | 451 | 221 | 5 | 25 | 473 | 1 | 361 | Phage tail sheath protein | Phage tail sheath protein | | afdb-uniprot50 | AF-A0A7W7Z2X1-F1-MODEL\_V4 | 1.0 | 3.186e-42 | 1468 | 0.307 | 485 | 310 | 16 | 1 | 474 | 1 | 470 | Uncharacterized protein | Uncharacterized protein | | afdb-uniprot50 | AF-A0A2D9ER23-F1-MODEL\_V4 | 1.0 | 2.131e-38 | 1458 | 0.298 | 476 | 240 | 7 | 3 | 474 | 1 | 386 | Phage tail protein | Phage tail protein | | afdb-uniprot50 | AF-A0A377H6F5-F1-MODEL\_V4 | 1.0 | 6.054e-39 | 1457 | 0.306 | 483 | 234 | 8 | 3 | 474 | 2 | 394 | Phage tail sheath protein | Phage tail sheath protein | | afdb-uniprot50 | AF-A0A1T4W573-F1-MODEL\_V4 | 1.0 | 1.601e-38 | 1456 | 0.292 | 478 | 239 | 7 | 3 | 474 | 2 | 386 | Uncharacterized protein | Uncharacterized protein | | afdb-uniprot50 | AF-A0A379C9Z4-F1-MODEL\_V4 | 1.0 | 1.136e-38 | 1453 | 0.272 | 473 | 251 | 5 | 3 | 474 | 2 | 382 | Phage tail sheath protein | Phage tail sheath protein | | afdb-uniprot50 | AF-A0A017HDD9-F1-MODEL\_V4 | 1.0 | 8.678e-44 | 1451 | 0.334 | 479 | 290 | 15 | 3 | 474 | 2 | 458 | Phage\_sheath\_1 domain-containing protein | Phage\_sheath\_1 domain-containing protein | | afdb-uniprot50 | AF-A0A545T5U0-F1-MODEL\_V4 | 1.0 | 7.096e-42 | 1449 | 0.344 | 540 | 182 | 9 | 5 | 475 | 3 | 439 | Phage tail protein | Phage tail protein | | afdb-uniprot50 | AF-A0A7W2BMI1-F1-MODEL\_V4 | 1.0 | 1.625e-39 | 1441 | 0.287 | 487 | 245 | 10 | 3 | 474 | 2 | 401 | Phage tail sheath subtilisin-like domain-containing protein | Phage tail sheath subtilisin-like domain-containing protein | | afdb-uniprot50 | AF-A0A7W3DV09-F1-MODEL\_V4 | 1.0 | 1.013e-38 | 1439 | 0.283 | 484 | 243 | 9 | 3 | 474 | 2 | 393 | Phage tail sheath subtilisin-like domain-containing protein | Phage tail sheath subtilisin-like domain-containing protein | | afdb-uniprot50 | AF-A0A8B3UBL2-F1-MODEL\_V4 | 1.0 | 2.162e-39 | 1435 | 0.3 | 482 | 234 | 10 | 1 | 474 | 16 | 402 | Major tail sheath protein FI | Major tail sheath protein FI | | afdb-uniprot50 | AF-A0A0Q8AN78-F1-MODEL\_V4 | 1.0 | 6.054e-39 | 1425 | 0.267 | 486 | 258 | 8 | 1 | 474 | 1 | 400 | Uncharacterized protein | Uncharacterized protein | | afdb-uniprot50 | AF-A0A3P8KCN2-F1-MODEL\_V4 | 1.0 | 6.316e-38 | 1408 | 0.301 | 478 | 236 | 9 | 3 | 474 | 2 | 387 | Phage tail sheath protein | Phage tail sheath protein | | afdb-uniprot50 | AF-A0A779QIX5-F1-MODEL\_V4 | 1.0 | 6.688e-38 | 1404 | 0.303 | 464 | 222 | 10 | 19 | 474 | 2 | 372 | Phage tail protein | Phage tail protein | | afdb-uniprot50 | AF-A0A2W5GCK7-F1-MODEL\_V4 | 1.0 | 2.016e-42 | 1404 | 0.375 | 421 | 245 | 8 | 55 | 474 | 4 | 407 | Phage tail protein | Phage tail protein | | afdb-uniprot50 | AF-A0A286BYN5-F1-MODEL\_V4 | 1.0 | 1.105e-40 | 1404 | 0.324 | 472 | 227 | 6 | 3 | 472 | 1 | 382 | Phage tail tape measure protein, TP901 family, core region | Phage tail tape measure protein, TP901 family, core region | | afdb-uniprot50 | AF-A0A7X3ZJQ5-F1-MODEL\_V4 | 1.0 | 2.016e-42 | 1398 | 0.304 | 598 | 205 | 14 | 6 | 475 | 1 | 515 | Phage tail sheath family protein | Phage tail sheath family protein | | afdb-uniprot50 | AF-A0A084ZPR3-F1-MODEL\_V4 | 1.0 | 6.054e-39 | 1394 | 0.311 | 481 | 231 | 12 | 1 | 474 | 17 | 404 | Phage tail sheath monomer | Phage tail sheath monomer | | afdb-uniprot50 | AF-A0A0P6WF34-F1-MODEL\_V4 | 1.0 | 2.227e-41 | 1383 | 0.268 | 502 | 328 | 12 | 1 | 474 | 1 | 491 | Uncharacterized protein | Uncharacterized protein | | afdb-uniprot50 | AF-A0A8B5G355-F1-MODEL\_V4 | 1.0 | 4.675e-37 | 1376 | 0.332 | 445 | 199 | 8 | 3 | 439 | 1 | 355 | Phage tail protein | Phage tail protein | | afdb-uniprot50 | AF-A0A4R7GG91-F1-MODEL\_V4 | 1.0 | 3.317e-37 | 1376 | 0.275 | 483 | 248 | 10 | 3 | 474 | 2 | 393 | Phage\_sheath\_1 domain-containing protein | Phage\_sheath\_1 domain-containing protein | | afdb-uniprot50 | AF-A0A5T6QRP6-F1-MODEL\_V4 | 1.0 | 2.456e-36 | 1375 | 0.328 | 439 | 203 | 6 | 3 | 439 | 2 | 350 | Phage tail protein | Phage tail protein | | afdb-uniprot50 | AF-A0A4P9VGL6-F1-MODEL\_V4 | 1.0 | 1.218e-35 | 1365 | 0.614 | 244 | 93 | 1 | 231 | 473 | 12 | 255 | Uncharacterized protein | Uncharacterized protein | | afdb-uniprot50 | AF-A0A2X5SGI3-F1-MODEL\_V4 | 1.0 | 1.292e-39 | 1364 | 0.275 | 573 | 212 | 8 | 5 | 474 | 2 | 474 | Phage tail sheath protein | Phage tail sheath protein | | afdb-uniprot50 | AF-V4PWM7-F1-MODEL\_V4 | 1.0 | 5.469e-36 | 1363 | 0.28 | 470 | 231 | 7 | 6 | 474 | 1 | 364 | Tail sheath protein | Tail sheath protein | | afdb-uniprot50 | AF-A0A794DQD8-F1-MODEL\_V4 | 1.0 | 3.275e-40 | 1363 | 0.282 | 592 | 207 | 10 | 1 | 474 | 1 | 492 | Phage tail protein | Phage tail protein | | afdb-uniprot50 | AF-A0A2A4XUJ8-F1-MODEL\_V4 | 1.0 | 4.675e-37 | 1362 | 0.238 | 477 | 273 | 9 | 3 | 474 | 4 | 395 | Uncharacterized protein | Uncharacterized protein | | afdb-uniprot50 | AF-A0A7W6F1M4-F1-MODEL\_V4 | 1.0 | 6.59e-37 | 1361 | 0.243 | 476 | 265 | 7 | 3 | 474 | 1 | 385 | Phage\_sheath\_1 domain-containing protein | Phage\_sheath\_1 domain-containing protein | | afdb-uniprot50 | AF-A0A6N7F0I1-F1-MODEL\_V4 | 1.0 | 1.15e-35 | 1350 | 0.25 | 471 | 254 | 5 | 3 | 472 | 2 | 374 | Phage tail protein | Phage tail protein | | afdb-uniprot50 | AF-A0A375AAB9-F1-MODEL\_V4 | 1.0 | 1.203e-38 | 1349 | 0.307 | 478 | 235 | 9 | 1 | 474 | 47 | 432 | Phage tail sheath monomer | Phage tail sheath monomer | | afdb-uniprot50 | AF-A0A767WHR7-F1-MODEL\_V4 | 1.0 | 5.165e-36 | 1348 | 0.331 | 432 | 191 | 8 | 20 | 443 | 1 | 342 | Phage tail sheath protein | Phage tail sheath protein | | afdb-uniprot50 | AF-A0A0S2G9I8-F1-MODEL\_V4 | 1.0 | 2.038e-35 | 1348 | 0.315 | 444 | 211 | 5 | 33 | 474 | 2 | 354 | Major tail sheath protein | Major tail sheath protein | | afdb-uniprot50 | AF-A0A4R7SNF9-F1-MODEL\_V4 | 1.0 | 1.119e-37 | 1347 | 0.286 | 481 | 246 | 11 | 1 | 474 | 1 | 391 | Phage\_sheath\_1 domain-containing protein | Phage\_sheath\_1 domain-containing protein | | afdb-uniprot50 | AF-A0A1I6JLA7-F1-MODEL\_V4 | 1.0 | 1.645e-36 | 1346 | 0.288 | 472 | 241 | 7 | 5 | 474 | 2 | 380 | Phage\_sheath\_1 domain-containing protein | Phage\_sheath\_1 domain-containing protein | | afdb-uniprot50 | AF-A0A717IAE2-F1-MODEL\_V4 | 1.0 | 5.165e-36 | 1345 | 0.317 | 438 | 207 | 5 | 39 | 474 | 2 | 349 | Phage tail sheath protein | Phage tail sheath protein | | afdb-uniprot50 | AF-A0A1Y3NT02-F1-MODEL\_V4 | 1.0 | 2.753e-36 | 1339 | 0.352 | 454 | 186 | 11 | 1 | 443 | 1 | 357 | Phage tail protein | Phage tail protein | | afdb-uniprot50 | AF-A0A6N8Q1J1-F1-MODEL\_V4 | 1.0 | 4.287e-35 | 1337 | 0.32 | 424 | 196 | 5 | 3 | 424 | 2 | 335 | Phage tail protein | Phage tail protein | | afdb-uniprot50 | AF-A0A8A7KVG0-F1-MODEL\_V4 | 1.0 | 1.218e-35 | 1335 | 0.275 | 475 | 248 | 7 | 4 | 474 | 5 | 387 | Phage tail protein | Phage tail protein | | afdb-uniprot50 | AF-A0A4R3EE36-F1-MODEL\_V4 | 1.0 | 2.19e-36 | 1324 | 0.301 | 485 | 234 | 16 | 1 | 474 | 1 | 391 | Uncharacterized protein | Uncharacterized protein | | afdb-uniprot50 | AF-A0A6I2IBC8-F1-MODEL\_V4 | 1.0 | 7.187e-39 | 1321 | 0.277 | 590 | 208 | 10 | 5 | 474 | 1 | 491 | Phage tail protein | Phage tail protein | | afdb-uniprot50 | AF-A0A6L8HKK1-F1-MODEL\_V4 | 1.0 | 6.519e-44 | 1309 | 0.361 | 534 | 206 | 15 | 3 | 475 | 1 | 460 | Phage tail protein | Phage tail protein | | afdb-uniprot50 | AF-A0A7V8DAB9-F1-MODEL\_V4 | 1.0 | 1.925e-35 | 1308 | 0.274 | 474 | 240 | 8 | 5 | 474 | 2 | 375 | Phage tail sheath monomer | Phage tail sheath monomer | | afdb-uniprot50 | AF-A0A528LBW2-F1-MODEL\_V4 | 1.0 | 4.548e-39 | 1304 | 0.281 | 501 | 317 | 20 | 1 | 474 | 1 | 485 | Phage tail protein | Phage tail protein | | afdb-uniprot50 | AF-A0A212S7J7-F1-MODEL\_V4 | 1.0 | 3.18e-38 | 1303 | 0.29 | 493 | 310 | 18 | 3 | 474 | 1 | 474 | Uncharacterized protein | Uncharacterized protein | | afdb-uniprot50 | AF-A0A2S8RS47-F1-MODEL\_V4 | 1.0 | 2.959e-37 | 1302 | 0.297 | 470 | 234 | 8 | 1 | 468 | 1 | 376 | Uncharacterized protein | Uncharacterized protein | | afdb-uniprot50 | AF-A0A1I7J4F6-F1-MODEL\_V4 | 1.0 | 5.32e-38 | 1301 | 0.277 | 588 | 220 | 9 | 1 | 474 | 1 | 497 | Uncharacterized protein | Uncharacterized protein | | afdb-uniprot50 | AF-A0A704CVA7-F1-MODEL\_V4 | 1.0 | 1.2e-34 | 1297 | 0.315 | 437 | 207 | 5 | 40 | 474 | 1 | 347 | Phage tail sheath protein | Phage tail sheath protein | | afdb-uniprot50 | AF-A0A256CAX3-F1-MODEL\_V4 | 1.0 | 1.133e-34 | 1292 | 0.247 | 477 | 264 | 9 | 3 | 474 | 2 | 388 | Phage\_sheath\_1 domain-containing protein | Phage\_sheath\_1 domain-containing protein | | afdb-uniprot50 | AF-U4T476-F1-MODEL\_V4 | 1.0 | 1.531e-35 | 1283 | 0.253 | 489 | 253 | 13 | 3 | 473 | 1 | 395 | Major tail sheath protein | Major tail sheath protein | | afdb-uniprot50 | AF-A0A1D2QMV4-F1-MODEL\_V4 | 1.0 | 1.791e-34 | 1281 | 0.273 | 476 | 247 | 10 | 1 | 474 | 1 | 379 | Uncharacterized protein | Uncharacterized protein | | afdb-uniprot50 | AF-A0A2T5J1F6-F1-MODEL\_V4 | 1.0 | 8.902e-38 | 1278 | 0.256 | 600 | 228 | 8 | 3 | 474 | 2 | 511 | Uncharacterized protein DUF3383 | Uncharacterized protein DUF3383 | | afdb-uniprot50 | AF-A0A7Y1CWN9-F1-MODEL\_V4 | 1.0 | 3.989e-34 | 1268 | 0.384 | 385 | 151 | 3 | 92 | 475 | 7 | 306 | Phage tail sheath family protein | Phage tail sheath family protein | | afdb-uniprot50 | AF-A0A7C2J5J0-F1-MODEL\_V4 | 1.0 | 1.49e-37 | 1265 | 0.275 | 559 | 226 | 16 | 1 | 475 | 1 | 464 | Phage tail sheath family protein | Phage tail sheath family protein | | afdb-uniprot50 | AF-A0A5M7LJZ8-F1-MODEL\_V4 | 1.0 | 3.719e-37 | 1248 | 0.29 | 486 | 240 | 11 | 1 | 474 | 82 | 474 | Phage tail protein | Phage tail protein | | afdb-uniprot50 | AF-E2CFK5-F1-MODEL\_V4 | 1.0 | 7.067e-34 | 1246 | 0.253 | 478 | 260 | 13 | 1 | 474 | 1 | 385 | Putative tail sheath protein | Putative tail sheath protein | | afdb-uniprot50 | AF-A0A7V8JNR8-F1-MODEL\_V4 | 1.0 | 3.461e-36 | 1245 | 0.628 | 296 | 99 | 1 | 190 | 474 | 1 | 296 | Putative prophage major tail sheath protein | Putative prophage major tail sheath protein | | afdb-uniprot50 | AF-A0A246KG12-F1-MODEL\_V4 | 1.0 | 3.36e-34 | 1237 | 0.261 | 482 | 243 | 11 | 1 | 474 | 1 | 377 | Phage tail sheath protein | Phage tail sheath protein | | afdb-uniprot50 | AF-A0A2W6XD46-F1-MODEL\_V4 | 1.0 | 9.96e-34 | 1228 | 0.252 | 476 | 258 | 7 | 1 | 474 | 1 | 380 | Phage tail protein | Phage tail protein | | afdb-uniprot50 | AF-A0A2W5VPE1-F1-MODEL\_V4 | 1.0 | 1.117e-33 | 1227 | 0.244 | 483 | 245 | 11 | 3 | 474 | 2 | 375 | Phage tail protein | Phage tail protein | | afdb-uniprot50 | AF-A0A2E4CSI8-F1-MODEL\_V4 | 1.0 | 4.607e-36 | 1221 | 0.244 | 585 | 232 | 7 | 5 | 474 | 2 | 491 | Phage tail protein | Phage tail protein | | afdb-uniprot50 | AF-A0A4D7B771-F1-MODEL\_V4 | 1.0 | 3.311e-33 | 1217 | 0.232 | 486 | 274 | 10 | 1 | 474 | 1 | 399 | Uncharacterized protein | Uncharacterized protein | | afdb-uniprot50 | AF-A0A6C1BRA6-F1-MODEL\_V4 | 1.0 | 3.41e-35 | 1216 | 0.274 | 507 | 245 | 16 | 3 | 474 | 1 | 419 | Phage tail sheath family protein | Phage tail sheath family protein | | afdb-uniprot50 | AF-A0A6L5IG60-F1-MODEL\_V4 | 1.0 | 1.618e-31 | 1205 | 0.5 | 246 | 122 | 1 | 230 | 474 | 24 | 269 | Phage tail sheath family protein | Phage tail sheath family protein | | afdb-uniprot50 | AF-A0A4D7DC88-F1-MODEL\_V4 | 1.0 | 1.979e-33 | 1204 | 0.273 | 482 | 229 | 10 | 6 | 474 | 1 | 374 | Phage tail protein | Phage tail protein | | afdb-uniprot50 | AF-A0A2X5PXW7-F1-MODEL\_V4 | 1.0 | 1.642e-32 | 1186 | 0.311 | 433 | 200 | 8 | 50 | 474 | 1 | 343 | Major tail sheath protein FI | Major tail sheath protein FI | | afdb-uniprot50 | AF-A0A7B6D7K3-F1-MODEL\_V4 | 1.0 | 7.708e-36 | 1185 | 0.265 | 564 | 229 | 15 | 1 | 472 | 1 | 470 | Phage tail sheath family protein | Phage tail sheath family protein | | afdb-uniprot50 | AF-A0A4U0YPU6-F1-MODEL\_V4 | 1.0 | 1.893e-30 | 1184 | 0.502 | 245 | 121 | 1 | 231 | 474 | 2 | 246 | Phage tail protein | Phage tail protein | | afdb-uniprot50 | AF-A0A3D9UGV7-F1-MODEL\_V4 | 1.0 | 1.618e-31 | 1180 | 0.312 | 400 | 187 | 4 | 77 | 474 | 2 | 315 | Uncharacterized protein | Uncharacterized protein | | afdb-uniprot50 | AF-A0A2X2RMN3-F1-MODEL\_V4 | 1.0 | 1.921e-31 | 1176 | 0.309 | 426 | 193 | 7 | 50 | 474 | 1 | 326 | Phage tail sheath protein | Phage tail sheath protein | | afdb-uniprot50 | AF-A0A336QN02-F1-MODEL\_V4 | 1.0 | 6.303e-34 | 1174 | 0.32 | 434 | 194 | 6 | 42 | 474 | 67 | 400 | Phage tail sheath protein | Phage tail sheath protein | | afdb-uniprot50 | AF-A0A4V3BMQ0-F1-MODEL\_V4 | 1.0 | 6.889e-40 | 1172 | 0.25 | 660 | 290 | 21 | 3 | 474 | 1 | 643 | Phage tail sheath protein FI | Phage tail sheath protein FI | | afdb-uniprot50 | AF-A0A6J4N9R5-F1-MODEL\_V4 | 1.0 | 2.872e-35 | 1168 | 0.254 | 565 | 239 | 15 | 1 | 471 | 1 | 477 | Phage tail sheath monomer | Phage tail sheath monomer | | afdb-uniprot50 | AF-A0A376ZHD7-F1-MODEL\_V4 | 1.0 | 1.252e-33 | 1164 | 0.296 | 479 | 229 | 14 | 1 | 468 | 1 | 382 | Major tail sheath protein FI | Major tail sheath protein FI | | afdb-uniprot50 | AF-A0A7Z7Y9X6-F1-MODEL\_V4 | 1.0 | 1.509e-34 | 1160 | 0.26 | 591 | 225 | 12 | 1 | 475 | 1 | 495 | Phage tail protein | Phage tail protein | | afdb-uniprot50 | AF-A0A6M1HTF4-F1-MODEL\_V4 | 1.0 | 6.384e-31 | 1150 | 0.333 | 393 | 167 | 8 | 3 | 390 | 1 | 303 | Phage tail sheath protein | Phage tail sheath protein | | afdb-uniprot50 | AF-A0A371WU78-F1-MODEL\_V4 | 1.0 | 7.067e-34 | 1144 | 0.208 | 608 | 329 | 20 | 3 | 474 | 1 | 591 | Uncharacterized protein | Uncharacterized protein | | afdb-uniprot50 | AF-A0A1M5AJX8-F1-MODEL\_V4 | 1.0 | 3.454e-32 | 1141 | 0.245 | 480 | 253 | 12 | 4 | 474 | 5 | 384 | Uncharacterized protein | Uncharacterized protein | | afdb-uniprot50 | AF-A0A444K860-F1-MODEL\_V4 | 1.0 | 1.2e-34 | 1141 | 0.257 | 498 | 298 | 14 | 3 | 474 | 1 | 452 | Phage tail protein | Phage tail protein | | afdb-uniprot50 | AF-A0A3M2ZQ51-F1-MODEL\_V4 | 1.0 | 3.603e-31 | 1139 | 0.345 | 400 | 168 | 7 | 5 | 399 | 2 | 312 | Phage\_sheath\_1 domain-containing protein | Phage\_sheath\_1 domain-containing protein | | afdb-uniprot50 | AF-A0A136QAK0-F1-MODEL\_V4 | 1.0 | 1.114e-29 | 1135 | 0.51 | 243 | 118 | 1 | 233 | 474 | 3 | 245 | Uncharacterized protein | Uncharacterized protein | | afdb-uniprot50 | AF-A0A2G2D3E0-F1-MODEL\_V4 | 1.0 | 3.872e-32 | 1133 | 0.258 | 500 | 251 | 15 | 3 | 474 | 2 | 409 | Phage tail protein | Phage tail protein | | afdb-uniprot50 | AF-A0A7Y7RNU4-F1-MODEL\_V4 | 1.0 | 6.861e-32 | 1130 | 0.236 | 483 | 261 | 11 | 1 | 474 | 1 | 384 | Phage tail protein | Phage tail protein | | afdb-uniprot50 | AF-A0A485D547-F1-MODEL\_V4 | 1.0 | 6.76e-31 | 1125 | 0.297 | 393 | 183 | 6 | 89 | 474 | 2 | 308 | Phage tail sheath protein | Phage tail sheath protein | | afdb-uniprot50 | AF-A0A061JGM3-F1-MODEL\_V4 | 1.0 | 1.921e-31 | 1124 | 0.498 | 331 | 82 | 4 | 3 | 333 | 2 | 248 | Major tail sheath protein | Major tail sheath protein | | afdb-uniprot50 | AF-A0A2A5BNT9-F1-MODEL\_V4 | 1.0 | 2.866e-31 | 1117 | 0.25 | 476 | 246 | 13 | 5 | 475 | 2 | 371 | Phage tail protein | Phage tail protein | | afdb-uniprot50 | AF-A0A2X3JUE5-F1-MODEL\_V4 | 1.0 | 3.214e-31 | 1116 | 0.309 | 423 | 194 | 11 | 1 | 417 | 1 | 331 | Major tail sheath protein FI | Major tail sheath protein FI | | afdb-uniprot50 | AF-B3QTJ6-F1-MODEL\_V4 | 1.0 | 3.712e-33 | 1108 | 0.249 | 521 | 236 | 17 | 1 | 475 | 1 | 412 | Tail sheath protein | Tail sheath protein | | afdb-uniprot50 | AF-A0A285NJC0-F1-MODEL\_V4 | 1.0 | 2.034e-31 | 1104 | 0.221 | 496 | 257 | 12 | 3 | 474 | 1 | 391 | Phage\_sheath\_1 domain-containing protein | Phage\_sheath\_1 domain-containing protein | | afdb-uniprot50 | AF-A0A1J5HQE5-F1-MODEL\_V4 | 1.0 | 2.122e-30 | 1102 | 0.244 | 491 | 257 | 13 | 1 | 474 | 1 | 394 | Phage\_sheath\_1C domain-containing protein | Phage\_sheath\_1C domain-containing protein | | afdb-uniprot50 | AF-A0A1T4UYU3-F1-MODEL\_V4 | 1.0 | 6.384e-31 | 1101 | 0.215 | 483 | 268 | 14 | 1 | 474 | 5 | 385 | Phage tail sheath protein | Phage tail sheath protein | | afdb-uniprot50 | AF-A0A7K1PIA1-F1-MODEL\_V4 | 1.0 | 2.28e-31 | 1098 | 0.253 | 498 | 259 | 14 | 3 | 474 | 1 | 411 | Phage tail protein | Phage tail protein | | afdb-uniprot50 | AF-A0A3D1NSE5-F1-MODEL\_V4 | 1.0 | 9.527e-31 | 1096 | 0.253 | 494 | 248 | 14 | 3 | 471 | 1 | 398 | Phage\_sheath\_1 domain-containing protein | Phage\_sheath\_1 domain-containing protein | | afdb-uniprot50 | AF-A0A839IZ43-F1-MODEL\_V4 | 1.0 | 1.528e-31 | 1093 | 0.234 | 482 | 255 | 12 | 1 | 474 | 9 | 384 | Phage tail protein | Phage tail protein | | afdb-uniprot50 | AF-A0A5M8P666-F1-MODEL\_V4 | 1.0 | 1.443e-31 | 1086 | 0.232 | 507 | 268 | 15 | 3 | 474 | 1 | 421 | Phage tail protein | Phage tail protein | | afdb-uniprot50 | AF-A0A355TSY3-F1-MODEL\_V4 | 1.0 | 7.373e-33 | 1086 | 0.238 | 557 | 228 | 13 | 20 | 471 | 2 | 467 | Phage tail protein | Phage tail protein | | afdb-uniprot50 | AF-A0A0Q3EW34-F1-MODEL\_V4 | 1.0 | 8.498e-31 | 1078 | 0.242 | 483 | 254 | 19 | 3 | 474 | 2 | 383 | Phage\_sheath\_1 domain-containing protein | Phage\_sheath\_1 domain-containing protein | | afdb-uniprot50 | AF-A0A846VLL6-F1-MODEL\_V4 | 1.0 | 7.907e-30 | 1075 | 0.218 | 495 | 279 | 14 | 1 | 474 | 1 | 408 | Uncharacterized protein | Uncharacterized protein | | afdb-uniprot50 | AF-A0A3L7AKN3-F1-MODEL\_V4 | 1.0 | 3.815e-31 | 1064 | 0.259 | 498 | 257 | 15 | 3 | 474 | 1 | 412 | Phage tail protein | Phage tail protein | | afdb-uniprot50 | AF-A0A2D2CYV4-F1-MODEL\_V4 | 1.0 | 1.896e-34 | 1053 | 0.231 | 610 | 325 | 21 | 3 | 474 | 1 | 604 | Uncharacterized protein | Uncharacterized protein | | afdb-uniprot50 | AF-A0A5S9NEJ8-F1-MODEL\_V4 | 1.0 | 3.55e-30 | 1052 | 0.239 | 505 | 264 | 14 | 3 | 474 | 2 | 419 | Prophage major tail sheath protein | Prophage major tail sheath protein | | afdb-uniprot50 | AF-A0A318TT75-F1-MODEL\_V4 | 1.0 | 1.323e-29 | 1049 | 0.22 | 494 | 279 | 13 | 3 | 474 | 1 | 410 | Uncharacterized protein | Uncharacterized protein | | afdb-uniprot50 | AF-A0A022PIB5-F1-MODEL\_V4 | 1.0 | 1.36e-27 | 1044 | 0.373 | 246 | 152 | 2 | 231 | 474 | 28 | 273 | Phage tail sheath protein FI | Phage tail sheath protein FI | | afdb-uniprot50 | AF-A0A3S4JT82-F1-MODEL\_V4 | 1.0 | 1.735e-28 | 1043 | 0.309 | 378 | 169 | 5 | 3 | 378 | 1 | 288 | Phage tail sheath protein | Phage tail sheath protein | | afdb-uniprot50 | AF-A0A0Q2Y3L7-F1-MODEL\_V4 | 1.0 | 7.053e-30 | 1034 | 0.215 | 505 | 275 | 16 | 1 | 473 | 1 | 416 | Uncharacterized protein | Uncharacterized protein | | afdb-uniprot50 | AF-A0A3A6S7R0-F1-MODEL\_V4 | 1.0 | 7.791e-29 | 1030 | 0.31 | 400 | 178 | 11 | 1 | 394 | 1 | 308 | Phage tail sheath family protein | Phage tail sheath family protein | | afdb-uniprot50 | AF-A0A1T4WVG7-F1-MODEL\_V4 | 1.0 | 1.098e-28 | 1028 | 0.224 | 489 | 253 | 13 | 3 | 475 | 2 | 380 | Phage\_sheath\_1 domain-containing protein | Phage\_sheath\_1 domain-containing protein | | afdb-uniprot50 | AF-A0A4R6E0C1-F1-MODEL\_V4 | 1.0 | 1.917e-27 | 1026 | 0.298 | 355 | 163 | 3 | 119 | 472 | 8 | 277 | Tail sheath protein | Tail sheath protein | | afdb-uniprot50 | AF-A0A0Q1F5U2-F1-MODEL\_V4 | 1.0 | 5.378e-31 | 1024 | 0.234 | 562 | 243 | 18 | 5 | 473 | 2 | 469 | Phage\_sheath\_1 domain-containing protein | Phage\_sheath\_1 domain-containing protein | | afdb-uniprot50 | AF-A0A5P0JHK8-F1-MODEL\_V4 | 1.0 | 1.066e-26 | 1020 | 0.371 | 242 | 150 | 2 | 235 | 474 | 3 | 244 | Phage tail protein | Phage tail protein | | afdb-uniprot50 | AF-A0A2R8CQQ9-F1-MODEL\_V4 | 1.0 | 2.31e-28 | 1018 | 0.243 | 477 | 191 | 7 | 1 | 474 | 1 | 310 | Prophage major tail sheath protein | Prophage major tail sheath protein | | afdb-uniprot50 | AF-A0A2Y0G935-F1-MODEL\_V4 | 1.0 | 1.945e-28 | 1017 | 0.28 | 396 | 186 | 9 | 88 | 474 | 2 | 307 | Major tail sheath protein FI | Major tail sheath protein FI | | afdb-uniprot50 | AF-A0A0T7FB95-F1-MODEL\_V4 | 1.0 | 4.93e-29 | 1011 | 0.233 | 489 | 268 | 18 | 1 | 473 | 1 | 398 | Phage tail sheath protein | Phage tail sheath protein | | afdb-uniprot50 | AF-A0A6A1TYH7-F1-MODEL\_V4 | 1.0 | 2.154e-31 | 1007 | 0.371 | 320 | 190 | 7 | 157 | 474 | 3 | 313 | Uncharacterized protein | Uncharacterized protein | | afdb-uniprot50 | AF-A0A2S5J5L4-F1-MODEL\_V4 | 1.0 | 1.231e-28 | 1000 | 0.21 | 493 | 259 | 15 | 1 | 474 | 1 | 382 | Phage tail protein | Phage tail protein | | afdb-uniprot50 | AF-S6SL27-F1-MODEL\_V4 | 1.0 | 2e-26 | 999 | 0.531 | 224 | 104 | 1 | 253 | 475 | 1 | 224 | Major tail sheath protein | Major tail sheath protein | | afdb-uniprot50 | AF-A0A437NT20-F1-MODEL\_V4 | 1.0 | 9.249e-29 | 999 | 0.191 | 497 | 292 | 14 | 3 | 474 | 1 | 412 | Uncharacterized protein | Uncharacterized protein | | afdb-uniprot50 | AF-A0A327QFK8-F1-MODEL\_V4 | 1.0 | 1.787e-30 | 996 | 0.22 | 566 | 252 | 16 | 3 | 472 | 2 | 474 | Phage\_sheath\_1 domain-containing protein | Phage\_sheath\_1 domain-containing protein | | afdb-uniprot50 | AF-A0A7K1RD86-F1-MODEL\_V4 | 1.0 | 4.796e-31 | 996 | 0.245 | 506 | 264 | 15 | 1 | 474 | 328 | 747 | Uncharacterized protein | Uncharacterized protein | | afdb-uniprot50 | AF-A0A6D0KAC9-F1-MODEL\_V4 | 1.0 | 8.498e-31 | 985 | 0.257 | 525 | 174 | 8 | 5 | 412 | 2 | 427 | Phage tail protein | Phage tail protein | | afdb-uniprot50 | AF-A0A4U8UCG0-F1-MODEL\_V4 | 1.0 | 6.198e-29 | 979 | 0.25 | 496 | 245 | 22 | 1 | 474 | 1 | 391 | Phage tail sheath family protein | Phage tail sheath family protein | | afdb-uniprot50 | AF-A0A2X3F9K0-F1-MODEL\_V4 | 1.0 | 1.398e-25 | 978 | 0.357 | 238 | 151 | 2 | 239 | 474 | 2 | 239 | Phage tail sheath protein | Phage tail sheath protein | | afdb-uniprot50 | AF-A0A4R2GWM4-F1-MODEL\_V4 | 1.0 | 4.092e-28 | 970 | 0.199 | 511 | 285 | 18 | 1 | 474 | 1 | 424 | Phage\_sheath\_1 domain-containing protein | Phage\_sheath\_1 domain-containing protein | | afdb-uniprot50 | AF-A0A2C9D5D6-F1-MODEL\_V4 | 1.0 | 1.591e-26 | 965 | 0.202 | 490 | 279 | 15 | 3 | 474 | 1 | 396 | Major tail sheath protein | Major tail sheath protein | | afdb-uniprot50 | AF-A0A418G9S2-F1-MODEL\_V4 | 1.0 | 6.746e-27 | 964 | 0.277 | 393 | 183 | 8 | 95 | 472 | 1 | 307 | Phage tail sheath family protein | Phage tail sheath family protein | | afdb-uniprot50 | AF-A0A7W4PVA6-F1-MODEL\_V4 | 1.0 | 3.914e-25 | 948 | 0.34 | 247 | 160 | 3 | 230 | 474 | 11 | 256 | Phage tail sheath family protein | Phage tail sheath family protein | | afdb-uniprot50 | AF-A0A1F1HV50-F1-MODEL\_V4 | 1.0 | 5.221e-29 | 944 | 0.209 | 562 | 256 | 18 | 1 | 474 | 1 | 461 | Phage tail protein | Phage tail protein | | afdb-uniprot50 | AF-A0A379CWG5-F1-MODEL\_V4 | 1.0 | 2.118e-26 | 943 | 0.315 | 365 | 152 | 7 | 3 | 359 | 1 | 275 | Phage tail sheath protein | Phage tail sheath protein | | afdb-uniprot50 | AF-A0A4D9Y687-F1-MODEL\_V4 | 1.0 | 4.716e-26 | 942 | 0.324 | 373 | 154 | 8 | 3 | 367 | 1 | 283 | Phage tail protein | Phage tail protein | | afdb-uniprot50 | AF-A0A1M7RIE6-F1-MODEL\_V4 | 1.0 | 7.358e-29 | 941 | 0.215 | 566 | 257 | 19 | 1 | 473 | 1 | 472 | Phage\_sheath\_1 domain-containing protein | Phage\_sheath\_1 domain-containing protein | | afdb-uniprot50 | AF-A0A611ESW8-F1-MODEL\_V4 | 1.0 | 4.269e-27 | 939 | 0.306 | 382 | 167 | 8 | 35 | 408 | 2 | 293 | Phage tail protein | Phage tail protein | | afdb-uniprot50 | AF-A0A369RI45-F1-MODEL\_V4 | 1.0 | 2.897e-24 | 938 | 0.723 | 177 | 49 | 0 | 291 | 467 | 12 | 188 | Phage tail sheath protein | Phage tail sheath protein | | afdb-uniprot50 | AF-A0A4Q6D3G5-F1-MODEL\_V4 | 1.0 | 3.491e-25 | 931 | 0.386 | 251 | 144 | 4 | 227 | 475 | 4 | 246 | Phage\_sheath\_1 domain-containing protein | Phage\_sheath\_1 domain-containing protein | | afdb-uniprot50 | AF-A0A327J8X6-F1-MODEL\_V4 | 1.0 | 1.462e-28 | 928 | 0.172 | 561 | 285 | 16 | 6 | 474 | 1 | 473 | Phage\_sheath\_1 domain-containing protein | Phage\_sheath\_1 domain-containing protein | | afdb-uniprot50 | AF-A0A531KHM3-F1-MODEL\_V4 | 1.0 | 1.437e-23 | 921 | 0.685 | 175 | 55 | 0 | 301 | 475 | 2 | 176 | Phage tail protein | Phage tail protein | | afdb-uniprot50 | AF-A0A833LH34-F1-MODEL\_V4 | 1.0 | 1.459e-24 | 913 | 0.328 | 262 | 161 | 4 | 227 | 474 | 27 | 287 | Phage tail protein | Phage tail protein | | afdb-uniprot50 | AF-F4QJB7-F1-MODEL\_V4 | 1.0 | 3.44e-24 | 911 | 0.333 | 237 | 150 | 2 | 239 | 474 | 4 | 233 | Major tail sheath protein | Major tail sheath protein | | afdb-uniprot50 | AF-A0A0T9LR91-F1-MODEL\_V4 | 1.0 | 1.096e-24 | 901 | 0.248 | 474 | 166 | 5 | 1 | 474 | 1 | 284 | Major tail sheath protein | Major tail sheath protein | | afdb-uniprot50 | AF-A0A2D3VMA0-F1-MODEL\_V4 | 1.0 | 4.144e-25 | 896 | 0.207 | 477 | 265 | 14 | 5 | 474 | 2 | 372 | Phage tail protein | Phage tail protein | | afdb-uniprot50 | AF-A0A7L5XUF0-F1-MODEL\_V4 | 1.0 | 5.682e-27 | 896 | 0.196 | 519 | 281 | 18 | 3 | 474 | 1 | 430 | Phage tail protein | Phage tail protein | | afdb-uniprot50 | AF-A0A836P084-F1-MODEL\_V4 | 1.0 | 9.095e-24 | 892 | 0.339 | 224 | 147 | 1 | 231 | 453 | 8 | 231 | Tail sheath protein | Tail sheath protein | | afdb-uniprot50 | AF-A0A496L9D9-F1-MODEL\_V4 | 1.0 | 1.889e-26 | 885 | 0.257 | 443 | 196 | 7 | 7 | 415 | 1 | 344 | Phage tail protein | Phage tail protein | | afdb-uniprot50 | AF-A0A266LLW9-F1-MODEL\_V4 | 1.0 | 4.388e-25 | 880 | 0.292 | 366 | 162 | 7 | 1 | 362 | 1 | 273 | Phage tail protein | Phage tail protein | | afdb-uniprot50 | AF-A0A1H0YQB3-F1-MODEL\_V4 | 1.0 | 1.112e-25 | 877 | 0.172 | 515 | 295 | 14 | 3 | 474 | 1 | 427 | Uncharacterized protein | Uncharacterized protein | | afdb-uniprot50 | AF-A0A3M4S0Q3-F1-MODEL\_V4 | 1.0 | 2.897e-24 | 871 | 0.304 | 364 | 144 | 8 | 10 | 372 | 1 | 256 | Major tail sheath protein | Major tail sheath protein | | afdb-uniprot50 | AF-A0A0H3ZR03-F1-MODEL\_V4 | 1.0 | 9.23e-25 | 864 | 0.185 | 479 | 278 | 15 | 1 | 474 | 1 | 372 | Phage tail sheath monomer | Phage tail sheath monomer | | afdb-uniprot50 | AF-A0A7C4DFG7-F1-MODEL\_V4 | 1.0 | 5.599e-26 | 864 | 0.222 | 503 | 262 | 18 | 5 | 475 | 2 | 407 | Phage tail sheath protein | Phage tail sheath protein | | afdb-uniprot50 | AF-A0A0D6ARN2-F1-MODEL\_V4 | 1.0 | 5.694e-31 | 864 | 0.235 | 590 | 299 | 26 | 5 | 474 | 2 | 559 | Phage tail sheath monomer | Phage tail sheath monomer | | afdb-uniprot50 | AF-A0A6L5VN99-F1-MODEL\_V4 | 1.0 | 1.502e-26 | 862 | 0.283 | 385 | 175 | 8 | 103 | 472 | 89 | 387 | Phage tail sheath family protein | Phage tail sheath family protein | | afdb-uniprot50 | AF-A0A2T4JE69-F1-MODEL\_V4 | 1.0 | 1.357e-23 | 860 | 0.361 | 293 | 104 | 5 | 100 | 391 | 2 | 212 | Phage tail protein | Phage tail protein | | afdb-uniprot50 | AF-A0A1E7Q659-F1-MODEL\_V4 | 1.0 | 2.941e-25 | 860 | 0.19 | 498 | 267 | 16 | 3 | 467 | 2 | 396 | Uncharacterized protein | Uncharacterized protein | | afdb-uniprot50 | AF-A0A2X4TXD9-F1-MODEL\_V4 | 1.0 | 1.377e-24 | 857 | 0.288 | 357 | 162 | 5 | 5 | 359 | 3 | 269 | Phage tail sheath protein | Phage tail sheath protein | | afdb-uniprot50 | AF-A0A6H7P0U8-F1-MODEL\_V4 | 1.0 | 7.891e-26 | 856 | 0.198 | 509 | 276 | 12 | 5 | 474 | 2 | 417 | Phage tail sheath family protein | Phage tail sheath family protein | | afdb-uniprot50 | AF-V0YRL2-F1-MODEL\_V4 | 1.0 | 1.78e-22 | 855 | 0.405 | 202 | 119 | 1 | 274 | 474 | 2 | 203 | Phage tail sheath protein | Phage tail sheath protein | | afdb-uniprot50 | AF-A0A6L8LZU4-F1-MODEL\_V4 | 1.0 | 2.941e-25 | 854 | 0.185 | 481 | 278 | 16 | 3 | 474 | 4 | 379 | Uncharacterized protein | Uncharacterized protein | | afdb-uniprot50 | AF-A0A8B2TR80-F1-MODEL\_V4 | 1.0 | 1.066e-26 | 854 | 0.185 | 550 | 246 | 14 | 28 | 474 | 7 | 457 | Uncharacterized protein | Uncharacterized protein | | afdb-uniprot50 | AF-A0A1V4ZC03-F1-MODEL\_V4 | 1.0 | 3.857e-24 | 848 | 0.161 | 478 | 271 | 13 | 3 | 468 | 2 | 361 | Phage tail sheath protein | Phage tail sheath protein | | afdb-uniprot50 | AF-D1P897-F1-MODEL\_V4 | 1.0 | 4.445e-22 | 847 | 0.368 | 209 | 131 | 1 | 267 | 474 | 1 | 209 | Phage tail sheath protein | Phage tail sheath protein | | afdb-uniprot50 | AF-A0A7X6FQ87-F1-MODEL\_V4 | 1.0 | 3.339e-22 | 844 | 0.386 | 215 | 129 | 2 | 262 | 474 | 4 | 217 | Phage\_sheath\_1 domain-containing protein | Phage\_sheath\_1 domain-containing protein | | afdb-uniprot50 | AF-A0A5R8Y7Q1-F1-MODEL\_V4 | 1.0 | 1.096e-24 | 840 | 0.198 | 499 | 286 | 20 | 3 | 474 | 1 | 412 | Uncharacterized protein | Uncharacterized protein | | afdb-uniprot50 | AF-A0A715MBH1-F1-MODEL\_V4 | 1.0 | 2.509e-22 | 835 | 0.297 | 339 | 146 | 6 | 39 | 375 | 1 | 249 | Phage tail protein | Phage tail protein | | afdb-uniprot50 | AF-A0A690AYC2-F1-MODEL\_V4 | 1.0 | 9.368e-26 | 833 | 0.18 | 515 | 282 | 15 | 5 | 474 | 2 | 421 | Phage tail sheath family protein | Phage tail sheath family protein | | afdb-uniprot50 | AF-A0A2A5M6V2-F1-MODEL\_V4 | 1.0 | 5.755e-24 | 828 | 0.207 | 468 | 238 | 12 | 33 | 474 | 2 | 362 | Phage tail protein | Phage tail protein | | afdb-uniprot50 | AF-A0A7I9TNA5-F1-MODEL\_V4 | 1.0 | 5.21e-25 | 821 | 0.202 | 519 | 268 | 16 | 1 | 474 | 1 | 418 | Phage tail sheath family protein | Phage tail sheath family protein | | afdb-uniprot50 | AF-A0A824RG65-F1-MODEL\_V4 | 1.0 | 1.282e-23 | 817 | 0.226 | 407 | 215 | 7 | 71 | 474 | 30 | 339 | Phage tail sheath family protein | Phage tail sheath family protein | | afdb-uniprot50 | AF-A0A7K3ZN16-F1-MODEL\_V4 | 1.0 | 1.143e-23 | 817 | 0.19 | 478 | 251 | 14 | 2 | 467 | 3 | 356 | Phage tail sheath family protein | Phage tail sheath family protein | | afdb-uniprot50 | AF-A0A446CQT9-F1-MODEL\_V4 | 1.0 | 8.589e-24 | 815 | 0.188 | 483 | 257 | 14 | 1 | 466 | 1 | 365 | Prophage major tail sheath protein | Prophage major tail sheath protein | | afdb-uniprot50 | AF-A0A6G5QYL8-F1-MODEL\_V4 | 1.0 | 3.154e-22 | 814 | 0.307 | 247 | 164 | 5 | 231 | 474 | 411 | 653 | Phage tail sheath protein FI (Integrase domain) | Phage tail sheath protein FI (Integrase domain) | | afdb-uniprot50 | AF-A0A534UC15-F1-MODEL\_V4 | 1.0 | 1.611e-23 | 812 | 0.175 | 473 | 281 | 16 | 1 | 460 | 1 | 377 | Phage tail sheath family protein | Phage tail sheath family protein | | afdb-uniprot50 | AF-A0A6V6ZU17-F1-MODEL\_V4 | 1.0 | 3.815e-31 | 810 | 0.294 | 434 | 248 | 14 | 1 | 419 | 1 | 391 | Uncharacterized protein | Uncharacterized protein | | afdb-uniprot50 | AF-A0A4Q6CEC2-F1-MODEL\_V4 | 1.0 | 1.318e-21 | 805 | 0.366 | 224 | 135 | 3 | 254 | 475 | 11 | 229 | Phage tail protein | Phage tail protein | | afdb-uniprot50 | AF-A0A1Z4PN29-F1-MODEL\_V4 | 1.0 | 1.282e-23 | 805 | 0.179 | 478 | 269 | 14 | 5 | 466 | 2 | 372 | Putative prophage major tail sheath protein | Putative prophage major tail sheath protein | | afdb-uniprot50 | AF-A0A521PE06-F1-MODEL\_V4 | 1.0 | 8.112e-24 | 804 | 0.185 | 485 | 263 | 17 | 3 | 468 | 2 | 373 | Phage tail sheath family protein | Phage tail sheath family protein | | afdb-uniprot50 | AF-A0A349XNZ8-F1-MODEL\_V4 | 1.0 | 1.522e-23 | 803 | 0.255 | 399 | 193 | 12 | 93 | 474 | 14 | 325 | Phage tail protein | Phage tail protein | | afdb-uniprot50 | AF-A0A1J5D0C9-F1-MODEL\_V4 | 1.0 | 1.941e-24 | 801 | 0.207 | 492 | 255 | 19 | 1 | 466 | 1 | 383 | Phage tail protein | Phage tail protein | | afdb-uniprot50 | AF-A0A6I7PVR7-F1-MODEL\_V4 | 1.0 | 5.355e-23 | 800 | 0.175 | 483 | 274 | 17 | 3 | 468 | 2 | 377 | Phage tail sheath family protein | Phage tail sheath family protein | | afdb-uniprot50 | AF-A0A6G3I5H9-F1-MODEL\_V4 | 1.0 | 4.379e-21 | 798 | 0.378 | 214 | 127 | 3 | 265 | 472 | 15 | 228 | Phage tail sheath family protein | Phage tail sheath family protein | | afdb-uniprot50 | AF-A0A4Q0ZK21-F1-MODEL\_V4 | 1.0 | 1.885e-22 | 795 | 0.182 | 478 | 272 | 14 | 2 | 474 | 5 | 368 | Phage tail protein | Phage tail protein | | afdb-uniprot50 | AF-A0A6M0L0M0-F1-MODEL\_V4 | 1.0 | 5.058e-23 | 794 | 0.181 | 491 | 271 | 20 | 3 | 468 | 2 | 386 | Phage tail sheath family protein | Phage tail sheath family protein | | afdb-uniprot50 | AF-A0A6A4R6J7-F1-MODEL\_V4 | 1.0 | 8.961e-23 | 792 | 0.218 | 407 | 211 | 10 | 1 | 398 | 1 | 309 | Phage tail protein | Phage tail protein | | afdb-uniprot50 | AF-A0A1I6LKB0-F1-MODEL\_V4 | 1.0 | 2.696e-23 | 788 | 0.187 | 480 | 252 | 11 | 5 | 467 | 3 | 361 | Phage tail sheath protein | Phage tail sheath protein | | afdb-uniprot50 | AF-A0A1H2U7X5-F1-MODEL\_V4 | 1.0 | 1.706e-23 | 788 | 0.183 | 486 | 259 | 18 | 4 | 468 | 5 | 373 | Uncharacterized protein | Uncharacterized protein | | afdb-uniprot50 | AF-A0A823XP83-F1-MODEL\_V4 | 1.0 | 1.048e-21 | 786 | 0.217 | 377 | 196 | 6 | 100 | 474 | 3 | 282 | Phage tail sheath family protein | Phage tail sheath family protein | | afdb-uniprot50 | AF-A0A1E3GNZ3-F1-MODEL\_V4 | 1.0 | 2.271e-23 | 786 | 0.173 | 485 | 268 | 14 | 3 | 466 | 2 | 374 | Phage tail sheath protein | Phage tail sheath protein | | afdb-uniprot50 | AF-A0A285MD48-F1-MODEL\_V4 | 1.0 | 1.211e-23 | 785 | 0.165 | 503 | 295 | 20 | 3 | 474 | 2 | 410 | Uncharacterized protein | Uncharacterized protein | | afdb-uniprot50 | AF-A0A5T0J8E0-F1-MODEL\_V4 | 1.0 | 4.379e-21 | 777 | 0.28 | 246 | 172 | 3 | 231 | 474 | 26 | 268 | Phage tail sheath family protein | Phage tail sheath family protein | | afdb-uniprot50 | AF-A0A1I5RNY6-F1-MODEL\_V4 | 1.0 | 2.546e-23 | 773 | 0.17 | 493 | 278 | 16 | 5 | 474 | 6 | 390 | Uncharacterized protein | Uncharacterized protein | | afdb-uniprot50 | AF-W9GDP8-F1-MODEL\_V4 | 1.0 | 5.133e-24 | 772 | 0.188 | 472 | 253 | 16 | 19 | 466 | 3 | 368 | Phage tail sheath protein | Phage tail sheath protein | | afdb-uniprot50 | AF-A0A5Y3QFL3-F1-MODEL\_V4 | 1.0 | 2.472e-21 | 768 | 0.308 | 327 | 132 | 7 | 76 | 394 | 2 | 242 | Phage tail protein | Phage tail protein | | afdb-uniprot50 | AF-A0A0F3RPG1-F1-MODEL\_V4 | 1.0 | 2.771e-21 | 765 | 0.439 | 273 | 71 | 4 | 3 | 275 | 2 | 192 | Phage tail sheath family protein | Phage tail sheath family protein | | afdb-uniprot50 | AF-A0A1G8HX37-F1-MODEL\_V4 | 1.0 | 2.205e-21 | 763 | 0.205 | 408 | 219 | 8 | 75 | 474 | 2 | 312 | Uncharacterized protein | Uncharacterized protein | | afdb-uniprot50 | AF-A0A436QUU1-F1-MODEL\_V4 | 1.0 | 1.632e-20 | 762 | 0.288 | 260 | 171 | 4 | 228 | 474 | 23 | 281 | Phage tail protein | Phage tail protein | | afdb-uniprot50 | AF-A0A6L3F9E2-F1-MODEL\_V4 | 1.0 | 2.271e-23 | 762 | 0.209 | 502 | 253 | 20 | 1 | 468 | 7 | 398 | Phage tail sheath family protein | Phage tail sheath family protein | | afdb-uniprot50 | AF-A0A7T8BA55-F1-MODEL\_V4 | 1.0 | 1.913e-23 | 759 | 0.173 | 513 | 269 | 20 | 3 | 468 | 2 | 406 | Phage tail sheath family protein | Phage tail sheath family protein | | afdb-uniprot50 | AF-A0A2U2MY54-F1-MODEL\_V4 | 1.0 | 1.611e-23 | 757 | 0.182 | 500 | 273 | 16 | 3 | 467 | 2 | 400 | Uncharacterized protein | Uncharacterized protein | | afdb-uniprot50 | AF-E6X1M8-F1-MODEL\_V4 | 1.0 | 3.29e-21 | 756 | 0.191 | 485 | 261 | 17 | 5 | 474 | 2 | 370 | Major tail sheath protein | Major tail sheath protein | | afdb-uniprot50 | AF-A0A530RAK9-F1-MODEL\_V4 | 1.0 | 1.519e-19 | 754 | 0.688 | 154 | 48 | 0 | 320 | 473 | 1 | 154 | Phage tail protein | Phage tail protein | | afdb-uniprot50 | AF-A0A7C3RPS7-F1-MODEL\_V4 | 1.0 | 5.755e-24 | 754 | 0.235 | 441 | 205 | 11 | 81 | 475 | 9 | 363 | Phage tail sheath family protein | Phage tail sheath family protein | | afdb-uniprot50 | AF-A0A7V8JNF3-F1-MODEL\_V4 | 1.0 | 1.861e-25 | 753 | 0.57 | 228 | 92 | 3 | 3 | 226 | 2 | 227 | Putative prophage major tail sheath protein | Putative prophage major tail sheath protein | | afdb-uniprot50 | AF-A0A1M6GFB7-F1-MODEL\_V4 | 1.0 | 3.589e-23 | 753 | 0.198 | 524 | 245 | 18 | 3 | 467 | 2 | 409 | Uncharacterized protein | Uncharacterized protein | | afdb-uniprot50 | AF-A0A537ZZS7-F1-MODEL\_V4 | 1.0 | 1.754e-21 | 750 | 0.196 | 463 | 234 | 14 | 3 | 456 | 2 | 335 | Phage tail sheath family protein | Phage tail sheath family protein | | afdb-uniprot50 | AF-A0A6G5QNE9-F1-MODEL\_V4 | 1.0 | 7.024e-22 | 750 | 0.169 | 478 | 276 | 15 | 5 | 474 | 2 | 366 | Phage tail sheath protein FI | Phage tail sheath protein FI | | afdb-uniprot50 | AF-A0A3S7DS92-F1-MODEL\_V4 | 1.0 | 2.276e-27 | 750 | 0.191 | 621 | 304 | 28 | 3 | 474 | 1 | 572 | Phage tail protein | Phage tail protein | | afdb-uniprot50 | AF-A0A6N8U467-F1-MODEL\_V4 | 1.0 | 8.463e-23 | 748 | 0.186 | 483 | 259 | 16 | 7 | 467 | 1 | 371 | Uncharacterized protein | Uncharacterized protein | | afdb-uniprot50 | AF-A0A7M3MAW8-F1-MODEL\_V4 | 1.0 | 2.025e-23 | 748 | 0.205 | 512 | 269 | 19 | 3 | 475 | 1 | 413 | Phage tail sheath family protein | Phage tail sheath family protein | | afdb-uniprot50 | AF-A0A6L9FQ12-F1-MODEL\_V4 | 1.0 | 7.676e-28 | 748 | 0.184 | 623 | 326 | 30 | 3 | 474 | 1 | 592 | Phage tail protein | Phage tail protein | | afdb-uniprot50 | AF-A0A7Y4DJI9-F1-MODEL\_V4 | 1.0 | 1.996e-22 | 746 | 0.14 | 478 | 295 | 15 | 1 | 474 | 3 | 368 | Uncharacterized protein | Uncharacterized protein | | afdb-uniprot50 | AF-A0A524IP71-F1-MODEL\_V4 | 1.0 | 1.996e-22 | 744 | 0.177 | 489 | 268 | 16 | 1 | 466 | 1 | 378 | Phage tail sheath family protein | Phage tail sheath family protein | | afdb-uniprot50 | AF-A0A1C0V8T7-F1-MODEL\_V4 | 1.0 | 4.52e-27 | 744 | 0.187 | 730 | 287 | 30 | 1 | 471 | 2 | 684 | Phage\_sheath\_1 domain-containing protein | Phage\_sheath\_1 domain-containing protein | | afdb-uniprot50 | AF-A0A1Q7X247-F1-MODEL\_V4 | 1.0 | 6.732e-23 | 741 | 0.188 | 473 | 243 | 16 | 3 | 464 | 24 | 366 | Uncharacterized protein | Uncharacterized protein | | afdb-uniprot50 | AF-A0A432UGQ1-F1-MODEL\_V4 | 1.0 | 2.405e-23 | 737 | 0.204 | 571 | 257 | 22 | 5 | 475 | 2 | 474 | Phage tail sheath family protein | Phage tail sheath family protein | | afdb-uniprot50 | AF-A0A1Q7MAI6-F1-MODEL\_V4 | 1.0 | 1.02e-23 | 735 | 0.171 | 497 | 293 | 18 | 1 | 474 | 1 | 401 | Uncharacterized protein | Uncharacterized protein | | afdb-uniprot50 | AF-A0A377I6M5-F1-MODEL\_V4 | 1.0 | 2.978e-22 | 732 | 0.238 | 398 | 193 | 12 | 93 | 473 | 31 | 335 | Tail sheath-like protein | Tail sheath-like protein | | afdb-uniprot50 | AF-A0A6P0MAY6-F1-MODEL\_V4 | 1.0 | 1.78e-22 | 730 | 0.18 | 481 | 262 | 17 | 5 | 466 | 2 | 369 | Uncharacterized protein | Uncharacterized protein | | afdb-uniprot50 | AF-A0A1J4YMP9-F1-MODEL\_V4 | 1.0 | 1.564e-21 | 727 | 0.171 | 485 | 270 | 21 | 5 | 468 | 3 | 376 | Uncharacterized protein | Uncharacterized protein | | afdb-uniprot50 | AF-A0A376YN89-F1-MODEL\_V4 | 1.0 | 1.541e-20 | 724 | 0.372 | 196 | 122 | 1 | 259 | 453 | 2 | 197 | Major tail sheath protein FI | Major tail sheath protein FI | | afdb-uniprot50 | AF-A0A660N068-F1-MODEL\_V4 | 1.0 | 3.8e-23 | 723 | 0.207 | 507 | 217 | 16 | 1 | 419 | 1 | 410 | Phage tail protein | Phage tail protein | | afdb-uniprot50 | AF-A0A4R9F3E7-F1-MODEL\_V4 | 1.0 | 2.771e-21 | 720 | 0.187 | 469 | 250 | 19 | 15 | 468 | 2 | 354 | Phage tail sheath family protein | Phage tail sheath family protein | | afdb-uniprot50 | AF-A0A0F0GLD7-F1-MODEL\_V4 | 1.0 | 3.543e-26 | 720 | 0.197 | 548 | 304 | 18 | 3 | 467 | 2 | 496 | Tail protein | Tail protein | | afdb-uniprot50 | AF-A0A353GA80-F1-MODEL\_V4 | 1.0 | 6.633e-22 | 719 | 0.251 | 414 | 188 | 15 | 1 | 382 | 1 | 324 | Phage tail protein | Phage tail protein | | afdb-uniprot50 | AF-A0A403T4T3-F1-MODEL\_V4 | 1.0 | 4.454e-26 | 719 | 0.256 | 449 | 208 | 9 | 1 | 419 | 1 | 353 | Phage\_sheath\_1 domain-containing protein | Phage\_sheath\_1 domain-containing protein | | afdb-uniprot50 | AF-A0A6B9YRM7-F1-MODEL\_V4 | 1.0 | 5.277e-22 | 715 | 0.158 | 506 | 278 | 22 | 1 | 468 | 8 | 403 | Phage tail sheath family protein | Phage tail sheath family protein | | afdb-uniprot50 | AF-B6WRQ3-F1-MODEL\_V4 | 1.0 | 1.416e-22 | 715 | 0.171 | 590 | 279 | 20 | 1 | 475 | 1 | 495 | Uncharacterized protein | Uncharacterized protein | | afdb-uniprot50 | AF-A0A0D0X3W4-F1-MODEL\_V4 | 1.0 | 1.784e-26 | 715 | 0.201 | 556 | 307 | 25 | 3 | 468 | 2 | 510 | Tail protein | Tail protein | | afdb-uniprot50 | AF-A0A523TY78-F1-MODEL\_V4 | 1.0 | 9.919e-26 | 712 | 0.173 | 547 | 314 | 23 | 1 | 468 | 1 | 488 | Phage tail sheath family protein | Phage tail sheath family protein | | afdb-uniprot50 | AF-A0A7Y5XG39-F1-MODEL\_V4 | 1.0 | 3.062e-20 | 711 | 0.277 | 252 | 162 | 8 | 230 | 467 | 73 | 318 | Phage tail sheath family protein | Phage tail sheath family protein | | afdb-uniprot50 | AF-A0A369BHJ8-F1-MODEL\_V4 | 1.0 | 1.126e-22 | 710 | 0.197 | 506 | 270 | 25 | 5 | 474 | 2 | 407 | Phage tail sheath protein FI | Phage tail sheath protein FI | | afdb-uniprot50 | AF-A0A535YX28-F1-MODEL\_V4 | 1.0 | 1.112e-25 | 706 | 0.2 | 565 | 286 | 24 | 3 | 467 | 2 | 500 | Phage tail sheath family protein | Phage tail sheath family protein | | afdb-uniprot50 | AF-A0A2X2BJR7-F1-MODEL\_V4 | 1.0 | 1.279e-19 | 704 | 0.383 | 193 | 117 | 2 | 230 | 420 | 27 | 219 | Major tail sheath protein | Major tail sheath protein | | afdb-uniprot50 | AF-A0A1Q9Q7N6-F1-MODEL\_V4 | 1.0 | 4.637e-21 | 703 | 0.184 | 505 | 254 | 13 | 3 | 468 | 2 | 387 | Uncharacterized protein | Uncharacterized protein | | afdb-uniprot50 | AF-A0A7Y1Y9C5-F1-MODEL\_V4 | 1.0 | 1.754e-21 | 700 | 0.165 | 490 | 277 | 20 | 1 | 466 | 3 | 384 | Phage tail sheath family protein | Phage tail sheath family protein | | afdb-uniprot50 | AF-A0A3P1YCU6-F1-MODEL\_V4 | 1.0 | 2.109e-18 | 699 | 0.336 | 202 | 132 | 2 | 274 | 474 | 1 | 201 | Phage tail sheath family protein | Phage tail sheath family protein | | afdb-uniprot50 | AF-A0A774NAH9-F1-MODEL\_V4 | 1.0 | 5.424e-20 | 699 | 0.269 | 341 | 154 | 7 | 88 | 420 | 2 | 255 | Phage tail protein | Phage tail protein | | afdb-uniprot50 | AF-A0A7H8K818-F1-MODEL\_V4 | 1.0 | 1.05e-25 | 698 | 0.199 | 556 | 309 | 21 | 3 | 468 | 2 | 511 | Phage tail sheath family protein | Phage tail sheath family protein | | afdb-uniprot50 | AF-A0A1X7M8N6-F1-MODEL\_V4 | 1.0 | 1.992e-18 | 697 | 0.397 | 254 | 65 | 2 | 30 | 283 | 2 | 167 | Phage major tail sheath protein | Phage major tail sheath protein | | afdb-uniprot50 | AF-A0A177NQ44-F1-MODEL\_V4 | 1.0 | 3.107e-21 | 697 | 0.197 | 502 | 251 | 18 | 3 | 467 | 2 | 388 | Phage tail protein | Phage tail protein | | afdb-uniprot50 | AF-A0A7K1H3K3-F1-MODEL\_V4 | 1.0 | 8.355e-26 | 697 | 0.184 | 557 | 314 | 24 | 3 | 468 | 2 | 508 | Phage tail sheath family protein | Phage tail sheath family protein | | afdb-uniprot50 | AF-A0A849Q2Q2-F1-MODEL\_V4 | 1.0 | 3.536e-22 | 693 | 0.17 | 504 | 262 | 22 | 1 | 467 | 1 | 385 | Uncharacterized protein | Uncharacterized protein | | afdb-uniprot50 | AF-A0A0G9K7V9-F1-MODEL\_V4 | 1.0 | 8.095e-20 | 693 | 0.152 | 491 | 285 | 17 | 5 | 474 | 2 | 382 | Tail protein | Tail protein | | afdb-uniprot50 | AF-A0A833EFL3-F1-MODEL\_V4 | 1.0 | 1.158e-20 | 693 | 0.195 | 502 | 269 | 20 | 3 | 475 | 1 | 396 | Phage tail sheath protein | Phage tail sheath protein | | afdb-uniprot50 | AF-A0A4Q6GVN0-F1-MODEL\_V4 | 1.0 | 4.379e-21 | 692 | 0.154 | 499 | 277 | 19 | 1 | 468 | 2 | 386 | Phage tail sheath family protein | Phage tail sheath family protein | | afdb-uniprot50 | AF-A0A6G7Z242-F1-MODEL\_V4 | 1.0 | 6.185e-25 | 692 | 0.193 | 573 | 300 | 23 | 3 | 468 | 2 | 519 | Phage tail sheath family protein | Phage tail sheath family protein | | afdb-uniprot50 | AF-A0A1E4GSC1-F1-MODEL\_V4 | 1.0 | 3.906e-21 | 691 | 0.184 | 487 | 266 | 18 | 3 | 468 | 2 | 378 | Uncharacterized protein | Uncharacterized protein | | afdb-uniprot50 | AF-A0A538DVN2-F1-MODEL\_V4 | 1.0 | 2.935e-21 | 689 | 0.195 | 492 | 260 | 20 | 3 | 468 | 2 | 383 | Phage tail sheath family protein | Phage tail sheath family protein | | afdb-uniprot50 | AF-L1P591-F1-MODEL\_V4 | 1.0 | 7.532e-19 | 687 | 0.215 | 376 | 188 | 10 | 113 | 474 | 2 | 284 | Phage\_sheath\_1 domain-containing protein | Phage\_sheath\_1 domain-containing protein | | afdb-uniprot50 | AF-A0A2E3R271-F1-MODEL\_V4 | 1.0 | 4.706e-22 | 687 | 0.176 | 521 | 283 | 24 | 3 | 475 | 2 | 424 | Phage\_sheath\_1 domain-containing protein | Phage\_sheath\_1 domain-containing protein | | afdb-uniprot50 | AF-A0A812WA44-F1-MODEL\_V4 | 1.0 | 1.247e-25 | 687 | 0.207 | 569 | 290 | 28 | 3 | 467 | 2 | 513 | 18 protein | 18 protein | | afdb-uniprot50 | AF-A0A7X5APW2-F1-MODEL\_V4 | 1.0 | 3.29e-21 | 685 | 0.171 | 496 | 264 | 21 | 3 | 466 | 2 | 382 | Phage\_sheath\_1 domain-containing protein | Phage\_sheath\_1 domain-containing protein | | afdb-uniprot50 | AF-A0A5Q4D9T1-F1-MODEL\_V4 | 1.0 | 7.875e-22 | 685 | 0.182 | 499 | 269 | 23 | 1 | 466 | 7 | 399 | Phage tail sheath family protein | Phage tail sheath family protein | | afdb-uniprot50 | AF-A0A859FDD1-F1-MODEL\_V4 | 1.0 | 5.671e-23 | 685 | 0.178 | 576 | 263 | 21 | 5 | 474 | 2 | 473 | Phage tail sheath family protein | Phage tail sheath family protein | | afdb-uniprot50 | AF-A0A3N5RBQ4-F1-MODEL\_V4 | 1.0 | 1.97e-25 | 685 | 0.188 | 561 | 305 | 26 | 1 | 467 | 1 | 504 | Phage tail sheath family protein | Phage tail sheath family protein | | afdb-uniprot50 | AF-A0A2W5Z8T4-F1-MODEL\_V4 | 1.0 | 1.588e-22 | 685 | 0.186 | 494 | 257 | 17 | 1 | 467 | 1 | 376 | AAA domain-containing protein | AAA domain-containing protein | | afdb-uniprot50 | AF-A0A150R6N1-F1-MODEL\_V4 | 1.0 | 7.22e-20 | 685 | 0.281 | 252 | 160 | 9 | 231 | 468 | 690 | 934 | Uncharacterized protein | Uncharacterized protein | | afdb-uniprot50 | AF-A0A1X4I4Z3-F1-MODEL\_V4 | 1.0 | 1.279e-19 | 684 | 0.3 | 253 | 156 | 9 | 230 | 467 | 65 | 311 | Phage tail protein | Phage tail protein | | afdb-uniprot50 | AF-A0A3B9JCN2-F1-MODEL\_V4 | 1.0 | 4.252e-19 | 682 | 0.277 | 252 | 162 | 8 | 230 | 467 | 104 | 349 | Phage tail protein | Phage tail protein | | afdb-uniprot50 | AF-A0A3S0CXJ3-F1-MODEL\_V4 | 1.0 | 2.021e-19 | 682 | 0.266 | 251 | 165 | 8 | 231 | 468 | 111 | 355 | Phage tail sheath family protein | Phage tail sheath family protein | | afdb-uniprot50 | AF-D4K2N0-F1-MODEL\_V4 | 1.0 | 2.3e-20 | 682 | 0.165 | 494 | 272 | 17 | 5 | 472 | 1 | 380 | Phage tail sheath protein FI | Phage tail sheath protein FI | | afdb-uniprot50 | AF-A0A4R5TTN3-F1-MODEL\_V4 | 1.0 | 3.689e-21 | 681 | 0.188 | 503 | 257 | 18 | 3 | 468 | 2 | 390 | Phage tail sheath family protein | Phage tail sheath family protein | | afdb-uniprot50 | AF-A0A2X0WI40-F1-MODEL\_V4 | 1.0 | 4.983e-22 | 680 | 0.164 | 594 | 275 | 26 | 3 | 475 | 1 | 493 | Phage tail sheath protein | Phage tail sheath protein | | afdb-uniprot50 | AF-A0A1G5B506-F1-MODEL\_V4 | 1.0 | 3.536e-22 | 679 | 0.174 | 583 | 264 | 21 | 5 | 475 | 2 | 478 | Uncharacterized protein | Uncharacterized protein | | afdb-uniprot50 | AF-A0A0S8BQ23-F1-MODEL\_V4 | 1.0 | 2.477e-25 | 678 | 0.202 | 563 | 302 | 27 | 1 | 467 | 1 | 512 | Uncharacterized protein | Uncharacterized protein | | afdb-uniprot50 | AF-V2YG99-F1-MODEL\_V4 | 1.0 | 5.047e-19 | 676 | 0.258 | 251 | 167 | 7 | 231 | 468 | 88 | 332 | Uncharacterized protein | Uncharacterized protein | | afdb-uniprot50 | AF-V6U633-F1-MODEL\_V4 | 1.0 | 3.581e-19 | 676 | 0.28 | 253 | 162 | 8 | 230 | 468 | 135 | 381 | Putative phage tail sheath protein | Putative phage tail sheath protein | | afdb-uniprot50 | AF-A0A497BTG9-F1-MODEL\_V4 | 1.0 | 1.459e-24 | 676 | 0.175 | 580 | 297 | 21 | 1 | 468 | 1 | 511 | Phage tail sheath family protein | Phage tail sheath family protein | | afdb-uniprot50 | AF-A0A7C5IAH6-F1-MODEL\_V4 | 1.0 | 7.976e-19 | 675 | 0.235 | 251 | 174 | 7 | 230 | 469 | 168 | 411 | Phage tail sheath family protein | Phage tail sheath family protein | | afdb-uniprot50 | AF-A0A0Q7MZB4-F1-MODEL\_V4 | 1.0 | 8.233e-25 | 675 | 0.186 | 575 | 299 | 25 | 1 | 468 | 1 | 513 | Phage tail protein | Phage tail protein | | afdb-uniprot50 | AF-A0A6G2PQT4-F1-MODEL\_V4 | 1.0 | 3.017e-19 | 674 | 0.273 | 252 | 161 | 7 | 230 | 468 | 48 | 290 | Phage tail sheath family protein | Phage tail sheath family protein | | afdb-uniprot50 | AF-A0A848Y6F5-F1-MODEL\_V4 | 1.0 | 3.581e-19 | 674 | 0.259 | 258 | 166 | 7 | 230 | 468 | 66 | 317 | Phage tail sheath family protein | Phage tail sheath family protein | | afdb-uniprot50 | AF-M0BGK5-F1-MODEL\_V4 | 1.0 | 2.14e-19 | 673 | 0.256 | 250 | 165 | 7 | 231 | 468 | 268 | 508 | Phage tail sheath protein FI | Phage tail sheath protein FI | | afdb-uniprot50 | AF-A0A651E436-F1-MODEL\_V4 | 1.0 | 5.659e-19 | 673 | 0.274 | 251 | 164 | 7 | 230 | 468 | 288 | 532 | Phage tail sheath family protein | Phage tail sheath family protein | | afdb-uniprot50 | AF-A0A5J6QAB9-F1-MODEL\_V4 | 1.0 | 3.696e-25 | 673 | 0.169 | 633 | 314 | 29 | 1 | 468 | 1 | 586 | Uncharacterized protein | Uncharacterized protein | | afdb-uniprot50 | AF-A0A6G9ZDV3-F1-MODEL\_V4 | 1.0 | 1.499e-22 | 672 | 0.177 | 534 | 265 | 18 | 5 | 468 | 1 | 430 | Uncharacterized protein | Uncharacterized protein | | afdb-uniprot50 | AF-A0A4U7JHW2-F1-MODEL\_V4 | 1.0 | 5.992e-19 | 671 | 0.261 | 252 | 167 | 8 | 230 | 468 | 311 | 556 | Phage tail sheath family protein | Phage tail sheath family protein | | afdb-uniprot50 | AF-A0A7V3SDC0-F1-MODEL\_V4 | 1.0 | 6.833e-24 | 669 | 0.168 | 576 | 303 | 23 | 1 | 468 | 1 | 508 | Phage tail sheath family protein | Phage tail sheath family protein | | afdb-uniprot50 | AF-A0A447JQB1-F1-MODEL\_V4 | 1.0 | 2.731e-20 | 667 | 0.268 | 358 | 161 | 8 | 90 | 431 | 19 | 291 | Tail protein | Tail protein | | afdb-uniprot50 | AF-A0A1V3NDB0-F1-MODEL\_V4 | 1.0 | 3.242e-20 | 666 | 0.175 | 473 | 256 | 14 | 9 | 468 | 31 | 382 | Phage\_sheath\_1C domain-containing protein | Phage\_sheath\_1C domain-containing protein | | afdb-uniprot50 | AF-A0A7C7E3G3-F1-MODEL\_V4 | 1.0 | 4.015e-19 | 665 | 0.254 | 255 | 168 | 9 | 230 | 468 | 158 | 406 | Phage tail sheath family protein | Phage tail sheath family protein | | afdb-uniprot50 | AF-A0A1V4VN97-F1-MODEL\_V4 | 1.0 | 8.233e-25 | 665 | 0.179 | 567 | 294 | 24 | 5 | 467 | 3 | 501 | Phage tail sheath protein | Phage tail sheath protein | | afdb-uniprot50 | AF-A0A2V7W469-F1-MODEL\_V4 | 1.0 | 9.63e-24 | 663 | 0.152 | 598 | 306 | 19 | 3 | 468 | 2 | 530 | Uncharacterized protein | Uncharacterized protein | | afdb-uniprot50 | AF-A0A656TYE7-F1-MODEL\_V4 | 1.0 | 7.976e-19 | 662 | 0.257 | 256 | 165 | 7 | 232 | 468 | 12 | 261 | Uncharacterized protein | Uncharacterized protein | | afdb-uniprot50 | AF-A0A1Q7S8Q3-F1-MODEL\_V4 | 1.0 | 1.062e-18 | 662 | 0.268 | 253 | 164 | 8 | 230 | 467 | 162 | 408 | Phage tail protein | Phage tail protein | | afdb-uniprot50 | AF-A0A1S2NW23-F1-MODEL\_V4 | 1.0 | 2.082e-21 | 662 | 0.185 | 495 | 268 | 20 | 20 | 466 | 2 | 409 | Uncharacterized protein | Uncharacterized protein | | afdb-uniprot50 | AF-A0A2S5F8Z6-F1-MODEL\_V4 | 1.0 | 6.264e-22 | 661 | 0.215 | 408 | 201 | 9 | 86 | 474 | 155 | 462 | Phage tail protein | Phage tail protein | | afdb-uniprot50 | AF-A0A1Z8SQC7-F1-MODEL\_V4 | 1.0 | 3.642e-24 | 660 | 0.165 | 669 | 328 | 25 | 3 | 473 | 2 | 637 | Uncharacterized protein | Uncharacterized protein | | afdb-uniprot50 | AF-A0A6V8M398-F1-MODEL\_V4 | 1.0 | 6.345e-19 | 659 | 0.269 | 252 | 166 | 8 | 229 | 468 | 73 | 318 | Uncharacterized protein | Uncharacterized protein | | afdb-uniprot50 | AF-B3PD96-F1-MODEL\_V4 | 1.0 | 2.177e-24 | 658 | 0.166 | 589 | 304 | 26 | 3 | 468 | 2 | 526 | Phage tail sheath protein | Phage tail sheath protein | | afdb-uniprot50 | AF-A0A7U6QNP6-F1-MODEL\_V4 | 1.0 | 2.33e-17 | 657 | 0.312 | 205 | 133 | 4 | 276 | 473 | 5 | 208 | Phage\_sheath\_1 domain-containing protein | Phage\_sheath\_1 domain-containing protein | | afdb-uniprot50 | AF-A0A2P1PP13-F1-MODEL\_V4 | 1.0 | 1.703e-19 | 657 | 0.154 | 484 | 275 | 21 | 11 | 468 | 4 | 379 | Uncharacterized protein | Uncharacterized protein | | afdb-uniprot50 | AF-A0A1Y3X9S1-F1-MODEL\_V4 | 1.0 | 1.459e-24 | 657 | 0.161 | 562 | 306 | 20 | 11 | 468 | 1 | 500 | Uncharacterized protein | Uncharacterized protein | | afdb-uniprot50 | AF-A0A3G2R5A9-F1-MODEL\_V4 | 1.0 | 4.084e-24 | 657 | 0.169 | 550 | 324 | 26 | 5 | 466 | 3 | 507 | Phage tail sheath family protein | Phage tail sheath family protein | | afdb-uniprot50 | AF-A0A7X0FS83-F1-MODEL\_V4 | 1.0 | 2.266e-19 | 656 | 0.171 | 456 | 238 | 18 | 28 | 466 | 4 | 336 | Uncharacterized protein | Uncharacterized protein | | afdb-uniprot50 | AF-E1QHA9-F1-MODEL\_V4 | 1.0 | 1.11e-21 | 655 | 0.188 | 583 | 260 | 24 | 3 | 474 | 1 | 481 | Uncharacterized protein | Uncharacterized protein | | afdb-uniprot50 | AF-A0A1H8VSD3-F1-MODEL\_V4 | 1.0 | 1.094e-20 | 654 | 0.192 | 578 | 262 | 22 | 5 | 475 | 2 | 481 | Uncharacterized protein | Uncharacterized protein | | afdb-uniprot50 | AF-A5UTU6-F1-MODEL\_V4 | 1.0 | 4.848e-24 | 653 | 0.185 | 581 | 290 | 28 | 5 | 467 | 3 | 517 | Phage tail sheath protein | Phage tail sheath protein | | afdb-uniprot50 | AF-A0A351G7S8-F1-MODEL\_V4 | 1.0 | 1.301e-24 | 653 | 0.178 | 600 | 294 | 29 | 5 | 467 | 3 | 540 | Phage tail protein | Phage tail protein | | afdb-uniprot50 | AF-A0A4Q3UGF8-F1-MODEL\_V4 | 1.0 | 1.062e-18 | 652 | 0.226 | 260 | 171 | 8 | 231 | 467 | 118 | 370 | Phage tail sheath family protein | Phage tail sheath family protein | | afdb-uniprot50 | AF-A0A7C3BTZ0-F1-MODEL\_V4 | 1.0 | 1.992e-18 | 652 | 0.265 | 252 | 165 | 8 | 230 | 467 | 207 | 452 | Phage tail sheath family protein | Phage tail sheath family protein | | afdb-uniprot50 | AF-A0A0A2WM66-F1-MODEL\_V4 | 1.0 | 4.848e-24 | 651 | 0.177 | 559 | 306 | 20 | 1 | 468 | 4 | 499 | Phage tail sheath protein FI | Phage tail sheath protein FI | | afdb-uniprot50 | AF-A0A2W4URD9-F1-MODEL\_V4 | 1.0 | 4.569e-20 | 650 | 0.191 | 486 | 249 | 16 | 3 | 467 | 2 | 364 | Phage tail sheath family protein | Phage tail sheath family protein | | afdb-uniprot50 | AF-A0A5E4K8W2-F1-MODEL\_V4 | 1.0 | 3.147e-18 | 649 | 0.242 | 252 | 172 | 7 | 230 | 468 | 59 | 304 | Phage tail sheath protein subtilisin-like domain protein | Phage tail sheath protein subtilisin-like domain protein | | afdb-uniprot50 | AF-A0A2V8QNT6-F1-MODEL\_V4 | 1.0 | 1.26e-18 | 649 | 0.238 | 260 | 168 | 8 | 231 | 467 | 58 | 310 | Uncharacterized protein | Uncharacterized protein | | afdb-uniprot50 | AF-A0A811B248-F1-MODEL\_V4 | 1.0 | 9.095e-24 | 649 | 0.171 | 576 | 301 | 21 | 5 | 468 | 3 | 513 | Tail protein | Tail protein | | afdb-uniprot50 | AF-A0A150MJ89-F1-MODEL\_V4 | 1.0 | 2.3e-20 | 648 | 0.156 | 580 | 282 | 19 | 5 | 475 | 2 | 482 | Uncharacterized protein | Uncharacterized protein | | afdb-uniprot50 | AF-U3TVR5-F1-MODEL\_V4 | 1.0 | 3.681e-17 | 647 | 0.364 | 203 | 121 | 4 | 278 | 473 | 2 | 203 | Tail sheath protein | Tail sheath protein | | afdb-uniprot50 | AF-G9PUI4-F1-MODEL\_V4 | 1.0 | 2.172e-20 | 646 | 0.173 | 588 | 271 | 23 | 5 | 475 | 2 | 491 | Uncharacterized protein | Uncharacterized protein | | afdb-uniprot50 | AF-A0A261QLY6-F1-MODEL\_V4 | 1.0 | 1.244e-21 | 645 | 0.262 | 454 | 150 | 17 | 3 | 365 | 2 | 361 | Phage tail protein | Phage tail protein | | afdb-uniprot50 | AF-A0A0M2VSK3-F1-MODEL\_V4 | 1.0 | 4.983e-22 | 645 | 0.186 | 578 | 260 | 23 | 5 | 472 | 2 | 479 | Phage tail protein | Phage tail protein | | afdb-uniprot50 | AF-A0A1B6ASG7-F1-MODEL\_V4 | 1.0 | 3.068e-24 | 645 | 0.19 | 625 | 300 | 27 | 3 | 467 | 2 | 580 | Putative phage tail sheath protein | Putative phage tail sheath protein | | afdb-uniprot50 | AF-A0A4R1QQL4-F1-MODEL\_V4 | 1.0 | 2.855e-23 | 644 | 0.155 | 565 | 317 | 27 | 1 | 468 | 10 | 511 | Uncharacterized protein | Uncharacterized protein | | afdb-uniprot50 | AF-A0A7X5DZ34-F1-MODEL\_V4 | 1.0 | 9.095e-24 | 643 | 0.162 | 629 | 304 | 26 | 3 | 468 | 1 | 569 | Phage tail sheath family protein | Phage tail sheath family protein | | afdb-uniprot50 | AF-A0A3M1GT53-F1-MODEL\_V4 | 1.0 | 5.516e-25 | 643 | 0.144 | 621 | 333 | 27 | 3 | 467 | 2 | 579 | Phage tail sheath family protein | Phage tail sheath family protein | | afdb-uniprot50 | AF-A0A523UW76-F1-MODEL\_V4 | 1.0 | 9.63e-24 | 642 | 0.189 | 554 | 296 | 23 | 5 | 468 | 3 | 493 | Phage tail sheath family protein | Phage tail sheath family protein | | afdb-uniprot50 | AF-A0A1G6NJX4-F1-MODEL\_V4 | 1.0 | 1.124e-18 | 641 | 0.261 | 249 | 165 | 8 | 231 | 468 | 165 | 405 | Uncharacterized protein | Uncharacterized protein | | afdb-uniprot50 | AF-A0A1C0AIV2-F1-MODEL\_V4 | 1.0 | 6.44e-20 | 640 | 0.214 | 467 | 229 | 21 | 10 | 457 | 1 | 348 | Phage\_sheath\_1 domain-containing protein | Phage\_sheath\_1 domain-containing protein | | afdb-uniprot50 | AF-A0A7H8TM34-F1-MODEL\_V4 | 1.0 | 5.266e-18 | 640 | 0.259 | 254 | 167 | 8 | 230 | 468 | 289 | 536 | Phage tail sheath family protein | Phage tail sheath family protein | | afdb-uniprot50 | AF-A0A1P8MVD1-F1-MODEL\_V4 | 1.0 | 1.678e-18 | 640 | 0.258 | 263 | 164 | 9 | 230 | 468 | 871 | 1126 | Uncharacterized protein | Uncharacterized protein | | afdb-uniprot50 | AF-A0A0B2YW47-F1-MODEL\_V4 | 1.0 | 3.062e-20 | 639 | 0.187 | 490 | 260 | 23 | 1 | 461 | 1 | 381 | Uncharacterized protein | Uncharacterized protein | | afdb-uniprot50 | AF-A0A417HJA2-F1-MODEL\_V4 | 1.0 | 1.632e-20 | 639 | 0.175 | 574 | 270 | 22 | 3 | 473 | 1 | 473 | Phage tail protein | Phage tail protein | | afdb-uniprot50 | AF-A0A2E8RZ20-F1-MODEL\_V4 | 1.0 | 1.282e-23 | 638 | 0.159 | 632 | 316 | 29 | 1 | 468 | 2 | 581 | Uncharacterized protein | Uncharacterized protein | | afdb-uniprot50 | AF-A0A448QV01-F1-MODEL\_V4 | 1.0 | 1.474e-17 | 637 | 0.273 | 249 | 161 | 8 | 239 | 474 | 2 | 243 | Phage tail sheath protein | Phage tail sheath protein | | afdb-uniprot50 | AF-A0A844UR49-F1-MODEL\_V4 | 1.0 | 7.743e-17 | 636 | 0.328 | 198 | 131 | 2 | 278 | 474 | 2 | 198 | Uncharacterized protein | Uncharacterized protein | | afdb-uniprot50 | AF-A0A3M1TA51-F1-MODEL\_V4 | 1.0 | 7.235e-24 | 636 | 0.17 | 634 | 310 | 31 | 1 | 471 | 66 | 646 | Phage tail sheath family protein | Phage tail sheath family protein | | afdb-uniprot50 | AF-A0A4Q7FYR0-F1-MODEL\_V4 | 1.0 | 1.357e-23 | 635 | 0.237 | 433 | 218 | 18 | 5 | 337 | 3 | 423 | Uncharacterized protein | Uncharacterized protein | | afdb-uniprot50 | AF-A0A3N5NWC7-F1-MODEL\_V4 | 1.0 | 7.422e-18 | 634 | 0.288 | 253 | 160 | 8 | 230 | 468 | 21 | 267 | Phage tail sheath family protein | Phage tail sheath family protein | | afdb-uniprot50 | AF-A0A349GR39-F1-MODEL\_V4 | 1.0 | 3.906e-21 | 634 | 0.19 | 576 | 259 | 26 | 5 | 474 | 1 | 474 | Phage tail protein | Phage tail protein | | afdb-uniprot50 | AF-A0A4P8XM20-F1-MODEL\_V4 | 1.0 | 5.829e-21 | 633 | 0.17 | 582 | 272 | 22 | 1 | 475 | 1 | 478 | Uncharacterized protein | Uncharacterized protein | | afdb-uniprot50 | AF-F2NYF2-F1-MODEL\_V4 | 1.0 | 4.023e-23 | 633 | 0.163 | 556 | 303 | 23 | 5 | 468 | 1 | 486 | Tail sheath protein | Tail sheath protein | | afdb-uniprot50 | AF-I5B7I5-F1-MODEL\_V4 | 1.0 | 5.435e-24 | 633 | 0.179 | 625 | 326 | 32 | 5 | 468 | 3 | 601 | Phage tail sheath protein FI | Phage tail sheath protein FI | | afdb-uniprot50 | AF-A0A1T5DNU0-F1-MODEL\_V4 | 1.0 | 1.437e-23 | 632 | 0.166 | 638 | 314 | 26 | 1 | 467 | 9 | 599 | Uncharacterized protein | Uncharacterized protein | | afdb-uniprot50 | AF-A0A1B8VUJ7-F1-MODEL\_V4 | 1.0 | 9.754e-21 | 631 | 0.173 | 582 | 271 | 23 | 6 | 475 | 1 | 484 | Phage tail protein | Phage tail protein | | afdb-uniprot50 | AF-A0A6L3F7Z3-F1-MODEL\_V4 | 1.0 | 1.653e-17 | 630 | 0.26 | 250 | 167 | 7 | 231 | 468 | 288 | 531 | Uncharacterized protein | Uncharacterized protein | | afdb-uniprot50 | AF-A0A124U4Q1-F1-MODEL\_V4 | 1.0 | 4.777e-23 | 629 | 0.158 | 586 | 313 | 28 | 1 | 466 | 1 | 526 | Phage tail protein | Phage tail protein | | afdb-uniprot50 | AF-A0A524Q8Z7-F1-MODEL\_V4 | 1.0 | 5.576e-18 | 628 | 0.248 | 250 | 165 | 8 | 231 | 467 | 87 | 326 | Phage tail sheath family protein | Phage tail sheath family protein | | afdb-uniprot50 | AF-A0A2E4F959-F1-MODEL\_V4 | 1.0 | 5.755e-24 | 628 | 0.189 | 622 | 307 | 26 | 3 | 470 | 2 | 580 | Uncharacterized protein | Uncharacterized protein | | afdb-uniprot50 | AF-A0A1V4IW92-F1-MODEL\_V4 | 1.0 | 1.094e-20 | 626 | 0.156 | 583 | 279 | 20 | 5 | 474 | 2 | 484 | Uncharacterized protein | Uncharacterized protein | | afdb-uniprot50 | AF-A0A6B8RL73-F1-MODEL\_V4 | 1.0 | 1.033e-20 | 623 | 0.166 | 571 | 278 | 21 | 5 | 474 | 2 | 475 | Phage tail sheath protein | Phage tail sheath protein | | afdb-uniprot50 | AF-A0A318XBR6-F1-MODEL\_V4 | 1.0 | 7.548e-23 | 623 | 0.168 | 593 | 305 | 24 | 1 | 468 | 22 | 551 | Uncharacterized protein | Uncharacterized protein | | afdb-uniprot50 | AF-A0A1G9NRA0-F1-MODEL\_V4 | 1.0 | 6.732e-23 | 623 | 0.165 | 573 | 308 | 28 | 1 | 467 | 64 | 572 | Uncharacterized protein | Uncharacterized protein | | afdb-uniprot50 | AF-S4XSN5-F1-MODEL\_V4 | 1.0 | 2.271e-23 | 623 | 0.166 | 765 | 288 | 32 | 3 | 468 | 2 | 715 | Uncharacterized protein | Uncharacterized protein | | afdb-uniprot50 | AF-A0A1I6IEN1-F1-MODEL\_V4 | 1.0 | 1.474e-17 | 623 | 0.235 | 251 | 171 | 7 | 229 | 467 | 734 | 975 | Phage tail sheath protein FI | Phage tail sheath protein FI | | afdb-uniprot50 | AF-A0A810LEP1-F1-MODEL\_V4 | 1.0 | 4.569e-20 | 622 | 0.202 | 480 | 272 | 23 | 4 | 468 | 1 | 384 | Uncharacterized protein | Uncharacterized protein | | afdb-uniprot50 | AF-A0A413ED89-F1-MODEL\_V4 | 1.0 | 5.743e-20 | 622 | 0.169 | 585 | 273 | 25 | 1 | 475 | 1 | 482 | Phage tail sheath family protein | Phage tail sheath family protein | | afdb-uniprot50 | AF-A0A0Q7BE77-F1-MODEL\_V4 | 1.0 | 9.63e-24 | 622 | 0.169 | 648 | 292 | 28 | 5 | 468 | 3 | 588 | Uncharacterized protein | Uncharacterized protein | | afdb-uniprot50 | AF-R5BL49-F1-MODEL\_V4 | 1.0 | 1.519e-19 | 621 | 0.184 | 580 | 264 | 21 | 5 | 474 | 2 | 482 | Uncharacterized protein | Uncharacterized protein | | afdb-uniprot50 | AF-A0A7C5ZLD0-F1-MODEL\_V4 | 1.0 | 5.355e-23 | 620 | 0.17 | 594 | 310 | 22 | 1 | 468 | 1 | 537 | Phage tail sheath family protein | Phage tail sheath family protein | | afdb-uniprot50 | AF-A0A6N7AQF3-F1-MODEL\_V4 | 1.0 | 4.127e-17 | 619 | 0.611 | 152 | 59 | 0 | 324 | 475 | 3 | 154 | Major tail sheath protein | Major tail sheath protein | | afdb-uniprot50 | AF-A0A414Q2K8-F1-MODEL\_V4 | 1.0 | 1.728e-20 | 618 | 0.166 | 571 | 273 | 26 | 5 | 474 | 2 | 470 | Phage tail sheath family protein | Phage tail sheath family protein | | afdb-uniprot50 | AF-A0A851IXL8-F1-MODEL\_V4 | 1.0 | 3.635e-20 | 618 | 0.145 | 569 | 293 | 19 | 5 | 474 | 2 | 476 | Phage tail sheath protein | Phage tail sheath protein | | afdb-uniprot50 | AF-A0A4U9UQL5-F1-MODEL\_V4 | 1.0 | 2.435e-20 | 617 | 0.165 | 567 | 259 | 20 | 23 | 475 | 1 | 467 | Phage-related contractile tail sheath protein | Phage-related contractile tail sheath protein | | afdb-uniprot50 | AF-A0A7Z9GAM2-F1-MODEL\_V4 | 1.0 | 1.437e-23 | 617 | 0.152 | 634 | 316 | 30 | 1 | 467 | 1 | 579 | Phage tail sheath family protein | Phage tail sheath family protein | | afdb-uniprot50 | AF-A0A4Q7AXR1-F1-MODEL\_V4 | 1.0 | 1.277e-15 | 615 | 0.32 | 181 | 123 | 0 | 294 | 474 | 15 | 195 | Phage tail protein | Phage tail protein | | afdb-uniprot50 | AF-A0A853IM30-F1-MODEL\_V4 | 1.0 | 3.898e-17 | 615 | 0.28 | 300 | 124 | 6 | 102 | 399 | 2 | 211 | Phage tail sheath subtilisin-like domain-containing protein | Phage tail sheath subtilisin-like domain-containing protein | | afdb-uniprot50 | AF-A0A3N5AEN8-F1-MODEL\_V4 | 1.0 | 7.114e-19 | 615 | 0.182 | 494 | 249 | 22 | 1 | 468 | 1 | 365 | Uncharacterized protein | Uncharacterized protein | | afdb-uniprot50 | AF-A0A454VXY5-F1-MODEL\_V4 | 1.0 | 2.467e-17 | 614 | 0.275 | 247 | 159 | 8 | 230 | 462 | 19 | 259 | Phage tail sheath family protein | Phage tail sheath family protein | | afdb-uniprot50 | AF-F8KPL7-F1-MODEL\_V4 | 1.0 | 3.101e-17 | 614 | 0.218 | 252 | 189 | 5 | 225 | 474 | 27 | 272 | Major tail sheath protein | Major tail sheath protein | | afdb-uniprot50 | AF-A0A1C6BNB5-F1-MODEL\_V4 | 1.0 | 2.3e-20 | 614 | 0.166 | 566 | 279 | 20 | 5 | 474 | 2 | 470 | Uncharacterized protein | Uncharacterized protein | | afdb-uniprot50 | AF-A0A3C0WX80-F1-MODEL\_V4 | 1.0 | 3.642e-24 | 614 | 0.167 | 555 | 302 | 27 | 5 | 468 | 3 | 488 | Phage tail sheath family protein | Phage tail sheath family protein | | afdb-uniprot50 | AF-A0A1N6X8E9-F1-MODEL\_V4 | 1.0 | 5.671e-23 | 614 | 0.166 | 614 | 330 | 26 | 1 | 468 | 3 | 580 | Uncharacterized protein | Uncharacterized protein | | afdb-uniprot50 | AF-A0A7X3MK04-F1-MODEL\_V4 | 1.0 | 8.463e-23 | 612 | 0.168 | 634 | 300 | 27 | 3 | 468 | 2 | 576 | Phage tail sheath family protein | Phage tail sheath family protein | | afdb-uniprot50 | AF-A0A2W6ZWR9-F1-MODEL\_V4 | 1.0 | 3.635e-20 | 612 | 0.21 | 385 | 191 | 12 | 102 | 468 | 562 | 851 | Phage tail protein | Phage tail protein | | afdb-uniprot50 | AF-A0A1A1YQW6-F1-MODEL\_V4 | 1.0 | 1.392e-17 | 612 | 0.241 | 261 | 168 | 8 | 231 | 468 | 857 | 1110 | Uncharacterized protein | Uncharacterized protein | | afdb-uniprot50 | AF-A0A2E1AI36-F1-MODEL\_V4 | 1.0 | 3.589e-23 | 611 | 0.182 | 575 | 287 | 29 | 5 | 468 | 3 | 505 | Phage tail protein | Phage tail protein | | afdb-uniprot50 | AF-A0A1Y4HZF3-F1-MODEL\_V4 | 1.0 | 3.201e-23 | 611 | 0.164 | 609 | 314 | 29 | 3 | 467 | 2 | 559 | Phage tail protein | Phage tail protein | | afdb-uniprot50 | AF-A0A2N2DHN9-F1-MODEL\_V4 | 1.0 | 4.023e-23 | 610 | 0.168 | 619 | 320 | 31 | 5 | 467 | 3 | 582 | Phage tail protein | Phage tail protein | | afdb-uniprot50 | AF-A0A7V9U445-F1-MODEL\_V4 | 1.0 | 3.898e-17 | 609 | 0.254 | 236 | 158 | 8 | 245 | 468 | 12 | 241 | Phage tail sheath family protein | Phage tail sheath family protein | | afdb-uniprot50 | AF-X0PXM3-F1-MODEL\_V4 | 1.0 | 2.2e-17 | 609 | 0.222 | 261 | 173 | 8 | 231 | 468 | 870 | 1123 | Uncharacterized protein | Uncharacterized protein | | afdb-uniprot50 | AF-A0A3A6RI96-F1-MODEL\_V4 | 1.0 | 9.192e-17 | 608 | 0.276 | 297 | 121 | 7 | 77 | 366 | 2 | 211 | Phage tail sheath family protein | Phage tail sheath family protein | | afdb-uniprot50 | AF-A0A416S5I6-F1-MODEL\_V4 | 1.0 | 1.416e-22 | 608 | 0.165 | 557 | 312 | 27 | 3 | 468 | 2 | 496 | Phage tail sheath family protein | Phage tail sheath family protein | | afdb-uniprot50 | AF-A0A5C5ZX69-F1-MODEL\_V4 | 1.0 | 3.589e-23 | 608 | 0.167 | 789 | 320 | 34 | 3 | 468 | 2 | 776 | Phage tail sheath protein | Phage tail sheath protein | | afdb-uniprot50 | AF-A0A836ZRP0-F1-MODEL\_V4 | 1.0 | 1.224e-16 | 607 | 0.32 | 175 | 118 | 1 | 231 | 404 | 8 | 182 | Tail sheath protein | Tail sheath protein | | afdb-uniprot50 | AF-A0A133NQP6-F1-MODEL\_V4 | 1.0 | 8.095e-20 | 606 | 0.165 | 579 | 277 | 21 | 3 | 474 | 1 | 480 | Uncharacterized protein | Uncharacterized protein | | afdb-uniprot50 | AF-A0A7C1G8D3-F1-MODEL\_V4 | 1.0 | 1.885e-22 | 606 | 0.187 | 596 | 295 | 24 | 1 | 468 | 1 | 534 | Phage tail sheath family protein | Phage tail sheath family protein | | afdb-uniprot50 | AF-A0A0J6J0B1-F1-MODEL\_V4 | 1.0 | 4.777e-23 | 606 | 0.169 | 591 | 306 | 23 | 1 | 468 | 14 | 542 | Uncharacterized protein | Uncharacterized protein | | afdb-uniprot50 | AF-A0A6L9A8T7-F1-MODEL\_V4 | 1.0 | 3.433e-20 | 605 | 0.174 | 556 | 253 | 17 | 30 | 475 | 2 | 461 | Phage tail sheath family protein | Phage tail sheath family protein | | afdb-uniprot50 | AF-A0A3A0B079-F1-MODEL\_V4 | 1.0 | 4.628e-17 | 605 | 0.226 | 256 | 174 | 6 | 231 | 468 | 242 | 491 | Uncharacterized protein | Uncharacterized protein | | afdb-uniprot50 | AF-A0A535I9P1-F1-MODEL\_V4 | 1.0 | 2.369e-22 | 604 | 0.193 | 588 | 281 | 24 | 1 | 468 | 1 | 514 | Phage tail sheath family protein | Phage tail sheath family protein | | afdb-uniprot50 | AF-A0A2N0KBH5-F1-MODEL\_V4 | 1.0 | 1.996e-22 | 603 | 0.17 | 635 | 299 | 30 | 3 | 468 | 1 | 576 | Phage tail sheath family protein | Phage tail sheath family protein | | afdb-uniprot50 | AF-A0A2E1AI38-F1-MODEL\_V4 | 1.0 | 6.633e-22 | 602 | 0.135 | 603 | 319 | 24 | 3 | 467 | 2 | 539 | Uncharacterized protein | Uncharacterized protein | | afdb-uniprot50 | AF-A0A7I8MYS9-F1-MODEL\_V4 | 1.0 | 1.499e-22 | 601 | 0.158 | 656 | 320 | 33 | 3 | 468 | 2 | 615 | Phage tail sheath protein FI | Phage tail sheath protein FI | | afdb-uniprot50 | AF-A0A2V9PGB7-F1-MODEL\_V4 | 1.0 | 1.156e-16 | 600 | 0.233 | 231 | 161 | 5 | 245 | 466 | 12 | 235 | Uncharacterized protein | Uncharacterized protein | | afdb-uniprot50 | AF-A0A660S5R2-F1-MODEL\_V4 | 1.0 | 1.108e-17 | 600 | 0.137 | 481 | 259 | 19 | 1 | 466 | 1 | 340 | Phage tail sheath protein | Phage tail sheath protein | | afdb-uniprot50 | AF-A0A7W7XTS9-F1-MODEL\_V4 | 1.0 | 1.909e-19 | 600 | 0.163 | 477 | 266 | 15 | 3 | 455 | 2 | 369 | Uncharacterized protein | Uncharacterized protein | | afdb-uniprot50 | AF-Q2N8P2-F1-MODEL\_V4 | 1.0 | 1.588e-22 | 600 | 0.168 | 658 | 315 | 33 | 3 | 468 | 2 | 619 | Phage tail sheath protein, putative | Phage tail sheath protein, putative | | afdb-uniprot50 | AF-A0A1Y6BIP2-F1-MODEL\_V4 | 1.0 | 8.463e-23 | 600 | 0.172 | 679 | 304 | 28 | 1 | 468 | 1 | 632 | Phage\_sheath\_1C domain-containing protein | Phage\_sheath\_1C domain-containing protein | | afdb-uniprot50 | AF-A0A1M5CL98-F1-MODEL\_V4 | 1.0 | 1.126e-22 | 599 | 0.175 | 594 | 292 | 26 | 3 | 468 | 2 | 525 | Uncharacterized protein | Uncharacterized protein | | afdb-uniprot50 | AF-A0A497VVB2-F1-MODEL\_V4 | 1.0 | 1.416e-22 | 599 | 0.148 | 658 | 309 | 30 | 5 | 468 | 3 | 602 | Uncharacterized protein | Uncharacterized protein | | afdb-uniprot50 | AF-A0A1H8WFQ1-F1-MODEL\_V4 | 1.0 | 9.33e-18 | 599 | 0.241 | 253 | 166 | 9 | 231 | 468 | 736 | 977 | Phage tail sheath protein FI | Phage tail sheath protein FI | | afdb-uniprot50 | AF-A0A351BHJ8-F1-MODEL\_V4 | 1.0 | 4.37e-17 | 598 | 0.266 | 259 | 164 | 8 | 230 | 468 | 31 | 283 | Phage tail sheath family protein | Phage tail sheath family protein | | afdb-uniprot50 | AF-A0A143PL89-F1-MODEL\_V4 | 1.0 | 5.916e-22 | 598 | 0.153 | 579 | 312 | 22 | 5 | 468 | 3 | 518 | Major tail sheath protein | Major tail sheath protein | | afdb-uniprot50 | AF-A0A117NJJ3-F1-MODEL\_V4 | 1.0 | 1.193e-22 | 598 | 0.164 | 577 | 322 | 27 | 3 | 470 | 1 | 526 | Uncharacterized protein | Uncharacterized protein | | afdb-uniprot50 | AF-A0A2E5HMA5-F1-MODEL\_V4 | 1.0 | 1.499e-22 | 598 | 0.149 | 647 | 312 | 29 | 3 | 468 | 2 | 590 | Uncharacterized protein | Uncharacterized protein | | afdb-uniprot50 | AF-A0A1H6TSH5-F1-MODEL\_V4 | 1.0 | 1.885e-22 | 598 | 0.169 | 671 | 301 | 31 | 3 | 468 | 2 | 620 | Uncharacterized protein | Uncharacterized protein | | afdb-uniprot50 | AF-A0A3A8B9H2-F1-MODEL\_V4 | 1.0 | 1.263e-22 | 598 | 0.152 | 710 | 327 | 30 | 3 | 468 | 2 | 680 | Uncharacterized protein | Uncharacterized protein | | afdb-uniprot50 | AF-A0A7Z7I241-F1-MODEL\_V4 | 1.0 | 2.33e-17 | 598 | 0.255 | 262 | 164 | 10 | 231 | 468 | 895 | 1149 | Phage tail sheath protein FI | Phage tail sheath protein FI | | afdb-uniprot50 | AF-A0A7S9UQU7-F1-MODEL\_V4 | 1.0 | 7.313e-17 | 597 | 0.234 | 260 | 169 | 8 | 231 | 467 | 266 | 518 | Phage tail sheath family protein | Phage tail sheath family protein | | afdb-uniprot50 | AF-A0A1Q3SJK3-F1-MODEL\_V4 | 1.0 | 5.355e-23 | 597 | 0.161 | 637 | 330 | 25 | 10 | 468 | 1 | 611 | Uncharacterized protein | Uncharacterized protein | | afdb-uniprot50 | AF-W4RWM2-F1-MODEL\_V4 | 1.0 | 3.574e-15 | 596 | 0.331 | 166 | 111 | 0 | 309 | 474 | 2 | 167 | Tail sheath protein | Tail sheath protein | | afdb-uniprot50 | AF-A0A3S9PC88-F1-MODEL\_V4 | 1.0 | 3.898e-17 | 595 | 0.25 | 256 | 169 | 9 | 230 | 468 | 144 | 393 | Phage tail sheath family protein | Phage tail sheath family protein | | afdb-uniprot50 | AF-A0A2K4ZHW7-F1-MODEL\_V4 | 1.0 | 2.172e-20 | 594 | 0.175 | 586 | 261 | 28 | 5 | 473 | 2 | 482 | Uncharacterized protein | Uncharacterized protein | | afdb-uniprot50 | AF-A0A7V6E9A4-F1-MODEL\_V4 | 1.0 | 1.996e-22 | 594 | 0.133 | 624 | 343 | 27 | 1 | 467 | 6 | 588 | Phage tail sheath family protein | Phage tail sheath family protein | | afdb-uniprot50 | AF-C7RKK7-F1-MODEL\_V4 | 1.0 | 2.978e-22 | 594 | 0.171 | 654 | 316 | 34 | 3 | 468 | 2 | 617 | Tail sheath protein | Tail sheath protein | | afdb-uniprot50 | AF-A0A1Q7BLH0-F1-MODEL\_V4 | 1.0 | 1.126e-22 | 594 | 0.139 | 746 | 319 | 29 | 5 | 468 | 3 | 707 | Uncharacterized protein | Uncharacterized protein | | afdb-uniprot50 | AF-A0A1L6L570-F1-MODEL\_V4 | 1.0 | 4.983e-22 | 593 | 0.156 | 689 | 311 | 31 | 1 | 468 | 145 | 783 | Phage tail sheath protein FI | Phage tail sheath protein FI | | afdb-uniprot50 | AF-A0A011MDR6-F1-MODEL\_V4 | 1.0 | 1.608e-19 | 593 | 0.206 | 387 | 196 | 12 | 102 | 468 | 706 | 1001 | Major tail sheath protein | Major tail sheath protein | | afdb-uniprot50 | AF-A0A317HHF4-F1-MODEL\_V4 | 1.0 | 3.792e-19 | 592 | 0.148 | 491 | 280 | 19 | 3 | 467 | 15 | 393 | Phage tail sheath family protein | Phage tail sheath family protein | | afdb-uniprot50 | AF-A0A158AZ89-F1-MODEL\_V4 | 1.0 | 2.14e-19 | 592 | 0.165 | 509 | 275 | 17 | 1 | 468 | 15 | 414 | Tail sheath protein | Tail sheath protein | | afdb-uniprot50 | AF-A0A3N5PYF2-F1-MODEL\_V4 | 1.0 | 3.681e-17 | 592 | 0.242 | 260 | 168 | 9 | 231 | 468 | 360 | 612 | Phage tail sheath family protein | Phage tail sheath family protein | | afdb-uniprot50 | AF-A0A1V0Q0C4-F1-MODEL\_V4 | 1.0 | 3.107e-21 | 592 | 0.139 | 730 | 335 | 25 | 5 | 467 | 1 | 703 | Phage tail sheath protein FI | Phage tail sheath protein FI | | afdb-uniprot50 | AF-A0A7W0WXT9-F1-MODEL\_V4 | 1.0 | 1.413e-18 | 591 | 0.2 | 429 | 205 | 16 | 1 | 399 | 1 | 321 | Phage tail sheath family protein | Phage tail sheath family protein | | afdb-uniprot50 | AF-A0A1X4H0I2-F1-MODEL\_V4 | 1.0 | 4.9e-17 | 591 | 0.213 | 258 | 177 | 8 | 230 | 467 | 99 | 350 | Uncharacterized protein | Uncharacterized protein | | afdb-uniprot50 | AF-A0A1W9H7R4-F1-MODEL\_V4 | 1.0 | 1.046e-17 | 590 | 0.192 | 446 | 233 | 20 | 46 | 466 | 1 | 344 | Phage\_sheath\_1C domain-containing protein | Phage\_sheath\_1C domain-containing protein | | afdb-uniprot50 | AF-A0A525C5G4-F1-MODEL\_V4 | 1.0 | 1.78e-22 | 590 | 0.158 | 581 | 307 | 26 | 5 | 466 | 3 | 520 | Phage tail sheath family protein | Phage tail sheath family protein | | afdb-uniprot50 | AF-D0LXB9-F1-MODEL\_V4 | 1.0 | 6.264e-22 | 590 | 0.158 | 624 | 326 | 30 | 1 | 467 | 2 | 583 | Tail sheath protein | Tail sheath protein | | afdb-uniprot50 | AF-A0A845WCQ0-F1-MODEL\_V4 | 1.0 | 5.344e-19 | 589 | 0.162 | 485 | 268 | 21 | 1 | 466 | 1 | 366 | Phage tail sheath protein | Phage tail sheath protein | | afdb-uniprot50 | AF-A0A6G4XFE5-F1-MODEL\_V4 | 1.0 | 1.885e-22 | 589 | 0.155 | 567 | 303 | 18 | 1 | 467 | 7 | 497 | Phage tail sheath family protein | Phage tail sheath family protein | | afdb-uniprot50 | AF-A0A1H2U4F8-F1-MODEL\_V4 | 1.0 | 1.588e-22 | 589 | 0.178 | 661 | 310 | 32 | 3 | 468 | 1 | 623 | Uncharacterized protein | Uncharacterized protein | | afdb-uniprot50 | AF-X5R2Z1-F1-MODEL\_V4 | 1.0 | 2.813e-22 | 587 | 0.176 | 579 | 294 | 23 | 3 | 468 | 2 | 510 | Tail protein | Tail protein | | afdb-uniprot50 | AF-A0A4D9Y1N5-F1-MODEL\_V4 | 1.0 | 6.332e-15 | 585 | 0.416 | 149 | 87 | 0 | 326 | 474 | 1 | 149 | Phage tail protein | Phage tail protein | | afdb-uniprot50 | AF-A0A7T9VKM0-F1-MODEL\_V4 | 1.0 | 9.59e-16 | 585 | 0.327 | 186 | 120 | 2 | 294 | 474 | 7 | 192 | Phage tail protein | Phage tail protein | | afdb-uniprot50 | AF-A0A3M1BL67-F1-MODEL\_V4 | 1.0 | 2.233e-18 | 585 | 0.247 | 380 | 167 | 15 | 3 | 351 | 1 | 292 | Phage tail sheath family protein | Phage tail sheath family protein | | afdb-uniprot50 | AF-A0A562GH79-F1-MODEL\_V4 | 1.0 | 1.244e-21 | 585 | 0.181 | 534 | 305 | 23 | 1 | 468 | 1 | 468 | Phage\_sheath\_1 domain-containing protein | Phage\_sheath\_1 domain-containing protein | | afdb-uniprot50 | AF-A0A1V5CVP8-F1-MODEL\_V4 | 1.0 | 5.188e-17 | 584 | 0.273 | 256 | 160 | 11 | 231 | 466 | 177 | 426 | Phage tail sheath protein | Phage tail sheath protein | | afdb-uniprot50 | AF-A0A5Q0GXI4-F1-MODEL\_V4 | 1.0 | 8.338e-22 | 584 | 0.16 | 581 | 314 | 20 | 3 | 467 | 15 | 537 | Phage tail sheath family protein | Phage tail sheath family protein | | afdb-uniprot50 | AF-A0A4Q9KMC0-F1-MODEL\_V4 | 1.0 | 1.395e-21 | 584 | 0.147 | 707 | 336 | 30 | 3 | 468 | 2 | 682 | Phage tail protein | Phage tail protein | | afdb-uniprot50 | AF-I5B7I6-F1-MODEL\_V4 | 1.0 | 2.509e-22 | 584 | 0.163 | 732 | 311 | 33 | 3 | 468 | 2 | 697 | Phage tail sheath protein FI | Phage tail sheath protein FI | | afdb-uniprot50 | AF-A0A1M6SAY6-F1-MODEL\_V4 | 1.0 | 8.942e-19 | 583 | 0.155 | 586 | 276 | 25 | 4 | 475 | 1 | 481 | Uncharacterized protein | Uncharacterized protein | | afdb-uniprot50 | AF-R7Z895-F1-MODEL\_V4 | 1.0 | 5.123e-20 | 582 | 0.173 | 581 | 266 | 27 | 5 | 475 | 1 | 477 | Uncharacterized protein | Uncharacterized protein | | afdb-uniprot50 | AF-A0A1V4GEV1-F1-MODEL\_V4 | 1.0 | 6.44e-20 | 582 | 0.168 | 589 | 264 | 25 | 5 | 474 | 2 | 483 | Uncharacterized protein | Uncharacterized protein | | afdb-uniprot50 | AF-A0A4Q3UKU1-F1-MODEL\_V4 | 1.0 | 8.338e-22 | 582 | 0.151 | 653 | 317 | 29 | 3 | 467 | 2 | 605 | Phage\_sheath\_1C domain-containing protein | Phage\_sheath\_1C domain-containing protein | | afdb-uniprot50 | AF-A0A2V2S183-F1-MODEL\_V4 | 1.0 | 2.509e-22 | 582 | 0.157 | 712 | 323 | 32 | 5 | 467 | 3 | 686 | Uncharacterized protein | Uncharacterized protein | | afdb-uniprot50 | AF-A0A2S0JKR3-F1-MODEL\_V4 | 1.0 | 9.076e-20 | 581 | 0.175 | 581 | 267 | 26 | 1 | 475 | 1 | 475 | Phage tail protein | Phage tail protein | | afdb-uniprot50 | AF-A0A2R6JS05-F1-MODEL\_V4 | 1.0 | 8.338e-22 | 580 | 0.153 | 625 | 307 | 29 | 5 | 468 | 3 | 566 | Phage tail protein | Phage tail protein | | afdb-uniprot50 | AF-A0A4P5W0E4-F1-MODEL\_V4 | 1.0 | 3.792e-19 | 579 | 0.171 | 496 | 275 | 21 | 6 | 468 | 1 | 393 | Uncharacterized protein | Uncharacterized protein | | afdb-uniprot50 | AF-A0A524Q0G6-F1-MODEL\_V4 | 1.0 | 1.933e-16 | 578 | 0.25 | 244 | 164 | 7 | 235 | 467 | 3 | 238 | Phage tail sheath family protein | Phage tail sheath family protein | | afdb-uniprot50 | AF-A0A6S7AUX4-F1-MODEL\_V4 | 1.0 | 4.706e-22 | 578 | 0.153 | 769 | 323 | 32 | 3 | 468 | 2 | 745 | Uncharacterized protein | Uncharacterized protein | | afdb-uniprot50 | AF-A0A496NWC5-F1-MODEL\_V4 | 1.0 | 3.194e-19 | 577 | 0.153 | 580 | 283 | 20 | 3 | 474 | 1 | 480 | Phage tail sheath family protein | Phage tail sheath family protein | | afdb-uniprot50 | AF-A0A7W0PUB1-F1-MODEL\_V4 | 1.0 | 6.805e-16 | 577 | 0.23 | 234 | 165 | 5 | 231 | 455 | 272 | 499 | Phage tail sheath family protein | Phage tail sheath family protein | | afdb-uniprot50 | AF-A0A827A5S8-F1-MODEL\_V4 | 1.0 | 2.69e-19 | 576 | 0.23 | 456 | 135 | 8 | 5 | 343 | 2 | 358 | Phage tail protein | Phage tail protein | | afdb-uniprot50 | AF-A0A5A8F1E7-F1-MODEL\_V4 | 1.0 | 3.147e-18 | 576 | 0.174 | 499 | 260 | 22 | 5 | 474 | 2 | 377 | Uncharacterized protein | Uncharacterized protein | | afdb-uniprot50 | AF-A0A849EW92-F1-MODEL\_V4 | 1.0 | 5.188e-17 | 576 | 0.254 | 259 | 166 | 10 | 231 | 468 | 507 | 759 | Phage tail sheath family protein | Phage tail sheath family protein | | afdb-uniprot50 | AF-A0A4Q9TLA8-F1-MODEL\_V4 | 1.0 | 5.344e-19 | 575 | 0.157 | 576 | 281 | 25 | 1 | 475 | 1 | 472 | Phage tail sheath family protein | Phage tail sheath family protein | | afdb-uniprot50 | AF-J2WTG1-F1-MODEL\_V4 | 1.0 | 1.091e-16 | 575 | 0.217 | 248 | 177 | 7 | 230 | 468 | 322 | 561 | Phage tail sheath protein FI | Phage tail sheath protein FI | | afdb-uniprot50 | AF-S7TBH7-F1-MODEL\_V4 | 1.0 | 3.906e-21 | 574 | 0.159 | 557 | 332 | 26 | 3 | 473 | 2 | 508 | Phage tail sheath protein | Phage tail sheath protein | | afdb-uniprot50 | AF-A0A7I8MZ51-F1-MODEL\_V4 | 1.0 | 3.898e-17 | 574 | 0.247 | 246 | 168 | 8 | 234 | 468 | 272 | 511 | Phage\_sheath\_1C domain-containing protein | Phage\_sheath\_1C domain-containing protein | | afdb-uniprot50 | AF-A0A1N6L5N5-F1-MODEL\_V4 | 1.0 | 7.875e-22 | 573 | 0.166 | 560 | 313 | 22 | 1 | 466 | 1 | 500 | Uncharacterized protein | Uncharacterized protein | | afdb-uniprot50 | AF-A0A4Q7LV90-F1-MODEL\_V4 | 1.0 | 3.906e-21 | 573 | 0.185 | 603 | 304 | 28 | 1 | 467 | 11 | 562 | Uncharacterized protein | Uncharacterized protein | | afdb-uniprot50 | AF-A0A7T9J4P3-F1-MODEL\_V4 | 1.0 | 3.29e-21 | 572 | 0.148 | 560 | 326 | 27 | 1 | 472 | 3 | 499 | Phage tail sheath family protein | Phage tail sheath family protein | | afdb-uniprot50 | AF-A0A661R710-F1-MODEL\_V4 | 1.0 | 3.536e-22 | 572 | 0.16 | 637 | 333 | 33 | 3 | 468 | 2 | 607 | Phage tail sheath family protein | Phage tail sheath family protein | | afdb-uniprot50 | AF-A0A7Y5WMY0-F1-MODEL\_V4 | 1.0 | 6.633e-22 | 572 | 0.157 | 717 | 333 | 32 | 3 | 467 | 1 | 698 | Uncharacterized protein | Uncharacterized protein | | afdb-uniprot50 | AF-I4IAB3-F1-MODEL\_V4 | 1.0 | 3.477e-17 | 572 | 0.225 | 257 | 172 | 8 | 231 | 466 | 687 | 937 | Putative phage tail sheath protein (Modular protein) | Putative phage tail sheath protein (Modular protein) | | afdb-uniprot50 | AF-A0A5T1QWS3-F1-MODEL\_V4 | 1.0 | 2.2e-17 | 571 | 0.242 | 330 | 152 | 6 | 71 | 397 | 51 | 285 | Phage tail sheath family protein | Phage tail sheath family protein | | afdb-uniprot50 | AF-A0A2E7CK62-F1-MODEL\_V4 | 1.0 | 2.109e-18 | 571 | 0.153 | 489 | 294 | 21 | 1 | 467 | 2 | 392 | Uncharacterized protein | Uncharacterized protein | | afdb-uniprot50 | AF-A0A5M9U7D7-F1-MODEL\_V4 | 1.0 | 1.26e-18 | 571 | 0.154 | 583 | 284 | 19 | 3 | 475 | 1 | 484 | Phage tail sheath family protein | Phage tail sheath family protein | | afdb-uniprot50 | AF-A0A7V6Q5J0-F1-MODEL\_V4 | 1.0 | 1.141e-19 | 570 | 0.18 | 586 | 259 | 28 | 1 | 474 | 1 | 476 | Uncharacterized protein | Uncharacterized protein | | afdb-uniprot50 | AF-A0A2E0KUM3-F1-MODEL\_V4 | 1.0 | 2.205e-21 | 570 | 0.153 | 698 | 318 | 31 | 3 | 468 | 1 | 657 | Uncharacterized protein | Uncharacterized protein | | afdb-uniprot50 | AF-A0A1C3FCE3-F1-MODEL\_V4 | 1.0 | 7.099e-15 | 569 | 0.375 | 181 | 106 | 3 | 299 | 473 | 2 | 181 | Phage tail sheath monomer | Phage tail sheath monomer | | afdb-uniprot50 | AF-A0A3R8ZYP4-F1-MODEL\_V4 | 1.0 | 1.538e-16 | 569 | 0.333 | 186 | 120 | 3 | 254 | 438 | 44 | 226 | Phage\_sheath\_1 domain-containing protein | Phage\_sheath\_1 domain-containing protein | | afdb-uniprot50 | AF-A0A7W4H212-F1-MODEL\_V4 | 1.0 | 4.767e-19 | 569 | 0.198 | 463 | 237 | 19 | 11 | 456 | 3 | 348 | Phage tail sheath subtilisin-like domain-containing protein | Phage tail sheath subtilisin-like domain-containing protein | | afdb-uniprot50 | AF-A0A7T7XS11-F1-MODEL\_V4 | 1.0 | 4.828e-16 | 569 | 0.264 | 242 | 158 | 7 | 230 | 457 | 228 | 463 | Phage tail sheath family protein | Phage tail sheath family protein | | afdb-uniprot50 | AF-A0A2N1QG40-F1-MODEL\_V4 | 1.0 | 5.047e-19 | 569 | 0.164 | 584 | 274 | 22 | 5 | 474 | 2 | 485 | Phage tail protein | Phage tail protein | | afdb-uniprot50 | AF-A0A494XZK3-F1-MODEL\_V4 | 1.0 | 3.681e-17 | 568 | 0.15 | 492 | 250 | 20 | 1 | 468 | 1 | 348 | Phage tail sheath family protein | Phage tail sheath family protein | | afdb-uniprot50 | AF-A0A4R1HQ95-F1-MODEL\_V4 | 1.0 | 6.264e-22 | 568 | 0.146 | 671 | 327 | 36 | 1 | 467 | 1 | 629 | Uncharacterized protein | Uncharacterized protein | | afdb-uniprot50 | AF-A0A5X8YUE9-F1-MODEL\_V4 | 1.0 | 1.001e-14 | 567 | 0.329 | 173 | 115 | 1 | 302 | 474 | 2 | 173 | Phage tail sheath family protein | Phage tail sheath family protein | | afdb-uniprot50 | AF-A0A3M1CYL5-F1-MODEL\_V4 | 1.0 | 4.252e-19 | 566 | 0.148 | 498 | 283 | 22 | 7 | 468 | 1 | 393 | Phage tail sheath family protein | Phage tail sheath family protein | | afdb-uniprot50 | AF-A0A832W4U3-F1-MODEL\_V4 | 1.0 | 2.364e-18 | 566 | 0.192 | 384 | 209 | 7 | 95 | 467 | 239 | 532 | Phage tail sheath family protein | Phage tail sheath family protein | | afdb-uniprot50 | AF-A0A6G9ZD31-F1-MODEL\_V4 | 1.0 | 4.306e-16 | 565 | 0.241 | 253 | 171 | 8 | 231 | 468 | 157 | 403 | Uncharacterized protein | Uncharacterized protein | | afdb-uniprot50 | AF-A0A7V1ZW97-F1-MODEL\_V4 | 1.0 | 8.829e-22 | 565 | 0.147 | 625 | 326 | 28 | 3 | 468 | 2 | 578 | Phage tail sheath family protein | Phage tail sheath family protein | | afdb-uniprot50 | AF-W5WF51-F1-MODEL\_V4 | 1.0 | 1.966e-21 | 565 | 0.148 | 652 | 329 | 28 | 1 | 468 | 2 | 610 | Uncharacterized protein | Uncharacterized protein | | afdb-uniprot50 | AF-A0A3A0GJR6-F1-MODEL\_V4 | 1.0 | 2.771e-21 | 565 | 0.153 | 744 | 302 | 37 | 1 | 466 | 1 | 694 | Phage tail sheath family protein | Phage tail sheath family protein | | afdb-uniprot50 | AF-A0A6P0TJE0-F1-MODEL\_V4 | 1.0 | 1.724e-16 | 564 | 0.203 | 275 | 176 | 9 | 231 | 468 | 240 | 508 | Phage tail sheath family protein | Phage tail sheath family protein | | afdb-uniprot50 | AF-A0A7V5V361-F1-MODEL\_V4 | 1.0 | 1.477e-21 | 564 | 0.177 | 661 | 318 | 34 | 5 | 466 | 3 | 636 | Phage tail sheath family protein | Phage tail sheath family protein | | afdb-uniprot50 | AF-A0A2K8MIB7-F1-MODEL\_V4 | 1.0 | 1.678e-18 | 564 | 0.212 | 381 | 188 | 12 | 101 | 467 | 647 | 929 | Uncharacterized protein | Uncharacterized protein | | afdb-uniprot50 | AF-A0A0Q6V5H7-F1-MODEL\_V4 | 1.0 | 1.11e-21 | 563 | 0.144 | 673 | 343 | 31 | 1 | 467 | 1 | 646 | Uncharacterized protein | Uncharacterized protein | | afdb-uniprot50 | AF-A0A1Q6YDS8-F1-MODEL\_V4 | 1.0 | 1.656e-21 | 563 | 0.158 | 731 | 321 | 32 | 3 | 468 | 2 | 702 | Uncharacterized protein | Uncharacterized protein | | afdb-uniprot50 | AF-A0A826YSY8-F1-MODEL\_V4 | 1.0 | 1.206e-15 | 562 | 0.424 | 158 | 90 | 1 | 272 | 428 | 5 | 162 | Phage tail protein | Phage tail protein | | afdb-uniprot50 | AF-A0A511MZM7-F1-MODEL\_V4 | 1.0 | 1.564e-21 | 562 | 0.145 | 733 | 322 | 35 | 3 | 468 | 2 | 696 | Uncharacterized protein | Uncharacterized protein | | afdb-uniprot50 | AF-A0A4Y8ND74-F1-MODEL\_V4 | 1.0 | 2.364e-18 | 561 | 0.182 | 389 | 208 | 11 | 93 | 468 | 226 | 517 | Phage tail sheath family protein | Phage tail sheath family protein | | afdb-uniprot50 | AF-A0A7U3YQ73-F1-MODEL\_V4 | 1.0 | 7.875e-22 | 561 | 0.17 | 661 | 316 | 40 | 3 | 466 | 1 | 625 | Phage tail sheath protein | Phage tail sheath protein | | afdb-uniprot50 | AF-A0A6J4YL04-F1-MODEL\_V4 | 1.0 | 6.906e-17 | 561 | 0.249 | 269 | 163 | 10 | 231 | 466 | 739 | 1001 | Phage tail sheath protein FI | Phage tail sheath protein FI | | afdb-uniprot50 | AF-A0A2U3LUJ3-F1-MODEL\_V4 | 1.0 | 1.724e-16 | 560 | 0.241 | 265 | 172 | 9 | 227 | 468 | 96 | 354 | Phage tail sheath protein | Phage tail sheath protein | | afdb-uniprot50 | AF-A0A7V8YCS0-F1-MODEL\_V4 | 1.0 | 1.992e-18 | 560 | 0.131 | 503 | 291 | 26 | 1 | 467 | 2 | 394 | Phage tail sheath family protein | Phage tail sheath family protein | | afdb-uniprot50 | AF-A0A1Q4ZX14-F1-MODEL\_V4 | 1.0 | 1.372e-16 | 560 | 0.251 | 262 | 165 | 9 | 230 | 466 | 727 | 982 | Uncharacterized protein | Uncharacterized protein | | afdb-uniprot50 | AF-A0A1G9EWK1-F1-MODEL\_V4 | 1.0 | 1.564e-21 | 559 | 0.145 | 696 | 313 | 35 | 1 | 467 | 1 | 643 | Uncharacterized protein | Uncharacterized protein | | afdb-uniprot50 | AF-A0A1Q6YE31-F1-MODEL\_V4 | 1.0 | 7.437e-22 | 558 | 0.156 | 709 | 328 | 35 | 3 | 467 | 2 | 684 | Uncharacterized protein | Uncharacterized protein | | afdb-uniprot50 | AF-A0A399ZLA4-F1-MODEL\_V4 | 1.0 | 3.689e-21 | 558 | 0.141 | 867 | 315 | 34 | 5 | 468 | 3 | 842 | Uncharacterized protein | Uncharacterized protein | | afdb-uniprot50 | AF-A0A1Y3SWA1-F1-MODEL\_V4 | 1.0 | 1.26e-18 | 556 | 0.172 | 575 | 271 | 25 | 6 | 474 | 1 | 476 | Phage tail protein | Phage tail protein | | afdb-uniprot50 | AF-A0A6N8HUV9-F1-MODEL\_V4 | 1.0 | 2.771e-21 | 556 | 0.184 | 557 | 284 | 29 | 12 | 463 | 1 | 491 | Phage tail sheath protein subtilisin-like domain protein | Phage tail sheath protein subtilisin-like domain protein | | afdb-uniprot50 | AF-A0A7C6A7S9-F1-MODEL\_V4 | 1.0 | 4.637e-21 | 555 | 0.157 | 551 | 308 | 25 | 1 | 468 | 1 | 478 | Phage\_sheath\_1C domain-containing protein | Phage\_sheath\_1C domain-containing protein | | afdb-uniprot50 | AF-C4ZMC0-F1-MODEL\_V4 | 1.0 | 9.754e-21 | 555 | 0.149 | 770 | 303 | 37 | 3 | 468 | 2 | 723 | Tail sheath protein | Tail sheath protein | | afdb-uniprot50 | AF-A0A3B0QYN2-F1-MODEL\_V4 | 1.0 | 1.937e-20 | 554 | 0.148 | 572 | 314 | 24 | 3 | 467 | 1 | 506 | Phage tail sheath protein FI | Phage tail sheath protein FI | | afdb-uniprot50 | AF-A0A1I3I3B9-F1-MODEL\_V4 | 1.0 | 2.205e-21 | 554 | 0.165 | 628 | 304 | 32 | 3 | 468 | 2 | 571 | Uncharacterized protein | Uncharacterized protein | | afdb-uniprot50 | AF-A0A0S4LDL9-F1-MODEL\_V4 | 1.0 | 3.107e-21 | 554 | 0.149 | 696 | 324 | 34 | 1 | 467 | 1 | 657 | Putative Phage tail sheath protein | Putative Phage tail sheath protein | | afdb-uniprot50 | AF-A0A6C9SSS4-F1-MODEL\_V4 | 1.0 | 4.18e-14 | 553 | 0.421 | 147 | 85 | 0 | 328 | 474 | 1 | 147 | Phage tail protein | Phage tail protein | | afdb-uniprot50 | AF-A0A5F1HSS1-F1-MODEL\_V4 | 1.0 | 2.078e-17 | 553 | 0.156 | 493 | 255 | 20 | 1 | 468 | 11 | 367 | Phage tail protein | Phage tail protein | | afdb-uniprot50 | AF-A0A5C8AYR4-F1-MODEL\_V4 | 1.0 | 7.976e-19 | 553 | 0.156 | 580 | 279 | 25 | 3 | 474 | 1 | 477 | Uncharacterized protein | Uncharacterized protein | | afdb-uniprot50 | AF-A0A401Z4M6-F1-MODEL\_V4 | 1.0 | 1.315e-17 | 553 | 0.231 | 281 | 166 | 9 | 230 | 466 | 462 | 736 | Tail protein | Tail protein | | afdb-uniprot50 | AF-A0A3A9D0K2-F1-MODEL\_V4 | 1.0 | 1.335e-18 | 552 | 0.153 | 591 | 280 | 27 | 3 | 474 | 2 | 490 | Phage tail sheath family protein | Phage tail sheath family protein | | afdb-uniprot50 | AF-A0A3D4UZL2-F1-MODEL\_V4 | 1.0 | 2.017e-15 | 552 | 0.184 | 249 | 187 | 8 | 230 | 468 | 248 | 490 | Uncharacterized protein | Uncharacterized protein | | afdb-uniprot50 | AF-A0A329LW71-F1-MODEL\_V4 | 1.0 | 2.771e-21 | 552 | 0.145 | 832 | 325 | 33 | 5 | 468 | 3 | 816 | Phage tail protein | Phage tail protein | | afdb-uniprot50 | AF-A0A4Q3MWA4-F1-MODEL\_V4 | 1.0 | 3.484e-21 | 551 | 0.16 | 616 | 296 | 31 | 9 | 468 | 8 | 558 | Phage tail sheath family protein | Phage tail sheath family protein | | afdb-uniprot50 | AF-A0A7S9UGE8-F1-MODEL\_V4 | 1.0 | 1.728e-20 | 551 | 0.136 | 702 | 322 | 24 | 9 | 468 | 8 | 666 | Phage tail sheath family protein | Phage tail sheath family protein | | afdb-uniprot50 | AF-A0A3N5L8K0-F1-MODEL\_V4 | 1.0 | 8.216e-21 | 551 | 0.157 | 755 | 308 | 34 | 5 | 459 | 3 | 729 | Phage tail protein | Phage tail protein | | afdb-uniprot50 | AF-A0A1Y4I4D9-F1-MODEL\_V4 | 1.0 | 5.829e-21 | 550 | 0.185 | 540 | 302 | 28 | 5 | 475 | 2 | 472 | Uncharacterized protein | Uncharacterized protein | | afdb-uniprot50 | AF-A0A2S7K9H5-F1-MODEL\_V4 | 1.0 | 1.909e-19 | 550 | 0.195 | 482 | 247 | 22 | 9 | 467 | 108 | 471 | Uncharacterized protein | Uncharacterized protein | | afdb-uniprot50 | AF-A0A1P8MV71-F1-MODEL\_V4 | 1.0 | 1.226e-20 | 550 | 0.131 | 708 | 314 | 24 | 3 | 468 | 2 | 650 | Uncharacterized protein | Uncharacterized protein | | afdb-uniprot50 | AF-A0A4Q7YEW2-F1-MODEL\_V4 | 1.0 | 2.771e-21 | 549 | 0.155 | 559 | 320 | 22 | 1 | 468 | 6 | 503 | Phage\_sheath\_1 domain-containing protein | Phage\_sheath\_1 domain-containing protein | | afdb-uniprot50 | AF-A0A5B7V9X8-F1-MODEL\_V4 | 1.0 | 3.792e-19 | 549 | 0.193 | 429 | 215 | 14 | 56 | 468 | 230 | 543 | Phage tail sheath protein | Phage tail sheath protein | | afdb-uniprot50 | AF-A0A0D8CYY8-F1-MODEL\_V4 | 1.0 | 8.338e-22 | 549 | 0.17 | 647 | 325 | 37 | 3 | 466 | 1 | 618 | Uncharacterized protein | Uncharacterized protein | | afdb-uniprot50 | AF-E3HBL6-F1-MODEL\_V4 | 1.0 | 5.904e-18 | 548 | 0.15 | 590 | 274 | 24 | 5 | 475 | 1 | 482 | Uncharacterized protein | Uncharacterized protein | | afdb-uniprot50 | AF-A0A7Z6LAT1-F1-MODEL\_V4 | 1.0 | 3.689e-21 | 547 | 0.266 | 364 | 198 | 14 | 162 | 474 | 91 | 436 | Phage tail protein | Phage tail protein | | afdb-uniprot50 | AF-A0A4V6JGZ6-F1-MODEL\_V4 | 1.0 | 1.19e-18 | 546 | 0.24 | 429 | 179 | 12 | 2 | 398 | 46 | 359 | Phage tail sheath protein | Phage tail sheath protein | | afdb-uniprot50 | AF-A0A1L6M114-F1-MODEL\_V4 | 1.0 | 2.766e-17 | 545 | 0.164 | 500 | 267 | 17 | 2 | 468 | 7 | 388 | Phage tail sheath protein FI | Phage tail sheath protein FI | | afdb-uniprot50 | AF-A0A0Q8V3E8-F1-MODEL\_V4 | 1.0 | 7.759e-21 | 545 | 0.152 | 675 | 317 | 30 | 3 | 466 | 2 | 632 | Uncharacterized protein | Uncharacterized protein | | afdb-uniprot50 | AF-A0A5C6M4S1-F1-MODEL\_V4 | 1.0 | 9.754e-21 | 544 | 0.134 | 715 | 327 | 37 | 1 | 467 | 1 | 671 | Tail protein | Tail protein | | afdb-uniprot50 | AF-A0A1L6L1T8-F1-MODEL\_V4 | 1.0 | 2.082e-21 | 544 | 0.141 | 784 | 337 | 33 | 3 | 467 | 1 | 767 | Phage tail sheath protein FI | Phage tail sheath protein FI | | afdb-uniprot50 | AF-A0A3N5XL57-F1-MODEL\_V4 | 1.0 | 5.505e-21 | 544 | 0.128 | 795 | 337 | 35 | 5 | 468 | 3 | 772 | Uncharacterized protein | Uncharacterized protein | | afdb-uniprot50 | AF-U2DD43-F1-MODEL\_V4 | 1.0 | 1.992e-18 | 543 | 0.149 | 575 | 288 | 20 | 5 | 474 | 1 | 479 | Uncharacterized protein | Uncharacterized protein | | afdb-uniprot50 | AF-A0A4P7HLX1-F1-MODEL\_V4 | 1.0 | 6.264e-22 | 543 | 0.181 | 595 | 302 | 26 | 3 | 468 | 2 | 540 | Phage tail sheath family protein | Phage tail sheath family protein | | afdb-uniprot50 | AF-A0A2V6EZ80-F1-MODEL\_V4 | 1.0 | 3.29e-21 | 543 | 0.149 | 797 | 327 | 37 | 5 | 467 | 3 | 782 | Uncharacterized protein | Uncharacterized protein | | afdb-uniprot50 | AF-A0A428MM72-F1-MODEL\_V4 | 1.0 | 5.199e-21 | 542 | 0.149 | 717 | 324 | 33 | 3 | 468 | 2 | 683 | Uncharacterized protein | Uncharacterized protein | | afdb-uniprot50 | AF-A0A7W1N6A9-F1-MODEL\_V4 | 1.0 | 6.252e-18 | 541 | 0.143 | 502 | 280 | 22 | 6 | 468 | 1 | 391 | Phage tail sheath family protein | Phage tail sheath family protein | | afdb-uniprot50 | AF-A0A1U7IY13-F1-MODEL\_V4 | 1.0 | 7.976e-19 | 541 | 0.169 | 519 | 259 | 25 | 1 | 462 | 1 | 404 | Phage\_sheath\_1 domain-containing protein | Phage\_sheath\_1 domain-containing protein | | afdb-uniprot50 | AF-A0A4R1J9T0-F1-MODEL\_V4 | 1.0 | 2.435e-20 | 541 | 0.144 | 604 | 320 | 33 | 1 | 466 | 1 | 545 | Uncharacterized protein | Uncharacterized protein | | afdb-uniprot50 | AF-A0A7Y4NFZ4-F1-MODEL\_V4 | 1.0 | 5.199e-21 | 541 | 0.144 | 788 | 333 | 34 | 3 | 467 | 1 | 769 | Uncharacterized protein | Uncharacterized protein | | afdb-uniprot50 | AF-A0A656TEJ8-F1-MODEL\_V4 | 1.0 | 3.574e-15 | 540 | 0.292 | 212 | 134 | 6 | 267 | 468 | 1 | 206 | Tail protein | Tail protein | | afdb-uniprot50 | AF-A0A2T9JXK8-F1-MODEL\_V4 | 1.0 | 4.445e-22 | 540 | 0.15 | 718 | 310 | 30 | 1 | 468 | 18 | 685 | Uncharacterized protein | Uncharacterized protein | | afdb-uniprot50 | AF-A0A3G7BLM4-F1-MODEL\_V4 | 1.0 | 2.651e-18 | 539 | 0.153 | 554 | 265 | 24 | 3 | 468 | 2 | 439 | Phage tail sheath protein FI | Phage tail sheath protein FI | | afdb-uniprot50 | AF-A0A1M7R6C0-F1-MODEL\_V4 | 1.0 | 1.564e-21 | 539 | 0.146 | 798 | 314 | 38 | 1 | 466 | 1 | 763 | Uncharacterized protein | Uncharacterized protein | | afdb-uniprot50 | AF-A0A5S4GTI6-F1-MODEL\_V4 | 1.0 | 3.062e-20 | 538 | 0.153 | 643 | 325 | 28 | 1 | 468 | 44 | 641 | Phage tail sheath family protein | Phage tail sheath family protein | | afdb-uniprot50 | AF-M2QID3-F1-MODEL\_V4 | 1.0 | 8.095e-20 | 538 | 0.131 | 798 | 309 | 28 | 5 | 468 | 3 | 750 | Phage tail sheath protein FI | Phage tail sheath protein FI | | afdb-uniprot50 | AF-A0A7W7LTD8-F1-MODEL\_V4 | 1.0 | 3.529e-18 | 537 | 0.192 | 421 | 222 | 14 | 68 | 468 | 158 | 480 | Uncharacterized protein | Uncharacterized protein | | afdb-uniprot50 | AF-A0A538QBQ6-F1-MODEL\_V4 | 1.0 | 6.819e-20 | 537 | 0.167 | 579 | 324 | 31 | 1 | 474 | 1 | 526 | Phage\_sheath\_1 domain-containing protein | Phage\_sheath\_1 domain-containing protein | | afdb-uniprot50 | AF-A0A6I8LL75-F1-MODEL\_V4 | 1.0 | 5.199e-21 | 537 | 0.178 | 660 | 322 | 31 | 3 | 463 | 2 | 639 | Phage tail sheath protein FI | Phage tail sheath protein FI | | afdb-uniprot50 | AF-A0A839MYJ4-F1-MODEL\_V4 | 1.0 | 3.849e-20 | 537 | 0.16 | 686 | 319 | 27 | 2 | 468 | 4 | 651 | Uncharacterized protein | Uncharacterized protein | | afdb-uniprot50 | AF-D1JAL9-F1-MODEL\_V4 | 1.0 | 5.829e-21 | 537 | 0.127 | 899 | 329 | 35 | 3 | 468 | 2 | 877 | Uncharacterized protein | Uncharacterized protein | | afdb-uniprot50 | AF-A0A6G9ZDL1-F1-MODEL\_V4 | 1.0 | 6.62e-18 | 536 | 0.182 | 421 | 227 | 18 | 72 | 467 | 247 | 575 | Uncharacterized protein | Uncharacterized protein | | afdb-uniprot50 | AF-J2GKS0-F1-MODEL\_V4 | 1.0 | 3.242e-20 | 536 | 0.137 | 771 | 323 | 31 | 3 | 468 | 2 | 735 | Phage tail sheath protein FI | Phage tail sheath protein FI | | afdb-uniprot50 | AF-A0A7Y9CZI0-F1-MODEL\_V4 | 1.0 | 3.956e-18 | 534 | 0.166 | 576 | 271 | 27 | 5 | 474 | 2 | 474 | Phage tail sheath protein FI | Phage tail sheath protein FI | | afdb-uniprot50 | AF-A0A661ZAW2-F1-MODEL\_V4 | 1.0 | 3.235e-16 | 534 | 0.219 | 260 | 181 | 8 | 231 | 468 | 332 | 591 | Uncharacterized protein | Uncharacterized protein | | afdb-uniprot50 | AF-A0A4Z0M8Z8-F1-MODEL\_V4 | 1.0 | 1.937e-20 | 534 | 0.159 | 697 | 316 | 29 | 3 | 468 | 1 | 658 | Uncharacterized protein | Uncharacterized protein | | afdb-uniprot50 | AF-A0A1L6LX19-F1-MODEL\_V4 | 1.0 | 8.321e-18 | 533 | 0.147 | 521 | 275 | 20 | 5 | 468 | 1 | 409 | Phage tail sheath protein FI | Phage tail sheath protein FI | | afdb-uniprot50 | AF-A0A1C6D4Q3-F1-MODEL\_V4 | 1.0 | 8.216e-21 | 532 | 0.183 | 546 | 292 | 30 | 6 | 473 | 1 | 470 | Phage tail sheath protein | Phage tail sheath protein | | afdb-uniprot50 | AF-E3IXB2-F1-MODEL\_V4 | 1.0 | 5.576e-18 | 531 | 0.148 | 506 | 273 | 19 | 6 | 467 | 1 | 392 | Phage tail sheath protein fi-like protein | Phage tail sheath protein fi-like protein | | afdb-uniprot50 | AF-A0A399IIQ1-F1-MODEL\_V4 | 1.0 | 2.651e-18 | 531 | 0.146 | 566 | 277 | 22 | 17 | 474 | 2 | 469 | Phage tail sheath protein | Phage tail sheath protein | | afdb-uniprot50 | AF-A0A3D8JWB0-F1-MODEL\_V4 | 1.0 | 3.062e-20 | 531 | 0.144 | 628 | 332 | 31 | 3 | 466 | 2 | 587 | Phage tail sheath family protein | Phage tail sheath family protein | | afdb-uniprot50 | AF-A0A2V6FAV4-F1-MODEL\_V4 | 1.0 | 4.569e-20 | 531 | 0.127 | 811 | 337 | 30 | 3 | 468 | 2 | 786 | Uncharacterized protein | Uncharacterized protein | | afdb-uniprot50 | AF-A0A4R5H846-F1-MODEL\_V4 | 1.0 | 2.731e-20 | 531 | 0.133 | 846 | 315 | 36 | 3 | 468 | 2 | 809 | Phage tail sheath protein | Phage tail sheath protein | | afdb-uniprot50 | AF-A0A2T1ENU3-F1-MODEL\_V4 | 1.0 | 1.031e-16 | 530 | 0.158 | 474 | 262 | 19 | 1 | 455 | 2 | 357 | Phage tail sheath family protein | Phage tail sheath family protein | | afdb-uniprot50 | AF-A0A1M6SKF9-F1-MODEL\_V4 | 1.0 | 8.095e-20 | 530 | 0.181 | 550 | 299 | 31 | 5 | 475 | 2 | 479 | Uncharacterized protein | Uncharacterized protein | | afdb-uniprot50 | AF-A0A2L2XDC6-F1-MODEL\_V4 | 1.0 | 7.645e-20 | 528 | 0.178 | 510 | 281 | 23 | 25 | 466 | 1 | 440 | Phage tail sheath protein FI | Phage tail sheath protein FI | | afdb-uniprot50 | AF-A0A630BY26-F1-MODEL\_V4 | 1.0 | 3.326e-14 | 527 | 0.273 | 201 | 141 | 3 | 276 | 474 | 4 | 201 | Phage tail sheath family protein | Phage tail sheath family protein | | afdb-uniprot50 | AF-A0A828D4K5-F1-MODEL\_V4 | 1.0 | 1.001e-14 | 527 | 0.225 | 301 | 135 | 6 | 75 | 372 | 16 | 221 | Phage tail sheath family protein | Phage tail sheath family protein | | afdb-uniprot50 | AF-A0A6H9V561-F1-MODEL\_V4 | 1.0 | 2.731e-20 | 526 | 0.13 | 895 | 320 | 34 | 1 | 468 | 1 | 863 | Uncharacterized protein | Uncharacterized protein | | afdb-uniprot50 | AF-A0A1L6M381-F1-MODEL\_V4 | 1.0 | 2.2e-17 | 525 | 0.152 | 513 | 266 | 23 | 1 | 467 | 2 | 391 | Phage tail sheath protein FI | Phage tail sheath protein FI | | afdb-uniprot50 | AF-R7HVY5-F1-MODEL\_V4 | 1.0 | 2.467e-17 | 525 | 0.148 | 572 | 286 | 22 | 5 | 474 | 1 | 473 | Uncharacterized protein | Uncharacterized protein | | afdb-uniprot50 | AF-A0A5B7V907-F1-MODEL\_V4 | 1.0 | 5.732e-16 | 525 | 0.223 | 260 | 171 | 9 | 230 | 466 | 230 | 481 | Phage tail sheath protein | Phage tail sheath protein | | afdb-uniprot50 | AF-A0A150TH68-F1-MODEL\_V4 | 1.0 | 1.298e-20 | 525 | 0.187 | 596 | 293 | 26 | 6 | 468 | 1 | 537 | Uncharacterized protein | Uncharacterized protein | | afdb-uniprot50 | AF-A0A2D6MDR9-F1-MODEL\_V4 | 1.0 | 1.909e-19 | 525 | 0.145 | 727 | 309 | 28 | 3 | 474 | 2 | 670 | Uncharacterized protein | Uncharacterized protein | | afdb-uniprot50 | AF-A0A1H6TUH7-F1-MODEL\_V4 | 1.0 | 1.909e-19 | 524 | 0.148 | 699 | 312 | 26 | 1 | 468 | 6 | 651 | Uncharacterized protein | Uncharacterized protein | | afdb-uniprot50 | AF-A0A832EAA6-F1-MODEL\_V4 | 1.0 | 1.298e-20 | 524 | 0.135 | 776 | 333 | 33 | 1 | 466 | 1 | 748 | Uncharacterized protein | Uncharacterized protein | | afdb-uniprot50 | AF-Q1D3W0-F1-MODEL\_V4 | 1.0 | 3.849e-20 | 524 | 0.152 | 822 | 309 | 38 | 1 | 468 | 4 | 791 | Putative Phage tail sheath protein | Putative Phage tail sheath protein | | afdb-uniprot50 | AF-A0A376J7I4-F1-MODEL\_V4 | 1.0 | 8.304e-14 | 523 | 0.363 | 146 | 90 | 3 | 238 | 381 | 2 | 146 | Major tail sheath protein FI | Major tail sheath protein FI | | afdb-uniprot50 | AF-A0A3D4L0R8-F1-MODEL\_V4 | 1.0 | 8.095e-20 | 523 | 0.16 | 550 | 305 | 29 | 5 | 474 | 2 | 474 | Phage tail protein | Phage tail protein | | afdb-uniprot50 | AF-A0A6G9ZDK1-F1-MODEL\_V4 | 1.0 | 4.973e-18 | 523 | 0.176 | 419 | 228 | 16 | 70 | 468 | 372 | 693 | Uncharacterized protein | Uncharacterized protein | | afdb-uniprot50 | AF-A4X0M3-F1-MODEL\_V4 | 1.0 | 6.44e-20 | 523 | 0.153 | 842 | 315 | 35 | 1 | 468 | 1 | 818 | Uncharacterized protein | Uncharacterized protein | | afdb-uniprot50 | AF-A0A0F9KEX1-F1-MODEL\_V4 | 1.0 | 2.017e-15 | 523 | 0.207 | 260 | 180 | 9 | 231 | 468 | 667 | 922 | Phage\_sheath\_1C domain-containing protein | Phage\_sheath\_1C domain-containing protein | | afdb-uniprot50 | AF-A0A690ZQB1-F1-MODEL\_V4 | 1.0 | 7.406e-14 | 522 | 0.35 | 154 | 97 | 2 | 230 | 381 | 38 | 190 | Phage tail sheath family protein | Phage tail sheath family protein | | afdb-uniprot50 | AF-A0A1H0JRF2-F1-MODEL\_V4 | 1.0 | 2.891e-20 | 522 | 0.154 | 621 | 305 | 34 | 9 | 466 | 8 | 571 | Uncharacterized protein | Uncharacterized protein | | afdb-uniprot50 | AF-A0A266LKS8-F1-MODEL\_V4 | 1.0 | 7.842e-14 | 521 | 0.507 | 136 | 67 | 0 | 339 | 474 | 2 | 137 | Phage tail protein | Phage tail protein | | afdb-uniprot50 | AF-A0A378N170-F1-MODEL\_V4 | 1.0 | 5.255e-14 | 521 | 0.317 | 173 | 114 | 3 | 304 | 474 | 10 | 180 | Phage tail sheath protein | Phage tail sheath protein | | afdb-uniprot50 | AF-A0A6N7C2F0-F1-MODEL\_V4 | 1.0 | 2.645e-14 | 521 | 0.239 | 234 | 161 | 5 | 245 | 467 | 6 | 233 | Phage\_sheath\_1C domain-containing protein | Phage\_sheath\_1C domain-containing protein | | afdb-uniprot50 | AF-A0A1I3W1P1-F1-MODEL\_V4 | 1.0 | 5.424e-20 | 521 | 0.146 | 794 | 334 | 26 | 3 | 468 | 2 | 779 | Uncharacterized protein | Uncharacterized protein | | afdb-uniprot50 | AF-A0A365VQG7-F1-MODEL\_V4 | 1.0 | 7.842e-14 | 520 | 0.335 | 143 | 93 | 2 | 231 | 371 | 51 | 193 | Phage tail protein | Phage tail protein | | afdb-uniprot50 | AF-A0A160T8X3-F1-MODEL\_V4 | 1.0 | 1.608e-19 | 520 | 0.156 | 583 | 297 | 25 | 3 | 455 | 2 | 519 | Putative Tail sheath protein | Putative Tail sheath protein | | afdb-uniprot50 | AF-A0A840BSA8-F1-MODEL\_V4 | 1.0 | 1.018e-19 | 520 | 0.138 | 778 | 339 | 35 | 3 | 468 | 2 | 759 | Uncharacterized protein | Uncharacterized protein | | afdb-uniprot50 | AF-A0A5X8YWD7-F1-MODEL\_V4 | 1.0 | 2.196e-13 | 519 | 0.333 | 165 | 109 | 1 | 310 | 474 | 3 | 166 | Phage tail sheath family protein | Phage tail sheath family protein | | afdb-uniprot50 | AF-A0A1G5B734-F1-MODEL\_V4 | 1.0 | 1.046e-17 | 519 | 0.142 | 532 | 268 | 19 | 1 | 457 | 8 | 426 | Phage\_sheath\_1 domain-containing protein | Phage\_sheath\_1 domain-containing protein | | afdb-uniprot50 | AF-A0A661RQ90-F1-MODEL\_V4 | 1.0 | 2.651e-18 | 519 | 0.144 | 519 | 281 | 23 | 1 | 467 | 10 | 417 | LTD domain-containing protein | LTD domain-containing protein | | afdb-uniprot50 | AF-A9CK92-F1-MODEL\_V4 | 1.0 | 3.107e-21 | 519 | 0.284 | 355 | 207 | 12 | 77 | 419 | 2 | 321 | Uncharacterized protein | Uncharacterized protein | | afdb-uniprot50 | AF-A0A2T5JUP8-F1-MODEL\_V4 | 1.0 | 2.504e-18 | 519 | 0.13 | 757 | 336 | 28 | 1 | 468 | 1 | 723 | Tail sheath protein | Tail sheath protein | | afdb-uniprot50 | AF-A0A5J6PCS3-F1-MODEL\_V4 | 1.0 | 1.018e-19 | 519 | 0.135 | 784 | 322 | 35 | 3 | 468 | 1 | 746 | Phage tail protein | Phage tail protein | | afdb-uniprot50 | AF-A0A662Q4X3-F1-MODEL\_V4 | 1.0 | 3.635e-20 | 518 | 0.169 | 579 | 302 | 31 | 1 | 468 | 1 | 511 | Phage\_sheath\_1 domain-containing protein | Phage\_sheath\_1 domain-containing protein | | afdb-uniprot50 | AF-A0A417XSR8-F1-MODEL\_V4 | 1.0 | 2.541e-19 | 518 | 0.132 | 901 | 303 | 34 | 3 | 468 | 1 | 857 | Uncharacterized protein | Uncharacterized protein | | afdb-uniprot50 | AF-A0A351U536-F1-MODEL\_V4 | 1.0 | 1.001e-14 | 516 | 0.28 | 221 | 138 | 8 | 260 | 465 | 6 | 220 | Phage tail protein | Phage tail protein | | afdb-uniprot50 | AF-A0A074LXD5-F1-MODEL\_V4 | 1.0 | 4.315e-20 | 516 | 0.189 | 538 | 297 | 28 | 5 | 474 | 1 | 467 | Uncharacterized protein | Uncharacterized protein | | afdb-uniprot50 | AF-A0A261GTB9-F1-MODEL\_V4 | 1.0 | 5.344e-19 | 516 | 0.151 | 593 | 313 | 32 | 1 | 467 | 1 | 529 | Uncharacterized protein | Uncharacterized protein | | afdb-uniprot50 | AF-A0A6L9G0N3-F1-MODEL\_V4 | 1.0 | 7.976e-19 | 516 | 0.131 | 769 | 335 | 28 | 1 | 468 | 1 | 737 | Phage tail sheath protein | Phage tail sheath protein | | afdb-uniprot50 | AF-A0A3A8JXS8-F1-MODEL\_V4 | 1.0 | 2.541e-19 | 515 | 0.158 | 601 | 304 | 31 | 5 | 467 | 2 | 538 | Phage tail sheath family protein | Phage tail sheath family protein | | afdb-uniprot50 | AF-A0A7G9YSQ3-F1-MODEL\_V4 | 1.0 | 1.803e-19 | 515 | 0.132 | 818 | 326 | 32 | 3 | 467 | 2 | 788 | Uncharacterized protein | Uncharacterized protein | | afdb-uniprot50 | AF-A0A1F7S379-F1-MODEL\_V4 | 1.0 | 5.647e-15 | 514 | 0.186 | 263 | 190 | 9 | 230 | 468 | 186 | 448 | Uncharacterized protein | Uncharacterized protein | | afdb-uniprot50 | AF-A0A2W5QE72-F1-MODEL\_V4 | 1.0 | 7.22e-20 | 514 | 0.157 | 624 | 320 | 31 | 1 | 467 | 32 | 606 | Phage tail sheath family protein | Phage tail sheath family protein | | afdb-uniprot50 | AF-A0A7Y6QLZ2-F1-MODEL\_V4 | 1.0 | 2.467e-17 | 514 | 0.187 | 411 | 210 | 12 | 84 | 467 | 733 | 1046 | Phage tail sheath subtilisin-like domain-containing protein | Phage tail sheath subtilisin-like domain-containing protein | | afdb-uniprot50 | AF-U2KP13-F1-MODEL\_V4 | 1.0 | 9.211e-21 | 512 | 0.161 | 612 | 296 | 31 | 5 | 455 | 3 | 558 | Phage tail sheath protein | Phage tail sheath protein | | afdb-uniprot50 | AF-A0A090AMB4-F1-MODEL\_V4 | 1.0 | 1.018e-19 | 512 | 0.158 | 613 | 336 | 32 | 3 | 467 | 2 | 582 | Putative phage tail sheath protein | Putative phage tail sheath protein | | afdb-uniprot50 | AF-A0A4Y6PV73-F1-MODEL\_V4 | 1.0 | 1.803e-19 | 512 | 0.143 | 821 | 326 | 35 | 5 | 468 | 3 | 802 | Uncharacterized protein | Uncharacterized protein | | afdb-uniprot50 | AF-A0A7G9WG91-F1-MODEL\_V4 | 1.0 | 1.75e-17 | 511 | 0.138 | 583 | 296 | 26 | 1 | 475 | 1 | 484 | Phage tail sheath family protein | Phage tail sheath family protein | | afdb-uniprot50 | AF-A0A2T5RE85-F1-MODEL\_V4 | 1.0 | 2.4e-19 | 511 | 0.144 | 669 | 321 | 31 | 3 | 474 | 2 | 615 | Tail sheath protein | Tail sheath protein | | afdb-uniprot50 | AF-A0A2K8U9B6-F1-MODEL\_V4 | 1.0 | 2.541e-19 | 510 | 0.141 | 712 | 327 | 29 | 1 | 468 | 2 | 672 | Uncharacterized protein | Uncharacterized protein | | afdb-uniprot50 | AF-A0A2E4ZEU9-F1-MODEL\_V4 | 1.0 | 1.608e-19 | 509 | 0.156 | 556 | 307 | 24 | 3 | 468 | 1 | 484 | Uncharacterized protein | Uncharacterized protein | | afdb-uniprot50 | AF-J9Z8U4-F1-MODEL\_V4 | 1.0 | 1.124e-18 | 509 | 0.145 | 551 | 328 | 22 | 1 | 468 | 4 | 494 | Uncharacterized protein | Uncharacterized protein | | afdb-uniprot50 | AF-A0A1D2QTZ4-F1-MODEL\_V4 | 1.0 | 1.354e-19 | 508 | 0.141 | 709 | 333 | 30 | 1 | 468 | 1 | 674 | Phage\_sheath\_1C domain-containing protein | Phage\_sheath\_1C domain-containing protein | | afdb-uniprot50 | AF-A0A2X4UYA7-F1-MODEL\_V4 | 1.0 | 8.554e-16 | 505 | 0.154 | 484 | 258 | 22 | 1 | 466 | 1 | 351 | Phage tail sheath protein | Phage tail sheath protein | | afdb-uniprot50 | AF-A0A1C6IJ19-F1-MODEL\_V4 | 1.0 | 1.208e-19 | 505 | 0.172 | 555 | 302 | 31 | 5 | 475 | 2 | 482 | Phage tail sheath protein | Phage tail sheath protein | | afdb-uniprot50 | AF-A0A1V5QKC2-F1-MODEL\_V4 | 1.0 | 1.674e-14 | 505 | 0.182 | 268 | 194 | 10 | 226 | 474 | 225 | 486 | Phage tail sheath protein | Phage tail sheath protein | | afdb-uniprot50 | AF-A0A1S6FHM7-F1-MODEL\_V4 | 1.0 | 2.972e-18 | 505 | 0.121 | 740 | 356 | 33 | 1 | 467 | 1 | 719 | Phage\_sheath\_1C domain-containing protein | Phage\_sheath\_1C domain-containing protein | | afdb-uniprot50 | AF-A0A2R2Q2H2-F1-MODEL\_V4 | 1.0 | 4.697e-18 | 504 | 0.159 | 572 | 255 | 22 | 1 | 468 | 1 | 450 | AfpX3 | AfpX3 | | afdb-uniprot50 | AF-A0A845SZJ5-F1-MODEL\_V4 | 1.0 | 8.942e-19 | 504 | 0.167 | 556 | 300 | 31 | 1 | 475 | 1 | 474 | Phage tail protein | Phage tail protein | | afdb-uniprot50 | AF-A0A8A6HCG5-F1-MODEL\_V4 | 1.0 | 5.047e-19 | 504 | 0.148 | 689 | 332 | 30 | 2 | 468 | 4 | 659 | Uncharacterized protein | Uncharacterized protein | | afdb-uniprot50 | AF-A0A4R8CM40-F1-MODEL\_V4 | 1.0 | 5.047e-19 | 504 | 0.138 | 700 | 326 | 31 | 2 | 468 | 9 | 664 | Uncharacterized protein | Uncharacterized protein | | afdb-uniprot50 | AF-A0A523INP5-F1-MODEL\_V4 | 1.0 | 1.703e-19 | 504 | 0.155 | 668 | 324 | 32 | 10 | 464 | 1 | 641 | Phage tail sheath family protein | Phage tail sheath family protein | | afdb-uniprot50 | AF-A0A522C7H8-F1-MODEL\_V4 | 1.0 | 3.898e-17 | 503 | 0.143 | 510 | 278 | 24 | 1 | 468 | 7 | 399 | Phage tail sheath family protein | Phage tail sheath family protein | | afdb-uniprot50 | AF-A0A382G038-F1-MODEL\_V4 | 1.0 | 9.449e-15 | 503 | 0.178 | 247 | 189 | 7 | 231 | 468 | 186 | 427 | Uncharacterized protein | Uncharacterized protein | | afdb-uniprot50 | AF-A0A266LLW5-F1-MODEL\_V4 | 1.0 | 1.221e-12 | 502 | 0.504 | 121 | 59 | 1 | 243 | 362 | 2 | 122 | Phage tail protein | Phage tail protein | | afdb-uniprot50 | AF-V0XQG6-F1-MODEL\_V4 | 1.0 | 2.196e-13 | 502 | 0.272 | 257 | 100 | 4 | 113 | 367 | 2 | 173 | Phage\_sheath\_1 domain-containing protein | Phage\_sheath\_1 domain-containing protein | | afdb-uniprot50 | AF-A0A1W0CS69-F1-MODEL\_V4 | 1.0 | 4.007e-15 | 501 | 0.158 | 487 | 250 | 19 | 1 | 468 | 1 | 346 | Phage tail protein | Phage tail protein | | afdb-uniprot50 | AF-U2PM90-F1-MODEL\_V4 | 1.0 | 8.554e-16 | 500 | 0.169 | 406 | 226 | 12 | 90 | 474 | 19 | 334 | Uncharacterized protein | Uncharacterized protein | | afdb-uniprot50 | AF-A0A1J5DEX6-F1-MODEL\_V4 | 1.0 | 7.645e-20 | 499 | 0.148 | 614 | 314 | 30 | 3 | 457 | 2 | 565 | Uncharacterized protein | Uncharacterized protein | | afdb-uniprot50 | AF-A0A850NPQ6-F1-MODEL\_V4 | 1.0 | 7.532e-19 | 498 | 0.149 | 550 | 325 | 28 | 2 | 468 | 6 | 495 | Phage tail protein | Phage tail protein | | afdb-uniprot50 | AF-D5CT51-F1-MODEL\_V4 | 1.0 | 2.233e-18 | 498 | 0.144 | 554 | 328 | 23 | 2 | 468 | 10 | 504 | Uncharacterized protein | Uncharacterized protein | | afdb-uniprot50 | AF-A0A4Y6PVA2-F1-MODEL\_V4 | 1.0 | 2.364e-18 | 498 | 0.137 | 686 | 333 | 30 | 1 | 455 | 1 | 658 | Phage tail sheath family protein | Phage tail sheath family protein | | afdb-uniprot50 | AF-A0A662L329-F1-MODEL\_V4 | 1.0 | 6.819e-20 | 497 | 0.187 | 495 | 267 | 20 | 3 | 421 | 2 | 437 | Phage tail protein | Phage tail protein | | afdb-uniprot50 | AF-A0A3T1B9R3-F1-MODEL\_V4 | 1.0 | 4.315e-20 | 497 | 0.143 | 598 | 337 | 23 | 1 | 466 | 4 | 558 | Uncharacterized protein | Uncharacterized protein | | afdb-uniprot50 | AF-A0A2S3UN30-F1-MODEL\_V4 | 1.0 | 2.4e-19 | 497 | 0.143 | 642 | 328 | 28 | 5 | 466 | 2 | 601 | Tail sheath protein | Tail sheath protein | | afdb-uniprot50 | AF-A0A7Z7I242-F1-MODEL\_V4 | 1.0 | 9.469e-19 | 497 | 0.141 | 728 | 315 | 28 | 6 | 468 | 1 | 683 | Phage tail sheath protein | Phage tail sheath protein | | afdb-uniprot50 | AF-A0A1Y4T0D0-F1-MODEL\_V4 | 1.0 | 6.069e-16 | 496 | 0.186 | 413 | 212 | 15 | 90 | 474 | 44 | 360 | Uncharacterized protein | Uncharacterized protein | | afdb-uniprot50 | AF-A0A4C2EPL5-F1-MODEL\_V4 | 1.0 | 3.581e-19 | 496 | 0.13 | 888 | 335 | 34 | 3 | 468 | 2 | 873 | Uncharacterized protein | Uncharacterized protein | | afdb-uniprot50 | AF-A0A430VBI2-F1-MODEL\_V4 | 1.0 | 2.228e-14 | 496 | 0.141 | 255 | 194 | 13 | 231 | 468 | 705 | 951 | Uncharacterized protein | Uncharacterized protein | | afdb-uniprot50 | AF-A0A1C5SXM5-F1-MODEL\_V4 | 1.0 | 9.61e-20 | 495 | 0.162 | 608 | 279 | 25 | 23 | 463 | 2 | 545 | Phage tail sheath protein | Phage tail sheath protein | | afdb-uniprot50 | AF-A0A2T6N8W8-F1-MODEL\_V4 | 1.0 | 9.076e-20 | 495 | 0.144 | 759 | 317 | 31 | 3 | 468 | 13 | 731 | Phage\_sheath\_1C domain-containing protein | Phage\_sheath\_1C domain-containing protein | | afdb-uniprot50 | AF-A0A1V5BE78-F1-MODEL\_V4 | 1.0 | 9.61e-20 | 495 | 0.131 | 824 | 333 | 37 | 1 | 466 | 1 | 799 | Phage tail sheath protein | Phage tail sheath protein | | afdb-uniprot50 | AF-A0A4Q5RRV7-F1-MODEL\_V4 | 1.0 | 3.235e-16 | 494 | 0.135 | 634 | 274 | 24 | 1 | 466 | 1 | 528 | Phage tail sheath family protein | Phage tail sheath family protein | | afdb-uniprot50 | AF-A0A6N8I017-F1-MODEL\_V4 | 1.0 | 5.743e-20 | 493 | 0.162 | 559 | 303 | 31 | 1 | 474 | 1 | 479 | Uncharacterized protein | Uncharacterized protein | | afdb-uniprot50 | AF-A0A3C1WS05-F1-MODEL\_V4 | 1.0 | 1.629e-16 | 493 | 0.151 | 588 | 284 | 22 | 1 | 475 | 1 | 486 | Phage tail protein | Phage tail protein | | afdb-uniprot50 | AF-A0A840MS80-F1-MODEL\_V4 | 1.0 | 1.992e-18 | 493 | 0.135 | 687 | 341 | 27 | 1 | 468 | 5 | 657 | Uncharacterized protein | Uncharacterized protein | | afdb-uniprot50 | AF-A0A7J4LL39-F1-MODEL\_V4 | 1.0 | 5.047e-19 | 493 | 0.15 | 710 | 333 | 39 | 5 | 471 | 3 | 685 | Phage tail sheath family protein | Phage tail sheath family protein | | afdb-uniprot50 | AF-A0A447PAZ2-F1-MODEL\_V4 | 1.0 | 5.178e-13 | 492 | 0.375 | 144 | 88 | 2 | 230 | 371 | 8 | 151 | Major tail sheath protein | Major tail sheath protein | | afdb-uniprot50 | AF-A0A382DIG5-F1-MODEL\_V4 | 1.0 | 2.645e-14 | 492 | 0.176 | 266 | 192 | 11 | 230 | 474 | 60 | 319 | Uncharacterized protein | Uncharacterized protein | | afdb-uniprot50 | AF-A0A2G2EDZ2-F1-MODEL\_V4 | 1.0 | 1.538e-16 | 492 | 0.145 | 577 | 282 | 27 | 1 | 468 | 1 | 475 | Uncharacterized protein | Uncharacterized protein | | afdb-uniprot50 | AF-A0A2D7CYR9-F1-MODEL\_V4 | 1.0 | 1.26e-18 | 492 | 0.151 | 642 | 318 | 34 | 3 | 475 | 2 | 585 | Uncharacterized protein | Uncharacterized protein | | afdb-uniprot50 | AF-A0A518BPN9-F1-MODEL\_V4 | 1.0 | 2.266e-19 | 492 | 0.137 | 757 | 349 | 31 | 3 | 467 | 2 | 746 | Phage tail sheath protein | Phage tail sheath protein | | afdb-uniprot50 | AF-A0A525HH54-F1-MODEL\_V4 | 1.0 | 1.258e-14 | 492 | 0.215 | 264 | 172 | 8 | 230 | 465 | 606 | 862 | Uncharacterized protein | Uncharacterized protein | | afdb-uniprot50 | AF-A0A064A4E1-F1-MODEL\_V4 | 1.0 | 1.988e-14 | 491 | 0.282 | 237 | 141 | 11 | 254 | 474 | 26 | 249 | Phage tail protein | Phage tail protein | | afdb-uniprot50 | AF-A0A2D5FCB7-F1-MODEL\_V4 | 1.0 | 2.105e-14 | 491 | 0.202 | 262 | 184 | 10 | 226 | 468 | 34 | 289 | Uncharacterized protein | Uncharacterized protein | | afdb-uniprot50 | AF-A0A3M0WVE2-F1-MODEL\_V4 | 1.0 | 4.315e-20 | 491 | 0.178 | 511 | 260 | 22 | 1 | 415 | 1 | 447 | Phage tail sheath family protein | Phage tail sheath family protein | | afdb-uniprot50 | AF-A0A4U3M6X8-F1-MODEL\_V4 | 1.0 | 6.345e-19 | 491 | 0.132 | 567 | 329 | 24 | 3 | 468 | 2 | 506 | Phage tail sheath family protein | Phage tail sheath family protein | | afdb-uniprot50 | AF-A0A2D7XGG3-F1-MODEL\_V4 | 1.0 | 6.332e-15 | 491 | 0.166 | 277 | 193 | 9 | 229 | 473 | 522 | 792 | Uncharacterized protein | Uncharacterized protein | | afdb-uniprot50 | AF-A0A2E0KX46-F1-MODEL\_V4 | 1.0 | 1.332e-14 | 491 | 0.176 | 261 | 186 | 9 | 229 | 466 | 571 | 825 | Uncharacterized protein | Uncharacterized protein | | afdb-uniprot50 | AF-A0A2D6ANC4-F1-MODEL\_V4 | 1.0 | 1.493e-14 | 491 | 0.13 | 275 | 207 | 8 | 225 | 473 | 853 | 1121 | Uncharacterized protein | Uncharacterized protein | | afdb-uniprot50 | AF-A0A368A7G8-F1-MODEL\_V4 | 1.0 | 2.536e-15 | 490 | 0.288 | 353 | 98 | 7 | 1 | 352 | 1 | 201 | Phage tail sheath protein FI | Phage tail sheath protein FI | | afdb-uniprot50 | AF-A0A2E4W7V1-F1-MODEL\_V4 | 1.0 | 1.853e-17 | 490 | 0.11 | 727 | 326 | 29 | 1 | 475 | 1 | 658 | Phage tail protein | Phage tail protein | | afdb-uniprot50 | AF-A0A2W5F6C6-F1-MODEL\_V4 | 1.0 | 4.252e-19 | 490 | 0.151 | 705 | 310 | 30 | 1 | 467 | 1 | 654 | Uncharacterized protein | Uncharacterized protein | | afdb-uniprot50 | AF-A0A7Y5LPM5-F1-MODEL\_V4 | 1.0 | 2.4e-19 | 490 | 0.141 | 684 | 332 | 30 | 1 | 459 | 1 | 653 | Phage tail sheath family protein | Phage tail sheath family protein | | afdb-uniprot50 | AF-A0A399YVR8-F1-MODEL\_V4 | 1.0 | 2.849e-19 | 490 | 0.142 | 838 | 325 | 40 | 1 | 466 | 1 | 816 | Uncharacterized protein | Uncharacterized protein | | afdb-uniprot50 | AF-A0A329WRQ2-F1-MODEL\_V4 | 1.0 | 8.681e-17 | 489 | 0.145 | 579 | 263 | 23 | 1 | 467 | 1 | 459 | Phage tail protein | Phage tail protein | | afdb-uniprot50 | AF-A0A0F9KVW3-F1-MODEL\_V4 | 1.0 | 4.493e-15 | 489 | 0.191 | 261 | 188 | 8 | 231 | 468 | 814 | 1074 | Phage\_sheath\_1C domain-containing protein | Phage\_sheath\_1C domain-containing protein | | afdb-uniprot50 | AF-A8T9K8-F1-MODEL\_V4 | 1.0 | 1.206e-15 | 488 | 0.158 | 497 | 246 | 22 | 1 | 468 | 1 | 354 | Phage\_sheath\_1C domain-containing protein | Phage\_sheath\_1C domain-containing protein | | afdb-uniprot50 | AF-R7MS92-F1-MODEL\_V4 | 1.0 | 1.206e-15 | 488 | 0.179 | 413 | 216 | 14 | 85 | 475 | 68 | 379 | Uncharacterized protein | Uncharacterized protein | | afdb-uniprot50 | AF-A0A4P8QGP8-F1-MODEL\_V4 | 1.0 | 6.345e-19 | 488 | 0.142 | 568 | 319 | 28 | 1 | 468 | 4 | 503 | Phage tail protein | Phage tail protein | | afdb-uniprot50 | AF-A0A0F5K3T8-F1-MODEL\_V4 | 1.0 | 6.805e-16 | 486 | 0.147 | 516 | 261 | 23 | 3 | 468 | 2 | 388 | Uncharacterized protein | Uncharacterized protein | | afdb-uniprot50 | AF-A0A842J0D4-F1-MODEL\_V4 | 1.0 | 6.718e-19 | 486 | 0.178 | 561 | 290 | 36 | 5 | 475 | 2 | 481 | Phage tail protein | Phage tail protein | | afdb-uniprot50 | AF-A0A143HDI3-F1-MODEL\_V4 | 1.0 | 2.725e-16 | 486 | 0.165 | 586 | 273 | 25 | 1 | 474 | 1 | 482 | Uncharacterized protein | Uncharacterized protein | | afdb-uniprot50 | AF-A0A257PQZ1-F1-MODEL\_V4 | 1.0 | 8.942e-19 | 485 | 0.137 | 540 | 308 | 28 | 19 | 468 | 28 | 499 | Uncharacterized protein | Uncharacterized protein | | afdb-uniprot50 | AF-A0A3N5PP70-F1-MODEL\_V4 | 1.0 | 4.015e-19 | 484 | 0.15 | 679 | 282 | 27 | 3 | 442 | 2 | 624 | Phage tail sheath family protein | Phage tail sheath family protein | | afdb-uniprot50 | AF-A0A2D6E403-F1-MODEL\_V4 | 1.0 | 1.877e-14 | 484 | 0.134 | 268 | 201 | 9 | 225 | 467 | 773 | 1034 | Uncharacterized protein | Uncharacterized protein | | afdb-uniprot50 | AF-A0A7J4GTR8-F1-MODEL\_V4 | 1.0 | 1.312e-13 | 483 | 0.16 | 255 | 188 | 9 | 241 | 475 | 150 | 398 | Uncharacterized protein | Uncharacterized protein | | afdb-uniprot50 | AF-K6Y8P6-F1-MODEL\_V4 | 1.0 | 1.19e-18 | 483 | 0.135 | 709 | 346 | 33 | 1 | 468 | 1 | 683 | Uncharacterized protein | Uncharacterized protein | | afdb-uniprot50 | AF-A0A2D9CBG6-F1-MODEL\_V4 | 1.0 | 3.095e-13 | 482 | 0.157 | 247 | 192 | 7 | 231 | 467 | 133 | 373 | Phage\_sheath\_1 domain-containing protein | Phage\_sheath\_1 domain-containing protein | | afdb-uniprot50 | AF-A0A7Y4SSP2-F1-MODEL\_V4 | 1.0 | 7.22e-20 | 482 | 0.172 | 667 | 317 | 29 | 5 | 465 | 3 | 640 | Phage tail protein | Phage tail protein | | afdb-uniprot50 | AF-A0A3N5STT9-F1-MODEL\_V4 | 1.0 | 1.41e-14 | 481 | 0.186 | 241 | 175 | 8 | 231 | 466 | 208 | 432 | Phage tail protein | Phage tail protein | | afdb-uniprot50 | AF-A0A4U7J714-F1-MODEL\_V4 | 1.0 | 7.532e-19 | 481 | 0.169 | 568 | 298 | 32 | 1 | 475 | 1 | 487 | Uncharacterized protein | Uncharacterized protein | | afdb-uniprot50 | AF-A0A432LJ81-F1-MODEL\_V4 | 1.0 | 2.295e-16 | 481 | 0.132 | 610 | 281 | 22 | 1 | 468 | 1 | 504 | Phage tail sheath family protein | Phage tail sheath family protein | | afdb-uniprot50 | AF-A0A7X8L470-F1-MODEL\_V4 | 1.0 | 6.345e-19 | 481 | 0.134 | 579 | 315 | 24 | 1 | 466 | 54 | 559 | Phage tail sheath family protein | Phage tail sheath family protein | | afdb-uniprot50 | AF-A0A4Q7WMN5-F1-MODEL\_V4 | 1.0 | 2.972e-18 | 481 | 0.155 | 688 | 323 | 33 | 1 | 467 | 3 | 653 | Phage\_sheath\_1 domain-containing protein | Phage\_sheath\_1 domain-containing protein | | afdb-uniprot50 | AF-A0A2D9C4T6-F1-MODEL\_V4 | 1.0 | 2.364e-18 | 481 | 0.143 | 737 | 341 | 34 | 1 | 468 | 1 | 715 | Phage\_sheath\_1C domain-containing protein | Phage\_sheath\_1C domain-containing protein | | afdb-uniprot50 | AF-A0A2E7WA37-F1-MODEL\_V4 | 1.0 | 2.645e-14 | 481 | 0.149 | 268 | 198 | 8 | 225 | 468 | 703 | 964 | Uncharacterized protein | Uncharacterized protein | | afdb-uniprot50 | AF-A0A832XY63-F1-MODEL\_V4 | 1.0 | 1.988e-14 | 480 | 0.169 | 266 | 194 | 9 | 230 | 474 | 82 | 341 | Uncharacterized protein | Uncharacterized protein | | afdb-uniprot50 | AF-A0A2V9GSQ5-F1-MODEL\_V4 | 1.0 | 4.189e-18 | 480 | 0.14 | 535 | 328 | 29 | 1 | 468 | 1 | 470 | Phage tail sheath protein | Phage tail sheath protein | | afdb-uniprot50 | AF-A0A7G7M251-F1-MODEL\_V4 | 1.0 | 3.141e-14 | 479 | 0.167 | 461 | 155 | 4 | 5 | 465 | 2 | 233 | Phage\_sheath\_1C domain-containing protein | Phage\_sheath\_1C domain-containing protein | | afdb-uniprot50 | AF-A0A5F1HY87-F1-MODEL\_V4 | 1.0 | 1.372e-16 | 479 | 0.155 | 509 | 264 | 24 | 1 | 466 | 66 | 451 | Phage tail protein | Phage tail protein | | afdb-uniprot50 | AF-A0A4Y9M3D3-F1-MODEL\_V4 | 1.0 | 4.973e-18 | 479 | 0.136 | 694 | 329 | 29 | 1 | 468 | 1 | 649 | Phage tail sheath family protein | Phage tail sheath family protein | | afdb-uniprot50 | AF-A0A1W1X8C9-F1-MODEL\_V4 | 1.0 | 5.992e-19 | 479 | 0.134 | 828 | 323 | 36 | 1 | 466 | 1 | 796 | Uncharacterized protein | Uncharacterized protein | | afdb-uniprot50 | AF-A0A2E1QDI5-F1-MODEL\_V4 | 1.0 | 7.099e-15 | 479 | 0.172 | 273 | 194 | 8 | 231 | 473 | 811 | 1081 | Phage\_sheath\_1C domain-containing protein | Phage\_sheath\_1C domain-containing protein | | afdb-uniprot50 | AF-A0A2E6H1W9-F1-MODEL\_V4 | 1.0 | 3.729e-14 | 479 | 0.144 | 270 | 199 | 8 | 225 | 468 | 915 | 1178 | Phage\_sheath\_1 domain-containing protein | Phage\_sheath\_1 domain-containing protein | | afdb-uniprot50 | AF-A0A7Y6QLZ9-F1-MODEL\_V4 | 1.0 | 1.584e-18 | 478 | 0.153 | 677 | 311 | 28 | 5 | 455 | 4 | 644 | Phage tail sheath family protein | Phage tail sheath family protein | | afdb-uniprot50 | AF-M0DAR5-F1-MODEL\_V4 | 1.0 | 2.504e-18 | 478 | 0.137 | 785 | 327 | 34 | 3 | 468 | 2 | 755 | Phage tail sheath protein FI | Phage tail sheath protein FI | | afdb-uniprot50 | AF-A0A3D3QRZ8-F1-MODEL\_V4 | 1.0 | 4.973e-18 | 476 | 0.218 | 366 | 201 | 20 | 166 | 468 | 26 | 369 | Uncharacterized protein | Uncharacterized protein | | afdb-uniprot50 | AF-A0A7K0CGL4-F1-MODEL\_V4 | 1.0 | 3.332e-18 | 475 | 0.154 | 701 | 305 | 26 | 1 | 468 | 1 | 646 | Phage\_sheath\_1 domain-containing protein | Phage\_sheath\_1 domain-containing protein | | afdb-uniprot50 | AF-A0A651HNK2-F1-MODEL\_V4 | 1.0 | 2.504e-18 | 475 | 0.149 | 754 | 317 | 31 | 3 | 468 | 1 | 717 | Phage tail sheath protein | Phage tail sheath protein | | afdb-uniprot50 | AF-A0A7W4X2D6-F1-MODEL\_V4 | 1.0 | 4.55e-12 | 474 | 0.789 | 95 | 20 | 0 | 381 | 475 | 2 | 96 | Phage tail sheath protein FI | Phage tail sheath protein FI | | afdb-uniprot50 | AF-A0A3P6KDJ0-F1-MODEL\_V4 | 1.0 | 5.732e-16 | 474 | 0.185 | 427 | 216 | 15 | 84 | 474 | 32 | 362 | Phage tail sheath protein FI | Phage tail sheath protein FI | | afdb-uniprot50 | AF-K4ZEC4-F1-MODEL\_V4 | 1.0 | 3.332e-18 | 474 | 0.215 | 394 | 217 | 19 | 148 | 475 | 64 | 431 | Phage tail sheath protein FI | Phage tail sheath protein FI | | afdb-uniprot50 | AF-A0A0Q9UDN4-F1-MODEL\_V4 | 1.0 | 1.124e-18 | 474 | 0.138 | 723 | 324 | 32 | 1 | 468 | 3 | 681 | Uncharacterized protein | Uncharacterized protein | | afdb-uniprot50 | AF-A0A2D7BTI7-F1-MODEL\_V4 | 1.0 | 1.471e-13 | 473 | 0.17 | 276 | 194 | 10 | 231 | 475 | 558 | 829 | Phage\_sheath\_1C domain-containing protein | Phage\_sheath\_1C domain-containing protein | | afdb-uniprot50 | AF-A0A7C3F0E2-F1-MODEL\_V4 | 1.0 | 1.105e-13 | 473 | 0.145 | 261 | 194 | 8 | 231 | 468 | 642 | 896 | Phage\_sheath\_1C domain-containing protein | Phage\_sheath\_1C domain-containing protein | | afdb-uniprot50 | AF-A0A3C1Q7E4-F1-MODEL\_V4 | 1.0 | 1.17e-13 | 471 | 0.202 | 247 | 168 | 11 | 245 | 472 | 15 | 251 | Uncharacterized protein | Uncharacterized protein | | afdb-uniprot50 | AF-A0A7Z9UQ35-F1-MODEL\_V4 | 1.0 | 1.105e-13 | 471 | 0.18 | 260 | 185 | 10 | 230 | 468 | 136 | 388 | Uncharacterized protein | Uncharacterized protein | | afdb-uniprot50 | AF-A0A382CH64-F1-MODEL\_V4 | 1.0 | 1.558e-13 | 471 | 0.201 | 238 | 176 | 7 | 229 | 457 | 417 | 649 | Uncharacterized protein | Uncharacterized protein | | afdb-uniprot50 | AF-A0A545UE66-F1-MODEL\_V4 | 1.0 | 5.047e-19 | 471 | 0.14 | 834 | 332 | 36 | 1 | 467 | 1 | 816 | Uncharacterized protein | Uncharacterized protein | | afdb-uniprot50 | AF-A0A484YNN0-F1-MODEL\_V4 | 1.0 | 2.074e-13 | 470 | 0.3 | 170 | 117 | 2 | 232 | 399 | 2 | 171 | Tail Sheath protein | Tail Sheath protein | | afdb-uniprot50 | AF-M1YXK8-F1-MODEL\_V4 | 1.0 | 5.266e-18 | 470 | 0.151 | 819 | 310 | 40 | 5 | 466 | 3 | 793 | Uncharacterized protein | Uncharacterized protein | | afdb-uniprot50 | AF-A0A2A4T3R4-F1-MODEL\_V4 | 1.0 | 1.17e-13 | 470 | 0.169 | 253 | 189 | 11 | 231 | 468 | 712 | 958 | Uncharacterized protein | Uncharacterized protein | | afdb-uniprot50 | AF-A0A7C7C6D0-F1-MODEL\_V4 | 1.0 | 8.304e-14 | 470 | 0.137 | 270 | 195 | 8 | 231 | 468 | 697 | 960 | Uncharacterized protein | Uncharacterized protein | | afdb-uniprot50 | AF-A0A2D6E3Y1-F1-MODEL\_V4 | 1.0 | 8.304e-14 | 470 | 0.141 | 269 | 200 | 9 | 225 | 468 | 787 | 1049 | Phage\_sheath\_1 domain-containing protein | Phage\_sheath\_1 domain-containing protein | | afdb-uniprot50 | AF-A0A1C5DEJ3-F1-MODEL\_V4 | 1.0 | 4.767e-19 | 469 | 0.194 | 489 | 263 | 22 | 71 | 467 | 3 | 452 | Uncharacterized protein | Uncharacterized protein | | afdb-uniprot50 | AF-A0A401XA82-F1-MODEL\_V4 | 1.0 | 2.2e-17 | 469 | 0.144 | 545 | 310 | 26 | 7 | 468 | 15 | 485 | Phage tail sheath protein | Phage tail sheath protein | | afdb-uniprot50 | AF-A0A382AQG4-F1-MODEL\_V4 | 1.0 | 4.687e-14 | 469 | 0.205 | 248 | 184 | 9 | 229 | 466 | 269 | 513 | Uncharacterized protein | Uncharacterized protein | | afdb-uniprot50 | AF-A0A1Z8SSC5-F1-MODEL\_V4 | 1.0 | 2.462e-13 | 469 | 0.162 | 258 | 191 | 8 | 231 | 467 | 634 | 887 | Phage\_sheath\_1C domain-containing protein | Phage\_sheath\_1C domain-containing protein | | afdb-uniprot50 | AF-A0A2V9GXL0-F1-MODEL\_V4 | 1.0 | 5.266e-18 | 468 | 0.143 | 797 | 312 | 36 | 3 | 468 | 1 | 757 | Phage tail protein | Phage tail protein | | afdb-uniprot50 | AF-A0A2E8NWC6-F1-MODEL\_V4 | 1.0 | 1.258e-14 | 468 | 0.208 | 268 | 181 | 8 | 231 | 468 | 794 | 1060 | Phage\_sheath\_1C domain-containing protein | Phage\_sheath\_1C domain-containing protein | | afdb-uniprot50 | AF-X0TSA3-F1-MODEL\_V4 | 1.0 | 1.044e-13 | 467 | 0.176 | 272 | 192 | 12 | 228 | 474 | 107 | 371 | Uncharacterized protein | Uncharacterized protein | | afdb-uniprot50 | AF-A0A7C5MPJ1-F1-MODEL\_V4 | 1.0 | 9.469e-19 | 467 | 0.154 | 616 | 312 | 26 | 3 | 466 | 2 | 560 | Phage tail sheath protein | Phage tail sheath protein | | afdb-uniprot50 | AF-A0A0F9MLP2-F1-MODEL\_V4 | 1.0 | 1.471e-13 | 467 | 0.149 | 247 | 196 | 7 | 231 | 468 | 343 | 584 | Phage\_sheath\_1 domain-containing protein | Phage\_sheath\_1 domain-containing protein | | afdb-uniprot50 | AF-A0A7K4MRN1-F1-MODEL\_V4 | 1.0 | 3.47e-13 | 466 | 0.159 | 269 | 198 | 12 | 227 | 474 | 241 | 502 | Phage tail sheath subtilisin-like domain-containing protein | Phage tail sheath subtilisin-like domain-containing protein | | afdb-uniprot50 | AF-A0A1Z9M029-F1-MODEL\_V4 | 1.0 | 3.521e-14 | 466 | 0.187 | 278 | 192 | 10 | 231 | 475 | 868 | 1144 | Phage\_sheath\_1C domain-containing protein | Phage\_sheath\_1C domain-containing protein | | afdb-uniprot50 | AF-A0A497PR65-F1-MODEL\_V4 | 1.0 | 1.221e-12 | 465 | 0.194 | 237 | 169 | 7 | 247 | 467 | 13 | 243 | Phage\_sheath\_1 domain-containing protein | Phage\_sheath\_1 domain-containing protein | | afdb-uniprot50 | AF-A0A2D4QWX6-F1-MODEL\_V4 | 1.0 | 6.252e-18 | 465 | 0.16 | 609 | 342 | 33 | 1 | 474 | 4 | 577 | Uncharacterized protein | Uncharacterized protein | | afdb-uniprot50 | AF-A0A538QL19-F1-MODEL\_V4 | 1.0 | 1.496e-18 | 465 | 0.131 | 847 | 325 | 41 | 1 | 468 | 1 | 815 | Phage tail sheath family protein | Phage tail sheath family protein | | afdb-uniprot50 | AF-A0A3S4W452-F1-MODEL\_V4 | 1.0 | 4.243e-15 | 463 | 0.198 | 352 | 159 | 7 | 71 | 391 | 15 | 274 | Major tail sheath protein | Major tail sheath protein | | afdb-uniprot50 | AF-A0A0N7JW40-F1-MODEL\_V4 | 1.0 | 1.799e-15 | 463 | 0.145 | 515 | 282 | 24 | 1 | 457 | 6 | 420 | Phage tail sheath protein FI | Phage tail sheath protein FI | | afdb-uniprot50 | AF-A0A831UJ70-F1-MODEL\_V4 | 1.0 | 1.678e-18 | 463 | 0.163 | 662 | 322 | 39 | 5 | 465 | 3 | 633 | Phage tail sheath family protein | Phage tail sheath family protein | | afdb-uniprot50 | AF-A0A2D7XGW4-F1-MODEL\_V4 | 1.0 | 6.995e-14 | 463 | 0.166 | 277 | 193 | 10 | 229 | 473 | 381 | 651 | Uncharacterized protein | Uncharacterized protein | | afdb-uniprot50 | AF-A0A5C7XHQ9-F1-MODEL\_V4 | 1.0 | 1.392e-17 | 463 | 0.129 | 885 | 336 | 39 | 3 | 468 | 1 | 869 | Uncharacterized protein | Uncharacterized protein | | afdb-uniprot50 | AF-A0A1X7MDT6-F1-MODEL\_V4 | 1.0 | 1.389e-13 | 462 | 0.708 | 120 | 35 | 0 | 301 | 420 | 2 | 121 | Phage tail sheath protein | Phage tail sheath protein | | afdb-uniprot50 | AF-A0A7Y2ZSL3-F1-MODEL\_V4 | 1.0 | 1.584e-18 | 462 | 0.143 | 669 | 334 | 37 | 1 | 473 | 2 | 627 | Uncharacterized protein | Uncharacterized protein | | afdb-uniprot50 | AF-A0A2D7M0S1-F1-MODEL\_V4 | 1.0 | 9.879e-18 | 462 | 0.135 | 714 | 318 | 25 | 1 | 467 | 1 | 661 | Uncharacterized protein | Uncharacterized protein | | afdb-uniprot50 | AF-J0UZD7-F1-MODEL\_V4 | 1.0 | 2.074e-13 | 462 | 0.15 | 253 | 191 | 10 | 233 | 468 | 424 | 669 | Phage tail sheath protein FI | Phage tail sheath protein FI | | afdb-uniprot50 | AF-A0A848UQ52-F1-MODEL\_V4 | 1.0 | 2.233e-18 | 461 | 0.14 | 614 | 330 | 32 | 1 | 467 | 1 | 563 | Uncharacterized protein | Uncharacterized protein | | afdb-uniprot50 | AF-A0A356TQ48-F1-MODEL\_V4 | 1.0 | 6.906e-17 | 461 | 0.132 | 769 | 323 | 32 | 5 | 474 | 2 | 725 | Phage tail protein | Phage tail protein | | afdb-uniprot50 | AF-A0A524BCG6-F1-MODEL\_V4 | 1.0 | 3.89e-13 | 460 | 0.169 | 254 | 186 | 8 | 230 | 468 | 282 | 525 | Uncharacterized protein | Uncharacterized protein | | afdb-uniprot50 | AF-A0A356KTV5-F1-MODEL\_V4 | 1.0 | 2.541e-19 | 460 | 0.147 | 588 | 279 | 25 | 1 | 424 | 1 | 529 | Uncharacterized protein | Uncharacterized protein | | afdb-uniprot50 | AF-A0A5J4SH86-F1-MODEL\_V4 | 1.0 | 9.858e-14 | 460 | 0.142 | 260 | 192 | 10 | 231 | 468 | 783 | 1033 | Uncharacterized protein | Uncharacterized protein | | afdb-uniprot50 | AF-A0A497CSG5-F1-MODEL\_V4 | 1.0 | 6.509e-13 | 459 | 0.205 | 219 | 159 | 7 | 265 | 474 | 3 | 215 | Phage tail protein | Phage tail protein | | afdb-uniprot50 | AF-A0A2P5K775-F1-MODEL\_V4 | 1.0 | 6.805e-16 | 459 | 0.146 | 512 | 277 | 28 | 3 | 468 | 2 | 399 | Tail sheath protein | Tail sheath protein | | afdb-uniprot50 | AF-A0A3B8M4T7-F1-MODEL\_V4 | 1.0 | 7.532e-19 | 458 | 0.183 | 435 | 232 | 20 | 144 | 467 | 12 | 434 | Phage tail sheath family protein | Phage tail sheath family protein | | afdb-uniprot50 | AF-A0A6M3K7K1-F1-MODEL\_V4 | 1.0 | 4.127e-17 | 458 | 0.12 | 615 | 355 | 35 | 3 | 474 | 2 | 573 | Putative tail sheath protein | Putative tail sheath protein | | afdb-uniprot50 | AF-A0A660SQZ6-F1-MODEL\_V4 | 1.0 | 2.074e-13 | 457 | 0.146 | 260 | 197 | 10 | 228 | 468 | 88 | 341 | Uncharacterized protein | Uncharacterized protein | | afdb-uniprot50 | AF-Q1H2H8-F1-MODEL\_V4 | 1.0 | 1.206e-15 | 457 | 0.131 | 563 | 280 | 25 | 3 | 468 | 2 | 452 | Phage\_sheath\_1C domain-containing protein | Phage\_sheath\_1C domain-containing protein | | afdb-uniprot50 | AF-A0A1Z8S9S0-F1-MODEL\_V4 | 1.0 | 2.462e-13 | 456 | 0.174 | 270 | 187 | 9 | 231 | 468 | 735 | 1000 | Phage\_sheath\_1C domain-containing protein | Phage\_sheath\_1C domain-containing protein | | afdb-uniprot50 | AF-A0A8B2R094-F1-MODEL\_V4 | 1.0 | 2.257e-11 | 455 | 0.409 | 127 | 75 | 0 | 348 | 474 | 1 | 127 | Phage tail protein | Phage tail protein | | afdb-uniprot50 | AF-A0A1I3LPK1-F1-MODEL\_V4 | 1.0 | 1.015e-15 | 455 | 0.133 | 583 | 258 | 23 | 1 | 468 | 1 | 451 | Uncharacterized protein | Uncharacterized protein | | afdb-uniprot50 | AF-A0A176RSK4-F1-MODEL\_V4 | 1.0 | 1.105e-13 | 454 | 0.188 | 244 | 180 | 9 | 231 | 466 | 4 | 237 | Phage tail sheath protein | Phage tail sheath protein | | afdb-uniprot50 | AF-A0A4R2C040-F1-MODEL\_V4 | 1.0 | 1.962e-17 | 454 | 0.14 | 684 | 304 | 29 | 2 | 455 | 3 | 632 | Uncharacterized protein | Uncharacterized protein | | afdb-uniprot50 | AF-A0A5P3VE82-F1-MODEL\_V4 | 1.0 | 8.321e-18 | 454 | 0.129 | 728 | 322 | 32 | 6 | 468 | 1 | 681 | Phage tail sheath family protein | Phage tail sheath family protein | | afdb-uniprot50 | AF-A0A2E7N1B0-F1-MODEL\_V4 | 1.0 | 7.406e-14 | 453 | 0.168 | 273 | 199 | 7 | 231 | 475 | 227 | 499 | Phage\_sheath\_1C domain-containing protein | Phage\_sheath\_1C domain-containing protein | | afdb-uniprot50 | AF-S4XY80-F1-MODEL\_V4 | 1.0 | 4.189e-18 | 453 | 0.19 | 702 | 313 | 29 | 5 | 468 | 3 | 686 | Uncharacterized protein | Uncharacterized protein | | afdb-uniprot50 | AF-A0A2D7XGK1-F1-MODEL\_V4 | 1.0 | 1.849e-13 | 453 | 0.151 | 278 | 196 | 9 | 225 | 468 | 833 | 1104 | Uncharacterized protein | Uncharacterized protein | | afdb-uniprot50 | AF-A0A3B8QSR2-F1-MODEL\_V4 | 1.0 | 1.65e-13 | 452 | 0.143 | 279 | 198 | 9 | 225 | 468 | 108 | 380 | Uncharacterized protein | Uncharacterized protein | | afdb-uniprot50 | AF-A0A6N0ZC76-F1-MODEL\_V4 | 1.0 | 1.933e-16 | 452 | 0.142 | 561 | 325 | 31 | 7 | 467 | 1 | 505 | Phage tail protein | Phage tail protein | | afdb-uniprot50 | AF-A0A4Q9KLK9-F1-MODEL\_V4 | 1.0 | 2.504e-18 | 452 | 0.155 | 680 | 328 | 28 | 1 | 456 | 1 | 657 | Phage tail sheath family protein | Phage tail sheath family protein | | afdb-uniprot50 | AF-A0A7J4ULP8-F1-MODEL\_V4 | 1.0 | 5.178e-13 | 451 | 0.174 | 264 | 186 | 8 | 231 | 468 | 124 | 381 | Phage tail protein | Phage tail protein | | afdb-uniprot50 | AF-A0A842PA51-F1-MODEL\_V4 | 1.0 | 2.607e-13 | 451 | 0.167 | 275 | 192 | 11 | 230 | 473 | 280 | 548 | Phage tail sheath subtilisin-like domain-containing protein | Phage tail sheath subtilisin-like domain-containing protein | | afdb-uniprot50 | AF-A0A524MJ07-F1-MODEL\_V4 | 1.0 | 1.992e-18 | 451 | 0.152 | 623 | 323 | 31 | 1 | 468 | 1 | 573 | Phage\_sheath\_1C domain-containing protein | Phage\_sheath\_1C domain-containing protein | | afdb-uniprot50 | AF-A0A1Z9EFV8-F1-MODEL\_V4 | 1.0 | 5.805e-13 | 451 | 0.168 | 267 | 195 | 11 | 230 | 475 | 663 | 923 | Uncharacterized protein | Uncharacterized protein | | afdb-uniprot50 | AF-A0A2D8CBU3-F1-MODEL\_V4 | 1.0 | 3.674e-13 | 451 | 0.155 | 276 | 195 | 9 | 225 | 468 | 861 | 1130 | Phage\_sheath\_1C domain-containing protein | Phage\_sheath\_1C domain-containing protein | | afdb-uniprot50 | AF-A0A660RI94-F1-MODEL\_V4 | 1.0 | 4.127e-17 | 450 | 0.16 | 510 | 304 | 21 | 3 | 456 | 1 | 442 | Phage\_sheath\_1C domain-containing protein | Phage\_sheath\_1C domain-containing protein | | afdb-uniprot50 | AF-A0A2D8ENN3-F1-MODEL\_V4 | 1.0 | 3.89e-13 | 450 | 0.145 | 289 | 196 | 10 | 231 | 474 | 308 | 590 | Uncharacterized protein | Uncharacterized protein | | afdb-uniprot50 | AF-A0A7Z2GQZ2-F1-MODEL\_V4 | 1.0 | 2.078e-17 | 449 | 0.193 | 423 | 263 | 27 | 68 | 461 | 135 | 508 | Uncharacterized protein | Uncharacterized protein | | afdb-uniprot50 | AF-A0A2E9U1Y6-F1-MODEL\_V4 | 1.0 | 1.962e-17 | 449 | 0.125 | 748 | 355 | 34 | 1 | 475 | 1 | 722 | Phage tail protein | Phage tail protein | | afdb-uniprot50 | AF-A0A376ZSE8-F1-MODEL\_V4 | 1.0 | 2.291e-12 | 448 | 0.344 | 148 | 92 | 3 | 252 | 398 | 7 | 150 | Major tail sheath protein FI | Major tail sheath protein FI | | afdb-uniprot50 | AF-A0A849TMA6-F1-MODEL\_V4 | 1.0 | 9.733e-17 | 448 | 0.148 | 558 | 307 | 34 | 7 | 468 | 14 | 499 | Phage tail protein | Phage tail protein | | afdb-uniprot50 | AF-A0A353BET0-F1-MODEL\_V4 | 1.0 | 1.075e-15 | 448 | 0.17 | 423 | 210 | 16 | 73 | 468 | 424 | 732 | Uncharacterized protein | Uncharacterized protein | | afdb-uniprot50 | AF-A0A511H440-F1-MODEL\_V4 | 1.0 | 7.422e-18 | 448 | 0.158 | 794 | 290 | 36 | 25 | 468 | 1 | 765 | Uncharacterized protein | Uncharacterized protein | | afdb-uniprot50 | AF-A0A6C9Q6A8-F1-MODEL\_V4 | 1.0 | 8.906e-11 | 447 | 0.41 | 117 | 69 | 0 | 358 | 474 | 3 | 119 | Phage tail protein | Phage tail protein | | afdb-uniprot50 | AF-A0A3N5HQ21-F1-MODEL\_V4 | 1.0 | 4.015e-19 | 447 | 0.177 | 534 | 270 | 26 | 3 | 420 | 1 | 481 | Uncharacterized protein | Uncharacterized protein | | afdb-uniprot50 | AF-A0A519CSN0-F1-MODEL\_V4 | 1.0 | 1.881e-18 | 447 | 0.127 | 907 | 342 | 35 | 3 | 468 | 2 | 899 | Phage\_sheath\_1C domain-containing protein | Phage\_sheath\_1C domain-containing protein | | afdb-uniprot50 | AF-A0A3D1A0D5-F1-MODEL\_V4 | 1.0 | 2.196e-13 | 447 | 0.154 | 278 | 195 | 9 | 225 | 468 | 751 | 1022 | Uncharacterized protein | Uncharacterized protein | | afdb-uniprot50 | AF-A0A2E6A8L5-F1-MODEL\_V4 | 1.0 | 2.462e-13 | 447 | 0.156 | 275 | 195 | 9 | 225 | 468 | 766 | 1034 | Uncharacterized protein | Uncharacterized protein | | afdb-uniprot50 | AF-A0A3C2A712-F1-MODEL\_V4 | 1.0 | 7.406e-14 | 446 | 0.178 | 241 | 177 | 10 | 230 | 466 | 84 | 307 | Phage tail protein | Phage tail protein | | afdb-uniprot50 | AF-L9PQK2-F1-MODEL\_V4 | 1.0 | 2.928e-17 | 446 | 0.143 | 647 | 293 | 28 | 19 | 468 | 28 | 610 | Uncharacterized protein | Uncharacterized protein | | afdb-uniprot50 | AF-A0A2D5MP23-F1-MODEL\_V4 | 1.0 | 1.653e-17 | 446 | 0.129 | 679 | 347 | 38 | 3 | 474 | 2 | 643 | Uncharacterized protein | Uncharacterized protein | | afdb-uniprot50 | AF-A0A2T1DKD7-F1-MODEL\_V4 | 1.0 | 6.62e-18 | 445 | 0.142 | 610 | 293 | 27 | 3 | 452 | 2 | 541 | Phage tail sheath protein | Phage tail sheath protein | | afdb-uniprot50 | AF-A0A5C5ZXM5-F1-MODEL\_V4 | 1.0 | 3.529e-18 | 445 | 0.144 | 597 | 321 | 25 | 1 | 455 | 1 | 549 | Phage tail sheath protein | Phage tail sheath protein | | afdb-uniprot50 | AF-A0A2D6FIM5-F1-MODEL\_V4 | 1.0 | 2.76e-13 | 444 | 0.145 | 274 | 196 | 9 | 227 | 468 | 700 | 967 | Phage\_sheath\_1 domain-containing protein | Phage\_sheath\_1 domain-containing protein | | afdb-uniprot50 | AF-A0A5D0S8K9-F1-MODEL\_V4 | 1.0 | 1.453e-16 | 443 | 0.202 | 385 | 226 | 16 | 114 | 468 | 423 | 756 | Phage tail sheath family protein | Phage tail sheath family protein | | afdb-uniprot50 | AF-A0A1X7MDW3-F1-MODEL\_V4 | 1.0 | 1.428e-11 | 442 | 0.705 | 95 | 28 | 0 | 379 | 473 | 2 | 96 | Phage-related contractile tail sheath protein | Phage-related contractile tail sheath protein | | afdb-uniprot50 | AF-A0A1M4WNH5-F1-MODEL\_V4 | 1.0 | 7.743e-17 | 442 | 0.14 | 570 | 320 | 27 | 1 | 468 | 1 | 502 | Phage tail sheath protein | Phage tail sheath protein | | afdb-uniprot50 | AF-A0A2D5XZB8-F1-MODEL\_V4 | 1.0 | 2.364e-18 | 442 | 0.153 | 643 | 351 | 38 | 3 | 468 | 2 | 627 | Phage\_sheath\_1C domain-containing protein | Phage\_sheath\_1C domain-containing protein | | afdb-uniprot50 | AF-A0A2D5YHB3-F1-MODEL\_V4 | 1.0 | 1.826e-16 | 442 | 0.11 | 766 | 322 | 32 | 5 | 474 | 2 | 703 | Uncharacterized protein | Uncharacterized protein | | afdb-uniprot50 | AF-A0A5C7LNH5-F1-MODEL\_V4 | 1.0 | 6.509e-13 | 442 | 0.13 | 269 | 203 | 9 | 225 | 468 | 730 | 992 | Phage\_sheath\_1 domain-containing protein | Phage\_sheath\_1 domain-containing protein | | afdb-uniprot50 | AF-A0A376X2C2-F1-MODEL\_V4 | 1.0 | 2.257e-11 | 441 | 0.351 | 148 | 91 | 2 | 330 | 472 | 1 | 148 | Phage-related contractile tail sheath protein | Phage-related contractile tail sheath protein | | afdb-uniprot50 | AF-A0A1V5Z4B1-F1-MODEL\_V4 | 1.0 | 1.046e-17 | 441 | 0.149 | 496 | 289 | 20 | 46 | 453 | 1 | 451 | Phage tail sheath protein | Phage tail sheath protein | | afdb-uniprot50 | AF-G4KQ75-F1-MODEL\_V4 | 1.0 | 3.283e-17 | 441 | 0.172 | 556 | 297 | 31 | 5 | 474 | 2 | 480 | Putative tail sheath protein | Putative tail sheath protein | | afdb-uniprot50 | AF-A0A7Z1V658-F1-MODEL\_V4 | 1.0 | 2.928e-17 | 441 | 0.136 | 535 | 307 | 31 | 19 | 468 | 28 | 492 | Phage tail protein | Phage tail protein | | afdb-uniprot50 | AF-A0A2A4ZDU8-F1-MODEL\_V4 | 1.0 | 8.199e-17 | 441 | 0.133 | 673 | 347 | 35 | 3 | 475 | 2 | 638 | Uncharacterized protein | Uncharacterized protein | | afdb-uniprot50 | AF-A0A2E6PTS5-F1-MODEL\_V4 | 1.0 | 4.361e-13 | 441 | 0.141 | 275 | 196 | 10 | 228 | 468 | 708 | 976 | Uncharacterized protein | Uncharacterized protein | | afdb-uniprot50 | AF-A0A2D6X5Z5-F1-MODEL\_V4 | 1.0 | 2.922e-13 | 441 | 0.161 | 278 | 192 | 8 | 225 | 467 | 726 | 997 | Uncharacterized protein | Uncharacterized protein | | afdb-uniprot50 | AF-A0A5C7LM99-F1-MODEL\_V4 | 1.0 | 5.483e-13 | 441 | 0.133 | 270 | 202 | 11 | 225 | 468 | 748 | 1011 | Uncharacterized protein | Uncharacterized protein | | afdb-uniprot50 | AF-A0A6C7KTB5-F1-MODEL\_V4 | 1.0 | 1.428e-11 | 438 | 0.279 | 186 | 129 | 3 | 291 | 474 | 8 | 190 | Phage tail sheath family protein | Phage tail sheath family protein | | afdb-uniprot50 | AF-A0A382KT14-F1-MODEL\_V4 | 1.0 | 1.153e-12 | 438 | 0.18 | 260 | 186 | 10 | 237 | 475 | 3 | 256 | Uncharacterized protein | Uncharacterized protein | | afdb-uniprot50 | AF-A0A011RAT7-F1-MODEL\_V4 | 1.0 | 5.413e-16 | 438 | 0.138 | 570 | 321 | 32 | 7 | 467 | 1 | 509 | Phage tail sheath protein | Phage tail sheath protein | | afdb-uniprot50 | AF-A0A099PD65-F1-MODEL\_V4 | 1.0 | 1.933e-16 | 438 | 0.153 | 417 | 264 | 19 | 119 | 468 | 234 | 628 | Phage\_sheath\_1C domain-containing protein | Phage\_sheath\_1C domain-containing protein | | afdb-uniprot50 | AF-A0A1Z9F154-F1-MODEL\_V4 | 1.0 | 6.16e-17 | 438 | 0.127 | 829 | 347 | 34 | 3 | 474 | 2 | 810 | Uncharacterized protein | Uncharacterized protein | | afdb-uniprot50 | AF-A0A563CW44-F1-MODEL\_V4 | 1.0 | 2.607e-13 | 438 | 0.165 | 260 | 186 | 12 | 231 | 468 | 910 | 1160 | Phage\_sheath\_1 domain-containing protein | Phage\_sheath\_1 domain-containing protein | | afdb-uniprot50 | AF-A0A0Q9M0T6-F1-MODEL\_V4 | 1.0 | 2.2e-17 | 437 | 0.144 | 685 | 320 | 31 | 1 | 455 | 1 | 649 | Uncharacterized protein | Uncharacterized protein | | afdb-uniprot50 | AF-A0A810LBR4-F1-MODEL\_V4 | 1.0 | 1.156e-16 | 437 | 0.126 | 932 | 316 | 37 | 3 | 467 | 1 | 900 | Uncharacterized protein | Uncharacterized protein | | afdb-uniprot50 | AF-A0A5N9VYH6-F1-MODEL\_V4 | 1.0 | 6.057e-12 | 436 | 0.429 | 135 | 77 | 0 | 303 | 437 | 1 | 135 | Phage tail protein | Phage tail protein | | afdb-uniprot50 | AF-A0A5C7LEF6-F1-MODEL\_V4 | 1.0 | 1.153e-12 | 436 | 0.13 | 269 | 203 | 8 | 225 | 468 | 740 | 1002 | Phage\_sheath\_1 domain-containing protein | Phage\_sheath\_1 domain-containing protein | | afdb-uniprot50 | AF-A0A800EES6-F1-MODEL\_V4 | 1.0 | 5.483e-13 | 435 | 0.133 | 278 | 201 | 10 | 225 | 468 | 79 | 350 | Phage\_sheath\_1C domain-containing protein | Phage\_sheath\_1C domain-containing protein | | afdb-uniprot50 | AF-A0A842WMT0-F1-MODEL\_V4 | 1.0 | 1.674e-14 | 434 | 0.138 | 398 | 221 | 15 | 95 | 468 | 633 | 932 | Uncharacterized protein | Uncharacterized protein | | afdb-uniprot50 | AF-A0A2D6E5M5-F1-MODEL\_V4 | 1.0 | 1.65e-13 | 434 | 0.153 | 281 | 196 | 11 | 225 | 468 | 750 | 1025 | Uncharacterized protein | Uncharacterized protein | | afdb-uniprot50 | AF-A0A521CKY5-F1-MODEL\_V4 | 1.0 | 1.224e-16 | 433 | 0.136 | 556 | 309 | 26 | 9 | 468 | 2 | 482 | Uncharacterized protein | Uncharacterized protein | | afdb-uniprot50 | AF-A0A2D5F6D1-F1-MODEL\_V4 | 1.0 | 1.372e-16 | 433 | 0.11 | 752 | 321 | 33 | 1 | 468 | 2 | 689 | Uncharacterized protein | Uncharacterized protein | | afdb-uniprot50 | AF-A0A3D5RSJ9-F1-MODEL\_V4 | 1.0 | 3.277e-13 | 433 | 0.127 | 275 | 200 | 10 | 228 | 468 | 599 | 867 | Uncharacterized protein | Uncharacterized protein | | afdb-uniprot50 | AF-A0A3N5GCM3-F1-MODEL\_V4 | 1.0 | 7.298e-13 | 432 | 0.194 | 221 | 151 | 8 | 259 | 466 | 11 | 217 | Phage tail sheath protein | Phage tail sheath protein | | afdb-uniprot50 | AF-A0A6I5RGG6-F1-MODEL\_V4 | 1.0 | 4.56e-16 | 432 | 0.15 | 586 | 316 | 30 | 1 | 468 | 1 | 522 | Uncharacterized protein | Uncharacterized protein | | afdb-uniprot50 | AF-A0A7J4UP87-F1-MODEL\_V4 | 1.0 | 5.817e-17 | 432 | 0.141 | 698 | 348 | 33 | 3 | 474 | 2 | 673 | Uncharacterized protein | Uncharacterized protein | | afdb-uniprot50 | AF-A0A2K3J0B0-F1-MODEL\_V4 | 1.0 | 8.681e-17 | 432 | 0.134 | 704 | 348 | 36 | 3 | 473 | 4 | 678 | Uncharacterized protein | Uncharacterized protein | | afdb-uniprot50 | AF-A0A3D4UU50-F1-MODEL\_V4 | 1.0 | 5.494e-17 | 431 | 0.128 | 750 | 335 | 38 | 1 | 468 | 1 | 713 | Uncharacterized protein | Uncharacterized protein | | afdb-uniprot50 | AF-A0A1V5KBQ7-F1-MODEL\_V4 | 1.0 | 1.877e-14 | 431 | 0.159 | 401 | 209 | 14 | 94 | 466 | 583 | 883 | Phage tail sheath protein | Phage tail sheath protein | | afdb-uniprot50 | AF-A0A0Q8V0M7-F1-MODEL\_V4 | 1.0 | 8.681e-17 | 429 | 0.121 | 747 | 337 | 32 | 1 | 468 | 1 | 706 | Phage\_sheath\_1C domain-containing protein | Phage\_sheath\_1C domain-containing protein | | afdb-uniprot50 | AF-A0A101JEY1-F1-MODEL\_V4 | 1.0 | 1.826e-16 | 429 | 0.127 | 745 | 344 | 33 | 5 | 468 | 2 | 721 | Phage\_sheath\_1 domain-containing protein | Phage\_sheath\_1 domain-containing protein | | afdb-uniprot50 | AF-A0A696JDI9-F1-MODEL\_V4 | 1.0 | 4.234e-11 | 428 | 0.255 | 168 | 122 | 2 | 307 | 474 | 2 | 166 | Phage tail sheath family protein | Phage tail sheath family protein | | afdb-uniprot50 | AF-A0A0F0HL05-F1-MODEL\_V4 | 1.0 | 9.174e-13 | 428 | 0.195 | 266 | 177 | 9 | 233 | 467 | 17 | 276 | Uncharacterized protein | Uncharacterized protein | | afdb-uniprot50 | AF-A0A7X7WC07-F1-MODEL\_V4 | 1.0 | 1.296e-16 | 428 | 0.165 | 526 | 303 | 25 | 1 | 468 | 3 | 450 | Phage\_sheath\_1C domain-containing protein | Phage\_sheath\_1C domain-containing protein | | afdb-uniprot50 | AF-K8GN70-F1-MODEL\_V4 | 1.0 | 2.568e-12 | 428 | 0.213 | 253 | 174 | 9 | 231 | 468 | 686 | 928 | Phage tail sheath protein FI | Phage tail sheath protein FI | | afdb-uniprot50 | AF-A0A2D6WY17-F1-MODEL\_V4 | 1.0 | 1.352e-15 | 427 | 0.114 | 828 | 325 | 30 | 3 | 475 | 2 | 775 | Uncharacterized protein | Uncharacterized protein | | afdb-uniprot50 | AF-A0A2D6MI23-F1-MODEL\_V4 | 1.0 | 8.182e-13 | 427 | 0.147 | 278 | 197 | 10 | 225 | 468 | 773 | 1044 | Uncharacterized protein | Uncharacterized protein | | afdb-uniprot50 | AF-A0A6P2CYQ9-F1-MODEL\_V4 | 1.0 | 2.078e-17 | 426 | 0.14 | 610 | 329 | 31 | 3 | 459 | 2 | 568 | Phage\_sheath\_1C domain-containing protein | Phage\_sheath\_1C domain-containing protein | | afdb-uniprot50 | AF-A0A1A9F3T7-F1-MODEL\_V4 | 1.0 | 1.296e-16 | 425 | 0.146 | 790 | 314 | 32 | 3 | 463 | 1 | 758 | Uncharacterized protein | Uncharacterized protein | | afdb-uniprot50 | AF-A0A833DC75-F1-MODEL\_V4 | 1.0 | 6.906e-17 | 424 | 0.128 | 683 | 352 | 34 | 1 | 474 | 4 | 651 | Uncharacterized protein | Uncharacterized protein | | afdb-uniprot50 | AF-A0A800EC41-F1-MODEL\_V4 | 1.0 | 2.291e-12 | 423 | 0.14 | 277 | 199 | 9 | 225 | 468 | 132 | 402 | Phage\_sheath\_1C domain-containing protein | Phage\_sheath\_1C domain-containing protein | | afdb-uniprot50 | AF-A0A6W2FV74-F1-MODEL\_V4 | 1.0 | 8.536e-12 | 422 | 0.225 | 270 | 112 | 5 | 92 | 359 | 9 | 183 | Phage tail sheath family protein | Phage tail sheath family protein | | afdb-uniprot50 | AF-D4EA08-F1-MODEL\_V4 | 1.0 | 1.699e-15 | 422 | 0.133 | 639 | 273 | 24 | 1 | 468 | 1 | 529 | Phage\_sheath\_1C domain-containing protein | Phage\_sheath\_1C domain-containing protein | | afdb-uniprot50 | AF-A0A2D6E5Y4-F1-MODEL\_V4 | 1.0 | 5.178e-13 | 422 | 0.164 | 274 | 187 | 10 | 231 | 468 | 762 | 1029 | Phage\_sheath\_1 domain-containing protein | Phage\_sheath\_1 domain-containing protein | | afdb-uniprot50 | AF-A0A661IMA0-F1-MODEL\_V4 | 1.0 | 7.406e-14 | 421 | 0.117 | 475 | 283 | 23 | 6 | 468 | 1 | 351 | Uncharacterized protein | Uncharacterized protein | | afdb-uniprot50 | AF-A0A2D6E608-F1-MODEL\_V4 | 1.0 | 4.89e-13 | 421 | 0.147 | 284 | 198 | 13 | 230 | 475 | 616 | 893 | Phage\_sheath\_1C domain-containing protein | Phage\_sheath\_1C domain-containing protein | | afdb-uniprot50 | AF-A0A3C0GEV4-F1-MODEL\_V4 | 1.0 | 1.822e-12 | 421 | 0.145 | 274 | 192 | 10 | 225 | 462 | 640 | 907 | Phage\_sheath\_1C domain-containing protein | Phage\_sheath\_1C domain-containing protein | | afdb-uniprot50 | AF-A0A1N7K568-F1-MODEL\_V4 | 1.0 | 7.959e-15 | 420 | 0.133 | 563 | 280 | 24 | 1 | 466 | 16 | 467 | Uncharacterized protein | Uncharacterized protein | | afdb-uniprot50 | AF-C7BIE9-F1-MODEL\_V4 | 1.0 | 8.554e-16 | 420 | 0.127 | 614 | 266 | 23 | 1 | 468 | 1 | 490 | Uncharacterized protein | Uncharacterized protein | | afdb-uniprot50 | AF-A0A7C1VR73-F1-MODEL\_V4 | 1.0 | 4.56e-16 | 420 | 0.129 | 631 | 336 | 37 | 1 | 468 | 1 | 580 | Phage\_sheath\_1 domain-containing protein | Phage\_sheath\_1 domain-containing protein | | afdb-uniprot50 | AF-A0A2H3KZU4-F1-MODEL\_V4 | 1.0 | 1.724e-16 | 420 | 0.153 | 503 | 292 | 24 | 46 | 467 | 167 | 616 | Uncharacterized protein | Uncharacterized protein | | afdb-uniprot50 | AF-A0A5C7JJ84-F1-MODEL\_V4 | 1.0 | 2.43e-16 | 420 | 0.129 | 712 | 320 | 31 | 1 | 468 | 5 | 660 | Phage tail sheath family protein | Phage tail sheath family protein | | afdb-uniprot50 | AF-A0A3M2DCM6-F1-MODEL\_V4 | 1.0 | 1.075e-15 | 419 | 0.127 | 541 | 331 | 25 | 7 | 460 | 1 | 487 | Phage tail sheath protein | Phage tail sheath protein | | afdb-uniprot50 | AF-A0A5C7LE80-F1-MODEL\_V4 | 1.0 | 2.568e-12 | 419 | 0.129 | 270 | 203 | 9 | 225 | 468 | 456 | 719 | Uncharacterized protein | Uncharacterized protein | | afdb-uniprot50 | AF-A0A3M2VUF5-F1-MODEL\_V4 | 1.0 | 2.531e-11 | 418 | 0.284 | 165 | 111 | 3 | 312 | 474 | 1 | 160 | Putative Phage tail sheath protein | Putative Phage tail sheath protein | | afdb-uniprot50 | AF-A0A350YB92-F1-MODEL\_V4 | 1.0 | 9.733e-17 | 418 | 0.148 | 525 | 301 | 22 | 3 | 452 | 2 | 455 | Phage tail protein | Phage tail protein | | afdb-uniprot50 | AF-A0A5R8TFK6-F1-MODEL\_V4 | 1.0 | 6.704e-15 | 418 | 0.137 | 587 | 267 | 26 | 5 | 468 | 2 | 471 | Phage tail sheath family protein | Phage tail sheath family protein | | afdb-uniprot50 | AF-A0A381RCS0-F1-MODEL\_V4 | 1.0 | 5.732e-16 | 418 | 0.121 | 776 | 357 | 42 | 1 | 475 | 1 | 752 | Uncharacterized protein | Uncharacterized protein | | afdb-uniprot50 | AF-A0A094J1V3-F1-MODEL\_V4 | 1.0 | 8.554e-16 | 417 | 0.12 | 800 | 323 | 31 | 3 | 474 | 2 | 748 | Phage tail sheath protein | Phage tail sheath protein | | afdb-uniprot50 | AF-A0A6N8K8V9-F1-MODEL\_V4 | 1.0 | 1.105e-13 | 416 | 0.268 | 287 | 108 | 6 | 43 | 326 | 127 | 314 | Phage tail protein | Phage tail protein | | afdb-uniprot50 | AF-A0A524QSS2-F1-MODEL\_V4 | 1.0 | 1.089e-12 | 416 | 0.181 | 265 | 179 | 12 | 232 | 468 | 208 | 462 | Uncharacterized protein | Uncharacterized protein | | afdb-uniprot50 | AF-A0A2D6F2P0-F1-MODEL\_V4 | 1.0 | 1.153e-12 | 416 | 0.16 | 268 | 195 | 9 | 225 | 468 | 337 | 598 | Phage\_sheath\_1C domain-containing protein | Phage\_sheath\_1C domain-containing protein | | afdb-uniprot50 | AF-A0A7C3HFY0-F1-MODEL\_V4 | 1.0 | 1.139e-15 | 415 | 0.144 | 588 | 328 | 27 | 2 | 467 | 51 | 585 | Uncharacterized protein | Uncharacterized protein | | afdb-uniprot50 | AF-A0A2A5GVC5-F1-MODEL\_V4 | 1.0 | 2.43e-16 | 415 | 0.125 | 692 | 352 | 36 | 3 | 473 | 4 | 663 | Uncharacterized protein | Uncharacterized protein | | afdb-uniprot50 | AF-A0A1L6L565-F1-MODEL\_V4 | 1.0 | 1.853e-17 | 415 | 0.149 | 462 | 277 | 25 | 100 | 468 | 493 | 931 | Phage tail sheath protein FI | Phage tail sheath protein FI | | afdb-uniprot50 | AF-R6PS34-F1-MODEL\_V4 | 1.0 | 1.45e-12 | 414 | 0.21 | 252 | 173 | 9 | 245 | 475 | 15 | 261 | Uncharacterized protein | Uncharacterized protein | | afdb-uniprot50 | AF-A0A2E6W8A2-F1-MODEL\_V4 | 1.0 | 4.058e-12 | 413 | 0.133 | 278 | 201 | 8 | 225 | 468 | 363 | 634 | Uncharacterized protein | Uncharacterized protein | | afdb-uniprot50 | AF-A0A1Z8RYI1-F1-MODEL\_V4 | 1.0 | 3.228e-12 | 411 | 0.144 | 270 | 199 | 11 | 225 | 468 | 400 | 663 | Phage\_sheath\_1 domain-containing protein | Phage\_sheath\_1 domain-containing protein | | afdb-uniprot50 | AF-A0A1Q7BLH3-F1-MODEL\_V4 | 1.0 | 5.494e-17 | 410 | 0.172 | 550 | 282 | 32 | 3 | 455 | 2 | 475 | Phage\_sheath\_1 domain-containing protein | Phage\_sheath\_1 domain-containing protein | | afdb-uniprot50 | AF-A0A8A6KD18-F1-MODEL\_V4 | 1.0 | 4.56e-16 | 410 | 0.108 | 781 | 344 | 33 | 5 | 468 | 2 | 746 | Phage tail sheath family protein | Phage tail sheath family protein | | afdb-uniprot50 | AF-A0A849QUP3-F1-MODEL\_V4 | 1.0 | 4.9e-17 | 409 | 0.163 | 490 | 259 | 25 | 3 | 398 | 2 | 434 | Phage tail sheath family protein | Phage tail sheath family protein | | afdb-uniprot50 | AF-A0A2D8FVW0-F1-MODEL\_V4 | 1.0 | 3.419e-12 | 409 | 0.136 | 279 | 200 | 8 | 225 | 468 | 579 | 851 | Phage\_sheath\_1C domain-containing protein | Phage\_sheath\_1C domain-containing protein | | afdb-uniprot50 | AF-A0A5F1HTD7-F1-MODEL\_V4 | 1.0 | 4.618e-13 | 408 | 0.19 | 315 | 162 | 12 | 231 | 468 | 61 | 359 | Phage\_sheath\_1C domain-containing protein | Phage\_sheath\_1C domain-containing protein | | afdb-uniprot50 | AF-A0A3M2BQ74-F1-MODEL\_V4 | 1.0 | 5.72e-12 | 408 | 0.141 | 276 | 194 | 9 | 231 | 467 | 508 | 779 | Uncharacterized protein | Uncharacterized protein | | afdb-uniprot50 | AF-A0A382PEL7-F1-MODEL\_V4 | 1.0 | 4.483e-11 | 407 | 0.169 | 230 | 175 | 7 | 255 | 474 | 8 | 231 | Uncharacterized protein | Uncharacterized protein | | afdb-uniprot50 | AF-A0A853I305-F1-MODEL\_V4 | 1.0 | 4.426e-14 | 407 | 0.121 | 594 | 295 | 25 | 3 | 468 | 2 | 496 | Phage tail sheath family protein | Phage tail sheath family protein | | afdb-uniprot50 | AF-A0A1Z8THJ1-F1-MODEL\_V4 | 1.0 | 5.72e-12 | 407 | 0.168 | 273 | 192 | 11 | 231 | 474 | 289 | 555 | Uncharacterized protein | Uncharacterized protein | | afdb-uniprot50 | AF-A0A2E7TM91-F1-MODEL\_V4 | 1.0 | 5.402e-12 | 407 | 0.184 | 265 | 191 | 10 | 230 | 474 | 410 | 669 | Uncharacterized protein | Uncharacterized protein | | afdb-uniprot50 | AF-A0A2E3KCZ5-F1-MODEL\_V4 | 1.0 | 1.629e-16 | 407 | 0.124 | 770 | 351 | 31 | 1 | 473 | 1 | 743 | Uncharacterized protein | Uncharacterized protein | | afdb-uniprot50 | AF-A0A3S4VXL9-F1-MODEL\_V4 | 1.0 | 8.182e-13 | 406 | 0.195 | 251 | 115 | 3 | 119 | 367 | 64 | 229 | Major tail sheath protein | Major tail sheath protein | | afdb-uniprot50 | AF-A0A2D6AJR1-F1-MODEL\_V4 | 1.0 | 4.687e-14 | 406 | 0.133 | 523 | 274 | 25 | 5 | 468 | 1 | 403 | Uncharacterized protein | Uncharacterized protein | | afdb-uniprot50 | AF-A0A7V8IUR4-F1-MODEL\_V4 | 1.0 | 2.612e-17 | 406 | 0.155 | 534 | 266 | 25 | 1 | 413 | 1 | 470 | Phage tail sheath protein | Phage tail sheath protein | | afdb-uniprot50 | AF-A0A1Z9GEN2-F1-MODEL\_V4 | 1.0 | 1.156e-16 | 406 | 0.112 | 687 | 361 | 41 | 1 | 474 | 1 | 651 | Uncharacterized protein | Uncharacterized protein | | afdb-uniprot50 | AF-A0A2N6N302-F1-MODEL\_V4 | 1.0 | 3.62e-12 | 406 | 0.247 | 202 | 129 | 9 | 283 | 467 | 477 | 672 | Phage tail protein | Phage tail protein | | afdb-uniprot50 | AF-A0A1R3VFK8-F1-MODEL\_V4 | 1.0 | 2.725e-16 | 406 | 0.139 | 783 | 317 | 32 | 5 | 457 | 4 | 759 | Putative Phage tail sheath protein | Putative Phage tail sheath protein | | afdb-uniprot50 | AF-A0A0R0ABK8-F1-MODEL\_V4 | 1.0 | 3.088e-09 | 405 | 0.54 | 87 | 38 | 2 | 140 | 226 | 103 | 187 | Uncharacterized protein | Uncharacterized protein | | afdb-uniprot50 | AF-A0A7Y6NZ27-F1-MODEL\_V4 | 1.0 | 6.069e-16 | 404 | 0.106 | 674 | 350 | 38 | 1 | 474 | 1 | 621 | Phage tail sheath subtilisin-like domain-containing protein | Phage tail sheath subtilisin-like domain-containing protein | | afdb-uniprot50 | AF-A0A4P2R373-F1-MODEL\_V4 | 1.0 | 1.799e-15 | 402 | 0.131 | 561 | 312 | 26 | 10 | 468 | 65 | 551 | Uncharacterized protein | Uncharacterized protein | | afdb-uniprot50 | AF-A0A2D8EUZ2-F1-MODEL\_V4 | 1.0 | 6.426e-16 | 401 | 0.115 | 703 | 366 | 37 | 3 | 474 | 2 | 679 | Uncharacterized protein | Uncharacterized protein | | afdb-uniprot50 | AF-A0A1H9D327-F1-MODEL\_V4 | 1.0 | 1.156e-16 | 401 | 0.15 | 477 | 272 | 26 | 99 | 468 | 462 | 912 | IPT/TIG domain-containing protein | IPT/TIG domain-containing protein | | afdb-uniprot50 | AF-A0A2V2GPW4-F1-MODEL\_V4 | 1.0 | 6.16e-17 | 400 | 0.171 | 560 | 295 | 29 | 5 | 474 | 2 | 482 | Phage tail protein | Phage tail protein | | afdb-uniprot50 | AF-A0A7V9L2C0-F1-MODEL\_V4 | 1.0 | 2.33e-17 | 400 | 0.158 | 548 | 272 | 28 | 1 | 401 | 2 | 507 | Phage tail sheath family protein | Phage tail sheath family protein | | afdb-uniprot50 | AF-A0A0F9QR86-F1-MODEL\_V4 | 1.0 | 1.255e-10 | 399 | 0.375 | 152 | 93 | 2 | 324 | 474 | 19 | 169 | Phage\_sheath\_1C domain-containing protein | Phage\_sheath\_1C domain-containing protein | | afdb-uniprot50 | AF-A0A1E7IPR4-F1-MODEL\_V4 | 1.0 | 1.091e-16 | 399 | 0.137 | 711 | 341 | 37 | 5 | 466 | 3 | 689 | Phage\_sheath\_1C domain-containing protein | Phage\_sheath\_1C domain-containing protein | | afdb-uniprot50 | AF-A0A370LRA8-F1-MODEL\_V4 | 1.0 | 4.828e-16 | 399 | 0.125 | 824 | 341 | 39 | 3 | 474 | 10 | 805 | Uncharacterized protein | Uncharacterized protein | | afdb-uniprot50 | AF-A0A3M1ZRW3-F1-MODEL\_V4 | 1.0 | 5.494e-17 | 398 | 0.174 | 470 | 240 | 19 | 141 | 471 | 241 | 701 | Phage tail sheath family protein | Phage tail sheath family protein | | afdb-uniprot50 | AF-A0A7V7ZI79-F1-MODEL\_V4 | 1.0 | 1.001e-14 | 398 | 0.144 | 366 | 237 | 16 | 151 | 468 | 570 | 907 | Phage tail sheath family protein | Phage tail sheath family protein | | afdb-uniprot50 | AF-A0A327JRX8-F1-MODEL\_V4 | 1.0 | 4.677e-10 | 396 | 0.255 | 168 | 123 | 1 | 307 | 474 | 5 | 170 | Phage\_sheath\_1C domain-containing protein | Phage\_sheath\_1C domain-containing protein | | afdb-uniprot50 | AF-A0A7C7GUK5-F1-MODEL\_V4 | 1.0 | 1.139e-15 | 394 | 0.132 | 740 | 349 | 38 | 3 | 473 | 2 | 717 | Uncharacterized protein | Uncharacterized protein | | afdb-uniprot50 | AF-A0A843FVB4-F1-MODEL\_V4 | 1.0 | 1.601e-11 | 394 | 0.14 | 263 | 199 | 10 | 227 | 468 | 619 | 875 | Uncharacterized protein | Uncharacterized protein | | afdb-uniprot50 | AF-A0A0Q8FLN8-F1-MODEL\_V4 | 1.0 | 1.826e-16 | 394 | 0.15 | 498 | 276 | 25 | 96 | 468 | 436 | 911 | Phage\_sheath\_1C domain-containing protein | Phage\_sheath\_1C domain-containing protein | | afdb-uniprot50 | AF-A0A4Q9KAR5-F1-MODEL\_V4 | 1.0 | 2.725e-16 | 393 | 0.118 | 752 | 326 | 35 | 1 | 468 | 6 | 704 | Phage tail sheath protein | Phage tail sheath protein | | afdb-uniprot50 | AF-J2ZV19-F1-MODEL\_V4 | 1.0 | 6.069e-16 | 392 | 0.141 | 475 | 285 | 20 | 100 | 468 | 465 | 922 | Phage tail sheath protein FI | Phage tail sheath protein FI | | afdb-uniprot50 | AF-A0A813AAM4-F1-MODEL\_V4 | 1.0 | 6.426e-16 | 392 | 0.133 | 796 | 334 | 34 | 1 | 468 | 499 | 1266 | 18 protein | 18 protein | | afdb-uniprot50 | AF-A0A2D6PBJ4-F1-MODEL\_V4 | 1.0 | 6.805e-16 | 391 | 0.127 | 598 | 313 | 29 | 1 | 453 | 1 | 534 | Uncharacterized protein | Uncharacterized protein | | afdb-uniprot50 | AF-A0A2W4LBW3-F1-MODEL\_V4 | 1.0 | 7.959e-15 | 391 | 0.16 | 506 | 290 | 25 | 46 | 468 | 101 | 554 | Phage tail sheath protein | Phage tail sheath protein | | afdb-uniprot50 | AF-A0A2D9Z9K2-F1-MODEL\_V4 | 1.0 | 5.98e-15 | 391 | 0.102 | 781 | 364 | 34 | 1 | 475 | 1 | 750 | Uncharacterized protein | Uncharacterized protein | | afdb-uniprot50 | AF-A0A1I3BRN0-F1-MODEL\_V4 | 1.0 | 1.224e-16 | 390 | 0.132 | 765 | 331 | 39 | 3 | 467 | 2 | 733 | Phage\_sheath\_1 domain-containing protein | Phage\_sheath\_1 domain-containing protein | | afdb-uniprot50 | AF-A0A2E3G893-F1-MODEL\_V4 | 1.0 | 3.188e-15 | 389 | 0.13 | 619 | 350 | 32 | 3 | 474 | 2 | 578 | Uncharacterized protein | Uncharacterized protein | | afdb-uniprot50 | AF-A0A662ABX1-F1-MODEL\_V4 | 1.0 | 4.243e-15 | 388 | 0.118 | 710 | 320 | 28 | 1 | 468 | 1 | 646 | Uncharacterized protein | Uncharacterized protein | | afdb-uniprot50 | AF-A0A7J4UPE8-F1-MODEL\_V4 | 1.0 | 2.136e-15 | 388 | 0.133 | 698 | 338 | 36 | 3 | 475 | 2 | 657 | Uncharacterized protein | Uncharacterized protein | | afdb-uniprot50 | AF-A0A356X586-F1-MODEL\_V4 | 1.0 | 1.153e-12 | 388 | 0.141 | 304 | 195 | 14 | 225 | 468 | 400 | 697 | Phage\_sheath\_1C domain-containing protein | Phage\_sheath\_1C domain-containing protein | | afdb-uniprot50 | AF-A0A849BHN9-F1-MODEL\_V4 | 1.0 | 4.05e-08 | 388 | 0.551 | 78 | 34 | 1 | 147 | 224 | 595 | 671 | Uncharacterized protein | Uncharacterized protein | | afdb-uniprot50 | AF-A0A6G2FUR4-F1-MODEL\_V4 | 1.0 | 5.112e-16 | 386 | 0.181 | 419 | 242 | 18 | 138 | 463 | 14 | 424 | Uncharacterized protein | Uncharacterized protein | | afdb-uniprot50 | AF-A0A2V8SH17-F1-MODEL\_V4 | 1.0 | 2.168e-16 | 386 | 0.168 | 468 | 245 | 19 | 132 | 467 | 28 | 483 | Phage tail sheath family protein | Phage tail sheath family protein | | afdb-uniprot50 | AF-A0A292SAA6-F1-MODEL\_V4 | 1.0 | 6.057e-12 | 385 | 0.17 | 287 | 185 | 9 | 230 | 468 | 161 | 442 | Phage\_sheath\_1C domain-containing protein | Phage\_sheath\_1C domain-containing protein | | afdb-uniprot50 | AF-A0A2A7RYU0-F1-MODEL\_V4 | 1.0 | 4.747e-11 | 384 | 0.199 | 316 | 161 | 9 | 107 | 418 | 2 | 229 | Phage\_sheath\_1 domain-containing protein | Phage\_sheath\_1 domain-containing protein | | afdb-uniprot50 | AF-A0A0F0HLZ5-F1-MODEL\_V4 | 1.0 | 3.776e-11 | 383 | 0.282 | 195 | 125 | 4 | 283 | 468 | 3 | 191 | Tail protein | Tail protein | | afdb-uniprot50 | AF-A0A7I8DID5-F1-MODEL\_V4 | 1.0 | 2.136e-15 | 383 | 0.144 | 559 | 311 | 30 | 6 | 475 | 1 | 480 | Uncharacterized protein | Uncharacterized protein | | afdb-uniprot50 | AF-A0A1X7MP25-F1-MODEL\_V4 | 1.0 | 1.31e-09 | 381 | 0.678 | 84 | 27 | 0 | 390 | 473 | 1 | 84 | Phage-related contractile tail sheath protein | Phage-related contractile tail sheath protein | | afdb-uniprot50 | AF-A0A800EG04-F1-MODEL\_V4 | 1.0 | 2.531e-11 | 381 | 0.129 | 248 | 186 | 10 | 245 | 468 | 5 | 246 | Phage\_sheath\_1 domain-containing protein | Phage\_sheath\_1 domain-containing protein | | afdb-uniprot50 | AF-A0A5C7JAB2-F1-MODEL\_V4 | 1.0 | 2.685e-15 | 381 | 0.128 | 636 | 334 | 36 | 3 | 468 | 2 | 586 | Phage\_sheath\_1C domain-containing protein | Phage\_sheath\_1C domain-containing protein | | afdb-uniprot50 | AF-A0A736IA12-F1-MODEL\_V4 | 1.0 | 8.775e-10 | 378 | 0.351 | 131 | 82 | 2 | 345 | 474 | 2 | 130 | Phage tail sheath family protein | Phage tail sheath family protein | | afdb-uniprot50 | AF-A0A1Q7YAH8-F1-MODEL\_V4 | 1.0 | 1.258e-14 | 378 | 0.136 | 511 | 296 | 24 | 46 | 468 | 197 | 649 | Uncharacterized protein | Uncharacterized protein | | afdb-uniprot50 | AF-A0A730JWC2-F1-MODEL\_V4 | 1.0 | 4.953e-10 | 375 | 0.472 | 110 | 58 | 0 | 340 | 449 | 2 | 111 | Phage tail protein | Phage tail protein | | afdb-uniprot50 | AF-A0A6V8EZA9-F1-MODEL\_V4 | 1.0 | 7.099e-15 | 375 | 0.1 | 624 | 349 | 34 | 15 | 466 | 2 | 584 | Uncharacterized protein | Uncharacterized protein | | afdb-uniprot50 | AF-A0A6N6X9E8-F1-MODEL\_V4 | 1.0 | 8.646e-09 | 374 | 0.432 | 111 | 63 | 0 | 364 | 474 | 1 | 111 | Phage tail protein | Phage tail protein | | afdb-uniprot50 | AF-A0A2E6EJU7-F1-MODEL\_V4 | 1.0 | 2.136e-15 | 372 | 0.137 | 590 | 274 | 23 | 1 | 468 | 8 | 484 | Uncharacterized protein | Uncharacterized protein | | afdb-uniprot50 | AF-A0A559QHL7-F1-MODEL\_V4 | 1.0 | 8.793e-14 | 372 | 0.134 | 578 | 325 | 30 | 1 | 468 | 1 | 512 | Phage tail sheath protein | Phage tail sheath protein | | afdb-uniprot50 | AF-A0A651EMP1-F1-MODEL\_V4 | 1.0 | 1.849e-13 | 372 | 0.159 | 383 | 259 | 14 | 99 | 455 | 460 | 805 | Uncharacterized protein | Uncharacterized protein | | afdb-uniprot50 | AF-I4HNE8-F1-MODEL\_V4 | 1.0 | 4.007e-15 | 371 | 0.147 | 399 | 237 | 19 | 142 | 466 | 6 | 375 | Uncharacterized protein | Uncharacterized protein | | afdb-uniprot50 | AF-A0A193QGV7-F1-MODEL\_V4 | 1.0 | 3.141e-14 | 370 | 0.285 | 217 | 138 | 2 | 259 | 474 | 2 | 202 | Phage tail sheath protein | Phage tail sheath protein | | afdb-uniprot50 | AF-A0A3N8BHF0-F1-MODEL\_V4 | 1.0 | 4.067e-16 | 370 | 0.152 | 557 | 299 | 31 | 5 | 468 | 2 | 478 | Phage tail sheath family protein | Phage tail sheath family protein | | afdb-uniprot50 | AF-Q2N8P0-F1-MODEL\_V4 | 1.0 | 3.784e-15 | 370 | 0.159 | 470 | 255 | 23 | 113 | 468 | 482 | 925 | Phage\_sheath\_1C domain-containing protein | Phage\_sheath\_1C domain-containing protein | | afdb-uniprot50 | AF-A0A2D6WY00-F1-MODEL\_V4 | 1.0 | 7.406e-14 | 368 | 0.128 | 568 | 279 | 23 | 5 | 468 | 1 | 456 | Uncharacterized protein | Uncharacterized protein | | afdb-uniprot50 | AF-A0A450ZA88-F1-MODEL\_V4 | 1.0 | 1.49e-10 | 367 | 0.214 | 256 | 114 | 3 | 121 | 375 | 3 | 172 | Phage\_sheath\_1 domain-containing protein | Phage\_sheath\_1 domain-containing protein | | afdb-uniprot50 | AF-A0A3D4UXI7-F1-MODEL\_V4 | 1.0 | 5.805e-13 | 366 | 0.121 | 534 | 267 | 24 | 5 | 468 | 1 | 402 | Uncharacterized protein | Uncharacterized protein | | afdb-uniprot50 | AF-A0A5J6PAG1-F1-MODEL\_V4 | 1.0 | 7.175e-08 | 365 | 0.44 | 84 | 45 | 2 | 144 | 226 | 120 | 202 | Phage tail protein | Phage tail protein | | afdb-uniprot50 | AF-A0A823Z769-F1-MODEL\_V4 | 1.0 | 1.073e-11 | 365 | 0.192 | 358 | 164 | 12 | 1 | 336 | 1 | 255 | Phage tail sheath family protein | Phage tail sheath family protein | | afdb-uniprot50 | AF-A0A011NTT3-F1-MODEL\_V4 | 1.0 | 4.243e-15 | 365 | 0.117 | 683 | 323 | 30 | 3 | 452 | 2 | 637 | Phage tail sheath protein | Phage tail sheath protein | | afdb-uniprot50 | AF-A0A0F9G5K0-F1-MODEL\_V4 | 1.0 | 6.319e-11 | 364 | 0.141 | 269 | 194 | 13 | 233 | 473 | 207 | 466 | Uncharacterized protein | Uncharacterized protein | | afdb-uniprot50 | AF-A0A3C1WGA5-F1-MODEL\_V4 | 1.0 | 7.598e-08 | 364 | 0.542 | 83 | 37 | 1 | 142 | 224 | 114 | 195 | Uncharacterized protein | Uncharacterized protein | | afdb-uniprot50 | AF-T5KPS4-F1-MODEL\_V4 | 1.0 | 2.563e-08 | 363 | 0.494 | 87 | 38 | 3 | 139 | 224 | 13 | 94 | Uncharacterized protein | Uncharacterized protein | | afdb-uniprot50 | AF-A0A2V8S9Y8-F1-MODEL\_V4 | 1.0 | 8.906e-11 | 363 | 0.17 | 247 | 176 | 10 | 231 | 456 | 228 | 466 | Uncharacterized protein | Uncharacterized protein | | afdb-uniprot50 | AF-A0A2E3GP57-F1-MODEL\_V4 | 1.0 | 3.521e-14 | 363 | 0.132 | 725 | 327 | 33 | 3 | 473 | 4 | 680 | Uncharacterized protein | Uncharacterized protein | | afdb-uniprot50 | AF-A0A4Q3AL90-F1-MODEL\_V4 | 1.0 | 3.134e-10 | 362 | 0.173 | 219 | 161 | 7 | 265 | 468 | 3 | 216 | Phage\_sheath\_1C domain-containing protein | Phage\_sheath\_1C domain-containing protein | | afdb-uniprot50 | AF-A0A843FPA9-F1-MODEL\_V4 | 1.0 | 2.493e-10 | 362 | 0.144 | 250 | 187 | 10 | 234 | 468 | 801 | 1038 | Uncharacterized protein | Uncharacterized protein | | afdb-uniprot50 | AF-A0A2E7URM1-F1-MODEL\_V4 | 1.0 | 2.498e-14 | 361 | 0.158 | 447 | 227 | 21 | 72 | 466 | 277 | 626 | Uncharacterized protein | Uncharacterized protein | | afdb-uniprot50 | AF-A0A1Z9STS7-F1-MODEL\_V4 | 1.0 | 3.729e-14 | 360 | 0.118 | 774 | 361 | 36 | 3 | 474 | 2 | 755 | Uncharacterized protein | Uncharacterized protein | | afdb-uniprot50 | AF-E3BL79-F1-MODEL\_V4 | 1.0 | 8.062e-12 | 359 | 0.101 | 510 | 279 | 23 | 1 | 466 | 3 | 377 | Uncharacterized protein | Uncharacterized protein | | afdb-uniprot50 | AF-A0A2D8ERL6-F1-MODEL\_V4 | 1.0 | 1.17e-13 | 359 | 0.124 | 531 | 268 | 26 | 15 | 466 | 11 | 423 | Phage\_sheath\_1C domain-containing protein | Phage\_sheath\_1C domain-containing protein | | afdb-uniprot50 | AF-A0A2S3UNF6-F1-MODEL\_V4 | 1.0 | 4.18e-14 | 359 | 0.11 | 802 | 351 | 36 | 5 | 466 | 1 | 779 | Phage tail sheath protein FI | Phage tail sheath protein FI | | afdb-uniprot50 | AF-A0A496KHE0-F1-MODEL\_V4 | 1.0 | 1.622e-08 | 358 | 0.436 | 94 | 51 | 2 | 136 | 227 | 663 | 756 | Uncharacterized protein | Uncharacterized protein | | afdb-uniprot50 | AF-A0A7Y5K224-F1-MODEL\_V4 | 1.0 | 5.636e-11 | 357 | 0.195 | 235 | 153 | 9 | 253 | 467 | 20 | 238 | Phage tail sheath family protein | Phage tail sheath family protein | | afdb-uniprot50 | AF-I5B7I1-F1-MODEL\_V4 | 1.0 | 2.536e-15 | 357 | 0.152 | 624 | 291 | 39 | 5 | 437 | 1 | 577 | Phage tail sheath protein FI | Phage tail sheath protein FI | | afdb-uniprot50 | AF-A0A1A7PRB7-F1-MODEL\_V4 | 1.0 | 2.457e-09 | 355 | 0.318 | 154 | 95 | 4 | 327 | 474 | 1 | 150 | Phage tail protein | Phage tail protein | | afdb-uniprot50 | AF-D3AR67-F1-MODEL\_V4 | 1.0 | 4.56e-16 | 355 | 0.16 | 493 | 253 | 23 | 121 | 463 | 14 | 495 | Phage tail sheath protein | Phage tail sheath protein | | afdb-uniprot50 | AF-A0A2E6FCL3-F1-MODEL\_V4 | 1.0 | 3.948e-14 | 355 | 0.133 | 622 | 354 | 36 | 3 | 473 | 2 | 589 | Uncharacterized protein | Uncharacterized protein | | afdb-uniprot50 | AF-A0A382CP73-F1-MODEL\_V4 | 1.0 | 3.326e-14 | 355 | 0.111 | 655 | 343 | 35 | 3 | 445 | 2 | 629 | Uncharacterized protein | Uncharacterized protein | | afdb-uniprot50 | AF-A0A2E0HZL7-F1-MODEL\_V4 | 1.0 | 1.17e-13 | 355 | 0.123 | 832 | 340 | 41 | 1 | 474 | 1 | 800 | Uncharacterized protein | Uncharacterized protein | | afdb-uniprot50 | AF-A0A6P1B3L2-F1-MODEL\_V4 | 1.0 | 1.717e-08 | 354 | 0.802 | 86 | 17 | 0 | 362 | 447 | 1 | 86 | Phage tail sheath family protein | Phage tail sheath family protein | | afdb-uniprot50 | AF-A0A1G8EJS8-F1-MODEL\_V4 | 1.0 | 1.332e-14 | 354 | 0.12 | 549 | 327 | 22 | 1 | 468 | 13 | 486 | Phage tail sheath protein | Phage tail sheath protein | | afdb-uniprot50 | AF-A0A328FDX7-F1-MODEL\_V4 | 1.0 | 1.747e-13 | 353 | 0.125 | 572 | 337 | 28 | 7 | 468 | 1 | 519 | Uncharacterized protein | Uncharacterized protein | | afdb-uniprot50 | AF-A0A2D0N1H2-F1-MODEL\_V4 | 1.0 | 1.203e-11 | 353 | 0.097 | 798 | 287 | 24 | 1 | 466 | 3 | 698 | Uncharacterized protein | Uncharacterized protein | | afdb-uniprot50 | AF-A0A7K4MQW1-F1-MODEL\_V4 | 1.0 | 9.31e-14 | 353 | 0.1 | 768 | 376 | 39 | 3 | 474 | 2 | 750 | Phage tail sheath subtilisin-like domain-containing protein | Phage tail sheath subtilisin-like domain-containing protein | | afdb-uniprot50 | AF-A0A2N2MEV8-F1-MODEL\_V4 | 1.0 | 9.449e-15 | 351 | 0.14 | 492 | 261 | 21 | 102 | 455 | 500 | 967 | Uncharacterized protein | Uncharacterized protein | | afdb-uniprot50 | AF-A0A3A8PQG2-F1-MODEL\_V4 | 1.0 | 6.147e-13 | 350 | 0.153 | 392 | 233 | 18 | 159 | 468 | 23 | 397 | Uncharacterized protein | Uncharacterized protein | | afdb-uniprot50 | AF-A0A418HAB9-F1-MODEL\_V4 | 1.0 | 1.468e-09 | 349 | 0.436 | 119 | 66 | 1 | 278 | 395 | 1 | 119 | Phage tail protein | Phage tail protein | | afdb-uniprot50 | AF-A0A0Q0D1V1-F1-MODEL\_V4 | 1.0 | 1.026e-08 | 347 | 0.484 | 99 | 51 | 0 | 376 | 474 | 2 | 100 | Major tail sheath protein | Major tail sheath protein | | afdb-uniprot50 | AF-A0A3M1BNH9-F1-MODEL\_V4 | 1.0 | 3.27e-09 | 347 | 0.333 | 135 | 82 | 4 | 346 | 474 | 2 | 134 | Phage tail sheath family protein | Phage tail sheath family protein | | afdb-uniprot50 | AF-A0A3M1QC03-F1-MODEL\_V4 | 1.0 | 2.191e-09 | 346 | 0.238 | 180 | 129 | 2 | 291 | 468 | 9 | 182 | Phage tail sheath family protein | Phage tail sheath family protein | | afdb-uniprot50 | AF-A0A6M1Q203-F1-MODEL\_V4 | 1.0 | 2.998e-07 | 345 | 0.415 | 77 | 43 | 1 | 148 | 224 | 2 | 76 | RagB/SusD family nutrient uptake outer membrane protein | RagB/SusD family nutrient uptake outer membrane protein | | afdb-uniprot50 | AF-A0A383D6D3-F1-MODEL\_V4 | 1.0 | 1.255e-10 | 345 | 0.176 | 204 | 152 | 7 | 229 | 424 | 39 | 234 | Phage\_sheath\_1 domain-containing protein | Phage\_sheath\_1 domain-containing protein | | afdb-uniprot50 | AF-A0A2D0AK03-F1-MODEL\_V4 | 1.0 | 5.091e-08 | 345 | 0.5 | 86 | 41 | 2 | 140 | 224 | 99 | 183 | Phage tail protein | Phage tail protein | | afdb-uniprot50 | AF-A0A5E4M1E6-F1-MODEL\_V4 | 1.0 | 3.095e-13 | 345 | 0.136 | 448 | 228 | 16 | 84 | 468 | 699 | 1050 | Phage\_sheath\_1C domain-containing protein | Phage\_sheath\_1C domain-containing protein | | afdb-uniprot50 | AF-A0A845AZ49-F1-MODEL\_V4 | 1.0 | 2.607e-13 | 343 | 0.122 | 603 | 327 | 33 | 7 | 467 | 3 | 545 | Uncharacterized protein | Uncharacterized protein | | afdb-uniprot50 | AF-A0A7V2ED64-F1-MODEL\_V4 | 1.0 | 5.027e-11 | 343 | 0.148 | 296 | 198 | 12 | 227 | 473 | 859 | 1149 | Phage\_sheath\_1C domain-containing protein | Phage\_sheath\_1C domain-containing protein | | afdb-uniprot50 | AF-A0A1S6HM92-F1-MODEL\_V4 | 1.0 | 5.255e-14 | 341 | 0.178 | 542 | 283 | 27 | 1 | 468 | 1 | 453 | Phage tail sheath protein | Phage tail sheath protein | | afdb-uniprot50 | AF-R9L6C8-F1-MODEL\_V4 | 1.0 | 3.088e-09 | 339 | 0.235 | 208 | 137 | 9 | 278 | 472 | 2 | 200 | Uncharacterized protein | Uncharacterized protein | | afdb-uniprot50 | AF-A0A7C5N1C4-F1-MODEL\_V4 | 1.0 | 1.103e-09 | 338 | 0.21 | 195 | 128 | 8 | 288 | 468 | 461 | 643 | Phage tail sheath family protein | Phage tail sheath family protein | | afdb-uniprot50 | AF-A0A854M7T7-F1-MODEL\_V4 | 1.0 | 2.966e-14 | 336 | 0.123 | 687 | 332 | 34 | 3 | 474 | 4 | 635 | Uncharacterized protein | Uncharacterized protein | | afdb-uniprot50 | AF-A0A7X7X247-F1-MODEL\_V4 | 1.0 | 5.255e-14 | 335 | 0.14 | 528 | 280 | 26 | 1 | 424 | 44 | 501 | Uncharacterized protein | Uncharacterized protein | | afdb-uniprot50 | AF-A0A1F1BZS2-F1-MODEL\_V4 | 1.0 | 1.071e-07 | 335 | 0.369 | 92 | 56 | 2 | 137 | 226 | 361 | 452 | Uncharacterized protein | Uncharacterized protein | | afdb-uniprot50 | AF-N6YII7-F1-MODEL\_V4 | 1.0 | 4.172e-10 | 334 | 0.134 | 261 | 185 | 9 | 231 | 452 | 131 | 389 | Uncharacterized protein | Uncharacterized protein | | afdb-uniprot50 | AF-A0A1H8JA80-F1-MODEL\_V4 | 1.0 | 7.614e-12 | 334 | 0.14 | 519 | 222 | 17 | 68 | 468 | 251 | 663 | Phage\_sheath\_1C domain-containing protein | Phage\_sheath\_1C domain-containing protein | | afdb-uniprot50 | AF-A0A496KYN9-F1-MODEL\_V4 | 1.0 | 4.541e-08 | 333 | 0.385 | 96 | 57 | 2 | 133 | 226 | 712 | 807 | Uncharacterized protein | Uncharacterized protein | | afdb-uniprot50 | AF-A0A2E0S344-F1-MODEL\_V4 | 1.0 | 6.509e-13 | 332 | 0.116 | 809 | 342 | 41 | 1 | 474 | 1 | 771 | Uncharacterized protein | Uncharacterized protein | | afdb-uniprot50 | AF-A0A1U7CXB8-F1-MODEL\_V4 | 1.0 | 1.134e-07 | 331 | 0.505 | 89 | 39 | 2 | 140 | 224 | 79 | 166 | Uncharacterized protein | Uncharacterized protein | | afdb-uniprot50 | AF-A0A3N5W9U7-F1-MODEL\_V4 | 1.0 | 7.927e-07 | 331 | 0.53 | 83 | 38 | 1 | 144 | 226 | 212 | 293 | C-type lectin domain-containing protein | C-type lectin domain-containing protein | | afdb-uniprot50 | AF-A0A4P5QN36-F1-MODEL\_V4 | 1.0 | 3.361e-07 | 330 | 0.463 | 82 | 42 | 2 | 142 | 223 | 123 | 202 | Uncharacterized protein | Uncharacterized protein | | afdb-uniprot50 | AF-A0A3N5MEB4-F1-MODEL\_V4 | 1.0 | 1.326e-06 | 330 | 0.576 | 78 | 32 | 1 | 147 | 224 | 323 | 399 | Fibronectin type-III domain-containing protein | Fibronectin type-III domain-containing protein | | afdb-uniprot50 | AF-A0A1F1HV94-F1-MODEL\_V4 | 1.0 | 4.808e-08 | 330 | 0.387 | 98 | 56 | 3 | 134 | 227 | 631 | 728 | Uncharacterized protein | Uncharacterized protein | | afdb-uniprot50 | AF-A0A419GQ10-F1-MODEL\_V4 | 1.0 | 1.792e-07 | 327 | 0.522 | 90 | 40 | 2 | 139 | 225 | 96 | 185 | Uncharacterized protein | Uncharacterized protein | | afdb-uniprot50 | AF-A0A6L6S241-F1-MODEL\_V4 | 1.0 | 3.833e-12 | 327 | 0.114 | 444 | 235 | 16 | 83 | 467 | 541 | 885 | Phage\_sheath\_1C domain-containing protein | Phage\_sheath\_1C domain-containing protein | | afdb-uniprot50 | AF-A0A843GVB5-F1-MODEL\_V4 | 1.0 | 2.1e-10 | 326 | 0.158 | 253 | 174 | 10 | 245 | 468 | 12 | 254 | Uncharacterized protein | Uncharacterized protein | | afdb-uniprot50 | AF-A0A2D8EN41-F1-MODEL\_V4 | 1.0 | 1.329e-10 | 323 | 0.099 | 522 | 282 | 18 | 2 | 468 | 7 | 395 | Uncharacterized protein | Uncharacterized protein | | afdb-uniprot50 | AF-A0A450Z311-F1-MODEL\_V4 | 1.0 | 6.4e-08 | 322 | 0.376 | 93 | 58 | 0 | 381 | 473 | 2 | 94 | Phage\_sheath\_1C domain-containing protein | Phage\_sheath\_1C domain-containing protein | | afdb-uniprot50 | AF-X0YSG3-F1-MODEL\_V4 | 1.0 | 4.11e-09 | 322 | 0.185 | 200 | 146 | 9 | 231 | 419 | 11 | 204 | Phage\_sheath\_1 domain-containing protein | Phage\_sheath\_1 domain-containing protein | | afdb-uniprot50 | AF-A0A2W7DZU1-F1-MODEL\_V4 | 1.0 | 7.943e-11 | 321 | 0.197 | 309 | 150 | 12 | 1 | 300 | 1 | 220 | Uncharacterized protein | Uncharacterized protein | | afdb-uniprot50 | AF-A0A3S0E3K7-F1-MODEL\_V4 | 1.0 | 2.219e-06 | 321 | 0.448 | 78 | 42 | 1 | 147 | 224 | 119 | 195 | Fibronectin type-III domain-containing protein | Fibronectin type-III domain-containing protein | | afdb-uniprot50 | AF-A0A1G3MC68-F1-MODEL\_V4 | 1.0 | 6.509e-13 | 319 | 0.121 | 427 | 264 | 18 | 140 | 474 | 376 | 783 | Uncharacterized protein | Uncharacterized protein | | afdb-uniprot50 | AF-A0A1F7WZM2-F1-MODEL\_V4 | 1.0 | 9.713e-13 | 318 | 0.114 | 532 | 326 | 24 | 1 | 455 | 1 | 464 | Uncharacterized protein | Uncharacterized protein | | afdb-uniprot50 | AF-A0A661IMM3-F1-MODEL\_V4 | 1.0 | 3.134e-10 | 318 | 0.131 | 266 | 186 | 12 | 231 | 466 | 463 | 713 | Uncharacterized protein | Uncharacterized protein | | afdb-uniprot50 | AF-A0A1Y6CY82-F1-MODEL\_V4 | 1.0 | 6.044e-08 | 317 | 0.373 | 99 | 61 | 1 | 376 | 473 | 2 | 100 | Phage tail sheath protein | Phage tail sheath protein | | afdb-uniprot50 | AF-A0A7V8NSJ0-F1-MODEL\_V4 | 1.0 | 3.277e-13 | 317 | 0.142 | 542 | 284 | 29 | 48 | 456 | 2 | 495 | Phage tail sheath subtilisin-like domain-containing protein | Phage tail sheath subtilisin-like domain-containing protein | | afdb-uniprot50 | AF-A0A0G1W3T5-F1-MODEL\_V4 | 1.0 | 3.411e-08 | 316 | 0.398 | 103 | 57 | 2 | 145 | 243 | 182 | 283 | Autotransporter-associated beta strand repeat protein | Autotransporter-associated beta strand repeat protein | | afdb-uniprot50 | AF-A0A2V3RDB6-F1-MODEL\_V4 | 1.0 | 1.897e-07 | 315 | 0.305 | 118 | 77 | 2 | 358 | 471 | 2 | 118 | Tail sheath protein | Tail sheath protein | | afdb-uniprot50 | AF-A0A225DFW4-F1-MODEL\_V4 | 1.0 | 1.598e-07 | 315 | 0.425 | 94 | 46 | 3 | 140 | 226 | 191 | 283 | Putative major tail protein | Putative major tail protein | | afdb-uniprot50 | AF-A0A7X1EF97-F1-MODEL\_V4 | 1.0 | 4.541e-08 | 314 | 0.346 | 101 | 65 | 1 | 260 | 359 | 2 | 102 | Phage tail sheath subtilisin-like domain-containing protein | Phage tail sheath subtilisin-like domain-containing protein | | afdb-uniprot50 | AF-A0A1I1EU22-F1-MODEL\_V4 | 1.0 | 7.842e-14 | 313 | 0.112 | 533 | 311 | 30 | 1 | 466 | 4 | 441 | Phage tail sheath protein | Phage tail sheath protein | | afdb-uniprot50 | AF-A0A1H2UW00-F1-MODEL\_V4 | 1.0 | 3.174e-07 | 312 | 0.363 | 99 | 63 | 0 | 376 | 474 | 7 | 105 | Phage tail sheath protein | Phage tail sheath protein | | afdb-uniprot50 | AF-A0A2D6XA84-F1-MODEL\_V4 | 1.0 | 8.393e-07 | 310 | 0.487 | 80 | 39 | 2 | 147 | 224 | 21 | 100 | Uncharacterized protein | Uncharacterized protein | | afdb-uniprot50 | AF-A0A4T3EYF2-F1-MODEL\_V4 | 1.0 | 1.293e-12 | 310 | 0.172 | 429 | 243 | 21 | 79 | 457 | 386 | 752 | Phage\_sheath\_1 domain-containing protein | Phage\_sheath\_1 domain-containing protein | | afdb-uniprot50 | AF-A0A3N7HIZ4-F1-MODEL\_V4 | 1.0 | 4.297e-12 | 309 | 0.134 | 497 | 274 | 23 | 72 | 467 | 201 | 642 | Uncharacterized protein | Uncharacterized protein | | afdb-uniprot50 | AF-A0A2T6GH30-F1-MODEL\_V4 | 1.0 | 2.563e-08 | 306 | 0.423 | 118 | 45 | 5 | 109 | 226 | 99 | 193 | Phage tail protein | Phage tail protein | | afdb-uniprot50 | AF-A0A1Z9B2F7-F1-MODEL\_V4 | 1.0 | 3.128e-06 | 306 | 0.382 | 81 | 48 | 1 | 146 | 226 | 613 | 691 | Uncharacterized protein | Uncharacterized protein | | afdb-uniprot50 | AF-A0A381W6D4-F1-MODEL\_V4 | 1.0 | 1.487e-06 | 303 | 0.38 | 84 | 51 | 1 | 142 | 224 | 134 | 217 | DUF4815 domain-containing protein | DUF4815 domain-containing protein | | afdb-uniprot50 | AF-A0A7Y7P2S0-F1-MODEL\_V4 | 1.0 | 1.535e-12 | 303 | 0.131 | 823 | 324 | 37 | 1 | 468 | 1 | 787 | Phage tail sheath subtilisin-like domain-containing protein | Phage tail sheath subtilisin-like domain-containing protein | | afdb-uniprot50 | AF-A0A540WH21-F1-MODEL\_V4 | 1.0 | 4.474e-07 | 302 | 0.306 | 98 | 68 | 0 | 377 | 474 | 4 | 101 | Phage\_sheath\_1C domain-containing protein | Phage\_sheath\_1C domain-containing protein | | afdb-uniprot50 | AF-A1B8I0-F1-MODEL\_V4 | 1.0 | 1.692e-07 | 299 | 0.447 | 96 | 50 | 2 | 132 | 225 | 384 | 478 | Uncharacterized protein | Uncharacterized protein | | afdb-uniprot50 | AF-A0A7W0VBV0-F1-MODEL\_V4 | 1.0 | 8.287e-10 | 298 | 0.169 | 366 | 176 | 13 | 1 | 342 | 1 | 262 | Phage tail sheath family protein | Phage tail sheath family protein | | afdb-uniprot50 | AF-A0A2V7WD79-F1-MODEL\_V4 | 1.0 | 1.509e-07 | 298 | 0.444 | 99 | 44 | 2 | 139 | 227 | 294 | 391 | Uncharacterized protein | Uncharacterized protein | | afdb-uniprot50 | AF-A0A3P6JQ12-F1-MODEL\_V4 | 1.0 | 2.039e-08 | 297 | 0.208 | 192 | 131 | 6 | 290 | 467 | 7 | 191 | Phage tail sheath protein FI | Phage tail sheath protein FI | | afdb-uniprot50 | AF-A0A3N5G9W8-F1-MODEL\_V4 | 1.0 | 1.312e-13 | 296 | 0.152 | 426 | 228 | 18 | 145 | 452 | 36 | 446 | Uncharacterized protein | Uncharacterized protein | | afdb-uniprot50 | AF-A0A1F5C1Z1-F1-MODEL\_V4 | 1.0 | 4.11e-09 | 295 | 0.134 | 253 | 187 | 11 | 232 | 466 | 6 | 244 | Uncharacterized protein | Uncharacterized protein | | afdb-uniprot50 | AF-G4KPJ2-F1-MODEL\_V4 | 1.0 | 3.612e-08 | 293 | 0.235 | 178 | 118 | 4 | 308 | 474 | 2 | 172 | Putative tail sheath protein | Putative tail sheath protein | | afdb-uniprot50 | AF-A0A843GHW6-F1-MODEL\_V4 | 1.0 | 4.808e-08 | 290 | 0.135 | 236 | 173 | 9 | 254 | 467 | 2 | 228 | Uncharacterized protein | Uncharacterized protein | | afdb-uniprot50 | AF-A0A4Q3NY20-F1-MODEL\_V4 | 1.0 | 1.822e-12 | 290 | 0.147 | 624 | 351 | 37 | 1 | 474 | 1 | 593 | Uncharacterized protein | Uncharacterized protein | | afdb-uniprot50 | AF-I0QL77-F1-MODEL\_V4 | 1.0 | 1.201e-07 | 289 | 0.471 | 87 | 45 | 1 | 291 | 376 | 8 | 94 | Major tail sheath protein | Major tail sheath protein | | afdb-uniprot50 | AF-A0A6V8GYL1-F1-MODEL\_V4 | 1.0 | 2.831e-07 | 288 | 0.232 | 129 | 97 | 1 | 346 | 474 | 5 | 131 | Phage tail sheath family protein | Phage tail sheath family protein | | afdb-uniprot50 | AF-A0A3S0LFZ3-F1-MODEL\_V4 | 1.0 | 6.057e-12 | 288 | 0.136 | 446 | 260 | 21 | 116 | 455 | 346 | 772 | Uncharacterized protein | Uncharacterized protein | | afdb-uniprot50 | AF-A0A2D6U3C2-F1-MODEL\_V4 | 1.0 | 3.932e-06 | 286 | 0.385 | 83 | 49 | 1 | 145 | 225 | 55 | 137 | Phage tail protein | Phage tail protein | | afdb-uniprot50 | AF-A0A842NAQ9-F1-MODEL\_V4 | 1.0 | 5.402e-12 | 286 | 0.112 | 731 | 318 | 37 | 3 | 423 | 2 | 711 | Phage tail sheath subtilisin-like domain-containing protein | Phage tail sheath subtilisin-like domain-containing protein | | afdb-uniprot50 | AF-A0A2E4BMY0-F1-MODEL\_V4 | 1.0 | 2.043e-12 | 286 | 0.121 | 445 | 265 | 21 | 145 | 473 | 543 | 977 | Uncharacterized protein | Uncharacterized protein | | afdb-uniprot50 | AF-A0A0Q4JAE9-F1-MODEL\_V4 | 1.0 | 2.096e-06 | 284 | 0.206 | 97 | 77 | 0 | 377 | 473 | 13 | 109 | Uncharacterized protein | Uncharacterized protein | | afdb-uniprot50 | AF-A0A825L246-F1-MODEL\_V4 | 1.0 | 1.349e-11 | 284 | 0.137 | 408 | 242 | 25 | 138 | 457 | 174 | 559 | Uncharacterized protein | Uncharacterized protein | | afdb-uniprot50 | AF-A0A1Z8Q4K5-F1-MODEL\_V4 | 1.0 | 4.163e-06 | 282 | 0.481 | 81 | 40 | 1 | 146 | 224 | 1010 | 1090 | Uncharacterized protein | Uncharacterized protein | | afdb-uniprot50 | AF-A0A2E7T9B3-F1-MODEL\_V4 | 1.0 | 9.411e-07 | 281 | 0.413 | 92 | 49 | 2 | 136 | 225 | 128 | 216 | Uncharacterized protein | Uncharacterized protein | | afdb-uniprot50 | AF-A0A661GUA0-F1-MODEL\_V4 | 1.0 | 2.291e-12 | 280 | 0.13 | 498 | 300 | 30 | 48 | 465 | 9 | 453 | Uncharacterized protein | Uncharacterized protein | | afdb-uniprot50 | AF-A0A2V8RQP8-F1-MODEL\_V4 | 1.0 | 4.474e-07 | 280 | 0.423 | 104 | 54 | 4 | 126 | 226 | 167 | 267 | Uncharacterized protein | Uncharacterized protein | | afdb-uniprot50 | AF-A0A3B9Z7Q0-F1-MODEL\_V4 | 1.0 | 7.614e-12 | 279 | 0.104 | 844 | 354 | 39 | 2 | 468 | 10 | 828 | Phage\_sheath\_1C domain-containing protein | Phage\_sheath\_1C domain-containing protein | | afdb-uniprot50 | AF-G5LWT8-F1-MODEL\_V4 | 1.0 | 3.312e-06 | 278 | 0.293 | 92 | 65 | 0 | 382 | 473 | 8 | 99 | Major tail sheath protein | Major tail sheath protein | | afdb-uniprot50 | AF-A0A5U2F9H2-F1-MODEL\_V4 | 1.0 | 3.312e-06 | 277 | 0.323 | 99 | 67 | 0 | 376 | 474 | 12 | 110 | Phage tail sheath family protein | Phage tail sheath family protein | | afdb-uniprot50 | AF-A0A1V5B852-F1-MODEL\_V4 | 1.0 | 1.822e-12 | 276 | 0.126 | 666 | 317 | 38 | 20 | 468 | 28 | 645 | Phage tail sheath protein | Phage tail sheath protein | | afdb-uniprot50 | AF-A0A7Z1HP85-F1-MODEL\_V4 | 1.0 | 3.507e-06 | 274 | 0.383 | 86 | 53 | 0 | 389 | 474 | 2 | 87 | Phage tail protein | Phage tail protein | | afdb-uniprot50 | AF-A0A3S4J167-F1-MODEL\_V4 | 1.0 | 1.487e-06 | 272 | 0.428 | 98 | 56 | 0 | 350 | 447 | 2 | 99 | Major tail sheath protein | Major tail sheath protein | | afdb-uniprot50 | AF-R7JPV7-F1-MODEL\_V4 | 1.0 | 1.667e-06 | 272 | 0.286 | 129 | 86 | 3 | 350 | 473 | 1 | 128 | Phage-related contractile tail sheath protein | Phage-related contractile tail sheath protein | | afdb-uniprot50 | AF-A0A0B0HIM0-F1-MODEL\_V4 | 1.0 | 8.27e-06 | 270 | 0.305 | 95 | 66 | 0 | 381 | 475 | 5 | 99 | Uncharacterized protein | Uncharacterized protein | | afdb-uniprot50 | AF-A0A447T5K7-F1-MODEL\_V4 | 1.0 | 6.226e-10 | 270 | 0.225 | 270 | 108 | 7 | 85 | 344 | 82 | 260 | Phage tail sheath protein | Phage tail sheath protein | | afdb-uniprot50 | AF-A0A7Z2GRK3-F1-MODEL\_V4 | 1.0 | 1.49e-10 | 270 | 0.192 | 359 | 166 | 15 | 1 | 275 | 1 | 319 | Uncharacterized protein | Uncharacterized protein | | afdb-uniprot50 | AF-A0A376YIM8-F1-MODEL\_V4 | 1.0 | 2.596e-05 | 269 | 0.354 | 79 | 51 | 0 | 376 | 454 | 9 | 87 | Phage-related contractile tail sheath protein | Phage-related contractile tail sheath protein | | afdb-uniprot50 | AF-A0A0C2Z8E2-F1-MODEL\_V4 | 1.0 | 8.887e-07 | 269 | 0.258 | 124 | 90 | 1 | 351 | 474 | 1 | 122 | Uncharacterized protein | Uncharacterized protein | | afdb-uniprot50 | AF-A0A2N7QTB6-F1-MODEL\_V4 | 1.0 | 4.474e-07 | 266 | 0.369 | 111 | 51 | 5 | 129 | 225 | 516 | 621 | Beta\_helix domain-containing protein | Beta\_helix domain-containing protein | | afdb-uniprot50 | AF-A0A5U2F6F1-F1-MODEL\_V4 | 1.0 | 1.667e-06 | 265 | 0.336 | 98 | 64 | 1 | 260 | 356 | 2 | 99 | Phage tail sheath family protein | Phage tail sheath family protein | | afdb-uniprot50 | AF-A0A2V8JS33-F1-MODEL\_V4 | 1.0 | 2.596e-05 | 263 | 0.433 | 83 | 44 | 3 | 145 | 226 | 225 | 305 | Uncharacterized protein | Uncharacterized protein | | afdb-uniprot50 | AF-A0A4Q3L8D9-F1-MODEL\_V4 | 1.0 | 6.592e-10 | 262 | 0.138 | 333 | 220 | 15 | 186 | 474 | 462 | 771 | Uncharacterized protein | Uncharacterized protein | | afdb-uniprot50 | AF-A0A644XLY2-F1-MODEL\_V4 | 1.0 | 1.04e-05 | 258 | 0.214 | 121 | 91 | 1 | 358 | 474 | 8 | 128 | Uncharacterized protein | Uncharacterized protein | | afdb-uniprot50 | AF-A0A2S9JTK1-F1-MODEL\_V4 | 1.0 | 1.425e-07 | 258 | 0.303 | 132 | 59 | 5 | 116 | 225 | 394 | 514 | RagB/SusD family nutrient uptake outer membrane protein | RagB/SusD family nutrient uptake outer membrane protein | | afdb-uniprot50 | AF-A0A7K4MSZ8-F1-MODEL\_V4 | 1.0 | 6.677e-07 | 257 | 0.316 | 120 | 56 | 4 | 109 | 224 | 117 | 214 | DUF4815 domain-containing protein | DUF4815 domain-containing protein | | afdb-uniprot50 | AF-A0A5D4H7Y3-F1-MODEL\_V4 | 1.0 | 2.009e-07 | 256 | 0.278 | 133 | 61 | 5 | 116 | 225 | 358 | 478 | RagB/SusD family nutrient uptake outer membrane protein | RagB/SusD family nutrient uptake outer membrane protein | | afdb-uniprot50 | AF-A0A3S4F1N2-F1-MODEL\_V4 | 1.0 | 7.376e-06 | 255 | 0.38 | 84 | 52 | 0 | 390 | 473 | 1 | 84 | Major tail sheath protein | Major tail sheath protein | | afdb-uniprot50 | AF-A0A382MVY2-F1-MODEL\_V4 | 1.0 | 6.213e-06 | 255 | 0.42 | 88 | 46 | 1 | 142 | 224 | 39 | 126 | Uncharacterized protein | Uncharacterized protein | | afdb-uniprot50 | AF-A0A5E9SB03-F1-MODEL\_V4 | 1.0 | 4.943e-06 | 253 | 0.3 | 90 | 63 | 0 | 385 | 474 | 2 | 91 | Phage tail sheath family protein | Phage tail sheath family protein | | afdb-uniprot50 | AF-A0A0L1JK15-F1-MODEL\_V4 | 1.0 | 2.187e-05 | 252 | 0.411 | 85 | 47 | 3 | 140 | 222 | 374 | 457 | Uncharacterized protein | Uncharacterized protein | | afdb-uniprot50 | AF-A0A2G4KB82-F1-MODEL\_V4 | 1.0 | 3.874e-05 | 249 | 0.437 | 80 | 45 | 0 | 144 | 223 | 269 | 348 | Uncharacterized protein | Uncharacterized protein | | afdb-uniprot50 | AF-A0A1W9F593-F1-MODEL\_V4 | 1.0 | 9.964e-07 | 247 | 0.448 | 98 | 54 | 0 | 321 | 418 | 2 | 99 | Phage\_sheath\_1C domain-containing protein | Phage\_sheath\_1C domain-containing protein | | afdb-uniprot50 | AF-A0A4Q7FYN1-F1-MODEL\_V4 | 1.0 | 3.312e-06 | 245 | 0.26 | 150 | 97 | 2 | 326 | 474 | 1 | 137 | Uncharacterized protein | Uncharacterized protein | | afdb-uniprot50 | AF-B5JF22-F1-MODEL\_V4 | 1.0 | 3.082e-05 | 244 | 0.494 | 91 | 38 | 3 | 140 | 224 | 641 | 729 | Uncharacterized protein | Uncharacterized protein | | afdb-uniprot50 | AF-A0A495J6Q7-F1-MODEL\_V4 | 1.0 | 2.488e-06 | 243 | 0.348 | 109 | 56 | 2 | 119 | 226 | 412 | 506 | Putative outer membrane starch-binding protein | Putative outer membrane starch-binding protein | | afdb-uniprot50 | AF-A0A0F8VRZ6-F1-MODEL\_V4 | 1.0 | 2.91e-05 | 241 | 0.241 | 112 | 84 | 1 | 358 | 468 | 4 | 115 | Phage\_sheath\_1C domain-containing protein | Phage\_sheath\_1C domain-containing protein | | afdb-uniprot50 | AF-A0A2D6AVV5-F1-MODEL\_V4 | 1.0 | 2.096e-06 | 241 | 0.192 | 166 | 124 | 4 | 308 | 468 | 2 | 162 | Uncharacterized protein | Uncharacterized protein | | afdb-uniprot50 | AF-A0A4R4G448-F1-MODEL\_V4 | 1.0 | 6.966e-06 | 239 | 0.467 | 77 | 41 | 0 | 398 | 474 | 1 | 77 | Phage tail sheath family protein | Phage tail sheath family protein | | afdb-uniprot50 | AF-A0A6M1E7R6-F1-MODEL\_V4 | 1.0 | 1.447e-08 | 239 | 0.284 | 158 | 76 | 6 | 116 | 242 | 379 | 530 | RagB/SusD family nutrient uptake outer membrane protein | RagB/SusD family nutrient uptake outer membrane protein | | afdb-uniprot50 | AF-G5R8L3-F1-MODEL\_V4 | 1.0 | 2.452e-05 | 237 | 0.32 | 81 | 55 | 0 | 393 | 473 | 1 | 81 | Major tail sheath protein | Major tail sheath protein | | afdb-uniprot50 | AF-A0A377U4U1-F1-MODEL\_V4 | 1.0 | 2.315e-05 | 237 | 0.35 | 80 | 52 | 0 | 395 | 474 | 1 | 80 | Phage tail sheath protein | Phage tail sheath protein | | afdb-uniprot50 | AF-A0A356X1N2-F1-MODEL\_V4 | 1.0 | 6.579e-06 | 235 | 0.179 | 184 | 137 | 5 | 295 | 468 | 2 | 181 | Phage tail protein | Phage tail protein | | afdb-uniprot50 | AF-A0A827DWH9-F1-MODEL\_V4 | 1.0 | 1.384e-05 | 234 | 0.402 | 72 | 43 | 0 | 403 | 474 | 4 | 75 | Phage tail protein | Phage tail protein | | afdb-uniprot50 | AF-A0A7K4MS80-F1-MODEL\_V4 | 1.0 | 4.541e-08 | 231 | 0.286 | 164 | 63 | 8 | 93 | 231 | 84 | 218 | DUF4815 domain-containing protein | DUF4815 domain-containing protein | | afdb-uniprot50 | AF-A0A7X5N4I8-F1-MODEL\_V4 | 1.0 | 5.542e-06 | 228 | 0.34 | 97 | 64 | 0 | 327 | 423 | 1 | 97 | Phage tail protein | Phage tail protein | | afdb-uniprot50 | AF-A0A7C3WVH1-F1-MODEL\_V4 | 1.0 | 6.226e-10 | 228 | 0.118 | 488 | 312 | 32 | 49 | 466 | 519 | 958 | Uncharacterized protein | Uncharacterized protein | | afdb-uniprot50 | AF-A0A317II01-F1-MODEL\_V4 | 1.0 | 8.757e-06 | 225 | 0.317 | 104 | 61 | 3 | 121 | 223 | 57 | 151 | Uncharacterized protein | Uncharacterized protein | | afdb-uniprot50 | AF-A0A350DHD4-F1-MODEL\_V4 | 1.0 | 3.263e-05 | 224 | 0.388 | 72 | 44 | 0 | 403 | 474 | 5 | 76 | Phage tail protein | Phage tail protein | | afdb-uniprot50 | AF-A0A3A9EM69-F1-MODEL\_V4 | 1.0 | 0.0002154 | 218 | 0.208 | 96 | 76 | 0 | 378 | 473 | 2 | 97 | Uncharacterized protein | Uncharacterized protein | | afdb-uniprot50 | AF-R6I7Y6-F1-MODEL\_V4 | 1.0 | 0.0001714 | 215 | 0.236 | 93 | 71 | 0 | 382 | 474 | 6 | 98 | Uncharacterized protein | Uncharacterized protein | | afdb-uniprot50 | AF-A0A653AK93-F1-MODEL\_V4 | 1.0 | 5.781e-05 | 214 | 0.206 | 131 | 99 | 3 | 349 | 474 | 2 | 132 | Tail sheath protein | Tail sheath protein | | afdb-uniprot50 | AF-A0A4U9HES6-F1-MODEL\_V4 | 1.0 | 2.749e-05 | 211 | 0.523 | 65 | 31 | 0 | 410 | 474 | 2 | 66 | Phage tail sheath protein | Phage tail sheath protein | | afdb-uniprot50 | AF-U2KYU4-F1-MODEL\_V4 | 1.0 | 3.514e-10 | 210 | 0.101 | 643 | 327 | 31 | 30 | 473 | 3 | 593 | Uncharacterized protein | Uncharacterized protein | | afdb-uniprot50 | AF-A0A6C7S0W9-F1-MODEL\_V4 | 1.0 | 0.0002558 | 209 | 0.209 | 110 | 85 | 1 | 361 | 470 | 2 | 109 | Phage tail sheath family protein | Phage tail sheath family protein | | afdb-uniprot50 | AF-A0A1W1Z5G4-F1-MODEL\_V4 | 1.0 | 5.016e-07 | 209 | 0.264 | 155 | 75 | 6 | 116 | 243 | 385 | 527 | Starch-binding associating with outer membrane | Starch-binding associating with outer membrane | | afdb-uniprot50 | AF-A0A382Q741-F1-MODEL\_V4 | 1.0 | 0.0001714 | 207 | 0.182 | 126 | 101 | 2 | 350 | 473 | 1 | 126 | Phage\_sheath\_1C domain-containing protein | Phage\_sheath\_1C domain-containing protein | | afdb-uniprot50 | AF-A0A0F8XJ22-F1-MODEL\_V4 | 1.0 | 0.0001529 | 206 | 0.145 | 131 | 109 | 2 | 341 | 468 | 2 | 132 | Uncharacterized protein | Uncharacterized protein | | afdb-uniprot50 | AF-A0A519WUA4-F1-MODEL\_V4 | 1.0 | 2.525e-07 | 205 | 0.26 | 161 | 74 | 9 | 116 | 243 | 388 | 536 | RagB/SusD family nutrient uptake outer membrane protein | RagB/SusD family nutrient uptake outer membrane protein | | afdb-uniprot50 | AF-A0A376KHV7-F1-MODEL\_V4 | 1.0 | 1.166e-05 | 204 | 0.275 | 120 | 62 | 2 | 273 | 367 | 3 | 122 | Major tail sheath protein FI | Major tail sheath protein FI | | afdb-uniprot50 | AF-X0XYY1-F1-MODEL\_V4 | 1.0 | 5.46e-05 | 204 | 0.155 | 148 | 122 | 2 | 324 | 468 | 1 | 148 | Phage\_sheath\_1C domain-containing protein | Phage\_sheath\_1C domain-containing protein | | afdb-uniprot50 | AF-A0A6M1N703-F1-MODEL\_V4 | 1.0 | 1.219e-08 | 197 | 0.241 | 207 | 98 | 12 | 61 | 243 | 377 | 548 | RagB/SusD family nutrient uptake outer membrane protein | RagB/SusD family nutrient uptake outer membrane protein | | afdb-uniprot50 | AF-A0A353G6H1-F1-MODEL\_V4 | 1.0 | 0.0002154 | 196 | 0.365 | 82 | 52 | 0 | 393 | 474 | 4 | 85 | Phage tail protein | Phage tail protein | | afdb-uniprot50 | AF-A0A1V6IIL8-F1-MODEL\_V4 | 1.0 | 9.674e-05 | 196 | 0.134 | 194 | 158 | 6 | 282 | 470 | 246 | 434 | Uncharacterized protein | Uncharacterized protein | | afdb-uniprot50 | AF-A0A495J3X6-F1-MODEL\_V4 | 1.0 | 1.692e-07 | 196 | 0.3 | 180 | 81 | 7 | 83 | 243 | 356 | 509 | Putative outer membrane starch-binding protein | Putative outer membrane starch-binding protein | | afdb-uniprot50 | AF-U2QTT3-F1-MODEL\_V4 | 1.0 | 1.575e-06 | 192 | 0.109 | 501 | 282 | 28 | 4 | 466 | 7 | 381 | Uncharacterized protein | Uncharacterized protein | | afdb-uniprot50 | AF-A8GDF6-F1-MODEL\_V4 | 1.0 | 4.737e-07 | 190 | 0.21 | 247 | 97 | 3 | 94 | 339 | 1 | 150 | Phage tail sheath protein FI-like protein | Phage tail sheath protein FI-like protein | | afdb-uniprot50 | AF-A0A5Z1TET5-F1-MODEL\_V4 | 1.0 | 0.0009531 | 189 | 0.177 | 96 | 77 | 1 | 379 | 474 | 2 | 95 | Phage tail sheath family protein | Phage tail sheath family protein | | afdb-uniprot50 | AF-A0A7C2S225-F1-MODEL\_V4 | 1.0 | 0.0009002 | 186 | 0.193 | 119 | 93 | 2 | 358 | 473 | 3 | 121 | Phage tail protein | Phage tail protein | | afdb-uniprot50 | AF-A0A350PJK4-F1-MODEL\_V4 | 1.0 | 0.0008501 | 185 | 0.169 | 112 | 92 | 1 | 358 | 468 | 2 | 113 | Phage\_sheath\_1C domain-containing protein | Phage\_sheath\_1C domain-containing protein | | afdb-uniprot50 | AF-A0A356WZX7-F1-MODEL\_V4 | 1.0 | 0.0003036 | 185 | 0.154 | 136 | 107 | 3 | 341 | 468 | 3 | 138 | Uncharacterized protein | Uncharacterized protein | | afdb-uniprot50 | AF-A0A519XZM0-F1-MODEL\_V4 | 1.0 | 8.887e-07 | 183 | 0.256 | 179 | 81 | 7 | 61 | 226 | 139 | 278 | RagB/SusD family nutrient uptake outer membrane protein | RagB/SusD family nutrient uptake outer membrane protein | | afdb-uniprot50 | AF-A0A519SBQ2-F1-MODEL\_V4 | 1.0 | 9.964e-07 | 183 | 0.245 | 179 | 83 | 7 | 61 | 226 | 361 | 500 | DUF5017 domain-containing protein | DUF5017 domain-containing protein | | afdb-uniprot50 | AF-A0A519MRJ7-F1-MODEL\_V4 | 1.0 | 1.055e-06 | 179 | 0.245 | 179 | 83 | 8 | 61 | 226 | 280 | 419 | RagB/SusD family nutrient uptake outer membrane protein | RagB/SusD family nutrient uptake outer membrane protein | | afdb-uniprot50 | AF-A0A7W4KR57-F1-MODEL\_V4 | 1.0 | 0.0002558 | 178 | 0.325 | 80 | 54 | 0 | 382 | 461 | 2 | 81 | Phage tail sheath family protein | Phage tail sheath family protein | | afdb-uniprot50 | AF-A0A248LIR3-F1-MODEL\_V4 | 1.0 | 0.0001529 | 177 | 0.426 | 61 | 35 | 0 | 413 | 473 | 28 | 88 | Phage tail sheath protein | Phage tail sheath protein | | afdb-uniprot50 | AF-A0A350PHK9-F1-MODEL\_V4 | 1.0 | 0.001689 | 173 | 0.178 | 95 | 78 | 0 | 374 | 468 | 5 | 99 | Phage tail protein | Phage tail protein | | afdb-uniprot50 | AF-A0A4R0MPD7-F1-MODEL\_V4 | 1.0 | 3.043e-08 | 170 | 0.225 | 231 | 104 | 11 | 113 | 288 | 399 | 609 | RagB/SusD family nutrient uptake outer membrane protein | RagB/SusD family nutrient uptake outer membrane protein | | afdb-uniprot50 | AF-A0A0F9LR86-F1-MODEL\_V4 | 1.0 | 0.003761 | 168 | 0.156 | 115 | 96 | 1 | 353 | 466 | 6 | 120 | Uncharacterized protein | Uncharacterized protein | | afdb-uniprot50 | AF-A0A496KP74-F1-MODEL\_V4 | 1.0 | 0.001343 | 166 | 0.277 | 72 | 52 | 0 | 403 | 474 | 4 | 75 | Phage tail protein | Phage tail protein | |
| Top keywords  (threshold 1.00e-02 (evalue)) | **tail, Phage, sheath, domain\_containing, Phage\_sheath\_1C, Phage\_sheath\_1, FI, Major, Putative, subtilisin\_like** |
| Output files | ../../similar\_structures/18\_FANPEZAQ\_CDS\_0018\_afdb-proteome\_foldseek.tsv ../../similar\_structures/18\_FANPEZAQ\_CDS\_0018\_afdb-uniprot50\_foldseek.tsv ../../similar\_structures/18\_FANPEZAQ\_CDS\_0018\_merged.svg ../../similar\_structures/18\_FANPEZAQ\_CDS\_0018\_pdb\_foldseek.tsv |

  
  
  

Return to summary | Go to previous | Go to next

  


---

**Sequence/structure alignments coloring**  
Each object in the alignment figures is colored according to its E-value following this color coding:

1e-100
10

**References:**  
1) Steinegger M, Meier M, Mirdita M, Vöhringer H, Haunsberger S J, and Söding J (2019) HH-suite3 for fast remote homology detection and deep protein annotation, BMC Bioinformatics, 473. doi: 10.1186/s12859-019-3019-7  
2) Jumper J, Evans R, Pritzel A, ..., Hassabis D (2021) Highly accurate protein structure prediction with AlphaFold, Nature, 596. doi: 10.1038/s41586-021-03819-2  
3) van Kempen M, Kim S, Tumescheit C, Mirdita M, Lee J, Gilchrist CLM, Söding J, and Steinegger M (2023) Fast and accurate protein structure search with Foldseek. Nature Biotechnology. doi: 10.1038/s41587-023-01773-0
