# Supplementary material for: Completing the BASEL phage collection to unlock hidden diversity for systematic exploration of phage–host interactions
Source: PLoS Biol. 2025 Apr 7;23(4):e3003063. doi: 10.1371/journal.pbio.3003063 (PMC11990801; doi:10.1371/journal.pbio.3003063)
Supplement: S2 Data — (ZIP) [file pbio.3003063.s009.zip › entries/19.html]

FANPEZAQ\_CDS\_0019


Return to summary | Go to previous | Go to next

|  |  |
| --- | --- |
| FANPEZAQ\_CDS\_0019 Page creation date: 02 Sep 2024, 12:00  Project folder: n/a  Input sequences file: Escherichia\_virus\_HeidiAbel.gb | tail phage tube major fii putative fragment contractile prophage p2 sheath bacteriophage hypothetical core head closure domain\_containing from structural tp901\_1 connector mumc02 phage\_like probable protoporphyrinogen oxidase pyocin bacteriocin tail\_like gpfi fii\_like afp1 baseplate hcp gp53 fels\_2 p2w3 conserved region |

### Sequence information

|  |  |
| --- | --- |
| Name | FANPEZAQ\_CDS\_0019  19\_FANPEZAQ\_CDS\_0019 (pipeline id) |
| Imported annotations | Escherichia\_virus\_HeidiAbel Bas97 |
| Protein sequence | MAARDVLKNINLFVDGRGYAGQVESVTPPTLTLQTEEFRAGGMDAPIDVTLGMEKLEASF ALRAYDSDVLALFGLSEGASVPLTFRGALESYDGSVKAVVMNMRGKITSMEPGTWTPGEL SSLNVTVSLNYFKQQHNGKTIHEIDVENMVRIINGVDTLAAIRSALAM |
| Number of residues | 168 |
| Molecular weight (Da) | 18113.49 |
| Output files | ../../query\_sequences/19\_FANPEZAQ\_CDS\_0019.fasta |

### Putative domain architecture and protein family

#### Search results (HHblits)1

|  |  |
| --- | --- |
| Domain family databases searched | Pfam, Ncbi-cd, Cath, Phrogs |
| Results, scheme(s)  (Top layers only; threshold 1.00e-03 (evalue)) | xml version="1.0" encoding="utf-8" standalone="no"?       2024-09-02T21:08:16.313371 image/svg+xml   Matplotlib v3.7.2, https://matplotlib.org/ |
| Results, table  (E-value ≤ 1.00e-03 (evalue)) | | db | id | prob | evalue | pvalue | score | cols | query | query\_len | template | template\_len | name | description | | --- | --- | --- | --- | --- | --- | --- | --- | --- | --- | --- | --- | --- | | pfam | PF04985 | 100.0 | 1.3e-42 | 2.5e-46 | 248.0 | 159 | (5, 167) | 168 | (2, 161) | 161 | Phage\_tube | Phage tail tube protein FII | | phrogs | 55 | 100.0 | 3e-52 | 3.9e-56 | 320.5 | 166 | (2, 168) | 168 | (1, 171) | 172 | head closure | head closure; Category: connector; p363376 VI\_00073 | | phrogs | 13676 | 99.9 | 2.7e-27 | 3e-31 | 157.0 | 89 | (80, 168) | 168 | (3, 92) | 92 | head closure | head closure; Category: connector; p268213 VI\_02784 | | phrogs | 15831 | 99.2 | 6.8e-16 | 7.8e-20 | 89.6 | 45 | (1, 45) | 168 | (1, 45) | 46 | head closure | head closure; Category: connector; p388731 VI\_08178 | |
| Top keywords  (threshold 1.00e-03 (evalue)) | **head, closure, connector, Phage, tail, tube, FII, p363376, VI\_00073, p268213** |
| Output files | ../../domain\_architecture/19\_FANPEZAQ\_CDS\_0019\_cath.hhr ../../domain\_architecture/19\_FANPEZAQ\_CDS\_0019\_merged.svg ../../domain\_architecture/19\_FANPEZAQ\_CDS\_0019\_ncbi-cd.hhr ../../domain\_architecture/19\_FANPEZAQ\_CDS\_0019\_pfam.hhr ../../domain\_architecture/19\_FANPEZAQ\_CDS\_0019\_phrogs.hhr |

### Identical protein sequences/structures

#### Search results

|  |  |
| --- | --- |
| Protein sequence databases searched | Pdb, Swissprot, Refseq |
| Identical proteins found | Refseq  - YP\_009168894.1: Head closure (Escherichia phage vB\_EcoM\_ECO1230-10) - YP\_009600711.1: Head closure (Escherichia phage vB\_EcoM\_ECOO78) - ADE87917.1: Putative phage tail protein (Escherichia phage vB\_EcoM\_ECO1230-10) - ARM70443.1: Putative tail protein (Escherichia phage vB\_EcoM\_ECOO78) - QIG65610.1: Major tail tube protein (Salmonella phage PT1) |
| Top keywords | **tail, Head, closure, Putative, phage, Major, tube** |
| Output files | ../../identical\_sequences/19\_FANPEZAQ\_CDS\_0019\_refseq.fasta |

### Similar protein sequences/structures

#### Sequence similarity search results (HHblits)1

|  |  |
| --- | --- |
| Sequence databases searched | Uniclust, Pdb70 |
| Results, scheme(s)  (Top layers only, threshold 1.00e-03 (evalue)) | xml version="1.0" encoding="utf-8" standalone="no"?       2024-09-02T21:08:37.339295 image/svg+xml   Matplotlib v3.7.2, https://matplotlib.org/ |
| Results, table(s)  (threshold 1.00e-03 (evalue)) | | db | id | prob | evalue | pvalue | score | cols | query | query\_len | template | template\_len | name | description | | --- | --- | --- | --- | --- | --- | --- | --- | --- | --- | --- | --- | --- | | uniclust | UniRef100\_A0A0B4BLQ2 | 100.0 | 3.6e-56 | 7.9e-62 | 333.3 | 168 | (1, 168) | 168 | (8, 175) | 189 | Major tail tube protein | Major tail tube protein | | uniclust | UniRef100\_A0A024E873 | 100.0 | 6.1e-54 | 1.4e-59 | 328.6 | 168 | (1, 168) | 168 | (22, 189) | 201 | Major tail tube protein | Major tail tube protein | | uniclust | UniRef100\_A0A009PKY6 | 100.0 | 2.6e-52 | 5.5e-58 | 321.7 | 168 | (1, 168) | 168 | (44, 214) | 239 | Phage major tail tube protein | Phage major tail tube protein | | uniclust | UniRef100\_A0A017HBX3 | 100.0 | 2.8e-47 | 6.1e-53 | 285.6 | 165 | (2, 168) | 168 | (26, 190) | 192 | Phage major tail tube protein | Phage major tail tube protein | | uniclust | UniRef100\_A0A075WT57 | 100.0 | 7.7e-46 | 1.6e-51 | 273.1 | 167 | (1, 168) | 168 | (3, 173) | 174 | Tail protein | Tail protein | | uniclust | UniRef100\_A0A0U1DAP0 | 100.0 | 5.5e-45 | 1e-50 | 261.6 | 167 | (2, 168) | 168 | (16, 183) | 183 | Phage tail tube protein | Phage tail tube protein | | uniclust | UniRef100\_A0A011NCB3 | 100.0 | 8.1e-45 | 1.7e-50 | 273.2 | 164 | (4, 168) | 168 | (24, 191) | 201 | Phage major tail tube protein | Phage major tail tube protein | | uniclust | UniRef100\_A0A061NPQ1 | 100.0 | 3.3e-43 | 7.4e-49 | 267.6 | 164 | (1, 168) | 168 | (20, 190) | 192 | Phage major tail tube protein | Phage major tail tube protein | | uniclust | UniRef100\_A0A0X1U7S9 | 100.0 | 1.2e-41 | 2.6e-47 | 254.4 | 166 | (1, 168) | 168 | (11, 183) | 183 | Phage tail tube protein FII | Phage tail tube protein FII | | uniclust | UniRef100\_A0A0E3Y6F4 | 100.0 | 1.6e-41 | 3.5e-47 | 252.4 | 164 | (1, 168) | 168 | (7, 177) | 178 | Major tail tube protein | Major tail tube protein | | uniclust | UniRef100\_A0A0P7HQ39 | 100.0 | 1.2e-40 | 2.6e-46 | 234.2 | 129 | (40, 168) | 168 | (1, 130) | 131 | Major tail tube protein | Major tail tube protein | | uniclust | UniRef100\_A0A8X6GSQ5 | 100.0 | 1.9e-40 | 3.5e-46 | 244.0 | 162 | (2, 163) | 168 | (1, 163) | 234 | Phage tail tube protein FII | Phage tail tube protein FII | | uniclust | UniRef100\_A0A165XH78 | 100.0 | 3.2e-40 | 6.7e-46 | 244.8 | 166 | (3, 168) | 168 | (10, 178) | 180 | Phage tail tube protein FII | Phage tail tube protein FII | | uniclust | UniRef100\_A0A0D7WZ75 | 100.0 | 3.8e-40 | 8.6e-46 | 248.3 | 164 | (1, 168) | 168 | (9, 179) | 181 | Phage tail protein | Phage tail protein | | uniclust | UniRef100\_A0A066RR28 | 100.0 | 9e-39 | 1.8e-44 | 231.1 | 168 | (1, 168) | 168 | (2, 171) | 172 | Major tail tube protein | Major tail tube protein | | uniclust | UniRef100\_A0A081MYF2 | 100.0 | 8e-37 | 1.6e-42 | 220.8 | 166 | (2, 168) | 168 | (4, 170) | 170 | Phage major tail tube protein | Phage major tail tube protein | | uniclust | UniRef100\_A0A2M7MWC6 | 100.0 | 1.2e-36 | 2.1e-42 | 223.1 | 166 | (2, 167) | 168 | (1, 166) | 235 | Phage major tail tube protein (Fragment) | Phage major tail tube protein (Fragment) | | uniclust | UniRef100\_A0A2X2BQ70 | 100.0 | 2e-36 | 4.1e-42 | 215.5 | 139 | (1, 140) | 168 | (1, 142) | 145 | Major tail tube protein | Major tail tube protein | | uniclust | UniRef100\_A0A450Z351 | 100.0 | 4.7e-36 | 9.4e-42 | 208.8 | 125 | (1, 125) | 168 | (3, 127) | 129 | Phage tail tube protein FII | Phage tail tube protein FII | | uniclust | UniRef100\_A0A8X6MUK6 | 100.0 | 6.6e-36 | 1.2e-41 | 238.1 | 165 | (3, 167) | 168 | (264, 429) | 498 | Putative prophage major tail sheath protein | Putative prophage major tail sheath protein | | uniclust | UniRef100\_A0A3G2IAH9 | 100.0 | 1.7e-35 | 3.3e-41 | 215.9 | 162 | (4, 167) | 168 | (9, 173) | 176 | Phage tail protein | Phage tail protein | | uniclust | UniRef100\_A0A061JHN2 | 100.0 | 2.2e-34 | 4e-40 | 207.1 | 146 | (2, 148) | 168 | (1, 146) | 201 | Major tail tube protein | Major tail tube protein | | uniclust | UniRef100\_A0A1Y6CXU4 | 100.0 | 2.4e-34 | 4.7e-40 | 207.7 | 168 | (1, 168) | 168 | (3, 172) | 173 | Phage major tail tube protein | Phage major tail tube protein | | uniclust | UniRef100\_A0A087M4E0 | 100.0 | 4.8e-34 | 1e-39 | 214.5 | 165 | (4, 168) | 168 | (8, 179) | 185 | Tail protein | Tail protein | | uniclust | UniRef100\_A0A061K3S3 | 100.0 | 8.2e-34 | 1.7e-39 | 209.2 | 166 | (1, 168) | 168 | (6, 173) | 174 | Phage tail protein | Phage tail protein | | uniclust | UniRef100\_A0A1V3VT23 | 99.9 | 2.8e-32 | 5.4e-38 | 196.8 | 166 | (1, 168) | 168 | (1, 169) | 171 | Phage tail protein (Fragment) | Phage tail protein (Fragment) | | uniclust | UniRef100\_A0A376W1N8 | 99.9 | 5.3e-32 | 1e-37 | 192.1 | 148 | (21, 168) | 168 | (2, 153) | 153 | Major tail sheath protein FII from prophage | Major tail sheath protein FII from prophage | | uniclust | UniRef100\_A0A2W6PC71 | 99.9 | 2.6e-31 | 5.1e-37 | 186.4 | 129 | (38, 168) | 168 | (1, 132) | 134 | Phage tail protein (Fragment) | Phage tail protein (Fragment) | | uniclust | UniRef100\_A0A8X6WS87 | 99.9 | 1.4e-30 | 2.5e-36 | 191.1 | 144 | (25, 168) | 168 | (84, 228) | 228 | Tail tube protein | Tail tube protein | | uniclust | UniRef100\_A0A261QLN0 | 99.9 | 1.8e-30 | 3.6e-36 | 184.9 | 139 | (28, 167) | 168 | (1, 143) | 148 | Phage major tail tube protein (Fragment) | Phage major tail tube protein (Fragment) | | uniclust | UniRef100\_A0A1A9VKH7 | 99.9 | 2.2e-30 | 4e-36 | 208.7 | 152 | (17, 168) | 168 | (383, 535) | 535 | Phage tail protein | Phage tail protein | | uniclust | UniRef100\_A0A0F4NK74 | 99.9 | 6.8e-30 | 1.4e-35 | 187.7 | 163 | (1, 167) | 168 | (7, 172) | 173 | Phage major tail tube protein | Phage major tail tube protein | | uniclust | UniRef100\_A0A0C2UPV3 | 99.9 | 9.3e-30 | 1.9e-35 | 186.4 | 156 | (1, 166) | 168 | (4, 160) | 161 | Major tail tube protein | Major tail tube protein | | uniclust | UniRef100\_A0A085AFM9 | 99.9 | 1.1e-29 | 2.4e-35 | 188.6 | 167 | (1, 168) | 168 | (1, 167) | 168 | Phage major tail tube protein | Phage major tail tube protein | | uniclust | UniRef100\_A0A0A8H9N3 | 99.9 | 8.3e-29 | 1.6e-34 | 180.1 | 164 | (2, 168) | 168 | (8, 174) | 174 | Phage contractile tail tube protein, P2 family | Phage contractile tail tube protein, P2 family | | uniclust | UniRef100\_A0A031FRM0 | 99.9 | 1.7e-28 | 3.4e-34 | 181.0 | 166 | (1, 167) | 168 | (2, 167) | 170 | Putative phage tail-like protein | Putative phage tail-like protein | | uniclust | UniRef100\_A0A095WG55 | 99.9 | 2e-28 | 4e-34 | 178.4 | 162 | (2, 168) | 168 | (6, 175) | 176 | Phage major tail tube protein | Phage major tail tube protein | | uniclust | UniRef100\_A0A0Q6RZW5 | 99.9 | 5.3e-28 | 1.1e-33 | 180.4 | 166 | (2, 167) | 168 | (4, 171) | 184 | Phage tail protein | Phage tail protein | | uniclust | UniRef100\_UPI00031064D1 | 99.9 | 9.2e-28 | 1.7e-33 | 163.4 | 123 | (2, 126) | 168 | (1, 123) | 125 | phage major tail tube protein | phage major tail tube protein | | uniclust | UniRef100\_A0A0S2ZDF0 | 99.9 | 2.2e-27 | 4.5e-33 | 174.0 | 161 | (1, 167) | 168 | (1, 169) | 169 | Phage tail protein | Phage tail protein | | uniclust | UniRef100\_E1SFY3 | 99.9 | 2.6e-27 | 4.8e-33 | 182.6 | 153 | (1, 156) | 168 | (1, 156) | 335 | Major tail tube protein (Protein FII) | Major tail tube protein (Protein FII) | | uniclust | UniRef100\_A0A345DE54 | 99.9 | 1.1e-26 | 1.9e-32 | 164.5 | 167 | (2, 168) | 168 | (1, 169) | 170 | Phage tail tube protein FII | Phage tail tube protein FII | | uniclust | UniRef100\_A0A1S6TP66 | 99.9 | 1.4e-26 | 2.7e-32 | 171.3 | 161 | (3, 167) | 168 | (7, 168) | 175 | Phage major tail tube protein | Phage major tail tube protein | | uniclust | UniRef100\_A0A450Z316 | 99.9 | 2.1e-26 | 4.2e-32 | 151.2 | 82 | (87, 168) | 168 | (2, 84) | 85 | Phage tail tube protein FII | Phage tail tube protein FII | | uniclust | UniRef100\_A0A0C1J3Q2 | 99.9 | 2.7e-26 | 5.8e-32 | 171.7 | 156 | (6, 162) | 168 | (9, 173) | 174 | Uncharacterized protein | Uncharacterized protein | | uniclust | UniRef100\_A0A059IUF5 | 99.9 | 3.2e-26 | 6.1e-32 | 153.4 | 106 | (2, 107) | 168 | (1, 107) | 110 | Contractile tail tube protein | Contractile tail tube protein | | uniclust | UniRef100\_A0A0A8ILB8 | 99.9 | 3.1e-26 | 6.5e-32 | 171.0 | 149 | (16, 168) | 168 | (21, 172) | 178 | Tail tube | Tail tube | | uniclust | UniRef100\_A0A173R5V1 | 99.9 | 5e-26 | 1e-31 | 168.0 | 155 | (5, 166) | 168 | (10, 170) | 170 | Phage major tail tube protein | Phage major tail tube protein | | uniclust | UniRef100\_A0A0S4XLP2 | 99.9 | 6.7e-26 | 1.4e-31 | 167.2 | 159 | (5, 166) | 168 | (10, 169) | 172 | Phage major tail tube protein | Phage major tail tube protein | | uniclust | UniRef100\_A0A248LI16 | 99.9 | 9.6e-26 | 1.8e-31 | 148.1 | 104 | (2, 168) | 168 | (1, 104) | 104 | Phage tail protein | Phage tail protein | | uniclust | UniRef100\_Q2NU86 | 99.9 | 1.2e-25 | 2.5e-31 | 154.9 | 112 | (1, 114) | 168 | (1, 114) | 115 | Hypothetical phage protein | Hypothetical phage protein | | uniclust | UniRef100\_J0PYV7 | 99.8 | 5.5e-25 | 1e-30 | 150.8 | 130 | (36, 168) | 168 | (2, 131) | 131 | Phage major tail tube protein | Phage major tail tube protein | | uniclust | UniRef100\_A0A379YDV4 | 99.8 | 2.1e-24 | 4.3e-30 | 161.8 | 156 | (3, 160) | 168 | (1, 159) | 191 | Phage major tail tube protein | Phage major tail tube protein | | uniclust | UniRef100\_A0A8D8JHV8 | 99.8 | 3.8e-24 | 7.3e-30 | 143.7 | 105 | (64, 168) | 168 | (3, 107) | 107 | Tail tube protein | Tail tube protein | | uniclust | UniRef100\_A0A1H2FLU6 | 99.8 | 7.1e-24 | 1.4e-29 | 156.9 | 167 | (1, 167) | 168 | (3, 174) | 188 | Phage tail tube protein FII | Phage tail tube protein FII | | uniclust | UniRef100\_UPI00082ED17C | 99.8 | 1e-23 | 2e-29 | 151.5 | 163 | (3, 168) | 168 | (2, 167) | 168 | phage major tail tube protein | phage major tail tube protein | | uniclust | UniRef100\_A0A1E7NW44 | 99.8 | 1.6e-23 | 3.2e-29 | 153.5 | 162 | (1, 168) | 168 | (3, 166) | 166 | Phage tail protein | Phage tail protein | | uniclust | UniRef100\_A0A645DSK8 | 99.8 | 3.8e-23 | 7.6e-29 | 141.1 | 90 | (79, 168) | 168 | (13, 107) | 107 | Phage tail tube protein FII | Phage tail tube protein FII | | uniclust | UniRef100\_UPI001FCC480A | 99.8 | 4.6e-23 | 8.4e-29 | 154.3 | 143 | (23, 167) | 168 | (106, 248) | 249 | phage major tail tube protein | phage major tail tube protein | | uniclust | UniRef100\_UPI000B6EE99C | 99.8 | 5.5e-23 | 1.1e-28 | 141.0 | 113 | (1, 113) | 168 | (1, 116) | 120 | phage major tail tube protein | phage major tail tube protein | | uniclust | UniRef100\_A0A173SC52 | 99.8 | 2.8e-22 | 5.1e-28 | 150.4 | 157 | (7, 167) | 168 | (65, 227) | 251 | Phage major tail tube protein | Phage major tail tube protein | | uniclust | UniRef100\_A0A2A4XT18 | 99.8 | 5.2e-22 | 9.8e-28 | 143.0 | 166 | (3, 168) | 168 | (2, 167) | 167 | Phage tail protein | Phage tail protein | | uniclust | UniRef100\_A0A1N7LR57 | 99.8 | 1.2e-21 | 2.3e-27 | 140.4 | 160 | (6, 168) | 168 | (12, 172) | 173 | Phage major tail tube protein | Phage major tail tube protein | | uniclust | UniRef100\_A0A378PU11 | 99.7 | 1.9e-21 | 3.4e-27 | 136.4 | 115 | (51, 166) | 168 | (12, 127) | 152 | Phage major tail tube protein | Phage major tail tube protein | | uniclust | UniRef100\_D1AFA7 | 99.7 | 3.6e-21 | 6.7e-27 | 137.6 | 161 | (3, 166) | 168 | (5, 172) | 173 | Major tail tube protein | Major tail tube protein | | uniclust | UniRef100\_A0A1E3G652 | 99.7 | 3.6e-21 | 6.7e-27 | 136.9 | 166 | (1, 168) | 168 | (1, 166) | 167 | Phage major tail tube protein | Phage major tail tube protein | | uniclust | UniRef100\_UPI001F575EED | 99.7 | 4e-21 | 7.6e-27 | 138.7 | 166 | (1, 168) | 168 | (3, 170) | 171 | phage major tail tube protein | phage major tail tube protein | | uniclust | UniRef100\_A0A7I8DLE1 | 99.7 | 6.2e-21 | 1.2e-26 | 138.6 | 143 | (22, 167) | 168 | (26, 174) | 175 | Bacteriophage protein | Bacteriophage protein | | uniclust | UniRef100\_A0A9E6RJX5 | 99.7 | 1.1e-20 | 2e-26 | 134.9 | 162 | (4, 167) | 168 | (3, 168) | 169 | Phage major tail tube protein | Phage major tail tube protein | | uniclust | UniRef100\_A0A1D2QMU1 | 99.7 | 1.6e-20 | 3e-26 | 136.2 | 164 | (2, 167) | 168 | (4, 168) | 172 | Phage major tail tube protein | Phage major tail tube protein | | uniclust | UniRef100\_A0A1Y1QXH8 | 99.7 | 1.9e-20 | 3.4e-26 | 133.9 | 162 | (2, 167) | 168 | (8, 170) | 171 | Phage tail protein | Phage tail protein | | uniclust | UniRef100\_A0A2T5U148 | 99.7 | 2.6e-20 | 5.2e-26 | 120.3 | 73 | (96, 168) | 168 | (3, 75) | 76 | P2 family phage contractile tail tube protein | P2 family phage contractile tail tube protein | | uniclust | UniRef100\_A0A0B6D149 | 99.7 | 6.6e-20 | 1.2e-25 | 131.2 | 159 | (5, 168) | 168 | (11, 171) | 171 | Phage tail tube FII family protein | Phage tail tube FII family protein | | uniclust | UniRef100\_A0A6P0MC46 | 99.7 | 9.9e-20 | 2e-25 | 135.8 | 163 | (5, 168) | 168 | (8, 176) | 178 | Phage tail protein | Phage tail protein | | uniclust | UniRef100\_A0A1X7MCI7 | 99.7 | 1.1e-19 | 2.2e-25 | 119.2 | 67 | (2, 69) | 168 | (15, 81) | 85 | Phage major tail tube protein (Fragment) | Phage major tail tube protein (Fragment) | | uniclust | UniRef100\_A0A090KJ19 | 99.7 | 1.3e-19 | 2.8e-25 | 136.0 | 158 | (1, 162) | 168 | (3, 160) | 165 | Putative phage tail tube protein | Putative phage tail tube protein | | uniclust | UniRef100\_A0A328TPU4 | 99.7 | 1.7e-19 | 3.3e-25 | 127.1 | 119 | (4, 123) | 168 | (7, 129) | 133 | Phage major tail tube protein | Phage major tail tube protein | | uniclust | UniRef100\_A0A2E3R286 | 99.7 | 2.1e-19 | 3.9e-25 | 128.8 | 162 | (3, 166) | 168 | (4, 172) | 172 | Phage tail protein | Phage tail protein | | uniclust | UniRef100\_A0A812QV43 | 99.6 | 5.4e-19 | 1e-24 | 153.1 | 143 | (18, 160) | 168 | (388, 531) | 1104 | GpFI protein | GpFI protein | | uniclust | UniRef100\_A0A2A5BNC9 | 99.6 | 6.7e-19 | 1.2e-24 | 126.4 | 166 | (3, 168) | 168 | (3, 171) | 173 | Phage tail protein | Phage tail protein | | uniclust | UniRef100\_A0A376FP60 | 99.6 | 1.1e-18 | 2.1e-24 | 108.9 | 66 | (103, 168) | 168 | (1, 66) | 66 | Phage major tail tube protein | Phage major tail tube protein | | uniclust | UniRef100\_A0A2X2J512 | 99.6 | 1.9e-18 | 3.6e-24 | 114.1 | 73 | (96, 168) | 168 | (11, 84) | 87 | Tail tube protein | Tail tube protein | | uniclust | UniRef100\_A0A0T7E1W1 | 99.6 | 1.9e-18 | 3.7e-24 | 125.4 | 158 | (9, 168) | 168 | (8, 165) | 166 | Phage tail protein | Phage tail protein | | uniclust | UniRef100\_UPI00056FD72B | 99.6 | 2.3e-18 | 4.3e-24 | 123.3 | 140 | (1, 141) | 168 | (1, 144) | 150 | phage major tail tube protein | phage major tail tube protein | | uniclust | UniRef100\_A0A7R9HA11 | 99.6 | 2.5e-18 | 4.7e-24 | 151.4 | 166 | (2, 168) | 168 | (953, 1133) | 1133 | Uncharacterized protein | Uncharacterized protein | | uniclust | UniRef100\_A0A369RL94 | 99.6 | 6.5e-18 | 1.2e-23 | 120.3 | 128 | (40, 168) | 168 | (22, 151) | 151 | Phage major tail tube protein | Phage major tail tube protein | | uniclust | UniRef100\_A0A078LLW6 | 99.6 | 7.2e-18 | 1.3e-23 | 122.9 | 104 | (1, 104) | 168 | (1, 107) | 189 | Phage P2 FII-like protein | Phage P2 FII-like protein | | uniclust | UniRef100\_A0A0F9Z9G8 | 99.6 | 8e-18 | 1.5e-23 | 118.6 | 111 | (4, 115) | 168 | (6, 120) | 137 | Prophage MuMc02, major tail tube protein | Prophage MuMc02, major tail tube protein | | uniclust | UniRef100\_UPI001FFFF203 | 99.5 | 2.3e-17 | 4.3e-23 | 110.3 | 95 | (3, 97) | 168 | (5, 99) | 107 | phage major tail tube protein | phage major tail tube protein | | uniclust | UniRef100\_UPI0021E519AF | 99.5 | 3e-17 | 5.4e-23 | 107.1 | 77 | (92, 168) | 168 | (16, 92) | 92 | phage major tail tube protein | phage major tail tube protein | | uniclust | UniRef100\_A0A450SXT5 | 99.5 | 3.1e-17 | 5.7e-23 | 110.6 | 85 | (43, 127) | 168 | (1, 85) | 112 | Uncharacterized protein | Uncharacterized protein | | uniclust | UniRef100\_A0A2I7R3K6 | 99.5 | 4.5e-17 | 8.3e-23 | 117.1 | 166 | (2, 168) | 168 | (3, 170) | 170 | Tail tube protein | Tail tube protein | | uniclust | UniRef100\_A0A485CDJ1 | 99.5 | 8e-17 | 1.5e-22 | 120.4 | 105 | (61, 165) | 168 | (2, 107) | 225 | Phage major tail tube protein | Phage major tail tube protein | | uniclust | UniRef100\_A0A327JAQ8 | 99.5 | 9.4e-17 | 1.7e-22 | 115.5 | 164 | (3, 168) | 168 | (1, 169) | 170 | Phage tail protein | Phage tail protein | | uniclust | UniRef100\_A0A022PER4 | 99.5 | 9.3e-17 | 1.9e-22 | 106.7 | 65 | (1, 65) | 168 | (10, 76) | 83 | Phage tail tube protein FII | Phage tail tube protein FII | | uniclust | UniRef100\_A0A0N1CCE3 | 99.5 | 1.1e-16 | 2.1e-22 | 119.1 | 157 | (5, 161) | 168 | (3, 163) | 177 | Phage tail protein | Phage tail protein | | uniclust | UniRef100\_A0A645CLD3 | 99.5 | 2.1e-16 | 3.8e-22 | 107.4 | 110 | (56, 168) | 168 | (1, 115) | 116 | Phage tail tube protein FII | Phage tail tube protein FII | | uniclust | UniRef100\_UPI001F3D1B67 | 99.5 | 2e-16 | 3.8e-22 | 115.1 | 165 | (1, 166) | 168 | (1, 165) | 166 | phage major tail tube protein | phage major tail tube protein | | uniclust | UniRef100\_A0A1I1EZL7 | 99.4 | 2.3e-16 | 4.6e-22 | 118.1 | 160 | (6, 168) | 168 | (8, 179) | 181 | Phage tail tube protein FII | Phage tail tube protein FII | | uniclust | UniRef100\_A0A285IVC6 | 99.4 | 2.9e-16 | 5.7e-22 | 96.5 | 54 | (1, 54) | 168 | (2, 55) | 58 | Phage contractile tail tube protein, P2 family (Fragment) | Phage contractile tail tube protein, P2 family (Fragment) | | uniclust | UniRef100\_A0A1Q6PWR7 | 99.4 | 3e-16 | 5.9e-22 | 116.0 | 152 | (9, 166) | 168 | (11, 170) | 171 | Phage tail protein | Phage tail protein | | uniclust | UniRef100\_A0A355TQV7 | 99.4 | 4.9e-16 | 9e-22 | 115.7 | 162 | (5, 167) | 168 | (48, 214) | 214 | Phage tail protein | Phage tail protein | | uniclust | UniRef100\_A0A0T7DUY0 | 99.4 | 4.9e-16 | 1e-21 | 117.8 | 147 | (6, 155) | 168 | (15, 167) | 180 | Phage tail protein | Phage tail protein | | uniclust | UniRef100\_UPI0021B36975 | 99.4 | 7.3e-16 | 1.3e-21 | 105.6 | 94 | (23, 116) | 168 | (19, 113) | 121 | phage major tail tube protein | phage major tail tube protein | | uniclust | UniRef100\_A0A1I5H9X3 | 99.4 | 7.8e-16 | 1.5e-21 | 114.0 | 147 | (17, 168) | 168 | (22, 172) | 173 | Phage tail tube protein FII | Phage tail tube protein FII | | uniclust | UniRef100\_UPI00201CE701 | 99.4 | 1.3e-15 | 2.4e-21 | 106.2 | 130 | (37, 167) | 168 | (2, 134) | 135 | phage major tail tube protein | phage major tail tube protein | | uniclust | UniRef100\_E8KG78 | 99.4 | 2.5e-15 | 4.7e-21 | 102.7 | 89 | (4, 93) | 168 | (6, 94) | 109 | Putative phage major tail tube protein | Putative phage major tail tube protein | | uniclust | UniRef100\_A4P152 | 99.3 | 3.4e-15 | 6.3e-21 | 100.9 | 91 | (1, 92) | 168 | (1, 91) | 110 | Uncharacterized protein | Uncharacterized protein | | uniclust | UniRef100\_UPI0015930F13 | 99.3 | 4.3e-15 | 8.2e-21 | 97.2 | 80 | (10, 90) | 168 | (1, 80) | 82 | phage major tail tube protein | phage major tail tube protein | | uniclust | UniRef100\_A0A930QI81 | 99.3 | 8.3e-15 | 1.5e-20 | 91.6 | 70 | (99, 168) | 168 | (1, 70) | 70 | Phage major tail tube protein | Phage major tail tube protein | | uniclust | UniRef100\_A0A1Y0FNC6 | 99.3 | 8.1e-15 | 1.6e-20 | 95.3 | 72 | (96, 167) | 168 | (5, 76) | 78 | FAD-dependent pyridine nucleotide-disulfide oxidoreductase | FAD-dependent pyridine nucleotide-disulfide oxidoreductase | | uniclust | UniRef100\_A0A7J6YK69 | 99.3 | 1e-14 | 1.9e-20 | 133.5 | 86 | (7, 92) | 168 | (1664, 1749) | 2523 | Uncharacterized protein | Uncharacterized protein | | uniclust | UniRef100\_UPI001904BA8E | 99.3 | 1.1e-14 | 2e-20 | 92.9 | 69 | (4, 72) | 168 | (7, 75) | 78 | phage major tail tube protein | phage major tail tube protein | | uniclust | UniRef100\_UPI00036492D7 | 99.3 | 1.5e-14 | 2.7e-20 | 93.9 | 62 | (1, 62) | 168 | (1, 62) | 86 | phage major tail tube protein | phage major tail tube protein | | uniclust | UniRef100\_A0A962FNC8 | 99.3 | 1.6e-14 | 3e-20 | 104.6 | 163 | (5, 168) | 168 | (3, 168) | 170 | Phage major tail tube protein | Phage major tail tube protein | | uniclust | UniRef100\_A0A377U0N6 | 99.3 | 2.2e-14 | 4.1e-20 | 91.5 | 65 | (1, 65) | 168 | (1, 67) | 70 | Phage major tail tube protein | Phage major tail tube protein | | uniclust | UniRef100\_A0A1T4WVE5 | 99.3 | 2.9e-14 | 5.3e-20 | 102.7 | 145 | (2, 147) | 168 | (4, 151) | 163 | Phage tail tube protein FII | Phage tail tube protein FII | | uniclust | UniRef100\_UPI000971A697 | 99.2 | 4e-14 | 7.3e-20 | 102.6 | 167 | (2, 168) | 168 | (1, 169) | 170 | phage major tail tube protein | phage major tail tube protein | | uniclust | UniRef100\_A0A8B2NVA0 | 99.2 | 7.3e-14 | 1.3e-19 | 101.5 | 156 | (4, 162) | 168 | (8, 166) | 172 | Phage major tail tube protein | Phage major tail tube protein | | uniclust | UniRef100\_A0A2T4JJM7 | 99.2 | 7.6e-14 | 1.4e-19 | 90.6 | 74 | (95, 168) | 168 | (12, 85) | 85 | Phage major tail tube protein (Fragment) | Phage major tail tube protein (Fragment) | | uniclust | UniRef100\_A0A9E6DQQ8 | 99.2 | 1.2e-13 | 2.2e-19 | 99.8 | 156 | (3, 160) | 168 | (4, 163) | 165 | Phage major tail tube protein | Phage major tail tube protein | | uniclust | UniRef100\_A0A4Q6CYM3 | 99.1 | 2e-13 | 3.7e-19 | 93.2 | 108 | (61, 168) | 168 | (6, 114) | 114 | Phage major tail tube protein | Phage major tail tube protein | | uniclust | UniRef100\_UPI00226CFAB6 | 99.1 | 2.4e-13 | 4.4e-19 | 97.7 | 156 | (10, 168) | 168 | (1, 158) | 158 | phage major tail tube protein | phage major tail tube protein | | uniclust | UniRef100\_A0A836RL47 | 99.1 | 2.9e-13 | 5.3e-19 | 90.5 | 89 | (1, 93) | 168 | (1, 91) | 100 | Phage tail protein (Fragment) | Phage tail protein (Fragment) | | uniclust | UniRef100\_UPI001BB6B4AF | 99.1 | 3.1e-13 | 5.7e-19 | 94.9 | 129 | (35, 168) | 168 | (1, 135) | 135 | phage major tail tube protein | phage major tail tube protein | | uniclust | UniRef100\_A0A418HAQ3 | 99.1 | 5.2e-13 | 9.8e-19 | 80.6 | 53 | (116, 168) | 168 | (1, 53) | 53 | Phage tail protein (Fragment) | Phage tail protein (Fragment) | | uniclust | UniRef100\_UPI001E503DE8 | 99.1 | 7.7e-13 | 1.4e-18 | 87.3 | 87 | (82, 168) | 168 | (2, 92) | 92 | phage major tail tube protein | phage major tail tube protein | | uniclust | UniRef100\_UPI002284FB3C | 99.1 | 8.2e-13 | 1.5e-18 | 102.4 | 128 | (7, 138) | 168 | (138, 273) | 273 | phage major tail tube protein | phage major tail tube protein | | uniclust | UniRef100\_UPI0021F6DC2B | 99.1 | 9e-13 | 1.6e-18 | 86.2 | 69 | (100, 168) | 168 | (19, 87) | 87 | phage major tail tube protein | phage major tail tube protein | | uniclust | UniRef100\_A0A377U351 | 99.0 | 1.7e-12 | 3.1e-18 | 81.9 | 68 | (82, 149) | 168 | (2, 70) | 71 | Phage major tail tube protein | Phage major tail tube protein | | uniclust | UniRef100\_A0A6L3XLT6 | 99.0 | 1.9e-12 | 3.8e-18 | 92.8 | 110 | (3, 114) | 168 | (5, 114) | 128 | Phage tail protein (Fragment) | Phage tail protein (Fragment) | | uniclust | UniRef100\_A0A2G6CDE2 | 99.0 | 3.7e-12 | 6.8e-18 | 86.8 | 103 | (43, 149) | 168 | (1, 104) | 109 | Uncharacterized protein | Uncharacterized protein | | uniclust | UniRef100\_A0A376X2D4 | 99.0 | 4.1e-12 | 7.7e-18 | 78.9 | 54 | (5, 58) | 168 | (8, 61) | 62 | Phage major tail tube protein | Phage major tail tube protein | | uniclust | UniRef100\_A0A840FUU9 | 99.0 | 4.2e-12 | 7.8e-18 | 79.4 | 61 | (108, 168) | 168 | (2, 62) | 67 | P2 family phage contractile tail tube protein | P2 family phage contractile tail tube protein | | uniclust | UniRef100\_Q31HT1 | 99.0 | 5.2e-12 | 9.5e-18 | 92.0 | 159 | (7, 168) | 168 | (9, 167) | 167 | Phage tail tube protein | Phage tail tube protein | | uniclust | UniRef100\_UPI0022E25BA4 | 98.9 | 5.6e-12 | 1e-17 | 80.1 | 71 | (98, 168) | 168 | (2, 72) | 73 | phage major tail tube protein | phage major tail tube protein | | uniclust | UniRef100\_A0A4Q6CFC6 | 98.9 | 9.1e-12 | 1.7e-17 | 83.9 | 75 | (1, 75) | 168 | (1, 75) | 101 | Uncharacterized protein (Fragment) | Uncharacterized protein (Fragment) | | uniclust | UniRef100\_UPI00037521CA | 98.9 | 9.9e-12 | 1.8e-17 | 86.9 | 116 | (3, 118) | 168 | (2, 120) | 121 | phage major tail tube protein | phage major tail tube protein | | uniclust | UniRef100\_A0A5E6QXM5 | 98.9 | 1e-11 | 1.9e-17 | 84.7 | 92 | (77, 168) | 168 | (4, 99) | 100 | Uncharacterized protein | Uncharacterized protein | | uniclust | UniRef100\_A0A4D7B2K3 | 98.9 | 1.1e-11 | 2e-17 | 90.7 | 157 | (7, 164) | 168 | (7, 166) | 171 | Phage tail protein | Phage tail protein | | uniclust | UniRef100\_A0A317H401 | 98.9 | 1.1e-11 | 2.1e-17 | 88.7 | 121 | (46, 166) | 168 | (4, 126) | 148 | Uncharacterized protein (Fragment) | Uncharacterized protein (Fragment) | | uniclust | UniRef100\_UPI0021B4D849 | 98.9 | 1.2e-11 | 2.1e-17 | 90.2 | 155 | (8, 168) | 168 | (10, 165) | 166 | phage major tail tube protein | phage major tail tube protein | | uniclust | UniRef100\_A0A656GGP8 | 98.9 | 1.9e-11 | 3.5e-17 | 72.4 | 41 | (128, 168) | 168 | (10, 50) | 50 | Major tail tube protein (Fragment) | Major tail tube protein (Fragment) | | uniclust | UniRef100\_A0A0H3PCB3 | 98.8 | 3.6e-11 | 6.9e-17 | 70.8 | 42 | (126, 167) | 168 | (2, 43) | 44 | Uncharacterized protein | Uncharacterized protein | | uniclust | UniRef100\_A0A376J2P6 | 98.8 | 3.8e-11 | 7.2e-17 | 71.9 | 43 | (1, 43) | 168 | (1, 43) | 49 | Phage major tail tube protein FII | Phage major tail tube protein FII | | uniclust | UniRef100\_A0A6M0FV91 | 98.8 | 4.8e-11 | 9e-17 | 89.0 | 142 | (18, 160) | 168 | (26, 174) | 176 | DUF1326 domain-containing protein | DUF1326 domain-containing protein | | uniclust | UniRef100\_A0A0H3G198 | 98.8 | 4.8e-11 | 9.2e-17 | 87.2 | 139 | (1, 168) | 168 | (1, 140) | 148 | Major tail tube protein | Major tail tube protein | | uniclust | UniRef100\_UPI000E0283AC | 98.7 | 1e-10 | 1.9e-16 | 78.6 | 69 | (2, 71) | 168 | (7, 77) | 90 | phage major tail tube protein | phage major tail tube protein | | uniclust | UniRef100\_A0A285NFT0 | 98.7 | 1.4e-10 | 2.6e-16 | 85.6 | 140 | (10, 151) | 168 | (12, 158) | 164 | Phage tail tube protein FII | Phage tail tube protein FII | | uniclust | UniRef100\_A0A451A1E0 | 98.7 | 1.8e-10 | 3.3e-16 | 67.4 | 42 | (127, 168) | 168 | (5, 46) | 46 | Phage tail tube protein FII | Phage tail tube protein FII | | uniclust | UniRef100\_A0A376P9I8 | 98.7 | 2.3e-10 | 4.3e-16 | 73.0 | 50 | (1, 50) | 168 | (1, 50) | 72 | Major tail tube protein FII | Major tail tube protein FII | | uniclust | UniRef100\_UPI00204C4744 | 98.7 | 2.4e-10 | 4.4e-16 | 74.7 | 61 | (1, 61) | 168 | (1, 61) | 82 | phage major tail tube protein | phage major tail tube protein | | uniclust | UniRef100\_A0A419A4I5 | 98.7 | 2.5e-10 | 4.6e-16 | 81.2 | 106 | (10, 132) | 168 | (3, 108) | 136 | Phage tail protein | Phage tail protein | | uniclust | UniRef100\_UPI000D9096AA | 98.6 | 2.8e-10 | 5.2e-16 | 84.8 | 134 | (2, 137) | 168 | (2, 137) | 186 | phage major tail tube protein | phage major tail tube protein | | uniclust | UniRef100\_UPI00159035F8 | 98.6 | 3e-10 | 5.5e-16 | 77.8 | 103 | (65, 167) | 168 | (2, 106) | 108 | phage major tail tube protein | phage major tail tube protein | | uniclust | UniRef100\_UPI001E291166 | 98.6 | 3.6e-10 | 6.7e-16 | 79.0 | 83 | (2, 88) | 168 | (38, 122) | 122 | phage major tail tube protein | phage major tail tube protein | | uniclust | UniRef100\_UPI001C69230E | 98.6 | 5e-10 | 9.2e-16 | 75.6 | 93 | (76, 168) | 168 | (4, 98) | 98 | phage major tail tube protein | phage major tail tube protein | | uniclust | UniRef100\_A0A6L5WKW9 | 98.6 | 5.5e-10 | 1e-15 | 79.1 | 115 | (49, 163) | 168 | (12, 129) | 131 | Phage tail protein (Fragment) | Phage tail protein (Fragment) | | uniclust | UniRef100\_UPI000CDE1566 | 98.5 | 8.7e-10 | 1.6e-15 | 71.1 | 61 | (107, 167) | 168 | (10, 70) | 71 | phage major tail tube protein | phage major tail tube protein | | uniclust | UniRef100\_UPI001BA0B277 | 98.5 | 9.6e-10 | 1.8e-15 | 68.0 | 59 | (109, 167) | 168 | (1, 59) | 60 | phage major tail tube protein | phage major tail tube protein | | uniclust | UniRef100\_A0A3A9EEY6 | 98.5 | 1.3e-09 | 2.4e-15 | 69.9 | 67 | (101, 167) | 168 | (6, 72) | 73 | Phage tail protein (Fragment) | Phage tail protein (Fragment) | | uniclust | UniRef100\_A0A954BDX2 | 98.5 | 1.5e-09 | 2.8e-15 | 77.4 | 121 | (47, 167) | 168 | (4, 128) | 135 | Phage major tail tube protein | Phage major tail tube protein | | uniclust | UniRef100\_A0A4Q0U6A3 | 98.4 | 2.3e-09 | 4.3e-15 | 76.2 | 111 | (53, 166) | 168 | (17, 132) | 132 | Phage tail protein | Phage tail protein | | uniclust | UniRef100\_A0A376DH64 | 98.4 | 4.8e-09 | 8.9e-15 | 63.1 | 46 | (123, 168) | 168 | (2, 48) | 49 | Major tail sheath protein FII from prophage | Major tail sheath protein FII from prophage | | uniclust | UniRef100\_UPI00048CC744 | 98.4 | 4.9e-09 | 9.1e-15 | 70.3 | 86 | (3, 88) | 168 | (1, 86) | 92 | phage major tail tube protein | phage major tail tube protein | | uniclust | UniRef100\_UPI002357F360 | 98.3 | 7.5e-09 | 1.4e-14 | 69.1 | 78 | (38, 116) | 168 | (8, 89) | 89 | phage major tail tube protein | phage major tail tube protein | | uniclust | UniRef100\_E2CFK4 | 98.3 | 7.4e-09 | 1.4e-14 | 77.6 | 159 | (6, 167) | 168 | (6, 167) | 169 | Uncharacterized protein | Uncharacterized protein | | uniclust | UniRef100\_A0A090SU12 | 98.3 | 8.2e-09 | 1.5e-14 | 72.9 | 75 | (3, 77) | 168 | (1, 75) | 124 | Uncharacterized protein | Uncharacterized protein | | uniclust | UniRef100\_A0A6D1AG81 | 98.3 | 9.4e-09 | 1.7e-14 | 63.7 | 41 | (120, 160) | 168 | (2, 42) | 59 | Phage tail protein (Fragment) | Phage tail protein (Fragment) | | uniclust | UniRef100\_A0A2W5HIC4 | 98.3 | 1.1e-08 | 2e-14 | 61.9 | 41 | (1, 41) | 168 | (1, 41) | 52 | Phage tail protein (Fragment) | Phage tail protein (Fragment) | | uniclust | UniRef100\_A0A7Y5Z3W4 | 98.2 | 1.5e-08 | 2.8e-14 | 55.7 | 33 | (136, 168) | 168 | (1, 33) | 33 | Phage tail protein | Phage tail protein | | uniclust | UniRef100\_UPI00190410E6 | 98.2 | 2e-08 | 3.7e-14 | 65.5 | 41 | (128, 168) | 168 | (37, 77) | 77 | phage major tail tube protein | phage major tail tube protein | | uniclust | UniRef100\_A0A376J1C5 | 98.2 | 2.8e-08 | 5.2e-14 | 68.5 | 81 | (44, 125) | 168 | (2, 86) | 99 | Phage major tail tube protein FII | Phage major tail tube protein FII | | uniclust | UniRef100\_UPI000421B9C0 | 98.2 | 3.2e-08 | 6e-14 | 65.2 | 65 | (28, 93) | 168 | (1, 65) | 76 | phage major tail tube protein | phage major tail tube protein | | uniclust | UniRef100\_UPI0021195812 | 98.1 | 4.1e-08 | 7.6e-14 | 67.0 | 94 | (29, 123) | 168 | (1, 98) | 99 | phage major tail tube protein | phage major tail tube protein | | uniclust | UniRef100\_A0A0T6ZCB2 | 98.1 | 4.9e-08 | 8.9e-14 | 69.4 | 116 | (46, 166) | 168 | (3, 126) | 126 | Uncharacterized protein | Uncharacterized protein | | uniclust | UniRef100\_UPI00201D4D24 | 98.1 | 5e-08 | 9.2e-14 | 60.9 | 52 | (24, 75) | 168 | (2, 55) | 60 | phage major tail tube protein | phage major tail tube protein | | uniclust | UniRef100\_UPI001A9CAF53 | 98.1 | 5.4e-08 | 9.9e-14 | 64.8 | 75 | (94, 168) | 168 | (11, 85) | 85 | phage major tail tube protein | phage major tail tube protein | | uniclust | UniRef100\_UPI0012C62FDD | 98.1 | 6.3e-08 | 1.2e-13 | 64.1 | 70 | (97, 166) | 168 | (7, 78) | 80 | phage major tail tube protein | phage major tail tube protein | | uniclust | UniRef100\_A0A3P6JSU6 | 98.0 | 8.2e-08 | 1.5e-13 | 65.1 | 72 | (4, 75) | 168 | (2, 78) | 94 | Putative phage major tail tube protein | Putative phage major tail tube protein | | uniclust | UniRef100\_A0A448QUZ8 | 98.0 | 1e-07 | 1.9e-13 | 59.6 | 46 | (4, 49) | 168 | (5, 50) | 60 | Phage major tail tube protein | Phage major tail tube protein | | uniclust | UniRef100\_UPI0019699564 | 98.0 | 1.2e-07 | 2.2e-13 | 62.4 | 65 | (103, 167) | 168 | (4, 70) | 72 | phage major tail tube protein | phage major tail tube protein | | uniclust | UniRef100\_U2EIY6 | 98.0 | 1.4e-07 | 2.5e-13 | 57.9 | 53 | (115, 167) | 168 | (1, 53) | 54 | Uncharacterized protein | Uncharacterized protein | | uniclust | UniRef100\_UPI001E51626C | 97.9 | 1.7e-07 | 3.2e-13 | 60.7 | 56 | (19, 74) | 168 | (9, 64) | 72 | phage major tail tube protein | phage major tail tube protein | | uniclust | UniRef100\_UPI0013ED546A | 97.9 | 2.4e-07 | 4.4e-13 | 70.6 | 112 | (24, 137) | 168 | (4, 122) | 195 | phage major tail tube protein | phage major tail tube protein | | uniclust | UniRef100\_UPI0013D30FFB | 97.9 | 3.1e-07 | 5.6e-13 | 55.8 | 48 | (121, 168) | 168 | (4, 51) | 51 | phage major tail tube protein | phage major tail tube protein | | uniclust | UniRef100\_A0A2X3LT05 | 97.8 | 3.7e-07 | 6.8e-13 | 53.3 | 40 | (103, 142) | 168 | (1, 40) | 42 | Major tail tube protein FII | Major tail tube protein FII | | uniclust | UniRef100\_UPI000F6D0689 | 97.8 | 4.3e-07 | 7.9e-13 | 55.6 | 45 | (124, 168) | 168 | (8, 52) | 53 | phage major tail tube protein | phage major tail tube protein | | uniclust | UniRef100\_UPI000360283B | 97.8 | 5.6e-07 | 1e-12 | 56.7 | 40 | (1, 40) | 168 | (1, 40) | 61 | phage major tail tube protein | phage major tail tube protein | | uniclust | UniRef100\_UPI000F0131DD | 97.8 | 5.8e-07 | 1.1e-12 | 63.2 | 108 | (57, 164) | 168 | (1, 108) | 113 | phage major tail tube protein | phage major tail tube protein | | uniclust | UniRef100\_A0A840E036 | 97.8 | 7e-07 | 1.3e-12 | 62.7 | 105 | (64, 168) | 168 | (4, 111) | 111 | Uncharacterized protein | Uncharacterized protein | | uniclust | UniRef100\_A0A109RVA8 | 97.8 | 7.1e-07 | 1.3e-12 | 57.6 | 43 | (125, 167) | 168 | (26, 68) | 69 | Uncharacterized protein | Uncharacterized protein | | uniclust | UniRef100\_A0A376YLN8 | 97.7 | 8.6e-07 | 1.6e-12 | 48.3 | 30 | (1, 30) | 168 | (1, 30) | 30 | Phage major tail tube protein FII | Phage major tail tube protein FII | | uniclust | UniRef100\_UPI00210EA4CD | 97.7 | 8.6e-07 | 1.6e-12 | 61.2 | 79 | (34, 113) | 168 | (4, 83) | 100 | phage major tail tube protein | phage major tail tube protein | | uniclust | UniRef100\_UPI001C8BCB0F | 97.7 | 8.8e-07 | 1.6e-12 | 57.7 | 64 | (7, 73) | 168 | (5, 69) | 72 | phage major tail tube protein | phage major tail tube protein | | uniclust | UniRef100\_UPI0009B7D3B4 | 97.7 | 1.1e-06 | 1.9e-12 | 56.1 | 39 | (24, 62) | 168 | (2, 40) | 64 | phage major tail tube protein | phage major tail tube protein | | uniclust | UniRef100\_A0A853IEB6 | 97.7 | 1.1e-06 | 2.1e-12 | 51.2 | 36 | (133, 168) | 168 | (6, 41) | 41 | Phage major tail tube protein | Phage major tail tube protein | | uniclust | UniRef100\_UPI0021C9E4C1 | 97.7 | 1.3e-06 | 2.4e-12 | 58.1 | 72 | (23, 94) | 168 | (3, 74) | 81 | phage major tail tube protein | phage major tail tube protein | | uniclust | UniRef100\_A0A7H4Z562 | 97.6 | 1.5e-06 | 2.8e-12 | 59.4 | 82 | (4, 88) | 168 | (6, 90) | 94 | Phage tail protein (Fragment) | Phage tail protein (Fragment) | | uniclust | UniRef100\_A0A4Y8VEF2 | 97.6 | 1.7e-06 | 3e-12 | 59.6 | 55 | (113, 167) | 168 | (27, 81) | 97 | DUF2790 domain-containing protein | DUF2790 domain-containing protein | | uniclust | UniRef100\_A0A7U9RYY6 | 97.6 | 2e-06 | 3.6e-12 | 62.5 | 119 | (45, 166) | 168 | (9, 134) | 134 | Uncharacterized protein | Uncharacterized protein | | uniclust | UniRef100\_UPI00215029C9 | 97.5 | 2.9e-06 | 5.4e-12 | 56.1 | 67 | (61, 127) | 168 | (4, 71) | 77 | phage major tail tube protein | phage major tail tube protein | | uniclust | UniRef100\_A0A143DGN7 | 97.5 | 3e-06 | 5.5e-12 | 56.9 | 41 | (1, 41) | 168 | (1, 41) | 84 | Uncharacterized protein | Uncharacterized protein | | uniclust | UniRef100\_A0A378TY62 | 97.5 | 3.3e-06 | 6.1e-12 | 48.0 | 33 | (1, 33) | 168 | (1, 33) | 36 | Phage-like protein | Phage-like protein | | uniclust | UniRef100\_A0A527W089 | 97.5 | 4.3e-06 | 7.8e-12 | 56.3 | 75 | (94, 168) | 168 | (8, 83) | 84 | Uncharacterized protein | Uncharacterized protein | | uniclust | UniRef100\_A0A2G2DI71 | 97.5 | 4.5e-06 | 8.3e-12 | 56.9 | 74 | (93, 166) | 168 | (14, 88) | 90 | Uncharacterized protein (Fragment) | Uncharacterized protein (Fragment) | | uniclust | UniRef100\_UPI000B1E361C | 97.4 | 5.1e-06 | 9.4e-12 | 53.7 | 55 | (4, 66) | 168 | (6, 60) | 67 | phage major tail tube protein | phage major tail tube protein | | uniclust | UniRef100\_UPI000AF19DF4 | 97.4 | 5.8e-06 | 1.1e-11 | 60.0 | 67 | (24, 90) | 168 | (51, 118) | 131 | phage major tail tube protein | phage major tail tube protein | | uniclust | UniRef100\_UPI001E2BAB95 | 97.4 | 6.4e-06 | 1.2e-11 | 53.3 | 58 | (45, 103) | 168 | (2, 63) | 66 | phage major tail tube protein | phage major tail tube protein | | uniclust | UniRef100\_A0A1T3BR98 | 97.4 | 8.5e-06 | 1.6e-11 | 52.0 | 47 | (3, 49) | 168 | (6, 52) | 62 | Uncharacterized protein | Uncharacterized protein | | uniclust | UniRef100\_I0QL77 | 97.3 | 9.6e-06 | 1.8e-11 | 59.6 | 44 | (123, 166) | 168 | (95, 138) | 140 | Major tail sheath protein | Major tail sheath protein | | uniclust | UniRef100\_A0A850F557 | 97.3 | 1.1e-05 | 2e-11 | 57.0 | 86 | (79, 165) | 168 | (11, 98) | 101 | Phage major tail tube protein | Phage major tail tube protein | | uniclust | UniRef100\_UPI001F4B2576 | 97.3 | 1.2e-05 | 2.2e-11 | 54.1 | 72 | (95, 166) | 168 | (11, 82) | 82 | phage major tail tube protein | phage major tail tube protein | | uniclust | UniRef100\_A0A377HW70 | 97.3 | 1.4e-05 | 2.5e-11 | 51.6 | 47 | (1, 47) | 168 | (1, 47) | 65 | Phage major tail tube protein | Phage major tail tube protein | | uniclust | UniRef100\_UPI0022470942 | 97.2 | 2.3e-05 | 4.2e-11 | 50.3 | 36 | (1, 36) | 168 | (1, 36) | 63 | phage major tail tube protein | phage major tail tube protein | | uniclust | UniRef100\_A0A3A8EJ92 | 97.1 | 2.6e-05 | 5e-11 | 60.1 | 86 | (83, 168) | 168 | (65, 150) | 150 | Phage tail protein (Fragment) | Phage tail protein (Fragment) | | uniclust | UniRef100\_A0A448QV03 | 97.1 | 3.9e-05 | 7.2e-11 | 53.5 | 92 | (50, 142) | 168 | (2, 97) | 98 | Phage major tail tube protein | Phage major tail tube protein | | uniclust | UniRef100\_A0A8S0FSJ8 | 97.1 | 4e-05 | 7.3e-11 | 49.6 | 62 | (1, 63) | 168 | (1, 62) | 65 | Uncharacterized protein | Uncharacterized protein | | uniclust | UniRef100\_UPI00116EFC7B | 97.0 | 4.5e-05 | 8.3e-11 | 53.3 | 45 | (124, 168) | 168 | (50, 94) | 99 | phage major tail tube protein | phage major tail tube protein | | uniclust | UniRef100\_A0A836W881 | 97.0 | 4.9e-05 | 9e-11 | 48.5 | 57 | (3, 60) | 168 | (4, 60) | 60 | Phage tail protein (Fragment) | Phage tail protein (Fragment) | | uniclust | UniRef100\_A0A6L5WIA9 | 97.0 | 5e-05 | 9.2e-11 | 46.3 | 39 | (3, 41) | 168 | (5, 43) | 48 | Phage tail protein | Phage tail protein | | uniclust | UniRef100\_UPI001F0E249E | 97.0 | 6.4e-05 | 1.2e-10 | 46.7 | 35 | (133, 167) | 168 | (2, 36) | 52 | phage major tail tube protein | phage major tail tube protein | | uniclust | UniRef100\_A0A853IE67 | 97.0 | 6.5e-05 | 1.2e-10 | 42.4 | 33 | (136, 168) | 168 | (1, 33) | 33 | Phage major tail tube protein | Phage major tail tube protein | | uniclust | UniRef100\_R9J2X8 | 96.9 | 9.1e-05 | 1.7e-10 | 51.2 | 72 | (79, 150) | 168 | (13, 89) | 91 | Uncharacterized protein | Uncharacterized protein | | uniclust | UniRef100\_UPI0005CAA7C2 | 96.8 | 0.00011 | 2.1e-10 | 54.1 | 88 | (5, 94) | 168 | (8, 96) | 134 | phage major tail tube protein | phage major tail tube protein | | uniclust | UniRef100\_R7BVV5 | 96.8 | 0.00015 | 2.7e-10 | 47.1 | 38 | (131, 168) | 168 | (24, 61) | 62 | Major tail tube protein | Major tail tube protein | | uniclust | UniRef100\_A0A4Q3YXT5 | 96.8 | 0.00015 | 2.7e-10 | 41.6 | 33 | (136, 168) | 168 | (3, 35) | 35 | Uncharacterized protein | Uncharacterized protein | | uniclust | UniRef100\_UPI0018E48B0B | 96.7 | 0.00019 | 3.4e-10 | 47.6 | 60 | (101, 160) | 168 | (8, 67) | 71 | phage major tail tube protein | phage major tail tube protein | | uniclust | UniRef100\_A0A6I4YK80 | 96.7 | 0.00022 | 3.9e-10 | 53.0 | 47 | (122, 168) | 168 | (92, 138) | 138 | GIY-YIG nuclease family protein | GIY-YIG nuclease family protein | | uniclust | UniRef100\_A0A0H3ZYX4 | 96.6 | 0.00035 | 6.5e-10 | 50.3 | 106 | (48, 156) | 168 | (4, 109) | 113 | Uncharacterized protein | Uncharacterized protein | | uniclust | UniRef100\_UPI000870CDAC | 96.6 | 0.00036 | 6.7e-10 | 53.2 | 64 | (97, 160) | 168 | (96, 161) | 162 | phage major tail tube protein | phage major tail tube protein | | uniclust | UniRef100\_J9FNQ2 | 96.6 | 0.00038 | 6.9e-10 | 47.3 | 70 | (97, 166) | 168 | (10, 79) | 80 | Phage major tail tube protein (Fragment) | Phage major tail tube protein (Fragment) | | uniclust | UniRef100\_UPI0014783C6E | 96.5 | 0.00041 | 7.5e-10 | 49.9 | 58 | (111, 168) | 168 | (53, 111) | 111 | phage major tail tube protein | phage major tail tube protein | | uniclust | UniRef100\_A0A376J940 | 96.5 | 0.00049 | 9e-10 | 46.6 | 67 | (60, 128) | 168 | (4, 70) | 78 | Major tail tube protein FII | Major tail tube protein FII | | uniclust | UniRef100\_UPI0021BD40D7 | 96.5 | 0.00055 | 1e-09 | 50.7 | 58 | (36, 93) | 168 | (60, 119) | 131 | phage major tail tube protein | phage major tail tube protein | | pdb70 | 3J9Q\_l | 100.0 | 3.3e-49 | 2.8e-53 | 292.7 | 168 | (1, 168) | 168 | (1, 168) | 168 | sheath, tube | 3J9Q\_l sheath, tube pyocin, bacteriocin, sheath, tube, STRUCTURAL | | pdb70 | 5W5E\_c | 100.0 | 3.3e-49 | 2.8e-53 | 292.7 | 168 | (1, 168) | 168 | (1, 168) | 168 | FIIR2 protein | 5W5E\_c FIIR2 protein pyocin, bacteriocin, STRUCTURAL PROTEIN | |
| Top keywords  (threshold 1.00e-03 (evalue)) | **tail, Phage, tube, major, FII, Fragment, sheath, Putative, contractile, P2** |
| Output files | ../../similar\_sequences/19\_FANPEZAQ\_CDS\_0019\_merged.svg ../../similar\_sequences/19\_FANPEZAQ\_CDS\_0019\_pdb70.a3m ../../similar\_sequences/19\_FANPEZAQ\_CDS\_0019\_pdb70.hhr ../../similar\_sequences/19\_FANPEZAQ\_CDS\_0019\_uniclust.a3m ../../similar\_sequences/19\_FANPEZAQ\_CDS\_0019\_uniclust.hhr |

#### Structure prediction (AlphaFold)2

|  |  |
| --- | --- |
| Stats | xml version="1.0" encoding="utf-8" standalone="no"?       2024-09-02T21:09:18.882232 image/svg+xml   Matplotlib v3.7.2, https://matplotlib.org/ |
| Predicted structure | **NGL Viewer Controls:**  - Center: *Left-Click* - Rotate: *Left-Click + Drag* - Translate: *Right-Click + Drag* - Zoom: *Shift + Left-Click + Drag* |
| Output files | ../../predicted\_structures/19\_FANPEZAQ\_CDS\_0019/features.pkl ../../predicted\_structures/19\_FANPEZAQ\_CDS\_0019/ranked\_0.pdb ../../predicted\_structures/19\_FANPEZAQ\_CDS\_0019/ranked\_0\_plots.svg ../../predicted\_structures/19\_FANPEZAQ\_CDS\_0019/result\_model\_1\_ptm\_pred\_0.pkl |

#### Structure similarity search results (Foldseek)3

|  |  |
| --- | --- |
| Structure databases searched | Pdb, Afdb-proteome, Afdb-uniprot50 |
| Results, scheme(s)  (Top layers only, threshold 1.00e-02 (evalue)) | xml version="1.0" encoding="utf-8" standalone="no"?       2024-09-02T21:10:44.149803 image/svg+xml   Matplotlib v3.7.2, https://matplotlib.org/ |
| Results, table  (threshold 1.00e-02 (evalue)) | | db | id | prob | evalue | bits | fident | alnlen | mismatch | gapopen | qstart | qend | tstart | tend | name | description | | --- | --- | --- | --- | --- | --- | --- | --- | --- | --- | --- | --- | --- | --- | --- | | pdb | 6U5F\_M | 1.0 | 7.011e-16 | 693 | 0.393 | 165 | 96 | 2 | 6 | 168 | 4 | 166 | Tube PA0623 | Tube PA0623 | | pdb | 7B5H\_AN | 1.0 | 0.0001664 | 174 | 0.123 | 138 | 104 | 10 | 9 | 135 | 9 | 140 | All3324 protein | All3324 protein | | pdb | 6RAO\_A | 1.0 | 0.0002933 | 163 | 0.117 | 136 | 105 | 9 | 9 | 135 | 17 | 146 | Afp1 | Afp1 | | pdb | 7AEB\_Y | 1.0 | 0.0003424 | 151 | 0.166 | 144 | 99 | 9 | 7 | 135 | 7 | 144 | Phospholipid/glycerol acyltransferase | Phospholipid/glycerol acyltransferase | | pdb | 5IV5\_W | 1.0 | 0.0001501 | 145 | 0.161 | 136 | 86 | 8 | 22 | 136 | 62 | 190 | Baseplate wedge protein gp7 | Baseplate wedge protein gp7 | | pdb | 7ADZ\_1A | 1.0 | 0.002081 | 133 | 0.087 | 148 | 111 | 10 | 1 | 135 | 3 | 139 | Phage tail protein | Phage tail protein | | pdb | 6J0N\_N | 1.0 | 0.000912 | 131 | 0.102 | 137 | 109 | 8 | 10 | 136 | 11 | 143 | Pvc5 | Pvc5 | | pdb | 7KJK\_A7 | 1.0 | 0.003485 | 123 | 0.138 | 144 | 104 | 10 | 6 | 135 | 4 | 141 | Tail tube protein | Tail tube protein | | pdb | 8GRA\_C | 1.0 | 0.001065 | 122 | 0.136 | 132 | 93 | 10 | 9 | 135 | 1 | 116 | Bacterodales T6SS protein TssD (Hcp) | Bacterodales T6SS protein TssD (Hcp) | | pdb | 6BDC\_A | 1.0 | 0.002835 | 121 | 0.156 | 134 | 94 | 10 | 10 | 140 | 3 | 120 | Hcp1 | Hcp1 | | pdb | 6V8I\_EA | 1.0 | 0.005 | 111 | 0.094 | 148 | 105 | 11 | 5 | 136 | 6 | 140 | Major Tail Protein, gp53 | Major Tail Protein, gp53 | | pdb | 6RAO\_F | 1.0 | 0.001011 | 109 | 0.109 | 164 | 109 | 11 | 1 | 147 | 5 | 148 | Afp5 | Afp5 | | pdb | 2X8K\_B | 1.0 | 0.006812 | 106 | 0.114 | 148 | 91 | 8 | 8 | 135 | 1 | 128 | HYPOTHETICAL PROTEIN 19.1 | HYPOTHETICAL PROTEIN 19.1 | | pdb | 2X8K\_C | 1.0 | 0.007552 | 105 | 0.126 | 142 | 97 | 8 | 8 | 135 | 1 | 129 | HYPOTHETICAL PROTEIN 19.1 | HYPOTHETICAL PROTEIN 19.1 | | pdb | 6V8I\_EB | 1.0 | 0.003863 | 104 | 0.108 | 147 | 112 | 11 | 1 | 136 | 2 | 140 | Major Tail Protein, gp53 | Major Tail Protein, gp53 | | pdb | 5IV5\_R | 1.0 | 0.002429 | 103 | 0.138 | 159 | 99 | 13 | 1 | 132 | 14 | 161 | Tail tube protein gp19 | Tail tube protein gp19 | | pdb | 8JAN\_L | 0.998 | 0.007552 | 95 | 0.13 | 146 | 107 | 9 | 1 | 129 | 13 | 155 | BplB | BplB | | pdb | 6J0N\_B | 0.998 | 0.003143 | 95 | 0.094 | 159 | 105 | 11 | 1 | 129 | 1 | 150 | Pvc7 | Pvc7 | | pdb | 7FCF\_B | 0.996 | 0.008816 | 90 | 0.122 | 163 | 104 | 12 | 9 | 152 | 1 | 143 | Fimbrial protein | Fimbrial protein | | pdb | 7AEB\_M | 0.981 | 0.009773 | 79 | 0.088 | 159 | 97 | 11 | 7 | 129 | 5 | 151 | Phage tail protein | Phage tail protein | | pdb | 7ZQP\_D | 0.887 | 0.006145 | 66 | 0.177 | 180 | 91 | 15 | 4 | 160 | 46 | 191 | Probable baseplate hub protein | Probable baseplate hub protein | | pdb | 8BFK\_L | 0.855 | 0.005543 | 64 | 0.089 | 191 | 112 | 12 | 3 | 136 | 45 | 230 | Putative virion structural protein | Putative virion structural protein | | afdb-proteome | AF-Q9I5S9-F1-MODEL\_V4 | 1.0 | 1.257e-17 | 817 | 0.392 | 168 | 101 | 1 | 1 | 168 | 1 | 167 | Probable bacteriophage protein | Probable bacteriophage protein | | afdb-proteome | AF-Q4AAY4-F1-MODEL\_V4 | 1.0 | 1.493e-16 | 751 | 0.356 | 171 | 107 | 2 | 1 | 168 | 1 | 171 | Fels-2 prophage protein | Fels-2 prophage protein | | afdb-proteome | AF-A0A0H3GX02-F1-MODEL\_V4 | 1.0 | 3.778e-16 | 737 | 0.343 | 172 | 108 | 4 | 1 | 168 | 1 | 171 | Putative prophage tail tube protein | Putative prophage tail tube protein | | afdb-proteome | AF-A0A0H3GQH1-F1-MODEL\_V4 | 1.0 | 5.706e-16 | 733 | 0.309 | 171 | 115 | 2 | 1 | 168 | 1 | 171 | Phage major tail tube protein | Phage major tail tube protein | | afdb-proteome | AF-Q8ZKJ3-F1-MODEL\_V4 | 1.0 | 6.661e-16 | 672 | 0.24 | 175 | 125 | 4 | 1 | 168 | 1 | 174 | Putative phage tail core protein | Putative phage tail core protein | | afdb-proteome | AF-G3XD38-F1-MODEL\_V4 | 1.0 | 3.543e-05 | 182 | 0.146 | 157 | 103 | 10 | 8 | 135 | 6 | 160 | Uncharacterized protein | Uncharacterized protein | | afdb-proteome | AF-A0A3P7DZT8-F1-MODEL\_V4 | 1.0 | 0.0003425 | 144 | 0.12 | 158 | 106 | 12 | 1 | 135 | 13 | 160 | Uncharacterized protein | Uncharacterized protein | | afdb-proteome | AF-A0A133CKK6-F1-MODEL\_V4 | 1.0 | 0.0004914 | 125 | 0.147 | 142 | 92 | 8 | 10 | 135 | 5 | 133 | Phage tail protein | Phage tail protein | | afdb-proteome | AF-P71389-F1-MODEL\_V4 | 1.0 | 0.007175 | 106 | 0.102 | 137 | 95 | 8 | 1 | 129 | 1 | 117 | Mu-like prophage FluMu DNA circularization protein | Mu-like prophage FluMu DNA circularization protein | | afdb-proteome | AF-Q2FX62-F1-MODEL\_V4 | 1.0 | 0.008818 | 102 | 0.127 | 149 | 99 | 13 | 5 | 136 | 9 | 143 | Phage structural protein, putative | Phage structural protein, putative | | afdb-proteome | AF-Q2FYC9-F1-MODEL\_V4 | 0.996 | 0.008818 | 89 | 0.11 | 145 | 92 | 10 | 10 | 133 | 5 | 133 | Holin-like protein | Holin-like protein | | afdb-uniprot50 | AF-A0A0M1I4B3-F1-MODEL\_V4 | 1.0 | 2.239e-23 | 1126 | 0.566 | 166 | 72 | 0 | 3 | 168 | 5 | 170 | Major tail tube protein | Major tail tube protein | | afdb-uniprot50 | AF-A0A1H2ZHT2-F1-MODEL\_V4 | 1.0 | 3.442e-22 | 1057 | 0.505 | 168 | 82 | 1 | 1 | 168 | 1 | 167 | Uncharacterized protein | Uncharacterized protein | | afdb-uniprot50 | AF-A0A6A4RBN6-F1-MODEL\_V4 | 1.0 | 5.475e-22 | 1056 | 0.523 | 168 | 80 | 0 | 1 | 168 | 1 | 168 | Phage major tail tube protein | Phage major tail tube protein | | afdb-uniprot50 | AF-A0A1T4WZ67-F1-MODEL\_V4 | 1.0 | 1.315e-21 | 1022 | 0.517 | 168 | 81 | 0 | 1 | 168 | 1 | 168 | Uncharacterized protein | Uncharacterized protein | | afdb-uniprot50 | AF-A0A1H9YDK4-F1-MODEL\_V4 | 1.0 | 3.327e-21 | 1022 | 0.452 | 168 | 92 | 0 | 1 | 168 | 1 | 168 | Uncharacterized protein | Uncharacterized protein | | afdb-uniprot50 | AF-A0A4T2A7H2-F1-MODEL\_V4 | 1.0 | 1.563e-20 | 995 | 0.449 | 169 | 92 | 1 | 1 | 168 | 1 | 169 | Phage major tail tube protein | Phage major tail tube protein | | afdb-uniprot50 | AF-A0A7C8LY52-F1-MODEL\_V4 | 1.0 | 1.824e-20 | 978 | 0.476 | 168 | 87 | 1 | 1 | 167 | 1 | 168 | Uncharacterized protein | Uncharacterized protein | | afdb-uniprot50 | AF-A0A7S8C720-F1-MODEL\_V4 | 1.0 | 2.242e-20 | 976 | 0.464 | 168 | 89 | 1 | 1 | 168 | 1 | 167 | Phage major tail tube protein | Phage major tail tube protein | | afdb-uniprot50 | AF-Q73HI5-F1-MODEL\_V4 | 1.0 | 1.089e-20 | 974 | 0.47 | 168 | 86 | 2 | 1 | 168 | 1 | 165 | Prophage P2W3, contractile tail tube protein | Prophage P2W3, contractile tail tube protein | | afdb-uniprot50 | AF-A0A7Y6Z4U2-F1-MODEL\_V4 | 1.0 | 1.271e-20 | 968 | 0.485 | 169 | 86 | 1 | 1 | 168 | 1 | 169 | Phage major tail tube protein | Phage major tail tube protein | | afdb-uniprot50 | AF-A0A7Z0MZH7-F1-MODEL\_V4 | 1.0 | 4.614e-20 | 967 | 0.415 | 166 | 96 | 1 | 4 | 168 | 3 | 168 | Phage major tail tube protein | Phage major tail tube protein | | afdb-uniprot50 | AF-A0A149SW17-F1-MODEL\_V4 | 1.0 | 3.953e-20 | 963 | 0.386 | 168 | 103 | 0 | 1 | 168 | 2 | 169 | Uncharacterized protein | Uncharacterized protein | | afdb-uniprot50 | AF-A0A4U8YKH6-F1-MODEL\_V4 | 1.0 | 3.754e-20 | 958 | 0.45 | 162 | 89 | 0 | 7 | 168 | 8 | 169 | Tail tube: phage major tail tube protein | Tail tube: phage major tail tube protein | | afdb-uniprot50 | AF-A0A2D8Q7A4-F1-MODEL\_V4 | 1.0 | 3.386e-20 | 948 | 0.416 | 168 | 96 | 2 | 1 | 168 | 2 | 167 | Phage major tail tube protein | Phage major tail tube protein | | afdb-uniprot50 | AF-A0A178GTC8-F1-MODEL\_V4 | 1.0 | 2.901e-20 | 945 | 0.456 | 171 | 89 | 3 | 1 | 168 | 1 | 170 | Phage tail protein | Phage tail protein | | afdb-uniprot50 | AF-A0A081N7T9-F1-MODEL\_V4 | 1.0 | 7.727e-20 | 936 | 0.402 | 169 | 99 | 2 | 1 | 168 | 1 | 168 | Uncharacterized protein | Uncharacterized protein | | afdb-uniprot50 | AF-A0A6I2KEU0-F1-MODEL\_V4 | 1.0 | 1.053e-19 | 933 | 0.534 | 144 | 67 | 0 | 2 | 145 | 3 | 146 | Phage major tail tube protein | Phage major tail tube protein | | afdb-uniprot50 | AF-A0A6M5E4Y8-F1-MODEL\_V4 | 1.0 | 6.97e-20 | 932 | 0.476 | 170 | 85 | 3 | 1 | 168 | 1 | 168 | Phage major tail tube protein | Phage major tail tube protein | | afdb-uniprot50 | AF-A0A0H3K123-F1-MODEL\_V4 | 1.0 | 1.59e-19 | 930 | 0.463 | 166 | 89 | 0 | 3 | 168 | 2 | 167 | Phage tail tube protein FII | Phage tail tube protein FII | | afdb-uniprot50 | AF-B8GS08-F1-MODEL\_V4 | 1.0 | 1.763e-19 | 923 | 0.446 | 168 | 89 | 2 | 1 | 168 | 1 | 164 | Contractile tail tube protein | Contractile tail tube protein | | afdb-uniprot50 | AF-A0A853ILE9-F1-MODEL\_V4 | 1.0 | 1.59e-19 | 923 | 0.392 | 168 | 101 | 1 | 1 | 168 | 1 | 167 | Phage major tail tube protein | Phage major tail tube protein | | afdb-uniprot50 | AF-A0A3N4UQP0-F1-MODEL\_V4 | 1.0 | 2.529e-19 | 917 | 0.437 | 169 | 93 | 2 | 1 | 168 | 1 | 168 | Uncharacterized protein | Uncharacterized protein | | afdb-uniprot50 | AF-A0A4R3LLQ7-F1-MODEL\_V4 | 1.0 | 5.772e-19 | 900 | 0.464 | 168 | 90 | 0 | 1 | 168 | 2 | 169 | Uncharacterized protein | Uncharacterized protein | | afdb-uniprot50 | AF-A0A6L2ZQ79-F1-MODEL\_V4 | 1.0 | 4.945e-19 | 896 | 0.423 | 170 | 95 | 2 | 1 | 168 | 2 | 170 | Phage major tail tube protein | Phage major tail tube protein | | afdb-uniprot50 | AF-A0A450W6Q5-F1-MODEL\_V4 | 1.0 | 4.023e-19 | 894 | 0.423 | 170 | 95 | 2 | 1 | 168 | 1 | 169 | Uncharacterized protein | Uncharacterized protein | | afdb-uniprot50 | AF-A0A4Q4L4P4-F1-MODEL\_V4 | 1.0 | 1.128e-18 | 893 | 0.41 | 168 | 98 | 1 | 1 | 168 | 1 | 167 | Phage major tail tube protein | Phage major tail tube protein | | afdb-uniprot50 | AF-A0A7X2D4H3-F1-MODEL\_V4 | 1.0 | 1.794e-18 | 889 | 0.44 | 161 | 89 | 1 | 7 | 167 | 14 | 173 | Phage major tail tube protein | Phage major tail tube protein | | afdb-uniprot50 | AF-A0A7X3TYV3-F1-MODEL\_V4 | 1.0 | 6.077e-19 | 888 | 0.422 | 168 | 95 | 2 | 2 | 168 | 3 | 169 | Phage major tail tube protein | Phage major tail tube protein | | afdb-uniprot50 | AF-A0A3N8AXB8-F1-MODEL\_V4 | 1.0 | 1.188e-18 | 887 | 0.446 | 168 | 92 | 1 | 1 | 168 | 1 | 167 | Phage major tail tube protein | Phage major tail tube protein | | afdb-uniprot50 | AF-A0A3N2E2A2-F1-MODEL\_V4 | 1.0 | 1.317e-18 | 880 | 0.416 | 168 | 93 | 2 | 1 | 168 | 2 | 164 | Uncharacterized protein | Uncharacterized protein | | afdb-uniprot50 | AF-A0A423MS82-F1-MODEL\_V4 | 1.0 | 1.704e-18 | 880 | 0.404 | 168 | 99 | 1 | 1 | 168 | 1 | 167 | Phage major tail tube protein | Phage major tail tube protein | | afdb-uniprot50 | AF-A0A836CEB5-F1-MODEL\_V4 | 1.0 | 1.794e-18 | 877 | 0.301 | 166 | 114 | 1 | 2 | 167 | 5 | 168 | Phage major tail tube protein | Phage major tail tube protein | | afdb-uniprot50 | AF-A0A257ZWP2-F1-MODEL\_V4 | 1.0 | 1.072e-18 | 874 | 0.335 | 167 | 109 | 1 | 2 | 168 | 4 | 168 | Phage tail protein | Phage tail protein | | afdb-uniprot50 | AF-A0A081B6C6-F1-MODEL\_V4 | 1.0 | 1.704e-18 | 873 | 0.384 | 169 | 99 | 3 | 1 | 168 | 1 | 165 | Phage major tail tube protein | Phage major tail tube protein | | afdb-uniprot50 | AF-A0A5A9EMS2-F1-MODEL\_V4 | 1.0 | 1.704e-18 | 871 | 0.41 | 168 | 98 | 1 | 1 | 168 | 1 | 167 | Phage major tail tube protein | Phage major tail tube protein | | afdb-uniprot50 | AF-A0A1E3G6Z6-F1-MODEL\_V4 | 1.0 | 1.989e-18 | 870 | 0.386 | 168 | 99 | 2 | 1 | 168 | 3 | 166 | Phage major tail tube protein | Phage major tail tube protein | | afdb-uniprot50 | AF-A0A2V3UAX1-F1-MODEL\_V4 | 1.0 | 2.574e-18 | 869 | 0.373 | 166 | 104 | 0 | 3 | 168 | 1 | 166 | P2 family phage contractile tail tube protein | P2 family phage contractile tail tube protein | | afdb-uniprot50 | AF-A0A1M7YZ05-F1-MODEL\_V4 | 1.0 | 2.445e-18 | 856 | 0.438 | 171 | 90 | 3 | 1 | 167 | 1 | 169 | Phage tail tube protein FII | Phage tail tube protein FII | | afdb-uniprot50 | AF-A0A0D8CN41-F1-MODEL\_V4 | 1.0 | 1.46e-18 | 853 | 0.366 | 169 | 104 | 3 | 1 | 168 | 1 | 167 | Tail protein | Tail protein | | afdb-uniprot50 | AF-A0A348FYG6-F1-MODEL\_V4 | 1.0 | 9.343e-18 | 853 | 0.353 | 167 | 108 | 0 | 2 | 168 | 3 | 169 | Tail protein | Tail protein | | afdb-uniprot50 | AF-A0A5P9F0T7-F1-MODEL\_V4 | 1.0 | 3.331e-18 | 851 | 0.388 | 170 | 101 | 3 | 1 | 168 | 1 | 169 | Phage tail tube protein FII | Phage tail tube protein FII | | afdb-uniprot50 | AF-A0A0J1K2H5-F1-MODEL\_V4 | 1.0 | 4.311e-18 | 849 | 0.372 | 169 | 104 | 1 | 1 | 167 | 1 | 169 | Major tail tube protein | Major tail tube protein | | afdb-uniprot50 | AF-A0A2G6CS51-F1-MODEL\_V4 | 1.0 | 5.874e-18 | 848 | 0.366 | 169 | 104 | 3 | 1 | 168 | 1 | 167 | Phage major tail tube protein | Phage major tail tube protein | | afdb-uniprot50 | AF-A0A7Y8ENP0-F1-MODEL\_V4 | 1.0 | 1.209e-17 | 842 | 0.392 | 168 | 101 | 1 | 1 | 168 | 1 | 167 | Phage major tail tube protein | Phage major tail tube protein | | afdb-uniprot50 | AF-A0A4P9VQM1-F1-MODEL\_V4 | 1.0 | 5.874e-18 | 841 | 0.385 | 171 | 102 | 2 | 1 | 168 | 2 | 172 | Phage major tail tube protein | Phage major tail tube protein | | afdb-uniprot50 | AF-A0A2W5DEX1-F1-MODEL\_V4 | 1.0 | 1.148e-17 | 840 | 0.416 | 168 | 98 | 0 | 1 | 168 | 1 | 168 | Phage major tail tube protein | Phage major tail tube protein | | afdb-uniprot50 | AF-A0A7Y8ED06-F1-MODEL\_V4 | 1.0 | 1.411e-17 | 839 | 0.41 | 168 | 98 | 1 | 1 | 168 | 1 | 167 | Phage major tail tube protein | Phage major tail tube protein | | afdb-uniprot50 | AF-A0A2A7UY84-F1-MODEL\_V4 | 1.0 | 8.004e-18 | 839 | 0.382 | 170 | 102 | 3 | 1 | 168 | 1 | 169 | Phage major tail tube protein | Phage major tail tube protein | | afdb-uniprot50 | AF-A0A3S4BVE8-F1-MODEL\_V4 | 1.0 | 8.004e-18 | 838 | 0.446 | 168 | 90 | 1 | 1 | 168 | 3 | 167 | Phage major tail tube protein | Phage major tail tube protein | | afdb-uniprot50 | AF-A0A4R2P5B2-F1-MODEL\_V4 | 1.0 | 1.036e-17 | 837 | 0.388 | 170 | 99 | 3 | 1 | 168 | 1 | 167 | Uncharacterized protein | Uncharacterized protein | | afdb-uniprot50 | AF-A0A522WEK0-F1-MODEL\_V4 | 1.0 | 1.273e-17 | 833 | 0.375 | 168 | 101 | 2 | 2 | 168 | 3 | 167 | Phage major tail tube protein | Phage major tail tube protein | | afdb-uniprot50 | AF-A0A2E9SIR0-F1-MODEL\_V4 | 1.0 | 1.209e-17 | 831 | 0.415 | 171 | 96 | 3 | 1 | 168 | 1 | 170 | Phage major tail tube protein | Phage major tail tube protein | | afdb-uniprot50 | AF-A0A2G6IRW7-F1-MODEL\_V4 | 1.0 | 1.036e-17 | 830 | 0.284 | 165 | 115 | 3 | 6 | 168 | 4 | 167 | Uncharacterized protein | Uncharacterized protein | | afdb-uniprot50 | AF-A0A2S6N2V7-F1-MODEL\_V4 | 1.0 | 1.647e-17 | 827 | 0.325 | 163 | 110 | 0 | 3 | 165 | 5 | 167 | Uncharacterized protein | Uncharacterized protein | | afdb-uniprot50 | AF-A0A0J9EDK5-F1-MODEL\_V4 | 1.0 | 2.62e-17 | 824 | 0.351 | 168 | 107 | 2 | 3 | 168 | 1 | 168 | Phage major tail tube protein | Phage major tail tube protein | | afdb-uniprot50 | AF-A0A7X3ZJY9-F1-MODEL\_V4 | 1.0 | 1.036e-17 | 824 | 0.417 | 170 | 96 | 3 | 1 | 168 | 1 | 169 | Phage major tail tube protein | Phage major tail tube protein | | afdb-uniprot50 | AF-S6I331-F1-MODEL\_V4 | 1.0 | 2.905e-17 | 816 | 0.408 | 169 | 98 | 2 | 1 | 168 | 1 | 168 | Phage major tail tube protein | Phage major tail tube protein | | afdb-uniprot50 | AF-A0A7T8NU05-F1-MODEL\_V4 | 1.0 | 3.759e-17 | 814 | 0.343 | 169 | 107 | 2 | 1 | 168 | 3 | 168 | Phage major tail tube protein | Phage major tail tube protein | | afdb-uniprot50 | AF-A0A7X9ZX79-F1-MODEL\_V4 | 1.0 | 5.979e-17 | 813 | 0.343 | 169 | 110 | 1 | 1 | 168 | 1 | 169 | Phage major tail tube protein | Phage major tail tube protein | | afdb-uniprot50 | AF-A0A432QTQ3-F1-MODEL\_V4 | 1.0 | 3.958e-17 | 813 | 0.293 | 167 | 118 | 0 | 1 | 167 | 2 | 168 | Uncharacterized protein | Uncharacterized protein | | afdb-uniprot50 | AF-A0A1I5DR52-F1-MODEL\_V4 | 1.0 | 4.388e-17 | 807 | 0.37 | 170 | 104 | 3 | 1 | 168 | 1 | 169 | Uncharacterized protein | Uncharacterized protein | | afdb-uniprot50 | AF-A0A4V1IPB1-F1-MODEL\_V4 | 1.0 | 3.22e-17 | 803 | 0.396 | 164 | 96 | 3 | 6 | 168 | 4 | 165 | Phage major tail tube protein | Phage major tail tube protein | | afdb-uniprot50 | AF-A0A379C9M6-F1-MODEL\_V4 | 1.0 | 1.169e-16 | 802 | 0.319 | 169 | 114 | 1 | 1 | 168 | 1 | 169 | Phage major tail tube protein | Phage major tail tube protein | | afdb-uniprot50 | AF-A0A3D9EDI9-F1-MODEL\_V4 | 1.0 | 6.628e-17 | 801 | 0.368 | 171 | 105 | 2 | 1 | 168 | 1 | 171 | Uncharacterized protein | Uncharacterized protein | | afdb-uniprot50 | AF-A0A1Y6CXV9-F1-MODEL\_V4 | 1.0 | 2.363e-17 | 800 | 0.329 | 170 | 111 | 2 | 1 | 168 | 1 | 169 | Uncharacterized protein | Uncharacterized protein | | afdb-uniprot50 | AF-A0A524RVY1-F1-MODEL\_V4 | 1.0 | 5.678e-17 | 798 | 0.396 | 169 | 96 | 4 | 1 | 168 | 4 | 167 | Phage major tail tube protein | Phage major tail tube protein | | afdb-uniprot50 | AF-A0A2J8GXU0-F1-MODEL\_V4 | 1.0 | 1.486e-17 | 797 | 0.41 | 168 | 93 | 4 | 3 | 168 | 5 | 168 | Phage major tail tube protein | Phage major tail tube protein | | afdb-uniprot50 | AF-C6ABW7-F1-MODEL\_V4 | 1.0 | 6.979e-17 | 796 | 0.363 | 168 | 104 | 1 | 1 | 168 | 4 | 168 | Phage tail tube protein FII | Phage tail tube protein FII | | afdb-uniprot50 | AF-A0A6B8KGJ9-F1-MODEL\_V4 | 1.0 | 1.512e-16 | 792 | 0.278 | 169 | 121 | 1 | 1 | 168 | 5 | 173 | Uncharacterized protein | Uncharacterized protein | | afdb-uniprot50 | AF-A0A061JHN2-F1-MODEL\_V4 | 1.0 | 5.979e-17 | 784 | 0.442 | 147 | 80 | 2 | 1 | 147 | 1 | 145 | Major tail tube protein | Major tail tube protein | | afdb-uniprot50 | AF-A0A2M7MWC6-F1-MODEL\_V4 | 1.0 | 1.765e-16 | 783 | 0.391 | 166 | 100 | 1 | 1 | 166 | 1 | 165 | Phage major tail tube protein | Phage major tail tube protein | | afdb-uniprot50 | AF-A0A3D1C6N3-F1-MODEL\_V4 | 1.0 | 2.285e-16 | 779 | 0.333 | 171 | 111 | 2 | 1 | 168 | 1 | 171 | Phage major tail tube protein | Phage major tail tube protein | | afdb-uniprot50 | AF-A0A0C5VGC6-F1-MODEL\_V4 | 1.0 | 1.923e-17 | 779 | 0.381 | 173 | 91 | 3 | 1 | 158 | 1 | 172 | Phage tail tube protein FII | Phage tail tube protein FII | | afdb-uniprot50 | AF-A0A6H9GVB3-F1-MODEL\_V4 | 1.0 | 1.364e-16 | 778 | 0.35 | 171 | 106 | 3 | 1 | 168 | 1 | 169 | Major tail tube protein | Major tail tube protein | | afdb-uniprot50 | AF-A0A6F8NL50-F1-MODEL\_V4 | 1.0 | 1.957e-16 | 777 | 0.329 | 164 | 109 | 1 | 6 | 168 | 11 | 174 | Bacteriophage P2 major tail sheath protein GPFII | Bacteriophage P2 major tail sheath protein GPFII | | afdb-uniprot50 | AF-A0A4U0YZA9-F1-MODEL\_V4 | 1.0 | 2.17e-16 | 775 | 0.325 | 169 | 109 | 2 | 1 | 168 | 1 | 165 | Phage major tail tube protein | Phage major tail tube protein | | afdb-uniprot50 | AF-A0A1E3G652-F1-MODEL\_V4 | 1.0 | 1.436e-16 | 773 | 0.281 | 167 | 117 | 3 | 1 | 167 | 2 | 165 | Uncharacterized protein | Uncharacterized protein | | afdb-uniprot50 | AF-A0A7K0GNR9-F1-MODEL\_V4 | 1.0 | 7.348e-17 | 772 | 0.459 | 148 | 79 | 1 | 21 | 168 | 1 | 147 | Phage major tail tube protein | Phage major tail tube protein | | afdb-uniprot50 | AF-A0A1A9VKH7-F1-MODEL\_V4 | 1.0 | 4.388e-17 | 772 | 0.45 | 153 | 83 | 1 | 17 | 168 | 383 | 535 | Uncharacterized protein | Uncharacterized protein | | afdb-uniprot50 | AF-A0A1M3PG22-F1-MODEL\_V4 | 1.0 | 2.17e-16 | 771 | 0.333 | 171 | 111 | 2 | 1 | 168 | 1 | 171 | Phage major tail tube protein | Phage major tail tube protein | | afdb-uniprot50 | AF-A0A537MFH6-F1-MODEL\_V4 | 1.0 | 3.826e-16 | 769 | 0.335 | 167 | 109 | 2 | 1 | 166 | 1 | 166 | Phage major tail tube protein | Phage major tail tube protein | | afdb-uniprot50 | AF-A0A380TT15-F1-MODEL\_V4 | 1.0 | 4.951e-16 | 768 | 0.331 | 169 | 111 | 2 | 1 | 168 | 1 | 168 | Phage major tail tube protein | Phage major tail tube protein | | afdb-uniprot50 | AF-A0A1A9RES3-F1-MODEL\_V4 | 1.0 | 1.512e-16 | 768 | 0.319 | 169 | 112 | 3 | 1 | 168 | 2 | 168 | Phage major tail tube protein | Phage major tail tube protein | | afdb-uniprot50 | AF-A0A840G0D8-F1-MODEL\_V4 | 1.0 | 3.826e-16 | 767 | 0.347 | 170 | 108 | 3 | 1 | 168 | 1 | 169 | Uncharacterized protein | Uncharacterized protein | | afdb-uniprot50 | AF-A0A212KMZ0-F1-MODEL\_V4 | 1.0 | 9.509e-17 | 763 | 0.361 | 163 | 97 | 4 | 7 | 168 | 10 | 166 | Prophage P2W3, contractile tail tube protein | Prophage P2W3, contractile tail tube protein | | afdb-uniprot50 | AF-A0A5S9Q1W1-F1-MODEL\_V4 | 1.0 | 5.489e-16 | 763 | 0.335 | 170 | 111 | 1 | 1 | 168 | 2 | 171 | Uncharacterized protein | Uncharacterized protein | | afdb-uniprot50 | AF-A0A5M6I344-F1-MODEL\_V4 | 1.0 | 3.278e-16 | 760 | 0.345 | 168 | 107 | 2 | 1 | 166 | 23 | 189 | Phage major tail tube protein | Phage major tail tube protein | | afdb-uniprot50 | AF-C8N757-F1-MODEL\_V4 | 1.0 | 6.746e-16 | 757 | 0.359 | 167 | 105 | 2 | 1 | 166 | 1 | 166 | Phage major tail tube protein | Phage major tail tube protein | | afdb-uniprot50 | AF-A0A2A5BNC9-F1-MODEL\_V4 | 1.0 | 4.702e-16 | 757 | 0.272 | 169 | 120 | 3 | 1 | 166 | 1 | 169 | Uncharacterized protein | Uncharacterized protein | | afdb-uniprot50 | AF-A0A3N2E0L8-F1-MODEL\_V4 | 1.0 | 1.11e-16 | 756 | 0.361 | 163 | 98 | 3 | 1 | 158 | 1 | 162 | Uncharacterized protein | Uncharacterized protein | | afdb-uniprot50 | AF-A0A318L0T4-F1-MODEL\_V4 | 1.0 | 6.407e-16 | 755 | 0.284 | 169 | 120 | 1 | 1 | 168 | 1 | 169 | Uncharacterized protein | Uncharacterized protein | | afdb-uniprot50 | AF-A0A1Z3N265-F1-MODEL\_V4 | 1.0 | 8.73e-16 | 755 | 0.321 | 171 | 113 | 2 | 1 | 168 | 1 | 171 | Phage major tail tube protein | Phage major tail tube protein | | afdb-uniprot50 | AF-A0A161YC88-F1-MODEL\_V4 | 1.0 | 4.028e-16 | 755 | 0.313 | 172 | 112 | 4 | 1 | 168 | 2 | 171 | Major tail tube protein | Major tail tube protein | | afdb-uniprot50 | AF-A0A2E5BU07-F1-MODEL\_V4 | 1.0 | 7.874e-16 | 754 | 0.305 | 170 | 115 | 2 | 1 | 168 | 1 | 169 | Phage major tail tube protein | Phage major tail tube protein | | afdb-uniprot50 | AF-A0A0K1NFI5-F1-MODEL\_V4 | 1.0 | 8.73e-16 | 753 | 0.313 | 169 | 114 | 2 | 1 | 168 | 1 | 168 | Phage tail protein | Phage tail protein | | afdb-uniprot50 | AF-A0A857E651-F1-MODEL\_V4 | 1.0 | 3.451e-16 | 753 | 0.323 | 170 | 110 | 4 | 1 | 167 | 1 | 168 | Phage tail protein | Phage tail protein | | afdb-uniprot50 | AF-A0A4Q3U3Y4-F1-MODEL\_V4 | 1.0 | 7.479e-16 | 751 | 0.309 | 168 | 113 | 3 | 1 | 166 | 1 | 167 | Phage major tail tube protein | Phage major tail tube protein | | afdb-uniprot50 | AF-Q4AAY4-F1-MODEL\_V4 | 1.0 | 5.489e-16 | 751 | 0.356 | 171 | 107 | 2 | 1 | 168 | 1 | 171 | Fels-2 prophage protein | Fels-2 prophage protein | | afdb-uniprot50 | AF-W9BWM4-F1-MODEL\_V4 | 1.0 | 1.073e-15 | 747 | 0.339 | 171 | 108 | 3 | 1 | 168 | 1 | 169 | Major tail tube protein | Major tail tube protein | | afdb-uniprot50 | AF-A0A837E5C9-F1-MODEL\_V4 | 1.0 | 6.407e-16 | 746 | 0.301 | 169 | 113 | 2 | 1 | 168 | 2 | 166 | Uncharacterized protein | Uncharacterized protein | | afdb-uniprot50 | AF-A0A6N7JGS1-F1-MODEL\_V4 | 1.0 | 9.192e-16 | 746 | 0.301 | 169 | 113 | 3 | 1 | 168 | 3 | 167 | Uncharacterized protein | Uncharacterized protein | | afdb-uniprot50 | AF-A0A326GGT4-F1-MODEL\_V4 | 1.0 | 1.189e-15 | 745 | 0.297 | 168 | 116 | 2 | 1 | 167 | 1 | 167 | Phage major tail tube protein | Phage major tail tube protein | | afdb-uniprot50 | AF-A0A659Q6F3-F1-MODEL\_V4 | 1.0 | 7.874e-16 | 743 | 0.323 | 173 | 110 | 4 | 1 | 168 | 1 | 171 | Phage major tail tube protein | Phage major tail tube protein | | afdb-uniprot50 | AF-A0A3R8V2Y2-F1-MODEL\_V4 | 1.0 | 1.539e-15 | 743 | 0.315 | 171 | 114 | 2 | 1 | 168 | 1 | 171 | Phage major tail tube protein | Phage major tail tube protein | | afdb-uniprot50 | AF-A0A2E4CSP6-F1-MODEL\_V4 | 1.0 | 1.621e-15 | 742 | 0.325 | 169 | 112 | 2 | 1 | 168 | 1 | 168 | Phage major tail tube protein | Phage major tail tube protein | | afdb-uniprot50 | AF-H6SQI7-F1-MODEL\_V4 | 1.0 | 1.319e-15 | 742 | 0.352 | 170 | 105 | 4 | 1 | 167 | 1 | 168 | Phage major tail tube protein (Probable bacteriophage tail tube protein FII) | Phage major tail tube protein (Probable bacteriophage tail tube protein FII) | | afdb-uniprot50 | AF-A0A3V8HSW3-F1-MODEL\_V4 | 1.0 | 1.621e-15 | 742 | 0.343 | 172 | 109 | 3 | 1 | 168 | 1 | 172 | Phage major tail tube protein | Phage major tail tube protein | | afdb-uniprot50 | AF-A0A2R7NI64-F1-MODEL\_V4 | 1.0 | 1.621e-15 | 740 | 0.313 | 172 | 113 | 4 | 1 | 168 | 2 | 172 | Phage major tail tube protein | Phage major tail tube protein | | afdb-uniprot50 | AF-A0A5S9HN48-F1-MODEL\_V4 | 1.0 | 1.252e-15 | 739 | 0.295 | 169 | 118 | 1 | 1 | 168 | 4 | 172 | Major tail tube protein | Major tail tube protein | | afdb-uniprot50 | AF-A0A1D2QSB5-F1-MODEL\_V4 | 1.0 | 1.436e-16 | 739 | 0.347 | 187 | 97 | 4 | 1 | 163 | 1 | 186 | Uncharacterized protein | Uncharacterized protein | | afdb-uniprot50 | AF-A0A345DE54-F1-MODEL\_V4 | 1.0 | 5.489e-16 | 738 | 0.266 | 169 | 121 | 3 | 1 | 167 | 1 | 168 | Uncharacterized protein | Uncharacterized protein | | afdb-uniprot50 | AF-A0A326L1L7-F1-MODEL\_V4 | 1.0 | 3.009e-15 | 738 | 0.305 | 170 | 116 | 2 | 1 | 168 | 1 | 170 | Phage major tail tube protein | Phage major tail tube protein | | afdb-uniprot50 | AF-A0A7X4FXW3-F1-MODEL\_V4 | 1.0 | 5.779e-16 | 737 | 0.313 | 172 | 111 | 5 | 1 | 168 | 1 | 169 | Phage major tail tube protein | Phage major tail tube protein | | afdb-uniprot50 | AF-A0A0H3GX02-F1-MODEL\_V4 | 1.0 | 1.388e-15 | 737 | 0.343 | 172 | 108 | 4 | 1 | 168 | 1 | 171 | Putative prophage tail tube protein | Putative prophage tail tube protein | | afdb-uniprot50 | AF-A0A3B7Q0S0-F1-MODEL\_V4 | 1.0 | 2.097e-15 | 732 | 0.323 | 170 | 111 | 4 | 1 | 168 | 1 | 168 | Phage major tail tube protein | Phage major tail tube protein | | afdb-uniprot50 | AF-A0A5E4XFQ3-F1-MODEL\_V4 | 1.0 | 1.436e-16 | 732 | 0.343 | 169 | 108 | 2 | 2 | 168 | 4 | 171 | Phage major tail tube protein | Phage major tail tube protein | | afdb-uniprot50 | AF-A0A0Q2YZZ0-F1-MODEL\_V4 | 1.0 | 1.892e-15 | 729 | 0.269 | 167 | 118 | 2 | 2 | 168 | 4 | 166 | Uncharacterized protein | Uncharacterized protein | | afdb-uniprot50 | AF-E2CJT1-F1-MODEL\_V4 | 1.0 | 2.448e-15 | 728 | 0.267 | 168 | 122 | 1 | 1 | 168 | 1 | 167 | Phage tail tube protein FII | Phage tail tube protein FII | | afdb-uniprot50 | AF-A0A379YDU2-F1-MODEL\_V4 | 1.0 | 1.073e-15 | 727 | 0.329 | 170 | 109 | 4 | 1 | 167 | 1 | 168 | Phage major tail tube protein | Phage major tail tube protein | | afdb-uniprot50 | AF-A0A2A2B589-F1-MODEL\_V4 | 1.0 | 1.189e-15 | 723 | 0.304 | 171 | 116 | 2 | 1 | 168 | 1 | 171 | Phage major tail tube protein | Phage major tail tube protein | | afdb-uniprot50 | AF-A0A2I1RIY9-F1-MODEL\_V4 | 1.0 | 2.325e-15 | 720 | 0.345 | 171 | 107 | 4 | 1 | 167 | 1 | 170 | Phage major tail tube protein | Phage major tail tube protein | | afdb-uniprot50 | AF-A0A743CC81-F1-MODEL\_V4 | 1.0 | 1.13e-15 | 719 | 0.349 | 169 | 107 | 3 | 1 | 167 | 1 | 168 | Phage tail protein | Phage tail protein | | afdb-uniprot50 | AF-F4BFQ1-F1-MODEL\_V4 | 1.0 | 1.992e-15 | 717 | 0.291 | 161 | 109 | 3 | 3 | 162 | 2 | 158 | Phage major tail tube protein | Phage major tail tube protein | | afdb-uniprot50 | AF-A0A246KGG0-F1-MODEL\_V4 | 1.0 | 8.438e-15 | 714 | 0.275 | 167 | 120 | 1 | 1 | 166 | 1 | 167 | Phage major tail tube protein | Phage major tail tube protein | | afdb-uniprot50 | AF-A0A2G6EWZ7-F1-MODEL\_V4 | 1.0 | 6.193e-15 | 714 | 0.272 | 169 | 122 | 1 | 1 | 168 | 1 | 169 | Phage major tail tube protein | Phage major tail tube protein | | afdb-uniprot50 | AF-A0A0U1DAP0-F1-MODEL\_V4 | 1.0 | 1.992e-15 | 712 | 0.446 | 141 | 77 | 1 | 29 | 168 | 2 | 142 | Phage tail tube protein | Phage tail tube protein | | afdb-uniprot50 | AF-A0A359KE96-F1-MODEL\_V4 | 1.0 | 6.193e-15 | 712 | 0.278 | 169 | 117 | 3 | 1 | 166 | 1 | 167 | Phage major tail tube protein | Phage major tail tube protein | | afdb-uniprot50 | AF-A0A7Y3N2Q7-F1-MODEL\_V4 | 1.0 | 3.009e-15 | 710 | 0.272 | 169 | 121 | 2 | 1 | 168 | 3 | 170 | Phage major tail tube protein | Phage major tail tube protein | | afdb-uniprot50 | AF-A0A1X3I4U3-F1-MODEL\_V4 | 1.0 | 6.866e-15 | 709 | 0.304 | 171 | 116 | 2 | 1 | 168 | 3 | 173 | Phage major tail tube protein | Phage major tail tube protein | | afdb-uniprot50 | AF-A0A7C2APA8-F1-MODEL\_V4 | 1.0 | 7.479e-16 | 702 | 0.293 | 174 | 114 | 5 | 1 | 168 | 1 | 171 | Phage major tail tube protein | Phage major tail tube protein | | afdb-uniprot50 | AF-A0A258L6Q4-F1-MODEL\_V4 | 1.0 | 1.13e-15 | 701 | 0.301 | 169 | 112 | 3 | 2 | 168 | 5 | 169 | Phage tail protein | Phage tail protein | | afdb-uniprot50 | AF-R7JNY0-F1-MODEL\_V4 | 1.0 | 2.448e-15 | 700 | 0.283 | 166 | 114 | 3 | 7 | 168 | 6 | 170 | Phage tail core protein | Phage tail core protein | | afdb-uniprot50 | AF-A0A7G7RNU7-F1-MODEL\_V4 | 1.0 | 2.448e-15 | 700 | 0.335 | 173 | 108 | 3 | 1 | 168 | 1 | 171 | Phage major tail tube protein | Phage major tail tube protein | | afdb-uniprot50 | AF-A0A6M8T223-F1-MODEL\_V4 | 1.0 | 2.325e-15 | 699 | 0.225 | 173 | 127 | 4 | 1 | 168 | 1 | 171 | Phage major tail tube protein | Phage major tail tube protein | | afdb-uniprot50 | AF-A0A0F9QWB3-F1-MODEL\_V4 | 1.0 | 8.438e-15 | 698 | 0.253 | 166 | 122 | 1 | 2 | 167 | 4 | 167 | Uncharacterized protein | Uncharacterized protein | | afdb-uniprot50 | AF-A0A089PTU9-F1-MODEL\_V4 | 1.0 | 4.317e-15 | 696 | 0.343 | 172 | 106 | 6 | 1 | 168 | 1 | 169 | Tail protein | Tail protein | | afdb-uniprot50 | AF-A0A6C1BNV4-F1-MODEL\_V4 | 1.0 | 3.336e-15 | 695 | 0.242 | 165 | 121 | 3 | 7 | 168 | 7 | 170 | Phage major tail tube protein | Phage major tail tube protein | | afdb-uniprot50 | AF-A0A827QKM1-F1-MODEL\_V4 | 1.0 | 4.317e-15 | 694 | 0.305 | 170 | 113 | 4 | 1 | 167 | 1 | 168 | Phage major tail tube protein | Phage major tail tube protein | | afdb-uniprot50 | AF-A0A2D5S2E1-F1-MODEL\_V4 | 1.0 | 7.612e-15 | 693 | 0.282 | 170 | 117 | 5 | 2 | 168 | 3 | 170 | Uncharacterized protein | Uncharacterized protein | | afdb-uniprot50 | AF-A0A2E6BPJ5-F1-MODEL\_V4 | 1.0 | 1.275e-14 | 690 | 0.295 | 169 | 117 | 2 | 1 | 168 | 1 | 168 | Phage major tail tube protein | Phage major tail tube protein | | afdb-uniprot50 | AF-A0A806CP35-F1-MODEL\_V4 | 1.0 | 6.866e-15 | 688 | 0.3 | 170 | 114 | 4 | 1 | 167 | 1 | 168 | Phage major tail tube protein | Phage major tail tube protein | | afdb-uniprot50 | AF-A0A450YW94-F1-MODEL\_V4 | 1.0 | 1.621e-15 | 684 | 0.24 | 166 | 121 | 3 | 7 | 168 | 10 | 174 | Uncharacterized protein | Uncharacterized protein | | afdb-uniprot50 | AF-A0A0Q8B4N4-F1-MODEL\_V4 | 1.0 | 6.521e-15 | 679 | 0.255 | 168 | 121 | 4 | 3 | 168 | 1 | 166 | Uncharacterized protein | Uncharacterized protein | | afdb-uniprot50 | AF-A0A809HDP3-F1-MODEL\_V4 | 1.0 | 8.438e-15 | 677 | 0.27 | 170 | 120 | 3 | 1 | 168 | 1 | 168 | Phage tail protein | Phage tail protein | | afdb-uniprot50 | AF-A0A1N7LR57-F1-MODEL\_V4 | 1.0 | 1.829e-14 | 676 | 0.251 | 167 | 120 | 3 | 2 | 167 | 9 | 171 | Uncharacterized protein | Uncharacterized protein | | afdb-uniprot50 | AF-A0A7W6RG44-F1-MODEL\_V4 | 1.0 | 2.624e-14 | 673 | 0.254 | 169 | 123 | 3 | 3 | 168 | 1 | 169 | Uncharacterized protein | Uncharacterized protein | | afdb-uniprot50 | AF-A0A4R6E272-F1-MODEL\_V4 | 1.0 | 1.925e-14 | 672 | 0.295 | 169 | 114 | 4 | 1 | 167 | 1 | 166 | Uncharacterized protein | Uncharacterized protein | | afdb-uniprot50 | AF-R7MC56-F1-MODEL\_V4 | 1.0 | 6.866e-15 | 672 | 0.229 | 174 | 127 | 4 | 1 | 168 | 1 | 173 | Prophage MuMc02 major tail tube protein | Prophage MuMc02 major tail tube protein | | afdb-uniprot50 | AF-Q8ZKJ3-F1-MODEL\_V4 | 1.0 | 2.448e-15 | 672 | 0.24 | 175 | 125 | 4 | 1 | 168 | 1 | 174 | Putative phage tail core protein | Putative phage tail core protein | | afdb-uniprot50 | AF-A0A085AFM9-F1-MODEL\_V4 | 1.0 | 2.909e-14 | 670 | 0.245 | 167 | 124 | 2 | 1 | 167 | 2 | 166 | Phage major tail tube protein | Phage major tail tube protein | | afdb-uniprot50 | AF-A0A4Q6D1V8-F1-MODEL\_V4 | 1.0 | 1.275e-14 | 666 | 0.29 | 155 | 108 | 1 | 1 | 153 | 1 | 155 | Uncharacterized protein | Uncharacterized protein | | afdb-uniprot50 | AF-Q31HT1-F1-MODEL\_V4 | 1.0 | 2.366e-14 | 666 | 0.265 | 162 | 116 | 3 | 7 | 168 | 9 | 167 | Phage tail tube protein | Phage tail tube protein | | afdb-uniprot50 | AF-A0A1C0V8D9-F1-MODEL\_V4 | 1.0 | 9.85e-15 | 666 | 0.224 | 165 | 123 | 3 | 7 | 167 | 7 | 170 | Uncharacterized protein | Uncharacterized protein | | afdb-uniprot50 | AF-A0A482IPK0-F1-MODEL\_V4 | 1.0 | 1.488e-14 | 664 | 0.226 | 172 | 127 | 4 | 1 | 168 | 4 | 173 | Phage major tail tube protein | Phage major tail tube protein | | afdb-uniprot50 | AF-A0A4Q0GQ17-F1-MODEL\_V4 | 1.0 | 2.909e-14 | 664 | 0.374 | 155 | 94 | 2 | 17 | 168 | 33 | 187 | Phage major tail tube protein | Phage major tail tube protein | | afdb-uniprot50 | AF-F2K1Q9-F1-MODEL\_V4 | 1.0 | 1.342e-14 | 663 | 0.236 | 169 | 122 | 6 | 1 | 168 | 3 | 165 | Major tail tube protein | Major tail tube protein | | afdb-uniprot50 | AF-U0ZR36-F1-MODEL\_V4 | 1.0 | 5.039e-15 | 663 | 0.236 | 173 | 125 | 4 | 1 | 168 | 1 | 171 | Tail protein | Tail protein | | afdb-uniprot50 | AF-A0A256CAU6-F1-MODEL\_V4 | 1.0 | 1.567e-14 | 662 | 0.307 | 169 | 114 | 3 | 1 | 167 | 3 | 170 | Uncharacterized protein | Uncharacterized protein | | afdb-uniprot50 | AF-E2CN26-F1-MODEL\_V4 | 1.0 | 1.037e-14 | 660 | 0.264 | 170 | 120 | 5 | 1 | 167 | 1 | 168 | Phage tail tube protein FII | Phage tail tube protein FII | | afdb-uniprot50 | AF-A0A1J5HZS6-F1-MODEL\_V4 | 1.0 | 1.737e-14 | 660 | 0.224 | 174 | 127 | 5 | 1 | 167 | 1 | 173 | Uncharacterized protein | Uncharacterized protein | | afdb-uniprot50 | AF-A0A1B9NSN8-F1-MODEL\_V4 | 1.0 | 9.043e-14 | 659 | 0.343 | 172 | 107 | 4 | 1 | 168 | 1 | 170 | Phage major tail tube protein | Phage major tail tube protein | | afdb-uniprot50 | AF-A0A1H2FLU6-F1-MODEL\_V4 | 1.0 | 2.492e-14 | 659 | 0.232 | 168 | 124 | 5 | 6 | 168 | 4 | 171 | Uncharacterized protein | Uncharacterized protein | | afdb-uniprot50 | AF-A0A1E4AKG0-F1-MODEL\_V4 | 1.0 | 7.229e-15 | 659 | 0.274 | 175 | 119 | 4 | 1 | 168 | 1 | 174 | Phage major tail tube protein | Phage major tail tube protein | | afdb-uniprot50 | AF-J5KT56-F1-MODEL\_V4 | 1.0 | 3.575e-14 | 658 | 0.209 | 167 | 126 | 4 | 1 | 166 | 2 | 163 | Phage tail tube protein FII | Phage tail tube protein FII | | afdb-uniprot50 | AF-A0A3S5C3B8-F1-MODEL\_V4 | 1.0 | 2.624e-14 | 655 | 0.197 | 167 | 129 | 3 | 6 | 168 | 7 | 172 | Phage-like protein | Phage-like protein | | afdb-uniprot50 | AF-W6W214-F1-MODEL\_V4 | 1.0 | 1.092e-14 | 652 | 0.298 | 171 | 113 | 5 | 3 | 168 | 1 | 169 | Major tail tube protein | Major tail tube protein | | afdb-uniprot50 | AF-A0A1C3EBQ2-F1-MODEL\_V4 | 1.0 | 5.128e-14 | 650 | 0.263 | 167 | 117 | 6 | 6 | 168 | 8 | 172 | Uncharacterized protein | Uncharacterized protein | | afdb-uniprot50 | AF-A0A327Q9I2-F1-MODEL\_V4 | 1.0 | 2.762e-14 | 649 | 0.227 | 167 | 123 | 4 | 7 | 168 | 8 | 173 | Uncharacterized protein | Uncharacterized protein | | afdb-uniprot50 | AF-E1SFY3-F1-MODEL\_V4 | 1.0 | 6.637e-14 | 649 | 0.35 | 157 | 96 | 3 | 1 | 154 | 1 | 154 | Major tail tube protein (Protein FII) | Major tail tube protein (Protein FII) | | afdb-uniprot50 | AF-B6JEF9-F1-MODEL\_V4 | 1.0 | 1.003e-13 | 645 | 0.289 | 169 | 113 | 4 | 6 | 167 | 4 | 172 | Putative phage tail tube protein FII | Putative phage tail tube protein FII | | afdb-uniprot50 | AF-A0A163V2U5-F1-MODEL\_V4 | 1.0 | 1.366e-13 | 643 | 0.212 | 165 | 128 | 2 | 6 | 168 | 4 | 168 | Uncharacterized protein | Uncharacterized protein | | afdb-uniprot50 | AF-A0A377JJD6-F1-MODEL\_V4 | 1.0 | 5.128e-14 | 643 | 0.184 | 173 | 135 | 4 | 1 | 168 | 1 | 172 | Tail core-like protein | Tail core-like protein | | afdb-uniprot50 | AF-B6IMG1-F1-MODEL\_V4 | 1.0 | 2.624e-14 | 643 | 0.218 | 174 | 129 | 5 | 1 | 168 | 1 | 173 | Phage major tail tube protein | Phage major tail tube protein | | afdb-uniprot50 | AF-A0A1T2YJS6-F1-MODEL\_V4 | 1.0 | 9.043e-14 | 642 | 0.232 | 168 | 127 | 2 | 1 | 168 | 2 | 167 | Phage tail protein | Phage tail protein | | afdb-uniprot50 | AF-A0A4V6YSQ4-F1-MODEL\_V4 | 1.0 | 5.686e-14 | 642 | 0.209 | 167 | 127 | 4 | 1 | 166 | 7 | 169 | Uncharacterized protein | Uncharacterized protein | | afdb-uniprot50 | AF-A0A1N6M6E1-F1-MODEL\_V4 | 1.0 | 6.637e-14 | 641 | 0.216 | 166 | 128 | 2 | 2 | 167 | 3 | 166 | Major tail tube protein | Major tail tube protein | | afdb-uniprot50 | AF-A0A7C4AK38-F1-MODEL\_V4 | 1.0 | 1.567e-14 | 640 | 0.228 | 175 | 126 | 6 | 1 | 168 | 2 | 174 | Uncharacterized protein | Uncharacterized protein | | afdb-uniprot50 | AF-A0A7L5Y1E9-F1-MODEL\_V4 | 1.0 | 1.514e-13 | 640 | 0.207 | 169 | 130 | 3 | 3 | 167 | 1 | 169 | Phage major tail tube protein | Phage major tail tube protein | | afdb-uniprot50 | AF-A0A1R4LKF5-F1-MODEL\_V4 | 1.0 | 1.003e-13 | 639 | 0.221 | 167 | 128 | 2 | 2 | 168 | 3 | 167 | Phage tail tube protein FII | Phage tail tube protein FII | | afdb-uniprot50 | AF-A0A379AQE4-F1-MODEL\_V4 | 1.0 | 5.128e-14 | 639 | 0.18 | 166 | 131 | 3 | 7 | 168 | 9 | 173 | Phage major tail tube protein | Phage major tail tube protein | | afdb-uniprot50 | AF-A0A348HHH9-F1-MODEL\_V4 | 1.0 | 1.056e-13 | 638 | 0.233 | 163 | 124 | 1 | 6 | 168 | 7 | 168 | Phage tail tube protein FII | Phage tail tube protein FII | | afdb-uniprot50 | AF-A0A3G2IKK8-F1-MODEL\_V4 | 1.0 | 7.358e-14 | 637 | 0.253 | 158 | 114 | 3 | 6 | 160 | 3 | 159 | Uncharacterized protein | Uncharacterized protein | | afdb-uniprot50 | AF-A0A7C3RQ40-F1-MODEL\_V4 | 1.0 | 1.413e-14 | 633 | 0.238 | 176 | 122 | 6 | 1 | 168 | 1 | 172 | Phage major tail tube protein | Phage major tail tube protein | | afdb-uniprot50 | AF-A0A1Y1QXH8-F1-MODEL\_V4 | 1.0 | 2.288e-13 | 631 | 0.251 | 163 | 117 | 2 | 7 | 168 | 13 | 171 | Uncharacterized protein | Uncharacterized protein | | afdb-uniprot50 | AF-A0A7W2BMA7-F1-MODEL\_V4 | 1.0 | 9.521e-14 | 631 | 0.258 | 174 | 121 | 7 | 1 | 168 | 1 | 172 | Phage major tail tube protein | Phage major tail tube protein | | afdb-uniprot50 | AF-G2HXA5-F1-MODEL\_V4 | 1.0 | 3.395e-14 | 630 | 0.285 | 161 | 108 | 5 | 7 | 166 | 10 | 164 | Phage tail protein | Phage tail protein | | afdb-uniprot50 | AF-A0A3G2IAV8-F1-MODEL\_V4 | 1.0 | 7.747e-14 | 630 | 0.269 | 163 | 115 | 3 | 7 | 167 | 5 | 165 | Uncharacterized protein | Uncharacterized protein | | afdb-uniprot50 | AF-A0A1G3UAL9-F1-MODEL\_V4 | 1.0 | 1.96e-13 | 628 | 0.191 | 162 | 127 | 3 | 6 | 166 | 9 | 167 | Uncharacterized protein | Uncharacterized protein | | afdb-uniprot50 | AF-A0A3T0L2U1-F1-MODEL\_V4 | 1.0 | 7.358e-14 | 625 | 0.21 | 166 | 125 | 4 | 1 | 166 | 1 | 160 | Uncharacterized protein | Uncharacterized protein | | afdb-uniprot50 | AF-A0A6I0CJR1-F1-MODEL\_V4 | 1.0 | 6.303e-14 | 625 | 0.216 | 171 | 128 | 4 | 1 | 167 | 4 | 172 | Phage major tail tube protein | Phage major tail tube protein | | afdb-uniprot50 | AF-A0A1R1MK86-F1-MODEL\_V4 | 1.0 | 5.128e-14 | 624 | 0.268 | 175 | 119 | 6 | 1 | 168 | 2 | 174 | Uncharacterized protein | Uncharacterized protein | | afdb-uniprot50 | AF-A0A2N3KSL5-F1-MODEL\_V4 | 1.0 | 1.111e-13 | 622 | 0.269 | 171 | 116 | 6 | 1 | 168 | 1 | 165 | Uncharacterized protein | Uncharacterized protein | | afdb-uniprot50 | AF-A0A327JM09-F1-MODEL\_V4 | 1.0 | 1.297e-13 | 622 | 0.204 | 171 | 130 | 6 | 1 | 166 | 1 | 170 | Phage tail protein | Phage tail protein | | afdb-uniprot50 | AF-A0A828HN09-F1-MODEL\_V4 | 1.0 | 4.033e-13 | 621 | 0.26 | 173 | 119 | 5 | 1 | 168 | 1 | 169 | Phage tail protein | Phage tail protein | | afdb-uniprot50 | AF-A0A7U7YUD0-F1-MODEL\_V4 | 1.0 | 1.17e-13 | 621 | 0.244 | 172 | 120 | 6 | 2 | 168 | 5 | 171 | Phage tail protein | Phage tail protein | | afdb-uniprot50 | AF-A0A075KC25-F1-MODEL\_V4 | 1.0 | 4.173e-14 | 620 | 0.231 | 173 | 122 | 6 | 2 | 167 | 6 | 174 | Major tail tube protein | Major tail tube protein | | afdb-uniprot50 | AF-A0A1T4W4F8-F1-MODEL\_V4 | 1.0 | 3.117e-13 | 619 | 0.227 | 167 | 125 | 3 | 3 | 168 | 2 | 165 | Uncharacterized protein | Uncharacterized protein | | afdb-uniprot50 | AF-A0A6A4R9K0-F1-MODEL\_V4 | 1.0 | 1.232e-13 | 619 | 0.232 | 168 | 127 | 2 | 1 | 168 | 2 | 167 | Phage tail protein | Phage tail protein | | afdb-uniprot50 | AF-A0A2W5VKB0-F1-MODEL\_V4 | 1.0 | 3.638e-13 | 619 | 0.264 | 170 | 117 | 5 | 1 | 166 | 2 | 167 | Phage tail protein | Phage tail protein | | afdb-uniprot50 | AF-G4CJF7-F1-MODEL\_V4 | 1.0 | 9.521e-14 | 618 | 0.208 | 173 | 131 | 4 | 1 | 168 | 1 | 172 | Phage tail core protein | Phage tail core protein | | afdb-uniprot50 | AF-E0NXJ4-F1-MODEL\_V4 | 1.0 | 6.637e-14 | 618 | 0.291 | 175 | 111 | 6 | 2 | 168 | 18 | 187 | Phage tail tube protein FII | Phage tail tube protein FII | | afdb-uniprot50 | AF-A0A6G5QXU3-F1-MODEL\_V4 | 1.0 | 2.063e-13 | 617 | 0.247 | 170 | 121 | 5 | 2 | 168 | 6 | 171 | Phage tail tube protein FII | Phage tail tube protein FII | | afdb-uniprot50 | AF-A0A1T4PVU5-F1-MODEL\_V4 | 1.0 | 5.686e-14 | 617 | 0.255 | 176 | 118 | 5 | 1 | 168 | 1 | 171 | Uncharacterized protein | Uncharacterized protein | | afdb-uniprot50 | AF-C9MV95-F1-MODEL\_V4 | 1.0 | 1.65e-14 | 617 | 0.209 | 172 | 125 | 5 | 4 | 168 | 7 | 174 | Putative phage major tail tube protein | Putative phage major tail tube protein | | afdb-uniprot50 | AF-A0A5S9QVA0-F1-MODEL\_V4 | 1.0 | 2.67e-13 | 615 | 0.291 | 168 | 115 | 3 | 1 | 167 | 1 | 165 | Uncharacterized protein | Uncharacterized protein | | afdb-uniprot50 | AF-R5BQ89-F1-MODEL\_V4 | 1.0 | 3.764e-14 | 615 | 0.248 | 177 | 120 | 7 | 1 | 168 | 1 | 173 | Major tail tube protein | Major tail tube protein | | afdb-uniprot50 | AF-A0A377AJ06-F1-MODEL\_V4 | 1.0 | 3.575e-14 | 615 | 0.324 | 179 | 107 | 6 | 1 | 167 | 1 | 177 | Phage major tail tube protein | Phage major tail tube protein | | afdb-uniprot50 | AF-A0A316Q9G3-F1-MODEL\_V4 | 1.0 | 3.575e-14 | 612 | 0.211 | 175 | 128 | 4 | 1 | 168 | 4 | 175 | Phage tail protein | Phage tail protein | | afdb-uniprot50 | AF-A0A414Q2K2-F1-MODEL\_V4 | 1.0 | 5.986e-14 | 611 | 0.24 | 175 | 123 | 5 | 1 | 168 | 1 | 172 | Phage tail protein | Phage tail protein | | afdb-uniprot50 | AF-A0A1Q8Q269-F1-MODEL\_V4 | 1.0 | 8.588e-14 | 611 | 0.227 | 176 | 122 | 7 | 1 | 168 | 6 | 175 | Uncharacterized protein | Uncharacterized protein | | afdb-uniprot50 | AF-A0A0T9RBM0-F1-MODEL\_V4 | 1.0 | 1.514e-13 | 610 | 0.339 | 174 | 109 | 3 | 1 | 168 | 1 | 174 | Major tail tube protein | Major tail tube protein | | afdb-uniprot50 | AF-A0A376JS17-F1-MODEL\_V4 | 1.0 | 3.282e-13 | 608 | 0.333 | 153 | 96 | 5 | 1 | 148 | 1 | 152 | Phage major tail tube protein FII | Phage major tail tube protein FII | | afdb-uniprot50 | AF-A0A839IVJ8-F1-MODEL\_V4 | 1.0 | 4.472e-13 | 608 | 0.239 | 167 | 124 | 3 | 2 | 168 | 3 | 166 | Phage major tail tube protein | Phage major tail tube protein | | afdb-uniprot50 | AF-A0A7X5EZ69-F1-MODEL\_V4 | 1.0 | 1.96e-13 | 608 | 0.171 | 181 | 135 | 4 | 3 | 168 | 1 | 181 | Uncharacterized protein | Uncharacterized protein | | afdb-uniprot50 | AF-A0A2P8EI37-F1-MODEL\_V4 | 1.0 | 7.747e-14 | 607 | 0.222 | 175 | 123 | 7 | 1 | 168 | 1 | 169 | Uncharacterized protein | Uncharacterized protein | | afdb-uniprot50 | AF-A0A417ISQ5-F1-MODEL\_V4 | 1.0 | 1.438e-13 | 606 | 0.245 | 175 | 121 | 5 | 1 | 168 | 1 | 171 | Phage tail protein | Phage tail protein | | afdb-uniprot50 | AF-A0A7U7ZB02-F1-MODEL\_V4 | 1.0 | 2.96e-13 | 606 | 0.244 | 172 | 120 | 6 | 1 | 168 | 4 | 169 | Phage tail protein | Phage tail protein | | afdb-uniprot50 | AF-A0A6N7VZ75-F1-MODEL\_V4 | 1.0 | 1.438e-13 | 605 | 0.264 | 174 | 115 | 5 | 1 | 167 | 2 | 169 | Phage tail protein | Phage tail protein | | afdb-uniprot50 | AF-A0A7K1RPH2-F1-MODEL\_V4 | 1.0 | 4.472e-13 | 604 | 0.2 | 175 | 132 | 5 | 1 | 167 | 1 | 175 | Phage tail protein | Phage tail protein | | afdb-uniprot50 | AF-A0A4P7L0L1-F1-MODEL\_V4 | 1.0 | 4.957e-13 | 603 | 0.348 | 149 | 94 | 2 | 23 | 168 | 2 | 150 | Phage tail tube protein FII | Phage tail tube protein FII | | afdb-uniprot50 | AF-A0A4U8S350-F1-MODEL\_V4 | 1.0 | 1.514e-13 | 603 | 0.218 | 169 | 124 | 5 | 1 | 166 | 3 | 166 | Phage tail protein | Phage tail protein | | afdb-uniprot50 | AF-A0A7C8KSM3-F1-MODEL\_V4 | 1.0 | 1.679e-13 | 603 | 0.217 | 175 | 127 | 4 | 1 | 168 | 4 | 175 | Phage tail protein | Phage tail protein | | afdb-uniprot50 | AF-A0A4V2ZH72-F1-MODEL\_V4 | 1.0 | 4.033e-13 | 602 | 0.222 | 166 | 127 | 2 | 1 | 166 | 2 | 165 | Phage tail protein | Phage tail protein | | afdb-uniprot50 | AF-A0A0B6D149-F1-MODEL\_V4 | 1.0 | 1.438e-13 | 600 | 0.264 | 170 | 117 | 6 | 1 | 168 | 8 | 171 | Phage tail tube FII family protein | Phage tail tube FII family protein | | afdb-uniprot50 | AF-A0A4U8YRD6-F1-MODEL\_V4 | 1.0 | 1.679e-13 | 600 | 0.232 | 176 | 123 | 7 | 1 | 168 | 1 | 172 | Tail tube protein | Tail tube protein | | afdb-uniprot50 | AF-A0A413EDA9-F1-MODEL\_V4 | 1.0 | 8.157e-14 | 600 | 0.262 | 175 | 118 | 5 | 1 | 168 | 2 | 172 | Phage tail protein | Phage tail protein | | afdb-uniprot50 | AF-A0A662Z5W3-F1-MODEL\_V4 | 1.0 | 1.594e-13 | 599 | 0.232 | 176 | 122 | 6 | 1 | 168 | 5 | 175 | Uncharacterized protein | Uncharacterized protein | | afdb-uniprot50 | AF-A0A840PWU5-F1-MODEL\_V4 | 1.0 | 3.282e-13 | 598 | 0.211 | 175 | 126 | 5 | 1 | 168 | 6 | 175 | Uncharacterized protein | Uncharacterized protein | | afdb-uniprot50 | AF-A0A7W6IIC9-F1-MODEL\_V4 | 1.0 | 6.415e-13 | 597 | 0.231 | 177 | 125 | 7 | 1 | 168 | 1 | 175 | Uncharacterized protein | Uncharacterized protein | | afdb-uniprot50 | AF-A0A212LD32-F1-MODEL\_V4 | 1.0 | 7.488e-13 | 596 | 0.192 | 166 | 131 | 3 | 6 | 168 | 8 | 173 | Uncharacterized protein | Uncharacterized protein | | afdb-uniprot50 | AF-R5BKU1-F1-MODEL\_V4 | 1.0 | 2.67e-13 | 596 | 0.255 | 176 | 117 | 6 | 2 | 168 | 5 | 175 | Uncharacterized protein | Uncharacterized protein | | afdb-uniprot50 | AF-A0A7X2TC80-F1-MODEL\_V4 | 1.0 | 6.637e-14 | 594 | 0.255 | 172 | 119 | 5 | 1 | 165 | 1 | 170 | Phage tail protein | Phage tail protein | | afdb-uniprot50 | AF-A0A0D6ARX0-F1-MODEL\_V4 | 1.0 | 9.043e-14 | 593 | 0.23 | 156 | 114 | 4 | 7 | 157 | 5 | 159 | Putative phage tail core protein | Putative phage tail core protein | | afdb-uniprot50 | AF-A0A268TWR0-F1-MODEL\_V4 | 1.0 | 8.302e-13 | 593 | 0.17 | 170 | 132 | 5 | 1 | 166 | 6 | 170 | Uncharacterized protein | Uncharacterized protein | | afdb-uniprot50 | AF-A0A1E4M047-F1-MODEL\_V4 | 1.0 | 8.741e-13 | 592 | 0.213 | 169 | 126 | 4 | 6 | 167 | 6 | 174 | Uncharacterized protein | Uncharacterized protein | | afdb-uniprot50 | AF-A0A376RTK4-F1-MODEL\_V4 | 1.0 | 1.02e-12 | 591 | 0.324 | 151 | 95 | 4 | 1 | 146 | 1 | 149 | Phage major tail tube protein | Phage major tail tube protein | | afdb-uniprot50 | AF-A0A2A4XT18-F1-MODEL\_V4 | 1.0 | 4.957e-13 | 591 | 0.282 | 170 | 116 | 4 | 1 | 168 | 1 | 166 | Uncharacterized protein | Uncharacterized protein | | afdb-uniprot50 | AF-A0A413FZE2-F1-MODEL\_V4 | 1.0 | 2.536e-13 | 591 | 0.226 | 172 | 122 | 5 | 1 | 166 | 4 | 170 | Uncharacterized protein | Uncharacterized protein | | afdb-uniprot50 | AF-A0A1M7RI12-F1-MODEL\_V4 | 1.0 | 1.366e-13 | 591 | 0.205 | 170 | 123 | 5 | 7 | 168 | 9 | 174 | Uncharacterized protein | Uncharacterized protein | | afdb-uniprot50 | AF-B0UK47-F1-MODEL\_V4 | 1.0 | 8.741e-13 | 588 | 0.228 | 175 | 128 | 5 | 1 | 168 | 1 | 175 | Major tail tube protein | Major tail tube protein | | afdb-uniprot50 | AF-A0A2G1CVI0-F1-MODEL\_V4 | 1.0 | 1.438e-13 | 587 | 0.233 | 171 | 120 | 6 | 1 | 166 | 7 | 171 | Uncharacterized protein | Uncharacterized protein | | afdb-uniprot50 | AF-U4T7P1-F1-MODEL\_V4 | 1.0 | 2.173e-13 | 587 | 0.248 | 165 | 117 | 4 | 7 | 166 | 11 | 173 | Putative bacteriophage tail core protein | Putative bacteriophage tail core protein | | afdb-uniprot50 | AF-A0A355TQV7-F1-MODEL\_V4 | 1.0 | 1.861e-13 | 586 | 0.242 | 165 | 119 | 4 | 7 | 166 | 50 | 213 | Uncharacterized protein | Uncharacterized protein | | afdb-uniprot50 | AF-A0A1A6FJS5-F1-MODEL\_V4 | 1.0 | 1.464e-12 | 584 | 0.215 | 176 | 130 | 5 | 1 | 168 | 1 | 176 | Uncharacterized protein | Uncharacterized protein | | afdb-uniprot50 | AF-A0A385Q1F7-F1-MODEL\_V4 | 1.0 | 1.111e-13 | 583 | 0.255 | 172 | 119 | 4 | 1 | 165 | 1 | 170 | Phage tail protein | Phage tail protein | | afdb-uniprot50 | AF-A0A1Q5XTX7-F1-MODEL\_V4 | 1.0 | 3.117e-13 | 582 | 0.238 | 176 | 121 | 6 | 1 | 168 | 1 | 171 | Phage tail protein | Phage tail protein | | afdb-uniprot50 | AF-A0A1L8CRR8-F1-MODEL\_V4 | 1.0 | 3.455e-13 | 582 | 0.219 | 173 | 124 | 5 | 3 | 168 | 4 | 172 | Phage tail protein | Phage tail protein | | afdb-uniprot50 | AF-A0A4Q2SZY2-F1-MODEL\_V4 | 1.0 | 1.995e-12 | 582 | 0.228 | 175 | 125 | 6 | 1 | 167 | 1 | 173 | Uncharacterized protein | Uncharacterized protein | | afdb-uniprot50 | AF-A0A098MDI9-F1-MODEL\_V4 | 1.0 | 2.96e-13 | 581 | 0.227 | 176 | 121 | 7 | 1 | 168 | 4 | 172 | Tail protein | Tail protein | | afdb-uniprot50 | AF-A0A1S0V4H2-F1-MODEL\_V4 | 1.0 | 1.894e-12 | 580 | 0.474 | 118 | 62 | 0 | 51 | 168 | 1 | 118 | Uncharacterized protein | Uncharacterized protein | | afdb-uniprot50 | AF-A0A7M3MBM7-F1-MODEL\_V4 | 1.0 | 3.282e-13 | 580 | 0.222 | 175 | 123 | 7 | 1 | 167 | 2 | 171 | Phage tail protein | Phage tail protein | | afdb-uniprot50 | AF-A0A3L7AKR1-F1-MODEL\_V4 | 1.0 | 3.703e-12 | 579 | 0.21 | 176 | 130 | 5 | 1 | 168 | 4 | 178 | Phage tail protein | Phage tail protein | | afdb-uniprot50 | AF-A0A2D3WHP5-F1-MODEL\_V4 | 1.0 | 9.204e-13 | 577 | 0.213 | 169 | 126 | 5 | 1 | 166 | 5 | 169 | Uncharacterized protein | Uncharacterized protein | | afdb-uniprot50 | AF-A0A2N0CX46-F1-MODEL\_V4 | 1.0 | 6.093e-13 | 576 | 0.295 | 169 | 108 | 5 | 1 | 167 | 1 | 160 | Phage tail protein | Phage tail protein | | afdb-uniprot50 | AF-A0A2E3R286-F1-MODEL\_V4 | 1.0 | 1.056e-13 | 574 | 0.192 | 171 | 131 | 4 | 1 | 165 | 2 | 171 | Uncharacterized protein | Uncharacterized protein | | afdb-uniprot50 | AF-B3QTJ5-F1-MODEL\_V4 | 1.0 | 3.638e-13 | 573 | 0.237 | 164 | 119 | 4 | 1 | 160 | 4 | 165 | Phage major tail tube protein | Phage major tail tube protein | | afdb-uniprot50 | AF-A0A1D2QMU1-F1-MODEL\_V4 | 1.0 | 2.1e-12 | 573 | 0.222 | 171 | 126 | 4 | 1 | 168 | 3 | 169 | Uncharacterized protein | Uncharacterized protein | | afdb-uniprot50 | AF-A0A064AK82-F1-MODEL\_V4 | 1.0 | 2.96e-13 | 573 | 0.218 | 174 | 124 | 5 | 1 | 166 | 2 | 171 | Major tail tube protein | Major tail tube protein | | afdb-uniprot50 | AF-D1AFA7-F1-MODEL\_V4 | 1.0 | 2.811e-13 | 573 | 0.208 | 173 | 127 | 4 | 1 | 166 | 3 | 172 | Major tail tube protein | Major tail tube protein | | afdb-uniprot50 | AF-A0A1Q6JPJ0-F1-MODEL\_V4 | 1.0 | 1.366e-13 | 573 | 0.263 | 171 | 116 | 5 | 1 | 165 | 8 | 174 | Phage tail protein | Phage tail protein | | afdb-uniprot50 | AF-G1UXP6-F1-MODEL\_V4 | 1.0 | 8.741e-13 | 572 | 0.217 | 179 | 124 | 7 | 1 | 168 | 5 | 178 | Uncharacterized protein | Uncharacterized protein | | afdb-uniprot50 | AF-A0A496JWV3-F1-MODEL\_V4 | 1.0 | 1.623e-12 | 571 | 0.267 | 146 | 103 | 3 | 25 | 168 | 2 | 145 | Phage tail protein | Phage tail protein | | afdb-uniprot50 | AF-A0A2P7V3M3-F1-MODEL\_V4 | 1.0 | 1.131e-12 | 570 | 0.228 | 175 | 122 | 6 | 2 | 168 | 5 | 174 | Phage tail protein | Phage tail protein | | afdb-uniprot50 | AF-A0A7C3GHU1-F1-MODEL\_V4 | 1.0 | 3.117e-13 | 570 | 0.224 | 174 | 128 | 5 | 1 | 168 | 4 | 176 | Phage major tail tube protein | Phage major tail tube protein | | afdb-uniprot50 | AF-A0A2E2N2N1-F1-MODEL\_V4 | 1.0 | 5.22e-13 | 569 | 0.209 | 172 | 125 | 5 | 1 | 165 | 2 | 169 | Phage tail protein | Phage tail protein | | afdb-uniprot50 | AF-A0A0C2UPV3-F1-MODEL\_V4 | 1.0 | 2.536e-13 | 568 | 0.233 | 163 | 111 | 7 | 6 | 166 | 9 | 159 | Uncharacterized protein | Uncharacterized protein | | afdb-uniprot50 | AF-A0A5N3PH51-F1-MODEL\_V4 | 1.0 | 2.581e-12 | 567 | 0.204 | 176 | 128 | 6 | 1 | 167 | 4 | 176 | Uncharacterized protein | Uncharacterized protein | | afdb-uniprot50 | AF-A0A3G2R410-F1-MODEL\_V4 | 1.0 | 7.885e-13 | 566 | 0.208 | 173 | 126 | 6 | 1 | 166 | 1 | 169 | Phage tail protein | Phage tail protein | | afdb-uniprot50 | AF-A0A4P8G1X0-F1-MODEL\_V4 | 1.0 | 2.328e-12 | 565 | 0.226 | 159 | 122 | 1 | 9 | 167 | 1 | 158 | Uncharacterized protein | Uncharacterized protein | | afdb-uniprot50 | AF-A0A0X1U7S9-F1-MODEL\_V4 | 1.0 | 2.96e-13 | 563 | 0.267 | 172 | 116 | 6 | 1 | 165 | 3 | 171 | Phage tail tube protein FII | Phage tail tube protein FII | | afdb-uniprot50 | AF-A0A8B2NVA0-F1-MODEL\_V4 | 1.0 | 2.211e-12 | 563 | 0.203 | 172 | 126 | 5 | 2 | 168 | 6 | 171 | Uncharacterized protein | Uncharacterized protein | | afdb-uniprot50 | AF-A0A0D7X7K2-F1-MODEL\_V4 | 1.0 | 5.22e-13 | 563 | 0.232 | 176 | 120 | 8 | 1 | 168 | 6 | 174 | Uncharacterized protein | Uncharacterized protein | | afdb-uniprot50 | AF-B6WRQ4-F1-MODEL\_V4 | 1.0 | 3.282e-13 | 560 | 0.145 | 172 | 139 | 4 | 1 | 167 | 22 | 190 | Putative phage major tail tube protein | Putative phage major tail tube protein | | afdb-uniprot50 | AF-A0A316DHI7-F1-MODEL\_V4 | 1.0 | 9.69e-13 | 558 | 0.215 | 176 | 123 | 8 | 1 | 168 | 4 | 172 | Uncharacterized protein | Uncharacterized protein | | afdb-uniprot50 | AF-A0A812QV43-F1-MODEL\_V4 | 1.0 | 3.517e-12 | 558 | 0.345 | 136 | 88 | 1 | 23 | 157 | 393 | 528 | GpFI protein | GpFI protein | | afdb-uniprot50 | AF-A0A327JAQ8-F1-MODEL\_V4 | 1.0 | 1.995e-12 | 557 | 0.22 | 168 | 122 | 6 | 6 | 167 | 4 | 168 | Uncharacterized protein | Uncharacterized protein | | afdb-uniprot50 | AF-A0A7J0BHY9-F1-MODEL\_V4 | 1.0 | 1.39e-12 | 557 | 0.193 | 176 | 130 | 6 | 1 | 168 | 1 | 172 | Uncharacterized protein | Uncharacterized protein | | afdb-uniprot50 | AF-A0A5N3S1K2-F1-MODEL\_V4 | 1.0 | 1.541e-12 | 555 | 0.179 | 162 | 130 | 2 | 1 | 162 | 1 | 159 | Uncharacterized protein | Uncharacterized protein | | afdb-uniprot50 | AF-A0A373CZ49-F1-MODEL\_V4 | 1.0 | 6.093e-13 | 555 | 0.273 | 172 | 116 | 5 | 1 | 165 | 4 | 173 | Phage tail protein | Phage tail protein | | afdb-uniprot50 | AF-D6KLG9-F1-MODEL\_V4 | 1.0 | 1.799e-12 | 554 | 0.221 | 176 | 122 | 6 | 3 | 168 | 1 | 171 | Putative phage major tail tube protein | Putative phage major tail tube protein | | afdb-uniprot50 | AF-A0A1V4GES7-F1-MODEL\_V4 | 1.0 | 8.302e-13 | 554 | 0.238 | 176 | 120 | 6 | 1 | 167 | 2 | 172 | Uncharacterized protein | Uncharacterized protein | | afdb-uniprot50 | AF-A0A285MEY3-F1-MODEL\_V4 | 1.0 | 2.861e-12 | 553 | 0.226 | 168 | 121 | 4 | 6 | 168 | 8 | 171 | Phage tail tube protein FII | Phage tail tube protein FII | | afdb-uniprot50 | AF-A0A268ELC0-F1-MODEL\_V4 | 1.0 | 1.074e-12 | 553 | 0.215 | 176 | 124 | 7 | 1 | 167 | 1 | 171 | Uncharacterized protein | Uncharacterized protein | | afdb-uniprot50 | AF-A0A329UB98-F1-MODEL\_V4 | 1.0 | 8.741e-13 | 553 | 0.17 | 170 | 133 | 4 | 2 | 165 | 9 | 176 | Phage tail protein | Phage tail protein | | afdb-uniprot50 | AF-A0A845STF4-F1-MODEL\_V4 | 1.0 | 2.581e-12 | 550 | 0.205 | 175 | 126 | 5 | 1 | 168 | 4 | 172 | Phage tail protein | Phage tail protein | | afdb-uniprot50 | AF-A0A2N0CX99-F1-MODEL\_V4 | 1.0 | 2.328e-12 | 548 | 0.301 | 166 | 105 | 5 | 1 | 164 | 1 | 157 | Phage tail protein | Phage tail protein | | afdb-uniprot50 | AF-A0A1B9NZR8-F1-MODEL\_V4 | 1.0 | 2.581e-12 | 548 | 0.175 | 165 | 133 | 2 | 1 | 165 | 1 | 162 | Uncharacterized protein | Uncharacterized protein | | afdb-uniprot50 | AF-A0A0M7ALA7-F1-MODEL\_V4 | 1.0 | 7.621e-12 | 548 | 0.173 | 173 | 132 | 6 | 2 | 168 | 3 | 170 | Major tail tube protein | Major tail tube protein | | afdb-uniprot50 | AF-A0A6L9HFW7-F1-MODEL\_V4 | 1.0 | 2.718e-12 | 547 | 0.194 | 175 | 130 | 6 | 1 | 168 | 2 | 172 | Phage tail protein | Phage tail protein | | afdb-uniprot50 | AF-A0A2Y0TYM0-F1-MODEL\_V4 | 1.0 | 1.709e-12 | 547 | 0.278 | 169 | 109 | 4 | 1 | 165 | 1 | 160 | Major tail tube protein FII | Major tail tube protein FII | | afdb-uniprot50 | AF-A0A1B8VX25-F1-MODEL\_V4 | 1.0 | 2.718e-12 | 546 | 0.181 | 176 | 132 | 6 | 1 | 168 | 1 | 172 | Uncharacterized protein | Uncharacterized protein | | afdb-uniprot50 | AF-A0A5A8F086-F1-MODEL\_V4 | 1.0 | 1.32e-12 | 545 | 0.173 | 173 | 136 | 5 | 1 | 168 | 1 | 171 | Uncharacterized protein | Uncharacterized protein | | afdb-uniprot50 | AF-G4KQ74-F1-MODEL\_V4 | 1.0 | 2.1e-12 | 544 | 0.205 | 175 | 124 | 6 | 1 | 165 | 2 | 171 | Putative tail tube protein | Putative tail tube protein | | afdb-uniprot50 | AF-A0A1N7A8E0-F1-MODEL\_V4 | 1.0 | 1.995e-12 | 544 | 0.194 | 175 | 126 | 7 | 1 | 167 | 4 | 171 | Uncharacterized protein | Uncharacterized protein | | afdb-uniprot50 | AF-A0A5S4VNC9-F1-MODEL\_V4 | 1.0 | 2.96e-13 | 543 | 0.229 | 170 | 124 | 4 | 1 | 165 | 3 | 170 | Phage tail protein | Phage tail protein | | afdb-uniprot50 | AF-A0A163TB63-F1-MODEL\_V4 | 1.0 | 4.551e-12 | 543 | 0.217 | 175 | 126 | 6 | 1 | 168 | 1 | 171 | Bacteriophage protein | Bacteriophage protein | | afdb-uniprot50 | AF-R5GLL1-F1-MODEL\_V4 | 1.0 | 3.34e-12 | 543 | 0.202 | 173 | 128 | 5 | 1 | 167 | 2 | 170 | Putative phage major tail tube protein | Putative phage major tail tube protein | | afdb-uniprot50 | AF-A0A7X6JBC6-F1-MODEL\_V4 | 1.0 | 5.593e-12 | 542 | 0.383 | 133 | 79 | 1 | 36 | 168 | 2 | 131 | Phage major tail tube protein | Phage major tail tube protein | | afdb-uniprot50 | AF-E6X1M7-F1-MODEL\_V4 | 1.0 | 2.718e-12 | 542 | 0.218 | 165 | 118 | 5 | 6 | 166 | 9 | 166 | Major tail tube protein | Major tail tube protein | | afdb-uniprot50 | AF-A0A3D5LTH1-F1-MODEL\_V4 | 1.0 | 2.288e-13 | 542 | 0.217 | 170 | 126 | 5 | 2 | 165 | 5 | 173 | Phage tail protein | Phage tail protein | | afdb-uniprot50 | AF-A0A3C1WQU9-F1-MODEL\_V4 | 1.0 | 1.131e-12 | 542 | 0.232 | 172 | 122 | 5 | 2 | 165 | 10 | 179 | Phage tail protein | Phage tail protein | | afdb-uniprot50 | AF-A0A611ERL2-F1-MODEL\_V4 | 1.0 | 3.172e-12 | 541 | 0.34 | 150 | 91 | 5 | 1 | 144 | 1 | 148 | Phage major tail tube protein | Phage major tail tube protein | | afdb-uniprot50 | AF-D6LHL5-F1-MODEL\_V4 | 1.0 | 4.957e-13 | 541 | 0.212 | 174 | 123 | 8 | 1 | 166 | 1 | 168 | Phage-related contractile tail tube protein | Phage-related contractile tail tube protein | | afdb-uniprot50 | AF-A0A833A242-F1-MODEL\_V4 | 1.0 | 1.32e-12 | 540 | 0.195 | 174 | 131 | 5 | 1 | 168 | 2 | 172 | Phage tail protein | Phage tail protein | | afdb-uniprot50 | AF-A0A844Q9G1-F1-MODEL\_V4 | 1.0 | 3.013e-12 | 538 | 0.259 | 177 | 116 | 8 | 1 | 167 | 1 | 172 | Phage tail protein | Phage tail protein | | afdb-uniprot50 | AF-A0A1T4WVE5-F1-MODEL\_V4 | 1.0 | 9.367e-12 | 537 | 0.201 | 149 | 115 | 4 | 1 | 146 | 3 | 150 | Uncharacterized protein | Uncharacterized protein | | afdb-uniprot50 | AF-A0A1I5MNT8-F1-MODEL\_V4 | 1.0 | 4.033e-13 | 537 | 0.235 | 170 | 124 | 4 | 1 | 165 | 1 | 169 | Uncharacterized protein | Uncharacterized protein | | afdb-uniprot50 | AF-A0A174PJS3-F1-MODEL\_V4 | 1.0 | 1.541e-12 | 536 | 0.217 | 175 | 123 | 8 | 1 | 165 | 1 | 171 | Phage major tail tube protein | Phage major tail tube protein | | afdb-uniprot50 | AF-A0A660NLN2-F1-MODEL\_V4 | 1.0 | 1.415e-11 | 534 | 0.663 | 116 | 39 | 0 | 1 | 116 | 1 | 116 | Phage major tail tube protein | Phage major tail tube protein | | afdb-uniprot50 | AF-A0A2X1PJG7-F1-MODEL\_V4 | 1.0 | 3.703e-12 | 534 | 0.312 | 157 | 101 | 5 | 15 | 167 | 2 | 155 | Major tail tube protein FII | Major tail tube protein FII | | afdb-uniprot50 | AF-A0A1A9AX50-F1-MODEL\_V4 | 1.0 | 2.766e-11 | 532 | 0.216 | 162 | 124 | 3 | 2 | 162 | 3 | 162 | Phage tail protein | Phage tail protein | | afdb-uniprot50 | AF-A0A842IW21-F1-MODEL\_V4 | 1.0 | 8.302e-13 | 532 | 0.215 | 172 | 126 | 5 | 1 | 168 | 2 | 168 | Phage major tail tube protein | Phage major tail tube protein | | afdb-uniprot50 | AF-A0A078MHI9-F1-MODEL\_V4 | 1.0 | 3.703e-12 | 532 | 0.184 | 173 | 129 | 6 | 3 | 168 | 1 | 168 | Phage tail tube protein FII | Phage tail tube protein FII | | afdb-uniprot50 | AF-A0A2D3W2J9-F1-MODEL\_V4 | 1.0 | 3.703e-12 | 528 | 0.26 | 161 | 108 | 6 | 7 | 166 | 10 | 160 | Uncharacterized protein | Uncharacterized protein | | afdb-uniprot50 | AF-A0A061NPQ1-F1-MODEL\_V4 | 1.0 | 2.451e-12 | 528 | 0.238 | 172 | 118 | 6 | 1 | 165 | 4 | 169 | Phage major tail tube protein | Phage major tail tube protein | | afdb-uniprot50 | AF-A0A4V2KL57-F1-MODEL\_V4 | 1.0 | 1.212e-11 | 528 | 0.217 | 175 | 125 | 6 | 1 | 168 | 2 | 171 | Phage tail protein | Phage tail protein | | afdb-uniprot50 | AF-G9PUI5-F1-MODEL\_V4 | 1.0 | 6.201e-12 | 528 | 0.195 | 174 | 129 | 6 | 1 | 167 | 2 | 171 | Phage major tail tube protein | Phage major tail tube protein | | afdb-uniprot50 | AF-A0A5T0IXF5-F1-MODEL\_V4 | 1.0 | 1.831e-11 | 526 | 0.245 | 155 | 108 | 5 | 18 | 168 | 2 | 151 | Phage tail protein | Phage tail protein | | afdb-uniprot50 | AF-A0A6L5TDP9-F1-MODEL\_V4 | 1.0 | 2.451e-12 | 526 | 0.235 | 170 | 121 | 5 | 2 | 165 | 5 | 171 | Phage tail protein | Phage tail protein | | afdb-uniprot50 | AF-A0A6N8K4V4-F1-MODEL\_V4 | 1.0 | 1.928e-11 | 525 | 0.331 | 145 | 90 | 4 | 29 | 168 | 1 | 143 | Phage major tail tube protein | Phage major tail tube protein | | afdb-uniprot50 | AF-R7HWW0-F1-MODEL\_V4 | 1.0 | 2.451e-12 | 525 | 0.201 | 174 | 128 | 5 | 1 | 165 | 1 | 172 | Phage tail tube protein FII | Phage tail tube protein FII | | afdb-uniprot50 | AF-A0A2V2FVX9-F1-MODEL\_V4 | 1.0 | 1.276e-11 | 525 | 0.198 | 176 | 129 | 6 | 1 | 168 | 2 | 173 | Uncharacterized protein | Uncharacterized protein | | afdb-uniprot50 | AF-A0A0D2GJP7-F1-MODEL\_V4 | 1.0 | 1.569e-11 | 525 | 0.22 | 172 | 123 | 4 | 4 | 168 | 7 | 174 | Tail protein | Tail protein | | afdb-uniprot50 | AF-A0A1T2XA25-F1-MODEL\_V4 | 1.0 | 8.449e-12 | 524 | 0.193 | 176 | 128 | 7 | 1 | 168 | 6 | 175 | Phage tail protein | Phage tail protein | | afdb-uniprot50 | AF-E3HBL5-F1-MODEL\_V4 | 1.0 | 7.621e-12 | 521 | 0.232 | 172 | 120 | 6 | 4 | 167 | 5 | 172 | Major tail tube protein | Major tail tube protein | | afdb-uniprot50 | AF-A0A7I8DLE1-F1-MODEL\_V4 | 1.0 | 7.621e-12 | 521 | 0.203 | 177 | 129 | 6 | 1 | 168 | 2 | 175 | Bacteriophage protein | Bacteriophage protein | | afdb-uniprot50 | AF-A0A8B2TUX0-F1-MODEL\_V4 | 1.0 | 5.593e-12 | 520 | 0.252 | 170 | 121 | 3 | 1 | 168 | 1 | 166 | Phage tail protein | Phage tail protein | | afdb-uniprot50 | AF-E5VKJ4-F1-MODEL\_V4 | 1.0 | 1.464e-12 | 520 | 0.222 | 171 | 127 | 4 | 1 | 165 | 2 | 172 | Phage major tail tube protein | Phage major tail tube protein | | afdb-uniprot50 | AF-A0A3D4L176-F1-MODEL\_V4 | 1.0 | 8.896e-12 | 516 | 0.204 | 176 | 127 | 7 | 2 | 168 | 4 | 175 | Phage tail protein | Phage tail protein | | afdb-uniprot50 | AF-A0A6I0DW72-F1-MODEL\_V4 | 1.0 | 3.399e-11 | 514 | 0.207 | 169 | 124 | 5 | 6 | 168 | 6 | 170 | Uncharacterized protein | Uncharacterized protein | | afdb-uniprot50 | AF-A0A087M4E0-F1-MODEL\_V4 | 1.0 | 5.994e-11 | 514 | 0.174 | 178 | 134 | 7 | 1 | 167 | 1 | 176 | Uncharacterized protein | Uncharacterized protein | | afdb-uniprot50 | AF-A0A7T5JQ37-F1-MODEL\_V4 | 1.0 | 2.137e-11 | 511 | 0.237 | 177 | 121 | 6 | 1 | 168 | 2 | 173 | Phage major tail tube protein | Phage major tail tube protein | | afdb-uniprot50 | AF-D1Y708-F1-MODEL\_V4 | 1.0 | 1.739e-11 | 510 | 0.241 | 174 | 119 | 6 | 1 | 166 | 2 | 170 | Putative phage major tail tube protein | Putative phage major tail tube protein | | afdb-uniprot50 | AF-A0A7X4ZQ89-F1-MODEL\_V4 | 1.0 | 1.212e-11 | 509 | 0.223 | 152 | 108 | 4 | 22 | 165 | 4 | 153 | Phage tail protein | Phage tail protein | | afdb-uniprot50 | AF-A0A5C8BE13-F1-MODEL\_V4 | 1.0 | 1.541e-12 | 506 | 0.191 | 172 | 130 | 6 | 1 | 165 | 1 | 170 | Uncharacterized protein | Uncharacterized protein | | afdb-uniprot50 | AF-A0A6P0MHV0-F1-MODEL\_V4 | 1.0 | 4.632e-11 | 506 | 0.193 | 176 | 132 | 5 | 1 | 168 | 1 | 174 | Uncharacterized protein | Uncharacterized protein | | afdb-uniprot50 | AF-E6L5Z4-F1-MODEL\_V4 | 1.0 | 3.579e-11 | 505 | 0.207 | 169 | 124 | 7 | 3 | 168 | 1 | 162 | Phage major tail tube protein | Phage major tail tube protein | | afdb-uniprot50 | AF-A0A349HHP9-F1-MODEL\_V4 | 1.0 | 1.928e-11 | 505 | 0.168 | 178 | 133 | 8 | 1 | 168 | 1 | 173 | Phage tail protein | Phage tail protein | | afdb-uniprot50 | AF-A0A3A9BH43-F1-MODEL\_V4 | 1.0 | 1.344e-11 | 505 | 0.175 | 171 | 129 | 6 | 4 | 165 | 9 | 176 | Uncharacterized protein | Uncharacterized protein | | afdb-uniprot50 | AF-A0A4R2GWP5-F1-MODEL\_V4 | 1.0 | 6.875e-12 | 504 | 0.215 | 167 | 120 | 6 | 2 | 158 | 3 | 168 | Phage tail tube protein FII | Phage tail tube protein FII | | afdb-uniprot50 | AF-A0A173SC52-F1-MODEL\_V4 | 1.0 | 1.831e-11 | 504 | 0.207 | 169 | 124 | 5 | 4 | 166 | 62 | 226 | Phage major tail tube protein | Phage major tail tube protein | | afdb-uniprot50 | AF-A0A1W2EJX0-F1-MODEL\_V4 | 1.0 | 4.632e-11 | 502 | 0.182 | 175 | 130 | 7 | 1 | 168 | 4 | 172 | Uncharacterized protein | Uncharacterized protein | | afdb-uniprot50 | AF-E2CJG9-F1-MODEL\_V4 | 1.0 | 2.495e-11 | 501 | 0.18 | 166 | 125 | 6 | 10 | 168 | 4 | 165 | Major tail tube protein | Major tail tube protein | | afdb-uniprot50 | AF-A0A139D6K0-F1-MODEL\_V4 | 1.0 | 4.551e-12 | 501 | 0.188 | 170 | 132 | 4 | 1 | 165 | 3 | 171 | Major tail tube protein | Major tail tube protein | | afdb-uniprot50 | AF-A0A5M9TYS6-F1-MODEL\_V4 | 1.0 | 2.627e-11 | 501 | 0.175 | 177 | 132 | 7 | 1 | 168 | 1 | 172 | Uncharacterized protein | Uncharacterized protein | | afdb-uniprot50 | AF-A0A1V3VT23-F1-MODEL\_V4 | 1.0 | 4.877e-11 | 500 | 0.288 | 135 | 91 | 3 | 21 | 152 | 2 | 134 | Phage tail protein | Phage tail protein | | afdb-uniprot50 | AF-S9TYX5-F1-MODEL\_V4 | 1.0 | 3.399e-11 | 500 | 0.165 | 175 | 132 | 7 | 3 | 168 | 5 | 174 | Major tail tube protein | Major tail tube protein | | afdb-uniprot50 | AF-A0A1I5RNP2-F1-MODEL\_V4 | 1.0 | 4.399e-11 | 499 | 0.203 | 167 | 123 | 6 | 1 | 166 | 1 | 158 | Phage contractile tail tube protein, P2 family | Phage contractile tail tube protein, P2 family | | afdb-uniprot50 | AF-A0A553SNF7-F1-MODEL\_V4 | 1.0 | 1.276e-11 | 499 | 0.209 | 172 | 126 | 4 | 1 | 165 | 1 | 169 | Phage tail protein | Phage tail protein | | afdb-uniprot50 | AF-A0A6N9P8Y1-F1-MODEL\_V4 | 1.0 | 7.621e-12 | 499 | 0.196 | 173 | 132 | 4 | 1 | 168 | 1 | 171 | Uncharacterized protein | Uncharacterized protein | | afdb-uniprot50 | AF-A0A7W6PS12-F1-MODEL\_V4 | 1.0 | 6.997e-11 | 498 | 0.184 | 168 | 126 | 4 | 6 | 168 | 8 | 169 | Uncharacterized protein | Uncharacterized protein | | afdb-uniprot50 | AF-A0A316ML96-F1-MODEL\_V4 | 1.0 | 4.551e-12 | 498 | 0.156 | 172 | 139 | 4 | 1 | 167 | 1 | 171 | Phage tail protein | Phage tail protein | | afdb-uniprot50 | AF-E2CFK4-F1-MODEL\_V4 | 1.0 | 2.137e-11 | 495 | 0.192 | 171 | 130 | 5 | 1 | 167 | 1 | 167 | Uncharacterized protein | Uncharacterized protein | | afdb-uniprot50 | AF-A0A7G9GXI4-F1-MODEL\_V4 | 1.0 | 7.238e-12 | 495 | 0.189 | 174 | 128 | 7 | 1 | 166 | 1 | 169 | Phage major tail tube protein | Phage major tail tube protein | | afdb-uniprot50 | AF-A0A369BH51-F1-MODEL\_V4 | 1.0 | 1.212e-11 | 495 | 0.18 | 177 | 131 | 6 | 1 | 166 | 1 | 174 | Uncharacterized protein | Uncharacterized protein | | afdb-uniprot50 | AF-A0A7J5WEE2-F1-MODEL\_V4 | 1.0 | 7.757e-11 | 495 | 0.222 | 175 | 122 | 8 | 2 | 167 | 6 | 175 | Major tail tube | Major tail tube | | afdb-uniprot50 | AF-A0A285M2K2-F1-MODEL\_V4 | 1.0 | 3.399e-11 | 492 | 0.238 | 176 | 119 | 8 | 1 | 168 | 1 | 169 | Phage contractile tail tube protein, P2 family | Phage contractile tail tube protein, P2 family | | afdb-uniprot50 | AF-A0A3A9CS87-F1-MODEL\_V4 | 1.0 | 4.551e-12 | 492 | 0.2 | 170 | 125 | 8 | 3 | 165 | 7 | 172 | Phage tail protein | Phage tail protein | | afdb-uniprot50 | AF-F5S5D1-F1-MODEL\_V4 | 1.0 | 1.299e-10 | 491 | 0.294 | 136 | 94 | 2 | 1 | 135 | 1 | 135 | Uncharacterized protein | Uncharacterized protein | | afdb-uniprot50 | AF-A0A7C4DIZ6-F1-MODEL\_V4 | 1.0 | 3.579e-11 | 491 | 0.236 | 148 | 105 | 4 | 28 | 168 | 2 | 148 | Uncharacterized protein | Uncharacterized protein | | afdb-uniprot50 | AF-A0A1Q6PWR7-F1-MODEL\_V4 | 1.0 | 1.739e-11 | 491 | 0.209 | 172 | 122 | 6 | 3 | 166 | 5 | 170 | Uncharacterized protein | Uncharacterized protein | | afdb-uniprot50 | AF-A0A3M4BUI2-F1-MODEL\_V4 | 1.0 | 3.46e-10 | 484 | 0.383 | 120 | 74 | 0 | 49 | 168 | 2 | 121 | Major tail tube protein | Major tail tube protein | | afdb-uniprot50 | AF-A0A4V6EN13-F1-MODEL\_V4 | 1.0 | 1.739e-11 | 484 | 0.19 | 173 | 131 | 6 | 1 | 165 | 1 | 172 | Phage major tail tube protein | Phage major tail tube protein | | afdb-uniprot50 | AF-A0A1M3AIS0-F1-MODEL\_V4 | 1.0 | 3.46e-10 | 483 | 0.201 | 154 | 117 | 3 | 8 | 155 | 2 | 155 | Phage tail protein | Phage tail protein | | afdb-uniprot50 | AF-A0A1H1D1J4-F1-MODEL\_V4 | 1.0 | 3.399e-11 | 481 | 0.132 | 166 | 133 | 6 | 2 | 158 | 6 | 169 | Uncharacterized protein | Uncharacterized protein | | afdb-uniprot50 | AF-A0A143HCW7-F1-MODEL\_V4 | 1.0 | 2.451e-12 | 480 | 0.224 | 169 | 125 | 4 | 2 | 165 | 4 | 171 | Uncharacterized protein | Uncharacterized protein | | afdb-uniprot50 | AF-A0A0S4XLP2-F1-MODEL\_V4 | 1.0 | 4.399e-11 | 478 | 0.177 | 169 | 127 | 5 | 3 | 168 | 5 | 164 | Uncharacterized protein | Uncharacterized protein | | afdb-uniprot50 | AF-A0A4D7B2K3-F1-MODEL\_V4 | 1.0 | 4.399e-11 | 478 | 0.222 | 171 | 127 | 6 | 1 | 167 | 1 | 169 | Uncharacterized protein | Uncharacterized protein | | afdb-uniprot50 | AF-A0A5M8P5M4-F1-MODEL\_V4 | 1.0 | 4.399e-11 | 476 | 0.175 | 160 | 123 | 5 | 7 | 158 | 10 | 168 | Uncharacterized protein | Uncharacterized protein | | afdb-uniprot50 | AF-A0A1Q6KUB1-F1-MODEL\_V4 | 1.0 | 9.863e-12 | 476 | 0.173 | 173 | 133 | 5 | 2 | 165 | 5 | 176 | Uncharacterized protein | Uncharacterized protein | | afdb-uniprot50 | AF-A0A5R8Y7D8-F1-MODEL\_V4 | 1.0 | 1.113e-10 | 473 | 0.18 | 177 | 128 | 9 | 1 | 167 | 1 | 170 | Uncharacterized protein | Uncharacterized protein | | afdb-uniprot50 | AF-A0A285M8B1-F1-MODEL\_V4 | 1.0 | 2.29e-10 | 469 | 0.177 | 175 | 130 | 8 | 1 | 166 | 1 | 170 | Phage tail tube protein FII | Phage tail tube protein FII | | afdb-uniprot50 | AF-A0A4R3EGB1-F1-MODEL\_V4 | 1.0 | 5.994e-11 | 469 | 0.205 | 180 | 126 | 7 | 1 | 167 | 1 | 176 | Uncharacterized protein | Uncharacterized protein | | afdb-uniprot50 | AF-A0A0Q7A9A4-F1-MODEL\_V4 | 1.0 | 3.643e-10 | 468 | 0.173 | 179 | 132 | 8 | 1 | 167 | 1 | 175 | Uncharacterized protein | Uncharacterized protein | | afdb-uniprot50 | AF-A0A448J7T4-F1-MODEL\_V4 | 1.0 | 5.794e-10 | 459 | 0.222 | 144 | 104 | 5 | 1 | 140 | 4 | 143 | Tail protein | Tail protein | | afdb-uniprot50 | AF-A0A268TJI4-F1-MODEL\_V4 | 1.0 | 8.312e-10 | 447 | 0.161 | 149 | 117 | 5 | 1 | 146 | 5 | 148 | Uncharacterized protein | Uncharacterized protein | | afdb-uniprot50 | AF-A0A1A9G1E2-F1-MODEL\_V4 | 1.0 | 1.057e-10 | 446 | 0.173 | 167 | 127 | 6 | 1 | 158 | 1 | 165 | Phage tail protein | Phage tail protein | | afdb-uniprot50 | AF-A0A1C6BND7-F1-MODEL\_V4 | 1.0 | 2.815e-10 | 442 | 0.192 | 171 | 126 | 7 | 2 | 165 | 5 | 170 | Phage major tail tube protein | Phage major tail tube protein | | afdb-uniprot50 | AF-A0A7Y9D078-F1-MODEL\_V4 | 1.0 | 3.769e-11 | 440 | 0.215 | 172 | 124 | 6 | 2 | 165 | 3 | 171 | P2 family phage contractile tail tube protein | P2 family phage contractile tail tube protein | | afdb-uniprot50 | AF-A0A0Q2UUR3-F1-MODEL\_V4 | 1.0 | 1.516e-10 | 439 | 0.161 | 167 | 130 | 5 | 1 | 158 | 1 | 166 | Uncharacterized protein | Uncharacterized protein | | afdb-uniprot50 | AF-A0A413PG50-F1-MODEL\_V4 | 1.0 | 2.29e-10 | 438 | 0.173 | 173 | 127 | 8 | 1 | 165 | 3 | 167 | Protoporphyrinogen oxidase | Protoporphyrinogen oxidase | | afdb-uniprot50 | AF-E3H9B4-F1-MODEL\_V4 | 1.0 | 5.503e-10 | 438 | 0.16 | 168 | 128 | 5 | 1 | 159 | 3 | 166 | Major tail tube protein | Major tail tube protein | | afdb-uniprot50 | AF-A0A2G6EZB0-F1-MODEL\_V4 | 1.0 | 1.597e-10 | 436 | 0.224 | 178 | 110 | 9 | 3 | 166 | 1 | 164 | Phage tail protein | Phage tail protein | | afdb-uniprot50 | AF-A0A2K1DEW6-F1-MODEL\_V4 | 1.0 | 7.498e-10 | 434 | 0.212 | 160 | 115 | 7 | 1 | 157 | 1 | 152 | Uncharacterized protein | Uncharacterized protein | | afdb-uniprot50 | AF-J0PYV7-F1-MODEL\_V4 | 1.0 | 3.344e-09 | 431 | 0.341 | 126 | 80 | 1 | 43 | 168 | 1 | 123 | Phage major tail tube protein | Phage major tail tube protein | | afdb-uniprot50 | AF-G1WGC6-F1-MODEL\_V4 | 1.0 | 1.172e-10 | 431 | 0.192 | 171 | 125 | 8 | 1 | 165 | 4 | 167 | Uncharacterized protein | Uncharacterized protein | | afdb-uniprot50 | AF-A0A345J3J5-F1-MODEL\_V4 | 1.0 | 2.455e-09 | 430 | 0.208 | 168 | 124 | 7 | 1 | 166 | 1 | 161 | Phage tail tube protein | Phage tail tube protein | | afdb-uniprot50 | AF-A0A0C1J3Q2-F1-MODEL\_V4 | 1.0 | 1.322e-09 | 428 | 0.156 | 160 | 125 | 5 | 8 | 158 | 8 | 166 | Uncharacterized protein | Uncharacterized protein | | afdb-uniprot50 | AF-A0A379X2H6-F1-MODEL\_V4 | 1.0 | 2.103e-09 | 426 | 0.242 | 140 | 101 | 3 | 33 | 168 | 1 | 139 | Tail protein | Tail protein | | afdb-uniprot50 | AF-A0A377HSE9-F1-MODEL\_V4 | 1.0 | 3.904e-09 | 425 | 0.392 | 125 | 71 | 3 | 47 | 168 | 1 | 123 | Phage major tail tube protein | Phage major tail tube protein | | afdb-uniprot50 | AF-A0A370DZ67-F1-MODEL\_V4 | 1.0 | 4.11e-09 | 420 | 0.46 | 102 | 55 | 0 | 67 | 168 | 7 | 108 | Uncharacterized protein | Uncharacterized protein | | afdb-uniprot50 | AF-A0A6L8LZW8-F1-MODEL\_V4 | 1.0 | 1.466e-09 | 420 | 0.183 | 158 | 119 | 8 | 3 | 157 | 2 | 152 | Uncharacterized protein | Uncharacterized protein | | afdb-uniprot50 | AF-A8TC06-F1-MODEL\_V4 | 1.0 | 4.328e-09 | 418 | 0.195 | 138 | 110 | 1 | 30 | 167 | 1 | 137 | Uncharacterized protein | Uncharacterized protein | | afdb-uniprot50 | AF-A0A7Y3Z3M6-F1-MODEL\_V4 | 1.0 | 1.543e-09 | 413 | 0.178 | 157 | 123 | 5 | 1 | 157 | 1 | 151 | Uncharacterized protein | Uncharacterized protein | | afdb-uniprot50 | AF-A0A2N8HQH0-F1-MODEL\_V4 | 1.0 | 4.039e-10 | 413 | 0.213 | 173 | 120 | 10 | 1 | 165 | 4 | 168 | Protoporphyrinogen oxidase | Protoporphyrinogen oxidase | | afdb-uniprot50 | AF-A0A5S9NBV0-F1-MODEL\_V4 | 1.0 | 1.322e-09 | 409 | 0.193 | 160 | 119 | 5 | 8 | 158 | 10 | 168 | Uncharacterized protein | Uncharacterized protein | | afdb-uniprot50 | AF-A0A285NFT0-F1-MODEL\_V4 | 1.0 | 9.215e-10 | 405 | 0.197 | 162 | 118 | 7 | 1 | 153 | 1 | 159 | Phage tail tube protein FII | Phage tail tube protein FII | | afdb-uniprot50 | AF-A0A378URC9-F1-MODEL\_V4 | 1.0 | 3.708e-09 | 404 | 0.151 | 139 | 115 | 2 | 33 | 168 | 3 | 141 | Phage-like protein | Phage-like protein | | afdb-uniprot50 | AF-A0A845Y1I5-F1-MODEL\_V4 | 1.0 | 3.836e-10 | 404 | 0.174 | 172 | 124 | 7 | 6 | 160 | 4 | 174 | Uncharacterized protein | Uncharacterized protein | | afdb-uniprot50 | AF-A0A2W7RI33-F1-MODEL\_V4 | 1.0 | 2.14e-08 | 401 | 0.297 | 121 | 80 | 2 | 49 | 168 | 1 | 117 | Uncharacterized protein | Uncharacterized protein | | afdb-uniprot50 | AF-F8KPL6-F1-MODEL\_V4 | 1.0 | 1.625e-09 | 399 | 0.181 | 143 | 112 | 3 | 27 | 166 | 2 | 142 | Uncharacterized protein | Uncharacterized protein | | afdb-uniprot50 | AF-A0A7G9WG90-F1-MODEL\_V4 | 1.0 | 7.498e-10 | 397 | 0.142 | 176 | 128 | 6 | 1 | 165 | 6 | 169 | Phage major tail tube protein | Phage major tail tube protein | | afdb-uniprot50 | AF-A0A2J8HSA6-F1-MODEL\_V4 | 1.0 | 2.214e-09 | 393 | 0.215 | 158 | 116 | 5 | 1 | 157 | 1 | 151 | Uncharacterized protein | Uncharacterized protein | | afdb-uniprot50 | AF-A0A735IT95-F1-MODEL\_V4 | 1.0 | 2.63e-08 | 390 | 0.305 | 118 | 78 | 4 | 54 | 168 | 1 | 117 | Phage major tail tube protein | Phage major tail tube protein | | afdb-uniprot50 | AF-A0A348SUU8-F1-MODEL\_V4 | 1.0 | 2.77e-08 | 390 | 0.272 | 125 | 87 | 4 | 47 | 168 | 1 | 124 | Phage tail protein | Phage tail protein | | afdb-uniprot50 | AF-A0A358PQ20-F1-MODEL\_V4 | 1.0 | 1.193e-09 | 390 | 0.189 | 174 | 124 | 8 | 1 | 165 | 5 | 170 | Protoporphyrinogen oxidase | Protoporphyrinogen oxidase | | afdb-uniprot50 | AF-A0A6L9ABJ3-F1-MODEL\_V4 | 1.0 | 1.214e-08 | 387 | 0.209 | 129 | 97 | 3 | 7 | 131 | 9 | 136 | Phage major tail tube protein | Phage major tail tube protein | | afdb-uniprot50 | AF-A0A3A9EJ85-F1-MODEL\_V4 | 1.0 | 4.11e-09 | 387 | 0.175 | 148 | 116 | 3 | 25 | 167 | 1 | 147 | Phage tail protein | Phage tail protein | | afdb-uniprot50 | AF-A0A1C6JGP5-F1-MODEL\_V4 | 1.0 | 1.322e-09 | 387 | 0.136 | 169 | 135 | 7 | 1 | 164 | 6 | 168 | Phage tail tube protein FII | Phage tail tube protein FII | | afdb-uniprot50 | AF-A0A378PU11-F1-MODEL\_V4 | 1.0 | 6.884e-09 | 378 | 0.34 | 132 | 81 | 4 | 38 | 167 | 1 | 128 | Phage major tail tube protein | Phage major tail tube protein | | afdb-uniprot50 | AF-A0A3E2BFZ8-F1-MODEL\_V4 | 1.0 | 4.405e-08 | 376 | 0.255 | 133 | 94 | 4 | 1 | 129 | 1 | 132 | Phage tail protein | Phage tail protein | | afdb-uniprot50 | AF-A0A1Z4PN25-F1-MODEL\_V4 | 1.0 | 3.176e-09 | 375 | 0.174 | 189 | 123 | 5 | 1 | 168 | 1 | 177 | Uncharacterized protein | Uncharacterized protein | | afdb-uniprot50 | AF-A0A541BHJ3-F1-MODEL\_V4 | 1.0 | 7.248e-09 | 367 | 0.2 | 165 | 115 | 9 | 1 | 158 | 1 | 155 | Uncharacterized protein | Uncharacterized protein | | afdb-uniprot50 | AF-A0A2X2BQ70-F1-MODEL\_V4 | 1.0 | 3.584e-08 | 365 | 0.296 | 118 | 76 | 4 | 23 | 135 | 4 | 119 | Major tail tube protein | Major tail tube protein | | afdb-uniprot50 | AF-A0A419A4I5-F1-MODEL\_V4 | 1.0 | 2.373e-08 | 361 | 0.403 | 129 | 60 | 2 | 8 | 136 | 1 | 112 | Phage tail protein | Phage tail protein | | afdb-uniprot50 | AF-A0A5M7P9N9-F1-MODEL\_V4 | 1.0 | 9.546e-08 | 354 | 0.341 | 129 | 75 | 4 | 1 | 124 | 1 | 124 | Phage tail protein | Phage tail protein | | afdb-uniprot50 | AF-A0A5Y7FW73-F1-MODEL\_V4 | 1.0 | 3.29e-07 | 346 | 0.227 | 132 | 92 | 6 | 1 | 128 | 4 | 129 | Phage tail protein | Phage tail protein | | afdb-uniprot50 | AF-A0A317H401-F1-MODEL\_V4 | 1.0 | 7.767e-08 | 341 | 0.236 | 127 | 87 | 4 | 47 | 167 | 5 | 127 | Uncharacterized protein | Uncharacterized protein | | afdb-uniprot50 | AF-A0A645CLD3-F1-MODEL\_V4 | 1.0 | 1.37e-07 | 334 | 0.284 | 116 | 75 | 3 | 57 | 167 | 2 | 114 | Uncharacterized protein | Uncharacterized protein | | afdb-uniprot50 | AF-A0A059IUF5-F1-MODEL\_V4 | 1.0 | 4.97e-07 | 331 | 0.585 | 82 | 34 | 0 | 6 | 87 | 5 | 86 | Contractile tail tube protein | Contractile tail tube protein | | afdb-uniprot50 | AF-A0A607GZE9-F1-MODEL\_V4 | 1.0 | 1.965e-07 | 331 | 0.242 | 136 | 91 | 5 | 39 | 168 | 1 | 130 | Phage tail protein | Phage tail protein | | afdb-uniprot50 | AF-A0A6L5WKW9-F1-MODEL\_V4 | 1.0 | 1.37e-07 | 331 | 0.223 | 130 | 98 | 3 | 38 | 164 | 1 | 130 | Phage tail protein | Phage tail protein | | afdb-uniprot50 | AF-A0A450SXT5-F1-MODEL\_V4 | 1.0 | 1.866e-07 | 330 | 0.505 | 93 | 46 | 0 | 43 | 135 | 1 | 93 | Uncharacterized protein | Uncharacterized protein | | afdb-uniprot50 | AF-A0A376DET1-F1-MODEL\_V4 | 1.0 | 1.023e-06 | 328 | 0.327 | 122 | 74 | 4 | 1 | 118 | 1 | 118 | Major tail sheath protein FII from prophage | Major tail sheath protein FII from prophage | | afdb-uniprot50 | AF-A0A8A8MDP2-F1-MODEL\_V4 | 1.0 | 1.023e-06 | 321 | 0.211 | 137 | 101 | 5 | 36 | 168 | 2 | 135 | Uncharacterized protein | Uncharacterized protein | | afdb-uniprot50 | AF-A0A3A6RI32-F1-MODEL\_V4 | 1.0 | 1.627e-06 | 318 | 0.297 | 121 | 81 | 3 | 1 | 119 | 1 | 119 | Phage tail protein | Phage tail protein | | afdb-uniprot50 | AF-A0A6S6NIH2-F1-MODEL\_V4 | 1.0 | 6.654e-08 | 318 | 0.159 | 138 | 108 | 4 | 29 | 158 | 2 | 139 | Uncharacterized protein | Uncharacterized protein | | afdb-uniprot50 | AF-A0A5C9AFX2-F1-MODEL\_V4 | 1.0 | 8.764e-07 | 316 | 0.33 | 106 | 66 | 3 | 66 | 168 | 1 | 104 | Phage tail protein | Phage tail protein | | afdb-uniprot50 | AF-A0A1C6YVF4-F1-MODEL\_V4 | 1.0 | 2.178e-07 | 314 | 0.321 | 115 | 75 | 2 | 53 | 165 | 1 | 114 | Uncharacterized protein | Uncharacterized protein | | afdb-uniprot50 | AF-A0A379ZTI5-F1-MODEL\_V4 | 1.0 | 1.545e-06 | 311 | 0.322 | 124 | 77 | 4 | 1 | 119 | 1 | 122 | Phage major tail tube protein FII | Phage major tail tube protein FII | | afdb-uniprot50 | AF-A0A369RL94-F1-MODEL\_V4 | 1.0 | 6.319e-08 | 309 | 0.367 | 106 | 62 | 2 | 64 | 168 | 46 | 147 | Phage major tail tube protein | Phage major tail tube protein | | afdb-uniprot50 | AF-A0A4Q6CYM3-F1-MODEL\_V4 | 1.0 | 1.194e-06 | 302 | 0.208 | 115 | 88 | 2 | 55 | 168 | 2 | 114 | Uncharacterized protein | Uncharacterized protein | | afdb-uniprot50 | AF-A0A7I6S727-F1-MODEL\_V4 | 1.0 | 6.217e-06 | 301 | 0.333 | 117 | 75 | 2 | 1 | 114 | 1 | 117 | Uncharacterized protein | Uncharacterized protein | | afdb-uniprot50 | AF-A0A078LLW6-F1-MODEL\_V4 | 1.0 | 1.899e-06 | 288 | 0.382 | 123 | 65 | 6 | 1 | 118 | 1 | 117 | Phage P2 FII-like protein | Phage P2 FII-like protein | | afdb-uniprot50 | AF-A0A5Y6SC09-F1-MODEL\_V4 | 1.0 | 4.334e-06 | 287 | 0.24 | 100 | 73 | 2 | 72 | 168 | 2 | 101 | Phage tail protein | Phage tail protein | | afdb-uniprot50 | AF-A0A4Z0KQR6-F1-MODEL\_V4 | 1.0 | 1.419e-05 | 284 | 0.375 | 72 | 45 | 0 | 97 | 168 | 4 | 75 | Phage tail protein | Phage tail protein | | afdb-uniprot50 | AF-A0A828B488-F1-MODEL\_V4 | 1.0 | 8.045e-06 | 284 | 0.313 | 102 | 66 | 3 | 1 | 100 | 1 | 100 | Phage tail protein | Phage tail protein | | afdb-uniprot50 | AF-A0A2T4JJM7-F1-MODEL\_V4 | 1.0 | 1.096e-05 | 282 | 0.309 | 84 | 57 | 1 | 86 | 168 | 2 | 85 | Phage major tail tube protein | Phage major tail tube protein | | afdb-uniprot50 | AF-A0A485CDJ1-F1-MODEL\_V4 | 1.0 | 9.227e-07 | 281 | 0.271 | 114 | 72 | 6 | 59 | 167 | 2 | 109 | Phage major tail tube protein | Phage major tail tube protein | | afdb-uniprot50 | AF-A0A244C1R5-F1-MODEL\_V4 | 1.0 | 2.725e-06 | 279 | 0.438 | 89 | 50 | 0 | 3 | 91 | 2 | 90 | Phage tail protein | Phage tail protein | | afdb-uniprot50 | AF-A0A7I9KEL5-F1-MODEL\_V4 | 1.0 | 1.836e-05 | 272 | 0.24 | 108 | 77 | 3 | 64 | 168 | 3 | 108 | Phage tail protein | Phage tail protein | | afdb-uniprot50 | AF-A0A3P6KKF7-F1-MODEL\_V4 | 1.0 | 3.909e-06 | 269 | 0.311 | 106 | 72 | 1 | 64 | 168 | 6 | 111 | Major tail tube protein | Major tail tube protein | | afdb-uniprot50 | AF-A0A0F9Z9G8-F1-MODEL\_V4 | 1.0 | 7.641e-06 | 268 | 0.241 | 112 | 79 | 4 | 7 | 114 | 9 | 118 | Prophage MuMc02, major tail tube protein | Prophage MuMc02, major tail tube protein | | afdb-uniprot50 | AF-A0A4Q0U6A3-F1-MODEL\_V4 | 1.0 | 1.804e-06 | 268 | 0.19 | 131 | 102 | 3 | 38 | 165 | 2 | 131 | Uncharacterized protein | Uncharacterized protein | | afdb-uniprot50 | AF-A4P152-F1-MODEL\_V4 | 1.0 | 1.494e-05 | 262 | 0.402 | 92 | 54 | 1 | 1 | 92 | 1 | 91 | Uncharacterized protein | Uncharacterized protein | | afdb-uniprot50 | AF-A0A829E329-F1-MODEL\_V4 | 1.0 | 1.096e-05 | 262 | 0.181 | 116 | 90 | 3 | 33 | 144 | 1 | 115 | Phage major tail tube protein | Phage major tail tube protein | | afdb-uniprot50 | AF-A0A7Z7RJ53-F1-MODEL\_V4 | 1.0 | 4.644e-05 | 258 | 0.292 | 82 | 57 | 1 | 87 | 167 | 2 | 83 | Phage major tail tube protein | Phage major tail tube protein | | afdb-uniprot50 | AF-A0A2G6CDE2-F1-MODEL\_V4 | 1.0 | 2.256e-05 | 253 | 0.241 | 112 | 78 | 3 | 43 | 153 | 1 | 106 | Uncharacterized protein | Uncharacterized protein | | afdb-uniprot50 | AF-A0A7U9RYY6-F1-MODEL\_V4 | 1.0 | 7.257e-06 | 252 | 0.181 | 138 | 102 | 7 | 34 | 165 | 1 | 133 | Uncharacterized protein | Uncharacterized protein | | afdb-uniprot50 | AF-A0A607GXK8-F1-MODEL\_V4 | 1.0 | 3.978e-05 | 251 | 0.25 | 112 | 77 | 5 | 59 | 168 | 3 | 109 | Phage tail protein | Phage tail protein | | afdb-uniprot50 | AF-A0A7W4KR61-F1-MODEL\_V4 | 1.0 | 5.708e-05 | 247 | 0.344 | 93 | 58 | 2 | 1 | 91 | 1 | 92 | Phage major tail tube protein | Phage major tail tube protein | | afdb-uniprot50 | AF-A0A645DSK8-F1-MODEL\_V4 | 1.0 | 2.035e-05 | 245 | 0.276 | 94 | 64 | 2 | 76 | 165 | 3 | 96 | Uncharacterized protein | Uncharacterized protein | | afdb-uniprot50 | AF-A0A826K908-F1-MODEL\_V4 | 1.0 | 0.0001116 | 237 | 0.333 | 96 | 61 | 2 | 1 | 93 | 1 | 96 | Phage tail protein | Phage tail protein | | afdb-uniprot50 | AF-A0A2W6PC71-F1-MODEL\_V4 | 1.0 | 8.188e-05 | 236 | 0.264 | 106 | 73 | 4 | 64 | 167 | 4 | 106 | Phage tail protein | Phage tail protein | | afdb-uniprot50 | AF-A0A0D1VL43-F1-MODEL\_V4 | 1.0 | 4.804e-06 | 235 | 0.151 | 145 | 107 | 8 | 1 | 135 | 4 | 142 | Phage tail tube protein | Phage tail tube protein | | afdb-uniprot50 | AF-A0A7K0ZTX3-F1-MODEL\_V4 | 1.0 | 5.326e-06 | 234 | 0.147 | 142 | 103 | 6 | 1 | 132 | 1 | 134 | Uncharacterized protein | Uncharacterized protein | | afdb-uniprot50 | AF-A0A163TDC7-F1-MODEL\_V4 | 1.0 | 0.0001685 | 231 | 0.238 | 84 | 62 | 2 | 87 | 168 | 2 | 85 | Bacteriophage protein | Bacteriophage protein | | afdb-uniprot50 | AF-A0A0T6ZCB2-F1-MODEL\_V4 | 1.0 | 1.419e-05 | 229 | 0.2 | 130 | 95 | 6 | 40 | 165 | 1 | 125 | Uncharacterized protein | Uncharacterized protein | | afdb-uniprot50 | AF-A0A1S7MQV2-F1-MODEL\_V4 | 1.0 | 7.015e-05 | 228 | 0.195 | 123 | 84 | 4 | 61 | 168 | 2 | 124 | Uncharacterized protein | Uncharacterized protein | | afdb-uniprot50 | AF-A0A450Z316-F1-MODEL\_V4 | 1.0 | 0.0002972 | 225 | 0.467 | 62 | 33 | 0 | 107 | 168 | 2 | 63 | Uncharacterized protein | Uncharacterized protein | | afdb-uniprot50 | AF-A0A799JWC1-F1-MODEL\_V4 | 1.0 | 0.0001175 | 225 | 0.174 | 109 | 85 | 3 | 64 | 168 | 2 | 109 | Phage major tail tube protein | Phage major tail tube protein | | afdb-uniprot50 | AF-A0A645IWR8-F1-MODEL\_V4 | 1.0 | 5.421e-05 | 221 | 0.277 | 90 | 62 | 2 | 79 | 165 | 7 | 96 | Uncharacterized protein | Uncharacterized protein | | afdb-uniprot50 | AF-A0A850F557-F1-MODEL\_V4 | 1.0 | 7.015e-05 | 221 | 0.229 | 96 | 71 | 2 | 75 | 168 | 7 | 101 | Phage major tail tube protein | Phage major tail tube protein | | afdb-uniprot50 | AF-A0A0H3PB23-F1-MODEL\_V4 | 1.0 | 0.0001371 | 219 | 0.185 | 124 | 95 | 4 | 1 | 119 | 1 | 123 | Uncharacterized protein | Uncharacterized protein | | afdb-uniprot50 | AF-Q2NU86-F1-MODEL\_V4 | 1.0 | 0.0004049 | 216 | 0.237 | 101 | 73 | 3 | 1 | 98 | 1 | 100 | Hypothetical phage protein | Hypothetical phage protein | | afdb-uniprot50 | AF-A0A376ZNY1-F1-MODEL\_V4 | 1.0 | 0.000152 | 213 | 0.273 | 95 | 65 | 3 | 74 | 167 | 3 | 94 | Major tail tube protein FII | Major tail tube protein FII | | afdb-uniprot50 | AF-A0A447QPL3-F1-MODEL\_V4 | 1.0 | 0.0008775 | 212 | 0.378 | 66 | 41 | 0 | 103 | 168 | 1 | 66 | Phage major tail tube protein | Phage major tail tube protein | | afdb-uniprot50 | AF-A0A7C5N8L8-F1-MODEL\_V4 | 1.0 | 0.0001371 | 212 | 0.154 | 142 | 103 | 7 | 3 | 135 | 2 | 135 | Uncharacterized protein | Uncharacterized protein | | afdb-uniprot50 | AF-A0A2G2DI71-F1-MODEL\_V4 | 1.0 | 0.0003652 | 211 | 0.229 | 87 | 65 | 2 | 84 | 168 | 4 | 90 | Uncharacterized protein | Uncharacterized protein | | afdb-uniprot50 | AF-A0A2U2AQA0-F1-MODEL\_V4 | 1.0 | 1.419e-05 | 210 | 0.109 | 155 | 108 | 9 | 1 | 129 | 1 | 151 | Uncharacterized protein | Uncharacterized protein | | afdb-uniprot50 | AF-A0A6L3XLT6-F1-MODEL\_V4 | 1.0 | 0.0002296 | 209 | 0.277 | 90 | 64 | 1 | 1 | 90 | 1 | 89 | Phage tail protein | Phage tail protein | | afdb-uniprot50 | AF-A0A376JCW1-F1-MODEL\_V4 | 1.0 | 0.0002296 | 209 | 0.205 | 117 | 88 | 3 | 7 | 119 | 9 | 124 | Phage major tail tube protein | Phage major tail tube protein | | afdb-uniprot50 | AF-A0A519ED62-F1-MODEL\_V4 | 1.0 | 4.89e-05 | 209 | 0.139 | 151 | 112 | 7 | 1 | 136 | 1 | 148 | Uncharacterized protein | Uncharacterized protein | | afdb-uniprot50 | AF-A0A7W4VT46-F1-MODEL\_V4 | 1.0 | 3.408e-05 | 204 | 0.174 | 143 | 98 | 9 | 1 | 132 | 1 | 134 | Uncharacterized protein | Uncharacterized protein | | afdb-uniprot50 | AF-A0A1E5G2B2-F1-MODEL\_V4 | 1.0 | 6.328e-05 | 204 | 0.109 | 146 | 109 | 10 | 1 | 135 | 8 | 143 | Uncharacterized protein | Uncharacterized protein | | afdb-uniprot50 | AF-A0A376ZEC3-F1-MODEL\_V4 | 1.0 | 0.0007915 | 203 | 0.297 | 74 | 51 | 1 | 95 | 167 | 3 | 76 | Major tail tube protein FII | Major tail tube protein FII | | afdb-uniprot50 | AF-A0A538F034-F1-MODEL\_V4 | 1.0 | 3.978e-05 | 201 | 0.124 | 137 | 105 | 9 | 9 | 135 | 13 | 144 | Phage tail protein | Phage tail protein | | afdb-uniprot50 | AF-E8KG78-F1-MODEL\_V4 | 1.0 | 0.000152 | 199 | 0.188 | 106 | 82 | 3 | 1 | 104 | 4 | 107 | Putative phage major tail tube protein | Putative phage major tail tube protein | | afdb-uniprot50 | AF-A0A1I4SH27-F1-MODEL\_V4 | 1.0 | 2.256e-05 | 199 | 0.164 | 140 | 88 | 8 | 9 | 129 | 7 | 136 | Uncharacterized protein | Uncharacterized protein | | afdb-uniprot50 | AF-A0A561K6F5-F1-MODEL\_V4 | 1.0 | 0.0008334 | 198 | 0.362 | 80 | 50 | 1 | 1 | 79 | 1 | 80 | P2 family phage contractile tail tube protein | P2 family phage contractile tail tube protein | | afdb-uniprot50 | AF-A0A4D9X464-F1-MODEL\_V4 | 1.0 | 0.0002296 | 198 | 0.244 | 90 | 65 | 2 | 7 | 94 | 9 | 97 | Phage major tail tube protein | Phage major tail tube protein | | afdb-uniprot50 | AF-A0A1F2QQE5-F1-MODEL\_V4 | 1.0 | 0.0001868 | 193 | 0.16 | 143 | 100 | 10 | 1 | 135 | 1 | 131 | Uncharacterized protein | Uncharacterized protein | | afdb-uniprot50 | AF-A0A448QV03-F1-MODEL\_V4 | 1.0 | 0.0008775 | 192 | 0.195 | 97 | 73 | 3 | 49 | 141 | 1 | 96 | Phage major tail tube protein | Phage major tail tube protein | | afdb-uniprot50 | AF-A0A0F9F7Z1-F1-MODEL\_V4 | 1.0 | 0.0001371 | 192 | 0.136 | 147 | 102 | 9 | 1 | 136 | 1 | 133 | Uncharacterized protein | Uncharacterized protein | | afdb-uniprot50 | AF-A0A3C1F3W3-F1-MODEL\_V4 | 1.0 | 0.0002296 | 192 | 0.165 | 145 | 99 | 9 | 1 | 136 | 1 | 132 | Uncharacterized protein | Uncharacterized protein | | afdb-uniprot50 | AF-A0A2T5U148-F1-MODEL\_V4 | 1.0 | 0.003025 | 191 | 0.323 | 65 | 44 | 0 | 103 | 167 | 1 | 65 | P2 family phage contractile tail tube protein | P2 family phage contractile tail tube protein | | afdb-uniprot50 | AF-A0A2W7QPU3-F1-MODEL\_V4 | 1.0 | 0.002002 | 191 | 0.384 | 78 | 48 | 0 | 1 | 78 | 1 | 78 | Uncharacterized protein | Uncharacterized protein | | afdb-uniprot50 | AF-A0A4T0UJL6-F1-MODEL\_V4 | 1.0 | 0.0001175 | 190 | 0.132 | 143 | 103 | 8 | 1 | 132 | 1 | 133 | Uncharacterized protein | Uncharacterized protein | | afdb-uniprot50 | AF-A0A0H3ZYX4-F1-MODEL\_V4 | 1.0 | 8.622e-05 | 189 | 0.173 | 115 | 90 | 4 | 43 | 157 | 1 | 110 | Uncharacterized protein | Uncharacterized protein | | afdb-uniprot50 | AF-A0A1S2JZI5-F1-MODEL\_V4 | 1.0 | 0.0002546 | 189 | 0.158 | 145 | 104 | 9 | 1 | 135 | 1 | 137 | Uncharacterized protein | Uncharacterized protein | | afdb-uniprot50 | AF-A0A176XNG2-F1-MODEL\_V4 | 1.0 | 9.078e-05 | 188 | 0.168 | 148 | 94 | 8 | 1 | 129 | 1 | 138 | Uncharacterized protein | Uncharacterized protein | | afdb-uniprot50 | AF-A0A3A9WR79-F1-MODEL\_V4 | 1.0 | 0.001136 | 186 | 0.102 | 137 | 108 | 9 | 9 | 137 | 18 | 147 | Phage tail protein | Phage tail protein | | afdb-uniprot50 | AF-A0A1G6LSJ2-F1-MODEL\_V4 | 1.0 | 0.0002071 | 185 | 0.131 | 144 | 103 | 9 | 1 | 132 | 1 | 134 | Uncharacterized protein | Uncharacterized protein | | afdb-uniprot50 | AF-A0A1I0EEG8-F1-MODEL\_V4 | 1.0 | 0.0008334 | 185 | 0.103 | 145 | 109 | 10 | 1 | 135 | 1 | 134 | Uncharacterized protein | Uncharacterized protein | | afdb-uniprot50 | AF-A0A1Z8QIQ5-F1-MODEL\_V4 | 1.0 | 0.001078 | 185 | 0.123 | 138 | 104 | 9 | 10 | 135 | 23 | 155 | Uncharacterized protein | Uncharacterized protein | | afdb-uniprot50 | AF-R6J7P7-F1-MODEL\_V4 | 1.0 | 0.0002181 | 184 | 0.151 | 145 | 110 | 8 | 1 | 136 | 1 | 141 | Uncharacterized protein | Uncharacterized protein | | afdb-uniprot50 | AF-A0A377U351-F1-MODEL\_V4 | 1.0 | 0.002461 | 183 | 0.347 | 69 | 44 | 1 | 82 | 149 | 2 | 70 | Phage major tail tube protein | Phage major tail tube protein | | afdb-uniprot50 | AF-A0A527W089-F1-MODEL\_V4 | 1.0 | 0.003914 | 183 | 0.16 | 81 | 66 | 2 | 89 | 167 | 2 | 82 | Uncharacterized protein | Uncharacterized protein | | afdb-uniprot50 | AF-A0A1U7GJ66-F1-MODEL\_V4 | 1.0 | 0.0002681 | 183 | 0.163 | 147 | 107 | 7 | 1 | 136 | 1 | 142 | Uncharacterized protein | Uncharacterized protein | | afdb-uniprot50 | AF-A0A376W1N8-F1-MODEL\_V4 | 1.0 | 0.001902 | 181 | 0.216 | 97 | 70 | 4 | 43 | 136 | 1 | 94 | Major tail sheath protein FII from prophage | Major tail sheath protein FII from prophage | | afdb-uniprot50 | AF-A1ZC06-F1-MODEL\_V4 | 1.0 | 0.0003846 | 181 | 0.17 | 135 | 98 | 8 | 9 | 136 | 18 | 145 | Afp1 | Afp1 | | afdb-uniprot50 | AF-A0A1S1NS96-F1-MODEL\_V4 | 1.0 | 6.662e-05 | 181 | 0.133 | 180 | 117 | 11 | 1 | 160 | 1 | 161 | Uncharacterized protein | Uncharacterized protein | | afdb-uniprot50 | AF-A0A354NH67-F1-MODEL\_V4 | 1.0 | 0.0008775 | 180 | 0.139 | 136 | 103 | 7 | 9 | 135 | 8 | 138 | Uncharacterized protein | Uncharacterized protein | | afdb-uniprot50 | AF-A0A3S9YSX8-F1-MODEL\_V4 | 1.0 | 0.0004489 | 178 | 0.13 | 153 | 107 | 8 | 1 | 135 | 1 | 145 | Uncharacterized protein | Uncharacterized protein | | afdb-uniprot50 | AF-A0A416PS04-F1-MODEL\_V4 | 1.0 | 0.0001371 | 178 | 0.163 | 141 | 100 | 10 | 8 | 139 | 17 | 148 | Uncharacterized protein | Uncharacterized protein | | afdb-uniprot50 | AF-A0A1Q7Q1G9-F1-MODEL\_V4 | 1.0 | 0.0002681 | 178 | 0.091 | 142 | 115 | 9 | 1 | 136 | 21 | 154 | Uncharacterized protein | Uncharacterized protein | | afdb-uniprot50 | AF-A0A0F9HCI3-F1-MODEL\_V4 | 1.0 | 0.001629 | 177 | 0.122 | 139 | 104 | 10 | 10 | 136 | 15 | 147 | Uncharacterized protein | Uncharacterized protein | | afdb-uniprot50 | AF-A0A2N2JDW8-F1-MODEL\_V4 | 1.0 | 0.001902 | 177 | 0.08 | 136 | 110 | 8 | 9 | 135 | 68 | 197 | Phage tail protein | Phage tail protein | | afdb-uniprot50 | AF-A0A3A9EEY6-F1-MODEL\_V4 | 1.0 | 0.008931 | 176 | 0.25 | 68 | 51 | 0 | 101 | 168 | 6 | 73 | Phage tail protein | Phage tail protein | | afdb-uniprot50 | AF-A0A261SH18-F1-MODEL\_V4 | 1.0 | 0.0001967 | 176 | 0.156 | 147 | 97 | 8 | 1 | 129 | 1 | 138 | Uncharacterized protein | Uncharacterized protein | | afdb-uniprot50 | AF-A0A7Y0JM30-F1-MODEL\_V4 | 1.0 | 0.0008334 | 175 | 0.16 | 143 | 100 | 9 | 3 | 135 | 2 | 134 | Uncharacterized protein | Uncharacterized protein | | afdb-uniprot50 | AF-A0A2L2BC49-F1-MODEL\_V4 | 1.0 | 0.0001175 | 175 | 0.143 | 146 | 93 | 9 | 3 | 129 | 5 | 137 | Uncharacterized protein | Uncharacterized protein | | afdb-uniprot50 | AF-A0A3A9VWV0-F1-MODEL\_V4 | 1.0 | 0.001902 | 175 | 0.109 | 137 | 107 | 9 | 9 | 137 | 18 | 147 | Phage tail protein | Phage tail protein | | afdb-uniprot50 | AF-A0A1S1HPU9-F1-MODEL\_V4 | 1.0 | 0.0007517 | 175 | 0.08 | 161 | 117 | 8 | 1 | 141 | 1 | 150 | Phage tail protein | Phage tail protein | | afdb-uniprot50 | AF-A0A413W1T0-F1-MODEL\_V4 | 1.0 | 0.0003652 | 174 | 0.136 | 146 | 110 | 9 | 1 | 135 | 1 | 141 | Phage tail protein | Phage tail protein | | afdb-uniprot50 | AF-A0A8B5RKL2-F1-MODEL\_V4 | 1.0 | 0.009403 | 173 | 0.285 | 63 | 45 | 0 | 106 | 168 | 1 | 63 | Phage tail protein | Phage tail protein | | afdb-uniprot50 | AF-A0A5W2M6M4-F1-MODEL\_V4 | 1.0 | 0.007267 | 173 | 0.343 | 67 | 43 | 1 | 103 | 168 | 3 | 69 | Phage tail protein | Phage tail protein | | afdb-uniprot50 | AF-A0A7Y2IRB2-F1-MODEL\_V4 | 1.0 | 0.0005809 | 173 | 0.155 | 135 | 94 | 9 | 10 | 135 | 9 | 132 | Uncharacterized protein | Uncharacterized protein | | afdb-uniprot50 | AF-A0A6G9IB21-F1-MODEL\_V4 | 1.0 | 0.0005809 | 173 | 0.09 | 155 | 110 | 10 | 1 | 136 | 1 | 143 | Phage tail protein | Phage tail protein | | afdb-uniprot50 | AF-A0A4S1E497-F1-MODEL\_V4 | 1.0 | 0.0004489 | 173 | 0.12 | 149 | 101 | 6 | 1 | 129 | 1 | 139 | Uncharacterized protein | Uncharacterized protein | | afdb-uniprot50 | AF-X1I0E2-F1-MODEL\_V4 | 1.0 | 0.0004726 | 172 | 0.125 | 144 | 106 | 7 | 3 | 135 | 2 | 136 | Uncharacterized protein | Uncharacterized protein | | afdb-uniprot50 | AF-A0A5V4ZED2-F1-MODEL\_V4 | 1.0 | 0.0007915 | 171 | 0.107 | 139 | 106 | 7 | 9 | 137 | 12 | 142 | Uncharacterized protein | Uncharacterized protein | | afdb-uniprot50 | AF-A0A1L6M2V1-F1-MODEL\_V4 | 1.0 | 0.001396 | 171 | 0.101 | 138 | 107 | 8 | 9 | 135 | 22 | 153 | Uncharacterized protein | Uncharacterized protein | | afdb-uniprot50 | AF-A0A327S9J4-F1-MODEL\_V4 | 1.0 | 0.001469 | 170 | 0.104 | 144 | 109 | 12 | 1 | 135 | 1 | 133 | TP901-1 family phage major tail protein | TP901-1 family phage major tail protein | | afdb-uniprot50 | AF-A0A2E6EJY7-F1-MODEL\_V4 | 1.0 | 0.002728 | 169 | 0.071 | 139 | 114 | 8 | 9 | 135 | 15 | 150 | Phage tail protein | Phage tail protein | | afdb-uniprot50 | AF-A0A6D1E7W5-F1-MODEL\_V4 | 1.0 | 0.001078 | 168 | 0.071 | 154 | 112 | 9 | 1 | 136 | 2 | 142 | Phage tail protein | Phage tail protein | | afdb-uniprot50 | AF-A0A2W4S886-F1-MODEL\_V4 | 1.0 | 0.00222 | 167 | 0.094 | 138 | 108 | 10 | 9 | 135 | 11 | 142 | Phage tail protein | Phage tail protein | | afdb-uniprot50 | AF-A0A853IAX0-F1-MODEL\_V4 | 1.0 | 0.0004049 | 167 | 0.109 | 146 | 111 | 11 | 1 | 136 | 17 | 153 | Phage tail protein | Phage tail protein | | afdb-uniprot50 | AF-A0A0G3CNN1-F1-MODEL\_V4 | 1.0 | 0.0009239 | 167 | 0.108 | 156 | 107 | 9 | 1 | 136 | 1 | 144 | Uncharacterized protein | Uncharacterized protein | | afdb-uniprot50 | AF-A0A4R5EZG6-F1-MODEL\_V4 | 1.0 | 0.0004489 | 165 | 0.176 | 147 | 95 | 12 | 1 | 135 | 1 | 133 | Uncharacterized protein | Uncharacterized protein | | afdb-uniprot50 | AF-T0QQH1-F1-MODEL\_V4 | 1.0 | 0.001806 | 165 | 0.114 | 131 | 109 | 5 | 10 | 136 | 9 | 136 | Uncharacterized protein | Uncharacterized protein | | afdb-uniprot50 | AF-A0A534PKH6-F1-MODEL\_V4 | 1.0 | 0.0007517 | 165 | 0.117 | 145 | 108 | 9 | 1 | 135 | 8 | 142 | Phage tail protein | Phage tail protein | | afdb-uniprot50 | AF-A0A497Y9K5-F1-MODEL\_V4 | 1.0 | 0.003185 | 165 | 0.104 | 134 | 108 | 8 | 9 | 136 | 25 | 152 | Phage tail protein | Phage tail protein | | afdb-uniprot50 | AF-A0A2E0EK74-F1-MODEL\_V4 | 1.0 | 0.001259 | 165 | 0.106 | 159 | 113 | 9 | 1 | 135 | 1 | 154 | Uncharacterized protein | Uncharacterized protein | | afdb-uniprot50 | AF-A0A090SU12-F1-MODEL\_V4 | 1.0 | 0.008931 | 162 | 0.293 | 75 | 51 | 1 | 1 | 75 | 1 | 73 | Uncharacterized protein | Uncharacterized protein | | afdb-uniprot50 | AF-A0A1C6AJU2-F1-MODEL\_V4 | 1.0 | 0.001469 | 161 | 0.158 | 139 | 107 | 7 | 5 | 136 | 11 | 146 | Conserved hypothetical phage tail region protein | Conserved hypothetical phage tail region protein | | afdb-uniprot50 | AF-A0A7V6WL49-F1-MODEL\_V4 | 1.0 | 0.001547 | 160 | 0.16 | 125 | 88 | 8 | 22 | 136 | 30 | 147 | Uncharacterized protein | Uncharacterized protein | | afdb-uniprot50 | AF-A0A419GEK8-F1-MODEL\_V4 | 1.0 | 0.001259 | 160 | 0.131 | 183 | 106 | 9 | 5 | 136 | 5 | 185 | Uncharacterized protein | Uncharacterized protein | | afdb-uniprot50 | AF-A0A3G9IH27-F1-MODEL\_V4 | 1.0 | 0.001469 | 159 | 0.143 | 139 | 105 | 7 | 1 | 132 | 1 | 132 | Uncharacterized protein | Uncharacterized protein | | afdb-uniprot50 | AF-A0A3R0Y9I4-F1-MODEL\_V4 | 1.0 | 0.002108 | 159 | 0.108 | 138 | 105 | 7 | 10 | 137 | 13 | 142 | Big-1 domain-containing protein | Big-1 domain-containing protein | | afdb-uniprot50 | AF-A0A4P5Y9X1-F1-MODEL\_V4 | 1.0 | 0.003717 | 158 | 0.101 | 138 | 113 | 6 | 1 | 135 | 4 | 133 | Uncharacterized protein | Uncharacterized protein | | afdb-uniprot50 | AF-A0A7X9N1M4-F1-MODEL\_V4 | 1.0 | 0.0003295 | 157 | 0.138 | 173 | 106 | 12 | 10 | 167 | 13 | 157 | Phage major tail protein, TP901-1 family | Phage major tail protein, TP901-1 family | | afdb-uniprot50 | AF-A0A2E7URN5-F1-MODEL\_V4 | 1.0 | 0.002337 | 156 | 0.096 | 155 | 108 | 10 | 1 | 135 | 7 | 149 | Uncharacterized protein | Uncharacterized protein | | afdb-uniprot50 | AF-E8X0R1-F1-MODEL\_V4 | 1.0 | 0.003185 | 153 | 0.17 | 141 | 102 | 6 | 9 | 135 | 16 | 155 | Uncharacterized protein | Uncharacterized protein | | afdb-uniprot50 | AF-A0A6P2BDC7-F1-MODEL\_V4 | 1.0 | 0.002873 | 152 | 0.136 | 132 | 104 | 6 | 9 | 135 | 10 | 136 | Uncharacterized protein | Uncharacterized protein | | afdb-uniprot50 | AF-A0A6M1N9D7-F1-MODEL\_V4 | 1.0 | 0.00353 | 152 | 0.095 | 146 | 108 | 9 | 10 | 136 | 9 | 149 | Uncharacterized protein | Uncharacterized protein | | afdb-uniprot50 | AF-A0A1Y4GV23-F1-MODEL\_V4 | 1.0 | 0.0009239 | 152 | 0.121 | 140 | 101 | 8 | 1 | 135 | 1 | 123 | Uncharacterized protein | Uncharacterized protein | | afdb-uniprot50 | AF-A0A0T2MKH8-F1-MODEL\_V4 | 1.0 | 0.00222 | 151 | 0.151 | 132 | 98 | 9 | 10 | 136 | 9 | 131 | Uncharacterized protein | Uncharacterized protein | | afdb-uniprot50 | AF-A0A1V6HRI2-F1-MODEL\_V4 | 1.0 | 0.003025 | 150 | 0.165 | 139 | 93 | 10 | 3 | 129 | 2 | 129 | Uncharacterized protein | Uncharacterized protein | | afdb-uniprot50 | AF-A0A521U4D5-F1-MODEL\_V4 | 1.0 | 0.002873 | 149 | 0.173 | 150 | 107 | 10 | 1 | 141 | 1 | 142 | Uncharacterized protein | Uncharacterized protein | | afdb-uniprot50 | AF-A0A0F5Q2G1-F1-MODEL\_V4 | 1.0 | 0.0004726 | 149 | 0.147 | 149 | 102 | 11 | 1 | 141 | 31 | 162 | Uncharacterized protein | Uncharacterized protein | | afdb-uniprot50 | AF-A0A2A7U7Q2-F1-MODEL\_V4 | 1.0 | 0.008482 | 149 | 0.063 | 141 | 110 | 8 | 5 | 129 | 8 | 142 | Uncharacterized protein | Uncharacterized protein | | afdb-uniprot50 | AF-A0A840ENA2-F1-MODEL\_V4 | 1.0 | 0.003717 | 147 | 0.108 | 138 | 100 | 10 | 10 | 140 | 11 | 132 | Uncharacterized protein | Uncharacterized protein | | afdb-uniprot50 | AF-A0A6B9HSX7-F1-MODEL\_V4 | 1.0 | 0.001902 | 147 | 0.131 | 145 | 94 | 13 | 9 | 134 | 12 | 143 | Phage major tail protein, TP901-1 family | Phage major tail protein, TP901-1 family | | afdb-uniprot50 | AF-A0A2D7ZXH8-F1-MODEL\_V4 | 1.0 | 0.001715 | 146 | 0.14 | 142 | 100 | 10 | 1 | 135 | 1 | 127 | Uncharacterized protein | Uncharacterized protein | | afdb-uniprot50 | AF-F6GGQ2-F1-MODEL\_V4 | 1.0 | 0.001547 | 146 | 0.069 | 144 | 112 | 12 | 7 | 147 | 4 | 128 | Uncharacterized protein | Uncharacterized protein | | afdb-uniprot50 | AF-A0A7W7CHS3-F1-MODEL\_V4 | 1.0 | 0.0005517 | 146 | 0.13 | 138 | 103 | 9 | 1 | 129 | 9 | 138 | Phage tail-like protein | Phage tail-like protein | | afdb-uniprot50 | AF-A0A1E3GXP7-F1-MODEL\_V4 | 1.0 | 0.008056 | 146 | 0.082 | 146 | 113 | 5 | 9 | 136 | 7 | 149 | Uncharacterized protein | Uncharacterized protein | | afdb-uniprot50 | AF-D9WWW1-F1-MODEL\_V4 | 1.0 | 0.003353 | 146 | 0.125 | 152 | 103 | 9 | 10 | 140 | 117 | 259 | Uncharacterized protein | Uncharacterized protein | | afdb-uniprot50 | AF-A0A7L5UII7-F1-MODEL\_V4 | 1.0 | 0.002002 | 145 | 0.104 | 143 | 106 | 13 | 5 | 136 | 12 | 143 | Phage tail protein | Phage tail protein | | afdb-uniprot50 | AF-A0A376W7L3-F1-MODEL\_V4 | 1.0 | 0.00222 | 144 | 0.095 | 157 | 105 | 10 | 1 | 136 | 2 | 142 | Major tail subunit encoded within prophage CP-933V | Major tail subunit encoded within prophage CP-933V | | afdb-uniprot50 | AF-A0A1F9ZG88-F1-MODEL\_V4 | 1.0 | 0.002461 | 144 | 0.096 | 145 | 111 | 9 | 1 | 135 | 15 | 149 | Uncharacterized protein | Uncharacterized protein | | afdb-uniprot50 | AF-A0A1H7T315-F1-MODEL\_V4 | 1.0 | 0.003185 | 144 | 0.14 | 128 | 95 | 9 | 10 | 135 | 5 | 119 | Uncharacterized protein | Uncharacterized protein | | afdb-uniprot50 | AF-A0A0K5ZHP5-F1-MODEL\_V4 | 1.0 | 0.004339 | 144 | 0.071 | 154 | 111 | 11 | 1 | 136 | 20 | 159 | Phage major tail subunit | Phage major tail subunit | | afdb-uniprot50 | AF-A0A521X7C1-F1-MODEL\_V4 | 1.0 | 0.0002546 | 143 | 0.148 | 141 | 99 | 9 | 6 | 140 | 3 | 128 | Uncharacterized protein | Uncharacterized protein | | afdb-uniprot50 | AF-A0A0S7YR27-F1-MODEL\_V4 | 1.0 | 0.001902 | 143 | 0.126 | 142 | 104 | 7 | 1 | 129 | 1 | 135 | Uncharacterized protein | Uncharacterized protein | | afdb-uniprot50 | AF-A0A3M2CPG9-F1-MODEL\_V4 | 1.0 | 0.003717 | 143 | 0.162 | 129 | 95 | 9 | 8 | 129 | 1 | 123 | Uncharacterized protein | Uncharacterized protein | | afdb-uniprot50 | AF-A0A1G2VKT4-F1-MODEL\_V4 | 1.0 | 0.005912 | 143 | 0.102 | 136 | 112 | 8 | 1 | 129 | 633 | 765 | Kazal-like domain-containing protein | Kazal-like domain-containing protein | | afdb-uniprot50 | AF-A0A2N1DEF5-F1-MODEL\_V4 | 1.0 | 0.002108 | 142 | 0.104 | 144 | 111 | 7 | 1 | 136 | 9 | 142 | Phage tail protein | Phage tail protein | | afdb-uniprot50 | AF-A0A396BX50-F1-MODEL\_V4 | 1.0 | 0.0007517 | 141 | 0.118 | 135 | 98 | 11 | 10 | 140 | 5 | 122 | Type VI secretion system needle protein Hcp | Type VI secretion system needle protein Hcp | | afdb-uniprot50 | AF-A0A7Y1UT33-F1-MODEL\_V4 | 1.0 | 0.008482 | 141 | 0.105 | 133 | 110 | 7 | 9 | 135 | 18 | 147 | Phage tail protein | Phage tail protein | | afdb-uniprot50 | AF-E6M101-F1-MODEL\_V4 | 1.0 | 0.002108 | 141 | 0.098 | 153 | 99 | 8 | 10 | 137 | 10 | 148 | Uncharacterized protein | Uncharacterized protein | | afdb-uniprot50 | AF-A0A1G7J1E5-F1-MODEL\_V4 | 1.0 | 0.0008775 | 141 | 0.093 | 149 | 107 | 11 | 1 | 144 | 1 | 126 | Uncharacterized protein | Uncharacterized protein | | afdb-uniprot50 | AF-A0A4U7JH93-F1-MODEL\_V4 | 1.0 | 0.004121 | 140 | 0.088 | 135 | 110 | 7 | 1 | 129 | 6 | 133 | Phage tail protein | Phage tail protein | | afdb-uniprot50 | AF-A0A6J4RD76-F1-MODEL\_V4 | 1.0 | 0.002591 | 140 | 0.167 | 131 | 95 | 8 | 5 | 129 | 11 | 133 | Uncharacterized protein | Uncharacterized protein | | afdb-uniprot50 | AF-A0A852QIZ0-F1-MODEL\_V4 | 1.0 | 0.005065 | 139 | 0.08 | 124 | 105 | 4 | 18 | 135 | 49 | 169 | Uncharacterized protein | Uncharacterized protein | | afdb-uniprot50 | AF-A0A1E8CW82-F1-MODEL\_V4 | 1.0 | 0.0003652 | 139 | 0.178 | 140 | 93 | 11 | 6 | 136 | 1 | 127 | ATPase\_AAA\_core domain-containing protein | ATPase\_AAA\_core domain-containing protein | | afdb-uniprot50 | AF-A0A2G6JA31-F1-MODEL\_V4 | 1.0 | 0.006901 | 138 | 0.076 | 144 | 115 | 9 | 1 | 135 | 10 | 144 | Phage tail protein | Phage tail protein | | afdb-uniprot50 | AF-A0A5E6QXM5-F1-MODEL\_V4 | 1.0 | 0.007267 | 137 | 0.204 | 83 | 64 | 1 | 88 | 168 | 2 | 84 | Uncharacterized protein | Uncharacterized protein | | afdb-uniprot50 | AF-A0A831QR42-F1-MODEL\_V4 | 1.0 | 0.002591 | 137 | 0.148 | 141 | 90 | 13 | 1 | 134 | 1 | 118 | Uncharacterized protein | Uncharacterized protein | | afdb-uniprot50 | AF-A0A1N6FWD3-F1-MODEL\_V4 | 1.0 | 0.004121 | 137 | 0.138 | 130 | 103 | 7 | 10 | 135 | 9 | 133 | Conserved hypothetical phage tail region protein | Conserved hypothetical phage tail region protein | | afdb-uniprot50 | AF-A0A5C5ZW62-F1-MODEL\_V4 | 1.0 | 0.004569 | 137 | 0.128 | 132 | 95 | 8 | 10 | 130 | 9 | 131 | Uncharacterized protein | Uncharacterized protein | | afdb-uniprot50 | AF-A0A366ZZU0-F1-MODEL\_V4 | 1.0 | 0.007267 | 137 | 0.131 | 122 | 94 | 5 | 18 | 135 | 42 | 155 | Uncharacterized protein | Uncharacterized protein | | afdb-uniprot50 | AF-A0A1H7UBE6-F1-MODEL\_V4 | 1.0 | 0.003914 | 136 | 0.104 | 143 | 104 | 11 | 10 | 144 | 30 | 156 | Uncharacterized protein | Uncharacterized protein | | afdb-uniprot50 | AF-A0A6H1ZDK9-F1-MODEL\_V4 | 1.0 | 0.003914 | 135 | 0.14 | 142 | 102 | 10 | 3 | 136 | 5 | 134 | Uncharacterized protein | Uncharacterized protein | | afdb-uniprot50 | AF-A0A1Y4DW26-F1-MODEL\_V4 | 1.0 | 0.004121 | 135 | 0.139 | 151 | 97 | 11 | 6 | 138 | 2 | 137 | Uncharacterized protein | Uncharacterized protein | | afdb-uniprot50 | AF-A0A1G6Z760-F1-MODEL\_V4 | 1.0 | 0.001259 | 135 | 0.166 | 144 | 90 | 16 | 10 | 146 | 5 | 125 | Uncharacterized protein | Uncharacterized protein | | afdb-uniprot50 | AF-A0A3N0E5J3-F1-MODEL\_V4 | 1.0 | 0.003185 | 134 | 0.116 | 137 | 100 | 11 | 7 | 140 | 4 | 122 | Uncharacterized protein | Uncharacterized protein | | afdb-uniprot50 | AF-A0A2W7NBR6-F1-MODEL\_V4 | 1.0 | 0.004121 | 132 | 0.098 | 183 | 122 | 12 | 1 | 168 | 3 | 157 | TP901-1 family phage major tail protein | TP901-1 family phage major tail protein | | afdb-uniprot50 | AF-A0A553DU56-F1-MODEL\_V4 | 1.0 | 0.003717 | 131 | 0.125 | 144 | 100 | 14 | 1 | 140 | 1 | 122 | Uncharacterized protein | Uncharacterized protein | | afdb-uniprot50 | AF-A0A5C1AH35-F1-MODEL\_V4 | 1.0 | 0.003914 | 130 | 0.137 | 124 | 89 | 6 | 22 | 135 | 22 | 137 | Uncharacterized protein | Uncharacterized protein | | afdb-uniprot50 | AF-A0A4P6U525-F1-MODEL\_V4 | 1.0 | 0.007267 | 130 | 0.141 | 141 | 93 | 7 | 10 | 129 | 15 | 148 | Uncharacterized protein | Uncharacterized protein | | afdb-uniprot50 | AF-A0A6B8KDY4-F1-MODEL\_V4 | 1.0 | 0.008056 | 129 | 0.081 | 159 | 111 | 11 | 1 | 135 | 3 | 150 | Uncharacterized protein | Uncharacterized protein | | afdb-uniprot50 | AF-A0A0F9A0R1-F1-MODEL\_V4 | 1.0 | 0.001469 | 128 | 0.153 | 150 | 96 | 13 | 1 | 136 | 1 | 133 | Uncharacterized protein | Uncharacterized protein | | afdb-uniprot50 | AF-A0A6N7RZ20-F1-MODEL\_V4 | 1.0 | 0.0007517 | 128 | 0.121 | 173 | 127 | 10 | 1 | 160 | 1 | 161 | Uncharacterized protein | Uncharacterized protein | | afdb-uniprot50 | AF-A0A4P7X061-F1-MODEL\_V4 | 1.0 | 0.004339 | 128 | 0.079 | 139 | 105 | 9 | 10 | 143 | 5 | 125 | Uncharacterized protein | Uncharacterized protein | | afdb-uniprot50 | AF-A0A329N2I3-F1-MODEL\_V4 | 1.0 | 0.005333 | 126 | 0.138 | 144 | 98 | 14 | 1 | 140 | 1 | 122 | Uncharacterized protein | Uncharacterized protein | | afdb-uniprot50 | AF-A0A4P6YRT8-F1-MODEL\_V4 | 1.0 | 0.006901 | 125 | 0.094 | 137 | 101 | 8 | 9 | 135 | 11 | 134 | Phage tail protein | Phage tail protein | | afdb-uniprot50 | AF-A0A662BGP6-F1-MODEL\_V4 | 1.0 | 0.007267 | 124 | 0.117 | 145 | 108 | 9 | 1 | 135 | 42 | 176 | Uncharacterized protein | Uncharacterized protein | | afdb-uniprot50 | AF-A0A7H8PN01-F1-MODEL\_V4 | 1.0 | 0.008931 | 122 | 0.14 | 135 | 94 | 11 | 10 | 136 | 5 | 125 | Phage tail protein | Phage tail protein | | afdb-uniprot50 | AF-A0A7Y2EDK0-F1-MODEL\_V4 | 1.0 | 0.005333 | 122 | 0.127 | 149 | 94 | 12 | 1 | 135 | 11 | 137 | Phage tail protein | Phage tail protein | | afdb-uniprot50 | AF-A0A1F1DKW7-F1-MODEL\_V4 | 1.0 | 0.007651 | 122 | 0.096 | 156 | 100 | 10 | 1 | 129 | 7 | 148 | Uncharacterized protein | Uncharacterized protein | | afdb-uniprot50 | AF-A0A540QCB7-F1-MODEL\_V4 | 1.0 | 0.002728 | 121 | 0.122 | 196 | 121 | 11 | 1 | 168 | 4 | 176 | Uncharacterized protein | Uncharacterized protein | | afdb-uniprot50 | AF-A0A1K1N5G7-F1-MODEL\_V4 | 1.0 | 0.005333 | 120 | 0.109 | 137 | 101 | 10 | 7 | 140 | 4 | 122 | Uncharacterized protein | Uncharacterized protein | | afdb-uniprot50 | AF-A0A1G7HEH5-F1-MODEL\_V4 | 1.0 | 0.006555 | 119 | 0.123 | 138 | 98 | 12 | 7 | 140 | 4 | 122 | Uncharacterized protein | Uncharacterized protein | | afdb-uniprot50 | AF-A0A2D6AJM6-F1-MODEL\_V4 | 1.0 | 0.007267 | 119 | 0.125 | 152 | 109 | 12 | 1 | 140 | 1 | 140 | Uncharacterized protein | Uncharacterized protein | | afdb-uniprot50 | AF-A0A3A9VUY5-F1-MODEL\_V4 | 1.0 | 0.00222 | 119 | 0.157 | 140 | 95 | 10 | 5 | 140 | 30 | 150 | Uncharacterized protein | Uncharacterized protein | | afdb-uniprot50 | AF-A0A3A6P734-F1-MODEL\_V4 | 1.0 | 0.008056 | 118 | 0.108 | 138 | 98 | 11 | 4 | 135 | 7 | 125 | Uncharacterized protein | Uncharacterized protein | | afdb-uniprot50 | AF-A0A1J0LW85-F1-MODEL\_V4 | 1.0 | 0.008056 | 117 | 0.167 | 137 | 95 | 11 | 4 | 132 | 9 | 134 | Uncharacterized protein | Uncharacterized protein | | afdb-uniprot50 | AF-A0A2D6X4M8-F1-MODEL\_V4 | 1.0 | 0.008931 | 113 | 0.138 | 152 | 94 | 12 | 1 | 129 | 4 | 141 | Uncharacterized protein | Uncharacterized protein | | afdb-uniprot50 | AF-A0A1I0J0M0-F1-MODEL\_V4 | 1.0 | 0.006555 | 113 | 0.124 | 153 | 104 | 11 | 6 | 135 | 9 | 154 | Phage tail tube protein | Phage tail tube protein | | afdb-uniprot50 | AF-A0A0K1EBJ0-F1-MODEL\_V4 | 1.0 | 0.00481 | 113 | 0.14 | 142 | 87 | 7 | 11 | 136 | 13 | 135 | Uncharacterized protein | Uncharacterized protein | | afdb-uniprot50 | AF-A0A7U9NPC4-F1-MODEL\_V4 | 1.0 | 0.008931 | 113 | 0.167 | 143 | 91 | 9 | 7 | 133 | 4 | 134 | Uncharacterized protein | Uncharacterized protein | | afdb-uniprot50 | AF-A0A0F9EL08-F1-MODEL\_V4 | 1.0 | 0.006225 | 111 | 0.138 | 152 | 110 | 11 | 1 | 136 | 79 | 225 | Uncharacterized protein | Uncharacterized protein | | afdb-uniprot50 | AF-I0K9T2-F1-MODEL\_V4 | 1.0 | 0.002728 | 110 | 0.187 | 144 | 83 | 12 | 1 | 132 | 1 | 122 | PaaR repeat-containing protein | PaaR repeat-containing protein | | afdb-uniprot50 | AF-A0A2W2GU04-F1-MODEL\_V4 | 1.0 | 0.009901 | 109 | 0.134 | 156 | 95 | 9 | 1 | 129 | 6 | 148 | Uncharacterized protein | Uncharacterized protein | | afdb-uniprot50 | AF-A0A7L4Q6N2-F1-MODEL\_V4 | 1.0 | 0.008056 | 108 | 0.106 | 132 | 90 | 8 | 11 | 129 | 5 | 121 | Uncharacterized protein | Uncharacterized protein | | afdb-uniprot50 | AF-A0A021VU20-F1-MODEL\_V4 | 1.0 | 0.009403 | 108 | 0.131 | 152 | 93 | 10 | 10 | 137 | 18 | 154 | Uncharacterized protein | Uncharacterized protein | | afdb-uniprot50 | AF-A0A6M3IF35-F1-MODEL\_V4 | 1.0 | 0.00481 | 107 | 0.12 | 150 | 106 | 11 | 1 | 135 | 1 | 139 | Uncharacterized protein | Uncharacterized protein | | afdb-uniprot50 | AF-A0A518ICN5-F1-MODEL\_V4 | 0.999 | 0.004569 | 99 | 0.131 | 145 | 87 | 7 | 1 | 129 | 1 | 122 | Uncharacterized protein | Uncharacterized protein | | afdb-uniprot50 | AF-A0A512H4S7-F1-MODEL\_V4 | 0.998 | 0.008931 | 94 | 0.129 | 154 | 104 | 11 | 4 | 135 | 11 | 156 | Uncharacterized protein | Uncharacterized protein | |
| Top keywords  (threshold 1.00e-02 (evalue)) | **tail, Phage, tube, major, FII, Putative, prophage, contractile, P2, Bacteriophage** |
| Output files | ../../similar\_structures/19\_FANPEZAQ\_CDS\_0019\_afdb-proteome\_foldseek.tsv ../../similar\_structures/19\_FANPEZAQ\_CDS\_0019\_afdb-uniprot50\_foldseek.tsv ../../similar\_structures/19\_FANPEZAQ\_CDS\_0019\_merged.svg ../../similar\_structures/19\_FANPEZAQ\_CDS\_0019\_pdb\_foldseek.tsv |

  
  
  

Return to summary | Go to previous | Go to next

  


---

**Sequence/structure alignments coloring**  
Each object in the alignment figures is colored according to its E-value following this color coding:

1e-100
10

**References:**  
1) Steinegger M, Meier M, Mirdita M, Vöhringer H, Haunsberger S J, and Söding J (2019) HH-suite3 for fast remote homology detection and deep protein annotation, BMC Bioinformatics, 473. doi: 10.1186/s12859-019-3019-7  
2) Jumper J, Evans R, Pritzel A, ..., Hassabis D (2021) Highly accurate protein structure prediction with AlphaFold, Nature, 596. doi: 10.1038/s41586-021-03819-2  
3) van Kempen M, Kim S, Tumescheit C, Mirdita M, Lee J, Gilchrist CLM, Söding J, and Steinegger M (2023) Fast and accurate protein structure search with Foldseek. Nature Biotechnology. doi: 10.1038/s41587-023-01773-0
