# Supplementary material for: Completing the BASEL phage collection to unlock hidden diversity for systematic exploration of phage–host interactions
Source: PLoS Biol. 2025 Apr 7;23(4):e3003063. doi: 10.1371/journal.pbio.3003063 (PMC11990801; doi:10.1371/journal.pbio.3003063)
Supplement: S2 Data — (ZIP) [file pbio.3003063.s009.zip › entries/20.html]

FANPEZAQ\_CDS\_0020


Return to summary | Go to previous | Go to next

|  |  |
| --- | --- |
| FANPEZAQ\_CDS\_0020 Page creation date: 02 Sep 2024, 12:00  Project folder: n/a  Input sequences file: Escherichia\_virus\_HeidiAbel.gb | tail phage assembly or e chaperone 14\_like gp41 flumu mu\_like prophage putative fragment bacteriophage hypothetical related phage\_related domain\_containing tube small phage\_like phage\_tac\_7 containing hypothethical major whole genome shotgun sequence arsr transcriptional regulator |

### Sequence information

|  |  |
| --- | --- |
| Name | FANPEZAQ\_CDS\_0020  20\_FANPEZAQ\_CDS\_0020 (pipeline id) |
| Imported annotations | Escherichia\_virus\_HeidiAbel Bas97 |
| Protein sequence | MAKLPDYLKFNHETGHCDITLSRPLKIAGVDVPVLRMREPTVADQEIASDMGGSDAAREI QTFANLCEQAPDDIRRLPLKDYKRLQAAYVNFID |
| Number of residues | 94 |
| Molecular weight (Da) | 10588.98 |
| Output files | ../../query\_sequences/20\_FANPEZAQ\_CDS\_0020.fasta |

### Putative domain architecture and protein family

#### Search results (HHblits)1

|  |  |
| --- | --- |
| Domain family databases searched | Pfam, Ncbi-cd, Cath, Phrogs |
| Results, scheme(s)  (Top layers only; threshold 1.00e-03 (evalue)) | xml version="1.0" encoding="utf-8" standalone="no"?       2024-09-02T21:08:16.522205 image/svg+xml   Matplotlib v3.7.2, https://matplotlib.org/ |
| Results, table  (E-value ≤ 1.00e-03 (evalue)) | | db | id | prob | evalue | pvalue | score | cols | query | query\_len | template | template\_len | name | description | | --- | --- | --- | --- | --- | --- | --- | --- | --- | --- | --- | --- | --- | | pfam | PF10109 | 99.5 | 1.1e-19 | 2.2e-23 | 102.9 | 77 | (18, 94) | 94 | (1, 84) | 84 | Phage\_TAC\_7 | Phage tail assembly chaperone proteins, E, or 41 or 14 | | phrogs | 103 | 99.7 | 3.3e-23 | 4.1e-27 | 128.6 | 84 | (11, 94) | 94 | (10, 97) | 108 | tail protein | tail protein; Category: tail; NC\_003278\_p26 | | phrogs | 166 | 99.6 | 4.9e-21 | 6.4e-25 | 116.5 | 79 | (16, 94) | 94 | (2, 89) | 98 | tail protein | tail protein; Category: tail; p131196 VI\_08606 | | phrogs | 25559 | 99.1 | 1.3e-14 | 1.5e-18 | 89.6 | 92 | (1, 93) | 94 | (19, 121) | 131 | NA | NA; Category: unknown function; p249034 VI\_04203 | | phrogs | 3140 | 99.0 | 3.8e-14 | 4.6e-18 | 84.7 | 77 | (17, 93) | 94 | (2, 90) | 92 | tail assembly chaperone | tail assembly chaperone; Category: tail; NC\_024369\_p21 | | phrogs | 4648 | 98.0 | 2.2e-09 | 2.6e-13 | 67.4 | 81 | (13, 93) | 94 | (53, 139) | 139 | NA | NA; Category: unknown function; KY421186\_p67 | | phrogs | 17026 | 97.9 | 3.4e-09 | 3.9e-13 | 68.0 | 77 | (12, 94) | 94 | (94, 170) | 180 | NA | NA; Category: unknown function; p161962 VI\_09828 | | phrogs | 7608 | 97.5 | 5e-08 | 5.7e-12 | 57.4 | 72 | (17, 91) | 94 | (2, 81) | 100 | NA | NA; Category: unknown function; p416168 VI\_03036 | | phrogs | 9738 | 96.4 | 1e-05 | 1.1e-09 | 50.0 | 82 | (13, 94) | 94 | (6, 131) | 155 | NA | NA; Category: unknown function; p338877 VI\_04185 | | phrogs | 30387 | 96.3 | 1.3e-05 | 1.5e-09 | 49.1 | 74 | (7, 84) | 94 | (63, 136) | 151 | NA | NA; Category: unknown function; p193301 VI\_04290 | | phrogs | 24302 | 96.1 | 2.6e-05 | 3e-09 | 45.6 | 78 | (16, 93) | 94 | (3, 94) | 106 | NA | NA; Category: unknown function; NC\_025427\_p15 | | phrogs | 30394 | 95.2 | 0.00024 | 2.7e-08 | 36.2 | 30 | (64, 93) | 94 | (2, 31) | 56 | NA | NA; Category: unknown function; p376042 VI\_09042 | |
| Top keywords  (threshold 1.00e-03 (evalue)) | **tail, assembly, chaperone, or, Phage, proteins, E, NC\_003278\_p26, p131196, VI\_08606** |
| Output files | ../../domain\_architecture/20\_FANPEZAQ\_CDS\_0020\_cath.hhr ../../domain\_architecture/20\_FANPEZAQ\_CDS\_0020\_merged.svg ../../domain\_architecture/20\_FANPEZAQ\_CDS\_0020\_ncbi-cd.hhr ../../domain\_architecture/20\_FANPEZAQ\_CDS\_0020\_pfam.hhr ../../domain\_architecture/20\_FANPEZAQ\_CDS\_0020\_phrogs.hhr |

### Identical protein sequences/structures

#### Search results

|  |  |
| --- | --- |
| Protein sequence databases searched | Pdb, Swissprot, Refseq |
| Identical proteins found | -- |
| Top keywords | -- |
| Output files | -- |

### Similar protein sequences/structures

#### Sequence similarity search results (HHblits)1

|  |  |
| --- | --- |
| Sequence databases searched | Uniclust, Pdb70 |
| Results, scheme(s)  (Top layers only, threshold 1.00e-03 (evalue)) | xml version="1.0" encoding="utf-8" standalone="no"?       2024-09-02T21:08:37.930161 image/svg+xml   Matplotlib v3.7.2, https://matplotlib.org/ |
| Results, table(s)  (threshold 1.00e-03 (evalue)) | | db | id | prob | evalue | pvalue | score | cols | query | query\_len | template | template\_len | name | description | | --- | --- | --- | --- | --- | --- | --- | --- | --- | --- | --- | --- | --- | | uniclust | UniRef100\_A0A023Q1E1 | 99.9 | 1.9e-26 | 4.1e-32 | 146.9 | 91 | (2, 94) | 94 | (16, 106) | 137 | Phage tail assembly protein | Phage tail assembly protein | | uniclust | UniRef100\_A0A0K2IH21 | 99.8 | 8.1e-24 | 1.7e-29 | 133.4 | 92 | (2, 94) | 94 | (16, 107) | 133 | Phage tail assembly protein | Phage tail assembly protein | | uniclust | UniRef100\_A0A069PKK6 | 99.8 | 1.3e-23 | 2.6e-29 | 131.8 | 82 | (13, 94) | 94 | (20, 102) | 129 | Phage tail assembly protein | Phage tail assembly protein | | uniclust | UniRef100\_A0A024HBU0 | 99.8 | 6e-23 | 1.2e-28 | 128.1 | 90 | (2, 93) | 94 | (14, 103) | 122 | Phage tail assembly protein | Phage tail assembly protein | | uniclust | UniRef100\_A0A060H358 | 99.8 | 8e-23 | 1.6e-28 | 126.7 | 93 | (1, 94) | 94 | (29, 121) | 126 | Phage tail assembly protein | Phage tail assembly protein | | uniclust | UniRef100\_A0A3M1X2P8 | 99.8 | 7.9e-23 | 1.6e-28 | 122.4 | 82 | (13, 94) | 94 | (6, 87) | 95 | Phage tail assembly protein | Phage tail assembly protein | | uniclust | UniRef100\_A0A0P8Y9Q0 | 99.8 | 1.2e-22 | 2.5e-28 | 121.6 | 93 | (1, 94) | 94 | (3, 96) | 96 | Phage tail assembly protein | Phage tail assembly protein | | uniclust | UniRef100\_A0A1G7S922 | 99.7 | 7.5e-21 | 1.5e-26 | 115.8 | 83 | (12, 94) | 94 | (5, 87) | 103 | Phage tail assembly chaperone protein, E, or 41 or 14 | Phage tail assembly chaperone protein, E, or 41 or 14 | | uniclust | UniRef100\_A0A212KJV9 | 99.7 | 1.9e-20 | 4.2e-26 | 113.5 | 82 | (12, 94) | 94 | (2, 84) | 96 | Phage tail assembly protein | Phage tail assembly protein | | uniclust | UniRef100\_A0A0C1H2X0 | 99.7 | 4.1e-20 | 8.3e-26 | 115.0 | 79 | (14, 93) | 94 | (28, 107) | 122 | Phage tail assembly protein | Phage tail assembly protein | | uniclust | UniRef100\_A0A0M4L5V1 | 99.7 | 5.2e-20 | 1.1e-25 | 110.8 | 80 | (13, 94) | 94 | (7, 86) | 99 | Uncharacterized protein | Uncharacterized protein | | uniclust | UniRef100\_A0A021XB90 | 99.7 | 1.2e-19 | 2.6e-25 | 115.8 | 82 | (12, 94) | 94 | (19, 100) | 137 | Mu-like prophage FluMu protein gp41 | Mu-like prophage FluMu protein gp41 | | uniclust | UniRef100\_A0A024E9W1 | 99.6 | 2.6e-19 | 5.3e-25 | 112.3 | 88 | (2, 91) | 94 | (18, 106) | 125 | Phage tail assembly protein | Phage tail assembly protein | | uniclust | UniRef100\_A0A011LYC3 | 99.6 | 5.4e-19 | 1.1e-24 | 111.6 | 79 | (14, 92) | 94 | (18, 97) | 129 | Phage tail protein | Phage tail protein | | uniclust | UniRef100\_A0A239LH38 | 99.6 | 7.3e-19 | 1.5e-24 | 105.6 | 80 | (15, 94) | 94 | (4, 84) | 93 | Phage tail assembly chaperone protein, E, or 41 or 14 | Phage tail assembly chaperone protein, E, or 41 or 14 | | uniclust | UniRef100\_A0A024E9U8 | 99.6 | 8.7e-19 | 1.8e-24 | 111.1 | 82 | (12, 93) | 94 | (8, 98) | 128 | Sigma-factor domain-containing protein | Sigma-factor domain-containing protein | | uniclust | UniRef100\_A0A1X7MB04 | 99.6 | 1e-18 | 1.9e-24 | 100.5 | 79 | (16, 94) | 94 | (3, 81) | 85 | Uncharacterized protein (Fragment) | Uncharacterized protein (Fragment) | | uniclust | UniRef100\_A0A085ARU0 | 99.6 | 1e-18 | 2.1e-24 | 110.8 | 82 | (12, 93) | 94 | (17, 99) | 136 | Phage tail assembly protein | Phage tail assembly protein | | uniclust | UniRef100\_A0A143DC33 | 99.6 | 1.1e-18 | 2.2e-24 | 106.7 | 81 | (14, 94) | 94 | (3, 90) | 104 | Phage tail protein | Phage tail protein | | uniclust | UniRef100\_A0A023WUD5 | 99.6 | 1.1e-18 | 2.3e-24 | 112.4 | 82 | (13, 94) | 94 | (37, 126) | 141 | Phage tail assembly protein | Phage tail assembly protein | | uniclust | UniRef100\_A0A0P1FH37 | 99.6 | 1.2e-18 | 2.4e-24 | 105.8 | 83 | (12, 94) | 94 | (3, 88) | 106 | Phage tail protein E | Phage tail protein E | | uniclust | UniRef100\_A0A0K1JXV3 | 99.6 | 6.9e-18 | 1.4e-23 | 105.5 | 88 | (3, 92) | 94 | (6, 95) | 123 | Phage tail assembly protein | Phage tail assembly protein | | uniclust | UniRef100\_A0A0Q4II11 | 99.6 | 7.1e-18 | 1.5e-23 | 104.1 | 87 | (4, 94) | 94 | (9, 95) | 107 | Phage tail protein | Phage tail protein | | uniclust | UniRef100\_A0A239C819 | 99.6 | 8.3e-18 | 1.6e-23 | 101.9 | 80 | (15, 94) | 94 | (26, 105) | 110 | Phage tail assembly chaperone protein, E, or 41 or 14 | Phage tail assembly chaperone protein, E, or 41 or 14 | | uniclust | UniRef100\_A0A4U0QBM5 | 99.5 | 1.1e-17 | 2.3e-23 | 99.7 | 82 | (12, 94) | 94 | (4, 85) | 93 | Phage tail assembly protein | Phage tail assembly protein | | uniclust | UniRef100\_A0A0C5VFB7 | 99.5 | 1.6e-17 | 3e-23 | 100.7 | 82 | (13, 94) | 94 | (18, 99) | 106 | Mu-like prophage FluMu protein gp41 | Mu-like prophage FluMu protein gp41 | | uniclust | UniRef100\_A0A7L8G6A2 | 99.5 | 1.6e-17 | 3.1e-23 | 104.2 | 91 | (2, 93) | 94 | (6, 96) | 145 | Tape measure chaperone | Tape measure chaperone | | uniclust | UniRef100\_A0A0R3MUP2 | 99.5 | 1.6e-17 | 3.1e-23 | 101.8 | 79 | (15, 93) | 94 | (19, 97) | 109 | Phage tail assembly protein | Phage tail assembly protein | | uniclust | UniRef100\_A0A022PMF2 | 99.5 | 1.5e-17 | 3.1e-23 | 104.6 | 81 | (13, 93) | 94 | (15, 96) | 123 | Mu-like prophage FluMu protein gp41 | Mu-like prophage FluMu protein gp41 | | uniclust | UniRef100\_A0A212KXH5 | 99.5 | 3.2e-17 | 6.4e-23 | 101.5 | 81 | (13, 93) | 94 | (3, 83) | 119 | Phage tail assembly protein | Phage tail assembly protein | | uniclust | UniRef100\_A0A0N0XKQ2 | 99.4 | 3.8e-16 | 7.4e-22 | 94.2 | 78 | (16, 93) | 94 | (3, 89) | 99 | Phage tail protein E | Phage tail protein E | | uniclust | UniRef100\_A0A011UPH2 | 99.4 | 4.1e-16 | 8.3e-22 | 96.5 | 82 | (12, 93) | 94 | (9, 90) | 111 | Phage tail assembly protein | Phage tail assembly protein | | uniclust | UniRef100\_A0A017HBQ7 | 99.4 | 4.2e-16 | 8.6e-22 | 97.1 | 81 | (13, 94) | 94 | (8, 88) | 115 | Phage tail assembly protein | Phage tail assembly protein | | uniclust | UniRef100\_A0A149SWH3 | 99.4 | 5e-16 | 9.6e-22 | 94.5 | 92 | (2, 93) | 94 | (14, 110) | 111 | Phage tail assembly protein | Phage tail assembly protein | | uniclust | UniRef100\_A0A143DG95 | 99.4 | 8.3e-16 | 1.7e-21 | 95.6 | 79 | (16, 94) | 94 | (9, 91) | 115 | Phage tail protein | Phage tail protein | | uniclust | UniRef100\_A0A1I3SW87 | 99.4 | 8.4e-16 | 1.7e-21 | 92.1 | 80 | (14, 93) | 94 | (3, 84) | 93 | Phage tail assembly chaperone protein, E, or 41 or 14 | Phage tail assembly chaperone protein, E, or 41 or 14 | | uniclust | UniRef100\_A0A1M5PX63 | 99.4 | 8.5e-16 | 1.7e-21 | 94.2 | 83 | (12, 94) | 94 | (11, 94) | 103 | Phage tail assembly chaperone protein, E, or 41 or 14 | Phage tail assembly chaperone protein, E, or 41 or 14 | | uniclust | UniRef100\_A0A4S5JA13 | 99.4 | 1.7e-15 | 3.4e-21 | 98.3 | 80 | (13, 94) | 94 | (37, 116) | 148 | Phage tail assembly protein | Phage tail assembly protein | | uniclust | UniRef100\_A0A0U5I607 | 99.4 | 2e-15 | 3.8e-21 | 93.8 | 83 | (12, 94) | 94 | (6, 89) | 119 | Uncharacterized protein | Uncharacterized protein | | uniclust | UniRef100\_A0A2W5N4W8 | 99.4 | 2.8e-15 | 5.1e-21 | 91.5 | 89 | (3, 93) | 94 | (35, 123) | 124 | Phage tail assembly protein | Phage tail assembly protein | | uniclust | UniRef100\_A0A066ZM25 | 99.4 | 2.9e-15 | 5.6e-21 | 90.6 | 81 | (13, 93) | 94 | (14, 96) | 99 | Phage tail assembly protein | Phage tail assembly protein | | uniclust | UniRef100\_A0A2J8GXT3 | 99.3 | 3.3e-15 | 6.6e-21 | 92.4 | 78 | (16, 93) | 94 | (10, 100) | 113 | Phage tail assembly protein | Phage tail assembly protein | | uniclust | UniRef100\_A0A0H3WRU3 | 99.3 | 4.7e-15 | 9.4e-21 | 92.7 | 80 | (13, 93) | 94 | (18, 101) | 117 | Phage tail assembly protein | Phage tail assembly protein | | uniclust | UniRef100\_A0A031IT93 | 99.3 | 5.3e-15 | 1.1e-20 | 99.5 | 90 | (2, 93) | 94 | (8, 98) | 200 | Phage tail assembly protein | Phage tail assembly protein | | uniclust | UniRef100\_A0A0C4YA46 | 99.3 | 5.4e-15 | 1.1e-20 | 96.3 | 81 | (13, 93) | 94 | (38, 122) | 144 | Tail protein | Tail protein | | uniclust | UniRef100\_A0A2G6CS65 | 99.3 | 7.9e-15 | 1.6e-20 | 85.9 | 71 | (15, 94) | 94 | (3, 73) | 79 | Phage tail assembly protein | Phage tail assembly protein | | uniclust | UniRef100\_A0A6I4YRP3 | 99.3 | 1.1e-14 | 2.1e-20 | 90.4 | 91 | (2, 94) | 94 | (8, 108) | 115 | Phage tail assembly protein | Phage tail assembly protein | | uniclust | UniRef100\_A0A031IT93 | 99.3 | 1.1e-14 | 2.2e-20 | 98.0 | 88 | (2, 91) | 94 | (105, 193) | 200 | Phage tail assembly protein | Phage tail assembly protein | | uniclust | UniRef100\_A0A151FEY6 | 99.3 | 1.1e-14 | 2.3e-20 | 92.0 | 81 | (13, 93) | 94 | (19, 110) | 126 | Phage tail assembly protein | Phage tail assembly protein | | uniclust | UniRef100\_A0A2V3VR35 | 99.3 | 1.2e-14 | 2.4e-20 | 89.0 | 82 | (13, 94) | 94 | (9, 94) | 103 | Tail assembly chaperone E/41/14-like protein | Tail assembly chaperone E/41/14-like protein | | uniclust | UniRef100\_A0A1I3TBZ1 | 99.3 | 1.5e-14 | 2.9e-20 | 92.4 | 81 | (13, 93) | 94 | (28, 125) | 138 | Phage tail assembly chaperone protein, E, or 41 or 14 | Phage tail assembly chaperone protein, E, or 41 or 14 | | uniclust | UniRef100\_A0A061YFP8 | 99.3 | 1.6e-14 | 3.1e-20 | 86.5 | 77 | (16, 92) | 94 | (8, 84) | 97 | Phage tail assembly protein | Phage tail assembly protein | | uniclust | UniRef100\_A0A014N9C7 | 99.3 | 1.5e-14 | 3.2e-20 | 96.7 | 78 | (16, 93) | 94 | (55, 136) | 174 | Mu-like prophage FluMu gp41 family protein | Mu-like prophage FluMu gp41 family protein | | uniclust | UniRef100\_A0A1G3UAH2 | 99.3 | 1.8e-14 | 3.5e-20 | 83.8 | 66 | (25, 94) | 94 | (8, 73) | 75 | Phage tail protein | Phage tail protein | | uniclust | UniRef100\_A0A066TS77 | 99.3 | 1.9e-14 | 3.7e-20 | 90.6 | 75 | (16, 90) | 94 | (39, 115) | 125 | Phage tail assembly protein | Phage tail assembly protein | | uniclust | UniRef100\_A0A098TF13 | 99.3 | 2.2e-14 | 4.6e-20 | 92.2 | 83 | (12, 94) | 94 | (12, 107) | 132 | Uncharacterized protein | Uncharacterized protein | | uniclust | UniRef100\_A0A0A1HVW3 | 99.3 | 2.4e-14 | 4.9e-20 | 92.9 | 79 | (15, 93) | 94 | (36, 118) | 141 | Putative phage tail protein | Putative phage tail protein | | uniclust | UniRef100\_A0A1G5SVM9 | 99.3 | 2.5e-14 | 5e-20 | 89.7 | 79 | (16, 94) | 94 | (25, 103) | 117 | Phage tail assembly chaperone protein, E, or 41 or 14 | Phage tail assembly chaperone protein, E, or 41 or 14 | | uniclust | UniRef100\_A0A259MGY1 | 99.2 | 4.5e-14 | 8.9e-20 | 86.4 | 78 | (16, 93) | 94 | (2, 82) | 101 | Phage tail assembly protein | Phage tail assembly protein | | uniclust | UniRef100\_A0A009QFF4 | 99.2 | 4.8e-14 | 9.8e-20 | 92.9 | 82 | (13, 94) | 94 | (41, 126) | 157 | Mu-like prophage FluMu gp41 family protein | Mu-like prophage FluMu gp41 family protein | | uniclust | UniRef100\_A0A165RMJ9 | 99.2 | 5e-14 | 9.9e-20 | 86.6 | 80 | (13, 93) | 94 | (5, 84) | 107 | Phage tail assembly chaperone protein, E, or 41 or 14 | Phage tail assembly chaperone protein, E, or 41 or 14 | | uniclust | UniRef100\_A0A7J0BVD3 | 99.2 | 5.4e-14 | 1e-19 | 86.6 | 82 | (13, 94) | 94 | (25, 106) | 129 | Phage tail assembly protein | Phage tail assembly protein | | uniclust | UniRef100\_A0A084EX45 | 99.2 | 5.5e-14 | 1.1e-19 | 91.1 | 82 | (13, 94) | 94 | (41, 126) | 146 | Phage tail protein E | Phage tail protein E | | uniclust | UniRef100\_A0A2T1B5W6 | 99.2 | 6e-14 | 1.2e-19 | 86.0 | 81 | (14, 94) | 94 | (15, 101) | 111 | Tail assembly chaperone E/41/14-like protein | Tail assembly chaperone E/41/14-like protein | | uniclust | UniRef100\_A0A087NA98 | 99.2 | 5.5e-14 | 1.2e-19 | 90.3 | 79 | (15, 93) | 94 | (26, 108) | 130 | Phage-related tail protein | Phage-related tail protein | | uniclust | UniRef100\_A0A1F8WN21 | 99.2 | 6.9e-14 | 1.3e-19 | 83.9 | 79 | (14, 94) | 94 | (4, 82) | 94 | Phage tail assembly protein | Phage tail assembly protein | | uniclust | UniRef100\_A0A248LIV2 | 99.2 | 8.8e-14 | 1.6e-19 | 82.2 | 77 | (15, 91) | 94 | (2, 78) | 98 | Phage\_TAC\_7 domain containing protein | Phage\_TAC\_7 domain containing protein | | uniclust | UniRef100\_A0A1Y2K105 | 99.2 | 1e-13 | 1.9e-19 | 79.2 | 78 | (16, 94) | 94 | (3, 80) | 80 | Phage tail assembly protein | Phage tail assembly protein | | uniclust | UniRef100\_A0A1A9R7D8 | 99.2 | 1.1e-13 | 2.3e-19 | 88.2 | 80 | (14, 93) | 94 | (23, 106) | 129 | Phage tail protein | Phage tail protein | | uniclust | UniRef100\_A0A370DZC3 | 99.2 | 1.3e-13 | 2.5e-19 | 82.9 | 77 | (16, 93) | 94 | (24, 100) | 100 | Phage tail assembly protein | Phage tail assembly protein | | uniclust | UniRef100\_A0A5E4XFR1 | 99.2 | 1.3e-13 | 2.5e-19 | 84.9 | 80 | (15, 94) | 94 | (27, 110) | 119 | Phage tail assembly protein | Phage tail assembly protein | | uniclust | UniRef100\_A0A9D2KQ00 | 99.2 | 1.4e-13 | 2.6e-19 | 81.1 | 79 | (15, 94) | 94 | (2, 81) | 90 | Phage tail assembly protein | Phage tail assembly protein | | uniclust | UniRef100\_A0A071MEA2 | 99.2 | 1.3e-13 | 2.6e-19 | 91.2 | 79 | (15, 93) | 94 | (67, 149) | 165 | Phage tail protein | Phage tail protein | | uniclust | UniRef100\_UPI00192BA721 | 99.2 | 1.5e-13 | 2.8e-19 | 85.0 | 82 | (12, 93) | 94 | (3, 84) | 123 | phage tail assembly protein | phage tail assembly protein | | uniclust | UniRef100\_A0A1B8QCY3 | 99.2 | 1.4e-13 | 2.9e-19 | 86.5 | 83 | (12, 94) | 94 | (15, 101) | 117 | Phage tail protein E | Phage tail protein E | | uniclust | UniRef100\_A0A0M7MLL6 | 99.1 | 2.1e-13 | 4.2e-19 | 86.4 | 80 | (15, 94) | 94 | (15, 101) | 124 | Phage tail assembly protein | Phage tail assembly protein | | uniclust | UniRef100\_A0A074LRJ5 | 99.1 | 2.1e-13 | 4.5e-19 | 88.3 | 80 | (14, 93) | 94 | (21, 112) | 131 | Phage tail assembly protein | Phage tail assembly protein | | uniclust | UniRef100\_A0A1I7ND34 | 99.1 | 2.7e-13 | 5e-19 | 84.5 | 78 | (16, 94) | 94 | (4, 81) | 137 | Phage tail assembly chaperone protein, E, or 41 or 14 | Phage tail assembly chaperone protein, E, or 41 or 14 | | uniclust | UniRef100\_A0A068Z8E9 | 99.1 | 2.8e-13 | 5.7e-19 | 89.0 | 82 | (13, 94) | 94 | (48, 133) | 154 | Phage tail assembly protein | Phage tail assembly protein | | uniclust | UniRef100\_A0A1M6QZE2 | 99.1 | 3.1e-13 | 5.7e-19 | 83.6 | 78 | (15, 92) | 94 | (30, 108) | 127 | Phage tail assembly chaperone protein, E, or 41 or 14 | Phage tail assembly chaperone protein, E, or 41 or 14 | | uniclust | UniRef100\_A0A1F3A562 | 99.1 | 4.2e-13 | 8e-19 | 87.3 | 81 | (14, 94) | 94 | (8, 101) | 165 | Phage tail assembly protein | Phage tail assembly protein | | uniclust | UniRef100\_A0A450ZAK9 | 99.1 | 4.5e-13 | 8.3e-19 | 80.4 | 82 | (13, 94) | 94 | (14, 99) | 107 | Phage tail assembly chaperone protein, E, or 41 or 14 | Phage tail assembly chaperone protein, E, or 41 or 14 | | uniclust | UniRef100\_A0A0J6K5A0 | 99.1 | 5.7e-13 | 1.1e-18 | 79.6 | 80 | (15, 94) | 94 | (12, 93) | 93 | Phage tail assembly protein | Phage tail assembly protein | | uniclust | UniRef100\_A0A067ZIM0 | 99.1 | 6.2e-13 | 1.3e-18 | 85.1 | 78 | (16, 93) | 94 | (4, 93) | 123 | Phage tail assembly protein | Phage tail assembly protein | | uniclust | UniRef100\_A0A0N1C7F5 | 99.1 | 6.1e-13 | 1.3e-18 | 90.0 | 80 | (14, 94) | 94 | (65, 144) | 177 | Phage tail assembly protein | Phage tail assembly protein | | uniclust | UniRef100\_A0A1H7TYI9 | 99.1 | 6.6e-13 | 1.3e-18 | 83.7 | 81 | (13, 93) | 94 | (12, 94) | 124 | Phage tail assembly chaperone protein, E, or 41 or 14 | Phage tail assembly chaperone protein, E, or 41 or 14 | | uniclust | UniRef100\_A0A068T8B4 | 99.1 | 6.4e-13 | 1.3e-18 | 90.3 | 82 | (13, 94) | 94 | (76, 157) | 187 | Phage tail assembly protein | Phage tail assembly protein | | uniclust | UniRef100\_A0A1M3BPS8 | 99.1 | 7.1e-13 | 1.5e-18 | 84.1 | 80 | (15, 94) | 94 | (20, 104) | 121 | Phage tail assembly protein | Phage tail assembly protein | | uniclust | UniRef100\_A0A524RVY5 | 99.1 | 8.6e-13 | 1.6e-18 | 80.3 | 92 | (3, 94) | 94 | (9, 104) | 116 | Phage tail assembly protein | Phage tail assembly protein | | uniclust | UniRef100\_A0A016XHR3 | 99.1 | 9.8e-13 | 1.9e-18 | 84.0 | 80 | (14, 93) | 94 | (32, 115) | 132 | Phage tail assembly protein | Phage tail assembly protein | | uniclust | UniRef100\_A0A0F3IN91 | 99.1 | 1e-12 | 2e-18 | 79.2 | 80 | (14, 94) | 94 | (2, 83) | 95 | Phage tail assembly protein (Fragment) | Phage tail assembly protein (Fragment) | | uniclust | UniRef100\_UPI000B7E693B | 99.0 | 1.2e-12 | 2.3e-18 | 76.3 | 74 | (20, 93) | 94 | (2, 83) | 86 | phage tail assembly protein | phage tail assembly protein | | uniclust | UniRef100\_A0A0A3ASC6 | 99.0 | 1.2e-12 | 2.5e-18 | 81.7 | 77 | (16, 92) | 94 | (19, 100) | 111 | Phage tail protein | Phage tail protein | | uniclust | UniRef100\_A0A3N7CHT4 | 99.0 | 1.3e-12 | 2.5e-18 | 79.4 | 82 | (13, 94) | 94 | (5, 93) | 101 | Phage tail assembly protein | Phage tail assembly protein | | uniclust | UniRef100\_A0A0G9KCB9 | 99.0 | 1.4e-12 | 2.8e-18 | 80.3 | 78 | (16, 94) | 94 | (30, 107) | 109 | Phage tail assembly protein | Phage tail assembly protein | | uniclust | UniRef100\_UPI0021AA529F | 99.0 | 1.7e-12 | 3.1e-18 | 83.0 | 79 | (12, 90) | 94 | (14, 92) | 156 | phage tail assembly protein | phage tail assembly protein | | uniclust | UniRef100\_UPI00082D28E9 | 99.0 | 1.7e-12 | 3.2e-18 | 76.7 | 81 | (12, 93) | 94 | (3, 83) | 96 | phage tail assembly protein | phage tail assembly protein | | uniclust | UniRef100\_A0A137SPX7 | 99.0 | 2.6e-12 | 5.3e-18 | 85.1 | 79 | (15, 93) | 94 | (52, 144) | 157 | Phage tail assembly protein | Phage tail assembly protein | | uniclust | UniRef100\_A0A0N0MC10 | 99.0 | 3.4e-12 | 6.6e-18 | 83.3 | 79 | (16, 94) | 94 | (47, 125) | 152 | Uncharacterized protein | Uncharacterized protein | | uniclust | UniRef100\_A0A502FV57 | 99.0 | 4e-12 | 7.7e-18 | 76.9 | 76 | (16, 93) | 94 | (11, 86) | 95 | Phage tail assembly protein | Phage tail assembly protein | | uniclust | UniRef100\_A0A1V0DX85 | 99.0 | 4e-12 | 8.1e-18 | 85.0 | 82 | (13, 94) | 94 | (25, 110) | 167 | Tapemeasure chaperone | Tapemeasure chaperone | | uniclust | UniRef100\_A0A650EN83 | 99.0 | 4.3e-12 | 8.6e-18 | 79.0 | 77 | (18, 94) | 94 | (3, 83) | 111 | Phage tail assembly protein | Phage tail assembly protein | | uniclust | UniRef100\_A0A554XFV5 | 99.0 | 4.9e-12 | 9e-18 | 74.9 | 87 | (1, 91) | 94 | (1, 88) | 96 | Phage tail assembly chaperone protein, E, or 41 or 14 | Phage tail assembly chaperone protein, E, or 41 or 14 | | uniclust | UniRef100\_A0A0Q4I3S2 | 99.0 | 4.6e-12 | 9.4e-18 | 84.3 | 78 | (16, 93) | 94 | (55, 136) | 163 | Phage tail protein | Phage tail protein | | uniclust | UniRef100\_UPI0009D9DDE6 | 99.0 | 5.2e-12 | 9.6e-18 | 75.9 | 89 | (3, 92) | 94 | (5, 99) | 101 | phage tail assembly protein | phage tail assembly protein | | uniclust | UniRef100\_A0A066RUL2 | 98.9 | 5.3e-12 | 1e-17 | 81.4 | 80 | (14, 93) | 94 | (10, 98) | 141 | Phage tail assembly protein | Phage tail assembly protein | | uniclust | UniRef100\_Q65WG3 | 98.9 | 7.7e-12 | 1.4e-17 | 77.4 | 75 | (17, 92) | 94 | (46, 121) | 127 | Phage tail assembly protein | Phage tail assembly protein | | uniclust | UniRef100\_A0A070A7W1 | 98.9 | 6.8e-12 | 1.4e-17 | 81.4 | 91 | (2, 94) | 94 | (22, 124) | 133 | Phage tail assembly protein | Phage tail assembly protein | | uniclust | UniRef100\_A0A1A9VKI4 | 98.9 | 8.6e-12 | 1.6e-17 | 82.2 | 81 | (14, 94) | 94 | (111, 191) | 197 | Phage tail assembly protein | Phage tail assembly protein | | uniclust | UniRef100\_A0A366IL82 | 98.9 | 8.9e-12 | 1.7e-17 | 78.6 | 79 | (15, 93) | 94 | (17, 99) | 125 | Tail assembly chaperone E/41/14-like protein | Tail assembly chaperone E/41/14-like protein | | uniclust | UniRef100\_A0A1H9Q8X8 | 98.9 | 1e-11 | 1.9e-17 | 76.1 | 81 | (13, 93) | 94 | (14, 105) | 111 | Phage tail assembly chaperone protein, E, or 41 or 14 | Phage tail assembly chaperone protein, E, or 41 or 14 | | uniclust | UniRef100\_A0A1A6FM26 | 98.9 | 1e-11 | 2.2e-17 | 82.5 | 79 | (12, 94) | 94 | (33, 112) | 152 | Uncharacterized protein | Uncharacterized protein | | uniclust | UniRef100\_A0A089JI40 | 98.9 | 1.1e-11 | 2.3e-17 | 80.0 | 78 | (16, 93) | 94 | (24, 116) | 131 | Phage tail assembly protein | Phage tail assembly protein | | uniclust | UniRef100\_A0A2P5MUZ4 | 98.9 | 1.1e-11 | 2.3e-17 | 77.9 | 74 | (17, 92) | 94 | (3, 77) | 110 | Phage tail assembly protein | Phage tail assembly protein | | uniclust | UniRef100\_UPI000C6D6F4B | 98.9 | 1.3e-11 | 2.4e-17 | 74.8 | 78 | (17, 94) | 94 | (2, 80) | 105 | phage tail assembly protein | phage tail assembly protein | | uniclust | UniRef100\_UPI001F58CA0F | 98.9 | 1.4e-11 | 2.6e-17 | 76.8 | 81 | (13, 93) | 94 | (11, 93) | 120 | phage tail assembly protein | phage tail assembly protein | | uniclust | UniRef100\_A0A0B4B3T3 | 98.9 | 1.4e-11 | 2.6e-17 | 73.6 | 89 | (2, 90) | 94 | (3, 94) | 98 | Phage tail assembly protein | Phage tail assembly protein | | uniclust | UniRef100\_A0A5S9R620 | 98.9 | 1.4e-11 | 2.7e-17 | 73.8 | 77 | (18, 94) | 94 | (4, 82) | 94 | Phage tail assembly protein | Phage tail assembly protein | | uniclust | UniRef100\_A0A1N7LRA5 | 98.9 | 1.6e-11 | 3.1e-17 | 77.5 | 85 | (8, 93) | 94 | (29, 113) | 129 | Phage tail assembly chaperone protein, E, or 41 or 14 | Phage tail assembly chaperone protein, E, or 41 or 14 | | uniclust | UniRef100\_A0A077KTP9 | 98.9 | 1.7e-11 | 3.4e-17 | 78.2 | 81 | (13, 93) | 94 | (28, 112) | 126 | Phage tail assembly protein | Phage tail assembly protein | | uniclust | UniRef100\_A0A212KBP9 | 98.9 | 1.9e-11 | 3.6e-17 | 75.2 | 77 | (17, 93) | 94 | (5, 84) | 112 | Uncharacterized protein | Uncharacterized protein | | uniclust | UniRef100\_A0A8S5PW02 | 98.9 | 1.9e-11 | 3.6e-17 | 76.4 | 78 | (13, 90) | 94 | (3, 86) | 115 | Tail assembly chaperone protein | Tail assembly chaperone protein | | uniclust | UniRef100\_UPI0013ED4072 | 98.9 | 2.1e-11 | 3.8e-17 | 72.8 | 82 | (12, 93) | 94 | (13, 94) | 99 | phage tail assembly protein | phage tail assembly protein | | uniclust | UniRef100\_A0A087KRL7 | 98.8 | 2.1e-11 | 4e-17 | 75.1 | 78 | (17, 94) | 94 | (2, 87) | 107 | Phage small tail protein E | Phage small tail protein E | | uniclust | UniRef100\_A0A174U989 | 98.8 | 2.1e-11 | 4.1e-17 | 80.6 | 80 | (14, 93) | 94 | (56, 147) | 157 | Phage tail assembly protein | Phage tail assembly protein | | uniclust | UniRef100\_A0A0K6HHB3 | 98.8 | 2.2e-11 | 4.8e-17 | 81.1 | 83 | (12, 94) | 94 | (24, 120) | 153 | Uncharacterized protein | Uncharacterized protein | | uniclust | UniRef100\_A0A085G9Z8 | 98.8 | 3e-11 | 6e-17 | 76.8 | 80 | (15, 94) | 94 | (22, 105) | 124 | Tail protein | Tail protein | | uniclust | UniRef100\_A0A916UDS7 | 98.8 | 3.4e-11 | 6.4e-17 | 73.2 | 79 | (16, 94) | 94 | (19, 98) | 102 | Uncharacterized protein | Uncharacterized protein | | uniclust | UniRef100\_A0A6M8HND0 | 98.8 | 3.6e-11 | 6.8e-17 | 74.1 | 82 | (13, 94) | 94 | (16, 103) | 111 | Phage tail assembly protein | Phage tail assembly protein | | uniclust | UniRef100\_A0A5A8F0S7 | 98.8 | 3.9e-11 | 7.4e-17 | 70.7 | 69 | (20, 93) | 94 | (6, 77) | 84 | Phage tail assembly protein | Phage tail assembly protein | | uniclust | UniRef100\_S7TEH7 | 98.8 | 4.1e-11 | 7.5e-17 | 70.2 | 76 | (18, 93) | 94 | (6, 84) | 88 | Mu-like prophage FluMu protein gp41 | Mu-like prophage FluMu protein gp41 | | uniclust | UniRef100\_A0A6N8BZ30 | 98.8 | 4.3e-11 | 8.4e-17 | 73.2 | 75 | (16, 94) | 94 | (16, 92) | 100 | Phage tail assembly protein | Phage tail assembly protein | | uniclust | UniRef100\_UPI00138AC42E | 98.8 | 5.4e-11 | 9.9e-17 | 69.1 | 76 | (18, 94) | 94 | (4, 79) | 83 | phage tail assembly protein | phage tail assembly protein | | uniclust | UniRef100\_A0A2A4R5T3 | 98.8 | 4.8e-11 | 1e-16 | 80.1 | 89 | (3, 94) | 94 | (50, 151) | 163 | Phage tail assembly protein | Phage tail assembly protein | | uniclust | UniRef100\_A0A431JGG1 | 98.8 | 5.3e-11 | 1e-16 | 74.6 | 79 | (14, 94) | 94 | (41, 119) | 119 | Phage tail assembly protein | Phage tail assembly protein | | uniclust | UniRef100\_A0A1Y6CZP6 | 98.8 | 5.3e-11 | 1e-16 | 75.4 | 79 | (16, 94) | 94 | (4, 91) | 121 | Phage tail assembly chaperone protein, E, or 41 or 14 | Phage tail assembly chaperone protein, E, or 41 or 14 | | uniclust | UniRef100\_UPI0006B454B7 | 98.8 | 6.7e-11 | 1.2e-16 | 70.1 | 80 | (13, 93) | 94 | (4, 83) | 94 | phage tail assembly protein | phage tail assembly protein | | uniclust | UniRef100\_A0A8X6G6V4 | 98.8 | 6.8e-11 | 1.3e-16 | 76.1 | 80 | (15, 94) | 94 | (69, 148) | 154 | Phage tail assembly protein | Phage tail assembly protein | | uniclust | UniRef100\_A0A1J5B7U6 | 98.8 | 6.7e-11 | 1.3e-16 | 70.6 | 76 | (16, 91) | 94 | (3, 81) | 87 | Phage tail assembly protein | Phage tail assembly protein | | uniclust | UniRef100\_A0A0J7LYN2 | 98.8 | 6.4e-11 | 1.3e-16 | 77.2 | 80 | (14, 94) | 94 | (40, 126) | 138 | Mu-like prophage FluMu protein gp41 | Mu-like prophage FluMu protein gp41 | | uniclust | UniRef100\_A0A3M5CVP7 | 98.8 | 7.3e-11 | 1.4e-16 | 67.2 | 64 | (2, 67) | 94 | (5, 69) | 70 | Uncharacterized protein | Uncharacterized protein | | uniclust | UniRef100\_A0A0D2HN99 | 98.7 | 8e-11 | 1.5e-16 | 68.1 | 77 | (17, 93) | 94 | (3, 80) | 81 | Phage tail assembly protein | Phage tail assembly protein | | uniclust | UniRef100\_A0A0W1G7Y5 | 98.7 | 9.6e-11 | 1.9e-16 | 72.1 | 77 | (16, 93) | 94 | (6, 89) | 99 | Phage tail assembly protein | Phage tail assembly protein | | uniclust | UniRef100\_A0A1N6NVV8 | 98.7 | 9.8e-11 | 1.9e-16 | 77.3 | 90 | (2, 94) | 94 | (46, 148) | 157 | Phage tail assembly chaperone protein, E, or 41 or 14 | Phage tail assembly chaperone protein, E, or 41 or 14 | | uniclust | UniRef100\_A0A0B0HZT8 | 98.7 | 1e-10 | 2e-16 | 74.5 | 79 | (16, 94) | 94 | (25, 120) | 123 | Phage tail assembly protein | Phage tail assembly protein | | uniclust | UniRef100\_A0A0D8ID76 | 98.7 | 9.7e-11 | 2e-16 | 76.6 | 81 | (13, 93) | 94 | (34, 127) | 134 | Mu-like prophage FluMu protein gp41 | Mu-like prophage FluMu protein gp41 | | uniclust | UniRef100\_A0A317H3T5 | 98.7 | 1.1e-10 | 2.2e-16 | 76.8 | 78 | (16, 93) | 94 | (57, 144) | 153 | PqqD family protein | PqqD family protein | | uniclust | UniRef100\_A0A059V670 | 98.7 | 1.2e-10 | 2.3e-16 | 76.2 | 88 | (3, 92) | 94 | (7, 95) | 150 | Phage tail assembly protein | Phage tail assembly protein | | uniclust | UniRef100\_A0A068Z6X2 | 98.7 | 1.2e-10 | 2.5e-16 | 75.5 | 81 | (14, 94) | 94 | (23, 109) | 130 | Phage tail assembly protein | Phage tail assembly protein | | uniclust | UniRef100\_A0A0D5LRC8 | 98.7 | 1.3e-10 | 2.6e-16 | 78.3 | 90 | (2, 94) | 94 | (58, 159) | 172 | Tail assembly chaperone E/41/14-like protein | Tail assembly chaperone E/41/14-like protein | | uniclust | UniRef100\_UPI0013D24EDB | 98.7 | 1.6e-10 | 3e-16 | 71.5 | 79 | (13, 91) | 94 | (19, 97) | 122 | phage tail assembly protein | phage tail assembly protein | | uniclust | UniRef100\_A0A2S6QN92 | 98.7 | 1.7e-10 | 3e-16 | 73.4 | 71 | (15, 85) | 94 | (3, 74) | 147 | Uncharacterized protein (Fragment) | Uncharacterized protein (Fragment) | | uniclust | UniRef100\_A0A349HHP8 | 98.7 | 1.7e-10 | 3.1e-16 | 71.9 | 79 | (12, 90) | 94 | (20, 100) | 128 | Phage tail assembly protein | Phage tail assembly protein | | uniclust | UniRef100\_A0A378YUI0 | 98.7 | 1.7e-10 | 3.2e-16 | 76.3 | 78 | (16, 93) | 94 | (3, 90) | 176 | Phage tail assembly protein | Phage tail assembly protein | | uniclust | UniRef100\_UPI001CCE5A3E | 98.7 | 1.8e-10 | 3.2e-16 | 73.2 | 77 | (16, 92) | 94 | (10, 86) | 145 | phage tail assembly protein | phage tail assembly protein | | uniclust | UniRef100\_A0A151FHH4 | 98.7 | 1.7e-10 | 3.5e-16 | 73.4 | 81 | (12, 92) | 94 | (4, 99) | 114 | Phage tail assembly protein | Phage tail assembly protein | | uniclust | UniRef100\_A0A336NB71 | 98.7 | 1.8e-10 | 3.5e-16 | 80.5 | 73 | (17, 91) | 94 | (7, 79) | 248 | Phage related protein | Phage related protein | | uniclust | UniRef100\_UPI002247CADB | 98.7 | 2.1e-10 | 3.9e-16 | 71.7 | 82 | (13, 94) | 94 | (41, 122) | 130 | phage tail assembly protein | phage tail assembly protein | | uniclust | UniRef100\_A0A0J8DFL1 | 98.7 | 2e-10 | 4.1e-16 | 75.0 | 78 | (16, 93) | 94 | (26, 115) | 134 | Uncharacterized protein | Uncharacterized protein | | uniclust | UniRef100\_A0A139D6Z5 | 98.7 | 2e-10 | 4.1e-16 | 75.0 | 79 | (15, 93) | 94 | (41, 132) | 137 | Phage tail assembly protein | Phage tail assembly protein | | uniclust | UniRef100\_A0A0S4VLK3 | 98.7 | 2.2e-10 | 4.2e-16 | 70.2 | 78 | (16, 93) | 94 | (6, 84) | 109 | Phage tail assembly protein | Phage tail assembly protein | | uniclust | UniRef100\_A0A133ZYZ6 | 98.7 | 2e-10 | 4.2e-16 | 74.6 | 78 | (16, 93) | 94 | (33, 123) | 130 | Phage tail assembly protein | Phage tail assembly protein | | uniclust | UniRef100\_A0A2E6KNH6 | 98.7 | 2.4e-10 | 4.4e-16 | 67.4 | 79 | (16, 94) | 94 | (7, 87) | 90 | Phage tail assembly protein | Phage tail assembly protein | | uniclust | UniRef100\_UPI002101B5F4 | 98.7 | 2.4e-10 | 4.5e-16 | 75.2 | 82 | (12, 93) | 94 | (59, 141) | 187 | phage tail assembly protein | phage tail assembly protein | | uniclust | UniRef100\_A0A158D0Y5 | 98.7 | 2.4e-10 | 4.6e-16 | 77.2 | 78 | (14, 92) | 94 | (2, 83) | 187 | Phage tail assembly protein | Phage tail assembly protein | | uniclust | UniRef100\_A0A212KMT6 | 98.6 | 2.6e-10 | 4.8e-16 | 74.3 | 78 | (16, 93) | 94 | (84, 161) | 174 | Phage tail assembly protein | Phage tail assembly protein | | uniclust | UniRef100\_UPI001FF44E79 | 98.6 | 2.6e-10 | 4.9e-16 | 67.5 | 76 | (15, 90) | 94 | (3, 79) | 92 | phage tail assembly protein | phage tail assembly protein | | uniclust | UniRef100\_A0A0Q4XXX4 | 98.6 | 2.5e-10 | 5.1e-16 | 74.7 | 82 | (13, 94) | 94 | (10, 105) | 134 | Uncharacterized protein | Uncharacterized protein | | uniclust | UniRef100\_A0A0Q7A331 | 98.6 | 2.9e-10 | 5.7e-16 | 73.4 | 82 | (12, 94) | 94 | (22, 105) | 129 | Phage tail assembly protein | Phage tail assembly protein | | uniclust | UniRef100\_UPI0022384EAA | 98.6 | 3.2e-10 | 5.8e-16 | 71.3 | 82 | (12, 93) | 94 | (38, 120) | 134 | phage tail assembly protein | phage tail assembly protein | | uniclust | UniRef100\_A0A212LR71 | 98.6 | 2.9e-10 | 5.8e-16 | 75.6 | 81 | (12, 94) | 94 | (43, 130) | 156 | Uncharacterized protein | Uncharacterized protein | | uniclust | UniRef100\_A0A1E2RY06 | 98.6 | 3.2e-10 | 6.2e-16 | 72.0 | 83 | (12, 94) | 94 | (27, 121) | 125 | Phage tail protein E | Phage tail protein E | | uniclust | UniRef100\_A0A102DFQ3 | 98.6 | 3.3e-10 | 6.3e-16 | 70.9 | 79 | (14, 94) | 94 | (42, 120) | 120 | Phage tail assembly protein | Phage tail assembly protein | | uniclust | UniRef100\_A0A935YDJ1 | 98.6 | 4e-10 | 7.3e-16 | 67.9 | 80 | (15, 94) | 94 | (6, 93) | 102 | Phage tail assembly protein | Phage tail assembly protein | | uniclust | UniRef100\_A0A1G7D511 | 98.6 | 3.7e-10 | 7.3e-16 | 70.8 | 82 | (13, 94) | 94 | (4, 90) | 110 | Phage tail assembly chaperone protein, E, or 41 or 14 | Phage tail assembly chaperone protein, E, or 41 or 14 | | uniclust | UniRef100\_UPI00197C731A | 98.6 | 4e-10 | 7.4e-16 | 70.6 | 82 | (12, 93) | 94 | (25, 109) | 131 | phage tail assembly protein | phage tail assembly protein | | uniclust | UniRef100\_A0A1C7L9J9 | 98.6 | 4e-10 | 8e-16 | 72.7 | 80 | (14, 93) | 94 | (31, 114) | 127 | Tail E family protein | Tail E family protein | | uniclust | UniRef100\_A0A7M3MAZ0 | 98.6 | 4.4e-10 | 8.2e-16 | 68.2 | 76 | (15, 90) | 94 | (6, 84) | 102 | Phage tail assembly protein | Phage tail assembly protein | | uniclust | UniRef100\_A0A849MU39 | 98.6 | 4.4e-10 | 8.2e-16 | 68.3 | 80 | (15, 94) | 94 | (14, 99) | 107 | Phage tail assembly protein | Phage tail assembly protein | | uniclust | UniRef100\_UPI0020CEC014 | 98.6 | 4.8e-10 | 8.8e-16 | 69.1 | 86 | (8, 93) | 94 | (21, 110) | 117 | phage tail assembly protein | phage tail assembly protein | | uniclust | UniRef100\_A0A929HY00 | 98.6 | 4.8e-10 | 8.8e-16 | 66.1 | 78 | (13, 92) | 94 | (11, 88) | 89 | Phage tail assembly protein | Phage tail assembly protein | | uniclust | UniRef100\_A0A0A2SKX1 | 98.6 | 4.5e-10 | 9e-16 | 72.9 | 79 | (15, 93) | 94 | (35, 124) | 137 | Phage tail assembly protein | Phage tail assembly protein | | uniclust | UniRef100\_A0A094ZEZ2 | 98.6 | 4.4e-10 | 9.1e-16 | 79.1 | 75 | (18, 92) | 94 | (31, 111) | 223 | Phage protein | Phage protein | | uniclust | UniRef100\_A0A2R8CKK2 | 98.6 | 5.1e-10 | 9.5e-16 | 73.3 | 78 | (16, 93) | 94 | (71, 152) | 162 | Phage tail protein E | Phage tail protein E | | uniclust | UniRef100\_UPI00156FF9AB | 98.6 | 5.3e-10 | 1e-15 | 68.6 | 82 | (13, 94) | 94 | (11, 96) | 105 | phage tail assembly protein | phage tail assembly protein | | uniclust | UniRef100\_A0A077PQ35 | 98.6 | 5.4e-10 | 1e-15 | 68.1 | 80 | (14, 93) | 94 | (3, 86) | 96 | Phage tail assembly protein | Phage tail assembly protein | | uniclust | UniRef100\_A0A212S820 | 98.6 | 5.3e-10 | 1e-15 | 72.9 | 82 | (13, 94) | 94 | (30, 116) | 141 | Phage tail assembly chaperone protein, E, or 41 or 14 | Phage tail assembly chaperone protein, E, or 41 or 14 | | uniclust | UniRef100\_A0A031FSF6 | 98.6 | 5e-10 | 1.1e-15 | 78.4 | 78 | (16, 94) | 94 | (116, 193) | 209 | Phage-like protein | Phage-like protein | | uniclust | UniRef100\_UPI001364C58B | 98.6 | 6.1e-10 | 1.1e-15 | 67.4 | 78 | (14, 92) | 94 | (7, 88) | 95 | phage tail assembly protein | phage tail assembly protein | | uniclust | UniRef100\_A0A4Q8MDQ4 | 98.6 | 6e-10 | 1.2e-15 | 68.1 | 77 | (17, 93) | 94 | (2, 86) | 95 | Phage tail assembly protein | Phage tail assembly protein | | uniclust | UniRef100\_A0A5C7NIW4 | 98.6 | 6.9e-10 | 1.3e-15 | 66.2 | 77 | (17, 94) | 94 | (2, 86) | 95 | Phage tail assembly protein | Phage tail assembly protein | | uniclust | UniRef100\_A0A074TD79 | 98.6 | 7.1e-10 | 1.4e-15 | 68.7 | 74 | (16, 90) | 94 | (4, 77) | 102 | Phage tail assembly protein | Phage tail assembly protein | | uniclust | UniRef100\_A0A0A8GWA2 | 98.6 | 7.2e-10 | 1.4e-15 | 66.0 | 62 | (33, 94) | 94 | (12, 73) | 82 | Phage tail assembly protein | Phage tail assembly protein | | uniclust | UniRef100\_A0A1H6QNL8 | 98.5 | 8.1e-10 | 1.5e-15 | 73.8 | 77 | (15, 92) | 94 | (3, 83) | 186 | Phage tail assembly chaperone protein, E, or 41 or 14 | Phage tail assembly chaperone protein, E, or 41 or 14 | | uniclust | UniRef100\_A0A7W4ICA4 | 98.5 | 7.7e-10 | 1.5e-15 | 73.1 | 90 | (2, 93) | 94 | (38, 139) | 147 | Phage tail assembly protein | Phage tail assembly protein | | uniclust | UniRef100\_A0A0C6FCI1 | 98.5 | 8.7e-10 | 1.7e-15 | 74.6 | 83 | (12, 94) | 94 | (70, 154) | 182 | Uncharacterized protein | Uncharacterized protein | | uniclust | UniRef100\_A0A173SBH0 | 98.5 | 9e-10 | 1.8e-15 | 72.4 | 79 | (15, 93) | 94 | (54, 144) | 149 | Phage tail assembly protein | Phage tail assembly protein | | uniclust | UniRef100\_A0A4Z0E5Q8 | 98.5 | 1.1e-09 | 2e-15 | 64.9 | 76 | (15, 90) | 94 | (3, 78) | 83 | Phage tail assembly protein | Phage tail assembly protein | | uniclust | UniRef100\_A0A016XIN1 | 98.5 | 1.1e-09 | 2.1e-15 | 67.7 | 79 | (13, 93) | 94 | (8, 86) | 119 | Phage tail assembly protein | Phage tail assembly protein | | uniclust | UniRef100\_A0A925L1X7 | 98.5 | 1.1e-09 | 2.1e-15 | 69.2 | 86 | (8, 94) | 94 | (14, 100) | 128 | Phage tail assembly protein | Phage tail assembly protein | | uniclust | UniRef100\_A0A094ZEZ2 | 98.5 | 1.3e-09 | 2.6e-15 | 76.9 | 78 | (16, 93) | 94 | (128, 210) | 223 | Phage protein | Phage protein | | uniclust | UniRef100\_A0NQ99 | 98.5 | 1.5e-09 | 2.7e-15 | 67.8 | 79 | (14, 93) | 94 | (5, 84) | 121 | Tail assembly chaperone | Tail assembly chaperone | | uniclust | UniRef100\_A0A090G422 | 98.5 | 1.5e-09 | 2.8e-15 | 69.6 | 72 | (16, 91) | 94 | (49, 120) | 134 | Phage tail assembly protein | Phage tail assembly protein | | uniclust | UniRef100\_UPI0013D729BF | 98.5 | 1.5e-09 | 2.9e-15 | 66.9 | 78 | (16, 93) | 94 | (10, 97) | 106 | phage tail assembly protein | phage tail assembly protein | | uniclust | UniRef100\_A0A8J2Z5X2 | 98.5 | 1.6e-09 | 3e-15 | 65.9 | 74 | (17, 91) | 94 | (4, 78) | 91 | Tail assembly chaperone E/41/14-like protein | Tail assembly chaperone E/41/14-like protein | | uniclust | UniRef100\_A0A7J5WEE9 | 98.5 | 1.7e-09 | 3.1e-15 | 65.5 | 73 | (18, 90) | 94 | (25, 98) | 103 | Phage tail assembly protein | Phage tail assembly protein | | uniclust | UniRef100\_A0A423PRR2 | 98.5 | 1.7e-09 | 3.3e-15 | 68.2 | 80 | (15, 94) | 94 | (28, 109) | 116 | Phage tail assembly protein | Phage tail assembly protein | | uniclust | UniRef100\_A0A0H5CY95 | 98.5 | 1.7e-09 | 3.4e-15 | 69.5 | 79 | (15, 93) | 94 | (3, 102) | 122 | Phage tail assembly protein | Phage tail assembly protein | | uniclust | UniRef100\_A0A1G9AZL3 | 98.5 | 2e-09 | 3.8e-15 | 67.9 | 88 | (3, 91) | 94 | (10, 108) | 122 | Phage tail assembly chaperone protein, E, or 41 or 14 | Phage tail assembly chaperone protein, E, or 41 or 14 | | uniclust | UniRef100\_A0A5P9JUS1 | 98.5 | 2e-09 | 3.8e-15 | 64.6 | 80 | (14, 93) | 94 | (5, 86) | 90 | Uncharacterized protein | Uncharacterized protein | | uniclust | UniRef100\_A0A061NN23 | 98.5 | 2e-09 | 3.8e-15 | 67.4 | 79 | (16, 94) | 94 | (25, 115) | 116 | Phage protein | Phage protein | | uniclust | UniRef100\_A0A940MVV2 | 98.5 | 2.1e-09 | 4e-15 | 66.5 | 70 | (23, 93) | 94 | (26, 95) | 105 | Phage tail assembly protein | Phage tail assembly protein | | uniclust | UniRef100\_A0A4D7BGG9 | 98.5 | 2.1e-09 | 4e-15 | 69.5 | 81 | (13, 93) | 94 | (7, 101) | 133 | Phage tail assembly protein | Phage tail assembly protein | | uniclust | UniRef100\_UPI0004B2DC3D | 98.4 | 2.6e-09 | 4.7e-15 | 67.6 | 76 | (17, 92) | 94 | (3, 82) | 136 | phage tail assembly protein | phage tail assembly protein | | uniclust | UniRef100\_UPI00143B4262 | 98.4 | 2.5e-09 | 4.7e-15 | 66.0 | 76 | (16, 91) | 94 | (3, 86) | 110 | phage tail assembly protein | phage tail assembly protein | | uniclust | UniRef100\_A0A1I7NC51 | 98.4 | 2.7e-09 | 5.3e-15 | 69.1 | 83 | (12, 94) | 94 | (21, 105) | 135 | Phage tail assembly chaperone protein, E, or 41 or 14 | Phage tail assembly chaperone protein, E, or 41 or 14 | | uniclust | UniRef100\_A0A1B9VJR2 | 98.4 | 2.8e-09 | 5.6e-15 | 69.0 | 90 | (4, 94) | 94 | (16, 117) | 130 | Mu-like prophage FluMu protein gp41 | Mu-like prophage FluMu protein gp41 | | uniclust | UniRef100\_A0A1M6ZC09 | 98.4 | 3.1e-09 | 6e-15 | 63.2 | 76 | (18, 94) | 94 | (2, 79) | 83 | Phage tail assembly chaperone protein, E, or 41 or 14 | Phage tail assembly chaperone protein, E, or 41 or 14 | | uniclust | UniRef100\_A0A0U3UX96 | 98.4 | 3.4e-09 | 6.4e-15 | 66.1 | 79 | (16, 94) | 94 | (5, 91) | 111 | Phage small tail protein E | Phage small tail protein E | | uniclust | UniRef100\_A0A2S4LWK7 | 98.4 | 4.6e-09 | 8.5e-15 | 69.1 | 79 | (15, 93) | 94 | (83, 165) | 179 | Tail assembly chaperone E/41/14-like protein | Tail assembly chaperone E/41/14-like protein | | uniclust | UniRef100\_A0A098ARP9 | 98.4 | 4.9e-09 | 9e-15 | 58.2 | 57 | (18, 74) | 94 | (3, 59) | 61 | Putative phage related protein | Putative phage related protein | | uniclust | UniRef100\_A0A2E2IRC7 | 98.4 | 5.1e-09 | 1e-14 | 65.9 | 75 | (16, 90) | 94 | (5, 93) | 108 | Phage tail assembly protein | Phage tail assembly protein | | uniclust | UniRef100\_UPI001BCB7C8E | 98.4 | 5.6e-09 | 1.1e-14 | 64.4 | 83 | (12, 94) | 94 | (3, 95) | 103 | phage tail assembly protein | phage tail assembly protein | | uniclust | UniRef100\_A0A1I2QQM4 | 98.3 | 5.8e-09 | 1.1e-14 | 64.3 | 82 | (13, 94) | 94 | (16, 98) | 107 | Phage tail assembly chaperone protein, E, or 41 or 14 | Phage tail assembly chaperone protein, E, or 41 or 14 | | uniclust | UniRef100\_A0A8J7LX76 | 98.3 | 6e-09 | 1.1e-14 | 67.1 | 83 | (12, 94) | 94 | (29, 115) | 137 | Phage tail assembly protein | Phage tail assembly protein | | uniclust | UniRef100\_A0A031FSF6 | 98.3 | 5.6e-09 | 1.2e-14 | 73.5 | 79 | (15, 93) | 94 | (18, 98) | 209 | Phage-like protein | Phage-like protein | | uniclust | UniRef100\_A0A349GR41 | 98.3 | 6.3e-09 | 1.2e-14 | 66.7 | 81 | (13, 93) | 94 | (30, 123) | 125 | Phage tail assembly protein | Phage tail assembly protein | | uniclust | UniRef100\_A0A2S6HV68 | 98.3 | 6.2e-09 | 1.3e-14 | 67.1 | 78 | (16, 93) | 94 | (25, 116) | 120 | Tail assembly chaperone E/41/14-like protein | Tail assembly chaperone E/41/14-like protein | | uniclust | UniRef100\_UPI0007377FCF | 98.3 | 6.6e-09 | 1.3e-14 | 63.5 | 70 | (13, 82) | 94 | (18, 91) | 92 | phage tail assembly protein | phage tail assembly protein | | uniclust | UniRef100\_UPI001FB7CC94 | 98.3 | 7e-09 | 1.3e-14 | 63.6 | 76 | (17, 92) | 94 | (8, 86) | 109 | phage tail assembly protein | phage tail assembly protein | | uniclust | UniRef100\_A0A256CAU0 | 98.3 | 7.1e-09 | 1.3e-14 | 63.7 | 79 | (15, 93) | 94 | (3, 85) | 100 | Phage tail protein | Phage tail protein | | uniclust | UniRef100\_UPI0014125629 | 98.3 | 7.2e-09 | 1.3e-14 | 65.2 | 82 | (12, 94) | 94 | (23, 113) | 121 | hypothetical protein | hypothetical protein | | uniclust | UniRef100\_A0A1V4VYZ4 | 98.3 | 7e-09 | 1.4e-14 | 65.6 | 77 | (17, 93) | 94 | (3, 91) | 110 | Phage tail protein E | Phage tail protein E | | uniclust | UniRef100\_A0A1X3S1E1 | 98.3 | 7.6e-09 | 1.5e-14 | 66.5 | 79 | (16, 94) | 94 | (5, 91) | 133 | Phage tail protein | Phage tail protein | | uniclust | UniRef100\_A0A7X4CN54 | 98.3 | 8.3e-09 | 1.5e-14 | 63.5 | 78 | (16, 93) | 94 | (33, 111) | 112 | Phage tail assembly protein | Phage tail assembly protein | | uniclust | UniRef100\_E2CN25 | 98.3 | 8.2e-09 | 1.6e-14 | 65.3 | 79 | (16, 94) | 94 | (4, 87) | 123 | Phage-related protein | Phage-related protein | | uniclust | UniRef100\_A0A1P8QQ42 | 98.3 | 9e-09 | 1.7e-14 | 63.4 | 82 | (12, 93) | 94 | (29, 110) | 112 | Uncharacterized protein | Uncharacterized protein | | uniclust | UniRef100\_A0A1H2FLU3 | 98.3 | 8.8e-09 | 1.7e-14 | 67.4 | 83 | (12, 94) | 94 | (7, 129) | 141 | Phage tail assembly chaperone protein, E, or 41 or 14 | Phage tail assembly chaperone protein, E, or 41 or 14 | | uniclust | UniRef100\_A0A0C2VFH9 | 98.3 | 9.5e-09 | 1.7e-14 | 60.7 | 77 | (15, 94) | 94 | (4, 82) | 86 | Phage tail assembly protein | Phage tail assembly protein | | uniclust | UniRef100\_A0A922XTU6 | 98.3 | 9.8e-09 | 1.8e-14 | 62.2 | 82 | (13, 94) | 94 | (10, 92) | 101 | Uncharacterized protein | Uncharacterized protein | | uniclust | UniRef100\_A0A2S5N1P1 | 98.3 | 9.3e-09 | 1.8e-14 | 68.3 | 82 | (13, 94) | 94 | (54, 136) | 157 | Phage tail assembly protein | Phage tail assembly protein | | uniclust | UniRef100\_A0A6L6YK12 | 98.3 | 1.1e-08 | 2e-14 | 62.3 | 79 | (16, 94) | 94 | (3, 86) | 96 | Phage tail assembly protein | Phage tail assembly protein | | uniclust | UniRef100\_A0A2E2N165 | 98.3 | 1.1e-08 | 2e-14 | 65.7 | 79 | (15, 93) | 94 | (2, 93) | 125 | Phage tail assembly protein | Phage tail assembly protein | | uniclust | UniRef100\_A0A1M3BNG8 | 98.3 | 1.1e-08 | 2.1e-14 | 66.3 | 79 | (12, 93) | 94 | (39, 117) | 126 | Phage tail assembly protein | Phage tail assembly protein | | uniclust | UniRef100\_A0A2N6FYM4 | 98.3 | 1.2e-08 | 2.1e-14 | 60.1 | 77 | (16, 93) | 94 | (5, 83) | 84 | Phage tail assembly protein | Phage tail assembly protein | | uniclust | UniRef100\_UPI0003802596 | 98.3 | 1.2e-08 | 2.2e-14 | 60.0 | 78 | (17, 94) | 94 | (2, 80) | 84 | phage tail assembly protein | phage tail assembly protein | | uniclust | UniRef100\_A0A844Q6C1 | 98.3 | 1.2e-08 | 2.3e-14 | 63.6 | 77 | (17, 93) | 94 | (2, 92) | 105 | Phage tail assembly protein | Phage tail assembly protein | | uniclust | UniRef100\_A0A1I5RNJ4 | 98.3 | 1.3e-08 | 2.4e-14 | 61.1 | 80 | (15, 94) | 94 | (2, 89) | 89 | Phage tail assembly chaperone protein, E, or 41 or 14 | Phage tail assembly chaperone protein, E, or 41 or 14 | | uniclust | UniRef100\_A0A0D8L4Q2 | 98.3 | 1.3e-08 | 2.5e-14 | 64.4 | 88 | (4, 93) | 94 | (17, 108) | 120 | Phage tail protein | Phage tail protein | | uniclust | UniRef100\_A0A1Y1S1F4 | 98.3 | 1.3e-08 | 2.5e-14 | 63.6 | 81 | (12, 94) | 94 | (42, 123) | 123 | Phage tail protein | Phage tail protein | | uniclust | UniRef100\_UPI0018AD3EFF | 98.3 | 1.4e-08 | 2.6e-14 | 55.9 | 52 | (38, 90) | 94 | (1, 52) | 58 | phage tail assembly protein | phage tail assembly protein | | uniclust | UniRef100\_A0A0C3RQ40 | 98.3 | 1.2e-08 | 2.6e-14 | 66.7 | 80 | (12, 91) | 94 | (17, 106) | 126 | Phage protein | Phage protein | | uniclust | UniRef100\_UPI00037665EA | 98.2 | 1.5e-08 | 2.8e-14 | 63.6 | 74 | (17, 90) | 94 | (4, 78) | 113 | phage tail assembly protein | phage tail assembly protein | | uniclust | UniRef100\_A0A2D8T8N2 | 98.2 | 1.5e-08 | 2.9e-14 | 64.1 | 85 | (3, 93) | 94 | (11, 99) | 109 | Phage tail assembly protein | Phage tail assembly protein | | uniclust | UniRef100\_UPI000D381B68 | 98.2 | 1.6e-08 | 2.9e-14 | 62.1 | 62 | (33, 94) | 94 | (43, 104) | 109 | phage tail assembly protein | phage tail assembly protein | | uniclust | UniRef100\_UPI00094A100B | 98.2 | 1.6e-08 | 3e-14 | 66.2 | 79 | (15, 93) | 94 | (59, 139) | 149 | phage tail assembly protein | phage tail assembly protein | | uniclust | UniRef100\_A0A4R5W1E0 | 98.2 | 1.6e-08 | 3e-14 | 62.9 | 78 | (13, 90) | 94 | (10, 88) | 118 | Phage tail assembly protein | Phage tail assembly protein | | uniclust | UniRef100\_UPI001030D5AE | 98.2 | 1.6e-08 | 3e-14 | 62.1 | 76 | (15, 91) | 94 | (29, 104) | 109 | phage tail assembly protein | phage tail assembly protein | | uniclust | UniRef100\_UPI001903CD40 | 98.2 | 1.8e-08 | 3.3e-14 | 62.7 | 76 | (17, 93) | 94 | (13, 88) | 118 | phage tail assembly protein | phage tail assembly protein | | uniclust | UniRef100\_UPI0002A3D1A3 | 98.2 | 1.8e-08 | 3.5e-14 | 60.7 | 62 | (32, 93) | 94 | (14, 75) | 86 | phage tail assembly protein | phage tail assembly protein | | uniclust | UniRef100\_A0A7J6YK69 | 98.2 | 2.1e-08 | 3.9e-14 | 83.0 | 75 | (16, 90) | 94 | (1760, 1834) | 2523 | Uncharacterized protein | Uncharacterized protein | | uniclust | UniRef100\_A0A8G2CCC5 | 98.2 | 2.2e-08 | 4.1e-14 | 61.5 | 76 | (16, 91) | 94 | (6, 84) | 109 | Phage tail assembly chaperone protein, E, or 41 or 14 | Phage tail assembly chaperone protein, E, or 41 or 14 | | uniclust | UniRef100\_A0A1I9KFV2 | 98.2 | 2.2e-08 | 4.4e-14 | 70.7 | 75 | (18, 94) | 94 | (127, 203) | 216 | Uncharacterized protein | Uncharacterized protein | | uniclust | UniRef100\_A0A066QH52 | 98.2 | 2.6e-08 | 5.1e-14 | 65.5 | 79 | (16, 94) | 94 | (27, 111) | 136 | Phage tail E family protein | Phage tail E family protein | | uniclust | UniRef100\_A0A1X7I5S5 | 98.2 | 2.9e-08 | 5.2e-14 | 62.1 | 74 | (20, 93) | 94 | (13, 94) | 121 | Phage tail assembly chaperone protein, E, or 41 or 14 | Phage tail assembly chaperone protein, E, or 41 or 14 | | uniclust | UniRef100\_A0A4P9VF09 | 98.2 | 3e-08 | 5.5e-14 | 54.0 | 40 | (18, 57) | 94 | (4, 43) | 54 | Phage tail assembly protein | Phage tail assembly protein | | uniclust | UniRef100\_UPI001FD41C6C | 98.2 | 3.2e-08 | 5.8e-14 | 58.8 | 73 | (16, 89) | 94 | (3, 75) | 88 | phage tail assembly protein | phage tail assembly protein | | uniclust | UniRef100\_A0A258L5N6 | 98.2 | 3.1e-08 | 6.1e-14 | 69.4 | 78 | (16, 94) | 94 | (115, 192) | 223 | Phage tail assembly protein | Phage tail assembly protein | | uniclust | UniRef100\_Q31HT0 | 98.1 | 3.5e-08 | 6.4e-14 | 56.1 | 64 | (30, 94) | 94 | (5, 68) | 68 | Phage tail assembly protein | Phage tail assembly protein | | uniclust | UniRef100\_A0A2J0QXA4 | 98.1 | 3.5e-08 | 6.6e-14 | 61.0 | 79 | (16, 94) | 94 | (20, 102) | 107 | Phage tail assembly protein | Phage tail assembly protein | | uniclust | UniRef100\_A0A0M7ALB4 | 98.1 | 3.9e-08 | 7.2e-14 | 59.9 | 80 | (14, 93) | 94 | (19, 99) | 102 | Tail assembly chaperone E/41/14-like protein | Tail assembly chaperone E/41/14-like protein | | uniclust | UniRef100\_A0A9D1VF36 | 98.1 | 4.2e-08 | 7.8e-14 | 60.5 | 79 | (13, 93) | 94 | (21, 100) | 110 | Phage tail assembly protein | Phage tail assembly protein | | uniclust | UniRef100\_A0A3A6N335 | 98.1 | 4.1e-08 | 7.9e-14 | 61.9 | 82 | (13, 94) | 94 | (6, 97) | 108 | Phage tail assembly protein | Phage tail assembly protein | | uniclust | UniRef100\_A0A661TQP7 | 98.1 | 4.7e-08 | 8.7e-14 | 59.5 | 81 | (13, 93) | 94 | (3, 93) | 101 | Phage tail assembly protein | Phage tail assembly protein | | uniclust | UniRef100\_A0A2T1B6V7 | 98.1 | 5e-08 | 9.4e-14 | 66.3 | 79 | (15, 93) | 94 | (95, 175) | 205 | Tail assembly chaperone E/41/14-like protein | Tail assembly chaperone E/41/14-like protein | | uniclust | UniRef100\_A0A1X0SVA2 | 98.1 | 4.8e-08 | 9.5e-14 | 62.4 | 80 | (15, 94) | 94 | (2, 90) | 115 | Uncharacterized protein | Uncharacterized protein | | uniclust | UniRef100\_A0A0Q8AP47 | 98.1 | 5.3e-08 | 1e-13 | 61.0 | 75 | (16, 90) | 94 | (3, 80) | 108 | Phage tail protein | Phage tail protein | | uniclust | UniRef100\_A0A0G0BNF2 | 98.1 | 5.5e-08 | 1.1e-13 | 61.3 | 62 | (31, 92) | 94 | (15, 83) | 106 | Phage tail assembly protein | Phage tail assembly protein | | uniclust | UniRef100\_A0A212KMT6 | 98.1 | 6.1e-08 | 1.1e-13 | 64.0 | 75 | (18, 92) | 94 | (3, 77) | 174 | Phage tail assembly protein | Phage tail assembly protein | | uniclust | UniRef100\_A0A7X6FP61 | 98.1 | 6.2e-08 | 1.1e-13 | 54.4 | 56 | (15, 71) | 94 | (6, 61) | 63 | Phage tail assembly protein | Phage tail assembly protein | | uniclust | UniRef100\_A0A3N2E0T6 | 98.1 | 6.4e-08 | 1.2e-13 | 59.1 | 69 | (15, 83) | 94 | (20, 89) | 103 | Tail assembly chaperone E/41/14-like protein | Tail assembly chaperone E/41/14-like protein | | uniclust | UniRef100\_A0A961TLJ2 | 98.1 | 6.7e-08 | 1.2e-13 | 60.6 | 78 | (15, 93) | 94 | (27, 104) | 121 | Phage tail assembly protein | Phage tail assembly protein | | uniclust | UniRef100\_A0A1B1KPJ6 | 98.1 | 7.1e-08 | 1.3e-13 | 61.3 | 78 | (16, 93) | 94 | (25, 106) | 120 | Tail protein | Tail protein | | uniclust | UniRef100\_A0A1H1G2N2 | 98.1 | 7e-08 | 1.3e-13 | 61.3 | 79 | (16, 94) | 94 | (3, 86) | 118 | Phage tail assembly chaperone protein, E, or 41 or 14 | Phage tail assembly chaperone protein, E, or 41 or 14 | | uniclust | UniRef100\_A0A1V3RSD9 | 98.1 | 7.6e-08 | 1.4e-13 | 58.7 | 76 | (16, 91) | 94 | (16, 97) | 102 | Phage tail assembly protein | Phage tail assembly protein | | uniclust | UniRef100\_A0A6B8KJI9 | 98.0 | 8e-08 | 1.5e-13 | 60.6 | 75 | (16, 94) | 94 | (52, 126) | 126 | Phage tail assembly protein | Phage tail assembly protein | | uniclust | UniRef100\_A0A266LMF2 | 98.0 | 7.9e-08 | 1.5e-13 | 57.2 | 64 | (28, 91) | 94 | (3, 67) | 79 | Phage tail assembly protein (Fragment) | Phage tail assembly protein (Fragment) | | uniclust | UniRef100\_UPI0009EC53EA | 98.0 | 8.7e-08 | 1.6e-13 | 68.9 | 70 | (15, 84) | 94 | (21, 101) | 344 | phage tail assembly protein | phage tail assembly protein | | uniclust | UniRef100\_UPI00056E07A5 | 98.0 | 8.7e-08 | 1.6e-13 | 55.3 | 56 | (16, 72) | 94 | (16, 72) | 73 | phage tail assembly protein | phage tail assembly protein | | uniclust | UniRef100\_A0A2I7R3J1 | 98.0 | 8.8e-08 | 1.6e-13 | 56.5 | 80 | (15, 94) | 94 | (2, 83) | 83 | Putative tail protein | Putative tail protein | | uniclust | UniRef100\_A0A1G7ZBM7 | 98.0 | 8.7e-08 | 1.7e-13 | 65.5 | 79 | (13, 92) | 94 | (13, 91) | 196 | Phage tail assembly chaperone protein, E, or 41 or 14 | Phage tail assembly chaperone protein, E, or 41 or 14 | | uniclust | UniRef100\_A0A7G6RKV0 | 98.0 | 9.1e-08 | 1.8e-13 | 60.1 | 74 | (16, 93) | 94 | (19, 97) | 107 | Phage tail assembly protein | Phage tail assembly protein | | uniclust | UniRef100\_A0A0P7I4K8 | 98.0 | 9.9e-08 | 1.8e-13 | 63.2 | 81 | (14, 94) | 94 | (28, 108) | 162 | Tail assembly chaperone | Tail assembly chaperone | | uniclust | UniRef100\_UPI00234F299C | 98.0 | 1.1e-07 | 2e-13 | 59.3 | 79 | (15, 93) | 94 | (3, 85) | 117 | phage tail assembly protein | phage tail assembly protein | | uniclust | UniRef100\_A0A2D3VZ53 | 98.0 | 1.2e-07 | 2.1e-13 | 58.1 | 59 | (33, 92) | 94 | (38, 96) | 103 | Phage tail assembly protein | Phage tail assembly protein | | uniclust | UniRef100\_A0A0A8IK84 | 98.0 | 1.1e-07 | 2.2e-13 | 64.3 | 70 | (16, 89) | 94 | (73, 142) | 153 | Uncharacterized protein | Uncharacterized protein | | uniclust | UniRef100\_F3YY69 | 98.0 | 1.2e-07 | 2.2e-13 | 58.7 | 75 | (16, 90) | 94 | (4, 86) | 111 | Phage tail assembly protein | Phage tail assembly protein | | uniclust | UniRef100\_A0A2N9Y8Z8 | 98.0 | 1.2e-07 | 2.4e-13 | 63.8 | 81 | (14, 94) | 94 | (8, 127) | 151 | Phage tail assembly protein | Phage tail assembly protein | | uniclust | UniRef100\_A0A9E7YFR7 | 98.0 | 1.3e-07 | 2.4e-13 | 60.3 | 76 | (14, 93) | 94 | (16, 91) | 135 | Uncharacterized protein | Uncharacterized protein | | uniclust | UniRef100\_A0A7J0BJG9 | 98.0 | 1.3e-07 | 2.4e-13 | 58.8 | 76 | (15, 90) | 94 | (4, 82) | 112 | Phage tail assembly protein | Phage tail assembly protein | | uniclust | UniRef100\_UPI0012BCC767 | 98.0 | 1.3e-07 | 2.4e-13 | 56.9 | 79 | (16, 94) | 94 | (4, 82) | 93 | phage tail assembly protein | phage tail assembly protein | | uniclust | UniRef100\_UPI002147F80F | 98.0 | 1.4e-07 | 2.5e-13 | 54.5 | 61 | (33, 94) | 94 | (4, 64) | 73 | phage tail assembly protein | phage tail assembly protein | | uniclust | UniRef100\_A0A4R3J9N6 | 98.0 | 1.5e-07 | 2.8e-13 | 56.9 | 78 | (17, 94) | 94 | (5, 92) | 96 | Tail assembly chaperone E/41/14-like protein | Tail assembly chaperone E/41/14-like protein | | uniclust | UniRef100\_A0A258L5N6 | 98.0 | 1.5e-07 | 2.9e-13 | 66.2 | 77 | (15, 91) | 94 | (21, 99) | 223 | Phage tail assembly protein | Phage tail assembly protein | | uniclust | UniRef100\_UPI00174E383D | 98.0 | 1.7e-07 | 3.1e-13 | 59.0 | 78 | (17, 94) | 94 | (4, 94) | 122 | phage tail assembly protein | phage tail assembly protein | | uniclust | UniRef100\_UPI002100D467 | 97.9 | 1.7e-07 | 3.1e-13 | 58.5 | 81 | (13, 94) | 94 | (16, 96) | 108 | phage tail assembly protein | phage tail assembly protein | | uniclust | UniRef100\_A0A077L031 | 97.9 | 1.7e-07 | 3.2e-13 | 58.7 | 79 | (16, 94) | 94 | (17, 98) | 104 | Phage tail assembly protein | Phage tail assembly protein | | uniclust | UniRef100\_A0A1S6U833 | 97.9 | 1.7e-07 | 3.4e-13 | 56.7 | 61 | (33, 94) | 94 | (13, 73) | 82 | Putative Mu-like phage protein | Putative Mu-like phage protein | | uniclust | UniRef100\_A0A1G8JZK0 | 97.9 | 1.8e-07 | 3.4e-13 | 61.3 | 78 | (16, 93) | 94 | (53, 134) | 147 | Phage tail assembly chaperone protein, E, or 41 or 14 | Phage tail assembly chaperone protein, E, or 41 or 14 | | uniclust | UniRef100\_A0A0P0URT8 | 97.9 | 2e-07 | 3.7e-13 | 58.2 | 74 | (16, 89) | 94 | (3, 78) | 117 | Phage-related terminase | Phage-related terminase | | uniclust | UniRef100\_A0A1X7MF74 | 97.9 | 2.1e-07 | 3.9e-13 | 50.2 | 42 | (53, 94) | 94 | (9, 50) | 50 | Phage tail assembly chaperone protein, E, or 41 or 14 | Phage tail assembly chaperone protein, E, or 41 or 14 | | uniclust | UniRef100\_A0A7W6WBB0 | 97.9 | 2.2e-07 | 4.1e-13 | 57.7 | 76 | (16, 91) | 94 | (13, 91) | 112 | Tail assembly chaperone E/41/14-like protein | Tail assembly chaperone E/41/14-like protein | | uniclust | UniRef100\_A0A068CE07 | 97.9 | 2.1e-07 | 4.2e-13 | 61.9 | 82 | (13, 94) | 94 | (6, 101) | 142 | Uncharacterized protein | Uncharacterized protein | | uniclust | UniRef100\_A0A095CPF9 | 97.9 | 2.4e-07 | 4.4e-13 | 58.0 | 75 | (14, 89) | 94 | (14, 88) | 112 | Uncharacterized protein | Uncharacterized protein | | uniclust | UniRef100\_A0A0K1NGL7 | 97.9 | 2.4e-07 | 4.6e-13 | 55.6 | 66 | (28, 93) | 94 | (3, 72) | 83 | Phage tail protein | Phage tail protein | | uniclust | UniRef100\_A0A1I1EZQ6 | 97.9 | 2.5e-07 | 4.7e-13 | 60.4 | 78 | (16, 93) | 94 | (49, 126) | 145 | Phage tail assembly protein | Phage tail assembly protein | | uniclust | UniRef100\_UPI00131AA224 | 97.9 | 2.8e-07 | 5.1e-13 | 56.4 | 78 | (13, 90) | 94 | (7, 95) | 102 | phage tail assembly protein | phage tail assembly protein | | uniclust | UniRef100\_A0A151FYQ4 | 97.9 | 3.1e-07 | 5.9e-13 | 60.1 | 78 | (16, 93) | 94 | (52, 139) | 146 | Phage tail assembly protein | Phage tail assembly protein | | uniclust | UniRef100\_A0A380ALS8 | 97.9 | 3.3e-07 | 6.1e-13 | 50.4 | 39 | (13, 51) | 94 | (14, 52) | 55 | Uncharacterized protein | Uncharacterized protein | | uniclust | UniRef100\_UPI00137719BA | 97.8 | 3.5e-07 | 6.5e-13 | 56.2 | 74 | (16, 90) | 94 | (6, 83) | 104 | phage tail assembly protein | phage tail assembly protein | | uniclust | UniRef100\_A0A380ZEC4 | 97.8 | 3.5e-07 | 6.6e-13 | 51.2 | 46 | (21, 68) | 94 | (11, 57) | 57 | Uncharacterized protein | Uncharacterized protein | | uniclust | UniRef100\_A0A1V5WR09 | 97.8 | 3.6e-07 | 6.6e-13 | 55.3 | 78 | (17, 94) | 94 | (7, 84) | 95 | Phage tail protein E | Phage tail protein E | | uniclust | UniRef100\_A0A418VWW7 | 97.8 | 3.7e-07 | 6.9e-13 | 54.0 | 62 | (32, 93) | 94 | (2, 67) | 77 | Phage tail assembly protein | Phage tail assembly protein | | uniclust | UniRef100\_UPI002243AD49 | 97.8 | 3.8e-07 | 7e-13 | 61.3 | 79 | (15, 93) | 94 | (96, 178) | 190 | phage tail assembly protein | phage tail assembly protein | | uniclust | UniRef100\_A0A395D213 | 97.8 | 3.7e-07 | 7.1e-13 | 57.9 | 78 | (12, 92) | 94 | (32, 109) | 116 | Uncharacterized protein | Uncharacterized protein | | uniclust | UniRef100\_A0A9E3GWE3 | 97.8 | 4e-07 | 7.4e-13 | 56.1 | 76 | (18, 93) | 94 | (3, 79) | 105 | Phage tail assembly protein | Phage tail assembly protein | | uniclust | UniRef100\_UPI001CD5C949 | 97.8 | 4.2e-07 | 7.7e-13 | 59.2 | 83 | (12, 94) | 94 | (16, 98) | 152 | phage tail assembly protein | phage tail assembly protein | | uniclust | UniRef100\_A0A5C7PFE2 | 97.8 | 4.4e-07 | 8e-13 | 56.2 | 80 | (15, 94) | 94 | (13, 95) | 109 | Phage tail assembly protein | Phage tail assembly protein | | uniclust | UniRef100\_A0A1M6LDM2 | 97.8 | 4.6e-07 | 8.9e-13 | 57.6 | 69 | (24, 93) | 94 | (26, 94) | 108 | Phage tail assembly chaperone protein, E, or 41 or 14 | Phage tail assembly chaperone protein, E, or 41 or 14 | | uniclust | UniRef100\_UPI001FEFFE9E | 97.8 | 5.1e-07 | 9.4e-13 | 60.5 | 78 | (16, 93) | 94 | (42, 129) | 186 | phage tail assembly protein | phage tail assembly protein | | uniclust | UniRef100\_A0A0D0GXK6 | 97.8 | 5e-07 | 9.7e-13 | 56.9 | 80 | (15, 94) | 94 | (13, 105) | 105 | Mu-like prophage FluMu gp41 family protein | Mu-like prophage FluMu gp41 family protein | | uniclust | UniRef100\_UPI00226FF933 | 97.8 | 5.4e-07 | 1e-12 | 59.0 | 80 | (12, 92) | 94 | (68, 147) | 157 | phage tail assembly protein | phage tail assembly protein | | uniclust | UniRef100\_A0A2S7JR59 | 97.8 | 5.6e-07 | 1e-12 | 56.3 | 80 | (14, 93) | 94 | (12, 93) | 110 | Phage tail assembly protein | Phage tail assembly protein | | uniclust | UniRef100\_A0A011LWS3 | 97.8 | 5.5e-07 | 1.1e-12 | 57.1 | 77 | (16, 92) | 94 | (4, 84) | 108 | Tail protein | Tail protein | | uniclust | UniRef100\_A0A6M3ZU73 | 97.8 | 5.8e-07 | 1.1e-12 | 60.0 | 74 | (20, 93) | 94 | (2, 84) | 174 | Phage tail assembly protein | Phage tail assembly protein | | uniclust | UniRef100\_A0A1Q6KUF5 | 97.8 | 5.8e-07 | 1.1e-12 | 59.6 | 80 | (13, 92) | 94 | (38, 130) | 136 | Uncharacterized protein | Uncharacterized protein | | uniclust | UniRef100\_UPI002094E750 | 97.8 | 6.3e-07 | 1.2e-12 | 54.1 | 73 | (18, 90) | 94 | (3, 84) | 92 | phage tail assembly protein | phage tail assembly protein | | uniclust | UniRef100\_A0A1L4D148 | 97.8 | 6.4e-07 | 1.2e-12 | 55.6 | 78 | (16, 93) | 94 | (19, 96) | 106 | Phage tail assembly protein | Phage tail assembly protein | | uniclust | UniRef100\_A6WZ02 | 97.8 | 6.6e-07 | 1.2e-12 | 56.0 | 73 | (17, 89) | 94 | (2, 92) | 110 | Phage tail assembly protein | Phage tail assembly protein | | uniclust | UniRef100\_A0A1X7MFT2 | 97.8 | 7.1e-07 | 1.3e-12 | 46.2 | 35 | (16, 50) | 94 | (3, 37) | 40 | Phage tail assembly protein (Fragment) | Phage tail assembly protein (Fragment) | | uniclust | UniRef100\_UPI001CBE614B | 97.7 | 7.1e-07 | 1.3e-12 | 53.7 | 61 | (2, 64) | 94 | (5, 66) | 90 | phage tail assembly protein | phage tail assembly protein | | uniclust | UniRef100\_UPI0021F431C5 | 97.7 | 7.3e-07 | 1.3e-12 | 57.9 | 78 | (13, 90) | 94 | (36, 114) | 148 | phage tail assembly protein | phage tail assembly protein | | uniclust | UniRef100\_A0A944GS05 | 97.7 | 7.5e-07 | 1.4e-12 | 58.5 | 80 | (12, 92) | 94 | (57, 136) | 158 | Phage tail assembly protein | Phage tail assembly protein | | uniclust | UniRef100\_A0A512JPF4 | 97.7 | 7.3e-07 | 1.4e-12 | 59.4 | 83 | (12, 94) | 94 | (52, 137) | 159 | Uncharacterized protein | Uncharacterized protein | | uniclust | UniRef100\_UPI0022DE3CC5 | 97.7 | 7.8e-07 | 1.4e-12 | 49.2 | 56 | (37, 94) | 94 | (1, 56) | 56 | phage tail assembly protein | phage tail assembly protein | | uniclust | UniRef100\_A0A257KLL7 | 97.7 | 7.5e-07 | 1.4e-12 | 55.4 | 76 | (13, 93) | 94 | (8, 83) | 95 | Phage tail assembly protein | Phage tail assembly protein | | uniclust | UniRef100\_A0A431IB45 | 97.7 | 8e-07 | 1.5e-12 | 57.7 | 76 | (12, 90) | 94 | (66, 141) | 147 | Phage tail assembly protein | Phage tail assembly protein | | uniclust | UniRef100\_A0A0U2ATQ0 | 97.7 | 7.9e-07 | 1.5e-12 | 55.1 | 81 | (14, 94) | 94 | (7, 91) | 101 | Phage tail assembly protein | Phage tail assembly protein | | uniclust | UniRef100\_UPI0013C53023 | 97.7 | 8.4e-07 | 1.5e-12 | 50.5 | 58 | (16, 73) | 94 | (7, 64) | 65 | phage tail assembly protein | phage tail assembly protein | | uniclust | UniRef100\_A0A1X9SVU8 | 97.7 | 8.1e-07 | 1.6e-12 | 55.7 | 62 | (33, 94) | 94 | (40, 102) | 102 | FluMu gp41 family protein | FluMu gp41 family protein | | uniclust | UniRef100\_A0A094ZPX7 | 97.7 | 8.2e-07 | 1.6e-12 | 57.9 | 82 | (12, 94) | 94 | (22, 109) | 125 | Phage tail assembly protein | Phage tail assembly protein | | uniclust | UniRef100\_A0A6N9P3E0 | 97.7 | 9e-07 | 1.7e-12 | 58.8 | 80 | (14, 93) | 94 | (76, 167) | 172 | Phage tail assembly protein | Phage tail assembly protein | | uniclust | UniRef100\_A0A1D8UTE9 | 97.7 | 8.8e-07 | 1.7e-12 | 56.6 | 81 | (13, 93) | 94 | (13, 102) | 115 | Phage tail assembly protein | Phage tail assembly protein | | uniclust | UniRef100\_E9L500 | 97.7 | 9.2e-07 | 1.7e-12 | 54.8 | 61 | (33, 93) | 94 | (37, 97) | 104 | Phage tail assembly protein | Phage tail assembly protein | | uniclust | UniRef100\_A0A948AK24 | 97.7 | 9.3e-07 | 1.7e-12 | 56.9 | 81 | (13, 93) | 94 | (42, 126) | 138 | Phage tail assembly protein | Phage tail assembly protein | | uniclust | UniRef100\_A0A176Z3V3 | 97.7 | 9.4e-07 | 1.8e-12 | 56.2 | 76 | (16, 91) | 94 | (3, 93) | 110 | Phage tail assembly protein | Phage tail assembly protein | | uniclust | UniRef100\_K0JHK7 | 97.7 | 1e-06 | 2e-12 | 54.8 | 81 | (13, 93) | 94 | (4, 85) | 101 | Phage protein | Phage protein | | uniclust | UniRef100\_A0A0J9EDU6 | 97.7 | 1.1e-06 | 2e-12 | 51.3 | 59 | (35, 94) | 94 | (1, 59) | 75 | Phage protein | Phage protein | | uniclust | UniRef100\_A0A7L5Y296 | 97.7 | 1.1e-06 | 2.1e-12 | 60.7 | 77 | (16, 94) | 94 | (77, 153) | 185 | Uncharacterized protein | Uncharacterized protein | | uniclust | UniRef100\_A0A1Q9A2K6 | 97.7 | 1.1e-06 | 2.1e-12 | 58.5 | 75 | (14, 91) | 94 | (73, 147) | 157 | Phage tail assembly protein | Phage tail assembly protein | | uniclust | UniRef100\_UPI0003955618 | 97.7 | 1.2e-06 | 2.2e-12 | 55.7 | 81 | (13, 93) | 94 | (31, 115) | 126 | phage tail assembly protein | phage tail assembly protein | | uniclust | UniRef100\_UPI001F249746 | 97.7 | 1.2e-06 | 2.2e-12 | 55.1 | 62 | (18, 80) | 94 | (2, 64) | 107 | phage tail assembly protein | phage tail assembly protein | | uniclust | UniRef100\_A0A0S4V3E5 | 97.7 | 1.3e-06 | 2.4e-12 | 56.3 | 74 | (16, 90) | 94 | (6, 83) | 131 | Hypothethical protein | Hypothethical protein | | uniclust | UniRef100\_R7I6D8 | 97.6 | 1.4e-06 | 2.6e-12 | 53.4 | 79 | (16, 94) | 94 | (3, 87) | 95 | Phage tail assembly protein | Phage tail assembly protein | | uniclust | UniRef100\_UPI0021CEA9E1 | 97.6 | 1.4e-06 | 2.6e-12 | 52.7 | 74 | (18, 91) | 94 | (2, 75) | 92 | phage tail assembly protein | phage tail assembly protein | | uniclust | UniRef100\_A0A968GD13 | 97.6 | 1.3e-06 | 2.6e-12 | 58.7 | 85 | (9, 93) | 94 | (62, 149) | 151 | Uncharacterized protein | Uncharacterized protein | | uniclust | UniRef100\_A0A8I2FQ78 | 97.6 | 1.4e-06 | 2.6e-12 | 53.2 | 81 | (13, 94) | 94 | (17, 98) | 98 | Phage tail assembly protein | Phage tail assembly protein | | uniclust | UniRef100\_A0A1M5RFG1 | 97.6 | 1.4e-06 | 2.7e-12 | 52.7 | 76 | (18, 94) | 94 | (2, 78) | 87 | Phage tail assembly chaperone protein, E, or 41 or 14 | Phage tail assembly chaperone protein, E, or 41 or 14 | | uniclust | UniRef100\_A0A7V8JT65 | 97.6 | 1.4e-06 | 2.7e-12 | 53.5 | 78 | (16, 94) | 94 | (6, 83) | 95 | Phage tail assembly protein | Phage tail assembly protein | | uniclust | UniRef100\_A0A3A6P6S0 | 97.6 | 1.5e-06 | 2.8e-12 | 54.8 | 83 | (12, 94) | 94 | (14, 107) | 120 | Phage tail assembly protein | Phage tail assembly protein | | uniclust | UniRef100\_A0A437N1X6 | 97.6 | 1.5e-06 | 2.8e-12 | 53.9 | 77 | (17, 93) | 94 | (12, 92) | 108 | Phage tail assembly protein | Phage tail assembly protein | | uniclust | UniRef100\_A0A3A9B6H8 | 97.6 | 1.5e-06 | 2.9e-12 | 56.5 | 82 | (12, 93) | 94 | (24, 118) | 122 | Phage tail assembly protein | Phage tail assembly protein | | uniclust | UniRef100\_A0A9D8STM0 | 97.6 | 1.6e-06 | 3e-12 | 52.8 | 76 | (18, 93) | 94 | (2, 78) | 89 | Uncharacterized protein | Uncharacterized protein | | uniclust | UniRef100\_A0A7H1NUI4 | 97.6 | 1.8e-06 | 3.4e-12 | 55.5 | 80 | (14, 93) | 94 | (26, 110) | 123 | Phage tail assembly chaperone protein | Phage tail assembly chaperone protein | | uniclust | UniRef100\_A0A225SNI8 | 97.6 | 1.9e-06 | 3.6e-12 | 54.0 | 76 | (18, 93) | 94 | (28, 105) | 115 | Phage tail assembly protein | Phage tail assembly protein | | uniclust | UniRef100\_A0A1F4HNB5 | 97.6 | 2e-06 | 3.7e-12 | 54.0 | 77 | (18, 94) | 94 | (24, 107) | 115 | Phage tail protein | Phage tail protein | | uniclust | UniRef100\_A0A8J7UJ23 | 97.6 | 2e-06 | 3.7e-12 | 51.4 | 74 | (18, 91) | 94 | (3, 78) | 85 | Phage tail assembly protein | Phage tail assembly protein | | uniclust | UniRef100\_UPI001D10B551 | 97.6 | 2.1e-06 | 3.8e-12 | 57.4 | 79 | (16, 94) | 94 | (71, 153) | 176 | phage tail assembly protein | phage tail assembly protein | | uniclust | UniRef100\_A0A1G3JSY5 | 97.6 | 1.9e-06 | 3.9e-12 | 53.8 | 65 | (30, 94) | 94 | (8, 76) | 90 | Phage tail protein | Phage tail protein | | uniclust | UniRef100\_A0A433X0G4 | 97.6 | 2.1e-06 | 3.9e-12 | 55.9 | 90 | (2, 93) | 94 | (38, 139) | 147 | Phage tail protein | Phage tail protein | | uniclust | UniRef100\_A0A076LV38 | 97.6 | 2.1e-06 | 3.9e-12 | 53.4 | 81 | (14, 94) | 94 | (5, 89) | 98 | Putative bacteriophage protein | Putative bacteriophage protein | | uniclust | UniRef100\_A0A0D2JFD8 | 97.6 | 2.2e-06 | 4e-12 | 52.2 | 79 | (15, 93) | 94 | (4, 92) | 95 | Phage tail assembly protein | Phage tail assembly protein | | uniclust | UniRef100\_A0A174TN41 | 97.6 | 2e-06 | 4e-12 | 57.5 | 81 | (13, 93) | 94 | (33, 134) | 139 | Uncharacterized protein | Uncharacterized protein | | uniclust | UniRef100\_A0A5M9U1M0 | 97.6 | 2.1e-06 | 4.1e-12 | 55.5 | 78 | (16, 93) | 94 | (20, 114) | 118 | Phage tail assembly protein | Phage tail assembly protein | | uniclust | UniRef100\_A0A165XHA6 | 97.6 | 2.3e-06 | 4.2e-12 | 56.7 | 75 | (13, 94) | 94 | (70, 149) | 165 | Phage tail assembly protein | Phage tail assembly protein | | uniclust | UniRef100\_UPI0016750E23 | 97.6 | 2.6e-06 | 4.7e-12 | 54.0 | 82 | (12, 93) | 94 | (27, 112) | 121 | phage tail assembly protein | phage tail assembly protein | | uniclust | UniRef100\_A0A5N8AC27 | 97.6 | 2.6e-06 | 4.8e-12 | 55.1 | 78 | (16, 93) | 94 | (43, 124) | 139 | Phage tail assembly protein | Phage tail assembly protein | | uniclust | UniRef100\_A0A367Q8P7 | 97.5 | 2.8e-06 | 5.3e-12 | 59.1 | 82 | (2, 93) | 94 | (109, 197) | 203 | Uncharacterized protein | Uncharacterized protein | | uniclust | UniRef100\_A0A0Q6BTN7 | 97.5 | 2.8e-06 | 5.5e-12 | 52.3 | 66 | (17, 86) | 94 | (11, 76) | 89 | Phage tail assembly protein | Phage tail assembly protein | | uniclust | UniRef100\_A0A6L8HXB9 | 97.5 | 3.1e-06 | 5.7e-12 | 60.1 | 77 | (16, 93) | 94 | (22, 99) | 280 | SMP-30/Gluconolactonase/LRE-like region domain-containing protein | SMP-30/Gluconolactonase/LRE-like region domain-containing protein | | uniclust | UniRef100\_UPI001378214B | 97.5 | 3.1e-06 | 5.7e-12 | 54.2 | 80 | (14, 93) | 94 | (26, 109) | 129 | phage tail assembly protein | phage tail assembly protein | | uniclust | UniRef100\_A0A9E9B835 | 97.5 | 3.2e-06 | 5.8e-12 | 55.0 | 74 | (14, 91) | 94 | (62, 135) | 143 | Phage tail assembly protein | Phage tail assembly protein | | uniclust | UniRef100\_S9S610 | 97.5 | 3.3e-06 | 6.1e-12 | 52.6 | 77 | (16, 92) | 94 | (14, 92) | 109 | Putative phage related protein | Putative phage related protein | | uniclust | UniRef100\_A0A4S8Q2I0 | 97.5 | 3.3e-06 | 6.3e-12 | 52.1 | 77 | (17, 93) | 94 | (2, 78) | 93 | Uncharacterized protein | Uncharacterized protein | | uniclust | UniRef100\_A0A564WJQ0 | 97.5 | 3.5e-06 | 6.4e-12 | 52.1 | 79 | (16, 94) | 94 | (2, 85) | 103 | Uncharacterized protein | Uncharacterized protein | | uniclust | UniRef100\_A0A485CDJ1 | 97.5 | 3.6e-06 | 6.7e-12 | 58.2 | 81 | (13, 93) | 94 | (129, 213) | 225 | Phage major tail tube protein | Phage major tail tube protein | | uniclust | UniRef100\_A0A0B1YRZ3 | 97.5 | 3.5e-06 | 6.7e-12 | 53.4 | 81 | (13, 93) | 94 | (3, 92) | 108 | Phage tail assembly protein | Phage tail assembly protein | | uniclust | UniRef100\_A0A961Y6K2 | 97.5 | 3.9e-06 | 7.2e-12 | 54.1 | 73 | (18, 94) | 94 | (5, 81) | 128 | Phage tail assembly protein | Phage tail assembly protein | | uniclust | UniRef100\_A0A176EWX2 | 97.5 | 4.3e-06 | 7.9e-12 | 50.4 | 72 | (18, 89) | 94 | (3, 75) | 86 | Phage tail protein | Phage tail protein | | uniclust | UniRef100\_A0A1I4TJ36 | 97.5 | 4e-06 | 8e-12 | 55.3 | 77 | (16, 93) | 94 | (3, 96) | 127 | Uncharacterized protein | Uncharacterized protein | | uniclust | UniRef100\_A0A7W6RF20 | 97.5 | 4.3e-06 | 8.1e-12 | 54.7 | 83 | (12, 94) | 94 | (15, 102) | 132 | Tail assembly chaperone E/41/14-like protein | Tail assembly chaperone E/41/14-like protein | | uniclust | UniRef100\_A0A1X3I4M1 | 97.4 | 5e-06 | 9.3e-12 | 52.8 | 80 | (15, 94) | 94 | (27, 112) | 113 | Phage tail protein E | Phage tail protein E | | uniclust | UniRef100\_A0A8B2QYK0 | 97.4 | 5.1e-06 | 9.4e-12 | 41.3 | 32 | (62, 93) | 94 | (1, 32) | 32 | Phage tail assembly protein | Phage tail assembly protein | | uniclust | UniRef100\_A0A1E3G5Y7 | 97.4 | 5.8e-06 | 1.1e-11 | 48.9 | 72 | (17, 93) | 94 | (3, 74) | 78 | Phage tail assembly protein | Phage tail assembly protein | | uniclust | UniRef100\_A0A4D7YHU8 | 97.4 | 5.8e-06 | 1.1e-11 | 48.2 | 60 | (33, 93) | 94 | (2, 61) | 72 | Phage tail assembly protein | Phage tail assembly protein | | uniclust | UniRef100\_UPI0006863B40 | 97.4 | 6.2e-06 | 1.1e-11 | 56.4 | 76 | (16, 93) | 94 | (113, 188) | 203 | phage tail assembly protein | phage tail assembly protein | | uniclust | UniRef100\_UPI0015D41224 | 97.4 | 6.3e-06 | 1.2e-11 | 52.5 | 73 | (18, 92) | 94 | (2, 75) | 123 | phage tail assembly protein | phage tail assembly protein | | uniclust | UniRef100\_A0A1X0WB04 | 97.4 | 5.9e-06 | 1.2e-11 | 57.2 | 83 | (12, 94) | 94 | (83, 169) | 183 | Uncharacterized protein | Uncharacterized protein | | uniclust | UniRef100\_A0A2S4MDR7 | 97.4 | 6.2e-06 | 1.2e-11 | 52.9 | 79 | (16, 94) | 94 | (23, 107) | 118 | Tail assembly chaperone E/41/14-like protein | Tail assembly chaperone E/41/14-like protein | | uniclust | UniRef100\_A0A2A4XUJ7 | 97.4 | 6.3e-06 | 1.2e-11 | 52.0 | 77 | (17, 93) | 94 | (4, 89) | 106 | Phage tail assembly protein | Phage tail assembly protein | | uniclust | UniRef100\_UPI001FB9F85B | 97.4 | 6.6e-06 | 1.2e-11 | 57.4 | 82 | (13, 94) | 94 | (113, 200) | 226 | hypothetical protein | hypothetical protein | | uniclust | UniRef100\_A0A087M272 | 97.4 | 6.7e-06 | 1.3e-11 | 51.3 | 78 | (15, 93) | 94 | (13, 91) | 100 | Phage tail assembly protein | Phage tail assembly protein | | uniclust | UniRef100\_A0A097P6T1 | 97.4 | 7.1e-06 | 1.3e-11 | 53.7 | 77 | (18, 94) | 94 | (3, 91) | 147 | Tail assembly chaperone | Tail assembly chaperone | | uniclust | UniRef100\_A0A7C1VCE0 | 97.4 | 7.3e-06 | 1.3e-11 | 52.8 | 79 | (15, 93) | 94 | (53, 133) | 133 | Phage tail assembly protein | Phage tail assembly protein | | uniclust | UniRef100\_A0A150HJQ6 | 97.4 | 7.2e-06 | 1.4e-11 | 53.1 | 80 | (15, 94) | 94 | (29, 114) | 126 | Phage tail protein E | Phage tail protein E | | uniclust | UniRef100\_A0A1T4WVF8 | 97.4 | 8.1e-06 | 1.5e-11 | 55.4 | 70 | (17, 92) | 94 | (113, 183) | 189 | Phage tail assembly chaperone protein, E, or 41 or 14 | Phage tail assembly chaperone protein, E, or 41 or 14 | | uniclust | UniRef100\_A0A078L7Q4 | 97.4 | 8.1e-06 | 1.5e-11 | 55.5 | 80 | (14, 93) | 94 | (28, 115) | 167 | Phage tail assembly protein | Phage tail assembly protein | | uniclust | UniRef100\_A0A0A3TNQ9 | 97.4 | 8.2e-06 | 1.6e-11 | 50.9 | 77 | (17, 93) | 94 | (2, 91) | 97 | Phage tail assembly protein | Phage tail assembly protein | | uniclust | UniRef100\_A0A4R2P5C3 | 97.4 | 8.6e-06 | 1.6e-11 | 51.4 | 81 | (13, 93) | 94 | (22, 106) | 115 | Tail assembly chaperone E/41/14-like protein | Tail assembly chaperone E/41/14-like protein | | uniclust | UniRef100\_D8JWC8 | 97.3 | 8.8e-06 | 1.6e-11 | 52.6 | 74 | (19, 92) | 94 | (34, 126) | 134 | Phage protein | Phage protein | | uniclust | UniRef100\_UPI0013EA8D57 | 97.3 | 9e-06 | 1.6e-11 | 51.9 | 79 | (13, 92) | 94 | (35, 123) | 123 | phage tail assembly protein | phage tail assembly protein | | uniclust | UniRef100\_A0A2S6N2U8 | 97.3 | 9.5e-06 | 1.7e-11 | 50.8 | 74 | (17, 92) | 94 | (33, 106) | 109 | Phage tail assembly protein | Phage tail assembly protein | | uniclust | UniRef100\_A0A4Q2U0L3 | 97.3 | 9.5e-06 | 1.8e-11 | 51.7 | 80 | (14, 93) | 94 | (11, 102) | 122 | Phage tail assembly protein | Phage tail assembly protein | | uniclust | UniRef100\_A0A0U1DB82 | 97.3 | 9.5e-06 | 1.8e-11 | 45.1 | 45 | (50, 94) | 94 | (5, 49) | 55 | Putative phage related protein | Putative phage related protein | | uniclust | UniRef100\_S5Y192 | 97.3 | 9.6e-06 | 1.8e-11 | 51.0 | 73 | (18, 90) | 94 | (2, 80) | 112 | Uncharacterized protein | Uncharacterized protein | | uniclust | UniRef100\_A0A560T0I5 | 97.3 | 1.1e-05 | 2.1e-11 | 50.6 | 76 | (16, 91) | 94 | (4, 80) | 104 | Tail assembly chaperone E/41/14-like protein | Tail assembly chaperone E/41/14-like protein | | uniclust | UniRef100\_A0A7W6EB82 | 97.3 | 1.2e-05 | 2.1e-11 | 53.1 | 68 | (16, 87) | 94 | (75, 142) | 154 | Phage tail assembly protein | Phage tail assembly protein | | uniclust | UniRef100\_UPI001EDF0F93 | 97.3 | 1.2e-05 | 2.1e-11 | 46.4 | 56 | (37, 92) | 94 | (1, 56) | 67 | phage tail assembly protein | phage tail assembly protein | | uniclust | UniRef100\_UPI00051682BF | 97.3 | 1.2e-05 | 2.1e-11 | 46.2 | 46 | (3, 50) | 94 | (12, 57) | 65 | phage tail assembly protein | phage tail assembly protein | | uniclust | UniRef100\_UPI002226FE03 | 97.3 | 1.2e-05 | 2.1e-11 | 51.9 | 77 | (16, 92) | 94 | (31, 118) | 132 | phage tail assembly protein | phage tail assembly protein | | uniclust | UniRef100\_A0A2A5EJW3 | 97.3 | 1.2e-05 | 2.3e-11 | 54.4 | 77 | (15, 91) | 94 | (5, 96) | 186 | Phage tail assembly protein | Phage tail assembly protein | | uniclust | UniRef100\_A0A7X6FRD4 | 97.3 | 1.3e-05 | 2.3e-11 | 47.3 | 33 | (18, 50) | 94 | (3, 35) | 76 | Phage tail assembly protein | Phage tail assembly protein | | uniclust | UniRef100\_A0A0S4XLQ8 | 97.3 | 1.3e-05 | 2.4e-11 | 49.5 | 61 | (33, 94) | 94 | (21, 81) | 90 | Phage tail assembly protein | Phage tail assembly protein | | uniclust | UniRef100\_A0A526RPY9 | 97.3 | 1.4e-05 | 2.5e-11 | 48.6 | 70 | (17, 90) | 94 | (6, 75) | 90 | Phage tail assembly protein (Fragment) | Phage tail assembly protein (Fragment) | | uniclust | UniRef100\_A0A2G1CU23 | 97.3 | 1.4e-05 | 2.5e-11 | 49.1 | 77 | (16, 93) | 94 | (13, 89) | 96 | Phage tail assembly protein | Phage tail assembly protein | | uniclust | UniRef100\_UPI000277D392 | 97.3 | 1.4e-05 | 2.5e-11 | 51.1 | 39 | (15, 53) | 94 | (2, 40) | 123 | phage tail assembly protein | phage tail assembly protein | | uniclust | UniRef100\_A0A317H578 | 97.3 | 1.4e-05 | 2.6e-11 | 53.4 | 76 | (13, 91) | 94 | (8, 83) | 167 | Phage tail assembly protein | Phage tail assembly protein | | uniclust | UniRef100\_A0A2S3W4R7 | 97.2 | 1.5e-05 | 2.8e-11 | 55.5 | 80 | (14, 93) | 94 | (97, 182) | 191 | Uncharacterized protein | Uncharacterized protein | | uniclust | UniRef100\_UPI0006528B58 | 97.2 | 1.6e-05 | 2.9e-11 | 46.2 | 34 | (18, 51) | 94 | (3, 36) | 69 | phage tail assembly protein | phage tail assembly protein | | uniclust | UniRef100\_A0A1C6E0X6 | 97.2 | 1.5e-05 | 3e-11 | 57.5 | 78 | (17, 94) | 94 | (45, 137) | 237 | Phage tail assembly protein | Phage tail assembly protein | | uniclust | UniRef100\_A0A8X6GSQ5 | 97.2 | 1.6e-05 | 3e-11 | 56.0 | 65 | (30, 94) | 94 | (164, 228) | 234 | Phage tail tube protein FII | Phage tail tube protein FII | | uniclust | UniRef100\_UPI001C12BF21 | 97.2 | 1.8e-05 | 3.3e-11 | 47.9 | 72 | (21, 93) | 94 | (1, 73) | 87 | phage tail assembly protein | phage tail assembly protein | | uniclust | UniRef100\_A0A0B7J8H1 | 97.2 | 1.7e-05 | 3.3e-11 | 55.8 | 80 | (14, 94) | 94 | (100, 180) | 196 | Phage tail assembly protein | Phage tail assembly protein | | uniclust | UniRef100\_A0A859FB61 | 97.2 | 1.9e-05 | 3.4e-11 | 52.7 | 81 | (14, 94) | 94 | (71, 163) | 164 | Phage tail assembly protein | Phage tail assembly protein | | uniclust | UniRef100\_A0A1G7PX14 | 97.2 | 1.9e-05 | 3.4e-11 | 51.5 | 81 | (12, 94) | 94 | (33, 114) | 139 | Phage tail assembly chaperone protein, E, or 41 or 14 | Phage tail assembly chaperone protein, E, or 41 or 14 | | uniclust | UniRef100\_UPI00186A80F2 | 97.2 | 1.9e-05 | 3.4e-11 | 48.1 | 73 | (17, 90) | 94 | (3, 75) | 91 | phage tail assembly protein | phage tail assembly protein | | uniclust | UniRef100\_A0A085AFM8 | 97.2 | 1.9e-05 | 3.6e-11 | 55.6 | 76 | (16, 91) | 94 | (4, 87) | 209 | Phage protein | Phage protein | | uniclust | UniRef100\_A0A812QV43 | 97.2 | 2.1e-05 | 3.8e-11 | 63.9 | 75 | (16, 91) | 94 | (1023, 1097) | 1104 | GpFI protein | GpFI protein | | uniclust | UniRef100\_UPI00235E67D6 | 97.2 | 2.2e-05 | 4e-11 | 47.7 | 74 | (17, 91) | 94 | (2, 75) | 89 | phage tail assembly protein | phage tail assembly protein | | uniclust | UniRef100\_A0A1L3SPW8 | 97.2 | 2.2e-05 | 4.2e-11 | 51.1 | 79 | (16, 94) | 94 | (2, 94) | 119 | Uncharacterized protein | Uncharacterized protein | | uniclust | UniRef100\_A0A067W7B7 | 97.1 | 2.6e-05 | 4.7e-11 | 45.5 | 56 | (25, 82) | 94 | (15, 70) | 70 | Uncharacterized protein | Uncharacterized protein | | uniclust | UniRef100\_A0A6I3KEY4 | 97.1 | 2.6e-05 | 4.8e-11 | 46.4 | 63 | (16, 88) | 94 | (3, 66) | 79 | Phage tail assembly chaperone protein, E, or 41 or 14 | Phage tail assembly chaperone protein, E, or 41 or 14 | | uniclust | UniRef100\_A0A261QN74 | 97.1 | 2.9e-05 | 5.5e-11 | 48.2 | 39 | (56, 94) | 94 | (25, 63) | 89 | Phage tail assembly protein | Phage tail assembly protein | | uniclust | UniRef100\_A0A9D1NLP2 | 97.1 | 3e-05 | 5.5e-11 | 47.7 | 74 | (17, 90) | 94 | (3, 80) | 95 | Phage tail assembly protein | Phage tail assembly protein | | uniclust | UniRef100\_A0A8S7UTI4 | 97.1 | 3.1e-05 | 5.6e-11 | 41.6 | 35 | (17, 51) | 94 | (2, 36) | 45 | Phage tail assembly protein (Fragment) | Phage tail assembly protein (Fragment) | | uniclust | UniRef100\_A0A1W0CCH5 | 97.1 | 3.4e-05 | 6.3e-11 | 47.4 | 77 | (16, 92) | 94 | (3, 89) | 95 | Phage tail assembly protein | Phage tail assembly protein | | uniclust | UniRef100\_A0A2U1TJU4 | 97.1 | 3.4e-05 | 6.4e-11 | 48.9 | 80 | (15, 94) | 94 | (5, 90) | 105 | Phage tail protein | Phage tail protein | | uniclust | UniRef100\_A0A094JIN5 | 97.1 | 3.5e-05 | 6.5e-11 | 45.6 | 62 | (18, 79) | 94 | (2, 73) | 76 | Uncharacterized protein | Uncharacterized protein | | uniclust | UniRef100\_A0A6L3YW32 | 97.1 | 3.7e-05 | 6.9e-11 | 51.5 | 33 | (18, 50) | 94 | (3, 35) | 155 | Phage tail assembly protein | Phage tail assembly protein | | uniclust | UniRef100\_A0A9E8T1G1 | 97.1 | 3.8e-05 | 7e-11 | 50.7 | 74 | (18, 91) | 94 | (41, 116) | 149 | Phage tail assembly protein | Phage tail assembly protein | | uniclust | UniRef100\_A0A0H3ZVR6 | 97.1 | 3.5e-05 | 7.1e-11 | 54.9 | 61 | (33, 94) | 94 | (130, 190) | 203 | Phage tail assembly protein | Phage tail assembly protein | | uniclust | UniRef100\_A0A255XY73 | 97.1 | 4e-05 | 7.3e-11 | 59.5 | 81 | (13, 94) | 94 | (505, 587) | 595 | Uncharacterized protein | Uncharacterized protein | | uniclust | UniRef100\_UPI00082FD7B7 | 97.1 | 4e-05 | 7.3e-11 | 50.0 | 73 | (16, 88) | 94 | (5, 85) | 137 | phage tail assembly protein | phage tail assembly protein | | uniclust | UniRef100\_A0A2H5DVZ8 | 97.1 | 4e-05 | 7.3e-11 | 51.0 | 78 | (13, 90) | 94 | (64, 151) | 158 | Phage tail assembly protein | Phage tail assembly protein | | uniclust | UniRef100\_UPI001CC07461 | 97.1 | 4.1e-05 | 7.6e-11 | 49.9 | 75 | (16, 90) | 94 | (4, 79) | 137 | phage tail assembly protein | phage tail assembly protein | | uniclust | UniRef100\_A0A252BR55 | 97.1 | 4e-05 | 7.7e-11 | 51.2 | 88 | (2, 94) | 94 | (33, 124) | 140 | Phage tail assembly protein | Phage tail assembly protein | | uniclust | UniRef100\_A0A482IXJ4 | 97.1 | 4.3e-05 | 7.8e-11 | 50.5 | 74 | (17, 90) | 94 | (42, 116) | 149 | Phage tail assembly protein | Phage tail assembly protein | | uniclust | UniRef100\_UPI0022AFC0B4 | 97.0 | 4.3e-05 | 7.9e-11 | 48.6 | 80 | (14, 94) | 94 | (28, 107) | 116 | hypothetical protein | hypothetical protein | | uniclust | UniRef100\_A0A2R7ILE3 | 97.0 | 4.5e-05 | 8.4e-11 | 49.1 | 76 | (14, 90) | 94 | (2, 87) | 114 | Phage tail assembly protein | Phage tail assembly protein | | uniclust | UniRef100\_UPI00226FC3D2 | 97.0 | 4.6e-05 | 8.5e-11 | 50.1 | 79 | (15, 93) | 94 | (3, 98) | 145 | hypothetical protein | hypothetical protein | | uniclust | UniRef100\_A0A1G0GGY7 | 97.0 | 4.5e-05 | 8.8e-11 | 49.6 | 78 | (14, 93) | 94 | (4, 91) | 113 | Phage tail assembly protein | Phage tail assembly protein | | uniclust | UniRef100\_UPI000A356193 | 97.0 | 4.9e-05 | 9e-11 | 43.3 | 59 | (36, 94) | 94 | (2, 61) | 61 | phage tail assembly protein | phage tail assembly protein | | uniclust | UniRef100\_A0A072TPP1 | 97.0 | 5e-05 | 9.1e-11 | 60.0 | 80 | (14, 93) | 94 | (625, 710) | 726 | Bacteriophage mu tail sheath protein | Bacteriophage mu tail sheath protein | | uniclust | UniRef100\_A0A0B1Q7C3 | 97.0 | 4.9e-05 | 9.5e-11 | 49.2 | 78 | (17, 94) | 94 | (3, 88) | 113 | Uncharacterized protein | Uncharacterized protein | | uniclust | UniRef100\_A0A9D9A8Q3 | 97.0 | 5.5e-05 | 1e-10 | 50.0 | 78 | (16, 94) | 94 | (43, 121) | 148 | Phage tail assembly protein | Phage tail assembly protein | | uniclust | UniRef100\_A0A444L4U4 | 97.0 | 5.6e-05 | 1e-10 | 47.4 | 67 | (20, 86) | 94 | (2, 84) | 105 | Phage tail assembly protein | Phage tail assembly protein | | uniclust | UniRef100\_A0A858X005 | 97.0 | 6.1e-05 | 1.2e-10 | 49.2 | 77 | (17, 93) | 94 | (2, 101) | 125 | Phage tail assembly protein | Phage tail assembly protein | | uniclust | UniRef100\_A0A1J5HSC3 | 97.0 | 6.4e-05 | 1.2e-10 | 48.2 | 79 | (16, 94) | 94 | (6, 102) | 121 | Phage tail protein | Phage tail protein | | uniclust | UniRef100\_UPI000E25BC78 | 97.0 | 6.5e-05 | 1.2e-10 | 48.2 | 73 | (16, 89) | 94 | (4, 76) | 122 | hypothetical protein | hypothetical protein | | uniclust | UniRef100\_UPI00082C10DB | 97.0 | 6.6e-05 | 1.2e-10 | 52.9 | 77 | (17, 93) | 94 | (45, 126) | 239 | phage tail assembly protein | phage tail assembly protein | | uniclust | UniRef100\_A0A432QTZ1 | 97.0 | 6.7e-05 | 1.2e-10 | 51.0 | 77 | (16, 92) | 94 | (11, 88) | 182 | Phage tail assembly protein | Phage tail assembly protein | | uniclust | UniRef100\_A0A1Z4RAB7 | 97.0 | 6.8e-05 | 1.2e-10 | 46.9 | 60 | (35, 94) | 94 | (33, 100) | 103 | Phage tail assembly protein | Phage tail assembly protein | | uniclust | UniRef100\_A0A1E3WJ29 | 97.0 | 6.8e-05 | 1.2e-10 | 47.6 | 77 | (17, 93) | 94 | (27, 105) | 114 | Phage tail assembly protein | Phage tail assembly protein | | uniclust | UniRef100\_UPI001BA5F6AD | 97.0 | 6.9e-05 | 1.3e-10 | 48.0 | 73 | (13, 86) | 94 | (34, 106) | 121 | hypothetical protein | hypothetical protein | | uniclust | UniRef100\_UPI00234EDE79 | 96.9 | 7e-05 | 1.3e-10 | 50.6 | 89 | (3, 94) | 94 | (65, 166) | 173 | phage tail assembly protein | phage tail assembly protein | | uniclust | UniRef100\_A0A4Q3U4W6 | 96.9 | 7.2e-05 | 1.3e-10 | 48.7 | 77 | (17, 93) | 94 | (40, 120) | 133 | Phage tail assembly protein | Phage tail assembly protein | | uniclust | UniRef100\_UPI001A5E140C | 96.9 | 7.4e-05 | 1.4e-10 | 48.5 | 78 | (16, 93) | 94 | (27, 123) | 130 | phage tail assembly protein | phage tail assembly protein | | uniclust | UniRef100\_A0A3G7TJ80 | 96.9 | 7.6e-05 | 1.4e-10 | 49.0 | 76 | (15, 90) | 94 | (3, 79) | 136 | Phage tail assembly protein | Phage tail assembly protein | | uniclust | UniRef100\_A0A2W5AE53 | 96.9 | 7.9e-05 | 1.4e-10 | 46.2 | 77 | (17, 93) | 94 | (3, 85) | 98 | Phage tail assembly protein | Phage tail assembly protein | | uniclust | UniRef100\_A0A5C7JB85 | 96.9 | 8.7e-05 | 1.6e-10 | 45.7 | 74 | (18, 92) | 94 | (5, 79) | 94 | Phage tail assembly protein | Phage tail assembly protein | | uniclust | UniRef100\_V4NZH5 | 96.9 | 8.8e-05 | 1.6e-10 | 46.0 | 74 | (16, 90) | 94 | (13, 95) | 97 | Phage protein | Phage protein | | uniclust | UniRef100\_UPI0016521F9B | 96.9 | 8.8e-05 | 1.6e-10 | 51.5 | 79 | (15, 93) | 94 | (77, 156) | 210 | phage tail assembly protein | phage tail assembly protein | | uniclust | UniRef100\_A0A090IQA6 | 96.9 | 8.5e-05 | 1.6e-10 | 53.6 | 80 | (14, 94) | 94 | (143, 223) | 236 | Uncharacterized phage protein | Uncharacterized phage protein | | uniclust | UniRef100\_A0A268TN79 | 96.9 | 8.5e-05 | 1.6e-10 | 46.1 | 62 | (33, 94) | 94 | (18, 81) | 86 | Phage tail assembly protein | Phage tail assembly protein | | uniclust | UniRef100\_A0RRS4 | 96.9 | 9e-05 | 1.7e-10 | 47.2 | 62 | (33, 94) | 94 | (46, 108) | 108 | Phage tail assembly protein | Phage tail assembly protein | | uniclust | UniRef100\_A0A2W6XD89 | 96.9 | 9.7e-05 | 1.8e-10 | 47.9 | 78 | (16, 93) | 94 | (30, 111) | 124 | Phage tail assembly protein | Phage tail assembly protein | | uniclust | UniRef100\_X1CAN2 | 96.9 | 9.8e-05 | 1.8e-10 | 46.9 | 80 | (13, 93) | 94 | (32, 113) | 113 | Uncharacterized protein | Uncharacterized protein | | uniclust | UniRef100\_A0A358C7S8 | 96.9 | 9.9e-05 | 1.8e-10 | 46.5 | 79 | (16, 94) | 94 | (5, 91) | 107 | Phage tail assembly protein | Phage tail assembly protein | | uniclust | UniRef100\_UPI00082C10DB | 96.9 | 0.0001 | 1.9e-10 | 52.0 | 79 | (15, 93) | 94 | (137, 219) | 239 | phage tail assembly protein | phage tail assembly protein | | uniclust | UniRef100\_UPI00200A87DE | 96.9 | 0.00011 | 2e-10 | 50.1 | 77 | (14, 92) | 94 | (94, 170) | 180 | phage tail assembly protein | phage tail assembly protein | |
| Top keywords  (threshold 1.00e-03 (evalue)) | **tail, Phage, assembly, or, E, chaperone, 14\_like, Mu\_like, FluMu, gp41** |
| Output files | ../../similar\_sequences/20\_FANPEZAQ\_CDS\_0020\_merged.svg ../../similar\_sequences/20\_FANPEZAQ\_CDS\_0020\_pdb70.a3m ../../similar\_sequences/20\_FANPEZAQ\_CDS\_0020\_pdb70.hhr ../../similar\_sequences/20\_FANPEZAQ\_CDS\_0020\_uniclust.a3m ../../similar\_sequences/20\_FANPEZAQ\_CDS\_0020\_uniclust.hhr |

#### Structure prediction (AlphaFold)2

|  |  |
| --- | --- |
| Stats | xml version="1.0" encoding="utf-8" standalone="no"?       2024-09-02T21:09:19.650353 image/svg+xml   Matplotlib v3.7.2, https://matplotlib.org/ |
| Predicted structure | **NGL Viewer Controls:**  - Center: *Left-Click* - Rotate: *Left-Click + Drag* - Translate: *Right-Click + Drag* - Zoom: *Shift + Left-Click + Drag* |
| Output files | ../../predicted\_structures/20\_FANPEZAQ\_CDS\_0020/features.pkl ../../predicted\_structures/20\_FANPEZAQ\_CDS\_0020/ranked\_0.pdb ../../predicted\_structures/20\_FANPEZAQ\_CDS\_0020/ranked\_0\_plots.svg ../../predicted\_structures/20\_FANPEZAQ\_CDS\_0020/result\_model\_1\_ptm\_pred\_0.pkl |

#### Structure similarity search results (Foldseek)3

|  |  |
| --- | --- |
| Structure databases searched | Pdb, Afdb-proteome, Afdb-uniprot50 |
| Results, scheme(s)  (Top layers only, threshold 1.00e-02 (evalue)) | xml version="1.0" encoding="utf-8" standalone="no"?       2024-09-02T21:10:44.797899 image/svg+xml   Matplotlib v3.7.2, https://matplotlib.org/ |
| Results, table  (threshold 1.00e-02 (evalue)) | | db | id | prob | evalue | bits | fident | alnlen | mismatch | gapopen | qstart | qend | tstart | tend | name | description | | --- | --- | --- | --- | --- | --- | --- | --- | --- | --- | --- | --- | --- | --- | --- | | afdb-proteome | AF-G3XCX2-F1-MODEL\_V4 | 1.0 | 1.424e-08 | 314 | 0.387 | 93 | 54 | 2 | 2 | 93 | 5 | 95 | Uncharacterized protein | Uncharacterized protein | | afdb-proteome | AF-Q8ZMV1-F1-MODEL\_V4 | 1.0 | 0.00306 | 127 | 0.13 | 92 | 70 | 3 | 10 | 94 | 1 | 89 | Fels-2 prophage protein | Fels-2 prophage protein | | afdb-proteome | AF-A0A0H3GM31-F1-MODEL\_V4 | 1.0 | 0.003962 | 125 | 0.147 | 88 | 63 | 3 | 15 | 94 | 5 | 88 | Phage tail protein E | Phage tail protein E | | afdb-uniprot50 | AF-A0A653KXL9-F1-MODEL\_V4 | 1.0 | 9.457e-13 | 514 | 0.663 | 95 | 30 | 2 | 1 | 94 | 1 | 94 | Phage tail assembly protein | Phage tail assembly protein | | afdb-uniprot50 | AF-A0A7R8GJ05-F1-MODEL\_V4 | 1.0 | 2.337e-12 | 506 | 0.623 | 93 | 34 | 1 | 2 | 94 | 25 | 116 | Uncharacterized protein | Uncharacterized protein | | afdb-uniprot50 | AF-K5YQ81-F1-MODEL\_V4 | 1.0 | 2.019e-10 | 422 | 0.494 | 93 | 45 | 1 | 2 | 94 | 16 | 106 | Uncharacterized protein | Uncharacterized protein | | afdb-uniprot50 | AF-A0A2Z3I5X9-F1-MODEL\_V4 | 1.0 | 3.174e-10 | 408 | 0.489 | 96 | 45 | 2 | 1 | 94 | 1 | 94 | Phage tail assembly protein | Phage tail assembly protein | | afdb-uniprot50 | AF-A0A0D0QRI8-F1-MODEL\_V4 | 1.0 | 7.352e-10 | 399 | 0.616 | 86 | 32 | 1 | 9 | 94 | 2 | 86 | Contig\_41, whole genome shotgun sequence | Contig\_41, whole genome shotgun sequence | | afdb-uniprot50 | AF-A0A315BIP8-F1-MODEL\_V4 | 1.0 | 1.817e-09 | 398 | 0.56 | 82 | 36 | 0 | 13 | 94 | 10 | 91 | Uncharacterized protein | Uncharacterized protein | | afdb-uniprot50 | AF-A0A7V8FKG1-F1-MODEL\_V4 | 1.0 | 1.083e-09 | 393 | 0.473 | 95 | 48 | 2 | 1 | 94 | 1 | 94 | Uncharacterized protein | Uncharacterized protein | | afdb-uniprot50 | AF-A0A158E8B3-F1-MODEL\_V4 | 1.0 | 7.352e-10 | 391 | 0.482 | 85 | 42 | 2 | 6 | 90 | 14 | 96 | Uncharacterized protein | Uncharacterized protein | | afdb-uniprot50 | AF-B8KZU3-F1-MODEL\_V4 | 1.0 | 1.938e-09 | 386 | 0.473 | 93 | 47 | 1 | 2 | 94 | 6 | 96 | Uncharacterized protein | Uncharacterized protein | | afdb-uniprot50 | AF-A0A2W5N4W8-F1-MODEL\_V4 | 1.0 | 7.058e-09 | 368 | 0.478 | 92 | 46 | 2 | 3 | 94 | 35 | 124 | Phage tail assembly protein | Phage tail assembly protein | | afdb-uniprot50 | AF-A0A143DHP2-F1-MODEL\_V4 | 1.0 | 1.04e-08 | 359 | 0.518 | 79 | 38 | 0 | 16 | 94 | 5 | 83 | Uncharacterized protein | Uncharacterized protein | | afdb-uniprot50 | AF-A0A410UF81-F1-MODEL\_V4 | 1.0 | 4.31e-08 | 334 | 0.384 | 91 | 54 | 1 | 2 | 92 | 30 | 118 | Phage tail assembly protein | Phage tail assembly protein | | afdb-uniprot50 | AF-A0A7G7TJ95-F1-MODEL\_V4 | 1.0 | 6.352e-08 | 333 | 0.354 | 93 | 58 | 1 | 2 | 94 | 5 | 95 | Phage tail assembly protein | Phage tail assembly protein | | afdb-uniprot50 | AF-A0A2U1XZ20-F1-MODEL\_V4 | 1.0 | 1.674e-07 | 329 | 0.384 | 78 | 48 | 0 | 15 | 92 | 5 | 82 | Uncharacterized protein | Uncharacterized protein | | afdb-uniprot50 | AF-A0A2K4GEU8-F1-MODEL\_V4 | 1.0 | 4.598e-08 | 327 | 0.378 | 95 | 56 | 2 | 1 | 94 | 8 | 100 | Phage tail assembly protein | Phage tail assembly protein | | afdb-uniprot50 | AF-A0A5S9Q2R5-F1-MODEL\_V4 | 1.0 | 1.293e-07 | 321 | 0.412 | 80 | 47 | 0 | 15 | 94 | 4 | 83 | Uncharacterized protein | Uncharacterized protein | | afdb-uniprot50 | AF-A0A7Y7J7Q5-F1-MODEL\_V4 | 1.0 | 6.352e-08 | 321 | 0.34 | 94 | 59 | 2 | 2 | 94 | 5 | 96 | Phage tail assembly protein | Phage tail assembly protein | | afdb-uniprot50 | AF-A0A4Q3YYA7-F1-MODEL\_V4 | 1.0 | 5.232e-08 | 319 | 0.397 | 93 | 51 | 3 | 3 | 94 | 10 | 98 | Phage tail assembly protein | Phage tail assembly protein | | afdb-uniprot50 | AF-A0A2E3N1U5-F1-MODEL\_V4 | 1.0 | 1.905e-07 | 314 | 0.373 | 83 | 52 | 0 | 12 | 94 | 4 | 86 | Uncharacterized protein | Uncharacterized protein | | afdb-uniprot50 | AF-A0A0M1I5Y4-F1-MODEL\_V4 | 1.0 | 9.985e-08 | 314 | 0.442 | 95 | 49 | 3 | 2 | 94 | 4 | 96 | Uncharacterized protein | Uncharacterized protein | | afdb-uniprot50 | AF-A0A2K9M0P8-F1-MODEL\_V4 | 1.0 | 9.36e-08 | 313 | 0.308 | 94 | 62 | 2 | 2 | 94 | 4 | 95 | Phage tail assembly protein | Phage tail assembly protein | | afdb-uniprot50 | AF-A0A291LZ34-F1-MODEL\_V4 | 1.0 | 2.033e-07 | 312 | 0.519 | 77 | 37 | 0 | 16 | 92 | 4 | 80 | Uncharacterized protein | Uncharacterized protein | | afdb-uniprot50 | AF-A0A149SWH3-F1-MODEL\_V4 | 1.0 | 1.379e-07 | 310 | 0.309 | 97 | 62 | 2 | 3 | 94 | 6 | 102 | Uncharacterized protein | Uncharacterized protein | | afdb-uniprot50 | AF-A0A0P8Y9Q0-F1-MODEL\_V4 | 1.0 | 9.985e-08 | 307 | 0.41 | 95 | 53 | 3 | 1 | 94 | 1 | 93 | Uncharacterized protein | Uncharacterized protein | | afdb-uniprot50 | AF-A0A6I2J072-F1-MODEL\_V4 | 1.0 | 2.808e-07 | 306 | 0.341 | 85 | 56 | 0 | 10 | 94 | 1 | 85 | Phage tail assembly protein | Phage tail assembly protein | | afdb-uniprot50 | AF-A0A443ZHA6-F1-MODEL\_V4 | 1.0 | 1.57e-07 | 306 | 0.382 | 94 | 55 | 2 | 2 | 94 | 5 | 96 | Phage tail assembly protein | Phage tail assembly protein | | afdb-uniprot50 | AF-A0A1G6JG81-F1-MODEL\_V4 | 1.0 | 1.905e-07 | 305 | 0.423 | 92 | 49 | 2 | 5 | 94 | 11 | 100 | Phage tail assembly chaperone protein, E, or 41 or 14 | Phage tail assembly chaperone protein, E, or 41 or 14 | | afdb-uniprot50 | AF-A0A3M4SLW6-F1-MODEL\_V4 | 1.0 | 1.786e-07 | 304 | 0.351 | 94 | 58 | 2 | 2 | 94 | 7 | 98 | Uncharacterized protein | Uncharacterized protein | | afdb-uniprot50 | AF-A0A3M2V2F9-F1-MODEL\_V4 | 1.0 | 2.168e-07 | 304 | 0.329 | 94 | 60 | 2 | 2 | 94 | 11 | 102 | Uncharacterized protein | Uncharacterized protein | | afdb-uniprot50 | AF-B7RNN7-F1-MODEL\_V4 | 1.0 | 3.636e-07 | 301 | 0.414 | 94 | 53 | 2 | 2 | 94 | 4 | 96 | Uncharacterized protein | Uncharacterized protein | | afdb-uniprot50 | AF-A0A515BE98-F1-MODEL\_V4 | 1.0 | 2.995e-07 | 300 | 0.378 | 95 | 55 | 3 | 2 | 94 | 3 | 95 | Phage tail assembly protein | Phage tail assembly protein | | afdb-uniprot50 | AF-A0A1T4W2E5-F1-MODEL\_V4 | 1.0 | 2.995e-07 | 298 | 0.379 | 79 | 49 | 0 | 16 | 94 | 2 | 80 | Phage tail assembly chaperone protein, E, or 41 or 14 | Phage tail assembly chaperone protein, E, or 41 or 14 | | afdb-uniprot50 | AF-A0A1N7LRA5-F1-MODEL\_V4 | 1.0 | 5.023e-07 | 295 | 0.318 | 91 | 61 | 1 | 3 | 93 | 5 | 94 | Phage tail assembly chaperone protein, E, or 41 or 14 | Phage tail assembly chaperone protein, E, or 41 or 14 | | afdb-uniprot50 | AF-A0A0C1KHK2-F1-MODEL\_V4 | 1.0 | 2.168e-07 | 294 | 0.268 | 93 | 65 | 2 | 2 | 93 | 7 | 97 | Uncharacterized protein | Uncharacterized protein | | afdb-uniprot50 | AF-A0A554X0U8-F1-MODEL\_V4 | 1.0 | 8.985e-07 | 291 | 0.389 | 77 | 47 | 0 | 18 | 94 | 7 | 83 | Phage tail assembly chaperone protein E, or 41 or 14 | Phage tail assembly chaperone protein E, or 41 or 14 | | afdb-uniprot50 | AF-A0A6A4RGH5-F1-MODEL\_V4 | 1.0 | 7.896e-07 | 291 | 0.336 | 95 | 61 | 2 | 1 | 94 | 1 | 94 | Phage tail assembly protein | Phage tail assembly protein | | afdb-uniprot50 | AF-A0A0Q8Z8Q7-F1-MODEL\_V4 | 1.0 | 5.023e-07 | 289 | 0.4 | 90 | 51 | 2 | 4 | 92 | 1 | 88 | Uncharacterized protein | Uncharacterized protein | | afdb-uniprot50 | AF-A0A2S0PEE6-F1-MODEL\_V4 | 1.0 | 3.195e-07 | 289 | 0.447 | 76 | 42 | 0 | 18 | 93 | 4 | 79 | Phage tail assembly protein | Phage tail assembly protein | | afdb-uniprot50 | AF-A0A266NDD3-F1-MODEL\_V4 | 1.0 | 4.709e-07 | 289 | 0.351 | 94 | 58 | 2 | 2 | 94 | 7 | 98 | Uncharacterized protein | Uncharacterized protein | | afdb-uniprot50 | AF-A0A2N8BUS6-F1-MODEL\_V4 | 1.0 | 7.402e-07 | 288 | 0.358 | 92 | 56 | 2 | 2 | 92 | 7 | 96 | Phage tail assembly protein | Phage tail assembly protein | | afdb-uniprot50 | AF-A0A0C5VFB7-F1-MODEL\_V4 | 1.0 | 1.023e-06 | 287 | 0.369 | 84 | 53 | 0 | 11 | 94 | 15 | 98 | Uncharacterized protein | Uncharacterized protein | | afdb-uniprot50 | AF-A0A1Q6U7M3-F1-MODEL\_V4 | 1.0 | 7.896e-07 | 286 | 0.35 | 80 | 52 | 0 | 15 | 94 | 3 | 82 | Uncharacterized protein | Uncharacterized protein | | afdb-uniprot50 | AF-A0A2R3H0M0-F1-MODEL\_V4 | 1.0 | 8.423e-07 | 286 | 0.36 | 86 | 54 | 1 | 10 | 94 | 1 | 86 | Uncharacterized protein | Uncharacterized protein | | afdb-uniprot50 | AF-A0A4V6IKT7-F1-MODEL\_V4 | 1.0 | 5.023e-07 | 284 | 0.441 | 77 | 43 | 0 | 18 | 94 | 5 | 81 | Bacteriophage tail protein gp41 putative | Bacteriophage tail protein gp41 putative | | afdb-uniprot50 | AF-A0A2V4FUR3-F1-MODEL\_V4 | 1.0 | 6.939e-07 | 284 | 0.312 | 96 | 62 | 3 | 1 | 94 | 1 | 94 | Phage tail assembly protein | Phage tail assembly protein | | afdb-uniprot50 | AF-A0A7J0BX99-F1-MODEL\_V4 | 1.0 | 1.164e-06 | 283 | 0.454 | 77 | 42 | 0 | 18 | 94 | 4 | 80 | Uncharacterized protein | Uncharacterized protein | | afdb-uniprot50 | AF-A0A248LIV2-F1-MODEL\_V4 | 1.0 | 1.413e-06 | 280 | 0.379 | 79 | 49 | 0 | 16 | 94 | 3 | 81 | Phage\_TAC\_7 domain containing protein | Phage\_TAC\_7 domain containing protein | | afdb-uniprot50 | AF-A0A239C819-F1-MODEL\_V4 | 1.0 | 1.951e-06 | 276 | 0.341 | 79 | 52 | 0 | 16 | 94 | 2 | 80 | Phage tail assembly chaperone protein, E, or 41 or 14 | Phage tail assembly chaperone protein, E, or 41 or 14 | | afdb-uniprot50 | AF-H0SKG3-F1-MODEL\_V4 | 1.0 | 3.272e-06 | 276 | 0.346 | 78 | 51 | 0 | 15 | 92 | 11 | 88 | Uncharacterized protein | Uncharacterized protein | | afdb-uniprot50 | AF-A0A5E7KKS2-F1-MODEL\_V4 | 1.0 | 2.369e-06 | 276 | 0.284 | 95 | 65 | 2 | 1 | 94 | 3 | 95 | Uncharacterized protein | Uncharacterized protein | | afdb-uniprot50 | AF-A0A7W5B8R9-F1-MODEL\_V4 | 1.0 | 1.413e-06 | 275 | 0.347 | 92 | 57 | 2 | 3 | 93 | 2 | 91 | Uncharacterized protein | Uncharacterized protein | | afdb-uniprot50 | AF-A0A4Y6UC53-F1-MODEL\_V4 | 1.0 | 1.507e-06 | 275 | 0.31 | 100 | 61 | 4 | 2 | 94 | 14 | 112 | Phage tail assembly protein | Phage tail assembly protein | | afdb-uniprot50 | AF-A0A743CDA4-F1-MODEL\_V4 | 1.0 | 2.369e-06 | 274 | 0.27 | 85 | 62 | 0 | 10 | 94 | 1 | 85 | Phage tail assembly protein | Phage tail assembly protein | | afdb-uniprot50 | AF-A0A4U1KTT9-F1-MODEL\_V4 | 1.0 | 1.091e-06 | 272 | 0.329 | 91 | 58 | 2 | 3 | 92 | 10 | 98 | Phage tail assembly protein | Phage tail assembly protein | | afdb-uniprot50 | AF-C6BVX2-F1-MODEL\_V4 | 1.0 | 2.369e-06 | 270 | 0.415 | 77 | 45 | 0 | 18 | 94 | 3 | 79 | Uncharacterized protein | Uncharacterized protein | | afdb-uniprot50 | AF-A0A849VJT3-F1-MODEL\_V4 | 1.0 | 4.52e-06 | 267 | 0.428 | 77 | 44 | 0 | 18 | 94 | 5 | 81 | Phage tail assembly protein | Phage tail assembly protein | | afdb-uniprot50 | AF-A0A7C8LWZ0-F1-MODEL\_V4 | 1.0 | 1.829e-06 | 266 | 0.329 | 79 | 52 | 1 | 16 | 94 | 3 | 80 | Uncharacterized protein | Uncharacterized protein | | afdb-uniprot50 | AF-A0A8A5W0U8-F1-MODEL\_V4 | 1.0 | 5.853e-06 | 266 | 0.337 | 77 | 51 | 0 | 18 | 94 | 3 | 79 | Phage tail assembly protein | Phage tail assembly protein | | afdb-uniprot50 | AF-A0A1Y3NT57-F1-MODEL\_V4 | 1.0 | 5.144e-06 | 261 | 0.279 | 93 | 64 | 2 | 2 | 93 | 12 | 102 | Uncharacterized protein | Uncharacterized protein | | afdb-uniprot50 | AF-A0A554XCC7-F1-MODEL\_V4 | 1.0 | 4.52e-06 | 260 | 0.32 | 78 | 52 | 1 | 16 | 93 | 2 | 78 | Phage tail assembly chaperone protein, E, or 41 or 14 | Phage tail assembly chaperone protein, E, or 41 or 14 | | afdb-uniprot50 | AF-A0A853I2L0-F1-MODEL\_V4 | 1.0 | 7.58e-06 | 260 | 0.289 | 76 | 54 | 0 | 18 | 93 | 4 | 79 | Phage tail assembly protein | Phage tail assembly protein | | afdb-uniprot50 | AF-A0A516SAU7-F1-MODEL\_V4 | 1.0 | 2.527e-06 | 260 | 0.279 | 93 | 63 | 2 | 1 | 93 | 1 | 89 | Phage tail assembly protein | Phage tail assembly protein | | afdb-uniprot50 | AF-A0A1H9YDQ2-F1-MODEL\_V4 | 1.0 | 4.52e-06 | 258 | 0.367 | 79 | 50 | 0 | 16 | 94 | 3 | 81 | Phage tail assembly chaperone protein, E, or 41 or 14 | Phage tail assembly chaperone protein, E, or 41 or 14 | | afdb-uniprot50 | AF-A0A3A9HX51-F1-MODEL\_V4 | 1.0 | 6.244e-06 | 258 | 0.381 | 76 | 47 | 0 | 18 | 93 | 6 | 81 | Phage tail assembly protein | Phage tail assembly protein | | afdb-uniprot50 | AF-A0A161YCG8-F1-MODEL\_V4 | 1.0 | 8.626e-06 | 258 | 0.342 | 76 | 50 | 0 | 18 | 93 | 4 | 79 | Uncharacterized protein | Uncharacterized protein | | afdb-uniprot50 | AF-A1W3J4-F1-MODEL\_V4 | 1.0 | 1.164e-06 | 258 | 0.326 | 101 | 61 | 1 | 1 | 94 | 1 | 101 | Uncharacterized protein | Uncharacterized protein | | afdb-uniprot50 | AF-A0A3G2IAI1-F1-MODEL\_V4 | 1.0 | 7.106e-06 | 258 | 0.302 | 86 | 59 | 1 | 10 | 94 | 1 | 86 | Phage tail assembly protein | Phage tail assembly protein | | afdb-uniprot50 | AF-A0A2N0CXC7-F1-MODEL\_V4 | 1.0 | 6.244e-06 | 254 | 0.302 | 86 | 59 | 1 | 10 | 94 | 1 | 86 | Phage tail assembly protein | Phage tail assembly protein | | afdb-uniprot50 | AF-A0A5A9EPG0-F1-MODEL\_V4 | 1.0 | 5.144e-06 | 252 | 0.487 | 80 | 38 | 1 | 18 | 94 | 2 | 81 | Phage tail assembly protein | Phage tail assembly protein | | afdb-uniprot50 | AF-A0A212KXH5-F1-MODEL\_V4 | 1.0 | 5.144e-06 | 252 | 0.32 | 78 | 53 | 0 | 15 | 92 | 5 | 82 | Uncharacterized protein | Uncharacterized protein | | afdb-uniprot50 | AF-A0A1N6I0V2-F1-MODEL\_V4 | 1.0 | 5.144e-06 | 251 | 0.329 | 79 | 52 | 1 | 16 | 94 | 3 | 80 | Phage tail assembly chaperone protein, E, or 41 or 14 | Phage tail assembly chaperone protein, E, or 41 or 14 | | afdb-uniprot50 | AF-A0A6N7JJ06-F1-MODEL\_V4 | 1.0 | 8.086e-06 | 249 | 0.341 | 79 | 52 | 0 | 16 | 94 | 2 | 80 | Phage tail assembly protein | Phage tail assembly protein | | afdb-uniprot50 | AF-A0A5Q0THE6-F1-MODEL\_V4 | 1.0 | 2.527e-06 | 249 | 0.255 | 94 | 70 | 0 | 1 | 94 | 5 | 98 | Uncharacterized protein | Uncharacterized protein | | afdb-uniprot50 | AF-A0A2W4T4A0-F1-MODEL\_V4 | 1.0 | 9.202e-06 | 247 | 0.192 | 83 | 67 | 0 | 11 | 93 | 1 | 83 | Uncharacterized protein | Uncharacterized protein | | afdb-uniprot50 | AF-G4CJF6-F1-MODEL\_V4 | 1.0 | 9.202e-06 | 247 | 0.313 | 83 | 55 | 2 | 13 | 94 | 13 | 94 | Uncharacterized protein | Uncharacterized protein | | afdb-uniprot50 | AF-A0A1E3LH51-F1-MODEL\_V4 | 1.0 | 9.816e-06 | 244 | 0.395 | 81 | 48 | 1 | 13 | 92 | 6 | 86 | Uncharacterized protein | Uncharacterized protein | | afdb-uniprot50 | AF-A0A125GZ96-F1-MODEL\_V4 | 1.0 | 1.271e-05 | 243 | 0.412 | 80 | 46 | 1 | 16 | 94 | 3 | 82 | Uncharacterized protein | Uncharacterized protein | | afdb-uniprot50 | AF-A0A5E4XFR1-F1-MODEL\_V4 | 1.0 | 3.491e-06 | 242 | 0.27 | 100 | 67 | 2 | 1 | 94 | 10 | 109 | Uncharacterized protein | Uncharacterized protein | | afdb-uniprot50 | AF-A0A7X3U083-F1-MODEL\_V4 | 1.0 | 6.661e-06 | 241 | 0.325 | 83 | 55 | 1 | 10 | 92 | 1 | 82 | Phage tail assembly protein | Phage tail assembly protein | | afdb-uniprot50 | AF-A0A5V6NLU6-F1-MODEL\_V4 | 1.0 | 2.426e-05 | 241 | 0.275 | 80 | 57 | 1 | 16 | 94 | 9 | 88 | Phage tail assembly protein | Phage tail assembly protein | | afdb-uniprot50 | AF-A0A7Y8GD21-F1-MODEL\_V4 | 1.0 | 1.271e-05 | 241 | 0.311 | 93 | 61 | 3 | 1 | 92 | 6 | 96 | Phage tail assembly protein | Phage tail assembly protein | | afdb-uniprot50 | AF-A0A1C3ELA5-F1-MODEL\_V4 | 1.0 | 4.339e-05 | 239 | 0.223 | 76 | 59 | 0 | 18 | 93 | 3 | 78 | Uncharacterized protein | Uncharacterized protein | | afdb-uniprot50 | AF-A0A103RA62-F1-MODEL\_V4 | 1.0 | 1.873e-05 | 238 | 0.412 | 80 | 46 | 1 | 16 | 94 | 3 | 82 | Uncharacterized protein | Uncharacterized protein | | afdb-uniprot50 | AF-A0A345DE52-F1-MODEL\_V4 | 1.0 | 8.086e-06 | 237 | 0.255 | 86 | 64 | 0 | 9 | 94 | 1 | 86 | Uncharacterized protein | Uncharacterized protein | | afdb-uniprot50 | AF-A0A495BJ37-F1-MODEL\_V4 | 1.0 | 5.487e-06 | 237 | 0.41 | 78 | 45 | 1 | 16 | 92 | 2 | 79 | Tail assembly chaperone E/41/14-like protein | Tail assembly chaperone E/41/14-like protein | | afdb-uniprot50 | AF-V4PY80-F1-MODEL\_V4 | 1.0 | 1.446e-05 | 236 | 0.309 | 84 | 56 | 2 | 10 | 91 | 1 | 84 | Phage tail protein | Phage tail protein | | afdb-uniprot50 | AF-A0A5P9F0G6-F1-MODEL\_V4 | 1.0 | 7.58e-06 | 236 | 0.279 | 86 | 53 | 1 | 18 | 94 | 5 | 90 | Uncharacterized protein | Uncharacterized protein | | afdb-uniprot50 | AF-A0A2P5LZ45-F1-MODEL\_V4 | 1.0 | 5.853e-06 | 235 | 0.407 | 81 | 45 | 2 | 14 | 94 | 3 | 80 | Uncharacterized protein | Uncharacterized protein | | afdb-uniprot50 | AF-A0A1X7L2K2-F1-MODEL\_V4 | 1.0 | 9.816e-06 | 235 | 0.289 | 83 | 55 | 1 | 16 | 94 | 2 | 84 | Phage tail assembly chaperone protein, E, or 41 or 14 | Phage tail assembly chaperone protein, E, or 41 or 14 | | afdb-uniprot50 | AF-G1USF7-F1-MODEL\_V4 | 1.0 | 2.274e-05 | 234 | 0.294 | 78 | 55 | 0 | 15 | 92 | 5 | 82 | Uncharacterized protein | Uncharacterized protein | | afdb-uniprot50 | AF-A0A4T2ACC4-F1-MODEL\_V4 | 1.0 | 2.76e-05 | 234 | 0.32 | 81 | 55 | 0 | 14 | 94 | 4 | 84 | Phage tail assembly protein | Phage tail assembly protein | | afdb-uniprot50 | AF-A0A0W0IQA4-F1-MODEL\_V4 | 1.0 | 1.543e-05 | 233 | 0.352 | 85 | 54 | 1 | 10 | 93 | 1 | 85 | Uncharacterized protein | Uncharacterized protein | | afdb-uniprot50 | AF-A0A3G2IKE7-F1-MODEL\_V4 | 1.0 | 1.047e-05 | 232 | 0.313 | 99 | 63 | 1 | 1 | 94 | 1 | 99 | Phage tail assembly protein | Phage tail assembly protein | | afdb-uniprot50 | AF-A0A2S2E568-F1-MODEL\_V4 | 1.0 | 5.268e-05 | 231 | 0.363 | 77 | 49 | 0 | 18 | 94 | 9 | 85 | Uncharacterized protein | Uncharacterized protein | | afdb-uniprot50 | AF-F2BEW6-F1-MODEL\_V4 | 1.0 | 3.575e-05 | 230 | 0.277 | 83 | 58 | 2 | 13 | 94 | 28 | 109 | Uncharacterized protein | Uncharacterized protein | | afdb-uniprot50 | AF-A0A853I978-F1-MODEL\_V4 | 1.0 | 6.821e-05 | 229 | 0.253 | 79 | 59 | 0 | 16 | 94 | 2 | 80 | Phage tail assembly protein | Phage tail assembly protein | | afdb-uniprot50 | AF-A0A644XKN4-F1-MODEL\_V4 | 1.0 | 2.945e-05 | 229 | 0.256 | 78 | 58 | 0 | 15 | 92 | 5 | 82 | Uncharacterized protein | Uncharacterized protein | | afdb-uniprot50 | AF-A0A554XFV5-F1-MODEL\_V4 | 1.0 | 8.086e-06 | 229 | 0.354 | 93 | 55 | 2 | 1 | 92 | 1 | 89 | Phage tail assembly chaperone protein, E, or 41 or 14 | Phage tail assembly chaperone protein, E, or 41 or 14 | | afdb-uniprot50 | AF-A0A1B6VVH9-F1-MODEL\_V4 | 1.0 | 2.76e-05 | 228 | 0.337 | 83 | 53 | 2 | 13 | 94 | 13 | 94 | Uncharacterized protein | Uncharacterized protein | | afdb-uniprot50 | AF-A8U2I1-F1-MODEL\_V4 | 1.0 | 4.938e-05 | 227 | 0.237 | 80 | 61 | 0 | 14 | 93 | 9 | 88 | Uncharacterized protein | Uncharacterized protein | | afdb-uniprot50 | AF-A0A1A9VKI4-F1-MODEL\_V4 | 1.0 | 7.762e-05 | 227 | 0.298 | 77 | 54 | 0 | 16 | 92 | 113 | 189 | Uncharacterized protein | Uncharacterized protein | | afdb-uniprot50 | AF-A0A1H8TDI4-F1-MODEL\_V4 | 1.0 | 9.202e-06 | 226 | 0.315 | 95 | 62 | 2 | 1 | 94 | 1 | 93 | Phage tail assembly chaperone protein, E, or 41 or 14 | Phage tail assembly chaperone protein, E, or 41 or 14 | | afdb-uniprot50 | AF-A0A198XEB0-F1-MODEL\_V4 | 1.0 | 6.394e-05 | 224 | 0.303 | 79 | 53 | 2 | 15 | 92 | 18 | 95 | Uncharacterized protein | Uncharacterized protein | | afdb-uniprot50 | AF-G3IRG0-F1-MODEL\_V4 | 1.0 | 6.661e-06 | 224 | 0.322 | 93 | 52 | 3 | 2 | 94 | 6 | 87 | Uncharacterized protein | Uncharacterized protein | | afdb-uniprot50 | AF-A0A0J1HAC3-F1-MODEL\_V4 | 1.0 | 2.76e-05 | 222 | 0.297 | 84 | 59 | 0 | 9 | 92 | 1 | 84 | Uncharacterized protein | Uncharacterized protein | | afdb-uniprot50 | AF-A0A4Y9WRE6-F1-MODEL\_V4 | 1.0 | 0.0001144 | 221 | 0.262 | 80 | 58 | 1 | 16 | 94 | 11 | 90 | Phage tail assembly protein | Phage tail assembly protein | | afdb-uniprot50 | AF-A0A316MPU8-F1-MODEL\_V4 | 1.0 | 2.76e-05 | 221 | 0.341 | 85 | 50 | 2 | 16 | 94 | 4 | 88 | Uncharacterized protein | Uncharacterized protein | | afdb-uniprot50 | AF-A0A1F1HUP3-F1-MODEL\_V4 | 1.0 | 5.619e-05 | 220 | 0.294 | 78 | 53 | 2 | 18 | 94 | 16 | 92 | Uncharacterized protein | Uncharacterized protein | | afdb-uniprot50 | AF-A0A7J0BVD3-F1-MODEL\_V4 | 1.0 | 4.629e-05 | 220 | 0.337 | 77 | 51 | 0 | 18 | 94 | 30 | 106 | Uncharacterized protein | Uncharacterized protein | | afdb-uniprot50 | AF-A0A1W0CDM4-F1-MODEL\_V4 | 1.0 | 4.339e-05 | 219 | 0.407 | 76 | 44 | 1 | 18 | 92 | 8 | 83 | Uncharacterized protein | Uncharacterized protein | | afdb-uniprot50 | AF-A0A258KUC8-F1-MODEL\_V4 | 1.0 | 0.0001389 | 219 | 0.215 | 79 | 60 | 1 | 16 | 92 | 3 | 81 | Uncharacterized protein | Uncharacterized protein | | afdb-uniprot50 | AF-A0A7S6MHP3-F1-MODEL\_V4 | 1.0 | 4.629e-05 | 219 | 0.317 | 82 | 55 | 1 | 12 | 92 | 12 | 93 | Phage tail assembly protein | Phage tail assembly protein | | afdb-uniprot50 | AF-A0A2G6CS65-F1-MODEL\_V4 | 1.0 | 4.938e-05 | 218 | 0.35 | 77 | 40 | 2 | 18 | 94 | 4 | 70 | Uncharacterized protein | Uncharacterized protein | | afdb-uniprot50 | AF-A0A0D2HN99-F1-MODEL\_V4 | 1.0 | 8.833e-05 | 218 | 0.307 | 78 | 53 | 1 | 18 | 94 | 4 | 81 | Uncharacterized protein | Uncharacterized protein | | afdb-uniprot50 | AF-A0A399Q037-F1-MODEL\_V4 | 1.0 | 6.821e-05 | 218 | 0.345 | 81 | 52 | 1 | 14 | 93 | 5 | 85 | Phage tail assembly protein | Phage tail assembly protein | | afdb-uniprot50 | AF-A0A0F0DXF6-F1-MODEL\_V4 | 1.0 | 4.629e-05 | 218 | 0.379 | 79 | 48 | 1 | 16 | 93 | 3 | 81 | Uncharacterized protein | Uncharacterized protein | | afdb-uniprot50 | AF-A0A228R688-F1-MODEL\_V4 | 1.0 | 7.277e-05 | 216 | 0.333 | 78 | 51 | 1 | 16 | 92 | 3 | 80 | Uncharacterized protein | Uncharacterized protein | | afdb-uniprot50 | AF-A0A2V3R3G9-F1-MODEL\_V4 | 1.0 | 1.356e-05 | 216 | 0.263 | 95 | 69 | 1 | 1 | 94 | 6 | 100 | Tail assembly chaperone E/41/14-like protein | Tail assembly chaperone E/41/14-like protein | | afdb-uniprot50 | AF-A0A6L6JDE6-F1-MODEL\_V4 | 1.0 | 2.76e-05 | 215 | 0.361 | 94 | 48 | 4 | 1 | 94 | 7 | 88 | Uncharacterized protein | Uncharacterized protein | | afdb-uniprot50 | AF-A0A1N7DI63-F1-MODEL\_V4 | 1.0 | 0.0001144 | 215 | 0.32 | 78 | 51 | 2 | 16 | 92 | 22 | 98 | Phage tail assembly chaperone protein, E, or 41 or 14 | Phage tail assembly chaperone protein, E, or 41 or 14 | | afdb-uniprot50 | AF-A0A081N7U0-F1-MODEL\_V4 | 1.0 | 7.277e-05 | 214 | 0.207 | 77 | 60 | 1 | 18 | 94 | 6 | 81 | Uncharacterized protein | Uncharacterized protein | | afdb-uniprot50 | AF-A0A258KTP8-F1-MODEL\_V4 | 1.0 | 0.0002183 | 214 | 0.227 | 79 | 59 | 1 | 16 | 92 | 4 | 82 | Uncharacterized protein | Uncharacterized protein | | afdb-uniprot50 | AF-A0A450W6P6-F1-MODEL\_V4 | 1.0 | 2.76e-05 | 214 | 0.25 | 92 | 61 | 1 | 11 | 94 | 22 | 113 | Phage tail assembly chaperone protein, E, or 41 or 14 | Phage tail assembly chaperone protein, E, or 41 or 14 | | afdb-uniprot50 | AF-A0A4P5VJP3-F1-MODEL\_V4 | 1.0 | 2.274e-05 | 213 | 0.25 | 92 | 64 | 2 | 1 | 92 | 2 | 88 | Uncharacterized protein | Uncharacterized protein | | afdb-uniprot50 | AF-A0A1B4S7U1-F1-MODEL\_V4 | 1.0 | 3.813e-05 | 213 | 0.349 | 83 | 47 | 2 | 16 | 94 | 3 | 82 | Uncharacterized protein | Uncharacterized protein | | afdb-uniprot50 | AF-A0A1N7JL23-F1-MODEL\_V4 | 1.0 | 9.202e-06 | 213 | 0.297 | 94 | 58 | 1 | 1 | 94 | 9 | 94 | Phage tail assembly chaperone protein, E, or 41 or 14 | Phage tail assembly chaperone protein, E, or 41 or 14 | | afdb-uniprot50 | AF-A0A349HHP8-F1-MODEL\_V4 | 1.0 | 4.068e-05 | 213 | 0.294 | 78 | 53 | 1 | 15 | 90 | 23 | 100 | Uncharacterized protein | Uncharacterized protein | | afdb-uniprot50 | AF-A0A2N1WD23-F1-MODEL\_V4 | 1.0 | 4.339e-05 | 212 | 0.292 | 89 | 58 | 2 | 11 | 94 | 3 | 91 | Phage tail assembly protein | Phage tail assembly protein | | afdb-uniprot50 | AF-A0A6L8HHU5-F1-MODEL\_V4 | 1.0 | 7.277e-05 | 212 | 0.333 | 75 | 49 | 1 | 18 | 92 | 25 | 98 | Phage tail assembly protein | Phage tail assembly protein | | afdb-uniprot50 | AF-A0A6B3LE99-F1-MODEL\_V4 | 1.0 | 1.356e-05 | 211 | 0.293 | 92 | 57 | 1 | 1 | 92 | 4 | 87 | Phage tail assembly protein | Phage tail assembly protein | | afdb-uniprot50 | AF-A0A081B6C5-F1-MODEL\_V4 | 1.0 | 0.0001144 | 209 | 0.285 | 77 | 54 | 1 | 18 | 94 | 3 | 78 | Conserved protein | Conserved protein | | afdb-uniprot50 | AF-A0A378WGB6-F1-MODEL\_V4 | 1.0 | 0.0001389 | 209 | 0.271 | 81 | 57 | 2 | 15 | 94 | 13 | 92 | Phage protein | Phage protein | | afdb-uniprot50 | AF-A0A4R1K4S3-F1-MODEL\_V4 | 1.0 | 0.0001302 | 209 | 0.258 | 85 | 63 | 0 | 10 | 94 | 1 | 85 | Tail assembly chaperone E/41/14-like protein | Tail assembly chaperone E/41/14-like protein | | afdb-uniprot50 | AF-A0A376BTM2-F1-MODEL\_V4 | 1.0 | 0.0001144 | 209 | 0.285 | 84 | 57 | 3 | 13 | 94 | 11 | 93 | Uncharacterized protein | Uncharacterized protein | | afdb-uniprot50 | AF-W0IYF1-F1-MODEL\_V4 | 1.0 | 0.0001072 | 208 | 0.333 | 78 | 51 | 1 | 18 | 94 | 3 | 80 | Uncharacterized protein | Uncharacterized protein | | afdb-uniprot50 | AF-A0A3S1CIX7-F1-MODEL\_V4 | 1.0 | 7.762e-05 | 208 | 0.209 | 86 | 66 | 1 | 10 | 93 | 1 | 86 | Phage tail assembly protein | Phage tail assembly protein | | afdb-uniprot50 | AF-A0A522WEL1-F1-MODEL\_V4 | 1.0 | 0.0001302 | 208 | 0.337 | 80 | 52 | 1 | 16 | 94 | 2 | 81 | Phage tail assembly protein | Phage tail assembly protein | | afdb-uniprot50 | AF-A0A1B4SJY4-F1-MODEL\_V4 | 1.0 | 0.0002329 | 208 | 0.311 | 77 | 52 | 1 | 18 | 93 | 2 | 78 | Uncharacterized protein | Uncharacterized protein | | afdb-uniprot50 | AF-A0A420FTB7-F1-MODEL\_V4 | 1.0 | 1.543e-05 | 208 | 0.285 | 98 | 66 | 2 | 1 | 94 | 1 | 98 | Uncharacterized protein | Uncharacterized protein | | afdb-uniprot50 | AF-Q65WG3-F1-MODEL\_V4 | 1.0 | 0.000158 | 208 | 0.337 | 77 | 49 | 2 | 18 | 93 | 47 | 122 | Uncharacterized protein | Uncharacterized protein | | afdb-uniprot50 | AF-A0A3G4V6S2-F1-MODEL\_V4 | 1.0 | 7.762e-05 | 207 | 0.295 | 88 | 53 | 1 | 16 | 94 | 6 | 93 | Phage tail assembly protein | Phage tail assembly protein | | afdb-uniprot50 | AF-A0A2W4TDV9-F1-MODEL\_V4 | 1.0 | 0.0002484 | 206 | 0.246 | 77 | 58 | 0 | 18 | 94 | 5 | 81 | Phage tail assembly protein | Phage tail assembly protein | | afdb-uniprot50 | AF-A0A7M3MAZ0-F1-MODEL\_V4 | 1.0 | 4.629e-05 | 206 | 0.257 | 97 | 60 | 2 | 1 | 94 | 1 | 88 | Uncharacterized protein | Uncharacterized protein | | afdb-uniprot50 | AF-A0A2R2IRA1-F1-MODEL\_V4 | 1.0 | 4.068e-05 | 205 | 0.255 | 94 | 69 | 1 | 1 | 93 | 1 | 94 | Uncharacterized protein | Uncharacterized protein | | afdb-uniprot50 | AF-A0A1A9KIB7-F1-MODEL\_V4 | 1.0 | 6.821e-05 | 205 | 0.311 | 93 | 59 | 3 | 1 | 93 | 1 | 88 | Uncharacterized protein | Uncharacterized protein | | afdb-uniprot50 | AF-A0A5A8F0S7-F1-MODEL\_V4 | 1.0 | 0.0002329 | 204 | 0.265 | 79 | 54 | 1 | 16 | 94 | 2 | 76 | Phage tail assembly protein | Phage tail assembly protein | | afdb-uniprot50 | AF-A0A3D5IQS9-F1-MODEL\_V4 | 1.0 | 4.629e-05 | 204 | 0.223 | 85 | 65 | 1 | 9 | 93 | 5 | 88 | Phage tail assembly protein | Phage tail assembly protein | | afdb-uniprot50 | AF-A0A2N3KSE8-F1-MODEL\_V4 | 1.0 | 5.268e-05 | 203 | 0.229 | 96 | 64 | 3 | 2 | 93 | 23 | 112 | Uncharacterized protein | Uncharacterized protein | | afdb-uniprot50 | AF-E6L5Z3-F1-MODEL\_V4 | 1.0 | 3.141e-05 | 202 | 0.285 | 84 | 59 | 1 | 9 | 92 | 7 | 89 | Uncharacterized protein | Uncharacterized protein | | afdb-uniprot50 | AF-A0A5C4PTD6-F1-MODEL\_V4 | 1.0 | 0.000265 | 202 | 0.324 | 77 | 50 | 2 | 18 | 93 | 13 | 88 | Phage tail assembly protein | Phage tail assembly protein | | afdb-uniprot50 | AF-A0A0U2B5L0-F1-MODEL\_V4 | 1.0 | 4.629e-05 | 202 | 0.257 | 101 | 67 | 3 | 1 | 94 | 1 | 100 | Uncharacterized protein | Uncharacterized protein | | afdb-uniprot50 | AF-A0A0A2YRD7-F1-MODEL\_V4 | 1.0 | 0.0001072 | 201 | 0.289 | 83 | 58 | 1 | 9 | 90 | 2 | 84 | Uncharacterized protein | Uncharacterized protein | | afdb-uniprot50 | AF-A0A348FYG5-F1-MODEL\_V4 | 1.0 | 0.0002329 | 201 | 0.22 | 77 | 60 | 0 | 18 | 94 | 6 | 82 | Uncharacterized protein | Uncharacterized protein | | afdb-uniprot50 | AF-A0A7J0BJG9-F1-MODEL\_V4 | 1.0 | 6.394e-05 | 201 | 0.41 | 78 | 41 | 2 | 16 | 89 | 5 | 81 | Uncharacterized protein | Uncharacterized protein | | afdb-uniprot50 | AF-A0A524RVY5-F1-MODEL\_V4 | 1.0 | 5.994e-05 | 201 | 0.247 | 105 | 67 | 5 | 1 | 94 | 1 | 104 | Phage tail assembly protein | Phage tail assembly protein | | afdb-uniprot50 | AF-A0A165NWZ7-F1-MODEL\_V4 | 1.0 | 0.0001686 | 200 | 0.269 | 78 | 57 | 0 | 15 | 92 | 2 | 79 | Uncharacterized protein | Uncharacterized protein | | afdb-uniprot50 | AF-A0A0U5I607-F1-MODEL\_V4 | 1.0 | 2.76e-05 | 200 | 0.258 | 93 | 61 | 2 | 4 | 94 | 1 | 87 | Uncharacterized protein | Uncharacterized protein | | afdb-uniprot50 | AF-A0A1Y1S1F4-F1-MODEL\_V4 | 1.0 | 0.0001918 | 200 | 0.179 | 89 | 70 | 3 | 7 | 94 | 37 | 123 | Uncharacterized protein | Uncharacterized protein | | afdb-uniprot50 | AF-A0A1Y2K105-F1-MODEL\_V4 | 1.0 | 0.0001918 | 199 | 0.272 | 77 | 55 | 1 | 18 | 94 | 5 | 80 | Uncharacterized protein | Uncharacterized protein | | afdb-uniprot50 | AF-A0A1M6YPI3-F1-MODEL\_V4 | 1.0 | 4.938e-05 | 198 | 0.257 | 97 | 69 | 2 | 1 | 94 | 2 | 98 | Phage tail assembly chaperone protein, E, or 41 or 14 | Phage tail assembly chaperone protein, E, or 41 or 14 | | afdb-uniprot50 | AF-A0A2A5DQ32-F1-MODEL\_V4 | 1.0 | 0.0005057 | 197 | 0.151 | 79 | 66 | 1 | 16 | 94 | 3 | 80 | Uncharacterized protein | Uncharacterized protein | | afdb-uniprot50 | AF-A0A837E6Q2-F1-MODEL\_V4 | 1.0 | 0.0002329 | 195 | 0.346 | 78 | 50 | 1 | 18 | 94 | 2 | 79 | Uncharacterized protein | Uncharacterized protein | | afdb-uniprot50 | AF-A0A1M3AJN0-F1-MODEL\_V4 | 1.0 | 0.000158 | 195 | 0.282 | 78 | 55 | 1 | 16 | 93 | 3 | 79 | Uncharacterized protein | Uncharacterized protein | | afdb-uniprot50 | AF-E4ZB37-F1-MODEL\_V4 | 1.0 | 0.0002046 | 194 | 0.262 | 80 | 57 | 2 | 16 | 94 | 33 | 111 | Uncharacterized protein | Uncharacterized protein | | afdb-uniprot50 | AF-A0A1I7ND34-F1-MODEL\_V4 | 1.0 | 0.0001798 | 194 | 0.298 | 77 | 53 | 1 | 16 | 92 | 4 | 79 | Phage tail assembly chaperone protein, E, or 41 or 14 | Phage tail assembly chaperone protein, E, or 41 or 14 | | afdb-uniprot50 | AF-A0A212KMT6-F1-MODEL\_V4 | 1.0 | 0.0004166 | 194 | 0.293 | 75 | 53 | 0 | 20 | 94 | 88 | 162 | Uncharacterized protein | Uncharacterized protein | | afdb-uniprot50 | AF-A0A4P9VU53-F1-MODEL\_V4 | 1.0 | 0.0005754 | 193 | 0.219 | 73 | 57 | 0 | 16 | 88 | 2 | 74 | Phage tail assembly protein | Phage tail assembly protein | | afdb-uniprot50 | AF-S7TEH7-F1-MODEL\_V4 | 1.0 | 0.000122 | 193 | 0.321 | 87 | 54 | 3 | 11 | 94 | 1 | 85 | Mu-like prophage FluMu protein gp41 | Mu-like prophage FluMu protein gp41 | | afdb-uniprot50 | AF-A0A6L5JVI4-F1-MODEL\_V4 | 1.0 | 0.0001072 | 193 | 0.226 | 84 | 64 | 1 | 10 | 93 | 1 | 83 | Uncharacterized protein | Uncharacterized protein | | afdb-uniprot50 | AF-A0A6I3KFP2-F1-MODEL\_V4 | 1.0 | 0.0002329 | 191 | 0.237 | 80 | 60 | 1 | 14 | 93 | 4 | 82 | Uncharacterized protein | Uncharacterized protein | | afdb-uniprot50 | AF-A0A2D2C1C9-F1-MODEL\_V4 | 1.0 | 0.0002183 | 191 | 0.217 | 78 | 60 | 1 | 15 | 92 | 4 | 80 | Uncharacterized protein | Uncharacterized protein | | afdb-uniprot50 | AF-A0A212KBP9-F1-MODEL\_V4 | 1.0 | 0.000265 | 191 | 0.378 | 82 | 48 | 3 | 14 | 92 | 2 | 83 | Uncharacterized protein | Uncharacterized protein | | afdb-uniprot50 | AF-J0QC11-F1-MODEL\_V4 | 1.0 | 0.0004166 | 190 | 0.189 | 79 | 62 | 1 | 15 | 93 | 5 | 81 | Uncharacterized protein | Uncharacterized protein | | afdb-uniprot50 | AF-A0A450ZAK9-F1-MODEL\_V4 | 1.0 | 7.277e-05 | 190 | 0.232 | 86 | 58 | 3 | 15 | 94 | 16 | 99 | Phage tail assembly chaperone protein, E, or 41 or 14 | Phage tail assembly chaperone protein, E, or 41 or 14 | | afdb-uniprot50 | AF-L0R6A7-F1-MODEL\_V4 | 1.0 | 0.0004166 | 189 | 0.337 | 80 | 44 | 3 | 15 | 94 | 2 | 72 | Uncharacterized protein | Uncharacterized protein | | afdb-uniprot50 | AF-A0A4R6U4B4-F1-MODEL\_V4 | 1.0 | 0.0002827 | 189 | 0.269 | 78 | 56 | 1 | 18 | 94 | 2 | 79 | Tail assembly chaperone E/41/14-like protein | Tail assembly chaperone E/41/14-like protein | | afdb-uniprot50 | AF-A0A445MWH4-F1-MODEL\_V4 | 1.0 | 0.0002484 | 188 | 0.23 | 78 | 58 | 2 | 18 | 94 | 8 | 84 | Uncharacterized protein | Uncharacterized protein | | afdb-uniprot50 | AF-A0A522IQG1-F1-MODEL\_V4 | 1.0 | 0.000848 | 188 | 0.294 | 78 | 54 | 1 | 18 | 94 | 2 | 79 | Phage tail assembly protein | Phage tail assembly protein | | afdb-uniprot50 | AF-A0A809Z9Z9-F1-MODEL\_V4 | 1.0 | 0.0001798 | 188 | 0.211 | 85 | 66 | 1 | 10 | 93 | 1 | 85 | Uncharacterized protein | Uncharacterized protein | | afdb-uniprot50 | AF-A0A837E323-F1-MODEL\_V4 | 1.0 | 0.0007452 | 188 | 0.197 | 76 | 60 | 1 | 18 | 93 | 6 | 80 | Uncharacterized protein | Uncharacterized protein | | afdb-uniprot50 | AF-G1UYU1-F1-MODEL\_V4 | 1.0 | 0.0005754 | 187 | 0.253 | 83 | 56 | 1 | 16 | 92 | 3 | 85 | Uncharacterized protein | Uncharacterized protein | | afdb-uniprot50 | AF-A0A3M4ZXY2-F1-MODEL\_V4 | 1.0 | 0.0003905 | 186 | 0.43 | 72 | 40 | 1 | 23 | 93 | 1 | 72 | Uncharacterized protein | Uncharacterized protein | | afdb-uniprot50 | AF-Q602Z2-F1-MODEL\_V4 | 1.0 | 0.0004444 | 186 | 0.41 | 78 | 45 | 1 | 18 | 94 | 3 | 80 | Uncharacterized protein | Uncharacterized protein | | afdb-uniprot50 | AF-A0A1W2F849-F1-MODEL\_V4 | 1.0 | 0.0003432 | 185 | 0.3 | 70 | 48 | 1 | 18 | 87 | 5 | 73 | Phage tail assembly chaperone protein, E, or 41 or 14 | Phage tail assembly chaperone protein, E, or 41 or 14 | | afdb-uniprot50 | AF-A0A0J6K5A0-F1-MODEL\_V4 | 1.0 | 0.0001072 | 183 | 0.234 | 94 | 62 | 3 | 2 | 92 | 4 | 90 | Uncharacterized protein | Uncharacterized protein | | afdb-uniprot50 | AF-A0A1H1FGP4-F1-MODEL\_V4 | 1.0 | 0.000474 | 183 | 0.177 | 79 | 64 | 1 | 10 | 88 | 1 | 78 | Phage tail assembly chaperone protein, E, or 41 or 14 | Phage tail assembly chaperone protein, E, or 41 or 14 | | afdb-uniprot50 | AF-A0A066RUL2-F1-MODEL\_V4 | 1.0 | 0.0004444 | 183 | 0.275 | 87 | 53 | 2 | 18 | 94 | 9 | 95 | Uncharacterized protein | Uncharacterized protein | | afdb-uniprot50 | AF-A0A317H578-F1-MODEL\_V4 | 1.0 | 0.0001798 | 183 | 0.2 | 85 | 62 | 3 | 8 | 91 | 4 | 83 | Uncharacterized protein | Uncharacterized protein | | afdb-uniprot50 | AF-A0A7W8GZY1-F1-MODEL\_V4 | 1.0 | 0.0006986 | 181 | 0.24 | 75 | 56 | 1 | 18 | 92 | 8 | 81 | Uncharacterized protein | Uncharacterized protein | | afdb-uniprot50 | AF-A0A2G1CU23-F1-MODEL\_V4 | 1.0 | 0.0002046 | 181 | 0.202 | 89 | 70 | 1 | 4 | 92 | 1 | 88 | Uncharacterized protein | Uncharacterized protein | | afdb-uniprot50 | AF-A0A7J5WEE9-F1-MODEL\_V4 | 1.0 | 9.423e-05 | 181 | 0.244 | 90 | 67 | 1 | 1 | 89 | 8 | 97 | Uncharacterized protein | Uncharacterized protein | | afdb-uniprot50 | AF-A0A1F3A562-F1-MODEL\_V4 | 1.0 | 0.000848 | 181 | 0.23 | 78 | 59 | 1 | 16 | 92 | 5 | 82 | Uncharacterized protein | Uncharacterized protein | | afdb-uniprot50 | AF-A0A833IQP1-F1-MODEL\_V4 | 1.0 | 0.0001481 | 180 | 0.204 | 93 | 63 | 3 | 1 | 93 | 1 | 82 | Phage tail assembly protein | Phage tail assembly protein | | afdb-uniprot50 | AF-A0A6N8BZ30-F1-MODEL\_V4 | 1.0 | 0.0005057 | 180 | 0.176 | 85 | 69 | 1 | 10 | 93 | 1 | 85 | Phage tail assembly protein | Phage tail assembly protein | | afdb-uniprot50 | AF-A0A6P2SQ52-F1-MODEL\_V4 | 1.0 | 0.001333 | 179 | 0.269 | 78 | 56 | 1 | 18 | 94 | 2 | 79 | Uncharacterized protein | Uncharacterized protein | | afdb-uniprot50 | AF-A0A5C8S8T5-F1-MODEL\_V4 | 1.0 | 0.0002484 | 179 | 0.139 | 86 | 73 | 1 | 9 | 93 | 1 | 86 | Phage tail assembly protein | Phage tail assembly protein | | afdb-uniprot50 | AF-A0A6L9FIH6-F1-MODEL\_V4 | 1.0 | 0.000474 | 179 | 0.271 | 92 | 52 | 2 | 18 | 94 | 3 | 94 | Uncharacterized protein | Uncharacterized protein | | afdb-uniprot50 | AF-A0A4V2RF86-F1-MODEL\_V4 | 1.0 | 0.0003905 | 178 | 0.246 | 77 | 57 | 1 | 16 | 92 | 2 | 77 | Tail assembly chaperone E/41/14-like protein | Tail assembly chaperone E/41/14-like protein | | afdb-uniprot50 | AF-A0A4U8YLQ2-F1-MODEL\_V4 | 1.0 | 0.0005754 | 178 | 0.253 | 79 | 57 | 2 | 16 | 93 | 2 | 79 | Bacteriophage tail protein gp41 putative | Bacteriophage tail protein gp41 putative | | afdb-uniprot50 | AF-A0A0E3BR39-F1-MODEL\_V4 | 1.0 | 0.001098 | 177 | 0.231 | 82 | 62 | 1 | 12 | 92 | 5 | 86 | Uncharacterized protein | Uncharacterized protein | | afdb-uniprot50 | AF-Q1QI92-F1-MODEL\_V4 | 1.0 | 0.0005394 | 177 | 0.155 | 77 | 64 | 1 | 16 | 92 | 5 | 80 | Uncharacterized protein | Uncharacterized protein | | afdb-uniprot50 | AF-A0A4R2GHJ3-F1-MODEL\_V4 | 1.0 | 0.0005394 | 176 | 0.25 | 76 | 56 | 1 | 18 | 93 | 3 | 77 | Tail assembly chaperone E/41/14-like protein | Tail assembly chaperone E/41/14-like protein | | afdb-uniprot50 | AF-A0A1H9Q8X8-F1-MODEL\_V4 | 1.0 | 0.0002827 | 176 | 0.188 | 106 | 74 | 2 | 1 | 94 | 1 | 106 | Phage tail assembly chaperone protein, E, or 41 or 14 | Phage tail assembly chaperone protein, E, or 41 or 14 | | afdb-uniprot50 | AF-A0A6M7VG49-F1-MODEL\_V4 | 1.0 | 0.001333 | 175 | 0.162 | 86 | 71 | 1 | 9 | 94 | 1 | 85 | Phage tail assembly protein | Phage tail assembly protein | | afdb-uniprot50 | AF-A0A1A9VZ72-F1-MODEL\_V4 | 1.0 | 0.001171 | 175 | 0.189 | 79 | 63 | 1 | 16 | 93 | 4 | 82 | Uncharacterized protein | Uncharacterized protein | | afdb-uniprot50 | AF-A0A1M4WE05-F1-MODEL\_V4 | 1.0 | 0.0009046 | 175 | 0.189 | 74 | 59 | 1 | 15 | 88 | 7 | 79 | Phage tail assembly chaperone protein, E, or 41 or 14 | Phage tail assembly chaperone protein, E, or 41 or 14 | | afdb-uniprot50 | AF-A0A2N6FYM4-F1-MODEL\_V4 | 1.0 | 0.0002183 | 174 | 0.182 | 93 | 65 | 3 | 4 | 94 | 1 | 84 | Uncharacterized protein | Uncharacterized protein | | afdb-uniprot50 | AF-A0A4Q0USL0-F1-MODEL\_V4 | 1.0 | 0.000122 | 174 | 0.191 | 94 | 75 | 1 | 1 | 94 | 5 | 97 | Uncharacterized protein | Uncharacterized protein | | afdb-uniprot50 | AF-A0A2D3VZ53-F1-MODEL\_V4 | 1.0 | 0.0003432 | 174 | 0.28 | 82 | 50 | 3 | 13 | 94 | 26 | 98 | Uncharacterized protein | Uncharacterized protein | | afdb-uniprot50 | AF-A0A837JCA3-F1-MODEL\_V4 | 1.0 | 0.000265 | 174 | 0.241 | 91 | 62 | 2 | 2 | 92 | 22 | 105 | Uncharacterized protein | Uncharacterized protein | | afdb-uniprot50 | AF-F3YY69-F1-MODEL\_V4 | 1.0 | 0.0007452 | 174 | 0.267 | 86 | 55 | 2 | 14 | 91 | 2 | 87 | Uncharacterized protein | Uncharacterized protein | | afdb-uniprot50 | AF-A0A847KS38-F1-MODEL\_V4 | 1.0 | 0.000848 | 173 | 0.227 | 79 | 60 | 1 | 16 | 94 | 3 | 80 | Phage tail assembly protein | Phage tail assembly protein | | afdb-uniprot50 | AF-A0A212KJV9-F1-MODEL\_V4 | 1.0 | 0.0005754 | 172 | 0.262 | 80 | 57 | 1 | 16 | 93 | 4 | 83 | Uncharacterized protein | Uncharacterized protein | | afdb-uniprot50 | AF-D5RTF6-F1-MODEL\_V4 | 1.0 | 0.0002827 | 172 | 0.197 | 86 | 68 | 1 | 9 | 94 | 1 | 85 | Uncharacterized protein | Uncharacterized protein | | afdb-uniprot50 | AF-A0A7R7YEW5-F1-MODEL\_V4 | 1.0 | 0.0004166 | 171 | 0.233 | 90 | 57 | 2 | 16 | 93 | 6 | 95 | Uncharacterized protein | Uncharacterized protein | | afdb-uniprot50 | AF-E5Y5W3-F1-MODEL\_V4 | 1.0 | 0.000965 | 171 | 0.265 | 83 | 55 | 1 | 16 | 92 | 4 | 86 | Uncharacterized protein | Uncharacterized protein | | afdb-uniprot50 | AF-A0A2N3KJK8-F1-MODEL\_V4 | 1.0 | 0.0007452 | 171 | 0.186 | 102 | 70 | 5 | 3 | 92 | 11 | 111 | Uncharacterized protein | Uncharacterized protein | | afdb-uniprot50 | AF-U6ZUR1-F1-MODEL\_V4 | 1.0 | 0.001841 | 171 | 0.194 | 77 | 61 | 1 | 18 | 94 | 102 | 177 | Uncharacterized protein | Uncharacterized protein | | afdb-uniprot50 | AF-A0A2P7PD21-F1-MODEL\_V4 | 1.0 | 0.001422 | 170 | 0.194 | 77 | 60 | 1 | 16 | 92 | 5 | 79 | Phage tail assembly protein | Phage tail assembly protein | | afdb-uniprot50 | AF-A0A2C5TPJ7-F1-MODEL\_V4 | 1.0 | 0.0006139 | 169 | 0.188 | 85 | 64 | 2 | 15 | 94 | 3 | 87 | Phage tail assembly protein | Phage tail assembly protein | | afdb-uniprot50 | AF-A0A847G972-F1-MODEL\_V4 | 1.0 | 0.001171 | 169 | 0.272 | 77 | 53 | 2 | 16 | 89 | 18 | 94 | Phage tail assembly protein | Phage tail assembly protein | | afdb-uniprot50 | AF-A0A165RMJ9-F1-MODEL\_V4 | 1.0 | 0.001029 | 169 | 0.171 | 76 | 62 | 1 | 10 | 85 | 1 | 75 | Uncharacterized protein | Uncharacterized protein | | afdb-uniprot50 | AF-A0A827DRS4-F1-MODEL\_V4 | 1.0 | 0.0006548 | 168 | 0.244 | 86 | 56 | 2 | 1 | 85 | 4 | 81 | Phage tail assembly protein | Phage tail assembly protein | | afdb-uniprot50 | AF-U3TX64-F1-MODEL\_V4 | 1.0 | 0.001517 | 168 | 0.215 | 79 | 61 | 1 | 16 | 93 | 2 | 80 | Uncharacterized protein | Uncharacterized protein | | afdb-uniprot50 | AF-F7U3Q5-F1-MODEL\_V4 | 1.0 | 0.000965 | 168 | 0.181 | 77 | 62 | 1 | 16 | 92 | 2 | 77 | Uncharacterized protein | Uncharacterized protein | | afdb-uniprot50 | AF-A0A827G1L9-F1-MODEL\_V4 | 1.0 | 0.0001686 | 168 | 0.197 | 91 | 65 | 3 | 9 | 93 | 2 | 90 | Phage tail assembly protein | Phage tail assembly protein | | afdb-uniprot50 | AF-A0A087KTW1-F1-MODEL\_V4 | 1.0 | 0.001171 | 168 | 0.204 | 83 | 62 | 2 | 15 | 93 | 4 | 86 | Uncharacterized protein | Uncharacterized protein | | afdb-uniprot50 | AF-A0A1D9BCU0-F1-MODEL\_V4 | 1.0 | 0.0006139 | 168 | 0.235 | 85 | 58 | 2 | 16 | 93 | 2 | 86 | Uncharacterized protein | Uncharacterized protein | | afdb-uniprot50 | AF-A0A7W6WBB0-F1-MODEL\_V4 | 1.0 | 0.0002827 | 168 | 0.235 | 89 | 63 | 2 | 10 | 93 | 5 | 93 | Uncharacterized protein | Uncharacterized protein | | afdb-uniprot50 | AF-A0A3M1X2P8-F1-MODEL\_V4 | 1.0 | 0.0006139 | 167 | 0.164 | 91 | 67 | 2 | 3 | 93 | 1 | 82 | Phage tail assembly protein | Phage tail assembly protein | | afdb-uniprot50 | AF-A0A4D8R9U9-F1-MODEL\_V4 | 1.0 | 0.0002183 | 167 | 0.223 | 94 | 62 | 2 | 1 | 94 | 1 | 83 | Phage tail assembly protein | Phage tail assembly protein | | afdb-uniprot50 | AF-A0A7U0N692-F1-MODEL\_V4 | 1.0 | 0.001618 | 167 | 0.172 | 81 | 65 | 1 | 15 | 93 | 6 | 86 | Phage tail assembly protein | Phage tail assembly protein | | afdb-uniprot50 | AF-A0A017HBQ7-F1-MODEL\_V4 | 1.0 | 0.000848 | 166 | 0.157 | 76 | 63 | 1 | 10 | 85 | 1 | 75 | Uncharacterized protein | Uncharacterized protein | | afdb-uniprot50 | AF-A0A6J5FMZ1-F1-MODEL\_V4 | 1.0 | 0.001098 | 166 | 0.212 | 80 | 61 | 1 | 15 | 92 | 3 | 82 | Uncharacterized protein | Uncharacterized protein | | afdb-uniprot50 | AF-A0A7C1VCE0-F1-MODEL\_V4 | 1.0 | 0.001333 | 166 | 0.164 | 85 | 69 | 2 | 7 | 89 | 45 | 129 | Phage tail assembly protein | Phage tail assembly protein | | afdb-uniprot50 | AF-A0A4U0QBM5-F1-MODEL\_V4 | 1.0 | 0.001098 | 165 | 0.266 | 75 | 54 | 1 | 18 | 92 | 10 | 83 | Phage tail assembly protein | Phage tail assembly protein | | afdb-uniprot50 | AF-A0A840C379-F1-MODEL\_V4 | 1.0 | 0.002544 | 165 | 0.189 | 79 | 62 | 1 | 16 | 92 | 2 | 80 | Uncharacterized protein | Uncharacterized protein | | afdb-uniprot50 | AF-A0A6F8URG2-F1-MODEL\_V4 | 1.0 | 0.001171 | 165 | 0.202 | 79 | 62 | 1 | 10 | 88 | 1 | 78 | Uncharacterized protein | Uncharacterized protein | | afdb-uniprot50 | AF-A0A855R6X1-F1-MODEL\_V4 | 1.0 | 0.001098 | 165 | 0.243 | 78 | 57 | 1 | 15 | 90 | 3 | 80 | Phage tail assembly protein | Phage tail assembly protein | | afdb-uniprot50 | AF-A0A484GB85-F1-MODEL\_V4 | 1.0 | 0.0006986 | 165 | 0.259 | 81 | 54 | 2 | 18 | 93 | 3 | 82 | Phage tail assembly protein | Phage tail assembly protein | | afdb-uniprot50 | AF-A0A2S9IP70-F1-MODEL\_V4 | 1.0 | 0.002235 | 165 | 0.202 | 69 | 54 | 1 | 15 | 83 | 5 | 72 | Phage tail assembly protein | Phage tail assembly protein | | afdb-uniprot50 | AF-A0A0Q2UGR9-F1-MODEL\_V4 | 1.0 | 0.0005754 | 165 | 0.191 | 94 | 70 | 2 | 1 | 94 | 1 | 88 | Uncharacterized protein | Uncharacterized protein | | afdb-uniprot50 | AF-A0A4R2D341-F1-MODEL\_V4 | 1.0 | 0.001726 | 164 | 0.197 | 76 | 60 | 1 | 18 | 93 | 3 | 77 | Tail assembly chaperone E/41/14-like protein | Tail assembly chaperone E/41/14-like protein | | afdb-uniprot50 | AF-A0A1F8WN21-F1-MODEL\_V4 | 1.0 | 0.001098 | 164 | 0.2 | 80 | 60 | 2 | 16 | 94 | 3 | 79 | Uncharacterized protein | Uncharacterized protein | | afdb-uniprot50 | AF-A0A2U1TU93-F1-MODEL\_V4 | 1.0 | 0.001841 | 164 | 0.168 | 83 | 65 | 2 | 15 | 93 | 4 | 86 | Phage tail assembly protein | Phage tail assembly protein | | afdb-uniprot50 | AF-H8FUZ8-F1-MODEL\_V4 | 1.0 | 0.0005754 | 164 | 0.176 | 85 | 63 | 1 | 16 | 93 | 5 | 89 | Putative bacteriophage protein | Putative bacteriophage protein | | afdb-uniprot50 | AF-A0A4Z0W7G4-F1-MODEL\_V4 | 1.0 | 0.001964 | 164 | 0.197 | 81 | 63 | 1 | 15 | 93 | 27 | 107 | Phage tail assembly protein | Phage tail assembly protein | | afdb-uniprot50 | AF-A0A4R5W1E0-F1-MODEL\_V4 | 1.0 | 0.004551 | 164 | 0.271 | 81 | 58 | 1 | 15 | 94 | 12 | 92 | Phage tail assembly protein | Phage tail assembly protein | | afdb-uniprot50 | AF-A0A8B3NM35-F1-MODEL\_V4 | 1.0 | 0.0005754 | 164 | 0.213 | 89 | 65 | 2 | 6 | 94 | 24 | 107 | Phage tail assembly protein | Phage tail assembly protein | | afdb-uniprot50 | AF-A0A854G7K2-F1-MODEL\_V4 | 1.0 | 0.005524 | 163 | 0.246 | 77 | 57 | 1 | 18 | 93 | 2 | 78 | Uncharacterized protein | Uncharacterized protein | | afdb-uniprot50 | AF-A0A2T3GP98-F1-MODEL\_V4 | 1.0 | 0.001726 | 162 | 0.194 | 77 | 61 | 1 | 16 | 92 | 4 | 79 | Phage tail assembly protein | Phage tail assembly protein | | afdb-uniprot50 | AF-A0A344UIJ8-F1-MODEL\_V4 | 1.0 | 0.003514 | 162 | 0.259 | 77 | 56 | 1 | 18 | 93 | 2 | 78 | Phage tail assembly protein | Phage tail assembly protein | | afdb-uniprot50 | AF-A0A346QYG8-F1-MODEL\_V4 | 1.0 | 0.0004166 | 162 | 0.216 | 83 | 60 | 2 | 14 | 92 | 4 | 85 | Phage tail assembly protein | Phage tail assembly protein | | afdb-uniprot50 | AF-A0A2K9P758-F1-MODEL\_V4 | 1.0 | 0.001517 | 162 | 0.136 | 88 | 72 | 2 | 10 | 93 | 1 | 88 | Uncharacterized protein | Uncharacterized protein | | afdb-uniprot50 | AF-A0A7Z0QG19-F1-MODEL\_V4 | 1.0 | 0.0006548 | 162 | 0.225 | 93 | 61 | 1 | 11 | 92 | 7 | 99 | Phage tail assembly protein | Phage tail assembly protein | | afdb-uniprot50 | AF-A0A1K0J3G7-F1-MODEL\_V4 | 1.0 | 0.002096 | 162 | 0.2 | 80 | 63 | 1 | 14 | 92 | 4 | 83 | Uncharacterized protein | Uncharacterized protein | | afdb-uniprot50 | AF-W0BWL8-F1-MODEL\_V4 | 1.0 | 0.0005394 | 162 | 0.189 | 95 | 69 | 3 | 4 | 93 | 1 | 92 | Uncharacterized protein | Uncharacterized protein | | afdb-uniprot50 | AF-A0A2S7JR59-F1-MODEL\_V4 | 1.0 | 0.0003661 | 162 | 0.229 | 96 | 70 | 2 | 1 | 94 | 2 | 95 | Phage tail assembly protein | Phage tail assembly protein | | afdb-uniprot50 | AF-A0A349GR41-F1-MODEL\_V4 | 1.0 | 0.000474 | 162 | 0.198 | 101 | 66 | 4 | 6 | 93 | 25 | 123 | Uncharacterized protein | Uncharacterized protein | | afdb-uniprot50 | AF-A0A5Q8CAB4-F1-MODEL\_V4 | 1.0 | 0.0004444 | 162 | 0.225 | 93 | 63 | 3 | 2 | 94 | 32 | 115 | Phage tail assembly protein | Phage tail assembly protein | | afdb-uniprot50 | AF-A0A2V3UB04-F1-MODEL\_V4 | 1.0 | 0.003749 | 161 | 0.216 | 74 | 58 | 0 | 18 | 91 | 3 | 76 | Tail assembly chaperone E/41/14-like protein | Tail assembly chaperone E/41/14-like protein | | afdb-uniprot50 | AF-A0A564G557-F1-MODEL\_V4 | 1.0 | 0.001029 | 161 | 0.197 | 76 | 60 | 1 | 18 | 92 | 14 | 89 | Uncharacterized protein | Uncharacterized protein | | afdb-uniprot50 | AF-A0A6L9MMR9-F1-MODEL\_V4 | 1.0 | 0.002235 | 161 | 0.189 | 79 | 63 | 1 | 10 | 88 | 1 | 78 | Phage tail assembly protein | Phage tail assembly protein | | afdb-uniprot50 | AF-A0A853T2H5-F1-MODEL\_V4 | 1.0 | 0.0009046 | 161 | 0.222 | 72 | 53 | 2 | 15 | 85 | 14 | 83 | Phage tail assembly chaperone protein, E, or 41 or 14 | Phage tail assembly chaperone protein, E, or 41 or 14 | | afdb-uniprot50 | AF-A0A2J8GXT3-F1-MODEL\_V4 | 1.0 | 0.002385 | 161 | 0.162 | 86 | 63 | 1 | 18 | 94 | 3 | 88 | Uncharacterized protein | Uncharacterized protein | | afdb-uniprot50 | AF-A0A3N2E0T6-F1-MODEL\_V4 | 1.0 | 0.0009046 | 161 | 0.253 | 79 | 54 | 2 | 18 | 91 | 23 | 101 | Tail assembly chaperone E/41/14-like protein | Tail assembly chaperone E/41/14-like protein | | afdb-uniprot50 | AF-A0A4Z1R4X7-F1-MODEL\_V4 | 1.0 | 0.0004166 | 161 | 0.224 | 98 | 69 | 2 | 2 | 94 | 4 | 99 | Phage tail assembly protein | Phage tail assembly protein | | afdb-uniprot50 | AF-E7H537-F1-MODEL\_V4 | 1.0 | 0.00125 | 160 | 0.226 | 84 | 57 | 1 | 18 | 93 | 2 | 85 | Uncharacterized protein | Uncharacterized protein | | afdb-uniprot50 | AF-A0A102DFQ3-F1-MODEL\_V4 | 1.0 | 0.0002329 | 160 | 0.21 | 100 | 69 | 3 | 1 | 94 | 4 | 99 | Uncharacterized protein | Uncharacterized protein | | afdb-uniprot50 | AF-A0A7L9Q1H1-F1-MODEL\_V4 | 1.0 | 0.000965 | 159 | 0.252 | 95 | 66 | 3 | 1 | 94 | 1 | 91 | Phage tail assembly protein | Phage tail assembly protein | | afdb-uniprot50 | AF-X0YLY6-F1-MODEL\_V4 | 1.0 | 0.0007949 | 159 | 0.151 | 86 | 71 | 1 | 9 | 94 | 2 | 85 | Uncharacterized protein | Uncharacterized protein | | afdb-uniprot50 | AF-A0A1S7Q6W0-F1-MODEL\_V4 | 1.0 | 0.002235 | 159 | 0.223 | 76 | 59 | 0 | 18 | 93 | 3 | 78 | Uncharacterized protein | Uncharacterized protein | | afdb-uniprot50 | AF-A0A143DC33-F1-MODEL\_V4 | 1.0 | 0.001098 | 159 | 0.252 | 87 | 58 | 2 | 14 | 93 | 3 | 89 | Uncharacterized protein | Uncharacterized protein | | afdb-uniprot50 | AF-A0A502GP93-F1-MODEL\_V4 | 1.0 | 0.0007949 | 159 | 0.218 | 96 | 70 | 3 | 3 | 93 | 37 | 132 | Phage tail assembly protein | Phage tail assembly protein | | afdb-uniprot50 | AF-A0A7V8JT65-F1-MODEL\_V4 | 1.0 | 0.003088 | 158 | 0.126 | 79 | 68 | 1 | 14 | 92 | 4 | 81 | Uncharacterized protein | Uncharacterized protein | | afdb-uniprot50 | AF-A0A258KWE3-F1-MODEL\_V4 | 1.0 | 0.003749 | 158 | 0.16 | 81 | 66 | 1 | 16 | 94 | 3 | 83 | Uncharacterized protein | Uncharacterized protein | | afdb-uniprot50 | AF-A0A323UKS3-F1-MODEL\_V4 | 1.0 | 0.000474 | 158 | 0.18 | 94 | 73 | 2 | 1 | 94 | 1 | 90 | Phage tail assembly protein | Phage tail assembly protein | | afdb-uniprot50 | AF-A0A7W4ICA4-F1-MODEL\_V4 | 1.0 | 0.0006548 | 158 | 0.25 | 104 | 65 | 5 | 3 | 94 | 36 | 138 | Phage tail assembly protein | Phage tail assembly protein | | afdb-uniprot50 | AF-A0A316GTU0-F1-MODEL\_V4 | 1.0 | 0.001841 | 157 | 0.207 | 77 | 60 | 1 | 18 | 93 | 3 | 79 | Tail assembly chaperone E/41/14-like protein | Tail assembly chaperone E/41/14-like protein | | afdb-uniprot50 | AF-R5EZ88-F1-MODEL\_V4 | 1.0 | 0.001333 | 157 | 0.178 | 84 | 61 | 2 | 18 | 93 | 7 | 90 | Putative bacteriophage protein | Putative bacteriophage protein | | afdb-uniprot50 | AF-A0A4Q1B5V9-F1-MODEL\_V4 | 1.0 | 0.004266 | 157 | 0.189 | 79 | 63 | 1 | 16 | 93 | 15 | 93 | Uncharacterized protein | Uncharacterized protein | | afdb-uniprot50 | AF-A0A0M3AY55-F1-MODEL\_V4 | 1.0 | 0.003999 | 156 | 0.166 | 72 | 59 | 1 | 16 | 87 | 5 | 75 | Uncharacterized protein | Uncharacterized protein | | afdb-uniprot50 | AF-A0A1X7F9Z0-F1-MODEL\_V4 | 1.0 | 0.001618 | 156 | 0.192 | 78 | 62 | 1 | 15 | 92 | 10 | 86 | Phage tail assembly chaperone protein, E, or 41 or 14 | Phage tail assembly chaperone protein, E, or 41 or 14 | | afdb-uniprot50 | AF-A0A629KGM0-F1-MODEL\_V4 | 1.0 | 0.003749 | 156 | 0.246 | 77 | 57 | 1 | 18 | 93 | 8 | 84 | Phage tail assembly protein | Phage tail assembly protein | | afdb-uniprot50 | AF-A0A2A2H0C8-F1-MODEL\_V4 | 1.0 | 0.003749 | 156 | 0.197 | 81 | 64 | 1 | 14 | 93 | 3 | 83 | Uncharacterized protein | Uncharacterized protein | | afdb-uniprot50 | AF-A0A847S1J7-F1-MODEL\_V4 | 1.0 | 0.004551 | 156 | 0.259 | 77 | 56 | 1 | 18 | 93 | 2 | 78 | Phage tail assembly protein | Phage tail assembly protein | | afdb-uniprot50 | AF-A0A109JFT6-F1-MODEL\_V4 | 1.0 | 0.001029 | 156 | 0.232 | 86 | 55 | 1 | 18 | 92 | 14 | 99 | Uncharacterized protein | Uncharacterized protein | | afdb-uniprot50 | AF-E3H9B2-F1-MODEL\_V4 | 1.0 | 0.0007949 | 156 | 0.15 | 106 | 75 | 4 | 1 | 93 | 7 | 110 | Uncharacterized protein | Uncharacterized protein | | afdb-uniprot50 | AF-A0A1Y2JNK8-F1-MODEL\_V4 | 1.0 | 0.001726 | 156 | 0.168 | 95 | 68 | 1 | 11 | 94 | 21 | 115 | Uncharacterized protein | Uncharacterized protein | | afdb-uniprot50 | AF-A0A0J7LYN2-F1-MODEL\_V4 | 1.0 | 0.001517 | 156 | 0.128 | 101 | 74 | 5 | 3 | 92 | 16 | 113 | Mu-like prophage FluMu protein gp41 | Mu-like prophage FluMu protein gp41 | | afdb-uniprot50 | AF-A0A5Y9K6W5-F1-MODEL\_V4 | 1.0 | 0.002544 | 155 | 0.302 | 76 | 45 | 2 | 18 | 93 | 5 | 72 | Phage tail assembly protein | Phage tail assembly protein | | afdb-uniprot50 | AF-I3D8M1-F1-MODEL\_V4 | 1.0 | 0.002235 | 155 | 0.202 | 79 | 58 | 3 | 16 | 92 | 3 | 78 | Phage tail protein E | Phage tail protein E | | afdb-uniprot50 | AF-A0A0D2JFD8-F1-MODEL\_V4 | 1.0 | 0.0007949 | 155 | 0.238 | 88 | 57 | 3 | 16 | 93 | 5 | 92 | Uncharacterized protein | Uncharacterized protein | | afdb-uniprot50 | AF-A0A418W4D9-F1-MODEL\_V4 | 1.0 | 0.001098 | 155 | 0.202 | 89 | 67 | 2 | 9 | 93 | 2 | 90 | Phage tail assembly protein | Phage tail assembly protein | | afdb-uniprot50 | AF-A0A258L5N6-F1-MODEL\_V4 | 1.0 | 0.005178 | 155 | 0.189 | 79 | 63 | 1 | 15 | 93 | 122 | 199 | Uncharacterized protein | Uncharacterized protein | | afdb-uniprot50 | AF-A0A1G3UAH2-F1-MODEL\_V4 | 1.0 | 0.002544 | 154 | 0.285 | 77 | 45 | 3 | 18 | 94 | 3 | 69 | Uncharacterized protein | Uncharacterized protein | | afdb-uniprot50 | AF-A0A3G2V508-F1-MODEL\_V4 | 1.0 | 0.0005394 | 154 | 0.223 | 94 | 66 | 2 | 4 | 93 | 1 | 91 | Phage tail assembly protein | Phage tail assembly protein | | afdb-uniprot50 | AF-A0A850I2F0-F1-MODEL\_V4 | 1.0 | 0.000848 | 154 | 0.192 | 104 | 65 | 2 | 2 | 94 | 34 | 129 | Phage tail assembly protein | Phage tail assembly protein | | afdb-uniprot50 | AF-A0A2W6YN90-F1-MODEL\_V4 | 1.0 | 0.0006986 | 154 | 0.193 | 93 | 73 | 1 | 2 | 92 | 38 | 130 | Uncharacterized protein | Uncharacterized protein | | afdb-uniprot50 | AF-A0A0F4NK84-F1-MODEL\_V4 | 1.0 | 0.002544 | 153 | 0.277 | 72 | 48 | 2 | 23 | 94 | 2 | 69 | Uncharacterized protein | Uncharacterized protein | | afdb-uniprot50 | AF-Q7NY03-F1-MODEL\_V4 | 1.0 | 0.009882 | 153 | 0.22 | 77 | 59 | 1 | 18 | 93 | 3 | 79 | Uncharacterized protein | Uncharacterized protein | | afdb-uniprot50 | AF-A0A5P9JUS1-F1-MODEL\_V4 | 1.0 | 0.002385 | 153 | 0.188 | 85 | 65 | 2 | 10 | 91 | 1 | 84 | Uncharacterized protein | Uncharacterized protein | | afdb-uniprot50 | AF-A0A430BCQ8-F1-MODEL\_V4 | 1.0 | 0.0006548 | 153 | 0.186 | 91 | 67 | 2 | 4 | 94 | 7 | 90 | Uncharacterized protein | Uncharacterized protein | | afdb-uniprot50 | AF-A0A077PQ35-F1-MODEL\_V4 | 1.0 | 0.003999 | 153 | 0.18 | 83 | 64 | 2 | 15 | 93 | 4 | 86 | Uncharacterized protein | Uncharacterized protein | | afdb-uniprot50 | AF-A0A6B2KN65-F1-MODEL\_V4 | 1.0 | 0.001422 | 153 | 0.147 | 88 | 65 | 1 | 16 | 93 | 3 | 90 | Phage tail assembly protein | Phage tail assembly protein | | afdb-uniprot50 | AF-S9S610-F1-MODEL\_V4 | 1.0 | 0.0009046 | 153 | 0.266 | 90 | 59 | 3 | 8 | 92 | 5 | 92 | Putative phage related protein | Putative phage related protein | | afdb-uniprot50 | AF-A0A423PRR2-F1-MODEL\_V4 | 1.0 | 0.004854 | 153 | 0.175 | 80 | 63 | 2 | 15 | 92 | 28 | 106 | Uncharacterized protein | Uncharacterized protein | | afdb-uniprot50 | AF-A0A5M8ZBZ0-F1-MODEL\_V4 | 1.0 | 0.0005754 | 153 | 0.206 | 97 | 67 | 6 | 6 | 94 | 11 | 105 | Uncharacterized protein | Uncharacterized protein | | afdb-uniprot50 | AF-A0A7W8PBC6-F1-MODEL\_V4 | 1.0 | 0.005178 | 152 | 0.185 | 81 | 60 | 3 | 16 | 94 | 2 | 78 | Uncharacterized protein | Uncharacterized protein | | afdb-uniprot50 | AF-I9WY64-F1-MODEL\_V4 | 1.0 | 0.002895 | 152 | 0.246 | 77 | 56 | 1 | 18 | 94 | 6 | 80 | Uncharacterized protein | Uncharacterized protein | | afdb-uniprot50 | AF-A0A1Y5TZY3-F1-MODEL\_V4 | 1.0 | 0.003294 | 152 | 0.246 | 81 | 54 | 2 | 16 | 92 | 5 | 82 | Phage tail protein E | Phage tail protein E | | afdb-uniprot50 | AF-A0A2I8DJT9-F1-MODEL\_V4 | 1.0 | 0.004551 | 152 | 0.202 | 79 | 63 | 0 | 16 | 94 | 4 | 82 | Phage tail assembly protein | Phage tail assembly protein | | afdb-uniprot50 | AF-A0A5E5PBU8-F1-MODEL\_V4 | 1.0 | 0.003514 | 152 | 0.214 | 84 | 62 | 1 | 15 | 94 | 5 | 88 | Phage tail assembly protein | Phage tail assembly protein | | afdb-uniprot50 | AF-A0A645HXF0-F1-MODEL\_V4 | 1.0 | 0.003749 | 152 | 0.222 | 81 | 60 | 2 | 14 | 92 | 2 | 81 | Uncharacterized protein | Uncharacterized protein | | afdb-uniprot50 | AF-A0A521D126-F1-MODEL\_V4 | 1.0 | 0.001618 | 152 | 0.193 | 93 | 64 | 2 | 3 | 92 | 1 | 85 | Phage tail assembly chaperone protein, E, or 41 or 14 | Phage tail assembly chaperone protein, E, or 41 or 14 | | afdb-uniprot50 | AF-A0A5B9CYR7-F1-MODEL\_V4 | 1.0 | 0.003749 | 152 | 0.219 | 73 | 55 | 1 | 16 | 88 | 6 | 76 | Phage tail assembly protein | Phage tail assembly protein | | afdb-uniprot50 | AF-A0A5N7YGG3-F1-MODEL\_V4 | 1.0 | 0.006286 | 151 | 0.421 | 57 | 33 | 0 | 37 | 93 | 3 | 59 | Phage tail assembly protein | Phage tail assembly protein | | afdb-uniprot50 | AF-A0A258D055-F1-MODEL\_V4 | 1.0 | 0.003749 | 151 | 0.202 | 69 | 53 | 2 | 16 | 83 | 4 | 71 | Uncharacterized protein | Uncharacterized protein | | afdb-uniprot50 | AF-A0A1P8QQ42-F1-MODEL\_V4 | 1.0 | 0.001171 | 151 | 0.189 | 95 | 74 | 1 | 1 | 92 | 1 | 95 | Uncharacterized protein | Uncharacterized protein | | afdb-uniprot50 | AF-A0A516W9Z3-F1-MODEL\_V4 | 1.0 | 0.002235 | 151 | 0.159 | 88 | 65 | 1 | 14 | 92 | 2 | 89 | Phage tail assembly protein | Phage tail assembly protein | | afdb-uniprot50 | AF-A0A411WIN1-F1-MODEL\_V4 | 1.0 | 0.002895 | 150 | 0.301 | 83 | 51 | 2 | 1 | 82 | 1 | 77 | Phage tail assembly protein | Phage tail assembly protein | | afdb-uniprot50 | AF-A0A2M8G048-F1-MODEL\_V4 | 1.0 | 0.002714 | 150 | 0.209 | 81 | 59 | 2 | 15 | 93 | 4 | 81 | Phage tail assembly protein | Phage tail assembly protein | | afdb-uniprot50 | AF-A0A4Y9GJY5-F1-MODEL\_V4 | 1.0 | 0.007631 | 150 | 0.217 | 78 | 60 | 1 | 13 | 89 | 11 | 88 | Phage tail assembly protein | Phage tail assembly protein | | afdb-uniprot50 | AF-A0A7Y3JAD0-F1-MODEL\_V4 | 1.0 | 0.0007949 | 150 | 0.206 | 92 | 64 | 3 | 2 | 92 | 4 | 87 | Phage tail assembly protein | Phage tail assembly protein | | afdb-uniprot50 | AF-A0A849MU39-F1-MODEL\_V4 | 1.0 | 0.00125 | 150 | 0.19 | 84 | 62 | 3 | 16 | 93 | 15 | 98 | Phage tail assembly protein | Phage tail assembly protein | | afdb-uniprot50 | AF-A0A2U8WC22-F1-MODEL\_V4 | 1.0 | 0.0006548 | 150 | 0.223 | 103 | 64 | 3 | 4 | 92 | 1 | 101 | Uncharacterized protein | Uncharacterized protein | | afdb-uniprot50 | AF-A0A482IXJ4-F1-MODEL\_V4 | 1.0 | 0.003088 | 150 | 0.295 | 71 | 48 | 2 | 18 | 86 | 43 | 113 | Phage tail assembly protein | Phage tail assembly protein | | afdb-uniprot50 | AF-A0A7W6EB82-F1-MODEL\_V4 | 1.0 | 0.0005394 | 150 | 0.202 | 94 | 66 | 3 | 1 | 92 | 61 | 147 | Uncharacterized protein | Uncharacterized protein | | afdb-uniprot50 | AF-A0A4Q0ZNJ1-F1-MODEL\_V4 | 1.0 | 0.001964 | 149 | 0.267 | 71 | 48 | 2 | 24 | 94 | 6 | 72 | Uncharacterized protein | Uncharacterized protein | | afdb-uniprot50 | AF-A0A2A7V0W4-F1-MODEL\_V4 | 1.0 | 0.002895 | 149 | 0.17 | 88 | 64 | 1 | 15 | 93 | 2 | 89 | Phage tail assembly protein | Phage tail assembly protein | | afdb-uniprot50 | AF-A0A2N5C424-F1-MODEL\_V4 | 1.0 | 0.002385 | 149 | 0.202 | 84 | 63 | 2 | 15 | 94 | 11 | 94 | Phage tail assembly protein | Phage tail assembly protein | | afdb-uniprot50 | AF-A0A4U5ZRD0-F1-MODEL\_V4 | 1.0 | 0.007154 | 149 | 0.171 | 70 | 57 | 1 | 18 | 87 | 56 | 124 | Phage tail assembly protein | Phage tail assembly protein | | afdb-uniprot50 | AF-A0A6S5Y2Z1-F1-MODEL\_V4 | 1.0 | 0.001422 | 148 | 0.234 | 94 | 68 | 2 | 1 | 92 | 1 | 92 | Uncharacterized protein | Uncharacterized protein | | afdb-uniprot50 | AF-A0A4R7WAS9-F1-MODEL\_V4 | 1.0 | 0.001726 | 148 | 0.2 | 85 | 59 | 1 | 18 | 93 | 6 | 90 | Tail assembly chaperone E/41/14-like protein | Tail assembly chaperone E/41/14-like protein | | afdb-uniprot50 | AF-A0A1W1Z5N4-F1-MODEL\_V4 | 1.0 | 0.001726 | 148 | 0.119 | 84 | 66 | 3 | 4 | 87 | 1 | 76 | Phage tail assembly chaperone protein, E, or 41 or 14 | Phage tail assembly chaperone protein, E, or 41 or 14 | | afdb-uniprot50 | AF-A0A4S5JA13-F1-MODEL\_V4 | 1.0 | 0.001333 | 148 | 0.195 | 92 | 72 | 1 | 1 | 92 | 30 | 119 | Phage tail assembly protein | Phage tail assembly protein | | afdb-uniprot50 | AF-A0A174ERE2-F1-MODEL\_V4 | 1.0 | 0.000265 | 148 | 0.231 | 108 | 68 | 4 | 1 | 93 | 36 | 143 | Uncharacterized protein | Uncharacterized protein | | afdb-uniprot50 | AF-A0A154IH79-F1-MODEL\_V4 | 1.0 | 0.004854 | 147 | 0.197 | 76 | 60 | 1 | 18 | 93 | 3 | 77 | Uncharacterized protein | Uncharacterized protein | | afdb-uniprot50 | AF-A0A6L5PED5-F1-MODEL\_V4 | 1.0 | 0.002895 | 147 | 0.148 | 81 | 65 | 2 | 15 | 91 | 4 | 84 | Uncharacterized protein | Uncharacterized protein | | afdb-uniprot50 | AF-B6WS21-F1-MODEL\_V4 | 1.0 | 0.002096 | 147 | 0.23 | 78 | 56 | 3 | 18 | 93 | 5 | 80 | Phage tail protein E | Phage tail protein E | | afdb-uniprot50 | AF-A0A6N9TJJ6-F1-MODEL\_V4 | 1.0 | 0.002714 | 147 | 0.228 | 83 | 57 | 2 | 15 | 93 | 2 | 81 | Phage tail assembly protein | Phage tail assembly protein | | afdb-uniprot50 | AF-A0A2U0TF96-F1-MODEL\_V4 | 1.0 | 0.008141 | 147 | 0.2 | 75 | 59 | 1 | 18 | 92 | 6 | 79 | Tail assembly chaperone E/41/14-like protein | Tail assembly chaperone E/41/14-like protein | | afdb-uniprot50 | AF-E9CK62-F1-MODEL\_V4 | 1.0 | 0.002544 | 147 | 0.209 | 86 | 60 | 1 | 16 | 93 | 2 | 87 | Uncharacterized protein | Uncharacterized protein | | afdb-uniprot50 | AF-A0A1E2RY06-F1-MODEL\_V4 | 1.0 | 0.001333 | 147 | 0.209 | 105 | 71 | 2 | 1 | 93 | 2 | 106 | Uncharacterized protein | Uncharacterized protein | | afdb-uniprot50 | AF-A0A4V2RX89-F1-MODEL\_V4 | 1.0 | 0.0009046 | 147 | 0.191 | 94 | 73 | 1 | 2 | 92 | 31 | 124 | Tail assembly chaperone E/41/14-like protein | Tail assembly chaperone E/41/14-like protein | | afdb-uniprot50 | AF-A0A600X9S7-F1-MODEL\_V4 | 1.0 | 0.004266 | 146 | 0.233 | 77 | 58 | 1 | 18 | 93 | 4 | 80 | Phage tail assembly protein | Phage tail assembly protein | | afdb-uniprot50 | AF-A0A5E4Z4W9-F1-MODEL\_V4 | 1.0 | 0.005893 | 145 | 0.214 | 84 | 62 | 1 | 15 | 94 | 5 | 88 | Phage tail assembly protein | Phage tail assembly protein | | afdb-uniprot50 | AF-A0A2G0Q146-F1-MODEL\_V4 | 1.0 | 0.004854 | 145 | 0.129 | 85 | 68 | 2 | 15 | 93 | 3 | 87 | Uncharacterized protein | Uncharacterized protein | | afdb-uniprot50 | AF-A0A2M9VHD7-F1-MODEL\_V4 | 1.0 | 0.002544 | 145 | 0.183 | 87 | 62 | 1 | 16 | 93 | 3 | 89 | Phage tail assembly protein | Phage tail assembly protein | | afdb-uniprot50 | AF-A0A1X0TET7-F1-MODEL\_V4 | 1.0 | 0.003999 | 145 | 0.244 | 90 | 57 | 3 | 16 | 94 | 3 | 92 | Uncharacterized protein | Uncharacterized protein | | afdb-uniprot50 | AF-A0A2A5EJW3-F1-MODEL\_V4 | 1.0 | 0.002714 | 145 | 0.189 | 95 | 62 | 2 | 11 | 90 | 1 | 95 | Uncharacterized protein | Uncharacterized protein | | afdb-uniprot50 | AF-A0A1G7ZBM7-F1-MODEL\_V4 | 1.0 | 0.0009046 | 145 | 0.186 | 91 | 71 | 3 | 3 | 92 | 2 | 90 | Phage tail assembly chaperone protein, E, or 41 or 14 | Phage tail assembly chaperone protein, E, or 41 or 14 | | afdb-uniprot50 | AF-A0A098ARP9-F1-MODEL\_V4 | 1.0 | 0.009264 | 144 | 0.339 | 56 | 37 | 0 | 18 | 73 | 3 | 58 | Putative phage related protein | Putative phage related protein | | afdb-uniprot50 | AF-A0A6G5QYA4-F1-MODEL\_V4 | 1.0 | 0.0006548 | 144 | 0.214 | 98 | 65 | 4 | 1 | 94 | 1 | 90 | Uncharacterized protein | Uncharacterized protein | | afdb-uniprot50 | AF-A0A1C3WNV3-F1-MODEL\_V4 | 1.0 | 0.002714 | 144 | 0.191 | 89 | 61 | 1 | 15 | 92 | 7 | 95 | Phage tail assembly chaperone protein, E, or 41 or 14 | Phage tail assembly chaperone protein, E, or 41 or 14 | | afdb-uniprot50 | AF-A0A743BZ38-F1-MODEL\_V4 | 1.0 | 0.005178 | 144 | 0.233 | 77 | 58 | 1 | 18 | 93 | 4 | 80 | Phage tail assembly protein | Phage tail assembly protein | | afdb-uniprot50 | AF-A0A0A8JLJ0-F1-MODEL\_V4 | 1.0 | 0.002235 | 144 | 0.221 | 95 | 61 | 4 | 12 | 93 | 25 | 119 | Uncharacterized protein | Uncharacterized protein | | afdb-uniprot50 | AF-A0A4R3EF76-F1-MODEL\_V4 | 1.0 | 0.000848 | 144 | 0.206 | 92 | 71 | 2 | 2 | 92 | 31 | 121 | Tail assembly chaperone E/41/14-like protein | Tail assembly chaperone E/41/14-like protein | | afdb-uniprot50 | AF-R9K258-F1-MODEL\_V4 | 1.0 | 0.0007452 | 144 | 0.2 | 105 | 71 | 4 | 1 | 93 | 39 | 142 | Uncharacterized protein | Uncharacterized protein | | afdb-uniprot50 | AF-A0A259CE66-F1-MODEL\_V4 | 1.0 | 0.007631 | 143 | 0.213 | 75 | 58 | 1 | 18 | 92 | 3 | 76 | Uncharacterized protein | Uncharacterized protein | | afdb-uniprot50 | AF-A0A2S7JEN3-F1-MODEL\_V4 | 1.0 | 0.002385 | 143 | 0.159 | 88 | 62 | 2 | 18 | 93 | 6 | 93 | Phage tail assembly protein | Phage tail assembly protein | | afdb-uniprot50 | AF-A0A0D1LLV6-F1-MODEL\_V4 | 1.0 | 0.003088 | 143 | 0.195 | 87 | 61 | 1 | 16 | 93 | 3 | 89 | ArsR family transcriptional regulator | ArsR family transcriptional regulator | | afdb-uniprot50 | AF-A0A509MUH0-F1-MODEL\_V4 | 1.0 | 0.006286 | 143 | 0.127 | 94 | 74 | 2 | 7 | 94 | 31 | 122 | Uncharacterized protein | Uncharacterized protein | | afdb-uniprot50 | AF-A0A840C0Q8-F1-MODEL\_V4 | 1.0 | 0.003088 | 143 | 0.225 | 93 | 71 | 1 | 1 | 92 | 27 | 119 | Uncharacterized protein | Uncharacterized protein | | afdb-uniprot50 | AF-A0A606FEL0-F1-MODEL\_V4 | 1.0 | 0.005893 | 142 | 0.236 | 76 | 57 | 1 | 18 | 92 | 4 | 79 | Phage tail assembly protein | Phage tail assembly protein | | afdb-uniprot50 | AF-A0A547PS60-F1-MODEL\_V4 | 1.0 | 0.008684 | 142 | 0.185 | 70 | 56 | 1 | 18 | 87 | 6 | 74 | Uncharacterized protein | Uncharacterized protein | | afdb-uniprot50 | AF-A0A502FV57-F1-MODEL\_V4 | 1.0 | 0.000965 | 142 | 0.206 | 92 | 69 | 2 | 4 | 94 | 1 | 89 | Phage tail assembly protein | Phage tail assembly protein | | afdb-uniprot50 | AF-A0A2V3VR35-F1-MODEL\_V4 | 1.0 | 0.004266 | 142 | 0.193 | 88 | 62 | 2 | 14 | 92 | 2 | 89 | Tail assembly chaperone E/41/14-like protein | Tail assembly chaperone E/41/14-like protein | | afdb-uniprot50 | AF-A0A249A392-F1-MODEL\_V4 | 1.0 | 0.007631 | 142 | 0.197 | 86 | 64 | 3 | 13 | 93 | 12 | 97 | Phage tail assembly protein | Phage tail assembly protein | | afdb-uniprot50 | AF-A0A2C9D5V1-F1-MODEL\_V4 | 1.0 | 0.003088 | 142 | 0.176 | 85 | 63 | 4 | 15 | 94 | 5 | 87 | Uncharacterized protein | Uncharacterized protein | | afdb-uniprot50 | AF-A0A7X5CQL9-F1-MODEL\_V4 | 1.0 | 0.0009046 | 142 | 0.237 | 97 | 62 | 3 | 9 | 93 | 34 | 130 | Phage tail assembly protein | Phage tail assembly protein | | afdb-uniprot50 | AF-A0A174U989-F1-MODEL\_V4 | 1.0 | 0.0006139 | 142 | 0.144 | 104 | 77 | 3 | 2 | 93 | 28 | 131 | Uncharacterized protein | Uncharacterized protein | | afdb-uniprot50 | AF-A0A4Y9VR20-F1-MODEL\_V4 | 1.0 | 0.003999 | 141 | 0.209 | 86 | 58 | 2 | 18 | 93 | 2 | 87 | Phage tail assembly protein | Phage tail assembly protein | | afdb-uniprot50 | AF-A0A450UE46-F1-MODEL\_V4 | 1.0 | 0.005893 | 141 | 0.223 | 76 | 58 | 1 | 18 | 93 | 3 | 77 | Mu-like prophage FluMu protein gp41 | Mu-like prophage FluMu protein gp41 | | afdb-uniprot50 | AF-A0A5E8GXQ8-F1-MODEL\_V4 | 1.0 | 0.0009046 | 141 | 0.206 | 97 | 72 | 3 | 1 | 92 | 5 | 101 | Uncharacterized protein | Uncharacterized protein | | afdb-uniprot50 | AF-A0A2S1BAT4-F1-MODEL\_V4 | 1.0 | 0.009264 | 140 | 0.166 | 78 | 60 | 2 | 16 | 88 | 4 | 81 | Phage tail assembly protein | Phage tail assembly protein | | afdb-uniprot50 | AF-A0A1F9FW38-F1-MODEL\_V4 | 1.0 | 0.008684 | 140 | 0.219 | 73 | 53 | 2 | 18 | 89 | 7 | 76 | Uncharacterized protein | Uncharacterized protein | | afdb-uniprot50 | AF-A0A837WJ78-F1-MODEL\_V4 | 1.0 | 0.005524 | 140 | 0.188 | 85 | 61 | 2 | 16 | 92 | 3 | 87 | Uncharacterized protein | Uncharacterized protein | | afdb-uniprot50 | AF-A0A2A5DI31-F1-MODEL\_V4 | 1.0 | 0.004266 | 140 | 0.13 | 84 | 63 | 2 | 15 | 93 | 8 | 86 | Uncharacterized protein | Uncharacterized protein | | afdb-uniprot50 | AF-A0A6L7G199-F1-MODEL\_V4 | 1.0 | 0.007154 | 140 | 0.171 | 76 | 60 | 2 | 18 | 91 | 6 | 80 | Uncharacterized protein | Uncharacterized protein | | afdb-uniprot50 | AF-A0A4Y8RG68-F1-MODEL\_V4 | 1.0 | 0.001964 | 140 | 0.155 | 90 | 68 | 3 | 1 | 90 | 1 | 82 | Phage tail assembly protein | Phage tail assembly protein | | afdb-uniprot50 | AF-A0A7H1NUI4-F1-MODEL\_V4 | 1.0 | 0.002385 | 140 | 0.163 | 98 | 71 | 2 | 1 | 93 | 15 | 106 | Phage tail assembly chaperone protein | Phage tail assembly chaperone protein | | afdb-uniprot50 | AF-A8I7U6-F1-MODEL\_V4 | 1.0 | 0.0005394 | 140 | 0.178 | 95 | 71 | 3 | 2 | 92 | 48 | 139 | Uncharacterized protein | Uncharacterized protein | | afdb-uniprot50 | AF-A0A4P2SNB1-F1-MODEL\_V4 | 1.0 | 0.004854 | 139 | 0.182 | 93 | 68 | 1 | 10 | 94 | 1 | 93 | Uncharacterized protein | Uncharacterized protein | | afdb-uniprot50 | AF-A0A1H7ZZV8-F1-MODEL\_V4 | 1.0 | 0.001618 | 139 | 0.142 | 105 | 75 | 4 | 1 | 93 | 4 | 105 | Uncharacterized protein | Uncharacterized protein | | afdb-uniprot50 | AF-A0A1X7I5S5-F1-MODEL\_V4 | 1.0 | 0.003088 | 139 | 0.176 | 85 | 61 | 4 | 18 | 93 | 10 | 94 | Phage tail assembly chaperone protein, E, or 41 or 14 | Phage tail assembly chaperone protein, E, or 41 or 14 | | afdb-uniprot50 | AF-A0A1M3E4Y4-F1-MODEL\_V4 | 1.0 | 0.003088 | 139 | 0.166 | 90 | 71 | 4 | 7 | 94 | 45 | 132 | Uncharacterized protein | Uncharacterized protein | | afdb-uniprot50 | AF-A0A4R6EIC9-F1-MODEL\_V4 | 1.0 | 0.003514 | 138 | 0.139 | 93 | 68 | 3 | 10 | 94 | 1 | 89 | Tail assembly chaperone E/41/14-like protein | Tail assembly chaperone E/41/14-like protein | | afdb-uniprot50 | AF-A0A4R5M9C3-F1-MODEL\_V4 | 1.0 | 0.008141 | 138 | 0.173 | 104 | 75 | 4 | 1 | 93 | 3 | 106 | Phage tail assembly protein | Phage tail assembly protein | | afdb-uniprot50 | AF-X1XFW3-F1-MODEL\_V4 | 1.0 | 0.005893 | 138 | 0.211 | 85 | 59 | 1 | 18 | 94 | 101 | 185 | DNA\_circ\_N domain-containing protein | DNA\_circ\_N domain-containing protein | | afdb-uniprot50 | AF-A0A806VH15-F1-MODEL\_V4 | 1.0 | 0.002096 | 137 | 0.204 | 93 | 65 | 3 | 1 | 91 | 8 | 93 | Uncharacterized protein | Uncharacterized protein | | afdb-uniprot50 | AF-A0A0Q8AKH2-F1-MODEL\_V4 | 1.0 | 0.008141 | 137 | 0.188 | 85 | 60 | 1 | 18 | 93 | 4 | 88 | ArsR family transcriptional regulator | ArsR family transcriptional regulator | | afdb-uniprot50 | AF-A0A509BPY4-F1-MODEL\_V4 | 1.0 | 0.003749 | 137 | 0.225 | 93 | 68 | 2 | 2 | 93 | 11 | 100 | Uncharacterized protein | Uncharacterized protein | | afdb-uniprot50 | AF-A0A2D0IHJ0-F1-MODEL\_V4 | 1.0 | 0.003999 | 137 | 0.211 | 90 | 58 | 2 | 18 | 94 | 5 | 94 | Uncharacterized protein | Uncharacterized protein | | afdb-uniprot50 | AF-A0A1H1JSE7-F1-MODEL\_V4 | 1.0 | 0.003088 | 137 | 0.149 | 107 | 77 | 4 | 1 | 94 | 1 | 106 | Phage tail assembly chaperone protein, E, or 41 or 14 | Phage tail assembly chaperone protein, E, or 41 or 14 | | afdb-uniprot50 | AF-A0A3A6P0B7-F1-MODEL\_V4 | 1.0 | 0.004854 | 137 | 0.178 | 101 | 70 | 6 | 3 | 92 | 1 | 99 | Uncharacterized protein | Uncharacterized protein | | afdb-uniprot50 | AF-A0A1Z1SQQ7-F1-MODEL\_V4 | 1.0 | 0.008684 | 137 | 0.16 | 100 | 72 | 4 | 2 | 94 | 10 | 104 | Phage tail protein | Phage tail protein | | afdb-uniprot50 | AF-A0A3A6P6S0-F1-MODEL\_V4 | 1.0 | 0.005178 | 137 | 0.166 | 90 | 64 | 2 | 15 | 93 | 17 | 106 | Phage tail assembly protein | Phage tail assembly protein | | afdb-uniprot50 | AF-A0A5M8C2B4-F1-MODEL\_V4 | 1.0 | 0.001171 | 137 | 0.183 | 98 | 67 | 4 | 9 | 93 | 30 | 127 | Phage tail assembly protein | Phage tail assembly protein | | afdb-uniprot50 | AF-A0A658Z224-F1-MODEL\_V4 | 1.0 | 0.005178 | 137 | 0.159 | 88 | 66 | 1 | 14 | 93 | 3 | 90 | Bacteriophage protein | Bacteriophage protein | | afdb-uniprot50 | AF-A0A4U8UC82-F1-MODEL\_V4 | 1.0 | 0.005893 | 136 | 0.319 | 72 | 45 | 2 | 23 | 94 | 3 | 70 | Phage tail assembly protein | Phage tail assembly protein | | afdb-uniprot50 | AF-A0A5U1J7E5-F1-MODEL\_V4 | 1.0 | 0.008684 | 136 | 0.232 | 73 | 55 | 1 | 18 | 89 | 6 | 78 | Phage tail assembly protein | Phage tail assembly protein | | afdb-uniprot50 | AF-A0A5E7ADD5-F1-MODEL\_V4 | 1.0 | 0.006286 | 136 | 0.2 | 85 | 59 | 1 | 18 | 93 | 6 | 90 | Uncharacterized protein | Uncharacterized protein | | afdb-uniprot50 | AF-E5F1U9-F1-MODEL\_V4 | 1.0 | 0.006286 | 136 | 0.291 | 72 | 48 | 1 | 22 | 93 | 29 | 97 | Uncharacterized protein | Uncharacterized protein | | afdb-uniprot50 | AF-A0A7W9QVC4-F1-MODEL\_V4 | 1.0 | 0.006286 | 136 | 0.208 | 96 | 66 | 5 | 2 | 93 | 8 | 97 | Uncharacterized protein | Uncharacterized protein | | afdb-uniprot50 | AF-A0A411T3J1-F1-MODEL\_V4 | 1.0 | 0.003749 | 136 | 0.279 | 86 | 52 | 2 | 18 | 93 | 9 | 94 | Phage tail assembly protein | Phage tail assembly protein | | afdb-uniprot50 | AF-A0A0E1GP01-F1-MODEL\_V4 | 1.0 | 0.001726 | 136 | 0.175 | 97 | 69 | 4 | 1 | 94 | 20 | 108 | Uncharacterized protein | Uncharacterized protein | | afdb-uniprot50 | AF-A0A431JGG1-F1-MODEL\_V4 | 1.0 | 0.003294 | 136 | 0.16 | 87 | 70 | 2 | 9 | 94 | 33 | 117 | Phage tail assembly protein | Phage tail assembly protein | | afdb-uniprot50 | AF-A0A4Q2SXK7-F1-MODEL\_V4 | 1.0 | 0.001618 | 136 | 0.21 | 95 | 68 | 3 | 2 | 94 | 65 | 154 | Phage tail assembly protein | Phage tail assembly protein | | afdb-uniprot50 | AF-A0A1S1D167-F1-MODEL\_V4 | 1.0 | 0.006706 | 135 | 0.195 | 87 | 59 | 2 | 15 | 93 | 2 | 85 | Uncharacterized protein | Uncharacterized protein | | afdb-uniprot50 | AF-A0A650EN83-F1-MODEL\_V4 | 1.0 | 0.004551 | 135 | 0.172 | 81 | 63 | 1 | 18 | 94 | 7 | 87 | Uncharacterized protein | Uncharacterized protein | | afdb-uniprot50 | AF-A0A7U4R2Y7-F1-MODEL\_V4 | 1.0 | 0.005178 | 135 | 0.165 | 103 | 77 | 3 | 1 | 94 | 1 | 103 | Uncharacterized protein | Uncharacterized protein | | afdb-uniprot50 | AF-A0A239JSH3-F1-MODEL\_V4 | 1.0 | 0.001618 | 135 | 0.172 | 110 | 72 | 6 | 1 | 92 | 1 | 109 | Phage tail assembly chaperone protein, E, or 41 or 14 | Phage tail assembly chaperone protein, E, or 41 or 14 | | afdb-uniprot50 | AF-A0A504U8T9-F1-MODEL\_V4 | 1.0 | 0.005178 | 135 | 0.141 | 78 | 64 | 1 | 15 | 92 | 35 | 109 | Phage tail assembly protein | Phage tail assembly protein | | afdb-uniprot50 | AF-H4FBM5-F1-MODEL\_V4 | 1.0 | 0.002544 | 135 | 0.184 | 92 | 67 | 3 | 1 | 92 | 28 | 111 | Uncharacterized protein | Uncharacterized protein | | afdb-uniprot50 | AF-A0A0F6B300-F1-MODEL\_V4 | 1.0 | 0.001964 | 135 | 0.197 | 86 | 59 | 4 | 16 | 92 | 5 | 89 | Uncharacterized protein | Uncharacterized protein | | afdb-uniprot50 | AF-A0A431MY95-F1-MODEL\_V4 | 1.0 | 0.001964 | 134 | 0.209 | 86 | 61 | 3 | 9 | 94 | 2 | 80 | Phage tail assembly protein | Phage tail assembly protein | | afdb-uniprot50 | AF-R7I6D8-F1-MODEL\_V4 | 1.0 | 0.005178 | 134 | 0.21 | 76 | 54 | 3 | 16 | 85 | 3 | 78 | Uncharacterized protein | Uncharacterized protein | | afdb-uniprot50 | AF-A0A345CNU2-F1-MODEL\_V4 | 1.0 | 0.003514 | 134 | 0.149 | 87 | 64 | 3 | 15 | 94 | 7 | 90 | Phage tail assembly protein | Phage tail assembly protein | | afdb-uniprot50 | AF-K0JHK7-F1-MODEL\_V4 | 1.0 | 0.004266 | 134 | 0.159 | 88 | 69 | 2 | 10 | 94 | 1 | 86 | Uncharacterized protein | Uncharacterized protein | | afdb-uniprot50 | AF-A0A418X1D6-F1-MODEL\_V4 | 1.0 | 0.005178 | 134 | 0.183 | 87 | 59 | 2 | 18 | 92 | 2 | 88 | Phage tail assembly protein | Phage tail assembly protein | | afdb-uniprot50 | AF-A0A0S4V3E5-F1-MODEL\_V4 | 1.0 | 0.009264 | 134 | 0.172 | 81 | 60 | 3 | 16 | 91 | 6 | 84 | Hypothethical protein | Hypothethical protein | | afdb-uniprot50 | AF-A0A1I1EZQ6-F1-MODEL\_V4 | 1.0 | 0.005178 | 134 | 0.141 | 85 | 73 | 0 | 3 | 87 | 36 | 120 | Uncharacterized protein | Uncharacterized protein | | afdb-uniprot50 | AF-A0A0Q6C6X2-F1-MODEL\_V4 | 1.0 | 0.003514 | 134 | 0.172 | 93 | 65 | 3 | 1 | 92 | 67 | 148 | Uncharacterized protein | Uncharacterized protein | | afdb-uniprot50 | AF-A0A7G6U2B7-F1-MODEL\_V4 | 1.0 | 0.006706 | 133 | 0.157 | 76 | 59 | 2 | 18 | 93 | 6 | 76 | Phage tail assembly protein | Phage tail assembly protein | | afdb-uniprot50 | AF-A0A522K8G1-F1-MODEL\_V4 | 1.0 | 0.007154 | 133 | 0.193 | 93 | 71 | 2 | 3 | 92 | 2 | 93 | Phage tail assembly protein | Phage tail assembly protein | | afdb-uniprot50 | AF-A0A5M8P9I7-F1-MODEL\_V4 | 1.0 | 0.005178 | 133 | 0.157 | 121 | 64 | 4 | 10 | 92 | 1 | 121 | Uncharacterized protein | Uncharacterized protein | | afdb-uniprot50 | AF-A0A285T5R0-F1-MODEL\_V4 | 1.0 | 0.00125 | 133 | 0.202 | 99 | 69 | 3 | 1 | 92 | 40 | 135 | Uncharacterized protein | Uncharacterized protein | | afdb-uniprot50 | AF-A0A7W7KJ99-F1-MODEL\_V4 | 1.0 | 0.007154 | 132 | 0.202 | 84 | 62 | 3 | 15 | 94 | 3 | 85 | Uncharacterized protein | Uncharacterized protein | | afdb-uniprot50 | AF-A0A291PD75-F1-MODEL\_V4 | 1.0 | 0.003088 | 132 | 0.177 | 96 | 69 | 5 | 5 | 93 | 11 | 103 | Uncharacterized protein | Uncharacterized protein | | afdb-uniprot50 | AF-A0A3R9CVH5-F1-MODEL\_V4 | 1.0 | 0.008141 | 132 | 0.17 | 88 | 61 | 3 | 15 | 94 | 18 | 101 | Phage tail assembly protein | Phage tail assembly protein | | afdb-uniprot50 | AF-A0A016XIN1-F1-MODEL\_V4 | 1.0 | 0.009882 | 132 | 0.157 | 89 | 69 | 3 | 9 | 94 | 2 | 87 | Uncharacterized protein | Uncharacterized protein | | afdb-uniprot50 | AF-A0A3R7HUR4-F1-MODEL\_V4 | 1.0 | 0.006706 | 132 | 0.243 | 82 | 58 | 3 | 15 | 94 | 5 | 84 | Tail assembly chaperone E/41/14-like protein | Tail assembly chaperone E/41/14-like protein | | afdb-uniprot50 | AF-A0A7Y6Z4K4-F1-MODEL\_V4 | 1.0 | 0.003514 | 131 | 0.172 | 93 | 67 | 4 | 9 | 94 | 2 | 91 | Phage tail assembly protein | Phage tail assembly protein | | afdb-uniprot50 | AF-A0A2W5S0Y9-F1-MODEL\_V4 | 1.0 | 0.009264 | 131 | 0.175 | 91 | 63 | 3 | 12 | 94 | 14 | 100 | Phage tail assembly protein | Phage tail assembly protein | | afdb-uniprot50 | AF-A0A225SNI8-F1-MODEL\_V4 | 1.0 | 0.003294 | 131 | 0.2 | 95 | 70 | 4 | 2 | 93 | 14 | 105 | Uncharacterized protein | Uncharacterized protein | | afdb-uniprot50 | AF-A0A7Y6T7N0-F1-MODEL\_V4 | 1.0 | 0.003749 | 131 | 0.205 | 102 | 67 | 2 | 2 | 92 | 12 | 110 | Phage tail assembly protein | Phage tail assembly protein | | afdb-uniprot50 | AF-A0A3A9EEN6-F1-MODEL\_V4 | 1.0 | 0.001517 | 131 | 0.154 | 97 | 70 | 4 | 9 | 93 | 35 | 131 | Phage tail assembly protein | Phage tail assembly protein | | afdb-uniprot50 | AF-A0A485CDJ1-F1-MODEL\_V4 | 1.0 | 0.004854 | 131 | 0.148 | 101 | 75 | 4 | 1 | 94 | 118 | 214 | Phage major tail tube protein | Phage major tail tube protein | | afdb-uniprot50 | AF-A0A0D0GXK6-F1-MODEL\_V4 | 1.0 | 0.005524 | 130 | 0.175 | 108 | 73 | 4 | 1 | 94 | 1 | 106 | KY49.ctg7180000000025\_quiver, whole genome shotgun sequence | KY49.ctg7180000000025\_quiver, whole genome shotgun sequence | | afdb-uniprot50 | AF-N8V954-F1-MODEL\_V4 | 1.0 | 0.009264 | 130 | 0.188 | 90 | 61 | 3 | 13 | 94 | 14 | 99 | Uncharacterized protein | Uncharacterized protein | | afdb-uniprot50 | AF-E2CHK2-F1-MODEL\_V4 | 1.0 | 0.001333 | 130 | 0.195 | 97 | 69 | 3 | 2 | 92 | 25 | 118 | Uncharacterized protein | Uncharacterized protein | | afdb-uniprot50 | AF-A0A5P8MUZ6-F1-MODEL\_V4 | 1.0 | 0.001841 | 130 | 0.178 | 95 | 72 | 3 | 1 | 92 | 48 | 139 | Uncharacterized protein | Uncharacterized protein | | afdb-uniprot50 | AF-A0A6I5WQG3-F1-MODEL\_V4 | 1.0 | 0.008684 | 129 | 0.162 | 86 | 67 | 3 | 13 | 93 | 14 | 99 | Phage tail assembly protein | Phage tail assembly protein | | afdb-uniprot50 | AF-A0A6N9P3E0-F1-MODEL\_V4 | 1.0 | 0.001964 | 129 | 0.2 | 105 | 69 | 5 | 1 | 93 | 66 | 167 | Phage tail assembly protein | Phage tail assembly protein | | afdb-uniprot50 | AF-A0A1X9T1B4-F1-MODEL\_V4 | 1.0 | 0.006286 | 128 | 0.189 | 95 | 65 | 4 | 1 | 94 | 1 | 84 | FluMu gp41 family protein | FluMu gp41 family protein | | afdb-uniprot50 | AF-A0A0Q4II11-F1-MODEL\_V4 | 1.0 | 0.003294 | 128 | 0.177 | 96 | 75 | 2 | 1 | 94 | 2 | 95 | Uncharacterized protein | Uncharacterized protein | | afdb-uniprot50 | AF-A0A841S0F6-F1-MODEL\_V4 | 1.0 | 0.007154 | 128 | 0.147 | 102 | 75 | 3 | 1 | 94 | 8 | 105 | Uncharacterized protein | Uncharacterized protein | | afdb-uniprot50 | AF-A0A6A1TS96-F1-MODEL\_V4 | 1.0 | 0.003749 | 128 | 0.186 | 102 | 70 | 2 | 1 | 92 | 45 | 143 | Phage tail assembly protein | Phage tail assembly protein | | afdb-uniprot50 | AF-A0A2W6TML0-F1-MODEL\_V4 | 1.0 | 0.004551 | 128 | 0.184 | 92 | 74 | 1 | 2 | 92 | 67 | 158 | Uncharacterized protein | Uncharacterized protein | | afdb-uniprot50 | AF-A0A736RG71-F1-MODEL\_V4 | 1.0 | 0.003999 | 127 | 0.195 | 87 | 60 | 4 | 16 | 93 | 2 | 87 | Phage tail assembly protein | Phage tail assembly protein | | afdb-uniprot50 | AF-A0A1P8JYY7-F1-MODEL\_V4 | 1.0 | 0.005893 | 127 | 0.177 | 96 | 66 | 3 | 9 | 93 | 5 | 98 | Uncharacterized protein | Uncharacterized protein | | afdb-uniprot50 | AF-A0A2U1SE14-F1-MODEL\_V4 | 1.0 | 0.008684 | 127 | 0.142 | 98 | 71 | 3 | 9 | 93 | 2 | 99 | Uncharacterized protein | Uncharacterized protein | | afdb-uniprot50 | AF-A0A0D7PAE5-F1-MODEL\_V4 | 1.0 | 0.007154 | 127 | 0.166 | 108 | 76 | 3 | 1 | 94 | 1 | 108 | Uncharacterized protein | Uncharacterized protein | | afdb-uniprot50 | AF-A0A7U5GYV5-F1-MODEL\_V4 | 1.0 | 0.001726 | 127 | 0.135 | 111 | 75 | 5 | 1 | 93 | 1 | 108 | Uncharacterized protein | Uncharacterized protein | | afdb-uniprot50 | AF-L0NDQ1-F1-MODEL\_V4 | 1.0 | 0.005524 | 127 | 0.223 | 94 | 67 | 3 | 2 | 92 | 31 | 121 | Uncharacterized protein | Uncharacterized protein | | afdb-uniprot50 | AF-A0A0P6VMZ8-F1-MODEL\_V4 | 1.0 | 0.003088 | 127 | 0.191 | 94 | 64 | 4 | 1 | 88 | 1 | 88 | Uncharacterized protein | Uncharacterized protein | | afdb-uniprot50 | AF-A0A432UNV4-F1-MODEL\_V4 | 1.0 | 0.009264 | 126 | 0.155 | 90 | 68 | 2 | 10 | 94 | 1 | 87 | Phage tail assembly protein | Phage tail assembly protein | | afdb-uniprot50 | AF-A0A6M8HND0-F1-MODEL\_V4 | 1.0 | 0.003999 | 126 | 0.175 | 97 | 66 | 3 | 4 | 94 | 8 | 96 | Phage tail assembly protein | Phage tail assembly protein | | afdb-uniprot50 | AF-A0A1Q6JQ84-F1-MODEL\_V4 | 1.0 | 0.002235 | 126 | 0.196 | 107 | 73 | 4 | 1 | 94 | 27 | 133 | Uncharacterized protein | Uncharacterized protein | | afdb-uniprot50 | AF-A0A829WCQ7-F1-MODEL\_V4 | 1.0 | 0.001964 | 126 | 0.194 | 108 | 71 | 5 | 2 | 93 | 30 | 137 | Uncharacterized protein | Uncharacterized protein | | afdb-uniprot50 | AF-A0A2G6IRS6-F1-MODEL\_V4 | 1.0 | 0.003514 | 126 | 0.229 | 96 | 66 | 3 | 2 | 92 | 14 | 106 | Uncharacterized protein | Uncharacterized protein | | afdb-uniprot50 | AF-A0A257KLL7-F1-MODEL\_V4 | 1.0 | 0.006706 | 125 | 0.207 | 77 | 56 | 3 | 18 | 94 | 2 | 73 | Uncharacterized protein | Uncharacterized protein | | afdb-uniprot50 | AF-A0A378Q4F7-F1-MODEL\_V4 | 1.0 | 0.007631 | 125 | 0.2 | 90 | 58 | 4 | 14 | 94 | 7 | 91 | Phage tail protein E | Phage tail protein E | | afdb-uniprot50 | AF-A0A2N3D8J3-F1-MODEL\_V4 | 1.0 | 0.009264 | 125 | 0.148 | 101 | 72 | 4 | 2 | 94 | 3 | 97 | Phage tail assembly protein | Phage tail assembly protein | | afdb-uniprot50 | AF-A0A2S6QN92-F1-MODEL\_V4 | 1.0 | 0.004551 | 125 | 0.271 | 70 | 50 | 1 | 14 | 82 | 2 | 71 | Uncharacterized protein | Uncharacterized protein | | afdb-uniprot50 | AF-A0A327K060-F1-MODEL\_V4 | 1.0 | 0.003294 | 124 | 0.244 | 94 | 64 | 3 | 3 | 92 | 2 | 92 | Uncharacterized protein | Uncharacterized protein | | afdb-uniprot50 | AF-C1DBJ2-F1-MODEL\_V4 | 1.0 | 0.005893 | 123 | 0.172 | 87 | 62 | 3 | 15 | 94 | 2 | 85 | Uncharacterized protein | Uncharacterized protein | | afdb-uniprot50 | AF-A0A547PW59-F1-MODEL\_V4 | 1.0 | 0.005524 | 123 | 0.207 | 106 | 66 | 5 | 1 | 94 | 3 | 102 | Phage tail assembly protein | Phage tail assembly protein | | afdb-uniprot50 | AF-A0A7T3DHK5-F1-MODEL\_V4 | 1.0 | 0.008141 | 123 | 0.128 | 101 | 76 | 5 | 1 | 94 | 27 | 122 | Phage tail assembly protein | Phage tail assembly protein | | afdb-uniprot50 | AF-A0A348HI85-F1-MODEL\_V4 | 1.0 | 0.008141 | 122 | 0.128 | 101 | 71 | 4 | 1 | 93 | 1 | 92 | Small-conductance mechanosensitive channel | Small-conductance mechanosensitive channel | | afdb-uniprot50 | AF-A0A3L7JF17-F1-MODEL\_V4 | 1.0 | 0.004551 | 122 | 0.215 | 93 | 65 | 4 | 2 | 92 | 50 | 136 | Phage tail assembly protein | Phage tail assembly protein | | afdb-uniprot50 | AF-A0A2C9D6L2-F1-MODEL\_V4 | 1.0 | 0.007631 | 122 | 0.18 | 94 | 70 | 3 | 3 | 92 | 61 | 151 | Uncharacterized protein | Uncharacterized protein | | afdb-uniprot50 | AF-A0A2N3KX48-F1-MODEL\_V4 | 1.0 | 0.005178 | 121 | 0.197 | 96 | 57 | 4 | 10 | 93 | 1 | 88 | Phage tail protein | Phage tail protein | | afdb-uniprot50 | AF-A0A806LZ96-F1-MODEL\_V4 | 1.0 | 0.007154 | 121 | 0.178 | 95 | 59 | 4 | 15 | 93 | 16 | 107 | Uncharacterized protein | Uncharacterized protein | | afdb-uniprot50 | AF-A0NQ99-F1-MODEL\_V4 | 1.0 | 0.003749 | 121 | 0.185 | 97 | 65 | 3 | 10 | 93 | 1 | 96 | Uncharacterized protein | Uncharacterized protein | | afdb-uniprot50 | AF-A0A2W5EHM5-F1-MODEL\_V4 | 1.0 | 0.008684 | 121 | 0.187 | 96 | 69 | 3 | 1 | 92 | 50 | 140 | Uncharacterized protein | Uncharacterized protein | | afdb-uniprot50 | AF-A0A7W4JQX5-F1-MODEL\_V4 | 1.0 | 0.003514 | 121 | 0.25 | 96 | 64 | 3 | 2 | 91 | 14 | 107 | Phage tail assembly protein | Phage tail assembly protein | | afdb-uniprot50 | AF-A0A410VCZ1-F1-MODEL\_V4 | 1.0 | 0.009264 | 120 | 0.228 | 83 | 59 | 3 | 12 | 94 | 7 | 84 | Uncharacterized protein | Uncharacterized protein | | afdb-uniprot50 | AF-A0A653HUE2-F1-MODEL\_V4 | 1.0 | 0.009264 | 120 | 0.166 | 90 | 65 | 3 | 12 | 94 | 16 | 102 | Uncharacterized protein | Uncharacterized protein | | afdb-uniprot50 | AF-A0A1M5NLQ3-F1-MODEL\_V4 | 1.0 | 0.004266 | 120 | 0.141 | 106 | 78 | 4 | 1 | 93 | 12 | 117 | Uncharacterized protein | Uncharacterized protein | | afdb-uniprot50 | AF-A0A212LD12-F1-MODEL\_V4 | 1.0 | 0.005893 | 120 | 0.147 | 95 | 73 | 3 | 2 | 92 | 44 | 134 | Uncharacterized protein | Uncharacterized protein | | afdb-uniprot50 | AF-A0A2E4CSJ3-F1-MODEL\_V4 | 1.0 | 0.008684 | 119 | 0.158 | 101 | 67 | 4 | 2 | 94 | 15 | 105 | Phage tail protein | Phage tail protein | | afdb-uniprot50 | AF-A0A6B8KJI9-F1-MODEL\_V4 | 1.0 | 0.004266 | 119 | 0.19 | 100 | 69 | 4 | 1 | 94 | 33 | 126 | Uncharacterized protein | Uncharacterized protein | | afdb-uniprot50 | AF-A0A7T9VKM3-F1-MODEL\_V4 | 1.0 | 0.008141 | 119 | 0.161 | 93 | 68 | 3 | 9 | 94 | 25 | 114 | Phage tail assembly protein | Phage tail assembly protein | | afdb-uniprot50 | AF-A0A7L5Y296-F1-MODEL\_V4 | 1.0 | 0.008684 | 119 | 0.153 | 98 | 75 | 3 | 1 | 93 | 34 | 128 | Uncharacterized protein | Uncharacterized protein | | afdb-uniprot50 | AF-A0A6N7EZF4-F1-MODEL\_V4 | 1.0 | 0.009264 | 118 | 0.186 | 102 | 65 | 4 | 1 | 94 | 12 | 103 | Phage tail assembly protein | Phage tail assembly protein | | afdb-uniprot50 | AF-A0A2X0YJY8-F1-MODEL\_V4 | 1.0 | 0.005524 | 118 | 0.166 | 108 | 74 | 4 | 2 | 93 | 16 | 123 | Uncharacterized protein | Uncharacterized protein | | afdb-uniprot50 | AF-A0A2M9WHJ3-F1-MODEL\_V4 | 1.0 | 0.009264 | 118 | 0.182 | 93 | 65 | 4 | 11 | 92 | 1 | 93 | Uncharacterized protein | Uncharacterized protein | | afdb-uniprot50 | AF-B1HTN1-F1-MODEL\_V4 | 1.0 | 0.008141 | 118 | 0.144 | 104 | 78 | 2 | 1 | 93 | 26 | 129 | Uncharacterized protein | Uncharacterized protein | | afdb-uniprot50 | AF-A0A4Q3U4W6-F1-MODEL\_V4 | 1.0 | 0.003294 | 117 | 0.186 | 102 | 69 | 7 | 2 | 94 | 25 | 121 | Phage tail assembly protein | Phage tail assembly protein | | afdb-uniprot50 | AF-A0A2W5H3D6-F1-MODEL\_V4 | 1.0 | 0.009882 | 117 | 0.159 | 94 | 71 | 3 | 3 | 92 | 65 | 154 | Uncharacterized protein | Uncharacterized protein | | afdb-uniprot50 | AF-A0A1M7ZLP0-F1-MODEL\_V4 | 1.0 | 0.005524 | 116 | 0.241 | 91 | 56 | 4 | 16 | 93 | 4 | 94 | Uncharacterized protein | Uncharacterized protein | | afdb-uniprot50 | AF-A0A4U2PVC1-F1-MODEL\_V4 | 1.0 | 0.002895 | 116 | 0.155 | 103 | 74 | 5 | 2 | 92 | 8 | 109 | Phage tail assembly protein | Phage tail assembly protein | | afdb-uniprot50 | AF-A0A0P7I4K8-F1-MODEL\_V4 | 1.0 | 0.007154 | 115 | 0.206 | 97 | 66 | 5 | 2 | 92 | 5 | 96 | Uncharacterized protein | Uncharacterized protein | | afdb-uniprot50 | AF-E2CI76-F1-MODEL\_V4 | 1.0 | 0.005524 | 115 | 0.183 | 98 | 67 | 5 | 2 | 92 | 42 | 133 | Uncharacterized protein | Uncharacterized protein | | afdb-uniprot50 | AF-A0A7S8IJ10-F1-MODEL\_V4 | 1.0 | 0.007631 | 113 | 0.18 | 100 | 70 | 3 | 2 | 92 | 14 | 110 | Uncharacterized protein | Uncharacterized protein | | afdb-uniprot50 | AF-A0A7Z0LWV3-F1-MODEL\_V4 | 1.0 | 0.009264 | 112 | 0.148 | 101 | 70 | 4 | 1 | 94 | 16 | 107 | Phage tail assembly protein | Phage tail assembly protein | | afdb-uniprot50 | AF-A0A1L5P7E5-F1-MODEL\_V4 | 1.0 | 0.009264 | 111 | 0.189 | 95 | 68 | 4 | 2 | 92 | 60 | 149 | Mu-like prophage FluMu protein | Mu-like prophage FluMu protein | | afdb-uniprot50 | AF-A0A2A2IKT4-F1-MODEL\_V4 | 1.0 | 0.009882 | 110 | 0.229 | 87 | 55 | 3 | 18 | 92 | 2 | 88 | Uncharacterized protein | Uncharacterized protein | | afdb-uniprot50 | AF-A0A2T6LZL0-F1-MODEL\_V4 | 1.0 | 0.008141 | 110 | 0.171 | 99 | 72 | 3 | 1 | 92 | 1 | 96 | Tail assembly chaperone E/41/14-like protein | Tail assembly chaperone E/41/14-like protein | | afdb-uniprot50 | AF-A0A2V2FWG8-F1-MODEL\_V4 | 1.0 | 0.008141 | 108 | 0.185 | 113 | 72 | 6 | 1 | 93 | 1 | 113 | Uncharacterized protein | Uncharacterized protein | | afdb-uniprot50 | AF-A0A2D9ER84-F1-MODEL\_V4 | 1.0 | 0.008684 | 106 | 0.113 | 106 | 79 | 4 | 1 | 94 | 8 | 110 | Phage tail protein | Phage tail protein | | afdb-uniprot50 | AF-A0A137SPX7-F1-MODEL\_V4 | 1.0 | 0.009264 | 106 | 0.145 | 110 | 77 | 4 | 1 | 93 | 6 | 115 | Uncharacterized protein | Uncharacterized protein | |
| Top keywords  (threshold 1.00e-02 (evalue)) | **tail, assembly, Phage, or, E, chaperone, 14\_like, gp41, Putative, prophage** |
| Output files | ../../similar\_structures/20\_FANPEZAQ\_CDS\_0020\_afdb-proteome\_foldseek.tsv ../../similar\_structures/20\_FANPEZAQ\_CDS\_0020\_afdb-uniprot50\_foldseek.tsv ../../similar\_structures/20\_FANPEZAQ\_CDS\_0020\_merged.svg ../../similar\_structures/20\_FANPEZAQ\_CDS\_0020\_pdb\_foldseek.tsv |

  
  
  

Return to summary | Go to previous | Go to next

  


---

**Sequence/structure alignments coloring**  
Each object in the alignment figures is colored according to its E-value following this color coding:

1e-100
10

**References:**  
1) Steinegger M, Meier M, Mirdita M, Vöhringer H, Haunsberger S J, and Söding J (2019) HH-suite3 for fast remote homology detection and deep protein annotation, BMC Bioinformatics, 473. doi: 10.1186/s12859-019-3019-7  
2) Jumper J, Evans R, Pritzel A, ..., Hassabis D (2021) Highly accurate protein structure prediction with AlphaFold, Nature, 596. doi: 10.1038/s41586-021-03819-2  
3) van Kempen M, Kim S, Tumescheit C, Mirdita M, Lee J, Gilchrist CLM, Söding J, and Steinegger M (2023) Fast and accurate protein structure search with Foldseek. Nature Biotechnology. doi: 10.1038/s41587-023-01773-0
