# Supplementary material for: Completing the BASEL phage collection to unlock hidden diversity for systematic exploration of phage–host interactions
Source: PLoS Biol. 2025 Apr 7;23(4):e3003063. doi: 10.1371/journal.pbio.3003063 (PMC11990801; doi:10.1371/journal.pbio.3003063)
Supplement: S2 Data — (ZIP) [file pbio.3003063.s009.zip › entries/21.html]

FANPEZAQ\_CDS\_0021


Return to summary | Go to previous | Go to next

|  |  |
| --- | --- |
| FANPEZAQ\_CDS\_0021 Page creation date: 02 Sep 2024, 12:00  Project folder: n/a  Input sequences file: Escherichia\_virus\_HeidiAbel.gb | phage gpe tail p2 tape measure chaperone hypothetical |

### Sequence information

|  |  |
| --- | --- |
| Name | FANPEZAQ\_CDS\_0021  21\_FANPEZAQ\_CDS\_0021 (pipeline id) |
| Imported annotations | Escherichia\_virus\_HeidiAbel Bas97 |
| Protein sequence | MALASHTGWQLSEIQRLRVSRFVWWLDGLPKKQN |
| Number of residues | 34 |
| Molecular weight (Da) | 4052.66 |
| Output files | ../../query\_sequences/21\_FANPEZAQ\_CDS\_0021.fasta |

### Putative domain architecture and protein family

#### Search results (HHblits)1

|  |  |
| --- | --- |
| Domain family databases searched | Pfam, Ncbi-cd, Cath, Phrogs |
| Results, scheme(s)  (Top layers only; threshold 1.00e-03 (evalue)) | xml version="1.0" encoding="utf-8" standalone="no"?       2024-09-02T21:08:16.703320 image/svg+xml   Matplotlib v3.7.2, https://matplotlib.org/ |
| Results, table  (E-value ≤ 1.00e-03 (evalue)) | -- |
| Top keywords  (threshold 1.00e-03 (evalue)) | -- |
| Output files | ../../domain\_architecture/21\_FANPEZAQ\_CDS\_0021\_cath.hhr ../../domain\_architecture/21\_FANPEZAQ\_CDS\_0021\_merged.svg ../../domain\_architecture/21\_FANPEZAQ\_CDS\_0021\_ncbi-cd.hhr ../../domain\_architecture/21\_FANPEZAQ\_CDS\_0021\_pfam.hhr ../../domain\_architecture/21\_FANPEZAQ\_CDS\_0021\_phrogs.hhr |

### Identical protein sequences/structures

#### Search results

|  |  |
| --- | --- |
| Protein sequence databases searched | Pdb, Swissprot, Refseq |
| Identical proteins found | -- |
| Top keywords | -- |
| Output files | -- |

### Similar protein sequences/structures

#### Sequence similarity search results (HHblits)1

|  |  |
| --- | --- |
| Sequence databases searched | Uniclust, Pdb70 |
| Results, scheme(s)  (Top layers only, threshold 1.00e-03 (evalue)) | xml version="1.0" encoding="utf-8" standalone="no"?       2024-09-02T21:08:38.105559 image/svg+xml   Matplotlib v3.7.2, https://matplotlib.org/ |
| Results, table(s)  (threshold 1.00e-03 (evalue)) | | db | id | prob | evalue | pvalue | score | cols | query | query\_len | template | template\_len | name | description | | --- | --- | --- | --- | --- | --- | --- | --- | --- | --- | --- | --- | --- | | uniclust | UniRef100\_A0A5C2H343 | 99.4 | 4.6e-16 | 9.2e-22 | 91.7 | 33 | (1, 33) | 34 | (39, 71) | 72 | Uncharacterized protein | Uncharacterized protein | | uniclust | UniRef100\_A0A060H7C1 | 99.4 | 2.8e-15 | 5.7e-21 | 82.7 | 33 | (1, 33) | 34 | (13, 45) | 46 | Uncharacterized protein | Uncharacterized protein | | uniclust | UniRef100\_A0A858NPH9 | 99.3 | 3.6e-15 | 7e-21 | 78.7 | 33 | (1, 33) | 34 | (2, 34) | 35 | Uncharacterized protein | Uncharacterized protein | | uniclust | UniRef100\_A0A7L8G6A2 | 99.2 | 1.3e-13 | 2.5e-19 | 89.7 | 33 | (1, 33) | 34 | (108, 140) | 145 | Tape measure chaperone | Tape measure chaperone | | uniclust | UniRef100\_A0A975AA02 | 99.0 | 3e-12 | 5.4e-18 | 71.0 | 34 | (1, 34) | 34 | (12, 45) | 45 | GpE family phage tail protein | GpE family phage tail protein | | uniclust | UniRef100\_A0A0P9QIX4 | 98.8 | 2.1e-11 | 4.4e-17 | 67.0 | 26 | (2, 27) | 34 | (16, 41) | 42 | Uncharacterized protein | Uncharacterized protein | | uniclust | UniRef100\_A0A8S5RUP2 | 98.5 | 1.9e-09 | 3.5e-15 | 58.5 | 31 | (1, 31) | 34 | (2, 32) | 37 | Phage protein | Phage protein | | uniclust | UniRef100\_UPI001F35B4CE | 98.4 | 2.6e-09 | 4.8e-15 | 61.5 | 33 | (1, 33) | 34 | (19, 51) | 53 | hypothetical protein | hypothetical protein | | uniclust | UniRef100\_R9TPW5 | 98.4 | 2.7e-09 | 4.9e-15 | 62.9 | 33 | (2, 34) | 34 | (29, 61) | 62 | Uncharacterized protein | Uncharacterized protein | | uniclust | UniRef100\_A0A1H4SBZ9 | 98.1 | 6.1e-08 | 1.2e-13 | 58.3 | 26 | (2, 27) | 34 | (42, 67) | 68 | Uncharacterized protein | Uncharacterized protein | | uniclust | UniRef100\_A0A0C5VIR9 | 97.8 | 5.3e-07 | 1e-12 | 51.0 | 31 | (1, 31) | 34 | (6, 36) | 44 | GpE family phage tail protein | GpE family phage tail protein | | uniclust | UniRef100\_A0A6I5RSZ0 | 97.8 | 5.8e-07 | 1.1e-12 | 51.0 | 26 | (2, 27) | 34 | (19, 44) | 45 | Uncharacterized protein | Uncharacterized protein | | uniclust | UniRef100\_A0A1T4WVU1 | 97.7 | 8.6e-07 | 1.6e-12 | 51.6 | 27 | (2, 28) | 34 | (24, 50) | 52 | Phage P2 GpE | Phage P2 GpE | | uniclust | UniRef100\_A0A023Q0E1 | 97.7 | 9.6e-07 | 1.8e-12 | 49.5 | 29 | (1, 29) | 34 | (3, 31) | 41 | GpE family phage tail protein | GpE family phage tail protein | | uniclust | UniRef100\_A0A1I7J3I1 | 97.2 | 1.6e-05 | 3.1e-11 | 46.1 | 30 | (2, 31) | 34 | (15, 44) | 48 | Uncharacterized protein | Uncharacterized protein | | uniclust | UniRef100\_E6VU91 | 97.1 | 3.8e-05 | 6.9e-11 | 43.5 | 29 | (1, 29) | 34 | (12, 40) | 41 | Uncharacterized protein | Uncharacterized protein | | uniclust | UniRef100\_A0A024HBK0 | 96.7 | 0.00016 | 3.2e-10 | 41.2 | 26 | (2, 27) | 34 | (12, 37) | 41 | Uncharacterized protein | Uncharacterized protein | | uniclust | UniRef100\_A0A450ZF00 | 96.4 | 0.00056 | 1e-09 | 38.9 | 28 | (3, 30) | 34 | (5, 32) | 39 | Phage P2 GpE | Phage P2 GpE | |
| Top keywords  (threshold 1.00e-03 (evalue)) | **phage, GpE, tail, P2, Tape, measure, chaperone, hypothetical** |
| Output files | ../../similar\_sequences/21\_FANPEZAQ\_CDS\_0021\_merged.svg ../../similar\_sequences/21\_FANPEZAQ\_CDS\_0021\_pdb70.a3m ../../similar\_sequences/21\_FANPEZAQ\_CDS\_0021\_pdb70.hhr ../../similar\_sequences/21\_FANPEZAQ\_CDS\_0021\_uniclust.a3m ../../similar\_sequences/21\_FANPEZAQ\_CDS\_0021\_uniclust.hhr |

#### Structure prediction (AlphaFold)2

|  |  |
| --- | --- |
| Stats | xml version="1.0" encoding="utf-8" standalone="no"?       2024-09-02T21:09:20.281047 image/svg+xml   Matplotlib v3.7.2, https://matplotlib.org/ |
| Predicted structure | **NGL Viewer Controls:**  - Center: *Left-Click* - Rotate: *Left-Click + Drag* - Translate: *Right-Click + Drag* - Zoom: *Shift + Left-Click + Drag* |
| Output files | ../../predicted\_structures/21\_FANPEZAQ\_CDS\_0021/features.pkl ../../predicted\_structures/21\_FANPEZAQ\_CDS\_0021/ranked\_0.pdb ../../predicted\_structures/21\_FANPEZAQ\_CDS\_0021/ranked\_0\_plots.svg ../../predicted\_structures/21\_FANPEZAQ\_CDS\_0021/result\_model\_1\_ptm\_pred\_0.pkl |

#### Structure similarity search results (Foldseek)3

|  |  |
| --- | --- |
| Structure databases searched | Pdb, Afdb-proteome, Afdb-uniprot50 |
| Results, scheme(s)  (Top layers only, threshold 1.00e-02 (evalue)) | xml version="1.0" encoding="utf-8" standalone="no"?       2024-09-02T21:10:44.906509 image/svg+xml   Matplotlib v3.7.2, https://matplotlib.org/ |
| Results, table  (threshold 1.00e-02 (evalue)) | -- |
| Top keywords  (threshold 1.00e-02 (evalue)) | -- |
| Output files | ../../similar\_structures/21\_FANPEZAQ\_CDS\_0021\_afdb-proteome\_foldseek.tsv ../../similar\_structures/21\_FANPEZAQ\_CDS\_0021\_afdb-uniprot50\_foldseek.tsv ../../similar\_structures/21\_FANPEZAQ\_CDS\_0021\_merged.svg ../../similar\_structures/21\_FANPEZAQ\_CDS\_0021\_pdb\_foldseek.tsv |

  
  
  

Return to summary | Go to previous | Go to next

  


---

**Sequence/structure alignments coloring**  
Each object in the alignment figures is colored according to its E-value following this color coding:

1e-100
10

**References:**  
1) Steinegger M, Meier M, Mirdita M, Vöhringer H, Haunsberger S J, and Söding J (2019) HH-suite3 for fast remote homology detection and deep protein annotation, BMC Bioinformatics, 473. doi: 10.1186/s12859-019-3019-7  
2) Jumper J, Evans R, Pritzel A, ..., Hassabis D (2021) Highly accurate protein structure prediction with AlphaFold, Nature, 596. doi: 10.1038/s41586-021-03819-2  
3) van Kempen M, Kim S, Tumescheit C, Mirdita M, Lee J, Gilchrist CLM, Söding J, and Steinegger M (2023) Fast and accurate protein structure search with Foldseek. Nature Biotechnology. doi: 10.1038/s41587-023-01773-0
