# Supplementary material for: Completing the BASEL phage collection to unlock hidden diversity for systematic exploration of phage–host interactions
Source: PLoS Biol. 2025 Apr 7;23(4):e3003063. doi: 10.1371/journal.pbio.3003063 (PMC11990801; doi:10.1371/journal.pbio.3003063)
Supplement: S2 Data — (ZIP) [file pbio.3003063.s009.zip › entries/22.html]

FANPEZAQ\_CDS\_0022


Return to summary | Go to previous | Go to next

|  |  |
| --- | --- |
| FANPEZAQ\_CDS\_0022 Page creation date: 02 Sep 2024, 12:00  Project folder: n/a  Input sequences file: Escherichia\_virus\_HeidiAbel.gb | tail tape measure phage tp901 domain\_containing core region phagemin\_tail length phage\_related minor putative fragment tape\_measure |

### Sequence information

|  |  |
| --- | --- |
| Name | FANPEZAQ\_CDS\_0022  22\_FANPEZAQ\_CDS\_0022 (pipeline id) |
| Imported annotations | Escherichia\_virus\_HeidiAbel Bas97 |
| Protein sequence | MANKKLNATITIGGAVSSSLKGAFGTVKSSVEQVGAAMAKLEREQRTLTNAIQTFGRQGK NVDSLRAKYAANVAAVDKLRTATERLKRVEDARERNLAKREQYKDGIMGTIALGATIAAP IKAAMDFESTVADIKKVVDAPKGADEDTFFKAISKQVLDMSKTLPMTANEIGRIFALGGQ SGIATEDLGKFTESAVKMGVAFDVSAEQAGQSMAELRSAFGMTQDQVNTLADQMNYLDNN GSAAAKDIMEIVQRIGPLAKVAGVSAGQVAALGSTLRGMGVQNEIAATGIKNLFLTLASG DAATKKQKEVFKQLGYTSKEVSKSMQLDAEKTMTVILKQISKLPKHAQAAALTELFGKEV VGSIAPLMTRLGELQKNFDAVADRTKYAGSMQKEFEARAATTANQLILFRNQITGLGVTI GSVLLPAVNSVLQTVGPWIGKITELADAHPVVTKAIVATAGALITMRIATFAAGFAFTYL RGGALRVVGALAGARAQMALTAVSARAVGAAATVANGGLVGMASRGIPAVVTGVRAIGAA FISTGIGALITGLALGGLWIYRNWAGVKAFMTGTLQGIQQGLQPVTEKFQALWDRLGPVK TAFQWVADAAGKAWDWFTKLAEPVSYSGEELKKAGDAGQQFGKALAAGIDYVLGPIQFLI DKIIWVSDNIGALTQKAVEFKNSVSDMAGGAWQKTKDFFSNPFGSDDVPEGNTLATPLTP NNALPAPALANRGGSTYTDSSTTTIQVTQRPGENNADLAKRIADEQERRRQVRQRSMMND GVTAP |
| Number of residues | 785 |
| Molecular weight (Da) | 82722.95 |
| Output files | ../../query\_sequences/22\_FANPEZAQ\_CDS\_0022.fasta |

### Putative domain architecture and protein family

#### Search results (HHblits)1

|  |  |
| --- | --- |
| Domain family databases searched | Pfam, Ncbi-cd, Cath, Phrogs |
| Results, scheme(s)  (Top layers only; threshold 1.00e-03 (evalue)) | xml version="1.0" encoding="utf-8" standalone="no"?       2024-09-02T21:08:16.903637 image/svg+xml   Matplotlib v3.7.2, https://matplotlib.org/ |
| Results, table  (E-value ≤ 1.00e-03 (evalue)) | | db | id | prob | evalue | pvalue | score | cols | query | query\_len | template | template\_len | name | description | | --- | --- | --- | --- | --- | --- | --- | --- | --- | --- | --- | --- | --- | | pfam | PF10145 | 95.2 | 9.7e-05 | 2.8e-08 | 57.2 | 90 | (206, 297) | 785 | (55, 146) | 200 | PhageMin\_Tail | Phage-related minor tail protein | | phrogs | 28 | 100.0 | 4.4e-44 | 5.6e-48 | 424.9 | 628 | (1, 665) | 785 | (1, 648) | 820 | tail length tape measure protein | tail length tape measure protein; Category: tail; p348226 VI\_02582 | | phrogs | 991 | 100.0 | 2e-42 | 2.5e-46 | 400.6 | 467 | (107, 706) | 785 | (155, 628) | 767 | tail length tape measure protein | tail length tape measure protein; Category: tail; p290120 VI\_00584 | | phrogs | 4704 | 100.0 | 2.5e-39 | 2.9e-43 | 342.8 | 420 | (15, 455) | 785 | (31, 496) | 576 | tail length tape measure protein | tail length tape measure protein; Category: tail; p73956 VI\_01292 | | phrogs | 1293 | 100.0 | 2.6e-37 | 3.1e-41 | 363.6 | 430 | (15, 464) | 785 | (30, 504) | 1391 | tail length tape measure protein | tail length tape measure protein; Category: tail; p160953 VI\_04898 | | phrogs | 5218 | 100.0 | 2.1e-34 | 2.5e-38 | 330.9 | 432 | (2, 455) | 785 | (12, 495) | 1296 | NA | NA; Category: unknown function; p362597 VI\_05060 | | phrogs | 1272 | 99.9 | 6.9e-31 | 8.3e-35 | 298.3 | 436 | (112, 596) | 785 | (45, 513) | 878 | tail length tape measure protein | tail length tape measure protein; Category: tail; p177841 VI\_05919 | | phrogs | 5493 | 99.9 | 8.1e-31 | 9.4e-35 | 290.2 | 324 | (105, 444) | 785 | (46, 388) | 1111 | tail length tape measure protein | tail length tape measure protein; Category: tail; p353434 VI\_05194 | | phrogs | 1533 | 99.9 | 1.1e-29 | 1.3e-33 | 289.9 | 267 | (181, 461) | 785 | (2, 291) | 954 | tail length tape measure protein | tail length tape measure protein; Category: tail; p53969 VI\_05779 | | phrogs | 19074 | 99.9 | 2e-28 | 2.3e-32 | 263.2 | 309 | (112, 439) | 785 | (273, 589) | 1750 | NA | NA; Category: unknown function; p33998 VI\_01128 | | phrogs | 16692 | 99.8 | 2.3e-26 | 2.6e-30 | 244.2 | 323 | (107, 448) | 785 | (123, 454) | 947 | NA | NA; Category: unknown function; p85627 VI\_04017 | | phrogs | 794 | 99.8 | 3.3e-24 | 4e-28 | 240.7 | 340 | (112, 471) | 785 | (137, 535) | 698 | tail length tape measure protein | tail length tape measure protein; Category: tail; p192014 VI\_06934 | | phrogs | 5690 | 99.7 | 1.4e-22 | 1.7e-26 | 199.6 | 138 | (537, 681) | 785 | (40, 177) | 347 | NA | NA; Category: unknown function; p270016 VI\_07747 | | phrogs | 3654 | 99.7 | 1.7e-22 | 1.9e-26 | 210.3 | 95 | (2, 96) | 785 | (3, 97) | 612 | NA | NA; Category: unknown function; p148691 VI\_02621 | | phrogs | 9228 | 99.7 | 2e-22 | 2.3e-26 | 224.5 | 435 | (110, 590) | 785 | (208, 659) | 1359 | tail length tape measure protein | tail length tape measure protein; Category: tail; NC\_009993\_p20 | | phrogs | 4211 | 99.7 | 1.3e-21 | 1.6e-25 | 230.1 | 318 | (109, 447) | 785 | (346, 673) | 2292 | tail length tape measure protein | tail length tape measure protein; Category: tail; p43110 VI\_00473 | | phrogs | 5448 | 99.6 | 9.5e-21 | 1.1e-24 | 216.7 | 347 | (106, 460) | 785 | (448, 829) | 3327 | tail length tape measure protein | tail length tape measure protein; Category: tail; NC\_030924\_p25 | | phrogs | 19282 | 99.6 | 6.7e-20 | 7.6e-24 | 178.8 | 310 | (6, 324) | 785 | (1, 336) | 434 | NA | NA; Category: unknown function; p400074 VI\_09057 | | phrogs | 28420 | 99.6 | 1.6e-19 | 1.8e-23 | 183.8 | 303 | (113, 443) | 785 | (47, 350) | 873 | tail length tape measure protein | tail length tape measure protein; Category: tail; p93440 VI\_05600 | | phrogs | 36688 | 99.5 | 1.9e-19 | 2.1e-23 | 188.5 | 325 | (112, 453) | 785 | (62, 388) | 1303 | tail length tape measure protein | tail length tape measure protein; Category: tail; KP836355\_p32 | | phrogs | 5755 | 99.5 | 3.5e-19 | 4e-23 | 175.7 | 382 | (1, 423) | 785 | (1, 436) | 441 | tail length tape measure protein | tail length tape measure protein; Category: tail; p325579 VI\_04535 | | phrogs | 34270 | 99.5 | 4.1e-19 | 4.6e-23 | 175.3 | 318 | (108, 440) | 785 | (61, 386) | 606 | tail length tape measure protein | tail length tape measure protein; Category: tail; NC\_025427\_p16 | | phrogs | 14917 | 99.5 | 7.7e-19 | 8.7e-23 | 190.8 | 306 | (101, 424) | 785 | (314, 624) | 2249 | tail length tape measure protein | tail length tape measure protein; Category: tail; NC\_022774\_p53 | | phrogs | 37649 | 99.4 | 4.5e-18 | 5.1e-22 | 182.1 | 317 | (108, 444) | 785 | (332, 650) | 1827 | tail length tape measure protein | tail length tape measure protein; Category: tail; NC\_029075\_p20 | | phrogs | 36752 | 99.4 | 4.6e-18 | 5.2e-22 | 174.1 | 326 | (119, 471) | 785 | (71, 406) | 1076 | NA | NA; Category: unknown function; p212730 VI\_04312 | | phrogs | 10030 | 99.3 | 5.6e-17 | 6.4e-21 | 178.5 | 352 | (107, 465) | 785 | (171, 536) | 2102 | tail length tape measure protein | tail length tape measure protein; Category: tail; MG757153\_p31 | | phrogs | 35707 | 99.3 | 7.1e-17 | 8e-21 | 161.5 | 324 | (114, 443) | 785 | (72, 398) | 715 | tail length tape measure protein | tail length tape measure protein; Category: tail; p193300 VI\_04290 | | phrogs | 17727 | 99.3 | 7.6e-17 | 8.6e-21 | 155.9 | 283 | (277, 627) | 785 | (53, 341) | 448 | tail length tape measure protein | tail length tape measure protein; Category: tail; p417437 VI\_07567 | | phrogs | 8462 | 99.3 | 9.4e-17 | 1.1e-20 | 168.7 | 235 | (347, 595) | 785 | (171, 418) | 671 | tail length tape measure protein | tail length tape measure protein; Category: tail; p424879 VI\_01041 | | phrogs | 10780 | 99.3 | 1.1e-16 | 1.3e-20 | 179.7 | 311 | (108, 439) | 785 | (63, 385) | 1884 | tail length tape measure protein | tail length tape measure protein; Category: tail; NC\_030942\_p18 | | phrogs | 2723 | 99.3 | 1.7e-16 | 2e-20 | 168.5 | 346 | (111, 469) | 785 | (162, 608) | 1577 | tail length tape measure protein | tail length tape measure protein; Category: tail; JN006062\_p23 | | phrogs | 15478 | 99.3 | 1.8e-16 | 2e-20 | 154.1 | 325 | (286, 706) | 785 | (3, 341) | 473 | tail length tape measure protein | tail length tape measure protein; Category: tail; NC\_019506\_p24 | | phrogs | 973 | 99.3 | 2.2e-16 | 2.7e-20 | 171.3 | 297 | (119, 440) | 785 | (71, 375) | 583 | tail length tape measure protein | tail length tape measure protein; Category: tail; p232472 VI\_03267 | | phrogs | 38390 | 99.2 | 9.7e-16 | 1.1e-19 | 154.8 | 294 | (112, 423) | 785 | (48, 341) | 1029 | tail length tape measure protein | tail length tape measure protein; Category: tail; NC\_015466\_p80 | | phrogs | 30543 | 99.2 | 1.5e-15 | 1.7e-19 | 154.6 | 325 | (108, 432) | 785 | (52, 381) | 1167 | tail length tape measure protein | tail length tape measure protein; Category: tail; MG711466\_p67 | | phrogs | 8125 | 99.2 | 3.1e-15 | 3.5e-19 | 157.3 | 373 | (251, 701) | 785 | (210, 597) | 662 | tail length tape measure protein | tail length tape measure protein; Category: tail; p114197 VI\_11397 | | phrogs | 23013 | 99.1 | 1.1e-14 | 1.3e-18 | 151.2 | 333 | (121, 471) | 785 | (132, 467) | 1379 | tail length tape measure protein | tail length tape measure protein; Category: tail; JF713456\_p61 | | phrogs | 4200 | 99.0 | 2.6e-14 | 3e-18 | 153.0 | 107 | (422, 584) | 785 | (141, 247) | 810 | tail length tape measure protein | tail length tape measure protein; Category: tail; p335142 VI\_05069 | | phrogs | 339 | 99.0 | 3.9e-14 | 4.7e-18 | 163.4 | 307 | (108, 447) | 785 | (76, 387) | 1128 | tail length tape measure protein | tail length tape measure protein; Category: tail; p256393 VI\_10266 | | phrogs | 1068 | 98.9 | 1.1e-13 | 1.3e-17 | 152.2 | 191 | (17, 217) | 785 | (11, 208) | 788 | tail length tape measure protein | tail length tape measure protein; Category: tail; p214035 VI\_06128 | | phrogs | 17594 | 98.9 | 2.8e-13 | 3.1e-17 | 144.4 | 339 | (112, 456) | 785 | (166, 513) | 1580 | tail length tape measure protein | tail length tape measure protein; Category: tail; MF185731\_p36 | | phrogs | 3336 | 98.8 | 4.7e-13 | 5.6e-17 | 149.9 | 313 | (108, 595) | 785 | (185, 508) | 1052 | tail length tape measure protein | tail length tape measure protein; Category: tail; NC\_029097\_p21 | | phrogs | 26705 | 98.8 | 7.4e-13 | 8.3e-17 | 136.9 | 298 | (119, 443) | 785 | (301, 599) | 1303 | NA | NA; Category: unknown function; p49245 VI\_05491 | | phrogs | 58 | 98.5 | 1.6e-11 | 2e-15 | 145.7 | 58 | (538, 595) | 785 | (663, 723) | 1263 | tail length tape measure protein | tail length tape measure protein; Category: tail; p145088 VI\_05474 | | phrogs | 9696 | 98.5 | 2.2e-11 | 2.6e-15 | 108.6 | 112 | (107, 223) | 785 | (64, 175) | 175 | tail length tape measure protein | tail length tape measure protein; Category: tail; p98305 VI\_00740 | | phrogs | 3824 | 98.5 | 2.8e-11 | 3.2e-15 | 126.5 | 192 | (247, 458) | 785 | (71, 272) | 682 | NA | NA; Category: unknown function; NC\_031117\_p21 | | phrogs | 17321 | 98.4 | 4.4e-11 | 4.9e-15 | 123.2 | 194 | (108, 301) | 785 | (74, 269) | 1484 | NA | NA; Category: unknown function; p152511 VI\_00610 | | phrogs | 17114 | 98.3 | 1.1e-10 | 1.3e-14 | 106.6 | 147 | (538, 691) | 785 | (24, 170) | 369 | NA | NA; Category: unknown function; p385881 VI\_10695 | | phrogs | 23108 | 98.2 | 4.2e-10 | 4.8e-14 | 120.8 | 324 | (109, 443) | 785 | (307, 643) | 1401 | tail length tape measure protein | tail length tape measure protein; Category: tail; NC\_025463\_p37 | | phrogs | 13339 | 98.1 | 6.8e-10 | 7.7e-14 | 91.2 | 130 | (161, 290) | 785 | (2, 131) | 133 | tail length tape measure protein | tail length tape measure protein; Category: tail; p142514 VI\_00726 | | phrogs | 4200 | 98.0 | 2.2e-09 | 2.5e-13 | 114.5 | 145 | (554, 707) | 785 | (459, 616) | 810 | tail length tape measure protein | tail length tape measure protein; Category: tail; p335142 VI\_05069 | | phrogs | 3981 | 97.9 | 2.9e-09 | 3.4e-13 | 112.3 | 48 | (396, 444) | 785 | (257, 304) | 730 | tail length tape measure protein | tail length tape measure protein; Category: tail; p408809 VI\_04997 | | phrogs | 14441 | 97.9 | 3e-09 | 3.5e-13 | 92.0 | 108 | (101, 211) | 785 | (56, 163) | 164 | tail length tape measure protein | tail length tape measure protein; Category: tail; p275139 VI\_05876 | | phrogs | 4200 | 97.9 | 3.5e-09 | 4e-13 | 112.8 | 137 | (555, 710) | 785 | (394, 531) | 810 | tail length tape measure protein | tail length tape measure protein; Category: tail; p335142 VI\_05069 | | phrogs | 4197 | 97.8 | 5.6e-09 | 6.5e-13 | 118.6 | 59 | (535, 593) | 785 | (950, 1011) | 1729 | tail length tape measure protein | tail length tape measure protein; Category: tail; p189664 VI\_00038 | | phrogs | 34405 | 97.8 | 6.7e-09 | 7.4e-13 | 109.9 | 295 | (116, 422) | 785 | (162, 475) | 2675 | tail length tape measure protein | tail length tape measure protein; Category: tail; MF001361\_p11 | | phrogs | 4372 | 97.6 | 3.1e-08 | 3.6e-12 | 112.1 | 104 | (116, 219) | 785 | (187, 291) | 1826 | tail length tape measure protein | tail length tape measure protein; Category: tail; MF467948\_p23 | | phrogs | 6090 | 97.5 | 4.5e-08 | 5.3e-12 | 110.7 | 52 | (393, 444) | 785 | (640, 691) | 1808 | tail length tape measure protein | tail length tape measure protein; Category: tail; MF919510\_p42 | | phrogs | 32669 | 97.5 | 5.7e-08 | 6.3e-12 | 97.3 | 141 | (536, 678) | 785 | (693, 873) | 1036 | NA | NA; Category: unknown function; p276907 VI\_03881 | | phrogs | 27190 | 97.5 | 7.4e-08 | 8.3e-12 | 97.3 | 128 | (83, 217) | 785 | (75, 206) | 1014 | tail length tape measure protein | tail length tape measure protein; Category: tail; p213007 VI\_01051 | | phrogs | 1686 | 97.3 | 1.8e-07 | 2.1e-11 | 103.2 | 57 | (539, 595) | 785 | (681, 741) | 1788 | tail length tape measure protein | tail length tape measure protein; Category: tail; NC\_024384\_p40 | | phrogs | 34082 | 97.2 | 2.6e-07 | 3e-11 | 88.4 | 131 | (537, 706) | 785 | (290, 421) | 529 | tail length tape measure protein | tail length tape measure protein; Category: tail; MH019216\_p23 | | phrogs | 21157 | 97.2 | 2.8e-07 | 3.2e-11 | 93.4 | 141 | (419, 581) | 785 | (575, 719) | 768 | minor tail protein | minor tail protein; Category: tail; NC\_002668\_p50 | | phrogs | 1204 | 97.2 | 3.1e-07 | 3.7e-11 | 105.3 | 126 | (122, 271) | 785 | (79, 205) | 1355 | tail protein | tail protein; Category: tail; p387522 VI\_00450 | | phrogs | 8927 | 97.1 | 6.2e-07 | 7.2e-11 | 100.4 | 146 | (148, 303) | 785 | (237, 395) | 1569 | tail length tape measure protein | tail length tape measure protein; Category: tail; KY203335\_p44 | | phrogs | 16297 | 97.0 | 7.6e-07 | 8.6e-11 | 89.1 | 108 | (108, 221) | 785 | (77, 188) | 776 | tail length tape measure protein | tail length tape measure protein; Category: tail; p238715 VI\_06415 | | phrogs | 13137 | 97.0 | 7.9e-07 | 8.8e-11 | 86.0 | 161 | (112, 273) | 785 | (345, 526) | 541 | tail length tape measure protein | tail length tape measure protein; Category: tail; p31102 VI\_05860 | | phrogs | 971 | 96.9 | 1.1e-06 | 1.3e-10 | 98.5 | 43 | (530, 572) | 785 | (416, 461) | 1164 | endolysin | endolysin; Category: lysis; NC\_031076\_p13 | | phrogs | 29312 | 96.9 | 1.5e-06 | 1.6e-10 | 82.8 | 242 | (22, 278) | 785 | (305, 547) | 558 | tail length tape measure protein | tail length tape measure protein; Category: tail; NC\_007581\_p131 | | phrogs | 2335 | 96.8 | 1.8e-06 | 2.1e-10 | 97.7 | 143 | (61, 213) | 785 | (55, 201) | 1433 | tail length tape measure protein | tail length tape measure protein; Category: tail; p247749 VI\_04302 | | phrogs | 3519 | 96.8 | 1.8e-06 | 2.2e-10 | 88.1 | 104 | (111, 220) | 785 | (75, 182) | 448 | tail length tape measure protein | tail length tape measure protein; Category: tail; p424447 VI\_08065 | | phrogs | 10613 | 96.7 | 3.6e-06 | 4.2e-10 | 71.5 | 84 | (3, 89) | 785 | (2, 97) | 138 | tail length tape measure protein | tail length tape measure protein; Category: tail; p365128 VI\_01223 | | phrogs | 8927 | 96.6 | 5.4e-06 | 6.2e-10 | 92.8 | 39 | (211, 249) | 785 | (223, 261) | 1569 | tail length tape measure protein | tail length tape measure protein; Category: tail; KY203335\_p44 | | phrogs | 1535 | 96.5 | 6.7e-06 | 7.9e-10 | 92.6 | 58 | (533, 590) | 785 | (845, 905) | 1217 | tail length tape measure protein | tail length tape measure protein; Category: tail; p395424 VI\_04399 | | phrogs | 9388 | 96.4 | 1.1e-05 | 1.3e-09 | 81.6 | 44 | (535, 578) | 785 | (399, 442) | 629 | NA | NA; Category: unknown function; p379982 VI\_11797 | | phrogs | 38386 | 96.4 | 1.2e-05 | 1.3e-09 | 82.8 | 134 | (536, 677) | 785 | (771, 941) | 1703 | NA | NA; Category: unknown function; p413626 VI\_12283 | | phrogs | 26805 | 96.4 | 1.2e-05 | 1.3e-09 | 70.5 | 56 | (537, 592) | 785 | (167, 222) | 253 | NA | NA; Category: unknown function; p228647 VI\_05149 | | phrogs | 12798 | 96.3 | 1.4e-05 | 1.6e-09 | 86.0 | 112 | (110, 227) | 785 | (53, 178) | 1052 | tail length tape measure protein | tail length tape measure protein; Category: tail; p167255 VI\_04008 | | phrogs | 8010 | 96.1 | 2.9e-05 | 3.4e-09 | 74.1 | 107 | (108, 220) | 785 | (72, 182) | 325 | tail length tape measure protein | tail length tape measure protein; Category: tail; p301250 VI\_08079 | | phrogs | 38258 | 96.0 | 3.8e-05 | 4.2e-09 | 70.3 | 63 | (533, 595) | 785 | (165, 237) | 427 | NA | NA; Category: unknown function; p292485 VI\_04908 | | phrogs | 26804 | 95.9 | 4.7e-05 | 5.2e-09 | 76.3 | 73 | (398, 470) | 785 | (291, 363) | 1065 | NA | NA; Category: unknown function; p121571 VI\_12455 | | phrogs | 34923 | 95.2 | 0.00022 | 2.4e-08 | 67.4 | 179 | (260, 455) | 785 | (145, 330) | 537 | tail length tape measure protein | tail length tape measure protein; Category: tail; MG675557\_p40 | | phrogs | 19166 | 95.2 | 0.00024 | 2.8e-08 | 61.2 | 111 | (111, 227) | 785 | (47, 159) | 186 | NA | NA; Category: unknown function; p255465 VI\_06352 | | phrogs | 305 | 95.1 | 0.00027 | 3.3e-08 | 79.2 | 69 | (644, 712) | 785 | (797, 867) | 1015 | tail length tape measure protein | tail length tape measure protein; Category: tail; MF975638\_p8 | | phrogs | 17020 | 95.0 | 0.00033 | 3.8e-08 | 62.2 | 25 | (651, 675) | 785 | (80, 104) | 238 | NA | NA; Category: unknown function; p388498 VI\_05144 | | phrogs | 15002 | 94.8 | 0.00044 | 5e-08 | 56.7 | 88 | (1, 95) | 785 | (1, 119) | 144 | NA | NA; Category: unknown function; p434568 VI\_05139 | | phrogs | 9959 | 94.8 | 0.00044 | 5.1e-08 | 76.5 | 93 | (108, 206) | 785 | (59, 151) | 1333 | tail length tape measure protein | tail length tape measure protein; Category: tail; p39219 VI\_01014 | | phrogs | 7740 | 94.5 | 0.00064 | 7.5e-08 | 63.4 | 144 | (108, 272) | 785 | (72, 220) | 255 | tail length tape measure protein | tail length tape measure protein; Category: tail; p336818 VI\_11619 | | phrogs | 33100 | 94.5 | 0.00068 | 7.5e-08 | 50.8 | 75 | (148, 222) | 785 | (13, 87) | 109 | NA | NA; Category: unknown function; p346208 VI\_06081 | | phrogs | 25337 | 94.2 | 0.001 | 1.2e-07 | 68.4 | 174 | (119, 299) | 785 | (190, 372) | 1419 | tail length tape measure protein | tail length tape measure protein; Category: tail; NC\_031926\_p4 | |
| Top keywords  (threshold 1.00e-03 (evalue)) | **tail, length, tape, measure, p335142, VI\_05069, minor, KY203335\_p44, Phage\_related, p348226** |
| Output files | ../../domain\_architecture/22\_FANPEZAQ\_CDS\_0022\_cath.hhr ../../domain\_architecture/22\_FANPEZAQ\_CDS\_0022\_merged.svg ../../domain\_architecture/22\_FANPEZAQ\_CDS\_0022\_ncbi-cd.hhr ../../domain\_architecture/22\_FANPEZAQ\_CDS\_0022\_pfam.hhr ../../domain\_architecture/22\_FANPEZAQ\_CDS\_0022\_phrogs.hhr |

### Identical protein sequences/structures

#### Search results

|  |  |
| --- | --- |
| Protein sequence databases searched | Pdb, Swissprot, Refseq |
| Identical proteins found | -- |
| Top keywords | -- |
| Output files | -- |

### Similar protein sequences/structures

#### Sequence similarity search results (HHblits)1

|  |  |
| --- | --- |
| Sequence databases searched | Uniclust, Pdb70 |
| Results, scheme(s)  (Top layers only, threshold 1.00e-03 (evalue)) | xml version="1.0" encoding="utf-8" standalone="no"?       2024-09-02T21:08:38.711127 image/svg+xml   Matplotlib v3.7.2, https://matplotlib.org/ |
| Results, table(s)  (threshold 1.00e-03 (evalue)) | | db | id | prob | evalue | pvalue | score | cols | query | query\_len | template | template\_len | name | description | | --- | --- | --- | --- | --- | --- | --- | --- | --- | --- | --- | --- | --- | | uniclust | UniRef100\_A0A088FV68 | 100.0 | 9.9e-84 | 1.8e-89 | 618.7 | 770 | (1, 785) | 785 | (6, 777) | 777 | Tail protein | Tail protein | | uniclust | UniRef100\_A0A060GZT2 | 100.0 | 4.9e-79 | 8.6e-85 | 629.3 | 762 | (1, 785) | 785 | (66, 836) | 925 | Phage tail protein | Phage tail protein | | uniclust | UniRef100\_A0A011NAN7 | 100.0 | 1.5e-71 | 2.7e-77 | 556.5 | 756 | (4, 782) | 785 | (90, 925) | 925 | Tail protein | Tail protein | | uniclust | UniRef100\_A0A061JKM9 | 100.0 | 1.9e-70 | 3.4e-76 | 574.7 | 735 | (4, 784) | 785 | (79, 856) | 1009 | Phage tail tape measure protein domain-containing protein | Phage tail tape measure protein domain-containing protein | | uniclust | UniRef100\_A0A126R353 | 100.0 | 2.4e-70 | 4.2e-76 | 557.8 | 731 | (5, 783) | 785 | (80, 845) | 851 | Putative phage-related membrane protein | Putative phage-related membrane protein | | uniclust | UniRef100\_A0A031GY25 | 100.0 | 9.5e-70 | 1.7e-75 | 572.0 | 743 | (4, 783) | 785 | (116, 902) | 907 | Bacteriophage-related tail transmembrane protein | Bacteriophage-related tail transmembrane protein | | uniclust | UniRef100\_A0A0C3DQL6 | 100.0 | 3.4e-68 | 6e-74 | 554.7 | 562 | (4, 589) | 785 | (78, 658) | 946 | Phage tail tape measure protein domain-containing protein | Phage tail tape measure protein domain-containing protein | | uniclust | UniRef100\_A0A081MYF0 | 100.0 | 9.2e-66 | 1.6e-71 | 538.1 | 741 | (4, 784) | 785 | (78, 852) | 862 | Phage tail tape measure protein domain-containing protein | Phage tail tape measure protein domain-containing protein | | uniclust | UniRef100\_A0A0M1HSJ1 | 100.0 | 1.9e-65 | 3.3e-71 | 536.8 | 565 | (1, 586) | 785 | (79, 654) | 877 | Membrane protein | Membrane protein | | uniclust | UniRef100\_A0A0A6TUZ1 | 100.0 | 1e-63 | 1.8e-69 | 487.5 | 611 | (4, 633) | 785 | (79, 699) | 966 | Phage-related tail protein | Phage-related tail protein | | uniclust | UniRef100\_A0A062I8W3 | 100.0 | 1.8e-62 | 3.1e-68 | 493.3 | 732 | (17, 783) | 785 | (89, 849) | 851 | Phage tail tape measure protein, TP901 family, core region (Fragment) | Phage tail tape measure protein, TP901 family, core region (Fragment) | | uniclust | UniRef100\_A0A077L0E5 | 100.0 | 3.5e-61 | 6.2e-67 | 487.1 | 634 | (4, 646) | 785 | (61, 729) | 1047 | Phage tail tape measure protein domain-containing protein | Phage tail tape measure protein domain-containing protein | | uniclust | UniRef100\_A0A077NKH9 | 100.0 | 1.1e-60 | 2e-66 | 490.4 | 650 | (5, 674) | 785 | (86, 747) | 984 | Putative bacteriophage tail fiber protein T | Putative bacteriophage tail fiber protein T | | uniclust | UniRef100\_A0A1G6JE81 | 100.0 | 5.9e-60 | 1e-65 | 468.8 | 714 | (1, 785) | 785 | (1, 724) | 725 | Phage tail tape measure protein, TP901 family, core region | Phage tail tape measure protein, TP901 family, core region | | uniclust | UniRef100\_A0A081N7U2 | 100.0 | 9.9e-60 | 1.8e-65 | 467.2 | 569 | (4, 579) | 785 | (74, 662) | 806 | Phage tail tape measure protein domain-containing protein | Phage tail tape measure protein domain-containing protein | | uniclust | UniRef100\_A0A0A1HVB3 | 100.0 | 1e-59 | 1.9e-65 | 481.5 | 736 | (4, 783) | 785 | (134, 941) | 944 | Phage tail tape measure protein, TP901 family | Phage tail tape measure protein, TP901 family | | uniclust | UniRef100\_A0A1E3G7C9 | 100.0 | 3e-59 | 5.4e-65 | 461.1 | 558 | (4, 586) | 785 | (79, 644) | 783 | Phage tail tape measure protein | Phage tail tape measure protein | | uniclust | UniRef100\_A0A0A2XLQ8 | 100.0 | 3.9e-59 | 7e-65 | 470.3 | 579 | (106, 703) | 785 | (60, 678) | 811 | Phage tail protein | Phage tail protein | | uniclust | UniRef100\_A0A0D7V1W3 | 100.0 | 2.3e-58 | 4.1e-64 | 464.5 | 732 | (4, 782) | 785 | (78, 887) | 887 | Phage tail tape measure protein domain-containing protein | Phage tail tape measure protein domain-containing protein | | uniclust | UniRef100\_A0A2P5N6P9 | 100.0 | 4.1e-58 | 7.3e-64 | 445.5 | 531 | (1, 588) | 785 | (23, 562) | 676 | Phage tail tape measure protein | Phage tail tape measure protein | | uniclust | UniRef100\_A0A022PGH9 | 100.0 | 6.5e-58 | 1.2e-63 | 453.7 | 629 | (118, 783) | 785 | (208, 887) | 889 | Phage tail tape measure protein, TP901 family | Phage tail tape measure protein, TP901 family | | uniclust | UniRef100\_A0A2P8QYQ4 | 100.0 | 3.1e-57 | 5.5e-63 | 461.0 | 600 | (63, 672) | 785 | (95, 715) | 833 | Phage tail tape measure protein | Phage tail tape measure protein | | uniclust | UniRef100\_A0A080IDD0 | 100.0 | 4.4e-55 | 7.9e-61 | 433.4 | 621 | (5, 675) | 785 | (78, 720) | 944 | Phage tail tape measure protein, TP901 family, core region | Phage tail tape measure protein, TP901 family, core region | | uniclust | UniRef100\_A0A009QNM6 | 100.0 | 5.2e-54 | 9.2e-60 | 442.3 | 724 | (4, 784) | 785 | (80, 893) | 893 | Putative bacteriophage tail protein gpT | Putative bacteriophage tail protein gpT | | uniclust | UniRef100\_A0A014N9D9 | 100.0 | 6.3e-54 | 1.1e-59 | 451.1 | 623 | (4, 668) | 785 | (80, 729) | 921 | Tail protein (Fragment) | Tail protein (Fragment) | | uniclust | UniRef100\_A0A017HCD3 | 100.0 | 1.2e-53 | 2.2e-59 | 449.9 | 482 | (111, 598) | 785 | (175, 678) | 941 | Phage tail length tape-measure protein | Phage tail length tape-measure protein | | uniclust | UniRef100\_A0A080ITW0 | 100.0 | 1.6e-53 | 2.8e-59 | 424.5 | 655 | (5, 674) | 785 | (95, 779) | 953 | Phage tail tape measure protein, TP901 family, core region | Phage tail tape measure protein, TP901 family, core region | | uniclust | UniRef100\_A0A0G9K5Q8 | 100.0 | 3e-53 | 5.3e-59 | 420.1 | 552 | (4, 585) | 785 | (73, 625) | 782 | Tail tape measure protein | Tail tape measure protein | | uniclust | UniRef100\_A0A0D0HTB3 | 100.0 | 3.8e-53 | 6.8e-59 | 422.4 | 529 | (17, 594) | 785 | (169, 731) | 1047 | Phage-related minor tail protein | Phage-related minor tail protein | | uniclust | UniRef100\_A0A0E2H9C3 | 100.0 | 4e-53 | 7.1e-59 | 455.9 | 471 | (110, 626) | 785 | (392, 872) | 1237 | Phage tail tape measure protein, TP901 family, core region | Phage tail tape measure protein, TP901 family, core region | | uniclust | UniRef100\_A0A0A0GNB0 | 100.0 | 7.3e-53 | 1.3e-58 | 438.6 | 643 | (20, 674) | 785 | (108, 773) | 998 | Phage tail tape measure protein domain-containing protein | Phage tail tape measure protein domain-containing protein | | uniclust | UniRef100\_A0A167GZN6 | 100.0 | 8.7e-53 | 1.6e-58 | 429.0 | 591 | (61, 660) | 785 | (210, 821) | 1235 | Transglycosylase | Transglycosylase | | uniclust | UniRef100\_A0A059V4B6 | 100.0 | 1.1e-52 | 2e-58 | 434.9 | 670 | (1, 702) | 785 | (134, 854) | 972 | Bacteriophage tail protein | Bacteriophage tail protein | | uniclust | UniRef100\_A0A0Q4IFW9 | 100.0 | 2.6e-52 | 4.7e-58 | 411.3 | 707 | (5, 783) | 785 | (80, 840) | 845 | Phage tail tape measure protein | Phage tail tape measure protein | | uniclust | UniRef100\_A0A0A3A3N3 | 100.0 | 3.8e-52 | 6.9e-58 | 409.4 | 562 | (4, 589) | 785 | (80, 663) | 778 | Tail protein (Fragment) | Tail protein (Fragment) | | uniclust | UniRef100\_A0A1M6SBV5 | 100.0 | 1.4e-51 | 2.4e-57 | 406.4 | 460 | (1, 465) | 785 | (75, 540) | 851 | Phage tail tape measure protein, TP901 family, core region (Fragment) | Phage tail tape measure protein, TP901 family, core region (Fragment) | | uniclust | UniRef100\_A0A075KAY1 | 100.0 | 1.5e-51 | 2.6e-57 | 420.2 | 561 | (1, 585) | 785 | (2, 574) | 760 | Phage tail tape measure protein, TP901 family | Phage tail tape measure protein, TP901 family | | uniclust | UniRef100\_A0A1V3IZV8 | 100.0 | 3.1e-51 | 5.5e-57 | 403.4 | 570 | (4, 583) | 785 | (78, 688) | 717 | Phage tail tape measure protein | Phage tail tape measure protein | | uniclust | UniRef100\_A0A0L1KF90 | 100.0 | 3.2e-51 | 5.6e-57 | 410.6 | 709 | (1, 784) | 785 | (1, 743) | 746 | Tp901 family phage tail tape measure protein | Tp901 family phage tail tape measure protein | | uniclust | UniRef100\_A0A496N6E3 | 100.0 | 2.3e-50 | 4.2e-56 | 388.0 | 568 | (26, 596) | 785 | (26, 644) | 844 | Phage tail tape measure protein (Fragment) | Phage tail tape measure protein (Fragment) | | uniclust | UniRef100\_A0A0K6GTP1 | 100.0 | 3.5e-50 | 6.3e-56 | 390.0 | 724 | (4, 782) | 785 | (79, 883) | 883 | Phage tail tape measure protein, TP901 family, core region | Phage tail tape measure protein, TP901 family, core region | | uniclust | UniRef100\_A0A062IV08 | 100.0 | 1.9e-49 | 3.4e-55 | 382.9 | 554 | (4, 584) | 785 | (79, 656) | 687 | Phage tail tape measure protein, TP901 family, core region (Fragment) | Phage tail tape measure protein, TP901 family, core region (Fragment) | | uniclust | UniRef100\_A0A059UU34 | 100.0 | 3.2e-49 | 5.7e-55 | 395.5 | 414 | (110, 584) | 785 | (122, 535) | 729 | Phage tail protein | Phage tail protein | | uniclust | UniRef100\_A0A099I5C4 | 100.0 | 4.3e-49 | 7.6e-55 | 410.4 | 551 | (1, 594) | 785 | (26, 628) | 1011 | Phage tail length tape measure protein | Phage tail length tape measure protein | | uniclust | UniRef100\_A0A2W6QW57 | 100.0 | 9.4e-49 | 1.7e-54 | 379.1 | 523 | (119, 675) | 785 | (196, 720) | 936 | Phage tail tape measure protein (Fragment) | Phage tail tape measure protein (Fragment) | | uniclust | UniRef100\_A0A095YJN6 | 100.0 | 1.3e-48 | 2.4e-54 | 389.2 | 375 | (109, 491) | 785 | (193, 567) | 1004 | Phage tail tape measure protein, TP901 family, core region | Phage tail tape measure protein, TP901 family, core region | | uniclust | UniRef100\_A0A6L5WL94 | 100.0 | 2.8e-48 | 5.1e-54 | 382.3 | 476 | (115, 596) | 785 | (106, 614) | 807 | Phage tail tape measure protein | Phage tail tape measure protein | | uniclust | UniRef100\_A0A484GDB7 | 100.0 | 6.8e-48 | 1.2e-53 | 370.5 | 648 | (4, 673) | 785 | (9, 728) | 902 | Phage tail tape measure protein | Phage tail tape measure protein | | uniclust | UniRef100\_A0A554WXF0 | 100.0 | 1.8e-47 | 3.3e-53 | 358.2 | 561 | (4, 594) | 785 | (72, 634) | 749 | Phage tail tape measure protein | Phage tail tape measure protein | | uniclust | UniRef100\_A0A5S9Q3L2 | 100.0 | 2.6e-47 | 4.8e-53 | 361.8 | 611 | (112, 782) | 785 | (173, 795) | 795 | Phage tail tape measure protein domain-containing protein | Phage tail tape measure protein domain-containing protein | | uniclust | UniRef100\_A0A022PG58 | 100.0 | 5.5e-47 | 9.7e-53 | 400.4 | 655 | (4, 674) | 785 | (163, 923) | 1097 | Phage tail tape measure protein, TP901 family | Phage tail tape measure protein, TP901 family | | uniclust | UniRef100\_A0A0H3ZXY1 | 100.0 | 5.4e-47 | 9.7e-53 | 382.2 | 733 | (4, 783) | 785 | (86, 881) | 939 | Phage tail length tape-measure protein | Phage tail length tape-measure protein | | uniclust | UniRef100\_A0A133QGT8 | 100.0 | 6.3e-47 | 1.1e-52 | 378.4 | 541 | (20, 593) | 785 | (47, 623) | 1124 | Phage tail tape measure protein, TP901 family | Phage tail tape measure protein, TP901 family | | uniclust | UniRef100\_A0A0B7D869 | 100.0 | 6.4e-47 | 1.2e-52 | 366.0 | 524 | (111, 695) | 785 | (110, 650) | 786 | Phage-related minor tail protein | Phage-related minor tail protein | | uniclust | UniRef100\_A0A2J4VH89 | 100.0 | 2.5e-46 | 4.5e-52 | 356.5 | 616 | (5, 670) | 785 | (78, 715) | 815 | Phage tail tape measure protein (Fragment) | Phage tail tape measure protein (Fragment) | | uniclust | UniRef100\_A0A0B5A7A7 | 100.0 | 2.8e-46 | 5.1e-52 | 380.4 | 420 | (111, 590) | 785 | (158, 580) | 994 | Membrane protein P6 | Membrane protein P6 | | uniclust | UniRef100\_A0A1B9KNC1 | 100.0 | 3.5e-46 | 6.5e-52 | 348.5 | 496 | (111, 625) | 785 | (51, 547) | 725 | Phage tail tape measure protein | Phage tail tape measure protein | | uniclust | UniRef100\_A0A1H0P6W7 | 100.0 | 4.9e-46 | 8.9e-52 | 378.7 | 464 | (1, 471) | 785 | (29, 496) | 1223 | Phage tail tape measure protein, TP901 family, core region | Phage tail tape measure protein, TP901 family, core region | | uniclust | UniRef100\_A0A0M4SUP3 | 100.0 | 2.1e-45 | 3.7e-51 | 357.8 | 478 | (113, 596) | 785 | (77, 588) | 749 | Phage tail tape measure protein, TP901 family | Phage tail tape measure protein, TP901 family | | uniclust | UniRef100\_A0A1H2M3U9 | 100.0 | 3.5e-45 | 6.5e-51 | 353.3 | 486 | (117, 617) | 785 | (332, 828) | 1105 | Phage tail tape measure protein, TP901 family, core region | Phage tail tape measure protein, TP901 family, core region | | uniclust | UniRef100\_A0A137SPW2 | 100.0 | 6.8e-45 | 1.2e-50 | 376.3 | 466 | (4, 488) | 785 | (81, 552) | 1180 | Phage tail tape measure protein, TP901 family | Phage tail tape measure protein, TP901 family | | uniclust | UniRef100\_A0A0R1KHJ8 | 100.0 | 1e-44 | 1.8e-50 | 377.2 | 457 | (110, 589) | 785 | (274, 737) | 1166 | Phage tail tape measure protein domain-containing protein | Phage tail tape measure protein domain-containing protein | | uniclust | UniRef100\_A0A0N8W5Z8 | 100.0 | 1.5e-44 | 2.8e-50 | 345.1 | 478 | (108, 622) | 785 | (57, 539) | 708 | Phage tail tape measure protein domain-containing protein | Phage tail tape measure protein domain-containing protein | | uniclust | UniRef100\_A0A844HR58 | 100.0 | 1.5e-44 | 2.8e-50 | 337.8 | 522 | (113, 667) | 785 | (11, 545) | 719 | Phage tail tape measure protein | Phage tail tape measure protein | | uniclust | UniRef100\_A0A0B5ATH8 | 100.0 | 1.7e-44 | 2.9e-50 | 374.6 | 543 | (4, 593) | 785 | (16, 597) | 973 | Phage tail tape measure protein domain-containing protein | Phage tail tape measure protein domain-containing protein | | uniclust | UniRef100\_A0A3A6WDZ8 | 100.0 | 2.1e-44 | 3.8e-50 | 355.7 | 561 | (1, 583) | 785 | (74, 661) | 853 | Phage tail tape measure protein | Phage tail tape measure protein | | uniclust | UniRef100\_A0A212KXI4 | 100.0 | 2.5e-44 | 4.4e-50 | 353.0 | 568 | (1, 576) | 785 | (1, 617) | 766 | Phage tail tape measure protein domain-containing protein | Phage tail tape measure protein domain-containing protein | | uniclust | UniRef100\_A0A143WZ13 | 100.0 | 2.9e-44 | 5.1e-50 | 374.1 | 423 | (112, 591) | 785 | (153, 581) | 890 | Phage-related minor tail protein | Phage-related minor tail protein | | uniclust | UniRef100\_A0A268TAT5 | 100.0 | 3.8e-44 | 6.7e-50 | 362.4 | 535 | (115, 664) | 785 | (119, 703) | 790 | Phage tail tape measure protein | Phage tail tape measure protein | | uniclust | UniRef100\_A0A375H2Y0 | 100.0 | 4.2e-44 | 7.6e-50 | 331.1 | 430 | (156, 594) | 785 | (4, 443) | 585 | Phage tail tape measure protein domain-containing protein | Phage tail tape measure protein domain-containing protein | | uniclust | UniRef100\_A0A376SBX2 | 100.0 | 4.8e-44 | 8.6e-50 | 338.9 | 508 | (117, 674) | 785 | (19, 533) | 630 | Tail length tape measure protein | Tail length tape measure protein | | uniclust | UniRef100\_A0A2X0VQT4 | 100.0 | 5.8e-44 | 1.1e-49 | 346.3 | 572 | (4, 624) | 785 | (73, 654) | 817 | Phage-related minor tail protein | Phage-related minor tail protein | | uniclust | UniRef100\_A0A068ZAA7 | 100.0 | 7.6e-44 | 1.4e-49 | 356.5 | 637 | (5, 675) | 785 | (79, 753) | 911 | Uncharacterized protein | Uncharacterized protein | | uniclust | UniRef100\_A0A0D0GQG2 | 100.0 | 8.7e-44 | 1.6e-49 | 366.3 | 746 | (4, 784) | 785 | (225, 1106) | 1154 | Uncharacterized protein | Uncharacterized protein | | uniclust | UniRef100\_A0A061P2R0 | 100.0 | 2e-43 | 3.6e-49 | 357.6 | 441 | (111, 593) | 785 | (162, 604) | 834 | Phage tail length tape-measure protein | Phage tail length tape-measure protein | | uniclust | UniRef100\_A0A069F3H5 | 100.0 | 2.2e-43 | 3.9e-49 | 363.5 | 431 | (111, 593) | 785 | (157, 590) | 899 | Phage tail tape measure protein domain-containing protein | Phage tail tape measure protein domain-containing protein | | uniclust | UniRef100\_A0A0R6PDG4 | 100.0 | 2.5e-43 | 4.4e-49 | 367.7 | 434 | (112, 592) | 785 | (158, 594) | 1290 | Tail protein | Tail protein | | uniclust | UniRef100\_A0A3B9Q8T3 | 100.0 | 3.7e-43 | 6.6e-49 | 341.7 | 572 | (4, 587) | 785 | (73, 656) | 788 | Phage tail tape measure protein | Phage tail tape measure protein | | uniclust | UniRef100\_A0A1I7FWK0 | 100.0 | 3.9e-43 | 7e-49 | 366.6 | 335 | (112, 462) | 785 | (202, 537) | 1046 | Phage tail tape measure protein, TP901 family, core region | Phage tail tape measure protein, TP901 family, core region | | uniclust | UniRef100\_A0A081RQL7 | 100.0 | 4.2e-43 | 7.6e-49 | 336.7 | 544 | (17, 587) | 785 | (88, 641) | 647 | Phage-related minor tail protein | Phage-related minor tail protein | | uniclust | UniRef100\_A0A1S6TMZ4 | 100.0 | 6.7e-43 | 1.2e-48 | 331.3 | 475 | (109, 595) | 785 | (74, 576) | 765 | Phage tail tape measure protein, TP901 family | Phage tail tape measure protein, TP901 family | | uniclust | UniRef100\_A0A0A8H9G3 | 100.0 | 7.3e-43 | 1.3e-48 | 331.4 | 476 | (115, 595) | 785 | (87, 575) | 740 | Phage tail tape measure protein, TP901 family | Phage tail tape measure protein, TP901 family | | uniclust | UniRef100\_A0A379VM56 | 100.0 | 1.1e-42 | 2e-48 | 336.5 | 659 | (6, 674) | 785 | (80, 848) | 1068 | Phage tail tape measure protein | Phage tail tape measure protein | | uniclust | UniRef100\_A0A2N1WF95 | 100.0 | 1.6e-42 | 2.8e-48 | 313.6 | 502 | (258, 784) | 785 | (4, 506) | 507 | Phage tail tape measure protein (Fragment) | Phage tail tape measure protein (Fragment) | | uniclust | UniRef100\_A0A317PGW3 | 100.0 | 2.2e-42 | 4e-48 | 322.8 | 413 | (114, 586) | 785 | (50, 462) | 551 | TP901 family phage tail tape measure protein (Fragment) | TP901 family phage tail tape measure protein (Fragment) | | uniclust | UniRef100\_A0A450W6Y7 | 100.0 | 3.9e-42 | 7.1e-48 | 327.0 | 652 | (12, 673) | 785 | (84, 737) | 1065 | Phage tail tape measure protein, TP901 family, core region | Phage tail tape measure protein, TP901 family, core region | | uniclust | UniRef100\_A0A109WD97 | 100.0 | 4.5e-42 | 8.1e-48 | 351.6 | 458 | (3, 471) | 785 | (105, 626) | 1175 | Phage tail tape measure protein domain-containing protein | Phage tail tape measure protein domain-containing protein | | uniclust | UniRef100\_A0A2C6C9V6 | 100.0 | 4.6e-42 | 8.4e-48 | 333.7 | 499 | (109, 619) | 785 | (174, 695) | 855 | Phage tail tape measure protein | Phage tail tape measure protein | | uniclust | UniRef100\_A0A135IJM6 | 100.0 | 5e-42 | 8.9e-48 | 343.8 | 488 | (106, 626) | 785 | (64, 562) | 933 | Phage tail tape measure protein domain-containing protein | Phage tail tape measure protein domain-containing protein | | uniclust | UniRef100\_A0A077K8S2 | 100.0 | 5.4e-42 | 9.5e-48 | 355.7 | 729 | (1, 784) | 785 | (82, 896) | 899 | Uncharacterized protein | Uncharacterized protein | | uniclust | UniRef100\_A0A132P719 | 100.0 | 5.9e-42 | 1e-47 | 370.8 | 351 | (110, 472) | 785 | (155, 506) | 1062 | Phage tail protein | Phage tail protein | | uniclust | UniRef100\_A0A358PSL6 | 100.0 | 6.2e-42 | 1.1e-47 | 337.6 | 441 | (111, 594) | 785 | (149, 595) | 971 | Phage tail tape measure protein | Phage tail tape measure protein | | uniclust | UniRef100\_A0A1Q1PVX7 | 100.0 | 8e-42 | 1.4e-47 | 363.1 | 441 | (23, 474) | 785 | (385, 871) | 2029 | Tail tape measure protein | Tail tape measure protein | | uniclust | UniRef100\_A0A0R2DDY7 | 100.0 | 8e-42 | 1.5e-47 | 339.5 | 518 | (111, 705) | 785 | (280, 807) | 1044 | Phage tail tape measure protein domain-containing protein | Phage tail tape measure protein domain-containing protein | | uniclust | UniRef100\_A0A2S0M4J9 | 100.0 | 8.3e-42 | 1.5e-47 | 340.0 | 469 | (112, 595) | 785 | (73, 567) | 814 | Phage tail tape measure protein | Phage tail tape measure protein | | uniclust | UniRef100\_A0A323UJ65 | 100.0 | 1.3e-41 | 2.4e-47 | 341.6 | 507 | (109, 621) | 785 | (54, 595) | 891 | Phage tail tape measure protein | Phage tail tape measure protein | | uniclust | UniRef100\_A0A1C4CAH5 | 100.0 | 1.3e-41 | 2.4e-47 | 343.4 | 422 | (112, 584) | 785 | (225, 648) | 1275 | Phage tail tape measure protein | Phage tail tape measure protein | | uniclust | UniRef100\_A0A1A9RNP4 | 100.0 | 1.4e-41 | 2.5e-47 | 344.9 | 615 | (2, 624) | 785 | (1, 660) | 899 | Phage tail tape measure protein | Phage tail tape measure protein | | uniclust | UniRef100\_A0A064AK64 | 100.0 | 2.2e-41 | 4e-47 | 340.7 | 460 | (114, 586) | 785 | (263, 730) | 951 | Phage tail tape measure protein domain-containing protein | Phage tail tape measure protein domain-containing protein | | uniclust | UniRef100\_A0A021X9J0 | 100.0 | 3e-41 | 5.2e-47 | 346.8 | 499 | (109, 621) | 785 | (88, 626) | 838 | TP901 family phage tail tape measure protein | TP901 family phage tail tape measure protein | | uniclust | UniRef100\_A0A022PJ34 | 100.0 | 2.9e-41 | 5.3e-47 | 327.4 | 667 | (4, 702) | 785 | (22, 735) | 840 | Phage tail tape measure protein, TP901 family | Phage tail tape measure protein, TP901 family | | uniclust | UniRef100\_UPI00137359FD | 100.0 | 3.2e-41 | 5.8e-47 | 327.6 | 583 | (2, 595) | 785 | (1, 632) | 661 | phage tail tape measure protein | phage tail tape measure protein | | uniclust | UniRef100\_A0A136C2Y5 | 100.0 | 3.9e-41 | 7e-47 | 352.3 | 418 | (54, 478) | 785 | (211, 651) | 1262 | Phage tail tape measure protein | Phage tail tape measure protein | | uniclust | UniRef100\_A0A1G8XEH5 | 100.0 | 4.5e-41 | 8.1e-47 | 337.6 | 339 | (257, 612) | 785 | (261, 623) | 860 | Phage-related minor tail protein | Phage-related minor tail protein | | uniclust | UniRef100\_A0A5P0YP70 | 100.0 | 5.5e-41 | 9.7e-47 | 338.9 | 460 | (111, 624) | 785 | (75, 544) | 762 | Phage tail tape measure protein | Phage tail tape measure protein | | uniclust | UniRef100\_A0A0A7RU22 | 100.0 | 6.1e-41 | 1.1e-46 | 334.7 | 331 | (111, 453) | 785 | (64, 395) | 699 | Tail tape measure protein | Tail tape measure protein | | uniclust | UniRef100\_A0A060LZ15 | 100.0 | 2.1e-40 | 3.8e-46 | 357.9 | 370 | (110, 492) | 785 | (173, 547) | 1524 | Metalloprotease | Metalloprotease | | uniclust | UniRef100\_A0A1T5K1D4 | 100.0 | 2.9e-40 | 5.2e-46 | 344.0 | 427 | (110, 575) | 785 | (159, 589) | 1280 | Phage tail tape measure protein, TP901 family, core region | Phage tail tape measure protein, TP901 family, core region | | uniclust | UniRef100\_A0A286BYN5 | 100.0 | 2.9e-40 | 5.2e-46 | 315.2 | 510 | (118, 674) | 785 | (265, 778) | 879 | Phage tail tape measure protein, TP901 family, core region | Phage tail tape measure protein, TP901 family, core region | | uniclust | UniRef100\_A0A2X4YRE2 | 100.0 | 3.1e-40 | 5.6e-46 | 313.2 | 497 | (112, 625) | 785 | (35, 594) | 676 | Phage protein | Phage protein | | uniclust | UniRef100\_A0A4V2PT27 | 100.0 | 3.1e-40 | 5.7e-46 | 318.8 | 497 | (120, 623) | 785 | (71, 574) | 1021 | TP901 family phage tail tape measure protein | TP901 family phage tail tape measure protein | | uniclust | UniRef100\_A0A1M5Z9G5 | 100.0 | 3.8e-40 | 6.9e-46 | 323.3 | 617 | (7, 657) | 785 | (137, 774) | 1021 | Phage-related minor tail protein | Phage-related minor tail protein | | uniclust | UniRef100\_A0A0F6XYW3 | 100.0 | 5.2e-40 | 9.4e-46 | 326.6 | 536 | (113, 705) | 785 | (240, 818) | 1005 | Phage tail tape measure protein domain-containing protein | Phage tail tape measure protein domain-containing protein | | uniclust | UniRef100\_A0A0D1X6H1 | 100.0 | 6.9e-40 | 1.2e-45 | 344.7 | 429 | (111, 591) | 785 | (161, 657) | 1211 | Phage tail tape measure protein, TP901 family, core region | Phage tail tape measure protein, TP901 family, core region | | uniclust | UniRef100\_A0A8X6G804 | 100.0 | 7.9e-40 | 1.4e-45 | 310.8 | 580 | (5, 587) | 785 | (70, 656) | 850 | Phage tail tape measure protein | Phage tail tape measure protein | | uniclust | UniRef100\_A0A1V9DAU4 | 100.0 | 9.2e-40 | 1.7e-45 | 314.8 | 710 | (6, 782) | 785 | (102, 840) | 842 | Phage tail tape measure protein | Phage tail tape measure protein | | uniclust | UniRef100\_A0A0F4VRI4 | 100.0 | 9.6e-40 | 1.7e-45 | 342.3 | 448 | (118, 594) | 785 | (174, 632) | 998 | Phage tail length tape-measure protein | Phage tail length tape-measure protein | | uniclust | UniRef100\_A0A1F0GHX6 | 100.0 | 9.6e-40 | 1.7e-45 | 330.8 | 465 | (111, 620) | 785 | (90, 560) | 880 | Phage tail tape measure protein domain-containing protein | Phage tail tape measure protein domain-containing protein | | uniclust | UniRef100\_H9A124 | 100.0 | 1.3e-39 | 2.4e-45 | 313.6 | 471 | (110, 592) | 785 | (168, 671) | 848 | Tail tape measure protein | Tail tape measure protein | | uniclust | UniRef100\_A0A6M0FWE7 | 100.0 | 1.6e-39 | 2.9e-45 | 324.6 | 346 | (110, 460) | 785 | (76, 422) | 751 | Phage tail tape measure protein | Phage tail tape measure protein | | uniclust | UniRef100\_A0A174B5Q2 | 100.0 | 2.2e-39 | 3.9e-45 | 342.4 | 443 | (111, 595) | 785 | (106, 553) | 1168 | Phage-related minor tail protein | Phage-related minor tail protein | | uniclust | UniRef100\_A0A1G3M6W5 | 100.0 | 2.2e-39 | 4e-45 | 334.0 | 494 | (110, 628) | 785 | (59, 569) | 767 | Phage tail tape measure protein | Phage tail tape measure protein | | uniclust | UniRef100\_A0A0U4IIN6 | 100.0 | 2.2e-39 | 4e-45 | 335.9 | 439 | (111, 596) | 785 | (243, 685) | 905 | Putative tail tape measure protein | Putative tail tape measure protein | | uniclust | UniRef100\_UPI0002AF4875 | 100.0 | 2.5e-39 | 4.5e-45 | 298.8 | 563 | (179, 782) | 785 | (15, 619) | 619 | phage tail tape measure protein | phage tail tape measure protein | | uniclust | UniRef100\_A0A3L2QDC9 | 100.0 | 3.4e-39 | 6.1e-45 | 329.3 | 535 | (116, 670) | 785 | (112, 722) | 978 | Phage tail tape measure protein (Fragment) | Phage tail tape measure protein (Fragment) | | uniclust | UniRef100\_A0A0E3K1L2 | 100.0 | 3.5e-39 | 6.4e-45 | 308.6 | 533 | (111, 703) | 785 | (72, 609) | 723 | Phage protein | Phage protein | | uniclust | UniRef100\_A0A411WHS8 | 100.0 | 5.2e-39 | 9.4e-45 | 316.6 | 680 | (2, 697) | 785 | (1, 717) | 937 | Phage tail tape measure protein | Phage tail tape measure protein | | uniclust | UniRef100\_A0A1A9RDG3 | 100.0 | 5.7e-39 | 1e-44 | 308.4 | 589 | (6, 623) | 785 | (79, 692) | 900 | Phage tail tape measure protein | Phage tail tape measure protein | | uniclust | UniRef100\_A0A1T1J9S7 | 100.0 | 7.2e-39 | 1.3e-44 | 317.6 | 426 | (98, 589) | 785 | (163, 596) | 798 | Phage tail tape measure protein | Phage tail tape measure protein | | uniclust | UniRef100\_A0A1X3LEP7 | 100.0 | 9.9e-39 | 1.8e-44 | 321.2 | 438 | (121, 619) | 785 | (253, 703) | 891 | Phage tail tape measure protein, TP901 family, core region | Phage tail tape measure protein, TP901 family, core region | | uniclust | UniRef100\_A0A1I4GFH8 | 100.0 | 1e-38 | 1.8e-44 | 295.4 | 453 | (113, 618) | 785 | (61, 518) | 598 | Phage tail tape measure protein, TP901 family, core region | Phage tail tape measure protein, TP901 family, core region | | uniclust | UniRef100\_A0A0S4TWE8 | 100.0 | 2.3e-38 | 4.1e-44 | 295.8 | 446 | (279, 782) | 785 | (5, 487) | 488 | Phage-related tail transmembrane protein | Phage-related tail transmembrane protein | | uniclust | UniRef100\_A0A173R5S4 | 100.0 | 2.7e-38 | 4.8e-44 | 327.2 | 311 | (115, 437) | 785 | (232, 543) | 1265 | Phage-related minor tail protein | Phage-related minor tail protein | | uniclust | UniRef100\_A0A1C5YW05 | 100.0 | 2.9e-38 | 5.2e-44 | 310.0 | 426 | (111, 596) | 785 | (61, 490) | 868 | Phage-related minor tail protein | Phage-related minor tail protein | | uniclust | UniRef100\_A0A072YR64 | 100.0 | 3.3e-38 | 6.1e-44 | 307.7 | 443 | (114, 584) | 785 | (61, 521) | 940 | Tail tape measure protein | Tail tape measure protein | | uniclust | UniRef100\_UPI00068C4C6A | 100.0 | 3.6e-38 | 6.7e-44 | 297.1 | 479 | (110, 593) | 785 | (90, 588) | 933 | phage tail tape measure protein | phage tail tape measure protein | | uniclust | UniRef100\_A0A2G6CRZ3 | 100.0 | 5.6e-38 | 1e-43 | 299.4 | 556 | (1, 567) | 785 | (1, 574) | 707 | Phage tail tape measure protein | Phage tail tape measure protein | | uniclust | UniRef100\_UPI001EF7523E | 100.0 | 5.5e-38 | 1e-43 | 291.4 | 528 | (117, 647) | 785 | (5, 649) | 795 | phage tail tape measure protein | phage tail tape measure protein | | uniclust | UniRef100\_A0A069RFS6 | 100.0 | 6.9e-38 | 1.2e-43 | 316.1 | 439 | (111, 593) | 785 | (165, 610) | 1085 | TP901 family phage tail tape measure protein | TP901 family phage tail tape measure protein | | uniclust | UniRef100\_A0A064AM58 | 100.0 | 8.2e-38 | 1.5e-43 | 318.1 | 296 | (118, 424) | 785 | (194, 497) | 925 | Phage tail tape measure protein domain-containing protein | Phage tail tape measure protein domain-containing protein | | uniclust | UniRef100\_A0A3S0BBD4 | 100.0 | 8.7e-38 | 1.6e-43 | 302.6 | 461 | (112, 597) | 785 | (83, 546) | 668 | Phage tail tape measure protein | Phage tail tape measure protein | | uniclust | UniRef100\_A0A0H3J8I0 | 100.0 | 1e-37 | 1.8e-43 | 332.0 | 306 | (125, 449) | 785 | (252, 565) | 1065 | Minor tail protein | Minor tail protein | | uniclust | UniRef100\_A0A0U2B0N6 | 100.0 | 1.1e-37 | 2e-43 | 309.2 | 600 | (4, 623) | 785 | (110, 759) | 1243 | Phage tail tape measure protein domain-containing protein | Phage tail tape measure protein domain-containing protein | | uniclust | UniRef100\_A0A1H2FMR0 | 100.0 | 1.8e-37 | 3.2e-43 | 315.2 | 478 | (106, 594) | 785 | (72, 587) | 825 | Phage tail tape measure protein, TP901 family, core region | Phage tail tape measure protein, TP901 family, core region | | uniclust | UniRef100\_A0A1Y4I868 | 100.0 | 2e-37 | 3.6e-43 | 320.3 | 526 | (6, 594) | 785 | (9, 594) | 825 | Phage tail tape measure protein (Fragment) | Phage tail tape measure protein (Fragment) | | uniclust | UniRef100\_A0A5A9EN03 | 100.0 | 2e-37 | 3.7e-43 | 288.3 | 536 | (77, 631) | 785 | (142, 684) | 808 | Phage tail tape measure protein (Fragment) | Phage tail tape measure protein (Fragment) | | uniclust | UniRef100\_A0A3Q8WGQ2 | 100.0 | 2.4e-37 | 4.4e-43 | 310.4 | 544 | (30, 601) | 785 | (159, 723) | 1541 | Phage tail tape measure protein | Phage tail tape measure protein | | uniclust | UniRef100\_A0A0F7PRC0 | 100.0 | 3.3e-37 | 5.9e-43 | 321.3 | 435 | (115, 592) | 785 | (275, 724) | 1436 | Phage tail tape mesure protein | Phage tail tape mesure protein | | uniclust | UniRef100\_A0A252F2N6 | 100.0 | 3.8e-37 | 6.8e-43 | 304.6 | 528 | (15, 596) | 785 | (117, 680) | 922 | Phage tail tape measure protein domain-containing protein | Phage tail tape measure protein domain-containing protein | | uniclust | UniRef100\_A0A077RGZ1 | 100.0 | 4.3e-37 | 7.6e-43 | 339.4 | 466 | (110, 593) | 785 | (435, 918) | 2100 | lysostaphin | lysostaphin | | uniclust | UniRef100\_A0A7Z0HAF9 | 100.0 | 4.7e-37 | 8.5e-43 | 310.1 | 432 | (111, 587) | 785 | (83, 521) | 1141 | Phage tail tape measure protein | Phage tail tape measure protein | | uniclust | UniRef100\_A0A3A6P993 | 100.0 | 5.7e-37 | 1e-42 | 295.3 | 572 | (11, 622) | 785 | (27, 637) | 754 | Phage tail tape measure protein | Phage tail tape measure protein | | uniclust | UniRef100\_A0A0Q2XJT6 | 100.0 | 7.4e-37 | 1.4e-42 | 290.1 | 492 | (111, 610) | 785 | (69, 579) | 764 | Phage tail tape measure protein domain-containing protein | Phage tail tape measure protein domain-containing protein | | uniclust | UniRef100\_A0A349GR42 | 100.0 | 1e-36 | 1.9e-42 | 296.3 | 437 | (112, 572) | 785 | (188, 625) | 826 | Phage tail tape measure protein | Phage tail tape measure protein | | uniclust | UniRef100\_A0A0R1H356 | 100.0 | 1.2e-36 | 2e-42 | 322.2 | 410 | (32, 463) | 785 | (186, 625) | 1227 | Tape measure protein | Tape measure protein | | uniclust | UniRef100\_A0A371IQX0 | 100.0 | 1.2e-36 | 2.1e-42 | 306.6 | 555 | (3, 594) | 785 | (65, 642) | 1121 | Phage tail tape measure protein | Phage tail tape measure protein | | uniclust | UniRef100\_A0A081NWQ5 | 100.0 | 1.3e-36 | 2.2e-42 | 318.0 | 316 | (113, 445) | 785 | (133, 456) | 854 | Phage tail tape measure protein domain-containing protein | Phage tail tape measure protein domain-containing protein | | uniclust | UniRef100\_A0A0D1IZE2 | 100.0 | 1.4e-36 | 2.6e-42 | 327.1 | 432 | (111, 594) | 785 | (130, 570) | 1126 | Phage tail tape measure protein domain-containing protein | Phage tail tape measure protein domain-containing protein | | uniclust | UniRef100\_A0A855AEA3 | 100.0 | 1.8e-36 | 3.3e-42 | 307.1 | 456 | (111, 623) | 785 | (61, 530) | 1054 | Phage tail tape measure protein domain-containing protein | Phage tail tape measure protein domain-containing protein | | uniclust | UniRef100\_E1QHA5 | 100.0 | 2.3e-36 | 4.1e-42 | 293.7 | 523 | (62, 589) | 785 | (97, 633) | 993 | Phage tail tape measure protein, TP901 family | Phage tail tape measure protein, TP901 family | | uniclust | UniRef100\_A0A176EZ66 | 100.0 | 2.5e-36 | 4.4e-42 | 311.2 | 362 | (108, 474) | 785 | (70, 431) | 1055 | Phage tail tape measure protein domain-containing protein | Phage tail tape measure protein domain-containing protein | | uniclust | UniRef100\_A0A381EBY2 | 100.0 | 2.9e-36 | 5.2e-42 | 294.3 | 539 | (17, 582) | 785 | (93, 667) | 874 | Uncharacterized conserved protein | Uncharacterized conserved protein | | uniclust | UniRef100\_A0A2X1LDV0 | 100.0 | 3e-36 | 5.4e-42 | 287.1 | 417 | (147, 587) | 785 | (60, 478) | 492 | Tail protein (Modular protein) | Tail protein (Modular protein) | | uniclust | UniRef100\_A0A7V7G2N6 | 100.0 | 3.3e-36 | 5.9e-42 | 287.0 | 651 | (1, 783) | 785 | (2, 729) | 730 | Phage tail tape measure protein | Phage tail tape measure protein | | uniclust | UniRef100\_A0A0A2XR61 | 100.0 | 3.7e-36 | 6.7e-42 | 298.6 | 608 | (1, 619) | 785 | (110, 769) | 1042 | Phage tail tape measure protein domain-containing protein | Phage tail tape measure protein domain-containing protein | | uniclust | UniRef100\_A0A2K1FR02 | 100.0 | 4.7e-36 | 8.6e-42 | 277.1 | 452 | (118, 589) | 785 | (51, 508) | 657 | Phage tail tape measure protein | Phage tail tape measure protein | | uniclust | UniRef100\_A0A0M0KJB6 | 100.0 | 5.1e-36 | 9e-42 | 318.0 | 440 | (6, 460) | 785 | (35, 499) | 1120 | Phage tail tape measure protein domain-containing protein | Phage tail tape measure protein domain-containing protein | | uniclust | UniRef100\_A0A510Y3F9 | 100.0 | 6.4e-36 | 1.2e-41 | 309.2 | 335 | (111, 457) | 785 | (73, 408) | 1202 | Phage tail tape measure protein domain-containing protein | Phage tail tape measure protein domain-containing protein | | uniclust | UniRef100\_A0A4D6ALN7 | 100.0 | 7.6e-36 | 1.4e-41 | 286.6 | 457 | (111, 620) | 785 | (75, 547) | 887 | Tail length tape-measure protein | Tail length tape-measure protein | | uniclust | UniRef100\_UPI00036738C7 | 100.0 | 8.2e-36 | 1.5e-41 | 277.6 | 540 | (100, 653) | 785 | (121, 686) | 801 | phage tail tape measure protein | phage tail tape measure protein | | uniclust | UniRef100\_A0A0E0TET7 | 100.0 | 1e-35 | 1.8e-41 | 331.7 | 492 | (56, 592) | 785 | (212, 731) | 1632 | Tail tape measure protein TP901 core region protein | Tail tape measure protein TP901 core region protein | | uniclust | UniRef100\_A0A1G5JIN5 | 100.0 | 1.2e-35 | 2.1e-41 | 300.9 | 338 | (115, 466) | 785 | (61, 399) | 812 | Phage tail tape measure protein, TP901 family, core region | Phage tail tape measure protein, TP901 family, core region | | uniclust | UniRef100\_A0A3T0L2L7 | 100.0 | 1.3e-35 | 2.4e-41 | 277.4 | 505 | (70, 594) | 785 | (126, 634) | 836 | Phage tail tape measure protein | Phage tail tape measure protein | | uniclust | UniRef100\_A0A812RFT8 | 100.0 | 1.4e-35 | 2.6e-41 | 295.3 | 470 | (103, 592) | 785 | (168, 637) | 1893 | site-specific DNA-methyltransferase (adenine-specific) | site-specific DNA-methyltransferase (adenine-specific) | | uniclust | UniRef100\_A0A061NKC8 | 100.0 | 1.6e-35 | 2.9e-41 | 311.9 | 340 | (112, 463) | 785 | (61, 401) | 968 | Phage tail length tape-measure protein | Phage tail length tape-measure protein | | uniclust | UniRef100\_A0A345CNQ0 | 100.0 | 1.8e-35 | 3.3e-41 | 282.4 | 587 | (3, 600) | 785 | (2, 639) | 716 | Phage tail tape measure protein | Phage tail tape measure protein | | uniclust | UniRef100\_A0A099WJF5 | 100.0 | 3e-35 | 5.3e-41 | 304.8 | 326 | (118, 470) | 785 | (258, 590) | 952 | Phage tail protein | Phage tail protein | | uniclust | UniRef100\_A0A2R3MZN4 | 100.0 | 3.3e-35 | 6e-41 | 287.2 | 458 | (112, 596) | 785 | (202, 665) | 911 | Phage tail tape measure protein | Phage tail tape measure protein | | uniclust | UniRef100\_A0A0V9UNT3 | 100.0 | 3.5e-35 | 6.1e-41 | 300.4 | 316 | (114, 441) | 785 | (70, 388) | 809 | Phage tail tape measure protein domain-containing protein | Phage tail tape measure protein domain-containing protein | | uniclust | UniRef100\_A0A059N3D7 | 100.0 | 3.6e-35 | 6.3e-41 | 312.5 | 478 | (110, 671) | 785 | (242, 744) | 920 | Phage tail tape measure protein, TP901 family | Phage tail tape measure protein, TP901 family | | uniclust | UniRef100\_A0A157BCK0 | 100.0 | 4.1e-35 | 7.5e-41 | 271.9 | 450 | (178, 674) | 785 | (16, 472) | 568 | Phage Tail Tape Measure protein | Phage Tail Tape Measure protein | | uniclust | UniRef100\_UPI000935C447 | 100.0 | 4.4e-35 | 8.1e-41 | 272.5 | 580 | (4, 601) | 785 | (77, 677) | 792 | phage tail tape measure protein | phage tail tape measure protein | | uniclust | UniRef100\_A0A085AFM7 | 100.0 | 5.2e-35 | 9.3e-41 | 300.9 | 450 | (21, 474) | 785 | (169, 624) | 945 | Phage tail tape measure protein domain-containing protein | Phage tail tape measure protein domain-containing protein | | uniclust | UniRef100\_E6TVF9 | 100.0 | 5.7e-35 | 1e-40 | 292.1 | 424 | (111, 591) | 785 | (163, 595) | 950 | Phage tail tape measure protein, TP901 family | Phage tail tape measure protein, TP901 family | | uniclust | UniRef100\_A0A133ZYY1 | 100.0 | 5.9e-35 | 1.1e-40 | 302.5 | 319 | (111, 439) | 785 | (60, 383) | 1184 | Phage tail tape measure protein, TP901 family | Phage tail tape measure protein, TP901 family | | uniclust | UniRef100\_A0A0A0IDQ6 | 100.0 | 6.2e-35 | 1.1e-40 | 303.8 | 423 | (111, 595) | 785 | (165, 599) | 876 | TP901 family phage tail tape measure protein | TP901 family phage tail tape measure protein | | uniclust | UniRef100\_A0A0F0CCS2 | 100.0 | 6.3e-35 | 1.1e-40 | 307.9 | 351 | (111, 473) | 785 | (229, 671) | 1807 | Phage-related minor tail protein | Phage-related minor tail protein | | uniclust | UniRef100\_A0A024QH21 | 100.0 | 7.1e-35 | 1.3e-40 | 293.7 | 398 | (111, 589) | 785 | (50, 457) | 763 | Phage tail tape measure protein, TP901 family, core region | Phage tail tape measure protein, TP901 family, core region | | uniclust | UniRef100\_A0A1K1MLR7 | 100.0 | 7.2e-35 | 1.3e-40 | 312.4 | 322 | (112, 445) | 785 | (164, 487) | 1544 | Phage tail tape measure protein, TP901 family, core region | Phage tail tape measure protein, TP901 family, core region | | uniclust | UniRef100\_A0A5T0QQZ5 | 100.0 | 7.5e-35 | 1.4e-40 | 267.5 | 455 | (115, 574) | 785 | (85, 552) | 554 | Phage tail tape measure protein (Fragment) | Phage tail tape measure protein (Fragment) | | uniclust | UniRef100\_A0A6N7JJS5 | 100.0 | 8e-35 | 1.5e-40 | 285.6 | 428 | (23, 456) | 785 | (357, 788) | 1494 | Phage tail tape measure protein | Phage tail tape measure protein | | uniclust | UniRef100\_A0A160M9X1 | 100.0 | 8.8e-35 | 1.6e-40 | 306.8 | 442 | (23, 475) | 785 | (176, 646) | 1265 | Phage tail tape measure protein domain-containing protein | Phage tail tape measure protein domain-containing protein | | uniclust | UniRef100\_UPI001FF55D27 | 100.0 | 9.6e-35 | 1.8e-40 | 273.7 | 482 | (106, 593) | 785 | (221, 719) | 902 | phage tail tape measure protein | phage tail tape measure protein | | uniclust | UniRef100\_A0A090IMA5 | 100.0 | 1.1e-34 | 1.9e-40 | 302.6 | 461 | (5, 470) | 785 | (136, 603) | 920 | Phage-related tail protein | Phage-related tail protein | | uniclust | UniRef100\_A0A0Q7K0A6 | 100.0 | 1.2e-34 | 2.1e-40 | 302.6 | 526 | (118, 701) | 785 | (84, 621) | 889 | Phage tail tape measure protein domain-containing protein | Phage tail tape measure protein domain-containing protein | | uniclust | UniRef100\_A0A0B3BNP9 | 100.0 | 1.3e-34 | 2.3e-40 | 286.3 | 538 | (63, 621) | 785 | (109, 660) | 800 | Phage tail tape measure protein, TP901 family | Phage tail tape measure protein, TP901 family | | uniclust | UniRef100\_A0A0T1XR25 | 100.0 | 1.3e-34 | 2.4e-40 | 299.6 | 331 | (110, 461) | 785 | (58, 390) | 826 | Phage tail tape measure protein domain-containing protein | Phage tail tape measure protein domain-containing protein | | uniclust | UniRef100\_A0A080KEC1 | 100.0 | 1.5e-34 | 2.8e-40 | 273.1 | 481 | (115, 647) | 785 | (74, 567) | 777 | Phage-related minor tail protein | Phage-related minor tail protein | | uniclust | UniRef100\_A0A075X0G2 | 100.0 | 1.6e-34 | 2.9e-40 | 286.5 | 473 | (114, 587) | 785 | (74, 604) | 778 | Phage tail tape measure protein domain-containing protein | Phage tail tape measure protein domain-containing protein | | uniclust | UniRef100\_A0A060PHZ7 | 100.0 | 2.6e-34 | 4.7e-40 | 308.3 | 436 | (23, 470) | 785 | (51, 514) | 1297 | Phage tail tape measure protein domain-containing protein | Phage tail tape measure protein domain-containing protein | | uniclust | UniRef100\_A0A8S5PUM7 | 100.0 | 3.7e-34 | 6.7e-40 | 280.6 | 576 | (9, 595) | 785 | (79, 697) | 1042 | Minor tail protein (Fragment) | Minor tail protein (Fragment) | | uniclust | UniRef100\_A0A143DED3 | 100.0 | 3.9e-34 | 6.9e-40 | 277.2 | 486 | (1, 621) | 785 | (2, 496) | 606 | Phage tail tape measure protein | Phage tail tape measure protein | | uniclust | UniRef100\_UPI0005E6ED2E | 100.0 | 5.3e-34 | 9.6e-40 | 270.2 | 474 | (4, 494) | 785 | (166, 659) | 732 | phage tail tape measure protein | phage tail tape measure protein | | uniclust | UniRef100\_UPI0022E9545A | 100.0 | 6.1e-34 | 1.1e-39 | 268.5 | 478 | (103, 592) | 785 | (308, 786) | 902 | phage tail tape measure protein | phage tail tape measure protein | | uniclust | UniRef100\_A0A367EFZ0 | 100.0 | 7e-34 | 1.3e-39 | 281.8 | 414 | (112, 586) | 785 | (70, 488) | 857 | Phage tail tape measure protein | Phage tail tape measure protein | | uniclust | UniRef100\_A0A269PKX6 | 100.0 | 7.5e-34 | 1.4e-39 | 283.2 | 454 | (110, 590) | 785 | (52, 528) | 1007 | Phage tail tape measure protein | Phage tail tape measure protein | | uniclust | UniRef100\_A0A073KF69 | 100.0 | 7.8e-34 | 1.4e-39 | 301.0 | 332 | (109, 458) | 785 | (71, 406) | 1013 | Phage tail tape measure protein domain-containing protein | Phage tail tape measure protein domain-containing protein | | uniclust | UniRef100\_D5EFA3 | 100.0 | 8.1e-34 | 1.5e-39 | 264.9 | 557 | (5, 591) | 785 | (73, 636) | 810 | Phage tail tape measure protein, TP901 family | Phage tail tape measure protein, TP901 family | | uniclust | UniRef100\_A0A076LFK6 | 100.0 | 9e-34 | 1.6e-39 | 259.3 | 354 | (111, 468) | 785 | (81, 434) | 466 | Phage tail length tape-measure protein | Phage tail length tape-measure protein | | uniclust | UniRef100\_A0A0D0IGY2 | 100.0 | 9.3e-34 | 1.7e-39 | 281.4 | 336 | (433, 782) | 785 | (494, 892) | 892 | Phage-related minor tail protein | Phage-related minor tail protein | | uniclust | UniRef100\_A0A0Q0F7A5 | 100.0 | 1.1e-33 | 2e-39 | 269.8 | 513 | (113, 691) | 785 | (52, 581) | 720 | Prophage PSSSM-04, tail length tape-measure protein | Prophage PSSSM-04, tail length tape-measure protein | | uniclust | UniRef100\_A0A0U4NFW6 | 100.0 | 1.1e-33 | 2.1e-39 | 286.9 | 418 | (111, 583) | 785 | (213, 634) | 1751 | Chromosome partition protein Smc | Chromosome partition protein Smc | | uniclust | UniRef100\_A0A090IER3 | 100.0 | 1.2e-33 | 2.1e-39 | 290.5 | 454 | (102, 578) | 785 | (44, 512) | 747 | Phage tail tape measure protein, TP901 family, core region,Phage-related minor tail protein | Phage tail tape measure protein, TP901 family, core region,Phage-related minor tail protein | | uniclust | UniRef100\_A0A5B0BRS7 | 100.0 | 1.3e-33 | 2.3e-39 | 293.4 | 421 | (112, 581) | 785 | (170, 597) | 1481 | Phage tail tape measure protein | Phage tail tape measure protein | | uniclust | UniRef100\_A0A1C2K2M3 | 100.0 | 1.3e-33 | 2.4e-39 | 280.2 | 470 | (4, 473) | 785 | (127, 609) | 884 | Phage tail tape measure protein (Fragment) | Phage tail tape measure protein (Fragment) | | uniclust | UniRef100\_A0A2T3FUX6 | 100.0 | 1.4e-33 | 2.5e-39 | 287.3 | 448 | (1, 464) | 785 | (1, 495) | 1969 | Phage tail tape measure protein | Phage tail tape measure protein | | uniclust | UniRef100\_UPI00069E9770 | 100.0 | 1.5e-33 | 2.7e-39 | 260.4 | 545 | (117, 674) | 785 | (31, 653) | 730 | phage tail tape measure protein | phage tail tape measure protein | | uniclust | UniRef100\_A0A1X3JZY9 | 100.0 | 1.6e-33 | 2.9e-39 | 276.2 | 488 | (184, 702) | 785 | (81, 613) | 727 | Putative phage tail protein | Putative phage tail protein | | uniclust | UniRef100\_A0A1L7N5W8 | 100.0 | 1.9e-33 | 3.5e-39 | 283.5 | 579 | (5, 610) | 785 | (79, 692) | 1199 | Phage tail tape measure protein domain-containing protein | Phage tail tape measure protein domain-containing protein | | uniclust | UniRef100\_A0A4Y5WA60 | 100.0 | 2.2e-33 | 3.9e-39 | 279.3 | 526 | (239, 782) | 785 | (200, 807) | 807 | Phage tail tape measure protein | Phage tail tape measure protein | | uniclust | UniRef100\_A0A1V0P119 | 100.0 | 2.7e-33 | 4.9e-39 | 288.1 | 440 | (6, 453) | 785 | (58, 522) | 1114 | Phage tail component | Phage tail component | | uniclust | UniRef100\_D3FXW3 | 100.0 | 3e-33 | 5.4e-39 | 269.0 | 416 | (111, 581) | 785 | (56, 475) | 718 | TP901 family phage tail tape measure protein | TP901 family phage tail tape measure protein | | uniclust | UniRef100\_A0A072YD48 | 100.0 | 3.7e-33 | 6.6e-39 | 300.1 | 341 | (110, 465) | 785 | (283, 624) | 1179 | Phage tail tape measure protein domain-containing protein | Phage tail tape measure protein domain-containing protein | | uniclust | UniRef100\_A0A142XX90 | 100.0 | 3.7e-33 | 6.6e-39 | 286.5 | 349 | (111, 474) | 785 | (60, 408) | 1044 | Phage-related minor tail protein | Phage-related minor tail protein | | uniclust | UniRef100\_A0A369RL83 | 100.0 | 4e-33 | 7.3e-39 | 259.4 | 459 | (13, 477) | 785 | (78, 536) | 574 | Phage tail tape measure protein, TP901 family, core region | Phage tail tape measure protein, TP901 family, core region | | uniclust | UniRef100\_A0A073JQH9 | 100.0 | 4.5e-33 | 8.1e-39 | 282.2 | 488 | (111, 627) | 785 | (68, 571) | 980 | Phage tail length tape measure protein | Phage tail length tape measure protein | | uniclust | UniRef100\_A0A2T0BLK1 | 100.0 | 5e-33 | 9e-39 | 281.2 | 328 | (116, 455) | 785 | (128, 456) | 1128 | Phage-related minor tail protein | Phage-related minor tail protein | | uniclust | UniRef100\_A0A1I4SVB4 | 100.0 | 5.3e-33 | 9.5e-39 | 287.8 | 359 | (98, 461) | 785 | (132, 490) | 1153 | Phage tail tape measure protein, TP901 family, core region | Phage tail tape measure protein, TP901 family, core region | | uniclust | UniRef100\_A0A3B8TYN9 | 100.0 | 6e-33 | 1.1e-38 | 275.2 | 452 | (57, 596) | 785 | (103, 573) | 737 | Phage tail tape measure protein domain-containing protein | Phage tail tape measure protein domain-containing protein | | uniclust | UniRef100\_A0A6N9Q7V1 | 100.0 | 6.7e-33 | 1.2e-38 | 274.2 | 427 | (112, 591) | 785 | (158, 596) | 874 | Phage tail tape measure protein | Phage tail tape measure protein | | uniclust | UniRef100\_UPI00117E3743 | 100.0 | 9.3e-33 | 1.7e-38 | 252.0 | 422 | (59, 490) | 785 | (33, 467) | 486 | phage tail tape measure protein | phage tail tape measure protein | | uniclust | UniRef100\_A0A072Y8W2 | 100.0 | 1.2e-32 | 2.1e-38 | 280.0 | 310 | (116, 437) | 785 | (96, 406) | 1003 | Phage tail tape measure protein | Phage tail tape measure protein | | uniclust | UniRef100\_A0A4P9VH79 | 100.0 | 1.2e-32 | 2.3e-38 | 263.9 | 352 | (254, 624) | 785 | (159, 514) | 665 | Phage tail tape measure protein | Phage tail tape measure protein | | uniclust | UniRef100\_A0A358QWZ2 | 100.0 | 1.3e-32 | 2.3e-38 | 281.2 | 324 | (116, 451) | 785 | (182, 506) | 1247 | Phage tail tape measure protein | Phage tail tape measure protein | | uniclust | UniRef100\_A0A2T4MNE9 | 100.0 | 1.3e-32 | 2.3e-38 | 285.7 | 331 | (111, 457) | 785 | (137, 468) | 1547 | Probable transglycosylase IsaA | Probable transglycosylase IsaA | | uniclust | UniRef100\_UPI000A7274A8 | 100.0 | 1.3e-32 | 2.4e-38 | 258.4 | 480 | (275, 782) | 785 | (7, 561) | 561 | phage tail tape measure protein | phage tail tape measure protein | | uniclust | UniRef100\_A0A2D5RV15 | 100.0 | 1.4e-32 | 2.5e-38 | 267.0 | 525 | (109, 662) | 785 | (51, 590) | 804 | Phage tail tape measure protein | Phage tail tape measure protein | | uniclust | UniRef100\_A0A1F9II93 | 100.0 | 2.2e-32 | 4e-38 | 263.2 | 422 | (337, 782) | 785 | (77, 567) | 567 | Phage tail tape measure protein (Fragment) | Phage tail tape measure protein (Fragment) | | uniclust | UniRef100\_A0A218KMB6 | 100.0 | 2.6e-32 | 4.7e-38 | 254.5 | 377 | (191, 587) | 785 | (3, 379) | 520 | Phage tail tape measure protein | Phage tail tape measure protein | | uniclust | UniRef100\_UPI0009B38736 | 100.0 | 2.6e-32 | 4.7e-38 | 259.6 | 439 | (113, 596) | 785 | (96, 541) | 612 | phage tail tape measure protein | phage tail tape measure protein | | uniclust | UniRef100\_A0A1S2U586 | 99.9 | 2.7e-32 | 4.9e-38 | 254.8 | 482 | (69, 593) | 785 | (34, 516) | 654 | Phage tail tape measure protein | Phage tail tape measure protein | | uniclust | UniRef100\_A0A1M7N201 | 99.9 | 2.9e-32 | 5.1e-38 | 277.4 | 414 | (112, 585) | 785 | (52, 478) | 870 | Phage tail tape measure protein, TP901 family, core region | Phage tail tape measure protein, TP901 family, core region | | uniclust | UniRef100\_A0A084ABK3 | 99.9 | 2.9e-32 | 5.2e-38 | 297.8 | 341 | (110, 460) | 785 | (224, 574) | 1524 | Phage tail length tape-measure protein | Phage tail length tape-measure protein | | uniclust | UniRef100\_A0A0H3NQN0 | 99.9 | 3.2e-32 | 5.9e-38 | 247.4 | 418 | (213, 674) | 785 | (3, 424) | 515 | Phage protein | Phage protein | | uniclust | UniRef100\_A0A7C4AJ87 | 99.9 | 4.2e-32 | 7.7e-38 | 258.0 | 537 | (23, 594) | 785 | (20, 564) | 747 | Phage tail tape measure protein | Phage tail tape measure protein | | uniclust | UniRef100\_A0A031IV85 | 99.9 | 6.6e-32 | 1.2e-37 | 283.8 | 432 | (99, 573) | 785 | (48, 492) | 856 | Prophage PSPPH06, TP901 family tail tape measure protein | Prophage PSPPH06, TP901 family tail tape measure protein | | uniclust | UniRef100\_A0A090YKY6 | 99.9 | 7.1e-32 | 1.3e-37 | 273.9 | 437 | (112, 596) | 785 | (103, 549) | 1340 | Phage tail tape measure protein, TP901 family, core region | Phage tail tape measure protein, TP901 family, core region | | uniclust | UniRef100\_A0A098AYW7 | 99.9 | 9.7e-32 | 1.7e-37 | 272.0 | 426 | (1, 441) | 785 | (2, 430) | 944 | Phage-related minor tail protein | Phage-related minor tail protein | | uniclust | UniRef100\_A0A1C2DED9 | 99.9 | 1.2e-31 | 2.1e-37 | 279.9 | 452 | (114, 621) | 785 | (52, 544) | 778 | Phage tail tape measure protein domain-containing protein | Phage tail tape measure protein domain-containing protein | | uniclust | UniRef100\_A0A0H2YTM6 | 99.9 | 1.2e-31 | 2.1e-37 | 280.5 | 456 | (112, 582) | 785 | (208, 677) | 1086 | Phage tail tape measure protein, TP901 family | Phage tail tape measure protein, TP901 family | | uniclust | UniRef100\_A0A0E2H1X6 | 99.9 | 1.4e-31 | 2.5e-37 | 272.4 | 464 | (113, 596) | 785 | (65, 570) | 815 | Phage tail tape measure protein, TP901 family, core region (Fragment) | Phage tail tape measure protein, TP901 family, core region (Fragment) | | uniclust | UniRef100\_UPI000EFC6317 | 99.9 | 1.4e-31 | 2.5e-37 | 251.9 | 466 | (119, 631) | 785 | (24, 493) | 747 | phage tail tape measure protein | phage tail tape measure protein | | uniclust | UniRef100\_A0A1H3I7B8 | 99.9 | 1.5e-31 | 2.6e-37 | 268.2 | 463 | (111, 593) | 785 | (52, 555) | 823 | Phage-related minor tail protein | Phage-related minor tail protein | | uniclust | UniRef100\_A0A7U5BUK8 | 99.9 | 1.5e-31 | 2.6e-37 | 268.7 | 434 | (124, 593) | 785 | (72, 512) | 937 | Phage tail tape measure protein domain-containing protein | Phage tail tape measure protein domain-containing protein | | uniclust | UniRef100\_A0A0R2JK06 | 99.9 | 1.7e-31 | 3.1e-37 | 283.5 | 341 | (111, 465) | 785 | (221, 561) | 1705 | TP901 family phage tail tape measure protein | TP901 family phage tail tape measure protein | | uniclust | UniRef100\_A0A497UTH2 | 99.9 | 1.8e-31 | 3.2e-37 | 253.6 | 490 | (109, 624) | 785 | (62, 574) | 727 | TP901 family phage tail tape measure protein | TP901 family phage tail tape measure protein | | uniclust | UniRef100\_A0A1Q4L0K8 | 99.9 | 1.8e-31 | 3.2e-37 | 261.5 | 327 | (111, 449) | 785 | (68, 395) | 847 | Phage tail tape measure protein | Phage tail tape measure protein | | uniclust | UniRef100\_A0A2E7EBE4 | 99.9 | 1.9e-31 | 3.4e-37 | 278.3 | 353 | (100, 457) | 785 | (259, 611) | 1382 | Phage tail tape measure protein | Phage tail tape measure protein | | uniclust | UniRef100\_A0A150K6J4 | 99.9 | 1.9e-31 | 3.4e-37 | 273.2 | 422 | (117, 593) | 785 | (175, 605) | 1428 | Phage tail tape measure protein domain-containing protein | Phage tail tape measure protein domain-containing protein | | uniclust | UniRef100\_A0A0A1GX62 | 99.9 | 2e-31 | 3.5e-37 | 293.5 | 376 | (110, 492) | 785 | (162, 547) | 1536 | Phage tail tape measure protein | Phage tail tape measure protein | | uniclust | UniRef100\_A0A0U3NKE8 | 99.9 | 2.5e-31 | 4.4e-37 | 288.9 | 337 | (111, 461) | 785 | (233, 569) | 1515 | Phage tail length tape-measure protein | Phage tail length tape-measure protein | | uniclust | UniRef100\_A0A1K1LB63 | 99.9 | 2.5e-31 | 4.6e-37 | 265.8 | 327 | (116, 446) | 785 | (61, 387) | 1011 | Phage tape measure | Phage tape measure | | uniclust | UniRef100\_A0A256CBP9 | 99.9 | 2.9e-31 | 5.2e-37 | 248.2 | 504 | (22, 592) | 785 | (19, 524) | 726 | Phage tail tape measure protein | Phage tail tape measure protein | | uniclust | UniRef100\_A0A0J1IJR7 | 99.9 | 3.5e-31 | 6.1e-37 | 285.9 | 401 | (53, 473) | 785 | (245, 663) | 1200 | Phage tail tape measure protein domain-containing protein | Phage tail tape measure protein domain-containing protein | | uniclust | UniRef100\_UPI00210E7B92 | 99.9 | 3.3e-31 | 6.1e-37 | 245.7 | 426 | (107, 593) | 785 | (139, 565) | 740 | phage tail tape measure protein | phage tail tape measure protein | | uniclust | UniRef100\_A0A0R3MWR6 | 99.9 | 4.6e-31 | 8.3e-37 | 265.3 | 601 | (2, 664) | 785 | (25, 733) | 876 | Phage tail protein | Phage tail protein | | uniclust | UniRef100\_A0A8H9QJE6 | 99.9 | 4.7e-31 | 8.3e-37 | 277.4 | 377 | (85, 473) | 785 | (199, 578) | 1231 | Phage tail tape measure protein | Phage tail tape measure protein | | uniclust | UniRef100\_A0A0F9I3U5 | 99.9 | 4.8e-31 | 8.7e-37 | 259.5 | 472 | (112, 593) | 785 | (67, 567) | 655 | Phage tail tape measure protein domain-containing protein | Phage tail tape measure protein domain-containing protein | | uniclust | UniRef100\_A0A1G8PK41 | 99.9 | 5.4e-31 | 9.5e-37 | 272.4 | 421 | (117, 580) | 785 | (110, 543) | 727 | Phage-related minor tail protein | Phage-related minor tail protein | | uniclust | UniRef100\_A0A0E2E9Q1 | 99.9 | 5.4e-31 | 9.7e-37 | 257.5 | 451 | (112, 594) | 785 | (67, 537) | 653 | Phage tail tape measure protein, TP901 family, core region | Phage tail tape measure protein, TP901 family, core region | | uniclust | UniRef100\_A0A1W1VPP1 | 99.9 | 5.5e-31 | 9.9e-37 | 257.9 | 447 | (111, 596) | 785 | (63, 524) | 946 | Phage tail tape measure protein, TP901 family, core region | Phage tail tape measure protein, TP901 family, core region | | uniclust | UniRef100\_A0A0C7QMD9 | 99.9 | 5.6e-31 | 1e-36 | 272.6 | 349 | (111, 474) | 785 | (234, 583) | 1211 | Phage tail tape measure protein | Phage tail tape measure protein | | uniclust | UniRef100\_A0A078MEL8 | 99.9 | 7.1e-31 | 1.3e-36 | 269.8 | 364 | (63, 448) | 785 | (24, 391) | 1010 | Phage-related minor tail protein | Phage-related minor tail protein | | uniclust | UniRef100\_A0A917G2Q0 | 99.9 | 6.9e-31 | 1.3e-36 | 251.0 | 490 | (1, 494) | 785 | (1, 494) | 1013 | Phage tail tape measure protein domain-containing protein | Phage tail tape measure protein domain-containing protein | | uniclust | UniRef100\_A0A239C875 | 99.9 | 7.8e-31 | 1.4e-36 | 251.1 | 468 | (101, 582) | 785 | (137, 619) | 800 | Phage tail tape measure protein, TP901 family, core region | Phage tail tape measure protein, TP901 family, core region | | uniclust | UniRef100\_A0A3C0QQX1 | 99.9 | 8.1e-31 | 1.5e-36 | 262.6 | 452 | (114, 595) | 785 | (64, 525) | 866 | Phage tail tape measure protein (Fragment) | Phage tail tape measure protein (Fragment) | | uniclust | UniRef100\_A0A1B9LEZ4 | 99.9 | 8.6e-31 | 1.6e-36 | 249.1 | 509 | (114, 632) | 785 | (203, 731) | 955 | Phage tail tape measure protein | Phage tail tape measure protein | | uniclust | UniRef100\_A0A0S4UKB0 | 99.9 | 9.2e-31 | 1.7e-36 | 255.2 | 729 | (4, 784) | 785 | (79, 889) | 901 | Phage-related tail transmembrane protein | Phage-related tail transmembrane protein | | uniclust | UniRef100\_A0A7J6YK69 | 99.9 | 9.4e-31 | 1.7e-36 | 266.0 | 440 | (110, 580) | 785 | (1938, 2378) | 2523 | Uncharacterized protein | Uncharacterized protein | | uniclust | UniRef100\_A0A1Q6K3L0 | 99.9 | 9.7e-31 | 1.8e-36 | 256.5 | 308 | (111, 433) | 785 | (159, 470) | 1048 | Phage tail tape measure protein | Phage tail tape measure protein | | uniclust | UniRef100\_A0A099WNM6 | 99.9 | 1.1e-30 | 1.9e-36 | 272.8 | 337 | (111, 460) | 785 | (72, 413) | 1584 | Phage tail tape measure protein domain-containing protein | Phage tail tape measure protein domain-containing protein | | uniclust | UniRef100\_A0A4S3L0X3 | 99.9 | 1.1e-30 | 2e-36 | 246.1 | 469 | (1, 473) | 785 | (1, 471) | 778 | Phage tail tape measure protein | Phage tail tape measure protein | | uniclust | UniRef100\_A0A0C2R458 | 99.9 | 1.2e-30 | 2.1e-36 | 296.2 | 324 | (111, 451) | 785 | (196, 523) | 1391 | Peptidase M23 domain-containing protein | Peptidase M23 domain-containing protein | | uniclust | UniRef100\_A0A074LW09 | 99.9 | 1.4e-30 | 2.5e-36 | 273.3 | 315 | (117, 448) | 785 | (241, 563) | 962 | Phage tail tape measure protein domain-containing protein | Phage tail tape measure protein domain-containing protein | | uniclust | UniRef100\_A0A1B8Q889 | 99.9 | 1.4e-30 | 2.5e-36 | 280.5 | 348 | (107, 459) | 785 | (245, 592) | 1395 | Phage tail tape measure protein | Phage tail tape measure protein | | uniclust | UniRef100\_A0A133YZY4 | 99.9 | 1.5e-30 | 2.6e-36 | 287.0 | 359 | (111, 477) | 785 | (357, 723) | 1470 | Phage tail tape measure protein domain-containing protein | Phage tail tape measure protein domain-containing protein | | uniclust | UniRef100\_A0A0G3WGN7 | 99.9 | 1.7e-30 | 2.9e-36 | 285.2 | 321 | (113, 444) | 785 | (133, 453) | 1035 | Tape measure protein | Tape measure protein | | uniclust | UniRef100\_A0A090FCA3 | 99.9 | 1.7e-30 | 3e-36 | 270.4 | 399 | (110, 580) | 785 | (80, 480) | 887 | Phage tail tape measure protein domain-containing protein | Phage tail tape measure protein domain-containing protein | | uniclust | UniRef100\_A0A919ZAN7 | 99.9 | 1.8e-30 | 3.2e-36 | 258.6 | 349 | (111, 465) | 785 | (151, 558) | 1016 | Phage tail tape measure protein domain-containing protein | Phage tail tape measure protein domain-containing protein | | uniclust | UniRef100\_A0A0F5ARQ0 | 99.9 | 1.9e-30 | 3.4e-36 | 260.7 | 430 | (104, 575) | 785 | (50, 492) | 806 | Phage tail tape measure protein domain-containing protein | Phage tail tape measure protein domain-containing protein | | uniclust | UniRef100\_A0A1H1G2L2 | 99.9 | 1.9e-30 | 3.5e-36 | 255.2 | 458 | (5, 472) | 785 | (94, 579) | 991 | Phage tail tape measure protein, TP901 family, core region | Phage tail tape measure protein, TP901 family, core region | | uniclust | UniRef100\_UPI001C007827 | 99.9 | 2e-30 | 3.7e-36 | 244.5 | 448 | (114, 594) | 785 | (232, 683) | 870 | phage tail tape measure protein | phage tail tape measure protein | | uniclust | UniRef100\_A0A1C6E246 | 99.9 | 2.2e-30 | 4e-36 | 250.3 | 331 | (111, 454) | 785 | (153, 483) | 566 | Phage-related minor tail protein | Phage-related minor tail protein | | uniclust | UniRef100\_A0A1H0GBX4 | 99.9 | 2.3e-30 | 4.2e-36 | 265.2 | 461 | (5, 469) | 785 | (67, 566) | 1109 | Phage tail tape measure protein, TP901 family, core region | Phage tail tape measure protein, TP901 family, core region | | uniclust | UniRef100\_UPI001F56DA87 | 99.9 | 2.5e-30 | 4.5e-36 | 250.4 | 395 | (60, 464) | 785 | (76, 476) | 902 | phage tail tape measure protein | phage tail tape measure protein | | uniclust | UniRef100\_A0A1I5RNE0 | 99.9 | 3e-30 | 5.5e-36 | 254.9 | 427 | (109, 584) | 785 | (203, 656) | 792 | Phage tail tape measure protein, TP901 family, core region (Fragment) | Phage tail tape measure protein, TP901 family, core region (Fragment) | | uniclust | UniRef100\_A0A430RXV9 | 99.9 | 3.2e-30 | 5.9e-36 | 234.4 | 354 | (111, 469) | 785 | (56, 409) | 486 | Phage tail tape measure protein (Fragment) | Phage tail tape measure protein (Fragment) | | uniclust | UniRef100\_UPI0010102470 | 99.9 | 3.3e-30 | 6e-36 | 231.1 | 485 | (234, 781) | 785 | (4, 496) | 497 | phage tail tape measure protein | phage tail tape measure protein | | uniclust | UniRef100\_A0A098F348 | 99.9 | 4.5e-30 | 8e-36 | 275.5 | 411 | (28, 443) | 785 | (57, 496) | 1182 | Phage tail tape measure protein, family, core region | Phage tail tape measure protein, family, core region | | uniclust | UniRef100\_A0A2X1J0M0 | 99.9 | 4.5e-30 | 8.2e-36 | 240.7 | 559 | (6, 574) | 785 | (80, 669) | 684 | TP901 family phage tail tape measure protein | TP901 family phage tail tape measure protein | | uniclust | UniRef100\_A0A1V4SUX2 | 99.9 | 4.8e-30 | 8.6e-36 | 270.8 | 345 | (110, 466) | 785 | (344, 699) | 1239 | Chromosome partition protein Smc | Chromosome partition protein Smc | | uniclust | UniRef100\_A0A1C5VU61 | 99.9 | 4.9e-30 | 8.8e-36 | 258.1 | 388 | (51, 448) | 785 | (79, 486) | 883 | Phage-related protein | Phage-related protein | | uniclust | UniRef100\_A0A0C3I468 | 99.9 | 5.5e-30 | 1e-35 | 271.7 | 343 | (111, 468) | 785 | (235, 578) | 1721 | Tail tape measure protein | Tail tape measure protein | | uniclust | UniRef100\_UPI0021053B5F | 99.9 | 6.2e-30 | 1.1e-35 | 243.7 | 331 | (113, 443) | 785 | (31, 363) | 967 | phage tail tape measure protein | phage tail tape measure protein | | uniclust | UniRef100\_A0A174TRV8 | 99.9 | 6.8e-30 | 1.2e-35 | 269.1 | 349 | (117, 470) | 785 | (202, 555) | 1242 | Phage-related minor tail protein | Phage-related minor tail protein | | uniclust | UniRef100\_A0A068QV54 | 99.9 | 7.6e-30 | 1.3e-35 | 263.0 | 555 | (103, 702) | 785 | (41, 612) | 715 | Putative phage protein | Putative phage protein | | uniclust | UniRef100\_A0A7W3T073 | 99.9 | 7.5e-30 | 1.3e-35 | 261.8 | 338 | (111, 469) | 785 | (68, 407) | 1083 | Phage tail tape measure protein | Phage tail tape measure protein | | uniclust | UniRef100\_A0A0R2FUF3 | 99.9 | 7.7e-30 | 1.4e-35 | 270.2 | 333 | (111, 450) | 785 | (172, 514) | 1325 | TP901 family phage tail tape measure protein | TP901 family phage tail tape measure protein | | uniclust | UniRef100\_A0A1I6URJ1 | 99.9 | 8.1e-30 | 1.5e-35 | 270.7 | 333 | (110, 456) | 785 | (325, 665) | 1585 | Phage tail tape measure protein, TP901 family, core region | Phage tail tape measure protein, TP901 family, core region | | uniclust | UniRef100\_A0A1A7PS85 | 99.9 | 8.1e-30 | 1.5e-35 | 244.8 | 585 | (3, 595) | 785 | (2, 621) | 696 | Phage tail tape measure protein domain-containing protein (Fragment) | Phage tail tape measure protein domain-containing protein (Fragment) | | uniclust | UniRef100\_A0A1B9AU19 | 99.9 | 9.1e-30 | 1.7e-35 | 246.6 | 423 | (119, 581) | 785 | (222, 672) | 899 | Phage tail tape measure protein | Phage tail tape measure protein | | uniclust | UniRef100\_A0A081R6K7 | 99.9 | 1.1e-29 | 1.9e-35 | 282.1 | 355 | (111, 469) | 785 | (201, 564) | 1424 | Phage tail tape measure protein, TP901 family, core region | Phage tail tape measure protein, TP901 family, core region | | uniclust | UniRef100\_UPI001F397BD3 | 99.9 | 1.2e-29 | 2.1e-35 | 250.4 | 318 | (116, 445) | 785 | (163, 494) | 914 | phage tail tape measure protein | phage tail tape measure protein | | uniclust | UniRef100\_A0A090I2H9 | 99.9 | 1.5e-29 | 2.6e-35 | 248.8 | 417 | (111, 587) | 785 | (84, 503) | 665 | Phage tail tape measure protein, TP901 family | Phage tail tape measure protein, TP901 family | | uniclust | UniRef100\_A0A011T795 | 99.9 | 1.5e-29 | 2.6e-35 | 257.7 | 330 | (112, 447) | 785 | (81, 410) | 753 | Tail protein | Tail protein | | uniclust | UniRef100\_A0A0B4BHD1 | 99.9 | 1.8e-29 | 3.2e-35 | 250.9 | 469 | (1, 594) | 785 | (3, 480) | 851 | Phage tail length tape measure protein | Phage tail length tape measure protein | | uniclust | UniRef100\_A0A0E3GSM8 | 99.9 | 1.9e-29 | 3.4e-35 | 259.9 | 321 | (243, 609) | 785 | (213, 549) | 994 | Phage-like protein | Phage-like protein | | uniclust | UniRef100\_A0A412CHP1 | 99.9 | 1.9e-29 | 3.5e-35 | 242.6 | 460 | (118, 594) | 785 | (67, 551) | 835 | Phage tail tape measure protein | Phage tail tape measure protein | | uniclust | UniRef100\_UPI000933C153 | 99.9 | 2.2e-29 | 4.1e-35 | 244.5 | 509 | (103, 628) | 785 | (168, 710) | 1200 | phage tail tape measure protein | phage tail tape measure protein | | uniclust | UniRef100\_A0A060PPH4 | 99.9 | 2.5e-29 | 4.4e-35 | 255.9 | 333 | (110, 457) | 785 | (57, 390) | 937 | Phage tail tape measure protein domain-containing protein | Phage tail tape measure protein domain-containing protein | | uniclust | UniRef100\_A0A023CIR9 | 99.9 | 2.6e-29 | 4.6e-35 | 265.8 | 423 | (14, 451) | 785 | (154, 607) | 1850 | Bacteriophage-related protein | Bacteriophage-related protein | | uniclust | UniRef100\_A0A2Z6DXQ1 | 99.9 | 2.8e-29 | 5.1e-35 | 249.9 | 577 | (6, 594) | 785 | (5, 652) | 1035 | Phage tail tape measure protein | Phage tail tape measure protein | | uniclust | UniRef100\_A0A178HLZ5 | 99.9 | 2.9e-29 | 5.3e-35 | 239.5 | 466 | (115, 595) | 785 | (64, 555) | 977 | Phage tail tape measure protein domain-containing protein | Phage tail tape measure protein domain-containing protein | | uniclust | UniRef100\_A0A143XDR8 | 99.9 | 3e-29 | 5.4e-35 | 260.9 | 321 | (241, 626) | 785 | (350, 720) | 1063 | Phage-related minor tail protein | Phage-related minor tail protein | | uniclust | UniRef100\_A0A7D4BEW5 | 99.9 | 3.6e-29 | 6.5e-35 | 252.8 | 399 | (115, 584) | 785 | (54, 453) | 805 | Phage tail tape measure protein | Phage tail tape measure protein | | uniclust | UniRef100\_A0A0B0SDM8 | 99.9 | 4.3e-29 | 7.8e-35 | 245.0 | 468 | (111, 585) | 785 | (58, 540) | 966 | Phage tail tape measure protein domain-containing protein | Phage tail tape measure protein domain-containing protein | | uniclust | UniRef100\_A0A973FFH6 | 99.9 | 4.3e-29 | 7.9e-35 | 229.8 | 342 | (118, 460) | 785 | (78, 421) | 672 | Phage tail tape measure protein | Phage tail tape measure protein | | uniclust | UniRef100\_A0A1R1MK84 | 99.9 | 4.7e-29 | 8.6e-35 | 232.7 | 469 | (114, 595) | 785 | (61, 553) | 767 | Phage tail tape measure protein | Phage tail tape measure protein | | uniclust | UniRef100\_A0A1I1GTR9 | 99.9 | 4.9e-29 | 8.7e-35 | 262.6 | 329 | (111, 450) | 785 | (192, 523) | 1274 | Phage tail tape measure protein, TP901 family, core region | Phage tail tape measure protein, TP901 family, core region | | uniclust | UniRef100\_A0A024Q9Y0 | 99.9 | 5.4e-29 | 9.6e-35 | 266.7 | 325 | (113, 447) | 785 | (67, 401) | 1226 | Phage tail tape measure protein, TP901 family, core region | Phage tail tape measure protein, TP901 family, core region | | uniclust | UniRef100\_A0A4R3J9G1 | 99.9 | 5.5e-29 | 9.9e-35 | 236.8 | 410 | (111, 574) | 785 | (74, 493) | 600 | TP901 family phage tail tape measure protein | TP901 family phage tail tape measure protein | | uniclust | UniRef100\_A0A135YQ01 | 99.9 | 6.4e-29 | 1.2e-34 | 240.4 | 335 | (111, 460) | 785 | (66, 401) | 777 | Phage tail tape measure protein, TP901 family | Phage tail tape measure protein, TP901 family | | uniclust | UniRef100\_A0A072NTF3 | 99.9 | 6.8e-29 | 1.2e-34 | 272.0 | 627 | (4, 705) | 785 | (212, 894) | 1237 | Phage-related minor tail protein | Phage-related minor tail protein | | uniclust | UniRef100\_UPI001CFF0720 | 99.9 | 7e-29 | 1.3e-34 | 243.6 | 492 | (68, 592) | 785 | (13, 509) | 1008 | phage tail tape measure protein | phage tail tape measure protein | | uniclust | UniRef100\_A0A1E7Q036 | 99.9 | 7.2e-29 | 1.3e-34 | 241.5 | 312 | (255, 575) | 785 | (258, 578) | 749 | Phage tail tape measure protein | Phage tail tape measure protein | | uniclust | UniRef100\_A0A916IVX2 | 99.9 | 9.2e-29 | 1.7e-34 | 236.4 | 553 | (3, 580) | 785 | (48, 653) | 763 | Phage tail tape measure protein domain-containing protein | Phage tail tape measure protein domain-containing protein | | uniclust | UniRef100\_A0A0L0W671 | 99.9 | 1e-28 | 1.8e-34 | 269.3 | 324 | (111, 451) | 785 | (220, 546) | 1170 | Phage-like minor tail protein (Fragment) | Phage-like minor tail protein (Fragment) | | uniclust | UniRef100\_A0A3P6KZI8 | 99.9 | 1.1e-28 | 1.9e-34 | 228.8 | 459 | (5, 476) | 785 | (78, 558) | 568 | Tail fiber protein T (Tape measure) (GpT) | Tail fiber protein T (Tape measure) (GpT) | | uniclust | UniRef100\_UPI000CDD6178 | 99.9 | 1.3e-28 | 2.4e-34 | 223.3 | 415 | (118, 572) | 785 | (89, 507) | 508 | phage tail tape measure protein | phage tail tape measure protein | | uniclust | UniRef100\_A0A0F9FT22 | 99.9 | 1.4e-28 | 2.5e-34 | 233.1 | 397 | (117, 585) | 785 | (56, 457) | 578 | Phage tail tape measure protein domain-containing protein (Fragment) | Phage tail tape measure protein domain-containing protein (Fragment) | | uniclust | UniRef100\_A0A495V9N5 | 99.9 | 1.4e-28 | 2.6e-34 | 237.2 | 450 | (118, 590) | 785 | (62, 512) | 1090 | TP901 family phage tail tape measure protein | TP901 family phage tail tape measure protein | | uniclust | UniRef100\_UPI0008A95C93 | 99.9 | 1.4e-28 | 2.6e-34 | 247.6 | 327 | (111, 445) | 785 | (510, 844) | 1366 | phage tail tape measure protein | phage tail tape measure protein | | uniclust | UniRef100\_A0A8T8DYB4 | 99.9 | 1.5e-28 | 2.7e-34 | 240.9 | 442 | (111, 577) | 785 | (59, 503) | 877 | Phage tail tape measure protein | Phage tail tape measure protein | | uniclust | UniRef100\_A0A080IQ97 | 99.9 | 1.6e-28 | 2.9e-34 | 239.3 | 440 | (111, 621) | 785 | (68, 518) | 633 | Phage tail tape measure protein, TP901 family, core region | Phage tail tape measure protein, TP901 family, core region | | uniclust | UniRef100\_A0A1S1V6R2 | 99.9 | 1.6e-28 | 2.9e-34 | 248.2 | 420 | (119, 592) | 785 | (111, 544) | 833 | Phage-related minor tail protein | Phage-related minor tail protein | | uniclust | UniRef100\_A0A378E4L1 | 99.9 | 1.7e-28 | 3.1e-34 | 238.9 | 538 | (4, 571) | 785 | (78, 644) | 690 | Phage tail tape measure protein, family | Phage tail tape measure protein, family | | uniclust | UniRef100\_A0A1Y5SUX3 | 99.9 | 1.9e-28 | 3.6e-34 | 239.1 | 469 | (115, 594) | 785 | (64, 554) | 1263 | Phage-related minor tail protein | Phage-related minor tail protein | | uniclust | UniRef100\_A0A1G3UAI7 | 99.9 | 2e-28 | 3.6e-34 | 238.6 | 592 | (1, 625) | 785 | (1, 617) | 850 | Phage tail tape measure protein | Phage tail tape measure protein | | uniclust | UniRef100\_A0A0D0FE37 | 99.9 | 2.1e-28 | 3.6e-34 | 262.3 | 320 | (116, 449) | 785 | (64, 383) | 1025 | Tape measure protein N-terminal domain-containing protein | Tape measure protein N-terminal domain-containing protein | | uniclust | UniRef100\_A0A2N9Y986 | 99.9 | 2.2e-28 | 3.9e-34 | 226.1 | 350 | (105, 464) | 785 | (67, 416) | 444 | Phage tail tape measure protein (Fragment) | Phage tail tape measure protein (Fragment) | | uniclust | UniRef100\_A0A0U5MPA6 | 99.9 | 2.2e-28 | 4e-34 | 232.4 | 409 | (118, 596) | 785 | (60, 475) | 598 | Phage tail tape measure protein domain-containing protein | Phage tail tape measure protein domain-containing protein | | uniclust | UniRef100\_A0A1H8A1R0 | 99.9 | 2.5e-28 | 4.6e-34 | 241.6 | 463 | (4, 469) | 785 | (75, 544) | 1108 | Phage tail tape measure protein, TP901 family, core region | Phage tail tape measure protein, TP901 family, core region | | uniclust | UniRef100\_A0A158S093 | 99.9 | 3.1e-28 | 5.5e-34 | 238.2 | 292 | (398, 783) | 785 | (302, 605) | 606 | Phage tail tape measure protein | Phage tail tape measure protein | | uniclust | UniRef100\_A0A024QBD0 | 99.9 | 3.1e-28 | 5.6e-34 | 260.5 | 332 | (111, 460) | 785 | (172, 508) | 1287 | Chromosome segregation protein SMC | Chromosome segregation protein SMC | | uniclust | UniRef100\_UPI00191A810C | 99.9 | 3.1e-28 | 5.6e-34 | 209.9 | 378 | (2, 383) | 785 | (3, 380) | 388 | phage tail tape measure protein | phage tail tape measure protein | | uniclust | UniRef100\_A0A1L8I8U0 | 99.9 | 3.3e-28 | 5.9e-34 | 242.0 | 345 | (111, 460) | 785 | (176, 525) | 653 | Phage tail tape measure protein (Fragment) | Phage tail tape measure protein (Fragment) | | uniclust | UniRef100\_UPI000AEE5402 | 99.9 | 3.3e-28 | 6e-34 | 241.7 | 472 | (109, 589) | 785 | (356, 1001) | 1155 | phage tail tape measure protein | phage tail tape measure protein | | uniclust | UniRef100\_A0A4R2N8L9 | 99.9 | 3.3e-28 | 6.1e-34 | 220.7 | 440 | (23, 466) | 785 | (16, 461) | 585 | TP901 family phage tail tape measure protein | TP901 family phage tail tape measure protein | | uniclust | UniRef100\_A0A069D3M4 | 99.9 | 3.7e-28 | 6.6e-34 | 263.9 | 300 | (111, 419) | 785 | (128, 439) | 1113 | Minor tail protein GP26-like protein | Minor tail protein GP26-like protein | | uniclust | UniRef100\_A0A133MP93 | 99.9 | 3.9e-28 | 7e-34 | 246.1 | 328 | (111, 456) | 785 | (209, 538) | 992 | Phage tail tape measure protein, TP901 family | Phage tail tape measure protein, TP901 family | | uniclust | UniRef100\_A0A0H4TEZ5 | 99.9 | 4.4e-28 | 8e-34 | 240.1 | 327 | (115, 446) | 785 | (159, 490) | 975 | Tail tape measure protein | Tail tape measure protein | | uniclust | UniRef100\_A0A5H2QW59 | 99.9 | 4.4e-28 | 8.1e-34 | 234.0 | 435 | (117, 595) | 785 | (54, 496) | 727 | Tail length tape-measure protein | Tail length tape-measure protein | | uniclust | UniRef100\_A0A0K1LJH9 | 99.9 | 4.6e-28 | 8.2e-34 | 247.9 | 448 | (1, 594) | 785 | (61, 521) | 657 | Putative tail protein | Putative tail protein | | uniclust | UniRef100\_UPI00097D468B | 99.9 | 5.8e-28 | 1.1e-33 | 238.7 | 461 | (114, 583) | 785 | (484, 948) | 1083 | phage tail tape measure protein | phage tail tape measure protein | | uniclust | UniRef100\_A0A1W7LHP8 | 99.9 | 5.9e-28 | 1.1e-33 | 259.2 | 329 | (111, 450) | 785 | (203, 531) | 1810 | Phage tail tape measure protein domain-containing protein | Phage tail tape measure protein domain-containing protein | | uniclust | UniRef100\_A0A024YKU6 | 99.9 | 6.6e-28 | 1.2e-33 | 250.3 | 414 | (111, 593) | 785 | (79, 507) | 782 | Phage tail tape measure protein domain-containing protein (Fragment) | Phage tail tape measure protein domain-containing protein (Fragment) | | uniclust | UniRef100\_A0A386PK18 | 99.9 | 7.4e-28 | 1.3e-33 | 243.0 | 323 | (112, 446) | 785 | (48, 371) | 871 | Phage tail tape measure protein | Phage tail tape measure protein | | uniclust | UniRef100\_UPI00042A37CB | 99.9 | 7.9e-28 | 1.4e-33 | 238.9 | 568 | (4, 595) | 785 | (44, 668) | 994 | phage tail tape measure protein | phage tail tape measure protein | | uniclust | UniRef100\_UPI001FCE6FAE | 99.9 | 8.2e-28 | 1.5e-33 | 207.3 | 352 | (407, 783) | 785 | (17, 369) | 371 | hypothetical protein | hypothetical protein | | uniclust | UniRef100\_A0A380FL26 | 99.9 | 1e-27 | 1.9e-33 | 246.6 | 442 | (113, 587) | 785 | (172, 620) | 1292 | Phage tail tape measure protein, TP901 family, core region | Phage tail tape measure protein, TP901 family, core region | | uniclust | UniRef100\_A0A377U3B0 | 99.9 | 1.1e-27 | 1.9e-33 | 225.7 | 290 | (276, 591) | 785 | (43, 338) | 555 | Phage tail tape measure protein, family | Phage tail tape measure protein, family | | uniclust | UniRef100\_A0A379TRM7 | 99.9 | 1.2e-27 | 2.2e-33 | 231.0 | 672 | (4, 703) | 785 | (3, 766) | 874 | Putative bacteriophage protein | Putative bacteriophage protein | | uniclust | UniRef100\_A0A0D0Q2R1 | 99.9 | 1.2e-27 | 2.2e-33 | 251.0 | 360 | (111, 483) | 785 | (266, 690) | 1291 | Tail tape measure protein | Tail tape measure protein | | uniclust | UniRef100\_A0A285NFD1 | 99.9 | 1.2e-27 | 2.2e-33 | 226.2 | 349 | (114, 471) | 785 | (63, 412) | 806 | Phage tail tape measure protein, TP901 family, core region | Phage tail tape measure protein, TP901 family, core region | | uniclust | UniRef100\_UPI001EFBD742 | 99.9 | 1.3e-27 | 2.4e-33 | 222.3 | 548 | (116, 703) | 785 | (53, 617) | 694 | phage tail tape measure protein | phage tail tape measure protein | | uniclust | UniRef100\_UPI001918F45D | 99.9 | 1.4e-27 | 2.5e-33 | 209.9 | 239 | (352, 594) | 785 | (4, 243) | 375 | phage tail tape measure protein | phage tail tape measure protein | | uniclust | UniRef100\_A0A0J9EDB0 | 99.9 | 1.4e-27 | 2.5e-33 | 236.7 | 413 | (188, 619) | 785 | (130, 564) | 1019 | Phage tail length tape-measure protein | Phage tail length tape-measure protein | | uniclust | UniRef100\_A0A0D0P0W2 | 99.9 | 1.6e-27 | 2.9e-33 | 233.3 | 427 | (102, 573) | 785 | (42, 480) | 633 | Contig\_52, whole genome shotgun sequence | Contig\_52, whole genome shotgun sequence | | uniclust | UniRef100\_A0A2X1MWP2 | 99.9 | 1.6e-27 | 2.9e-33 | 216.1 | 448 | (119, 616) | 785 | (39, 493) | 493 | Phage related tail protein | Phage related tail protein | | uniclust | UniRef100\_A0A136H2X7 | 99.9 | 1.8e-27 | 3.1e-33 | 241.1 | 203 | (4, 214) | 785 | (77, 292) | 717 | Tape measure protein N-terminal domain-containing protein | Tape measure protein N-terminal domain-containing protein | | uniclust | UniRef100\_A0A0A6VGD4 | 99.9 | 1.8e-27 | 3.2e-33 | 239.5 | 393 | (115, 589) | 785 | (261, 664) | 842 | Phage tail tape measure protein domain-containing protein | Phage tail tape measure protein domain-containing protein | | uniclust | UniRef100\_UPI0021BF577E | 99.9 | 2.1e-27 | 3.8e-33 | 230.2 | 425 | (112, 595) | 785 | (236, 674) | 999 | phage tail tape measure protein | phage tail tape measure protein | | uniclust | UniRef100\_A0A401UU86 | 99.9 | 2.2e-27 | 4.1e-33 | 244.6 | 428 | (111, 585) | 785 | (155, 592) | 1714 | Phage tail tape measure protein domain-containing protein | Phage tail tape measure protein domain-containing protein | | uniclust | UniRef100\_A0A379S656 | 99.9 | 2.2e-27 | 4.1e-33 | 219.6 | 331 | (196, 583) | 785 | (4, 334) | 517 | Transglycosylase | Transglycosylase | | uniclust | UniRef100\_A0A1Q6JQ74 | 99.9 | 2.5e-27 | 4.6e-33 | 217.5 | 453 | (5, 472) | 785 | (75, 533) | 643 | Phage tail tape measure protein | Phage tail tape measure protein | | uniclust | UniRef100\_A0A0R8VA05 | 99.9 | 3e-27 | 5.4e-33 | 247.3 | 405 | (118, 591) | 785 | (220, 629) | 1657 | Tail tape measure protein | Tail tape measure protein | | uniclust | UniRef100\_A0A084E6E2 | 99.9 | 3.1e-27 | 5.6e-33 | 251.3 | 322 | (111, 444) | 785 | (78, 401) | 1183 | Phage tail tape measure protein, TP901 family, core region | Phage tail tape measure protein, TP901 family, core region | | uniclust | UniRef100\_A0A0J0VAR5 | 99.9 | 3.2e-27 | 5.7e-33 | 241.2 | 319 | (205, 592) | 785 | (331, 658) | 941 | Replication protein | Replication protein | | uniclust | UniRef100\_UPI0015D2B999 | 99.9 | 3.1e-27 | 5.8e-33 | 203.6 | 378 | (303, 702) | 785 | (7, 384) | 385 | phage tail tape measure protein | phage tail tape measure protein | | uniclust | UniRef100\_A0A1B2DVD5 | 99.9 | 3.3e-27 | 5.9e-33 | 252.1 | 330 | (113, 462) | 785 | (170, 561) | 1489 | Phage tail tape measure protein domain-containing protein | Phage tail tape measure protein domain-containing protein | | uniclust | UniRef100\_I0GWR9 | 99.9 | 3.4e-27 | 6.2e-33 | 225.3 | 464 | (120, 618) | 785 | (68, 546) | 951 | Putative tail tape measure protein | Putative tail tape measure protein | | uniclust | UniRef100\_A0A484YXF6 | 99.9 | 3.6e-27 | 6.6e-33 | 208.3 | 313 | (157, 469) | 785 | (5, 317) | 362 | Phage tail tape measure protein | Phage tail tape measure protein | | uniclust | UniRef100\_A0A235CJC1 | 99.9 | 3.6e-27 | 6.6e-33 | 232.3 | 486 | (147, 674) | 785 | (81, 584) | 952 | Phage tail protein | Phage tail protein | | uniclust | UniRef100\_A0A072YJ62 | 99.9 | 4e-27 | 7.2e-33 | 237.0 | 456 | (111, 594) | 785 | (67, 643) | 859 | Phage tail tape measure protein | Phage tail tape measure protein | | uniclust | UniRef100\_A0A2J0QV17 | 99.9 | 4.7e-27 | 8.5e-33 | 227.3 | 472 | (163, 674) | 785 | (12, 501) | 602 | Phage tail tape measure protein (Fragment) | Phage tail tape measure protein (Fragment) | | uniclust | UniRef100\_A0A1I6REX1 | 99.9 | 4.8e-27 | 8.6e-33 | 233.4 | 328 | (111, 456) | 785 | (56, 385) | 761 | Phage tail tape measure protein, TP901 family, core region | Phage tail tape measure protein, TP901 family, core region | | uniclust | UniRef100\_A0A1U7M6P2 | 99.9 | 5e-27 | 9e-33 | 250.1 | 361 | (111, 477) | 785 | (194, 609) | 1210 | Chromosome partition protein Smc | Chromosome partition protein Smc | | uniclust | UniRef100\_A0A5C4YDP2 | 99.9 | 5e-27 | 9.2e-33 | 215.8 | 475 | (116, 595) | 785 | (86, 575) | 602 | Phage tail tape measure protein (Fragment) | Phage tail tape measure protein (Fragment) | | uniclust | UniRef100\_UPI0018EADAE6 | 99.9 | 5.3e-27 | 9.7e-33 | 223.4 | 480 | (109, 622) | 785 | (50, 545) | 705 | phage tail tape measure protein | phage tail tape measure protein | | uniclust | UniRef100\_A0A1Z4PN55 | 99.9 | 6e-27 | 1.1e-32 | 225.5 | 444 | (110, 596) | 785 | (53, 505) | 775 | Putative bacteriophage protein | Putative bacteriophage protein | | uniclust | UniRef100\_A0A1C3H4A0 | 99.9 | 6.9e-27 | 1.3e-32 | 240.5 | 349 | (108, 461) | 785 | (342, 690) | 1661 | Phage tail length tape-measure protein | Phage tail length tape-measure protein | | uniclust | UniRef100\_UPI0005878020 | 99.9 | 7.2e-27 | 1.3e-32 | 218.0 | 529 | (30, 600) | 785 | (159, 736) | 745 | phage tail tape measure protein | phage tail tape measure protein | | uniclust | UniRef100\_A0A0Q1AXG2 | 99.9 | 7.8e-27 | 1.4e-32 | 248.8 | 330 | (112, 455) | 785 | (171, 509) | 1635 | Putative membrane protein | Putative membrane protein | | uniclust | UniRef100\_A0A089HYG9 | 99.9 | 8.1e-27 | 1.4e-32 | 244.1 | 308 | (116, 447) | 785 | (128, 441) | 838 | Phage tail tape measure protein domain-containing protein | Phage tail tape measure protein domain-containing protein | | uniclust | UniRef100\_C9MV98 | 99.9 | 1e-26 | 1.9e-32 | 212.0 | 372 | (110, 485) | 785 | (194, 565) | 600 | Phage tail tape measure protein, TP901 family | Phage tail tape measure protein, TP901 family | | uniclust | UniRef100\_H0U9Y6 | 99.9 | 1e-26 | 1.9e-32 | 219.1 | 413 | (118, 591) | 785 | (82, 503) | 654 | Phage tail tape measure protein, TP901 family, core region | Phage tail tape measure protein, TP901 family, core region | | uniclust | UniRef100\_A0A1M6KNC3 | 99.9 | 1.1e-26 | 1.9e-32 | 235.6 | 367 | (111, 484) | 785 | (76, 492) | 865 | Phage tail tape measure protein, TP901 family, core region (Fragment) | Phage tail tape measure protein, TP901 family, core region (Fragment) | | uniclust | UniRef100\_A0A0J1FKM1 | 99.9 | 1.2e-26 | 2.1e-32 | 235.5 | 336 | (111, 460) | 785 | (63, 409) | 839 | Phage-related minor tail protein | Phage-related minor tail protein | | uniclust | UniRef100\_UPI001495E0A7 | 99.9 | 1.2e-26 | 2.1e-32 | 220.6 | 325 | (113, 449) | 785 | (53, 378) | 738 | phage tail tape measure protein | phage tail tape measure protein | | uniclust | UniRef100\_A0A1X3ITD6 | 99.9 | 1.3e-26 | 2.4e-32 | 207.2 | 340 | (118, 467) | 785 | (43, 384) | 403 | Phage tail tape measure protein, TP901 family, core region (Fragment) | Phage tail tape measure protein, TP901 family, core region (Fragment) | | uniclust | UniRef100\_A0A2M7BJQ9 | 99.9 | 1.4e-26 | 2.5e-32 | 215.9 | 352 | (100, 456) | 785 | (231, 582) | 615 | Phage tail tape measure protein (Fragment) | Phage tail tape measure protein (Fragment) | | uniclust | UniRef100\_UPI001EF72727 | 99.9 | 1.5e-26 | 2.6e-32 | 234.4 | 406 | (146, 587) | 785 | (11, 420) | 975 | phage tail tape measure protein | phage tail tape measure protein | | uniclust | UniRef100\_A0A380DUS3 | 99.9 | 1.4e-26 | 2.6e-32 | 235.8 | 326 | (111, 455) | 785 | (326, 655) | 1604 | TP901 family prophage L54a | TP901 family prophage L54a | | uniclust | UniRef100\_UPI0018A2AB22 | 99.9 | 1.5e-26 | 2.7e-32 | 233.2 | 537 | (20, 587) | 785 | (241, 823) | 1216 | hypothetical protein | hypothetical protein | | uniclust | UniRef100\_A0A0N7I3V5 | 99.9 | 1.9e-26 | 3.3e-32 | 236.2 | 379 | (121, 593) | 785 | (73, 459) | 762 | Phage tail tape measure protein domain-containing protein | Phage tail tape measure protein domain-containing protein | | uniclust | UniRef100\_A0A1C6H3B5 | 99.9 | 1.9e-26 | 3.4e-32 | 243.1 | 328 | (112, 451) | 785 | (195, 523) | 1278 | Phage-related minor tail protein | Phage-related minor tail protein | | uniclust | UniRef100\_A0A343TII8 | 99.9 | 1.9e-26 | 3.5e-32 | 233.8 | 363 | (114, 489) | 785 | (52, 419) | 943 | Phage tail tape measure protein | Phage tail tape measure protein | | uniclust | UniRef100\_UPI001EFFEF77 | 99.9 | 1.9e-26 | 3.5e-32 | 234.4 | 344 | (111, 466) | 785 | (151, 495) | 1485 | phage tail tape measure protein | phage tail tape measure protein | | uniclust | UniRef100\_A0A174N846 | 99.9 | 2e-26 | 3.6e-32 | 238.1 | 425 | (116, 596) | 785 | (61, 499) | 1019 | Phage-related minor tail protein | Phage-related minor tail protein | | uniclust | UniRef100\_A0A377ALG4 | 99.9 | 2e-26 | 3.7e-32 | 223.6 | 478 | (186, 673) | 785 | (59, 618) | 646 | TP901 family phage tail tape measure protein | TP901 family phage tail tape measure protein | | uniclust | UniRef100\_A0A0A7RUN0 | 99.9 | 2.1e-26 | 3.8e-32 | 232.6 | 329 | (110, 460) | 785 | (66, 398) | 757 | Tail-tape measure protein | Tail-tape measure protein | | uniclust | UniRef100\_UPI00065279AA | 99.9 | 2.2e-26 | 4e-32 | 224.8 | 296 | (115, 422) | 785 | (94, 390) | 779 | phage tail tape measure protein | phage tail tape measure protein | | uniclust | UniRef100\_A0A1H6HI37 | 99.9 | 2.5e-26 | 4.6e-32 | 223.4 | 471 | (5, 488) | 785 | (87, 590) | 838 | Phage tail tape measure protein, TP901 family, core region | Phage tail tape measure protein, TP901 family, core region | | uniclust | UniRef100\_UPI00079563B0 | 99.9 | 2.6e-26 | 4.7e-32 | 221.4 | 442 | (111, 581) | 785 | (47, 505) | 668 | phage tail tape measure protein | phage tail tape measure protein | | uniclust | UniRef100\_A0A1Q9TM56 | 99.9 | 2.6e-26 | 4.7e-32 | 218.3 | 358 | (5, 369) | 785 | (78, 450) | 505 | Phage tail tape measure protein | Phage tail tape measure protein | | uniclust | UniRef100\_A0A3R9NYT5 | 99.9 | 2.6e-26 | 4.8e-32 | 218.4 | 305 | (119, 441) | 785 | (62, 367) | 714 | Phage tail tape measure protein | Phage tail tape measure protein | | uniclust | UniRef100\_A0A848AWP4 | 99.9 | 2.7e-26 | 4.8e-32 | 223.2 | 532 | (112, 671) | 785 | (53, 656) | 774 | Phage tail tape measure protein domain-containing protein | Phage tail tape measure protein domain-containing protein | | uniclust | UniRef100\_A0A0J6WUL5 | 99.9 | 3.1e-26 | 5.5e-32 | 242.0 | 323 | (109, 447) | 785 | (148, 480) | 1003 | Phage tail tape measure protein domain-containing protein | Phage tail tape measure protein domain-containing protein | | uniclust | UniRef100\_UPI0003B73CF5 | 99.9 | 3.2e-26 | 5.9e-32 | 218.0 | 542 | (37, 586) | 785 | (103, 675) | 913 | phage tail tape measure protein | phage tail tape measure protein | | uniclust | UniRef100\_A0A1C9C1A8 | 99.9 | 3.4e-26 | 6e-32 | 245.2 | 291 | (247, 592) | 785 | (486, 788) | 1575 | Phage protein | Phage protein | | uniclust | UniRef100\_A0A4Y8RH88 | 99.9 | 3.3e-26 | 6.1e-32 | 226.2 | 441 | (114, 574) | 785 | (59, 500) | 1437 | Phage tail tape measure protein | Phage tail tape measure protein | | uniclust | UniRef100\_A0A1T4WD18 | 99.9 | 3.7e-26 | 6.7e-32 | 221.4 | 424 | (113, 592) | 785 | (63, 490) | 712 | Phage tail tape measure protein, TP901 family, core region | Phage tail tape measure protein, TP901 family, core region | | uniclust | UniRef100\_A0A0J1IMP2 | 99.9 | 4.1e-26 | 7.3e-32 | 246.6 | 329 | (111, 461) | 785 | (299, 641) | 1304 | Phage tail tape measure protein domain-containing protein | Phage tail tape measure protein domain-containing protein | | uniclust | UniRef100\_A0A0C3M8Z2 | 99.9 | 4.2e-26 | 7.5e-32 | 246.1 | 340 | (110, 464) | 785 | (322, 715) | 1915 | Tail protein | Tail protein | | uniclust | UniRef100\_E2CFK1 | 99.9 | 4.2e-26 | 7.6e-32 | 221.7 | 462 | (103, 593) | 785 | (73, 545) | 796 | Putative phage tail tape measure protein, core region | Putative phage tail tape measure protein, core region | | uniclust | UniRef100\_A0A259MGX1 | 99.9 | 4.2e-26 | 7.8e-32 | 214.9 | 454 | (113, 585) | 785 | (70, 523) | 813 | Phage tail tape measure protein | Phage tail tape measure protein | | uniclust | UniRef100\_UPI00039627E7 | 99.9 | 4.7e-26 | 8.6e-32 | 213.3 | 541 | (110, 702) | 785 | (77, 634) | 764 | phage tail tape measure protein | phage tail tape measure protein | | uniclust | UniRef100\_C9RFS8 | 99.9 | 4.8e-26 | 8.8e-32 | 216.2 | 289 | (113, 421) | 785 | (62, 351) | 730 | Phage tail tape measure protein, TP901 family | Phage tail tape measure protein, TP901 family | | uniclust | UniRef100\_A0A086F263 | 99.9 | 4.9e-26 | 8.9e-32 | 220.0 | 563 | (17, 591) | 785 | (11, 620) | 839 | Tail protein | Tail protein | | uniclust | UniRef100\_UPI000949D917 | 99.9 | 5.3e-26 | 9.8e-32 | 198.5 | 341 | (118, 462) | 785 | (73, 413) | 419 | phage tail tape measure protein | phage tail tape measure protein | | uniclust | UniRef100\_A0A2Z4PV63 | 99.9 | 5.6e-26 | 1e-31 | 224.0 | 389 | (80, 472) | 785 | (8, 406) | 729 | Phage tail tape measure protein | Phage tail tape measure protein | | uniclust | UniRef100\_A0A0F7L6E4 | 99.9 | 5.9e-26 | 1e-31 | 236.7 | 395 | (111, 580) | 785 | (50, 448) | 846 | TP901 family phage tail tape measure protein | TP901 family phage tail tape measure protein | | uniclust | UniRef100\_A0A1X0A497 | 99.9 | 6.2e-26 | 1.1e-31 | 238.9 | 319 | (118, 448) | 785 | (330, 651) | 1468 | Phage tail tape measure protein | Phage tail tape measure protein | | uniclust | UniRef100\_A0A660N6E2 | 99.9 | 6.1e-26 | 1.1e-31 | 202.2 | 347 | (118, 468) | 785 | (40, 391) | 491 | Phage tail tape measure protein (Fragment) | Phage tail tape measure protein (Fragment) | | uniclust | UniRef100\_A0A1C6EPE4 | 99.9 | 6.7e-26 | 1.2e-31 | 243.0 | 398 | (4, 443) | 785 | (19, 416) | 1134 | Phage-related protein | Phage-related protein | | uniclust | UniRef100\_A0A2S9YZW5 | 99.9 | 7.8e-26 | 1.4e-31 | 244.2 | 334 | (115, 464) | 785 | (248, 595) | 2271 | Phage tail tape measure protein | Phage tail tape measure protein | | uniclust | UniRef100\_A0A7Z7J1X9 | 99.9 | 8.7e-26 | 1.6e-31 | 205.2 | 310 | (304, 628) | 785 | (4, 315) | 577 | Phage-related tail protein | Phage-related tail protein | | uniclust | UniRef100\_A0A1C6F7V7 | 99.9 | 8.9e-26 | 1.6e-31 | 239.6 | 421 | (26, 463) | 785 | (123, 552) | 1280 | Chromosome segregation protein | Chromosome segregation protein | | uniclust | UniRef100\_A0A071MDL4 | 99.9 | 9.4e-26 | 1.7e-31 | 223.7 | 478 | (240, 780) | 785 | (328, 850) | 850 | Membrane protein | Membrane protein | | uniclust | UniRef100\_A0A0J6WRI9 | 99.9 | 1.1e-25 | 2e-31 | 219.3 | 430 | (115, 584) | 785 | (84, 518) | 737 | Phage tail tape measure protein domain-containing protein | Phage tail tape measure protein domain-containing protein | | uniclust | UniRef100\_A0A3D1NR59 | 99.9 | 1.1e-25 | 2e-31 | 215.1 | 406 | (260, 702) | 785 | (86, 502) | 686 | Phage tail tape measure protein | Phage tail tape measure protein | | uniclust | UniRef100\_A0A829MBP5 | 99.9 | 1.2e-25 | 2.1e-31 | 224.9 | 454 | (117, 591) | 785 | (215, 675) | 1046 | Phage tail tape measure protein, TP901 family, core region | Phage tail tape measure protein, TP901 family, core region | | uniclust | UniRef100\_A0A100JLS2 | 99.9 | 1.2e-25 | 2.2e-31 | 235.2 | 415 | (110, 620) | 785 | (85, 517) | 702 | Phage-related minor tail protein | Phage-related minor tail protein | | uniclust | UniRef100\_A0A2I0DVT3 | 99.9 | 1.2e-25 | 2.2e-31 | 221.9 | 426 | (103, 573) | 785 | (42, 479) | 735 | Phage tail tape measure protein | Phage tail tape measure protein | | uniclust | UniRef100\_A0A0F9MG89 | 99.9 | 1.3e-25 | 2.3e-31 | 210.1 | 327 | (119, 457) | 785 | (104, 431) | 489 | Phage tail tape measure protein domain-containing protein (Fragment) | Phage tail tape measure protein domain-containing protein (Fragment) | | uniclust | UniRef100\_UPI001878DDDC | 99.9 | 1.3e-25 | 2.3e-31 | 206.9 | 349 | (115, 467) | 785 | (53, 401) | 619 | phage tail tape measure protein | phage tail tape measure protein | | uniclust | UniRef100\_A0A2J4RFS2 | 99.9 | 1.3e-25 | 2.3e-31 | 212.4 | 347 | (4, 357) | 785 | (78, 432) | 434 | Phage tail tape measure protein (Fragment) | Phage tail tape measure protein (Fragment) | | uniclust | UniRef100\_A0A9D5UUJ2 | 99.9 | 1.3e-25 | 2.4e-31 | 226.7 | 451 | (5, 459) | 785 | (80, 581) | 1509 | Phage tail tape measure protein | Phage tail tape measure protein | | uniclust | UniRef100\_A0A1C0TVM0 | 99.9 | 1.4e-25 | 2.4e-31 | 216.7 | 344 | (101, 467) | 785 | (43, 395) | 486 | Phage tail tape measure protein | Phage tail tape measure protein | | uniclust | UniRef100\_A0A355D6E1 | 99.9 | 1.5e-25 | 2.6e-31 | 226.9 | 343 | (111, 464) | 785 | (64, 406) | 1089 | Phage tail tape measure protein | Phage tail tape measure protein | | uniclust | UniRef100\_A0A081L6U1 | 99.9 | 1.5e-25 | 2.7e-31 | 248.8 | 317 | (117, 450) | 785 | (304, 628) | 1423 | Transglycosylase SLT domain-containing protein | Transglycosylase SLT domain-containing protein | | uniclust | UniRef100\_A0A1V3J0L3 | 99.9 | 1.6e-25 | 3e-31 | 201.2 | 330 | (430, 782) | 785 | (3, 404) | 404 | Phage tail tape measure protein | Phage tail tape measure protein | | uniclust | UniRef100\_A0A1C6E118 | 99.9 | 1.7e-25 | 3.1e-31 | 232.9 | 327 | (114, 440) | 785 | (58, 399) | 1176 | Phage-related protein | Phage-related protein | | uniclust | UniRef100\_UPI002227BD23 | 99.9 | 1.7e-25 | 3.1e-31 | 214.8 | 413 | (117, 586) | 785 | (96, 517) | 984 | phage tail tape measure protein | phage tail tape measure protein | | uniclust | UniRef100\_A0A4Q4AZ49 | 99.9 | 1.7e-25 | 3.1e-31 | 202.4 | 451 | (282, 782) | 785 | (7, 464) | 464 | Phage tail tape measure protein | Phage tail tape measure protein | | uniclust | UniRef100\_A0A143Z8T0 | 99.9 | 1.9e-25 | 3.5e-31 | 220.3 | 427 | (111, 597) | 785 | (153, 588) | 858 | Phage tail tape measure protein | Phage tail tape measure protein | | uniclust | UniRef100\_A0A1X7MGH5 | 99.9 | 2.1e-25 | 3.9e-31 | 199.4 | 314 | (265, 594) | 785 | (2, 321) | 448 | Phage tail tape measure protein, family, core region | Phage tail tape measure protein, family, core region | | uniclust | UniRef100\_A0A143HC28 | 99.9 | 2.2e-25 | 3.9e-31 | 238.9 | 373 | (4, 444) | 785 | (5, 385) | 1162 | Phage tail tape measure protein domain-containing protein | Phage tail tape measure protein domain-containing protein | | uniclust | UniRef100\_A0A1V0UQY8 | 99.9 | 2.5e-25 | 4.6e-31 | 222.5 | 324 | (110, 445) | 785 | (61, 385) | 954 | Phage tail tape measure protein | Phage tail tape measure protein | | uniclust | UniRef100\_A0A0H3U2U8 | 99.9 | 2.6e-25 | 4.8e-31 | 216.8 | 417 | (118, 594) | 785 | (180, 605) | 951 | Tail length tape measure | Tail length tape measure | | uniclust | UniRef100\_A0A1I5H9Z3 | 99.9 | 2.7e-25 | 5e-31 | 213.3 | 471 | (5, 485) | 785 | (79, 564) | 874 | Phage tail tape measure protein, TP901 family, core region | Phage tail tape measure protein, TP901 family, core region | | uniclust | UniRef100\_A0A7W9YIK7 | 99.9 | 2.8e-25 | 5e-31 | 228.3 | 451 | (118, 589) | 785 | (230, 715) | 1645 | TP901 family phage tail tape measure protein | TP901 family phage tail tape measure protein | | uniclust | UniRef100\_A0A0U4II47 | 99.9 | 3e-25 | 5.3e-31 | 228.2 | 319 | (115, 449) | 785 | (146, 467) | 753 | Tail tape measure protein | Tail tape measure protein | | uniclust | UniRef100\_A0A099S5E7 | 99.9 | 3.1e-25 | 5.4e-31 | 234.5 | 273 | (393, 701) | 785 | (328, 638) | 875 | Phage-like protein | Phage-like protein | | uniclust | UniRef100\_A0A2P5GLT3 | 99.9 | 3.2e-25 | 5.8e-31 | 212.5 | 517 | (124, 670) | 785 | (90, 646) | 761 | Phage tail tape measure protein | Phage tail tape measure protein | | uniclust | UniRef100\_A0A1S1YB82 | 99.9 | 3.4e-25 | 6.2e-31 | 219.9 | 421 | (111, 593) | 785 | (50, 486) | 784 | Phage tail tape measure protein domain-containing protein | Phage tail tape measure protein domain-containing protein | | uniclust | UniRef100\_A0A1E3GZT2 | 99.9 | 3.7e-25 | 6.6e-31 | 219.7 | 332 | (112, 462) | 785 | (62, 403) | 766 | Phage-related minor tail protein | Phage-related minor tail protein | | uniclust | UniRef100\_A0A2T4MMQ9 | 99.9 | 3.8e-25 | 7e-31 | 208.6 | 309 | (112, 432) | 785 | (170, 479) | 541 | Phage tail tape measure protein (Fragment) | Phage tail tape measure protein (Fragment) | | uniclust | UniRef100\_A0A0R1GKD1 | 99.9 | 3.9e-25 | 7e-31 | 242.2 | 347 | (110, 466) | 785 | (303, 659) | 2004 | NlpC/P60 domain-containing protein | NlpC/P60 domain-containing protein | | uniclust | UniRef100\_A0A1A7KG91 | 99.9 | 4e-25 | 7.1e-31 | 222.7 | 468 | (118, 622) | 785 | (67, 554) | 793 | Phage-related minor tail protein | Phage-related minor tail protein | | uniclust | UniRef100\_A0A173RDD3 | 99.9 | 4.2e-25 | 7.4e-31 | 242.2 | 323 | (111, 444) | 785 | (82, 404) | 1392 | Phage-related protein | Phage-related protein | | uniclust | UniRef100\_A0A4T2BU47 | 99.9 | 4.2e-25 | 7.5e-31 | 234.5 | 327 | (119, 457) | 785 | (252, 667) | 1102 | Phage tail tape measure protein | Phage tail tape measure protein | | uniclust | UniRef100\_A0A4Z0GIX0 | 99.9 | 4.2e-25 | 7.6e-31 | 226.2 | 326 | (110, 445) | 785 | (272, 598) | 1299 | Phage tail tape measure protein (Fragment) | Phage tail tape measure protein (Fragment) | | uniclust | UniRef100\_A0A5C5TFK9 | 99.9 | 4.2e-25 | 7.6e-31 | 207.0 | 423 | (19, 448) | 785 | (47, 494) | 535 | Phage tail tape measure protein | Phage tail tape measure protein | | uniclust | UniRef100\_A0A143ZV16 | 99.9 | 4.8e-25 | 8.4e-31 | 238.9 | 321 | (111, 443) | 785 | (46, 367) | 1213 | Phage-related minor tail protein | Phage-related minor tail protein | | uniclust | UniRef100\_A0A239EK42 | 99.9 | 4.6e-25 | 8.5e-31 | 215.5 | 445 | (107, 582) | 785 | (63, 512) | 1202 | Phage tail tape measure protein, TP901 family, core region | Phage tail tape measure protein, TP901 family, core region | | uniclust | UniRef100\_A0A291LZ62 | 99.9 | 4.7e-25 | 8.6e-31 | 206.3 | 423 | (4, 439) | 785 | (5, 437) | 746 | Phage tail tape measure protein | Phage tail tape measure protein | | uniclust | UniRef100\_UPI001118EFF6 | 99.9 | 5.2e-25 | 9.5e-31 | 193.5 | 316 | (208, 580) | 785 | (3, 318) | 377 | phage tail tape measure protein | phage tail tape measure protein | | uniclust | UniRef100\_A0A432QRK4 | 99.9 | 5.2e-25 | 9.5e-31 | 210.0 | 348 | (116, 472) | 785 | (9, 359) | 908 | Phage tail tape measure protein (Fragment) | Phage tail tape measure protein (Fragment) | | uniclust | UniRef100\_A0A7C4LLA7 | 99.9 | 5.3e-25 | 9.7e-31 | 215.0 | 347 | (115, 473) | 785 | (57, 404) | 926 | Phage tail tape measure protein | Phage tail tape measure protein | | uniclust | UniRef100\_A0A3R6NIQ7 | 99.9 | 5.4e-25 | 9.9e-31 | 220.3 | 320 | (111, 448) | 785 | (111, 432) | 1172 | Phage tail tape measure protein | Phage tail tape measure protein | | uniclust | UniRef100\_A0A0K1Y580 | 99.8 | 5.8e-25 | 1e-30 | 237.1 | 433 | (114, 594) | 785 | (69, 531) | 1587 | Phage tail tape measure protein domain-containing protein | Phage tail tape measure protein domain-containing protein | | uniclust | UniRef100\_A0A806D8P0 | 99.8 | 5.8e-25 | 1.1e-30 | 209.0 | 468 | (120, 594) | 785 | (204, 704) | 876 | Phage tail tape measure protein, TP901 family | Phage tail tape measure protein, TP901 family | | uniclust | UniRef100\_A0A7X8V2E7 | 99.8 | 6.9e-25 | 1.3e-30 | 194.7 | 288 | (278, 588) | 785 | (9, 296) | 471 | Phage tail tape measure protein | Phage tail tape measure protein | | uniclust | UniRef100\_A0A2W4KBS4 | 99.8 | 7.1e-25 | 1.3e-30 | 212.3 | 346 | (112, 463) | 785 | (84, 439) | 738 | Phage tail tape measure protein (Fragment) | Phage tail tape measure protein (Fragment) | | uniclust | UniRef100\_A0A3N4UR92 | 99.8 | 7.3e-25 | 1.3e-30 | 217.4 | 448 | (121, 592) | 785 | (124, 616) | 812 | TP901 family phage tail tape measure protein | TP901 family phage tail tape measure protein | | uniclust | UniRef100\_A0A0A0YSZ0 | 99.8 | 7.7e-25 | 1.4e-30 | 216.1 | 409 | (109, 572) | 785 | (60, 481) | 637 | Phage tail tape measure protein domain-containing protein | Phage tail tape measure protein domain-containing protein | | uniclust | UniRef100\_A0A5C7JCR9 | 99.8 | 7.6e-25 | 1.4e-30 | 199.4 | 344 | (115, 464) | 785 | (85, 429) | 580 | Phage tail tape measure protein (Fragment) | Phage tail tape measure protein (Fragment) | | uniclust | UniRef100\_A0A8T4G949 | 99.8 | 8.2e-25 | 1.5e-30 | 208.2 | 306 | (117, 439) | 785 | (47, 353) | 713 | Phage tail tape measure protein | Phage tail tape measure protein | | uniclust | UniRef100\_A0A8I1W8W3 | 99.8 | 8.2e-25 | 1.5e-30 | 210.5 | 438 | (24, 466) | 785 | (174, 617) | 802 | Phage tail tape measure protein | Phage tail tape measure protein | | pdb70 | 6V8I\_CF | 99.2 | 9.4e-16 | 1.3e-19 | 163.0 | 95 | (155, 250) | 785 | (175, 269) | 1154 | Distal Tail Protein, gp58, Tail-Associated | 6V8I\_CF Distal Tail Protein, gp58, Tail-Associated phage tail, tail tip, tape | |
| Top keywords  (threshold 1.00e-03 (evalue)) | **tail, Phage, tape, measure, TP901, domain\_containing, core, region, Phage\_related, Fragment** |
| Output files | ../../similar\_sequences/22\_FANPEZAQ\_CDS\_0022\_merged.svg ../../similar\_sequences/22\_FANPEZAQ\_CDS\_0022\_pdb70.a3m ../../similar\_sequences/22\_FANPEZAQ\_CDS\_0022\_pdb70.hhr ../../similar\_sequences/22\_FANPEZAQ\_CDS\_0022\_uniclust.a3m ../../similar\_sequences/22\_FANPEZAQ\_CDS\_0022\_uniclust.hhr |

#### Structure prediction (AlphaFold)2

|  |  |
| --- | --- |
| Stats | xml version="1.0" encoding="utf-8" standalone="no"?       2024-09-02T21:09:22.305241 image/svg+xml   Matplotlib v3.7.2, https://matplotlib.org/ |
| Predicted structure | **NGL Viewer Controls:**  - Center: *Left-Click* - Rotate: *Left-Click + Drag* - Translate: *Right-Click + Drag* - Zoom: *Shift + Left-Click + Drag* |
| Output files | ../../predicted\_structures/22\_FANPEZAQ\_CDS\_0022/features.pkl ../../predicted\_structures/22\_FANPEZAQ\_CDS\_0022/ranked\_0.pdb ../../predicted\_structures/22\_FANPEZAQ\_CDS\_0022/ranked\_0\_plots.svg ../../predicted\_structures/22\_FANPEZAQ\_CDS\_0022/result\_model\_1\_ptm\_pred\_0.pkl |

#### Structure similarity search results (Foldseek)3

|  |  |
| --- | --- |
| Structure databases searched | Pdb, Afdb-proteome, Afdb-uniprot50 |
| Results, scheme(s)  (Top layers only, threshold 1.00e-02 (evalue)) | xml version="1.0" encoding="utf-8" standalone="no"?       2024-09-02T21:10:49.053219 image/svg+xml   Matplotlib v3.7.2, https://matplotlib.org/ |
| Results, table  (threshold 1.00e-02 (evalue)) | | db | id | prob | evalue | bits | fident | alnlen | mismatch | gapopen | qstart | qend | tstart | tend | name | description | | --- | --- | --- | --- | --- | --- | --- | --- | --- | --- | --- | --- | --- | --- | --- | | afdb-proteome | AF-Q8ZMV3-F1-MODEL\_V4 | 1.0 | 1.278e-23 | 829 | 0.198 | 920 | 552 | 21 | 19 | 785 | 48 | 934 | Fels-2 prophage protein | Fels-2 prophage protein | | afdb-proteome | AF-A0A0H3H1R5-F1-MODEL\_V4 | 1.0 | 3.576e-18 | 539 | 0.202 | 846 | 510 | 25 | 19 | 781 | 50 | 813 | Putative prophage tail length determinator | Putative prophage tail length determinator | | afdb-proteome | AF-A0A0H3GQS8-F1-MODEL\_V4 | 1.0 | 3.039e-14 | 434 | 0.163 | 883 | 530 | 30 | 15 | 785 | 11 | 796 | Phage tail tape measure protein, family | Phage tail tape measure protein, family | | afdb-proteome | AF-A0A5P3FW64-F1-MODEL\_V4 | 1.0 | 3.051e-11 | 405 | 0.176 | 589 | 352 | 14 | 1 | 459 | 1 | 586 | Phage tail tape measure protein | Phage tail tape measure protein | | afdb-proteome | AF-Q2FWU3-F1-MODEL\_V4 | 1.0 | 1.937e-12 | 377 | 0.142 | 921 | 545 | 39 | 1 | 702 | 1 | 895 | Lysostaphin | Lysostaphin | | afdb-proteome | AF-Q8ZKJ4-F1-MODEL\_V4 | 1.0 | 4.604e-13 | 352 | 0.174 | 785 | 510 | 32 | 35 | 754 | 3 | 714 | Putative phage tail protein | Putative phage tail protein | | afdb-proteome | AF-Q2FYC8-F1-MODEL\_V4 | 1.0 | 4.623e-10 | 349 | 0.142 | 631 | 368 | 19 | 19 | 519 | 127 | 714 | Lysostaphin | Lysostaphin | | afdb-proteome | AF-G3XD83-F1-MODEL\_V4 | 1.0 | 1.953e-06 | 208 | 0.157 | 513 | 341 | 19 | 2 | 469 | 1 | 467 | Uncharacterized protein | Uncharacterized protein | | afdb-proteome | AF-A0A5K4F6A0-F1-MODEL\_V4 | 0.382 | 0.007632 | 47 | 0.088 | 782 | 393 | 30 | 103 | 785 | 14 | 574 | G\_PROTEIN\_RECEP\_F1\_2 domain-containing protein | G\_PROTEIN\_RECEP\_F1\_2 domain-containing protein | | afdb-uniprot50 | AF-A0A5U1APE0-F1-MODEL\_V4 | 1.0 | 4.447e-25 | 1458 | 0.456 | 353 | 189 | 1 | 117 | 469 | 12 | 361 | Phage tail tape measure protein | Phage tail tape measure protein | | afdb-uniprot50 | AF-A0A5U1APS5-F1-MODEL\_V4 | 1.0 | 1.639e-26 | 1366 | 0.423 | 418 | 216 | 5 | 61 | 469 | 1 | 402 | Phage tail tape measure protein | Phage tail tape measure protein | | afdb-uniprot50 | AF-A0A761LW72-F1-MODEL\_V4 | 1.0 | 9.78e-26 | 1344 | 0.432 | 414 | 210 | 5 | 61 | 465 | 1 | 398 | Phage tail tape measure protein | Phage tail tape measure protein | | afdb-uniprot50 | AF-A0A5Y1GPG4-F1-MODEL\_V4 | 1.0 | 6.507e-21 | 1221 | 0.496 | 298 | 147 | 1 | 133 | 430 | 2 | 296 | Phage tail tape measure protein | Phage tail tape measure protein | | afdb-uniprot50 | AF-A0A7T7R8S8-F1-MODEL\_V4 | 1.0 | 2.979e-34 | 1216 | 0.314 | 796 | 504 | 9 | 1 | 785 | 1 | 765 | Phage tail tape measure protein | Phage tail tape measure protein | | afdb-uniprot50 | AF-A0A450W6Y7-F1-MODEL\_V4 | 1.0 | 1.115e-33 | 1146 | 0.316 | 791 | 442 | 10 | 21 | 755 | 50 | 797 | Phage tail tape measure protein, TP901 family, core region | Phage tail tape measure protein, TP901 family, core region | | afdb-uniprot50 | AF-A0A5T2W9F8-F1-MODEL\_V4 | 1.0 | 1.945e-24 | 1138 | 0.334 | 577 | 278 | 10 | 118 | 687 | 2 | 479 | Phage tail tape measure protein | Phage tail tape measure protein | | afdb-uniprot50 | AF-A0A630L2F6-F1-MODEL\_V4 | 1.0 | 1.745e-18 | 1128 | 0.496 | 266 | 131 | 1 | 134 | 399 | 2 | 264 | Phage tail tape measure protein | Phage tail tape measure protein | | afdb-uniprot50 | AF-A0A5Z1EPF8-F1-MODEL\_V4 | 1.0 | 9.86e-20 | 1127 | 0.344 | 348 | 219 | 3 | 117 | 464 | 12 | 350 | Phage tail tape measure protein | Phage tail tape measure protein | | afdb-uniprot50 | AF-A0A4P6BLS6-F1-MODEL\_V4 | 1.0 | 1.278e-28 | 1087 | 0.309 | 795 | 390 | 16 | 18 | 777 | 17 | 687 | PhageMin\_Tail domain-containing protein | PhageMin\_Tail domain-containing protein | | afdb-uniprot50 | AF-A0A2J7U4C6-F1-MODEL\_V4 | 1.0 | 1.249e-26 | 1075 | 0.347 | 656 | 337 | 17 | 61 | 692 | 1 | 589 | Phage tail tape measure protein | Phage tail tape measure protein | | afdb-uniprot50 | AF-A0A5Z1ENM2-F1-MODEL\_V4 | 1.0 | 1.23e-18 | 1070 | 0.331 | 347 | 224 | 4 | 117 | 461 | 12 | 352 | Phage tail tape measure protein | Phage tail tape measure protein | | afdb-uniprot50 | AF-A0A601P240-F1-MODEL\_V4 | 1.0 | 3.662e-25 | 1069 | 0.323 | 631 | 320 | 13 | 117 | 729 | 12 | 553 | Phage tail tape measure protein | Phage tail tape measure protein | | afdb-uniprot50 | AF-A0A754TYG6-F1-MODEL\_V4 | 1.0 | 1.002e-17 | 1045 | 0.513 | 251 | 122 | 0 | 180 | 430 | 1 | 251 | Phage tail tape measure protein | Phage tail tape measure protein | | afdb-uniprot50 | AF-A0A5T1M5R4-F1-MODEL\_V4 | 1.0 | 2.144e-19 | 1034 | 0.332 | 388 | 248 | 4 | 79 | 464 | 21 | 399 | Phage tail tape measure protein | Phage tail tape measure protein | | afdb-uniprot50 | AF-F7U3Q6-F1-MODEL\_V4 | 1.0 | 7.336e-28 | 1033 | 0.269 | 789 | 432 | 18 | 63 | 782 | 2 | 714 | Family phage tail tape measure protein | Family phage tail tape measure protein | | afdb-uniprot50 | AF-A0A560D0Q3-F1-MODEL\_V4 | 1.0 | 5.173e-28 | 1015 | 0.33 | 727 | 362 | 19 | 70 | 752 | 7 | 652 | TP901 family phage tail tape measure protein | TP901 family phage tail tape measure protein | | afdb-uniprot50 | AF-A0A7U3GJM6-F1-MODEL\_V4 | 1.0 | 3.593e-20 | 1009 | 0.343 | 405 | 231 | 7 | 39 | 433 | 8 | 387 | Phage tail tape measure protein | Phage tail tape measure protein | | afdb-uniprot50 | AF-A0A4R2N8L9-F1-MODEL\_V4 | 1.0 | 3.677e-22 | 1007 | 0.34 | 487 | 305 | 6 | 6 | 484 | 1 | 479 | TP901 family phage tail tape measure protein | TP901 family phage tail tape measure protein | | afdb-uniprot50 | AF-A0A7X8PBI2-F1-MODEL\_V4 | 1.0 | 4.48e-29 | 999 | 0.284 | 826 | 471 | 20 | 1 | 782 | 1 | 750 | Phage tail tape measure protein | Phage tail tape measure protein | | afdb-uniprot50 | AF-A0A430S197-F1-MODEL\_V4 | 1.0 | 5.571e-21 | 993 | 0.329 | 465 | 282 | 8 | 62 | 508 | 4 | 456 | Phage tail tape measure protein | Phage tail tape measure protein | | afdb-uniprot50 | AF-A0A4P7EI80-F1-MODEL\_V4 | 1.0 | 3.677e-22 | 982 | 0.302 | 505 | 290 | 7 | 19 | 466 | 51 | 550 | Phage tail tape measure protein | Phage tail tape measure protein | | afdb-uniprot50 | AF-A0A1M6LSY3-F1-MODEL\_V4 | 1.0 | 3.689e-29 | 981 | 0.256 | 829 | 467 | 17 | 19 | 702 | 45 | 868 | Phage tail tape measure protein, TP901 family, core region | Phage tail tape measure protein, TP901 family, core region | | afdb-uniprot50 | AF-F5SA52-F1-MODEL\_V4 | 1.0 | 8.378e-16 | 980 | 0.554 | 222 | 99 | 0 | 155 | 376 | 3 | 224 | Tail tape measure protein | Tail tape measure protein | | afdb-uniprot50 | AF-A0A2A2IKN4-F1-MODEL\_V4 | 1.0 | 2.856e-27 | 977 | 0.289 | 797 | 404 | 20 | 19 | 785 | 20 | 683 | Phage tail tape measure protein | Phage tail tape measure protein | | afdb-uniprot50 | AF-A0A823TKD2-F1-MODEL\_V4 | 1.0 | 9.337e-22 | 973 | 0.267 | 527 | 330 | 8 | 29 | 552 | 2 | 475 | Phage tail tape measure protein | Phage tail tape measure protein | | afdb-uniprot50 | AF-A0A315BPW6-F1-MODEL\_V4 | 1.0 | 1.283e-25 | 963 | 0.271 | 789 | 424 | 11 | 1 | 785 | 8 | 649 | Phage tail tape measure protein | Phage tail tape measure protein | | afdb-uniprot50 | AF-A0A745AEG2-F1-MODEL\_V4 | 1.0 | 2.07e-16 | 961 | 0.501 | 251 | 122 | 1 | 118 | 368 | 33 | 280 | Phage tail tape measure protein | Phage tail tape measure protein | | afdb-uniprot50 | AF-A0A4Q0GN92-F1-MODEL\_V4 | 1.0 | 1.298e-26 | 961 | 0.268 | 823 | 412 | 18 | 22 | 782 | 50 | 744 | Phage tail tape measure protein | Phage tail tape measure protein | | afdb-uniprot50 | AF-A0A484YXF6-F1-MODEL\_V4 | 1.0 | 1.25e-16 | 958 | 0.522 | 241 | 115 | 0 | 190 | 430 | 2 | 242 | Phage tail tape measure protein | Phage tail tape measure protein | | afdb-uniprot50 | AF-Q4EB59-F1-MODEL\_V4 | 1.0 | 1.799e-24 | 957 | 0.279 | 636 | 378 | 8 | 24 | 619 | 4 | 598 | Phage tail tape meausure protein, TP901 family, putative | Phage tail tape meausure protein, TP901 family, putative | | afdb-uniprot50 | AF-A0A751FK49-F1-MODEL\_V4 | 1.0 | 5.594e-18 | 955 | 0.467 | 276 | 147 | 0 | 190 | 465 | 3 | 278 | Phage tail tape measure protein | Phage tail tape measure protein | | afdb-uniprot50 | AF-G3IRG2-F1-MODEL\_V4 | 1.0 | 2.571e-28 | 952 | 0.315 | 842 | 423 | 22 | 1 | 780 | 1 | 750 | Phage tail tape measure protein, TP901 family | Phage tail tape measure protein, TP901 family | | afdb-uniprot50 | AF-A0A8A6KE23-F1-MODEL\_V4 | 1.0 | 1.499e-25 | 943 | 0.266 | 789 | 481 | 18 | 1 | 784 | 1 | 696 | Phage tail tape measure protein | Phage tail tape measure protein | | afdb-uniprot50 | AF-B6WUA5-F1-MODEL\_V4 | 1.0 | 3.311e-23 | 940 | 0.274 | 677 | 350 | 16 | 1 | 672 | 1 | 540 | Phage tail tape measure protein, TP901 family | Phage tail tape measure protein, TP901 family | | afdb-uniprot50 | AF-A0A430B9P2-F1-MODEL\_V4 | 1.0 | 2.592e-22 | 935 | 0.311 | 575 | 322 | 14 | 70 | 620 | 2 | 526 | Phage tail tape measure protein | Phage tail tape measure protein | | afdb-uniprot50 | AF-A0A2M7BJQ9-F1-MODEL\_V4 | 1.0 | 4.196e-20 | 929 | 0.272 | 480 | 313 | 6 | 19 | 480 | 61 | 522 | Phage tail tape measure protein | Phage tail tape measure protein | | afdb-uniprot50 | AF-A0A8B0U511-F1-MODEL\_V4 | 1.0 | 1.35e-26 | 927 | 0.267 | 821 | 483 | 23 | 1 | 785 | 1 | 738 | Phage tail tape measure protein | Phage tail tape measure protein | | afdb-uniprot50 | AF-A0A259MGX1-F1-MODEL\_V4 | 1.0 | 5.59e-28 | 922 | 0.29 | 799 | 443 | 25 | 13 | 782 | 1 | 704 | Phage tail tape measure protein | Phage tail tape measure protein | | afdb-uniprot50 | AF-A0A846BM80-F1-MODEL\_V4 | 1.0 | 6.358e-19 | 921 | 0.325 | 446 | 255 | 7 | 117 | 562 | 38 | 437 | Phage tail tape measure protein | Phage tail tape measure protein | | afdb-uniprot50 | AF-A0A350LX86-F1-MODEL\_V4 | 1.0 | 4.362e-20 | 918 | 0.294 | 455 | 296 | 7 | 66 | 499 | 15 | 465 | Phage tail tape measure protein | Phage tail tape measure protein | | afdb-uniprot50 | AF-A0A844HR58-F1-MODEL\_V4 | 1.0 | 3.958e-25 | 915 | 0.271 | 767 | 395 | 18 | 116 | 783 | 14 | 715 | Phage tail tape measure protein | Phage tail tape measure protein | | afdb-uniprot50 | AF-A0A660N6E2-F1-MODEL\_V4 | 1.0 | 5.359e-21 | 913 | 0.303 | 530 | 286 | 11 | 46 | 539 | 5 | 487 | Phage tail tape measure protein | Phage tail tape measure protein | | afdb-uniprot50 | AF-A0A348AJ09-F1-MODEL\_V4 | 1.0 | 7.539e-27 | 911 | 0.262 | 843 | 486 | 16 | 19 | 785 | 11 | 793 | Phage-related minor tail protein | Phage-related minor tail protein | | afdb-uniprot50 | AF-A0A2W5VBB3-F1-MODEL\_V4 | 1.0 | 5.972e-27 | 911 | 0.286 | 839 | 461 | 22 | 19 | 783 | 52 | 826 | Phage tail tape measure protein | Phage tail tape measure protein | | afdb-uniprot50 | AF-A0A7W5RGC2-F1-MODEL\_V4 | 1.0 | 6.454e-27 | 907 | 0.284 | 847 | 478 | 19 | 1 | 784 | 2 | 783 | TP901 family phage tail tape measure protein | TP901 family phage tail tape measure protein | | afdb-uniprot50 | AF-A0A1S9ZKE4-F1-MODEL\_V4 | 1.0 | 3.259e-25 | 906 | 0.287 | 695 | 432 | 14 | 36 | 702 | 2 | 660 | Phage tail tape measure protein | Phage tail tape measure protein | | afdb-uniprot50 | AF-G2HXA7-F1-MODEL\_V4 | 1.0 | 7.168e-26 | 903 | 0.272 | 799 | 458 | 21 | 3 | 785 | 1 | 691 | Phage tail tape measure protein | Phage tail tape measure protein | | afdb-uniprot50 | AF-G4CJF3-F1-MODEL\_V4 | 1.0 | 1.029e-26 | 903 | 0.255 | 758 | 471 | 19 | 19 | 710 | 21 | 750 | Phage tail tape measure protein | Phage tail tape measure protein | | afdb-uniprot50 | AF-A0A1M3AG88-F1-MODEL\_V4 | 1.0 | 1.156e-26 | 901 | 0.26 | 920 | 459 | 23 | 13 | 785 | 1 | 845 | Phage tail tape measure protein | Phage tail tape measure protein | | afdb-uniprot50 | AF-A0A359KC48-F1-MODEL\_V4 | 1.0 | 8.776e-20 | 894 | 0.346 | 513 | 266 | 12 | 22 | 475 | 53 | 554 | Phage tail tape measure protein | Phage tail tape measure protein | | afdb-uniprot50 | AF-A0A5M7PDK9-F1-MODEL\_V4 | 1.0 | 3.272e-22 | 891 | 0.279 | 644 | 362 | 13 | 70 | 706 | 7 | 555 | Phage tail tape measure protein | Phage tail tape measure protein | | afdb-uniprot50 | AF-A0A1I3JGJ4-F1-MODEL\_V4 | 1.0 | 1.771e-26 | 890 | 0.319 | 793 | 435 | 21 | 62 | 784 | 14 | 771 | Phage tail tape measure protein, TP901 family, core region | Phage tail tape measure protein, TP901 family, core region | | afdb-uniprot50 | AF-A0A5C7R6S2-F1-MODEL\_V4 | 1.0 | 1.108e-19 | 888 | 0.313 | 492 | 317 | 6 | 1 | 480 | 1 | 483 | Phage tail tape measure protein | Phage tail tape measure protein | | afdb-uniprot50 | AF-A0A2G0WA81-F1-MODEL\_V4 | 1.0 | 1.289e-22 | 888 | 0.272 | 729 | 371 | 16 | 71 | 750 | 2 | 619 | PhageMin\_Tail domain-containing protein | PhageMin\_Tail domain-containing protein | | afdb-uniprot50 | AF-A0A378UD30-F1-MODEL\_V4 | 1.0 | 6.71e-27 | 887 | 0.257 | 793 | 466 | 17 | 18 | 710 | 20 | 789 | Putative phage-related tail protein | Putative phage-related tail protein | | afdb-uniprot50 | AF-W4M094-F1-MODEL\_V4 | 1.0 | 5.529e-17 | 886 | 0.341 | 340 | 219 | 3 | 143 | 480 | 6 | 342 | PhageMin\_Tail domain-containing protein | PhageMin\_Tail domain-containing protein | | afdb-uniprot50 | AF-A0A5A9EN03-F1-MODEL\_V4 | 1.0 | 4.327e-26 | 886 | 0.269 | 762 | 457 | 14 | 19 | 700 | 32 | 773 | Phage tail tape measure protein | Phage tail tape measure protein | | afdb-uniprot50 | AF-K8AC29-F1-MODEL\_V4 | 1.0 | 5.702e-23 | 882 | 0.254 | 711 | 402 | 19 | 71 | 754 | 2 | 611 | Phage tail length tape-measure protein | Phage tail length tape-measure protein | | afdb-uniprot50 | AF-A0A1V0BIB8-F1-MODEL\_V4 | 1.0 | 4.327e-26 | 881 | 0.227 | 985 | 507 | 19 | 19 | 782 | 28 | 979 | Phage tail tape measure protein | Phage tail tape measure protein | | afdb-uniprot50 | AF-A0A2X4DBT0-F1-MODEL\_V4 | 1.0 | 2.684e-25 | 880 | 0.234 | 891 | 510 | 22 | 19 | 780 | 64 | 910 | Phage-like tail protein | Phage-like tail protein | | afdb-uniprot50 | AF-A0A606E632-F1-MODEL\_V4 | 1.0 | 4.449e-15 | 879 | 0.493 | 243 | 120 | 1 | 115 | 357 | 1 | 240 | Phage tail tape measure protein | Phage tail tape measure protein | | afdb-uniprot50 | AF-A0A5M7L7B0-F1-MODEL\_V4 | 1.0 | 7.779e-23 | 878 | 0.259 | 689 | 388 | 17 | 87 | 755 | 3 | 588 | Phage tail tape measure protein | Phage tail tape measure protein | | afdb-uniprot50 | AF-A0A2U0TFG7-F1-MODEL\_V4 | 1.0 | 8.054e-26 | 874 | 0.249 | 763 | 449 | 16 | 7 | 754 | 1 | 654 | TP901 family phage tail tape measure protein | TP901 family phage tail tape measure protein | | afdb-uniprot50 | AF-A0A423XU08-F1-MODEL\_V4 | 1.0 | 2.16e-23 | 873 | 0.257 | 733 | 398 | 20 | 74 | 754 | 2 | 639 | Phage tail tape measure protein | Phage tail tape measure protein | | afdb-uniprot50 | AF-A0A6I2KDC7-F1-MODEL\_V4 | 1.0 | 1.23e-18 | 869 | 0.301 | 478 | 292 | 10 | 120 | 572 | 4 | 464 | Phage tail tape measure protein | Phage tail tape measure protein | | afdb-uniprot50 | AF-A0A5C4PN41-F1-MODEL\_V4 | 1.0 | 1.967e-25 | 866 | 0.313 | 720 | 416 | 14 | 14 | 702 | 12 | 683 | Phage tail tape measure protein | Phage tail tape measure protein | | afdb-uniprot50 | AF-A0A1M5CNY5-F1-MODEL\_V4 | 1.0 | 2.633e-20 | 864 | 0.268 | 536 | 345 | 9 | 5 | 508 | 2 | 522 | Phage tail tape measure protein, TP901 family, core region | Phage tail tape measure protein, TP901 family, core region | | afdb-uniprot50 | AF-A0A0F9TCL8-F1-MODEL\_V4 | 1.0 | 8.981e-22 | 863 | 0.286 | 608 | 342 | 11 | 19 | 558 | 55 | 638 | PhageMin\_Tail domain-containing protein | PhageMin\_Tail domain-containing protein | | afdb-uniprot50 | AF-A0A0X1U7S0-F1-MODEL\_V4 | 1.0 | 1.583e-23 | 862 | 0.264 | 832 | 393 | 24 | 14 | 776 | 1 | 682 | Phage-related minor tail protein | Phage-related minor tail protein | | afdb-uniprot50 | AF-A0A259CEH9-F1-MODEL\_V4 | 1.0 | 6.38e-26 | 862 | 0.271 | 814 | 458 | 15 | 46 | 780 | 3 | 760 | Phage tail tape measure protein | Phage tail tape measure protein | | afdb-uniprot50 | AF-A0A2N9ASI3-F1-MODEL\_V4 | 1.0 | 2.686e-15 | 861 | 0.229 | 340 | 254 | 3 | 117 | 455 | 138 | 470 | PhageMin\_Tail domain-containing protein | PhageMin\_Tail domain-containing protein | | afdb-uniprot50 | AF-A0A1T4V251-F1-MODEL\_V4 | 1.0 | 4.1e-18 | 859 | 0.301 | 451 | 272 | 10 | 91 | 519 | 9 | 438 | Phage-related minor tail protein | Phage-related minor tail protein | | afdb-uniprot50 | AF-A0A519ZJF7-F1-MODEL\_V4 | 1.0 | 5.215e-22 | 858 | 0.226 | 690 | 426 | 16 | 119 | 782 | 38 | 645 | Phage tail tape measure protein | Phage tail tape measure protein | | afdb-uniprot50 | AF-A0A6N8NKN5-F1-MODEL\_V4 | 1.0 | 3.272e-22 | 857 | 0.311 | 642 | 299 | 13 | 156 | 749 | 2 | 548 | Phage tail tape measure protein | Phage tail tape measure protein | | afdb-uniprot50 | AF-A0A0Q6ZZL4-F1-MODEL\_V4 | 1.0 | 7.658e-25 | 857 | 0.246 | 820 | 489 | 20 | 1 | 780 | 1 | 731 | PhageMin\_Tail domain-containing protein | PhageMin\_Tail domain-containing protein | | afdb-uniprot50 | AF-A0A254UT32-F1-MODEL\_V4 | 1.0 | 6.066e-25 | 856 | 0.249 | 857 | 452 | 22 | 27 | 785 | 9 | 771 | Phage tail tape measure protein | Phage tail tape measure protein | | afdb-uniprot50 | AF-V5SJ01-F1-MODEL\_V4 | 1.0 | 3.297e-26 | 854 | 0.258 | 836 | 486 | 23 | 13 | 785 | 1 | 765 | PhageMin\_Tail domain-containing protein | PhageMin\_Tail domain-containing protein | | afdb-uniprot50 | AF-A0A5F1B8J0-F1-MODEL\_V4 | 1.0 | 4.067e-24 | 852 | 0.244 | 844 | 449 | 15 | 6 | 782 | 2 | 723 | Phage tail tape measure protein | Phage tail tape measure protein | | afdb-uniprot50 | AF-A0A3P6KAY6-F1-MODEL\_V4 | 1.0 | 2.389e-25 | 852 | 0.249 | 818 | 463 | 20 | 66 | 776 | 5 | 778 | Phage-like protein | Phage-like protein | | afdb-uniprot50 | AF-S5XQ18-F1-MODEL\_V4 | 1.0 | 1.283e-25 | 852 | 0.258 | 877 | 499 | 24 | 1 | 784 | 1 | 818 | Phage-related tail protein | Phage-related tail protein | | afdb-uniprot50 | AF-A0A2W5DL54-F1-MODEL\_V4 | 1.0 | 7.452e-26 | 852 | 0.223 | 941 | 515 | 20 | 22 | 776 | 54 | 964 | Phage tail tape measure protein | Phage tail tape measure protein | | afdb-uniprot50 | AF-A0A378QQC7-F1-MODEL\_V4 | 1.0 | 1.16e-23 | 852 | 0.301 | 684 | 388 | 16 | 39 | 702 | 2 | 615 | Phage-related minor tail protein | Phage-related minor tail protein | | afdb-uniprot50 | AF-A0A521D146-F1-MODEL\_V4 | 1.0 | 5.835e-25 | 850 | 0.288 | 790 | 454 | 20 | 26 | 759 | 1 | 738 | Phage tail tape measure protein, TP901 family, core region | Phage tail tape measure protein, TP901 family, core region | | afdb-uniprot50 | AF-A0A1Y3P9U4-F1-MODEL\_V4 | 1.0 | 7.281e-24 | 845 | 0.263 | 759 | 445 | 20 | 64 | 755 | 2 | 713 | PhageMin\_Tail domain-containing protein | PhageMin\_Tail domain-containing protein | | afdb-uniprot50 | AF-A0A7W6A8D0-F1-MODEL\_V4 | 1.0 | 8.604e-25 | 844 | 0.241 | 845 | 477 | 24 | 22 | 785 | 53 | 814 | TP901 family phage tail tape measure protein | TP901 family phage tail tape measure protein | | afdb-uniprot50 | AF-A0A3N1J100-F1-MODEL\_V4 | 1.0 | 9.446e-23 | 840 | 0.252 | 757 | 390 | 22 | 32 | 754 | 5 | 619 | TP901 family phage tail tape measure protein | TP901 family phage tail tape measure protein | | afdb-uniprot50 | AF-A0A1I4C772-F1-MODEL\_V4 | 1.0 | 3.016e-25 | 840 | 0.253 | 895 | 476 | 22 | 1 | 785 | 1 | 812 | Phage tail tape measure protein, TP901 family, core region | Phage tail tape measure protein, TP901 family, core region | | afdb-uniprot50 | AF-A0A4P8FYQ0-F1-MODEL\_V4 | 1.0 | 3.299e-16 | 838 | 0.363 | 333 | 205 | 1 | 155 | 480 | 3 | 335 | Phage tail tape measure protein | Phage tail tape measure protein | | afdb-uniprot50 | AF-A0A2K2G0G8-F1-MODEL\_V4 | 1.0 | 1.684e-25 | 833 | 0.274 | 849 | 444 | 27 | 19 | 782 | 58 | 819 | Phage tail tape measure protein | Phage tail tape measure protein | | afdb-uniprot50 | AF-A0A5C4LLJ5-F1-MODEL\_V4 | 1.0 | 1.894e-15 | 831 | 0.231 | 371 | 259 | 5 | 109 | 461 | 132 | 494 | Phage tail tape measure protein | Phage tail tape measure protein | | afdb-uniprot50 | AF-A0A0Q2UDJ2-F1-MODEL\_V4 | 1.0 | 9.086e-23 | 831 | 0.231 | 854 | 488 | 20 | 37 | 773 | 2 | 803 | PhageMin\_Tail domain-containing protein | PhageMin\_Tail domain-containing protein | | afdb-uniprot50 | AF-A0A1V3YRW6-F1-MODEL\_V4 | 1.0 | 1.535e-17 | 826 | 0.245 | 432 | 276 | 8 | 61 | 457 | 3 | 419 | Phage tail tape measure protein | Phage tail tape measure protein | | afdb-uniprot50 | AF-A0A212KXI4-F1-MODEL\_V4 | 1.0 | 2.758e-24 | 824 | 0.258 | 865 | 432 | 24 | 1 | 775 | 1 | 745 | PhageMin\_Tail domain-containing protein | PhageMin\_Tail domain-containing protein | | afdb-uniprot50 | AF-A0A6F9MD22-F1-MODEL\_V4 | 1.0 | 1.799e-24 | 823 | 0.244 | 776 | 491 | 18 | 55 | 784 | 13 | 738 | Phage tail tape measure protein | Phage tail tape measure protein | | afdb-uniprot50 | AF-B9D1S6-F1-MODEL\_V4 | 1.0 | 5.613e-25 | 820 | 0.253 | 881 | 484 | 25 | 1 | 764 | 1 | 824 | Phage tail tape measure protein, TP901 family | Phage tail tape measure protein, TP901 family | | afdb-uniprot50 | AF-A0A6P0VE51-F1-MODEL\_V4 | 1.0 | 1.061e-22 | 819 | 0.235 | 753 | 467 | 16 | 71 | 782 | 2 | 686 | Phage tail tape measure protein | Phage tail tape measure protein | | afdb-uniprot50 | AF-A0A1W1Z477-F1-MODEL\_V4 | 1.0 | 5.613e-25 | 819 | 0.246 | 901 | 463 | 26 | 36 | 783 | 3 | 840 | Phage tail tape measure protein, TP901 family, core region | Phage tail tape measure protein, TP901 family, core region | | afdb-uniprot50 | AF-A0A285V0T5-F1-MODEL\_V4 | 1.0 | 1.22e-24 | 819 | 0.243 | 787 | 460 | 20 | 1 | 713 | 1 | 725 | TP901 family phage tail tape measure protein | TP901 family phage tail tape measure protein | | afdb-uniprot50 | AF-B3R3J6-F1-MODEL\_V4 | 1.0 | 2.271e-24 | 815 | 0.203 | 926 | 557 | 26 | 19 | 782 | 59 | 965 | Bacteriophage P2 tail protein GPT putative tail length determinator | Bacteriophage P2 tail protein GPT putative tail length determinator | | afdb-uniprot50 | AF-B6IMG4-F1-MODEL\_V4 | 1.0 | 5.613e-25 | 811 | 0.235 | 914 | 486 | 26 | 63 | 782 | 2 | 896 | Phage tail tape measure protein, TP901 family, core region | Phage tail tape measure protein, TP901 family, core region | | afdb-uniprot50 | AF-A0A017HCD3-F1-MODEL\_V4 | 1.0 | 2.867e-24 | 808 | 0.22 | 900 | 502 | 23 | 1 | 782 | 1 | 818 | Phage tail length tape-measure protein | Phage tail length tape-measure protein | | afdb-uniprot50 | AF-A0A285P0E0-F1-MODEL\_V4 | 1.0 | 2.769e-21 | 807 | 0.268 | 718 | 381 | 19 | 71 | 750 | 2 | 612 | Phage tail tape measure protein, TP901 family, core region | Phage tail tape measure protein, TP901 family, core region | | afdb-uniprot50 | AF-A0A836WW58-F1-MODEL\_V4 | 1.0 | 1.108e-19 | 805 | 0.294 | 605 | 339 | 16 | 36 | 613 | 4 | 547 | Phage tail tape measure protein | Phage tail tape measure protein | | afdb-uniprot50 | AF-A0A1E3VZJ0-F1-MODEL\_V4 | 1.0 | 3.899e-17 | 802 | 0.243 | 469 | 326 | 10 | 63 | 519 | 2 | 453 | PhageMin\_Tail domain-containing protein | PhageMin\_Tail domain-containing protein | | afdb-uniprot50 | AF-A0A378QE64-F1-MODEL\_V4 | 1.0 | 7.57e-24 | 802 | 0.262 | 808 | 427 | 19 | 39 | 779 | 2 | 707 | Phage-related minor tail protein | Phage-related minor tail protein | | afdb-uniprot50 | AF-A0A1G8HWZ8-F1-MODEL\_V4 | 1.0 | 2.177e-17 | 801 | 0.331 | 456 | 274 | 7 | 42 | 480 | 168 | 609 | Phage tail tape measure protein, TP901 family, core region | Phage tail tape measure protein, TP901 family, core region | | afdb-uniprot50 | AF-A0A7C3GEH9-F1-MODEL\_V4 | 1.0 | 7.311e-21 | 800 | 0.246 | 642 | 377 | 18 | 29 | 655 | 2 | 551 | Phage tail tape measure protein | Phage tail tape measure protein | | afdb-uniprot50 | AF-A0A4R2D627-F1-MODEL\_V4 | 1.0 | 3.763e-24 | 799 | 0.251 | 792 | 432 | 21 | 3 | 726 | 1 | 699 | TP901 family phage tail tape measure protein | TP901 family phage tail tape measure protein | | afdb-uniprot50 | AF-A0A8B2QSJ7-F1-MODEL\_V4 | 1.0 | 3.867e-23 | 795 | 0.228 | 792 | 484 | 21 | 19 | 776 | 12 | 710 | Phage tail tape measure protein | Phage tail tape measure protein | | afdb-uniprot50 | AF-A0A386UJJ2-F1-MODEL\_V4 | 1.0 | 4.067e-24 | 788 | 0.234 | 914 | 483 | 27 | 1 | 782 | 1 | 829 | Phage tail tape measure protein | Phage tail tape measure protein | | afdb-uniprot50 | AF-A0A6G5QFI2-F1-MODEL\_V4 | 1.0 | 2.427e-23 | 784 | 0.246 | 817 | 503 | 21 | 1 | 764 | 1 | 757 | Phage tail tape measure protein, TP901 family | Phage tail tape measure protein, TP901 family | | afdb-uniprot50 | AF-A0A074U4R1-F1-MODEL\_V4 | 1.0 | 1.665e-24 | 783 | 0.238 | 992 | 495 | 29 | 19 | 780 | 51 | 1011 | Tail protein | Tail protein | | afdb-uniprot50 | AF-A0A753E0J5-F1-MODEL\_V4 | 1.0 | 7.119e-12 | 782 | 0.505 | 180 | 89 | 0 | 220 | 399 | 1 | 180 | Phage tail tape measure protein | Phage tail tape measure protein | | afdb-uniprot50 | AF-A0A135IJM6-F1-MODEL\_V4 | 1.0 | 2.16e-23 | 781 | 0.212 | 965 | 506 | 19 | 46 | 784 | 9 | 945 | PhageMin\_Tail domain-containing protein | PhageMin\_Tail domain-containing protein | | afdb-uniprot50 | AF-A0A1H8A1R0-F1-MODEL\_V4 | 1.0 | 4.228e-24 | 781 | 0.211 | 1063 | 535 | 26 | 19 | 779 | 38 | 1098 | Phage tail tape measure protein, TP901 family, core region | Phage tail tape measure protein, TP901 family, core region | | afdb-uniprot50 | AF-A0A2P5N6P9-F1-MODEL\_V4 | 1.0 | 2.738e-20 | 780 | 0.218 | 772 | 429 | 19 | 25 | 780 | 2 | 614 | Phage tail tape measure protein | Phage tail tape measure protein | | afdb-uniprot50 | AF-A0A1E3G7C9-F1-MODEL\_V4 | 1.0 | 1.922e-23 | 780 | 0.24 | 820 | 439 | 17 | 22 | 785 | 53 | 744 | Phage tail tape measure protein | Phage tail tape measure protein | | afdb-uniprot50 | AF-A0A5C7E451-F1-MODEL\_V4 | 1.0 | 1.021e-22 | 779 | 0.212 | 775 | 511 | 19 | 39 | 779 | 21 | 729 | Phage tail tape measure protein | Phage tail tape measure protein | | afdb-uniprot50 | AF-A0A268TUE9-F1-MODEL\_V4 | 1.0 | 1.254e-23 | 779 | 0.243 | 818 | 498 | 22 | 6 | 764 | 1 | 756 | Phage tail tape measure protein | Phage tail tape measure protein | | afdb-uniprot50 | AF-A0A239C875-F1-MODEL\_V4 | 1.0 | 8.407e-23 | 778 | 0.267 | 804 | 464 | 19 | 27 | 779 | 4 | 733 | Phage tail tape measure protein, TP901 family, core region | Phage tail tape measure protein, TP901 family, core region | | afdb-uniprot50 | AF-A0A1M4WE74-F1-MODEL\_V4 | 1.0 | 5.702e-23 | 777 | 0.245 | 876 | 468 | 21 | 13 | 782 | 1 | 789 | Phage tail tape measure protein, TP901 family, core region | Phage tail tape measure protein, TP901 family, core region | | afdb-uniprot50 | AF-A0A2P8QYQ4-F1-MODEL\_V4 | 1.0 | 1.061e-22 | 776 | 0.228 | 822 | 485 | 20 | 16 | 780 | 1 | 729 | Phage tail tape measure protein | Phage tail tape measure protein | | afdb-uniprot50 | AF-A0A845ZTZ3-F1-MODEL\_V4 | 1.0 | 9.086e-23 | 773 | 0.243 | 794 | 478 | 27 | 63 | 781 | 2 | 747 | Phage tail tape measure protein | Phage tail tape measure protein | | afdb-uniprot50 | AF-A0A349GR42-F1-MODEL\_V4 | 1.0 | 1.183e-18 | 772 | 0.274 | 532 | 307 | 9 | 5 | 461 | 2 | 529 | Phage tail tape measure protein | Phage tail tape measure protein | | afdb-uniprot50 | AF-A0A0U1DA91-F1-MODEL\_V4 | 1.0 | 1.454e-19 | 771 | 0.251 | 695 | 354 | 12 | 132 | 785 | 2 | 570 | Phage tail tape measure protein | Phage tail tape measure protein | | afdb-uniprot50 | AF-A0A3P4AP62-F1-MODEL\_V4 | 1.0 | 2.623e-23 | 771 | 0.242 | 828 | 460 | 18 | 81 | 762 | 25 | 830 | PhageMin\_Tail domain-containing protein | PhageMin\_Tail domain-containing protein | | afdb-uniprot50 | AF-W4SK08-F1-MODEL\_V4 | 1.0 | 4.789e-18 | 769 | 0.359 | 414 | 200 | 7 | 19 | 375 | 49 | 454 | Phage-related tail protein | Phage-related tail protein | | afdb-uniprot50 | AF-A0A3B0IZ82-F1-MODEL\_V4 | 1.0 | 4.734e-17 | 766 | 0.289 | 439 | 258 | 7 | 19 | 411 | 39 | 469 | PhageMin\_Tail domain-containing protein | PhageMin\_Tail domain-containing protein | | afdb-uniprot50 | AF-G1UYT9-F1-MODEL\_V4 | 1.0 | 7.341e-18 | 766 | 0.317 | 539 | 311 | 12 | 18 | 516 | 184 | 705 | PhageMin\_Tail domain-containing protein | PhageMin\_Tail domain-containing protein | | afdb-uniprot50 | AF-A0A4S3L0X3-F1-MODEL\_V4 | 1.0 | 1.608e-21 | 766 | 0.217 | 763 | 484 | 15 | 18 | 708 | 18 | 739 | Phage tail tape measure protein | Phage tail tape measure protein | | afdb-uniprot50 | AF-A0A7C5VJK5-F1-MODEL\_V4 | 1.0 | 9.707e-22 | 765 | 0.258 | 703 | 425 | 14 | 62 | 690 | 4 | 683 | Phage tail tape measure protein | Phage tail tape measure protein | | afdb-uniprot50 | AF-N6VPY0-F1-MODEL\_V4 | 1.0 | 1.773e-16 | 764 | 0.28 | 453 | 305 | 7 | 39 | 487 | 4 | 439 | Phage tail protein | Phage tail protein | | afdb-uniprot50 | AF-A0A327Q7T9-F1-MODEL\_V4 | 1.0 | 3.928e-21 | 762 | 0.208 | 768 | 471 | 22 | 47 | 780 | 4 | 668 | TP901 family phage tail tape measure protein | TP901 family phage tail tape measure protein | | afdb-uniprot50 | AF-A0A2Z4PV63-F1-MODEL\_V4 | 1.0 | 1.087e-14 | 761 | 0.328 | 317 | 209 | 1 | 160 | 472 | 5 | 321 | Phage tail tape measure protein | Phage tail tape measure protein | | afdb-uniprot50 | AF-A0A161V1N0-F1-MODEL\_V4 | 1.0 | 5.859e-22 | 758 | 0.224 | 875 | 470 | 22 | 1 | 782 | 1 | 759 | Phage-related minor tail protein | Phage-related minor tail protein | | afdb-uniprot50 | AF-A0A1B9MVQ4-F1-MODEL\_V4 | 1.0 | 9.446e-23 | 757 | 0.282 | 764 | 421 | 22 | 79 | 756 | 6 | 727 | Phage tail tape measure protein | Phage tail tape measure protein | | afdb-uniprot50 | AF-A0A807AA81-F1-MODEL\_V4 | 1.0 | 7.869e-24 | 757 | 0.207 | 905 | 544 | 23 | 19 | 781 | 50 | 922 | Phage tail tape measure protein | Phage tail tape measure protein | | afdb-uniprot50 | AF-A0A751FIF2-F1-MODEL\_V4 | 1.0 | 2.381e-18 | 756 | 0.322 | 483 | 242 | 8 | 193 | 675 | 2 | 399 | Phage tail tape measure protein | Phage tail tape measure protein | | afdb-uniprot50 | AF-A0A6L7FWV9-F1-MODEL\_V4 | 1.0 | 1.711e-23 | 756 | 0.249 | 858 | 457 | 27 | 20 | 785 | 46 | 808 | Phage tail tape measure protein | Phage tail tape measure protein | | afdb-uniprot50 | AF-A0A0K1NEY0-F1-MODEL\_V4 | 1.0 | 5.421e-22 | 754 | 0.232 | 847 | 532 | 22 | 22 | 781 | 53 | 868 | PhageMin\_Tail domain-containing protein | PhageMin\_Tail domain-containing protein | | afdb-uniprot50 | AF-A0A7T5EN76-F1-MODEL\_V4 | 1.0 | 1.953e-21 | 753 | 0.238 | 797 | 470 | 22 | 18 | 776 | 18 | 715 | Phage tail tape measure protein | Phage tail tape measure protein | | afdb-uniprot50 | AF-A0A2N3KSG5-F1-MODEL\_V4 | 1.0 | 2.494e-22 | 752 | 0.221 | 809 | 471 | 14 | 19 | 772 | 51 | 755 | Phage tail tape measure protein | Phage tail tape measure protein | | afdb-uniprot50 | AF-A0A7M1L7G1-F1-MODEL\_V4 | 1.0 | 9.086e-23 | 752 | 0.22 | 917 | 523 | 28 | 19 | 782 | 35 | 912 | Phage tail tape measure protein | Phage tail tape measure protein | | afdb-uniprot50 | AF-A0A2N9Y986-F1-MODEL\_V4 | 1.0 | 3.137e-15 | 748 | 0.254 | 432 | 275 | 7 | 16 | 440 | 1 | 392 | Phage tail tape measure protein | Phage tail tape measure protein | | afdb-uniprot50 | AF-A0A1J5HRD0-F1-MODEL\_V4 | 1.0 | 4.295e-22 | 748 | 0.219 | 913 | 480 | 25 | 2 | 783 | 1 | 811 | Phage tail tape measure protein | Phage tail tape measure protein | | afdb-uniprot50 | AF-A0A1T0CPH3-F1-MODEL\_V4 | 1.0 | 1.393e-22 | 746 | 0.24 | 879 | 485 | 18 | 36 | 777 | 2 | 834 | Phage tail tape measure protein | Phage tail tape measure protein | | afdb-uniprot50 | AF-A0A6Y5D9Q6-F1-MODEL\_V4 | 1.0 | 7.999e-12 | 745 | 0.51 | 182 | 89 | 0 | 187 | 368 | 1 | 182 | Phage tail tape measure protein | Phage tail tape measure protein | | afdb-uniprot50 | AF-A0A7Y8PBA4-F1-MODEL\_V4 | 1.0 | 2.334e-23 | 745 | 0.242 | 808 | 530 | 21 | 1 | 780 | 1 | 754 | Phage tail tape measure protein | Phage tail tape measure protein | | afdb-uniprot50 | AF-G1USF9-F1-MODEL\_V4 | 1.0 | 1.289e-22 | 744 | 0.216 | 863 | 487 | 21 | 2 | 777 | 1 | 760 | PhageMin\_Tail domain-containing protein | PhageMin\_Tail domain-containing protein | | afdb-uniprot50 | AF-A0A2E6Y2Q7-F1-MODEL\_V4 | 1.0 | 5.075e-23 | 744 | 0.228 | 872 | 526 | 25 | 13 | 784 | 2 | 826 | Phage tail tape measure protein | Phage tail tape measure protein | | afdb-uniprot50 | AF-A0A076LFK6-F1-MODEL\_V4 | 1.0 | 2.047e-15 | 743 | 0.437 | 318 | 159 | 6 | 76 | 383 | 6 | 313 | Phage tail length tape-measure protein | Phage tail length tape-measure protein | | afdb-uniprot50 | AF-A0A7W8HL33-F1-MODEL\_V4 | 1.0 | 5.155e-21 | 741 | 0.228 | 789 | 447 | 24 | 64 | 783 | 6 | 701 | TP901 family phage tail tape measure protein | TP901 family phage tail tape measure protein | | afdb-uniprot50 | AF-L0R7N5-F1-MODEL\_V4 | 1.0 | 1.324e-21 | 736 | 0.241 | 849 | 453 | 23 | 15 | 782 | 14 | 752 | PhageMin\_Tail domain-containing protein | PhageMin\_Tail domain-containing protein | | afdb-uniprot50 | AF-A0A6M3KKZ6-F1-MODEL\_V4 | 1.0 | 2.563e-21 | 736 | 0.197 | 881 | 533 | 22 | 19 | 782 | 34 | 857 | Putative tail protein | Putative tail protein | | afdb-uniprot50 | AF-A0A6L5WL94-F1-MODEL\_V4 | 1.0 | 1.23e-18 | 735 | 0.251 | 613 | 413 | 12 | 25 | 628 | 4 | 579 | Phage tail tape measure protein | Phage tail tape measure protein | | afdb-uniprot50 | AF-A0A0C1HF59-F1-MODEL\_V4 | 1.0 | 2.993e-21 | 734 | 0.248 | 769 | 444 | 23 | 80 | 780 | 11 | 713 | Phage tail tape measure protein, TP901 family | Phage tail tape measure protein, TP901 family | | afdb-uniprot50 | AF-A0A840X1L3-F1-MODEL\_V4 | 1.0 | 5.636e-22 | 734 | 0.264 | 799 | 392 | 24 | 15 | 712 | 27 | 730 | TP901 family phage tail tape measure protein | TP901 family phage tail tape measure protein | | afdb-uniprot50 | AF-A0A753E616-F1-MODEL\_V4 | 1.0 | 6.485e-14 | 732 | 0.43 | 260 | 140 | 1 | 220 | 471 | 1 | 260 | Phage tail tape measure protein | Phage tail tape measure protein | | afdb-uniprot50 | AF-A0A7G5EHY0-F1-MODEL\_V4 | 1.0 | 5.016e-22 | 732 | 0.213 | 874 | 499 | 23 | 19 | 782 | 47 | 841 | Phage tail tape measure protein | Phage tail tape measure protein | | afdb-uniprot50 | AF-A0A2T3NIG3-F1-MODEL\_V4 | 1.0 | 1.202e-16 | 732 | 0.298 | 510 | 305 | 12 | 42 | 519 | 167 | 655 | Phage tail tape measure protein | Phage tail tape measure protein | | afdb-uniprot50 | AF-A0A660MJK7-F1-MODEL\_V4 | 1.0 | 2.238e-16 | 730 | 0.254 | 503 | 289 | 7 | 19 | 440 | 69 | 566 | Phage tail tape measure protein | Phage tail tape measure protein | | afdb-uniprot50 | AF-A7MW86-F1-MODEL\_V4 | 1.0 | 7.544e-17 | 728 | 0.326 | 499 | 306 | 7 | 42 | 519 | 168 | 657 | PhageMin\_Tail domain-containing protein | PhageMin\_Tail domain-containing protein | | afdb-uniprot50 | AF-A0A4P5QGR5-F1-MODEL\_V4 | 1.0 | 2.264e-17 | 728 | 0.24 | 598 | 346 | 9 | 19 | 513 | 60 | 652 | Phage tail tape measure protein | Phage tail tape measure protein | | afdb-uniprot50 | AF-A0A0U2B0N6-F1-MODEL\_V4 | 1.0 | 5.215e-22 | 728 | 0.235 | 837 | 465 | 22 | 1 | 712 | 1 | 787 | PhageMin\_Tail domain-containing protein | PhageMin\_Tail domain-containing protein | | afdb-uniprot50 | AF-A0A1E3XJQ2-F1-MODEL\_V4 | 1.0 | 9.123e-20 | 727 | 0.252 | 718 | 397 | 20 | 16 | 678 | 1 | 633 | Phage tail tape measure protein | Phage tail tape measure protein | | afdb-uniprot50 | AF-A0A1I5ZGS7-F1-MODEL\_V4 | 1.0 | 2.03e-21 | 725 | 0.255 | 762 | 421 | 20 | 36 | 733 | 5 | 683 | Phage tail tape measure protein, TP901 family, core region | Phage tail tape measure protein, TP901 family, core region | | afdb-uniprot50 | AF-A0A369YAP6-F1-MODEL\_V4 | 1.0 | 6.41e-13 | 723 | 0.329 | 282 | 166 | 6 | 122 | 392 | 73 | 342 | Phage tail tape measure protein | Phage tail tape measure protein | | afdb-uniprot50 | AF-A0A0N0ZNR6-F1-MODEL\_V4 | 1.0 | 5.859e-22 | 723 | 0.216 | 1000 | 498 | 25 | 36 | 783 | 2 | 967 | PhageMin\_Tail domain-containing protein | PhageMin\_Tail domain-containing protein | | afdb-uniprot50 | AF-E5Y5W4-F1-MODEL\_V4 | 1.0 | 5.215e-22 | 722 | 0.259 | 827 | 452 | 23 | 19 | 743 | 36 | 803 | Phage tail tape measure protein, TP901 family, core region | Phage tail tape measure protein, TP901 family, core region | | afdb-uniprot50 | AF-A0A0F8XZV2-F1-MODEL\_V4 | 1.0 | 1.946e-14 | 719 | 0.236 | 422 | 285 | 7 | 46 | 464 | 2 | 389 | PhageMin\_Tail domain-containing protein | PhageMin\_Tail domain-containing protein | | afdb-uniprot50 | AF-A0A2W5GET3-F1-MODEL\_V4 | 1.0 | 4.535e-20 | 718 | 0.198 | 730 | 369 | 17 | 110 | 784 | 35 | 603 | Phage tail tape measure protein | Phage tail tape measure protein | | afdb-uniprot50 | AF-A0A504UBL4-F1-MODEL\_V4 | 1.0 | 2.281e-21 | 718 | 0.196 | 860 | 499 | 26 | 18 | 785 | 4 | 763 | Phage tail tape measure protein | Phage tail tape measure protein | | afdb-uniprot50 | AF-A0A5S9Q3L2-F1-MODEL\_V4 | 1.0 | 6.844e-22 | 718 | 0.216 | 827 | 538 | 17 | 19 | 782 | 14 | 793 | PhageMin\_Tail domain-containing protein | PhageMin\_Tail domain-containing protein | | afdb-uniprot50 | AF-A0A2C6C9V6-F1-MODEL\_V4 | 1.0 | 2.494e-22 | 718 | 0.198 | 966 | 567 | 26 | 1 | 778 | 5 | 950 | Phage tail tape measure protein | Phage tail tape measure protein | | afdb-uniprot50 | AF-A0A0D0J8M0-F1-MODEL\_V4 | 1.0 | 2.169e-20 | 714 | 0.237 | 788 | 445 | 19 | 62 | 780 | 13 | 713 | Phage tail tape measure protein, family | Phage tail tape measure protein, family | | afdb-uniprot50 | AF-A0A4Q8ME49-F1-MODEL\_V4 | 1.0 | 1.529e-20 | 712 | 0.237 | 807 | 446 | 26 | 47 | 780 | 9 | 718 | Phage tail tape measure protein | Phage tail tape measure protein | | afdb-uniprot50 | AF-A0A7X6JUT3-F1-MODEL\_V4 | 1.0 | 1.738e-21 | 711 | 0.205 | 1002 | 504 | 27 | 19 | 780 | 27 | 975 | Phage tail tape measure protein | Phage tail tape measure protein | | afdb-uniprot50 | AF-A0A5C8WLB6-F1-MODEL\_V4 | 1.0 | 1.188e-15 | 709 | 0.217 | 474 | 295 | 9 | 27 | 444 | 3 | 456 | Phage tail tape measure protein | Phage tail tape measure protein | | afdb-uniprot50 | AF-B7AAN0-F1-MODEL\_V4 | 1.0 | 2.664e-21 | 705 | 0.224 | 932 | 480 | 18 | 39 | 754 | 4 | 908 | Phage tail tape measure protein, TP901 family | Phage tail tape measure protein, TP901 family | | afdb-uniprot50 | AF-A0A7X6NRZ0-F1-MODEL\_V4 | 1.0 | 3.444e-13 | 704 | 0.228 | 342 | 241 | 6 | 117 | 443 | 6 | 339 | Phage tail tape measure protein | Phage tail tape measure protein | | afdb-uniprot50 | AF-A0A5P8MUD0-F1-MODEL\_V4 | 1.0 | 3.99e-19 | 704 | 0.21 | 808 | 455 | 21 | 31 | 785 | 13 | 690 | Phage tail tape measure protein | Phage tail tape measure protein | | afdb-uniprot50 | AF-B9NP85-F1-MODEL\_V4 | 1.0 | 3.928e-21 | 704 | 0.209 | 917 | 470 | 27 | 19 | 785 | 51 | 862 | Tail tape measure protein | Tail tape measure protein | | afdb-uniprot50 | AF-A0A316GV47-F1-MODEL\_V4 | 1.0 | 3.076e-20 | 703 | 0.219 | 841 | 470 | 23 | 1 | 785 | 1 | 710 | TP901 family phage tail tape measure protein | TP901 family phage tail tape measure protein | | afdb-uniprot50 | AF-A0A806J4A9-F1-MODEL\_V4 | 1.0 | 4.958e-21 | 703 | 0.193 | 862 | 548 | 25 | 28 | 781 | 5 | 826 | Bacteriophage P2 tail protein GPT putative tail length determinator | Bacteriophage P2 tail protein GPT putative tail length determinator | | afdb-uniprot50 | AF-A0A3D3B5Z8-F1-MODEL\_V4 | 1.0 | 3.112e-21 | 702 | 0.22 | 757 | 480 | 17 | 16 | 714 | 1 | 705 | Phage tail tape measure protein | Phage tail tape measure protein | | afdb-uniprot50 | AF-A0A840BWZ4-F1-MODEL\_V4 | 1.0 | 9.055e-16 | 697 | 0.23 | 499 | 317 | 10 | 27 | 467 | 6 | 495 | TP901 family phage tail tape measure protein | TP901 family phage tail tape measure protein | | afdb-uniprot50 | AF-E3H9B1-F1-MODEL\_V4 | 1.0 | 1.511e-19 | 697 | 0.235 | 714 | 407 | 17 | 1 | 633 | 1 | 656 | Phage tail tape measure protein, TP901 family | Phage tail tape measure protein, TP901 family | | afdb-uniprot50 | AF-A0A0H3ZYV9-F1-MODEL\_V4 | 1.0 | 2.655e-14 | 696 | 0.226 | 445 | 308 | 10 | 98 | 519 | 5 | 436 | Phage tail length tape-measure protein | Phage tail length tape-measure protein | | afdb-uniprot50 | AF-A0A1H9HD03-F1-MODEL\_V4 | 1.0 | 1.121e-20 | 692 | 0.221 | 823 | 503 | 23 | 34 | 784 | 6 | 762 | Phage tail tape measure protein, TP901 family, core region | Phage tail tape measure protein, TP901 family, core region | | afdb-uniprot50 | AF-A0A855G9Q1-F1-MODEL\_V4 | 1.0 | 7.033e-21 | 692 | 0.216 | 837 | 420 | 20 | 1 | 785 | 1 | 653 | Phage tail tape measure protein | Phage tail tape measure protein | | afdb-uniprot50 | AF-A0A660MG03-F1-MODEL\_V4 | 1.0 | 1.765e-19 | 692 | 0.237 | 694 | 431 | 13 | 40 | 702 | 60 | 685 | Phage tail tape measure protein | Phage tail tape measure protein | | afdb-uniprot50 | AF-A0A6B8RHZ8-F1-MODEL\_V4 | 1.0 | 3.456e-20 | 688 | 0.206 | 769 | 470 | 24 | 19 | 713 | 15 | 716 | Phage tail tape measure protein | Phage tail tape measure protein | | afdb-uniprot50 | AF-A0A4R2MR69-F1-MODEL\_V4 | 1.0 | 2.633e-20 | 688 | 0.226 | 844 | 506 | 21 | 19 | 784 | 28 | 802 | TP901 family phage tail tape measure protein | TP901 family phage tail tape measure protein | | afdb-uniprot50 | AF-A0A315BEH9-F1-MODEL\_V4 | 1.0 | 8.878e-21 | 686 | 0.192 | 836 | 526 | 20 | 19 | 755 | 45 | 830 | Phage tail tape measure protein | Phage tail tape measure protein | | afdb-uniprot50 | AF-A0A1C3E538-F1-MODEL\_V4 | 1.0 | 6.384e-16 | 685 | 0.304 | 542 | 289 | 12 | 19 | 477 | 76 | 612 | Phage tail tape measure protein | Phage tail tape measure protein | | afdb-uniprot50 | AF-A0A369Y1L1-F1-MODEL\_V4 | 1.0 | 2.846e-20 | 685 | 0.209 | 853 | 464 | 25 | 19 | 784 | 77 | 805 | Phage tail tape measure protein | Phage tail tape measure protein | | afdb-uniprot50 | AF-A0A2G6CD52-F1-MODEL\_V4 | 1.0 | 1.454e-19 | 683 | 0.229 | 786 | 418 | 23 | 47 | 785 | 10 | 654 | Phage tail tape measure protein | Phage tail tape measure protein | | afdb-uniprot50 | AF-A0A0F8XNY0-F1-MODEL\_V4 | 1.0 | 4.753e-14 | 682 | 0.22 | 404 | 281 | 10 | 126 | 519 | 2 | 381 | PhageMin\_Tail domain-containing protein | PhageMin\_Tail domain-containing protein | | afdb-uniprot50 | AF-A0A1Q6PXA2-F1-MODEL\_V4 | 1.0 | 1.108e-19 | 677 | 0.223 | 702 | 422 | 18 | 79 | 708 | 21 | 671 | Phage tail tape measure protein | Phage tail tape measure protein | | afdb-uniprot50 | AF-A0A379CMS6-F1-MODEL\_V4 | 1.0 | 3.607e-17 | 674 | 0.265 | 598 | 327 | 12 | 27 | 538 | 113 | 683 | Phage tail tape measure protein, TP901 family, core region | Phage tail tape measure protein, TP901 family, core region | | afdb-uniprot50 | AF-A0A635X8N3-F1-MODEL\_V4 | 1.0 | 1.477e-17 | 673 | 0.276 | 567 | 297 | 11 | 214 | 755 | 2 | 479 | Phage tail tape measure protein | Phage tail tape measure protein | | afdb-uniprot50 | AF-A0A2G6FTD7-F1-MODEL\_V4 | 1.0 | 9.86e-20 | 672 | 0.202 | 787 | 511 | 15 | 26 | 785 | 1 | 697 | Phage tail tape measure protein | Phage tail tape measure protein | | afdb-uniprot50 | AF-A0A354YUM2-F1-MODEL\_V4 | 1.0 | 2.055e-12 | 671 | 0.228 | 315 | 229 | 6 | 109 | 416 | 31 | 338 | Phage tail tape measure protein | Phage tail tape measure protein | | afdb-uniprot50 | AF-A0A4R5HEK1-F1-MODEL\_V4 | 1.0 | 3.299e-16 | 671 | 0.252 | 595 | 347 | 9 | 19 | 538 | 77 | 648 | Phage tail tape measure protein | Phage tail tape measure protein | | afdb-uniprot50 | AF-A0A2J9QJ08-F1-MODEL\_V4 | 1.0 | 4.196e-20 | 670 | 0.223 | 852 | 479 | 24 | 16 | 777 | 1 | 759 | Phage tail tape measure protein | Phage tail tape measure protein | | afdb-uniprot50 | AF-A0A1C2K2M3-F1-MODEL\_V4 | 1.0 | 2.418e-16 | 668 | 0.255 | 587 | 327 | 11 | 19 | 514 | 77 | 644 | Phage tail tape measure protein | Phage tail tape measure protein | | afdb-uniprot50 | AF-A0A248UM23-F1-MODEL\_V4 | 1.0 | 8.214e-21 | 668 | 0.189 | 886 | 510 | 23 | 1 | 783 | 1 | 780 | Phage tail tape measure protein, TP901 family, core region | Phage tail tape measure protein, TP901 family, core region | | afdb-uniprot50 | AF-A0A1H0GBX4-F1-MODEL\_V4 | 1.0 | 4.083e-21 | 668 | 0.192 | 997 | 544 | 27 | 3 | 785 | 1 | 949 | Phage tail tape measure protein, TP901 family, core region | Phage tail tape measure protein, TP901 family, core region | | afdb-uniprot50 | AF-D3UG43-F1-MODEL\_V4 | 1.0 | 1.59e-20 | 667 | 0.207 | 832 | 509 | 24 | 6 | 785 | 1 | 733 | Putative phage tape-measure protein | Putative phage tape-measure protein | | afdb-uniprot50 | AF-A0A4V2PT27-F1-MODEL\_V4 | 1.0 | 5.359e-21 | 666 | 0.224 | 852 | 513 | 25 | 16 | 785 | 1 | 786 | TP901 family phage tail tape measure protein | TP901 family phage tail tape measure protein | | afdb-uniprot50 | AF-A0A2X3K7Y1-F1-MODEL\_V4 | 1.0 | 3.365e-11 | 665 | 0.554 | 184 | 82 | 0 | 177 | 360 | 4 | 187 | Putative phage tail tape measure protein | Putative phage tail tape measure protein | | afdb-uniprot50 | AF-B6WRQ7-F1-MODEL\_V4 | 1.0 | 4.362e-20 | 664 | 0.218 | 850 | 505 | 18 | 19 | 784 | 3 | 776 | Phage tail tape measure protein, TP901 family | Phage tail tape measure protein, TP901 family | | afdb-uniprot50 | AF-A0A0F2N598-F1-MODEL\_V4 | 1.0 | 5.236e-19 | 664 | 0.219 | 737 | 430 | 18 | 39 | 750 | 209 | 824 | PhageMin\_Tail domain-containing protein | PhageMin\_Tail domain-containing protein | | afdb-uniprot50 | AF-A0A6N2Z5X0-F1-MODEL\_V4 | 1.0 | 3.593e-20 | 663 | 0.231 | 817 | 475 | 26 | 27 | 776 | 124 | 854 | Chromosome partition protein Smc | Chromosome partition protein Smc | | afdb-uniprot50 | AF-A0A4Y9VRB4-F1-MODEL\_V4 | 1.0 | 7.144e-19 | 661 | 0.191 | 831 | 472 | 21 | 22 | 782 | 53 | 753 | Phage tail tape measure protein | Phage tail tape measure protein | | afdb-uniprot50 | AF-A0A0N8W5Z8-F1-MODEL\_V4 | 1.0 | 1.415e-20 | 660 | 0.194 | 835 | 492 | 23 | 63 | 779 | 2 | 773 | PhageMin\_Tail domain-containing protein | PhageMin\_Tail domain-containing protein | | afdb-uniprot50 | AF-A0A1C6E118-F1-MODEL\_V4 | 1.0 | 8.12e-20 | 660 | 0.248 | 749 | 436 | 22 | 53 | 734 | 9 | 697 | Phage-related protein | Phage-related protein | | afdb-uniprot50 | AF-A0A1I4SVB4-F1-MODEL\_V4 | 1.0 | 6.432e-20 | 660 | 0.211 | 857 | 491 | 22 | 1 | 716 | 1 | 813 | Phage tail tape measure protein, TP901 family, core region | Phage tail tape measure protein, TP901 family, core region | | afdb-uniprot50 | AF-A0A378QS12-F1-MODEL\_V4 | 1.0 | 6.687e-20 | 659 | 0.2 | 798 | 476 | 22 | 27 | 701 | 6 | 764 | Phage-related minor tail protein | Phage-related minor tail protein | | afdb-uniprot50 | AF-A0A3G8M4E6-F1-MODEL\_V4 | 1.0 | 1.006e-14 | 656 | 0.232 | 478 | 321 | 13 | 18 | 469 | 15 | 472 | Phage tail tape measure protein | Phage tail tape measure protein | | afdb-uniprot50 | AF-A0A7X3TZT8-F1-MODEL\_V4 | 1.0 | 3.838e-19 | 654 | 0.22 | 872 | 521 | 25 | 19 | 785 | 34 | 851 | Phage tail tape measure protein | Phage tail tape measure protein | | afdb-uniprot50 | AF-A0A323TXZ9-F1-MODEL\_V4 | 1.0 | 1.165e-20 | 654 | 0.214 | 928 | 520 | 24 | 3 | 785 | 1 | 864 | Phage tail tape measure protein | Phage tail tape measure protein | | afdb-uniprot50 | AF-A0A1I3T075-F1-MODEL\_V4 | 1.0 | 5.725e-20 | 653 | 0.209 | 810 | 462 | 23 | 1 | 785 | 1 | 656 | Phage tail tape measure protein, TP901 family, core region | Phage tail tape measure protein, TP901 family, core region | | afdb-uniprot50 | AF-A0A1T4QE70-F1-MODEL\_V4 | 1.0 | 6.952e-20 | 651 | 0.184 | 949 | 514 | 28 | 3 | 782 | 1 | 858 | Phage tail tape measure protein, TP901 family, core region | Phage tail tape measure protein, TP901 family, core region | | afdb-uniprot50 | AF-A0A5C7P5Y8-F1-MODEL\_V4 | 1.0 | 1.398e-19 | 649 | 0.201 | 848 | 412 | 21 | 19 | 735 | 28 | 740 | Phage tail tape measure protein | Phage tail tape measure protein | | afdb-uniprot50 | AF-A0A0A3A3N3-F1-MODEL\_V4 | 1.0 | 9.9e-17 | 648 | 0.207 | 627 | 386 | 16 | 19 | 570 | 57 | 647 | Tail protein | Tail protein | | afdb-uniprot50 | AF-A0A373FPP0-F1-MODEL\_V4 | 1.0 | 3.076e-20 | 648 | 0.196 | 1035 | 538 | 30 | 19 | 782 | 41 | 1052 | Phage tail tape measure protein | Phage tail tape measure protein | | afdb-uniprot50 | AF-A0A448V531-F1-MODEL\_V4 | 1.0 | 6.187e-20 | 647 | 0.215 | 830 | 515 | 21 | 16 | 776 | 1 | 762 | Phage-related minor tail protein | Phage-related minor tail protein | | afdb-uniprot50 | AF-A0A5N7YE88-F1-MODEL\_V4 | 1.0 | 5.976e-17 | 645 | 0.328 | 493 | 251 | 10 | 193 | 685 | 3 | 415 | Phage tail tape measure protein | Phage tail tape measure protein | | afdb-uniprot50 | AF-A0A840DVF7-F1-MODEL\_V4 | 1.0 | 3.51e-18 | 642 | 0.232 | 732 | 423 | 21 | 16 | 661 | 1 | 679 | TP901 family phage tail tape measure protein | TP901 family phage tail tape measure protein | | afdb-uniprot50 | AF-A0A376ZDS5-F1-MODEL\_V4 | 1.0 | 5.815e-18 | 641 | 0.189 | 690 | 466 | 15 | 118 | 785 | 4 | 621 | Tail protein (Modular protein) | Tail protein (Modular protein) | | afdb-uniprot50 | AF-A0A366CXV4-F1-MODEL\_V4 | 1.0 | 2.903e-15 | 641 | 0.251 | 588 | 312 | 9 | 20 | 483 | 124 | 707 | TP901 family phage tail tape measure protein | TP901 family phage tail tape measure protein | | afdb-uniprot50 | AF-A0A6B8M5R6-F1-MODEL\_V4 | 1.0 | 2.76e-14 | 640 | 0.244 | 462 | 315 | 10 | 4 | 450 | 10 | 452 | Phage tail tape measure protein | Phage tail tape measure protein | | afdb-uniprot50 | AF-G1UXP3-F1-MODEL\_V4 | 1.0 | 5.725e-20 | 638 | 0.253 | 813 | 472 | 26 | 18 | 754 | 28 | 781 | PhageMin\_Tail domain-containing protein | PhageMin\_Tail domain-containing protein | | afdb-uniprot50 | AF-A0A2S9IP78-F1-MODEL\_V4 | 1.0 | 1.506e-12 | 635 | 0.365 | 309 | 185 | 3 | 51 | 358 | 2 | 300 | Phage tail tape measure protein | Phage tail tape measure protein | | afdb-uniprot50 | AF-E1QHA5-F1-MODEL\_V4 | 1.0 | 4.362e-20 | 633 | 0.197 | 1022 | 525 | 26 | 19 | 782 | 10 | 993 | Phage tail tape measure protein, TP901 family | Phage tail tape measure protein, TP901 family | | afdb-uniprot50 | AF-A0A3P3EPW8-F1-MODEL\_V4 | 1.0 | 1.156e-16 | 630 | 0.165 | 745 | 403 | 20 | 116 | 785 | 1 | 601 | Phage tail tape measure protein | Phage tail tape measure protein | | afdb-uniprot50 | AF-A0A1E2RY73-F1-MODEL\_V4 | 1.0 | 1.041e-17 | 627 | 0.204 | 762 | 440 | 28 | 49 | 785 | 6 | 625 | Phage-related minor tail protein | Phage-related minor tail protein | | afdb-uniprot50 | AF-A0A286BYN5-F1-MODEL\_V4 | 1.0 | 5.176e-18 | 627 | 0.196 | 708 | 451 | 16 | 109 | 782 | 372 | 995 | Phage tail tape measure protein, TP901 family, core region | Phage tail tape measure protein, TP901 family, core region | | afdb-uniprot50 | AF-A0A2M8RTD1-F1-MODEL\_V4 | 1.0 | 2.228e-19 | 625 | 0.216 | 785 | 507 | 22 | 19 | 734 | 16 | 761 | Phage tail tape measure protein | Phage tail tape measure protein | | afdb-uniprot50 | AF-A0A7C7D7E0-F1-MODEL\_V4 | 1.0 | 1.732e-14 | 624 | 0.215 | 479 | 331 | 13 | 21 | 465 | 16 | 483 | Phage tail tape measure protein | Phage tail tape measure protein | | afdb-uniprot50 | AF-A0A2W4IRV0-F1-MODEL\_V4 | 1.0 | 8.186e-14 | 623 | 0.202 | 519 | 345 | 13 | 6 | 516 | 1 | 458 | Phage tail tape measure protein | Phage tail tape measure protein | | afdb-uniprot50 | AF-A0A510KEQ7-F1-MODEL\_V4 | 1.0 | 6.358e-19 | 623 | 0.196 | 834 | 517 | 22 | 19 | 715 | 48 | 864 | Phage tail tape measure protein, TP901 family | Phage tail tape measure protein, TP901 family | | afdb-uniprot50 | AF-A0A1L9CWC2-F1-MODEL\_V4 | 1.0 | 8.987e-12 | 621 | 0.227 | 339 | 245 | 7 | 91 | 422 | 3 | 331 | Phage tail tape measure protein | Phage tail tape measure protein | | afdb-uniprot50 | AF-A0A2T3R1S0-F1-MODEL\_V4 | 1.0 | 3.854e-16 | 619 | 0.265 | 594 | 337 | 13 | 19 | 519 | 69 | 655 | Phage tail tape measure protein | Phage tail tape measure protein | | afdb-uniprot50 | AF-A0A1B8Q889-F1-MODEL\_V4 | 1.0 | 3.99e-19 | 617 | 0.207 | 795 | 464 | 19 | 20 | 704 | 14 | 752 | Phage tail tape measure protein | Phage tail tape measure protein | | afdb-uniprot50 | AF-A0A1Q8Q232-F1-MODEL\_V4 | 1.0 | 5.236e-19 | 616 | 0.177 | 1005 | 553 | 26 | 19 | 781 | 19 | 991 | Phage tail tape measure protein | Phage tail tape measure protein | | afdb-uniprot50 | AF-A0A7W2GS65-F1-MODEL\_V4 | 1.0 | 2.675e-18 | 612 | 0.212 | 770 | 461 | 19 | 14 | 757 | 1 | 650 | Phage tail tape measure protein | Phage tail tape measure protein | | afdb-uniprot50 | AF-A0A238HFL8-F1-MODEL\_V4 | 1.0 | 1.494e-18 | 612 | 0.175 | 874 | 545 | 27 | 24 | 785 | 55 | 864 | Phage-related minor tail protein | Phage-related minor tail protein | | afdb-uniprot50 | AF-T4VE82-F1-MODEL\_V4 | 1.0 | 2.177e-17 | 611 | 0.192 | 750 | 466 | 22 | 64 | 782 | 4 | 644 | Phage tail tape measure protein, TP901 family, core region | Phage tail tape measure protein, TP901 family, core region | | afdb-uniprot50 | AF-A0A2G2Q4G5-F1-MODEL\_V4 | 1.0 | 1.013e-18 | 611 | 0.21 | 789 | 485 | 20 | 2 | 705 | 1 | 736 | Phage tail tape measure protein | Phage tail tape measure protein | | afdb-uniprot50 | AF-A0A327JMP0-F1-MODEL\_V4 | 1.0 | 1.17e-17 | 605 | 0.177 | 872 | 548 | 25 | 8 | 783 | 1 | 798 | Phage tail tape measure protein | Phage tail tape measure protein | | afdb-uniprot50 | AF-J0Q8Z0-F1-MODEL\_V4 | 1.0 | 2.079e-13 | 604 | 0.273 | 355 | 242 | 6 | 155 | 508 | 3 | 342 | Phage tail tape measure protein, TP901 family, core region | Phage tail tape measure protein, TP901 family, core region | | afdb-uniprot50 | AF-A0A852QWW4-F1-MODEL\_V4 | 1.0 | 1.006e-14 | 604 | 0.214 | 518 | 311 | 15 | 18 | 526 | 16 | 446 | TP901 family phage tail tape measure protein | TP901 family phage tail tape measure protein | | afdb-uniprot50 | AF-R5BHC1-F1-MODEL\_V4 | 1.0 | 2.514e-16 | 604 | 0.227 | 694 | 416 | 22 | 132 | 781 | 2 | 619 | Tail tape measure protein TIGR01760 family | Tail tape measure protein TIGR01760 family | | afdb-uniprot50 | AF-A0A844UUJ6-F1-MODEL\_V4 | 1.0 | 1.314e-17 | 604 | 0.187 | 780 | 461 | 21 | 129 | 785 | 2 | 731 | Phage tail tape measure protein | Phage tail tape measure protein | | afdb-uniprot50 | AF-A0A2D5PEJ4-F1-MODEL\_V4 | 1.0 | 1.082e-17 | 604 | 0.209 | 820 | 391 | 18 | 19 | 755 | 74 | 718 | Phage tail tape measure protein | Phage tail tape measure protein | | afdb-uniprot50 | AF-A0A485DIL8-F1-MODEL\_V4 | 1.0 | 9.267e-18 | 603 | 0.213 | 796 | 506 | 21 | 21 | 780 | 2 | 713 | Phage protein | Phage protein | | afdb-uniprot50 | AF-A0A8B2NXI7-F1-MODEL\_V4 | 1.0 | 5.659e-19 | 603 | 0.218 | 781 | 439 | 29 | 98 | 785 | 27 | 728 | Phage tail tape measure protein | Phage tail tape measure protein | | afdb-uniprot50 | AF-U6Q3L1-F1-MODEL\_V4 | 1.0 | 2.015e-17 | 602 | 0.214 | 778 | 499 | 23 | 1 | 702 | 1 | 741 | Phage tail tape measure protein, TP901 family, core region | Phage tail tape measure protein, TP901 family, core region | | afdb-uniprot50 | AF-A0A1F1HUA7-F1-MODEL\_V4 | 1.0 | 5.116e-17 | 601 | 0.197 | 679 | 428 | 20 | 21 | 628 | 48 | 680 | PhageMin\_Tail domain-containing protein | PhageMin\_Tail domain-containing protein | | afdb-uniprot50 | AF-A0A4Y6KLY6-F1-MODEL\_V4 | 1.0 | 2.573e-18 | 600 | 0.165 | 989 | 560 | 24 | 40 | 785 | 94 | 1059 | Phage tail tape measure protein | Phage tail tape measure protein | | afdb-uniprot50 | AF-A0A2G1QM28-F1-MODEL\_V4 | 1.0 | 3.96e-15 | 599 | 0.185 | 701 | 405 | 14 | 40 | 719 | 20 | 575 | Phage tail tape measure protein | Phage tail tape measure protein | | afdb-uniprot50 | AF-A0A2J9QPE7-F1-MODEL\_V4 | 1.0 | 1.398e-19 | 599 | 0.188 | 906 | 538 | 23 | 19 | 782 | 44 | 893 | Phage tail tape measure protein | Phage tail tape measure protein | | afdb-uniprot50 | AF-A0A0M9GN37-F1-MODEL\_V4 | 1.0 | 9.267e-18 | 597 | 0.179 | 779 | 442 | 17 | 39 | 785 | 3 | 615 | PhageMin\_Tail domain-containing protein | PhageMin\_Tail domain-containing protein | | afdb-uniprot50 | AF-A0A2X7FXT8-F1-MODEL\_V4 | 1.0 | 9.375e-19 | 597 | 0.187 | 940 | 531 | 24 | 23 | 785 | 50 | 933 | Tail protein (Modular protein) | Tail protein (Modular protein) | | afdb-uniprot50 | AF-A0A2P1S7S0-F1-MODEL\_V4 | 1.0 | 1.961e-18 | 597 | 0.21 | 939 | 521 | 20 | 18 | 776 | 108 | 1005 | Phage tail tape measure protein | Phage tail tape measure protein | | afdb-uniprot50 | AF-A0A7X5J7E5-F1-MODEL\_V4 | 1.0 | 3.444e-13 | 596 | 0.2 | 445 | 326 | 9 | 13 | 432 | 47 | 486 | Phage tail tape measure protein | Phage tail tape measure protein | | afdb-uniprot50 | AF-A0A846BMG1-F1-MODEL\_V4 | 1.0 | 1.432e-11 | 595 | 0.309 | 317 | 195 | 6 | 64 | 371 | 4 | 305 | Phage tail tape measure protein | Phage tail tape measure protein | | afdb-uniprot50 | AF-A0A496JTX2-F1-MODEL\_V4 | 1.0 | 2.119e-18 | 595 | 0.218 | 842 | 448 | 25 | 19 | 710 | 34 | 815 | Phage tail tape measure protein | Phage tail tape measure protein | | afdb-uniprot50 | AF-V7ZKH4-F1-MODEL\_V4 | 1.0 | 6.587e-12 | 594 | 0.197 | 385 | 242 | 8 | 117 | 436 | 20 | 402 | PhageMin\_Tail domain-containing protein | PhageMin\_Tail domain-containing protein | | afdb-uniprot50 | AF-A0A486PRA3-F1-MODEL\_V4 | 1.0 | 6.046e-18 | 594 | 0.18 | 925 | 555 | 29 | 19 | 785 | 32 | 911 | Putative prophage tail length determinator | Putative prophage tail length determinator | | afdb-uniprot50 | AF-A0A414PRA3-F1-MODEL\_V4 | 1.0 | 1.615e-18 | 594 | 0.209 | 917 | 507 | 28 | 27 | 782 | 88 | 947 | Phage tail tape measure protein | Phage tail tape measure protein | | afdb-uniprot50 | AF-A0A1Q8Z201-F1-MODEL\_V4 | 1.0 | 2.706e-19 | 594 | 0.167 | 980 | 565 | 27 | 21 | 785 | 48 | 991 | Phage tail tape measure protein | Phage tail tape measure protein | | afdb-uniprot50 | AF-A0A0R2D4C9-F1-MODEL\_V4 | 1.0 | 7.341e-18 | 590 | 0.199 | 717 | 486 | 21 | 12 | 702 | 141 | 795 | PhageMin\_Tail domain-containing protein | PhageMin\_Tail domain-containing protein | | afdb-uniprot50 | AF-A0A286IFD9-F1-MODEL\_V4 | 1.0 | 2.675e-18 | 588 | 0.207 | 816 | 504 | 27 | 3 | 785 | 1 | 706 | TP901 family phage tail tape measure protein | TP901 family phage tail tape measure protein | | afdb-uniprot50 | AF-A0A2V4L3Y4-F1-MODEL\_V4 | 1.0 | 5.815e-18 | 588 | 0.211 | 902 | 446 | 25 | 19 | 783 | 28 | 800 | Phage tail tape measure protein | Phage tail tape measure protein | | afdb-uniprot50 | AF-A0A376TE73-F1-MODEL\_V4 | 1.0 | 3.124e-18 | 587 | 0.188 | 833 | 526 | 25 | 1 | 781 | 15 | 749 | Phage tail tape measure protein | Phage tail tape measure protein | | afdb-uniprot50 | AF-A0A843BA66-F1-MODEL\_V4 | 1.0 | 8.575e-18 | 585 | 0.225 | 695 | 415 | 19 | 165 | 780 | 2 | 651 | Phage tail tape measure protein | Phage tail tape measure protein | | afdb-uniprot50 | AF-A0A744CZM2-F1-MODEL\_V4 | 1.0 | 5.529e-17 | 583 | 0.202 | 731 | 447 | 19 | 120 | 774 | 26 | 696 | Phage tail tape measure protein | Phage tail tape measure protein | | afdb-uniprot50 | AF-A0A414Q2W1-F1-MODEL\_V4 | 1.0 | 1.814e-18 | 582 | 0.212 | 942 | 504 | 29 | 19 | 776 | 81 | 968 | Phage tail tape measure protein | Phage tail tape measure protein | | afdb-uniprot50 | AF-U6Q3L9-F1-MODEL\_V4 | 1.0 | 6.459e-17 | 579 | 0.22 | 767 | 505 | 19 | 1 | 701 | 1 | 740 | Phage tail tape measure protein, TP901 family, core region | Phage tail tape measure protein, TP901 family, core region | | afdb-uniprot50 | AF-A0A826S7R5-F1-MODEL\_V4 | 1.0 | 8.575e-18 | 578 | 0.175 | 887 | 545 | 20 | 17 | 785 | 1 | 818 | Phage tail tape measure protein | Phage tail tape measure protein | | afdb-uniprot50 | AF-A0A3L9GV37-F1-MODEL\_V4 | 1.0 | 2.038e-18 | 578 | 0.195 | 863 | 506 | 27 | 21 | 735 | 48 | 869 | Phage tail tape measure protein | Phage tail tape measure protein | | afdb-uniprot50 | AF-A0A168UYB6-F1-MODEL\_V4 | 1.0 | 1.006e-14 | 577 | 0.202 | 672 | 374 | 17 | 117 | 715 | 14 | 596 | PhageMin\_Tail domain-containing protein | PhageMin\_Tail domain-containing protein | | afdb-uniprot50 | AF-A0A7W6N9T0-F1-MODEL\_V4 | 1.0 | 4.165e-16 | 577 | 0.174 | 783 | 484 | 18 | 46 | 785 | 2 | 664 | TP901 family phage tail tape measure protein | TP901 family phage tail tape measure protein | | afdb-uniprot50 | AF-A0A846VL41-F1-MODEL\_V4 | 1.0 | 2.936e-16 | 577 | 0.19 | 820 | 486 | 24 | 13 | 784 | 1 | 690 | TP901 family phage tail tape measure protein | TP901 family phage tail tape measure protein | | afdb-uniprot50 | AF-A0A504KM96-F1-MODEL\_V4 | 1.0 | 1.299e-16 | 577 | 0.18 | 802 | 407 | 23 | 104 | 782 | 56 | 729 | Phage tail tape measure protein | Phage tail tape measure protein | | afdb-uniprot50 | AF-A0A679GE32-F1-MODEL\_V4 | 1.0 | 1.421e-17 | 576 | 0.209 | 773 | 503 | 16 | 19 | 702 | 45 | 798 | PhageMin\_Tail domain-containing protein | PhageMin\_Tail domain-containing protein | | afdb-uniprot50 | AF-A0A731W7G9-F1-MODEL\_V4 | 1.0 | 4.999e-15 | 575 | 0.186 | 639 | 427 | 14 | 105 | 722 | 7 | 573 | Phage tail tape measure protein | Phage tail tape measure protein | | afdb-uniprot50 | AF-V4N3Q7-F1-MODEL\_V4 | 1.0 | 1.518e-16 | 574 | 0.199 | 731 | 451 | 20 | 89 | 749 | 4 | 669 | Tail protein | Tail protein | | afdb-uniprot50 | AF-A0A411WMH1-F1-MODEL\_V4 | 1.0 | 1.314e-17 | 574 | 0.163 | 930 | 574 | 25 | 19 | 781 | 35 | 927 | Phage tail tape measure protein | Phage tail tape measure protein | | afdb-uniprot50 | AF-A0A1W2EJS2-F1-MODEL\_V4 | 1.0 | 7.457e-16 | 572 | 0.155 | 859 | 480 | 25 | 1 | 785 | 1 | 687 | Phage tail tape measure protein, TP901 family, core region | Phage tail tape measure protein, TP901 family, core region | | afdb-uniprot50 | AF-A0A2S9GTE6-F1-MODEL\_V4 | 1.0 | 1.17e-17 | 572 | 0.17 | 919 | 549 | 29 | 19 | 780 | 56 | 917 | Phage tail tape measure protein, TP901 family, core region | Phage tail tape measure protein, TP901 family, core region | | afdb-uniprot50 | AF-A0A0T9KM18-F1-MODEL\_V4 | 1.0 | 3.794e-18 | 570 | 0.171 | 950 | 569 | 26 | 22 | 781 | 54 | 974 | Tail fiber component T of bacteriophage P2 | Tail fiber component T of bacteriophage P2 | | afdb-uniprot50 | AF-A0A432WB56-F1-MODEL\_V4 | 1.0 | 3.51e-18 | 570 | 0.182 | 778 | 484 | 20 | 19 | 698 | 37 | 760 | Phage tail tape measure protein | Phage tail tape measure protein | | afdb-uniprot50 | AF-U7T719-F1-MODEL\_V4 | 1.0 | 7.061e-18 | 568 | 0.196 | 913 | 524 | 29 | 1 | 778 | 2 | 839 | Phage tail tape measure protein, TP901 family, core region | Phage tail tape measure protein, TP901 family, core region | | afdb-uniprot50 | AF-A0A1N6MRW7-F1-MODEL\_V4 | 1.0 | 5.319e-17 | 566 | 0.177 | 829 | 538 | 22 | 19 | 754 | 45 | 822 | PhageMin\_Tail domain-containing protein | PhageMin\_Tail domain-containing protein | | afdb-uniprot50 | AF-A0A375H2Y0-F1-MODEL\_V4 | 1.0 | 4.165e-16 | 565 | 0.264 | 579 | 316 | 9 | 206 | 784 | 7 | 475 | PhageMin\_Tail domain-containing protein | PhageMin\_Tail domain-containing protein | | afdb-uniprot50 | AF-A0A1F1DK89-F1-MODEL\_V4 | 1.0 | 1.341e-12 | 565 | 0.234 | 474 | 308 | 13 | 1 | 466 | 1 | 427 | PhageMin\_Tail domain-containing protein | PhageMin\_Tail domain-containing protein | | afdb-uniprot50 | AF-A0A7X7BBI3-F1-MODEL\_V4 | 1.0 | 6.166e-13 | 564 | 0.18 | 516 | 347 | 9 | 19 | 463 | 15 | 525 | Phage tail tape measure protein | Phage tail tape measure protein | | afdb-uniprot50 | AF-A0A3B8S463-F1-MODEL\_V4 | 1.0 | 3.39e-15 | 563 | 0.215 | 593 | 359 | 24 | 50 | 564 | 4 | 567 | Phage tail tape measure protein | Phage tail tape measure protein | | afdb-uniprot50 | AF-A0A7C7DFP0-F1-MODEL\_V4 | 1.0 | 8.153e-17 | 563 | 0.188 | 802 | 477 | 31 | 1 | 750 | 1 | 680 | Phage tail tape measure protein | Phage tail tape measure protein | | afdb-uniprot50 | AF-A0A826YZU6-F1-MODEL\_V4 | 1.0 | 5.137e-14 | 560 | 0.195 | 589 | 374 | 15 | 88 | 644 | 5 | 525 | Phage tail tape measure protein | Phage tail tape measure protein | | afdb-uniprot50 | AF-A0A3L2QDC9-F1-MODEL\_V4 | 1.0 | 1.299e-16 | 560 | 0.206 | 737 | 453 | 19 | 132 | 785 | 2 | 689 | Phage tail tape measure protein | Phage tail tape measure protein | | afdb-uniprot50 | AF-A0A547PW98-F1-MODEL\_V4 | 1.0 | 7.632e-18 | 560 | 0.197 | 890 | 498 | 25 | 49 | 784 | 6 | 832 | Phage tail tape measure protein | Phage tail tape measure protein | | afdb-uniprot50 | AF-A0A2E1XVX6-F1-MODEL\_V4 | 1.0 | 2.186e-14 | 557 | 0.22 | 650 | 386 | 14 | 1 | 628 | 1 | 551 | Phage tail tape measure protein | Phage tail tape measure protein | | afdb-uniprot50 | AF-A0A0N1N416-F1-MODEL\_V4 | 1.0 | 5.403e-15 | 557 | 0.174 | 733 | 465 | 17 | 33 | 721 | 2 | 638 | PhageMin\_Tail domain-containing protein | PhageMin\_Tail domain-containing protein | | afdb-uniprot50 | AF-A0A174F216-F1-MODEL\_V4 | 1.0 | 6.848e-12 | 557 | 0.21 | 433 | 321 | 8 | 42 | 460 | 199 | 624 | Phage tail tape measure protein, family | Phage tail tape measure protein, family | | afdb-uniprot50 | AF-A0A6P1K7K2-F1-MODEL\_V4 | 1.0 | 2.177e-17 | 553 | 0.179 | 945 | 535 | 26 | 23 | 785 | 50 | 935 | Phage tail tape measure protein | Phage tail tape measure protein | | afdb-uniprot50 | AF-A0A7T4T096-F1-MODEL\_V4 | 1.0 | 5.529e-17 | 552 | 0.181 | 745 | 535 | 20 | 12 | 702 | 169 | 892 | Phage tail tape measure protein | Phage tail tape measure protein | | afdb-uniprot50 | AF-A0A0F3RNQ7-F1-MODEL\_V4 | 1.0 | 3.553e-09 | 551 | 0.317 | 227 | 151 | 2 | 132 | 358 | 2 | 224 | Phage tail tape measure protein, TP901 family, core region | Phage tail tape measure protein, TP901 family, core region | | afdb-uniprot50 | AF-A0A2W6QW57-F1-MODEL\_V4 | 1.0 | 7.875e-14 | 549 | 0.22 | 550 | 379 | 15 | 132 | 672 | 2 | 510 | Phage tail tape measure protein | Phage tail tape measure protein | | afdb-uniprot50 | AF-A0A6P1Q224-F1-MODEL\_V4 | 1.0 | 3.899e-17 | 549 | 0.183 | 813 | 513 | 23 | 19 | 782 | 13 | 723 | PhageMin\_Tail domain-containing protein | PhageMin\_Tail domain-containing protein | | afdb-uniprot50 | AF-Q5D5E9-F1-MODEL\_V4 | 1.0 | 2.815e-09 | 547 | 0.345 | 223 | 144 | 1 | 116 | 338 | 8 | 228 | Prophage P2W3, tail tape measure protein | Prophage P2W3, tail tape measure protein | | afdb-uniprot50 | AF-A0A7V5PAS2-F1-MODEL\_V4 | 1.0 | 5.575e-11 | 547 | 0.175 | 411 | 314 | 8 | 70 | 463 | 4 | 406 | Phage tail tape measure protein | Phage tail tape measure protein | | afdb-uniprot50 | AF-A0A827RXC1-F1-MODEL\_V4 | 1.0 | 1.156e-16 | 547 | 0.193 | 782 | 472 | 21 | 120 | 785 | 36 | 774 | Phage tail tape measure protein | Phage tail tape measure protein | | afdb-uniprot50 | AF-A8I7U7-F1-MODEL\_V4 | 1.0 | 4.679e-16 | 546 | 0.186 | 844 | 469 | 25 | 14 | 784 | 38 | 736 | Putative phage-related tail protein | Putative phage-related tail protein | | afdb-uniprot50 | AF-A0A2D9FXU5-F1-MODEL\_V4 | 1.0 | 1.705e-16 | 545 | 0.187 | 730 | 454 | 25 | 76 | 780 | 18 | 633 | Phage tail tape measure protein | Phage tail tape measure protein | | afdb-uniprot50 | AF-A0A3A6S8Q3-F1-MODEL\_V4 | 1.0 | 2.971e-17 | 544 | 0.198 | 784 | 480 | 22 | 21 | 717 | 48 | 769 | Phage tail tape measure protein | Phage tail tape measure protein | | afdb-uniprot50 | AF-A0A2U1TTC3-F1-MODEL\_V4 | 1.0 | 1.535e-17 | 544 | 0.167 | 955 | 559 | 24 | 19 | 785 | 47 | 952 | Phage tail tape measure protein | Phage tail tape measure protein | | afdb-uniprot50 | AF-A0A2T4M4G0-F1-MODEL\_V4 | 1.0 | 3.275e-12 | 543 | 0.198 | 513 | 348 | 15 | 19 | 480 | 324 | 824 | Phage tail tape measure protein | Phage tail tape measure protein | | afdb-uniprot50 | AF-A0A1B9AKU2-F1-MODEL\_V4 | 1.0 | 3.338e-17 | 543 | 0.207 | 760 | 478 | 22 | 27 | 702 | 60 | 778 | Phage tail tape measure protein | Phage tail tape measure protein | | afdb-uniprot50 | AF-A0A1X3ITD6-F1-MODEL\_V4 | 1.0 | 1.591e-10 | 542 | 0.174 | 412 | 277 | 10 | 36 | 442 | 2 | 355 | Phage tail tape measure protein, TP901 family, core region | Phage tail tape measure protein, TP901 family, core region | | afdb-uniprot50 | AF-S4FQD2-F1-MODEL\_V4 | 1.0 | 1.193e-12 | 542 | 0.205 | 443 | 269 | 9 | 118 | 479 | 2 | 442 | Phage tail tape measure protein, TP901 family | Phage tail tape measure protein, TP901 family | | afdb-uniprot50 | AF-A0A484GDB7-F1-MODEL\_V4 | 1.0 | 2.103e-14 | 542 | 0.17 | 579 | 436 | 8 | 125 | 690 | 2 | 548 | Phage tail tape measure protein | Phage tail tape measure protein | | afdb-uniprot50 | AF-A0A4U3CH96-F1-MODEL\_V4 | 1.0 | 1.773e-16 | 542 | 0.2 | 759 | 464 | 26 | 1 | 702 | 1 | 673 | PhageMin\_Tail domain-containing protein | PhageMin\_Tail domain-containing protein | | afdb-uniprot50 | AF-A0A2T5UQZ4-F1-MODEL\_V4 | 1.0 | 1.725e-17 | 541 | 0.199 | 879 | 459 | 32 | 1 | 785 | 1 | 728 | TP901 family phage tail tape measure protein | TP901 family phage tail tape measure protein | | afdb-uniprot50 | AF-A0A348FYG3-F1-MODEL\_V4 | 1.0 | 2.152e-16 | 539 | 0.189 | 761 | 483 | 22 | 13 | 750 | 1 | 650 | PhageMin\_Tail domain-containing protein | PhageMin\_Tail domain-containing protein | | afdb-uniprot50 | AF-A0A0H3H1R5-F1-MODEL\_V4 | 1.0 | 1.314e-17 | 539 | 0.202 | 846 | 510 | 25 | 19 | 781 | 50 | 813 | Putative prophage tail length determinator | Putative prophage tail length determinator | | afdb-uniprot50 | AF-A0A078LGR8-F1-MODEL\_V4 | 1.0 | 5.976e-17 | 538 | 0.192 | 841 | 506 | 28 | 19 | 755 | 43 | 813 | Phage tail tape measure protein | Phage tail tape measure protein | | afdb-uniprot50 | AF-A0A8B5P8D7-F1-MODEL\_V4 | 1.0 | 8.282e-15 | 537 | 0.199 | 658 | 422 | 18 | 120 | 754 | 12 | 587 | Phage tail tape measure protein | Phage tail tape measure protein | | afdb-uniprot50 | AF-A0A126NVC8-F1-MODEL\_V4 | 1.0 | 7.371e-15 | 535 | 0.163 | 778 | 453 | 21 | 36 | 785 | 5 | 612 | PhageMin\_Tail domain-containing protein | PhageMin\_Tail domain-containing protein | | afdb-uniprot50 | AF-A0A1C4WZH7-F1-MODEL\_V4 | 1.0 | 2.238e-16 | 534 | 0.187 | 741 | 466 | 28 | 2 | 700 | 1 | 647 | Phage tail tape measure protein, TP901 family, core region | Phage tail tape measure protein, TP901 family, core region | | afdb-uniprot50 | AF-A0A653HUE7-F1-MODEL\_V4 | 1.0 | 1.299e-16 | 534 | 0.179 | 915 | 563 | 22 | 19 | 785 | 29 | 903 | PhageMin\_Tail domain-containing protein | PhageMin\_Tail domain-containing protein | | afdb-uniprot50 | AF-A0A1F1C065-F1-MODEL\_V4 | 1.0 | 6.98e-17 | 532 | 0.182 | 878 | 494 | 26 | 21 | 781 | 48 | 818 | PhageMin\_Tail domain-containing protein | PhageMin\_Tail domain-containing protein | | afdb-uniprot50 | AF-A0A3D4QR57-F1-MODEL\_V4 | 1.0 | 3.275e-12 | 531 | 0.176 | 471 | 312 | 10 | 19 | 422 | 34 | 495 | Phage tail tape measure protein | Phage tail tape measure protein | | afdb-uniprot50 | AF-A0A6B4AIV4-F1-MODEL\_V4 | 1.0 | 3.679e-12 | 530 | 0.213 | 482 | 301 | 13 | 49 | 464 | 203 | 672 | Phage tail tape measure protein | Phage tail tape measure protein | | afdb-uniprot50 | AF-A0A0B0SDM8-F1-MODEL\_V4 | 1.0 | 2.015e-17 | 530 | 0.224 | 837 | 421 | 19 | 152 | 762 | 2 | 835 | PhageMin\_Tail domain-containing protein | PhageMin\_Tail domain-containing protein | | afdb-uniprot50 | AF-F9PY49-F1-MODEL\_V4 | 1.0 | 1.5e-15 | 528 | 0.198 | 724 | 463 | 24 | 19 | 668 | 3 | 682 | Phage tail tape measure protein, TP901 family | Phage tail tape measure protein, TP901 family | | afdb-uniprot50 | AF-A0A6I6AWA7-F1-MODEL\_V4 | 1.0 | 4.38e-17 | 528 | 0.194 | 838 | 496 | 25 | 19 | 758 | 37 | 793 | Phage tail tape measure protein | Phage tail tape measure protein | | afdb-uniprot50 | AF-A0A2D1KVM8-F1-MODEL\_V4 | 1.0 | 2.614e-16 | 528 | 0.183 | 778 | 524 | 16 | 18 | 702 | 122 | 880 | Phage tail tape measure protein | Phage tail tape measure protein | | afdb-uniprot50 | AF-A0A268IIP2-F1-MODEL\_V4 | 1.0 | 5.466e-16 | 527 | 0.212 | 705 | 465 | 19 | 19 | 669 | 52 | 719 | Phage tail tape measure protein | Phage tail tape measure protein | | afdb-uniprot50 | AF-A0A1Q6U7T4-F1-MODEL\_V4 | 1.0 | 1.864e-17 | 526 | 0.179 | 858 | 507 | 23 | 19 | 781 | 40 | 795 | Phage tail tape measure protein | Phage tail tape measure protein | | afdb-uniprot50 | AF-A0A1B9NQD5-F1-MODEL\_V4 | 1.0 | 1.916e-16 | 526 | 0.16 | 978 | 561 | 25 | 19 | 782 | 43 | 973 | Phage tail tape measure protein | Phage tail tape measure protein | | afdb-uniprot50 | AF-A0A1E4UTG7-F1-MODEL\_V4 | 1.0 | 3.053e-16 | 525 | 0.215 | 936 | 475 | 26 | 18 | 783 | 76 | 921 | Phage tail tape measure protein | Phage tail tape measure protein | | afdb-uniprot50 | AF-A0A1W2EA98-F1-MODEL\_V4 | 1.0 | 3.664e-15 | 523 | 0.186 | 729 | 461 | 20 | 39 | 754 | 2 | 611 | Phage tail tape measure protein, TP901 family, core region | Phage tail tape measure protein, TP901 family, core region | | afdb-uniprot50 | AF-A0A845QX85-F1-MODEL\_V4 | 1.0 | 2.804e-12 | 522 | 0.2 | 515 | 321 | 13 | 20 | 460 | 68 | 565 | Phage tail tape measure protein | Phage tail tape measure protein | | afdb-uniprot50 | AF-A0A8B5S3L7-F1-MODEL\_V4 | 1.0 | 1.916e-16 | 521 | 0.154 | 933 | 591 | 23 | 19 | 782 | 22 | 925 | Phage tail tape measure protein | Phage tail tape measure protein | | afdb-uniprot50 | AF-A0A2D5B2W5-F1-MODEL\_V4 | 1.0 | 1.752e-15 | 520 | 0.185 | 729 | 486 | 26 | 35 | 700 | 13 | 696 | Phage tail tape measure protein | Phage tail tape measure protein | | afdb-uniprot50 | AF-A0A7V9PI93-F1-MODEL\_V4 | 1.0 | 2.583e-15 | 520 | 0.19 | 721 | 456 | 19 | 67 | 702 | 2 | 679 | Phage tail tape measure protein | Phage tail tape measure protein | | afdb-uniprot50 | AF-A0A5J6LCN0-F1-MODEL\_V4 | 1.0 | 1.559e-15 | 519 | 0.168 | 869 | 581 | 22 | 19 | 782 | 58 | 889 | Phage tail tape measure protein | Phage tail tape measure protein | | afdb-uniprot50 | AF-A0A0F9RHG1-F1-MODEL\_V4 | 1.0 | 1.087e-14 | 519 | 0.183 | 699 | 461 | 27 | 70 | 700 | 23 | 679 | PhageMin\_Tail domain-containing protein | PhageMin\_Tail domain-containing protein | | afdb-uniprot50 | AF-A0A1H8B8F5-F1-MODEL\_V4 | 1.0 | 7.606e-11 | 518 | 0.175 | 444 | 290 | 9 | 43 | 466 | 17 | 404 | Phage tail tape measure protein, TP901 family, core region | Phage tail tape measure protein, TP901 family, core region | | afdb-uniprot50 | AF-A0A3T0L2L7-F1-MODEL\_V4 | 1.0 | 3.299e-16 | 517 | 0.188 | 759 | 494 | 26 | 4 | 710 | 42 | 730 | Phage tail tape measure protein | Phage tail tape measure protein | | afdb-uniprot50 | AF-A0A841GB24-F1-MODEL\_V4 | 1.0 | 1.801e-14 | 516 | 0.161 | 773 | 444 | 23 | 62 | 781 | 9 | 629 | TP901 family phage tail tape measure protein | TP901 family phage tail tape measure protein | | afdb-uniprot50 | AF-A0A2J9H2B8-F1-MODEL\_V4 | 1.0 | 4.006e-16 | 515 | 0.178 | 859 | 514 | 25 | 22 | 778 | 50 | 818 | Phage tail tape measure protein | Phage tail tape measure protein | | afdb-uniprot50 | AF-A0A2N6UUM1-F1-MODEL\_V4 | 1.0 | 2.238e-16 | 515 | 0.161 | 976 | 549 | 33 | 63 | 785 | 3 | 961 | Phage tail tape measure protein | Phage tail tape measure protein | | afdb-uniprot50 | AF-A0A1M3MNY4-F1-MODEL\_V4 | 1.0 | 5.682e-16 | 514 | 0.202 | 819 | 511 | 24 | 51 | 780 | 2 | 766 | Phage tail tape measure protein | Phage tail tape measure protein | | afdb-uniprot50 | AF-A0A1V5V4L3-F1-MODEL\_V4 | 1.0 | 8.059e-16 | 514 | 0.207 | 742 | 441 | 23 | 65 | 716 | 10 | 694 | Phage-related minor tail protein | Phage-related minor tail protein | | afdb-uniprot50 | AF-G8PUN4-F1-MODEL\_V4 | 1.0 | 5.403e-15 | 514 | 0.182 | 1019 | 516 | 31 | 20 | 782 | 93 | 1049 | Phage-related tail protein | Phage-related tail protein | | afdb-uniprot50 | AF-A0A0F9CXC0-F1-MODEL\_V4 | 1.0 | 3.484e-14 | 513 | 0.202 | 607 | 433 | 18 | 122 | 702 | 5 | 586 | PhageMin\_Tail domain-containing protein | PhageMin\_Tail domain-containing protein | | afdb-uniprot50 | AF-A0A2S5N2H2-F1-MODEL\_V4 | 1.0 | 7.966e-15 | 513 | 0.163 | 831 | 475 | 25 | 4 | 781 | 65 | 727 | Phage tail tape measure protein | Phage tail tape measure protein | | afdb-uniprot50 | AF-A0A5E9SAB7-F1-MODEL\_V4 | 1.0 | 9.198e-14 | 512 | 0.206 | 629 | 408 | 20 | 32 | 623 | 2 | 576 | Phage tail tape measure protein | Phage tail tape measure protein | | afdb-uniprot50 | AF-A0A0M7AQ48-F1-MODEL\_V4 | 1.0 | 1.752e-15 | 512 | 0.182 | 794 | 458 | 25 | 1 | 766 | 1 | 631 | Phage tail tape measure protein, TP901 family, core region | Phage tail tape measure protein, TP901 family, core region | | afdb-uniprot50 | AF-A0A1F4X418-F1-MODEL\_V4 | 1.0 | 3.722e-13 | 511 | 0.147 | 727 | 447 | 21 | 35 | 750 | 7 | 571 | Phage tail tape measure protein | Phage tail tape measure protein | | afdb-uniprot50 | AF-A0A1G9I6R0-F1-MODEL\_V4 | 1.0 | 4.753e-14 | 511 | 0.192 | 581 | 396 | 16 | 27 | 566 | 62 | 610 | Phage tail tape measure protein, TP901 family, core region | Phage tail tape measure protein, TP901 family, core region | | afdb-uniprot50 | AF-A0A6L8LYC8-F1-MODEL\_V4 | 1.0 | 4.626e-15 | 511 | 0.161 | 871 | 532 | 26 | 6 | 785 | 1 | 763 | Phage tail tape measure protein | Phage tail tape measure protein | | afdb-uniprot50 | AF-A0A0N1LUL1-F1-MODEL\_V4 | 1.0 | 1.5e-15 | 510 | 0.172 | 864 | 451 | 27 | 19 | 785 | 66 | 762 | PhageMin\_Tail domain-containing protein | PhageMin\_Tail domain-containing protein | | afdb-uniprot50 | AF-A0A0U5L5G9-F1-MODEL\_V4 | 1.0 | 4.33e-16 | 510 | 0.184 | 845 | 526 | 22 | 19 | 781 | 50 | 812 | Tail protein | Tail protein | | afdb-uniprot50 | AF-A0A0F9RC03-F1-MODEL\_V4 | 1.0 | 4.039e-10 | 509 | 0.179 | 424 | 273 | 10 | 27 | 433 | 5 | 370 | PhageMin\_Tail domain-containing protein | PhageMin\_Tail domain-containing protein | | afdb-uniprot50 | AF-A0A268U7H8-F1-MODEL\_V4 | 1.0 | 1.082e-17 | 508 | 0.197 | 826 | 510 | 24 | 2 | 720 | 1 | 780 | Phage tail tape measure protein | Phage tail tape measure protein | | afdb-uniprot50 | AF-A0A417HJE2-F1-MODEL\_V4 | 1.0 | 1.916e-16 | 508 | 0.181 | 844 | 497 | 25 | 19 | 702 | 10 | 819 | Phage tail tape measure protein | Phage tail tape measure protein | | afdb-uniprot50 | AF-A0A517IVI2-F1-MODEL\_V4 | 1.0 | 6.769e-11 | 506 | 0.154 | 427 | 317 | 10 | 28 | 443 | 10 | 403 | Phage tail tape measure protein | Phage tail tape measure protein | | afdb-uniprot50 | AF-A0A1X0SVA8-F1-MODEL\_V4 | 1.0 | 3.351e-14 | 506 | 0.16 | 761 | 435 | 18 | 70 | 785 | 2 | 603 | Phage tail tape measure protein | Phage tail tape measure protein | | afdb-uniprot50 | AF-A0A559QNE5-F1-MODEL\_V4 | 1.0 | 3.018e-15 | 506 | 0.184 | 833 | 551 | 26 | 31 | 780 | 164 | 950 | TP901 family phage tail tape measure protein | TP901 family phage tail tape measure protein | | afdb-uniprot50 | AF-K0ZBN7-F1-MODEL\_V4 | 1.0 | 5.403e-15 | 505 | 0.183 | 745 | 448 | 28 | 1 | 655 | 1 | 674 | Phage tail tape measure protein, TP901 family, core region | Phage tail tape measure protein, TP901 family, core region | | afdb-uniprot50 | AF-A0A2T6LZL7-F1-MODEL\_V4 | 1.0 | 6.384e-16 | 505 | 0.18 | 809 | 560 | 24 | 1 | 785 | 1 | 730 | TP901 family phage tail tape measure protein | TP901 family phage tail tape measure protein | | afdb-uniprot50 | AF-A0A0F9T8W6-F1-MODEL\_V4 | 1.0 | 6.436e-10 | 504 | 0.17 | 392 | 293 | 10 | 86 | 465 | 18 | 389 | PhageMin\_Tail domain-containing protein | PhageMin\_Tail domain-containing protein | | afdb-uniprot50 | AF-A0A175R8T9-F1-MODEL\_V4 | 1.0 | 5.617e-15 | 504 | 0.18 | 782 | 480 | 19 | 2 | 702 | 1 | 702 | PhageMin\_Tail domain-containing protein | PhageMin\_Tail domain-containing protein | | afdb-uniprot50 | AF-W7CZ00-F1-MODEL\_V4 | 1.0 | 7.457e-16 | 504 | 0.2 | 687 | 449 | 25 | 20 | 662 | 55 | 684 | Tail tape measure protein | Tail tape measure protein | | afdb-uniprot50 | AF-A0A4R8HHY1-F1-MODEL\_V4 | 1.0 | 3.809e-15 | 504 | 0.177 | 884 | 506 | 26 | 27 | 780 | 20 | 811 | TP901 family phage tail tape measure protein | TP901 family phage tail tape measure protein | | afdb-uniprot50 | AF-A0A1R1MK84-F1-MODEL\_V4 | 1.0 | 7.457e-16 | 503 | 0.197 | 809 | 516 | 27 | 55 | 773 | 2 | 766 | Phage tail tape measure protein | Phage tail tape measure protein | | afdb-uniprot50 | AF-A0A4R2GR49-F1-MODEL\_V4 | 1.0 | 5.682e-16 | 501 | 0.186 | 847 | 503 | 26 | 21 | 764 | 24 | 787 | TP901 family phage tail tape measure protein | TP901 family phage tail tape measure protein | | afdb-uniprot50 | AF-A0A231UT38-F1-MODEL\_V4 | 1.0 | 5.617e-15 | 501 | 0.172 | 765 | 449 | 24 | 49 | 785 | 76 | 684 | Phage tail tape measure protein | Phage tail tape measure protein | | afdb-uniprot50 | AF-A0A268TWS7-F1-MODEL\_V4 | 1.0 | 1.202e-16 | 500 | 0.186 | 813 | 527 | 24 | 27 | 785 | 16 | 747 | Phage tail tape measure protein | Phage tail tape measure protein | | afdb-uniprot50 | AF-A0A1W2BBP6-F1-MODEL\_V4 | 1.0 | 2.903e-15 | 500 | 0.161 | 799 | 487 | 22 | 26 | 785 | 1 | 655 | Phage tail tape measure protein, TP901 family, core region | Phage tail tape measure protein, TP901 family, core region | | afdb-uniprot50 | AF-A0A349HHP7-F1-MODEL\_V4 | 1.0 | 8.645e-12 | 499 | 0.175 | 523 | 360 | 8 | 12 | 475 | 40 | 550 | Phage tail tape measure protein | Phage tail tape measure protein | | afdb-uniprot50 | AF-D5EFA3-F1-MODEL\_V4 | 1.0 | 1.143e-15 | 499 | 0.163 | 846 | 549 | 25 | 19 | 782 | 42 | 810 | Phage tail tape measure protein, TP901 family | Phage tail tape measure protein, TP901 family | | afdb-uniprot50 | AF-A0A077L0E5-F1-MODEL\_V4 | 1.0 | 9.413e-16 | 499 | 0.177 | 841 | 589 | 24 | 19 | 783 | 22 | 835 | PhageMin\_Tail domain-containing protein | PhageMin\_Tail domain-containing protein | | afdb-uniprot50 | AF-A0A0B1Q8A4-F1-MODEL\_V4 | 1.0 | 1.006e-14 | 498 | 0.171 | 799 | 501 | 24 | 11 | 754 | 1 | 693 | PhageMin\_Tail domain-containing protein | PhageMin\_Tail domain-containing protein | | afdb-uniprot50 | AF-A0A4Z0DLG0-F1-MODEL\_V4 | 1.0 | 3.211e-17 | 497 | 0.201 | 887 | 533 | 28 | 1 | 782 | 1 | 816 | Phage tail tape measure protein | Phage tail tape measure protein | | afdb-uniprot50 | AF-K8EVH7-F1-MODEL\_V4 | 1.0 | 1.29e-12 | 495 | 0.176 | 539 | 343 | 15 | 12 | 461 | 45 | 571 | Phage tail tape measure protein, TP901 family,core region | Phage tail tape measure protein, TP901 family,core region | | afdb-uniprot50 | AF-A0A660ZYG0-F1-MODEL\_V4 | 1.0 | 3.261e-15 | 495 | 0.175 | 736 | 479 | 25 | 16 | 689 | 1 | 670 | Phage tail tape measure protein | Phage tail tape measure protein | | afdb-uniprot50 | AF-A0A3B0B7K3-F1-MODEL\_V4 | 1.0 | 2.212e-15 | 495 | 0.151 | 997 | 549 | 28 | 1 | 785 | 1 | 912 | Phage tail tape measure protein | Phage tail tape measure protein | | afdb-uniprot50 | AF-A0A7X3HJK4-F1-MODEL\_V4 | 1.0 | 4.117e-15 | 494 | 0.176 | 875 | 550 | 29 | 19 | 782 | 42 | 856 | Phage tail tape measure protein | Phage tail tape measure protein | | afdb-uniprot50 | AF-A0A1Q3NZU9-F1-MODEL\_V4 | 1.0 | 4.773e-11 | 493 | 0.192 | 453 | 323 | 10 | 35 | 474 | 7 | 429 | Phage tail tape measure protein | Phage tail tape measure protein | | afdb-uniprot50 | AF-A0A7W6BZB0-F1-MODEL\_V4 | 1.0 | 5.137e-14 | 493 | 0.177 | 798 | 463 | 26 | 1 | 785 | 1 | 617 | TP901 family phage tail tape measure protein | TP901 family phage tail tape measure protein | | afdb-uniprot50 | AF-A0A839Y122-F1-MODEL\_V4 | 1.0 | 1.969e-15 | 493 | 0.17 | 867 | 491 | 27 | 50 | 750 | 2 | 805 | TP901 family phage tail tape measure protein | TP901 family phage tail tape measure protein | | afdb-uniprot50 | AF-A0A642C2T3-F1-MODEL\_V4 | 1.0 | 7.726e-09 | 492 | 0.137 | 327 | 256 | 10 | 117 | 433 | 4 | 314 | Phage tail tape measure protein | Phage tail tape measure protein | | afdb-uniprot50 | AF-A0A7W5WZ30-F1-MODEL\_V4 | 1.0 | 6e-14 | 492 | 0.191 | 794 | 475 | 23 | 1 | 728 | 1 | 693 | TP901 family phage tail tape measure protein | TP901 family phage tail tape measure protein | | afdb-uniprot50 | AF-A0A1W1ZU85-F1-MODEL\_V4 | 1.0 | 4.28e-15 | 492 | 0.2 | 808 | 464 | 25 | 31 | 785 | 13 | 691 | Phage tail tape measure protein, TP901 family, core region | Phage tail tape measure protein, TP901 family, core region | | afdb-uniprot50 | AF-S3XXC4-F1-MODEL\_V4 | 1.0 | 1.443e-15 | 492 | 0.182 | 941 | 530 | 37 | 19 | 785 | 10 | 884 | Phage tail tape measure protein, TP901 family, core region | Phage tail tape measure protein, TP901 family, core region | | afdb-uniprot50 | AF-A0A1B8P3Y8-F1-MODEL\_V4 | 1.0 | 1.346e-09 | 491 | 0.182 | 356 | 260 | 9 | 77 | 426 | 18 | 348 | Phage-related minor tail protein | Phage-related minor tail protein | | afdb-uniprot50 | AF-A0A829CHN2-F1-MODEL\_V4 | 1.0 | 1.489e-11 | 491 | 0.193 | 516 | 342 | 12 | 23 | 480 | 50 | 549 | Phage tail tape measure protein, TP901 family, core region | Phage tail tape measure protein, TP901 family, core region | | afdb-uniprot50 | AF-A0A0Q4C350-F1-MODEL\_V4 | 1.0 | 6.384e-16 | 491 | 0.196 | 825 | 537 | 29 | 2 | 781 | 1 | 744 | PhageMin\_Tail domain-containing protein | PhageMin\_Tail domain-containing protein | | afdb-uniprot50 | AF-A0A1S1V6R2-F1-MODEL\_V4 | 1.0 | 8.61e-15 | 491 | 0.194 | 795 | 487 | 29 | 62 | 785 | 8 | 719 | Phage-related minor tail protein | Phage-related minor tail protein | | afdb-uniprot50 | AF-A0A1Q6JQ74-F1-MODEL\_V4 | 1.0 | 1.226e-11 | 489 | 0.205 | 506 | 322 | 12 | 19 | 466 | 44 | 527 | Phage tail tape measure protein | Phage tail tape measure protein | | afdb-uniprot50 | AF-A0A2M8WDP6-F1-MODEL\_V4 | 1.0 | 1.894e-15 | 489 | 0.173 | 838 | 561 | 29 | 1 | 781 | 1 | 763 | Phage tail tape measure protein | Phage tail tape measure protein | | afdb-uniprot50 | AF-A0A2W6YTE4-F1-MODEL\_V4 | 1.0 | 9.305e-15 | 489 | 0.176 | 854 | 504 | 30 | 15 | 784 | 13 | 750 | Phage tail tape measure protein | Phage tail tape measure protein | | afdb-uniprot50 | AF-A0A1L3SQ06-F1-MODEL\_V4 | 1.0 | 2.023e-14 | 488 | 0.176 | 767 | 463 | 23 | 26 | 723 | 1 | 667 | Phage tail tape measure protein | Phage tail tape measure protein | | afdb-uniprot50 | AF-H6U8K4-F1-MODEL\_V4 | 1.0 | 2.825e-16 | 488 | 0.221 | 766 | 404 | 24 | 166 | 776 | 1 | 728 | Phage tail tape measure protein TP901 family core region | Phage tail tape measure protein TP901 family core region | | afdb-uniprot50 | AF-A0A291LZ62-F1-MODEL\_V4 | 1.0 | 1.372e-14 | 488 | 0.178 | 855 | 519 | 24 | 1 | 785 | 2 | 742 | Phage tail tape measure protein | Phage tail tape measure protein | | afdb-uniprot50 | AF-A0A380JYP8-F1-MODEL\_V4 | 1.0 | 6e-14 | 488 | 0.19 | 678 | 443 | 19 | 67 | 702 | 15 | 628 | Phage protein | Phage protein | | afdb-uniprot50 | AF-A0A4R3NZQ3-F1-MODEL\_V4 | 1.0 | 1.752e-15 | 487 | 0.205 | 795 | 497 | 29 | 39 | 776 | 4 | 720 | TP901 family phage tail tape measure protein | TP901 family phage tail tape measure protein | | afdb-uniprot50 | AF-A0A379YC51-F1-MODEL\_V4 | 1.0 | 1.335e-15 | 487 | 0.183 | 800 | 499 | 25 | 19 | 690 | 48 | 820 | Phage tail tape measure protein, TP901 family, core region | Phage tail tape measure protein, TP901 family, core region | | afdb-uniprot50 | AF-A0A101IPF4-F1-MODEL\_V4 | 1.0 | 8.447e-10 | 486 | 0.175 | 358 | 265 | 11 | 70 | 422 | 10 | 342 | Phage tail tape measure protein, TP901 family, core region | Phage tail tape measure protein, TP901 family, core region | | afdb-uniprot50 | AF-A0A5R8LBZ4-F1-MODEL\_V4 | 1.0 | 1.32e-14 | 486 | 0.186 | 741 | 457 | 22 | 35 | 711 | 14 | 672 | Phage tail tape measure protein | Phage tail tape measure protein | | afdb-uniprot50 | AF-A0A2X2BQ74-F1-MODEL\_V4 | 1.0 | 4.117e-15 | 485 | 0.168 | 823 | 516 | 28 | 81 | 784 | 3 | 775 | Phage tail fiber protein | Phage tail fiber protein | | afdb-uniprot50 | AF-W7BV28-F1-MODEL\_V4 | 1.0 | 1.909e-09 | 484 | 0.189 | 328 | 206 | 8 | 137 | 408 | 4 | 327 | TP901 family phage tail tape measure protein | TP901 family phage tail tape measure protein | | afdb-uniprot50 | AF-A0A6M3L5E0-F1-MODEL\_V4 | 1.0 | 1.512e-09 | 484 | 0.135 | 392 | 306 | 11 | 88 | 469 | 6 | 374 | Putative tail protein | Putative tail protein | | afdb-uniprot50 | AF-A0A2D6X7M0-F1-MODEL\_V4 | 1.0 | 1.732e-14 | 484 | 0.188 | 784 | 494 | 27 | 66 | 785 | 3 | 707 | Phage tail tape measure protein | Phage tail tape measure protein | | afdb-uniprot50 | AF-A0A4R7DE84-F1-MODEL\_V4 | 1.0 | 8.951e-15 | 484 | 0.179 | 813 | 546 | 28 | 1 | 781 | 1 | 724 | TP901 family phage tail tape measure protein | TP901 family phage tail tape measure protein | | afdb-uniprot50 | AF-R2T824-F1-MODEL\_V4 | 1.0 | 7.457e-16 | 483 | 0.192 | 775 | 513 | 23 | 19 | 702 | 123 | 875 | Phage tail tape measure protein, TP901 family, core region | Phage tail tape measure protein, TP901 family, core region | | afdb-uniprot50 | AF-A0A021VU74-F1-MODEL\_V4 | 1.0 | 4.449e-15 | 482 | 0.161 | 977 | 554 | 30 | 14 | 785 | 1 | 916 | PhageMin\_Tail domain-containing protein | PhageMin\_Tail domain-containing protein | | afdb-uniprot50 | AF-A0A0C5XG03-F1-MODEL\_V4 | 1.0 | 2.212e-15 | 482 | 0.174 | 913 | 527 | 32 | 61 | 785 | 7 | 880 | Phage tail length tape-measure protein | Phage tail length tape-measure protein | | afdb-uniprot50 | AF-A0A3R8SD78-F1-MODEL\_V4 | 1.0 | 1.969e-15 | 482 | 0.163 | 915 | 547 | 30 | 39 | 765 | 2 | 885 | Phage tail tape measure protein | Phage tail tape measure protein | | afdb-uniprot50 | AF-A0A7C5G4Y5-F1-MODEL\_V4 | 1.0 | 4.039e-10 | 481 | 0.179 | 424 | 313 | 14 | 62 | 473 | 8 | 408 | Phage tail tape measure protein | Phage tail tape measure protein | | afdb-uniprot50 | AF-A0A0Q5FLM7-F1-MODEL\_V4 | 1.0 | 3.622e-14 | 481 | 0.157 | 923 | 528 | 26 | 76 | 785 | 14 | 899 | PhageMin\_Tail domain-containing protein | PhageMin\_Tail domain-containing protein | | afdb-uniprot50 | AF-A0A1I5W4T8-F1-MODEL\_V4 | 1.0 | 3.224e-14 | 481 | 0.222 | 637 | 406 | 20 | 109 | 710 | 50 | 631 | Phage tail tape measure protein, TP901 family, core region | Phage tail tape measure protein, TP901 family, core region | | afdb-uniprot50 | AF-A0A6B4JNP3-F1-MODEL\_V4 | 1.0 | 1.673e-11 | 481 | 0.178 | 488 | 336 | 12 | 1 | 459 | 1 | 452 | Phage tail tape measure protein | Phage tail tape measure protein | | afdb-uniprot50 | AF-A0A0Q5GQH4-F1-MODEL\_V4 | 1.0 | 7.457e-16 | 480 | 0.18 | 896 | 548 | 24 | 1 | 780 | 1 | 825 | PhageMin\_Tail domain-containing protein | PhageMin\_Tail domain-containing protein | | afdb-uniprot50 | AF-A0A6G3L4W1-F1-MODEL\_V4 | 1.0 | 1.666e-14 | 480 | 0.186 | 921 | 515 | 25 | 42 | 785 | 167 | 1029 | Phage tail tape measure protein | Phage tail tape measure protein | | afdb-uniprot50 | AF-X0RKC4-F1-MODEL\_V4 | 1.0 | 2.686e-15 | 480 | 0.183 | 772 | 496 | 27 | 27 | 706 | 53 | 781 | PhageMin\_Tail domain-containing protein | PhageMin\_Tail domain-containing protein | | afdb-uniprot50 | AF-A0A376KHT3-F1-MODEL\_V4 | 1.0 | 2.554e-14 | 479 | 0.193 | 697 | 424 | 19 | 21 | 639 | 48 | 684 | Tail protein (Modular protein) | Tail protein (Modular protein) | | afdb-uniprot50 | AF-A0A0Q0XGG6-F1-MODEL\_V4 | 1.0 | 1.946e-14 | 479 | 0.17 | 778 | 508 | 27 | 51 | 780 | 9 | 696 | PhageMin\_Tail domain-containing protein | PhageMin\_Tail domain-containing protein | | afdb-uniprot50 | AF-E2S7P3-F1-MODEL\_V4 | 1.0 | 4.626e-15 | 478 | 0.175 | 808 | 497 | 27 | 61 | 750 | 11 | 766 | Phage tail tape measure protein, TP901 family | Phage tail tape measure protein, TP901 family | | afdb-uniprot50 | AF-A0A3A9ICH5-F1-MODEL\_V4 | 1.0 | 4.572e-14 | 478 | 0.176 | 917 | 585 | 26 | 3 | 784 | 1 | 881 | Phage tail tape measure protein | Phage tail tape measure protein | | afdb-uniprot50 | AF-A0A2E3N1K5-F1-MODEL\_V4 | 1.0 | 1.372e-14 | 477 | 0.163 | 868 | 510 | 30 | 1 | 780 | 1 | 739 | Phage tail tape measure protein | Phage tail tape measure protein | | afdb-uniprot50 | AF-A0A0X8GNA4-F1-MODEL\_V4 | 1.0 | 2.363e-14 | 477 | 0.188 | 869 | 546 | 29 | 19 | 782 | 49 | 862 | PhageMin\_Tail domain-containing protein | PhageMin\_Tail domain-containing protein | | afdb-uniprot50 | AF-J3DZE3-F1-MODEL\_V4 | 1.0 | 2.869e-14 | 476 | 0.192 | 862 | 456 | 25 | 1 | 783 | 26 | 726 | Phage tail tape measure protein, TP901 family | Phage tail tape measure protein, TP901 family | | afdb-uniprot50 | AF-A0A836WW60-F1-MODEL\_V4 | 1.0 | 7.173e-16 | 476 | 0.152 | 906 | 584 | 31 | 28 | 780 | 4 | 878 | Phage tail tape measure protein | Phage tail tape measure protein | | afdb-uniprot50 | AF-A0A7X9B3Q6-F1-MODEL\_V4 | 1.0 | 2.848e-10 | 475 | 0.159 | 421 | 310 | 11 | 77 | 489 | 20 | 404 | Phage tail tape measure protein | Phage tail tape measure protein | | afdb-uniprot50 | AF-A0A5C7JCR9-F1-MODEL\_V4 | 1.0 | 2.055e-12 | 475 | 0.174 | 619 | 429 | 19 | 47 | 625 | 2 | 578 | Phage tail tape measure protein | Phage tail tape measure protein | | afdb-uniprot50 | AF-A0A7C4AJ87-F1-MODEL\_V4 | 1.0 | 2.363e-14 | 475 | 0.174 | 795 | 502 | 25 | 30 | 785 | 6 | 684 | Phage tail tape measure protein | Phage tail tape measure protein | | afdb-uniprot50 | AF-R9J8Y0-F1-MODEL\_V4 | 1.0 | 2.023e-14 | 475 | 0.161 | 683 | 481 | 21 | 39 | 702 | 3 | 612 | Phage tail tape measure protein, TP901 family, core region | Phage tail tape measure protein, TP901 family, core region | | afdb-uniprot50 | AF-K6DYV4-F1-MODEL\_V4 | 1.0 | 1.732e-14 | 474 | 0.193 | 744 | 452 | 27 | 1 | 701 | 1 | 639 | PhageMin\_Tail domain-containing protein | PhageMin\_Tail domain-containing protein | | afdb-uniprot50 | AF-A0A377ALG4-F1-MODEL\_V4 | 1.0 | 3.237e-11 | 473 | 0.231 | 384 | 254 | 7 | 182 | 556 | 11 | 362 | TP901 family phage tail tape measure protein | TP901 family phage tail tape measure protein | | afdb-uniprot50 | AF-A0A2N3J4A2-F1-MODEL\_V4 | 1.0 | 3.261e-15 | 473 | 0.165 | 859 | 530 | 30 | 19 | 782 | 46 | 812 | Phage tail protein | Phage tail protein | | afdb-uniprot50 | AF-A0A6G2L5R9-F1-MODEL\_V4 | 1.0 | 3.664e-15 | 473 | 0.171 | 852 | 524 | 20 | 1 | 679 | 1 | 843 | Phage tail tape measure protein | Phage tail tape measure protein | | afdb-uniprot50 | AF-A0A061P2R0-F1-MODEL\_V4 | 1.0 | 1.692e-12 | 472 | 0.171 | 607 | 393 | 20 | 5 | 541 | 2 | 568 | Phage tail length tape-measure protein | Phage tail length tape-measure protein | | afdb-uniprot50 | AF-F2J629-F1-MODEL\_V4 | 1.0 | 9.413e-16 | 472 | 0.188 | 835 | 516 | 32 | 13 | 760 | 1 | 760 | Phage tail tape measure protein, TP901 family, core region | Phage tail tape measure protein, TP901 family, core region | | afdb-uniprot50 | AF-A0A653LRG4-F1-MODEL\_V4 | 1.0 | 5.839e-15 | 472 | 0.184 | 785 | 504 | 26 | 19 | 708 | 41 | 783 | PhageMin\_Tail domain-containing protein | PhageMin\_Tail domain-containing protein | | afdb-uniprot50 | AF-A0A6V6Y4C0-F1-MODEL\_V4 | 1.0 | 1.045e-14 | 471 | 0.176 | 835 | 534 | 22 | 49 | 782 | 3 | 784 | Phage tail tape measure protein, TP901 family | Phage tail tape measure protein, TP901 family | | afdb-uniprot50 | AF-I3DCP6-F1-MODEL\_V4 | 1.0 | 4.449e-15 | 471 | 0.16 | 876 | 548 | 26 | 19 | 782 | 16 | 815 | Phage tail tape measure protein, TP901 family | Phage tail tape measure protein, TP901 family | | afdb-uniprot50 | AF-A0A1B3E8J2-F1-MODEL\_V4 | 1.0 | 3.664e-15 | 471 | 0.169 | 873 | 554 | 26 | 20 | 782 | 36 | 847 | Tail tape measure protein | Tail tape measure protein | | afdb-uniprot50 | AF-A0A852Q908-F1-MODEL\_V4 | 1.0 | 4.999e-15 | 471 | 0.184 | 927 | 514 | 30 | 21 | 780 | 39 | 890 | Phage tail tape measure protein | Phage tail tape measure protein | | afdb-uniprot50 | AF-A0A839FTM8-F1-MODEL\_V4 | 1.0 | 2.363e-14 | 471 | 0.177 | 753 | 465 | 25 | 7 | 708 | 1 | 649 | TP901 family phage tail tape measure protein | TP901 family phage tail tape measure protein | | afdb-uniprot50 | AF-A0A446I8U4-F1-MODEL\_V4 | 1.0 | 2.023e-14 | 471 | 0.161 | 835 | 521 | 26 | 19 | 702 | 74 | 879 | Phage tail tape measure protein | Phage tail tape measure protein | | afdb-uniprot50 | AF-A0A7X7XZN6-F1-MODEL\_V4 | 1.0 | 8.61e-15 | 470 | 0.183 | 756 | 506 | 20 | 27 | 708 | 53 | 770 | Phage tail tape measure protein | Phage tail tape measure protein | | afdb-uniprot50 | AF-A0A2E5BTY7-F1-MODEL\_V4 | 1.0 | 6.56e-15 | 470 | 0.177 | 864 | 539 | 27 | 18 | 780 | 49 | 841 | Phage tail tape measure protein | Phage tail tape measure protein | | afdb-uniprot50 | AF-A0A6N6NKD3-F1-MODEL\_V4 | 1.0 | 6.485e-14 | 470 | 0.182 | 749 | 486 | 23 | 1 | 654 | 1 | 717 | Phage tail tape measure protein | Phage tail tape measure protein | | afdb-uniprot50 | AF-A0A1E4AIA1-F1-MODEL\_V4 | 1.0 | 1.541e-14 | 469 | 0.182 | 806 | 484 | 26 | 35 | 776 | 8 | 702 | Phage tail tape measure protein | Phage tail tape measure protein | | afdb-uniprot50 | AF-F1Z9C8-F1-MODEL\_V4 | 1.0 | 8.282e-15 | 469 | 0.173 | 828 | 525 | 33 | 1 | 776 | 1 | 720 | TP901 family phage tail tape measure protein | TP901 family phage tail tape measure protein | | afdb-uniprot50 | AF-A0A075JTQ7-F1-MODEL\_V4 | 1.0 | 3.622e-14 | 469 | 0.151 | 811 | 532 | 26 | 69 | 785 | 18 | 765 | PhageMin\_Tail domain-containing protein | PhageMin\_Tail domain-containing protein | | afdb-uniprot50 | AF-A0A556S942-F1-MODEL\_V4 | 1.0 | 8.61e-15 | 469 | 0.16 | 851 | 533 | 17 | 19 | 702 | 36 | 871 | Phage tail tape measure protein | Phage tail tape measure protein | | afdb-uniprot50 | AF-A0A258KVP7-F1-MODEL\_V4 | 1.0 | 3.622e-14 | 468 | 0.18 | 769 | 497 | 26 | 96 | 781 | 8 | 725 | Phage tail tape measure protein | Phage tail tape measure protein | | afdb-uniprot50 | AF-A0A6G6ZVQ1-F1-MODEL\_V4 | 1.0 | 1.483e-14 | 468 | 0.176 | 906 | 475 | 28 | 1 | 783 | 22 | 778 | Phage tail tape measure protein | Phage tail tape measure protein | | afdb-uniprot50 | AF-A0A6N2ZLI6-F1-MODEL\_V4 | 1.0 | 1.006e-14 | 468 | 0.151 | 837 | 565 | 22 | 19 | 782 | 19 | 782 | Phage-related minor tail protein | Phage-related minor tail protein | | afdb-uniprot50 | AF-S6GUR2-F1-MODEL\_V4 | 1.0 | 1.045e-14 | 468 | 0.176 | 883 | 546 | 26 | 19 | 782 | 57 | 876 | PhageMin\_Tail domain-containing protein | PhageMin\_Tail domain-containing protein | | afdb-uniprot50 | AF-A0A1D9FNG0-F1-MODEL\_V4 | 1.0 | 1.85e-13 | 467 | 0.15 | 773 | 507 | 28 | 60 | 783 | 9 | 680 | Phage tail tape measure protein | Phage tail tape measure protein | | afdb-uniprot50 | AF-A0A157SJY1-F1-MODEL\_V4 | 1.0 | 7.966e-15 | 467 | 0.169 | 957 | 572 | 25 | 22 | 785 | 53 | 979 | Bacteriophage protein | Bacteriophage protein | | afdb-uniprot50 | AF-A0A072Y8W2-F1-MODEL\_V4 | 1.0 | 2.008e-10 | 467 | 0.165 | 471 | 304 | 12 | 1 | 465 | 102 | 489 | Phage tail tape measure protein | Phage tail tape measure protein | | afdb-uniprot50 | AF-A0A2W4M4D8-F1-MODEL\_V4 | 1.0 | 4.591e-11 | 466 | 0.161 | 507 | 356 | 12 | 16 | 510 | 1 | 450 | Phage tail tape measure protein | Phage tail tape measure protein | | afdb-uniprot50 | AF-A0A7V8U794-F1-MODEL\_V4 | 1.0 | 3.809e-15 | 466 | 0.178 | 814 | 550 | 26 | 1 | 781 | 1 | 728 | Phage tail tape measure protein | Phage tail tape measure protein | | afdb-uniprot50 | AF-A0A7I0K7H0-F1-MODEL\_V4 | 1.0 | 4.626e-15 | 466 | 0.188 | 813 | 534 | 26 | 47 | 782 | 3 | 766 | Phage tail tape measure protein | Phage tail tape measure protein | | afdb-uniprot50 | AF-A0A0F7LMA5-F1-MODEL\_V4 | 1.0 | 2.103e-14 | 466 | 0.153 | 878 | 577 | 23 | 19 | 785 | 62 | 883 | Tail protein | Tail protein | | afdb-uniprot50 | AF-A0A223S641-F1-MODEL\_V4 | 1.0 | 1.087e-14 | 466 | 0.162 | 912 | 568 | 32 | 35 | 785 | 5 | 881 | Phage tail tape measure protein | Phage tail tape measure protein | | afdb-uniprot50 | AF-A0A1V3VRH9-F1-MODEL\_V4 | 1.0 | 8.781e-10 | 465 | 0.17 | 429 | 314 | 10 | 32 | 439 | 4 | 411 | Phage tail tape measure protein | Phage tail tape measure protein | | afdb-uniprot50 | AF-A0A0F9TFS1-F1-MODEL\_V4 | 1.0 | 1.879e-11 | 464 | 0.177 | 541 | 354 | 20 | 42 | 572 | 5 | 464 | PhageMin\_Tail domain-containing protein | PhageMin\_Tail domain-containing protein | | afdb-uniprot50 | AF-A0A632WVI2-F1-MODEL\_V4 | 1.0 | 2.4e-12 | 464 | 0.162 | 700 | 455 | 21 | 118 | 781 | 31 | 634 | Phage tail tape measure protein | Phage tail tape measure protein | | afdb-uniprot50 | AF-Q30VW6-F1-MODEL\_V4 | 1.0 | 4.753e-14 | 464 | 0.155 | 859 | 519 | 25 | 1 | 785 | 1 | 726 | Phage tail tape measure protein, TP901 family | Phage tail tape measure protein, TP901 family | | afdb-uniprot50 | AF-A0A1W9UFW0-F1-MODEL\_V4 | 1.0 | 1.872e-14 | 464 | 0.183 | 821 | 534 | 22 | 61 | 785 | 3 | 782 | Phage tail tape measure protein | Phage tail tape measure protein | | afdb-uniprot50 | AF-A0A316QC74-F1-MODEL\_V4 | 1.0 | 2e-13 | 463 | 0.154 | 720 | 518 | 20 | 19 | 702 | 19 | 683 | Phage tail tape measure protein | Phage tail tape measure protein | | afdb-uniprot50 | AF-A0A497UTH2-F1-MODEL\_V4 | 1.0 | 8.847e-14 | 461 | 0.175 | 771 | 511 | 23 | 64 | 776 | 11 | 714 | TP901 family phage tail tape measure protein | TP901 family phage tail tape measure protein | | afdb-uniprot50 | AF-W6N4J1-F1-MODEL\_V4 | 1.0 | 7.286e-14 | 461 | 0.16 | 771 | 518 | 23 | 75 | 780 | 19 | 724 | Phage tail length tape-measure protein | Phage tail length tape-measure protein | | afdb-uniprot50 | AF-A0A1I4ZMH4-F1-MODEL\_V4 | 1.0 | 4.999e-15 | 461 | 0.16 | 998 | 585 | 31 | 12 | 785 | 175 | 1143 | Phage tail tape measure protein, TP901 family, core region | Phage tail tape measure protein, TP901 family, core region | | afdb-uniprot50 | AF-A0A2U1BMI5-F1-MODEL\_V4 | 1.0 | 4.231e-14 | 460 | 0.162 | 851 | 563 | 25 | 22 | 780 | 53 | 845 | TP901 family phage tail tape measure protein | TP901 family phage tail tape measure protein | | afdb-uniprot50 | AF-B6XAD2-F1-MODEL\_V4 | 1.0 | 1.074e-13 | 460 | 0.149 | 898 | 548 | 22 | 19 | 784 | 57 | 870 | Phage tail tape measure protein, TP901 family | Phage tail tape measure protein, TP901 family | | afdb-uniprot50 | AF-A0A6L5FX98-F1-MODEL\_V4 | 1.0 | 2.161e-13 | 459 | 0.173 | 776 | 493 | 30 | 63 | 781 | 16 | 699 | Phage tail tape measure protein | Phage tail tape measure protein | | afdb-uniprot50 | AF-A0A7Y4MDX7-F1-MODEL\_V4 | 1.0 | 5.137e-14 | 459 | 0.186 | 747 | 452 | 27 | 47 | 711 | 2 | 674 | Phage tail tape measure protein | Phage tail tape measure protein | | afdb-uniprot50 | AF-A0A4U7BTH2-F1-MODEL\_V4 | 1.0 | 1.726e-07 | 458 | 0.202 | 252 | 195 | 3 | 126 | 376 | 5 | 251 | Phage tail tape measure protein | Phage tail tape measure protein | | afdb-uniprot50 | AF-A0A081RQL7-F1-MODEL\_V4 | 1.0 | 5.575e-11 | 458 | 0.135 | 540 | 382 | 12 | 19 | 498 | 48 | 562 | Phage-related minor tail protein | Phage-related minor tail protein | | afdb-uniprot50 | AF-A0A3S8ZPZ1-F1-MODEL\_V4 | 1.0 | 6.238e-14 | 458 | 0.158 | 971 | 580 | 30 | 19 | 782 | 46 | 985 | Phage tail tape measure protein | Phage tail tape measure protein | | afdb-uniprot50 | AF-A0A662KJ26-F1-MODEL\_V4 | 1.0 | 4.848e-09 | 456 | 0.175 | 398 | 288 | 12 | 61 | 441 | 1 | 375 | Phage tail tape measure protein | Phage tail tape measure protein | | afdb-uniprot50 | AF-A0A833NTL2-F1-MODEL\_V4 | 1.0 | 1.977e-12 | 456 | 0.19 | 620 | 387 | 17 | 21 | 570 | 48 | 622 | Phage tail tape measure protein | Phage tail tape measure protein | | afdb-uniprot50 | AF-A0A256CBP9-F1-MODEL\_V4 | 1.0 | 1.466e-13 | 456 | 0.14 | 816 | 514 | 24 | 19 | 785 | 9 | 685 | Phage tail tape measure protein | Phage tail tape measure protein | | afdb-uniprot50 | AF-A0A1I7FWK0-F1-MODEL\_V4 | 1.0 | 2.554e-14 | 456 | 0.144 | 899 | 585 | 26 | 17 | 781 | 3 | 850 | Phage tail tape measure protein, TP901 family, core region | Phage tail tape measure protein, TP901 family, core region | | afdb-uniprot50 | AF-A0A658JFX6-F1-MODEL\_V4 | 1.0 | 2.063e-09 | 455 | 0.157 | 419 | 315 | 12 | 35 | 441 | 2 | 394 | Phage tail tape measure protein | Phage tail tape measure protein | | afdb-uniprot50 | AF-A0A081MYF0-F1-MODEL\_V4 | 1.0 | 2.728e-13 | 455 | 0.173 | 812 | 471 | 21 | 27 | 776 | 60 | 732 | PhageMin\_Tail domain-containing protein | PhageMin\_Tail domain-containing protein | | afdb-uniprot50 | AF-X1H190-F1-MODEL\_V4 | 1.0 | 1.026e-09 | 454 | 0.162 | 443 | 288 | 13 | 19 | 447 | 7 | 380 | PhageMin\_Tail domain-containing protein | PhageMin\_Tail domain-containing protein | | afdb-uniprot50 | AF-X1FNH0-F1-MODEL\_V4 | 1.0 | 5.24e-09 | 453 | 0.162 | 407 | 303 | 10 | 119 | 508 | 2 | 387 | PhageMin\_Tail domain-containing protein | PhageMin\_Tail domain-containing protein | | afdb-uniprot50 | AF-A0A1H1RSV7-F1-MODEL\_V4 | 1.0 | 2.554e-14 | 453 | 0.174 | 888 | 472 | 24 | 1 | 783 | 22 | 753 | Phage tail tape measure protein, TP901 family, core region | Phage tail tape measure protein, TP901 family, core region | | afdb-uniprot50 | AF-A0A416DY15-F1-MODEL\_V4 | 1.0 | 2.554e-14 | 453 | 0.169 | 909 | 564 | 36 | 1 | 785 | 1 | 842 | Phage tail tape measure protein | Phage tail tape measure protein | | afdb-uniprot50 | AF-A0A0F9L912-F1-MODEL\_V4 | 1.0 | 1.014e-08 | 452 | 0.192 | 390 | 276 | 12 | 70 | 443 | 4 | 370 | PhageMin\_Tail domain-containing protein | PhageMin\_Tail domain-containing protein | | afdb-uniprot50 | AF-A0A1E4MVY1-F1-MODEL\_V4 | 1.0 | 4.572e-14 | 452 | 0.166 | 812 | 496 | 27 | 1 | 784 | 1 | 659 | Phage tail tape measure protein | Phage tail tape measure protein | | afdb-uniprot50 | AF-A0A239A9Y9-F1-MODEL\_V4 | 1.0 | 1.117e-13 | 452 | 0.171 | 907 | 541 | 29 | 19 | 785 | 33 | 868 | Phage tail tape measure protein, TP901 family, core region | Phage tail tape measure protein, TP901 family, core region | | afdb-uniprot50 | AF-A0A268TN51-F1-MODEL\_V4 | 1.0 | 2.728e-13 | 451 | 0.172 | 804 | 501 | 22 | 6 | 764 | 1 | 684 | Phage tail tape measure protein | Phage tail tape measure protein | | afdb-uniprot50 | AF-A0A5S5CR07-F1-MODEL\_V4 | 1.0 | 5.403e-15 | 451 | 0.162 | 1014 | 554 | 36 | 1 | 784 | 1 | 948 | TP901 family phage tail tape measure protein | TP901 family phage tail tape measure protein | | afdb-uniprot50 | AF-A0A6M3XFH2-F1-MODEL\_V4 | 1.0 | 2.635e-10 | 450 | 0.203 | 535 | 320 | 20 | 46 | 570 | 3 | 441 | Putative tail protein | Putative tail protein | | afdb-uniprot50 | AF-A0A235B980-F1-MODEL\_V4 | 1.0 | 2.079e-13 | 450 | 0.16 | 705 | 485 | 23 | 50 | 702 | 2 | 651 | Phage tail tape measure protein | Phage tail tape measure protein | | afdb-uniprot50 | AF-A0A7V2AMB8-F1-MODEL\_V4 | 1.0 | 7.009e-14 | 450 | 0.177 | 739 | 490 | 23 | 1 | 676 | 1 | 684 | Phage tail tape measure protein | Phage tail tape measure protein | | afdb-uniprot50 | AF-A0A371PCG1-F1-MODEL\_V4 | 1.0 | 2.655e-14 | 450 | 0.16 | 784 | 506 | 21 | 1 | 701 | 27 | 740 | Phage tail tape measure protein | Phage tail tape measure protein | | afdb-uniprot50 | AF-A0A840DK27-F1-MODEL\_V4 | 1.0 | 1.524e-13 | 449 | 0.17 | 756 | 479 | 27 | 1 | 701 | 1 | 663 | TP901 family phage tail tape measure protein | TP901 family phage tail tape measure protein | | afdb-uniprot50 | AF-A0A3D3CGB4-F1-MODEL\_V4 | 1.0 | 1.13e-14 | 448 | 0.168 | 892 | 525 | 35 | 52 | 782 | 13 | 848 | Phage tail tape measure protein | Phage tail tape measure protein | | afdb-uniprot50 | AF-A0A166IL48-F1-MODEL\_V4 | 1.0 | 2.728e-13 | 448 | 0.156 | 839 | 422 | 21 | 19 | 755 | 56 | 710 | PhageMin\_Tail domain-containing protein | PhageMin\_Tail domain-containing protein | | afdb-uniprot50 | AF-A0A7U9XL26-F1-MODEL\_V4 | 1.0 | 3.224e-14 | 448 | 0.17 | 761 | 493 | 25 | 19 | 702 | 79 | 777 | Chromosome partition protein Smc | Chromosome partition protein Smc | | afdb-uniprot50 | AF-A0A0F9GIW1-F1-MODEL\_V4 | 1.0 | 7.316e-11 | 447 | 0.225 | 444 | 287 | 15 | 166 | 604 | 2 | 393 | PhageMin\_Tail domain-containing protein | PhageMin\_Tail domain-containing protein | | afdb-uniprot50 | AF-A0A1I7G2Q8-F1-MODEL\_V4 | 1.0 | 3.885e-10 | 447 | 0.156 | 461 | 317 | 14 | 27 | 439 | 72 | 508 | Phage tail tape measure protein, TP901 family, core region | Phage tail tape measure protein, TP901 family, core region | | afdb-uniprot50 | AF-A0A6M3J046-F1-MODEL\_V4 | 1.0 | 9.601e-11 | 447 | 0.176 | 709 | 366 | 26 | 91 | 782 | 24 | 531 | Putative tail protein | Putative tail protein | | afdb-uniprot50 | AF-A0A7Y6Z4I9-F1-MODEL\_V4 | 1.0 | 2.055e-12 | 447 | 0.173 | 768 | 448 | 26 | 17 | 765 | 1 | 600 | Phage tail tape measure protein | Phage tail tape measure protein | | afdb-uniprot50 | AF-A0A1X3I4F2-F1-MODEL\_V4 | 1.0 | 5.795e-11 | 447 | 0.161 | 527 | 359 | 14 | 19 | 480 | 47 | 555 | Putative tail protein | Putative tail protein | | afdb-uniprot50 | AF-A0A4S3LKS8-F1-MODEL\_V4 | 1.0 | 4.023e-13 | 446 | 0.158 | 758 | 503 | 20 | 20 | 715 | 41 | 725 | Phage tail tape measure protein | Phage tail tape measure protein | | afdb-uniprot50 | AF-H4FBM6-F1-MODEL\_V4 | 1.0 | 3.484e-14 | 446 | 0.165 | 847 | 541 | 22 | 19 | 785 | 3 | 763 | Phage tail tape measure protein, TP901 family | Phage tail tape measure protein, TP901 family | | afdb-uniprot50 | AF-A0A5Q2N0Q6-F1-MODEL\_V4 | 1.0 | 5.552e-14 | 446 | 0.177 | 886 | 579 | 35 | 2 | 783 | 1 | 840 | Phage tail tape measure protein, TP901 family protein | Phage tail tape measure protein, TP901 family protein | | afdb-uniprot50 | AF-A0A410QG87-F1-MODEL\_V4 | 1.0 | 8.186e-14 | 445 | 0.149 | 910 | 576 | 34 | 19 | 783 | 49 | 905 | Phage tail tape measure protein | Phage tail tape measure protein | | afdb-uniprot50 | AF-A0A7H0U435-F1-MODEL\_V4 | 1.0 | 4.069e-14 | 445 | 0.158 | 917 | 575 | 27 | 19 | 783 | 39 | 910 | Phage tail tape measure protein | Phage tail tape measure protein | | afdb-uniprot50 | AF-A0A1M6EWV2-F1-MODEL\_V4 | 1.0 | 1.245e-09 | 445 | 0.191 | 439 | 300 | 15 | 52 | 472 | 18 | 419 | Phage tail tape measure protein, TP901 family, core region | Phage tail tape measure protein, TP901 family, core region | | afdb-uniprot50 | AF-A0A024Q9Y0-F1-MODEL\_V4 | 1.0 | 1.117e-13 | 445 | 0.187 | 698 | 446 | 25 | 69 | 702 | 16 | 656 | Phage tail tape measure protein, TP901 family, core region | Phage tail tape measure protein, TP901 family, core region | | afdb-uniprot50 | AF-A0A5M6DGJ8-F1-MODEL\_V4 | 1.0 | 2e-13 | 445 | 0.164 | 819 | 531 | 26 | 63 | 785 | 2 | 762 | Phage tail tape measure protein | Phage tail tape measure protein | | afdb-uniprot50 | AF-A0A3D4MZY3-F1-MODEL\_V4 | 1.0 | 1.584e-13 | 445 | 0.166 | 847 | 506 | 22 | 22 | 702 | 50 | 862 | Phage tail tape measure protein | Phage tail tape measure protein | | afdb-uniprot50 | AF-A0A0D7V1W3-F1-MODEL\_V4 | 1.0 | 2.336e-13 | 444 | 0.175 | 861 | 552 | 26 | 22 | 780 | 54 | 858 | PhageMin\_Tail domain-containing protein | PhageMin\_Tail domain-containing protein | | afdb-uniprot50 | AF-A0A5Y1L0N9-F1-MODEL\_V4 | 1.0 | 6.166e-13 | 443 | 0.202 | 692 | 460 | 23 | 34 | 701 | 10 | 633 | Phage tail tape measure protein | Phage tail tape measure protein | | afdb-uniprot50 | AF-A0A374E9U4-F1-MODEL\_V4 | 1.0 | 6.956e-10 | 441 | 0.197 | 486 | 342 | 15 | 13 | 466 | 47 | 516 | Phage tail tape measure protein | Phage tail tape measure protein | | afdb-uniprot50 | AF-A0A5P9CRD9-F1-MODEL\_V4 | 1.0 | 1.924e-13 | 441 | 0.187 | 801 | 490 | 23 | 19 | 700 | 56 | 814 | Chromosome segregation protein | Chromosome segregation protein | | afdb-uniprot50 | AF-A0A2I1IQK4-F1-MODEL\_V4 | 1.0 | 9.562e-14 | 441 | 0.171 | 793 | 498 | 29 | 6 | 702 | 2 | 731 | Phage tail tape measure protein | Phage tail tape measure protein | | afdb-uniprot50 | AF-A0A3L7JE15-F1-MODEL\_V4 | 1.0 | 1.85e-13 | 441 | 0.156 | 833 | 474 | 23 | 19 | 753 | 85 | 786 | Phage tail tape measure protein | Phage tail tape measure protein | | afdb-uniprot50 | AF-A0A0B1QAA3-F1-MODEL\_V4 | 1.0 | 1.584e-13 | 440 | 0.192 | 804 | 504 | 26 | 2 | 734 | 1 | 729 | PhageMin\_Tail domain-containing protein | PhageMin\_Tail domain-containing protein | | afdb-uniprot50 | AF-A0A3S2TUB2-F1-MODEL\_V4 | 1.0 | 6.742e-14 | 440 | 0.171 | 753 | 502 | 23 | 77 | 746 | 19 | 732 | Phage tail tape measure protein | Phage tail tape measure protein | | afdb-uniprot50 | AF-A0A425XBK8-F1-MODEL\_V4 | 1.0 | 9.198e-14 | 440 | 0.163 | 797 | 536 | 28 | 1 | 713 | 1 | 750 | Phage tail tape measure protein | Phage tail tape measure protein | | afdb-uniprot50 | AF-A0A5F1PHV9-F1-MODEL\_V4 | 1.0 | 5.863e-12 | 439 | 0.183 | 644 | 399 | 20 | 23 | 590 | 50 | 642 | Phage tail tape measure protein | Phage tail tape measure protein | | afdb-uniprot50 | AF-A0A841TAB5-F1-MODEL\_V4 | 1.0 | 4.885e-13 | 439 | 0.169 | 763 | 462 | 23 | 49 | 755 | 3 | 649 | Phage tail tape measure protein | Phage tail tape measure protein | | afdb-uniprot50 | AF-A0A6L6J9S0-F1-MODEL\_V4 | 1.0 | 9.94e-14 | 439 | 0.188 | 917 | 515 | 31 | 1 | 779 | 2 | 826 | Phage tail tape measure protein | Phage tail tape measure protein | | afdb-uniprot50 | AF-A0A0F9VTR3-F1-MODEL\_V4 | 1.0 | 1.584e-13 | 439 | 0.185 | 783 | 452 | 27 | 27 | 775 | 8 | 638 | PhageMin\_Tail domain-containing protein | PhageMin\_Tail domain-containing protein | | afdb-uniprot50 | AF-C7MM53-F1-MODEL\_V4 | 1.0 | 7.875e-14 | 439 | 0.174 | 740 | 478 | 22 | 27 | 701 | 11 | 682 | Phage tail tape measure protein, TP901 family | Phage tail tape measure protein, TP901 family | | afdb-uniprot50 | AF-A0A2G6CBN5-F1-MODEL\_V4 | 1.0 | 1.699e-09 | 438 | 0.175 | 398 | 284 | 10 | 19 | 392 | 11 | 388 | Phage tail tape measure protein | Phage tail tape measure protein | | afdb-uniprot50 | AF-A0A0F9ELH6-F1-MODEL\_V4 | 1.0 | 1.198e-09 | 438 | 0.148 | 432 | 306 | 15 | 61 | 467 | 4 | 398 | PhageMin\_Tail domain-containing protein | PhageMin\_Tail domain-containing protein | | afdb-uniprot50 | AF-T2RFP8-F1-MODEL\_V4 | 1.0 | 4.265e-08 | 437 | 0.123 | 349 | 266 | 10 | 125 | 456 | 2 | 327 | Phage tail tape measure protein, TP901 family, core region | Phage tail tape measure protein, TP901 family, core region | | afdb-uniprot50 | AF-A0A2X3H9A1-F1-MODEL\_V4 | 1.0 | 1.01e-11 | 437 | 0.178 | 621 | 404 | 17 | 19 | 570 | 35 | 618 | Putative prophage tail length determinator | Putative prophage tail length determinator | | afdb-uniprot50 | AF-A0A258E9Z4-F1-MODEL\_V4 | 1.0 | 7.575e-14 | 437 | 0.176 | 759 | 529 | 20 | 67 | 781 | 2 | 708 | Phage tail tape measure protein | Phage tail tape measure protein | | afdb-uniprot50 | AF-A0A7T9D1E2-F1-MODEL\_V4 | 1.0 | 1.712e-13 | 437 | 0.159 | 765 | 485 | 26 | 118 | 768 | 2 | 722 | Phage tail tape measure protein | Phage tail tape measure protein | | afdb-uniprot50 | AF-D8HAR8-F1-MODEL\_V4 | 1.0 | 5.552e-14 | 437 | 0.166 | 770 | 545 | 29 | 51 | 785 | 3 | 710 | Putative phage tail tape measure protein | Putative phage tail tape measure protein | | afdb-uniprot50 | AF-A0A1C6J674-F1-MODEL\_V4 | 1.0 | 8.51e-14 | 437 | 0.16 | 833 | 555 | 34 | 49 | 785 | 2 | 785 | Phage-related minor tail protein | Phage-related minor tail protein | | afdb-uniprot50 | AF-A0A1F8UPJ4-F1-MODEL\_V4 | 1.0 | 4.753e-14 | 437 | 0.156 | 809 | 520 | 29 | 4 | 702 | 6 | 761 | Phage tail tape measure protein | Phage tail tape measure protein | | afdb-uniprot50 | AF-A0A7C4LLA7-F1-MODEL\_V4 | 1.0 | 4.753e-14 | 437 | 0.164 | 768 | 505 | 26 | 73 | 755 | 9 | 724 | Phage tail tape measure protein | Phage tail tape measure protein | | afdb-uniprot50 | AF-A0A367YQV0-F1-MODEL\_V4 | 1.0 | 2.247e-13 | 437 | 0.176 | 797 | 464 | 24 | 1 | 701 | 38 | 737 | Phage tail tape measure protein | Phage tail tape measure protein | | afdb-uniprot50 | AF-F4A0G3-F1-MODEL\_V4 | 1.0 | 8.51e-14 | 436 | 0.183 | 830 | 490 | 31 | 32 | 782 | 2 | 722 | Phage tail tape measure protein, TP901 family | Phage tail tape measure protein, TP901 family | | afdb-uniprot50 | AF-A0A7V5P9D7-F1-MODEL\_V4 | 1.0 | 1.022e-12 | 436 | 0.163 | 649 | 429 | 24 | 77 | 702 | 18 | 575 | Phage tail tape measure protein | Phage tail tape measure protein | | afdb-uniprot50 | AF-A0A7X1ZGF5-F1-MODEL\_V4 | 1.0 | 1.924e-13 | 436 | 0.175 | 904 | 507 | 33 | 19 | 777 | 56 | 865 | Phage tail tape measure protein | Phage tail tape measure protein | | afdb-uniprot50 | AF-A0A1Q8Q338-F1-MODEL\_V4 | 1.0 | 2.336e-13 | 436 | 0.17 | 743 | 483 | 23 | 13 | 702 | 84 | 745 | Phage tail tape measure protein | Phage tail tape measure protein | | afdb-uniprot50 | AF-A0A845R2K9-F1-MODEL\_V4 | 1.0 | 1.074e-13 | 436 | 0.141 | 806 | 540 | 21 | 19 | 702 | 42 | 817 | Phage tail tape measure protein | Phage tail tape measure protein | | afdb-uniprot50 | AF-A0A2E4CSW6-F1-MODEL\_V4 | 1.0 | 2.103e-14 | 435 | 0.152 | 844 | 568 | 27 | 1 | 781 | 1 | 759 | Phage tail tape measure protein | Phage tail tape measure protein | | afdb-uniprot50 | AF-A0A1C3K3I9-F1-MODEL\_V4 | 1.0 | 2.186e-14 | 435 | 0.17 | 914 | 555 | 32 | 7 | 782 | 1 | 848 | Phage tail length tape-measure protein | Phage tail length tape-measure protein | | afdb-uniprot50 | AF-I0GWR9-F1-MODEL\_V4 | 1.0 | 2.804e-12 | 435 | 0.193 | 681 | 463 | 18 | 62 | 702 | 5 | 638 | Putative tail tape measure protein | Putative tail tape measure protein | | afdb-uniprot50 | AF-A0A4Y8K9M9-F1-MODEL\_V4 | 1.0 | 5.552e-14 | 435 | 0.152 | 997 | 556 | 30 | 46 | 784 | 3 | 968 | Phage tail tape measure protein | Phage tail tape measure protein | | afdb-uniprot50 | AF-A0A1Y5SUX3-F1-MODEL\_V4 | 1.0 | 8.987e-12 | 435 | 0.201 | 609 | 404 | 19 | 63 | 629 | 2 | 569 | Phage-related minor tail protein | Phage-related minor tail protein | | afdb-uniprot50 | AF-A0A7B5ZAU9-F1-MODEL\_V4 | 1.0 | 8.781e-10 | 434 | 0.178 | 476 | 308 | 11 | 26 | 434 | 1 | 460 | Phage tail tape measure protein | Phage tail tape measure protein | | afdb-uniprot50 | AF-A0A1M3PG12-F1-MODEL\_V4 | 1.0 | 1.666e-14 | 433 | 0.18 | 844 | 528 | 29 | 1 | 782 | 1 | 742 | Phage tail tape measure protein | Phage tail tape measure protein | | afdb-uniprot50 | AF-A0A2D8YMB0-F1-MODEL\_V4 | 1.0 | 3.15e-12 | 433 | 0.187 | 662 | 483 | 19 | 62 | 702 | 7 | 634 | Phage tail tape measure protein | Phage tail tape measure protein | | afdb-uniprot50 | AF-A0A069F3H5-F1-MODEL\_V4 | 1.0 | 1.712e-13 | 433 | 0.151 | 812 | 525 | 29 | 77 | 778 | 17 | 774 | PhageMin\_Tail domain-containing protein | PhageMin\_Tail domain-containing protein | | afdb-uniprot50 | AF-A0A2D5HGI6-F1-MODEL\_V4 | 1.0 | 3.15e-12 | 432 | 0.187 | 652 | 442 | 23 | 1 | 620 | 1 | 596 | Phage tail tape measure protein | Phage tail tape measure protein | | afdb-uniprot50 | AF-A0A847KSJ3-F1-MODEL\_V4 | 1.0 | 1.924e-13 | 432 | 0.173 | 832 | 513 | 31 | 1 | 785 | 14 | 717 | Phage tail tape measure protein | Phage tail tape measure protein | | afdb-uniprot50 | AF-A0A3P1S6F0-F1-MODEL\_V4 | 1.0 | 1.074e-13 | 432 | 0.145 | 996 | 561 | 30 | 9 | 776 | 1 | 933 | Phage tail tape measure protein | Phage tail tape measure protein | | afdb-uniprot50 | AF-A0A352TM73-F1-MODEL\_V4 | 1.0 | 8.186e-14 | 432 | 0.174 | 791 | 515 | 26 | 1 | 702 | 1 | 742 | Phage tail tape measure protein | Phage tail tape measure protein | | afdb-uniprot50 | AF-A0A1R4FZY8-F1-MODEL\_V4 | 1.0 | 2.161e-13 | 432 | 0.181 | 776 | 482 | 26 | 1 | 701 | 1 | 697 | Phage tail length tape-measure protein | Phage tail length tape-measure protein | | afdb-uniprot50 | AF-A0A315X605-F1-MODEL\_V4 | 1.0 | 8.092e-13 | 432 | 0.161 | 731 | 447 | 22 | 43 | 712 | 21 | 646 | Phage tail tape measure protein | Phage tail tape measure protein | | afdb-uniprot50 | AF-A0A417T5R9-F1-MODEL\_V4 | 1.0 | 3.722e-13 | 431 | 0.167 | 760 | 524 | 25 | 19 | 702 | 22 | 748 | Phage tail tape measure protein | Phage tail tape measure protein | | afdb-uniprot50 | AF-A0A0V0PX43-F1-MODEL\_V4 | 1.0 | 5.137e-14 | 431 | 0.177 | 846 | 558 | 29 | 36 | 776 | 7 | 819 | PhageMin\_Tail domain-containing protein | PhageMin\_Tail domain-containing protein | | afdb-uniprot50 | AF-A0A4Q7RAQ5-F1-MODEL\_V4 | 1.0 | 8.412e-13 | 431 | 0.189 | 744 | 476 | 23 | 3 | 719 | 203 | 846 | Lambda family phage tail tape measure protein/TP901 family phage tail tape measure protein | Lambda family phage tail tape measure protein/TP901 family phage tail tape measure protein | | afdb-uniprot50 | AF-A0A373ZD47-F1-MODEL\_V4 | 1.0 | 4.52e-13 | 430 | 0.152 | 881 | 571 | 33 | 2 | 784 | 1 | 803 | Phage tail tape measure protein | Phage tail tape measure protein | | afdb-uniprot50 | AF-A0A558GXF9-F1-MODEL\_V4 | 1.0 | 6.664e-13 | 430 | 0.179 | 960 | 533 | 32 | 19 | 755 | 9 | 936 | Phage tail tape measure protein | Phage tail tape measure protein | | afdb-uniprot50 | AF-A0A5J6SLX3-F1-MODEL\_V4 | 1.0 | 7.009e-14 | 430 | 0.18 | 831 | 465 | 26 | 1 | 667 | 1 | 779 | Phage tail tape measure protein | Phage tail tape measure protein | | afdb-uniprot50 | AF-A0A5U3G3I6-F1-MODEL\_V4 | 1.0 | 7.518e-10 | 429 | 0.164 | 524 | 343 | 13 | 118 | 626 | 31 | 474 | Phage tail tape measure protein | Phage tail tape measure protein | | afdb-uniprot50 | AF-A0A6G3HIH4-F1-MODEL\_V4 | 1.0 | 1.284e-15 | 429 | 0.215 | 725 | 406 | 16 | 211 | 785 | 2 | 713 | Phage tail tape measure protein | Phage tail tape measure protein | | afdb-uniprot50 | AF-A0A1I2YB35-F1-MODEL\_V4 | 1.0 | 5.552e-14 | 429 | 0.162 | 862 | 530 | 29 | 62 | 785 | 4 | 811 | Phage tail tape measure protein, TP901 family, core region | Phage tail tape measure protein, TP901 family, core region | | afdb-uniprot50 | AF-A0A7S8E854-F1-MODEL\_V4 | 1.0 | 2.836e-13 | 428 | 0.174 | 850 | 523 | 32 | 61 | 776 | 1 | 805 | Phage tail tape measure protein | Phage tail tape measure protein | | afdb-uniprot50 | AF-A0A1C5PPL3-F1-MODEL\_V4 | 1.0 | 1.628e-12 | 428 | 0.148 | 701 | 510 | 23 | 53 | 702 | 2 | 666 | Phage-related minor tail protein | Phage-related minor tail protein | | afdb-uniprot50 | AF-A0A5M7L9A2-F1-MODEL\_V4 | 1.0 | 4.182e-13 | 428 | 0.151 | 816 | 509 | 26 | 19 | 755 | 34 | 744 | Phage tail tape measure protein | Phage tail tape measure protein | | afdb-uniprot50 | AF-A0A6G8J7Q0-F1-MODEL\_V4 | 1.0 | 4.699e-13 | 428 | 0.153 | 880 | 569 | 26 | 19 | 780 | 54 | 875 | Phage tail tape measure protein | Phage tail tape measure protein | | afdb-uniprot50 | AF-A0A7W4EBY4-F1-MODEL\_V4 | 1.0 | 6.485e-14 | 428 | 0.16 | 842 | 525 | 25 | 35 | 781 | 2 | 756 | Phage tail tape measure protein | Phage tail tape measure protein | | afdb-uniprot50 | AF-E6MGX4-F1-MODEL\_V4 | 1.0 | 1.41e-13 | 427 | 0.141 | 907 | 585 | 31 | 1 | 785 | 1 | 835 | Phage tail tape measure protein, TP901 family | Phage tail tape measure protein, TP901 family | | afdb-uniprot50 | AF-A0A2N2CA29-F1-MODEL\_V4 | 1.0 | 2.428e-13 | 427 | 0.18 | 761 | 503 | 29 | 1 | 702 | 1 | 699 | Phage tail tape measure protein | Phage tail tape measure protein | | afdb-uniprot50 | AF-B8FNZ4-F1-MODEL\_V4 | 1.0 | 9.94e-14 | 427 | 0.183 | 790 | 439 | 25 | 21 | 662 | 77 | 808 | Phage tail tape measure protein, TP901 family | Phage tail tape measure protein, TP901 family | | afdb-uniprot50 | AF-A0A2G0E7P5-F1-MODEL\_V4 | 1.0 | 5.931e-13 | 426 | 0.174 | 687 | 398 | 19 | 1 | 532 | 1 | 673 | Phage tail tape measure protein | Phage tail tape measure protein | | afdb-uniprot50 | AF-A0A6B4HS58-F1-MODEL\_V4 | 1.0 | 6.41e-13 | 426 | 0.15 | 729 | 501 | 26 | 27 | 702 | 61 | 723 | Phage tail tape measure protein | Phage tail tape measure protein | | afdb-uniprot50 | AF-A0A239GVA7-F1-MODEL\_V4 | 1.0 | 3.042e-09 | 425 | 0.332 | 304 | 171 | 5 | 26 | 321 | 1 | 280 | Phage tail tape measure protein, TP901 family, core region | Phage tail tape measure protein, TP901 family, core region | | afdb-uniprot50 | AF-A0A7V6Y6C8-F1-MODEL\_V4 | 1.0 | 2.524e-13 | 425 | 0.159 | 859 | 536 | 32 | 19 | 781 | 25 | 793 | Phage tail tape measure protein | Phage tail tape measure protein | | afdb-uniprot50 | AF-A0A5E4XFP1-F1-MODEL\_V4 | 1.0 | 2.961e-10 | 425 | 0.153 | 553 | 380 | 11 | 19 | 519 | 46 | 562 | Phage tail tape measure protein | Phage tail tape measure protein | | afdb-uniprot50 | AF-A0A838LDI5-F1-MODEL\_V4 | 1.0 | 1.356e-13 | 425 | 0.152 | 995 | 515 | 29 | 1 | 784 | 1 | 877 | Phage tail tape measure protein | Phage tail tape measure protein | | afdb-uniprot50 | AF-A0A552V6Z6-F1-MODEL\_V4 | 1.0 | 1.712e-13 | 424 | 0.155 | 867 | 585 | 30 | 9 | 767 | 1 | 827 | Phage tail tape measure protein | Phage tail tape measure protein | | afdb-uniprot50 | AF-A0A484ZHI6-F1-MODEL\_V4 | 1.0 | 1.466e-13 | 424 | 0.161 | 860 | 565 | 26 | 19 | 782 | 58 | 856 | Phage-related minor tail protein | Phage-related minor tail protein | | afdb-uniprot50 | AF-A0A350TW28-F1-MODEL\_V4 | 1.0 | 3.58e-13 | 424 | 0.164 | 789 | 506 | 30 | 1 | 709 | 1 | 715 | Phage tail tape measure protein | Phage tail tape measure protein | | afdb-uniprot50 | AF-A0A3N5VV08-F1-MODEL\_V4 | 1.0 | 5.863e-12 | 423 | 0.145 | 640 | 444 | 17 | 117 | 702 | 24 | 614 | Phage tail tape measure protein | Phage tail tape measure protein | | afdb-uniprot50 | AF-A0A7Z9YAJ2-F1-MODEL\_V4 | 1.0 | 1.432e-11 | 423 | 0.173 | 653 | 445 | 19 | 76 | 702 | 5 | 588 | Phage tail tape measure protein | Phage tail tape measure protein | | afdb-uniprot50 | AF-A0A4R3JH78-F1-MODEL\_V4 | 1.0 | 1.179e-11 | 423 | 0.153 | 647 | 469 | 17 | 87 | 701 | 23 | 622 | TP901 family phage tail tape measure protein | TP901 family phage tail tape measure protein | | afdb-uniprot50 | AF-A0A4Y3W6Q9-F1-MODEL\_V4 | 1.0 | 3.065e-13 | 423 | 0.148 | 803 | 533 | 23 | 1 | 748 | 2 | 708 | PhageMin\_Tail domain-containing protein | PhageMin\_Tail domain-containing protein | | afdb-uniprot50 | AF-A0A3A9AGY9-F1-MODEL\_V4 | 1.0 | 5.137e-14 | 422 | 0.167 | 793 | 513 | 28 | 19 | 702 | 24 | 777 | Phage tail tape measure protein | Phage tail tape measure protein | | afdb-uniprot50 | AF-A0A1D3N8J3-F1-MODEL\_V4 | 1.0 | 4.52e-13 | 422 | 0.175 | 759 | 517 | 21 | 19 | 702 | 85 | 809 | Putative tail tape measure protein | Putative tail tape measure protein | | afdb-uniprot50 | AF-R7FHV4-F1-MODEL\_V4 | 1.0 | 8.847e-14 | 422 | 0.162 | 915 | 518 | 32 | 4 | 702 | 6 | 887 | Phage tail tape measure protein family | Phage tail tape measure protein family | | afdb-uniprot50 | AF-A0A2T2UAU0-F1-MODEL\_V4 | 1.0 | 1.954e-11 | 421 | 0.151 | 725 | 432 | 31 | 1 | 714 | 21 | 572 | Phage tail tape measure protein | Phage tail tape measure protein | | afdb-uniprot50 | AF-A6TR98-F1-MODEL\_V4 | 1.0 | 2.161e-13 | 421 | 0.16 | 873 | 556 | 31 | 1 | 779 | 4 | 793 | Phage tail tape measure protein, TP901 family | Phage tail tape measure protein, TP901 family | | afdb-uniprot50 | AF-A0A374VKX6-F1-MODEL\_V4 | 1.0 | 1.712e-13 | 421 | 0.176 | 767 | 503 | 25 | 19 | 702 | 22 | 742 | Phage tail tape measure protein | Phage tail tape measure protein | | afdb-uniprot50 | AF-D4M9M3-F1-MODEL\_V4 | 1.0 | 1.524e-13 | 421 | 0.171 | 917 | 531 | 30 | 3 | 780 | 1 | 827 | Phage tail tape measure protein, TP901 family, core region | Phage tail tape measure protein, TP901 family, core region | | afdb-uniprot50 | AF-A0A178HLZ5-F1-MODEL\_V4 | 1.0 | 1.062e-12 | 421 | 0.173 | 766 | 468 | 20 | 50 | 706 | 2 | 711 | PhageMin\_Tail domain-containing protein | PhageMin\_Tail domain-containing protein | | afdb-uniprot50 | AF-A0A7C1VR42-F1-MODEL\_V4 | 1.0 | 6.41e-13 | 421 | 0.134 | 922 | 581 | 27 | 2 | 776 | 1 | 852 | Phage tail tape measure protein | Phage tail tape measure protein | | afdb-uniprot50 | AF-A0A4E0QXR2-F1-MODEL\_V4 | 1.0 | 4.134e-12 | 420 | 0.174 | 663 | 425 | 27 | 81 | 701 | 25 | 606 | Phage tail tape measure protein | Phage tail tape measure protein | | afdb-uniprot50 | AF-A0A7W2CT18-F1-MODEL\_V4 | 1.0 | 1.117e-13 | 420 | 0.162 | 888 | 543 | 28 | 1 | 779 | 1 | 796 | Phage tail tape measure protein | Phage tail tape measure protein | | afdb-uniprot50 | AF-A0A5K1ITD8-F1-MODEL\_V4 | 1.0 | 1.628e-12 | 420 | 0.171 | 730 | 434 | 24 | 116 | 785 | 83 | 701 | Phage-related minor tail protein | Phage-related minor tail protein | | afdb-uniprot50 | AF-A0A0F9IXZ9-F1-MODEL\_V4 | 1.0 | 1.466e-13 | 419 | 0.172 | 755 | 454 | 31 | 64 | 700 | 11 | 712 | PhageMin\_Tail domain-containing protein | PhageMin\_Tail domain-containing protein | | afdb-uniprot50 | AF-A0A3M2BDP3-F1-MODEL\_V4 | 1.0 | 3.15e-12 | 419 | 0.161 | 670 | 449 | 24 | 53 | 702 | 33 | 609 | Phage tail tape measure protein | Phage tail tape measure protein | | afdb-uniprot50 | AF-A0A542YF06-F1-MODEL\_V4 | 1.0 | 3.065e-13 | 419 | 0.174 | 787 | 507 | 22 | 10 | 704 | 1 | 736 | TP901 family phage tail tape measure protein | TP901 family phage tail tape measure protein | | afdb-uniprot50 | AF-A0A1F4QW09-F1-MODEL\_V4 | 1.0 | 2.477e-08 | 418 | 0.17 | 435 | 274 | 15 | 14 | 433 | 1 | 363 | Phage tail tape measure protein | Phage tail tape measure protein | | afdb-uniprot50 | AF-Q7NAC3-F1-MODEL\_V4 | 1.0 | 5.279e-13 | 418 | 0.141 | 875 | 576 | 23 | 18 | 784 | 6 | 812 | PhageMin\_Tail domain-containing protein | PhageMin\_Tail domain-containing protein | | afdb-uniprot50 | AF-R6LX22-F1-MODEL\_V4 | 1.0 | 3.187e-13 | 418 | 0.162 | 825 | 500 | 26 | 32 | 712 | 8 | 785 | Phage tail tape measure protein TP901 family | Phage tail tape measure protein TP901 family | | afdb-uniprot50 | AF-A0A376SKG7-F1-MODEL\_V4 | 1.0 | 1.245e-09 | 417 | 0.177 | 456 | 294 | 15 | 23 | 417 | 50 | 485 | Tail fiber component of prophage CP-933T | Tail fiber component of prophage CP-933T | | afdb-uniprot50 | AF-A0A661DYN7-F1-MODEL\_V4 | 1.0 | 3.595e-10 | 417 | 0.146 | 663 | 422 | 21 | 46 | 687 | 2 | 541 | Phage tail tape measure protein | Phage tail tape measure protein | | afdb-uniprot50 | AF-A0A6N9QF02-F1-MODEL\_V4 | 1.0 | 4.182e-13 | 417 | 0.168 | 741 | 480 | 25 | 1 | 710 | 1 | 635 | Phage tail tape measure protein | Phage tail tape measure protein | | afdb-uniprot50 | AF-A0A0B3BNP9-F1-MODEL\_V4 | 1.0 | 5.705e-13 | 417 | 0.167 | 872 | 506 | 31 | 1 | 782 | 1 | 742 | Phage tail tape measure protein, TP901 family | Phage tail tape measure protein, TP901 family | | afdb-uniprot50 | AF-A0A1M5S5P3-F1-MODEL\_V4 | 1.0 | 2.728e-13 | 417 | 0.17 | 810 | 483 | 30 | 25 | 783 | 16 | 687 | Phage tail tape measure protein, TP901 family, core region | Phage tail tape measure protein, TP901 family, core region | | afdb-uniprot50 | AF-A0A2A2P5S7-F1-MODEL\_V4 | 1.0 | 1.548e-11 | 417 | 0.157 | 629 | 453 | 19 | 108 | 702 | 63 | 648 | Phage tail tape measure protein | Phage tail tape measure protein | | afdb-uniprot50 | AF-W0FHT5-F1-MODEL\_V4 | 1.0 | 9.94e-14 | 417 | 0.153 | 911 | 529 | 31 | 1 | 701 | 1 | 878 | Phage tail tape measure protein, family, core region | Phage tail tape measure protein, family, core region | | afdb-uniprot50 | AF-W7ZHM9-F1-MODEL\_V4 | 1.0 | 4.086e-11 | 416 | 0.159 | 591 | 401 | 23 | 75 | 637 | 7 | 529 | Phage tail length tape-measure protein | Phage tail length tape-measure protein | | afdb-uniprot50 | AF-A0A2J6HSG0-F1-MODEL\_V4 | 1.0 | 5.019e-12 | 416 | 0.162 | 701 | 485 | 22 | 39 | 696 | 3 | 644 | Phage tail tape measure protein | Phage tail tape measure protein | | afdb-uniprot50 | AF-Q896A9-F1-MODEL\_V4 | 1.0 | 1.062e-12 | 415 | 0.137 | 801 | 533 | 28 | 8 | 779 | 1 | 672 | Phage protein | Phage protein | | afdb-uniprot50 | AF-C3X1X5-F1-MODEL\_V4 | 1.0 | 5.078e-13 | 415 | 0.159 | 855 | 544 | 22 | 19 | 781 | 34 | 805 | Phage tail tape measure protein, TP901 family, core region | Phage tail tape measure protein, TP901 family, core region | | afdb-uniprot50 | AF-A0A1Q3R2P7-F1-MODEL\_V4 | 1.0 | 4.347e-13 | 414 | 0.2 | 698 | 440 | 24 | 148 | 782 | 12 | 654 | Phage tail tape measure protein | Phage tail tape measure protein | | afdb-uniprot50 | AF-A0A7W9VFJ5-F1-MODEL\_V4 | 1.0 | 4.231e-14 | 414 | 0.178 | 831 | 536 | 31 | 1 | 750 | 2 | 766 | TP901 family phage tail tape measure protein | TP901 family phage tail tape measure protein | | afdb-uniprot50 | AF-A0A644VK59-F1-MODEL\_V4 | 1.0 | 6.41e-13 | 414 | 0.143 | 752 | 524 | 28 | 27 | 702 | 61 | 768 | PhageMin\_Tail domain-containing protein | PhageMin\_Tail domain-containing protein | | afdb-uniprot50 | AF-A0A0F9M5T9-F1-MODEL\_V4 | 1.0 | 2.256e-10 | 413 | 0.149 | 741 | 419 | 22 | 50 | 785 | 6 | 539 | PhageMin\_Tail domain-containing protein | PhageMin\_Tail domain-containing protein | | afdb-uniprot50 | AF-A0A6I6WW05-F1-MODEL\_V4 | 1.0 | 7.784e-13 | 413 | 0.159 | 878 | 521 | 30 | 1 | 769 | 1 | 770 | Phage tail tape measure protein | Phage tail tape measure protein | | afdb-uniprot50 | AF-A0A0H3F7C7-F1-MODEL\_V4 | 1.0 | 5.931e-13 | 413 | 0.164 | 905 | 525 | 26 | 19 | 781 | 49 | 863 | Phage tail tape measure protein, TP901 family | Phage tail tape measure protein, TP901 family | | afdb-uniprot50 | AF-H1D5F2-F1-MODEL\_V4 | 1.0 | 2.524e-13 | 412 | 0.229 | 627 | 358 | 18 | 207 | 785 | 4 | 553 | Phage tail tape measure protein, TP901 family, core region | Phage tail tape measure protein, TP901 family, core region | | afdb-uniprot50 | AF-A0A3R9NYT5-F1-MODEL\_V4 | 1.0 | 3.539e-12 | 412 | 0.146 | 804 | 499 | 25 | 65 | 777 | 5 | 711 | Phage tail tape measure protein | Phage tail tape measure protein | | afdb-uniprot50 | AF-A0A124GKD2-F1-MODEL\_V4 | 1.0 | 3.58e-13 | 412 | 0.145 | 751 | 512 | 23 | 117 | 779 | 37 | 745 | PhageMin\_Tail domain-containing protein | PhageMin\_Tail domain-containing protein | | afdb-uniprot50 | AF-A0A3D5P8N1-F1-MODEL\_V4 | 1.0 | 5.078e-13 | 412 | 0.139 | 972 | 595 | 31 | 1 | 749 | 1 | 953 | Phage tail tape measure protein | Phage tail tape measure protein | | afdb-uniprot50 | AF-A0A7Y5FSF4-F1-MODEL\_V4 | 1.0 | 5.341e-14 | 412 | 0.179 | 803 | 517 | 30 | 19 | 765 | 11 | 727 | Phage tail tape measure protein | Phage tail tape measure protein | | afdb-uniprot50 | AF-A0A1M5PWE6-F1-MODEL\_V4 | 1.0 | 8.092e-13 | 412 | 0.16 | 899 | 573 | 29 | 19 | 783 | 146 | 996 | Phage tail tape measure protein, TP901 family, core region | Phage tail tape measure protein, TP901 family, core region | | afdb-uniprot50 | AF-A0A2G2JMR9-F1-MODEL\_V4 | 1.0 | 3.825e-12 | 411 | 0.155 | 686 | 465 | 28 | 63 | 702 | 10 | 626 | Phage tail tape measure protein | Phage tail tape measure protein | | afdb-uniprot50 | AF-A0A7V6YSF2-F1-MODEL\_V4 | 1.0 | 6.166e-13 | 411 | 0.166 | 783 | 490 | 26 | 63 | 747 | 5 | 722 | Phage tail tape measure protein | Phage tail tape measure protein | | afdb-uniprot50 | AF-A0A2W4KBI5-F1-MODEL\_V4 | 1.0 | 4.182e-13 | 411 | 0.158 | 894 | 551 | 26 | 35 | 776 | 2 | 845 | Phage tail tape measure protein | Phage tail tape measure protein | | afdb-uniprot50 | AF-F4HB27-F1-MODEL\_V4 | 1.0 | 8.746e-13 | 411 | 0.176 | 940 | 529 | 28 | 19 | 781 | 77 | 947 | Phage-related minor tail protein | Phage-related minor tail protein | | afdb-uniprot50 | AF-A0A1P8WB67-F1-MODEL\_V4 | 1.0 | 1.654e-10 | 410 | 0.174 | 557 | 373 | 17 | 32 | 556 | 4 | 505 | Phage tail tape measure protein, TP901 family, core region | Phage tail tape measure protein, TP901 family, core region | | afdb-uniprot50 | AF-A0A7Y2L7P9-F1-MODEL\_V4 | 1.0 | 7.606e-11 | 410 | 0.183 | 633 | 368 | 19 | 2 | 620 | 1 | 498 | Phage tail tape measure protein | Phage tail tape measure protein | | afdb-uniprot50 | AF-A0A1I3C2N2-F1-MODEL\_V4 | 1.0 | 6.095e-12 | 410 | 0.197 | 593 | 384 | 16 | 160 | 702 | 1 | 551 | Phage tail tape measure protein, TP901 family, core region | Phage tail tape measure protein, TP901 family, core region | | afdb-uniprot50 | AF-A0A2A4V258-F1-MODEL\_V4 | 1.0 | 1.449e-12 | 410 | 0.164 | 722 | 492 | 23 | 46 | 732 | 18 | 662 | Phage tail tape measure protein | Phage tail tape measure protein | | afdb-uniprot50 | AF-A0A1Q6K3L0-F1-MODEL\_V4 | 1.0 | 1.24e-12 | 410 | 0.154 | 895 | 562 | 32 | 19 | 785 | 81 | 908 | Phage tail tape measure protein | Phage tail tape measure protein | | afdb-uniprot50 | AF-A0A1D9FP83-F1-MODEL\_V4 | 1.0 | 9.826e-13 | 410 | 0.147 | 740 | 486 | 26 | 1 | 702 | 1 | 633 | Phage tail tape measure protein | Phage tail tape measure protein | | afdb-uniprot50 | AF-A0A4V6M1R7-F1-MODEL\_V4 | 1.0 | 7.487e-13 | 409 | 0.182 | 784 | 502 | 29 | 88 | 784 | 35 | 766 | PhageMin\_Tail domain-containing protein | PhageMin\_Tail domain-containing protein | | afdb-uniprot50 | AF-A0A6S4UXC8-F1-MODEL\_V4 | 1.0 | 1.022e-12 | 409 | 0.158 | 977 | 523 | 31 | 19 | 782 | 56 | 945 | PhageMin\_Tail domain-containing protein | PhageMin\_Tail domain-containing protein | | afdb-uniprot50 | AF-A0A6D2F7P3-F1-MODEL\_V4 | 1.0 | 1.198e-09 | 408 | 0.165 | 509 | 332 | 13 | 19 | 452 | 44 | 534 | Phage tail tape measure protein | Phage tail tape measure protein | | afdb-uniprot50 | AF-A0A285M600-F1-MODEL\_V4 | 1.0 | 2.221e-12 | 408 | 0.153 | 842 | 522 | 23 | 19 | 780 | 71 | 801 | Phage tail tape measure protein, TP901 family, core region | Phage tail tape measure protein, TP901 family, core region | | afdb-uniprot50 | AF-A0A7U6KQY0-F1-MODEL\_V4 | 1.0 | 3.065e-13 | 408 | 0.162 | 868 | 553 | 23 | 19 | 782 | 49 | 846 | PhageMin\_Tail domain-containing protein | PhageMin\_Tail domain-containing protein | | afdb-uniprot50 | AF-A0A2U3F164-F1-MODEL\_V4 | 1.0 | 4.699e-13 | 408 | 0.153 | 918 | 543 | 28 | 19 | 781 | 47 | 884 | Phage tail tape measure protein | Phage tail tape measure protein | | afdb-uniprot50 | AF-A0A402A530-F1-MODEL\_V4 | 1.0 | 5.575e-11 | 407 | 0.174 | 649 | 444 | 21 | 112 | 702 | 53 | 667 | PhageMin\_Tail domain-containing protein | PhageMin\_Tail domain-containing protein | | afdb-uniprot50 | AF-J3UKH6-F1-MODEL\_V4 | 1.0 | 4.885e-13 | 407 | 0.133 | 879 | 586 | 27 | 20 | 785 | 2 | 817 | TP901 family phage tail tape measure protein | TP901 family phage tail tape measure protein | | afdb-uniprot50 | AF-A0A2C9CPT9-F1-MODEL\_V4 | 1.0 | 4.828e-12 | 407 | 0.152 | 741 | 493 | 26 | 83 | 780 | 2 | 650 | Phage tail tape measure protein, lambda family/phage tail tape measure protein, TP901 family, core region | Phage tail tape measure protein, lambda family/phage tail tape measure protein, TP901 family, core region | | afdb-uniprot50 | AF-A0A433KZN9-F1-MODEL\_V4 | 1.0 | 3.418e-09 | 407 | 0.181 | 496 | 331 | 16 | 19 | 458 | 78 | 554 | Phage tail tape measure protein | Phage tail tape measure protein | | afdb-uniprot50 | AF-A0A1H0P6W7-F1-MODEL\_V4 | 1.0 | 5.019e-12 | 407 | 0.176 | 730 | 498 | 18 | 19 | 702 | 19 | 690 | Phage tail tape measure protein, TP901 family, core region | Phage tail tape measure protein, TP901 family, core region | | afdb-uniprot50 | AF-A0A765X8R7-F1-MODEL\_V4 | 1.0 | 1.179e-11 | 406 | 0.155 | 783 | 506 | 25 | 80 | 781 | 3 | 710 | Phage tail tape measure protein | Phage tail tape measure protein | | afdb-uniprot50 | AF-A0A150ISK2-F1-MODEL\_V4 | 1.0 | 3.93e-11 | 406 | 0.171 | 641 | 400 | 24 | 115 | 702 | 47 | 609 | Phage-related minor tail protein | Phage-related minor tail protein | | afdb-uniprot50 | AF-A0A1Y6FRA0-F1-MODEL\_V4 | 1.0 | 2.221e-12 | 406 | 0.16 | 814 | 519 | 31 | 1 | 702 | 1 | 761 | Phage tail tape measure protein, TP901 family, core region | Phage tail tape measure protein, TP901 family, core region | | afdb-uniprot50 | AF-Q4E9A1-F1-MODEL\_V4 | 1.0 | 2.42e-06 | 405 | 0.317 | 195 | 132 | 1 | 144 | 338 | 2 | 195 | PhageMin\_Tail domain-containing protein | PhageMin\_Tail domain-containing protein | | afdb-uniprot50 | AF-A0A497GWY3-F1-MODEL\_V4 | 1.0 | 7.316e-11 | 405 | 0.148 | 547 | 384 | 18 | 2 | 509 | 1 | 504 | Phage tail tape measure protein | Phage tail tape measure protein | | afdb-uniprot50 | AF-W7PY06-F1-MODEL\_V4 | 1.0 | 4.416e-11 | 405 | 0.15 | 730 | 492 | 23 | 39 | 741 | 2 | 630 | PhageMin\_Tail domain-containing protein | PhageMin\_Tail domain-containing protein | | afdb-uniprot50 | AF-A0A2S8FUK5-F1-MODEL\_V4 | 1.0 | 4.086e-11 | 405 | 0.16 | 711 | 467 | 22 | 83 | 774 | 18 | 617 | Phage tail tape measure protein | Phage tail tape measure protein | | afdb-uniprot50 | AF-A0A1F3BA55-F1-MODEL\_V4 | 1.0 | 2.195e-11 | 405 | 0.161 | 695 | 468 | 23 | 48 | 703 | 3 | 621 | Phage tail tape measure protein | Phage tail tape measure protein | | afdb-uniprot50 | AF-A0A5P3FW64-F1-MODEL\_V4 | 1.0 | 1.121e-10 | 405 | 0.176 | 589 | 352 | 14 | 1 | 459 | 1 | 586 | Phage tail tape measure protein | Phage tail tape measure protein | | afdb-uniprot50 | AF-A0A7V2ILC6-F1-MODEL\_V4 | 1.0 | 2.292e-08 | 404 | 0.146 | 437 | 327 | 12 | 39 | 472 | 11 | 404 | Phage tail tape measure protein | Phage tail tape measure protein | | afdb-uniprot50 | AF-A0A2M8E3Z2-F1-MODEL\_V4 | 1.0 | 9.866e-10 | 404 | 0.171 | 573 | 378 | 23 | 97 | 651 | 9 | 502 | Phage tail tape measure protein | Phage tail tape measure protein | | afdb-uniprot50 | AF-A0A523V8K2-F1-MODEL\_V4 | 1.0 | 3.404e-12 | 403 | 0.165 | 712 | 483 | 21 | 35 | 702 | 17 | 660 | Phage tail tape measure protein | Phage tail tape measure protein | | afdb-uniprot50 | AF-A0A7W8U3E0-F1-MODEL\_V4 | 1.0 | 2.221e-12 | 403 | 0.177 | 806 | 545 | 24 | 35 | 782 | 231 | 976 | TP901 family phage tail tape measure protein | TP901 family phage tail tape measure protein | | afdb-uniprot50 | AF-A0A3S0BBD4-F1-MODEL\_V4 | 1.0 | 1.212e-10 | 402 | 0.181 | 720 | 434 | 20 | 35 | 716 | 5 | 606 | Phage tail tape measure protein | Phage tail tape measure protein | | afdb-uniprot50 | AF-A0A5P0YP70-F1-MODEL\_V4 | 1.0 | 2.495e-12 | 402 | 0.162 | 796 | 484 | 32 | 41 | 767 | 13 | 694 | Phage tail tape measure protein | Phage tail tape measure protein | | afdb-uniprot50 | AF-M0EPI4-F1-MODEL\_V4 | 1.0 | 2.221e-12 | 402 | 0.192 | 750 | 452 | 35 | 63 | 783 | 5 | 629 | Phage tail tape measure protein, TP901 family | Phage tail tape measure protein, TP901 family | | afdb-uniprot50 | AF-A0A7Z7QQC7-F1-MODEL\_V4 | 1.0 | 2.594e-12 | 402 | 0.171 | 768 | 508 | 27 | 1 | 701 | 1 | 706 | Phage tail tape measure protein, TP901 family, core region | Phage tail tape measure protein, TP901 family, core region | | afdb-uniprot50 | AF-A0A1R0WG41-F1-MODEL\_V4 | 1.0 | 4.182e-13 | 402 | 0.156 | 1005 | 571 | 31 | 1 | 784 | 3 | 951 | Phage tail tape measure protein | Phage tail tape measure protein | | afdb-uniprot50 | AF-A0A135YZ87-F1-MODEL\_V4 | 1.0 | 2.948e-13 | 402 | 0.183 | 833 | 510 | 28 | 19 | 709 | 164 | 967 | Phage tail tape measure protein, TP901 family | Phage tail tape measure protein, TP901 family | | afdb-uniprot50 | AF-A0A0F9TKT3-F1-MODEL\_V4 | 1.0 | 3.365e-11 | 401 | 0.159 | 690 | 464 | 25 | 61 | 704 | 3 | 622 | PhageMin\_Tail domain-containing protein | PhageMin\_Tail domain-containing protein | | afdb-uniprot50 | AF-K4Z9C6-F1-MODEL\_V4 | 1.0 | 4.885e-13 | 401 | 0.152 | 806 | 547 | 28 | 46 | 776 | 3 | 747 | Phage tail tape measure protein, TP901 family | Phage tail tape measure protein, TP901 family | | afdb-uniprot50 | AF-G5S7F4-F1-MODEL\_V4 | 1.0 | 1.449e-12 | 401 | 0.16 | 873 | 479 | 26 | 1 | 755 | 11 | 747 | Phage tail tape measure protein, TP901 family | Phage tail tape measure protein, TP901 family | | afdb-uniprot50 | AF-A0A371IQX0-F1-MODEL\_V4 | 1.0 | 9.826e-13 | 401 | 0.167 | 762 | 471 | 27 | 6 | 701 | 3 | 666 | Phage tail tape measure protein | Phage tail tape measure protein | | afdb-uniprot50 | AF-A0A5C5XSP4-F1-MODEL\_V4 | 1.0 | 2.915e-12 | 400 | 0.16 | 849 | 514 | 31 | 6 | 785 | 2 | 720 | Phage-related minor tail protein | Phage-related minor tail protein | | afdb-uniprot50 | AF-C8WM92-F1-MODEL\_V4 | 1.0 | 1.01e-11 | 400 | 0.199 | 676 | 453 | 26 | 61 | 702 | 13 | 634 | Phage tail tape measure protein, TP901 family | Phage tail tape measure protein, TP901 family | | afdb-uniprot50 | AF-F3ZVD4-F1-MODEL\_V4 | 1.0 | 8.316e-12 | 400 | 0.173 | 703 | 474 | 24 | 46 | 702 | 2 | 643 | Phage tail tape measure protein, TP901 family | Phage tail tape measure protein, TP901 family | | afdb-uniprot50 | AF-A0A1H1G2L2-F1-MODEL\_V4 | 1.0 | 2.495e-12 | 400 | 0.169 | 908 | 565 | 32 | 19 | 785 | 65 | 923 | Phage tail tape measure protein, TP901 family, core region | Phage tail tape measure protein, TP901 family, core region | | afdb-uniprot50 | AF-A0A378AFF2-F1-MODEL\_V4 | 1.0 | 4.663e-09 | 399 | 0.172 | 487 | 321 | 12 | 20 | 438 | 45 | 517 | Putative prophage tail length determinator | Putative prophage tail length determinator | | afdb-uniprot50 | AF-A0A5M6CYT6-F1-MODEL\_V4 | 1.0 | 2.466e-11 | 399 | 0.14 | 776 | 463 | 22 | 26 | 777 | 1 | 596 | Phage tail tape measure protein | Phage tail tape measure protein | | afdb-uniprot50 | AF-A0A4R3J9G1-F1-MODEL\_V4 | 1.0 | 9.235e-11 | 399 | 0.154 | 732 | 446 | 24 | 36 | 735 | 5 | 595 | TP901 family phage tail tape measure protein | TP901 family phage tail tape measure protein | | afdb-uniprot50 | AF-A0A379FY57-F1-MODEL\_V4 | 1.0 | 1.079e-10 | 399 | 0.153 | 663 | 430 | 15 | 21 | 604 | 39 | 648 | Chromosome segregation protein | Chromosome segregation protein | | afdb-uniprot50 | AF-A0A537MFF8-F1-MODEL\_V4 | 1.0 | 3.065e-13 | 399 | 0.168 | 891 | 526 | 32 | 22 | 781 | 53 | 859 | Phage tail tape measure protein | Phage tail tape measure protein | | afdb-uniprot50 | AF-A0A506TXK5-F1-MODEL\_V4 | 1.0 | 1.901e-12 | 399 | 0.159 | 801 | 506 | 24 | 6 | 774 | 1 | 665 | Phage tail tape measure protein | Phage tail tape measure protein | | afdb-uniprot50 | AF-A0A354PGC8-F1-MODEL\_V4 | 1.0 | 8.987e-12 | 399 | 0.165 | 724 | 465 | 23 | 83 | 702 | 18 | 705 | Phage tail tape measure protein | Phage tail tape measure protein | | afdb-uniprot50 | AF-A0A7J2N4A4-F1-MODEL\_V4 | 1.0 | 1.148e-12 | 399 | 0.168 | 790 | 477 | 23 | 19 | 716 | 207 | 908 | Phage tail tape measure protein | Phage tail tape measure protein | | afdb-uniprot50 | AF-A0A268TN88-F1-MODEL\_V4 | 1.0 | 3.379e-08 | 398 | 0.164 | 371 | 239 | 4 | 27 | 362 | 9 | 343 | Phage tail tape measure protein | Phage tail tape measure protein | | afdb-uniprot50 | AF-A0A2Z4WE31-F1-MODEL\_V4 | 1.0 | 1.28e-08 | 398 | 0.175 | 417 | 265 | 13 | 80 | 444 | 2 | 391 | Phage tail tape measure protein | Phage tail tape measure protein | | afdb-uniprot50 | AF-A0A346QYG9-F1-MODEL\_V4 | 1.0 | 1.022e-12 | 398 | 0.199 | 667 | 387 | 23 | 166 | 754 | 8 | 605 | Phage tail tape measure protein | Phage tail tape measure protein | | afdb-uniprot50 | AF-A0A6B5AE33-F1-MODEL\_V4 | 1.0 | 4.885e-13 | 398 | 0.151 | 910 | 575 | 40 | 1 | 783 | 1 | 839 | Phage tail tape measure protein | Phage tail tape measure protein | | afdb-uniprot50 | AF-A0A1Y3CT02-F1-MODEL\_V4 | 1.0 | 6.485e-14 | 398 | 0.164 | 862 | 565 | 28 | 19 | 785 | 56 | 856 | Phage tail tape measure protein | Phage tail tape measure protein | | afdb-uniprot50 | AF-A0A1I5H9Z3-F1-MODEL\_V4 | 1.0 | 7.487e-13 | 398 | 0.173 | 861 | 551 | 28 | 19 | 785 | 51 | 844 | Phage tail tape measure protein, TP901 family, core region | Phage tail tape measure protein, TP901 family, core region | | afdb-uniprot50 | AF-A0A0P0EGD7-F1-MODEL\_V4 | 1.0 | 1.78e-13 | 398 | 0.156 | 1005 | 555 | 39 | 39 | 785 | 43 | 1012 | PhageMin\_Tail domain-containing protein | PhageMin\_Tail domain-containing protein | | afdb-uniprot50 | AF-A0A380DV55-F1-MODEL\_V4 | 1.0 | 1.566e-12 | 398 | 0.158 | 790 | 534 | 31 | 1 | 702 | 1 | 747 | Phage tail tape measure protein, TP901 family, core region | Phage tail tape measure protein, TP901 family, core region | | afdb-uniprot50 | AF-A0A523CMP8-F1-MODEL\_V4 | 1.0 | 2.995e-11 | 397 | 0.126 | 829 | 488 | 21 | 6 | 785 | 3 | 643 | Phage tail tape measure protein | Phage tail tape measure protein | | afdb-uniprot50 | AF-A0A3E0KFX5-F1-MODEL\_V4 | 1.0 | 9.826e-13 | 397 | 0.192 | 741 | 478 | 25 | 35 | 708 | 23 | 709 | Phage tail tape measure protein | Phage tail tape measure protein | | afdb-uniprot50 | AF-A0A0M6WZJ0-F1-MODEL\_V4 | 1.0 | 6.336e-12 | 397 | 0.157 | 728 | 524 | 21 | 27 | 701 | 62 | 752 | PhageMin\_Tail domain-containing protein | PhageMin\_Tail domain-containing protein | | afdb-uniprot50 | AF-A0A0D0HTB3-F1-MODEL\_V4 | 1.0 | 1.977e-12 | 397 | 0.166 | 900 | 590 | 27 | 8 | 776 | 157 | 1026 | Phage-related minor tail protein | Phage-related minor tail protein | | afdb-uniprot50 | AF-A0A3A9F184-F1-MODEL\_V4 | 1.0 | 1.739e-11 | 397 | 0.178 | 667 | 463 | 23 | 77 | 701 | 3 | 626 | Phage tail tape measure protein | Phage tail tape measure protein | | afdb-uniprot50 | AF-A0A523V4W2-F1-MODEL\_V4 | 1.0 | 5.729e-10 | 396 | 0.149 | 576 | 385 | 19 | 61 | 627 | 1 | 480 | Phage tail tape measure protein | Phage tail tape measure protein | | afdb-uniprot50 | AF-A0A0A8FI92-F1-MODEL\_V4 | 1.0 | 5.384e-08 | 396 | 0.157 | 369 | 280 | 13 | 89 | 445 | 97 | 446 | PhageMin\_Tail domain-containing protein | PhageMin\_Tail domain-containing protein | | afdb-uniprot50 | AF-A0A6M0SSJ1-F1-MODEL\_V4 | 1.0 | 2.309e-12 | 396 | 0.149 | 869 | 527 | 30 | 1 | 775 | 1 | 750 | Phage tail tape measure protein | Phage tail tape measure protein | | afdb-uniprot50 | AF-A0A3D4VTZ8-F1-MODEL\_V4 | 1.0 | 8.092e-13 | 396 | 0.165 | 892 | 552 | 35 | 8 | 782 | 8 | 823 | Phage tail tape measure protein | Phage tail tape measure protein | | afdb-uniprot50 | AF-A0A7C6ZVA5-F1-MODEL\_V4 | 1.0 | 2.564e-11 | 396 | 0.164 | 676 | 450 | 20 | 46 | 700 | 2 | 583 | Phage tail tape measure protein | Phage tail tape measure protein | | afdb-uniprot50 | AF-A0A6N9P328-F1-MODEL\_V4 | 1.0 | 3.976e-12 | 396 | 0.215 | 705 | 454 | 24 | 53 | 704 | 3 | 661 | Phage tail tape measure protein | Phage tail tape measure protein | | afdb-uniprot50 | AF-R9MQH3-F1-MODEL\_V4 | 1.0 | 1.449e-12 | 396 | 0.155 | 823 | 519 | 28 | 1 | 702 | 22 | 789 | Phage tail tape measure protein, TP901 family, core region | Phage tail tape measure protein, TP901 family, core region | | afdb-uniprot50 | AF-A0A354XNE6-F1-MODEL\_V4 | 1.0 | 1.109e-09 | 395 | 0.164 | 596 | 398 | 16 | 22 | 552 | 54 | 614 | Phage tail tape measure protein | Phage tail tape measure protein | | afdb-uniprot50 | AF-A0A7X2D754-F1-MODEL\_V4 | 1.0 | 1.038e-10 | 395 | 0.15 | 690 | 480 | 23 | 42 | 684 | 2 | 632 | Phage tail tape measure protein | Phage tail tape measure protein | | afdb-uniprot50 | AF-A0A1S7PL82-F1-MODEL\_V4 | 1.0 | 2.771e-11 | 395 | 0.173 | 776 | 448 | 26 | 2 | 723 | 1 | 636 | Putative Phage tail tape measure protein, family, core region | Putative Phage tail tape measure protein, family, core region | | afdb-uniprot50 | AF-A0A0F9IVD7-F1-MODEL\_V4 | 1.0 | 1.325e-11 | 395 | 0.206 | 620 | 426 | 26 | 96 | 702 | 5 | 571 | PhageMin\_Tail domain-containing protein | PhageMin\_Tail domain-containing protein | | afdb-uniprot50 | AF-C9RFS8-F1-MODEL\_V4 | 1.0 | 7.784e-13 | 395 | 0.154 | 788 | 514 | 31 | 63 | 772 | 17 | 729 | Phage tail tape measure protein, TP901 family | Phage tail tape measure protein, TP901 family | | afdb-uniprot50 | AF-A0A839XX57-F1-MODEL\_V4 | 1.0 | 8.746e-13 | 395 | 0.156 | 928 | 563 | 32 | 2 | 785 | 8 | 859 | TP901 family phage tail tape measure protein | TP901 family phage tail tape measure protein | | afdb-uniprot50 | AF-A0A2E0VFX7-F1-MODEL\_V4 | 1.0 | 6.336e-12 | 395 | 0.137 | 790 | 484 | 27 | 49 | 785 | 5 | 649 | Phage tail tape measure protein | Phage tail tape measure protein | | afdb-uniprot50 | AF-A0A6N7XH90-F1-MODEL\_V4 | 1.0 | 7.119e-12 | 394 | 0.161 | 747 | 493 | 29 | 109 | 785 | 52 | 734 | Phage tail tape measure protein | Phage tail tape measure protein | | afdb-uniprot50 | AF-A0A5S4Z6P0-F1-MODEL\_V4 | 1.0 | 2.697e-12 | 394 | 0.157 | 787 | 494 | 33 | 70 | 784 | 6 | 695 | Phage tail tape measure protein | Phage tail tape measure protein | | afdb-uniprot50 | AF-E5Y2G1-F1-MODEL\_V4 | 1.0 | 1.506e-12 | 394 | 0.163 | 917 | 522 | 30 | 1 | 785 | 1 | 803 | Phage tail tape measure protein, TP901 family, core region | Phage tail tape measure protein, TP901 family, core region | | afdb-uniprot50 | AF-A0A7Y0EFA1-F1-MODEL\_V4 | 1.0 | 1.879e-11 | 394 | 0.137 | 712 | 488 | 18 | 77 | 702 | 21 | 692 | Phage tail tape measure protein | Phage tail tape measure protein | | afdb-uniprot50 | AF-A0A5K7WX33-F1-MODEL\_V4 | 1.0 | 8.412e-13 | 394 | 0.16 | 936 | 560 | 27 | 27 | 784 | 62 | 949 | PhageMin\_Tail domain-containing protein | PhageMin\_Tail domain-containing protein | | afdb-uniprot50 | AF-A0A380FL26-F1-MODEL\_V4 | 1.0 | 5.863e-12 | 394 | 0.153 | 731 | 506 | 27 | 26 | 702 | 51 | 722 | Phage tail tape measure protein, TP901 family, core region | Phage tail tape measure protein, TP901 family, core region | | afdb-uniprot50 | AF-A0A7X7IZG9-F1-MODEL\_V4 | 1.0 | 1.829e-12 | 393 | 0.163 | 803 | 507 | 29 | 79 | 774 | 22 | 766 | Phage tail tape measure protein | Phage tail tape measure protein | | afdb-uniprot50 | AF-A0A2S0JRG7-F1-MODEL\_V4 | 1.0 | 1.148e-12 | 393 | 0.155 | 798 | 492 | 28 | 19 | 778 | 23 | 676 | Phage tail tape measure protein | Phage tail tape measure protein | | afdb-uniprot50 | AF-A0A5D4RWQ3-F1-MODEL\_V4 | 1.0 | 8.645e-12 | 393 | 0.16 | 699 | 467 | 22 | 70 | 701 | 4 | 649 | Phage tail tape measure protein | Phage tail tape measure protein | | afdb-uniprot50 | AF-A0A4Q2R8E7-F1-MODEL\_V4 | 1.0 | 3.114e-11 | 393 | 0.151 | 839 | 452 | 21 | 32 | 755 | 192 | 885 | Phage tail tape measure protein | Phage tail tape measure protein | | afdb-uniprot50 | AF-A0A5Y5IG90-F1-MODEL\_V4 | 1.0 | 2.635e-10 | 392 | 0.284 | 436 | 217 | 9 | 250 | 682 | 3 | 346 | Phage tail tape measure protein | Phage tail tape measure protein | | afdb-uniprot50 | AF-A0A3R6KUE5-F1-MODEL\_V4 | 1.0 | 2.112e-11 | 392 | 0.16 | 716 | 451 | 23 | 19 | 675 | 45 | 669 | Phage tail tape measure protein | Phage tail tape measure protein | | afdb-uniprot50 | AF-A0A4R1Z735-F1-MODEL\_V4 | 1.0 | 3.404e-12 | 392 | 0.161 | 857 | 525 | 24 | 32 | 781 | 3 | 772 | TP901 family phage tail tape measure protein | TP901 family phage tail tape measure protein | | afdb-uniprot50 | AF-A0A1Y3TQP8-F1-MODEL\_V4 | 1.0 | 7.037e-11 | 392 | 0.16 | 637 | 437 | 22 | 77 | 702 | 22 | 571 | Phage tail tape measure protein | Phage tail tape measure protein | | afdb-uniprot50 | AF-A0A3Q9B6I7-F1-MODEL\_V4 | 1.0 | 9.601e-11 | 391 | 0.153 | 721 | 465 | 29 | 90 | 785 | 17 | 616 | Phage tail tape measure protein | Phage tail tape measure protein | | afdb-uniprot50 | AF-S9TGE4-F1-MODEL\_V4 | 1.0 | 2.031e-11 | 391 | 0.162 | 775 | 459 | 21 | 5 | 750 | 2 | 615 | TP901 family phage tail tape measure protein | TP901 family phage tail tape measure protein | | afdb-uniprot50 | AF-A0A846I152-F1-MODEL\_V4 | 1.0 | 3.404e-12 | 391 | 0.169 | 736 | 492 | 27 | 1 | 701 | 1 | 651 | Phage tail tape measure protein | Phage tail tape measure protein | | afdb-uniprot50 | AF-A0A371IJP1-F1-MODEL\_V4 | 1.0 | 1.901e-12 | 391 | 0.152 | 916 | 529 | 30 | 27 | 785 | 130 | 954 | Phage tail tape measure protein | Phage tail tape measure protein | | afdb-uniprot50 | AF-A0A0D1X6H1-F1-MODEL\_V4 | 1.0 | 1.566e-12 | 391 | 0.137 | 970 | 586 | 30 | 19 | 784 | 40 | 962 | Phage tail tape measure protein, TP901 family, core region | Phage tail tape measure protein, TP901 family, core region | | afdb-uniprot50 | AF-V4PS02-F1-MODEL\_V4 | 1.0 | 1.506e-12 | 390 | 0.171 | 849 | 573 | 27 | 1 | 782 | 1 | 785 | PhageMin\_Tail domain-containing protein | PhageMin\_Tail domain-containing protein | | afdb-uniprot50 | AF-A0A1C3EE79-F1-MODEL\_V4 | 1.0 | 1.759e-12 | 390 | 0.179 | 880 | 536 | 24 | 19 | 782 | 57 | 866 | Phage tail tape measure protein | Phage tail tape measure protein | | afdb-uniprot50 | AF-A0A2A4H5A1-F1-MODEL\_V4 | 1.0 | 4.644e-12 | 390 | 0.151 | 790 | 515 | 25 | 1 | 702 | 1 | 722 | Phage tail tape measure protein | Phage tail tape measure protein | | afdb-uniprot50 | AF-A0A1W9SV37-F1-MODEL\_V4 | 1.0 | 4.297e-12 | 389 | 0.17 | 751 | 502 | 24 | 46 | 722 | 5 | 708 | Phage tail tape measure protein | Phage tail tape measure protein | | afdb-uniprot50 | AF-A0A1X9MF98-F1-MODEL\_V4 | 1.0 | 5.64e-12 | 389 | 0.185 | 819 | 504 | 31 | 1 | 702 | 1 | 773 | Phage-related minor tail protein | Phage-related minor tail protein | | afdb-uniprot50 | AF-A0A6M0STF1-F1-MODEL\_V4 | 1.0 | 2.848e-10 | 388 | 0.148 | 794 | 445 | 28 | 1 | 785 | 1 | 572 | Phage tail tape measure protein | Phage tail tape measure protein | | afdb-uniprot50 | AF-A0A4D7BBQ9-F1-MODEL\_V4 | 1.0 | 2.466e-11 | 388 | 0.155 | 664 | 425 | 20 | 116 | 755 | 29 | 580 | Phage tail tape measure protein | Phage tail tape measure protein | | afdb-uniprot50 | AF-A0A6P0MHK5-F1-MODEL\_V4 | 1.0 | 4.828e-12 | 388 | 0.159 | 805 | 527 | 29 | 70 | 784 | 4 | 748 | Phage tail tape measure protein | Phage tail tape measure protein | | afdb-uniprot50 | AF-A0A661DRW5-F1-MODEL\_V4 | 1.0 | 1.179e-11 | 388 | 0.151 | 772 | 461 | 25 | 1 | 702 | 6 | 653 | Phage tail tape measure protein | Phage tail tape measure protein | | afdb-uniprot50 | AF-A0A5C6C1J2-F1-MODEL\_V4 | 1.0 | 1.325e-11 | 388 | 0.177 | 777 | 467 | 25 | 1 | 705 | 1 | 677 | Phage-related minor tail protein | Phage-related minor tail protein | | afdb-uniprot50 | AF-G4Q995-F1-MODEL\_V4 | 1.0 | 5.51e-10 | 388 | 0.163 | 537 | 335 | 17 | 1 | 463 | 1 | 497 | Phage tail tape measure protein, TP901 family, core region domain protein | Phage tail tape measure protein, TP901 family, core region domain protein | | afdb-uniprot50 | AF-A0A0P6WTM8-F1-MODEL\_V4 | 1.0 | 6.664e-13 | 388 | 0.168 | 730 | 460 | 26 | 62 | 705 | 5 | 673 | PhageMin\_Tail domain-containing protein | PhageMin\_Tail domain-containing protein | | afdb-uniprot50 | AF-A0A502CNZ0-F1-MODEL\_V4 | 1.0 | 1.506e-12 | 388 | 0.163 | 1003 | 572 | 34 | 19 | 785 | 39 | 1009 | Phage tail tape measure protein | Phage tail tape measure protein | | afdb-uniprot50 | AF-A0A417X761-F1-MODEL\_V4 | 1.0 | 1.455e-09 | 387 | 0.145 | 618 | 389 | 20 | 81 | 662 | 5 | 519 | Phage tail tape measure protein | Phage tail tape measure protein | | afdb-uniprot50 | AF-A0A4Q0YND4-F1-MODEL\_V4 | 1.0 | 1.985e-09 | 387 | 0.179 | 564 | 369 | 16 | 19 | 508 | 57 | 600 | Phage tail tape measure protein | Phage tail tape measure protein | | afdb-uniprot50 | AF-A0A841GQ15-F1-MODEL\_V4 | 1.0 | 3.825e-12 | 387 | 0.148 | 739 | 493 | 18 | 1 | 701 | 2 | 641 | TP901 family phage tail tape measure protein | TP901 family phage tail tape measure protein | | afdb-uniprot50 | AF-A0A5T7NPI6-F1-MODEL\_V4 | 1.0 | 4.086e-11 | 386 | 0.17 | 700 | 411 | 19 | 19 | 623 | 44 | 668 | Phage tail tape measure protein | Phage tail tape measure protein | | afdb-uniprot50 | AF-A0A0F9R9A9-F1-MODEL\_V4 | 1.0 | 5.64e-12 | 386 | 0.183 | 794 | 500 | 29 | 63 | 764 | 5 | 741 | PhageMin\_Tail domain-containing protein | PhageMin\_Tail domain-containing protein | | afdb-uniprot50 | AF-A0A6L9H1L1-F1-MODEL\_V4 | 1.0 | 8.545e-11 | 386 | 0.172 | 609 | 415 | 20 | 113 | 702 | 1 | 539 | Phage tail tape measure protein | Phage tail tape measure protein | | afdb-uniprot50 | AF-A0A4S2HCP5-F1-MODEL\_V4 | 1.0 | 4.644e-12 | 386 | 0.146 | 825 | 524 | 20 | 22 | 702 | 50 | 837 | Phage tail tape measure protein | Phage tail tape measure protein | | afdb-uniprot50 | AF-A0A417YR53-F1-MODEL\_V4 | 1.0 | 5.279e-13 | 386 | 0.158 | 1074 | 560 | 37 | 19 | 785 | 131 | 1167 | Phage tail tape measure protein | Phage tail tape measure protein | | afdb-uniprot50 | AF-A0A5C7LFW0-F1-MODEL\_V4 | 1.0 | 1.416e-10 | 385 | 0.131 | 645 | 438 | 17 | 70 | 700 | 21 | 556 | Phage tail tape measure protein | Phage tail tape measure protein | | afdb-uniprot50 | AF-A0A2E8I968-F1-MODEL\_V4 | 1.0 | 2.666e-11 | 385 | 0.159 | 791 | 446 | 30 | 1 | 750 | 1 | 613 | Phage tail tape measure protein | Phage tail tape measure protein | | afdb-uniprot50 | AF-A0A3C0IJN3-F1-MODEL\_V4 | 1.0 | 1.109e-09 | 385 | 0.178 | 573 | 361 | 19 | 19 | 519 | 91 | 625 | Phage tail tape measure protein | Phage tail tape measure protein | | afdb-uniprot50 | AF-A0A0F9KJV0-F1-MODEL\_V4 | 1.0 | 6.025e-11 | 385 | 0.152 | 707 | 485 | 24 | 13 | 677 | 2 | 635 | PhageMin\_Tail domain-containing protein | PhageMin\_Tail domain-containing protein | | afdb-uniprot50 | AF-A0A2N5EEH0-F1-MODEL\_V4 | 1.0 | 2.804e-12 | 385 | 0.178 | 783 | 459 | 24 | 42 | 776 | 9 | 654 | Phage tail tape measure protein | Phage tail tape measure protein | | afdb-uniprot50 | AF-A0A7C6HAK4-F1-MODEL\_V4 | 1.0 | 9.235e-11 | 385 | 0.145 | 661 | 386 | 20 | 108 | 754 | 111 | 606 | Phage tail tape measure protein | Phage tail tape measure protein | | afdb-uniprot50 | AF-A0A1F4MH22-F1-MODEL\_V4 | 1.0 | 7.606e-11 | 385 | 0.164 | 640 | 443 | 25 | 76 | 702 | 6 | 566 | Phage tail tape measure protein | Phage tail tape measure protein | | afdb-uniprot50 | AF-A0A2I6QM47-F1-MODEL\_V4 | 1.0 | 4.134e-12 | 385 | 0.153 | 919 | 582 | 28 | 6 | 785 | 1 | 862 | Phage tail length tape-measure protein | Phage tail length tape-measure protein | | afdb-uniprot50 | AF-A0A7X5L8G2-F1-MODEL\_V4 | 1.0 | 3.679e-12 | 385 | 0.158 | 777 | 494 | 32 | 2 | 702 | 3 | 695 | Phage tail tape measure protein | Phage tail tape measure protein | | afdb-uniprot50 | AF-A0A660IYM3-F1-MODEL\_V4 | 1.0 | 1.466e-13 | 385 | 0.155 | 876 | 490 | 29 | 9 | 650 | 1 | 860 | Phage tail tape measure protein | Phage tail tape measure protein | | afdb-uniprot50 | AF-A0A7G2KFG2-F1-MODEL\_V4 | 1.0 | 2.594e-12 | 384 | 0.233 | 549 | 329 | 12 | 212 | 678 | 2 | 540 | Phage tail tape measure protein, TP901 family, core region | Phage tail tape measure protein, TP901 family, core region | | afdb-uniprot50 | AF-F4N710-F1-MODEL\_V4 | 1.0 | 4.591e-11 | 384 | 0.143 | 731 | 489 | 23 | 9 | 687 | 1 | 646 | PhageMin\_Tail domain-containing protein | PhageMin\_Tail domain-containing protein | | afdb-uniprot50 | AF-A0A2G3DYB1-F1-MODEL\_V4 | 1.0 | 1.05e-11 | 384 | 0.14 | 860 | 541 | 32 | 1 | 782 | 1 | 739 | Phage tail tape measure protein | Phage tail tape measure protein | | afdb-uniprot50 | AF-E6U1K4-F1-MODEL\_V4 | 1.0 | 4.248e-11 | 384 | 0.16 | 680 | 446 | 21 | 38 | 702 | 11 | 580 | Phage tail tape measure protein, TP901 family | Phage tail tape measure protein, TP901 family | | afdb-uniprot50 | AF-A0A2E0SQ31-F1-MODEL\_V4 | 1.0 | 3.03e-12 | 384 | 0.151 | 925 | 558 | 31 | 19 | 785 | 26 | 881 | Phage tail tape measure protein | Phage tail tape measure protein | | afdb-uniprot50 | AF-A0A845QYL5-F1-MODEL\_V4 | 1.0 | 1.362e-10 | 384 | 0.165 | 669 | 435 | 24 | 118 | 708 | 52 | 674 | Phage tail tape measure protein | Phage tail tape measure protein | | afdb-uniprot50 | AF-B9CLJ5-F1-MODEL\_V4 | 1.0 | 1.759e-12 | 384 | 0.161 | 844 | 541 | 24 | 1 | 702 | 1 | 819 | Phage tail tape measure protein, TP901 family | Phage tail tape measure protein, TP901 family | | afdb-uniprot50 | AF-A0A830K262-F1-MODEL\_V4 | 1.0 | 4.365e-10 | 383 | 0.239 | 422 | 283 | 8 | 222 | 617 | 1 | 410 | Phage tail tape measure protein | Phage tail tape measure protein | | afdb-uniprot50 | AF-A0A1V3IZV8-F1-MODEL\_V4 | 1.0 | 1.399e-09 | 383 | 0.153 | 614 | 421 | 16 | 19 | 555 | 49 | 640 | Phage tail tape measure protein | Phage tail tape measure protein | | afdb-uniprot50 | AF-A0A7Y9Z9G2-F1-MODEL\_V4 | 1.0 | 1.166e-10 | 383 | 0.162 | 783 | 484 | 27 | 37 | 784 | 2 | 647 | TP901 family phage tail tape measure protein | TP901 family phage tail tape measure protein | | afdb-uniprot50 | AF-A0A0F9T832-F1-MODEL\_V4 | 1.0 | 9.981e-11 | 383 | 0.162 | 659 | 465 | 19 | 64 | 702 | 2 | 593 | PhageMin\_Tail domain-containing protein | PhageMin\_Tail domain-containing protein | | afdb-uniprot50 | AF-R7IG02-F1-MODEL\_V4 | 1.0 | 2.136e-12 | 383 | 0.158 | 845 | 563 | 34 | 1 | 767 | 1 | 775 | Phage tail tape measure protein TP901 family core region | Phage tail tape measure protein TP901 family core region | | afdb-uniprot50 | AF-A0A0R2KRB1-F1-MODEL\_V4 | 1.0 | 3.275e-12 | 383 | 0.159 | 805 | 537 | 26 | 19 | 716 | 22 | 793 | PhageMin\_Tail domain-containing protein | PhageMin\_Tail domain-containing protein | | afdb-uniprot50 | AF-A0A3A6JVW7-F1-MODEL\_V4 | 1.0 | 2.961e-10 | 382 | 0.177 | 635 | 468 | 21 | 94 | 702 | 19 | 624 | Phage tail tape measure protein | Phage tail tape measure protein | | afdb-uniprot50 | AF-A0A5C5X117-F1-MODEL\_V4 | 1.0 | 6.095e-12 | 382 | 0.164 | 789 | 520 | 24 | 63 | 776 | 4 | 727 | Phage-related minor tail protein | Phage-related minor tail protein | | afdb-uniprot50 | AF-A0A1T4MQV6-F1-MODEL\_V4 | 1.0 | 8.645e-12 | 382 | 0.143 | 872 | 576 | 29 | 27 | 785 | 59 | 872 | Phage tail tape measure protein, TP901 family, core region | Phage tail tape measure protein, TP901 family, core region | | afdb-uniprot50 | AF-A0A6N7TNS1-F1-MODEL\_V4 | 1.0 | 7.907e-11 | 382 | 0.176 | 623 | 440 | 19 | 117 | 708 | 48 | 628 | Phage tail tape measure protein | Phage tail tape measure protein | | afdb-uniprot50 | AF-A0A0F9HCP7-F1-MODEL\_V4 | 1.0 | 9.981e-11 | 381 | 0.151 | 744 | 467 | 31 | 39 | 748 | 2 | 614 | PhageMin\_Tail domain-containing protein | PhageMin\_Tail domain-containing protein | | afdb-uniprot50 | AF-A0A0A0CMA7-F1-MODEL\_V4 | 1.0 | 1.179e-11 | 381 | 0.131 | 963 | 588 | 30 | 19 | 784 | 42 | 952 | PhageMin\_Tail domain-containing protein | PhageMin\_Tail domain-containing protein | | afdb-uniprot50 | AF-A0A235F9B5-F1-MODEL\_V4 | 1.0 | 1.134e-11 | 381 | 0.151 | 764 | 487 | 30 | 19 | 689 | 40 | 734 | Phage tail tape measure protein | Phage tail tape measure protein | | afdb-uniprot50 | AF-A0A1E8RGF4-F1-MODEL\_V4 | 1.0 | 9.452e-13 | 381 | 0.142 | 952 | 520 | 32 | 27 | 702 | 172 | 1102 | PhageMin\_Tail domain-containing protein | PhageMin\_Tail domain-containing protein | | afdb-uniprot50 | AF-A0A1U7PT90-F1-MODEL\_V4 | 1.0 | 4.468e-12 | 380 | 0.145 | 795 | 502 | 26 | 5 | 702 | 2 | 715 | Phage tail tape measure protein, TP901 family, core region | Phage tail tape measure protein, TP901 family, core region | | afdb-uniprot50 | AF-E7GJW0-F1-MODEL\_V4 | 1.0 | 3.825e-12 | 379 | 0.183 | 772 | 492 | 30 | 10 | 702 | 10 | 721 | TMP repeat-containing protein | TMP repeat-containing protein | | afdb-uniprot50 | AF-A0A1M4VAW5-F1-MODEL\_V4 | 1.0 | 3.976e-12 | 378 | 0.152 | 976 | 576 | 30 | 16 | 776 | 1 | 939 | Phage tail tape measure protein, TP901 family, core region | Phage tail tape measure protein, TP901 family, core region | | afdb-uniprot50 | AF-A1SE57-F1-MODEL\_V4 | 1.0 | 6.025e-11 | 378 | 0.168 | 651 | 443 | 24 | 118 | 724 | 41 | 636 | Phage tail tape measure protein, TP901 family | Phage tail tape measure protein, TP901 family | | afdb-uniprot50 | AF-A0A7W1XA84-F1-MODEL\_V4 | 1.0 | 2.666e-11 | 378 | 0.146 | 738 | 496 | 20 | 37 | 702 | 2 | 677 | Phage tail tape measure protein | Phage tail tape measure protein | | afdb-uniprot50 | AF-A0A1H0MFG2-F1-MODEL\_V4 | 1.0 | 1.325e-11 | 377 | 0.155 | 734 | 452 | 27 | 1 | 641 | 1 | 659 | Phage tail tape measure protein, TP901 family, core region | Phage tail tape measure protein, TP901 family, core region | | afdb-uniprot50 | AF-A5ZRU7-F1-MODEL\_V4 | 1.0 | 3.93e-11 | 377 | 0.16 | 754 | 510 | 29 | 27 | 702 | 78 | 786 | Phage tail tape measure protein, TP901 family | Phage tail tape measure protein, TP901 family | | afdb-uniprot50 | AF-Q2FWU3-F1-MODEL\_V4 | 1.0 | 7.119e-12 | 377 | 0.142 | 921 | 545 | 39 | 1 | 702 | 1 | 895 | Lysostaphin | Lysostaphin | | afdb-uniprot50 | AF-A0A7Y8S2G1-F1-MODEL\_V4 | 1.0 | 2.256e-10 | 376 | 0.155 | 636 | 421 | 20 | 1 | 551 | 1 | 605 | Phage tail tape measure protein | Phage tail tape measure protein | | afdb-uniprot50 | AF-A1VPH8-F1-MODEL\_V4 | 1.0 | 1.166e-10 | 376 | 0.155 | 655 | 442 | 24 | 76 | 702 | 20 | 591 | Phage tail tape measure protein, TP901 family | Phage tail tape measure protein, TP901 family | | afdb-uniprot50 | AF-A0A6I2HFM4-F1-MODEL\_V4 | 1.0 | 2.995e-11 | 376 | 0.16 | 735 | 488 | 22 | 50 | 702 | 16 | 703 | Phage tail tape measure protein | Phage tail tape measure protein | | afdb-uniprot50 | AF-A0A6N8HZ99-F1-MODEL\_V4 | 1.0 | 2.771e-11 | 376 | 0.152 | 754 | 527 | 22 | 13 | 702 | 42 | 747 | Phage-related minor tail protein | Phage-related minor tail protein | | afdb-uniprot50 | AF-A0A1Q3LGX9-F1-MODEL\_V4 | 1.0 | 4.644e-12 | 376 | 0.179 | 741 | 466 | 29 | 19 | 707 | 144 | 794 | Phage tail tape measure protein | Phage tail tape measure protein | | afdb-uniprot50 | AF-A0A0F9FLG5-F1-MODEL\_V4 | 1.0 | 6.049e-08 | 375 | 0.167 | 425 | 291 | 15 | 16 | 420 | 1 | 382 | PhageMin\_Tail domain-containing protein | PhageMin\_Tail domain-containing protein | | afdb-uniprot50 | AF-A0A087M271-F1-MODEL\_V4 | 1.0 | 6.025e-11 | 375 | 0.159 | 829 | 485 | 30 | 1 | 783 | 1 | 663 | PhageMin\_Tail domain-containing protein | PhageMin\_Tail domain-containing protein | | afdb-uniprot50 | AF-K5D6H4-F1-MODEL\_V4 | 1.0 | 6.848e-12 | 375 | 0.15 | 842 | 529 | 31 | 47 | 779 | 4 | 767 | Family phage tail tape measure protein | Family phage tail tape measure protein | | afdb-uniprot50 | AF-A0A806NJH9-F1-MODEL\_V4 | 1.0 | 1.325e-11 | 375 | 0.158 | 752 | 482 | 24 | 1 | 702 | 1 | 651 | Tail protein | Tail protein | | afdb-uniprot50 | AF-A0A354SEK7-F1-MODEL\_V4 | 1.0 | 6.587e-12 | 375 | 0.157 | 920 | 515 | 36 | 13 | 779 | 2 | 813 | Phage tail tape measure protein | Phage tail tape measure protein | | afdb-uniprot50 | AF-A0A7U5GYW1-F1-MODEL\_V4 | 1.0 | 8.645e-12 | 375 | 0.15 | 808 | 504 | 22 | 18 | 785 | 18 | 682 | Phage tail tape measure protein | Phage tail tape measure protein | | afdb-uniprot50 | AF-A0A5E8P1F1-F1-MODEL\_V4 | 1.0 | 1.931e-10 | 374 | 0.173 | 670 | 399 | 20 | 19 | 599 | 35 | 638 | Phage tail tape measure protein | Phage tail tape measure protein | | afdb-uniprot50 | AF-A0A6B4V7Z7-F1-MODEL\_V4 | 1.0 | 2.031e-11 | 374 | 0.141 | 856 | 539 | 32 | 1 | 774 | 1 | 742 | Phage tail tape measure protein | Phage tail tape measure protein | | afdb-uniprot50 | AF-A0A2I1RIZ6-F1-MODEL\_V4 | 1.0 | 5.279e-13 | 374 | 0.179 | 880 | 563 | 32 | 19 | 782 | 56 | 892 | Phage tail tape measure protein | Phage tail tape measure protein | | afdb-uniprot50 | AF-A0A3A9ETB4-F1-MODEL\_V4 | 1.0 | 5.795e-11 | 374 | 0.163 | 668 | 455 | 19 | 70 | 702 | 6 | 604 | Phage tail tape measure protein | Phage tail tape measure protein | | afdb-uniprot50 | AF-A0A4U9RAU9-F1-MODEL\_V4 | 1.0 | 3.694e-09 | 374 | 0.154 | 504 | 332 | 15 | 19 | 441 | 70 | 560 | Phage tail tape measure protein, family, core region | Phage tail tape measure protein, family, core region | | afdb-uniprot50 | AF-A0A6N9B9M4-F1-MODEL\_V4 | 1.0 | 6.511e-11 | 373 | 0.156 | 641 | 410 | 22 | 81 | 696 | 4 | 538 | Phage tail tape measure protein | Phage tail tape measure protein | | afdb-uniprot50 | AF-A0A5M5BW70-F1-MODEL\_V4 | 1.0 | 1.152e-09 | 373 | 0.112 | 659 | 405 | 16 | 110 | 750 | 211 | 707 | Phage tail tape measure protein | Phage tail tape measure protein | | afdb-uniprot50 | AF-A0A7J5WEE1-F1-MODEL\_V4 | 1.0 | 3.237e-11 | 373 | 0.158 | 870 | 512 | 27 | 1 | 777 | 1 | 742 | Family phage tail tape measure | Family phage tail tape measure | | afdb-uniprot50 | AF-A0A496UIZ6-F1-MODEL\_V4 | 1.0 | 3.781e-11 | 373 | 0.142 | 716 | 479 | 27 | 28 | 702 | 5 | 626 | Phage tail tape measure protein | Phage tail tape measure protein | | afdb-uniprot50 | AF-A0A2E2FJ28-F1-MODEL\_V4 | 1.0 | 5.64e-12 | 373 | 0.168 | 771 | 484 | 30 | 6 | 702 | 2 | 689 | Phage tail tape measure protein | Phage tail tape measure protein | | afdb-uniprot50 | AF-A0A5B9MJG5-F1-MODEL\_V4 | 1.0 | 8.987e-12 | 373 | 0.154 | 868 | 524 | 29 | 1 | 785 | 1 | 741 | Phage-related minor tail protein | Phage-related minor tail protein | | afdb-uniprot50 | AF-A0A535HRI6-F1-MODEL\_V4 | 1.0 | 6.691e-10 | 373 | 0.179 | 631 | 442 | 20 | 117 | 702 | 16 | 615 | Phage tail tape measure protein | Phage tail tape measure protein | | afdb-uniprot50 | AF-A0A848EQ20-F1-MODEL\_V4 | 1.0 | 6.511e-11 | 372 | 0.154 | 833 | 501 | 37 | 19 | 784 | 11 | 706 | Phage tail tape measure protein | Phage tail tape measure protein | | afdb-uniprot50 | AF-A0A661KKY6-F1-MODEL\_V4 | 1.0 | 1.226e-11 | 372 | 0.137 | 821 | 550 | 29 | 64 | 785 | 4 | 764 | Phage tail tape measure protein | Phage tail tape measure protein | | afdb-uniprot50 | AF-A0A7M2B111-F1-MODEL\_V4 | 1.0 | 1.275e-11 | 372 | 0.14 | 800 | 535 | 22 | 21 | 701 | 83 | 848 | Phage tail tape measure protein | Phage tail tape measure protein | | afdb-uniprot50 | AF-A0A0F9SUJ2-F1-MODEL\_V4 | 1.0 | 4.644e-12 | 372 | 0.167 | 778 | 486 | 25 | 32 | 702 | 15 | 737 | Uncharacterized protein | Uncharacterized protein | | afdb-uniprot50 | AF-A0A101W526-F1-MODEL\_V4 | 1.0 | 7.316e-11 | 371 | 0.147 | 732 | 500 | 24 | 80 | 772 | 18 | 664 | PhageMin\_Tail domain-containing protein | PhageMin\_Tail domain-containing protein | | afdb-uniprot50 | AF-A0A2R4MEE8-F1-MODEL\_V4 | 1.0 | 6.848e-12 | 371 | 0.152 | 750 | 484 | 30 | 49 | 783 | 20 | 632 | Tape measure protein | Tape measure protein | | afdb-uniprot50 | AF-A0A1N7MQX6-F1-MODEL\_V4 | 1.0 | 7.037e-11 | 371 | 0.146 | 717 | 466 | 31 | 60 | 702 | 2 | 646 | Phage tail tape measure protein, TP901 family, core region | Phage tail tape measure protein, TP901 family, core region | | afdb-uniprot50 | AF-A0A3N0IV62-F1-MODEL\_V4 | 1.0 | 4.134e-12 | 371 | 0.168 | 850 | 499 | 32 | 1 | 701 | 1 | 791 | Phage tail tape measure protein | Phage tail tape measure protein | | afdb-uniprot50 | AF-W9V4X2-F1-MODEL\_V4 | 1.0 | 1.449e-12 | 371 | 0.169 | 827 | 476 | 30 | 63 | 705 | 6 | 805 | PhageMin\_Tail domain-containing protein | PhageMin\_Tail domain-containing protein | | afdb-uniprot50 | AF-A0A3C1WR03-F1-MODEL\_V4 | 1.0 | 3.93e-11 | 371 | 0.157 | 700 | 482 | 22 | 50 | 700 | 7 | 647 | Phage tail tape measure protein | Phage tail tape measure protein | | afdb-uniprot50 | AF-A0A7G9W8A8-F1-MODEL\_V4 | 1.0 | 6.095e-12 | 371 | 0.15 | 757 | 494 | 30 | 1 | 667 | 3 | 700 | Phage tail tape measure protein | Phage tail tape measure protein | | afdb-uniprot50 | AF-K9EC41-F1-MODEL\_V4 | 1.0 | 1.038e-10 | 371 | 0.16 | 661 | 449 | 24 | 64 | 702 | 19 | 595 | Phage tail tape measure protein, TP901 family, core region | Phage tail tape measure protein, TP901 family, core region | | afdb-uniprot50 | AF-A0A553KRV1-F1-MODEL\_V4 | 1.0 | 1.179e-11 | 371 | 0.176 | 778 | 503 | 26 | 18 | 706 | 42 | 770 | Phage tail tape measure protein | Phage tail tape measure protein | | afdb-uniprot50 | AF-A0A078MEL8-F1-MODEL\_V4 | 1.0 | 1.166e-10 | 371 | 0.159 | 669 | 450 | 24 | 49 | 702 | 3 | 573 | Phage-related minor tail protein | Phage-related minor tail protein | | afdb-uniprot50 | AF-A0A1T4QWG9-F1-MODEL\_V4 | 1.0 | 8.545e-11 | 370 | 0.136 | 791 | 466 | 27 | 32 | 781 | 49 | 663 | Phage tail tape measure protein, TP901 family, core region | Phage tail tape measure protein, TP901 family, core region | | afdb-uniprot50 | AF-A0A7Y5QHS0-F1-MODEL\_V4 | 1.0 | 4.248e-11 | 370 | 0.171 | 763 | 522 | 29 | 74 | 773 | 14 | 728 | Phage tail tape measure protein | Phage tail tape measure protein | | afdb-uniprot50 | AF-A0A662HX82-F1-MODEL\_V4 | 1.0 | 2.995e-11 | 370 | 0.148 | 809 | 527 | 28 | 46 | 772 | 8 | 736 | Phage tail tape measure protein | Phage tail tape measure protein | | afdb-uniprot50 | AF-A0A350XS20-F1-MODEL\_V4 | 1.0 | 5.575e-11 | 370 | 0.148 | 786 | 504 | 22 | 1 | 711 | 1 | 695 | Phage tail tape measure protein | Phage tail tape measure protein | | afdb-uniprot50 | AF-A0A416ZSH5-F1-MODEL\_V4 | 1.0 | 4.962e-11 | 370 | 0.182 | 723 | 466 | 25 | 17 | 712 | 34 | 658 | Phage tail tape measure protein | Phage tail tape measure protein | | afdb-uniprot50 | AF-A0A2A6Z7M7-F1-MODEL\_V4 | 1.0 | 7.606e-11 | 370 | 0.151 | 694 | 409 | 26 | 97 | 755 | 90 | 638 | Phage tail tape measure protein | Phage tail tape measure protein | | afdb-uniprot50 | AF-A0A846T1D6-F1-MODEL\_V4 | 1.0 | 3.781e-11 | 369 | 0.204 | 582 | 324 | 19 | 214 | 743 | 2 | 496 | Phage tail tape measure protein | Phage tail tape measure protein | | afdb-uniprot50 | AF-H3SBJ7-F1-MODEL\_V4 | 1.0 | 1.378e-11 | 369 | 0.15 | 955 | 584 | 32 | 19 | 785 | 88 | 1002 | Tail tape measure protein TP901 core region protein | Tail tape measure protein TP901 core region protein | | afdb-uniprot50 | AF-A0A2V3W552-F1-MODEL\_V4 | 1.0 | 1.808e-11 | 369 | 0.147 | 781 | 501 | 25 | 14 | 685 | 1 | 725 | TP901 family phage tail tape measure protein | TP901 family phage tail tape measure protein | | afdb-uniprot50 | AF-R9LVJ9-F1-MODEL\_V4 | 1.0 | 3.637e-11 | 369 | 0.172 | 706 | 480 | 22 | 63 | 702 | 5 | 671 | Phage tail tape measure protein, TP901 family, core region | Phage tail tape measure protein, TP901 family, core region | | afdb-uniprot50 | AF-A0A4Q2B6C1-F1-MODEL\_V4 | 1.0 | 2.466e-11 | 369 | 0.158 | 755 | 505 | 27 | 36 | 702 | 10 | 721 | Phage tail tape measure protein | Phage tail tape measure protein | | afdb-uniprot50 | AF-A0A497MDG9-F1-MODEL\_V4 | 1.0 | 1.226e-11 | 368 | 0.152 | 831 | 511 | 29 | 28 | 774 | 12 | 732 | Phage tail tape measure protein | Phage tail tape measure protein | | afdb-uniprot50 | AF-A0A243J227-F1-MODEL\_V4 | 1.0 | 9.343e-12 | 368 | 0.122 | 971 | 573 | 34 | 1 | 765 | 1 | 897 | Phage tail tape measure protein | Phage tail tape measure protein | | afdb-uniprot50 | AF-A0A431JPH2-F1-MODEL\_V4 | 1.0 | 5.019e-12 | 368 | 0.198 | 895 | 521 | 33 | 9 | 780 | 1 | 821 | Phage tail tape measure protein | Phage tail tape measure protein | | afdb-uniprot50 | AF-A0A120A0Q4-F1-MODEL\_V4 | 1.0 | 1.787e-10 | 368 | 0.106 | 694 | 421 | 21 | 109 | 785 | 216 | 727 | Phage-related tail protein | Phage-related tail protein | | afdb-uniprot50 | AF-A0A7Z7J4C2-F1-MODEL\_V4 | 1.0 | 3.288e-09 | 367 | 0.324 | 348 | 166 | 6 | 19 | 302 | 39 | 381 | Putative enzyme | Putative enzyme | | afdb-uniprot50 | AF-A0A1H9SQJ9-F1-MODEL\_V4 | 1.0 | 1.325e-11 | 367 | 0.15 | 815 | 501 | 28 | 104 | 778 | 15 | 777 | Phage tail tape measure protein, TP901 family, core region | Phage tail tape measure protein, TP901 family, core region | | afdb-uniprot50 | AF-A0A2E2B3T2-F1-MODEL\_V4 | 1.0 | 2.031e-11 | 367 | 0.131 | 834 | 524 | 29 | 76 | 785 | 28 | 784 | Phage tail tape measure protein | Phage tail tape measure protein | | afdb-uniprot50 | AF-A0A2T4Z920-F1-MODEL\_V4 | 1.0 | 6.025e-11 | 367 | 0.152 | 729 | 518 | 21 | 44 | 702 | 8 | 706 | TP901 family phage tail tape measure protein | TP901 family phage tail tape measure protein | | afdb-uniprot50 | AF-A0A328KRU5-F1-MODEL\_V4 | 1.0 | 2.087e-10 | 367 | 0.167 | 667 | 461 | 22 | 61 | 702 | 13 | 609 | Phage tail tape measure protein | Phage tail tape measure protein | | afdb-uniprot50 | AF-E6LKB1-F1-MODEL\_V4 | 1.0 | 2.666e-11 | 367 | 0.137 | 692 | 472 | 23 | 90 | 748 | 29 | 628 | Phage tail tape measure protein, TP901 family | Phage tail tape measure protein, TP901 family | | afdb-uniprot50 | AF-A0A243K0L1-F1-MODEL\_V4 | 1.0 | 1.325e-11 | 367 | 0.108 | 1037 | 567 | 28 | 1 | 785 | 1 | 931 | Phage tail tape measure protein | Phage tail tape measure protein | | afdb-uniprot50 | AF-A0A3A9I4F3-F1-MODEL\_V4 | 1.0 | 2.594e-12 | 367 | 0.165 | 829 | 533 | 29 | 18 | 750 | 224 | 989 | Phage tail tape measure protein | Phage tail tape measure protein | | afdb-uniprot50 | AF-A0A2G8BY92-F1-MODEL\_V4 | 1.0 | 4.248e-11 | 366 | 0.153 | 900 | 539 | 26 | 22 | 785 | 54 | 866 | Phage tail tape measure protein | Phage tail tape measure protein | | afdb-uniprot50 | AF-A0A0F9QC99-F1-MODEL\_V4 | 1.0 | 2.466e-11 | 366 | 0.143 | 780 | 445 | 19 | 50 | 783 | 3 | 605 | PhageMin\_Tail domain-containing protein | PhageMin\_Tail domain-containing protein | | afdb-uniprot50 | AF-A0A1T1AVS0-F1-MODEL\_V4 | 1.0 | 4.086e-11 | 366 | 0.177 | 745 | 433 | 30 | 70 | 772 | 20 | 626 | Phage tail tape measure protein | Phage tail tape measure protein | | afdb-uniprot50 | AF-A0A1I2DTC2-F1-MODEL\_V4 | 1.0 | 1.566e-12 | 366 | 0.141 | 905 | 562 | 35 | 19 | 785 | 25 | 852 | Phage tail tape measure protein, TP901 family, core region | Phage tail tape measure protein, TP901 family, core region | | afdb-uniprot50 | AF-A0A4U1KS68-F1-MODEL\_V4 | 1.0 | 3.15e-12 | 366 | 0.15 | 951 | 557 | 32 | 1 | 785 | 1 | 866 | Phage tail tape measure protein | Phage tail tape measure protein | | afdb-uniprot50 | AF-A0A510Y3F9-F1-MODEL\_V4 | 1.0 | 8.884e-11 | 366 | 0.154 | 705 | 478 | 18 | 59 | 699 | 5 | 655 | PhageMin\_Tail domain-containing protein | PhageMin\_Tail domain-containing protein | | afdb-uniprot50 | AF-V1GPQ7-F1-MODEL\_V4 | 1.0 | 7.999e-12 | 365 | 0.153 | 873 | 521 | 29 | 19 | 781 | 54 | 818 | TP901 family phage tail tape measure protein | TP901 family phage tail tape measure protein | | afdb-uniprot50 | AF-A0A0F9UQC3-F1-MODEL\_V4 | 1.0 | 3.637e-11 | 365 | 0.136 | 799 | 529 | 26 | 46 | 780 | 4 | 705 | PhageMin\_Tail domain-containing protein | PhageMin\_Tail domain-containing protein | | afdb-uniprot50 | AF-A0A3D5P8F7-F1-MODEL\_V4 | 1.0 | 8.316e-12 | 365 | 0.15 | 976 | 580 | 33 | 1 | 776 | 1 | 926 | Phage tail tape measure protein | Phage tail tape measure protein | | afdb-uniprot50 | AF-A0A829ZJ99-F1-MODEL\_V4 | 1.0 | 2.031e-11 | 365 | 0.144 | 952 | 530 | 29 | 19 | 755 | 62 | 943 | Chromosome partition protein Smc | Chromosome partition protein Smc | | afdb-uniprot50 | AF-A0A0B2A639-F1-MODEL\_V4 | 1.0 | 6.336e-12 | 365 | 0.163 | 800 | 493 | 28 | 18 | 705 | 24 | 758 | PhageMin\_Tail domain-containing protein | PhageMin\_Tail domain-containing protein | | afdb-uniprot50 | AF-A0A380ED76-F1-MODEL\_V4 | 1.0 | 3.993e-09 | 365 | 0.15 | 591 | 350 | 19 | 19 | 492 | 127 | 682 | Phage protein | Phage protein | | afdb-uniprot50 | AF-A0A3A8WM39-F1-MODEL\_V4 | 1.0 | 5.425e-12 | 364 | 0.182 | 716 | 483 | 20 | 19 | 703 | 18 | 661 | Phage tail tape measure protein | Phage tail tape measure protein | | afdb-uniprot50 | AF-A0A2N6UJH0-F1-MODEL\_V4 | 1.0 | 1.325e-11 | 364 | 0.16 | 773 | 480 | 26 | 10 | 701 | 8 | 692 | Phage tail tape measure protein | Phage tail tape measure protein | | afdb-uniprot50 | AF-A0A1I4HAG3-F1-MODEL\_V4 | 1.0 | 2.466e-11 | 364 | 0.144 | 849 | 513 | 32 | 7 | 701 | 1 | 789 | Phage tail tape measure protein, TP901 family, core region | Phage tail tape measure protein, TP901 family, core region | | afdb-uniprot50 | AF-A0A6I1NYL3-F1-MODEL\_V4 | 1.0 | 1.134e-11 | 364 | 0.173 | 794 | 510 | 27 | 19 | 701 | 35 | 792 | Phage tail tape measure protein | Phage tail tape measure protein | | afdb-uniprot50 | AF-A0A0F9JQN3-F1-MODEL\_V4 | 1.0 | 4.538e-10 | 363 | 0.155 | 706 | 427 | 22 | 40 | 702 | 2 | 580 | PhageMin\_Tail domain-containing protein | PhageMin\_Tail domain-containing protein | | afdb-uniprot50 | AF-A0A2W6TNQ0-F1-MODEL\_V4 | 1.0 | 2.635e-10 | 363 | 0.143 | 803 | 500 | 24 | 14 | 785 | 1 | 646 | PhageMin\_Tail domain-containing protein | PhageMin\_Tail domain-containing protein | | afdb-uniprot50 | AF-A0A1C5VU61-F1-MODEL\_V4 | 1.0 | 1.858e-10 | 363 | 0.13 | 772 | 496 | 27 | 117 | 784 | 19 | 718 | Phage-related protein | Phage-related protein | | afdb-uniprot50 | AF-A0A859DR59-F1-MODEL\_V4 | 1.0 | 2.282e-11 | 363 | 0.149 | 878 | 516 | 28 | 19 | 702 | 81 | 921 | Phage tail tape measure protein | Phage tail tape measure protein | | afdb-uniprot50 | AF-A0A2B0C957-F1-MODEL\_V4 | 1.0 | 1.01e-11 | 363 | 0.172 | 796 | 473 | 29 | 6 | 702 | 15 | 723 | Phage tail tape measure protein | Phage tail tape measure protein | | afdb-uniprot50 | AF-A0A1I0H884-F1-MODEL\_V4 | 1.0 | 2.282e-11 | 363 | 0.148 | 857 | 486 | 31 | 13 | 702 | 2 | 781 | Phage tail tape measure protein, TP901 family, core region | Phage tail tape measure protein, TP901 family, core region | | afdb-uniprot50 | AF-A0A822P8U8-F1-MODEL\_V4 | 1.0 | 2.564e-11 | 363 | 0.142 | 1057 | 577 | 41 | 19 | 785 | 74 | 1090 | Phage tail tape measure protein | Phage tail tape measure protein | | afdb-uniprot50 | AF-B1V2P5-F1-MODEL\_V4 | 1.0 | 1.548e-11 | 363 | 0.134 | 1001 | 602 | 39 | 21 | 785 | 216 | 1187 | Putative phage tail tape measure protein, family, core region domain protein | Putative phage tail tape measure protein, family, core region domain protein | | afdb-uniprot50 | AF-A0A543NFS2-F1-MODEL\_V4 | 1.0 | 3.03e-12 | 363 | 0.146 | 948 | 533 | 33 | 50 | 785 | 2 | 884 | TP901 family phage tail tape measure protein | TP901 family phage tail tape measure protein | | afdb-uniprot50 | AF-A0A7Z1FTZ1-F1-MODEL\_V4 | 1.0 | 1.739e-11 | 362 | 0.118 | 989 | 582 | 30 | 61 | 783 | 8 | 972 | Phage tail tape measure protein | Phage tail tape measure protein | | afdb-uniprot50 | AF-A0A6N9P0N2-F1-MODEL\_V4 | 1.0 | 1.954e-11 | 362 | 0.158 | 774 | 464 | 31 | 19 | 700 | 44 | 721 | Phage tail tape measure protein | Phage tail tape measure protein | | afdb-uniprot50 | AF-D8F206-F1-MODEL\_V4 | 1.0 | 6.263e-11 | 361 | 0.157 | 744 | 457 | 29 | 35 | 718 | 19 | 652 | Phage tail tape measure protein, TP901 family | Phage tail tape measure protein, TP901 family | | afdb-uniprot50 | AF-A0A1Y0EH74-F1-MODEL\_V4 | 1.0 | 1.01e-11 | 361 | 0.133 | 810 | 520 | 29 | 6 | 735 | 1 | 708 | Phage-related minor tail protein | Phage-related minor tail protein | | afdb-uniprot50 | AF-A0A7V1ZUT4-F1-MODEL\_V4 | 1.0 | 3.781e-11 | 360 | 0.152 | 694 | 486 | 20 | 61 | 702 | 4 | 646 | Phage tail tape measure protein | Phage tail tape measure protein | | afdb-uniprot50 | AF-A0A7V6XHU3-F1-MODEL\_V4 | 1.0 | 1.489e-11 | 360 | 0.157 | 843 | 510 | 37 | 1 | 702 | 1 | 783 | Phage tail tape measure protein | Phage tail tape measure protein | | afdb-uniprot50 | AF-A0A0R2DDY7-F1-MODEL\_V4 | 1.0 | 8.22e-11 | 360 | 0.14 | 902 | 530 | 26 | 1 | 702 | 1 | 856 | PhageMin\_Tail domain-containing protein | PhageMin\_Tail domain-containing protein | | afdb-uniprot50 | AF-F6AX89-F1-MODEL\_V4 | 1.0 | 1.609e-11 | 359 | 0.141 | 790 | 500 | 28 | 19 | 702 | 33 | 749 | Phage tail tape measure protein, TP901 family | Phage tail tape measure protein, TP901 family | | afdb-uniprot50 | AF-A0A3D1WP41-F1-MODEL\_V4 | 1.0 | 4.086e-11 | 359 | 0.142 | 767 | 515 | 28 | 19 | 702 | 91 | 797 | Phage tail tape measure protein | Phage tail tape measure protein | | afdb-uniprot50 | AF-A0A497QGF2-F1-MODEL\_V4 | 1.0 | 8.884e-11 | 358 | 0.151 | 728 | 497 | 22 | 19 | 702 | 15 | 665 | Phage tail tape measure protein | Phage tail tape measure protein | | afdb-uniprot50 | AF-A0A496UZA7-F1-MODEL\_V4 | 1.0 | 9.981e-11 | 358 | 0.155 | 669 | 480 | 19 | 81 | 708 | 17 | 641 | Phage tail tape measure protein | Phage tail tape measure protein | | afdb-uniprot50 | AF-A0A354UB38-F1-MODEL\_V4 | 1.0 | 1.295e-09 | 358 | 0.145 | 619 | 435 | 14 | 115 | 701 | 109 | 665 | Phage tail tape measure protein | Phage tail tape measure protein | | afdb-uniprot50 | AF-A0A1G6LVA0-F1-MODEL\_V4 | 1.0 | 1.609e-11 | 358 | 0.17 | 810 | 546 | 31 | 49 | 785 | 6 | 762 | Phage tail tape measure protein, TP901 family, core region | Phage tail tape measure protein, TP901 family, core region | | afdb-uniprot50 | AF-A0A151BBD5-F1-MODEL\_V4 | 1.0 | 3.2e-10 | 357 | 0.157 | 747 | 451 | 29 | 19 | 718 | 10 | 624 | Phage-like protein | Phage-like protein | | afdb-uniprot50 | AF-A0A1I6REX1-F1-MODEL\_V4 | 1.0 | 2.535e-10 | 357 | 0.143 | 803 | 441 | 27 | 6 | 783 | 3 | 583 | Phage tail tape measure protein, TP901 family, core region | Phage tail tape measure protein, TP901 family, core region | | afdb-uniprot50 | AF-A0A6G7JLG1-F1-MODEL\_V4 | 1.0 | 4.086e-11 | 357 | 0.135 | 859 | 550 | 27 | 1 | 783 | 1 | 742 | Phage tail tape measure protein | Phage tail tape measure protein | | afdb-uniprot50 | AF-A0A1H5Z5X2-F1-MODEL\_V4 | 1.0 | 5.955e-10 | 357 | 0.145 | 666 | 446 | 24 | 49 | 702 | 7 | 561 | Phage tail length tape-measure protein | Phage tail length tape-measure protein | | afdb-uniprot50 | AF-A0A0F9TFD4-F1-MODEL\_V4 | 1.0 | 2.564e-11 | 356 | 0.14 | 781 | 501 | 24 | 1 | 702 | 1 | 689 | PhageMin\_Tail domain-containing protein | PhageMin\_Tail domain-containing protein | | afdb-uniprot50 | AF-A0A098AYW7-F1-MODEL\_V4 | 1.0 | 4.773e-11 | 355 | 0.138 | 785 | 520 | 24 | 18 | 785 | 18 | 662 | Phage-related minor tail protein | Phage-related minor tail protein | | afdb-uniprot50 | AF-A0A489F6U0-F1-MODEL\_V4 | 1.0 | 1.654e-10 | 355 | 0.157 | 703 | 444 | 23 | 67 | 662 | 3 | 663 | Phage protein, tail length tape measure protein | Phage protein, tail length tape measure protein | | afdb-uniprot50 | AF-A0A7U6KQJ0-F1-MODEL\_V4 | 1.0 | 2.575e-08 | 354 | 0.289 | 321 | 207 | 5 | 1 | 311 | 1 | 310 | PhageMin\_Tail domain-containing protein | PhageMin\_Tail domain-containing protein | | afdb-uniprot50 | AF-A0A354KNI6-F1-MODEL\_V4 | 1.0 | 1.673e-11 | 354 | 0.154 | 746 | 469 | 26 | 35 | 701 | 6 | 668 | Phage tail tape measure protein | Phage tail tape measure protein | | afdb-uniprot50 | AF-A0A847STA2-F1-MODEL\_V4 | 1.0 | 2.771e-11 | 354 | 0.146 | 791 | 494 | 28 | 1 | 703 | 1 | 697 | Phage tail tape measure protein | Phage tail tape measure protein | | afdb-uniprot50 | AF-A0A7T0D4M4-F1-MODEL\_V4 | 1.0 | 1.739e-11 | 354 | 0.161 | 797 | 534 | 23 | 19 | 701 | 76 | 851 | Phage tail tape measure protein | Phage tail tape measure protein | | afdb-uniprot50 | AF-E0MRI5-F1-MODEL\_V4 | 1.0 | 7.037e-11 | 353 | 0.161 | 807 | 438 | 29 | 36 | 785 | 11 | 635 | Phage tail tape measure protein, family, core region | Phage tail tape measure protein, family, core region | | afdb-uniprot50 | AF-A0A1X7HG53-F1-MODEL\_V4 | 1.0 | 2.17e-10 | 353 | 0.154 | 731 | 444 | 29 | 70 | 702 | 12 | 666 | Phage tail tape measure protein, TP901 family, core region | Phage tail tape measure protein, TP901 family, core region | | afdb-uniprot50 | AF-A0A6M3L4K5-F1-MODEL\_V4 | 1.0 | 9.981e-11 | 353 | 0.163 | 790 | 443 | 29 | 61 | 784 | 1 | 638 | Putative tail protein | Putative tail protein | | afdb-uniprot50 | AF-H0U9Y6-F1-MODEL\_V4 | 1.0 | 1.109e-09 | 352 | 0.144 | 814 | 477 | 26 | 16 | 772 | 1 | 651 | Phage tail tape measure protein, TP901 family, core region | Phage tail tape measure protein, TP901 family, core region | | afdb-uniprot50 | AF-A0A1M6L0R6-F1-MODEL\_V4 | 1.0 | 4.468e-12 | 352 | 0.162 | 737 | 505 | 26 | 80 | 772 | 25 | 692 | Phage tail tape measure protein, TP901 family, core region | Phage tail tape measure protein, TP901 family, core region | | afdb-uniprot50 | AF-A0A660UYP3-F1-MODEL\_V4 | 1.0 | 7.037e-11 | 352 | 0.139 | 755 | 470 | 28 | 109 | 716 | 63 | 784 | Phage tail tape measure protein | Phage tail tape measure protein | | afdb-uniprot50 | AF-R7JDX5-F1-MODEL\_V4 | 1.0 | 5.64e-12 | 352 | 0.155 | 851 | 545 | 27 | 29 | 776 | 3 | 782 | Phage tail tape measure protein TP901 family | Phage tail tape measure protein TP901 family | | afdb-uniprot50 | AF-A0A518GEF8-F1-MODEL\_V4 | 1.0 | 9.235e-11 | 352 | 0.155 | 879 | 530 | 29 | 70 | 785 | 8 | 836 | Phage-related minor tail protein | Phage-related minor tail protein | | afdb-uniprot50 | AF-A0A2I0FHY8-F1-MODEL\_V4 | 1.0 | 4.248e-11 | 352 | 0.151 | 919 | 545 | 36 | 61 | 785 | 4 | 881 | Phage tail tape measure protein | Phage tail tape measure protein | | afdb-uniprot50 | AF-U6SUL4-F1-MODEL\_V4 | 1.0 | 3.637e-11 | 352 | 0.138 | 836 | 526 | 25 | 3 | 702 | 1 | 777 | PhageMin\_Tail domain-containing protein | PhageMin\_Tail domain-containing protein | | afdb-uniprot50 | AF-A0A349YIH1-F1-MODEL\_V4 | 1.0 | 1.416e-10 | 352 | 0.122 | 1103 | 538 | 31 | 19 | 785 | 79 | 1087 | Phage tail tape measure protein | Phage tail tape measure protein | | afdb-uniprot50 | AF-A0A2E7ZRD4-F1-MODEL\_V4 | 1.0 | 1.152e-09 | 351 | 0.14 | 706 | 469 | 26 | 80 | 713 | 16 | 655 | Phage tail tape measure protein | Phage tail tape measure protein | | afdb-uniprot50 | AF-D0GNX1-F1-MODEL\_V4 | 1.0 | 4.416e-11 | 351 | 0.137 | 885 | 546 | 34 | 2 | 785 | 1 | 768 | Phage tail tape measure protein, TP901 family | Phage tail tape measure protein, TP901 family | | afdb-uniprot50 | AF-A0A150KS49-F1-MODEL\_V4 | 1.0 | 7.316e-11 | 351 | 0.151 | 767 | 501 | 23 | 18 | 759 | 18 | 659 | PhageMin\_Tail domain-containing protein | PhageMin\_Tail domain-containing protein | | afdb-uniprot50 | AF-A0A3T1DR99-F1-MODEL\_V4 | 1.0 | 9.024e-09 | 349 | 0.161 | 673 | 423 | 27 | 39 | 690 | 2 | 553 | PhageMin\_Tail domain-containing protein | PhageMin\_Tail domain-containing protein | | afdb-uniprot50 | AF-A0A512CS43-F1-MODEL\_V4 | 1.0 | 1.858e-10 | 349 | 0.157 | 750 | 499 | 24 | 1 | 708 | 1 | 659 | PhageMin\_Tail domain-containing protein | PhageMin\_Tail domain-containing protein | | afdb-uniprot50 | AF-F2JRA3-F1-MODEL\_V4 | 1.0 | 1.673e-11 | 349 | 0.145 | 781 | 545 | 27 | 1 | 719 | 1 | 720 | Phage tail tape measure protein, TP901 family | Phage tail tape measure protein, TP901 family | | afdb-uniprot50 | AF-A0A1B7LM07-F1-MODEL\_V4 | 1.0 | 1.166e-10 | 349 | 0.131 | 910 | 499 | 34 | 19 | 783 | 74 | 836 | PhageMin\_Tail domain-containing protein | PhageMin\_Tail domain-containing protein | | afdb-uniprot50 | AF-A0A2E1AEH8-F1-MODEL\_V4 | 1.0 | 3.781e-11 | 349 | 0.133 | 896 | 544 | 34 | 1 | 748 | 9 | 819 | Phage tail tape measure protein | Phage tail tape measure protein | | afdb-uniprot50 | AF-A0A847H9X0-F1-MODEL\_V4 | 1.0 | 3.237e-11 | 349 | 0.137 | 893 | 551 | 37 | 48 | 785 | 4 | 831 | Phage tail tape measure protein | Phage tail tape measure protein | | afdb-uniprot50 | AF-G9RPT7-F1-MODEL\_V4 | 1.0 | 5.158e-11 | 349 | 0.155 | 886 | 525 | 35 | 4 | 702 | 6 | 854 | Phage tail tape measure protein, TP901 family, core region | Phage tail tape measure protein, TP901 family, core region | | afdb-uniprot50 | AF-Q2FYC8-F1-MODEL\_V4 | 1.0 | 1.699e-09 | 349 | 0.142 | 631 | 368 | 19 | 19 | 519 | 127 | 714 | Lysostaphin | Lysostaphin | | afdb-uniprot50 | AF-A0A7H0RK43-F1-MODEL\_V4 | 1.0 | 1.472e-10 | 348 | 0.17 | 686 | 423 | 22 | 139 | 706 | 36 | 693 | Phage tail tape measure protein | Phage tail tape measure protein | | afdb-uniprot50 | AF-A0A2W4GQJ4-F1-MODEL\_V4 | 1.0 | 1.954e-11 | 348 | 0.157 | 823 | 533 | 26 | 19 | 785 | 34 | 751 | Phage tail tape measure protein | Phage tail tape measure protein | | afdb-uniprot50 | AF-A0A841RZ32-F1-MODEL\_V4 | 1.0 | 2.708e-09 | 348 | 0.161 | 594 | 385 | 27 | 117 | 702 | 43 | 531 | TP901 family phage tail tape measure protein | TP901 family phage tail tape measure protein | | afdb-uniprot50 | AF-A0A2W4GR67-F1-MODEL\_V4 | 1.0 | 1.739e-11 | 348 | 0.14 | 942 | 550 | 24 | 50 | 785 | 17 | 904 | Phage tail tape measure protein | Phage tail tape measure protein | | afdb-uniprot50 | AF-A0A0A8B9B9-F1-MODEL\_V4 | 1.0 | 4.416e-11 | 348 | 0.135 | 1033 | 582 | 33 | 14 | 785 | 13 | 994 | PhageMin\_Tail domain-containing protein | PhageMin\_Tail domain-containing protein | | afdb-uniprot50 | AF-A0A0J6WRI9-F1-MODEL\_V4 | 1.0 | 1.53e-10 | 347 | 0.133 | 841 | 535 | 34 | 2 | 782 | 1 | 707 | PhageMin\_Tail domain-containing protein | PhageMin\_Tail domain-containing protein | | afdb-uniprot50 | AF-E6SKI6-F1-MODEL\_V4 | 1.0 | 2.848e-10 | 347 | 0.176 | 718 | 473 | 23 | 36 | 708 | 2 | 645 | Phage tail tape measure protein, TP901 family | Phage tail tape measure protein, TP901 family | | afdb-uniprot50 | AF-A0A843HVN5-F1-MODEL\_V4 | 1.0 | 1.038e-10 | 347 | 0.164 | 841 | 492 | 27 | 19 | 702 | 119 | 905 | Phage tail tape measure protein | Phage tail tape measure protein | | afdb-uniprot50 | AF-A0A3A9G8N3-F1-MODEL\_V4 | 1.0 | 3.163e-09 | 346 | 0.142 | 686 | 475 | 19 | 28 | 685 | 121 | 720 | Phage tail tape measure protein | Phage tail tape measure protein | | afdb-uniprot50 | AF-A0A2M9KTE8-F1-MODEL\_V4 | 1.0 | 1.489e-11 | 346 | 0.163 | 950 | 543 | 36 | 1 | 785 | 1 | 863 | Phage tail tape measure protein | Phage tail tape measure protein | | afdb-uniprot50 | AF-A0A2E1Y1Q6-F1-MODEL\_V4 | 1.0 | 7.606e-11 | 346 | 0.159 | 783 | 498 | 26 | 35 | 706 | 5 | 737 | Phage tail tape measure protein | Phage tail tape measure protein | | afdb-uniprot50 | AF-A0A3R6GFF2-F1-MODEL\_V4 | 1.0 | 2.112e-11 | 345 | 0.158 | 871 | 519 | 34 | 1 | 785 | 1 | 743 | Phage tail tape measure protein | Phage tail tape measure protein | | afdb-uniprot50 | AF-A0A2I5TL78-F1-MODEL\_V4 | 1.0 | 1.31e-10 | 345 | 0.154 | 805 | 451 | 28 | 1 | 779 | 167 | 767 | Phage tail tape measure protein | Phage tail tape measure protein | | afdb-uniprot50 | AF-A0A069AZP6-F1-MODEL\_V4 | 1.0 | 5.099e-10 | 345 | 0.15 | 719 | 483 | 26 | 18 | 668 | 213 | 871 | Putative phage tail tape measure protein, family, core region domain protein | Putative phage tail tape measure protein, family, core region domain protein | | afdb-uniprot50 | AF-A0A840PRY4-F1-MODEL\_V4 | 1.0 | 7.316e-11 | 345 | 0.14 | 806 | 471 | 26 | 18 | 759 | 13 | 660 | TP901 family phage tail tape measure protein | TP901 family phage tail tape measure protein | | afdb-uniprot50 | AF-A0A848AWW3-F1-MODEL\_V4 | 1.0 | 1.416e-10 | 345 | 0.168 | 734 | 446 | 24 | 83 | 681 | 24 | 727 | Phage tail tape measure protein | Phage tail tape measure protein | | afdb-uniprot50 | AF-A0A4Y3TD25-F1-MODEL\_V4 | 1.0 | 2.535e-10 | 345 | 0.136 | 761 | 511 | 26 | 117 | 785 | 65 | 770 | PhageMin\_Tail domain-containing protein | PhageMin\_Tail domain-containing protein | | afdb-uniprot50 | AF-A0A419HC04-F1-MODEL\_V4 | 1.0 | 1.091e-11 | 345 | 0.151 | 966 | 532 | 32 | 36 | 785 | 10 | 903 | Phage tail tape measure protein | Phage tail tape measure protein | | afdb-uniprot50 | AF-A0A6N6XWZ4-F1-MODEL\_V4 | 1.0 | 6.769e-11 | 345 | 0.162 | 961 | 533 | 37 | 19 | 781 | 44 | 930 | Phage tail tape measure protein | Phage tail tape measure protein | | afdb-uniprot50 | AF-A0A368U1R4-F1-MODEL\_V4 | 1.0 | 2.848e-10 | 345 | 0.158 | 718 | 504 | 22 | 39 | 705 | 2 | 669 | Phage tail tape measure protein | Phage tail tape measure protein | | afdb-uniprot50 | AF-A0A3M2MF09-F1-MODEL\_V4 | 1.0 | 5.795e-11 | 345 | 0.154 | 887 | 554 | 33 | 15 | 785 | 28 | 834 | Phage tail tape measure protein | Phage tail tape measure protein | | afdb-uniprot50 | AF-A0A1W7LS78-F1-MODEL\_V4 | 1.0 | 7.316e-11 | 345 | 0.141 | 892 | 522 | 28 | 1 | 710 | 4 | 833 | PhageMin\_Tail domain-containing protein | PhageMin\_Tail domain-containing protein | | afdb-uniprot50 | AF-A0A7D4GDK8-F1-MODEL\_V4 | 1.0 | 1.31e-10 | 344 | 0.161 | 781 | 508 | 28 | 1 | 702 | 1 | 713 | Phage tail tape measure protein | Phage tail tape measure protein | | afdb-uniprot50 | AF-A0A496XFC7-F1-MODEL\_V4 | 1.0 | 3.595e-10 | 343 | 0.183 | 724 | 482 | 27 | 17 | 686 | 1 | 668 | Phage tail tape measure protein | Phage tail tape measure protein | | afdb-uniprot50 | AF-A0A1G0RDW7-F1-MODEL\_V4 | 1.0 | 4.591e-11 | 343 | 0.143 | 834 | 533 | 30 | 61 | 785 | 11 | 771 | Phage tail tape measure protein | Phage tail tape measure protein | | afdb-uniprot50 | AF-A0A7Y0ENB1-F1-MODEL\_V4 | 1.0 | 1.166e-10 | 343 | 0.151 | 781 | 462 | 23 | 19 | 662 | 79 | 795 | Phage tail tape measure protein | Phage tail tape measure protein | | afdb-uniprot50 | AF-A0A143ZV16-F1-MODEL\_V4 | 1.0 | 7.431e-09 | 343 | 0.17 | 667 | 453 | 19 | 97 | 701 | 29 | 656 | Phage-related minor tail protein | Phage-related minor tail protein | | afdb-uniprot50 | AF-D8J9U6-F1-MODEL\_V4 | 1.0 | 9.713e-12 | 343 | 0.155 | 861 | 476 | 28 | 1 | 708 | 8 | 769 | Prophage pi3 protein 14 | Prophage pi3 protein 14 | | afdb-uniprot50 | AF-A0A174ERH0-F1-MODEL\_V4 | 1.0 | 3.237e-11 | 343 | 0.157 | 864 | 520 | 30 | 21 | 701 | 64 | 902 | Phage tail tape measure protein, TP901 family | Phage tail tape measure protein, TP901 family | | afdb-uniprot50 | AF-A0A369RL83-F1-MODEL\_V4 | 1.0 | 7.726e-09 | 342 | 0.251 | 394 | 225 | 10 | 6 | 332 | 1 | 391 | Phage tail tape measure protein, TP901 family, core region | Phage tail tape measure protein, TP901 family, core region | | afdb-uniprot50 | AF-A0A352HSY4-F1-MODEL\_V4 | 1.0 | 2.345e-10 | 342 | 0.162 | 714 | 448 | 29 | 61 | 727 | 25 | 635 | Phage tail tape measure protein | Phage tail tape measure protein | | afdb-uniprot50 | AF-A0A1G5EF29-F1-MODEL\_V4 | 1.0 | 6.691e-10 | 342 | 0.157 | 724 | 485 | 22 | 62 | 702 | 4 | 685 | Phage tail tape measure protein, TP901 family, core region | Phage tail tape measure protein, TP901 family, core region | | afdb-uniprot50 | AF-A0A2K3J8S3-F1-MODEL\_V4 | 1.0 | 1.879e-11 | 342 | 0.151 | 902 | 559 | 32 | 11 | 785 | 10 | 831 | Phage tail tape measure protein | Phage tail tape measure protein | | afdb-uniprot50 | AF-A0A285Q2V0-F1-MODEL\_V4 | 1.0 | 5.362e-11 | 342 | 0.159 | 815 | 466 | 27 | 13 | 702 | 1 | 721 | Phage tail tape measure protein, TP901 family, core region | Phage tail tape measure protein, TP901 family, core region | | afdb-uniprot50 | AF-J4WBN7-F1-MODEL\_V4 | 1.0 | 7.907e-11 | 342 | 0.159 | 869 | 559 | 24 | 19 | 782 | 125 | 926 | Tail tape measure protein, TIGR01760 family | Tail tape measure protein, TIGR01760 family | | afdb-uniprot50 | AF-A0A859FEZ2-F1-MODEL\_V4 | 1.0 | 9.49e-10 | 342 | 0.168 | 688 | 466 | 24 | 80 | 702 | 19 | 665 | Phage tail tape measure protein | Phage tail tape measure protein | | afdb-uniprot50 | AF-A0A1E5JY92-F1-MODEL\_V4 | 1.0 | 4.718e-10 | 342 | 0.151 | 800 | 520 | 29 | 1 | 700 | 1 | 741 | Phage tail tape measure protein | Phage tail tape measure protein | | afdb-uniprot50 | AF-A0A174GWB8-F1-MODEL\_V4 | 1.0 | 1.362e-10 | 342 | 0.118 | 803 | 486 | 28 | 27 | 649 | 72 | 832 | TP901 family phage tail tape measure protein | TP901 family phage tail tape measure protein | | afdb-uniprot50 | AF-A0A1G2Z547-F1-MODEL\_V4 | 1.0 | 1.591e-10 | 341 | 0.153 | 802 | 521 | 33 | 91 | 785 | 38 | 788 | Phage tail tape measure protein | Phage tail tape measure protein | | afdb-uniprot50 | AF-A0A524AE58-F1-MODEL\_V4 | 1.0 | 6.691e-10 | 341 | 0.142 | 721 | 497 | 27 | 60 | 702 | 15 | 691 | Phage tail tape measure protein | Phage tail tape measure protein | | afdb-uniprot50 | AF-A0A194AHA9-F1-MODEL\_V4 | 1.0 | 1.591e-10 | 341 | 0.142 | 742 | 494 | 26 | 1 | 702 | 1 | 639 | PhageMin\_Tail domain-containing protein | PhageMin\_Tail domain-containing protein | | afdb-uniprot50 | AF-A0A6M3IS27-F1-MODEL\_V4 | 1.0 | 7.518e-10 | 339 | 0.169 | 761 | 462 | 24 | 61 | 722 | 1 | 690 | Putative tail protein | Putative tail protein | | afdb-uniprot50 | AF-A0A1V6CLC6-F1-MODEL\_V4 | 1.0 | 2.087e-10 | 339 | 0.135 | 892 | 550 | 34 | 1 | 785 | 1 | 777 | Phage-related minor tail protein | Phage-related minor tail protein | | afdb-uniprot50 | AF-D7K0E2-F1-MODEL\_V4 | 1.0 | 5.24e-09 | 339 | 0.102 | 694 | 422 | 20 | 117 | 776 | 4 | 530 | Putative bacteriophage tail fiber protein T (Tape measure) | Putative bacteriophage tail fiber protein T (Tape measure) | | afdb-uniprot50 | AF-A0A6N9NRD9-F1-MODEL\_V4 | 1.0 | 2.063e-09 | 339 | 0.154 | 694 | 447 | 19 | 120 | 779 | 4 | 591 | Phage tail tape measure protein | Phage tail tape measure protein | | afdb-uniprot50 | AF-A0A4Q5NKK3-F1-MODEL\_V4 | 1.0 | 1.315e-07 | 338 | 0.208 | 365 | 262 | 9 | 9 | 355 | 69 | 424 | Phage tail tape measure protein | Phage tail tape measure protein | | afdb-uniprot50 | AF-A0A517QH69-F1-MODEL\_V4 | 1.0 | 3.458e-10 | 338 | 0.141 | 807 | 499 | 23 | 1 | 782 | 3 | 640 | Phage-related minor tail protein | Phage-related minor tail protein | | afdb-uniprot50 | AF-A0A285PTB9-F1-MODEL\_V4 | 1.0 | 1.079e-10 | 338 | 0.149 | 785 | 496 | 26 | 105 | 772 | 41 | 770 | Phage tail tape measure protein | Phage tail tape measure protein | | afdb-uniprot50 | AF-G5FA09-F1-MODEL\_V4 | 1.0 | 4.773e-11 | 338 | 0.143 | 1021 | 593 | 37 | 1 | 784 | 1 | 976 | PhageMin\_Tail domain-containing protein | PhageMin\_Tail domain-containing protein | | afdb-uniprot50 | AF-A0A2C3G875-F1-MODEL\_V4 | 1.0 | 3.737e-10 | 338 | 0.138 | 860 | 526 | 24 | 1 | 702 | 1 | 803 | Phage tail tape measure protein | Phage tail tape measure protein | | afdb-uniprot50 | AF-A0A0Q6RQW9-F1-MODEL\_V4 | 1.0 | 2.318e-09 | 337 | 0.14 | 830 | 476 | 26 | 32 | 785 | 14 | 681 | PhageMin\_Tail domain-containing protein | PhageMin\_Tail domain-containing protein | | afdb-uniprot50 | AF-A0A7W3T073-F1-MODEL\_V4 | 1.0 | 7.518e-10 | 337 | 0.184 | 725 | 466 | 31 | 53 | 702 | 2 | 675 | Phage tail tape measure protein | Phage tail tape measure protein | | afdb-uniprot50 | AF-A0A2E7QIC9-F1-MODEL\_V4 | 1.0 | 7.606e-11 | 337 | 0.161 | 767 | 493 | 27 | 28 | 702 | 4 | 711 | Phage tail tape measure protein | Phage tail tape measure protein | | afdb-uniprot50 | AF-A0A735D9H4-F1-MODEL\_V4 | 1.0 | 1.489e-11 | 336 | 0.262 | 525 | 285 | 17 | 250 | 755 | 3 | 444 | Phage tail tape measure protein | Phage tail tape measure protein | | afdb-uniprot50 | AF-A0A1G4U6I3-F1-MODEL\_V4 | 1.0 | 2.926e-09 | 336 | 0.166 | 793 | 455 | 34 | 5 | 746 | 2 | 639 | Phage tail tape measure protein, TP901 family, core region | Phage tail tape measure protein, TP901 family, core region | | afdb-uniprot50 | AF-A0A060PHZ7-F1-MODEL\_V4 | 1.0 | 1.654e-10 | 336 | 0.151 | 791 | 476 | 27 | 19 | 700 | 21 | 724 | PhageMin\_Tail domain-containing protein | PhageMin\_Tail domain-containing protein | | afdb-uniprot50 | AF-A0A847KXS4-F1-MODEL\_V4 | 1.0 | 9.49e-10 | 335 | 0.136 | 820 | 508 | 26 | 3 | 750 | 1 | 692 | Phage tail tape measure protein | Phage tail tape measure protein | | afdb-uniprot50 | AF-A0A521F9Y7-F1-MODEL\_V4 | 1.0 | 1.931e-10 | 335 | 0.134 | 926 | 520 | 33 | 77 | 784 | 20 | 881 | Phage tail tape measure protein, TP901 family, core region | Phage tail tape measure protein, TP901 family, core region | | afdb-uniprot50 | AF-A0A7X5KST3-F1-MODEL\_V4 | 1.0 | 5.469e-06 | 335 | 0.144 | 375 | 276 | 9 | 87 | 437 | 21 | 374 | Phage tail tape measure protein | Phage tail tape measure protein | | afdb-uniprot50 | AF-A0A2E2MD95-F1-MODEL\_V4 | 1.0 | 1.265e-07 | 334 | 0.178 | 455 | 288 | 17 | 1 | 439 | 1 | 385 | Phage tail tape measure protein | Phage tail tape measure protein | | afdb-uniprot50 | AF-A0A6B4SLJ1-F1-MODEL\_V4 | 1.0 | 6.191e-10 | 334 | 0.157 | 735 | 415 | 31 | 19 | 620 | 22 | 684 | Phage tail tape measure protein | Phage tail tape measure protein | | afdb-uniprot50 | AF-A0A859FFP0-F1-MODEL\_V4 | 1.0 | 1.038e-10 | 334 | 0.178 | 837 | 510 | 34 | 62 | 776 | 8 | 788 | Phage tail tape measure protein | Phage tail tape measure protein | | afdb-uniprot50 | AF-A0A354P661-F1-MODEL\_V4 | 1.0 | 1.26e-10 | 334 | 0.172 | 834 | 459 | 29 | 1 | 702 | 1 | 734 | Phage tail tape measure protein | Phage tail tape measure protein | | afdb-uniprot50 | AF-A0A653R895-F1-MODEL\_V4 | 1.0 | 3.326e-10 | 334 | 0.154 | 766 | 462 | 24 | 5 | 702 | 2 | 649 | Putative Phage tail tape measure protein | Putative Phage tail tape measure protein | | afdb-uniprot50 | AF-A0A1C3H6T6-F1-MODEL\_V4 | 1.0 | 5.955e-10 | 334 | 0.148 | 708 | 496 | 26 | 27 | 702 | 13 | 645 | Phage tail length tape-measure protein | Phage tail length tape-measure protein | | afdb-uniprot50 | AF-R6CVJ2-F1-MODEL\_V4 | 1.0 | 2.635e-10 | 334 | 0.132 | 847 | 544 | 27 | 21 | 709 | 83 | 896 | Phage tail tape measure protein TP901 family core region | Phage tail tape measure protein TP901 family core region | | afdb-uniprot50 | AF-A0A662GNB5-F1-MODEL\_V4 | 1.0 | 1.472e-10 | 333 | 0.168 | 725 | 461 | 24 | 117 | 773 | 59 | 709 | Phage tail tape measure protein | Phage tail tape measure protein | | afdb-uniprot50 | AF-A0A3L8PL57-F1-MODEL\_V4 | 1.0 | 8.22e-11 | 333 | 0.155 | 783 | 512 | 27 | 91 | 760 | 20 | 765 | Phage tail tape measure protein | Phage tail tape measure protein | | afdb-uniprot50 | AF-A0A0F9GIC6-F1-MODEL\_V4 | 1.0 | 4.538e-10 | 333 | 0.164 | 758 | 461 | 23 | 26 | 702 | 8 | 673 | PhageMin\_Tail domain-containing protein | PhageMin\_Tail domain-containing protein | | afdb-uniprot50 | AF-R6MP60-F1-MODEL\_V4 | 1.0 | 1.121e-10 | 333 | 0.157 | 819 | 502 | 28 | 1 | 708 | 1 | 742 | Phage tail tape measure protein TP901 family | Phage tail tape measure protein TP901 family | | afdb-uniprot50 | AF-A0A7X9BRP2-F1-MODEL\_V4 | 1.0 | 1.121e-10 | 333 | 0.139 | 844 | 511 | 34 | 1 | 689 | 1 | 783 | Phage tail tape measure protein | Phage tail tape measure protein | | afdb-uniprot50 | AF-A0A173WSY1-F1-MODEL\_V4 | 1.0 | 9.601e-11 | 332 | 0.132 | 799 | 483 | 31 | 39 | 784 | 5 | 645 | Phage-related minor tail protein | Phage-related minor tail protein | | afdb-uniprot50 | AF-D5RM85-F1-MODEL\_V4 | 1.0 | 4.718e-10 | 331 | 0.174 | 717 | 474 | 31 | 61 | 702 | 3 | 676 | Phage tail tape measure protein, TP901 family | Phage tail tape measure protein, TP901 family | | afdb-uniprot50 | AF-A0A2I6S817-F1-MODEL\_V4 | 1.0 | 1.245e-09 | 330 | 0.147 | 645 | 453 | 31 | 75 | 677 | 6 | 595 | Phage tail tape measure protein | Phage tail tape measure protein | | afdb-uniprot50 | AF-A0A5J6LAV7-F1-MODEL\_V4 | 1.0 | 1.591e-10 | 330 | 0.138 | 797 | 487 | 28 | 117 | 784 | 45 | 770 | Phage tail tape measure protein | Phage tail tape measure protein | | afdb-uniprot50 | AF-A0A4R8I8J4-F1-MODEL\_V4 | 1.0 | 6.691e-10 | 329 | 0.136 | 825 | 518 | 29 | 70 | 779 | 3 | 747 | TP901 family phage tail tape measure protein | TP901 family phage tail tape measure protein | | afdb-uniprot50 | AF-A0A073KF69-F1-MODEL\_V4 | 1.0 | 1.152e-09 | 329 | 0.164 | 772 | 459 | 27 | 1 | 702 | 1 | 656 | PhageMin\_Tail domain-containing protein | PhageMin\_Tail domain-containing protein | | afdb-uniprot50 | AF-A0A661VDL1-F1-MODEL\_V4 | 1.0 | 2.41e-09 | 328 | 0.155 | 806 | 482 | 30 | 19 | 785 | 2 | 647 | Phage tail tape measure protein | Phage tail tape measure protein | | afdb-uniprot50 | AF-A0A3E3EFA4-F1-MODEL\_V4 | 1.0 | 8.22e-11 | 328 | 0.157 | 799 | 510 | 33 | 94 | 771 | 18 | 773 | Phage tail tape measure protein | Phage tail tape measure protein | | afdb-uniprot50 | AF-A0A3R6RKG2-F1-MODEL\_V4 | 1.0 | 4.962e-11 | 328 | 0.136 | 955 | 555 | 33 | 1 | 767 | 1 | 873 | Phage tail tape measure protein | Phage tail tape measure protein | | afdb-uniprot50 | AF-A0A497QP70-F1-MODEL\_V4 | 1.0 | 3.326e-10 | 328 | 0.154 | 840 | 536 | 34 | 13 | 779 | 1 | 738 | Phage tail tape measure protein | Phage tail tape measure protein | | afdb-uniprot50 | AF-A0A6B2YV45-F1-MODEL\_V4 | 1.0 | 3.93e-11 | 328 | 0.16 | 995 | 560 | 30 | 14 | 785 | 1 | 942 | Phage tail tape measure protein | Phage tail tape measure protein | | afdb-uniprot50 | AF-U2Q4W9-F1-MODEL\_V4 | 1.0 | 1.985e-09 | 328 | 0.151 | 674 | 472 | 21 | 80 | 702 | 19 | 643 | Phage tail tape measure protein, TP901 family | Phage tail tape measure protein, TP901 family | | afdb-uniprot50 | AF-A0A2S5M680-F1-MODEL\_V4 | 1.0 | 4.538e-10 | 327 | 0.131 | 850 | 468 | 32 | 19 | 785 | 25 | 686 | Phage tail tape measure protein | Phage tail tape measure protein | | afdb-uniprot50 | AF-A0A6N8H0F9-F1-MODEL\_V4 | 1.0 | 2.848e-10 | 327 | 0.147 | 730 | 478 | 19 | 117 | 770 | 65 | 725 | Phage tail tape measure protein | Phage tail tape measure protein | | afdb-uniprot50 | AF-A0A7C4CJ38-F1-MODEL\_V4 | 1.0 | 8.158e-07 | 326 | 0.137 | 401 | 281 | 15 | 64 | 437 | 10 | 372 | Phage tail tape measure protein | Phage tail tape measure protein | | afdb-uniprot50 | AF-M0BS65-F1-MODEL\_V4 | 1.0 | 7.518e-10 | 326 | 0.177 | 760 | 439 | 30 | 6 | 701 | 3 | 640 | Phage tail tape measure protein, TP901 family | Phage tail tape measure protein, TP901 family | | afdb-uniprot50 | AF-A0A2R3MZN4-F1-MODEL\_V4 | 1.0 | 3.458e-10 | 326 | 0.155 | 861 | 542 | 34 | 19 | 785 | 106 | 874 | Phage tail tape measure protein | Phage tail tape measure protein | | afdb-uniprot50 | AF-A0A495R7S4-F1-MODEL\_V4 | 1.0 | 1.26e-10 | 326 | 0.141 | 933 | 511 | 31 | 81 | 768 | 22 | 909 | TP901 family phage tail tape measure protein | TP901 family phage tail tape measure protein | | afdb-uniprot50 | AF-A0A0F3FS32-F1-MODEL\_V4 | 1.0 | 3.595e-10 | 326 | 0.162 | 743 | 504 | 25 | 19 | 701 | 77 | 760 | PhageMin\_Tail domain-containing protein | PhageMin\_Tail domain-containing protein | | afdb-uniprot50 | AF-A0A7W3T5L2-F1-MODEL\_V4 | 1.0 | 8.884e-11 | 326 | 0.183 | 734 | 460 | 31 | 4 | 701 | 66 | 695 | Phage tail tape measure protein | Phage tail tape measure protein | | afdb-uniprot50 | AF-A0A1I5WTC2-F1-MODEL\_V4 | 1.0 | 1.673e-11 | 325 | 0.143 | 835 | 515 | 34 | 77 | 785 | 18 | 777 | Phage tail tape measure protein, TP901 family, core region | Phage tail tape measure protein, TP901 family, core region | | afdb-uniprot50 | AF-A0A1C6GH83-F1-MODEL\_V4 | 1.0 | 2.438e-10 | 325 | 0.118 | 811 | 504 | 30 | 1 | 706 | 1 | 705 | Phage-related minor tail protein | Phage-related minor tail protein | | afdb-uniprot50 | AF-D3I9W8-F1-MODEL\_V4 | 1.0 | 2.438e-10 | 324 | 0.182 | 712 | 460 | 32 | 70 | 706 | 9 | 673 | Phage tail tape measure protein, TP901 family, core region | Phage tail tape measure protein, TP901 family, core region | | afdb-uniprot50 | AF-A0A221KDC2-F1-MODEL\_V4 | 1.0 | 5.51e-10 | 323 | 0.148 | 767 | 446 | 24 | 118 | 713 | 46 | 776 | Phage tail tape measure protein | Phage tail tape measure protein | | afdb-uniprot50 | AF-A0A402CRD8-F1-MODEL\_V4 | 1.0 | 2.961e-10 | 323 | 0.136 | 806 | 514 | 31 | 65 | 785 | 7 | 715 | PhageMin\_Tail domain-containing protein | PhageMin\_Tail domain-containing protein | | afdb-uniprot50 | AF-A0A6L8JRL2-F1-MODEL\_V4 | 1.0 | 1.121e-10 | 323 | 0.156 | 772 | 500 | 28 | 34 | 702 | 4 | 726 | Phage tail tape measure protein | Phage tail tape measure protein | | afdb-uniprot50 | AF-A0A1Q7RJA2-F1-MODEL\_V4 | 1.0 | 2.256e-10 | 322 | 0.155 | 861 | 499 | 34 | 97 | 781 | 16 | 823 | Phage tail tape measure protein | Phage tail tape measure protein | | afdb-uniprot50 | AF-A0A3N2KV37-F1-MODEL\_V4 | 1.0 | 6.511e-11 | 321 | 0.139 | 938 | 549 | 34 | 1 | 785 | 1 | 832 | Phage tail tape measure protein | Phage tail tape measure protein | | afdb-uniprot50 | AF-A0A5M8P9Z2-F1-MODEL\_V4 | 1.0 | 2.23e-09 | 320 | 0.148 | 653 | 438 | 25 | 114 | 708 | 42 | 634 | Phage tail tape measure protein | Phage tail tape measure protein | | afdb-uniprot50 | AF-U5BPA9-F1-MODEL\_V4 | 1.0 | 3.84e-09 | 320 | 0.15 | 705 | 458 | 26 | 118 | 781 | 56 | 660 | Uncharacterized protein | Uncharacterized protein | | afdb-uniprot50 | AF-A0A7U3G9U5-F1-MODEL\_V4 | 1.0 | 1.699e-09 | 320 | 0.138 | 709 | 421 | 29 | 118 | 699 | 44 | 689 | Phage tail tape measure protein | Phage tail tape measure protein | | afdb-uniprot50 | AF-A0A6H1ZVM3-F1-MODEL\_V4 | 1.0 | 1.931e-10 | 320 | 0.141 | 926 | 532 | 33 | 62 | 785 | 5 | 869 | Putative tail protein | Putative tail protein | | afdb-uniprot50 | AF-D8JAW3-F1-MODEL\_V4 | 1.0 | 5.51e-10 | 319 | 0.167 | 662 | 427 | 24 | 132 | 702 | 2 | 630 | Phage tail tape measure protein, TP901 family | Phage tail tape measure protein, TP901 family | | afdb-uniprot50 | AF-A0A6B1XSV2-F1-MODEL\_V4 | 1.0 | 9.866e-10 | 318 | 0.133 | 841 | 539 | 32 | 12 | 772 | 32 | 762 | Phage tail tape measure protein | Phage tail tape measure protein | | afdb-uniprot50 | AF-V7HXZ1-F1-MODEL\_V4 | 1.0 | 1.512e-09 | 318 | 0.148 | 672 | 462 | 20 | 81 | 702 | 3 | 613 | PhageMin\_Tail domain-containing protein | PhageMin\_Tail domain-containing protein | | afdb-uniprot50 | AF-A0A7C6P3T7-F1-MODEL\_V4 | 1.0 | 5.729e-10 | 317 | 0.148 | 803 | 499 | 34 | 110 | 779 | 50 | 800 | Phage tail tape measure protein | Phage tail tape measure protein | | afdb-uniprot50 | AF-K9DZ49-F1-MODEL\_V4 | 1.0 | 3.418e-09 | 317 | 0.142 | 675 | 431 | 27 | 90 | 706 | 30 | 614 | Phage tail tape measure protein, TP901 family, core region | Phage tail tape measure protein, TP901 family, core region | | afdb-uniprot50 | AF-A0A2P9EK54-F1-MODEL\_V4 | 1.0 | 5.179e-08 | 316 | 0.158 | 619 | 404 | 17 | 19 | 613 | 142 | 667 | Putative phage minor tail protein | Putative phage minor tail protein | | afdb-uniprot50 | AF-A0A7C2SUJ3-F1-MODEL\_V4 | 1.0 | 2.505e-09 | 316 | 0.137 | 828 | 503 | 24 | 53 | 785 | 6 | 716 | Phage tail tape measure protein | Phage tail tape measure protein | | afdb-uniprot50 | AF-A0A497ITP3-F1-MODEL\_V4 | 1.0 | 3.885e-10 | 316 | 0.138 | 854 | 468 | 29 | 4 | 677 | 171 | 936 | Phage tail tape measure protein | Phage tail tape measure protein | | afdb-uniprot50 | AF-A0A1Q6KUJ6-F1-MODEL\_V4 | 1.0 | 1.198e-09 | 316 | 0.158 | 727 | 447 | 26 | 86 | 719 | 25 | 679 | Phage tail tape measure protein | Phage tail tape measure protein | | afdb-uniprot50 | AF-A0A5R1ZFV2-F1-MODEL\_V4 | 1.0 | 4.848e-09 | 315 | 0.145 | 717 | 437 | 22 | 18 | 677 | 141 | 738 | Phage tail tape measure protein | Phage tail tape measure protein | | afdb-uniprot50 | AF-A0A351TP38-F1-MODEL\_V4 | 1.0 | 5.51e-10 | 315 | 0.153 | 774 | 512 | 29 | 19 | 701 | 92 | 812 | Phage tail tape measure protein | Phage tail tape measure protein | | afdb-uniprot50 | AF-A0A3D9L500-F1-MODEL\_V4 | 1.0 | 1.121e-10 | 315 | 0.141 | 942 | 566 | 33 | 1 | 759 | 1 | 882 | TP901 family phage tail tape measure protein | TP901 family phage tail tape measure protein | | afdb-uniprot50 | AF-A0A0F9U446-F1-MODEL\_V4 | 1.0 | 9.129e-10 | 313 | 0.163 | 782 | 448 | 33 | 6 | 709 | 1 | 653 | PhageMin\_Tail domain-containing protein | PhageMin\_Tail domain-containing protein | | afdb-uniprot50 | AF-A0A6A7URX7-F1-MODEL\_V4 | 1.0 | 4.905e-10 | 313 | 0.131 | 889 | 532 | 34 | 43 | 783 | 27 | 822 | Phage tail tape measure protein | Phage tail tape measure protein | | afdb-uniprot50 | AF-R6FSK8-F1-MODEL\_V4 | 1.0 | 7.816e-10 | 313 | 0.133 | 905 | 502 | 32 | 21 | 701 | 67 | 912 | Phage tail tape measure protein TP901 family core region | Phage tail tape measure protein TP901 family core region | | afdb-uniprot50 | AF-A0A2G6ZIE4-F1-MODEL\_V4 | 1.0 | 4.365e-10 | 312 | 0.175 | 780 | 442 | 31 | 70 | 702 | 4 | 729 | TP901 family phage tail tape measure protein | TP901 family phage tail tape measure protein | | afdb-uniprot50 | AF-A0A255T722-F1-MODEL\_V4 | 1.0 | 7.816e-10 | 312 | 0.154 | 697 | 456 | 29 | 46 | 707 | 27 | 624 | Phage tail tape measure protein | Phage tail tape measure protein | | afdb-uniprot50 | AF-A0A0Q8PQD2-F1-MODEL\_V4 | 1.0 | 1.512e-09 | 311 | 0.148 | 807 | 486 | 35 | 80 | 712 | 14 | 792 | PhageMin\_Tail domain-containing protein | PhageMin\_Tail domain-containing protein | | afdb-uniprot50 | AF-A0A6I4YMZ5-F1-MODEL\_V4 | 1.0 | 2.708e-09 | 310 | 0.151 | 821 | 506 | 26 | 14 | 752 | 81 | 792 | Phage tail tape measure protein | Phage tail tape measure protein | | afdb-uniprot50 | AF-J8W777-F1-MODEL\_V4 | 1.0 | 4.905e-10 | 310 | 0.136 | 914 | 564 | 32 | 16 | 778 | 20 | 858 | Tail tape measure protein, TIGR01760 family | Tail tape measure protein, TIGR01760 family | | afdb-uniprot50 | AF-A0A1W2GQA3-F1-MODEL\_V4 | 1.0 | 1.699e-09 | 309 | 0.151 | 752 | 495 | 26 | 45 | 702 | 18 | 720 | Phage tail tape measure protein, TP901 family, core region | Phage tail tape measure protein, TP901 family, core region | | afdb-uniprot50 | AF-A0A1I2G2M1-F1-MODEL\_V4 | 1.0 | 7.148e-09 | 309 | 0.168 | 694 | 437 | 20 | 87 | 702 | 19 | 650 | Phage tail tape measure protein, TP901 family, core region | Phage tail tape measure protein, TP901 family, core region | | afdb-uniprot50 | AF-A0A3C0QQX1-F1-MODEL\_V4 | 1.0 | 1.152e-09 | 308 | 0.164 | 742 | 471 | 30 | 80 | 702 | 15 | 726 | Phage tail tape measure protein | Phage tail tape measure protein | | afdb-uniprot50 | AF-A0A6B4MZE8-F1-MODEL\_V4 | 1.0 | 4.039e-10 | 308 | 0.156 | 822 | 513 | 30 | 21 | 702 | 73 | 853 | Phage tail tape measure protein | Phage tail tape measure protein | | afdb-uniprot50 | AF-A0A1S6GAN8-F1-MODEL\_V4 | 1.0 | 2.41e-09 | 308 | 0.148 | 793 | 480 | 31 | 51 | 702 | 2 | 739 | Phage tail tape measure protein | Phage tail tape measure protein | | afdb-uniprot50 | AF-A0A1I4PAD3-F1-MODEL\_V4 | 1.0 | 1.512e-09 | 308 | 0.136 | 745 | 509 | 30 | 19 | 728 | 67 | 711 | Phage tail tape measure protein, TP901 family, core region | Phage tail tape measure protein, TP901 family, core region | | afdb-uniprot50 | AF-A0A1D7XZW9-F1-MODEL\_V4 | 1.0 | 7.148e-09 | 308 | 0.169 | 666 | 430 | 24 | 117 | 714 | 60 | 670 | Phage tail length tape-measure protein | Phage tail length tape-measure protein | | afdb-uniprot50 | AF-A0A410MCA2-F1-MODEL\_V4 | 1.0 | 5.955e-10 | 307 | 0.138 | 801 | 481 | 32 | 2 | 748 | 161 | 806 | Phage tail tape measure protein | Phage tail tape measure protein | | afdb-uniprot50 | AF-A0A774SDM2-F1-MODEL\_V4 | 1.0 | 2.328e-06 | 306 | 0.154 | 448 | 340 | 12 | 19 | 443 | 142 | 573 | Phage tail tape measure protein | Phage tail tape measure protein | | afdb-uniprot50 | AF-A0A4U9R711-F1-MODEL\_V4 | 1.0 | 2.345e-10 | 306 | 0.126 | 886 | 509 | 31 | 81 | 782 | 16 | 820 | Phage protein | Phage protein | | afdb-uniprot50 | AF-F7SRZ5-F1-MODEL\_V4 | 1.0 | 8.125e-10 | 306 | 0.16 | 850 | 481 | 30 | 46 | 702 | 6 | 815 | Phage tail tape measure protein, TP901 family, core region | Phage tail tape measure protein, TP901 family, core region | | afdb-uniprot50 | AF-B2TQU2-F1-MODEL\_V4 | 1.0 | 1.673e-11 | 306 | 0.152 | 850 | 509 | 35 | 9 | 701 | 7 | 801 | Tail tape measure protein | Tail tape measure protein | | afdb-uniprot50 | AF-F9MRG1-F1-MODEL\_V4 | 1.0 | 1.054e-08 | 305 | 0.162 | 790 | 461 | 33 | 32 | 782 | 27 | 654 | Phage tail tape measure protein, TP901 family | Phage tail tape measure protein, TP901 family | | afdb-uniprot50 | AF-A0A1N7LR98-F1-MODEL\_V4 | 1.0 | 3.885e-10 | 305 | 0.201 | 799 | 483 | 32 | 46 | 785 | 196 | 898 | Phage tail tape measure protein, TP901 family, core region | Phage tail tape measure protein, TP901 family, core region | | afdb-uniprot50 | AF-A0A7J2RGM5-F1-MODEL\_V4 | 1.0 | 1.331e-08 | 305 | 0.138 | 709 | 479 | 22 | 14 | 706 | 1 | 593 | Phage tail tape measure protein | Phage tail tape measure protein | | afdb-uniprot50 | AF-A0A414RNY6-F1-MODEL\_V4 | 1.0 | 5.729e-10 | 305 | 0.143 | 768 | 480 | 30 | 63 | 785 | 8 | 642 | Phage tail tape measure protein | Phage tail tape measure protein | | afdb-uniprot50 | AF-A0A6M1QSR6-F1-MODEL\_V4 | 1.0 | 2.23e-09 | 304 | 0.156 | 967 | 542 | 38 | 52 | 785 | 9 | 934 | Phage tail tape measure protein | Phage tail tape measure protein | | afdb-uniprot50 | AF-B9DJ04-F1-MODEL\_V4 | 1.0 | 1.66e-07 | 302 | 0.151 | 587 | 356 | 15 | 19 | 483 | 127 | 693 | Truncated phiSLT orf2067-like protein (Fragment 1) | Truncated phiSLT orf2067-like protein (Fragment 1) | | afdb-uniprot50 | AF-A0A6M3KH64-F1-MODEL\_V4 | 1.0 | 1.985e-09 | 302 | 0.139 | 730 | 512 | 28 | 14 | 702 | 14 | 667 | Putative tail protein | Putative tail protein | | afdb-uniprot50 | AF-A0A1W6WWI4-F1-MODEL\_V4 | 1.0 | 1.367e-07 | 302 | 0.18 | 621 | 424 | 18 | 118 | 702 | 17 | 588 | Phage tail tape measure protein | Phage tail tape measure protein | | afdb-uniprot50 | AF-A0A2H5YGJ4-F1-MODEL\_V4 | 1.0 | 2.41e-09 | 301 | 0.153 | 880 | 500 | 38 | 36 | 785 | 2 | 766 | PhageMin\_Tail domain-containing protein | PhageMin\_Tail domain-containing protein | | afdb-uniprot50 | AF-A0A6M3M5N5-F1-MODEL\_V4 | 1.0 | 1.634e-09 | 301 | 0.145 | 741 | 491 | 26 | 87 | 755 | 27 | 696 | Putative tail protein | Putative tail protein | | afdb-uniprot50 | AF-U5RUF6-F1-MODEL\_V4 | 1.0 | 1.572e-09 | 301 | 0.143 | 869 | 517 | 27 | 13 | 702 | 62 | 881 | Phage tail tape measure protein, TP901 family | Phage tail tape measure protein, TP901 family | | afdb-uniprot50 | AF-A0A2D6FNM6-F1-MODEL\_V4 | 1.0 | 9.752e-09 | 300 | 0.146 | 723 | 476 | 22 | 34 | 702 | 10 | 645 | Phage tail tape measure protein | Phage tail tape measure protein | | afdb-uniprot50 | AF-A0A4R4D151-F1-MODEL\_V4 | 1.0 | 3.042e-09 | 299 | 0.148 | 776 | 471 | 30 | 98 | 785 | 31 | 704 | Phage tail tape measure protein | Phage tail tape measure protein | | afdb-uniprot50 | AF-A0A6M3J350-F1-MODEL\_V4 | 1.0 | 5.955e-10 | 299 | 0.158 | 771 | 503 | 30 | 79 | 767 | 22 | 728 | Putative tail protein | Putative tail protein | | afdb-uniprot50 | AF-A0A518I8T5-F1-MODEL\_V4 | 1.0 | 9.381e-09 | 299 | 0.168 | 716 | 490 | 25 | 62 | 707 | 5 | 684 | Phage-related minor tail protein | Phage-related minor tail protein | | afdb-uniprot50 | AF-A0A497SD96-F1-MODEL\_V4 | 1.0 | 1.699e-09 | 298 | 0.144 | 876 | 533 | 33 | 1 | 772 | 4 | 766 | Phage tail tape measure protein | Phage tail tape measure protein | | afdb-uniprot50 | AF-A0A7U9SJK2-F1-MODEL\_V4 | 1.0 | 1.909e-09 | 298 | 0.16 | 790 | 489 | 30 | 117 | 785 | 40 | 775 | PhageMin\_Tail domain-containing protein | PhageMin\_Tail domain-containing protein | | afdb-uniprot50 | AF-J9GWP7-F1-MODEL\_V4 | 1.0 | 3.126e-08 | 297 | 0.166 | 572 | 372 | 19 | 151 | 677 | 3 | 514 | Protein containing Phage tail tape measure protein TP901, core region domain protein | Protein containing Phage tail tape measure protein TP901, core region domain protein | | afdb-uniprot50 | AF-A0A822VLY9-F1-MODEL\_V4 | 1.0 | 2.17e-10 | 297 | 0.139 | 875 | 547 | 32 | 21 | 785 | 69 | 847 | Prophage LambdaSa04, TP901 family tail tape measure protein | Prophage LambdaSa04, TP901 family tail tape measure protein | | afdb-uniprot50 | AF-A0A1H3EM43-F1-MODEL\_V4 | 1.0 | 1.495e-08 | 296 | 0.152 | 749 | 497 | 32 | 35 | 744 | 11 | 660 | Phage tail tape measure protein, TP901 family, core region | Phage tail tape measure protein, TP901 family, core region | | afdb-uniprot50 | AF-A0A3C1GIL4-F1-MODEL\_V4 | 1.0 | 7.148e-09 | 296 | 0.165 | 805 | 483 | 32 | 13 | 785 | 1 | 648 | Phage tail tape measure protein | Phage tail tape measure protein | | afdb-uniprot50 | AF-A0A658JZU8-F1-MODEL\_V4 | 1.0 | 2.23e-09 | 295 | 0.154 | 855 | 521 | 31 | 19 | 712 | 49 | 862 | Phage tail tape measure protein | Phage tail tape measure protein | | afdb-uniprot50 | AF-A0A3E5B8P9-F1-MODEL\_V4 | 1.0 | 2.204e-08 | 294 | 0.126 | 729 | 460 | 25 | 32 | 719 | 27 | 619 | Phage tail tape measure protein | Phage tail tape measure protein | | afdb-uniprot50 | AF-A0A098YPR7-F1-MODEL\_V4 | 1.0 | 6.614e-09 | 292 | 0.164 | 693 | 408 | 28 | 41 | 713 | 29 | 570 | PhageMin\_Tail domain-containing protein | PhageMin\_Tail domain-containing protein | | afdb-uniprot50 | AF-A0A0F9U574-F1-MODEL\_V4 | 1.0 | 2.145e-09 | 291 | 0.15 | 753 | 496 | 31 | 53 | 706 | 7 | 714 | PhageMin\_Tail domain-containing protein | PhageMin\_Tail domain-containing protein | | afdb-uniprot50 | AF-M1ZYX2-F1-MODEL\_V4 | 1.0 | 3.694e-09 | 291 | 0.13 | 791 | 480 | 23 | 19 | 749 | 127 | 769 | Phage protein | Phage protein | | afdb-uniprot50 | AF-A0A094IDZ6-F1-MODEL\_V4 | 1.0 | 1.026e-09 | 291 | 0.129 | 779 | 527 | 30 | 19 | 702 | 5 | 726 | Phage-related minor tail protein | Phage-related minor tail protein | | afdb-uniprot50 | AF-A0A3D5S7X8-F1-MODEL\_V4 | 1.0 | 1.198e-09 | 291 | 0.15 | 817 | 545 | 31 | 14 | 716 | 38 | 819 | Phage tail tape measure protein | Phage tail tape measure protein | | afdb-uniprot50 | AF-N0BGY2-F1-MODEL\_V4 | 1.0 | 1.766e-09 | 291 | 0.131 | 951 | 559 | 29 | 74 | 785 | 16 | 938 | Phage tail tape measure protein, TP901 family, core region | Phage tail tape measure protein, TP901 family, core region | | afdb-uniprot50 | AF-G4WKD4-F1-MODEL\_V4 | 1.0 | 5.04e-09 | 291 | 0.14 | 792 | 504 | 31 | 49 | 706 | 7 | 755 | Putative tail tape measure protein | Putative tail tape measure protein | | afdb-uniprot50 | AF-A0A1N6R4M9-F1-MODEL\_V4 | 1.0 | 6.614e-09 | 290 | 0.155 | 747 | 498 | 23 | 64 | 747 | 2 | 678 | Phage tail tape measure protein, TP901 family, core region | Phage tail tape measure protein, TP901 family, core region | | afdb-uniprot50 | AF-A0A3N5RQ82-F1-MODEL\_V4 | 1.0 | 4.103e-08 | 290 | 0.147 | 714 | 480 | 24 | 16 | 701 | 1 | 613 | Phage tail tape measure protein | Phage tail tape measure protein | | afdb-uniprot50 | AF-A0A6M3K6S3-F1-MODEL\_V4 | 1.0 | 7.431e-09 | 290 | 0.13 | 868 | 567 | 24 | 46 | 784 | 140 | 948 | Putative tail protein | Putative tail protein | | afdb-uniprot50 | AF-A0A1C5SWG1-F1-MODEL\_V4 | 1.0 | 1.438e-08 | 290 | 0.154 | 685 | 445 | 29 | 81 | 702 | 6 | 618 | Phage tail tape measure protein, TP901 family, core region | Phage tail tape measure protein, TP901 family, core region | | afdb-uniprot50 | AF-A0A7C5LDN6-F1-MODEL\_V4 | 1.0 | 3.163e-09 | 289 | 0.14 | 900 | 510 | 33 | 1 | 772 | 1 | 764 | Phage tail tape measure protein | Phage tail tape measure protein | | afdb-uniprot50 | AF-A0A221KA30-F1-MODEL\_V4 | 1.0 | 1.634e-09 | 289 | 0.139 | 887 | 504 | 28 | 97 | 755 | 8 | 862 | Phage tail tape measure protein | Phage tail tape measure protein | | afdb-uniprot50 | AF-A0A2E9DMI6-F1-MODEL\_V4 | 1.0 | 1.383e-08 | 289 | 0.145 | 731 | 435 | 23 | 79 | 780 | 22 | 591 | Phage tail tape measure protein | Phage tail tape measure protein | | afdb-uniprot50 | AF-A0A3A9H1N1-F1-MODEL\_V4 | 1.0 | 8.481e-07 | 288 | 0.139 | 486 | 326 | 15 | 19 | 442 | 192 | 647 | Phage tail tape measure protein | Phage tail tape measure protein | | afdb-uniprot50 | AF-A0A0F9RZT3-F1-MODEL\_V4 | 1.0 | 1.455e-09 | 288 | 0.166 | 746 | 509 | 21 | 19 | 702 | 23 | 717 | PhageMin\_Tail domain-containing protein | PhageMin\_Tail domain-containing protein | | afdb-uniprot50 | AF-A0A5B0D944-F1-MODEL\_V4 | 1.0 | 5.795e-11 | 286 | 0.293 | 538 | 281 | 14 | 264 | 753 | 4 | 490 | Phage tail tape measure protein | Phage tail tape measure protein | | afdb-uniprot50 | AF-K1ZEP0-F1-MODEL\_V4 | 1.0 | 1.352e-06 | 286 | 0.147 | 469 | 314 | 13 | 1 | 455 | 1 | 397 | PhageMin\_Tail domain-containing protein | PhageMin\_Tail domain-containing protein | | afdb-uniprot50 | AF-A0A7K0ATB2-F1-MODEL\_V4 | 1.0 | 4.663e-09 | 286 | 0.149 | 803 | 497 | 32 | 60 | 780 | 24 | 722 | Phage tail tape measure protein | Phage tail tape measure protein | | afdb-uniprot50 | AF-A0A6I4Z5B8-F1-MODEL\_V4 | 1.0 | 5.597e-08 | 285 | 0.128 | 804 | 484 | 22 | 14 | 785 | 208 | 826 | Phage tail tape measure protein | Phage tail tape measure protein | | afdb-uniprot50 | AF-A0A0N9MLM3-F1-MODEL\_V4 | 1.0 | 7.907e-11 | 285 | 0.15 | 1043 | 538 | 38 | 61 | 781 | 7 | 1022 | Phage tail tape measure protein, TP901 family | Phage tail tape measure protein, TP901 family | | afdb-uniprot50 | AF-A0A2P8EI71-F1-MODEL\_V4 | 1.0 | 1.096e-08 | 285 | 0.108 | 883 | 562 | 25 | 18 | 709 | 43 | 890 | TP901 family phage tail tape measure protein | TP901 family phage tail tape measure protein | | afdb-uniprot50 | AF-R5IIS8-F1-MODEL\_V4 | 1.0 | 9.381e-09 | 283 | 0.131 | 827 | 523 | 30 | 19 | 714 | 87 | 848 | Phage tail tape measure protein TP901 family core region | Phage tail tape measure protein TP901 family core region | | afdb-uniprot50 | AF-A0A085EXN0-F1-MODEL\_V4 | 1.0 | 1.217e-07 | 282 | 0.154 | 732 | 473 | 28 | 46 | 723 | 2 | 641 | Putative bacteriophage protein | Putative bacteriophage protein | | afdb-uniprot50 | AF-A0A6M0RDA6-F1-MODEL\_V4 | 1.0 | 2.677e-08 | 282 | 0.128 | 909 | 527 | 30 | 3 | 785 | 7 | 775 | Phage tail tape measure protein | Phage tail tape measure protein | | afdb-uniprot50 | AF-A0A7X7EMK5-F1-MODEL\_V4 | 1.0 | 4.486e-09 | 282 | 0.115 | 939 | 552 | 33 | 42 | 770 | 18 | 887 | Phage tail tape measure protein | Phage tail tape measure protein | | afdb-uniprot50 | AF-A0A2G6ESM5-F1-MODEL\_V4 | 1.0 | 1.331e-08 | 282 | 0.129 | 763 | 532 | 27 | 19 | 707 | 11 | 714 | Phage tail tape measure protein | Phage tail tape measure protein | | afdb-uniprot50 | AF-A0A416BKF0-F1-MODEL\_V4 | 1.0 | 7.346e-08 | 280 | 0.152 | 682 | 432 | 27 | 111 | 686 | 17 | 658 | Phage tail tape measure protein | Phage tail tape measure protein | | afdb-uniprot50 | AF-A0A0F9MJ74-F1-MODEL\_V4 | 1.0 | 4.663e-09 | 280 | 0.145 | 793 | 547 | 25 | 1 | 701 | 2 | 755 | PhageMin\_Tail domain-containing protein | PhageMin\_Tail domain-containing protein | | afdb-uniprot50 | AF-A0A853EHV0-F1-MODEL\_V4 | 1.0 | 1.31e-10 | 279 | 0.146 | 893 | 563 | 37 | 1 | 746 | 1 | 840 | Phage tail tape measure protein | Phage tail tape measure protein | | afdb-uniprot50 | AF-A0A180FCQ4-F1-MODEL\_V4 | 1.0 | 6.049e-08 | 279 | 0.136 | 654 | 437 | 19 | 77 | 719 | 15 | 551 | Phage tail tape measure protein, TP901 family, core region | Phage tail tape measure protein, TP901 family, core region | | afdb-uniprot50 | AF-A0A3D0Y8L6-F1-MODEL\_V4 | 1.0 | 5.04e-09 | 279 | 0.133 | 891 | 560 | 36 | 1 | 784 | 2 | 787 | Phage tail tape measure protein | Phage tail tape measure protein | | afdb-uniprot50 | AF-A0A1Q7RI84-F1-MODEL\_V4 | 1.0 | 6.614e-09 | 279 | 0.16 | 858 | 526 | 31 | 21 | 702 | 91 | 929 | Phage tail tape measure protein | Phage tail tape measure protein | | afdb-uniprot50 | AF-A0A1S7MQ78-F1-MODEL\_V4 | 1.0 | 9.273e-08 | 277 | 0.147 | 850 | 461 | 28 | 24 | 785 | 90 | 763 | PhageMin\_Tail domain-containing protein | PhageMin\_Tail domain-containing protein | | afdb-uniprot50 | AF-A0A2U2DG04-F1-MODEL\_V4 | 1.0 | 1.139e-08 | 277 | 0.138 | 908 | 475 | 33 | 15 | 783 | 39 | 777 | PhageMin\_Tail domain-containing protein | PhageMin\_Tail domain-containing protein | | afdb-uniprot50 | AF-A0A2K8U7C3-F1-MODEL\_V4 | 1.0 | 2.646e-07 | 276 | 0.155 | 668 | 454 | 26 | 64 | 702 | 7 | 593 | Phage tail tape measure protein | Phage tail tape measure protein | | afdb-uniprot50 | AF-A0A661TQT9-F1-MODEL\_V4 | 1.0 | 8.68e-09 | 276 | 0.162 | 738 | 469 | 31 | 46 | 700 | 2 | 673 | Phage tail tape measure protein | Phage tail tape measure protein | | afdb-uniprot50 | AF-A0A3A6RBH3-F1-MODEL\_V4 | 1.0 | 5.04e-09 | 276 | 0.138 | 996 | 576 | 38 | 14 | 785 | 141 | 1077 | Phage tail tape measure protein | Phage tail tape measure protein | | afdb-uniprot50 | AF-A0A2D7ZNU8-F1-MODEL\_V4 | 1.0 | 3.84e-09 | 274 | 0.143 | 774 | 470 | 26 | 118 | 750 | 87 | 808 | Phage tail tape measure protein | Phage tail tape measure protein | | afdb-uniprot50 | AF-A0A8B5R2V2-F1-MODEL\_V4 | 1.0 | 5.099e-10 | 274 | 0.171 | 705 | 450 | 30 | 66 | 701 | 3 | 642 | Phage tail tape measure protein | Phage tail tape measure protein | | afdb-uniprot50 | AF-A0A2G2J0Y5-F1-MODEL\_V4 | 1.0 | 4.103e-08 | 274 | 0.147 | 718 | 487 | 27 | 39 | 712 | 14 | 650 | Phage tail tape measure protein | Phage tail tape measure protein | | afdb-uniprot50 | AF-A0A6M3KIG7-F1-MODEL\_V4 | 1.0 | 4.792e-08 | 273 | 0.132 | 746 | 515 | 28 | 1 | 702 | 1 | 657 | Putative tail protein | Putative tail protein | | afdb-uniprot50 | AF-E2CI78-F1-MODEL\_V4 | 1.0 | 3.652e-08 | 271 | 0.153 | 875 | 458 | 31 | 14 | 784 | 38 | 733 | Putative phage tail tape measure protein, core region | Putative phage tail tape measure protein, core region | | afdb-uniprot50 | AF-A0A7Z8JJT8-F1-MODEL\_V4 | 1.0 | 1.495e-08 | 271 | 0.147 | 740 | 481 | 28 | 1 | 702 | 1 | 628 | Phage tail tape measure protein | Phage tail tape measure protein | | afdb-uniprot50 | AF-A0A1W9TY32-F1-MODEL\_V4 | 1.0 | 1.331e-08 | 271 | 0.137 | 732 | 473 | 28 | 1 | 702 | 1 | 603 | Phage tail tape measure protein | Phage tail tape measure protein | | afdb-uniprot50 | AF-A0A7J3WET8-F1-MODEL\_V4 | 1.0 | 1.383e-08 | 269 | 0.142 | 889 | 532 | 35 | 1 | 777 | 3 | 772 | Phage tail tape measure protein | Phage tail tape measure protein | | afdb-uniprot50 | AF-D1Y710-F1-MODEL\_V4 | 1.0 | 5.819e-08 | 268 | 0.153 | 683 | 449 | 21 | 20 | 625 | 41 | 670 | Phage tail tape measure protein, TP901 family | Phage tail tape measure protein, TP901 family | | afdb-uniprot50 | AF-A0A662BU24-F1-MODEL\_V4 | 1.0 | 9.381e-09 | 268 | 0.126 | 792 | 519 | 26 | 19 | 704 | 12 | 736 | PhageMin\_Tail domain-containing protein | PhageMin\_Tail domain-containing protein | | afdb-uniprot50 | AF-A0A1Q6LLB6-F1-MODEL\_V4 | 1.0 | 3.752e-07 | 268 | 0.139 | 695 | 446 | 24 | 100 | 699 | 40 | 677 | Phage tail tape measure protein | Phage tail tape measure protein | | afdb-uniprot50 | AF-A0A2E0R799-F1-MODEL\_V4 | 1.0 | 1.265e-07 | 267 | 0.228 | 503 | 263 | 16 | 13 | 465 | 17 | 444 | Phage tail tape measure protein | Phage tail tape measure protein | | afdb-uniprot50 | AF-A0A4Y9G537-F1-MODEL\_V4 | 1.0 | 1.641e-06 | 267 | 0.205 | 429 | 230 | 9 | 18 | 345 | 54 | 472 | Phage tail tape measure protein | Phage tail tape measure protein | | afdb-uniprot50 | AF-A0A2T7H7Y6-F1-MODEL\_V4 | 1.0 | 1.939e-07 | 267 | 0.136 | 798 | 468 | 24 | 39 | 782 | 75 | 705 | PhageMin\_Tail domain-containing protein | PhageMin\_Tail domain-containing protein | | afdb-uniprot50 | AF-A0A4Q6Y918-F1-MODEL\_V4 | 1.0 | 2.12e-08 | 266 | 0.168 | 794 | 515 | 32 | 18 | 772 | 6 | 692 | Phage tail tape measure protein | Phage tail tape measure protein | | afdb-uniprot50 | AF-A0A367HZ43-F1-MODEL\_V4 | 1.0 | 2.04e-08 | 265 | 0.138 | 1094 | 550 | 35 | 32 | 785 | 2 | 1042 | Phage tail tape measure protein | Phage tail tape measure protein | | afdb-uniprot50 | AF-A0A3D2IQU7-F1-MODEL\_V4 | 1.0 | 3.947e-08 | 264 | 0.137 | 763 | 487 | 26 | 42 | 704 | 4 | 695 | Phage tail tape measure protein | Phage tail tape measure protein | | afdb-uniprot50 | AF-A0A6I4XSS1-F1-MODEL\_V4 | 1.0 | 4.737e-07 | 263 | 0.181 | 447 | 253 | 16 | 1 | 423 | 2 | 359 | Phage tail tape measure protein | Phage tail tape measure protein | | afdb-uniprot50 | AF-A0A412EM13-F1-MODEL\_V4 | 1.0 | 3.694e-09 | 263 | 0.142 | 808 | 537 | 25 | 1 | 713 | 1 | 747 | Phage tail tape measure protein | Phage tail tape measure protein | | afdb-uniprot50 | AF-A0A2G2DHY8-F1-MODEL\_V4 | 1.0 | 3.752e-07 | 262 | 0.133 | 809 | 494 | 26 | 32 | 784 | 13 | 670 | Phage tail tape measure protein | Phage tail tape measure protein | | afdb-uniprot50 | AF-A0A2P8NKI0-F1-MODEL\_V4 | 1.0 | 5.98e-07 | 261 | 0.146 | 722 | 457 | 27 | 49 | 731 | 6 | 606 | Phage tail tape measure protein | Phage tail tape measure protein | | afdb-uniprot50 | AF-A0A2M8NE79-F1-MODEL\_V4 | 1.0 | 4.103e-08 | 260 | 0.125 | 837 | 526 | 32 | 19 | 704 | 28 | 809 | Phage tail tape measure protein | Phage tail tape measure protein | | afdb-uniprot50 | AF-A0A6N8TKC3-F1-MODEL\_V4 | 1.0 | 2.096e-07 | 259 | 0.131 | 838 | 455 | 27 | 19 | 783 | 52 | 689 | Phage tail tape measure protein | Phage tail tape measure protein | | afdb-uniprot50 | AF-A0A7J2HKH6-F1-MODEL\_V4 | 1.0 | 2.893e-08 | 259 | 0.129 | 862 | 527 | 28 | 109 | 785 | 23 | 845 | Phage tail tape measure protein | Phage tail tape measure protein | | afdb-uniprot50 | AF-A0A6M3KID6-F1-MODEL\_V4 | 1.0 | 3.513e-08 | 254 | 0.122 | 757 | 470 | 27 | 31 | 702 | 83 | 729 | Putative tail protein | Putative tail protein | | afdb-uniprot50 | AF-A0A349NKI0-F1-MODEL\_V4 | 1.0 | 2.265e-07 | 252 | 0.131 | 593 | 392 | 17 | 155 | 702 | 2 | 516 | Phage tail tape measure protein | Phage tail tape measure protein | | afdb-uniprot50 | AF-A0A4Z0V6T5-F1-MODEL\_V4 | 1.0 | 4.925e-07 | 252 | 0.168 | 664 | 433 | 25 | 70 | 690 | 30 | 617 | Phage tail tape measure protein | Phage tail tape measure protein | | afdb-uniprot50 | AF-A0A3E4VC51-F1-MODEL\_V4 | 1.0 | 1.794e-07 | 251 | 0.142 | 807 | 462 | 34 | 49 | 684 | 5 | 752 | Phage tail tape measure protein | Phage tail tape measure protein | | afdb-uniprot50 | AF-A4W8K8-F1-MODEL\_V4 | 1.0 | 1.28e-08 | 248 | 0.2 | 614 | 354 | 15 | 243 | 785 | 2 | 549 | Phage tail-like protein | Phage tail-like protein | | afdb-uniprot50 | AF-A0A662F5X9-F1-MODEL\_V4 | 1.0 | 5.322e-07 | 248 | 0.123 | 735 | 470 | 25 | 10 | 680 | 1 | 624 | Phage tail tape measure protein | Phage tail tape measure protein | | afdb-uniprot50 | AF-A0A2W5EKK6-F1-MODEL\_V4 | 1.0 | 5.533e-07 | 247 | 0.137 | 691 | 389 | 24 | 109 | 785 | 200 | 697 | PhageMin\_Tail domain-containing protein | PhageMin\_Tail domain-containing protein | | afdb-uniprot50 | AF-A0A1H6JMQ9-F1-MODEL\_V4 | 1.0 | 3.007e-08 | 247 | 0.14 | 740 | 445 | 28 | 50 | 674 | 10 | 673 | Phage tail tape measure protein, TP901 family, core region | Phage tail tape measure protein, TP901 family, core region | | afdb-uniprot50 | AF-A0A662PR09-F1-MODEL\_V4 | 1.0 | 4.737e-07 | 246 | 0.148 | 672 | 437 | 21 | 66 | 701 | 2 | 573 | Phage tail tape measure protein | Phage tail tape measure protein | | afdb-uniprot50 | AF-H1PPE3-F1-MODEL\_V4 | 1.0 | 1.519e-06 | 244 | 0.111 | 664 | 486 | 22 | 109 | 702 | 54 | 683 | Phage tail tape measure protein, TP901 family, core region | Phage tail tape measure protein, TP901 family, core region | | afdb-uniprot50 | AF-A0A376FD60-F1-MODEL\_V4 | 1.0 | 3.568e-06 | 243 | 0.146 | 513 | 300 | 14 | 19 | 458 | 34 | 481 | Phage Tail Tape Measure protein | Phage Tail Tape Measure protein | | afdb-uniprot50 | AF-A0A1C5ZH20-F1-MODEL\_V4 | 1.0 | 4.737e-07 | 243 | 0.156 | 775 | 447 | 32 | 103 | 702 | 37 | 779 | Phage-related protein | Phage-related protein | | afdb-uniprot50 | AF-A0A662Q4G6-F1-MODEL\_V4 | 1.0 | 8.716e-06 | 242 | 0.138 | 506 | 367 | 13 | 10 | 485 | 313 | 779 | Phage tail tape measure protein | Phage tail tape measure protein | | afdb-uniprot50 | AF-A0A2N6Q6V0-F1-MODEL\_V4 | 1.0 | 4.982e-08 | 242 | 0.125 | 836 | 503 | 33 | 44 | 780 | 29 | 735 | Phage tail tape measure protein | Phage tail tape measure protein | | afdb-uniprot50 | AF-A0A212L7A0-F1-MODEL\_V4 | 1.0 | 5.752e-07 | 237 | 0.12 | 797 | 460 | 31 | 1 | 776 | 1 | 577 | PhageMin\_Tail domain-containing protein | PhageMin\_Tail domain-containing protein | | afdb-uniprot50 | AF-A0A1Y0C490-F1-MODEL\_V4 | 1.0 | 5.819e-08 | 203 | 0.155 | 836 | 516 | 32 | 4 | 755 | 39 | 768 | Phage tail tape measure protein | Phage tail tape measure protein | | afdb-uniprot50 | AF-A0A7X7BVE1-F1-MODEL\_V4 | 1.0 | 9.68e-05 | 170 | 0.223 | 466 | 289 | 9 | 239 | 702 | 3 | 397 | Phage tail tape measure protein | Phage tail tape measure protein | |
| Top keywords  (threshold 1.00e-02 (evalue)) | **tail, Phage, tape, measure, TP901, domain\_containing, PhageMin\_Tail, core, region, Phage\_related** |
| Output files | ../../similar\_structures/22\_FANPEZAQ\_CDS\_0022\_afdb-proteome\_foldseek.tsv ../../similar\_structures/22\_FANPEZAQ\_CDS\_0022\_afdb-uniprot50\_foldseek.tsv ../../similar\_structures/22\_FANPEZAQ\_CDS\_0022\_merged.svg ../../similar\_structures/22\_FANPEZAQ\_CDS\_0022\_pdb\_foldseek.tsv |

  
  
  

Return to summary | Go to previous | Go to next

  


---

**Sequence/structure alignments coloring**  
Each object in the alignment figures is colored according to its E-value following this color coding:

1e-100
10

**References:**  
1) Steinegger M, Meier M, Mirdita M, Vöhringer H, Haunsberger S J, and Söding J (2019) HH-suite3 for fast remote homology detection and deep protein annotation, BMC Bioinformatics, 473. doi: 10.1186/s12859-019-3019-7  
2) Jumper J, Evans R, Pritzel A, ..., Hassabis D (2021) Highly accurate protein structure prediction with AlphaFold, Nature, 596. doi: 10.1038/s41586-021-03819-2  
3) van Kempen M, Kim S, Tumescheit C, Mirdita M, Lee J, Gilchrist CLM, Söding J, and Steinegger M (2023) Fast and accurate protein structure search with Foldseek. Nature Biotechnology. doi: 10.1038/s41587-023-01773-0
