# Supplementary material for: Completing the BASEL phage collection to unlock hidden diversity for systematic exploration of phage–host interactions
Source: PLoS Biol. 2025 Apr 7;23(4):e3003063. doi: 10.1371/journal.pbio.3003063 (PMC11990801; doi:10.1371/journal.pbio.3003063)
Supplement: S2 Data — (ZIP) [file pbio.3003063.s009.zip › entries/23.html]

FANPEZAQ\_CDS\_0023


Return to summary | Go to previous | Go to next

|  |  |
| --- | --- |
| FANPEZAQ\_CDS\_0023 Page creation date: 02 Sep 2024, 12:00  Project folder: n/a  Input sequences file: Escherichia\_virus\_HeidiAbel.gb | phage tail domain\_containing gpu lysm p2 u tube contractile system injection n\_terminal oxidoreductase peptidoglycan\_binding putative fragment bacteriophage hypothetical phage\_related dna fiber prophage |

### Sequence information

|  |  |
| --- | --- |
| Name | FANPEZAQ\_CDS\_0023  23\_FANPEZAQ\_CDS\_0023 (pipeline id) |
| Imported annotations | Escherichia\_virus\_HeidiAbel Bas97 |
| Protein sequence | MTSFTILTGQTNIMMMLGGFQFCILTAAYQELNRHSEYKWPSQHRFGQRPSSQFVGIGEE TITLAGIIYPEYRGGFQQVEQMRSMAGRGQPLLLVDGLGKLWGRWVIEVIDEKQSLFAAF GAPRKQEFNLQLRRFN |
| Number of residues | 136 |
| Molecular weight (Da) | 15595.79 |
| Output files | ../../query\_sequences/23\_FANPEZAQ\_CDS\_0023.fasta |

### Putative domain architecture and protein family

#### Search results (HHblits)1

|  |  |
| --- | --- |
| Domain family databases searched | Pfam, Ncbi-cd, Cath, Phrogs |
| Results, scheme(s)  (Top layers only; threshold 1.00e-03 (evalue)) | xml version="1.0" encoding="utf-8" standalone="no"?       2024-09-02T21:08:17.150768 image/svg+xml   Matplotlib v3.7.2, https://matplotlib.org/ |
| Results, table  (E-value ≤ 1.00e-03 (evalue)) | | db | id | prob | evalue | pvalue | score | cols | query | query\_len | template | template\_len | name | description | | --- | --- | --- | --- | --- | --- | --- | --- | --- | --- | --- | --- | --- | | pfam | PF06995 | 99.7 | 1e-22 | 1.8e-26 | 126.0 | 118 | (18, 135) | 136 | (1, 118) | 120 | Phage\_P2\_GpU | Phage P2 GpU | | pfam | PF19266 | 99.5 | 5.8e-19 | 1.1e-22 | 114.2 | 112 | (21, 135) | 136 | (22, 151) | 159 | CIS\_tube | Contractile injection system tube protein | | pfam | PF19512 | 99.4 | 2.7e-17 | 4.8e-21 | 103.9 | 104 | (28, 135) | 136 | (4, 125) | 137 | DUF6046 | Domain of unknown function (DUF6046) | | pfam | PF07157 | 96.9 | 8.2e-07 | 1.5e-10 | 49.2 | 75 | (16, 97) | 136 | (5, 80) | 86 | DNA\_circ\_N | DNA circularisation protein N-terminus | | phrogs | 76 | 100.0 | 9.5e-35 | 1.2e-38 | 205.3 | 124 | (13, 136) | 136 | (1, 124) | 162 | tail protein | tail protein; Category: tail; p258311 VI\_01887 | | phrogs | 788 | 99.9 | 3.8e-29 | 4.5e-33 | 189.2 | 122 | (13, 135) | 136 | (1, 122) | 298 | tail protein | tail protein; Category: tail; p182850 VI\_05566 | | phrogs | 4094 | 99.7 | 2.3e-23 | 2.7e-27 | 151.9 | 121 | (16, 136) | 136 | (167, 287) | 293 | NA | NA; Category: unknown function; p422642 VI\_05571 | | phrogs | 380 | 99.6 | 2.2e-20 | 2.8e-24 | 137.2 | 119 | (13, 136) | 136 | (5, 132) | 230 | endolysin | endolysin; Category: lysis; NC\_005294\_p57 | | phrogs | 5184 | 99.2 | 1.1e-15 | 1.3e-19 | 103.5 | 109 | (19, 136) | 136 | (16, 136) | 145 | NA | NA; Category: unknown function; p243320 VI\_01361 | | phrogs | 32879 | 99.1 | 6.3e-15 | 7e-19 | 103.7 | 124 | (11, 135) | 136 | (164, 291) | 292 | NA | NA; Category: unknown function; p423527 VI\_06236 | | phrogs | 29415 | 98.7 | 4.2e-12 | 4.7e-16 | 70.2 | 58 | (13, 70) | 136 | (1, 58) | 59 | NA | NA; Category: unknown function; p408258 VI\_11010 | | phrogs | 21362 | 98.2 | 2.9e-10 | 3.4e-14 | 82.8 | 111 | (3, 118) | 136 | (9, 119) | 329 | NA | NA; Category: unknown function; p152991 VI\_08595 | | phrogs | 23201 | 96.2 | 1.9e-05 | 2.1e-09 | 53.6 | 105 | (26, 136) | 136 | (17, 129) | 222 | NA | NA; Category: unknown function; p369082 VI\_04056 | | phrogs | 27169 | 96.2 | 2.5e-05 | 2.8e-09 | 43.5 | 35 | (101, 135) | 136 | (2, 36) | 72 | NA | NA; Category: unknown function; p366908 VI\_01591 | | phrogs | 1517 | 96.1 | 2.8e-05 | 3.5e-09 | 53.6 | 99 | (31, 134) | 136 | (22, 133) | 194 | endolysin | endolysin; Category: lysis; NC\_020843\_p101 | | phrogs | 817 | 95.3 | 0.00018 | 2.2e-08 | 48.1 | 91 | (40, 135) | 136 | (4, 110) | 165 | tail fiber protein | tail fiber protein; Category: tail; p327551 VI\_10369 | |
| Top keywords  (threshold 1.00e-03 (evalue)) | **tail, endolysin, lysis, Phage, P2, GpU, Contractile, injection, system, tube** |
| Output files | ../../domain\_architecture/23\_FANPEZAQ\_CDS\_0023\_cath.hhr ../../domain\_architecture/23\_FANPEZAQ\_CDS\_0023\_merged.svg ../../domain\_architecture/23\_FANPEZAQ\_CDS\_0023\_ncbi-cd.hhr ../../domain\_architecture/23\_FANPEZAQ\_CDS\_0023\_pfam.hhr ../../domain\_architecture/23\_FANPEZAQ\_CDS\_0023\_phrogs.hhr |

### Identical protein sequences/structures

#### Search results

|  |  |
| --- | --- |
| Protein sequence databases searched | Pdb, Swissprot, Refseq |
| Identical proteins found | -- |
| Top keywords | -- |
| Output files | -- |

### Similar protein sequences/structures

#### Sequence similarity search results (HHblits)1

|  |  |
| --- | --- |
| Sequence databases searched | Uniclust, Pdb70 |
| Results, scheme(s)  (Top layers only, threshold 1.00e-03 (evalue)) | xml version="1.0" encoding="utf-8" standalone="no"?       2024-09-02T21:08:39.322659 image/svg+xml   Matplotlib v3.7.2, https://matplotlib.org/ |
| Results, table(s)  (threshold 1.00e-03 (evalue)) | | db | id | prob | evalue | pvalue | score | cols | query | query\_len | template | template\_len | name | description | | --- | --- | --- | --- | --- | --- | --- | --- | --- | --- | --- | --- | --- | | uniclust | UniRef100\_A0A009PIW6 | 100.0 | 7.9e-42 | 1.7e-47 | 246.7 | 136 | (1, 136) | 136 | (1, 136) | 202 | Phage P2 GpU family protein | Phage P2 GpU family protein | | uniclust | UniRef100\_A0A061YJX4 | 100.0 | 1.9e-40 | 4.3e-46 | 238.6 | 134 | (3, 136) | 136 | (29, 162) | 187 | Phage tail protein | Phage tail protein | | uniclust | UniRef100\_A0A011NB97 | 100.0 | 1.4e-38 | 2.9e-44 | 233.6 | 131 | (6, 136) | 136 | (24, 154) | 235 | Oxidoreductase | Oxidoreductase | | uniclust | UniRef100\_A0A158E8Q2 | 100.0 | 3.3e-38 | 6.7e-44 | 238.3 | 133 | (4, 136) | 136 | (2, 134) | 311 | Bacteriophage tail-related protein | Bacteriophage tail-related protein | | uniclust | UniRef100\_A0A060H3V2 | 100.0 | 7.7e-37 | 1.6e-42 | 216.2 | 130 | (7, 136) | 136 | (29, 158) | 175 | Tail protein | Tail protein | | uniclust | UniRef100\_A0A022PJH1 | 100.0 | 9.8e-37 | 2e-42 | 218.3 | 128 | (9, 136) | 136 | (6, 133) | 211 | Phage protein U | Phage protein U | | uniclust | UniRef100\_A0A074LRJ1 | 100.0 | 2.1e-35 | 4.5e-41 | 211.6 | 131 | (5, 136) | 136 | (21, 157) | 188 | Phage tail protein | Phage tail protein | | uniclust | UniRef100\_A0A059WRP1 | 100.0 | 2.8e-35 | 5.9e-41 | 207.2 | 125 | (11, 136) | 136 | (1, 129) | 165 | Tail protein | Tail protein | | uniclust | UniRef100\_A0A098B0B0 | 100.0 | 3.4e-34 | 7.5e-40 | 197.6 | 125 | (11, 136) | 136 | (1, 129) | 138 | Phage P2 GpU | Phage P2 GpU | | uniclust | UniRef100\_A0A066RZT5 | 100.0 | 2.8e-33 | 5.6e-39 | 194.1 | 132 | (5, 136) | 136 | (24, 156) | 162 | p2 GpU family protein | p2 GpU family protein | | uniclust | UniRef100\_A0A022PDG1 | 100.0 | 1.1e-32 | 2.1e-38 | 194.0 | 125 | (11, 135) | 136 | (6, 130) | 190 | Phage protein U | Phage protein U | | uniclust | UniRef100\_A0A024L4N8 | 100.0 | 1.3e-32 | 2.5e-38 | 193.4 | 128 | (9, 136) | 136 | (38, 165) | 206 | Bacteriophage tail protein GpU | Bacteriophage tail protein GpU | | uniclust | UniRef100\_A0A017HDE4 | 99.9 | 2.3e-32 | 4.9e-38 | 197.0 | 131 | (6, 136) | 136 | (27, 157) | 194 | Phage P2 GpU | Phage P2 GpU | | uniclust | UniRef100\_A0A064AE81 | 99.9 | 3e-32 | 6.2e-38 | 190.2 | 127 | (9, 136) | 136 | (22, 154) | 160 | Phage tail protein | Phage tail protein | | uniclust | UniRef100\_A0A4R3LHW8 | 99.9 | 7.4e-32 | 1.4e-37 | 199.4 | 128 | (9, 136) | 136 | (1, 128) | 288 | Phage protein U | Phage protein U | | uniclust | UniRef100\_A0A137SQ39 | 99.9 | 2.2e-31 | 4.4e-37 | 186.3 | 126 | (10, 136) | 136 | (19, 150) | 165 | Phage P2 GpU | Phage P2 GpU | | uniclust | UniRef100\_A0A134C9G6 | 99.9 | 2.4e-31 | 4.9e-37 | 185.2 | 127 | (9, 136) | 136 | (1, 133) | 165 | Phage tail protein (Fragment) | Phage tail protein (Fragment) | | uniclust | UniRef100\_A0A011VGA6 | 99.9 | 3.5e-31 | 7.5e-37 | 189.5 | 131 | (6, 136) | 136 | (17, 147) | 183 | Tail protein | Tail protein | | uniclust | UniRef100\_A0A120KRM4 | 99.9 | 4.8e-31 | 1e-36 | 189.5 | 125 | (10, 135) | 136 | (46, 176) | 197 | Phage tail protein | Phage tail protein | | uniclust | UniRef100\_A0A0G0CA59 | 99.9 | 1.1e-30 | 2.3e-36 | 185.2 | 125 | (11, 136) | 136 | (1, 125) | 176 | Phage protein U | Phage protein U | | uniclust | UniRef100\_A0A348FYG2 | 99.9 | 3.9e-30 | 7.7e-36 | 179.7 | 130 | (7, 136) | 136 | (21, 150) | 176 | Oxidoreductase | Oxidoreductase | | uniclust | UniRef100\_A0A022PKF1 | 99.9 | 5.6e-30 | 1.2e-35 | 197.9 | 127 | (9, 136) | 136 | (19, 145) | 322 | Phage protein U | Phage protein U | | uniclust | UniRef100\_A0A098F6K3 | 99.9 | 7.3e-30 | 1.5e-35 | 186.9 | 129 | (7, 136) | 136 | (3, 137) | 239 | SH3 domain-containing protein | SH3 domain-containing protein | | uniclust | UniRef100\_A0A0H3HPD4 | 99.9 | 1.5e-29 | 2.9e-35 | 176.7 | 126 | (11, 136) | 136 | (20, 145) | 189 | Phage-related tail protein | Phage-related tail protein | | uniclust | UniRef100\_A0A1C0A2B2 | 99.9 | 1.8e-29 | 3.6e-35 | 186.3 | 125 | (11, 136) | 136 | (26, 150) | 247 | Phage tail protein | Phage tail protein | | uniclust | UniRef100\_A0A1X3HX14 | 99.9 | 3.1e-29 | 6.1e-35 | 167.8 | 124 | (12, 135) | 136 | (5, 128) | 131 | Putative bacteriophage tail fiber protein U | Putative bacteriophage tail fiber protein U | | uniclust | UniRef100\_A0A1T4WWY7 | 99.9 | 2.1e-28 | 4.1e-34 | 169.6 | 128 | (9, 136) | 136 | (26, 156) | 169 | Phage P2 GpU | Phage P2 GpU | | uniclust | UniRef100\_A0A8J7IC90 | 99.9 | 2.4e-28 | 4.7e-34 | 175.1 | 129 | (7, 135) | 136 | (33, 161) | 222 | Phage tail protein | Phage tail protein | | uniclust | UniRef100\_A0A4P5VNR5 | 99.9 | 9.2e-28 | 1.8e-33 | 178.9 | 127 | (10, 136) | 136 | (1, 127) | 308 | Phage tail protein | Phage tail protein | | uniclust | UniRef100\_UPI0013D8B061 | 99.9 | 1.1e-27 | 2e-33 | 158.3 | 117 | (20, 136) | 136 | (17, 133) | 139 | phage tail protein | phage tail protein | | uniclust | UniRef100\_A0A0E3BS28 | 99.9 | 1.4e-27 | 2.9e-33 | 182.9 | 127 | (9, 136) | 136 | (19, 145) | 327 | Phage P2 GpU family protein | Phage P2 GpU family protein | | uniclust | UniRef100\_A0A3A9CQH5 | 99.9 | 2e-27 | 3.9e-33 | 165.8 | 126 | (10, 136) | 136 | (41, 172) | 172 | Phage tail protein | Phage tail protein | | uniclust | UniRef100\_A0A104N430 | 99.9 | 4.1e-27 | 7.9e-33 | 169.4 | 123 | (13, 135) | 136 | (1, 124) | 236 | Phage tail protein | Phage tail protein | | uniclust | UniRef100\_UPI0018EE9540 | 99.9 | 4.7e-27 | 8.6e-33 | 174.3 | 123 | (14, 136) | 136 | (1, 123) | 363 | phage tail protein | phage tail protein | | uniclust | UniRef100\_A0A0D2JH39 | 99.9 | 7e-27 | 1.5e-32 | 160.7 | 127 | (8, 136) | 136 | (1, 127) | 142 | Uncharacterized protein | Uncharacterized protein | | uniclust | UniRef100\_A0A0A8H9Q9 | 99.9 | 1e-26 | 2.1e-32 | 157.7 | 122 | (12, 135) | 136 | (1, 122) | 134 | Phage P2 GpU family protein | Phage P2 GpU family protein | | uniclust | UniRef100\_A0A0A3TKD9 | 99.9 | 1.1e-26 | 2.2e-32 | 159.5 | 126 | (10, 136) | 136 | (1, 130) | 157 | Phage protein U | Phage protein U | | uniclust | UniRef100\_A0A072R0Y2 | 99.9 | 3e-26 | 6.1e-32 | 160.3 | 126 | (10, 135) | 136 | (1, 132) | 168 | Uncharacterized protein | Uncharacterized protein | | uniclust | UniRef100\_A0A143HCV2 | 99.9 | 4.4e-26 | 8.7e-32 | 167.9 | 127 | (10, 136) | 136 | (12, 143) | 260 | SH3b domain-containing protein | SH3b domain-containing protein | | uniclust | UniRef100\_UPI002227D0FF | 99.9 | 9.2e-26 | 1.7e-31 | 156.0 | 125 | (11, 135) | 136 | (2, 126) | 199 | phage tail protein | phage tail protein | | uniclust | UniRef100\_B6IMG5 | 99.9 | 9.8e-26 | 1.9e-31 | 155.5 | 123 | (13, 136) | 136 | (1, 123) | 162 | Phage P2 GpU | Phage P2 GpU | | uniclust | UniRef100\_A0A2E1R8X5 | 99.9 | 1.9e-25 | 3.4e-31 | 151.8 | 132 | (5, 136) | 136 | (2, 133) | 174 | Phage tail protein | Phage tail protein | | uniclust | UniRef100\_A0A024E920 | 99.9 | 3.9e-25 | 8.1e-31 | 171.3 | 118 | (19, 136) | 136 | (214, 331) | 339 | Phage tail protein | Phage tail protein | | uniclust | UniRef100\_N9WEM7 | 99.8 | 5.6e-25 | 1.1e-30 | 152.4 | 123 | (13, 135) | 136 | (62, 184) | 186 | Phage P2 GpU protein | Phage P2 GpU protein | | uniclust | UniRef100\_A0A0M1I1F2 | 99.8 | 1.1e-24 | 2.1e-30 | 146.1 | 128 | (9, 136) | 136 | (7, 134) | 153 | Phage tail protein | Phage tail protein | | uniclust | UniRef100\_UPI001F442EBF | 99.8 | 1.9e-24 | 3.4e-30 | 153.9 | 126 | (11, 136) | 136 | (118, 243) | 246 | phage tail protein | phage tail protein | | uniclust | UniRef100\_A0A1M7R7F9 | 99.8 | 5.3e-24 | 1.1e-29 | 161.0 | 124 | (12, 136) | 136 | (1, 124) | 284 | Phage protein U | Phage protein U | | uniclust | UniRef100\_I7LIF3 | 99.8 | 6.5e-24 | 1.2e-29 | 145.5 | 122 | (13, 135) | 136 | (1, 126) | 183 | Phage protein U | Phage protein U | | uniclust | UniRef100\_A0A1H5DBV8 | 99.8 | 1.1e-23 | 2.2e-29 | 151.2 | 122 | (13, 135) | 136 | (1, 122) | 213 | Phage protein U | Phage protein U | | uniclust | UniRef100\_A0A239C7Y7 | 99.8 | 1.2e-23 | 2.4e-29 | 160.0 | 125 | (11, 136) | 136 | (172, 300) | 300 | Phage P2 GpU | Phage P2 GpU | | uniclust | UniRef100\_A0A7Z0SJA2 | 99.8 | 2e-23 | 3.8e-29 | 153.4 | 127 | (10, 136) | 136 | (109, 235) | 273 | Phage tail protein | Phage tail protein | | uniclust | UniRef100\_UPI001D0D63E4 | 99.8 | 2.5e-23 | 4.6e-29 | 141.9 | 116 | (20, 135) | 136 | (2, 117) | 176 | phage tail protein | phage tail protein | | uniclust | UniRef100\_A0A4P9VIK5 | 99.8 | 2.6e-23 | 4.7e-29 | 142.0 | 119 | (18, 136) | 136 | (23, 142) | 172 | Phage tail protein | Phage tail protein | | uniclust | UniRef100\_A0A1H1G2U6 | 99.8 | 3e-23 | 5.9e-29 | 139.7 | 126 | (10, 135) | 136 | (1, 127) | 136 | Phage tail protein | Phage tail protein | | uniclust | UniRef100\_A0A0S2F794 | 99.8 | 3.2e-23 | 6.3e-29 | 137.2 | 92 | (44, 135) | 136 | (2, 93) | 120 | Phage P2 GpU family protein | Phage P2 GpU family protein | | uniclust | UniRef100\_A0A0T7DUZ2 | 99.8 | 3.1e-23 | 6.4e-29 | 144.8 | 132 | (2, 135) | 136 | (17, 148) | 156 | Phage tail protein | Phage tail protein | | uniclust | UniRef100\_A0A2I0GV19 | 99.8 | 3.5e-23 | 6.8e-29 | 134.5 | 89 | (48, 136) | 136 | (1, 89) | 105 | Phage tail protein | Phage tail protein | | uniclust | UniRef100\_A0A522WZG7 | 99.8 | 3.7e-23 | 7.2e-29 | 133.0 | 100 | (9, 108) | 136 | (2, 102) | 104 | Phage tail protein (Fragment) | Phage tail protein (Fragment) | | uniclust | UniRef100\_A0A0C1G7H4 | 99.8 | 6e-23 | 1.2e-28 | 145.1 | 126 | (11, 136) | 136 | (1, 128) | 164 | Tail protein | Tail protein | | uniclust | UniRef100\_A0A069PK85 | 99.8 | 7.2e-23 | 1.4e-28 | 153.1 | 117 | (20, 136) | 136 | (170, 286) | 292 | Oxidoreductase | Oxidoreductase | | uniclust | UniRef100\_A0A1J5BJG5 | 99.8 | 7.4e-23 | 1.4e-28 | 147.9 | 122 | (14, 136) | 136 | (45, 166) | 218 | Phage tail protein | Phage tail protein | | uniclust | UniRef100\_A0A1U7MFM3 | 99.8 | 1.1e-22 | 2.1e-28 | 150.0 | 123 | (12, 135) | 136 | (1, 127) | 286 | Phage P2 GpU | Phage P2 GpU | | uniclust | UniRef100\_A0A345DE50 | 99.8 | 1.2e-22 | 2.2e-28 | 139.4 | 126 | (11, 136) | 136 | (36, 161) | 182 | Phage tail protein | Phage tail protein | | uniclust | UniRef100\_A0A369RKP4 | 99.8 | 1.7e-22 | 3.2e-28 | 134.1 | 114 | (14, 136) | 136 | (1, 114) | 138 | Phage tail protein GpU | Phage tail protein GpU | | uniclust | UniRef100\_UPI00227ED58D | 99.8 | 1.8e-22 | 3.4e-28 | 144.6 | 126 | (10, 135) | 136 | (2, 127) | 254 | phage tail protein | phage tail protein | | uniclust | UniRef100\_A0A9D5ZW77 | 99.8 | 2.6e-22 | 4.9e-28 | 152.2 | 122 | (13, 135) | 136 | (1, 122) | 361 | Phage tail protein | Phage tail protein | | uniclust | UniRef100\_A0A9D8DA11 | 99.8 | 2.9e-22 | 5.5e-28 | 124.4 | 83 | (9, 91) | 136 | (1, 83) | 83 | Phage tail protein (Fragment) | Phage tail protein (Fragment) | | uniclust | UniRef100\_A0A4Q4GV95 | 99.8 | 3e-22 | 5.5e-28 | 135.0 | 122 | (13, 134) | 136 | (1, 123) | 149 | Oxidoreductase | Oxidoreductase | | uniclust | UniRef100\_UPI00027C90E7 | 99.8 | 4.2e-22 | 7.9e-28 | 157.6 | 123 | (13, 135) | 136 | (1, 123) | 563 | phage tail protein | phage tail protein | | uniclust | UniRef100\_A0A0N4UTZ6 | 99.8 | 9.4e-22 | 1.7e-27 | 145.3 | 123 | (13, 135) | 136 | (1, 123) | 326 | Phage tail protein | Phage tail protein | | uniclust | UniRef100\_A0A7X3TZ69 | 99.8 | 9.8e-22 | 1.9e-27 | 147.5 | 127 | (10, 136) | 136 | (31, 158) | 327 | Phage tail protein | Phage tail protein | | uniclust | UniRef100\_A0A379YFH8 | 99.8 | 1.1e-21 | 2.1e-27 | 140.8 | 125 | (12, 136) | 136 | (25, 166) | 207 | Phage protein U | Phage protein U | | uniclust | UniRef100\_A0A2D8Q6P1 | 99.8 | 1.3e-21 | 2.4e-27 | 148.4 | 126 | (11, 136) | 136 | (282, 407) | 416 | Phage tail protein | Phage tail protein | | uniclust | UniRef100\_A0A7K0GNT9 | 99.8 | 1.3e-21 | 2.5e-27 | 133.5 | 127 | (10, 136) | 136 | (37, 163) | 173 | Phage tail protein | Phage tail protein | | uniclust | UniRef100\_A0A430HF79 | 99.7 | 1.6e-21 | 3e-27 | 142.9 | 125 | (11, 135) | 136 | (2, 126) | 302 | Phage tail protein | Phage tail protein | | uniclust | UniRef100\_A0A8D4LNE0 | 99.7 | 2.1e-21 | 3.9e-27 | 135.9 | 124 | (12, 135) | 136 | (5, 128) | 208 | Phage tail protein | Phage tail protein | | uniclust | UniRef100\_A0A0H3GS01 | 99.7 | 2.2e-21 | 4.3e-27 | 130.8 | 84 | (53, 136) | 136 | (1, 84) | 134 | Putative prophage tail protein | Putative prophage tail protein | | uniclust | UniRef100\_A0A2M7G5P3 | 99.7 | 2.7e-21 | 5.5e-27 | 147.8 | 125 | (11, 136) | 136 | (1, 125) | 306 | Phage tail protein | Phage tail protein | | uniclust | UniRef100\_A0A495YAP1 | 99.7 | 4e-21 | 7.8e-27 | 134.2 | 118 | (19, 136) | 136 | (45, 162) | 170 | Oxidoreductase | Oxidoreductase | | uniclust | UniRef100\_A0A1V3RQN1 | 99.7 | 5.3e-21 | 1e-26 | 139.0 | 126 | (9, 136) | 136 | (54, 179) | 233 | Phage tail protein | Phage tail protein | | uniclust | UniRef100\_A0A1Q6KUC1 | 99.7 | 5.8e-21 | 1.1e-26 | 138.8 | 127 | (9, 136) | 136 | (14, 150) | 228 | LysM domain-containing protein | LysM domain-containing protein | | uniclust | UniRef100\_UPI0011303F02 | 99.7 | 7.3e-21 | 1.4e-26 | 125.1 | 118 | (13, 131) | 136 | (1, 118) | 120 | phage tail protein | phage tail protein | | uniclust | UniRef100\_A0A075KG62 | 99.7 | 7.3e-21 | 1.4e-26 | 134.4 | 109 | (28, 136) | 136 | (70, 182) | 189 | p2 GpU family protein | p2 GpU family protein | | uniclust | UniRef100\_A0A4Q6D3J7 | 99.7 | 8.4e-21 | 1.6e-26 | 121.4 | 85 | (52, 136) | 136 | (2, 86) | 99 | Phage tail protein (Fragment) | Phage tail protein (Fragment) | | uniclust | UniRef100\_A0A0Q8AZW1 | 99.7 | 9e-21 | 1.7e-26 | 127.6 | 124 | (12, 135) | 136 | (1, 126) | 141 | Phage tail protein | Phage tail protein | | uniclust | UniRef100\_A0A0F4NJK3 | 99.7 | 1.4e-20 | 2.9e-26 | 131.4 | 122 | (11, 136) | 136 | (10, 131) | 150 | Tail protein | Tail protein | | uniclust | UniRef100\_A0A0E4G101 | 99.7 | 2.5e-20 | 4.8e-26 | 130.0 | 118 | (19, 136) | 136 | (19, 150) | 171 | Phage tail protein | Phage tail protein | | uniclust | UniRef100\_A0A853I3C0 | 99.7 | 2.7e-20 | 5e-26 | 132.0 | 117 | (20, 136) | 136 | (25, 142) | 227 | Phage tail protein | Phage tail protein | | uniclust | UniRef100\_A0A011NCS8 | 99.7 | 2.8e-20 | 5.8e-26 | 143.1 | 124 | (11, 135) | 136 | (1, 124) | 313 | Phage tail protein | Phage tail protein | | uniclust | UniRef100\_A0A256CB29 | 99.7 | 3.1e-20 | 5.9e-26 | 125.1 | 124 | (12, 135) | 136 | (2, 125) | 144 | Oxidoreductase | Oxidoreductase | | uniclust | UniRef100\_UPI0013EEEE0C | 99.7 | 3.4e-20 | 6.2e-26 | 138.3 | 124 | (13, 136) | 136 | (1, 124) | 347 | phage tail protein | phage tail protein | | uniclust | UniRef100\_A0A940VSA1 | 99.7 | 4.2e-20 | 7.7e-26 | 126.4 | 124 | (12, 136) | 136 | (1, 130) | 173 | Uncharacterized protein | Uncharacterized protein | | uniclust | UniRef100\_A0A0J8DFL5 | 99.7 | 6.1e-20 | 1.3e-25 | 135.3 | 128 | (8, 136) | 136 | (11, 144) | 212 | Uncharacterized protein | Uncharacterized protein | | uniclust | UniRef100\_A0A4P9VGW4 | 99.7 | 8.3e-20 | 1.5e-25 | 135.1 | 126 | (11, 136) | 136 | (184, 309) | 320 | Phage tail protein | Phage tail protein | | uniclust | UniRef100\_A0A1Q6LL69 | 99.7 | 8.4e-20 | 1.6e-25 | 133.2 | 124 | (11, 135) | 136 | (1, 134) | 232 | LysM domain-containing protein | LysM domain-containing protein | | uniclust | UniRef100\_UPI00135C3713 | 99.7 | 1.3e-19 | 2.5e-25 | 129.6 | 128 | (9, 136) | 136 | (22, 166) | 205 | phage tail protein | phage tail protein | | uniclust | UniRef100\_A0A2H4J1T3 | 99.7 | 1.5e-19 | 3e-25 | 119.5 | 103 | (34, 136) | 136 | (3, 105) | 116 | Tail protein | Tail protein | | uniclust | UniRef100\_A0A8S0FTG4 | 99.7 | 2e-19 | 3.9e-25 | 117.6 | 101 | (36, 136) | 136 | (2, 104) | 110 | Phage tail protein | Phage tail protein | | uniclust | UniRef100\_A0A2W5VBP0 | 99.7 | 2.4e-19 | 4.3e-25 | 122.4 | 123 | (13, 135) | 136 | (3, 125) | 168 | Oxidoreductase | Oxidoreductase | | uniclust | UniRef100\_A0A8S5U2I9 | 99.6 | 2.4e-19 | 4.5e-25 | 126.9 | 124 | (12, 136) | 136 | (19, 149) | 221 | Uncharacterized protein | Uncharacterized protein | | uniclust | UniRef100\_A0A0H5AI46 | 99.6 | 2.6e-19 | 4.9e-25 | 132.0 | 118 | (19, 136) | 136 | (163, 280) | 286 | Phage tail protein | Phage tail protein | | uniclust | UniRef100\_A0A8J3E9E2 | 99.6 | 3e-19 | 5.7e-25 | 121.2 | 121 | (10, 136) | 136 | (1, 122) | 140 | Phage tail protein | Phage tail protein | | uniclust | UniRef100\_A0A064AGR8 | 99.6 | 3.6e-19 | 7.3e-25 | 131.1 | 123 | (12, 135) | 136 | (30, 157) | 219 | Phage tail protein | Phage tail protein | | uniclust | UniRef100\_A0A926ZYZ2 | 99.6 | 3.9e-19 | 7.4e-25 | 122.1 | 124 | (11, 135) | 136 | (16, 139) | 159 | Phage tail protein | Phage tail protein | | uniclust | UniRef100\_A0A4Y6UBW6 | 99.6 | 4.5e-19 | 8.2e-25 | 121.9 | 126 | (10, 136) | 136 | (22, 147) | 176 | Phage tail protein | Phage tail protein | | uniclust | UniRef100\_UPI00217550BA | 99.6 | 4.8e-19 | 8.8e-25 | 114.1 | 88 | (13, 100) | 136 | (1, 88) | 114 | phage tail protein | phage tail protein | | uniclust | UniRef100\_A0A4P7KXL2 | 99.6 | 6.4e-19 | 1.2e-24 | 119.7 | 122 | (13, 135) | 136 | (1, 122) | 162 | Phage P2 GpU | Phage P2 GpU | | uniclust | UniRef100\_A0A5T7Y1R6 | 99.6 | 9.7e-19 | 1.8e-24 | 121.9 | 129 | (8, 136) | 136 | (58, 188) | 194 | Phage tail protein | Phage tail protein | | uniclust | UniRef100\_A0A0S3TZR2 | 99.6 | 9.3e-19 | 1.9e-24 | 129.7 | 108 | (28, 136) | 136 | (66, 190) | 220 | Contractile injection system tube protein N-terminal domain-containing protein | Contractile injection system tube protein N-terminal domain-containing protein | | uniclust | UniRef100\_A0A0X8GMF9 | 99.6 | 9.9e-19 | 1.9e-24 | 132.4 | 116 | (20, 136) | 136 | (183, 298) | 304 | Phage tail protein | Phage tail protein | | uniclust | UniRef100\_A0A6L9ABV2 | 99.6 | 1.3e-18 | 2.4e-24 | 110.5 | 95 | (13, 108) | 136 | (1, 95) | 95 | Phage tail protein (Fragment) | Phage tail protein (Fragment) | | uniclust | UniRef100\_A0A0E1UDG7 | 99.6 | 1.6e-18 | 2.9e-24 | 129.5 | 118 | (19, 136) | 136 | (173, 290) | 296 | Phage P2 GpU family protein | Phage P2 GpU family protein | | uniclust | UniRef100\_A0A2X0VB68 | 99.6 | 1.6e-18 | 3e-24 | 118.5 | 127 | (9, 136) | 136 | (1, 135) | 152 | Phage protein U | Phage protein U | | uniclust | UniRef100\_A0A095WG46 | 99.6 | 1.5e-18 | 3.1e-24 | 123.8 | 124 | (12, 136) | 136 | (1, 132) | 174 | Uncharacterized protein | Uncharacterized protein | | uniclust | UniRef100\_A0A2D5PEI7 | 99.6 | 1.9e-18 | 3.7e-24 | 114.7 | 123 | (11, 136) | 136 | (1, 124) | 125 | Phage tail protein | Phage tail protein | | uniclust | UniRef100\_R5BE61 | 99.6 | 2.7e-18 | 4.9e-24 | 119.5 | 123 | (12, 135) | 136 | (36, 164) | 191 | p2 GpU family protein | p2 GpU family protein | | uniclust | UniRef100\_A0A377DJJ0 | 99.6 | 2.5e-18 | 4.9e-24 | 117.6 | 90 | (47, 136) | 136 | (15, 104) | 142 | Tail fiber protein | Tail fiber protein | | uniclust | UniRef100\_A0A0S4RVH7 | 99.6 | 4.1e-18 | 8e-24 | 116.4 | 123 | (11, 135) | 136 | (12, 134) | 138 | Phage tail protein | Phage tail protein | | uniclust | UniRef100\_A0A014P2G8 | 99.6 | 5.1e-18 | 1.1e-23 | 130.0 | 107 | (28, 135) | 136 | (63, 183) | 266 | Peptidoglycan-binding protein | Peptidoglycan-binding protein | | uniclust | UniRef100\_A0A524RVY3 | 99.6 | 5.9e-18 | 1.1e-23 | 122.5 | 125 | (12, 136) | 136 | (3, 127) | 257 | Phage tail protein | Phage tail protein | | uniclust | UniRef100\_A0A7V8U724 | 99.6 | 6.1e-18 | 1.1e-23 | 116.8 | 124 | (12, 135) | 136 | (55, 178) | 179 | Phage tail protein | Phage tail protein | | uniclust | UniRef100\_A0A075WU95 | 99.6 | 9.4e-18 | 1.8e-23 | 118.1 | 121 | (12, 135) | 136 | (3, 124) | 175 | Phage tail protein | Phage tail protein | | uniclust | UniRef100\_A0A162QMP6 | 99.6 | 1e-17 | 1.9e-23 | 121.1 | 122 | (13, 135) | 136 | (1, 128) | 254 | Putative peptidoglycan binding domain protein | Putative peptidoglycan binding domain protein | | uniclust | UniRef100\_UPI001F3F7F85 | 99.5 | 1.3e-17 | 2.4e-23 | 126.4 | 122 | (13, 135) | 136 | (1, 122) | 383 | phage tail protein | phage tail protein | | uniclust | UniRef100\_A0A022MNT4 | 99.5 | 1.1e-17 | 2.5e-23 | 131.4 | 108 | (27, 135) | 136 | (86, 202) | 302 | Peptidase M23B | Peptidase M23B | | uniclust | UniRef100\_A0A6J4N4J5 | 99.5 | 1.6e-17 | 2.9e-23 | 114.6 | 124 | (11, 135) | 136 | (1, 124) | 176 | Phage protein U | Phage protein U | | uniclust | UniRef100\_A0A0N1FE70 | 99.5 | 1.7e-17 | 3.3e-23 | 113.0 | 127 | (9, 135) | 136 | (1, 132) | 142 | Phage tail protein | Phage tail protein | | uniclust | UniRef100\_UPI0000380ED2 | 99.5 | 1.8e-17 | 3.4e-23 | 108.7 | 105 | (3, 107) | 136 | (18, 122) | 126 | phage tail protein | phage tail protein | | uniclust | UniRef100\_UPI00197DB023 | 99.5 | 2.3e-17 | 4.2e-23 | 112.8 | 124 | (11, 136) | 136 | (36, 165) | 165 | phage tail protein | phage tail protein | | uniclust | UniRef100\_A0A0Q0VG01 | 99.5 | 2.2e-17 | 4.3e-23 | 124.6 | 123 | (13, 136) | 136 | (1, 124) | 284 | Phage tail protein | Phage tail protein | | uniclust | UniRef100\_A0A085AFM6 | 99.5 | 2.3e-17 | 4.5e-23 | 117.4 | 123 | (12, 136) | 136 | (24, 147) | 184 | Uncharacterized protein | Uncharacterized protein | | uniclust | UniRef100\_A0A6L9HIG7 | 99.5 | 2.7e-17 | 4.9e-23 | 113.5 | 123 | (14, 136) | 136 | (48, 176) | 176 | Phage tail protein | Phage tail protein | | uniclust | UniRef100\_A0A068T9F9 | 99.5 | 3.1e-17 | 6.3e-23 | 117.3 | 131 | (3, 134) | 136 | (9, 144) | 168 | Phage tail protein | Phage tail protein | | uniclust | UniRef100\_A0A285NP30 | 99.5 | 3.3e-17 | 6.4e-23 | 111.1 | 124 | (10, 135) | 136 | (3, 127) | 134 | Phage protein U | Phage protein U | | uniclust | UniRef100\_A0A4S2HDA3 | 99.5 | 3.7e-17 | 6.7e-23 | 112.3 | 124 | (12, 136) | 136 | (40, 168) | 170 | Phage tail protein | Phage tail protein | | uniclust | UniRef100\_A0A1R1MK76 | 99.5 | 3.8e-17 | 7.1e-23 | 107.0 | 123 | (13, 136) | 136 | (1, 124) | 124 | Phage tail protein | Phage tail protein | | uniclust | UniRef100\_A0A179CYQ5 | 99.5 | 3.8e-17 | 7.6e-23 | 125.4 | 122 | (13, 136) | 136 | (1, 122) | 316 | Tail protein | Tail protein | | uniclust | UniRef100\_UPI000427E38B | 99.5 | 4.5e-17 | 8.5e-23 | 111.2 | 123 | (12, 136) | 136 | (2, 124) | 148 | phage tail protein | phage tail protein | | uniclust | UniRef100\_UPI000764B768 | 99.5 | 6e-17 | 1.1e-22 | 106.6 | 123 | (12, 135) | 136 | (3, 125) | 127 | phage tail protein | phage tail protein | | uniclust | UniRef100\_A0A951W1P6 | 99.5 | 9.2e-17 | 1.7e-22 | 109.1 | 123 | (13, 136) | 136 | (2, 125) | 157 | Phage tail protein | Phage tail protein | | uniclust | UniRef100\_UPI001ABB63DB | 99.5 | 1.1e-16 | 2e-22 | 108.5 | 124 | (11, 134) | 136 | (2, 131) | 148 | phage tail protein | phage tail protein | | uniclust | UniRef100\_UPI00223F0CB2 | 99.5 | 1.6e-16 | 3e-22 | 113.7 | 118 | (19, 136) | 136 | (105, 222) | 228 | phage tail protein | phage tail protein | | uniclust | UniRef100\_A0A812RFT8 | 99.5 | 1.8e-16 | 3.4e-22 | 135.6 | 108 | (29, 136) | 136 | (746, 853) | 1893 | site-specific DNA-methyltransferase (adenine-specific) | site-specific DNA-methyltransferase (adenine-specific) | | uniclust | UniRef100\_A0A7X7NI24 | 99.4 | 2.8e-16 | 5.1e-22 | 108.7 | 121 | (13, 135) | 136 | (1, 121) | 176 | Phage tail protein | Phage tail protein | | uniclust | UniRef100\_A0A376J404 | 99.4 | 2.6e-16 | 5.2e-22 | 100.6 | 80 | (12, 91) | 136 | (2, 81) | 85 | GpU phage protein | GpU phage protein | | uniclust | UniRef100\_UPI001F36ED2C | 99.4 | 3.1e-16 | 5.7e-22 | 119.0 | 124 | (12, 135) | 136 | (8, 143) | 375 | phage tail protein | phage tail protein | | uniclust | UniRef100\_A0A620N5X6 | 99.4 | 4e-16 | 7.3e-22 | 121.6 | 122 | (13, 135) | 136 | (1, 122) | 488 | Phage protein D | Phage protein D | | uniclust | UniRef100\_A0A1C5WMN6 | 99.4 | 4.1e-16 | 7.8e-22 | 114.3 | 124 | (12, 136) | 136 | (114, 244) | 244 | Bacterial SH3 domain | Bacterial SH3 domain | | uniclust | UniRef100\_A0A3P6JH52 | 99.4 | 4.5e-16 | 8.9e-22 | 108.7 | 124 | (12, 136) | 136 | (1, 130) | 147 | Phage tail protein | Phage tail protein | | uniclust | UniRef100\_UPI001A8E6AE5 | 99.4 | 4.9e-16 | 9e-22 | 118.2 | 110 | (27, 136) | 136 | (235, 344) | 383 | N-acetylmuramidase domain-containing protein | N-acetylmuramidase domain-containing protein | | uniclust | UniRef100\_A0A1G3UAP7 | 99.4 | 8.6e-16 | 1.7e-21 | 103.9 | 120 | (13, 135) | 136 | (2, 121) | 124 | Phage tail protein | Phage tail protein | | uniclust | UniRef100\_A0A318KUR9 | 99.4 | 9.9e-16 | 1.8e-21 | 113.7 | 122 | (13, 135) | 136 | (1, 122) | 303 | Phage protein U | Phage protein U | | uniclust | UniRef100\_A0A2W5H3R5 | 99.4 | 1.2e-15 | 2.2e-21 | 104.5 | 95 | (16, 110) | 136 | (1, 95) | 163 | Phage tail protein | Phage tail protein | | uniclust | UniRef100\_A0A482IL72 | 99.4 | 1.6e-15 | 2.9e-21 | 113.0 | 123 | (12, 136) | 136 | (1, 123) | 311 | Phage tail protein | Phage tail protein | | uniclust | UniRef100\_A0A024YYK6 | 99.4 | 1.6e-15 | 3.6e-21 | 119.1 | 109 | (27, 136) | 136 | (58, 184) | 297 | Peptidase M23B | Peptidase M23B | | uniclust | UniRef100\_A0A2W5C6U1 | 99.4 | 2.2e-15 | 4.3e-21 | 102.3 | 120 | (11, 135) | 136 | (1, 124) | 128 | Phage tail protein | Phage tail protein | | uniclust | UniRef100\_UPI0005B3BCC5 | 99.4 | 2.4e-15 | 4.4e-21 | 93.0 | 82 | (13, 94) | 136 | (1, 82) | 85 | phage tail protein | phage tail protein | | uniclust | UniRef100\_A0A9D1QXB7 | 99.4 | 2.4e-15 | 4.5e-21 | 112.3 | 124 | (11, 135) | 136 | (2, 125) | 317 | Phage tail protein | Phage tail protein | | uniclust | UniRef100\_A0A084T293 | 99.4 | 2.2e-15 | 4.5e-21 | 117.0 | 109 | (27, 136) | 136 | (50, 193) | 283 | LysM domain-containing protein | LysM domain-containing protein | | uniclust | UniRef100\_A0A9E8EL20 | 99.4 | 2.8e-15 | 5.1e-21 | 103.3 | 122 | (13, 135) | 136 | (1, 122) | 168 | Phage tail protein | Phage tail protein | | uniclust | UniRef100\_UPI0005609077 | 99.4 | 2.8e-15 | 5.2e-21 | 94.8 | 93 | (43, 136) | 136 | (2, 94) | 97 | phage tail protein | phage tail protein | | uniclust | UniRef100\_E2CFJ9 | 99.4 | 3.1e-15 | 5.9e-21 | 103.9 | 123 | (11, 135) | 136 | (1, 123) | 159 | Putative phage tail protein | Putative phage tail protein | | uniclust | UniRef100\_A0A8S5NYM1 | 99.3 | 3.5e-15 | 6.5e-21 | 103.9 | 124 | (11, 135) | 136 | (1, 130) | 182 | Uncharacterized protein | Uncharacterized protein | | uniclust | UniRef100\_A0A1I5H9R0 | 99.3 | 3.6e-15 | 6.9e-21 | 103.6 | 124 | (11, 134) | 136 | (1, 124) | 149 | Phage protein U | Phage protein U | | uniclust | UniRef100\_A0A2P1VUY8 | 99.3 | 4.4e-15 | 8.3e-21 | 101.1 | 122 | (13, 136) | 136 | (2, 124) | 143 | Phage protein U | Phage protein U | | uniclust | UniRef100\_A0A970G5W5 | 99.3 | 4.6e-15 | 8.5e-21 | 108.8 | 125 | (12, 136) | 136 | (1, 129) | 270 | Phage tail protein | Phage tail protein | | uniclust | UniRef100\_A0A3D1NSN0 | 99.3 | 5.6e-15 | 1.1e-20 | 110.6 | 122 | (13, 135) | 136 | (1, 122) | 280 | Phage tail protein | Phage tail protein | | uniclust | UniRef100\_A0A165W2G2 | 99.3 | 7.4e-15 | 1.5e-20 | 102.0 | 124 | (9, 135) | 136 | (4, 128) | 142 | Phage tail protein | Phage tail protein | | uniclust | UniRef100\_UPI002094A02D | 99.3 | 8.5e-15 | 1.6e-20 | 98.4 | 90 | (46, 135) | 136 | (1, 90) | 141 | phage tail protein | phage tail protein | | uniclust | UniRef100\_A0A150S1J3 | 99.3 | 7.9e-15 | 1.6e-20 | 110.0 | 115 | (21, 136) | 136 | (27, 155) | 231 | LysM domain-containing protein | LysM domain-containing protein | | uniclust | UniRef100\_A0A090ILA7 | 99.3 | 8.8e-15 | 1.7e-20 | 99.3 | 120 | (11, 135) | 136 | (1, 121) | 127 | Phage U-like protein | Phage U-like protein | | uniclust | UniRef100\_UPI000CF8F1C8 | 99.3 | 1.2e-14 | 2.2e-20 | 102.1 | 123 | (12, 135) | 136 | (59, 190) | 191 | phage tail protein | phage tail protein | | uniclust | UniRef100\_A0A5S4YPE8 | 99.3 | 1.3e-14 | 2.4e-20 | 107.0 | 118 | (19, 136) | 136 | (126, 257) | 279 | Phage tail protein | Phage tail protein | | uniclust | UniRef100\_UPI00215D9118 | 99.3 | 1.6e-14 | 2.9e-20 | 94.8 | 109 | (13, 122) | 136 | (1, 109) | 120 | phage tail protein | phage tail protein | | uniclust | UniRef100\_A0A1S0V6E3 | 99.3 | 1.8e-14 | 3.3e-20 | 86.6 | 56 | (80, 135) | 136 | (1, 56) | 66 | Tail protein (Fragment) | Tail protein (Fragment) | | uniclust | UniRef100\_E5VKI8 | 99.3 | 2e-14 | 3.6e-20 | 99.1 | 125 | (11, 136) | 136 | (1, 131) | 166 | Phage P2 GpU | Phage P2 GpU | | uniclust | UniRef100\_A0A1C6BMU7 | 99.3 | 2.3e-14 | 4.3e-20 | 96.5 | 122 | (13, 135) | 136 | (1, 129) | 129 | Phage-related protein | Phage-related protein | | uniclust | UniRef100\_R7HX73 | 99.3 | 2.8e-14 | 5.1e-20 | 100.5 | 116 | (20, 136) | 136 | (71, 192) | 193 | Phage protein U | Phage protein U | | uniclust | UniRef100\_UPI001270A914 | 99.2 | 2.9e-14 | 5.5e-20 | 82.9 | 56 | (13, 68) | 136 | (1, 56) | 57 | phage tail protein | phage tail protein | | uniclust | UniRef100\_UPI00068D9337 | 99.2 | 4e-14 | 7.3e-20 | 105.3 | 122 | (13, 135) | 136 | (1, 122) | 295 | phage tail protein | phage tail protein | | uniclust | UniRef100\_UPI0005EE32AE | 99.2 | 4.8e-14 | 9e-20 | 91.1 | 79 | (10, 88) | 136 | (21, 99) | 100 | phage tail protein | phage tail protein | | uniclust | UniRef100\_A0A423PRP6 | 99.2 | 5.4e-14 | 9.9e-20 | 91.3 | 79 | (11, 89) | 136 | (31, 109) | 111 | Tail protein (Fragment) | Tail protein (Fragment) | | uniclust | UniRef100\_A0A2Z4RAD7 | 99.2 | 5.7e-14 | 1.1e-19 | 105.8 | 122 | (13, 135) | 136 | (1, 122) | 258 | Phage protein U | Phage protein U | | uniclust | UniRef100\_A0A0D6KHC7 | 99.2 | 8.2e-14 | 1.7e-19 | 106.1 | 112 | (22, 135) | 136 | (28, 156) | 238 | LysM domain protein | LysM domain protein | | uniclust | UniRef100\_A0A3G2V4J2 | 99.2 | 1e-13 | 1.9e-19 | 98.4 | 127 | (10, 136) | 136 | (1, 146) | 196 | Phage tail protein | Phage tail protein | | uniclust | UniRef100\_A0A126S8J7 | 99.2 | 1.1e-13 | 2.2e-19 | 86.7 | 67 | (70, 136) | 136 | (3, 69) | 76 | Phage protein U | Phage protein U | | uniclust | UniRef100\_A0A840MJS2 | 99.2 | 1.4e-13 | 2.5e-19 | 107.4 | 123 | (12, 135) | 136 | (4, 126) | 451 | Phage protein U | Phage protein U | | uniclust | UniRef100\_A0A1H7YIQ2 | 99.2 | 1.5e-13 | 2.9e-19 | 99.0 | 124 | (12, 136) | 136 | (1, 134) | 180 | Phage P2 GpU | Phage P2 GpU | | uniclust | UniRef100\_A0A6C1BSU7 | 99.2 | 1.6e-13 | 3e-19 | 95.0 | 121 | (13, 136) | 136 | (2, 122) | 168 | Phage tail protein | Phage tail protein | | uniclust | UniRef100\_A0A7G7ZA20 | 99.1 | 2.5e-13 | 4.6e-19 | 88.7 | 80 | (56, 135) | 136 | (11, 90) | 114 | Phage tail protein | Phage tail protein | | uniclust | UniRef100\_A0A2W4LKE1 | 99.1 | 2.7e-13 | 5.2e-19 | 96.1 | 109 | (27, 136) | 136 | (23, 142) | 169 | Peptidoglycan-binding protein (Fragment) | Peptidoglycan-binding protein (Fragment) | | uniclust | UniRef100\_A0A1T4W4X0 | 99.1 | 3e-13 | 5.5e-19 | 91.7 | 126 | (11, 136) | 136 | (1, 133) | 145 | Phage P2 GpU | Phage P2 GpU | | uniclust | UniRef100\_A0A3C1WUQ3 | 99.1 | 3.2e-13 | 5.8e-19 | 90.8 | 123 | (13, 135) | 136 | (3, 136) | 137 | Phage tail protein | Phage tail protein | | uniclust | UniRef100\_A0A836CGV5 | 99.1 | 3.3e-13 | 6.1e-19 | 113.2 | 107 | (30, 136) | 136 | (703, 809) | 1085 | Phage tail tape measure protein domain-containing protein | Phage tail tape measure protein domain-containing protein | | uniclust | UniRef100\_A0A8J6Y7F4 | 99.1 | 3.6e-13 | 6.5e-19 | 94.4 | 122 | (13, 135) | 136 | (18, 161) | 181 | Phage tail protein | Phage tail protein | | uniclust | UniRef100\_A0A062UU40 | 99.1 | 3.2e-13 | 6.6e-19 | 103.7 | 103 | (27, 135) | 136 | (49, 161) | 258 | Contractile injection system tube protein N-terminal domain-containing protein | Contractile injection system tube protein N-terminal domain-containing protein | | uniclust | UniRef100\_UPI0021562D4A | 99.1 | 4.5e-13 | 8.3e-19 | 88.2 | 86 | (11, 96) | 136 | (27, 113) | 119 | phage tail protein | phage tail protein | | uniclust | UniRef100\_A0A1B6BCU7 | 99.1 | 4.2e-13 | 9e-19 | 104.3 | 113 | (21, 135) | 136 | (45, 175) | 265 | Contractile injection system tube protein N-terminal domain-containing protein | Contractile injection system tube protein N-terminal domain-containing protein | | uniclust | UniRef100\_A0A066U3S9 | 99.1 | 4.5e-13 | 9.8e-19 | 107.1 | 113 | (20, 136) | 136 | (66, 194) | 325 | Peptidase M23B | Peptidase M23B | | uniclust | UniRef100\_A0A173SAB9 | 99.1 | 5.7e-13 | 1.1e-18 | 91.6 | 123 | (12, 135) | 136 | (29, 157) | 158 | Phage protein U | Phage protein U | | uniclust | UniRef100\_UPI000B16A0A2 | 99.1 | 9.3e-13 | 1.7e-18 | 95.0 | 122 | (13, 135) | 136 | (2, 123) | 223 | phage tail protein | phage tail protein | | uniclust | UniRef100\_Q602Z4 | 99.1 | 1.1e-12 | 2.1e-18 | 92.6 | 122 | (13, 135) | 136 | (2, 123) | 189 | Conserved domain protein | Conserved domain protein | | uniclust | UniRef100\_A0A450YW55 | 99.0 | 1.5e-12 | 2.7e-18 | 98.2 | 121 | (13, 135) | 136 | (2, 122) | 319 | Phage P2 GpU | Phage P2 GpU | | uniclust | UniRef100\_A0A3S4YZC4 | 99.0 | 1.7e-12 | 3.1e-18 | 85.6 | 104 | (11, 114) | 136 | (2, 111) | 114 | Phage protein U | Phage protein U | | uniclust | UniRef100\_A0A2M8PTG2 | 99.0 | 1.6e-12 | 3.2e-18 | 96.1 | 109 | (27, 136) | 136 | (27, 143) | 221 | Peptidoglycan-binding protein | Peptidoglycan-binding protein | | uniclust | UniRef100\_UPI00190F23EE | 99.0 | 1.8e-12 | 3.4e-18 | 79.2 | 73 | (33, 105) | 136 | (3, 75) | 76 | phage tail protein | phage tail protein | | uniclust | UniRef100\_A0A7Z0UY93 | 99.0 | 2e-12 | 3.7e-18 | 86.6 | 71 | (65, 135) | 136 | (2, 72) | 131 | Phage tail protein | Phage tail protein | | uniclust | UniRef100\_Q31HS8 | 99.0 | 2.7e-12 | 4.9e-18 | 87.1 | 124 | (12, 136) | 136 | (5, 139) | 142 | Phage P2 GpU family protein | Phage P2 GpU family protein | | uniclust | UniRef100\_A0A8X6WSB0 | 99.0 | 2.8e-12 | 5.2e-18 | 96.5 | 108 | (28, 135) | 136 | (10, 117) | 309 | Phage late control gene d protein GpD | Phage late control gene d protein GpD | | uniclust | UniRef100\_A0A317H5Z8 | 99.0 | 3.3e-12 | 6e-18 | 90.5 | 125 | (11, 135) | 136 | (47, 171) | 192 | Phage tail protein | Phage tail protein | | uniclust | UniRef100\_UPI001F273361 | 99.0 | 3.4e-12 | 6.4e-18 | 86.9 | 120 | (14, 136) | 136 | (8, 129) | 131 | phage tail protein | phage tail protein | | uniclust | UniRef100\_A0A0D6ARK4 | 99.0 | 4e-12 | 7.3e-18 | 86.9 | 124 | (11, 135) | 136 | (1, 127) | 148 | Phage protein | Phage protein | | uniclust | UniRef100\_UPI0015C58154 | 99.0 | 4.1e-12 | 7.5e-18 | 79.8 | 84 | (13, 96) | 136 | (3, 86) | 88 | phage tail protein | phage tail protein | | uniclust | UniRef100\_UPI0021794BC2 | 99.0 | 4.2e-12 | 7.7e-18 | 83.5 | 88 | (47, 135) | 136 | (2, 90) | 116 | phage tail protein | phage tail protein | | uniclust | UniRef100\_A0A0B1YHP6 | 98.9 | 5e-12 | 1.1e-17 | 101.2 | 119 | (4, 133) | 136 | (11, 129) | 335 | DNA circulation N-terminal domain-containing protein | DNA circulation N-terminal domain-containing protein | | uniclust | UniRef100\_UPI0020C15A30 | 98.9 | 5.8e-12 | 1.1e-17 | 82.4 | 89 | (47, 135) | 136 | (2, 90) | 112 | phage tail protein | phage tail protein | | uniclust | UniRef100\_UPI00224967BF | 98.9 | 6.1e-12 | 1.1e-17 | 88.6 | 107 | (27, 133) | 136 | (54, 160) | 183 | phage tail protein | phage tail protein | | uniclust | UniRef100\_UPI001F07EFB3 | 98.9 | 7.1e-12 | 1.3e-17 | 103.9 | 88 | (48, 135) | 136 | (722, 809) | 847 | phage tail tape measure protein | phage tail tape measure protein | | uniclust | UniRef100\_UPI001FCD6EEF | 98.9 | 9.2e-12 | 1.7e-17 | 80.1 | 86 | (9, 94) | 136 | (7, 94) | 99 | phage tail protein | phage tail protein | | uniclust | UniRef100\_A0A016QLM3 | 98.9 | 1e-11 | 2e-17 | 92.3 | 109 | (27, 135) | 136 | (34, 167) | 213 | Peptidoglycan-binding LysM | Peptidoglycan-binding LysM | | uniclust | UniRef100\_A0A3A4NRP5 | 98.9 | 1.4e-11 | 2.7e-17 | 93.1 | 107 | (28, 135) | 136 | (23, 139) | 292 | Contractile injection system tube protein N-terminal domain-containing protein | Contractile injection system tube protein N-terminal domain-containing protein | | uniclust | UniRef100\_A0A014NMA0 | 98.9 | 1.3e-11 | 2.8e-17 | 98.5 | 107 | (28, 135) | 136 | (78, 202) | 294 | Peptidoglycan-binding protein | Peptidoglycan-binding protein | | uniclust | UniRef100\_A0A174T297 | 98.9 | 1.5e-11 | 3e-17 | 91.5 | 125 | (9, 136) | 136 | (10, 140) | 195 | Phage tail protein | Phage tail protein | | uniclust | UniRef100\_A0A0P6Y0I8 | 98.9 | 1.5e-11 | 3.3e-17 | 98.8 | 112 | (21, 135) | 136 | (30, 154) | 330 | Contractile injection system tube protein N-terminal domain-containing protein | Contractile injection system tube protein N-terminal domain-containing protein | | uniclust | UniRef100\_A0A0S4XMR6 | 98.9 | 1.8e-11 | 3.5e-17 | 82.3 | 117 | (13, 135) | 136 | (1, 117) | 119 | Phage tail protein | Phage tail protein | | uniclust | UniRef100\_UPI0018835B78 | 98.9 | 2e-11 | 3.7e-17 | 78.1 | 60 | (12, 71) | 136 | (27, 86) | 93 | phage tail protein | phage tail protein | | uniclust | UniRef100\_UPI000583C80F | 98.9 | 2e-11 | 3.8e-17 | 74.4 | 60 | (13, 73) | 136 | (1, 60) | 66 | phage tail protein | phage tail protein | | uniclust | UniRef100\_A0A0L6JHX4 | 98.8 | 2.2e-11 | 4.5e-17 | 92.7 | 110 | (27, 136) | 136 | (52, 176) | 233 | Contractile injection system tube protein N-terminal domain-containing protein | Contractile injection system tube protein N-terminal domain-containing protein | | uniclust | UniRef100\_A0A2N8GSE5 | 98.8 | 2.7e-11 | 5e-17 | 78.6 | 76 | (19, 94) | 136 | (22, 97) | 100 | Phage tail protein (Fragment) | Phage tail protein (Fragment) | | uniclust | UniRef100\_A0A1C0V8H7 | 98.8 | 3.8e-11 | 7.1e-17 | 83.5 | 121 | (13, 134) | 136 | (5, 125) | 163 | Phage tail protein | Phage tail protein | | uniclust | UniRef100\_A0A945U371 | 98.8 | 3.8e-11 | 7.1e-17 | 85.2 | 117 | (18, 136) | 136 | (17, 157) | 168 | Peptidoglycan-binding protein (Fragment) | Peptidoglycan-binding protein (Fragment) | | uniclust | UniRef100\_A0A842IWC8 | 98.8 | 3.9e-11 | 7.2e-17 | 86.5 | 124 | (12, 136) | 136 | (31, 159) | 200 | Phage tail protein | Phage tail protein | | uniclust | UniRef100\_A0A327Q841 | 98.8 | 5.7e-11 | 1e-16 | 88.8 | 122 | (13, 135) | 136 | (1, 122) | 280 | GpU protein | GpU protein | | uniclust | UniRef100\_UPI000629E4F5 | 98.8 | 6.6e-11 | 1.2e-16 | 85.4 | 63 | (74, 136) | 136 | (113, 175) | 212 | phage regulatory CII family protein | phage regulatory CII family protein | | uniclust | UniRef100\_A0A976C8U3 | 98.8 | 6.7e-11 | 1.2e-16 | 88.5 | 123 | (13, 136) | 136 | (1, 123) | 280 | Uncharacterized protein | Uncharacterized protein | | uniclust | UniRef100\_A0A2G2BDF5 | 98.8 | 6.7e-11 | 1.3e-16 | 76.3 | 59 | (78, 136) | 136 | (11, 69) | 92 | Phage tail protein | Phage tail protein | | uniclust | UniRef100\_A0A378TUW3 | 98.8 | 7.2e-11 | 1.3e-16 | 96.1 | 121 | (13, 134) | 136 | (1, 121) | 606 | Putative phage tail fiber protein | Putative phage tail fiber protein | | uniclust | UniRef100\_A0A8S5UB81 | 98.7 | 8.9e-11 | 1.6e-16 | 80.4 | 120 | (13, 133) | 136 | (2, 130) | 145 | Uncharacterized protein | Uncharacterized protein | | uniclust | UniRef100\_A0A0V7ZMG3 | 98.7 | 8e-11 | 1.6e-16 | 91.1 | 107 | (28, 134) | 136 | (66, 187) | 256 | Contractile injection system tube protein N-terminal domain-containing protein | Contractile injection system tube protein N-terminal domain-containing protein | | uniclust | UniRef100\_A0A8E3ZHR9 | 98.7 | 8.8e-11 | 1.7e-16 | 85.1 | 57 | (13, 69) | 136 | (1, 57) | 196 | Late control gene D protein | Late control gene D protein | | uniclust | UniRef100\_A0A3S4FEQ5 | 98.7 | 9e-11 | 1.7e-16 | 68.7 | 56 | (13, 68) | 136 | (1, 56) | 57 | Phage tail protein | Phage tail protein | | uniclust | UniRef100\_A0A2S7JR76 | 98.7 | 9.1e-11 | 1.7e-16 | 86.6 | 118 | (16, 136) | 136 | (63, 180) | 250 | Phage tail protein | Phage tail protein | | uniclust | UniRef100\_UPI002364701C | 98.7 | 1.1e-10 | 2e-16 | 83.3 | 122 | (13, 136) | 136 | (64, 186) | 193 | phage tail protein | phage tail protein | | uniclust | UniRef100\_A0A011PSV8 | 98.7 | 9.4e-11 | 2e-16 | 91.2 | 107 | (28, 135) | 136 | (39, 160) | 244 | Contractile injection system tube protein N-terminal domain-containing protein | Contractile injection system tube protein N-terminal domain-containing protein | | uniclust | UniRef100\_UPI000829C05D | 98.7 | 1.2e-10 | 2.2e-16 | 83.3 | 122 | (12, 135) | 136 | (2, 124) | 196 | phage tail protein | phage tail protein | | uniclust | UniRef100\_A0A377K9Z3 | 98.7 | 1.3e-10 | 2.4e-16 | 88.6 | 75 | (45, 119) | 136 | (247, 321) | 328 | Putative phage tail tape measure protein | Putative phage tail tape measure protein | | uniclust | UniRef100\_A0A1E2WLU3 | 98.7 | 1.3e-10 | 2.5e-16 | 88.9 | 108 | (28, 136) | 136 | (40, 161) | 259 | Contractile injection system tube protein N-terminal domain-containing protein | Contractile injection system tube protein N-terminal domain-containing protein | | uniclust | UniRef100\_A0A0P9CJQ9 | 98.7 | 1.7e-10 | 3.1e-16 | 74.7 | 86 | (50, 135) | 136 | (2, 89) | 101 | Oxidoreductase (Fragment) | Oxidoreductase (Fragment) | | uniclust | UniRef100\_A0A377I4B1 | 98.7 | 2.1e-10 | 3.9e-16 | 73.6 | 84 | (13, 98) | 136 | (1, 84) | 91 | Putative bacteriophage V tail protein | Putative bacteriophage V tail protein | | uniclust | UniRef100\_A0A177M7D1 | 98.7 | 1.9e-10 | 4e-16 | 95.1 | 108 | (27, 135) | 136 | (42, 161) | 428 | Contractile injection system tube protein N-terminal domain-containing protein | Contractile injection system tube protein N-terminal domain-containing protein | | uniclust | UniRef100\_A0A0Q6MK11 | 98.7 | 1.9e-10 | 4e-16 | 90.9 | 107 | (28, 135) | 136 | (58, 178) | 288 | Contractile injection system tube protein N-terminal domain-containing protein | Contractile injection system tube protein N-terminal domain-containing protein | | uniclust | UniRef100\_B9NM79 | 98.6 | 2.6e-10 | 4.8e-16 | 72.4 | 75 | (62, 136) | 136 | (2, 76) | 90 | Putative phage tail protein U | Putative phage tail protein U | | uniclust | UniRef100\_A0A0B6D192 | 98.6 | 2.9e-10 | 5.3e-16 | 75.6 | 116 | (15, 136) | 136 | (2, 117) | 118 | Phage P2 GpU family protein | Phage P2 GpU family protein | | uniclust | UniRef100\_A0A1Q7YAK2 | 98.6 | 2.6e-10 | 5.5e-16 | 95.3 | 105 | (27, 135) | 136 | (43, 156) | 447 | Contractile injection system tube protein N-terminal domain-containing protein | Contractile injection system tube protein N-terminal domain-containing protein | | uniclust | UniRef100\_A0A7X6FT88 | 98.6 | 3.4e-10 | 6.3e-16 | 68.1 | 61 | (12, 72) | 136 | (1, 61) | 66 | Uncharacterized protein | Uncharacterized protein | | uniclust | UniRef100\_UPI00192B53BF | 98.6 | 3.6e-10 | 6.7e-16 | 71.6 | 62 | (75, 136) | 136 | (9, 70) | 88 | phage tail protein | phage tail protein | | uniclust | UniRef100\_UPI0021B6119D | 98.6 | 3.7e-10 | 6.8e-16 | 75.2 | 115 | (13, 135) | 136 | (2, 116) | 119 | phage tail protein | phage tail protein | | uniclust | UniRef100\_A0A9E6URY0 | 98.6 | 3.7e-10 | 6.8e-16 | 89.2 | 115 | (20, 135) | 136 | (263, 377) | 447 | Phage tail protein | Phage tail protein | | uniclust | UniRef100\_A0A1B9SFL0 | 98.6 | 3.3e-10 | 7.2e-16 | 90.0 | 116 | (7, 133) | 136 | (16, 131) | 285 | Phage tail protein | Phage tail protein | | uniclust | UniRef100\_A0A0F0HZW4 | 98.6 | 3.4e-10 | 7.3e-16 | 94.3 | 107 | (27, 135) | 136 | (38, 157) | 443 | Contractile injection system tube protein N-terminal domain-containing protein | Contractile injection system tube protein N-terminal domain-containing protein | | uniclust | UniRef100\_A0A0H3ZMX6 | 98.6 | 4.9e-10 | 8.9e-16 | 72.3 | 72 | (18, 89) | 136 | (17, 88) | 98 | Uncharacterized protein | Uncharacterized protein | | uniclust | UniRef100\_UPI000E5BC930 | 98.6 | 5.4e-10 | 1e-15 | 72.8 | 98 | (13, 112) | 136 | (1, 98) | 98 | phage tail protein | phage tail protein | | uniclust | UniRef100\_A0A1H8C8S6 | 98.6 | 6.5e-10 | 1.3e-15 | 86.8 | 113 | (21, 135) | 136 | (50, 181) | 266 | Contractile injection system tube protein N-terminal domain-containing protein | Contractile injection system tube protein N-terminal domain-containing protein | | uniclust | UniRef100\_A0A536CDU8 | 98.6 | 7.7e-10 | 1.4e-15 | 77.1 | 115 | (18, 135) | 136 | (16, 149) | 159 | Contractile injection system tube protein N-terminal domain-containing protein (Fragment) | Contractile injection system tube protein N-terminal domain-containing protein (Fragment) | | uniclust | UniRef100\_A0A0D6P4T5 | 98.5 | 7e-10 | 1.5e-15 | 87.6 | 119 | (7, 132) | 136 | (18, 136) | 269 | Phage protein | Phage protein | | uniclust | UniRef100\_A0A2X1L4N2 | 98.5 | 8.2e-10 | 1.5e-15 | 73.4 | 69 | (68, 136) | 136 | (1, 69) | 108 | Tail fiber protein | Tail fiber protein | | uniclust | UniRef100\_UPI001F155EF8 | 98.5 | 8.5e-10 | 1.6e-15 | 77.9 | 108 | (20, 130) | 136 | (55, 162) | 174 | phage tail protein | phage tail protein | | uniclust | UniRef100\_A0A545SBB2 | 98.5 | 8.8e-10 | 1.6e-15 | 65.0 | 58 | (11, 68) | 136 | (2, 59) | 59 | Oxidoreductase (Fragment) | Oxidoreductase (Fragment) | | uniclust | UniRef100\_A0A2A2HJB8 | 98.5 | 1e-09 | 1.9e-15 | 67.1 | 48 | (89, 136) | 136 | (1, 48) | 66 | Phage tail protein (Fragment) | Phage tail protein (Fragment) | | uniclust | UniRef100\_A0A080IDD0 | 98.5 | 9.8e-10 | 1.9e-15 | 96.6 | 87 | (50, 136) | 136 | (814, 900) | 944 | Phage tail tape measure protein, TP901 family, core region | Phage tail tape measure protein, TP901 family, core region | | uniclust | UniRef100\_UPI001ABB4451 | 98.5 | 1.1e-09 | 2e-15 | 65.9 | 62 | (11, 72) | 136 | (2, 63) | 65 | phage tail protein | phage tail protein | | uniclust | UniRef100\_A0A3E0DS09 | 98.5 | 1.1e-09 | 2e-15 | 70.9 | 78 | (11, 90) | 136 | (1, 79) | 92 | GpU protein | GpU protein | | uniclust | UniRef100\_A0A074LNR3 | 98.5 | 1e-09 | 2.1e-15 | 82.5 | 121 | (5, 135) | 136 | (6, 126) | 211 | LysM domain-containing protein | LysM domain-containing protein | | uniclust | UniRef100\_A0A661D5Z7 | 98.5 | 1.3e-09 | 2.5e-15 | 79.6 | 113 | (21, 135) | 136 | (28, 154) | 170 | Contractile injection system tube protein N-terminal domain-containing protein (Fragment) | Contractile injection system tube protein N-terminal domain-containing protein (Fragment) | | uniclust | UniRef100\_A0A022PIZ6 | 98.5 | 1.2e-09 | 2.6e-15 | 86.4 | 107 | (28, 134) | 136 | (39, 161) | 281 | Contractile injection system tube protein N-terminal domain-containing protein | Contractile injection system tube protein N-terminal domain-containing protein | | uniclust | UniRef100\_A0A959IW21 | 98.5 | 1.5e-09 | 2.9e-15 | 76.5 | 125 | (8, 133) | 136 | (3, 148) | 149 | Contractile injection system tube protein N-terminal domain-containing protein (Fragment) | Contractile injection system tube protein N-terminal domain-containing protein (Fragment) | | uniclust | UniRef100\_A0A0F5IZK2 | 98.5 | 1.5e-09 | 3e-15 | 82.7 | 107 | (28, 135) | 136 | (36, 157) | 230 | Contractile injection system tube protein N-terminal domain-containing protein | Contractile injection system tube protein N-terminal domain-containing protein | | uniclust | UniRef100\_UPI001BD310B7 | 98.5 | 1.9e-09 | 3.4e-15 | 73.9 | 91 | (18, 108) | 136 | (40, 139) | 140 | phage tail protein | phage tail protein | | uniclust | UniRef100\_A0A011NTK7 | 98.5 | 1.7e-09 | 3.5e-15 | 86.0 | 107 | (27, 134) | 136 | (34, 161) | 293 | Uncharacterized protein | Uncharacterized protein | | uniclust | UniRef100\_A0A0A7HAL1 | 98.5 | 1.8e-09 | 3.8e-15 | 83.6 | 114 | (20, 135) | 136 | (47, 164) | 248 | Peptidoglycan-binding protein LysM | Peptidoglycan-binding protein LysM | | uniclust | UniRef100\_A0A812QV43 | 98.4 | 3.2e-09 | 5.9e-15 | 90.9 | 92 | (36, 127) | 136 | (530, 633) | 1104 | GpFI protein | GpFI protein | | uniclust | UniRef100\_A0A656GLE8 | 98.4 | 4.1e-09 | 7.5e-15 | 64.7 | 71 | (35, 105) | 136 | (2, 72) | 72 | Uncharacterized protein (Fragment) | Uncharacterized protein (Fragment) | | uniclust | UniRef100\_A0A8T5Z531 | 98.4 | 3.9e-09 | 7.5e-15 | 66.5 | 62 | (75, 136) | 136 | (7, 68) | 74 | Phage tail protein | Phage tail protein | | uniclust | UniRef100\_UPI0006D28F55 | 98.4 | 4.2e-09 | 7.8e-15 | 69.4 | 99 | (13, 111) | 136 | (2, 104) | 108 | phage tail protein | phage tail protein | | uniclust | UniRef100\_A0A101JEW8 | 98.4 | 3.9e-09 | 7.9e-15 | 79.9 | 108 | (27, 135) | 136 | (30, 176) | 203 | Contractile injection system tube protein N-terminal domain-containing protein | Contractile injection system tube protein N-terminal domain-containing protein | | uniclust | UniRef100\_UPI001E4C1914 | 98.4 | 4.6e-09 | 8.4e-15 | 66.3 | 73 | (63, 135) | 136 | (2, 74) | 84 | phage tail protein | phage tail protein | | uniclust | UniRef100\_A0A0X8JTK8 | 98.4 | 5.2e-09 | 9.7e-15 | 78.9 | 122 | (13, 135) | 136 | (1, 132) | 263 | Phage tail protein | Phage tail protein | | uniclust | UniRef100\_UPI000B1FC26E | 98.4 | 5.7e-09 | 1e-14 | 65.0 | 57 | (6, 62) | 136 | (21, 77) | 77 | phage tail protein | phage tail protein | | uniclust | UniRef100\_A0A4V1M2L6 | 98.4 | 5.7e-09 | 1e-14 | 72.8 | 73 | (13, 86) | 136 | (1, 73) | 155 | Uncharacterized protein | Uncharacterized protein | | uniclust | UniRef100\_UPI001483AD28 | 98.3 | 6.2e-09 | 1.1e-14 | 66.8 | 50 | (87, 136) | 136 | (2, 51) | 88 | phage tail protein | phage tail protein | | uniclust | UniRef100\_A0A6P0M9Y5 | 98.3 | 8.7e-09 | 1.6e-14 | 69.6 | 118 | (12, 134) | 136 | (1, 122) | 125 | Phage tail protein | Phage tail protein | | uniclust | UniRef100\_UPI000AA14590 | 98.3 | 9e-09 | 1.7e-14 | 70.5 | 68 | (48, 115) | 136 | (68, 135) | 136 | phage tail protein | phage tail protein | | uniclust | UniRef100\_A0A011MMX4 | 98.3 | 9.6e-09 | 2e-14 | 83.5 | 112 | (19, 134) | 136 | (25, 145) | 352 | Contractile injection system tube protein N-terminal domain-containing protein | Contractile injection system tube protein N-terminal domain-containing protein | | uniclust | UniRef100\_A0A2M8WDQ8 | 98.3 | 1.2e-08 | 2.1e-14 | 67.5 | 79 | (57, 135) | 136 | (29, 107) | 108 | Uncharacterized protein | Uncharacterized protein | | uniclust | UniRef100\_A0A496NCJ1 | 98.3 | 1.2e-08 | 2.2e-14 | 65.5 | 89 | (48, 136) | 136 | (1, 91) | 91 | Phage tail protein (Fragment) | Phage tail protein (Fragment) | | uniclust | UniRef100\_A0A936B9Z8 | 98.2 | 1.4e-08 | 2.8e-14 | 80.0 | 112 | (22, 135) | 136 | (75, 215) | 288 | Contractile injection system tube protein N-terminal domain-containing protein | Contractile injection system tube protein N-terminal domain-containing protein | | uniclust | UniRef100\_UPI00211C54AE | 98.2 | 1.6e-08 | 2.9e-14 | 69.4 | 106 | (25, 132) | 136 | (2, 107) | 136 | phage tail protein | phage tail protein | | uniclust | UniRef100\_A0A4C4L837 | 98.2 | 1.7e-08 | 3.2e-14 | 65.8 | 55 | (10, 64) | 136 | (27, 81) | 100 | Uncharacterized protein | Uncharacterized protein | | uniclust | UniRef100\_UPI001F061B49 | 98.2 | 2e-08 | 3.7e-14 | 68.8 | 78 | (57, 134) | 136 | (8, 85) | 135 | phage tail protein | phage tail protein | | uniclust | UniRef100\_UPI00146D20ED | 98.2 | 2.1e-08 | 3.8e-14 | 60.4 | 56 | (81, 136) | 136 | (1, 56) | 63 | phage tail protein | phage tail protein | | uniclust | UniRef100\_A0A1Q8A398 | 98.2 | 2.1e-08 | 4e-14 | 70.5 | 103 | (27, 135) | 136 | (30, 140) | 148 | Contractile injection system tube protein N-terminal domain-containing protein | Contractile injection system tube protein N-terminal domain-containing protein | | uniclust | UniRef100\_A0A7S4ZV00 | 98.2 | 2.1e-08 | 4.1e-14 | 69.5 | 112 | (14, 125) | 136 | (6, 120) | 131 | Phage P2 GpU family protein | Phage P2 GpU family protein | | uniclust | UniRef100\_UPI001374B094 | 98.2 | 2.2e-08 | 4.1e-14 | 68.4 | 60 | (76, 135) | 136 | (59, 118) | 133 | phage tail protein | phage tail protein | | uniclust | UniRef100\_A0A0M0SE01 | 98.2 | 2e-08 | 4.2e-14 | 80.5 | 116 | (18, 136) | 136 | (49, 176) | 296 | Contractile injection system tube protein N-terminal domain-containing protein | Contractile injection system tube protein N-terminal domain-containing protein | | uniclust | UniRef100\_A0A2I7R3K3 | 98.2 | 2.5e-08 | 4.6e-14 | 68.1 | 121 | (14, 135) | 136 | (6, 129) | 131 | Uncharacterized protein | Uncharacterized protein | | uniclust | UniRef100\_A0A2G6EZG3 | 98.2 | 2.6e-08 | 4.8e-14 | 75.5 | 123 | (12, 135) | 136 | (31, 159) | 246 | Phage tail protein | Phage tail protein | | uniclust | UniRef100\_A0A853IPJ9 | 98.2 | 2.7e-08 | 4.9e-14 | 62.8 | 73 | (64, 136) | 136 | (2, 77) | 82 | Phage tail protein | Phage tail protein | | uniclust | UniRef100\_UPI0018F0E200 | 98.1 | 3.6e-08 | 6.6e-14 | 59.2 | 57 | (80, 136) | 136 | (3, 59) | 62 | phage tail protein | phage tail protein | | uniclust | UniRef100\_A0A1Q7YH54 | 98.1 | 3.5e-08 | 6.7e-14 | 71.0 | 114 | (18, 135) | 136 | (17, 137) | 157 | Contractile injection system tube protein N-terminal domain-containing protein | Contractile injection system tube protein N-terminal domain-containing protein | | uniclust | UniRef100\_A0A484X1E5 | 98.1 | 3.6e-08 | 6.8e-14 | 61.6 | 60 | (13, 72) | 136 | (1, 60) | 74 | GpU phage protein | GpU phage protein | | uniclust | UniRef100\_A0A1V5LS04 | 98.1 | 3.9e-08 | 7.5e-14 | 72.1 | 105 | (28, 135) | 136 | (43, 161) | 174 | Contractile injection system tube protein N-terminal domain-containing protein | Contractile injection system tube protein N-terminal domain-containing protein | | uniclust | UniRef100\_A0A7W0VBY2 | 98.1 | 4.5e-08 | 8.2e-14 | 67.4 | 87 | (49, 135) | 136 | (21, 127) | 137 | Contractile injection system tube protein N-terminal domain-containing protein | Contractile injection system tube protein N-terminal domain-containing protein | | uniclust | UniRef100\_UPI001EE21EE9 | 98.1 | 4.5e-08 | 8.2e-14 | 63.6 | 77 | (13, 89) | 136 | (1, 80) | 96 | phage tail protein | phage tail protein | | uniclust | UniRef100\_UPI0007EEB1E3 | 98.1 | 4.6e-08 | 8.5e-14 | 63.2 | 83 | (15, 102) | 136 | (2, 84) | 88 | phage tail protein | phage tail protein | | uniclust | UniRef100\_UPI0020223C4F | 98.1 | 4.8e-08 | 8.8e-14 | 61.5 | 68 | (68, 136) | 136 | (3, 71) | 80 | phage tail protein | phage tail protein | | uniclust | UniRef100\_A0A0F9K3Y5 | 98.1 | 4.6e-08 | 9.2e-14 | 76.6 | 108 | (28, 135) | 136 | (33, 151) | 272 | Tip attachment protein J domain-containing protein | Tip attachment protein J domain-containing protein | | uniclust | UniRef100\_A0A366DKR3 | 98.1 | 5.3e-08 | 9.8e-14 | 62.6 | 64 | (13, 76) | 136 | (1, 64) | 90 | GpU protein | GpU protein | | uniclust | UniRef100\_A0A099ICH7 | 98.1 | 4.9e-08 | 1e-13 | 74.2 | 110 | (20, 133) | 136 | (20, 149) | 202 | Contractile injection system tube protein N-terminal domain-containing protein | Contractile injection system tube protein N-terminal domain-containing protein | | uniclust | UniRef100\_A0A947CVW6 | 98.1 | 5.5e-08 | 1e-13 | 70.1 | 117 | (18, 135) | 136 | (16, 142) | 187 | Contractile injection system tube protein N-terminal domain-containing protein | Contractile injection system tube protein N-terminal domain-containing protein | | uniclust | UniRef100\_UPI001FC8D6E6 | 98.1 | 5.6e-08 | 1e-13 | 59.9 | 51 | (86, 136) | 136 | (2, 52) | 71 | phage tail protein | phage tail protein | | uniclust | UniRef100\_A0A968ZAB9 | 98.1 | 5.7e-08 | 1.1e-13 | 73.2 | 107 | (28, 135) | 136 | (39, 166) | 263 | Protein kinase domain-containing protein | Protein kinase domain-containing protein | | uniclust | UniRef100\_A0A6B2WT17 | 98.1 | 5.6e-08 | 1.1e-13 | 70.4 | 114 | (22, 136) | 136 | (39, 170) | 173 | Peptidase M23 (Fragment) | Peptidase M23 (Fragment) | | uniclust | UniRef100\_A0A928YH49 | 98.1 | 5.8e-08 | 1.1e-13 | 70.0 | 121 | (12, 135) | 136 | (2, 128) | 166 | Uncharacterized protein | Uncharacterized protein | | uniclust | UniRef100\_A0A0Q9U2K6 | 98.1 | 5.6e-08 | 1.2e-13 | 82.5 | 107 | (27, 135) | 136 | (31, 151) | 489 | Contractile injection system tube protein N-terminal domain-containing protein | Contractile injection system tube protein N-terminal domain-containing protein | | uniclust | UniRef100\_A0A7J0CYE3 | 98.1 | 6.5e-08 | 1.2e-13 | 74.6 | 108 | (27, 135) | 136 | (38, 163) | 319 | LysM domain-containing protein | LysM domain-containing protein | | uniclust | UniRef100\_A0A7V9L357 | 98.0 | 9.6e-08 | 1.8e-13 | 70.2 | 121 | (14, 135) | 136 | (40, 183) | 214 | Contractile injection system tube protein N-terminal domain-containing protein | Contractile injection system tube protein N-terminal domain-containing protein | | uniclust | UniRef100\_A0A9D1LT46 | 98.0 | 1e-07 | 1.9e-13 | 69.6 | 122 | (13, 135) | 136 | (2, 129) | 202 | Phage tail protein | Phage tail protein | | uniclust | UniRef100\_UPI00202512B1 | 98.0 | 1.1e-07 | 2e-13 | 56.0 | 47 | (90, 136) | 136 | (3, 49) | 55 | phage tail protein | phage tail protein | | uniclust | UniRef100\_A0A433VYQ6 | 98.0 | 1.1e-07 | 2e-13 | 70.6 | 109 | (28, 136) | 136 | (24, 157) | 197 | Contractile injection system tube protein N-terminal domain-containing protein | Contractile injection system tube protein N-terminal domain-containing protein | | uniclust | UniRef100\_A0A0H3NMP3 | 98.0 | 1.3e-07 | 2.4e-13 | 59.8 | 43 | (94, 136) | 136 | (1, 43) | 73 | Phage-related tail protein | Phage-related tail protein | | uniclust | UniRef100\_A0A1L6I298 | 98.0 | 1.3e-07 | 2.6e-13 | 69.8 | 113 | (9, 131) | 136 | (4, 116) | 165 | Uncharacterized protein | Uncharacterized protein | | uniclust | UniRef100\_A0A2V2A0Z3 | 98.0 | 1.6e-07 | 3e-13 | 62.0 | 85 | (52, 136) | 136 | (16, 102) | 104 | GpU protein | GpU protein | | uniclust | UniRef100\_A0A3M1ER04 | 97.9 | 1.8e-07 | 3.4e-13 | 65.8 | 91 | (27, 118) | 136 | (21, 130) | 131 | Peptigoglycan-binding protein LysM (Fragment) | Peptigoglycan-binding protein LysM (Fragment) | | uniclust | UniRef100\_A0A853IKN5 | 97.9 | 2.2e-07 | 4.1e-13 | 54.2 | 43 | (94, 136) | 136 | (2, 44) | 52 | Phage tail protein | Phage tail protein | | uniclust | UniRef100\_UPI000BA48FEE | 97.9 | 2.3e-07 | 4.2e-13 | 73.6 | 70 | (19, 88) | 136 | (158, 227) | 392 | phage tail protein | phage tail protein | | uniclust | UniRef100\_UPI001BD14020 | 97.9 | 2.4e-07 | 4.3e-13 | 62.7 | 59 | (77, 135) | 136 | (21, 79) | 119 | phage tail protein | phage tail protein | | uniclust | UniRef100\_UPI001FE72D82 | 97.9 | 2.4e-07 | 4.5e-13 | 67.8 | 109 | (27, 136) | 136 | (24, 147) | 204 | hypothetical protein | hypothetical protein | | uniclust | UniRef100\_UPI001F18FFEC | 97.9 | 2.9e-07 | 5.4e-13 | 64.7 | 88 | (42, 135) | 136 | (42, 129) | 152 | phage tail protein | phage tail protein | | uniclust | UniRef100\_A0A3E4VCF7 | 97.9 | 3e-07 | 5.8e-13 | 56.8 | 61 | (75, 136) | 136 | (3, 65) | 65 | Uncharacterized protein | Uncharacterized protein | | uniclust | UniRef100\_UPI001F4C87A2 | 97.9 | 3.2e-07 | 5.9e-13 | 53.3 | 41 | (96, 136) | 136 | (2, 42) | 51 | phage tail protein | phage tail protein | | uniclust | UniRef100\_A0A1Z4PN67 | 97.8 | 3.7e-07 | 6.9e-13 | 63.6 | 118 | (13, 133) | 136 | (4, 127) | 131 | Uncharacterized protein | Uncharacterized protein | | uniclust | UniRef100\_UPI0022B72820 | 97.8 | 4.2e-07 | 7.7e-13 | 61.8 | 51 | (86, 136) | 136 | (2, 52) | 122 | phage tail protein | phage tail protein | | uniclust | UniRef100\_UPI001F173E80 | 97.8 | 4.5e-07 | 8.4e-13 | 58.5 | 69 | (14, 82) | 136 | (6, 74) | 83 | phage tail protein | phage tail protein | | uniclust | UniRef100\_UPI0021C3E1B0 | 97.8 | 4.7e-07 | 8.6e-13 | 62.1 | 61 | (13, 74) | 136 | (1, 61) | 129 | phage tail protein | phage tail protein | | uniclust | UniRef100\_UPI001C999792 | 97.8 | 4.8e-07 | 8.9e-13 | 59.9 | 90 | (11, 100) | 136 | (2, 91) | 103 | phage tail protein | phage tail protein | | uniclust | UniRef100\_A0A0F9VF18 | 97.8 | 4.7e-07 | 9.3e-13 | 68.1 | 109 | (27, 135) | 136 | (32, 159) | 187 | Contractile injection system tube protein N-terminal domain-containing protein | Contractile injection system tube protein N-terminal domain-containing protein | | uniclust | UniRef100\_A0A0H3ZVP6 | 97.8 | 5.5e-07 | 1e-12 | 55.6 | 58 | (78, 135) | 136 | (3, 60) | 69 | Phage tail protein | Phage tail protein | | uniclust | UniRef100\_H1D5E7 | 97.8 | 5.6e-07 | 1e-12 | 56.8 | 62 | (12, 73) | 136 | (1, 65) | 78 | Uncharacterized protein | Uncharacterized protein | | uniclust | UniRef100\_A0A935R531 | 97.8 | 5.7e-07 | 1.1e-12 | 65.0 | 112 | (20, 133) | 136 | (27, 148) | 169 | Contractile injection system tube protein N-terminal domain-containing protein | Contractile injection system tube protein N-terminal domain-containing protein | | uniclust | UniRef100\_A0A4Q3K0V1 | 97.8 | 5.9e-07 | 1.1e-12 | 51.7 | 48 | (85, 132) | 136 | (1, 48) | 48 | Oxidoreductase (Fragment) | Oxidoreductase (Fragment) | | uniclust | UniRef100\_UPI001B7CF361 | 97.8 | 6e-07 | 1.1e-12 | 67.9 | 109 | (27, 136) | 136 | (79, 202) | 254 | hypothetical protein | hypothetical protein | | uniclust | UniRef100\_A0A3S5K2M8 | 97.8 | 6e-07 | 1.1e-12 | 71.7 | 109 | (27, 136) | 136 | (35, 156) | 408 | Contractile injection system tube protein N-terminal domain-containing protein | Contractile injection system tube protein N-terminal domain-containing protein | | uniclust | UniRef100\_A0A524GYW0 | 97.8 | 6e-07 | 1.2e-12 | 66.3 | 58 | (77, 135) | 136 | (13, 75) | 159 | LysM peptidoglycan-binding domain-containing protein | LysM peptidoglycan-binding domain-containing protein | | uniclust | UniRef100\_A0A378MZA4 | 97.7 | 7.7e-07 | 1.4e-12 | 57.2 | 54 | (80, 133) | 136 | (4, 58) | 86 | Phage protein U | Phage protein U | | uniclust | UniRef100\_UPI000AA7DEF5 | 97.7 | 7.7e-07 | 1.4e-12 | 53.6 | 55 | (81, 136) | 136 | (3, 59) | 60 | phage tail protein | phage tail protein | | uniclust | UniRef100\_A0A447N5T3 | 97.7 | 7.8e-07 | 1.4e-12 | 50.4 | 41 | (13, 53) | 136 | (1, 41) | 44 | Gp25 | Gp25 | | uniclust | UniRef100\_A0A074LMC3 | 97.7 | 7.8e-07 | 1.5e-12 | 69.1 | 113 | (9, 133) | 136 | (1, 113) | 243 | LysM domain-containing protein | LysM domain-containing protein | | uniclust | UniRef100\_UPI0009BF56FE | 97.7 | 8.6e-07 | 1.6e-12 | 69.2 | 63 | (11, 73) | 136 | (2, 64) | 318 | contractile injection system protein, VgrG/Pvc8 family | contractile injection system protein, VgrG/Pvc8 family | | uniclust | UniRef100\_A0A1S6HMA4 | 97.7 | 8.5e-07 | 1.6e-12 | 64.1 | 108 | (28, 135) | 136 | (30, 154) | 167 | Contractile injection system tube protein N-terminal domain-containing protein | Contractile injection system tube protein N-terminal domain-containing protein | | uniclust | UniRef100\_A0A2Z3EGZ9 | 97.7 | 1e-06 | 1.8e-12 | 63.1 | 115 | (20, 135) | 136 | (28, 151) | 167 | Contractile injection system tube protein N-terminal domain-containing protein | Contractile injection system tube protein N-terminal domain-containing protein | | uniclust | UniRef100\_A0A060BT42 | 97.7 | 9.6e-07 | 1.9e-12 | 65.3 | 80 | (56, 136) | 136 | (6, 102) | 177 | CAZy families CBM50 protein (Fragment) | CAZy families CBM50 protein (Fragment) | | uniclust | UniRef100\_A0A174USZ0 | 97.7 | 9.1e-07 | 1.9e-12 | 63.6 | 117 | (10, 135) | 136 | (1, 118) | 129 | Minor capsid protein | Minor capsid protein | | uniclust | UniRef100\_A0A073CFQ3 | 97.7 | 9.9e-07 | 2e-12 | 70.9 | 109 | (28, 136) | 136 | (118, 295) | 314 | Contractile injection system tube protein N-terminal domain-containing protein | Contractile injection system tube protein N-terminal domain-containing protein | | uniclust | UniRef100\_UPI001659B0F1 | 97.7 | 1.1e-06 | 2e-12 | 58.1 | 57 | (79, 135) | 136 | (2, 58) | 97 | phage tail protein | phage tail protein | | uniclust | UniRef100\_A0A2D7W1J1 | 97.7 | 1e-06 | 2e-12 | 74.9 | 124 | (10, 136) | 136 | (255, 408) | 515 | Contractile injection system tube protein N-terminal domain-containing protein | Contractile injection system tube protein N-terminal domain-containing protein | | uniclust | UniRef100\_A0A061NGS6 | 97.7 | 9.6e-07 | 2.1e-12 | 70.8 | 126 | (5, 135) | 136 | (19, 156) | 255 | Phage-like element PBSX protein XkdP | Phage-like element PBSX protein XkdP | | uniclust | UniRef100\_A0A2A7WUN3 | 97.7 | 1.2e-06 | 2.3e-12 | 67.3 | 107 | (28, 135) | 136 | (38, 147) | 227 | LysM domain-containing protein | LysM domain-containing protein | | uniclust | UniRef100\_R6PMQ9 | 97.7 | 1.3e-06 | 2.4e-12 | 56.5 | 68 | (68, 135) | 136 | (3, 70) | 88 | Uncharacterized protein | Uncharacterized protein | | uniclust | UniRef100\_S5MV70 | 97.7 | 1.3e-06 | 2.4e-12 | 52.6 | 57 | (68, 124) | 136 | (2, 58) | 60 | Phage tail protein | Phage tail protein | | uniclust | UniRef100\_A0A105T8J5 | 97.7 | 1.3e-06 | 2.4e-12 | 64.1 | 57 | (19, 75) | 136 | (142, 198) | 200 | Phage protein U | Phage protein U | | uniclust | UniRef100\_UPI00227354E9 | 97.7 | 1.3e-06 | 2.4e-12 | 59.7 | 111 | (13, 124) | 136 | (3, 116) | 123 | phage tail protein | phage tail protein | | uniclust | UniRef100\_A0A1J0AH11 | 97.7 | 1.3e-06 | 2.5e-12 | 67.7 | 116 | (20, 136) | 136 | (17, 150) | 263 | Contractile injection system tube protein N-terminal domain-containing protein | Contractile injection system tube protein N-terminal domain-containing protein | | uniclust | UniRef100\_UPI00157B406D | 97.6 | 1.4e-06 | 2.6e-12 | 51.6 | 36 | (100, 135) | 136 | (19, 54) | 55 | phage tail protein | phage tail protein | | uniclust | UniRef100\_A0A846T4R7 | 97.6 | 1.6e-06 | 2.8e-12 | 53.3 | 60 | (13, 72) | 136 | (1, 60) | 66 | Uncharacterized protein | Uncharacterized protein | | uniclust | UniRef100\_A0A318ENP1 | 97.6 | 1.7e-06 | 3e-12 | 56.3 | 70 | (14, 83) | 136 | (3, 77) | 91 | GpU protein | GpU protein | | uniclust | UniRef100\_A0A1F3S067 | 97.6 | 1.7e-06 | 3.4e-12 | 67.4 | 105 | (28, 134) | 136 | (40, 162) | 233 | Contractile injection system tube protein N-terminal domain-containing protein (Fragment) | Contractile injection system tube protein N-terminal domain-containing protein (Fragment) | | uniclust | UniRef100\_A0A2N5DAB4 | 97.6 | 2.1e-06 | 4.1e-12 | 73.2 | 108 | (27, 135) | 136 | (37, 166) | 496 | Contractile injection system tube protein N-terminal domain-containing protein | Contractile injection system tube protein N-terminal domain-containing protein | | uniclust | UniRef100\_UPI001F0A3F5E | 97.6 | 2.3e-06 | 4.2e-12 | 51.9 | 39 | (39, 77) | 136 | (4, 43) | 61 | phage tail protein | phage tail protein | | uniclust | UniRef100\_UPI0022A7BF70 | 97.5 | 2.9e-06 | 5.3e-12 | 54.9 | 54 | (83, 136) | 136 | (4, 57) | 87 | phage tail protein | phage tail protein | | uniclust | UniRef100\_A0A955R5H5 | 97.5 | 3e-06 | 5.5e-12 | 66.9 | 116 | (18, 135) | 136 | (16, 146) | 350 | LysM peptidoglycan-binding domain-containing protein | LysM peptidoglycan-binding domain-containing protein | | uniclust | UniRef100\_A0A1C6JGS8 | 97.5 | 3.4e-06 | 6.4e-12 | 62.8 | 121 | (12, 136) | 136 | (2, 129) | 201 | Phage protein U | Phage protein U | | uniclust | UniRef100\_A0A1M6M9A3 | 97.5 | 3.3e-06 | 6.5e-12 | 64.5 | 114 | (10, 133) | 136 | (1, 115) | 199 | LysM domain-containing protein | LysM domain-containing protein | | uniclust | UniRef100\_U2F2B2 | 97.5 | 3.6e-06 | 6.7e-12 | 52.2 | 61 | (74, 134) | 136 | (6, 66) | 69 | Phage tail protein | Phage tail protein | | uniclust | UniRef100\_UPI0009EDCD06 | 97.5 | 3.8e-06 | 7.2e-12 | 54.9 | 78 | (14, 99) | 136 | (1, 78) | 85 | phage tail protein | phage tail protein | | uniclust | UniRef100\_A0A935P3X5 | 97.5 | 3.9e-06 | 7.3e-12 | 63.3 | 106 | (28, 135) | 136 | (56, 178) | 238 | Contractile injection system tube protein N-terminal domain-containing protein | Contractile injection system tube protein N-terminal domain-containing protein | | uniclust | UniRef100\_A0A1Q3WFK6 | 97.5 | 3.6e-06 | 7.4e-12 | 70.9 | 106 | (28, 134) | 136 | (29, 147) | 414 | Contractile injection system tube protein N-terminal domain-containing protein | Contractile injection system tube protein N-terminal domain-containing protein | | uniclust | UniRef100\_A0A084SKL0 | 97.5 | 3.6e-06 | 7.6e-12 | 68.9 | 107 | (28, 135) | 136 | (23, 153) | 306 | Contractile injection system tube protein N-terminal domain-containing protein | Contractile injection system tube protein N-terminal domain-containing protein | | uniclust | UniRef100\_UPI001E5FDDE9 | 97.5 | 4.3e-06 | 7.9e-12 | 49.0 | 40 | (10, 49) | 136 | (8, 47) | 51 | phage tail protein | phage tail protein | | uniclust | UniRef100\_A0A9D1ATR1 | 97.5 | 4.6e-06 | 8.4e-12 | 66.4 | 113 | (21, 135) | 136 | (42, 171) | 372 | LysM peptidoglycan-binding domain-containing protein (Fragment) | LysM peptidoglycan-binding domain-containing protein (Fragment) | | uniclust | UniRef100\_UPI001F06D85D | 97.5 | 4.6e-06 | 8.4e-12 | 54.2 | 59 | (76, 135) | 136 | (3, 61) | 89 | phage tail protein | phage tail protein | | uniclust | UniRef100\_A0A1X7MDE4 | 97.4 | 5.2e-06 | 9.5e-12 | 45.9 | 35 | (12, 46) | 136 | (3, 37) | 38 | [weak similarity to] phage P2 GpU (Fragment) | [weak similarity to] phage P2 GpU (Fragment) | | uniclust | UniRef100\_A0A4P5W163 | 97.4 | 5.3e-06 | 9.8e-12 | 64.0 | 115 | (19, 135) | 136 | (20, 155) | 283 | Contractile injection system tube protein N-terminal domain-containing protein | Contractile injection system tube protein N-terminal domain-containing protein | | uniclust | UniRef100\_A0A0Q4KRA9 | 97.4 | 4.9e-06 | 1e-11 | 66.4 | 116 | (17, 135) | 136 | (35, 215) | 264 | Contractile injection system tube protein N-terminal domain-containing protein | Contractile injection system tube protein N-terminal domain-containing protein | | uniclust | UniRef100\_A0A0K9NAG5 | 97.4 | 5.1e-06 | 1e-11 | 62.4 | 114 | (19, 134) | 136 | (23, 143) | 169 | Contractile injection system tube protein N-terminal domain-containing protein | Contractile injection system tube protein N-terminal domain-containing protein | | uniclust | UniRef100\_A0A7X6JBU4 | 97.4 | 6e-06 | 1.1e-11 | 54.8 | 59 | (78, 136) | 136 | (3, 64) | 100 | Phage tail protein | Phage tail protein | | uniclust | UniRef100\_N8S9H2 | 97.4 | 6.3e-06 | 1.2e-11 | 47.0 | 40 | (13, 52) | 136 | (1, 40) | 44 | Uncharacterized protein | Uncharacterized protein | | uniclust | UniRef100\_UPI0018EF83E4 | 97.4 | 7e-06 | 1.3e-11 | 70.6 | 108 | (28, 136) | 136 | (27, 147) | 834 | VgrG-related protein | VgrG-related protein | | uniclust | UniRef100\_UPI001E41EFA3 | 97.4 | 7.2e-06 | 1.3e-11 | 49.2 | 34 | (102, 135) | 136 | (2, 35) | 57 | phage tail protein | phage tail protein | | uniclust | UniRef100\_A0A941G883 | 97.4 | 7.4e-06 | 1.4e-11 | 56.1 | 69 | (68, 136) | 136 | (1, 69) | 120 | Phage tail protein | Phage tail protein | | uniclust | UniRef100\_A0A014LIK0 | 97.4 | 7.2e-06 | 1.5e-11 | 67.8 | 105 | (28, 133) | 136 | (112, 250) | 323 | Signal peptide protein | Signal peptide protein | | uniclust | UniRef100\_A0A3S0DNP6 | 97.3 | 8.6e-06 | 1.7e-11 | 62.3 | 105 | (27, 134) | 136 | (33, 146) | 202 | Contractile injection system tube protein N-terminal domain-containing protein (Fragment) | Contractile injection system tube protein N-terminal domain-containing protein (Fragment) | | uniclust | UniRef100\_A0A2W4LKG2 | 97.3 | 8.7e-06 | 1.7e-11 | 58.0 | 45 | (91, 136) | 136 | (2, 47) | 124 | Peptidoglycan-binding protein (Fragment) | Peptidoglycan-binding protein (Fragment) | | uniclust | UniRef100\_A0A2D4SL56 | 97.3 | 8.9e-06 | 1.7e-11 | 63.5 | 106 | (27, 133) | 136 | (39, 192) | 245 | Contractile injection system tube protein N-terminal domain-containing protein | Contractile injection system tube protein N-terminal domain-containing protein | | uniclust | UniRef100\_A0A069A2Y0 | 97.3 | 8.5e-06 | 1.8e-11 | 64.9 | 122 | (8, 136) | 136 | (18, 149) | 233 | LysM domain protein | LysM domain protein | | uniclust | UniRef100\_A0A173TJ12 | 97.3 | 1e-05 | 1.9e-11 | 60.7 | 115 | (17, 135) | 136 | (21, 150) | 218 | LysM domain-containing protein | LysM domain-containing protein | | uniclust | UniRef100\_UPI00049A5F88 | 97.3 | 1.2e-05 | 2.2e-11 | 57.1 | 122 | (13, 135) | 136 | (1, 126) | 150 | phage tail protein | phage tail protein | | uniclust | UniRef100\_A0A173R5A8 | 97.3 | 1.2e-05 | 2.4e-11 | 62.6 | 123 | (11, 136) | 136 | (1, 128) | 227 | LysM domain-containing protein | LysM domain-containing protein | | uniclust | UniRef100\_A0A075RDD3 | 97.3 | 1.3e-05 | 2.6e-11 | 61.6 | 110 | (15, 135) | 136 | (3, 112) | 198 | LysM domain/BON superfamily protein | LysM domain/BON superfamily protein | | uniclust | UniRef100\_UPI0013FA864C | 97.2 | 1.6e-05 | 2.9e-11 | 50.3 | 69 | (29, 99) | 136 | (5, 73) | 74 | phage tail protein | phage tail protein | | uniclust | UniRef100\_UPI001BDA1730 | 97.2 | 1.7e-05 | 3.1e-11 | 52.2 | 81 | (54, 134) | 136 | (8, 88) | 92 | phage tail protein | phage tail protein | | uniclust | UniRef100\_A0A1N7BSF8 | 97.2 | 1.6e-05 | 3.1e-11 | 63.1 | 108 | (27, 135) | 136 | (31, 169) | 266 | Contractile injection system tube protein N-terminal domain-containing protein | Contractile injection system tube protein N-terminal domain-containing protein | | uniclust | UniRef100\_A0A7V8YCD3 | 97.2 | 1.7e-05 | 3.2e-11 | 62.0 | 113 | (20, 134) | 136 | (20, 149) | 303 | LysM peptidoglycan-binding domain-containing protein | LysM peptidoglycan-binding domain-containing protein | | uniclust | UniRef100\_UPI001FE103EA | 97.2 | 1.8e-05 | 3.2e-11 | 51.6 | 71 | (20, 90) | 136 | (2, 72) | 87 | phage tail protein | phage tail protein | | uniclust | UniRef100\_A0A1T4KBT6 | 97.2 | 1.8e-05 | 3.7e-11 | 61.0 | 123 | (1, 134) | 136 | (1, 123) | 184 | LysM domain-containing protein | LysM domain-containing protein | | uniclust | UniRef100\_UPI001F05C40E | 97.2 | 2.1e-05 | 3.8e-11 | 60.6 | 108 | (28, 136) | 136 | (22, 147) | 265 | hypothetical protein | hypothetical protein | | uniclust | UniRef100\_A0A1Z4HE74 | 97.2 | 2.1e-05 | 4e-11 | 58.2 | 108 | (28, 136) | 136 | (35, 161) | 191 | Contractile injection system tube protein N-terminal domain-containing protein | Contractile injection system tube protein N-terminal domain-containing protein | | uniclust | UniRef100\_A0A1V2H6I8 | 97.2 | 2.4e-05 | 4.5e-11 | 62.2 | 97 | (12, 113) | 136 | (45, 141) | 291 | Phage tail protein | Phage tail protein | | uniclust | UniRef100\_UPI002019748F | 97.1 | 2.6e-05 | 4.7e-11 | 50.1 | 48 | (48, 95) | 136 | (5, 52) | 80 | phage tail protein | phage tail protein | | uniclust | UniRef100\_A0A376J1X2 | 97.1 | 2.6e-05 | 4.8e-11 | 45.5 | 38 | (81, 118) | 136 | (3, 40) | 48 | GpU phage protein | GpU phage protein | | uniclust | UniRef100\_A0A0B0HFG9 | 97.1 | 2.7e-05 | 4.9e-11 | 47.1 | 50 | (86, 136) | 136 | (2, 53) | 58 | Uncharacterized protein | Uncharacterized protein | | uniclust | UniRef100\_A0A0F3RPF7 | 97.1 | 2.7e-05 | 5e-11 | 53.9 | 44 | (93, 136) | 136 | (80, 123) | 125 | Phage tail tape measure protein, TP901 family, core region | Phage tail tape measure protein, TP901 family, core region | | uniclust | UniRef100\_C8PII4 | 97.1 | 3.5e-05 | 6.5e-11 | 44.4 | 40 | (95, 134) | 136 | (2, 41) | 45 | Phage P2 GpU | Phage P2 GpU | | uniclust | UniRef100\_A0A399IQQ4 | 97.1 | 3.5e-05 | 6.7e-11 | 58.3 | 111 | (12, 134) | 136 | (2, 112) | 196 | LysM peptidoglycan-binding domain-containing protein | LysM peptidoglycan-binding domain-containing protein | | uniclust | UniRef100\_A0A090T0X2 | 97.1 | 3.7e-05 | 6.8e-11 | 58.6 | 107 | (28, 135) | 136 | (24, 150) | 241 | Contractile injection system tube protein N-terminal domain-containing protein | Contractile injection system tube protein N-terminal domain-containing protein | | uniclust | UniRef100\_A0A497PS16 | 97.1 | 3.9e-05 | 7.3e-11 | 60.1 | 107 | (28, 135) | 136 | (59, 212) | 258 | Uncharacterized protein | Uncharacterized protein | | uniclust | UniRef100\_E2CCF4 | 97.1 | 4.1e-05 | 7.5e-11 | 45.8 | 38 | (99, 136) | 136 | (4, 41) | 54 | Phage-related tail protein | Phage-related tail protein | | uniclust | UniRef100\_A0A017RV10 | 97.1 | 3.7e-05 | 7.6e-11 | 62.7 | 127 | (3, 135) | 136 | (33, 171) | 282 | LysM domain-containing protein | LysM domain-containing protein | | uniclust | UniRef100\_A0A952YFU8 | 97.1 | 4.2e-05 | 7.7e-11 | 47.8 | 50 | (87, 136) | 136 | (4, 53) | 68 | Phage tail protein | Phage tail protein | | uniclust | UniRef100\_A0A3E4VD00 | 97.1 | 4.2e-05 | 7.8e-11 | 56.0 | 62 | (12, 73) | 136 | (109, 174) | 179 | SH3b domain-containing protein | SH3b domain-containing protein | | uniclust | UniRef100\_A0A6B3MKD8 | 97.0 | 4e-05 | 8.2e-11 | 58.0 | 115 | (17, 135) | 136 | (32, 154) | 165 | Uncharacterized protein | Uncharacterized protein | | uniclust | UniRef100\_A0A8S0FXT8 | 97.0 | 4.5e-05 | 8.3e-11 | 54.1 | 48 | (88, 135) | 136 | (3, 50) | 144 | Phage tail protein | Phage tail protein | | uniclust | UniRef100\_A0A1G6LTE8 | 97.0 | 4.3e-05 | 8.6e-11 | 58.7 | 113 | (20, 136) | 136 | (19, 145) | 186 | Uncharacterized protein | Uncharacterized protein | | uniclust | UniRef100\_UPI00207C8BD9 | 97.0 | 4.7e-05 | 8.6e-11 | 45.4 | 30 | (106, 135) | 136 | (1, 30) | 53 | phage tail protein | phage tail protein | | uniclust | UniRef100\_UPI00202188DC | 97.0 | 4.8e-05 | 8.8e-11 | 49.7 | 51 | (27, 77) | 136 | (7, 57) | 87 | phage tail protein | phage tail protein | | uniclust | UniRef100\_A0A6I0EJT0 | 97.0 | 5.3e-05 | 9.8e-11 | 44.8 | 37 | (100, 136) | 136 | (15, 51) | 51 | Uncharacterized protein | Uncharacterized protein | | uniclust | UniRef100\_UPI002153A50E | 97.0 | 5.4e-05 | 9.9e-11 | 47.1 | 64 | (28, 91) | 136 | (2, 65) | 66 | phage tail protein | phage tail protein | | uniclust | UniRef100\_UPI001D1340C6 | 97.0 | 5.4e-05 | 1e-10 | 56.3 | 110 | (12, 131) | 136 | (3, 112) | 198 | hypothetical protein | hypothetical protein | | uniclust | UniRef100\_A0A8G2HV68 | 97.0 | 5.8e-05 | 1.1e-10 | 48.8 | 44 | (92, 136) | 136 | (8, 51) | 79 | Uncharacterized protein | Uncharacterized protein | | uniclust | UniRef100\_A0A951MSF9 | 97.0 | 5.8e-05 | 1.1e-10 | 58.4 | 107 | (28, 135) | 136 | (55, 179) | 254 | Contractile injection system tube protein N-terminal domain-containing protein | Contractile injection system tube protein N-terminal domain-containing protein | | uniclust | UniRef100\_A0A1Z8QW35 | 96.9 | 6.1e-05 | 1.3e-10 | 60.0 | 108 | (28, 135) | 136 | (32, 161) | 230 | Contractile injection system tube protein N-terminal domain-containing protein | Contractile injection system tube protein N-terminal domain-containing protein | | uniclust | UniRef100\_E6X1M4 | 96.9 | 7.3e-05 | 1.3e-10 | 52.6 | 117 | (13, 135) | 136 | (1, 117) | 135 | Uncharacterized protein | Uncharacterized protein | | uniclust | UniRef100\_A0A011PPH1 | 96.9 | 6.9e-05 | 1.4e-10 | 58.3 | 101 | (29, 136) | 136 | (13, 117) | 197 | Mu-like prophage DNA circulation protein | Mu-like prophage DNA circulation protein | | uniclust | UniRef100\_A0A935YIZ6 | 96.9 | 8.4e-05 | 1.5e-10 | 54.5 | 117 | (17, 135) | 136 | (28, 147) | 178 | Phage tail protein | Phage tail protein | | uniclust | UniRef100\_A0A0C1UAF4 | 96.9 | 7.1e-05 | 1.6e-10 | 60.9 | 121 | (10, 136) | 136 | (28, 157) | 248 | LysM domain protein | LysM domain protein | | uniclust | UniRef100\_UPI001FE4EA06 | 96.8 | 0.00013 | 2.4e-10 | 49.7 | 70 | (66, 135) | 136 | (13, 85) | 109 | phage tail protein | phage tail protein | | uniclust | UniRef100\_A0A069A1N0 | 96.8 | 0.00011 | 2.5e-10 | 61.1 | 128 | (5, 135) | 136 | (24, 165) | 294 | LysM domain protein | LysM domain protein | | uniclust | UniRef100\_A0A081R180 | 96.8 | 0.00012 | 2.6e-10 | 59.6 | 111 | (20, 135) | 136 | (47, 168) | 262 | Phage protein | Phage protein | | uniclust | UniRef100\_A6VY17 | 96.8 | 0.00014 | 2.6e-10 | 48.0 | 79 | (44, 124) | 136 | (2, 80) | 90 | Uncharacterized protein | Uncharacterized protein | | uniclust | UniRef100\_F4BFP8 | 96.8 | 0.00015 | 2.7e-10 | 47.5 | 67 | (63, 135) | 136 | (2, 69) | 86 | Uncharacterized protein | Uncharacterized protein | | uniclust | UniRef100\_A0A6I7NZD3 | 96.8 | 0.00015 | 2.8e-10 | 60.5 | 104 | (27, 134) | 136 | (42, 161) | 393 | Contractile injection system tube protein N-terminal domain-containing protein | Contractile injection system tube protein N-terminal domain-containing protein | | uniclust | UniRef100\_A0A0D1XSK1 | 96.8 | 0.00014 | 2.9e-10 | 58.8 | 102 | (28, 135) | 136 | (44, 154) | 248 | Uncharacterized protein | Uncharacterized protein | | uniclust | UniRef100\_A0A3M3B317 | 96.7 | 0.00017 | 3.2e-10 | 51.8 | 55 | (19, 73) | 136 | (71, 125) | 142 | Uncharacterized protein (Fragment) | Uncharacterized protein (Fragment) | | uniclust | UniRef100\_A0A3C0WX82 | 96.7 | 0.00018 | 3.3e-10 | 57.6 | 108 | (27, 135) | 136 | (62, 180) | 340 | Contractile injection system tube protein N-terminal domain-containing protein | Contractile injection system tube protein N-terminal domain-containing protein | | uniclust | UniRef100\_A0A6J7DL88 | 96.7 | 0.0002 | 3.6e-10 | 57.0 | 122 | (12, 136) | 136 | (57, 215) | 320 | Unannotated protein | Unannotated protein | | uniclust | UniRef100\_A0A6I5PEH0 | 96.7 | 0.0002 | 3.6e-10 | 57.4 | 108 | (28, 136) | 136 | (24, 150) | 341 | Beta/gamma crystallin 'Greek key' domain-containing protein | Beta/gamma crystallin 'Greek key' domain-containing protein | | uniclust | UniRef100\_A0A1U7IQP6 | 96.7 | 0.0002 | 3.8e-10 | 58.3 | 109 | (28, 136) | 136 | (149, 273) | 395 | Contractile injection system tube protein N-terminal domain-containing protein | Contractile injection system tube protein N-terminal domain-containing protein | | uniclust | UniRef100\_A0A853I0P3 | 96.7 | 0.00021 | 4e-10 | 55.5 | 107 | (28, 135) | 136 | (32, 150) | 225 | LysM peptidoglycan-binding domain-containing protein | LysM peptidoglycan-binding domain-containing protein | | uniclust | UniRef100\_A0A6M0RCM0 | 96.7 | 0.00021 | 4.1e-10 | 54.8 | 115 | (11, 135) | 136 | (1, 115) | 184 | DNA-binding protein | DNA-binding protein | | uniclust | UniRef100\_A0A061N9X2 | 96.7 | 0.00022 | 4.6e-10 | 55.7 | 112 | (10, 135) | 136 | (1, 112) | 190 | Uncharacterized protein | Uncharacterized protein | | uniclust | UniRef100\_A0A377B712 | 96.6 | 0.00026 | 4.8e-10 | 45.5 | 50 | (41, 90) | 136 | (12, 61) | 76 | Putative phage P2 GpU family protein | Putative phage P2 GpU family protein | | uniclust | UniRef100\_A0A0E2HQC6 | 96.6 | 0.00023 | 4.8e-10 | 58.6 | 107 | (20, 135) | 136 | (45, 164) | 276 | LysM domain-containing protein | LysM domain-containing protein | | uniclust | UniRef100\_A0A072NRR3 | 96.6 | 0.00023 | 4.8e-10 | 58.3 | 104 | (28, 136) | 136 | (49, 165) | 262 | LysM domain-containing protein | LysM domain-containing protein | | uniclust | UniRef100\_UPI00055A433D | 96.6 | 0.00026 | 4.8e-10 | 58.9 | 107 | (20, 134) | 136 | (23, 155) | 456 | hypothetical protein | hypothetical protein | | uniclust | UniRef100\_A0A8S0FZB8 | 96.6 | 0.00028 | 5.2e-10 | 46.4 | 42 | (13, 55) | 136 | (1, 42) | 86 | Uncharacterized protein | Uncharacterized protein | | uniclust | UniRef100\_UPI001F192CD3 | 96.6 | 0.00029 | 5.2e-10 | 53.8 | 109 | (28, 136) | 136 | (34, 209) | 226 | hypothetical protein | hypothetical protein | | uniclust | UniRef100\_UPI001E4DB374 | 96.6 | 0.0003 | 5.5e-10 | 41.9 | 40 | (94, 133) | 136 | (3, 42) | 51 | phage tail protein | phage tail protein | | uniclust | UniRef100\_UPI001E431A0C | 96.6 | 0.00031 | 5.8e-10 | 50.8 | 110 | (15, 135) | 136 | (3, 112) | 155 | hypothetical protein | hypothetical protein | | uniclust | UniRef100\_UPI0022B5F671 | 96.6 | 0.00032 | 5.8e-10 | 43.2 | 43 | (27, 69) | 136 | (5, 47) | 60 | phage tail protein | phage tail protein | | uniclust | UniRef100\_UPI001F2F5FEE | 96.6 | 0.00032 | 5.9e-10 | 47.1 | 46 | (20, 65) | 136 | (2, 47) | 97 | phage tail protein | phage tail protein | | uniclust | UniRef100\_A0A1I5USI5 | 96.6 | 0.00032 | 5.9e-10 | 48.6 | 115 | (13, 135) | 136 | (2, 116) | 118 | Phage P2 GpU | Phage P2 GpU | | uniclust | UniRef100\_A0A327JCS4 | 96.6 | 0.00033 | 6.1e-10 | 53.8 | 104 | (30, 135) | 136 | (11, 114) | 237 | CYTH domain-containing protein | CYTH domain-containing protein | | uniclust | UniRef100\_A0A2T4UGD8 | 96.6 | 0.00033 | 6.1e-10 | 59.8 | 116 | (18, 135) | 136 | (410, 544) | 622 | LysM domain-containing protein | LysM domain-containing protein | | uniclust | UniRef100\_UPI0008A65577 | 96.6 | 0.00038 | 6.9e-10 | 48.2 | 48 | (48, 95) | 136 | (5, 52) | 116 | phage tail protein | phage tail protein | | uniclust | UniRef100\_UPI0018970A81 | 96.5 | 0.00038 | 7e-10 | 44.2 | 53 | (83, 135) | 136 | (3, 55) | 71 | phage tail protein | phage tail protein | | uniclust | UniRef100\_UPI00126FC877 | 96.5 | 0.0004 | 7.4e-10 | 45.4 | 60 | (76, 135) | 136 | (3, 62) | 83 | phage tail protein | phage tail protein | | uniclust | UniRef100\_UPI0019A7B555 | 96.5 | 0.00041 | 7.5e-10 | 43.9 | 41 | (11, 51) | 136 | (1, 42) | 69 | hypothetical protein | hypothetical protein | | uniclust | UniRef100\_UPI001F2E8B4F | 96.5 | 0.00043 | 7.9e-10 | 41.2 | 45 | (91, 135) | 136 | (5, 49) | 50 | phage tail protein | phage tail protein | | uniclust | UniRef100\_A0A0P7ZF50 | 96.5 | 0.00045 | 8.2e-10 | 46.2 | 35 | (100, 135) | 136 | (11, 45) | 94 | Contractile injection system tube protein N-terminal domain-containing protein | Contractile injection system tube protein N-terminal domain-containing protein | | uniclust | UniRef100\_UPI0002DAFDF3 | 96.5 | 0.00052 | 9.6e-10 | 53.9 | 119 | (13, 135) | 136 | (2, 129) | 280 | hypothetical protein | hypothetical protein | | uniclust | UniRef100\_A0A448QV13 | 96.5 | 0.00053 | 9.7e-10 | 45.8 | 47 | (89, 135) | 136 | (8, 54) | 93 | Phage P2 GpU | Phage P2 GpU | | uniclust | UniRef100\_A0A432DSZ4 | 96.5 | 0.00049 | 9.9e-10 | 53.4 | 82 | (53, 135) | 136 | (16, 115) | 189 | LysM peptidoglycan-binding domain-containing protein | LysM peptidoglycan-binding domain-containing protein | | uniclust | UniRef100\_A0A2E3R265 | 96.5 | 0.00054 | 9.9e-10 | 49.9 | 106 | (20, 129) | 136 | (14, 130) | 160 | Uncharacterized protein | Uncharacterized protein | | uniclust | UniRef100\_A0A958XQD9 | 96.5 | 0.00055 | 1e-09 | 52.9 | 107 | (28, 134) | 136 | (41, 159) | 233 | Contractile injection system tube protein N-terminal domain-containing protein | Contractile injection system tube protein N-terminal domain-containing protein | | uniclust | UniRef100\_A0A6P0YVG0 | 96.4 | 0.00055 | 1e-09 | 44.8 | 46 | (90, 135) | 136 | (10, 55) | 83 | Contractile injection system tube protein N-terminal domain-containing protein (Fragment) | Contractile injection system tube protein N-terminal domain-containing protein (Fragment) | | uniclust | UniRef100\_A0A1C4SHA2 | 96.4 | 0.00052 | 1e-09 | 52.9 | 71 | (64, 135) | 136 | (8, 94) | 186 | LysM domain-containing protein (Fragment) | LysM domain-containing protein (Fragment) | | uniclust | UniRef100\_A0A761H723 | 96.4 | 0.00058 | 1.1e-09 | 45.9 | 48 | (13, 61) | 136 | (1, 48) | 96 | Uncharacterized protein | Uncharacterized protein | | uniclust | UniRef100\_A0A0D6PEJ5 | 96.4 | 0.00057 | 1.1e-09 | 55.2 | 85 | (28, 116) | 136 | (39, 123) | 274 | Phage protein | Phage protein | | uniclust | UniRef100\_UPI0011AF9C52 | 96.4 | 0.00063 | 1.1e-09 | 50.9 | 122 | (11, 134) | 136 | (10, 139) | 191 | hypothetical protein | hypothetical protein | | uniclust | UniRef100\_A0A1G9JFT4 | 96.4 | 0.0006 | 1.2e-09 | 54.2 | 115 | (11, 135) | 136 | (4, 121) | 225 | DNA circularisation protein N-terminus | DNA circularisation protein N-terminus | | uniclust | UniRef100\_UPI000D8ED903 | 96.4 | 0.00065 | 1.2e-09 | 44.3 | 52 | (20, 71) | 136 | (16, 70) | 81 | hypothetical protein | hypothetical protein | | uniclust | UniRef100\_A0A1G5H8K2 | 96.4 | 0.00061 | 1.3e-09 | 57.7 | 59 | (76, 135) | 136 | (216, 275) | 329 | Contractile injection system tube protein N-terminal domain-containing protein | Contractile injection system tube protein N-terminal domain-containing protein | | uniclust | UniRef100\_A0A1Q7LVG0 | 96.4 | 0.00067 | 1.3e-09 | 56.0 | 101 | (28, 135) | 136 | (37, 141) | 287 | Carboxypeptidase regulatory-like domain-containing protein | Carboxypeptidase regulatory-like domain-containing protein | | uniclust | UniRef100\_UPI002252A225 | 96.4 | 0.00075 | 1.4e-09 | 48.2 | 52 | (18, 69) | 136 | (81, 132) | 139 | phage tail protein | phage tail protein | | uniclust | UniRef100\_A0A081C209 | 96.4 | 0.00066 | 1.4e-09 | 54.0 | 103 | (29, 136) | 136 | (15, 120) | 204 | Uncharacterized protein | Uncharacterized protein | | uniclust | UniRef100\_A0A3M0WJW7 | 96.4 | 0.00075 | 1.4e-09 | 50.0 | 107 | (28, 135) | 136 | (25, 144) | 156 | Uncharacterized protein | Uncharacterized protein | | uniclust | UniRef100\_A0A9D9KBG8 | 96.4 | 0.00076 | 1.4e-09 | 52.8 | 108 | (28, 135) | 136 | (23, 145) | 238 | Uncharacterized protein | Uncharacterized protein | | uniclust | UniRef100\_A0A2E3A8K4 | 96.3 | 0.00086 | 1.6e-09 | 48.1 | 99 | (28, 132) | 136 | (27, 135) | 137 | Contractile injection system tube protein N-terminal domain-containing protein | Contractile injection system tube protein N-terminal domain-containing protein | | uniclust | UniRef100\_A0A7C6PPU2 | 96.3 | 0.00087 | 1.6e-09 | 50.2 | 109 | (28, 136) | 136 | (42, 163) | 191 | Contractile injection system tube protein N-terminal domain-containing protein | Contractile injection system tube protein N-terminal domain-containing protein | | uniclust | UniRef100\_A0A061NTU5 | 96.3 | 0.00089 | 1.6e-09 | 43.2 | 61 | (74, 135) | 136 | (5, 67) | 75 | Uncharacterized protein | Uncharacterized protein | | uniclust | UniRef100\_A0A1C7GNG6 | 96.3 | 0.0008 | 1.7e-09 | 52.4 | 103 | (4, 112) | 136 | (1, 103) | 177 | LysM domain-containing protein | LysM domain-containing protein | | pdb70 | 6J0N\_d | 99.3 | 5.1e-16 | 4.4e-20 | 109.7 | 112 | (23, 135) | 136 | (28, 155) | 229 | Pvc1, Pvc9, Pvc11, Pvc12, Pvc4 | 6J0N\_d Pvc1, Pvc9, Pvc11, Pvc12, Pvc4 assembly, Photorhabdus asymbiotica, PVC, contractile | | pdb70 | 6RAO\_G | 99.2 | 1.1e-15 | 9.2e-20 | 107.8 | 112 | (23, 135) | 136 | (28, 155) | 229 | Afp1, Afp2, Afp3, Afp5, Afp9 | 6RAO\_G Afp1, Afp2, Afp3, Afp5, Afp9 Anti-feeding prophage, secretion system, AFP | |
| Top keywords  (threshold 1.00e-03 (evalue)) | **Phage, tail, domain\_containing, Contractile, system, injection, N\_terminal, tube, GpU, LysM** |
| Output files | ../../similar\_sequences/23\_FANPEZAQ\_CDS\_0023\_merged.svg ../../similar\_sequences/23\_FANPEZAQ\_CDS\_0023\_pdb70.a3m ../../similar\_sequences/23\_FANPEZAQ\_CDS\_0023\_pdb70.hhr ../../similar\_sequences/23\_FANPEZAQ\_CDS\_0023\_uniclust.a3m ../../similar\_sequences/23\_FANPEZAQ\_CDS\_0023\_uniclust.hhr |

#### Structure prediction (AlphaFold)2

|  |  |
| --- | --- |
| Stats | xml version="1.0" encoding="utf-8" standalone="no"?       2024-09-02T21:09:23.942820 image/svg+xml   Matplotlib v3.7.2, https://matplotlib.org/ |
| Predicted structure | **NGL Viewer Controls:**  - Center: *Left-Click* - Rotate: *Left-Click + Drag* - Translate: *Right-Click + Drag* - Zoom: *Shift + Left-Click + Drag* |
| Output files | ../../predicted\_structures/23\_FANPEZAQ\_CDS\_0023/features.pkl ../../predicted\_structures/23\_FANPEZAQ\_CDS\_0023/ranked\_0.pdb ../../predicted\_structures/23\_FANPEZAQ\_CDS\_0023/ranked\_0\_plots.svg ../../predicted\_structures/23\_FANPEZAQ\_CDS\_0023/result\_model\_1\_ptm\_pred\_0.pkl |

#### Structure similarity search results (Foldseek)3

|  |  |
| --- | --- |
| Structure databases searched | Pdb, Afdb-proteome, Afdb-uniprot50 |
| Results, scheme(s)  (Top layers only, threshold 1.00e-02 (evalue)) | xml version="1.0" encoding="utf-8" standalone="no"?       2024-09-02T21:10:52.344980 image/svg+xml   Matplotlib v3.7.2, https://matplotlib.org/ |
| Results, table  (threshold 1.00e-02 (evalue)) | | db | id | prob | evalue | bits | fident | alnlen | mismatch | gapopen | qstart | qend | tstart | tend | name | description | | --- | --- | --- | --- | --- | --- | --- | --- | --- | --- | --- | --- | --- | --- | --- | | pdb | 6U5B\_H | 1.0 | 1.055e-11 | 483 | 0.378 | 140 | 71 | 3 | 13 | 136 | 144 | 283 | Ripcord PA0626 | Ripcord PA0626 | | pdb | 6J0N\_B | 1.0 | 2.363e-05 | 199 | 0.147 | 122 | 87 | 6 | 29 | 134 | 32 | 152 | Pvc7 | Pvc7 | | pdb | 7AEB\_M | 1.0 | 2.494e-05 | 173 | 0.114 | 149 | 106 | 6 | 13 | 136 | 8 | 155 | Phage tail protein | Phage tail protein | | pdb | 7B5H\_AK | 1.0 | 2.928e-05 | 169 | 0.126 | 158 | 102 | 9 | 13 | 136 | 5 | 160 | All3321 protein | All3321 protein | | pdb | 2X8K\_C | 1.0 | 0.0002495 | 169 | 0.097 | 133 | 99 | 8 | 13 | 136 | 3 | 123 | HYPOTHETICAL PROTEIN 19.1 | HYPOTHETICAL PROTEIN 19.1 | | pdb | 6RBK\_A | 1.0 | 0.000146 | 160 | 0.127 | 118 | 88 | 4 | 30 | 132 | 34 | 151 | Afp7 | Afp7 | | pdb | 8EON\_C | 1.0 | 0.0008551 | 153 | 0.096 | 135 | 110 | 5 | 4 | 136 | 5 | 129 | Baseplate component gp37 | Baseplate component gp37 | | pdb | 6TUI\_G | 1.0 | 0.0007282 | 150 | 0.125 | 136 | 95 | 7 | 12 | 136 | 6 | 128 | Tail tube protein Rcc01691 | Tail tube protein Rcc01691 | | pdb | 7YFZ\_B | 1.0 | 0.001244 | 147 | 0.112 | 124 | 98 | 4 | 16 | 136 | 1 | 115 | Pam3 tube initiator gp17 | Pam3 tube initiator gp17 | | pdb | 6V8I\_AC | 1.0 | 0.001004 | 121 | 0.081 | 172 | 100 | 9 | 13 | 136 | 3 | 164 | Distal Tail Protein, gp58 | Distal Tail Protein, gp58 | | pdb | 6TBA\_5A | 1.0 | 0.003263 | 117 | 0.127 | 141 | 96 | 10 | 6 | 136 | 1 | 124 | Phage major tail protein, TP901-1 family | Phage major tail protein, TP901-1 family | | pdb | 8HDR\_1 | 1.0 | 0.004264 | 109 | 0.107 | 140 | 102 | 8 | 6 | 134 | 4 | 131 | pam3 tube protein | pam3 tube protein | | pdb | 6V8I\_EA | 1.0 | 0.006204 | 109 | 0.094 | 148 | 102 | 11 | 7 | 136 | 1 | 134 | Major Tail Protein, gp53 | Major Tail Protein, gp53 | | pdb | 6RAO\_A | 1.0 | 0.00191 | 104 | 0.113 | 150 | 102 | 9 | 2 | 136 | 9 | 142 | Afp1 | Afp1 | | pdb | 5IV5\_U | 1.0 | 0.003831 | 100 | 0.11 | 127 | 78 | 9 | 37 | 136 | 41 | 159 | Baseplate tail-tube protein gp48 | Baseplate tail-tube protein gp48 | | pdb | 6J0F\_A | 0.998 | 0.005881 | 95 | 0.108 | 147 | 100 | 9 | 1 | 131 | 1 | 132 | Pvc1 | Pvc1 | | pdb | 6J0B\_A | 0.998 | 0.001313 | 94 | 0.124 | 145 | 104 | 9 | 2 | 134 | 9 | 142 | Pvc1 | Pvc1 | | pdb | 6V8I\_EB | 0.998 | 0.004499 | 93 | 0.121 | 148 | 93 | 14 | 10 | 136 | 3 | 134 | Major Tail Protein, gp53 | Major Tail Protein, gp53 | | pdb | 8BL4\_P | 0.988 | 0.006546 | 82 | 0.091 | 131 | 88 | 10 | 29 | 136 | 24 | 146 | Phage tail protein | Phage tail protein | | pdb | 6RAO\_F | 0.923 | 0.005881 | 69 | 0.133 | 142 | 93 | 10 | 13 | 136 | 12 | 141 | Afp5 | Afp5 | | afdb-proteome | AF-Q8ZMV4-F1-MODEL\_V4 | 1.0 | 7.529e-13 | 587 | 0.346 | 124 | 81 | 0 | 13 | 136 | 1 | 124 | Fels-2 prophage protein | Fels-2 prophage protein | | afdb-proteome | AF-A0A0H3GQH5-F1-MODEL\_V4 | 1.0 | 6.411e-13 | 582 | 0.338 | 124 | 82 | 0 | 13 | 136 | 1 | 124 | Putative bacteriophage tail protein | Putative bacteriophage tail protein | | afdb-proteome | AF-G3XD65-F1-MODEL\_V4 | 1.0 | 7.943e-13 | 536 | 0.35 | 154 | 81 | 5 | 2 | 136 | 131 | 284 | Uncharacterized protein | Uncharacterized protein | | afdb-proteome | AF-Q8ZKJ5-F1-MODEL\_V4 | 1.0 | 2.727e-09 | 403 | 0.227 | 123 | 94 | 1 | 13 | 135 | 1 | 122 | Putative methyl-accepting chemotaxis protein | Putative methyl-accepting chemotaxis protein | | afdb-proteome | AF-A0A0H3GS01-F1-MODEL\_V4 | 1.0 | 5.783e-05 | 222 | 0.424 | 73 | 42 | 0 | 64 | 136 | 1 | 73 | Putative prophage tail protein | Putative prophage tail protein | | afdb-proteome | AF-P71389-F1-MODEL\_V4 | 1.0 | 4.668e-05 | 199 | 0.099 | 131 | 106 | 5 | 7 | 136 | 1 | 120 | Mu-like prophage FluMu DNA circularization protein | Mu-like prophage FluMu DNA circularization protein | | afdb-proteome | AF-A0A3P7DQ66-F1-MODEL\_V4 | 1.0 | 0.0002884 | 154 | 0.11 | 163 | 100 | 10 | 13 | 136 | 328 | 484 | Uncharacterized protein | Uncharacterized protein | | afdb-proteome | AF-A0A0H3GKS4-F1-MODEL\_V4 | 1.0 | 0.000377 | 145 | 0.091 | 153 | 101 | 10 | 13 | 136 | 31 | 174 | Uncharacterized protein | Uncharacterized protein | | afdb-proteome | AF-A0A0H3H550-F1-MODEL\_V4 | 1.0 | 0.006798 | 131 | 0.118 | 110 | 82 | 5 | 30 | 136 | 12 | 109 | Minor tail fiber protein M | Minor tail fiber protein M | | afdb-proteome | AF-A0A133CKK6-F1-MODEL\_V4 | 1.0 | 0.003212 | 121 | 0.156 | 128 | 94 | 7 | 13 | 133 | 5 | 125 | Phage tail protein | Phage tail protein | | afdb-proteome | AF-Q57791-F1-MODEL\_V4 | 1.0 | 0.002592 | 121 | 0.086 | 127 | 95 | 7 | 13 | 136 | 209 | 317 | Uncharacterized protein MJ0345 | Uncharacterized protein MJ0345 | | afdb-proteome | AF-Q8ZQ89-F1-MODEL\_V4 | 1.0 | 0.002592 | 114 | 0.086 | 162 | 105 | 12 | 1 | 136 | 6 | 150 | Gifsy-2 prophage probable major tail protein | Gifsy-2 prophage probable major tail protein | | afdb-proteome | AF-Q2FX66-F1-MODEL\_V4 | 1.0 | 0.002592 | 110 | 0.108 | 157 | 82 | 9 | 27 | 136 | 20 | 165 | Conserved hypothetical phage protein | Conserved hypothetical phage protein | | afdb-proteome | AF-Q2FYC9-F1-MODEL\_V4 | 1.0 | 0.003044 | 109 | 0.09 | 132 | 99 | 9 | 13 | 133 | 5 | 126 | Holin-like protein | Holin-like protein | | afdb-proteome | AF-A0A077ZLB1-F1-MODEL\_V4 | 1.0 | 0.006798 | 101 | 0.118 | 143 | 96 | 11 | 19 | 134 | 23 | 162 | Uncharacterized protein | Uncharacterized protein | | afdb-proteome | AF-Q2FX62-F1-MODEL\_V4 | 0.998 | 0.008886 | 94 | 0.106 | 141 | 90 | 11 | 13 | 134 | 12 | 135 | Phage structural protein, putative | Phage structural protein, putative | | afdb-uniprot50 | AF-A0A1V2DRL6-F1-MODEL\_V4 | 1.0 | 2.002e-17 | 820 | 0.559 | 127 | 56 | 0 | 10 | 136 | 1 | 127 | Phage tail protein | Phage tail protein | | afdb-uniprot50 | AF-A0A6M4Y8X7-F1-MODEL\_V4 | 1.0 | 1.304e-17 | 816 | 0.564 | 131 | 57 | 0 | 6 | 136 | 3 | 133 | Phage tail protein | Phage tail protein | | afdb-uniprot50 | AF-A0A660NPZ3-F1-MODEL\_V4 | 1.0 | 1.053e-16 | 794 | 0.559 | 127 | 56 | 0 | 10 | 136 | 8 | 134 | Phage tail protein | Phage tail protein | | afdb-uniprot50 | AF-A0A2W5L0T9-F1-MODEL\_V4 | 1.0 | 8.057e-17 | 790 | 0.58 | 124 | 52 | 0 | 13 | 136 | 10 | 133 | Uncharacterized protein | Uncharacterized protein | | afdb-uniprot50 | AF-A0A1G6JEA6-F1-MODEL\_V4 | 1.0 | 6.503e-17 | 788 | 0.492 | 130 | 66 | 0 | 7 | 136 | 5 | 134 | Uncharacterized protein | Uncharacterized protein | | afdb-uniprot50 | AF-A0A2Z3IG89-F1-MODEL\_V4 | 1.0 | 2.228e-17 | 782 | 0.544 | 136 | 59 | 1 | 1 | 136 | 1 | 133 | Phage tail protein | Phage tail protein | | afdb-uniprot50 | AF-A0A315BRY9-F1-MODEL\_V4 | 1.0 | 6.164e-17 | 768 | 0.588 | 136 | 53 | 1 | 1 | 136 | 2 | 134 | Uncharacterized protein | Uncharacterized protein | | afdb-uniprot50 | AF-A0A410UF80-F1-MODEL\_V4 | 1.0 | 2.914e-16 | 767 | 0.434 | 129 | 73 | 0 | 8 | 136 | 25 | 153 | Phage tail protein | Phage tail protein | | afdb-uniprot50 | AF-A0A2Z6AYW2-F1-MODEL\_V4 | 1.0 | 2.762e-16 | 766 | 0.496 | 127 | 64 | 0 | 10 | 136 | 1 | 127 | P2 GpU family protein | P2 GpU family protein | | afdb-uniprot50 | AF-A0A081N7U3-F1-MODEL\_V4 | 1.0 | 3.422e-16 | 762 | 0.417 | 127 | 74 | 0 | 10 | 136 | 1 | 127 | Tail protein | Tail protein | | afdb-uniprot50 | AF-A0A1N6I0S3-F1-MODEL\_V4 | 1.0 | 5.846e-16 | 758 | 0.472 | 127 | 67 | 0 | 10 | 136 | 10 | 136 | Uncharacterized protein | Uncharacterized protein | | afdb-uniprot50 | AF-A0A291LZ32-F1-MODEL\_V4 | 1.0 | 3.422e-16 | 757 | 0.425 | 127 | 73 | 0 | 10 | 136 | 1 | 127 | Phage tail protein | Phage tail protein | | afdb-uniprot50 | AF-A0A212KMX5-F1-MODEL\_V4 | 1.0 | 7.641e-16 | 756 | 0.511 | 127 | 62 | 0 | 10 | 136 | 1 | 127 | Uncharacterized protein | Uncharacterized protein | | afdb-uniprot50 | AF-A0A5A9EN75-F1-MODEL\_V4 | 1.0 | 8.505e-16 | 756 | 0.409 | 127 | 75 | 0 | 10 | 136 | 1 | 127 | Phage tail protein | Phage tail protein | | afdb-uniprot50 | AF-A0A212KBL8-F1-MODEL\_V4 | 1.0 | 8.973e-16 | 754 | 0.488 | 127 | 65 | 0 | 10 | 136 | 1 | 127 | Uncharacterized protein | Uncharacterized protein | | afdb-uniprot50 | AF-A0A4Z0WCE3-F1-MODEL\_V4 | 1.0 | 1.706e-15 | 747 | 0.451 | 124 | 68 | 0 | 13 | 136 | 31 | 154 | Phage tail protein | Phage tail protein | | afdb-uniprot50 | AF-A0A430HF79-F1-MODEL\_V4 | 1.0 | 1.453e-15 | 746 | 0.456 | 127 | 69 | 0 | 10 | 136 | 1 | 127 | Phage tail protein | Phage tail protein | | afdb-uniprot50 | AF-A0A4U8YGX8-F1-MODEL\_V4 | 1.0 | 1.305e-15 | 739 | 0.484 | 126 | 65 | 0 | 11 | 136 | 17 | 142 | Myoviridae gpu | Myoviridae gpu | | afdb-uniprot50 | AF-A0A7C8M0S1-F1-MODEL\_V4 | 1.0 | 5.846e-16 | 738 | 0.462 | 132 | 71 | 0 | 5 | 136 | 20 | 151 | Uncharacterized protein | Uncharacterized protein | | afdb-uniprot50 | AF-A0A291G0E7-F1-MODEL\_V4 | 1.0 | 1.706e-15 | 737 | 0.48 | 127 | 66 | 0 | 10 | 136 | 2 | 128 | Phage-related tail protein (GpU) | Phage-related tail protein (GpU) | | afdb-uniprot50 | AF-A0A345DE50-F1-MODEL\_V4 | 1.0 | 1.533e-15 | 736 | 0.382 | 128 | 79 | 0 | 9 | 136 | 34 | 161 | Uncharacterized protein | Uncharacterized protein | | afdb-uniprot50 | AF-Q31Q86-F1-MODEL\_V4 | 1.0 | 2.619e-15 | 736 | 0.464 | 127 | 68 | 0 | 10 | 136 | 1 | 127 | Uncharacterized protein | Uncharacterized protein | | afdb-uniprot50 | AF-B8GS04-F1-MODEL\_V4 | 1.0 | 2.114e-15 | 730 | 0.419 | 124 | 72 | 0 | 13 | 136 | 1 | 124 | Putative phage tail protein U | Putative phage tail protein U | | afdb-uniprot50 | AF-A0A1S1TJY4-F1-MODEL\_V4 | 1.0 | 1.377e-15 | 730 | 0.472 | 125 | 66 | 0 | 12 | 136 | 2 | 126 | Uncharacterized protein | Uncharacterized protein | | afdb-uniprot50 | AF-A0A1G7SAR7-F1-MODEL\_V4 | 1.0 | 1.706e-15 | 727 | 0.46 | 128 | 68 | 1 | 10 | 136 | 1 | 128 | Uncharacterized protein | Uncharacterized protein | | afdb-uniprot50 | AF-A0A118DSW1-F1-MODEL\_V4 | 1.0 | 8.505e-16 | 720 | 0.448 | 136 | 74 | 1 | 1 | 136 | 1 | 135 | Uncharacterized protein | Uncharacterized protein | | afdb-uniprot50 | AF-A0A8A6KDU1-F1-MODEL\_V4 | 1.0 | 4.475e-15 | 718 | 0.537 | 121 | 56 | 0 | 14 | 134 | 1 | 121 | Phage tail protein | Phage tail protein | | afdb-uniprot50 | AF-A0A6S6Y3Q4-F1-MODEL\_V4 | 1.0 | 3.245e-15 | 715 | 0.433 | 127 | 72 | 0 | 10 | 136 | 2 | 128 | Phage tail protein | Phage tail protein | | afdb-uniprot50 | AF-A0A1L2AZL7-F1-MODEL\_V4 | 1.0 | 1.305e-15 | 713 | 0.419 | 136 | 79 | 0 | 1 | 136 | 16 | 151 | Phage tail protein | Phage tail protein | | afdb-uniprot50 | AF-C6BVX0-F1-MODEL\_V4 | 1.0 | 5.544e-15 | 712 | 0.395 | 124 | 75 | 0 | 13 | 136 | 2 | 125 | p2 GpU family protein | p2 GpU family protein | | afdb-uniprot50 | AF-A0A7K0GNT9-F1-MODEL\_V4 | 1.0 | 6.167e-16 | 707 | 0.422 | 135 | 78 | 0 | 2 | 136 | 29 | 163 | Uncharacterized protein | Uncharacterized protein | | afdb-uniprot50 | AF-K5Z1D9-F1-MODEL\_V4 | 1.0 | 1.706e-15 | 707 | 0.448 | 136 | 74 | 1 | 1 | 136 | 5 | 139 | Phage P2 GpU | Phage P2 GpU | | afdb-uniprot50 | AF-A0A239EIZ1-F1-MODEL\_V4 | 1.0 | 2.915e-15 | 705 | 0.438 | 130 | 73 | 0 | 7 | 136 | 1 | 130 | Uncharacterized protein | Uncharacterized protein | | afdb-uniprot50 | AF-A0A1C3ELB9-F1-MODEL\_V4 | 1.0 | 6.51e-15 | 703 | 0.491 | 124 | 63 | 0 | 13 | 136 | 1 | 124 | Phage tail protein | Phage tail protein | | afdb-uniprot50 | AF-A0A1C3K3E1-F1-MODEL\_V4 | 1.0 | 6.868e-15 | 703 | 0.42 | 126 | 73 | 0 | 11 | 136 | 3 | 128 | Phage tail protein | Phage tail protein | | afdb-uniprot50 | AF-A0A4P5VNR5-F1-MODEL\_V4 | 1.0 | 8.065e-15 | 702 | 0.416 | 125 | 73 | 0 | 12 | 136 | 2 | 126 | Uncharacterized protein | Uncharacterized protein | | afdb-uniprot50 | AF-A0A6B3LC06-F1-MODEL\_V4 | 1.0 | 8.065e-15 | 700 | 0.343 | 128 | 83 | 1 | 10 | 136 | 1 | 128 | Phage tail protein | Phage tail protein | | afdb-uniprot50 | AF-A0A3N2E1A2-F1-MODEL\_V4 | 1.0 | 3.81e-15 | 700 | 0.395 | 134 | 81 | 0 | 3 | 136 | 15 | 148 | Uncharacterized protein | Uncharacterized protein | | afdb-uniprot50 | AF-A0A285D6T1-F1-MODEL\_V4 | 1.0 | 1.054e-14 | 696 | 0.373 | 126 | 79 | 0 | 10 | 135 | 1 | 126 | Uncharacterized protein | Uncharacterized protein | | afdb-uniprot50 | AF-A0A348HI90-F1-MODEL\_V4 | 1.0 | 8.065e-15 | 695 | 0.425 | 127 | 73 | 0 | 10 | 136 | 2 | 128 | Phage protein U | Phage protein U | | afdb-uniprot50 | AF-A0A3G6WB93-F1-MODEL\_V4 | 1.0 | 1.534e-14 | 689 | 0.428 | 126 | 72 | 0 | 10 | 135 | 1 | 126 | Phage tail protein | Phage tail protein | | afdb-uniprot50 | AF-A0A2E3N1P0-F1-MODEL\_V4 | 1.0 | 5.254e-15 | 686 | 0.425 | 127 | 73 | 0 | 10 | 136 | 2 | 128 | Phage tail protein | Phage tail protein | | afdb-uniprot50 | AF-A0A1Y2K0S7-F1-MODEL\_V4 | 1.0 | 5.544e-15 | 685 | 0.431 | 132 | 74 | 1 | 6 | 136 | 2 | 133 | Putative P2 GpU family protein | Putative P2 GpU family protein | | afdb-uniprot50 | AF-A0A158E8Q2-F1-MODEL\_V4 | 1.0 | 8.977e-15 | 683 | 0.426 | 129 | 74 | 0 | 8 | 136 | 5 | 133 | Bacteriophage tail-related protein | Bacteriophage tail-related protein | | afdb-uniprot50 | AF-A0A1E3G8A9-F1-MODEL\_V4 | 1.0 | 1.618e-14 | 682 | 0.401 | 127 | 76 | 0 | 10 | 136 | 2 | 128 | Uncharacterized protein | Uncharacterized protein | | afdb-uniprot50 | AF-J7SX82-F1-MODEL\_V4 | 1.0 | 6.51e-15 | 681 | 0.401 | 132 | 79 | 0 | 5 | 136 | 19 | 150 | Uncharacterized protein | Uncharacterized protein | | afdb-uniprot50 | AF-A0A6G8F2C6-F1-MODEL\_V4 | 1.0 | 4.02e-15 | 681 | 0.413 | 133 | 76 | 1 | 6 | 136 | 18 | 150 | Uncharacterized protein | Uncharacterized protein | | afdb-uniprot50 | AF-A0A4R3LHW8-F1-MODEL\_V4 | 1.0 | 1.801e-14 | 680 | 0.42 | 126 | 73 | 0 | 11 | 136 | 3 | 128 | Uncharacterized protein | Uncharacterized protein | | afdb-uniprot50 | AF-A0A349FC45-F1-MODEL\_V4 | 1.0 | 3.077e-14 | 679 | 0.33 | 127 | 85 | 0 | 10 | 136 | 1 | 127 | Phage tail protein | Phage tail protein | | afdb-uniprot50 | AF-A0A7Y4DZ99-F1-MODEL\_V4 | 1.0 | 3.246e-14 | 679 | 0.328 | 128 | 85 | 1 | 10 | 136 | 1 | 128 | Phage tail protein | Phage tail protein | | afdb-uniprot50 | AF-A0A4R3L5P7-F1-MODEL\_V4 | 1.0 | 4.721e-15 | 679 | 0.433 | 136 | 66 | 2 | 12 | 136 | 2 | 137 | Uncharacterized protein | Uncharacterized protein | | afdb-uniprot50 | AF-A0A4P9VGW4-F1-MODEL\_V4 | 1.0 | 3.613e-14 | 678 | 0.37 | 124 | 78 | 0 | 13 | 136 | 186 | 309 | Uncharacterized protein | Uncharacterized protein | | afdb-uniprot50 | AF-A0A2S2E5C8-F1-MODEL\_V4 | 1.0 | 2.917e-14 | 676 | 0.359 | 128 | 82 | 0 | 9 | 136 | 2 | 129 | Putative tape measure protein | Putative tape measure protein | | afdb-uniprot50 | AF-A0A1X7Q475-F1-MODEL\_V4 | 1.0 | 1.707e-14 | 673 | 0.409 | 127 | 75 | 0 | 10 | 136 | 2 | 128 | Uncharacterized protein | Uncharacterized protein | | afdb-uniprot50 | AF-A0A066RZT5-F1-MODEL\_V4 | 1.0 | 1.618e-14 | 672 | 0.359 | 128 | 81 | 1 | 10 | 136 | 1 | 128 | p2 GpU family protein | p2 GpU family protein | | afdb-uniprot50 | AF-I3TTC4-F1-MODEL\_V4 | 1.0 | 5.257e-14 | 672 | 0.406 | 123 | 73 | 0 | 14 | 136 | 285 | 407 | Uncharacterized protein | Uncharacterized protein | | afdb-uniprot50 | AF-A0A1Y1QYE0-F1-MODEL\_V4 | 1.0 | 2.231e-14 | 670 | 0.437 | 128 | 68 | 2 | 13 | 136 | 1 | 128 | Uncharacterized protein | Uncharacterized protein | | afdb-uniprot50 | AF-A0A135IJT4-F1-MODEL\_V4 | 1.0 | 3.613e-14 | 665 | 0.44 | 127 | 71 | 0 | 10 | 136 | 2 | 128 | Phage tail protein | Phage tail protein | | afdb-uniprot50 | AF-A0A145VQC9-F1-MODEL\_V4 | 1.0 | 6.868e-15 | 665 | 0.398 | 148 | 68 | 2 | 10 | 136 | 1 | 148 | Uncharacterized protein | Uncharacterized protein | | afdb-uniprot50 | AF-A0A149SP38-F1-MODEL\_V4 | 1.0 | 3.812e-14 | 662 | 0.341 | 129 | 85 | 0 | 8 | 136 | 21 | 149 | Uncharacterized protein | Uncharacterized protein | | afdb-uniprot50 | AF-A0A3R8L896-F1-MODEL\_V4 | 1.0 | 5.257e-14 | 661 | 0.39 | 128 | 77 | 1 | 10 | 136 | 1 | 128 | Phage tail protein | Phage tail protein | | afdb-uniprot50 | AF-A0A4P7EJT5-F1-MODEL\_V4 | 1.0 | 2.354e-14 | 661 | 0.423 | 130 | 75 | 0 | 7 | 136 | 1 | 130 | Phage tail protein | Phage tail protein | | afdb-uniprot50 | AF-A0A0F9VVN6-F1-MODEL\_V4 | 1.0 | 6.173e-14 | 658 | 0.475 | 124 | 65 | 0 | 13 | 136 | 1 | 124 | Uncharacterized protein | Uncharacterized protein | | afdb-uniprot50 | AF-A0A376TF55-F1-MODEL\_V4 | 1.0 | 8.982e-14 | 658 | 0.443 | 124 | 69 | 0 | 13 | 136 | 1 | 124 | GpU phage protein | GpU phage protein | | afdb-uniprot50 | AF-A0A554X131-F1-MODEL\_V4 | 1.0 | 4.983e-14 | 657 | 0.388 | 126 | 77 | 0 | 11 | 136 | 3 | 128 | Phage P2 GpU | Phage P2 GpU | | afdb-uniprot50 | AF-A0A4S5DES3-F1-MODEL\_V4 | 1.0 | 4.022e-14 | 655 | 0.362 | 127 | 81 | 0 | 10 | 136 | 1 | 127 | Phage tail protein | Phage tail protein | | afdb-uniprot50 | AF-A0A4R3LF92-F1-MODEL\_V4 | 1.0 | 5.546e-14 | 654 | 0.433 | 127 | 72 | 0 | 10 | 136 | 1 | 127 | Uncharacterized protein | Uncharacterized protein | | afdb-uniprot50 | AF-A0A849B434-F1-MODEL\_V4 | 1.0 | 9.476e-14 | 654 | 0.411 | 124 | 73 | 0 | 13 | 136 | 1 | 124 | Oxidoreductase | Oxidoreductase | | afdb-uniprot50 | AF-A0A554XB21-F1-MODEL\_V4 | 1.0 | 5.257e-14 | 652 | 0.433 | 127 | 72 | 0 | 10 | 136 | 1 | 127 | Phage P2 GpU | Phage P2 GpU | | afdb-uniprot50 | AF-A0A7S8C741-F1-MODEL\_V4 | 1.0 | 5.257e-14 | 650 | 0.385 | 127 | 78 | 0 | 10 | 136 | 1 | 127 | Phage tail protein | Phage tail protein | | afdb-uniprot50 | AF-V4P7Q6-F1-MODEL\_V4 | 1.0 | 1.055e-13 | 649 | 0.411 | 124 | 73 | 0 | 13 | 136 | 2 | 125 | Oxidoreductase | Oxidoreductase | | afdb-uniprot50 | AF-A0A326L399-F1-MODEL\_V4 | 1.0 | 1.534e-13 | 649 | 0.336 | 125 | 83 | 0 | 12 | 136 | 2 | 126 | Oxidoreductase | Oxidoreductase | | afdb-uniprot50 | AF-A0A6L2ZPM8-F1-MODEL\_V4 | 1.0 | 1.534e-13 | 647 | 0.427 | 124 | 71 | 0 | 13 | 136 | 1 | 124 | Tail protein | Tail protein | | afdb-uniprot50 | AF-A0A4P9VFE7-F1-MODEL\_V4 | 1.0 | 1.708e-13 | 647 | 0.365 | 126 | 80 | 0 | 11 | 136 | 3 | 128 | Phage tail protein | Phage tail protein | | afdb-uniprot50 | AF-A0A1N6Q3K9-F1-MODEL\_V4 | 1.0 | 9.476e-14 | 646 | 0.403 | 124 | 74 | 0 | 13 | 136 | 1 | 124 | Uncharacterized protein | Uncharacterized protein | | afdb-uniprot50 | AF-A0A3M3TVG5-F1-MODEL\_V4 | 1.0 | 8.513e-14 | 643 | 0.381 | 131 | 74 | 1 | 13 | 136 | 23 | 153 | Uncharacterized protein | Uncharacterized protein | | afdb-uniprot50 | AF-A0A7V8FKF8-F1-MODEL\_V4 | 1.0 | 3.812e-14 | 642 | 0.387 | 129 | 79 | 0 | 8 | 136 | 23 | 151 | Uncharacterized protein | Uncharacterized protein | | afdb-uniprot50 | AF-A0A6L9FGD7-F1-MODEL\_V4 | 1.0 | 5.852e-14 | 642 | 0.375 | 136 | 84 | 1 | 2 | 136 | 17 | 152 | Uncharacterized protein | Uncharacterized protein | | afdb-uniprot50 | AF-N9WEM7-F1-MODEL\_V4 | 1.0 | 1.174e-13 | 642 | 0.387 | 129 | 79 | 0 | 8 | 136 | 57 | 185 | Phage P2 GpU protein | Phage P2 GpU protein | | afdb-uniprot50 | AF-A0A429LGV1-F1-MODEL\_V4 | 1.0 | 2.232e-13 | 641 | 0.443 | 124 | 69 | 0 | 13 | 136 | 1 | 124 | Phage tail protein | Phage tail protein | | afdb-uniprot50 | AF-A0A524RVY3-F1-MODEL\_V4 | 1.0 | 1.802e-13 | 641 | 0.307 | 127 | 88 | 0 | 10 | 136 | 1 | 127 | Phage tail protein | Phage tail protein | | afdb-uniprot50 | AF-A0A7W6RFB2-F1-MODEL\_V4 | 1.0 | 4.022e-14 | 640 | 0.4 | 135 | 73 | 3 | 10 | 136 | 1 | 135 | Uncharacterized protein | Uncharacterized protein | | afdb-uniprot50 | AF-A0A1D2QSE6-F1-MODEL\_V4 | 1.0 | 5.257e-14 | 640 | 0.443 | 133 | 65 | 1 | 13 | 136 | 1 | 133 | Uncharacterized protein | Uncharacterized protein | | afdb-uniprot50 | AF-A0A395R3N0-F1-MODEL\_V4 | 1.0 | 1.454e-13 | 638 | 0.443 | 124 | 69 | 0 | 13 | 136 | 1 | 124 | Oxidoreductase | Oxidoreductase | | afdb-uniprot50 | AF-A0A1W1XJU9-F1-MODEL\_V4 | 1.0 | 5.546e-14 | 636 | 0.382 | 136 | 84 | 0 | 1 | 136 | 16 | 151 | Phage protein U | Phage protein U | | afdb-uniprot50 | AF-A0A4Q0YJQ4-F1-MODEL\_V4 | 1.0 | 2.232e-13 | 635 | 0.475 | 124 | 65 | 0 | 13 | 136 | 1 | 124 | Oxidoreductase | Oxidoreductase | | afdb-uniprot50 | AF-A0A1C6YVI2-F1-MODEL\_V4 | 1.0 | 9.476e-14 | 635 | 0.335 | 128 | 85 | 0 | 8 | 135 | 24 | 151 | Uncharacterized protein | Uncharacterized protein | | afdb-uniprot50 | AF-B6I123-F1-MODEL\_V4 | 1.0 | 1.379e-13 | 634 | 0.346 | 130 | 83 | 1 | 9 | 136 | 9 | 138 | Uncharacterized protein | Uncharacterized protein | | afdb-uniprot50 | AF-A0A6L8HM89-F1-MODEL\_V4 | 1.0 | 2.006e-13 | 633 | 0.404 | 126 | 75 | 0 | 11 | 136 | 3 | 128 | Phage tail protein | Phage tail protein | | afdb-uniprot50 | AF-A0A2C9B8U0-F1-MODEL\_V4 | 1.0 | 1.802e-13 | 633 | 0.395 | 124 | 75 | 0 | 13 | 136 | 1 | 124 | Uncharacterized protein | Uncharacterized protein | | afdb-uniprot50 | AF-A0A375FXQ5-F1-MODEL\_V4 | 1.0 | 4.245e-13 | 633 | 0.395 | 124 | 75 | 0 | 13 | 136 | 1 | 124 | Bacteriophage P2 tail protein GPU | Bacteriophage P2 tail protein GPU | | afdb-uniprot50 | AF-A0A396RZM8-F1-MODEL\_V4 | 1.0 | 2.622e-13 | 633 | 0.435 | 124 | 70 | 0 | 13 | 136 | 1 | 124 | Oxidoreductase | Oxidoreductase | | afdb-uniprot50 | AF-A0A1G8XD62-F1-MODEL\_V4 | 1.0 | 2.232e-13 | 632 | 0.411 | 124 | 73 | 0 | 13 | 136 | 1 | 124 | Uncharacterized protein | Uncharacterized protein | | afdb-uniprot50 | AF-A0A1Y6CS08-F1-MODEL\_V4 | 1.0 | 1.055e-13 | 631 | 0.35 | 134 | 83 | 2 | 6 | 136 | 4 | 136 | Uncharacterized protein | Uncharacterized protein | | afdb-uniprot50 | AF-Q1QXT0-F1-MODEL\_V4 | 1.0 | 3.079e-13 | 630 | 0.419 | 124 | 72 | 0 | 13 | 136 | 1 | 124 | Phage P2 GpU | Phage P2 GpU | | afdb-uniprot50 | AF-C1D6Q1-F1-MODEL\_V4 | 1.0 | 2.006e-13 | 629 | 0.39 | 128 | 77 | 1 | 10 | 136 | 2 | 129 | Phage P2 GpU | Phage P2 GpU | | afdb-uniprot50 | AF-A0A318KNF0-F1-MODEL\_V4 | 1.0 | 2.766e-13 | 628 | 0.376 | 125 | 77 | 1 | 13 | 136 | 2 | 126 | Uncharacterized protein | Uncharacterized protein | | afdb-uniprot50 | AF-A0A1K0JN57-F1-MODEL\_V4 | 1.0 | 5.26e-13 | 628 | 0.395 | 124 | 75 | 0 | 13 | 136 | 1 | 124 | Bacteriophage P2 tail protein GPU | Bacteriophage P2 tail protein GPU | | afdb-uniprot50 | AF-A0A0P6X2A9-F1-MODEL\_V4 | 1.0 | 3.079e-13 | 626 | 0.322 | 127 | 86 | 0 | 10 | 136 | 2 | 128 | Uncharacterized protein | Uncharacterized protein | | afdb-uniprot50 | AF-A0A3N1XDI5-F1-MODEL\_V4 | 1.0 | 3.427e-13 | 626 | 0.443 | 124 | 69 | 0 | 13 | 136 | 1 | 124 | Uncharacterized protein | Uncharacterized protein | | afdb-uniprot50 | AF-D4M9M2-F1-MODEL\_V4 | 1.0 | 6.875e-13 | 624 | 0.376 | 125 | 77 | 1 | 13 | 136 | 1 | 125 | Phage protein U | Phage protein U | | afdb-uniprot50 | AF-A0A1E3G6A9-F1-MODEL\_V4 | 1.0 | 2.766e-13 | 624 | 0.447 | 123 | 66 | 2 | 14 | 136 | 1 | 121 | Uncharacterized protein | Uncharacterized protein | | afdb-uniprot50 | AF-A0A4Y7X8T9-F1-MODEL\_V4 | 1.0 | 7.253e-13 | 624 | 0.403 | 124 | 74 | 0 | 13 | 136 | 1 | 124 | Phage tail protein | Phage tail protein | | afdb-uniprot50 | AF-A0A1H1SML2-F1-MODEL\_V4 | 1.0 | 4.479e-13 | 622 | 0.435 | 124 | 70 | 0 | 13 | 136 | 1 | 124 | Uncharacterized protein | Uncharacterized protein | | afdb-uniprot50 | AF-A0A7V8INR8-F1-MODEL\_V4 | 1.0 | 3.248e-13 | 621 | 0.387 | 124 | 76 | 0 | 13 | 136 | 1 | 124 | Tail formation protein phage P2 GpU | Tail formation protein phage P2 GpU | | afdb-uniprot50 | AF-A0A1X3JWZ1-F1-MODEL\_V4 | 1.0 | 1.379e-13 | 619 | 0.34 | 138 | 87 | 3 | 1 | 136 | 1 | 136 | Tail formation protein | Tail formation protein | | afdb-uniprot50 | AF-A0A7V8U724-F1-MODEL\_V4 | 1.0 | 1.307e-13 | 619 | 0.348 | 132 | 86 | 0 | 5 | 136 | 48 | 179 | Uncharacterized protein | Uncharacterized protein | | afdb-uniprot50 | AF-A0A0N4UTZ6-F1-MODEL\_V4 | 1.0 | 7.253e-13 | 617 | 0.435 | 124 | 70 | 0 | 13 | 136 | 1 | 124 | Uncharacterized protein | Uncharacterized protein | | afdb-uniprot50 | AF-A0A0H3NZQ0-F1-MODEL\_V4 | 1.0 | 6.875e-13 | 614 | 0.395 | 124 | 75 | 0 | 13 | 136 | 1 | 124 | Phage-related tail protein | Phage-related tail protein | | afdb-uniprot50 | AF-A0A853I3C0-F1-MODEL\_V4 | 1.0 | 1.379e-13 | 614 | 0.381 | 144 | 79 | 4 | 1 | 136 | 1 | 142 | Phage tail protein | Phage tail protein | | afdb-uniprot50 | AF-A0A2C6DLN0-F1-MODEL\_V4 | 1.0 | 4.985e-13 | 613 | 0.37 | 124 | 78 | 0 | 13 | 136 | 1 | 124 | Phage protein U | Phage protein U | | afdb-uniprot50 | AF-A0A8A7WU58-F1-MODEL\_V4 | 1.0 | 7.652e-13 | 612 | 0.411 | 124 | 73 | 0 | 13 | 136 | 1 | 124 | Phage tail protein | Phage tail protein | | afdb-uniprot50 | AF-A0A2N3KSK9-F1-MODEL\_V4 | 1.0 | 8.982e-14 | 611 | 0.353 | 130 | 83 | 1 | 7 | 136 | 1 | 129 | Phage tail protein | Phage tail protein | | afdb-uniprot50 | AF-A0A2D0KY36-F1-MODEL\_V4 | 1.0 | 6.875e-13 | 611 | 0.403 | 124 | 74 | 0 | 13 | 136 | 1 | 124 | Tail assembly protein | Tail assembly protein | | afdb-uniprot50 | AF-A0A2Z4UEV7-F1-MODEL\_V4 | 1.0 | 8.517e-13 | 609 | 0.395 | 124 | 75 | 0 | 13 | 136 | 1 | 124 | Phage P2 GpU | Phage P2 GpU | | afdb-uniprot50 | AF-A0A0T9UNC2-F1-MODEL\_V4 | 1.0 | 6.177e-13 | 609 | 0.379 | 124 | 77 | 0 | 13 | 136 | 1 | 124 | Phage-related tail protein | Phage-related tail protein | | afdb-uniprot50 | AF-A0A5P9F1S5-F1-MODEL\_V4 | 1.0 | 3.079e-13 | 609 | 0.311 | 135 | 93 | 0 | 2 | 136 | 16 | 150 | Phage P2 GpU | Phage P2 GpU | | afdb-uniprot50 | AF-A0A5U3EXN1-F1-MODEL\_V4 | 1.0 | 1.055e-12 | 609 | 0.395 | 124 | 75 | 0 | 13 | 136 | 1 | 124 | Phage tail protein | Phage tail protein | | afdb-uniprot50 | AF-A0A2W5DF00-F1-MODEL\_V4 | 1.0 | 8.073e-13 | 607 | 0.403 | 124 | 74 | 0 | 13 | 136 | 1 | 124 | Oxidoreductase | Oxidoreductase | | afdb-uniprot50 | AF-A0A5E4XFM2-F1-MODEL\_V4 | 1.0 | 4.477e-14 | 607 | 0.394 | 142 | 80 | 1 | 1 | 136 | 1 | 142 | Oxidoreductase | Oxidoreductase | | afdb-uniprot50 | AF-A0A5J2S1K9-F1-MODEL\_V4 | 1.0 | 6.875e-13 | 607 | 0.346 | 124 | 81 | 0 | 13 | 136 | 1 | 124 | Phage tail protein | Phage tail protein | | afdb-uniprot50 | AF-A0A1I0YHT2-F1-MODEL\_V4 | 1.0 | 6.516e-13 | 607 | 0.387 | 124 | 76 | 0 | 13 | 136 | 1 | 124 | Uncharacterized protein | Uncharacterized protein | | afdb-uniprot50 | AF-A0A7S5LS35-F1-MODEL\_V4 | 1.0 | 7.652e-13 | 607 | 0.358 | 131 | 77 | 1 | 13 | 136 | 48 | 178 | Phage tail protein | Phage tail protein | | afdb-uniprot50 | AF-A0A2G3K2E9-F1-MODEL\_V4 | 1.0 | 1.175e-12 | 606 | 0.403 | 124 | 74 | 0 | 13 | 136 | 1 | 124 | Oxidoreductase | Oxidoreductase | | afdb-uniprot50 | AF-A0A7C8LYL7-F1-MODEL\_V4 | 1.0 | 6.173e-14 | 606 | 0.383 | 146 | 75 | 3 | 6 | 136 | 1 | 146 | Uncharacterized protein | Uncharacterized protein | | afdb-uniprot50 | AF-A0A157QP36-F1-MODEL\_V4 | 1.0 | 8.517e-13 | 606 | 0.379 | 124 | 77 | 0 | 13 | 136 | 1 | 124 | Phage protein U | Phage protein U | | afdb-uniprot50 | AF-Q87ZN2-F1-MODEL\_V4 | 1.0 | 9.481e-13 | 606 | 0.335 | 131 | 80 | 1 | 13 | 136 | 118 | 248 | Uncharacterized protein | Uncharacterized protein | | afdb-uniprot50 | AF-H8L2K9-F1-MODEL\_V4 | 1.0 | 8.982e-14 | 606 | 0.382 | 136 | 79 | 1 | 6 | 136 | 139 | 274 | Phage protein U | Phage protein U | | afdb-uniprot50 | AF-A0A7X3TZ69-F1-MODEL\_V4 | 1.0 | 3.615e-13 | 606 | 0.346 | 130 | 84 | 1 | 6 | 134 | 24 | 153 | Uncharacterized protein | Uncharacterized protein | | afdb-uniprot50 | AF-A0A329B3N7-F1-MODEL\_V4 | 1.0 | 6.516e-13 | 605 | 0.395 | 124 | 75 | 0 | 13 | 136 | 1 | 124 | Uncharacterized protein | Uncharacterized protein | | afdb-uniprot50 | AF-A0A258E9H2-F1-MODEL\_V4 | 1.0 | 4.479e-13 | 605 | 0.308 | 136 | 94 | 0 | 1 | 136 | 19 | 154 | Uncharacterized protein | Uncharacterized protein | | afdb-uniprot50 | AF-A0A564M9A9-F1-MODEL\_V4 | 1.0 | 7.652e-13 | 605 | 0.346 | 124 | 81 | 0 | 13 | 136 | 1 | 124 | Uncharacterized protein | Uncharacterized protein | | afdb-uniprot50 | AF-A0A0M0P397-F1-MODEL\_V4 | 1.0 | 6.875e-13 | 604 | 0.36 | 122 | 78 | 0 | 13 | 134 | 1 | 122 | Oxidoreductase | Oxidoreductase | | afdb-uniprot50 | AF-A0A271U8M1-F1-MODEL\_V4 | 1.0 | 1.307e-12 | 604 | 0.411 | 124 | 73 | 0 | 13 | 136 | 1 | 124 | Oxidoreductase | Oxidoreductase | | afdb-uniprot50 | AF-A0A4Q3KFT0-F1-MODEL\_V4 | 1.0 | 1.455e-12 | 604 | 0.427 | 124 | 71 | 0 | 13 | 136 | 1 | 124 | Phage tail protein | Phage tail protein | | afdb-uniprot50 | AF-A0A838C006-F1-MODEL\_V4 | 1.0 | 8.986e-13 | 604 | 0.346 | 124 | 81 | 0 | 13 | 136 | 1 | 124 | Phage tail protein | Phage tail protein | | afdb-uniprot50 | AF-A0A4R0GC61-F1-MODEL\_V4 | 1.0 | 8.073e-13 | 604 | 0.354 | 124 | 80 | 0 | 13 | 136 | 1 | 124 | Phage tail protein | Phage tail protein | | afdb-uniprot50 | AF-A0A1I5KZ84-F1-MODEL\_V4 | 1.0 | 7.253e-13 | 604 | 0.333 | 135 | 85 | 1 | 7 | 136 | 152 | 286 | Uncharacterized protein | Uncharacterized protein | | afdb-uniprot50 | AF-A0A537MFJ2-F1-MODEL\_V4 | 1.0 | 1e-12 | 603 | 0.346 | 124 | 81 | 0 | 13 | 136 | 2 | 125 | Oxidoreductase | Oxidoreductase | | afdb-uniprot50 | AF-A0A1M7YZC4-F1-MODEL\_V4 | 1.0 | 1.534e-13 | 603 | 0.363 | 143 | 83 | 3 | 1 | 136 | 5 | 146 | Phage P2 GpU | Phage P2 GpU | | afdb-uniprot50 | AF-A0A239CL96-F1-MODEL\_V4 | 1.0 | 7.652e-13 | 602 | 0.393 | 127 | 77 | 0 | 10 | 136 | 1 | 127 | Uncharacterized protein | Uncharacterized protein | | afdb-uniprot50 | AF-A0A1Q6U7S4-F1-MODEL\_V4 | 1.0 | 2.918e-13 | 602 | 0.43 | 123 | 70 | 0 | 14 | 136 | 1 | 123 | Uncharacterized protein | Uncharacterized protein | | afdb-uniprot50 | AF-A0A3M5X4R0-F1-MODEL\_V4 | 1.0 | 1.113e-12 | 601 | 0.418 | 117 | 68 | 0 | 20 | 136 | 11 | 127 | Uncharacterized protein | Uncharacterized protein | | afdb-uniprot50 | AF-A0A7X6X091-F1-MODEL\_V4 | 1.0 | 1.709e-12 | 601 | 0.419 | 124 | 72 | 0 | 13 | 136 | 1 | 124 | Phage tail protein | Phage tail protein | | afdb-uniprot50 | AF-A0A5S3YY93-F1-MODEL\_V4 | 1.0 | 2.485e-13 | 601 | 0.38 | 142 | 76 | 3 | 7 | 136 | 3 | 144 | Uncharacterized protein | Uncharacterized protein | | afdb-uniprot50 | AF-A0A0H3NJV7-F1-MODEL\_V4 | 1.0 | 9.481e-13 | 601 | 0.379 | 124 | 77 | 0 | 13 | 136 | 1 | 124 | Phage-related tail protein | Phage-related tail protein | | afdb-uniprot50 | AF-F5S5D7-F1-MODEL\_V4 | 1.0 | 1e-12 | 601 | 0.37 | 124 | 78 | 0 | 13 | 136 | 2 | 125 | Phage P2 GpU | Phage P2 GpU | | afdb-uniprot50 | AF-A0A3Q9W528-F1-MODEL\_V4 | 1.0 | 1.535e-12 | 600 | 0.379 | 124 | 77 | 0 | 13 | 136 | 1 | 124 | Oxidoreductase | Oxidoreductase | | afdb-uniprot50 | AF-A0A7X0AIN6-F1-MODEL\_V4 | 1.0 | 1.902e-12 | 600 | 0.37 | 124 | 78 | 0 | 13 | 136 | 1 | 124 | Uncharacterized protein | Uncharacterized protein | | afdb-uniprot50 | AF-A0A6P2P2T0-F1-MODEL\_V4 | 1.0 | 1.379e-12 | 600 | 0.338 | 124 | 82 | 0 | 13 | 136 | 1 | 124 | Oxidoreductase | Oxidoreductase | | afdb-uniprot50 | AF-A0A090SIS3-F1-MODEL\_V4 | 1.0 | 8.073e-13 | 599 | 0.346 | 127 | 80 | 1 | 13 | 136 | 4 | 130 | Phage tail protein | Phage tail protein | | afdb-uniprot50 | AF-A0A4Q3U4S0-F1-MODEL\_V4 | 1.0 | 1.379e-12 | 599 | 0.327 | 122 | 82 | 0 | 13 | 134 | 2 | 123 | Phage tail protein | Phage tail protein | | afdb-uniprot50 | AF-A0A2D0ITE4-F1-MODEL\_V4 | 1.0 | 1.055e-12 | 599 | 0.403 | 124 | 74 | 0 | 13 | 136 | 1 | 124 | Tail assembly protein | Tail assembly protein | | afdb-uniprot50 | AF-C3X1X4-F1-MODEL\_V4 | 1.0 | 1.175e-12 | 599 | 0.37 | 124 | 78 | 0 | 13 | 136 | 1 | 124 | Uncharacterized protein | Uncharacterized protein | | afdb-uniprot50 | AF-A0A376RU30-F1-MODEL\_V4 | 1.0 | 1.055e-12 | 599 | 0.346 | 124 | 81 | 0 | 13 | 136 | 1 | 124 | Bacteriophage tail protein GpU | Bacteriophage tail protein GpU | | afdb-uniprot50 | AF-G0AIR7-F1-MODEL\_V4 | 1.0 | 1.379e-12 | 599 | 0.379 | 124 | 77 | 0 | 13 | 136 | 1 | 124 | Phage P2 GpU | Phage P2 GpU | | afdb-uniprot50 | AF-A0A1H8J1Q9-F1-MODEL\_V4 | 1.0 | 1.708e-13 | 599 | 0.352 | 136 | 83 | 1 | 6 | 136 | 140 | 275 | Uncharacterized protein | Uncharacterized protein | | afdb-uniprot50 | AF-A0A7X3NEZ9-F1-MODEL\_V4 | 1.0 | 1.113e-12 | 598 | 0.338 | 124 | 82 | 0 | 13 | 136 | 1 | 124 | Phage tail protein | Phage tail protein | | afdb-uniprot50 | AF-A0A519MN22-F1-MODEL\_V4 | 1.0 | 1.175e-12 | 598 | 0.379 | 124 | 77 | 0 | 13 | 136 | 1 | 124 | Phage tail protein | Phage tail protein | | afdb-uniprot50 | AF-A0A376HJR0-F1-MODEL\_V4 | 1.0 | 1.709e-12 | 598 | 0.373 | 123 | 77 | 0 | 14 | 136 | 1 | 123 | Tail fiber protein | Tail fiber protein | | afdb-uniprot50 | AF-A0A4Q9EHW1-F1-MODEL\_V4 | 1.0 | 1.307e-12 | 598 | 0.362 | 124 | 79 | 0 | 13 | 136 | 1 | 124 | Phage tail protein | Phage tail protein | | afdb-uniprot50 | AF-A0A5T5B4P6-F1-MODEL\_V4 | 1.0 | 1.055e-12 | 598 | 0.346 | 124 | 81 | 0 | 13 | 136 | 1 | 124 | Phage tail protein | Phage tail protein | | afdb-uniprot50 | AF-A0A5M7L4M4-F1-MODEL\_V4 | 1.0 | 1.902e-12 | 597 | 0.354 | 124 | 80 | 0 | 13 | 136 | 1 | 124 | Phage tail protein | Phage tail protein | | afdb-uniprot50 | AF-A0A1B7IQW7-F1-MODEL\_V4 | 1.0 | 1.113e-12 | 597 | 0.403 | 124 | 74 | 0 | 13 | 136 | 1 | 124 | Putative phage tail protein | Putative phage tail protein | | afdb-uniprot50 | AF-A0A6L3ZUK1-F1-MODEL\_V4 | 1.0 | 1.379e-12 | 596 | 0.39 | 123 | 75 | 0 | 14 | 136 | 1 | 123 | Oxidoreductase | Oxidoreductase | | afdb-uniprot50 | AF-A0A7Y0I342-F1-MODEL\_V4 | 1.0 | 2.622e-13 | 596 | 0.355 | 138 | 86 | 1 | 2 | 136 | 14 | 151 | Uncharacterized protein | Uncharacterized protein | | afdb-uniprot50 | AF-A0A0P7LSF8-F1-MODEL\_V4 | 1.0 | 1.379e-12 | 596 | 0.346 | 124 | 81 | 0 | 13 | 136 | 1 | 124 | Phage tail protein | Phage tail protein | | afdb-uniprot50 | AF-A0A485Z843-F1-MODEL\_V4 | 1.0 | 1.307e-12 | 596 | 0.346 | 124 | 81 | 0 | 13 | 136 | 1 | 124 | Phage tail protein | Phage tail protein | | afdb-uniprot50 | AF-A0A7W6WBR9-F1-MODEL\_V4 | 1.0 | 1.455e-12 | 596 | 0.368 | 125 | 77 | 2 | 13 | 136 | 2 | 125 | Uncharacterized protein | Uncharacterized protein | | afdb-uniprot50 | AF-A0A702B4K5-F1-MODEL\_V4 | 1.0 | 2.007e-12 | 596 | 0.37 | 124 | 78 | 0 | 13 | 136 | 1 | 124 | Phage tail protein | Phage tail protein | | afdb-uniprot50 | AF-A0A6L5PEK8-F1-MODEL\_V4 | 1.0 | 2.355e-13 | 595 | 0.33 | 127 | 84 | 1 | 10 | 136 | 1 | 126 | Phage tail protein | Phage tail protein | | afdb-uniprot50 | AF-A0A250DSZ6-F1-MODEL\_V4 | 1.0 | 1.62e-12 | 595 | 0.382 | 123 | 76 | 0 | 14 | 136 | 1 | 123 | Oxidoreductase | Oxidoreductase | | afdb-uniprot50 | AF-A0A024L4N8-F1-MODEL\_V4 | 1.0 | 1.379e-12 | 595 | 0.346 | 124 | 81 | 0 | 13 | 136 | 1 | 124 | Bacteriophage tail protein GpU | Bacteriophage tail protein GpU | | afdb-uniprot50 | AF-A0A8A5ILR9-F1-MODEL\_V4 | 1.0 | 1.113e-12 | 595 | 0.346 | 124 | 81 | 0 | 13 | 136 | 1 | 124 | Phage tail protein | Phage tail protein | | afdb-uniprot50 | AF-A0A2W5VBP0-F1-MODEL\_V4 | 1.0 | 1.62e-12 | 595 | 0.296 | 125 | 88 | 0 | 12 | 136 | 2 | 126 | Oxidoreductase | Oxidoreductase | | afdb-uniprot50 | AF-A0A7Z0SJA2-F1-MODEL\_V4 | 1.0 | 7.652e-13 | 595 | 0.333 | 135 | 90 | 0 | 2 | 136 | 101 | 235 | Phage tail protein | Phage tail protein | | afdb-uniprot50 | AF-A0A4E0GYK4-F1-MODEL\_V4 | 1.0 | 1.175e-12 | 594 | 0.346 | 124 | 81 | 0 | 13 | 136 | 1 | 124 | Phage tail protein | Phage tail protein | | afdb-uniprot50 | AF-A0A246KFY5-F1-MODEL\_V4 | 1.0 | 2.007e-12 | 593 | 0.33 | 124 | 83 | 0 | 13 | 136 | 1 | 124 | Phage protein U | Phage protein U | | afdb-uniprot50 | AF-A0A5U9GBD7-F1-MODEL\_V4 | 1.0 | 1.455e-12 | 593 | 0.346 | 124 | 81 | 0 | 13 | 136 | 1 | 124 | Phage tail protein | Phage tail protein | | afdb-uniprot50 | AF-A0A495Y9Q8-F1-MODEL\_V4 | 1.0 | 1e-12 | 593 | 0.342 | 140 | 87 | 1 | 2 | 136 | 150 | 289 | Oxidoreductase | Oxidoreductase | | afdb-uniprot50 | AF-M1PBX3-F1-MODEL\_V4 | 1.0 | 4.024e-13 | 592 | 0.403 | 124 | 73 | 1 | 13 | 136 | 1 | 123 | Phage tail protein U | Phage tail protein U | | afdb-uniprot50 | AF-E2CJS7-F1-MODEL\_V4 | 1.0 | 5.26e-13 | 592 | 0.343 | 131 | 85 | 1 | 6 | 136 | 1 | 130 | p2 GpU family protein | p2 GpU family protein | | afdb-uniprot50 | AF-A0A660MIA9-F1-MODEL\_V4 | 1.0 | 1.902e-12 | 592 | 0.384 | 125 | 75 | 2 | 13 | 136 | 1 | 124 | Phage tail protein | Phage tail protein | | afdb-uniprot50 | AF-A0A2D5S2F6-F1-MODEL\_V4 | 1.0 | 3.814e-13 | 591 | 0.313 | 134 | 88 | 3 | 7 | 136 | 1 | 134 | Oxidoreductase | Oxidoreductase | | afdb-uniprot50 | AF-A0A241WFD3-F1-MODEL\_V4 | 1.0 | 6.516e-13 | 591 | 0.392 | 125 | 75 | 1 | 13 | 136 | 1 | 125 | Oxidoreductase | Oxidoreductase | | afdb-uniprot50 | AF-A0A1I4NEJ2-F1-MODEL\_V4 | 1.0 | 9.481e-13 | 591 | 0.306 | 137 | 90 | 1 | 5 | 136 | 151 | 287 | Uncharacterized protein | Uncharacterized protein | | afdb-uniprot50 | AF-A0A1M3HI07-F1-MODEL\_V4 | 1.0 | 9.481e-13 | 590 | 0.384 | 125 | 76 | 1 | 13 | 136 | 1 | 125 | Uncharacterized protein | Uncharacterized protein | | afdb-uniprot50 | AF-A0A5V0HLG2-F1-MODEL\_V4 | 1.0 | 1.902e-12 | 590 | 0.346 | 124 | 81 | 0 | 13 | 136 | 1 | 124 | Phage tail protein | Phage tail protein | | afdb-uniprot50 | AF-A0A838CHN8-F1-MODEL\_V4 | 1.0 | 1.175e-12 | 590 | 0.321 | 140 | 90 | 1 | 2 | 136 | 92 | 231 | Phage tail protein | Phage tail protein | | afdb-uniprot50 | AF-A0A2G2NAY4-F1-MODEL\_V4 | 1.0 | 7.253e-13 | 589 | 0.513 | 111 | 54 | 0 | 26 | 136 | 3 | 113 | Phage tail protein | Phage tail protein | | afdb-uniprot50 | AF-A0A6C9KZW5-F1-MODEL\_V4 | 1.0 | 2.117e-12 | 589 | 0.338 | 124 | 82 | 0 | 13 | 136 | 1 | 124 | Phage tail protein | Phage tail protein | | afdb-uniprot50 | AF-A0A2G0Q023-F1-MODEL\_V4 | 1.0 | 1.902e-12 | 589 | 0.395 | 124 | 75 | 0 | 13 | 136 | 1 | 124 | Tail assembly protein | Tail assembly protein | | afdb-uniprot50 | AF-A0A1G5TM27-F1-MODEL\_V4 | 1.0 | 1.62e-12 | 587 | 0.416 | 125 | 71 | 2 | 13 | 136 | 1 | 124 | Uncharacterized protein | Uncharacterized protein | | afdb-uniprot50 | AF-A0A659PR58-F1-MODEL\_V4 | 1.0 | 2.234e-12 | 586 | 0.33 | 124 | 83 | 0 | 13 | 136 | 1 | 124 | Phage tail protein | Phage tail protein | | afdb-uniprot50 | AF-A0A1B9LPX2-F1-MODEL\_V4 | 1.0 | 2.234e-12 | 586 | 0.344 | 122 | 80 | 0 | 13 | 134 | 1 | 122 | Uncharacterized protein | Uncharacterized protein | | afdb-uniprot50 | AF-A0A486S2L3-F1-MODEL\_V4 | 1.0 | 2.92e-12 | 586 | 0.346 | 124 | 81 | 0 | 13 | 136 | 1 | 124 | Putative bacteriophage tail protein | Putative bacteriophage tail protein | | afdb-uniprot50 | AF-B7LNA5-F1-MODEL\_V4 | 1.0 | 6.875e-13 | 586 | 0.309 | 126 | 87 | 0 | 11 | 136 | 25 | 150 | Putative Phage-related tail protein | Putative Phage-related tail protein | | afdb-uniprot50 | AF-D4GLP7-F1-MODEL\_V4 | 1.0 | 2.767e-12 | 586 | 0.379 | 124 | 77 | 0 | 13 | 136 | 1 | 124 | Uncharacterized protein | Uncharacterized protein | | afdb-uniprot50 | AF-A0A833KUX0-F1-MODEL\_V4 | 1.0 | 2.117e-12 | 586 | 0.346 | 124 | 81 | 0 | 13 | 136 | 1 | 124 | Phage tail protein | Phage tail protein | | afdb-uniprot50 | AF-A0A6P1Q0K5-F1-MODEL\_V4 | 1.0 | 3.617e-12 | 585 | 0.322 | 124 | 84 | 0 | 13 | 136 | 1 | 124 | Uncharacterized protein | Uncharacterized protein | | afdb-uniprot50 | AF-A0A1E7WJB8-F1-MODEL\_V4 | 1.0 | 2.007e-12 | 585 | 0.377 | 122 | 76 | 0 | 13 | 134 | 1 | 122 | Phage P2 GpU | Phage P2 GpU | | afdb-uniprot50 | AF-A0A4P0TK52-F1-MODEL\_V4 | 1.0 | 8.073e-13 | 585 | 0.381 | 139 | 71 | 3 | 13 | 136 | 114 | 252 | Putative tail formation protein | Putative tail formation protein | | afdb-uniprot50 | AF-A0A4V2B685-F1-MODEL\_V4 | 1.0 | 6.875e-13 | 584 | 0.305 | 134 | 90 | 1 | 6 | 136 | 3 | 136 | Phage tail protein | Phage tail protein | | afdb-uniprot50 | AF-A0A3B0TD64-F1-MODEL\_V4 | 1.0 | 1.239e-12 | 584 | 0.362 | 127 | 79 | 1 | 12 | 136 | 2 | 128 | Uncharacterized protein | Uncharacterized protein | | afdb-uniprot50 | AF-A0A2W4T7M1-F1-MODEL\_V4 | 1.0 | 8.517e-13 | 584 | 0.354 | 127 | 81 | 1 | 10 | 136 | 2 | 127 | Phage tail protein | Phage tail protein | | afdb-uniprot50 | AF-A0A515CWV7-F1-MODEL\_V4 | 1.0 | 1.709e-12 | 584 | 0.379 | 124 | 77 | 0 | 13 | 136 | 1 | 124 | Phage tail protein | Phage tail protein | | afdb-uniprot50 | AF-F4VCL7-F1-MODEL\_V4 | 1.0 | 2.234e-12 | 584 | 0.346 | 124 | 81 | 0 | 13 | 136 | 1 | 124 | Putative bacteriophage tail protein | Putative bacteriophage tail protein | | afdb-uniprot50 | AF-A0A248LHR8-F1-MODEL\_V4 | 1.0 | 3.25e-12 | 582 | 0.333 | 126 | 84 | 0 | 11 | 136 | 3 | 128 | Phage protein U | Phage protein U | | afdb-uniprot50 | AF-A0A1I1Q5P9-F1-MODEL\_V4 | 1.0 | 6.516e-13 | 582 | 0.356 | 143 | 80 | 3 | 6 | 136 | 1 | 143 | Uncharacterized protein | Uncharacterized protein | | afdb-uniprot50 | AF-A0A4R9NLD4-F1-MODEL\_V4 | 1.0 | 3.617e-12 | 582 | 0.346 | 124 | 81 | 0 | 13 | 136 | 1 | 124 | Phage tail protein | Phage tail protein | | afdb-uniprot50 | AF-A0A0Q4MMS8-F1-MODEL\_V4 | 1.0 | 4.728e-12 | 582 | 0.362 | 124 | 79 | 0 | 13 | 136 | 1 | 124 | Phage tail protein | Phage tail protein | | afdb-uniprot50 | AF-A0A4Y6UBW6-F1-MODEL\_V4 | 1.0 | 3.615e-13 | 582 | 0.294 | 136 | 95 | 1 | 1 | 136 | 13 | 147 | Phage tail protein | Phage tail protein | | afdb-uniprot50 | AF-A0A1I0AWP0-F1-MODEL\_V4 | 1.0 | 2.117e-12 | 582 | 0.357 | 126 | 78 | 2 | 13 | 136 | 1 | 125 | Uncharacterized protein | Uncharacterized protein | | afdb-uniprot50 | AF-A0A516SM14-F1-MODEL\_V4 | 1.0 | 1.055e-12 | 582 | 0.354 | 144 | 84 | 2 | 2 | 136 | 118 | 261 | Phage tail protein | Phage tail protein | | afdb-uniprot50 | AF-A0A7X9X5I9-F1-MODEL\_V4 | 1.0 | 2.357e-12 | 581 | 0.36 | 122 | 78 | 0 | 13 | 134 | 2 | 123 | Phage tail protein | Phage tail protein | | afdb-uniprot50 | AF-A0A2V3UC18-F1-MODEL\_V4 | 1.0 | 4.248e-12 | 580 | 0.299 | 127 | 89 | 0 | 10 | 136 | 1 | 127 | Uncharacterized protein | Uncharacterized protein | | afdb-uniprot50 | AF-A0A853IGN0-F1-MODEL\_V4 | 1.0 | 4.026e-12 | 580 | 0.322 | 127 | 86 | 0 | 10 | 136 | 1 | 127 | Phage tail protein | Phage tail protein | | afdb-uniprot50 | AF-A0A7T5QS73-F1-MODEL\_V4 | 1.0 | 4.026e-12 | 580 | 0.379 | 124 | 77 | 0 | 13 | 136 | 1 | 124 | Phage tail protein | Phage tail protein | | afdb-uniprot50 | AF-A0A2A7V0X6-F1-MODEL\_V4 | 1.0 | 3.08e-12 | 580 | 0.395 | 124 | 75 | 0 | 13 | 136 | 1 | 124 | Oxidoreductase | Oxidoreductase | | afdb-uniprot50 | AF-A0A0P7PMR1-F1-MODEL\_V4 | 1.0 | 4.481e-12 | 580 | 0.354 | 124 | 80 | 0 | 13 | 136 | 1 | 124 | Oxidoreductase | Oxidoreductase | | afdb-uniprot50 | AF-A0A222EWH4-F1-MODEL\_V4 | 1.0 | 2.234e-12 | 579 | 0.362 | 124 | 79 | 0 | 13 | 136 | 1 | 124 | Oxidoreductase | Oxidoreductase | | afdb-uniprot50 | AF-A0A0G3S980-F1-MODEL\_V4 | 1.0 | 4.026e-12 | 579 | 0.346 | 124 | 81 | 0 | 13 | 136 | 1 | 124 | Tail protein | Tail protein | | afdb-uniprot50 | AF-A0A1I1UCM4-F1-MODEL\_V4 | 1.0 | 3.08e-12 | 578 | 0.317 | 126 | 84 | 1 | 13 | 136 | 3 | 128 | Uncharacterized protein | Uncharacterized protein | | afdb-uniprot50 | AF-A0A356D2V6-F1-MODEL\_V4 | 1.0 | 5.857e-12 | 577 | 0.346 | 124 | 81 | 0 | 13 | 136 | 1 | 124 | Oxidoreductase | Oxidoreductase | | afdb-uniprot50 | AF-A0A559QNF7-F1-MODEL\_V4 | 1.0 | 5.552e-12 | 577 | 0.379 | 124 | 77 | 0 | 13 | 136 | 1 | 124 | Uncharacterized protein | Uncharacterized protein | | afdb-uniprot50 | AF-A0A5E5ANB0-F1-MODEL\_V4 | 1.0 | 4.481e-12 | 577 | 0.338 | 124 | 82 | 0 | 13 | 136 | 1 | 124 | Phage tail protein | Phage tail protein | | afdb-uniprot50 | AF-T0QJT7-F1-MODEL\_V4 | 1.0 | 2.623e-12 | 576 | 0.37 | 124 | 78 | 0 | 13 | 136 | 1 | 124 | Uncharacterized protein | Uncharacterized protein | | afdb-uniprot50 | AF-A0A1C7BUG8-F1-MODEL\_V4 | 1.0 | 1.379e-12 | 576 | 0.375 | 141 | 72 | 3 | 12 | 136 | 144 | 284 | Uncharacterized protein | Uncharacterized protein | | afdb-uniprot50 | AF-A0A348FYG2-F1-MODEL\_V4 | 1.0 | 1.62e-12 | 575 | 0.325 | 129 | 81 | 2 | 12 | 136 | 2 | 128 | Oxidoreductase | Oxidoreductase | | afdb-uniprot50 | AF-A0A3S4IAG6-F1-MODEL\_V4 | 1.0 | 6.18e-12 | 575 | 0.346 | 124 | 81 | 0 | 13 | 136 | 1 | 124 | Phage tail protein | Phage tail protein | | afdb-uniprot50 | AF-A0A256CB29-F1-MODEL\_V4 | 1.0 | 4.988e-12 | 574 | 0.311 | 122 | 84 | 0 | 13 | 134 | 3 | 124 | Uncharacterized protein | Uncharacterized protein | | afdb-uniprot50 | AF-A0A1Z9Z2S2-F1-MODEL\_V4 | 1.0 | 5.262e-12 | 574 | 0.352 | 125 | 80 | 1 | 13 | 136 | 1 | 125 | Oxidoreductase | Oxidoreductase | | afdb-uniprot50 | AF-A0A212J3T6-F1-MODEL\_V4 | 1.0 | 4.728e-12 | 573 | 0.36 | 125 | 78 | 2 | 13 | 136 | 1 | 124 | Phage P2 GpU protein | Phage P2 GpU protein | | afdb-uniprot50 | AF-A0A3D9UGE8-F1-MODEL\_V4 | 1.0 | 4.026e-12 | 572 | 0.37 | 124 | 78 | 0 | 13 | 136 | 1 | 124 | Uncharacterized protein | Uncharacterized protein | | afdb-uniprot50 | AF-A0A0H3KJY5-F1-MODEL\_V4 | 1.0 | 6.52e-12 | 571 | 0.37 | 124 | 78 | 0 | 13 | 136 | 1 | 124 | Probable bacteriophage protein U | Probable bacteriophage protein U | | afdb-uniprot50 | AF-A0A853X1L4-F1-MODEL\_V4 | 1.0 | 4.026e-12 | 569 | 0.357 | 126 | 78 | 2 | 13 | 136 | 1 | 125 | Oxidoreductase | Oxidoreductase | | afdb-uniprot50 | AF-A0A516S9T9-F1-MODEL\_V4 | 1.0 | 3.08e-12 | 569 | 0.344 | 145 | 86 | 2 | 1 | 136 | 154 | 298 | Phage tail protein | Phage tail protein | | afdb-uniprot50 | AF-A0A855M4M1-F1-MODEL\_V4 | 1.0 | 8.522e-12 | 568 | 0.338 | 124 | 82 | 0 | 13 | 136 | 1 | 124 | Phage tail protein | Phage tail protein | | afdb-uniprot50 | AF-A0A228PZF6-F1-MODEL\_V4 | 1.0 | 4.026e-12 | 567 | 0.373 | 126 | 76 | 2 | 13 | 136 | 1 | 125 | Oxidoreductase | Oxidoreductase | | afdb-uniprot50 | AF-A0A7X1ZI31-F1-MODEL\_V4 | 1.0 | 2.766e-13 | 567 | 0.383 | 146 | 75 | 5 | 1 | 136 | 16 | 156 | Phage tail protein | Phage tail protein | | afdb-uniprot50 | AF-A0A4D7AYY7-F1-MODEL\_V4 | 1.0 | 4.481e-12 | 566 | 0.251 | 127 | 93 | 2 | 12 | 136 | 2 | 128 | Phage tail protein | Phage tail protein | | afdb-uniprot50 | AF-A0A5E4YCW2-F1-MODEL\_V4 | 1.0 | 8.991e-12 | 566 | 0.338 | 124 | 82 | 0 | 13 | 136 | 1 | 124 | Phage tail protein | Phage tail protein | | afdb-uniprot50 | AF-A0A7U3R1Q9-F1-MODEL\_V4 | 1.0 | 4.728e-12 | 566 | 0.29 | 141 | 94 | 2 | 1 | 136 | 164 | 303 | Uncharacterized protein | Uncharacterized protein | | afdb-uniprot50 | AF-A0A7X4CLP0-F1-MODEL\_V4 | 1.0 | 4.026e-12 | 564 | 0.366 | 131 | 76 | 2 | 13 | 136 | 1 | 131 | Phage tail protein | Phage tail protein | | afdb-uniprot50 | AF-A0A263HHG5-F1-MODEL\_V4 | 1.0 | 2.92e-12 | 564 | 0.335 | 131 | 86 | 1 | 7 | 136 | 1 | 131 | Oxidoreductase | Oxidoreductase | | afdb-uniprot50 | AF-A0A740LUZ9-F1-MODEL\_V4 | 1.0 | 7.652e-13 | 564 | 0.313 | 134 | 90 | 1 | 5 | 136 | 17 | 150 | Phage tail protein | Phage tail protein | | afdb-uniprot50 | AF-A0A376ZJI1-F1-MODEL\_V4 | 1.0 | 1.056e-11 | 564 | 0.314 | 124 | 85 | 0 | 13 | 136 | 1 | 124 | Tail fiber protein | Tail fiber protein | | afdb-uniprot50 | AF-A0A2E3Q565-F1-MODEL\_V4 | 1.0 | 5.552e-12 | 562 | 0.318 | 135 | 85 | 2 | 8 | 136 | 178 | 311 | Uncharacterized protein | Uncharacterized protein | | afdb-uniprot50 | AF-A0A7Z0EYT7-F1-MODEL\_V4 | 1.0 | 8.991e-12 | 561 | 0.346 | 124 | 81 | 0 | 13 | 136 | 1 | 124 | Uncharacterized protein | Uncharacterized protein | | afdb-uniprot50 | AF-A0A5T7Y1R6-F1-MODEL\_V4 | 1.0 | 1e-12 | 561 | 0.272 | 143 | 97 | 2 | 1 | 136 | 43 | 185 | Phage tail protein | Phage tail protein | | afdb-uniprot50 | AF-A0A4V5PSP7-F1-MODEL\_V4 | 1.0 | 5.552e-12 | 560 | 0.312 | 144 | 91 | 2 | 1 | 136 | 147 | 290 | Phage tail protein | Phage tail protein | | afdb-uniprot50 | AF-D6I841-F1-MODEL\_V4 | 1.0 | 3.816e-12 | 559 | 0.31 | 129 | 86 | 2 | 11 | 136 | 24 | 152 | Predicted protein | Predicted protein | | afdb-uniprot50 | AF-A0A4P7QXJ5-F1-MODEL\_V4 | 1.0 | 6.878e-12 | 557 | 0.36 | 125 | 78 | 2 | 13 | 136 | 1 | 124 | Phage tail protein | Phage tail protein | | afdb-uniprot50 | AF-A0A4R6E0B6-F1-MODEL\_V4 | 1.0 | 3.615e-13 | 557 | 0.293 | 150 | 92 | 3 | 1 | 136 | 12 | 161 | Uncharacterized protein | Uncharacterized protein | | afdb-uniprot50 | AF-A0A377Q627-F1-MODEL\_V4 | 1.0 | 1.001e-11 | 556 | 0.384 | 125 | 75 | 2 | 13 | 136 | 1 | 124 | Phage protein U | Phage protein U | | afdb-uniprot50 | AF-A0A080KNK2-F1-MODEL\_V4 | 1.0 | 7.656e-12 | 556 | 0.32 | 125 | 83 | 2 | 13 | 136 | 1 | 124 | Phage protein U | Phage protein U | | afdb-uniprot50 | AF-A0A2C5TNN0-F1-MODEL\_V4 | 1.0 | 8.522e-12 | 556 | 0.354 | 124 | 80 | 0 | 13 | 136 | 2 | 125 | Phage tail protein | Phage tail protein | | afdb-uniprot50 | AF-A0A0X8GMF9-F1-MODEL\_V4 | 1.0 | 5.552e-12 | 556 | 0.321 | 140 | 87 | 3 | 2 | 136 | 134 | 270 | Uncharacterized protein | Uncharacterized protein | | afdb-uniprot50 | AF-A0A4Q0GPA2-F1-MODEL\_V4 | 1.0 | 8.522e-12 | 555 | 0.306 | 124 | 86 | 0 | 13 | 136 | 1 | 124 | Phage tail protein | Phage tail protein | | afdb-uniprot50 | AF-A0A516SHQ7-F1-MODEL\_V4 | 1.0 | 1.621e-11 | 552 | 0.37 | 124 | 77 | 1 | 14 | 136 | 1 | 124 | Phage tail protein | Phage tail protein | | afdb-uniprot50 | AF-A0A024HAP1-F1-MODEL\_V4 | 1.0 | 1.803e-12 | 552 | 0.364 | 140 | 80 | 2 | 6 | 136 | 140 | 279 | Pyocin R2\_PP, tail formation protein | Pyocin R2\_PP, tail formation protein | | afdb-uniprot50 | AF-A0A4P9VIK5-F1-MODEL\_V4 | 1.0 | 8.517e-13 | 551 | 0.381 | 144 | 79 | 3 | 1 | 136 | 1 | 142 | Uncharacterized protein | Uncharacterized protein | | afdb-uniprot50 | AF-A0A381GQM7-F1-MODEL\_V4 | 1.0 | 1.114e-11 | 550 | 0.336 | 122 | 81 | 0 | 13 | 134 | 1 | 122 | Phage tail protein | Phage tail protein | | afdb-uniprot50 | AF-A0A3N2E0P3-F1-MODEL\_V4 | 1.0 | 1e-12 | 549 | 0.367 | 136 | 77 | 1 | 10 | 136 | 1 | 136 | Uncharacterized protein | Uncharacterized protein | | afdb-uniprot50 | AF-D4BTZ4-F1-MODEL\_V4 | 1.0 | 2.624e-11 | 548 | 0.362 | 124 | 79 | 0 | 13 | 136 | 1 | 124 | Phage P2 GpU | Phage P2 GpU | | afdb-uniprot50 | AF-A0A4Y6W0D4-F1-MODEL\_V4 | 1.0 | 1.308e-11 | 547 | 0.355 | 121 | 78 | 0 | 16 | 136 | 2 | 122 | Oxidoreductase | Oxidoreductase | | afdb-uniprot50 | AF-A0A2W6Y6Z1-F1-MODEL\_V4 | 1.0 | 3.251e-11 | 546 | 0.306 | 124 | 86 | 0 | 13 | 136 | 1 | 124 | Oxidoreductase | Oxidoreductase | | afdb-uniprot50 | AF-A0A0Q8AZW1-F1-MODEL\_V4 | 1.0 | 1.621e-11 | 546 | 0.301 | 126 | 86 | 2 | 13 | 136 | 2 | 127 | Uncharacterized protein | Uncharacterized protein | | afdb-uniprot50 | AF-A0A2Y0TQQ1-F1-MODEL\_V4 | 1.0 | 2.487e-11 | 545 | 0.306 | 124 | 86 | 0 | 13 | 136 | 1 | 124 | Phage tail protein | Phage tail protein | | afdb-uniprot50 | AF-A0A1V9UC49-F1-MODEL\_V4 | 1.0 | 1.239e-12 | 544 | 0.297 | 148 | 91 | 2 | 2 | 136 | 147 | 294 | Uncharacterized protein | Uncharacterized protein | | afdb-uniprot50 | AF-A0A369YGR3-F1-MODEL\_V4 | 1.0 | 1.903e-11 | 542 | 0.343 | 128 | 83 | 1 | 10 | 136 | 3 | 130 | Oxidoreductase | Oxidoreductase | | afdb-uniprot50 | AF-A0A085ARU2-F1-MODEL\_V4 | 1.0 | 2.234e-12 | 542 | 0.287 | 139 | 95 | 1 | 2 | 136 | 17 | 155 | Phage tail protein | Phage tail protein | | afdb-uniprot50 | AF-A0A7S6MLX5-F1-MODEL\_V4 | 1.0 | 2.008e-11 | 536 | 0.307 | 140 | 88 | 3 | 5 | 136 | 119 | 257 | Phage tail protein | Phage tail protein | | afdb-uniprot50 | AF-A0A4R1TKW3-F1-MODEL\_V4 | 1.0 | 2.624e-11 | 536 | 0.303 | 135 | 87 | 3 | 8 | 136 | 182 | 315 | Uncharacterized protein | Uncharacterized protein | | afdb-uniprot50 | AF-A0A7S8IIF9-F1-MODEL\_V4 | 1.0 | 3.251e-11 | 533 | 0.301 | 126 | 86 | 1 | 13 | 136 | 1 | 126 | Uncharacterized protein | Uncharacterized protein | | afdb-uniprot50 | AF-A0A1C3EBL2-F1-MODEL\_V4 | 1.0 | 6.52e-12 | 533 | 0.251 | 139 | 101 | 2 | 1 | 136 | 5 | 143 | Uncharacterized protein | Uncharacterized protein | | afdb-uniprot50 | AF-A0A1W0CDP0-F1-MODEL\_V4 | 1.0 | 1.38e-11 | 533 | 0.313 | 137 | 88 | 3 | 5 | 136 | 134 | 269 | Uncharacterized protein | Uncharacterized protein | | afdb-uniprot50 | AF-A0A7Z1Y6H2-F1-MODEL\_V4 | 1.0 | 2.118e-11 | 532 | 0.308 | 123 | 85 | 0 | 14 | 136 | 1 | 123 | Phage tail protein | Phage tail protein | | afdb-uniprot50 | AF-A0A1B6NQL9-F1-MODEL\_V4 | 1.0 | 4.25e-11 | 531 | 0.33 | 124 | 83 | 0 | 13 | 136 | 1 | 124 | Phage P2 GpU | Phage P2 GpU | | afdb-uniprot50 | AF-A0A2A4XTZ4-F1-MODEL\_V4 | 1.0 | 3.619e-11 | 529 | 0.27 | 122 | 89 | 0 | 13 | 134 | 1 | 122 | Uncharacterized protein | Uncharacterized protein | | afdb-uniprot50 | AF-A0A4R7RZR3-F1-MODEL\_V4 | 1.0 | 2.624e-11 | 528 | 0.309 | 126 | 85 | 2 | 10 | 134 | 1 | 125 | Uncharacterized protein | Uncharacterized protein | | afdb-uniprot50 | AF-A0A0H5CXN7-F1-MODEL\_V4 | 1.0 | 3.43e-11 | 528 | 0.282 | 124 | 89 | 0 | 13 | 136 | 5 | 128 | Phage protein U | Phage protein U | | afdb-uniprot50 | AF-A0A0K1K621-F1-MODEL\_V4 | 1.0 | 2.769e-11 | 528 | 0.309 | 142 | 91 | 2 | 2 | 136 | 107 | 248 | Uncharacterized protein | Uncharacterized protein | | afdb-uniprot50 | AF-A0A1T4W4X0-F1-MODEL\_V4 | 1.0 | 8.991e-12 | 525 | 0.272 | 132 | 87 | 4 | 13 | 136 | 3 | 133 | Uncharacterized protein | Uncharacterized protein | | afdb-uniprot50 | AF-A0A2X3DKM3-F1-MODEL\_V4 | 1.0 | 7.26e-11 | 523 | 0.298 | 124 | 87 | 0 | 13 | 136 | 1 | 124 | Putative bacteriophage tail protein | Putative bacteriophage tail protein | | afdb-uniprot50 | AF-A0A2S6N2W6-F1-MODEL\_V4 | 1.0 | 3.818e-11 | 522 | 0.293 | 126 | 87 | 2 | 13 | 136 | 2 | 127 | Uncharacterized protein | Uncharacterized protein | | afdb-uniprot50 | AF-A0A6L3Y3D4-F1-MODEL\_V4 | 1.0 | 2.769e-11 | 522 | 0.338 | 124 | 82 | 0 | 13 | 136 | 3 | 126 | Phage tail protein | Phage tail protein | | afdb-uniprot50 | AF-A0A6B3IYA5-F1-MODEL\_V4 | 1.0 | 1.71e-11 | 522 | 0.248 | 145 | 92 | 1 | 9 | 136 | 22 | 166 | Uncharacterized protein | Uncharacterized protein | | afdb-uniprot50 | AF-A0A425B1Z8-F1-MODEL\_V4 | 1.0 | 8.081e-11 | 520 | 0.282 | 124 | 89 | 0 | 13 | 136 | 1 | 124 | Uncharacterized protein | Uncharacterized protein | | afdb-uniprot50 | AF-A0A317H5Z8-F1-MODEL\_V4 | 1.0 | 3.818e-11 | 520 | 0.219 | 132 | 103 | 0 | 5 | 136 | 41 | 172 | Phage tail protein | Phage tail protein | | afdb-uniprot50 | AF-A0A844Z703-F1-MODEL\_V4 | 1.0 | 4.99e-11 | 519 | 0.371 | 121 | 76 | 0 | 16 | 136 | 2 | 122 | Oxidoreductase | Oxidoreductase | | afdb-uniprot50 | AF-A0A2D2CYK8-F1-MODEL\_V4 | 1.0 | 6.183e-11 | 518 | 0.256 | 125 | 91 | 2 | 13 | 136 | 3 | 126 | Phage tail protein | Phage tail protein | | afdb-uniprot50 | AF-M5JLR0-F1-MODEL\_V4 | 1.0 | 1.001e-10 | 517 | 0.241 | 124 | 94 | 0 | 13 | 136 | 1 | 124 | Fels-2 prophage protein | Fels-2 prophage protein | | afdb-uniprot50 | AF-Q31HS8-F1-MODEL\_V4 | 1.0 | 8.991e-12 | 517 | 0.273 | 139 | 89 | 2 | 9 | 136 | 2 | 139 | Phage P2 GpU family protein | Phage P2 GpU family protein | | afdb-uniprot50 | AF-A0A0P6W270-F1-MODEL\_V4 | 1.0 | 1.114e-10 | 516 | 0.233 | 124 | 94 | 1 | 13 | 136 | 1 | 123 | Uncharacterized protein | Uncharacterized protein | | afdb-uniprot50 | AF-A0A3S0GWK0-F1-MODEL\_V4 | 1.0 | 1.536e-11 | 516 | 0.338 | 127 | 81 | 1 | 13 | 136 | 3 | 129 | Phage tail protein | Phage tail protein | | afdb-uniprot50 | AF-A0A6B8KGJ5-F1-MODEL\_V4 | 1.0 | 3.619e-11 | 516 | 0.263 | 129 | 93 | 1 | 10 | 136 | 1 | 129 | Uncharacterized protein | Uncharacterized protein | | afdb-uniprot50 | AF-A0A318TZH9-F1-MODEL\_V4 | 1.0 | 8.995e-11 | 515 | 0.217 | 124 | 97 | 0 | 13 | 136 | 2 | 125 | Uncharacterized protein | Uncharacterized protein | | afdb-uniprot50 | AF-A0A0J5GKQ4-F1-MODEL\_V4 | 1.0 | 3.251e-11 | 515 | 0.297 | 141 | 92 | 4 | 1 | 136 | 104 | 242 | Uncharacterized protein | Uncharacterized protein | | afdb-uniprot50 | AF-A0A7W2GS23-F1-MODEL\_V4 | 1.0 | 6.523e-11 | 513 | 0.328 | 131 | 84 | 1 | 10 | 136 | 1 | 131 | Phage tail protein | Phage tail protein | | afdb-uniprot50 | AF-A0A143I3T8-F1-MODEL\_V4 | 1.0 | 1.381e-10 | 513 | 0.363 | 121 | 77 | 0 | 16 | 136 | 2 | 122 | Uncharacterized protein | Uncharacterized protein | | afdb-uniprot50 | AF-A0A8B2NS16-F1-MODEL\_V4 | 1.0 | 1.114e-10 | 507 | 0.262 | 122 | 90 | 0 | 14 | 135 | 6 | 127 | Oxidoreductase | Oxidoreductase | | afdb-uniprot50 | AF-A0A2T5UQY9-F1-MODEL\_V4 | 1.0 | 1.904e-10 | 506 | 0.33 | 121 | 81 | 0 | 16 | 136 | 2 | 122 | Uncharacterized protein | Uncharacterized protein | | afdb-uniprot50 | AF-A0A2W7APL3-F1-MODEL\_V4 | 1.0 | 8.526e-11 | 504 | 0.268 | 123 | 88 | 2 | 13 | 134 | 1 | 122 | Phage tail protein | Phage tail protein | | afdb-uniprot50 | AF-A0A037US03-F1-MODEL\_V4 | 1.0 | 1.537e-10 | 504 | 0.185 | 124 | 101 | 0 | 13 | 136 | 1 | 124 | Tail protein | Tail protein | | afdb-uniprot50 | AF-A0A7X2IMF3-F1-MODEL\_V4 | 1.0 | 1.056e-10 | 502 | 0.297 | 141 | 87 | 2 | 8 | 136 | 158 | 298 | Uncharacterized protein | Uncharacterized protein | | afdb-uniprot50 | AF-A0A847KTZ8-F1-MODEL\_V4 | 1.0 | 8.995e-11 | 494 | 0.292 | 130 | 92 | 0 | 7 | 136 | 10 | 139 | Phage tail protein | Phage tail protein | | afdb-uniprot50 | AF-A0A6I6AWA7-F1-MODEL\_V4 | 1.0 | 1.904e-10 | 494 | 0.385 | 109 | 67 | 0 | 28 | 136 | 797 | 905 | Phage tail tape measure protein | Phage tail tape measure protein | | afdb-uniprot50 | AF-A0A1Q8D7K3-F1-MODEL\_V4 | 1.0 | 2.489e-10 | 491 | 0.33 | 121 | 80 | 1 | 13 | 133 | 1 | 120 | Phage tail protein | Phage tail protein | | afdb-uniprot50 | AF-A0A0D0K1T5-F1-MODEL\_V4 | 1.0 | 1.457e-10 | 491 | 0.289 | 128 | 88 | 2 | 10 | 134 | 2 | 129 | Tail protein | Tail protein | | afdb-uniprot50 | AF-A0A6M3KNW0-F1-MODEL\_V4 | 1.0 | 2.236e-10 | 489 | 0.349 | 126 | 79 | 2 | 12 | 136 | 2 | 125 | Uncharacterized protein | Uncharacterized protein | | afdb-uniprot50 | AF-A0A5S9QVS9-F1-MODEL\_V4 | 1.0 | 6.523e-11 | 489 | 0.257 | 128 | 91 | 2 | 13 | 136 | 3 | 130 | Uncharacterized protein | Uncharacterized protein | | afdb-uniprot50 | AF-A0A833LH97-F1-MODEL\_V4 | 1.0 | 4.993e-10 | 488 | 0.233 | 124 | 95 | 0 | 13 | 136 | 1 | 124 | Phage tail protein | Phage tail protein | | afdb-uniprot50 | AF-A0A212LR60-F1-MODEL\_V4 | 1.0 | 1.457e-10 | 488 | 0.293 | 126 | 84 | 4 | 13 | 133 | 1 | 126 | Phage protein U | Phage protein U | | afdb-uniprot50 | AF-A0A0R3KUL0-F1-MODEL\_V4 | 1.0 | 5.863e-10 | 487 | 0.225 | 124 | 96 | 0 | 13 | 136 | 1 | 124 | Uncharacterized protein | Uncharacterized protein | | afdb-uniprot50 | AF-A0A4Z1QXY8-F1-MODEL\_V4 | 1.0 | 4.252e-10 | 482 | 0.212 | 127 | 97 | 1 | 11 | 134 | 22 | 148 | Phage tail protein | Phage tail protein | | afdb-uniprot50 | AF-A0A1M5AJI1-F1-MODEL\_V4 | 1.0 | 1.904e-10 | 481 | 0.33 | 127 | 78 | 4 | 13 | 136 | 3 | 125 | Uncharacterized protein | Uncharacterized protein | | afdb-uniprot50 | AF-A0A165XHF3-F1-MODEL\_V4 | 1.0 | 8.081e-11 | 481 | 0.296 | 128 | 86 | 3 | 12 | 136 | 2 | 128 | Phage P2 GpU | Phage P2 GpU | | afdb-uniprot50 | AF-A0A1S2U5I4-F1-MODEL\_V4 | 1.0 | 2.236e-10 | 480 | 0.264 | 125 | 89 | 2 | 13 | 136 | 1 | 123 | Phage tail protein | Phage tail protein | | afdb-uniprot50 | AF-M5K5B9-F1-MODEL\_V4 | 1.0 | 8.53e-10 | 480 | 0.274 | 124 | 90 | 0 | 13 | 136 | 1 | 124 | Fels-2 prophage protein | Fels-2 prophage protein | | afdb-uniprot50 | AF-A0A3L7AM95-F1-MODEL\_V4 | 1.0 | 3.82e-10 | 480 | 0.278 | 122 | 88 | 0 | 13 | 134 | 1 | 122 | Phage tail protein | Phage tail protein | | afdb-uniprot50 | AF-A0A4R2GR57-F1-MODEL\_V4 | 1.0 | 3.253e-10 | 479 | 0.234 | 132 | 96 | 1 | 10 | 136 | 1 | 132 | Phage protein U | Phage protein U | | afdb-uniprot50 | AF-A0A371WU12-F1-MODEL\_V4 | 1.0 | 9.495e-10 | 479 | 0.245 | 122 | 92 | 0 | 13 | 134 | 1 | 122 | Phage tail protein | Phage tail protein | | afdb-uniprot50 | AF-A0A1T4WWY7-F1-MODEL\_V4 | 1.0 | 1.537e-10 | 477 | 0.323 | 130 | 82 | 3 | 13 | 136 | 2 | 131 | Uncharacterized protein | Uncharacterized protein | | afdb-uniprot50 | AF-A0A2X4TYK3-F1-MODEL\_V4 | 1.0 | 4.252e-10 | 473 | 0.413 | 104 | 61 | 0 | 33 | 136 | 2 | 105 | Phage protein U | Phage protein U | | afdb-uniprot50 | AF-A0A7W6WAV6-F1-MODEL\_V4 | 1.0 | 3.083e-10 | 472 | 0.261 | 130 | 88 | 4 | 13 | 136 | 3 | 130 | Uncharacterized protein | Uncharacterized protein | | afdb-uniprot50 | AF-A0A5E7CNT0-F1-MODEL\_V4 | 1.0 | 5.863e-10 | 471 | 0.256 | 125 | 90 | 2 | 13 | 136 | 3 | 125 | Uncharacterized protein | Uncharacterized protein | | afdb-uniprot50 | AF-A0A4Q7FRM2-F1-MODEL\_V4 | 1.0 | 4.252e-10 | 471 | 0.207 | 135 | 99 | 1 | 10 | 136 | 1 | 135 | Uncharacterized protein | Uncharacterized protein | | afdb-uniprot50 | AF-A0A5Y6QJ23-F1-MODEL\_V4 | 1.0 | 6.186e-10 | 470 | 0.256 | 125 | 90 | 2 | 13 | 136 | 1 | 123 | Phage protein U | Phage protein U | | afdb-uniprot50 | AF-A0A218KPW4-F1-MODEL\_V4 | 1.0 | 6.526e-10 | 469 | 0.333 | 123 | 77 | 1 | 14 | 136 | 1 | 118 | Phage tail protein | Phage tail protein | | afdb-uniprot50 | AF-A0A5C8S9X4-F1-MODEL\_V4 | 1.0 | 2.119e-10 | 469 | 0.248 | 141 | 92 | 3 | 10 | 136 | 1 | 141 | Phage tail protein | Phage tail protein | | afdb-uniprot50 | AF-A0A3G2V4J2-F1-MODEL\_V4 | 1.0 | 1.537e-10 | 468 | 0.23 | 143 | 91 | 3 | 13 | 136 | 4 | 146 | Uncharacterized protein | Uncharacterized protein | | afdb-uniprot50 | AF-A0A6N6MG51-F1-MODEL\_V4 | 1.0 | 4.03e-10 | 467 | 0.255 | 129 | 91 | 2 | 13 | 136 | 1 | 129 | Phage tail protein | Phage tail protein | | afdb-uniprot50 | AF-A0A285V255-F1-MODEL\_V4 | 1.0 | 1.309e-09 | 465 | 0.258 | 124 | 92 | 0 | 13 | 136 | 1 | 124 | Uncharacterized protein | Uncharacterized protein | | afdb-uniprot50 | AF-A0A1G7PUT3-F1-MODEL\_V4 | 1.0 | 9e-10 | 464 | 0.258 | 124 | 90 | 1 | 13 | 134 | 1 | 124 | Phage P2 GpU | Phage P2 GpU | | afdb-uniprot50 | AF-A0A377LS59-F1-MODEL\_V4 | 1.0 | 8.526e-11 | 464 | 0.278 | 147 | 95 | 1 | 1 | 136 | 19 | 165 | Phage P2 GpU family protein | Phage P2 GpU family protein | | afdb-uniprot50 | AF-A0A522WZG7-F1-MODEL\_V4 | 1.0 | 4.486e-10 | 462 | 0.535 | 99 | 46 | 0 | 10 | 108 | 1 | 99 | Phage tail protein | Phage tail protein | | afdb-uniprot50 | AF-A0A5N3PHE3-F1-MODEL\_V4 | 1.0 | 5.268e-10 | 462 | 0.232 | 125 | 93 | 1 | 13 | 134 | 1 | 125 | Uncharacterized protein | Uncharacterized protein | | afdb-uniprot50 | AF-A0A1S2E7C7-F1-MODEL\_V4 | 1.0 | 1.622e-09 | 461 | 0.225 | 124 | 95 | 1 | 13 | 136 | 1 | 123 | Phage tail protein | Phage tail protein | | afdb-uniprot50 | AF-A0A1W2EJV4-F1-MODEL\_V4 | 1.0 | 1.711e-09 | 461 | 0.196 | 122 | 98 | 0 | 13 | 134 | 1 | 122 | Uncharacterized protein | Uncharacterized protein | | afdb-uniprot50 | AF-A0A4Y8RF33-F1-MODEL\_V4 | 1.0 | 1.905e-09 | 460 | 0.219 | 123 | 96 | 0 | 13 | 135 | 5 | 127 | Oxidoreductase | Oxidoreductase | | afdb-uniprot50 | AF-A0A346QYH1-F1-MODEL\_V4 | 1.0 | 3.083e-10 | 460 | 0.31 | 119 | 82 | 0 | 18 | 136 | 20 | 138 | Oxidoreductase | Oxidoreductase | | afdb-uniprot50 | AF-A0A485ALS4-F1-MODEL\_V4 | 1.0 | 1.309e-09 | 460 | 0.366 | 109 | 69 | 0 | 28 | 136 | 6 | 114 | Phage protein U | Phage protein U | | afdb-uniprot50 | AF-A0A846VJH3-F1-MODEL\_V4 | 1.0 | 1.381e-09 | 459 | 0.187 | 128 | 100 | 1 | 13 | 136 | 4 | 131 | Phage protein U | Phage protein U | | afdb-uniprot50 | AF-A0A7L5Y3W4-F1-MODEL\_V4 | 1.0 | 6.526e-10 | 459 | 0.195 | 133 | 98 | 2 | 13 | 136 | 4 | 136 | Phage tail protein | Phage tail protein | | afdb-uniprot50 | AF-A0A0M7ANC8-F1-MODEL\_V4 | 1.0 | 1.905e-09 | 459 | 0.209 | 124 | 98 | 0 | 13 | 136 | 1 | 124 | Phage protein U | Phage protein U | | afdb-uniprot50 | AF-A0A1H0KW29-F1-MODEL\_V4 | 1.0 | 1.309e-09 | 458 | 0.288 | 125 | 86 | 2 | 13 | 136 | 3 | 125 | Phage tail protein | Phage tail protein | | afdb-uniprot50 | AF-A0A4Q6DUT2-F1-MODEL\_V4 | 1.0 | 6.186e-10 | 457 | 0.325 | 123 | 81 | 2 | 13 | 134 | 1 | 122 | Uncharacterized protein | Uncharacterized protein | | afdb-uniprot50 | AF-A0A549T0T0-F1-MODEL\_V4 | 1.0 | 2.237e-09 | 457 | 0.217 | 124 | 97 | 0 | 13 | 136 | 1 | 124 | Phage tail protein | Phage tail protein | | afdb-uniprot50 | AF-A0A0G0CA59-F1-MODEL\_V4 | 1.0 | 1.711e-09 | 455 | 0.161 | 124 | 103 | 1 | 13 | 136 | 1 | 123 | Uncharacterized protein | Uncharacterized protein | | afdb-uniprot50 | AF-A0A6I1JKM9-F1-MODEL\_V4 | 1.0 | 1.905e-09 | 454 | 0.25 | 124 | 93 | 0 | 13 | 136 | 1 | 124 | Putative phage tail assembly protein | Putative phage tail assembly protein | | afdb-uniprot50 | AF-A0A1L9QZK1-F1-MODEL\_V4 | 1.0 | 1.176e-09 | 453 | 0.286 | 122 | 85 | 1 | 13 | 134 | 1 | 120 | Uncharacterized protein | Uncharacterized protein | | afdb-uniprot50 | AF-A0A1N7LQZ5-F1-MODEL\_V4 | 1.0 | 3.083e-10 | 453 | 0.281 | 128 | 90 | 2 | 10 | 136 | 2 | 128 | Phage protein U | Phage protein U | | afdb-uniprot50 | AF-A0A369RKP4-F1-MODEL\_V4 | 1.0 | 5.555e-11 | 453 | 0.39 | 123 | 66 | 3 | 14 | 136 | 1 | 114 | Phage tail protein GpU | Phage tail protein GpU | | afdb-uniprot50 | AF-A0A7C4AK56-F1-MODEL\_V4 | 1.0 | 8.085e-10 | 452 | 0.244 | 127 | 92 | 3 | 13 | 136 | 2 | 127 | Phage tail protein | Phage tail protein | | afdb-uniprot50 | AF-A0A377NJ59-F1-MODEL\_V4 | 1.0 | 6.526e-10 | 451 | 0.319 | 119 | 78 | 1 | 21 | 136 | 11 | 129 | Phage protein U | Phage protein U | | afdb-uniprot50 | AF-A0A7C4EKB5-F1-MODEL\_V4 | 1.0 | 3.085e-09 | 451 | 0.193 | 124 | 99 | 1 | 13 | 136 | 1 | 123 | Phage tail protein | Phage tail protein | | afdb-uniprot50 | AF-A0A0M3AYT1-F1-MODEL\_V4 | 1.0 | 3.085e-09 | 450 | 0.185 | 124 | 101 | 0 | 13 | 136 | 1 | 124 | Tail protein | Tail protein | | afdb-uniprot50 | AF-A0A1E4M031-F1-MODEL\_V4 | 1.0 | 3.085e-09 | 449 | 0.233 | 124 | 94 | 1 | 13 | 136 | 1 | 123 | Uncharacterized protein | Uncharacterized protein | | afdb-uniprot50 | AF-A0A3S0YGQ2-F1-MODEL\_V4 | 1.0 | 2.489e-10 | 449 | 0.233 | 137 | 100 | 3 | 3 | 136 | 14 | 148 | Phage tail protein | Phage tail protein | | afdb-uniprot50 | AF-A0A512JPP3-F1-MODEL\_V4 | 1.0 | 4.488e-09 | 448 | 0.177 | 124 | 102 | 0 | 13 | 136 | 1 | 124 | Uncharacterized protein | Uncharacterized protein | | afdb-uniprot50 | AF-F8ESZ6-F1-MODEL\_V4 | 1.0 | 5.265e-11 | 448 | 0.255 | 184 | 88 | 4 | 1 | 136 | 2 | 184 | p2 GpU family protein | p2 GpU family protein | | afdb-uniprot50 | AF-A0A7W9D3K1-F1-MODEL\_V4 | 1.0 | 1.711e-09 | 447 | 0.286 | 122 | 84 | 2 | 16 | 134 | 2 | 123 | Uncharacterized protein | Uncharacterized protein | | afdb-uniprot50 | AF-A0A7X5J7J9-F1-MODEL\_V4 | 1.0 | 7.664e-10 | 446 | 0.211 | 137 | 102 | 3 | 5 | 136 | 2 | 137 | Uncharacterized protein | Uncharacterized protein | | afdb-uniprot50 | AF-A0A379QTF1-F1-MODEL\_V4 | 1.0 | 2.49e-09 | 445 | 0.227 | 123 | 94 | 1 | 13 | 135 | 1 | 122 | P2 GpU | P2 GpU | | afdb-uniprot50 | AF-A0A2L0IJT3-F1-MODEL\_V4 | 1.0 | 1.115e-09 | 442 | 0.415 | 113 | 66 | 0 | 24 | 136 | 26 | 138 | Phage tail protein | Phage tail protein | | afdb-uniprot50 | AF-Q602Z4-F1-MODEL\_V4 | 1.0 | 2.36e-09 | 442 | 0.201 | 124 | 98 | 1 | 13 | 136 | 2 | 124 | Conserved domain protein | Conserved domain protein | | afdb-uniprot50 | AF-A0A017HDE4-F1-MODEL\_V4 | 1.0 | 2.12e-09 | 441 | 0.325 | 123 | 79 | 2 | 17 | 136 | 3 | 124 | Phage P2 GpU | Phage P2 GpU | | afdb-uniprot50 | AF-A0A847GAS6-F1-MODEL\_V4 | 1.0 | 4.254e-09 | 441 | 0.216 | 125 | 96 | 2 | 13 | 136 | 2 | 125 | Phage tail protein | Phage tail protein | | afdb-uniprot50 | AF-E2CHK4-F1-MODEL\_V4 | 1.0 | 1.905e-09 | 440 | 0.275 | 127 | 88 | 3 | 13 | 136 | 1 | 126 | Putative phage tail protein U | Putative phage tail protein U | | afdb-uniprot50 | AF-A0A3G8MA71-F1-MODEL\_V4 | 1.0 | 4.254e-09 | 439 | 0.157 | 127 | 104 | 1 | 13 | 136 | 5 | 131 | Uncharacterized protein | Uncharacterized protein | | afdb-uniprot50 | AF-B6IMG5-F1-MODEL\_V4 | 1.0 | 2.36e-09 | 439 | 0.219 | 123 | 94 | 1 | 13 | 135 | 1 | 121 | Uncharacterized protein | Uncharacterized protein | | afdb-uniprot50 | AF-A0A397LXE3-F1-MODEL\_V4 | 1.0 | 5.56e-09 | 439 | 0.241 | 124 | 94 | 0 | 13 | 136 | 1 | 124 | Uncharacterized protein | Uncharacterized protein | | afdb-uniprot50 | AF-A0A651FVX8-F1-MODEL\_V4 | 1.0 | 2.12e-09 | 438 | 0.225 | 124 | 95 | 1 | 13 | 136 | 1 | 123 | Phage tail protein | Phage tail protein | | afdb-uniprot50 | AF-R5HI01-F1-MODEL\_V4 | 1.0 | 2.771e-09 | 438 | 0.193 | 124 | 99 | 1 | 13 | 136 | 1 | 123 | Putative phage-related protein | Putative phage-related protein | | afdb-uniprot50 | AF-E2CJH4-F1-MODEL\_V4 | 1.0 | 8.089e-09 | 438 | 0.217 | 124 | 97 | 0 | 13 | 136 | 1 | 124 | p2 GpU family protein | p2 GpU family protein | | afdb-uniprot50 | AF-A0A258L8T5-F1-MODEL\_V4 | 1.0 | 1.176e-09 | 438 | 0.259 | 135 | 98 | 1 | 1 | 135 | 1 | 133 | Uncharacterized protein | Uncharacterized protein | | afdb-uniprot50 | AF-R8AR70-F1-MODEL\_V4 | 1.0 | 3.822e-09 | 436 | 0.24 | 125 | 92 | 2 | 13 | 136 | 2 | 124 | GpU family protein | GpU family protein | | afdb-uniprot50 | AF-A0NQA4-F1-MODEL\_V4 | 1.0 | 3.822e-09 | 436 | 0.204 | 122 | 97 | 0 | 13 | 134 | 1 | 122 | Putative bacteriophage tail protein | Putative bacteriophage tail protein | | afdb-uniprot50 | AF-A0A7C7PSI2-F1-MODEL\_V4 | 1.0 | 2.771e-09 | 436 | 0.217 | 124 | 94 | 2 | 13 | 136 | 4 | 124 | Phage tail protein | Phage tail protein | | afdb-uniprot50 | AF-A0A376MUH3-F1-MODEL\_V4 | 1.0 | 9e-10 | 434 | 0.285 | 126 | 86 | 2 | 13 | 136 | 1 | 124 | Tail fiber protein | Tail fiber protein | | afdb-uniprot50 | AF-A0A806DA75-F1-MODEL\_V4 | 1.0 | 3.621e-10 | 434 | 0.255 | 168 | 80 | 3 | 13 | 136 | 1 | 167 | P2 GpU family protein | P2 GpU family protein | | afdb-uniprot50 | AF-A0A2G1CVT3-F1-MODEL\_V4 | 1.0 | 5.27e-09 | 432 | 0.233 | 124 | 91 | 2 | 13 | 136 | 2 | 121 | Uncharacterized protein | Uncharacterized protein | | afdb-uniprot50 | AF-G2HXA8-F1-MODEL\_V4 | 1.0 | 6.529e-09 | 431 | 0.209 | 124 | 94 | 2 | 13 | 136 | 1 | 120 | Hypothetical phage protein | Hypothetical phage protein | | afdb-uniprot50 | AF-A0A7U9JER2-F1-MODEL\_V4 | 1.0 | 4.254e-09 | 431 | 0.336 | 104 | 69 | 0 | 33 | 136 | 2 | 105 | Oxidoreductase | Oxidoreductase | | afdb-uniprot50 | AF-U1ASZ1-F1-MODEL\_V4 | 1.0 | 5.56e-09 | 431 | 0.225 | 124 | 95 | 1 | 13 | 136 | 1 | 123 | Phage tail protein | Phage tail protein | | afdb-uniprot50 | AF-A0A7Y4XET2-F1-MODEL\_V4 | 1.0 | 2.12e-09 | 431 | 0.167 | 131 | 108 | 1 | 6 | 136 | 9 | 138 | Phage tail protein | Phage tail protein | | afdb-uniprot50 | AF-A0A437NT45-F1-MODEL\_V4 | 1.0 | 1.623e-08 | 430 | 0.217 | 124 | 97 | 0 | 13 | 136 | 1 | 124 | Phage tail protein | Phage tail protein | | afdb-uniprot50 | AF-A0A2A3D6X4-F1-MODEL\_V4 | 1.0 | 6.529e-09 | 429 | 0.209 | 124 | 97 | 1 | 13 | 136 | 1 | 123 | Phage tail protein | Phage tail protein | | afdb-uniprot50 | AF-A0A411WI49-F1-MODEL\_V4 | 1.0 | 5.27e-09 | 429 | 0.224 | 125 | 96 | 1 | 12 | 136 | 2 | 125 | Phage tail protein | Phage tail protein | | afdb-uniprot50 | AF-A0A6D0J052-F1-MODEL\_V4 | 1.0 | 7.668e-09 | 428 | 0.353 | 116 | 75 | 0 | 13 | 128 | 1 | 116 | Phage tail protein | Phage tail protein | | afdb-uniprot50 | AF-L0NDK6-F1-MODEL\_V4 | 1.0 | 2.627e-09 | 428 | 0.246 | 126 | 90 | 4 | 16 | 136 | 2 | 127 | Putative Phage P2 GpU | Putative Phage P2 GpU | | afdb-uniprot50 | AF-A0A327K1Q9-F1-MODEL\_V4 | 1.0 | 3.085e-09 | 427 | 0.265 | 128 | 85 | 4 | 13 | 136 | 3 | 125 | Uncharacterized protein | Uncharacterized protein | | afdb-uniprot50 | AF-A0A2D5PEI7-F1-MODEL\_V4 | 1.0 | 2.771e-09 | 426 | 0.244 | 127 | 90 | 5 | 12 | 136 | 2 | 124 | Uncharacterized protein | Uncharacterized protein | | afdb-uniprot50 | AF-A0A0Q6ZRY4-F1-MODEL\_V4 | 1.0 | 5.56e-09 | 423 | 0.23 | 126 | 94 | 3 | 13 | 136 | 1 | 125 | Uncharacterized protein | Uncharacterized protein | | afdb-uniprot50 | AF-A0A5M8P9Q4-F1-MODEL\_V4 | 1.0 | 6.189e-09 | 423 | 0.246 | 134 | 93 | 4 | 10 | 136 | 1 | 133 | Phage tail protein | Phage tail protein | | afdb-uniprot50 | AF-A0A4P7KXL2-F1-MODEL\_V4 | 1.0 | 2.924e-09 | 423 | 0.219 | 123 | 95 | 1 | 13 | 135 | 1 | 122 | Phage P2 GpU | Phage P2 GpU | | afdb-uniprot50 | AF-A0A125QFM2-F1-MODEL\_V4 | 1.0 | 1.242e-08 | 422 | 0.201 | 124 | 98 | 1 | 13 | 136 | 1 | 123 | Phage P2 GpU | Phage P2 GpU | | afdb-uniprot50 | AF-A0A7C3M160-F1-MODEL\_V4 | 1.0 | 1.309e-09 | 421 | 0.22 | 136 | 100 | 4 | 1 | 136 | 1 | 130 | Uncharacterized protein | Uncharacterized protein | | afdb-uniprot50 | AF-A0A432QVN4-F1-MODEL\_V4 | 1.0 | 5.557e-10 | 420 | 0.27 | 137 | 87 | 3 | 13 | 136 | 1 | 137 | Uncharacterized protein | Uncharacterized protein | | afdb-uniprot50 | AF-A0A4P5QXL4-F1-MODEL\_V4 | 1.0 | 1.176e-09 | 420 | 0.211 | 137 | 105 | 2 | 1 | 136 | 7 | 141 | Uncharacterized protein | Uncharacterized protein | | afdb-uniprot50 | AF-A0A377PAA7-F1-MODEL\_V4 | 1.0 | 4.254e-09 | 420 | 0.227 | 123 | 94 | 1 | 13 | 135 | 1 | 122 | Phage protein U | Phage protein U | | afdb-uniprot50 | AF-A0A221T8K1-F1-MODEL\_V4 | 1.0 | 4.735e-09 | 419 | 0.211 | 123 | 96 | 1 | 13 | 135 | 1 | 122 | Phage tail protein | Phage tail protein | | afdb-uniprot50 | AF-A0A085AFM6-F1-MODEL\_V4 | 1.0 | 1.309e-09 | 418 | 0.206 | 145 | 104 | 3 | 1 | 136 | 1 | 143 | Uncharacterized protein | Uncharacterized protein | | afdb-uniprot50 | AF-A0A4R6M7R2-F1-MODEL\_V4 | 1.0 | 1.177e-08 | 417 | 0.217 | 124 | 93 | 2 | 13 | 136 | 1 | 120 | Phage protein U | Phage protein U | | afdb-uniprot50 | AF-A0A6N8TE18-F1-MODEL\_V4 | 1.0 | 4.032e-09 | 417 | 0.224 | 129 | 94 | 3 | 13 | 136 | 9 | 136 | Uncharacterized protein | Uncharacterized protein | | afdb-uniprot50 | AF-A0A7A6ZBU5-F1-MODEL\_V4 | 1.0 | 8.535e-09 | 416 | 0.211 | 123 | 96 | 1 | 13 | 135 | 1 | 122 | Phage tail protein | Phage tail protein | | afdb-uniprot50 | AF-A0A7X7NI24-F1-MODEL\_V4 | 1.0 | 2.121e-08 | 415 | 0.169 | 124 | 101 | 1 | 13 | 136 | 1 | 122 | Phage tail protein | Phage tail protein | | afdb-uniprot50 | AF-A0A3B9Q8H5-F1-MODEL\_V4 | 1.0 | 4.488e-09 | 414 | 0.179 | 134 | 105 | 4 | 7 | 136 | 1 | 133 | Uncharacterized protein | Uncharacterized protein | | afdb-uniprot50 | AF-A0A829E1U0-F1-MODEL\_V4 | 1.0 | 9.5e-09 | 414 | 0.203 | 123 | 97 | 1 | 13 | 135 | 1 | 122 | Phage P2 GpU family protein | Phage P2 GpU family protein | | afdb-uniprot50 | AF-A0A1C3E6K8-F1-MODEL\_V4 | 1.0 | 4.735e-09 | 413 | 0.206 | 126 | 94 | 4 | 12 | 136 | 2 | 122 | Uncharacterized protein | Uncharacterized protein | | afdb-uniprot50 | AF-A0A1M7R7F9-F1-MODEL\_V4 | 1.0 | 1.242e-08 | 413 | 0.233 | 124 | 94 | 1 | 13 | 136 | 1 | 123 | Phage protein U | Phage protein U | | afdb-uniprot50 | AF-A0A2M7MVA7-F1-MODEL\_V4 | 1.0 | 2.238e-08 | 413 | 0.185 | 124 | 100 | 1 | 13 | 136 | 2 | 124 | Uncharacterized protein | Uncharacterized protein | | afdb-uniprot50 | AF-A0A0B6D192-F1-MODEL\_V4 | 1.0 | 5.866e-09 | 412 | 0.186 | 123 | 94 | 3 | 14 | 136 | 1 | 117 | Phage P2 GpU family protein | Phage P2 GpU family protein | | afdb-uniprot50 | AF-A0A8B2QSL7-F1-MODEL\_V4 | 1.0 | 9.004e-09 | 412 | 0.243 | 123 | 89 | 2 | 13 | 134 | 1 | 120 | Oxidoreductase | Oxidoreductase | | afdb-uniprot50 | AF-A0A855HFC3-F1-MODEL\_V4 | 1.0 | 6.529e-09 | 411 | 0.466 | 103 | 54 | 1 | 8 | 109 | 5 | 107 | Phage tail protein | Phage tail protein | | afdb-uniprot50 | AF-A0A1J5BJG5-F1-MODEL\_V4 | 1.0 | 9.5e-09 | 410 | 0.182 | 126 | 101 | 2 | 9 | 134 | 26 | 149 | Uncharacterized protein | Uncharacterized protein | | afdb-uniprot50 | AF-A0A0F4NJK3-F1-MODEL\_V4 | 1.0 | 1.712e-08 | 409 | 0.204 | 122 | 93 | 2 | 13 | 134 | 1 | 118 | Uncharacterized protein | Uncharacterized protein | | afdb-uniprot50 | AF-A0A7J4ETH5-F1-MODEL\_V4 | 1.0 | 1.177e-08 | 409 | 0.266 | 124 | 87 | 3 | 13 | 136 | 1 | 120 | Uncharacterized protein | Uncharacterized protein | | afdb-uniprot50 | AF-A0A2M7G5P3-F1-MODEL\_V4 | 1.0 | 9.004e-09 | 409 | 0.169 | 124 | 102 | 1 | 12 | 135 | 2 | 124 | Uncharacterized protein | Uncharacterized protein | | afdb-uniprot50 | AF-A0A6L5BH81-F1-MODEL\_V4 | 1.0 | 2.628e-08 | 407 | 0.169 | 124 | 102 | 1 | 13 | 136 | 1 | 123 | Uncharacterized protein | Uncharacterized protein | | afdb-uniprot50 | AF-A0A2X2GG49-F1-MODEL\_V4 | 1.0 | 4.032e-09 | 406 | 0.25 | 108 | 81 | 0 | 28 | 135 | 9 | 116 | Phage protein U | Phage protein U | | afdb-uniprot50 | AF-A0A4R5HEI0-F1-MODEL\_V4 | 1.0 | 4.254e-09 | 406 | 0.274 | 124 | 87 | 2 | 14 | 136 | 3 | 124 | Phage tail protein | Phage tail protein | | afdb-uniprot50 | AF-A0A6F8NNB5-F1-MODEL\_V4 | 1.0 | 1.806e-09 | 406 | 0.266 | 120 | 79 | 2 | 26 | 136 | 37 | 156 | Bacteriophage P2 tail protein GPU | Bacteriophage P2 tail protein GPU | | afdb-uniprot50 | AF-A0A1G3UAP7-F1-MODEL\_V4 | 1.0 | 9.5e-09 | 405 | 0.233 | 124 | 92 | 1 | 13 | 136 | 2 | 122 | Uncharacterized protein | Uncharacterized protein | | afdb-uniprot50 | AF-A0A2T5NQ46-F1-MODEL\_V4 | 1.0 | 1.623e-08 | 405 | 0.233 | 124 | 94 | 1 | 13 | 136 | 1 | 123 | Uncharacterized protein | Uncharacterized protein | | afdb-uniprot50 | AF-A0A5C7EXC1-F1-MODEL\_V4 | 1.0 | 1.538e-08 | 405 | 0.186 | 123 | 99 | 1 | 13 | 135 | 3 | 124 | Phage tail protein | Phage tail protein | | afdb-uniprot50 | AF-A0A0M1VTG3-F1-MODEL\_V4 | 1.0 | 1.002e-08 | 402 | 0.257 | 128 | 90 | 4 | 13 | 136 | 2 | 128 | Uncharacterized protein | Uncharacterized protein | | afdb-uniprot50 | AF-A0A7U9G627-F1-MODEL\_V4 | 1.0 | 2.238e-08 | 402 | 0.203 | 123 | 97 | 1 | 13 | 135 | 1 | 122 | Putative phage-related protein | Putative phage-related protein | | afdb-uniprot50 | AF-A0A1X0TI83-F1-MODEL\_V4 | 1.0 | 3.256e-08 | 402 | 0.211 | 123 | 96 | 1 | 13 | 135 | 1 | 122 | Phage tail protein | Phage tail protein | | afdb-uniprot50 | AF-A0A7Z1M3M8-F1-MODEL\_V4 | 1.0 | 1.806e-08 | 402 | 0.193 | 124 | 99 | 1 | 13 | 136 | 2 | 124 | Phage protein U | Phage protein U | | afdb-uniprot50 | AF-A0A1R1MK76-F1-MODEL\_V4 | 1.0 | 9.004e-09 | 399 | 0.188 | 127 | 97 | 3 | 13 | 136 | 1 | 124 | Uncharacterized protein | Uncharacterized protein | | afdb-uniprot50 | AF-E8KG72-F1-MODEL\_V4 | 1.0 | 3.435e-08 | 398 | 0.169 | 124 | 102 | 1 | 13 | 136 | 3 | 125 | Uncharacterized protein | Uncharacterized protein | | afdb-uniprot50 | AF-A0A6J4N4J5-F1-MODEL\_V4 | 1.0 | 2.361e-08 | 398 | 0.201 | 124 | 98 | 1 | 13 | 136 | 3 | 125 | Uncharacterized protein | Uncharacterized protein | | afdb-uniprot50 | AF-A0A1M7RIG2-F1-MODEL\_V4 | 1.0 | 3.086e-08 | 398 | 0.225 | 124 | 95 | 1 | 13 | 136 | 1 | 123 | Phage protein U | Phage protein U | | afdb-uniprot50 | AF-A0A432PWE8-F1-MODEL\_V4 | 1.0 | 1.538e-08 | 396 | 0.288 | 125 | 79 | 5 | 13 | 136 | 1 | 116 | Uncharacterized protein | Uncharacterized protein | | afdb-uniprot50 | AF-A0A379WIT7-F1-MODEL\_V4 | 1.0 | 1.242e-08 | 396 | 0.219 | 123 | 95 | 1 | 13 | 135 | 1 | 122 | Methyl-accepting chemotaxis protein | Methyl-accepting chemotaxis protein | | afdb-uniprot50 | AF-A0A836CGV5-F1-MODEL\_V4 | 1.0 | 1.457e-09 | 396 | 0.274 | 135 | 89 | 3 | 8 | 135 | 676 | 808 | Uncharacterized protein | Uncharacterized protein | | afdb-uniprot50 | AF-A0A1M5PY95-F1-MODEL\_V4 | 1.0 | 4.486e-10 | 395 | 0.237 | 143 | 89 | 4 | 8 | 136 | 14 | 150 | Uncharacterized protein | Uncharacterized protein | | afdb-uniprot50 | AF-A0A1T4QF76-F1-MODEL\_V4 | 1.0 | 3.824e-08 | 394 | 0.14 | 128 | 105 | 4 | 13 | 136 | 2 | 128 | Uncharacterized protein | Uncharacterized protein | | afdb-uniprot50 | AF-A0A1Y5SUW4-F1-MODEL\_V4 | 1.0 | 2.925e-08 | 394 | 0.169 | 124 | 102 | 1 | 13 | 136 | 1 | 123 | Phage P2 GpU | Phage P2 GpU | | afdb-uniprot50 | AF-A0A659PNI1-F1-MODEL\_V4 | 1.0 | 2.361e-08 | 393 | 0.323 | 102 | 69 | 0 | 35 | 136 | 2 | 103 | Phage tail protein | Phage tail protein | | afdb-uniprot50 | AF-A0A745FX58-F1-MODEL\_V4 | 1.0 | 4.256e-08 | 393 | 0.343 | 102 | 67 | 0 | 35 | 136 | 1 | 102 | Phage tail protein | Phage tail protein | | afdb-uniprot50 | AF-A0A5S4YPE8-F1-MODEL\_V4 | 1.0 | 3.434e-09 | 393 | 0.275 | 120 | 78 | 2 | 26 | 136 | 138 | 257 | Phage tail protein | Phage tail protein | | afdb-uniprot50 | AF-A0A5V6NIV2-F1-MODEL\_V4 | 1.0 | 1.458e-08 | 392 | 0.187 | 128 | 99 | 4 | 13 | 136 | 2 | 128 | Uncharacterized protein | Uncharacterized protein | | afdb-uniprot50 | AF-A0A1C0V8H7-F1-MODEL\_V4 | 1.0 | 1.382e-08 | 392 | 0.193 | 129 | 100 | 4 | 10 | 136 | 2 | 128 | Uncharacterized protein | Uncharacterized protein | | afdb-uniprot50 | AF-K1JX06-F1-MODEL\_V4 | 1.0 | 1.906e-08 | 391 | 0.171 | 128 | 101 | 4 | 13 | 136 | 7 | 133 | Uncharacterized protein | Uncharacterized protein | | afdb-uniprot50 | AF-A0A2T7H7Y5-F1-MODEL\_V4 | 1.0 | 2.773e-08 | 390 | 0.226 | 128 | 91 | 4 | 13 | 134 | 3 | 128 | Uncharacterized protein | Uncharacterized protein | | afdb-uniprot50 | AF-A0A7Z0SRH5-F1-MODEL\_V4 | 1.0 | 6.192e-08 | 390 | 0.217 | 124 | 96 | 1 | 13 | 136 | 1 | 123 | Phage tail protein | Phage tail protein | | afdb-uniprot50 | AF-A0A4R5W1S1-F1-MODEL\_V4 | 1.0 | 3.256e-08 | 390 | 0.209 | 124 | 97 | 1 | 12 | 135 | 2 | 124 | Phage tail protein | Phage tail protein | | afdb-uniprot50 | AF-A0A1U7MFM3-F1-MODEL\_V4 | 1.0 | 1.382e-08 | 389 | 0.2 | 130 | 95 | 5 | 13 | 136 | 2 | 128 | Phage P2 GpU | Phage P2 GpU | | afdb-uniprot50 | AF-A0A2G3J6J3-F1-MODEL\_V4 | 1.0 | 4.256e-08 | 389 | 0.217 | 124 | 96 | 1 | 13 | 136 | 1 | 123 | Uncharacterized protein | Uncharacterized protein | | afdb-uniprot50 | AF-E2CFJ9-F1-MODEL\_V4 | 1.0 | 3.086e-08 | 387 | 0.259 | 127 | 88 | 2 | 13 | 136 | 3 | 126 | Putative phage tail protein | Putative phage tail protein | | afdb-uniprot50 | AF-A0A2D3VW39-F1-MODEL\_V4 | 1.0 | 3.435e-08 | 386 | 0.193 | 124 | 96 | 2 | 13 | 136 | 1 | 120 | Uncharacterized protein | Uncharacterized protein | | afdb-uniprot50 | AF-A0A7V8L4R9-F1-MODEL\_V4 | 1.0 | 1.382e-08 | 386 | 0.17 | 123 | 101 | 1 | 13 | 135 | 1 | 122 | Phage P2 GpU family protein | Phage P2 GpU family protein | | afdb-uniprot50 | AF-I7LIF3-F1-MODEL\_V4 | 1.0 | 2.121e-08 | 385 | 0.207 | 130 | 94 | 4 | 13 | 136 | 1 | 127 | Uncharacterized protein | Uncharacterized protein | | afdb-uniprot50 | AF-A0A4V3D5Q8-F1-MODEL\_V4 | 1.0 | 2.121e-08 | 385 | 0.162 | 123 | 102 | 1 | 12 | 134 | 2 | 123 | Phage protein U | Phage protein U | | afdb-uniprot50 | AF-Q3RD92-F1-MODEL\_V4 | 1.0 | 3.086e-08 | 384 | 0.424 | 99 | 57 | 0 | 38 | 136 | 1 | 99 | Phage P2 GpU | Phage P2 GpU | | afdb-uniprot50 | AF-A0A1V3RQN1-F1-MODEL\_V4 | 1.0 | 1.002e-08 | 384 | 0.173 | 127 | 103 | 2 | 10 | 136 | 1 | 125 | Uncharacterized protein | Uncharacterized protein | | afdb-uniprot50 | AF-D8IV23-F1-MODEL\_V4 | 1.0 | 1.906e-08 | 384 | 0.171 | 134 | 110 | 1 | 3 | 136 | 13 | 145 | Uncharacterized protein | Uncharacterized protein | | afdb-uniprot50 | AF-A0A318KUR9-F1-MODEL\_V4 | 1.0 | 4.998e-08 | 384 | 0.245 | 122 | 91 | 1 | 13 | 134 | 1 | 121 | Phage protein U | Phage protein U | | afdb-uniprot50 | AF-A0A0E4CZC6-F1-MODEL\_V4 | 1.0 | 7.268e-09 | 382 | 0.181 | 149 | 101 | 6 | 1 | 136 | 17 | 157 | Uncharacterized protein | Uncharacterized protein | | afdb-uniprot50 | AF-A0A432PWN2-F1-MODEL\_V4 | 1.0 | 6.533e-08 | 381 | 0.233 | 124 | 90 | 3 | 13 | 136 | 1 | 119 | Uncharacterized protein | Uncharacterized protein | | afdb-uniprot50 | AF-A0A3D1NSN0-F1-MODEL\_V4 | 1.0 | 1.058e-07 | 381 | 0.18 | 122 | 99 | 1 | 13 | 134 | 1 | 121 | Uncharacterized protein | Uncharacterized protein | | afdb-uniprot50 | AF-A0A662Z774-F1-MODEL\_V4 | 1.0 | 2.773e-08 | 380 | 0.187 | 128 | 99 | 4 | 13 | 136 | 6 | 132 | Uncharacterized protein | Uncharacterized protein | | afdb-uniprot50 | AF-A0A541BHI4-F1-MODEL\_V4 | 1.0 | 6.533e-08 | 379 | 0.193 | 124 | 98 | 2 | 13 | 136 | 2 | 123 | Uncharacterized protein | Uncharacterized protein | | afdb-uniprot50 | AF-A0A1H5DBV8-F1-MODEL\_V4 | 1.0 | 1.058e-07 | 379 | 0.153 | 124 | 104 | 1 | 13 | 136 | 1 | 123 | Phage protein U | Phage protein U | | afdb-uniprot50 | AF-A0A6I2ITM1-F1-MODEL\_V4 | 1.0 | 2.238e-08 | 378 | 0.4 | 105 | 63 | 0 | 2 | 106 | 17 | 121 | Phage tail protein | Phage tail protein | | afdb-uniprot50 | AF-A0A1L9Q5U4-F1-MODEL\_V4 | 1.0 | 9.009e-08 | 378 | 0.178 | 129 | 100 | 3 | 13 | 136 | 3 | 130 | Uncharacterized protein | Uncharacterized protein | | afdb-uniprot50 | AF-E0NXI7-F1-MODEL\_V4 | 1.0 | 1.242e-08 | 378 | 0.207 | 135 | 96 | 5 | 10 | 136 | 39 | 170 | Uncharacterized protein | Uncharacterized protein | | afdb-uniprot50 | AF-A0A3G3HJJ9-F1-MODEL\_V4 | 1.0 | 4.737e-08 | 377 | 0.178 | 123 | 100 | 1 | 13 | 135 | 1 | 122 | Uncharacterized protein | Uncharacterized protein | | afdb-uniprot50 | AF-A0A2N6FYN8-F1-MODEL\_V4 | 1.0 | 4.737e-08 | 376 | 0.208 | 125 | 93 | 4 | 13 | 136 | 1 | 120 | Uncharacterized protein | Uncharacterized protein | | afdb-uniprot50 | AF-A0A559IZL2-F1-MODEL\_V4 | 1.0 | 1.31e-08 | 376 | 0.207 | 135 | 90 | 6 | 13 | 136 | 1 | 129 | Phage tail protein | Phage tail protein | | afdb-uniprot50 | AF-A0A822TT98-F1-MODEL\_V4 | 1.0 | 1.458e-08 | 376 | 0.186 | 123 | 99 | 1 | 13 | 135 | 1 | 122 | Phage tail protein | Phage tail protein | | afdb-uniprot50 | AF-A0A5M8P636-F1-MODEL\_V4 | 1.0 | 3.824e-08 | 375 | 0.147 | 129 | 105 | 1 | 13 | 136 | 2 | 130 | Uncharacterized protein | Uncharacterized protein | | afdb-uniprot50 | AF-A0A561QKN3-F1-MODEL\_V4 | 1.0 | 1.538e-08 | 374 | 0.227 | 132 | 91 | 6 | 13 | 136 | 1 | 129 | Uncharacterized protein | Uncharacterized protein | | afdb-uniprot50 | AF-U4T472-F1-MODEL\_V4 | 1.0 | 1.624e-07 | 372 | 0.177 | 124 | 101 | 1 | 13 | 136 | 1 | 123 | Putative methyl-accepting chemotaxis protein | Putative methyl-accepting chemotaxis protein | | afdb-uniprot50 | AF-A0A622TXM7-F1-MODEL\_V4 | 1.0 | 1.623e-08 | 371 | 0.195 | 128 | 94 | 5 | 13 | 134 | 2 | 126 | Phage tail protein | Phage tail protein | | afdb-uniprot50 | AF-A0A150KS36-F1-MODEL\_V4 | 1.0 | 2.361e-08 | 371 | 0.25 | 132 | 88 | 6 | 13 | 136 | 3 | 131 | SH3b domain-containing protein | SH3b domain-containing protein | | afdb-uniprot50 | AF-E5VKI8-F1-MODEL\_V4 | 1.0 | 2.491e-08 | 370 | 0.159 | 132 | 100 | 7 | 13 | 136 | 3 | 131 | Uncharacterized protein | Uncharacterized protein | | afdb-uniprot50 | AF-A0A2Z4RAD7-F1-MODEL\_V4 | 1.0 | 1.003e-07 | 370 | 0.184 | 125 | 99 | 2 | 13 | 136 | 1 | 123 | Uncharacterized protein | Uncharacterized protein | | afdb-uniprot50 | AF-W4EGN0-F1-MODEL\_V4 | 1.0 | 1.906e-08 | 369 | 0.163 | 147 | 106 | 6 | 1 | 136 | 5 | 145 | Uncharacterized protein | Uncharacterized protein | | afdb-uniprot50 | AF-A0A2W5JTV2-F1-MODEL\_V4 | 1.0 | 3.824e-08 | 368 | 0.209 | 124 | 94 | 2 | 13 | 136 | 2 | 121 | Uncharacterized protein | Uncharacterized protein | | afdb-uniprot50 | AF-A0A0Q0VG01-F1-MODEL\_V4 | 1.0 | 1.459e-07 | 368 | 0.2 | 125 | 98 | 2 | 13 | 136 | 1 | 124 | Uncharacterized protein | Uncharacterized protein | | afdb-uniprot50 | AF-A0A7J0BHU5-F1-MODEL\_V4 | 1.0 | 4.034e-08 | 368 | 0.165 | 139 | 111 | 4 | 2 | 136 | 151 | 288 | Uncharacterized protein | Uncharacterized protein | | afdb-uniprot50 | AF-D2ZHP5-F1-MODEL\_V4 | 1.0 | 1.623e-08 | 368 | 0.195 | 123 | 98 | 1 | 13 | 135 | 1 | 122 | Phage P2 GpU | Phage P2 GpU | | afdb-uniprot50 | AF-A0A7C3GV02-F1-MODEL\_V4 | 1.0 | 6.892e-08 | 366 | 0.207 | 130 | 96 | 3 | 12 | 136 | 2 | 129 | Phage tail protein | Phage tail protein | | afdb-uniprot50 | AF-A0A3A9CQH5-F1-MODEL\_V4 | 1.0 | 1.806e-08 | 366 | 0.2 | 145 | 104 | 7 | 1 | 136 | 25 | 166 | Phage tail protein | Phage tail protein | | afdb-uniprot50 | AF-A0A521DSD1-F1-MODEL\_V4 | 1.0 | 1.178e-07 | 365 | 0.237 | 122 | 91 | 2 | 13 | 134 | 1 | 120 | Phage protein U | Phage protein U | | afdb-uniprot50 | AF-A0A859FBV8-F1-MODEL\_V4 | 1.0 | 2.491e-08 | 365 | 0.189 | 132 | 96 | 6 | 13 | 136 | 1 | 129 | Phage tail protein | Phage tail protein | | afdb-uniprot50 | AF-A0A2W6YBV7-F1-MODEL\_V4 | 1.0 | 9.009e-08 | 365 | 0.185 | 124 | 97 | 3 | 13 | 133 | 1 | 123 | Uncharacterized protein | Uncharacterized protein | | afdb-uniprot50 | AF-S7UNK7-F1-MODEL\_V4 | 1.0 | 4.737e-08 | 365 | 0.17 | 123 | 101 | 1 | 13 | 135 | 3 | 124 | p2 GpU family protein | p2 GpU family protein | | afdb-uniprot50 | AF-A0A450WGZ2-F1-MODEL\_V4 | 1.0 | 5.563e-08 | 365 | 0.209 | 124 | 97 | 1 | 13 | 136 | 2 | 124 | Phage protein U | Phage protein U | | afdb-uniprot50 | AF-A0A5B8JQ08-F1-MODEL\_V4 | 1.0 | 2.361e-08 | 364 | 0.204 | 137 | 100 | 5 | 7 | 136 | 1 | 135 | Phage tail protein | Phage tail protein | | afdb-uniprot50 | AF-A0A0D6ARK4-F1-MODEL\_V4 | 1.0 | 2.925e-08 | 364 | 0.267 | 127 | 89 | 2 | 12 | 135 | 2 | 127 | Phage protein | Phage protein | | afdb-uniprot50 | AF-A0A7J5WF49-F1-MODEL\_V4 | 1.0 | 3.824e-08 | 364 | 0.164 | 134 | 106 | 5 | 7 | 136 | 164 | 295 | Uncharacterized protein | Uncharacterized protein | | afdb-uniprot50 | AF-A0A6N7B4J7-F1-MODEL\_V4 | 1.0 | 1.003e-07 | 364 | 0.163 | 122 | 100 | 1 | 13 | 134 | 2 | 121 | Uncharacterized protein | Uncharacterized protein | | afdb-uniprot50 | AF-A0A1Y3SYD9-F1-MODEL\_V4 | 1.0 | 2.925e-08 | 363 | 0.21 | 133 | 93 | 7 | 13 | 136 | 2 | 131 | Uncharacterized protein | Uncharacterized protein | | afdb-uniprot50 | AF-R5BE61-F1-MODEL\_V4 | 1.0 | 6.192e-08 | 363 | 0.181 | 132 | 97 | 6 | 11 | 134 | 35 | 163 | p2 GpU family protein | p2 GpU family protein | | afdb-uniprot50 | AF-A0A316MLA5-F1-MODEL\_V4 | 1.0 | 4.49e-08 | 359 | 0.218 | 142 | 91 | 7 | 13 | 136 | 2 | 141 | Uncharacterized protein | Uncharacterized protein | | afdb-uniprot50 | AF-A0A4U8YXW6-F1-MODEL\_V4 | 1.0 | 5.273e-08 | 358 | 0.201 | 134 | 88 | 5 | 14 | 136 | 3 | 128 | Myoviridae gpu | Myoviridae gpu | | afdb-uniprot50 | AF-A0A511Z868-F1-MODEL\_V4 | 1.0 | 5.273e-08 | 358 | 0.174 | 132 | 98 | 6 | 13 | 136 | 1 | 129 | Uncharacterized protein | Uncharacterized protein | | afdb-uniprot50 | AF-F3YY63-F1-MODEL\_V4 | 1.0 | 1.907e-07 | 357 | 0.14 | 128 | 105 | 4 | 13 | 136 | 2 | 128 | Uncharacterized protein | Uncharacterized protein | | afdb-uniprot50 | AF-A0A2P8EI36-F1-MODEL\_V4 | 1.0 | 1.459e-07 | 357 | 0.147 | 129 | 103 | 5 | 13 | 136 | 2 | 128 | GpU protein | GpU protein | | afdb-uniprot50 | AF-B3QTJ0-F1-MODEL\_V4 | 1.0 | 6.533e-08 | 357 | 0.201 | 129 | 91 | 4 | 13 | 136 | 1 | 122 | Uncharacterized protein | Uncharacterized protein | | afdb-uniprot50 | AF-A0A855IT43-F1-MODEL\_V4 | 1.0 | 1.116e-07 | 356 | 0.148 | 128 | 99 | 4 | 13 | 136 | 2 | 123 | Uncharacterized protein | Uncharacterized protein | | afdb-uniprot50 | AF-A0A416B7P4-F1-MODEL\_V4 | 1.0 | 5.273e-08 | 356 | 0.18 | 133 | 97 | 7 | 13 | 136 | 3 | 132 | Uncharacterized protein | Uncharacterized protein | | afdb-uniprot50 | AF-A0A840MJS2-F1-MODEL\_V4 | 1.0 | 5.563e-08 | 356 | 0.211 | 123 | 96 | 1 | 13 | 135 | 5 | 126 | Phage protein U | Phage protein U | | afdb-uniprot50 | AF-A0A620N5X6-F1-MODEL\_V4 | 1.0 | 3.256e-08 | 356 | 0.219 | 123 | 95 | 1 | 13 | 135 | 1 | 122 | Uncharacterized protein | Uncharacterized protein | | afdb-uniprot50 | AF-A0A833UXN4-F1-MODEL\_V4 | 1.0 | 1.459e-07 | 355 | 0.32 | 128 | 82 | 1 | 2 | 124 | 16 | 143 | Uncharacterized protein | Uncharacterized protein | | afdb-uniprot50 | AF-A0A3A9GXW5-F1-MODEL\_V4 | 1.0 | 3.435e-08 | 354 | 0.201 | 134 | 91 | 6 | 13 | 136 | 3 | 130 | Phage tail protein | Phage tail protein | | afdb-uniprot50 | AF-A0A6D2GEA9-F1-MODEL\_V4 | 1.0 | 4.737e-08 | 354 | 0.203 | 123 | 97 | 1 | 13 | 135 | 1 | 122 | Methyl-accepting chemotaxis protein | Methyl-accepting chemotaxis protein | | afdb-uniprot50 | AF-J5HM67-F1-MODEL\_V4 | 1.0 | 4.256e-08 | 354 | 0.2 | 135 | 92 | 6 | 13 | 136 | 20 | 149 | Phage P2 GpU | Phage P2 GpU | | afdb-uniprot50 | AF-A0A4S2HDA3-F1-MODEL\_V4 | 1.0 | 4.256e-08 | 353 | 0.179 | 134 | 94 | 6 | 13 | 136 | 41 | 168 | Phage tail protein | Phage tail protein | | afdb-uniprot50 | AF-E5Y5W8-F1-MODEL\_V4 | 1.0 | 9.504e-08 | 352 | 0.215 | 130 | 94 | 4 | 13 | 136 | 3 | 130 | Uncharacterized protein | Uncharacterized protein | | afdb-uniprot50 | AF-A0A4U7J7L0-F1-MODEL\_V4 | 1.0 | 1.178e-07 | 352 | 0.155 | 129 | 103 | 4 | 13 | 136 | 2 | 129 | Phage tail protein | Phage tail protein | | afdb-uniprot50 | AF-A0A450WK91-F1-MODEL\_V4 | 1.0 | 7.271e-08 | 352 | 0.177 | 124 | 101 | 1 | 13 | 136 | 2 | 124 | Phage P2 GpU | Phage P2 GpU | | afdb-uniprot50 | AF-E1QHA1-F1-MODEL\_V4 | 1.0 | 1.624e-07 | 351 | 0.173 | 127 | 101 | 3 | 13 | 136 | 5 | 130 | Uncharacterized protein | Uncharacterized protein | | afdb-uniprot50 | AF-A0A1M6GF68-F1-MODEL\_V4 | 1.0 | 2.238e-08 | 351 | 0.179 | 134 | 101 | 5 | 10 | 136 | 20 | 151 | Uncharacterized protein | Uncharacterized protein | | afdb-uniprot50 | AF-A0A497UT05-F1-MODEL\_V4 | 1.0 | 1.907e-07 | 351 | 0.186 | 123 | 98 | 2 | 13 | 134 | 1 | 122 | Phage protein U | Phage protein U | | afdb-uniprot50 | AF-A0A2J9QJ37-F1-MODEL\_V4 | 1.0 | 1.383e-07 | 351 | 0.188 | 122 | 98 | 1 | 14 | 135 | 3 | 123 | Phage tail protein | Phage tail protein | | afdb-uniprot50 | AF-A0A327JMS0-F1-MODEL\_V4 | 1.0 | 1.003e-07 | 350 | 0.258 | 112 | 77 | 1 | 28 | 133 | 4 | 115 | Uncharacterized protein | Uncharacterized protein | | afdb-uniprot50 | AF-E5WEF4-F1-MODEL\_V4 | 1.0 | 6.892e-08 | 350 | 0.204 | 132 | 94 | 6 | 13 | 136 | 2 | 130 | Uncharacterized protein | Uncharacterized protein | | afdb-uniprot50 | AF-U2BMK3-F1-MODEL\_V4 | 1.0 | 3.435e-08 | 348 | 0.2 | 135 | 91 | 6 | 13 | 136 | 6 | 134 | Uncharacterized protein | Uncharacterized protein | | afdb-uniprot50 | AF-A0A0P0A749-F1-MODEL\_V4 | 1.0 | 9.009e-08 | 348 | 0.198 | 126 | 90 | 3 | 13 | 133 | 1 | 120 | Uncharacterized protein | Uncharacterized protein | | afdb-uniprot50 | AF-A0A376P7R0-F1-MODEL\_V4 | 1.0 | 6.896e-07 | 347 | 0.294 | 112 | 79 | 0 | 13 | 124 | 1 | 112 | Tail fiber protein | Tail fiber protein | | afdb-uniprot50 | AF-A0A6L9HIG7-F1-MODEL\_V4 | 1.0 | 1.712e-08 | 347 | 0.187 | 144 | 102 | 7 | 6 | 136 | 35 | 176 | Phage tail protein | Phage tail protein | | afdb-uniprot50 | AF-A0A4U8UH88-F1-MODEL\_V4 | 1.0 | 3.626e-07 | 346 | 0.209 | 124 | 95 | 1 | 13 | 136 | 2 | 122 | Uncharacterized protein | Uncharacterized protein | | afdb-uniprot50 | AF-N2AGK0-F1-MODEL\_V4 | 1.0 | 1.311e-07 | 345 | 0.157 | 133 | 100 | 7 | 13 | 136 | 3 | 132 | Uncharacterized protein | Uncharacterized protein | | afdb-uniprot50 | AF-A0A2D3WLM9-F1-MODEL\_V4 | 1.0 | 2.362e-07 | 345 | 0.206 | 121 | 92 | 2 | 13 | 133 | 1 | 117 | Uncharacterized protein | Uncharacterized protein | | afdb-uniprot50 | AF-A0A212KXX9-F1-MODEL\_V4 | 1.0 | 1.539e-07 | 344 | 0.121 | 132 | 104 | 6 | 13 | 136 | 2 | 129 | Uncharacterized protein | Uncharacterized protein | | afdb-uniprot50 | AF-A0A5P9CRA6-F1-MODEL\_V4 | 1.0 | 2.63e-07 | 344 | 0.16 | 125 | 101 | 3 | 13 | 136 | 1 | 122 | Phage P2 GpU | Phage P2 GpU | | afdb-uniprot50 | AF-A0A1I7ND59-F1-MODEL\_V4 | 1.0 | 3.258e-07 | 343 | 0.25 | 104 | 78 | 0 | 33 | 136 | 2 | 105 | Uncharacterized protein | Uncharacterized protein | | afdb-uniprot50 | AF-A0A8B3A6D8-F1-MODEL\_V4 | 1.0 | 1.003e-07 | 343 | 0.181 | 132 | 97 | 6 | 13 | 136 | 1 | 129 | DNA-packaging protein | DNA-packaging protein | | afdb-uniprot50 | AF-E2CGG6-F1-MODEL\_V4 | 1.0 | 5.872e-07 | 343 | 0.133 | 127 | 107 | 2 | 13 | 136 | 2 | 128 | Gp45 | Gp45 | | afdb-uniprot50 | AF-A0A075WU95-F1-MODEL\_V4 | 1.0 | 1.311e-07 | 343 | 0.217 | 124 | 93 | 3 | 14 | 136 | 3 | 123 | Uncharacterized protein | Uncharacterized protein | | afdb-uniprot50 | AF-A0A377ICM6-F1-MODEL\_V4 | 1.0 | 3.824e-08 | 343 | 0.185 | 124 | 99 | 1 | 13 | 136 | 1 | 122 | Putative bacteriophage V tail protein | Putative bacteriophage V tail protein | | afdb-uniprot50 | AF-A0A3A9CSQ2-F1-MODEL\_V4 | 1.0 | 3.258e-07 | 342 | 0.186 | 129 | 99 | 5 | 13 | 136 | 2 | 129 | Phage tail protein | Phage tail protein | | afdb-uniprot50 | AF-A0A1H1FLQ9-F1-MODEL\_V4 | 1.0 | 3.258e-07 | 342 | 0.2 | 110 | 86 | 2 | 28 | 136 | 7 | 115 | Uncharacterized protein | Uncharacterized protein | | afdb-uniprot50 | AF-A0A7V6Q5B3-F1-MODEL\_V4 | 1.0 | 1.459e-07 | 342 | 0.181 | 132 | 97 | 6 | 13 | 136 | 2 | 130 | Uncharacterized protein | Uncharacterized protein | | afdb-uniprot50 | AF-A0A327Q841-F1-MODEL\_V4 | 1.0 | 6.195e-07 | 342 | 0.137 | 124 | 106 | 1 | 13 | 136 | 1 | 123 | GpU protein | GpU protein | | afdb-uniprot50 | AF-A0A165W2G2-F1-MODEL\_V4 | 1.0 | 1.807e-07 | 341 | 0.155 | 135 | 104 | 4 | 6 | 136 | 1 | 129 | Uncharacterized protein | Uncharacterized protein | | afdb-uniprot50 | AF-A0A6C1BSU7-F1-MODEL\_V4 | 1.0 | 1.178e-07 | 341 | 0.161 | 124 | 101 | 2 | 13 | 136 | 2 | 122 | Phage tail protein | Phage tail protein | | afdb-uniprot50 | AF-A0A450YW55-F1-MODEL\_V4 | 1.0 | 2.927e-07 | 341 | 0.169 | 124 | 101 | 1 | 13 | 136 | 2 | 123 | Phage P2 GpU | Phage P2 GpU | | afdb-uniprot50 | AF-A0A385Q1W0-F1-MODEL\_V4 | 1.0 | 1.807e-07 | 340 | 0.195 | 133 | 95 | 7 | 13 | 136 | 3 | 132 | Phage tail protein | Phage tail protein | | afdb-uniprot50 | AF-A0A413BFN5-F1-MODEL\_V4 | 1.0 | 1.459e-07 | 340 | 0.175 | 137 | 101 | 7 | 9 | 136 | 82 | 215 | Uncharacterized protein | Uncharacterized protein | | afdb-uniprot50 | AF-A0A239C7Y7-F1-MODEL\_V4 | 1.0 | 4.998e-08 | 340 | 0.169 | 142 | 102 | 6 | 4 | 136 | 170 | 304 | Phage P2 GpU | Phage P2 GpU | | afdb-uniprot50 | AF-A0A7C2IU81-F1-MODEL\_V4 | 1.0 | 7.675e-07 | 339 | 0.161 | 130 | 101 | 3 | 13 | 136 | 1 | 128 | Uncharacterized protein | Uncharacterized protein | | afdb-uniprot50 | AF-A0A3M4ZPG5-F1-MODEL\_V4 | 1.0 | 7.675e-07 | 338 | 0.404 | 84 | 50 | 0 | 53 | 136 | 8 | 91 | Uncharacterized protein | Uncharacterized protein | | afdb-uniprot50 | AF-A0A828ULQ4-F1-MODEL\_V4 | 1.0 | 1.624e-07 | 338 | 0.279 | 118 | 77 | 2 | 13 | 130 | 1 | 110 | Phage P2 GpU | Phage P2 GpU | | afdb-uniprot50 | AF-A0A5A8F1S4-F1-MODEL\_V4 | 1.0 | 2.012e-07 | 338 | 0.183 | 136 | 87 | 6 | 13 | 136 | 2 | 125 | Uncharacterized protein | Uncharacterized protein | | afdb-uniprot50 | AF-G4KPI6-F1-MODEL\_V4 | 1.0 | 1.242e-07 | 338 | 0.149 | 134 | 100 | 5 | 13 | 136 | 1 | 130 | Uncharacterized protein | Uncharacterized protein | | afdb-uniprot50 | AF-A0A1M6SJI7-F1-MODEL\_V4 | 1.0 | 7.671e-08 | 338 | 0.222 | 135 | 88 | 6 | 13 | 136 | 2 | 130 | Uncharacterized protein | Uncharacterized protein | | afdb-uniprot50 | AF-K4ZKW4-F1-MODEL\_V4 | 1.0 | 1.907e-07 | 337 | 0.204 | 132 | 94 | 6 | 13 | 136 | 1 | 129 | Phage P2 GpU | Phage P2 GpU | | afdb-uniprot50 | AF-A0A1Q6PU11-F1-MODEL\_V4 | 1.0 | 1.459e-07 | 337 | 0.2 | 135 | 91 | 5 | 13 | 136 | 2 | 130 | Uncharacterized protein | Uncharacterized protein | | afdb-uniprot50 | AF-A0A2A2ILD3-F1-MODEL\_V4 | 1.0 | 8.094e-08 | 337 | 0.223 | 134 | 89 | 4 | 13 | 136 | 2 | 130 | Uncharacterized protein | Uncharacterized protein | | afdb-uniprot50 | AF-A0A173SAB9-F1-MODEL\_V4 | 1.0 | 1.242e-07 | 337 | 0.164 | 146 | 105 | 6 | 2 | 136 | 19 | 158 | Phage protein U | Phage protein U | | afdb-uniprot50 | AF-W6THI8-F1-MODEL\_V4 | 1.0 | 4.258e-07 | 336 | 0.488 | 84 | 43 | 0 | 53 | 136 | 3 | 86 | Phage protein U | Phage protein U | | afdb-uniprot50 | AF-A0A2N2EIQ3-F1-MODEL\_V4 | 1.0 | 8.539e-08 | 336 | 0.143 | 132 | 102 | 6 | 13 | 136 | 1 | 129 | Uncharacterized protein | Uncharacterized protein | | afdb-uniprot50 | AF-A0A3P6KUM2-F1-MODEL\_V4 | 1.0 | 3.824e-08 | 336 | 0.171 | 140 | 105 | 6 | 5 | 136 | 31 | 167 | Phage protein U | Phage protein U | | afdb-uniprot50 | AF-A0A7M3MAX1-F1-MODEL\_V4 | 1.0 | 1.003e-06 | 335 | 0.157 | 127 | 102 | 3 | 13 | 136 | 2 | 126 | Uncharacterized protein | Uncharacterized protein | | afdb-uniprot50 | AF-A0A369Y010-F1-MODEL\_V4 | 1.0 | 2.63e-07 | 335 | 0.165 | 133 | 102 | 3 | 13 | 136 | 1 | 133 | Uncharacterized protein | Uncharacterized protein | | afdb-uniprot50 | AF-A0A3S0JS18-F1-MODEL\_V4 | 1.0 | 4.256e-08 | 334 | 0.189 | 132 | 96 | 6 | 13 | 136 | 1 | 129 | Phage tail protein | Phage tail protein | | afdb-uniprot50 | AF-A0A414ZQ39-F1-MODEL\_V4 | 1.0 | 5.273e-08 | 334 | 0.165 | 145 | 105 | 5 | 1 | 136 | 14 | 151 | Uncharacterized protein | Uncharacterized protein | | afdb-uniprot50 | AF-A0A482IL72-F1-MODEL\_V4 | 1.0 | 8.539e-08 | 334 | 0.185 | 124 | 99 | 1 | 13 | 136 | 2 | 123 | Phage tail protein | Phage tail protein | | afdb-uniprot50 | AF-A0A8B2E404-F1-MODEL\_V4 | 1.0 | 8.094e-08 | 334 | 0.196 | 127 | 94 | 2 | 13 | 136 | 1 | 122 | Phage tail protein | Phage tail protein | | afdb-uniprot50 | AF-A0A6N9P7S3-F1-MODEL\_V4 | 1.0 | 4.258e-07 | 333 | 0.152 | 131 | 103 | 6 | 13 | 136 | 2 | 131 | Phage tail protein | Phage tail protein | | afdb-uniprot50 | AF-A0A4P8G292-F1-MODEL\_V4 | 1.0 | 5e-07 | 332 | 0.201 | 109 | 83 | 2 | 29 | 136 | 27 | 132 | Uncharacterized protein | Uncharacterized protein | | afdb-uniprot50 | AF-J0QKB2-F1-MODEL\_V4 | 1.0 | 1.539e-07 | 332 | 0.223 | 112 | 81 | 2 | 31 | 136 | 16 | 127 | Uncharacterized protein | Uncharacterized protein | | afdb-uniprot50 | AF-A8SB39-F1-MODEL\_V4 | 1.0 | 2.492e-07 | 331 | 0.157 | 133 | 100 | 6 | 13 | 136 | 1 | 130 | Uncharacterized protein | Uncharacterized protein | | afdb-uniprot50 | AF-A0A7U6KE04-F1-MODEL\_V4 | 1.0 | 4.737e-08 | 331 | 0.185 | 135 | 94 | 5 | 13 | 136 | 50 | 179 | Uncharacterized protein | Uncharacterized protein | | afdb-uniprot50 | AF-A0A2N1QG54-F1-MODEL\_V4 | 1.0 | 1.539e-07 | 329 | 0.143 | 132 | 102 | 6 | 13 | 136 | 1 | 129 | Uncharacterized protein | Uncharacterized protein | | afdb-uniprot50 | AF-A0A3A9EU93-F1-MODEL\_V4 | 1.0 | 1.311e-07 | 329 | 0.159 | 132 | 100 | 6 | 13 | 136 | 3 | 131 | Uncharacterized protein | Uncharacterized protein | | afdb-uniprot50 | AF-A0A0C2YV44-F1-MODEL\_V4 | 1.0 | 1.907e-07 | 329 | 0.172 | 133 | 102 | 4 | 6 | 136 | 2 | 128 | Uncharacterized protein | Uncharacterized protein | | afdb-uniprot50 | AF-A0A7X5C9Z1-F1-MODEL\_V4 | 1.0 | 5.563e-08 | 329 | 0.149 | 134 | 103 | 6 | 11 | 136 | 41 | 171 | Phage tail protein | Phage tail protein | | afdb-uniprot50 | AF-A0A2E2N194-F1-MODEL\_V4 | 1.0 | 9.009e-08 | 328 | 0.212 | 132 | 89 | 5 | 13 | 135 | 1 | 126 | Uncharacterized protein | Uncharacterized protein | | afdb-uniprot50 | AF-A0A2S7JR76-F1-MODEL\_V4 | 1.0 | 2.492e-07 | 328 | 0.151 | 132 | 109 | 2 | 5 | 136 | 52 | 180 | Uncharacterized protein | Uncharacterized protein | | afdb-uniprot50 | AF-A0A3D4L0U5-F1-MODEL\_V4 | 1.0 | 6.195e-07 | 327 | 0.161 | 130 | 102 | 5 | 13 | 136 | 2 | 130 | Uncharacterized protein | Uncharacterized protein | | afdb-uniprot50 | AF-A0A5R8Y635-F1-MODEL\_V4 | 1.0 | 2.63e-07 | 327 | 0.181 | 127 | 98 | 3 | 13 | 136 | 3 | 126 | Uncharacterized protein | Uncharacterized protein | | afdb-uniprot50 | AF-A0A1A9G3Z3-F1-MODEL\_V4 | 1.0 | 7.275e-07 | 327 | 0.169 | 124 | 99 | 3 | 13 | 133 | 1 | 123 | Uncharacterized protein | Uncharacterized protein | | afdb-uniprot50 | AF-A0A064AGR8-F1-MODEL\_V4 | 1.0 | 6.195e-07 | 327 | 0.154 | 136 | 107 | 6 | 7 | 136 | 22 | 155 | Uncharacterized protein | Uncharacterized protein | | afdb-uniprot50 | AF-A0A1C0A2B2-F1-MODEL\_V4 | 1.0 | 8.094e-08 | 326 | 0.198 | 136 | 95 | 4 | 9 | 136 | 24 | 153 | Uncharacterized protein | Uncharacterized protein | | afdb-uniprot50 | AF-A0A416GNR4-F1-MODEL\_V4 | 1.0 | 3.826e-07 | 326 | 0.184 | 130 | 94 | 7 | 16 | 136 | 114 | 240 | SH3b domain-containing protein | SH3b domain-containing protein | | afdb-uniprot50 | AF-A0A6N9K7R1-F1-MODEL\_V4 | 1.0 | 2.362e-07 | 325 | 0.167 | 137 | 96 | 7 | 12 | 136 | 2 | 132 | Uncharacterized protein | Uncharacterized protein | | afdb-uniprot50 | AF-A0A3C1WUQ3-F1-MODEL\_V4 | 1.0 | 3.626e-07 | 325 | 0.176 | 136 | 99 | 6 | 13 | 136 | 3 | 137 | Uncharacterized protein | Uncharacterized protein | | afdb-uniprot50 | AF-A0A1V4IUT3-F1-MODEL\_V4 | 1.0 | 1.713e-07 | 324 | 0.181 | 132 | 97 | 6 | 13 | 136 | 1 | 129 | Phage P2 GpU | Phage P2 GpU | | afdb-uniprot50 | AF-A0A162QMP6-F1-MODEL\_V4 | 1.0 | 1.116e-07 | 324 | 0.198 | 131 | 94 | 6 | 13 | 135 | 1 | 128 | Putative peptidoglycan binding domain protein | Putative peptidoglycan binding domain protein | | afdb-uniprot50 | AF-A0A329UAJ4-F1-MODEL\_V4 | 1.0 | 9.509e-07 | 323 | 0.1 | 130 | 110 | 5 | 13 | 136 | 2 | 130 | Uncharacterized protein | Uncharacterized protein | | afdb-uniprot50 | AF-A0A7K1PMP8-F1-MODEL\_V4 | 1.0 | 9.509e-07 | 322 | 0.193 | 124 | 96 | 3 | 13 | 133 | 1 | 123 | Uncharacterized protein | Uncharacterized protein | | afdb-uniprot50 | AF-A0A5Y3VZG7-F1-MODEL\_V4 | 1.0 | 9.013e-07 | 322 | 0.184 | 125 | 96 | 3 | 13 | 135 | 1 | 121 | Uncharacterized protein | Uncharacterized protein | | afdb-uniprot50 | AF-A0A0X1U7T4-F1-MODEL\_V4 | 1.0 | 8.543e-07 | 321 | 0.166 | 132 | 99 | 6 | 13 | 136 | 1 | 129 | Phage P2 GpU | Phage P2 GpU | | afdb-uniprot50 | AF-A0A6N2ZLQ2-F1-MODEL\_V4 | 1.0 | 4.74e-07 | 321 | 0.192 | 135 | 98 | 6 | 10 | 136 | 56 | 187 | Phage P2 GpU | Phage P2 GpU | | afdb-uniprot50 | AF-I0GRI8-F1-MODEL\_V4 | 1.0 | 2.774e-07 | 320 | 0.222 | 144 | 90 | 7 | 6 | 136 | 28 | 162 | Putative phage protein | Putative phage protein | | afdb-uniprot50 | AF-A0A369RIA8-F1-MODEL\_V4 | 1.0 | 3.088e-07 | 319 | 0.321 | 115 | 73 | 1 | 14 | 128 | 1 | 110 | Phage protein U | Phage protein U | | afdb-uniprot50 | AF-A0A7C8KPW6-F1-MODEL\_V4 | 1.0 | 4.74e-07 | 319 | 0.212 | 132 | 93 | 6 | 13 | 136 | 3 | 131 | Phage tail protein | Phage tail protein | | afdb-uniprot50 | AF-A0A1B1NT78-F1-MODEL\_V4 | 1.0 | 3.437e-07 | 319 | 0.167 | 137 | 108 | 4 | 1 | 136 | 2 | 133 | Uncharacterized protein | Uncharacterized protein | | afdb-uniprot50 | AF-A0A261E7R4-F1-MODEL\_V4 | 1.0 | 2.012e-07 | 319 | 0.159 | 138 | 112 | 3 | 1 | 136 | 14 | 149 | Uncharacterized protein | Uncharacterized protein | | afdb-uniprot50 | AF-R7HX73-F1-MODEL\_V4 | 1.0 | 4.258e-07 | 319 | 0.143 | 146 | 109 | 5 | 2 | 136 | 52 | 192 | Uncharacterized protein | Uncharacterized protein | | afdb-uniprot50 | AF-W7S4G8-F1-MODEL\_V4 | 1.0 | 4.036e-07 | 319 | 0.183 | 131 | 96 | 6 | 13 | 135 | 3 | 130 | SH3b domain-containing protein | SH3b domain-containing protein | | afdb-uniprot50 | AF-A0A6L9GVJ5-F1-MODEL\_V4 | 1.0 | 4.492e-07 | 318 | 0.177 | 135 | 94 | 6 | 13 | 136 | 3 | 131 | Phage tail protein | Phage tail protein | | afdb-uniprot50 | AF-R5GZY0-F1-MODEL\_V4 | 1.0 | 4.036e-07 | 317 | 0.171 | 134 | 96 | 6 | 13 | 136 | 2 | 130 | p2 GpU family protein | p2 GpU family protein | | afdb-uniprot50 | AF-A0A1H7YIQ2-F1-MODEL\_V4 | 1.0 | 2.012e-07 | 317 | 0.125 | 135 | 101 | 6 | 13 | 136 | 1 | 129 | Phage P2 GpU | Phage P2 GpU | | afdb-uniprot50 | AF-A0A1T4PTF8-F1-MODEL\_V4 | 1.0 | 1.54e-06 | 317 | 0.121 | 132 | 110 | 4 | 10 | 136 | 28 | 158 | Phage P2 GpU | Phage P2 GpU | | afdb-uniprot50 | AF-A0A1Q6KUC1-F1-MODEL\_V4 | 1.0 | 1.907e-07 | 317 | 0.222 | 135 | 90 | 6 | 13 | 135 | 3 | 134 | LysM domain-containing protein | LysM domain-containing protein | | afdb-uniprot50 | AF-A0A4Q2LYV9-F1-MODEL\_V4 | 1.0 | 2.927e-07 | 316 | 0.152 | 131 | 102 | 5 | 13 | 136 | 1 | 129 | Phage tail protein | Phage tail protein | | afdb-uniprot50 | AF-A0A377A2A2-F1-MODEL\_V4 | 1.0 | 1.311e-06 | 316 | 0.322 | 96 | 65 | 0 | 41 | 136 | 1 | 96 | Tail fiber protein | Tail fiber protein | | afdb-uniprot50 | AF-A0A765XB11-F1-MODEL\_V4 | 1.0 | 3.828e-06 | 314 | 0.293 | 109 | 77 | 0 | 13 | 121 | 1 | 109 | Phage tail protein | Phage tail protein | | afdb-uniprot50 | AF-A0A4Q2B414-F1-MODEL\_V4 | 1.0 | 5.872e-07 | 314 | 0.155 | 135 | 97 | 6 | 13 | 136 | 1 | 129 | Phage tail protein | Phage tail protein | | afdb-uniprot50 | AF-A0A5D8QDT5-F1-MODEL\_V4 | 1.0 | 6.195e-07 | 314 | 0.115 | 130 | 108 | 5 | 13 | 136 | 2 | 130 | Uncharacterized protein | Uncharacterized protein | | afdb-uniprot50 | AF-A0A2V2FV54-F1-MODEL\_V4 | 1.0 | 1.003e-06 | 313 | 0.138 | 130 | 105 | 4 | 13 | 136 | 2 | 130 | Uncharacterized protein | Uncharacterized protein | | afdb-uniprot50 | AF-A0A316QBZ5-F1-MODEL\_V4 | 1.0 | 3.258e-07 | 313 | 0.17 | 135 | 95 | 6 | 13 | 136 | 2 | 130 | Uncharacterized protein | Uncharacterized protein | | afdb-uniprot50 | AF-A0A0P8WV76-F1-MODEL\_V4 | 1.0 | 4.036e-07 | 312 | 0.155 | 135 | 97 | 6 | 13 | 136 | 2 | 130 | Uncharacterized protein | Uncharacterized protein | | afdb-uniprot50 | AF-A0A078MHP7-F1-MODEL\_V4 | 1.0 | 3.258e-07 | 311 | 0.124 | 137 | 99 | 6 | 13 | 136 | 1 | 129 | Phage P2 GpU | Phage P2 GpU | | afdb-uniprot50 | AF-I9DAV5-F1-MODEL\_V4 | 1.0 | 4.036e-07 | 311 | 0.179 | 134 | 95 | 4 | 13 | 136 | 1 | 129 | p2 GpU family protein | p2 GpU family protein | | afdb-uniprot50 | AF-A0A7I9SHL0-F1-MODEL\_V4 | 1.0 | 2.494e-06 | 310 | 0.252 | 103 | 75 | 2 | 33 | 134 | 1 | 102 | Phage tail protein | Phage tail protein | | afdb-uniprot50 | AF-A0A4Q9TKB8-F1-MODEL\_V4 | 1.0 | 6.195e-07 | 310 | 0.143 | 132 | 102 | 6 | 13 | 136 | 1 | 129 | Phage tail protein | Phage tail protein | | afdb-uniprot50 | AF-A0A496P7D4-F1-MODEL\_V4 | 1.0 | 2.927e-07 | 310 | 0.2 | 135 | 91 | 6 | 13 | 136 | 2 | 130 | Phage tail protein | Phage tail protein | | afdb-uniprot50 | AF-R6LLD3-F1-MODEL\_V4 | 1.0 | 1.625e-06 | 309 | 0.148 | 135 | 98 | 5 | 13 | 136 | 2 | 130 | Uncharacterized protein | Uncharacterized protein | | afdb-uniprot50 | AF-K9D590-F1-MODEL\_V4 | 1.0 | 4.036e-07 | 309 | 0.198 | 131 | 96 | 5 | 13 | 136 | 2 | 130 | Uncharacterized protein | Uncharacterized protein | | afdb-uniprot50 | AF-A0A3R6V8J2-F1-MODEL\_V4 | 1.0 | 4.492e-07 | 308 | 0.151 | 132 | 101 | 6 | 13 | 136 | 1 | 129 | Uncharacterized protein | Uncharacterized protein | | afdb-uniprot50 | AF-A0A7T1BPS3-F1-MODEL\_V4 | 1.0 | 8.543e-07 | 308 | 0.153 | 124 | 103 | 2 | 13 | 136 | 9 | 130 | Phage tail protein | Phage tail protein | | afdb-uniprot50 | AF-E3HBK8-F1-MODEL\_V4 | 1.0 | 2.927e-07 | 308 | 0.165 | 133 | 96 | 5 | 13 | 136 | 1 | 127 | Uncharacterized protein | Uncharacterized protein | | afdb-uniprot50 | AF-A0A553SNJ4-F1-MODEL\_V4 | 1.0 | 3.437e-07 | 308 | 0.201 | 139 | 98 | 8 | 6 | 135 | 7 | 141 | Phage tail protein | Phage tail protein | | afdb-uniprot50 | AF-A0A6I2R3F1-F1-MODEL\_V4 | 1.0 | 6.896e-07 | 307 | 0.17 | 135 | 95 | 6 | 13 | 136 | 3 | 131 | Uncharacterized protein | Uncharacterized protein | | afdb-uniprot50 | AF-D1AFB3-F1-MODEL\_V4 | 1.0 | 4.492e-07 | 307 | 0.159 | 132 | 98 | 6 | 13 | 136 | 2 | 128 | Uncharacterized protein | Uncharacterized protein | | afdb-uniprot50 | AF-A0A198XEA5-F1-MODEL\_V4 | 1.0 | 3.437e-07 | 307 | 0.177 | 124 | 99 | 3 | 13 | 135 | 1 | 122 | Uncharacterized protein | Uncharacterized protein | | afdb-uniprot50 | AF-A0A2X0YZL3-F1-MODEL\_V4 | 1.0 | 6.195e-07 | 306 | 0.176 | 130 | 98 | 5 | 13 | 135 | 3 | 130 | N-acetylmuramoyl-L-alanine amidase | N-acetylmuramoyl-L-alanine amidase | | afdb-uniprot50 | AF-A0A1M6N828-F1-MODEL\_V4 | 1.0 | 1.117e-06 | 305 | 0.169 | 130 | 101 | 5 | 13 | 136 | 2 | 130 | Uncharacterized protein | Uncharacterized protein | | afdb-uniprot50 | AF-G2FT53-F1-MODEL\_V4 | 1.0 | 5.276e-07 | 305 | 0.11 | 136 | 103 | 6 | 13 | 136 | 2 | 131 | Uncharacterized protein | Uncharacterized protein | | afdb-uniprot50 | AF-A0A0J8DFL5-F1-MODEL\_V4 | 1.0 | 1.058e-06 | 304 | 0.143 | 132 | 102 | 7 | 13 | 136 | 2 | 130 | Uncharacterized protein | Uncharacterized protein | | afdb-uniprot50 | AF-A0A150MJG2-F1-MODEL\_V4 | 1.0 | 6.896e-07 | 304 | 0.183 | 131 | 96 | 6 | 13 | 135 | 3 | 130 | SH3b domain-containing protein | SH3b domain-containing protein | | afdb-uniprot50 | AF-B9NM79-F1-MODEL\_V4 | 1.0 | 2.631e-06 | 303 | 0.472 | 74 | 39 | 0 | 63 | 136 | 3 | 76 | Putative phage tail protein U | Putative phage tail protein U | | afdb-uniprot50 | AF-A0A7G9GXJ0-F1-MODEL\_V4 | 1.0 | 4.495e-06 | 303 | 0.131 | 129 | 106 | 3 | 13 | 136 | 24 | 151 | Phage tail protein | Phage tail protein | | afdb-uniprot50 | AF-E6LKB5-F1-MODEL\_V4 | 1.0 | 1.383e-06 | 302 | 0.143 | 132 | 102 | 8 | 13 | 136 | 3 | 131 | Uncharacterized protein | Uncharacterized protein | | afdb-uniprot50 | AF-A0A399IPH0-F1-MODEL\_V4 | 1.0 | 3.089e-06 | 302 | 0.145 | 131 | 104 | 5 | 13 | 136 | 2 | 131 | Uncharacterized protein | Uncharacterized protein | | afdb-uniprot50 | AF-A0A4U1VS70-F1-MODEL\_V4 | 1.0 | 1.311e-06 | 300 | 0.179 | 134 | 104 | 4 | 5 | 136 | 2 | 131 | Uncharacterized protein | Uncharacterized protein | | afdb-uniprot50 | AF-A0A1C6BMU7-F1-MODEL\_V4 | 1.0 | 3.828e-06 | 299 | 0.178 | 129 | 98 | 5 | 13 | 134 | 1 | 128 | Uncharacterized protein | Uncharacterized protein | | afdb-uniprot50 | AF-A0A6M0SNX6-F1-MODEL\_V4 | 1.0 | 8.543e-07 | 299 | 0.148 | 135 | 98 | 5 | 13 | 136 | 2 | 130 | Uncharacterized protein | Uncharacterized protein | | afdb-uniprot50 | AF-A0A7C9LIZ2-F1-MODEL\_V4 | 1.0 | 1.311e-06 | 297 | 0.133 | 135 | 100 | 6 | 13 | 136 | 1 | 129 | Uncharacterized protein | Uncharacterized protein | | afdb-uniprot50 | AF-A0A844Q469-F1-MODEL\_V4 | 1.0 | 7.275e-07 | 296 | 0.162 | 129 | 99 | 5 | 13 | 134 | 5 | 131 | Uncharacterized protein | Uncharacterized protein | | afdb-uniprot50 | AF-A0A2D3P2W1-F1-MODEL\_V4 | 1.0 | 4.038e-06 | 296 | 0.13 | 138 | 113 | 5 | 4 | 136 | 15 | 150 | Uncharacterized protein | Uncharacterized protein | | afdb-uniprot50 | AF-A0A3A6JMF6-F1-MODEL\_V4 | 1.0 | 1.625e-06 | 295 | 0.098 | 132 | 108 | 6 | 13 | 136 | 5 | 133 | Uncharacterized protein | Uncharacterized protein | | afdb-uniprot50 | AF-A0A3C1Q6J4-F1-MODEL\_V4 | 1.0 | 2.927e-07 | 295 | 0.16 | 143 | 102 | 6 | 6 | 136 | 23 | 159 | Uncharacterized protein | Uncharacterized protein | | afdb-uniprot50 | AF-A0A3G2Q678-F1-MODEL\_V4 | 1.0 | 7.275e-07 | 294 | 0.195 | 133 | 103 | 3 | 6 | 136 | 1 | 131 | Uncharacterized protein | Uncharacterized protein | | afdb-uniprot50 | AF-A0A699YKY6-F1-MODEL\_V4 | 1.0 | 5.003e-06 | 293 | 0.428 | 84 | 48 | 0 | 53 | 136 | 5 | 88 | Uncharacterized protein | Uncharacterized protein | | afdb-uniprot50 | AF-A0A0G3WAU0-F1-MODEL\_V4 | 1.0 | 4.74e-07 | 291 | 0.174 | 132 | 95 | 6 | 16 | 136 | 1 | 129 | Putative phage P2 GpU family protein | Putative phage P2 GpU family protein | | afdb-uniprot50 | AF-G9PUJ3-F1-MODEL\_V4 | 1.0 | 1.058e-06 | 290 | 0.186 | 118 | 84 | 3 | 25 | 136 | 9 | 120 | Uncharacterized protein | Uncharacterized protein | | afdb-uniprot50 | AF-G4KQ70-F1-MODEL\_V4 | 1.0 | 3.259e-06 | 290 | 0.113 | 132 | 106 | 7 | 13 | 136 | 2 | 130 | Uncharacterized protein | Uncharacterized protein | | afdb-uniprot50 | AF-A0A2W5H3R5-F1-MODEL\_V4 | 1.0 | 1.243e-06 | 289 | 0.37 | 100 | 62 | 1 | 16 | 114 | 1 | 100 | Uncharacterized protein | Uncharacterized protein | | afdb-uniprot50 | AF-U2PM82-F1-MODEL\_V4 | 1.0 | 4.038e-06 | 289 | 0.156 | 134 | 102 | 7 | 13 | 136 | 1 | 133 | Uncharacterized protein | Uncharacterized protein | | afdb-uniprot50 | AF-A0A285M8K9-F1-MODEL\_V4 | 1.0 | 1.54e-06 | 288 | 0.15 | 126 | 101 | 3 | 14 | 136 | 4 | 126 | Uncharacterized protein | Uncharacterized protein | | afdb-uniprot50 | AF-A0A379ZRU9-F1-MODEL\_V4 | 1.0 | 5.569e-06 | 288 | 0.342 | 76 | 50 | 0 | 61 | 136 | 51 | 126 | Phage tail protein | Phage tail protein | | afdb-uniprot50 | AF-A0A2X1LFE2-F1-MODEL\_V4 | 1.0 | 1.312e-05 | 287 | 0.312 | 96 | 66 | 0 | 13 | 108 | 1 | 96 | Tail fiber protein | Tail fiber protein | | afdb-uniprot50 | AF-A0A836RHU7-F1-MODEL\_V4 | 1.0 | 3.828e-06 | 287 | 0.135 | 133 | 100 | 5 | 13 | 136 | 3 | 129 | Uncharacterized protein | Uncharacterized protein | | afdb-uniprot50 | AF-A0A1Q6LL69-F1-MODEL\_V4 | 1.0 | 2.123e-06 | 287 | 0.155 | 122 | 96 | 4 | 18 | 136 | 36 | 153 | LysM domain-containing protein | LysM domain-containing protein | | afdb-uniprot50 | AF-A0A1Q6JPI8-F1-MODEL\_V4 | 1.0 | 1.178e-06 | 285 | 0.136 | 132 | 100 | 5 | 16 | 136 | 1 | 129 | Uncharacterized protein | Uncharacterized protein | | afdb-uniprot50 | AF-A0A2X1KFU1-F1-MODEL\_V4 | 1.0 | 5.875e-06 | 285 | 0.452 | 84 | 46 | 0 | 53 | 136 | 20 | 103 | GpU phage protein | GpU phage protein | | afdb-uniprot50 | AF-A0A3M3AZU3-F1-MODEL\_V4 | 1.0 | 1.715e-05 | 284 | 0.402 | 72 | 43 | 0 | 64 | 135 | 1 | 72 | Uncharacterized protein | Uncharacterized protein | | afdb-uniprot50 | AF-A0A3P6KJ42-F1-MODEL\_V4 | 1.0 | 2.24e-06 | 284 | 0.149 | 134 | 97 | 6 | 13 | 135 | 2 | 129 | Uncharacterized protein | Uncharacterized protein | | afdb-uniprot50 | AF-A0A0C1G7H4-F1-MODEL\_V4 | 1.0 | 6.899e-06 | 284 | 0.14 | 107 | 91 | 1 | 29 | 134 | 18 | 124 | Uncharacterized protein | Uncharacterized protein | | afdb-uniprot50 | AF-A0A644SV89-F1-MODEL\_V4 | 1.0 | 2.631e-06 | 283 | 0.164 | 134 | 97 | 5 | 13 | 136 | 1 | 129 | Uncharacterized protein | Uncharacterized protein | | afdb-uniprot50 | AF-A0A3D1M336-F1-MODEL\_V4 | 1.0 | 3.628e-06 | 283 | 0.185 | 113 | 87 | 2 | 29 | 136 | 84 | 196 | Uncharacterized protein | Uncharacterized protein | | afdb-uniprot50 | AF-A0A2V2GP09-F1-MODEL\_V4 | 1.0 | 1.54e-06 | 282 | 0.103 | 135 | 104 | 6 | 13 | 136 | 2 | 130 | Uncharacterized protein | Uncharacterized protein | | afdb-uniprot50 | AF-A0A827SX25-F1-MODEL\_V4 | 1.0 | 6.539e-06 | 281 | 0.355 | 90 | 58 | 0 | 47 | 136 | 1 | 90 | Phage tail protein | Phage tail protein | | afdb-uniprot50 | AF-A0A4R4E4X3-F1-MODEL\_V4 | 1.0 | 2.24e-06 | 281 | 0.125 | 135 | 101 | 6 | 13 | 136 | 2 | 130 | Uncharacterized protein | Uncharacterized protein | | afdb-uniprot50 | AF-A0A6D1AID6-F1-MODEL\_V4 | 1.0 | 1.179e-05 | 280 | 0.451 | 93 | 51 | 0 | 31 | 123 | 1 | 93 | Phage tail protein | Phage tail protein | | afdb-uniprot50 | AF-A0A2G6EZG3-F1-MODEL\_V4 | 1.0 | 1.541e-05 | 279 | 0.115 | 130 | 108 | 5 | 13 | 136 | 32 | 160 | Uncharacterized protein | Uncharacterized protein | | afdb-uniprot50 | AF-A0A7I8DHZ1-F1-MODEL\_V4 | 1.0 | 6.539e-06 | 278 | 0.139 | 129 | 103 | 5 | 13 | 136 | 2 | 127 | Uncharacterized protein | Uncharacterized protein | | afdb-uniprot50 | AF-A0A5S9NAT8-F1-MODEL\_V4 | 1.0 | 1.244e-05 | 277 | 0.14 | 128 | 102 | 4 | 13 | 133 | 1 | 127 | Uncharacterized protein | Uncharacterized protein | | afdb-uniprot50 | AF-A4P157-F1-MODEL\_V4 | 1.0 | 1.059e-05 | 274 | 0.319 | 97 | 65 | 1 | 9 | 104 | 2 | 98 | Probable phage-related tail protein | Probable phage-related tail protein | | afdb-uniprot50 | AF-A0A510JEI6-F1-MODEL\_V4 | 1.0 | 8.102e-06 | 271 | 0.156 | 134 | 102 | 7 | 13 | 136 | 1 | 133 | Uncharacterized protein | Uncharacterized protein | | afdb-uniprot50 | AF-A0A0S2F794-F1-MODEL\_V4 | 1.0 | 1.004e-05 | 265 | 0.373 | 91 | 55 | 2 | 47 | 136 | 2 | 91 | Phage P2 GpU family protein | Phage P2 GpU family protein | | afdb-uniprot50 | AF-A0A7G7ZA20-F1-MODEL\_V4 | 1.0 | 2.93e-05 | 264 | 0.379 | 79 | 49 | 0 | 58 | 136 | 13 | 91 | Phage tail protein | Phage tail protein | | afdb-uniprot50 | AF-A0A3C1LVB9-F1-MODEL\_V4 | 1.0 | 1.809e-05 | 264 | 0.125 | 128 | 102 | 4 | 10 | 136 | 1 | 119 | Uncharacterized protein | Uncharacterized protein | | afdb-uniprot50 | AF-A0A842IWC8-F1-MODEL\_V4 | 1.0 | 1.004e-05 | 264 | 0.142 | 133 | 100 | 5 | 13 | 136 | 29 | 156 | Phage tail protein | Phage tail protein | | afdb-uniprot50 | AF-A0A351VDG0-F1-MODEL\_V4 | 1.0 | 1.384e-05 | 263 | 0.121 | 140 | 100 | 6 | 13 | 136 | 3 | 135 | LysM domain-containing protein | LysM domain-containing protein | | afdb-uniprot50 | AF-A0A7X3VGF6-F1-MODEL\_V4 | 1.0 | 2.365e-05 | 261 | 0.457 | 70 | 38 | 0 | 67 | 136 | 4 | 73 | Uncharacterized protein | Uncharacterized protein | | afdb-uniprot50 | AF-A0A4D7AUF4-F1-MODEL\_V4 | 1.0 | 1.625e-05 | 261 | 0.178 | 112 | 78 | 4 | 33 | 136 | 2 | 107 | Phage tail protein | Phage tail protein | | afdb-uniprot50 | AF-A0A4S3LUR7-F1-MODEL\_V4 | 1.0 | 4.262e-05 | 259 | 0.405 | 79 | 47 | 0 | 58 | 136 | 1 | 79 | Oxidoreductase | Oxidoreductase | | afdb-uniprot50 | AF-A0A6N8HZ03-F1-MODEL\_V4 | 1.0 | 7.279e-06 | 259 | 0.119 | 134 | 106 | 6 | 13 | 136 | 2 | 133 | Uncharacterized protein | Uncharacterized protein | | afdb-uniprot50 | AF-A0A846M0R6-F1-MODEL\_V4 | 1.0 | 4.497e-05 | 258 | 0.392 | 79 | 48 | 0 | 58 | 136 | 2 | 80 | Phage protein U | Phage protein U | | afdb-uniprot50 | AF-A0A2B7YL04-F1-MODEL\_V4 | 1.0 | 2.632e-05 | 258 | 0.157 | 133 | 101 | 7 | 13 | 136 | 1 | 131 | Uncharacterized protein | Uncharacterized protein | | afdb-uniprot50 | AF-A0A132MI06-F1-MODEL\_V4 | 1.0 | 2.123e-06 | 258 | 0.165 | 145 | 103 | 8 | 1 | 135 | 20 | 156 | LysM domain-containing protein | LysM domain-containing protein | | afdb-uniprot50 | AF-A0A316S5B6-F1-MODEL\_V4 | 1.0 | 1.625e-05 | 257 | 0.205 | 112 | 75 | 4 | 33 | 136 | 2 | 107 | Uncharacterized protein | Uncharacterized protein | | afdb-uniprot50 | AF-V5TVJ3-F1-MODEL\_V4 | 1.0 | 4.262e-05 | 257 | 0.379 | 79 | 49 | 0 | 58 | 136 | 2 | 80 | Uncharacterized protein | Uncharacterized protein | | afdb-uniprot50 | AF-A0A6P0MS31-F1-MODEL\_V4 | 1.0 | 1.059e-05 | 257 | 0.134 | 134 | 102 | 6 | 13 | 136 | 3 | 132 | Uncharacterized protein | Uncharacterized protein | | afdb-uniprot50 | AF-A0A1F0F4I9-F1-MODEL\_V4 | 1.0 | 4.742e-06 | 257 | 0.177 | 141 | 96 | 6 | 5 | 136 | 8 | 137 | Uncharacterized protein | Uncharacterized protein | | afdb-uniprot50 | AF-A0A1I1D0Z6-F1-MODEL\_V4 | 1.0 | 3.439e-06 | 256 | 0.172 | 145 | 99 | 7 | 1 | 136 | 3 | 135 | Uncharacterized protein | Uncharacterized protein | | afdb-uniprot50 | AF-A0A1F0F528-F1-MODEL\_V4 | 1.0 | 6.198e-06 | 256 | 0.172 | 133 | 92 | 5 | 13 | 136 | 14 | 137 | Uncharacterized protein | Uncharacterized protein | | afdb-uniprot50 | AF-A0A6P0RQA4-F1-MODEL\_V4 | 1.0 | 6.539e-06 | 250 | 0.171 | 128 | 95 | 5 | 14 | 136 | 3 | 124 | Uncharacterized protein | Uncharacterized protein | | afdb-uniprot50 | AF-A0A4R4CZH4-F1-MODEL\_V4 | 1.0 | 1.244e-05 | 249 | 0.151 | 132 | 101 | 5 | 10 | 136 | 2 | 127 | LysM domain-containing protein | LysM domain-containing protein | | afdb-uniprot50 | AF-C4GFW6-F1-MODEL\_V4 | 1.0 | 4.038e-06 | 249 | 0.173 | 144 | 94 | 7 | 2 | 136 | 23 | 150 | Uncharacterized protein | Uncharacterized protein | | afdb-uniprot50 | AF-A0A1C5WXV0-F1-MODEL\_V4 | 1.0 | 3.261e-05 | 246 | 0.169 | 124 | 92 | 5 | 13 | 136 | 3 | 115 | LysM domain-containing protein | LysM domain-containing protein | | afdb-uniprot50 | AF-A0A011PPH1-F1-MODEL\_V4 | 1.0 | 1.625e-05 | 245 | 0.132 | 128 | 96 | 6 | 13 | 136 | 1 | 117 | Mu-like prophage DNA circulation protein | Mu-like prophage DNA circulation protein | | afdb-uniprot50 | AF-A0A1Q6PWR2-F1-MODEL\_V4 | 1.0 | 6.201e-05 | 245 | 0.116 | 129 | 108 | 4 | 13 | 136 | 3 | 130 | Uncharacterized protein | Uncharacterized protein | | afdb-uniprot50 | AF-A0A6L5BK85-F1-MODEL\_V4 | 1.0 | 5.281e-05 | 243 | 0.348 | 89 | 52 | 2 | 54 | 136 | 1 | 89 | Uncharacterized protein | Uncharacterized protein | | afdb-uniprot50 | AF-R9L5R6-F1-MODEL\_V4 | 1.0 | 1.004e-05 | 243 | 0.107 | 130 | 107 | 7 | 13 | 136 | 3 | 129 | Uncharacterized protein | Uncharacterized protein | | afdb-uniprot50 | AF-A0A3D4CGN3-F1-MODEL\_V4 | 1.0 | 5.571e-05 | 242 | 0.106 | 122 | 98 | 5 | 13 | 134 | 5 | 115 | Uncharacterized protein | Uncharacterized protein | | afdb-uniprot50 | AF-N8SB57-F1-MODEL\_V4 | 1.0 | 0.0001461 | 239 | 0.364 | 74 | 47 | 0 | 63 | 136 | 3 | 76 | Uncharacterized protein | Uncharacterized protein | | afdb-uniprot50 | AF-A0A6N2U2G7-F1-MODEL\_V4 | 1.0 | 4.04e-05 | 239 | 0.132 | 121 | 95 | 5 | 14 | 133 | 3 | 114 | Uncharacterized protein | Uncharacterized protein | | afdb-uniprot50 | AF-A0A5Q0GYR9-F1-MODEL\_V4 | 1.0 | 4.26e-06 | 239 | 0.207 | 140 | 93 | 5 | 13 | 136 | 6 | 143 | Peptidoglycan-binding protein | Peptidoglycan-binding protein | | afdb-uniprot50 | AF-R6UJW8-F1-MODEL\_V4 | 1.0 | 5.005e-05 | 238 | 0.112 | 125 | 100 | 5 | 10 | 134 | 1 | 114 | LysM domain-containing protein | LysM domain-containing protein | | afdb-uniprot50 | AF-A0A358PQ27-F1-MODEL\_V4 | 1.0 | 6.899e-06 | 238 | 0.143 | 146 | 110 | 10 | 1 | 136 | 1 | 141 | Uncharacterized protein | Uncharacterized protein | | afdb-uniprot50 | AF-A0A848D1N5-F1-MODEL\_V4 | 1.0 | 1.179e-05 | 238 | 0.144 | 145 | 101 | 7 | 10 | 136 | 1 | 140 | LysM peptidoglycan-binding domain-containing protein | LysM peptidoglycan-binding domain-containing protein | | afdb-uniprot50 | AF-A0A6N2T955-F1-MODEL\_V4 | 1.0 | 1.244e-05 | 238 | 0.149 | 134 | 95 | 7 | 14 | 136 | 4 | 129 | Uncharacterized protein | Uncharacterized protein | | afdb-uniprot50 | AF-A0A7G9WG84-F1-MODEL\_V4 | 1.0 | 6.201e-05 | 237 | 0.137 | 131 | 100 | 6 | 13 | 135 | 2 | 127 | Uncharacterized protein | Uncharacterized protein | | afdb-uniprot50 | AF-A0A6N6VMR6-F1-MODEL\_V4 | 1.0 | 1.715e-05 | 237 | 0.112 | 142 | 104 | 5 | 8 | 136 | 7 | 139 | Uncharacterized protein | Uncharacterized protein | | afdb-uniprot50 | AF-A0A1C6JGS8-F1-MODEL\_V4 | 1.0 | 3.829e-05 | 237 | 0.16 | 131 | 97 | 7 | 14 | 136 | 1 | 126 | Uncharacterized protein | Uncharacterized protein | | afdb-uniprot50 | AF-A0A842J060-F1-MODEL\_V4 | 1.0 | 3.091e-05 | 237 | 0.128 | 132 | 103 | 4 | 13 | 136 | 29 | 156 | Phage tail protein | Phage tail protein | | afdb-uniprot50 | AF-A0A7C6A8Q0-F1-MODEL\_V4 | 1.0 | 1.715e-05 | 237 | 0.184 | 125 | 88 | 7 | 13 | 136 | 1 | 112 | LysM domain-containing protein | LysM domain-containing protein | | afdb-uniprot50 | AF-A0A2T6G9C8-F1-MODEL\_V4 | 1.0 | 2.241e-05 | 235 | 0.138 | 137 | 100 | 4 | 13 | 136 | 1 | 132 | Terminase | Terminase | | afdb-uniprot50 | AF-A0A3S9SLX9-F1-MODEL\_V4 | 1.0 | 2.014e-05 | 235 | 0.165 | 139 | 96 | 6 | 7 | 136 | 20 | 147 | Uncharacterized protein | Uncharacterized protein | | afdb-uniprot50 | AF-A0A1H8DLN7-F1-MODEL\_V4 | 1.0 | 5.878e-05 | 234 | 0.123 | 121 | 95 | 5 | 14 | 134 | 4 | 113 | DNA circularisation protein N-terminus | DNA circularisation protein N-terminus | | afdb-uniprot50 | AF-A0A2X4V2I9-F1-MODEL\_V4 | 1.0 | 1.384e-05 | 234 | 0.145 | 158 | 103 | 6 | 2 | 136 | 6 | 154 | Uncharacterized protein | Uncharacterized protein | | afdb-uniprot50 | AF-A0A7Z0UY93-F1-MODEL\_V4 | 1.0 | 0.0001716 | 233 | 0.356 | 73 | 47 | 0 | 64 | 136 | 1 | 73 | Phage tail protein | Phage tail protein | | afdb-uniprot50 | AF-A0A7W0G3T0-F1-MODEL\_V4 | 1.0 | 2.241e-05 | 233 | 0.18 | 144 | 98 | 9 | 7 | 136 | 1 | 138 | Uncharacterized protein | Uncharacterized protein | | afdb-uniprot50 | AF-A0A7X3MI16-F1-MODEL\_V4 | 1.0 | 5.878e-05 | 232 | 0.093 | 128 | 108 | 6 | 14 | 136 | 1 | 125 | Uncharacterized protein | Uncharacterized protein | | afdb-uniprot50 | AF-A0A417HQS1-F1-MODEL\_V4 | 1.0 | 6.543e-05 | 231 | 0.122 | 131 | 104 | 7 | 13 | 136 | 3 | 129 | Uncharacterized protein | Uncharacterized protein | | afdb-uniprot50 | AF-A9I936-F1-MODEL\_V4 | 1.0 | 2.365e-05 | 231 | 0.131 | 145 | 107 | 7 | 1 | 136 | 1 | 135 | Uncharacterized protein | Uncharacterized protein | | afdb-uniprot50 | AF-A0A0P0RPN1-F1-MODEL\_V4 | 1.0 | 2.632e-05 | 230 | 0.129 | 124 | 93 | 3 | 28 | 136 | 28 | 151 | LysM domain | LysM domain | | afdb-uniprot50 | AF-A0A0F5EPQ5-F1-MODEL\_V4 | 1.0 | 4.744e-05 | 229 | 0.128 | 132 | 97 | 4 | 14 | 136 | 15 | 137 | Uncharacterized protein | Uncharacterized protein | | afdb-uniprot50 | AF-A0A2M8P9D4-F1-MODEL\_V4 | 1.0 | 2.632e-05 | 229 | 0.157 | 159 | 110 | 6 | 1 | 136 | 1 | 158 | Uncharacterized protein | Uncharacterized protein | | afdb-uniprot50 | AF-A0A2E8DR59-F1-MODEL\_V4 | 1.0 | 0.0001244 | 229 | 0.131 | 122 | 98 | 4 | 20 | 136 | 80 | 198 | Uncharacterized protein | Uncharacterized protein | | afdb-uniprot50 | AF-A0A174T7I7-F1-MODEL\_V4 | 1.0 | 3.261e-05 | 229 | 0.148 | 128 | 98 | 7 | 13 | 135 | 3 | 124 | Phage protein D | Phage protein D | | afdb-uniprot50 | AF-A0A2I0GV19-F1-MODEL\_V4 | 1.0 | 0.0002015 | 228 | 0.309 | 84 | 58 | 0 | 53 | 136 | 2 | 85 | Uncharacterized protein | Uncharacterized protein | | afdb-uniprot50 | AF-A0A562GHF5-F1-MODEL\_V4 | 1.0 | 3.261e-05 | 228 | 0.153 | 137 | 94 | 7 | 12 | 136 | 2 | 128 | Uncharacterized protein | Uncharacterized protein | | afdb-uniprot50 | AF-A0A7X1U0D0-F1-MODEL\_V4 | 1.0 | 2.632e-05 | 227 | 0.175 | 131 | 90 | 5 | 21 | 136 | 19 | 146 | LysM peptidoglycan-binding domain-containing protein | LysM peptidoglycan-binding domain-containing protein | | afdb-uniprot50 | AF-G1WGB9-F1-MODEL\_V4 | 1.0 | 1.625e-05 | 226 | 0.119 | 134 | 102 | 9 | 13 | 136 | 3 | 130 | Uncharacterized protein | Uncharacterized protein | | afdb-uniprot50 | AF-A0A412CI02-F1-MODEL\_V4 | 1.0 | 6.201e-05 | 226 | 0.15 | 146 | 105 | 7 | 2 | 136 | 17 | 154 | Uncharacterized protein | Uncharacterized protein | | afdb-uniprot50 | AF-A0A436RST7-F1-MODEL\_V4 | 1.0 | 0.0002778 | 225 | 0.232 | 99 | 75 | 1 | 13 | 111 | 1 | 98 | Phage tail protein | Phage tail protein | | afdb-uniprot50 | AF-A0A285NP30-F1-MODEL\_V4 | 1.0 | 8.106e-05 | 225 | 0.13 | 130 | 100 | 5 | 13 | 136 | 1 | 123 | Phage protein U | Phage protein U | | afdb-uniprot50 | AF-A0A2V4VHT5-F1-MODEL\_V4 | 1.0 | 2.125e-05 | 225 | 0.153 | 150 | 101 | 9 | 5 | 136 | 2 | 143 | LysM domain-containing protein | LysM domain-containing protein | | afdb-uniprot50 | AF-A0A630BA39-F1-MODEL\_V4 | 1.0 | 0.000191 | 224 | 0.215 | 88 | 69 | 0 | 47 | 134 | 2 | 89 | Phage tail protein | Phage tail protein | | afdb-uniprot50 | AF-A0A1H9SSE7-F1-MODEL\_V4 | 1.0 | 2.93e-05 | 224 | 0.148 | 141 | 102 | 7 | 10 | 136 | 1 | 137 | LysM domain-containing protein | LysM domain-containing protein | | afdb-uniprot50 | AF-A0A125V4N9-F1-MODEL\_V4 | 1.0 | 3.091e-05 | 223 | 0.127 | 133 | 101 | 4 | 13 | 136 | 3 | 129 | Phage-like element pbsx protein xkdP | Phage-like element pbsx protein xkdP | | afdb-uniprot50 | AF-A0A1W9V6L5-F1-MODEL\_V4 | 1.0 | 2.93e-05 | 223 | 0.115 | 147 | 104 | 7 | 6 | 136 | 1 | 137 | Uncharacterized protein | Uncharacterized protein | | afdb-uniprot50 | AF-A0A1H7MLA5-F1-MODEL\_V4 | 1.0 | 4.497e-05 | 223 | 0.13 | 123 | 92 | 4 | 29 | 136 | 34 | 156 | LysM domain-containing protein | LysM domain-containing protein | | afdb-uniprot50 | AF-A0A6L5WH99-F1-MODEL\_V4 | 1.0 | 0.0001461 | 222 | 0.239 | 92 | 70 | 0 | 43 | 134 | 3 | 94 | Oxidoreductase | Oxidoreductase | | afdb-uniprot50 | AF-A0A0P9CJQ9-F1-MODEL\_V4 | 1.0 | 0.0001059 | 222 | 0.321 | 87 | 57 | 1 | 50 | 134 | 2 | 88 | Uncharacterized protein | Uncharacterized protein | | afdb-uniprot50 | AF-A0A0H3GS01-F1-MODEL\_V4 | 1.0 | 0.0002126 | 222 | 0.424 | 73 | 42 | 0 | 64 | 136 | 1 | 73 | Putative prophage tail protein | Putative prophage tail protein | | afdb-uniprot50 | AF-R6N2M2-F1-MODEL\_V4 | 1.0 | 0.0001004 | 222 | 0.12 | 124 | 98 | 5 | 13 | 136 | 4 | 116 | LysM domain protein | LysM domain protein | | afdb-uniprot50 | AF-A0A2E8S0C4-F1-MODEL\_V4 | 1.0 | 4.497e-05 | 222 | 0.176 | 153 | 103 | 13 | 3 | 136 | 30 | 178 | LysM domain-containing protein | LysM domain-containing protein | | afdb-uniprot50 | AF-A0A1Y4MDW2-F1-MODEL\_V4 | 1.0 | 0.0001716 | 221 | 0.147 | 129 | 103 | 5 | 13 | 136 | 3 | 129 | Uncharacterized protein | Uncharacterized protein | | afdb-uniprot50 | AF-A0A143HGM4-F1-MODEL\_V4 | 1.0 | 5.878e-05 | 221 | 0.153 | 130 | 95 | 7 | 10 | 136 | 1 | 118 | LysM domain-containing protein | LysM domain-containing protein | | afdb-uniprot50 | AF-A0A5T2A9N3-F1-MODEL\_V4 | 1.0 | 0.0006204 | 220 | 0.252 | 107 | 76 | 3 | 13 | 118 | 1 | 104 | Phage tail protein | Phage tail protein | | afdb-uniprot50 | AF-A0A1W6WBE5-F1-MODEL\_V4 | 1.0 | 0.0001626 | 220 | 0.107 | 130 | 99 | 5 | 10 | 136 | 1 | 116 | LysM domain-containing protein | LysM domain-containing protein | | afdb-uniprot50 | AF-A0A3S4Z3K3-F1-MODEL\_V4 | 1.0 | 0.0001461 | 219 | 0.252 | 111 | 77 | 1 | 13 | 117 | 4 | 114 | Phage protein U | Phage protein U | | afdb-uniprot50 | AF-A0A075KG62-F1-MODEL\_V4 | 1.0 | 3.63e-05 | 218 | 0.133 | 142 | 105 | 5 | 1 | 136 | 45 | 174 | p2 GpU family protein | p2 GpU family protein | | afdb-uniprot50 | AF-V2YJG8-F1-MODEL\_V4 | 1.0 | 7.282e-05 | 218 | 0.104 | 125 | 95 | 5 | 13 | 134 | 4 | 114 | LysM domain-containing protein | LysM domain-containing protein | | afdb-uniprot50 | AF-A0A1R0FQC3-F1-MODEL\_V4 | 1.0 | 4.744e-05 | 218 | 0.081 | 159 | 116 | 7 | 1 | 136 | 13 | 164 | Uncharacterized protein | Uncharacterized protein | | afdb-uniprot50 | AF-A0A562HK82-F1-MODEL\_V4 | 1.0 | 6.903e-05 | 218 | 0.102 | 146 | 114 | 7 | 2 | 136 | 12 | 151 | LysM domain-containing protein | LysM domain-containing protein | | afdb-uniprot50 | AF-A0A1V5KAZ2-F1-MODEL\_V4 | 1.0 | 0.0001385 | 217 | 0.161 | 130 | 93 | 5 | 13 | 136 | 1 | 120 | Uncharacterized protein | Uncharacterized protein | | afdb-uniprot50 | AF-A0A1H7XXV0-F1-MODEL\_V4 | 1.0 | 3.261e-05 | 217 | 0.107 | 140 | 104 | 7 | 6 | 136 | 7 | 134 | Uncharacterized protein | Uncharacterized protein | | afdb-uniprot50 | AF-A0A4R6IUX9-F1-MODEL\_V4 | 1.0 | 3.829e-05 | 217 | 0.15 | 159 | 105 | 6 | 5 | 134 | 6 | 163 | LysM domain-containing protein | LysM domain-containing protein | | afdb-uniprot50 | AF-A0A6N7W5B6-F1-MODEL\_V4 | 1.0 | 6.903e-05 | 216 | 0.169 | 130 | 92 | 8 | 13 | 136 | 3 | 122 | Uncharacterized protein | Uncharacterized protein | | afdb-uniprot50 | AF-A0A416C480-F1-MODEL\_V4 | 1.0 | 8.106e-05 | 216 | 0.15 | 120 | 87 | 4 | 20 | 136 | 13 | 120 | Uncharacterized protein | Uncharacterized protein | | afdb-uniprot50 | AF-A0A5B8K6Q3-F1-MODEL\_V4 | 1.0 | 4.744e-05 | 216 | 0.158 | 139 | 94 | 7 | 13 | 136 | 1 | 131 | LysM peptidoglycan-binding domain-containing protein | LysM peptidoglycan-binding domain-containing protein | | afdb-uniprot50 | AF-A0A374UJF1-F1-MODEL\_V4 | 1.0 | 0.000181 | 215 | 0.133 | 127 | 95 | 5 | 10 | 134 | 1 | 114 | DNA\_circ\_N domain-containing protein | DNA\_circ\_N domain-containing protein | | afdb-uniprot50 | AF-A0A3M1D9D3-F1-MODEL\_V4 | 1.0 | 3.44e-05 | 215 | 0.123 | 154 | 109 | 5 | 6 | 136 | 2 | 152 | LysM peptidoglycan-binding domain-containing protein | LysM peptidoglycan-binding domain-containing protein | | afdb-uniprot50 | AF-A0A3N8BG80-F1-MODEL\_V4 | 1.0 | 7.683e-05 | 215 | 0.119 | 159 | 110 | 8 | 5 | 136 | 2 | 157 | Uncharacterized protein | Uncharacterized protein | | afdb-uniprot50 | AF-A0A6I4VAC5-F1-MODEL\_V4 | 1.0 | 0.0002366 | 215 | 0.08 | 125 | 103 | 4 | 13 | 136 | 18 | 131 | DNA circulation protein | DNA circulation protein | | afdb-uniprot50 | AF-A0A1V2YD69-F1-MODEL\_V4 | 1.0 | 5.571e-05 | 214 | 0.102 | 137 | 102 | 7 | 13 | 136 | 3 | 131 | Uncharacterized protein | Uncharacterized protein | | afdb-uniprot50 | AF-A0A6N9NZL1-F1-MODEL\_V4 | 1.0 | 9.519e-05 | 214 | 0.105 | 133 | 100 | 6 | 14 | 136 | 6 | 129 | Uncharacterized protein | Uncharacterized protein | | afdb-uniprot50 | AF-A0A3P3U9B4-F1-MODEL\_V4 | 1.0 | 5.878e-05 | 214 | 0.136 | 139 | 98 | 7 | 13 | 136 | 1 | 132 | LysM peptidoglycan-binding domain-containing protein | LysM peptidoglycan-binding domain-containing protein | | afdb-uniprot50 | AF-A0A2M8WDQ8-F1-MODEL\_V4 | 1.0 | 4.744e-05 | 213 | 0.261 | 107 | 72 | 1 | 37 | 136 | 2 | 108 | Uncharacterized protein | Uncharacterized protein | | afdb-uniprot50 | AF-A0A0S8GTR5-F1-MODEL\_V4 | 1.0 | 0.0001004 | 213 | 0.139 | 129 | 105 | 5 | 10 | 136 | 1 | 125 | Uncharacterized protein | Uncharacterized protein | | afdb-uniprot50 | AF-A0A497RM57-F1-MODEL\_V4 | 1.0 | 4.04e-05 | 213 | 0.151 | 132 | 85 | 6 | 13 | 136 | 1 | 113 | Uncharacterized protein | Uncharacterized protein | | afdb-uniprot50 | AF-A0A1Q6J5B4-F1-MODEL\_V4 | 1.0 | 0.0001244 | 213 | 0.106 | 131 | 98 | 7 | 10 | 136 | 1 | 116 | Uncharacterized protein | Uncharacterized protein | | afdb-uniprot50 | AF-A0A8B5L9S7-F1-MODEL\_V4 | 1.0 | 8.552e-05 | 213 | 0.135 | 133 | 100 | 4 | 13 | 136 | 3 | 129 | LysM peptidoglycan-binding domain-containing protein | LysM peptidoglycan-binding domain-containing protein | | afdb-uniprot50 | AF-A0A849CUH9-F1-MODEL\_V4 | 1.0 | 7.282e-05 | 211 | 0.116 | 129 | 99 | 8 | 13 | 136 | 3 | 121 | Uncharacterized protein | Uncharacterized protein | | afdb-uniprot50 | AF-A0A327JCS4-F1-MODEL\_V4 | 1.0 | 0.0004747 | 211 | 0.173 | 104 | 84 | 2 | 31 | 134 | 12 | 113 | Uncharacterized protein | Uncharacterized protein | | afdb-uniprot50 | AF-A0A1H1IJ14-F1-MODEL\_V4 | 1.0 | 9.519e-05 | 211 | 0.128 | 132 | 97 | 6 | 10 | 136 | 1 | 119 | Uncharacterized protein | Uncharacterized protein | | afdb-uniprot50 | AF-A0A542AM64-F1-MODEL\_V4 | 1.0 | 0.0005283 | 210 | 0.072 | 125 | 101 | 5 | 14 | 136 | 5 | 116 | LysM domain-containing protein | LysM domain-containing protein | | afdb-uniprot50 | AF-A0A352UR19-F1-MODEL\_V4 | 1.0 | 4.497e-05 | 210 | 0.133 | 142 | 100 | 10 | 13 | 136 | 2 | 138 | LysM domain-containing protein | LysM domain-containing protein | | afdb-uniprot50 | AF-A0A061NGS6-F1-MODEL\_V4 | 1.0 | 1.312e-05 | 210 | 0.147 | 136 | 100 | 6 | 13 | 136 | 8 | 139 | Phage-like element PBSX protein XkdP | Phage-like element PBSX protein XkdP | | afdb-uniprot50 | AF-A0A1Y4EYM2-F1-MODEL\_V4 | 1.0 | 0.0001059 | 210 | 0.124 | 137 | 105 | 7 | 2 | 134 | 53 | 178 | LysM domain-containing protein | LysM domain-containing protein | | afdb-uniprot50 | AF-A0A479ZZ86-F1-MODEL\_V4 | 1.0 | 6.543e-05 | 209 | 0.134 | 156 | 100 | 9 | 7 | 136 | 1 | 147 | Uncharacterized protein | Uncharacterized protein | | afdb-uniprot50 | AF-A0A8A7K8X4-F1-MODEL\_V4 | 1.0 | 0.0001385 | 209 | 0.104 | 125 | 100 | 5 | 14 | 136 | 3 | 117 | Uncharacterized protein | Uncharacterized protein | | afdb-uniprot50 | AF-A0A5D8QJ58-F1-MODEL\_V4 | 1.0 | 6.903e-05 | 209 | 0.132 | 136 | 96 | 9 | 13 | 136 | 5 | 130 | Uncharacterized protein | Uncharacterized protein | | afdb-uniprot50 | AF-C7RHB7-F1-MODEL\_V4 | 1.0 | 5.281e-05 | 209 | 0.128 | 132 | 101 | 5 | 6 | 136 | 1 | 119 | Uncharacterized protein | Uncharacterized protein | | afdb-uniprot50 | AF-A0A423PRP6-F1-MODEL\_V4 | 1.0 | 0.0001385 | 208 | 0.465 | 86 | 46 | 0 | 5 | 90 | 25 | 110 | Tail protein | Tail protein | | afdb-uniprot50 | AF-G9YSH0-F1-MODEL\_V4 | 1.0 | 0.0004265 | 208 | 0.1 | 119 | 96 | 4 | 16 | 134 | 2 | 109 | LysM domain protein | LysM domain protein | | afdb-uniprot50 | AF-C0ED24-F1-MODEL\_V4 | 1.0 | 6.903e-05 | 208 | 0.086 | 138 | 106 | 6 | 13 | 136 | 1 | 132 | LysM domain protein | LysM domain protein | | afdb-uniprot50 | AF-A0A5C7J2Y4-F1-MODEL\_V4 | 1.0 | 7.282e-05 | 208 | 0.095 | 157 | 112 | 7 | 1 | 136 | 3 | 150 | Uncharacterized protein | Uncharacterized protein | | afdb-uniprot50 | AF-A0A3D1ACM7-F1-MODEL\_V4 | 1.0 | 3.091e-05 | 208 | 0.125 | 128 | 99 | 4 | 21 | 136 | 12 | 138 | Peptidoglycan-binding protein | Peptidoglycan-binding protein | | afdb-uniprot50 | AF-A0A498CN55-F1-MODEL\_V4 | 1.0 | 0.0001461 | 207 | 0.114 | 131 | 102 | 5 | 13 | 136 | 6 | 129 | LysM peptidoglycan-binding domain-containing protein | LysM peptidoglycan-binding domain-containing protein | | afdb-uniprot50 | AF-D3EHD2-F1-MODEL\_V4 | 1.0 | 0.0001244 | 207 | 0.122 | 139 | 102 | 6 | 13 | 136 | 1 | 134 | Peptidoglycan-binding lysin domain protein | Peptidoglycan-binding lysin domain protein | | afdb-uniprot50 | AF-A0A497FQZ2-F1-MODEL\_V4 | 1.0 | 0.0001626 | 206 | 0.104 | 124 | 93 | 5 | 13 | 134 | 1 | 108 | Uncharacterized protein | Uncharacterized protein | | afdb-uniprot50 | AF-A0A661D5Z7-F1-MODEL\_V4 | 1.0 | 5.571e-05 | 206 | 0.118 | 152 | 109 | 7 | 7 | 136 | 1 | 149 | Uncharacterized protein | Uncharacterized protein | | afdb-uniprot50 | AF-A0A5P8WCD5-F1-MODEL\_V4 | 1.0 | 0.0001004 | 206 | 0.121 | 157 | 113 | 10 | 1 | 136 | 3 | 155 | Uncharacterized protein | Uncharacterized protein | | afdb-uniprot50 | AF-A0A2L2NSD1-F1-MODEL\_V4 | 1.0 | 3.44e-05 | 206 | 0.195 | 138 | 76 | 10 | 21 | 136 | 22 | 146 | Uncharacterized protein | Uncharacterized protein | | afdb-uniprot50 | AF-A0A6H2GWJ3-F1-MODEL\_V4 | 1.0 | 6.903e-05 | 206 | 0.136 | 147 | 97 | 7 | 10 | 136 | 1 | 137 | LysM peptidoglycan-binding domain-containing protein | LysM peptidoglycan-binding domain-containing protein | | afdb-uniprot50 | AF-D4LIC3-F1-MODEL\_V4 | 1.0 | 0.0001541 | 206 | 0.108 | 138 | 100 | 8 | 13 | 136 | 3 | 131 | LysM domain | LysM domain | | afdb-uniprot50 | AF-A0A2D5YZE5-F1-MODEL\_V4 | 1.0 | 9.022e-05 | 205 | 0.14 | 128 | 98 | 8 | 14 | 136 | 1 | 121 | Uncharacterized protein | Uncharacterized protein | | afdb-uniprot50 | AF-A0A3D3FCD8-F1-MODEL\_V4 | 1.0 | 0.0001541 | 205 | 0.1 | 130 | 100 | 6 | 10 | 136 | 1 | 116 | DNA\_circ\_N domain-containing protein | DNA\_circ\_N domain-containing protein | | afdb-uniprot50 | AF-L9WCV5-F1-MODEL\_V4 | 1.0 | 6.201e-05 | 205 | 0.115 | 138 | 110 | 6 | 1 | 135 | 1 | 129 | Uncharacterized protein | Uncharacterized protein | | afdb-uniprot50 | AF-A0A0S2ZJ55-F1-MODEL\_V4 | 1.0 | 0.0001244 | 205 | 0.104 | 134 | 104 | 6 | 6 | 136 | 3 | 123 | Uncharacterized protein | Uncharacterized protein | | afdb-uniprot50 | AF-A0A1X0SYI6-F1-MODEL\_V4 | 1.0 | 0.0004265 | 204 | 0.081 | 123 | 100 | 5 | 14 | 136 | 13 | 122 | DNA circulation family protein | DNA circulation family protein | | afdb-uniprot50 | AF-A0A2V2E9H6-F1-MODEL\_V4 | 1.0 | 0.0002243 | 204 | 0.12 | 125 | 99 | 4 | 14 | 136 | 4 | 119 | Uncharacterized protein | Uncharacterized protein | | afdb-uniprot50 | AF-A0A1C5SWE6-F1-MODEL\_V4 | 1.0 | 0.000191 | 204 | 0.088 | 136 | 106 | 5 | 14 | 136 | 4 | 134 | LysM domain/BON superfamily protein | LysM domain/BON superfamily protein | | afdb-uniprot50 | AF-A0A838RHN1-F1-MODEL\_V4 | 1.0 | 7.683e-05 | 204 | 0.102 | 147 | 107 | 8 | 13 | 136 | 9 | 153 | LysM peptidoglycan-binding domain-containing protein | LysM peptidoglycan-binding domain-containing protein | | afdb-uniprot50 | AF-A0A1H8U727-F1-MODEL\_V4 | 1.0 | 7.282e-05 | 204 | 0.112 | 160 | 108 | 11 | 3 | 136 | 40 | 191 | Uncharacterized protein | Uncharacterized protein | | afdb-uniprot50 | AF-A0A5E6YD38-F1-MODEL\_V4 | 1.0 | 0.0008556 | 203 | 0.308 | 94 | 58 | 1 | 10 | 96 | 3 | 96 | Uncharacterized protein | Uncharacterized protein | | afdb-uniprot50 | AF-A0A6M3M430-F1-MODEL\_V4 | 1.0 | 0.0001244 | 203 | 0.113 | 159 | 111 | 7 | 1 | 136 | 26 | 177 | Uncharacterized protein | Uncharacterized protein | | afdb-uniprot50 | AF-A0A845W825-F1-MODEL\_V4 | 1.0 | 2.632e-05 | 203 | 0.108 | 193 | 114 | 10 | 1 | 136 | 20 | 211 | LysM peptidoglycan-binding domain-containing protein | LysM peptidoglycan-binding domain-containing protein | | afdb-uniprot50 | AF-A0A349URD4-F1-MODEL\_V4 | 1.0 | 0.0001461 | 202 | 0.157 | 127 | 97 | 7 | 14 | 136 | 1 | 121 | Uncharacterized protein | Uncharacterized protein | | afdb-uniprot50 | AF-A0A3A6HVN4-F1-MODEL\_V4 | 1.0 | 0.0002366 | 202 | 0.139 | 129 | 96 | 4 | 13 | 136 | 1 | 119 | Uncharacterized protein | Uncharacterized protein | | afdb-uniprot50 | AF-A0A1C5ZE84-F1-MODEL\_V4 | 1.0 | 0.0004499 | 202 | 0.115 | 113 | 90 | 4 | 24 | 136 | 17 | 119 | Uncharacterized protein | Uncharacterized protein | | afdb-uniprot50 | AF-A0A0R2DDQ3-F1-MODEL\_V4 | 1.0 | 0.0001716 | 202 | 0.147 | 115 | 81 | 5 | 27 | 136 | 234 | 336 | Uncharacterized protein | Uncharacterized protein | | afdb-uniprot50 | AF-A0A2C8EVW5-F1-MODEL\_V4 | 1.0 | 0.001245 | 201 | 0.396 | 63 | 38 | 0 | 73 | 135 | 3 | 65 | Phage protein U | Phage protein U | | afdb-uniprot50 | AF-A0A7X0IVG1-F1-MODEL\_V4 | 1.0 | 9.022e-05 | 201 | 0.196 | 112 | 85 | 3 | 28 | 136 | 4 | 113 | Uncharacterized protein | Uncharacterized protein | | afdb-uniprot50 | AF-A0A1V5B941-F1-MODEL\_V4 | 1.0 | 3.829e-05 | 201 | 0.156 | 134 | 98 | 8 | 13 | 136 | 8 | 136 | LysM domain/BON superfamily protein | LysM domain/BON superfamily protein | | afdb-uniprot50 | AF-A0A562STB2-F1-MODEL\_V4 | 1.0 | 8.552e-05 | 201 | 0.166 | 120 | 86 | 4 | 29 | 134 | 30 | 149 | LysM domain-containing protein | LysM domain-containing protein | | afdb-uniprot50 | AF-A0A661PHT0-F1-MODEL\_V4 | 1.0 | 0.0002366 | 201 | 0.136 | 154 | 91 | 7 | 15 | 136 | 16 | 159 | Uncharacterized protein | Uncharacterized protein | | afdb-uniprot50 | AF-A0A1Z5IQW4-F1-MODEL\_V4 | 1.0 | 0.0002243 | 201 | 0.095 | 115 | 97 | 3 | 27 | 136 | 140 | 252 | LysM domain protein | LysM domain protein | | afdb-uniprot50 | AF-A0A0C1PPW5-F1-MODEL\_V4 | 1.0 | 0.0001626 | 201 | 0.068 | 131 | 109 | 5 | 11 | 136 | 219 | 341 | Muramidase (Flagellum-specific) | Muramidase (Flagellum-specific) | | afdb-uniprot50 | AF-A0A5C4LKH5-F1-MODEL\_V4 | 1.0 | 0.0005283 | 200 | 0.128 | 109 | 86 | 3 | 28 | 136 | 4 | 103 | Uncharacterized protein | Uncharacterized protein | | afdb-uniprot50 | AF-A0A4R2CQ90-F1-MODEL\_V4 | 1.0 | 0.0002778 | 200 | 0.161 | 136 | 95 | 7 | 10 | 136 | 1 | 126 | Uncharacterized protein | Uncharacterized protein | | afdb-uniprot50 | AF-A0A3B7MQB9-F1-MODEL\_V4 | 1.0 | 9.519e-05 | 200 | 0.111 | 143 | 99 | 5 | 21 | 136 | 32 | 173 | LysM peptidoglycan-binding domain-containing protein | LysM peptidoglycan-binding domain-containing protein | | afdb-uniprot50 | AF-A0A7W1N1F9-F1-MODEL\_V4 | 1.0 | 8.106e-05 | 200 | 0.107 | 168 | 102 | 9 | 7 | 136 | 1 | 158 | Uncharacterized protein | Uncharacterized protein | | afdb-uniprot50 | AF-A0A2E7P7M5-F1-MODEL\_V4 | 1.0 | 0.0001004 | 200 | 0.121 | 140 | 107 | 7 | 1 | 136 | 1 | 128 | DNA\_circ\_N domain-containing protein | DNA\_circ\_N domain-containing protein | | afdb-uniprot50 | AF-B0PCG8-F1-MODEL\_V4 | 1.0 | 0.0004265 | 199 | 0.119 | 126 | 93 | 6 | 16 | 136 | 2 | 114 | Uncharacterized protein | Uncharacterized protein | | afdb-uniprot50 | AF-A0A5C7QIA0-F1-MODEL\_V4 | 1.0 | 7.282e-05 | 199 | 0.143 | 132 | 94 | 8 | 14 | 136 | 1 | 122 | Uncharacterized protein | Uncharacterized protein | | afdb-uniprot50 | AF-A0A133ZRU1-F1-MODEL\_V4 | 1.0 | 0.0002633 | 199 | 0.126 | 134 | 97 | 7 | 14 | 136 | 8 | 132 | Uncharacterized protein | Uncharacterized protein | | afdb-uniprot50 | AF-A0A6M3XXM1-F1-MODEL\_V4 | 1.0 | 6.903e-05 | 198 | 0.106 | 141 | 108 | 8 | 6 | 136 | 3 | 135 | Uncharacterized protein | Uncharacterized protein | | afdb-uniprot50 | AF-A0A6L9ZDI7-F1-MODEL\_V4 | 1.0 | 8.106e-05 | 198 | 0.15 | 159 | 107 | 8 | 1 | 134 | 1 | 156 | LysM domain-containing protein | LysM domain-containing protein | | afdb-uniprot50 | AF-A0A416BNH2-F1-MODEL\_V4 | 1.0 | 0.0001385 | 198 | 0.092 | 130 | 105 | 5 | 14 | 136 | 4 | 127 | LysM peptidoglycan-binding domain-containing protein | LysM peptidoglycan-binding domain-containing protein | | afdb-uniprot50 | AF-A0A072R0Y2-F1-MODEL\_V4 | 1.0 | 0.001717 | 197 | 0.162 | 117 | 92 | 1 | 10 | 120 | 1 | 117 | Uncharacterized protein | Uncharacterized protein | | afdb-uniprot50 | AF-A0A6B2FIX0-F1-MODEL\_V4 | 1.0 | 0.0003831 | 197 | 0.07 | 142 | 107 | 5 | 11 | 136 | 9 | 141 | Uncharacterized protein | Uncharacterized protein | | afdb-uniprot50 | AF-A0A357B502-F1-MODEL\_V4 | 1.0 | 0.0007286 | 197 | 0.084 | 119 | 98 | 4 | 16 | 134 | 2 | 109 | Peptidoglycan-binding protein LysM | Peptidoglycan-binding protein LysM | | afdb-uniprot50 | AF-A0A7U0I7H4-F1-MODEL\_V4 | 1.0 | 0.0001179 | 196 | 0.158 | 151 | 94 | 8 | 13 | 136 | 4 | 148 | Uncharacterized protein | Uncharacterized protein | | afdb-uniprot50 | AF-A0A7S9CMN8-F1-MODEL\_V4 | 1.0 | 0.0002931 | 196 | 0.115 | 130 | 104 | 6 | 10 | 136 | 37 | 158 | Uncharacterized protein | Uncharacterized protein | | afdb-uniprot50 | AF-A0A2W6YVY0-F1-MODEL\_V4 | 1.0 | 0.0002778 | 196 | 0.1 | 129 | 99 | 7 | 11 | 136 | 3 | 117 | DNA\_circ\_N domain-containing protein | DNA\_circ\_N domain-containing protein | | afdb-uniprot50 | AF-A0A174LM59-F1-MODEL\_V4 | 1.0 | 0.000181 | 195 | 0.124 | 129 | 96 | 3 | 20 | 136 | 13 | 136 | LysM domain/BON superfamily protein | LysM domain/BON superfamily protein | | afdb-uniprot50 | AF-A0A412CG89-F1-MODEL\_V4 | 1.0 | 0.0004042 | 195 | 0.137 | 153 | 105 | 7 | 2 | 136 | 82 | 225 | Uncharacterized protein | Uncharacterized protein | | afdb-uniprot50 | AF-A0A140PTD7-F1-MODEL\_V4 | 1.0 | 0.0002778 | 194 | 0.116 | 129 | 98 | 6 | 11 | 136 | 17 | 132 | Uncharacterized protein | Uncharacterized protein | | afdb-uniprot50 | AF-M9LYN7-F1-MODEL\_V4 | 1.0 | 0.0004747 | 194 | 0.087 | 137 | 106 | 7 | 13 | 136 | 1 | 131 | LysM domain-containing protein | LysM domain-containing protein | | afdb-uniprot50 | AF-A0A246IW45-F1-MODEL\_V4 | 1.0 | 3.63e-05 | 194 | 0.176 | 147 | 98 | 6 | 13 | 136 | 9 | 155 | Uncharacterized protein | Uncharacterized protein | | afdb-uniprot50 | AF-A0A542ARR8-F1-MODEL\_V4 | 1.0 | 0.0002243 | 194 | 0.087 | 137 | 103 | 8 | 11 | 136 | 3 | 128 | Uncharacterized protein | Uncharacterized protein | | afdb-uniprot50 | AF-A0A3S5K2M8-F1-MODEL\_V4 | 1.0 | 7.683e-05 | 194 | 0.104 | 162 | 110 | 11 | 1 | 136 | 4 | 156 | Uncharacterized protein | Uncharacterized protein | | afdb-uniprot50 | AF-A0A7Z6QNR3-F1-MODEL\_V4 | 1.0 | 0.0002778 | 194 | 0.083 | 131 | 105 | 7 | 13 | 136 | 197 | 319 | LysM peptidoglycan-binding domain-containing protein | LysM peptidoglycan-binding domain-containing protein | | afdb-uniprot50 | AF-A0A2P5GVD3-F1-MODEL\_V4 | 1.0 | 0.0004265 | 193 | 0.164 | 134 | 99 | 7 | 6 | 136 | 1 | 124 | Uncharacterized protein | Uncharacterized protein | | afdb-uniprot50 | AF-A0A653QVV7-F1-MODEL\_V4 | 1.0 | 0.0001313 | 193 | 0.082 | 134 | 107 | 7 | 8 | 136 | 9 | 131 | Uncharacterized protein | Uncharacterized protein | | afdb-uniprot50 | AF-A0A1C6CLA3-F1-MODEL\_V4 | 1.0 | 0.0001461 | 193 | 0.159 | 138 | 98 | 8 | 10 | 136 | 1 | 131 | LysM domain/BON superfamily protein | LysM domain/BON superfamily protein | | afdb-uniprot50 | AF-A0A1B9AUD6-F1-MODEL\_V4 | 1.0 | 0.0001385 | 193 | 0.124 | 137 | 103 | 6 | 13 | 136 | 4 | 136 | LysM domain-containing protein | LysM domain-containing protein | | afdb-uniprot50 | AF-R8W901-F1-MODEL\_V4 | 1.0 | 0.0001118 | 193 | 0.147 | 142 | 96 | 7 | 14 | 136 | 3 | 138 | LysM domain-containing protein | LysM domain-containing protein | | afdb-uniprot50 | AF-A0A3N9F9V1-F1-MODEL\_V4 | 1.0 | 0.0003442 | 193 | 0.119 | 184 | 108 | 7 | 6 | 136 | 2 | 184 | Uncharacterized protein | Uncharacterized protein | | afdb-uniprot50 | AF-J4UC17-F1-MODEL\_V4 | 1.0 | 0.0002243 | 192 | 0.095 | 136 | 102 | 7 | 14 | 136 | 3 | 130 | Uncharacterized protein | Uncharacterized protein | | afdb-uniprot50 | AF-K1JXW6-F1-MODEL\_V4 | 1.0 | 0.0001004 | 192 | 0.122 | 131 | 99 | 7 | 13 | 136 | 3 | 124 | Uncharacterized protein | Uncharacterized protein | | afdb-uniprot50 | AF-A0A2P5KA36-F1-MODEL\_V4 | 1.0 | 0.0003093 | 192 | 0.148 | 141 | 103 | 8 | 1 | 136 | 2 | 130 | Uncharacterized protein | Uncharacterized protein | | afdb-uniprot50 | AF-A0A7X0Y1A5-F1-MODEL\_V4 | 1.0 | 0.0003632 | 192 | 0.094 | 127 | 96 | 5 | 14 | 136 | 1 | 112 | LysM domain-containing protein | LysM domain-containing protein | | afdb-uniprot50 | AF-A0A373VS92-F1-MODEL\_V4 | 1.0 | 0.0006204 | 192 | 0.111 | 135 | 101 | 7 | 14 | 136 | 1 | 128 | LysM domain-containing protein | LysM domain-containing protein | | afdb-uniprot50 | AF-A0A0N0E150-F1-MODEL\_V4 | 1.0 | 0.0005008 | 192 | 0.069 | 130 | 111 | 5 | 12 | 136 | 49 | 173 | Uncharacterized protein | Uncharacterized protein | | afdb-uniprot50 | AF-A0A849CT25-F1-MODEL\_V4 | 1.0 | 0.0006204 | 191 | 0.207 | 101 | 74 | 3 | 40 | 136 | 11 | 109 | Phage tail protein | Phage tail protein | | afdb-uniprot50 | AF-A0A413PG52-F1-MODEL\_V4 | 1.0 | 0.0003263 | 191 | 0.1 | 129 | 101 | 8 | 13 | 136 | 3 | 121 | Uncharacterized protein | Uncharacterized protein | | afdb-uniprot50 | AF-A0A176XP81-F1-MODEL\_V4 | 1.0 | 0.0006204 | 191 | 0.077 | 129 | 110 | 4 | 9 | 136 | 11 | 131 | Uncharacterized protein | Uncharacterized protein | | afdb-uniprot50 | AF-E5Y1X5-F1-MODEL\_V4 | 1.0 | 0.0004265 | 191 | 0.125 | 144 | 108 | 7 | 1 | 136 | 5 | 138 | Uncharacterized protein | Uncharacterized protein | | afdb-uniprot50 | AF-A0A6M8UDV6-F1-MODEL\_V4 | 1.0 | 0.0002126 | 191 | 0.113 | 158 | 108 | 10 | 1 | 136 | 6 | 153 | Uncharacterized protein | Uncharacterized protein | | afdb-uniprot50 | AF-C0Q8T6-F1-MODEL\_V4 | 1.0 | 6.543e-05 | 191 | 0.156 | 128 | 94 | 4 | 20 | 136 | 27 | 151 | LysM domain-containing protein | LysM domain-containing protein | | afdb-uniprot50 | AF-A0A193QH76-F1-MODEL\_V4 | 1.0 | 0.0003831 | 191 | 0.132 | 128 | 91 | 5 | 24 | 136 | 49 | 171 | Uncharacterized protein | Uncharacterized protein | | afdb-uniprot50 | AF-A0A0R2CKH2-F1-MODEL\_V4 | 1.0 | 0.0003263 | 191 | 0.089 | 112 | 93 | 5 | 27 | 136 | 143 | 247 | Uncharacterized protein | Uncharacterized protein | | afdb-uniprot50 | AF-A0A7C6NZD4-F1-MODEL\_V4 | 1.0 | 0.0002778 | 190 | 0.076 | 144 | 107 | 6 | 13 | 136 | 6 | 143 | LysM peptidoglycan-binding domain-containing protein | LysM peptidoglycan-binding domain-containing protein | | afdb-uniprot50 | AF-A0A350PIS1-F1-MODEL\_V4 | 1.0 | 0.0003442 | 190 | 0.092 | 163 | 113 | 7 | 5 | 133 | 17 | 178 | Uncharacterized protein | Uncharacterized protein | | afdb-uniprot50 | AF-A0A348HFQ1-F1-MODEL\_V4 | 1.0 | 0.0003632 | 190 | 0.094 | 127 | 108 | 5 | 13 | 136 | 61 | 183 | Glutamate decarboxylase | Glutamate decarboxylase | | afdb-uniprot50 | AF-Z9JLS4-F1-MODEL\_V4 | 1.0 | 0.0005283 | 189 | 0.328 | 73 | 49 | 0 | 64 | 136 | 1 | 73 | Uncharacterized protein | Uncharacterized protein | | afdb-uniprot50 | AF-A0A0F9D999-F1-MODEL\_V4 | 1.0 | 0.0004747 | 189 | 0.141 | 127 | 99 | 5 | 13 | 136 | 1 | 120 | Uncharacterized protein | Uncharacterized protein | | afdb-uniprot50 | AF-A0A7C7EU00-F1-MODEL\_V4 | 1.0 | 0.0005283 | 189 | 0.075 | 132 | 101 | 6 | 7 | 134 | 1 | 115 | LysM peptidoglycan-binding domain-containing protein | LysM peptidoglycan-binding domain-containing protein | | afdb-uniprot50 | AF-A0A5Q4Z738-F1-MODEL\_V4 | 1.0 | 0.0007286 | 189 | 0.112 | 116 | 91 | 4 | 28 | 136 | 25 | 135 | Uncharacterized protein | Uncharacterized protein | | afdb-uniprot50 | AF-A0A317H4C1-F1-MODEL\_V4 | 1.0 | 0.0002778 | 189 | 0.125 | 144 | 107 | 9 | 1 | 136 | 1 | 133 | DNA\_circ\_N domain-containing protein | DNA\_circ\_N domain-containing protein | | afdb-uniprot50 | AF-A0A174KNK1-F1-MODEL\_V4 | 1.0 | 0.0003093 | 189 | 0.1 | 140 | 103 | 8 | 11 | 136 | 3 | 133 | Uncharacterized protein | Uncharacterized protein | | afdb-uniprot50 | AF-A0A5M6IU86-F1-MODEL\_V4 | 1.0 | 0.0006906 | 189 | 0.135 | 133 | 101 | 6 | 4 | 132 | 18 | 140 | Uncharacterized protein | Uncharacterized protein | | afdb-uniprot50 | AF-A0A178TQB9-F1-MODEL\_V4 | 1.0 | 0.0002243 | 189 | 0.136 | 132 | 104 | 5 | 13 | 136 | 148 | 277 | Uncharacterized protein | Uncharacterized protein | | afdb-uniprot50 | AF-A0A2G5KZW3-F1-MODEL\_V4 | 1.0 | 0.0002633 | 188 | 0.074 | 147 | 107 | 7 | 13 | 136 | 2 | 142 | Uncharacterized protein | Uncharacterized protein | | afdb-uniprot50 | AF-A0A1H9HQS4-F1-MODEL\_V4 | 1.0 | 0.0008556 | 188 | 0.09 | 155 | 108 | 6 | 6 | 136 | 10 | 155 | Uncharacterized protein | Uncharacterized protein | | afdb-uniprot50 | AF-A0A410WT00-F1-MODEL\_V4 | 1.0 | 0.0003093 | 188 | 0.113 | 132 | 107 | 6 | 13 | 136 | 147 | 276 | Uncharacterized protein | Uncharacterized protein | | afdb-uniprot50 | AF-A0A0N0UTD2-F1-MODEL\_V4 | 1.0 | 0.0004499 | 188 | 0.085 | 128 | 103 | 7 | 14 | 136 | 188 | 306 | Uncharacterized protein | Uncharacterized protein | | afdb-uniprot50 | AF-A0A0G3CNH3-F1-MODEL\_V4 | 1.0 | 0.0004265 | 187 | 0.11 | 127 | 89 | 4 | 29 | 136 | 33 | 154 | Uncharacterized protein | Uncharacterized protein | | afdb-uniprot50 | AF-A0A1Q3NUK7-F1-MODEL\_V4 | 1.0 | 0.0006204 | 187 | 0.097 | 133 | 104 | 7 | 6 | 134 | 1 | 121 | DNA\_circ\_N domain-containing protein | DNA\_circ\_N domain-containing protein | | afdb-uniprot50 | AF-A0A763M6N7-F1-MODEL\_V4 | 1.0 | 0.001245 | 187 | 0.238 | 88 | 67 | 0 | 48 | 135 | 513 | 600 | Phage tail tape measure protein | Phage tail tape measure protein | | afdb-uniprot50 | AF-A0A3C0RSF2-F1-MODEL\_V4 | 1.0 | 0.002016 | 186 | 0.148 | 121 | 88 | 5 | 13 | 124 | 3 | 117 | Uncharacterized protein | Uncharacterized protein | | afdb-uniprot50 | AF-A0A133NAR5-F1-MODEL\_V4 | 1.0 | 0.0002015 | 186 | 0.145 | 131 | 92 | 5 | 11 | 136 | 21 | 136 | Uncharacterized protein | Uncharacterized protein | | afdb-uniprot50 | AF-A0A6N1B959-F1-MODEL\_V4 | 1.0 | 0.0005008 | 186 | 0.072 | 152 | 110 | 7 | 7 | 136 | 1 | 143 | Uncharacterized protein | Uncharacterized protein | | afdb-uniprot50 | AF-A0A1Z5IUN3-F1-MODEL\_V4 | 1.0 | 0.0005283 | 186 | 0.097 | 134 | 104 | 6 | 9 | 136 | 198 | 320 | LysM domain-containing protein | LysM domain-containing protein | | afdb-uniprot50 | AF-A0A3C0YSN0-F1-MODEL\_V4 | 1.0 | 0.0002931 | 185 | 0.093 | 128 | 105 | 4 | 13 | 136 | 1 | 121 | Uncharacterized protein | Uncharacterized protein | | afdb-uniprot50 | AF-A0A401X7L6-F1-MODEL\_V4 | 1.0 | 0.0004265 | 185 | 0.092 | 130 | 113 | 4 | 8 | 136 | 59 | 184 | Uncharacterized protein | Uncharacterized protein | | afdb-uniprot50 | AF-A0A3R6PJA9-F1-MODEL\_V4 | 1.0 | 0.0004265 | 185 | 0.129 | 139 | 103 | 7 | 8 | 136 | 23 | 153 | LysM peptidoglycan-binding domain-containing protein | LysM peptidoglycan-binding domain-containing protein | | afdb-uniprot50 | AF-R0EE86-F1-MODEL\_V4 | 1.0 | 0.0003093 | 185 | 0.12 | 150 | 104 | 10 | 4 | 136 | 28 | 166 | Mu-like prophage DNA circulation protein | Mu-like prophage DNA circulation protein | | afdb-uniprot50 | AF-A0A2V2A0Z3-F1-MODEL\_V4 | 1.0 | 0.003833 | 184 | 0.25 | 76 | 55 | 1 | 61 | 136 | 29 | 102 | GpU protein | GpU protein | | afdb-uniprot50 | AF-A0A317HDG5-F1-MODEL\_V4 | 1.0 | 0.0003263 | 184 | 0.117 | 145 | 108 | 6 | 1 | 136 | 16 | 149 | Uncharacterized protein | Uncharacterized protein | | afdb-uniprot50 | AF-A0A352S729-F1-MODEL\_V4 | 1.0 | 0.0003831 | 184 | 0.107 | 139 | 99 | 6 | 13 | 136 | 39 | 167 | DNA circulation family protein | DNA circulation family protein | | afdb-uniprot50 | AF-A0A5M6IYQ8-F1-MODEL\_V4 | 1.0 | 0.0007286 | 183 | 0.156 | 134 | 89 | 8 | 10 | 136 | 1 | 117 | Uncharacterized protein | Uncharacterized protein | | afdb-uniprot50 | AF-A0A661VCX6-F1-MODEL\_V4 | 1.0 | 0.0005283 | 183 | 0.121 | 123 | 96 | 6 | 13 | 133 | 7 | 119 | DNA\_circ\_N domain-containing protein | DNA\_circ\_N domain-containing protein | | afdb-uniprot50 | AF-A0A447N0F3-F1-MODEL\_V4 | 1.0 | 0.004044 | 182 | 0.378 | 82 | 51 | 0 | 13 | 94 | 1 | 82 | Gp25 | Gp25 | | afdb-uniprot50 | AF-A0A349YQ66-F1-MODEL\_V4 | 1.0 | 0.001118 | 182 | 0.088 | 124 | 100 | 5 | 14 | 136 | 1 | 112 | LysM domain-containing protein | LysM domain-containing protein | | afdb-uniprot50 | AF-A0A2E5S6T5-F1-MODEL\_V4 | 1.0 | 0.0003831 | 182 | 0.126 | 150 | 108 | 8 | 1 | 136 | 6 | 146 | Uncharacterized protein | Uncharacterized protein | | afdb-uniprot50 | AF-A0A4R7PWP3-F1-MODEL\_V4 | 1.0 | 0.001005 | 182 | 0.097 | 144 | 100 | 5 | 14 | 136 | 12 | 146 | Uncharacterized protein | Uncharacterized protein | | afdb-uniprot50 | AF-A0A3B7DR28-F1-MODEL\_V4 | 1.0 | 0.0004499 | 182 | 0.119 | 142 | 110 | 8 | 1 | 136 | 35 | 167 | Uncharacterized protein | Uncharacterized protein | | afdb-uniprot50 | AF-A0A2W4KCL7-F1-MODEL\_V4 | 1.0 | 0.001118 | 182 | 0.065 | 137 | 107 | 6 | 14 | 136 | 3 | 132 | Peptidoglycan-binding protein | Peptidoglycan-binding protein | | afdb-uniprot50 | AF-A0A661FPN4-F1-MODEL\_V4 | 1.0 | 0.0002633 | 182 | 0.138 | 130 | 90 | 4 | 29 | 136 | 38 | 167 | Peptidoglycan-binding protein | Peptidoglycan-binding protein | | afdb-uniprot50 | AF-A0A7C9KHI7-F1-MODEL\_V4 | 1.0 | 0.0003263 | 181 | 0.128 | 132 | 101 | 6 | 13 | 136 | 3 | 128 | Uncharacterized protein | Uncharacterized protein | | afdb-uniprot50 | AF-A0A3D3SD07-F1-MODEL\_V4 | 1.0 | 0.0006204 | 181 | 0.107 | 139 | 113 | 6 | 1 | 136 | 1 | 131 | Uncharacterized protein | Uncharacterized protein | | afdb-uniprot50 | AF-A0A2Z3I6W3-F1-MODEL\_V4 | 1.0 | 0.0002243 | 181 | 0.161 | 136 | 99 | 8 | 5 | 136 | 4 | 128 | Uncharacterized protein | Uncharacterized protein | | afdb-uniprot50 | AF-A0A0M0X770-F1-MODEL\_V4 | 1.0 | 0.0008556 | 181 | 0.137 | 131 | 96 | 5 | 10 | 136 | 2 | 119 | LysM domain-containing protein | LysM domain-containing protein | | afdb-uniprot50 | AF-A0A495RHL5-F1-MODEL\_V4 | 1.0 | 0.0007687 | 180 | 0.117 | 205 | 103 | 5 | 1 | 136 | 4 | 199 | Uncharacterized protein | Uncharacterized protein | | afdb-uniprot50 | AF-A0A2T6LTV8-F1-MODEL\_V4 | 1.0 | 0.001717 | 179 | 0.096 | 125 | 102 | 6 | 13 | 136 | 5 | 119 | Uncharacterized protein | Uncharacterized protein | | afdb-uniprot50 | AF-A0A2T0QI76-F1-MODEL\_V4 | 1.0 | 0.000811 | 179 | 0.094 | 137 | 101 | 6 | 14 | 136 | 27 | 154 | Uncharacterized protein | Uncharacterized protein | | afdb-uniprot50 | AF-A0A2G3Q549-F1-MODEL\_V4 | 1.0 | 0.0006204 | 179 | 0.11 | 127 | 96 | 5 | 23 | 136 | 10 | 132 | Peptidoglycan-binding protein | Peptidoglycan-binding protein | | afdb-uniprot50 | AF-A0A4P5VDU8-F1-MODEL\_V4 | 1.0 | 0.0001179 | 179 | 0.138 | 130 | 87 | 7 | 29 | 134 | 39 | 167 | Uncharacterized protein | Uncharacterized protein | | afdb-uniprot50 | AF-A0A2D9CHW4-F1-MODEL\_V4 | 1.0 | 0.001811 | 178 | 0.089 | 112 | 94 | 4 | 28 | 136 | 28 | 134 | Uncharacterized protein | Uncharacterized protein | | afdb-uniprot50 | AF-A0A2B7YZ51-F1-MODEL\_V4 | 1.0 | 0.0003263 | 178 | 0.121 | 132 | 100 | 6 | 8 | 136 | 5 | 123 | Uncharacterized protein | Uncharacterized protein | | afdb-uniprot50 | AF-A0A1H8DHX8-F1-MODEL\_V4 | 1.0 | 0.0007286 | 178 | 0.109 | 164 | 106 | 9 | 2 | 136 | 57 | 209 | Uncharacterized protein | Uncharacterized protein | | afdb-uniprot50 | AF-A0A0A0IRV2-F1-MODEL\_V4 | 1.0 | 0.0004265 | 178 | 0.141 | 134 | 87 | 10 | 12 | 136 | 2 | 116 | Uncharacterized protein | Uncharacterized protein | | afdb-uniprot50 | AF-A0A6L9ABV2-F1-MODEL\_V4 | 1.0 | 0.002016 | 177 | 0.229 | 96 | 73 | 1 | 13 | 108 | 1 | 95 | Uncharacterized protein | Uncharacterized protein | | afdb-uniprot50 | AF-A0A257VSV7-F1-MODEL\_V4 | 1.0 | 0.001386 | 177 | 0.094 | 127 | 102 | 4 | 14 | 136 | 18 | 135 | Uncharacterized protein | Uncharacterized protein | | afdb-uniprot50 | AF-A0A5Q2VGY7-F1-MODEL\_V4 | 1.0 | 0.0008556 | 177 | 0.097 | 164 | 110 | 8 | 1 | 136 | 1 | 154 | Uncharacterized protein | Uncharacterized protein | | afdb-uniprot50 | AF-A0A158D0R9-F1-MODEL\_V4 | 1.0 | 0.0006546 | 177 | 0.117 | 136 | 111 | 5 | 4 | 136 | 41 | 170 | Uncharacterized protein | Uncharacterized protein | | afdb-uniprot50 | AF-A0A843GUK1-F1-MODEL\_V4 | 1.0 | 0.0007286 | 176 | 0.102 | 136 | 104 | 6 | 10 | 136 | 60 | 186 | Uncharacterized protein | Uncharacterized protein | | afdb-uniprot50 | AF-A0A097R3F3-F1-MODEL\_V4 | 1.0 | 0.0006906 | 176 | 0.117 | 153 | 101 | 9 | 8 | 136 | 12 | 154 | Uncharacterized protein | Uncharacterized protein | | afdb-uniprot50 | AF-A0A239MZ86-F1-MODEL\_V4 | 1.0 | 0.0009027 | 175 | 0.145 | 137 | 108 | 5 | 4 | 136 | 41 | 172 | Uncharacterized protein | Uncharacterized protein | | afdb-uniprot50 | AF-A0A5M5NPH4-F1-MODEL\_V4 | 1.0 | 0.0001626 | 175 | 0.152 | 157 | 103 | 7 | 6 | 136 | 1 | 153 | LysM peptidoglycan-binding domain-containing protein | LysM peptidoglycan-binding domain-containing protein | | afdb-uniprot50 | AF-A0A0R2FPR3-F1-MODEL\_V4 | 1.0 | 0.0003831 | 175 | 0.104 | 143 | 90 | 8 | 27 | 136 | 20 | 157 | Muramidase (Flagellum-specific) | Muramidase (Flagellum-specific) | | afdb-uniprot50 | AF-A0A0M7MQ27-F1-MODEL\_V4 | 1.0 | 0.002367 | 174 | 0.081 | 110 | 95 | 4 | 28 | 136 | 63 | 167 | Uncharacterized protein | Uncharacterized protein | | afdb-uniprot50 | AF-A0A1E5QLG5-F1-MODEL\_V4 | 1.0 | 9.022e-05 | 172 | 0.174 | 132 | 89 | 9 | 20 | 136 | 15 | 141 | Uncharacterized protein | Uncharacterized protein | | afdb-uniprot50 | AF-A0A1Y4GLM1-F1-MODEL\_V4 | 1.0 | 0.0002778 | 172 | 0.108 | 138 | 104 | 10 | 7 | 136 | 1 | 127 | Uncharacterized protein | Uncharacterized protein | | afdb-uniprot50 | AF-R6NK38-F1-MODEL\_V4 | 1.0 | 0.0009523 | 172 | 0.094 | 138 | 100 | 7 | 14 | 136 | 7 | 134 | Uncharacterized protein | Uncharacterized protein | | afdb-uniprot50 | AF-A0A2N9X683-F1-MODEL\_V4 | 1.0 | 0.001627 | 172 | 0.131 | 129 | 99 | 7 | 13 | 136 | 41 | 161 | Uncharacterized protein | Uncharacterized protein | | afdb-uniprot50 | AF-A0A267S2K4-F1-MODEL\_V4 | 1.0 | 0.001811 | 172 | 0.129 | 124 | 87 | 5 | 28 | 136 | 48 | 165 | Uncharacterized protein | Uncharacterized protein | | afdb-uniprot50 | AF-A0A7C8AGL9-F1-MODEL\_V4 | 1.0 | 0.0006546 | 171 | 0.139 | 129 | 97 | 10 | 13 | 136 | 1 | 120 | Uncharacterized protein | Uncharacterized protein | | afdb-uniprot50 | AF-A0A661DK94-F1-MODEL\_V4 | 1.0 | 0.0006906 | 171 | 0.1 | 129 | 91 | 3 | 28 | 136 | 21 | 144 | Uncharacterized protein | Uncharacterized protein | | afdb-uniprot50 | AF-A0A212KEY3-F1-MODEL\_V4 | 1.0 | 0.001313 | 171 | 0.136 | 139 | 100 | 6 | 2 | 136 | 3 | 125 | Uncharacterized protein | Uncharacterized protein | | afdb-uniprot50 | AF-A0A371YK61-F1-MODEL\_V4 | 1.0 | 0.001313 | 171 | 0.097 | 113 | 89 | 4 | 28 | 136 | 38 | 141 | Uncharacterized protein | Uncharacterized protein | | afdb-uniprot50 | AF-A0A378E5K6-F1-MODEL\_V4 | 1.0 | 0.006549 | 170 | 0.311 | 77 | 53 | 0 | 16 | 92 | 4 | 80 | Phage tail protein | Phage tail protein | | afdb-uniprot50 | AF-A0A497FI72-F1-MODEL\_V4 | 1.0 | 0.0006204 | 170 | 0.109 | 128 | 103 | 4 | 13 | 136 | 1 | 121 | Uncharacterized protein | Uncharacterized protein | | afdb-uniprot50 | AF-G1UUM6-F1-MODEL\_V4 | 1.0 | 0.003264 | 170 | 0.129 | 139 | 104 | 7 | 1 | 136 | 1 | 125 | Uncharacterized protein | Uncharacterized protein | | afdb-uniprot50 | AF-A0A542BN30-F1-MODEL\_V4 | 1.0 | 0.002497 | 170 | 0.106 | 132 | 109 | 6 | 6 | 136 | 1 | 124 | Uncharacterized protein | Uncharacterized protein | | afdb-uniprot50 | AF-A0A7R6YQJ1-F1-MODEL\_V4 | 1.0 | 0.002244 | 170 | 0.093 | 129 | 112 | 3 | 8 | 136 | 20 | 143 | Uncharacterized protein | Uncharacterized protein | | afdb-uniprot50 | AF-A0A0B6S238-F1-MODEL\_V4 | 1.0 | 0.001386 | 170 | 0.089 | 146 | 101 | 6 | 14 | 136 | 15 | 151 | Uncharacterized protein | Uncharacterized protein | | afdb-uniprot50 | AF-A0A2E7CJN2-F1-MODEL\_V4 | 1.0 | 0.0004499 | 170 | 0.125 | 151 | 93 | 7 | 15 | 135 | 6 | 147 | LysM domain-containing protein | LysM domain-containing protein | | afdb-uniprot50 | AF-A0A7H9S354-F1-MODEL\_V4 | 1.0 | 0.00106 | 170 | 0.161 | 124 | 82 | 5 | 28 | 136 | 52 | 168 | Uncharacterized protein | Uncharacterized protein | | afdb-uniprot50 | AF-A0A6N8MHG0-F1-MODEL\_V4 | 1.0 | 0.0009523 | 170 | 0.144 | 125 | 83 | 7 | 28 | 136 | 73 | 189 | Uncharacterized protein | Uncharacterized protein | | afdb-uniprot50 | AF-A0A3D5P8C3-F1-MODEL\_V4 | 1.0 | 0.001462 | 170 | 0.057 | 139 | 108 | 6 | 14 | 136 | 3 | 134 | Uncharacterized protein | Uncharacterized protein | | afdb-uniprot50 | AF-A0A0S8DK82-F1-MODEL\_V4 | 1.0 | 0.0001716 | 169 | 0.133 | 165 | 104 | 10 | 6 | 136 | 1 | 160 | Uncharacterized protein | Uncharacterized protein | | afdb-uniprot50 | AF-A0A4D0QGZ7-F1-MODEL\_V4 | 1.0 | 0.0007286 | 169 | 0.079 | 139 | 103 | 6 | 14 | 136 | 12 | 141 | Uncharacterized protein | Uncharacterized protein | | afdb-uniprot50 | AF-A0A1V3IL17-F1-MODEL\_V4 | 1.0 | 0.002127 | 169 | 0.123 | 113 | 88 | 5 | 28 | 136 | 54 | 159 | Uncharacterized protein | Uncharacterized protein | | afdb-uniprot50 | AF-A0A1U9NH92-F1-MODEL\_V4 | 1.0 | 0.001811 | 168 | 0.079 | 126 | 106 | 5 | 14 | 136 | 3 | 121 | Uncharacterized protein | Uncharacterized protein | | afdb-uniprot50 | AF-A0A7T9IJ30-F1-MODEL\_V4 | 1.0 | 0.0005283 | 168 | 0.148 | 128 | 99 | 6 | 14 | 136 | 1 | 123 | Uncharacterized protein | Uncharacterized protein | | afdb-uniprot50 | AF-A0A5C7NN52-F1-MODEL\_V4 | 1.0 | 0.002367 | 168 | 0.083 | 132 | 103 | 7 | 13 | 136 | 2 | 123 | Uncharacterized protein | Uncharacterized protein | | afdb-uniprot50 | AF-A0A4V5MR96-F1-MODEL\_V4 | 1.0 | 0.001542 | 168 | 0.068 | 161 | 114 | 7 | 1 | 136 | 3 | 152 | Uncharacterized protein | Uncharacterized protein | | afdb-uniprot50 | AF-A0A3A9BCQ7-F1-MODEL\_V4 | 1.0 | 0.0006546 | 168 | 0.088 | 135 | 102 | 8 | 13 | 136 | 7 | 131 | LysM peptidoglycan-binding domain-containing protein | LysM peptidoglycan-binding domain-containing protein | | afdb-uniprot50 | AF-A0A354M6D3-F1-MODEL\_V4 | 1.0 | 0.0003263 | 168 | 0.13 | 161 | 100 | 9 | 2 | 136 | 65 | 211 | Uncharacterized protein | Uncharacterized protein | | afdb-uniprot50 | AF-A0A1H0K7P7-F1-MODEL\_V4 | 1.0 | 0.003264 | 168 | 0.1 | 140 | 112 | 6 | 1 | 136 | 3 | 132 | Uncharacterized protein | Uncharacterized protein | | afdb-uniprot50 | AF-A0A354WTT6-F1-MODEL\_V4 | 1.0 | 0.002635 | 167 | 0.09 | 121 | 93 | 4 | 28 | 136 | 69 | 184 | Uncharacterized protein | Uncharacterized protein | | afdb-uniprot50 | AF-A0A202DYA2-F1-MODEL\_V4 | 1.0 | 0.0002778 | 167 | 0.122 | 131 | 96 | 5 | 20 | 134 | 27 | 154 | LysM domain-containing protein | LysM domain-containing protein | | afdb-uniprot50 | AF-A0A6N3BSE7-F1-MODEL\_V4 | 1.0 | 0.002244 | 167 | 0.088 | 158 | 99 | 5 | 15 | 136 | 64 | 212 | Uncharacterized protein | Uncharacterized protein | | afdb-uniprot50 | AF-A0A0F9NHG4-F1-MODEL\_V4 | 1.0 | 0.001386 | 166 | 0.115 | 139 | 107 | 8 | 1 | 132 | 12 | 141 | Uncharacterized protein | Uncharacterized protein | | afdb-uniprot50 | AF-A0A1C7W148-F1-MODEL\_V4 | 1.0 | 0.002933 | 166 | 0.106 | 132 | 109 | 6 | 6 | 136 | 1 | 124 | Uncharacterized protein | Uncharacterized protein | | afdb-uniprot50 | AF-N6U770-F1-MODEL\_V4 | 1.0 | 0.00278 | 166 | 0.102 | 136 | 115 | 5 | 2 | 136 | 35 | 164 | Uncharacterized protein | Uncharacterized protein | | afdb-uniprot50 | AF-A0A379DMP2-F1-MODEL\_V4 | 1.0 | 0.001313 | 166 | 0.084 | 154 | 100 | 6 | 2 | 136 | 24 | 155 | Uncharacterized protein | Uncharacterized protein | | afdb-uniprot50 | AF-A0A7X8YZY7-F1-MODEL\_V4 | 1.0 | 0.0004499 | 166 | 0.112 | 133 | 103 | 6 | 11 | 134 | 149 | 275 | Uncharacterized protein | Uncharacterized protein | | afdb-uniprot50 | AF-A0A3G2V8Z8-F1-MODEL\_V4 | 1.0 | 0.001627 | 166 | 0.086 | 138 | 107 | 7 | 7 | 136 | 38 | 164 | Uncharacterized protein | Uncharacterized protein | | afdb-uniprot50 | AF-A0A494R194-F1-MODEL\_V4 | 1.0 | 0.003264 | 165 | 0.301 | 73 | 44 | 1 | 63 | 135 | 3 | 68 | Uncharacterized protein | Uncharacterized protein | | afdb-uniprot50 | AF-A0A366MWP2-F1-MODEL\_V4 | 1.0 | 0.002244 | 165 | 0.083 | 131 | 103 | 8 | 13 | 136 | 3 | 123 | Uncharacterized protein | Uncharacterized protein | | afdb-uniprot50 | AF-A0A2P1S0D5-F1-MODEL\_V4 | 1.0 | 0.001911 | 165 | 0.108 | 129 | 93 | 8 | 14 | 136 | 23 | 135 | Uncharacterized protein | Uncharacterized protein | | afdb-uniprot50 | AF-A0A3D0E3C9-F1-MODEL\_V4 | 1.0 | 0.001811 | 165 | 0.129 | 139 | 97 | 9 | 13 | 136 | 1 | 130 | LysM peptidoglycan-binding domain-containing protein | LysM peptidoglycan-binding domain-containing protein | | afdb-uniprot50 | AF-A0A060QFF2-F1-MODEL\_V4 | 1.0 | 0.004749 | 165 | 0.082 | 170 | 113 | 6 | 1 | 136 | 1 | 161 | Putative bacteriophage protein | Putative bacteriophage protein | | afdb-uniprot50 | AF-U2M6B1-F1-MODEL\_V4 | 1.0 | 0.0005008 | 165 | 0.119 | 168 | 113 | 9 | 3 | 136 | 165 | 331 | Uncharacterized protein | Uncharacterized protein | | afdb-uniprot50 | AF-A0A0F8XZK0-F1-MODEL\_V4 | 1.0 | 0.001245 | 164 | 0.105 | 151 | 108 | 8 | 5 | 136 | 3 | 145 | Uncharacterized protein | Uncharacterized protein | | afdb-uniprot50 | AF-H6U8J6-F1-MODEL\_V4 | 1.0 | 0.006549 | 164 | 0.224 | 89 | 67 | 2 | 48 | 135 | 3 | 90 | GpU | GpU | | afdb-uniprot50 | AF-A0A3M3XDC7-F1-MODEL\_V4 | 1.0 | 0.004749 | 163 | 0.095 | 178 | 106 | 6 | 1 | 136 | 1 | 165 | Uncharacterized protein | Uncharacterized protein | | afdb-uniprot50 | AF-A0A496CXI1-F1-MODEL\_V4 | 1.0 | 0.0002496 | 163 | 0.12 | 124 | 87 | 7 | 29 | 134 | 30 | 149 | LysM domain-containing protein | LysM domain-containing protein | | afdb-uniprot50 | AF-A0A7Y8YBS2-F1-MODEL\_V4 | 1.0 | 0.002933 | 163 | 0.046 | 151 | 120 | 6 | 4 | 136 | 20 | 164 | Uncharacterized protein | Uncharacterized protein | | afdb-uniprot50 | AF-A0A5C7BSU6-F1-MODEL\_V4 | 1.0 | 0.002016 | 162 | 0.107 | 130 | 90 | 5 | 28 | 136 | 22 | 146 | Uncharacterized protein | Uncharacterized protein | | afdb-uniprot50 | AF-A0A6B5QZN2-F1-MODEL\_V4 | 1.0 | 0.001386 | 162 | 0.082 | 145 | 105 | 6 | 8 | 136 | 9 | 141 | Uncharacterized protein | Uncharacterized protein | | afdb-uniprot50 | AF-A0A528TS09-F1-MODEL\_V4 | 1.0 | 0.003833 | 161 | 0.101 | 128 | 110 | 3 | 9 | 136 | 21 | 143 | Uncharacterized protein | Uncharacterized protein | | afdb-uniprot50 | AF-A0A661R841-F1-MODEL\_V4 | 1.0 | 0.001386 | 161 | 0.102 | 146 | 101 | 7 | 9 | 136 | 9 | 142 | Uncharacterized protein | Uncharacterized protein | | afdb-uniprot50 | AF-A0A1X0TE28-F1-MODEL\_V4 | 1.0 | 0.002497 | 161 | 0.13 | 130 | 96 | 7 | 16 | 136 | 40 | 161 | Uncharacterized protein | Uncharacterized protein | | afdb-uniprot50 | AF-H3K667-F1-MODEL\_V4 | 1.0 | 0.001118 | 161 | 0.069 | 144 | 115 | 7 | 2 | 136 | 18 | 151 | Uncharacterized protein | Uncharacterized protein | | afdb-uniprot50 | AF-A0A497RKB8-F1-MODEL\_V4 | 1.0 | 0.002016 | 160 | 0.187 | 133 | 90 | 7 | 13 | 136 | 5 | 128 | Uncharacterized protein | Uncharacterized protein | | afdb-uniprot50 | AF-I2IJR8-F1-MODEL\_V4 | 1.0 | 0.001717 | 160 | 0.109 | 146 | 114 | 8 | 1 | 136 | 33 | 172 | Uncharacterized protein | Uncharacterized protein | | afdb-uniprot50 | AF-A0A497PXB8-F1-MODEL\_V4 | 1.0 | 0.001462 | 160 | 0.085 | 117 | 89 | 5 | 26 | 132 | 74 | 182 | Uncharacterized protein | Uncharacterized protein | | afdb-uniprot50 | AF-A0A261SPV5-F1-MODEL\_V4 | 1.0 | 0.002635 | 159 | 0.087 | 114 | 90 | 6 | 28 | 136 | 6 | 110 | Uncharacterized protein | Uncharacterized protein | | afdb-uniprot50 | AF-J6H051-F1-MODEL\_V4 | 1.0 | 0.0006204 | 159 | 0.15 | 133 | 85 | 7 | 13 | 136 | 23 | 136 | Uncharacterized protein | Uncharacterized protein | | afdb-uniprot50 | AF-H0TLJ4-F1-MODEL\_V4 | 1.0 | 0.004501 | 159 | 0.12 | 133 | 110 | 6 | 5 | 136 | 30 | 156 | Uncharacterized protein | Uncharacterized protein | | afdb-uniprot50 | AF-A0A5B0HDI0-F1-MODEL\_V4 | 1.0 | 0.005884 | 159 | 0.1 | 109 | 95 | 1 | 28 | 136 | 53 | 158 | Uncharacterized protein | Uncharacterized protein | | afdb-uniprot50 | AF-A0A419QIV5-F1-MODEL\_V4 | 1.0 | 0.00118 | 159 | 0.168 | 125 | 87 | 6 | 13 | 133 | 4 | 115 | Uncharacterized protein | Uncharacterized protein | | afdb-uniprot50 | AF-A0A7C5SLD3-F1-MODEL\_V4 | 1.0 | 0.002635 | 158 | 0.106 | 131 | 105 | 7 | 13 | 136 | 1 | 126 | Uncharacterized protein | Uncharacterized protein | | afdb-uniprot50 | AF-A0A316J465-F1-MODEL\_V4 | 1.0 | 0.001313 | 158 | 0.116 | 146 | 106 | 9 | 2 | 136 | 22 | 155 | Uncharacterized protein | Uncharacterized protein | | afdb-uniprot50 | AF-A0A3E2KNQ6-F1-MODEL\_V4 | 1.0 | 0.002244 | 158 | 0.097 | 133 | 105 | 9 | 8 | 136 | 25 | 146 | Uncharacterized protein | Uncharacterized protein | | afdb-uniprot50 | AF-A0A261UEK8-F1-MODEL\_V4 | 1.0 | 0.001811 | 158 | 0.062 | 129 | 107 | 6 | 13 | 136 | 48 | 167 | Uncharacterized protein | Uncharacterized protein | | afdb-uniprot50 | AF-A0A6S7DDP2-F1-MODEL\_V4 | 1.0 | 0.006208 | 158 | 0.084 | 119 | 102 | 5 | 19 | 136 | 55 | 167 | Uncharacterized protein | Uncharacterized protein | | afdb-uniprot50 | AF-A0A2A7QBL7-F1-MODEL\_V4 | 1.0 | 0.002497 | 158 | 0.112 | 142 | 106 | 10 | 3 | 134 | 10 | 141 | Uncharacterized protein | Uncharacterized protein | | afdb-uniprot50 | AF-A0A0S8ER01-F1-MODEL\_V4 | 1.0 | 0.002933 | 158 | 0.133 | 127 | 98 | 6 | 15 | 136 | 162 | 281 | Uncharacterized protein | Uncharacterized protein | | afdb-uniprot50 | AF-A0A537KYH8-F1-MODEL\_V4 | 1.0 | 0.001245 | 157 | 0.116 | 146 | 106 | 7 | 6 | 133 | 15 | 155 | Uncharacterized protein | Uncharacterized protein | | afdb-uniprot50 | AF-A0A4Q3P5X9-F1-MODEL\_V4 | 1.0 | 0.002016 | 157 | 0.1 | 180 | 96 | 10 | 14 | 136 | 34 | 204 | Uncharacterized protein | Uncharacterized protein | | afdb-uniprot50 | AF-A0A2N0CX77-F1-MODEL\_V4 | 1.0 | 0.0004747 | 156 | 0.206 | 121 | 82 | 4 | 28 | 136 | 6 | 124 | Uncharacterized protein | Uncharacterized protein | | afdb-uniprot50 | AF-A0A6I7P5X6-F1-MODEL\_V4 | 1.0 | 0.000811 | 156 | 0.125 | 136 | 86 | 7 | 29 | 136 | 34 | 164 | Peptidoglycan-binding protein | Peptidoglycan-binding protein | | afdb-uniprot50 | AF-A0A7H1NU42-F1-MODEL\_V4 | 1.0 | 0.00278 | 156 | 0.086 | 116 | 92 | 5 | 26 | 136 | 83 | 189 | Uncharacterized protein | Uncharacterized protein | | afdb-uniprot50 | AF-A0A5C7M9Y1-F1-MODEL\_V4 | 1.0 | 0.003633 | 156 | 0.1 | 140 | 107 | 12 | 7 | 136 | 1 | 131 | Uncharacterized protein | Uncharacterized protein | | afdb-uniprot50 | AF-A0A3D0M341-F1-MODEL\_V4 | 1.0 | 0.003264 | 155 | 0.074 | 135 | 107 | 7 | 7 | 134 | 1 | 124 | Uncharacterized protein | Uncharacterized protein | | afdb-uniprot50 | AF-A0A1Y4D3Z5-F1-MODEL\_V4 | 1.0 | 0.001627 | 155 | 0.137 | 131 | 95 | 9 | 13 | 136 | 12 | 131 | Uncharacterized protein | Uncharacterized protein | | afdb-uniprot50 | AF-A0A6V8IG24-F1-MODEL\_V4 | 1.0 | 0.002933 | 155 | 0.074 | 148 | 108 | 7 | 13 | 136 | 56 | 198 | Uncharacterized protein | Uncharacterized protein | | afdb-uniprot50 | AF-W7DNG3-F1-MODEL\_V4 | 1.0 | 0.005577 | 155 | 0.096 | 114 | 90 | 4 | 27 | 136 | 98 | 202 | Uncharacterized protein | Uncharacterized protein | | afdb-uniprot50 | AF-A0A2T0BLH2-F1-MODEL\_V4 | 1.0 | 0.004044 | 155 | 0.08 | 125 | 108 | 6 | 14 | 133 | 3 | 125 | Phage tail protein | Phage tail protein | | afdb-uniprot50 | AF-A0A1Y2SAM4-F1-MODEL\_V4 | 1.0 | 0.00501 | 154 | 0.138 | 130 | 95 | 7 | 16 | 136 | 37 | 158 | Uncharacterized protein | Uncharacterized protein | | afdb-uniprot50 | AF-A0A561JSY6-F1-MODEL\_V4 | 1.0 | 0.001386 | 154 | 0.111 | 144 | 109 | 8 | 2 | 136 | 39 | 172 | Uncharacterized protein | Uncharacterized protein | | afdb-uniprot50 | AF-A0A7I7AC95-F1-MODEL\_V4 | 1.0 | 0.004501 | 154 | 0.128 | 125 | 85 | 8 | 28 | 136 | 55 | 171 | Uncharacterized protein | Uncharacterized protein | | afdb-uniprot50 | AF-A0A3S0C2K4-F1-MODEL\_V4 | 1.0 | 0.001313 | 153 | 0.146 | 116 | 83 | 6 | 27 | 136 | 42 | 147 | Uncharacterized protein | Uncharacterized protein | | afdb-uniprot50 | AF-A0A166CB51-F1-MODEL\_V4 | 1.0 | 0.003094 | 153 | 0.055 | 127 | 106 | 5 | 14 | 136 | 4 | 120 | Putative peptidoglycan binding domain protein | Putative peptidoglycan binding domain protein | | afdb-uniprot50 | AF-A0A1M7AAE4-F1-MODEL\_V4 | 1.0 | 0.0004747 | 153 | 0.157 | 146 | 89 | 6 | 20 | 134 | 35 | 177 | Uncharacterized protein | Uncharacterized protein | | afdb-uniprot50 | AF-A0A840C384-F1-MODEL\_V4 | 1.0 | 0.00856 | 152 | 0.061 | 130 | 109 | 4 | 11 | 136 | 12 | 132 | Uncharacterized protein | Uncharacterized protein | | afdb-uniprot50 | AF-A0A4P7WFQ6-F1-MODEL\_V4 | 1.0 | 0.0007286 | 152 | 0.097 | 133 | 94 | 5 | 25 | 136 | 69 | 196 | Uncharacterized protein | Uncharacterized protein | | afdb-uniprot50 | AF-A0A2J9H268-F1-MODEL\_V4 | 1.0 | 0.002497 | 152 | 0.088 | 159 | 94 | 6 | 20 | 136 | 57 | 206 | Uncharacterized protein | Uncharacterized protein | | afdb-uniprot50 | AF-A0A246Q0L6-F1-MODEL\_V4 | 1.0 | 0.007691 | 151 | 0.056 | 125 | 96 | 5 | 28 | 136 | 30 | 148 | Uncharacterized protein | Uncharacterized protein | | afdb-uniprot50 | AF-A0A7H1NTS9-F1-MODEL\_V4 | 1.0 | 0.006549 | 150 | 0.041 | 144 | 97 | 4 | 28 | 136 | 26 | 163 | Uncharacterized protein | Uncharacterized protein | | afdb-uniprot50 | AF-A0A0U5F1N5-F1-MODEL\_V4 | 1.0 | 0.0008556 | 150 | 0.073 | 150 | 108 | 8 | 13 | 136 | 55 | 199 | Burkholderia phage Bcep781 gp51 | Burkholderia phage Bcep781 gp51 | | afdb-uniprot50 | AF-M7CDH5-F1-MODEL\_V4 | 1.0 | 0.005286 | 150 | 0.116 | 129 | 99 | 6 | 16 | 136 | 86 | 207 | Uncharacterized protein | Uncharacterized protein | | afdb-uniprot50 | AF-A0A7X5QF72-F1-MODEL\_V4 | 1.0 | 0.005577 | 150 | 0.062 | 143 | 108 | 6 | 13 | 136 | 319 | 454 | Uncharacterized protein | Uncharacterized protein | | afdb-uniprot50 | AF-A0A0H3B1Y1-F1-MODEL\_V4 | 1.0 | 0.004749 | 149 | 0.081 | 135 | 107 | 9 | 7 | 136 | 1 | 123 | Uncharacterized protein | Uncharacterized protein | | afdb-uniprot50 | AF-A0A1J5RYQ4-F1-MODEL\_V4 | 1.0 | 0.006549 | 149 | 0.069 | 130 | 114 | 5 | 8 | 136 | 35 | 158 | Uncharacterized protein | Uncharacterized protein | | afdb-uniprot50 | AF-A0A420W5B5-F1-MODEL\_V4 | 1.0 | 0.003264 | 149 | 0.13 | 130 | 96 | 8 | 16 | 136 | 37 | 158 | Uncharacterized protein | Uncharacterized protein | | afdb-uniprot50 | AF-A0A1Q8E695-F1-MODEL\_V4 | 1.0 | 0.004267 | 149 | 0.075 | 132 | 106 | 8 | 14 | 136 | 4 | 128 | Uncharacterized protein | Uncharacterized protein | | afdb-uniprot50 | AF-A0A0C1PS50-F1-MODEL\_V4 | 1.0 | 0.002635 | 149 | 0.063 | 142 | 111 | 9 | 5 | 136 | 195 | 324 | N-acetylmuramoyl-L-alanine amidase, family 4 | N-acetylmuramoyl-L-alanine amidase, family 4 | | afdb-uniprot50 | AF-A0A1U7GIE9-F1-MODEL\_V4 | 1.0 | 0.003833 | 148 | 0.128 | 132 | 97 | 7 | 14 | 136 | 1 | 123 | Uncharacterized protein | Uncharacterized protein | | afdb-uniprot50 | AF-A0A0D6N7K5-F1-MODEL\_V4 | 1.0 | 0.003094 | 148 | 0.076 | 156 | 114 | 8 | 2 | 136 | 29 | 175 | Uncharacterized protein | Uncharacterized protein | | afdb-uniprot50 | AF-A0A3A1YWM1-F1-MODEL\_V4 | 1.0 | 0.00501 | 148 | 0.084 | 166 | 103 | 7 | 11 | 136 | 8 | 164 | Uncharacterized protein | Uncharacterized protein | | afdb-uniprot50 | AF-A0A3A9FPZ3-F1-MODEL\_V4 | 1.0 | 0.002635 | 148 | 0.142 | 140 | 108 | 8 | 1 | 132 | 8 | 143 | Uncharacterized protein | Uncharacterized protein | | afdb-uniprot50 | AF-A0A239HRH5-F1-MODEL\_V4 | 1.0 | 0.00856 | 147 | 0.1 | 110 | 87 | 4 | 30 | 136 | 12 | 112 | Phage-related protein | Phage-related protein | | afdb-uniprot50 | AF-A0A7X9XB50-F1-MODEL\_V4 | 1.0 | 0.001386 | 147 | 0.067 | 149 | 111 | 6 | 13 | 136 | 3 | 148 | Uncharacterized protein | Uncharacterized protein | | afdb-uniprot50 | AF-A0A4R1FNK5-F1-MODEL\_V4 | 1.0 | 0.003094 | 147 | 0.084 | 130 | 102 | 6 | 16 | 136 | 38 | 159 | Uncharacterized protein | Uncharacterized protein | | afdb-uniprot50 | AF-A0A563VQY9-F1-MODEL\_V4 | 1.0 | 0.0002243 | 147 | 0.161 | 130 | 90 | 6 | 21 | 136 | 22 | 146 | Rhs element Vgr protein (Modular protein) | Rhs element Vgr protein (Modular protein) | | afdb-uniprot50 | AF-A0A6A7KA75-F1-MODEL\_V4 | 1.0 | 0.002367 | 146 | 0.121 | 132 | 94 | 8 | 13 | 135 | 2 | 120 | Uncharacterized protein | Uncharacterized protein | | afdb-uniprot50 | AF-A0A4Z0RKI1-F1-MODEL\_V4 | 1.0 | 0.004044 | 146 | 0.103 | 116 | 93 | 5 | 25 | 136 | 12 | 120 | LysM domain-containing protein | LysM domain-containing protein | | afdb-uniprot50 | AF-A0A2D8LFG3-F1-MODEL\_V4 | 1.0 | 0.002016 | 144 | 0.063 | 141 | 107 | 9 | 7 | 136 | 1 | 127 | Uncharacterized protein | Uncharacterized protein | | afdb-uniprot50 | AF-A0A346PHI6-F1-MODEL\_V4 | 1.0 | 0.009031 | 144 | 0.131 | 114 | 86 | 6 | 29 | 136 | 14 | 120 | Phage associated protein | Phage associated protein | | afdb-uniprot50 | AF-A0A379S622-F1-MODEL\_V4 | 1.0 | 0.004267 | 143 | 0.066 | 150 | 112 | 11 | 6 | 136 | 19 | 159 | Uncharacterized protein | Uncharacterized protein | | afdb-uniprot50 | AF-A0A1B9Y3L0-F1-MODEL\_V4 | 1.0 | 0.0009027 | 142 | 0.104 | 143 | 97 | 8 | 7 | 136 | 5 | 129 | Uncharacterized protein | Uncharacterized protein | | afdb-uniprot50 | AF-A0A3C1Y293-F1-MODEL\_V4 | 1.0 | 0.005884 | 142 | 0.145 | 117 | 86 | 6 | 25 | 136 | 11 | 118 | Uncharacterized protein | Uncharacterized protein | | afdb-uniprot50 | AF-A0A1V6D5U1-F1-MODEL\_V4 | 1.0 | 0.009528 | 141 | 0.069 | 130 | 103 | 11 | 14 | 136 | 4 | 122 | Uncharacterized protein | Uncharacterized protein | | afdb-uniprot50 | AF-A0A257QIG8-F1-MODEL\_V4 | 1.0 | 0.006909 | 140 | 0.105 | 151 | 108 | 9 | 2 | 136 | 31 | 170 | Uncharacterized protein | Uncharacterized protein | | afdb-uniprot50 | AF-A0A654D8R3-F1-MODEL\_V4 | 1.0 | 0.002127 | 139 | 0.091 | 142 | 112 | 10 | 3 | 136 | 2 | 134 | Uncharacterized protein | Uncharacterized protein | | afdb-uniprot50 | AF-A0A412UZK8-F1-MODEL\_V4 | 1.0 | 0.00118 | 139 | 0.137 | 124 | 87 | 5 | 18 | 134 | 18 | 128 | Uncharacterized protein | Uncharacterized protein | | afdb-uniprot50 | AF-A0A6M3IHI8-F1-MODEL\_V4 | 1.0 | 0.003633 | 139 | 0.116 | 112 | 83 | 5 | 30 | 136 | 148 | 248 | Uncharacterized protein | Uncharacterized protein | | afdb-uniprot50 | AF-A0A3A2J2V4-F1-MODEL\_V4 | 1.0 | 0.00856 | 138 | 0.094 | 127 | 97 | 8 | 20 | 133 | 39 | 160 | Uncharacterized protein | Uncharacterized protein | | afdb-uniprot50 | AF-A0A1H0N722-F1-MODEL\_V4 | 1.0 | 0.005884 | 138 | 0.073 | 123 | 106 | 5 | 16 | 134 | 2 | 120 | Phage-related protein | Phage-related protein | | afdb-uniprot50 | AF-A0A2L0WYZ1-F1-MODEL\_V4 | 1.0 | 0.006549 | 137 | 0.115 | 130 | 99 | 8 | 15 | 136 | 52 | 173 | Uncharacterized protein | Uncharacterized protein | | afdb-uniprot50 | AF-A0A7Y7ISW7-F1-MODEL\_V4 | 1.0 | 0.007691 | 137 | 0.067 | 163 | 119 | 10 | 2 | 136 | 32 | 189 | Uncharacterized protein | Uncharacterized protein | | afdb-uniprot50 | AF-A0A270BII5-F1-MODEL\_V4 | 1.0 | 0.003444 | 136 | 0.08 | 137 | 97 | 5 | 25 | 136 | 68 | 200 | Uncharacterized protein | Uncharacterized protein | | afdb-uniprot50 | AF-A0A644T6N0-F1-MODEL\_V4 | 1.0 | 0.001245 | 136 | 0.129 | 155 | 95 | 9 | 12 | 136 | 8 | 152 | Uncharacterized protein | Uncharacterized protein | | afdb-uniprot50 | AF-A0A497RPK8-F1-MODEL\_V4 | 1.0 | 0.009528 | 136 | 0.107 | 112 | 86 | 4 | 29 | 132 | 123 | 228 | Uncharacterized protein | Uncharacterized protein | | afdb-uniprot50 | AF-A0A6L4BC15-F1-MODEL\_V4 | 1.0 | 0.009528 | 135 | 0.08 | 112 | 94 | 4 | 30 | 136 | 13 | 120 | Phage tail protein | Phage tail protein | | afdb-uniprot50 | AF-A0A1M5UNR8-F1-MODEL\_V4 | 1.0 | 0.0007687 | 135 | 0.101 | 158 | 95 | 8 | 21 | 136 | 25 | 177 | LysM domain-containing protein | LysM domain-containing protein | | afdb-uniprot50 | AF-A0A4Y3TK87-F1-MODEL\_V4 | 1.0 | 0.00729 | 135 | 0.071 | 140 | 100 | 6 | 23 | 136 | 65 | 200 | Uncharacterized protein | Uncharacterized protein | | afdb-uniprot50 | AF-X8IK28-F1-MODEL\_V4 | 1.0 | 0.008114 | 134 | 0.129 | 124 | 96 | 5 | 11 | 133 | 17 | 129 | Uncharacterized protein | Uncharacterized protein | | afdb-uniprot50 | AF-A0A7X7YWV6-F1-MODEL\_V4 | 1.0 | 0.009528 | 134 | 0.136 | 125 | 100 | 5 | 13 | 134 | 1 | 120 | Uncharacterized protein | Uncharacterized protein | | afdb-uniprot50 | AF-A0A0F9ES18-F1-MODEL\_V4 | 1.0 | 0.00856 | 134 | 0.093 | 139 | 110 | 9 | 1 | 133 | 33 | 161 | Uncharacterized protein | Uncharacterized protein | | afdb-uniprot50 | AF-A0A1J5KCD7-F1-MODEL\_V4 | 1.0 | 0.002497 | 132 | 0.095 | 147 | 99 | 11 | 6 | 136 | 1 | 129 | Uncharacterized protein | Uncharacterized protein | | afdb-uniprot50 | AF-A0A0R1L1L1-F1-MODEL\_V4 | 1.0 | 0.007691 | 132 | 0.095 | 136 | 93 | 10 | 13 | 136 | 122 | 239 | Mn2+ Fe2+ transporter | Mn2+ Fe2+ transporter | | afdb-uniprot50 | AF-B6WQ21-F1-MODEL\_V4 | 1.0 | 0.005884 | 131 | 0.069 | 130 | 96 | 7 | 19 | 136 | 82 | 198 | Uncharacterized protein | Uncharacterized protein | | afdb-uniprot50 | AF-A0A2T4TTF0-F1-MODEL\_V4 | 1.0 | 0.003094 | 130 | 0.107 | 139 | 97 | 8 | 18 | 136 | 22 | 153 | Uncharacterized protein | Uncharacterized protein | | afdb-uniprot50 | AF-A0A316LZT1-F1-MODEL\_V4 | 1.0 | 0.006909 | 130 | 0.105 | 114 | 94 | 5 | 25 | 134 | 11 | 120 | Phage tail protein | Phage tail protein | | afdb-uniprot50 | AF-A0A2V7R7U0-F1-MODEL\_V4 | 1.0 | 0.001462 | 130 | 0.113 | 150 | 103 | 11 | 5 | 134 | 2 | 141 | Uncharacterized protein | Uncharacterized protein | | afdb-uniprot50 | AF-A0A6I4YV07-F1-MODEL\_V4 | 1.0 | 0.002933 | 129 | 0.128 | 140 | 95 | 12 | 13 | 136 | 31 | 159 | Uncharacterized protein | Uncharacterized protein | | afdb-uniprot50 | AF-A0A2W4ILC1-F1-MODEL\_V4 | 1.0 | 0.007691 | 129 | 0.101 | 158 | 102 | 10 | 1 | 134 | 5 | 146 | Uncharacterized protein | Uncharacterized protein | | afdb-uniprot50 | AF-A0A352SX32-F1-MODEL\_V4 | 1.0 | 0.00856 | 128 | 0.075 | 132 | 98 | 10 | 13 | 135 | 2 | 118 | Uncharacterized protein | Uncharacterized protein | | afdb-uniprot50 | AF-A0A2D6MH57-F1-MODEL\_V4 | 1.0 | 0.009031 | 128 | 0.091 | 120 | 91 | 5 | 23 | 132 | 118 | 229 | Uncharacterized protein | Uncharacterized protein | | afdb-uniprot50 | AF-A0A2X3JWJ8-F1-MODEL\_V4 | 1.0 | 0.002016 | 125 | 0.083 | 143 | 95 | 9 | 5 | 133 | 15 | 135 | Putative DNA circulation protein | Putative DNA circulation protein | | afdb-uniprot50 | AF-A0A497RR60-F1-MODEL\_V4 | 1.0 | 0.00856 | 124 | 0.128 | 125 | 81 | 6 | 23 | 132 | 144 | 255 | Uncharacterized protein | Uncharacterized protein | | afdb-uniprot50 | AF-A0A1Z4HHM5-F1-MODEL\_V4 | 1.0 | 0.006208 | 119 | 0.11 | 172 | 90 | 7 | 13 | 136 | 6 | 162 | Peptidoglycan-binding LysM | Peptidoglycan-binding LysM | | afdb-uniprot50 | AF-A0A0F9EZB2-F1-MODEL\_V4 | 1.0 | 0.00501 | 115 | 0.154 | 136 | 83 | 12 | 14 | 133 | 23 | 142 | Uncharacterized protein | Uncharacterized protein | | afdb-uniprot50 | AF-A0A2T0YM39-F1-MODEL\_V4 | 1.0 | 0.005577 | 114 | 0.079 | 151 | 109 | 9 | 6 | 136 | 1 | 141 | Uncharacterized protein | Uncharacterized protein | | afdb-uniprot50 | AF-G5H934-F1-MODEL\_V4 | 1.0 | 0.00729 | 111 | 0.156 | 147 | 93 | 9 | 1 | 133 | 6 | 135 | Uncharacterized protein | Uncharacterized protein | | afdb-uniprot50 | AF-A0A136Q8M2-F1-MODEL\_V4 | 1.0 | 0.006208 | 107 | 0.095 | 126 | 95 | 7 | 14 | 136 | 20 | 129 | Uncharacterized protein | Uncharacterized protein | |
| Top keywords  (threshold 1.00e-02 (evalue)) | **Phage, tail, GpU, LysM, domain\_containing, P2, U, Oxidoreductase, Putative, peptidoglycan\_binding** |
| Output files | ../../similar\_structures/23\_FANPEZAQ\_CDS\_0023\_afdb-proteome\_foldseek.tsv ../../similar\_structures/23\_FANPEZAQ\_CDS\_0023\_afdb-uniprot50\_foldseek.tsv ../../similar\_structures/23\_FANPEZAQ\_CDS\_0023\_merged.svg ../../similar\_structures/23\_FANPEZAQ\_CDS\_0023\_pdb\_foldseek.tsv |

  
  
  

Return to summary | Go to previous | Go to next

  


---

**Sequence/structure alignments coloring**  
Each object in the alignment figures is colored according to its E-value following this color coding:

1e-100
10

**References:**  
1) Steinegger M, Meier M, Mirdita M, Vöhringer H, Haunsberger S J, and Söding J (2019) HH-suite3 for fast remote homology detection and deep protein annotation, BMC Bioinformatics, 473. doi: 10.1186/s12859-019-3019-7  
2) Jumper J, Evans R, Pritzel A, ..., Hassabis D (2021) Highly accurate protein structure prediction with AlphaFold, Nature, 596. doi: 10.1038/s41586-021-03819-2  
3) van Kempen M, Kim S, Tumescheit C, Mirdita M, Lee J, Gilchrist CLM, Söding J, and Steinegger M (2023) Fast and accurate protein structure search with Foldseek. Nature Biotechnology. doi: 10.1038/s41587-023-01773-0
