# Supplementary material for: Completing the BASEL phage collection to unlock hidden diversity for systematic exploration of phage–host interactions
Source: PLoS Biol. 2025 Apr 7;23(4):e3003063. doi: 10.1371/journal.pbio.3003063 (PMC11990801; doi:10.1371/journal.pbio.3003063)
Supplement: S2 Data — (ZIP) [file pbio.3003063.s009.zip › entries/24.html]

FANPEZAQ\_CDS\_0024


Return to summary | Go to previous | Go to next

|  |  |
| --- | --- |
| FANPEZAQ\_CDS\_0024 Page creation date: 02 Sep 2024, 12:00  Project folder: n/a  Input sequences file: Escherichia\_virus\_HeidiAbel.gb | lysm domain\_containing tail phage x peptidoglycan\_binding prophage p2\_like fragment bon superfamily putative spore coat assembly morphogenetic safa associated with spovid cazy families cbm50 lectin membrane |

### Sequence information

|  |  |
| --- | --- |
| Name | FANPEZAQ\_CDS\_0024  24\_FANPEZAQ\_CDS\_0024 (pipeline id) |
| Imported annotations | Escherichia\_virus\_HeidiAbel Bas97 |
| Protein sequence | MQTYITKDGDTADYIAWKYYGNQDAGTVEALVDANKGLADRGPVLPAGLVINLPEITTPA TTQGVKLWD |
| Number of residues | 69 |
| Molecular weight (Da) | 7426.24 |
| Output files | ../../query\_sequences/24\_FANPEZAQ\_CDS\_0024.fasta |

### Putative domain architecture and protein family

#### Search results (HHblits)1

|  |  |
| --- | --- |
| Domain family databases searched | Pfam, Ncbi-cd, Cath, Phrogs |
| Results, scheme(s)  (Top layers only; threshold 1.00e-03 (evalue)) | xml version="1.0" encoding="utf-8" standalone="no"?       2024-09-02T21:08:17.350755 image/svg+xml   Matplotlib v3.7.2, https://matplotlib.org/ |
| Results, table  (E-value ≤ 1.00e-03 (evalue)) | | db | id | prob | evalue | pvalue | score | cols | query | query\_len | template | template\_len | name | description | | --- | --- | --- | --- | --- | --- | --- | --- | --- | --- | --- | --- | --- | | phrogs | 46 | 99.8 | 1.4e-26 | 1.9e-30 | 132.6 | 66 | (1, 69) | 69 | (9, 76) | 85 | baseplate hub | baseplate hub; Category: tail; p199511 VI\_11453 | | phrogs | 7145 | 98.7 | 1.3e-12 | 1.6e-16 | 75.2 | 55 | (3, 59) | 69 | (38, 93) | 97 | baseplate wedge subunit | baseplate wedge subunit; Category: tail; MF360958\_p187 | | phrogs | 21966 | 98.4 | 5.5e-11 | 6.3e-15 | 69.9 | 51 | (2, 54) | 69 | (78, 128) | 128 | NA | NA; Category: unknown function; AY855346\_p20 | | phrogs | 7348 | 98.2 | 3.7e-10 | 4.3e-14 | 64.9 | 53 | (2, 58) | 69 | (5, 57) | 104 | endolysin | endolysin; Category: lysis; MG428991\_p108 | | phrogs | 8272 | 96.9 | 1.6e-06 | 1.8e-10 | 47.8 | 53 | (2, 56) | 69 | (38, 90) | 93 | tail protein | tail protein; Category: tail; NC\_029013\_p171 | | phrogs | 14367 | 96.5 | 6.8e-06 | 8e-10 | 45.8 | 54 | (3, 58) | 69 | (36, 91) | 93 | NA | NA; Category: unknown function; p23038 VI\_12315 | | phrogs | 975 | 96.0 | 3.9e-05 | 4.8e-09 | 48.8 | 56 | (2, 59) | 69 | (5, 72) | 236 | baseplate protein | baseplate protein; Category: tail; NC\_020871\_p93 | | phrogs | 380 | 95.9 | 4.5e-05 | 5.9e-09 | 47.7 | 39 | (2, 58) | 69 | (162, 203) | 230 | endolysin | endolysin; Category: lysis; NC\_005294\_p57 | | phrogs | 232 | 95.0 | 0.00032 | 4.1e-08 | 43.3 | 35 | (2, 38) | 69 | (49, 83) | 209 | baseplate wedge subunit | baseplate wedge subunit; Category: tail; NC\_027132\_p2 | | phrogs | 4033 | 94.2 | 0.001 | 1.2e-07 | 43.3 | 51 | (2, 57) | 69 | (133, 183) | 320 | NA | NA; Category: unknown function; p55048 VI\_12418 | |
| Top keywords  (threshold 1.00e-03 (evalue)) | **tail, baseplate, wedge, endolysin, lysis, hub, p199511, VI\_11453, MF360958\_p187, AY855346\_p20** |
| Output files | ../../domain\_architecture/24\_FANPEZAQ\_CDS\_0024\_cath.hhr ../../domain\_architecture/24\_FANPEZAQ\_CDS\_0024\_merged.svg ../../domain\_architecture/24\_FANPEZAQ\_CDS\_0024\_ncbi-cd.hhr ../../domain\_architecture/24\_FANPEZAQ\_CDS\_0024\_pfam.hhr ../../domain\_architecture/24\_FANPEZAQ\_CDS\_0024\_phrogs.hhr |

### Identical protein sequences/structures

#### Search results

|  |  |
| --- | --- |
| Protein sequence databases searched | Pdb, Swissprot, Refseq |
| Identical proteins found | -- |
| Top keywords | -- |
| Output files | -- |

### Similar protein sequences/structures

#### Sequence similarity search results (HHblits)1

|  |  |
| --- | --- |
| Sequence databases searched | Uniclust, Pdb70 |
| Results, scheme(s)  (Top layers only, threshold 1.00e-03 (evalue)) | xml version="1.0" encoding="utf-8" standalone="no"?       2024-09-02T21:08:39.917925 image/svg+xml   Matplotlib v3.7.2, https://matplotlib.org/ |
| Results, table(s)  (threshold 1.00e-03 (evalue)) | | db | id | prob | evalue | pvalue | score | cols | query | query\_len | template | template\_len | name | description | | --- | --- | --- | --- | --- | --- | --- | --- | --- | --- | --- | --- | --- | | uniclust | UniRef100\_A0A0B4B3T0 | 99.8 | 7.2e-24 | 1.4e-29 | 113.1 | 69 | (1, 69) | 69 | (12, 80) | 83 | p2-like protein prophage tail protein X-like protein | p2-like protein prophage tail protein X-like protein | | uniclust | UniRef100\_A0A009QNP1 | 99.8 | 8.2e-23 | 1.8e-28 | 112.9 | 68 | (1, 69) | 69 | (16, 83) | 92 | Phage Tail Protein X family protein | Phage Tail Protein X family protein | | uniclust | UniRef100\_A0A074TD60 | 99.8 | 2.4e-22 | 4.9e-28 | 108.7 | 68 | (1, 69) | 69 | (3, 70) | 86 | Tail protein | Tail protein | | uniclust | UniRef100\_A0A085ARU3 | 99.8 | 7.6e-22 | 1.5e-27 | 105.2 | 69 | (1, 69) | 69 | (7, 75) | 78 | Phage tail protein | Phage tail protein | | uniclust | UniRef100\_A0A165XHI0 | 99.8 | 8e-22 | 1.7e-27 | 107.2 | 68 | (1, 69) | 69 | (15, 83) | 85 | Phage Tail Protein X | Phage Tail Protein X | | uniclust | UniRef100\_A0A069CJM3 | 99.8 | 1.1e-21 | 2.2e-27 | 108.0 | 68 | (1, 69) | 69 | (10, 77) | 95 | Phage tail protein | Phage tail protein | | uniclust | UniRef100\_A0A1Q8SPL8 | 99.7 | 1.5e-21 | 3e-27 | 101.8 | 68 | (1, 69) | 69 | (4, 72) | 72 | Phage tail protein | Phage tail protein | | uniclust | UniRef100\_A0A060GYH3 | 99.7 | 2.9e-21 | 5.9e-27 | 101.5 | 69 | (1, 69) | 69 | (5, 73) | 73 | Tail protein | Tail protein | | uniclust | UniRef100\_A0A0B6D337 | 99.7 | 5.9e-21 | 1.2e-26 | 103.4 | 66 | (2, 69) | 69 | (18, 84) | 86 | Phage Tail Protein X family protein | Phage Tail Protein X family protein | | uniclust | UniRef100\_A0A069PL70 | 99.7 | 3e-20 | 6.3e-26 | 101.0 | 66 | (2, 69) | 69 | (18, 83) | 84 | Phage tail protein | Phage tail protein | | uniclust | UniRef100\_A0A024E969 | 99.7 | 3.6e-20 | 7.4e-26 | 100.8 | 67 | (1, 69) | 69 | (17, 83) | 86 | Tail protein X-related protein | Tail protein X-related protein | | uniclust | UniRef100\_Q94MD7 | 99.7 | 5e-20 | 1e-25 | 97.4 | 68 | (1, 69) | 69 | (7, 74) | 74 | Tail component protein (Fragment) | Tail component protein (Fragment) | | uniclust | UniRef100\_A0A077KTR2 | 99.7 | 9.6e-20 | 2e-25 | 96.3 | 67 | (1, 69) | 69 | (7, 73) | 73 | Phage tail protein | Phage tail protein | | uniclust | UniRef100\_A0A0N1C803 | 99.6 | 3.1e-19 | 6.5e-25 | 96.3 | 68 | (2, 69) | 69 | (8, 75) | 78 | Phage tail protein | Phage tail protein | | uniclust | UniRef100\_A0A0C5VGD3 | 99.6 | 5.8e-19 | 1.2e-24 | 99.8 | 68 | (2, 69) | 69 | (3, 106) | 107 | p2-like prophage tail protein X | p2-like prophage tail protein X | | uniclust | UniRef100\_A0A0E4BWV6 | 99.6 | 1.3e-18 | 2.6e-24 | 93.7 | 69 | (1, 69) | 69 | (7, 79) | 79 | Phage tail protein | Phage tail protein | | uniclust | UniRef100\_A0A0D8L3F8 | 99.6 | 1.2e-18 | 2.6e-24 | 93.7 | 67 | (2, 69) | 69 | (9, 77) | 77 | Phage tail protein | Phage tail protein | | uniclust | UniRef100\_A0A0E3UN65 | 99.6 | 4.7e-18 | 9.3e-24 | 97.0 | 56 | (2, 59) | 69 | (59, 114) | 120 | LysM domain-containing protein | LysM domain-containing protein | | uniclust | UniRef100\_A0A1R4JJ60 | 99.5 | 1.1e-17 | 2.3e-23 | 91.9 | 56 | (1, 58) | 69 | (32, 87) | 90 | LysM domain-containing protein | LysM domain-containing protein | | uniclust | UniRef100\_A0A0F8ZZ29 | 99.5 | 1.3e-17 | 2.7e-23 | 88.2 | 55 | (2, 58) | 69 | (14, 69) | 73 | LysM domain-containing protein (Fragment) | LysM domain-containing protein (Fragment) | | uniclust | UniRef100\_A0A0A2WI51 | 99.5 | 1.4e-17 | 2.7e-23 | 96.5 | 55 | (3, 59) | 69 | (62, 116) | 129 | Peptidoglycan-binding lysin domain-containing protein | Peptidoglycan-binding lysin domain-containing protein | | uniclust | UniRef100\_A0A1B8SM38 | 99.5 | 1.7e-17 | 3.4e-23 | 88.1 | 54 | (2, 57) | 69 | (18, 71) | 75 | LysM domain-containing protein | LysM domain-containing protein | | uniclust | UniRef100\_A0A023XZ78 | 99.5 | 1.8e-17 | 3.7e-23 | 96.2 | 56 | (2, 59) | 69 | (65, 120) | 131 | LysM peptidoglycan-binding domain-containing protein | LysM peptidoglycan-binding domain-containing protein | | uniclust | UniRef100\_A0A219Y8Z5 | 99.5 | 2.4e-17 | 5e-23 | 90.4 | 67 | (2, 69) | 69 | (17, 84) | 88 | Tail protein | Tail protein | | uniclust | UniRef100\_A0A031FQW4 | 99.5 | 2.5e-17 | 5.2e-23 | 90.4 | 69 | (1, 69) | 69 | (18, 87) | 87 | Phage tail X | Phage tail X | | uniclust | UniRef100\_A0A3B9ZHN5 | 99.5 | 2.9e-17 | 6e-23 | 94.0 | 56 | (2, 59) | 69 | (55, 110) | 114 | LysM domain-containing protein | LysM domain-containing protein | | uniclust | UniRef100\_A0A535FPJ6 | 99.5 | 3.2e-17 | 6.5e-23 | 84.4 | 53 | (2, 58) | 69 | (7, 59) | 61 | LysM peptidoglycan-binding domain-containing protein | LysM peptidoglycan-binding domain-containing protein | | uniclust | UniRef100\_A0A011MIB9 | 99.5 | 4.7e-17 | 9.9e-23 | 90.8 | 64 | (2, 68) | 69 | (17, 80) | 95 | Membrane protein | Membrane protein | | uniclust | UniRef100\_A0A0C2Z8D7 | 99.5 | 6.1e-17 | 1.2e-22 | 85.2 | 68 | (1, 69) | 69 | (1, 68) | 68 | Phage tail protein | Phage tail protein | | uniclust | UniRef100\_A0A0S4XN94 | 99.5 | 7e-17 | 1.5e-22 | 85.3 | 66 | (1, 69) | 69 | (2, 68) | 68 | Putative Phage Tail Protein X | Putative Phage Tail Protein X | | uniclust | UniRef100\_A0A0D0S6Z4 | 99.5 | 8.5e-17 | 1.7e-22 | 93.4 | 54 | (2, 57) | 69 | (70, 123) | 136 | NODE\_43, whole genome shotgun sequence | NODE\_43, whole genome shotgun sequence | | uniclust | UniRef100\_A0A1A9RBR4 | 99.5 | 1.1e-16 | 2e-22 | 84.7 | 66 | (3, 69) | 69 | (10, 77) | 77 | Phage tail protein | Phage tail protein | | uniclust | UniRef100\_A0A3A9HNX8 | 99.5 | 1.2e-16 | 2.3e-22 | 83.6 | 66 | (1, 69) | 69 | (6, 71) | 71 | Phage tail protein | Phage tail protein | | uniclust | UniRef100\_A0A0F9YGF8 | 99.5 | 1.6e-16 | 3.3e-22 | 93.7 | 50 | (2, 55) | 69 | (92, 141) | 142 | LysM domain-containing protein | LysM domain-containing protein | | uniclust | UniRef100\_A0A0D3LEX1 | 99.4 | 2.3e-16 | 4.6e-22 | 88.3 | 54 | (2, 57) | 69 | (43, 96) | 97 | Putative phospholipid-binding protein,LysM domain-containing protein | Putative phospholipid-binding protein,LysM domain-containing protein | | uniclust | UniRef100\_A0A0A8HA04 | 99.4 | 2.3e-16 | 4.7e-22 | 82.1 | 64 | (1, 69) | 69 | (2, 65) | 65 | Phage tail protein X family protein | Phage tail protein X family protein | | uniclust | UniRef100\_A0A7C2W7N3 | 99.4 | 2.6e-16 | 5.3e-22 | 92.2 | 53 | (2, 56) | 69 | (82, 134) | 135 | LysM peptidoglycan-binding domain-containing protein | LysM peptidoglycan-binding domain-containing protein | | uniclust | UniRef100\_A0A0D0RZI1 | 99.4 | 2.7e-16 | 5.6e-22 | 95.0 | 54 | (2, 57) | 69 | (112, 165) | 167 | LysM domain protein | LysM domain protein | | uniclust | UniRef100\_A0A1I4XDP5 | 99.4 | 3.6e-16 | 6.9e-22 | 85.3 | 55 | (2, 58) | 69 | (35, 89) | 92 | LysM domain-containing protein | LysM domain-containing protein | | uniclust | UniRef100\_A0A521DSA7 | 99.4 | 3.6e-16 | 7.2e-22 | 84.6 | 66 | (1, 69) | 69 | (12, 77) | 80 | Phage Tail Protein X | Phage Tail Protein X | | uniclust | UniRef100\_A0A1F8QSY2 | 99.4 | 4.9e-16 | 9.9e-22 | 81.8 | 56 | (1, 56) | 69 | (11, 67) | 68 | LysM domain-containing protein | LysM domain-containing protein | | uniclust | UniRef100\_A0A0Q8DXV3 | 99.4 | 5.6e-16 | 1.1e-21 | 86.5 | 53 | (2, 56) | 69 | (48, 100) | 101 | Peptidoglycan-binding protein | Peptidoglycan-binding protein | | uniclust | UniRef100\_A0A3M1G446 | 99.4 | 6.9e-16 | 1.3e-21 | 78.1 | 52 | (2, 55) | 69 | (10, 61) | 62 | LysM peptidoglycan-binding domain-containing protein (Fragment) | LysM peptidoglycan-binding domain-containing protein (Fragment) | | uniclust | UniRef100\_A0A060BNX4 | 99.4 | 7.1e-16 | 1.4e-21 | 80.6 | 55 | (1, 57) | 69 | (11, 65) | 68 | CAZy families CBM50 protein | CAZy families CBM50 protein | | uniclust | UniRef100\_A0A0G3EL81 | 99.4 | 6.7e-16 | 1.4e-21 | 100.6 | 55 | (2, 58) | 69 | (230, 284) | 286 | LysM domain/BON superfamily protein | LysM domain/BON superfamily protein | | uniclust | UniRef100\_A0A1W1D095 | 99.4 | 9.1e-16 | 1.8e-21 | 90.8 | 52 | (1, 55) | 69 | (96, 147) | 147 | Ferric siderophore transport system, periplasmic binding protein TonB | Ferric siderophore transport system, periplasmic binding protein TonB | | uniclust | UniRef100\_A0A0S8E6Q2 | 99.4 | 9.8e-16 | 2.1e-21 | 94.9 | 54 | (2, 58) | 69 | (123, 177) | 191 | LysM domain-containing protein | LysM domain-containing protein | | uniclust | UniRef100\_A0A011UMN3 | 99.4 | 1e-15 | 2.2e-21 | 83.5 | 67 | (3, 69) | 69 | (10, 78) | 80 | Phage tail protein | Phage tail protein | | uniclust | UniRef100\_A0A1I7HNC1 | 99.4 | 1.4e-15 | 2.6e-21 | 86.6 | 66 | (3, 69) | 69 | (73, 138) | 138 | P2-like prophage tail protein X | P2-like prophage tail protein X | | uniclust | UniRef100\_A0A1G0ZIW6 | 99.4 | 1.2e-15 | 2.6e-21 | 94.8 | 55 | (2, 58) | 69 | (136, 190) | 192 | LysM domain-containing protein | LysM domain-containing protein | | uniclust | UniRef100\_A0A0E3ZHJ8 | 99.4 | 1.3e-15 | 2.7e-21 | 87.4 | 55 | (2, 58) | 69 | (63, 118) | 120 | Peptidoglycan-binding protein | Peptidoglycan-binding protein | | uniclust | UniRef100\_A0A1G1PBG2 | 99.4 | 1.5e-15 | 3.1e-21 | 85.1 | 53 | (2, 56) | 69 | (43, 96) | 97 | LysM domain-containing protein | LysM domain-containing protein | | uniclust | UniRef100\_A0A290XBI2 | 99.4 | 1.7e-15 | 3.3e-21 | 82.1 | 57 | (1, 59) | 69 | (26, 82) | 87 | LysM domain-containing protein | LysM domain-containing protein | | uniclust | UniRef100\_A0A1W1BHR3 | 99.4 | 2e-15 | 4.1e-21 | 99.5 | 57 | (1, 59) | 69 | (254, 310) | 321 | LysM domain-containing protein | LysM domain-containing protein | | uniclust | UniRef100\_D2ZKL4 | 99.4 | 2.3e-15 | 4.2e-21 | 82.9 | 64 | (5, 69) | 69 | (46, 109) | 109 | Phage Tail Protein X | Phage Tail Protein X | | uniclust | UniRef100\_A0A7C3XNE8 | 99.4 | 2.5e-15 | 4.8e-21 | 86.9 | 50 | (2, 55) | 69 | (81, 130) | 130 | LysM domain-containing protein | LysM domain-containing protein | | uniclust | UniRef100\_A0A061NN19 | 99.4 | 2.4e-15 | 5e-21 | 86.7 | 64 | (1, 68) | 69 | (18, 81) | 114 | Phage tail protein | Phage tail protein | | uniclust | UniRef100\_A0A6I0F174 | 99.4 | 2.6e-15 | 5.1e-21 | 86.6 | 52 | (2, 55) | 69 | (75, 126) | 127 | LysM peptidoglycan-binding domain-containing protein | LysM peptidoglycan-binding domain-containing protein | | uniclust | UniRef100\_A0A085ZVS9 | 99.4 | 2.5e-15 | 5.1e-21 | 90.6 | 55 | (2, 58) | 69 | (103, 157) | 161 | Peptidoglycan-binding protein | Peptidoglycan-binding protein | | uniclust | UniRef100\_A0A0B5FQ72 | 99.3 | 3.1e-15 | 6.4e-21 | 84.3 | 53 | (2, 58) | 69 | (39, 91) | 99 | LysM domain-containing protein | LysM domain-containing protein | | uniclust | UniRef100\_A0A1G0XZP4 | 99.3 | 5e-15 | 9.5e-21 | 80.5 | 53 | (2, 56) | 69 | (35, 87) | 88 | LysM domain-containing protein | LysM domain-containing protein | | uniclust | UniRef100\_A0A2A4RLX5 | 99.3 | 5.1e-15 | 1.1e-20 | 94.5 | 55 | (2, 58) | 69 | (179, 233) | 246 | LysM domain-containing protein | LysM domain-containing protein | | uniclust | UniRef100\_UPI0011B4CCD0 | 99.3 | 5.9e-15 | 1.1e-20 | 73.3 | 51 | (1, 55) | 69 | (2, 52) | 53 | LysM domain-containing protein | LysM domain-containing protein | | uniclust | UniRef100\_A0A0R2XHY5 | 99.3 | 5.7e-15 | 1.2e-20 | 96.9 | 55 | (2, 58) | 69 | (249, 303) | 305 | LysM domain-containing protein | LysM domain-containing protein | | uniclust | UniRef100\_A0A098PW87 | 99.3 | 6.3e-15 | 1.2e-20 | 83.3 | 53 | (2, 56) | 69 | (60, 112) | 113 | LysM peptidoglycan-binding domain-containing protein | LysM peptidoglycan-binding domain-containing protein | | uniclust | UniRef100\_A0A009H911 | 99.3 | 6.4e-15 | 1.3e-20 | 93.2 | 56 | (2, 59) | 69 | (150, 205) | 227 | LysM domain-containing protein | LysM domain-containing protein | | uniclust | UniRef100\_A0A023BS42 | 99.3 | 7.6e-15 | 1.5e-20 | 90.1 | 56 | (2, 59) | 69 | (99, 154) | 184 | Peptidase M23B | Peptidase M23B | | uniclust | UniRef100\_A0A060C391 | 99.3 | 7.9e-15 | 1.6e-20 | 87.4 | 53 | (2, 56) | 69 | (97, 149) | 150 | CAZy families CBM50 protein (Fragment) | CAZy families CBM50 protein (Fragment) | | uniclust | UniRef100\_A0A0A1FIF4 | 99.3 | 8.8e-15 | 1.8e-20 | 89.3 | 53 | (2, 56) | 69 | (128, 180) | 182 | BON domain protein | BON domain protein | | uniclust | UniRef100\_A0A0D2JA12 | 99.3 | 8.6e-15 | 1.8e-20 | 80.1 | 64 | (1, 68) | 69 | (11, 74) | 81 | Tail protein | Tail protein | | uniclust | UniRef100\_A0A1V5UVW7 | 99.3 | 9.1e-15 | 1.8e-20 | 81.6 | 53 | (3, 58) | 69 | (44, 96) | 98 | LysM domain-containing protein | LysM domain-containing protein | | uniclust | UniRef100\_A0A3M1EEN8 | 99.3 | 9.3e-15 | 1.8e-20 | 85.5 | 56 | (2, 59) | 69 | (74, 129) | 139 | LysM peptidoglycan-binding domain-containing protein | LysM peptidoglycan-binding domain-containing protein | | uniclust | UniRef100\_A0A060BU78 | 99.3 | 9.8e-15 | 1.9e-20 | 79.9 | 53 | (2, 56) | 69 | (35, 87) | 89 | CAZy families CBM50 protein (Fragment) | CAZy families CBM50 protein (Fragment) | | uniclust | UniRef100\_A0A1U9NM08 | 99.3 | 1.1e-14 | 2.2e-20 | 88.4 | 55 | (1, 57) | 69 | (117, 171) | 172 | LysM domain/BON superfamily protein | LysM domain/BON superfamily protein | | uniclust | UniRef100\_A0A7X6LA34 | 99.3 | 1.2e-14 | 2.3e-20 | 76.7 | 54 | (2, 59) | 69 | (9, 62) | 70 | LysM peptidoglycan-binding domain-containing protein | LysM peptidoglycan-binding domain-containing protein | | uniclust | UniRef100\_A0A6G5QG46 | 99.3 | 1.3e-14 | 2.5e-20 | 75.5 | 67 | (1, 69) | 69 | (2, 68) | 68 | Phage tail protein X family protein | Phage tail protein X family protein | | uniclust | UniRef100\_A0A1R4HC34 | 99.3 | 1.4e-14 | 2.8e-20 | 93.1 | 52 | (2, 55) | 69 | (237, 288) | 288 | LysM domain-containing protein | LysM domain-containing protein | | uniclust | UniRef100\_A0A0P6WT71 | 99.3 | 1.5e-14 | 3.2e-20 | 88.9 | 58 | (2, 59) | 69 | (113, 171) | 177 | LysM domain-containing protein | LysM domain-containing protein | | uniclust | UniRef100\_E5AKR2 | 99.3 | 2e-14 | 3.6e-20 | 77.1 | 67 | (2, 69) | 69 | (23, 90) | 90 | Tail protein X | Tail protein X | | uniclust | UniRef100\_A0A1D2UDF4 | 99.3 | 2e-14 | 3.9e-20 | 85.4 | 51 | (3, 57) | 69 | (95, 145) | 146 | Peptidoglycan-binding protein | Peptidoglycan-binding protein | | uniclust | UniRef100\_A0A9D8L854 | 99.3 | 2.3e-14 | 4.3e-20 | 76.0 | 66 | (3, 69) | 69 | (12, 78) | 78 | Tail protein X | Tail protein X | | uniclust | UniRef100\_A0A346Y079 | 99.3 | 2.2e-14 | 4.5e-20 | 89.2 | 54 | (1, 58) | 69 | (138, 191) | 194 | LysM domain-containing protein | LysM domain-containing protein | | uniclust | UniRef100\_A0A087N8U9 | 99.3 | 2.3e-14 | 4.5e-20 | 77.3 | 66 | (3, 69) | 69 | (11, 79) | 80 | Phage tail protein | Phage tail protein | | uniclust | UniRef100\_A0A0S8KMS1 | 99.3 | 2.3e-14 | 4.6e-20 | 83.8 | 56 | (2, 57) | 69 | (74, 130) | 131 | LysM domain-containing protein | LysM domain-containing protein | | uniclust | UniRef100\_A0A0P7CPN1 | 99.3 | 2.3e-14 | 4.7e-20 | 85.2 | 53 | (2, 58) | 69 | (82, 134) | 141 | LysM domain-containing protein | LysM domain-containing protein | | uniclust | UniRef100\_A0A023BZG8 | 99.3 | 2.5e-14 | 5e-20 | 86.2 | 56 | (2, 59) | 69 | (104, 159) | 163 | Peptidoglycan-binding protein | Peptidoglycan-binding protein | | uniclust | UniRef100\_A0A011M151 | 99.3 | 2.6e-14 | 5.3e-20 | 88.3 | 55 | (3, 59) | 69 | (109, 163) | 197 | Peptidoglycan-binding protein LysM | Peptidoglycan-binding protein LysM | | uniclust | UniRef100\_A0A068MT20 | 99.2 | 2.6e-14 | 5.4e-20 | 89.7 | 54 | (3, 58) | 69 | (144, 197) | 208 | LysM domain-containing protein | LysM domain-containing protein | | uniclust | UniRef100\_A0A1Q7YIP8 | 99.2 | 3.1e-14 | 5.9e-20 | 74.6 | 53 | (2, 56) | 69 | (17, 69) | 71 | LysM domain-containing protein | LysM domain-containing protein | | uniclust | UniRef100\_A0A4R5HGF7 | 99.2 | 3e-14 | 6e-20 | 75.3 | 68 | (2, 69) | 69 | (3, 70) | 70 | Phage tail protein | Phage tail protein | | uniclust | UniRef100\_A0A0M9U3I1 | 99.2 | 3.2e-14 | 6.1e-20 | 77.9 | 57 | (3, 59) | 69 | (31, 88) | 91 | LysM domain-containing protein | LysM domain-containing protein | | uniclust | UniRef100\_A0A0P8WJ67 | 99.2 | 3.1e-14 | 6.2e-20 | 78.8 | 63 | (3, 68) | 69 | (17, 79) | 91 | Phage tail protein X | Phage tail protein X | | uniclust | UniRef100\_A0A0H5RIV1 | 99.2 | 3.4e-14 | 6.4e-20 | 71.9 | 50 | (2, 55) | 69 | (11, 60) | 60 | Mannose-binding lectin | Mannose-binding lectin | | uniclust | UniRef100\_A0A0K0Y197 | 99.2 | 3.3e-14 | 6.5e-20 | 92.7 | 55 | (2, 58) | 69 | (247, 301) | 303 | LysM domain/BON superfamily protein | LysM domain/BON superfamily protein | | uniclust | UniRef100\_A0A1V6GXV1 | 99.2 | 3.2e-14 | 6.7e-20 | 95.1 | 53 | (2, 56) | 69 | (310, 362) | 362 | LysM domain/BON superfamily protein | LysM domain/BON superfamily protein | | uniclust | UniRef100\_A0A1G3Z8F3 | 99.2 | 3.7e-14 | 7.5e-20 | 90.9 | 52 | (2, 57) | 69 | (198, 249) | 251 | LysM domain-containing protein | LysM domain-containing protein | | uniclust | UniRef100\_A0A2N3IK62 | 99.2 | 4.3e-14 | 8.6e-20 | 83.6 | 53 | (2, 56) | 69 | (83, 136) | 137 | LysM domain | LysM domain | | uniclust | UniRef100\_A0A0B0VWC3 | 99.2 | 4.8e-14 | 9e-20 | 76.8 | 64 | (3, 69) | 69 | (7, 70) | 95 | Phage tail protein | Phage tail protein | | uniclust | UniRef100\_A0A948S9G9 | 99.2 | 5.2e-14 | 9.5e-20 | 73.0 | 51 | (2, 54) | 69 | (23, 73) | 73 | LysM peptidoglycan-binding domain-containing protein | LysM peptidoglycan-binding domain-containing protein | | uniclust | UniRef100\_A0A024K1G4 | 99.2 | 5.6e-14 | 1.1e-19 | 88.5 | 53 | (2, 58) | 69 | (174, 226) | 237 | Mannose-binding lectin | Mannose-binding lectin | | uniclust | UniRef100\_A0A0Q4L1W6 | 99.2 | 5.7e-14 | 1.2e-19 | 80.4 | 54 | (2, 59) | 69 | (43, 96) | 107 | LysM domain-containing protein | LysM domain-containing protein | | uniclust | UniRef100\_A0A1G0YUF0 | 99.2 | 6.1e-14 | 1.3e-19 | 91.7 | 56 | (2, 59) | 69 | (239, 294) | 298 | LysM domain-containing protein | LysM domain-containing protein | | uniclust | UniRef100\_A0A2V8QG62 | 99.2 | 7.4e-14 | 1.4e-19 | 83.8 | 54 | (2, 57) | 69 | (108, 161) | 163 | Peptidoglycan-binding protein | Peptidoglycan-binding protein | | uniclust | UniRef100\_A0A1G8UZV7 | 99.2 | 7.2e-14 | 1.4e-19 | 85.3 | 52 | (2, 55) | 69 | (124, 176) | 176 | LysM domain-containing protein | LysM domain-containing protein | | uniclust | UniRef100\_A0A3M2CM87 | 99.2 | 7.4e-14 | 1.5e-19 | 88.7 | 54 | (2, 57) | 69 | (190, 243) | 249 | LysM domain-containing protein | LysM domain-containing protein | | uniclust | UniRef100\_A0A1G1LW77 | 99.2 | 7.7e-14 | 1.5e-19 | 87.3 | 56 | (2, 59) | 69 | (158, 213) | 218 | LysM domain-containing protein | LysM domain-containing protein | | uniclust | UniRef100\_A0A3M1PZI9 | 99.2 | 8.5e-14 | 1.7e-19 | 93.3 | 52 | (2, 57) | 69 | (224, 275) | 405 | LysM peptidoglycan-binding domain-containing protein | LysM peptidoglycan-binding domain-containing protein | | uniclust | UniRef100\_A0A1I3PDS2 | 99.2 | 8.8e-14 | 1.8e-19 | 88.9 | 53 | (2, 56) | 69 | (123, 175) | 249 | LysM repeat-containing protein | LysM repeat-containing protein | | uniclust | UniRef100\_A0A016QMV4 | 99.2 | 9e-14 | 1.8e-19 | 89.3 | 53 | (2, 58) | 69 | (214, 266) | 273 | Peptidoglycan-binding LysM | Peptidoglycan-binding LysM | | uniclust | UniRef100\_A0A024YYK6 | 99.2 | 8.5e-14 | 1.8e-19 | 91.6 | 54 | (2, 59) | 69 | (201, 254) | 297 | Peptidase M23B | Peptidase M23B | | uniclust | UniRef100\_A0A084T293 | 99.2 | 8.9e-14 | 1.8e-19 | 90.6 | 54 | (2, 59) | 69 | (210, 263) | 283 | LysM domain-containing protein | LysM domain-containing protein | | uniclust | UniRef100\_A0A069A2Y0 | 99.2 | 9.5e-14 | 2e-19 | 88.7 | 52 | (2, 55) | 69 | (182, 233) | 233 | LysM domain protein | LysM domain protein | | uniclust | UniRef100\_A0A962ECX3 | 99.2 | 1.1e-13 | 2.1e-19 | 81.3 | 56 | (2, 59) | 69 | (92, 147) | 152 | LysM peptidoglycan-binding domain-containing protein | LysM peptidoglycan-binding domain-containing protein | | uniclust | UniRef100\_A0A0B1KPG3 | 99.2 | 1.2e-13 | 2.3e-19 | 72.1 | 68 | (2, 69) | 69 | (3, 72) | 72 | Phage tail protein | Phage tail protein | | uniclust | UniRef100\_A0A1G1N886 | 99.2 | 1.2e-13 | 2.4e-19 | 86.6 | 54 | (2, 57) | 69 | (132, 185) | 201 | LysM domain-containing protein | LysM domain-containing protein | | uniclust | UniRef100\_A0A086ML12 | 99.2 | 1.2e-13 | 2.5e-19 | 87.9 | 55 | (2, 58) | 69 | (165, 219) | 230 | Peptigoglycan-binding protein LysM | Peptigoglycan-binding protein LysM | | uniclust | UniRef100\_A0A0N7LZZ4 | 99.2 | 1.3e-13 | 2.6e-19 | 86.1 | 53 | (2, 56) | 69 | (154, 206) | 207 | LysM domain/BON superfamily protein | LysM domain/BON superfamily protein | | uniclust | UniRef100\_A0A4P5YH32 | 99.2 | 1.6e-13 | 3.1e-19 | 86.8 | 52 | (2, 57) | 69 | (178, 229) | 231 | LysM domain-containing protein | LysM domain-containing protein | | uniclust | UniRef100\_A0A1Z9KPR5 | 99.2 | 1.5e-13 | 3.1e-19 | 87.4 | 54 | (3, 59) | 69 | (175, 228) | 235 | LysM domain-containing protein | LysM domain-containing protein | | uniclust | UniRef100\_A0A2H0LH77 | 99.2 | 1.7e-13 | 3.4e-19 | 85.6 | 55 | (2, 58) | 69 | (132, 188) | 204 | LysM domain-containing protein | LysM domain-containing protein | | uniclust | UniRef100\_A0A399ZZW7 | 99.2 | 1.7e-13 | 3.4e-19 | 93.3 | 56 | (2, 59) | 69 | (373, 428) | 435 | LysM domain-containing protein | LysM domain-containing protein | | uniclust | UniRef100\_A0A0S7WJV5 | 99.2 | 1.7e-13 | 3.5e-19 | 82.7 | 56 | (3, 58) | 69 | (89, 146) | 149 | LysM domain-containing protein | LysM domain-containing protein | | uniclust | UniRef100\_A0A1X7MBB4 | 99.1 | 2e-13 | 4e-19 | 78.1 | 53 | (2, 56) | 69 | (59, 111) | 113 | LysM domain/BON superfamily protein | LysM domain/BON superfamily protein | | uniclust | UniRef100\_A0A2E8Y824 | 99.1 | 2.1e-13 | 4e-19 | 74.1 | 53 | (1, 55) | 69 | (31, 84) | 84 | LysM domain-containing protein | LysM domain-containing protein | | uniclust | UniRef100\_A0A2A4RLX5 | 99.1 | 2e-13 | 4e-19 | 87.6 | 53 | (2, 58) | 69 | (105, 157) | 246 | LysM domain-containing protein | LysM domain-containing protein | | uniclust | UniRef100\_A0A358MIT7 | 99.1 | 2.4e-13 | 4.4e-19 | 69.3 | 51 | (3, 55) | 69 | (14, 64) | 65 | LysM domain-containing protein | LysM domain-containing protein | | uniclust | UniRef100\_A0A0H3L797 | 99.1 | 2.5e-13 | 4.6e-19 | 79.2 | 64 | (5, 69) | 69 | (93, 156) | 156 | Phage tail protein X | Phage tail protein X | | uniclust | UniRef100\_A0A022MNT4 | 99.1 | 2.2e-13 | 4.7e-19 | 90.1 | 53 | (2, 58) | 69 | (221, 273) | 302 | Peptidase M23B | Peptidase M23B | | uniclust | UniRef100\_A0A143HC07 | 99.1 | 2.4e-13 | 4.8e-19 | 77.5 | 55 | (1, 58) | 69 | (2, 56) | 108 | Phage tail protein | Phage tail protein | | uniclust | UniRef100\_A0A0A0HQW3 | 99.1 | 2.5e-13 | 5.1e-19 | 87.1 | 54 | (2, 57) | 69 | (191, 244) | 246 | LysM domain protein | LysM domain protein | | uniclust | UniRef100\_A0A6C0GK95 | 99.1 | 2.8e-13 | 5.2e-19 | 76.9 | 55 | (2, 58) | 69 | (53, 107) | 119 | LysM peptidoglycan-binding domain-containing protein | LysM peptidoglycan-binding domain-containing protein | | uniclust | UniRef100\_A0A098B9N3 | 99.1 | 2.7e-13 | 5.4e-19 | 88.9 | 53 | (2, 58) | 69 | (193, 245) | 309 | Peptidoglycan-binding LysM | Peptidoglycan-binding LysM | | uniclust | UniRef100\_A0A2G2AA53 | 99.1 | 2.8e-13 | 5.5e-19 | 82.8 | 54 | (2, 57) | 69 | (124, 177) | 178 | LysM domain-containing protein | LysM domain-containing protein | | uniclust | UniRef100\_A0A0V0PS59 | 99.1 | 2.9e-13 | 5.6e-19 | 76.3 | 50 | (1, 57) | 69 | (3, 52) | 103 | Peptidoglycan-binding protein | Peptidoglycan-binding protein | | uniclust | UniRef100\_A0A1M9YTZ0 | 99.1 | 3.2e-13 | 6e-19 | 79.7 | 50 | (2, 55) | 69 | (103, 152) | 152 | Putative mannose-specific lectin | Putative mannose-specific lectin | | uniclust | UniRef100\_A0A1G1IT91 | 99.1 | 3.2e-13 | 6.6e-19 | 85.7 | 55 | (2, 58) | 69 | (156, 210) | 223 | LysM domain-containing protein | LysM domain-containing protein | | uniclust | UniRef100\_A0A2V2D9M4 | 99.1 | 3.5e-13 | 6.8e-19 | 68.5 | 49 | (1, 56) | 69 | (7, 55) | 57 | LysM domain-containing protein | LysM domain-containing protein | | uniclust | UniRef100\_A0A2D5WBI1 | 99.1 | 3.4e-13 | 6.9e-19 | 87.1 | 52 | (1, 58) | 69 | (139, 190) | 266 | LysM domain-containing protein | LysM domain-containing protein | | uniclust | UniRef100\_A0A0N1F3Q5 | 99.1 | 3.5e-13 | 6.9e-19 | 73.0 | 65 | (5, 69) | 69 | (12, 79) | 79 | Phage tail protein | Phage tail protein | | uniclust | UniRef100\_A0A0E3XT95 | 99.1 | 3.9e-13 | 7.8e-19 | 86.7 | 54 | (1, 58) | 69 | (38, 91) | 278 | Mannose-binding protein | Mannose-binding protein | | uniclust | UniRef100\_A0A2W4LKG2 | 99.1 | 4.1e-13 | 8e-19 | 77.9 | 53 | (2, 58) | 69 | (66, 118) | 124 | Peptidoglycan-binding protein (Fragment) | Peptidoglycan-binding protein (Fragment) | | uniclust | UniRef100\_A0A9E4JKM9 | 99.1 | 4.6e-13 | 8.4e-19 | 80.3 | 59 | (1, 59) | 69 | (24, 83) | 192 | LysM peptidoglycan-binding domain-containing protein | LysM peptidoglycan-binding domain-containing protein | | uniclust | UniRef100\_A0A1V6BES0 | 99.1 | 4.4e-13 | 8.7e-19 | 70.6 | 48 | (3, 57) | 69 | (15, 62) | 67 | Gamma-D-glutamyl-L-diamino acid endopeptidase 1 | Gamma-D-glutamyl-L-diamino acid endopeptidase 1 | | uniclust | UniRef100\_A0A7C1AWR5 | 99.1 | 4.9e-13 | 9.4e-19 | 76.3 | 56 | (2, 59) | 69 | (54, 109) | 115 | LysM peptidoglycan-binding domain-containing protein | LysM peptidoglycan-binding domain-containing protein | | uniclust | UniRef100\_A0A959MSN4 | 99.1 | 5.1e-13 | 9.4e-19 | 68.5 | 56 | (2, 59) | 69 | (5, 60) | 67 | LysM peptidoglycan-binding domain-containing protein | LysM peptidoglycan-binding domain-containing protein | | uniclust | UniRef100\_A0A7C5GIV3 | 99.1 | 5e-13 | 9.5e-19 | 80.0 | 53 | (1, 55) | 69 | (111, 163) | 163 | LysM peptidoglycan-binding domain-containing protein | LysM peptidoglycan-binding domain-containing protein | | uniclust | UniRef100\_A0A1D7YSS0 | 99.1 | 4.7e-13 | 9.6e-19 | 83.7 | 56 | (2, 59) | 69 | (140, 195) | 199 | LysM domain-containing protein | LysM domain-containing protein | | uniclust | UniRef100\_A0A849XXF2 | 99.1 | 5.4e-13 | 9.9e-19 | 69.7 | 51 | (2, 55) | 69 | (25, 75) | 75 | LysM peptidoglycan-binding domain-containing protein | LysM peptidoglycan-binding domain-containing protein | | uniclust | UniRef100\_A0A3M1E5Y8 | 99.1 | 5.5e-13 | 1e-18 | 87.7 | 55 | (2, 58) | 69 | (323, 377) | 378 | LysM peptidoglycan-binding domain-containing protein | LysM peptidoglycan-binding domain-containing protein | | uniclust | UniRef100\_A0A2E2NN19 | 99.1 | 5.6e-13 | 1.1e-18 | 87.5 | 55 | (2, 58) | 69 | (271, 325) | 327 | LysM domain-containing protein | LysM domain-containing protein | | uniclust | UniRef100\_A0A0F9II26 | 99.1 | 5.8e-13 | 1.1e-18 | 71.0 | 50 | (2, 55) | 69 | (26, 76) | 76 | LysM domain-containing protein (Fragment) | LysM domain-containing protein (Fragment) | | uniclust | UniRef100\_A0A351VDG3 | 99.1 | 5.5e-13 | 1.1e-18 | 75.2 | 56 | (1, 59) | 69 | (3, 58) | 99 | Phage tail protein | Phage tail protein | | uniclust | UniRef100\_A0A2A2TBS7 | 99.1 | 5.8e-13 | 1.1e-18 | 70.4 | 49 | (2, 56) | 69 | (5, 53) | 73 | LysM domain-containing protein | LysM domain-containing protein | | uniclust | UniRef100\_A0A142X5D6 | 99.1 | 5.6e-13 | 1.1e-18 | 88.2 | 54 | (2, 57) | 69 | (183, 236) | 334 | LysM domain/BON superfamily protein | LysM domain/BON superfamily protein | | uniclust | UniRef100\_A0A3A4QHX3 | 99.1 | 5.6e-13 | 1.2e-18 | 90.2 | 58 | (2, 59) | 69 | (210, 267) | 387 | LysM peptidoglycan-binding domain-containing protein | LysM peptidoglycan-binding domain-containing protein | | uniclust | UniRef100\_A0A2D5B1A9 | 99.1 | 5.5e-13 | 1.2e-18 | 89.2 | 53 | (2, 58) | 69 | (181, 233) | 333 | LysM domain-containing protein | LysM domain-containing protein | | uniclust | UniRef100\_A0A1C3E468 | 99.1 | 5.7e-13 | 1.2e-18 | 90.3 | 52 | (1, 54) | 69 | (210, 261) | 392 | LysM domain-containing protein | LysM domain-containing protein | | uniclust | UniRef100\_A0A7Y2EMS1 | 99.1 | 6.5e-13 | 1.2e-18 | 72.6 | 53 | (2, 56) | 69 | (39, 91) | 92 | LysM peptidoglycan-binding domain-containing protein | LysM peptidoglycan-binding domain-containing protein | | uniclust | UniRef100\_A0A074LNR3 | 99.1 | 6.6e-13 | 1.3e-18 | 82.9 | 51 | (1, 55) | 69 | (161, 211) | 211 | LysM domain-containing protein | LysM domain-containing protein | | uniclust | UniRef100\_A0A937VFL9 | 99.1 | 7e-13 | 1.3e-18 | 76.1 | 51 | (2, 54) | 69 | (78, 128) | 128 | LysM peptidoglycan-binding domain-containing protein | LysM peptidoglycan-binding domain-containing protein | | uniclust | UniRef100\_A0A177Q767 | 99.1 | 6.6e-13 | 1.3e-18 | 86.2 | 55 | (2, 58) | 69 | (225, 279) | 282 | LysM domain-containing protein | LysM domain-containing protein | | uniclust | UniRef100\_A0A1G2XHC5 | 99.1 | 6.7e-13 | 1.3e-18 | 80.2 | 55 | (2, 58) | 69 | (106, 160) | 164 | LysM domain-containing protein | LysM domain-containing protein | | uniclust | UniRef100\_A0A069DLW2 | 99.1 | 6.2e-13 | 1.4e-18 | 94.0 | 51 | (1, 57) | 69 | (25, 75) | 570 | LysM domain-containing protein | LysM domain-containing protein | | uniclust | UniRef100\_A0A1H6F920 | 99.1 | 7e-13 | 1.4e-18 | 89.4 | 53 | (2, 56) | 69 | (334, 386) | 386 | LysM domain/BON superfamily protein | LysM domain/BON superfamily protein | | uniclust | UniRef100\_A0A0F0HL11 | 99.1 | 7.6e-13 | 1.5e-18 | 74.4 | 51 | (3, 56) | 69 | (45, 95) | 97 | LysM domain-containing protein | LysM domain-containing protein | | uniclust | UniRef100\_A0A098MA02 | 99.1 | 7.8e-13 | 1.5e-18 | 76.6 | 49 | (2, 57) | 69 | (8, 56) | 120 | LysM domain-containing protein | LysM domain-containing protein | | uniclust | UniRef100\_A0A3C0A1K5 | 99.1 | 8.2e-13 | 1.6e-18 | 71.1 | 53 | (2, 56) | 69 | (26, 78) | 80 | LysM domain-containing protein (Fragment) | LysM domain-containing protein (Fragment) | | uniclust | UniRef100\_A0A7X7RKI7 | 99.1 | 8.2e-13 | 1.6e-18 | 83.0 | 56 | (2, 57) | 69 | (180, 235) | 236 | LysM peptidoglycan-binding domain-containing protein | LysM peptidoglycan-binding domain-containing protein | | uniclust | UniRef100\_A0A0G0VNZ0 | 99.1 | 8e-13 | 1.6e-18 | 78.9 | 51 | (2, 56) | 69 | (88, 138) | 143 | LysM domain-containing protein | LysM domain-containing protein | | uniclust | UniRef100\_A0A0P6YX09 | 99.1 | 9e-13 | 1.6e-18 | 67.5 | 52 | (2, 59) | 69 | (11, 62) | 66 | LysM domain-containing protein | LysM domain-containing protein | | uniclust | UniRef100\_A0A1Q7A1S2 | 99.1 | 9.7e-13 | 1.8e-18 | 77.3 | 55 | (3, 59) | 69 | (24, 79) | 150 | LysM domain-containing protein | LysM domain-containing protein | | uniclust | UniRef100\_A0A2D5Z103 | 99.1 | 9.7e-13 | 1.8e-18 | 78.6 | 49 | (2, 54) | 69 | (119, 167) | 167 | LysM domain-containing protein | LysM domain-containing protein | | uniclust | UniRef100\_A0A066U3S9 | 99.1 | 8.7e-13 | 1.9e-18 | 87.9 | 54 | (2, 59) | 69 | (211, 264) | 325 | Peptidase M23B | Peptidase M23B | | uniclust | UniRef100\_A0A1J5SZ61 | 99.1 | 9.4e-13 | 1.9e-18 | 87.7 | 55 | (2, 58) | 69 | (297, 351) | 353 | Chromosome partition protein Smc | Chromosome partition protein Smc | | uniclust | UniRef100\_A0A7V3PXX5 | 99.1 | 1.1e-12 | 2e-18 | 66.4 | 54 | (3, 58) | 69 | (1, 54) | 62 | LysM peptidoglycan-binding domain-containing protein | LysM peptidoglycan-binding domain-containing protein | | uniclust | UniRef100\_UPI0002EF452D | 99.0 | 1.1e-12 | 2.1e-18 | 70.3 | 56 | (2, 58) | 69 | (17, 74) | 80 | LysM peptidoglycan-binding domain-containing protein | LysM peptidoglycan-binding domain-containing protein | | uniclust | UniRef100\_A0A1G6JKH2 | 99.0 | 1e-12 | 2.1e-18 | 86.9 | 52 | (1, 58) | 69 | (3, 54) | 325 | LysM domain-containing protein | LysM domain-containing protein | | uniclust | UniRef100\_A0A136P411 | 99.0 | 1.1e-12 | 2.1e-18 | 68.7 | 51 | (2, 54) | 69 | (26, 76) | 76 | LysM domain/BON superfamily protein | LysM domain/BON superfamily protein | | uniclust | UniRef100\_A0A2E0Y0Y8 | 99.0 | 1.1e-12 | 2.2e-18 | 85.0 | 52 | (2, 57) | 69 | (188, 239) | 312 | LysM domain-containing protein | LysM domain-containing protein | | uniclust | UniRef100\_A0A520XTY3 | 99.0 | 1.1e-12 | 2.2e-18 | 81.7 | 55 | (2, 58) | 69 | (141, 195) | 213 | Peptidoglycan-binding protein LysM | Peptidoglycan-binding protein LysM | | uniclust | UniRef100\_A0A356KNA6 | 99.0 | 1.2e-12 | 2.3e-18 | 70.9 | 55 | (2, 58) | 69 | (30, 84) | 87 | LysM domain-containing protein (Fragment) | LysM domain-containing protein (Fragment) | | uniclust | UniRef100\_A0A6J5KM05 | 99.0 | 1.1e-12 | 2.3e-18 | 73.6 | 51 | (3, 56) | 69 | (42, 92) | 94 | LysM domain | LysM domain | | uniclust | UniRef100\_A0A7X1TZH6 | 99.0 | 1.3e-12 | 2.3e-18 | 66.0 | 52 | (3, 58) | 69 | (6, 57) | 61 | LysM peptidoglycan-binding domain-containing protein | LysM peptidoglycan-binding domain-containing protein | | uniclust | UniRef100\_A0A014P2G8 | 99.0 | 1.1e-12 | 2.4e-18 | 85.3 | 54 | (2, 59) | 69 | (202, 255) | 266 | Peptidoglycan-binding protein | Peptidoglycan-binding protein | | uniclust | UniRef100\_A0A2E2N8R8 | 99.0 | 1.1e-12 | 2.4e-18 | 87.9 | 54 | (2, 57) | 69 | (291, 344) | 347 | LysM domain-containing protein | LysM domain-containing protein | | uniclust | UniRef100\_A0A1W9UYL2 | 99.0 | 1.3e-12 | 2.4e-18 | 83.9 | 54 | (2, 57) | 69 | (235, 288) | 289 | LysM domain-containing protein | LysM domain-containing protein | | uniclust | UniRef100\_A0A1H2WQU9 | 99.0 | 1.2e-12 | 2.4e-18 | 85.0 | 50 | (3, 54) | 69 | (219, 268) | 268 | LysM domain-containing protein | LysM domain-containing protein | | uniclust | UniRef100\_A0A1Z9QC33 | 99.0 | 1.2e-12 | 2.5e-18 | 86.2 | 57 | (1, 59) | 69 | (233, 289) | 293 | LysM domain-containing protein | LysM domain-containing protein | | uniclust | UniRef100\_A0A1W1CAC6 | 99.0 | 1.3e-12 | 2.5e-18 | 71.4 | 53 | (1, 55) | 69 | (38, 90) | 90 | LysM domain-containing protein | LysM domain-containing protein | | uniclust | UniRef100\_A0A1H7C4U6 | 99.0 | 1.3e-12 | 2.5e-18 | 70.8 | 53 | (2, 56) | 69 | (33, 85) | 86 | LysM domain-containing protein | LysM domain-containing protein | | uniclust | UniRef100\_A0A0C2VQS2 | 99.0 | 1.2e-12 | 2.5e-18 | 81.3 | 53 | (2, 56) | 69 | (137, 189) | 190 | LysM domain-containing protein | LysM domain-containing protein | | uniclust | UniRef100\_A0A6I7PWC1 | 99.0 | 1.3e-12 | 2.7e-18 | 86.8 | 51 | (2, 54) | 69 | (302, 352) | 352 | LysM peptidoglycan-binding domain-containing protein | LysM peptidoglycan-binding domain-containing protein | | uniclust | UniRef100\_A0A1V4QZM9 | 99.0 | 1.3e-12 | 2.7e-18 | 85.4 | 56 | (2, 57) | 69 | (229, 284) | 288 | LysM domain-containing protein | LysM domain-containing protein | | uniclust | UniRef100\_A0A151Z132 | 99.0 | 1.3e-12 | 2.7e-18 | 84.1 | 51 | (1, 57) | 69 | (38, 88) | 243 | LysM domain-containing protein | LysM domain-containing protein | | uniclust | UniRef100\_A0A376KHT5 | 99.0 | 1.5e-12 | 2.8e-18 | 74.9 | 63 | (4, 69) | 69 | (3, 65) | 139 | Tail protein X (GpX) | Tail protein X (GpX) | | uniclust | UniRef100\_A0A348PCI9 | 99.0 | 1.5e-12 | 2.9e-18 | 85.5 | 53 | (2, 58) | 69 | (174, 226) | 301 | LysM domain-containing protein | LysM domain-containing protein | | uniclust | UniRef100\_A0A3M1EYD0 | 99.0 | 1.5e-12 | 3e-18 | 78.9 | 53 | (2, 57) | 69 | (121, 174) | 175 | LysM peptidoglycan-binding domain-containing protein | LysM peptidoglycan-binding domain-containing protein | | uniclust | UniRef100\_A0A1M6M9A3 | 99.0 | 1.5e-12 | 3e-18 | 80.8 | 50 | (2, 55) | 69 | (149, 198) | 199 | LysM domain-containing protein | LysM domain-containing protein | | uniclust | UniRef100\_A0A0B3RRN2 | 99.0 | 1.7e-12 | 3.4e-18 | 85.0 | 54 | (3, 58) | 69 | (261, 314) | 316 | LysM domain protein | LysM domain protein | | uniclust | UniRef100\_A0A0C2W9T9 | 99.0 | 1.9e-12 | 3.8e-18 | 82.3 | 51 | (1, 57) | 69 | (1, 51) | 247 | LysM domain-containing protein | LysM domain-containing protein | | uniclust | UniRef100\_A0A0Q9LMA6 | 99.0 | 1.8e-12 | 3.8e-18 | 93.1 | 49 | (1, 55) | 69 | (22, 70) | 708 | LysM domain-containing protein | LysM domain-containing protein | | uniclust | UniRef100\_A0A015KHE2 | 99.0 | 1.9e-12 | 3.9e-18 | 92.0 | 50 | (1, 56) | 69 | (93, 142) | 640 | LysM domain-containing protein | LysM domain-containing protein | | uniclust | UniRef100\_A0A1G1NHF1 | 99.0 | 2.1e-12 | 4e-18 | 71.3 | 54 | (3, 58) | 69 | (22, 77) | 95 | LysM domain-containing protein | LysM domain-containing protein | | uniclust | UniRef100\_A0A0A3HNG1 | 99.0 | 1.9e-12 | 4.1e-18 | 83.8 | 50 | (2, 56) | 69 | (194, 243) | 244 | LysM domain-containing protein | LysM domain-containing protein | | uniclust | UniRef100\_A0A2E2B0B4 | 99.0 | 2e-12 | 4.1e-18 | 86.4 | 56 | (2, 59) | 69 | (297, 353) | 362 | LysM domain-containing protein | LysM domain-containing protein | | uniclust | UniRef100\_A0A1G0XXQ9 | 99.0 | 2.2e-12 | 4.4e-18 | 84.0 | 56 | (2, 59) | 69 | (214, 269) | 282 | LysM domain-containing protein | LysM domain-containing protein | | uniclust | UniRef100\_A0A3M1ZW81 | 99.0 | 2.3e-12 | 4.5e-18 | 75.6 | 55 | (2, 56) | 69 | (83, 138) | 139 | LysM peptidoglycan-binding domain-containing protein | LysM peptidoglycan-binding domain-containing protein | | uniclust | UniRef100\_A0A177Q305 | 99.0 | 2.4e-12 | 4.6e-18 | 75.1 | 56 | (2, 59) | 69 | (75, 130) | 134 | LysM domain-containing protein | LysM domain-containing protein | | uniclust | UniRef100\_A0A1P8WSI9 | 99.0 | 2.2e-12 | 4.7e-18 | 86.3 | 56 | (1, 58) | 69 | (207, 262) | 337 | LysM domain/BON superfamily protein | LysM domain/BON superfamily protein | | uniclust | UniRef100\_A0A0G0CX04 | 99.0 | 2.4e-12 | 4.8e-18 | 82.3 | 49 | (3, 55) | 69 | (183, 231) | 232 | LysM domain-containing protein | LysM domain-containing protein | | uniclust | UniRef100\_A0A7C4EC90 | 99.0 | 2.5e-12 | 4.9e-18 | 84.8 | 53 | (2, 58) | 69 | (193, 245) | 332 | LysM peptidoglycan-binding domain-containing protein | LysM peptidoglycan-binding domain-containing protein | | uniclust | UniRef100\_A0A061NGS6 | 99.0 | 2.3e-12 | 5e-18 | 83.7 | 56 | (2, 59) | 69 | (186, 242) | 255 | Phage-like element PBSX protein XkdP | Phage-like element PBSX protein XkdP | | uniclust | UniRef100\_A0A2D5B1A9 | 99.0 | 2.3e-12 | 5e-18 | 86.4 | 53 | (2, 58) | 69 | (258, 310) | 333 | LysM domain-containing protein | LysM domain-containing protein | | uniclust | UniRef100\_A0A0E3UH91 | 99.0 | 2.5e-12 | 5.1e-18 | 84.7 | 53 | (1, 55) | 69 | (261, 313) | 313 | LysM domain-containing protein | LysM domain-containing protein | | uniclust | UniRef100\_A0A7C1J8S6 | 99.0 | 2.8e-12 | 5.1e-18 | 67.3 | 48 | (3, 54) | 69 | (29, 76) | 76 | LysM peptidoglycan-binding domain-containing protein | LysM peptidoglycan-binding domain-containing protein | | uniclust | UniRef100\_A0A7J9WBE9 | 99.0 | 2.6e-12 | 5.1e-18 | 78.3 | 53 | (2, 58) | 69 | (119, 171) | 173 | LysM peptidoglycan-binding domain-containing protein | LysM peptidoglycan-binding domain-containing protein | | uniclust | UniRef100\_A0A0T7DV60 | 99.0 | 2.6e-12 | 5.2e-18 | 69.6 | 68 | (1, 68) | 69 | (5, 75) | 77 | Phage tail protein | Phage tail protein | | uniclust | UniRef100\_A0A0G0HFV7 | 99.0 | 2.6e-12 | 5.2e-18 | 79.0 | 50 | (3, 56) | 69 | (131, 180) | 182 | D-mannose binding lectin | D-mannose binding lectin | | uniclust | UniRef100\_A0A0P0FPI5 | 99.0 | 2.7e-12 | 5.3e-18 | 82.3 | 55 | (2, 58) | 69 | (200, 254) | 256 | LysM domain/BON superfamily protein | LysM domain/BON superfamily protein | | uniclust | UniRef100\_A0A1M4LC88 | 99.0 | 2.9e-12 | 5.3e-18 | 83.0 | 67 | (2, 69) | 69 | (301, 368) | 368 | Phage tail tape measure protein domain-containing protein | Phage tail tape measure protein domain-containing protein | | uniclust | UniRef100\_A0A1I2AZK8 | 99.0 | 2.6e-12 | 5.4e-18 | 88.3 | 52 | (1, 58) | 69 | (4, 55) | 439 | Morphogenetic protein associated with SpoVID (Fragment) | Morphogenetic protein associated with SpoVID (Fragment) | | uniclust | UniRef100\_UPI0003462B98 | 99.0 | 3e-12 | 5.5e-18 | 68.2 | 58 | (2, 59) | 69 | (4, 62) | 83 | LysM peptidoglycan-binding domain-containing protein | LysM peptidoglycan-binding domain-containing protein | | uniclust | UniRef100\_A0A0F9YZQ3 | 99.0 | 2.7e-12 | 5.6e-18 | 81.6 | 53 | (2, 58) | 69 | (92, 144) | 217 | Cell-wall-binding phage-encoded protein | Cell-wall-binding phage-encoded protein | | uniclust | UniRef100\_A0A0B1YCV7 | 99.0 | 2.8e-12 | 5.6e-18 | 85.8 | 53 | (1, 59) | 69 | (19, 71) | 363 | Morphogenetic protein associated with SpoVID | Morphogenetic protein associated with SpoVID | | uniclust | UniRef100\_A0A0S8ICA2 | 99.0 | 2.9e-12 | 5.6e-18 | 72.2 | 56 | (2, 57) | 69 | (45, 101) | 103 | LysM domain-containing protein | LysM domain-containing protein | | uniclust | UniRef100\_A0A0B8MZE7 | 99.0 | 3e-12 | 5.7e-18 | 79.4 | 49 | (2, 54) | 69 | (162, 210) | 210 | 5'-nucleotidase | 5'-nucleotidase | | uniclust | UniRef100\_A0A080LZ55 | 99.0 | 2.8e-12 | 5.8e-18 | 73.5 | 56 | (2, 59) | 69 | (40, 99) | 105 | LysM domain-containing protein | LysM domain-containing protein | | uniclust | UniRef100\_A0A517QRQ4 | 99.0 | 3.1e-12 | 6e-18 | 82.3 | 52 | (2, 56) | 69 | (215, 266) | 267 | LysM domain/BON superfamily protein | LysM domain/BON superfamily protein | | uniclust | UniRef100\_A0A2D7F4L0 | 99.0 | 3.3e-12 | 6.1e-18 | 68.5 | 51 | (2, 54) | 69 | (32, 82) | 82 | Peptidoglycan-binding protein | Peptidoglycan-binding protein | | uniclust | UniRef100\_A0A496RP82 | 99.0 | 3.2e-12 | 6.2e-18 | 76.7 | 55 | (2, 58) | 69 | (87, 141) | 156 | LysM peptidoglycan-binding domain-containing protein | LysM peptidoglycan-binding domain-containing protein | | uniclust | UniRef100\_A0A1V5WI12 | 99.0 | 3.2e-12 | 6.3e-18 | 71.3 | 52 | (2, 56) | 69 | (43, 94) | 97 | LysM domain/BON superfamily protein | LysM domain/BON superfamily protein | | uniclust | UniRef100\_A0A068TBM2 | 99.0 | 3.2e-12 | 6.3e-18 | 68.4 | 68 | (1, 68) | 69 | (1, 72) | 73 | Phage tail protein | Phage tail protein | | uniclust | UniRef100\_A0A1F8UA20 | 99.0 | 3.1e-12 | 6.5e-18 | 88.2 | 54 | (2, 59) | 69 | (398, 451) | 455 | LysM domain-containing protein | LysM domain-containing protein | | uniclust | UniRef100\_A0A1F4ZYX1 | 99.0 | 3.6e-12 | 6.6e-18 | 69.1 | 51 | (3, 55) | 69 | (42, 92) | 92 | LysM domain-containing protein | LysM domain-containing protein | | uniclust | UniRef100\_A0A1G2ZRS0 | 99.0 | 3.1e-12 | 6.6e-18 | 81.9 | 55 | (2, 58) | 69 | (138, 192) | 227 | LysM domain-containing protein | LysM domain-containing protein | | uniclust | UniRef100\_A0A1G9Q464 | 99.0 | 3.7e-12 | 6.8e-18 | 72.5 | 55 | (2, 58) | 69 | (42, 96) | 128 | LysM domain-containing protein | LysM domain-containing protein | | uniclust | UniRef100\_A0A0C7NBC1 | 99.0 | 3.5e-12 | 6.9e-18 | 81.4 | 50 | (3, 58) | 69 | (90, 139) | 255 | Spore coat assembly protein SafA | Spore coat assembly protein SafA | | uniclust | UniRef100\_A0A090ZT01 | 99.0 | 3.3e-12 | 7e-18 | 83.8 | 54 | (2, 57) | 69 | (212, 266) | 278 | LysM domain protein | LysM domain protein | | uniclust | UniRef100\_A0A6L3F9N8 | 99.0 | 3.8e-12 | 7.4e-18 | 82.8 | 52 | (2, 55) | 69 | (260, 311) | 312 | LysM peptidoglycan-binding domain-containing protein | LysM peptidoglycan-binding domain-containing protein | | uniclust | UniRef100\_A0A1A5XTK2 | 99.0 | 3.9e-12 | 7.8e-18 | 72.7 | 51 | (1, 58) | 69 | (1, 51) | 107 | Peptidoglycan-binding protein LysM | Peptidoglycan-binding protein LysM | | uniclust | UniRef100\_A0A2E2N8R8 | 99.0 | 3.9e-12 | 8.3e-18 | 85.5 | 52 | (2, 55) | 69 | (207, 258) | 347 | LysM domain-containing protein | LysM domain-containing protein | | uniclust | UniRef100\_X0VWZ0 | 99.0 | 4.6e-12 | 8.5e-18 | 76.2 | 53 | (2, 58) | 69 | (58, 110) | 191 | LysM domain-containing protein (Fragment) | LysM domain-containing protein (Fragment) | | uniclust | UniRef100\_A0A0F9QKI0 | 99.0 | 4.1e-12 | 8.6e-18 | 72.9 | 53 | (3, 58) | 69 | (37, 89) | 103 | LysM domain-containing protein | LysM domain-containing protein | | uniclust | UniRef100\_A0A1F9QVD6 | 99.0 | 4.2e-12 | 8.8e-18 | 88.2 | 57 | (1, 59) | 69 | (428, 484) | 487 | LysM domain-containing protein | LysM domain-containing protein | | uniclust | UniRef100\_A0A348PCI9 | 99.0 | 4.4e-12 | 8.9e-18 | 83.3 | 52 | (3, 56) | 69 | (248, 300) | 301 | LysM domain-containing protein | LysM domain-containing protein | | uniclust | UniRef100\_A0A2A5DCA3 | 99.0 | 4.3e-12 | 8.9e-18 | 82.7 | 53 | (2, 58) | 69 | (135, 187) | 264 | LysM domain-containing protein | LysM domain-containing protein | | uniclust | UniRef100\_A0A0A2SNG7 | 99.0 | 4.2e-12 | 8.9e-18 | 89.8 | 51 | (1, 57) | 69 | (31, 81) | 565 | Peptidoglycan-binding protein | Peptidoglycan-binding protein | | uniclust | UniRef100\_A0A0P1EW10 | 99.0 | 4.5e-12 | 9e-18 | 69.4 | 63 | (7, 69) | 69 | (13, 77) | 80 | Phage Tail Protein X | Phage Tail Protein X | | uniclust | UniRef100\_A0A1C3WM59 | 99.0 | 4.9e-12 | 9.1e-18 | 68.3 | 67 | (2, 69) | 69 | (23, 90) | 90 | p2-like prophage tail protein X | p2-like prophage tail protein X | | uniclust | UniRef100\_UPI001373773E | 99.0 | 4.8e-12 | 9.1e-18 | 71.8 | 54 | (2, 57) | 69 | (60, 113) | 114 | LysM peptidoglycan-binding domain-containing protein | LysM peptidoglycan-binding domain-containing protein | | uniclust | UniRef100\_A0A2E8CYE4 | 99.0 | 4.6e-12 | 9.2e-18 | 81.2 | 55 | (2, 58) | 69 | (158, 212) | 243 | LysM domain-containing protein | LysM domain-containing protein | | uniclust | UniRef100\_A0A0S8DVM3 | 98.9 | 5.2e-12 | 9.9e-18 | 68.9 | 54 | (2, 57) | 69 | (21, 74) | 86 | LysM domain-containing protein | LysM domain-containing protein | | uniclust | UniRef100\_A0A524HEJ6 | 98.9 | 5.4e-12 | 9.9e-18 | 67.0 | 53 | (2, 56) | 69 | (27, 80) | 81 | LysM domain-containing protein | LysM domain-containing protein | | uniclust | UniRef100\_A0A9D7QCG6 | 98.9 | 5.5e-12 | 1e-17 | 63.6 | 51 | (2, 54) | 69 | (10, 60) | 60 | LysM peptidoglycan-binding domain-containing protein | LysM peptidoglycan-binding domain-containing protein | | uniclust | UniRef100\_A0A0H5SYF8 | 98.9 | 4.6e-12 | 1e-17 | 89.0 | 52 | (2, 57) | 69 | (460, 511) | 513 | Putative membrane protein | Putative membrane protein | | uniclust | UniRef100\_A0A1F2Y198 | 98.9 | 5.2e-12 | 1e-17 | 70.3 | 51 | (2, 56) | 69 | (37, 88) | 89 | LysM domain-containing protein | LysM domain-containing protein | | uniclust | UniRef100\_A0A080KJZ3 | 98.9 | 5.5e-12 | 1e-17 | 65.7 | 62 | (7, 69) | 69 | (6, 67) | 67 | p2-like prophage tail protein X | p2-like prophage tail protein X | | uniclust | UniRef100\_A0A1Q2SNZ6 | 98.9 | 5.4e-12 | 1.1e-17 | 82.9 | 53 | (2, 56) | 69 | (257, 309) | 311 | Peptidoglycan-binding LysM | Peptidoglycan-binding LysM | | uniclust | UniRef100\_A0A293NIF4 | 98.9 | 5.2e-12 | 1.1e-17 | 83.7 | 54 | (1, 57) | 69 | (216, 270) | 298 | LysM domain-containing protein | LysM domain-containing protein | | uniclust | UniRef100\_A0A089HPX8 | 98.9 | 5.4e-12 | 1.1e-17 | 89.4 | 53 | (1, 59) | 69 | (58, 110) | 610 | LysM domain-containing protein | LysM domain-containing protein | | uniclust | UniRef100\_A0A5C5VW67 | 98.9 | 5.9e-12 | 1.1e-17 | 75.3 | 51 | (2, 54) | 69 | (106, 156) | 156 | LysM domain/BON superfamily protein | LysM domain/BON superfamily protein | | uniclust | UniRef100\_A0A1C6BB91 | 98.9 | 5.6e-12 | 1.1e-17 | 86.9 | 50 | (2, 55) | 69 | (424, 473) | 474 | LysM domain/BON superfamily protein | LysM domain/BON superfamily protein | | uniclust | UniRef100\_A0A0D6KHC7 | 98.9 | 5.5e-12 | 1.1e-17 | 81.0 | 54 | (3, 60) | 69 | (173, 226) | 238 | LysM domain protein | LysM domain protein | | uniclust | UniRef100\_E2CFJ8 | 98.9 | 6e-12 | 1.1e-17 | 66.7 | 57 | (1, 59) | 69 | (5, 61) | 73 | Phage Tail Protein X | Phage Tail Protein X | | uniclust | UniRef100\_A0A1W1BYN0 | 98.9 | 5.6e-12 | 1.1e-17 | 84.3 | 57 | (2, 60) | 69 | (236, 292) | 346 | LysM domain-containing protein | LysM domain-containing protein | | uniclust | UniRef100\_A0A838EI61 | 98.9 | 6e-12 | 1.1e-17 | 72.2 | 52 | (2, 55) | 69 | (65, 117) | 118 | LysM peptidoglycan-binding domain-containing protein | LysM peptidoglycan-binding domain-containing protein | | uniclust | UniRef100\_A0A2A4M5P7 | 98.9 | 5.6e-12 | 1.2e-17 | 83.6 | 51 | (2, 59) | 69 | (247, 297) | 303 | LysM domain-containing protein | LysM domain-containing protein | | uniclust | UniRef100\_A0A1C0AD30 | 98.9 | 6.1e-12 | 1.2e-17 | 80.2 | 47 | (1, 54) | 69 | (17, 63) | 231 | LysM domain-containing protein | LysM domain-containing protein | | uniclust | UniRef100\_A0A2A4RPQ5 | 98.9 | 5.9e-12 | 1.3e-17 | 84.9 | 55 | (1, 57) | 69 | (212, 266) | 354 | LysM domain-containing protein | LysM domain-containing protein | | uniclust | UniRef100\_A0A2N9MSL1 | 98.9 | 6.6e-12 | 1.3e-17 | 71.7 | 54 | (2, 59) | 69 | (50, 103) | 111 | Peptidoglycan-binding LysM | Peptidoglycan-binding LysM | | uniclust | UniRef100\_A0A1F2RWZ5 | 98.9 | 6.9e-12 | 1.3e-17 | 72.0 | 54 | (3, 59) | 69 | (9, 62) | 122 | LysM domain-containing protein | LysM domain-containing protein | | uniclust | UniRef100\_A0A037ZGX1 | 98.9 | 6.2e-12 | 1.3e-17 | 82.5 | 53 | (3, 57) | 69 | (27, 79) | 277 | LysM domain-containing protein | LysM domain-containing protein | | uniclust | UniRef100\_A0A288TXV0 | 98.9 | 7.2e-12 | 1.3e-17 | 62.3 | 51 | (2, 55) | 69 | (1, 53) | 54 | LysM domain-containing protein | LysM domain-containing protein | | uniclust | UniRef100\_A0A0A8JME8 | 98.9 | 6.5e-12 | 1.4e-17 | 85.5 | 50 | (1, 56) | 69 | (26, 75) | 392 | LysM domain-containing protein | LysM domain-containing protein | | uniclust | UniRef100\_A0A937KBK3 | 98.9 | 7.3e-12 | 1.4e-17 | 64.9 | 54 | (2, 57) | 69 | (13, 66) | 67 | LysM peptidoglycan-binding domain-containing protein | LysM peptidoglycan-binding domain-containing protein | | uniclust | UniRef100\_A0A1Q3SS48 | 98.9 | 7.3e-12 | 1.4e-17 | 79.3 | 52 | (2, 55) | 69 | (201, 252) | 252 | LysM domain-containing protein | LysM domain-containing protein | | uniclust | UniRef100\_A0A924W8C7 | 98.9 | 7.2e-12 | 1.4e-17 | 79.7 | 55 | (2, 58) | 69 | (200, 254) | 256 | LysM peptidoglycan-binding domain-containing protein | LysM peptidoglycan-binding domain-containing protein | | uniclust | UniRef100\_A0A8J3HSV3 | 98.9 | 7.5e-12 | 1.4e-17 | 73.9 | 50 | (2, 55) | 69 | (100, 149) | 149 | LysM domain-containing protein | LysM domain-containing protein | | uniclust | UniRef100\_A0A7Y7XD64 | 98.9 | 7.7e-12 | 1.4e-17 | 68.7 | 52 | (2, 55) | 69 | (42, 93) | 93 | LysM peptidoglycan-binding domain-containing protein | LysM peptidoglycan-binding domain-containing protein | | uniclust | UniRef100\_A0A1H3K5G8 | 98.9 | 7.1e-12 | 1.4e-17 | 85.1 | 53 | (1, 59) | 69 | (49, 101) | 400 | Morphogenetic protein associated with SpoVID | Morphogenetic protein associated with SpoVID | | uniclust | UniRef100\_A0A2E2B0B4 | 98.9 | 7.2e-12 | 1.4e-17 | 84.0 | 53 | (2, 58) | 69 | (229, 281) | 362 | LysM domain-containing protein | LysM domain-containing protein | | uniclust | UniRef100\_A0A1Z9QC33 | 98.9 | 7e-12 | 1.5e-17 | 82.8 | 54 | (2, 58) | 69 | (157, 211) | 293 | LysM domain-containing protein | LysM domain-containing protein | | uniclust | UniRef100\_A0A4P5YIC6 | 98.9 | 7.5e-12 | 1.5e-17 | 86.7 | 52 | (2, 57) | 69 | (346, 397) | 510 | LysM domain-containing protein | LysM domain-containing protein | | uniclust | UniRef100\_A0A1G0Y4L6 | 98.9 | 7.8e-12 | 1.5e-17 | 80.3 | 57 | (1, 59) | 69 | (196, 252) | 255 | LysM domain-containing protein | LysM domain-containing protein | | uniclust | UniRef100\_A0A0P1GCB9 | 98.9 | 8.2e-12 | 1.7e-17 | 78.5 | 51 | (3, 55) | 69 | (152, 202) | 203 | LysM domain/BON superfamily protein | LysM domain/BON superfamily protein | | uniclust | UniRef100\_A0A9D0S2Q7 | 98.9 | 9e-12 | 1.7e-17 | 78.5 | 51 | (2, 54) | 69 | (204, 254) | 254 | LysM peptidoglycan-binding domain-containing protein | LysM peptidoglycan-binding domain-containing protein | | uniclust | UniRef100\_A0A2K2TWU4 | 98.9 | 9.1e-12 | 1.7e-17 | 72.8 | 51 | (2, 54) | 69 | (87, 137) | 137 | LysM domain-containing protein | LysM domain-containing protein | | uniclust | UniRef100\_A0A0K2SFQ4 | 98.9 | 8.9e-12 | 1.8e-17 | 77.6 | 51 | (2, 58) | 69 | (21, 71) | 199 | LysM domain-containing protein | LysM domain-containing protein | | uniclust | UniRef100\_A0A953GLI6 | 98.9 | 9.5e-12 | 1.8e-17 | 76.2 | 52 | (2, 55) | 69 | (143, 194) | 195 | LysM peptidoglycan-binding domain-containing protein | LysM peptidoglycan-binding domain-containing protein | | uniclust | UniRef100\_A0A3M2EYF6 | 98.9 | 9.9e-12 | 1.8e-17 | 64.3 | 51 | (2, 54) | 69 | (19, 69) | 69 | LysM peptidoglycan-binding domain-containing protein | LysM peptidoglycan-binding domain-containing protein | | uniclust | UniRef100\_A0A135L2D6 | 98.9 | 8.8e-12 | 1.8e-17 | 82.9 | 52 | (1, 58) | 69 | (1, 52) | 317 | LysM domain-containing protein | LysM domain-containing protein | | uniclust | UniRef100\_A0A0D6KAX3 | 98.9 | 9.9e-12 | 1.9e-17 | 66.0 | 51 | (2, 56) | 69 | (22, 72) | 74 | LysM domain protein | LysM domain protein | | uniclust | UniRef100\_A0A524GYW0 | 98.9 | 9.4e-12 | 1.9e-17 | 75.6 | 54 | (2, 59) | 69 | (94, 147) | 159 | LysM peptidoglycan-binding domain-containing protein | LysM peptidoglycan-binding domain-containing protein | | uniclust | UniRef100\_A0A956BD51 | 98.9 | 1e-11 | 1.9e-17 | 74.8 | 53 | (2, 58) | 69 | (107, 159) | 176 | LysM peptidoglycan-binding domain-containing protein | LysM peptidoglycan-binding domain-containing protein | | uniclust | UniRef100\_A0A0S2W5J5 | 98.9 | 1e-11 | 1.9e-17 | 79.0 | 53 | (2, 58) | 69 | (160, 212) | 278 | LysM domain-containing protein | LysM domain-containing protein | | uniclust | UniRef100\_A0A1G8DH49 | 98.9 | 9.8e-12 | 2e-17 | 82.7 | 53 | (1, 59) | 69 | (1, 53) | 323 | Morphogenetic protein associated with SpoVID | Morphogenetic protein associated with SpoVID | | uniclust | UniRef100\_A0A086YNE6 | 98.9 | 9.6e-12 | 2.1e-17 | 88.7 | 47 | (2, 55) | 69 | (515, 561) | 618 | Peptidoglycan-binding protein | Peptidoglycan-binding protein | | uniclust | UniRef100\_A0A7V3QFU2 | 98.9 | 1.1e-11 | 2.1e-17 | 62.2 | 54 | (2, 57) | 69 | (4, 57) | 58 | LysM domain-containing protein | LysM domain-containing protein | | uniclust | UniRef100\_A0A533Z935 | 98.9 | 1.1e-11 | 2.1e-17 | 77.2 | 55 | (1, 59) | 69 | (4, 58) | 237 | LysM peptidoglycan-binding domain-containing protein | LysM peptidoglycan-binding domain-containing protein | | uniclust | UniRef100\_A0A178IH20 | 98.9 | 1.1e-11 | 2.1e-17 | 88.9 | 51 | (2, 54) | 69 | (729, 779) | 779 | LysM domain-containing protein | LysM domain-containing protein | | uniclust | UniRef100\_A0A1V2SEX1 | 98.9 | 1.1e-11 | 2.2e-17 | 75.0 | 53 | (2, 56) | 69 | (94, 154) | 156 | LysM domain-containing protein (Fragment) | LysM domain-containing protein (Fragment) | | uniclust | UniRef100\_A0A0B0ELG5 | 98.9 | 1.1e-11 | 2.2e-17 | 81.8 | 54 | (2, 57) | 69 | (260, 313) | 315 | LysM domain-containing protein | LysM domain-containing protein | | uniclust | UniRef100\_A0A2A5FW86 | 98.9 | 1.2e-11 | 2.4e-17 | 79.4 | 51 | (1, 54) | 69 | (216, 266) | 267 | LysM domain-containing protein | LysM domain-containing protein | | uniclust | UniRef100\_A0A2P5MCF5 | 98.9 | 1.2e-11 | 2.4e-17 | 82.1 | 53 | (3, 59) | 69 | (281, 333) | 338 | LysM domain-containing protein | LysM domain-containing protein | | uniclust | UniRef100\_A0A0G0JZZ0 | 98.9 | 1.1e-11 | 2.4e-17 | 79.4 | 53 | (2, 58) | 69 | (90, 142) | 227 | LysM domain-containing protein | LysM domain-containing protein | | uniclust | UniRef100\_A0A1N6VA67 | 98.9 | 1.2e-11 | 2.4e-17 | 82.6 | 54 | (2, 58) | 69 | (35, 88) | 329 | LysM domain-containing protein | LysM domain-containing protein | | uniclust | UniRef100\_A0A0K9YT35 | 98.9 | 1.3e-11 | 2.4e-17 | 74.2 | 51 | (1, 58) | 69 | (27, 77) | 157 | Peptidoglycan-binding protein LysM | Peptidoglycan-binding protein LysM | | uniclust | UniRef100\_A0A2N3DL64 | 98.9 | 1.3e-11 | 2.5e-17 | 64.5 | 60 | (6, 69) | 69 | (10, 70) | 70 | Phage tail protein | Phage tail protein | | uniclust | UniRef100\_A0A4Y9QV55 | 98.9 | 1.4e-11 | 2.5e-17 | 71.6 | 49 | (2, 54) | 69 | (96, 144) | 144 | LysM domain-containing protein | LysM domain-containing protein | | uniclust | UniRef100\_A0A7C5KF11 | 98.9 | 1.4e-11 | 2.5e-17 | 65.3 | 53 | (3, 58) | 69 | (18, 70) | 79 | LysM domain-containing protein | LysM domain-containing protein | | uniclust | UniRef100\_A0A1W9WAD3 | 98.9 | 1.4e-11 | 2.6e-17 | 59.6 | 46 | (7, 54) | 69 | (2, 47) | 47 | LysM domain-containing protein | LysM domain-containing protein | | uniclust | UniRef100\_A0A350UMH8 | 98.9 | 1.3e-11 | 2.6e-17 | 80.2 | 54 | (1, 58) | 69 | (11, 64) | 297 | LysM domain-containing protein | LysM domain-containing protein | | uniclust | UniRef100\_A0A024QAB1 | 98.9 | 1.3e-11 | 2.7e-17 | 82.9 | 53 | (1, 59) | 69 | (29, 81) | 347 | Spore coat assembly protein ExsA | Spore coat assembly protein ExsA | | uniclust | UniRef100\_A0A0S8C8Z0 | 98.9 | 1.3e-11 | 2.7e-17 | 84.3 | 53 | (2, 57) | 69 | (71, 123) | 397 | LysM domain-containing protein | LysM domain-containing protein | | uniclust | UniRef100\_A0A2D5NJU9 | 98.9 | 1.3e-11 | 2.8e-17 | 80.1 | 54 | (1, 58) | 69 | (124, 177) | 250 | LysM domain-containing protein | LysM domain-containing protein | | uniclust | UniRef100\_A0A081LB99 | 98.9 | 1.3e-11 | 2.8e-17 | 86.9 | 53 | (1, 59) | 69 | (14, 66) | 538 | SpoVID-associated morphogenetic protein | SpoVID-associated morphogenetic protein | | uniclust | UniRef100\_A0A084SYV7 | 98.9 | 1.3e-11 | 2.8e-17 | 85.5 | 51 | (2, 55) | 69 | (72, 122) | 435 | Peptidoglycan-binding protein | Peptidoglycan-binding protein | | uniclust | UniRef100\_A0A838HEQ8 | 98.9 | 1.5e-11 | 2.8e-17 | 61.6 | 51 | (2, 54) | 69 | (7, 57) | 57 | LysM peptidoglycan-binding domain-containing protein | LysM peptidoglycan-binding domain-containing protein | | uniclust | UniRef100\_A0A078MBJ7 | 98.9 | 1.4e-11 | 2.8e-17 | 80.1 | 51 | (2, 55) | 69 | (205, 256) | 257 | LysM domain protein | LysM domain protein | | uniclust | UniRef100\_A0A2T6CMD0 | 98.9 | 1.4e-11 | 2.9e-17 | 89.4 | 51 | (2, 54) | 69 | (820, 870) | 870 | LysM domain-containing protein | LysM domain-containing protein | | uniclust | UniRef100\_A0A0A3I541 | 98.9 | 1.5e-11 | 3e-17 | 83.6 | 50 | (1, 56) | 69 | (3, 52) | 415 | Uncharacterized protein | Uncharacterized protein | | uniclust | UniRef100\_A0A0G0CX04 | 98.9 | 1.4e-11 | 3e-17 | 78.9 | 54 | (2, 59) | 69 | (109, 162) | 232 | LysM domain-containing protein | LysM domain-containing protein | | uniclust | UniRef100\_A0A0U1NKI6 | 98.9 | 1.4e-11 | 3e-17 | 83.1 | 52 | (2, 55) | 69 | (303, 354) | 355 | LysM domain/BON superfamily protein | LysM domain/BON superfamily protein | | uniclust | UniRef100\_A0A2P5LPX3 | 98.9 | 1.7e-11 | 3.2e-17 | 69.6 | 57 | (2, 58) | 69 | (42, 98) | 118 | Peptidoglycan-binding protein | Peptidoglycan-binding protein | | uniclust | UniRef100\_A0A074LMC3 | 98.9 | 1.6e-11 | 3.2e-17 | 78.2 | 53 | (2, 58) | 69 | (149, 201) | 243 | LysM domain-containing protein | LysM domain-containing protein | | uniclust | UniRef100\_A0A1W9VZ66 | 98.9 | 1.6e-11 | 3.2e-17 | 81.0 | 53 | (2, 58) | 69 | (75, 127) | 327 | LysM domain-containing protein | LysM domain-containing protein | | uniclust | UniRef100\_A0A379LXN4 | 98.9 | 1.7e-11 | 3.2e-17 | 69.5 | 52 | (2, 57) | 69 | (55, 106) | 108 | LysM domain/BON superfamily protein | LysM domain/BON superfamily protein | | uniclust | UniRef100\_A0A2A4XSY9 | 98.9 | 1.7e-11 | 3.3e-17 | 65.9 | 68 | (2, 69) | 69 | (7, 77) | 77 | Phage tail protein | Phage tail protein | | uniclust | UniRef100\_A0A1I7J0W0 | 98.9 | 1.7e-11 | 3.3e-17 | 82.3 | 53 | (1, 59) | 69 | (34, 86) | 372 | LysM repeat-containing protein | LysM repeat-containing protein | | uniclust | UniRef100\_A0A0G0YVI2 | 98.9 | 1.8e-11 | 3.3e-17 | 66.3 | 49 | (3, 55) | 69 | (36, 84) | 84 | LysM domain-containing protein | LysM domain-containing protein | | uniclust | UniRef100\_A0A1X1NF73 | 98.9 | 1.7e-11 | 3.3e-17 | 68.1 | 52 | (2, 56) | 69 | (40, 91) | 92 | LysM domain-containing protein | LysM domain-containing protein | | uniclust | UniRef100\_A0A345ZPL7 | 98.9 | 1.8e-11 | 3.4e-17 | 71.2 | 54 | (2, 57) | 69 | (84, 137) | 139 | LysM peptidoglycan-binding domain-containing protein | LysM peptidoglycan-binding domain-containing protein | | uniclust | UniRef100\_A0A0G1F008 | 98.9 | 1.8e-11 | 3.5e-17 | 63.0 | 52 | (3, 56) | 69 | (8, 59) | 60 | LysM domain-containing protein (Fragment) | LysM domain-containing protein (Fragment) | | uniclust | UniRef100\_A0A0X8HDA8 | 98.9 | 1.8e-11 | 3.6e-17 | 77.4 | 51 | (3, 55) | 69 | (174, 224) | 224 | LysM domain/BON superfamily protein | LysM domain/BON superfamily protein | | uniclust | UniRef100\_UPI000665D35C | 98.9 | 2e-11 | 3.6e-17 | 63.4 | 69 | (1, 69) | 69 | (1, 70) | 70 | tail protein X | tail protein X | | uniclust | UniRef100\_A0A097ARJ0 | 98.9 | 1.9e-11 | 3.7e-17 | 74.1 | 49 | (2, 56) | 69 | (33, 81) | 166 | Spore coat assembly protein SafA | Spore coat assembly protein SafA | | uniclust | UniRef100\_A0A1F9QAA3 | 98.9 | 1.9e-11 | 3.7e-17 | 76.8 | 54 | (2, 58) | 69 | (152, 205) | 219 | LysM domain-containing protein | LysM domain-containing protein | | uniclust | UniRef100\_A0A143XDP1 | 98.9 | 1.8e-11 | 3.8e-17 | 84.7 | 52 | (3, 58) | 69 | (397, 448) | 451 | LysM domain/BON superfamily protein | LysM domain/BON superfamily protein | | uniclust | UniRef100\_A0A7V2K829 | 98.9 | 2.1e-11 | 3.8e-17 | 68.9 | 57 | (2, 60) | 69 | (54, 110) | 119 | LysM peptidoglycan-binding domain-containing protein | LysM peptidoglycan-binding domain-containing protein | | uniclust | UniRef100\_A0A7C4EC90 | 98.9 | 2e-11 | 3.9e-17 | 80.8 | 57 | (2, 60) | 69 | (269, 326) | 332 | LysM peptidoglycan-binding domain-containing protein | LysM peptidoglycan-binding domain-containing protein | | uniclust | UniRef100\_A0A2R7Z0I0 | 98.9 | 1.9e-11 | 3.9e-17 | 72.6 | 47 | (2, 55) | 69 | (85, 131) | 132 | LysM domain-containing protein | LysM domain-containing protein | | uniclust | UniRef100\_A0A0A0HMA7 | 98.9 | 1.8e-11 | 3.9e-17 | 83.9 | 55 | (2, 58) | 69 | (71, 126) | 411 | Amino acid ABC transporter substrate-binding protein, PAAT family | Amino acid ABC transporter substrate-binding protein, PAAT family | | uniclust | UniRef100\_A0A0A7HAL1 | 98.8 | 2e-11 | 4e-17 | 78.7 | 54 | (2, 57) | 69 | (192, 245) | 248 | Peptidoglycan-binding protein LysM | Peptidoglycan-binding protein LysM | | uniclust | UniRef100\_A0A7W3WB71 | 98.8 | 2.2e-11 | 4e-17 | 61.6 | 53 | (2, 56) | 69 | (7, 59) | 60 | LysM peptidoglycan-binding domain-containing protein | LysM peptidoglycan-binding domain-containing protein | | uniclust | UniRef100\_A0A1G0ZHH7 | 98.8 | 2e-11 | 4.1e-17 | 77.9 | 48 | (2, 56) | 69 | (173, 220) | 220 | LysM domain-containing protein | LysM domain-containing protein | | uniclust | UniRef100\_A0A1Q7T6T4 | 98.8 | 2.1e-11 | 4.1e-17 | 65.3 | 52 | (2, 55) | 69 | (22, 74) | 75 | LysM domain-containing protein | LysM domain-containing protein | | uniclust | UniRef100\_A0A1R4IUY4 | 98.8 | 2.1e-11 | 4.2e-17 | 76.3 | 47 | (2, 55) | 69 | (145, 191) | 192 | TolA protein | TolA protein | | uniclust | UniRef100\_S3J5W6 | 98.8 | 2.3e-11 | 4.2e-17 | 68.8 | 55 | (1, 59) | 69 | (1, 55) | 120 | LysM domain-containing protein | LysM domain-containing protein | | uniclust | UniRef100\_A0A1I3WQT9 | 98.8 | 2.1e-11 | 4.3e-17 | 84.9 | 51 | (1, 57) | 69 | (12, 62) | 522 | Spore coat assembly protein SafA | Spore coat assembly protein SafA | | uniclust | UniRef100\_A0A6I0F283 | 98.8 | 2.2e-11 | 4.3e-17 | 64.7 | 50 | (3, 59) | 69 | (15, 64) | 68 | LysM peptidoglycan-binding domain-containing protein | LysM peptidoglycan-binding domain-containing protein | | uniclust | UniRef100\_A0A925ALC7 | 98.8 | 2.3e-11 | 4.4e-17 | 70.8 | 49 | (2, 54) | 69 | (85, 133) | 133 | LysM peptidoglycan-binding domain-containing protein | LysM peptidoglycan-binding domain-containing protein | | uniclust | UniRef100\_A0A3C0VA40 | 98.8 | 2.4e-11 | 4.5e-17 | 75.7 | 53 | (2, 56) | 69 | (185, 237) | 238 | LysM domain-containing protein | LysM domain-containing protein | | uniclust | UniRef100\_A0A2H5Z0M6 | 98.8 | 2.2e-11 | 4.5e-17 | 72.6 | 50 | (2, 58) | 69 | (88, 137) | 138 | N-acetylmuramoyl-L-alanine amidase XlyA | N-acetylmuramoyl-L-alanine amidase XlyA | | uniclust | UniRef100\_A0A0G0VNZ0 | 98.8 | 2.3e-11 | 4.6e-17 | 73.1 | 53 | (2, 58) | 69 | (18, 70) | 143 | LysM domain-containing protein | LysM domain-containing protein | | uniclust | UniRef100\_A0A1R0F851 | 98.8 | 2.3e-11 | 4.6e-17 | 81.0 | 56 | (2, 59) | 69 | (265, 320) | 335 | Nucleoid-associated protein YgaU, contains BON and LysM domains | Nucleoid-associated protein YgaU, contains BON and LysM domains | | uniclust | UniRef100\_A0A2W5H3R5 | 98.8 | 2.5e-11 | 4.6e-17 | 71.7 | 68 | (2, 69) | 69 | (95, 163) | 163 | Phage tail protein | Phage tail protein | | uniclust | UniRef100\_A0A534MS82 | 98.8 | 2.5e-11 | 4.6e-17 | 71.2 | 54 | (2, 57) | 69 | (86, 139) | 142 | LysM peptidoglycan-binding domain-containing protein (Fragment) | LysM peptidoglycan-binding domain-containing protein (Fragment) | | uniclust | UniRef100\_A0A357KMI0 | 98.8 | 2.6e-11 | 4.7e-17 | 67.8 | 49 | (3, 54) | 69 | (61, 109) | 110 | LysM domain-containing protein | LysM domain-containing protein | | uniclust | UniRef100\_A0A2D6N5F9 | 98.8 | 2.4e-11 | 4.7e-17 | 67.4 | 55 | (2, 58) | 69 | (18, 73) | 90 | LysM domain-containing protein | LysM domain-containing protein | | uniclust | UniRef100\_A0A2D5XPP0 | 98.8 | 2.4e-11 | 4.7e-17 | 74.3 | 56 | (2, 59) | 69 | (98, 153) | 165 | LysM domain-containing protein | LysM domain-containing protein | | uniclust | UniRef100\_A0A7C5HFW9 | 98.8 | 2.6e-11 | 4.8e-17 | 72.8 | 56 | (1, 58) | 69 | (122, 177) | 184 | LysM peptidoglycan-binding domain-containing protein | LysM peptidoglycan-binding domain-containing protein | | uniclust | UniRef100\_A0A1F7R001 | 98.8 | 2.3e-11 | 4.9e-17 | 77.5 | 52 | (2, 59) | 69 | (52, 103) | 217 | LysM domain-containing protein | LysM domain-containing protein | | uniclust | UniRef100\_A0A109MUW9 | 98.8 | 2.5e-11 | 5e-17 | 85.8 | 53 | (1, 59) | 69 | (11, 63) | 595 | Uncharacterized protein (Fragment) | Uncharacterized protein (Fragment) | | uniclust | UniRef100\_UPI00117B2ACC | 98.8 | 2.8e-11 | 5.1e-17 | 61.1 | 53 | (1, 59) | 69 | (1, 53) | 59 | SafA/ExsA family spore coat assembly protein | SafA/ExsA family spore coat assembly protein | | uniclust | UniRef100\_A0A1Z9KPR5 | 98.8 | 2.5e-11 | 5.2e-17 | 77.9 | 54 | (2, 59) | 69 | (110, 163) | 235 | LysM domain-containing protein | LysM domain-containing protein | | uniclust | UniRef100\_A0A202DNT9 | 98.8 | 2.7e-11 | 5.2e-17 | 81.5 | 53 | (2, 56) | 69 | (365, 417) | 419 | LysM domain-containing protein | LysM domain-containing protein | | uniclust | UniRef100\_A0A8J3GFQ9 | 98.8 | 2.8e-11 | 5.2e-17 | 65.7 | 51 | (2, 55) | 69 | (40, 90) | 91 | LysM domain-containing protein | LysM domain-containing protein | | uniclust | UniRef100\_A0A2T7TER6 | 98.8 | 2.7e-11 | 5.3e-17 | 67.2 | 52 | (2, 57) | 69 | (30, 83) | 92 | LysM domain-containing protein | LysM domain-containing protein | | uniclust | UniRef100\_A0A2A5DCA3 | 98.8 | 2.5e-11 | 5.3e-17 | 79.3 | 52 | (2, 55) | 69 | (211, 263) | 264 | LysM domain-containing protein | LysM domain-containing protein | | uniclust | UniRef100\_A0A0P6YH39 | 98.8 | 2.9e-11 | 5.3e-17 | 66.9 | 52 | (2, 55) | 69 | (52, 103) | 103 | LysM domain-containing protein | LysM domain-containing protein | | uniclust | UniRef100\_A0A432QVU7 | 98.8 | 2.9e-11 | 5.4e-17 | 63.6 | 69 | (1, 69) | 69 | (1, 71) | 75 | Phage tail protein | Phage tail protein | | uniclust | UniRef100\_A0A2V5Q5V7 | 98.8 | 2.7e-11 | 5.4e-17 | 83.1 | 55 | (2, 58) | 69 | (406, 460) | 465 | LysM domain-containing protein | LysM domain-containing protein | | uniclust | UniRef100\_A0A120MKI8 | 98.8 | 2.7e-11 | 5.4e-17 | 82.2 | 50 | (2, 55) | 69 | (335, 384) | 385 | LysM domain/BON superfamily protein | LysM domain/BON superfamily protein | | uniclust | UniRef100\_A0A2E5G5P4 | 98.8 | 3e-11 | 5.5e-17 | 70.4 | 54 | (2, 59) | 69 | (86, 139) | 146 | LysM domain-containing protein | LysM domain-containing protein | | uniclust | UniRef100\_A0A961BZ49 | 98.8 | 3.1e-11 | 5.7e-17 | 73.2 | 50 | (2, 55) | 69 | (136, 185) | 185 | LysM peptidoglycan-binding domain-containing protein | LysM peptidoglycan-binding domain-containing protein | | uniclust | UniRef100\_A0A7X4CNS4 | 98.8 | 3.2e-11 | 5.8e-17 | 62.7 | 67 | (3, 69) | 69 | (4, 70) | 70 | Phage tail protein | Phage tail protein | | uniclust | UniRef100\_A0A353M381 | 98.8 | 2.9e-11 | 5.8e-17 | 80.3 | 53 | (2, 58) | 69 | (269, 321) | 324 | LysM peptidoglycan-binding domain-containing protein | LysM peptidoglycan-binding domain-containing protein | | uniclust | UniRef100\_A0A099S5L2 | 98.8 | 3e-11 | 6e-17 | 74.3 | 49 | (1, 55) | 69 | (1, 49) | 179 | Peptidoglycan-binding protein | Peptidoglycan-binding protein | | uniclust | UniRef100\_A0A7C1J0I8 | 98.8 | 3.2e-11 | 6.1e-17 | 71.2 | 53 | (3, 57) | 69 | (86, 139) | 140 | LysM peptidoglycan-binding domain-containing protein | LysM peptidoglycan-binding domain-containing protein | | uniclust | UniRef100\_A0A084JKQ0 | 98.8 | 3e-11 | 6.3e-17 | 85.2 | 54 | (2, 59) | 69 | (486, 539) | 549 | Peptidoglycan-binding protein | Peptidoglycan-binding protein | | uniclust | UniRef100\_A0A2E2MDG1 | 98.8 | 3.3e-11 | 6.3e-17 | 79.1 | 50 | (3, 56) | 69 | (274, 323) | 324 | LysM domain-containing protein | LysM domain-containing protein | | uniclust | UniRef100\_A0A1F9NKC6 | 98.8 | 3.1e-11 | 6.3e-17 | 78.7 | 61 | (2, 68) | 69 | (116, 176) | 263 | Endolytic peptidoglycan transglycosylase RlpA | Endolytic peptidoglycan transglycosylase RlpA | | uniclust | UniRef100\_A0A3M1Q3A8 | 98.8 | 3.2e-11 | 6.5e-17 | 81.5 | 56 | (2, 57) | 69 | (204, 259) | 370 | LysM domain-containing protein | LysM domain-containing protein | | uniclust | UniRef100\_UPI001FC8EFCB | 98.8 | 3.6e-11 | 6.6e-17 | 62.5 | 51 | (3, 55) | 69 | (18, 68) | 70 | LysM peptidoglycan-binding domain-containing protein | LysM peptidoglycan-binding domain-containing protein | | uniclust | UniRef100\_A0A064AM34 | 98.8 | 3.4e-11 | 6.7e-17 | 64.8 | 56 | (1, 59) | 69 | (9, 64) | 75 | Tail protein | Tail protein | | uniclust | UniRef100\_A0A2D5NJ77 | 98.8 | 3.3e-11 | 6.8e-17 | 80.2 | 55 | (3, 59) | 69 | (254, 309) | 314 | LysM domain-containing protein | LysM domain-containing protein | | uniclust | UniRef100\_A0A916G808 | 98.8 | 3.5e-11 | 6.9e-17 | 77.6 | 54 | (2, 59) | 69 | (99, 152) | 258 | LysM domain-containing protein | LysM domain-containing protein | | uniclust | UniRef100\_A0A072P1Q2 | 98.8 | 3.3e-11 | 6.9e-17 | 84.2 | 52 | (1, 58) | 69 | (16, 67) | 504 | Spore coat assembly protein SafA | Spore coat assembly protein SafA | | uniclust | UniRef100\_A0A0M0LN26 | 98.8 | 3.6e-11 | 7e-17 | 82.0 | 52 | (1, 58) | 69 | (15, 66) | 456 | LysM domain-containing protein | LysM domain-containing protein | | uniclust | UniRef100\_A0A535BUW1 | 98.8 | 3.7e-11 | 7.1e-17 | 66.0 | 52 | (2, 55) | 69 | (38, 90) | 90 | LysM peptidoglycan-binding domain-containing protein | LysM peptidoglycan-binding domain-containing protein | | uniclust | UniRef100\_A0A1G2Y5P4 | 98.8 | 3.5e-11 | 7.3e-17 | 80.9 | 57 | (2, 58) | 69 | (190, 249) | 340 | LysM domain-containing protein | LysM domain-containing protein | | uniclust | UniRef100\_A0A2V8X4F0 | 98.8 | 3.9e-11 | 7.3e-17 | 64.3 | 52 | (2, 56) | 69 | (26, 77) | 80 | Peptidoglycan-binding protein LysM (Fragment) | Peptidoglycan-binding protein LysM (Fragment) | | uniclust | UniRef100\_A0A969XUJ1 | 98.8 | 4e-11 | 7.4e-17 | 69.3 | 51 | (3, 55) | 69 | (88, 138) | 138 | LysM peptidoglycan-binding domain-containing protein | LysM peptidoglycan-binding domain-containing protein | | uniclust | UniRef100\_A0A4P5YIC6 | 98.8 | 3.7e-11 | 7.5e-17 | 83.5 | 53 | (2, 58) | 69 | (276, 328) | 510 | LysM domain-containing protein | LysM domain-containing protein | | uniclust | UniRef100\_A0A533ZFK9 | 98.8 | 4.1e-11 | 7.6e-17 | 67.5 | 54 | (1, 58) | 69 | (1, 54) | 116 | LysM peptidoglycan-binding domain-containing protein | LysM peptidoglycan-binding domain-containing protein | | uniclust | UniRef100\_A0A097ARJ0 | 98.8 | 4e-11 | 7.8e-17 | 72.8 | 49 | (2, 56) | 69 | (91, 139) | 166 | Spore coat assembly protein SafA | Spore coat assembly protein SafA | | uniclust | UniRef100\_A0A2A5A5I2 | 98.8 | 4e-11 | 7.8e-17 | 77.8 | 52 | (2, 55) | 69 | (232, 283) | 283 | LysM domain-containing protein | LysM domain-containing protein | | uniclust | UniRef100\_A0A1H0NML7 | 98.8 | 4.3e-11 | 7.9e-17 | 65.0 | 54 | (5, 59) | 69 | (4, 57) | 91 | P2-like prophage tail protein X | P2-like prophage tail protein X | | uniclust | UniRef100\_A0A7C5FFV2 | 98.8 | 4.4e-11 | 8.2e-17 | 78.9 | 56 | (1, 58) | 69 | (310, 365) | 372 | LysM peptidoglycan-binding domain-containing protein | LysM peptidoglycan-binding domain-containing protein | | uniclust | UniRef100\_A0A0F9YZQ3 | 98.8 | 4e-11 | 8.2e-17 | 76.6 | 49 | (3, 55) | 69 | (168, 216) | 217 | Cell-wall-binding phage-encoded protein | Cell-wall-binding phage-encoded protein | | uniclust | UniRef100\_A0A085LA63 | 98.8 | 4.2e-11 | 8.3e-17 | 75.8 | 50 | (2, 58) | 69 | (138, 187) | 215 | LysM domain-containing protein | LysM domain-containing protein | | uniclust | UniRef100\_A0A0P7KLW9 | 98.8 | 4.5e-11 | 8.4e-17 | 68.2 | 55 | (2, 58) | 69 | (62, 116) | 118 | LysM domain-containing protein | LysM domain-containing protein | | uniclust | UniRef100\_A0A0S8HL66 | 98.8 | 4.3e-11 | 8.4e-17 | 78.9 | 54 | (2, 59) | 69 | (139, 193) | 312 | LysM domain-containing protein | LysM domain-containing protein | | uniclust | UniRef100\_A0A8T3PST5 | 98.8 | 4.6e-11 | 8.5e-17 | 66.2 | 52 | (3, 58) | 69 | (49, 100) | 103 | LysM peptidoglycan-binding domain-containing protein | LysM peptidoglycan-binding domain-containing protein | | uniclust | UniRef100\_A0A1A2S6J1 | 98.8 | 4.7e-11 | 8.6e-17 | 72.3 | 53 | (2, 58) | 69 | (3, 55) | 195 | LysM domain-containing protein | LysM domain-containing protein | | uniclust | UniRef100\_A0A1B6BCU7 | 98.8 | 4.1e-11 | 8.6e-17 | 78.5 | 54 | (2, 59) | 69 | (194, 247) | 265 | Contractile injection system tube protein N-terminal domain-containing protein | Contractile injection system tube protein N-terminal domain-containing protein | | uniclust | UniRef100\_A0A090IT45 | 98.8 | 4.2e-11 | 8.6e-17 | 64.4 | 67 | (1, 69) | 69 | (2, 69) | 69 | Putative phage tail X protein | Putative phage tail X protein | | uniclust | UniRef100\_A0A0S8BGR5 | 98.8 | 4.2e-11 | 8.7e-17 | 75.2 | 49 | (2, 57) | 69 | (140, 188) | 190 | LysM domain-containing protein | LysM domain-containing protein | | uniclust | UniRef100\_A0A7X7N646 | 98.8 | 4.8e-11 | 8.8e-17 | 63.8 | 51 | (2, 54) | 69 | (32, 82) | 82 | LysM peptidoglycan-binding domain-containing protein | LysM peptidoglycan-binding domain-containing protein | | uniclust | UniRef100\_A0A966QMG2 | 98.8 | 4.8e-11 | 8.9e-17 | 69.3 | 51 | (2, 54) | 69 | (91, 142) | 143 | LysM peptidoglycan-binding domain-containing protein | LysM peptidoglycan-binding domain-containing protein | | uniclust | UniRef100\_A0A9D8NX83 | 98.8 | 4.9e-11 | 9e-17 | 69.6 | 54 | (3, 56) | 69 | (93, 147) | 148 | LysM peptidoglycan-binding domain-containing protein | LysM peptidoglycan-binding domain-containing protein | | uniclust | UniRef100\_A0A1Q9QNI0 | 98.8 | 4.7e-11 | 9e-17 | 68.1 | 55 | (2, 59) | 69 | (40, 109) | 111 | LysM domain-containing protein | LysM domain-containing protein | | uniclust | UniRef100\_A0A1V6D8Z8 | 98.8 | 4.6e-11 | 9.2e-17 | 81.6 | 53 | (2, 58) | 69 | (321, 374) | 417 | LysM domain/BON superfamily protein | LysM domain/BON superfamily protein | | uniclust | UniRef100\_A0A0A0J9G3 | 98.8 | 4.7e-11 | 9.4e-17 | 72.5 | 47 | (2, 55) | 69 | (109, 155) | 156 | LysM domain-containing protein | LysM domain-containing protein | | uniclust | UniRef100\_A0A1F7FIL3 | 98.8 | 4.8e-11 | 9.4e-17 | 76.2 | 50 | (2, 55) | 69 | (186, 235) | 236 | LysM domain-containing protein | LysM domain-containing protein | | uniclust | UniRef100\_A0A1F9Q953 | 98.8 | 4.7e-11 | 9.5e-17 | 83.6 | 55 | (2, 59) | 69 | (468, 522) | 528 | LysM domain-containing protein | LysM domain-containing protein | | uniclust | UniRef100\_A0A292YEE7 | 98.8 | 4.9e-11 | 9.5e-17 | 78.9 | 50 | (1, 56) | 69 | (87, 136) | 342 | LysM domain-containing protein | LysM domain-containing protein | | uniclust | UniRef100\_A0A1G6JHR3 | 98.8 | 4.9e-11 | 9.6e-17 | 74.0 | 54 | (3, 58) | 69 | (129, 182) | 187 | BON domain-containing protein | BON domain-containing protein | | uniclust | UniRef100\_A0A2E8JYY7 | 98.8 | 4.8e-11 | 9.7e-17 | 77.7 | 53 | (2, 56) | 69 | (137, 189) | 269 | LysM domain-containing protein | LysM domain-containing protein | | uniclust | UniRef100\_A0A017RV10 | 98.8 | 4.8e-11 | 9.7e-17 | 78.4 | 52 | (2, 56) | 69 | (203, 257) | 282 | LysM domain-containing protein | LysM domain-containing protein | | uniclust | UniRef100\_A0A7X8VU95 | 98.8 | 5.4e-11 | 9.9e-17 | 65.2 | 53 | (2, 55) | 69 | (43, 95) | 96 | LysM peptidoglycan-binding domain-containing protein | LysM peptidoglycan-binding domain-containing protein | | uniclust | UniRef100\_A0A918W949 | 98.8 | 5.5e-11 | 1e-16 | 70.5 | 54 | (2, 57) | 69 | (109, 162) | 165 | LysM domain-containing protein | LysM domain-containing protein | | uniclust | UniRef100\_A0A024YSH0 | 98.8 | 5.3e-11 | 1e-16 | 73.9 | 53 | (2, 56) | 69 | (148, 201) | 204 | LysM domain-containing protein | LysM domain-containing protein | | uniclust | UniRef100\_A0A1M7PRK3 | 98.8 | 5e-11 | 1e-16 | 74.4 | 53 | (2, 59) | 69 | (127, 179) | 187 | LysM domain-containing protein | LysM domain-containing protein | | uniclust | UniRef100\_A0A2D5AB17 | 98.8 | 5.2e-11 | 1e-16 | 83.3 | 51 | (2, 54) | 69 | (497, 548) | 548 | LysM domain-containing protein | LysM domain-containing protein | | uniclust | UniRef100\_A0A2E1VR95 | 98.8 | 5.4e-11 | 1.1e-16 | 76.6 | 52 | (3, 58) | 69 | (203, 254) | 259 | LysM domain-containing protein | LysM domain-containing protein | | uniclust | UniRef100\_A0A3M0ZNV8 | 98.8 | 5.8e-11 | 1.1e-16 | 61.0 | 49 | (3, 58) | 69 | (10, 58) | 65 | LysM domain-containing protein (Fragment) | LysM domain-containing protein (Fragment) | | uniclust | UniRef100\_A0A0C2M3Y0 | 98.8 | 5.6e-11 | 1.1e-16 | 64.5 | 50 | (2, 57) | 69 | (11, 60) | 82 | LysM peptidoglycan-binding domain-containing protein | LysM peptidoglycan-binding domain-containing protein | | uniclust | UniRef100\_A0A257ECJ0 | 98.8 | 5.9e-11 | 1.1e-16 | 75.4 | 52 | (2, 55) | 69 | (218, 269) | 269 | LysM domain-containing protein | LysM domain-containing protein | | uniclust | UniRef100\_A0A1W9P516 | 98.8 | 5.9e-11 | 1.1e-16 | 66.4 | 56 | (2, 59) | 69 | (22, 79) | 100 | LysM domain-containing protein | LysM domain-containing protein | | uniclust | UniRef100\_A0A1L9QA14 | 98.8 | 5.9e-11 | 1.1e-16 | 63.2 | 69 | (1, 69) | 69 | (1, 73) | 74 | Phage tail protein | Phage tail protein | | uniclust | UniRef100\_A0A1Q6PX12 | 98.8 | 5.4e-11 | 1.1e-16 | 65.0 | 55 | (2, 59) | 69 | (7, 61) | 76 | Phage tail protein | Phage tail protein | | uniclust | UniRef100\_A0A0S7ZZD8 | 98.8 | 5.2e-11 | 1.1e-16 | 82.7 | 53 | (2, 56) | 69 | (85, 137) | 446 | LysM domain-containing protein | LysM domain-containing protein | | uniclust | UniRef100\_A0A081BQ49 | 98.8 | 5.8e-11 | 1.1e-16 | 82.2 | 54 | (1, 58) | 69 | (1, 54) | 533 | LysM domain-containing protein | LysM domain-containing protein | | uniclust | UniRef100\_UPI0020B20EE2 | 98.8 | 6.1e-11 | 1.1e-16 | 60.4 | 51 | (2, 54) | 69 | (12, 62) | 62 | LysM peptidoglycan-binding domain-containing protein | LysM peptidoglycan-binding domain-containing protein | | uniclust | UniRef100\_A0A0F8XQA1 | 98.8 | 6e-11 | 1.1e-16 | 73.3 | 51 | (2, 54) | 69 | (147, 198) | 198 | LysM domain-containing protein (Fragment) | LysM domain-containing protein (Fragment) | | uniclust | UniRef100\_A0A376EW91 | 98.8 | 6.1e-11 | 1.1e-16 | 59.9 | 50 | (2, 54) | 69 | (3, 52) | 56 | Inner membrane protein | Inner membrane protein | | uniclust | UniRef100\_A0A1V4QZM9 | 98.8 | 5.6e-11 | 1.1e-16 | 78.2 | 56 | (2, 57) | 69 | (128, 183) | 288 | LysM domain-containing protein | LysM domain-containing protein | | uniclust | UniRef100\_A0A351FFR9 | 98.8 | 6.3e-11 | 1.2e-16 | 66.9 | 55 | (2, 58) | 69 | (61, 115) | 117 | LysM domain-containing protein (Fragment) | LysM domain-containing protein (Fragment) | | uniclust | UniRef100\_A0A0F2PY82 | 98.8 | 5.8e-11 | 1.2e-16 | 75.3 | 47 | (2, 54) | 69 | (20, 66) | 216 | LysM domain-containing protein | LysM domain-containing protein | | uniclust | UniRef100\_A0A356JRW9 | 98.8 | 6.7e-11 | 1.2e-16 | 66.5 | 52 | (2, 55) | 69 | (61, 112) | 113 | LysM peptidoglycan-binding domain-containing protein | LysM peptidoglycan-binding domain-containing protein | | uniclust | UniRef100\_UPI00117BF9B8 | 98.8 | 6.7e-11 | 1.2e-16 | 63.7 | 54 | (2, 59) | 69 | (12, 65) | 86 | LysM peptidoglycan-binding domain-containing protein | LysM peptidoglycan-binding domain-containing protein | | uniclust | UniRef100\_A0A7X7ENM6 | 98.8 | 6.6e-11 | 1.2e-16 | 76.4 | 53 | (2, 58) | 69 | (166, 218) | 298 | LysM peptidoglycan-binding domain-containing protein | LysM peptidoglycan-binding domain-containing protein | | uniclust | UniRef100\_A0A136KIR5 | 98.8 | 6.5e-11 | 1.2e-16 | 74.3 | 51 | (2, 56) | 69 | (165, 215) | 218 | LysM domain/BON superfamily protein | LysM domain/BON superfamily protein | | uniclust | UniRef100\_A0A2A2RQ54 | 98.8 | 6.5e-11 | 1.2e-16 | 74.8 | 51 | (2, 54) | 69 | (181, 231) | 231 | LysM domain-containing protein | LysM domain-containing protein | | uniclust | UniRef100\_A0A060BXH1 | 98.8 | 6.4e-11 | 1.3e-16 | 73.4 | 54 | (2, 59) | 69 | (12, 65) | 184 | CAZy families CBM50|CE1 protein (Fragment) | CAZy families CBM50|CE1 protein (Fragment) | | uniclust | UniRef100\_A0A7X8YZD2 | 98.8 | 6.7e-11 | 1.3e-16 | 68.9 | 50 | (2, 55) | 69 | (82, 131) | 132 | LysM peptidoglycan-binding domain-containing protein | LysM peptidoglycan-binding domain-containing protein | | uniclust | UniRef100\_A0A286RCX4 | 98.8 | 6.6e-11 | 1.3e-16 | 75.8 | 55 | (2, 58) | 69 | (165, 219) | 261 | Peptidoglycan-binding LysM | Peptidoglycan-binding LysM | | uniclust | UniRef100\_A0A4Z0QZ87 | 98.8 | 6.5e-11 | 1.3e-16 | 75.2 | 52 | (2, 59) | 69 | (1, 52) | 227 | LysM peptidoglycan-binding domain-containing protein | LysM peptidoglycan-binding domain-containing protein | | uniclust | UniRef100\_A0A096B6Z9 | 98.8 | 6.1e-11 | 1.3e-16 | 75.4 | 48 | (3, 56) | 69 | (155, 202) | 204 | LysM domain-containing protein | LysM domain-containing protein | | uniclust | UniRef100\_A0A935WEF4 | 98.8 | 7.1e-11 | 1.3e-16 | 67.2 | 53 | (1, 55) | 69 | (70, 122) | 123 | LysM peptidoglycan-binding domain-containing protein | LysM peptidoglycan-binding domain-containing protein | | uniclust | UniRef100\_A0A350XTG1 | 98.8 | 7.2e-11 | 1.3e-16 | 65.5 | 52 | (2, 55) | 69 | (52, 103) | 104 | LysM domain-containing protein | LysM domain-containing protein | | uniclust | UniRef100\_A0A938DEZ6 | 98.8 | 7.3e-11 | 1.3e-16 | 64.7 | 52 | (2, 57) | 69 | (43, 94) | 96 | LysM peptidoglycan-binding domain-containing protein | LysM peptidoglycan-binding domain-containing protein | | uniclust | UniRef100\_A0A7C5W2S4 | 98.8 | 7.3e-11 | 1.3e-16 | 68.4 | 51 | (1, 55) | 69 | (89, 139) | 140 | LysM peptidoglycan-binding domain-containing protein | LysM peptidoglycan-binding domain-containing protein | | uniclust | UniRef100\_A0A078M1Q6 | 98.8 | 6.7e-11 | 1.3e-16 | 79.8 | 51 | (2, 55) | 69 | (314, 364) | 364 | Elastin-binding protein EbpS | Elastin-binding protein EbpS | | uniclust | UniRef100\_A0A0E2ELW5 | 98.8 | 6.7e-11 | 1.4e-16 | 81.3 | 53 | (1, 58) | 69 | (14, 66) | 427 | LysM domain-containing protein | LysM domain-containing protein | | uniclust | UniRef100\_A0A0J1DQ06 | 98.8 | 6.6e-11 | 1.4e-16 | 70.4 | 55 | (2, 59) | 69 | (60, 115) | 128 | LysM domain-containing protein | LysM domain-containing protein | | uniclust | UniRef100\_A0A2D7GZ53 | 98.8 | 7.2e-11 | 1.4e-16 | 75.2 | 51 | (1, 55) | 69 | (185, 235) | 236 | LysM domain-containing protein | LysM domain-containing protein | | uniclust | UniRef100\_A0A7X8UXT2 | 98.8 | 7.5e-11 | 1.4e-16 | 70.1 | 51 | (2, 54) | 69 | (103, 154) | 154 | LysM peptidoglycan-binding domain-containing protein | LysM peptidoglycan-binding domain-containing protein | | uniclust | UniRef100\_A0A7K0PL28 | 98.8 | 7.1e-11 | 1.4e-16 | 68.1 | 48 | (2, 56) | 69 | (59, 106) | 108 | LysM peptidoglycan-binding domain-containing protein | LysM peptidoglycan-binding domain-containing protein | | uniclust | UniRef100\_A0A1G7JKS9 | 98.7 | 7.3e-11 | 1.4e-16 | 79.3 | 52 | (1, 58) | 69 | (1, 52) | 367 | Morphogenetic protein associated with SpoVID (Fragment) | Morphogenetic protein associated with SpoVID (Fragment) | | uniclust | UniRef100\_A0A0A3IYD3 | 98.7 | 7.4e-11 | 1.4e-16 | 77.2 | 50 | (1, 56) | 69 | (1, 50) | 298 | Morphogenetic protein associated with SpoVID | Morphogenetic protein associated with SpoVID | | uniclust | UniRef100\_A0A1V4MSH9 | 98.7 | 7.9e-11 | 1.5e-16 | 69.9 | 57 | (2, 58) | 69 | (106, 162) | 166 | LysM domain-containing protein | LysM domain-containing protein | | uniclust | UniRef100\_A0A1N7P9I2 | 98.7 | 7.4e-11 | 1.5e-16 | 76.2 | 52 | (1, 58) | 69 | (1, 52) | 262 | LysM domain-containing protein | LysM domain-containing protein | | uniclust | UniRef100\_A0A2W4JPI4 | 98.7 | 7.3e-11 | 1.5e-16 | 76.2 | 56 | (2, 59) | 69 | (174, 229) | 243 | LysM domain-containing protein | LysM domain-containing protein | | uniclust | UniRef100\_A0A1V2MFZ6 | 98.7 | 7.6e-11 | 1.5e-16 | 69.2 | 52 | (2, 55) | 69 | (78, 129) | 130 | LysM domain-containing protein | LysM domain-containing protein | | uniclust | UniRef100\_A0A202DMI1 | 98.7 | 7.6e-11 | 1.5e-16 | 74.9 | 55 | (2, 58) | 69 | (173, 227) | 228 | LysM domain-containing protein | LysM domain-containing protein | | uniclust | UniRef100\_A0A9E7IT89 | 98.7 | 7.8e-11 | 1.5e-16 | 61.3 | 52 | (2, 56) | 69 | (8, 59) | 61 | LysM peptidoglycan-binding domain-containing protein | LysM peptidoglycan-binding domain-containing protein | | uniclust | UniRef100\_A0A080LV77 | 98.7 | 7.1e-11 | 1.5e-16 | 68.0 | 52 | (3, 58) | 69 | (44, 95) | 101 | LysM domain-containing protein | LysM domain-containing protein | | uniclust | UniRef100\_A0A060CH00 | 98.7 | 7.6e-11 | 1.5e-16 | 70.5 | 48 | (2, 56) | 69 | (85, 132) | 136 | CAZy families CE4|CBM50 protein (Fragment) | CAZy families CE4|CBM50 protein (Fragment) | | uniclust | UniRef100\_A0A0C1UAF4 | 98.7 | 7.1e-11 | 1.5e-16 | 77.1 | 54 | (2, 58) | 69 | (186, 239) | 248 | LysM domain protein | LysM domain protein | | uniclust | UniRef100\_A0A069A1N0 | 98.7 | 7.3e-11 | 1.6e-16 | 78.5 | 54 | (2, 58) | 69 | (201, 255) | 294 | LysM domain protein | LysM domain protein | | uniclust | UniRef100\_A0A0G0JZZ0 | 98.7 | 7.5e-11 | 1.6e-16 | 75.9 | 53 | (3, 59) | 69 | (165, 217) | 227 | LysM domain-containing protein | LysM domain-containing protein | | uniclust | UniRef100\_A0A0J1FA30 | 98.7 | 8.1e-11 | 1.6e-16 | 61.0 | 48 | (1, 54) | 69 | (7, 54) | 59 | LysM domain protein | LysM domain protein | | uniclust | UniRef100\_X0S693 | 98.7 | 8.6e-11 | 1.6e-16 | 69.3 | 57 | (3, 59) | 69 | (4, 60) | 158 | LysM domain-containing protein (Fragment) | LysM domain-containing protein (Fragment) | | uniclust | UniRef100\_A0A0B0I074 | 98.7 | 9e-11 | 1.7e-16 | 65.2 | 53 | (1, 59) | 69 | (1, 53) | 104 | Putative peptidoglycan endopeptidase LytE | Putative peptidoglycan endopeptidase LytE | | uniclust | UniRef100\_A0A957XDI4 | 98.7 | 9.1e-11 | 1.7e-16 | 70.9 | 54 | (2, 57) | 69 | (135, 188) | 189 | LysM peptidoglycan-binding domain-containing protein | LysM peptidoglycan-binding domain-containing protein | | uniclust | UniRef100\_A0A2V5KHA2 | 98.7 | 8.9e-11 | 1.7e-16 | 70.6 | 52 | (2, 55) | 69 | (114, 165) | 166 | LysM domain-containing protein (Fragment) | LysM domain-containing protein (Fragment) | | uniclust | UniRef100\_A0A1C4SHA2 | 98.7 | 8.8e-11 | 1.7e-16 | 72.7 | 54 | (2, 59) | 69 | (112, 165) | 186 | LysM domain-containing protein (Fragment) | LysM domain-containing protein (Fragment) | | uniclust | UniRef100\_A0A058ZQ48 | 98.7 | 8.4e-11 | 1.8e-16 | 73.1 | 55 | (3, 59) | 69 | (97, 151) | 174 | Uncharacterized protein | Uncharacterized protein | | uniclust | UniRef100\_A0A0Q9NJ51 | 98.7 | 8.6e-11 | 1.8e-16 | 81.3 | 52 | (1, 58) | 69 | (21, 72) | 464 | LysM domain-containing protein | LysM domain-containing protein | | uniclust | UniRef100\_A0A090AGM7 | 98.7 | 8.6e-11 | 1.8e-16 | 74.3 | 42 | (2, 46) | 69 | (42, 83) | 203 | Uncharacterized protein | Uncharacterized protein | | uniclust | UniRef100\_A0A1F7G2S0 | 98.7 | 9.3e-11 | 1.8e-16 | 78.1 | 54 | (2, 57) | 69 | (311, 364) | 368 | LysM domain-containing protein | LysM domain-containing protein | | uniclust | UniRef100\_A0A7V9DN14 | 98.7 | 8.9e-11 | 1.8e-16 | 69.6 | 51 | (2, 59) | 69 | (58, 108) | 130 | LysM peptidoglycan-binding domain-containing protein | LysM peptidoglycan-binding domain-containing protein | | uniclust | UniRef100\_A0A0E2BBV5 | 98.7 | 9e-11 | 1.8e-16 | 70.4 | 56 | (1, 59) | 69 | (8, 63) | 141 | Phage tail protein X | Phage tail protein X | | uniclust | UniRef100\_UPI001F499B57 | 98.7 | 1e-10 | 1.9e-16 | 62.8 | 62 | (1, 68) | 69 | (23, 84) | 84 | tail protein X | tail protein X | | uniclust | UniRef100\_A0A2S5MYD0 | 98.7 | 1e-10 | 1.9e-16 | 68.1 | 52 | (2, 57) | 69 | (91, 142) | 144 | Heavy metal transporter (Fragment) | Heavy metal transporter (Fragment) | | uniclust | UniRef100\_A0A1Z8RYG9 | 98.7 | 9.1e-11 | 1.9e-16 | 67.2 | 52 | (2, 57) | 69 | (40, 91) | 99 | LysM domain-containing protein | LysM domain-containing protein | | uniclust | UniRef100\_A0A1V6DV92 | 98.7 | 9.2e-11 | 1.9e-16 | 76.2 | 55 | (2, 58) | 69 | (192, 246) | 248 | LysM domain protein | LysM domain protein | | uniclust | UniRef100\_A0A1W9R610 | 98.7 | 1e-10 | 1.9e-16 | 57.6 | 49 | (7, 57) | 69 | (2, 50) | 51 | LysM domain-containing protein | LysM domain-containing protein | | uniclust | UniRef100\_A0A7C1HTJ5 | 98.7 | 1e-10 | 1.9e-16 | 70.3 | 53 | (2, 56) | 69 | (112, 164) | 164 | BON domain-containing protein (Fragment) | BON domain-containing protein (Fragment) | | uniclust | UniRef100\_A0A1Z9KNB0 | 98.7 | 1e-10 | 2e-16 | 78.6 | 55 | (1, 58) | 69 | (239, 293) | 364 | LysM domain-containing protein | LysM domain-containing protein | | uniclust | UniRef100\_UPI001F1763FC | 98.7 | 1.1e-10 | 2e-16 | 62.2 | 54 | (1, 60) | 69 | (1, 54) | 80 | LysM domain-containing protein | LysM domain-containing protein | | uniclust | UniRef100\_A0A972MN72 | 98.7 | 1.1e-10 | 2e-16 | 71.8 | 52 | (1, 54) | 69 | (168, 219) | 219 | LysM peptidoglycan-binding domain-containing protein | LysM peptidoglycan-binding domain-containing protein | | uniclust | UniRef100\_A0A933KDG5 | 98.7 | 1.1e-10 | 2e-16 | 64.8 | 55 | (2, 58) | 69 | (43, 97) | 104 | LysM peptidoglycan-binding domain-containing protein (Fragment) | LysM peptidoglycan-binding domain-containing protein (Fragment) | | uniclust | UniRef100\_A0A935LSQ5 | 98.7 | 1.1e-10 | 2e-16 | 58.0 | 49 | (3, 55) | 69 | (2, 50) | 51 | LysM peptidoglycan-binding domain-containing protein | LysM peptidoglycan-binding domain-containing protein | | uniclust | UniRef100\_A0A1R0XIF0 | 98.7 | 1.1e-10 | 2.1e-16 | 72.8 | 52 | (1, 58) | 69 | (1, 52) | 217 | LysM domain-containing protein (Fragment) | LysM domain-containing protein (Fragment) | | uniclust | UniRef100\_A0A098F1J4 | 98.7 | 1e-10 | 2.1e-16 | 84.8 | 52 | (1, 58) | 69 | (12, 63) | 807 | Spore coat assembly protein SafA | Spore coat assembly protein SafA | | uniclust | UniRef100\_A0A7C6PRM4 | 98.7 | 1.1e-10 | 2.1e-16 | 57.9 | 47 | (2, 55) | 69 | (3, 49) | 49 | LysM peptidoglycan-binding domain-containing protein | LysM peptidoglycan-binding domain-containing protein | | uniclust | UniRef100\_A0A0C9PWS5 | 98.7 | 1.1e-10 | 2.1e-16 | 78.2 | 54 | (1, 58) | 69 | (243, 296) | 377 | LysM domain-containing protein | LysM domain-containing protein | |
| Top keywords  (threshold 1.00e-03 (evalue)) | **LysM, domain\_containing, peptidoglycan\_binding, tail, Phage, Fragment, BON, X, superfamily, Spore** |
| Output files | ../../similar\_sequences/24\_FANPEZAQ\_CDS\_0024\_merged.svg ../../similar\_sequences/24\_FANPEZAQ\_CDS\_0024\_pdb70.a3m ../../similar\_sequences/24\_FANPEZAQ\_CDS\_0024\_pdb70.hhr ../../similar\_sequences/24\_FANPEZAQ\_CDS\_0024\_uniclust.a3m ../../similar\_sequences/24\_FANPEZAQ\_CDS\_0024\_uniclust.hhr |

#### Structure prediction (AlphaFold)2

|  |  |
| --- | --- |
| Stats | xml version="1.0" encoding="utf-8" standalone="no"?       2024-09-02T21:09:24.638686 image/svg+xml   Matplotlib v3.7.2, https://matplotlib.org/ |
| Predicted structure | **NGL Viewer Controls:**  - Center: *Left-Click* - Rotate: *Left-Click + Drag* - Translate: *Right-Click + Drag* - Zoom: *Shift + Left-Click + Drag* |
| Output files | ../../predicted\_structures/24\_FANPEZAQ\_CDS\_0024/features.pkl ../../predicted\_structures/24\_FANPEZAQ\_CDS\_0024/ranked\_0.pdb ../../predicted\_structures/24\_FANPEZAQ\_CDS\_0024/ranked\_0\_plots.svg ../../predicted\_structures/24\_FANPEZAQ\_CDS\_0024/result\_model\_1\_ptm\_pred\_0.pkl |

#### Structure similarity search results (Foldseek)3

|  |  |
| --- | --- |
| Structure databases searched | Pdb, Afdb-proteome, Afdb-uniprot50 |
| Results, scheme(s)  (Top layers only, threshold 1.00e-02 (evalue)) | xml version="1.0" encoding="utf-8" standalone="no"?       2024-09-02T21:10:52.721427 image/svg+xml   Matplotlib v3.7.2, https://matplotlib.org/ |
| Results, table  (threshold 1.00e-02 (evalue)) | | db | id | prob | evalue | bits | fident | alnlen | mismatch | gapopen | qstart | qend | tstart | tend | name | description | | --- | --- | --- | --- | --- | --- | --- | --- | --- | --- | --- | --- | --- | --- | --- | | pdb | 6U5B\_6 | 1.0 | 0.0003522 | 149 | 0.436 | 55 | 30 | 1 | 2 | 56 | 1 | 54 | Glue PA0627 | Glue PA0627 | | pdb | 2LTF\_A | 1.0 | 0.0001977 | 139 | 0.397 | 68 | 40 | 1 | 2 | 69 | 1 | 67 | Tail protein X | Tail protein X | | pdb | 2LTF\_A | 1.0 | 0.0001378 | 135 | 0.397 | 68 | 40 | 1 | 2 | 69 | 1 | 67 | Tail protein X | Tail protein X | | pdb | 2LTF\_A | 1.0 | 0.0003785 | 127 | 0.397 | 68 | 40 | 1 | 2 | 69 | 1 | 67 | Tail protein X | Tail protein X | | afdb-proteome | AF-A0A0H3GM11-F1-MODEL\_V4 | 1.0 | 6.871e-09 | 283 | 0.441 | 68 | 37 | 1 | 2 | 69 | 1 | 67 | Uncharacterized protein | Uncharacterized protein | | afdb-proteome | AF-Q8ZMT5-F1-MODEL\_V4 | 1.0 | 1.53e-07 | 244 | 0.426 | 68 | 38 | 1 | 2 | 69 | 1 | 67 | Fels-2 prophage protein | Fels-2 prophage protein | | afdb-proteome | AF-A0A0H3GWZ2-F1-MODEL\_V4 | 1.0 | 4.516e-07 | 232 | 0.397 | 68 | 40 | 1 | 2 | 69 | 1 | 67 | Putative prophage tail component protein | Putative prophage tail component protein | | afdb-proteome | AF-Q8ZKJ6-F1-MODEL\_V4 | 1.0 | 0.0001938 | 145 | 0.354 | 62 | 37 | 2 | 2 | 63 | 4 | 62 | Putative inner membrane protein | Putative inner membrane protein | | afdb-proteome | AF-G3XD62-F1-MODEL\_V4 | 1.0 | 0.0006607 | 139 | 0.411 | 68 | 38 | 2 | 2 | 69 | 3 | 68 | Uncharacterized protein | Uncharacterized protein | | afdb-uniprot50 | AF-A0A212KMZ3-F1-MODEL\_V4 | 1.0 | 9.95e-08 | 267 | 0.5 | 70 | 32 | 2 | 2 | 69 | 3 | 71 | Tail protein X | Tail protein X | | afdb-uniprot50 | AF-A0A3G6WBC5-F1-MODEL\_V4 | 1.0 | 6.004e-08 | 264 | 0.608 | 69 | 27 | 0 | 1 | 69 | 1 | 69 | Phage tail protein | Phage tail protein | | afdb-uniprot50 | AF-A0A4R0EZY9-F1-MODEL\_V4 | 1.0 | 1.069e-07 | 260 | 0.414 | 70 | 39 | 2 | 1 | 69 | 1 | 69 | Phage tail protein | Phage tail protein | | afdb-uniprot50 | AF-A0A1Y3CEI3-F1-MODEL\_V4 | 1.0 | 4.867e-07 | 260 | 0.478 | 69 | 36 | 0 | 1 | 69 | 1 | 69 | Uncharacterized protein | Uncharacterized protein | | afdb-uniprot50 | AF-A0A450SYU4-F1-MODEL\_V4 | 1.0 | 1.427e-07 | 260 | 0.507 | 69 | 32 | 2 | 2 | 69 | 3 | 70 | P2-like prophage tail protein X | P2-like prophage tail protein X | | afdb-uniprot50 | AF-A0A1G5ACY5-F1-MODEL\_V4 | 1.0 | 1.427e-07 | 258 | 0.521 | 69 | 32 | 1 | 1 | 69 | 1 | 68 | P2-like prophage tail protein X | P2-like prophage tail protein X | | afdb-uniprot50 | AF-A0A136HGF2-F1-MODEL\_V4 | 1.0 | 1.328e-07 | 258 | 0.528 | 70 | 31 | 2 | 1 | 69 | 1 | 69 | Phage tail protein | Phage tail protein | | afdb-uniprot50 | AF-A0A2W5H3R5-F1-MODEL\_V4 | 1.0 | 1.534e-07 | 254 | 0.521 | 69 | 32 | 1 | 2 | 69 | 95 | 163 | Uncharacterized protein | Uncharacterized protein | | afdb-uniprot50 | AF-A0A2U1XYZ3-F1-MODEL\_V4 | 1.0 | 1.149e-07 | 250 | 0.536 | 69 | 31 | 1 | 1 | 69 | 1 | 68 | Phage tail protein | Phage tail protein | | afdb-uniprot50 | AF-D2ZKL4-F1-MODEL\_V4 | 1.0 | 1.076e-06 | 243 | 0.455 | 68 | 36 | 1 | 2 | 69 | 43 | 109 | Phage Tail Protein X | Phage Tail Protein X | | afdb-uniprot50 | AF-A0A4Q0YIT0-F1-MODEL\_V4 | 1.0 | 1.076e-06 | 239 | 0.5 | 68 | 32 | 2 | 2 | 69 | 1 | 66 | Phage tail protein | Phage tail protein | | afdb-uniprot50 | AF-F4BFP7-F1-MODEL\_V4 | 1.0 | 6.044e-07 | 237 | 0.455 | 68 | 35 | 1 | 1 | 68 | 1 | 66 | Uncharacterized protein | Uncharacterized protein | | afdb-uniprot50 | AF-A0A1Q5TNQ8-F1-MODEL\_V4 | 1.0 | 1.244e-06 | 237 | 0.455 | 68 | 36 | 1 | 2 | 69 | 1 | 67 | Tail protein X | Tail protein X | | afdb-uniprot50 | AF-B8GS03-F1-MODEL\_V4 | 1.0 | 1.337e-06 | 237 | 0.426 | 68 | 37 | 2 | 1 | 67 | 1 | 67 | P2-like protein prophage tail protein X-like protein | P2-like protein prophage tail protein X-like protein | | afdb-uniprot50 | AF-A0A846VV59-F1-MODEL\_V4 | 1.0 | 2.381e-06 | 236 | 0.323 | 68 | 46 | 0 | 2 | 69 | 7 | 74 | Phage tail protein | Phage tail protein | | afdb-uniprot50 | AF-C0B391-F1-MODEL\_V4 | 1.0 | 5.231e-07 | 234 | 0.391 | 69 | 40 | 2 | 2 | 69 | 1 | 68 | Phage Tail Protein X | Phage Tail Protein X | | afdb-uniprot50 | AF-A0A348FYG1-F1-MODEL\_V4 | 1.0 | 6.044e-07 | 234 | 0.528 | 70 | 31 | 2 | 1 | 69 | 1 | 69 | Tail protein X | Tail protein X | | afdb-uniprot50 | AF-A0A2J0SUU1-F1-MODEL\_V4 | 1.0 | 9.318e-07 | 234 | 0.536 | 69 | 31 | 1 | 2 | 69 | 3 | 71 | Phage tail protein | Phage tail protein | | afdb-uniprot50 | AF-A0A7U4KP86-F1-MODEL\_V4 | 1.0 | 9.318e-07 | 233 | 0.397 | 68 | 40 | 1 | 2 | 69 | 1 | 67 | Tail protein X | Tail protein X | | afdb-uniprot50 | AF-R9VJF3-F1-MODEL\_V4 | 1.0 | 1.784e-06 | 233 | 0.426 | 68 | 38 | 1 | 2 | 69 | 1 | 67 | Tail protein X | Tail protein X | | afdb-uniprot50 | AF-A0A5D4YFH7-F1-MODEL\_V4 | 1.0 | 2.061e-06 | 233 | 0.397 | 68 | 40 | 1 | 2 | 69 | 1 | 67 | Phage tail protein | Phage tail protein | | afdb-uniprot50 | AF-A0A502FVN3-F1-MODEL\_V4 | 1.0 | 1.437e-06 | 232 | 0.367 | 68 | 42 | 1 | 2 | 69 | 1 | 67 | Phage tail protein | Phage tail protein | | afdb-uniprot50 | AF-A0A239EHV7-F1-MODEL\_V4 | 1.0 | 4.867e-07 | 230 | 0.478 | 69 | 34 | 1 | 1 | 69 | 1 | 67 | P2-like prophage tail protein X | P2-like prophage tail protein X | | afdb-uniprot50 | AF-A0A0G4QHL0-F1-MODEL\_V4 | 1.0 | 9.318e-07 | 229 | 0.391 | 69 | 40 | 2 | 2 | 69 | 1 | 68 | Phage tail protein X | Phage tail protein X | | afdb-uniprot50 | AF-A0A2W4T7L0-F1-MODEL\_V4 | 1.0 | 4.899e-06 | 228 | 0.455 | 68 | 36 | 1 | 2 | 69 | 3 | 69 | Phage tail protein | Phage tail protein | | afdb-uniprot50 | AF-A0A4Y6U9C4-F1-MODEL\_V4 | 1.0 | 1.076e-06 | 228 | 0.394 | 71 | 40 | 1 | 2 | 69 | 1 | 71 | Phage tail protein | Phage tail protein | | afdb-uniprot50 | AF-C9XYT5-F1-MODEL\_V4 | 1.0 | 1.917e-06 | 226 | 0.405 | 69 | 38 | 3 | 2 | 69 | 1 | 67 | Tail protein X | Tail protein X | | afdb-uniprot50 | AF-A0A1X7Q4C3-F1-MODEL\_V4 | 1.0 | 5.231e-07 | 226 | 0.565 | 69 | 27 | 3 | 2 | 69 | 3 | 69 | p2-like prophage tail protein X | p2-like prophage tail protein X | | afdb-uniprot50 | AF-A0A1T4WWY3-F1-MODEL\_V4 | 1.0 | 2.381e-06 | 224 | 0.428 | 70 | 38 | 2 | 1 | 69 | 1 | 69 | p2-like prophage tail protein X | p2-like prophage tail protein X | | afdb-uniprot50 | AF-A0A2D3TFL6-F1-MODEL\_V4 | 1.0 | 2.215e-06 | 224 | 0.406 | 64 | 37 | 1 | 2 | 65 | 1 | 63 | Phage tail protein | Phage tail protein | | afdb-uniprot50 | AF-A0A6G8F2N9-F1-MODEL\_V4 | 1.0 | 4.241e-06 | 223 | 0.5 | 68 | 33 | 1 | 1 | 68 | 1 | 67 | Uncharacterized protein | Uncharacterized protein | | afdb-uniprot50 | AF-A0A2X3CVI6-F1-MODEL\_V4 | 1.0 | 3.178e-06 | 223 | 0.382 | 68 | 41 | 1 | 2 | 69 | 1 | 67 | Tail component protein | Tail component protein | | afdb-uniprot50 | AF-A0A496K2J9-F1-MODEL\_V4 | 1.0 | 2.381e-06 | 221 | 0.436 | 71 | 36 | 2 | 2 | 69 | 1 | 70 | Phage tail protein | Phage tail protein | | afdb-uniprot50 | AF-A0A1C3WM59-F1-MODEL\_V4 | 1.0 | 2.559e-06 | 220 | 0.405 | 69 | 39 | 2 | 2 | 69 | 23 | 90 | p2-like prophage tail protein X | p2-like prophage tail protein X | | afdb-uniprot50 | AF-A0A738YWL5-F1-MODEL\_V4 | 1.0 | 7.028e-06 | 219 | 0.367 | 68 | 42 | 1 | 2 | 69 | 1 | 67 | Phage tail protein | Phage tail protein | | afdb-uniprot50 | AF-A0A8A5W2Y0-F1-MODEL\_V4 | 1.0 | 3.946e-06 | 219 | 0.441 | 68 | 37 | 1 | 2 | 69 | 3 | 69 | Tail protein X | Tail protein X | | afdb-uniprot50 | AF-R9AS75-F1-MODEL\_V4 | 1.0 | 2.381e-06 | 219 | 0.376 | 69 | 41 | 2 | 2 | 69 | 3 | 70 | Uncharacterized protein | Uncharacterized protein | | afdb-uniprot50 | AF-A0A426CLA1-F1-MODEL\_V4 | 1.0 | 5.266e-06 | 218 | 0.417 | 67 | 37 | 2 | 2 | 68 | 6 | 70 | Phage tail protein | Phage tail protein | | afdb-uniprot50 | AF-A0A841ILH7-F1-MODEL\_V4 | 1.0 | 3.671e-06 | 217 | 0.397 | 68 | 40 | 1 | 2 | 69 | 1 | 67 | Phage tail protein X | Phage tail protein X | | afdb-uniprot50 | AF-A0A1X3I4F6-F1-MODEL\_V4 | 1.0 | 5.266e-06 | 217 | 0.411 | 68 | 39 | 1 | 2 | 69 | 19 | 85 | Uncharacterized protein | Uncharacterized protein | | afdb-uniprot50 | AF-S6GQ04-F1-MODEL\_V4 | 1.0 | 8.119e-06 | 216 | 0.347 | 69 | 43 | 2 | 2 | 69 | 1 | 68 | Gp8 | Gp8 | | afdb-uniprot50 | AF-A0A6M3KNH7-F1-MODEL\_V4 | 1.0 | 9.38e-06 | 216 | 0.304 | 69 | 48 | 0 | 1 | 69 | 1 | 69 | Putative tail protein | Putative tail protein | | afdb-uniprot50 | AF-A0A432QVU7-F1-MODEL\_V4 | 1.0 | 2.061e-06 | 216 | 0.338 | 71 | 45 | 1 | 1 | 69 | 1 | 71 | Phage tail protein | Phage tail protein | | afdb-uniprot50 | AF-A0A809H9W3-F1-MODEL\_V4 | 1.0 | 2.751e-06 | 215 | 0.397 | 68 | 40 | 1 | 2 | 69 | 1 | 67 | Phage tail protein | Phage tail protein | | afdb-uniprot50 | AF-A0A0Q7XZV6-F1-MODEL\_V4 | 1.0 | 2.956e-06 | 214 | 0.441 | 68 | 36 | 2 | 2 | 68 | 3 | 69 | Phage tail protein | Phage tail protein | | afdb-uniprot50 | AF-E2CFJ8-F1-MODEL\_V4 | 1.0 | 7.554e-06 | 213 | 0.43 | 65 | 35 | 1 | 1 | 65 | 1 | 63 | Phage Tail Protein X | Phage Tail Protein X | | afdb-uniprot50 | AF-A0A6L2ZQ46-F1-MODEL\_V4 | 1.0 | 5.266e-06 | 213 | 0.428 | 70 | 39 | 1 | 1 | 69 | 1 | 70 | Tail protein X | Tail protein X | | afdb-uniprot50 | AF-A0A6H1ZLH4-F1-MODEL\_V4 | 1.0 | 2.215e-06 | 213 | 0.38 | 71 | 41 | 1 | 2 | 69 | 1 | 71 | Putative tail protein | Putative tail protein | | afdb-uniprot50 | AF-I7E1A5-F1-MODEL\_V4 | 1.0 | 2.061e-06 | 212 | 0.5 | 68 | 34 | 0 | 2 | 69 | 6 | 73 | Phage tail protein X | Phage tail protein X | | afdb-uniprot50 | AF-A0A7L9REI3-F1-MODEL\_V4 | 1.0 | 5.66e-06 | 211 | 0.338 | 68 | 44 | 1 | 2 | 69 | 1 | 67 | Phage tail protein | Phage tail protein | | afdb-uniprot50 | AF-A0A0H3L797-F1-MODEL\_V4 | 1.0 | 4.899e-06 | 210 | 0.397 | 68 | 40 | 1 | 2 | 69 | 90 | 156 | Phage tail protein X | Phage tail protein X | | afdb-uniprot50 | AF-A0A6L9FJD9-F1-MODEL\_V4 | 1.0 | 3.946e-06 | 209 | 0.411 | 68 | 38 | 1 | 2 | 69 | 3 | 68 | Phage tail protein | Phage tail protein | | afdb-uniprot50 | AF-A0A7Z7RKR3-F1-MODEL\_V4 | 1.0 | 9.38e-06 | 209 | 0.388 | 67 | 39 | 2 | 1 | 66 | 1 | 66 | Phage Tail Protein X | Phage Tail Protein X | | afdb-uniprot50 | AF-A0A1H0NML7-F1-MODEL\_V4 | 1.0 | 1.252e-05 | 209 | 0.468 | 64 | 33 | 1 | 2 | 65 | 1 | 63 | P2-like prophage tail protein X | P2-like prophage tail protein X | | afdb-uniprot50 | AF-A0A484S6Y7-F1-MODEL\_V4 | 1.0 | 3.671e-06 | 208 | 0.405 | 69 | 39 | 2 | 2 | 69 | 1 | 68 | Phage tail completion protein | Phage tail completion protein | | afdb-uniprot50 | AF-A0A659L5Z7-F1-MODEL\_V4 | 1.0 | 1.446e-05 | 208 | 0.367 | 68 | 42 | 1 | 2 | 69 | 1 | 67 | Phage tail protein | Phage tail protein | | afdb-uniprot50 | AF-A0A143DFK3-F1-MODEL\_V4 | 1.0 | 8.727e-06 | 208 | 0.514 | 68 | 32 | 1 | 2 | 69 | 3 | 69 | Uncharacterized protein | Uncharacterized protein | | afdb-uniprot50 | AF-E5AKR2-F1-MODEL\_V4 | 1.0 | 5.266e-06 | 207 | 0.463 | 69 | 35 | 2 | 2 | 69 | 23 | 90 | Tail protein X | Tail protein X | | afdb-uniprot50 | AF-A0A7K0GNQ8-F1-MODEL\_V4 | 1.0 | 3.415e-06 | 206 | 0.442 | 70 | 35 | 2 | 2 | 69 | 1 | 68 | Phage tail protein | Phage tail protein | | afdb-uniprot50 | AF-Q31Q85-F1-MODEL\_V4 | 1.0 | 7.028e-06 | 206 | 0.434 | 69 | 38 | 1 | 1 | 69 | 1 | 68 | Uncharacterized protein | Uncharacterized protein | | afdb-uniprot50 | AF-A0A1T4W4K7-F1-MODEL\_V4 | 1.0 | 1.084e-05 | 206 | 0.414 | 70 | 37 | 3 | 2 | 69 | 4 | 71 | p2-like prophage tail protein X | p2-like prophage tail protein X | | afdb-uniprot50 | AF-A0A6I4T3J1-F1-MODEL\_V4 | 1.0 | 4.558e-06 | 206 | 0.478 | 69 | 34 | 2 | 2 | 69 | 4 | 71 | Phage tail protein | Phage tail protein | | afdb-uniprot50 | AF-A0A248LIW4-F1-MODEL\_V4 | 1.0 | 7.028e-06 | 205 | 0.463 | 69 | 35 | 2 | 2 | 69 | 1 | 68 | Tail protein X | Tail protein X | | afdb-uniprot50 | AF-A0A4R5HGF7-F1-MODEL\_V4 | 1.0 | 1.93e-05 | 205 | 0.405 | 69 | 41 | 0 | 1 | 69 | 1 | 69 | Phage tail protein | Phage tail protein | | afdb-uniprot50 | AF-G9PUI9-F1-MODEL\_V4 | 1.0 | 2.075e-05 | 205 | 0.362 | 58 | 35 | 2 | 1 | 57 | 1 | 57 | Uncharacterized protein | Uncharacterized protein | | afdb-uniprot50 | AF-A0A1S0V5S2-F1-MODEL\_V4 | 1.0 | 5.66e-06 | 205 | 0.492 | 69 | 34 | 1 | 2 | 69 | 4 | 72 | Tail protein | Tail protein | | afdb-uniprot50 | AF-A0A2N0DX25-F1-MODEL\_V4 | 1.0 | 2.075e-05 | 205 | 0.369 | 65 | 39 | 2 | 2 | 66 | 3 | 65 | Phage tail protein X | Phage tail protein X | | afdb-uniprot50 | AF-M4Z448-F1-MODEL\_V4 | 1.0 | 1.446e-05 | 204 | 0.397 | 68 | 40 | 1 | 2 | 69 | 3 | 69 | Putative phage tail protein | Putative phage tail protein | | afdb-uniprot50 | AF-A0A616MEN0-F1-MODEL\_V4 | 1.0 | 1.671e-05 | 204 | 0.376 | 69 | 42 | 1 | 2 | 69 | 3 | 71 | Phage tail protein | Phage tail protein | | afdb-uniprot50 | AF-A0A350LX84-F1-MODEL\_V4 | 1.0 | 4.899e-06 | 204 | 0.45 | 71 | 35 | 2 | 1 | 69 | 1 | 69 | Phage tail protein | Phage tail protein | | afdb-uniprot50 | AF-A0A165XHI0-F1-MODEL\_V4 | 1.0 | 4.558e-06 | 204 | 0.417 | 67 | 38 | 1 | 3 | 69 | 12 | 77 | Phage Tail Protein X | Phage Tail Protein X | | afdb-uniprot50 | AF-F4V8N9-F1-MODEL\_V4 | 1.0 | 1.671e-05 | 203 | 0.391 | 69 | 41 | 1 | 1 | 69 | 23 | 90 | Conserved domain protein | Conserved domain protein | | afdb-uniprot50 | AF-A0A292AJG7-F1-MODEL\_V4 | 1.0 | 7.554e-06 | 202 | 0.391 | 69 | 39 | 3 | 2 | 69 | 1 | 67 | Phage tail protein | Phage tail protein | | afdb-uniprot50 | AF-A0A3Q9W3P2-F1-MODEL\_V4 | 1.0 | 1.93e-05 | 202 | 0.382 | 68 | 41 | 1 | 2 | 69 | 5 | 71 | Phage tail protein | Phage tail protein | | afdb-uniprot50 | AF-A0A2S6N2T4-F1-MODEL\_V4 | 1.0 | 1.93e-05 | 202 | 0.428 | 70 | 38 | 2 | 2 | 69 | 5 | 74 | Uncharacterized protein | Uncharacterized protein | | afdb-uniprot50 | AF-A0A7W6WBC8-F1-MODEL\_V4 | 1.0 | 3.415e-06 | 201 | 0.457 | 70 | 34 | 2 | 3 | 69 | 15 | 83 | Phage tail protein X | Phage tail protein X | | afdb-uniprot50 | AF-A0A1K0IP73-F1-MODEL\_V4 | 1.0 | 1.252e-05 | 200 | 0.47 | 68 | 35 | 1 | 2 | 69 | 1 | 67 | Tail protein X | Tail protein X | | afdb-uniprot50 | AF-A0A2S9IC13-F1-MODEL\_V4 | 1.0 | 1.165e-05 | 199 | 0.426 | 68 | 38 | 1 | 2 | 69 | 1 | 67 | Phage tail protein | Phage tail protein | | afdb-uniprot50 | AF-A0A541BL22-F1-MODEL\_V4 | 1.0 | 1.008e-05 | 199 | 0.4 | 70 | 38 | 3 | 2 | 69 | 1 | 68 | Phage tail protein | Phage tail protein | | afdb-uniprot50 | AF-A0A1H9YCX3-F1-MODEL\_V4 | 1.0 | 6.539e-06 | 199 | 0.42 | 69 | 38 | 2 | 2 | 69 | 6 | 73 | P2-like prophage tail protein X | P2-like prophage tail protein X | | afdb-uniprot50 | AF-A0A142BH73-F1-MODEL\_V4 | 1.0 | 1.671e-05 | 198 | 0.441 | 68 | 36 | 1 | 2 | 69 | 1 | 66 | Tail X family protein | Tail X family protein | | afdb-uniprot50 | AF-C6DCA5-F1-MODEL\_V4 | 1.0 | 2.397e-05 | 198 | 0.382 | 68 | 41 | 1 | 2 | 69 | 1 | 67 | Tail X family protein | Tail X family protein | | afdb-uniprot50 | AF-A0A4P7LCT2-F1-MODEL\_V4 | 1.0 | 1.446e-05 | 198 | 0.347 | 69 | 43 | 2 | 2 | 69 | 1 | 68 | Phage Tail Protein X | Phage Tail Protein X | | afdb-uniprot50 | AF-A0A1S1U741-F1-MODEL\_V4 | 1.0 | 3.178e-06 | 198 | 0.43 | 72 | 36 | 2 | 2 | 69 | 1 | 71 | Uncharacterized protein | Uncharacterized protein | | afdb-uniprot50 | AF-A0A1N6I0M6-F1-MODEL\_V4 | 1.0 | 1.446e-05 | 197 | 0.417 | 67 | 38 | 1 | 2 | 68 | 3 | 68 | p2-like prophage tail protein X | p2-like prophage tail protein X | | afdb-uniprot50 | AF-A0A8B2TUJ1-F1-MODEL\_V4 | 1.0 | 2.576e-05 | 196 | 0.308 | 68 | 47 | 0 | 2 | 69 | 1 | 68 | Phage tail protein | Phage tail protein | | afdb-uniprot50 | AF-A0A1I1F5Z9-F1-MODEL\_V4 | 1.0 | 1.165e-05 | 196 | 0.375 | 72 | 42 | 2 | 1 | 69 | 1 | 72 | P2-like prophage tail protein X | P2-like prophage tail protein X | | afdb-uniprot50 | AF-A0A1X7MAH8-F1-MODEL\_V4 | 1.0 | 6.124e-05 | 195 | 0.384 | 65 | 39 | 1 | 1 | 65 | 1 | 64 | Phage tail protein X | Phage tail protein X | | afdb-uniprot50 | AF-A0A0H5CZ07-F1-MODEL\_V4 | 1.0 | 3.695e-05 | 194 | 0.367 | 68 | 42 | 1 | 2 | 69 | 3 | 69 | Phage Tail Protein X | Phage Tail Protein X | | afdb-uniprot50 | AF-N9SPH0-F1-MODEL\_V4 | 1.0 | 3.695e-05 | 194 | 0.371 | 70 | 42 | 2 | 1 | 69 | 1 | 69 | Uncharacterized protein | Uncharacterized protein | | afdb-uniprot50 | AF-A0A496AHX0-F1-MODEL\_V4 | 1.0 | 1.252e-05 | 193 | 0.246 | 69 | 52 | 0 | 1 | 69 | 1 | 69 | Uncharacterized protein | Uncharacterized protein | | afdb-uniprot50 | AF-A0A285VTB6-F1-MODEL\_V4 | 1.0 | 2.075e-05 | 193 | 0.449 | 69 | 36 | 2 | 2 | 69 | 3 | 70 | P2-like prophage tail protein X | P2-like prophage tail protein X | | afdb-uniprot50 | AF-A0A2T5UR74-F1-MODEL\_V4 | 1.0 | 1.446e-05 | 193 | 0.449 | 69 | 36 | 2 | 2 | 69 | 3 | 70 | Phage tail protein X | Phage tail protein X | | afdb-uniprot50 | AF-A0A1V0BI36-F1-MODEL\_V4 | 1.0 | 1.554e-05 | 193 | 0.426 | 68 | 38 | 1 | 2 | 69 | 4 | 70 | Phage tail protein | Phage tail protein | | afdb-uniprot50 | AF-A0A149SNW1-F1-MODEL\_V4 | 1.0 | 1.671e-05 | 193 | 0.464 | 71 | 36 | 2 | 1 | 69 | 1 | 71 | Uncharacterized protein | Uncharacterized protein | | afdb-uniprot50 | AF-A0A1A9VKI1-F1-MODEL\_V4 | 1.0 | 2.576e-05 | 193 | 0.447 | 67 | 36 | 1 | 3 | 69 | 342 | 407 | Uncharacterized protein | Uncharacterized protein | | afdb-uniprot50 | AF-A0A367WTU8-F1-MODEL\_V4 | 1.0 | 1.93e-05 | 192 | 0.434 | 69 | 36 | 2 | 1 | 69 | 1 | 66 | Tail protein X | Tail protein X | | afdb-uniprot50 | AF-A0A439F0D7-F1-MODEL\_V4 | 1.0 | 1.554e-05 | 192 | 0.441 | 68 | 37 | 1 | 2 | 69 | 1 | 67 | Phage tail protein | Phage tail protein | | afdb-uniprot50 | AF-A0A167GZN0-F1-MODEL\_V4 | 1.0 | 3.972e-05 | 192 | 0.42 | 69 | 38 | 2 | 2 | 69 | 1 | 68 | Tail protein | Tail protein | | afdb-uniprot50 | AF-A0A1X3DKC3-F1-MODEL\_V4 | 1.0 | 2.976e-05 | 192 | 0.362 | 69 | 42 | 2 | 2 | 69 | 6 | 73 | Phage tail protein | Phage tail protein | | afdb-uniprot50 | AF-A0A376SBV1-F1-MODEL\_V4 | 1.0 | 0.0002245 | 192 | 0.428 | 56 | 31 | 1 | 2 | 57 | 1 | 55 | Phage tail protein X | Phage tail protein X | | afdb-uniprot50 | AF-V0XX61-F1-MODEL\_V4 | 1.0 | 0.0002413 | 190 | 0.421 | 57 | 32 | 1 | 2 | 58 | 1 | 56 | Phage Tail Protein X | Phage Tail Protein X | | afdb-uniprot50 | AF-A0A7X5THN7-F1-MODEL\_V4 | 1.0 | 3.438e-05 | 190 | 0.397 | 68 | 40 | 1 | 2 | 69 | 1 | 67 | Phage tail protein | Phage tail protein | | afdb-uniprot50 | AF-A0A2T4JP28-F1-MODEL\_V4 | 1.0 | 2.075e-05 | 188 | 0.382 | 68 | 40 | 1 | 2 | 69 | 6 | 71 | Phage tail protein | Phage tail protein | | afdb-uniprot50 | AF-A0A1I7HNC1-F1-MODEL\_V4 | 1.0 | 2.576e-05 | 188 | 0.426 | 68 | 38 | 1 | 2 | 69 | 72 | 138 | P2-like prophage tail protein X | P2-like prophage tail protein X | | afdb-uniprot50 | AF-A0A840C414-F1-MODEL\_V4 | 1.0 | 2.075e-05 | 187 | 0.449 | 69 | 37 | 1 | 1 | 69 | 1 | 68 | Phage tail protein X | Phage tail protein X | | afdb-uniprot50 | AF-A0A7Z0SIU9-F1-MODEL\_V4 | 1.0 | 3.972e-05 | 187 | 0.352 | 68 | 41 | 2 | 2 | 68 | 3 | 68 | Tail protein X | Tail protein X | | afdb-uniprot50 | AF-L8MGI2-F1-MODEL\_V4 | 1.0 | 2.976e-05 | 186 | 0.376 | 69 | 42 | 1 | 1 | 69 | 1 | 68 | Phage-related tail protein | Phage-related tail protein | | afdb-uniprot50 | AF-A0A7Y6Z4E9-F1-MODEL\_V4 | 1.0 | 0.000126 | 185 | 0.375 | 64 | 39 | 1 | 2 | 65 | 5 | 67 | Tail protein X | Tail protein X | | afdb-uniprot50 | AF-A0A3S4CJZ7-F1-MODEL\_V4 | 1.0 | 2.976e-05 | 184 | 0.376 | 69 | 41 | 1 | 1 | 69 | 1 | 67 | Phage Tail Protein X | Phage Tail Protein X | | afdb-uniprot50 | AF-G0AIP8-F1-MODEL\_V4 | 1.0 | 3.438e-05 | 184 | 0.4 | 70 | 39 | 2 | 2 | 69 | 1 | 69 | Putative phage tail protein | Putative phage tail protein | | afdb-uniprot50 | AF-A0A1Q9PIY2-F1-MODEL\_V4 | 1.0 | 8.785e-05 | 184 | 0.344 | 58 | 35 | 2 | 1 | 58 | 1 | 55 | Uncharacterized protein | Uncharacterized protein | | afdb-uniprot50 | AF-A0A5B9YAQ9-F1-MODEL\_V4 | 1.0 | 0.000126 | 184 | 0.306 | 62 | 40 | 2 | 2 | 63 | 4 | 62 | Phage tail protein | Phage tail protein | | afdb-uniprot50 | AF-A0A1R4LK82-F1-MODEL\_V4 | 1.0 | 4.269e-05 | 183 | 0.42 | 69 | 40 | 0 | 1 | 69 | 1 | 69 | Phage Tail Protein X | Phage Tail Protein X | | afdb-uniprot50 | AF-H6SQI2-F1-MODEL\_V4 | 1.0 | 3.199e-05 | 183 | 0.397 | 68 | 38 | 2 | 2 | 69 | 5 | 69 | Uncharacterized protein | Uncharacterized protein | | afdb-uniprot50 | AF-A0A6P2ZWQ5-F1-MODEL\_V4 | 1.0 | 1.346e-05 | 183 | 0.352 | 71 | 42 | 2 | 2 | 69 | 1 | 70 | Tail X family protein | Tail X family protein | | afdb-uniprot50 | AF-A0A3N2E0P9-F1-MODEL\_V4 | 1.0 | 1.084e-05 | 183 | 0.375 | 72 | 38 | 2 | 2 | 69 | 3 | 71 | Phage tail protein X | Phage tail protein X | | afdb-uniprot50 | AF-A0A1R3W3I9-F1-MODEL\_V4 | 1.0 | 3.695e-05 | 182 | 0.42 | 69 | 38 | 2 | 2 | 69 | 1 | 68 | p2-like prophage tail protein X | p2-like prophage tail protein X | | afdb-uniprot50 | AF-A0A2K2G664-F1-MODEL\_V4 | 1.0 | 2.576e-05 | 182 | 0.428 | 70 | 37 | 2 | 2 | 69 | 3 | 71 | Phage tail protein | Phage tail protein | | afdb-uniprot50 | AF-A0A6G5QMP5-F1-MODEL\_V4 | 1.0 | 3.695e-05 | 180 | 0.352 | 68 | 43 | 1 | 2 | 69 | 1 | 67 | Phage tail protein X family protein | Phage tail protein X family protein | | afdb-uniprot50 | AF-A0A5D0CMM3-F1-MODEL\_V4 | 1.0 | 0.0001456 | 180 | 0.254 | 59 | 41 | 2 | 2 | 60 | 1 | 56 | Phage tail protein | Phage tail protein | | afdb-uniprot50 | AF-A0A7T8QHI0-F1-MODEL\_V4 | 1.0 | 3.695e-05 | 180 | 0.449 | 69 | 37 | 1 | 1 | 69 | 12 | 79 | Tail protein X | Tail protein X | | afdb-uniprot50 | AF-A0A4Q8MDG8-F1-MODEL\_V4 | 1.0 | 3.199e-05 | 180 | 0.426 | 68 | 37 | 2 | 3 | 69 | 18 | 84 | Phage tail protein | Phage tail protein | | afdb-uniprot50 | AF-A0A0J5WBN4-F1-MODEL\_V4 | 1.0 | 4.932e-05 | 178 | 0.405 | 69 | 39 | 2 | 2 | 69 | 1 | 68 | Tail protein | Tail protein | | afdb-uniprot50 | AF-A0A7X4CNS4-F1-MODEL\_V4 | 1.0 | 0.0001943 | 178 | 0.338 | 68 | 45 | 0 | 2 | 69 | 3 | 70 | Phage tail protein | Phage tail protein | | afdb-uniprot50 | AF-A0A2E2MZM5-F1-MODEL\_V4 | 1.0 | 8.785e-05 | 178 | 0.309 | 71 | 46 | 2 | 1 | 69 | 1 | 70 | Phage tail protein | Phage tail protein | | afdb-uniprot50 | AF-A0A6C1BPB3-F1-MODEL\_V4 | 1.0 | 0.0001015 | 177 | 0.373 | 67 | 39 | 2 | 2 | 68 | 1 | 64 | Uncharacterized protein | Uncharacterized protein | | afdb-uniprot50 | AF-A0A2N3KSN4-F1-MODEL\_V4 | 1.0 | 0.0001456 | 177 | 0.308 | 68 | 47 | 0 | 2 | 69 | 1 | 68 | Phage tail protein | Phage tail protein | | afdb-uniprot50 | AF-A0A6L5PED9-F1-MODEL\_V4 | 1.0 | 0.0001354 | 176 | 0.424 | 66 | 37 | 1 | 4 | 69 | 1 | 65 | Phage tail protein | Phage tail protein | | afdb-uniprot50 | AF-A0A5C7CK72-F1-MODEL\_V4 | 1.0 | 5.697e-05 | 176 | 0.391 | 69 | 39 | 3 | 2 | 69 | 1 | 67 | Phage tail protein | Phage tail protein | | afdb-uniprot50 | AF-A0A7X6X0F0-F1-MODEL\_V4 | 1.0 | 2.576e-05 | 176 | 0.4 | 70 | 37 | 3 | 2 | 69 | 1 | 67 | Phage tail protein | Phage tail protein | | afdb-uniprot50 | AF-A0A1E3G6A1-F1-MODEL\_V4 | 1.0 | 0.0001565 | 176 | 0.294 | 68 | 47 | 1 | 2 | 69 | 3 | 69 | Uncharacterized protein | Uncharacterized protein | | afdb-uniprot50 | AF-A0A7W6DBB8-F1-MODEL\_V4 | 1.0 | 4.588e-05 | 176 | 0.485 | 68 | 33 | 2 | 3 | 69 | 17 | 83 | Phage tail protein X | Phage tail protein X | | afdb-uniprot50 | AF-A0A4P9VJG3-F1-MODEL\_V4 | 1.0 | 0.0001354 | 176 | 0.382 | 68 | 39 | 2 | 2 | 69 | 38 | 102 | Uncharacterized protein | Uncharacterized protein | | afdb-uniprot50 | AF-A0A249A0N5-F1-MODEL\_V4 | 1.0 | 2.23e-05 | 175 | 0.361 | 72 | 41 | 3 | 1 | 69 | 1 | 70 | Phage Tail Protein X | Phage Tail Protein X | | afdb-uniprot50 | AF-A0A849VAT6-F1-MODEL\_V4 | 1.0 | 7.604e-05 | 175 | 0.323 | 71 | 44 | 2 | 1 | 68 | 1 | 70 | Uncharacterized protein | Uncharacterized protein | | afdb-uniprot50 | AF-A0A498RQG7-F1-MODEL\_V4 | 1.0 | 4.932e-05 | 174 | 0.347 | 69 | 43 | 2 | 2 | 69 | 1 | 68 | Phage Tail Protein X | Phage Tail Protein X | | afdb-uniprot50 | AF-A0A0F2RD92-F1-MODEL\_V4 | 1.0 | 2.976e-05 | 174 | 0.411 | 68 | 38 | 2 | 2 | 69 | 3 | 68 | Uncharacterized protein | Uncharacterized protein | | afdb-uniprot50 | AF-A0A1M4LC88-F1-MODEL\_V4 | 1.0 | 8.173e-05 | 174 | 0.42 | 69 | 38 | 2 | 2 | 69 | 301 | 368 | PhageMin\_Tail domain-containing protein | PhageMin\_Tail domain-containing protein | | afdb-uniprot50 | AF-A0A4U2PVD7-F1-MODEL\_V4 | 1.0 | 0.0002787 | 173 | 0.35 | 57 | 34 | 2 | 1 | 57 | 1 | 54 | Phage tail protein | Phage tail protein | | afdb-uniprot50 | AF-A0A495YI39-F1-MODEL\_V4 | 1.0 | 7.604e-05 | 173 | 0.463 | 69 | 35 | 2 | 2 | 69 | 6 | 73 | Phage tail protein | Phage tail protein | | afdb-uniprot50 | AF-A0A1A9RBR4-F1-MODEL\_V4 | 1.0 | 4.932e-05 | 173 | 0.385 | 70 | 39 | 2 | 3 | 69 | 10 | 78 | Uncharacterized protein | Uncharacterized protein | | afdb-uniprot50 | AF-A0A854I2Y0-F1-MODEL\_V4 | 1.0 | 9.442e-05 | 173 | 0.296 | 64 | 42 | 2 | 2 | 65 | 1 | 61 | Uncharacterized protein | Uncharacterized protein | | afdb-uniprot50 | AF-A0A1Y6HDB3-F1-MODEL\_V4 | 1.0 | 7.604e-05 | 171 | 0.414 | 70 | 38 | 2 | 2 | 69 | 1 | 69 | Phage-related tail protein | Phage-related tail protein | | afdb-uniprot50 | AF-J8W766-F1-MODEL\_V4 | 1.0 | 0.0001172 | 171 | 0.372 | 59 | 32 | 3 | 1 | 57 | 1 | 56 | Phage tail protein X | Phage tail protein X | | afdb-uniprot50 | AF-A0A261E6J5-F1-MODEL\_V4 | 1.0 | 0.0001172 | 171 | 0.348 | 66 | 41 | 1 | 2 | 65 | 6 | 71 | Uncharacterized protein | Uncharacterized protein | | afdb-uniprot50 | AF-A0A2S4U2P3-F1-MODEL\_V4 | 1.0 | 8.173e-05 | 170 | 0.367 | 68 | 42 | 1 | 2 | 69 | 1 | 67 | Phage tail protein | Phage tail protein | | afdb-uniprot50 | AF-A0A095GKT5-F1-MODEL\_V4 | 1.0 | 9.442e-05 | 170 | 0.318 | 69 | 45 | 2 | 2 | 69 | 1 | 68 | Phage tail protein | Phage tail protein | | afdb-uniprot50 | AF-A0A545T5R5-F1-MODEL\_V4 | 1.0 | 0.000126 | 170 | 0.317 | 104 | 35 | 2 | 2 | 69 | 3 | 106 | Uncharacterized protein | Uncharacterized protein | | afdb-uniprot50 | AF-A0A2G6EI15-F1-MODEL\_V4 | 1.0 | 6.582e-05 | 169 | 0.333 | 69 | 42 | 3 | 2 | 69 | 1 | 66 | Phage tail protein | Phage tail protein | | afdb-uniprot50 | AF-A0A165NX17-F1-MODEL\_V4 | 1.0 | 0.0002787 | 169 | 0.333 | 69 | 42 | 3 | 2 | 69 | 3 | 68 | Phage Tail Protein X | Phage Tail Protein X | | afdb-uniprot50 | AF-A0A2D3VR18-F1-MODEL\_V4 | 1.0 | 0.0001015 | 169 | 0.376 | 69 | 41 | 2 | 1 | 68 | 1 | 68 | Phage tail protein | Phage tail protein | | afdb-uniprot50 | AF-A0A376WTB8-F1-MODEL\_V4 | 1.0 | 0.0002787 | 169 | 0.369 | 65 | 39 | 2 | 2 | 65 | 1 | 64 | Phage tail protein X | Phage tail protein X | | afdb-uniprot50 | AF-A0A3A9IJM0-F1-MODEL\_V4 | 1.0 | 0.0001682 | 169 | 0.306 | 62 | 40 | 2 | 1 | 61 | 1 | 60 | Phage tail protein | Phage tail protein | | afdb-uniprot50 | AF-A0A7X1YEJ4-F1-MODEL\_V4 | 1.0 | 0.0004297 | 169 | 0.428 | 63 | 35 | 1 | 2 | 64 | 3 | 64 | Phage tail protein | Phage tail protein | | afdb-uniprot50 | AF-A0A6L9JTD6-F1-MODEL\_V4 | 1.0 | 0.0002245 | 168 | 0.308 | 68 | 45 | 2 | 2 | 69 | 1 | 66 | Phage tail protein | Phage tail protein | | afdb-uniprot50 | AF-A0A2G6DPR2-F1-MODEL\_V4 | 1.0 | 4.588e-05 | 168 | 0.309 | 71 | 44 | 4 | 1 | 69 | 1 | 68 | Phage tail protein | Phage tail protein | | afdb-uniprot50 | AF-A0A246KGZ2-F1-MODEL\_V4 | 1.0 | 6.124e-05 | 168 | 0.361 | 72 | 42 | 3 | 1 | 69 | 1 | 71 | Phage Tail Protein X | Phage Tail Protein X | | afdb-uniprot50 | AF-A0A5Q3RUR6-F1-MODEL\_V4 | 1.0 | 0.0002413 | 168 | 0.357 | 70 | 41 | 2 | 2 | 69 | 5 | 72 | Phage tail protein | Phage tail protein | | afdb-uniprot50 | AF-A0A1Y3SU18-F1-MODEL\_V4 | 1.0 | 0.0004619 | 168 | 0.41 | 56 | 30 | 2 | 1 | 56 | 1 | 53 | Phage tail protein | Phage tail protein | | afdb-uniprot50 | AF-A0A376J471-F1-MODEL\_V4 | 1.0 | 0.0001943 | 168 | 0.367 | 68 | 42 | 1 | 2 | 69 | 24 | 90 | Tail protein X (GpX) | Tail protein X (GpX) | | afdb-uniprot50 | AF-A0A7X3TZ69-F1-MODEL\_V4 | 1.0 | 8.785e-05 | 168 | 0.397 | 68 | 37 | 2 | 4 | 69 | 259 | 324 | Uncharacterized protein | Uncharacterized protein | | afdb-uniprot50 | AF-A0A1C3EE73-F1-MODEL\_V4 | 1.0 | 0.0001565 | 167 | 0.294 | 68 | 46 | 2 | 2 | 69 | 1 | 66 | Uncharacterized protein | Uncharacterized protein | | afdb-uniprot50 | AF-A0A3G2IBA3-F1-MODEL\_V4 | 1.0 | 0.0001456 | 167 | 0.388 | 67 | 39 | 1 | 2 | 68 | 3 | 67 | Phage tail protein | Phage tail protein | | afdb-uniprot50 | AF-A0A7Z1A3P8-F1-MODEL\_V4 | 1.0 | 0.0001456 | 167 | 0.289 | 69 | 45 | 2 | 2 | 69 | 3 | 68 | Uncharacterized protein | Uncharacterized protein | | afdb-uniprot50 | AF-A0A1T4QDP8-F1-MODEL\_V4 | 1.0 | 0.0002593 | 167 | 0.298 | 67 | 44 | 2 | 2 | 68 | 3 | 66 | Phage Tail Protein X | Phage Tail Protein X | | afdb-uniprot50 | AF-B7MRX8-F1-MODEL\_V4 | 1.0 | 0.0001943 | 167 | 0.318 | 69 | 43 | 4 | 2 | 69 | 24 | 89 | Tail protein X from prophage | Tail protein X from prophage | | afdb-uniprot50 | AF-A0A0S4XN94-F1-MODEL\_V4 | 1.0 | 8.785e-05 | 166 | 0.357 | 70 | 41 | 3 | 1 | 69 | 1 | 67 | Putative Phage Tail Protein X | Putative Phage Tail Protein X | | afdb-uniprot50 | AF-A0A1K2ALR6-F1-MODEL\_V4 | 1.0 | 0.0001943 | 166 | 0.411 | 68 | 38 | 2 | 2 | 69 | 3 | 68 | p2-like prophage tail protein X | p2-like prophage tail protein X | | afdb-uniprot50 | AF-A0A7S8EHN3-F1-MODEL\_V4 | 1.0 | 0.0001808 | 166 | 0.417 | 67 | 38 | 1 | 3 | 69 | 17 | 82 | Phage tail protein | Phage tail protein | | afdb-uniprot50 | AF-A0A1B8QCU9-F1-MODEL\_V4 | 1.0 | 0.0001565 | 165 | 0.323 | 68 | 44 | 2 | 2 | 69 | 5 | 70 | Uncharacterized protein | Uncharacterized protein | | afdb-uniprot50 | AF-A0A2D5PEH8-F1-MODEL\_V4 | 1.0 | 0.0001354 | 165 | 0.294 | 68 | 48 | 0 | 2 | 69 | 4 | 71 | Phage tail protein | Phage tail protein | | afdb-uniprot50 | AF-A0A317PI36-F1-MODEL\_V4 | 1.0 | 0.0002245 | 165 | 0.367 | 68 | 43 | 0 | 2 | 69 | 6 | 73 | Phage tail protein X | Phage tail protein X | | afdb-uniprot50 | AF-A0A2Z4UEW3-F1-MODEL\_V4 | 1.0 | 8.173e-05 | 165 | 0.361 | 72 | 37 | 3 | 3 | 69 | 13 | 80 | Phage tail protein X | Phage tail protein X | | afdb-uniprot50 | AF-A0A2N1QH04-F1-MODEL\_V4 | 1.0 | 0.0002413 | 164 | 0.292 | 65 | 43 | 2 | 1 | 65 | 1 | 62 | Phage tail protein | Phage tail protein | | afdb-uniprot50 | AF-A0A3Q8DHW5-F1-MODEL\_V4 | 1.0 | 0.0001808 | 163 | 0.347 | 69 | 42 | 2 | 2 | 68 | 6 | 73 | Uncharacterized protein | Uncharacterized protein | | afdb-uniprot50 | AF-A0A6L9HTM9-F1-MODEL\_V4 | 1.0 | 0.0001015 | 163 | 0.297 | 74 | 44 | 3 | 1 | 69 | 1 | 71 | Phage tail protein | Phage tail protein | | afdb-uniprot50 | AF-A0A3A8YFV1-F1-MODEL\_V4 | 1.0 | 0.0001943 | 163 | 0.384 | 65 | 36 | 3 | 2 | 65 | 4 | 65 | Phage tail protein | Phage tail protein | | afdb-uniprot50 | AF-A0A250DS69-F1-MODEL\_V4 | 1.0 | 0.0002245 | 162 | 0.362 | 69 | 42 | 2 | 2 | 69 | 3 | 70 | Phage tail protein | Phage tail protein | | afdb-uniprot50 | AF-A0A1S1CG91-F1-MODEL\_V4 | 1.0 | 0.0002088 | 162 | 0.388 | 72 | 39 | 3 | 2 | 69 | 1 | 71 | LysM domain-containing protein | LysM domain-containing protein | | afdb-uniprot50 | AF-A0A1G8NEF7-F1-MODEL\_V4 | 1.0 | 0.0001682 | 161 | 0.411 | 68 | 38 | 1 | 2 | 69 | 1 | 66 | P2-like prophage tail protein X | P2-like prophage tail protein X | | afdb-uniprot50 | AF-A0A6L5YES5-F1-MODEL\_V4 | 1.0 | 0.0007121 | 161 | 0.359 | 64 | 40 | 1 | 2 | 65 | 7 | 69 | Phage tail protein | Phage tail protein | | afdb-uniprot50 | AF-A0A806CIL3-F1-MODEL\_V4 | 1.0 | 0.000126 | 161 | 0.362 | 69 | 42 | 2 | 2 | 69 | 3 | 70 | Tail X family protein | Tail X family protein | | afdb-uniprot50 | AF-A0A142YH45-F1-MODEL\_V4 | 1.0 | 0.0007654 | 161 | 0.345 | 55 | 34 | 1 | 2 | 56 | 221 | 273 | Phage Tail Protein X | Phage Tail Protein X | | afdb-uniprot50 | AF-A0A066RM09-F1-MODEL\_V4 | 1.0 | 0.0001354 | 160 | 0.449 | 69 | 37 | 1 | 1 | 69 | 1 | 68 | Tail protein | Tail protein | | afdb-uniprot50 | AF-A0A4R2MR54-F1-MODEL\_V4 | 1.0 | 0.0001943 | 159 | 0.371 | 70 | 40 | 3 | 2 | 69 | 1 | 68 | Phage tail protein X | Phage tail protein X | | afdb-uniprot50 | AF-A0A4R3ABI5-F1-MODEL\_V4 | 1.0 | 0.000372 | 159 | 0.318 | 69 | 45 | 2 | 2 | 69 | 3 | 70 | Phage tail protein X | Phage tail protein X | | afdb-uniprot50 | AF-A0A840FZY4-F1-MODEL\_V4 | 1.0 | 0.000322 | 159 | 0.42 | 69 | 38 | 2 | 2 | 69 | 3 | 70 | Phage tail protein X | Phage tail protein X | | afdb-uniprot50 | AF-C2BG19-F1-MODEL\_V4 | 1.0 | 0.0002593 | 159 | 0.294 | 68 | 42 | 3 | 2 | 69 | 3 | 64 | Uncharacterized protein | Uncharacterized protein | | afdb-uniprot50 | AF-A0A2S8YUV6-F1-MODEL\_V4 | 1.0 | 0.0002787 | 158 | 0.455 | 68 | 35 | 2 | 2 | 69 | 3 | 68 | Phage tail protein | Phage tail protein | | afdb-uniprot50 | AF-A0A5P8MUY6-F1-MODEL\_V4 | 1.0 | 0.000322 | 158 | 0.4 | 70 | 40 | 2 | 2 | 69 | 6 | 75 | Phage tail protein | Phage tail protein | | afdb-uniprot50 | AF-A0A5M6I273-F1-MODEL\_V4 | 1.0 | 0.0001682 | 157 | 0.385 | 70 | 39 | 2 | 1 | 68 | 1 | 68 | Phage tail protein | Phage tail protein | | afdb-uniprot50 | AF-A0A6A8A117-F1-MODEL\_V4 | 1.0 | 0.0002593 | 157 | 0.323 | 68 | 43 | 2 | 2 | 69 | 3 | 67 | Phage tail protein | Phage tail protein | | afdb-uniprot50 | AF-A0A397PIN3-F1-MODEL\_V4 | 1.0 | 0.0002088 | 157 | 0.42 | 69 | 38 | 2 | 2 | 69 | 3 | 70 | Phage tail protein X | Phage tail protein X | | afdb-uniprot50 | AF-A0A7W9RBM3-F1-MODEL\_V4 | 1.0 | 0.0001354 | 157 | 0.385 | 70 | 40 | 2 | 2 | 69 | 4 | 72 | Phage tail protein X | Phage tail protein X | | afdb-uniprot50 | AF-A0A554XC87-F1-MODEL\_V4 | 1.0 | 0.0006164 | 156 | 0.4 | 65 | 34 | 3 | 1 | 60 | 2 | 66 | Phage Tail Protein X | Phage Tail Protein X | | afdb-uniprot50 | AF-A0A1G5MFC1-F1-MODEL\_V4 | 1.0 | 0.0002996 | 156 | 0.367 | 68 | 43 | 0 | 2 | 69 | 3 | 70 | P2-like prophage tail protein X | P2-like prophage tail protein X | | afdb-uniprot50 | AF-A0A4Q3U2H3-F1-MODEL\_V4 | 1.0 | 0.0002413 | 156 | 0.318 | 69 | 45 | 2 | 2 | 69 | 9 | 76 | Phage tail protein | Phage tail protein | | afdb-uniprot50 | AF-A0A6B4FAW5-F1-MODEL\_V4 | 1.0 | 0.0005735 | 155 | 0.275 | 69 | 46 | 3 | 1 | 69 | 1 | 65 | LysM domain-containing protein | LysM domain-containing protein | | afdb-uniprot50 | AF-A0A7H1VPF4-F1-MODEL\_V4 | 1.0 | 0.001098 | 155 | 0.258 | 62 | 43 | 2 | 2 | 63 | 8 | 66 | Tail protein X | Tail protein X | | afdb-uniprot50 | AF-A0A512IX42-F1-MODEL\_V4 | 1.0 | 0.0004297 | 155 | 0.363 | 77 | 37 | 2 | 2 | 69 | 4 | 77 | Uncharacterized protein | Uncharacterized protein | | afdb-uniprot50 | AF-V4JIT5-F1-MODEL\_V4 | 1.0 | 0.0001456 | 155 | 0.333 | 105 | 32 | 4 | 2 | 69 | 3 | 106 | Tail protein X | Tail protein X | | afdb-uniprot50 | AF-A0A315BEJ3-F1-MODEL\_V4 | 1.0 | 0.0002996 | 154 | 0.352 | 68 | 41 | 3 | 2 | 69 | 10 | 74 | Uncharacterized protein | Uncharacterized protein | | afdb-uniprot50 | AF-A0A5E4XFK5-F1-MODEL\_V4 | 1.0 | 0.0006164 | 154 | 0.382 | 68 | 40 | 2 | 2 | 69 | 11 | 76 | Phage tail protein | Phage tail protein | | afdb-uniprot50 | AF-A0A4Y6VUF0-F1-MODEL\_V4 | 1.0 | 0.0001808 | 154 | 0.375 | 72 | 40 | 2 | 2 | 69 | 22 | 92 | Phage tail protein | Phage tail protein | | afdb-uniprot50 | AF-A0A7W0S7Q0-F1-MODEL\_V4 | 1.0 | 0.0007654 | 154 | 0.333 | 54 | 34 | 1 | 2 | 55 | 137 | 188 | Lectin | Lectin | | afdb-uniprot50 | AF-A0A7Y7V0E2-F1-MODEL\_V4 | 1.0 | 0.0003998 | 153 | 0.357 | 70 | 44 | 1 | 1 | 69 | 1 | 70 | Tail protein X | Tail protein X | | afdb-uniprot50 | AF-A0A653HUK6-F1-MODEL\_V4 | 1.0 | 0.0003461 | 153 | 0.333 | 69 | 44 | 2 | 2 | 69 | 5 | 72 | Phage Tail Protein X | Phage Tail Protein X | | afdb-uniprot50 | AF-A0A0T7FB11-F1-MODEL\_V4 | 1.0 | 0.000372 | 153 | 0.356 | 73 | 43 | 3 | 1 | 69 | 1 | 73 | Uncharacterized protein | Uncharacterized protein | | afdb-uniprot50 | AF-A0A1L9QA14-F1-MODEL\_V4 | 1.0 | 0.0004297 | 153 | 0.281 | 71 | 49 | 1 | 1 | 69 | 1 | 71 | Uncharacterized protein | Uncharacterized protein | | afdb-uniprot50 | AF-A0A0E4BWV6-F1-MODEL\_V4 | 1.0 | 0.0005336 | 153 | 0.402 | 72 | 39 | 1 | 2 | 69 | 4 | 75 | Uncharacterized protein | Uncharacterized protein | | afdb-uniprot50 | AF-L0NDQ6-F1-MODEL\_V4 | 1.0 | 0.002102 | 153 | 0.396 | 58 | 34 | 1 | 3 | 60 | 54 | 110 | Phage tail protein X (Modular protein) | Phage tail protein X (Modular protein) | | afdb-uniprot50 | AF-A0A812QV43-F1-MODEL\_V4 | 1.0 | 0.0003461 | 153 | 0.353 | 65 | 40 | 1 | 1 | 65 | 631 | 693 | GpFI protein | GpFI protein | | afdb-uniprot50 | AF-A0A3A9ERJ5-F1-MODEL\_V4 | 1.0 | 0.0006164 | 152 | 0.301 | 63 | 41 | 2 | 1 | 63 | 1 | 60 | Phage tail protein | Phage tail protein | | afdb-uniprot50 | AF-A0A833GDU7-F1-MODEL\_V4 | 1.0 | 0.0002787 | 152 | 0.376 | 69 | 41 | 2 | 2 | 69 | 3 | 70 | Phage tail protein | Phage tail protein | | afdb-uniprot50 | AF-N9PQN2-F1-MODEL\_V4 | 1.0 | 0.0001456 | 151 | 0.347 | 69 | 43 | 1 | 1 | 69 | 1 | 67 | Uncharacterized protein | Uncharacterized protein | | afdb-uniprot50 | AF-A0A842IYZ7-F1-MODEL\_V4 | 1.0 | 0.0007121 | 151 | 0.268 | 67 | 46 | 2 | 2 | 68 | 7 | 70 | Tail protein X | Tail protein X | | afdb-uniprot50 | AF-R7HY28-F1-MODEL\_V4 | 1.0 | 0.001363 | 151 | 0.321 | 56 | 35 | 2 | 2 | 57 | 3 | 55 | Phage Tail Protein X | Phage Tail Protein X | | afdb-uniprot50 | AF-A0A366DKA8-F1-MODEL\_V4 | 1.0 | 0.0004619 | 151 | 0.397 | 68 | 39 | 2 | 3 | 69 | 14 | 80 | Phage tail protein X | Phage tail protein X | | afdb-uniprot50 | AF-A0A4P5SNE7-F1-MODEL\_V4 | 1.0 | 0.0005735 | 151 | 0.357 | 56 | 29 | 3 | 2 | 55 | 119 | 169 | Uncharacterized protein | Uncharacterized protein | | afdb-uniprot50 | AF-A0A7W6RH01-F1-MODEL\_V4 | 1.0 | 0.0005336 | 150 | 0.385 | 70 | 39 | 2 | 1 | 68 | 10 | 77 | Phage tail protein X | Phage tail protein X | | afdb-uniprot50 | AF-M5JVY0-F1-MODEL\_V4 | 1.0 | 0.0002413 | 150 | 0.347 | 72 | 43 | 2 | 2 | 69 | 8 | 79 | p2-like prophage tail protein X | p2-like prophage tail protein X | | afdb-uniprot50 | AF-A0A0C5VGD3-F1-MODEL\_V4 | 1.0 | 0.0006164 | 150 | 0.33 | 103 | 34 | 3 | 2 | 69 | 3 | 105 | p2-like prophage tail protein X | p2-like prophage tail protein X | | afdb-uniprot50 | AF-A0A2J9QNL2-F1-MODEL\_V4 | 1.0 | 0.0004964 | 149 | 0.289 | 69 | 46 | 3 | 2 | 69 | 1 | 67 | Phage tail protein | Phage tail protein | | afdb-uniprot50 | AF-A0A7Y0MMX1-F1-MODEL\_V4 | 1.0 | 0.0003998 | 149 | 0.314 | 70 | 44 | 3 | 2 | 69 | 1 | 68 | Phage tail protein | Phage tail protein | | afdb-uniprot50 | AF-A0A2N3D6G0-F1-MODEL\_V4 | 1.0 | 0.0004619 | 149 | 0.342 | 70 | 42 | 3 | 2 | 69 | 4 | 71 | Phage tail protein | Phage tail protein | | afdb-uniprot50 | AF-A0A1V3DCJ7-F1-MODEL\_V4 | 1.0 | 0.0004619 | 149 | 0.375 | 72 | 40 | 3 | 2 | 69 | 1 | 71 | Phage tail protein | Phage tail protein | | afdb-uniprot50 | AF-A0A806LPR1-F1-MODEL\_V4 | 1.0 | 0.001268 | 149 | 0.301 | 63 | 41 | 2 | 1 | 63 | 1 | 60 | Phage tail protein | Phage tail protein | | afdb-uniprot50 | AF-A0A1T3BRC0-F1-MODEL\_V4 | 1.0 | 0.0002996 | 148 | 0.492 | 67 | 31 | 2 | 5 | 69 | 1 | 66 | p2-like prophage tail protein X | p2-like prophage tail protein X | | afdb-uniprot50 | AF-A0A0B6D337-F1-MODEL\_V4 | 1.0 | 0.0006626 | 148 | 0.281 | 71 | 49 | 2 | 1 | 69 | 1 | 71 | Phage Tail Protein X family protein | Phage Tail Protein X family protein | | afdb-uniprot50 | AF-A0A7L5ZYB4-F1-MODEL\_V4 | 1.0 | 0.0005735 | 148 | 0.276 | 65 | 44 | 2 | 1 | 65 | 1 | 62 | LysM domain-containing protein | LysM domain-containing protein | | afdb-uniprot50 | AF-A0A537KXH0-F1-MODEL\_V4 | 1.0 | 0.0004619 | 147 | 0.323 | 68 | 45 | 1 | 2 | 69 | 1 | 67 | Phage tail protein | Phage tail protein | | afdb-uniprot50 | AF-R9B495-F1-MODEL\_V4 | 1.0 | 0.0002413 | 147 | 0.362 | 69 | 42 | 1 | 1 | 69 | 1 | 67 | Uncharacterized protein | Uncharacterized protein | | afdb-uniprot50 | AF-A0A379KDD4-F1-MODEL\_V4 | 1.0 | 0.0006626 | 147 | 0.303 | 66 | 45 | 1 | 2 | 67 | 1 | 65 | Phage Tail Protein X | Phage Tail Protein X | | afdb-uniprot50 | AF-A0A1N7LQZ2-F1-MODEL\_V4 | 1.0 | 0.0005735 | 146 | 0.388 | 72 | 40 | 3 | 1 | 69 | 1 | 71 | p2-like prophage tail protein X | p2-like prophage tail protein X | | afdb-uniprot50 | AF-A0A4R2T1X8-F1-MODEL\_V4 | 1.0 | 0.0003998 | 146 | 0.328 | 70 | 44 | 2 | 2 | 69 | 3 | 71 | Phage tail protein X | Phage tail protein X | | afdb-uniprot50 | AF-A0A345DE49-F1-MODEL\_V4 | 1.0 | 0.0007654 | 146 | 0.279 | 68 | 47 | 1 | 2 | 69 | 3 | 68 | Uncharacterized protein | Uncharacterized protein | | afdb-uniprot50 | AF-A0A529HSU4-F1-MODEL\_V4 | 1.0 | 0.000372 | 146 | 0.405 | 69 | 39 | 2 | 2 | 69 | 16 | 83 | Phage tail protein | Phage tail protein | | afdb-uniprot50 | AF-A0A5U3G616-F1-MODEL\_V4 | 1.0 | 0.0005735 | 145 | 0.333 | 66 | 40 | 3 | 1 | 65 | 1 | 63 | Phage tail protein | Phage tail protein | | afdb-uniprot50 | AF-A0A7U9H248-F1-MODEL\_V4 | 1.0 | 0.001268 | 145 | 0.258 | 62 | 43 | 2 | 2 | 63 | 6 | 64 | Uncharacterized protein | Uncharacterized protein | | afdb-uniprot50 | AF-A0A363RF60-F1-MODEL\_V4 | 1.0 | 0.0008843 | 144 | 0.308 | 68 | 45 | 2 | 2 | 69 | 1 | 66 | Phage tail protein | Phage tail protein | | afdb-uniprot50 | AF-A0A371J2Y1-F1-MODEL\_V4 | 1.0 | 0.0007121 | 144 | 0.323 | 65 | 41 | 2 | 1 | 65 | 1 | 62 | Phage tail protein | Phage tail protein | | afdb-uniprot50 | AF-A0A0L0WAM0-F1-MODEL\_V4 | 1.0 | 0.002102 | 144 | 0.213 | 61 | 45 | 2 | 2 | 62 | 8 | 65 | Phage tail protein X | Phage tail protein X | | afdb-uniprot50 | AF-A0A3G2VEY6-F1-MODEL\_V4 | 1.0 | 0.001465 | 144 | 0.338 | 71 | 43 | 1 | 3 | 69 | 7 | 77 | Phage tail protein | Phage tail protein | | afdb-uniprot50 | AF-A0A2D3WQQ6-F1-MODEL\_V4 | 1.0 | 0.0004619 | 143 | 0.333 | 69 | 42 | 3 | 1 | 68 | 1 | 66 | Phage tail protein | Phage tail protein | | afdb-uniprot50 | AF-A0A857E7Y2-F1-MODEL\_V4 | 1.0 | 0.0008843 | 143 | 0.328 | 70 | 43 | 3 | 2 | 69 | 1 | 68 | Phage tail protein | Phage tail protein | | afdb-uniprot50 | AF-A0A7X2H1T7-F1-MODEL\_V4 | 1.0 | 0.001268 | 143 | 0.292 | 65 | 43 | 2 | 1 | 65 | 1 | 62 | Phage tail protein | Phage tail protein | | afdb-uniprot50 | AF-E2CJS6-F1-MODEL\_V4 | 1.0 | 0.001098 | 143 | 0.328 | 70 | 46 | 1 | 1 | 69 | 1 | 70 | Putative tail protein X | Putative tail protein X | | afdb-uniprot50 | AF-A0A7U9X278-F1-MODEL\_V4 | 1.0 | 0.000322 | 143 | 0.328 | 64 | 38 | 3 | 3 | 64 | 5 | 65 | Uncharacterized protein | Uncharacterized protein | | afdb-uniprot50 | AF-E2CN21-F1-MODEL\_V4 | 1.0 | 0.0008227 | 143 | 0.378 | 74 | 40 | 3 | 2 | 69 | 5 | 78 | Phage Tail Protein X | Phage Tail Protein X | | afdb-uniprot50 | AF-A0A2K3TWI8-F1-MODEL\_V4 | 1.0 | 0.0008227 | 142 | 0.323 | 68 | 45 | 1 | 2 | 69 | 1 | 67 | Phage tail protein | Phage tail protein | | afdb-uniprot50 | AF-A0A2G6EZ32-F1-MODEL\_V4 | 1.0 | 0.0008227 | 142 | 0.25 | 68 | 46 | 3 | 2 | 69 | 6 | 68 | Phage tail protein | Phage tail protein | | afdb-uniprot50 | AF-A0A4Q0GTV4-F1-MODEL\_V4 | 1.0 | 0.00118 | 142 | 0.246 | 69 | 50 | 2 | 2 | 69 | 1 | 68 | Phage tail protein | Phage tail protein | | afdb-uniprot50 | AF-B6JEG3-F1-MODEL\_V4 | 1.0 | 0.0003998 | 142 | 0.394 | 71 | 39 | 2 | 2 | 69 | 4 | 73 | Putative phage tail protein | Putative phage tail protein | | afdb-uniprot50 | AF-A0A2G2DHZ7-F1-MODEL\_V4 | 1.0 | 0.001956 | 142 | 0.394 | 71 | 41 | 2 | 1 | 69 | 6 | 76 | Phage tail protein | Phage tail protein | | afdb-uniprot50 | AF-A0A377A283-F1-MODEL\_V4 | 1.0 | 0.001022 | 142 | 0.308 | 68 | 44 | 3 | 2 | 69 | 166 | 230 | Head completion/stabilization protein L | Head completion/stabilization protein L | | afdb-uniprot50 | AF-A0A7C5AES2-F1-MODEL\_V4 | 1.0 | 0.004997 | 142 | 0.29 | 55 | 39 | 0 | 2 | 56 | 306 | 360 | LysM peptidoglycan-binding domain-containing protein | LysM peptidoglycan-binding domain-containing protein | | afdb-uniprot50 | AF-A0A7I8DM73-F1-MODEL\_V4 | 1.0 | 0.001956 | 141 | 0.274 | 62 | 42 | 2 | 2 | 63 | 3 | 61 | Uncharacterized protein | Uncharacterized protein | | afdb-uniprot50 | AF-A0A0P6VPS0-F1-MODEL\_V4 | 1.0 | 0.004649 | 141 | 0.298 | 57 | 39 | 1 | 1 | 56 | 1 | 57 | Uncharacterized protein | Uncharacterized protein | | afdb-uniprot50 | AF-A0A6B8RNW6-F1-MODEL\_V4 | 1.0 | 0.004326 | 140 | 0.233 | 60 | 43 | 2 | 2 | 61 | 5 | 61 | LysM domain-containing protein | LysM domain-containing protein | | afdb-uniprot50 | AF-A0A3L7AJJ2-F1-MODEL\_V4 | 1.0 | 0.0005735 | 140 | 0.371 | 70 | 42 | 2 | 2 | 69 | 4 | 73 | Phage tail protein | Phage tail protein | | afdb-uniprot50 | AF-A0A3N2E0V9-F1-MODEL\_V4 | 1.0 | 0.0006626 | 140 | 0.287 | 101 | 39 | 3 | 2 | 69 | 3 | 103 | Phage tail protein X | Phage tail protein X | | afdb-uniprot50 | AF-A0A2E4ZYN4-F1-MODEL\_V4 | 1.0 | 0.005371 | 140 | 0.258 | 58 | 43 | 0 | 2 | 59 | 192 | 249 | LysM domain-containing protein | LysM domain-containing protein | | afdb-uniprot50 | AF-A0A537MKE3-F1-MODEL\_V4 | 1.0 | 0.001098 | 139 | 0.338 | 68 | 40 | 3 | 4 | 69 | 1 | 65 | Phage tail protein | Phage tail protein | | afdb-uniprot50 | AF-A0A2P1VUX4-F1-MODEL\_V4 | 1.0 | 0.001268 | 139 | 0.304 | 69 | 48 | 0 | 1 | 69 | 1 | 69 | Phage Tail Protein X | Phage Tail Protein X | | afdb-uniprot50 | AF-A0A1C0V8F9-F1-MODEL\_V4 | 1.0 | 0.001956 | 139 | 0.338 | 65 | 40 | 2 | 2 | 65 | 4 | 66 | Uncharacterized protein | Uncharacterized protein | | afdb-uniprot50 | AF-A0A4Q5K7X7-F1-MODEL\_V4 | 1.0 | 0.001465 | 139 | 0.253 | 67 | 45 | 2 | 1 | 65 | 1 | 64 | Uncharacterized protein | Uncharacterized protein | | afdb-uniprot50 | AF-A0A3G2Q4R3-F1-MODEL\_V4 | 1.0 | 0.00182 | 139 | 0.217 | 69 | 52 | 1 | 2 | 68 | 1 | 69 | Uncharacterized protein | Uncharacterized protein | | afdb-uniprot50 | AF-A0A087N8U9-F1-MODEL\_V4 | 1.0 | 0.0004619 | 139 | 0.347 | 72 | 42 | 3 | 2 | 69 | 5 | 75 | Uncharacterized protein | Uncharacterized protein | | afdb-uniprot50 | AF-A0A0M4SYX6-F1-MODEL\_V4 | 1.0 | 0.001022 | 138 | 0.279 | 68 | 48 | 1 | 2 | 69 | 8 | 74 | Uncharacterized protein | Uncharacterized protein | | afdb-uniprot50 | AF-A0A7W7INH6-F1-MODEL\_V4 | 1.0 | 0.001465 | 138 | 0.277 | 72 | 46 | 3 | 1 | 69 | 7 | 75 | Phage tail protein X | Phage tail protein X | | afdb-uniprot50 | AF-A0A6N2Z4M7-F1-MODEL\_V4 | 1.0 | 0.002102 | 137 | 0.281 | 64 | 43 | 2 | 2 | 65 | 3 | 63 | Uncharacterized protein | Uncharacterized protein | | afdb-uniprot50 | AF-A0A2W6YQP5-F1-MODEL\_V4 | 1.0 | 0.001022 | 137 | 0.405 | 74 | 37 | 3 | 1 | 69 | 1 | 72 | Phage tail protein | Phage tail protein | | afdb-uniprot50 | AF-A0A6P1LZV2-F1-MODEL\_V4 | 1.0 | 0.0008843 | 136 | 0.294 | 68 | 46 | 1 | 2 | 69 | 3 | 68 | Uncharacterized protein | Uncharacterized protein | | afdb-uniprot50 | AF-E1W2C5-F1-MODEL\_V4 | 1.0 | 0.001022 | 136 | 0.267 | 71 | 47 | 3 | 2 | 69 | 1 | 69 | Putative tail synthesis protein | Putative tail synthesis protein | | afdb-uniprot50 | AF-A0A7V8RB52-F1-MODEL\_V4 | 1.0 | 0.0008227 | 135 | 0.406 | 64 | 35 | 2 | 8 | 69 | 2 | 64 | Phage tail protein | Phage tail protein | | afdb-uniprot50 | AF-A0A411WHQ6-F1-MODEL\_V4 | 1.0 | 0.001022 | 135 | 0.283 | 67 | 45 | 2 | 2 | 68 | 6 | 69 | Uncharacterized protein | Uncharacterized protein | | afdb-uniprot50 | AF-A0A8B3RD81-F1-MODEL\_V4 | 1.0 | 0.00182 | 133 | 0.414 | 70 | 36 | 4 | 1 | 68 | 1 | 67 | Phage tail protein | Phage tail protein | | afdb-uniprot50 | AF-A0A3G8M8D8-F1-MODEL\_V4 | 1.0 | 0.00182 | 133 | 0.308 | 68 | 47 | 0 | 2 | 69 | 1 | 68 | Phage tail protein | Phage tail protein | | afdb-uniprot50 | AF-A0A318KRC1-F1-MODEL\_V4 | 1.0 | 0.00182 | 133 | 0.323 | 71 | 45 | 3 | 1 | 69 | 1 | 70 | Phage tail protein X | Phage tail protein X | | afdb-uniprot50 | AF-A0A1G8HXC6-F1-MODEL\_V4 | 1.0 | 0.002806 | 133 | 0.25 | 72 | 51 | 1 | 1 | 69 | 1 | 72 | Phage Tail Protein X | Phage Tail Protein X | | afdb-uniprot50 | AF-A0A842J0J6-F1-MODEL\_V4 | 1.0 | 0.003744 | 132 | 0.264 | 68 | 47 | 2 | 2 | 69 | 7 | 71 | Tail protein X | Tail protein X | | afdb-uniprot50 | AF-B3QTI9-F1-MODEL\_V4 | 1.0 | 0.001465 | 132 | 0.271 | 81 | 44 | 3 | 2 | 69 | 5 | 83 | Putative inner membrane protein | Putative inner membrane protein | | afdb-uniprot50 | AF-A0A064AM34-F1-MODEL\_V4 | 1.0 | 0.002259 | 131 | 0.23 | 65 | 47 | 2 | 2 | 66 | 10 | 71 | Tail protein | Tail protein | | afdb-uniprot50 | AF-A0A3B0MEG3-F1-MODEL\_V4 | 1.0 | 0.001268 | 131 | 0.342 | 73 | 38 | 3 | 2 | 69 | 118 | 185 | Uncharacterized protein | Uncharacterized protein | | afdb-uniprot50 | AF-A0A7V8A966-F1-MODEL\_V4 | 1.0 | 0.003241 | 129 | 0.242 | 70 | 49 | 3 | 2 | 69 | 1 | 68 | Uncharacterized protein | Uncharacterized protein | | afdb-uniprot50 | AF-A0A1I1XSG4-F1-MODEL\_V4 | 1.0 | 0.0005336 | 128 | 0.394 | 71 | 35 | 4 | 2 | 69 | 8 | 73 | P2-like prophage tail protein X | P2-like prophage tail protein X | | afdb-uniprot50 | AF-A0A845R965-F1-MODEL\_V4 | 1.0 | 0.002428 | 127 | 0.323 | 65 | 39 | 3 | 3 | 67 | 4 | 63 | Uncharacterized protein | Uncharacterized protein | | afdb-uniprot50 | AF-A0A5M8P5L1-F1-MODEL\_V4 | 1.0 | 0.00261 | 126 | 0.323 | 65 | 38 | 3 | 2 | 62 | 4 | 66 | Uncharacterized protein | Uncharacterized protein | | afdb-uniprot50 | AF-Q0FZ17-F1-MODEL\_V4 | 1.0 | 0.003015 | 125 | 0.391 | 69 | 38 | 3 | 4 | 69 | 2 | 69 | Uncharacterized protein | Uncharacterized protein | | afdb-uniprot50 | AF-A0A3L7JDR3-F1-MODEL\_V4 | 1.0 | 0.004326 | 124 | 0.319 | 72 | 46 | 1 | 1 | 69 | 1 | 72 | Uncharacterized protein | Uncharacterized protein | | afdb-uniprot50 | AF-A0A4Q3Q4D9-F1-MODEL\_V4 | 1.0 | 0.0005735 | 124 | 0.352 | 71 | 38 | 3 | 2 | 69 | 7 | 72 | Phage tail protein | Phage tail protein | | afdb-uniprot50 | AF-A8SB42-F1-MODEL\_V4 | 1.0 | 0.0006626 | 124 | 0.376 | 69 | 34 | 4 | 2 | 64 | 10 | 75 | Phage Tail Protein X | Phage Tail Protein X | | afdb-uniprot50 | AF-A0A1Y4BIR9-F1-MODEL\_V4 | 1.0 | 0.004326 | 124 | 0.281 | 64 | 43 | 2 | 2 | 65 | 11 | 71 | Uncharacterized protein | Uncharacterized protein | | afdb-uniprot50 | AF-A0A7C3CH29-F1-MODEL\_V4 | 1.0 | 0.008281 | 124 | 0.315 | 57 | 38 | 1 | 1 | 56 | 270 | 326 | LysM peptidoglycan-binding domain-containing protein | LysM peptidoglycan-binding domain-containing protein | | afdb-uniprot50 | AF-M5JRD9-F1-MODEL\_V4 | 1.0 | 0.004997 | 123 | 0.314 | 70 | 47 | 1 | 1 | 69 | 1 | 70 | Tail X family protein | Tail X family protein | | afdb-uniprot50 | AF-A0A318EKS8-F1-MODEL\_V4 | 1.0 | 0.004025 | 123 | 0.312 | 64 | 41 | 2 | 1 | 64 | 18 | 78 | Uncharacterized protein | Uncharacterized protein | | afdb-uniprot50 | AF-A0A643EZQ1-F1-MODEL\_V4 | 1.0 | 0.004997 | 122 | 0.3 | 70 | 48 | 1 | 1 | 69 | 1 | 70 | Phage tail protein | Phage tail protein | | afdb-uniprot50 | AF-A0A6N9P0A8-F1-MODEL\_V4 | 1.0 | 0.001956 | 121 | 0.303 | 66 | 43 | 2 | 1 | 65 | 1 | 64 | Phage tail protein | Phage tail protein | | afdb-uniprot50 | AF-A0A7C2KV08-F1-MODEL\_V4 | 1.0 | 0.008281 | 120 | 0.292 | 65 | 45 | 1 | 2 | 66 | 185 | 248 | LysM peptidoglycan-binding domain-containing protein | LysM peptidoglycan-binding domain-containing protein | | afdb-uniprot50 | AF-A0A285MC51-F1-MODEL\_V4 | 1.0 | 0.004997 | 119 | 0.271 | 70 | 47 | 3 | 2 | 69 | 3 | 70 | P2-like prophage tail protein X | P2-like prophage tail protein X | | afdb-uniprot50 | AF-A0A7Y3K1T7-F1-MODEL\_V4 | 1.0 | 0.007169 | 119 | 0.271 | 70 | 48 | 2 | 2 | 69 | 149 | 217 | LysM peptidoglycan-binding domain-containing protein | LysM peptidoglycan-binding domain-containing protein | | afdb-uniprot50 | AF-A0A2K2G0B1-F1-MODEL\_V4 | 1.0 | 0.008901 | 118 | 0.281 | 71 | 47 | 3 | 2 | 69 | 1 | 70 | Phage tail protein | Phage tail protein | | afdb-uniprot50 | AF-A0A258L1B2-F1-MODEL\_V4 | 1.0 | 0.003744 | 116 | 0.342 | 70 | 43 | 2 | 2 | 68 | 6 | 75 | Phage tail protein | Phage tail protein | | afdb-uniprot50 | AF-A0A329U822-F1-MODEL\_V4 | 1.0 | 0.009567 | 115 | 0.296 | 64 | 40 | 3 | 2 | 65 | 3 | 61 | Phage tail protein | Phage tail protein | | afdb-uniprot50 | AF-A0A4V2V312-F1-MODEL\_V4 | 1.0 | 0.003015 | 115 | 0.341 | 79 | 41 | 4 | 2 | 69 | 7 | 85 | Phage tail protein X | Phage tail protein X | | afdb-uniprot50 | AF-A0A7C6DLC9-F1-MODEL\_V4 | 1.0 | 0.007169 | 115 | 0.234 | 64 | 45 | 1 | 2 | 61 | 203 | 266 | LysM peptidoglycan-binding domain-containing protein | LysM peptidoglycan-binding domain-containing protein | | afdb-uniprot50 | AF-A0A7V4P1T4-F1-MODEL\_V4 | 1.0 | 0.007705 | 115 | 0.323 | 68 | 46 | 0 | 2 | 69 | 207 | 274 | LysM peptidoglycan-binding domain-containing protein | LysM peptidoglycan-binding domain-containing protein | | afdb-uniprot50 | AF-A0A4R2GR23-F1-MODEL\_V4 | 1.0 | 0.005773 | 113 | 0.346 | 75 | 40 | 5 | 2 | 69 | 1 | 73 | Phage tail protein X | Phage tail protein X | | afdb-uniprot50 | AF-A0A414D8J5-F1-MODEL\_V4 | 1.0 | 0.003241 | 113 | 0.257 | 66 | 38 | 3 | 2 | 59 | 8 | 70 | LysM domain-containing protein | LysM domain-containing protein | | afdb-uniprot50 | AF-A0A1V5X535-F1-MODEL\_V4 | 1.0 | 0.008901 | 113 | 0.296 | 64 | 43 | 1 | 2 | 65 | 113 | 174 | LysM domain/BON superfamily protein | LysM domain/BON superfamily protein | | afdb-uniprot50 | AF-A0A7W6IJI9-F1-MODEL\_V4 | 1.0 | 0.002259 | 112 | 0.356 | 73 | 42 | 2 | 1 | 69 | 1 | 72 | Phage tail protein X | Phage tail protein X | | afdb-uniprot50 | AF-F4HB38-F1-MODEL\_V4 | 1.0 | 0.004025 | 112 | 0.271 | 70 | 47 | 3 | 2 | 69 | 8 | 75 | Phage Tail Protein X | Phage Tail Protein X | | afdb-uniprot50 | AF-A0A2W6Z0D8-F1-MODEL\_V4 | 1.0 | 0.003241 | 112 | 0.369 | 73 | 41 | 4 | 2 | 69 | 4 | 76 | Phage tail protein | Phage tail protein | | afdb-uniprot50 | AF-A0A0T6ZD76-F1-MODEL\_V4 | 1.0 | 0.005371 | 112 | 0.22 | 77 | 48 | 3 | 2 | 69 | 1 | 74 | Uncharacterized protein | Uncharacterized protein | | afdb-uniprot50 | AF-A0A1C3E6J5-F1-MODEL\_V4 | 1.0 | 0.007705 | 111 | 0.217 | 69 | 51 | 2 | 2 | 69 | 1 | 67 | Uncharacterized protein | Uncharacterized protein | | afdb-uniprot50 | AF-A0A7C3XER5-F1-MODEL\_V4 | 1.0 | 0.008901 | 111 | 0.313 | 67 | 41 | 2 | 2 | 63 | 183 | 249 | LysM peptidoglycan-binding domain-containing protein | LysM peptidoglycan-binding domain-containing protein | | afdb-uniprot50 | AF-A0A371WU87-F1-MODEL\_V4 | 1.0 | 0.005371 | 107 | 0.281 | 71 | 48 | 2 | 2 | 69 | 4 | 74 | Phage tail protein | Phage tail protein | | afdb-uniprot50 | AF-A0A6I1JS86-F1-MODEL\_V4 | 1.0 | 0.004997 | 103 | 0.324 | 74 | 45 | 3 | 1 | 69 | 1 | 74 | Phage tail protein | Phage tail protein | | afdb-uniprot50 | AF-A0A174T8M1-F1-MODEL\_V4 | 0.998 | 0.006205 | 94 | 0.217 | 69 | 41 | 4 | 1 | 59 | 1 | 66 | LysM domain-containing protein | LysM domain-containing protein | | afdb-uniprot50 | AF-A0A256CAT5-F1-MODEL\_V4 | 0.995 | 0.006669 | 88 | 0.295 | 71 | 45 | 3 | 1 | 69 | 1 | 68 | Uncharacterized protein | Uncharacterized protein | |
| Top keywords  (threshold 1.00e-02 (evalue)) | **tail, Phage, X, prophage, P2\_like, domain\_containing, LysM, Putative, peptidoglycan\_binding, component** |
| Output files | ../../similar\_structures/24\_FANPEZAQ\_CDS\_0024\_afdb-proteome\_foldseek.tsv ../../similar\_structures/24\_FANPEZAQ\_CDS\_0024\_afdb-uniprot50\_foldseek.tsv ../../similar\_structures/24\_FANPEZAQ\_CDS\_0024\_merged.svg ../../similar\_structures/24\_FANPEZAQ\_CDS\_0024\_pdb\_foldseek.tsv |

  
  
  

Return to summary | Go to previous | Go to next

  


---

**Sequence/structure alignments coloring**  
Each object in the alignment figures is colored according to its E-value following this color coding:

1e-100
10

**References:**  
1) Steinegger M, Meier M, Mirdita M, Vöhringer H, Haunsberger S J, and Söding J (2019) HH-suite3 for fast remote homology detection and deep protein annotation, BMC Bioinformatics, 473. doi: 10.1186/s12859-019-3019-7  
2) Jumper J, Evans R, Pritzel A, ..., Hassabis D (2021) Highly accurate protein structure prediction with AlphaFold, Nature, 596. doi: 10.1038/s41586-021-03819-2  
3) van Kempen M, Kim S, Tumescheit C, Mirdita M, Lee J, Gilchrist CLM, Söding J, and Steinegger M (2023) Fast and accurate protein structure search with Foldseek. Nature Biotechnology. doi: 10.1038/s41587-023-01773-0
