# Supplementary material for: Completing the BASEL phage collection to unlock hidden diversity for systematic exploration of phage–host interactions
Source: PLoS Biol. 2025 Apr 7;23(4):e3003063. doi: 10.1371/journal.pbio.3003063 (PMC11990801; doi:10.1371/journal.pbio.3003063)
Supplement: S2 Data — (ZIP) [file pbio.3003063.s009.zip › entries/25.html]

FANPEZAQ\_CDS\_0025


Return to summary | Go to previous | Go to next

|  |  |
| --- | --- |
| FANPEZAQ\_CDS\_0025 Page creation date: 02 Sep 2024, 12:00  Project folder: n/a  Input sequences file: Escherichia\_virus\_HeidiAbel.gb | d phage late control type secretion vi system vgr vgrg rhs domain\_containing tip tail element fragment gene ob\_fold gp5 iv phage\_base\_v gpd baseplate putative assembly prophage vgrg\_related in kda tssi phage\_related vgrg1 bacteriophage beta impa muso2 and fold conserved lysm roll hub structural spike escherichia coli regulatory engineered yes organism\_taxid |

### Sequence information

|  |  |
| --- | --- |
| Name | FANPEZAQ\_CDS\_0025  25\_FANPEZAQ\_CDS\_0025 (pipeline id) |
| Imported annotations | Escherichia\_virus\_HeidiAbel Bas97 |
| Protein sequence | MPETGAELELFLGYDNWTQRMGLFICDEVELAGWPGTMTIRARAATYDKSKGGKTDLQTQ KSRSWTKGTKFGDMVKKIASEHGMESAVSESMAKITLPHTDQADESDLNLLLRLAKKYDG VVKPSGGKITVTKRGEGKTASGEELPTITLTAQDCTAFRLTSQKRENAGTVVAYYQMTKK AARNEVKVGEGEPVKRIGKYYPTQAMALDAAKAELAKRQRNQVTVAVTLPGDPSIAAECK LVLVGFRDGIDGEWLITRADHRLDDNGYSCDVEATKPNSEEEPKVEVIERKKPQESDPPD DDGPGHVIEEK |
| Number of residues | 311 |
| Molecular weight (Da) | 34138.16 |
| Output files | ../../query\_sequences/25\_FANPEZAQ\_CDS\_0025.fasta |

### Putative domain architecture and protein family

#### Search results (HHblits)1

|  |  |
| --- | --- |
| Domain family databases searched | Pfam, Ncbi-cd, Cath, Phrogs |
| Results, scheme(s)  (Top layers only; threshold 1.00e-03 (evalue)) | xml version="1.0" encoding="utf-8" standalone="no"?       2024-09-02T21:08:17.614933 image/svg+xml   Matplotlib v3.7.2, https://matplotlib.org/ |
| Results, table  (E-value ≤ 1.00e-03 (evalue)) | | db | id | prob | evalue | pvalue | score | cols | query | query\_len | template | template\_len | name | description | | --- | --- | --- | --- | --- | --- | --- | --- | --- | --- | --- | --- | --- | | pfam | PF05954 | 99.4 | 7.3e-18 | 1.2e-21 | 135.2 | 250 | (3, 266) | 311 | (27, 297) | 298 | Phage\_GPD | Phage tail baseplate hub (GPD) | | pfam | PF14594 | 98.9 | 6e-14 | 1e-17 | 114.4 | 244 | (20, 275) | 311 | (54, 343) | 343 | Sipho\_Gp37 | Siphovirus ReqiPepy6 Gp37-like protein | | pfam | PF06605 | 98.7 | 1.9e-12 | 3.2e-16 | 101.0 | 205 | (62, 278) | 311 | (12, 253) | 263 | Prophage\_tail | Prophage endopeptidase tail | | pfam | PF13550 | 97.9 | 3.2e-09 | 5.3e-13 | 75.0 | 146 | (104, 260) | 311 | (3, 163) | 164 | Phage-tail\_3 | Putative phage tail protein | | pfam | PF16467 | 97.8 | 6.9e-09 | 1.2e-12 | 67.6 | 74 | (201, 276) | 311 | (24, 97) | 102 | DUF5048 | Domain of unknown function (DUF5048) | | cath | 1wruA01 | 98.5 | 1.3e-11 | 2.2e-15 | 102.5 | 64 | (218, 281) | 311 | (107, 177) | 177 | 43 kda tail protein | CATHCODE: 2.30.300.10 NAME: 43 kda tail protein. Chain: a. Synonym: gpp, gene product 44. Engineered: yes SOURCE: Enterobacteria phage mu. Organism\_taxid: 10677. Gene: 44. Expressed in: escherichia coli. Expression\_system\_taxid: 562. CLASS: Mainly Beta, ARCH: Roll, TOPOL: Phage tail proteins - horseshoe like beta roll fold , HOMOL: Baseplate protein-like domain - beta roll fold | | cath | 3cddA01 | 98.3 | 8.7e-11 | 1.5e-14 | 97.4 | 59 | (225, 283) | 311 | (121, 185) | 187 | Prophage muso2, 43 kda tail protein | CATHCODE: 2.30.300.10 NAME: Prophage muso2, 43 kda tail protein. Chain: a, b, c, d, e, f. Engineered: yes SOURCE: Shewanella oneidensis. Organism\_taxid: 211586. Strain: mr-1. Gene: so\_2699. Expressed in: escherichia coli bl21(de3). Expression\_system\_taxid: 469008. CLASS: Mainly Beta, ARCH: Roll, TOPOL: Phage tail proteins - horseshoe like beta roll fold , HOMOL: Baseplate protein-like domain - beta roll fold | | cath | 3d37B01 | 98.2 | 2.5e-10 | 4.3e-14 | 93.2 | 61 | (221, 281) | 311 | (122, 188) | 188 | Tail protein, 43 kda | CATHCODE: 2.30.300.10 NAME: Tail protein, 43 kda. Chain: a, b. Engineered: yes SOURCE: Neisseria meningitidis mc58. Organism\_taxid: 122586. Strain: mc58 / serogroup b. Gene: gi:7226348, nmb1110. Expressed in: escherichia coli. CLASS: Mainly Beta, ARCH: Roll, TOPOL: Phage tail proteins - horseshoe like beta roll fold , HOMOL: Baseplate protein-like domain - beta roll fold | | cath | 4uhvA02 | 97.7 | 1.2e-08 | 2.3e-12 | 69.6 | 78 | (58, 136) | 311 | (8, 90) | 92 | Vgrg1, valine-glycine repeat protein g1 | CATHCODE: 3.55.50.10 NAME: Vgrg1, valine-glycine repeat protein g1. Chain: a, b. Engineered: yes SOURCE: Pseudomonas aeruginosa. Organism\_taxid: 208964. Strain: pao1. Expressed in: escherichia coli. Expression\_system\_taxid: 469008. CLASS: Alpha Beta, ARCH: 3-Layer(bab) Sandwich, TOPOL: Phage tail protein beta-alpha-beta fold, HOMOL: Baseplate protein-like domains | | cath | 1wruA02 | 97.6 | 1.4e-08 | 2.6e-12 | 70.6 | 71 | (61, 132) | 311 | (13, 87) | 88 | 43 kda tail protein | CATHCODE: 3.55.50.10 NAME: 43 kda tail protein. Chain: a. Synonym: gpp, gene product 44. Engineered: yes SOURCE: Enterobacteria phage mu. Organism\_taxid: 10677. Gene: 44. Expressed in: escherichia coli. Expression\_system\_taxid: 562. CLASS: Alpha Beta, ARCH: 3-Layer(bab) Sandwich, TOPOL: Phage tail protein beta-alpha-beta fold, HOMOL: Baseplate protein-like domains | | cath | 3d37A02 | 97.5 | 3.5e-08 | 6.6e-12 | 66.8 | 71 | (61, 132) | 311 | (11, 84) | 85 | Tail protein, 43 kda | CATHCODE: 3.55.50.10 NAME: Tail protein, 43 kda. Chain: a, b. Engineered: yes SOURCE: Neisseria meningitidis mc58. Organism\_taxid: 122586. Strain: mc58 / serogroup b. Gene: gi:7226348, nmb1110. Expressed in: escherichia coli. CLASS: Alpha Beta, ARCH: 3-Layer(bab) Sandwich, TOPOL: Phage tail protein beta-alpha-beta fold, HOMOL: Baseplate protein-like domains | | cath | 2p5zX02 | 97.4 | 8.1e-08 | 1.6e-11 | 65.6 | 76 | (59, 135) | 311 | (9, 94) | 97 | Type vi secretion system component | CATHCODE: 3.55.50.10 NAME: Type vi secretion system component. Chain: x. Fragment: n-terminal domain. Engineered: yes SOURCE: Escherichia coli o6. Organism\_taxid: 217992. Strain: o6:h1, cft073, upec. Atcc: 700928. Gene: c3393. Expressed in: escherichia coli bl21(de3). Expression\_system\_taxid: 469008. CLASS: Alpha Beta, ARCH: 3-Layer(bab) Sandwich, TOPOL: Phage tail protein beta-alpha-beta fold, HOMOL: Baseplate protein-like domains | | phrogs | 52 | 100.0 | 4.8e-40 | 5.8e-44 | 290.6 | 275 | (1, 283) | 311 | (52, 328) | 333 | tail protein | tail protein; Category: tail; KY290949\_p37 | | phrogs | 177 | 99.9 | 1e-33 | 1.2e-37 | 252.2 | 269 | (3, 293) | 311 | (49, 352) | 374 | baseplate hub | baseplate hub; Category: tail; p166740 VI\_07350 | | phrogs | 400 | 99.9 | 2.6e-28 | 3.1e-32 | 212.7 | 250 | (2, 276) | 311 | (50, 322) | 324 | tail protein | tail protein; Category: tail; p235698 VI\_00931 | | phrogs | 1887 | 99.5 | 3.2e-19 | 3.8e-23 | 154.6 | 258 | (5, 274) | 311 | (23, 312) | 316 | tail protein | tail protein; Category: tail; NC\_022766\_p29 | | phrogs | 4930 | 99.5 | 5.5e-19 | 6.3e-23 | 146.7 | 255 | (3, 278) | 311 | (50, 337) | 363 | baseplate hub | baseplate hub; Category: tail; p87959 VI\_12426 | | phrogs | 11191 | 99.3 | 2.2e-16 | 2.5e-20 | 121.9 | 159 | (3, 182) | 311 | (52, 221) | 262 | NA | NA; Category: unknown function; p213285 VI\_11403 | | phrogs | 196 | 99.0 | 2.3e-14 | 2.8e-18 | 124.3 | 214 | (4, 277) | 311 | (102, 331) | 333 | baseplate hub | baseplate hub; Category: tail; NC\_020843\_p99 | | phrogs | 1390 | 98.7 | 2.4e-12 | 2.8e-16 | 106.8 | 243 | (4, 277) | 311 | (70, 336) | 338 | tail protein | tail protein; Category: tail; p10114 VI\_10453 | | phrogs | 4456 | 98.6 | 5.4e-12 | 6e-16 | 91.5 | 107 | (1, 117) | 311 | (57, 172) | 173 | NA | NA; Category: unknown function; p151864 VI\_00910 | | phrogs | 20686 | 98.6 | 1.1e-11 | 1.3e-15 | 82.1 | 80 | (198, 278) | 311 | (12, 92) | 97 | NA | NA; Category: unknown function; p199509 VI\_11453 | | phrogs | 12925 | 98.3 | 1.1e-10 | 1.3e-14 | 77.6 | 87 | (192, 278) | 311 | (6, 94) | 102 | tail protein | tail protein; Category: tail; p409900 VI\_07763 | | phrogs | 350 | 97.1 | 4.3e-07 | 5.2e-11 | 79.5 | 80 | (204, 283) | 311 | (324, 409) | 412 | tail protein | tail protein; Category: tail; MF448340\_p356 | | phrogs | 9932 | 97.1 | 5.2e-07 | 5.8e-11 | 63.2 | 96 | (191, 286) | 311 | (12, 120) | 148 | NA | NA; Category: unknown function; p278086 VI\_09755 | | phrogs | 33062 | 96.4 | 1.1e-05 | 1.2e-09 | 45.1 | 47 | (230, 277) | 311 | (2, 49) | 54 | NA | NA; Category: unknown function; p376724 VI\_10043 | | phrogs | 24827 | 96.2 | 2.1e-05 | 2.4e-09 | 60.3 | 72 | (208, 279) | 311 | (204, 286) | 288 | tail protein | tail protein; Category: tail; NC\_019443\_p15 | | phrogs | 6616 | 95.9 | 5e-05 | 5.7e-09 | 68.3 | 222 | (18, 265) | 311 | (121, 354) | 873 | tail protein | tail protein; Category: tail; p153366 VI\_00729 | | phrogs | 21793 | 95.9 | 5.3e-05 | 6e-09 | 61.8 | 150 | (103, 264) | 311 | (301, 465) | 476 | NA | NA; Category: unknown function; NC\_021327\_p131 | | phrogs | 33423 | 94.7 | 0.00052 | 5.8e-08 | 51.0 | 100 | (4, 117) | 311 | (52, 157) | 265 | NA | NA; Category: unknown function; p240160 VI\_01320 | | phrogs | 5313 | 94.6 | 0.00062 | 7.3e-08 | 63.0 | 209 | (69, 286) | 311 | (571, 805) | 903 | NA | NA; Category: unknown function; p230345 VI\_04092 | | phrogs | 13879 | 94.3 | 0.00092 | 1.1e-07 | 57.2 | 224 | (3, 246) | 311 | (252, 489) | 519 | NA | NA; Category: unknown function; p308556 VI\_00914 | | phrogs | 326 | 94.2 | 0.0009 | 1.1e-07 | 58.4 | 187 | (69, 264) | 311 | (107, 317) | 486 | tail protein with endopeptidase domain | tail protein with endopeptidase domain; Category: other; p133814 VI\_05051 | |
| Top keywords  (threshold 1.00e-03 (evalue)) | **tail, Beta, Phage, Baseplate, fold, roll, Gene, escherichia, coli, Engineered** |
| Output files | ../../domain\_architecture/25\_FANPEZAQ\_CDS\_0025\_cath.hhr ../../domain\_architecture/25\_FANPEZAQ\_CDS\_0025\_merged.svg ../../domain\_architecture/25\_FANPEZAQ\_CDS\_0025\_ncbi-cd.hhr ../../domain\_architecture/25\_FANPEZAQ\_CDS\_0025\_pfam.hhr ../../domain\_architecture/25\_FANPEZAQ\_CDS\_0025\_phrogs.hhr |

### Identical protein sequences/structures

#### Search results

|  |  |
| --- | --- |
| Protein sequence databases searched | Pdb, Swissprot, Refseq |
| Identical proteins found | -- |
| Top keywords | -- |
| Output files | -- |

### Similar protein sequences/structures

#### Sequence similarity search results (HHblits)1

|  |  |
| --- | --- |
| Sequence databases searched | Uniclust, Pdb70 |
| Results, scheme(s)  (Top layers only, threshold 1.00e-03 (evalue)) | xml version="1.0" encoding="utf-8" standalone="no"?       2024-09-02T21:08:40.558087 image/svg+xml   Matplotlib v3.7.2, https://matplotlib.org/ |
| Results, table(s)  (threshold 1.00e-03 (evalue)) | | db | id | prob | evalue | pvalue | score | cols | query | query\_len | template | template\_len | name | description | | --- | --- | --- | --- | --- | --- | --- | --- | --- | --- | --- | --- | --- | | uniclust | UniRef100\_A0A0S8GYZ7 | 100.0 | 4.5e-41 | 8.2e-47 | 294.5 | 290 | (4, 307) | 311 | (143, 471) | 781 | Type VI secretion protein ImpA (Fragment) | Type VI secretion protein ImpA (Fragment) | | uniclust | UniRef100\_A0A166E304 | 100.0 | 9.2e-41 | 1.7e-46 | 278.7 | 287 | (4, 305) | 311 | (62, 380) | 520 | Phage-related baseplate assembly protein | Phage-related baseplate assembly protein | | uniclust | UniRef100\_A0A062V571 | 100.0 | 2.5e-40 | 4.6e-46 | 280.6 | 268 | (2, 285) | 311 | (84, 378) | 510 | Phage protein D | Phage protein D | | uniclust | UniRef100\_A0A074LMF2 | 100.0 | 6e-40 | 1.1e-45 | 277.6 | 265 | (3, 283) | 311 | (99, 389) | 421 | Phage late control D family protein | Phage late control D family protein | | uniclust | UniRef100\_A0A011P577 | 100.0 | 8e-40 | 1.5e-45 | 279.3 | 273 | (1, 280) | 311 | (89, 368) | 445 | Tail protein | Tail protein | | uniclust | UniRef100\_A0A017T2T3 | 100.0 | 1.1e-39 | 2e-45 | 297.7 | 289 | (4, 306) | 311 | (93, 422) | 905 | VgrG protein | VgrG protein | | uniclust | UniRef100\_A0A021XHC5 | 100.0 | 1.5e-39 | 2.8e-45 | 299.4 | 289 | (4, 306) | 311 | (168, 495) | 1005 | Gp5/Type VI secretion system Vgr protein OB-fold domain-containing protein | Gp5/Type VI secretion system Vgr protein OB-fold domain-containing protein | | uniclust | UniRef100\_A0A022FQY4 | 100.0 | 3.1e-39 | 5.6e-45 | 298.6 | 290 | (4, 306) | 311 | (158, 483) | 1025 | ImpA family type VI secretion-associated protein | ImpA family type VI secretion-associated protein | | uniclust | UniRef100\_A0A1I4TS69 | 100.0 | 7.1e-39 | 1.3e-44 | 282.9 | 287 | (4, 304) | 311 | (70, 392) | 713 | Type VI secretion system secreted protein VgrG | Type VI secretion system secreted protein VgrG | | uniclust | UniRef100\_A0A1W0CDX9 | 100.0 | 1.9e-38 | 3.5e-44 | 279.7 | 288 | (4, 305) | 311 | (65, 395) | 944 | Gp5/Type VI secretion system Vgr protein OB-fold domain-containing protein | Gp5/Type VI secretion system Vgr protein OB-fold domain-containing protein | | uniclust | UniRef100\_A0A010RV36 | 100.0 | 3.8e-38 | 6.9e-44 | 286.3 | 288 | (4, 305) | 311 | (182, 508) | 910 | Type IV secretion protein Rhs | Type IV secretion protein Rhs | | uniclust | UniRef100\_A0A011MPL4 | 100.0 | 7.8e-38 | 1.4e-43 | 277.8 | 288 | (4, 305) | 311 | (94, 421) | 797 | Type VI secretion system Vgr family protein | Type VI secretion system Vgr family protein | | uniclust | UniRef100\_A0A071LQ37 | 100.0 | 7.8e-38 | 1.4e-43 | 285.2 | 289 | (3, 305) | 311 | (134, 464) | 1175 | Type IV secretion protein Rhs | Type IV secretion protein Rhs | | uniclust | UniRef100\_A0A017HSZ5 | 100.0 | 1.3e-37 | 2.5e-43 | 280.2 | 289 | (4, 306) | 311 | (192, 520) | 1037 | VgrG protein | VgrG protein | | uniclust | UniRef100\_A0A1H0PUY2 | 100.0 | 2.2e-37 | 3.9e-43 | 266.0 | 288 | (3, 305) | 311 | (59, 397) | 619 | Rhs element Vgr protein | Rhs element Vgr protein | | uniclust | UniRef100\_A0A017SWS8 | 100.0 | 2.3e-37 | 4.2e-43 | 288.6 | 289 | (4, 307) | 311 | (139, 471) | 957 | VgrG protein | VgrG protein | | uniclust | UniRef100\_A0A0S8A627 | 100.0 | 3.3e-37 | 6.1e-43 | 252.4 | 291 | (3, 307) | 311 | (56, 387) | 431 | Type VI secretion protein ImpA (Fragment) | Type VI secretion protein ImpA (Fragment) | | uniclust | UniRef100\_A0A0K1E835 | 100.0 | 3.4e-37 | 6.3e-43 | 275.4 | 284 | (4, 303) | 311 | (199, 522) | 1030 | Gp5/Type VI secretion system Vgr protein OB-fold domain-containing protein | Gp5/Type VI secretion system Vgr protein OB-fold domain-containing protein | | uniclust | UniRef100\_A0A011NCB8 | 100.0 | 7.6e-37 | 1.4e-42 | 261.9 | 289 | (1, 304) | 311 | (119, 422) | 496 | Regulatory protein | Regulatory protein | | uniclust | UniRef100\_A0A075K3F1 | 100.0 | 8.3e-37 | 1.5e-42 | 273.1 | 291 | (4, 309) | 311 | (183, 520) | 918 | Type IV secretion protein Rhs | Type IV secretion protein Rhs | | uniclust | UniRef100\_A0A081P6T1 | 100.0 | 8.5e-37 | 1.6e-42 | 243.3 | 260 | (3, 278) | 311 | (91, 372) | 374 | Phage protein D | Phage protein D | | uniclust | UniRef100\_A0A1E7J776 | 100.0 | 1.3e-36 | 2.3e-42 | 248.7 | 268 | (3, 282) | 311 | (80, 383) | 395 | Phage late control D family protein | Phage late control D family protein | | uniclust | UniRef100\_A0A014PYH8 | 100.0 | 1.3e-36 | 2.4e-42 | 263.6 | 277 | (1, 284) | 311 | (169, 469) | 561 | Late control protein D | Late control protein D | | uniclust | UniRef100\_A0A2E5HMJ2 | 100.0 | 1.5e-36 | 2.7e-42 | 248.8 | 264 | (2, 280) | 311 | (64, 366) | 374 | Phage late control D family protein | Phage late control D family protein | | uniclust | UniRef100\_A0A023XMA1 | 100.0 | 1.5e-36 | 2.8e-42 | 271.9 | 286 | (3, 303) | 311 | (141, 470) | 845 | Type VI secretion protein, VrgS/VgrG family | Type VI secretion protein, VrgS/VgrG family | | uniclust | UniRef100\_A0A2U2N2P6 | 100.0 | 1.7e-36 | 3e-42 | 265.2 | 295 | (4, 310) | 311 | (113, 446) | 873 | Type VI secretion system tip protein VgrG | Type VI secretion system tip protein VgrG | | uniclust | UniRef100\_A0A0E1SGK6 | 100.0 | 1.8e-36 | 3.3e-42 | 259.8 | 288 | (4, 305) | 311 | (70, 399) | 741 | Rhs element Vgr protein | Rhs element Vgr protein | | uniclust | UniRef100\_A0A0N1N1R4 | 100.0 | 4.6e-36 | 8.4e-42 | 246.0 | 275 | (1, 282) | 311 | (67, 349) | 394 | Late control protein | Late control protein | | uniclust | UniRef100\_A0A017T4K1 | 100.0 | 4.7e-36 | 8.5e-42 | 274.3 | 286 | (4, 304) | 311 | (197, 518) | 895 | VgrG protein | VgrG protein | | uniclust | UniRef100\_A0A016XIQ1 | 100.0 | 4.7e-36 | 8.6e-42 | 250.5 | 273 | (1, 280) | 311 | (92, 367) | 458 | Phage late control protein | Phage late control protein | | uniclust | UniRef100\_A0A060H9H7 | 100.0 | 1e-35 | 1.9e-41 | 234.5 | 267 | (11, 284) | 311 | (2, 270) | 292 | Phage tail protein | Phage tail protein | | uniclust | UniRef100\_A0A0F7PH30 | 100.0 | 1.5e-35 | 2.8e-41 | 242.6 | 289 | (4, 306) | 311 | (59, 389) | 485 | Rhs element Vgr protein | Rhs element Vgr protein | | uniclust | UniRef100\_A0A0S8CC80 | 100.0 | 2.7e-35 | 4.9e-41 | 251.9 | 261 | (2, 278) | 311 | (192, 482) | 728 | Gp5/Type VI secretion system Vgr protein OB-fold domain-containing protein | Gp5/Type VI secretion system Vgr protein OB-fold domain-containing protein | | uniclust | UniRef100\_A0A0P8A270 | 100.0 | 4e-35 | 7.4e-41 | 236.6 | 260 | (3, 278) | 311 | (65, 349) | 448 | Phage late controlD protein (GPD) | Phage late controlD protein (GPD) | | uniclust | UniRef100\_A0A0C5VFD9 | 100.0 | 5.2e-35 | 9.6e-41 | 232.9 | 268 | (1, 279) | 311 | (69, 342) | 350 | Phage protein D | Phage protein D | | uniclust | UniRef100\_A0A016QM46 | 100.0 | 7.6e-35 | 1.4e-40 | 265.6 | 265 | (3, 278) | 311 | (240, 532) | 899 | Rhs element Vgr protein | Rhs element Vgr protein | | uniclust | UniRef100\_A0A011PNN2 | 100.0 | 9.9e-35 | 1.8e-40 | 242.0 | 263 | (3, 281) | 311 | (88, 377) | 383 | Phage protein D | Phage protein D | | uniclust | UniRef100\_A0A010SW75 | 100.0 | 1.5e-34 | 2.7e-40 | 263.0 | 285 | (4, 305) | 311 | (147, 477) | 1128 | Type VI secretion protein ImpA | Type VI secretion protein ImpA | | uniclust | UniRef100\_A0A162LXF1 | 100.0 | 2.2e-34 | 4e-40 | 250.7 | 287 | (4, 305) | 311 | (70, 395) | 919 | Gp5/Type VI secretion system Vgr protein OB-fold domain-containing protein | Gp5/Type VI secretion system Vgr protein OB-fold domain-containing protein | | uniclust | UniRef100\_A0A068QS18 | 100.0 | 2.7e-34 | 5e-40 | 252.1 | 287 | (4, 305) | 311 | (76, 405) | 832 | SURP motif domain-containing protein | SURP motif domain-containing protein | | uniclust | UniRef100\_A0A2E2NRL3 | 100.0 | 2.8e-34 | 5.1e-40 | 229.9 | 264 | (3, 283) | 311 | (63, 349) | 370 | Phage late control D family protein | Phage late control D family protein | | uniclust | UniRef100\_A0A0A2WGR2 | 100.0 | 3.4e-34 | 6.3e-40 | 241.3 | 265 | (3, 281) | 311 | (99, 402) | 415 | Phage protein D | Phage protein D | | uniclust | UniRef100\_A0A061Q576 | 100.0 | 3.8e-34 | 6.9e-40 | 252.9 | 291 | (4, 308) | 311 | (72, 399) | 848 | VgrG protein | VgrG protein | | uniclust | UniRef100\_A0A0T5NZY3 | 100.0 | 3.8e-34 | 7e-40 | 246.9 | 289 | (2, 308) | 311 | (75, 386) | 591 | Gp5/Type VI secretion system Vgr protein OB-fold domain-containing protein | Gp5/Type VI secretion system Vgr protein OB-fold domain-containing protein | | uniclust | UniRef100\_A0A0C1ITH4 | 100.0 | 5.4e-34 | 9.9e-40 | 231.2 | 263 | (2, 279) | 311 | (103, 376) | 414 | Phage protein D | Phage protein D | | uniclust | UniRef100\_A0A022PIB0 | 100.0 | 8.1e-34 | 1.5e-39 | 233.1 | 275 | (1, 282) | 311 | (69, 384) | 479 | Phage protein D | Phage protein D | | uniclust | UniRef100\_A0A0A8H9K0 | 100.0 | 9.8e-34 | 1.8e-39 | 231.7 | 265 | (2, 277) | 311 | (107, 376) | 381 | Phage protein D | Phage protein D | | uniclust | UniRef100\_A0A023PRE5 | 100.0 | 1.2e-33 | 2.1e-39 | 258.5 | 288 | (6, 309) | 311 | (104, 432) | 1093 | Gp5/Type VI secretion system Vgr protein OB-fold domain-containing protein | Gp5/Type VI secretion system Vgr protein OB-fold domain-containing protein | | uniclust | UniRef100\_A0A084A131 | 100.0 | 1.2e-33 | 2.1e-39 | 244.4 | 288 | (4, 304) | 311 | (62, 390) | 809 | Baseplate hub subunit and tail lysozyme | Baseplate hub subunit and tail lysozyme | | uniclust | UniRef100\_A0A080M5H4 | 100.0 | 1.3e-33 | 2.4e-39 | 217.1 | 245 | (20, 279) | 311 | (4, 272) | 275 | Tail protein | Tail protein | | uniclust | UniRef100\_A0A2S5R6X2 | 100.0 | 1.4e-33 | 2.5e-39 | 216.6 | 250 | (22, 278) | 311 | (2, 251) | 260 | Phage late control D protein (GPD) | Phage late control D protein (GPD) | | uniclust | UniRef100\_A0A1W9R245 | 100.0 | 1.6e-33 | 2.9e-39 | 235.0 | 286 | (3, 306) | 311 | (72, 380) | 431 | Type IV secretion protein Rhs (Fragment) | Type IV secretion protein Rhs (Fragment) | | uniclust | UniRef100\_A0A1F5AGG1 | 100.0 | 1.7e-33 | 3.2e-39 | 255.3 | 270 | (6, 289) | 311 | (72, 381) | 834 | Gp5/Type VI secretion system Vgr protein OB-fold domain-containing protein | Gp5/Type VI secretion system Vgr protein OB-fold domain-containing protein | | uniclust | UniRef100\_A0A178ILA2 | 100.0 | 1.8e-33 | 3.2e-39 | 244.5 | 288 | (4, 305) | 311 | (58, 392) | 883 | Gp5/Type VI secretion system Vgr protein OB-fold domain-containing protein | Gp5/Type VI secretion system Vgr protein OB-fold domain-containing protein | | uniclust | UniRef100\_A0A063B487 | 100.0 | 1.9e-33 | 3.4e-39 | 217.7 | 266 | (20, 295) | 311 | (3, 274) | 300 | Phage late control D family protein | Phage late control D family protein | | uniclust | UniRef100\_A0A0F9ANU3 | 100.0 | 2.2e-33 | 4e-39 | 241.6 | 285 | (5, 306) | 311 | (131, 456) | 708 | Gp5/Type VI secretion system Vgr protein OB-fold domain-containing protein (Fragment) | Gp5/Type VI secretion system Vgr protein OB-fold domain-containing protein (Fragment) | | uniclust | UniRef100\_A0A2T5PAE8 | 100.0 | 2.2e-33 | 4e-39 | 232.7 | 287 | (4, 304) | 311 | (108, 434) | 606 | Type VI secretion system tip protein VgrG (Fragment) | Type VI secretion system tip protein VgrG (Fragment) | | uniclust | UniRef100\_A0A0B5KGS8 | 100.0 | 2.6e-33 | 4.8e-39 | 244.0 | 291 | (4, 307) | 311 | (259, 591) | 848 | ImpA family type VI secretion-associated protein | ImpA family type VI secretion-associated protein | | uniclust | UniRef100\_A0A022FWW3 | 100.0 | 3.7e-33 | 6.8e-39 | 260.4 | 288 | (4, 305) | 311 | (143, 472) | 1100 | Type IV secretion protein Rhs | Type IV secretion protein Rhs | | uniclust | UniRef100\_A0A257YB17 | 100.0 | 4.6e-33 | 8.4e-39 | 226.9 | 290 | (4, 307) | 311 | (65, 392) | 401 | Gp5/Type VI secretion system Vgr protein OB-fold domain-containing protein (Fragment) | Gp5/Type VI secretion system Vgr protein OB-fold domain-containing protein (Fragment) | | uniclust | UniRef100\_A0A0G3Q9L4 | 100.0 | 5.1e-33 | 9.4e-39 | 243.2 | 291 | (4, 305) | 311 | (65, 395) | 915 | ImpA family type VI secretion-associated protein | ImpA family type VI secretion-associated protein | | uniclust | UniRef100\_A0A014LZB8 | 100.0 | 5.6e-33 | 1e-38 | 248.0 | 289 | (2, 308) | 311 | (88, 399) | 640 | Type VI secretion protein VgrG | Type VI secretion protein VgrG | | uniclust | UniRef100\_A0A2M6UWZ5 | 100.0 | 9.9e-33 | 1.8e-38 | 217.1 | 268 | (1, 283) | 311 | (56, 329) | 373 | Late control protein (Fragment) | Late control protein (Fragment) | | uniclust | UniRef100\_A0A1H8RSK6 | 100.0 | 1.4e-32 | 2.6e-38 | 215.2 | 267 | (1, 278) | 311 | (53, 320) | 325 | Phage protein D | Phage protein D | | uniclust | UniRef100\_A0A093RA64 | 100.0 | 1.5e-32 | 2.8e-38 | 225.8 | 285 | (7, 308) | 311 | (144, 469) | 516 | Type IV secretion protein Rhs (Fragment) | Type IV secretion protein Rhs (Fragment) | | uniclust | UniRef100\_A0A1H2FLK2 | 100.0 | 1.9e-32 | 3.5e-38 | 214.3 | 266 | (1, 282) | 311 | (58, 328) | 335 | Phage protein D | Phage protein D | | uniclust | UniRef100\_A0A0A7JME1 | 100.0 | 1.9e-32 | 3.5e-38 | 212.9 | 270 | (1, 280) | 311 | (51, 327) | 354 | Phage late control protein | Phage late control protein | | uniclust | UniRef100\_A0A066RIQ8 | 100.0 | 2.3e-32 | 4.3e-38 | 237.6 | 290 | (3, 307) | 311 | (57, 380) | 666 | Type IV secretion protein Rhs | Type IV secretion protein Rhs | | uniclust | UniRef100\_A0A2V0QT28 | 100.0 | 2.6e-32 | 4.8e-38 | 227.5 | 291 | (4, 307) | 311 | (83, 415) | 528 | Uncharacterized conserved protein | Uncharacterized conserved protein | | uniclust | UniRef100\_A0A0C2D2R7 | 99.9 | 3e-32 | 5.5e-38 | 236.8 | 291 | (4, 308) | 311 | (53, 384) | 900 | Putative VGR-RELATED protein | Putative VGR-RELATED protein | | uniclust | UniRef100\_A0A1N6KVX8 | 99.9 | 3.5e-32 | 6.4e-38 | 246.6 | 286 | (4, 305) | 311 | (69, 421) | 1049 | Rhs element Vgr protein | Rhs element Vgr protein | | uniclust | UniRef100\_A0A084A0B4 | 99.9 | 4e-32 | 7.4e-38 | 235.0 | 285 | (3, 301) | 311 | (53, 380) | 880 | Type VI secretion system Vgr family protein | Type VI secretion system Vgr family protein | | uniclust | UniRef100\_A0A162L1Y1 | 99.9 | 6e-32 | 1.1e-37 | 235.8 | 288 | (3, 306) | 311 | (73, 399) | 702 | Gp5/Type VI secretion system Vgr protein OB-fold domain-containing protein | Gp5/Type VI secretion system Vgr protein OB-fold domain-containing protein | | uniclust | UniRef100\_A0A3B9Q8K7 | 99.9 | 7.8e-32 | 1.4e-37 | 212.8 | 268 | (2, 280) | 311 | (64, 377) | 378 | Late control D family protein | Late control D family protein | | uniclust | UniRef100\_A0A023D7D4 | 99.9 | 8.8e-32 | 1.6e-37 | 233.3 | 287 | (4, 305) | 311 | (79, 402) | 675 | Secretion system Type VI Rhs element Vgr | Secretion system Type VI Rhs element Vgr | | uniclust | UniRef100\_A0A017T2T0 | 99.9 | 1e-31 | 1.9e-37 | 238.7 | 273 | (4, 288) | 311 | (127, 436) | 662 | VgrG protein | VgrG protein | | uniclust | UniRef100\_A0A0C1GDZ6 | 99.9 | 1.4e-31 | 2.6e-37 | 235.9 | 288 | (4, 304) | 311 | (64, 392) | 871 | Type IV secretion protein Rhs | Type IV secretion protein Rhs | | uniclust | UniRef100\_A0A072N5Y6 | 99.9 | 1.5e-31 | 2.7e-37 | 233.6 | 285 | (4, 305) | 311 | (94, 422) | 761 | VgrG protein | VgrG protein | | uniclust | UniRef100\_A0A0N1LTT8 | 99.9 | 1.5e-31 | 2.7e-37 | 219.2 | 274 | (1, 281) | 311 | (64, 345) | 373 | Late control protein | Late control protein | | uniclust | UniRef100\_A0A074VFM2 | 99.9 | 1.6e-31 | 3e-37 | 208.0 | 272 | (25, 310) | 311 | (3, 291) | 300 | Phage protein D (Fragment) | Phage protein D (Fragment) | | uniclust | UniRef100\_A0A4U1GXL6 | 99.9 | 1.7e-31 | 3e-37 | 228.6 | 286 | (5, 305) | 311 | (83, 405) | 858 | Type VI secretion system tip protein VgrG | Type VI secretion system tip protein VgrG | | uniclust | UniRef100\_A0A2D3T999 | 99.9 | 1.7e-31 | 3.2e-37 | 208.3 | 254 | (21, 281) | 311 | (3, 258) | 292 | Phage tail protein | Phage tail protein | | uniclust | UniRef100\_A0A099EW46 | 99.9 | 1.8e-31 | 3.3e-37 | 233.0 | 283 | (4, 301) | 311 | (75, 399) | 762 | Type IV secretion protein Rhs | Type IV secretion protein Rhs | | uniclust | UniRef100\_A0A1E4HSI3 | 99.9 | 2.2e-31 | 3.9e-37 | 227.3 | 289 | (4, 305) | 311 | (66, 393) | 642 | Gp5/Type VI secretion system Vgr protein OB-fold domain-containing protein | Gp5/Type VI secretion system Vgr protein OB-fold domain-containing protein | | uniclust | UniRef100\_A0A175QC54 | 99.9 | 2.4e-31 | 4.5e-37 | 213.6 | 291 | (4, 305) | 311 | (88, 418) | 470 | Type IV secretion protein Rhs (Fragment) | Type IV secretion protein Rhs (Fragment) | | uniclust | UniRef100\_A0A0X8EV94 | 99.9 | 3.4e-31 | 6.1e-37 | 230.3 | 292 | (4, 309) | 311 | (62, 393) | 708 | Gp5/Type VI secretion system Vgr protein OB-fold domain-containing protein | Gp5/Type VI secretion system Vgr protein OB-fold domain-containing protein | | uniclust | UniRef100\_A0A017SVJ8 | 99.9 | 3.6e-31 | 6.6e-37 | 240.2 | 288 | (4, 305) | 311 | (137, 478) | 967 | VgrG protein | VgrG protein | | uniclust | UniRef100\_A0A087NA94 | 99.9 | 3.6e-31 | 6.7e-37 | 210.9 | 272 | (1, 279) | 311 | (81, 382) | 398 | Uncharacterized protein | Uncharacterized protein | | uniclust | UniRef100\_A0A1V6ENZ6 | 99.9 | 4.1e-31 | 7.5e-37 | 215.4 | 244 | (4, 261) | 311 | (84, 353) | 383 | Phage-related baseplate assembly protein | Phage-related baseplate assembly protein | | uniclust | UniRef100\_A0A6N2ELR8 | 99.9 | 4.8e-31 | 8.7e-37 | 225.4 | 279 | (17, 308) | 311 | (82, 397) | 828 | Type VI secretion system tip protein VgrG (Fragment) | Type VI secretion system tip protein VgrG (Fragment) | | uniclust | UniRef100\_A0A1L3I5I6 | 99.9 | 5.6e-31 | 1e-36 | 204.0 | 271 | (1, 279) | 311 | (54, 325) | 349 | Putative phage late control gene D protein | Putative phage late control gene D protein | | uniclust | UniRef100\_A0A377AJP2 | 99.9 | 5.8e-31 | 1.1e-36 | 212.6 | 245 | (26, 283) | 311 | (3, 263) | 295 | Phage late control D family protein | Phage late control D family protein | | uniclust | UniRef100\_A0A023PRF0 | 99.9 | 6.1e-31 | 1.1e-36 | 219.4 | 276 | (17, 308) | 311 | (86, 400) | 562 | VgrG protein | VgrG protein | | uniclust | UniRef100\_A0A064AHQ8 | 99.9 | 6.3e-31 | 1.2e-36 | 213.4 | 268 | (1, 282) | 311 | (70, 352) | 356 | Late control protein D | Late control protein D | | uniclust | UniRef100\_A0A432PWE3 | 99.9 | 6.8e-31 | 1.3e-36 | 204.2 | 262 | (1, 276) | 311 | (59, 326) | 330 | Phage late control D family protein | Phage late control D family protein | | uniclust | UniRef100\_A0A1G4UZT6 | 99.9 | 7.1e-31 | 1.3e-36 | 214.5 | 243 | (57, 305) | 311 | (20, 299) | 427 | Rhs element Vgr protein (Fragment) | Rhs element Vgr protein (Fragment) | | uniclust | UniRef100\_A0A0E3VX40 | 99.9 | 1.5e-30 | 2.7e-36 | 214.2 | 275 | (2, 284) | 311 | (75, 360) | 419 | Uncharacterized protein | Uncharacterized protein | | uniclust | UniRef100\_A0A072N097 | 99.9 | 1.5e-30 | 2.8e-36 | 229.1 | 274 | (17, 307) | 311 | (200, 511) | 1019 | Lysozyme | Lysozyme | | uniclust | UniRef100\_A0A399WGD3 | 99.9 | 1.8e-30 | 3.3e-36 | 229.6 | 287 | (4, 305) | 311 | (62, 411) | 1055 | Uncharacterized protein | Uncharacterized protein | | uniclust | UniRef100\_A0A932J5G0 | 99.9 | 2.2e-30 | 4.1e-36 | 211.1 | 289 | (4, 306) | 311 | (70, 402) | 500 | Type VI secretion system tip protein VgrG | Type VI secretion system tip protein VgrG | | uniclust | UniRef100\_A0A063BIY2 | 99.9 | 2.4e-30 | 4.4e-36 | 218.9 | 280 | (17, 309) | 311 | (126, 457) | 622 | Type VI secretion system Vgr family protein (Fragment) | Type VI secretion system Vgr family protein (Fragment) | | uniclust | UniRef100\_A0A017STW5 | 99.9 | 2.8e-30 | 5.2e-36 | 235.4 | 290 | (3, 307) | 311 | (192, 528) | 951 | VgrG protein | VgrG protein | | uniclust | UniRef100\_A0A0N0VWG2 | 99.9 | 3.1e-30 | 5.6e-36 | 220.6 | 272 | (17, 305) | 311 | (69, 383) | 777 | Rhs element Vgr protein | Rhs element Vgr protein | | uniclust | UniRef100\_A0A1Q3H876 | 99.9 | 3.4e-30 | 6.2e-36 | 223.9 | 291 | (4, 308) | 311 | (61, 395) | 1047 | Gp5/Type VI secretion system Vgr protein OB-fold domain-containing protein | Gp5/Type VI secretion system Vgr protein OB-fold domain-containing protein | | uniclust | UniRef100\_A0A0B1XX70 | 99.9 | 3.7e-30 | 6.7e-36 | 224.5 | 266 | (2, 283) | 311 | (68, 357) | 589 | Type VI secretion protein VgrG | Type VI secretion protein VgrG | | uniclust | UniRef100\_A0A066PR67 | 99.9 | 4e-30 | 7.3e-36 | 223.7 | 288 | (4, 304) | 311 | (77, 407) | 673 | Putative type VI secretion system protein VgrGB | Putative type VI secretion system protein VgrGB | | uniclust | UniRef100\_A0A085WRZ9 | 99.9 | 4.6e-30 | 8.4e-36 | 221.9 | 271 | (4, 289) | 311 | (169, 478) | 618 | VgrG protein | VgrG protein | | uniclust | UniRef100\_A0A1H4BCX2 | 99.9 | 4.8e-30 | 8.7e-36 | 219.8 | 289 | (2, 308) | 311 | (70, 382) | 583 | Rhs element Vgr protein | Rhs element Vgr protein | | uniclust | UniRef100\_A0A085ZBA7 | 99.9 | 4.9e-30 | 8.9e-36 | 236.0 | 281 | (4, 305) | 311 | (170, 487) | 858 | Rhs element Vgr protein | Rhs element Vgr protein | | uniclust | UniRef100\_A0A0B6XFU3 | 99.9 | 5.3e-30 | 9.7e-36 | 225.6 | 277 | (17, 310) | 311 | (171, 484) | 836 | Gp5/Type VI secretion system Vgr protein OB-fold domain-containing protein | Gp5/Type VI secretion system Vgr protein OB-fold domain-containing protein | | uniclust | UniRef100\_A0A0Q4EHZ4 | 99.9 | 5.3e-30 | 9.8e-36 | 217.7 | 294 | (1, 308) | 311 | (49, 380) | 676 | ImpA family type VI secretion-associated protein | ImpA family type VI secretion-associated protein | | uniclust | UniRef100\_A0A3N5LHC6 | 99.9 | 5.5e-30 | 1e-35 | 210.3 | 260 | (3, 277) | 311 | (90, 378) | 394 | Phage late control D family protein | Phage late control D family protein | | uniclust | UniRef100\_A0A1E7HX75 | 99.9 | 5.9e-30 | 1.1e-35 | 229.0 | 263 | (17, 291) | 311 | (69, 374) | 831 | Rhs element Vgr protein | Rhs element Vgr protein | | uniclust | UniRef100\_A0A1R1JJ15 | 99.9 | 6.3e-30 | 1.1e-35 | 223.0 | 276 | (17, 304) | 311 | (121, 442) | 903 | Type IV secretion protein Rhs | Type IV secretion protein Rhs | | uniclust | UniRef100\_A0A009Q2V4 | 99.9 | 7.5e-30 | 1.4e-35 | 214.6 | 269 | (1, 277) | 311 | (96, 370) | 585 | Putative phage late control D protein | Putative phage late control D protein | | uniclust | UniRef100\_A0A1E4J654 | 99.9 | 7.6e-30 | 1.4e-35 | 220.0 | 290 | (4, 305) | 311 | (49, 391) | 1057 | Type IV secretion protein Rhs | Type IV secretion protein Rhs | | uniclust | UniRef100\_A0A1N6X884 | 99.9 | 8.3e-30 | 1.5e-35 | 201.9 | 262 | (3, 280) | 311 | (62, 350) | 351 | Phage protein D | Phage protein D | | uniclust | UniRef100\_A0A177Q3K8 | 99.9 | 9.3e-30 | 1.7e-35 | 219.7 | 286 | (4, 307) | 311 | (65, 419) | 570 | Gp5/Type VI secretion system Vgr protein OB-fold domain-containing protein | Gp5/Type VI secretion system Vgr protein OB-fold domain-containing protein | | uniclust | UniRef100\_A0A090G3M4 | 99.9 | 9.4e-30 | 1.7e-35 | 213.7 | 276 | (1, 285) | 311 | (129, 417) | 463 | Late control D family protein | Late control D family protein | | uniclust | UniRef100\_A0A023Q190 | 99.9 | 9.5e-30 | 1.7e-35 | 228.0 | 267 | (4, 286) | 311 | (127, 430) | 766 | Type IV secretion protein Rhs | Type IV secretion protein Rhs | | uniclust | UniRef100\_A0A022PJC1 | 99.9 | 1.2e-29 | 2.1e-35 | 222.3 | 273 | (17, 306) | 311 | (210, 521) | 860 | Rhs element Vgr protein | Rhs element Vgr protein | | uniclust | UniRef100\_A0A5M7LZ08 | 99.9 | 1.2e-29 | 2.2e-35 | 197.4 | 272 | (4, 286) | 311 | (54, 362) | 374 | Type VI secretion system tip protein VgrG (Fragment) | Type VI secretion system tip protein VgrG (Fragment) | | uniclust | UniRef100\_A0A538CK92 | 99.9 | 1.3e-29 | 2.3e-35 | 194.6 | 229 | (35, 279) | 311 | (9, 261) | 266 | Phage late control D family protein | Phage late control D family protein | | uniclust | UniRef100\_A0A1C6AKC2 | 99.9 | 1.3e-29 | 2.3e-35 | 208.8 | 257 | (3, 277) | 311 | (98, 373) | 375 | Phage protein D | Phage protein D | | uniclust | UniRef100\_A0A017TAU5 | 99.9 | 1.3e-29 | 2.4e-35 | 221.8 | 290 | (4, 304) | 311 | (63, 381) | 749 | VgrG protein | VgrG protein | | uniclust | UniRef100\_A0A061PST6 | 99.9 | 1.6e-29 | 2.9e-35 | 222.4 | 286 | (3, 304) | 311 | (70, 393) | 676 | VgrG protein | VgrG protein | | uniclust | UniRef100\_A0A088UGI5 | 99.9 | 1.6e-29 | 3e-35 | 232.7 | 291 | (4, 306) | 311 | (80, 417) | 2022 | Rhs element Vgr family protein | Rhs element Vgr family protein | | uniclust | UniRef100\_A0A069BDU2 | 99.9 | 1.8e-29 | 3.3e-35 | 220.9 | 287 | (4, 304) | 311 | (59, 387) | 874 | Type IV secretion protein Rhs | Type IV secretion protein Rhs | | uniclust | UniRef100\_A0A0H2LRL4 | 99.9 | 2.1e-29 | 3.8e-35 | 236.7 | 291 | (4, 305) | 311 | (72, 402) | 2113 | Putative deoxyribonuclease RhsC | Putative deoxyribonuclease RhsC | | uniclust | UniRef100\_A0A1G3DZZ5 | 99.9 | 2.3e-29 | 4.3e-35 | 204.7 | 275 | (17, 305) | 311 | (68, 364) | 517 | Type IV secretion protein Rhs (Fragment) | Type IV secretion protein Rhs (Fragment) | | uniclust | UniRef100\_A0A2G6L1C0 | 99.9 | 2.5e-29 | 4.6e-35 | 204.6 | 275 | (17, 306) | 311 | (70, 390) | 481 | Type VI secretion system tip protein VgrG (Fragment) | Type VI secretion system tip protein VgrG (Fragment) | | uniclust | UniRef100\_A0A4Q6CNI6 | 99.9 | 2.5e-29 | 4.6e-35 | 195.0 | 265 | (2, 276) | 311 | (51, 315) | 332 | Phage late control D family protein | Phage late control D family protein | | uniclust | UniRef100\_A0A0E3V6K5 | 99.9 | 2.8e-29 | 5.1e-35 | 221.3 | 282 | (4, 305) | 311 | (60, 381) | 610 | Gp5/Type VI secretion system Vgr protein OB-fold domain-containing protein | Gp5/Type VI secretion system Vgr protein OB-fold domain-containing protein | | uniclust | UniRef100\_A0A356TQN0 | 99.9 | 2.8e-29 | 5.2e-35 | 220.4 | 293 | (3, 308) | 311 | (62, 390) | 856 | Gp5/Type VI secretion system Vgr protein OB-fold domain-containing protein | Gp5/Type VI secretion system Vgr protein OB-fold domain-containing protein | | uniclust | UniRef100\_A0A0Q4RTT5 | 99.9 | 3.2e-29 | 5.9e-35 | 211.5 | 287 | (4, 304) | 311 | (49, 384) | 848 | Type IV secretion protein Rhs | Type IV secretion protein Rhs | | uniclust | UniRef100\_A0A0K8MDT9 | 99.9 | 3.3e-29 | 6e-35 | 219.9 | 287 | (4, 304) | 311 | (243, 572) | 1067 | Phage-related baseplate assembly protein | Phage-related baseplate assembly protein | | uniclust | UniRef100\_A0A086ELE6 | 99.9 | 3.4e-29 | 6.2e-35 | 217.6 | 258 | (38, 310) | 311 | (98, 391) | 470 | Type VI secretion system tip protein VgrG (Fragment) | Type VI secretion system tip protein VgrG (Fragment) | | uniclust | UniRef100\_A0A017TBW0 | 99.9 | 3.7e-29 | 6.8e-35 | 220.6 | 257 | (37, 304) | 311 | (91, 375) | 825 | VgrG protein | VgrG protein | | uniclust | UniRef100\_A0A077PRC6 | 99.9 | 3.7e-29 | 6.8e-35 | 213.4 | 274 | (17, 307) | 311 | (90, 406) | 664 | Putative VrgG protein | Putative VrgG protein | | uniclust | UniRef100\_A0A085GIL1 | 99.9 | 4.1e-29 | 7.5e-35 | 219.3 | 274 | (17, 304) | 311 | (101, 410) | 1073 | Gp5/Type VI secretion system Vgr protein OB-fold domain-containing protein | Gp5/Type VI secretion system Vgr protein OB-fold domain-containing protein | | uniclust | UniRef100\_A0A017T830 | 99.9 | 7.2e-29 | 1.3e-34 | 194.6 | 248 | (53, 306) | 311 | (39, 329) | 367 | VgrG protein | VgrG protein | | uniclust | UniRef100\_A0A0G3A0D9 | 99.9 | 7.7e-29 | 1.4e-34 | 216.5 | 260 | (4, 280) | 311 | (94, 374) | 568 | Phage baseplate assembly protein V | Phage baseplate assembly protein V | | uniclust | UniRef100\_A0A356KF96 | 99.9 | 7.9e-29 | 1.5e-34 | 201.4 | 264 | (3, 282) | 311 | (68, 360) | 454 | Phage late control D family protein | Phage late control D family protein | | uniclust | UniRef100\_A0A090JZ97 | 99.9 | 8.2e-29 | 1.5e-34 | 211.9 | 274 | (18, 307) | 311 | (81, 393) | 742 | VgrG protein | VgrG protein | | uniclust | UniRef100\_A0A1H1G3E9 | 99.9 | 9.5e-29 | 1.7e-34 | 192.7 | 270 | (1, 277) | 311 | (57, 331) | 333 | Phage protein D | Phage protein D | | uniclust | UniRef100\_A0A0A6D7Z2 | 99.9 | 1e-28 | 1.9e-34 | 203.1 | 269 | (20, 305) | 311 | (95, 409) | 550 | Type IV secretion protein Rhs (Fragment) | Type IV secretion protein Rhs (Fragment) | | uniclust | UniRef100\_A0A1V1VC84 | 99.9 | 1.1e-28 | 2.1e-34 | 198.0 | 277 | (17, 307) | 311 | (79, 392) | 455 | Type VI secretion system tip protein VgrG | Type VI secretion system tip protein VgrG | | uniclust | UniRef100\_A0A0D0GR35 | 99.9 | 1.2e-28 | 2.2e-34 | 198.2 | 272 | (1, 279) | 311 | (120, 416) | 428 | Phage late control D family protein | Phage late control D family protein | | uniclust | UniRef100\_A0A1C5CUP6 | 99.9 | 1.2e-28 | 2.2e-34 | 203.4 | 264 | (4, 277) | 311 | (62, 356) | 521 | Uncharacterized conserved protein, implicated in type VI secretion and phage assembly (Fragment) | Uncharacterized conserved protein, implicated in type VI secretion and phage assembly (Fragment) | | uniclust | UniRef100\_A0A0S9QJG9 | 99.9 | 1.7e-28 | 3.1e-34 | 214.8 | 240 | (58, 305) | 311 | (2, 277) | 679 | Gp5/Type VI secretion system Vgr protein OB-fold domain-containing protein (Fragment) | Gp5/Type VI secretion system Vgr protein OB-fold domain-containing protein (Fragment) | | uniclust | UniRef100\_A0A0B5KGT4 | 99.9 | 2e-28 | 3.7e-34 | 204.2 | 288 | (4, 305) | 311 | (48, 376) | 644 | Rhs element Vgr protein | Rhs element Vgr protein | | uniclust | UniRef100\_A0A2K9EFG9 | 99.9 | 2.1e-28 | 3.9e-34 | 212.0 | 283 | (4, 301) | 311 | (64, 388) | 726 | Type VI secretion system tip protein VgrG | Type VI secretion system tip protein VgrG | | uniclust | UniRef100\_A0A0F0H101 | 99.9 | 2.2e-28 | 3.9e-34 | 215.2 | 263 | (3, 279) | 311 | (85, 403) | 653 | Gp5/Type VI secretion system Vgr protein OB-fold domain-containing protein | Gp5/Type VI secretion system Vgr protein OB-fold domain-containing protein | | uniclust | UniRef100\_A0A0F2S4F5 | 99.9 | 2.4e-28 | 4.3e-34 | 209.2 | 287 | (4, 304) | 311 | (59, 384) | 742 | Gp5/Type VI secretion system Vgr protein OB-fold domain-containing protein | Gp5/Type VI secretion system Vgr protein OB-fold domain-containing protein | | uniclust | UniRef100\_A0A174TRQ2 | 99.9 | 2.4e-28 | 4.4e-34 | 205.8 | 256 | (17, 285) | 311 | (191, 457) | 530 | Phage protein D | Phage protein D | | uniclust | UniRef100\_A0A1I3SYP4 | 99.9 | 2.5e-28 | 4.6e-34 | 191.2 | 270 | (1, 277) | 311 | (52, 328) | 381 | Phage protein D | Phage protein D | | uniclust | UniRef100\_A0A2J7TZ74 | 99.9 | 2.5e-28 | 4.7e-34 | 212.9 | 272 | (18, 307) | 311 | (148, 471) | 978 | Gp5/Type VI secretion system Vgr protein OB-fold domain-containing protein | Gp5/Type VI secretion system Vgr protein OB-fold domain-containing protein | | uniclust | UniRef100\_A0A0C1KLU7 | 99.9 | 2.7e-28 | 5e-34 | 211.3 | 288 | (4, 304) | 311 | (88, 417) | 969 | Type IV secretion protein Rhs | Type IV secretion protein Rhs | | uniclust | UniRef100\_A0A1F9MHR6 | 99.9 | 2.7e-28 | 5e-34 | 201.4 | 232 | (36, 283) | 311 | (97, 364) | 446 | Late control protein D | Late control protein D | | uniclust | UniRef100\_A0A060I7P9 | 99.9 | 2.8e-28 | 5.1e-34 | 216.6 | 287 | (5, 304) | 311 | (83, 414) | 930 | Type VI secretion system Rhs element Vgr family protein | Type VI secretion system Rhs element Vgr family protein | | uniclust | UniRef100\_A0A143XPS7 | 99.9 | 3.5e-28 | 6.4e-34 | 206.5 | 268 | (1, 282) | 311 | (98, 373) | 483 | Phage late control gene D protein (GPD) | Phage late control gene D protein (GPD) | | uniclust | UniRef100\_A0A2A2HZ20 | 99.9 | 3.6e-28 | 6.6e-34 | 201.5 | 285 | (6, 306) | 311 | (42, 367) | 607 | Type IV secretion protein Rhs (Fragment) | Type IV secretion protein Rhs (Fragment) | | uniclust | UniRef100\_A0A090SM11 | 99.9 | 3.9e-28 | 7.2e-34 | 208.3 | 242 | (56, 305) | 311 | (9, 293) | 644 | Probable vgr related protein | Probable vgr related protein | | uniclust | UniRef100\_A0A0M0SQ28 | 99.9 | 4e-28 | 7.3e-34 | 203.5 | 260 | (2, 278) | 311 | (64, 349) | 572 | Gp5/Type VI secretion system Vgr protein OB-fold domain-containing protein | Gp5/Type VI secretion system Vgr protein OB-fold domain-containing protein | | uniclust | UniRef100\_A0A1S8DCS2 | 99.9 | 4.7e-28 | 8.6e-34 | 191.2 | 253 | (37, 304) | 311 | (14, 309) | 414 | Type VI secretion system tip protein VgrG (Fragment) | Type VI secretion system tip protein VgrG (Fragment) | | uniclust | UniRef100\_A0A031FRL6 | 99.9 | 5.4e-28 | 9.9e-34 | 194.6 | 272 | (1, 280) | 311 | (59, 345) | 355 | Phage protein D-like protein | Phage protein D-like protein | | uniclust | UniRef100\_A0A947C8Q0 | 99.9 | 5.8e-28 | 1.1e-33 | 194.0 | 291 | (4, 307) | 311 | (57, 397) | 449 | Type VI secretion system tip protein VgrG (Fragment) | Type VI secretion system tip protein VgrG (Fragment) | | uniclust | UniRef100\_A0A133ZYU8 | 99.9 | 7.6e-28 | 1.4e-33 | 201.2 | 267 | (1, 281) | 311 | (83, 360) | 445 | Phage late control D protein | Phage late control D protein | | uniclust | UniRef100\_A0A0C1ZDR4 | 99.9 | 7.8e-28 | 1.4e-33 | 220.0 | 290 | (4, 305) | 311 | (278, 613) | 980 | VgrG protein | VgrG protein | | uniclust | UniRef100\_A0A2A5C5S4 | 99.9 | 8e-28 | 1.5e-33 | 209.8 | 288 | (5, 305) | 311 | (58, 382) | 816 | Gp5/Type VI secretion system Vgr protein OB-fold domain-containing protein | Gp5/Type VI secretion system Vgr protein OB-fold domain-containing protein | | uniclust | UniRef100\_A0A349VLS7 | 99.9 | 8.1e-28 | 1.5e-33 | 189.7 | 211 | (4, 227) | 311 | (57, 294) | 300 | Type VI secretion system tip protein VgrG (Fragment) | Type VI secretion system tip protein VgrG (Fragment) | | uniclust | UniRef100\_A0A212RQ57 | 99.9 | 8.8e-28 | 1.6e-33 | 206.3 | 294 | (2, 305) | 311 | (76, 445) | 695 | Type VI secretion system secreted protein VgrG | Type VI secretion system secreted protein VgrG | | uniclust | UniRef100\_A0A016A3P0 | 99.9 | 1e-27 | 1.9e-33 | 216.3 | 282 | (4, 305) | 311 | (100, 418) | 721 | Phage late control D family protein | Phage late control D family protein | | uniclust | UniRef100\_A0A193QJR7 | 99.9 | 1.1e-27 | 1.9e-33 | 188.1 | 271 | (1, 278) | 311 | (63, 360) | 374 | Phage late control gene D protein (GPD) | Phage late control gene D protein (GPD) | | uniclust | UniRef100\_A0A4U1HBY7 | 99.9 | 1.3e-27 | 2.3e-33 | 192.2 | 289 | (4, 305) | 311 | (52, 377) | 501 | Type VI secretion system tip protein VgrG | Type VI secretion system tip protein VgrG | | uniclust | UniRef100\_A0A0Q8PZU1 | 99.9 | 1.4e-27 | 2.5e-33 | 207.8 | 290 | (4, 305) | 311 | (294, 629) | 937 | Gp5/Type VI secretion system Vgr protein OB-fold domain-containing protein | Gp5/Type VI secretion system Vgr protein OB-fold domain-containing protein | | uniclust | UniRef100\_A0A2N1PDD4 | 99.9 | 1.5e-27 | 2.7e-33 | 210.4 | 281 | (3, 304) | 311 | (108, 425) | 648 | Gp5/Type VI secretion system Vgr protein OB-fold domain-containing protein | Gp5/Type VI secretion system Vgr protein OB-fold domain-containing protein | | uniclust | UniRef100\_A0A0P9T8U5 | 99.9 | 1.7e-27 | 3.2e-33 | 203.3 | 289 | (4, 305) | 311 | (71, 401) | 729 | Rhs element Vgr protein | Rhs element Vgr protein | | uniclust | UniRef100\_A0A081J2H0 | 99.9 | 2e-27 | 3.7e-33 | 184.7 | 246 | (4, 263) | 311 | (49, 322) | 331 | Type VI secretion system tip protein VgrG (Fragment) | Type VI secretion system tip protein VgrG (Fragment) | | uniclust | UniRef100\_A0A0E3KUE2 | 99.9 | 2.1e-27 | 3.9e-33 | 196.6 | 269 | (3, 285) | 311 | (76, 365) | 574 | VgrG protein | VgrG protein | | uniclust | UniRef100\_UPI000573CFEF | 99.9 | 2.2e-27 | 4.1e-33 | 191.4 | 289 | (4, 305) | 311 | (48, 377) | 477 | type VI secretion system tip protein TssI/VgrG | type VI secretion system tip protein TssI/VgrG | | uniclust | UniRef100\_A0A0K9N9X5 | 99.9 | 2.7e-27 | 4.9e-33 | 197.6 | 264 | (3, 280) | 311 | (112, 411) | 430 | Phage late control gene D protein (GPD) | Phage late control gene D protein (GPD) | | uniclust | UniRef100\_A0A524PWE2 | 99.9 | 2.7e-27 | 5e-33 | 177.2 | 244 | (17, 278) | 311 | (8, 274) | 277 | Phage late control D family protein (Fragment) | Phage late control D family protein (Fragment) | | uniclust | UniRef100\_A0A246IXC8 | 99.9 | 2.7e-27 | 5e-33 | 202.2 | 289 | (4, 305) | 311 | (72, 398) | 942 | Gp5/Type VI secretion system Vgr protein OB-fold domain-containing protein | Gp5/Type VI secretion system Vgr protein OB-fold domain-containing protein | | uniclust | UniRef100\_A0A085G1R1 | 99.9 | 2.8e-27 | 5.1e-33 | 209.3 | 270 | (17, 305) | 311 | (116, 427) | 925 | VgrG family protein | VgrG family protein | | uniclust | UniRef100\_A0A255HM11 | 99.9 | 2.9e-27 | 5.3e-33 | 216.6 | 267 | (7, 288) | 311 | (95, 407) | 1043 | Gp5/Type VI secretion system Vgr protein OB-fold domain-containing protein | Gp5/Type VI secretion system Vgr protein OB-fold domain-containing protein | | uniclust | UniRef100\_A0A0B3BQR0 | 99.9 | 3e-27 | 5.5e-33 | 212.4 | 283 | (6, 307) | 311 | (107, 433) | 1205 | Type VI secretion system secreted protein VgrG | Type VI secretion system secreted protein VgrG | | uniclust | UniRef100\_A0A0H3K4B3 | 99.9 | 3.1e-27 | 5.7e-33 | 188.5 | 271 | (1, 278) | 311 | (52, 329) | 480 | Phage late control gene D protein GPD | Phage late control gene D protein GPD | | uniclust | UniRef100\_A0A1W6LMB9 | 99.9 | 3.5e-27 | 6.5e-33 | 206.3 | 289 | (4, 305) | 311 | (55, 380) | 1193 | Type VI secretion system Vgr family protein | Type VI secretion system Vgr family protein | | uniclust | UniRef100\_A0A0C2EHH0 | 99.9 | 4e-27 | 7.4e-33 | 199.0 | 271 | (18, 305) | 311 | (101, 415) | 608 | VgrG protein | VgrG protein | | uniclust | UniRef100\_A0A0D8FQF9 | 99.9 | 5.2e-27 | 9.4e-33 | 206.6 | 259 | (3, 280) | 311 | (122, 401) | 645 | Phage-like baseplate assembly protein | Phage-like baseplate assembly protein | | uniclust | UniRef100\_A0A0Q8LLQ7 | 99.9 | 6.2e-27 | 1.1e-32 | 198.8 | 263 | (3, 283) | 311 | (75, 364) | 601 | Gp5/Type VI secretion system Vgr protein OB-fold domain-containing protein | Gp5/Type VI secretion system Vgr protein OB-fold domain-containing protein | | uniclust | UniRef100\_A0A0Q4S1L3 | 99.9 | 6.7e-27 | 1.2e-32 | 200.1 | 287 | (5, 305) | 311 | (50, 388) | 770 | ImpA family type VI secretion-associated protein (Fragment) | ImpA family type VI secretion-associated protein (Fragment) | | uniclust | UniRef100\_A0A031FNF9 | 99.9 | 8.6e-27 | 1.6e-32 | 211.3 | 279 | (17, 309) | 311 | (106, 424) | 1055 | Rhs element Vgr protein | Rhs element Vgr protein | | uniclust | UniRef100\_A0A017T1P1 | 99.9 | 9e-27 | 1.6e-32 | 213.9 | 290 | (5, 306) | 311 | (66, 387) | 1090 | VgrG protein | VgrG protein | | uniclust | UniRef100\_A0A268TJ33 | 99.9 | 9.6e-27 | 1.8e-32 | 185.4 | 256 | (1, 277) | 311 | (53, 308) | 314 | Phage tail protein | Phage tail protein | | uniclust | UniRef100\_A0A017TAS0 | 99.9 | 1e-26 | 1.9e-32 | 207.1 | 289 | (4, 303) | 311 | (89, 410) | 890 | VgrG protein | VgrG protein | | uniclust | UniRef100\_A0A518AUG2 | 99.9 | 1.1e-26 | 1.9e-32 | 209.9 | 288 | (4, 305) | 311 | (90, 451) | 1273 | Phage-related baseplate assembly protein | Phage-related baseplate assembly protein | | uniclust | UniRef100\_A0A0J6KFU2 | 99.9 | 1.2e-26 | 2.1e-32 | 211.9 | 286 | (4, 304) | 311 | (111, 470) | 1143 | Type VI secretion system protein | Type VI secretion system protein | | uniclust | UniRef100\_A0A0D0K4C1 | 99.9 | 1.4e-26 | 2.6e-32 | 198.7 | 290 | (4, 305) | 311 | (133, 468) | 722 | Type VI secretion system tip protein VgrG (Fragment) | Type VI secretion system tip protein VgrG (Fragment) | | uniclust | UniRef100\_A0A3D9MY61 | 99.9 | 1.7e-26 | 3e-32 | 213.2 | 278 | (17, 308) | 311 | (121, 452) | 1218 | Rhs element Vgr protein | Rhs element Vgr protein | | uniclust | UniRef100\_A0A374MIX7 | 99.9 | 1.8e-26 | 3.4e-32 | 195.5 | 280 | (3, 304) | 311 | (62, 373) | 476 | Type VI secretion system tip protein VgrG (Fragment) | Type VI secretion system tip protein VgrG (Fragment) | | uniclust | UniRef100\_A0A2K1QDW4 | 99.9 | 1.9e-26 | 3.5e-32 | 193.9 | 291 | (3, 305) | 311 | (160, 485) | 752 | Type VI secretion system tip protein VgrG (Fragment) | Type VI secretion system tip protein VgrG (Fragment) | | uniclust | UniRef100\_A0A150Q779 | 99.9 | 2.4e-26 | 4.4e-32 | 204.9 | 269 | (5, 287) | 311 | (75, 361) | 1028 | Gp5/Type VI secretion system Vgr protein OB-fold domain-containing protein | Gp5/Type VI secretion system Vgr protein OB-fold domain-containing protein | | uniclust | UniRef100\_A0A023Y2K3 | 99.9 | 2.4e-26 | 4.4e-32 | 212.7 | 288 | (3, 304) | 311 | (143, 522) | 1156 | Type IV secretion protein Rhs | Type IV secretion protein Rhs | | uniclust | UniRef100\_A0A3S1JAZ2 | 99.9 | 2.5e-26 | 4.5e-32 | 184.9 | 288 | (4, 305) | 311 | (52, 378) | 502 | Type VI secretion system tip protein VgrG (Fragment) | Type VI secretion system tip protein VgrG (Fragment) | | uniclust | UniRef100\_A0A085EST2 | 99.9 | 2.8e-26 | 5.2e-32 | 211.8 | 284 | (4, 303) | 311 | (107, 449) | 1280 | Type VI secretion system Vgr family protein | Type VI secretion system Vgr family protein | | uniclust | UniRef100\_A0A963HZS7 | 99.9 | 2.8e-26 | 5.2e-32 | 185.5 | 268 | (4, 285) | 311 | (51, 355) | 524 | Type VI secretion system tip protein VgrG | Type VI secretion system tip protein VgrG | | uniclust | UniRef100\_A0A0B4BFG3 | 99.9 | 2.9e-26 | 5.3e-32 | 200.8 | 287 | (4, 305) | 311 | (77, 402) | 769 | Gp5/Type VI secretion system Vgr protein OB-fold domain-containing protein | Gp5/Type VI secretion system Vgr protein OB-fold domain-containing protein | | uniclust | UniRef100\_A0A094QB96 | 99.9 | 3e-26 | 5.5e-32 | 189.8 | 259 | (2, 279) | 311 | (59, 348) | 596 | Gp5/Type VI secretion system Vgr protein OB-fold domain-containing protein | Gp5/Type VI secretion system Vgr protein OB-fold domain-containing protein | | uniclust | UniRef100\_A0A077KP81 | 99.9 | 3.1e-26 | 5.7e-32 | 213.8 | 281 | (4, 304) | 311 | (132, 450) | 1149 | Gp5/Type VI secretion system Vgr protein OB-fold domain-containing protein | Gp5/Type VI secretion system Vgr protein OB-fold domain-containing protein | | uniclust | UniRef100\_A0A327JI00 | 99.9 | 3.5e-26 | 6.4e-32 | 175.6 | 261 | (2, 276) | 311 | (59, 325) | 328 | Phage late control D family protein | Phage late control D family protein | | uniclust | UniRef100\_A0A022FT39 | 99.9 | 3.5e-26 | 6.4e-32 | 205.8 | 277 | (17, 305) | 311 | (135, 469) | 1045 | Type IV secretion protein Rhs | Type IV secretion protein Rhs | | uniclust | UniRef100\_A0A228HLH2 | 99.9 | 4e-26 | 7.2e-32 | 210.4 | 285 | (3, 305) | 311 | (69, 398) | 1515 | Gp5/Type VI secretion system Vgr protein OB-fold domain-containing protein | Gp5/Type VI secretion system Vgr protein OB-fold domain-containing protein | | uniclust | UniRef100\_A0A0E1SC86 | 99.9 | 4.3e-26 | 7.9e-32 | 207.0 | 258 | (18, 288) | 311 | (96, 398) | 1057 | Rhs element Vgr protein | Rhs element Vgr protein | | uniclust | UniRef100\_A0A0A1FJE6 | 99.9 | 4.7e-26 | 8.7e-32 | 198.9 | 275 | (17, 305) | 311 | (237, 549) | 1075 | VgrG protein | VgrG protein | | uniclust | UniRef100\_A0A0C2HQP1 | 99.9 | 5.3e-26 | 9.8e-32 | 186.8 | 258 | (2, 281) | 311 | (67, 347) | 361 | Phage protein D | Phage protein D | | uniclust | UniRef100\_A0A1I4YEC9 | 99.9 | 5.8e-26 | 1.1e-31 | 211.0 | 284 | (5, 303) | 311 | (67, 440) | 873 | Rhs element Vgr protein (Fragment) | Rhs element Vgr protein (Fragment) | | uniclust | UniRef100\_A0A6L4A988 | 99.9 | 5.9e-26 | 1.1e-31 | 184.7 | 209 | (61, 278) | 311 | (3, 237) | 487 | VgrG-related protein (Fragment) | VgrG-related protein (Fragment) | | uniclust | UniRef100\_A0A016CPP4 | 99.9 | 6.6e-26 | 1.2e-31 | 200.0 | 280 | (4, 304) | 311 | (91, 401) | 745 | Phage-related baseplate assembly family protein | Phage-related baseplate assembly family protein | | uniclust | UniRef100\_A0A084DLK1 | 99.9 | 7e-26 | 1.3e-31 | 210.8 | 279 | (17, 307) | 311 | (127, 458) | 1317 | Type IV secretion protein Rhs | Type IV secretion protein Rhs | | uniclust | UniRef100\_A0A3M1LWC1 | 99.9 | 9.3e-26 | 1.7e-31 | 189.8 | 292 | (5, 309) | 311 | (59, 387) | 681 | Type VI secretion system tip protein VgrG (Fragment) | Type VI secretion system tip protein VgrG (Fragment) | | uniclust | UniRef100\_A0A0P9ZJ72 | 99.9 | 9.9e-26 | 1.8e-31 | 185.0 | 271 | (18, 305) | 311 | (83, 399) | 602 | Rhs element Vgr protein (Fragment) | Rhs element Vgr protein (Fragment) | | uniclust | UniRef100\_A0A6A7XZM2 | 99.9 | 1.2e-25 | 2.1e-31 | 202.9 | 275 | (4, 289) | 311 | (144, 467) | 942 | Type VI secretion system tip protein VgrG | Type VI secretion system tip protein VgrG | | uniclust | UniRef100\_A0A198GE76 | 99.9 | 1.2e-25 | 2.2e-31 | 192.4 | 284 | (4, 305) | 311 | (67, 387) | 619 | VgrG family protein | VgrG family protein | | uniclust | UniRef100\_UPI000AA40F38 | 99.9 | 1.4e-25 | 2.5e-31 | 176.6 | 276 | (17, 304) | 311 | (16, 326) | 382 | type VI secretion system tip protein TssI/VgrG | type VI secretion system tip protein TssI/VgrG | | uniclust | UniRef100\_A0A1I7HLI2 | 99.9 | 1.5e-25 | 2.8e-31 | 202.5 | 267 | (4, 287) | 311 | (68, 386) | 863 | Type VI secretion system secreted protein VgrG | Type VI secretion system secreted protein VgrG | | uniclust | UniRef100\_A0A0M2S027 | 99.9 | 1.5e-25 | 2.8e-31 | 186.2 | 262 | (3, 276) | 311 | (61, 380) | 380 | Phage protein D | Phage protein D | | uniclust | UniRef100\_A0A015YA37 | 99.9 | 1.6e-25 | 2.9e-31 | 199.4 | 279 | (4, 304) | 311 | (86, 403) | 651 | Phage late control D family protein | Phage late control D family protein | | uniclust | UniRef100\_A0A7V1ZWM7 | 99.9 | 1.6e-25 | 3e-31 | 180.3 | 267 | (3, 285) | 311 | (71, 364) | 596 | Phage late control D family protein | Phage late control D family protein | | uniclust | UniRef100\_A0A2M8S304 | 99.9 | 1.8e-25 | 3.3e-31 | 196.3 | 241 | (58, 306) | 311 | (86, 362) | 579 | Type VI secretion system tip protein VgrG (Fragment) | Type VI secretion system tip protein VgrG (Fragment) | | uniclust | UniRef100\_A0A2A4Y9Q6 | 99.9 | 1.9e-25 | 3.4e-31 | 203.2 | 260 | (17, 289) | 311 | (73, 377) | 1037 | Gp5/Type VI secretion system Vgr protein OB-fold domain-containing protein | Gp5/Type VI secretion system Vgr protein OB-fold domain-containing protein | | uniclust | UniRef100\_A0A014MDT4 | 99.9 | 2.2e-25 | 4e-31 | 176.6 | 263 | (3, 277) | 311 | (79, 394) | 401 | Type IV secretion protein Rhs | Type IV secretion protein Rhs | | uniclust | UniRef100\_A0A2N8DM59 | 99.9 | 2.2e-25 | 4.1e-31 | 185.9 | 254 | (37, 305) | 311 | (88, 384) | 680 | Type VI secretion system tip protein VgrG (Fragment) | Type VI secretion system tip protein VgrG (Fragment) | | uniclust | UniRef100\_A0A7G8BMN3 | 99.9 | 2.5e-25 | 4.6e-31 | 191.1 | 293 | (4, 308) | 311 | (56, 402) | 986 | Type VI secretion system tip protein VgrG | Type VI secretion system tip protein VgrG | | uniclust | UniRef100\_A0A0A2ECB3 | 99.9 | 2.6e-25 | 4.8e-31 | 199.7 | 282 | (3, 304) | 311 | (163, 476) | 733 | Gp5/Type VI secretion system Vgr protein OB-fold domain-containing protein | Gp5/Type VI secretion system Vgr protein OB-fold domain-containing protein | | uniclust | UniRef100\_A0A0D0R540 | 99.9 | 2.7e-25 | 5e-31 | 196.9 | 287 | (4, 304) | 311 | (144, 515) | 939 | VgrG protein | VgrG protein | | uniclust | UniRef100\_A0A0M6XR59 | 99.9 | 2.7e-25 | 5e-31 | 181.3 | 284 | (4, 301) | 311 | (57, 384) | 468 | Type VI secretion system Vgr family protein | Type VI secretion system Vgr family protein | | uniclust | UniRef100\_A0A380U1Y3 | 99.9 | 2.8e-25 | 5.1e-31 | 189.5 | 274 | (17, 305) | 311 | (146, 456) | 778 | Uncharacterized protein conserved in bacteria | Uncharacterized protein conserved in bacteria | | uniclust | UniRef100\_A0A0T9NGE2 | 99.9 | 4e-25 | 7.4e-31 | 184.9 | 287 | (4, 304) | 311 | (56, 383) | 690 | Rhs element Vgr protein | Rhs element Vgr protein | | uniclust | UniRef100\_A0A933X3D3 | 99.9 | 4.1e-25 | 7.6e-31 | 189.4 | 278 | (16, 307) | 311 | (69, 385) | 1211 | Type VI secretion system tip protein VgrG | Type VI secretion system tip protein VgrG | | uniclust | UniRef100\_A0A085WSA7 | 99.9 | 4.5e-25 | 8.2e-31 | 188.4 | 273 | (17, 305) | 311 | (75, 373) | 1140 | VgrG protein | VgrG protein | | uniclust | UniRef100\_A0A077MYI5 | 99.9 | 4.7e-25 | 8.6e-31 | 196.6 | 290 | (4, 310) | 311 | (153, 475) | 942 | Gp5/Type VI secretion system Vgr protein OB-fold domain-containing protein | Gp5/Type VI secretion system Vgr protein OB-fold domain-containing protein | | uniclust | UniRef100\_A0A0N0UY75 | 99.9 | 5e-25 | 9.2e-31 | 186.0 | 264 | (3, 283) | 311 | (71, 358) | 595 | Gp5/Type VI secretion system Vgr protein OB-fold domain-containing protein | Gp5/Type VI secretion system Vgr protein OB-fold domain-containing protein | | uniclust | UniRef100\_A0A2E8S0G6 | 99.8 | 5.7e-25 | 1.1e-30 | 181.7 | 268 | (3, 283) | 311 | (75, 393) | 407 | Phage late control D family protein | Phage late control D family protein | | uniclust | UniRef100\_A0A068QXL9 | 99.8 | 5.9e-25 | 1.1e-30 | 179.8 | 273 | (1, 281) | 311 | (75, 480) | 498 | Putative phage protein (D protein) (Modular protein) | Putative phage protein (D protein) (Modular protein) | | uniclust | UniRef100\_A0A0X8HC04 | 99.8 | 6.2e-25 | 1.1e-30 | 192.5 | 274 | (17, 306) | 311 | (72, 400) | 1032 | Electron transfer flavoprotein subunit alpha | Electron transfer flavoprotein subunit alpha | | uniclust | UniRef100\_A0A090AED5 | 99.8 | 6.5e-25 | 1.2e-30 | 187.1 | 262 | (3, 280) | 311 | (69, 371) | 418 | Phage protein D | Phage protein D | | uniclust | UniRef100\_A0A1G0FFT8 | 99.8 | 8.3e-25 | 1.5e-30 | 180.9 | 281 | (4, 310) | 311 | (57, 361) | 542 | Gp5/Type VI secretion system Vgr protein OB-fold domain-containing protein | Gp5/Type VI secretion system Vgr protein OB-fold domain-containing protein | | uniclust | UniRef100\_A0A1B9SFQ1 | 99.8 | 1.1e-24 | 2.1e-30 | 178.9 | 268 | (17, 300) | 311 | (82, 377) | 394 | Phage tail protein | Phage tail protein | | uniclust | UniRef100\_A0A0B5FHG4 | 99.8 | 1.2e-24 | 2.2e-30 | 174.7 | 270 | (2, 288) | 311 | (61, 390) | 432 | Phage protein D-like protein | Phage protein D-like protein | | uniclust | UniRef100\_A0A178J7K0 | 99.8 | 1.3e-24 | 2.3e-30 | 194.3 | 285 | (7, 307) | 311 | (65, 390) | 1022 | Type IV secretion protein Rhs | Type IV secretion protein Rhs | | uniclust | UniRef100\_A0A257MWB5 | 99.8 | 1.3e-24 | 2.3e-30 | 174.8 | 289 | (4, 304) | 311 | (55, 403) | 462 | Rhs-family protein | Rhs-family protein | | uniclust | UniRef100\_A0A1X0TCA5 | 99.8 | 1.3e-24 | 2.3e-30 | 182.3 | 291 | (2, 305) | 311 | (222, 548) | 823 | Type VI secretion protein Vgr | Type VI secretion protein Vgr | | uniclust | UniRef100\_A0A099PCF2 | 99.8 | 1.3e-24 | 2.4e-30 | 177.7 | 260 | (3, 277) | 311 | (89, 387) | 391 | Tip attachment protein J domain-containing protein | Tip attachment protein J domain-containing protein | | uniclust | UniRef100\_A0A1I1H522 | 99.8 | 1.5e-24 | 2.7e-30 | 199.4 | 268 | (5, 286) | 311 | (62, 369) | 950 | Rhs element Vgr protein | Rhs element Vgr protein | | uniclust | UniRef100\_A0A0K9L316 | 99.8 | 1.5e-24 | 2.7e-30 | 170.0 | 262 | (1, 276) | 311 | (59, 335) | 393 | Phage late control gene D protein | Phage late control gene D protein | | uniclust | UniRef100\_A0A063BL00 | 99.8 | 1.8e-24 | 3.2e-30 | 194.3 | 270 | (17, 302) | 311 | (136, 437) | 985 | Rhs element Vgr protein | Rhs element Vgr protein | | uniclust | UniRef100\_A0A1B8H0M3 | 99.8 | 2.1e-24 | 3.8e-30 | 188.7 | 289 | (5, 310) | 311 | (107, 426) | 644 | Type VI secretion protein (Fragment) | Type VI secretion protein (Fragment) | | uniclust | UniRef100\_A0A0W8JA15 | 99.8 | 2.2e-24 | 4e-30 | 190.5 | 276 | (18, 307) | 311 | (90, 402) | 1057 | VgrG protein | VgrG protein | | uniclust | UniRef100\_A0A0X3APK5 | 99.8 | 2.3e-24 | 4.1e-30 | 193.3 | 279 | (4, 304) | 311 | (103, 423) | 813 | Uncharacterized conserved protein, implicated in type VI secretion and phage assembly | Uncharacterized conserved protein, implicated in type VI secretion and phage assembly | | uniclust | UniRef100\_A0A3M1QV85 | 99.8 | 2.7e-24 | 4.9e-30 | 168.1 | 275 | (17, 307) | 311 | (117, 442) | 446 | Type VI secretion system tip protein VgrG (Fragment) | Type VI secretion system tip protein VgrG (Fragment) | | uniclust | UniRef100\_A0A1Q7UPE7 | 99.8 | 2.8e-24 | 5.1e-30 | 171.7 | 263 | (3, 280) | 311 | (61, 349) | 437 | Phage late control D family protein (Fragment) | Phage late control D family protein (Fragment) | | uniclust | UniRef100\_A0A097QZD7 | 99.8 | 3e-24 | 5.5e-30 | 183.5 | 269 | (17, 304) | 311 | (111, 421) | 749 | Type IV secretion protein Rhs | Type IV secretion protein Rhs | | uniclust | UniRef100\_A0A2A6PZI3 | 99.8 | 3.2e-24 | 5.9e-30 | 179.8 | 281 | (6, 305) | 311 | (113, 435) | 637 | Type VI secretion system tip protein VgrG (Fragment) | Type VI secretion system tip protein VgrG (Fragment) | | uniclust | UniRef100\_A0A022PDU6 | 99.8 | 3.4e-24 | 6.1e-30 | 188.4 | 258 | (3, 275) | 311 | (73, 356) | 575 | Gp5/Type VI secretion system Vgr protein OB-fold domain-containing protein | Gp5/Type VI secretion system Vgr protein OB-fold domain-containing protein | | uniclust | UniRef100\_A0A0M2WHW5 | 99.8 | 4.2e-24 | 7.6e-30 | 194.8 | 287 | (4, 305) | 311 | (77, 457) | 1005 | Phage-like baseplate assembly protein | Phage-like baseplate assembly protein | | uniclust | UniRef100\_A0A518AYV3 | 99.8 | 4.9e-24 | 9e-30 | 193.7 | 284 | (4, 303) | 311 | (106, 451) | 787 | Phage-related baseplate assembly protein | Phage-related baseplate assembly protein | | uniclust | UniRef100\_A0A074M4V0 | 99.8 | 5.3e-24 | 9.7e-30 | 173.3 | 255 | (3, 277) | 311 | (53, 334) | 366 | Uncharacterized protein | Uncharacterized protein | | uniclust | UniRef100\_A0A0S4X111 | 99.8 | 5.8e-24 | 1.1e-29 | 186.6 | 289 | (4, 304) | 311 | (127, 510) | 899 | Vgr-related protein (Fragment) | Vgr-related protein (Fragment) | | uniclust | UniRef100\_A0A940QC34 | 99.8 | 6.6e-24 | 1.2e-29 | 163.6 | 260 | (3, 277) | 311 | (68, 350) | 352 | Uncharacterized protein | Uncharacterized protein | | uniclust | UniRef100\_A0A085U800 | 99.8 | 6.8e-24 | 1.3e-29 | 163.5 | 240 | (36, 282) | 311 | (11, 274) | 287 | Gene D protein | Gene D protein | | uniclust | UniRef100\_A0A3M1TEK0 | 99.8 | 7.5e-24 | 1.4e-29 | 168.4 | 266 | (2, 283) | 311 | (104, 395) | 516 | Phage late control D family protein | Phage late control D family protein | | uniclust | UniRef100\_A0A4U1HBW3 | 99.8 | 7.7e-24 | 1.4e-29 | 179.1 | 287 | (4, 305) | 311 | (68, 407) | 545 | Type VI secretion system tip protein VgrG | Type VI secretion system tip protein VgrG | | uniclust | UniRef100\_A0A522CBE1 | 99.8 | 8.6e-24 | 1.6e-29 | 164.0 | 245 | (5, 266) | 311 | (55, 339) | 356 | Type VI secretion system tip protein VgrG (Fragment) | Type VI secretion system tip protein VgrG (Fragment) | | uniclust | UniRef100\_A0A0Q6V477 | 99.8 | 8.7e-24 | 1.6e-29 | 175.2 | 262 | (3, 277) | 311 | (67, 388) | 417 | Phage tail protein | Phage tail protein | | uniclust | UniRef100\_A0A376EWE4 | 99.8 | 1e-23 | 1.8e-29 | 168.9 | 290 | (1, 304) | 311 | (74, 380) | 550 | Gene D protein | Gene D protein | | uniclust | UniRef100\_A0A090SLR1 | 99.8 | 1e-23 | 1.9e-29 | 165.7 | 128 | (3, 139) | 311 | (62, 198) | 266 | VgrG protein | VgrG protein | | uniclust | UniRef100\_I7IJM6 | 99.8 | 1.1e-23 | 2e-29 | 190.1 | 289 | (4, 305) | 311 | (72, 402) | 1638 | RHs family protein (Fragment) | RHs family protein (Fragment) | | uniclust | UniRef100\_A0A066RRK7 | 99.8 | 1.2e-23 | 2.1e-29 | 190.7 | 276 | (17, 305) | 311 | (68, 388) | 1616 | Gp5/Type VI secretion system Vgr protein OB-fold domain-containing protein | Gp5/Type VI secretion system Vgr protein OB-fold domain-containing protein | | uniclust | UniRef100\_A0A3N4GAW7 | 99.8 | 1.2e-23 | 2.1e-29 | 177.4 | 287 | (4, 304) | 311 | (63, 392) | 763 | Type VI secretion system tip protein VgrG | Type VI secretion system tip protein VgrG | | uniclust | UniRef100\_A0A356T4I1 | 99.8 | 1.2e-23 | 2.1e-29 | 172.4 | 257 | (17, 286) | 311 | (23, 316) | 500 | Type VI secretion system tip protein VgrG (Fragment) | Type VI secretion system tip protein VgrG (Fragment) | | uniclust | UniRef100\_A0A126ZFA3 | 99.8 | 1.2e-23 | 2.2e-29 | 172.9 | 269 | (3, 285) | 311 | (79, 422) | 466 | Type VI secretion protein | Type VI secretion protein | | uniclust | UniRef100\_A0A653V686 | 99.8 | 1.2e-23 | 2.2e-29 | 175.9 | 278 | (4, 302) | 311 | (82, 408) | 636 | Gp5/Type VI secretion system Vgr protein OB-fold domain-containing protein | Gp5/Type VI secretion system Vgr protein OB-fold domain-containing protein | | uniclust | UniRef100\_A0A1I3FF82 | 99.8 | 1.3e-23 | 2.3e-29 | 161.0 | 252 | (37, 301) | 311 | (21, 317) | 318 | Type VI secretion system secreted protein VgrG | Type VI secretion system secreted protein VgrG | | uniclust | UniRef100\_UPI000A45C3D4 | 99.8 | 1.3e-23 | 2.4e-29 | 152.5 | 223 | (58, 285) | 311 | (4, 244) | 251 | type VI secretion system tip protein TssI/VgrG | type VI secretion system tip protein TssI/VgrG | | uniclust | UniRef100\_A0A369UJ95 | 99.8 | 1.4e-23 | 2.6e-29 | 184.3 | 287 | (4, 303) | 311 | (62, 393) | 1177 | Type VI secretion system tip protein VgrG | Type VI secretion system tip protein VgrG | | uniclust | UniRef100\_A0A519EAL2 | 99.8 | 1.6e-23 | 3e-29 | 173.8 | 282 | (4, 302) | 311 | (57, 376) | 568 | Type VI secretion system tip protein VgrG (Fragment) | Type VI secretion system tip protein VgrG (Fragment) | | uniclust | UniRef100\_A0A084ZAN1 | 99.8 | 1.7e-23 | 3.2e-29 | 172.9 | 269 | (17, 305) | 311 | (70, 380) | 583 | Rhs element Vgr family protein (Fragment) | Rhs element Vgr family protein (Fragment) | | uniclust | UniRef100\_A0A177QF44 | 99.8 | 1.8e-23 | 3.3e-29 | 178.4 | 285 | (4, 304) | 311 | (56, 390) | 872 | Gp5/Type VI secretion system Vgr protein OB-fold domain-containing protein | Gp5/Type VI secretion system Vgr protein OB-fold domain-containing protein | | uniclust | UniRef100\_B8KYT8 | 99.8 | 1.9e-23 | 3.6e-29 | 166.7 | 248 | (4, 265) | 311 | (67, 348) | 387 | Type VI secretion system Vgr family protein | Type VI secretion system Vgr family protein | | uniclust | UniRef100\_A0A1H3JED8 | 99.8 | 2e-23 | 3.7e-29 | 179.8 | 260 | (3, 278) | 311 | (74, 363) | 567 | Rhs element Vgr protein | Rhs element Vgr protein | | uniclust | UniRef100\_A0A2D9TST2 | 99.8 | 2e-23 | 3.7e-29 | 183.4 | 275 | (17, 306) | 311 | (69, 382) | 1081 | Gp5/Type VI secretion system Vgr protein OB-fold domain-containing protein | Gp5/Type VI secretion system Vgr protein OB-fold domain-containing protein | | uniclust | UniRef100\_A0A077FEP7 | 99.8 | 2.2e-23 | 4.1e-29 | 170.5 | 272 | (19, 306) | 311 | (69, 382) | 531 | Rhs element Vgr protein | Rhs element Vgr protein | | uniclust | UniRef100\_A0A380U204 | 99.8 | 2.2e-23 | 4.1e-29 | 182.2 | 274 | (17, 305) | 311 | (215, 525) | 1051 | Extracellular phospholipase A1 | Extracellular phospholipase A1 | | uniclust | UniRef100\_A0A4Y9T3M5 | 99.8 | 2.5e-23 | 4.6e-29 | 174.7 | 279 | (17, 310) | 311 | (66, 390) | 898 | Type VI secretion system tip protein VgrG | Type VI secretion system tip protein VgrG | | uniclust | UniRef100\_A0A0F9V267 | 99.8 | 2.6e-23 | 4.9e-29 | 169.5 | 274 | (17, 307) | 311 | (73, 395) | 511 | Gp5/Type VI secretion system Vgr protein OB-fold domain-containing protein | Gp5/Type VI secretion system Vgr protein OB-fold domain-containing protein | | uniclust | UniRef100\_A0A0U2P820 | 99.8 | 2.9e-23 | 5.2e-29 | 179.5 | 254 | (17, 285) | 311 | (85, 366) | 692 | Gp5/Type VI secretion system Vgr protein OB-fold domain-containing protein | Gp5/Type VI secretion system Vgr protein OB-fold domain-containing protein | | uniclust | UniRef100\_A0A953GUI7 | 99.8 | 2.9e-23 | 5.4e-29 | 175.1 | 286 | (4, 301) | 311 | (50, 375) | 942 | Type VI secretion system tip protein VgrG | Type VI secretion system tip protein VgrG | | uniclust | UniRef100\_A0A2L1UY19 | 99.8 | 3e-23 | 5.6e-29 | 166.3 | 230 | (17, 264) | 311 | (98, 355) | 390 | Type IV secretion protein Rhs | Type IV secretion protein Rhs | | uniclust | UniRef100\_A0A078M1E5 | 99.8 | 3.6e-23 | 6.6e-29 | 163.9 | 214 | (19, 248) | 311 | (99, 336) | 341 | Rhs element Vgr protein | Rhs element Vgr protein | | uniclust | UniRef100\_A0A071M6W2 | 99.8 | 4e-23 | 7.4e-29 | 191.8 | 288 | (4, 306) | 311 | (156, 536) | 1273 | Type IV secretion protein Rhs | Type IV secretion protein Rhs | | uniclust | UniRef100\_A0A0D0GEX9 | 99.8 | 4.1e-23 | 7.5e-29 | 185.5 | 254 | (17, 285) | 311 | (172, 456) | 1050 | Rhs element Vgr family protein | Rhs element Vgr family protein | | uniclust | UniRef100\_A0A3B8ISA8 | 99.8 | 4.3e-23 | 7.8e-29 | 167.0 | 213 | (2, 230) | 311 | (73, 308) | 311 | Rhs element Vgr protein (Fragment) | Rhs element Vgr protein (Fragment) | | uniclust | UniRef100\_A0A1V5B929 | 99.8 | 4.3e-23 | 7.9e-29 | 160.6 | 259 | (2, 277) | 311 | (61, 350) | 355 | Phage late control gene D protein (GPD) | Phage late control gene D protein (GPD) | | uniclust | UniRef100\_A0A024E5Z2 | 99.8 | 4.4e-23 | 8.1e-29 | 178.4 | 279 | (6, 303) | 311 | (73, 383) | 626 | Putative Rhs element Vgr protein | Putative Rhs element Vgr protein | | uniclust | UniRef100\_A0A519M875 | 99.8 | 4.7e-23 | 8.6e-29 | 166.4 | 259 | (4, 283) | 311 | (64, 353) | 407 | Type VI secretion system tip protein VgrG (Fragment) | Type VI secretion system tip protein VgrG (Fragment) | | uniclust | UniRef100\_UPI00027C90E7 | 99.8 | 4.7e-23 | 8.6e-29 | 168.9 | 270 | (1, 277) | 311 | (243, 544) | 563 | phage tail protein | phage tail protein | | uniclust | UniRef100\_A0A1Z8A3M6 | 99.8 | 4.8e-23 | 8.8e-29 | 177.2 | 259 | (37, 308) | 311 | (90, 387) | 1207 | Gp5/Type VI secretion system Vgr protein OB-fold domain-containing protein | Gp5/Type VI secretion system Vgr protein OB-fold domain-containing protein | | uniclust | UniRef100\_A0A828PCY0 | 99.8 | 5.4e-23 | 9.9e-29 | 175.2 | 292 | (2, 305) | 311 | (159, 485) | 945 | Type VI secretion system tip protein VgrG | Type VI secretion system tip protein VgrG | | uniclust | UniRef100\_A0A2G0VNE3 | 99.8 | 5.5e-23 | 1e-28 | 156.3 | 124 | (3, 137) | 311 | (72, 206) | 213 | Type VI secretion system tip protein VgrG | Type VI secretion system tip protein VgrG | | uniclust | UniRef100\_UPI0020A7DE59 | 99.8 | 6e-23 | 1.1e-28 | 165.5 | 252 | (17, 281) | 311 | (71, 355) | 579 | type VI secretion system tip protein TssI/VgrG | type VI secretion system tip protein TssI/VgrG | | uniclust | UniRef100\_A0A066PIW0 | 99.8 | 6e-23 | 1.1e-28 | 173.4 | 247 | (17, 280) | 311 | (111, 380) | 397 | Phage late control protein D | Phage late control protein D | | uniclust | UniRef100\_A0A165W2E7 | 99.8 | 6.4e-23 | 1.2e-28 | 163.6 | 266 | (1, 278) | 311 | (50, 322) | 324 | Late control protein D | Late control protein D | | uniclust | UniRef100\_A0A0J6NA85 | 99.8 | 6.6e-23 | 1.2e-28 | 175.2 | 285 | (4, 304) | 311 | (69, 424) | 752 | Type VI secretion system protein (Fragment) | Type VI secretion system protein (Fragment) | | uniclust | UniRef100\_A0A2N2R031 | 99.8 | 6.6e-23 | 1.2e-28 | 170.2 | 265 | (3, 283) | 311 | (86, 407) | 454 | Type VI secretion system tip protein VgrG (Fragment) | Type VI secretion system tip protein VgrG (Fragment) | | uniclust | UniRef100\_A0A417HIZ6 | 99.8 | 6.7e-23 | 1.2e-28 | 162.1 | 259 | (3, 276) | 311 | (77, 364) | 367 | Phage late control D family protein | Phage late control D family protein | | uniclust | UniRef100\_A0A5N4FUG3 | 99.8 | 7.4e-23 | 1.4e-28 | 166.5 | 258 | (38, 307) | 311 | (98, 389) | 514 | Type VI secretion system tip protein VgrG (Fragment) | Type VI secretion system tip protein VgrG (Fragment) | | uniclust | UniRef100\_A0A849ZEB7 | 99.8 | 7.4e-23 | 1.4e-28 | 169.4 | 290 | (4, 308) | 311 | (84, 503) | 625 | Type VI secretion system tip protein VgrG (Fragment) | Type VI secretion system tip protein VgrG (Fragment) | | uniclust | UniRef100\_A0A071LW85 | 99.8 | 8e-23 | 1.5e-28 | 186.0 | 250 | (17, 279) | 311 | (142, 438) | 982 | Type IV secretion protein Rhs | Type IV secretion protein Rhs | | uniclust | UniRef100\_A0A158K0S5 | 99.8 | 8e-23 | 1.5e-28 | 181.6 | 253 | (17, 284) | 311 | (96, 379) | 863 | Rhs element Vgr protein | Rhs element Vgr protein | | uniclust | UniRef100\_A0A0E3ZX09 | 99.8 | 8.2e-23 | 1.5e-28 | 172.8 | 264 | (4, 285) | 311 | (61, 342) | 576 | Gp5/Type VI secretion system Vgr protein OB-fold domain-containing protein | Gp5/Type VI secretion system Vgr protein OB-fold domain-containing protein | | uniclust | UniRef100\_A0A147GLC0 | 99.8 | 8.3e-23 | 1.5e-28 | 165.9 | 239 | (17, 267) | 311 | (118, 389) | 398 | Type VI secretion protein ImpA (Fragment) | Type VI secretion protein ImpA (Fragment) | | uniclust | UniRef100\_A0A010SYM9 | 99.8 | 8.6e-23 | 1.6e-28 | 172.5 | 258 | (7, 279) | 311 | (126, 419) | 431 | Type IV secretion protein Rhs | Type IV secretion protein Rhs | | uniclust | UniRef100\_A0A069PJ07 | 99.8 | 8.9e-23 | 1.6e-28 | 187.6 | 253 | (18, 285) | 311 | (145, 424) | 988 | Type IV secretion protein Rhs | Type IV secretion protein Rhs | | uniclust | UniRef100\_A0A0G3BGP6 | 99.8 | 8.9e-23 | 1.6e-28 | 179.4 | 290 | (5, 306) | 311 | (89, 420) | 1041 | Type VI secretion system tip protein VgrG | Type VI secretion system tip protein VgrG | | uniclust | UniRef100\_A0A7W0Y6A5 | 99.8 | 9.5e-23 | 1.7e-28 | 158.4 | 262 | (3, 277) | 311 | (54, 351) | 356 | Phage late control D family protein | Phage late control D family protein | | uniclust | UniRef100\_A0A080M199 | 99.8 | 1e-22 | 1.8e-28 | 169.3 | 262 | (6, 282) | 311 | (79, 370) | 398 | Phage protein D | Phage protein D | | uniclust | UniRef100\_A0A1U7N292 | 99.8 | 1e-22 | 1.9e-28 | 165.1 | 250 | (3, 277) | 311 | (66, 337) | 347 | Phage protein D | Phage protein D | | uniclust | UniRef100\_UPI00056E02FB | 99.8 | 1.1e-22 | 2e-28 | 157.4 | 246 | (4, 262) | 311 | (50, 329) | 340 | type VI secretion system tip protein TssI/VgrG | type VI secretion system tip protein TssI/VgrG | | uniclust | UniRef100\_A0A954DSN9 | 99.8 | 1.1e-22 | 2.1e-28 | 171.4 | 285 | (4, 305) | 311 | (276, 607) | 923 | Type VI secretion system tip protein VgrG | Type VI secretion system tip protein VgrG | | uniclust | UniRef100\_A0A1H4H7U1 | 99.8 | 1.4e-22 | 2.6e-28 | 177.7 | 277 | (4, 300) | 311 | (94, 406) | 643 | Uncharacterized conserved protein, implicated in type VI secretion and phage assembly | Uncharacterized conserved protein, implicated in type VI secretion and phage assembly | | uniclust | UniRef100\_A0A0J6RCQ0 | 99.8 | 1.4e-22 | 2.6e-28 | 173.8 | 275 | (17, 304) | 311 | (85, 399) | 647 | Gp5/Type VI secretion system Vgr protein OB-fold domain-containing protein (Fragment) | Gp5/Type VI secretion system Vgr protein OB-fold domain-containing protein (Fragment) | | uniclust | UniRef100\_A0A9D5K2R1 | 99.8 | 1.5e-22 | 2.8e-28 | 149.0 | 225 | (58, 286) | 311 | (3, 249) | 275 | Type VI secretion system tip protein VgrG | Type VI secretion system tip protein VgrG | | uniclust | UniRef100\_A0A0A0EXH8 | 99.8 | 1.6e-22 | 2.9e-28 | 174.0 | 284 | (2, 304) | 311 | (67, 404) | 668 | Gp5/Type VI secretion system Vgr protein OB-fold domain-containing protein (Fragment) | Gp5/Type VI secretion system Vgr protein OB-fold domain-containing protein (Fragment) | | uniclust | UniRef100\_A0A2X1MAT3 | 99.8 | 1.6e-22 | 2.9e-28 | 163.4 | 174 | (1, 181) | 311 | (63, 237) | 280 | Phage protein D | Phage protein D | | uniclust | UniRef100\_UPI0003D2A615 | 99.8 | 1.7e-22 | 3.2e-28 | 156.5 | 255 | (1, 262) | 311 | (12, 309) | 310 | contractile injection system protein, VgrG/Pvc8 family | contractile injection system protein, VgrG/Pvc8 family | | uniclust | UniRef100\_A0A1A9VKH9 | 99.8 | 1.8e-22 | 3.3e-28 | 158.9 | 268 | (7, 281) | 311 | (49, 317) | 462 | Uncharacterized protein | Uncharacterized protein | | uniclust | UniRef100\_A0A0B1XTW2 | 99.8 | 1.8e-22 | 3.3e-28 | 177.6 | 287 | (3, 303) | 311 | (98, 442) | 1005 | Type VI secretion protein (Fragment) | Type VI secretion protein (Fragment) | | uniclust | UniRef100\_A0A1X3D1R8 | 99.8 | 1.9e-22 | 3.4e-28 | 169.3 | 274 | (17, 303) | 311 | (94, 399) | 534 | Gp5/Type VI secretion system Vgr protein OB-fold domain-containing protein (Fragment) | Gp5/Type VI secretion system Vgr protein OB-fold domain-containing protein (Fragment) | | uniclust | UniRef100\_UPI0003807C1F | 99.8 | 2e-22 | 3.7e-28 | 179.5 | 280 | (17, 307) | 311 | (68, 386) | 1399 | type VI secretion system tip protein TssI/VgrG | type VI secretion system tip protein TssI/VgrG | | uniclust | UniRef100\_A0A1H4SWS9 | 99.8 | 2.1e-22 | 3.9e-28 | 174.9 | 254 | (38, 304) | 311 | (89, 380) | 1019 | Rhs element Vgr protein | Rhs element Vgr protein | | uniclust | UniRef100\_A0A061NEY3 | 99.8 | 2.3e-22 | 4.1e-28 | 172.2 | 252 | (3, 278) | 311 | (79, 346) | 432 | Phage-like element PBSX protein XkdQ | Phage-like element PBSX protein XkdQ | | uniclust | UniRef100\_A0A534PM04 | 99.8 | 2.3e-22 | 4.3e-28 | 163.6 | 258 | (6, 278) | 311 | (53, 336) | 565 | Phage late control D family protein | Phage late control D family protein | | uniclust | UniRef100\_A0A8X6GUF6 | 99.8 | 2.4e-22 | 4.3e-28 | 160.7 | 264 | (8, 278) | 311 | (73, 337) | 529 | Phage tail protein | Phage tail protein | | uniclust | UniRef100\_A0A017T573 | 99.8 | 2.6e-22 | 4.8e-28 | 182.4 | 273 | (17, 304) | 311 | (95, 421) | 956 | VgrG protein | VgrG protein | | uniclust | UniRef100\_UPI000AD481B1 | 99.8 | 2.8e-22 | 5.1e-28 | 149.0 | 207 | (58, 268) | 311 | (8, 235) | 251 | type VI secretion system tip protein VgrG | type VI secretion system tip protein VgrG | | uniclust | UniRef100\_A0A085ULB1 | 99.8 | 2.8e-22 | 5.1e-28 | 152.2 | 201 | (56, 265) | 311 | (27, 250) | 263 | Type IV secretion protein Rhs (Fragment) | Type IV secretion protein Rhs (Fragment) | | uniclust | UniRef100\_A0A250KKR6 | 99.8 | 3e-22 | 5.6e-28 | 168.5 | 279 | (4, 304) | 311 | (59, 369) | 593 | Type IV secretion protein Rhs | Type IV secretion protein Rhs | | uniclust | UniRef100\_A0A0N0D4A4 | 99.8 | 3.1e-22 | 5.7e-28 | 156.3 | 167 | (2, 183) | 311 | (67, 250) | 301 | Rhs element Vgr protein, subgroup (Fragment) | Rhs element Vgr protein, subgroup (Fragment) | | uniclust | UniRef100\_A0A071M8E6 | 99.8 | 3.2e-22 | 5.9e-28 | 183.1 | 285 | (4, 306) | 311 | (122, 491) | 1175 | Rhs element Vgr protein | Rhs element Vgr protein | | uniclust | UniRef100\_A0A136Q991 | 99.8 | 3.2e-22 | 5.9e-28 | 156.3 | 211 | (1, 221) | 311 | (55, 272) | 292 | Phage late control protein (Fragment) | Phage late control protein (Fragment) | | uniclust | UniRef100\_A0A380U1Z0 | 99.8 | 3.6e-22 | 6.7e-28 | 181.9 | 273 | (17, 305) | 311 | (199, 507) | 2004 | Cell wall-associated polypeptide CWBP200 | Cell wall-associated polypeptide CWBP200 | | uniclust | UniRef100\_A0A105DDV7 | 99.8 | 4.6e-22 | 8.5e-28 | 184.0 | 272 | (17, 303) | 311 | (130, 434) | 1296 | Type IV secretion protein Rhs | Type IV secretion protein Rhs | | uniclust | UniRef100\_A0A069NIW4 | 99.8 | 5.5e-22 | 1e-27 | 172.3 | 276 | (17, 304) | 311 | (103, 430) | 789 | Type VI secretion protein ImpA (Fragment) | Type VI secretion protein ImpA (Fragment) | | uniclust | UniRef100\_A0A0T7DUV3 | 99.8 | 5.6e-22 | 1e-27 | 161.6 | 258 | (1, 277) | 311 | (69, 342) | 344 | Putative bacteriophage regulatory protein | Putative bacteriophage regulatory protein | | uniclust | UniRef100\_A0A0D0MWE3 | 99.8 | 5.6e-22 | 1e-27 | 168.6 | 285 | (4, 303) | 311 | (96, 442) | 636 | Gp5/Type VI secretion system Vgr protein OB-fold domain-containing protein (Fragment) | Gp5/Type VI secretion system Vgr protein OB-fold domain-containing protein (Fragment) | | uniclust | UniRef100\_A0A0H2LUU0 | 99.8 | 5.7e-22 | 1e-27 | 178.7 | 236 | (17, 265) | 311 | (72, 336) | 1189 | Phage-related baseplate assembly protein | Phage-related baseplate assembly protein | | uniclust | UniRef100\_A0A1Q7M720 | 99.8 | 6e-22 | 1.1e-27 | 159.6 | 259 | (3, 281) | 311 | (88, 365) | 448 | Phage late control D family protein | Phage late control D family protein | | uniclust | UniRef100\_A0A354P3G0 | 99.8 | 6.4e-22 | 1.2e-27 | 175.1 | 270 | (17, 301) | 311 | (101, 446) | 944 | Gp5/Type VI secretion system Vgr protein OB-fold domain-containing protein | Gp5/Type VI secretion system Vgr protein OB-fold domain-containing protein | | uniclust | UniRef100\_A0A2D4SL60 | 99.8 | 6.6e-22 | 1.2e-27 | 158.0 | 263 | (3, 282) | 311 | (61, 347) | 355 | Phage late control D family protein | Phage late control D family protein | | uniclust | UniRef100\_A0A011TTI3 | 99.8 | 7.1e-22 | 1.3e-27 | 161.3 | 274 | (1, 287) | 311 | (68, 353) | 397 | Late control D family protein | Late control D family protein | | uniclust | UniRef100\_UPI00129B7A46 | 99.8 | 7.4e-22 | 1.4e-27 | 151.9 | 232 | (17, 265) | 311 | (10, 271) | 286 | type VI secretion system tip protein TssI/VgrG | type VI secretion system tip protein TssI/VgrG | | uniclust | UniRef100\_A0A023Y2E8 | 99.8 | 7.8e-22 | 1.4e-27 | 184.5 | 288 | (4, 303) | 311 | (140, 520) | 1157 | Type IV secretion protein Rhs | Type IV secretion protein Rhs | | uniclust | UniRef100\_A0A0S3TZS7 | 99.8 | 8e-22 | 1.5e-27 | 166.9 | 244 | (18, 278) | 311 | (117, 394) | 810 | Rhs element Vgr protein | Rhs element Vgr protein | | uniclust | UniRef100\_A0A4U1HWQ4 | 99.8 | 9e-22 | 1.6e-27 | 165.4 | 256 | (37, 307) | 311 | (92, 376) | 643 | Type VI secretion system tip protein VgrG | Type VI secretion system tip protein VgrG | | uniclust | UniRef100\_A0A0Q8WLP0 | 99.8 | 9.3e-22 | 1.7e-27 | 165.8 | 276 | (17, 307) | 311 | (76, 392) | 558 | Type VI secretion protein VgrG | Type VI secretion protein VgrG | | uniclust | UniRef100\_A0A1Y5GX49 | 99.8 | 9.5e-22 | 1.7e-27 | 177.3 | 276 | (17, 307) | 311 | (73, 385) | 1309 | Type VI secretion system tip protein VgrG | Type VI secretion system tip protein VgrG | | uniclust | UniRef100\_A0A518AZS0 | 99.8 | 9.8e-22 | 1.8e-27 | 174.6 | 283 | (4, 302) | 311 | (59, 408) | 1054 | Phage-related baseplate assembly protein | Phage-related baseplate assembly protein | | uniclust | UniRef100\_A0A081C212 | 99.8 | 1.1e-21 | 2e-27 | 161.9 | 255 | (3, 279) | 311 | (84, 361) | 371 | Uncharacterized protein | Uncharacterized protein | | uniclust | UniRef100\_F2NUD2 | 99.8 | 1.1e-21 | 2e-27 | 165.0 | 259 | (3, 277) | 311 | (63, 348) | 619 | Uncharacterized protein | Uncharacterized protein | | uniclust | UniRef100\_A0A0X8C270 | 99.8 | 1.1e-21 | 2e-27 | 182.0 | 277 | (4, 302) | 311 | (101, 412) | 1104 | Gp5/Type VI secretion system Vgr protein OB-fold domain-containing protein | Gp5/Type VI secretion system Vgr protein OB-fold domain-containing protein | | uniclust | UniRef100\_A0A080IYI6 | 99.8 | 1.1e-21 | 2.1e-27 | 162.1 | 291 | (2, 304) | 311 | (154, 479) | 646 | Rhs element Vgr family protein | Rhs element Vgr family protein | | uniclust | UniRef100\_A0A4R4ILG7 | 99.8 | 1.2e-21 | 2.3e-27 | 158.0 | 256 | (37, 305) | 311 | (112, 401) | 496 | Type VI secretion system tip protein VgrG (Fragment) | Type VI secretion system tip protein VgrG (Fragment) | | uniclust | UniRef100\_A0A849Z2N6 | 99.7 | 1.4e-21 | 2.6e-27 | 158.1 | 257 | (36, 304) | 311 | (92, 375) | 492 | Type VI secretion system tip protein VgrG (Fragment) | Type VI secretion system tip protein VgrG (Fragment) | | uniclust | UniRef100\_A0A0J8DB12 | 99.7 | 1.4e-21 | 2.6e-27 | 161.8 | 253 | (2, 281) | 311 | (71, 327) | 330 | Uncharacterized protein | Uncharacterized protein | | uniclust | UniRef100\_A0A2V8RQE1 | 99.7 | 1.5e-21 | 2.7e-27 | 151.8 | 247 | (4, 265) | 311 | (62, 354) | 360 | Type VI secretion system tip protein VgrG (Fragment) | Type VI secretion system tip protein VgrG (Fragment) | | uniclust | UniRef100\_UPI001ED99487 | 99.7 | 1.5e-21 | 2.7e-27 | 170.6 | 287 | (4, 305) | 311 | (759, 1086) | 1451 | type VI secretion system tip protein TssI/VgrG | type VI secretion system tip protein TssI/VgrG | | uniclust | UniRef100\_UPI001ED99487 | 99.7 | 1.5e-21 | 2.7e-27 | 170.6 | 287 | (4, 305) | 311 | (197, 524) | 1451 | type VI secretion system tip protein TssI/VgrG | type VI secretion system tip protein TssI/VgrG | | uniclust | UniRef100\_A0A023Y2A2 | 99.7 | 1.5e-21 | 2.7e-27 | 177.8 | 284 | (2, 303) | 311 | (279, 612) | 1207 | Type IV secretion protein Rhs | Type IV secretion protein Rhs | | uniclust | UniRef100\_A0A2E7ADA3 | 99.7 | 1.5e-21 | 2.8e-27 | 168.6 | 275 | (16, 303) | 311 | (67, 374) | 975 | Gp5/Type VI secretion system Vgr protein OB-fold domain-containing protein | Gp5/Type VI secretion system Vgr protein OB-fold domain-containing protein | | uniclust | UniRef100\_A0A2D6B585 | 99.7 | 1.6e-21 | 3e-27 | 164.6 | 287 | (5, 303) | 311 | (266, 607) | 917 | Type VI secretion system tip protein VgrG | Type VI secretion system tip protein VgrG | | uniclust | UniRef100\_A0A150PRS2 | 99.7 | 1.6e-21 | 3e-27 | 169.3 | 288 | (4, 305) | 311 | (58, 382) | 732 | Gp5/Type VI secretion system Vgr protein OB-fold domain-containing protein (Fragment) | Gp5/Type VI secretion system Vgr protein OB-fold domain-containing protein (Fragment) | | uniclust | UniRef100\_A0A1H0U3P0 | 99.7 | 1.6e-21 | 3e-27 | 143.2 | 123 | (4, 135) | 311 | (50, 180) | 204 | Rhs element Vgr protein (Fragment) | Rhs element Vgr protein (Fragment) | | uniclust | UniRef100\_A0A2X3JV88 | 99.7 | 1.6e-21 | 3e-27 | 150.5 | 252 | (37, 305) | 311 | (31, 321) | 337 | Rhs element Vgr protein | Rhs element Vgr protein | | uniclust | UniRef100\_A0A068T9R7 | 99.7 | 1.7e-21 | 3.1e-27 | 154.8 | 272 | (2, 281) | 311 | (58, 339) | 355 | Phage late control D family protein | Phage late control D family protein | | uniclust | UniRef100\_A0A016XHX9 | 99.7 | 1.7e-21 | 3.2e-27 | 169.5 | 264 | (3, 282) | 311 | (89, 375) | 432 | Baseplate protein | Baseplate protein | | uniclust | UniRef100\_A0A2A4HGW6 | 99.7 | 1.8e-21 | 3.4e-27 | 153.1 | 250 | (17, 283) | 311 | (68, 357) | 370 | Type VI secretion system tip protein VgrG (Fragment) | Type VI secretion system tip protein VgrG (Fragment) | | uniclust | UniRef100\_UPI0018EF83E4 | 99.7 | 2e-21 | 3.7e-27 | 162.7 | 257 | (3, 277) | 311 | (262, 556) | 834 | VgrG-related protein | VgrG-related protein | | uniclust | UniRef100\_A0A010RVC8 | 99.7 | 2.1e-21 | 3.9e-27 | 175.4 | 274 | (18, 303) | 311 | (83, 417) | 709 | Type IV secretion protein Rhs | Type IV secretion protein Rhs | | uniclust | UniRef100\_A0A7J6YLS0 | 99.7 | 2.2e-21 | 4e-27 | 162.6 | 270 | (7, 283) | 311 | (73, 343) | 756 | phospholipase A2 | phospholipase A2 | | uniclust | UniRef100\_A0A351G4S7 | 99.7 | 2.3e-21 | 4.3e-27 | 149.0 | 247 | (17, 278) | 311 | (85, 362) | 374 | Phage late control D family protein | Phage late control D family protein | | uniclust | UniRef100\_A0A024L6E0 | 99.7 | 2.7e-21 | 4.9e-27 | 164.1 | 260 | (17, 308) | 311 | (81, 383) | 723 | Rhs element Vgr protein | Rhs element Vgr protein | | uniclust | UniRef100\_A0A3B0ZLP7 | 99.7 | 3e-21 | 5.5e-27 | 170.5 | 274 | (16, 303) | 311 | (81, 392) | 1041 | Gp5/Type VI secretion system Vgr protein OB-fold domain-containing protein | Gp5/Type VI secretion system Vgr protein OB-fold domain-containing protein | | uniclust | UniRef100\_A0A0F2QXK9 | 99.7 | 3.1e-21 | 5.7e-27 | 152.0 | 213 | (19, 248) | 311 | (85, 321) | 347 | Type IV secretion protein Rhs (Fragment) | Type IV secretion protein Rhs (Fragment) | | uniclust | UniRef100\_A0A1Q8BNB2 | 99.7 | 3.1e-21 | 5.8e-27 | 160.0 | 258 | (6, 278) | 311 | (64, 347) | 627 | Gp5/Type VI secretion system Vgr protein OB-fold domain-containing protein | Gp5/Type VI secretion system Vgr protein OB-fold domain-containing protein | | uniclust | UniRef100\_A0A074V5I6 | 99.7 | 3.9e-21 | 7.1e-27 | 167.4 | 273 | (19, 307) | 311 | (72, 420) | 847 | Gp5/Type VI secretion system Vgr protein OB-fold domain-containing protein | Gp5/Type VI secretion system Vgr protein OB-fold domain-containing protein | | uniclust | UniRef100\_A0A7V8K0W3 | 99.7 | 4e-21 | 7.3e-27 | 167.1 | 277 | (17, 305) | 311 | (91, 401) | 1332 | Actin cross-linking toxin VgrG1 | Actin cross-linking toxin VgrG1 | | uniclust | UniRef100\_A0A349NY43 | 99.7 | 4e-21 | 7.3e-27 | 147.8 | 240 | (2, 257) | 311 | (60, 326) | 328 | Rhs element Vgr protein (Fragment) | Rhs element Vgr protein (Fragment) | | uniclust | UniRef100\_UPI001FCF2336 | 99.7 | 4.2e-21 | 7.6e-27 | 165.4 | 292 | (2, 305) | 311 | (591, 917) | 1169 | type VI secretion system membrane subunit TssM | type VI secretion system membrane subunit TssM | | uniclust | UniRef100\_A0A661NEM7 | 99.7 | 4.3e-21 | 7.9e-27 | 157.4 | 278 | (17, 308) | 311 | (69, 376) | 561 | Type VI secretion system tip protein VgrG (Fragment) | Type VI secretion system tip protein VgrG (Fragment) | | uniclust | UniRef100\_A0A0Q7MJZ9 | 99.7 | 5e-21 | 9.2e-27 | 159.6 | 266 | (4, 283) | 311 | (60, 360) | 372 | Phage tail protein | Phage tail protein | | uniclust | UniRef100\_A0A126UXB1 | 99.7 | 5.3e-21 | 9.7e-27 | 161.4 | 208 | (94, 305) | 311 | (12, 253) | 540 | Gp5/Type VI secretion system Vgr protein OB-fold domain-containing protein | Gp5/Type VI secretion system Vgr protein OB-fold domain-containing protein | | uniclust | UniRef100\_A0A318UXY0 | 99.7 | 5.4e-21 | 9.9e-27 | 167.5 | 274 | (19, 306) | 311 | (79, 400) | 1054 | Rhs element Vgr protein (Fragment) | Rhs element Vgr protein (Fragment) | | uniclust | UniRef100\_A0A1E4W345 | 99.7 | 5.7e-21 | 1e-26 | 162.5 | 208 | (96, 306) | 311 | (2, 243) | 580 | Type VI secretion protein ImpA (Fragment) | Type VI secretion protein ImpA (Fragment) | | uniclust | UniRef100\_A0A059USW8 | 99.7 | 6.5e-21 | 1.2e-26 | 168.0 | 279 | (17, 307) | 311 | (92, 426) | 855 | Rhs element Vgr protein | Rhs element Vgr protein | | uniclust | UniRef100\_A0A7Y4J6W0 | 99.7 | 6.8e-21 | 1.3e-26 | 137.1 | 109 | (17, 134) | 311 | (12, 126) | 185 | Type VI secretion system tip protein VgrG (Fragment) | Type VI secretion system tip protein VgrG (Fragment) | | uniclust | UniRef100\_A0A1H2PKZ2 | 99.7 | 7e-21 | 1.3e-26 | 160.3 | 258 | (17, 288) | 311 | (77, 378) | 609 | Type VI secretion system secreted protein VgrG | Type VI secretion system secreted protein VgrG | | uniclust | UniRef100\_A0A017TA00 | 99.7 | 7.4e-21 | 1.4e-26 | 168.0 | 276 | (17, 309) | 311 | (68, 391) | 945 | VgrG protein | VgrG protein | | uniclust | UniRef100\_A0A1Y2SPT5 | 99.7 | 7.7e-21 | 1.4e-26 | 155.8 | 277 | (17, 310) | 311 | (74, 384) | 520 | Rhs element Vgr family protein | Rhs element Vgr family protein | | uniclust | UniRef100\_A0A356TFU1 | 99.7 | 8.2e-21 | 1.5e-26 | 164.5 | 291 | (5, 305) | 311 | (250, 581) | 934 | Gp5/Type VI secretion system Vgr protein OB-fold domain-containing protein | Gp5/Type VI secretion system Vgr protein OB-fold domain-containing protein | | uniclust | UniRef100\_A0A1E4LS02 | 99.7 | 9.4e-21 | 1.7e-26 | 149.1 | 260 | (18, 284) | 311 | (2, 269) | 317 | Late control protein (Fragment) | Late control protein (Fragment) | | uniclust | UniRef100\_A0A4U7JJ27 | 99.7 | 9.8e-21 | 1.8e-26 | 154.8 | 262 | (3, 279) | 311 | (91, 377) | 639 | Gp5/Type VI secretion system Vgr protein OB-fold domain-containing protein | Gp5/Type VI secretion system Vgr protein OB-fold domain-containing protein | | uniclust | UniRef100\_A0A086CZD6 | 99.7 | 1.1e-20 | 2e-26 | 166.3 | 286 | (5, 306) | 311 | (82, 450) | 859 | Type IV secretion protein Rhs (Fragment) | Type IV secretion protein Rhs (Fragment) | | uniclust | UniRef100\_A0A158G169 | 99.7 | 1.1e-20 | 2e-26 | 156.1 | 268 | (19, 302) | 311 | (101, 400) | 486 | Rhs element Vgr protein | Rhs element Vgr protein | | uniclust | UniRef100\_A0A563DMM3 | 99.7 | 1.1e-20 | 2.1e-26 | 153.5 | 257 | (4, 284) | 311 | (98, 398) | 453 | Uncharacterized protein (Fragment) | Uncharacterized protein (Fragment) | | uniclust | UniRef100\_A0A7S7SKC8 | 99.7 | 1.1e-20 | 2.1e-26 | 160.0 | 274 | (17, 302) | 311 | (72, 384) | 937 | Type VI secretion system tip protein VgrG | Type VI secretion system tip protein VgrG | | uniclust | UniRef100\_A0A376JF19 | 99.7 | 1.2e-20 | 2.2e-26 | 149.1 | 172 | (1, 185) | 311 | (74, 246) | 295 | Phage late control D family protein | Phage late control D family protein | | uniclust | UniRef100\_A0A812QV43 | 99.7 | 1.2e-20 | 2.2e-26 | 162.0 | 266 | (1, 273) | 311 | (734, 1005) | 1104 | GpFI protein | GpFI protein | | uniclust | UniRef100\_UPI00142415E8 | 99.7 | 1.3e-20 | 2.3e-26 | 159.2 | 290 | (5, 307) | 311 | (59, 399) | 901 | type VI secretion system tip protein TssI/VgrG | type VI secretion system tip protein TssI/VgrG | | uniclust | UniRef100\_UPI00079AD8BF | 99.7 | 1.3e-20 | 2.5e-26 | 154.7 | 270 | (1, 277) | 311 | (362, 658) | 665 | phage major capsid protein, P2 family | phage major capsid protein, P2 family | | uniclust | UniRef100\_A0A0N8P2U1 | 99.7 | 1.4e-20 | 2.5e-26 | 147.5 | 179 | (1, 186) | 311 | (59, 238) | 267 | Uncharacterized protein (Fragment) | Uncharacterized protein (Fragment) | | uniclust | UniRef100\_A0A3M1GYX0 | 99.7 | 1.6e-20 | 2.9e-26 | 145.4 | 265 | (3, 282) | 311 | (60, 356) | 389 | Phage late control D family protein (Fragment) | Phage late control D family protein (Fragment) | | uniclust | UniRef100\_UPI000B4AB36B | 99.7 | 1.7e-20 | 3.1e-26 | 153.2 | 286 | (4, 305) | 311 | (125, 489) | 625 | type VI secretion system tip protein TssI/VgrG | type VI secretion system tip protein TssI/VgrG | | uniclust | UniRef100\_UPI0005AAC8F8 | 99.7 | 1.8e-20 | 3.3e-26 | 150.1 | 256 | (18, 287) | 311 | (52, 334) | 432 | type VI secretion system tip protein VgrG | type VI secretion system tip protein VgrG | | uniclust | UniRef100\_A0A109PY16 | 99.7 | 1.9e-20 | 3.4e-26 | 155.4 | 254 | (4, 277) | 311 | (94, 379) | 493 | Gp5/Type VI secretion system Vgr protein OB-fold domain-containing protein | Gp5/Type VI secretion system Vgr protein OB-fold domain-containing protein | | uniclust | UniRef100\_UPI000AEA10C1 | 99.7 | 2e-20 | 3.6e-26 | 137.5 | 209 | (53, 268) | 311 | (6, 240) | 255 | type VI secretion system tip protein TssI/VgrG | type VI secretion system tip protein TssI/VgrG | | uniclust | UniRef100\_UPI0009F9D285 | 99.7 | 2e-20 | 3.7e-26 | 163.8 | 276 | (17, 305) | 311 | (67, 377) | 1451 | type VI secretion system tip protein TssI/VgrG | type VI secretion system tip protein TssI/VgrG | | uniclust | UniRef100\_A0A379AXR7 | 99.7 | 2.1e-20 | 3.8e-26 | 158.7 | 272 | (17, 305) | 311 | (80, 397) | 723 | Uncharacterized protein conserved in bacteria | Uncharacterized protein conserved in bacteria | | uniclust | UniRef100\_A0A840P1G0 | 99.7 | 2.1e-20 | 3.8e-26 | 155.9 | 265 | (1, 280) | 311 | (499, 769) | 775 | Phage protein D | Phage protein D | | uniclust | UniRef100\_A0A451AF14 | 99.7 | 2.1e-20 | 3.8e-26 | 137.1 | 202 | (22, 230) | 311 | (2, 208) | 211 | Phage late control gene D protein (GPD) | Phage late control gene D protein (GPD) | | uniclust | UniRef100\_A0A930IWN4 | 99.7 | 2.1e-20 | 3.9e-26 | 148.4 | 277 | (5, 302) | 311 | (60, 369) | 398 | Uncharacterized protein | Uncharacterized protein | | uniclust | UniRef100\_A0A4U9VH05 | 99.7 | 2.2e-20 | 4e-26 | 153.1 | 277 | (17, 310) | 311 | (69, 378) | 473 | Uncharacterized protein conserved in bacteria | Uncharacterized protein conserved in bacteria | | uniclust | UniRef100\_A0A0J5KJT2 | 99.7 | 2.3e-20 | 4.3e-26 | 159.9 | 235 | (65, 305) | 311 | (1, 269) | 787 | Type IV secretion protein Rhs (Fragment) | Type IV secretion protein Rhs (Fragment) | | uniclust | UniRef100\_UPI0003754FB8 | 99.7 | 2.4e-20 | 4.4e-26 | 147.5 | 239 | (5, 265) | 311 | (62, 330) | 335 | contractile injection system protein, VgrG/Pvc8 family | contractile injection system protein, VgrG/Pvc8 family | | uniclust | UniRef100\_A0A0U1PQF4 | 99.7 | 2.5e-20 | 4.5e-26 | 166.7 | 249 | (18, 281) | 311 | (93, 379) | 930 | Type IV secretion protein Rhs (Fragment) | Type IV secretion protein Rhs (Fragment) | | uniclust | UniRef100\_A0A4Q1SII8 | 99.7 | 2.5e-20 | 4.6e-26 | 160.0 | 261 | (4, 285) | 311 | (101, 390) | 596 | Type VI secretion system tip protein VgrG | Type VI secretion system tip protein VgrG | | uniclust | UniRef100\_A0A7K0IVA3 | 99.7 | 2.5e-20 | 4.7e-26 | 155.9 | 282 | (7, 305) | 311 | (80, 400) | 804 | Type VI secretion system tip protein VgrG | Type VI secretion system tip protein VgrG | | uniclust | UniRef100\_A0A0Q9QUG3 | 99.7 | 2.6e-20 | 4.7e-26 | 152.1 | 286 | (2, 304) | 311 | (86, 428) | 474 | Uncharacterized protein | Uncharacterized protein | | uniclust | UniRef100\_A0A916CQA2 | 99.7 | 2.6e-20 | 4.8e-26 | 159.5 | 273 | (17, 304) | 311 | (81, 393) | 949 | Actin cross-linking toxin VgrG1 | Actin cross-linking toxin VgrG1 | | uniclust | UniRef100\_A0A3B0Z0G3 | 99.7 | 2.7e-20 | 4.9e-26 | 166.6 | 266 | (4, 284) | 311 | (64, 376) | 913 | VgrG protein | VgrG protein | | uniclust | UniRef100\_A0A1Q7URD9 | 99.7 | 2.8e-20 | 5.1e-26 | 142.1 | 257 | (6, 277) | 311 | (58, 340) | 350 | Phage late control D family protein (Fragment) | Phage late control D family protein (Fragment) | | uniclust | UniRef100\_A0A956DL55 | 99.7 | 3.2e-20 | 5.8e-26 | 143.1 | 245 | (57, 305) | 311 | (104, 370) | 374 | Type VI secretion system tip protein VgrG (Fragment) | Type VI secretion system tip protein VgrG (Fragment) | | uniclust | UniRef100\_A0A376W2Q8 | 99.7 | 3.2e-20 | 5.9e-26 | 141.2 | 228 | (1, 235) | 311 | (13, 271) | 273 | Late control gene D protein from prophage | Late control gene D protein from prophage | | uniclust | UniRef100\_UPI00124E8932 | 99.7 | 3.3e-20 | 6.1e-26 | 161.6 | 288 | (5, 304) | 311 | (209, 542) | 999 | type VI secretion system tip protein TssI/VgrG | type VI secretion system tip protein TssI/VgrG | | uniclust | UniRef100\_UPI0006494E4F | 99.7 | 3.3e-20 | 6.1e-26 | 137.1 | 229 | (4, 243) | 311 | (3, 258) | 259 | type VI secretion system tip protein VgrG | type VI secretion system tip protein VgrG | | uniclust | UniRef100\_A0A1E5AAS2 | 99.7 | 3.5e-20 | 6.5e-26 | 152.9 | 161 | (4, 184) | 311 | (95, 263) | 332 | Uncharacterized protein | Uncharacterized protein | | uniclust | UniRef100\_A0A285CU56 | 99.7 | 3.7e-20 | 6.7e-26 | 155.9 | 219 | (80, 308) | 311 | (2, 240) | 456 | Gp5/Type VI secretion system Vgr protein OB-fold domain-containing protein | Gp5/Type VI secretion system Vgr protein OB-fold domain-containing protein | | uniclust | UniRef100\_E2CFJ7 | 99.7 | 4.2e-20 | 7.8e-26 | 143.6 | 272 | (2, 281) | 311 | (51, 331) | 335 | Phage late control D protein (GPD) | Phage late control D protein (GPD) | | uniclust | UniRef100\_A0A8S5UBA3 | 99.7 | 4.4e-20 | 8.1e-26 | 151.9 | 262 | (2, 277) | 311 | (61, 344) | 668 | Tail protein | Tail protein | | uniclust | UniRef100\_A0A9D8CM58 | 99.7 | 4.4e-20 | 8.1e-26 | 160.7 | 289 | (5, 304) | 311 | (704, 1015) | 1314 | Type VI secretion system tip protein VgrG | Type VI secretion system tip protein VgrG | | uniclust | UniRef100\_A0A257DQE2 | 99.7 | 4.4e-20 | 8.1e-26 | 157.8 | 278 | (17, 308) | 311 | (78, 395) | 710 | Type VI secretion protein VgrG (Fragment) | Type VI secretion protein VgrG (Fragment) | | uniclust | UniRef100\_A0A2S6N506 | 99.7 | 4.5e-20 | 8.2e-26 | 142.6 | 243 | (6, 265) | 311 | (58, 329) | 331 | Gp5/Type VI secretion system Vgr protein OB-fold domain-containing protein | Gp5/Type VI secretion system Vgr protein OB-fold domain-containing protein | | uniclust | UniRef100\_UPI00207C1DAF | 99.7 | 4.6e-20 | 8.5e-26 | 159.5 | 285 | (7, 308) | 311 | (58, 388) | 1198 | type VI secretion system tip protein TssI/VgrG | type VI secretion system tip protein TssI/VgrG | | uniclust | UniRef100\_A0A1I2UCB8 | 99.7 | 4.7e-20 | 8.6e-26 | 162.9 | 291 | (4, 306) | 311 | (70, 407) | 1076 | Rhs element Vgr protein | Rhs element Vgr protein | | uniclust | UniRef100\_A0A010SJZ7 | 99.7 | 4.9e-20 | 8.9e-26 | 160.0 | 220 | (18, 303) | 311 | (107, 346) | 548 | Type VI secretion protein | Type VI secretion protein | | uniclust | UniRef100\_A0A1V4Z8I8 | 99.7 | 4.9e-20 | 9e-26 | 158.7 | 251 | (3, 277) | 311 | (54, 323) | 707 | Chromosome segregation protein | Chromosome segregation protein | | uniclust | UniRef100\_A0A1G6Z7R3 | 99.7 | 4.9e-20 | 9.1e-26 | 162.1 | 274 | (3, 301) | 311 | (61, 361) | 627 | Uncharacterized conserved protein, implicated in type VI secretion and phage assembly | Uncharacterized conserved protein, implicated in type VI secretion and phage assembly | | uniclust | UniRef100\_A0A0K2RMD0 | 99.7 | 5.4e-20 | 9.8e-26 | 142.0 | 247 | (17, 280) | 311 | (8, 274) | 318 | Rhs element Vgr protein | Rhs element Vgr protein | | uniclust | UniRef100\_A0A850B9U5 | 99.7 | 5.7e-20 | 1.1e-25 | 161.0 | 284 | (5, 303) | 311 | (70, 408) | 1439 | Type VI secretion system tip protein VgrG | Type VI secretion system tip protein VgrG | | uniclust | UniRef100\_A0A0B4BF20 | 99.7 | 5.9e-20 | 1.1e-25 | 149.1 | 275 | (17, 304) | 311 | (83, 397) | 484 | Gp5/Type VI secretion system Vgr protein OB-fold domain-containing protein (Fragment) | Gp5/Type VI secretion system Vgr protein OB-fold domain-containing protein (Fragment) | | uniclust | UniRef100\_A0A3Q9JH54 | 99.7 | 6.1e-20 | 1.1e-25 | 157.3 | 245 | (56, 307) | 311 | (102, 410) | 912 | Type VI secretion system tip protein VgrG | Type VI secretion system tip protein VgrG | | uniclust | UniRef100\_A0A1H4XF09 | 99.7 | 6.3e-20 | 1.2e-25 | 155.2 | 252 | (37, 304) | 311 | (293, 588) | 900 | Type VI secretion system secreted protein VgrG | Type VI secretion system secreted protein VgrG | | uniclust | UniRef100\_A0A542MBF0 | 99.7 | 6.3e-20 | 1.2e-25 | 160.9 | 290 | (3, 304) | 311 | (106, 486) | 908 | Rhs element Vgr protein | Rhs element Vgr protein | | uniclust | UniRef100\_A0A518B2T4 | 99.7 | 6.3e-20 | 1.2e-25 | 153.8 | 272 | (17, 305) | 311 | (72, 409) | 812 | Phage-related baseplate assembly protein | Phage-related baseplate assembly protein | | uniclust | UniRef100\_A0A074LLZ7 | 99.7 | 6.4e-20 | 1.2e-25 | 149.0 | 263 | (7, 279) | 311 | (80, 375) | 385 | Uncharacterized protein | Uncharacterized protein | | uniclust | UniRef100\_A0A1W2F9M8 | 99.7 | 6.5e-20 | 1.2e-25 | 147.1 | 250 | (17, 281) | 311 | (90, 382) | 389 | Rhs element Vgr protein (Fragment) | Rhs element Vgr protein (Fragment) | | uniclust | UniRef100\_A0A023WVR6 | 99.7 | 6.5e-20 | 1.2e-25 | 156.0 | 265 | (3, 282) | 311 | (78, 363) | 435 | Baseplate protein | Baseplate protein | | uniclust | UniRef100\_A0A518B4Q1 | 99.7 | 6.5e-20 | 1.2e-25 | 156.4 | 282 | (6, 304) | 311 | (59, 413) | 995 | Phage-related baseplate assembly protein | Phage-related baseplate assembly protein | | uniclust | UniRef100\_A0A4P9VTF7 | 99.7 | 7e-20 | 1.3e-25 | 162.6 | 276 | (17, 306) | 311 | (69, 386) | 1114 | Type VI secretion system tip protein VgrG | Type VI secretion system tip protein VgrG | | uniclust | UniRef100\_A0A1Q9WIR8 | 99.7 | 7.1e-20 | 1.3e-25 | 140.8 | 248 | (53, 305) | 311 | (12, 298) | 365 | Type IV secretion protein Rhs (Fragment) | Type IV secretion protein Rhs (Fragment) | | uniclust | UniRef100\_A0A2J4Y4X1 | 99.7 | 7.3e-20 | 1.3e-25 | 153.6 | 248 | (17, 279) | 311 | (91, 375) | 515 | Type VI secretion system tip protein VgrG (Fragment) | Type VI secretion system tip protein VgrG (Fragment) | | uniclust | UniRef100\_A0A084ZJJ8 | 99.7 | 7.7e-20 | 1.4e-25 | 147.1 | 274 | (8, 310) | 311 | (115, 429) | 430 | VgrG family protein | VgrG family protein | | uniclust | UniRef100\_A0A150Q280 | 99.7 | 7.9e-20 | 1.5e-25 | 149.9 | 259 | (2, 282) | 311 | (53, 321) | 329 | Uncharacterized protein | Uncharacterized protein | | uniclust | UniRef100\_A0A1V3NAA1 | 99.7 | 8.3e-20 | 1.5e-25 | 154.3 | 255 | (17, 286) | 311 | (67, 358) | 641 | Gp5/Type VI secretion system Vgr protein OB-fold domain-containing protein (Fragment) | Gp5/Type VI secretion system Vgr protein OB-fold domain-containing protein (Fragment) | | uniclust | UniRef100\_A0A4P9VIU8 | 99.7 | 8.4e-20 | 1.5e-25 | 161.4 | 285 | (7, 307) | 311 | (58, 383) | 1432 | Type VI secretion system tip protein VgrG | Type VI secretion system tip protein VgrG | | uniclust | UniRef100\_A0A8J4ZPK6 | 99.7 | 8.9e-20 | 1.6e-25 | 164.3 | 259 | (38, 308) | 311 | (1038, 1334) | 2288 | Protein ClpV1 | Protein ClpV1 | | uniclust | UniRef100\_A0A084DHQ4 | 99.7 | 9.3e-20 | 1.7e-25 | 163.5 | 287 | (4, 305) | 311 | (116, 500) | 978 | Type IV secretion protein Rhs | Type IV secretion protein Rhs | | uniclust | UniRef100\_A0A3M2UZC3 | 99.7 | 9.5e-20 | 1.7e-25 | 144.4 | 226 | (3, 241) | 311 | (87, 340) | 341 | Type VI secretion system Vgr protein | Type VI secretion system Vgr protein | | uniclust | UniRef100\_A0A2T3WF36 | 99.7 | 1e-19 | 1.8e-25 | 152.1 | 246 | (3, 264) | 311 | (73, 358) | 466 | Type VI secretion system tip protein VgrG (Fragment) | Type VI secretion system tip protein VgrG (Fragment) | | uniclust | UniRef100\_A0A1T2CG57 | 99.7 | 1e-19 | 1.9e-25 | 132.8 | 165 | (109, 279) | 311 | (2, 166) | 173 | Phage late control D family protein (Fragment) | Phage late control D family protein (Fragment) | | uniclust | UniRef100\_UPI001FE95B91 | 99.7 | 1e-19 | 1.9e-25 | 153.5 | 289 | (4, 304) | 311 | (54, 382) | 675 | type VI secretion system tip protein TssI/VgrG | type VI secretion system tip protein TssI/VgrG | | uniclust | UniRef100\_A0A518AY46 | 99.7 | 1.1e-19 | 1.9e-25 | 157.9 | 286 | (4, 305) | 311 | (57, 413) | 1249 | Filamentous hemagglutinin | Filamentous hemagglutinin | | uniclust | UniRef100\_A0A1V2GC00 | 99.7 | 1.2e-19 | 2.1e-25 | 143.7 | 271 | (1, 278) | 311 | (62, 350) | 367 | Late control protein | Late control protein | | uniclust | UniRef100\_A0A0S4UVJ0 | 99.7 | 1.2e-19 | 2.2e-25 | 158.4 | 285 | (4, 306) | 311 | (107, 476) | 822 | Gp5/Type VI secretion system Vgr protein OB-fold domain-containing protein (Fragment) | Gp5/Type VI secretion system Vgr protein OB-fold domain-containing protein (Fragment) | | uniclust | UniRef100\_A0A2M8B170 | 99.7 | 1.3e-19 | 2.3e-25 | 134.0 | 126 | (4, 138) | 311 | (60, 196) | 202 | Type VI secretion system tip protein VgrG (Fragment) | Type VI secretion system tip protein VgrG (Fragment) | | uniclust | UniRef100\_A0A812RFT8 | 99.7 | 1.3e-19 | 2.3e-25 | 161.7 | 268 | (4, 278) | 311 | (1045, 1314) | 1893 | site-specific DNA-methyltransferase (adenine-specific) | site-specific DNA-methyltransferase (adenine-specific) | | uniclust | UniRef100\_A0A1G9SJN9 | 99.7 | 1.3e-19 | 2.4e-25 | 167.0 | 275 | (17, 308) | 311 | (68, 399) | 1956 | Rhs element Vgr protein | Rhs element Vgr protein | | uniclust | UniRef100\_A0A1B3JDP2 | 99.7 | 1.3e-19 | 2.5e-25 | 166.8 | 274 | (17, 307) | 311 | (199, 515) | 2127 | Uncharacterized protein | Uncharacterized protein | | uniclust | UniRef100\_A0A1M5NRK7 | 99.7 | 1.4e-19 | 2.5e-25 | 147.0 | 257 | (16, 290) | 311 | (73, 348) | 365 | Phage protein D | Phage protein D | | uniclust | UniRef100\_UPI0013A681D6 | 99.7 | 1.4e-19 | 2.5e-25 | 150.9 | 273 | (17, 307) | 311 | (198, 509) | 650 | type VI secretion system tip protein TssI/VgrG | type VI secretion system tip protein TssI/VgrG | | uniclust | UniRef100\_A0A1Q7JQQ3 | 99.7 | 1.4e-19 | 2.5e-25 | 163.8 | 264 | (2, 281) | 311 | (61, 350) | 1718 | Methyl-accepting transducer domain-containing protein | Methyl-accepting transducer domain-containing protein | | uniclust | UniRef100\_A0A0A1YX04 | 99.7 | 1.4e-19 | 2.6e-25 | 154.3 | 247 | (17, 278) | 311 | (105, 393) | 579 | Type IV secretion protein Rhs (Fragment) | Type IV secretion protein Rhs (Fragment) | | uniclust | UniRef100\_A0A8J4ZXS2 | 99.7 | 1.5e-19 | 2.7e-25 | 165.0 | 259 | (38, 308) | 311 | (1011, 1307) | 3044 | Protein ClpV1 | Protein ClpV1 | | uniclust | UniRef100\_A0A3N7E1V0 | 99.7 | 1.6e-19 | 2.9e-25 | 145.4 | 235 | (17, 263) | 311 | (101, 383) | 415 | Type VI secretion system tip protein VgrG (Fragment) | Type VI secretion system tip protein VgrG (Fragment) | | uniclust | UniRef100\_A0A853I7P2 | 99.7 | 1.7e-19 | 3e-25 | 137.5 | 187 | (1, 196) | 311 | (55, 241) | 248 | Uncharacterized protein (Fragment) | Uncharacterized protein (Fragment) | | uniclust | UniRef100\_A0A017RU14 | 99.7 | 1.7e-19 | 3e-25 | 154.0 | 252 | (3, 277) | 311 | (85, 354) | 396 | Uncharacterized protein | Uncharacterized protein | | uniclust | UniRef100\_A0A0D6BIP0 | 99.7 | 1.8e-19 | 3.2e-25 | 150.4 | 219 | (55, 304) | 311 | (114, 369) | 539 | ImpA family type VI secretion-associated protein | ImpA family type VI secretion-associated protein | | uniclust | UniRef100\_A0A285JXF0 | 99.7 | 1.8e-19 | 3.3e-25 | 146.3 | 262 | (9, 277) | 311 | (176, 481) | 494 | Gp5/Type VI secretion system Vgr protein OB-fold domain-containing protein | Gp5/Type VI secretion system Vgr protein OB-fold domain-containing protein | | uniclust | UniRef100\_UPI000A3B321B | 99.7 | 1.8e-19 | 3.4e-25 | 150.1 | 233 | (68, 305) | 311 | (1, 267) | 751 | type VI secretion system tip protein TssI/VgrG | type VI secretion system tip protein TssI/VgrG | | pdb70 | 4MTK\_A | 99.9 | 8.3e-29 | 6.2e-33 | 229.3 | 263 | (3, 278) | 311 | (51, 350) | 643 | VgrG1 | 4MTK\_A VgrG1 Beta-barrel, OB-fold, Beta-helix, Type VI HET: SO4, TAM | | pdb70 | 4MTK\_B | 99.9 | 8.3e-29 | 6.2e-33 | 229.3 | 263 | (3, 278) | 311 | (51, 350) | 643 | VgrG1 | 4MTK\_B VgrG1 Beta-barrel, OB-fold, Beta-helix, Type VI HET: TAM, SO4 | | pdb70 | 4MTK\_C | 99.9 | 8.3e-29 | 6.2e-33 | 229.3 | 263 | (3, 278) | 311 | (51, 350) | 643 | VgrG1 | 4MTK\_C VgrG1 Beta-barrel, OB-fold, Beta-helix, Type VI HET: TAM, SO4 | | pdb70 | 4MTK\_D | 99.9 | 8.3e-29 | 6.2e-33 | 229.3 | 263 | (3, 278) | 311 | (51, 350) | 643 | VgrG1 | 4MTK\_D VgrG1 Beta-barrel, OB-fold, Beta-helix, Type VI HET: SO4, TAM | | pdb70 | 4MTK\_E | 99.9 | 8.3e-29 | 6.2e-33 | 229.3 | 263 | (3, 278) | 311 | (51, 350) | 643 | VgrG1 | 4MTK\_E VgrG1 Beta-barrel, OB-fold, Beta-helix, Type VI HET: SO4, TAM | | pdb70 | 4MTK\_F | 99.9 | 8.3e-29 | 6.2e-33 | 229.3 | 263 | (3, 278) | 311 | (51, 350) | 643 | VgrG1 | 4MTK\_F VgrG1 Beta-barrel, OB-fold, Beta-helix, Type VI HET: SO4, TAM | | pdb70 | 4UHV\_A | 99.9 | 1e-28 | 7.8e-33 | 229.0 | 263 | (3, 278) | 311 | (51, 350) | 651 | VGRG1, VALINE-GLYCINE REPEAT PROTEIN G1 | 4UHV\_A VGRG1, VALINE-GLYCINE REPEAT PROTEIN G1 STRUCTURAL PROTEIN, VGRG1, VIRULENCE, TOXIN HET: NA, MSE, CL | | pdb70 | 4UHV\_B | 99.9 | 1e-28 | 7.8e-33 | 229.0 | 263 | (3, 278) | 311 | (51, 350) | 651 | VGRG1, VALINE-GLYCINE REPEAT PROTEIN G1 | 4UHV\_B VGRG1, VALINE-GLYCINE REPEAT PROTEIN G1 STRUCTURAL PROTEIN, VGRG1, VIRULENCE, TOXIN HET: CL, NA, MSE | | pdb70 | 2P5Z\_X | 99.8 | 1.3e-25 | 9.6e-30 | 201.6 | 261 | (3, 278) | 311 | (61, 368) | 491 | Type VI secretion system component | 2P5Z\_X Type VI secretion system component structural genomics, UNKNOWN FUNCTION, PSI-2 | | pdb70 | 6RBK\_C | 99.8 | 1.9e-25 | 1.4e-29 | 202.2 | 256 | (4, 277) | 311 | (59, 343) | 529 | Afp1, Afp2, Afp3, Afp16 | 6RBK\_C Afp1, Afp2, Afp3, Afp16 Anti-feeding prophage, secretion system, AFP | | pdb70 | 6J0M\_C | 99.8 | 2.1e-25 | 1.6e-29 | 202.3 | 259 | (3, 274) | 311 | (61, 348) | 538 | Pvc8 | 6J0M\_C Pvc8 assembly, Photorhabdus asymbiotica, PVC, contractile | | pdb70 | 1WRU\_A | 99.7 | 4.4e-23 | 3.3e-27 | 178.9 | 257 | (2, 279) | 311 | (48, 338) | 379 | 43 kDa tail protein | 1WRU\_A 43 kDa tail protein BACTERIOPHAGE MU, BASEPLATE, GENE PRODUCT | | pdb70 | 3D37\_A | 99.7 | 2.3e-22 | 1.7e-26 | 174.4 | 259 | (2, 279) | 311 | (53, 340) | 381 | Tail protein, 43 kDa | 3D37\_A Tail protein, 43 kDa tail protein, structural genomics, PSI | | pdb70 | 3D37\_B | 99.7 | 2.3e-22 | 1.7e-26 | 174.4 | 259 | (2, 279) | 311 | (53, 340) | 381 | Tail protein, 43 kDa | 3D37\_B Tail protein, 43 kDa tail protein, structural genomics, PSI | | pdb70 | 3CDD\_F | 99.7 | 1.4e-21 | 1e-25 | 168.0 | 259 | (3, 279) | 311 | (58, 343) | 361 | Prophage MuSo2, 43 kDa tail | 3CDD\_F Prophage MuSo2, 43 kDa tail MuSo2, Shewanella oneidensis MR-1, Structural HET: MSE | | pdb70 | 3CDD\_D | 99.7 | 1.4e-21 | 1e-25 | 168.0 | 259 | (3, 279) | 311 | (58, 343) | 361 | Prophage MuSo2, 43 kDa tail | 3CDD\_D Prophage MuSo2, 43 kDa tail MuSo2, Shewanella oneidensis MR-1, Structural | |
| Top keywords  (threshold 1.00e-03 (evalue)) | **Type, secretion, VI, system, VgrG, Vgr, Fragment, Rhs, Phage, tip** |
| Output files | ../../similar\_sequences/25\_FANPEZAQ\_CDS\_0025\_merged.svg ../../similar\_sequences/25\_FANPEZAQ\_CDS\_0025\_pdb70.a3m ../../similar\_sequences/25\_FANPEZAQ\_CDS\_0025\_pdb70.hhr ../../similar\_sequences/25\_FANPEZAQ\_CDS\_0025\_uniclust.a3m ../../similar\_sequences/25\_FANPEZAQ\_CDS\_0025\_uniclust.hhr |

#### Structure prediction (AlphaFold)2

|  |  |
| --- | --- |
| Stats | xml version="1.0" encoding="utf-8" standalone="no"?       2024-09-02T21:09:25.673745 image/svg+xml   Matplotlib v3.7.2, https://matplotlib.org/ |
| Predicted structure | **NGL Viewer Controls:**  - Center: *Left-Click* - Rotate: *Left-Click + Drag* - Translate: *Right-Click + Drag* - Zoom: *Shift + Left-Click + Drag* |
| Output files | ../../predicted\_structures/25\_FANPEZAQ\_CDS\_0025/features.pkl ../../predicted\_structures/25\_FANPEZAQ\_CDS\_0025/ranked\_0.pdb ../../predicted\_structures/25\_FANPEZAQ\_CDS\_0025/ranked\_0\_plots.svg ../../predicted\_structures/25\_FANPEZAQ\_CDS\_0025/result\_model\_1\_ptm\_pred\_0.pkl |

#### Structure similarity search results (Foldseek)3

|  |  |
| --- | --- |
| Structure databases searched | Pdb, Afdb-proteome, Afdb-uniprot50 |
| Results, scheme(s)  (Top layers only, threshold 1.00e-02 (evalue)) | xml version="1.0" encoding="utf-8" standalone="no"?       2024-09-02T21:10:56.958834 image/svg+xml   Matplotlib v3.7.2, https://matplotlib.org/ |
| Results, table  (threshold 1.00e-02 (evalue)) | | db | id | prob | evalue | bits | fident | alnlen | mismatch | gapopen | qstart | qend | tstart | tend | name | description | | --- | --- | --- | --- | --- | --- | --- | --- | --- | --- | --- | --- | --- | --- | --- | | pdb | 7B5H\_AJ | 1.0 | 2.032e-14 | 435 | 0.169 | 307 | 208 | 12 | 7 | 284 | 70 | 358 | All3320 protein | All3320 protein | | pdb | 7AEF\_Q | 1.0 | 2.392e-13 | 408 | 0.164 | 297 | 198 | 12 | 7 | 275 | 74 | 348 | Phosphoserine phosphatase SerB | Phosphoserine phosphatase SerB | | pdb | 6U5H\_C | 1.0 | 2.866e-14 | 395 | 0.261 | 298 | 164 | 17 | 1 | 275 | 51 | 315 | Probable bacteriophage protein Pyocin R2 | Probable bacteriophage protein Pyocin R2 | | pdb | 6J0M\_A | 1.0 | 1.239e-10 | 298 | 0.129 | 308 | 214 | 17 | 7 | 279 | 64 | 352 | Pvc8 | Pvc8 | | pdb | 3CDD\_B | 1.0 | 3.129e-11 | 287 | 0.146 | 280 | 193 | 17 | 19 | 277 | 66 | 320 | Prophage MuSo2, 43 kDa tail protein | Prophage MuSo2, 43 kDa tail protein | | pdb | 3CDD\_C | 1.0 | 2.79e-11 | 283 | 0.134 | 313 | 207 | 17 | 7 | 277 | 34 | 324 | Prophage MuSo2, 43 kDa tail protein | Prophage MuSo2, 43 kDa tail protein | | pdb | 3CDD\_D | 1.0 | 4.949e-11 | 269 | 0.141 | 317 | 204 | 20 | 7 | 277 | 33 | 327 | Prophage MuSo2, 43 kDa tail protein | Prophage MuSo2, 43 kDa tail protein | | pdb | 3D37\_A | 1.0 | 4.631e-10 | 267 | 0.134 | 283 | 200 | 17 | 19 | 276 | 54 | 316 | Tail protein, 43 kDa | Tail protein, 43 kDa | | pdb | 3CDD\_A | 1.0 | 1.747e-10 | 266 | 0.167 | 281 | 184 | 17 | 19 | 277 | 67 | 319 | Prophage MuSo2, 43 kDa tail protein | Prophage MuSo2, 43 kDa tail protein | | pdb | 3CDD\_F | 1.0 | 6.593e-11 | 258 | 0.146 | 313 | 200 | 18 | 7 | 277 | 32 | 319 | Prophage MuSo2, 43 kDa tail protein | Prophage MuSo2, 43 kDa tail protein | | pdb | 3CDD\_E | 1.0 | 6.169e-10 | 256 | 0.148 | 283 | 184 | 18 | 19 | 277 | 69 | 318 | Prophage MuSo2, 43 kDa tail protein | Prophage MuSo2, 43 kDa tail protein | | pdb | 3D37\_B | 1.0 | 3.864e-09 | 245 | 0.125 | 287 | 190 | 17 | 19 | 276 | 52 | 306 | Tail protein, 43 kDa | Tail protein, 43 kDa | | pdb | 8GRA\_G | 1.0 | 6.919e-10 | 242 | 0.116 | 317 | 187 | 24 | 17 | 279 | 75 | 352 | Type VI secretion system spike protein VgrG | Type VI secretion system spike protein VgrG | | pdb | 7Q5P\_B | 1.0 | 4.092e-09 | 239 | 0.1 | 320 | 216 | 24 | 5 | 278 | 49 | 342 | Type VI secretion protein VgrG | Type VI secretion protein VgrG | | pdb | 6RBK\_C | 1.0 | 3.415e-08 | 223 | 0.125 | 302 | 203 | 17 | 7 | 282 | 58 | 324 | Afp8 | Afp8 | | pdb | 6H3N\_B | 1.0 | 9.05e-08 | 218 | 0.117 | 314 | 210 | 24 | 5 | 277 | 49 | 336 | VgrG1 | VgrG1 | | pdb | 7YFZ\_V | 1.0 | 1.503e-06 | 185 | 0.093 | 289 | 167 | 17 | 8 | 276 | 2 | 215 | Pam3 hub gp19 | Pam3 hub gp19 | | pdb | 5IV5\_YD | 1.0 | 6.733e-07 | 177 | 0.096 | 322 | 224 | 20 | 7 | 289 | 68 | 361 | Baseplate hub protein gp27 | Baseplate hub protein gp27 | | pdb | 2Z6B\_D | 1.0 | 1.195e-06 | 168 | 0.09 | 309 | 206 | 19 | 16 | 281 | 78 | 354 | Baseplate structural protein Gp27 | Baseplate structural protein Gp27 | | pdb | 3GS9\_A | 1.0 | 1.065e-06 | 165 | 0.095 | 304 | 207 | 19 | 7 | 275 | 37 | 307 | Protein gp18 | Protein gp18 | | pdb | 1WTH\_D | 1.0 | 5.618e-06 | 157 | 0.089 | 313 | 215 | 17 | 16 | 291 | 78 | 357 | Baseplate structural protein Gp27 | Baseplate structural protein Gp27 | | pdb | 4UHV\_A | 1.0 | 1.118e-05 | 149 | 0.103 | 328 | 216 | 24 | 4 | 281 | 52 | 351 | VGRG1, VALINE-GLYCINE REPEAT PROTEIN G1 | VGRG1, VALINE-GLYCINE REPEAT PROTEIN G1 | | pdb | 7ZN2\_C | 1.0 | 7.483e-06 | 144 | 0.126 | 229 | 138 | 18 | 89 | 281 | 519 | 721 | Tail tube protein | Tail tube protein | | pdb | 8EON\_L | 1.0 | 7.853e-05 | 140 | 0.124 | 290 | 157 | 17 | 7 | 279 | 78 | 287 | Baseplate hub gp41 | Baseplate hub gp41 | | pdb | 6V8I\_BE | 1.0 | 0.000521 | 109 | 0.095 | 304 | 198 | 21 | 22 | 280 | 63 | 334 | Tail-Associated Lysin, gp59 | Tail-Associated Lysin, gp59 | | pdb | 2P5Z\_X | 1.0 | 0.0002334 | 105 | 0.112 | 302 | 168 | 24 | 9 | 280 | 49 | 280 | Type VI secretion system component | Type VI secretion system component | | pdb | 4V5I\_A0 | 0.999 | 1.769e-05 | 99 | 0.063 | 332 | 219 | 26 | 8 | 281 | 75 | 372 | ORF16 | ORF16 | | afdb-proteome | AF-G3XCU8-F1-MODEL\_V4 | 1.0 | 9.522e-25 | 896 | 0.29 | 282 | 183 | 5 | 1 | 275 | 51 | 322 | Uncharacterized protein | Uncharacterized protein | | afdb-proteome | AF-Q8ZMV5-F1-MODEL\_V4 | 1.0 | 1.912e-25 | 875 | 0.291 | 309 | 184 | 8 | 1 | 281 | 63 | 364 | Fels-2 prophage protein | Fels-2 prophage protein | | afdb-proteome | AF-A0A0H3GX06-F1-MODEL\_V4 | 1.0 | 8.017e-25 | 869 | 0.301 | 332 | 180 | 8 | 1 | 288 | 63 | 386 | Putative prophage tail protein | Putative prophage tail protein | | afdb-proteome | AF-A0A0H3GM37-F1-MODEL\_V4 | 1.0 | 2.125e-24 | 868 | 0.289 | 307 | 182 | 9 | 1 | 279 | 63 | 361 | Late control gene D protein (GpD) | Late control gene D protein (GpD) | | afdb-proteome | AF-Q8ZKJ7-F1-MODEL\_V4 | 1.0 | 3.213e-19 | 652 | 0.214 | 289 | 200 | 9 | 2 | 277 | 68 | 342 | Putative cytoplasmic protein | Putative cytoplasmic protein | | afdb-proteome | AF-Q32HG2-F1-MODEL\_V4 | 1.0 | 1.923e-13 | 430 | 0.301 | 196 | 126 | 4 | 1 | 187 | 30 | 223 | Uncharacterized protein | Uncharacterized protein | | afdb-proteome | AF-Q9I1A6-F1-MODEL\_V4 | 1.0 | 3.517e-10 | 280 | 0.129 | 316 | 211 | 19 | 4 | 283 | 54 | 341 | Phage\_base\_V domain-containing protein | Phage\_base\_V domain-containing protein | | afdb-proteome | AF-Q8ZRL1-F1-MODEL\_V4 | 1.0 | 2.471e-09 | 256 | 0.124 | 313 | 211 | 24 | 7 | 277 | 159 | 450 | Putative cytoplasmic protein | Putative cytoplasmic protein | | afdb-proteome | AF-Q9I741-F1-MODEL\_V4 | 1.0 | 2.448e-08 | 240 | 0.112 | 321 | 213 | 21 | 5 | 277 | 53 | 349 | Type VI secretion system spike protein VgrG1a | Type VI secretion system spike protein VgrG1a | | afdb-proteome | AF-Q9I6M7-F1-MODEL\_V4 | 1.0 | 7.778e-09 | 240 | 0.135 | 325 | 196 | 22 | 17 | 283 | 62 | 359 | Type VI secretion system spike protein VgrG2b | Type VI secretion system spike protein VgrG2b | | afdb-proteome | AF-Q9I737-F1-MODEL\_V4 | 1.0 | 5.159e-08 | 229 | 0.118 | 321 | 211 | 21 | 5 | 277 | 56 | 352 | Type VI secretion system spike protein VgrG1b | Type VI secretion system spike protein VgrG1b | | afdb-proteome | AF-Q9I3K1-F1-MODEL\_V4 | 1.0 | 1.461e-08 | 222 | 0.129 | 316 | 192 | 21 | 23 | 283 | 72 | 359 | Type VI secretion system spike protein VgrG2a | Type VI secretion system spike protein VgrG2a | | afdb-proteome | AF-Q9HYC3-F1-MODEL\_V4 | 1.0 | 4.384e-09 | 210 | 0.152 | 342 | 196 | 24 | 7 | 276 | 39 | 358 | Type VI secretion system spike protein VgrG4b | Type VI secretion system spike protein VgrG4b | | afdb-proteome | AF-Q9I0F3-F1-MODEL\_V4 | 1.0 | 1.72e-07 | 208 | 0.131 | 319 | 209 | 25 | 5 | 277 | 93 | 389 | Type VI secretion system spike protein VgrG1c | Type VI secretion system spike protein VgrG1c | | afdb-proteome | AF-Q9HYU9-F1-MODEL\_V4 | 1.0 | 1.23e-08 | 202 | 0.159 | 338 | 198 | 24 | 7 | 276 | 39 | 358 | Phage\_base\_V domain-containing protein | Phage\_base\_V domain-containing protein | | afdb-proteome | AF-Q5F989-F1-MODEL\_V4 | 1.0 | 1.689e-05 | 161 | 0.131 | 213 | 145 | 16 | 95 | 276 | 215 | 418 | Uncharacterized protein | Uncharacterized protein | | afdb-proteome | AF-Q2FX67-F1-MODEL\_V4 | 1.0 | 5.682e-06 | 151 | 0.12 | 298 | 201 | 19 | 20 | 280 | 62 | 335 | Phi ETA orf 55-like protein | Phi ETA orf 55-like protein | | afdb-proteome | AF-Q2FYD0-F1-MODEL\_V4 | 1.0 | 0.0001121 | 121 | 0.076 | 434 | 205 | 24 | 22 | 280 | 68 | 480 | SLT orf 527-like protein | SLT orf 527-like protein | | afdb-uniprot50 | AF-Q3RCU8-F1-MODEL\_V4 | 1.0 | 3.926e-43 | 1669 | 0.438 | 303 | 163 | 2 | 1 | 297 | 1 | 302 | Phage late control D | Phage late control D | | afdb-uniprot50 | AF-Q87CI2-F1-MODEL\_V4 | 1.0 | 6.459e-41 | 1576 | 0.442 | 303 | 162 | 2 | 1 | 297 | 55 | 356 | Phage-related tail protein | Phage-related tail protein | | afdb-uniprot50 | AF-A0A2J0SR82-F1-MODEL\_V4 | 1.0 | 4.806e-40 | 1538 | 0.486 | 296 | 146 | 1 | 1 | 290 | 57 | 352 | Phage tail protein | Phage tail protein | | afdb-uniprot50 | AF-K5ZNX9-F1-MODEL\_V4 | 1.0 | 2.956e-37 | 1478 | 0.491 | 281 | 142 | 1 | 1 | 280 | 57 | 337 | Uncharacterized protein | Uncharacterized protein | | afdb-uniprot50 | AF-A0A158E854-F1-MODEL\_V4 | 1.0 | 5.882e-37 | 1378 | 0.513 | 292 | 136 | 1 | 2 | 287 | 58 | 349 | Late control D family protein | Late control D family protein | | afdb-uniprot50 | AF-A0A1H2ZGX1-F1-MODEL\_V4 | 1.0 | 5.724e-34 | 1337 | 0.431 | 276 | 157 | 0 | 1 | 276 | 58 | 333 | Uncharacterized protein | Uncharacterized protein | | afdb-uniprot50 | AF-A0A1N6R0G0-F1-MODEL\_V4 | 1.0 | 1.206e-33 | 1312 | 0.41 | 280 | 157 | 3 | 1 | 279 | 1 | 273 | Uncharacterized protein | Uncharacterized protein | | afdb-uniprot50 | AF-A0A2G2N728-F1-MODEL\_V4 | 1.0 | 5.673e-33 | 1297 | 0.417 | 278 | 162 | 0 | 1 | 278 | 55 | 332 | Phage tail protein | Phage tail protein | | afdb-uniprot50 | AF-A0A5J5GRJ3-F1-MODEL\_V4 | 1.0 | 5.357e-33 | 1293 | 0.415 | 255 | 149 | 0 | 22 | 276 | 2 | 256 | Late control protein D | Late control protein D | | afdb-uniprot50 | AF-A0A4Q7BES0-F1-MODEL\_V4 | 1.0 | 5.673e-33 | 1275 | 0.457 | 280 | 145 | 2 | 1 | 279 | 54 | 327 | Phage tail protein | Phage tail protein | | afdb-uniprot50 | AF-A0A2N1ANJ8-F1-MODEL\_V4 | 1.0 | 6.062e-34 | 1271 | 0.445 | 285 | 152 | 2 | 1 | 279 | 55 | 339 | Phage tail protein | Phage tail protein | | afdb-uniprot50 | AF-A0A0P8YPJ6-F1-MODEL\_V4 | 1.0 | 1.702e-33 | 1266 | 0.361 | 277 | 177 | 0 | 2 | 278 | 8 | 284 | Uncharacterized protein | Uncharacterized protein | | afdb-uniprot50 | AF-A0A7Z1WDF0-F1-MODEL\_V4 | 1.0 | 2.668e-32 | 1209 | 0.459 | 272 | 140 | 2 | 12 | 282 | 1 | 266 | Phage tail protein | Phage tail protein | | afdb-uniprot50 | AF-A0A149SNT1-F1-MODEL\_V4 | 1.0 | 1.129e-32 | 1203 | 0.366 | 322 | 189 | 5 | 1 | 311 | 55 | 372 | Uncharacterized protein | Uncharacterized protein | | afdb-uniprot50 | AF-A0A2Z6AZ21-F1-MODEL\_V4 | 1.0 | 2.644e-31 | 1200 | 0.436 | 277 | 153 | 3 | 1 | 276 | 56 | 330 | Late control D family protein | Late control D family protein | | afdb-uniprot50 | AF-A0A145VT91-F1-MODEL\_V4 | 1.0 | 9.974e-32 | 1187 | 0.43 | 279 | 159 | 0 | 1 | 279 | 55 | 333 | Uncharacterized protein | Uncharacterized protein | | afdb-uniprot50 | AF-A0A143DDT4-F1-MODEL\_V4 | 1.0 | 5.26e-31 | 1179 | 0.414 | 285 | 159 | 3 | 1 | 279 | 55 | 337 | Uncharacterized protein | Uncharacterized protein | | afdb-uniprot50 | AF-A0A844HSX7-F1-MODEL\_V4 | 1.0 | 4.469e-32 | 1172 | 0.388 | 286 | 168 | 1 | 1 | 279 | 58 | 343 | Late control protein D | Late control protein D | | afdb-uniprot50 | AF-A0A7S8C754-F1-MODEL\_V4 | 1.0 | 1.463e-29 | 1106 | 0.389 | 280 | 163 | 3 | 1 | 279 | 51 | 323 | Phage late control D family protein | Phage late control D family protein | | afdb-uniprot50 | AF-A0A1H9YCN2-F1-MODEL\_V4 | 1.0 | 1.305e-29 | 1091 | 0.377 | 278 | 172 | 1 | 1 | 278 | 55 | 331 | Uncharacterized protein | Uncharacterized protein | | afdb-uniprot50 | AF-A0A1Z5H6V1-F1-MODEL\_V4 | 1.0 | 6.881e-29 | 1088 | 0.397 | 279 | 160 | 3 | 1 | 278 | 51 | 322 | Uncharacterized protein | Uncharacterized protein | | afdb-uniprot50 | AF-A0A2G6CS06-F1-MODEL\_V4 | 1.0 | 7.717e-29 | 1082 | 0.376 | 279 | 165 | 5 | 1 | 277 | 52 | 323 | Uncharacterized protein | Uncharacterized protein | | afdb-uniprot50 | AF-A0A2W4TBH4-F1-MODEL\_V4 | 1.0 | 1.369e-28 | 1080 | 0.358 | 282 | 171 | 4 | 1 | 279 | 55 | 329 | Uncharacterized protein | Uncharacterized protein | | afdb-uniprot50 | AF-B7RNP1-F1-MODEL\_V4 | 1.0 | 1.738e-29 | 1080 | 0.377 | 278 | 173 | 0 | 1 | 278 | 58 | 335 | Phage late control D | Phage late control D | | afdb-uniprot50 | AF-A0A4R3LEZ2-F1-MODEL\_V4 | 1.0 | 1.949e-29 | 1065 | 0.361 | 296 | 165 | 6 | 1 | 281 | 55 | 341 | Uncharacterized protein | Uncharacterized protein | | afdb-uniprot50 | AF-A0A0J1K2H0-F1-MODEL\_V4 | 1.0 | 3.056e-28 | 1064 | 0.371 | 283 | 171 | 4 | 1 | 279 | 54 | 333 | Tail protein | Tail protein | | afdb-uniprot50 | AF-A8U2S0-F1-MODEL\_V4 | 1.0 | 1.382e-29 | 1063 | 0.323 | 284 | 176 | 5 | 1 | 276 | 85 | 360 | Late control gene D protein | Late control gene D protein | | afdb-uniprot50 | AF-A0A4V1IMX3-F1-MODEL\_V4 | 1.0 | 5.421e-28 | 1051 | 0.35 | 277 | 172 | 3 | 1 | 276 | 50 | 319 | Phage late control D family protein | Phage late control D family protein | | afdb-uniprot50 | AF-A0A7C8M556-F1-MODEL\_V4 | 1.0 | 4.878e-29 | 1044 | 0.409 | 281 | 161 | 3 | 1 | 279 | 53 | 330 | Uncharacterized protein | Uncharacterized protein | | afdb-uniprot50 | AF-A0A1X7JLX1-F1-MODEL\_V4 | 1.0 | 2.315e-29 | 1037 | 0.375 | 280 | 161 | 5 | 1 | 275 | 56 | 326 | Uncharacterized protein | Uncharacterized protein | | afdb-uniprot50 | AF-A0A167H3I8-F1-MODEL\_V4 | 1.0 | 6.819e-28 | 1032 | 0.361 | 282 | 170 | 3 | 1 | 279 | 51 | 325 | Uncharacterized protein | Uncharacterized protein | | afdb-uniprot50 | AF-B2I805-F1-MODEL\_V4 | 1.0 | 3.843e-28 | 1030 | 0.4 | 225 | 129 | 1 | 79 | 297 | 2 | 226 | Late control D family protein | Late control D family protein | | afdb-uniprot50 | AF-A0A2U1XZ03-F1-MODEL\_V4 | 1.0 | 1.437e-27 | 1023 | 0.373 | 284 | 168 | 5 | 1 | 281 | 55 | 331 | Uncharacterized protein | Uncharacterized protein | | afdb-uniprot50 | AF-A0A3M6B5Q4-F1-MODEL\_V4 | 1.0 | 1.707e-27 | 1019 | 0.31 | 283 | 176 | 6 | 1 | 275 | 19 | 290 | Prophage PSPPH02, late control protein D protein | Prophage PSPPH02, late control protein D protein | | afdb-uniprot50 | AF-A0A0P9S9Z1-F1-MODEL\_V4 | 1.0 | 2.147e-27 | 1016 | 0.307 | 283 | 177 | 6 | 1 | 275 | 19 | 290 | Prophage PSPPH02, late control protein D protein | Prophage PSPPH02, late control protein D protein | | afdb-uniprot50 | AF-A0A329B6J9-F1-MODEL\_V4 | 1.0 | 1.437e-27 | 1015 | 0.315 | 285 | 181 | 7 | 2 | 280 | 16 | 292 | Uncharacterized protein | Uncharacterized protein | | afdb-uniprot50 | AF-A0A3N2E0R5-F1-MODEL\_V4 | 1.0 | 2.549e-27 | 1015 | 0.341 | 278 | 178 | 3 | 1 | 276 | 53 | 327 | Uncharacterized protein | Uncharacterized protein | | afdb-uniprot50 | AF-A0A5E4XF48-F1-MODEL\_V4 | 1.0 | 1.612e-27 | 1015 | 0.348 | 281 | 177 | 2 | 1 | 280 | 51 | 326 | Late control protein | Late control protein | | afdb-uniprot50 | AF-A0A210BC43-F1-MODEL\_V4 | 1.0 | 7.648e-28 | 1014 | 0.245 | 310 | 200 | 8 | 1 | 284 | 8 | 309 | Late control protein | Late control protein | | afdb-uniprot50 | AF-A0A4D9YTW0-F1-MODEL\_V4 | 1.0 | 1.221e-28 | 1014 | 0.286 | 332 | 181 | 12 | 2 | 288 | 14 | 334 | Phage late control D family protein | Phage late control D family protein | | afdb-uniprot50 | AF-A0A5A9EMZ8-F1-MODEL\_V4 | 1.0 | 2.912e-29 | 1013 | 0.349 | 312 | 178 | 7 | 1 | 304 | 97 | 391 | Uncharacterized protein | Uncharacterized protein | | afdb-uniprot50 | AF-A0A3S0U918-F1-MODEL\_V4 | 1.0 | 1.807e-27 | 1009 | 0.357 | 277 | 170 | 3 | 1 | 276 | 51 | 320 | Phage tail protein | Phage tail protein | | afdb-uniprot50 | AF-A0A4R1PBW7-F1-MODEL\_V4 | 1.0 | 4.79e-27 | 1004 | 0.353 | 286 | 171 | 7 | 1 | 280 | 41 | 318 | Uncharacterized protein | Uncharacterized protein | | afdb-uniprot50 | AF-A0A6G8F2E2-F1-MODEL\_V4 | 1.0 | 7.579e-27 | 999 | 0.347 | 282 | 177 | 5 | 1 | 278 | 51 | 329 | Phage late control protein | Phage late control protein | | afdb-uniprot50 | AF-A0A2T5J1F0-F1-MODEL\_V4 | 1.0 | 1.009e-26 | 999 | 0.364 | 280 | 167 | 6 | 2 | 277 | 56 | 328 | Uncharacterized protein | Uncharacterized protein | | afdb-uniprot50 | AF-A0A5S9Q3J3-F1-MODEL\_V4 | 1.0 | 9.532e-27 | 997 | 0.368 | 285 | 167 | 5 | 1 | 279 | 51 | 328 | Uncharacterized protein | Uncharacterized protein | | afdb-uniprot50 | AF-A0A5J6LDM7-F1-MODEL\_V4 | 1.0 | 3.997e-26 | 994 | 0.307 | 276 | 177 | 7 | 8 | 278 | 1 | 267 | Phage late control D family protein | Phage late control D family protein | | afdb-uniprot50 | AF-A0A496N5Y9-F1-MODEL\_V4 | 1.0 | 3.236e-28 | 994 | 0.28 | 332 | 181 | 10 | 1 | 282 | 15 | 338 | Phage late control D family protein | Phage late control D family protein | | afdb-uniprot50 | AF-A0A7Z0N080-F1-MODEL\_V4 | 1.0 | 1.522e-27 | 990 | 0.363 | 278 | 167 | 6 | 1 | 276 | 51 | 320 | Uncharacterized protein | Uncharacterized protein | | afdb-uniprot50 | AF-A0A7K0GN29-F1-MODEL\_V4 | 1.0 | 3.178e-26 | 989 | 0.403 | 238 | 133 | 4 | 39 | 274 | 2 | 232 | Phage late control D family protein | Phage late control D family protein | | afdb-uniprot50 | AF-A0A1H7EQQ5-F1-MODEL\_V4 | 1.0 | 1.597e-26 | 989 | 0.348 | 287 | 175 | 7 | 1 | 282 | 54 | 333 | Uncharacterized protein | Uncharacterized protein | | afdb-uniprot50 | AF-A0A809Z5I0-F1-MODEL\_V4 | 1.0 | 1.258e-25 | 988 | 0.306 | 281 | 187 | 3 | 1 | 280 | 72 | 345 | Regulator of late gene expression | Regulator of late gene expression | | afdb-uniprot50 | AF-A0A1Y6CY31-F1-MODEL\_V4 | 1.0 | 1.691e-26 | 985 | 0.329 | 279 | 177 | 5 | 1 | 279 | 51 | 319 | Uncharacterized protein | Uncharacterized protein | | afdb-uniprot50 | AF-A0A362XU25-F1-MODEL\_V4 | 1.0 | 2.526e-26 | 985 | 0.341 | 281 | 175 | 5 | 2 | 279 | 55 | 328 | Uncharacterized protein | Uncharacterized protein | | afdb-uniprot50 | AF-A0A7W6CNX6-F1-MODEL\_V4 | 1.0 | 1.508e-26 | 977 | 0.293 | 286 | 183 | 8 | 1 | 276 | 7 | 283 | Uncharacterized protein | Uncharacterized protein | | afdb-uniprot50 | AF-E5AKN5-F1-MODEL\_V4 | 1.0 | 1.188e-25 | 976 | 0.322 | 285 | 181 | 6 | 1 | 280 | 61 | 338 | Gene D protein | Gene D protein | | afdb-uniprot50 | AF-A0A2E3N1J0-F1-MODEL\_V4 | 1.0 | 5.324e-26 | 975 | 0.31 | 287 | 183 | 5 | 1 | 279 | 51 | 330 | Uncharacterized protein | Uncharacterized protein | | afdb-uniprot50 | AF-A0A4R2PFS0-F1-MODEL\_V4 | 1.0 | 1.791e-26 | 975 | 0.314 | 286 | 181 | 5 | 1 | 279 | 55 | 332 | Uncharacterized protein | Uncharacterized protein | | afdb-uniprot50 | AF-A0A4V3GTL6-F1-MODEL\_V4 | 1.0 | 2.407e-27 | 974 | 0.332 | 283 | 175 | 7 | 10 | 288 | 1 | 273 | Uncharacterized protein | Uncharacterized protein | | afdb-uniprot50 | AF-A0A8A7WWT4-F1-MODEL\_V4 | 1.0 | 3.001e-26 | 973 | 0.347 | 282 | 172 | 8 | 2 | 279 | 55 | 328 | Phage late control D family protein | Phage late control D family protein | | afdb-uniprot50 | AF-A0A4Q3Z0D3-F1-MODEL\_V4 | 1.0 | 2.676e-26 | 971 | 0.35 | 280 | 181 | 1 | 2 | 281 | 59 | 337 | Late control protein D | Late control protein D | | afdb-uniprot50 | AF-A0A318KN30-F1-MODEL\_V4 | 1.0 | 7.156e-27 | 968 | 0.333 | 300 | 173 | 8 | 1 | 280 | 64 | 356 | Uncharacterized protein | Uncharacterized protein | | afdb-uniprot50 | AF-A0A6L2ZP70-F1-MODEL\_V4 | 1.0 | 1.597e-26 | 966 | 0.346 | 283 | 168 | 7 | 1 | 276 | 52 | 324 | Phage late control gene D protein | Phage late control gene D protein | | afdb-uniprot50 | AF-A0A2V3Y0H4-F1-MODEL\_V4 | 1.0 | 2.676e-26 | 966 | 0.296 | 283 | 183 | 7 | 1 | 275 | 60 | 334 | Uncharacterized protein | Uncharacterized protein | | afdb-uniprot50 | AF-A0A368VC74-F1-MODEL\_V4 | 1.0 | 7.953e-26 | 965 | 0.334 | 281 | 177 | 5 | 2 | 279 | 55 | 328 | Uncharacterized protein | Uncharacterized protein | | afdb-uniprot50 | AF-A0A4U8YMH7-F1-MODEL\_V4 | 1.0 | 7.953e-26 | 964 | 0.306 | 284 | 186 | 6 | 1 | 281 | 51 | 326 | Phage late control gene d protein (Gpd) | Phage late control gene d protein (Gpd) | | afdb-uniprot50 | AF-L8MFF9-F1-MODEL\_V4 | 1.0 | 8.92e-26 | 964 | 0.352 | 284 | 172 | 6 | 2 | 281 | 57 | 332 | Phage late control D | Phage late control D | | afdb-uniprot50 | AF-A0A3N1QKI9-F1-MODEL\_V4 | 1.0 | 1.122e-25 | 963 | 0.345 | 281 | 174 | 6 | 2 | 279 | 55 | 328 | Uncharacterized protein | Uncharacterized protein | | afdb-uniprot50 | AF-A0A519ZAP9-F1-MODEL\_V4 | 1.0 | 2.009e-26 | 961 | 0.325 | 286 | 167 | 8 | 7 | 275 | 2 | 278 | Uncharacterized protein | Uncharacterized protein | | afdb-uniprot50 | AF-A0A2D8Q6U7-F1-MODEL\_V4 | 1.0 | 2.526e-26 | 961 | 0.339 | 286 | 174 | 6 | 1 | 280 | 51 | 327 | Phage tail protein | Phage tail protein | | afdb-uniprot50 | AF-A0A4Y6UAT9-F1-MODEL\_V4 | 1.0 | 2.253e-26 | 961 | 0.301 | 312 | 184 | 6 | 1 | 278 | 81 | 392 | Uncharacterized protein | Uncharacterized protein | | afdb-uniprot50 | AF-A0A256CB22-F1-MODEL\_V4 | 1.0 | 6.323e-26 | 957 | 0.342 | 283 | 176 | 5 | 1 | 281 | 56 | 330 | Uncharacterized protein | Uncharacterized protein | | afdb-uniprot50 | AF-A0A166Z710-F1-MODEL\_V4 | 1.0 | 4.483e-26 | 956 | 0.267 | 307 | 198 | 8 | 1 | 290 | 49 | 345 | Late control protein D | Late control protein D | | afdb-uniprot50 | AF-A0A1A9VKH9-F1-MODEL\_V4 | 1.0 | 1.508e-26 | 953 | 0.359 | 270 | 165 | 3 | 7 | 275 | 49 | 311 | Ankyrin\_rpt-contain\_dom domain-containing protein | Ankyrin\_rpt-contain\_dom domain-containing protein | | afdb-uniprot50 | AF-A0A7W4WU07-F1-MODEL\_V4 | 1.0 | 5.324e-26 | 952 | 0.309 | 284 | 182 | 10 | 2 | 279 | 22 | 297 | Phage protein D | Phage protein D | | afdb-uniprot50 | AF-A0A6L7X9K2-F1-MODEL\_V4 | 1.0 | 5.276e-25 | 951 | 0.377 | 225 | 137 | 3 | 57 | 279 | 5 | 228 | Uncharacterized protein | Uncharacterized protein | | afdb-uniprot50 | AF-A0A5K6V8S0-F1-MODEL\_V4 | 1.0 | 9.532e-27 | 950 | 0.353 | 283 | 170 | 4 | 1 | 276 | 53 | 329 | Late control protein | Late control protein | | afdb-uniprot50 | AF-A0A7W6E035-F1-MODEL\_V4 | 1.0 | 1.991e-25 | 949 | 0.322 | 276 | 180 | 2 | 1 | 276 | 56 | 324 | Uncharacterized protein | Uncharacterized protein | | afdb-uniprot50 | AF-A0A713UWD1-F1-MODEL\_V4 | 1.0 | 3.365e-26 | 948 | 0.3 | 313 | 179 | 11 | 1 | 282 | 18 | 321 | Phage late control D family protein | Phage late control D family protein | | afdb-uniprot50 | AF-A0A1I1UD34-F1-MODEL\_V4 | 1.0 | 5.324e-26 | 948 | 0.292 | 287 | 190 | 9 | 1 | 279 | 50 | 331 | Uncharacterized protein | Uncharacterized protein | | afdb-uniprot50 | AF-A0A5M6IGE8-F1-MODEL\_V4 | 1.0 | 4.483e-26 | 946 | 0.317 | 287 | 178 | 6 | 1 | 277 | 55 | 333 | Late control protein D | Late control protein D | | afdb-uniprot50 | AF-A0A840UI14-F1-MODEL\_V4 | 1.0 | 2.364e-25 | 945 | 0.327 | 281 | 179 | 5 | 2 | 279 | 55 | 328 | Uncharacterized protein | Uncharacterized protein | | afdb-uniprot50 | AF-A0A3G2VC57-F1-MODEL\_V4 | 1.0 | 3.178e-26 | 942 | 0.237 | 299 | 204 | 8 | 1 | 282 | 10 | 301 | Uncharacterized protein | Uncharacterized protein | | afdb-uniprot50 | AF-A0A3T0N1H3-F1-MODEL\_V4 | 1.0 | 3.997e-26 | 941 | 0.325 | 280 | 179 | 6 | 1 | 278 | 51 | 322 | Phage tail protein | Phage tail protein | | afdb-uniprot50 | AF-A0A1H8RSK6-F1-MODEL\_V4 | 1.0 | 6.323e-26 | 941 | 0.313 | 287 | 180 | 6 | 1 | 279 | 51 | 328 | Uncharacterized protein | Uncharacterized protein | | afdb-uniprot50 | AF-A0A519MN03-F1-MODEL\_V4 | 1.0 | 1e-25 | 940 | 0.371 | 285 | 167 | 7 | 2 | 282 | 65 | 341 | Phage late control D family protein | Phage late control D family protein | | afdb-uniprot50 | AF-A0A2E3M0S7-F1-MODEL\_V4 | 1.0 | 1.009e-26 | 939 | 0.329 | 282 | 179 | 5 | 1 | 280 | 63 | 336 | Phage tail protein | Phage tail protein | | afdb-uniprot50 | AF-A0A3G2FX49-F1-MODEL\_V4 | 1.0 | 8.423e-26 | 937 | 0.311 | 260 | 170 | 4 | 22 | 279 | 5 | 257 | Phage late control D family protein | Phage late control D family protein | | afdb-uniprot50 | AF-A0A381EBY3-F1-MODEL\_V4 | 1.0 | 2.232e-25 | 937 | 0.312 | 282 | 184 | 5 | 1 | 279 | 49 | 323 | Phage protein D | Phage protein D | | afdb-uniprot50 | AF-A0A847KNG7-F1-MODEL\_V4 | 1.0 | 1.88e-25 | 937 | 0.263 | 288 | 196 | 7 | 1 | 280 | 51 | 330 | Phage late control D family protein | Phage late control D family protein | | afdb-uniprot50 | AF-A0A1X7NEM5-F1-MODEL\_V4 | 1.0 | 9.446e-26 | 936 | 0.332 | 280 | 176 | 6 | 1 | 278 | 49 | 319 | Uncharacterized protein | Uncharacterized protein | | afdb-uniprot50 | AF-W6TE35-F1-MODEL\_V4 | 1.0 | 3.809e-27 | 936 | 0.33 | 312 | 191 | 4 | 1 | 311 | 51 | 345 | Gene D protein | Gene D protein | | afdb-uniprot50 | AF-A0A3E0X405-F1-MODEL\_V4 | 1.0 | 3.335e-25 | 932 | 0.323 | 281 | 180 | 6 | 1 | 278 | 59 | 332 | Late control protein | Late control protein | | afdb-uniprot50 | AF-G3IRG5-F1-MODEL\_V4 | 1.0 | 2.504e-25 | 931 | 0.313 | 281 | 178 | 9 | 1 | 275 | 51 | 322 | Late control D family protein | Late control D family protein | | afdb-uniprot50 | AF-A0A1G7PWP3-F1-MODEL\_V4 | 1.0 | 5.027e-26 | 931 | 0.259 | 293 | 197 | 8 | 1 | 282 | 50 | 333 | Uncharacterized protein | Uncharacterized protein | | afdb-uniprot50 | AF-A0A2E2MXP4-F1-MODEL\_V4 | 1.0 | 3.365e-26 | 930 | 0.305 | 314 | 199 | 8 | 1 | 311 | 67 | 364 | Late control protein | Late control protein | | afdb-uniprot50 | AF-A0A315BKV5-F1-MODEL\_V4 | 1.0 | 3.001e-26 | 928 | 0.302 | 324 | 198 | 9 | 2 | 310 | 61 | 371 | Uncharacterized protein | Uncharacterized protein | | afdb-uniprot50 | AF-A0A5P9F186-F1-MODEL\_V4 | 1.0 | 7.028e-25 | 927 | 0.316 | 281 | 181 | 5 | 1 | 278 | 50 | 322 | Phage late control gene D protein (GPD) | Phage late control gene D protein (GPD) | | afdb-uniprot50 | AF-A0A235EW76-F1-MODEL\_V4 | 1.0 | 3.149e-25 | 927 | 0.316 | 297 | 178 | 6 | 1 | 279 | 54 | 343 | Late control protein | Late control protein | | afdb-uniprot50 | AF-A0A3S4CKJ0-F1-MODEL\_V4 | 1.0 | 3.149e-25 | 925 | 0.307 | 280 | 184 | 5 | 2 | 279 | 52 | 323 | Phage late control gene D protein (GPD) | Phage late control gene D protein (GPD) | | afdb-uniprot50 | AF-A0A2T6F1B4-F1-MODEL\_V4 | 1.0 | 2.651e-25 | 925 | 0.266 | 304 | 205 | 7 | 1 | 296 | 54 | 347 | Late control protein | Late control protein | | afdb-uniprot50 | AF-A0A0Q3BFB6-F1-MODEL\_V4 | 1.0 | 7.028e-25 | 923 | 0.311 | 279 | 182 | 5 | 1 | 277 | 51 | 321 | Uncharacterized protein | Uncharacterized protein | | afdb-uniprot50 | AF-A0A7W6RE50-F1-MODEL\_V4 | 1.0 | 3.149e-25 | 922 | 0.278 | 294 | 197 | 10 | 1 | 288 | 61 | 345 | Uncharacterized protein | Uncharacterized protein | | afdb-uniprot50 | AF-A0A2N1WF73-F1-MODEL\_V4 | 1.0 | 5.917e-25 | 921 | 0.332 | 283 | 179 | 6 | 1 | 280 | 61 | 336 | Late control protein | Late control protein | | afdb-uniprot50 | AF-C3X1X3-F1-MODEL\_V4 | 1.0 | 1.568e-24 | 921 | 0.262 | 290 | 201 | 6 | 1 | 284 | 55 | 337 | Uncharacterized protein | Uncharacterized protein | | afdb-uniprot50 | AF-A0A1X7Q5G6-F1-MODEL\_V4 | 1.0 | 6.696e-26 | 921 | 0.296 | 293 | 183 | 6 | 1 | 278 | 56 | 340 | Uncharacterized protein | Uncharacterized protein | | afdb-uniprot50 | AF-A0A7Z0UXS5-F1-MODEL\_V4 | 1.0 | 1.676e-25 | 921 | 0.295 | 318 | 208 | 9 | 1 | 311 | 98 | 406 | Uncharacterized protein | Uncharacterized protein | | afdb-uniprot50 | AF-A0A0Q2Y5J5-F1-MODEL\_V4 | 1.0 | 4.442e-25 | 920 | 0.255 | 297 | 204 | 7 | 2 | 290 | 51 | 338 | Uncharacterized protein | Uncharacterized protein | | afdb-uniprot50 | AF-A0A096FN45-F1-MODEL\_V4 | 1.0 | 4.442e-25 | 920 | 0.267 | 310 | 195 | 7 | 2 | 287 | 49 | 350 | Tail protein | Tail protein | | afdb-uniprot50 | AF-A0A2E5BTI9-F1-MODEL\_V4 | 1.0 | 3.961e-25 | 918 | 0.29 | 286 | 189 | 7 | 1 | 279 | 51 | 329 | Late control protein | Late control protein | | afdb-uniprot50 | AF-A0A5B0VXU9-F1-MODEL\_V4 | 1.0 | 3.961e-25 | 918 | 0.273 | 296 | 191 | 7 | 1 | 279 | 63 | 351 | Phage late control D family protein | Phage late control D family protein | | afdb-uniprot50 | AF-A0A089PYY8-F1-MODEL\_V4 | 1.0 | 1.059e-25 | 918 | 0.304 | 305 | 198 | 6 | 2 | 295 | 54 | 355 | Uncharacterized protein | Uncharacterized protein | | afdb-uniprot50 | AF-A0A2N7P6U8-F1-MODEL\_V4 | 1.0 | 1.112e-24 | 917 | 0.307 | 280 | 184 | 5 | 1 | 278 | 52 | 323 | Uncharacterized protein | Uncharacterized protein | | afdb-uniprot50 | AF-A0A1G7D4E3-F1-MODEL\_V4 | 1.0 | 6.636e-25 | 917 | 0.287 | 289 | 194 | 7 | 1 | 284 | 51 | 332 | Uncharacterized protein | Uncharacterized protein | | afdb-uniprot50 | AF-A0A8B2NZ52-F1-MODEL\_V4 | 1.0 | 6.266e-25 | 916 | 0.292 | 284 | 184 | 8 | 2 | 277 | 54 | 328 | Late control protein D | Late control protein D | | afdb-uniprot50 | AF-A0A6L9FMV3-F1-MODEL\_V4 | 1.0 | 4.937e-24 | 916 | 0.291 | 285 | 194 | 6 | 1 | 279 | 54 | 336 | Late control protein D | Late control protein D | | afdb-uniprot50 | AF-A0A2S8R677-F1-MODEL\_V4 | 1.0 | 2.974e-25 | 916 | 0.283 | 307 | 184 | 10 | 1 | 280 | 62 | 359 | Late control protein D | Late control protein D | | afdb-uniprot50 | AF-A0A1D2QS63-F1-MODEL\_V4 | 1.0 | 7.092e-26 | 914 | 0.304 | 292 | 178 | 8 | 1 | 280 | 49 | 327 | Uncharacterized protein | Uncharacterized protein | | afdb-uniprot50 | AF-A0A345DE48-F1-MODEL\_V4 | 1.0 | 5.971e-26 | 914 | 0.353 | 283 | 169 | 8 | 1 | 277 | 49 | 323 | Uncharacterized protein | Uncharacterized protein | | afdb-uniprot50 | AF-A0A328X5L9-F1-MODEL\_V4 | 1.0 | 7.028e-25 | 911 | 0.324 | 287 | 177 | 8 | 2 | 280 | 64 | 341 | Uncharacterized protein | Uncharacterized protein | | afdb-uniprot50 | AF-A0A545T5X0-F1-MODEL\_V4 | 1.0 | 2.089e-24 | 908 | 0.279 | 283 | 189 | 5 | 1 | 279 | 74 | 345 | Uncharacterized protein | Uncharacterized protein | | afdb-uniprot50 | AF-A0A1W1Z5V8-F1-MODEL\_V4 | 1.0 | 8.839e-25 | 906 | 0.267 | 284 | 194 | 8 | 2 | 278 | 51 | 327 | Uncharacterized protein | Uncharacterized protein | | afdb-uniprot50 | AF-A0A1N6Q3I2-F1-MODEL\_V4 | 1.0 | 5.537e-24 | 905 | 0.301 | 282 | 185 | 6 | 1 | 278 | 55 | 328 | Uncharacterized protein | Uncharacterized protein | | afdb-uniprot50 | AF-A0A251ZIV1-F1-MODEL\_V4 | 1.0 | 8.423e-26 | 904 | 0.259 | 308 | 201 | 8 | 1 | 288 | 71 | 371 | Uncharacterized protein | Uncharacterized protein | | afdb-uniprot50 | AF-A0A143DG31-F1-MODEL\_V4 | 1.0 | 4.662e-24 | 903 | 0.29 | 282 | 187 | 8 | 1 | 276 | 50 | 324 | Uncharacterized protein | Uncharacterized protein | | afdb-uniprot50 | AF-A0A1E7Q062-F1-MODEL\_V4 | 1.0 | 2.628e-24 | 902 | 0.362 | 281 | 169 | 5 | 2 | 279 | 55 | 328 | Uncharacterized protein | Uncharacterized protein | | afdb-uniprot50 | AF-A0A7Z1UDY9-F1-MODEL\_V4 | 1.0 | 2.783e-24 | 902 | 0.223 | 326 | 208 | 8 | 1 | 289 | 67 | 384 | Uncharacterized protein | Uncharacterized protein | | afdb-uniprot50 | AF-A0A6H1S4A5-F1-MODEL\_V4 | 1.0 | 2.947e-24 | 902 | 0.225 | 328 | 207 | 8 | 1 | 289 | 69 | 388 | Late control D family protein | Late control D family protein | | afdb-uniprot50 | AF-A0A348HI91-F1-MODEL\_V4 | 1.0 | 5.587e-25 | 901 | 0.31 | 283 | 183 | 8 | 1 | 279 | 55 | 329 | Phage protein D | Phage protein D | | afdb-uniprot50 | AF-A0A350LX83-F1-MODEL\_V4 | 1.0 | 2.108e-25 | 900 | 0.314 | 283 | 184 | 6 | 1 | 281 | 51 | 325 | Phage tail protein | Phage tail protein | | afdb-uniprot50 | AF-A0A1E3G722-F1-MODEL\_V4 | 1.0 | 6.696e-26 | 899 | 0.345 | 278 | 175 | 2 | 2 | 279 | 54 | 324 | Uncharacterized protein | Uncharacterized protein | | afdb-uniprot50 | AF-A0A1M4WEC4-F1-MODEL\_V4 | 1.0 | 1.398e-24 | 897 | 0.269 | 286 | 194 | 9 | 1 | 278 | 51 | 329 | Uncharacterized protein | Uncharacterized protein | | afdb-uniprot50 | AF-G3XCU8-F1-MODEL\_V4 | 1.0 | 3.5e-24 | 896 | 0.29 | 282 | 183 | 5 | 1 | 275 | 51 | 322 | Uncharacterized protein | Uncharacterized protein | | afdb-uniprot50 | AF-A0A1Y2K0X3-F1-MODEL\_V4 | 1.0 | 1.424e-26 | 896 | 0.342 | 283 | 176 | 6 | 1 | 280 | 51 | 326 | Uncharacterized protein | Uncharacterized protein | | afdb-uniprot50 | AF-W0HLP1-F1-MODEL\_V4 | 1.0 | 4.704e-25 | 896 | 0.293 | 300 | 182 | 7 | 1 | 277 | 64 | 356 | Late control gene D protein | Late control gene D protein | | afdb-uniprot50 | AF-A0A1B4NNN3-F1-MODEL\_V4 | 1.0 | 7.51e-26 | 896 | 0.311 | 318 | 183 | 9 | 2 | 291 | 74 | 383 | Late control protein D | Late control protein D | | afdb-uniprot50 | AF-A0A840P1G0-F1-MODEL\_V4 | 1.0 | 2.232e-25 | 896 | 0.269 | 289 | 188 | 10 | 1 | 281 | 499 | 772 | Phage protein D | Phage protein D | | afdb-uniprot50 | AF-C5BEQ4-F1-MODEL\_V4 | 1.0 | 9.913e-25 | 895 | 0.311 | 299 | 179 | 8 | 1 | 280 | 59 | 349 | Phage late control gene D protein (GPD) | Phage late control gene D protein (GPD) | | afdb-uniprot50 | AF-A0A6D0JLG2-F1-MODEL\_V4 | 1.0 | 5.537e-24 | 895 | 0.284 | 288 | 192 | 7 | 1 | 277 | 56 | 340 | Late control protein | Late control protein | | afdb-uniprot50 | AF-C6C6Z8-F1-MODEL\_V4 | 1.0 | 1.247e-24 | 895 | 0.22 | 326 | 208 | 7 | 2 | 289 | 77 | 394 | Late control D family protein | Late control D family protein | | afdb-uniprot50 | AF-A0A166F5B7-F1-MODEL\_V4 | 1.0 | 2.089e-24 | 894 | 0.269 | 289 | 196 | 8 | 1 | 281 | 51 | 332 | Phage late control gene D protein (GPD) | Phage late control gene D protein (GPD) | | afdb-uniprot50 | AF-A0A212J3N4-F1-MODEL\_V4 | 1.0 | 8.839e-25 | 893 | 0.295 | 284 | 188 | 7 | 2 | 280 | 52 | 328 | Gene D protein | Gene D protein | | afdb-uniprot50 | AF-A0A7Z1Y516-F1-MODEL\_V4 | 1.0 | 2.783e-24 | 892 | 0.272 | 286 | 192 | 7 | 2 | 275 | 61 | 342 | Late control protein D | Late control protein D | | afdb-uniprot50 | AF-N6VTB1-F1-MODEL\_V4 | 1.0 | 3.564e-26 | 892 | 0.282 | 340 | 169 | 7 | 10 | 282 | 1 | 332 | Phage late control protein D | Phage late control protein D | | afdb-uniprot50 | AF-A0A4U3FAV9-F1-MODEL\_V4 | 1.0 | 2.089e-24 | 892 | 0.263 | 323 | 205 | 8 | 1 | 298 | 63 | 377 | Uncharacterized protein | Uncharacterized protein | | afdb-uniprot50 | AF-A0A844YYZ9-F1-MODEL\_V4 | 1.0 | 4.402e-24 | 889 | 0.277 | 288 | 192 | 7 | 1 | 279 | 11 | 291 | Phage late control D family protein | Phage late control D family protein | | afdb-uniprot50 | AF-A0A1N6M6E7-F1-MODEL\_V4 | 1.0 | 4.195e-25 | 889 | 0.272 | 294 | 195 | 9 | 1 | 281 | 33 | 320 | Phage late control gene D protein (GPD) | Phage late control gene D protein (GPD) | | afdb-uniprot50 | AF-A0A2D2C160-F1-MODEL\_V4 | 1.0 | 1.398e-24 | 889 | 0.267 | 284 | 194 | 7 | 2 | 278 | 51 | 327 | Late control protein D | Late control protein D | | afdb-uniprot50 | AF-A0A7Y6Z4E7-F1-MODEL\_V4 | 1.0 | 2.089e-24 | 889 | 0.273 | 285 | 193 | 7 | 1 | 279 | 51 | 327 | Uncharacterized protein | Uncharacterized protein | | afdb-uniprot50 | AF-S6I325-F1-MODEL\_V4 | 1.0 | 3.706e-24 | 889 | 0.283 | 293 | 192 | 7 | 1 | 282 | 54 | 339 | Uncharacterized protein | Uncharacterized protein | | afdb-uniprot50 | AF-A0A0A3ZBQ9-F1-MODEL\_V4 | 1.0 | 1.481e-24 | 889 | 0.264 | 306 | 192 | 6 | 1 | 280 | 58 | 356 | Late control protein D | Late control protein D | | afdb-uniprot50 | AF-A0A0S2F7D3-F1-MODEL\_V4 | 1.0 | 5.228e-24 | 888 | 0.333 | 282 | 176 | 8 | 2 | 279 | 31 | 304 | Phage late control gene D family protein | Phage late control gene D family protein | | afdb-uniprot50 | AF-A0A2G6RW27-F1-MODEL\_V4 | 1.0 | 6.21e-24 | 888 | 0.285 | 242 | 168 | 4 | 57 | 296 | 10 | 248 | Uncharacterized protein | Uncharacterized protein | | afdb-uniprot50 | AF-A0A2S5TZV2-F1-MODEL\_V4 | 1.0 | 1.568e-24 | 887 | 0.265 | 282 | 194 | 7 | 2 | 277 | 52 | 326 | Uncharacterized protein | Uncharacterized protein | | afdb-uniprot50 | AF-A0A3M2YE85-F1-MODEL\_V4 | 1.0 | 1.661e-24 | 887 | 0.28 | 282 | 186 | 6 | 1 | 275 | 85 | 356 | Tail protein D | Tail protein D | | afdb-uniprot50 | AF-A0A6G8AI05-F1-MODEL\_V4 | 1.0 | 4.442e-25 | 886 | 0.284 | 313 | 206 | 6 | 2 | 311 | 65 | 362 | Phage late control D family protein | Phage late control D family protein | | afdb-uniprot50 | AF-A0A378YM62-F1-MODEL\_V4 | 1.0 | 2.364e-25 | 886 | 0.311 | 315 | 171 | 10 | 1 | 279 | 73 | 377 | Phage protein D | Phage protein D | | afdb-uniprot50 | AF-D6JBF4-F1-MODEL\_V4 | 1.0 | 4.937e-24 | 886 | 0.244 | 311 | 203 | 7 | 1 | 286 | 110 | 413 | Uncharacterized protein | Uncharacterized protein | | afdb-uniprot50 | AF-A0A199YKM6-F1-MODEL\_V4 | 1.0 | 8.839e-25 | 884 | 0.308 | 279 | 183 | 5 | 1 | 276 | 51 | 322 | Uncharacterized protein | Uncharacterized protein | | afdb-uniprot50 | AF-A0A212KMW2-F1-MODEL\_V4 | 1.0 | 4.402e-24 | 884 | 0.341 | 287 | 173 | 9 | 1 | 278 | 51 | 330 | Late control D family protein | Late control D family protein | | afdb-uniprot50 | AF-A0A7W7KUK6-F1-MODEL\_V4 | 1.0 | 3.121e-24 | 883 | 0.347 | 279 | 171 | 7 | 2 | 276 | 55 | 326 | Uncharacterized protein | Uncharacterized protein | | afdb-uniprot50 | AF-A0A2G6EX41-F1-MODEL\_V4 | 1.0 | 1.167e-23 | 882 | 0.315 | 282 | 183 | 5 | 2 | 280 | 65 | 339 | Late control protein D | Late control protein D | | afdb-uniprot50 | AF-A0A4R3V673-F1-MODEL\_V4 | 1.0 | 2.089e-24 | 882 | 0.27 | 299 | 197 | 10 | 2 | 291 | 60 | 346 | Uncharacterized protein | Uncharacterized protein | | afdb-uniprot50 | AF-A0A7C8G8Q1-F1-MODEL\_V4 | 1.0 | 3.5e-24 | 882 | 0.261 | 310 | 199 | 8 | 1 | 288 | 63 | 364 | Phage late control D family protein | Phage late control D family protein | | afdb-uniprot50 | AF-A0A6N3HNE7-F1-MODEL\_V4 | 1.0 | 5.228e-24 | 882 | 0.25 | 311 | 201 | 6 | 1 | 286 | 109 | 412 | Phage late control gene D protein (GPD) | Phage late control gene D protein (GPD) | | afdb-uniprot50 | AF-A0A423PQL7-F1-MODEL\_V4 | 1.0 | 1e-25 | 881 | 0.299 | 284 | 182 | 6 | 1 | 281 | 29 | 298 | Uncharacterized protein | Uncharacterized protein | | afdb-uniprot50 | AF-A0A3R9DQ60-F1-MODEL\_V4 | 1.0 | 5.864e-24 | 881 | 0.309 | 284 | 183 | 7 | 1 | 277 | 52 | 329 | Phage late control D family protein | Phage late control D family protein | | afdb-uniprot50 | AF-A0A0T9KDK3-F1-MODEL\_V4 | 1.0 | 3.961e-25 | 880 | 0.254 | 310 | 195 | 8 | 1 | 282 | 130 | 431 | Late control gene D protein | Late control gene D protein | | afdb-uniprot50 | AF-A0A559QNE2-F1-MODEL\_V4 | 1.0 | 1.386e-23 | 878 | 0.303 | 280 | 185 | 6 | 1 | 277 | 56 | 328 | Uncharacterized protein | Uncharacterized protein | | afdb-uniprot50 | AF-A0A455W3W8-F1-MODEL\_V4 | 1.0 | 2.481e-24 | 876 | 0.255 | 321 | 189 | 9 | 1 | 279 | 63 | 375 | Regulator of late gene expression | Regulator of late gene expression | | afdb-uniprot50 | AF-Q8ZMV5-F1-MODEL\_V4 | 1.0 | 7.028e-25 | 875 | 0.291 | 309 | 184 | 8 | 1 | 281 | 63 | 364 | Fels-2 prophage protein | Fels-2 prophage protein | | afdb-uniprot50 | AF-A0A3A8E5R3-F1-MODEL\_V4 | 1.0 | 1.167e-23 | 874 | 0.318 | 283 | 181 | 7 | 2 | 279 | 54 | 329 | Uncharacterized protein | Uncharacterized protein | | afdb-uniprot50 | AF-A0A1S1NEL7-F1-MODEL\_V4 | 1.0 | 4.157e-24 | 874 | 0.267 | 288 | 191 | 6 | 1 | 276 | 54 | 333 | Phage tail protein | Phage tail protein | | afdb-uniprot50 | AF-A0A5V6NKW8-F1-MODEL\_V4 | 1.0 | 4.662e-24 | 873 | 0.278 | 284 | 195 | 3 | 1 | 275 | 61 | 343 | Late control protein D | Late control protein D | | afdb-uniprot50 | AF-A0A6L3Z4U3-F1-MODEL\_V4 | 1.0 | 9.361e-25 | 872 | 0.269 | 319 | 204 | 9 | 1 | 311 | 52 | 349 | Late control protein D | Late control protein D | | afdb-uniprot50 | AF-A0A198GQJ0-F1-MODEL\_V4 | 1.0 | 2.628e-24 | 871 | 0.264 | 306 | 192 | 6 | 1 | 280 | 61 | 359 | Phage late control gene D protein | Phage late control gene D protein | | afdb-uniprot50 | AF-A0A5U9S5Z9-F1-MODEL\_V4 | 1.0 | 4.402e-24 | 871 | 0.278 | 327 | 183 | 8 | 1 | 282 | 163 | 481 | Phage late control D family protein | Phage late control D family protein | | afdb-uniprot50 | AF-A0A221DKQ4-F1-MODEL\_V4 | 1.0 | 5.864e-24 | 868 | 0.266 | 330 | 193 | 10 | 1 | 290 | 63 | 383 | Uncharacterized protein | Uncharacterized protein | | afdb-uniprot50 | AF-A0A516W9U2-F1-MODEL\_V4 | 1.0 | 1.646e-23 | 867 | 0.306 | 284 | 186 | 6 | 1 | 280 | 59 | 335 | Phage late control D family protein | Phage late control D family protein | | afdb-uniprot50 | AF-A0A1C3EBM6-F1-MODEL\_V4 | 1.0 | 1.236e-23 | 866 | 0.301 | 279 | 187 | 3 | 1 | 278 | 52 | 323 | Uncharacterized protein | Uncharacterized protein | | afdb-uniprot50 | AF-B5JYF8-F1-MODEL\_V4 | 1.0 | 6.21e-24 | 866 | 0.297 | 279 | 186 | 6 | 2 | 277 | 57 | 328 | Phage late control D | Phage late control D | | afdb-uniprot50 | AF-A0A269PKZ6-F1-MODEL\_V4 | 1.0 | 5.864e-24 | 866 | 0.281 | 291 | 190 | 6 | 1 | 279 | 53 | 336 | Uncharacterized protein | Uncharacterized protein | | afdb-uniprot50 | AF-A0A7S6S5Y4-F1-MODEL\_V4 | 1.0 | 5.228e-24 | 866 | 0.271 | 287 | 190 | 7 | 1 | 278 | 51 | 327 | Late control protein | Late control protein | | afdb-uniprot50 | AF-A0A4Q6CNI6-F1-MODEL\_V4 | 1.0 | 1.646e-23 | 865 | 0.249 | 277 | 198 | 5 | 1 | 277 | 13 | 279 | Uncharacterized protein | Uncharacterized protein | | afdb-uniprot50 | AF-A0A381AML1-F1-MODEL\_V4 | 1.0 | 2.192e-23 | 865 | 0.275 | 287 | 191 | 7 | 2 | 279 | 35 | 313 | Phage protein D | Phage protein D | | afdb-uniprot50 | AF-A0A1C7L3E6-F1-MODEL\_V4 | 1.0 | 1.554e-23 | 865 | 0.283 | 282 | 192 | 5 | 1 | 279 | 59 | 333 | Phage late control protein | Phage late control protein | | afdb-uniprot50 | AF-A0A4S1DI58-F1-MODEL\_V4 | 1.0 | 1.167e-23 | 865 | 0.326 | 282 | 175 | 9 | 1 | 275 | 69 | 342 | DNA primase | DNA primase | | afdb-uniprot50 | AF-A0A0Q5LSP6-F1-MODEL\_V4 | 1.0 | 3.925e-24 | 864 | 0.264 | 314 | 208 | 6 | 1 | 311 | 61 | 354 | Late control protein | Late control protein | | afdb-uniprot50 | AF-A0A329B2U7-F1-MODEL\_V4 | 1.0 | 3.706e-24 | 863 | 0.303 | 264 | 171 | 6 | 22 | 280 | 4 | 259 | Uncharacterized protein | Uncharacterized protein | | afdb-uniprot50 | AF-A0A1M7YYR7-F1-MODEL\_V4 | 1.0 | 2.628e-24 | 863 | 0.278 | 294 | 185 | 6 | 1 | 274 | 51 | 337 | Phage late control gene D protein (GPD) | Phage late control gene D protein (GPD) | | afdb-uniprot50 | AF-Q1QKT4-F1-MODEL\_V4 | 1.0 | 5.864e-24 | 862 | 0.27 | 288 | 194 | 8 | 2 | 281 | 53 | 332 | Phage late control D | Phage late control D | | afdb-uniprot50 | AF-A0A1E3G781-F1-MODEL\_V4 | 1.0 | 1.167e-23 | 861 | 0.255 | 290 | 198 | 8 | 1 | 279 | 56 | 338 | Uncharacterized protein | Uncharacterized protein | | afdb-uniprot50 | AF-A0A5S9P2X8-F1-MODEL\_V4 | 1.0 | 3.121e-24 | 860 | 0.25 | 287 | 200 | 7 | 1 | 281 | 52 | 329 | Uncharacterized protein | Uncharacterized protein | | afdb-uniprot50 | AF-A0A292AJG1-F1-MODEL\_V4 | 1.0 | 9.361e-25 | 860 | 0.263 | 322 | 189 | 11 | 1 | 288 | 70 | 377 | Late control protein D | Late control protein D | | afdb-uniprot50 | AF-A0A395R2S1-F1-MODEL\_V4 | 1.0 | 1.177e-24 | 860 | 0.28 | 317 | 197 | 6 | 2 | 294 | 59 | 368 | Late control protein | Late control protein | | afdb-uniprot50 | AF-A0A2A2B6A0-F1-MODEL\_V4 | 1.0 | 4.402e-24 | 859 | 0.255 | 294 | 196 | 9 | 1 | 278 | 33 | 319 | Uncharacterized protein | Uncharacterized protein | | afdb-uniprot50 | AF-E2CJS5-F1-MODEL\_V4 | 1.0 | 7.442e-25 | 859 | 0.271 | 335 | 201 | 10 | 1 | 311 | 54 | 369 | Late control D protein | Late control D protein | | afdb-uniprot50 | AF-F4BFP6-F1-MODEL\_V4 | 1.0 | 8.839e-25 | 859 | 0.249 | 317 | 220 | 6 | 2 | 311 | 53 | 358 | Phage-related tail protein | Phage-related tail protein | | afdb-uniprot50 | AF-A0A1H0NNX3-F1-MODEL\_V4 | 1.0 | 6.636e-25 | 859 | 0.292 | 339 | 195 | 8 | 1 | 310 | 69 | 391 | Uncharacterized protein | Uncharacterized protein | | afdb-uniprot50 | AF-E2CN20-F1-MODEL\_V4 | 1.0 | 3.5e-24 | 858 | 0.327 | 281 | 177 | 7 | 2 | 279 | 56 | 327 | Putative bacteriophage late control D protein | Putative bacteriophage late control D protein | | afdb-uniprot50 | AF-A0A812QV43-F1-MODEL\_V4 | 1.0 | 1.398e-24 | 858 | 0.313 | 287 | 182 | 7 | 1 | 280 | 734 | 1012 | GpFI protein | GpFI protein | | afdb-uniprot50 | AF-A0A829E6A7-F1-MODEL\_V4 | 1.0 | 2.192e-23 | 857 | 0.252 | 297 | 208 | 9 | 2 | 292 | 60 | 348 | Uncharacterized protein | Uncharacterized protein | | afdb-uniprot50 | AF-A0A1T4W4M5-F1-MODEL\_V4 | 1.0 | 2.628e-24 | 856 | 0.3 | 290 | 182 | 9 | 1 | 278 | 51 | 331 | Uncharacterized protein | Uncharacterized protein | | afdb-uniprot50 | AF-A0A1S1TJX6-F1-MODEL\_V4 | 1.0 | 4.704e-25 | 856 | 0.261 | 333 | 206 | 9 | 1 | 309 | 62 | 378 | Uncharacterized protein | Uncharacterized protein | | afdb-uniprot50 | AF-A0A2W5XH22-F1-MODEL\_V4 | 1.0 | 3.093e-23 | 853 | 0.295 | 281 | 185 | 7 | 2 | 279 | 55 | 325 | Late control protein | Late control protein | | afdb-uniprot50 | AF-E3G761-F1-MODEL\_V4 | 1.0 | 2.459e-23 | 850 | 0.241 | 306 | 206 | 10 | 1 | 290 | 63 | 358 | Putative prophage tail protein | Putative prophage tail protein | | afdb-uniprot50 | AF-A0A4Q0GTT7-F1-MODEL\_V4 | 1.0 | 3.468e-23 | 850 | 0.27 | 288 | 193 | 8 | 2 | 279 | 78 | 358 | Phage late control D family protein | Phage late control D family protein | | afdb-uniprot50 | AF-A0A515D4Y2-F1-MODEL\_V4 | 1.0 | 6.964e-24 | 850 | 0.285 | 301 | 182 | 7 | 1 | 275 | 154 | 447 | Phage late control D family protein | Phage late control D family protein | | afdb-uniprot50 | AF-A0A2U9U220-F1-MODEL\_V4 | 1.0 | 3.468e-23 | 849 | 0.235 | 293 | 202 | 8 | 1 | 279 | 56 | 340 | Uncharacterized protein | Uncharacterized protein | | afdb-uniprot50 | AF-A0A841IGU3-F1-MODEL\_V4 | 1.0 | 1.743e-23 | 848 | 0.237 | 333 | 202 | 11 | 1 | 290 | 30 | 353 | Uncharacterized protein | Uncharacterized protein | | afdb-uniprot50 | AF-A0A377KBH9-F1-MODEL\_V4 | 1.0 | 2.604e-23 | 848 | 0.29 | 310 | 184 | 8 | 1 | 282 | 117 | 418 | Late control protein D protein | Late control protein D protein | | afdb-uniprot50 | AF-A0A853HRL3-F1-MODEL\_V4 | 1.0 | 6.266e-25 | 845 | 0.286 | 297 | 177 | 6 | 1 | 278 | 53 | 333 | Late control protein | Late control protein | | afdb-uniprot50 | AF-A0A5S3YZL2-F1-MODEL\_V4 | 1.0 | 4.892e-23 | 845 | 0.251 | 290 | 199 | 5 | 1 | 279 | 55 | 337 | Phage tail protein | Phage tail protein | | afdb-uniprot50 | AF-A0A4D7AWL8-F1-MODEL\_V4 | 1.0 | 1.454e-22 | 845 | 0.244 | 286 | 202 | 7 | 1 | 279 | 67 | 345 | Uncharacterized protein | Uncharacterized protein | | afdb-uniprot50 | AF-A0A1B6NQN7-F1-MODEL\_V4 | 1.0 | 2.192e-23 | 844 | 0.265 | 290 | 191 | 8 | 2 | 276 | 52 | 334 | Phage late control D family protein | Phage late control D family protein | | afdb-uniprot50 | AF-N9R5A0-F1-MODEL\_V4 | 1.0 | 1.236e-23 | 844 | 0.254 | 307 | 209 | 12 | 1 | 299 | 73 | 367 | Uncharacterized protein | Uncharacterized protein | | afdb-uniprot50 | AF-A0A7X2ILW0-F1-MODEL\_V4 | 1.0 | 4.362e-23 | 843 | 0.281 | 281 | 186 | 6 | 1 | 275 | 51 | 321 | Late control protein | Late control protein | | afdb-uniprot50 | AF-A0A827Q2Z4-F1-MODEL\_V4 | 1.0 | 9.276e-24 | 842 | 0.292 | 267 | 168 | 7 | 24 | 274 | 2 | 263 | Late control protein | Late control protein | | afdb-uniprot50 | AF-A0A379CYH0-F1-MODEL\_V4 | 1.0 | 1.468e-23 | 842 | 0.245 | 318 | 199 | 11 | 1 | 288 | 81 | 387 | Phage protein D | Phage protein D | | afdb-uniprot50 | AF-A0A2V3Q6R4-F1-MODEL\_V4 | 1.0 | 1.468e-23 | 841 | 0.253 | 328 | 199 | 8 | 1 | 291 | 65 | 383 | Uncharacterized protein | Uncharacterized protein | | afdb-uniprot50 | AF-A0A4Z0WCL9-F1-MODEL\_V4 | 1.0 | 1.04e-23 | 840 | 0.28 | 278 | 182 | 8 | 1 | 275 | 53 | 315 | Uncharacterized protein | Uncharacterized protein | | afdb-uniprot50 | AF-A0A4Q3Z459-F1-MODEL\_V4 | 1.0 | 3.89e-23 | 840 | 0.273 | 278 | 191 | 7 | 1 | 275 | 51 | 320 | Phage tail protein | Phage tail protein | | afdb-uniprot50 | AF-A0A348FYG0-F1-MODEL\_V4 | 1.0 | 2.628e-24 | 839 | 0.286 | 311 | 199 | 4 | 2 | 311 | 52 | 340 | Phage late control protein | Phage late control protein | | afdb-uniprot50 | AF-A0A1S1WTC3-F1-MODEL\_V4 | 1.0 | 3.5e-24 | 839 | 0.29 | 317 | 180 | 11 | 1 | 280 | 67 | 375 | Phage tail protein | Phage tail protein | | afdb-uniprot50 | AF-A0A6C8XZE7-F1-MODEL\_V4 | 1.0 | 3.093e-23 | 839 | 0.247 | 311 | 202 | 6 | 1 | 286 | 77 | 380 | Phage late control D family protein | Phage late control D family protein | | afdb-uniprot50 | AF-A0A7Z1M7H1-F1-MODEL\_V4 | 1.0 | 2.481e-24 | 838 | 0.249 | 305 | 204 | 8 | 2 | 291 | 52 | 346 | Uncharacterized protein | Uncharacterized protein | | afdb-uniprot50 | AF-A0A524RW11-F1-MODEL\_V4 | 1.0 | 1.468e-23 | 838 | 0.281 | 291 | 196 | 7 | 7 | 290 | 68 | 352 | Uncharacterized protein | Uncharacterized protein | | afdb-uniprot50 | AF-A0A3R8K6F9-F1-MODEL\_V4 | 1.0 | 3.5e-24 | 838 | 0.318 | 298 | 179 | 7 | 1 | 278 | 61 | 354 | Uncharacterized protein | Uncharacterized protein | | afdb-uniprot50 | AF-A0A6L3Y0N7-F1-MODEL\_V4 | 1.0 | 1.568e-24 | 837 | 0.265 | 305 | 195 | 9 | 21 | 308 | 3 | 295 | Late control protein D | Late control protein D | | afdb-uniprot50 | AF-A0A2A4XT50-F1-MODEL\_V4 | 1.0 | 3.275e-23 | 837 | 0.281 | 274 | 182 | 6 | 9 | 277 | 55 | 318 | Uncharacterized protein | Uncharacterized protein | | afdb-uniprot50 | AF-A0A7S8IIV4-F1-MODEL\_V4 | 1.0 | 9.735e-23 | 837 | 0.26 | 284 | 198 | 6 | 1 | 278 | 52 | 329 | Late control protein D | Late control protein D | | afdb-uniprot50 | AF-A0A2S8JPK2-F1-MODEL\_V4 | 1.0 | 2.604e-23 | 837 | 0.295 | 288 | 186 | 8 | 1 | 274 | 62 | 346 | Late control protein | Late control protein | | afdb-uniprot50 | AF-N9UTV2-F1-MODEL\_V4 | 1.0 | 5.811e-23 | 836 | 0.243 | 288 | 202 | 6 | 1 | 279 | 72 | 352 | Phage-related tail protein | Phage-related tail protein | | afdb-uniprot50 | AF-A0A836MBB7-F1-MODEL\_V4 | 1.0 | 1.398e-24 | 836 | 0.292 | 314 | 178 | 9 | 1 | 277 | 72 | 378 | Tail protein | Tail protein | | afdb-uniprot50 | AF-C1DBI8-F1-MODEL\_V4 | 1.0 | 5.537e-24 | 836 | 0.287 | 316 | 182 | 9 | 2 | 281 | 89 | 397 | Tail protein D | Tail protein D | | afdb-uniprot50 | AF-A0A1W9H416-F1-MODEL\_V4 | 1.0 | 4.892e-23 | 834 | 0.284 | 288 | 183 | 10 | 1 | 275 | 57 | 334 | Uncharacterized protein | Uncharacterized protein | | afdb-uniprot50 | AF-A0A2T5TZJ6-F1-MODEL\_V4 | 1.0 | 1.743e-23 | 834 | 0.299 | 284 | 184 | 6 | 1 | 276 | 66 | 342 | Uncharacterized protein | Uncharacterized protein | | afdb-uniprot50 | AF-A0A432WBJ7-F1-MODEL\_V4 | 1.0 | 1.846e-23 | 834 | 0.287 | 292 | 187 | 8 | 1 | 279 | 59 | 342 | Late control protein D | Late control protein D | | afdb-uniprot50 | AF-A0A516SAU9-F1-MODEL\_V4 | 1.0 | 7.309e-23 | 833 | 0.255 | 286 | 195 | 6 | 1 | 278 | 51 | 326 | Late control protein | Late control protein | | afdb-uniprot50 | AF-A0A2S6N2T0-F1-MODEL\_V4 | 1.0 | 2.192e-23 | 832 | 0.261 | 294 | 200 | 8 | 1 | 286 | 51 | 335 | Uncharacterized protein | Uncharacterized protein | | afdb-uniprot50 | AF-A0A326L1B4-F1-MODEL\_V4 | 1.0 | 7.309e-23 | 832 | 0.277 | 299 | 191 | 6 | 2 | 281 | 56 | 348 | Late control protein | Late control protein | | afdb-uniprot50 | AF-A0A846VID6-F1-MODEL\_V4 | 1.0 | 3.89e-23 | 831 | 0.25 | 287 | 200 | 6 | 1 | 279 | 56 | 335 | Uncharacterized protein | Uncharacterized protein | | afdb-uniprot50 | AF-A0A380ALZ0-F1-MODEL\_V4 | 1.0 | 8.271e-24 | 830 | 0.279 | 286 | 191 | 7 | 2 | 274 | 62 | 345 | Phage protein D | Phage protein D | | afdb-uniprot50 | AF-A0A484GCS1-F1-MODEL\_V4 | 1.0 | 4.62e-23 | 829 | 0.265 | 298 | 189 | 8 | 2 | 279 | 53 | 340 | Phage late control D family protein | Phage late control D family protein | | afdb-uniprot50 | AF-A0A1Z9Z2K2-F1-MODEL\_V4 | 1.0 | 1.373e-22 | 829 | 0.286 | 283 | 187 | 10 | 2 | 277 | 71 | 345 | Uncharacterized protein | Uncharacterized protein | | afdb-uniprot50 | AF-A0A2G6IRT2-F1-MODEL\_V4 | 1.0 | 8.839e-25 | 827 | 0.288 | 326 | 205 | 9 | 1 | 309 | 54 | 369 | Uncharacterized protein | Uncharacterized protein | | afdb-uniprot50 | AF-A0A4R5EG26-F1-MODEL\_V4 | 1.0 | 3.673e-23 | 826 | 0.234 | 286 | 204 | 8 | 1 | 279 | 52 | 329 | Late control protein D | Late control protein D | | afdb-uniprot50 | AF-A0A7X4GD43-F1-MODEL\_V4 | 1.0 | 5.811e-23 | 826 | 0.271 | 284 | 190 | 7 | 1 | 275 | 59 | 334 | Phage late control D family protein | Phage late control D family protein | | afdb-uniprot50 | AF-A0A222EYQ7-F1-MODEL\_V4 | 1.0 | 6.901e-23 | 825 | 0.291 | 285 | 184 | 7 | 2 | 275 | 38 | 315 | Late control protein | Late control protein | | afdb-uniprot50 | AF-A0A3G4VA69-F1-MODEL\_V4 | 1.0 | 3.065e-22 | 825 | 0.27 | 281 | 194 | 6 | 1 | 277 | 51 | 324 | Uncharacterized protein | Uncharacterized protein | | afdb-uniprot50 | AF-A0A7T9VKR8-F1-MODEL\_V4 | 1.0 | 1.04e-23 | 825 | 0.293 | 303 | 194 | 9 | 1 | 297 | 53 | 341 | Uncharacterized protein | Uncharacterized protein | | afdb-uniprot50 | AF-A0A506V2R9-F1-MODEL\_V4 | 1.0 | 1.955e-23 | 825 | 0.248 | 326 | 197 | 12 | 2 | 290 | 66 | 380 | Phage late control D family protein | Phage late control D family protein | | afdb-uniprot50 | AF-A0A840E0Y3-F1-MODEL\_V4 | 1.0 | 5.181e-23 | 824 | 0.271 | 273 | 178 | 9 | 12 | 278 | 1 | 258 | Uncharacterized protein | Uncharacterized protein | | afdb-uniprot50 | AF-A0A1G5AD05-F1-MODEL\_V4 | 1.0 | 6.964e-24 | 824 | 0.343 | 282 | 172 | 7 | 1 | 279 | 51 | 322 | Uncharacterized protein | Uncharacterized protein | | afdb-uniprot50 | AF-A0A2V3UNG3-F1-MODEL\_V4 | 1.0 | 1.454e-22 | 823 | 0.254 | 287 | 200 | 6 | 1 | 281 | 51 | 329 | Uncharacterized protein | Uncharacterized protein | | afdb-uniprot50 | AF-V5ZC41-F1-MODEL\_V4 | 1.0 | 5.181e-23 | 823 | 0.262 | 308 | 190 | 9 | 1 | 279 | 63 | 362 | Late control gene D protein GpD | Late control gene D protein GpD | | afdb-uniprot50 | AF-A0A839IXE6-F1-MODEL\_V4 | 1.0 | 6.21e-24 | 822 | 0.291 | 288 | 187 | 9 | 1 | 277 | 53 | 334 | Phage late control D family protein | Phage late control D family protein | | afdb-uniprot50 | AF-A0A833GCM2-F1-MODEL\_V4 | 1.0 | 3.275e-23 | 822 | 0.293 | 303 | 184 | 7 | 1 | 281 | 51 | 345 | Uncharacterized protein | Uncharacterized protein | | afdb-uniprot50 | AF-A0A379CBI1-F1-MODEL\_V4 | 1.0 | 4.119e-23 | 820 | 0.288 | 305 | 184 | 8 | 1 | 279 | 68 | 365 | Phage protein D | Phage protein D | | afdb-uniprot50 | AF-A0A1Y2S8A5-F1-MODEL\_V4 | 1.0 | 2.07e-23 | 819 | 0.258 | 333 | 188 | 8 | 1 | 282 | 75 | 399 | Phage protein | Phage protein | | afdb-uniprot50 | AF-A0A085A808-F1-MODEL\_V4 | 1.0 | 2.459e-23 | 818 | 0.253 | 272 | 187 | 8 | 22 | 282 | 5 | 271 | Phage late control gene D protein | Phage late control gene D protein | | afdb-uniprot50 | AF-A0A1Y1QXI4-F1-MODEL\_V4 | 1.0 | 6.154e-23 | 814 | 0.299 | 284 | 185 | 7 | 2 | 279 | 52 | 327 | Uncharacterized protein | Uncharacterized protein | | afdb-uniprot50 | AF-A0A3B0MDS7-F1-MODEL\_V4 | 1.0 | 3.437e-22 | 813 | 0.262 | 282 | 196 | 6 | 1 | 279 | 51 | 323 | Uncharacterized protein | Uncharacterized protein | | afdb-uniprot50 | AF-A0A2K3TWD5-F1-MODEL\_V4 | 1.0 | 4.119e-23 | 813 | 0.291 | 312 | 178 | 8 | 1 | 277 | 62 | 365 | Late control protein D | Late control protein D | | afdb-uniprot50 | AF-A0A0C5VFD9-F1-MODEL\_V4 | 1.0 | 1.031e-22 | 811 | 0.252 | 285 | 193 | 5 | 1 | 276 | 37 | 310 | Phage protein D | Phage protein D | | afdb-uniprot50 | AF-A0A2M8RTD2-F1-MODEL\_V4 | 1.0 | 1.236e-23 | 811 | 0.26 | 323 | 186 | 9 | 1 | 277 | 66 | 381 | Phage tail protein | Phage tail protein | | afdb-uniprot50 | AF-A0A7X6FQ70-F1-MODEL\_V4 | 1.0 | 4.119e-23 | 810 | 0.239 | 317 | 217 | 7 | 1 | 311 | 27 | 325 | Late control protein D | Late control protein D | | afdb-uniprot50 | AF-A0A2P1VV04-F1-MODEL\_V4 | 1.0 | 3.275e-23 | 809 | 0.298 | 288 | 184 | 10 | 1 | 276 | 53 | 334 | Phage tail protein | Phage tail protein | | afdb-uniprot50 | AF-J0ZBK7-F1-MODEL\_V4 | 1.0 | 9.824e-24 | 808 | 0.274 | 342 | 173 | 7 | 1 | 275 | 56 | 389 | Uncharacterized protein | Uncharacterized protein | | afdb-uniprot50 | AF-A0A2N3KSJ2-F1-MODEL\_V4 | 1.0 | 4.119e-23 | 807 | 0.318 | 289 | 179 | 10 | 2 | 281 | 54 | 333 | Uncharacterized protein | Uncharacterized protein | | afdb-uniprot50 | AF-A0A522JN25-F1-MODEL\_V4 | 1.0 | 3.065e-22 | 807 | 0.311 | 295 | 181 | 9 | 1 | 280 | 69 | 356 | Phage late control D family protein | Phage late control D family protein | | afdb-uniprot50 | AF-A0A1N6MRZ2-F1-MODEL\_V4 | 1.0 | 5.487e-23 | 807 | 0.237 | 312 | 205 | 7 | 1 | 286 | 86 | 390 | Late control gene D protein | Late control gene D protein | | afdb-uniprot50 | AF-A0A4Q0YKX8-F1-MODEL\_V4 | 1.0 | 9.735e-23 | 802 | 0.297 | 279 | 185 | 7 | 1 | 275 | 53 | 324 | Late control protein | Late control protein | | afdb-uniprot50 | AF-A0A6G0ZW07-F1-MODEL\_V4 | 1.0 | 4.805e-21 | 801 | 0.322 | 220 | 145 | 4 | 60 | 277 | 2 | 219 | Uncharacterized protein | Uncharacterized protein | | afdb-uniprot50 | AF-A0A2W6YRE0-F1-MODEL\_V4 | 1.0 | 7.74e-23 | 801 | 0.277 | 295 | 193 | 9 | 1 | 282 | 61 | 348 | Late control protein | Late control protein | | afdb-uniprot50 | AF-A0A8A6BLQ2-F1-MODEL\_V4 | 1.0 | 2.301e-22 | 800 | 0.271 | 287 | 191 | 5 | 1 | 276 | 54 | 333 | Phage tail protein | Phage tail protein | | afdb-uniprot50 | AF-A0A5S9R5J4-F1-MODEL\_V4 | 1.0 | 2.415e-21 | 799 | 0.245 | 224 | 165 | 3 | 57 | 277 | 1 | 223 | Uncharacterized protein | Uncharacterized protein | | afdb-uniprot50 | AF-A0A5N8AC16-F1-MODEL\_V4 | 1.0 | 4.848e-22 | 799 | 0.281 | 288 | 189 | 9 | 2 | 278 | 53 | 333 | Phage late control D family protein | Phage late control D family protein | | afdb-uniprot50 | AF-A0A3B0T6D5-F1-MODEL\_V4 | 1.0 | 3.673e-23 | 798 | 0.336 | 285 | 157 | 8 | 1 | 279 | 51 | 309 | Uncharacterized protein | Uncharacterized protein | | afdb-uniprot50 | AF-A0A2S2E755-F1-MODEL\_V4 | 1.0 | 8.602e-22 | 796 | 0.243 | 283 | 200 | 8 | 1 | 277 | 50 | 324 | Putative baseplate hub protein | Putative baseplate hub protein | | afdb-uniprot50 | AF-A0A258L5C4-F1-MODEL\_V4 | 1.0 | 2.58e-22 | 795 | 0.241 | 286 | 201 | 7 | 1 | 277 | 47 | 325 | Uncharacterized protein | Uncharacterized protein | | afdb-uniprot50 | AF-A0A4Q6D1D6-F1-MODEL\_V4 | 1.0 | 1.361e-21 | 792 | 0.246 | 280 | 199 | 5 | 1 | 279 | 50 | 318 | Uncharacterized protein | Uncharacterized protein | | afdb-uniprot50 | AF-B8GS02-F1-MODEL\_V4 | 1.0 | 1.224e-22 | 792 | 0.292 | 287 | 179 | 8 | 1 | 276 | 51 | 324 | Phage-related tail protein | Phage-related tail protein | | afdb-uniprot50 | AF-A0A6B8KJK1-F1-MODEL\_V4 | 1.0 | 6.839e-22 | 792 | 0.261 | 287 | 196 | 8 | 2 | 279 | 51 | 330 | Uncharacterized protein | Uncharacterized protein | | afdb-uniprot50 | AF-F7VG01-F1-MODEL\_V4 | 1.0 | 1.373e-22 | 791 | 0.266 | 270 | 173 | 6 | 41 | 292 | 2 | 264 | Phage late control D family protein | Phage late control D family protein | | afdb-uniprot50 | AF-A0A432QTK6-F1-MODEL\_V4 | 1.0 | 7.67e-22 | 791 | 0.286 | 279 | 183 | 6 | 1 | 276 | 51 | 316 | Uncharacterized protein | Uncharacterized protein | | afdb-uniprot50 | AF-A0A537MFG1-F1-MODEL\_V4 | 1.0 | 2.733e-22 | 789 | 0.269 | 304 | 186 | 9 | 2 | 276 | 57 | 353 | Phage late control D family protein | Phage late control D family protein | | afdb-uniprot50 | AF-A0A1I3SYP4-F1-MODEL\_V4 | 1.0 | 6.517e-23 | 788 | 0.24 | 320 | 220 | 10 | 1 | 311 | 52 | 357 | Uncharacterized protein | Uncharacterized protein | | afdb-uniprot50 | AF-A0A1M5PXU2-F1-MODEL\_V4 | 1.0 | 1.082e-21 | 787 | 0.237 | 286 | 200 | 9 | 1 | 277 | 56 | 332 | Phage protein D | Phage protein D | | afdb-uniprot50 | AF-A0A7W6WMD7-F1-MODEL\_V4 | 1.0 | 5.437e-22 | 786 | 0.256 | 292 | 195 | 7 | 1 | 281 | 52 | 332 | Uncharacterized protein | Uncharacterized protein | | afdb-uniprot50 | AF-A0A258E9L1-F1-MODEL\_V4 | 1.0 | 1.022e-21 | 784 | 0.278 | 284 | 189 | 6 | 1 | 275 | 54 | 330 | Uncharacterized protein | Uncharacterized protein | | afdb-uniprot50 | AF-A0A7X3TYP5-F1-MODEL\_V4 | 1.0 | 1.727e-22 | 783 | 0.278 | 291 | 189 | 9 | 2 | 279 | 53 | 335 | Uncharacterized protein | Uncharacterized protein | | afdb-uniprot50 | AF-J1JNX9-F1-MODEL\_V4 | 1.0 | 1.454e-22 | 782 | 0.272 | 283 | 185 | 10 | 1 | 277 | 43 | 310 | Uncharacterized protein | Uncharacterized protein | | afdb-uniprot50 | AF-A0A4Y7X954-F1-MODEL\_V4 | 1.0 | 9.193e-23 | 782 | 0.251 | 326 | 203 | 12 | 1 | 294 | 73 | 389 | DNA primase | DNA primase | | afdb-uniprot50 | AF-A0A5C8S7D7-F1-MODEL\_V4 | 1.0 | 1.082e-21 | 780 | 0.221 | 293 | 201 | 9 | 1 | 277 | 56 | 337 | Uncharacterized protein | Uncharacterized protein | | afdb-uniprot50 | AF-A0A6L9HG12-F1-MODEL\_V4 | 1.0 | 3.245e-22 | 775 | 0.181 | 319 | 238 | 10 | 2 | 311 | 1 | 305 | Uncharacterized protein | Uncharacterized protein | | afdb-uniprot50 | AF-A0A0Q8AN74-F1-MODEL\_V4 | 1.0 | 5.811e-23 | 775 | 0.242 | 297 | 203 | 6 | 18 | 311 | 84 | 361 | Uncharacterized protein | Uncharacterized protein | | afdb-uniprot50 | AF-A0A1C3HKM4-F1-MODEL\_V4 | 1.0 | 2.051e-22 | 773 | 0.229 | 335 | 207 | 9 | 2 | 310 | 64 | 373 | Phage late control gene D protein (GPD) | Phage late control gene D protein (GPD) | | afdb-uniprot50 | AF-A0A7G5CTF9-F1-MODEL\_V4 | 1.0 | 6.098e-22 | 765 | 0.22 | 331 | 207 | 9 | 1 | 288 | 62 | 384 | Phage late control D family protein | Phage late control D family protein | | afdb-uniprot50 | AF-A0A2N3DW94-F1-MODEL\_V4 | 1.0 | 5.042e-20 | 764 | 0.251 | 223 | 163 | 4 | 60 | 279 | 2 | 223 | Late control protein | Late control protein | | afdb-uniprot50 | AF-A0A852Y1I1-F1-MODEL\_V4 | 1.0 | 2.134e-20 | 764 | 0.257 | 284 | 195 | 7 | 1 | 276 | 55 | 330 | Uncharacterized protein | Uncharacterized protein | | afdb-uniprot50 | AF-A0A554XC54-F1-MODEL\_V4 | 1.0 | 5.758e-22 | 764 | 0.286 | 290 | 190 | 7 | 1 | 280 | 52 | 334 | Phage late control D protein (GPD) | Phage late control D protein (GPD) | | afdb-uniprot50 | AF-A0A7X3ZHS0-F1-MODEL\_V4 | 1.0 | 8.123e-22 | 762 | 0.26 | 280 | 195 | 6 | 1 | 276 | 51 | 322 | Uncharacterized protein | Uncharacterized protein | | afdb-uniprot50 | AF-A0A806CG79-F1-MODEL\_V4 | 1.0 | 3.82e-21 | 762 | 0.218 | 288 | 208 | 9 | 1 | 282 | 54 | 330 | Late control D family protein | Late control D family protein | | afdb-uniprot50 | AF-A0A317PFJ8-F1-MODEL\_V4 | 1.0 | 4.805e-21 | 762 | 0.212 | 292 | 209 | 9 | 1 | 279 | 55 | 338 | Uncharacterized protein | Uncharacterized protein | | afdb-uniprot50 | AF-A0A512IX34-F1-MODEL\_V4 | 1.0 | 2.733e-22 | 759 | 0.225 | 337 | 225 | 11 | 1 | 311 | 56 | 382 | Bacteriophage late control gene D protein | Bacteriophage late control gene D protein | | afdb-uniprot50 | AF-A0A1D2QMU3-F1-MODEL\_V4 | 1.0 | 2.393e-20 | 758 | 0.242 | 231 | 166 | 4 | 57 | 281 | 5 | 232 | Uncharacterized protein | Uncharacterized protein | | afdb-uniprot50 | AF-A0A1Y1R1L0-F1-MODEL\_V4 | 1.0 | 4.848e-22 | 758 | 0.261 | 271 | 184 | 6 | 13 | 278 | 3 | 262 | Uncharacterized protein | Uncharacterized protein | | afdb-uniprot50 | AF-A0A5V4G7N6-F1-MODEL\_V4 | 1.0 | 1.454e-22 | 758 | 0.285 | 298 | 162 | 7 | 32 | 286 | 1 | 290 | Phage late control D family protein | Phage late control D family protein | | afdb-uniprot50 | AF-A0A1N7LR18-F1-MODEL\_V4 | 1.0 | 6.266e-25 | 757 | 0.306 | 284 | 184 | 5 | 1 | 279 | 52 | 327 | Uncharacterized protein | Uncharacterized protein | | afdb-uniprot50 | AF-A0A4Q8MFD5-F1-MODEL\_V4 | 1.0 | 4.537e-21 | 757 | 0.256 | 285 | 196 | 6 | 1 | 277 | 52 | 328 | Uncharacterized protein | Uncharacterized protein | | afdb-uniprot50 | AF-J8V8B9-F1-MODEL\_V4 | 1.0 | 9.11e-22 | 757 | 0.28 | 285 | 178 | 10 | 1 | 276 | 51 | 317 | Tail protein D | Tail protein D | | afdb-uniprot50 | AF-V4JM50-F1-MODEL\_V4 | 1.0 | 3.89e-23 | 755 | 0.292 | 284 | 183 | 7 | 1 | 278 | 63 | 334 | Uncharacterized protein | Uncharacterized protein | | afdb-uniprot50 | AF-A0A1Y6HD93-F1-MODEL\_V4 | 1.0 | 3.855e-22 | 754 | 0.319 | 285 | 178 | 8 | 30 | 311 | 2 | 273 | Bacteriophage P2 gpD protein | Bacteriophage P2 gpD protein | | afdb-uniprot50 | AF-A0A2Z6GCV6-F1-MODEL\_V4 | 1.0 | 1.92e-21 | 752 | 0.221 | 398 | 185 | 8 | 1 | 280 | 54 | 444 | Late control D family protein | Late control D family protein | | afdb-uniprot50 | AF-F8KPK8-F1-MODEL\_V4 | 1.0 | 9.11e-22 | 751 | 0.222 | 283 | 200 | 12 | 1 | 281 | 45 | 309 | Phage late control D family protein | Phage late control D family protein | | afdb-uniprot50 | AF-A0A285M2K7-F1-MODEL\_V4 | 1.0 | 1.512e-20 | 751 | 0.239 | 284 | 201 | 8 | 2 | 278 | 54 | 329 | Phage late control gene D protein (GPD) | Phage late control gene D protein (GPD) | | afdb-uniprot50 | AF-A0A496KC99-F1-MODEL\_V4 | 1.0 | 3.855e-22 | 750 | 0.237 | 358 | 204 | 9 | 1 | 308 | 85 | 423 | Late control protein | Late control protein | | afdb-uniprot50 | AF-J0Q1E2-F1-MODEL\_V4 | 1.0 | 1.349e-20 | 749 | 0.281 | 224 | 149 | 5 | 57 | 277 | 70 | 284 | Uncharacterized protein | Uncharacterized protein | | afdb-uniprot50 | AF-A0A6N8TBC0-F1-MODEL\_V4 | 1.0 | 3.82e-21 | 749 | 0.236 | 288 | 202 | 11 | 2 | 279 | 54 | 333 | Uncharacterized protein | Uncharacterized protein | | afdb-uniprot50 | AF-A0A2G9WV33-F1-MODEL\_V4 | 1.0 | 2.033e-21 | 749 | 0.244 | 307 | 211 | 9 | 1 | 296 | 55 | 351 | Uncharacterized protein | Uncharacterized protein | | afdb-uniprot50 | AF-W1J5W8-F1-MODEL\_V4 | 1.0 | 6.458e-22 | 749 | 0.256 | 312 | 187 | 10 | 1 | 280 | 106 | 404 | Late control gene D protein | Late control gene D protein | | afdb-uniprot50 | AF-A0A4Y5WA89-F1-MODEL\_V4 | 1.0 | 2.173e-22 | 749 | 0.265 | 324 | 194 | 10 | 1 | 292 | 77 | 388 | Late control protein | Late control protein | | afdb-uniprot50 | AF-A0A2N2S1D8-F1-MODEL\_V4 | 1.0 | 1.441e-21 | 745 | 0.245 | 391 | 177 | 10 | 2 | 281 | 187 | 570 | Uncharacterized protein | Uncharacterized protein | | afdb-uniprot50 | AF-A0A2V4DTS8-F1-MODEL\_V4 | 1.0 | 4.537e-21 | 744 | 0.209 | 329 | 207 | 15 | 1 | 288 | 56 | 372 | Uncharacterized protein | Uncharacterized protein | | afdb-uniprot50 | AF-J0Q3C9-F1-MODEL\_V4 | 1.0 | 1.631e-22 | 744 | 0.266 | 338 | 174 | 7 | 1 | 272 | 56 | 385 | Uncharacterized protein | Uncharacterized protein | | afdb-uniprot50 | AF-A0A7I0BEW8-F1-MODEL\_V4 | 1.0 | 2.015e-20 | 740 | 0.26 | 230 | 156 | 6 | 57 | 278 | 15 | 238 | Late control protein | Late control protein | | afdb-uniprot50 | AF-E2CCF5-F1-MODEL\_V4 | 1.0 | 2.033e-21 | 740 | 0.203 | 330 | 222 | 10 | 1 | 311 | 51 | 358 | Phage late control D protein (GPD) | Phage late control D protein (GPD) | | afdb-uniprot50 | AF-A0A1G6QR90-F1-MODEL\_V4 | 1.0 | 8.602e-22 | 739 | 0.272 | 264 | 164 | 5 | 57 | 294 | 5 | 266 | Uncharacterized protein | Uncharacterized protein | | afdb-uniprot50 | AF-A0A2N5EEH1-F1-MODEL\_V4 | 1.0 | 3.406e-21 | 738 | 0.231 | 302 | 200 | 8 | 1 | 277 | 62 | 356 | Late control protein D | Late control protein D | | afdb-uniprot50 | AF-A0A327JNY9-F1-MODEL\_V4 | 1.0 | 3.574e-20 | 737 | 0.241 | 290 | 200 | 11 | 2 | 281 | 60 | 339 | Uncharacterized protein | Uncharacterized protein | | afdb-uniprot50 | AF-A0A3G2IAI4-F1-MODEL\_V4 | 1.0 | 5.864e-24 | 737 | 0.273 | 303 | 190 | 8 | 1 | 276 | 59 | 358 | Uncharacterized protein | Uncharacterized protein | | afdb-uniprot50 | AF-A0A1X3DKM2-F1-MODEL\_V4 | 1.0 | 4.848e-22 | 736 | 0.244 | 331 | 191 | 8 | 1 | 280 | 74 | 396 | Late control protein | Late control protein | | afdb-uniprot50 | AF-E3PAT4-F1-MODEL\_V4 | 1.0 | 3.187e-20 | 733 | 0.241 | 248 | 163 | 4 | 63 | 286 | 1 | 247 | Putative phage late gene regulator | Putative phage late gene regulator | | afdb-uniprot50 | AF-A0A4R3NVK0-F1-MODEL\_V4 | 1.0 | 1.349e-20 | 731 | 0.229 | 283 | 195 | 9 | 1 | 277 | 52 | 317 | Uncharacterized protein | Uncharacterized protein | | afdb-uniprot50 | AF-A0A5N3PH75-F1-MODEL\_V4 | 1.0 | 1.349e-20 | 731 | 0.243 | 292 | 199 | 11 | 1 | 279 | 54 | 336 | Late control protein | Late control protein | | afdb-uniprot50 | AF-A0A844H206-F1-MODEL\_V4 | 1.0 | 3.64e-22 | 729 | 0.267 | 318 | 208 | 7 | 1 | 310 | 51 | 351 | Phage tail protein | Phage tail protein | | afdb-uniprot50 | AF-A0A366DKG6-F1-MODEL\_V4 | 1.0 | 7.177e-21 | 726 | 0.238 | 319 | 223 | 6 | 1 | 309 | 52 | 360 | Phage protein D | Phage protein D | | afdb-uniprot50 | AF-E2CFJ7-F1-MODEL\_V4 | 1.0 | 1.003e-19 | 725 | 0.243 | 287 | 200 | 10 | 1 | 278 | 50 | 328 | Phage late control D protein (GPD) | Phage late control D protein (GPD) | | afdb-uniprot50 | AF-Q31Q84-F1-MODEL\_V4 | 1.0 | 2.415e-21 | 725 | 0.27 | 285 | 188 | 8 | 1 | 275 | 52 | 326 | Phage late control gene D protein GPD | Phage late control gene D protein GPD | | afdb-uniprot50 | AF-D4M9M1-F1-MODEL\_V4 | 1.0 | 6.777e-21 | 724 | 0.277 | 285 | 183 | 10 | 1 | 279 | 54 | 321 | Phage protein D | Phage protein D | | afdb-uniprot50 | AF-A0A175R4Y2-F1-MODEL\_V4 | 1.0 | 1.92e-21 | 724 | 0.25 | 324 | 222 | 12 | 1 | 311 | 51 | 366 | Uncharacterized protein | Uncharacterized protein | | afdb-uniprot50 | AF-A0A6L8M6A2-F1-MODEL\_V4 | 1.0 | 3.375e-20 | 722 | 0.244 | 278 | 201 | 6 | 1 | 277 | 47 | 316 | Uncharacterized protein | Uncharacterized protein | | afdb-uniprot50 | AF-A0A2C9D686-F1-MODEL\_V4 | 1.0 | 5.655e-20 | 721 | 0.228 | 289 | 205 | 10 | 1 | 279 | 55 | 335 | Tail protein | Tail protein | | afdb-uniprot50 | AF-A0A2D0L424-F1-MODEL\_V4 | 1.0 | 3.01e-20 | 720 | 0.188 | 414 | 193 | 8 | 1 | 279 | 75 | 480 | Phage protein | Phage protein | | afdb-uniprot50 | AF-U3BZ25-F1-MODEL\_V4 | 1.0 | 2.015e-20 | 719 | 0.25 | 283 | 199 | 8 | 1 | 279 | 47 | 320 | Uncharacterized protein | Uncharacterized protein | | afdb-uniprot50 | AF-A0A3N2E0P8-F1-MODEL\_V4 | 1.0 | 2.259e-20 | 716 | 0.236 | 288 | 194 | 10 | 1 | 277 | 75 | 347 | Phage protein D | Phage protein D | | afdb-uniprot50 | AF-A0A451AIR0-F1-MODEL\_V4 | 1.0 | 4.245e-20 | 715 | 0.377 | 244 | 143 | 4 | 1 | 242 | 51 | 287 | Uncharacterized protein | Uncharacterized protein | | afdb-uniprot50 | AF-A0A068T9R7-F1-MODEL\_V4 | 1.0 | 8.602e-22 | 715 | 0.236 | 296 | 201 | 14 | 1 | 281 | 53 | 338 | Phage late control D family protein | Phage late control D family protein | | afdb-uniprot50 | AF-A0A3M4MW84-F1-MODEL\_V4 | 1.0 | 1.885e-19 | 714 | 0.295 | 210 | 139 | 2 | 72 | 275 | 2 | 208 | Uncharacterized protein | Uncharacterized protein | | afdb-uniprot50 | AF-A0A2W6ZLW1-F1-MODEL\_V4 | 1.0 | 9.56e-21 | 714 | 0.236 | 326 | 223 | 12 | 1 | 311 | 54 | 368 | Late control protein | Late control protein | | afdb-uniprot50 | AF-A0A7W2GRY8-F1-MODEL\_V4 | 1.0 | 1.146e-21 | 713 | 0.275 | 283 | 191 | 8 | 1 | 277 | 51 | 325 | Late control protein | Late control protein | | afdb-uniprot50 | AF-A0A7L5XWR6-F1-MODEL\_V4 | 1.0 | 1.135e-20 | 712 | 0.323 | 229 | 148 | 2 | 84 | 311 | 1 | 223 | Uncharacterized protein | Uncharacterized protein | | afdb-uniprot50 | AF-A0A679JNK0-F1-MODEL\_V4 | 1.0 | 3.01e-20 | 712 | 0.216 | 296 | 200 | 12 | 1 | 279 | 57 | 337 | Uncharacterized protein | Uncharacterized protein | | afdb-uniprot50 | AF-A0A4Y9VRL2-F1-MODEL\_V4 | 1.0 | 4.045e-21 | 712 | 0.238 | 385 | 180 | 13 | 2 | 281 | 58 | 434 | Uncharacterized protein | Uncharacterized protein | | afdb-uniprot50 | AF-A0A379TSJ9-F1-MODEL\_V4 | 1.0 | 1.146e-21 | 708 | 0.223 | 331 | 199 | 13 | 5 | 292 | 2 | 317 | Putative bacteriophage regulatory protein | Putative bacteriophage regulatory protein | | afdb-uniprot50 | AF-Q0FZ18-F1-MODEL\_V4 | 1.0 | 1.512e-20 | 708 | 0.189 | 291 | 211 | 13 | 1 | 275 | 55 | 336 | Phage-related tail protein | Phage-related tail protein | | afdb-uniprot50 | AF-A0A0S4XLQ7-F1-MODEL\_V4 | 1.0 | 2.816e-19 | 703 | 0.266 | 278 | 186 | 9 | 1 | 275 | 50 | 312 | Putative phage tail protein D | Putative phage tail protein D | | afdb-uniprot50 | AF-U2FA85-F1-MODEL\_V4 | 1.0 | 4.761e-20 | 702 | 0.245 | 285 | 193 | 11 | 1 | 275 | 50 | 322 | Tail protein D, putative | Tail protein D, putative | | afdb-uniprot50 | AF-A0A6I1K313-F1-MODEL\_V4 | 1.0 | 6.716e-20 | 700 | 0.232 | 292 | 203 | 13 | 1 | 280 | 54 | 336 | Phage late control D family protein | Phage late control D family protein | | afdb-uniprot50 | AF-A0A3T0L2M0-F1-MODEL\_V4 | 1.0 | 7.532e-20 | 698 | 0.242 | 276 | 190 | 9 | 1 | 275 | 52 | 309 | Phage tail protein | Phage tail protein | | afdb-uniprot50 | AF-E6X1M2-F1-MODEL\_V4 | 1.0 | 7.532e-20 | 697 | 0.246 | 272 | 183 | 9 | 7 | 277 | 50 | 300 | Phage tail protein D | Phage tail protein D | | afdb-uniprot50 | AF-A0A165XHG8-F1-MODEL\_V4 | 1.0 | 5.34e-20 | 697 | 0.231 | 294 | 201 | 9 | 1 | 277 | 51 | 336 | Phage late control gene D protein (GPD) | Phage late control gene D protein (GPD) | | afdb-uniprot50 | AF-A0A1T4WXQ6-F1-MODEL\_V4 | 1.0 | 4.009e-20 | 695 | 0.241 | 277 | 191 | 8 | 7 | 275 | 57 | 322 | Uncharacterized protein | Uncharacterized protein | | afdb-uniprot50 | AF-A0A7Y3Y9A7-F1-MODEL\_V4 | 1.0 | 6.342e-20 | 691 | 0.23 | 282 | 203 | 8 | 2 | 279 | 46 | 317 | Uncharacterized protein | Uncharacterized protein | | afdb-uniprot50 | AF-A0A0B6CUQ7-F1-MODEL\_V4 | 1.0 | 7.601e-21 | 689 | 0.25 | 284 | 185 | 8 | 1 | 276 | 52 | 315 | Phage late control D family protein | Phage late control D family protein | | afdb-uniprot50 | AF-A0A651FN49-F1-MODEL\_V4 | 1.0 | 2.816e-19 | 687 | 0.212 | 292 | 210 | 9 | 1 | 280 | 56 | 339 | Late control D family protein | Late control D family protein | | afdb-uniprot50 | AF-A0A6L5YCW9-F1-MODEL\_V4 | 1.0 | 5.292e-19 | 685 | 0.235 | 289 | 199 | 10 | 1 | 281 | 54 | 328 | Uncharacterized protein | Uncharacterized protein | | afdb-uniprot50 | AF-D2U2W5-F1-MODEL\_V4 | 1.0 | 2.534e-20 | 685 | 0.217 | 299 | 198 | 10 | 2 | 278 | 38 | 322 | Phage transcriptional regulator | Phage transcriptional regulator | | afdb-uniprot50 | AF-A0A179SHY2-F1-MODEL\_V4 | 1.0 | 3.345e-19 | 684 | 0.221 | 289 | 204 | 10 | 1 | 275 | 55 | 336 | Uncharacterized protein | Uncharacterized protein | | afdb-uniprot50 | AF-A0A063B487-F1-MODEL\_V4 | 1.0 | 9.389e-19 | 681 | 0.268 | 212 | 142 | 4 | 72 | 275 | 2 | 208 | Uncharacterized protein | Uncharacterized protein | | afdb-uniprot50 | AF-A0A1I7E360-F1-MODEL\_V4 | 1.0 | 5.34e-20 | 681 | 0.273 | 256 | 146 | 4 | 72 | 289 | 265 | 518 | Phage tail tape measure protein, TP901 family, core region | Phage tail tape measure protein, TP901 family, core region | | afdb-uniprot50 | AF-A0A7X6WN95-F1-MODEL\_V4 | 1.0 | 1.403e-18 | 680 | 0.293 | 252 | 169 | 5 | 2 | 251 | 56 | 300 | Phage late control D family protein | Phage late control D family protein | | afdb-uniprot50 | AF-A0A8B4S4Z3-F1-MODEL\_V4 | 1.0 | 3.542e-19 | 678 | 0.266 | 255 | 155 | 8 | 26 | 277 | 2 | 227 | Phage protein D | Phage protein D | | afdb-uniprot50 | AF-A0A1I5USE1-F1-MODEL\_V4 | 1.0 | 1.273e-20 | 678 | 0.305 | 278 | 167 | 15 | 1 | 276 | 43 | 296 | Uncharacterized protein | Uncharacterized protein | | afdb-uniprot50 | AF-A0A7X5L5E7-F1-MODEL\_V4 | 1.0 | 1.499e-19 | 675 | 0.179 | 324 | 235 | 14 | 1 | 311 | 68 | 373 | Uncharacterized protein | Uncharacterized protein | | afdb-uniprot50 | AF-A0A7X3T0M9-F1-MODEL\_V4 | 1.0 | 3.973e-19 | 674 | 0.275 | 269 | 161 | 6 | 1 | 243 | 63 | 323 | Phage late control D family protein | Phage late control D family protein | | afdb-uniprot50 | AF-B6JEG4-F1-MODEL\_V4 | 1.0 | 4.997e-19 | 674 | 0.215 | 292 | 209 | 11 | 1 | 279 | 52 | 336 | Putative phage late control protein D | Putative phage late control protein D | | afdb-uniprot50 | AF-A0A109LFY2-F1-MODEL\_V4 | 1.0 | 3.345e-19 | 672 | 0.247 | 251 | 172 | 7 | 39 | 279 | 1 | 244 | Phage late control protein D protein (GPD) | Phage late control protein D protein (GPD) | | afdb-uniprot50 | AF-A0A7Y4XEN4-F1-MODEL\_V4 | 1.0 | 4.997e-19 | 672 | 0.208 | 288 | 204 | 10 | 2 | 279 | 59 | 332 | Cro/Cl family transcriptional regulator | Cro/Cl family transcriptional regulator | | afdb-uniprot50 | AF-A0A4Y8P2F6-F1-MODEL\_V4 | 1.0 | 2.766e-17 | 670 | 0.432 | 164 | 93 | 0 | 2 | 165 | 60 | 223 | Phage late control D family protein | Phage late control D family protein | | afdb-uniprot50 | AF-A0A4P8G0Y2-F1-MODEL\_V4 | 1.0 | 1.192e-19 | 669 | 0.234 | 294 | 203 | 10 | 1 | 279 | 51 | 337 | Phage late control D family protein | Phage late control D family protein | | afdb-uniprot50 | AF-A0A435G0Q0-F1-MODEL\_V4 | 1.0 | 1.403e-18 | 667 | 0.24 | 245 | 172 | 7 | 41 | 279 | 3 | 239 | Uncharacterized protein | Uncharacterized protein | | afdb-uniprot50 | AF-A0A4R7WMC5-F1-MODEL\_V4 | 1.0 | 1.587e-19 | 667 | 0.183 | 322 | 239 | 10 | 1 | 311 | 56 | 364 | Uncharacterized protein | Uncharacterized protein | | afdb-uniprot50 | AF-E5VKJ0-F1-MODEL\_V4 | 1.0 | 2.982e-19 | 664 | 0.212 | 292 | 207 | 10 | 1 | 284 | 66 | 342 | Uncharacterized protein | Uncharacterized protein | | afdb-uniprot50 | AF-A0A285V0M3-F1-MODEL\_V4 | 1.0 | 6.285e-19 | 662 | 0.215 | 288 | 212 | 8 | 1 | 279 | 53 | 335 | Uncharacterized protein | Uncharacterized protein | | afdb-uniprot50 | AF-C6LFY9-F1-MODEL\_V4 | 1.0 | 1.181e-18 | 657 | 0.188 | 265 | 197 | 9 | 18 | 279 | 89 | 338 | Uncharacterized protein | Uncharacterized protein | | afdb-uniprot50 | AF-A0A4R2GH06-F1-MODEL\_V4 | 1.0 | 1.053e-18 | 657 | 0.222 | 296 | 198 | 11 | 1 | 280 | 53 | 332 | Uncharacterized protein | Uncharacterized protein | | afdb-uniprot50 | AF-A0A1E4LS02-F1-MODEL\_V4 | 1.0 | 1.499e-19 | 656 | 0.209 | 291 | 196 | 12 | 26 | 299 | 5 | 278 | Uncharacterized protein | Uncharacterized protein | | afdb-uniprot50 | AF-A0A1A9WZ35-F1-MODEL\_V4 | 1.0 | 2.982e-19 | 656 | 0.201 | 352 | 229 | 11 | 2 | 311 | 278 | 619 | Uncharacterized protein | Uncharacterized protein | | afdb-uniprot50 | AF-A0A0J6NA89-F1-MODEL\_V4 | 1.0 | 1.115e-18 | 655 | 0.243 | 292 | 193 | 10 | 2 | 279 | 64 | 341 | Bacteriophage regulatory protein | Bacteriophage regulatory protein | | afdb-uniprot50 | AF-A0A2A2HM81-F1-MODEL\_V4 | 1.0 | 2.982e-19 | 654 | 0.222 | 301 | 194 | 10 | 2 | 278 | 67 | 351 | Phage protein D | Phage protein D | | afdb-uniprot50 | AF-A0A2G1CU53-F1-MODEL\_V4 | 1.0 | 1.063e-19 | 652 | 0.248 | 278 | 193 | 9 | 1 | 275 | 48 | 312 | Phage tail protein | Phage tail protein | | afdb-uniprot50 | AF-A0A3L7ALJ4-F1-MODEL\_V4 | 1.0 | 4.415e-18 | 652 | 0.201 | 288 | 211 | 9 | 1 | 277 | 51 | 330 | Uncharacterized protein | Uncharacterized protein | | afdb-uniprot50 | AF-Q8ZKJ7-F1-MODEL\_V4 | 1.0 | 1.181e-18 | 652 | 0.214 | 289 | 200 | 9 | 2 | 277 | 68 | 342 | Putative cytoplasmic protein | Putative cytoplasmic protein | | afdb-uniprot50 | AF-A0A022PIB0-F1-MODEL\_V4 | 1.0 | 1.764e-18 | 651 | 0.285 | 263 | 155 | 8 | 1 | 238 | 14 | 268 | Phage protein D | Phage protein D | | afdb-uniprot50 | AF-A0A1G3UAZ7-F1-MODEL\_V4 | 1.0 | 7.464e-19 | 651 | 0.228 | 280 | 196 | 10 | 1 | 275 | 50 | 314 | Uncharacterized protein | Uncharacterized protein | | afdb-uniprot50 | AF-A0A5R8Y6A7-F1-MODEL\_V4 | 1.0 | 1.262e-19 | 651 | 0.215 | 316 | 223 | 15 | 9 | 309 | 61 | 366 | Uncharacterized protein | Uncharacterized protein | | afdb-uniprot50 | AF-A0A6C8XQL5-F1-MODEL\_V4 | 1.0 | 6.716e-20 | 651 | 0.201 | 328 | 211 | 15 | 2 | 292 | 74 | 387 | Phage protein D | Phage protein D | | afdb-uniprot50 | AF-A0A7W4RH00-F1-MODEL\_V4 | 1.0 | 9.304e-18 | 649 | 0.311 | 202 | 133 | 6 | 84 | 280 | 1 | 201 | Phage protein D | Phage protein D | | afdb-uniprot50 | AF-A0A3R8K9T6-F1-MODEL\_V4 | 1.0 | 2.982e-19 | 649 | 0.227 | 308 | 204 | 11 | 2 | 292 | 75 | 365 | Phage late control D family protein | Phage late control D family protein | | afdb-uniprot50 | AF-A0A6C1BNV6-F1-MODEL\_V4 | 1.0 | 1.485e-18 | 647 | 0.196 | 285 | 205 | 12 | 2 | 280 | 64 | 330 | Uncharacterized protein | Uncharacterized protein | | afdb-uniprot50 | AF-A0A090G3M4-F1-MODEL\_V4 | 1.0 | 3.51e-18 | 647 | 0.221 | 294 | 210 | 10 | 1 | 284 | 52 | 336 | Uncharacterized protein | Uncharacterized protein | | afdb-uniprot50 | AF-A0A5W6TUF8-F1-MODEL\_V4 | 1.0 | 4.952e-18 | 646 | 0.245 | 281 | 180 | 7 | 1 | 256 | 66 | 339 | Phage late control D family protein | Phage late control D family protein | | afdb-uniprot50 | AF-A0A087KKM1-F1-MODEL\_V4 | 1.0 | 7.532e-20 | 646 | 0.238 | 327 | 198 | 13 | 2 | 292 | 75 | 386 | Bacteriophage regulatory protein | Bacteriophage regulatory protein | | afdb-uniprot50 | AF-A0A7X5I0X9-F1-MODEL\_V4 | 1.0 | 8.865e-19 | 645 | 0.192 | 301 | 213 | 11 | 15 | 310 | 78 | 353 | LysM peptidoglycan-binding domain-containing protein | LysM peptidoglycan-binding domain-containing protein | | afdb-uniprot50 | AF-A0A1B9JGB6-F1-MODEL\_V4 | 1.0 | 1.053e-18 | 644 | 0.175 | 400 | 198 | 9 | 2 | 277 | 57 | 448 | Uncharacterized protein | Uncharacterized protein | | afdb-uniprot50 | AF-A0A1H0W5E2-F1-MODEL\_V4 | 1.0 | 7.834e-18 | 641 | 0.267 | 239 | 155 | 5 | 72 | 308 | 2 | 222 | Phage late control gene D protein (GPD) | Phage late control gene D protein (GPD) | | afdb-uniprot50 | AF-A0A6P0VER2-F1-MODEL\_V4 | 1.0 | 1.666e-18 | 641 | 0.21 | 299 | 199 | 14 | 1 | 284 | 9 | 285 | Uncharacterized protein | Uncharacterized protein | | afdb-uniprot50 | AF-A0A743PFW0-F1-MODEL\_V4 | 1.0 | 8.946e-20 | 641 | 0.223 | 327 | 203 | 12 | 2 | 292 | 64 | 375 | Phage protein D | Phage protein D | | afdb-uniprot50 | AF-A0A7W3CBM1-F1-MODEL\_V4 | 1.0 | 1.78e-19 | 641 | 0.212 | 329 | 206 | 14 | 2 | 292 | 75 | 388 | Phage late control D family protein | Phage late control D family protein | | afdb-uniprot50 | AF-A0A7U6KDT6-F1-MODEL\_V4 | 1.0 | 2.791e-18 | 639 | 0.193 | 264 | 200 | 9 | 17 | 279 | 83 | 334 | Putative bacteriophage regulatory protein | Putative bacteriophage regulatory protein | | afdb-uniprot50 | AF-A0A1M3AKH3-F1-MODEL\_V4 | 1.0 | 7.464e-19 | 639 | 0.213 | 323 | 219 | 13 | 1 | 311 | 53 | 352 | Late control D family protein | Late control D family protein | | afdb-uniprot50 | AF-A0A1C6ENZ2-F1-MODEL\_V4 | 1.0 | 5.881e-18 | 639 | 0.223 | 264 | 191 | 7 | 17 | 279 | 83 | 333 | LysM domain/BON superfamily protein | LysM domain/BON superfamily protein | | afdb-uniprot50 | AF-A0A2A3D6W1-F1-MODEL\_V4 | 1.0 | 5.553e-18 | 638 | 0.203 | 295 | 214 | 11 | 1 | 284 | 52 | 336 | Uncharacterized protein | Uncharacterized protein | | afdb-uniprot50 | AF-A0A7X5CGI8-F1-MODEL\_V4 | 1.0 | 6.228e-18 | 638 | 0.16 | 299 | 226 | 11 | 1 | 289 | 58 | 341 | LysM peptidoglycan-binding domain-containing protein | LysM peptidoglycan-binding domain-containing protein | | afdb-uniprot50 | AF-A0A0R3KWX4-F1-MODEL\_V4 | 1.0 | 8.371e-19 | 637 | 0.222 | 328 | 220 | 14 | 1 | 311 | 55 | 364 | Uncharacterized protein | Uncharacterized protein | | afdb-uniprot50 | AF-A0A1J5I2G1-F1-MODEL\_V4 | 1.0 | 1.666e-18 | 636 | 0.18 | 294 | 209 | 13 | 1 | 277 | 54 | 332 | Uncharacterized protein | Uncharacterized protein | | afdb-uniprot50 | AF-A0A2D3T999-F1-MODEL\_V4 | 1.0 | 3.285e-17 | 635 | 0.339 | 165 | 109 | 0 | 113 | 277 | 3 | 167 | Uncharacterized protein | Uncharacterized protein | | afdb-uniprot50 | AF-A0A6L5TD89-F1-MODEL\_V4 | 1.0 | 5.604e-19 | 634 | 0.216 | 328 | 217 | 14 | 1 | 311 | 57 | 361 | Uncharacterized protein | Uncharacterized protein | | afdb-uniprot50 | AF-A0A1B4SFB1-F1-MODEL\_V4 | 1.0 | 1.324e-18 | 632 | 0.19 | 341 | 215 | 11 | 2 | 311 | 73 | 383 | Cro/Cl family transcriptional regulator | Cro/Cl family transcriptional regulator | | afdb-uniprot50 | AF-A0A1M3BPS0-F1-MODEL\_V4 | 1.0 | 6.596e-18 | 631 | 0.223 | 282 | 195 | 10 | 7 | 272 | 51 | 324 | Uncharacterized protein | Uncharacterized protein | | afdb-uniprot50 | AF-A8SB41-F1-MODEL\_V4 | 1.0 | 7.834e-18 | 631 | 0.188 | 260 | 199 | 6 | 18 | 276 | 82 | 330 | Uncharacterized protein | Uncharacterized protein | | afdb-uniprot50 | AF-R7JNW8-F1-MODEL\_V4 | 1.0 | 1.181e-18 | 630 | 0.216 | 291 | 202 | 10 | 2 | 279 | 59 | 336 | Phage late control gene D protein (GPD) putative | Phage late control gene D protein (GPD) putative | | afdb-uniprot50 | AF-A0A0P6VKL9-F1-MODEL\_V4 | 1.0 | 1.39e-17 | 629 | 0.205 | 282 | 206 | 8 | 9 | 279 | 72 | 346 | Uncharacterized protein | Uncharacterized protein | | afdb-uniprot50 | AF-A0A370X1T7-F1-MODEL\_V4 | 1.0 | 8.296e-18 | 628 | 0.304 | 240 | 149 | 5 | 74 | 310 | 38 | 262 | Uncharacterized protein | Uncharacterized protein | | afdb-uniprot50 | AF-A0A371J384-F1-MODEL\_V4 | 1.0 | 4.718e-19 | 628 | 0.184 | 314 | 213 | 15 | 2 | 296 | 61 | 350 | LysM peptidoglycan-binding domain-containing protein | LysM peptidoglycan-binding domain-containing protein | | afdb-uniprot50 | AF-A0A161VRP1-F1-MODEL\_V4 | 1.0 | 5.935e-19 | 627 | 0.185 | 297 | 204 | 13 | 2 | 279 | 19 | 296 | Late control D family protein | Late control D family protein | | afdb-uniprot50 | AF-A0A1M7LUC3-F1-MODEL\_V4 | 1.0 | 8.296e-18 | 627 | 0.197 | 289 | 207 | 12 | 2 | 283 | 58 | 328 | LysM domain-containing protein | LysM domain-containing protein | | afdb-uniprot50 | AF-A0A2V2GIE6-F1-MODEL\_V4 | 1.0 | 1.105e-17 | 627 | 0.208 | 264 | 195 | 7 | 18 | 278 | 79 | 331 | Uncharacterized protein | Uncharacterized protein | | afdb-uniprot50 | AF-A0A2U1CFJ9-F1-MODEL\_V4 | 1.0 | 1.666e-18 | 625 | 0.175 | 308 | 226 | 13 | 16 | 311 | 185 | 476 | Uncharacterized protein | Uncharacterized protein | | afdb-uniprot50 | AF-A0A4U8UEB9-F1-MODEL\_V4 | 1.0 | 1.181e-18 | 624 | 0.265 | 279 | 182 | 10 | 1 | 276 | 50 | 308 | Phage tail protein | Phage tail protein | | afdb-uniprot50 | AF-A0A0Q7A3Q8-F1-MODEL\_V4 | 1.0 | 8.785e-18 | 624 | 0.202 | 271 | 197 | 9 | 20 | 279 | 69 | 331 | Uncharacterized protein | Uncharacterized protein | | afdb-uniprot50 | AF-C1D832-F1-MODEL\_V4 | 1.0 | 1.764e-18 | 624 | 0.23 | 299 | 194 | 10 | 2 | 278 | 64 | 348 | Probable bacteriophage regulatory protein | Probable bacteriophage regulatory protein | | afdb-uniprot50 | AF-A0A3E2T6D6-F1-MODEL\_V4 | 1.0 | 1.17e-17 | 623 | 0.209 | 287 | 202 | 11 | 2 | 278 | 58 | 329 | Uncharacterized protein | Uncharacterized protein | | afdb-uniprot50 | AF-A0A4D7AV46-F1-MODEL\_V4 | 1.0 | 2.466e-17 | 622 | 0.211 | 284 | 202 | 10 | 2 | 276 | 62 | 332 | Late control protein D | Late control protein D | | afdb-uniprot50 | AF-A0A2U8VQW8-F1-MODEL\_V4 | 1.0 | 5.553e-18 | 622 | 0.217 | 303 | 205 | 10 | 1 | 280 | 52 | 345 | Uncharacterized protein | Uncharacterized protein | | afdb-uniprot50 | AF-A0A2U0TFH0-F1-MODEL\_V4 | 1.0 | 6.656e-19 | 620 | 0.188 | 302 | 220 | 10 | 20 | 311 | 72 | 358 | Uncharacterized protein | Uncharacterized protein | | afdb-uniprot50 | AF-A0A1R1MKJ1-F1-MODEL\_V4 | 1.0 | 9.943e-19 | 619 | 0.246 | 288 | 188 | 14 | 2 | 276 | 59 | 330 | Uncharacterized protein | Uncharacterized protein | | afdb-uniprot50 | AF-A0A385Q3K1-F1-MODEL\_V4 | 1.0 | 6.656e-19 | 619 | 0.181 | 297 | 216 | 8 | 16 | 310 | 72 | 343 | LysM peptidoglycan-binding domain-containing protein | LysM peptidoglycan-binding domain-containing protein | | afdb-uniprot50 | AF-A0A1S7Q5S5-F1-MODEL\_V4 | 1.0 | 1.651e-17 | 617 | 0.208 | 283 | 205 | 10 | 9 | 280 | 117 | 391 | Phage late control D family protein | Phage late control D family protein | | afdb-uniprot50 | AF-A0A4R6MB46-F1-MODEL\_V4 | 1.0 | 6.985e-18 | 616 | 0.2 | 279 | 198 | 8 | 1 | 279 | 50 | 303 | Uncharacterized protein | Uncharacterized protein | | afdb-uniprot50 | AF-A0A1X3HWQ9-F1-MODEL\_V4 | 1.0 | 3.479e-17 | 616 | 0.25 | 264 | 173 | 6 | 1 | 246 | 68 | 324 | Bacteriophage late protein control protein D | Bacteriophage late protein control protein D | | afdb-uniprot50 | AF-A0A285M8H1-F1-MODEL\_V4 | 1.0 | 1.764e-18 | 616 | 0.194 | 304 | 218 | 11 | 18 | 311 | 71 | 357 | Uncharacterized protein | Uncharacterized protein | | afdb-uniprot50 | AF-A0A451AF14-F1-MODEL\_V4 | 1.0 | 2.199e-17 | 615 | 0.416 | 197 | 108 | 2 | 22 | 218 | 2 | 191 | Phage late control gene D protein (GPD) | Phage late control gene D protein (GPD) | | afdb-uniprot50 | AF-A0A163TCJ4-F1-MODEL\_V4 | 1.0 | 1.403e-18 | 614 | 0.209 | 272 | 185 | 11 | 25 | 279 | 1 | 259 | Late control D family protein | Late control D family protein | | afdb-uniprot50 | AF-A0A268U3E7-F1-MODEL\_V4 | 1.0 | 3.51e-18 | 614 | 0.2 | 274 | 198 | 9 | 1 | 274 | 48 | 300 | Uncharacterized protein | Uncharacterized protein | | afdb-uniprot50 | AF-A0A2S9IK07-F1-MODEL\_V4 | 1.0 | 1.472e-17 | 614 | 0.21 | 295 | 203 | 11 | 1 | 279 | 53 | 333 | Late control D family protein | Late control D family protein | | afdb-uniprot50 | AF-A0A0H3ZXP3-F1-MODEL\_V4 | 1.0 | 3.102e-17 | 612 | 0.245 | 200 | 147 | 3 | 82 | 278 | 4 | 202 | Phage protein D | Phage protein D | | afdb-uniprot50 | AF-A0A037UNC0-F1-MODEL\_V4 | 1.0 | 5.553e-18 | 612 | 0.245 | 273 | 186 | 10 | 20 | 279 | 68 | 333 | Late control protein D | Late control protein D | | afdb-uniprot50 | AF-A0A2T7H7Y0-F1-MODEL\_V4 | 1.0 | 1.485e-18 | 611 | 0.217 | 294 | 206 | 9 | 1 | 279 | 54 | 338 | Uncharacterized protein | Uncharacterized protein | | afdb-uniprot50 | AF-A0A3P6KIP4-F1-MODEL\_V4 | 1.0 | 8.296e-18 | 611 | 0.184 | 293 | 210 | 10 | 2 | 281 | 86 | 362 | Phage protein D | Phage protein D | | afdb-uniprot50 | AF-A0A1B9LF16-F1-MODEL\_V4 | 1.0 | 2.114e-19 | 608 | 0.253 | 308 | 193 | 10 | 1 | 281 | 56 | 353 | Uncharacterized protein | Uncharacterized protein | | afdb-uniprot50 | AF-A0A6P0J3I4-F1-MODEL\_V4 | 1.0 | 2.488e-18 | 607 | 0.167 | 304 | 210 | 12 | 3 | 279 | 20 | 307 | Phage late control D family protein | Phage late control D family protein | | afdb-uniprot50 | AF-A0A3D0U3C5-F1-MODEL\_V4 | 1.0 | 9.943e-19 | 607 | 0.206 | 305 | 190 | 14 | 2 | 278 | 26 | 306 | Uncharacterized protein | Uncharacterized protein | | afdb-uniprot50 | AF-A0A212S2I3-F1-MODEL\_V4 | 1.0 | 9.853e-18 | 606 | 0.243 | 267 | 186 | 8 | 22 | 279 | 77 | 336 | Uncharacterized protein | Uncharacterized protein | | afdb-uniprot50 | AF-W4SDY7-F1-MODEL\_V4 | 1.0 | 2.588e-16 | 604 | 0.331 | 172 | 112 | 3 | 109 | 277 | 2 | 173 | Uncharacterized protein | Uncharacterized protein | | afdb-uniprot50 | AF-A0A6I0CE52-F1-MODEL\_V4 | 1.0 | 5.828e-17 | 603 | 0.206 | 295 | 202 | 11 | 2 | 279 | 64 | 343 | Late control protein D | Late control protein D | | afdb-uniprot50 | AF-A0A6L2ZNF4-F1-MODEL\_V4 | 1.0 | 3.479e-17 | 602 | 0.28 | 203 | 128 | 3 | 95 | 279 | 4 | 206 | Late control gene D protein | Late control gene D protein | | afdb-uniprot50 | AF-A0A7C2ARE1-F1-MODEL\_V4 | 1.0 | 1.239e-17 | 602 | 0.222 | 283 | 194 | 13 | 3 | 276 | 65 | 330 | Uncharacterized protein | Uncharacterized protein | | afdb-uniprot50 | AF-A0A376EWE4-F1-MODEL\_V4 | 1.0 | 2.488e-18 | 601 | 0.233 | 330 | 215 | 13 | 2 | 311 | 75 | 386 | Gene D protein | Gene D protein | | afdb-uniprot50 | AF-A0A3A6K9Q8-F1-MODEL\_V4 | 1.0 | 1.472e-17 | 600 | 0.179 | 268 | 197 | 11 | 15 | 276 | 81 | 331 | Preprotein translocase subunit TatB | Preprotein translocase subunit TatB | | afdb-uniprot50 | AF-E2CJH8-F1-MODEL\_V4 | 1.0 | 1.651e-17 | 600 | 0.211 | 274 | 194 | 12 | 19 | 279 | 70 | 334 | Late control D family protein | Late control D family protein | | afdb-uniprot50 | AF-A0NQA6-F1-MODEL\_V4 | 1.0 | 6.985e-18 | 595 | 0.214 | 280 | 186 | 12 | 19 | 279 | 67 | 331 | Uncharacterized protein | Uncharacterized protein | | afdb-uniprot50 | AF-M5K2K4-F1-MODEL\_V4 | 1.0 | 1.651e-17 | 595 | 0.22 | 327 | 224 | 15 | 1 | 311 | 53 | 364 | Phage late control D family protein | Phage late control D family protein | | afdb-uniprot50 | AF-A0A3A9CQQ7-F1-MODEL\_V4 | 1.0 | 1.651e-17 | 595 | 0.182 | 307 | 217 | 14 | 1 | 297 | 175 | 457 | LysM peptidoglycan-binding domain-containing protein | LysM peptidoglycan-binding domain-containing protein | | afdb-uniprot50 | AF-A0A3D2IA26-F1-MODEL\_V4 | 1.0 | 4.634e-17 | 592 | 0.195 | 332 | 224 | 19 | 1 | 311 | 58 | 367 | Uncharacterized protein | Uncharacterized protein | | afdb-uniprot50 | AF-A0A7X2NKR7-F1-MODEL\_V4 | 1.0 | 1.545e-16 | 592 | 0.194 | 293 | 204 | 10 | 1 | 280 | 57 | 330 | LysM peptidoglycan-binding domain-containing protein | LysM peptidoglycan-binding domain-containing protein | | afdb-uniprot50 | AF-A0A1G6EJ79-F1-MODEL\_V4 | 1.0 | 2.929e-17 | 591 | 0.219 | 292 | 200 | 13 | 1 | 279 | 53 | 329 | Uncharacterized protein | Uncharacterized protein | | afdb-uniprot50 | AF-A0A0X1U7U0-F1-MODEL\_V4 | 1.0 | 1.16e-16 | 590 | 0.178 | 269 | 199 | 9 | 15 | 276 | 74 | 327 | Phage late control protein D | Phage late control protein D | | afdb-uniprot50 | AF-A0A5R9GK92-F1-MODEL\_V4 | 1.0 | 2.35e-18 | 588 | 0.222 | 306 | 186 | 10 | 4 | 279 | 83 | 366 | Phage late control D family protein | Phage late control D family protein | | afdb-uniprot50 | AF-A0A318LI70-F1-MODEL\_V4 | 1.0 | 6.228e-18 | 587 | 0.233 | 334 | 215 | 14 | 2 | 311 | 62 | 378 | Uncharacterized protein | Uncharacterized protein | | afdb-uniprot50 | AF-A0A2D2CYN5-F1-MODEL\_V4 | 1.0 | 4.131e-17 | 585 | 0.193 | 300 | 212 | 12 | 2 | 279 | 54 | 345 | Uncharacterized protein | Uncharacterized protein | | afdb-uniprot50 | AF-A0A317H3Z9-F1-MODEL\_V4 | 1.0 | 1.39e-17 | 581 | 0.167 | 347 | 236 | 15 | 1 | 311 | 84 | 413 | Uncharacterized protein | Uncharacterized protein | | afdb-uniprot50 | AF-A0A434TY84-F1-MODEL\_V4 | 1.0 | 5.244e-18 | 580 | 0.213 | 295 | 201 | 12 | 26 | 310 | 8 | 281 | Uncharacterized protein | Uncharacterized protein | | afdb-uniprot50 | AF-A0A327KRF2-F1-MODEL\_V4 | 1.0 | 9.676e-16 | 574 | 0.301 | 189 | 128 | 3 | 93 | 277 | 1 | 189 | Uncharacterized protein | Uncharacterized protein | | afdb-uniprot50 | AF-A0A7W6PUG3-F1-MODEL\_V4 | 1.0 | 9.22e-17 | 574 | 0.203 | 275 | 188 | 12 | 19 | 277 | 82 | 341 | Uncharacterized protein | Uncharacterized protein | | afdb-uniprot50 | AF-A0A5E8H666-F1-MODEL\_V4 | 1.0 | 7.397e-18 | 572 | 0.21 | 309 | 207 | 12 | 20 | 311 | 69 | 357 | Phage protein D | Phage protein D | | afdb-uniprot50 | AF-A0A0D6KHQ0-F1-MODEL\_V4 | 1.0 | 4.907e-17 | 571 | 0.153 | 299 | 211 | 11 | 7 | 279 | 73 | 355 | Bacteriophage protein D | Bacteriophage protein D | | afdb-uniprot50 | AF-A0A2I8Q7T7-F1-MODEL\_V4 | 1.0 | 1.16e-16 | 571 | 0.231 | 272 | 178 | 7 | 2 | 249 | 81 | 345 | Uncharacterized protein | Uncharacterized protein | | afdb-uniprot50 | AF-A0A7B9FRA4-F1-MODEL\_V4 | 1.0 | 1.472e-17 | 570 | 0.268 | 246 | 134 | 7 | 89 | 291 | 1 | 243 | Phage late control D family protein | Phage late control D family protein | | afdb-uniprot50 | AF-A0A2C9D5M1-F1-MODEL\_V4 | 1.0 | 5.197e-17 | 569 | 0.2 | 279 | 192 | 12 | 20 | 279 | 68 | 334 | Tail protein | Tail protein | | afdb-uniprot50 | AF-A0A445ZUU9-F1-MODEL\_V4 | 1.0 | 1.312e-17 | 569 | 0.256 | 277 | 172 | 11 | 1 | 261 | 51 | 309 | Uncharacterized protein | Uncharacterized protein | | afdb-uniprot50 | AF-A0A2X2S5N7-F1-MODEL\_V4 | 1.0 | 6.536e-17 | 568 | 0.236 | 250 | 158 | 6 | 79 | 308 | 2 | 238 | Phage tail protein | Phage tail protein | | afdb-uniprot50 | AF-A0A833AM78-F1-MODEL\_V4 | 1.0 | 1.835e-16 | 568 | 0.198 | 247 | 164 | 8 | 57 | 278 | 9 | 246 | Uncharacterized protein | Uncharacterized protein | | afdb-uniprot50 | AF-A0A3B8Q684-F1-MODEL\_V4 | 1.0 | 5.503e-17 | 568 | 0.185 | 297 | 201 | 11 | 7 | 278 | 77 | 357 | Uncharacterized protein | Uncharacterized protein | | afdb-uniprot50 | AF-A0A0C1ITH4-F1-MODEL\_V4 | 1.0 | 1.545e-16 | 565 | 0.225 | 288 | 198 | 10 | 3 | 278 | 60 | 334 | Uncharacterized protein | Uncharacterized protein | | afdb-uniprot50 | AF-Q602Z6-F1-MODEL\_V4 | 1.0 | 4.863e-16 | 565 | 0.219 | 287 | 198 | 11 | 2 | 279 | 71 | 340 | Putative prophage MuMc02, late control gene D protein | Putative prophage MuMc02, late control gene D protein | | afdb-uniprot50 | AF-A0A1I3I3R8-F1-MODEL\_V4 | 1.0 | 2.588e-16 | 565 | 0.199 | 301 | 201 | 10 | 3 | 278 | 70 | 355 | Phage protein D | Phage protein D | | afdb-uniprot50 | AF-A0A2S5R6X2-F1-MODEL\_V4 | 1.0 | 3.046e-15 | 562 | 0.353 | 178 | 115 | 0 | 75 | 252 | 2 | 179 | Phage late control D protein (GPD) | Phage late control D protein (GPD) | | afdb-uniprot50 | AF-F3YY66-F1-MODEL\_V4 | 1.0 | 7.763e-17 | 561 | 0.237 | 278 | 183 | 11 | 15 | 279 | 76 | 337 | Late control D family protein | Late control D family protein | | afdb-uniprot50 | AF-A0A377YUK4-F1-MODEL\_V4 | 1.0 | 5.15e-16 | 558 | 0.261 | 210 | 130 | 4 | 95 | 279 | 1 | 210 | Late control gene D protein | Late control gene D protein | | afdb-uniprot50 | AF-A0A832EBM3-F1-MODEL\_V4 | 1.0 | 2.741e-16 | 558 | 0.212 | 296 | 191 | 11 | 9 | 278 | 82 | 361 | Uncharacterized protein | Uncharacterized protein | | afdb-uniprot50 | AF-A0A6G5QFX8-F1-MODEL\_V4 | 1.0 | 3.074e-16 | 557 | 0.172 | 255 | 195 | 7 | 20 | 274 | 67 | 305 | Phage protein D | Phage protein D | | afdb-uniprot50 | AF-A0A0S9PYR5-F1-MODEL\_V4 | 1.0 | 3.447e-16 | 557 | 0.169 | 295 | 215 | 12 | 1 | 278 | 52 | 333 | Uncharacterized protein | Uncharacterized protein | | afdb-uniprot50 | AF-A0A411WI36-F1-MODEL\_V4 | 1.0 | 1.651e-17 | 557 | 0.201 | 333 | 213 | 15 | 2 | 311 | 63 | 365 | Late control protein D | Late control protein D | | afdb-uniprot50 | AF-A0A0C2UPU9-F1-MODEL\_V4 | 1.0 | 1.251e-18 | 556 | 0.252 | 277 | 179 | 10 | 7 | 279 | 49 | 301 | Uncharacterized protein | Uncharacterized protein | | afdb-uniprot50 | AF-H1CIQ5-F1-MODEL\_V4 | 1.0 | 3.074e-16 | 556 | 0.182 | 324 | 225 | 17 | 1 | 308 | 58 | 357 | Uncharacterized protein | Uncharacterized protein | | afdb-uniprot50 | AF-A0A7Y5KGH0-F1-MODEL\_V4 | 1.0 | 1.651e-17 | 555 | 0.175 | 313 | 204 | 12 | 3 | 282 | 5 | 296 | Phage late control D family protein | Phage late control D family protein | | afdb-uniprot50 | AF-U3TRV7-F1-MODEL\_V4 | 1.0 | 4.131e-17 | 554 | 0.23 | 260 | 163 | 10 | 59 | 294 | 3 | 249 | Uncharacterized protein | Uncharacterized protein | | afdb-uniprot50 | AF-A0A1W2EJU3-F1-MODEL\_V4 | 1.0 | 1.377e-16 | 554 | 0.165 | 278 | 206 | 12 | 20 | 278 | 67 | 337 | Uncharacterized protein | Uncharacterized protein | | afdb-uniprot50 | AF-A0A2N9MSM3-F1-MODEL\_V4 | 1.0 | 6.536e-17 | 554 | 0.174 | 304 | 196 | 12 | 9 | 279 | 75 | 356 | Uncharacterized protein | Uncharacterized protein | | afdb-uniprot50 | AF-A0A6I3SN83-F1-MODEL\_V4 | 1.0 | 1.545e-16 | 552 | 0.161 | 297 | 202 | 9 | 9 | 279 | 92 | 367 | Uncharacterized protein | Uncharacterized protein | | afdb-uniprot50 | AF-A0A285MDK0-F1-MODEL\_V4 | 1.0 | 2.308e-16 | 551 | 0.196 | 275 | 191 | 10 | 20 | 279 | 68 | 327 | Uncharacterized protein | Uncharacterized protein | | afdb-uniprot50 | AF-A0A3M2ZMQ4-F1-MODEL\_V4 | 1.0 | 8.628e-16 | 550 | 0.297 | 232 | 146 | 5 | 1 | 225 | 53 | 274 | Tail protein D | Tail protein D | | afdb-uniprot50 | AF-A0A2V4UUX4-F1-MODEL\_V4 | 1.0 | 2.219e-18 | 550 | 0.272 | 279 | 165 | 8 | 12 | 280 | 68 | 318 | Phage baseplate assembly protein V | Phage baseplate assembly protein V | | afdb-uniprot50 | AF-A0A6H2NTK7-F1-MODEL\_V4 | 1.0 | 7.693e-16 | 549 | 0.3 | 213 | 141 | 4 | 7 | 218 | 49 | 254 | Phage tail protein | Phage tail protein | | afdb-uniprot50 | AF-A0A6P0MTJ9-F1-MODEL\_V4 | 1.0 | 1.301e-16 | 549 | 0.213 | 295 | 192 | 14 | 4 | 281 | 62 | 333 | Uncharacterized protein | Uncharacterized protein | | afdb-uniprot50 | AF-A0A1C6BMG6-F1-MODEL\_V4 | 1.0 | 1.095e-16 | 549 | 0.19 | 283 | 195 | 13 | 7 | 278 | 73 | 332 | Phage protein D | Phage protein D | | afdb-uniprot50 | AF-A0A6L5BH82-F1-MODEL\_V4 | 1.0 | 1.545e-16 | 547 | 0.191 | 323 | 223 | 11 | 2 | 308 | 68 | 368 | Uncharacterized protein | Uncharacterized protein | | afdb-uniprot50 | AF-A0A6P0L7N5-F1-MODEL\_V4 | 1.0 | 3.074e-16 | 546 | 0.166 | 301 | 206 | 13 | 7 | 279 | 73 | 356 | Phage late control D family protein | Phage late control D family protein | | afdb-uniprot50 | AF-A0A3M1TEK0-F1-MODEL\_V4 | 1.0 | 1.459e-16 | 546 | 0.184 | 331 | 219 | 15 | 7 | 308 | 109 | 417 | Phage late control D family protein | Phage late control D family protein | | afdb-uniprot50 | AF-A0A7X6ACD1-F1-MODEL\_V4 | 1.0 | 1.377e-16 | 544 | 0.166 | 306 | 210 | 14 | 3 | 279 | 44 | 333 | Uncharacterized protein | Uncharacterized protein | | afdb-uniprot50 | AF-A0A5B9EBV9-F1-MODEL\_V4 | 1.0 | 6.477e-16 | 544 | 0.187 | 299 | 201 | 10 | 7 | 279 | 74 | 356 | Phage late control D family protein | Phage late control D family protein | | afdb-uniprot50 | AF-A0A329TWB6-F1-MODEL\_V4 | 1.0 | 1.034e-16 | 544 | 0.19 | 309 | 204 | 13 | 2 | 282 | 65 | 355 | Preprotein translocase subunit TatB | Preprotein translocase subunit TatB | | afdb-uniprot50 | AF-A0A838CFT9-F1-MODEL\_V4 | 1.0 | 4.592e-16 | 543 | 0.235 | 208 | 134 | 3 | 113 | 307 | 2 | 197 | Late control protein | Late control protein | | afdb-uniprot50 | AF-A0A534NNF3-F1-MODEL\_V4 | 1.0 | 7.763e-17 | 543 | 0.196 | 320 | 201 | 13 | 7 | 304 | 80 | 365 | Phage late control D family protein | Phage late control D family protein | | afdb-uniprot50 | AF-A0A534TDB4-F1-MODEL\_V4 | 1.0 | 5.828e-17 | 543 | 0.181 | 331 | 217 | 15 | 7 | 311 | 51 | 353 | Uncharacterized protein | Uncharacterized protein | | afdb-uniprot50 | AF-A0A2J4V7J1-F1-MODEL\_V4 | 1.0 | 7.693e-16 | 541 | 0.266 | 229 | 126 | 3 | 95 | 281 | 2 | 230 | Uncharacterized protein | Uncharacterized protein | | afdb-uniprot50 | AF-A0A4R1J9U4-F1-MODEL\_V4 | 1.0 | 4.592e-16 | 541 | 0.144 | 298 | 209 | 11 | 7 | 279 | 69 | 345 | Phage protein D | Phage protein D | | afdb-uniprot50 | AF-A0A2T6G5A6-F1-MODEL\_V4 | 1.0 | 4.907e-17 | 540 | 0.185 | 296 | 199 | 9 | 7 | 278 | 84 | 361 | Uncharacterized protein | Uncharacterized protein | | afdb-uniprot50 | AF-A0A0P9CMZ8-F1-MODEL\_V4 | 1.0 | 2.444e-16 | 539 | 0.247 | 218 | 141 | 5 | 102 | 311 | 2 | 204 | Uncharacterized protein | Uncharacterized protein | | afdb-uniprot50 | AF-A0A0Q2UDB3-F1-MODEL\_V4 | 1.0 | 5.775e-16 | 539 | 0.211 | 289 | 197 | 13 | 2 | 276 | 54 | 325 | Uncharacterized protein | Uncharacterized protein | | afdb-uniprot50 | AF-A0A5M3Y7N1-F1-MODEL\_V4 | 1.0 | 6.477e-16 | 539 | 0.166 | 300 | 205 | 11 | 7 | 278 | 58 | 340 | Mlr6558 protein | Mlr6558 protein | | afdb-uniprot50 | AF-A0A7V1ZWM7-F1-MODEL\_V4 | 1.0 | 1.377e-16 | 539 | 0.218 | 325 | 199 | 15 | 9 | 305 | 77 | 374 | Phage late control D family protein | Phage late control D family protein | | afdb-uniprot50 | AF-A0A6M0FC52-F1-MODEL\_V4 | 1.0 | 5.454e-16 | 538 | 0.167 | 299 | 204 | 14 | 9 | 279 | 76 | 357 | Phage late control D family protein | Phage late control D family protein | | afdb-uniprot50 | AF-A0A6I7P4G6-F1-MODEL\_V4 | 1.0 | 2.058e-16 | 538 | 0.194 | 308 | 193 | 13 | 3 | 278 | 73 | 357 | Phage late control D family protein | Phage late control D family protein | | afdb-uniprot50 | AF-A0A850BD04-F1-MODEL\_V4 | 1.0 | 2.741e-16 | 537 | 0.188 | 302 | 196 | 13 | 7 | 279 | 68 | 349 | Phage late control D family protein | Phage late control D family protein | | afdb-uniprot50 | AF-A0A7W6JTC3-F1-MODEL\_V4 | 1.0 | 3.255e-16 | 537 | 0.196 | 300 | 191 | 12 | 9 | 278 | 77 | 356 | Phage protein D | Phage protein D | | afdb-uniprot50 | AF-A0A1V4SNX6-F1-MODEL\_V4 | 1.0 | 4.863e-16 | 535 | 0.18 | 304 | 195 | 13 | 7 | 279 | 66 | 346 | Phage late control protein Gpd | Phage late control protein Gpd | | afdb-uniprot50 | AF-A0A534WF19-F1-MODEL\_V4 | 1.0 | 3.447e-16 | 535 | 0.161 | 298 | 204 | 14 | 9 | 278 | 67 | 346 | Phage late control D family protein | Phage late control D family protein | | afdb-uniprot50 | AF-L9Z2P0-F1-MODEL\_V4 | 1.0 | 2.287e-15 | 534 | 0.164 | 285 | 218 | 9 | 3 | 279 | 37 | 309 | Phage protein D | Phage protein D | | afdb-uniprot50 | AF-A0A7Y3X8C2-F1-MODEL\_V4 | 1.0 | 5.15e-16 | 534 | 0.173 | 300 | 203 | 14 | 7 | 279 | 79 | 360 | Phage late control D family protein | Phage late control D family protein | | afdb-uniprot50 | AF-A0A7X5DYJ1-F1-MODEL\_V4 | 1.0 | 1.621e-15 | 533 | 0.164 | 274 | 212 | 9 | 17 | 287 | 80 | 339 | Uncharacterized protein | Uncharacterized protein | | afdb-uniprot50 | AF-A0A2E2G3Z9-F1-MODEL\_V4 | 1.0 | 2.308e-16 | 532 | 0.178 | 303 | 194 | 16 | 9 | 281 | 73 | 350 | Uncharacterized protein | Uncharacterized protein | | afdb-uniprot50 | AF-A0A2V8IMH5-F1-MODEL\_V4 | 1.0 | 7.693e-16 | 532 | 0.154 | 304 | 202 | 12 | 9 | 279 | 72 | 353 | Uncharacterized protein | Uncharacterized protein | | afdb-uniprot50 | AF-A0A6M0AY93-F1-MODEL\_V4 | 1.0 | 8.628e-16 | 531 | 0.169 | 283 | 191 | 12 | 24 | 279 | 6 | 271 | Phage late control D family protein | Phage late control D family protein | | afdb-uniprot50 | AF-A0A524PWE2-F1-MODEL\_V4 | 1.0 | 1.217e-15 | 531 | 0.163 | 281 | 193 | 9 | 24 | 279 | 11 | 274 | Phage late control D family protein | Phage late control D family protein | | afdb-uniprot50 | AF-A0A1V4VPA0-F1-MODEL\_V4 | 1.0 | 3.447e-16 | 531 | 0.161 | 303 | 201 | 10 | 9 | 279 | 78 | 359 | Phage late control gene D protein (GPD) | Phage late control gene D protein (GPD) | | afdb-uniprot50 | AF-A0A7X3GEI3-F1-MODEL\_V4 | 1.0 | 7.264e-16 | 531 | 0.185 | 302 | 201 | 11 | 7 | 279 | 76 | 361 | Uncharacterized protein | Uncharacterized protein | | afdb-uniprot50 | AF-A0A0Q6TBZ7-F1-MODEL\_V4 | 1.0 | 1.025e-15 | 531 | 0.186 | 305 | 199 | 12 | 3 | 278 | 76 | 360 | Uncharacterized protein | Uncharacterized protein | | afdb-uniprot50 | AF-A0A1R1C2A7-F1-MODEL\_V4 | 1.0 | 2.179e-16 | 531 | 0.177 | 299 | 196 | 11 | 9 | 279 | 88 | 364 | Uncharacterized protein | Uncharacterized protein | | afdb-uniprot50 | AF-A0A6L5TFX2-F1-MODEL\_V4 | 1.0 | 1.717e-15 | 529 | 0.191 | 293 | 209 | 13 | 1 | 281 | 57 | 333 | Preprotein translocase subunit TatB | Preprotein translocase subunit TatB | | afdb-uniprot50 | AF-A0A1L6M343-F1-MODEL\_V4 | 1.0 | 1.085e-15 | 528 | 0.167 | 299 | 204 | 12 | 7 | 279 | 76 | 355 | VgrG protein | VgrG protein | | afdb-uniprot50 | AF-A0A4R1J4F9-F1-MODEL\_V4 | 1.0 | 6.859e-16 | 528 | 0.165 | 303 | 204 | 14 | 7 | 279 | 73 | 356 | Phage protein D | Phage protein D | | afdb-uniprot50 | AF-A0A1E7J776-F1-MODEL\_V4 | 1.0 | 3.447e-16 | 528 | 0.16 | 305 | 198 | 13 | 9 | 279 | 80 | 360 | Uncharacterized protein | Uncharacterized protein | | afdb-uniprot50 | AF-A0A534XWT7-F1-MODEL\_V4 | 1.0 | 7.693e-16 | 528 | 0.161 | 297 | 205 | 13 | 9 | 278 | 67 | 346 | Uncharacterized protein | Uncharacterized protein | | afdb-uniprot50 | AF-A0A1C0TMK1-F1-MODEL\_V4 | 1.0 | 1.785e-13 | 527 | 0.239 | 146 | 111 | 0 | 134 | 279 | 13 | 158 | Phage tail protein | Phage tail protein | | afdb-uniprot50 | AF-A0A1I5AWE8-F1-MODEL\_V4 | 1.0 | 1.445e-15 | 524 | 0.243 | 234 | 127 | 3 | 99 | 282 | 2 | 235 | Uncharacterized protein | Uncharacterized protein | | afdb-uniprot50 | AF-A0A7V6P996-F1-MODEL\_V4 | 1.0 | 2.444e-16 | 524 | 0.18 | 294 | 201 | 12 | 1 | 279 | 55 | 323 | Late control D family protein | Late control D family protein | | afdb-uniprot50 | AF-A0A4Q7FRN2-F1-MODEL\_V4 | 1.0 | 1.217e-15 | 524 | 0.163 | 288 | 208 | 11 | 18 | 281 | 118 | 396 | Uncharacterized protein | Uncharacterized protein | | afdb-uniprot50 | AF-A0A6L6VHW7-F1-MODEL\_V4 | 1.0 | 5.723e-15 | 521 | 0.212 | 207 | 155 | 5 | 79 | 279 | 9 | 213 | Uncharacterized protein | Uncharacterized protein | | afdb-uniprot50 | AF-A0A0L6JHZ3-F1-MODEL\_V4 | 1.0 | 2.308e-16 | 519 | 0.189 | 301 | 191 | 11 | 9 | 279 | 87 | 364 | Uncharacterized protein | Uncharacterized protein | | afdb-uniprot50 | AF-A0A3A4NZJ3-F1-MODEL\_V4 | 1.0 | 1.925e-15 | 519 | 0.191 | 303 | 197 | 13 | 7 | 277 | 63 | 349 | Phage late control D family protein | Phage late control D family protein | | afdb-uniprot50 | AF-A0A3N5R5L3-F1-MODEL\_V4 | 1.0 | 2.588e-16 | 519 | 0.174 | 310 | 207 | 14 | 3 | 279 | 6 | 299 | Type IV secretion protein Rhs | Type IV secretion protein Rhs | | afdb-uniprot50 | AF-A0A840BJW7-F1-MODEL\_V4 | 1.0 | 6.859e-16 | 518 | 0.168 | 297 | 201 | 13 | 9 | 278 | 72 | 349 | Phage protein D | Phage protein D | | afdb-uniprot50 | AF-A0A535MWT5-F1-MODEL\_V4 | 1.0 | 1.025e-15 | 518 | 0.177 | 327 | 212 | 13 | 9 | 311 | 60 | 353 | Uncharacterized protein | Uncharacterized protein | | afdb-uniprot50 | AF-A0A1L6M143-F1-MODEL\_V4 | 1.0 | 1.445e-15 | 517 | 0.165 | 303 | 202 | 12 | 7 | 279 | 82 | 363 | Phage protein D | Phage protein D | | afdb-uniprot50 | AF-A0A328FDU1-F1-MODEL\_V4 | 1.0 | 1.818e-15 | 517 | 0.163 | 305 | 199 | 12 | 7 | 278 | 80 | 361 | Uncharacterized protein | Uncharacterized protein | | afdb-uniprot50 | AF-A0A7W6N992-F1-MODEL\_V4 | 1.0 | 3.255e-16 | 516 | 0.205 | 326 | 224 | 16 | 2 | 311 | 52 | 358 | Uncharacterized protein | Uncharacterized protein | | afdb-uniprot50 | AF-A0A7X5KSP1-F1-MODEL\_V4 | 1.0 | 1.085e-15 | 516 | 0.212 | 273 | 184 | 12 | 17 | 280 | 83 | 333 | Uncharacterized protein | Uncharacterized protein | | afdb-uniprot50 | AF-A0A1U7IXV3-F1-MODEL\_V4 | 1.0 | 2.039e-15 | 515 | 0.151 | 303 | 206 | 11 | 7 | 279 | 73 | 354 | Uncharacterized protein | Uncharacterized protein | | afdb-uniprot50 | AF-S4XY39-F1-MODEL\_V4 | 1.0 | 2.422e-15 | 515 | 0.177 | 305 | 199 | 11 | 9 | 277 | 69 | 357 | Uncharacterized protein | Uncharacterized protein | | afdb-uniprot50 | AF-A0A2R5EJX7-F1-MODEL\_V4 | 1.0 | 8.147e-16 | 515 | 0.194 | 298 | 193 | 12 | 7 | 277 | 80 | 357 | Uncharacterized protein | Uncharacterized protein | | afdb-uniprot50 | AF-A0A6J4XBG3-F1-MODEL\_V4 | 1.0 | 7.264e-16 | 515 | 0.197 | 309 | 184 | 13 | 7 | 277 | 76 | 358 | Uncharacterized protein | Uncharacterized protein | | afdb-uniprot50 | AF-A0A0Q9LKQ7-F1-MODEL\_V4 | 1.0 | 7.693e-16 | 515 | 0.182 | 296 | 198 | 11 | 9 | 279 | 86 | 362 | Uncharacterized protein | Uncharacterized protein | | afdb-uniprot50 | AF-A0A1H9D136-F1-MODEL\_V4 | 1.0 | 2.876e-15 | 515 | 0.191 | 303 | 190 | 12 | 9 | 278 | 78 | 358 | Phage late control gene D protein (GPD) | Phage late control gene D protein (GPD) | | afdb-uniprot50 | AF-A0A6L6WH01-F1-MODEL\_V4 | 1.0 | 6.477e-16 | 514 | 0.444 | 144 | 80 | 0 | 133 | 276 | 3 | 146 | Uncharacterized protein | Uncharacterized protein | | afdb-uniprot50 | AF-F0B7N5-F1-MODEL\_V4 | 1.0 | 4.509e-14 | 513 | 0.343 | 192 | 124 | 2 | 75 | 264 | 2 | 193 | Phage protein D | Phage protein D | | afdb-uniprot50 | AF-A0A848YEL6-F1-MODEL\_V4 | 1.0 | 1.717e-15 | 513 | 0.192 | 302 | 194 | 12 | 7 | 278 | 82 | 363 | Phage late control D family protein | Phage late control D family protein | | afdb-uniprot50 | AF-A0A534UFN3-F1-MODEL\_V4 | 1.0 | 1.289e-15 | 513 | 0.189 | 322 | 213 | 11 | 9 | 307 | 67 | 363 | Uncharacterized protein | Uncharacterized protein | | afdb-uniprot50 | AF-A0A534VJ89-F1-MODEL\_V4 | 1.0 | 5.454e-16 | 513 | 0.166 | 313 | 207 | 15 | 9 | 293 | 60 | 346 | Phage late control D family protein | Phage late control D family protein | | afdb-uniprot50 | AF-A0A833GXP7-F1-MODEL\_V4 | 1.0 | 8.073e-15 | 512 | 0.153 | 300 | 211 | 10 | 3 | 277 | 76 | 357 | Phage late control D family protein | Phage late control D family protein | | afdb-uniprot50 | AF-A0A520K035-F1-MODEL\_V4 | 1.0 | 4.297e-15 | 511 | 0.162 | 308 | 208 | 13 | 3 | 279 | 71 | 359 | Phage late control D family protein | Phage late control D family protein | | afdb-uniprot50 | AF-A0A1Q8A378-F1-MODEL\_V4 | 1.0 | 6.477e-16 | 510 | 0.185 | 307 | 199 | 15 | 3 | 279 | 69 | 354 | Uncharacterized protein | Uncharacterized protein | | afdb-uniprot50 | AF-A0A5S9ITQ6-F1-MODEL\_V4 | 1.0 | 3.046e-15 | 509 | 0.165 | 303 | 200 | 11 | 7 | 279 | 65 | 344 | Uncharacterized protein | Uncharacterized protein | | afdb-uniprot50 | AF-A0A2E0L150-F1-MODEL\_V4 | 1.0 | 1.531e-15 | 509 | 0.162 | 307 | 201 | 13 | 7 | 279 | 66 | 350 | Phage\_base\_V domain-containing protein | Phage\_base\_V domain-containing protein | | afdb-uniprot50 | AF-A0A165RMG4-F1-MODEL\_V4 | 1.0 | 4.297e-15 | 508 | 0.173 | 277 | 200 | 11 | 20 | 279 | 69 | 333 | Phage late control gene D protein (GPD) | Phage late control gene D protein (GPD) | | afdb-uniprot50 | AF-A0A1V4W1U0-F1-MODEL\_V4 | 1.0 | 4.336e-16 | 508 | 0.193 | 299 | 192 | 11 | 9 | 279 | 88 | 365 | Phage late control gene D protein (GPD) | Phage late control gene D protein (GPD) | | afdb-uniprot50 | AF-A0A401HTD7-F1-MODEL\_V4 | 1.0 | 3.618e-15 | 508 | 0.164 | 298 | 206 | 11 | 7 | 279 | 87 | 366 | Uncharacterized protein | Uncharacterized protein | | afdb-uniprot50 | AF-A0A0L8VEK1-F1-MODEL\_V4 | 1.0 | 1.531e-15 | 508 | 0.163 | 300 | 205 | 12 | 3 | 275 | 38 | 318 | Phage\_base\_V domain-containing protein | Phage\_base\_V domain-containing protein | | afdb-uniprot50 | AF-A0A285M2Q7-F1-MODEL\_V4 | 1.0 | 2.716e-15 | 507 | 0.213 | 286 | 200 | 13 | 2 | 276 | 59 | 330 | Uncharacterized protein | Uncharacterized protein | | afdb-uniprot50 | AF-A0A2N2D1G3-F1-MODEL\_V4 | 1.0 | 1.621e-15 | 507 | 0.187 | 298 | 198 | 12 | 7 | 279 | 88 | 366 | Uncharacterized protein | Uncharacterized protein | | afdb-uniprot50 | AF-A0A661RNA4-F1-MODEL\_V4 | 1.0 | 2.422e-15 | 505 | 0.195 | 302 | 198 | 14 | 7 | 279 | 73 | 358 | Uncharacterized protein | Uncharacterized protein | | afdb-uniprot50 | AF-A0A1Y3RUL2-F1-MODEL\_V4 | 1.0 | 6.797e-15 | 505 | 0.144 | 269 | 206 | 12 | 57 | 311 | 15 | 273 | Uncharacterized protein | Uncharacterized protein | | afdb-uniprot50 | AF-A0A7V6UDV8-F1-MODEL\_V4 | 1.0 | 5.404e-15 | 503 | 0.189 | 280 | 198 | 15 | 2 | 276 | 48 | 303 | Uncharacterized protein | Uncharacterized protein | | afdb-uniprot50 | AF-A0A6P1IRG3-F1-MODEL\_V4 | 1.0 | 8.628e-16 | 503 | 0.168 | 302 | 197 | 14 | 9 | 278 | 79 | 358 | Phage late control D family protein | Phage late control D family protein | | afdb-uniprot50 | AF-G8PLB4-F1-MODEL\_V4 | 1.0 | 1.925e-15 | 503 | 0.213 | 323 | 226 | 12 | 2 | 308 | 58 | 368 | Bacteriophage regulatory protein | Bacteriophage regulatory protein | | afdb-uniprot50 | AF-A0A376W2Q8-F1-MODEL\_V4 | 1.0 | 8.55e-15 | 502 | 0.228 | 223 | 145 | 5 | 1 | 203 | 11 | 226 | Late control gene D protein from prophage | Late control gene D protein from prophage | | afdb-uniprot50 | AF-A0A3N5XNR8-F1-MODEL\_V4 | 1.0 | 2.716e-15 | 502 | 0.136 | 307 | 207 | 11 | 7 | 279 | 68 | 350 | Phage late control D family protein | Phage late control D family protein | | afdb-uniprot50 | AF-A0A7W7MBI9-F1-MODEL\_V4 | 1.0 | 3.046e-15 | 500 | 0.17 | 300 | 200 | 10 | 9 | 278 | 69 | 349 | Phage protein D | Phage protein D | | afdb-uniprot50 | AF-U2LWR3-F1-MODEL\_V4 | 1.0 | 1.149e-15 | 498 | 0.12 | 315 | 241 | 11 | 7 | 311 | 64 | 352 | Uncharacterized protein | Uncharacterized protein | | afdb-uniprot50 | AF-A0A0E3KUE2-F1-MODEL\_V4 | 1.0 | 6.061e-15 | 497 | 0.179 | 295 | 204 | 12 | 7 | 279 | 78 | 356 | VgrG protein | VgrG protein | | afdb-uniprot50 | AF-A0A4R4G0N1-F1-MODEL\_V4 | 1.0 | 8.973e-14 | 494 | 0.273 | 219 | 140 | 6 | 10 | 220 | 2 | 209 | Late control protein | Late control protein | | afdb-uniprot50 | AF-A0A3M9Z207-F1-MODEL\_V4 | 1.0 | 1.217e-15 | 494 | 0.155 | 309 | 199 | 16 | 7 | 279 | 82 | 364 | Phage late control D family protein | Phage late control D family protein | | afdb-uniprot50 | AF-A0A3M1T127-F1-MODEL\_V4 | 1.0 | 4.55e-15 | 493 | 0.17 | 334 | 221 | 14 | 7 | 309 | 72 | 380 | Phage late control D family protein | Phage late control D family protein | | afdb-uniprot50 | AF-A0A4C2EPK7-F1-MODEL\_V4 | 1.0 | 1.075e-14 | 492 | 0.171 | 285 | 204 | 12 | 7 | 277 | 77 | 343 | Uncharacterized protein | Uncharacterized protein | | afdb-uniprot50 | AF-A0A7Y5L630-F1-MODEL\_V4 | 1.0 | 4.297e-15 | 492 | 0.173 | 305 | 194 | 12 | 9 | 279 | 73 | 353 | Phage late control D family protein | Phage late control D family protein | | afdb-uniprot50 | AF-F2NYF5-F1-MODEL\_V4 | 1.0 | 2.876e-15 | 492 | 0.148 | 309 | 208 | 16 | 7 | 282 | 66 | 352 | Uncharacterized protein | Uncharacterized protein | | afdb-uniprot50 | AF-G2G245-F1-MODEL\_V4 | 1.0 | 4.258e-14 | 491 | 0.199 | 246 | 166 | 6 | 57 | 279 | 1 | 238 | Uncharacterized protein | Uncharacterized protein | | afdb-uniprot50 | AF-A0A5U2MRG6-F1-MODEL\_V4 | 1.0 | 8.706e-17 | 491 | 0.168 | 302 | 197 | 12 | 7 | 277 | 2 | 280 | Phage late control D family protein | Phage late control D family protein | | afdb-uniprot50 | AF-A0A4R2R9S9-F1-MODEL\_V4 | 1.0 | 2.039e-15 | 490 | 0.157 | 299 | 208 | 15 | 9 | 279 | 88 | 370 | Uncharacterized protein | Uncharacterized protein | | afdb-uniprot50 | AF-A0A081N302-F1-MODEL\_V4 | 1.0 | 2.039e-15 | 487 | 0.378 | 169 | 103 | 1 | 113 | 279 | 1 | 169 | Uncharacterized protein | Uncharacterized protein | | afdb-uniprot50 | AF-A0A0F4NJB2-F1-MODEL\_V4 | 1.0 | 3.651e-16 | 487 | 0.207 | 284 | 191 | 11 | 1 | 279 | 53 | 307 | Uncharacterized protein | Uncharacterized protein | | afdb-uniprot50 | AF-A0A080M5H4-F1-MODEL\_V4 | 1.0 | 2.691e-14 | 486 | 0.181 | 248 | 169 | 10 | 57 | 279 | 10 | 248 | Tail protein | Tail protein | | afdb-uniprot50 | AF-A0A0V7ZK90-F1-MODEL\_V4 | 1.0 | 5.103e-15 | 486 | 0.156 | 325 | 219 | 12 | 7 | 290 | 77 | 387 | Uncharacterized protein | Uncharacterized protein | | afdb-uniprot50 | AF-A0A5B8ST50-F1-MODEL\_V4 | 1.0 | 1.769e-12 | 485 | 0.309 | 139 | 94 | 2 | 145 | 281 | 1 | 139 | Uncharacterized protein | Uncharacterized protein | | afdb-uniprot50 | AF-A0A521P0G8-F1-MODEL\_V4 | 1.0 | 3.585e-14 | 485 | 0.265 | 181 | 128 | 3 | 112 | 290 | 2 | 179 | Uncharacterized protein | Uncharacterized protein | | afdb-uniprot50 | AF-A0A545T2R6-F1-MODEL\_V4 | 1.0 | 6.419e-15 | 485 | 0.162 | 296 | 200 | 15 | 9 | 278 | 72 | 345 | Uncharacterized protein | Uncharacterized protein | | afdb-uniprot50 | AF-A0A410ULA3-F1-MODEL\_V4 | 1.0 | 3.797e-14 | 484 | 0.149 | 302 | 215 | 14 | 3 | 279 | 65 | 349 | Phage late control D family protein | Phage late control D family protein | | afdb-uniprot50 | AF-A0A1F8N4S5-F1-MODEL\_V4 | 1.0 | 1.149e-15 | 484 | 0.211 | 307 | 187 | 12 | 7 | 281 | 66 | 349 | Phage\_base\_V domain-containing protein | Phage\_base\_V domain-containing protein | | afdb-uniprot50 | AF-F2NYH1-F1-MODEL\_V4 | 1.0 | 3.416e-15 | 484 | 0.142 | 301 | 209 | 14 | 7 | 277 | 66 | 347 | Uncharacterized protein | Uncharacterized protein | | afdb-uniprot50 | AF-G4KQ66-F1-MODEL\_V4 | 1.0 | 1.353e-14 | 483 | 0.149 | 274 | 203 | 11 | 9 | 277 | 53 | 301 | Uncharacterized protein | Uncharacterized protein | | afdb-uniprot50 | AF-A0A1I6IQH4-F1-MODEL\_V4 | 1.0 | 5.103e-15 | 483 | 0.157 | 292 | 208 | 14 | 7 | 279 | 77 | 349 | Uncharacterized protein | Uncharacterized protein | | afdb-uniprot50 | AF-A0A534PEG0-F1-MODEL\_V4 | 1.0 | 7.199e-15 | 483 | 0.175 | 324 | 208 | 13 | 9 | 310 | 69 | 355 | Phage late control D family protein | Phage late control D family protein | | afdb-uniprot50 | AF-A0A3B9YXC3-F1-MODEL\_V4 | 1.0 | 7.623e-15 | 481 | 0.175 | 308 | 197 | 15 | 7 | 279 | 83 | 368 | Uncharacterized protein | Uncharacterized protein | | afdb-uniprot50 | AF-A0A7C6PS95-F1-MODEL\_V4 | 1.0 | 9.589e-15 | 481 | 0.183 | 310 | 197 | 14 | 3 | 278 | 62 | 349 | Phage late control D family protein | Phage late control D family protein | | afdb-uniprot50 | AF-A0A660RRK5-F1-MODEL\_V4 | 1.0 | 1.277e-14 | 481 | 0.164 | 304 | 207 | 14 | 7 | 281 | 65 | 350 | Type IV secretion protein Rhs | Type IV secretion protein Rhs | | afdb-uniprot50 | AF-A0A376YJ37-F1-MODEL\_V4 | 1.0 | 4.021e-14 | 480 | 0.235 | 242 | 153 | 6 | 1 | 218 | 29 | 262 | Gene late control D protein | Gene late control D protein | | afdb-uniprot50 | AF-A0A2N6CT05-F1-MODEL\_V4 | 1.0 | 1.908e-14 | 480 | 0.153 | 319 | 209 | 12 | 7 | 284 | 82 | 380 | Type IV secretion protein Rhs | Type IV secretion protein Rhs | | afdb-uniprot50 | AF-A0A6G3WNF9-F1-MODEL\_V4 | 1.0 | 7.623e-15 | 480 | 0.197 | 319 | 199 | 17 | 3 | 281 | 3 | 304 | VgrG-related protein | VgrG-related protein | | afdb-uniprot50 | AF-A0A0Q4KJV3-F1-MODEL\_V4 | 1.0 | 2.021e-14 | 479 | 0.188 | 297 | 201 | 12 | 7 | 279 | 63 | 343 | Uncharacterized protein | Uncharacterized protein | | afdb-uniprot50 | AF-B9NUV5-F1-MODEL\_V4 | 1.0 | 2.14e-14 | 479 | 0.176 | 301 | 204 | 11 | 7 | 279 | 85 | 369 | Uncharacterized protein | Uncharacterized protein | | afdb-uniprot50 | AF-A0A7V3SCS7-F1-MODEL\_V4 | 1.0 | 3.416e-15 | 479 | 0.162 | 308 | 197 | 12 | 9 | 281 | 67 | 348 | Type IV secretion protein Rhs | Type IV secretion protein Rhs | | afdb-uniprot50 | AF-A0A661I686-F1-MODEL\_V4 | 1.0 | 3.018e-14 | 478 | 0.172 | 301 | 202 | 12 | 7 | 278 | 60 | 342 | Uncharacterized protein | Uncharacterized protein | | afdb-uniprot50 | AF-A0A853R2R6-F1-MODEL\_V4 | 1.0 | 4.469e-13 | 477 | 0.333 | 198 | 129 | 3 | 1 | 197 | 55 | 250 | Late control protein D | Late control protein D | | afdb-uniprot50 | AF-A0A2R6EXY7-F1-MODEL\_V4 | 1.0 | 9.054e-15 | 477 | 0.174 | 287 | 202 | 13 | 7 | 279 | 72 | 337 | Uncharacterized protein | Uncharacterized protein | | afdb-uniprot50 | AF-A0A6N8BHD5-F1-MODEL\_V4 | 1.0 | 2.287e-15 | 476 | 0.164 | 304 | 205 | 9 | 7 | 279 | 79 | 364 | Phage late control D family protein | Phage late control D family protein | | afdb-uniprot50 | AF-F2NUD6-F1-MODEL\_V4 | 1.0 | 5.103e-15 | 476 | 0.152 | 309 | 207 | 15 | 7 | 282 | 66 | 352 | Uncharacterized protein | Uncharacterized protein | | afdb-uniprot50 | AF-A0A4R4G4D4-F1-MODEL\_V4 | 1.0 | 5.672e-14 | 475 | 0.28 | 228 | 145 | 8 | 1 | 220 | 51 | 267 | Late control protein | Late control protein | | afdb-uniprot50 | AF-A0A4P7HPH6-F1-MODEL\_V4 | 1.0 | 6.061e-15 | 474 | 0.156 | 314 | 204 | 11 | 9 | 280 | 82 | 376 | Phage late control D family protein | Phage late control D family protein | | afdb-uniprot50 | AF-X5J8E7-F1-MODEL\_V4 | 1.0 | 1.517e-14 | 474 | 0.145 | 296 | 205 | 12 | 9 | 276 | 70 | 345 | Rhs element Vgr protein | Rhs element Vgr protein | | afdb-uniprot50 | AF-A0A6S7B3U2-F1-MODEL\_V4 | 1.0 | 5.404e-15 | 473 | 0.149 | 308 | 213 | 15 | 7 | 278 | 81 | 375 | Uncharacterized protein | Uncharacterized protein | | afdb-uniprot50 | AF-A0A356KG57-F1-MODEL\_V4 | 1.0 | 1.015e-14 | 473 | 0.165 | 344 | 223 | 19 | 3 | 311 | 61 | 375 | Uncharacterized protein | Uncharacterized protein | | afdb-uniprot50 | AF-A0A418VPR5-F1-MODEL\_V4 | 1.0 | 1.075e-14 | 472 | 0.154 | 291 | 201 | 10 | 16 | 279 | 77 | 349 | Phage late control D family protein | Phage late control D family protein | | afdb-uniprot50 | AF-A0A3A0AIX8-F1-MODEL\_V4 | 1.0 | 2.266e-14 | 472 | 0.163 | 306 | 201 | 13 | 7 | 279 | 65 | 348 | Type IV secretion protein Rhs | Type IV secretion protein Rhs | | afdb-uniprot50 | AF-A0A5N8XB31-F1-MODEL\_V4 | 1.0 | 8.073e-15 | 472 | 0.146 | 307 | 209 | 12 | 9 | 284 | 72 | 356 | Phage\_base\_V domain-containing protein | Phage\_base\_V domain-containing protein | | afdb-uniprot50 | AF-A0A3A8NI17-F1-MODEL\_V4 | 1.0 | 9.589e-15 | 471 | 0.176 | 301 | 194 | 14 | 9 | 279 | 63 | 339 | Phage late control D family protein | Phage late control D family protein | | afdb-uniprot50 | AF-A0A7C7R7X2-F1-MODEL\_V4 | 1.0 | 4.775e-14 | 471 | 0.173 | 305 | 199 | 12 | 3 | 275 | 59 | 342 | Phage late control D family protein | Phage late control D family protein | | afdb-uniprot50 | AF-A0A3M1T848-F1-MODEL\_V4 | 1.0 | 2.021e-14 | 471 | 0.172 | 325 | 206 | 15 | 7 | 297 | 70 | 365 | Phage late control D family protein | Phage late control D family protein | | afdb-uniprot50 | AF-A0A7K0GTP6-F1-MODEL\_V4 | 1.0 | 2.4e-14 | 471 | 0.17 | 293 | 203 | 12 | 7 | 276 | 64 | 339 | Type VI secretion system tip protein VgrG | Type VI secretion system tip protein VgrG | | afdb-uniprot50 | AF-A0A255Z5N1-F1-MODEL\_V4 | 1.0 | 2.14e-14 | 471 | 0.182 | 301 | 196 | 11 | 7 | 279 | 69 | 347 | Type IV secretion protein Rhs | Type IV secretion protein Rhs | | afdb-uniprot50 | AF-A0A1H4FYY6-F1-MODEL\_V4 | 1.0 | 2.159e-15 | 470 | 0.167 | 322 | 195 | 15 | 7 | 277 | 93 | 392 | Phage protein D | Phage protein D | | afdb-uniprot50 | AF-A0A7Y2SMN3-F1-MODEL\_V4 | 1.0 | 3.797e-14 | 470 | 0.177 | 298 | 192 | 10 | 7 | 273 | 64 | 339 | VgrG-related protein | VgrG-related protein | | afdb-uniprot50 | AF-A0A442UMS5-F1-MODEL\_V4 | 1.0 | 1.606e-14 | 469 | 0.166 | 306 | 210 | 12 | 3 | 279 | 78 | 367 | Phage late control D family protein | Phage late control D family protein | | afdb-uniprot50 | AF-A0A3G6TJU6-F1-MODEL\_V4 | 1.0 | 8.55e-15 | 469 | 0.124 | 305 | 214 | 12 | 7 | 281 | 72 | 353 | Type VI secretion system tip protein VgrG | Type VI secretion system tip protein VgrG | | afdb-uniprot50 | AF-L8JQG5-F1-MODEL\_V4 | 1.0 | 1.206e-14 | 468 | 0.179 | 295 | 200 | 11 | 7 | 275 | 72 | 350 | VgrG protein | VgrG protein | | afdb-uniprot50 | AF-A0A497BPI8-F1-MODEL\_V4 | 1.0 | 1.353e-14 | 468 | 0.194 | 303 | 188 | 15 | 9 | 278 | 66 | 345 | Phage\_base\_V domain-containing protein | Phage\_base\_V domain-containing protein | | afdb-uniprot50 | AF-A8U2N1-F1-MODEL\_V4 | 1.0 | 2.85e-14 | 467 | 0.303 | 227 | 140 | 7 | 1 | 218 | 57 | 274 | Late control gene D protein | Late control gene D protein | | afdb-uniprot50 | AF-A0A7W7V134-F1-MODEL\_V4 | 1.0 | 8e-14 | 467 | 0.17 | 305 | 208 | 14 | 3 | 279 | 65 | 352 | Phage protein D | Phage protein D | | afdb-uniprot50 | AF-A0A1L6M2U5-F1-MODEL\_V4 | 1.0 | 2.4e-14 | 467 | 0.155 | 309 | 201 | 14 | 3 | 278 | 109 | 390 | Phage protein D | Phage protein D | | afdb-uniprot50 | AF-A0A0S2FQJ5-F1-MODEL\_V4 | 1.0 | 1.802e-14 | 466 | 0.147 | 292 | 194 | 15 | 20 | 278 | 82 | 351 | Phage late control gene D family protein | Phage late control gene D family protein | | afdb-uniprot50 | AF-A0A7J0CY33-F1-MODEL\_V4 | 1.0 | 3.385e-14 | 466 | 0.208 | 312 | 194 | 17 | 3 | 276 | 18 | 314 | Uncharacterized protein | Uncharacterized protein | | afdb-uniprot50 | AF-A0A353VJI0-F1-MODEL\_V4 | 1.0 | 1.802e-14 | 466 | 0.174 | 293 | 203 | 13 | 7 | 277 | 72 | 347 | Rhs element Vgr protein | Rhs element Vgr protein | | afdb-uniprot50 | AF-A0A7Y4QYQ7-F1-MODEL\_V4 | 1.0 | 2.266e-14 | 466 | 0.156 | 301 | 198 | 13 | 7 | 276 | 72 | 347 | Type VI secretion system tip protein VgrG | Type VI secretion system tip protein VgrG | | afdb-uniprot50 | AF-A0A6I5QAM8-F1-MODEL\_V4 | 1.0 | 7.555e-14 | 465 | 0.165 | 296 | 204 | 12 | 7 | 277 | 74 | 351 | Type VI secretion system tip protein VgrG | Type VI secretion system tip protein VgrG | | afdb-uniprot50 | AF-Q08VZ1-F1-MODEL\_V4 | 1.0 | 1.925e-15 | 463 | 0.152 | 375 | 222 | 15 | 9 | 307 | 198 | 552 | Uncharacterized protein | Uncharacterized protein | | afdb-uniprot50 | AF-A0A7X6GUB8-F1-MODEL\_V4 | 1.0 | 3.618e-15 | 462 | 0.145 | 282 | 220 | 9 | 39 | 311 | 1 | 270 | Uncharacterized protein | Uncharacterized protein | | afdb-uniprot50 | AF-A0A3A0A9Z2-F1-MODEL\_V4 | 1.0 | 8e-14 | 462 | 0.161 | 298 | 208 | 10 | 7 | 279 | 76 | 356 | Rhs element Vgr protein | Rhs element Vgr protein | | afdb-uniprot50 | AF-A0A5B8ATZ1-F1-MODEL\_V4 | 1.0 | 1.353e-14 | 460 | 0.155 | 348 | 198 | 16 | 7 | 277 | 63 | 391 | Uncharacterized protein | Uncharacterized protein | | afdb-uniprot50 | AF-A0A2S6NI59-F1-MODEL\_V4 | 1.0 | 3.618e-15 | 459 | 0.137 | 305 | 205 | 13 | 7 | 276 | 19 | 300 | Uncharacterized protein | Uncharacterized protein | | afdb-uniprot50 | AF-A0A1N6X884-F1-MODEL\_V4 | 1.0 | 4.258e-14 | 459 | 0.136 | 307 | 207 | 14 | 7 | 279 | 66 | 348 | Phage protein D | Phage protein D | | afdb-uniprot50 | AF-A0A6N7YBH3-F1-MODEL\_V4 | 1.0 | 3.018e-14 | 459 | 0.196 | 301 | 194 | 14 | 7 | 279 | 67 | 347 | VgrG-related protein | VgrG-related protein | | afdb-uniprot50 | AF-A0A4U7JJ27-F1-MODEL\_V4 | 1.0 | 1.701e-14 | 459 | 0.149 | 301 | 204 | 16 | 7 | 276 | 95 | 374 | Uncharacterized protein | Uncharacterized protein | | afdb-uniprot50 | AF-A0A7T8BBA2-F1-MODEL\_V4 | 1.0 | 1.006e-13 | 458 | 0.11 | 299 | 221 | 12 | 7 | 277 | 65 | 346 | Phage late control D family protein | Phage late control D family protein | | afdb-uniprot50 | AF-A0A3M1GYX0-F1-MODEL\_V4 | 1.0 | 1.802e-14 | 458 | 0.146 | 335 | 232 | 16 | 9 | 311 | 66 | 378 | Phage late control D family protein | Phage late control D family protein | | afdb-uniprot50 | AF-A0A4Z0M9I4-F1-MODEL\_V4 | 1.0 | 1.701e-14 | 458 | 0.149 | 321 | 210 | 12 | 7 | 280 | 112 | 416 | Phage late control D family protein | Phage late control D family protein | | afdb-uniprot50 | AF-A0A497AT53-F1-MODEL\_V4 | 1.0 | 4.509e-14 | 458 | 0.171 | 291 | 199 | 11 | 17 | 281 | 78 | 352 | Type IV secretion protein Rhs | Type IV secretion protein Rhs | | afdb-uniprot50 | AF-A0A7W1S8H0-F1-MODEL\_V4 | 1.0 | 1.908e-14 | 458 | 0.156 | 306 | 208 | 15 | 7 | 281 | 67 | 353 | VgrG-related protein | VgrG-related protein | | afdb-uniprot50 | AF-A0A318JR32-F1-MODEL\_V4 | 1.0 | 9.331e-12 | 457 | 0.293 | 150 | 103 | 3 | 135 | 281 | 4 | 153 | Late control gene D protein (GPD) | Late control gene D protein (GPD) | | afdb-uniprot50 | AF-J0ZTM2-F1-MODEL\_V4 | 1.0 | 2.496e-12 | 455 | 0.31 | 145 | 92 | 3 | 145 | 281 | 12 | 156 | Uncharacterized protein | Uncharacterized protein | | afdb-uniprot50 | AF-A0A7Y2WZB6-F1-MODEL\_V4 | 1.0 | 1.139e-14 | 455 | 0.175 | 290 | 185 | 13 | 20 | 281 | 72 | 335 | VgrG-related protein | VgrG-related protein | | afdb-uniprot50 | AF-A0A418QD23-F1-MODEL\_V4 | 1.0 | 1.266e-13 | 453 | 0.166 | 313 | 202 | 13 | 3 | 281 | 42 | 329 | Phage\_base\_V domain-containing protein | Phage\_base\_V domain-containing protein | | afdb-uniprot50 | AF-A0A2S6I9B9-F1-MODEL\_V4 | 1.0 | 1.006e-13 | 453 | 0.195 | 292 | 196 | 11 | 7 | 275 | 76 | 351 | Rhs element Vgr protein | Rhs element Vgr protein | | afdb-uniprot50 | AF-A0A1T2CG57-F1-MODEL\_V4 | 1.0 | 5.57e-12 | 452 | 0.362 | 127 | 81 | 0 | 153 | 279 | 3 | 129 | Uncharacterized protein | Uncharacterized protein | | afdb-uniprot50 | AF-A0A317N0I9-F1-MODEL\_V4 | 1.0 | 1.503e-13 | 452 | 0.18 | 294 | 191 | 12 | 9 | 278 | 70 | 337 | Phage protein D | Phage protein D | | afdb-uniprot50 | AF-A0A511MZG9-F1-MODEL\_V4 | 1.0 | 5.672e-14 | 452 | 0.191 | 293 | 181 | 12 | 19 | 278 | 75 | 344 | Uncharacterized protein | Uncharacterized protein | | afdb-uniprot50 | AF-A0A098LIX2-F1-MODEL\_V4 | 1.0 | 2.667e-13 | 452 | 0.133 | 293 | 213 | 9 | 7 | 276 | 74 | 348 | Rhs element Vgr protein | Rhs element Vgr protein | | afdb-uniprot50 | AF-A0A377A6L9-F1-MODEL\_V4 | 1.0 | 9.972e-13 | 451 | 0.232 | 219 | 144 | 3 | 68 | 262 | 2 | 220 | Putative phage late gene regulator | Putative phage late gene regulator | | afdb-uniprot50 | AF-A0A7V1N7G8-F1-MODEL\_V4 | 1.0 | 8.55e-15 | 451 | 0.157 | 324 | 196 | 14 | 24 | 285 | 12 | 320 | Phage late control D family protein | Phage late control D family protein | | afdb-uniprot50 | AF-A0A1M7TZB9-F1-MODEL\_V4 | 1.0 | 6.361e-14 | 451 | 0.186 | 295 | 197 | 16 | 9 | 277 | 82 | 359 | Phage protein D | Phage protein D | | afdb-uniprot50 | AF-A0A1G9EX68-F1-MODEL\_V4 | 1.0 | 3.018e-14 | 451 | 0.163 | 331 | 196 | 15 | 7 | 276 | 62 | 372 | Phage protein D | Phage protein D | | afdb-uniprot50 | AF-Q3A0I0-F1-MODEL\_V4 | 1.0 | 3.197e-14 | 451 | 0.18 | 293 | 196 | 16 | 9 | 281 | 62 | 330 | Phage protein D and tail spike protein, putative | Phage protein D and tail spike protein, putative | | afdb-uniprot50 | AF-R5H1V4-F1-MODEL\_V4 | 1.0 | 2.691e-14 | 450 | 0.145 | 302 | 208 | 14 | 7 | 279 | 72 | 352 | Uncharacterized protein | Uncharacterized protein | | afdb-uniprot50 | AF-A0A7C3CDK3-F1-MODEL\_V4 | 1.0 | 1.34e-13 | 450 | 0.174 | 293 | 196 | 12 | 17 | 281 | 76 | 350 | Phage late control D family protein | Phage late control D family protein | | afdb-uniprot50 | AF-A0A158HQY1-F1-MODEL\_V4 | 1.0 | 8e-14 | 449 | 0.148 | 331 | 206 | 14 | 7 | 277 | 64 | 378 | Phage late control gene D protein (GPD) | Phage late control gene D protein (GPD) | | afdb-uniprot50 | AF-N1V1U4-F1-MODEL\_V4 | 1.0 | 9.589e-15 | 449 | 0.179 | 296 | 202 | 14 | 1 | 273 | 1 | 278 | Phage\_base\_V domain-containing protein | Phage\_base\_V domain-containing protein | | afdb-uniprot50 | AF-A0A2G5L080-F1-MODEL\_V4 | 1.0 | 1.066e-13 | 449 | 0.134 | 305 | 208 | 11 | 3 | 276 | 68 | 347 | Phage\_base\_V domain-containing protein | Phage\_base\_V domain-containing protein | | afdb-uniprot50 | AF-A0A7X4YHH4-F1-MODEL\_V4 | 1.0 | 2.991e-13 | 448 | 0.123 | 291 | 202 | 12 | 20 | 278 | 84 | 353 | Uncharacterized protein | Uncharacterized protein | | afdb-uniprot50 | AF-A0A7J4PP46-F1-MODEL\_V4 | 1.0 | 8.473e-14 | 448 | 0.134 | 304 | 210 | 14 | 7 | 277 | 73 | 356 | Phage late control D family protein | Phage late control D family protein | | afdb-uniprot50 | AF-A0A1W9R245-F1-MODEL\_V4 | 1.0 | 1.686e-13 | 448 | 0.139 | 293 | 211 | 11 | 7 | 275 | 75 | 350 | Type IV secretion protein Rhs | Type IV secretion protein Rhs | | afdb-uniprot50 | AF-A0A6G6GPZ0-F1-MODEL\_V4 | 1.0 | 2.002e-13 | 448 | 0.155 | 302 | 212 | 12 | 3 | 279 | 68 | 351 | Type VI secretion system tip protein VgrG | Type VI secretion system tip protein VgrG | | afdb-uniprot50 | AF-A0A850P3R3-F1-MODEL\_V4 | 1.0 | 9.589e-15 | 447 | 0.135 | 303 | 221 | 14 | 7 | 277 | 27 | 320 | Uncharacterized protein | Uncharacterized protein | | afdb-uniprot50 | AF-A0A7U7FD77-F1-MODEL\_V4 | 1.0 | 5.197e-17 | 447 | 0.185 | 312 | 161 | 10 | 26 | 288 | 155 | 422 | Putative phage-related protein | Putative phage-related protein | | afdb-uniprot50 | AF-A0A0F9U2B2-F1-MODEL\_V4 | 1.0 | 4.509e-14 | 447 | 0.138 | 389 | 232 | 16 | 7 | 311 | 90 | 459 | Uncharacterized protein | Uncharacterized protein | | afdb-uniprot50 | AF-A0A620N5X6-F1-MODEL\_V4 | 1.0 | 3.762e-13 | 447 | 0.196 | 219 | 153 | 7 | 74 | 278 | 275 | 484 | Uncharacterized protein | Uncharacterized protein | | afdb-uniprot50 | AF-A0A2A3CLV9-F1-MODEL\_V4 | 1.0 | 3.553e-13 | 446 | 0.216 | 250 | 136 | 9 | 37 | 279 | 1 | 197 | Uncharacterized protein | Uncharacterized protein | | afdb-uniprot50 | AF-U1KQI4-F1-MODEL\_V4 | 1.0 | 2.002e-13 | 446 | 0.177 | 299 | 202 | 15 | 3 | 276 | 59 | 338 | Rhs element Vgr protein | Rhs element Vgr protein | | afdb-uniprot50 | AF-W7VHJ1-F1-MODEL\_V4 | 1.0 | 3.197e-14 | 445 | 0.178 | 269 | 184 | 12 | 26 | 273 | 6 | 258 | Phage protein | Phage protein | | afdb-uniprot50 | AF-A0A1M5CLP3-F1-MODEL\_V4 | 1.0 | 1.206e-14 | 445 | 0.151 | 304 | 210 | 12 | 3 | 276 | 74 | 359 | Phage protein D | Phage protein D | | afdb-uniprot50 | AF-A0A0F2NZ57-F1-MODEL\_V4 | 1.0 | 5.356e-14 | 445 | 0.173 | 306 | 199 | 13 | 7 | 282 | 72 | 353 | Phage\_base\_V domain-containing protein | Phage\_base\_V domain-containing protein | | afdb-uniprot50 | AF-A0A1Q3SKN4-F1-MODEL\_V4 | 1.0 | 7.134e-14 | 445 | 0.173 | 305 | 199 | 12 | 9 | 281 | 71 | 354 | Phage\_base\_V domain-containing protein | Phage\_base\_V domain-containing protein | | afdb-uniprot50 | AF-A0A7Y5SFG2-F1-MODEL\_V4 | 1.0 | 1.066e-13 | 444 | 0.156 | 313 | 207 | 15 | 7 | 281 | 65 | 358 | VgrG-related protein | VgrG-related protein | | afdb-uniprot50 | AF-A0A202DY36-F1-MODEL\_V4 | 1.0 | 1.891e-13 | 444 | 0.163 | 299 | 206 | 14 | 7 | 280 | 68 | 347 | Phage\_base\_V domain-containing protein | Phage\_base\_V domain-containing protein | | afdb-uniprot50 | AF-A0A826JQU7-F1-MODEL\_V4 | 1.0 | 3.553e-13 | 443 | 0.218 | 211 | 142 | 6 | 84 | 278 | 2 | 205 | Phage late control D family protein | Phage late control D family protein | | afdb-uniprot50 | AF-X5R3B7-F1-MODEL\_V4 | 1.0 | 6.006e-14 | 443 | 0.157 | 305 | 201 | 16 | 14 | 279 | 87 | 374 | Uncharacterized protein | Uncharacterized protein | | afdb-uniprot50 | AF-A0A7V5YZF1-F1-MODEL\_V4 | 1.0 | 7.555e-14 | 443 | 0.155 | 308 | 206 | 16 | 7 | 281 | 65 | 351 | Type IV secretion protein Rhs | Type IV secretion protein Rhs | | afdb-uniprot50 | AF-A0A4R1AAX1-F1-MODEL\_V4 | 1.0 | 4.775e-14 | 443 | 0.14 | 292 | 208 | 15 | 9 | 276 | 74 | 346 | Type VI secretion system tip protein VgrG | Type VI secretion system tip protein VgrG | | afdb-uniprot50 | AF-A0A3C2AA05-F1-MODEL\_V4 | 1.0 | 8.473e-14 | 443 | 0.158 | 296 | 204 | 12 | 7 | 276 | 71 | 347 | Phage\_base\_V domain-containing protein | Phage\_base\_V domain-containing protein | | afdb-uniprot50 | AF-A0A3M1GZV7-F1-MODEL\_V4 | 1.0 | 3.385e-14 | 442 | 0.169 | 295 | 197 | 14 | 15 | 280 | 73 | 348 | Phage late control D family protein | Phage late control D family protein | | afdb-uniprot50 | AF-A0A2P5MZM2-F1-MODEL\_V4 | 1.0 | 2.667e-13 | 441 | 0.156 | 288 | 200 | 14 | 15 | 278 | 76 | 344 | Uncharacterized protein | Uncharacterized protein | | afdb-uniprot50 | AF-A0A850ABE1-F1-MODEL\_V4 | 1.0 | 3.168e-13 | 441 | 0.159 | 307 | 207 | 15 | 7 | 279 | 78 | 367 | Phage late control D family protein | Phage late control D family protein | | afdb-uniprot50 | AF-A0A7C3CCR0-F1-MODEL\_V4 | 1.0 | 1.34e-13 | 441 | 0.133 | 299 | 208 | 11 | 7 | 276 | 74 | 350 | Type VI secretion system tip protein VgrG | Type VI secretion system tip protein VgrG | | afdb-uniprot50 | AF-A0A7V8CYH2-F1-MODEL\_V4 | 1.0 | 1.34e-13 | 441 | 0.185 | 285 | 187 | 14 | 20 | 279 | 74 | 338 | VgrG-related protein | VgrG-related protein | | afdb-uniprot50 | AF-A0A497TMB9-F1-MODEL\_V4 | 1.0 | 5.011e-13 | 441 | 0.17 | 294 | 202 | 12 | 7 | 276 | 75 | 350 | Rhs element Vgr protein | Rhs element Vgr protein | | afdb-uniprot50 | AF-A0A767SPK6-F1-MODEL\_V4 | 1.0 | 3.948e-12 | 440 | 0.342 | 181 | 109 | 4 | 1 | 179 | 24 | 196 | Phage late control D family protein | Phage late control D family protein | | afdb-uniprot50 | AF-J2D8Z2-F1-MODEL\_V4 | 1.0 | 1.503e-13 | 440 | 0.148 | 310 | 197 | 14 | 9 | 278 | 76 | 358 | Phage protein D | Phage protein D | | afdb-uniprot50 | AF-A0A2V8S145-F1-MODEL\_V4 | 1.0 | 9.054e-15 | 440 | 0.156 | 346 | 203 | 14 | 3 | 281 | 38 | 361 | Uncharacterized protein | Uncharacterized protein | | afdb-uniprot50 | AF-A0A447N5J0-F1-MODEL\_V4 | 1.0 | 1.108e-11 | 439 | 0.347 | 161 | 95 | 4 | 8 | 166 | 2 | 154 | Phage late control gene D | Phage late control gene D | | afdb-uniprot50 | AF-A0A5C1IFS9-F1-MODEL\_V4 | 1.0 | 4.22e-13 | 439 | 0.123 | 292 | 216 | 11 | 9 | 276 | 77 | 352 | Type VI secretion system tip protein VgrG | Type VI secretion system tip protein VgrG | | afdb-uniprot50 | AF-A0A2A5AR63-F1-MODEL\_V4 | 1.0 | 1.891e-13 | 439 | 0.172 | 301 | 192 | 14 | 7 | 275 | 72 | 347 | Type VI secretion protein VgrG | Type VI secretion protein VgrG | | afdb-uniprot50 | AF-Q09DF8-F1-MODEL\_V4 | 1.0 | 1.503e-13 | 439 | 0.175 | 302 | 202 | 14 | 7 | 282 | 73 | 353 | Rhs element Vgr protein | Rhs element Vgr protein | | afdb-uniprot50 | AF-A0A853I7P2-F1-MODEL\_V4 | 1.0 | 2.121e-13 | 438 | 0.341 | 205 | 118 | 5 | 1 | 203 | 49 | 238 | Uncharacterized protein | Uncharacterized protein | | afdb-uniprot50 | AF-A0A1M4WE38-F1-MODEL\_V4 | 1.0 | 1.066e-13 | 438 | 0.15 | 299 | 202 | 13 | 7 | 276 | 75 | 350 | Rhs element Vgr protein | Rhs element Vgr protein | | afdb-uniprot50 | AF-A0A315XAN7-F1-MODEL\_V4 | 1.0 | 2.991e-13 | 438 | 0.155 | 303 | 210 | 12 | 3 | 276 | 67 | 352 | Phage\_base\_V domain-containing protein | Phage\_base\_V domain-containing protein | | afdb-uniprot50 | AF-A0A2M9BS42-F1-MODEL\_V4 | 1.0 | 6.361e-14 | 438 | 0.18 | 300 | 193 | 14 | 7 | 276 | 68 | 344 | Rhs element Vgr protein | Rhs element Vgr protein | | afdb-uniprot50 | AF-A0A8B5FWM5-F1-MODEL\_V4 | 1.0 | 4.181e-12 | 437 | 0.338 | 195 | 119 | 4 | 1 | 193 | 63 | 249 | Uncharacterized protein | Uncharacterized protein | | afdb-uniprot50 | AF-A0A7V8YCD2-F1-MODEL\_V4 | 1.0 | 2.85e-14 | 437 | 0.133 | 353 | 226 | 13 | 7 | 311 | 47 | 367 | Phage late control D family protein | Phage late control D family protein | | afdb-uniprot50 | AF-A0A0F9SYY8-F1-MODEL\_V4 | 1.0 | 5.057e-14 | 437 | 0.192 | 306 | 188 | 16 | 9 | 278 | 67 | 349 | Uncharacterized protein | Uncharacterized protein | | afdb-uniprot50 | AF-A0A5K7S6F5-F1-MODEL\_V4 | 1.0 | 9.502e-14 | 437 | 0.163 | 300 | 202 | 14 | 7 | 278 | 74 | 352 | VgrG protein | VgrG protein | | afdb-uniprot50 | AF-A0A244EQM1-F1-MODEL\_V4 | 1.0 | 2.667e-13 | 437 | 0.177 | 293 | 201 | 13 | 7 | 276 | 69 | 344 | Type IV secretion protein Rhs | Type IV secretion protein Rhs | | afdb-uniprot50 | AF-B9M7I2-F1-MODEL\_V4 | 1.0 | 1.184e-12 | 436 | 0.148 | 296 | 211 | 10 | 3 | 274 | 45 | 323 | Phage protein D and tail spike protein, putative | Phage protein D and tail spike protein, putative | | afdb-uniprot50 | AF-A0A2E7CJ66-F1-MODEL\_V4 | 1.0 | 1.419e-13 | 435 | 0.164 | 291 | 189 | 14 | 22 | 278 | 72 | 342 | Uncharacterized protein | Uncharacterized protein | | afdb-uniprot50 | AF-A0A852ZLG6-F1-MODEL\_V4 | 1.0 | 3.168e-13 | 435 | 0.193 | 300 | 201 | 10 | 7 | 279 | 85 | 370 | Uncharacterized protein | Uncharacterized protein | | afdb-uniprot50 | AF-A0A0K2RMD0-F1-MODEL\_V4 | 1.0 | 2.021e-14 | 434 | 0.175 | 296 | 193 | 15 | 12 | 280 | 1 | 272 | Rhs element Vgr protein | Rhs element Vgr protein | | afdb-uniprot50 | AF-A0A535WQL9-F1-MODEL\_V4 | 1.0 | 2.246e-13 | 434 | 0.18 | 311 | 193 | 12 | 7 | 275 | 66 | 356 | Phage late control D family protein | Phage late control D family protein | | afdb-uniprot50 | AF-A0A5N7Z8R8-F1-MODEL\_V4 | 1.0 | 1.592e-13 | 434 | 0.162 | 296 | 202 | 16 | 7 | 276 | 75 | 350 | Type VI secretion system tip protein VgrG | Type VI secretion system tip protein VgrG | | afdb-uniprot50 | AF-A0A7C3NZA9-F1-MODEL\_V4 | 1.0 | 8.473e-14 | 434 | 0.173 | 311 | 196 | 12 | 7 | 281 | 67 | 352 | Phage late control D family protein | Phage late control D family protein | | afdb-uniprot50 | AF-A0A3D2C425-F1-MODEL\_V4 | 1.0 | 1.195e-13 | 433 | 0.175 | 290 | 187 | 13 | 19 | 278 | 74 | 341 | Uncharacterized protein | Uncharacterized protein | | afdb-uniprot50 | AF-A0A1M6GFP2-F1-MODEL\_V4 | 1.0 | 2.002e-13 | 433 | 0.201 | 283 | 191 | 15 | 1 | 273 | 52 | 309 | Uncharacterized conserved protein, implicated in type VI secretion and phage assembly | Uncharacterized conserved protein, implicated in type VI secretion and phage assembly | | afdb-uniprot50 | AF-A0A2E6F191-F1-MODEL\_V4 | 1.0 | 4.732e-13 | 433 | 0.136 | 293 | 213 | 10 | 7 | 276 | 64 | 339 | Phage\_base\_V domain-containing protein | Phage\_base\_V domain-containing protein | | afdb-uniprot50 | AF-A0A3M1XG71-F1-MODEL\_V4 | 1.0 | 6.361e-14 | 433 | 0.182 | 302 | 196 | 15 | 12 | 281 | 79 | 361 | Phage\_base\_V domain-containing protein | Phage\_base\_V domain-containing protein | | afdb-uniprot50 | AF-A0A420WUX9-F1-MODEL\_V4 | 1.0 | 5.15e-16 | 432 | 0.331 | 178 | 102 | 1 | 118 | 278 | 2 | 179 | Late control gene D protein (GPD) | Late control gene D protein (GPD) | | afdb-uniprot50 | AF-A0A538CK92-F1-MODEL\_V4 | 1.0 | 2.519e-13 | 432 | 0.176 | 261 | 168 | 9 | 62 | 278 | 16 | 273 | Phage late control D family protein | Phage late control D family protein | | afdb-uniprot50 | AF-A0A5R9JEF4-F1-MODEL\_V4 | 1.0 | 4.509e-14 | 432 | 0.149 | 315 | 205 | 13 | 7 | 279 | 38 | 331 | Uncharacterized protein | Uncharacterized protein | | afdb-uniprot50 | AF-A0A0C1QXT6-F1-MODEL\_V4 | 1.0 | 2.002e-13 | 432 | 0.187 | 288 | 189 | 14 | 19 | 278 | 88 | 358 | Uncharacterized protein | Uncharacterized protein | | afdb-uniprot50 | AF-A0A849TPI3-F1-MODEL\_V4 | 1.0 | 1.701e-14 | 432 | 0.162 | 332 | 217 | 14 | 9 | 311 | 62 | 361 | Uncharacterized protein | Uncharacterized protein | | afdb-uniprot50 | AF-A0A1I0S6T2-F1-MODEL\_V4 | 1.0 | 4.22e-13 | 432 | 0.136 | 300 | 214 | 15 | 7 | 279 | 75 | 356 | Rhs element Vgr protein | Rhs element Vgr protein | | afdb-uniprot50 | AF-A0A2K8QSP8-F1-MODEL\_V4 | 1.0 | 2.246e-13 | 432 | 0.153 | 299 | 209 | 14 | 7 | 279 | 72 | 352 | Rhs element Vgr protein | Rhs element Vgr protein | | afdb-uniprot50 | AF-A0A1Q3QSK3-F1-MODEL\_V4 | 1.0 | 5.356e-14 | 431 | 0.169 | 330 | 198 | 16 | 7 | 281 | 98 | 406 | Uncharacterized protein | Uncharacterized protein | | afdb-uniprot50 | AF-A0A3C2A1R6-F1-MODEL\_V4 | 1.0 | 8e-14 | 431 | 0.144 | 305 | 202 | 14 | 7 | 278 | 71 | 349 | Rhs element Vgr protein | Rhs element Vgr protein | | afdb-uniprot50 | AF-A0A399D1A6-F1-MODEL\_V4 | 1.0 | 2.121e-13 | 431 | 0.159 | 294 | 202 | 14 | 7 | 275 | 75 | 348 | Type VI secretion system tip protein VgrG | Type VI secretion system tip protein VgrG | | afdb-uniprot50 | AF-A0A1H8J908-F1-MODEL\_V4 | 1.0 | 7.069e-13 | 431 | 0.152 | 295 | 207 | 12 | 7 | 276 | 73 | 349 | Rhs element Vgr protein | Rhs element Vgr protein | | afdb-uniprot50 | AF-Q32HG2-F1-MODEL\_V4 | 1.0 | 7.069e-13 | 430 | 0.301 | 196 | 126 | 4 | 1 | 187 | 30 | 223 | Uncharacterized protein | Uncharacterized protein | | afdb-uniprot50 | AF-K5YW23-F1-MODEL\_V4 | 1.0 | 1.503e-13 | 430 | 0.169 | 289 | 196 | 11 | 7 | 276 | 59 | 322 | Uncharacterized protein | Uncharacterized protein | | afdb-uniprot50 | AF-A0A7V2RMU7-F1-MODEL\_V4 | 1.0 | 3.355e-13 | 430 | 0.142 | 301 | 204 | 12 | 7 | 276 | 76 | 353 | Phage late control D family protein | Phage late control D family protein | | afdb-uniprot50 | AF-A0A7S9UGH2-F1-MODEL\_V4 | 1.0 | 7.134e-14 | 430 | 0.17 | 328 | 201 | 15 | 7 | 281 | 97 | 406 | Phage late control D family protein | Phage late control D family protein | | afdb-uniprot50 | AF-A0A4R2IXT5-F1-MODEL\_V4 | 1.0 | 8.473e-14 | 430 | 0.188 | 308 | 202 | 14 | 7 | 281 | 64 | 356 | Phage protein D | Phage protein D | | afdb-uniprot50 | AF-A0A6C0GDX1-F1-MODEL\_V4 | 1.0 | 3.984e-13 | 430 | 0.157 | 298 | 203 | 12 | 7 | 276 | 72 | 349 | Type VI secretion system tip protein VgrG | Type VI secretion system tip protein VgrG | | afdb-uniprot50 | AF-A0A0Q0VUT6-F1-MODEL\_V4 | 1.0 | 1.686e-13 | 429 | 0.174 | 298 | 195 | 12 | 11 | 277 | 77 | 354 | Uncharacterized protein | Uncharacterized protein | | afdb-uniprot50 | AF-A0A3N5Q711-F1-MODEL\_V4 | 1.0 | 6.361e-14 | 429 | 0.195 | 292 | 183 | 16 | 20 | 279 | 67 | 338 | Phage late control D family protein | Phage late control D family protein | | afdb-uniprot50 | AF-A0A2N2JMI8-F1-MODEL\_V4 | 1.0 | 3.762e-13 | 429 | 0.142 | 330 | 212 | 15 | 3 | 279 | 64 | 375 | Uncharacterized protein | Uncharacterized protein | | afdb-uniprot50 | AF-D0LLS6-F1-MODEL\_V4 | 1.0 | 3.797e-14 | 429 | 0.173 | 300 | 198 | 11 | 22 | 287 | 85 | 368 | Putative phage late control gene D protein | Putative phage late control gene D protein | | afdb-uniprot50 | AF-H8GWZ7-F1-MODEL\_V4 | 1.0 | 7.928e-13 | 429 | 0.14 | 300 | 211 | 15 | 7 | 281 | 67 | 344 | Rhs element Vgr protein | Rhs element Vgr protein | | afdb-uniprot50 | AF-A0A136KQ05-F1-MODEL\_V4 | 1.0 | 8.473e-14 | 429 | 0.162 | 296 | 194 | 16 | 16 | 279 | 76 | 349 | Phage late controlD protein (GPD) | Phage late controlD protein (GPD) | | afdb-uniprot50 | AF-A0A5N5ZAG1-F1-MODEL\_V4 | 1.0 | 5.952e-13 | 429 | 0.136 | 294 | 211 | 12 | 7 | 276 | 76 | 350 | Type VI secretion system tip protein VgrG | Type VI secretion system tip protein VgrG | | afdb-uniprot50 | AF-A0A1Q3MS08-F1-MODEL\_V4 | 1.0 | 5.62e-13 | 429 | 0.15 | 305 | 212 | 14 | 7 | 281 | 64 | 351 | Phage\_base\_V domain-containing protein | Phage\_base\_V domain-containing protein | | afdb-uniprot50 | AF-A0A4Y9QX69-F1-MODEL\_V4 | 1.0 | 6.303e-13 | 428 | 0.105 | 302 | 223 | 13 | 7 | 281 | 70 | 351 | Type VI secretion system tip protein VgrG | Type VI secretion system tip protein VgrG | | afdb-uniprot50 | AF-U2J4H7-F1-MODEL\_V4 | 1.0 | 4.469e-13 | 428 | 0.153 | 307 | 210 | 12 | 7 | 282 | 61 | 348 | Phage\_base\_V domain-containing protein | Phage\_base\_V domain-containing protein | | afdb-uniprot50 | AF-A0A1F8LMF4-F1-MODEL\_V4 | 1.0 | 8.973e-14 | 428 | 0.198 | 313 | 194 | 14 | 7 | 281 | 65 | 358 | Phage\_base\_V domain-containing protein | Phage\_base\_V domain-containing protein | | afdb-uniprot50 | AF-A0A4R5H677-F1-MODEL\_V4 | 1.0 | 2.14e-14 | 427 | 0.213 | 314 | 195 | 14 | 3 | 278 | 86 | 385 | Phage late control D family protein | Phage late control D family protein | | afdb-uniprot50 | AF-A0A7I0NT04-F1-MODEL\_V4 | 1.0 | 1.34e-13 | 427 | 0.174 | 332 | 197 | 16 | 7 | 278 | 87 | 401 | Phage late control D family protein | Phage late control D family protein | | afdb-uniprot50 | AF-A0A1P8MVG2-F1-MODEL\_V4 | 1.0 | 9.972e-13 | 427 | 0.152 | 322 | 217 | 15 | 7 | 280 | 115 | 428 | Uncharacterized protein | Uncharacterized protein | | afdb-uniprot50 | AF-A0A3D0UKR5-F1-MODEL\_V4 | 1.0 | 3.762e-13 | 427 | 0.178 | 292 | 194 | 12 | 17 | 281 | 76 | 348 | Phage\_base\_V domain-containing protein | Phage\_base\_V domain-containing protein | | afdb-uniprot50 | AF-A0A2N6N316-F1-MODEL\_V4 | 1.0 | 3.168e-13 | 427 | 0.168 | 297 | 205 | 12 | 7 | 278 | 75 | 354 | Type IV secretion protein Rhs | Type IV secretion protein Rhs | | afdb-uniprot50 | AF-A0A4S3LP71-F1-MODEL\_V4 | 1.0 | 7.069e-13 | 426 | 0.268 | 246 | 133 | 6 | 1 | 207 | 63 | 300 | Phage late control D family protein | Phage late control D family protein | | afdb-uniprot50 | AF-B0P8P7-F1-MODEL\_V4 | 1.0 | 2.121e-13 | 426 | 0.151 | 298 | 210 | 14 | 7 | 278 | 96 | 376 | Uncharacterized protein | Uncharacterized protein | | afdb-uniprot50 | AF-A0A2T4X8Z9-F1-MODEL\_V4 | 1.0 | 8.892e-13 | 426 | 0.145 | 295 | 210 | 11 | 9 | 279 | 76 | 352 | Rhs element Vgr protein | Rhs element Vgr protein | | afdb-uniprot50 | AF-A0A1G9ZZY8-F1-MODEL\_V4 | 1.0 | 3.762e-13 | 426 | 0.145 | 296 | 208 | 16 | 7 | 276 | 74 | 350 | Rhs element Vgr protein | Rhs element Vgr protein | | afdb-uniprot50 | AF-A0A402CWS6-F1-MODEL\_V4 | 1.0 | 6.675e-13 | 426 | 0.131 | 304 | 216 | 14 | 9 | 281 | 67 | 353 | Phage\_base\_V domain-containing protein | Phage\_base\_V domain-containing protein | | afdb-uniprot50 | AF-A0A6I3ME80-F1-MODEL\_V4 | 1.0 | 7.134e-14 | 425 | 0.159 | 332 | 196 | 15 | 9 | 276 | 67 | 379 | Uncharacterized protein | Uncharacterized protein | | afdb-uniprot50 | AF-A0A553KYW3-F1-MODEL\_V4 | 1.0 | 7.928e-13 | 425 | 0.132 | 301 | 206 | 14 | 7 | 276 | 75 | 351 | Type VI secretion system tip protein VgrG | Type VI secretion system tip protein VgrG | | afdb-uniprot50 | AF-A0A1I0RS25-F1-MODEL\_V4 | 1.0 | 3.355e-13 | 425 | 0.175 | 297 | 204 | 12 | 7 | 279 | 72 | 351 | Rhs element Vgr protein | Rhs element Vgr protein | | afdb-uniprot50 | AF-A0A1V5B929-F1-MODEL\_V4 | 1.0 | 5.011e-13 | 424 | 0.187 | 304 | 195 | 13 | 7 | 277 | 66 | 350 | Phage late control gene D protein (GPD) | Phage late control gene D protein (GPD) | | afdb-uniprot50 | AF-A0A0A8WVU1-F1-MODEL\_V4 | 1.0 | 2.991e-13 | 424 | 0.151 | 311 | 202 | 15 | 7 | 279 | 76 | 362 | Phage late control gene D protein | Phage late control gene D protein | | afdb-uniprot50 | AF-A0A7Z7BGZ7-F1-MODEL\_V4 | 1.0 | 9.502e-14 | 424 | 0.176 | 317 | 199 | 16 | 7 | 279 | 82 | 380 | Uncharacterized protein | Uncharacterized protein | | afdb-uniprot50 | AF-A0A7J5ERL7-F1-MODEL\_V4 | 1.0 | 4.021e-14 | 424 | 0.157 | 331 | 207 | 14 | 22 | 311 | 81 | 380 | Phage late control D family protein | Phage late control D family protein | | afdb-uniprot50 | AF-A0A1M5XUS7-F1-MODEL\_V4 | 1.0 | 3.168e-13 | 424 | 0.165 | 303 | 206 | 15 | 7 | 282 | 71 | 353 | Rhs element Vgr protein | Rhs element Vgr protein | | afdb-uniprot50 | AF-A0A3N4MGZ2-F1-MODEL\_V4 | 1.0 | 2.825e-13 | 424 | 0.155 | 295 | 199 | 15 | 7 | 275 | 75 | 345 | Type VI secretion system tip protein VgrG | Type VI secretion system tip protein VgrG | | afdb-uniprot50 | AF-A0A497IHJ3-F1-MODEL\_V4 | 1.0 | 1.891e-13 | 423 | 0.173 | 306 | 198 | 15 | 9 | 279 | 75 | 360 | Uncharacterized protein | Uncharacterized protein | | afdb-uniprot50 | AF-A0A1B6BCT6-F1-MODEL\_V4 | 1.0 | 1.254e-12 | 423 | 0.186 | 301 | 199 | 16 | 7 | 279 | 82 | 364 | Phage protein D | Phage protein D | | afdb-uniprot50 | AF-A0A1I4DGB1-F1-MODEL\_V4 | 1.0 | 7.555e-14 | 423 | 0.169 | 318 | 203 | 17 | 7 | 280 | 89 | 389 | Phage protein D | Phage protein D | | afdb-uniprot50 | AF-A0A2E8S0G6-F1-MODEL\_V4 | 1.0 | 1.328e-12 | 423 | 0.118 | 362 | 236 | 19 | 3 | 309 | 74 | 407 | Uncharacterized protein | Uncharacterized protein | | afdb-uniprot50 | AF-A0A6L9YV50-F1-MODEL\_V4 | 1.0 | 1.686e-13 | 423 | 0.153 | 300 | 210 | 12 | 8 | 279 | 1 | 284 | Type VI secretion system tip protein VgrG | Type VI secretion system tip protein VgrG | | afdb-uniprot50 | AF-A0A5S3QJL7-F1-MODEL\_V4 | 1.0 | 5.011e-13 | 423 | 0.124 | 297 | 210 | 11 | 9 | 277 | 77 | 351 | Type VI secretion system tip protein VgrG | Type VI secretion system tip protein VgrG | | afdb-uniprot50 | AF-A0A5J6P870-F1-MODEL\_V4 | 1.0 | 2.002e-13 | 422 | 0.157 | 299 | 198 | 13 | 9 | 278 | 73 | 346 | Uncharacterized protein | Uncharacterized protein | | afdb-uniprot50 | AF-A0A3B8IAA2-F1-MODEL\_V4 | 1.0 | 8.473e-14 | 422 | 0.144 | 305 | 202 | 15 | 7 | 278 | 71 | 349 | Rhs element Vgr protein | Rhs element Vgr protein | | afdb-uniprot50 | AF-A0A368NIY7-F1-MODEL\_V4 | 1.0 | 1.407e-12 | 422 | 0.146 | 300 | 211 | 12 | 3 | 276 | 67 | 347 | Type VI secretion system tip protein VgrG | Type VI secretion system tip protein VgrG | | afdb-uniprot50 | AF-A0A317I459-F1-MODEL\_V4 | 1.0 | 2.4e-14 | 421 | 0.138 | 333 | 204 | 17 | 7 | 279 | 86 | 395 | Uncharacterized protein | Uncharacterized protein | | afdb-uniprot50 | AF-A0A1A1YQV5-F1-MODEL\_V4 | 1.0 | 1.066e-13 | 421 | 0.148 | 317 | 208 | 15 | 7 | 278 | 97 | 396 | Uncharacterized protein | Uncharacterized protein | | afdb-uniprot50 | AF-A0A1M7AA40-F1-MODEL\_V4 | 1.0 | 1.328e-12 | 421 | 0.132 | 294 | 214 | 12 | 7 | 276 | 81 | 357 | Rhs element Vgr protein | Rhs element Vgr protein | | afdb-uniprot50 | AF-A0A1H9K9T5-F1-MODEL\_V4 | 1.0 | 1.891e-13 | 421 | 0.157 | 304 | 202 | 17 | 9 | 282 | 74 | 353 | Rhs element Vgr protein | Rhs element Vgr protein | | afdb-uniprot50 | AF-A0A4R5VFK6-F1-MODEL\_V4 | 1.0 | 9.416e-13 | 421 | 0.152 | 296 | 206 | 14 | 9 | 278 | 76 | 352 | Type VI secretion system tip protein VgrG | Type VI secretion system tip protein VgrG | | afdb-uniprot50 | AF-A0A1J5Q762-F1-MODEL\_V4 | 1.0 | 1.266e-13 | 420 | 0.132 | 295 | 206 | 12 | 9 | 276 | 65 | 336 | Phage late control protein Gpd | Phage late control protein Gpd | | afdb-uniprot50 | AF-A0A846AR29-F1-MODEL\_V4 | 1.0 | 8.396e-13 | 420 | 0.155 | 303 | 206 | 15 | 3 | 275 | 38 | 320 | Type VI secretion system tip protein VgrG | Type VI secretion system tip protein VgrG | | afdb-uniprot50 | AF-A0A5M8QXA8-F1-MODEL\_V4 | 1.0 | 9.972e-13 | 420 | 0.156 | 306 | 208 | 11 | 3 | 279 | 62 | 346 | Type VI secretion system tip protein VgrG | Type VI secretion system tip protein VgrG | | afdb-uniprot50 | AF-A0A844MNW0-F1-MODEL\_V4 | 1.0 | 1.254e-12 | 420 | 0.145 | 295 | 209 | 12 | 7 | 276 | 74 | 350 | Type VI secretion system tip protein VgrG | Type VI secretion system tip protein VgrG | | afdb-uniprot50 | AF-A0A660LA17-F1-MODEL\_V4 | 1.0 | 2.121e-13 | 419 | 0.198 | 303 | 205 | 13 | 3 | 281 | 66 | 354 | Uncharacterized protein involved in type VI secretion and phage assembly | Uncharacterized protein involved in type VI secretion and phage assembly | | afdb-uniprot50 | AF-A0A7C6ZWP8-F1-MODEL\_V4 | 1.0 | 1.328e-12 | 419 | 0.168 | 297 | 194 | 12 | 16 | 281 | 75 | 349 | VgrG-related protein | VgrG-related protein | | afdb-uniprot50 | AF-A0A645AVJ7-F1-MODEL\_V4 | 1.0 | 6.736e-14 | 418 | 0.174 | 316 | 197 | 19 | 3 | 280 | 36 | 325 | Uncharacterized protein | Uncharacterized protein | | afdb-uniprot50 | AF-A0A2W2B7E0-F1-MODEL\_V4 | 1.0 | 2.121e-13 | 418 | 0.177 | 293 | 198 | 12 | 9 | 277 | 61 | 334 | Type IV secretion protein Rhs | Type IV secretion protein Rhs | | afdb-uniprot50 | AF-A0A7U8YTK7-F1-MODEL\_V4 | 1.0 | 2.991e-13 | 418 | 0.141 | 311 | 205 | 16 | 8 | 276 | 1 | 291 | Rhs element Vgr protein | Rhs element Vgr protein | | afdb-uniprot50 | AF-A0A369QEE2-F1-MODEL\_V4 | 1.0 | 1.328e-12 | 418 | 0.172 | 301 | 203 | 16 | 7 | 279 | 78 | 360 | Phage\_base\_V domain-containing protein | Phage\_base\_V domain-containing protein | | afdb-uniprot50 | AF-A0A4U9HGJ7-F1-MODEL\_V4 | 1.0 | 4.732e-13 | 416 | 0.271 | 217 | 141 | 4 | 1 | 204 | 59 | 271 | Phage protein D | Phage protein D | | afdb-uniprot50 | AF-A0A1I1ZGB1-F1-MODEL\_V4 | 1.0 | 2.496e-12 | 416 | 0.146 | 293 | 206 | 12 | 9 | 276 | 77 | 350 | Rhs element Vgr protein | Rhs element Vgr protein | | afdb-uniprot50 | AF-A0A6A8Q9P6-F1-MODEL\_V4 | 1.0 | 1.769e-12 | 416 | 0.148 | 296 | 204 | 11 | 7 | 275 | 73 | 347 | Type VI secretion system tip protein VgrG | Type VI secretion system tip protein VgrG | | afdb-uniprot50 | AF-A0A6P1ATK7-F1-MODEL\_V4 | 1.0 | 8.732e-11 | 415 | 0.515 | 126 | 61 | 0 | 1 | 126 | 45 | 170 | Phage tail protein | Phage tail protein | | afdb-uniprot50 | AF-A0A378MZ86-F1-MODEL\_V4 | 1.0 | 1.656e-11 | 415 | 0.331 | 172 | 102 | 5 | 1 | 166 | 14 | 178 | Phage protein D | Phage protein D | | afdb-uniprot50 | AF-A0A2X1MAT3-F1-MODEL\_V4 | 1.0 | 1.108e-11 | 415 | 0.343 | 172 | 103 | 4 | 10 | 179 | 1 | 164 | Phage protein D | Phage protein D | | afdb-uniprot50 | AF-A0A4V1UKC6-F1-MODEL\_V4 | 1.0 | 1.769e-12 | 415 | 0.148 | 243 | 171 | 12 | 57 | 275 | 7 | 237 | Type VI secretion system tip protein VgrG | Type VI secretion system tip protein VgrG | | afdb-uniprot50 | AF-A0A2P7THA8-F1-MODEL\_V4 | 1.0 | 4.469e-13 | 415 | 0.152 | 296 | 204 | 11 | 9 | 277 | 68 | 343 | Type VI secretion system tip protein VgrG | Type VI secretion system tip protein VgrG | | afdb-uniprot50 | AF-A0A375AES2-F1-MODEL\_V4 | 1.0 | 7.069e-13 | 415 | 0.148 | 296 | 210 | 14 | 7 | 277 | 72 | 350 | VgrG protein | VgrG protein | | afdb-uniprot50 | AF-N1ZL07-F1-MODEL\_V4 | 1.0 | 1.066e-13 | 414 | 0.181 | 309 | 196 | 13 | 9 | 279 | 84 | 373 | Uncharacterized protein | Uncharacterized protein | | afdb-uniprot50 | AF-G5IB76-F1-MODEL\_V4 | 1.0 | 3.553e-13 | 414 | 0.143 | 313 | 218 | 12 | 2 | 281 | 80 | 375 | Uncharacterized protein | Uncharacterized protein | | afdb-uniprot50 | AF-A0A7Y6QLZ6-F1-MODEL\_V4 | 1.0 | 3.018e-14 | 414 | 0.144 | 312 | 213 | 14 | 7 | 278 | 99 | 396 | Phage late control D family protein | Phage late control D family protein | | afdb-uniprot50 | AF-A0A2N5Z8J6-F1-MODEL\_V4 | 1.0 | 4.469e-13 | 414 | 0.162 | 295 | 203 | 14 | 7 | 277 | 70 | 344 | Rhs element Vgr protein | Rhs element Vgr protein | | afdb-uniprot50 | AF-A8LSA3-F1-MODEL\_V4 | 1.0 | 9.416e-13 | 414 | 0.181 | 297 | 193 | 15 | 7 | 275 | 72 | 346 | Phage\_base\_V domain-containing protein | Phage\_base\_V domain-containing protein | | afdb-uniprot50 | AF-A0A2N6A013-F1-MODEL\_V4 | 1.0 | 1.407e-12 | 414 | 0.141 | 297 | 212 | 12 | 7 | 278 | 73 | 351 | Rhs element Vgr protein | Rhs element Vgr protein | | afdb-uniprot50 | AF-A0A349D7K0-F1-MODEL\_V4 | 1.0 | 2.378e-13 | 414 | 0.157 | 298 | 203 | 14 | 7 | 275 | 72 | 350 | Rhs element Vgr protein | Rhs element Vgr protein | | afdb-uniprot50 | AF-A0A0N0UY75-F1-MODEL\_V4 | 1.0 | 1.769e-12 | 414 | 0.132 | 294 | 213 | 11 | 7 | 275 | 75 | 351 | Phage\_base\_V domain-containing protein | Phage\_base\_V domain-containing protein | | afdb-uniprot50 | AF-A0A351UYP7-F1-MODEL\_V4 | 1.0 | 7.928e-13 | 413 | 0.145 | 302 | 207 | 13 | 7 | 279 | 75 | 354 | Rhs element Vgr protein | Rhs element Vgr protein | | afdb-uniprot50 | AF-A0A651HNM5-F1-MODEL\_V4 | 1.0 | 1.517e-14 | 413 | 0.156 | 313 | 213 | 15 | 3 | 279 | 88 | 385 | Phage late control D family protein | Phage late control D family protein | | afdb-uniprot50 | AF-A0A538EV62-F1-MODEL\_V4 | 1.0 | 3.355e-13 | 413 | 0.16 | 275 | 179 | 11 | 32 | 275 | 58 | 311 | Phage\_base\_V domain-containing protein | Phage\_base\_V domain-containing protein | | afdb-uniprot50 | AF-A0A810LDR6-F1-MODEL\_V4 | 1.0 | 1.006e-13 | 412 | 0.18 | 326 | 192 | 17 | 3 | 279 | 64 | 363 | Uncharacterized protein | Uncharacterized protein | | afdb-uniprot50 | AF-A0A0M6ZMU0-F1-MODEL\_V4 | 1.0 | 7.486e-13 | 412 | 0.169 | 295 | 200 | 12 | 7 | 276 | 71 | 345 | Rhs element Vgr protein | Rhs element Vgr protein | | afdb-uniprot50 | AF-A0A369WSX9-F1-MODEL\_V4 | 1.0 | 3.948e-12 | 412 | 0.15 | 300 | 210 | 11 | 3 | 274 | 79 | 361 | Type VI secretion system tip protein VgrG | Type VI secretion system tip protein VgrG | | afdb-uniprot50 | AF-A0A7A6RA84-F1-MODEL\_V4 | 1.0 | 1.232e-10 | 411 | 0.365 | 167 | 96 | 4 | 1 | 165 | 63 | 221 | Phage late control D family protein | Phage late control D family protein | | afdb-uniprot50 | AF-A0A417HIZ6-F1-MODEL\_V4 | 1.0 | 1.592e-13 | 411 | 0.186 | 306 | 197 | 14 | 7 | 279 | 80 | 366 | Uncharacterized protein | Uncharacterized protein | | afdb-uniprot50 | AF-A0A8A6KDY8-F1-MODEL\_V4 | 1.0 | 2.85e-14 | 411 | 0.166 | 324 | 200 | 16 | 7 | 277 | 89 | 395 | Phage late control D family protein | Phage late control D family protein | | afdb-uniprot50 | AF-D1JAM5-F1-MODEL\_V4 | 1.0 | 4.732e-13 | 411 | 0.177 | 326 | 223 | 14 | 7 | 302 | 74 | 384 | Phage\_base\_V domain-containing protein | Phage\_base\_V domain-containing protein | | afdb-uniprot50 | AF-A0A2L2NSD3-F1-MODEL\_V4 | 1.0 | 9.972e-13 | 411 | 0.138 | 311 | 214 | 13 | 7 | 281 | 98 | 390 | Rhs element Vgr protein | Rhs element Vgr protein | | afdb-uniprot50 | AF-A0A381E6W6-F1-MODEL\_V4 | 1.0 | 8.32e-12 | 410 | 0.213 | 183 | 134 | 5 | 92 | 274 | 1 | 173 | Phage protein D | Phage protein D | | afdb-uniprot50 | AF-A0A512ISE5-F1-MODEL\_V4 | 1.0 | 1.066e-13 | 410 | 0.189 | 311 | 203 | 15 | 7 | 286 | 60 | 352 | Uncharacterized protein | Uncharacterized protein | | afdb-uniprot50 | AF-A0A7X5W735-F1-MODEL\_V4 | 1.0 | 6.736e-14 | 410 | 0.179 | 289 | 186 | 15 | 23 | 279 | 88 | 357 | Uncharacterized protein | Uncharacterized protein | | afdb-uniprot50 | AF-A0A0A2EIY0-F1-MODEL\_V4 | 1.0 | 6.675e-13 | 410 | 0.114 | 287 | 209 | 11 | 19 | 279 | 84 | 351 | Phage\_base\_V domain-containing protein | Phage\_base\_V domain-containing protein | | afdb-uniprot50 | AF-A0A4U1I5U2-F1-MODEL\_V4 | 1.0 | 8.892e-13 | 410 | 0.136 | 301 | 205 | 14 | 7 | 276 | 72 | 348 | Type VI secretion system tip protein VgrG | Type VI secretion system tip protein VgrG | | afdb-uniprot50 | AF-A0A2D0N1B1-F1-MODEL\_V4 | 1.0 | 5.011e-13 | 410 | 0.157 | 304 | 195 | 16 | 7 | 276 | 69 | 345 | Phage\_base\_V domain-containing protein | Phage\_base\_V domain-containing protein | | afdb-uniprot50 | AF-A0A4R7HI80-F1-MODEL\_V4 | 1.0 | 4.22e-13 | 410 | 0.188 | 307 | 206 | 15 | 7 | 281 | 64 | 359 | Uncharacterized protein involved in type VI secretion and phage assembly | Uncharacterized protein involved in type VI secretion and phage assembly | | afdb-uniprot50 | AF-A0A6G9ZEV2-F1-MODEL\_V4 | 1.0 | 5.952e-13 | 410 | 0.15 | 300 | 200 | 13 | 15 | 277 | 190 | 471 | VgrG-related protein | VgrG-related protein | | afdb-uniprot50 | AF-A0A2A3WJY1-F1-MODEL\_V4 | 1.0 | 9.331e-12 | 409 | 0.304 | 194 | 127 | 3 | 1 | 188 | 56 | 247 | Late control protein | Late control protein | | afdb-uniprot50 | AF-A0A7L4Q7F5-F1-MODEL\_V4 | 1.0 | 1.49e-12 | 409 | 0.174 | 310 | 201 | 14 | 7 | 278 | 73 | 365 | Phage late control D family protein | Phage late control D family protein | | afdb-uniprot50 | AF-C7XD81-F1-MODEL\_V4 | 1.0 | 4.69e-12 | 409 | 0.175 | 239 | 169 | 7 | 57 | 276 | 7 | 236 | Rhs element Vgr protein | Rhs element Vgr protein | | afdb-uniprot50 | AF-A0A1H3K869-F1-MODEL\_V4 | 1.0 | 5.57e-12 | 409 | 0.166 | 301 | 203 | 12 | 9 | 281 | 66 | 346 | Phage protein D | Phage protein D | | afdb-uniprot50 | AF-A0A563VQY9-F1-MODEL\_V4 | 1.0 | 2.964e-12 | 408 | 0.12 | 299 | 209 | 10 | 15 | 281 | 270 | 546 | Rhs element Vgr protein (Modular protein) | Rhs element Vgr protein (Modular protein) | | afdb-uniprot50 | AF-A0A7X8ICU0-F1-MODEL\_V4 | 1.0 | 6.361e-14 | 407 | 0.171 | 314 | 196 | 18 | 7 | 279 | 83 | 373 | Phage late control D family protein | Phage late control D family protein | | afdb-uniprot50 | AF-A0A7V2INA6-F1-MODEL\_V4 | 1.0 | 1.984e-12 | 407 | 0.156 | 344 | 209 | 18 | 7 | 287 | 64 | 389 | Phage late control D family protein | Phage late control D family protein | | afdb-uniprot50 | AF-A0A4Y8ABY2-F1-MODEL\_V4 | 1.0 | 6.675e-13 | 407 | 0.134 | 289 | 206 | 12 | 13 | 275 | 79 | 349 | Rhs element Vgr protein | Rhs element Vgr protein | | afdb-uniprot50 | AF-A0A495EIK4-F1-MODEL\_V4 | 1.0 | 8.396e-13 | 407 | 0.147 | 299 | 203 | 14 | 7 | 276 | 75 | 350 | Rhs element Vgr protein | Rhs element Vgr protein | | afdb-uniprot50 | AF-A0A7W9EDQ9-F1-MODEL\_V4 | 1.0 | 8.811e-12 | 406 | 0.38 | 189 | 109 | 3 | 2 | 189 | 55 | 236 | Uncharacterized protein | Uncharacterized protein | | afdb-uniprot50 | AF-A0A3P6KJ21-F1-MODEL\_V4 | 1.0 | 5.259e-12 | 406 | 0.162 | 289 | 206 | 12 | 7 | 278 | 103 | 372 | Phage protein D | Phage protein D | | afdb-uniprot50 | AF-A0A402CKJ8-F1-MODEL\_V4 | 1.0 | 7.555e-14 | 406 | 0.161 | 310 | 208 | 12 | 7 | 278 | 78 | 373 | Phage protein D | Phage protein D | | afdb-uniprot50 | AF-A0A5M6DLW7-F1-MODEL\_V4 | 1.0 | 1.874e-12 | 406 | 0.149 | 295 | 205 | 15 | 7 | 275 | 75 | 349 | Type VI secretion system tip protein VgrG | Type VI secretion system tip protein VgrG | | afdb-uniprot50 | AF-A0A412YNE6-F1-MODEL\_V4 | 1.0 | 1.118e-12 | 406 | 0.143 | 300 | 204 | 11 | 7 | 275 | 73 | 350 | Type VI secretion system tip protein VgrG | Type VI secretion system tip protein VgrG | | afdb-uniprot50 | AF-A0A258B3I5-F1-MODEL\_V4 | 1.0 | 1.407e-12 | 406 | 0.16 | 299 | 204 | 12 | 9 | 279 | 75 | 354 | Phage\_base\_V domain-containing protein | Phage\_base\_V domain-containing protein | | afdb-uniprot50 | AF-A0A316QJQ2-F1-MODEL\_V4 | 1.0 | 3.728e-12 | 405 | 0.176 | 300 | 203 | 14 | 7 | 279 | 83 | 365 | Uncharacterized protein | Uncharacterized protein | | afdb-uniprot50 | AF-A0A3A0B1P0-F1-MODEL\_V4 | 1.0 | 2.4e-14 | 405 | 0.18 | 316 | 201 | 12 | 9 | 280 | 83 | 384 | Uncharacterized protein | Uncharacterized protein | | afdb-uniprot50 | AF-A0A7Y2DHA3-F1-MODEL\_V4 | 1.0 | 9.416e-13 | 405 | 0.132 | 303 | 210 | 11 | 7 | 279 | 75 | 354 | Type VI secretion system tip protein VgrG | Type VI secretion system tip protein VgrG | | afdb-uniprot50 | AF-A0A838RL76-F1-MODEL\_V4 | 1.0 | 1.056e-12 | 405 | 0.168 | 297 | 193 | 15 | 19 | 281 | 77 | 353 | VgrG-related protein | VgrG-related protein | | afdb-uniprot50 | AF-A0A538PYD2-F1-MODEL\_V4 | 1.0 | 1.195e-13 | 404 | 0.173 | 328 | 210 | 13 | 22 | 309 | 83 | 389 | Phage late control D family protein | Phage late control D family protein | | afdb-uniprot50 | AF-A0A364Y3U2-F1-MODEL\_V4 | 1.0 | 1.874e-12 | 404 | 0.137 | 305 | 206 | 13 | 7 | 279 | 69 | 348 | Rhs element Vgr protein | Rhs element Vgr protein | | afdb-uniprot50 | AF-A0A447R078-F1-MODEL\_V4 | 1.0 | 8.732e-11 | 403 | 0.333 | 168 | 102 | 4 | 1 | 166 | 25 | 184 | Phage late control gene D | Phage late control gene D | | afdb-uniprot50 | AF-A0A3R6R790-F1-MODEL\_V4 | 1.0 | 1.056e-12 | 403 | 0.151 | 303 | 197 | 16 | 7 | 275 | 85 | 361 | Uncharacterized protein | Uncharacterized protein | | afdb-uniprot50 | AF-A0A2E2FL30-F1-MODEL\_V4 | 1.0 | 1.254e-12 | 403 | 0.158 | 315 | 201 | 16 | 7 | 278 | 57 | 350 | Uncharacterized protein | Uncharacterized protein | | afdb-uniprot50 | AF-A0A2K8QX00-F1-MODEL\_V4 | 1.0 | 5.952e-13 | 403 | 0.13 | 329 | 213 | 18 | 7 | 278 | 77 | 389 | Phage control protein (GPD) | Phage control protein (GPD) | | afdb-uniprot50 | AF-A0A1G3LKX5-F1-MODEL\_V4 | 1.0 | 8.892e-13 | 403 | 0.177 | 355 | 210 | 21 | 7 | 311 | 67 | 389 | Uncharacterized protein | Uncharacterized protein | | afdb-uniprot50 | AF-A0A841BZE2-F1-MODEL\_V4 | 1.0 | 1.34e-13 | 403 | 0.165 | 308 | 198 | 16 | 7 | 279 | 33 | 316 | Phage protein D/phage baseplate assembly protein gpV | Phage protein D/phage baseplate assembly protein gpV | | afdb-uniprot50 | AF-A0A2T0WPJ0-F1-MODEL\_V4 | 1.0 | 7.069e-13 | 402 | 0.14 | 307 | 209 | 13 | 7 | 279 | 72 | 357 | Phage protein D | Phage protein D | | afdb-uniprot50 | AF-A0A1I5CAJ2-F1-MODEL\_V4 | 1.0 | 4.732e-13 | 400 | 0.153 | 312 | 206 | 16 | 7 | 278 | 82 | 375 | Uncharacterized protein | Uncharacterized protein | | afdb-uniprot50 | AF-A0A4Q6ANH2-F1-MODEL\_V4 | 1.0 | 5.57e-12 | 400 | 0.148 | 296 | 208 | 14 | 7 | 276 | 75 | 352 | Type VI secretion system tip protein VgrG | Type VI secretion system tip protein VgrG | | afdb-uniprot50 | AF-A0A6N3K7N7-F1-MODEL\_V4 | 1.0 | 1.407e-12 | 400 | 0.159 | 301 | 202 | 14 | 9 | 281 | 57 | 334 | Type IV secretion protein Rhs | Type IV secretion protein Rhs | | afdb-uniprot50 | AF-A0A1Q7S8W7-F1-MODEL\_V4 | 1.0 | 1.328e-12 | 400 | 0.146 | 315 | 219 | 15 | 2 | 281 | 37 | 336 | Phage\_base\_V domain-containing protein | Phage\_base\_V domain-containing protein | | afdb-uniprot50 | AF-A0A6I7PB73-F1-MODEL\_V4 | 1.0 | 3.521e-12 | 400 | 0.131 | 296 | 214 | 13 | 7 | 276 | 71 | 349 | Type VI secretion system tip protein VgrG | Type VI secretion system tip protein VgrG | | afdb-uniprot50 | AF-A0A7H5F1M2-F1-MODEL\_V4 | 1.0 | 2.225e-12 | 400 | 0.163 | 293 | 204 | 12 | 7 | 275 | 74 | 349 | Type VI secretion system tip protein VgrG | Type VI secretion system tip protein VgrG | | afdb-uniprot50 | AF-A0A484HHC7-F1-MODEL\_V4 | 1.0 | 9.972e-13 | 400 | 0.157 | 298 | 211 | 14 | 7 | 276 | 66 | 351 | Phage\_base\_V domain-containing protein | Phage\_base\_V domain-containing protein | | afdb-uniprot50 | AF-A0A5P9HZY4-F1-MODEL\_V4 | 1.0 | 4.181e-12 | 399 | 0.151 | 298 | 204 | 12 | 7 | 278 | 72 | 346 | Phage late control gene D protein (GPD) | Phage late control gene D protein (GPD) | | afdb-uniprot50 | AF-A0A1L3I302-F1-MODEL\_V4 | 1.0 | 8.396e-13 | 398 | 0.261 | 176 | 124 | 3 | 137 | 310 | 45 | 216 | Phage late control gene D protein (GPD) | Phage late control gene D protein (GPD) | | afdb-uniprot50 | AF-A0A7C1JGV2-F1-MODEL\_V4 | 1.0 | 7.069e-13 | 398 | 0.171 | 297 | 193 | 15 | 16 | 281 | 78 | 352 | Phage\_base\_V domain-containing protein | Phage\_base\_V domain-containing protein | | afdb-uniprot50 | AF-A0A1E5G2C5-F1-MODEL\_V4 | 1.0 | 3.553e-13 | 397 | 0.174 | 310 | 191 | 17 | 9 | 279 | 88 | 371 | Uncharacterized protein | Uncharacterized protein | | afdb-uniprot50 | AF-A0A480AGP3-F1-MODEL\_V4 | 1.0 | 8.892e-13 | 397 | 0.158 | 303 | 195 | 17 | 7 | 275 | 63 | 339 | Phage\_base\_V domain-containing protein | Phage\_base\_V domain-containing protein | | afdb-uniprot50 | AF-A0A2M8Q5J0-F1-MODEL\_V4 | 1.0 | 8.396e-13 | 397 | 0.19 | 309 | 194 | 18 | 7 | 281 | 67 | 353 | Type IV secretion protein Rhs | Type IV secretion protein Rhs | | afdb-uniprot50 | AF-A0A2V2LGK4-F1-MODEL\_V4 | 1.0 | 6.615e-12 | 396 | 0.147 | 291 | 211 | 13 | 7 | 279 | 71 | 342 | Phage\_base\_V domain-containing protein | Phage\_base\_V domain-containing protein | | afdb-uniprot50 | AF-A0A1N6HBQ1-F1-MODEL\_V4 | 1.0 | 3.948e-12 | 396 | 0.132 | 295 | 213 | 14 | 7 | 276 | 74 | 350 | Rhs element Vgr protein | Rhs element Vgr protein | | afdb-uniprot50 | AF-A0A512BIY2-F1-MODEL\_V4 | 1.0 | 1.476e-11 | 395 | 0.16 | 292 | 205 | 10 | 9 | 276 | 76 | 351 | Type IV secretion protein Rhs | Type IV secretion protein Rhs | | afdb-uniprot50 | AF-A0A4R1YY35-F1-MODEL\_V4 | 1.0 | 6.675e-13 | 395 | 0.162 | 301 | 200 | 19 | 7 | 277 | 72 | 350 | Phage\_base\_V domain-containing protein | Phage\_base\_V domain-containing protein | | afdb-uniprot50 | AF-A0A3D9UDY1-F1-MODEL\_V4 | 1.0 | 5.519e-11 | 394 | 0.276 | 195 | 130 | 5 | 1 | 193 | 75 | 260 | Late control gene D protein (GPD) | Late control gene D protein (GPD) | | afdb-uniprot50 | AF-A0A7X9I2D4-F1-MODEL\_V4 | 1.0 | 1.753e-11 | 394 | 0.237 | 257 | 148 | 6 | 1 | 217 | 63 | 311 | Phage tail protein | Phage tail protein | | afdb-uniprot50 | AF-A0A424H668-F1-MODEL\_V4 | 1.0 | 5.898e-12 | 394 | 0.126 | 301 | 213 | 13 | 7 | 275 | 75 | 357 | Phage\_base\_V domain-containing protein | Phage\_base\_V domain-containing protein | | afdb-uniprot50 | AF-A0A1I3Q7R5-F1-MODEL\_V4 | 1.0 | 2.357e-12 | 393 | 0.138 | 297 | 208 | 18 | 7 | 276 | 77 | 352 | Rhs element Vgr protein | Rhs element Vgr protein | | afdb-uniprot50 | AF-A0A368JR84-F1-MODEL\_V4 | 1.0 | 3.324e-12 | 393 | 0.163 | 299 | 199 | 13 | 7 | 276 | 76 | 352 | Type VI secretion system tip protein VgrG | Type VI secretion system tip protein VgrG | | afdb-uniprot50 | AF-A0A2A2EVG0-F1-MODEL\_V4 | 1.0 | 7.419e-12 | 392 | 0.424 | 125 | 72 | 0 | 155 | 279 | 2 | 126 | Uncharacterized protein | Uncharacterized protein | | afdb-uniprot50 | AF-A0A257Q6I6-F1-MODEL\_V4 | 1.0 | 1.769e-12 | 392 | 0.134 | 290 | 192 | 11 | 14 | 276 | 68 | 325 | Uncharacterized protein | Uncharacterized protein | | afdb-uniprot50 | AF-A0A369ASP5-F1-MODEL\_V4 | 1.0 | 3.521e-12 | 392 | 0.154 | 297 | 201 | 14 | 7 | 275 | 91 | 365 | Phage protein D | Phage protein D | | afdb-uniprot50 | AF-A0A644Z3M6-F1-MODEL\_V4 | 1.0 | 1.184e-12 | 392 | 0.138 | 310 | 213 | 13 | 9 | 281 | 83 | 375 | Uncharacterized protein | Uncharacterized protein | | afdb-uniprot50 | AF-A0A1V0Q0E9-F1-MODEL\_V4 | 1.0 | 1.129e-13 | 392 | 0.156 | 320 | 205 | 14 | 9 | 279 | 94 | 397 | Phage protein D | Phage protein D | | afdb-uniprot50 | AF-A0A2S6I8V1-F1-MODEL\_V4 | 1.0 | 5.259e-12 | 392 | 0.14 | 298 | 205 | 13 | 7 | 276 | 76 | 350 | Rhs element Vgr protein | Rhs element Vgr protein | | afdb-uniprot50 | AF-A0A3M1YH05-F1-MODEL\_V4 | 1.0 | 5.307e-13 | 392 | 0.151 | 303 | 204 | 14 | 7 | 279 | 72 | 351 | Type VI secretion system tip protein VgrG | Type VI secretion system tip protein VgrG | | afdb-uniprot50 | AF-A0A1T5N4P7-F1-MODEL\_V4 | 1.0 | 4.428e-12 | 392 | 0.116 | 293 | 220 | 11 | 7 | 276 | 79 | 355 | Rhs element Vgr protein | Rhs element Vgr protein | | afdb-uniprot50 | AF-A0A165X1V4-F1-MODEL\_V4 | 1.0 | 2.225e-12 | 391 | 0.191 | 219 | 145 | 7 | 112 | 311 | 4 | 209 | Phage late control gene D protein (GPD) | Phage late control gene D protein (GPD) | | afdb-uniprot50 | AF-A0A5S9QSE6-F1-MODEL\_V4 | 1.0 | 7.419e-12 | 391 | 0.133 | 292 | 211 | 10 | 7 | 275 | 72 | 344 | Phage\_base\_V domain-containing protein | Phage\_base\_V domain-containing protein | | afdb-uniprot50 | AF-A0A2D0MYA0-F1-MODEL\_V4 | 1.0 | 2.101e-12 | 391 | 0.165 | 302 | 199 | 17 | 7 | 278 | 76 | 354 | Type IV secretion protein Rhs | Type IV secretion protein Rhs | | afdb-uniprot50 | AF-A0A559QPW3-F1-MODEL\_V4 | 1.0 | 1.328e-12 | 391 | 0.155 | 302 | 203 | 13 | 7 | 275 | 72 | 354 | Rhs element Vgr protein | Rhs element Vgr protein | | afdb-uniprot50 | AF-A0A850NG49-F1-MODEL\_V4 | 1.0 | 7.486e-13 | 390 | 0.138 | 297 | 211 | 13 | 7 | 276 | 61 | 339 | Uncharacterized protein | Uncharacterized protein | | afdb-uniprot50 | AF-R5JY02-F1-MODEL\_V4 | 1.0 | 6.247e-12 | 390 | 0.167 | 311 | 203 | 13 | 7 | 278 | 84 | 377 | Uncharacterized protein | Uncharacterized protein | | afdb-uniprot50 | AF-A0A3P1CVH5-F1-MODEL\_V4 | 1.0 | 3.728e-12 | 390 | 0.132 | 303 | 209 | 12 | 9 | 278 | 74 | 355 | Type VI secretion system tip protein VgrG | Type VI secretion system tip protein VgrG | | afdb-uniprot50 | AF-F2NUE0-F1-MODEL\_V4 | 1.0 | 3.521e-12 | 390 | 0.155 | 257 | 174 | 12 | 61 | 290 | 149 | 389 | Uncharacterized protein | Uncharacterized protein | | afdb-uniprot50 | AF-A0A2E7URL9-F1-MODEL\_V4 | 1.0 | 1.056e-12 | 389 | 0.132 | 303 | 204 | 15 | 9 | 278 | 36 | 312 | Uncharacterized protein | Uncharacterized protein | | afdb-uniprot50 | AF-A0A7U9SDK9-F1-MODEL\_V4 | 1.0 | 7.928e-13 | 389 | 0.13 | 315 | 212 | 16 | 9 | 281 | 83 | 377 | Uncharacterized protein | Uncharacterized protein | | afdb-uniprot50 | AF-A0A257KZG6-F1-MODEL\_V4 | 1.0 | 6.247e-12 | 389 | 0.136 | 294 | 204 | 14 | 9 | 274 | 75 | 346 | Type IV secretion protein Rhs | Type IV secretion protein Rhs | | afdb-uniprot50 | AF-A0A2D8HSQ5-F1-MODEL\_V4 | 1.0 | 9.331e-12 | 389 | 0.134 | 297 | 217 | 16 | 1 | 281 | 53 | 325 | Phage\_base\_V domain-containing protein | Phage\_base\_V domain-containing protein | | afdb-uniprot50 | AF-A0A136LBS7-F1-MODEL\_V4 | 1.0 | 4.428e-12 | 389 | 0.119 | 302 | 212 | 13 | 7 | 276 | 78 | 357 | Rhs element Vgr protein | Rhs element Vgr protein | | afdb-uniprot50 | AF-A0A853IAA6-F1-MODEL\_V4 | 1.0 | 1.769e-12 | 388 | 0.148 | 316 | 212 | 15 | 3 | 278 | 61 | 359 | Uncharacterized protein | Uncharacterized protein | | afdb-uniprot50 | AF-A0A6C2CEP6-F1-MODEL\_V4 | 1.0 | 1.174e-11 | 388 | 0.153 | 299 | 210 | 13 | 7 | 281 | 73 | 352 | Type VI secretion system tip protein VgrG | Type VI secretion system tip protein VgrG | | afdb-uniprot50 | AF-A0A518BPM5-F1-MODEL\_V4 | 1.0 | 1.874e-12 | 388 | 0.165 | 315 | 189 | 14 | 9 | 281 | 73 | 355 | Phage late control gene D protein (GPD) | Phage late control gene D protein (GPD) | | afdb-uniprot50 | AF-J0P5E3-F1-MODEL\_V4 | 1.0 | 2.799e-12 | 388 | 0.137 | 299 | 207 | 17 | 7 | 276 | 73 | 349 | Rhs element Vgr protein | Rhs element Vgr protein | | afdb-uniprot50 | AF-A0A2N0KBG2-F1-MODEL\_V4 | 1.0 | 2.496e-12 | 387 | 0.143 | 285 | 211 | 11 | 7 | 275 | 88 | 355 | Uncharacterized protein | Uncharacterized protein | | afdb-uniprot50 | AF-A0A1Y3U421-F1-MODEL\_V4 | 1.0 | 1.49e-12 | 387 | 0.166 | 295 | 197 | 16 | 7 | 275 | 85 | 356 | Uncharacterized protein | Uncharacterized protein | | afdb-uniprot50 | AF-A0A1Y3SD88-F1-MODEL\_V4 | 1.0 | 3.521e-12 | 387 | 0.168 | 309 | 202 | 16 | 3 | 279 | 79 | 364 | Uncharacterized protein | Uncharacterized protein | | afdb-uniprot50 | AF-A0A7Y9PD51-F1-MODEL\_V4 | 1.0 | 2.378e-13 | 387 | 0.181 | 342 | 209 | 20 | 7 | 311 | 51 | 358 | Phage protein D | Phage protein D | | afdb-uniprot50 | AF-A0A3D1AC35-F1-MODEL\_V4 | 1.0 | 4.966e-12 | 387 | 0.138 | 295 | 218 | 12 | 7 | 279 | 73 | 353 | Rhs element Vgr protein | Rhs element Vgr protein | | afdb-uniprot50 | AF-A0A379YH32-F1-MODEL\_V4 | 1.0 | 2.335e-11 | 386 | 0.309 | 194 | 125 | 4 | 1 | 193 | 63 | 248 | Phage protein D | Phage protein D | | afdb-uniprot50 | AF-A0A7G9WG86-F1-MODEL\_V4 | 1.0 | 2.496e-12 | 386 | 0.137 | 269 | 191 | 16 | 20 | 277 | 64 | 302 | Phage late control D family protein | Phage late control D family protein | | afdb-uniprot50 | AF-A0A3S0U1E7-F1-MODEL\_V4 | 1.0 | 3.728e-12 | 386 | 0.149 | 335 | 212 | 16 | 7 | 291 | 59 | 370 | Uncharacterized protein | Uncharacterized protein | | afdb-uniprot50 | AF-A0A2N5YLC6-F1-MODEL\_V4 | 1.0 | 2.082e-11 | 386 | 0.129 | 293 | 213 | 12 | 7 | 275 | 69 | 343 | Rhs element Vgr protein | Rhs element Vgr protein | | afdb-uniprot50 | AF-A0A4P5VE00-F1-MODEL\_V4 | 1.0 | 2.643e-12 | 386 | 0.126 | 293 | 205 | 14 | 13 | 276 | 72 | 342 | Type IV secretion protein Rhs | Type IV secretion protein Rhs | | afdb-uniprot50 | AF-A0A257QUN5-F1-MODEL\_V4 | 1.0 | 1.184e-12 | 385 | 0.148 | 282 | 187 | 12 | 19 | 275 | 82 | 335 | Uncharacterized protein | Uncharacterized protein | | afdb-uniprot50 | AF-A0A3D4KWM2-F1-MODEL\_V4 | 1.0 | 3.139e-12 | 385 | 0.126 | 325 | 209 | 14 | 7 | 275 | 83 | 388 | Uncharacterized protein | Uncharacterized protein | | afdb-uniprot50 | AF-A0A291INC8-F1-MODEL\_V4 | 1.0 | 5.259e-12 | 385 | 0.167 | 287 | 196 | 14 | 18 | 281 | 72 | 338 | Baseplate assembly protein V | Baseplate assembly protein V | | afdb-uniprot50 | AF-C7QK16-F1-MODEL\_V4 | 1.0 | 4.69e-12 | 385 | 0.163 | 311 | 207 | 14 | 3 | 276 | 60 | 354 | Rhs element Vgr protein | Rhs element Vgr protein | | afdb-uniprot50 | AF-A0A371WSZ2-F1-MODEL\_V4 | 1.0 | 8e-14 | 384 | 0.153 | 346 | 215 | 18 | 7 | 310 | 61 | 370 | Uncharacterized protein | Uncharacterized protein | | afdb-uniprot50 | AF-A0A661IT63-F1-MODEL\_V4 | 1.0 | 1.407e-12 | 384 | 0.133 | 314 | 207 | 16 | 7 | 277 | 94 | 385 | Uncharacterized protein | Uncharacterized protein | | afdb-uniprot50 | AF-A0A4Y6PVJ4-F1-MODEL\_V4 | 1.0 | 7.069e-13 | 384 | 0.141 | 339 | 201 | 17 | 9 | 278 | 79 | 396 | Phage late control D family protein | Phage late control D family protein | | afdb-uniprot50 | AF-A0A1M4ZE13-F1-MODEL\_V4 | 1.0 | 1.108e-11 | 384 | 0.126 | 293 | 218 | 14 | 7 | 279 | 76 | 350 | Uncharacterized conserved protein, implicated in type VI secretion and phage assembly | Uncharacterized conserved protein, implicated in type VI secretion and phage assembly | | afdb-uniprot50 | AF-A0A1G7A3Q8-F1-MODEL\_V4 | 1.0 | 6.247e-12 | 384 | 0.138 | 304 | 199 | 18 | 7 | 275 | 76 | 351 | Rhs element Vgr protein | Rhs element Vgr protein | | afdb-uniprot50 | AF-A0A6D1ICX5-F1-MODEL\_V4 | 1.0 | 3.324e-12 | 384 | 0.179 | 301 | 211 | 12 | 9 | 281 | 66 | 358 | Type IV secretion protein Rhs | Type IV secretion protein Rhs | | afdb-uniprot50 | AF-A0A7C6P4H4-F1-MODEL\_V4 | 1.0 | 2.799e-12 | 383 | 0.134 | 298 | 205 | 16 | 3 | 274 | 82 | 352 | Phage late control D family protein | Phage late control D family protein | | afdb-uniprot50 | AF-A0A1Q3UZH9-F1-MODEL\_V4 | 1.0 | 3.762e-13 | 383 | 0.138 | 332 | 202 | 16 | 7 | 290 | 61 | 356 | Uncharacterized protein | Uncharacterized protein | | afdb-uniprot50 | AF-A0A7Z9KYN7-F1-MODEL\_V4 | 1.0 | 1.476e-11 | 382 | 0.136 | 330 | 216 | 15 | 7 | 279 | 77 | 394 | Uncharacterized protein | Uncharacterized protein | | afdb-uniprot50 | AF-A0A4Q3UH64-F1-MODEL\_V4 | 1.0 | 2.378e-13 | 382 | 0.163 | 311 | 210 | 12 | 7 | 277 | 116 | 416 | Phage late control D family protein | Phage late control D family protein | | afdb-uniprot50 | AF-A0A4Y8NQP5-F1-MODEL\_V4 | 1.0 | 9.416e-13 | 382 | 0.142 | 322 | 199 | 13 | 18 | 278 | 106 | 411 | Uncharacterized protein | Uncharacterized protein | | afdb-uniprot50 | AF-A0A536PPK3-F1-MODEL\_V4 | 1.0 | 2.643e-12 | 382 | 0.136 | 308 | 216 | 16 | 8 | 281 | 1 | 292 | VgrG-related protein | VgrG-related protein | | afdb-uniprot50 | AF-A0A553FM29-F1-MODEL\_V4 | 1.0 | 7.928e-13 | 382 | 0.131 | 311 | 205 | 14 | 7 | 282 | 72 | 352 | Type VI secretion system tip protein VgrG | Type VI secretion system tip protein VgrG | | afdb-uniprot50 | AF-A0A1B9SFQ1-F1-MODEL\_V4 | 1.0 | 2.667e-13 | 381 | 0.17 | 311 | 192 | 16 | 9 | 282 | 62 | 343 | Uncharacterized protein | Uncharacterized protein | | afdb-uniprot50 | AF-A0A3D2CCS9-F1-MODEL\_V4 | 1.0 | 1.984e-12 | 381 | 0.139 | 301 | 204 | 15 | 9 | 278 | 55 | 331 | Uncharacterized protein | Uncharacterized protein | | afdb-uniprot50 | AF-A0A136LD44-F1-MODEL\_V4 | 1.0 | 6.247e-12 | 381 | 0.146 | 313 | 208 | 16 | 7 | 281 | 70 | 361 | Rhs element Vgr protein | Rhs element Vgr protein | | afdb-uniprot50 | AF-A0A0E3SC16-F1-MODEL\_V4 | 1.0 | 8.32e-12 | 380 | 0.132 | 286 | 201 | 11 | 22 | 277 | 90 | 358 | Phage protein D | Phage protein D | | afdb-uniprot50 | AF-A0A4R5WB95-F1-MODEL\_V4 | 1.0 | 2.225e-12 | 380 | 0.175 | 291 | 193 | 14 | 9 | 273 | 63 | 332 | Type IV secretion protein Rhs | Type IV secretion protein Rhs | | afdb-uniprot50 | AF-A0A2S6GL78-F1-MODEL\_V4 | 1.0 | 7.006e-12 | 380 | 0.147 | 305 | 207 | 13 | 7 | 279 | 63 | 346 | Rhs element Vgr protein | Rhs element Vgr protein | | afdb-uniprot50 | AF-A0A1W2GZA8-F1-MODEL\_V4 | 1.0 | 6.247e-12 | 380 | 0.156 | 300 | 198 | 15 | 7 | 276 | 76 | 350 | Rhs element Vgr protein | Rhs element Vgr protein | | afdb-uniprot50 | AF-A0A7G8V716-F1-MODEL\_V4 | 1.0 | 8.32e-12 | 380 | 0.135 | 296 | 211 | 15 | 7 | 276 | 74 | 350 | Type VI secretion system tip protein VgrG | Type VI secretion system tip protein VgrG | | afdb-uniprot50 | AF-A0A1T5DNZ3-F1-MODEL\_V4 | 1.0 | 3.139e-12 | 379 | 0.144 | 311 | 212 | 18 | 3 | 279 | 83 | 373 | Phage protein D | Phage protein D | | afdb-uniprot50 | AF-A0A7Z7N0R5-F1-MODEL\_V4 | 1.0 | 2.002e-13 | 379 | 0.157 | 331 | 202 | 14 | 9 | 281 | 102 | 413 | Phage protein D | Phage protein D | | afdb-uniprot50 | AF-A0A150S1I8-F1-MODEL\_V4 | 1.0 | 3.948e-12 | 379 | 0.134 | 380 | 207 | 15 | 4 | 278 | 88 | 450 | Uncharacterized protein | Uncharacterized protein | | afdb-uniprot50 | AF-A0A7Y0MDW7-F1-MODEL\_V4 | 1.0 | 1.984e-12 | 379 | 0.17 | 310 | 204 | 15 | 1 | 282 | 59 | 343 | Type IV secretion protein Rhs | Type IV secretion protein Rhs | | afdb-uniprot50 | AF-A0A1Z9NC79-F1-MODEL\_V4 | 1.0 | 4.69e-12 | 379 | 0.141 | 310 | 206 | 14 | 7 | 280 | 73 | 358 | Phage\_base\_V domain-containing protein | Phage\_base\_V domain-containing protein | | afdb-uniprot50 | AF-A0A377AID4-F1-MODEL\_V4 | 1.0 | 8.732e-11 | 378 | 0.263 | 171 | 101 | 3 | 113 | 258 | 2 | 172 | Late control protein D protein | Late control protein D protein | | afdb-uniprot50 | AF-A0A377AGI3-F1-MODEL\_V4 | 1.0 | 2.082e-11 | 378 | 0.247 | 230 | 158 | 4 | 1 | 222 | 49 | 271 | Gene late control D protein | Gene late control D protein | | afdb-uniprot50 | AF-A0A2T7QF39-F1-MODEL\_V4 | 1.0 | 6.247e-12 | 378 | 0.137 | 321 | 207 | 14 | 7 | 276 | 78 | 379 | Phage\_base\_V domain-containing protein | Phage\_base\_V domain-containing protein | | afdb-uniprot50 | AF-A0A856MT28-F1-MODEL\_V4 | 1.0 | 2.938e-11 | 378 | 0.122 | 295 | 213 | 12 | 15 | 281 | 98 | 374 | Type IV secretion protein Rhs | Type IV secretion protein Rhs | | afdb-uniprot50 | AF-A0A5C9AP84-F1-MODEL\_V4 | 1.0 | 5.165e-10 | 377 | 0.35 | 151 | 90 | 3 | 1 | 150 | 63 | 206 | Phage late control D family protein | Phage late control D family protein | | afdb-uniprot50 | AF-A0A0P8A270-F1-MODEL\_V4 | 1.0 | 7.006e-12 | 377 | 0.166 | 307 | 212 | 14 | 3 | 281 | 69 | 359 | Phage late controlD protein (GPD) | Phage late controlD protein (GPD) | | afdb-uniprot50 | AF-A0A8A3HJ98-F1-MODEL\_V4 | 1.0 | 2.082e-11 | 377 | 0.191 | 245 | 165 | 12 | 63 | 278 | 2 | 242 | VgrG-related protein | VgrG-related protein | | afdb-uniprot50 | AF-A0A2A2HU16-F1-MODEL\_V4 | 1.0 | 7.419e-12 | 377 | 0.163 | 312 | 199 | 11 | 7 | 281 | 63 | 349 | Phage\_base\_V domain-containing protein | Phage\_base\_V domain-containing protein | | afdb-uniprot50 | AF-A0A6P0W330-F1-MODEL\_V4 | 1.0 | 1.243e-11 | 377 | 0.138 | 267 | 200 | 13 | 20 | 275 | 93 | 340 | Phage\_base\_V domain-containing protein | Phage\_base\_V domain-containing protein | | afdb-uniprot50 | AF-B6XAD0-F1-MODEL\_V4 | 1.0 | 7.286e-10 | 376 | 0.299 | 167 | 107 | 4 | 1 | 165 | 59 | 217 | Phage late control gene D protein (GPD) | Phage late control gene D protein (GPD) | | afdb-uniprot50 | AF-W8ZT94-F1-MODEL\_V4 | 1.0 | 2.774e-11 | 376 | 0.295 | 196 | 127 | 3 | 1 | 187 | 62 | 255 | Uncharacterized protein | Uncharacterized protein | | afdb-uniprot50 | AF-A0A7Y5NVM8-F1-MODEL\_V4 | 1.0 | 3.728e-12 | 376 | 0.177 | 304 | 181 | 15 | 23 | 278 | 79 | 361 | Uncharacterized protein | Uncharacterized protein | | afdb-uniprot50 | AF-A0A6L6J115-F1-MODEL\_V4 | 1.0 | 3.457e-10 | 375 | 0.329 | 167 | 104 | 3 | 1 | 166 | 51 | 210 | Phage tail protein | Phage tail protein | | afdb-uniprot50 | AF-A0A840VPW3-F1-MODEL\_V4 | 1.0 | 9.416e-13 | 375 | 0.138 | 289 | 190 | 10 | 16 | 278 | 66 | 321 | Phage protein D | Phage protein D | | afdb-uniprot50 | AF-A0A1S6FJC9-F1-MODEL\_V4 | 1.0 | 2.357e-12 | 375 | 0.136 | 307 | 204 | 14 | 7 | 277 | 72 | 353 | Phage\_base\_V domain-containing protein | Phage\_base\_V domain-containing protein | | afdb-uniprot50 | AF-A0A530QSH5-F1-MODEL\_V4 | 1.0 | 3.762e-13 | 374 | 0.246 | 239 | 116 | 7 | 133 | 311 | 3 | 237 | Uncharacterized protein | Uncharacterized protein | | afdb-uniprot50 | AF-A0A349E0J1-F1-MODEL\_V4 | 1.0 | 1.243e-11 | 374 | 0.166 | 307 | 204 | 15 | 7 | 280 | 61 | 348 | Phage\_base\_V domain-containing protein | Phage\_base\_V domain-containing protein | | afdb-uniprot50 | AF-M4Z9U0-F1-MODEL\_V4 | 1.0 | 3.948e-12 | 374 | 0.185 | 308 | 202 | 17 | 7 | 284 | 65 | 353 | Phage\_base\_V domain-containing protein | Phage\_base\_V domain-containing protein | | afdb-uniprot50 | AF-A0A135IJV0-F1-MODEL\_V4 | 1.0 | 3.878e-10 | 373 | 0.292 | 123 | 85 | 2 | 157 | 278 | 3 | 124 | Phage tail protein | Phage tail protein | | afdb-uniprot50 | AF-A0A377Z1K9-F1-MODEL\_V4 | 1.0 | 5.165e-10 | 373 | 0.359 | 167 | 97 | 4 | 1 | 165 | 63 | 221 | Putative prophage tail protein | Putative prophage tail protein | | afdb-uniprot50 | AF-A0A1I0CAS2-F1-MODEL\_V4 | 1.0 | 1.769e-12 | 373 | 0.123 | 292 | 217 | 13 | 7 | 279 | 86 | 357 | Phage protein D | Phage protein D | | afdb-uniprot50 | AF-A0A101WDJ3-F1-MODEL\_V4 | 1.0 | 1.563e-11 | 373 | 0.164 | 298 | 205 | 14 | 7 | 278 | 84 | 363 | Uncharacterized protein | Uncharacterized protein | | afdb-uniprot50 | AF-A0A536GUG3-F1-MODEL\_V4 | 1.0 | 8.811e-12 | 373 | 0.146 | 294 | 202 | 15 | 17 | 276 | 74 | 352 | VgrG-related protein | VgrG-related protein | | afdb-uniprot50 | AF-A0A349BRR9-F1-MODEL\_V4 | 1.0 | 5.259e-12 | 372 | 0.122 | 302 | 214 | 16 | 7 | 275 | 76 | 359 | Uncharacterized protein | Uncharacterized protein | | afdb-uniprot50 | AF-J3UA61-F1-MODEL\_V4 | 1.0 | 1.769e-12 | 372 | 0.12 | 332 | 247 | 18 | 7 | 311 | 103 | 416 | Uncharacterized protein | Uncharacterized protein | | afdb-uniprot50 | AF-A0A7V7PRG0-F1-MODEL\_V4 | 1.0 | 1.394e-11 | 372 | 0.166 | 282 | 184 | 15 | 20 | 273 | 69 | 327 | Phage\_base\_V domain-containing protein | Phage\_base\_V domain-containing protein | | afdb-uniprot50 | AF-A0A6G3WKK2-F1-MODEL\_V4 | 1.0 | 1.316e-11 | 372 | 0.173 | 253 | 175 | 11 | 57 | 281 | 7 | 253 | VgrG-related protein | VgrG-related protein | | afdb-uniprot50 | AF-A0A1X4H071-F1-MODEL\_V4 | 1.0 | 2.643e-12 | 372 | 0.15 | 306 | 205 | 18 | 14 | 280 | 73 | 362 | Phage\_base\_V domain-containing protein | Phage\_base\_V domain-containing protein | | afdb-uniprot50 | AF-A0A257NMK5-F1-MODEL\_V4 | 1.0 | 1.874e-12 | 369 | 0.132 | 302 | 200 | 13 | 7 | 279 | 60 | 328 | Uncharacterized protein | Uncharacterized protein | | afdb-uniprot50 | AF-A0A1G5HB84-F1-MODEL\_V4 | 1.0 | 3.139e-12 | 369 | 0.137 | 341 | 209 | 16 | 7 | 281 | 85 | 406 | Uncharacterized protein | Uncharacterized protein | | afdb-uniprot50 | AF-A0A2E1UZT3-F1-MODEL\_V4 | 1.0 | 6.247e-12 | 368 | 0.147 | 311 | 202 | 16 | 7 | 279 | 75 | 360 | Phage\_base\_V domain-containing protein | Phage\_base\_V domain-containing protein | | afdb-uniprot50 | AF-A0A3D2XI45-F1-MODEL\_V4 | 1.0 | 3.111e-11 | 368 | 0.15 | 305 | 208 | 15 | 7 | 279 | 73 | 358 | Rhs element Vgr protein | Rhs element Vgr protein | | afdb-uniprot50 | AF-A0A1I3BRU1-F1-MODEL\_V4 | 1.0 | 3.553e-13 | 367 | 0.165 | 326 | 197 | 16 | 7 | 278 | 101 | 405 | Phage protein D | Phage protein D | | afdb-uniprot50 | AF-A0A1Q7VC12-F1-MODEL\_V4 | 1.0 | 1.118e-12 | 366 | 0.166 | 319 | 205 | 13 | 7 | 281 | 87 | 388 | Uncharacterized protein | Uncharacterized protein | | afdb-uniprot50 | AF-A0A850I9N9-F1-MODEL\_V4 | 1.0 | 4.428e-12 | 365 | 0.154 | 318 | 202 | 17 | 7 | 279 | 84 | 379 | Phage late control D family protein | Phage late control D family protein | | afdb-uniprot50 | AF-A0A7X1E8L6-F1-MODEL\_V4 | 1.0 | 3.913e-11 | 365 | 0.129 | 332 | 220 | 14 | 20 | 311 | 75 | 377 | Uncharacterized protein | Uncharacterized protein | | afdb-uniprot50 | AF-A0A150KDD5-F1-MODEL\_V4 | 1.0 | 1.266e-13 | 364 | 0.182 | 313 | 209 | 16 | 1 | 296 | 13 | 295 | Uncharacterized protein | Uncharacterized protein | | afdb-uniprot50 | AF-A0A6N2W373-F1-MODEL\_V4 | 1.0 | 4.428e-12 | 363 | 0.141 | 311 | 203 | 18 | 2 | 275 | 67 | 350 | Uncharacterized protein | Uncharacterized protein | | afdb-uniprot50 | AF-A0A7Y5K2K5-F1-MODEL\_V4 | 1.0 | 3.324e-12 | 363 | 0.146 | 328 | 207 | 19 | 18 | 284 | 147 | 462 | Uncharacterized protein | Uncharacterized protein | | afdb-uniprot50 | AF-A0A7X0CIA8-F1-MODEL\_V4 | 1.0 | 2.774e-11 | 363 | 0.131 | 304 | 215 | 16 | 7 | 282 | 63 | 345 | Rhs element Vgr protein | Rhs element Vgr protein | | afdb-uniprot50 | AF-G2ZT36-F1-MODEL\_V4 | 1.0 | 3.521e-12 | 362 | 0.157 | 317 | 205 | 14 | 7 | 280 | 71 | 368 | Uncharacterized protein | Uncharacterized protein | | afdb-uniprot50 | AF-A0A7Y5LNK5-F1-MODEL\_V4 | 1.0 | 2.774e-11 | 362 | 0.133 | 329 | 210 | 15 | 7 | 278 | 76 | 386 | Uncharacterized protein | Uncharacterized protein | | afdb-uniprot50 | AF-A0A6P1MD19-F1-MODEL\_V4 | 1.0 | 1.316e-11 | 361 | 0.145 | 295 | 208 | 12 | 3 | 274 | 83 | 356 | Uncharacterized protein | Uncharacterized protein | | afdb-uniprot50 | AF-A0A4R1HQU8-F1-MODEL\_V4 | 1.0 | 1.857e-11 | 361 | 0.18 | 304 | 201 | 10 | 7 | 281 | 63 | 347 | Phage protein D | Phage protein D | | afdb-uniprot50 | AF-A0A3A9G426-F1-MODEL\_V4 | 1.0 | 5.212e-11 | 360 | 0.145 | 309 | 206 | 18 | 2 | 276 | 74 | 358 | Phage late control D family protein | Phage late control D family protein | | afdb-uniprot50 | AF-A0A760MUK4-F1-MODEL\_V4 | 1.0 | 3.489e-11 | 357 | 0.24 | 229 | 142 | 6 | 1 | 205 | 72 | 292 | Phage late control D family protein | Phage late control D family protein | | afdb-uniprot50 | AF-A0A2C8EXW9-F1-MODEL\_V4 | 1.0 | 1.088e-09 | 356 | 0.353 | 167 | 100 | 3 | 1 | 166 | 77 | 236 | Phage protein D | Phage protein D | | afdb-uniprot50 | AF-A0A149USP9-F1-MODEL\_V4 | 1.0 | 1.966e-11 | 356 | 0.112 | 303 | 213 | 11 | 17 | 281 | 106 | 390 | Uncharacterized protein | Uncharacterized protein | | afdb-uniprot50 | AF-A0A1S9CQS2-F1-MODEL\_V4 | 1.0 | 3.728e-12 | 356 | 0.096 | 354 | 244 | 18 | 7 | 308 | 105 | 434 | NlpC/P60 domain-containing protein | NlpC/P60 domain-containing protein | | afdb-uniprot50 | AF-A0A6C8RYY1-F1-MODEL\_V4 | 1.0 | 1.221e-09 | 354 | 0.378 | 140 | 78 | 3 | 27 | 165 | 1 | 132 | Phage late control D family protein | Phage late control D family protein | | afdb-uniprot50 | AF-A0A6M8HQ44-F1-MODEL\_V4 | 1.0 | 4.966e-12 | 354 | 0.129 | 308 | 213 | 15 | 7 | 277 | 55 | 344 | Uncharacterized protein | Uncharacterized protein | | afdb-uniprot50 | AF-A0A3S0CSF5-F1-MODEL\_V4 | 1.0 | 3.913e-11 | 354 | 0.152 | 328 | 216 | 17 | 3 | 285 | 63 | 373 | Uncharacterized protein | Uncharacterized protein | | afdb-uniprot50 | AF-A0A2E7UTL5-F1-MODEL\_V4 | 1.0 | 8.32e-12 | 353 | 0.131 | 304 | 203 | 14 | 9 | 278 | 55 | 331 | Uncharacterized protein | Uncharacterized protein | | afdb-uniprot50 | AF-A0A6S5WYG3-F1-MODEL\_V4 | 1.0 | 3.913e-11 | 352 | 0.312 | 205 | 127 | 5 | 1 | 204 | 75 | 266 | Uncharacterized protein | Uncharacterized protein | | afdb-uniprot50 | AF-A0A1Y4S3J0-F1-MODEL\_V4 | 1.0 | 2.335e-11 | 352 | 0.147 | 291 | 212 | 13 | 3 | 275 | 79 | 351 | Uncharacterized protein | Uncharacterized protein | | afdb-uniprot50 | AF-A0A357YYD2-F1-MODEL\_V4 | 1.0 | 2.619e-11 | 352 | 0.149 | 294 | 195 | 16 | 9 | 273 | 64 | 331 | Phage\_base\_V domain-containing protein | Phage\_base\_V domain-containing protein | | afdb-uniprot50 | AF-A0A836CGV5-F1-MODEL\_V4 | 1.0 | 2.596e-10 | 352 | 0.269 | 189 | 129 | 4 | 1 | 187 | 895 | 1076 | Uncharacterized protein | Uncharacterized protein | | afdb-uniprot50 | AF-A0A257SB31-F1-MODEL\_V4 | 1.0 | 2.473e-11 | 351 | 0.154 | 285 | 198 | 10 | 12 | 276 | 71 | 332 | Uncharacterized protein | Uncharacterized protein | | afdb-uniprot50 | AF-A0A7X5INZ1-F1-MODEL\_V4 | 1.0 | 1.476e-11 | 350 | 0.131 | 304 | 201 | 13 | 9 | 276 | 78 | 354 | Uncharacterized protein | Uncharacterized protein | | afdb-uniprot50 | AF-A0A3N8BFJ3-F1-MODEL\_V4 | 1.0 | 3.695e-11 | 350 | 0.162 | 308 | 207 | 13 | 3 | 279 | 74 | 361 | Phage\_base\_V domain-containing protein | Phage\_base\_V domain-containing protein | | afdb-uniprot50 | AF-F3IS59-F1-MODEL\_V4 | 1.0 | 3.235e-09 | 349 | 0.246 | 126 | 89 | 1 | 156 | 275 | 1 | 126 | Tail protein D | Tail protein D | | afdb-uniprot50 | AF-A0A1I0IWR4-F1-MODEL\_V4 | 1.0 | 4.144e-11 | 348 | 0.105 | 293 | 224 | 16 | 3 | 275 | 78 | 352 | Phage protein D | Phage protein D | | afdb-uniprot50 | AF-A0A1Y4T7B9-F1-MODEL\_V4 | 1.0 | 2.335e-11 | 348 | 0.161 | 315 | 197 | 17 | 3 | 279 | 75 | 360 | Uncharacterized protein | Uncharacterized protein | | afdb-uniprot50 | AF-A0A192A527-F1-MODEL\_V4 | 1.0 | 8.32e-12 | 348 | 0.165 | 314 | 197 | 14 | 7 | 279 | 70 | 359 | Uncharacterized protein | Uncharacterized protein | | afdb-uniprot50 | AF-A0A0F9MJ86-F1-MODEL\_V4 | 1.0 | 1.966e-11 | 347 | 0.152 | 335 | 206 | 17 | 7 | 281 | 78 | 394 | Uncharacterized protein | Uncharacterized protein | | afdb-uniprot50 | AF-A0A2X4X699-F1-MODEL\_V4 | 1.0 | 3.426e-09 | 346 | 0.321 | 168 | 105 | 4 | 1 | 166 | 63 | 223 | Phage protein D | Phage protein D | | afdb-uniprot50 | AF-A0A090SGD1-F1-MODEL\_V4 | 1.0 | 1.037e-10 | 345 | 0.271 | 140 | 99 | 2 | 145 | 281 | 15 | 154 | Uncharacterized protein | Uncharacterized protein | | afdb-uniprot50 | AF-A0A1C6AKC2-F1-MODEL\_V4 | 1.0 | 9.331e-12 | 345 | 0.149 | 295 | 199 | 17 | 9 | 275 | 91 | 361 | Phage protein D | Phage protein D | | afdb-uniprot50 | AF-A0A7W4CKY1-F1-MODEL\_V4 | 1.0 | 7.857e-12 | 345 | 0.174 | 316 | 193 | 14 | 7 | 278 | 42 | 333 | Phage\_base\_V domain-containing protein | Phage\_base\_V domain-containing protein | | afdb-uniprot50 | AF-A0A4Q9KNI5-F1-MODEL\_V4 | 1.0 | 9.416e-13 | 344 | 0.158 | 329 | 194 | 21 | 9 | 281 | 87 | 388 | Phage late control D family protein | Phage late control D family protein | | afdb-uniprot50 | AF-A0A1R3VFD6-F1-MODEL\_V4 | 1.0 | 6.247e-12 | 344 | 0.137 | 319 | 215 | 14 | 7 | 278 | 82 | 387 | Phage protein D-like protein | Phage protein D-like protein | | afdb-uniprot50 | AF-A0A6G2IEN4-F1-MODEL\_V4 | 1.0 | 2.082e-11 | 344 | 0.145 | 310 | 201 | 15 | 9 | 281 | 79 | 361 | Phage\_base\_V domain-containing protein | Phage\_base\_V domain-containing protein | | afdb-uniprot50 | AF-A0A4D7ATE7-F1-MODEL\_V4 | 1.0 | 6.438e-09 | 343 | 0.169 | 136 | 110 | 3 | 143 | 278 | 11 | 143 | Uncharacterized protein | Uncharacterized protein | | afdb-uniprot50 | AF-A4X0M8-F1-MODEL\_V4 | 1.0 | 1.476e-11 | 343 | 0.168 | 320 | 200 | 18 | 3 | 276 | 89 | 388 | Uncharacterized protein | Uncharacterized protein | | afdb-uniprot50 | AF-Q479Z3-F1-MODEL\_V4 | 1.0 | 1.641e-10 | 343 | 0.142 | 294 | 202 | 14 | 9 | 274 | 75 | 346 | Rhs element Vgr protein | Rhs element Vgr protein | | afdb-uniprot50 | AF-A0A7G2TQH6-F1-MODEL\_V4 | 1.0 | 5.519e-11 | 342 | 0.15 | 305 | 192 | 15 | 7 | 275 | 72 | 345 | Type IV secretion protein Rhs | Type IV secretion protein Rhs | | afdb-uniprot50 | AF-A0A849VCJ4-F1-MODEL\_V4 | 1.0 | 3.294e-11 | 342 | 0.151 | 310 | 201 | 15 | 7 | 279 | 63 | 347 | Uncharacterized protein | Uncharacterized protein | | afdb-uniprot50 | AF-R7UKH8-F1-MODEL\_V4 | 1.0 | 4.069e-09 | 339 | 0.283 | 166 | 107 | 6 | 1 | 164 | 48 | 203 | Uncharacterized protein | Uncharacterized protein | | afdb-uniprot50 | AF-A0A0D6MQM0-F1-MODEL\_V4 | 1.0 | 1.163e-10 | 338 | 0.114 | 296 | 210 | 12 | 13 | 277 | 76 | 350 | Uncharacterized protein | Uncharacterized protein | | afdb-uniprot50 | AF-A0A6N8HUA9-F1-MODEL\_V4 | 1.0 | 9.247e-11 | 337 | 0.14 | 305 | 204 | 16 | 9 | 276 | 81 | 364 | Uncharacterized protein | Uncharacterized protein | | afdb-uniprot50 | AF-A0A1D9H202-F1-MODEL\_V4 | 1.0 | 9.331e-12 | 337 | 0.164 | 323 | 194 | 14 | 7 | 278 | 69 | 366 | Uncharacterized protein | Uncharacterized protein | | afdb-uniprot50 | AF-A0A0F9NFA5-F1-MODEL\_V4 | 1.0 | 1.563e-11 | 336 | 0.137 | 392 | 209 | 17 | 7 | 279 | 70 | 451 | Uncharacterized protein | Uncharacterized protein | | afdb-uniprot50 | AF-A0A401Z4L8-F1-MODEL\_V4 | 1.0 | 4.921e-11 | 336 | 0.128 | 319 | 209 | 16 | 13 | 280 | 82 | 382 | Type IV secretion protein Rhs | Type IV secretion protein Rhs | | afdb-uniprot50 | AF-A8T9K3-F1-MODEL\_V4 | 1.0 | 2.314e-10 | 335 | 0.116 | 301 | 215 | 16 | 7 | 275 | 233 | 514 | Phage\_base\_V domain-containing protein | Phage\_base\_V domain-containing protein | | afdb-uniprot50 | AF-A0A5U6FP48-F1-MODEL\_V4 | 1.0 | 3.235e-09 | 333 | 0.267 | 168 | 113 | 4 | 1 | 165 | 66 | 226 | Late control protein | Late control protein | | afdb-uniprot50 | AF-A0A074LLZ7-F1-MODEL\_V4 | 1.0 | 7.069e-13 | 333 | 0.145 | 317 | 200 | 12 | 7 | 279 | 79 | 368 | Uncharacterized protein | Uncharacterized protein | | afdb-uniprot50 | AF-A1ZC10-F1-MODEL\_V4 | 1.0 | 2.335e-11 | 332 | 0.165 | 321 | 203 | 19 | 7 | 280 | 62 | 364 | Rhs element Vgr protein | Rhs element Vgr protein | | afdb-uniprot50 | AF-A0A2Z5ZLK9-F1-MODEL\_V4 | 1.0 | 8.245e-11 | 329 | 0.138 | 317 | 206 | 15 | 7 | 279 | 69 | 362 | Phage late control protein D | Phage late control protein D | | afdb-uniprot50 | AF-C0BXW3-F1-MODEL\_V4 | 1.0 | 1.232e-10 | 329 | 0.137 | 312 | 212 | 13 | 9 | 281 | 84 | 377 | Uncharacterized protein | Uncharacterized protein | | afdb-uniprot50 | AF-A0A5Q4DFT5-F1-MODEL\_V4 | 1.0 | 1.463e-10 | 329 | 0.179 | 335 | 196 | 16 | 7 | 276 | 61 | 381 | Phage late control D family protein | Phage late control D family protein | | afdb-uniprot50 | AF-A0A2N0F3R2-F1-MODEL\_V4 | 1.0 | 9.882e-12 | 329 | 0.142 | 336 | 221 | 18 | 7 | 297 | 111 | 424 | Uncharacterized protein | Uncharacterized protein | | afdb-uniprot50 | AF-A0A430AW93-F1-MODEL\_V4 | 1.0 | 8.732e-11 | 329 | 0.137 | 312 | 215 | 16 | 7 | 277 | 107 | 405 | Flagellin | Flagellin | | afdb-uniprot50 | AF-A0A329MVT4-F1-MODEL\_V4 | 1.0 | 5.212e-11 | 329 | 0.122 | 310 | 211 | 20 | 7 | 279 | 466 | 751 | Uncharacterized protein | Uncharacterized protein | | afdb-uniprot50 | AF-A0A2R7SMS6-F1-MODEL\_V4 | 1.0 | 5.165e-10 | 328 | 0.204 | 181 | 118 | 4 | 148 | 311 | 23 | 194 | Late control protein D | Late control protein D | | afdb-uniprot50 | AF-A0A0F9U2F1-F1-MODEL\_V4 | 1.0 | 6.942e-11 | 328 | 0.165 | 302 | 187 | 17 | 22 | 281 | 105 | 383 | Uncharacterized protein | Uncharacterized protein | | afdb-uniprot50 | AF-A0A7Y5J5A2-F1-MODEL\_V4 | 1.0 | 3.083e-10 | 328 | 0.116 | 325 | 212 | 14 | 7 | 279 | 79 | 380 | Type VI secretion system tip protein VgrG | Type VI secretion system tip protein VgrG | | afdb-uniprot50 | AF-A0A1H5RLG9-F1-MODEL\_V4 | 1.0 | 2.473e-11 | 325 | 0.131 | 251 | 175 | 10 | 83 | 311 | 295 | 524 | Uncharacterized protein | Uncharacterized protein | | afdb-uniprot50 | AF-G2JAY7-F1-MODEL\_V4 | 1.0 | 3.265e-10 | 324 | 0.14 | 321 | 203 | 13 | 7 | 280 | 74 | 368 | Uncharacterized protein | Uncharacterized protein | | afdb-uniprot50 | AF-A0A3A5IJU8-F1-MODEL\_V4 | 1.0 | 3.695e-11 | 324 | 0.128 | 334 | 218 | 17 | 7 | 304 | 115 | 411 | Uncharacterized protein | Uncharacterized protein | | afdb-uniprot50 | AF-A0A827NCX0-F1-MODEL\_V4 | 1.0 | 2.724e-09 | 323 | 0.25 | 168 | 117 | 3 | 27 | 193 | 2 | 161 | Phage late control D family protein | Phage late control D family protein | | afdb-uniprot50 | AF-A0A7C3BT38-F1-MODEL\_V4 | 1.0 | 1.028e-09 | 322 | 0.16 | 225 | 154 | 7 | 83 | 281 | 4 | 219 | VgrG-related protein | VgrG-related protein | | afdb-uniprot50 | AF-A0A352UL14-F1-MODEL\_V4 | 1.0 | 6.555e-11 | 321 | 0.125 | 286 | 198 | 17 | 9 | 274 | 10 | 263 | Uncharacterized protein | Uncharacterized protein | | afdb-uniprot50 | AF-A0A497Y796-F1-MODEL\_V4 | 1.0 | 6.496e-10 | 320 | 0.11 | 290 | 212 | 16 | 13 | 275 | 72 | 342 | Phage\_base\_V domain-containing protein | Phage\_base\_V domain-containing protein | | afdb-uniprot50 | AF-A0A7C5MPP8-F1-MODEL\_V4 | 1.0 | 4.921e-11 | 318 | 0.133 | 321 | 215 | 14 | 7 | 279 | 68 | 373 | Phage\_base\_V domain-containing protein | Phage\_base\_V domain-containing protein | | afdb-uniprot50 | AF-A0A4Y7RGG6-F1-MODEL\_V4 | 1.0 | 5.519e-11 | 317 | 0.15 | 339 | 197 | 20 | 7 | 274 | 59 | 377 | Phage late control gene D protein (GPD) | Phage late control gene D protein (GPD) | | afdb-uniprot50 | AF-A0A7Y0GUL4-F1-MODEL\_V4 | 1.0 | 2.619e-11 | 315 | 0.158 | 322 | 200 | 16 | 7 | 280 | 69 | 367 | Uncharacterized protein | Uncharacterized protein | | afdb-uniprot50 | AF-A0A2N2FIC8-F1-MODEL\_V4 | 1.0 | 4.388e-11 | 315 | 0.14 | 334 | 208 | 18 | 7 | 288 | 69 | 375 | Uncharacterized protein | Uncharacterized protein | | afdb-uniprot50 | AF-A0A371J250-F1-MODEL\_V4 | 1.0 | 2.273e-08 | 314 | 0.153 | 124 | 103 | 2 | 156 | 279 | 2 | 123 | LysM peptidoglycan-binding domain-containing protein | LysM peptidoglycan-binding domain-containing protein | | afdb-uniprot50 | AF-G4Q993-F1-MODEL\_V4 | 1.0 | 6.19e-11 | 313 | 0.126 | 315 | 203 | 16 | 1 | 282 | 1 | 276 | Uncharacterized protein | Uncharacterized protein | | afdb-uniprot50 | AF-A0A7Z1V5Y3-F1-MODEL\_V4 | 1.0 | 2.619e-11 | 313 | 0.134 | 402 | 213 | 16 | 13 | 311 | 86 | 455 | Uncharacterized protein | Uncharacterized protein | | afdb-uniprot50 | AF-U2X5L0-F1-MODEL\_V4 | 1.0 | 9.793e-11 | 312 | 0.128 | 287 | 207 | 16 | 10 | 275 | 2 | 266 | Phage protein | Phage protein | | afdb-uniprot50 | AF-A0A654C107-F1-MODEL\_V4 | 1.0 | 3.913e-11 | 312 | 0.106 | 319 | 216 | 18 | 7 | 286 | 103 | 391 | Uncharacterized protein | Uncharacterized protein | | afdb-uniprot50 | AF-A0A2V2RGV6-F1-MODEL\_V4 | 1.0 | 3.055e-09 | 311 | 0.181 | 160 | 107 | 7 | 144 | 281 | 21 | 178 | Uncharacterized protein | Uncharacterized protein | | afdb-uniprot50 | AF-A0A060QI63-F1-MODEL\_V4 | 1.0 | 1.098e-10 | 310 | 0.138 | 304 | 197 | 19 | 13 | 279 | 10 | 285 | Uncharacterized protein | Uncharacterized protein | | afdb-uniprot50 | AF-A0A4D7QFP1-F1-MODEL\_V4 | 1.0 | 1.381e-10 | 310 | 0.108 | 305 | 225 | 17 | 7 | 279 | 21 | 310 | Uncharacterized protein | Uncharacterized protein | | afdb-uniprot50 | AF-A0A433X014-F1-MODEL\_V4 | 1.0 | 1.549e-10 | 308 | 0.136 | 300 | 196 | 15 | 18 | 279 | 81 | 355 | Uncharacterized protein | Uncharacterized protein | | afdb-uniprot50 | AF-A0A6P0SVX6-F1-MODEL\_V4 | 1.0 | 2.407e-08 | 305 | 0.161 | 155 | 107 | 4 | 148 | 279 | 14 | 168 | Uncharacterized protein | Uncharacterized protein | | afdb-uniprot50 | AF-A4N701-F1-MODEL\_V4 | 1.0 | 1.174e-11 | 305 | 0.251 | 282 | 156 | 9 | 2 | 281 | 67 | 295 | Bacteriophage P2 gpD protein | Bacteriophage P2 gpD protein | | afdb-uniprot50 | AF-A0A3D5LUE2-F1-MODEL\_V4 | 1.0 | 1.293e-09 | 304 | 0.202 | 168 | 116 | 5 | 148 | 309 | 9 | 164 | LysM domain-containing protein | LysM domain-containing protein | | afdb-uniprot50 | AF-A0A5E4KK76-F1-MODEL\_V4 | 1.0 | 2.146e-08 | 302 | 0.17 | 164 | 108 | 5 | 141 | 278 | 13 | 174 | Uncharacterized protein | Uncharacterized protein | | afdb-uniprot50 | AF-A0A011PC04-F1-MODEL\_V4 | 1.0 | 2.859e-08 | 300 | 0.135 | 155 | 111 | 5 | 146 | 277 | 10 | 164 | Phage protein D | Phage protein D | | afdb-uniprot50 | AF-A0A511BLX4-F1-MODEL\_V4 | 1.0 | 1.641e-10 | 298 | 0.114 | 305 | 200 | 13 | 17 | 279 | 80 | 356 | Uncharacterized protein | Uncharacterized protein | | afdb-uniprot50 | AF-A0A843SHV9-F1-MODEL\_V4 | 1.0 | 2.451e-10 | 298 | 0.171 | 320 | 196 | 16 | 7 | 279 | 69 | 366 | Uncharacterized protein | Uncharacterized protein | | afdb-uniprot50 | AF-A0A0C2UIQ9-F1-MODEL\_V4 | 1.0 | 4.106e-10 | 298 | 0.107 | 298 | 221 | 13 | 7 | 281 | 74 | 349 | Phage-like element PBSX protein xkdQ | Phage-like element PBSX protein xkdQ | | afdb-uniprot50 | AF-A0A089QIV9-F1-MODEL\_V4 | 1.0 | 2.911e-10 | 298 | 0.131 | 297 | 207 | 19 | 18 | 278 | 123 | 404 | Putative flagellin-like protein | Putative flagellin-like protein | | afdb-uniprot50 | AF-A0A0V0PZL6-F1-MODEL\_V4 | 1.0 | 3.596e-08 | 296 | 0.28 | 157 | 106 | 2 | 37 | 193 | 3 | 152 | Uncharacterized protein | Uncharacterized protein | | afdb-uniprot50 | AF-A0A1H9SQV1-F1-MODEL\_V4 | 1.0 | 1.037e-10 | 296 | 0.137 | 335 | 237 | 16 | 1 | 311 | 48 | 354 | Uncharacterized protein | Uncharacterized protein | | afdb-uniprot50 | AF-A0A257QRW0-F1-MODEL\_V4 | 1.0 | 4.349e-10 | 296 | 0.145 | 317 | 198 | 14 | 7 | 273 | 71 | 364 | Uncharacterized protein | Uncharacterized protein | | afdb-uniprot50 | AF-A0A376FE78-F1-MODEL\_V4 | 1.0 | 2.724e-09 | 295 | 0.195 | 179 | 107 | 5 | 150 | 292 | 1 | 178 | Gene D protein | Gene D protein | | afdb-uniprot50 | AF-A0A3G2ECV7-F1-MODEL\_V4 | 1.0 | 1.641e-10 | 294 | 0.157 | 323 | 197 | 16 | 7 | 280 | 69 | 365 | Uncharacterized protein | Uncharacterized protein | | afdb-uniprot50 | AF-R5RWZ2-F1-MODEL\_V4 | 1.0 | 7.286e-10 | 291 | 0.136 | 285 | 197 | 17 | 11 | 276 | 55 | 309 | Uncharacterized protein | Uncharacterized protein | | afdb-uniprot50 | AF-A0A510KG70-F1-MODEL\_V4 | 1.0 | 8.732e-11 | 289 | 0.155 | 327 | 212 | 14 | 9 | 311 | 47 | 333 | Uncharacterized protein | Uncharacterized protein | | afdb-uniprot50 | AF-A0A5R8Q954-F1-MODEL\_V4 | 1.0 | 4.106e-10 | 287 | 0.141 | 303 | 203 | 16 | 7 | 275 | 35 | 314 | Uncharacterized protein | Uncharacterized protein | | afdb-uniprot50 | AF-A0A4P9VGZ3-F1-MODEL\_V4 | 1.0 | 1.21e-08 | 286 | 0.361 | 141 | 83 | 3 | 26 | 166 | 6 | 139 | Uncharacterized protein | Uncharacterized protein | | afdb-uniprot50 | AF-A0A1M4LET7-F1-MODEL\_V4 | 1.0 | 3.055e-09 | 285 | 0.212 | 174 | 111 | 5 | 160 | 311 | 50 | 219 | Uncharacterized protein | Uncharacterized protein | | afdb-uniprot50 | AF-A0A536FPD7-F1-MODEL\_V4 | 1.0 | 3.628e-09 | 284 | 0.154 | 233 | 165 | 8 | 76 | 281 | 2 | 229 | VgrG-related protein | VgrG-related protein | | afdb-uniprot50 | AF-R0KRW8-F1-MODEL\_V4 | 1.0 | 1.132e-07 | 283 | 0.311 | 138 | 86 | 3 | 37 | 173 | 4 | 133 | Gene D protein | Gene D protein | | afdb-uniprot50 | AF-A0A556RSP6-F1-MODEL\_V4 | 1.0 | 5.42e-09 | 282 | 0.239 | 163 | 99 | 6 | 1 | 146 | 14 | 168 | Uncharacterized protein | Uncharacterized protein | | afdb-uniprot50 | AF-A0A7C5ITK4-F1-MODEL\_V4 | 1.0 | 4.523e-08 | 281 | 0.161 | 192 | 131 | 6 | 106 | 275 | 4 | 187 | Type VI secretion system tip protein VgrG | Type VI secretion system tip protein VgrG | | afdb-uniprot50 | AF-A0A522CNZ2-F1-MODEL\_V4 | 1.0 | 1.626e-09 | 279 | 0.143 | 279 | 204 | 17 | 9 | 279 | 52 | 303 | Uncharacterized protein | Uncharacterized protein | | afdb-uniprot50 | AF-A0A2C7A780-F1-MODEL\_V4 | 1.0 | 2.749e-10 | 278 | 0.134 | 431 | 222 | 14 | 7 | 311 | 69 | 474 | Uncharacterized protein | Uncharacterized protein | | afdb-uniprot50 | AF-A0A372IVE0-F1-MODEL\_V4 | 1.0 | 1.706e-08 | 277 | 0.198 | 207 | 138 | 7 | 102 | 281 | 10 | 215 | Type IV secretion protein Rhs | Type IV secretion protein Rhs | | afdb-uniprot50 | AF-A0A2S0JG92-F1-MODEL\_V4 | 1.0 | 4.605e-10 | 274 | 0.135 | 273 | 193 | 12 | 20 | 275 | 64 | 310 | Uncharacterized protein | Uncharacterized protein | | afdb-uniprot50 | AF-A0A7Z0PR13-F1-MODEL\_V4 | 1.0 | 1.641e-10 | 274 | 0.111 | 313 | 228 | 18 | 9 | 300 | 60 | 343 | Uncharacterized protein | Uncharacterized protein | | afdb-uniprot50 | AF-R5KQT4-F1-MODEL\_V4 | 1.0 | 2.596e-10 | 273 | 0.128 | 311 | 204 | 19 | 9 | 293 | 49 | 318 | Uncharacterized protein | Uncharacterized protein | | afdb-uniprot50 | AF-A0A520HRV1-F1-MODEL\_V4 | 1.0 | 3.027e-08 | 273 | 0.135 | 244 | 173 | 12 | 65 | 282 | 2 | 233 | Phage\_base\_V domain-containing protein | Phage\_base\_V domain-containing protein | | afdb-uniprot50 | AF-B9NXS0-F1-MODEL\_V4 | 1.0 | 1.931e-09 | 272 | 0.309 | 126 | 83 | 3 | 157 | 281 | 5 | 127 | Phage tail protein D | Phage tail protein D | | afdb-uniprot50 | AF-A0A7U6BTU5-F1-MODEL\_V4 | 1.0 | 5.47e-10 | 272 | 0.137 | 328 | 228 | 16 | 1 | 311 | 53 | 342 | NlpC/P60 domain-containing protein | NlpC/P60 domain-containing protein | | afdb-uniprot50 | AF-A0A6L4A988-F1-MODEL\_V4 | 1.0 | 3.206e-08 | 271 | 0.16 | 218 | 148 | 7 | 84 | 278 | 2 | 207 | VgrG-related protein | VgrG-related protein | | afdb-uniprot50 | AF-A0A3E2B4M2-F1-MODEL\_V4 | 1.0 | 2.294e-09 | 270 | 0.125 | 296 | 196 | 17 | 9 | 277 | 53 | 312 | Uncharacterized protein | Uncharacterized protein | | afdb-uniprot50 | AF-A0A535AFH3-F1-MODEL\_V4 | 1.0 | 7.22e-09 | 270 | 0.145 | 227 | 155 | 9 | 85 | 281 | 2 | 219 | VgrG-related protein | VgrG-related protein | | afdb-uniprot50 | AF-A0A0P9G0N8-F1-MODEL\_V4 | 1.0 | 3.177e-07 | 269 | 0.187 | 144 | 99 | 2 | 153 | 278 | 1 | 144 | Uncharacterized protein | Uncharacterized protein | | afdb-uniprot50 | AF-A0A1C2Y2A6-F1-MODEL\_V4 | 1.0 | 1.722e-09 | 269 | 0.142 | 288 | 191 | 16 | 20 | 276 | 66 | 328 | Uncharacterized protein | Uncharacterized protein | | afdb-uniprot50 | AF-U2C6T1-F1-MODEL\_V4 | 1.0 | 1.437e-08 | 266 | 0.148 | 235 | 161 | 13 | 61 | 277 | 18 | 231 | Uncharacterized protein | Uncharacterized protein | | afdb-uniprot50 | AF-A0A1C5QMY9-F1-MODEL\_V4 | 1.0 | 1.079e-08 | 265 | 0.113 | 290 | 202 | 19 | 11 | 277 | 55 | 312 | Mu-like prophage tail protein gpP | Mu-like prophage tail protein gpP | | afdb-uniprot50 | AF-A0A6B3GF16-F1-MODEL\_V4 | 1.0 | 6.38e-08 | 263 | 0.176 | 210 | 130 | 10 | 106 | 280 | 7 | 208 | VgrG-related protein | VgrG-related protein | | afdb-uniprot50 | AF-A0A3B8Q5W1-F1-MODEL\_V4 | 1.0 | 5.688e-08 | 263 | 0.101 | 177 | 138 | 5 | 106 | 275 | 1 | 163 | Rhs element Vgr protein | Rhs element Vgr protein | | afdb-uniprot50 | AF-Q67SN3-F1-MODEL\_V4 | 1.0 | 3.206e-08 | 262 | 0.092 | 388 | 221 | 22 | 7 | 279 | 309 | 680 | Uncharacterized protein | Uncharacterized protein | | afdb-uniprot50 | AF-A0A3P1WM06-F1-MODEL\_V4 | 1.0 | 8.838e-06 | 261 | 0.277 | 90 | 64 | 1 | 57 | 146 | 12 | 100 | Phage late control D family protein | Phage late control D family protein | | afdb-uniprot50 | AF-A0A1W9WK55-F1-MODEL\_V4 | 1.0 | 9.081e-09 | 261 | 0.147 | 305 | 174 | 17 | 9 | 281 | 259 | 509 | Phage\_base\_V domain-containing protein | Phage\_base\_V domain-containing protein | | afdb-uniprot50 | AF-A0A2G6AV19-F1-MODEL\_V4 | 1.0 | 4.605e-10 | 259 | 0.131 | 305 | 209 | 19 | 19 | 300 | 75 | 346 | NlpC/P60 domain-containing protein | NlpC/P60 domain-containing protein | | afdb-uniprot50 | AF-A0A1F7WZV6-F1-MODEL\_V4 | 1.0 | 1.21e-08 | 259 | 0.117 | 212 | 151 | 9 | 105 | 288 | 862 | 1065 | Uncharacterized protein | Uncharacterized protein | | afdb-uniprot50 | AF-A0A523ZZ81-F1-MODEL\_V4 | 1.0 | 6.695e-07 | 253 | 0.13 | 161 | 116 | 5 | 145 | 281 | 16 | 176 | Phage\_base\_V domain-containing protein | Phage\_base\_V domain-containing protein | | afdb-uniprot50 | AF-W4M5L5-F1-MODEL\_V4 | 1.0 | 2.232e-06 | 252 | 0.154 | 149 | 103 | 2 | 154 | 279 | 4 | 152 | Uncharacterized protein | Uncharacterized protein | | afdb-uniprot50 | AF-A0A2G2BDD8-F1-MODEL\_V4 | 1.0 | 2.294e-09 | 250 | 0.352 | 119 | 77 | 0 | 161 | 279 | 62 | 180 | Uncharacterized protein | Uncharacterized protein | | afdb-uniprot50 | AF-A0A539E6J3-F1-MODEL\_V4 | 1.0 | 1.344e-07 | 250 | 0.131 | 183 | 131 | 9 | 114 | 275 | 4 | 179 | Rhs element Vgr protein | Rhs element Vgr protein | | afdb-uniprot50 | AF-A0A3C1JKA1-F1-MODEL\_V4 | 1.0 | 9.53e-08 | 250 | 0.143 | 195 | 128 | 9 | 114 | 279 | 2 | 186 | Phage\_base\_V domain-containing protein | Phage\_base\_V domain-containing protein | | afdb-uniprot50 | AF-A0A0P0E7V7-F1-MODEL\_V4 | 1.0 | 2.859e-08 | 249 | 0.103 | 289 | 213 | 16 | 18 | 279 | 345 | 614 | Uncharacterized protein | Uncharacterized protein | | afdb-uniprot50 | AF-A0A7G9B2C8-F1-MODEL\_V4 | 1.0 | 1.088e-09 | 248 | 0.155 | 290 | 186 | 18 | 11 | 276 | 55 | 309 | Uncharacterized protein | Uncharacterized protein | | afdb-uniprot50 | AF-A0A859FGK9-F1-MODEL\_V4 | 1.0 | 3.055e-09 | 247 | 0.145 | 316 | 216 | 18 | 17 | 311 | 72 | 354 | Uncharacterized protein | Uncharacterized protein | | afdb-uniprot50 | AF-A0A220MH41-F1-MODEL\_V4 | 1.0 | 5.072e-08 | 246 | 0.108 | 285 | 202 | 16 | 19 | 276 | 65 | 324 | Uncharacterized protein | Uncharacterized protein | | afdb-uniprot50 | AF-D4CX49-F1-MODEL\_V4 | 1.0 | 1.931e-09 | 246 | 0.155 | 334 | 217 | 18 | 1 | 310 | 42 | 334 | Uncharacterized protein | Uncharacterized protein | | afdb-uniprot50 | AF-A0A318XLJ8-F1-MODEL\_V4 | 1.0 | 5.072e-08 | 245 | 0.091 | 296 | 213 | 19 | 18 | 279 | 99 | 372 | Uncharacterized protein | Uncharacterized protein | | afdb-uniprot50 | AF-A0A6I1I493-F1-MODEL\_V4 | 1.0 | 1.199e-07 | 244 | 0.328 | 146 | 89 | 3 | 22 | 166 | 2 | 139 | Uncharacterized protein | Uncharacterized protein | | afdb-uniprot50 | AF-A0A352X1L2-F1-MODEL\_V4 | 1.0 | 4.271e-08 | 244 | 0.129 | 293 | 192 | 25 | 14 | 279 | 53 | 309 | Uncharacterized protein | Uncharacterized protein | | afdb-uniprot50 | AF-J1H9W8-F1-MODEL\_V4 | 1.0 | 8.498e-08 | 242 | 0.14 | 220 | 162 | 9 | 73 | 275 | 2 | 211 | Uncharacterized protein | Uncharacterized protein | | afdb-uniprot50 | AF-A0A519GJE0-F1-MODEL\_V4 | 1.0 | 2.833e-07 | 241 | 0.171 | 198 | 133 | 9 | 106 | 280 | 4 | 193 | Type VI secretion system tip protein VgrG | Type VI secretion system tip protein VgrG | | afdb-uniprot50 | AF-A0A2E4F7X2-F1-MODEL\_V4 | 1.0 | 5.026e-07 | 239 | 0.143 | 174 | 116 | 5 | 141 | 281 | 3 | 176 | Uncharacterized protein | Uncharacterized protein | | afdb-uniprot50 | AF-A0A3A4U4Y7-F1-MODEL\_V4 | 1.0 | 3.808e-08 | 238 | 0.16 | 268 | 165 | 12 | 37 | 281 | 446 | 676 | Uncharacterized protein | Uncharacterized protein | | afdb-uniprot50 | AF-A0A7Z0MMM6-F1-MODEL\_V4 | 1.0 | 2.127e-07 | 235 | 0.117 | 179 | 134 | 7 | 110 | 276 | 26 | 192 | Uncharacterized protein | Uncharacterized protein | | afdb-uniprot50 | AF-A0A837LG98-F1-MODEL\_V4 | 1.0 | 6.438e-09 | 233 | 0.134 | 312 | 193 | 20 | 23 | 278 | 74 | 364 | Type IV secretion protein Rhs | Type IV secretion protein Rhs | | afdb-uniprot50 | AF-A0A0L6JT74-F1-MODEL\_V4 | 1.0 | 3.206e-08 | 233 | 0.102 | 381 | 213 | 18 | 11 | 279 | 451 | 814 | Uncharacterized protein | Uncharacterized protein | | afdb-uniprot50 | AF-A0A2E4HHK0-F1-MODEL\_V4 | 1.0 | 1.775e-06 | 232 | 0.168 | 154 | 104 | 6 | 144 | 275 | 7 | 158 | Phage\_base\_V domain-containing protein | Phage\_base\_V domain-containing protein | | afdb-uniprot50 | AF-A0A0P9YAT2-F1-MODEL\_V4 | 1.0 | 8.838e-06 | 231 | 0.263 | 110 | 75 | 2 | 172 | 275 | 2 | 111 | Tail protein D | Tail protein D | | afdb-uniprot50 | AF-A0A845L7G9-F1-MODEL\_V4 | 1.0 | 6.818e-09 | 229 | 0.118 | 305 | 205 | 17 | 7 | 274 | 44 | 321 | Uncharacterized protein | Uncharacterized protein | | afdb-uniprot50 | AF-A0A1H9HAT7-F1-MODEL\_V4 | 1.0 | 2.027e-08 | 228 | 0.111 | 313 | 207 | 18 | 3 | 276 | 26 | 306 | Uncharacterized protein | Uncharacterized protein | | afdb-uniprot50 | AF-A0A061P2C1-F1-MODEL\_V4 | 1.0 | 3.365e-07 | 222 | 0.101 | 305 | 215 | 21 | 8 | 275 | 28 | 310 | Uncharacterized protein | Uncharacterized protein | | afdb-uniprot50 | AF-A0A829CIG2-F1-MODEL\_V4 | 1.0 | 8.345e-06 | 221 | 0.272 | 125 | 86 | 4 | 63 | 185 | 1 | 122 | Phage late control D family protein | Phage late control D family protein | | afdb-uniprot50 | AF-A0A2V7V433-F1-MODEL\_V4 | 1.0 | 4.271e-08 | 214 | 0.18 | 205 | 124 | 8 | 105 | 277 | 1 | 193 | Phage\_base\_V domain-containing protein | Phage\_base\_V domain-containing protein | | afdb-uniprot50 | AF-A0A833G3K2-F1-MODEL\_V4 | 1.0 | 2.089e-05 | 209 | 0.248 | 137 | 92 | 6 | 20 | 152 | 66 | 195 | Late control D family protein | Late control D family protein | | afdb-uniprot50 | AF-A0A265Q3H3-F1-MODEL\_V4 | 1.0 | 2.675e-07 | 201 | 0.14 | 242 | 151 | 13 | 69 | 275 | 1 | 220 | Uncharacterized protein | Uncharacterized protein | | afdb-uniprot50 | AF-A0A4U9KC87-F1-MODEL\_V4 | 1.0 | 1.879e-06 | 201 | 0.139 | 165 | 108 | 12 | 144 | 282 | 17 | 173 | Phage protein D | Phage protein D | | afdb-uniprot50 | AF-A0A8B4Q938-F1-MODEL\_V4 | 1.0 | 1.508e-07 | 200 | 0.109 | 319 | 201 | 17 | 7 | 276 | 35 | 319 | Uncharacterized protein | Uncharacterized protein | | afdb-uniprot50 | AF-A0A2S5HQZ7-F1-MODEL\_V4 | 1.0 | 9.444e-07 | 199 | 0.118 | 363 | 205 | 23 | 9 | 278 | 808 | 1148 | Peptidase M23 | Peptidase M23 | | afdb-uniprot50 | AF-A0A2L2X7P9-F1-MODEL\_V4 | 1.0 | 1.188e-06 | 198 | 0.123 | 218 | 157 | 12 | 88 | 278 | 821 | 1031 | Peptidase, M23/M37 family | Peptidase, M23/M37 family | | afdb-uniprot50 | AF-A0A2G2BI84-F1-MODEL\_V4 | 1.0 | 6.265e-06 | 195 | 0.38 | 92 | 54 | 1 | 191 | 279 | 25 | 116 | Uncharacterized protein | Uncharacterized protein | | afdb-uniprot50 | AF-A0A3D0XCR8-F1-MODEL\_V4 | 1.0 | 3.773e-07 | 193 | 0.114 | 324 | 191 | 26 | 7 | 279 | 34 | 312 | Uncharacterized protein | Uncharacterized protein | | afdb-uniprot50 | AF-A0A7H0YH63-F1-MODEL\_V4 | 1.0 | 1.582e-06 | 192 | 0.098 | 355 | 213 | 19 | 9 | 279 | 687 | 1018 | M23 family metallopeptidase | M23 family metallopeptidase | | afdb-uniprot50 | AF-A0A3C1CUM3-F1-MODEL\_V4 | 1.0 | 3.334e-06 | 189 | 0.114 | 289 | 204 | 24 | 9 | 276 | 49 | 306 | Uncharacterized protein | Uncharacterized protein | | afdb-uniprot50 | AF-A0A7M2GS28-F1-MODEL\_V4 | 1.0 | 1e-06 | 187 | 0.169 | 165 | 108 | 8 | 147 | 284 | 157 | 319 | Uncharacterized protein | Uncharacterized protein | | afdb-uniprot50 | AF-A0A2R8BS21-F1-MODEL\_V4 | 1.0 | 0.0002603 | 177 | 0.268 | 93 | 68 | 0 | 189 | 281 | 11 | 103 | Uncharacterized protein | Uncharacterized protein | | afdb-uniprot50 | AF-A0A662L4J5-F1-MODEL\_V4 | 1.0 | 0.0001167 | 176 | 0.131 | 137 | 93 | 4 | 166 | 278 | 1 | 135 | Type IV secretion protein Rhs | Type IV secretion protein Rhs | | afdb-uniprot50 | AF-A0A0F9MNU2-F1-MODEL\_V4 | 1.0 | 5.863e-05 | 172 | 0.126 | 182 | 136 | 7 | 108 | 275 | 610 | 782 | Uncharacterized protein | Uncharacterized protein | | afdb-uniprot50 | AF-A0A1I3WTN1-F1-MODEL\_V4 | 1.0 | 0.0002458 | 164 | 0.149 | 154 | 108 | 3 | 146 | 277 | 83 | 235 | Phage\_base\_V domain-containing protein | Phage\_base\_V domain-containing protein | | afdb-uniprot50 | AF-A0A431HHS2-F1-MODEL\_V4 | 1.0 | 1.759e-05 | 161 | 0.095 | 325 | 204 | 21 | 22 | 275 | 728 | 1033 | Uncharacterized protein | Uncharacterized protein | | afdb-uniprot50 | AF-A0A6P0J1U6-F1-MODEL\_V4 | 1.0 | 0.0009733 | 145 | 0.141 | 134 | 87 | 3 | 171 | 279 | 2 | 132 | Uncharacterized protein | Uncharacterized protein | | afdb-uniprot50 | AF-A0A316PEN3-F1-MODEL\_V4 | 1.0 | 6.575e-05 | 128 | 0.126 | 142 | 106 | 5 | 153 | 291 | 340 | 466 | Uncharacterized protein | Uncharacterized protein | | afdb-uniprot50 | AF-A0A7C1Y5F9-F1-MODEL\_V4 | 1.0 | 0.0002757 | 125 | 0.152 | 197 | 117 | 6 | 157 | 311 | 2 | 190 | Uncharacterized protein | Uncharacterized protein | | afdb-uniprot50 | AF-A0A0F9L0Y6-F1-MODEL\_V4 | 1.0 | 0.001937 | 118 | 0.117 | 170 | 123 | 7 | 147 | 302 | 12 | 168 | Uncharacterized protein | Uncharacterized protein | | afdb-uniprot50 | AF-A0A1V5V4Y2-F1-MODEL\_V4 | 1.0 | 0.006838 | 115 | 0.215 | 93 | 65 | 4 | 224 | 311 | 74 | 163 | Uncharacterized protein | Uncharacterized protein | | afdb-uniprot50 | AF-A0A0F9QPT5-F1-MODEL\_V4 | 1.0 | 0.001373 | 110 | 0.078 | 395 | 169 | 16 | 59 | 275 | 13 | 390 | Uncharacterized protein | Uncharacterized protein | | afdb-uniprot50 | AF-A0A1V5LRQ3-F1-MODEL\_V4 | 1.0 | 0.005133 | 101 | 0.185 | 124 | 94 | 5 | 191 | 311 | 6 | 125 | Uncharacterized protein | Uncharacterized protein | |
| Top keywords  (threshold 1.00e-02 (evalue)) | **D, Phage, late, control, Type, secretion, Rhs, domain\_containing, tail, gene** |
| Output files | ../../similar\_structures/25\_FANPEZAQ\_CDS\_0025\_afdb-proteome\_foldseek.tsv ../../similar\_structures/25\_FANPEZAQ\_CDS\_0025\_afdb-uniprot50\_foldseek.tsv ../../similar\_structures/25\_FANPEZAQ\_CDS\_0025\_merged.svg ../../similar\_structures/25\_FANPEZAQ\_CDS\_0025\_pdb\_foldseek.tsv |

  
  
  

Return to summary | Go to previous | Go to next

  


---

**Sequence/structure alignments coloring**  
Each object in the alignment figures is colored according to its E-value following this color coding:

1e-100
10

**References:**  
1) Steinegger M, Meier M, Mirdita M, Vöhringer H, Haunsberger S J, and Söding J (2019) HH-suite3 for fast remote homology detection and deep protein annotation, BMC Bioinformatics, 473. doi: 10.1186/s12859-019-3019-7  
2) Jumper J, Evans R, Pritzel A, ..., Hassabis D (2021) Highly accurate protein structure prediction with AlphaFold, Nature, 596. doi: 10.1038/s41586-021-03819-2  
3) van Kempen M, Kim S, Tumescheit C, Mirdita M, Lee J, Gilchrist CLM, Söding J, and Steinegger M (2023) Fast and accurate protein structure search with Foldseek. Nature Biotechnology. doi: 10.1038/s41587-023-01773-0
