# Supplementary material for: Completing the BASEL phage collection to unlock hidden diversity for systematic exploration of phage–host interactions
Source: PLoS Biol. 2025 Apr 7;23(4):e3003063. doi: 10.1371/journal.pbio.3003063 (PMC11990801; doi:10.1371/journal.pbio.3003063)
Supplement: S2 Data — (ZIP) [file pbio.3003063.s009.zip › entries/27.html]

FANPEZAQ\_CDS\_0027


Return to summary | Go to previous | Go to next

|  |  |
| --- | --- |
| FANPEZAQ\_CDS\_0027 Page creation date: 02 Sep 2024, 12:00  Project folder: n/a  Input sequences file: Escherichia\_virus\_HeidiAbel.gb | hypothetical domain\_containing duf551 duf6378 morphogenetic fragment restriction alleviation lar ky705409\_p32 nc\_031918\_p6 mg676466\_p62 nc\_004333\_p63 mf153391\_p29 he956707\_p19 sav\_like kow hnh endonuclease duf3310 cupin duf3085 datp dgtp diphosphohydrolase n\_terminal duf4326 jacalin\_type lectin 3'\_phosphatase 5'\_polynucleotide kinase phage duf2203 |

### Sequence information

|  |  |
| --- | --- |
| Name | FANPEZAQ\_CDS\_0027  27\_FANPEZAQ\_CDS\_0027 (pipeline id) |
| Imported annotations | Escherichia\_virus\_HeidiAbel Bas97 |
| Protein sequence | MNTSNKTLLQILVEELPKRGGWPKGAVKIHQDHDGETWAWLNHDGTERLFKLDQVAKNAR QFAKDETEENMVTRDQYESALAEKEHPLPWDEKNTDADGWISWPGGECPVDGDAIVEVKY RKPNPYQFNNDRAGDFTWSHDGFDGDIIAYRLPHPHEVKTAGIDVQRDAVKSNDAQLEAD LNDCIGQPQSNDYARDEILYELANFAATTKTSLDIGVATEIAIWLSGKGYRKQ |
| Number of residues | 233 |
| Molecular weight (Da) | 26353.72 |
| Output files | ../../query\_sequences/27\_FANPEZAQ\_CDS\_0027.fasta |

### Putative domain architecture and protein family

#### Search results (HHblits)1

|  |  |
| --- | --- |
| Domain family databases searched | Pfam, Ncbi-cd, Cath, Phrogs |
| Results, scheme(s)  (Top layers only; threshold 1.00e-03 (evalue)) | xml version="1.0" encoding="utf-8" standalone="no"?       2024-09-02T21:08:18.031237 image/svg+xml   Matplotlib v3.7.2, https://matplotlib.org/ |
| Results, table  (E-value ≤ 1.00e-03 (evalue)) | | db | id | prob | evalue | pvalue | score | cols | query | query\_len | template | template\_len | name | description | | --- | --- | --- | --- | --- | --- | --- | --- | --- | --- | --- | --- | --- | | phrogs | 26870 | 100.0 | 2.9e-80 | 3.4e-84 | 531.8 | 218 | (1, 233) | 233 | (1, 233) | 233 | NA | NA; Category: unknown function; KY705409\_p32 | | phrogs | 7798 | 99.9 | 4.6e-34 | 5.7e-38 | 244.2 | 107 | (5, 126) | 233 | (10, 125) | 211 | NA | NA; Category: unknown function; NC\_031918\_p6 | | phrogs | 9174 | 99.9 | 7.4e-34 | 9.2e-38 | 226.9 | 104 | (92, 217) | 233 | (5, 115) | 124 | NA | NA; Category: unknown function; MG676466\_p62 | | phrogs | 16943 | 99.5 | 2.4e-18 | 2.7e-22 | 139.8 | 74 | (80, 156) | 233 | (92, 165) | 166 | NA | NA; Category: unknown function; NC\_004333\_p63 | | phrogs | 34383 | 96.6 | 4.9e-06 | 5.5e-10 | 60.3 | 34 | (6, 39) | 233 | (1, 34) | 85 | NA | NA; Category: unknown function; MF153391\_p29 | | phrogs | 13874 | 95.5 | 0.00013 | 1.6e-08 | 52.0 | 57 | (100, 157) | 233 | (4, 69) | 75 | NA | NA; Category: unknown function; HE956707\_p19 | |
| Top keywords  (threshold 1.00e-03 (evalue)) | **KY705409\_p32, NC\_031918\_p6, MG676466\_p62, NC\_004333\_p63, MF153391\_p29, HE956707\_p19** |
| Output files | ../../domain\_architecture/27\_FANPEZAQ\_CDS\_0027\_cath.hhr ../../domain\_architecture/27\_FANPEZAQ\_CDS\_0027\_merged.svg ../../domain\_architecture/27\_FANPEZAQ\_CDS\_0027\_ncbi-cd.hhr ../../domain\_architecture/27\_FANPEZAQ\_CDS\_0027\_pfam.hhr ../../domain\_architecture/27\_FANPEZAQ\_CDS\_0027\_phrogs.hhr |

### Identical protein sequences/structures

#### Search results

|  |  |
| --- | --- |
| Protein sequence databases searched | Pdb, Swissprot, Refseq |
| Identical proteins found | -- |
| Top keywords | -- |
| Output files | -- |

### Similar protein sequences/structures

#### Sequence similarity search results (HHblits)1

|  |  |
| --- | --- |
| Sequence databases searched | Uniclust, Pdb70 |
| Results, scheme(s)  (Top layers only, threshold 1.00e-03 (evalue)) | xml version="1.0" encoding="utf-8" standalone="no"?       2024-09-02T21:08:40.951344 image/svg+xml   Matplotlib v3.7.2, https://matplotlib.org/ |
| Results, table(s)  (threshold 1.00e-03 (evalue)) | | db | id | prob | evalue | pvalue | score | cols | query | query\_len | template | template\_len | name | description | | --- | --- | --- | --- | --- | --- | --- | --- | --- | --- | --- | --- | --- | | uniclust | UniRef100\_A0A193GZ03 | 100.0 | 1.6e-69 | 3.3e-75 | 467.6 | 217 | (1, 233) | 233 | (1, 230) | 230 | Uncharacterized protein | Uncharacterized protein | | uniclust | UniRef100\_A0A088FV72 | 100.0 | 1.4e-53 | 2.6e-59 | 347.9 | 141 | (93, 233) | 233 | (19, 159) | 159 | Uncharacterized protein | Uncharacterized protein | | uniclust | UniRef100\_A0A291AXI6 | 100.0 | 1.8e-50 | 4.8e-56 | 368.0 | 159 | (5, 191) | 233 | (4, 166) | 274 | Uncharacterized protein | Uncharacterized protein | | uniclust | UniRef100\_A0A173GC52 | 99.9 | 4.3e-31 | 1.3e-36 | 234.7 | 101 | (1, 123) | 233 | (7, 108) | 201 | Uncharacterized protein | Uncharacterized protein | | uniclust | UniRef100\_A0A7D7FL43 | 99.9 | 1.3e-30 | 2.4e-36 | 209.9 | 122 | (6, 153) | 233 | (1, 131) | 131 | Uncharacterized protein | Uncharacterized protein | | uniclust | UniRef100\_A0A1X1C2R2 | 99.8 | 4.6e-25 | 1.1e-30 | 184.9 | 88 | (69, 162) | 233 | (24, 112) | 132 | Uncharacterized protein | Uncharacterized protein | | uniclust | UniRef100\_A0A1I9SES2 | 99.8 | 6.6e-23 | 1.6e-28 | 180.1 | 95 | (6, 124) | 233 | (1, 99) | 185 | Uncharacterized protein | Uncharacterized protein | | uniclust | UniRef100\_UPI000A1DC278 | 99.7 | 5.3e-21 | 9.6e-27 | 156.3 | 101 | (6, 124) | 233 | (1, 106) | 133 | hypothetical protein | hypothetical protein | | uniclust | UniRef100\_UPI000F510781 | 99.6 | 1.3e-18 | 2.5e-24 | 135.8 | 86 | (6, 119) | 233 | (2, 87) | 95 | hypothetical protein | hypothetical protein | | uniclust | UniRef100\_A0A6G5Y9E3 | 99.5 | 2.2e-17 | 4.1e-23 | 141.5 | 89 | (75, 190) | 233 | (3, 91) | 181 | Uncharacterized protein | Uncharacterized protein | | uniclust | UniRef100\_A0A513ZYB6 | 99.5 | 2.6e-17 | 4.7e-23 | 136.9 | 85 | (76, 162) | 233 | (54, 141) | 143 | Uncharacterized protein | Uncharacterized protein | | uniclust | UniRef100\_A0A653TFN3 | 99.4 | 3.7e-16 | 7.7e-22 | 138.6 | 85 | (76, 162) | 233 | (113, 198) | 200 | Uncharacterized protein | Uncharacterized protein | | uniclust | UniRef100\_UPI0005C660F9 | 99.4 | 4.2e-16 | 8.5e-22 | 123.1 | 82 | (3, 87) | 233 | (1, 86) | 91 | hypothetical protein | hypothetical protein | | uniclust | UniRef100\_J3DEC6 | 99.4 | 4.3e-16 | 8.7e-22 | 133.0 | 86 | (71, 162) | 233 | (27, 149) | 151 | DUF551 domain-containing protein | DUF551 domain-containing protein | | uniclust | UniRef100\_A0A2E7P804 | 99.4 | 6.7e-16 | 1.5e-21 | 122.7 | 65 | (96, 162) | 233 | (12, 82) | 87 | Uncharacterized protein | Uncharacterized protein | | uniclust | UniRef100\_A0A3B7DLK8 | 99.4 | 6.9e-16 | 1.8e-21 | 119.9 | 62 | (93, 156) | 233 | (4, 71) | 74 | Uncharacterized protein | Uncharacterized protein | | uniclust | UniRef100\_UPI000D50E511 | 99.4 | 2.8e-15 | 5.1e-21 | 139.1 | 116 | (95, 216) | 233 | (120, 249) | 348 | hypothetical protein | hypothetical protein | | uniclust | UniRef100\_A0A5J6TWV0 | 99.3 | 9.6e-15 | 1.8e-20 | 129.9 | 190 | (11, 221) | 233 | (9, 211) | 231 | Uncharacterized protein | Uncharacterized protein | | uniclust | UniRef100\_A0A411H6I8 | 99.3 | 2.1e-14 | 4.2e-20 | 133.1 | 97 | (94, 216) | 233 | (63, 176) | 280 | DUF551 domain-containing protein | DUF551 domain-containing protein | | uniclust | UniRef100\_A0A6J5LK44 | 99.3 | 2.4e-14 | 4.6e-20 | 122.3 | 94 | (97, 205) | 233 | (5, 102) | 155 | SaV-like | SaV-like | | uniclust | UniRef100\_A0A7Y7SSS1 | 99.2 | 4.9e-14 | 9.5e-20 | 123.4 | 140 | (5, 158) | 233 | (32, 181) | 183 | Uncharacterized protein | Uncharacterized protein | | uniclust | UniRef100\_A0A6G5YAH6 | 99.2 | 5.3e-14 | 9.8e-20 | 116.6 | 62 | (92, 155) | 233 | (70, 131) | 131 | Uncharacterized protein | Uncharacterized protein | | uniclust | UniRef100\_A0A2S1PDQ1 | 99.2 | 5.5e-14 | 1e-19 | 132.1 | 150 | (11, 172) | 233 | (44, 215) | 347 | KOW domain-containing protein | KOW domain-containing protein | | uniclust | UniRef100\_UPI0018DF4812 | 99.2 | 6.3e-14 | 1.2e-19 | 118.6 | 97 | (98, 206) | 233 | (2, 100) | 144 | hypothetical protein | hypothetical protein | | uniclust | UniRef100\_A0A6A6K281 | 99.2 | 7.7e-14 | 1.4e-19 | 125.2 | 93 | (5, 123) | 233 | (65, 158) | 244 | Uncharacterized protein | Uncharacterized protein | | uniclust | UniRef100\_A0A2E7P1H3 | 99.2 | 8.7e-14 | 1.6e-19 | 109.2 | 62 | (95, 157) | 233 | (29, 90) | 91 | Uncharacterized protein | Uncharacterized protein | | uniclust | UniRef100\_UPI000681C15B | 99.2 | 1e-13 | 2.3e-19 | 124.3 | 92 | (6, 124) | 233 | (1, 92) | 190 | hypothetical protein | hypothetical protein | | uniclust | UniRef100\_UPI0023312D73 | 99.2 | 1.8e-13 | 3.3e-19 | 110.8 | 75 | (6, 81) | 233 | (1, 83) | 110 | hypothetical protein | hypothetical protein | | uniclust | UniRef100\_A0A6G5Y6M2 | 99.1 | 2.3e-13 | 4.2e-19 | 116.6 | 62 | (92, 155) | 233 | (104, 165) | 165 | Uncharacterized protein | Uncharacterized protein | | uniclust | UniRef100\_A0A6G5Y600 | 99.1 | 3.6e-13 | 6.6e-19 | 125.9 | 140 | (6, 159) | 233 | (1, 162) | 351 | Uncharacterized protein | Uncharacterized protein | | uniclust | UniRef100\_UPI00065FE3AD | 99.1 | 4.4e-13 | 9e-19 | 118.1 | 71 | (85, 158) | 233 | (107, 177) | 179 | hypothetical protein | hypothetical protein | | uniclust | UniRef100\_A0A0E2NX12 | 99.1 | 4.9e-13 | 1.1e-18 | 101.5 | 58 | (96, 156) | 233 | (6, 63) | 65 | Uncharacterized protein | Uncharacterized protein | | uniclust | UniRef100\_A0A1X3M1B6 | 99.1 | 1e-12 | 2e-18 | 122.2 | 105 | (5, 113) | 233 | (55, 171) | 287 | Morphogenetic protein | Morphogenetic protein | | uniclust | UniRef100\_A0A5B9N4V6 | 99.0 | 1.8e-12 | 3.4e-18 | 119.8 | 134 | (8, 158) | 233 | (22, 160) | 307 | Uncharacterized protein | Uncharacterized protein | | uniclust | UniRef100\_A0A127KN71 | 99.0 | 2.4e-12 | 5.3e-18 | 110.0 | 61 | (100, 162) | 233 | (69, 131) | 133 | Uncharacterized protein | Uncharacterized protein | | uniclust | UniRef100\_A0A950H461 | 99.0 | 3e-12 | 5.5e-18 | 110.0 | 60 | (95, 156) | 233 | (98, 157) | 163 | Uncharacterized protein | Uncharacterized protein | | uniclust | UniRef100\_A0A1X1D6V7 | 99.0 | 3.9e-12 | 7.2e-18 | 100.6 | 76 | (6, 81) | 233 | (1, 89) | 92 | DUF551 domain-containing protein | DUF551 domain-containing protein | | uniclust | UniRef100\_UPI001D1510C6 | 99.0 | 4e-12 | 7.4e-18 | 98.5 | 71 | (6, 76) | 233 | (1, 81) | 81 | hypothetical protein | hypothetical protein | | uniclust | UniRef100\_A0A2R3UAP6 | 98.9 | 5.1e-12 | 1.1e-17 | 118.5 | 73 | (6, 81) | 233 | (1, 82) | 269 | Uncharacterized protein | Uncharacterized protein | | uniclust | UniRef100\_UPI001CCC15E2 | 98.9 | 7.8e-12 | 1.4e-17 | 94.0 | 56 | (95, 154) | 233 | (12, 67) | 67 | hypothetical protein | hypothetical protein | | uniclust | UniRef100\_A0A127KN71 | 98.9 | 6.8e-12 | 1.5e-17 | 107.4 | 57 | (94, 153) | 233 | (7, 68) | 133 | Uncharacterized protein | Uncharacterized protein | | uniclust | UniRef100\_UPI0003EB40DC | 98.9 | 8.5e-12 | 1.6e-17 | 95.6 | 73 | (6, 85) | 233 | (1, 73) | 75 | hypothetical protein | hypothetical protein | | uniclust | UniRef100\_A0A1U9HYR5 | 98.9 | 1.3e-11 | 2.4e-17 | 97.0 | 77 | (6, 84) | 233 | (1, 82) | 85 | Uncharacterized protein | Uncharacterized protein | | uniclust | UniRef100\_UPI000FE13C13 | 98.8 | 2.9e-11 | 5.7e-17 | 96.0 | 61 | (96, 158) | 233 | (23, 83) | 85 | hypothetical protein | hypothetical protein | | uniclust | UniRef100\_A0A5C8J0V6 | 98.8 | 3.6e-11 | 6.6e-17 | 103.4 | 63 | (98, 162) | 233 | (8, 70) | 159 | Uncharacterized protein | Uncharacterized protein | | uniclust | UniRef100\_A0A6G5Y1Q1 | 98.8 | 4.9e-11 | 9.2e-17 | 92.3 | 36 | (5, 40) | 233 | (2, 37) | 76 | Uncharacterized protein | Uncharacterized protein | | uniclust | UniRef100\_UPI00226A3416 | 98.8 | 6.3e-11 | 1.2e-16 | 99.7 | 60 | (95, 156) | 233 | (73, 132) | 135 | hypothetical protein | hypothetical protein | | uniclust | UniRef100\_UPI00102A3018 | 98.8 | 6.6e-11 | 1.2e-16 | 87.8 | 34 | (6, 39) | 233 | (1, 34) | 61 | hypothetical protein | hypothetical protein | | uniclust | UniRef100\_A0A949XH11 | 98.7 | 8e-11 | 1.5e-16 | 97.4 | 66 | (91, 157) | 233 | (47, 114) | 119 | Uncharacterized protein | Uncharacterized protein | | uniclust | UniRef100\_UPI001E79528C | 98.7 | 8.2e-11 | 1.5e-16 | 108.9 | 68 | (6, 80) | 233 | (1, 72) | 291 | HNH endonuclease | HNH endonuclease | | uniclust | UniRef100\_A0A6G5Y609 | 98.7 | 9.6e-11 | 1.8e-16 | 96.0 | 66 | (88, 154) | 233 | (41, 111) | 112 | Uncharacterized protein | Uncharacterized protein | | uniclust | UniRef100\_A0A2X5UEE0 | 98.7 | 9.6e-11 | 1.8e-16 | 107.4 | 81 | (5, 87) | 233 | (51, 140) | 259 | Uncharacterized protein | Uncharacterized protein | | uniclust | UniRef100\_UPI001B343D12 | 98.7 | 9.8e-11 | 1.8e-16 | 102.6 | 64 | (96, 162) | 233 | (73, 136) | 182 | hypothetical protein | hypothetical protein | | uniclust | UniRef100\_A0A345MLB9 | 98.7 | 1e-10 | 1.9e-16 | 100.8 | 62 | (6, 85) | 233 | (1, 63) | 160 | DUF6378 domain-containing protein | DUF6378 domain-containing protein | | uniclust | UniRef100\_A0A7X2IEN7 | 98.7 | 2e-10 | 3.7e-16 | 97.0 | 63 | (95, 157) | 233 | (72, 135) | 136 | DUF3310 domain-containing protein | DUF3310 domain-containing protein | | uniclust | UniRef100\_UPI001E45F460 | 98.6 | 2.7e-10 | 5e-16 | 105.9 | 86 | (6, 121) | 233 | (1, 86) | 296 | hypothetical protein | hypothetical protein | | uniclust | UniRef100\_A0A2C9DUX5 | 98.6 | 3.7e-10 | 7.9e-16 | 92.8 | 64 | (95, 159) | 233 | (21, 90) | 99 | Uncharacterized protein | Uncharacterized protein | | uniclust | UniRef100\_UPI0013C3000B | 98.6 | 4.8e-10 | 9.4e-16 | 86.6 | 60 | (96, 157) | 233 | (4, 67) | 71 | hypothetical protein | hypothetical protein | | uniclust | UniRef100\_A0A246JRV2 | 98.6 | 6.1e-10 | 1.2e-15 | 86.5 | 62 | (97, 158) | 233 | (4, 71) | 73 | Uncharacterized protein | Uncharacterized protein | | uniclust | UniRef100\_A0A2E5T5S2 | 98.5 | 9.7e-10 | 1.9e-15 | 92.7 | 61 | (96, 157) | 233 | (52, 112) | 125 | Uncharacterized protein | Uncharacterized protein | | uniclust | UniRef100\_A0A1H8H2L7 | 98.5 | 8e-10 | 1.9e-15 | 93.8 | 66 | (97, 162) | 233 | (8, 73) | 114 | Uncharacterized protein | Uncharacterized protein | | uniclust | UniRef100\_A0A2S1GM15 | 98.5 | 1.1e-09 | 2e-15 | 91.4 | 64 | (91, 156) | 233 | (40, 103) | 122 | Uncharacterized protein | Uncharacterized protein | | uniclust | UniRef100\_UPI0019821726 | 98.5 | 1.3e-09 | 2.3e-15 | 96.5 | 58 | (95, 155) | 233 | (129, 187) | 187 | hypothetical protein | hypothetical protein | | uniclust | UniRef100\_UPI000DE54B3E | 98.5 | 1.4e-09 | 2.5e-15 | 99.7 | 61 | (96, 158) | 233 | (185, 247) | 249 | hypothetical protein | hypothetical protein | | uniclust | UniRef100\_A0A1E4GP42 | 98.5 | 1.4e-09 | 2.6e-15 | 92.3 | 57 | (96, 154) | 233 | (82, 138) | 138 | Uncharacterized protein | Uncharacterized protein | | uniclust | UniRef100\_A0A1B0VDS6 | 98.5 | 1.5e-09 | 2.8e-15 | 103.9 | 81 | (5, 87) | 233 | (51, 140) | 381 | Morphogenetic protein | Morphogenetic protein | | uniclust | UniRef100\_A0A386KMJ4 | 98.5 | 1.6e-09 | 3.1e-15 | 95.3 | 59 | (95, 155) | 233 | (109, 167) | 168 | Uncharacterized protein | Uncharacterized protein | | uniclust | UniRef100\_A0A8E7FPP9 | 98.5 | 1.7e-09 | 3.2e-15 | 97.7 | 96 | (6, 119) | 233 | (1, 111) | 201 | Uncharacterized protein | Uncharacterized protein | | uniclust | UniRef100\_A0A074JFI6 | 98.4 | 1.9e-09 | 4.3e-15 | 91.3 | 66 | (96, 162) | 233 | (12, 84) | 114 | Uncharacterized protein | Uncharacterized protein | | uniclust | UniRef100\_UPI00106EAB0B | 98.4 | 2.2e-09 | 4.4e-15 | 85.6 | 60 | (96, 157) | 233 | (17, 83) | 84 | hypothetical protein | hypothetical protein | | uniclust | UniRef100\_UPI001C915A73 | 98.4 | 2.7e-09 | 5e-15 | 85.9 | 78 | (69, 154) | 233 | (8, 94) | 95 | hypothetical protein | hypothetical protein | | uniclust | UniRef100\_UPI00096A5A9B | 98.4 | 2.7e-09 | 5e-15 | 95.5 | 57 | (95, 154) | 233 | (34, 93) | 204 | hypothetical protein | hypothetical protein | | uniclust | UniRef100\_UPI00143CFDF2 | 98.4 | 2.7e-09 | 5e-15 | 89.6 | 57 | (96, 154) | 233 | (65, 123) | 126 | hypothetical protein | hypothetical protein | | uniclust | UniRef100\_A0A0Q8RJ07 | 98.4 | 2.7e-09 | 5.2e-15 | 87.5 | 64 | (96, 160) | 233 | (9, 79) | 99 | Uncharacterized protein | Uncharacterized protein | | uniclust | UniRef100\_A0A386KLU1 | 98.4 | 2.8e-09 | 5.7e-15 | 92.7 | 58 | (96, 155) | 233 | (81, 140) | 141 | Uncharacterized protein | Uncharacterized protein | | uniclust | UniRef100\_A0A962YGS3 | 98.4 | 3.9e-09 | 7.2e-15 | 94.7 | 61 | (96, 158) | 233 | (109, 169) | 206 | Uncharacterized protein (Fragment) | Uncharacterized protein (Fragment) | | uniclust | UniRef100\_A0A941I2F7 | 98.4 | 4e-09 | 7.3e-15 | 89.9 | 70 | (84, 154) | 233 | (64, 139) | 139 | Uncharacterized protein | Uncharacterized protein | | uniclust | UniRef100\_A0A6G5YBR4 | 98.4 | 4.1e-09 | 7.5e-15 | 86.0 | 74 | (4, 84) | 233 | (23, 97) | 103 | Uncharacterized protein | Uncharacterized protein | | uniclust | UniRef100\_A0A1I3FVE1 | 98.4 | 5.3e-09 | 9.7e-15 | 89.0 | 126 | (7, 156) | 233 | (5, 136) | 137 | Uncharacterized protein | Uncharacterized protein | | uniclust | UniRef100\_A0A482MK44 | 98.4 | 5.2e-09 | 1e-14 | 93.3 | 60 | (92, 154) | 233 | (114, 174) | 174 | Uncharacterized protein | Uncharacterized protein | | uniclust | UniRef100\_A0A0C3RLK6 | 98.3 | 5.1e-09 | 1.1e-14 | 82.3 | 59 | (97, 157) | 233 | (4, 70) | 73 | Uncharacterized protein | Uncharacterized protein | | uniclust | UniRef100\_A0A4R5PK78 | 98.3 | 5.6e-09 | 1.1e-14 | 80.3 | 56 | (97, 154) | 233 | (6, 65) | 67 | Cupin | Cupin | | uniclust | UniRef100\_A0A6G5YPA9 | 98.3 | 7.7e-09 | 1.4e-14 | 96.0 | 137 | (6, 157) | 233 | (1, 155) | 271 | Uncharacterized protein | Uncharacterized protein | | uniclust | UniRef100\_A0A931HEC0 | 98.3 | 7.5e-09 | 1.5e-14 | 81.8 | 60 | (95, 157) | 233 | (8, 71) | 79 | Uncharacterized protein | Uncharacterized protein | | uniclust | UniRef100\_UPI000969E4E3 | 98.3 | 1.1e-08 | 2.1e-14 | 92.9 | 74 | (68, 155) | 233 | (129, 203) | 205 | hypothetical protein | hypothetical protein | | uniclust | UniRef100\_A0A385EED3 | 98.3 | 1.3e-08 | 2.5e-14 | 87.2 | 58 | (97, 156) | 233 | (69, 137) | 138 | Uncharacterized protein | Uncharacterized protein | | uniclust | UniRef100\_A0A6H1ZBT8 | 98.2 | 1.6e-08 | 2.9e-14 | 87.8 | 68 | (75, 155) | 233 | (82, 154) | 154 | Uncharacterized protein | Uncharacterized protein | | uniclust | UniRef100\_UPI00221E5426 | 98.2 | 1.7e-08 | 3.2e-14 | 93.7 | 62 | (96, 159) | 233 | (82, 143) | 267 | hypothetical protein | hypothetical protein | | uniclust | UniRef100\_A0A2D7XBX7 | 98.1 | 4.6e-08 | 8.5e-14 | 82.9 | 59 | (95, 154) | 233 | (70, 128) | 128 | DUF3085 domain-containing protein | DUF3085 domain-containing protein | | uniclust | UniRef100\_A0A6G5YJZ6 | 98.1 | 5e-08 | 9.2e-14 | 86.8 | 72 | (97, 172) | 233 | (18, 101) | 183 | dATP/dGTP diphosphohydrolase N-terminal domain-containing protein | dATP/dGTP diphosphohydrolase N-terminal domain-containing protein | | uniclust | UniRef100\_A0A0B3RIM0 | 98.1 | 4.1e-08 | 9.2e-14 | 81.7 | 64 | (96, 160) | 233 | (10, 82) | 98 | Uncharacterized protein | Uncharacterized protein | | uniclust | UniRef100\_A0A368TTC8 | 98.1 | 5.8e-08 | 1.1e-13 | 78.2 | 53 | (98, 153) | 233 | (38, 90) | 91 | Uncharacterized protein | Uncharacterized protein | | uniclust | UniRef100\_A0A0R3Q2Y3 | 98.1 | 6.3e-08 | 1.2e-13 | 79.9 | 53 | (96, 153) | 233 | (53, 105) | 106 | Uncharacterized protein | Uncharacterized protein | | uniclust | UniRef100\_A0A516MMQ1 | 98.1 | 7.6e-08 | 1.4e-13 | 88.6 | 62 | (94, 155) | 233 | (171, 233) | 239 | Uncharacterized protein | Uncharacterized protein | | uniclust | UniRef100\_A0A345BNB0 | 98.0 | 1.2e-07 | 2.2e-13 | 82.4 | 81 | (6, 90) | 233 | (1, 82) | 151 | Uncharacterized protein | Uncharacterized protein | | uniclust | UniRef100\_A0A481W720 | 98.0 | 1.3e-07 | 2.4e-13 | 86.7 | 63 | (96, 160) | 233 | (100, 163) | 229 | Uncharacterized protein | Uncharacterized protein | | uniclust | UniRef100\_A0A1H7CNT9 | 98.0 | 9.6e-08 | 2.5e-13 | 81.7 | 65 | (96, 160) | 233 | (11, 87) | 111 | Uncharacterized protein | Uncharacterized protein | | uniclust | UniRef100\_A0A4Q2SVH6 | 98.0 | 1.4e-07 | 2.5e-13 | 71.3 | 55 | (99, 156) | 233 | (2, 56) | 61 | Uncharacterized protein | Uncharacterized protein | | uniclust | UniRef100\_A0A0F5PG93 | 98.0 | 1.5e-07 | 3.1e-13 | 74.9 | 58 | (96, 155) | 233 | (11, 73) | 76 | Uncharacterized protein | Uncharacterized protein | | uniclust | UniRef100\_A0A7S5R7Q8 | 97.9 | 2.1e-07 | 3.8e-13 | 71.0 | 56 | (98, 155) | 233 | (2, 61) | 64 | Uncharacterized protein | Uncharacterized protein | | uniclust | UniRef100\_A0A246JRV1 | 97.9 | 2.2e-07 | 4.2e-13 | 75.7 | 61 | (96, 156) | 233 | (16, 87) | 90 | Uncharacterized protein | Uncharacterized protein | | uniclust | UniRef100\_UPI00192B0E5B | 97.9 | 2.3e-07 | 4.3e-13 | 75.9 | 44 | (96, 141) | 233 | (20, 65) | 98 | hypothetical protein | hypothetical protein | | uniclust | UniRef100\_N0DPA9 | 97.9 | 3.3e-07 | 6.1e-13 | 81.6 | 60 | (96, 156) | 233 | (65, 124) | 176 | Uncharacterized protein | Uncharacterized protein | | uniclust | UniRef100\_A0A516MMP7 | 97.9 | 3.5e-07 | 6.4e-13 | 82.3 | 89 | (6, 124) | 233 | (2, 93) | 191 | Uncharacterized protein | Uncharacterized protein | | uniclust | UniRef100\_A0A0F9BXC9 | 97.8 | 3.7e-07 | 6.8e-13 | 81.5 | 65 | (96, 162) | 233 | (49, 115) | 179 | Uncharacterized protein (Fragment) | Uncharacterized protein (Fragment) | | uniclust | UniRef100\_UPI0013B66D81 | 97.8 | 4.2e-07 | 7.7e-13 | 67.4 | 32 | (6, 37) | 233 | (1, 32) | 54 | hypothetical protein | hypothetical protein | | uniclust | UniRef100\_A0A2D2W544 | 97.8 | 4e-07 | 7.8e-13 | 81.0 | 59 | (97, 156) | 233 | (78, 136) | 160 | Uncharacterized protein | Uncharacterized protein | | uniclust | UniRef100\_A0A7W5S4A8 | 97.8 | 5e-07 | 9.2e-13 | 73.7 | 61 | (97, 160) | 233 | (9, 73) | 95 | Uncharacterized protein | Uncharacterized protein | | uniclust | UniRef100\_A0A1T4SRW2 | 97.8 | 6.3e-07 | 1.2e-12 | 67.0 | 54 | (98, 154) | 233 | (2, 55) | 56 | Uncharacterized protein | Uncharacterized protein | | uniclust | UniRef100\_A0A2W5IUS1 | 97.7 | 7.8e-07 | 1.4e-12 | 68.5 | 57 | (97, 155) | 233 | (2, 63) | 66 | Uncharacterized protein | Uncharacterized protein | | uniclust | UniRef100\_A0A431IDW0 | 97.7 | 8.5e-07 | 1.6e-12 | 83.2 | 61 | (96, 156) | 233 | (96, 170) | 264 | Uncharacterized protein | Uncharacterized protein | | uniclust | UniRef100\_A0A2E6JZ26 | 97.7 | 9.2e-07 | 1.7e-12 | 79.4 | 58 | (96, 155) | 233 | (119, 179) | 183 | Uncharacterized protein | Uncharacterized protein | | uniclust | UniRef100\_A0A976S1A0 | 97.7 | 9.8e-07 | 1.8e-12 | 76.1 | 80 | (69, 157) | 233 | (44, 134) | 136 | Uncharacterized protein | Uncharacterized protein | | uniclust | UniRef100\_UPI001F11253C | 97.7 | 1.2e-06 | 2.2e-12 | 73.3 | 28 | (5, 32) | 233 | (46, 73) | 108 | hypothetical protein | hypothetical protein | | uniclust | UniRef100\_A0A431RAX2 | 97.7 | 1.2e-06 | 2.3e-12 | 67.8 | 58 | (98, 155) | 233 | (5, 68) | 68 | Uncharacterized protein | Uncharacterized protein | | uniclust | UniRef100\_UPI002283A885 | 97.7 | 1.3e-06 | 2.4e-12 | 73.9 | 68 | (6, 83) | 233 | (5, 73) | 117 | hypothetical protein | hypothetical protein | | uniclust | UniRef100\_W0ADT7 | 97.6 | 1.6e-06 | 2.9e-12 | 74.9 | 71 | (73, 154) | 233 | (57, 133) | 135 | Uncharacterized protein | Uncharacterized protein | | uniclust | UniRef100\_UPI0008AB9E75 | 97.6 | 1.6e-06 | 2.9e-12 | 75.2 | 54 | (96, 152) | 233 | (82, 138) | 139 | DUF4326 domain-containing protein | DUF4326 domain-containing protein | | uniclust | UniRef100\_A0A4S0XR95 | 97.6 | 1.9e-06 | 3.6e-12 | 68.2 | 63 | (95, 157) | 233 | (4, 70) | 77 | Uncharacterized protein | Uncharacterized protein | | uniclust | UniRef100\_UPI002091CB86 | 97.6 | 2.2e-06 | 4.1e-12 | 77.0 | 68 | (87, 157) | 233 | (91, 166) | 168 | hypothetical protein | hypothetical protein | | uniclust | UniRef100\_UPI00111C8B24 | 97.6 | 2.3e-06 | 4.2e-12 | 71.1 | 72 | (6, 80) | 233 | (1, 73) | 103 | hypothetical protein | hypothetical protein | | uniclust | UniRef100\_A0A0A6CX03 | 97.5 | 2.5e-06 | 4.9e-12 | 69.1 | 58 | (96, 153) | 233 | (15, 81) | 82 | Uncharacterized protein | Uncharacterized protein | | uniclust | UniRef100\_A0A7S5SKF1 | 97.5 | 3e-06 | 5.5e-12 | 82.2 | 88 | (19, 124) | 233 | (12, 110) | 347 | Uncharacterized protein | Uncharacterized protein | | uniclust | UniRef100\_A0A6S7EY07 | 97.5 | 3.1e-06 | 5.7e-12 | 65.5 | 51 | (102, 156) | 233 | (11, 61) | 66 | Uncharacterized protein | Uncharacterized protein | | uniclust | UniRef100\_A0A3S9U7R6 | 97.5 | 3.2e-06 | 5.9e-12 | 79.4 | 65 | (96, 162) | 233 | (128, 192) | 258 | DUF6378 domain-containing protein | DUF6378 domain-containing protein | | uniclust | UniRef100\_A0A179BTQ8 | 97.5 | 3.2e-06 | 6.4e-12 | 67.9 | 57 | (97, 155) | 233 | (5, 75) | 77 | Uncharacterized protein | Uncharacterized protein | | uniclust | UniRef100\_A0A291GC54 | 97.5 | 2.7e-06 | 7e-12 | 72.3 | 66 | (96, 162) | 233 | (9, 84) | 101 | Uncharacterized protein | Uncharacterized protein | | uniclust | UniRef100\_UPI001294A8BB | 97.4 | 5.2e-06 | 9.6e-12 | 66.0 | 56 | (98, 155) | 233 | (12, 67) | 77 | hypothetical protein | hypothetical protein | | uniclust | UniRef100\_UPI000A11260D | 97.4 | 5.6e-06 | 1e-11 | 80.2 | 76 | (7, 85) | 233 | (136, 223) | 333 | hypothetical protein | hypothetical protein | | uniclust | UniRef100\_A0A839YDB8 | 97.4 | 6.2e-06 | 1.2e-11 | 65.1 | 58 | (97, 156) | 233 | (5, 65) | 70 | Uncharacterized protein | Uncharacterized protein | | uniclust | UniRef100\_A0A5C0ZYP1 | 97.4 | 7.8e-06 | 1.4e-11 | 84.0 | 69 | (96, 166) | 233 | (421, 502) | 630 | Uncharacterized protein | Uncharacterized protein | | uniclust | UniRef100\_A0A1P8DJC3 | 97.3 | 9.4e-06 | 1.7e-11 | 75.7 | 55 | (97, 154) | 233 | (170, 227) | 232 | Uncharacterized protein | Uncharacterized protein | | uniclust | UniRef100\_A0A6I1NN18 | 97.3 | 1.1e-05 | 2e-11 | 73.0 | 63 | (96, 160) | 233 | (112, 175) | 181 | Uncharacterized protein | Uncharacterized protein | | uniclust | UniRef100\_A0A1Z3U571 | 97.3 | 1.2e-05 | 2.2e-11 | 77.0 | 64 | (96, 162) | 233 | (71, 138) | 292 | Uncharacterized protein | Uncharacterized protein | | uniclust | UniRef100\_UPI0011D19E68 | 97.3 | 1.1e-05 | 2.3e-11 | 74.1 | 40 | (68, 123) | 233 | (47, 86) | 178 | hypothetical protein | hypothetical protein | | uniclust | UniRef100\_U5N140 | 97.3 | 1.4e-05 | 2.5e-11 | 59.2 | 42 | (110, 154) | 233 | (9, 50) | 50 | Uncharacterized protein | Uncharacterized protein | | uniclust | UniRef100\_UPI0008D812AA | 97.3 | 1.4e-05 | 2.5e-11 | 68.5 | 63 | (97, 159) | 233 | (12, 80) | 121 | hypothetical protein | hypothetical protein | | uniclust | UniRef100\_A0A2E2D777 | 97.2 | 1.3e-05 | 2.8e-11 | 75.0 | 57 | (96, 157) | 233 | (38, 95) | 199 | Uncharacterized protein | Uncharacterized protein | | uniclust | UniRef100\_A0A6J5RMM4 | 97.2 | 1.6e-05 | 2.9e-11 | 73.6 | 56 | (96, 154) | 233 | (160, 215) | 215 | DUF6378 domain-containing protein | DUF6378 domain-containing protein | | uniclust | UniRef100\_UPI0011408B34 | 97.2 | 1.9e-05 | 3.4e-11 | 64.2 | 55 | (103, 161) | 233 | (9, 63) | 85 | hypothetical protein | hypothetical protein | | uniclust | UniRef100\_A0A5D9C913 | 97.2 | 2e-05 | 3.9e-11 | 62.2 | 56 | (96, 154) | 233 | (4, 65) | 67 | Uncharacterized protein | Uncharacterized protein | | uniclust | UniRef100\_A0A7S5R948 | 97.2 | 2.1e-05 | 4.1e-11 | 69.5 | 55 | (96, 153) | 233 | (83, 142) | 142 | Uncharacterized protein | Uncharacterized protein | | uniclust | UniRef100\_UPI001CFF8498 | 97.1 | 3.1e-05 | 5.7e-11 | 69.2 | 60 | (98, 157) | 233 | (11, 73) | 161 | hypothetical protein | hypothetical protein | | uniclust | UniRef100\_A0A5C7PPU1 | 97.1 | 3.1e-05 | 5.8e-11 | 77.5 | 95 | (86, 184) | 233 | (245, 340) | 438 | Uncharacterized protein | Uncharacterized protein | | uniclust | UniRef100\_A0A2E0UI72 | 97.1 | 3.6e-05 | 6.5e-11 | 70.9 | 98 | (96, 200) | 233 | (62, 166) | 201 | Jacalin-type lectin domain-containing protein | Jacalin-type lectin domain-containing protein | | uniclust | UniRef100\_A0A0C3NUH1 | 97.1 | 3.6e-05 | 6.6e-11 | 64.9 | 55 | (98, 154) | 233 | (42, 102) | 106 | Uncharacterized protein | Uncharacterized protein | | uniclust | UniRef100\_A0A7L5BCL0 | 97.1 | 4.2e-05 | 7.6e-11 | 65.8 | 58 | (98, 157) | 233 | (61, 121) | 121 | Restriction alleviation protein, Lar family | Restriction alleviation protein, Lar family | | uniclust | UniRef100\_A0A3P3ZQD0 | 97.0 | 5.6e-05 | 1e-10 | 68.9 | 53 | (96, 154) | 233 | (123, 175) | 183 | Uncharacterized protein | Uncharacterized protein | | uniclust | UniRef100\_A0A385IKG2 | 97.0 | 5.8e-05 | 1.1e-10 | 73.1 | 65 | (92, 158) | 233 | (63, 129) | 277 | 3'-phosphatase, 5'-polynucleotide kinase | 3'-phosphatase, 5'-polynucleotide kinase | | uniclust | UniRef100\_A0A379CP65 | 96.9 | 7.7e-05 | 1.5e-10 | 69.6 | 149 | (15, 215) | 233 | (17, 172) | 199 | Uncharacterized protein | Uncharacterized protein | | uniclust | UniRef100\_UPI00070EF9DC | 96.9 | 8.6e-05 | 1.6e-10 | 66.9 | 65 | (98, 162) | 233 | (3, 68) | 165 | hypothetical protein | hypothetical protein | | uniclust | UniRef100\_A0A3B7DND1 | 96.9 | 8.6e-05 | 1.6e-10 | 69.5 | 59 | (93, 153) | 233 | (4, 64) | 222 | Uncharacterized protein | Uncharacterized protein | | uniclust | UniRef100\_UPI002022F4A1 | 96.8 | 0.00013 | 2.3e-10 | 74.4 | 60 | (96, 156) | 233 | (267, 329) | 487 | hypothetical protein | hypothetical protein | | uniclust | UniRef100\_B5AX45 | 96.8 | 0.00013 | 2.4e-10 | 72.1 | 61 | (96, 160) | 233 | (140, 200) | 353 | Uncharacterized protein | Uncharacterized protein | | uniclust | UniRef100\_UPI001C8B0C5D | 96.8 | 0.00014 | 2.5e-10 | 66.3 | 68 | (6, 86) | 233 | (1, 68) | 176 | hypothetical protein | hypothetical protein | | uniclust | UniRef100\_A0A0F6TI71 | 96.8 | 0.00015 | 2.7e-10 | 63.8 | 52 | (96, 151) | 233 | (74, 130) | 132 | Phage protein | Phage protein | | uniclust | UniRef100\_UPI000DF92DE2 | 96.8 | 0.00015 | 2.7e-10 | 56.7 | 52 | (101, 154) | 233 | (3, 61) | 64 | hypothetical protein | hypothetical protein | | uniclust | UniRef100\_A0A350UCK3 | 96.8 | 0.00016 | 3e-10 | 64.0 | 61 | (98, 161) | 233 | (2, 63) | 142 | Uncharacterized protein | Uncharacterized protein | | uniclust | UniRef100\_A0A516MIH6 | 96.7 | 0.00019 | 3.5e-10 | 53.7 | 48 | (102, 152) | 233 | (2, 49) | 50 | Uncharacterized protein | Uncharacterized protein | | uniclust | UniRef100\_A0A259NIN2 | 96.7 | 0.0002 | 3.7e-10 | 68.3 | 93 | (62, 162) | 233 | (39, 131) | 252 | Uncharacterized protein | Uncharacterized protein | | uniclust | UniRef100\_A0A2D9H0Z1 | 96.7 | 0.00023 | 4.3e-10 | 62.5 | 56 | (98, 156) | 233 | (2, 59) | 133 | Uncharacterized protein | Uncharacterized protein | | uniclust | UniRef100\_A0A2L1KEG8 | 96.6 | 0.00027 | 5e-10 | 61.7 | 56 | (98, 156) | 233 | (55, 119) | 126 | Uncharacterized protein | Uncharacterized protein | | uniclust | UniRef100\_A0A931MMN7 | 96.6 | 0.00029 | 5.3e-10 | 58.6 | 54 | (96, 152) | 233 | (33, 90) | 91 | Uncharacterized protein | Uncharacterized protein | | uniclust | UniRef100\_UPI002302FDAA | 96.6 | 0.00029 | 5.3e-10 | 66.6 | 63 | (95, 160) | 233 | (36, 99) | 230 | hypothetical protein | hypothetical protein | | uniclust | UniRef100\_UPI001124F1A2 | 96.6 | 0.00033 | 6.1e-10 | 61.4 | 67 | (87, 153) | 233 | (59, 129) | 129 | hypothetical protein | hypothetical protein | | uniclust | UniRef100\_A0A5C7LQ10 | 96.6 | 0.00034 | 6.2e-10 | 64.1 | 61 | (96, 156) | 233 | (108, 171) | 177 | DUF2203 family protein | DUF2203 family protein | | uniclust | UniRef100\_A0A6A6K0Z2 | 96.5 | 0.00044 | 8e-10 | 62.4 | 38 | (66, 118) | 233 | (29, 66) | 156 | Uncharacterized protein | Uncharacterized protein | | uniclust | UniRef100\_A0A7S5QYL1 | 96.5 | 0.00051 | 9.4e-10 | 55.6 | 59 | (98, 158) | 233 | (2, 69) | 76 | Uncharacterized protein | Uncharacterized protein | | uniclust | UniRef100\_A0A2D6BGZ8 | 96.4 | 0.00059 | 1.1e-09 | 57.5 | 55 | (98, 154) | 233 | (16, 82) | 94 | Uncharacterized protein | Uncharacterized protein | | uniclust | UniRef100\_A0A021XBI4 | 96.4 | 0.00065 | 1.2e-09 | 52.7 | 52 | (99, 155) | 233 | (2, 57) | 59 | Uncharacterized protein | Uncharacterized protein | | uniclust | UniRef100\_A0A1V0M647 | 96.3 | 0.00085 | 1.6e-09 | 61.3 | 56 | (98, 156) | 233 | (98, 162) | 169 | Uncharacterized protein | Uncharacterized protein | | uniclust | UniRef100\_W0A8K8 | 96.3 | 0.00092 | 1.7e-09 | 64.9 | 57 | (96, 157) | 233 | (82, 139) | 274 | Restriction alleviation protein Lar | Restriction alleviation protein Lar | |
| Top keywords  (threshold 1.00e-03 (evalue)) | **hypothetical, domain\_containing, DUF551, DUF6378, Morphogenetic, Fragment, Restriction, alleviation, Lar, SaV\_like** |
| Output files | ../../similar\_sequences/27\_FANPEZAQ\_CDS\_0027\_merged.svg ../../similar\_sequences/27\_FANPEZAQ\_CDS\_0027\_pdb70.a3m ../../similar\_sequences/27\_FANPEZAQ\_CDS\_0027\_pdb70.hhr ../../similar\_sequences/27\_FANPEZAQ\_CDS\_0027\_uniclust.a3m ../../similar\_sequences/27\_FANPEZAQ\_CDS\_0027\_uniclust.hhr |

#### Structure prediction (AlphaFold)2

|  |  |
| --- | --- |
| Stats | xml version="1.0" encoding="utf-8" standalone="no"?       2024-09-02T21:09:27.189806 image/svg+xml   Matplotlib v3.7.2, https://matplotlib.org/ |
| Predicted structure | **NGL Viewer Controls:**  - Center: *Left-Click* - Rotate: *Left-Click + Drag* - Translate: *Right-Click + Drag* - Zoom: *Shift + Left-Click + Drag* |
| Output files | ../../predicted\_structures/27\_FANPEZAQ\_CDS\_0027/features.pkl ../../predicted\_structures/27\_FANPEZAQ\_CDS\_0027/ranked\_0.pdb ../../predicted\_structures/27\_FANPEZAQ\_CDS\_0027/ranked\_0\_plots.svg ../../predicted\_structures/27\_FANPEZAQ\_CDS\_0027/result\_model\_1\_ptm\_pred\_0.pkl |

#### Structure similarity search results (Foldseek)3

|  |  |
| --- | --- |
| Structure databases searched | Pdb, Afdb-proteome, Afdb-uniprot50 |
| Results, scheme(s)  (Top layers only, threshold 1.00e-02 (evalue)) | xml version="1.0" encoding="utf-8" standalone="no"?       2024-09-02T21:10:57.290511 image/svg+xml   Matplotlib v3.7.2, https://matplotlib.org/ |
| Results, table  (threshold 1.00e-02 (evalue)) | | db | id | prob | evalue | bits | fident | alnlen | mismatch | gapopen | qstart | qend | tstart | tend | name | description | | --- | --- | --- | --- | --- | --- | --- | --- | --- | --- | --- | --- | --- | --- | --- | | afdb-uniprot50 | AF-A0A8B2FBR4-F1-MODEL\_V4 | 1.0 | 7.831e-07 | 146 | 0.197 | 203 | 111 | 11 | 6 | 186 | 2 | 174 | Uncharacterized protein | Uncharacterized protein | | afdb-uniprot50 | AF-A0A0F9BXC9-F1-MODEL\_V4 | 1.0 | 7.324e-05 | 120 | 0.174 | 166 | 111 | 6 | 78 | 230 | 27 | 179 | Uncharacterized protein | Uncharacterized protein | | afdb-uniprot50 | AF-A0A259NIN2-F1-MODEL\_V4 | 1.0 | 0.0004499 | 112 | 0.179 | 162 | 98 | 6 | 72 | 233 | 49 | 175 | Uncharacterized protein | Uncharacterized protein | | afdb-uniprot50 | AF-A0A7X5MK68-F1-MODEL\_V4 | 1.0 | 4.086e-05 | 104 | 0.229 | 170 | 78 | 10 | 9 | 158 | 57 | 193 | Uncharacterized protein | Uncharacterized protein | |
| Top keywords  (threshold 1.00e-02 (evalue)) | -- |
| Output files | ../../similar\_structures/27\_FANPEZAQ\_CDS\_0027\_afdb-proteome\_foldseek.tsv ../../similar\_structures/27\_FANPEZAQ\_CDS\_0027\_afdb-uniprot50\_foldseek.tsv ../../similar\_structures/27\_FANPEZAQ\_CDS\_0027\_merged.svg ../../similar\_structures/27\_FANPEZAQ\_CDS\_0027\_pdb\_foldseek.tsv |

  
  
  

Return to summary | Go to previous | Go to next

  


---

**Sequence/structure alignments coloring**  
Each object in the alignment figures is colored according to its E-value following this color coding:

1e-100
10

**References:**  
1) Steinegger M, Meier M, Mirdita M, Vöhringer H, Haunsberger S J, and Söding J (2019) HH-suite3 for fast remote homology detection and deep protein annotation, BMC Bioinformatics, 473. doi: 10.1186/s12859-019-3019-7  
2) Jumper J, Evans R, Pritzel A, ..., Hassabis D (2021) Highly accurate protein structure prediction with AlphaFold, Nature, 596. doi: 10.1038/s41586-021-03819-2  
3) van Kempen M, Kim S, Tumescheit C, Mirdita M, Lee J, Gilchrist CLM, Söding J, and Steinegger M (2023) Fast and accurate protein structure search with Foldseek. Nature Biotechnology. doi: 10.1038/s41587-023-01773-0
