# Supplementary material for: Completing the BASEL phage collection to unlock hidden diversity for systematic exploration of phage–host interactions
Source: PLoS Biol. 2025 Apr 7;23(4):e3003063. doi: 10.1371/journal.pbio.3003063 (PMC11990801; doi:10.1371/journal.pbio.3003063)
Supplement: S2 Data — (ZIP) [file pbio.3003063.s009.zip › entries/28.html]

FANPEZAQ\_CDS\_0028


Return to summary | Go to previous | Go to next

|  |  |
| --- | --- |
| FANPEZAQ\_CDS\_0028 Page creation date: 02 Sep 2024, 12:00  Project folder: n/a  Input sequences file: Escherichia\_virus\_HeidiAbel.gb | parb partition spo0j domain\_containing repb partitioning chromosome sulfiredoxin chromosome\_partitioning parb\_like plasmid probable fragment putative and dna nuclease dna\_binding stage sporulation j segregation transcriptional regulator hth nucleoid occlusion rna nucleotide metabolism n\_terminal cro c1\_type |

### Sequence information

|  |  |
| --- | --- |
| Name | FANPEZAQ\_CDS\_0028  28\_FANPEZAQ\_CDS\_0028 (pipeline id) |
| Imported annotations | Escherichia\_virus\_HeidiAbel Bas97 |
| Protein sequence | MATIGQLYKAGATEAKPRKVFFVPLAELYVEDGYNIRDVDQAHVEEFRDAYLAGEYVPPL TVEVTQQGIKVIDGHHRFAGAKMAAETGLELRLEVNDITGTSEADKIALMVTSSQGKPLE PLERAKAYARLKAQGWTNDEIARKVKRSPSDVANMLALAECPEPIKDMVRGGQMSYVTAT ELTRQHGTEAEAVAAQALEEAKAAGKDKVTKKFTEPKAPKAPKAKPEAIQPPFGKDKLTR LADLAVQIELVHSDDRLEDAEPDESMTLRAPAGVVREFMALVADYVGGVPE |
| Number of residues | 291 |
| Molecular weight (Da) | 31696.74 |
| Output files | ../../query\_sequences/28\_FANPEZAQ\_CDS\_0028.fasta |

### Putative domain architecture and protein family

#### Search results (HHblits)1

|  |  |
| --- | --- |
| Domain family databases searched | Pfam, Ncbi-cd, Cath, Phrogs |
| Results, scheme(s)  (Top layers only; threshold 1.00e-03 (evalue)) | xml version="1.0" encoding="utf-8" standalone="no"?       2024-09-02T21:08:18.303612 image/svg+xml   Matplotlib v3.7.2, https://matplotlib.org/ |
| Results, table  (E-value ≤ 1.00e-03 (evalue)) | | db | id | prob | evalue | pvalue | score | cols | query | query\_len | template | template\_len | name | description | | --- | --- | --- | --- | --- | --- | --- | --- | --- | --- | --- | --- | --- | | pfam | PF20188 | 98.6 | 3.8e-12 | 7.9e-16 | 107.2 | 113 | (31, 147) | 291 | (3, 118) | 242 | DUF6551 | Family of unknown function (DUF6551) | | pfam | PF08857 | 98.4 | 4.1e-11 | 8.1e-15 | 95.6 | 90 | (57, 151) | 291 | (38, 138) | 153 | ParBc\_2 | Putative ParB-like nuclease | | pfam | PF14072 | 97.9 | 2.2e-09 | 4.6e-13 | 93.6 | 94 | (28, 124) | 291 | (34, 159) | 337 | DndB | DNA-sulfur modification-associated | | pfam | PF07506 | 96.9 | 6.9e-07 | 1.4e-10 | 69.5 | 73 | (116, 188) | 291 | (2, 76) | 171 | RepB | RepB plasmid partitioning protein | | pfam | PF02195 | 96.8 | 1e-06 | 2.3e-10 | 57.8 | 70 | (23, 95) | 291 | (3, 74) | 91 | ParBc | ParB/Sulfiredoxin domain | | pfam | PF18090 | 95.8 | 3.5e-05 | 7.8e-09 | 47.8 | 69 | (119, 187) | 291 | (1, 72) | 75 | SoPB\_HTH | Centromere-binding protein HTH domain | | pfam | PF08535 | 95.3 | 0.0001 | 2.2e-08 | 48.9 | 56 | (134, 189) | 291 | (2, 58) | 92 | KorB | KorB domain | | ncbi-cd | cd16396 | 97.9 | 1.7e-09 | 4.6e-13 | 70.8 | 78 | (19, 96) | 291 | (2, 80) | 95 | Noc\_N | cd16396 Noc\_N; nucleoid occlusion protein, N-terminal domain, and related domains of the ParB partitioning protein family. | | ncbi-cd | cd16393 | 97.9 | 2.3e-09 | 6.3e-13 | 70.1 | 76 | (21, 96) | 291 | (2, 78) | 97 | SPO0J\_N | cd16393 SPO0J\_N; Thermus thermophilus stage 0 sporulation protein J-like N-terminal domain, ParB family member. | | ncbi-cd | cd16390 | 97.8 | 2.6e-09 | 6.5e-13 | 82.1 | 71 | (21, 96) | 291 | (2, 84) | 162 | ParB\_N\_Srx\_like | cd16390 ParB\_N\_Srx\_like; uncharacterized family distantly related to the N-terminal domain of the ParB/Srx superfamily. | | ncbi-cd | cd16402 | 97.5 | 2e-08 | 5.3e-12 | 64.9 | 67 | (25, 96) | 291 | (2, 68) | 87 | ParB\_N\_like\_MT | cd16402 ParB\_N\_like\_MT; ParB N-terminal-like domain, some attached to C-terminal S-adenosylmethionine-dependent methyltransferase domain. | | ncbi-cd | cd16397 | 97.3 | 6e-08 | 1.6e-11 | 64.5 | 77 | (19, 96) | 291 | (4, 87) | 100 | IbrB\_like | cd16397 IbrB\_like; immunoglobulin-binding regulator IbrB activates eib genes. IbrB (along with IbrA) activates immunoglobulin-binding eib genes in Escherichia coli. | | ncbi-cd | cd16403 | 97.3 | 6.1e-08 | 1.6e-11 | 62.8 | 68 | (25, 96) | 291 | (2, 69) | 88 | ParB\_N\_like\_MT | cd16403 ParB\_N\_like\_MT; ParB N-terminal-like domain, some attached to C-terminal S-adenosylmethionine-dependent methyltransferase. | | ncbi-cd | cd16408 | 97.3 | 6.1e-08 | 1.7e-11 | 61.6 | 58 | (39, 96) | 291 | (12, 70) | 84 | ParB\_N\_like | cd16408 ParB\_N\_like; ParB N-terminal, parA -binding, -like domain of bacterial and plasmid parABS partitioning systems. | | ncbi-cd | cd16405 | 97.3 | 6.8e-08 | 1.8e-11 | 62.9 | 72 | (24, 96) | 291 | (2, 78) | 91 | RepB\_like\_N | cd16405 RepB\_like\_N; plasmid segregation replication protein B like protein, N-terminal domain. RepB, found on plasmids and secondary chromosomes, works along with repA in directing plasmid segregation, and has been shown in Rhizobium etli to require the parS centromere-like sequence for full transcriptional repression of the repABC operon, inducing plasmid incompatibility. | | ncbi-cd | cd16410 | 97.3 | 7.5e-08 | 2e-11 | 62.1 | 65 | (40, 111) | 291 | (13, 77) | 80 | ParB\_N\_like | cd16410 ParB\_N\_like; ParB N-terminal, parA-binding, -like domain of bacterial and plasmid parABS partitioning systems. | | ncbi-cd | cd16407 | 97.2 | 1.1e-07 | 2.9e-11 | 60.6 | 70 | (27, 96) | 291 | (3, 73) | 86 | ParB\_N\_like | cd16407 ParB\_N\_like; ParB N-terminal, parA-binding, -like domain of bacterial and plasmid parABS partitioning systems. | | ncbi-cd | cd16398 | 97.1 | 2.4e-07 | 6.5e-11 | 60.3 | 70 | (27, 96) | 291 | (3, 75) | 91 | KorB\_N\_like | cd16398 KorB\_N\_like; ParB-like partition protein of low copy number plasmid RK2, N-terminal domain and related domains. | | ncbi-cd | cd16388 | 97.0 | 3.3e-07 | 9.2e-11 | 56.2 | 71 | (21, 96) | 291 | (2, 74) | 77 | SbnI\_like\_N | cd16388 SbnI\_like\_N; N-terminal domain of transcriptional regulators similar to SbnI. Siderophore staphylobactin biosynthesis protein SbnI of Staphylococcus aureus is a ParB/Spo0J like protein required for the expression of genes in the sbn operon, which is responsible for staphyloferrin B (SB) biosynthesis. | | ncbi-cd | cd16411 | 97.0 | 4.4e-07 | 1.2e-10 | 58.2 | 70 | (24, 96) | 291 | (2, 76) | 90 | ParB\_N\_like | cd16411 ParB\_N\_like; ParB N-terminal, parA -binding, domain of bacterial and plasmid parABS partitioning systems. | | ncbi-cd | cd16395 | 96.9 | 4.9e-07 | 1.3e-10 | 58.8 | 73 | (21, 96) | 291 | (2, 80) | 90 | Srx | cd16395 Srx; Sulfiredoxin reactivates peroxiredoxins after oxidative inactivation. Sulfiredoxin reduces and thereby re-activates 2-cys peroxiredoxins. | | ncbi-cd | cd16409 | 96.9 | 4.9e-07 | 1.3e-10 | 55.5 | 56 | (41, 96) | 291 | (3, 59) | 74 | ParB\_N\_like | cd16409 ParB\_N\_like; ParB N-terminal-like domain of bacterial and plasmid parABS partitioning systems. | | ncbi-cd | cd16404 | 96.9 | 5.4e-07 | 1.4e-10 | 55.7 | 55 | (39, 96) | 291 | (12, 66) | 69 | pNOB8\_ParB\_N\_like | cd16404 pNOB8\_ParB\_N\_like; pNOB8 ParB-like N-terminal domain, plasmid partitioning system protein domain. archaeal pNOB8 ParB acts in a plasmid partitioning system made up of 3 parts: AspA, ParA motor protein, and ParB, which links ParA to the protein-DNA superhelix. | | ncbi-cd | cd16394 | 96.9 | 7e-07 | 1.9e-10 | 54.3 | 55 | (39, 94) | 291 | (12, 66) | 67 | sopB\_N | cd16394 sopB\_N; N-terminal domain of sopB protein, which promotes proper partitioning of F1 plasmid. | | ncbi-cd | cd16400 | 96.7 | 1.6e-06 | 4.4e-10 | 53.2 | 67 | (23, 96) | 291 | (2, 69) | 72 | ParB\_Srx\_like\_nuclease | cd16400 ParB\_Srx\_like\_nuclease; ParB/Srx\_like nuclease and putative transcriptional regulators related to SbnI. This family contains a Pyrococcus Furiosus enzyme reported to have DNA nuclease activity and resembles the N-terminal domain of ParB proteins of the parABS bacterial chromosome partitioning system. | | ncbi-cd | cd16401 | 96.6 | 2e-06 | 5.4e-10 | 54.4 | 56 | (39, 96) | 291 | (12, 67) | 85 | ParB\_N\_like\_MT | cd16401 ParB\_N\_like\_MT; ParB N-terminal-like domain, some attached to C-terminal S-adenosylmethionine-dependent methyltransferase domain. | | ncbi-cd | cd16406 | 96.5 | 3e-06 | 8e-10 | 53.7 | 55 | (41, 95) | 291 | (3, 66) | 82 | ParB\_N\_like | cd16406 ParB\_N\_like; ParB N-terminal, parA-binding, -like domain of bacterial and plasmid parABS partitioning systems. | | ncbi-cd | cd16387 | 96.3 | 5.5e-06 | 1.5e-09 | 46.5 | 50 | (42, 91) | 291 | (3, 53) | 54 | ParB\_N\_Srx | cd16387 ParB\_N\_Srx; ParB N-terminal domain and sulfiredoxin protein-related families. The ParB N-terminal domain/Sulfiredoxin (Srx) superfamily contains proteins with diverse activities. | | ncbi-cd | cd16844 | 96.2 | 1e-05 | 2.7e-09 | 46.5 | 51 | (41, 94) | 291 | (3, 53) | 54 | ParB\_N\_like\_MT | cd16844 ParB\_N\_like\_MT; ParB N-terminal-like domain, some attached to C-terminal S-adenosylmethionine-dependent methyltransferase domain. | | ncbi-cd | cd16392 | 96.1 | 1.1e-05 | 3.1e-09 | 48.8 | 56 | (40, 95) | 291 | (10, 69) | 72 | toxin-ParB | cd16392 toxin-ParB; toxin domain of the ParB/Srx superfamily. toxin domain with similarity to the N-terminal domain of ParB, a DNA-binding component of the prokaryotic parABS partitioning system and related proteins. | | cath | 2hwjA01 | 98.3 | 8.8e-11 | 1.4e-14 | 92.2 | 92 | (16, 112) | 291 | (5, 105) | 134 | Hypothetical protein atu1540 | CATHCODE: 3.90.1530.10 NAME: Hypothetical protein atu1540. Chain: a, b, c, d, e, f. Synonym: agr\_c\_2837p. Engineered: yes SOURCE: Agrobacterium tumefaciens str. C58. Organism\_taxid: 176299. Strain: c58 / atcc 33970. Expressed in: escherichia coli. Expression\_system\_taxid: 562. CLASS: Alpha Beta, ARCH: Alpha-Beta Complex, TOPOL: Conserved hypothetical protein from pyrococcus furiosus pfu- 392566-001, ParB domain, HOMOL: Conserved hypothetical protein from pyrococcus furiosus pfu- 392566-001, ParB domain | | cath | 1vz0C02 | 97.8 | 5.4e-09 | 9.5e-13 | 73.9 | 84 | (106, 189) | 291 | (6, 89) | 113 | Chromosome-partitioning protein spo0j | CATHCODE: 1.10.10.2830 NAME: Chromosome-partitioning protein spo0j. Chain: a, b, c, d, e, f, g, h. Fragment: DNA-binding domain, residues 1-222. Engineered: yes SOURCE: Thermus thermophilus hb27. Organism\_taxid: 262724. Atcc: baa-163d. Gene: spo0c, parb, tt\_c1604. Expressed in: escherichia coli bl21(de3). Expression\_system\_taxid: 469008. Expression\_system\_variant: c41 CLASS: Mainly Alpha, ARCH: Orthogonal Bundle, TOPOL: Arc Repressor Mutant, subunit A, HOMOL: Arc Repressor Mutant, subunit A | | cath | 3mkyP00 | 97.7 | 9.5e-09 | 1.6e-12 | 75.8 | 73 | (118, 190) | 291 | (1, 76) | 114 | Dna (5'- d(\*cp\*tp\*gp\*gp\*gp\*ap\*cp\*cp\*ap\*tp\*gp\*gp\*tp\*cp\*cp\*cp\*ap\*g)-3') | CATHCODE: 1.10.10.2830 NAME: Dna (5'- d(\*cp\*tp\*gp\*gp\*gp\*ap\*cp\*cp\*ap\*tp\*gp\*gp\*tp\*cp\*cp\*cp\*ap\*g)-3').Chain: u, t. Engineered: yes. Protein sopb. Chain: b, p. Fragment: unp residues 155 to 323. Synonym: plasmid partition protein b. Engineered: yes SOURCE: Yes. Other\_details: the DNA was chemically synthesized.. CLASS: Mainly Alpha, ARCH: Orthogonal Bundle, TOPOL: Arc Repressor Mutant, subunit A, HOMOL: Arc Repressor Mutant, subunit A | | cath | 1xw3A01 | 97.5 | 5.2e-08 | 8.8e-12 | 67.8 | 74 | (19, 95) | 291 | (3, 82) | 96 | Sulfiredoxin | CATHCODE: 3.90.1530.10 NAME: Sulfiredoxin. Chain: a. Fragment: residues 32-137. Engineered: yes SOURCE: Homo sapiens. Human. Organism\_taxid: 9606. Expressed in: escherichia coli. Expression\_system\_taxid: 562. CLASS: Alpha Beta, ARCH: Alpha-Beta Complex, TOPOL: Conserved hypothetical protein from pyrococcus furiosus pfu- 392566-001, ParB domain, HOMOL: Conserved hypothetical protein from pyrococcus furiosus pfu- 392566-001, ParB domain | | cath | 1vk1A01 | 97.2 | 2.2e-07 | 3.7e-11 | 67.0 | 74 | (17, 95) | 291 | (15, 91) | 112 | Conserved hypothetical protein | CATHCODE: 3.90.1530.10 NAME: Conserved hypothetical protein. Chain: a. Engineered: yes SOURCE: Pyrococcus furiosus. Organism\_taxid: 2261. Expressed in: escherichia coli. Expression\_system\_taxid: 562 CLASS: Alpha Beta, ARCH: Alpha-Beta Complex, TOPOL: Conserved hypothetical protein from pyrococcus furiosus pfu- 392566-001, ParB domain, HOMOL: Conserved hypothetical protein from pyrococcus furiosus pfu- 392566-001, ParB domain | | cath | 1vz0A01 | 96.8 | 1.5e-06 | 2.6e-10 | 54.1 | 56 | (38, 93) | 291 | (5, 60) | 63 | Chromosome-partitioning protein spo0j | CATHCODE: 3.90.1530.30 NAME: Chromosome-partitioning protein spo0j. Chain: a, b, c, d, e, f, g, h. Fragment: DNA-binding domain, residues 1-222. Engineered: yes SOURCE: Thermus thermophilus hb27. Organism\_taxid: 262724. Atcc: baa-163d. Gene: spo0c, parb, tt\_c1604. Expressed in: escherichia coli bl21(de3). Expression\_system\_taxid: 469008. Expression\_system\_variant: c41 CLASS: Alpha Beta, ARCH: Alpha-Beta Complex, TOPOL: Conserved hypothetical protein from pyrococcus furiosus pfu- 392566-001, ParB domain, HOMOL: Conserved hypothetical protein from pyrococcus furiosus pfu- 392566-001, ParB domain | | phrogs | 1033 | 100.0 | 8.5e-40 | 1.1e-43 | 293.7 | 270 | (1, 286) | 291 | (3, 283) | 288 | ParB-like partition protein | ParB-like partition protein; Category: DNA, RNA and nucleotide metabolism; NC\_031091\_p46 | | phrogs | 4715 | 99.8 | 1.5e-25 | 1.8e-29 | 194.6 | 176 | (16, 196) | 291 | (3, 215) | 257 | ParB-like partition protein | ParB-like partition protein; Category: DNA, RNA and nucleotide metabolism; p121521 VI\_12455 | | phrogs | 620 | 99.8 | 7.8e-24 | 9.7e-28 | 177.3 | 134 | (13, 161) | 291 | (19, 152) | 191 | ParB-like partition protein | ParB-like partition protein; Category: DNA, RNA and nucleotide metabolism; MF189178\_p69 | | phrogs | 6218 | 99.7 | 4.8e-23 | 5.7e-27 | 189.8 | 164 | (19, 189) | 291 | (2, 173) | 502 | ParB-like partition protein | ParB-like partition protein; Category: DNA, RNA and nucleotide metabolism; KJ829260\_p91 | | phrogs | 2159 | 99.7 | 1.9e-22 | 2.3e-26 | 167.2 | 147 | (14, 161) | 291 | (48, 198) | 214 | ParB-like partition protein | ParB-like partition protein; Category: DNA, RNA and nucleotide metabolism; p9193 VI\_02947 | | phrogs | 498 | 99.7 | 4.5e-22 | 5.5e-26 | 183.2 | 130 | (15, 150) | 291 | (7, 145) | 419 | DNA methyltransferase | DNA methyltransferase; Category: other; p48104 VI\_12205 | | phrogs | 3951 | 99.6 | 1.5e-20 | 1.7e-24 | 161.8 | 175 | (16, 198) | 291 | (6, 185) | 300 | NA | NA; Category: unknown function; p296951 VI\_02154 | | phrogs | 3264 | 99.6 | 2.2e-20 | 2.8e-24 | 162.8 | 132 | (15, 154) | 291 | (7, 141) | 272 | ParB-like partition protein | ParB-like partition protein; Category: DNA, RNA and nucleotide metabolism; p313310 VI\_04191 | | phrogs | 15396 | 99.6 | 4.9e-20 | 5.5e-24 | 145.5 | 132 | (15, 158) | 291 | (1, 135) | 201 | ParB-like partition nuclease | ParB-like partition nuclease; Category: DNA, RNA and nucleotide metabolism; NC\_024369\_p1 | | phrogs | 18726 | 99.6 | 6.9e-20 | 7.7e-24 | 162.8 | 181 | (18, 200) | 291 | (1, 215) | 572 | NA | NA; Category: unknown function; p234010 VI\_05492 | | phrogs | 8071 | 99.6 | 7.4e-20 | 8.4e-24 | 159.7 | 129 | (15, 159) | 291 | (5, 133) | 394 | ParB-like partition protein | ParB-like partition protein; Category: DNA, RNA and nucleotide metabolism; KU886268\_p74 | | phrogs | 4272 | 99.6 | 1.2e-19 | 1.4e-23 | 166.0 | 173 | (17, 189) | 291 | (294, 474) | 658 | NA | NA; Category: unknown function; p55719 VI\_12435 | | phrogs | 723 | 99.5 | 1.5e-18 | 1.8e-22 | 145.9 | 133 | (15, 161) | 291 | (14, 149) | 219 | ParB-like partition protein | ParB-like partition protein; Category: DNA, RNA and nucleotide metabolism; NC\_031253\_p1 | | phrogs | 18056 | 99.4 | 4.4e-18 | 5e-22 | 121.1 | 77 | (18, 103) | 291 | (1, 77) | 94 | ParB-like partition nuclease | ParB-like partition nuclease; Category: DNA, RNA and nucleotide metabolism; NC\_007924\_p29 | | phrogs | 1146 | 99.3 | 2.3e-16 | 2.8e-20 | 138.7 | 167 | (15, 188) | 291 | (42, 219) | 333 | ParB-like partition protein | ParB-like partition protein; Category: DNA, RNA and nucleotide metabolism; p43547 VI\_12111 | | phrogs | 26387 | 99.3 | 3e-16 | 3.4e-20 | 127.6 | 148 | (16, 170) | 291 | (6, 162) | 252 | ParB-like partition nuclease | ParB-like partition nuclease; Category: DNA, RNA and nucleotide metabolism; NC\_031231\_p92 | | phrogs | 15982 | 99.3 | 3.3e-16 | 3.8e-20 | 137.6 | 178 | (16, 196) | 291 | (25, 226) | 452 | NA | NA; Category: unknown function; p176212 VI\_01150 | | phrogs | 9353 | 99.2 | 7.5e-16 | 8.8e-20 | 129.5 | 123 | (17, 142) | 291 | (8, 136) | 246 | NA | NA; Category: unknown function; p407464 VI\_01025 | | phrogs | 12047 | 99.2 | 1.1e-15 | 1.3e-19 | 131.9 | 157 | (22, 188) | 291 | (2, 188) | 359 | ParB-like partition nuclease | ParB-like partition nuclease; Category: DNA, RNA and nucleotide metabolism; NC\_007581\_p185 | | phrogs | 7783 | 99.1 | 1.5e-14 | 1.7e-18 | 131.1 | 186 | (17, 208) | 291 | (33, 224) | 669 | NA | NA; Category: unknown function; p235076 VI\_06411 | | phrogs | 3694 | 99.0 | 1.9e-14 | 2.2e-18 | 126.7 | 147 | (15, 163) | 291 | (10, 201) | 375 | NA | NA; Category: unknown function; p237513 VI\_09544 | | phrogs | 27290 | 99.0 | 7.5e-14 | 8.4e-18 | 121.9 | 172 | (15, 189) | 291 | (25, 214) | 517 | NA | NA; Category: unknown function; p222055 VI\_01195 | | phrogs | 7405 | 98.7 | 3.6e-12 | 4.2e-16 | 105.9 | 111 | (16, 132) | 291 | (21, 134) | 249 | NA | NA; Category: unknown function; p151533 VI\_12464 | | phrogs | 8071 | 98.5 | 2.4e-11 | 2.7e-15 | 105.3 | 122 | (15, 147) | 291 | (210, 336) | 394 | ParB-like partition protein | ParB-like partition protein; Category: DNA, RNA and nucleotide metabolism; KU886268\_p74 | | phrogs | 5475 | 98.4 | 4.4e-11 | 5e-15 | 106.3 | 98 | (20, 125) | 291 | (2, 100) | 519 | NA | NA; Category: unknown function; p62317 VI\_01138 | | phrogs | 32069 | 98.3 | 1.6e-10 | 1.8e-14 | 95.4 | 171 | (17, 188) | 291 | (39, 221) | 348 | ParB-like partition protein | ParB-like partition protein; Category: DNA, RNA and nucleotide metabolism; NC\_019713\_p89 | | phrogs | 18017 | 98.1 | 9.6e-10 | 1.1e-13 | 87.7 | 164 | (16, 189) | 291 | (3, 211) | 228 | NA | NA; Category: unknown function; p240016 VI\_08189 | | phrogs | 8947 | 98.1 | 1.2e-09 | 1.3e-13 | 86.5 | 130 | (18, 156) | 291 | (10, 146) | 216 | NA | NA; Category: unknown function; p43781 VI\_12381 | | phrogs | 6392 | 97.8 | 1.1e-08 | 1.3e-12 | 82.7 | 134 | (16, 158) | 291 | (6, 146) | 265 | ParB-like partition protein | ParB-like partition protein; Category: DNA, RNA and nucleotide metabolism; KX589269\_p1 | | phrogs | 26377 | 97.7 | 1.7e-08 | 1.9e-12 | 82.0 | 99 | (43, 148) | 291 | (29, 128) | 326 | ParB-like partition protein | ParB-like partition protein; Category: DNA, RNA and nucleotide metabolism; NC\_007409\_p70 | | phrogs | 17439 | 97.6 | 3.6e-08 | 4.1e-12 | 78.4 | 135 | (26, 167) | 291 | (22, 160) | 226 | NA | NA; Category: unknown function; p97272 VI\_12440 | | phrogs | 21315 | 97.4 | 8.7e-08 | 9.8e-12 | 82.3 | 88 | (39, 131) | 291 | (27, 117) | 450 | NA | NA; Category: unknown function; p108440 VI\_04298 | | phrogs | 5931 | 96.9 | 1.2e-06 | 1.4e-10 | 75.6 | 75 | (34, 117) | 291 | (46, 123) | 303 | NA | NA; Category: unknown function; NC\_023604\_p41 | | phrogs | 16545 | 96.9 | 1.6e-06 | 1.8e-10 | 63.4 | 134 | (21, 160) | 291 | (2, 136) | 154 | ParB-like partition protein | ParB-like partition protein; Category: DNA, RNA and nucleotide metabolism; KC821630\_p51 | | phrogs | 31451 | 96.8 | 1.9e-06 | 2.1e-10 | 68.8 | 139 | (42, 193) | 291 | (32, 175) | 298 | NA | NA; Category: unknown function; p274462 VI\_03342 | | phrogs | 2859 | 96.8 | 1.8e-06 | 2.2e-10 | 66.7 | 71 | (19, 100) | 291 | (53, 124) | 149 | NA | NA; Category: unknown function; KM209228\_p98 | | phrogs | 12305 | 96.7 | 2.7e-06 | 3.1e-10 | 69.7 | 62 | (54, 127) | 291 | (40, 102) | 265 | ParB-like partition protein | ParB-like partition protein; Category: DNA, RNA and nucleotide metabolism; KY065505\_p11 | | phrogs | 17622 | 96.6 | 5.7e-06 | 6.4e-10 | 63.6 | 85 | (26, 115) | 291 | (15, 104) | 218 | NA | NA; Category: unknown function; p234013 VI\_05492 | | phrogs | 21912 | 96.5 | 6.4e-06 | 7.2e-10 | 61.3 | 56 | (42, 101) | 291 | (52, 119) | 158 | DNA binding protein | DNA binding protein; Category: DNA, RNA and nucleotide metabolism; MH045566\_p52 | | phrogs | 26754 | 96.5 | 6.7e-06 | 7.5e-10 | 62.6 | 77 | (17, 102) | 291 | (67, 144) | 207 | ParB-like partition nuclease | ParB-like partition nuclease; Category: DNA, RNA and nucleotide metabolism; NC\_011421\_p55 | | phrogs | 26392 | 96.4 | 1.3e-05 | 1.4e-09 | 61.8 | 81 | (40, 131) | 291 | (4, 85) | 231 | NA | NA; Category: unknown function; KF005319\_p27 | | phrogs | 27360 | 96.3 | 1.5e-05 | 1.7e-09 | 64.4 | 83 | (47, 131) | 291 | (1, 89) | 280 | NA | NA; Category: unknown function; NC\_006268\_p50 | | phrogs | 26063 | 96.0 | 3.5e-05 | 4e-09 | 62.3 | 136 | (21, 160) | 291 | (6, 161) | 334 | NA | NA; Category: unknown function; p11818 VI\_06587 | | phrogs | 26588 | 95.9 | 5e-05 | 5.6e-09 | 67.4 | 76 | (17, 101) | 291 | (97, 173) | 932 | ParB-like partition nuclease | ParB-like partition nuclease; Category: DNA, RNA and nucleotide metabolism; NC\_025423\_p216 | | phrogs | 35656 | 95.5 | 0.00014 | 1.6e-08 | 61.1 | 140 | (50, 189) | 291 | (69, 238) | 432 | NA | NA; Category: unknown function; KU234533\_p86 | | phrogs | 30771 | 94.9 | 0.00038 | 4.3e-08 | 60.3 | 107 | (19, 126) | 291 | (40, 161) | 549 | NA | NA; Category: unknown function; p308958 VI\_06640 | | phrogs | 17442 | 94.7 | 0.00051 | 5.7e-08 | 50.9 | 97 | (13, 116) | 291 | (69, 165) | 185 | NA | NA; Category: unknown function; p407489 VI\_05339 | |
| Top keywords  (threshold 1.00e-03 (evalue)) | **and, ParB, DNA, ParB\_like, partition, RNA, nucleotide, metabolism, N\_terminal, plasmid** |
| Output files | ../../domain\_architecture/28\_FANPEZAQ\_CDS\_0028\_cath.hhr ../../domain\_architecture/28\_FANPEZAQ\_CDS\_0028\_merged.svg ../../domain\_architecture/28\_FANPEZAQ\_CDS\_0028\_ncbi-cd.hhr ../../domain\_architecture/28\_FANPEZAQ\_CDS\_0028\_pfam.hhr ../../domain\_architecture/28\_FANPEZAQ\_CDS\_0028\_phrogs.hhr |

### Identical protein sequences/structures

#### Search results

|  |  |
| --- | --- |
| Protein sequence databases searched | Pdb, Swissprot, Refseq |
| Identical proteins found | -- |
| Top keywords | -- |
| Output files | -- |

### Similar protein sequences/structures

#### Sequence similarity search results (HHblits)1

|  |  |
| --- | --- |
| Sequence databases searched | Uniclust, Pdb70 |
| Results, scheme(s)  (Top layers only, threshold 1.00e-03 (evalue)) | xml version="1.0" encoding="utf-8" standalone="no"?       2024-09-02T21:08:41.732191 image/svg+xml   Matplotlib v3.7.2, https://matplotlib.org/ |
| Results, table(s)  (threshold 1.00e-03 (evalue)) | | db | id | prob | evalue | pvalue | score | cols | query | query\_len | template | template\_len | name | description | | --- | --- | --- | --- | --- | --- | --- | --- | --- | --- | --- | --- | --- | | uniclust | UniRef100\_A0A071M4B4 | 100.0 | 4.4e-41 | 8.9e-47 | 295.9 | 270 | (1, 288) | 291 | (40, 315) | 388 | Chromosome partitioning protein ParB | Chromosome partitioning protein ParB | | uniclust | UniRef100\_A0A076G7J3 | 100.0 | 7.3e-40 | 1.4e-45 | 282.0 | 260 | (1, 290) | 291 | (19, 282) | 452 | ParB/Sulfiredoxin domain-containing protein | ParB/Sulfiredoxin domain-containing protein | | uniclust | UniRef100\_A0A0F3IEX4 | 100.0 | 8.8e-38 | 1.7e-43 | 249.2 | 170 | (17, 192) | 291 | (33, 205) | 231 | Probable chromosome-partitioning protein ParB (Fragment) | Probable chromosome-partitioning protein ParB (Fragment) | | uniclust | UniRef100\_A0A0J1DM72 | 100.0 | 4.8e-37 | 9.2e-43 | 261.2 | 179 | (16, 200) | 291 | (86, 268) | 389 | HTH cro/C1-type domain-containing protein | HTH cro/C1-type domain-containing protein | | uniclust | UniRef100\_A0A0Q8XC42 | 100.0 | 5.2e-37 | 9.8e-43 | 244.9 | 266 | (1, 285) | 291 | (13, 281) | 282 | ParB/Sulfiredoxin domain-containing protein | ParB/Sulfiredoxin domain-containing protein | | uniclust | UniRef100\_A0A073JT65 | 100.0 | 1e-36 | 2.1e-42 | 264.7 | 177 | (19, 201) | 291 | (65, 245) | 352 | Nucleoid occlusion protein | Nucleoid occlusion protein | | uniclust | UniRef100\_A0A011VWW9 | 100.0 | 1.3e-36 | 2.7e-42 | 263.8 | 177 | (18, 200) | 291 | (77, 258) | 349 | Stage 0 sporulation protein J | Stage 0 sporulation protein J | | uniclust | UniRef100\_A0A0Q4N015 | 100.0 | 1.4e-36 | 2.8e-42 | 264.4 | 266 | (1, 290) | 291 | (19, 287) | 512 | Chromosome partitioning protein ParB | Chromosome partitioning protein ParB | | uniclust | UniRef100\_A0A0G1XWI0 | 100.0 | 1.6e-36 | 3.2e-42 | 256.5 | 178 | (18, 201) | 291 | (43, 224) | 311 | ParB-like protein partition protein | ParB-like protein partition protein | | uniclust | UniRef100\_A0A0G3EIV5 | 100.0 | 1.6e-36 | 3.3e-42 | 262.9 | 176 | (18, 199) | 291 | (75, 254) | 355 | Putative chromosome-partitioning protein ParB | Putative chromosome-partitioning protein ParB | | uniclust | UniRef100\_A0A0G1TMM8 | 100.0 | 1.9e-36 | 3.7e-42 | 245.8 | 170 | (15, 190) | 291 | (29, 201) | 248 | ParB-like protein partition protein | ParB-like protein partition protein | | uniclust | UniRef100\_A0A097AUS5 | 100.0 | 2.7e-36 | 5.3e-42 | 251.7 | 178 | (17, 200) | 291 | (51, 233) | 313 | Nucleoid occlusion protein Noc | Nucleoid occlusion protein Noc | | uniclust | UniRef100\_A0A088SZT7 | 100.0 | 3.6e-36 | 7.3e-42 | 256.6 | 178 | (18, 201) | 291 | (42, 223) | 313 | Chromosome-partitioning protein Spo0J | Chromosome-partitioning protein Spo0J | | uniclust | UniRef100\_A0A0C2D3E5 | 100.0 | 3.9e-36 | 7.8e-42 | 265.3 | 176 | (18, 199) | 291 | (127, 306) | 405 | Chromosome (Plasmid) partitioning protein ParB | Chromosome (Plasmid) partitioning protein ParB | | uniclust | UniRef100\_A0A010PSR0 | 100.0 | 4.2e-36 | 8.7e-42 | 268.1 | 177 | (18, 200) | 291 | (96, 276) | 408 | Chromosome partitioning protein ParB | Chromosome partitioning protein ParB | | uniclust | UniRef100\_A0A257WQE5 | 100.0 | 4.8e-36 | 9.4e-42 | 239.7 | 169 | (17, 191) | 291 | (45, 217) | 229 | Chromosome partitioning protein ParB (Fragment) | Chromosome partitioning protein ParB (Fragment) | | uniclust | UniRef100\_A0A0D0QNA4 | 100.0 | 7.3e-36 | 1.4e-41 | 252.3 | 178 | (18, 201) | 291 | (73, 254) | 339 | Chromosome (Plasmid) partitioning protein ParB / Stage 0 sporulation protein J | Chromosome (Plasmid) partitioning protein ParB / Stage 0 sporulation protein J | | uniclust | UniRef100\_A0A0G0M240 | 100.0 | 8.9e-36 | 1.8e-41 | 252.3 | 178 | (18, 201) | 291 | (51, 233) | 318 | Chromosome segregation DNA-binding protein | Chromosome segregation DNA-binding protein | | uniclust | UniRef100\_A0A094S1U1 | 100.0 | 1.4e-35 | 2.7e-41 | 238.1 | 262 | (1, 288) | 291 | (20, 282) | 306 | DNA-binding protein | DNA-binding protein | | uniclust | UniRef100\_A0A011VV16 | 100.0 | 2e-35 | 4.1e-41 | 260.0 | 178 | (18, 201) | 291 | (92, 274) | 401 | Chromosome partitioning protein ParB | Chromosome partitioning protein ParB | | uniclust | UniRef100\_A0A010RRU4 | 100.0 | 2.3e-35 | 4.7e-41 | 267.7 | 177 | (18, 200) | 291 | (186, 366) | 515 | Probable chromosome-partitioning protein ParB | Probable chromosome-partitioning protein ParB | | uniclust | UniRef100\_A0A0D5LM02 | 100.0 | 2.6e-35 | 4.9e-41 | 245.5 | 174 | (19, 200) | 291 | (60, 239) | 325 | Chromosome partitioning protein ParB | Chromosome partitioning protein ParB | | uniclust | UniRef100\_A0A0A8B5V6 | 100.0 | 2.9e-35 | 5.7e-41 | 259.2 | 166 | (19, 190) | 291 | (181, 350) | 463 | Chromosome partitioning protein ParB | Chromosome partitioning protein ParB | | uniclust | UniRef100\_A0A009GHB1 | 100.0 | 3.3e-35 | 6.5e-41 | 249.2 | 179 | (18, 202) | 291 | (67, 249) | 342 | Probable chromosome-partitioning protein ParB | Probable chromosome-partitioning protein ParB | | uniclust | UniRef100\_A0A1I1P9F0 | 100.0 | 3.5e-35 | 7e-41 | 246.6 | 215 | (1, 216) | 291 | (18, 233) | 299 | ParB/RepB/Spo0J family partition protein | ParB/RepB/Spo0J family partition protein | | uniclust | UniRef100\_A0A1G9Q6I4 | 100.0 | 3.7e-35 | 7.2e-41 | 244.2 | 181 | (14, 199) | 291 | (24, 209) | 316 | Chromosome segregation DNA-binding protein | Chromosome segregation DNA-binding protein | | uniclust | UniRef100\_A0A014P5P6 | 100.0 | 3.7e-35 | 7.6e-41 | 265.9 | 176 | (19, 200) | 291 | (153, 333) | 490 | Chromosome partitioning protein ParB | Chromosome partitioning protein ParB | | uniclust | UniRef100\_A0A1Q6R1A4 | 100.0 | 4.3e-35 | 8.3e-41 | 241.5 | 178 | (18, 201) | 291 | (34, 215) | 316 | HTH cro/C1-type domain-containing protein | HTH cro/C1-type domain-containing protein | | uniclust | UniRef100\_A0A1G3VWE4 | 100.0 | 4.4e-35 | 8.4e-41 | 241.0 | 172 | (16, 193) | 291 | (45, 219) | 306 | ParB/Sulfiredoxin domain-containing protein | ParB/Sulfiredoxin domain-containing protein | | uniclust | UniRef100\_A0A011SW19 | 100.0 | 5.6e-35 | 1.1e-40 | 257.5 | 173 | (19, 199) | 291 | (84, 262) | 416 | Chromosome partitioning protein ParB | Chromosome partitioning protein ParB | | uniclust | UniRef100\_A0A1B1AIB8 | 100.0 | 1.2e-34 | 2.2e-40 | 249.5 | 174 | (19, 200) | 291 | (92, 271) | 432 | ParB/Sulfiredoxin domain-containing protein | ParB/Sulfiredoxin domain-containing protein | | uniclust | UniRef100\_A0A081DF57 | 100.0 | 1.9e-34 | 3.7e-40 | 239.8 | 176 | (19, 200) | 291 | (57, 237) | 313 | Chromosome (Plasmid) partitioning protein ParB | Chromosome (Plasmid) partitioning protein ParB | | uniclust | UniRef100\_A0A1F6CPK3 | 100.0 | 3.4e-34 | 6.7e-40 | 243.5 | 181 | (13, 199) | 291 | (16, 203) | 323 | ParB/Sulfiredoxin domain-containing protein | ParB/Sulfiredoxin domain-containing protein | | uniclust | UniRef100\_A0A094QAV1 | 100.0 | 4e-34 | 7.9e-40 | 248.1 | 177 | (19, 200) | 291 | (61, 241) | 402 | Chromosome partitioning protein ParB | Chromosome partitioning protein ParB | | uniclust | UniRef100\_A0A0G0YQH3 | 100.0 | 4.1e-34 | 8.3e-40 | 250.7 | 180 | (16, 200) | 291 | (122, 306) | 390 | ParB-like protein partition protein | ParB-like protein partition protein | | uniclust | UniRef100\_A0A3B8LAV2 | 100.0 | 7.7e-34 | 1.5e-39 | 241.6 | 176 | (18, 199) | 291 | (55, 234) | 332 | Chromosome partitioning protein ParB | Chromosome partitioning protein ParB | | uniclust | UniRef100\_A0A2D9PNL7 | 100.0 | 8.3e-34 | 1.6e-39 | 236.7 | 181 | (15, 201) | 291 | (44, 227) | 326 | Chromosome partitioning protein ParB | Chromosome partitioning protein ParB | | uniclust | UniRef100\_A0A117LBP9 | 100.0 | 1.2e-33 | 2.3e-39 | 231.6 | 178 | (18, 201) | 291 | (35, 216) | 299 | Chromosome-partitioning protein ParB | Chromosome-partitioning protein ParB | | uniclust | UniRef100\_A0A081B6M9 | 100.0 | 1.3e-33 | 2.5e-39 | 238.0 | 166 | (17, 188) | 291 | (65, 236) | 346 | ParB-like partition protein | ParB-like partition protein | | uniclust | UniRef100\_A0A142X869 | 100.0 | 1.3e-33 | 2.6e-39 | 243.6 | 169 | (18, 191) | 291 | (50, 224) | 331 | Chromosome-partitioning protein Spo0J | Chromosome-partitioning protein Spo0J | | uniclust | UniRef100\_A0A1C3K881 | 100.0 | 1.4e-33 | 2.7e-39 | 234.3 | 212 | (1, 215) | 291 | (7, 223) | 316 | Predicted transcriptional regulators | Predicted transcriptional regulators | | uniclust | UniRef100\_A0A1Q6QWS0 | 100.0 | 1.5e-33 | 2.9e-39 | 248.5 | 178 | (18, 201) | 291 | (20, 201) | 434 | HTH cro/C1-type domain-containing protein | HTH cro/C1-type domain-containing protein | | uniclust | UniRef100\_A0A1W9QJJ8 | 100.0 | 1.5e-33 | 3e-39 | 234.3 | 170 | (16, 191) | 291 | (56, 229) | 311 | ParB/Sulfiredoxin domain-containing protein (Fragment) | ParB/Sulfiredoxin domain-containing protein (Fragment) | | uniclust | UniRef100\_A0A1F5N9U7 | 100.0 | 1.6e-33 | 3.2e-39 | 238.8 | 181 | (15, 201) | 291 | (61, 246) | 333 | ParB/Sulfiredoxin domain-containing protein | ParB/Sulfiredoxin domain-containing protein | | uniclust | UniRef100\_A0A0F2IXB0 | 100.0 | 1.9e-33 | 3.7e-39 | 229.3 | 180 | (16, 201) | 291 | (19, 203) | 283 | Stage 0 sporulation protein J | Stage 0 sporulation protein J | | uniclust | UniRef100\_A0A101FLX1 | 100.0 | 1.9e-33 | 3.8e-39 | 235.7 | 183 | (13, 201) | 291 | (31, 216) | 319 | ParB-like partition protein | ParB-like partition protein | | uniclust | UniRef100\_A0A0F9YZP5 | 100.0 | 2.1e-33 | 4e-39 | 238.9 | 166 | (18, 189) | 291 | (33, 201) | 352 | ParB-like protein partition protein | ParB-like protein partition protein | | uniclust | UniRef100\_A0A074JJ97 | 100.0 | 2.7e-33 | 5.2e-39 | 242.8 | 164 | (18, 187) | 291 | (43, 212) | 480 | DNA-binding protein | DNA-binding protein | | uniclust | UniRef100\_A0A1F2X3R4 | 100.0 | 2.9e-33 | 5.7e-39 | 237.7 | 180 | (14, 199) | 291 | (29, 211) | 319 | ParB/Sulfiredoxin domain-containing protein | ParB/Sulfiredoxin domain-containing protein | | uniclust | UniRef100\_A0A024EM96 | 100.0 | 3.1e-33 | 5.9e-39 | 240.3 | 256 | (14, 287) | 291 | (78, 357) | 397 | ParB/Sulfiredoxin domain-containing protein | ParB/Sulfiredoxin domain-containing protein | | uniclust | UniRef100\_A0A0G0GAF4 | 100.0 | 7.5e-33 | 1.5e-38 | 238.3 | 178 | (18, 200) | 291 | (103, 292) | 387 | ParB-like protein partition protein | ParB-like protein partition protein | | uniclust | UniRef100\_A0A077ZKX5 | 100.0 | 9.6e-33 | 1.9e-38 | 247.8 | 179 | (16, 200) | 291 | (279, 463) | 547 | CbiA and ParBc and KorB domain containing protein | CbiA and ParBc and KorB domain containing protein | | uniclust | UniRef100\_A0A062WZP7 | 100.0 | 1.4e-32 | 2.6e-38 | 240.0 | 178 | (18, 200) | 291 | (136, 317) | 472 | Chromosome segregation DNA-binding protein | Chromosome segregation DNA-binding protein | | uniclust | UniRef100\_A0A0X7A5Y3 | 100.0 | 2.2e-32 | 4.3e-38 | 227.7 | 249 | (14, 283) | 291 | (30, 286) | 305 | ParB/Sulfiredoxin domain-containing protein | ParB/Sulfiredoxin domain-containing protein | | uniclust | UniRef100\_A0A0F8XGC3 | 99.9 | 2.5e-32 | 4.9e-38 | 238.1 | 176 | (13, 193) | 291 | (65, 242) | 379 | ParB/Sulfiredoxin domain-containing protein | ParB/Sulfiredoxin domain-containing protein | | uniclust | UniRef100\_A0A212AQY6 | 99.9 | 2.7e-32 | 5.2e-38 | 215.6 | 171 | (16, 192) | 291 | (46, 221) | 231 | ParB/Sulfiredoxin domain-containing protein | ParB/Sulfiredoxin domain-containing protein | | uniclust | UniRef100\_A0A0F9ICC8 | 99.9 | 2.7e-32 | 5.5e-38 | 232.8 | 179 | (16, 200) | 291 | (24, 209) | 306 | ParB/Sulfiredoxin domain-containing protein (Fragment) | ParB/Sulfiredoxin domain-containing protein (Fragment) | | uniclust | UniRef100\_A0A024BX61 | 99.9 | 2.9e-32 | 5.6e-38 | 242.7 | 178 | (18, 200) | 291 | (77, 267) | 498 | Chromosome partitioning protein ParB | Chromosome partitioning protein ParB | | uniclust | UniRef100\_A0A1V4VEC1 | 99.9 | 3.6e-32 | 7e-38 | 229.3 | 180 | (16, 200) | 291 | (65, 251) | 328 | Chromosome-partitioning protein Spo0J | Chromosome-partitioning protein Spo0J | | uniclust | UniRef100\_A0A0M7DV75 | 99.9 | 4.7e-32 | 8.9e-38 | 228.5 | 269 | (1, 286) | 291 | (8, 300) | 396 | ParB/RepB/Spo0J family partition protein | ParB/RepB/Spo0J family partition protein | | uniclust | UniRef100\_A0A257V0R5 | 99.9 | 7.2e-32 | 1.4e-37 | 213.8 | 175 | (16, 195) | 291 | (36, 214) | 237 | ParB/Sulfiredoxin domain-containing protein | ParB/Sulfiredoxin domain-containing protein | | uniclust | UniRef100\_A0A066UG41 | 99.9 | 9.6e-32 | 1.9e-37 | 232.3 | 194 | (17, 216) | 291 | (123, 329) | 403 | Probable chromosome-partitioning protein ParB | Probable chromosome-partitioning protein ParB | | uniclust | UniRef100\_A0A1C5QNA2 | 99.9 | 1.2e-31 | 2.4e-37 | 222.9 | 178 | (17, 201) | 291 | (6, 188) | 302 | Nucleoid occlusion protein | Nucleoid occlusion protein | | uniclust | UniRef100\_A0A011TZQ8 | 99.9 | 1.8e-31 | 3.5e-37 | 239.8 | 173 | (17, 194) | 291 | (212, 388) | 538 | Chromosome partitioning protein ParB | Chromosome partitioning protein ParB | | uniclust | UniRef100\_A0A929SNF7 | 99.9 | 1.8e-31 | 3.6e-37 | 243.5 | 178 | (18, 201) | 291 | (5, 186) | 584 | ParB/RepB/Spo0J family partition protein | ParB/RepB/Spo0J family partition protein | | uniclust | UniRef100\_A0A1A9KGS4 | 99.9 | 2e-31 | 3.7e-37 | 217.7 | 273 | (2, 290) | 291 | (71, 354) | 357 | ParB/Sulfiredoxin domain-containing protein | ParB/Sulfiredoxin domain-containing protein | | uniclust | UniRef100\_A0A2H0T7Y9 | 99.9 | 2.3e-31 | 4.4e-37 | 206.5 | 160 | (5, 170) | 291 | (15, 177) | 178 | Chromosome partitioning protein ParB (Fragment) | Chromosome partitioning protein ParB (Fragment) | | uniclust | UniRef100\_A0A068VSG1 | 99.9 | 2.6e-31 | 4.9e-37 | 234.7 | 179 | (17, 200) | 291 | (243, 425) | 533 | Chromosome partitioning protein | Chromosome partitioning protein | | uniclust | UniRef100\_A0A3A0CR42 | 99.9 | 2.5e-31 | 5e-37 | 225.4 | 170 | (15, 190) | 291 | (47, 220) | 333 | HTH cro/C1-type domain-containing protein | HTH cro/C1-type domain-containing protein | | uniclust | UniRef100\_A0A060DLG4 | 99.9 | 2.7e-31 | 5.5e-37 | 233.8 | 175 | (16, 198) | 291 | (76, 258) | 389 | ParB/Sulfiredoxin domain-containing protein | ParB/Sulfiredoxin domain-containing protein | | uniclust | UniRef100\_A0A022KT50 | 99.9 | 3.1e-31 | 6.2e-37 | 242.2 | 176 | (15, 196) | 291 | (302, 484) | 593 | Chromosome partitioning protein ParB | Chromosome partitioning protein ParB | | uniclust | UniRef100\_A0A2J0LBM3 | 99.9 | 9e-31 | 1.7e-36 | 212.2 | 166 | (17, 188) | 291 | (57, 225) | 269 | Chromosome partitioning protein ParB (Fragment) | Chromosome partitioning protein ParB (Fragment) | | uniclust | UniRef100\_A0A096HPB0 | 99.9 | 1.3e-30 | 2.6e-36 | 222.2 | 272 | (3, 286) | 291 | (17, 321) | 353 | Chromosome partitioning protein ParB | Chromosome partitioning protein ParB | | uniclust | UniRef100\_A0A022PNW0 | 99.9 | 1.6e-30 | 3e-36 | 227.1 | 269 | (13, 288) | 291 | (90, 371) | 486 | ParB/Sulfiredoxin domain-containing protein | ParB/Sulfiredoxin domain-containing protein | | uniclust | UniRef100\_A0A075GB43 | 99.9 | 2e-30 | 3.9e-36 | 222.7 | 178 | (17, 200) | 291 | (48, 229) | 359 | Transcriptional regulator (ParB, spo0J) | Transcriptional regulator (ParB, spo0J) | | uniclust | UniRef100\_A0A0F9U0H1 | 99.9 | 2.3e-30 | 4.7e-36 | 227.9 | 203 | (14, 216) | 291 | (72, 285) | 392 | ParB/Sulfiredoxin domain-containing protein | ParB/Sulfiredoxin domain-containing protein | | uniclust | UniRef100\_A0A930FAM5 | 99.9 | 2.8e-30 | 5.4e-36 | 194.7 | 160 | (18, 183) | 291 | (4, 166) | 167 | ParB/RepB/Spo0J family partition protein (Fragment) | ParB/RepB/Spo0J family partition protein (Fragment) | | uniclust | UniRef100\_A0A233HUD9 | 99.9 | 3.8e-30 | 7.4e-36 | 219.6 | 264 | (13, 287) | 291 | (50, 321) | 373 | Repressor KorB domain-containing protein | Repressor KorB domain-containing protein | | uniclust | UniRef100\_A0A382LW29 | 99.9 | 4e-30 | 7.5e-36 | 194.4 | 152 | (15, 172) | 291 | (19, 174) | 175 | ParB/Sulfiredoxin domain-containing protein (Fragment) | ParB/Sulfiredoxin domain-containing protein (Fragment) | | uniclust | UniRef100\_A0A7X9GCQ7 | 99.9 | 5.2e-30 | 9.9e-36 | 213.2 | 178 | (18, 201) | 291 | (8, 189) | 343 | ParB/RepB/Spo0J family partition protein | ParB/RepB/Spo0J family partition protein | | uniclust | UniRef100\_A0A4V1URX4 | 99.9 | 6.3e-30 | 1.2e-35 | 214.1 | 173 | (20, 197) | 291 | (49, 227) | 318 | ParB/RepB/Spo0J family partition protein | ParB/RepB/Spo0J family partition protein | | uniclust | UniRef100\_A0A021VUN1 | 99.9 | 7e-30 | 1.4e-35 | 213.3 | 167 | (15, 187) | 291 | (25, 192) | 297 | ParB/Sulfiredoxin domain-containing protein | ParB/Sulfiredoxin domain-containing protein | | uniclust | UniRef100\_A0A060DS69 | 99.9 | 7.4e-30 | 1.5e-35 | 222.6 | 173 | (18, 197) | 291 | (89, 267) | 361 | Chromosome partitioning protein | Chromosome partitioning protein | | uniclust | UniRef100\_A0A3L7VX77 | 99.9 | 7.8e-30 | 1.5e-35 | 200.8 | 165 | (21, 191) | 291 | (44, 213) | 216 | ParB/RepB/Spo0J family partition protein (Fragment) | ParB/RepB/Spo0J family partition protein (Fragment) | | uniclust | UniRef100\_A0A0L6JYD4 | 99.9 | 9.1e-30 | 1.8e-35 | 211.5 | 193 | (14, 214) | 291 | (31, 229) | 302 | ParB/Sulfiredoxin domain-containing protein | ParB/Sulfiredoxin domain-containing protein | | uniclust | UniRef100\_A0A0T5ZL74 | 99.9 | 9.4e-30 | 1.8e-35 | 215.1 | 173 | (16, 194) | 291 | (67, 243) | 379 | Chromosome partitioning protein ParB, chromosome partitioning protein, ParB family | Chromosome partitioning protein ParB, chromosome partitioning protein, ParB family | | uniclust | UniRef100\_A0A1F4I2D7 | 99.9 | 9.9e-30 | 1.9e-35 | 216.0 | 165 | (18, 187) | 291 | (48, 223) | 344 | ParB/Sulfiredoxin domain-containing protein | ParB/Sulfiredoxin domain-containing protein | | uniclust | UniRef100\_A0A2G2FUH5 | 99.9 | 1.2e-29 | 2.3e-35 | 214.2 | 171 | (16, 192) | 291 | (36, 211) | 342 | Chromosome partitioning protein ParB | Chromosome partitioning protein ParB | | uniclust | UniRef100\_A0A0F8XDG1 | 99.9 | 1.5e-29 | 2.9e-35 | 213.4 | 173 | (15, 193) | 291 | (10, 185) | 287 | ParB/Spo0J HTH domain-containing protein (Fragment) | ParB/Spo0J HTH domain-containing protein (Fragment) | | uniclust | UniRef100\_A0A139NDU2 | 99.9 | 1.6e-29 | 3e-35 | 194.0 | 178 | (18, 201) | 291 | (9, 189) | 209 | Chromosome (Plasmid) partitioning protein ParB / Stage 0 sporulation protein J | Chromosome (Plasmid) partitioning protein ParB / Stage 0 sporulation protein J | | uniclust | UniRef100\_A0A432HCS4 | 99.9 | 1.8e-29 | 3.3e-35 | 206.8 | 168 | (16, 188) | 291 | (59, 229) | 325 | Chromosome partitioning protein ParB | Chromosome partitioning protein ParB | | uniclust | UniRef100\_A0A090I4P8 | 99.9 | 1.7e-29 | 3.4e-35 | 219.0 | 180 | (17, 202) | 291 | (30, 214) | 392 | ParB/Sulfiredoxin domain-containing protein | ParB/Sulfiredoxin domain-containing protein | | uniclust | UniRef100\_A0A088C411 | 99.9 | 1.9e-29 | 3.6e-35 | 201.1 | 196 | (13, 214) | 291 | (20, 216) | 262 | ParB-like protein | ParB-like protein | | uniclust | UniRef100\_A0A0C1V2T0 | 99.9 | 2e-29 | 3.8e-35 | 221.3 | 180 | (1, 189) | 291 | (2, 184) | 540 | ParB/RepB/Spo0J family partition protein | ParB/RepB/Spo0J family partition protein | | uniclust | UniRef100\_A0A086DUB0 | 99.9 | 2.1e-29 | 4.1e-35 | 208.4 | 172 | (16, 192) | 291 | (111, 288) | 295 | Chromosome partitioning protein ParB (Fragment) | Chromosome partitioning protein ParB (Fragment) | | uniclust | UniRef100\_A0A3G8F6G3 | 99.9 | 2.1e-29 | 4.2e-35 | 219.0 | 173 | (17, 195) | 291 | (34, 210) | 361 | Chromosome (Plasmid) partitioning protein | Chromosome (Plasmid) partitioning protein | | uniclust | UniRef100\_A0A086D7K6 | 99.9 | 2.6e-29 | 5.1e-35 | 211.1 | 180 | (17, 202) | 291 | (49, 232) | 332 | Probable chromosome-partitioning protein ParB (Fragment) | Probable chromosome-partitioning protein ParB (Fragment) | | uniclust | UniRef100\_D1B9D9 | 99.9 | 3e-29 | 5.6e-35 | 209.0 | 178 | (17, 200) | 291 | (96, 276) | 368 | ParB-like partition protein | ParB-like partition protein | | uniclust | UniRef100\_A0A062Y0Q3 | 99.9 | 2.8e-29 | 5.6e-35 | 216.8 | 167 | (17, 191) | 291 | (65, 236) | 354 | ParB/Sulfiredoxin domain-containing protein | ParB/Sulfiredoxin domain-containing protein | | uniclust | UniRef100\_A0A1F9PB14 | 99.9 | 3.2e-29 | 6.1e-35 | 197.7 | 170 | (14, 188) | 291 | (53, 225) | 258 | Chromosome partitioning protein ParB (Fragment) | Chromosome partitioning protein ParB (Fragment) | | uniclust | UniRef100\_A0A2G4ISH6 | 99.9 | 3.6e-29 | 6.9e-35 | 213.9 | 165 | (18, 188) | 291 | (40, 210) | 432 | Chromosome partitioning protein ParB | Chromosome partitioning protein ParB | | uniclust | UniRef100\_A0A1P8WKH2 | 99.9 | 3.7e-29 | 7.2e-35 | 209.3 | 171 | (17, 191) | 291 | (35, 208) | 322 | Putative chromosome-partitioning protein ParB | Putative chromosome-partitioning protein ParB | | uniclust | UniRef100\_A0A0F9CE03 | 99.9 | 4e-29 | 7.9e-35 | 208.7 | 173 | (15, 193) | 291 | (11, 186) | 275 | ParB/Sulfiredoxin domain-containing protein | ParB/Sulfiredoxin domain-containing protein | | uniclust | UniRef100\_A0A0F9EZV6 | 99.9 | 4.6e-29 | 9e-35 | 223.9 | 177 | (15, 197) | 291 | (10, 192) | 536 | ParB/Sulfiredoxin domain-containing protein | ParB/Sulfiredoxin domain-containing protein | | uniclust | UniRef100\_A0A095WUL4 | 99.9 | 5.5e-29 | 1.1e-34 | 222.1 | 175 | (16, 195) | 291 | (194, 387) | 560 | Chromosome partitioning protein ParB | Chromosome partitioning protein ParB | | uniclust | UniRef100\_A0A0F2RAT7 | 99.9 | 5.5e-29 | 1.1e-34 | 212.0 | 174 | (18, 197) | 291 | (64, 241) | 332 | ParB/Sulfiredoxin domain-containing protein | ParB/Sulfiredoxin domain-containing protein | | uniclust | UniRef100\_A0A0G1ET61 | 99.9 | 6.4e-29 | 1.3e-34 | 219.6 | 180 | (15, 199) | 291 | (38, 232) | 479 | ParB-like protein partition protein | ParB-like protein partition protein | | uniclust | UniRef100\_A0A2A5EJ42 | 99.9 | 7.7e-29 | 1.5e-34 | 191.7 | 132 | (19, 156) | 291 | (38, 175) | 175 | Chromosome partitioning protein ParB (Fragment) | Chromosome partitioning protein ParB (Fragment) | | uniclust | UniRef100\_A0A7R8WWZ7 | 99.9 | 8.6e-29 | 1.6e-34 | 191.0 | 166 | (23, 194) | 291 | (6, 175) | 209 | ParB/Sulfiredoxin domain-containing protein (Fragment) | ParB/Sulfiredoxin domain-containing protein (Fragment) | | uniclust | UniRef100\_A0A2U3KH09 | 99.9 | 1e-28 | 1.9e-34 | 211.5 | 169 | (15, 189) | 291 | (147, 320) | 421 | Chromosome segregation DNA-binding protein | Chromosome segregation DNA-binding protein | | uniclust | UniRef100\_UPI0004B65802 | 99.9 | 1.2e-28 | 2.3e-34 | 197.3 | 269 | (2, 289) | 291 | (4, 286) | 289 | ParB N-terminal domain-containing protein | ParB N-terminal domain-containing protein | | uniclust | UniRef100\_A0A078MLS8 | 99.9 | 1.3e-28 | 2.4e-34 | 209.0 | 176 | (17, 198) | 291 | (123, 305) | 405 | Putative chromosome-partitioning protein ParB | Putative chromosome-partitioning protein ParB | | uniclust | UniRef100\_A0A085FMH6 | 99.9 | 1.3e-28 | 2.6e-34 | 211.3 | 203 | (14, 216) | 291 | (66, 280) | 357 | Uncharacterized protein | Uncharacterized protein | | uniclust | UniRef100\_A0A352SVJ6 | 99.9 | 1.7e-28 | 3.1e-34 | 185.7 | 162 | (13, 180) | 291 | (18, 183) | 183 | Nucleoid occlusion protein (Fragment) | Nucleoid occlusion protein (Fragment) | | uniclust | UniRef100\_A0A3M0WWG3 | 99.9 | 1.9e-28 | 3.6e-34 | 188.3 | 133 | (16, 154) | 291 | (28, 164) | 165 | ParB/RepB/Spo0J family partition protein (Fragment) | ParB/RepB/Spo0J family partition protein (Fragment) | | uniclust | UniRef100\_A0A5C6FSQ3 | 99.9 | 1.9e-28 | 3.7e-34 | 200.2 | 180 | (16, 201) | 291 | (45, 231) | 308 | Nucleoid occlusion protein | Nucleoid occlusion protein | | uniclust | UniRef100\_A0A517R8W8 | 99.9 | 2.1e-28 | 4.1e-34 | 202.9 | 178 | (18, 199) | 291 | (48, 231) | 308 | Putative chromosome-partitioning protein ParB | Putative chromosome-partitioning protein ParB | | uniclust | UniRef100\_A0A1V4R7Y3 | 99.9 | 2.4e-28 | 4.6e-34 | 204.8 | 164 | (22, 191) | 291 | (41, 210) | 298 | ParB/Sulfiredoxin domain-containing protein | ParB/Sulfiredoxin domain-containing protein | | uniclust | UniRef100\_A0A524ABT4 | 99.9 | 2.4e-28 | 4.6e-34 | 201.3 | 167 | (18, 190) | 291 | (2, 171) | 271 | ParB/RepB/Spo0J family partition protein | ParB/RepB/Spo0J family partition protein | | uniclust | UniRef100\_A0A2N8GU19 | 99.9 | 3.5e-28 | 6.5e-34 | 187.1 | 177 | (16, 198) | 291 | (39, 220) | 223 | Nucleoid occlusion protein (Fragment) | Nucleoid occlusion protein (Fragment) | | uniclust | UniRef100\_A0A4P7A9C7 | 99.9 | 4.2e-28 | 7.9e-34 | 200.2 | 212 | (3, 217) | 291 | (6, 225) | 319 | ParB family chromosome partitioning protein | ParB family chromosome partitioning protein | | uniclust | UniRef100\_A0A059W6T3 | 99.9 | 4.2e-28 | 8e-34 | 207.6 | 175 | (19, 198) | 291 | (33, 212) | 415 | Chromosome partitioning protein ParB | Chromosome partitioning protein ParB | | uniclust | UniRef100\_A0A0R2P7U3 | 99.9 | 4.6e-28 | 8.7e-34 | 198.2 | 179 | (16, 199) | 291 | (52, 233) | 313 | Probable chromosome-partitioning protein ParB | Probable chromosome-partitioning protein ParB | | uniclust | UniRef100\_A0A2D4RST1 | 99.9 | 5e-28 | 9.4e-34 | 194.5 | 172 | (16, 193) | 291 | (11, 189) | 291 | Chromosome partitioning protein ParB | Chromosome partitioning protein ParB | | uniclust | UniRef100\_A0A0F9CXE9 | 99.9 | 4.7e-28 | 9.4e-34 | 207.9 | 170 | (17, 192) | 291 | (29, 201) | 317 | ParB/Sulfiredoxin domain-containing protein | ParB/Sulfiredoxin domain-containing protein | | uniclust | UniRef100\_A0A0M9UL29 | 99.9 | 5.7e-28 | 1.1e-33 | 218.2 | 170 | (15, 190) | 291 | (401, 578) | 679 | Chromosome (Plasmid) partitioning protein ParB | Chromosome (Plasmid) partitioning protein ParB | | uniclust | UniRef100\_UPI0004B4361E | 99.9 | 5.9e-28 | 1.1e-33 | 200.5 | 185 | (18, 211) | 291 | (84, 274) | 338 | ParB/RepB/Spo0J family partition protein | ParB/RepB/Spo0J family partition protein | | uniclust | UniRef100\_A0A402D6S3 | 99.9 | 6e-28 | 1.1e-33 | 208.1 | 169 | (15, 189) | 291 | (72, 248) | 474 | Chromosome partitioning protein ParB | Chromosome partitioning protein ParB | | uniclust | UniRef100\_A0A497B792 | 99.9 | 7.9e-28 | 1.5e-33 | 199.3 | 170 | (16, 190) | 291 | (32, 203) | 314 | ParB/Sulfiredoxin domain-containing protein | ParB/Sulfiredoxin domain-containing protein | | uniclust | UniRef100\_A0A1F8TYH4 | 99.9 | 1e-27 | 2e-33 | 198.8 | 189 | (3, 199) | 291 | (8, 204) | 279 | Chromosome partitioning protein ParB | Chromosome partitioning protein ParB | | uniclust | UniRef100\_A0A0J5KM24 | 99.9 | 1.2e-27 | 2.2e-33 | 196.3 | 256 | (2, 286) | 291 | (4, 265) | 367 | Chromosome partitioning protein ParB | Chromosome partitioning protein ParB | | uniclust | UniRef100\_A0A2E6VPY2 | 99.9 | 1.8e-27 | 3.3e-33 | 195.9 | 168 | (17, 189) | 291 | (39, 211) | 370 | Chromosome partitioning protein ParB | Chromosome partitioning protein ParB | | uniclust | UniRef100\_A0A009RW17 | 99.9 | 1.6e-27 | 3.3e-33 | 209.9 | 161 | (21, 187) | 291 | (67, 231) | 362 | ParB/RepB/Spo0J family partition domain protein | ParB/RepB/Spo0J family partition domain protein | | uniclust | UniRef100\_A0A1E3ZL90 | 99.9 | 1.9e-27 | 3.7e-33 | 220.6 | 180 | (15, 200) | 291 | (82, 268) | 726 | ParB/Sulfiredoxin domain-containing protein | ParB/Sulfiredoxin domain-containing protein | | uniclust | UniRef100\_A0A060D5B2 | 99.9 | 2.1e-27 | 4.1e-33 | 199.5 | 182 | (2, 184) | 291 | (12, 208) | 296 | Uncharacterized protein | Uncharacterized protein | | uniclust | UniRef100\_A0A2E3TXI9 | 99.9 | 2.3e-27 | 4.3e-33 | 192.2 | 164 | (18, 187) | 291 | (35, 201) | 295 | Chromosome partitioning protein ParB | Chromosome partitioning protein ParB | | uniclust | UniRef100\_A0A171DQU0 | 99.9 | 2.6e-27 | 5e-33 | 198.9 | 178 | (17, 199) | 291 | (41, 234) | 313 | Chromosome partitioning protein ParB | Chromosome partitioning protein ParB | | uniclust | UniRef100\_A0A015USM3 | 99.9 | 2.5e-27 | 5e-33 | 221.2 | 178 | (17, 200) | 291 | (103, 284) | 724 | ParB/RepB/Spo0J family partition domain protein | ParB/RepB/Spo0J family partition domain protein | | uniclust | UniRef100\_A0A2N7QGC9 | 99.9 | 2.6e-27 | 5.1e-33 | 186.2 | 134 | (18, 157) | 291 | (54, 190) | 190 | ParB/Sulfiredoxin domain-containing protein | ParB/Sulfiredoxin domain-containing protein | | uniclust | UniRef100\_A0A023WZP1 | 99.9 | 2.6e-27 | 5.2e-33 | 205.4 | 166 | (18, 189) | 291 | (77, 249) | 349 | Partitioning protein | Partitioning protein | | uniclust | UniRef100\_A0A938R653 | 99.9 | 3.3e-27 | 6.2e-33 | 197.2 | 176 | (17, 198) | 291 | (60, 238) | 361 | ParB/RepB/Spo0J family partition protein | ParB/RepB/Spo0J family partition protein | | uniclust | UniRef100\_A0A0F5MQM1 | 99.9 | 3.8e-27 | 7.6e-33 | 198.2 | 173 | (17, 195) | 291 | (40, 219) | 284 | Probable chromosome-partitioning protein ParB | Probable chromosome-partitioning protein ParB | | uniclust | UniRef100\_A0A1F2QAI7 | 99.9 | 4.2e-27 | 8.1e-33 | 185.6 | 164 | (20, 190) | 291 | (32, 199) | 221 | ParB/Sulfiredoxin domain-containing protein (Fragment) | ParB/Sulfiredoxin domain-containing protein (Fragment) | | uniclust | UniRef100\_A0A1G2ESN9 | 99.9 | 4.2e-27 | 8.2e-33 | 199.8 | 182 | (13, 199) | 291 | (36, 228) | 339 | ParB/Sulfiredoxin domain-containing protein | ParB/Sulfiredoxin domain-containing protein | | uniclust | UniRef100\_A0A083UPG1 | 99.9 | 5.2e-27 | 1e-32 | 203.7 | 178 | (17, 200) | 291 | (91, 275) | 361 | Partitioning protein | Partitioning protein | | uniclust | UniRef100\_A0A0F9HNR6 | 99.9 | 5.6e-27 | 1.1e-32 | 183.0 | 128 | (17, 150) | 291 | (56, 187) | 187 | ParB/Sulfiredoxin domain-containing protein (Fragment) | ParB/Sulfiredoxin domain-containing protein (Fragment) | | uniclust | UniRef100\_A0A0F9MK30 | 99.9 | 5.8e-27 | 1.1e-32 | 193.8 | 171 | (14, 190) | 291 | (4, 177) | 276 | ParB/Sulfiredoxin domain-containing protein | ParB/Sulfiredoxin domain-containing protein | | uniclust | UniRef100\_A0A0A8WV70 | 99.9 | 5.8e-27 | 1.2e-32 | 201.9 | 168 | (13, 188) | 291 | (55, 226) | 325 | Putative chromosome-partitioning protein ParB | Putative chromosome-partitioning protein ParB | | uniclust | UniRef100\_A0A1F8L508 | 99.9 | 7.9e-27 | 1.5e-32 | 187.4 | 179 | (17, 201) | 291 | (28, 208) | 292 | ParB/Sulfiredoxin domain-containing protein | ParB/Sulfiredoxin domain-containing protein | | uniclust | UniRef100\_A0A0G1NIH2 | 99.9 | 8.2e-27 | 1.5e-32 | 200.1 | 182 | (17, 202) | 291 | (178, 363) | 463 | ParB-like protein partition protein | ParB-like protein partition protein | | uniclust | UniRef100\_A0A0U5F961 | 99.9 | 9.5e-27 | 1.8e-32 | 196.6 | 212 | (3, 216) | 291 | (19, 236) | 395 | FtsK gamma domain-containing protein | FtsK gamma domain-containing protein | | uniclust | UniRef100\_A0A142XAM1 | 99.9 | 1e-26 | 2e-32 | 194.5 | 173 | (16, 198) | 291 | (15, 192) | 283 | Putative chromosome-partitioning protein ParB | Putative chromosome-partitioning protein ParB | | uniclust | UniRef100\_A0A072MWG2 | 99.9 | 1.1e-26 | 2.1e-32 | 208.4 | 175 | (15, 195) | 291 | (232, 434) | 546 | Chromosome partitioning protein ParB | Chromosome partitioning protein ParB | | uniclust | UniRef100\_A0A2H5YTQ1 | 99.9 | 1.1e-26 | 2.2e-32 | 198.9 | 177 | (19, 200) | 291 | (53, 234) | 370 | Putative chromosome-partitioning protein ParB | Putative chromosome-partitioning protein ParB | | uniclust | UniRef100\_A0A3D3EDT8 | 99.9 | 1.1e-26 | 2.2e-32 | 182.2 | 144 | (18, 167) | 291 | (52, 201) | 206 | Chromosome partitioning protein ParB (Fragment) | Chromosome partitioning protein ParB (Fragment) | | uniclust | UniRef100\_A0A1G0G5V5 | 99.9 | 1.2e-26 | 2.3e-32 | 192.7 | 176 | (17, 199) | 291 | (22, 204) | 325 | Probable chromosome-partitioning protein ParB | Probable chromosome-partitioning protein ParB | | uniclust | UniRef100\_A0A081XJM3 | 99.9 | 1.2e-26 | 2.4e-32 | 203.7 | 177 | (20, 201) | 291 | (58, 252) | 408 | Plasmid partitioning protein | Plasmid partitioning protein | | uniclust | UniRef100\_A0A0Q6YVV5 | 99.9 | 1.6e-26 | 3e-32 | 198.2 | 180 | (17, 200) | 291 | (59, 243) | 414 | ParB/Sulfiredoxin domain-containing protein | ParB/Sulfiredoxin domain-containing protein | | uniclust | UniRef100\_A0A3A0DHT8 | 99.9 | 1.8e-26 | 3.3e-32 | 192.4 | 176 | (17, 198) | 291 | (61, 241) | 400 | Chromosome partitioning protein ParB | Chromosome partitioning protein ParB | | uniclust | UniRef100\_A0A1Y4U457 | 99.9 | 1.8e-26 | 3.5e-32 | 185.9 | 177 | (17, 199) | 291 | (72, 251) | 275 | Chromosome partitioning protein ParB (Fragment) | Chromosome partitioning protein ParB (Fragment) | | uniclust | UniRef100\_A0A0P6YU31 | 99.9 | 2e-26 | 3.8e-32 | 199.1 | 179 | (16, 200) | 291 | (46, 236) | 405 | Chromosome partitioning protein ParB | Chromosome partitioning protein ParB | | uniclust | UniRef100\_A0A1A5PA14 | 99.9 | 3.6e-26 | 6.9e-32 | 197.1 | 176 | (22, 202) | 291 | (58, 250) | 390 | ParB/Sulfiredoxin domain-containing protein | ParB/Sulfiredoxin domain-containing protein | | uniclust | UniRef100\_A0A517P8X0 | 99.9 | 3.8e-26 | 7.1e-32 | 191.6 | 177 | (19, 200) | 291 | (56, 234) | 407 | Chromosome-partitioning protein Spo0J | Chromosome-partitioning protein Spo0J | | uniclust | UniRef100\_A0A1F4ZSL5 | 99.9 | 3.7e-26 | 7.3e-32 | 211.1 | 174 | (14, 190) | 291 | (32, 214) | 610 | ParB/Sulfiredoxin domain-containing protein | ParB/Sulfiredoxin domain-containing protein | | uniclust | UniRef100\_A0A2S1XUK5 | 99.9 | 3.8e-26 | 7.5e-32 | 195.0 | 173 | (17, 195) | 291 | (52, 229) | 333 | Chromosome partitioning protein | Chromosome partitioning protein | | uniclust | UniRef100\_A0A356EUL3 | 99.9 | 4.7e-26 | 8.7e-32 | 191.9 | 170 | (16, 191) | 291 | (159, 331) | 442 | ParB/Sulfiredoxin domain-containing protein | ParB/Sulfiredoxin domain-containing protein | | uniclust | UniRef100\_A0A928HHV2 | 99.9 | 4.7e-26 | 8.8e-32 | 192.5 | 166 | (17, 188) | 291 | (172, 340) | 445 | ParB/RepB/Spo0J family partition protein | ParB/RepB/Spo0J family partition protein | | uniclust | UniRef100\_A0A0F5ILC4 | 99.9 | 4.7e-26 | 8.9e-32 | 184.5 | 178 | (17, 200) | 291 | (11, 194) | 268 | ParB-like partition protein | ParB-like partition protein | | uniclust | UniRef100\_A0A0Q5I357 | 99.9 | 4.7e-26 | 9.2e-32 | 195.1 | 175 | (20, 200) | 291 | (36, 241) | 337 | ParB/Sulfiredoxin domain-containing protein | ParB/Sulfiredoxin domain-containing protein | | uniclust | UniRef100\_A0A432MEU0 | 99.9 | 4.9e-26 | 9.3e-32 | 190.3 | 169 | (19, 191) | 291 | (58, 231) | 365 | ParB/RepB/Spo0J family partition protein | ParB/RepB/Spo0J family partition protein | | uniclust | UniRef100\_A0A015M8C0 | 99.9 | 5.3e-26 | 1.1e-31 | 203.5 | 176 | (17, 197) | 291 | (79, 274) | 424 | Plasmid stablization protein ParB | Plasmid stablization protein ParB | | uniclust | UniRef100\_A0A087ABQ3 | 99.9 | 5.3e-26 | 1.1e-31 | 207.8 | 167 | (16, 188) | 291 | (26, 195) | 529 | Chromosome partitioning protein parB | Chromosome partitioning protein parB | | uniclust | UniRef100\_A0A095TLQ3 | 99.9 | 5.5e-26 | 1.1e-31 | 201.7 | 174 | (17, 198) | 291 | (40, 222) | 415 | ParB-like partitioning protein | ParB-like partitioning protein | | uniclust | UniRef100\_UPI0004152AE1 | 99.9 | 5.8e-26 | 1.1e-31 | 198.7 | 178 | (19, 202) | 291 | (290, 471) | 573 | ParB/RepB/Spo0J family partition protein | ParB/RepB/Spo0J family partition protein | | uniclust | UniRef100\_A0A077XU49 | 99.9 | 6.1e-26 | 1.2e-31 | 210.4 | 181 | (17, 203) | 291 | (60, 243) | 659 | Putative ParB-like partition protein | Putative ParB-like partition protein | | uniclust | UniRef100\_A0A1G1ZRB4 | 99.9 | 6.7e-26 | 1.3e-31 | 189.1 | 174 | (15, 193) | 291 | (13, 197) | 366 | ParB/Sulfiredoxin domain-containing protein | ParB/Sulfiredoxin domain-containing protein | | uniclust | UniRef100\_A0A1C6R5S8 | 99.9 | 7.4e-26 | 1.4e-31 | 202.9 | 172 | (15, 193) | 291 | (103, 292) | 538 | ParB/RepB/Spo0J family partition protein | ParB/RepB/Spo0J family partition protein | | uniclust | UniRef100\_A0A024YNU6 | 99.9 | 7.6e-26 | 1.4e-31 | 193.0 | 180 | (16, 200) | 291 | (19, 204) | 402 | Chromosome partitioning protein | Chromosome partitioning protein | | uniclust | UniRef100\_A0A963MDU3 | 99.9 | 8.9e-26 | 1.6e-31 | 164.8 | 150 | (32, 186) | 291 | (3, 155) | 170 | ParB/RepB/Spo0J family partition protein (Fragment) | ParB/RepB/Spo0J family partition protein (Fragment) | | uniclust | UniRef100\_A0A1H6C3X3 | 99.9 | 8.5e-26 | 1.7e-31 | 202.7 | 186 | (14, 206) | 291 | (26, 218) | 555 | Chromosome partitioning protein, ParB family | Chromosome partitioning protein, ParB family | | uniclust | UniRef100\_A0A1F5DHD6 | 99.9 | 8.6e-26 | 1.7e-31 | 193.4 | 180 | (15, 200) | 291 | (15, 212) | 368 | ParB/Sulfiredoxin domain-containing protein | ParB/Sulfiredoxin domain-containing protein | | uniclust | UniRef100\_A0A1H6A853 | 99.9 | 9.7e-26 | 1.8e-31 | 182.0 | 165 | (15, 185) | 291 | (14, 182) | 273 | Chromosome partitioning protein, ParB family | Chromosome partitioning protein, ParB family | | uniclust | UniRef100\_A0A0U3N394 | 99.9 | 1.1e-25 | 2.1e-31 | 208.4 | 182 | (17, 200) | 291 | (81, 269) | 675 | ParB/Sulfiredoxin domain-containing protein | ParB/Sulfiredoxin domain-containing protein | | uniclust | UniRef100\_A0A1E7NI96 | 99.9 | 1.2e-25 | 2.2e-31 | 179.8 | 180 | (13, 198) | 291 | (24, 206) | 279 | Chromosome partitioning protein ParB | Chromosome partitioning protein ParB | | uniclust | UniRef100\_A0A073CBP1 | 99.9 | 1.3e-25 | 2.5e-31 | 201.1 | 173 | (21, 199) | 291 | (95, 306) | 444 | ParB | ParB | | uniclust | UniRef100\_A0A1N7ST09 | 99.9 | 1.5e-25 | 2.9e-31 | 186.8 | 202 | (16, 217) | 291 | (41, 251) | 347 | ParB-like partition protein | ParB-like partition protein | | uniclust | UniRef100\_A0A085FM87 | 99.9 | 1.5e-25 | 3e-31 | 195.6 | 165 | (15, 185) | 291 | (72, 243) | 354 | ParB-like partition protein | ParB-like partition protein | | uniclust | UniRef100\_A0A170PJZ6 | 99.9 | 1.6e-25 | 3e-31 | 193.1 | 180 | (15, 198) | 291 | (63, 266) | 410 | Putative ParB-like partition protein | Putative ParB-like partition protein | | uniclust | UniRef100\_A0A2E5HJM8 | 99.9 | 1.7e-25 | 3.1e-31 | 185.0 | 179 | (16, 199) | 291 | (52, 233) | 382 | Chromosome partitioning protein ParB | Chromosome partitioning protein ParB | | uniclust | UniRef100\_A0A014MAM2 | 99.9 | 1.6e-25 | 3.2e-31 | 216.0 | 177 | (16, 198) | 291 | (118, 314) | 921 | ParB/Sulfiredoxin domain-containing protein | ParB/Sulfiredoxin domain-containing protein | | uniclust | UniRef100\_A0A0G1YN89 | 99.9 | 1.9e-25 | 3.6e-31 | 183.7 | 177 | (18, 199) | 291 | (2, 191) | 315 | ParB-like protein partition protein | ParB-like protein partition protein | | uniclust | UniRef100\_A0A0D5A6I8 | 99.9 | 1.8e-25 | 3.6e-31 | 205.7 | 166 | (18, 189) | 291 | (109, 279) | 583 | Chromosome partitioning protein ParB | Chromosome partitioning protein ParB | | uniclust | UniRef100\_A0A0C1R7E8 | 99.9 | 1.8e-25 | 3.6e-31 | 192.7 | 177 | (19, 201) | 291 | (45, 260) | 342 | Plasmid partitioning protein ParB | Plasmid partitioning protein ParB | | uniclust | UniRef100\_A0A086PDT6 | 99.9 | 1.9e-25 | 3.7e-31 | 187.3 | 163 | (15, 185) | 291 | (23, 190) | 326 | ParB-like protein | ParB-like protein | | uniclust | UniRef100\_A0A022MM69 | 99.9 | 1.9e-25 | 3.7e-31 | 196.7 | 174 | (19, 198) | 291 | (54, 246) | 404 | ParB/Sulfiredoxin domain-containing protein | ParB/Sulfiredoxin domain-containing protein | | uniclust | UniRef100\_A0A015YUD5 | 99.9 | 2e-25 | 3.8e-31 | 179.5 | 198 | (15, 215) | 291 | (6, 214) | 273 | ParB/RepB/Spo0J family partition domain protein | ParB/RepB/Spo0J family partition domain protein | | uniclust | UniRef100\_A0A0D5YU03 | 99.9 | 2.2e-25 | 4.2e-31 | 199.2 | 176 | (16, 198) | 291 | (31, 208) | 600 | Putative partitioning protein | Putative partitioning protein | | uniclust | UniRef100\_A0A071MC08 | 99.9 | 2.3e-25 | 4.4e-31 | 198.6 | 234 | (13, 247) | 291 | (77, 327) | 558 | Pyridoxal phosphate biosynthetic protein PdxJ | Pyridoxal phosphate biosynthetic protein PdxJ | | uniclust | UniRef100\_A0A2D2LWZ4 | 99.9 | 2.7e-25 | 5.1e-31 | 183.7 | 166 | (18, 191) | 291 | (26, 196) | 321 | ParB/Sulfiredoxin domain-containing protein | ParB/Sulfiredoxin domain-containing protein | | uniclust | UniRef100\_A0A1B8RYV7 | 99.9 | 2.7e-25 | 5.1e-31 | 187.9 | 152 | (16, 172) | 291 | (34, 187) | 347 | ParB/Sulfiredoxin domain-containing protein | ParB/Sulfiredoxin domain-containing protein | | uniclust | UniRef100\_A0A0D6KEL4 | 99.9 | 2.6e-25 | 5.2e-31 | 191.5 | 176 | (20, 200) | 291 | (74, 287) | 362 | Partitioning protein ParB | Partitioning protein ParB | | uniclust | UniRef100\_E0NJQ3 | 99.9 | 3.2e-25 | 6e-31 | 181.5 | 168 | (16, 189) | 291 | (91, 261) | 346 | ParB-like protein | ParB-like protein | | uniclust | UniRef100\_A0A086P5Z5 | 99.9 | 3.1e-25 | 6e-31 | 192.4 | 165 | (17, 188) | 291 | (48, 215) | 368 | Chromosome partitioning protein ParB | Chromosome partitioning protein ParB | | uniclust | UniRef100\_A0A4Y8ZAY6 | 99.9 | 3.3e-25 | 6.1e-31 | 160.7 | 149 | (23, 176) | 291 | (1, 153) | 155 | ParB/RepB/Spo0J family partition protein (Fragment) | ParB/RepB/Spo0J family partition protein (Fragment) | | uniclust | UniRef100\_A0A1G3URH6 | 99.9 | 3.3e-25 | 6.4e-31 | 187.0 | 166 | (15, 188) | 291 | (31, 201) | 311 | ParB/Sulfiredoxin domain-containing protein | ParB/Sulfiredoxin domain-containing protein | | uniclust | UniRef100\_A0A1C7HFJ6 | 99.9 | 3.3e-25 | 6.6e-31 | 192.9 | 168 | (17, 189) | 291 | (25, 196) | 388 | ParB/Sulfiredoxin domain-containing protein | ParB/Sulfiredoxin domain-containing protein | | uniclust | UniRef100\_A0A069RGP5 | 99.9 | 3.5e-25 | 6.9e-31 | 187.0 | 238 | (18, 289) | 291 | (42, 298) | 304 | ParB-like protein | ParB-like protein | | uniclust | UniRef100\_A0A096XNS1 | 99.9 | 3.7e-25 | 7.5e-31 | 198.5 | 166 | (17, 190) | 291 | (35, 205) | 436 | ParB-like partition protein | ParB-like partition protein | | uniclust | UniRef100\_A0A0K2SP45 | 99.9 | 4e-25 | 7.9e-31 | 190.1 | 153 | (14, 172) | 291 | (11, 165) | 341 | Chromosome partitioning protein ParB | Chromosome partitioning protein ParB | | uniclust | UniRef100\_A0A140L4M6 | 99.9 | 4.2e-25 | 7.9e-31 | 186.4 | 174 | (18, 197) | 291 | (8, 197) | 358 | Chromosome-partitioning protein Spo0J | Chromosome-partitioning protein Spo0J | | uniclust | UniRef100\_A0A139KWY8 | 99.9 | 4.2e-25 | 8e-31 | 200.9 | 179 | (18, 199) | 291 | (2, 215) | 698 | Chromosome partitioning protein ParB | Chromosome partitioning protein ParB | | uniclust | UniRef100\_A0A8S9ARD5 | 99.9 | 5.2e-25 | 9.9e-31 | 184.3 | 175 | (16, 195) | 291 | (50, 236) | 373 | ParB/RepB/Spo0J family partition protein | ParB/RepB/Spo0J family partition protein | | uniclust | UniRef100\_A0A011N723 | 99.8 | 5.3e-25 | 1.1e-30 | 196.5 | 164 | (18, 189) | 291 | (78, 246) | 410 | Transcriptional repressor protein KorB | Transcriptional repressor protein KorB | | uniclust | UniRef100\_A0A2K0XC05 | 99.8 | 5.7e-25 | 1.1e-30 | 198.0 | 177 | (14, 196) | 291 | (8, 191) | 623 | ParB/Sulfiredoxin domain-containing protein | ParB/Sulfiredoxin domain-containing protein | | uniclust | UniRef100\_A0A011W170 | 99.8 | 5.6e-25 | 1.1e-30 | 205.1 | 168 | (15, 189) | 291 | (117, 291) | 608 | Chromosome partitioning protein ParB | Chromosome partitioning protein ParB | | uniclust | UniRef100\_A0A1D2R6B9 | 99.8 | 6e-25 | 1.2e-30 | 182.6 | 176 | (17, 197) | 291 | (9, 194) | 281 | ParB/Sulfiredoxin domain-containing protein | ParB/Sulfiredoxin domain-containing protein | | uniclust | UniRef100\_A0A3S0DWZ9 | 99.8 | 7e-25 | 1.3e-30 | 160.8 | 126 | (15, 146) | 291 | (17, 145) | 145 | ParB/RepB/Spo0J family partition protein (Fragment) | ParB/RepB/Spo0J family partition protein (Fragment) | | uniclust | UniRef100\_A0A1G6LR52 | 99.8 | 6.7e-25 | 1.3e-30 | 200.7 | 166 | (16, 187) | 291 | (12, 181) | 540 | ParB/RepB/Spo0J family partition protein | ParB/RepB/Spo0J family partition protein | | uniclust | UniRef100\_A0A0C5BR18 | 99.8 | 6.8e-25 | 1.4e-30 | 184.2 | 162 | (21, 187) | 291 | (15, 178) | 281 | ParB/Sulfiredoxin domain-containing protein | ParB/Sulfiredoxin domain-containing protein | | uniclust | UniRef100\_A0A0F9W5T8 | 99.8 | 7.3e-25 | 1.4e-30 | 177.0 | 165 | (21, 189) | 291 | (2, 171) | 296 | ParB/Sulfiredoxin domain-containing protein | ParB/Sulfiredoxin domain-containing protein | | uniclust | UniRef100\_A0A076ZED2 | 99.8 | 7.7e-25 | 1.5e-30 | 187.6 | 191 | (2, 197) | 291 | (42, 255) | 345 | ParB-like protein | ParB-like protein | | uniclust | UniRef100\_A0A1W9HD66 | 99.8 | 8.5e-25 | 1.6e-30 | 182.8 | 165 | (18, 188) | 291 | (102, 272) | 365 | ParB/Sulfiredoxin domain-containing protein | ParB/Sulfiredoxin domain-containing protein | | uniclust | UniRef100\_A0A1K1LLY6 | 99.8 | 9.6e-25 | 1.9e-30 | 183.9 | 226 | (19, 250) | 291 | (45, 291) | 323 | Chromosome partitioning protein, ParB family | Chromosome partitioning protein, ParB family | | uniclust | UniRef100\_A0A069IHU9 | 99.8 | 1.1e-24 | 2.2e-30 | 173.0 | 179 | (17, 202) | 291 | (8, 192) | 218 | ParB/Sulfiredoxin domain-containing protein (Fragment) | ParB/Sulfiredoxin domain-containing protein (Fragment) | | uniclust | UniRef100\_A0A061M5E0 | 99.8 | 1.2e-24 | 2.3e-30 | 188.0 | 163 | (17, 186) | 291 | (35, 199) | 349 | Transcriptional repressor protein korB | Transcriptional repressor protein korB | | uniclust | UniRef100\_A0A0C2LAE5 | 99.8 | 1.2e-24 | 2.3e-30 | 186.9 | 179 | (16, 200) | 291 | (39, 257) | 338 | ParB/RepB/Spo0J family partition protein | ParB/RepB/Spo0J family partition protein | | uniclust | UniRef100\_A0A075JJA6 | 99.8 | 1.2e-24 | 2.4e-30 | 201.0 | 166 | (18, 189) | 291 | (84, 267) | 552 | HTH cro/C1-type domain-containing protein | HTH cro/C1-type domain-containing protein | | uniclust | UniRef100\_A0A0F8X7Y3 | 99.8 | 1.3e-24 | 2.5e-30 | 169.8 | 144 | (18, 166) | 291 | (54, 204) | 206 | ParB/Sulfiredoxin domain-containing protein (Fragment) | ParB/Sulfiredoxin domain-containing protein (Fragment) | | uniclust | UniRef100\_A0A069P132 | 99.8 | 1.3e-24 | 2.7e-30 | 208.3 | 181 | (14, 201) | 291 | (111, 298) | 811 | Chromosome partitioning protein ParB | Chromosome partitioning protein ParB | | uniclust | UniRef100\_A0A0C5K7X7 | 99.8 | 1.4e-24 | 2.8e-30 | 191.4 | 219 | (17, 244) | 291 | (104, 333) | 386 | ParB/Sulfiredoxin domain-containing protein | ParB/Sulfiredoxin domain-containing protein | | uniclust | UniRef100\_A0A523DAK4 | 99.8 | 1.8e-24 | 3.4e-30 | 190.3 | 179 | (15, 199) | 291 | (14, 203) | 553 | ParB/RepB/Spo0J family partition protein | ParB/RepB/Spo0J family partition protein | | uniclust | UniRef100\_A0A4V2UP75 | 99.8 | 1.8e-24 | 3.5e-30 | 190.9 | 167 | (17, 189) | 291 | (81, 253) | 606 | ParB/RepB/Spo0J family partition protein | ParB/RepB/Spo0J family partition protein | | uniclust | UniRef100\_A0A1T1B7P7 | 99.8 | 1.8e-24 | 3.5e-30 | 184.3 | 178 | (17, 199) | 291 | (47, 256) | 352 | ParB/Sulfiredoxin domain-containing protein | ParB/Sulfiredoxin domain-containing protein | | uniclust | UniRef100\_A0A016XIJ2 | 99.8 | 1.8e-24 | 3.5e-30 | 195.8 | 169 | (16, 190) | 291 | (2, 185) | 612 | Chromosome partitioning protein ParB | Chromosome partitioning protein ParB | | uniclust | UniRef100\_A0A0F9LDT1 | 99.8 | 2e-24 | 3.8e-30 | 181.5 | 176 | (18, 197) | 291 | (71, 256) | 326 | ParB/Sulfiredoxin domain-containing protein | ParB/Sulfiredoxin domain-containing protein | | uniclust | UniRef100\_A0A015W8Q1 | 99.8 | 2.1e-24 | 3.9e-30 | 194.8 | 177 | (17, 200) | 291 | (67, 249) | 691 | ParB/RepB/Spo0J family partition domain protein | ParB/RepB/Spo0J family partition domain protein | | uniclust | UniRef100\_A0A1H5Z312 | 99.8 | 2.2e-24 | 4.1e-30 | 173.7 | 174 | (19, 198) | 291 | (9, 188) | 264 | ParB-like nuclease | ParB-like nuclease | | uniclust | UniRef100\_A0A1I6NRN6 | 99.8 | 2.2e-24 | 4.4e-30 | 201.4 | 176 | (18, 199) | 291 | (107, 285) | 681 | ParB/RepB/Spo0J family partition protein | ParB/RepB/Spo0J family partition protein | | uniclust | UniRef100\_A0A1V4W4D9 | 99.8 | 2.4e-24 | 4.5e-30 | 196.2 | 171 | (24, 201) | 291 | (30, 207) | 789 | Chromosome-partitioning protein Spo0J | Chromosome-partitioning protein Spo0J | | uniclust | UniRef100\_A0A0F8W8Q8 | 99.8 | 2.3e-24 | 4.5e-30 | 180.2 | 169 | (16, 188) | 291 | (29, 208) | 313 | ParB/Sulfiredoxin domain-containing protein (Fragment) | ParB/Sulfiredoxin domain-containing protein (Fragment) | | uniclust | UniRef100\_A0A0A8F238 | 99.8 | 2.3e-24 | 4.5e-30 | 183.4 | 174 | (18, 197) | 291 | (42, 234) | 349 | Putative plasmid partitioning protein, parb2 | Putative plasmid partitioning protein, parb2 | | uniclust | UniRef100\_A0A174EVK5 | 99.8 | 2.3e-24 | 4.6e-30 | 186.7 | 168 | (15, 189) | 291 | (3, 182) | 342 | Chromosome-partitioning protein parB | Chromosome-partitioning protein parB | | uniclust | UniRef100\_A0A254TB70 | 99.8 | 2.5e-24 | 4.9e-30 | 202.3 | 173 | (20, 198) | 291 | (56, 238) | 748 | ParB/Sulfiredoxin domain-containing protein | ParB/Sulfiredoxin domain-containing protein | | uniclust | UniRef100\_A0A143XPK3 | 99.8 | 2.6e-24 | 5e-30 | 180.6 | 179 | (15, 198) | 291 | (38, 238) | 325 | Chromosome-partitioning protein Spo0J | Chromosome-partitioning protein Spo0J | | uniclust | UniRef100\_A0A0G1AJS6 | 99.8 | 2.6e-24 | 5e-30 | 184.1 | 186 | (17, 207) | 291 | (31, 222) | 336 | Chromosome segregation DNA-binding protein | Chromosome segregation DNA-binding protein | | uniclust | UniRef100\_A0A0D6QE72 | 99.8 | 3e-24 | 5.8e-30 | 196.1 | 179 | (14, 198) | 291 | (40, 221) | 596 | Putative chromosome-partitioning protein ParB | Putative chromosome-partitioning protein ParB | | uniclust | UniRef100\_A0A1M5YKM5 | 99.8 | 4e-24 | 7.6e-30 | 180.1 | 267 | (5, 285) | 291 | (55, 336) | 348 | Chromosome partitioning protein, ParB family | Chromosome partitioning protein, ParB family | | uniclust | UniRef100\_A0A1Q3SMN2 | 99.8 | 4.1e-24 | 8e-30 | 185.3 | 167 | (18, 192) | 291 | (56, 228) | 375 | ParB/Sulfiredoxin domain-containing protein | ParB/Sulfiredoxin domain-containing protein | | uniclust | UniRef100\_X1CJ30 | 99.8 | 4.8e-24 | 8.8e-30 | 164.9 | 170 | (14, 189) | 291 | (41, 213) | 248 | ParB/Sulfiredoxin domain-containing protein (Fragment) | ParB/Sulfiredoxin domain-containing protein (Fragment) | | uniclust | UniRef100\_A0A0G1WPA1 | 99.8 | 5e-24 | 9.8e-30 | 180.6 | 184 | (13, 200) | 291 | (28, 220) | 324 | ParB/Sulfiredoxin domain-containing protein | ParB/Sulfiredoxin domain-containing protein | | uniclust | UniRef100\_A0A212L1T9 | 99.8 | 5.3e-24 | 1e-29 | 177.2 | 183 | (16, 201) | 291 | (34, 229) | 322 | Putative Nucleoid occlusion protein | Putative Nucleoid occlusion protein | | uniclust | UniRef100\_A0A9E2BEM2 | 99.8 | 5.5e-24 | 1e-29 | 172.1 | 166 | (17, 188) | 291 | (41, 209) | 302 | Chromosome-partitioning protein ParB | Chromosome-partitioning protein ParB | | uniclust | UniRef100\_A0A0F8ZIF1 | 99.8 | 5.7e-24 | 1.1e-29 | 176.2 | 189 | (15, 207) | 291 | (18, 216) | 298 | ParB/Sulfiredoxin domain-containing protein | ParB/Sulfiredoxin domain-containing protein | | uniclust | UniRef100\_A0A5N5XVG2 | 99.8 | 6.6e-24 | 1.3e-29 | 174.6 | 171 | (18, 193) | 291 | (6, 194) | 296 | ParB/RepB/Spo0J family partition protein | ParB/RepB/Spo0J family partition protein | | uniclust | UniRef100\_A0A069PKE4 | 99.8 | 7.2e-24 | 1.4e-29 | 181.9 | 204 | (13, 216) | 291 | (65, 278) | 404 | Transcriptional regulator | Transcriptional regulator | | uniclust | UniRef100\_A0A967V135 | 99.8 | 7.9e-24 | 1.5e-29 | 174.7 | 164 | (19, 188) | 291 | (25, 192) | 416 | ParB/RepB/Spo0J family partition protein | ParB/RepB/Spo0J family partition protein | | uniclust | UniRef100\_A0A1I4DE87 | 99.8 | 7.7e-24 | 1.5e-29 | 180.7 | 166 | (18, 189) | 291 | (143, 312) | 399 | ParB/RepB/Spo0J family partition protein | ParB/RepB/Spo0J family partition protein | | uniclust | UniRef100\_A0A3D1LXR1 | 99.8 | 8e-24 | 1.5e-29 | 178.2 | 175 | (17, 195) | 291 | (39, 224) | 313 | Chromosome partitioning protein ParB | Chromosome partitioning protein ParB | | uniclust | UniRef100\_A0A3A5APM2 | 99.8 | 8.4e-24 | 1.6e-29 | 175.1 | 171 | (15, 192) | 291 | (77, 251) | 381 | ParB/RepB/Spo0J family partition protein | ParB/RepB/Spo0J family partition protein | | uniclust | UniRef100\_A0A142KA42 | 99.8 | 9.1e-24 | 1.8e-29 | 190.6 | 170 | (13, 188) | 291 | (17, 190) | 527 | ParB-like nuclease domain protein | ParB-like nuclease domain protein | | uniclust | UniRef100\_A0A2N2QZ62 | 99.8 | 9.4e-24 | 1.8e-29 | 171.3 | 177 | (6, 189) | 291 | (30, 209) | 271 | Chromosome partitioning protein ParB (Fragment) | Chromosome partitioning protein ParB (Fragment) | | uniclust | UniRef100\_A0A318SDS1 | 99.8 | 1e-23 | 2e-29 | 177.8 | 175 | (18, 197) | 291 | (29, 232) | 316 | ParB family protein | ParB family protein | | uniclust | UniRef100\_A0A1F3KBJ9 | 99.8 | 1e-23 | 2e-29 | 190.3 | 177 | (17, 199) | 291 | (5, 189) | 583 | ParB/Sulfiredoxin domain-containing protein | ParB/Sulfiredoxin domain-containing protein | | uniclust | UniRef100\_A0A1E3S303 | 99.8 | 1e-23 | 2e-29 | 185.8 | 169 | (14, 188) | 291 | (20, 193) | 487 | ParB/Sulfiredoxin domain-containing protein | ParB/Sulfiredoxin domain-containing protein | | uniclust | UniRef100\_A0A0F7VPB0 | 99.8 | 1e-23 | 2e-29 | 187.8 | 181 | (13, 199) | 291 | (40, 240) | 409 | ParB | ParB | | uniclust | UniRef100\_A0A351H5H0 | 99.8 | 1.1e-23 | 2.1e-29 | 173.2 | 182 | (16, 202) | 291 | (34, 218) | 311 | Nucleoid occlusion protein | Nucleoid occlusion protein | | uniclust | UniRef100\_A0A959ZJQ8 | 99.8 | 1.1e-23 | 2.1e-29 | 185.4 | 159 | (17, 179) | 291 | (11, 172) | 544 | ParB/RepB/Spo0J family partition protein | ParB/RepB/Spo0J family partition protein | | uniclust | UniRef100\_UPI002072E589 | 99.8 | 1.2e-23 | 2.3e-29 | 178.4 | 162 | (21, 188) | 291 | (3, 176) | 428 | ParB/RepB/Spo0J family partition protein | ParB/RepB/Spo0J family partition protein | | uniclust | UniRef100\_A0A222WSL8 | 99.8 | 1.3e-23 | 2.4e-29 | 176.0 | 160 | (22, 187) | 291 | (4, 165) | 380 | ParB/Sulfiredoxin domain-containing protein | ParB/Sulfiredoxin domain-containing protein | | uniclust | UniRef100\_A0A4Y6UB58 | 99.8 | 1.3e-23 | 2.4e-29 | 171.3 | 163 | (20, 188) | 291 | (17, 204) | 323 | ParB/RepB/Spo0J family partition protein | ParB/RepB/Spo0J family partition protein | | uniclust | UniRef100\_A0A1C6HC15 | 99.8 | 1.6e-23 | 2.9e-29 | 179.7 | 173 | (21, 199) | 291 | (290, 466) | 549 | Probable chromosome-partitioning protein parB | Probable chromosome-partitioning protein parB | | uniclust | UniRef100\_A0A0C1MVS1 | 99.8 | 1.5e-23 | 3e-29 | 187.7 | 204 | (17, 246) | 291 | (79, 338) | 413 | Plasmid partitioning protein ParB | Plasmid partitioning protein ParB | | uniclust | UniRef100\_A0A3A4U2T7 | 99.8 | 1.8e-23 | 3.4e-29 | 174.8 | 172 | (13, 190) | 291 | (74, 248) | 407 | ParB/RepB/Spo0J family partition protein | ParB/RepB/Spo0J family partition protein | | uniclust | UniRef100\_A0A064CDN1 | 99.8 | 1.8e-23 | 3.5e-29 | 177.5 | 220 | (18, 248) | 291 | (54, 308) | 326 | Peptide transporter | Peptide transporter | | uniclust | UniRef100\_A0A086ZNU6 | 99.8 | 1.9e-23 | 3.7e-29 | 185.5 | 168 | (15, 188) | 291 | (80, 248) | 548 | ParB-like nuclease domain | ParB-like nuclease domain | | uniclust | UniRef100\_A0A352BEU1 | 99.8 | 2.2e-23 | 4.1e-29 | 154.9 | 147 | (17, 168) | 291 | (11, 174) | 175 | ParB/Sulfiredoxin domain-containing protein | ParB/Sulfiredoxin domain-containing protein | | uniclust | UniRef100\_A0A1U7J7S0 | 99.8 | 2.2e-23 | 4.5e-29 | 180.5 | 176 | (17, 198) | 291 | (44, 256) | 339 | Chromosome partitioning protein ParB | Chromosome partitioning protein ParB | | uniclust | UniRef100\_A0A016QJQ7 | 99.8 | 2.3e-23 | 4.5e-29 | 179.9 | 177 | (19, 201) | 291 | (44, 253) | 361 | ParB/Sulfiredoxin domain-containing protein | ParB/Sulfiredoxin domain-containing protein | | uniclust | UniRef100\_A0A2X1TFX5 | 99.8 | 2.4e-23 | 4.5e-29 | 160.8 | 162 | (54, 216) | 291 | (3, 166) | 211 | ParB/RepB/Spo0J family partition protein | ParB/RepB/Spo0J family partition protein | | uniclust | UniRef100\_A0A317EEA9 | 99.8 | 2.4e-23 | 4.6e-29 | 186.4 | 182 | (15, 198) | 291 | (115, 303) | 601 | ParB/Sulfiredoxin domain-containing protein | ParB/Sulfiredoxin domain-containing protein | | uniclust | UniRef100\_A0A016QT58 | 99.8 | 2.4e-23 | 4.6e-29 | 178.5 | 172 | (21, 199) | 291 | (59, 259) | 346 | ParB-like partition protein | ParB-like partition protein | | uniclust | UniRef100\_A0A5M8SQ23 | 99.8 | 2.6e-23 | 4.8e-29 | 167.8 | 185 | (15, 205) | 291 | (6, 196) | 286 | ParB/RepB/Spo0J family partition protein | ParB/RepB/Spo0J family partition protein | | uniclust | UniRef100\_A0A173SU81 | 99.8 | 2.5e-23 | 4.9e-29 | 177.5 | 179 | (15, 198) | 291 | (43, 242) | 351 | Probable chromosome-partitioning protein parB | Probable chromosome-partitioning protein parB | | uniclust | UniRef100\_A0A060ULH7 | 99.8 | 2.5e-23 | 4.9e-29 | 189.1 | 175 | (18, 200) | 291 | (30, 208) | 583 | ParB/Sulfiredoxin domain-containing protein | ParB/Sulfiredoxin domain-containing protein | | uniclust | UniRef100\_A0A0M0BQB3 | 99.8 | 2.7e-23 | 5.1e-29 | 165.4 | 178 | (20, 201) | 291 | (4, 184) | 249 | ParB/Sulfiredoxin domain-containing protein | ParB/Sulfiredoxin domain-containing protein | | uniclust | UniRef100\_A0A1H4K415 | 99.8 | 2.8e-23 | 5.3e-29 | 185.1 | 180 | (16, 201) | 291 | (19, 201) | 578 | Chromosome partitioning protein, ParB family | Chromosome partitioning protein, ParB family | | uniclust | UniRef100\_A0A2L2WSX3 | 99.8 | 3.2e-23 | 6.1e-29 | 180.8 | 179 | (15, 199) | 291 | (10, 193) | 515 | Chromosome partitioning protein, ParB family | Chromosome partitioning protein, ParB family | | uniclust | UniRef100\_A0A922VHK8 | 99.8 | 3.4e-23 | 6.3e-29 | 165.4 | 175 | (18, 197) | 291 | (27, 203) | 313 | Chromosome partitioning protein ParB | Chromosome partitioning protein ParB | | uniclust | UniRef100\_A0A1H8AXE2 | 99.8 | 3.2e-23 | 6.5e-29 | 178.8 | 176 | (17, 197) | 291 | (42, 227) | 326 | Chromosome partitioning protein, ParB family | Chromosome partitioning protein, ParB family | | uniclust | UniRef100\_A0A2E5S2I9 | 99.8 | 3.4e-23 | 6.5e-29 | 170.7 | 168 | (15, 188) | 291 | (19, 189) | 284 | Chromosome partitioning protein ParB | Chromosome partitioning protein ParB | | uniclust | UniRef100\_A0A660VCE9 | 99.8 | 3.6e-23 | 6.6e-29 | 167.2 | 162 | (14, 181) | 291 | (112, 275) | 344 | HTH cro/C1-type domain-containing protein | HTH cro/C1-type domain-containing protein | | uniclust | UniRef100\_A0A2D9HGI4 | 99.8 | 3.6e-23 | 6.6e-29 | 167.1 | 179 | (15, 198) | 291 | (51, 233) | 318 | ParB/Sulfiredoxin domain-containing protein | ParB/Sulfiredoxin domain-containing protein | | uniclust | UniRef100\_A0A3D3LYG7 | 99.8 | 3.6e-23 | 7e-29 | 182.2 | 169 | (14, 188) | 291 | (8, 182) | 487 | ParB/Sulfiredoxin domain-containing protein | ParB/Sulfiredoxin domain-containing protein | | uniclust | UniRef100\_A0A011NAJ4 | 99.8 | 4.4e-23 | 8.9e-29 | 182.5 | 165 | (18, 187) | 291 | (82, 255) | 373 | Chromosome-partitioning protein ParB | Chromosome-partitioning protein ParB | | uniclust | UniRef100\_A0A1G8EV61 | 99.8 | 4.8e-23 | 9.3e-29 | 177.0 | 156 | (21, 182) | 291 | (88, 250) | 352 | Chromosome partitioning protein, ParB family | Chromosome partitioning protein, ParB family | | uniclust | UniRef100\_C3NJE7 | 99.8 | 5.2e-23 | 9.6e-29 | 152.5 | 160 | (22, 187) | 291 | (2, 167) | 183 | ParB-like partition protein | ParB-like partition protein | | uniclust | UniRef100\_A0A2H9LMS7 | 99.8 | 5e-23 | 9.7e-29 | 169.0 | 164 | (19, 187) | 291 | (11, 179) | 265 | ParB/Sulfiredoxin domain-containing protein | ParB/Sulfiredoxin domain-containing protein | | uniclust | UniRef100\_A0A1Q3SGT3 | 99.8 | 5e-23 | 9.8e-29 | 174.0 | 137 | (18, 160) | 291 | (55, 197) | 296 | ParB/Sulfiredoxin domain-containing protein | ParB/Sulfiredoxin domain-containing protein | | uniclust | UniRef100\_A0A517T448 | 99.8 | 5.6e-23 | 1e-28 | 170.1 | 175 | (19, 199) | 291 | (48, 225) | 421 | Putative chromosome-partitioning protein ParB | Putative chromosome-partitioning protein ParB | | uniclust | UniRef100\_UPI000698FBAD | 99.8 | 5.5e-23 | 1e-28 | 177.9 | 162 | (20, 187) | 291 | (3, 166) | 497 | ParB/RepB/Spo0J family partition protein | ParB/RepB/Spo0J family partition protein | | uniclust | UniRef100\_A0A938L9U1 | 99.8 | 5.9e-23 | 1.1e-28 | 171.8 | 164 | (19, 187) | 291 | (197, 363) | 466 | ParB/RepB/Spo0J family partition protein | ParB/RepB/Spo0J family partition protein | | uniclust | UniRef100\_A0A3M1FTJ6 | 99.8 | 6.1e-23 | 1.1e-28 | 172.6 | 163 | (20, 188) | 291 | (227, 392) | 487 | ParB/RepB/Spo0J family partition protein | ParB/RepB/Spo0J family partition protein | | uniclust | UniRef100\_A0A1Q3T2X0 | 99.8 | 5.9e-23 | 1.1e-28 | 177.3 | 151 | (13, 168) | 291 | (31, 186) | 380 | ParB/Sulfiredoxin domain-containing protein | ParB/Sulfiredoxin domain-containing protein | | uniclust | UniRef100\_A0A1T4V321 | 99.8 | 6.3e-23 | 1.2e-28 | 176.7 | 168 | (15, 187) | 291 | (44, 213) | 561 | Probable chromosome-partitioning protein ParB | Probable chromosome-partitioning protein ParB | | uniclust | UniRef100\_A0A2N5KH80 | 99.8 | 6.3e-23 | 1.2e-28 | 175.0 | 168 | (17, 191) | 291 | (34, 206) | 325 | ParB/Sulfiredoxin domain-containing protein | ParB/Sulfiredoxin domain-containing protein | | uniclust | UniRef100\_A0A354BGG0 | 99.8 | 6.6e-23 | 1.3e-28 | 172.7 | 166 | (16, 188) | 291 | (87, 257) | 367 | ParB/Sulfiredoxin domain-containing protein | ParB/Sulfiredoxin domain-containing protein | | uniclust | UniRef100\_A0A1V4MDQ5 | 99.8 | 6.4e-23 | 1.3e-28 | 188.3 | 174 | (18, 199) | 291 | (64, 243) | 593 | site-specific DNA-methyltransferase (cytosine-N(4)-specific) | site-specific DNA-methyltransferase (cytosine-N(4)-specific) | | uniclust | UniRef100\_A0A2V2GRE7 | 99.8 | 6.6e-23 | 1.3e-28 | 172.6 | 182 | (15, 201) | 291 | (19, 207) | 327 | ParB/Sulfiredoxin domain-containing protein | ParB/Sulfiredoxin domain-containing protein | | uniclust | UniRef100\_A0A069P7S0 | 99.8 | 6.6e-23 | 1.3e-28 | 175.2 | 217 | (16, 242) | 291 | (64, 289) | 335 | Chromosome partitioning protein ParB | Chromosome partitioning protein ParB | | uniclust | UniRef100\_A0A166QSF2 | 99.8 | 8.5e-23 | 1.6e-28 | 165.7 | 166 | (18, 189) | 291 | (28, 197) | 290 | Probable chromosome-partitioning protein ParB | Probable chromosome-partitioning protein ParB | | uniclust | UniRef100\_UPI00069DE912 | 99.8 | 8.7e-23 | 1.6e-28 | 171.7 | 168 | (16, 187) | 291 | (42, 214) | 372 | ParB/RepB/Spo0J family partition protein | ParB/RepB/Spo0J family partition protein | | uniclust | UniRef100\_A0A060A0B8 | 99.8 | 8.4e-23 | 1.7e-28 | 183.1 | 169 | (17, 188) | 291 | (61, 248) | 483 | Chromosome (Plasmid) partitioning protein ParB / Stage 0 sporulation protein J | Chromosome (Plasmid) partitioning protein ParB / Stage 0 sporulation protein J | | uniclust | UniRef100\_A0A8J6TJ42 | 99.8 | 9.3e-23 | 1.8e-28 | 183.7 | 179 | (15, 202) | 291 | (168, 352) | 692 | ParB/RepB/Spo0J family partition protein | ParB/RepB/Spo0J family partition protein | | uniclust | UniRef100\_A0A535C431 | 99.8 | 9.8e-23 | 1.8e-28 | 168.0 | 167 | (17, 189) | 291 | (134, 303) | 406 | ParB/RepB/Spo0J family partition protein | ParB/RepB/Spo0J family partition protein | | uniclust | UniRef100\_A0A1Q9PA47 | 99.8 | 1e-22 | 1.9e-28 | 172.6 | 143 | (18, 166) | 291 | (3, 153) | 338 | Putative chromosome-partitioning protein ParB | Putative chromosome-partitioning protein ParB | | uniclust | UniRef100\_A0A0F9TED6 | 99.8 | 1.1e-22 | 2e-28 | 174.8 | 167 | (18, 189) | 291 | (31, 202) | 430 | ParB/Sulfiredoxin domain-containing protein | ParB/Sulfiredoxin domain-containing protein | | uniclust | UniRef100\_A0A6M5YN73 | 99.8 | 1.1e-22 | 2.1e-28 | 166.3 | 165 | (20, 189) | 291 | (51, 221) | 378 | Chromosome (Plasmid) partitioning protein ParB | Chromosome (Plasmid) partitioning protein ParB | | uniclust | UniRef100\_A0A0Q6VIP4 | 99.8 | 1.1e-22 | 2.1e-28 | 188.1 | 172 | (20, 197) | 291 | (40, 220) | 649 | ParB/Sulfiredoxin domain-containing protein | ParB/Sulfiredoxin domain-containing protein | | uniclust | UniRef100\_A0A084CND8 | 99.8 | 1.1e-22 | 2.1e-28 | 175.4 | 168 | (17, 187) | 291 | (53, 229) | 358 | Nucleoid occlusion protein | Nucleoid occlusion protein | | uniclust | UniRef100\_A0A0N1JNZ4 | 99.8 | 1.1e-22 | 2.2e-28 | 170.2 | 164 | (18, 188) | 291 | (71, 241) | 337 | Partitioning protein | Partitioning protein | | uniclust | UniRef100\_A0A4Q3NWG3 | 99.8 | 1.2e-22 | 2.2e-28 | 170.6 | 167 | (17, 188) | 291 | (36, 204) | 480 | Aldehyde dehydrogenase family protein | Aldehyde dehydrogenase family protein | | uniclust | UniRef100\_A0A1F4NGN6 | 99.8 | 1.1e-22 | 2.2e-28 | 176.3 | 168 | (17, 190) | 291 | (63, 235) | 329 | HTH cro/C1-type domain-containing protein | HTH cro/C1-type domain-containing protein | | uniclust | UniRef100\_A0A1K2ICX5 | 99.8 | 1.2e-22 | 2.2e-28 | 169.1 | 175 | (16, 197) | 291 | (30, 206) | 369 | Chromosome partitioning protein, ParB family | Chromosome partitioning protein, ParB family | | uniclust | UniRef100\_A0A0Q7DD19 | 99.8 | 1.1e-22 | 2.2e-28 | 174.8 | 176 | (15, 199) | 291 | (30, 210) | 354 | ParB/Sulfiredoxin domain-containing protein | ParB/Sulfiredoxin domain-containing protein | | uniclust | UniRef100\_A0A373ZZS3 | 99.8 | 1.2e-22 | 2.2e-28 | 186.3 | 173 | (20, 198) | 291 | (60, 236) | 738 | ParB/RepB/Spo0J family partition protein | ParB/RepB/Spo0J family partition protein | | uniclust | UniRef100\_A0A0A8WL90 | 99.8 | 1.2e-22 | 2.3e-28 | 163.8 | 170 | (16, 188) | 291 | (9, 186) | 229 | ParB/Sulfiredoxin domain-containing protein | ParB/Sulfiredoxin domain-containing protein | | uniclust | UniRef100\_A0A1F8SA75 | 99.8 | 1.4e-22 | 2.5e-28 | 159.7 | 168 | (16, 189) | 291 | (35, 206) | 245 | ParB/Sulfiredoxin domain-containing protein (Fragment) | ParB/Sulfiredoxin domain-containing protein (Fragment) | | uniclust | UniRef100\_A0A0E2B209 | 99.8 | 1.4e-22 | 2.7e-28 | 160.9 | 160 | (18, 183) | 291 | (21, 184) | 236 | ParB-like protein | ParB-like protein | | uniclust | UniRef100\_A0A7C6QZE1 | 99.8 | 1.6e-22 | 2.9e-28 | 170.8 | 164 | (17, 186) | 291 | (33, 198) | 502 | ParB/RepB/Spo0J family partition protein | ParB/RepB/Spo0J family partition protein | | uniclust | UniRef100\_A0A662TCT7 | 99.8 | 1.6e-22 | 2.9e-28 | 163.6 | 171 | (17, 193) | 291 | (5, 178) | 293 | ParB/Sulfiredoxin domain-containing protein | ParB/Sulfiredoxin domain-containing protein | | uniclust | UniRef100\_A0A1G2SN81 | 99.8 | 1.7e-22 | 3.1e-28 | 168.6 | 175 | (18, 197) | 291 | (11, 190) | 346 | ParB/Sulfiredoxin domain-containing protein | ParB/Sulfiredoxin domain-containing protein | | uniclust | UniRef100\_A0A2T5IQH8 | 99.8 | 1.7e-22 | 3.2e-28 | 164.7 | 180 | (17, 200) | 291 | (45, 231) | 313 | ParB/RepB/Spo0J family partition protein | ParB/RepB/Spo0J family partition protein | | uniclust | UniRef100\_A0A084SGC0 | 99.8 | 1.7e-22 | 3.2e-28 | 148.8 | 117 | (17, 139) | 291 | (11, 131) | 134 | ParB/Sulfiredoxin domain-containing protein (Fragment) | ParB/Sulfiredoxin domain-containing protein (Fragment) | | uniclust | UniRef100\_A0A1N0EX50 | 99.8 | 1.7e-22 | 3.3e-28 | 155.7 | 145 | (39, 188) | 291 | (2, 149) | 184 | Chromosome partitioning protein ParB | Chromosome partitioning protein ParB | | uniclust | UniRef100\_A0A069I5N8 | 99.8 | 1.7e-22 | 3.4e-28 | 197.0 | 178 | (17, 197) | 291 | (231, 414) | 944 | ParB/Sulfiredoxin domain-containing protein | ParB/Sulfiredoxin domain-containing protein | | uniclust | UniRef100\_A0A395X326 | 99.8 | 1.9e-22 | 3.6e-28 | 163.2 | 179 | (14, 197) | 291 | (27, 225) | 272 | ParB/RepB/Spo0J family partition protein (Fragment) | ParB/RepB/Spo0J family partition protein (Fragment) | | uniclust | UniRef100\_A0A0F6L5S3 | 99.8 | 1.9e-22 | 3.7e-28 | 180.2 | 147 | (19, 172) | 291 | (75, 227) | 448 | ParB/RepB/Spo0J family partition domain protein | ParB/RepB/Spo0J family partition domain protein | | uniclust | UniRef100\_A0A349FWC2 | 99.8 | 2e-22 | 3.8e-28 | 151.5 | 118 | (14, 137) | 291 | (32, 152) | 154 | ParB/Sulfiredoxin domain-containing protein | ParB/Sulfiredoxin domain-containing protein | | uniclust | UniRef100\_A0A1Q3SIH1 | 99.8 | 2e-22 | 3.8e-28 | 165.4 | 165 | (18, 189) | 291 | (38, 206) | 299 | ParB/Sulfiredoxin domain-containing protein | ParB/Sulfiredoxin domain-containing protein | | uniclust | UniRef100\_A0A0Q8BHN4 | 99.8 | 2e-22 | 3.8e-28 | 174.6 | 161 | (22, 190) | 291 | (36, 211) | 419 | ParB/Sulfiredoxin domain-containing protein | ParB/Sulfiredoxin domain-containing protein | | uniclust | UniRef100\_A6W8V9 | 99.8 | 2e-22 | 3.8e-28 | 169.8 | 164 | (16, 185) | 291 | (80, 245) | 398 | ParB-like partition protein | ParB-like partition protein | | uniclust | UniRef100\_A0A3D2QDE7 | 99.8 | 2e-22 | 3.8e-28 | 149.6 | 140 | (54, 198) | 291 | (2, 145) | 153 | Nucleoid occlusion protein (Fragment) | Nucleoid occlusion protein (Fragment) | | uniclust | UniRef100\_A0A258BLJ1 | 99.8 | 2e-22 | 3.9e-28 | 168.7 | 177 | (18, 196) | 291 | (18, 202) | 330 | Chromosome partitioning protein ParB (Fragment) | Chromosome partitioning protein ParB (Fragment) | | uniclust | UniRef100\_A0A7C6XDD3 | 99.8 | 2.1e-22 | 4e-28 | 166.1 | 175 | (20, 200) | 291 | (41, 219) | 344 | ParB/RepB/Spo0J family partition protein | ParB/RepB/Spo0J family partition protein | | uniclust | UniRef100\_A0A1C0B7H8 | 99.8 | 2.2e-22 | 4.2e-28 | 170.3 | 177 | (14, 198) | 291 | (26, 209) | 336 | Chromosome-partitioning protein Spo0J | Chromosome-partitioning protein Spo0J | | uniclust | UniRef100\_A0A0D6KAG1 | 99.8 | 2.2e-22 | 4.2e-28 | 171.7 | 174 | (21, 199) | 291 | (117, 328) | 402 | Partitioning protein ParB | Partitioning protein ParB | | uniclust | UniRef100\_UPI001BCFECD9 | 99.8 | 2.5e-22 | 4.5e-28 | 162.8 | 268 | (14, 288) | 291 | (42, 315) | 348 | hypothetical protein | hypothetical protein | | uniclust | UniRef100\_A0A1V5NPZ0 | 99.8 | 2.5e-22 | 4.6e-28 | 158.4 | 171 | (21, 198) | 291 | (3, 177) | 248 | Chromosome-partitioning protein Spo0J | Chromosome-partitioning protein Spo0J | | uniclust | UniRef100\_A0A064A7Z1 | 99.8 | 2.3e-22 | 4.7e-28 | 180.6 | 173 | (17, 193) | 291 | (66, 278) | 412 | Chromosome partitioning protein ParB | Chromosome partitioning protein ParB | | uniclust | UniRef100\_A0A2A6U4Q8 | 99.8 | 2.8e-22 | 5.1e-28 | 153.6 | 165 | (29, 199) | 291 | (2, 169) | 227 | Chromosome partitioning protein ParB (Fragment) | Chromosome partitioning protein ParB (Fragment) | | uniclust | UniRef100\_A0A1I9YVG4 | 99.8 | 2.6e-22 | 5.1e-28 | 173.5 | 192 | (15, 214) | 291 | (23, 232) | 385 | ParB/RepB/Spo0J family partition protein | ParB/RepB/Spo0J family partition protein | | uniclust | UniRef100\_A0A059G147 | 99.8 | 2.7e-22 | 5.1e-28 | 179.3 | 182 | (13, 195) | 291 | (82, 268) | 555 | Partitioning protein-like protein (Fragment) | Partitioning protein-like protein (Fragment) | | uniclust | UniRef100\_A0A0L0M783 | 99.8 | 2.8e-22 | 5.6e-28 | 174.2 | 177 | (20, 201) | 291 | (22, 203) | 361 | Chromosome (Plasmid) partitioning protein ParB | Chromosome (Plasmid) partitioning protein ParB | | uniclust | UniRef100\_A0A165R355 | 99.8 | 3.1e-22 | 5.7e-28 | 178.7 | 170 | (17, 192) | 291 | (126, 298) | 734 | ParB/Sulfiredoxin domain-containing protein (Fragment) | ParB/Sulfiredoxin domain-containing protein (Fragment) | | uniclust | UniRef100\_A0A097ENY6 | 99.8 | 3.1e-22 | 5.8e-28 | 164.2 | 169 | (14, 188) | 291 | (40, 213) | 305 | Chromosome partitioning protein ParB | Chromosome partitioning protein ParB | | uniclust | UniRef100\_A0A0F5JTH4 | 99.8 | 3e-22 | 5.9e-28 | 173.7 | 256 | (19, 289) | 291 | (82, 352) | 377 | ParB/Sulfiredoxin domain-containing protein | ParB/Sulfiredoxin domain-containing protein | | uniclust | UniRef100\_A0A0F9LWS5 | 99.8 | 3.4e-22 | 6.3e-28 | 154.7 | 166 | (18, 189) | 291 | (7, 175) | 202 | ParB/Sulfiredoxin domain-containing protein (Fragment) | ParB/Sulfiredoxin domain-containing protein (Fragment) | | uniclust | UniRef100\_A0A1F9UGF1 | 99.8 | 3.5e-22 | 6.4e-28 | 161.0 | 167 | (17, 189) | 291 | (8, 183) | 332 | ParB/Sulfiredoxin domain-containing protein | ParB/Sulfiredoxin domain-containing protein | | uniclust | UniRef100\_A0A4V2P9K0 | 99.8 | 3.5e-22 | 6.6e-28 | 178.5 | 179 | (18, 201) | 291 | (70, 269) | 577 | ParB/RepB/Spo0J family partition protein | ParB/RepB/Spo0J family partition protein | | uniclust | UniRef100\_A0A1N6KBF8 | 99.8 | 3.5e-22 | 6.8e-28 | 180.4 | 179 | (18, 201) | 291 | (26, 206) | 630 | ParB/RepB/Spo0J family partition protein | ParB/RepB/Spo0J family partition protein | | uniclust | UniRef100\_UPI0002BEB314 | 99.8 | 3.6e-22 | 6.8e-28 | 169.2 | 173 | (15, 193) | 291 | (47, 226) | 387 | ParB/RepB/Spo0J family partition protein | ParB/RepB/Spo0J family partition protein | | uniclust | UniRef100\_A0A1P8WH68 | 99.8 | 3.5e-22 | 6.9e-28 | 168.0 | 178 | (15, 198) | 291 | (18, 201) | 291 | Putative chromosome-partitioning protein ParB | Putative chromosome-partitioning protein ParB | | uniclust | UniRef100\_A0A3S3RBF0 | 99.8 | 3.9e-22 | 7.4e-28 | 148.1 | 123 | (15, 142) | 291 | (29, 153) | 155 | ParB/RepB/Spo0J family partition protein | ParB/RepB/Spo0J family partition protein | | uniclust | UniRef100\_A0A7X7KEX9 | 99.8 | 4.2e-22 | 7.7e-28 | 159.0 | 162 | (17, 184) | 291 | (142, 306) | 308 | ParB/RepB/Spo0J family partition protein (Fragment) | ParB/RepB/Spo0J family partition protein (Fragment) | | uniclust | UniRef100\_A0A066YG78 | 99.8 | 4.1e-22 | 7.9e-28 | 173.4 | 174 | (20, 202) | 291 | (58, 251) | 376 | ParB/Sulfiredoxin domain-containing protein | ParB/Sulfiredoxin domain-containing protein | | uniclust | UniRef100\_A0A3G9IH11 | 99.8 | 4.2e-22 | 8e-28 | 167.0 | 166 | (18, 188) | 291 | (106, 273) | 359 | ParB/Sulfiredoxin domain-containing protein | ParB/Sulfiredoxin domain-containing protein | | uniclust | UniRef100\_A0A7W1GI18 | 99.8 | 4.3e-22 | 8e-28 | 156.1 | 172 | (14, 190) | 291 | (11, 190) | 231 | ParB/RepB/Spo0J family partition protein | ParB/RepB/Spo0J family partition protein | | uniclust | UniRef100\_B6AP60 | 99.8 | 4.5e-22 | 8.4e-28 | 162.4 | 177 | (17, 199) | 291 | (29, 209) | 319 | ParB-like partition protein | ParB-like partition protein | | uniclust | UniRef100\_A0A0G0QGC9 | 99.8 | 4.6e-22 | 8.4e-28 | 162.0 | 182 | (13, 199) | 291 | (94, 278) | 361 | ParB-like protein partition protein | ParB-like protein partition protein | | uniclust | UniRef100\_A0A225D4E0 | 99.8 | 4.9e-22 | 9.4e-28 | 162.6 | 183 | (1, 188) | 291 | (1, 187) | 266 | ParB/Sulfiredoxin domain-containing protein | ParB/Sulfiredoxin domain-containing protein | | uniclust | UniRef100\_A0A2D9Y5I6 | 99.8 | 5.6e-22 | 1e-27 | 172.9 | 177 | (17, 198) | 291 | (6, 197) | 579 | ParB/Sulfiredoxin domain-containing protein | ParB/Sulfiredoxin domain-containing protein | | uniclust | UniRef100\_A0A0W7YQU1 | 99.8 | 5.6e-22 | 1.1e-27 | 174.9 | 180 | (17, 199) | 291 | (38, 234) | 446 | ParB/Sulfiredoxin domain-containing protein | ParB/Sulfiredoxin domain-containing protein | | uniclust | UniRef100\_A0A0J0YDN7 | 99.8 | 5.7e-22 | 1.1e-27 | 173.1 | 245 | (17, 290) | 291 | (62, 321) | 351 | ParB/Sulfiredoxin domain-containing protein | ParB/Sulfiredoxin domain-containing protein | | uniclust | UniRef100\_A0A3B9QB56 | 99.8 | 6e-22 | 1.1e-27 | 164.4 | 176 | (17, 199) | 291 | (28, 207) | 310 | ParB/Spo0J HTH domain-containing protein | ParB/Spo0J HTH domain-containing protein | | uniclust | UniRef100\_A0A2D5W999 | 99.8 | 6.4e-22 | 1.2e-27 | 165.8 | 168 | (18, 190) | 291 | (150, 323) | 419 | Chromosome partitioning protein ParB | Chromosome partitioning protein ParB | | uniclust | UniRef100\_A0A2R3UAM1 | 99.8 | 6.5e-22 | 1.2e-27 | 161.5 | 213 | (3, 216) | 291 | (4, 225) | 305 | Endonuclease | Endonuclease | | uniclust | UniRef100\_A0A0G1Q7M5 | 99.8 | 6.8e-22 | 1.2e-27 | 150.7 | 169 | (15, 188) | 291 | (31, 203) | 218 | Stage 0 sporulation protein J | Stage 0 sporulation protein J | | uniclust | UniRef100\_A0A1T1HAZ1 | 99.8 | 6.7e-22 | 1.3e-27 | 164.3 | 168 | (15, 188) | 291 | (32, 204) | 344 | ParB/Sulfiredoxin domain-containing protein | ParB/Sulfiredoxin domain-containing protein | | uniclust | UniRef100\_A0A259CJI7 | 99.8 | 6.8e-22 | 1.3e-27 | 162.2 | 157 | (13, 175) | 291 | (47, 209) | 299 | ParB/Sulfiredoxin domain-containing protein | ParB/Sulfiredoxin domain-containing protein | | uniclust | UniRef100\_UPI000B35E396 | 99.8 | 7.6e-22 | 1.4e-27 | 151.0 | 147 | (17, 168) | 291 | (31, 180) | 224 | ParB/RepB/Spo0J family partition protein | ParB/RepB/Spo0J family partition protein | | uniclust | UniRef100\_A0A1C6F112 | 99.8 | 7.3e-22 | 1.4e-27 | 165.8 | 168 | (21, 193) | 291 | (33, 211) | 321 | Nucleoid occlusion protein | Nucleoid occlusion protein | | uniclust | UniRef100\_A0A7Y9T2W1 | 99.8 | 7.6e-22 | 1.4e-27 | 161.3 | 178 | (17, 200) | 291 | (4, 184) | 326 | ParB/RepB/Spo0J family partition protein | ParB/RepB/Spo0J family partition protein | | uniclust | UniRef100\_A0A2I7QW51 | 99.8 | 7.7e-22 | 1.4e-27 | 159.0 | 271 | (2, 288) | 291 | (6, 282) | 286 | Uncharacterized protein | Uncharacterized protein | | uniclust | UniRef100\_A0A1G7A3Z4 | 99.8 | 7.7e-22 | 1.5e-27 | 169.2 | 170 | (16, 190) | 291 | (51, 223) | 388 | Chromosome partitioning protein, ParB family | Chromosome partitioning protein, ParB family | | uniclust | UniRef100\_A0A0P9EKV8 | 99.8 | 7.9e-22 | 1.5e-27 | 166.9 | 158 | (21, 187) | 291 | (3, 162) | 389 | Nucleoid occlusion protein | Nucleoid occlusion protein | | uniclust | UniRef100\_A0A1M6KVA2 | 99.8 | 8.8e-22 | 1.7e-27 | 161.8 | 162 | (20, 189) | 291 | (34, 200) | 304 | ParB/RepB/Spo0J family partition protein | ParB/RepB/Spo0J family partition protein | | uniclust | UniRef100\_F3AXU5 | 99.8 | 8.9e-22 | 1.7e-27 | 152.4 | 145 | (17, 169) | 291 | (3, 154) | 199 | ParB/Sulfiredoxin domain-containing protein | ParB/Sulfiredoxin domain-containing protein | | uniclust | UniRef100\_A0A1Q3SL05 | 99.8 | 8.4e-22 | 1.7e-27 | 173.1 | 178 | (16, 199) | 291 | (45, 231) | 373 | ParB/Sulfiredoxin domain-containing protein | ParB/Sulfiredoxin domain-containing protein | | uniclust | UniRef100\_A0A2E2NA70 | 99.8 | 9.1e-22 | 1.7e-27 | 165.0 | 167 | (16, 188) | 291 | (198, 368) | 466 | HTH cro/C1-type domain-containing protein | HTH cro/C1-type domain-containing protein | | uniclust | UniRef100\_A0A1V2SIP1 | 99.8 | 9.2e-22 | 1.7e-27 | 162.6 | 161 | (20, 186) | 291 | (3, 165) | 409 | ParB/Sulfiredoxin domain-containing protein | ParB/Sulfiredoxin domain-containing protein | | uniclust | UniRef100\_A0A087C1L2 | 99.8 | 9.4e-22 | 1.8e-27 | 177.3 | 172 | (17, 194) | 291 | (386, 604) | 710 | Chromosome partitioning protein ParB | Chromosome partitioning protein ParB | | uniclust | UniRef100\_A0A1V6MIC0 | 99.8 | 9.4e-22 | 1.8e-27 | 159.3 | 175 | (18, 199) | 291 | (39, 231) | 263 | Plasmid partitioning protein (Fragment) | Plasmid partitioning protein (Fragment) | | uniclust | UniRef100\_A0A1C5G7E9 | 99.8 | 9.4e-22 | 1.8e-27 | 180.5 | 163 | (20, 187) | 291 | (58, 223) | 580 | Chromosome partitioning protein, ParB family | Chromosome partitioning protein, ParB family | | uniclust | UniRef100\_A0A031HLV3 | 99.8 | 9.7e-22 | 1.9e-27 | 184.6 | 171 | (22, 197) | 291 | (115, 302) | 792 | ParB-like partition protein | ParB-like partition protein | | uniclust | UniRef100\_A0A1G5CHR0 | 99.8 | 1.2e-21 | 2.2e-27 | 162.4 | 165 | (17, 189) | 291 | (31, 197) | 354 | Chromosome partitioning protein, ParB family | Chromosome partitioning protein, ParB family | | uniclust | UniRef100\_UPI001607B1A3 | 99.8 | 1.2e-21 | 2.2e-27 | 152.4 | 195 | (69, 288) | 291 | (2, 197) | 209 | chromosome partitioning protein ParB | chromosome partitioning protein ParB | | uniclust | UniRef100\_A0A2L2NV26 | 99.8 | 1.1e-21 | 2.2e-27 | 167.3 | 164 | (16, 184) | 291 | (16, 184) | 291 | Chromosome/plasmid partitioning protein ParB | Chromosome/plasmid partitioning protein ParB | | uniclust | UniRef100\_A0A800ETG4 | 99.8 | 1.2e-21 | 2.3e-27 | 154.0 | 152 | (14, 171) | 291 | (31, 185) | 273 | ParB/RepB/Spo0J family partition protein (Fragment) | ParB/RepB/Spo0J family partition protein (Fragment) | | uniclust | UniRef100\_A0A7V9IA21 | 99.8 | 1.2e-21 | 2.3e-27 | 164.4 | 182 | (13, 200) | 291 | (32, 220) | 411 | ParB/RepB/Spo0J family partition protein | ParB/RepB/Spo0J family partition protein | | uniclust | UniRef100\_A0A7X1ZX84 | 99.8 | 1.3e-21 | 2.3e-27 | 158.8 | 169 | (15, 189) | 291 | (31, 202) | 305 | ParB/RepB/Spo0J family partition protein | ParB/RepB/Spo0J family partition protein | | uniclust | UniRef100\_A0A098MHM3 | 99.8 | 1.3e-21 | 2.4e-27 | 160.2 | 154 | (15, 174) | 291 | (19, 176) | 285 | Plasmid partitioning protein ParB | Plasmid partitioning protein ParB | | uniclust | UniRef100\_A0A1C5TTA9 | 99.8 | 1.3e-21 | 2.4e-27 | 161.1 | 178 | (15, 197) | 291 | (43, 240) | 289 | Chromosome-partitioning protein parB | Chromosome-partitioning protein parB | | uniclust | UniRef100\_A0A077FQM9 | 99.7 | 1.3e-21 | 2.5e-27 | 171.9 | 166 | (17, 188) | 291 | (71, 244) | 360 | Probable chromosome-partitioning protein ParB | Probable chromosome-partitioning protein ParB | | uniclust | UniRef100\_A0A173WSZ4 | 99.7 | 1.3e-21 | 2.6e-27 | 156.8 | 179 | (13, 196) | 291 | (27, 226) | 231 | Nucleoid occlusion protein | Nucleoid occlusion protein | | uniclust | UniRef100\_A0A0M4DV65 | 99.7 | 1.4e-21 | 2.6e-27 | 165.7 | 176 | (20, 199) | 291 | (36, 235) | 376 | ParB/Sulfiredoxin domain-containing protein | ParB/Sulfiredoxin domain-containing protein | | uniclust | UniRef100\_A0A0B3BFF3 | 99.7 | 1.3e-21 | 2.7e-27 | 178.8 | 171 | (20, 197) | 291 | (35, 235) | 489 | Putative transcriptional regulator | Putative transcriptional regulator | | uniclust | UniRef100\_A0A2M7TEW5 | 99.7 | 1.5e-21 | 2.8e-27 | 149.1 | 165 | (18, 188) | 291 | (4, 171) | 182 | ParB/Sulfiredoxin domain-containing protein | ParB/Sulfiredoxin domain-containing protein | | uniclust | UniRef100\_A0A0P9H9C4 | 99.7 | 1.5e-21 | 2.8e-27 | 147.9 | 164 | (19, 188) | 291 | (2, 176) | 188 | Chromosome partitioning protein ParB (Fragment) | Chromosome partitioning protein ParB (Fragment) | | uniclust | UniRef100\_A0A133XIV0 | 99.7 | 1.5e-21 | 2.8e-27 | 164.9 | 166 | (17, 187) | 291 | (43, 215) | 341 | Chromosome partitioning protein ParB | Chromosome partitioning protein ParB | | uniclust | UniRef100\_A0A519FZE1 | 99.7 | 1.5e-21 | 2.9e-27 | 176.2 | 171 | (17, 193) | 291 | (7, 185) | 686 | ParB/RepB/Spo0J family partition protein | ParB/RepB/Spo0J family partition protein | | uniclust | UniRef100\_A0A953F209 | 99.7 | 1.6e-21 | 3e-27 | 149.0 | 163 | (17, 185) | 291 | (32, 204) | 221 | ParB/RepB/Spo0J family partition protein (Fragment) | ParB/RepB/Spo0J family partition protein (Fragment) | | uniclust | UniRef100\_A0A0T7G344 | 99.7 | 1.6e-21 | 3e-27 | 180.1 | 181 | (15, 197) | 291 | (33, 220) | 667 | ParB domain protein nuclease (Fragment) | ParB domain protein nuclease (Fragment) | | uniclust | UniRef100\_A0A7C5GQE9 | 99.7 | 1.6e-21 | 3e-27 | 160.0 | 169 | (15, 189) | 291 | (33, 209) | 291 | ParB/RepB/Spo0J family partition protein | ParB/RepB/Spo0J family partition protein | | uniclust | UniRef100\_A0A2M7E1N5 | 99.7 | 1.6e-21 | 3e-27 | 161.1 | 165 | (15, 185) | 291 | (8, 186) | 278 | ParB/Sulfiredoxin domain-containing protein | ParB/Sulfiredoxin domain-containing protein | | uniclust | UniRef100\_A0A512MCK4 | 99.7 | 1.6e-21 | 3.1e-27 | 158.5 | 177 | (14, 196) | 291 | (26, 207) | 288 | ParB/Sulfiredoxin domain-containing protein | ParB/Sulfiredoxin domain-containing protein | | uniclust | UniRef100\_A0A5S4ZSV0 | 99.7 | 1.7e-21 | 3.2e-27 | 170.0 | 164 | (18, 187) | 291 | (2, 172) | 638 | ParB/RepB/Spo0J family partition protein | ParB/RepB/Spo0J family partition protein | | uniclust | UniRef100\_A0A0H3ZQX6 | 99.7 | 1.7e-21 | 3.2e-27 | 165.1 | 152 | (17, 177) | 291 | (31, 186) | 319 | Chromosome (Plasmid) partitioning protein ParB | Chromosome (Plasmid) partitioning protein ParB | | uniclust | UniRef100\_A0A1X7DWC0 | 99.7 | 1.7e-21 | 3.4e-27 | 167.0 | 179 | (16, 197) | 291 | (6, 193) | 370 | Chromosome partitioning protein, ParB family (Fragment) | Chromosome partitioning protein, ParB family (Fragment) | | uniclust | UniRef100\_A0A0N1NWJ0 | 99.7 | 1.8e-21 | 3.4e-27 | 183.2 | 174 | (20, 198) | 291 | (675, 854) | 1019 | ParB-like partition protein | ParB-like partition protein | | uniclust | UniRef100\_A0A0C2AKN6 | 99.7 | 1.8e-21 | 3.5e-27 | 172.9 | 168 | (15, 188) | 291 | (32, 221) | 476 | ParB/Sulfiredoxin domain-containing protein | ParB/Sulfiredoxin domain-containing protein | | uniclust | UniRef100\_A0A177NYB8 | 99.7 | 1.9e-21 | 3.7e-27 | 183.8 | 177 | (18, 196) | 291 | (7, 190) | 726 | ParB/Sulfiredoxin domain-containing protein | ParB/Sulfiredoxin domain-containing protein | | uniclust | UniRef100\_A0A1C4K463 | 99.7 | 1.9e-21 | 3.7e-27 | 170.0 | 174 | (21, 199) | 291 | (76, 272) | 431 | ParB/RepB/Spo0J family partition protein | ParB/RepB/Spo0J family partition protein | | uniclust | UniRef100\_A0A1F9UE43 | 99.7 | 2.1e-21 | 4e-27 | 161.9 | 167 | (18, 189) | 291 | (13, 183) | 369 | ParB/Sulfiredoxin domain-containing protein | ParB/Sulfiredoxin domain-containing protein | | uniclust | UniRef100\_A0A537LQR2 | 99.7 | 2.2e-21 | 4e-27 | 149.5 | 145 | (19, 169) | 291 | (85, 232) | 234 | ParB/RepB/Spo0J family partition protein (Fragment) | ParB/RepB/Spo0J family partition protein (Fragment) | | uniclust | UniRef100\_A0A352Z5M4 | 99.7 | 2.1e-21 | 4.1e-27 | 160.9 | 181 | (14, 199) | 291 | (17, 200) | 309 | ParB/Sulfiredoxin domain-containing protein | ParB/Sulfiredoxin domain-containing protein | | uniclust | UniRef100\_A0A497FIQ1 | 99.7 | 2.2e-21 | 4.2e-27 | 165.4 | 165 | (18, 188) | 291 | (3, 170) | 348 | ParB/Sulfiredoxin domain-containing protein | ParB/Sulfiredoxin domain-containing protein | | uniclust | UniRef100\_UPI00041C85F7 | 99.7 | 2.4e-21 | 4.4e-27 | 159.9 | 169 | (16, 189) | 291 | (24, 198) | 400 | ParB/RepB/Spo0J family partition protein | ParB/RepB/Spo0J family partition protein | | uniclust | UniRef100\_A0A257NIY2 | 99.7 | 2.4e-21 | 4.5e-27 | 145.5 | 152 | (43, 198) | 291 | (2, 156) | 180 | ParB/Sulfiredoxin domain-containing protein (Fragment) | ParB/Sulfiredoxin domain-containing protein (Fragment) | | uniclust | UniRef100\_A0A225DDB5 | 99.7 | 2.4e-21 | 4.5e-27 | 161.8 | 179 | (17, 201) | 291 | (44, 233) | 312 | Chromosome (Plasmid) partitioning protein ParB / Stage 0 sporulation protein J | Chromosome (Plasmid) partitioning protein ParB / Stage 0 sporulation protein J | | uniclust | UniRef100\_A0A075MDE3 | 99.7 | 2.4e-21 | 4.5e-27 | 149.0 | 145 | (20, 170) | 291 | (36, 183) | 188 | Chromosome (Plasmid) partitioning protein ParB/ Stage 0 sporulation protein J | Chromosome (Plasmid) partitioning protein ParB/ Stage 0 sporulation protein J | | uniclust | UniRef100\_A0A6N6T5G8 | 99.7 | 2.4e-21 | 4.5e-27 | 156.7 | 176 | (18, 194) | 291 | (5, 186) | 270 | ParB/RepB/Spo0J family partition protein | ParB/RepB/Spo0J family partition protein | | uniclust | UniRef100\_A0A3A4Z2I3 | 99.7 | 2.5e-21 | 4.7e-27 | 158.3 | 164 | (19, 187) | 291 | (38, 206) | 313 | ParB/RepB/Spo0J family partition protein | ParB/RepB/Spo0J family partition protein | | uniclust | UniRef100\_A0A950B1H5 | 99.7 | 2.6e-21 | 4.8e-27 | 156.9 | 165 | (20, 189) | 291 | (50, 218) | 345 | ParB/RepB/Spo0J family partition protein | ParB/RepB/Spo0J family partition protein | | uniclust | UniRef100\_A0A176EXX9 | 99.7 | 2.4e-21 | 4.8e-27 | 170.2 | 223 | (13, 247) | 291 | (29, 266) | 330 | ParB/Sulfiredoxin domain-containing protein | ParB/Sulfiredoxin domain-containing protein | | uniclust | UniRef100\_A0A0S8I9V8 | 99.7 | 2.6e-21 | 4.9e-27 | 160.2 | 168 | (18, 186) | 291 | (5, 178) | 291 | Chromosome partitioning protein ParB | Chromosome partitioning protein ParB | | uniclust | UniRef100\_A0A952V660 | 99.7 | 2.7e-21 | 5.1e-27 | 172.3 | 180 | (14, 199) | 291 | (25, 221) | 581 | ParB/RepB/Spo0J family partition protein | ParB/RepB/Spo0J family partition protein | | uniclust | UniRef100\_A0A1F8MEB9 | 99.7 | 2.8e-21 | 5.3e-27 | 156.1 | 176 | (21, 201) | 291 | (3, 183) | 259 | ParB/Sulfiredoxin domain-containing protein | ParB/Sulfiredoxin domain-containing protein | | uniclust | UniRef100\_A0A7X6ND40 | 99.7 | 2.9e-21 | 5.3e-27 | 168.9 | 179 | (16, 199) | 291 | (430, 620) | 706 | ParB/RepB/Spo0J family partition protein | ParB/RepB/Spo0J family partition protein | | uniclust | UniRef100\_A0A977UBZ6 | 99.7 | 3e-21 | 5.4e-27 | 145.0 | 162 | (23, 189) | 291 | (5, 171) | 196 | ParB/RepB/Spo0J family partition protein | ParB/RepB/Spo0J family partition protein | | uniclust | UniRef100\_A0A537PWK3 | 99.7 | 3e-21 | 5.5e-27 | 145.1 | 144 | (39, 187) | 291 | (4, 152) | 197 | ParB/RepB/Spo0J family partition protein (Fragment) | ParB/RepB/Spo0J family partition protein (Fragment) | | uniclust | UniRef100\_A0A0F9L3S6 | 99.7 | 3e-21 | 5.7e-27 | 167.9 | 180 | (14, 198) | 291 | (49, 237) | 403 | ParB/Sulfiredoxin domain-containing protein | ParB/Sulfiredoxin domain-containing protein | | uniclust | UniRef100\_A0A7V9GCS1 | 99.7 | 3.1e-21 | 5.8e-27 | 149.5 | 167 | (16, 189) | 291 | (27, 197) | 230 | ParB/RepB/Spo0J family partition protein (Fragment) | ParB/RepB/Spo0J family partition protein (Fragment) | | uniclust | UniRef100\_A0A0N8Q4D0 | 99.7 | 3e-21 | 5.9e-27 | 180.4 | 176 | (21, 201) | 291 | (63, 243) | 631 | Probable chromosome-partitioning protein ParB | Probable chromosome-partitioning protein ParB | | uniclust | UniRef100\_A0A0F4I4G6 | 99.7 | 3.2e-21 | 5.9e-27 | 150.2 | 164 | (30, 198) | 291 | (4, 184) | 221 | ParB/Sulfiredoxin domain-containing protein (Fragment) | ParB/Sulfiredoxin domain-containing protein (Fragment) | | uniclust | UniRef100\_A0A5Q4ZYV4 | 99.7 | 3.1e-21 | 6e-27 | 165.3 | 172 | (16, 188) | 291 | (32, 208) | 340 | ParB/Sulfiredoxin domain-containing protein | ParB/Sulfiredoxin domain-containing protein | | uniclust | UniRef100\_R7M4B8 | 99.7 | 3.3e-21 | 6.1e-27 | 160.9 | 174 | (20, 199) | 291 | (7, 184) | 444 | ParB-like partition protein | ParB-like partition protein | | uniclust | UniRef100\_A0A6L7GCN8 | 99.7 | 3.2e-21 | 6.1e-27 | 161.7 | 181 | (1, 187) | 291 | (1, 184) | 304 | ParB/RepB/Spo0J family partition protein | ParB/RepB/Spo0J family partition protein | | uniclust | UniRef100\_A0A1V2H5Y6 | 99.7 | 3.1e-21 | 6.1e-27 | 171.4 | 165 | (17, 187) | 291 | (132, 304) | 419 | Chromosome partitioning protein ParB | Chromosome partitioning protein ParB | | uniclust | UniRef100\_A0A0Q4MYY7 | 99.7 | 3.6e-21 | 7e-27 | 162.3 | 168 | (15, 188) | 291 | (35, 205) | 324 | ParB/Sulfiredoxin domain-containing protein | ParB/Sulfiredoxin domain-containing protein | | uniclust | UniRef100\_UPI00216A9ACB | 99.7 | 3.8e-21 | 7e-27 | 139.6 | 139 | (21, 165) | 291 | (3, 153) | 157 | ParB/RepB/Spo0J family partition protein | ParB/RepB/Spo0J family partition protein | | uniclust | UniRef100\_E7H2H0 | 99.7 | 3.9e-21 | 7.3e-27 | 163.3 | 167 | (16, 190) | 291 | (26, 200) | 388 | ParB/Sulfiredoxin domain-containing protein | ParB/Sulfiredoxin domain-containing protein | | uniclust | UniRef100\_A0A936A9A9 | 99.7 | 4e-21 | 7.4e-27 | 161.3 | 170 | (17, 191) | 291 | (33, 205) | 467 | Probable chromosome-partitioning protein ParB | Probable chromosome-partitioning protein ParB | | uniclust | UniRef100\_A0A060HQA9 | 99.7 | 3.9e-21 | 7.4e-27 | 156.0 | 172 | (18, 195) | 291 | (3, 182) | 257 | ParB/Sulfiredoxin domain-containing protein | ParB/Sulfiredoxin domain-containing protein | | uniclust | UniRef100\_A0A073IWS9 | 99.7 | 4e-21 | 8e-27 | 182.2 | 178 | (19, 198) | 291 | (62, 247) | 704 | ParB/Sulfiredoxin domain-containing protein | ParB/Sulfiredoxin domain-containing protein | | uniclust | UniRef100\_UPI00126055D0 | 99.7 | 4.4e-21 | 8.1e-27 | 149.1 | 166 | (18, 189) | 291 | (49, 220) | 248 | ParB/RepB/Spo0J family partition protein | ParB/RepB/Spo0J family partition protein | | uniclust | UniRef100\_A0A5F0LUW4 | 99.7 | 4.2e-21 | 8.1e-27 | 176.4 | 166 | (18, 189) | 291 | (37, 212) | 703 | ParB/RepB/Spo0J family partition protein | ParB/RepB/Spo0J family partition protein | | uniclust | UniRef100\_A0A1C5P2R5 | 99.7 | 4.4e-21 | 8.3e-27 | 159.0 | 176 | (14, 194) | 291 | (36, 220) | 309 | Probable chromosome-partitioning protein parB | Probable chromosome-partitioning protein parB | | uniclust | UniRef100\_UPI001F0133BB | 99.7 | 4.6e-21 | 8.5e-27 | 154.7 | 167 | (15, 187) | 291 | (29, 198) | 332 | ParB/RepB/Spo0J family partition protein | ParB/RepB/Spo0J family partition protein | | uniclust | UniRef100\_A0A962QQD6 | 99.7 | 4.5e-21 | 8.6e-27 | 161.1 | 149 | (19, 173) | 291 | (23, 176) | 342 | ParB/RepB/Spo0J family partition protein | ParB/RepB/Spo0J family partition protein | | uniclust | UniRef100\_A0A920G1W5 | 99.7 | 5.1e-21 | 9.4e-27 | 158.9 | 175 | (20, 199) | 291 | (171, 348) | 422 | Probable chromosome-partitioning protein ParB | Probable chromosome-partitioning protein ParB | | uniclust | UniRef100\_A0A840CS83 | 99.7 | 5.2e-21 | 1e-26 | 176.9 | 177 | (14, 196) | 291 | (294, 473) | 832 | ParB/RepB/Spo0J family partition protein | ParB/RepB/Spo0J family partition protein | | uniclust | UniRef100\_A0A021VU08 | 99.7 | 5.3e-21 | 1e-26 | 170.6 | 176 | (15, 196) | 291 | (40, 219) | 533 | Chromosomal partitioning protein ParB | Chromosomal partitioning protein ParB | | uniclust | UniRef100\_A0A011TXK3 | 99.7 | 5.1e-21 | 1e-26 | 167.8 | 186 | (14, 206) | 291 | (20, 210) | 362 | Plasmid stablization protein ParB | Plasmid stablization protein ParB | | uniclust | UniRef100\_A0A2T2VQX6 | 99.7 | 5.2e-21 | 1e-26 | 156.9 | 131 | (66, 201) | 291 | (6, 139) | 228 | ParB/Spo0J HTH domain-containing protein (Fragment) | ParB/Spo0J HTH domain-containing protein (Fragment) | | uniclust | UniRef100\_A0A1J9PXM3 | 99.7 | 5.5e-21 | 1e-26 | 163.3 | 235 | (13, 247) | 291 | (27, 290) | 453 | Pyridoxal phosphate biosynthetic protein PdxJ | Pyridoxal phosphate biosynthetic protein PdxJ | | uniclust | UniRef100\_A0A3M2CVE6 | 99.7 | 5.6e-21 | 1e-26 | 156.5 | 163 | (21, 190) | 291 | (31, 197) | 327 | ParB/RepB/Spo0J family partition protein | ParB/RepB/Spo0J family partition protein | | uniclust | UniRef100\_A0A173ZK65 | 99.7 | 5.5e-21 | 1e-26 | 172.6 | 176 | (17, 197) | 291 | (331, 531) | 660 | Probable chromosome-partitioning protein parB | Probable chromosome-partitioning protein parB | | uniclust | UniRef100\_A0A246BRZ4 | 99.7 | 5.5e-21 | 1.1e-26 | 162.3 | 175 | (16, 198) | 291 | (32, 233) | 315 | ParB/Sulfiredoxin domain-containing protein | ParB/Sulfiredoxin domain-containing protein | | uniclust | UniRef100\_A0A0F9U9W1 | 99.7 | 5.7e-21 | 1.1e-26 | 163.3 | 155 | (15, 176) | 291 | (35, 193) | 383 | ParB/Sulfiredoxin domain-containing protein | ParB/Sulfiredoxin domain-containing protein | | uniclust | UniRef100\_A0A011Q0A6 | 99.7 | 5.5e-21 | 1.1e-26 | 188.3 | 179 | (16, 197) | 291 | (179, 363) | 924 | Plasmid partitioning protein | Plasmid partitioning protein | | uniclust | UniRef100\_A0A352YU83 | 99.7 | 6e-21 | 1.1e-26 | 159.1 | 166 | (16, 187) | 291 | (33, 210) | 321 | ParB/Sulfiredoxin domain-containing protein | ParB/Sulfiredoxin domain-containing protein | | uniclust | UniRef100\_A0A7C3VM87 | 99.7 | 6.3e-21 | 1.2e-26 | 153.5 | 162 | (18, 184) | 291 | (5, 176) | 323 | ParB/RepB/Spo0J family partition protein | ParB/RepB/Spo0J family partition protein | | uniclust | UniRef100\_A0A1G0T9I7 | 99.7 | 6.3e-21 | 1.2e-26 | 160.2 | 176 | (19, 199) | 291 | (44, 224) | 466 | ParB/Sulfiredoxin domain-containing protein | ParB/Sulfiredoxin domain-containing protein | | uniclust | UniRef100\_A0A916DA64 | 99.7 | 6.1e-21 | 1.2e-26 | 159.4 | 171 | (19, 199) | 291 | (42, 213) | 333 | Chromosome-partitioning protein Spo0J | Chromosome-partitioning protein Spo0J | | uniclust | UniRef100\_A0A0A7PKV5 | 99.7 | 6e-21 | 1.2e-26 | 169.5 | 166 | (13, 183) | 291 | (45, 220) | 372 | ParB-like partition protein | ParB-like partition protein | | uniclust | UniRef100\_A0A357EIG5 | 99.7 | 6.6e-21 | 1.2e-26 | 152.6 | 165 | (18, 188) | 291 | (20, 190) | 281 | ParB/Sulfiredoxin domain-containing protein (Fragment) | ParB/Sulfiredoxin domain-containing protein (Fragment) | | uniclust | UniRef100\_A0A920UHB7 | 99.7 | 6.8e-21 | 1.3e-26 | 144.4 | 152 | (15, 173) | 291 | (10, 168) | 208 | ParB/Sulfiredoxin domain-containing protein | ParB/Sulfiredoxin domain-containing protein | | uniclust | UniRef100\_A0A1F3T0T4 | 99.7 | 7.1e-21 | 1.3e-26 | 157.5 | 173 | (18, 196) | 291 | (49, 226) | 308 | ParB/Sulfiredoxin domain-containing protein | ParB/Sulfiredoxin domain-containing protein | | uniclust | UniRef100\_A0A2E9QWR4 | 99.7 | 6.9e-21 | 1.3e-26 | 163.4 | 181 | (14, 200) | 291 | (73, 261) | 349 | ParB/Sulfiredoxin domain-containing protein | ParB/Sulfiredoxin domain-containing protein | | uniclust | UniRef100\_A0A1I7FCB2 | 99.7 | 7e-21 | 1.4e-26 | 165.6 | 170 | (18, 194) | 291 | (42, 221) | 384 | ParB/RepB/Spo0J family partition protein | ParB/RepB/Spo0J family partition protein | | uniclust | UniRef100\_A0A523SL81 | 99.7 | 7.5e-21 | 1.4e-26 | 169.9 | 175 | (17, 198) | 291 | (9, 186) | 623 | ParB/RepB/Spo0J family partition protein | ParB/RepB/Spo0J family partition protein | | uniclust | UniRef100\_A0A934DLA7 | 99.7 | 7.7e-21 | 1.4e-26 | 156.3 | 175 | (15, 196) | 291 | (29, 206) | 386 | ParB/RepB/Spo0J family partition protein | ParB/RepB/Spo0J family partition protein | | uniclust | UniRef100\_A0A1F6FNT4 | 99.7 | 7.6e-21 | 1.4e-26 | 168.6 | 178 | (16, 199) | 291 | (8, 194) | 627 | ParB/Sulfiredoxin domain-containing protein | ParB/Sulfiredoxin domain-containing protein | | uniclust | UniRef100\_A0A0P8BD48 | 99.7 | 7.3e-21 | 1.4e-26 | 164.7 | 180 | (16, 200) | 291 | (45, 266) | 349 | Chromosome partitioning protein, ParB family | Chromosome partitioning protein, ParB family | | uniclust | UniRef100\_X1ICS2 | 99.7 | 7.7e-21 | 1.5e-26 | 147.0 | 174 | (22, 199) | 291 | (8, 185) | 202 | ParB/Sulfiredoxin domain-containing protein (Fragment) | ParB/Sulfiredoxin domain-containing protein (Fragment) | | uniclust | UniRef100\_A0A7Y4RZN6 | 99.7 | 8e-21 | 1.5e-26 | 159.6 | 167 | (14, 188) | 291 | (31, 202) | 332 | ParB/RepB/Spo0J family partition protein | ParB/RepB/Spo0J family partition protein | | uniclust | UniRef100\_A0A068YQ66 | 99.7 | 8.1e-21 | 1.6e-26 | 164.0 | 169 | (14, 189) | 291 | (57, 226) | 381 | Chromosome (Plasmid) partitioning protein ParB / Stage 0 sporulation protein J | Chromosome (Plasmid) partitioning protein ParB / Stage 0 sporulation protein J | | uniclust | UniRef100\_A0A0Q5HQC1 | 99.7 | 8.3e-21 | 1.6e-26 | 165.0 | 166 | (17, 190) | 291 | (29, 205) | 372 | ParB/Sulfiredoxin domain-containing protein | ParB/Sulfiredoxin domain-containing protein | | uniclust | UniRef100\_A0A662E073 | 99.7 | 8.9e-21 | 1.6e-26 | 150.6 | 173 | (19, 197) | 291 | (2, 177) | 290 | ParB/Sulfiredoxin domain-containing protein | ParB/Sulfiredoxin domain-containing protein | | uniclust | UniRef100\_A0A940CRG9 | 99.7 | 9.7e-21 | 1.8e-26 | 162.0 | 162 | (19, 185) | 291 | (3, 172) | 555 | ParB/RepB/Spo0J family partition protein | ParB/RepB/Spo0J family partition protein | | uniclust | UniRef100\_A0A0L6J3Z5 | 99.7 | 9.1e-21 | 1.8e-26 | 178.3 | 178 | (19, 198) | 291 | (114, 304) | 786 | ParB/Sulfiredoxin domain-containing protein | ParB/Sulfiredoxin domain-containing protein | | uniclust | UniRef100\_A0A011U8K2 | 99.7 | 8.9e-21 | 1.8e-26 | 185.1 | 180 | (15, 197) | 291 | (144, 336) | 961 | DNA-binding protein | DNA-binding protein | | uniclust | UniRef100\_A0A5R8QHF2 | 99.7 | 1.1e-20 | 1.9e-26 | 164.8 | 168 | (19, 192) | 291 | (12, 183) | 675 | ParB/RepB/Spo0J family partition protein | ParB/RepB/Spo0J family partition protein | | uniclust | UniRef100\_UPI001A972BC8 | 99.7 | 1e-20 | 1.9e-26 | 156.8 | 164 | (19, 188) | 291 | (52, 226) | 367 | ParB/RepB/Spo0J family partition protein | ParB/RepB/Spo0J family partition protein | | uniclust | UniRef100\_A0A0C2R4G1 | 99.7 | 1e-20 | 2e-26 | 163.8 | 152 | (16, 172) | 291 | (4, 161) | 406 | ParB/Sulfiredoxin domain-containing protein | ParB/Sulfiredoxin domain-containing protein | | uniclust | UniRef100\_A0A1V5UI04 | 99.7 | 1.1e-20 | 2.1e-26 | 157.8 | 181 | (15, 200) | 291 | (35, 217) | 283 | Putative chromosome-partitioning protein ParB | Putative chromosome-partitioning protein ParB | | uniclust | UniRef100\_A0A067YD33 | 99.7 | 1.1e-20 | 2.1e-26 | 162.3 | 143 | (17, 165) | 291 | (45, 192) | 326 | Chromosome-partitioning protein ParB | Chromosome-partitioning protein ParB | | uniclust | UniRef100\_A0A6G8FUF2 | 99.7 | 1.2e-20 | 2.2e-26 | 152.9 | 166 | (14, 185) | 291 | (73, 240) | 330 | ParB/RepB/Spo0J family partition protein | ParB/RepB/Spo0J family partition protein | | uniclust | UniRef100\_A0A016QQ86 | 99.7 | 1.2e-20 | 2.3e-26 | 160.9 | 180 | (17, 201) | 291 | (29, 238) | 313 | ParB-like partition protein | ParB-like partition protein | | uniclust | UniRef100\_A0A6N7Z139 | 99.7 | 1.2e-20 | 2.3e-26 | 150.7 | 167 | (17, 188) | 291 | (32, 205) | 305 | ParB/RepB/Spo0J family partition protein | ParB/RepB/Spo0J family partition protein | | pdb70 | 6SDK\_B | 99.8 | 1.3e-25 | 1.2e-29 | 190.6 | 176 | (18, 199) | 291 | (2, 181) | 199 | Stage 0 sporulation protein J | 6SDK\_B Stage 0 sporulation protein J ParB, Chromosome segregation, Chromosome organization HET: MSE, CDP | | pdb70 | 6SDK\_C | 99.8 | 1.3e-25 | 1.2e-29 | 190.6 | 176 | (18, 199) | 291 | (2, 181) | 199 | Stage 0 sporulation protein J | 6SDK\_C Stage 0 sporulation protein J ParB, Chromosome segregation, Chromosome organization HET: MSE, CDP | | pdb70 | 1VZ0\_D | 99.8 | 2.7e-25 | 2.5e-29 | 193.9 | 178 | (16, 199) | 291 | (19, 198) | 230 | CHROMOSOME PARTITIONING PROTEIN PARB | 1VZ0\_D CHROMOSOME PARTITIONING PROTEIN PARB NUCLEAR PROTEIN, CHROMOSOME SEGREGATION, DNA-BINDING HET: CO | | pdb70 | 4UMK\_A | 99.8 | 2e-24 | 1.8e-28 | 189.5 | 179 | (14, 198) | 291 | (27, 208) | 240 | PROBABLE CHROMOSOME-PARTITIONING PROTEIN PARB | 4UMK\_A PROBABLE CHROMOSOME-PARTITIONING PROTEIN PARB DNA BINDING PROTEIN-DNA COMPLEX HET: SO4 | | pdb70 | 4UMK\_C | 99.8 | 2e-24 | 1.8e-28 | 189.5 | 179 | (14, 198) | 291 | (27, 208) | 240 | PROBABLE CHROMOSOME-PARTITIONING PROTEIN PARB | 4UMK\_C PROBABLE CHROMOSOME-PARTITIONING PROTEIN PARB DNA BINDING PROTEIN-DNA COMPLEX HET: SO4 | | pdb70 | 5K5A\_B | 99.7 | 3.1e-22 | 2.8e-26 | 181.6 | 166 | (14, 188) | 291 | (5, 230) | 300 | ParB domain protein nuclease | 5K5A\_B ParB domain protein nuclease ParB, pNOB8, partition, DNA segregation HET: MSE | | pdb70 | 5K5D\_C | 99.7 | 6.7e-22 | 6e-26 | 179.4 | 166 | (14, 188) | 291 | (5, 230) | 300 | ParB domain protein nuclease | 5K5D\_C ParB domain protein nuclease ParB-N, pnob8, partition, HYDROLASE HET: CIT, MSE | | pdb70 | 6RYK\_A | 99.6 | 7.8e-20 | 7.1e-24 | 159.2 | 159 | (14, 184) | 291 | (28, 189) | 229 | ParB-like nuclease domain protein | 6RYK\_A ParB-like nuclease domain protein ParABS, cytoskeleton, bactofilin, CTP, CELL HET: CTP | | pdb70 | 6KY4\_A | 99.0 | 6.9e-14 | 6.5e-18 | 106.9 | 87 | (17, 109) | 291 | (16, 102) | 112 | Sulfiredoxin, chloroplastic/mitochondrial (E.C.1.8.98.2) | 6KY4\_A Sulfiredoxin, chloroplastic/mitochondrial (E.C.1.8.98.2) sulfiredoxin, cysteine, sulfinic acid, peroxiredoxin HET: PO4, ADP | | pdb70 | 1R71\_B | 99.0 | 1e-13 | 9.5e-18 | 115.1 | 103 | (77, 187) | 291 | (1, 105) | 178 | Transcriptional repressor protein korB/DNA Complex | 1R71\_B Transcriptional repressor protein korB/DNA Complex IncP, plasmid partitioning, protein-DNA complex | | pdb70 | 1XW3\_A | 98.8 | 5.7e-13 | 5.4e-17 | 101.2 | 91 | (8, 106) | 291 | (2, 98) | 110 | SULFIREDOXIN | 1XW3\_A SULFIREDOXIN retroreduction, sulfinic acid, peroxiredoxin, OXIDOREDUCTASE | | pdb70 | 3CYI\_A | 98.8 | 1.2e-12 | 1.1e-16 | 99.2 | 91 | (11, 109) | 291 | (5, 101) | 110 | Sulfiredoxin-1 (E.C.1.8.98.2) | 3CYI\_A Sulfiredoxin-1 (E.C.1.8.98.2) Cys sulfinic acid reduction, Antioxidant HET: ATP | | pdb70 | 2B6F\_A | 98.8 | 1.4e-12 | 1.3e-16 | 100.9 | 81 | (12, 97) | 291 | (17, 103) | 121 | Sulfiredoxin (E.C.1.18.-.-) | 2B6F\_A Sulfiredoxin (E.C.1.18.-.-) PARB DOMAIN FOLD, OXIDOREDUCTASE HET: ATP, MG | | pdb70 | 1VK1\_A | 98.4 | 9e-11 | 8.3e-15 | 101.2 | 91 | (17, 117) | 291 | (15, 109) | 242 | PROTEIN | 1VK1\_A PROTEIN reductive methylation, dimethyl lysine, Pyrococcus | | pdb70 | 5X0J\_A | 98.1 | 7e-10 | 6.5e-14 | 95.4 | 91 | (17, 117) | 291 | (15, 109) | 242 | Free serine kinase | 5X0J\_A Free serine kinase Thermococcus kodakarensis, cysteine biosynthesis, TRANSFERASE HET: SEP, AMP | | pdb70 | 6NR6\_B | 98.1 | 8.8e-10 | 8.1e-14 | 95.8 | 90 | (17, 118) | 291 | (5, 97) | 254 | Bifunctional transcriptional regulator/O-phospho-L-serine synthase SbnI | 6NR6\_B Bifunctional transcriptional regulator/O-phospho-L-serine synthase SbnI free serine kinase, siderophore, ParB/Srx HET: ADP | | pdb70 | 3MKW\_B | 98.0 | 2.2e-09 | 2e-13 | 84.9 | 81 | (110, 190) | 291 | (15, 98) | 138 | PROTEIN/DNA Complex | 3MKW\_B PROTEIN/DNA Complex segregation, sopB, F plasmid, centromere HET: SO4 | | pdb70 | 5UJE\_A | 98.0 | 2.2e-09 | 2.1e-13 | 92.3 | 75 | (16, 97) | 291 | (6, 82) | 242 | SbnI protein | 5UJE\_A SbnI protein staphyloferrin B, heme, regulator, siderophore HET: GOL, MSE | | pdb70 | 3MKY\_B | 98.0 | 3.3e-09 | 3e-13 | 87.7 | 83 | (108, 190) | 291 | (12, 98) | 189 | PROTEIN/DNA Complex | 3MKY\_B PROTEIN/DNA Complex partition, F plasmid, sopB, centromere HET: SO4 | | pdb70 | 3MKY\_P | 97.9 | 4.9e-09 | 4.6e-13 | 86.6 | 81 | (110, 190) | 291 | (15, 98) | 189 | PROTEIN/DNA Complex | 3MKY\_P PROTEIN/DNA Complex partition, F plasmid, sopB, centromere HET: SO4 | | pdb70 | 3MKZ\_A | 97.7 | 2e-08 | 1.9e-12 | 76.7 | 80 | (115, 194) | 291 | (3, 85) | 121 | Protein sopB | 3MKZ\_A Protein sopB partition, SopB, F plasmid, centromere | | pdb70 | 3MKZ\_N | 97.7 | 2e-08 | 1.9e-12 | 76.7 | 80 | (115, 194) | 291 | (3, 85) | 121 | Protein sopB | 3MKZ\_N Protein sopB partition, SopB, F plasmid, centromere | | pdb70 | 2HWJ\_A | 97.6 | 5.1e-08 | 4.4e-12 | 82.2 | 76 | (15, 97) | 291 | (4, 89) | 205 | Hypothetical protein Atu1540 | 2HWJ\_A Hypothetical protein Atu1540 Agrobacterium tumefaciens, Hypothetical protein, Structural | | pdb70 | 2HWJ\_C | 97.6 | 5.1e-08 | 4.4e-12 | 82.2 | 76 | (15, 97) | 291 | (4, 89) | 205 | Hypothetical protein Atu1540 | 2HWJ\_C Hypothetical protein Atu1540 Agrobacterium tumefaciens, Hypothetical protein, Structural HET: MSE | | pdb70 | 1ZX4\_B | 97.0 | 1.2e-06 | 1.2e-10 | 71.3 | 74 | (115, 188) | 291 | (4, 78) | 192 | Plasmid Partition par B protein/DNA | 1ZX4\_B Plasmid Partition par B protein/DNA partition P1 plasmid HET: CIT, MSE | | pdb70 | 2NTZ\_A | 96.6 | 6.1e-06 | 5.8e-10 | 66.8 | 73 | (115, 187) | 291 | (4, 77) | 192 | ParB/DNA Complex | 2NTZ\_A ParB/DNA Complex partition, segregation, parb, para, CELL | |
| Top keywords  (threshold 1.00e-03 (evalue)) | **ParB, domain\_containing, Sulfiredoxin, partition, partitioning, Chromosome, Spo0J, RepB, Fragment, chromosome\_partitioning** |
| Output files | ../../similar\_sequences/28\_FANPEZAQ\_CDS\_0028\_merged.svg ../../similar\_sequences/28\_FANPEZAQ\_CDS\_0028\_pdb70.a3m ../../similar\_sequences/28\_FANPEZAQ\_CDS\_0028\_pdb70.hhr ../../similar\_sequences/28\_FANPEZAQ\_CDS\_0028\_uniclust.a3m ../../similar\_sequences/28\_FANPEZAQ\_CDS\_0028\_uniclust.hhr |

#### Structure prediction (AlphaFold)2

|  |  |
| --- | --- |
| Stats | xml version="1.0" encoding="utf-8" standalone="no"?       2024-09-02T21:09:28.200035 image/svg+xml   Matplotlib v3.7.2, https://matplotlib.org/ |
| Predicted structure | **NGL Viewer Controls:**  - Center: *Left-Click* - Rotate: *Left-Click + Drag* - Translate: *Right-Click + Drag* - Zoom: *Shift + Left-Click + Drag* |
| Output files | ../../predicted\_structures/28\_FANPEZAQ\_CDS\_0028/features.pkl ../../predicted\_structures/28\_FANPEZAQ\_CDS\_0028/ranked\_0.pdb ../../predicted\_structures/28\_FANPEZAQ\_CDS\_0028/ranked\_0\_plots.svg ../../predicted\_structures/28\_FANPEZAQ\_CDS\_0028/result\_model\_1\_ptm\_pred\_0.pkl |

#### Structure similarity search results (Foldseek)3

|  |  |
| --- | --- |
| Structure databases searched | Pdb, Afdb-proteome, Afdb-uniprot50 |
| Results, scheme(s)  (Top layers only, threshold 1.00e-02 (evalue)) | xml version="1.0" encoding="utf-8" standalone="no"?       2024-09-02T21:11:01.550994 image/svg+xml   Matplotlib v3.7.2, https://matplotlib.org/ |
| Results, table  (threshold 1.00e-02 (evalue)) | | db | id | prob | evalue | bits | fident | alnlen | mismatch | gapopen | qstart | qend | tstart | tend | name | description | | --- | --- | --- | --- | --- | --- | --- | --- | --- | --- | --- | --- | --- | --- | --- | | pdb | 6T1F\_D | 1.0 | 1.275e-08 | 286 | 0.182 | 186 | 134 | 9 | 21 | 201 | 2 | 174 | Chromosome-partitioning protein ParB | Chromosome-partitioning protein ParB | | pdb | 7NFU\_A | 1.0 | 2.384e-09 | 279 | 0.159 | 219 | 161 | 13 | 4 | 215 | 9 | 211 | Nucleoid occlusion protein | Nucleoid occlusion protein | | pdb | 7NFU\_B | 1.0 | 6.663e-09 | 276 | 0.178 | 202 | 148 | 12 | 19 | 215 | 15 | 203 | Nucleoid occlusion protein | Nucleoid occlusion protein | | pdb | 7BNK\_B | 1.0 | 9.502e-10 | 262 | 0.236 | 186 | 132 | 8 | 19 | 200 | 1 | 180 | ParB family protein | ParB family protein | | pdb | 7BNR\_A | 1.0 | 3.675e-09 | 249 | 0.227 | 185 | 135 | 6 | 19 | 200 | 1 | 180 | ParB family protein | ParB family protein | | pdb | 1VZ0\_C | 1.0 | 8.943e-08 | 243 | 0.257 | 171 | 113 | 5 | 41 | 203 | 22 | 186 | Chromosome-partitioning protein Spo0J | Chromosome-partitioning protein Spo0J | | pdb | 6SDK\_B | 1.0 | 3.298e-09 | 232 | 0.262 | 187 | 126 | 8 | 19 | 200 | 3 | 182 | Stage 0 sporulation protein J | Stage 0 sporulation protein J | | pdb | 7BM8\_B | 1.0 | 1.084e-08 | 231 | 0.202 | 183 | 125 | 8 | 18 | 195 | 1 | 167 | Chromosome-partitioning protein ParB | Chromosome-partitioning protein ParB | | pdb | 7O0N\_B | 1.0 | 1.027e-08 | 224 | 0.232 | 185 | 130 | 7 | 21 | 200 | 2 | 179 | ParB family protein | ParB family protein | | pdb | 7NG0\_A | 1.0 | 9.73e-09 | 224 | 0.167 | 185 | 144 | 8 | 18 | 198 | 1 | 179 | Nucleoid occlusion protein | Nucleoid occlusion protein | | pdb | 6T1F\_C | 1.0 | 5.941e-07 | 197 | 0.192 | 187 | 137 | 8 | 21 | 201 | 2 | 180 | Chromosome-partitioning protein ParB | Chromosome-partitioning protein ParB | | pdb | 7OL9\_A | 1.0 | 1.11e-07 | 194 | 0.166 | 186 | 145 | 7 | 21 | 201 | 2 | 182 | Nucleoid occlusion protein | Nucleoid occlusion protein | | pdb | 6T1F\_A | 1.0 | 1.137e-06 | 192 | 0.187 | 187 | 134 | 8 | 21 | 201 | 2 | 176 | Chromosome-partitioning protein ParB | Chromosome-partitioning protein ParB | | pdb | 6KY4\_A | 1.0 | 0.0002409 | 176 | 0.178 | 84 | 64 | 2 | 18 | 101 | 1 | 79 | Sulfiredoxin, chloroplastic/mitochondrial | Sulfiredoxin, chloroplastic/mitochondrial | | pdb | 6RYK\_B | 1.0 | 0.0002992 | 136 | 0.142 | 133 | 102 | 6 | 19 | 145 | 5 | 131 | ParB-like nuclease domain protein | ParB-like nuclease domain protein | | pdb | 5K5D\_B | 1.0 | 0.0001941 | 125 | 0.131 | 244 | 155 | 16 | 17 | 214 | 1 | 233 | ParB domain protein nuclease | ParB domain protein nuclease | | pdb | 6S6H\_A | 1.0 | 0.008564 | 123 | 0.258 | 120 | 79 | 3 | 107 | 225 | 6 | 116 | Chromosome-partitioning protein ParB | Chromosome-partitioning protein ParB | | pdb | 5X0G\_A | 1.0 | 0.006897 | 118 | 0.206 | 92 | 61 | 6 | 16 | 104 | 12 | 94 | Free serine kinase | Free serine kinase | | pdb | 6S6H\_B | 1.0 | 0.00619 | 117 | 0.248 | 141 | 93 | 4 | 107 | 243 | 6 | 137 | Chromosome-partitioning protein ParB | Chromosome-partitioning protein ParB | | pdb | 5X0F\_A | 1.0 | 0.008564 | 116 | 0.206 | 92 | 61 | 6 | 16 | 104 | 13 | 95 | Free serine kinase | Free serine kinase | | pdb | 4UMK\_B | 1.0 | 0.000638 | 115 | 0.204 | 166 | 123 | 6 | 31 | 194 | 2 | 160 | PROBABLE CHROMOSOME-PARTITIONING PROTEIN PARB | PROBABLE CHROMOSOME-PARTITIONING PROTEIN PARB | | pdb | 5K5A\_B | 1.0 | 0.0002992 | 113 | 0.135 | 236 | 141 | 17 | 15 | 206 | 2 | 218 | ParB domain protein nuclease | ParB domain protein nuclease | | pdb | 3HY2\_Y | 1.0 | 0.002749 | 110 | 0.2 | 110 | 73 | 6 | 16 | 119 | 1 | 101 | Sulfiredoxin-1 | Sulfiredoxin-1 | | pdb | 5K5D\_A | 1.0 | 0.0009318 | 106 | 0.128 | 233 | 138 | 17 | 17 | 205 | 1 | 212 | ParB domain protein nuclease | ParB domain protein nuclease | | pdb | 4UMK\_A | 1.0 | 0.004015 | 104 | 0.189 | 164 | 122 | 4 | 40 | 200 | 1 | 156 | PROBABLE CHROMOSOME-PARTITIONING PROTEIN PARB | PROBABLE CHROMOSOME-PARTITIONING PROTEIN PARB | | pdb | 2B6F\_A | 0.999 | 0.008564 | 96 | 0.216 | 97 | 61 | 7 | 11 | 98 | 14 | 104 | Sulfiredoxin | Sulfiredoxin | | pdb | 1VK1\_A | 0.995 | 0.0003714 | 88 | 0.15 | 212 | 102 | 15 | 16 | 160 | 4 | 204 | Conserved hypothetical protein | Conserved hypothetical protein | | pdb | 5X0K\_A | 0.99 | 0.006534 | 83 | 0.136 | 168 | 88 | 10 | 16 | 135 | 12 | 170 | Free serine kinase | Free serine kinase | | pdb | 5X0B\_A | 0.961 | 0.003064 | 74 | 0.133 | 224 | 105 | 14 | 16 | 172 | 13 | 214 | Free serine kinase | Free serine kinase | | afdb-proteome | AF-A0A077ZKX5-F1-MODEL\_V4 | 1.0 | 4.292e-13 | 349 | 0.201 | 307 | 199 | 15 | 4 | 291 | 250 | 529 | CbiA and ParBc and KorB domain containing protein | CbiA and ParBc and KorB domain containing protein | | afdb-proteome | AF-Q9HT12-F1-MODEL\_V4 | 1.0 | 1.2e-12 | 343 | 0.205 | 297 | 196 | 15 | 1 | 282 | 18 | 289 | Probable chromosome-partitioning protein ParB | Probable chromosome-partitioning protein ParB | | afdb-proteome | AF-A0A132Z8J9-F1-MODEL\_V4 | 1.0 | 8.215e-13 | 334 | 0.201 | 302 | 189 | 15 | 5 | 282 | 16 | 289 | Chromosome partitioning protein ParB | Chromosome partitioning protein ParB | | afdb-proteome | AF-P77174-F1-MODEL\_V4 | 1.0 | 2.393e-08 | 316 | 0.185 | 140 | 108 | 5 | 20 | 155 | 50 | 187 | Uncharacterized protein YbdM | Uncharacterized protein YbdM | | afdb-proteome | AF-Q8DMW1-F1-MODEL\_V4 | 1.0 | 2.92e-11 | 308 | 0.174 | 270 | 196 | 10 | 19 | 282 | 3 | 251 | Chromosome segregation protein | Chromosome segregation protein | | afdb-proteome | AF-Q5F4Z9-F1-MODEL\_V4 | 1.0 | 1.61e-11 | 304 | 0.194 | 303 | 194 | 14 | 7 | 291 | 15 | 285 | Chromosome partitioning protein ParB | Chromosome partitioning protein ParB | | afdb-proteome | AF-A0A0H3GZZ3-F1-MODEL\_V4 | 1.0 | 6.227e-11 | 294 | 0.199 | 286 | 178 | 14 | 1 | 272 | 6 | 254 | ParB family protein | ParB family protein | | afdb-proteome | AF-Q2G118-F1-MODEL\_V4 | 1.0 | 2.482e-11 | 290 | 0.187 | 310 | 199 | 14 | 1 | 287 | 1 | 280 | Chromosome partioning protein, ParB family, putative | Chromosome partioning protein, ParB family, putative | | afdb-proteome | AF-Q8ZR25-F1-MODEL\_V4 | 1.0 | 1.351e-07 | 287 | 0.157 | 140 | 112 | 5 | 20 | 155 | 46 | 183 | Putative transcriptional regulator | Putative transcriptional regulator | | afdb-proteome | AF-Q9I1Y9-F1-MODEL\_V4 | 1.0 | 2.321e-07 | 267 | 0.2 | 150 | 113 | 5 | 20 | 163 | 51 | 199 | ParB domain-containing protein | ParB domain-containing protein | | afdb-proteome | AF-K0EKP4-F1-MODEL\_V4 | 1.0 | 3.987e-07 | 266 | 0.202 | 148 | 104 | 6 | 17 | 160 | 18 | 155 | StrR-like regulatory protein | StrR-like regulatory protein | | afdb-proteome | AF-A0A0H3GPU5-F1-MODEL\_V4 | 1.0 | 6.147e-07 | 264 | 0.201 | 149 | 108 | 7 | 20 | 160 | 46 | 191 | ParB domain-containing protein | ParB domain-containing protein | | afdb-proteome | AF-Q2FUQ5-F1-MODEL\_V4 | 1.0 | 3.332e-10 | 260 | 0.184 | 287 | 188 | 17 | 4 | 271 | 18 | 277 | ParB domain-containing protein | ParB domain-containing protein | | afdb-proteome | AF-O25758-F1-MODEL\_V4 | 1.0 | 1.94e-10 | 260 | 0.189 | 296 | 204 | 13 | 1 | 282 | 15 | 288 | Probable chromosome-partitioning protein ParB | Probable chromosome-partitioning protein ParB | | afdb-proteome | AF-Q93GP5-F1-MODEL\_V4 | 1.0 | 1.47e-08 | 258 | 0.18 | 277 | 192 | 13 | 19 | 283 | 37 | 290 | Putative ParB-like nuclease domain | Putative ParB-like nuclease domain | | afdb-proteome | AF-Q9PJ25-F1-MODEL\_V4 | 1.0 | 1.36e-09 | 254 | 0.136 | 272 | 201 | 13 | 19 | 282 | 30 | 275 | Probable chromosome-partitioning protein ParB | Probable chromosome-partitioning protein ParB | | afdb-proteome | AF-K0F0D7-F1-MODEL\_V4 | 1.0 | 1.461e-06 | 243 | 0.135 | 192 | 154 | 7 | 14 | 198 | 42 | 228 | Putative Chromosome partitioning protein | Putative Chromosome partitioning protein | | afdb-proteome | AF-Q50201-F1-MODEL\_V4 | 1.0 | 3.712e-10 | 231 | 0.159 | 314 | 216 | 14 | 1 | 285 | 37 | 331 | Probable chromosome-partitioning protein ParB | Probable chromosome-partitioning protein ParB | | afdb-proteome | AF-P9WIJ9-F1-MODEL\_V4 | 1.0 | 8.358e-10 | 220 | 0.182 | 302 | 194 | 13 | 19 | 291 | 59 | 336 | Probable chromosome-partitioning protein ParB | Probable chromosome-partitioning protein ParB | | afdb-proteome | AF-K0F2G5-F1-MODEL\_V4 | 1.0 | 8.823e-10 | 215 | 0.184 | 309 | 195 | 17 | 10 | 282 | 30 | 317 | Stage 0 sporulation protein J, parB family protein | Stage 0 sporulation protein J, parB family protein | | afdb-proteome | AF-A0A0H3GXN9-F1-MODEL\_V4 | 1.0 | 6.488e-07 | 201 | 0.133 | 270 | 171 | 14 | 3 | 214 | 1 | 265 | Uncharacterized protein | Uncharacterized protein | | afdb-proteome | AF-P76068-F1-MODEL\_V4 | 1.0 | 0.0001375 | 178 | 0.228 | 92 | 63 | 4 | 15 | 106 | 2 | 85 | Uncharacterized protein YnaK | Uncharacterized protein YnaK | | afdb-proteome | AF-A0A0H3GWE9-F1-MODEL\_V4 | 1.0 | 6.147e-07 | 175 | 0.18 | 222 | 165 | 10 | 2 | 214 | 18 | 231 | DNA-binding protein | DNA-binding protein | | afdb-proteome | AF-Q45GC6-F1-MODEL\_V4 | 1.0 | 0.0002778 | 156 | 0.229 | 87 | 62 | 3 | 13 | 98 | 62 | 144 | Sulfiredoxin | Sulfiredoxin | | afdb-proteome | AF-Q8GY89-F1-MODEL\_V4 | 1.0 | 0.0003449 | 152 | 0.191 | 89 | 67 | 3 | 11 | 98 | 31 | 115 | Sulfiredoxin, chloroplastic/mitochondrial | Sulfiredoxin, chloroplastic/mitochondrial | | afdb-proteome | AF-I1NDB8-F1-MODEL\_V4 | 1.0 | 0.0009641 | 148 | 0.17 | 88 | 66 | 4 | 13 | 98 | 34 | 116 | Sulfiredoxin | Sulfiredoxin | | afdb-proteome | AF-Q32IW8-F1-MODEL\_V4 | 1.0 | 0.0001169 | 148 | 0.183 | 136 | 89 | 5 | 20 | 152 | 50 | 166 | ParB domain-containing protein | ParB domain-containing protein | | afdb-proteome | AF-Q93GQ7-F1-MODEL\_V4 | 1.0 | 2.586e-07 | 147 | 0.143 | 313 | 184 | 16 | 20 | 286 | 44 | 318 | Plasmid partition protein B | Plasmid partition protein B | | afdb-proteome | AF-Q327G3-F1-MODEL\_V4 | 1.0 | 7.632e-07 | 146 | 0.147 | 298 | 195 | 16 | 20 | 283 | 44 | 316 | Plasmid segregation protein | Plasmid segregation protein | | afdb-proteome | AF-A0A0H3H577-F1-MODEL\_V4 | 1.0 | 2.07e-05 | 139 | 0.121 | 272 | 183 | 14 | 19 | 287 | 14 | 232 | ParB domain-containing protein | ParB domain-containing protein | | afdb-proteome | AF-A0A0H3GZ74-F1-MODEL\_V4 | 1.0 | 5.823e-07 | 139 | 0.153 | 306 | 189 | 15 | 20 | 286 | 43 | 317 | Plasmid partition protein B | Plasmid partition protein B | | afdb-proteome | AF-Q326T4-F1-MODEL\_V4 | 1.0 | 2.252e-06 | 127 | 0.141 | 296 | 187 | 16 | 26 | 287 | 41 | 303 | VirB | VirB | | afdb-proteome | AF-A0A0H3GY80-F1-MODEL\_V4 | 1.0 | 3.368e-05 | 123 | 0.156 | 287 | 166 | 15 | 15 | 291 | 2 | 222 | ParB domain-containing protein | ParB domain-containing protein | | afdb-proteome | AF-X8FLN4-F1-MODEL\_V4 | 1.0 | 0.001334 | 122 | 0.262 | 122 | 76 | 7 | 63 | 176 | 2 | 117 | ParB/RepB/Spo0J family partition domain protein | ParB/RepB/Spo0J family partition domain protein | | afdb-proteome | AF-Q9D975-F1-MODEL\_V4 | 1.0 | 0.007533 | 110 | 0.214 | 107 | 69 | 6 | 19 | 119 | 39 | 136 | Sulfiredoxin-1 | Sulfiredoxin-1 | | afdb-proteome | AF-Q9BYN0-F1-MODEL\_V4 | 1.0 | 0.007136 | 104 | 0.198 | 116 | 74 | 7 | 10 | 119 | 35 | 137 | Sulfiredoxin-1 | Sulfiredoxin-1 | | afdb-proteome | AF-Q9VX10-F1-MODEL\_V4 | 0.999 | 0.008861 | 98 | 0.149 | 127 | 84 | 7 | 8 | 119 | 44 | 161 | Putative sulfiredoxin | Putative sulfiredoxin | | afdb-proteome | AF-K0F507-F1-MODEL\_V4 | 0.997 | 0.0004282 | 91 | 0.128 | 367 | 205 | 17 | 4 | 285 | 18 | 354 | Uncharacterized protein | Uncharacterized protein | | afdb-proteome | AF-Q54RQ8-F1-MODEL\_V4 | 0.993 | 0.004887 | 86 | 0.122 | 179 | 115 | 9 | 19 | 169 | 9 | 173 | BEACH domain-containing protein lvsE | BEACH domain-containing protein lvsE | | afdb-proteome | AF-Q7TP44-F1-MODEL\_V4 | 0.967 | 0.004154 | 75 | 0.167 | 215 | 120 | 9 | 19 | 195 | 39 | 232 | Sulfiredoxin | Sulfiredoxin | | afdb-uniprot50 | AF-A0A3S4JMI3-F1-MODEL\_V4 | 1.0 | 2.427e-28 | 1052 | 0.453 | 291 | 130 | 5 | 1 | 287 | 1 | 266 | ParB/RepB/Spo0J family partition protein | ParB/RepB/Spo0J family partition protein | | afdb-uniprot50 | AF-A0A0P0QGS8-F1-MODEL\_V4 | 1.0 | 7.16e-28 | 1028 | 0.46 | 291 | 128 | 5 | 1 | 287 | 1 | 266 | DNA-binding protein | DNA-binding protein | | afdb-uniprot50 | AF-A0A198GH96-F1-MODEL\_V4 | 1.0 | 8.171e-27 | 980 | 0.435 | 289 | 134 | 5 | 1 | 287 | 1 | 262 | Uncharacterized protein | Uncharacterized protein | | afdb-uniprot50 | AF-A0A318NRJ4-F1-MODEL\_V4 | 1.0 | 1.612e-27 | 979 | 0.443 | 291 | 133 | 5 | 1 | 287 | 1 | 266 | DNA-binding protein | DNA-binding protein | | afdb-uniprot50 | AF-A0A2S9I472-F1-MODEL\_V4 | 1.0 | 2.545e-26 | 946 | 0.461 | 297 | 125 | 7 | 1 | 291 | 1 | 268 | Chromosome partitioning protein ParB | Chromosome partitioning protein ParB | | afdb-uniprot50 | AF-A0A437T2I0-F1-MODEL\_V4 | 1.0 | 1.691e-25 | 898 | 0.433 | 307 | 134 | 8 | 1 | 289 | 1 | 285 | DUF550 domain-containing protein | DUF550 domain-containing protein | | afdb-uniprot50 | AF-A0A209B1U4-F1-MODEL\_V4 | 1.0 | 4.989e-25 | 897 | 0.419 | 293 | 139 | 7 | 1 | 287 | 1 | 268 | ParB domain-containing protein | ParB domain-containing protein | | afdb-uniprot50 | AF-A0A377XPM9-F1-MODEL\_V4 | 1.0 | 1.63e-22 | 892 | 0.477 | 249 | 110 | 4 | 1 | 245 | 1 | 233 | ParB/RepB/Spo0J family partition protein | ParB/RepB/Spo0J family partition protein | | afdb-uniprot50 | AF-A0A484A9Q6-F1-MODEL\_V4 | 1.0 | 1.773e-23 | 872 | 0.433 | 284 | 124 | 7 | 1 | 280 | 1 | 251 | DNA-binding plasmid partitioning protein | DNA-binding plasmid partitioning protein | | afdb-uniprot50 | AF-K8C8K5-F1-MODEL\_V4 | 1.0 | 4.695e-23 | 870 | 0.459 | 259 | 107 | 4 | 23 | 280 | 1 | 227 | Predicted transcriptional regulators | Predicted transcriptional regulators | | afdb-uniprot50 | AF-V5U266-F1-MODEL\_V4 | 1.0 | 8.065e-23 | 859 | 0.463 | 259 | 106 | 5 | 23 | 280 | 1 | 227 | Uncharacterized protein | Uncharacterized protein | | afdb-uniprot50 | AF-A0A561G4M7-F1-MODEL\_V4 | 1.0 | 8.458e-21 | 750 | 0.335 | 274 | 170 | 6 | 1 | 271 | 8 | 272 | ParB/RepB/Spo0J family partition protein | ParB/RepB/Spo0J family partition protein | | afdb-uniprot50 | AF-A0A378DHA4-F1-MODEL\_V4 | 1.0 | 1.128e-17 | 731 | 0.561 | 171 | 70 | 3 | 1 | 167 | 1 | 170 | ParB/RepB/Spo0J family partition protein | ParB/RepB/Spo0J family partition protein | | afdb-uniprot50 | AF-A0A5E7QKL3-F1-MODEL\_V4 | 1.0 | 6.609e-20 | 700 | 0.254 | 302 | 187 | 8 | 1 | 285 | 2 | 282 | ParB domain-containing protein | ParB domain-containing protein | | afdb-uniprot50 | AF-A0A6N8BW42-F1-MODEL\_V4 | 1.0 | 4.746e-18 | 691 | 0.329 | 237 | 143 | 4 | 14 | 246 | 9 | 233 | Uncharacterized protein | Uncharacterized protein | | afdb-uniprot50 | AF-A0A2A2GPN7-F1-MODEL\_V4 | 1.0 | 2.01e-20 | 686 | 0.274 | 302 | 187 | 12 | 1 | 287 | 38 | 322 | Uncharacterized protein | Uncharacterized protein | | afdb-uniprot50 | AF-A0A7W9JWW8-F1-MODEL\_V4 | 1.0 | 5.323e-20 | 679 | 0.278 | 305 | 171 | 9 | 1 | 282 | 3 | 281 | ParB-like chromosome segregation protein Spo0J | ParB-like chromosome segregation protein Spo0J | | afdb-uniprot50 | AF-A0A2L1KUX8-F1-MODEL\_V4 | 1.0 | 3.271e-20 | 678 | 0.285 | 301 | 181 | 11 | 4 | 287 | 40 | 323 | Uncharacterized protein | Uncharacterized protein | | afdb-uniprot50 | AF-A0A1I1VMS0-F1-MODEL\_V4 | 1.0 | 4.061e-20 | 674 | 0.298 | 291 | 184 | 8 | 1 | 287 | 7 | 281 | Chromosome partitioning protein, ParB family | Chromosome partitioning protein, ParB family | | afdb-uniprot50 | AF-A0A847BHV1-F1-MODEL\_V4 | 1.0 | 2.698e-19 | 673 | 0.27 | 307 | 185 | 11 | 1 | 291 | 5 | 288 | Uncharacterized protein | Uncharacterized protein | | afdb-uniprot50 | AF-A0A411X2Y8-F1-MODEL\_V4 | 1.0 | 3.453e-20 | 667 | 0.303 | 297 | 175 | 8 | 8 | 291 | 31 | 308 | Uncharacterized protein | Uncharacterized protein | | afdb-uniprot50 | AF-A9BYB8-F1-MODEL\_V4 | 1.0 | 3.536e-19 | 661 | 0.321 | 271 | 167 | 6 | 1 | 259 | 38 | 303 | Uncharacterized protein | Uncharacterized protein | | afdb-uniprot50 | AF-A0A1B8TI45-F1-MODEL\_V4 | 1.0 | 7.493e-17 | 660 | 0.324 | 228 | 135 | 7 | 1 | 213 | 24 | 247 | Uncharacterized protein | Uncharacterized protein | | afdb-uniprot50 | AF-A0A2W5FW74-F1-MODEL\_V4 | 1.0 | 4.635e-19 | 657 | 0.286 | 286 | 176 | 9 | 3 | 273 | 4 | 276 | ParB domain-containing protein | ParB domain-containing protein | | afdb-uniprot50 | AF-A0A0E3BYE5-F1-MODEL\_V4 | 1.0 | 7.542e-19 | 652 | 0.286 | 304 | 181 | 11 | 1 | 287 | 9 | 293 | Uncharacterized protein | Uncharacterized protein | | afdb-uniprot50 | AF-A0A2Z3I5V1-F1-MODEL\_V4 | 1.0 | 3.35e-19 | 649 | 0.289 | 311 | 184 | 11 | 1 | 287 | 1 | 298 | Uncharacterized protein | Uncharacterized protein | | afdb-uniprot50 | AF-A0A7W7JCN3-F1-MODEL\_V4 | 1.0 | 6.412e-19 | 646 | 0.263 | 304 | 189 | 9 | 1 | 289 | 3 | 286 | ParB-like chromosome segregation protein Spo0J | ParB-like chromosome segregation protein Spo0J | | afdb-uniprot50 | AF-A0A193GNC7-F1-MODEL\_V4 | 1.0 | 2.294e-19 | 642 | 0.289 | 287 | 175 | 10 | 16 | 288 | 25 | 296 | Uncharacterized protein | Uncharacterized protein | | afdb-uniprot50 | AF-A0A5P8XR09-F1-MODEL\_V4 | 1.0 | 3.94e-19 | 641 | 0.305 | 298 | 176 | 10 | 3 | 282 | 4 | 288 | ParB domain-containing protein | ParB domain-containing protein | | afdb-uniprot50 | AF-A0A0S4PBI2-F1-MODEL\_V4 | 1.0 | 2.987e-17 | 635 | 0.285 | 252 | 157 | 5 | 1 | 246 | 18 | 252 | Phage protein | Phage protein | | afdb-uniprot50 | AF-A0A5K7YEB8-F1-MODEL\_V4 | 1.0 | 7.542e-19 | 635 | 0.313 | 281 | 174 | 10 | 16 | 287 | 61 | 331 | Uncharacterized protein | Uncharacterized protein | | afdb-uniprot50 | AF-A0A7X0PCX7-F1-MODEL\_V4 | 1.0 | 2.917e-18 | 630 | 0.271 | 280 | 179 | 8 | 1 | 268 | 1 | 267 | ParB-like chromosome segregation protein Spo0J | ParB-like chromosome segregation protein Spo0J | | afdb-uniprot50 | AF-A0A484VAU1-F1-MODEL\_V4 | 1.0 | 2.618e-18 | 621 | 0.282 | 294 | 186 | 8 | 3 | 287 | 9 | 286 | Predicted transcriptional regulators | Predicted transcriptional regulators | | afdb-uniprot50 | AF-A0A556AID6-F1-MODEL\_V4 | 1.0 | 3.513e-17 | 618 | 0.281 | 259 | 165 | 9 | 1 | 246 | 16 | 266 | ParB/RepB/Spo0J family partition protein | ParB/RepB/Spo0J family partition protein | | afdb-uniprot50 | AF-A0A7U3GA52-F1-MODEL\_V4 | 1.0 | 1.938e-17 | 617 | 0.258 | 298 | 172 | 9 | 1 | 289 | 11 | 268 | ParB/RepB/Spo0J family partition protein | ParB/RepB/Spo0J family partition protein | | afdb-uniprot50 | AF-A0A6N4J675-F1-MODEL\_V4 | 1.0 | 3.079e-18 | 617 | 0.276 | 300 | 188 | 10 | 1 | 288 | 2 | 284 | ParB domain-containing protein | ParB domain-containing protein | | afdb-uniprot50 | AF-A0A4P7L9Q5-F1-MODEL\_V4 | 1.0 | 3.733e-19 | 617 | 0.281 | 295 | 181 | 11 | 7 | 287 | 45 | 322 | Uncharacterized protein | Uncharacterized protein | | afdb-uniprot50 | AF-A0A6M1CKP2-F1-MODEL\_V4 | 1.0 | 1.35e-14 | 608 | 0.57 | 149 | 59 | 3 | 1 | 145 | 1 | 148 | DNA-binding protein | DNA-binding protein | | afdb-uniprot50 | AF-A0A0P9Z862-F1-MODEL\_V4 | 1.0 | 1.012e-17 | 607 | 0.273 | 300 | 190 | 12 | 2 | 287 | 3 | 288 | Carbon storage regulator CsrA | Carbon storage regulator CsrA | | afdb-uniprot50 | AF-A0A1H2PPM0-F1-MODEL\_V4 | 1.0 | 4.232e-16 | 605 | 0.266 | 248 | 167 | 7 | 7 | 244 | 25 | 267 | Chromosome segregation protein Spo0J, contains ParB-like nuclease domain | Chromosome segregation protein Spo0J, contains ParB-like nuclease domain | | afdb-uniprot50 | AF-A0A5N7WBC4-F1-MODEL\_V4 | 1.0 | 4.035e-18 | 604 | 0.289 | 294 | 174 | 10 | 11 | 287 | 14 | 289 | Ftsk\_gamma domain-containing protein | Ftsk\_gamma domain-containing protein | | afdb-uniprot50 | AF-A0A4Q2V971-F1-MODEL\_V4 | 1.0 | 1.128e-17 | 602 | 0.262 | 293 | 187 | 9 | 6 | 287 | 16 | 290 | Uncharacterized protein | Uncharacterized protein | | afdb-uniprot50 | AF-A0A1Q9BSC2-F1-MODEL\_V4 | 1.0 | 7.961e-19 | 598 | 0.302 | 298 | 189 | 11 | 4 | 287 | 32 | 324 | Uncharacterized protein | Uncharacterized protein | | afdb-uniprot50 | AF-N2IPQ2-F1-MODEL\_V4 | 1.0 | 1.647e-17 | 596 | 0.291 | 278 | 167 | 8 | 1 | 265 | 1 | 261 | Uncharacterized protein | Uncharacterized protein | | afdb-uniprot50 | AF-A0A261UUL3-F1-MODEL\_V4 | 1.0 | 1.012e-17 | 594 | 0.238 | 306 | 189 | 12 | 2 | 288 | 9 | 289 | Uncharacterized protein | Uncharacterized protein | | afdb-uniprot50 | AF-A0A2R7NKF8-F1-MODEL\_V4 | 1.0 | 1.739e-17 | 592 | 0.263 | 300 | 177 | 10 | 8 | 287 | 15 | 290 | KorB domain-containing protein | KorB domain-containing protein | | afdb-uniprot50 | AF-A0A1C7LIL5-F1-MODEL\_V4 | 1.0 | 1.588e-14 | 591 | 0.317 | 205 | 129 | 5 | 5 | 202 | 22 | 222 | Uncharacterized protein | Uncharacterized protein | | afdb-uniprot50 | AF-A1VVC1-F1-MODEL\_V4 | 1.0 | 5.416e-17 | 591 | 0.268 | 264 | 164 | 10 | 1 | 246 | 33 | 285 | Uncharacterized protein | Uncharacterized protein | | afdb-uniprot50 | AF-A0A2C9D2B9-F1-MODEL\_V4 | 1.0 | 2.681e-17 | 590 | 0.27 | 300 | 186 | 10 | 1 | 291 | 1 | 276 | Plasmid partitioning protein | Plasmid partitioning protein | | afdb-uniprot50 | AF-A0A2S9P286-F1-MODEL\_V4 | 1.0 | 1.835e-17 | 588 | 0.256 | 300 | 193 | 12 | 1 | 284 | 9 | 294 | Chromosome partitioning protein ParB | Chromosome partitioning protein ParB | | afdb-uniprot50 | AF-B7XGN8-F1-MODEL\_V4 | 1.0 | 4.746e-18 | 588 | 0.249 | 309 | 188 | 10 | 1 | 286 | 7 | 294 | KorB domain-containing protein | KorB domain-containing protein | | afdb-uniprot50 | AF-A0A846VCZ1-F1-MODEL\_V4 | 1.0 | 5.717e-17 | 585 | 0.238 | 289 | 195 | 8 | 1 | 282 | 2 | 272 | ParB/RepB/Spo0J family partition protein | ParB/RepB/Spo0J family partition protein | | afdb-uniprot50 | AF-A0A426FNN8-F1-MODEL\_V4 | 1.0 | 2.83e-17 | 582 | 0.22 | 313 | 196 | 11 | 3 | 287 | 34 | 326 | ParB domain-containing protein | ParB domain-containing protein | | afdb-uniprot50 | AF-A0A6P1IN57-F1-MODEL\_V4 | 1.0 | 5.131e-17 | 582 | 0.257 | 299 | 182 | 10 | 1 | 287 | 54 | 324 | Uncharacterized protein | Uncharacterized protein | | afdb-uniprot50 | AF-A0A7Y8KX42-F1-MODEL\_V4 | 1.0 | 4.232e-16 | 581 | 0.278 | 273 | 169 | 8 | 1 | 247 | 22 | 292 | Uncharacterized protein | Uncharacterized protein | | afdb-uniprot50 | AF-A0A2D2DTI9-F1-MODEL\_V4 | 1.0 | 4.861e-17 | 580 | 0.268 | 287 | 168 | 9 | 11 | 291 | 5 | 255 | Uncharacterized protein | Uncharacterized protein | | afdb-uniprot50 | AF-A0A158EA19-F1-MODEL\_V4 | 1.0 | 1.835e-17 | 578 | 0.266 | 311 | 184 | 12 | 1 | 287 | 5 | 295 | Nucleoid occlusion protein | Nucleoid occlusion protein | | afdb-uniprot50 | AF-A0A4V1RYX4-F1-MODEL\_V4 | 1.0 | 3.513e-17 | 576 | 0.266 | 300 | 183 | 10 | 5 | 287 | 18 | 297 | Uncharacterized protein | Uncharacterized protein | | afdb-uniprot50 | AF-A0A6J5FJG2-F1-MODEL\_V4 | 1.0 | 8.813e-17 | 575 | 0.254 | 311 | 187 | 11 | 1 | 282 | 9 | 303 | ParB domain-containing protein | ParB domain-containing protein | | afdb-uniprot50 | AF-W7XAG2-F1-MODEL\_V4 | 1.0 | 2.539e-17 | 574 | 0.268 | 313 | 188 | 14 | 1 | 291 | 10 | 303 | Uncharacterized protein | Uncharacterized protein | | afdb-uniprot50 | AF-A0A3A6QPU2-F1-MODEL\_V4 | 1.0 | 1.287e-16 | 572 | 0.179 | 351 | 201 | 11 | 1 | 287 | 20 | 347 | Uncharacterized protein | Uncharacterized protein | | afdb-uniprot50 | AF-A0A1H9NHG0-F1-MODEL\_V4 | 1.0 | 3.709e-17 | 571 | 0.26 | 292 | 188 | 10 | 9 | 289 | 20 | 294 | ParB/RepB/Spo0J family partition protein | ParB/RepB/Spo0J family partition protein | | afdb-uniprot50 | AF-A0A1E5X7A6-F1-MODEL\_V4 | 1.0 | 5.416e-17 | 571 | 0.268 | 324 | 196 | 11 | 1 | 291 | 5 | 320 | ParB domain-containing protein | ParB domain-containing protein | | afdb-uniprot50 | AF-Q5NWX7-F1-MODEL\_V4 | 1.0 | 3.798e-16 | 570 | 0.305 | 259 | 156 | 9 | 1 | 246 | 15 | 262 | Uncharacterized protein | Uncharacterized protein | | afdb-uniprot50 | AF-A0A5E4SIJ2-F1-MODEL\_V4 | 1.0 | 6.724e-17 | 569 | 0.236 | 317 | 193 | 11 | 1 | 291 | 4 | 297 | Uncharacterized protein | Uncharacterized protein | | afdb-uniprot50 | AF-A0A5C7NQX4-F1-MODEL\_V4 | 1.0 | 1.55e-15 | 567 | 0.237 | 257 | 173 | 9 | 1 | 246 | 20 | 264 | Uncharacterized protein | Uncharacterized protein | | afdb-uniprot50 | AF-A0A427FDH5-F1-MODEL\_V4 | 1.0 | 3.915e-17 | 566 | 0.261 | 298 | 185 | 10 | 8 | 282 | 18 | 303 | Uncharacterized protein | Uncharacterized protein | | afdb-uniprot50 | AF-A0A3S7HQT3-F1-MODEL\_V4 | 1.0 | 4.132e-17 | 566 | 0.27 | 310 | 187 | 11 | 5 | 287 | 43 | 340 | Uncharacterized protein | Uncharacterized protein | | afdb-uniprot50 | AF-A0A6G8CZU1-F1-MODEL\_V4 | 1.0 | 2.539e-17 | 565 | 0.274 | 288 | 180 | 11 | 14 | 287 | 16 | 288 | Uncharacterized protein | Uncharacterized protein | | afdb-uniprot50 | AF-A0A6N8DQB4-F1-MODEL\_V4 | 1.0 | 6.329e-15 | 563 | 0.236 | 254 | 166 | 7 | 1 | 246 | 1 | 234 | Uncharacterized protein | Uncharacterized protein | | afdb-uniprot50 | AF-A0A443KCC4-F1-MODEL\_V4 | 1.0 | 1.88e-16 | 557 | 0.273 | 304 | 177 | 12 | 1 | 291 | 1 | 273 | ParB/RepB/Spo0J family partition protein | ParB/RepB/Spo0J family partition protein | | afdb-uniprot50 | AF-A0A0F2QVW0-F1-MODEL\_V4 | 1.0 | 1.824e-15 | 557 | 0.251 | 258 | 169 | 9 | 1 | 246 | 9 | 254 | Uncharacterized protein | Uncharacterized protein | | afdb-uniprot50 | AF-A0A1B8TLL9-F1-MODEL\_V4 | 1.0 | 1.514e-16 | 557 | 0.212 | 324 | 199 | 11 | 1 | 290 | 21 | 322 | ParB domain-containing protein | ParB domain-containing protein | | afdb-uniprot50 | AF-A0A2W4VYK9-F1-MODEL\_V4 | 1.0 | 9.82e-17 | 555 | 0.264 | 318 | 185 | 11 | 1 | 272 | 29 | 343 | Uncharacterized protein | Uncharacterized protein | | afdb-uniprot50 | AF-A0A1B8HMK7-F1-MODEL\_V4 | 1.0 | 1.327e-17 | 555 | 0.299 | 304 | 183 | 14 | 8 | 287 | 44 | 341 | Uncharacterized protein | Uncharacterized protein | | afdb-uniprot50 | AF-A0A7V8FVR6-F1-MODEL\_V4 | 1.0 | 5.131e-17 | 553 | 0.279 | 304 | 178 | 12 | 3 | 287 | 6 | 287 | Uncharacterized protein | Uncharacterized protein | | afdb-uniprot50 | AF-A0A502TIG2-F1-MODEL\_V4 | 1.0 | 6.524e-16 | 552 | 0.251 | 282 | 179 | 9 | 11 | 282 | 10 | 269 | Uncharacterized protein | Uncharacterized protein | | afdb-uniprot50 | AF-A0A022PNW0-F1-MODEL\_V4 | 1.0 | 7.909e-17 | 552 | 0.29 | 286 | 184 | 11 | 16 | 287 | 60 | 340 | Uncharacterized protein | Uncharacterized protein | | afdb-uniprot50 | AF-A0A7Z1BBA0-F1-MODEL\_V4 | 1.0 | 1.219e-16 | 550 | 0.264 | 302 | 185 | 11 | 1 | 291 | 39 | 314 | Uncharacterized protein | Uncharacterized protein | | afdb-uniprot50 | AF-A0A1N7P1X5-F1-MODEL\_V4 | 1.0 | 4.978e-16 | 548 | 0.225 | 297 | 204 | 11 | 1 | 282 | 21 | 306 | Uncharacterized protein | Uncharacterized protein | | afdb-uniprot50 | AF-A0A659AP85-F1-MODEL\_V4 | 1.0 | 1.868e-14 | 545 | 0.311 | 228 | 143 | 8 | 2 | 217 | 11 | 236 | Uncharacterized protein | Uncharacterized protein | | afdb-uniprot50 | AF-A0A1V0M6I1-F1-MODEL\_V4 | 1.0 | 2.898e-16 | 545 | 0.277 | 285 | 176 | 10 | 16 | 287 | 86 | 353 | Uncharacterized protein | Uncharacterized protein | | afdb-uniprot50 | AF-A0A4R3V4Q9-F1-MODEL\_V4 | 1.0 | 7.269e-16 | 545 | 0.275 | 279 | 167 | 10 | 1 | 247 | 14 | 289 | ParB-like chromosome segregation protein Spo0J | ParB-like chromosome segregation protein Spo0J | | afdb-uniprot50 | AF-A0A1H6NM10-F1-MODEL\_V4 | 1.0 | 1.094e-16 | 544 | 0.231 | 315 | 194 | 12 | 1 | 288 | 1 | 294 | Uncharacterized protein | Uncharacterized protein | | afdb-uniprot50 | AF-A0A2X1TFX5-F1-MODEL\_V4 | 1.0 | 7.673e-16 | 543 | 0.425 | 242 | 103 | 7 | 53 | 291 | 2 | 210 | ParB/RepB/Spo0J family partition protein | ParB/RepB/Spo0J family partition protein | | afdb-uniprot50 | AF-A0A5Q6PET4-F1-MODEL\_V4 | 1.0 | 7.493e-17 | 543 | 0.258 | 302 | 189 | 10 | 2 | 287 | 11 | 293 | Uncharacterized protein | Uncharacterized protein | | afdb-uniprot50 | AF-A0A069PKE4-F1-MODEL\_V4 | 1.0 | 4.978e-16 | 543 | 0.259 | 308 | 186 | 12 | 1 | 291 | 15 | 297 | Transcriptional regulator | Transcriptional regulator | | afdb-uniprot50 | AF-A0A2N5BQD3-F1-MODEL\_V4 | 1.0 | 8.349e-17 | 541 | 0.27 | 299 | 184 | 14 | 7 | 287 | 35 | 317 | Uncharacterized protein | Uncharacterized protein | | afdb-uniprot50 | AF-A0A7Y1B9U3-F1-MODEL\_V4 | 1.0 | 4.132e-17 | 541 | 0.242 | 363 | 180 | 13 | 3 | 291 | 15 | 356 | Chromosome partitioning protein ParB | Chromosome partitioning protein ParB | | afdb-uniprot50 | AF-A0A4Q2L955-F1-MODEL\_V4 | 1.0 | 2.464e-16 | 540 | 0.217 | 317 | 200 | 12 | 1 | 291 | 1 | 295 | Uncharacterized protein | Uncharacterized protein | | afdb-uniprot50 | AF-A0A7Y3JE79-F1-MODEL\_V4 | 1.0 | 4.575e-15 | 540 | 0.26 | 257 | 169 | 7 | 1 | 246 | 18 | 264 | ParB N-terminal domain-containing protein | ParB N-terminal domain-containing protein | | afdb-uniprot50 | AF-A0A840VDW9-F1-MODEL\_V4 | 1.0 | 5.546e-16 | 540 | 0.245 | 309 | 193 | 9 | 1 | 287 | 1 | 291 | Uncharacterized protein | Uncharacterized protein | | afdb-uniprot50 | AF-A0A2K4L1R5-F1-MODEL\_V4 | 1.0 | 6.524e-16 | 535 | 0.192 | 332 | 201 | 10 | 7 | 291 | 32 | 343 | Uncharacterized protein | Uncharacterized protein | | afdb-uniprot50 | AF-A0A2N8QQS3-F1-MODEL\_V4 | 1.0 | 5.546e-16 | 534 | 0.243 | 304 | 192 | 13 | 1 | 291 | 28 | 306 | Uncharacterized protein | Uncharacterized protein | | afdb-uniprot50 | AF-A0A157RM33-F1-MODEL\_V4 | 1.0 | 8.55e-16 | 534 | 0.267 | 277 | 170 | 10 | 1 | 247 | 13 | 286 | Protein of uncharacterized function (DUF550) | Protein of uncharacterized function (DUF550) | | afdb-uniprot50 | AF-A0A522ZXA9-F1-MODEL\_V4 | 1.0 | 6.886e-16 | 531 | 0.24 | 304 | 186 | 11 | 1 | 291 | 18 | 289 | Uncharacterized protein | Uncharacterized protein | | afdb-uniprot50 | AF-A0A1H2PMU7-F1-MODEL\_V4 | 1.0 | 9.026e-16 | 530 | 0.243 | 287 | 176 | 11 | 1 | 271 | 18 | 279 | ParB-like nuclease domain-containing protein | ParB-like nuclease domain-containing protein | | afdb-uniprot50 | AF-A0A178LKV7-F1-MODEL\_V4 | 1.0 | 1.006e-15 | 530 | 0.211 | 327 | 198 | 11 | 5 | 291 | 23 | 329 | Uncharacterized protein | Uncharacterized protein | | afdb-uniprot50 | AF-A0A6M5HTZ0-F1-MODEL\_V4 | 1.0 | 8.1e-16 | 529 | 0.21 | 295 | 198 | 10 | 1 | 287 | 1 | 268 | Chromosome partitioning protein ParB | Chromosome partitioning protein ParB | | afdb-uniprot50 | AF-A0A3D9P4K5-F1-MODEL\_V4 | 1.0 | 8.349e-17 | 529 | 0.247 | 311 | 199 | 12 | 2 | 287 | 6 | 306 | Uncharacterized protein | Uncharacterized protein | | afdb-uniprot50 | AF-A0A6L3Y803-F1-MODEL\_V4 | 1.0 | 7.222e-14 | 526 | 0.244 | 249 | 165 | 7 | 2 | 244 | 4 | 235 | Chromosome partitioning protein ParB | Chromosome partitioning protein ParB | | afdb-uniprot50 | AF-A0A502HT17-F1-MODEL\_V4 | 1.0 | 4.978e-16 | 526 | 0.221 | 303 | 198 | 10 | 2 | 288 | 5 | 285 | ParB domain-containing protein | ParB domain-containing protein | | afdb-uniprot50 | AF-A0A3N6N757-F1-MODEL\_V4 | 1.0 | 7.269e-16 | 525 | 0.28 | 324 | 179 | 11 | 1 | 283 | 15 | 325 | Uncharacterized protein | Uncharacterized protein | | afdb-uniprot50 | AF-A0A857JBI8-F1-MODEL\_V4 | 1.0 | 1.469e-15 | 524 | 0.231 | 303 | 187 | 10 | 1 | 289 | 1 | 271 | Uncharacterized protein | Uncharacterized protein | | afdb-uniprot50 | AF-A0A0F5LX83-F1-MODEL\_V4 | 1.0 | 2.523e-15 | 524 | 0.226 | 291 | 190 | 9 | 2 | 286 | 4 | 265 | Chromosome partitioning protein ParB | Chromosome partitioning protein ParB | | afdb-uniprot50 | AF-A0A0Q8XC42-F1-MODEL\_V4 | 1.0 | 5.346e-13 | 523 | 0.31 | 177 | 113 | 5 | 21 | 190 | 1 | 175 | Uncharacterized protein | Uncharacterized protein | | afdb-uniprot50 | AF-A0A601DCY2-F1-MODEL\_V4 | 1.0 | 3.889e-15 | 523 | 0.232 | 293 | 188 | 10 | 2 | 287 | 4 | 266 | Chromosome partitioning protein ParB | Chromosome partitioning protein ParB | | afdb-uniprot50 | AF-A0A4V3UTC2-F1-MODEL\_V4 | 1.0 | 4.106e-15 | 520 | 0.204 | 298 | 201 | 9 | 1 | 287 | 15 | 287 | ParB domain-containing protein | ParB domain-containing protein | | afdb-uniprot50 | AF-A0A6I4KMK4-F1-MODEL\_V4 | 1.0 | 1.287e-16 | 519 | 0.307 | 299 | 158 | 9 | 15 | 286 | 1 | 277 | ParB/RepB/Spo0J family partition protein | ParB/RepB/Spo0J family partition protein | | afdb-uniprot50 | AF-A0A1J5QET2-F1-MODEL\_V4 | 1.0 | 1.824e-15 | 517 | 0.25 | 304 | 190 | 12 | 1 | 291 | 36 | 314 | Uncharacterized protein | Uncharacterized protein | | afdb-uniprot50 | AF-A0A072TPP5-F1-MODEL\_V4 | 1.0 | 5.546e-16 | 517 | 0.271 | 280 | 170 | 10 | 23 | 291 | 1 | 257 | Curli production assembly/transport component CsgG | Curli production assembly/transport component CsgG | | afdb-uniprot50 | AF-A4JUB9-F1-MODEL\_V4 | 1.0 | 2.811e-15 | 516 | 0.223 | 295 | 195 | 9 | 1 | 283 | 23 | 295 | Uncharacterized protein | Uncharacterized protein | | afdb-uniprot50 | AF-U7GET7-F1-MODEL\_V4 | 1.0 | 1.469e-15 | 514 | 0.244 | 307 | 194 | 12 | 1 | 282 | 1 | 294 | Uncharacterized protein | Uncharacterized protein | | afdb-uniprot50 | AF-A0A5C7KRJ6-F1-MODEL\_V4 | 1.0 | 2.25e-13 | 514 | 0.288 | 218 | 142 | 8 | 2 | 207 | 22 | 238 | Uncharacterized protein | Uncharacterized protein | | afdb-uniprot50 | AF-A0A4R3Z3N6-F1-MODEL\_V4 | 1.0 | 2.663e-15 | 513 | 0.246 | 292 | 181 | 9 | 2 | 287 | 32 | 290 | Uncharacterized protein | Uncharacterized protein | | afdb-uniprot50 | AF-A0A837FD39-F1-MODEL\_V4 | 1.0 | 4.467e-16 | 509 | 0.314 | 312 | 176 | 10 | 1 | 291 | 1 | 295 | Chromosome partitioning protein ParB | Chromosome partitioning protein ParB | | afdb-uniprot50 | AF-A0A850QVK8-F1-MODEL\_V4 | 1.0 | 9.303e-17 | 509 | 0.259 | 335 | 185 | 13 | 2 | 287 | 23 | 343 | Uncharacterized protein | Uncharacterized protein | | afdb-uniprot50 | AF-A0A0P0RBG0-F1-MODEL\_V4 | 1.0 | 5.51e-14 | 509 | 0.252 | 257 | 175 | 8 | 1 | 247 | 4 | 253 | Transcriptional regulator | Transcriptional regulator | | afdb-uniprot50 | AF-A0A373FB27-F1-MODEL\_V4 | 1.0 | 3.598e-16 | 508 | 0.272 | 294 | 167 | 9 | 28 | 291 | 2 | 278 | Chromosome partitioning protein ParB | Chromosome partitioning protein ParB | | afdb-uniprot50 | AF-A0A0E3BGD4-F1-MODEL\_V4 | 1.0 | 4.467e-16 | 508 | 0.267 | 329 | 184 | 11 | 2 | 291 | 4 | 314 | Uncharacterized protein | Uncharacterized protein | | afdb-uniprot50 | AF-A0A7Z7BHH9-F1-MODEL\_V4 | 1.0 | 1.183e-15 | 507 | 0.2 | 315 | 204 | 12 | 1 | 287 | 2 | 296 | ParB-like nuclease domain-containing protein | ParB-like nuclease domain-containing protein | | afdb-uniprot50 | AF-A0A509JFZ1-F1-MODEL\_V4 | 1.0 | 1.425e-14 | 506 | 0.269 | 267 | 164 | 12 | 1 | 245 | 32 | 289 | Uncharacterized protein | Uncharacterized protein | | afdb-uniprot50 | AF-A0A1G0NK38-F1-MODEL\_V4 | 1.0 | 3.773e-14 | 505 | 0.238 | 260 | 174 | 10 | 1 | 246 | 15 | 264 | Uncharacterized protein | Uncharacterized protein | | afdb-uniprot50 | AF-A0A096HPB0-F1-MODEL\_V4 | 1.0 | 3.798e-16 | 499 | 0.25 | 312 | 175 | 11 | 20 | 291 | 6 | 298 | Uncharacterized protein | Uncharacterized protein | | afdb-uniprot50 | AF-A0A4R1MTX3-F1-MODEL\_V4 | 1.0 | 1.087e-14 | 496 | 0.238 | 294 | 185 | 11 | 1 | 282 | 15 | 281 | Uncharacterized protein | Uncharacterized protein | | afdb-uniprot50 | AF-A0A494XEZ0-F1-MODEL\_V4 | 1.0 | 8.295e-15 | 495 | 0.233 | 291 | 188 | 10 | 1 | 281 | 17 | 282 | Transcriptional regulator | Transcriptional regulator | | afdb-uniprot50 | AF-A0A7W9ADE5-F1-MODEL\_V4 | 1.0 | 4.945e-14 | 493 | 0.258 | 263 | 167 | 11 | 1 | 246 | 26 | 277 | ParB-like chromosome segregation protein Spo0J | ParB-like chromosome segregation protein Spo0J | | afdb-uniprot50 | AF-A0A4V3Z014-F1-MODEL\_V4 | 1.0 | 4.334e-15 | 492 | 0.217 | 358 | 190 | 14 | 1 | 291 | 20 | 354 | Uncharacterized protein | Uncharacterized protein | | afdb-uniprot50 | AF-A0A4Q3X4M1-F1-MODEL\_V4 | 1.0 | 1.959e-12 | 491 | 0.252 | 198 | 135 | 6 | 1 | 187 | 24 | 219 | Uncharacterized protein | Uncharacterized protein | | afdb-uniprot50 | AF-A0A4R5I757-F1-MODEL\_V4 | 1.0 | 6.482e-14 | 491 | 0.229 | 253 | 169 | 9 | 3 | 246 | 10 | 245 | Uncharacterized protein | Uncharacterized protein | | afdb-uniprot50 | AF-A0A240UI40-F1-MODEL\_V4 | 1.0 | 1.425e-14 | 489 | 0.258 | 298 | 191 | 10 | 2 | 272 | 30 | 324 | Uncharacterized protein | Uncharacterized protein | | afdb-uniprot50 | AF-A0A4V5T687-F1-MODEL\_V4 | 1.0 | 1.279e-14 | 487 | 0.209 | 296 | 196 | 10 | 2 | 287 | 3 | 270 | Chromosome partitioning protein ParB | Chromosome partitioning protein ParB | | afdb-uniprot50 | AF-A0A7U2LLI1-F1-MODEL\_V4 | 1.0 | 1.971e-14 | 487 | 0.225 | 302 | 195 | 12 | 1 | 290 | 55 | 329 | Uncharacterized protein | Uncharacterized protein | | afdb-uniprot50 | AF-A0A2T4MZN0-F1-MODEL\_V4 | 1.0 | 2.081e-14 | 486 | 0.194 | 339 | 200 | 11 | 1 | 287 | 19 | 336 | Uncharacterized protein | Uncharacterized protein | | afdb-uniprot50 | AF-A0A1E3FN09-F1-MODEL\_V4 | 1.0 | 4.106e-15 | 485 | 0.249 | 321 | 192 | 12 | 1 | 291 | 1 | 302 | Uncharacterized protein | Uncharacterized protein | | afdb-uniprot50 | AF-A0A1H9NKE0-F1-MODEL\_V4 | 1.0 | 1.769e-14 | 483 | 0.233 | 300 | 195 | 12 | 2 | 282 | 4 | 287 | Uncharacterized protein | Uncharacterized protein | | afdb-uniprot50 | AF-A0A6G9QRE5-F1-MODEL\_V4 | 1.0 | 2.464e-16 | 483 | 0.246 | 317 | 189 | 12 | 3 | 291 | 4 | 298 | Uncharacterized protein | Uncharacterized protein | | afdb-uniprot50 | AF-A0A2E3EM85-F1-MODEL\_V4 | 1.0 | 8.295e-15 | 480 | 0.228 | 298 | 187 | 11 | 10 | 287 | 4 | 278 | ParB domain-containing protein | ParB domain-containing protein | | afdb-uniprot50 | AF-A0A370N771-F1-MODEL\_V4 | 1.0 | 2.319e-14 | 480 | 0.215 | 306 | 197 | 12 | 1 | 289 | 15 | 294 | Transcriptional regulator | Transcriptional regulator | | afdb-uniprot50 | AF-A0A7X6KYA8-F1-MODEL\_V4 | 1.0 | 9.404e-12 | 479 | 0.302 | 172 | 113 | 5 | 18 | 188 | 1 | 166 | ParB/RepB/Spo0J family partition protein | ParB/RepB/Spo0J family partition protein | | afdb-uniprot50 | AF-A0A3D9EDD3-F1-MODEL\_V4 | 1.0 | 2.081e-14 | 479 | 0.21 | 290 | 184 | 8 | 5 | 289 | 23 | 272 | ParB/RepB/Spo0J family partition protein | ParB/RepB/Spo0J family partition protein | | afdb-uniprot50 | AF-A0A5E4SS88-F1-MODEL\_V4 | 1.0 | 1.113e-13 | 479 | 0.222 | 310 | 197 | 10 | 1 | 287 | 16 | 304 | Chromosome-partitioning protein Spo0J | Chromosome-partitioning protein Spo0J | | afdb-uniprot50 | AF-A0A106DRD1-F1-MODEL\_V4 | 1.0 | 9.243e-15 | 479 | 0.242 | 293 | 189 | 12 | 13 | 287 | 10 | 287 | ParB domain-containing protein | ParB domain-containing protein | | afdb-uniprot50 | AF-A0A0F9U0H1-F1-MODEL\_V4 | 1.0 | 3.208e-14 | 478 | 0.238 | 302 | 190 | 13 | 2 | 283 | 20 | 301 | Uncharacterized protein | Uncharacterized protein | | afdb-uniprot50 | AF-A0A4Q2ZCP1-F1-MODEL\_V4 | 1.0 | 8.909e-12 | 475 | 0.245 | 208 | 144 | 8 | 9 | 205 | 31 | 236 | ParB domain-containing protein | ParB domain-containing protein | | afdb-uniprot50 | AF-A0A7Z0F9Z9-F1-MODEL\_V4 | 1.0 | 3.386e-14 | 475 | 0.225 | 311 | 194 | 14 | 1 | 291 | 15 | 298 | ParB domain-containing protein | ParB domain-containing protein | | afdb-uniprot50 | AF-A0A522FGG9-F1-MODEL\_V4 | 1.0 | 3.983e-14 | 471 | 0.259 | 301 | 187 | 11 | 2 | 291 | 28 | 303 | ParB/RepB/Spo0J family partition protein | ParB/RepB/Spo0J family partition protein | | afdb-uniprot50 | AF-A0A7Z7NRH8-F1-MODEL\_V4 | 1.0 | 1.868e-14 | 471 | 0.212 | 292 | 204 | 10 | 3 | 282 | 10 | 287 | Uncharacterized protein | Uncharacterized protein | | afdb-uniprot50 | AF-A0A7U6D4Y3-F1-MODEL\_V4 | 1.0 | 4.334e-15 | 469 | 0.234 | 316 | 182 | 12 | 1 | 282 | 1 | 290 | Putative transcriptional regulator | Putative transcriptional regulator | | afdb-uniprot50 | AF-A0A7U9K830-F1-MODEL\_V4 | 1.0 | 1.676e-14 | 468 | 0.237 | 295 | 156 | 11 | 1 | 279 | 9 | 250 | Uncharacterized protein | Uncharacterized protein | | afdb-uniprot50 | AF-A0A4Z0IUQ2-F1-MODEL\_V4 | 1.0 | 1.148e-14 | 468 | 0.208 | 321 | 193 | 13 | 2 | 282 | 5 | 304 | KorB domain-containing protein | KorB domain-containing protein | | afdb-uniprot50 | AF-H0BRY7-F1-MODEL\_V4 | 1.0 | 1.31e-13 | 465 | 0.25 | 291 | 174 | 13 | 15 | 291 | 1 | 261 | Uncharacterized protein | Uncharacterized protein | | afdb-uniprot50 | AF-A0A7S6Z1Q0-F1-MODEL\_V4 | 1.0 | 5.064e-13 | 464 | 0.239 | 259 | 170 | 12 | 1 | 246 | 16 | 260 | ParB N-terminal domain-containing protein | ParB N-terminal domain-containing protein | | afdb-uniprot50 | AF-A0A085FMH6-F1-MODEL\_V4 | 1.0 | 4.685e-14 | 464 | 0.214 | 321 | 198 | 13 | 1 | 291 | 17 | 313 | Uncharacterized protein | Uncharacterized protein | | afdb-uniprot50 | AF-A0A377Q615-F1-MODEL\_V4 | 1.0 | 1.912e-13 | 461 | 0.218 | 321 | 190 | 15 | 1 | 291 | 13 | 302 | ParB/RepB/Spo0J family partition protein | ParB/RepB/Spo0J family partition protein | | afdb-uniprot50 | AF-A4JWD7-F1-MODEL\_V4 | 1.0 | 3.039e-14 | 458 | 0.203 | 305 | 201 | 9 | 7 | 291 | 11 | 293 | Uncharacterized protein | Uncharacterized protein | | afdb-uniprot50 | AF-A0A7X3HRP0-F1-MODEL\_V4 | 1.0 | 6.482e-14 | 456 | 0.207 | 313 | 206 | 11 | 1 | 291 | 19 | 311 | Uncharacterized protein | Uncharacterized protein | | afdb-uniprot50 | AF-A0A5K1NGU7-F1-MODEL\_V4 | 1.0 | 1.912e-13 | 455 | 0.192 | 302 | 207 | 10 | 3 | 291 | 32 | 309 | Uncharacterized protein | Uncharacterized protein | | afdb-uniprot50 | AF-A0A3D9VR34-F1-MODEL\_V4 | 1.0 | 2.507e-13 | 452 | 0.181 | 298 | 201 | 11 | 1 | 282 | 47 | 317 | Uncharacterized protein | Uncharacterized protein | | afdb-uniprot50 | AF-D3VM21-F1-MODEL\_V4 | 1.0 | 1.31e-13 | 449 | 0.221 | 294 | 158 | 10 | 1 | 278 | 7 | 245 | Uncharacterized protein | Uncharacterized protein | | afdb-uniprot50 | AF-A0A2E8AAK1-F1-MODEL\_V4 | 1.0 | 7.222e-14 | 448 | 0.267 | 292 | 191 | 10 | 13 | 287 | 21 | 306 | Uncharacterized protein | Uncharacterized protein | | afdb-uniprot50 | AF-A0A2D7YR92-F1-MODEL\_V4 | 1.0 | 1.382e-13 | 445 | 0.214 | 317 | 188 | 13 | 1 | 272 | 19 | 319 | Uncharacterized protein | Uncharacterized protein | | afdb-uniprot50 | AF-A0A1D3KAB3-F1-MODEL\_V4 | 1.0 | 1.626e-13 | 444 | 0.216 | 282 | 181 | 11 | 16 | 282 | 51 | 307 | Uncharacterized protein | Uncharacterized protein | | afdb-uniprot50 | AF-A0A645D0H4-F1-MODEL\_V4 | 1.0 | 1.382e-13 | 440 | 0.24 | 275 | 140 | 11 | 21 | 279 | 1 | 222 | Uncharacterized protein | Uncharacterized protein | | afdb-uniprot50 | AF-A0A6A6K0T1-F1-MODEL\_V4 | 1.0 | 1.062e-15 | 439 | 0.277 | 285 | 130 | 7 | 1 | 282 | 1 | 212 | Uncharacterized protein | Uncharacterized protein | | afdb-uniprot50 | AF-A0A077M210-F1-MODEL\_V4 | 1.0 | 2.359e-11 | 438 | 0.241 | 186 | 126 | 5 | 1 | 185 | 1 | 172 | ParB domain-containing protein | ParB domain-containing protein | | afdb-uniprot50 | AF-A0A2D0K822-F1-MODEL\_V4 | 1.0 | 2.375e-13 | 438 | 0.206 | 300 | 161 | 12 | 1 | 280 | 6 | 248 | Chromosome partitioning protein ParB | Chromosome partitioning protein ParB | | afdb-uniprot50 | AF-A0A2S4KFT6-F1-MODEL\_V4 | 1.0 | 1.055e-13 | 438 | 0.233 | 304 | 195 | 13 | 1 | 291 | 40 | 318 | Uncharacterized protein | Uncharacterized protein | | afdb-uniprot50 | AF-A0A021VUN1-F1-MODEL\_V4 | 1.0 | 9.124e-11 | 433 | 0.289 | 169 | 113 | 5 | 18 | 185 | 1 | 163 | ParB domain-containing protein | ParB domain-containing protein | | afdb-uniprot50 | AF-A0A1E3LIY1-F1-MODEL\_V4 | 1.0 | 7.396e-13 | 433 | 0.211 | 308 | 198 | 13 | 1 | 282 | 16 | 304 | Uncharacterized protein | Uncharacterized protein | | afdb-uniprot50 | AF-A0A220WPE6-F1-MODEL\_V4 | 1.0 | 7.007e-13 | 432 | 0.24 | 295 | 181 | 14 | 9 | 282 | 250 | 522 | Uncharacterized protein | Uncharacterized protein | | afdb-uniprot50 | AF-A0A6H1ZXB1-F1-MODEL\_V4 | 1.0 | 1.665e-12 | 429 | 0.167 | 280 | 179 | 8 | 15 | 285 | 9 | 243 | ParB domain-containing protein | ParB domain-containing protein | | afdb-uniprot50 | AF-A0A6N6T5G8-F1-MODEL\_V4 | 1.0 | 3.932e-10 | 428 | 0.224 | 183 | 137 | 4 | 18 | 196 | 1 | 182 | ParB/RepB/Spo0J family partition protein | ParB/RepB/Spo0J family partition protein | | afdb-uniprot50 | AF-A0A2A3UEJ5-F1-MODEL\_V4 | 1.0 | 8.241e-13 | 421 | 0.188 | 286 | 194 | 12 | 13 | 288 | 11 | 268 | ParB domain-containing protein | ParB domain-containing protein | | afdb-uniprot50 | AF-A0A1G3YGW4-F1-MODEL\_V4 | 1.0 | 2.929e-11 | 418 | 0.221 | 190 | 137 | 6 | 14 | 199 | 6 | 188 | ParB domain-containing protein | ParB domain-containing protein | | afdb-uniprot50 | AF-A0A1G5PGC5-F1-MODEL\_V4 | 1.0 | 1.812e-13 | 417 | 0.211 | 303 | 179 | 9 | 22 | 287 | 40 | 319 | Chromosome partitioning protein, ParB family | Chromosome partitioning protein, ParB family | | afdb-uniprot50 | AF-A0A542ZE30-F1-MODEL\_V4 | 1.0 | 1.946e-10 | 416 | 0.291 | 168 | 112 | 4 | 20 | 185 | 3 | 165 | ParB/RepB/Spo0J family partition protein | ParB/RepB/Spo0J family partition protein | | afdb-uniprot50 | AF-A0A373FCW8-F1-MODEL\_V4 | 1.0 | 1.204e-12 | 415 | 0.225 | 293 | 187 | 12 | 13 | 290 | 69 | 336 | Uncharacterized protein | Uncharacterized protein | | afdb-uniprot50 | AF-A0A420RV90-F1-MODEL\_V4 | 1.0 | 2.567e-12 | 413 | 0.189 | 290 | 197 | 10 | 9 | 283 | 36 | 302 | PAPS\_reduct domain-containing protein | PAPS\_reduct domain-containing protein | | afdb-uniprot50 | AF-A0A729D027-F1-MODEL\_V4 | 1.0 | 3.724e-10 | 412 | 0.205 | 190 | 139 | 2 | 19 | 196 | 7 | 196 | Uncharacterized protein | Uncharacterized protein | | afdb-uniprot50 | AF-A0A5R2ATN3-F1-MODEL\_V4 | 1.0 | 2.842e-10 | 407 | 0.216 | 171 | 123 | 5 | 13 | 172 | 241 | 411 | Chromosome partitioning protein ParB | Chromosome partitioning protein ParB | | afdb-uniprot50 | AF-A0A6N7YJC7-F1-MODEL\_V4 | 1.0 | 4.881e-10 | 406 | 0.237 | 194 | 136 | 8 | 19 | 204 | 68 | 257 | ParB/RepB/Spo0J family partition protein | ParB/RepB/Spo0J family partition protein | | afdb-uniprot50 | AF-A0A6L5X784-F1-MODEL\_V4 | 1.0 | 1.654e-10 | 404 | 0.188 | 207 | 148 | 8 | 18 | 214 | 1 | 197 | ParB/RepB/Spo0J family partition protein | ParB/RepB/Spo0J family partition protein | | afdb-uniprot50 | AF-A0A644X8U2-F1-MODEL\_V4 | 1.0 | 1.406e-10 | 404 | 0.202 | 188 | 140 | 6 | 13 | 197 | 1 | 181 | Nucleoid occlusion protein | Nucleoid occlusion protein | | afdb-uniprot50 | AF-A0A7X2BNE3-F1-MODEL\_V4 | 1.0 | 2.182e-12 | 395 | 0.182 | 345 | 204 | 17 | 2 | 291 | 22 | 343 | Uncharacterized protein | Uncharacterized protein | | afdb-uniprot50 | AF-A0A4R8EP77-F1-MODEL\_V4 | 1.0 | 7.396e-13 | 395 | 0.18 | 315 | 210 | 14 | 1 | 282 | 26 | 325 | Uncharacterized protein | Uncharacterized protein | | afdb-uniprot50 | AF-Q9LCY0-F1-MODEL\_V4 | 1.0 | 2.235e-11 | 389 | 0.231 | 203 | 147 | 5 | 1 | 201 | 5 | 200 | Putative partitioning protein | Putative partitioning protein | | afdb-uniprot50 | AF-X1RK19-F1-MODEL\_V4 | 1.0 | 1.041e-09 | 388 | 0.2 | 190 | 141 | 7 | 18 | 200 | 1 | 186 | ParB domain-containing protein | ParB domain-containing protein | | afdb-uniprot50 | AF-V0UPH2-F1-MODEL\_V4 | 1.0 | 1.341e-12 | 388 | 0.226 | 296 | 193 | 13 | 19 | 287 | 19 | 305 | Uncharacterized protein | Uncharacterized protein | | afdb-uniprot50 | AF-A0A1F7U682-F1-MODEL\_V4 | 1.0 | 2.117e-11 | 387 | 0.209 | 243 | 172 | 12 | 14 | 246 | 15 | 247 | ParB domain-containing protein | ParB domain-containing protein | | afdb-uniprot50 | AF-A0A2M9YIY9-F1-MODEL\_V4 | 1.0 | 9.863e-10 | 387 | 0.21 | 185 | 131 | 6 | 13 | 182 | 527 | 711 | Chromosome partitioning protein ParB | Chromosome partitioning protein ParB | | afdb-uniprot50 | AF-A0A1E3LMZ5-F1-MODEL\_V4 | 1.0 | 9.183e-13 | 386 | 0.211 | 335 | 192 | 13 | 4 | 288 | 1 | 313 | Uncharacterized protein | Uncharacterized protein | | afdb-uniprot50 | AF-A0A5I0PMP9-F1-MODEL\_V4 | 1.0 | 1.855e-12 | 386 | 0.233 | 295 | 191 | 10 | 19 | 287 | 55 | 340 | DNA-binding protein | DNA-binding protein | | afdb-uniprot50 | AF-A0A7Z0PSX1-F1-MODEL\_V4 | 1.0 | 1.16e-09 | 385 | 0.218 | 169 | 124 | 6 | 18 | 184 | 1 | 163 | ParB/RepB/Spo0J family partition protein | ParB/RepB/Spo0J family partition protein | | afdb-uniprot50 | AF-A0A1E3Y3Z3-F1-MODEL\_V4 | 1.0 | 9.343e-10 | 384 | 0.164 | 182 | 146 | 3 | 17 | 197 | 1 | 177 | ParB domain-containing protein | ParB domain-containing protein | | afdb-uniprot50 | AF-A0A6M3IM54-F1-MODEL\_V4 | 1.0 | 4.25e-09 | 383 | 0.155 | 186 | 149 | 4 | 18 | 199 | 1 | 182 | ParB domain-containing protein | ParB domain-containing protein | | afdb-uniprot50 | AF-A0A062XQP4-F1-MODEL\_V4 | 1.0 | 5.064e-13 | 382 | 0.212 | 297 | 199 | 11 | 1 | 287 | 3 | 274 | HTH cro/C1-type domain-containing protein | HTH cro/C1-type domain-containing protein | | afdb-uniprot50 | AF-A0A522PSB4-F1-MODEL\_V4 | 1.0 | 6.061e-10 | 382 | 0.207 | 198 | 140 | 8 | 13 | 199 | 6 | 197 | ParB/RepB/Spo0J family partition protein | ParB/RepB/Spo0J family partition protein | | afdb-uniprot50 | AF-A0A811H1P2-F1-MODEL\_V4 | 1.0 | 8.44e-12 | 381 | 0.246 | 256 | 149 | 6 | 53 | 287 | 2 | 234 | Uncharacterized protein | Uncharacterized protein | | afdb-uniprot50 | AF-A0A522RG87-F1-MODEL\_V4 | 1.0 | 3.092e-11 | 381 | 0.225 | 200 | 140 | 9 | 5 | 200 | 22 | 210 | ParB/RepB/Spo0J family partition protein | ParB/RepB/Spo0J family partition protein | | afdb-uniprot50 | AF-A0A6L6XV94-F1-MODEL\_V4 | 1.0 | 5.311e-11 | 380 | 0.205 | 200 | 132 | 8 | 19 | 210 | 63 | 243 | ParB/RepB/Spo0J family partition protein | ParB/RepB/Spo0J family partition protein | | afdb-uniprot50 | AF-A0A2A2M1Z6-F1-MODEL\_V4 | 1.0 | 9.863e-10 | 379 | 0.205 | 185 | 137 | 6 | 18 | 194 | 1 | 183 | ParB domain-containing protein | ParB domain-containing protein | | afdb-uniprot50 | AF-A0A098EFW5-F1-MODEL\_V4 | 1.0 | 4.306e-13 | 379 | 0.175 | 297 | 209 | 14 | 1 | 282 | 1 | 276 | Chromosome-partitioning protein Spo0J | Chromosome-partitioning protein Spo0J | | afdb-uniprot50 | AF-A0A1G2AS51-F1-MODEL\_V4 | 1.0 | 1.099e-09 | 379 | 0.216 | 189 | 134 | 8 | 19 | 200 | 20 | 201 | ParB domain-containing protein | ParB domain-containing protein | | afdb-uniprot50 | AF-A0A4R8JJU0-F1-MODEL\_V4 | 1.0 | 2.054e-10 | 379 | 0.177 | 203 | 152 | 8 | 19 | 214 | 8 | 202 | ParB/RepB/Spo0J family partition protein | ParB/RepB/Spo0J family partition protein | | afdb-uniprot50 | AF-A0A512RS84-F1-MODEL\_V4 | 1.0 | 1.52e-09 | 379 | 0.16 | 181 | 145 | 5 | 13 | 192 | 28 | 202 | ParB domain-containing protein | ParB domain-containing protein | | afdb-uniprot50 | AF-E2S7S5-F1-MODEL\_V4 | 1.0 | 2.842e-10 | 377 | 0.265 | 196 | 124 | 8 | 13 | 202 | 1 | 182 | ParB-like protein | ParB-like protein | | afdb-uniprot50 | AF-A0A6H1Z9T6-F1-MODEL\_V4 | 1.0 | 1.16e-09 | 377 | 0.163 | 202 | 150 | 8 | 19 | 214 | 6 | 194 | ParB domain-containing protein | ParB domain-containing protein | | afdb-uniprot50 | AF-A0A1M3EWF3-F1-MODEL\_V4 | 1.0 | 2.006e-11 | 377 | 0.226 | 238 | 142 | 13 | 19 | 246 | 33 | 238 | ParB domain-containing protein | ParB domain-containing protein | | afdb-uniprot50 | AF-Q141V7-F1-MODEL\_V4 | 1.0 | 9.863e-10 | 377 | 0.211 | 184 | 134 | 6 | 19 | 197 | 81 | 258 | ParB family protein | ParB family protein | | afdb-uniprot50 | AF-A0A2V9TQV9-F1-MODEL\_V4 | 1.0 | 1.758e-12 | 376 | 0.192 | 296 | 211 | 14 | 2 | 282 | 3 | 285 | Chromosome partitioning protein ParB | Chromosome partitioning protein ParB | | afdb-uniprot50 | AF-A0A6G9HIL3-F1-MODEL\_V4 | 1.0 | 8.909e-12 | 376 | 0.243 | 275 | 177 | 9 | 19 | 267 | 55 | 324 | Putative transcriptional regulator | Putative transcriptional regulator | | afdb-uniprot50 | AF-A0A1Q3THK1-F1-MODEL\_V4 | 1.0 | 3.343e-10 | 375 | 0.172 | 185 | 133 | 8 | 19 | 189 | 35 | 213 | ParB domain-containing protein | ParB domain-containing protein | | afdb-uniprot50 | AF-A0A1H2IE39-F1-MODEL\_V4 | 1.0 | 2.289e-10 | 374 | 0.237 | 236 | 138 | 10 | 20 | 246 | 26 | 228 | ParB-like nuclease domain-containing protein | ParB-like nuclease domain-containing protein | | afdb-uniprot50 | AF-Q72H91-F1-MODEL\_V4 | 1.0 | 2.71e-12 | 372 | 0.182 | 290 | 201 | 10 | 1 | 282 | 4 | 265 | Chromosome-partitioning protein Spo0J | Chromosome-partitioning protein Spo0J | | afdb-uniprot50 | AF-A0A6P1V5I1-F1-MODEL\_V4 | 1.0 | 7.175e-12 | 372 | 0.186 | 333 | 210 | 14 | 1 | 287 | 10 | 327 | DNA-binding protein | DNA-binding protein | | afdb-uniprot50 | AF-A0A1F4FSB8-F1-MODEL\_V4 | 1.0 | 2.344e-09 | 371 | 0.232 | 185 | 127 | 9 | 19 | 198 | 1 | 175 | BZIP domain-containing protein | BZIP domain-containing protein | | afdb-uniprot50 | AF-A0A239A1N1-F1-MODEL\_V4 | 1.0 | 3.724e-10 | 371 | 0.213 | 183 | 130 | 6 | 19 | 194 | 17 | 192 | ParB/RepB/Spo0J family partition protein | ParB/RepB/Spo0J family partition protein | | afdb-uniprot50 | AF-A0A5N9VV60-F1-MODEL\_V4 | 1.0 | 5.88e-09 | 370 | 0.202 | 178 | 130 | 2 | 26 | 191 | 2 | 179 | Uncharacterized protein | Uncharacterized protein | | afdb-uniprot50 | AF-A0A2A7RHV9-F1-MODEL\_V4 | 1.0 | 4.306e-13 | 370 | 0.193 | 310 | 203 | 16 | 1 | 291 | 13 | 294 | Chromosome partitioning protein ParB | Chromosome partitioning protein ParB | | afdb-uniprot50 | AF-A0A1F2QTS0-F1-MODEL\_V4 | 1.0 | 8.241e-13 | 369 | 0.171 | 286 | 203 | 11 | 13 | 282 | 38 | 305 | ParB domain-containing protein | ParB domain-containing protein | | afdb-uniprot50 | AF-A0A5Q4ZYV4-F1-MODEL\_V4 | 1.0 | 3.637e-11 | 369 | 0.191 | 297 | 185 | 12 | 20 | 282 | 32 | 307 | Uncharacterized protein | Uncharacterized protein | | afdb-uniprot50 | AF-A0A2P9H4Z5-F1-MODEL\_V4 | 1.0 | 3.423e-09 | 369 | 0.243 | 185 | 128 | 8 | 19 | 197 | 10 | 188 | Putative partition protein | Putative partition protein | | afdb-uniprot50 | AF-A0A3D2NSA5-F1-MODEL\_V4 | 1.0 | 5.153e-10 | 368 | 0.316 | 183 | 117 | 4 | 71 | 246 | 8 | 189 | Uncharacterized protein | Uncharacterized protein | | afdb-uniprot50 | AF-A0A2E4W5V6-F1-MODEL\_V4 | 1.0 | 1.946e-10 | 368 | 0.176 | 244 | 177 | 11 | 13 | 247 | 2 | 230 | ParB domain-containing protein | ParB domain-containing protein | | afdb-uniprot50 | AF-A0A378MEE2-F1-MODEL\_V4 | 1.0 | 8.188e-11 | 367 | 0.227 | 211 | 146 | 10 | 1 | 204 | 1 | 201 | Probable chromosome-partitioning protein parB | Probable chromosome-partitioning protein parB | | afdb-uniprot50 | AF-A0A6G7Z243-F1-MODEL\_V4 | 1.0 | 7.129e-10 | 367 | 0.267 | 191 | 125 | 7 | 17 | 198 | 1 | 185 | ParB/RepB/Spo0J family partition protein | ParB/RepB/Spo0J family partition protein | | afdb-uniprot50 | AF-A0A4S2FYS1-F1-MODEL\_V4 | 1.0 | 4.486e-09 | 367 | 0.167 | 185 | 144 | 6 | 19 | 198 | 14 | 193 | ParB/RepB/Spo0J family partition protein | ParB/RepB/Spo0J family partition protein | | afdb-uniprot50 | AF-A0A1J5D517-F1-MODEL\_V4 | 1.0 | 1.416e-12 | 366 | 0.204 | 299 | 196 | 12 | 1 | 282 | 7 | 280 | ParB domain-containing protein | ParB domain-containing protein | | afdb-uniprot50 | AF-A0A2V3UB25-F1-MODEL\_V4 | 1.0 | 7.525e-10 | 366 | 0.175 | 188 | 149 | 5 | 19 | 202 | 1 | 186 | ParB family chromosome partitioning protein | ParB family chromosome partitioning protein | | afdb-uniprot50 | AF-E8V178-F1-MODEL\_V4 | 1.0 | 1.993e-09 | 366 | 0.221 | 185 | 132 | 7 | 19 | 197 | 10 | 188 | ParB-like partition protein | ParB-like partition protein | | afdb-uniprot50 | AF-W7DNX5-F1-MODEL\_V4 | 1.0 | 4.624e-10 | 363 | 0.204 | 205 | 153 | 8 | 1 | 201 | 4 | 202 | ParB family chromosome partition protein | ParB family chromosome partition protein | | afdb-uniprot50 | AF-A0A1F4T8B3-F1-MODEL\_V4 | 1.0 | 1.577e-12 | 363 | 0.169 | 289 | 210 | 10 | 4 | 282 | 16 | 284 | ParB domain-containing protein | ParB domain-containing protein | | afdb-uniprot50 | AF-A0A7W2GL23-F1-MODEL\_V4 | 1.0 | 2.948e-13 | 363 | 0.193 | 310 | 200 | 15 | 1 | 287 | 5 | 287 | ParB/RepB/Spo0J family partition protein | ParB/RepB/Spo0J family partition protein | | afdb-uniprot50 | AF-A0A3B8Q0U2-F1-MODEL\_V4 | 1.0 | 2.304e-12 | 363 | 0.205 | 292 | 195 | 14 | 12 | 291 | 28 | 294 | Chromosome partitioning protein ParB | Chromosome partitioning protein ParB | | afdb-uniprot50 | AF-A0A1G2EPD7-F1-MODEL\_V4 | 1.0 | 1.788e-09 | 362 | 0.169 | 213 | 158 | 9 | 13 | 214 | 11 | 215 | ParB domain-containing protein | ParB domain-containing protein | | afdb-uniprot50 | AF-A0A1M3PL20-F1-MODEL\_V4 | 1.0 | 3.243e-09 | 362 | 0.201 | 194 | 146 | 8 | 19 | 205 | 1 | 192 | ParB domain-containing protein | ParB domain-containing protein | | afdb-uniprot50 | AF-M6UQM9-F1-MODEL\_V4 | 1.0 | 3.724e-10 | 362 | 0.196 | 219 | 138 | 8 | 13 | 193 | 352 | 570 | ParB-like protein | ParB-like protein | | afdb-uniprot50 | AF-A0A7X6YYW6-F1-MODEL\_V4 | 1.0 | 2.692e-10 | 361 | 0.22 | 177 | 129 | 6 | 19 | 192 | 35 | 205 | ParB/RepB/Spo0J family partition protein | ParB/RepB/Spo0J family partition protein | | afdb-uniprot50 | AF-A0A2H0SUV7-F1-MODEL\_V4 | 1.0 | 1.946e-10 | 361 | 0.225 | 191 | 138 | 8 | 15 | 201 | 2 | 186 | ParB domain-containing protein | ParB domain-containing protein | | afdb-uniprot50 | AF-A0A327KRU4-F1-MODEL\_V4 | 1.0 | 4.409e-12 | 361 | 0.22 | 281 | 175 | 13 | 21 | 282 | 5 | 260 | Chromosome partitioning protein ParB | Chromosome partitioning protein ParB | | afdb-uniprot50 | AF-A0A1I4DE87-F1-MODEL\_V4 | 1.0 | 9.631e-11 | 361 | 0.272 | 202 | 123 | 7 | 11 | 205 | 124 | 308 | ParB/RepB/Spo0J family partition protein | ParB/RepB/Spo0J family partition protein | | afdb-uniprot50 | AF-A0A357IVD1-F1-MODEL\_V4 | 1.0 | 1.16e-09 | 361 | 0.207 | 212 | 146 | 9 | 13 | 203 | 155 | 365 | Site-specific DNA-methyltransferase (adenine-specific) | Site-specific DNA-methyltransferase (adenine-specific) | | afdb-uniprot50 | AF-E9S8W9-F1-MODEL\_V4 | 1.0 | 6.753e-10 | 360 | 0.187 | 203 | 146 | 7 | 18 | 212 | 31 | 222 | ParB-like protein | ParB-like protein | | afdb-uniprot50 | AF-A0A7U6FS42-F1-MODEL\_V4 | 1.0 | 2.117e-11 | 360 | 0.201 | 318 | 203 | 11 | 5 | 291 | 20 | 317 | Uncharacterized protein | Uncharacterized protein | | afdb-uniprot50 | AF-A0A7Y9UCD1-F1-MODEL\_V4 | 1.0 | 1.705e-11 | 360 | 0.226 | 278 | 169 | 15 | 1 | 263 | 38 | 284 | ParB/RepB/Spo0J family partition protein | ParB/RepB/Spo0J family partition protein | | afdb-uniprot50 | AF-A0A3M1TME7-F1-MODEL\_V4 | 1.0 | 1.023e-12 | 359 | 0.213 | 295 | 186 | 14 | 14 | 291 | 31 | 296 | ParB/RepB/Spo0J family partition protein | ParB/RepB/Spo0J family partition protein | | afdb-uniprot50 | AF-A0A849PIM8-F1-MODEL\_V4 | 1.0 | 2.55e-10 | 359 | 0.189 | 216 | 143 | 10 | 13 | 205 | 1 | 207 | ParB/RepB/Spo0J family partition protein | ParB/RepB/Spo0J family partition protein | | afdb-uniprot50 | AF-A0A7C6QC06-F1-MODEL\_V4 | 1.0 | 2.757e-09 | 359 | 0.182 | 192 | 145 | 7 | 13 | 198 | 29 | 214 | ParB/RepB/Spo0J family partition protein | ParB/RepB/Spo0J family partition protein | | afdb-uniprot50 | AF-A0A839IZR4-F1-MODEL\_V4 | 1.0 | 7.083e-08 | 358 | 0.38 | 100 | 59 | 2 | 1 | 97 | 1 | 100 | Uncharacterized protein | Uncharacterized protein | | afdb-uniprot50 | AF-A0A380DKU1-F1-MODEL\_V4 | 1.0 | 1.843e-10 | 358 | 0.234 | 205 | 145 | 8 | 1 | 200 | 1 | 198 | ParB family partitioning protein | ParB family partitioning protein | | afdb-uniprot50 | AF-A0A1H6A853-F1-MODEL\_V4 | 1.0 | 7.129e-10 | 358 | 0.221 | 190 | 136 | 7 | 14 | 198 | 13 | 195 | Chromosome partitioning protein, ParB family | Chromosome partitioning protein, ParB family | | afdb-uniprot50 | AF-A0A1G5IQC4-F1-MODEL\_V4 | 1.0 | 1.16e-09 | 358 | 0.158 | 208 | 162 | 6 | 1 | 202 | 11 | 211 | ParB/RepB/Spo0J family partition protein | ParB/RepB/Spo0J family partition protein | | afdb-uniprot50 | AF-A0A7X6P274-F1-MODEL\_V4 | 1.0 | 3.724e-10 | 357 | 0.213 | 248 | 156 | 11 | 19 | 265 | 1 | 210 | ParB/RepB/Spo0J family partition protein | ParB/RepB/Spo0J family partition protein | | afdb-uniprot50 | AF-A0A511N9H7-F1-MODEL\_V4 | 1.0 | 1.615e-11 | 357 | 0.186 | 274 | 184 | 11 | 21 | 282 | 2 | 248 | Putative chromosome 1-partitioning protein ParB | Putative chromosome 1-partitioning protein ParB | | afdb-uniprot50 | AF-A0A381KQP5-F1-MODEL\_V4 | 1.0 | 2.929e-11 | 357 | 0.16 | 355 | 205 | 11 | 19 | 291 | 8 | 351 | ParB/RepB/Spo0J family partition protein | ParB/RepB/Spo0J family partition protein | | afdb-uniprot50 | AF-A0A1Q3PK79-F1-MODEL\_V4 | 1.0 | 5.311e-11 | 357 | 0.232 | 232 | 150 | 10 | 1 | 218 | 1 | 218 | ParB domain-containing protein | ParB domain-containing protein | | afdb-uniprot50 | AF-A0A521U4H3-F1-MODEL\_V4 | 1.0 | 9.064e-09 | 357 | 0.185 | 183 | 138 | 8 | 19 | 197 | 13 | 188 | ParB/RepB/Spo0J family partition protein | ParB/RepB/Spo0J family partition protein | | afdb-uniprot50 | AF-A0A3M1FTJ6-F1-MODEL\_V4 | 1.0 | 1.14e-12 | 356 | 0.205 | 282 | 197 | 11 | 14 | 282 | 218 | 485 | ParB/RepB/Spo0J family partition protein | ParB/RepB/Spo0J family partition protein | | afdb-uniprot50 | AF-R5J9W3-F1-MODEL\_V4 | 1.0 | 3.724e-10 | 354 | 0.183 | 191 | 148 | 6 | 13 | 200 | 2 | 187 | Nucleoid occlusion protein | Nucleoid occlusion protein | | afdb-uniprot50 | AF-A0A1G3IP66-F1-MODEL\_V4 | 1.0 | 4.079e-13 | 352 | 0.201 | 318 | 195 | 16 | 1 | 291 | 6 | 291 | Chromosome partitioning protein ParB | Chromosome partitioning protein ParB | | afdb-uniprot50 | AF-A0A1M6XZ26-F1-MODEL\_V4 | 1.0 | 1.694e-09 | 352 | 0.227 | 189 | 129 | 11 | 19 | 198 | 12 | 192 | ParB/RepB/Spo0J family partition protein | ParB/RepB/Spo0J family partition protein | | afdb-uniprot50 | AF-A0A7C3C974-F1-MODEL\_V4 | 1.0 | 9.631e-11 | 351 | 0.234 | 235 | 169 | 6 | 1 | 230 | 5 | 233 | ParB/RepB/Spo0J family partition protein | ParB/RepB/Spo0J family partition protein | | afdb-uniprot50 | AF-A0A2E9KZY7-F1-MODEL\_V4 | 1.0 | 2.359e-11 | 351 | 0.171 | 285 | 204 | 10 | 15 | 282 | 2 | 271 | Chromosome partitioning protein ParB | Chromosome partitioning protein ParB | | afdb-uniprot50 | AF-A0A516P4G1-F1-MODEL\_V4 | 1.0 | 1.099e-09 | 351 | 0.172 | 191 | 140 | 10 | 19 | 202 | 3 | 182 | ParB/RepB/Spo0J family partition protein | ParB/RepB/Spo0J family partition protein | | afdb-uniprot50 | AF-A0A536RK66-F1-MODEL\_V4 | 1.0 | 2.104e-09 | 350 | 0.201 | 174 | 130 | 6 | 1 | 172 | 24 | 190 | ParB/RepB/Spo0J family partition protein | ParB/RepB/Spo0J family partition protein | | afdb-uniprot50 | AF-A0A1G0HZB1-F1-MODEL\_V4 | 1.0 | 7.175e-12 | 350 | 0.195 | 296 | 207 | 13 | 5 | 291 | 19 | 292 | Probable chromosome-partitioning protein ParB | Probable chromosome-partitioning protein ParB | | afdb-uniprot50 | AF-A0A3D5A0J3-F1-MODEL\_V4 | 1.0 | 9.183e-13 | 350 | 0.191 | 298 | 212 | 12 | 1 | 282 | 23 | 307 | Stage 0 sporulation protein J | Stage 0 sporulation protein J | | afdb-uniprot50 | AF-A0A497JKX0-F1-MODEL\_V4 | 1.0 | 1.16e-09 | 350 | 0.205 | 200 | 147 | 6 | 4 | 200 | 59 | 249 | HTH cro/C1-type domain-containing protein | HTH cro/C1-type domain-containing protein | | afdb-uniprot50 | AF-A0A7C5RVI1-F1-MODEL\_V4 | 1.0 | 1.373e-11 | 349 | 0.195 | 261 | 184 | 9 | 15 | 271 | 17 | 255 | ParB/RepB/Spo0J family partition protein | ParB/RepB/Spo0J family partition protein | | afdb-uniprot50 | AF-A0A7V2YAS1-F1-MODEL\_V4 | 1.0 | 6.44e-12 | 349 | 0.187 | 293 | 209 | 12 | 7 | 291 | 11 | 282 | ParB/RepB/Spo0J family partition protein | ParB/RepB/Spo0J family partition protein | | afdb-uniprot50 | AF-A0A524MV92-F1-MODEL\_V4 | 1.0 | 2.22e-09 | 349 | 0.217 | 193 | 135 | 8 | 1 | 184 | 39 | 224 | ParB/RepB/Spo0J family partition protein | ParB/RepB/Spo0J family partition protein | | afdb-uniprot50 | AF-A0A495IX89-F1-MODEL\_V4 | 1.0 | 4.381e-10 | 349 | 0.192 | 203 | 142 | 8 | 3 | 189 | 23 | 219 | ParB/RepB/Spo0J family partition protein | ParB/RepB/Spo0J family partition protein | | afdb-uniprot50 | AF-A0A847JDU6-F1-MODEL\_V4 | 1.0 | 6.44e-12 | 348 | 0.218 | 293 | 185 | 14 | 13 | 288 | 8 | 273 | ParB/RepB/Spo0J family partition protein | ParB/RepB/Spo0J family partition protein | | afdb-uniprot50 | AF-E5C648-F1-MODEL\_V4 | 1.0 | 1.993e-09 | 348 | 0.192 | 192 | 139 | 9 | 14 | 199 | 30 | 211 | ParB-like partition protein | ParB-like partition protein | | afdb-uniprot50 | AF-A0A356HZ39-F1-MODEL\_V4 | 1.0 | 5.475e-12 | 347 | 0.15 | 293 | 216 | 12 | 2 | 282 | 8 | 279 | ParB domain-containing protein | ParB domain-containing protein | | afdb-uniprot50 | AF-A0A846QGQ9-F1-MODEL\_V4 | 1.0 | 1.27e-12 | 347 | 0.188 | 307 | 212 | 11 | 4 | 291 | 12 | 300 | ParB family chromosome partitioning protein | ParB family chromosome partitioning protein | | afdb-uniprot50 | AF-A0A519M6E1-F1-MODEL\_V4 | 1.0 | 6.753e-10 | 346 | 0.212 | 212 | 151 | 7 | 10 | 214 | 14 | 216 | ParB/RepB/Spo0J family partition protein | ParB/RepB/Spo0J family partition protein | | afdb-uniprot50 | AF-A0A1E4LQW4-F1-MODEL\_V4 | 1.0 | 1.301e-11 | 346 | 0.221 | 289 | 200 | 12 | 2 | 282 | 1 | 272 | ParB domain-containing protein | ParB domain-containing protein | | afdb-uniprot50 | AF-A0A430RCV0-F1-MODEL\_V4 | 1.0 | 1.406e-10 | 346 | 0.266 | 218 | 125 | 9 | 19 | 205 | 8 | 221 | ParB domain-containing protein | ParB domain-containing protein | | afdb-uniprot50 | AF-A0A562SHY2-F1-MODEL\_V4 | 1.0 | 9.863e-10 | 346 | 0.202 | 198 | 142 | 9 | 19 | 210 | 1 | 188 | ParB/RepB/Spo0J family partition protein | ParB/RepB/Spo0J family partition protein | | afdb-uniprot50 | AF-A0A4Y9IUR5-F1-MODEL\_V4 | 1.0 | 9.343e-10 | 346 | 0.189 | 206 | 152 | 8 | 18 | 214 | 1 | 200 | ParB/RepB/Spo0J family partition protein | ParB/RepB/Spo0J family partition protein | | afdb-uniprot50 | AF-A0A661BEU3-F1-MODEL\_V4 | 1.0 | 5.742e-10 | 345 | 0.212 | 174 | 131 | 4 | 13 | 184 | 15 | 184 | ParB domain-containing protein | ParB domain-containing protein | | afdb-uniprot50 | AF-A0A511FBI8-F1-MODEL\_V4 | 1.0 | 5.311e-11 | 345 | 0.225 | 280 | 171 | 16 | 18 | 287 | 1 | 244 | ParB family chromosome partitioning protein | ParB family chromosome partitioning protein | | afdb-uniprot50 | AF-A0A0U1QTP7-F1-MODEL\_V4 | 1.0 | 2.117e-11 | 345 | 0.193 | 330 | 218 | 12 | 5 | 287 | 17 | 345 | DNA-binding protein homolog | DNA-binding protein homolog | | afdb-uniprot50 | AF-A0A2E3VV26-F1-MODEL\_V4 | 1.0 | 8.851e-10 | 345 | 0.175 | 216 | 161 | 9 | 1 | 214 | 17 | 217 | Partitioning protein | Partitioning protein | | afdb-uniprot50 | AF-A0A512ICY0-F1-MODEL\_V4 | 1.0 | 2.359e-11 | 345 | 0.176 | 317 | 207 | 18 | 1 | 291 | 320 | 608 | Chromosome partitioning protein ParB | Chromosome partitioning protein ParB | | afdb-uniprot50 | AF-A0A520FL95-F1-MODEL\_V4 | 1.0 | 7.525e-10 | 344 | 0.217 | 170 | 125 | 5 | 18 | 183 | 1 | 166 | Chromosome partitioning protein ParB | Chromosome partitioning protein ParB | | afdb-uniprot50 | AF-A0A637WJU4-F1-MODEL\_V4 | 1.0 | 1.125e-08 | 344 | 0.229 | 187 | 128 | 4 | 1 | 172 | 34 | 219 | DNA-binding protein | DNA-binding protein | | afdb-uniprot50 | AF-A0A1M3DP83-F1-MODEL\_V4 | 1.0 | 4.25e-09 | 344 | 0.189 | 179 | 124 | 7 | 19 | 182 | 3 | 175 | ParB domain-containing protein | ParB domain-containing protein | | afdb-uniprot50 | AF-A0A3D5I6M5-F1-MODEL\_V4 | 1.0 | 5.88e-09 | 343 | 0.216 | 189 | 130 | 10 | 23 | 205 | 1 | 177 | Chromosome partitioning protein ParB | Chromosome partitioning protein ParB | | afdb-uniprot50 | AF-A0A726LUF6-F1-MODEL\_V4 | 1.0 | 1.262e-10 | 343 | 0.172 | 273 | 185 | 6 | 19 | 253 | 7 | 276 | Uncharacterized protein | Uncharacterized protein | | afdb-uniprot50 | AF-A0A2V7X017-F1-MODEL\_V4 | 1.0 | 2.49e-11 | 343 | 0.18 | 311 | 205 | 16 | 1 | 282 | 120 | 409 | ParB domain-containing protein | ParB domain-containing protein | | afdb-uniprot50 | AF-W4RPT4-F1-MODEL\_V4 | 1.0 | 1.406e-10 | 342 | 0.217 | 253 | 179 | 12 | 1 | 246 | 1 | 241 | Chromosome (Plasmid) partitioning protein ParB | Chromosome (Plasmid) partitioning protein ParB | | afdb-uniprot50 | AF-A0A4V2B1M2-F1-MODEL\_V4 | 1.0 | 1.225e-09 | 342 | 0.197 | 197 | 149 | 6 | 10 | 202 | 34 | 225 | ParB/RepB/Spo0J family partition protein | ParB/RepB/Spo0J family partition protein | | afdb-uniprot50 | AF-A0A5B8Z9Y2-F1-MODEL\_V4 | 1.0 | 2.629e-11 | 342 | 0.201 | 298 | 200 | 16 | 1 | 282 | 1 | 276 | ParB/RepB/Spo0J family partition protein | ParB/RepB/Spo0J family partition protein | | afdb-uniprot50 | AF-A0A829R838-F1-MODEL\_V4 | 1.0 | 7.574e-12 | 342 | 0.187 | 298 | 204 | 15 | 1 | 282 | 1 | 276 | ParB family chromosome partioning protein | ParB family chromosome partioning protein | | afdb-uniprot50 | AF-A0A4Z1A271-F1-MODEL\_V4 | 1.0 | 3e-10 | 342 | 0.18 | 233 | 155 | 7 | 5 | 203 | 350 | 580 | Chromosome partitioning protein ParB | Chromosome partitioning protein ParB | | afdb-uniprot50 | AF-A0A3M1MVL3-F1-MODEL\_V4 | 1.0 | 3.072e-09 | 341 | 0.2 | 195 | 143 | 8 | 13 | 201 | 30 | 217 | ParB/RepB/Spo0J family partition protein | ParB/RepB/Spo0J family partition protein | | afdb-uniprot50 | AF-A0A7C4YD08-F1-MODEL\_V4 | 1.0 | 3.957e-12 | 341 | 0.18 | 288 | 198 | 14 | 1 | 282 | 3 | 258 | ParB/RepB/Spo0J family partition protein | ParB/RepB/Spo0J family partition protein | | afdb-uniprot50 | AF-A0A4R9H704-F1-MODEL\_V4 | 1.0 | 3.724e-10 | 341 | 0.178 | 224 | 146 | 8 | 13 | 200 | 404 | 625 | Chromosome partitioning protein ParB | Chromosome partitioning protein ParB | | afdb-uniprot50 | AF-K1AA06-F1-MODEL\_V4 | 1.0 | 7.944e-10 | 340 | 0.228 | 166 | 121 | 5 | 19 | 182 | 3 | 163 | Chromosome partitioning protein | Chromosome partitioning protein | | afdb-uniprot50 | AF-A0A7C6LJS3-F1-MODEL\_V4 | 1.0 | 5.186e-12 | 340 | 0.2 | 310 | 206 | 14 | 1 | 291 | 4 | 290 | ParB/RepB/Spo0J family partition protein | ParB/RepB/Spo0J family partition protein | | afdb-uniprot50 | AF-A0A7W9ZEF2-F1-MODEL\_V4 | 1.0 | 9.927e-12 | 340 | 0.212 | 287 | 190 | 14 | 13 | 282 | 32 | 299 | ParB family chromosome partitioning protein | ParB family chromosome partitioning protein | | afdb-uniprot50 | AF-A0A069JDM1-F1-MODEL\_V4 | 1.0 | 5.607e-11 | 340 | 0.225 | 275 | 171 | 14 | 13 | 263 | 8 | 264 | ParB domain-containing protein | ParB domain-containing protein | | afdb-uniprot50 | AF-R5VX17-F1-MODEL\_V4 | 1.0 | 4.736e-09 | 340 | 0.155 | 212 | 151 | 8 | 19 | 210 | 3 | 206 | ParB-like partition protein | ParB-like partition protein | | afdb-uniprot50 | AF-A0A7V6Y4G7-F1-MODEL\_V4 | 1.0 | 6.44e-12 | 339 | 0.183 | 295 | 194 | 13 | 9 | 282 | 10 | 278 | ParB/RepB/Spo0J family partition protein | ParB/RepB/Spo0J family partition protein | | afdb-uniprot50 | AF-A0A3A9SIV7-F1-MODEL\_V4 | 1.0 | 6.797e-12 | 339 | 0.166 | 283 | 208 | 11 | 12 | 282 | 34 | 300 | ParB/RepB/Spo0J family partition protein | ParB/RepB/Spo0J family partition protein | | afdb-uniprot50 | AF-A0A2V9YQC4-F1-MODEL\_V4 | 1.0 | 1.106e-11 | 339 | 0.163 | 294 | 207 | 14 | 13 | 291 | 49 | 318 | Chromosome partitioning protein ParB | Chromosome partitioning protein ParB | | afdb-uniprot50 | AF-A0A363TPC3-F1-MODEL\_V4 | 1.0 | 4.913e-12 | 338 | 0.18 | 299 | 212 | 10 | 1 | 289 | 10 | 285 | ParB domain-containing protein | ParB domain-containing protein | | afdb-uniprot50 | AF-A0A7X8E5Z8-F1-MODEL\_V4 | 1.0 | 2.117e-11 | 338 | 0.164 | 286 | 206 | 11 | 9 | 282 | 21 | 285 | ParB/RepB/Spo0J family partition protein | ParB/RepB/Spo0J family partition protein | | afdb-uniprot50 | AF-A0A5J6WBF0-F1-MODEL\_V4 | 1.0 | 1.654e-10 | 337 | 0.155 | 283 | 203 | 11 | 19 | 291 | 4 | 260 | ParB/RepB/Spo0J family partition protein | ParB/RepB/Spo0J family partition protein | | afdb-uniprot50 | AF-A0A318EAH7-F1-MODEL\_V4 | 1.0 | 4.767e-11 | 337 | 0.171 | 298 | 194 | 11 | 13 | 291 | 3 | 266 | Probable chromosome-partitioning protein ParB | Probable chromosome-partitioning protein ParB | | afdb-uniprot50 | AF-A0A509J6R8-F1-MODEL\_V4 | 1.0 | 1.45e-11 | 337 | 0.179 | 296 | 192 | 19 | 13 | 282 | 6 | 276 | Probable chromosome-partitioning protein ParB | Probable chromosome-partitioning protein ParB | | afdb-uniprot50 | AF-H5SEN9-F1-MODEL\_V4 | 1.0 | 1.233e-11 | 337 | 0.192 | 302 | 212 | 10 | 1 | 290 | 5 | 286 | Chromosome partitioning protein, ParB family | Chromosome partitioning protein, ParB family | | afdb-uniprot50 | AF-A0A2E4E0Y8-F1-MODEL\_V4 | 1.0 | 1.048e-11 | 337 | 0.175 | 314 | 213 | 17 | 1 | 291 | 37 | 327 | Probable chromosome-partitioning protein ParB | Probable chromosome-partitioning protein ParB | | afdb-uniprot50 | AF-A0A7K2V6D7-F1-MODEL\_V4 | 1.0 | 2.359e-11 | 337 | 0.204 | 294 | 183 | 15 | 13 | 268 | 7 | 287 | ParB/RepB/Spo0J family partition protein | ParB/RepB/Spo0J family partition protein | | afdb-uniprot50 | AF-A0A7D7UY71-F1-MODEL\_V4 | 1.0 | 4.881e-10 | 337 | 0.238 | 235 | 145 | 13 | 19 | 247 | 8 | 214 | ParB/RepB/Spo0J family partition protein | ParB/RepB/Spo0J family partition protein | | afdb-uniprot50 | AF-A0A016BNU9-F1-MODEL\_V4 | 1.0 | 7.301e-09 | 337 | 0.198 | 191 | 140 | 9 | 13 | 196 | 7 | 191 | ParB/RepB/Spo0J family partition domain protein | ParB/RepB/Spo0J family partition domain protein | | afdb-uniprot50 | AF-A0A2D8R2Z7-F1-MODEL\_V4 | 1.0 | 7.757e-11 | 337 | 0.19 | 304 | 195 | 15 | 1 | 287 | 1 | 270 | ParB domain-containing protein | ParB domain-containing protein | | afdb-uniprot50 | AF-A0A0Q6Z6C7-F1-MODEL\_V4 | 1.0 | 1.9e-11 | 336 | 0.209 | 287 | 189 | 15 | 19 | 291 | 15 | 277 | Probable chromosome-partitioning protein ParB | Probable chromosome-partitioning protein ParB | | afdb-uniprot50 | AF-A0A3M1JT40-F1-MODEL\_V4 | 1.0 | 6.797e-12 | 336 | 0.187 | 282 | 196 | 10 | 14 | 282 | 22 | 283 | ParB/RepB/Spo0J family partition protein | ParB/RepB/Spo0J family partition protein | | afdb-uniprot50 | AF-A0A3M7LGN8-F1-MODEL\_V4 | 1.0 | 2.104e-09 | 336 | 0.167 | 203 | 145 | 9 | 19 | 204 | 13 | 208 | ParB/RepB/Spo0J family partition protein | ParB/RepB/Spo0J family partition protein | | afdb-uniprot50 | AF-A0A5V0QUK1-F1-MODEL\_V4 | 1.0 | 1.365e-09 | 335 | 0.221 | 208 | 141 | 11 | 1 | 195 | 1 | 200 | ParB/RepB/Spo0J family partition protein | ParB/RepB/Spo0J family partition protein | | afdb-uniprot50 | AF-A0A4P0XZV3-F1-MODEL\_V4 | 1.0 | 1.605e-09 | 335 | 0.182 | 219 | 156 | 10 | 3 | 205 | 1 | 212 | PRTRC system ParB family protein | PRTRC system ParB family protein | | afdb-uniprot50 | AF-A0A3N1JZ34-F1-MODEL\_V4 | 1.0 | 3.072e-09 | 335 | 0.182 | 192 | 144 | 7 | 13 | 197 | 3 | 188 | ParB/RepB/Spo0J family partition protein | ParB/RepB/Spo0J family partition protein | | afdb-uniprot50 | AF-A0A2K0XC05-F1-MODEL\_V4 | 1.0 | 1.735e-08 | 335 | 0.161 | 204 | 157 | 9 | 19 | 214 | 15 | 212 | ParB domain-containing protein | ParB domain-containing protein | | afdb-uniprot50 | AF-A0A0M0BQB3-F1-MODEL\_V4 | 1.0 | 3e-10 | 334 | 0.207 | 284 | 171 | 12 | 19 | 291 | 3 | 243 | ParB domain-containing protein | ParB domain-containing protein | | afdb-uniprot50 | AF-A0A1F2VSB4-F1-MODEL\_V4 | 1.0 | 3.264e-11 | 334 | 0.179 | 295 | 204 | 14 | 8 | 291 | 9 | 276 | Probable chromosome-partitioning protein ParB | Probable chromosome-partitioning protein ParB | | afdb-uniprot50 | AF-A0A6L6YJM1-F1-MODEL\_V4 | 1.0 | 1.332e-10 | 334 | 0.149 | 275 | 186 | 15 | 1 | 253 | 19 | 267 | ParB/RepB/Spo0J family partition protein | ParB/RepB/Spo0J family partition protein | | afdb-uniprot50 | AF-A0A7K3KR56-F1-MODEL\_V4 | 1.0 | 3.613e-09 | 334 | 0.165 | 206 | 157 | 8 | 18 | 214 | 1 | 200 | ParB/RepB/Spo0J family partition protein | ParB/RepB/Spo0J family partition protein | | afdb-uniprot50 | AF-A0A7W1RZL1-F1-MODEL\_V4 | 1.0 | 5.742e-10 | 333 | 0.25 | 172 | 123 | 4 | 13 | 182 | 22 | 189 | ParB/RepB/Spo0J family partition protein | ParB/RepB/Spo0J family partition protein | | afdb-uniprot50 | AF-C8WZ31-F1-MODEL\_V4 | 1.0 | 3.551e-12 | 333 | 0.172 | 325 | 209 | 15 | 1 | 291 | 7 | 305 | ParB-like partition protein | ParB-like partition protein | | afdb-uniprot50 | AF-A0A2L2XH21-F1-MODEL\_V4 | 1.0 | 1.53e-11 | 333 | 0.216 | 291 | 192 | 16 | 16 | 287 | 1 | 274 | Chromosome partitioning protein ParB | Chromosome partitioning protein ParB | | afdb-uniprot50 | AF-A0A3E0KLR5-F1-MODEL\_V4 | 1.0 | 7.757e-11 | 332 | 0.184 | 288 | 193 | 11 | 20 | 291 | 26 | 287 | Chromosome partitioning protein ParB | Chromosome partitioning protein ParB | | afdb-uniprot50 | AF-A0A358G3B0-F1-MODEL\_V4 | 1.0 | 9.343e-10 | 332 | 0.185 | 221 | 158 | 10 | 1 | 214 | 12 | 217 | Chromosome partitioning protein ParB | Chromosome partitioning protein ParB | | afdb-uniprot50 | AF-A0A7C7K384-F1-MODEL\_V4 | 1.0 | 4.026e-09 | 332 | 0.155 | 206 | 162 | 7 | 1 | 200 | 11 | 210 | PRTRC system ParB family protein | PRTRC system ParB family protein | | afdb-uniprot50 | AF-A0A382GYA7-F1-MODEL\_V4 | 1.0 | 1.066e-08 | 331 | 0.202 | 168 | 124 | 6 | 20 | 182 | 14 | 176 | ParB domain-containing protein | ParB domain-containing protein | | afdb-uniprot50 | AF-A0A4D7QF63-F1-MODEL\_V4 | 1.0 | 1.373e-11 | 331 | 0.191 | 308 | 192 | 14 | 4 | 288 | 18 | 291 | ParB/RepB/Spo0J family partition protein | ParB/RepB/Spo0J family partition protein | | afdb-uniprot50 | AF-A0A521WSH2-F1-MODEL\_V4 | 1.0 | 7.348e-11 | 331 | 0.15 | 300 | 206 | 15 | 13 | 291 | 26 | 297 | ParB/RepB/Spo0J family partition protein | ParB/RepB/Spo0J family partition protein | | afdb-uniprot50 | AF-A0A1G3HSG6-F1-MODEL\_V4 | 1.0 | 4.177e-12 | 331 | 0.199 | 301 | 201 | 12 | 1 | 282 | 6 | 285 | Chromosome partitioning protein ParB | Chromosome partitioning protein ParB | | afdb-uniprot50 | AF-A0A6D1VXK9-F1-MODEL\_V4 | 1.0 | 5.88e-09 | 331 | 0.18 | 194 | 147 | 9 | 13 | 201 | 5 | 191 | Chromosome partitioning protein ParB | Chromosome partitioning protein ParB | | afdb-uniprot50 | AF-A0A1N7S8P1-F1-MODEL\_V4 | 1.0 | 1.066e-08 | 331 | 0.207 | 202 | 138 | 7 | 1 | 197 | 1 | 185 | ParB family protein | ParB family protein | | afdb-uniprot50 | AF-A0A6L2LGI1-F1-MODEL\_V4 | 1.0 | 2.55e-10 | 331 | 0.192 | 250 | 157 | 9 | 12 | 251 | 312 | 526 | ParB domain-containing protein | ParB domain-containing protein | | afdb-uniprot50 | AF-A0A382I217-F1-MODEL\_V4 | 1.0 | 1.45e-11 | 330 | 0.194 | 278 | 185 | 12 | 4 | 269 | 16 | 266 | ParB domain-containing protein | ParB domain-containing protein | | afdb-uniprot50 | AF-A0A497SN10-F1-MODEL\_V4 | 1.0 | 6.71e-08 | 330 | 0.21 | 171 | 122 | 8 | 21 | 184 | 1 | 165 | ParB domain-containing protein | ParB domain-containing protein | | afdb-uniprot50 | AF-A0A4Y3KAD6-F1-MODEL\_V4 | 1.0 | 9.124e-11 | 330 | 0.169 | 313 | 195 | 11 | 1 | 290 | 13 | 283 | Chromosome partitioning protein ParB | Chromosome partitioning protein ParB | | afdb-uniprot50 | AF-K9H3Q4-F1-MODEL\_V4 | 1.0 | 9.404e-12 | 330 | 0.192 | 307 | 203 | 13 | 2 | 291 | 38 | 316 | Chromosome (Plasmid) partitioning protein ParB | Chromosome (Plasmid) partitioning protein ParB | | afdb-uniprot50 | AF-A0A2H1J509-F1-MODEL\_V4 | 1.0 | 3.839e-11 | 330 | 0.17 | 300 | 189 | 16 | 12 | 282 | 250 | 518 | Chromosome partitioning protein, ParB family | Chromosome partitioning protein, ParB family | | afdb-uniprot50 | AF-A0A6F9ZNA4-F1-MODEL\_V4 | 1.0 | 6.207e-09 | 329 | 0.162 | 197 | 146 | 8 | 1 | 189 | 2 | 187 | Chromosome-partitioning protein Spo0J | Chromosome-partitioning protein Spo0J | | afdb-uniprot50 | AF-K5DPP1-F1-MODEL\_V4 | 1.0 | 1.324e-08 | 328 | 0.197 | 187 | 141 | 7 | 13 | 192 | 1 | 185 | Transcriptional regulator | Transcriptional regulator | | afdb-uniprot50 | AF-A0A7C6WVD0-F1-MODEL\_V4 | 1.0 | 2.006e-11 | 328 | 0.181 | 292 | 187 | 12 | 13 | 282 | 2 | 263 | ParB/RepB/Spo0J family partition protein | ParB/RepB/Spo0J family partition protein | | afdb-uniprot50 | AF-A0A7R8B2R3-F1-MODEL\_V4 | 1.0 | 6.797e-12 | 328 | 0.224 | 289 | 190 | 12 | 12 | 287 | 42 | 309 | Chromosome partitioning protein ParB | Chromosome partitioning protein ParB | | afdb-uniprot50 | AF-A0A2V9VKL2-F1-MODEL\_V4 | 1.0 | 2.006e-11 | 328 | 0.2 | 300 | 202 | 14 | 3 | 282 | 116 | 397 | Chromosome partitioning protein ParB | Chromosome partitioning protein ParB | | afdb-uniprot50 | AF-A0A0F9I9R4-F1-MODEL\_V4 | 1.0 | 5.032e-11 | 328 | 0.188 | 286 | 153 | 12 | 14 | 283 | 9 | 231 | ParB domain-containing protein | ParB domain-containing protein | | afdb-uniprot50 | AF-A0A7V7CWH9-F1-MODEL\_V4 | 1.0 | 6.398e-10 | 327 | 0.257 | 198 | 129 | 8 | 13 | 205 | 38 | 222 | ParB/RepB/Spo0J family partition protein | ParB/RepB/Spo0J family partition protein | | afdb-uniprot50 | AF-A0A2W4SXL5-F1-MODEL\_V4 | 1.0 | 9.927e-12 | 327 | 0.208 | 298 | 178 | 13 | 13 | 282 | 6 | 273 | Chromosome partitioning protein ParB | Chromosome partitioning protein ParB | | afdb-uniprot50 | AF-A0A2V6T438-F1-MODEL\_V4 | 1.0 | 7.574e-12 | 327 | 0.207 | 289 | 190 | 13 | 15 | 291 | 19 | 280 | ParB domain-containing protein | ParB domain-containing protein | | afdb-uniprot50 | AF-A0A7D7ZNA8-F1-MODEL\_V4 | 1.0 | 2.182e-12 | 327 | 0.211 | 298 | 184 | 13 | 13 | 290 | 33 | 299 | Probable chromosome-partitioning protein ParB | Probable chromosome-partitioning protein ParB | | afdb-uniprot50 | AF-A0A2J0MU83-F1-MODEL\_V4 | 1.0 | 5.779e-12 | 327 | 0.178 | 292 | 207 | 12 | 1 | 282 | 33 | 301 | Chromosome partitioning protein ParB | Chromosome partitioning protein ParB | | afdb-uniprot50 | AF-A0A2E8PUX0-F1-MODEL\_V4 | 1.0 | 7.129e-10 | 327 | 0.167 | 275 | 182 | 13 | 13 | 246 | 155 | 423 | ParB domain-containing protein | ParB domain-containing protein | | afdb-uniprot50 | AF-A0A7U4TW42-F1-MODEL\_V4 | 1.0 | 6.398e-10 | 326 | 0.282 | 198 | 120 | 4 | 96 | 282 | 5 | 191 | Uncharacterized protein | Uncharacterized protein | | afdb-uniprot50 | AF-A0A4P1KK94-F1-MODEL\_V4 | 1.0 | 8.851e-10 | 326 | 0.253 | 189 | 129 | 6 | 12 | 195 | 28 | 209 | Chromosome-partitioning protein parB | Chromosome-partitioning protein parB | | afdb-uniprot50 | AF-A0A2D5ZN56-F1-MODEL\_V4 | 1.0 | 7.995e-12 | 326 | 0.182 | 291 | 207 | 11 | 1 | 282 | 8 | 276 | ParB domain-containing protein | ParB domain-containing protein | | afdb-uniprot50 | AF-A0A7G9A8K7-F1-MODEL\_V4 | 1.0 | 9.124e-11 | 326 | 0.23 | 265 | 174 | 10 | 41 | 287 | 14 | 266 | Putative transcriptional regulators | Putative transcriptional regulators | | afdb-uniprot50 | AF-A0A537KSY3-F1-MODEL\_V4 | 1.0 | 4.053e-11 | 326 | 0.202 | 306 | 204 | 15 | 4 | 291 | 15 | 298 | ParB/RepB/Spo0J family partition protein | ParB/RepB/Spo0J family partition protein | | afdb-uniprot50 | AF-A0A137SW11-F1-MODEL\_V4 | 1.0 | 2.117e-11 | 325 | 0.196 | 300 | 206 | 14 | 4 | 291 | 19 | 295 | Putative stage 0 sporulation protein J | Putative stage 0 sporulation protein J | | afdb-uniprot50 | AF-A0A4Q7IXJ8-F1-MODEL\_V4 | 1.0 | 1.168e-11 | 325 | 0.146 | 300 | 218 | 12 | 7 | 291 | 23 | 299 | ParB/RepB/Spo0J family partition protein | ParB/RepB/Spo0J family partition protein | | afdb-uniprot50 | AF-A0A3M2BDT7-F1-MODEL\_V4 | 1.0 | 2.929e-11 | 325 | 0.18 | 294 | 203 | 16 | 13 | 291 | 47 | 317 | ParB/RepB/Spo0J family partition protein | ParB/RepB/Spo0J family partition protein | | afdb-uniprot50 | AF-A0A7K0PKF7-F1-MODEL\_V4 | 1.0 | 1.406e-10 | 325 | 0.183 | 343 | 206 | 17 | 1 | 287 | 1 | 325 | ParB/RepB/Spo0J family partition protein | ParB/RepB/Spo0J family partition protein | | afdb-uniprot50 | AF-A0A2V8F9Y2-F1-MODEL\_V4 | 1.0 | 6.552e-09 | 324 | 0.256 | 164 | 113 | 6 | 14 | 172 | 18 | 177 | Stage 0 sporulation protein J | Stage 0 sporulation protein J | | afdb-uniprot50 | AF-A0A2S5UTJ6-F1-MODEL\_V4 | 1.0 | 9.343e-10 | 324 | 0.195 | 205 | 131 | 10 | 13 | 193 | 15 | 209 | ParB domain-containing protein | ParB domain-containing protein | | afdb-uniprot50 | AF-A0A839QSA6-F1-MODEL\_V4 | 1.0 | 3.092e-11 | 324 | 0.182 | 285 | 191 | 16 | 19 | 283 | 78 | 340 | ParB family chromosome partitioning protein | ParB family chromosome partitioning protein | | afdb-uniprot50 | AF-A0A4R6WAD4-F1-MODEL\_V4 | 1.0 | 2.612e-09 | 324 | 0.155 | 187 | 138 | 6 | 19 | 191 | 11 | 191 | ParB/RepB/Spo0J family partition protein | ParB/RepB/Spo0J family partition protein | | afdb-uniprot50 | AF-A0A2G8CD03-F1-MODEL\_V4 | 1.0 | 4.352e-08 | 323 | 0.149 | 194 | 151 | 7 | 19 | 205 | 6 | 192 | Chromosome partitioning protein ParB | Chromosome partitioning protein ParB | | afdb-uniprot50 | AF-A0A1T4NQJ2-F1-MODEL\_V4 | 1.0 | 2.775e-11 | 323 | 0.186 | 274 | 200 | 11 | 19 | 282 | 37 | 297 | Chromosome partitioning protein, ParB family | Chromosome partitioning protein, ParB family | | afdb-uniprot50 | AF-A0A518BT11-F1-MODEL\_V4 | 1.0 | 5.918e-11 | 323 | 0.192 | 306 | 198 | 14 | 10 | 283 | 10 | 298 | Putative chromosome-partitioning protein ParB | Putative chromosome-partitioning protein ParB | | afdb-uniprot50 | AF-A0A1N7JF37-F1-MODEL\_V4 | 1.0 | 5.475e-12 | 323 | 0.189 | 332 | 199 | 19 | 1 | 291 | 6 | 308 | ParB/RepB/Spo0J family partition protein | ParB/RepB/Spo0J family partition protein | | afdb-uniprot50 | AF-A0A496S0Y3-F1-MODEL\_V4 | 1.0 | 6.247e-11 | 322 | 0.17 | 299 | 203 | 11 | 1 | 282 | 5 | 275 | ParB domain-containing protein | ParB domain-containing protein | | afdb-uniprot50 | AF-A0A7X8DA56-F1-MODEL\_V4 | 1.0 | 7.574e-12 | 322 | 0.182 | 301 | 207 | 15 | 1 | 288 | 5 | 279 | ParB/RepB/Spo0J family partition protein | ParB/RepB/Spo0J family partition protein | | afdb-uniprot50 | AF-A0A3R7RVA8-F1-MODEL\_V4 | 1.0 | 8.643e-11 | 322 | 0.162 | 289 | 209 | 12 | 5 | 282 | 14 | 280 | ParB/RepB/Spo0J family partition protein | ParB/RepB/Spo0J family partition protein | | afdb-uniprot50 | AF-A0A522RQE6-F1-MODEL\_V4 | 1.0 | 2.775e-11 | 322 | 0.176 | 306 | 202 | 17 | 1 | 282 | 6 | 285 | ParB/RepB/Spo0J family partition protein | ParB/RepB/Spo0J family partition protein | | afdb-uniprot50 | AF-A0A3B9TXJ6-F1-MODEL\_V4 | 1.0 | 3.613e-09 | 321 | 0.217 | 175 | 125 | 6 | 14 | 182 | 75 | 243 | ParB domain-containing protein | ParB domain-containing protein | | afdb-uniprot50 | AF-A0A1F5PE80-F1-MODEL\_V4 | 1.0 | 1.373e-11 | 321 | 0.202 | 287 | 194 | 14 | 14 | 283 | 7 | 275 | ParB domain-containing protein | ParB domain-containing protein | | afdb-uniprot50 | AF-A0A2G9ZDA8-F1-MODEL\_V4 | 1.0 | 3.445e-11 | 321 | 0.202 | 287 | 200 | 11 | 3 | 282 | 13 | 277 | Chromosome partitioning protein ParB | Chromosome partitioning protein ParB | | afdb-uniprot50 | AF-A0A0F9XAK5-F1-MODEL\_V4 | 1.0 | 2.168e-10 | 321 | 0.186 | 301 | 202 | 15 | 5 | 291 | 11 | 282 | Uncharacterized protein | Uncharacterized protein | | afdb-uniprot50 | AF-A0A2A4LKK3-F1-MODEL\_V4 | 1.0 | 1.615e-11 | 321 | 0.19 | 310 | 197 | 16 | 4 | 288 | 18 | 298 | Chromosome partitioning protein ParB | Chromosome partitioning protein ParB | | afdb-uniprot50 | AF-A0A7H8KV52-F1-MODEL\_V4 | 1.0 | 2.629e-11 | 321 | 0.208 | 307 | 212 | 17 | 1 | 291 | 60 | 351 | ParB/RepB/Spo0J family partition protein | ParB/RepB/Spo0J family partition protein | | afdb-uniprot50 | AF-A0A4R7C1F4-F1-MODEL\_V4 | 1.0 | 1.946e-10 | 321 | 0.183 | 289 | 191 | 11 | 13 | 287 | 80 | 337 | ParB/RepB/Spo0J family partition protein | ParB/RepB/Spo0J family partition protein | | afdb-uniprot50 | AF-A0A7W8YCJ4-F1-MODEL\_V4 | 1.0 | 3.839e-11 | 321 | 0.185 | 286 | 189 | 15 | 19 | 283 | 359 | 621 | ParB/RepB/Spo0J family partition protein | ParB/RepB/Spo0J family partition protein | | afdb-uniprot50 | AF-A0A1V3YZ69-F1-MODEL\_V4 | 1.0 | 4.736e-09 | 320 | 0.205 | 219 | 149 | 11 | 1 | 203 | 1 | 210 | Uncharacterized protein | Uncharacterized protein | | afdb-uniprot50 | AF-A0A1G6F7D8-F1-MODEL\_V4 | 1.0 | 2.235e-11 | 320 | 0.184 | 309 | 204 | 11 | 1 | 282 | 7 | 294 | Chromosome partitioning protein, ParB family | Chromosome partitioning protein, ParB family | | afdb-uniprot50 | AF-U7UNL4-F1-MODEL\_V4 | 1.0 | 2.117e-11 | 320 | 0.156 | 287 | 197 | 12 | 19 | 291 | 60 | 315 | ParB-like protein | ParB-like protein | | afdb-uniprot50 | AF-A0A7X6XF92-F1-MODEL\_V4 | 1.0 | 1.406e-10 | 320 | 0.185 | 275 | 186 | 10 | 13 | 271 | 1 | 253 | ParB/RepB/Spo0J family partition protein | ParB/RepB/Spo0J family partition protein | | afdb-uniprot50 | AF-A0A0F9TED6-F1-MODEL\_V4 | 1.0 | 4.999e-09 | 320 | 0.208 | 211 | 145 | 10 | 2 | 200 | 10 | 210 | ParB domain-containing protein | ParB domain-containing protein | | afdb-uniprot50 | AF-A0A383DD87-F1-MODEL\_V4 | 1.0 | 1.788e-09 | 319 | 0.228 | 184 | 123 | 8 | 1 | 176 | 1 | 173 | ParB domain-containing protein | ParB domain-containing protein | | afdb-uniprot50 | AF-A0A527D7P3-F1-MODEL\_V4 | 1.0 | 4.352e-08 | 319 | 0.22 | 186 | 133 | 7 | 21 | 201 | 36 | 214 | ParB/RepB/Spo0J family partition protein | ParB/RepB/Spo0J family partition protein | | afdb-uniprot50 | AF-A0A3A4PA16-F1-MODEL\_V4 | 1.0 | 4.767e-11 | 319 | 0.146 | 307 | 224 | 12 | 1 | 290 | 5 | 290 | ParB/RepB/Spo0J family partition protein | ParB/RepB/Spo0J family partition protein | | afdb-uniprot50 | AF-A0A1F2SQJ3-F1-MODEL\_V4 | 1.0 | 2.117e-11 | 319 | 0.201 | 302 | 206 | 15 | 1 | 287 | 17 | 298 | ParB domain-containing protein | ParB domain-containing protein | | afdb-uniprot50 | AF-A0A7C3CPY4-F1-MODEL\_V4 | 1.0 | 6.398e-10 | 319 | 0.216 | 217 | 144 | 10 | 8 | 205 | 45 | 254 | ParB/RepB/Spo0J family partition protein | ParB/RepB/Spo0J family partition protein | | afdb-uniprot50 | AF-A0A4R2PPN9-F1-MODEL\_V4 | 1.0 | 6.595e-11 | 319 | 0.219 | 315 | 189 | 15 | 9 | 290 | 29 | 319 | ParB family chromosome partitioning protein | ParB family chromosome partitioning protein | | afdb-uniprot50 | AF-E5Y7U8-F1-MODEL\_V4 | 1.0 | 5.779e-12 | 319 | 0.19 | 310 | 191 | 16 | 13 | 291 | 37 | 317 | ParB-like partition protein | ParB-like partition protein | | afdb-uniprot50 | AF-A0A542JTD4-F1-MODEL\_V4 | 1.0 | 1.831e-08 | 319 | 0.215 | 204 | 140 | 11 | 19 | 215 | 150 | 340 | ParB family chromosome partitioning protein | ParB family chromosome partitioning protein | | afdb-uniprot50 | AF-A0A7W7FP95-F1-MODEL\_V4 | 1.0 | 4.624e-10 | 319 | 0.192 | 285 | 198 | 15 | 4 | 280 | 51 | 311 | ParB/RepB/Spo0J family partition protein | ParB/RepB/Spo0J family partition protein | | afdb-uniprot50 | AF-A0A7Y7ILR4-F1-MODEL\_V4 | 1.0 | 2.168e-10 | 319 | 0.187 | 283 | 179 | 13 | 19 | 284 | 3 | 251 | ParB/RepB/Spo0J family partition protein | ParB/RepB/Spo0J family partition protein | | afdb-uniprot50 | AF-A0A5D0XVA2-F1-MODEL\_V4 | 1.0 | 6.961e-11 | 319 | 0.17 | 287 | 190 | 16 | 19 | 282 | 221 | 482 | ParB/RepB/Spo0J family partition protein | ParB/RepB/Spo0J family partition protein | | afdb-uniprot50 | AF-A0A3N9WYB0-F1-MODEL\_V4 | 1.0 | 1.831e-08 | 319 | 0.224 | 196 | 137 | 9 | 10 | 199 | 21 | 207 | ParB domain-containing protein | ParB domain-containing protein | | afdb-uniprot50 | AF-A0A238XIP6-F1-MODEL\_V4 | 1.0 | 4.736e-09 | 319 | 0.153 | 247 | 184 | 10 | 5 | 247 | 72 | 297 | Chromosome partitioning protein, ParB family | Chromosome partitioning protein, ParB family | | afdb-uniprot50 | AF-A0A0F9T9X7-F1-MODEL\_V4 | 1.0 | 1.788e-09 | 318 | 0.188 | 270 | 171 | 12 | 22 | 282 | 16 | 246 | ParB domain-containing protein | ParB domain-containing protein | | afdb-uniprot50 | AF-R6QZ87-F1-MODEL\_V4 | 1.0 | 1.373e-11 | 318 | 0.167 | 298 | 208 | 11 | 1 | 282 | 21 | 294 | ParB partition protein | ParB partition protein | | afdb-uniprot50 | AF-A0A2N5IVP5-F1-MODEL\_V4 | 1.0 | 8.587e-09 | 318 | 0.24 | 204 | 128 | 12 | 19 | 205 | 5 | 198 | ParB-like protein | ParB-like protein | | afdb-uniprot50 | AF-A0A7R9L9W8-F1-MODEL\_V4 | 1.0 | 2.416e-10 | 318 | 0.153 | 286 | 202 | 11 | 19 | 291 | 282 | 540 | Hypothetical protein | Hypothetical protein | | afdb-uniprot50 | AF-A0A7C5XEJ0-F1-MODEL\_V4 | 1.0 | 3.166e-10 | 317 | 0.186 | 284 | 198 | 11 | 1 | 271 | 2 | 265 | ParB/RepB/Spo0J family partition protein | ParB/RepB/Spo0J family partition protein | | afdb-uniprot50 | AF-A0A7G9GAZ6-F1-MODEL\_V4 | 1.0 | 2.359e-11 | 317 | 0.172 | 290 | 211 | 10 | 4 | 282 | 38 | 309 | ParB/RepB/Spo0J family partition protein | ParB/RepB/Spo0J family partition protein | | afdb-uniprot50 | AF-A0A2D8HHY6-F1-MODEL\_V4 | 1.0 | 2.117e-11 | 317 | 0.198 | 302 | 209 | 10 | 7 | 291 | 22 | 307 | Chromosome partitioning protein ParB | Chromosome partitioning protein ParB | | afdb-uniprot50 | AF-A0A2E2FIX1-F1-MODEL\_V4 | 1.0 | 5.404e-08 | 317 | 0.147 | 183 | 146 | 6 | 19 | 198 | 3 | 178 | ParB domain-containing protein | ParB domain-containing protein | | afdb-uniprot50 | AF-A0A7K1TL10-F1-MODEL\_V4 | 1.0 | 9.568e-09 | 317 | 0.175 | 188 | 139 | 7 | 19 | 200 | 23 | 200 | ParB/RepB/Spo0J family partition protein | ParB/RepB/Spo0J family partition protein | | afdb-uniprot50 | AF-Q11ZU5-F1-MODEL\_V4 | 1.0 | 1.644e-08 | 317 | 0.2 | 165 | 123 | 5 | 13 | 169 | 4 | 167 | ParB family protein | ParB family protein | | afdb-uniprot50 | AF-A0A7T3CHN4-F1-MODEL\_V4 | 1.0 | 3.839e-11 | 317 | 0.173 | 289 | 197 | 13 | 19 | 287 | 307 | 573 | ParB/RepB/Spo0J family partition protein | ParB/RepB/Spo0J family partition protein | | afdb-uniprot50 | AF-A0A368UB33-F1-MODEL\_V4 | 1.0 | 2.823e-08 | 317 | 0.169 | 189 | 144 | 7 | 19 | 200 | 10 | 192 | PRTRC system ParB family protein | PRTRC system ParB family protein | | afdb-uniprot50 | AF-A0A434ELE8-F1-MODEL\_V4 | 1.0 | 6.552e-09 | 316 | 0.219 | 191 | 132 | 8 | 2 | 180 | 10 | 195 | ParB/RepB/Spo0J family partition protein | ParB/RepB/Spo0J family partition protein | | afdb-uniprot50 | AF-A0A2V8CRA1-F1-MODEL\_V4 | 1.0 | 1.041e-09 | 316 | 0.225 | 217 | 152 | 6 | 1 | 204 | 4 | 217 | Chromosome partitioning protein ParB | Chromosome partitioning protein ParB | | afdb-uniprot50 | AF-A0A552UA07-F1-MODEL\_V4 | 1.0 | 5.311e-11 | 316 | 0.232 | 301 | 187 | 16 | 4 | 291 | 9 | 278 | ParB/RepB/Spo0J family partition protein | ParB/RepB/Spo0J family partition protein | | afdb-uniprot50 | AF-A0A494TNC1-F1-MODEL\_V4 | 1.0 | 3.839e-11 | 316 | 0.159 | 308 | 216 | 13 | 1 | 291 | 12 | 293 | ParB/RepB/Spo0J family partition protein | ParB/RepB/Spo0J family partition protein | | afdb-uniprot50 | AF-A0A497RGL2-F1-MODEL\_V4 | 1.0 | 1.475e-08 | 316 | 0.194 | 185 | 143 | 4 | 19 | 201 | 6 | 186 | ParB domain-containing protein | ParB domain-containing protein | | afdb-uniprot50 | AF-A0A7K3LT60-F1-MODEL\_V4 | 1.0 | 1.293e-09 | 316 | 0.213 | 244 | 153 | 15 | 19 | 251 | 5 | 220 | ParB/RepB/Spo0J family partition protein | ParB/RepB/Spo0J family partition protein | | afdb-uniprot50 | AF-A0A4Z0V345-F1-MODEL\_V4 | 1.0 | 1.254e-08 | 316 | 0.186 | 209 | 137 | 9 | 18 | 195 | 1 | 207 | ParB domain-containing protein | ParB domain-containing protein | | afdb-uniprot50 | AF-A0A0R2F0I0-F1-MODEL\_V4 | 1.0 | 6.961e-11 | 315 | 0.198 | 292 | 185 | 16 | 19 | 291 | 1 | 262 | Chromosome partitioning protein ParB | Chromosome partitioning protein ParB | | afdb-uniprot50 | AF-A0A7V2T8H7-F1-MODEL\_V4 | 1.0 | 3.637e-11 | 315 | 0.215 | 283 | 191 | 11 | 11 | 283 | 24 | 285 | ParB/RepB/Spo0J family partition protein | ParB/RepB/Spo0J family partition protein | | afdb-uniprot50 | AF-A0A7V6W467-F1-MODEL\_V4 | 1.0 | 2.49e-11 | 315 | 0.161 | 304 | 209 | 14 | 1 | 282 | 9 | 288 | ParB/RepB/Spo0J family partition protein | ParB/RepB/Spo0J family partition protein | | afdb-uniprot50 | AF-A0A3M0ZST4-F1-MODEL\_V4 | 1.0 | 1.196e-10 | 315 | 0.177 | 288 | 188 | 13 | 20 | 282 | 31 | 294 | ParB/RepB/Spo0J family partition protein | ParB/RepB/Spo0J family partition protein | | afdb-uniprot50 | AF-A0A355T8A7-F1-MODEL\_V4 | 1.0 | 1.048e-11 | 315 | 0.177 | 309 | 206 | 14 | 1 | 282 | 10 | 297 | Chromosome partitioning protein ParB | Chromosome partitioning protein ParB | | afdb-uniprot50 | AF-A0A518C0J3-F1-MODEL\_V4 | 1.0 | 2.629e-11 | 315 | 0.209 | 296 | 195 | 13 | 12 | 291 | 36 | 308 | Putative chromosome-partitioning protein ParB | Putative chromosome-partitioning protein ParB | | afdb-uniprot50 | AF-A0A0S8F0I7-F1-MODEL\_V4 | 1.0 | 3.906e-08 | 315 | 0.18 | 183 | 136 | 8 | 20 | 195 | 64 | 239 | ParB domain-containing protein | ParB domain-containing protein | | afdb-uniprot50 | AF-A0A5F2BGZ2-F1-MODEL\_V4 | 1.0 | 1.073e-10 | 315 | 0.161 | 321 | 193 | 14 | 13 | 283 | 409 | 703 | Chromosome partitioning protein ParB | Chromosome partitioning protein ParB | | afdb-uniprot50 | AF-A0A7C1AMD4-F1-MODEL\_V4 | 1.0 | 1.788e-09 | 314 | 0.226 | 190 | 127 | 7 | 19 | 206 | 3 | 174 | ParB domain-containing protein | ParB domain-containing protein | | afdb-uniprot50 | AF-A0A7X7X790-F1-MODEL\_V4 | 1.0 | 7.757e-11 | 314 | 0.185 | 285 | 205 | 10 | 7 | 283 | 11 | 276 | ParB/RepB/Spo0J family partition protein | ParB/RepB/Spo0J family partition protein | | afdb-uniprot50 | AF-A0A2R4MAE9-F1-MODEL\_V4 | 1.0 | 3.092e-11 | 314 | 0.18 | 304 | 209 | 12 | 1 | 283 | 7 | 291 | Chromosome-partitioning protein ParB | Chromosome-partitioning protein ParB | | afdb-uniprot50 | AF-A0A0Q7JFS1-F1-MODEL\_V4 | 1.0 | 3.264e-11 | 314 | 0.212 | 311 | 192 | 19 | 9 | 291 | 84 | 369 | Chromosome partitioning protein ParB | Chromosome partitioning protein ParB | | afdb-uniprot50 | AF-A0A542ZDY3-F1-MODEL\_V4 | 1.0 | 1.073e-10 | 314 | 0.204 | 294 | 180 | 14 | 17 | 290 | 53 | 312 | ParB family chromosome partitioning protein | ParB family chromosome partitioning protein | | afdb-uniprot50 | AF-A0A165DRH5-F1-MODEL\_V4 | 1.0 | 1.406e-10 | 314 | 0.183 | 295 | 189 | 17 | 19 | 291 | 297 | 561 | Chromosome partitioning protein ParB | Chromosome partitioning protein ParB | | afdb-uniprot50 | AF-A0A1C2AW71-F1-MODEL\_V4 | 1.0 | 9.064e-09 | 313 | 0.19 | 200 | 148 | 7 | 7 | 200 | 15 | 206 | Plasmid partitioning protein ParB | Plasmid partitioning protein ParB | | afdb-uniprot50 | AF-A0A1F4YHQ1-F1-MODEL\_V4 | 1.0 | 2.86e-12 | 313 | 0.17 | 294 | 211 | 10 | 4 | 285 | 9 | 281 | ParB domain-containing protein | ParB domain-containing protein | | afdb-uniprot50 | AF-A0A2A4X9S6-F1-MODEL\_V4 | 1.0 | 4.881e-10 | 313 | 0.153 | 306 | 215 | 15 | 1 | 282 | 7 | 292 | Probable chromosome-partitioning protein ParB | Probable chromosome-partitioning protein ParB | | afdb-uniprot50 | AF-F4GVT0-F1-MODEL\_V4 | 1.0 | 2.49e-11 | 313 | 0.196 | 300 | 202 | 15 | 4 | 282 | 19 | 300 | Chromosome partitioning protein | Chromosome partitioning protein | | afdb-uniprot50 | AF-A0A543INT7-F1-MODEL\_V4 | 1.0 | 6.961e-11 | 313 | 0.192 | 311 | 201 | 16 | 1 | 291 | 41 | 321 | Chromosome segregation DNA-binding protein | Chromosome segregation DNA-binding protein | | afdb-uniprot50 | AF-A0A2V8TIC9-F1-MODEL\_V4 | 1.0 | 1.705e-11 | 313 | 0.183 | 295 | 204 | 13 | 1 | 283 | 57 | 326 | Chromosome partitioning protein ParB | Chromosome partitioning protein ParB | | afdb-uniprot50 | AF-A0A4Q3BDE3-F1-MODEL\_V4 | 1.0 | 5.404e-08 | 313 | 0.134 | 201 | 162 | 7 | 19 | 214 | 12 | 205 | ParB/RepB/Spo0J family partition protein | ParB/RepB/Spo0J family partition protein | | afdb-uniprot50 | AF-A0A1C9T8A9-F1-MODEL\_V4 | 1.0 | 4.381e-10 | 313 | 0.15 | 339 | 210 | 11 | 19 | 291 | 7 | 333 | DNA-binding plasmid partitioning protein | DNA-binding plasmid partitioning protein | | afdb-uniprot50 | AF-A0A519Z998-F1-MODEL\_V4 | 1.0 | 3.814e-09 | 313 | 0.204 | 205 | 141 | 8 | 19 | 214 | 61 | 252 | ParB/RepB/Spo0J family partition protein | ParB/RepB/Spo0J family partition protein | | afdb-uniprot50 | AF-A0A1Y0I2T9-F1-MODEL\_V4 | 1.0 | 3.637e-11 | 313 | 0.208 | 312 | 205 | 15 | 1 | 291 | 74 | 364 | Chromosome partitioning protein ParB | Chromosome partitioning protein ParB | | afdb-uniprot50 | AF-A0A7H2BDL5-F1-MODEL\_V4 | 1.0 | 2.054e-10 | 313 | 0.166 | 312 | 217 | 17 | 2 | 291 | 144 | 434 | ParB/RepB/Spo0J family partition protein | ParB/RepB/Spo0J family partition protein | | afdb-uniprot50 | AF-A0A6C0GBQ2-F1-MODEL\_V4 | 1.0 | 4.736e-09 | 313 | 0.197 | 182 | 133 | 7 | 16 | 192 | 1 | 174 | ParB/RepB/Spo0J family partition protein | ParB/RepB/Spo0J family partition protein | | afdb-uniprot50 | AF-A0A1U7GS74-F1-MODEL\_V4 | 1.0 | 1.262e-10 | 312 | 0.171 | 285 | 189 | 14 | 10 | 282 | 38 | 287 | ParB domain-containing protein | ParB domain-containing protein | | afdb-uniprot50 | AF-A0A194AGK5-F1-MODEL\_V4 | 1.0 | 1.9e-11 | 312 | 0.178 | 314 | 221 | 13 | 1 | 291 | 5 | 304 | Chromosome partitioning protein ParB | Chromosome partitioning protein ParB | | afdb-uniprot50 | AF-A0A5C7TZB4-F1-MODEL\_V4 | 1.0 | 1.843e-10 | 312 | 0.151 | 290 | 208 | 13 | 19 | 282 | 55 | 332 | ParB/RepB/Spo0J family partition protein | ParB/RepB/Spo0J family partition protein | | afdb-uniprot50 | AF-A0A2N0J7B4-F1-MODEL\_V4 | 1.0 | 7.476e-08 | 312 | 0.165 | 224 | 149 | 7 | 13 | 205 | 28 | 244 | ParB/RepB/Spo0J family partition protein | ParB/RepB/Spo0J family partition protein | | afdb-uniprot50 | AF-A0A416VSQ0-F1-MODEL\_V4 | 1.0 | 1.735e-08 | 312 | 0.202 | 183 | 135 | 6 | 19 | 196 | 4 | 180 | ParB/RepB/Spo0J family partition protein | ParB/RepB/Spo0J family partition protein | | afdb-uniprot50 | AF-A0A661EP20-F1-MODEL\_V4 | 1.0 | 2.359e-11 | 311 | 0.173 | 276 | 197 | 10 | 19 | 282 | 8 | 264 | Probable chromosome-partitioning protein ParB | Probable chromosome-partitioning protein ParB | | afdb-uniprot50 | AF-A0A1H0EQE3-F1-MODEL\_V4 | 1.0 | 6.595e-11 | 311 | 0.166 | 283 | 210 | 10 | 7 | 284 | 14 | 275 | Chromosome partitioning protein, ParB family | Chromosome partitioning protein, ParB family | | afdb-uniprot50 | AF-A0A2E9PTD0-F1-MODEL\_V4 | 1.0 | 3.445e-11 | 311 | 0.202 | 306 | 197 | 14 | 1 | 286 | 4 | 282 | Stage 0 sporulation protein J | Stage 0 sporulation protein J | | afdb-uniprot50 | AF-A0A0G2BIR2-F1-MODEL\_V4 | 1.0 | 5.607e-11 | 311 | 0.202 | 296 | 174 | 12 | 12 | 281 | 5 | 264 | Chromosome partitioning protein gp35 | Chromosome partitioning protein gp35 | | afdb-uniprot50 | AF-A0A1G1YI34-F1-MODEL\_V4 | 1.0 | 8.188e-11 | 311 | 0.176 | 289 | 205 | 11 | 4 | 282 | 25 | 290 | ParB domain-containing protein | ParB domain-containing protein | | afdb-uniprot50 | AF-A0A5Q4DNZ7-F1-MODEL\_V4 | 1.0 | 3.445e-11 | 311 | 0.182 | 296 | 207 | 14 | 5 | 290 | 19 | 289 | ParB/RepB/Spo0J family partition protein | ParB/RepB/Spo0J family partition protein | | afdb-uniprot50 | AF-A0A2D7MGH1-F1-MODEL\_V4 | 1.0 | 2.359e-11 | 311 | 0.169 | 307 | 216 | 13 | 1 | 282 | 1 | 293 | DNA-binding protein | DNA-binding protein | | afdb-uniprot50 | AF-A0A841QGG9-F1-MODEL\_V4 | 1.0 | 2.117e-11 | 311 | 0.201 | 298 | 197 | 12 | 7 | 283 | 22 | 299 | ParB family chromosome partitioning protein | ParB family chromosome partitioning protein | | afdb-uniprot50 | AF-A0A1F5DSG9-F1-MODEL\_V4 | 1.0 | 6.552e-09 | 311 | 0.182 | 208 | 145 | 9 | 18 | 206 | 1 | 202 | ParB domain-containing protein | ParB domain-containing protein | | afdb-uniprot50 | AF-A0A1G7MC72-F1-MODEL\_V4 | 1.0 | 7.348e-11 | 311 | 0.142 | 364 | 196 | 14 | 19 | 287 | 28 | 370 | ParB/RepB/Spo0J family partition protein | ParB/RepB/Spo0J family partition protein | | afdb-uniprot50 | AF-A0A261F4A2-F1-MODEL\_V4 | 1.0 | 4.881e-10 | 311 | 0.216 | 277 | 185 | 14 | 19 | 282 | 238 | 495 | ParB-like partition protein | ParB-like partition protein | | afdb-uniprot50 | AF-A0A556CDC8-F1-MODEL\_V4 | 1.0 | 3.724e-10 | 311 | 0.162 | 301 | 201 | 17 | 13 | 291 | 319 | 590 | ParB/RepB/Spo0J family partition protein | ParB/RepB/Spo0J family partition protein | | afdb-uniprot50 | AF-A0A3D9DRE9-F1-MODEL\_V4 | 1.0 | 4.486e-09 | 311 | 0.183 | 196 | 136 | 8 | 18 | 193 | 1 | 192 | ParB family chromosome partitioning protein | ParB family chromosome partitioning protein | | afdb-uniprot50 | AF-A0A5A9X8I3-F1-MODEL\_V4 | 1.0 | 1.605e-09 | 310 | 0.185 | 297 | 179 | 14 | 12 | 290 | 6 | 257 | Uncharacterized protein | Uncharacterized protein | | afdb-uniprot50 | AF-A0A7X9L0Y1-F1-MODEL\_V4 | 1.0 | 6.247e-11 | 310 | 0.164 | 280 | 190 | 14 | 20 | 282 | 25 | 277 | ParB/RepB/Spo0J family partition protein | ParB/RepB/Spo0J family partition protein | | afdb-uniprot50 | AF-A0A4Y6PLY5-F1-MODEL\_V4 | 1.0 | 1.023e-12 | 310 | 0.216 | 309 | 184 | 14 | 2 | 282 | 15 | 293 | ParB/RepB/Spo0J family partition protein | ParB/RepB/Spo0J family partition protein | | afdb-uniprot50 | AF-A0A7C7RWA4-F1-MODEL\_V4 | 1.0 | 4.624e-10 | 310 | 0.201 | 253 | 163 | 14 | 19 | 244 | 23 | 263 | ParB/RepB/Spo0J family partition protein | ParB/RepB/Spo0J family partition protein | | afdb-uniprot50 | AF-A0A2D6G3C5-F1-MODEL\_V4 | 1.0 | 1.45e-11 | 310 | 0.189 | 312 | 210 | 14 | 1 | 291 | 7 | 296 | Chromosome partitioning protein ParB | Chromosome partitioning protein ParB | | afdb-uniprot50 | AF-A0A1V4WRS0-F1-MODEL\_V4 | 1.0 | 2.823e-08 | 310 | 0.176 | 170 | 128 | 5 | 14 | 176 | 32 | 196 | Chromosome-partitioning protein Spo0J | Chromosome-partitioning protein Spo0J | | afdb-uniprot50 | AF-A0A2N1Q4Q7-F1-MODEL\_V4 | 1.0 | 6.207e-09 | 309 | 0.212 | 174 | 127 | 7 | 3 | 172 | 10 | 177 | ParB domain-containing protein | ParB domain-containing protein | | afdb-uniprot50 | AF-A0A1F4X477-F1-MODEL\_V4 | 1.0 | 1.373e-11 | 309 | 0.169 | 284 | 197 | 12 | 14 | 282 | 1 | 260 | Chromosome partitioning protein ParB | Chromosome partitioning protein ParB | | afdb-uniprot50 | AF-A0A2H6HTM0-F1-MODEL\_V4 | 1.0 | 5.918e-11 | 309 | 0.175 | 302 | 207 | 11 | 1 | 282 | 6 | 285 | Chromosome-partitioning protein Spo0J | Chromosome-partitioning protein Spo0J | | afdb-uniprot50 | AF-A0A227JND4-F1-MODEL\_V4 | 1.0 | 1.373e-11 | 309 | 0.182 | 312 | 199 | 14 | 1 | 282 | 1 | 286 | Chromosome partitioning protein ParB | Chromosome partitioning protein ParB | | afdb-uniprot50 | AF-A0A2N1V271-F1-MODEL\_V4 | 1.0 | 7.348e-11 | 309 | 0.149 | 288 | 206 | 11 | 13 | 282 | 28 | 294 | ParB domain-containing protein | ParB domain-containing protein | | afdb-uniprot50 | AF-U7D5A5-F1-MODEL\_V4 | 1.0 | 6.961e-11 | 309 | 0.158 | 302 | 220 | 10 | 1 | 287 | 10 | 292 | ParB-like partition protein | ParB-like partition protein | | afdb-uniprot50 | AF-A0A1E4BNQ1-F1-MODEL\_V4 | 1.0 | 2.692e-10 | 309 | 0.201 | 263 | 170 | 15 | 13 | 265 | 3 | 235 | ParB domain-containing protein | ParB domain-containing protein | | afdb-uniprot50 | AF-A0A4R3KGR8-F1-MODEL\_V4 | 1.0 | 1.373e-11 | 309 | 0.174 | 309 | 211 | 15 | 4 | 291 | 13 | 298 | ParB family chromosome partitioning protein | ParB family chromosome partitioning protein | | afdb-uniprot50 | AF-B8GW30-F1-MODEL\_V4 | 1.0 | 8.909e-12 | 309 | 0.174 | 310 | 207 | 13 | 4 | 289 | 20 | 304 | Chromosome-partitioning protein ParB | Chromosome-partitioning protein ParB | | afdb-uniprot50 | AF-A0A3C1SR32-F1-MODEL\_V4 | 1.0 | 1.017e-10 | 309 | 0.164 | 298 | 214 | 13 | 7 | 291 | 32 | 307 | Probable chromosome-partitioning protein ParB | Probable chromosome-partitioning protein ParB | | afdb-uniprot50 | AF-A0A7C1ED27-F1-MODEL\_V4 | 1.0 | 2.49e-11 | 309 | 0.161 | 310 | 208 | 12 | 6 | 282 | 22 | 312 | ParB/RepB/Spo0J family partition protein | ParB/RepB/Spo0J family partition protein | | afdb-uniprot50 | AF-A0A3D5NI57-F1-MODEL\_V4 | 1.0 | 4.486e-09 | 309 | 0.158 | 259 | 201 | 9 | 13 | 265 | 23 | 270 | ParB domain-containing protein | ParB domain-containing protein | | afdb-uniprot50 | AF-A0A4V2UP75-F1-MODEL\_V4 | 1.0 | 6.961e-11 | 309 | 0.188 | 318 | 209 | 15 | 1 | 291 | 7 | 302 | ParB/RepB/Spo0J family partition protein | ParB/RepB/Spo0J family partition protein | | afdb-uniprot50 | AF-A0A5C4X7V6-F1-MODEL\_V4 | 1.0 | 1.746e-10 | 309 | 0.166 | 312 | 210 | 17 | 1 | 291 | 323 | 605 | ParB/RepB/Spo0J family partition protein | ParB/RepB/Spo0J family partition protein | | afdb-uniprot50 | AF-A0A4Y4D3V1-F1-MODEL\_V4 | 1.0 | 3.528e-10 | 309 | 0.173 | 306 | 196 | 17 | 2 | 282 | 360 | 633 | Chromosome partitioning protein ParB | Chromosome partitioning protein ParB | | afdb-uniprot50 | AF-A0A352D877-F1-MODEL\_V4 | 1.0 | 9.568e-09 | 308 | 0.222 | 180 | 132 | 5 | 7 | 182 | 13 | 188 | ParB domain-containing protein | ParB domain-containing protein | | afdb-uniprot50 | AF-Q8DMW1-F1-MODEL\_V4 | 1.0 | 1.073e-10 | 308 | 0.174 | 270 | 196 | 10 | 19 | 282 | 3 | 251 | Chromosome segregation protein | Chromosome segregation protein | | afdb-uniprot50 | AF-A0A1F5JUW6-F1-MODEL\_V4 | 1.0 | 1.017e-10 | 308 | 0.202 | 292 | 191 | 12 | 19 | 287 | 4 | 276 | ParB domain-containing protein | ParB domain-containing protein | | afdb-uniprot50 | AF-A0A1F2XIK5-F1-MODEL\_V4 | 1.0 | 2.49e-11 | 308 | 0.187 | 304 | 197 | 17 | 1 | 282 | 5 | 280 | ParB domain-containing protein | ParB domain-containing protein | | afdb-uniprot50 | AF-A0A2D2D129-F1-MODEL\_V4 | 1.0 | 8.188e-11 | 308 | 0.191 | 319 | 197 | 17 | 1 | 288 | 1 | 289 | Chromosome partitioning protein ParB | Chromosome partitioning protein ParB | | afdb-uniprot50 | AF-F7SA52-F1-MODEL\_V4 | 1.0 | 5.607e-11 | 308 | 0.23 | 299 | 191 | 14 | 4 | 289 | 20 | 292 | ParB-like partition protein | ParB-like partition protein | | afdb-uniprot50 | AF-A0A7W0M8C8-F1-MODEL\_V4 | 1.0 | 3.166e-10 | 308 | 0.158 | 297 | 205 | 13 | 1 | 287 | 30 | 291 | ParB/RepB/Spo0J family partition protein | ParB/RepB/Spo0J family partition protein | | afdb-uniprot50 | AF-A0A3C0V9C7-F1-MODEL\_V4 | 1.0 | 1.332e-10 | 308 | 0.191 | 297 | 203 | 14 | 9 | 291 | 52 | 325 | HTH cro/C1-type domain-containing protein | HTH cro/C1-type domain-containing protein | | afdb-uniprot50 | AF-A0A7X7P058-F1-MODEL\_V4 | 1.0 | 2.49e-11 | 308 | 0.21 | 294 | 192 | 15 | 13 | 282 | 47 | 324 | ParB/RepB/Spo0J family partition protein | ParB/RepB/Spo0J family partition protein | | afdb-uniprot50 | AF-A0A512D7U3-F1-MODEL\_V4 | 1.0 | 5.918e-11 | 308 | 0.211 | 298 | 196 | 14 | 14 | 291 | 56 | 334 | Chromosome partitioning protein ParB | Chromosome partitioning protein ParB | | afdb-uniprot50 | AF-A0A7X5KQ06-F1-MODEL\_V4 | 1.0 | 3.146e-08 | 308 | 0.191 | 198 | 146 | 9 | 5 | 196 | 19 | 208 | ParB/RepB/Spo0J family partition protein | ParB/RepB/Spo0J family partition protein | | afdb-uniprot50 | AF-A0A286GTP8-F1-MODEL\_V4 | 1.0 | 6.961e-11 | 308 | 0.18 | 310 | 208 | 15 | 2 | 291 | 34 | 317 | Chromosome partitioning protein, ParB family | Chromosome partitioning protein, ParB family | | afdb-uniprot50 | AF-A0A540WI34-F1-MODEL\_V4 | 1.0 | 1.644e-08 | 308 | 0.182 | 197 | 138 | 8 | 13 | 193 | 5 | 194 | ParB/RepB/Spo0J family partition protein | ParB/RepB/Spo0J family partition protein | | afdb-uniprot50 | AF-A0A7V3ZSZ4-F1-MODEL\_V4 | 1.0 | 2.168e-10 | 307 | 0.177 | 299 | 202 | 13 | 1 | 291 | 2 | 264 | ParB/RepB/Spo0J family partition protein | ParB/RepB/Spo0J family partition protein | | afdb-uniprot50 | AF-O74066-F1-MODEL\_V4 | 1.0 | 1.788e-09 | 307 | 0.21 | 290 | 176 | 15 | 13 | 289 | 3 | 252 | ParB domain-containing protein | ParB domain-containing protein | | afdb-uniprot50 | AF-A0A7U4ZBY4-F1-MODEL\_V4 | 1.0 | 1.654e-10 | 307 | 0.202 | 282 | 191 | 14 | 15 | 283 | 16 | 276 | Chromosome-partitioning protein ParB | Chromosome-partitioning protein ParB | | afdb-uniprot50 | AF-S4NKJ4-F1-MODEL\_V4 | 1.0 | 5.439e-10 | 307 | 0.187 | 283 | 179 | 13 | 1 | 246 | 1 | 269 | ParB-like partition protein | ParB-like partition protein | | afdb-uniprot50 | AF-A0A4Y8I9C7-F1-MODEL\_V4 | 1.0 | 1.017e-10 | 307 | 0.186 | 295 | 209 | 10 | 1 | 282 | 7 | 283 | ParB/RepB/Spo0J family partition protein | ParB/RepB/Spo0J family partition protein | | afdb-uniprot50 | AF-A0A7X9AHL4-F1-MODEL\_V4 | 1.0 | 2.49e-11 | 307 | 0.18 | 305 | 201 | 14 | 3 | 282 | 9 | 289 | ParB/RepB/Spo0J family partition protein | ParB/RepB/Spo0J family partition protein | | afdb-uniprot50 | AF-A0A258L6L1-F1-MODEL\_V4 | 1.0 | 2.117e-11 | 307 | 0.189 | 296 | 198 | 15 | 13 | 291 | 26 | 296 | Chromosome partitioning protein ParB | Chromosome partitioning protein ParB | | afdb-uniprot50 | AF-A0A6M8HVA6-F1-MODEL\_V4 | 1.0 | 8.188e-11 | 307 | 0.182 | 307 | 210 | 13 | 1 | 282 | 6 | 296 | ParB/RepB/Spo0J family partition protein | ParB/RepB/Spo0J family partition protein | | afdb-uniprot50 | AF-H6SS19-F1-MODEL\_V4 | 1.0 | 8.188e-11 | 307 | 0.193 | 300 | 199 | 16 | 7 | 291 | 73 | 344 | Chromosome segregation DNA-binding protein | Chromosome segregation DNA-binding protein | | afdb-uniprot50 | AF-A0A7D7PZW5-F1-MODEL\_V4 | 1.0 | 5.742e-10 | 307 | 0.176 | 295 | 190 | 17 | 14 | 282 | 92 | 359 | Putative chromosome-partitioning protein ParB | Putative chromosome-partitioning protein ParB | | afdb-uniprot50 | AF-A0A2D5W999-F1-MODEL\_V4 | 1.0 | 7.757e-11 | 307 | 0.184 | 298 | 211 | 13 | 1 | 283 | 134 | 414 | Chromosome partitioning protein ParB | Chromosome partitioning protein ParB | | afdb-uniprot50 | AF-A0A239V883-F1-MODEL\_V4 | 1.0 | 2.842e-10 | 307 | 0.176 | 289 | 197 | 14 | 14 | 282 | 151 | 418 | Probable chromosome-partitioning protein parB | Probable chromosome-partitioning protein parB | | afdb-uniprot50 | AF-Q4JSC7-F1-MODEL\_V4 | 1.0 | 7.129e-10 | 307 | 0.163 | 287 | 195 | 13 | 19 | 282 | 239 | 503 | Chromosome partitioning protein ParB | Chromosome partitioning protein ParB | | afdb-uniprot50 | AF-A0A517SER9-F1-MODEL\_V4 | 1.0 | 4.999e-09 | 307 | 0.183 | 218 | 160 | 9 | 5 | 218 | 121 | 324 | Chromosome-partitioning protein Spo0J | Chromosome-partitioning protein Spo0J | | afdb-uniprot50 | AF-A0A382NUU8-F1-MODEL\_V4 | 1.0 | 1.933e-08 | 306 | 0.206 | 199 | 144 | 8 | 7 | 199 | 13 | 203 | ParB domain-containing protein | ParB domain-containing protein | | afdb-uniprot50 | AF-A0A1F7BL00-F1-MODEL\_V4 | 1.0 | 2.823e-08 | 306 | 0.179 | 184 | 129 | 9 | 23 | 205 | 2 | 164 | ParB domain-containing protein | ParB domain-containing protein | | afdb-uniprot50 | AF-A0A838NRV8-F1-MODEL\_V4 | 1.0 | 1.53e-11 | 306 | 0.17 | 288 | 206 | 14 | 13 | 282 | 25 | 297 | ParB/RepB/Spo0J family partition protein | ParB/RepB/Spo0J family partition protein | | afdb-uniprot50 | AF-A0A497Y0S2-F1-MODEL\_V4 | 1.0 | 3.724e-10 | 306 | 0.172 | 301 | 211 | 14 | 1 | 282 | 39 | 320 | ParB family chromosome partitioning protein | ParB family chromosome partitioning protein | | afdb-uniprot50 | AF-A0A2E2LB55-F1-MODEL\_V4 | 1.0 | 1.735e-08 | 306 | 0.201 | 203 | 143 | 11 | 5 | 201 | 70 | 259 | Chromosome partitioning protein | Chromosome partitioning protein | | afdb-uniprot50 | AF-A1VV97-F1-MODEL\_V4 | 1.0 | 4.594e-08 | 306 | 0.158 | 164 | 131 | 6 | 13 | 172 | 5 | 165 | ParB family protein | ParB family protein | | afdb-uniprot50 | AF-A0A2E0DRV3-F1-MODEL\_V4 | 1.0 | 1.01e-08 | 305 | 0.206 | 174 | 128 | 5 | 14 | 182 | 27 | 195 | Chromosome partitioning protein ParB | Chromosome partitioning protein ParB | | afdb-uniprot50 | AF-A0A3A4PV03-F1-MODEL\_V4 | 1.0 | 1.843e-10 | 305 | 0.173 | 294 | 217 | 11 | 1 | 289 | 7 | 279 | ParB/RepB/Spo0J family partition protein | ParB/RepB/Spo0J family partition protein | | afdb-uniprot50 | AF-A0A7V6VL57-F1-MODEL\_V4 | 1.0 | 9.568e-09 | 305 | 0.235 | 195 | 132 | 10 | 12 | 195 | 94 | 282 | ParB/RepB/Spo0J family partition protein | ParB/RepB/Spo0J family partition protein | | afdb-uniprot50 | AF-A0A7W1G0B4-F1-MODEL\_V4 | 1.0 | 9.124e-11 | 305 | 0.185 | 291 | 194 | 13 | 13 | 282 | 17 | 285 | ParB/RepB/Spo0J family partition protein | ParB/RepB/Spo0J family partition protein | | afdb-uniprot50 | AF-A0A1V5DMX0-F1-MODEL\_V4 | 1.0 | 3.613e-09 | 305 | 0.187 | 203 | 141 | 8 | 13 | 206 | 2 | 189 | Chromosome-partitioning protein Spo0J | Chromosome-partitioning protein Spo0J | | afdb-uniprot50 | AF-A0A1F6CPK3-F1-MODEL\_V4 | 1.0 | 2.168e-10 | 305 | 0.176 | 283 | 196 | 14 | 19 | 286 | 9 | 269 | ParB domain-containing protein | ParB domain-containing protein | | afdb-uniprot50 | AF-A0A7U7GA97-F1-MODEL\_V4 | 1.0 | 7.348e-11 | 305 | 0.163 | 311 | 210 | 15 | 2 | 287 | 21 | 306 | Probable chromosome-partitioning protein ParB | Probable chromosome-partitioning protein ParB | | afdb-uniprot50 | AF-A0A261QDL4-F1-MODEL\_V4 | 1.0 | 3.321e-08 | 305 | 0.164 | 188 | 145 | 8 | 19 | 200 | 5 | 186 | ParB domain-containing protein | ParB domain-containing protein | | afdb-uniprot50 | AF-A0A2D6BI86-F1-MODEL\_V4 | 1.0 | 3.839e-11 | 305 | 0.14 | 298 | 212 | 12 | 13 | 291 | 53 | 325 | Chromosome partitioning protein ParB | Chromosome partitioning protein ParB | | afdb-uniprot50 | AF-A0A495QZL2-F1-MODEL\_V4 | 1.0 | 1.406e-10 | 305 | 0.179 | 296 | 194 | 14 | 19 | 291 | 57 | 326 | ParB family chromosome partitioning protein | ParB family chromosome partitioning protein | | afdb-uniprot50 | AF-A0A7X3NCE7-F1-MODEL\_V4 | 1.0 | 3.724e-10 | 305 | 0.179 | 351 | 204 | 13 | 22 | 291 | 38 | 385 | Uncharacterized protein | Uncharacterized protein | | afdb-uniprot50 | AF-A0A448FNI8-F1-MODEL\_V4 | 1.0 | 2.98e-08 | 304 | 0.187 | 155 | 117 | 7 | 5 | 156 | 11 | 159 | ParB-like partition protein | ParB-like partition protein | | afdb-uniprot50 | AF-A0A2Z3HZR4-F1-MODEL\_V4 | 1.0 | 1.233e-11 | 304 | 0.192 | 312 | 191 | 14 | 1 | 282 | 4 | 284 | Chromosome partitioning protein ParB | Chromosome partitioning protein ParB | | afdb-uniprot50 | AF-A0A496NGH7-F1-MODEL\_V4 | 1.0 | 1.746e-10 | 304 | 0.158 | 291 | 206 | 13 | 14 | 282 | 48 | 321 | Probable chromosome-partitioning protein ParB | Probable chromosome-partitioning protein ParB | | afdb-uniprot50 | AF-A0A4P6F2X0-F1-MODEL\_V4 | 1.0 | 3.445e-11 | 304 | 0.19 | 320 | 199 | 17 | 1 | 291 | 60 | 348 | ParB/RepB/Spo0J family partition protein | ParB/RepB/Spo0J family partition protein | | afdb-uniprot50 | AF-A0A6N9H888-F1-MODEL\_V4 | 1.0 | 5.439e-10 | 304 | 0.167 | 299 | 201 | 15 | 14 | 291 | 172 | 443 | ParB/RepB/Spo0J family partition protein | ParB/RepB/Spo0J family partition protein | | afdb-uniprot50 | AF-E6JYG5-F1-MODEL\_V4 | 1.0 | 5.439e-10 | 304 | 0.189 | 291 | 195 | 14 | 14 | 285 | 248 | 516 | ParB-like protein | ParB-like protein | | afdb-uniprot50 | AF-A0A1I3C721-F1-MODEL\_V4 | 1.0 | 7.757e-11 | 303 | 0.172 | 278 | 207 | 11 | 13 | 282 | 29 | 291 | Chromosome partitioning protein, ParB family | Chromosome partitioning protein, ParB family | | afdb-uniprot50 | AF-A0A1G2HXV3-F1-MODEL\_V4 | 1.0 | 3.505e-08 | 303 | 0.204 | 161 | 116 | 6 | 14 | 169 | 19 | 172 | ParB domain-containing protein | ParB domain-containing protein | | afdb-uniprot50 | AF-A0A7X6H7L1-F1-MODEL\_V4 | 1.0 | 6.398e-10 | 303 | 0.177 | 288 | 187 | 17 | 19 | 282 | 87 | 348 | ParB/RepB/Spo0J family partition protein | ParB/RepB/Spo0J family partition protein | | afdb-uniprot50 | AF-A0A2J9SJJ5-F1-MODEL\_V4 | 1.0 | 2.612e-09 | 303 | 0.162 | 271 | 182 | 11 | 11 | 272 | 23 | 257 | ParB domain-containing protein | ParB domain-containing protein | | afdb-uniprot50 | AF-A0A4P6EPV7-F1-MODEL\_V4 | 1.0 | 2.054e-10 | 303 | 0.194 | 313 | 197 | 15 | 1 | 282 | 3 | 291 | ParB/RepB/Spo0J family partition protein | ParB/RepB/Spo0J family partition protein | | afdb-uniprot50 | AF-A0A139DP90-F1-MODEL\_V4 | 1.0 | 1.831e-08 | 303 | 0.168 | 196 | 153 | 6 | 10 | 200 | 6 | 196 | Probable chromosome-partitioning protein ParB | Probable chromosome-partitioning protein ParB | | afdb-uniprot50 | AF-A0A1F8UGV0-F1-MODEL\_V4 | 1.0 | 4.278e-11 | 302 | 0.211 | 303 | 198 | 13 | 2 | 288 | 4 | 281 | Chromosome partitioning protein ParB | Chromosome partitioning protein ParB | | afdb-uniprot50 | AF-A0A1C5TMF3-F1-MODEL\_V4 | 1.0 | 4.516e-11 | 302 | 0.186 | 284 | 205 | 12 | 7 | 282 | 32 | 297 | Probable chromosome-partitioning protein parB | Probable chromosome-partitioning protein parB | | afdb-uniprot50 | AF-A0A0S2KEG7-F1-MODEL\_V4 | 1.0 | 1.293e-09 | 302 | 0.169 | 266 | 182 | 15 | 12 | 263 | 9 | 249 | ParB-like partitioning protein | ParB-like partitioning protein | | afdb-uniprot50 | AF-A0A7L9BNL5-F1-MODEL\_V4 | 1.0 | 3.343e-10 | 302 | 0.165 | 296 | 206 | 12 | 11 | 282 | 35 | 313 | ParB/RepB/Spo0J family partition protein | ParB/RepB/Spo0J family partition protein | | afdb-uniprot50 | AF-A0A520Q7U8-F1-MODEL\_V4 | 1.0 | 2.006e-11 | 302 | 0.163 | 300 | 197 | 13 | 13 | 282 | 63 | 338 | ParB/RepB/Spo0J family partition protein | ParB/RepB/Spo0J family partition protein | | afdb-uniprot50 | AF-A0A0K2RK52-F1-MODEL\_V4 | 1.0 | 3.343e-10 | 302 | 0.169 | 313 | 196 | 20 | 10 | 291 | 66 | 345 | Probable chromosome-partitioning protein ParB | Probable chromosome-partitioning protein ParB | | afdb-uniprot50 | AF-A0A523V2R2-F1-MODEL\_V4 | 1.0 | 9.343e-10 | 302 | 0.149 | 321 | 205 | 17 | 13 | 291 | 4 | 298 | ParB/RepB/Spo0J family partition protein | ParB/RepB/Spo0J family partition protein | | afdb-uniprot50 | AF-I8ARF6-F1-MODEL\_V4 | 1.0 | 1.843e-10 | 302 | 0.178 | 292 | 196 | 15 | 19 | 291 | 96 | 362 | ParB-like protein | ParB-like protein | | afdb-uniprot50 | AF-A0A2G4ISH6-F1-MODEL\_V4 | 1.0 | 2.359e-11 | 302 | 0.19 | 310 | 207 | 12 | 1 | 290 | 8 | 293 | Chromosome partitioning protein ParB | Chromosome partitioning protein ParB | | afdb-uniprot50 | AF-A0A4Y1XBM5-F1-MODEL\_V4 | 1.0 | 5.404e-08 | 302 | 0.169 | 189 | 147 | 7 | 1 | 184 | 6 | 189 | Chromosome partitioning protein ParB | Chromosome partitioning protein ParB | | afdb-uniprot50 | AF-M6VZ53-F1-MODEL\_V4 | 1.0 | 1.843e-10 | 302 | 0.158 | 334 | 185 | 16 | 13 | 283 | 351 | 651 | ParB-like protein | ParB-like protein | | afdb-uniprot50 | AF-A0A150PXB6-F1-MODEL\_V4 | 1.0 | 6.398e-10 | 301 | 0.243 | 242 | 152 | 10 | 13 | 230 | 29 | 263 | Chromosome partitioning protein ParB | Chromosome partitioning protein ParB | | afdb-uniprot50 | AF-A0A7C7VJH1-F1-MODEL\_V4 | 1.0 | 8.643e-11 | 301 | 0.183 | 294 | 208 | 13 | 1 | 282 | 8 | 281 | ParB/RepB/Spo0J family partition protein | ParB/RepB/Spo0J family partition protein | | afdb-uniprot50 | AF-A0A2V8FIU9-F1-MODEL\_V4 | 1.0 | 2.235e-11 | 301 | 0.176 | 311 | 197 | 13 | 1 | 282 | 4 | 284 | Chromosome partitioning protein ParB | Chromosome partitioning protein ParB | | afdb-uniprot50 | AF-A0A7C2JKK4-F1-MODEL\_V4 | 1.0 | 5.311e-11 | 301 | 0.197 | 289 | 183 | 15 | 15 | 282 | 28 | 288 | ParB/RepB/Spo0J family partition protein | ParB/RepB/Spo0J family partition protein | | afdb-uniprot50 | AF-A0A7T9F7V5-F1-MODEL\_V4 | 1.0 | 2.55e-10 | 301 | 0.149 | 295 | 216 | 11 | 13 | 291 | 104 | 379 | ParB/RepB/Spo0J family partition protein | ParB/RepB/Spo0J family partition protein | | afdb-uniprot50 | AF-A0A350AES6-F1-MODEL\_V4 | 1.0 | 3.505e-08 | 300 | 0.185 | 156 | 117 | 5 | 13 | 163 | 11 | 161 | Chromosome partitioning protein ParB | Chromosome partitioning protein ParB | | afdb-uniprot50 | AF-A0A523CVJ9-F1-MODEL\_V4 | 1.0 | 1.654e-10 | 300 | 0.163 | 306 | 210 | 13 | 1 | 291 | 5 | 279 | ParB/RepB/Spo0J family partition protein | ParB/RepB/Spo0J family partition protein | | afdb-uniprot50 | AF-A0A7R9RA11-F1-MODEL\_V4 | 1.0 | 1.017e-10 | 300 | 0.187 | 294 | 204 | 12 | 1 | 282 | 3 | 273 | Stage 0 sporulation protein J | Stage 0 sporulation protein J | | afdb-uniprot50 | AF-A0A2H1J8P3-F1-MODEL\_V4 | 1.0 | 3.166e-10 | 300 | 0.174 | 315 | 202 | 18 | 2 | 291 | 38 | 319 | Chromosome partitioning protein, ParB family | Chromosome partitioning protein, ParB family | | afdb-uniprot50 | AF-A0A6C1SLD8-F1-MODEL\_V4 | 1.0 | 7.348e-11 | 300 | 0.182 | 291 | 186 | 12 | 20 | 291 | 35 | 292 | ParB/RepB/Spo0J family partition protein | ParB/RepB/Spo0J family partition protein | | afdb-uniprot50 | AF-A0A7X6L5B1-F1-MODEL\_V4 | 1.0 | 5.153e-10 | 300 | 0.17 | 316 | 209 | 15 | 1 | 291 | 49 | 336 | ParB/RepB/Spo0J family partition protein | ParB/RepB/Spo0J family partition protein | | afdb-uniprot50 | AF-A0A845MLR6-F1-MODEL\_V4 | 1.0 | 2.929e-11 | 300 | 0.203 | 300 | 194 | 17 | 7 | 282 | 22 | 300 | ParB/RepB/Spo0J family partition protein | ParB/RepB/Spo0J family partition protein | | afdb-uniprot50 | AF-A0A838LF52-F1-MODEL\_V4 | 1.0 | 1.567e-10 | 299 | 0.19 | 288 | 180 | 16 | 13 | 291 | 1 | 244 | ParB/RepB/Spo0J family partition protein | ParB/RepB/Spo0J family partition protein | | afdb-uniprot50 | AF-A0A520JRG8-F1-MODEL\_V4 | 1.0 | 1.644e-08 | 299 | 0.212 | 188 | 137 | 6 | 18 | 201 | 1 | 181 | Chromosome partitioning protein ParB | Chromosome partitioning protein ParB | | afdb-uniprot50 | AF-A0A4R8DHE2-F1-MODEL\_V4 | 1.0 | 6.753e-10 | 299 | 0.146 | 308 | 194 | 17 | 13 | 287 | 28 | 299 | ParB/RepB/Spo0J family partition protein | ParB/RepB/Spo0J family partition protein | | afdb-uniprot50 | AF-A0A382X411-F1-MODEL\_V4 | 1.0 | 3.613e-09 | 298 | 0.19 | 199 | 144 | 8 | 15 | 205 | 2 | 191 | HTH cro/C1-type domain-containing protein | HTH cro/C1-type domain-containing protein | | afdb-uniprot50 | AF-A0A357ZK67-F1-MODEL\_V4 | 1.0 | 2.474e-09 | 298 | 0.227 | 211 | 141 | 8 | 7 | 200 | 33 | 238 | ParB domain-containing protein | ParB domain-containing protein | | afdb-uniprot50 | AF-A0A1Y1RFK6-F1-MODEL\_V4 | 1.0 | 1.406e-10 | 298 | 0.172 | 301 | 217 | 11 | 1 | 290 | 13 | 292 | ParB domain-containing protein | ParB domain-containing protein | | afdb-uniprot50 | AF-A0A1M3IXE2-F1-MODEL\_V4 | 1.0 | 1.843e-10 | 298 | 0.196 | 295 | 195 | 15 | 14 | 290 | 27 | 297 | ParB domain-containing protein | ParB domain-containing protein | | afdb-uniprot50 | AF-A0A1I0F2H7-F1-MODEL\_V4 | 1.0 | 4.053e-11 | 298 | 0.182 | 301 | 213 | 11 | 5 | 290 | 22 | 304 | Chromosome partitioning protein, ParB family | Chromosome partitioning protein, ParB family | | afdb-uniprot50 | AF-A0A1F5AZ83-F1-MODEL\_V4 | 1.0 | 1.153e-07 | 298 | 0.157 | 197 | 151 | 10 | 18 | 206 | 24 | 213 | ParB domain-containing protein | ParB domain-containing protein | | afdb-uniprot50 | AF-C6HTU9-F1-MODEL\_V4 | 1.0 | 5.439e-10 | 298 | 0.194 | 262 | 175 | 12 | 11 | 265 | 21 | 253 | ParB-like partition protein | ParB-like partition protein | | afdb-uniprot50 | AF-A0A512H3L9-F1-MODEL\_V4 | 1.0 | 1.946e-10 | 298 | 0.193 | 294 | 207 | 13 | 7 | 291 | 49 | 321 | Chromosome partitioning protein ParB | Chromosome partitioning protein ParB | | afdb-uniprot50 | AF-A0A7K1A1N3-F1-MODEL\_V4 | 1.0 | 2.55e-10 | 298 | 0.201 | 248 | 143 | 12 | 13 | 247 | 1 | 206 | ParB/RepB/Spo0J family partition protein | ParB/RepB/Spo0J family partition protein | | afdb-uniprot50 | AF-A0A1G2VKP5-F1-MODEL\_V4 | 1.0 | 6.753e-10 | 297 | 0.209 | 196 | 139 | 7 | 14 | 200 | 5 | 193 | ParB domain-containing protein | ParB domain-containing protein | | afdb-uniprot50 | AF-A0A1X7KAE4-F1-MODEL\_V4 | 1.0 | 1.843e-10 | 297 | 0.189 | 306 | 201 | 13 | 1 | 290 | 4 | 278 | Chromosome partitioning protein, ParB family | Chromosome partitioning protein, ParB family | | afdb-uniprot50 | AF-A0A3D1U3G5-F1-MODEL\_V4 | 1.0 | 4.15e-10 | 297 | 0.198 | 287 | 199 | 12 | 1 | 277 | 12 | 277 | Stage 0 sporulation protein J | Stage 0 sporulation protein J | | afdb-uniprot50 | AF-E4TJE6-F1-MODEL\_V4 | 1.0 | 1.196e-10 | 297 | 0.183 | 294 | 193 | 13 | 7 | 282 | 11 | 275 | ParB-like partition protein | ParB-like partition protein | | afdb-uniprot50 | AF-A0A2G6LB17-F1-MODEL\_V4 | 1.0 | 6.247e-11 | 297 | 0.181 | 297 | 197 | 15 | 7 | 282 | 10 | 281 | Probable chromosome-partitioning protein ParB | Probable chromosome-partitioning protein ParB | | afdb-uniprot50 | AF-A0A0L0M783-F1-MODEL\_V4 | 1.0 | 9.283e-08 | 297 | 0.218 | 183 | 136 | 5 | 22 | 201 | 6 | 184 | Chromosome (Plasmid) partitioning protein ParB | Chromosome (Plasmid) partitioning protein ParB | | afdb-uniprot50 | AF-A0A2S6TU51-F1-MODEL\_V4 | 1.0 | 3.343e-10 | 297 | 0.152 | 315 | 215 | 15 | 1 | 291 | 9 | 295 | Chromosome-partitioning protein ParB | Chromosome-partitioning protein ParB | | afdb-uniprot50 | AF-A0A2W5A523-F1-MODEL\_V4 | 1.0 | 1.53e-11 | 297 | 0.178 | 330 | 203 | 17 | 1 | 291 | 4 | 304 | Chromosome partitioning protein ParB | Chromosome partitioning protein ParB | | afdb-uniprot50 | AF-A0A318B644-F1-MODEL\_V4 | 1.0 | 1.993e-09 | 296 | 0.2 | 215 | 150 | 8 | 1 | 201 | 7 | 213 | Chromosome partitioning protein ParB | Chromosome partitioning protein ParB | | afdb-uniprot50 | AF-A0A2N1PW21-F1-MODEL\_V4 | 1.0 | 3.423e-09 | 296 | 0.21 | 204 | 149 | 7 | 3 | 200 | 9 | 206 | Chromosome partitioning protein ParB | Chromosome partitioning protein ParB | | afdb-uniprot50 | AF-A0A1N6LGW4-F1-MODEL\_V4 | 1.0 | 3.166e-10 | 296 | 0.162 | 302 | 190 | 12 | 3 | 282 | 29 | 289 | Chromosome partitioning protein, ParB family | Chromosome partitioning protein, ParB family | | afdb-uniprot50 | AF-A0A2S6S2D6-F1-MODEL\_V4 | 1.0 | 2.692e-10 | 296 | 0.18 | 315 | 208 | 15 | 2 | 290 | 8 | 298 | Chromosome-partitioning protein ParB | Chromosome-partitioning protein ParB | | afdb-uniprot50 | AF-A0A1Z9K2S5-F1-MODEL\_V4 | 1.0 | 1.196e-10 | 296 | 0.177 | 293 | 190 | 14 | 13 | 282 | 36 | 300 | Probable chromosome-partitioning protein ParB | Probable chromosome-partitioning protein ParB | | afdb-uniprot50 | AF-A0A3M0Y0V7-F1-MODEL\_V4 | 1.0 | 1.017e-10 | 296 | 0.192 | 301 | 199 | 13 | 13 | 291 | 34 | 312 | ParB/RepB/Spo0J family partition protein | ParB/RepB/Spo0J family partition protein | | afdb-uniprot50 | AF-A0A6N7YJS6-F1-MODEL\_V4 | 1.0 | 9.863e-10 | 296 | 0.195 | 323 | 192 | 18 | 13 | 287 | 7 | 309 | ParB/RepB/Spo0J family partition protein | ParB/RepB/Spo0J family partition protein | | afdb-uniprot50 | AF-A0A095YDD6-F1-MODEL\_V4 | 1.0 | 5.742e-10 | 296 | 0.162 | 314 | 218 | 17 | 1 | 291 | 223 | 514 | ParB domain-containing protein | ParB domain-containing protein | | afdb-uniprot50 | AF-A0A1V9G4E3-F1-MODEL\_V4 | 1.0 | 5.404e-08 | 296 | 0.141 | 184 | 147 | 6 | 5 | 182 | 9 | 187 | ParB domain-containing protein | ParB domain-containing protein | | afdb-uniprot50 | AF-A0A2D8FML5-F1-MODEL\_V4 | 1.0 | 1.073e-10 | 295 | 0.225 | 279 | 172 | 16 | 23 | 282 | 1 | 254 | Chromosome partitioning protein ParB | Chromosome partitioning protein ParB | | afdb-uniprot50 | AF-A0A6P4HEC8-F1-MODEL\_V4 | 1.0 | 1.993e-09 | 295 | 0.218 | 252 | 161 | 12 | 13 | 246 | 33 | 266 | chromosome-partitioning protein ParB-like | chromosome-partitioning protein ParB-like | | afdb-uniprot50 | AF-A0A660TB83-F1-MODEL\_V4 | 1.0 | 7.995e-12 | 295 | 0.164 | 291 | 214 | 11 | 4 | 282 | 13 | 286 | Chromosome partitioning protein ParB | Chromosome partitioning protein ParB | | afdb-uniprot50 | AF-A0A2E6RNQ1-F1-MODEL\_V4 | 1.0 | 4.767e-11 | 295 | 0.154 | 298 | 210 | 14 | 4 | 291 | 26 | 291 | Chromosome partitioning protein ParB | Chromosome partitioning protein ParB | | afdb-uniprot50 | AF-A0A3D0RBB3-F1-MODEL\_V4 | 1.0 | 4.881e-10 | 295 | 0.172 | 279 | 195 | 14 | 20 | 282 | 38 | 296 | Chromosome partitioning protein ParB | Chromosome partitioning protein ParB | | afdb-uniprot50 | AF-A0A7T8SHN7-F1-MODEL\_V4 | 1.0 | 2.416e-10 | 295 | 0.209 | 311 | 187 | 14 | 4 | 282 | 54 | 337 | ParB/RepB/Spo0J family partition protein | ParB/RepB/Spo0J family partition protein | | afdb-uniprot50 | AF-A0A7C2KI98-F1-MODEL\_V4 | 1.0 | 1.511e-07 | 295 | 0.172 | 174 | 136 | 6 | 4 | 172 | 24 | 194 | ParB/RepB/Spo0J family partition protein | ParB/RepB/Spo0J family partition protein | | afdb-uniprot50 | AF-A0A2E5HJM8-F1-MODEL\_V4 | 1.0 | 2.117e-11 | 295 | 0.195 | 373 | 182 | 17 | 13 | 288 | 19 | 370 | Chromosome partitioning protein ParB | Chromosome partitioning protein ParB | | afdb-uniprot50 | AF-A0A199YNH2-F1-MODEL\_V4 | 1.0 | 1.262e-10 | 295 | 0.161 | 315 | 212 | 15 | 1 | 282 | 6 | 301 | Chromosome partitioning protein ParB | Chromosome partitioning protein ParB | | afdb-uniprot50 | AF-A0A2V2E4M2-F1-MODEL\_V4 | 1.0 | 1.293e-09 | 294 | 0.17 | 276 | 186 | 14 | 13 | 269 | 2 | 253 | ParB domain-containing protein | ParB domain-containing protein | | afdb-uniprot50 | AF-A0A800F760-F1-MODEL\_V4 | 1.0 | 1.196e-10 | 294 | 0.18 | 271 | 193 | 11 | 23 | 282 | 2 | 254 | ParB/RepB/Spo0J family partition protein | ParB/RepB/Spo0J family partition protein | | afdb-uniprot50 | AF-A0A4R8MPW6-F1-MODEL\_V4 | 1.0 | 8.851e-10 | 294 | 0.18 | 261 | 174 | 11 | 4 | 246 | 11 | 249 | ParB/RepB/Spo0J family partition protein | ParB/RepB/Spo0J family partition protein | | afdb-uniprot50 | AF-A0A1V2YCR6-F1-MODEL\_V4 | 1.0 | 2.168e-10 | 294 | 0.138 | 297 | 212 | 13 | 4 | 282 | 10 | 280 | ParB domain-containing protein | ParB domain-containing protein | | afdb-uniprot50 | AF-A0A1V5J2I1-F1-MODEL\_V4 | 1.0 | 3.839e-11 | 294 | 0.182 | 302 | 207 | 15 | 2 | 289 | 14 | 289 | Putative chromosome-partitioning protein ParB | Putative chromosome-partitioning protein ParB | | afdb-uniprot50 | AF-A0A7G8T7A8-F1-MODEL\_V4 | 1.0 | 6.061e-10 | 294 | 0.198 | 303 | 207 | 14 | 2 | 291 | 12 | 291 | ParB/RepB/Spo0J family partition protein | ParB/RepB/Spo0J family partition protein | | afdb-uniprot50 | AF-A0A7C8LUY7-F1-MODEL\_V4 | 1.0 | 7.574e-12 | 294 | 0.199 | 311 | 194 | 17 | 7 | 291 | 15 | 296 | Stage 0 sporulation protein J | Stage 0 sporulation protein J | | afdb-uniprot50 | AF-A0A2E3KDB6-F1-MODEL\_V4 | 1.0 | 9.124e-11 | 294 | 0.178 | 302 | 202 | 15 | 1 | 282 | 20 | 295 | Chromosome partitioning protein ParB | Chromosome partitioning protein ParB | | afdb-uniprot50 | AF-A0A1U7GAU7-F1-MODEL\_V4 | 1.0 | 1.485e-10 | 294 | 0.175 | 297 | 189 | 15 | 9 | 287 | 43 | 301 | HTH cro/C1-type domain-containing protein | HTH cro/C1-type domain-containing protein | | afdb-uniprot50 | AF-A0A2M7B686-F1-MODEL\_V4 | 1.0 | 2.55e-10 | 294 | 0.172 | 284 | 201 | 11 | 19 | 282 | 49 | 318 | ParB domain-containing protein | ParB domain-containing protein | | afdb-uniprot50 | AF-A0A1Z8NWP4-F1-MODEL\_V4 | 1.0 | 5.311e-11 | 294 | 0.179 | 301 | 211 | 13 | 7 | 291 | 28 | 308 | ParB domain-containing protein | ParB domain-containing protein | | afdb-uniprot50 | AF-A0A2E0P9T0-F1-MODEL\_V4 | 1.0 | 9.343e-10 | 294 | 0.179 | 279 | 197 | 17 | 20 | 284 | 81 | 341 | Chromosome partitioning protein ParB | Chromosome partitioning protein ParB | | afdb-uniprot50 | AF-A0A2I1YC93-F1-MODEL\_V4 | 1.0 | 2.55e-10 | 294 | 0.163 | 293 | 202 | 15 | 13 | 282 | 105 | 377 | Chromosome partitioning protein ParB | Chromosome partitioning protein ParB | | afdb-uniprot50 | AF-A0A6L9G7V0-F1-MODEL\_V4 | 1.0 | 1.694e-09 | 294 | 0.158 | 302 | 211 | 14 | 1 | 282 | 98 | 376 | ParB/RepB/Spo0J family partition protein | ParB/RepB/Spo0J family partition protein | | afdb-uniprot50 | AF-A0A7W8TTU9-F1-MODEL\_V4 | 1.0 | 2.55e-10 | 294 | 0.184 | 293 | 194 | 16 | 13 | 283 | 325 | 594 | ParB family chromosome partitioning protein | ParB family chromosome partitioning protein | | afdb-uniprot50 | AF-S5LZF8-F1-MODEL\_V4 | 1.0 | 8.643e-11 | 293 | 0.146 | 273 | 196 | 10 | 19 | 282 | 3 | 247 | Chromosome partitioning protein, DNA-binding protein | Chromosome partitioning protein, DNA-binding protein | | afdb-uniprot50 | AF-A0A512BQ88-F1-MODEL\_V4 | 1.0 | 3.932e-10 | 293 | 0.201 | 283 | 182 | 10 | 18 | 282 | 19 | 275 | Chromosome partitioning protein ParB | Chromosome partitioning protein ParB | | afdb-uniprot50 | AF-A0A7C7X6R7-F1-MODEL\_V4 | 1.0 | 6.398e-10 | 293 | 0.151 | 290 | 207 | 12 | 7 | 282 | 14 | 278 | ParB/RepB/Spo0J family partition protein | ParB/RepB/Spo0J family partition protein | | afdb-uniprot50 | AF-A0A1Q6RN12-F1-MODEL\_V4 | 1.0 | 2.168e-10 | 293 | 0.177 | 288 | 201 | 14 | 7 | 282 | 15 | 278 | HTH cro/C1-type domain-containing protein | HTH cro/C1-type domain-containing protein | | afdb-uniprot50 | AF-A0A2E0EKM2-F1-MODEL\_V4 | 1.0 | 2.842e-10 | 293 | 0.182 | 318 | 204 | 14 | 4 | 287 | 18 | 313 | Probable chromosome-partitioning protein ParB | Probable chromosome-partitioning protein ParB | | afdb-uniprot50 | AF-A0A3D5E876-F1-MODEL\_V4 | 1.0 | 6.595e-11 | 293 | 0.164 | 279 | 195 | 13 | 19 | 282 | 59 | 314 | Chromosome partitioning protein ParB | Chromosome partitioning protein ParB | | afdb-uniprot50 | AF-A0A7V2JJV2-F1-MODEL\_V4 | 1.0 | 2.054e-10 | 293 | 0.167 | 304 | 207 | 11 | 1 | 282 | 42 | 321 | ParB/RepB/Spo0J family partition protein | ParB/RepB/Spo0J family partition protein | | afdb-uniprot50 | AF-A0A1G6LR52-F1-MODEL\_V4 | 1.0 | 9.064e-09 | 293 | 0.228 | 197 | 130 | 9 | 13 | 195 | 3 | 191 | ParB/RepB/Spo0J family partition protein | ParB/RepB/Spo0J family partition protein | | afdb-uniprot50 | AF-A0A7X7TY23-F1-MODEL\_V4 | 1.0 | 7.892e-08 | 293 | 0.207 | 154 | 114 | 4 | 13 | 162 | 1 | 150 | ParB N-terminal domain-containing protein | ParB N-terminal domain-containing protein | | afdb-uniprot50 | AF-A0A1F7M1P9-F1-MODEL\_V4 | 1.0 | 3.092e-11 | 292 | 0.177 | 309 | 197 | 13 | 1 | 282 | 3 | 281 | HTH cro/C1-type domain-containing protein | HTH cro/C1-type domain-containing protein | | afdb-uniprot50 | AF-A0A651ECM7-F1-MODEL\_V4 | 1.0 | 2.289e-10 | 292 | 0.21 | 304 | 201 | 12 | 1 | 291 | 11 | 288 | ParB/RepB/Spo0J family partition protein | ParB/RepB/Spo0J family partition protein | | afdb-uniprot50 | AF-A0A3D2CD18-F1-MODEL\_V4 | 1.0 | 1.406e-10 | 292 | 0.19 | 283 | 194 | 14 | 14 | 282 | 29 | 290 | ParB domain-containing protein | ParB domain-containing protein | | afdb-uniprot50 | AF-A0A847AE66-F1-MODEL\_V4 | 1.0 | 1.301e-11 | 292 | 0.193 | 295 | 204 | 14 | 4 | 282 | 20 | 296 | ParB/RepB/Spo0J family partition protein | ParB/RepB/Spo0J family partition protein | | afdb-uniprot50 | AF-A0A1C5SVQ1-F1-MODEL\_V4 | 1.0 | 6.247e-11 | 292 | 0.181 | 292 | 213 | 12 | 2 | 282 | 22 | 298 | Probable chromosome-partitioning protein parB | Probable chromosome-partitioning protein parB | | afdb-uniprot50 | AF-A0A2W5PW74-F1-MODEL\_V4 | 1.0 | 3.839e-11 | 292 | 0.165 | 308 | 215 | 12 | 1 | 291 | 19 | 301 | Chromosome partitioning protein ParB | Chromosome partitioning protein ParB | | afdb-uniprot50 | AF-A0A024YNU6-F1-MODEL\_V4 | 1.0 | 4.486e-09 | 292 | 0.161 | 254 | 172 | 11 | 19 | 246 | 3 | 241 | Chromosome partitioning protein | Chromosome partitioning protein | | afdb-uniprot50 | AF-A0A7C3M1B3-F1-MODEL\_V4 | 1.0 | 1.225e-09 | 292 | 0.183 | 283 | 197 | 13 | 19 | 282 | 10 | 277 | ParB/RepB/Spo0J family partition protein | ParB/RepB/Spo0J family partition protein | | afdb-uniprot50 | AF-A0A133UNQ5-F1-MODEL\_V4 | 1.0 | 2.612e-09 | 292 | 0.161 | 304 | 204 | 14 | 14 | 282 | 4 | 291 | ParB domain-containing protein | ParB domain-containing protein | | afdb-uniprot50 | AF-A0A7X2FTF1-F1-MODEL\_V4 | 1.0 | 8.385e-10 | 292 | 0.174 | 310 | 198 | 16 | 2 | 280 | 14 | 296 | ParB/RepB/Spo0J family partition protein | ParB/RepB/Spo0J family partition protein | | afdb-uniprot50 | AF-A0A1V5LF66-F1-MODEL\_V4 | 1.0 | 1.332e-10 | 292 | 0.183 | 305 | 205 | 15 | 1 | 282 | 7 | 290 | Chromosome-partitioning protein ParB | Chromosome-partitioning protein ParB | | afdb-uniprot50 | AF-A0A2D6EYC8-F1-MODEL\_V4 | 1.0 | 3.724e-10 | 292 | 0.177 | 305 | 204 | 14 | 13 | 291 | 240 | 523 | ParB domain-containing protein | ParB domain-containing protein | | afdb-uniprot50 | AF-A0A0F9MIX9-F1-MODEL\_V4 | 1.0 | 3.321e-08 | 292 | 0.253 | 158 | 102 | 8 | 19 | 168 | 5 | 154 | ParB domain-containing protein | ParB domain-containing protein | | afdb-uniprot50 | AF-A0A3C1FMD8-F1-MODEL\_V4 | 1.0 | 3.072e-09 | 291 | 0.219 | 187 | 125 | 7 | 1 | 172 | 7 | 187 | Chromosome partitioning protein ParB | Chromosome partitioning protein ParB | | afdb-uniprot50 | AF-A0A5C7QJK5-F1-MODEL\_V4 | 1.0 | 4.85e-08 | 291 | 0.15 | 279 | 189 | 13 | 19 | 291 | 1 | 237 | ParB/RepB/Spo0J family partition protein | ParB/RepB/Spo0J family partition protein | | afdb-uniprot50 | AF-A0A1G0IPH5-F1-MODEL\_V4 | 1.0 | 7.944e-10 | 291 | 0.158 | 296 | 218 | 12 | 3 | 291 | 6 | 277 | Probable chromosome-partitioning protein ParB | Probable chromosome-partitioning protein ParB | | afdb-uniprot50 | AF-A0A485M023-F1-MODEL\_V4 | 1.0 | 2.235e-11 | 291 | 0.186 | 306 | 192 | 13 | 8 | 291 | 14 | 284 | Chromosome-partitioning protein Spo0J | Chromosome-partitioning protein Spo0J | | afdb-uniprot50 | AF-A0A2Z5R1P5-F1-MODEL\_V4 | 1.0 | 2.692e-10 | 291 | 0.17 | 287 | 190 | 15 | 19 | 282 | 39 | 300 | Chromosome (Plasmid) partitioning protein ParB | Chromosome (Plasmid) partitioning protein ParB | | afdb-uniprot50 | AF-A0A2H5V7K5-F1-MODEL\_V4 | 1.0 | 6.398e-10 | 291 | 0.147 | 291 | 195 | 12 | 19 | 287 | 37 | 296 | Putative chromosome-partitioning protein ParB | Putative chromosome-partitioning protein ParB | | afdb-uniprot50 | AF-A0A4P7WB27-F1-MODEL\_V4 | 1.0 | 9.863e-10 | 291 | 0.171 | 304 | 208 | 14 | 3 | 282 | 28 | 311 | ParB/RepB/Spo0J family partition protein | ParB/RepB/Spo0J family partition protein | | afdb-uniprot50 | AF-A0A535XXB1-F1-MODEL\_V4 | 1.0 | 8.851e-10 | 291 | 0.203 | 305 | 190 | 17 | 5 | 287 | 28 | 301 | ParB/RepB/Spo0J family partition protein | ParB/RepB/Spo0J family partition protein | | afdb-uniprot50 | AF-A0A142YPG3-F1-MODEL\_V4 | 1.0 | 3.528e-10 | 291 | 0.181 | 309 | 196 | 16 | 2 | 291 | 39 | 309 | Chromosome-partitioning protein Spo0J | Chromosome-partitioning protein Spo0J | | afdb-uniprot50 | AF-A0A366IM11-F1-MODEL\_V4 | 1.0 | 7.944e-10 | 291 | 0.173 | 294 | 194 | 17 | 11 | 282 | 58 | 324 | ParB family chromosome partitioning protein | ParB family chromosome partitioning protein | | afdb-uniprot50 | AF-A0A7W7T048-F1-MODEL\_V4 | 1.0 | 2.739e-07 | 291 | 0.195 | 189 | 141 | 6 | 19 | 202 | 8 | 190 | ParB family chromosome partitioning protein | ParB family chromosome partitioning protein | | afdb-uniprot50 | AF-A0A4R5AUM8-F1-MODEL\_V4 | 1.0 | 8.385e-10 | 290 | 0.189 | 274 | 176 | 12 | 19 | 287 | 3 | 235 | ParB/RepB/Spo0J family partition protein | ParB/RepB/Spo0J family partition protein | | afdb-uniprot50 | AF-A0A358UY81-F1-MODEL\_V4 | 1.0 | 7.129e-10 | 290 | 0.172 | 290 | 203 | 11 | 13 | 287 | 32 | 299 | Chromosome partitioning protein ParB | Chromosome partitioning protein ParB | | afdb-uniprot50 | AF-R5E743-F1-MODEL\_V4 | 1.0 | 6.247e-11 | 290 | 0.156 | 300 | 212 | 13 | 1 | 282 | 29 | 305 | ParB-like protein | ParB-like protein | | afdb-uniprot50 | AF-R6FU05-F1-MODEL\_V4 | 1.0 | 4.767e-11 | 290 | 0.161 | 291 | 218 | 11 | 4 | 284 | 36 | 310 | ParB-like protein | ParB-like protein | | afdb-uniprot50 | AF-A0A521ZYP4-F1-MODEL\_V4 | 1.0 | 6.961e-11 | 290 | 0.177 | 293 | 206 | 15 | 10 | 291 | 31 | 299 | ParB/RepB/Spo0J family partition protein | ParB/RepB/Spo0J family partition protein | | afdb-uniprot50 | AF-A0A662SF16-F1-MODEL\_V4 | 1.0 | 1.225e-09 | 290 | 0.169 | 289 | 194 | 13 | 18 | 287 | 1 | 262 | ParB domain-containing protein | ParB domain-containing protein | | afdb-uniprot50 | AF-A0A1M3HAQ9-F1-MODEL\_V4 | 1.0 | 1.557e-08 | 290 | 0.158 | 195 | 144 | 8 | 8 | 189 | 28 | 215 | ParB domain-containing protein | ParB domain-containing protein | | afdb-uniprot50 | AF-A0A355SEW4-F1-MODEL\_V4 | 1.0 | 5.918e-11 | 289 | 0.168 | 303 | 206 | 12 | 1 | 282 | 5 | 282 | Chromosome partitioning protein ParB | Chromosome partitioning protein ParB | | afdb-uniprot50 | AF-A0A0S9NZS1-F1-MODEL\_V4 | 1.0 | 1.225e-09 | 289 | 0.182 | 313 | 211 | 14 | 1 | 291 | 9 | 298 | Chromosome partitioning protein ParB | Chromosome partitioning protein ParB | | afdb-uniprot50 | AF-A0A3R6TEA5-F1-MODEL\_V4 | 1.0 | 3.445e-11 | 289 | 0.165 | 290 | 221 | 10 | 1 | 282 | 30 | 306 | ParB/RepB/Spo0J family partition protein | ParB/RepB/Spo0J family partition protein | | afdb-uniprot50 | AF-A0A1T4T4U9-F1-MODEL\_V4 | 1.0 | 2.55e-10 | 289 | 0.211 | 308 | 196 | 13 | 10 | 287 | 30 | 320 | Chromosome segregation DNA-binding protein | Chromosome segregation DNA-binding protein | | afdb-uniprot50 | AF-A0A3N1GT42-F1-MODEL\_V4 | 1.0 | 2.757e-09 | 289 | 0.168 | 249 | 173 | 9 | 19 | 246 | 6 | 241 | ParB/RepB/Spo0J family partition protein | ParB/RepB/Spo0J family partition protein | | afdb-uniprot50 | AF-A0A4P8SIY6-F1-MODEL\_V4 | 1.0 | 9.863e-10 | 289 | 0.167 | 298 | 196 | 14 | 1 | 272 | 37 | 308 | Chromosome partitioning protein ParB | Chromosome partitioning protein ParB | | afdb-uniprot50 | AF-A0A7U6Y6C4-F1-MODEL\_V4 | 1.0 | 6.398e-10 | 289 | 0.18 | 327 | 199 | 17 | 1 | 291 | 10 | 303 | Chromosome-partitioning protein ParB | Chromosome-partitioning protein ParB | | afdb-uniprot50 | AF-A0A0U3PNC0-F1-MODEL\_V4 | 1.0 | 1.188e-08 | 289 | 0.176 | 261 | 178 | 11 | 19 | 261 | 4 | 245 | ParB domain-containing protein | ParB domain-containing protein | | afdb-uniprot50 | AF-A0A021VXB0-F1-MODEL\_V4 | 1.0 | 1.397e-08 | 288 | 0.208 | 269 | 158 | 10 | 19 | 282 | 3 | 221 | ParB domain-containing protein | ParB domain-containing protein | | afdb-uniprot50 | AF-A0A3M1V3E9-F1-MODEL\_V4 | 1.0 | 2.289e-10 | 288 | 0.204 | 293 | 190 | 15 | 14 | 291 | 4 | 268 | Probable chromosome-partitioning protein ParB | Probable chromosome-partitioning protein ParB | | afdb-uniprot50 | AF-A0A2E8QXZ0-F1-MODEL\_V4 | 1.0 | 5.032e-11 | 288 | 0.221 | 307 | 186 | 15 | 1 | 282 | 15 | 293 | Chromosome partitioning protein ParB | Chromosome partitioning protein ParB | | afdb-uniprot50 | AF-A0A1E3G6T5-F1-MODEL\_V4 | 1.0 | 2.289e-10 | 288 | 0.163 | 288 | 182 | 15 | 1 | 272 | 4 | 248 | ParB domain-containing protein | ParB domain-containing protein | | afdb-uniprot50 | AF-A0A2E2QKV5-F1-MODEL\_V4 | 1.0 | 1.406e-10 | 288 | 0.209 | 286 | 177 | 15 | 20 | 282 | 35 | 294 | Chromosome partitioning protein ParB | Chromosome partitioning protein ParB | | afdb-uniprot50 | AF-R5LI34-F1-MODEL\_V4 | 1.0 | 1.52e-09 | 288 | 0.161 | 272 | 180 | 14 | 13 | 271 | 2 | 238 | Nucleoid occlusion protein | Nucleoid occlusion protein | | afdb-uniprot50 | AF-A0A7Y7A845-F1-MODEL\_V4 | 1.0 | 1.933e-08 | 287 | 0.222 | 166 | 117 | 6 | 13 | 172 | 32 | 191 | ParB/RepB/Spo0J family partition protein | ParB/RepB/Spo0J family partition protein | | afdb-uniprot50 | AF-A0A2H0CD87-F1-MODEL\_V4 | 1.0 | 2.49e-11 | 287 | 0.148 | 309 | 196 | 14 | 1 | 282 | 19 | 287 | ParB domain-containing protein | ParB domain-containing protein | | afdb-uniprot50 | AF-A0A521H3H6-F1-MODEL\_V4 | 1.0 | 1.567e-10 | 287 | 0.179 | 317 | 186 | 14 | 5 | 282 | 28 | 309 | ParB/RepB/Spo0J family partition protein | ParB/RepB/Spo0J family partition protein | | afdb-uniprot50 | AF-A0A2Z3J328-F1-MODEL\_V4 | 1.0 | 1.485e-10 | 287 | 0.208 | 293 | 182 | 12 | 8 | 282 | 62 | 322 | Probable chromosome-partitioning protein ParB | Probable chromosome-partitioning protein ParB | | afdb-uniprot50 | AF-A0A2D5RV49-F1-MODEL\_V4 | 1.0 | 8.851e-10 | 287 | 0.178 | 297 | 184 | 14 | 1 | 272 | 7 | 268 | Chromosome partitioning protein ParB | Chromosome partitioning protein ParB | | afdb-uniprot50 | AF-A0A259MJE9-F1-MODEL\_V4 | 1.0 | 2.534e-08 | 287 | 0.183 | 185 | 132 | 6 | 13 | 182 | 74 | 254 | HTH cro/C1-type domain-containing protein | HTH cro/C1-type domain-containing protein | | afdb-uniprot50 | AF-A0A0F9GST2-F1-MODEL\_V4 | 1.0 | 3.243e-09 | 287 | 0.149 | 315 | 207 | 18 | 4 | 287 | 1 | 285 | ParB domain-containing protein | ParB domain-containing protein | | afdb-uniprot50 | AF-A0A2E9CXQ8-F1-MODEL\_V4 | 1.0 | 1.188e-08 | 286 | 0.18 | 211 | 157 | 7 | 1 | 205 | 57 | 257 | Probable chromosome-partitioning protein ParB | Probable chromosome-partitioning protein ParB | | afdb-uniprot50 | AF-A0A2S8IL38-F1-MODEL\_V4 | 1.0 | 1.605e-09 | 286 | 0.179 | 301 | 187 | 16 | 1 | 271 | 1 | 271 | Uncharacterized protein | Uncharacterized protein | | afdb-uniprot50 | AF-A0A2E6W466-F1-MODEL\_V4 | 1.0 | 3.528e-10 | 286 | 0.171 | 298 | 209 | 14 | 2 | 282 | 5 | 281 | Chromosome partitioning protein ParB | Chromosome partitioning protein ParB | | afdb-uniprot50 | AF-Q1NN18-F1-MODEL\_V4 | 1.0 | 3.839e-11 | 286 | 0.187 | 309 | 197 | 11 | 1 | 282 | 6 | 287 | ParB-like partition protein | ParB-like partition protein | | afdb-uniprot50 | AF-A0A7D7ZD79-F1-MODEL\_V4 | 1.0 | 2.842e-10 | 286 | 0.15 | 306 | 224 | 12 | 2 | 290 | 6 | 292 | ParB/RepB/Spo0J family partition protein | ParB/RepB/Spo0J family partition protein | | afdb-uniprot50 | AF-A0A1Y5RLZ8-F1-MODEL\_V4 | 1.0 | 1.406e-10 | 286 | 0.189 | 290 | 190 | 14 | 21 | 291 | 36 | 299 | Chromosome-partitioning protein ParB | Chromosome-partitioning protein ParB | | afdb-uniprot50 | AF-A0A1F9UGF1-F1-MODEL\_V4 | 1.0 | 9.343e-10 | 286 | 0.209 | 306 | 191 | 15 | 13 | 291 | 4 | 285 | ParB domain-containing protein | ParB domain-containing protein | | afdb-uniprot50 | AF-A0A3A1YS35-F1-MODEL\_V4 | 1.0 | 5.153e-10 | 286 | 0.138 | 311 | 220 | 17 | 1 | 291 | 58 | 340 | ParB domain-containing protein | ParB domain-containing protein | | afdb-uniprot50 | AF-A0A7W9SVT8-F1-MODEL\_V4 | 1.0 | 5.439e-10 | 286 | 0.197 | 274 | 183 | 11 | 18 | 265 | 1 | 263 | ParB/RepB/Spo0J family partition protein | ParB/RepB/Spo0J family partition protein | | afdb-uniprot50 | AF-A0A0A8ESX7-F1-MODEL\_V4 | 1.0 | 6.552e-09 | 286 | 0.137 | 277 | 183 | 11 | 19 | 287 | 1 | 229 | Chromosome segregation DNA-binding protein | Chromosome segregation DNA-binding protein | | afdb-uniprot50 | AF-A0A3E0W7R3-F1-MODEL\_V4 | 1.0 | 2.823e-08 | 286 | 0.187 | 213 | 147 | 11 | 1 | 204 | 21 | 216 | ParB domain-containing protein | ParB domain-containing protein | | afdb-uniprot50 | AF-A0A2D6XAJ0-F1-MODEL\_V4 | 1.0 | 2.344e-09 | 285 | 0.176 | 306 | 180 | 14 | 16 | 291 | 1 | 264 | ParB domain-containing protein | ParB domain-containing protein | | afdb-uniprot50 | AF-A0A6N7AYY8-F1-MODEL\_V4 | 1.0 | 3.343e-10 | 285 | 0.155 | 295 | 208 | 13 | 6 | 283 | 11 | 281 | Chromosome partitioning protein ParB family | Chromosome partitioning protein ParB family | | afdb-uniprot50 | AF-A0A2D7IRV4-F1-MODEL\_V4 | 1.0 | 7.944e-10 | 285 | 0.139 | 294 | 229 | 12 | 1 | 282 | 7 | 288 | DNA-binding protein | DNA-binding protein | | afdb-uniprot50 | AF-A0A358ICQ6-F1-MODEL\_V4 | 1.0 | 3e-10 | 285 | 0.168 | 297 | 209 | 12 | 5 | 287 | 18 | 290 | ParB domain-containing protein | ParB domain-containing protein | | afdb-uniprot50 | AF-A0A6B1A4N4-F1-MODEL\_V4 | 1.0 | 4.053e-11 | 285 | 0.212 | 296 | 206 | 11 | 9 | 291 | 21 | 302 | ParB/RepB/Spo0J family partition protein | ParB/RepB/Spo0J family partition protein | | afdb-uniprot50 | AF-A0A5C7UBH5-F1-MODEL\_V4 | 1.0 | 2.534e-08 | 285 | 0.229 | 179 | 124 | 8 | 13 | 183 | 2 | 174 | PRTRC system ParB family protein | PRTRC system ParB family protein | | afdb-uniprot50 | AF-A0A1B9RA54-F1-MODEL\_V4 | 1.0 | 5.439e-10 | 285 | 0.161 | 321 | 188 | 16 | 13 | 272 | 1 | 301 | ParB domain-containing protein | ParB domain-containing protein | | afdb-uniprot50 | AF-D7ATR0-F1-MODEL\_V4 | 1.0 | 6.961e-11 | 284 | 0.164 | 310 | 203 | 16 | 1 | 282 | 1 | 282 | ParB-like partition protein | ParB-like partition protein | | afdb-uniprot50 | AF-A0A2K5ARA4-F1-MODEL\_V4 | 1.0 | 4.381e-10 | 284 | 0.152 | 308 | 202 | 15 | 3 | 287 | 1 | 272 | Chromosome-partitioning protein Spo0J | Chromosome-partitioning protein Spo0J | | afdb-uniprot50 | AF-A0A2E9E3N7-F1-MODEL\_V4 | 1.0 | 5.607e-11 | 284 | 0.165 | 314 | 196 | 16 | 1 | 282 | 6 | 285 | Chromosome partitioning protein ParB | Chromosome partitioning protein ParB | | afdb-uniprot50 | AF-A0A0D0QNA4-F1-MODEL\_V4 | 1.0 | 4.278e-11 | 284 | 0.145 | 296 | 197 | 11 | 13 | 282 | 28 | 293 | TWA4\_scaffold00001, whole genome shotgun sequence | TWA4\_scaffold00001, whole genome shotgun sequence | | afdb-uniprot50 | AF-L0DRM7-F1-MODEL\_V4 | 1.0 | 2.55e-10 | 284 | 0.182 | 296 | 197 | 14 | 1 | 287 | 37 | 296 | ParB-like partition protein | ParB-like partition protein | | afdb-uniprot50 | AF-R5JVN8-F1-MODEL\_V4 | 1.0 | 1.485e-10 | 284 | 0.174 | 281 | 203 | 11 | 14 | 282 | 52 | 315 | Stage 0 sporulation protein J | Stage 0 sporulation protein J | | afdb-uniprot50 | AF-L7LEH1-F1-MODEL\_V4 | 1.0 | 3.613e-09 | 284 | 0.171 | 304 | 176 | 14 | 20 | 272 | 4 | 282 | ParB domain-containing protein | ParB domain-containing protein | | afdb-uniprot50 | AF-A0A3M2CZK8-F1-MODEL\_V4 | 1.0 | 7.574e-12 | 283 | 0.161 | 309 | 198 | 12 | 1 | 282 | 4 | 278 | ParB/RepB/Spo0J family partition protein | ParB/RepB/Spo0J family partition protein | | afdb-uniprot50 | AF-A0A1Q2HRV3-F1-MODEL\_V4 | 1.0 | 7.348e-11 | 283 | 0.164 | 310 | 213 | 9 | 2 | 291 | 1 | 284 | Putative chromosome-partitioning protein ParB | Putative chromosome-partitioning protein ParB | | afdb-uniprot50 | AF-A0A373LA92-F1-MODEL\_V4 | 1.0 | 9.343e-10 | 283 | 0.178 | 285 | 190 | 13 | 12 | 272 | 19 | 283 | ParB/RepB/Spo0J family partition protein | ParB/RepB/Spo0J family partition protein | | afdb-uniprot50 | AF-A0A3A6JNC2-F1-MODEL\_V4 | 1.0 | 3.814e-09 | 283 | 0.18 | 277 | 188 | 15 | 3 | 246 | 22 | 292 | ParB/RepB/Spo0J family partition protein | ParB/RepB/Spo0J family partition protein | | afdb-uniprot50 | AF-A0A1G0G5V5-F1-MODEL\_V4 | 1.0 | 2.344e-09 | 283 | 0.192 | 260 | 171 | 13 | 19 | 271 | 15 | 242 | Probable chromosome-partitioning protein ParB | Probable chromosome-partitioning protein ParB | | afdb-uniprot50 | AF-A0A1I2HRP5-F1-MODEL\_V4 | 1.0 | 1.041e-09 | 283 | 0.207 | 313 | 200 | 19 | 1 | 291 | 73 | 359 | Chromosome segregation DNA-binding protein | Chromosome segregation DNA-binding protein | | afdb-uniprot50 | AF-A0A518GYN7-F1-MODEL\_V4 | 1.0 | 3.814e-09 | 283 | 0.171 | 286 | 199 | 8 | 11 | 287 | 74 | 330 | Putative chromosome-partitioning protein ParB | Putative chromosome-partitioning protein ParB | | afdb-uniprot50 | AF-A0A1V9VAD5-F1-MODEL\_V4 | 1.0 | 2.823e-08 | 282 | 0.169 | 177 | 135 | 6 | 1 | 169 | 8 | 180 | ParB domain-containing protein | ParB domain-containing protein | | afdb-uniprot50 | AF-A0A7X9CR71-F1-MODEL\_V4 | 1.0 | 6.595e-11 | 282 | 0.179 | 290 | 194 | 13 | 13 | 282 | 16 | 281 | ParB/RepB/Spo0J family partition protein | ParB/RepB/Spo0J family partition protein | | afdb-uniprot50 | AF-A0A847NG60-F1-MODEL\_V4 | 1.0 | 2.49e-11 | 282 | 0.185 | 281 | 192 | 12 | 14 | 282 | 25 | 280 | ParB/RepB/Spo0J family partition protein | ParB/RepB/Spo0J family partition protein | | afdb-uniprot50 | AF-A0A2N6AKC7-F1-MODEL\_V4 | 1.0 | 7.944e-10 | 282 | 0.155 | 289 | 206 | 12 | 8 | 282 | 24 | 288 | Chromosome partitioning protein ParB | Chromosome partitioning protein ParB | | afdb-uniprot50 | AF-A0A0Q7MU60-F1-MODEL\_V4 | 1.0 | 7.129e-10 | 282 | 0.189 | 311 | 193 | 16 | 13 | 291 | 59 | 342 | ParB domain-containing protein | ParB domain-containing protein | | afdb-uniprot50 | AF-A0A517VMS6-F1-MODEL\_V4 | 1.0 | 2.757e-09 | 282 | 0.167 | 293 | 193 | 17 | 12 | 281 | 3 | 267 | Nucleoid occlusion protein | Nucleoid occlusion protein | | afdb-uniprot50 | AF-A0A3B9JIH2-F1-MODEL\_V4 | 1.0 | 1.017e-10 | 281 | 0.18 | 300 | 205 | 13 | 1 | 282 | 4 | 280 | Chromosome partitioning protein ParB | Chromosome partitioning protein ParB | | afdb-uniprot50 | AF-A0A059FMC4-F1-MODEL\_V4 | 1.0 | 5.607e-11 | 281 | 0.199 | 296 | 192 | 11 | 14 | 291 | 17 | 285 | Chromosome partitioning protein ParB | Chromosome partitioning protein ParB | | afdb-uniprot50 | AF-A0A6N8V9B5-F1-MODEL\_V4 | 1.0 | 3e-10 | 281 | 0.165 | 314 | 201 | 15 | 1 | 282 | 11 | 295 | ParB/RepB/Spo0J family partition protein | ParB/RepB/Spo0J family partition protein | | afdb-uniprot50 | AF-A0A4Z0KDG3-F1-MODEL\_V4 | 1.0 | 2.474e-09 | 281 | 0.168 | 302 | 207 | 18 | 1 | 282 | 26 | 303 | ParB/RepB/Spo0J family partition protein | ParB/RepB/Spo0J family partition protein | | afdb-uniprot50 | AF-A0A5U1PXS1-F1-MODEL\_V4 | 1.0 | 4.381e-10 | 281 | 0.203 | 285 | 178 | 14 | 1 | 272 | 6 | 254 | ParB/RepB/Spo0J family partition protein | ParB/RepB/Spo0J family partition protein | | afdb-uniprot50 | AF-A0A411H8J1-F1-MODEL\_V4 | 1.0 | 3.072e-09 | 281 | 0.17 | 310 | 190 | 17 | 3 | 290 | 8 | 272 | ParB/RepB/Spo0J family partition protein | ParB/RepB/Spo0J family partition protein | | afdb-uniprot50 | AF-A0A7H2BGE9-F1-MODEL\_V4 | 1.0 | 1.788e-09 | 281 | 0.186 | 268 | 171 | 14 | 19 | 265 | 9 | 250 | ParB/RepB/Spo0J family partition protein | ParB/RepB/Spo0J family partition protein | | afdb-uniprot50 | AF-M4NG45-F1-MODEL\_V4 | 1.0 | 1.217e-07 | 281 | 0.175 | 188 | 144 | 6 | 19 | 200 | 14 | 196 | Probable chromosome-partitioning protein ParB | Probable chromosome-partitioning protein ParB | | afdb-uniprot50 | AF-A0A4P7D5W3-F1-MODEL\_V4 | 1.0 | 2.274e-08 | 280 | 0.181 | 204 | 152 | 8 | 13 | 205 | 6 | 205 | ParB/RepB/Spo0J family partition protein | ParB/RepB/Spo0J family partition protein | | afdb-uniprot50 | AF-A0A2S6QJ87-F1-MODEL\_V4 | 1.0 | 1.133e-10 | 280 | 0.184 | 315 | 201 | 14 | 1 | 290 | 6 | 289 | Chromosome-partitioning protein ParB | Chromosome-partitioning protein ParB | | afdb-uniprot50 | AF-A0A2H9T523-F1-MODEL\_V4 | 1.0 | 7.525e-10 | 280 | 0.179 | 301 | 197 | 17 | 5 | 273 | 8 | 290 | Putative chromosome-partitioning protein ParB | Putative chromosome-partitioning protein ParB | | afdb-uniprot50 | AF-A0A1F5FFJ9-F1-MODEL\_V4 | 1.0 | 8.385e-10 | 280 | 0.189 | 311 | 204 | 13 | 7 | 290 | 18 | 307 | HTH cro/C1-type domain-containing protein | HTH cro/C1-type domain-containing protein | | afdb-uniprot50 | AF-A0A2A3XKZ8-F1-MODEL\_V4 | 1.0 | 1.099e-09 | 280 | 0.173 | 288 | 190 | 17 | 19 | 283 | 184 | 446 | ParB domain-containing protein | ParB domain-containing protein | | afdb-uniprot50 | AF-A0A4P9VE58-F1-MODEL\_V4 | 1.0 | 4.486e-09 | 279 | 0.16 | 249 | 176 | 10 | 19 | 246 | 6 | 242 | ParB/RepB/Spo0J family partition protein | ParB/RepB/Spo0J family partition protein | | afdb-uniprot50 | AF-A0A1F2Z098-F1-MODEL\_V4 | 1.0 | 5.439e-10 | 279 | 0.155 | 303 | 212 | 13 | 13 | 291 | 46 | 328 | ParB domain-containing protein | ParB domain-containing protein | | afdb-uniprot50 | AF-A0A559UHF1-F1-MODEL\_V4 | 1.0 | 5.277e-09 | 279 | 0.17 | 294 | 204 | 15 | 8 | 287 | 44 | 311 | ParB family chromosome partitioning protein | ParB family chromosome partitioning protein | | afdb-uniprot50 | AF-A0A5F0M8I8-F1-MODEL\_V4 | 1.0 | 4.026e-09 | 279 | 0.165 | 290 | 207 | 14 | 11 | 282 | 211 | 483 | ParB/RepB/Spo0J family partition protein | ParB/RepB/Spo0J family partition protein | | afdb-uniprot50 | AF-A0A7C7PFL2-F1-MODEL\_V4 | 1.0 | 4.999e-09 | 278 | 0.223 | 215 | 148 | 9 | 1 | 201 | 1 | 210 | ParB/RepB/Spo0J family partition protein | ParB/RepB/Spo0J family partition protein | | afdb-uniprot50 | AF-A0A3N5NMG6-F1-MODEL\_V4 | 1.0 | 4.881e-10 | 278 | 0.2 | 279 | 170 | 11 | 3 | 256 | 10 | 260 | ParB/RepB/Spo0J family partition protein | ParB/RepB/Spo0J family partition protein | | afdb-uniprot50 | AF-A0A7X3YB26-F1-MODEL\_V4 | 1.0 | 1.225e-09 | 278 | 0.185 | 286 | 186 | 14 | 13 | 282 | 24 | 278 | ParB/RepB/Spo0J family partition protein | ParB/RepB/Spo0J family partition protein | | afdb-uniprot50 | AF-A0A7C7A9V0-F1-MODEL\_V4 | 1.0 | 5.032e-11 | 278 | 0.179 | 317 | 197 | 16 | 1 | 288 | 4 | 286 | ParB/RepB/Spo0J family partition protein | ParB/RepB/Spo0J family partition protein | | afdb-uniprot50 | AF-A0A7X5HWV5-F1-MODEL\_V4 | 1.0 | 4.767e-11 | 278 | 0.185 | 308 | 195 | 13 | 9 | 291 | 21 | 297 | ParB/RepB/Spo0J family partition protein | ParB/RepB/Spo0J family partition protein | | afdb-uniprot50 | AF-A0A539DA23-F1-MODEL\_V4 | 1.0 | 2.006e-11 | 278 | 0.211 | 298 | 181 | 12 | 14 | 288 | 51 | 317 | Chromosome partitioning protein ParB | Chromosome partitioning protein ParB | | afdb-uniprot50 | AF-A0A7K1LKD1-F1-MODEL\_V4 | 1.0 | 6.753e-10 | 278 | 0.183 | 299 | 201 | 16 | 13 | 289 | 105 | 382 | ParB/RepB/Spo0J family partition protein | ParB/RepB/Spo0J family partition protein | | afdb-uniprot50 | AF-A0A827LWJ5-F1-MODEL\_V4 | 1.0 | 6.916e-09 | 278 | 0.188 | 302 | 201 | 15 | 1 | 286 | 22 | 295 | ParB/RepB/Spo0J family partition protein | ParB/RepB/Spo0J family partition protein | | afdb-uniprot50 | AF-A0A366KCA2-F1-MODEL\_V4 | 1.0 | 2.22e-09 | 278 | 0.214 | 247 | 149 | 14 | 12 | 246 | 6 | 219 | ParB domain-containing protein | ParB domain-containing protein | | afdb-uniprot50 | AF-A0A538S316-F1-MODEL\_V4 | 1.0 | 4.85e-08 | 278 | 0.216 | 217 | 136 | 10 | 19 | 214 | 184 | 387 | ParB domain-containing protein | ParB domain-containing protein | | afdb-uniprot50 | AF-A0A7C6QZE1-F1-MODEL\_V4 | 1.0 | 4.624e-10 | 278 | 0.159 | 301 | 187 | 15 | 13 | 281 | 23 | 289 | ParB/RepB/Spo0J family partition protein | ParB/RepB/Spo0J family partition protein | | afdb-uniprot50 | AF-A0A3S0HPY2-F1-MODEL\_V4 | 1.0 | 8.331e-08 | 277 | 0.184 | 206 | 148 | 8 | 18 | 214 | 1 | 195 | ParB/RepB/Spo0J family partition protein | ParB/RepB/Spo0J family partition protein | | afdb-uniprot50 | AF-A0A2E6Z228-F1-MODEL\_V4 | 1.0 | 2.458e-07 | 277 | 0.134 | 178 | 146 | 5 | 19 | 192 | 6 | 179 | ParB domain-containing protein | ParB domain-containing protein | | afdb-uniprot50 | AF-A0A7C6P963-F1-MODEL\_V4 | 1.0 | 7.476e-08 | 277 | 0.216 | 157 | 112 | 5 | 18 | 170 | 1 | 150 | ParB/RepB/Spo0J family partition protein | ParB/RepB/Spo0J family partition protein | | afdb-uniprot50 | AF-A0A3D5L091-F1-MODEL\_V4 | 1.0 | 6.753e-10 | 277 | 0.151 | 290 | 188 | 13 | 20 | 282 | 55 | 313 | Chromosome partitioning protein ParB | Chromosome partitioning protein ParB | | afdb-uniprot50 | AF-A0A2G6DSE2-F1-MODEL\_V4 | 1.0 | 1.406e-10 | 277 | 0.164 | 280 | 193 | 13 | 19 | 282 | 71 | 325 | Chromosome partitioning protein ParB | Chromosome partitioning protein ParB | | afdb-uniprot50 | AF-A0A375JD00-F1-MODEL\_V4 | 1.0 | 3.613e-09 | 277 | 0.18 | 305 | 184 | 16 | 9 | 272 | 6 | 285 | ParB domain-containing protein | ParB domain-containing protein | | afdb-uniprot50 | AF-A0A5C5TNX6-F1-MODEL\_V4 | 1.0 | 2.117e-11 | 277 | 0.221 | 316 | 186 | 19 | 13 | 282 | 36 | 337 | ParB/RepB/Spo0J family partition protein | ParB/RepB/Spo0J family partition protein | | afdb-uniprot50 | AF-A0A6D1V7U6-F1-MODEL\_V4 | 1.0 | 7.476e-08 | 277 | 0.174 | 223 | 143 | 9 | 16 | 205 | 3 | 217 | ParB domain-containing protein | ParB domain-containing protein | | afdb-uniprot50 | AF-A0A7W6KP23-F1-MODEL\_V4 | 1.0 | 2.739e-07 | 276 | 0.173 | 190 | 144 | 8 | 19 | 199 | 5 | 190 | ParB family chromosome partitioning protein | ParB family chromosome partitioning protein | | afdb-uniprot50 | AF-A0A7C2K3S6-F1-MODEL\_V4 | 1.0 | 5.57e-09 | 276 | 0.156 | 300 | 201 | 13 | 18 | 291 | 4 | 277 | Probable chromosome-partitioning protein ParB | Probable chromosome-partitioning protein ParB | | afdb-uniprot50 | AF-A0A3D3IUN0-F1-MODEL\_V4 | 1.0 | 1.654e-10 | 276 | 0.165 | 315 | 207 | 15 | 4 | 288 | 37 | 325 | Chromosome partitioning protein ParB | Chromosome partitioning protein ParB | | afdb-uniprot50 | AF-D9Y7S8-F1-MODEL\_V4 | 1.0 | 1.788e-09 | 276 | 0.147 | 285 | 197 | 11 | 2 | 272 | 5 | 257 | ParB-like protein | ParB-like protein | | afdb-uniprot50 | AF-R6M2U8-F1-MODEL\_V4 | 1.0 | 9.799e-08 | 276 | 0.203 | 201 | 139 | 10 | 4 | 193 | 12 | 202 | ParB-like partition protein | ParB-like partition protein | | afdb-uniprot50 | AF-A0A3M1I3Z7-F1-MODEL\_V4 | 1.0 | 4.15e-10 | 275 | 0.22 | 299 | 188 | 15 | 1 | 282 | 4 | 274 | ParB/RepB/Spo0J family partition protein | ParB/RepB/Spo0J family partition protein | | afdb-uniprot50 | AF-A0A2A4QUW5-F1-MODEL\_V4 | 1.0 | 4.381e-10 | 275 | 0.171 | 320 | 208 | 15 | 1 | 291 | 22 | 313 | Chromosome partitioning protein ParB | Chromosome partitioning protein ParB | | afdb-uniprot50 | AF-A0A5C8PGP3-F1-MODEL\_V4 | 1.0 | 2.629e-11 | 275 | 0.227 | 317 | 184 | 18 | 13 | 282 | 42 | 344 | ParB/RepB/Spo0J family partition protein | ParB/RepB/Spo0J family partition protein | | afdb-uniprot50 | AF-A0A221Q2J2-F1-MODEL\_V4 | 1.0 | 2.91e-09 | 275 | 0.151 | 316 | 195 | 13 | 19 | 282 | 4 | 298 | ParB domain-containing protein | ParB domain-containing protein | | afdb-uniprot50 | AF-A0A2G9Y3U1-F1-MODEL\_V4 | 1.0 | 2.55e-10 | 274 | 0.148 | 303 | 207 | 14 | 1 | 282 | 2 | 274 | Chromosome partitioning protein ParB | Chromosome partitioning protein ParB | | afdb-uniprot50 | AF-A0A2A4U4T4-F1-MODEL\_V4 | 1.0 | 2.842e-10 | 274 | 0.195 | 302 | 198 | 14 | 4 | 282 | 8 | 287 | Chromosome partitioning protein ParB | Chromosome partitioning protein ParB | | afdb-uniprot50 | AF-A0A845Q5L7-F1-MODEL\_V4 | 1.0 | 6.398e-10 | 274 | 0.14 | 314 | 207 | 14 | 1 | 282 | 8 | 290 | ParB/RepB/Spo0J family partition protein | ParB/RepB/Spo0J family partition protein | | afdb-uniprot50 | AF-A0A4Q5VAH7-F1-MODEL\_V4 | 1.0 | 4.736e-09 | 274 | 0.152 | 288 | 213 | 15 | 19 | 291 | 4 | 275 | Uncharacterized protein | Uncharacterized protein | | afdb-uniprot50 | AF-A0A5C7QL10-F1-MODEL\_V4 | 1.0 | 3.814e-09 | 274 | 0.156 | 256 | 169 | 12 | 19 | 247 | 1 | 236 | ParB/RepB/Spo0J family partition protein | ParB/RepB/Spo0J family partition protein | | afdb-uniprot50 | AF-A0A075KIZ2-F1-MODEL\_V4 | 1.0 | 1.746e-10 | 274 | 0.159 | 294 | 219 | 12 | 1 | 282 | 8 | 285 | ParB-like partition protein | ParB-like partition protein | | afdb-uniprot50 | AF-G2DG78-F1-MODEL\_V4 | 1.0 | 4.624e-10 | 274 | 0.172 | 290 | 198 | 14 | 19 | 291 | 40 | 304 | Probable chromosome-partitioning protein ParB | Probable chromosome-partitioning protein ParB | | afdb-uniprot50 | AF-A0A561EN29-F1-MODEL\_V4 | 1.0 | 2.041e-08 | 274 | 0.192 | 250 | 169 | 11 | 19 | 246 | 1 | 239 | ParB family chromosome partitioning protein | ParB family chromosome partitioning protein | | afdb-uniprot50 | AF-A0A2X3KGI8-F1-MODEL\_V4 | 1.0 | 1.254e-08 | 273 | 0.18 | 238 | 151 | 10 | 19 | 216 | 35 | 268 | Probable chromosome-partitioning protein parB | Probable chromosome-partitioning protein parB | | afdb-uniprot50 | AF-A0A0G0LW51-F1-MODEL\_V4 | 1.0 | 1.946e-10 | 273 | 0.198 | 287 | 201 | 11 | 3 | 282 | 13 | 277 | Chromosome (Plasmid) partitioning protein ParB / Stage 0 sporulation protein J | Chromosome (Plasmid) partitioning protein ParB / Stage 0 sporulation protein J | | afdb-uniprot50 | AF-I8R461-F1-MODEL\_V4 | 1.0 | 6.595e-11 | 273 | 0.186 | 317 | 195 | 16 | 1 | 288 | 4 | 286 | ParB-like partition protein | ParB-like partition protein | | afdb-uniprot50 | AF-A0A2M8QGU9-F1-MODEL\_V4 | 1.0 | 1.332e-10 | 273 | 0.203 | 314 | 201 | 18 | 1 | 290 | 4 | 292 | Stage 0 sporulation protein J | Stage 0 sporulation protein J | | afdb-uniprot50 | AF-A0A3B9WD89-F1-MODEL\_V4 | 1.0 | 5.88e-09 | 273 | 0.167 | 299 | 194 | 16 | 12 | 282 | 54 | 325 | Chromosome partitioning protein ParB | Chromosome partitioning protein ParB | | afdb-uniprot50 | AF-A0A1H3WS16-F1-MODEL\_V4 | 1.0 | 3.932e-10 | 273 | 0.175 | 291 | 194 | 15 | 13 | 282 | 79 | 344 | Chromosome partitioning protein, ParB family | Chromosome partitioning protein, ParB family | | afdb-uniprot50 | AF-A0A1C3E499-F1-MODEL\_V4 | 1.0 | 1.475e-08 | 273 | 0.245 | 220 | 125 | 12 | 1 | 195 | 1 | 204 | ParB domain-containing protein | ParB domain-containing protein | | afdb-uniprot50 | AF-A0A1T4M6I0-F1-MODEL\_V4 | 1.0 | 1.735e-08 | 272 | 0.134 | 275 | 196 | 13 | 19 | 272 | 3 | 256 | Chromosome partitioning protein, ParB family | Chromosome partitioning protein, ParB family | | afdb-uniprot50 | AF-A0A2M7AKN5-F1-MODEL\_V4 | 1.0 | 6.44e-12 | 272 | 0.206 | 296 | 200 | 11 | 1 | 282 | 3 | 277 | ParB domain-containing protein | ParB domain-containing protein | | afdb-uniprot50 | AF-A0A3N5J5N6-F1-MODEL\_V4 | 1.0 | 7.757e-11 | 272 | 0.198 | 313 | 190 | 16 | 1 | 282 | 4 | 286 | ParB/RepB/Spo0J family partition protein | ParB/RepB/Spo0J family partition protein | | afdb-uniprot50 | AF-A0A7V8DCV6-F1-MODEL\_V4 | 1.0 | 6.398e-10 | 272 | 0.141 | 310 | 215 | 14 | 1 | 282 | 2 | 288 | Chromosome partitioning protein ParB family | Chromosome partitioning protein ParB family | | afdb-uniprot50 | AF-A0A1M6HWY9-F1-MODEL\_V4 | 1.0 | 4.85e-08 | 272 | 0.192 | 213 | 139 | 11 | 13 | 214 | 73 | 263 | ParB/RepB/Spo0J family partition protein | ParB/RepB/Spo0J family partition protein | | afdb-uniprot50 | AF-X0TEE3-F1-MODEL\_V4 | 1.0 | 6.509e-07 | 271 | 0.189 | 158 | 112 | 9 | 18 | 168 | 1 | 149 | ParB domain-containing protein | ParB domain-containing protein | | afdb-uniprot50 | AF-T0YE96-F1-MODEL\_V4 | 1.0 | 4.428e-05 | 271 | 0.373 | 91 | 57 | 0 | 123 | 213 | 2 | 92 | Uncharacterized protein | Uncharacterized protein | | afdb-uniprot50 | AF-A0A1F8H6F6-F1-MODEL\_V4 | 1.0 | 1.397e-08 | 271 | 0.154 | 291 | 198 | 14 | 13 | 282 | 8 | 271 | ParB domain-containing protein | ParB domain-containing protein | | afdb-uniprot50 | AF-A0A4R6UCT0-F1-MODEL\_V4 | 1.0 | 6.753e-10 | 271 | 0.207 | 308 | 186 | 15 | 22 | 289 | 36 | 325 | ParB family chromosome partitioning protein | ParB family chromosome partitioning protein | | afdb-uniprot50 | AF-A0A8B0SSF4-F1-MODEL\_V4 | 1.0 | 3.814e-09 | 271 | 0.186 | 279 | 171 | 14 | 5 | 267 | 16 | 254 | ParB/RepB/Spo0J family partition protein | ParB/RepB/Spo0J family partition protein | | afdb-uniprot50 | AF-A0A0D6P2K6-F1-MODEL\_V4 | 1.0 | 2.757e-09 | 270 | 0.2 | 264 | 180 | 10 | 26 | 282 | 2 | 241 | Chromosome partitioning nuclease protein ParB | Chromosome partitioning nuclease protein ParB | | afdb-uniprot50 | AF-A0A1Z9RIE5-F1-MODEL\_V4 | 1.0 | 8.643e-11 | 270 | 0.168 | 285 | 199 | 13 | 13 | 282 | 23 | 284 | Probable chromosome-partitioning protein ParB | Probable chromosome-partitioning protein ParB | | afdb-uniprot50 | AF-A0A3M1XFH8-F1-MODEL\_V4 | 1.0 | 2.55e-10 | 270 | 0.202 | 296 | 196 | 15 | 12 | 290 | 20 | 292 | ParB/RepB/Spo0J family partition protein | ParB/RepB/Spo0J family partition protein | | afdb-uniprot50 | AF-A0A1Y4DG68-F1-MODEL\_V4 | 1.0 | 3.423e-09 | 270 | 0.152 | 296 | 210 | 13 | 8 | 287 | 26 | 296 | ParB domain-containing protein | ParB domain-containing protein | | afdb-uniprot50 | AF-A0A7X1ZX84-F1-MODEL\_V4 | 1.0 | 9.343e-10 | 270 | 0.174 | 310 | 200 | 14 | 1 | 291 | 5 | 277 | ParB/RepB/Spo0J family partition protein | ParB/RepB/Spo0J family partition protein | | afdb-uniprot50 | AF-A0A2M6X6D0-F1-MODEL\_V4 | 1.0 | 1.946e-10 | 270 | 0.161 | 278 | 212 | 10 | 14 | 282 | 59 | 324 | Chromosome partitioning protein | Chromosome partitioning protein | | afdb-uniprot50 | AF-A0A370GYF5-F1-MODEL\_V4 | 1.0 | 1.993e-09 | 270 | 0.176 | 300 | 191 | 16 | 13 | 282 | 55 | 328 | Chromosome segregation DNA-binding protein | Chromosome segregation DNA-binding protein | | afdb-uniprot50 | AF-A0A1Q8LIG9-F1-MODEL\_V4 | 1.0 | 3.401e-07 | 270 | 0.194 | 216 | 146 | 10 | 2 | 196 | 16 | 224 | Chromosome (Plasmid) partitioning protein ParB / Stage 0 sporulation protein J | Chromosome (Plasmid) partitioning protein ParB / Stage 0 sporulation protein J | | afdb-uniprot50 | AF-A0A1Q8LHK6-F1-MODEL\_V4 | 1.0 | 1.475e-08 | 270 | 0.235 | 242 | 134 | 10 | 10 | 205 | 50 | 286 | Chromosome (Plasmid) partitioning protein ParB / Stage 0 sporulation protein J | Chromosome (Plasmid) partitioning protein ParB / Stage 0 sporulation protein J | | afdb-uniprot50 | AF-A0A7V4UP31-F1-MODEL\_V4 | 1.0 | 9.799e-08 | 269 | 0.218 | 165 | 120 | 5 | 2 | 163 | 5 | 163 | ParB/RepB/Spo0J family partition protein | ParB/RepB/Spo0J family partition protein | | afdb-uniprot50 | AF-A0A6L7X7V6-F1-MODEL\_V4 | 1.0 | 8.188e-11 | 269 | 0.209 | 282 | 195 | 9 | 21 | 291 | 2 | 266 | ParB/RepB/Spo0J family partition protein | ParB/RepB/Spo0J family partition protein | | afdb-uniprot50 | AF-A0A357IP86-F1-MODEL\_V4 | 1.0 | 7.301e-09 | 269 | 0.161 | 298 | 201 | 12 | 1 | 282 | 11 | 275 | ParB domain-containing protein | ParB domain-containing protein | | afdb-uniprot50 | AF-A0A2A1ZTU4-F1-MODEL\_V4 | 1.0 | 2.474e-09 | 269 | 0.155 | 295 | 211 | 14 | 1 | 283 | 6 | 274 | ParB domain-containing protein | ParB domain-containing protein | | afdb-uniprot50 | AF-R7KQ80-F1-MODEL\_V4 | 1.0 | 1.099e-09 | 269 | 0.143 | 306 | 232 | 15 | 1 | 291 | 1 | 291 | ParB domain-containing protein | ParB domain-containing protein | | afdb-uniprot50 | AF-A0A1G2U0D6-F1-MODEL\_V4 | 1.0 | 3.072e-09 | 269 | 0.147 | 291 | 196 | 12 | 19 | 282 | 34 | 299 | ParB domain-containing protein | ParB domain-containing protein | | afdb-uniprot50 | AF-A0A1Y6CZ00-F1-MODEL\_V4 | 1.0 | 1.475e-08 | 269 | 0.196 | 264 | 180 | 14 | 6 | 247 | 45 | 298 | ParB-like nuclease domain-containing protein | ParB-like nuclease domain-containing protein | | afdb-uniprot50 | AF-A0A1Y3M0Q7-F1-MODEL\_V4 | 1.0 | 1.788e-09 | 269 | 0.178 | 285 | 190 | 14 | 19 | 282 | 96 | 357 | Chromosome partitioning protein ParB | Chromosome partitioning protein ParB | | afdb-uniprot50 | AF-A0A7Z7VNP5-F1-MODEL\_V4 | 1.0 | 2.757e-09 | 269 | 0.15 | 280 | 192 | 13 | 1 | 272 | 33 | 274 | ParB/RepB/Spo0J family partition protein | ParB/RepB/Spo0J family partition protein | | afdb-uniprot50 | AF-A0A087D6M2-F1-MODEL\_V4 | 1.0 | 8.135e-09 | 269 | 0.181 | 297 | 190 | 20 | 13 | 290 | 3 | 265 | ParB domain-containing protein | ParB domain-containing protein | | afdb-uniprot50 | AF-A0A239PGG5-F1-MODEL\_V4 | 1.0 | 1.188e-08 | 269 | 0.171 | 285 | 171 | 12 | 1 | 246 | 9 | 267 | Chromosome partitioning protein, ParB family | Chromosome partitioning protein, ParB family | | afdb-uniprot50 | AF-A0A257WQE5-F1-MODEL\_V4 | 1.0 | 2.154e-08 | 268 | 0.213 | 173 | 119 | 7 | 1 | 162 | 1 | 167 | Chromosome partitioning protein ParB | Chromosome partitioning protein ParB | | afdb-uniprot50 | AF-A0A3A0A5C3-F1-MODEL\_V4 | 1.0 | 1.356e-07 | 268 | 0.2 | 170 | 126 | 6 | 5 | 169 | 27 | 191 | Stage 0 sporulation protein J | Stage 0 sporulation protein J | | afdb-uniprot50 | AF-A0A4Y6UB58-F1-MODEL\_V4 | 1.0 | 6.061e-10 | 268 | 0.194 | 308 | 190 | 16 | 14 | 291 | 10 | 289 | ParB/RepB/Spo0J family partition protein | ParB/RepB/Spo0J family partition protein | | afdb-uniprot50 | AF-A0A1W6N531-F1-MODEL\_V4 | 1.0 | 1.788e-09 | 268 | 0.186 | 305 | 215 | 16 | 1 | 291 | 7 | 292 | ParB domain-containing protein | ParB domain-containing protein | | afdb-uniprot50 | AF-A0A7Y3NTN7-F1-MODEL\_V4 | 1.0 | 4.516e-11 | 268 | 0.146 | 294 | 196 | 11 | 16 | 282 | 50 | 315 | ParB/RepB/Spo0J family partition protein | ParB/RepB/Spo0J family partition protein | | afdb-uniprot50 | AF-A0A1S9CBB3-F1-MODEL\_V4 | 1.0 | 1.567e-10 | 267 | 0.15 | 293 | 191 | 10 | 19 | 281 | 28 | 292 | ParB domain-containing protein | ParB domain-containing protein | | afdb-uniprot50 | AF-A0A7V8DGJ8-F1-MODEL\_V4 | 1.0 | 5.153e-10 | 267 | 0.186 | 300 | 199 | 14 | 15 | 291 | 22 | 299 | Chromosome partitioning protein ParB family | Chromosome partitioning protein ParB family | | afdb-uniprot50 | AF-A0A2D7FLY9-F1-MODEL\_V4 | 1.0 | 6.247e-11 | 267 | 0.2 | 304 | 191 | 13 | 13 | 291 | 70 | 346 | Chromosome partitioning protein ParB | Chromosome partitioning protein ParB | | afdb-uniprot50 | AF-A0A1V5R4B4-F1-MODEL\_V4 | 1.0 | 1.654e-10 | 266 | 0.204 | 313 | 192 | 14 | 1 | 291 | 5 | 282 | Putative chromosome-partitioning protein ParB | Putative chromosome-partitioning protein ParB | | afdb-uniprot50 | AF-A0A7C2F5M6-F1-MODEL\_V4 | 1.0 | 4.278e-11 | 266 | 0.164 | 310 | 200 | 13 | 1 | 282 | 5 | 283 | ParB/RepB/Spo0J family partition protein | ParB/RepB/Spo0J family partition protein | | afdb-uniprot50 | AF-E6NHU5-F1-MODEL\_V4 | 1.0 | 8.851e-10 | 266 | 0.191 | 297 | 202 | 15 | 1 | 282 | 15 | 288 | Plasmid replication-partition related protein | Plasmid replication-partition related protein | | afdb-uniprot50 | AF-A0A1H8CWV8-F1-MODEL\_V4 | 1.0 | 3.814e-09 | 266 | 0.139 | 329 | 186 | 16 | 13 | 287 | 22 | 307 | Chromosome partitioning protein, ParB family | Chromosome partitioning protein, ParB family | | afdb-uniprot50 | AF-A0A7Y8ME42-F1-MODEL\_V4 | 1.0 | 2.289e-10 | 266 | 0.182 | 307 | 195 | 13 | 13 | 290 | 60 | 339 | ParB/RepB/Spo0J family partition protein | ParB/RepB/Spo0J family partition protein | | afdb-uniprot50 | AF-A0A3A6KCT7-F1-MODEL\_V4 | 1.0 | 1.017e-10 | 266 | 0.168 | 291 | 217 | 11 | 1 | 282 | 118 | 392 | ParB/RepB/Spo0J family partition protein | ParB/RepB/Spo0J family partition protein | | afdb-uniprot50 | AF-A0A7C6TI73-F1-MODEL\_V4 | 1.0 | 2.206e-07 | 266 | 0.2 | 170 | 123 | 7 | 14 | 176 | 383 | 546 | ParB/RepB/Spo0J family partition protein | ParB/RepB/Spo0J family partition protein | | afdb-uniprot50 | AF-B4RER5-F1-MODEL\_V4 | 1.0 | 1.397e-08 | 266 | 0.189 | 296 | 201 | 14 | 1 | 282 | 52 | 322 | Predicted transcriptional regulator | Predicted transcriptional regulator | | afdb-uniprot50 | AF-M4S4L0-F1-MODEL\_V4 | 1.0 | 1.324e-08 | 265 | 0.191 | 261 | 171 | 12 | 13 | 267 | 2 | 228 | ParB family protein | ParB family protein | | afdb-uniprot50 | AF-A0A1M6YGD4-F1-MODEL\_V4 | 1.0 | 7.301e-09 | 265 | 0.187 | 278 | 175 | 15 | 13 | 282 | 2 | 236 | Chromosome partitioning protein, ParB family | Chromosome partitioning protein, ParB family | | afdb-uniprot50 | AF-A0A1V4AMK9-F1-MODEL\_V4 | 1.0 | 1.099e-09 | 265 | 0.18 | 305 | 221 | 13 | 1 | 291 | 5 | 294 | HTH cro/C1-type domain-containing protein | HTH cro/C1-type domain-containing protein | | afdb-uniprot50 | AF-A0A7C1FDR3-F1-MODEL\_V4 | 1.0 | 1.133e-10 | 265 | 0.201 | 302 | 193 | 14 | 12 | 291 | 31 | 306 | ParB/RepB/Spo0J family partition protein | ParB/RepB/Spo0J family partition protein | | afdb-uniprot50 | AF-A0A235F0V7-F1-MODEL\_V4 | 1.0 | 3.423e-09 | 265 | 0.166 | 312 | 211 | 17 | 2 | 291 | 39 | 323 | ParB domain-containing protein | ParB domain-containing protein | | afdb-uniprot50 | AF-A0A2W4UM90-F1-MODEL\_V4 | 1.0 | 1.694e-09 | 265 | 0.167 | 311 | 191 | 20 | 14 | 291 | 67 | 342 | Chromosome partitioning protein ParB | Chromosome partitioning protein ParB | | afdb-uniprot50 | AF-A0A0N8Q4D0-F1-MODEL\_V4 | 1.0 | 2.206e-07 | 265 | 0.135 | 177 | 141 | 7 | 13 | 183 | 3 | 173 | Probable chromosome-partitioning protein ParB | Probable chromosome-partitioning protein ParB | | afdb-uniprot50 | AF-C1AAS4-F1-MODEL\_V4 | 1.0 | 3.613e-09 | 265 | 0.164 | 291 | 187 | 15 | 14 | 284 | 27 | 281 | ParB-like partition protein family protein | ParB-like partition protein family protein | | afdb-uniprot50 | AF-A0A355U2X2-F1-MODEL\_V4 | 1.0 | 1.066e-08 | 264 | 0.167 | 281 | 197 | 13 | 4 | 280 | 23 | 270 | Chromosome partitioning protein ParB | Chromosome partitioning protein ParB | | afdb-uniprot50 | AF-I9WSL1-F1-MODEL\_V4 | 1.0 | 3.932e-10 | 264 | 0.166 | 306 | 199 | 14 | 1 | 282 | 15 | 288 | Transcriptional regulator involved in chromosome partitioning ParB | Transcriptional regulator involved in chromosome partitioning ParB | | afdb-uniprot50 | AF-E2SKW7-F1-MODEL\_V4 | 1.0 | 4.457e-07 | 264 | 0.13 | 199 | 150 | 6 | 20 | 200 | 25 | 218 | ParB-like protein | ParB-like protein | | afdb-uniprot50 | AF-A0A1Z9AP12-F1-MODEL\_V4 | 1.0 | 2.612e-09 | 264 | 0.184 | 288 | 194 | 16 | 3 | 272 | 8 | 272 | HTH cro/C1-type domain-containing protein | HTH cro/C1-type domain-containing protein | | afdb-uniprot50 | AF-A0A364VD40-F1-MODEL\_V4 | 1.0 | 3.072e-09 | 264 | 0.186 | 295 | 193 | 15 | 13 | 282 | 165 | 437 | Chromosome partitioning protein ParB | Chromosome partitioning protein ParB | | afdb-uniprot50 | AF-A0A2E0EQ09-F1-MODEL\_V4 | 1.0 | 4.736e-09 | 263 | 0.162 | 302 | 198 | 14 | 4 | 282 | 12 | 281 | Probable chromosome-partitioning protein ParB | Probable chromosome-partitioning protein ParB | | afdb-uniprot50 | AF-A0A0Q6PTA8-F1-MODEL\_V4 | 1.0 | 9.343e-10 | 263 | 0.182 | 329 | 202 | 17 | 1 | 291 | 9 | 308 | Chromosome partitioning protein ParB | Chromosome partitioning protein ParB | | afdb-uniprot50 | AF-A0A554R345-F1-MODEL\_V4 | 1.0 | 2.344e-09 | 263 | 0.188 | 323 | 183 | 18 | 13 | 291 | 2 | 289 | ParB/RepB/Spo0J family partition protein | ParB/RepB/Spo0J family partition protein | | afdb-uniprot50 | AF-A0A0D5A6I8-F1-MODEL\_V4 | 1.0 | 1.092e-07 | 263 | 0.176 | 227 | 146 | 11 | 13 | 205 | 9 | 228 | Chromosome partitioning protein ParB | Chromosome partitioning protein ParB | | afdb-uniprot50 | AF-A0A3D3EDT8-F1-MODEL\_V4 | 1.0 | 2.891e-07 | 262 | 0.198 | 161 | 111 | 7 | 11 | 158 | 20 | 175 | Chromosome partitioning protein ParB | Chromosome partitioning protein ParB | | afdb-uniprot50 | AF-A0A512HN72-F1-MODEL\_V4 | 1.0 | 1.511e-07 | 262 | 0.16 | 199 | 145 | 9 | 18 | 205 | 1 | 188 | ParB domain-containing protein | ParB domain-containing protein | | afdb-uniprot50 | AF-A0A496G6Q8-F1-MODEL\_V4 | 1.0 | 2.612e-09 | 262 | 0.172 | 296 | 203 | 15 | 4 | 282 | 18 | 288 | Chromosome partitioning protein ParB | Chromosome partitioning protein ParB | | afdb-uniprot50 | AF-B8D0M7-F1-MODEL\_V4 | 1.0 | 3.423e-09 | 262 | 0.115 | 268 | 186 | 11 | 18 | 282 | 1 | 220 | ParB-like partition protein | ParB-like partition protein | | afdb-uniprot50 | AF-Q120X6-F1-MODEL\_V4 | 1.0 | 2.474e-09 | 262 | 0.16 | 312 | 193 | 17 | 14 | 291 | 56 | 332 | ParB family protein | ParB family protein | | afdb-uniprot50 | AF-A0A2E2Q773-F1-MODEL\_V4 | 1.0 | 4.736e-09 | 262 | 0.137 | 327 | 188 | 20 | 12 | 271 | 16 | 315 | Nuclease | Nuclease | | afdb-uniprot50 | AF-A0A538CC36-F1-MODEL\_V4 | 1.0 | 2.22e-09 | 261 | 0.198 | 212 | 156 | 6 | 1 | 205 | 3 | 207 | ParB/RepB/Spo0J family partition protein | ParB/RepB/Spo0J family partition protein | | afdb-uniprot50 | AF-A0A7W4KCA4-F1-MODEL\_V4 | 1.0 | 4.624e-10 | 261 | 0.162 | 302 | 192 | 14 | 12 | 288 | 32 | 297 | ParB/RepB/Spo0J family partition protein | ParB/RepB/Spo0J family partition protein | | afdb-uniprot50 | AF-A0A3E0VBU9-F1-MODEL\_V4 | 1.0 | 1.557e-08 | 261 | 0.154 | 266 | 167 | 15 | 19 | 257 | 30 | 264 | ParB domain-containing protein | ParB domain-containing protein | | afdb-uniprot50 | AF-A0A3L7PQ84-F1-MODEL\_V4 | 1.0 | 7.525e-10 | 261 | 0.152 | 302 | 213 | 15 | 13 | 290 | 108 | 390 | ParB/RepB/Spo0J family partition protein | ParB/RepB/Spo0J family partition protein | | afdb-uniprot50 | AF-A0A382M7S9-F1-MODEL\_V4 | 1.0 | 5.57e-09 | 260 | 0.213 | 187 | 137 | 6 | 19 | 201 | 33 | 213 | ParB domain-containing protein | ParB domain-containing protein | | afdb-uniprot50 | AF-A0A836SUD4-F1-MODEL\_V4 | 1.0 | 3.814e-09 | 260 | 0.157 | 267 | 184 | 13 | 20 | 269 | 30 | 272 | ParB/RepB/Spo0J family partition protein | ParB/RepB/Spo0J family partition protein | | afdb-uniprot50 | AF-H5Y0W1-F1-MODEL\_V4 | 1.0 | 3.637e-11 | 260 | 0.14 | 306 | 221 | 13 | 1 | 291 | 3 | 281 | ParB-like partition protein | ParB-like partition protein | | afdb-uniprot50 | AF-A0A0F6VZF3-F1-MODEL\_V4 | 1.0 | 1.254e-08 | 260 | 0.181 | 292 | 178 | 13 | 19 | 291 | 3 | 252 | Partition protein, phage-associated | Partition protein, phage-associated | | afdb-uniprot50 | AF-A0A4Q1SR21-F1-MODEL\_V4 | 1.0 | 1.18e-06 | 260 | 0.2 | 155 | 108 | 7 | 19 | 166 | 3 | 148 | ParB domain-containing protein | ParB domain-containing protein | | afdb-uniprot50 | AF-A0A662F1F4-F1-MODEL\_V4 | 1.0 | 5.404e-08 | 260 | 0.154 | 278 | 192 | 12 | 1 | 246 | 377 | 643 | Uncharacterized protein | Uncharacterized protein | | afdb-uniprot50 | AF-A0A497AWF1-F1-MODEL\_V4 | 1.0 | 4.123e-08 | 259 | 0.163 | 269 | 187 | 13 | 1 | 263 | 7 | 243 | ParB domain-containing protein | ParB domain-containing protein | | afdb-uniprot50 | AF-A0A1F6GQ66-F1-MODEL\_V4 | 1.0 | 2.344e-09 | 259 | 0.187 | 278 | 191 | 15 | 20 | 282 | 30 | 287 | HTH cro/C1-type domain-containing protein | HTH cro/C1-type domain-containing protein | | afdb-uniprot50 | AF-A0A2A6SDX0-F1-MODEL\_V4 | 1.0 | 1.293e-09 | 259 | 0.172 | 296 | 209 | 12 | 1 | 282 | 15 | 288 | Chromosome partitioning protein ParB | Chromosome partitioning protein ParB | | afdb-uniprot50 | AF-A0A293UGH0-F1-MODEL\_V4 | 1.0 | 7.525e-10 | 259 | 0.169 | 295 | 211 | 12 | 1 | 282 | 15 | 288 | Chromosome partitioning protein ParB | Chromosome partitioning protein ParB | | afdb-uniprot50 | AF-A0A174SUC7-F1-MODEL\_V4 | 1.0 | 6.061e-10 | 259 | 0.141 | 303 | 196 | 16 | 8 | 282 | 22 | 288 | Chromosome-partitioning protein parB | Chromosome-partitioning protein parB | | afdb-uniprot50 | AF-A0A3S0DKL5-F1-MODEL\_V4 | 1.0 | 6.207e-09 | 259 | 0.173 | 311 | 189 | 17 | 13 | 287 | 190 | 468 | ParB/RepB/Spo0J family partition protein | ParB/RepB/Spo0J family partition protein | | afdb-uniprot50 | AF-A0A4Q6E257-F1-MODEL\_V4 | 1.0 | 3.166e-10 | 258 | 0.18 | 315 | 192 | 14 | 1 | 282 | 9 | 290 | ParB/RepB/Spo0J family partition protein | ParB/RepB/Spo0J family partition protein | | afdb-uniprot50 | AF-A0A1F6RNA7-F1-MODEL\_V4 | 1.0 | 3.423e-09 | 258 | 0.162 | 264 | 181 | 11 | 7 | 246 | 23 | 270 | ParB domain-containing protein | ParB domain-containing protein | | afdb-uniprot50 | AF-A0A1D8AU14-F1-MODEL\_V4 | 1.0 | 7.656e-07 | 258 | 0.18 | 150 | 105 | 4 | 20 | 156 | 81 | 225 | ParB-like nuclease domain protein | ParB-like nuclease domain protein | | afdb-uniprot50 | AF-A0A353CWW4-F1-MODEL\_V4 | 1.0 | 7.129e-10 | 258 | 0.184 | 304 | 205 | 16 | 13 | 291 | 167 | 452 | ParB domain-containing protein | ParB domain-containing protein | | afdb-uniprot50 | AF-A0A5C7LNT5-F1-MODEL\_V4 | 1.0 | 1.921e-06 | 257 | 0.152 | 144 | 115 | 5 | 20 | 160 | 27 | 166 | Chromosome partitioning protein ParB | Chromosome partitioning protein ParB | | afdb-uniprot50 | AF-A0A2M7AKL3-F1-MODEL\_V4 | 1.0 | 4.278e-11 | 257 | 0.208 | 307 | 192 | 17 | 7 | 291 | 6 | 283 | ParB domain-containing protein | ParB domain-containing protein | | afdb-uniprot50 | AF-A0A133UR66-F1-MODEL\_V4 | 1.0 | 6.022e-08 | 257 | 0.142 | 295 | 215 | 12 | 4 | 289 | 20 | 285 | ParB domain-containing protein | ParB domain-containing protein | | afdb-uniprot50 | AF-A0A2T6WAW2-F1-MODEL\_V4 | 1.0 | 6.398e-10 | 257 | 0.189 | 301 | 198 | 16 | 1 | 282 | 15 | 288 | Chromosome partitioning protein ParB | Chromosome partitioning protein ParB | | afdb-uniprot50 | AF-A0A7X7RYK0-F1-MODEL\_V4 | 1.0 | 1.694e-09 | 257 | 0.132 | 303 | 212 | 13 | 19 | 291 | 44 | 325 | ParB/RepB/Spo0J family partition protein | ParB/RepB/Spo0J family partition protein | | afdb-uniprot50 | AF-A0A1F0CX37-F1-MODEL\_V4 | 1.0 | 7.944e-10 | 257 | 0.183 | 311 | 200 | 13 | 13 | 290 | 67 | 356 | ParB domain-containing protein | ParB domain-containing protein | | afdb-uniprot50 | AF-A0A2N2T789-F1-MODEL\_V4 | 1.0 | 1.888e-09 | 256 | 0.205 | 273 | 171 | 13 | 39 | 287 | 1 | 251 | Chromosome partitioning protein ParB | Chromosome partitioning protein ParB | | afdb-uniprot50 | AF-A0A7C1ZJF9-F1-MODEL\_V4 | 1.0 | 7.944e-10 | 256 | 0.199 | 281 | 178 | 12 | 21 | 287 | 2 | 249 | ParB/RepB/Spo0J family partition protein | ParB/RepB/Spo0J family partition protein | | afdb-uniprot50 | AF-A0A0R1QC79-F1-MODEL\_V4 | 1.0 | 5.88e-09 | 256 | 0.181 | 287 | 190 | 15 | 13 | 271 | 12 | 281 | Chromosome partitioning protein, DNA-binding protein | Chromosome partitioning protein, DNA-binding protein | | afdb-uniprot50 | AF-A0A2E8TD00-F1-MODEL\_V4 | 1.0 | 9.863e-10 | 256 | 0.154 | 305 | 213 | 12 | 4 | 289 | 11 | 289 | ParB domain-containing protein | ParB domain-containing protein | | afdb-uniprot50 | AF-A0A2D7NEY1-F1-MODEL\_V4 | 1.0 | 8.135e-09 | 256 | 0.16 | 280 | 202 | 13 | 20 | 282 | 23 | 286 | Chromosome partitioning protein ParB | Chromosome partitioning protein ParB | | afdb-uniprot50 | AF-A0A4Q5N154-F1-MODEL\_V4 | 1.0 | 7.301e-09 | 256 | 0.147 | 319 | 193 | 15 | 12 | 271 | 7 | 305 | Nuclease | Nuclease | | afdb-uniprot50 | AF-A0A3D4V4B5-F1-MODEL\_V4 | 1.0 | 7.706e-09 | 256 | 0.164 | 292 | 190 | 15 | 12 | 284 | 143 | 399 | ParB/RepB/Spo0J family partition protein | ParB/RepB/Spo0J family partition protein | | afdb-uniprot50 | AF-A0A5E7N365-F1-MODEL\_V4 | 1.0 | 2.739e-07 | 255 | 0.261 | 191 | 116 | 5 | 98 | 287 | 7 | 173 | Uncharacterized protein | Uncharacterized protein | | afdb-uniprot50 | AF-A0A496RX12-F1-MODEL\_V4 | 1.0 | 1.125e-08 | 255 | 0.123 | 275 | 214 | 12 | 19 | 282 | 12 | 270 | Uncharacterized protein | Uncharacterized protein | | afdb-uniprot50 | AF-A0A4R1KCC4-F1-MODEL\_V4 | 1.0 | 1.233e-11 | 255 | 0.197 | 298 | 198 | 13 | 1 | 282 | 3 | 275 | ParB family chromosome partitioning protein | ParB family chromosome partitioning protein | | afdb-uniprot50 | AF-A0A134BLW4-F1-MODEL\_V4 | 1.0 | 6.398e-10 | 255 | 0.16 | 287 | 210 | 10 | 13 | 282 | 23 | 295 | Putative stage 0 sporulation protein J | Putative stage 0 sporulation protein J | | afdb-uniprot50 | AF-A0A7W1TPW5-F1-MODEL\_V4 | 1.0 | 3.423e-09 | 255 | 0.173 | 289 | 194 | 13 | 7 | 286 | 28 | 280 | ParB/RepB/Spo0J family partition protein | ParB/RepB/Spo0J family partition protein | | afdb-uniprot50 | AF-A0A3M2F058-F1-MODEL\_V4 | 1.0 | 5.153e-10 | 255 | 0.178 | 303 | 183 | 16 | 14 | 282 | 57 | 327 | ParB/RepB/Spo0J family partition protein | ParB/RepB/Spo0J family partition protein | | afdb-uniprot50 | AF-A0A2M7JUR1-F1-MODEL\_V4 | 1.0 | 2.041e-08 | 255 | 0.189 | 258 | 158 | 12 | 19 | 246 | 1 | 237 | ParB domain-containing protein | ParB domain-containing protein | | afdb-uniprot50 | AF-A0A850QHF8-F1-MODEL\_V4 | 1.0 | 2.274e-08 | 255 | 0.119 | 317 | 209 | 16 | 4 | 287 | 59 | 338 | ParB/RepB/Spo0J family partition protein | ParB/RepB/Spo0J family partition protein | | afdb-uniprot50 | AF-A0A1C3XLZ9-F1-MODEL\_V4 | 1.0 | 1.475e-08 | 255 | 0.153 | 287 | 201 | 13 | 18 | 291 | 1 | 258 | ParB/RepB/Spo0J family partition protein | ParB/RepB/Spo0J family partition protein | | afdb-uniprot50 | AF-A0A1F1V577-F1-MODEL\_V4 | 1.0 | 1.066e-08 | 255 | 0.175 | 296 | 181 | 15 | 19 | 282 | 156 | 420 | ParB domain-containing protein | ParB domain-containing protein | | afdb-uniprot50 | AF-A0A1G3M1J8-F1-MODEL\_V4 | 1.0 | 6.509e-07 | 254 | 0.161 | 161 | 123 | 7 | 13 | 168 | 17 | 170 | ParB domain-containing protein | ParB domain-containing protein | | afdb-uniprot50 | AF-A0A349XYG3-F1-MODEL\_V4 | 1.0 | 2.154e-08 | 254 | 0.166 | 289 | 194 | 15 | 18 | 291 | 1 | 257 | Plasmid partitioning protein | Plasmid partitioning protein | | afdb-uniprot50 | AF-A0A0F9RH44-F1-MODEL\_V4 | 1.0 | 1.315e-06 | 254 | 0.171 | 163 | 124 | 5 | 18 | 173 | 1 | 159 | Uncharacterized protein | Uncharacterized protein | | afdb-uniprot50 | AF-A0A1G5EUA4-F1-MODEL\_V4 | 1.0 | 9.568e-09 | 254 | 0.175 | 285 | 186 | 15 | 1 | 265 | 9 | 264 | Chromosome partitioning protein, ParB family | Chromosome partitioning protein, ParB family | | afdb-uniprot50 | AF-A0A853ERN6-F1-MODEL\_V4 | 1.0 | 1.993e-09 | 254 | 0.179 | 346 | 168 | 19 | 19 | 291 | 3 | 305 | ParB/RepB/Spo0J family partition protein | ParB/RepB/Spo0J family partition protein | | afdb-uniprot50 | AF-A0A5S4SWL2-F1-MODEL\_V4 | 1.0 | 1.993e-09 | 254 | 0.177 | 282 | 193 | 13 | 1 | 272 | 4 | 256 | ParB/RepB/Spo0J family partition protein | ParB/RepB/Spo0J family partition protein | | afdb-uniprot50 | AF-A0A536HIP7-F1-MODEL\_V4 | 1.0 | 2.274e-08 | 253 | 0.188 | 186 | 143 | 4 | 21 | 202 | 1 | 182 | ParB/RepB/Spo0J family partition protein | ParB/RepB/Spo0J family partition protein | | afdb-uniprot50 | AF-A0A846M3I8-F1-MODEL\_V4 | 1.0 | 1.694e-09 | 253 | 0.176 | 266 | 199 | 10 | 23 | 282 | 2 | 253 | ParB family chromosome partitioning protein | ParB family chromosome partitioning protein | | afdb-uniprot50 | AF-A0A5C0UI65-F1-MODEL\_V4 | 1.0 | 1.254e-08 | 253 | 0.126 | 292 | 188 | 12 | 13 | 287 | 31 | 272 | Probable chromosome-partitioning protein ParB | Probable chromosome-partitioning protein ParB | | afdb-uniprot50 | AF-A0A134AFD9-F1-MODEL\_V4 | 1.0 | 2.675e-08 | 253 | 0.124 | 273 | 202 | 11 | 1 | 246 | 4 | 266 | ParB-like protein | ParB-like protein | | afdb-uniprot50 | AF-A0A139JRJ0-F1-MODEL\_V4 | 1.0 | 3.528e-10 | 253 | 0.182 | 279 | 193 | 10 | 1 | 271 | 6 | 257 | ParB-like protein | ParB-like protein | | afdb-uniprot50 | AF-A0A3N4RJM6-F1-MODEL\_V4 | 1.0 | 1.485e-10 | 253 | 0.195 | 307 | 209 | 17 | 5 | 291 | 61 | 349 | ParB family chromosome partitioning protein | ParB family chromosome partitioning protein | | afdb-uniprot50 | AF-A0A1H0AXS4-F1-MODEL\_V4 | 1.0 | 8.794e-08 | 252 | 0.166 | 276 | 188 | 13 | 18 | 281 | 1 | 246 | ParB/RepB/Spo0J family partition protein | ParB/RepB/Spo0J family partition protein | | afdb-uniprot50 | AF-A0A2V7WSH1-F1-MODEL\_V4 | 1.0 | 1.946e-10 | 251 | 0.236 | 254 | 172 | 11 | 13 | 256 | 18 | 259 | Chromosome partitioning protein ParB | Chromosome partitioning protein ParB | | afdb-uniprot50 | AF-A0A1W9SAC9-F1-MODEL\_V4 | 1.0 | 1.993e-09 | 251 | 0.161 | 298 | 204 | 15 | 9 | 280 | 20 | 297 | Probable chromosome-partitioning protein ParB | Probable chromosome-partitioning protein ParB | | afdb-uniprot50 | AF-A0A7C6PVT3-F1-MODEL\_V4 | 1.0 | 7.944e-10 | 251 | 0.18 | 311 | 194 | 14 | 2 | 275 | 1 | 287 | ParB/RepB/Spo0J family partition protein | ParB/RepB/Spo0J family partition protein | | afdb-uniprot50 | AF-A0A2N2BDG8-F1-MODEL\_V4 | 1.0 | 3.906e-08 | 251 | 0.146 | 226 | 150 | 4 | 13 | 199 | 19 | 240 | Chromosome partitioning protein ParB | Chromosome partitioning protein ParB | | afdb-uniprot50 | AF-A0A1Q3TKN1-F1-MODEL\_V4 | 1.0 | 1.735e-08 | 251 | 0.177 | 281 | 180 | 15 | 19 | 283 | 44 | 289 | ParB domain-containing protein | ParB domain-containing protein | | afdb-uniprot50 | AF-A0A1R1LSW7-F1-MODEL\_V4 | 1.0 | 5.57e-09 | 251 | 0.189 | 327 | 164 | 14 | 1 | 273 | 1 | 280 | ParB domain-containing protein | ParB domain-containing protein | | afdb-uniprot50 | AF-A0A7X7NNE2-F1-MODEL\_V4 | 1.0 | 1.324e-08 | 251 | 0.18 | 255 | 160 | 10 | 13 | 247 | 50 | 275 | ParB/RepB/Spo0J family partition protein | ParB/RepB/Spo0J family partition protein | | afdb-uniprot50 | AF-A0A0F9PBG4-F1-MODEL\_V4 | 1.0 | 1.284e-07 | 250 | 0.133 | 233 | 178 | 8 | 18 | 245 | 1 | 214 | ParB domain-containing protein | ParB domain-containing protein | | afdb-uniprot50 | AF-A0A800BNS5-F1-MODEL\_V4 | 1.0 | 5.842e-07 | 249 | 0.225 | 155 | 107 | 8 | 1 | 148 | 4 | 152 | ParB/RepB/Spo0J family partition protein | ParB/RepB/Spo0J family partition protein | | afdb-uniprot50 | AF-A0A6M0L040-F1-MODEL\_V4 | 1.0 | 6.356e-08 | 249 | 0.175 | 205 | 156 | 9 | 4 | 200 | 14 | 213 | ParB/RepB/Spo0J family partition protein | ParB/RepB/Spo0J family partition protein | | afdb-uniprot50 | AF-A0A4Y4W985-F1-MODEL\_V4 | 1.0 | 8.385e-10 | 249 | 0.157 | 304 | 204 | 12 | 1 | 282 | 15 | 288 | Chromosome partitioning protein ParB | Chromosome partitioning protein ParB | | afdb-uniprot50 | AF-A0A3D8IE22-F1-MODEL\_V4 | 1.0 | 1.188e-08 | 249 | 0.142 | 308 | 210 | 15 | 4 | 282 | 18 | 300 | Chromosome partitioning protein ParB | Chromosome partitioning protein ParB | | afdb-uniprot50 | AF-A0A2H1L4V2-F1-MODEL\_V4 | 1.0 | 2.344e-09 | 249 | 0.189 | 316 | 203 | 17 | 1 | 291 | 27 | 314 | Chromosome partitioning protein, ParB family | Chromosome partitioning protein, ParB family | | afdb-uniprot50 | AF-A0A430DXP8-F1-MODEL\_V4 | 1.0 | 2.534e-08 | 249 | 0.142 | 295 | 204 | 14 | 19 | 290 | 51 | 319 | ParB domain-containing protein | ParB domain-containing protein | | afdb-uniprot50 | AF-A0A2E4QU58-F1-MODEL\_V4 | 1.0 | 2.842e-10 | 249 | 0.16 | 317 | 206 | 15 | 2 | 291 | 57 | 340 | Chromosome partitioning protein ParB | Chromosome partitioning protein ParB | | afdb-uniprot50 | AF-A0A7C3VNC3-F1-MODEL\_V4 | 1.0 | 1.724e-06 | 249 | 0.182 | 159 | 119 | 6 | 14 | 167 | 39 | 191 | ParB domain-containing protein | ParB domain-containing protein | | afdb-uniprot50 | AF-A0A7W6T8F3-F1-MODEL\_V4 | 1.0 | 7.706e-09 | 249 | 0.146 | 301 | 187 | 17 | 18 | 291 | 1 | 258 | ParB family chromosome partitioning protein | ParB family chromosome partitioning protein | | afdb-uniprot50 | AF-A0A7W4IY87-F1-MODEL\_V4 | 1.0 | 9.343e-10 | 248 | 0.178 | 292 | 186 | 13 | 14 | 283 | 34 | 293 | ParB/RepB/Spo0J family partition protein | ParB/RepB/Spo0J family partition protein | | afdb-uniprot50 | AF-A0A318SDS1-F1-MODEL\_V4 | 1.0 | 8.135e-09 | 248 | 0.2 | 274 | 158 | 15 | 13 | 245 | 24 | 277 | ParB family protein | ParB family protein | | afdb-uniprot50 | AF-A0A3C1FVK0-F1-MODEL\_V4 | 1.0 | 7.706e-09 | 248 | 0.144 | 304 | 203 | 16 | 6 | 287 | 30 | 298 | ParB domain-containing protein | ParB domain-containing protein | | afdb-uniprot50 | AF-A0A7K1PX19-F1-MODEL\_V4 | 1.0 | 1.831e-08 | 248 | 0.168 | 290 | 193 | 14 | 18 | 291 | 1 | 258 | ParB/RepB/Spo0J family partition protein | ParB/RepB/Spo0J family partition protein | | afdb-uniprot50 | AF-K9D005-F1-MODEL\_V4 | 1.0 | 9.799e-08 | 248 | 0.157 | 279 | 186 | 14 | 18 | 284 | 1 | 242 | ParB-like partition protein | ParB-like partition protein | | afdb-uniprot50 | AF-A0A318KWI1-F1-MODEL\_V4 | 1.0 | 7.476e-08 | 247 | 0.261 | 199 | 118 | 9 | 104 | 291 | 10 | 190 | KorB domain-containing protein | KorB domain-containing protein | | afdb-uniprot50 | AF-A0A646LUJ1-F1-MODEL\_V4 | 1.0 | 2.4e-08 | 247 | 0.127 | 275 | 199 | 12 | 19 | 282 | 7 | 251 | ParB/RepB/Spo0J family partition protein | ParB/RepB/Spo0J family partition protein | | afdb-uniprot50 | AF-K9F001-F1-MODEL\_V4 | 1.0 | 9.568e-09 | 247 | 0.143 | 328 | 210 | 15 | 4 | 291 | 17 | 313 | ParB-like partition protein | ParB-like partition protein | | afdb-uniprot50 | AF-A0A1Q3T1A0-F1-MODEL\_V4 | 1.0 | 1.788e-09 | 247 | 0.177 | 316 | 204 | 20 | 5 | 289 | 32 | 322 | ParB domain-containing protein | ParB domain-containing protein | | afdb-uniprot50 | AF-A0A3E0V9I7-F1-MODEL\_V4 | 1.0 | 4.352e-08 | 247 | 0.149 | 281 | 176 | 15 | 4 | 257 | 60 | 304 | ParB domain-containing protein | ParB domain-containing protein | | afdb-uniprot50 | AF-A0A2H9LML8-F1-MODEL\_V4 | 1.0 | 1.98e-07 | 246 | 0.13 | 300 | 168 | 18 | 16 | 291 | 1 | 231 | ParB domain-containing protein | ParB domain-containing protein | | afdb-uniprot50 | AF-A0A2T2QZM2-F1-MODEL\_V4 | 1.0 | 4.881e-10 | 246 | 0.196 | 290 | 190 | 13 | 13 | 284 | 34 | 298 | ParB domain-containing protein | ParB domain-containing protein | | afdb-uniprot50 | AF-A0A135IPZ6-F1-MODEL\_V4 | 1.0 | 3.146e-08 | 246 | 0.193 | 186 | 136 | 7 | 18 | 196 | 1 | 179 | ParB domain-containing protein | ParB domain-containing protein | | afdb-uniprot50 | AF-A0A3A6MUC0-F1-MODEL\_V4 | 1.0 | 2.385e-06 | 246 | 0.151 | 185 | 143 | 9 | 19 | 193 | 49 | 229 | ParB/RepB/Spo0J family partition protein | ParB/RepB/Spo0J family partition protein | | afdb-uniprot50 | AF-A0A521TB26-F1-MODEL\_V4 | 1.0 | 1.777e-07 | 245 | 0.129 | 271 | 172 | 12 | 19 | 282 | 7 | 220 | ParB/RepB/Spo0J family partition protein | ParB/RepB/Spo0J family partition protein | | afdb-uniprot50 | AF-A0A077FQM9-F1-MODEL\_V4 | 1.0 | 1.188e-08 | 245 | 0.155 | 308 | 180 | 17 | 5 | 278 | 62 | 323 | Probable chromosome-partitioning protein ParB | Probable chromosome-partitioning protein ParB | | afdb-uniprot50 | AF-A0A432MEU0-F1-MODEL\_V4 | 1.0 | 2.416e-10 | 245 | 0.167 | 329 | 210 | 13 | 1 | 291 | 37 | 339 | ParB/RepB/Spo0J family partition protein | ParB/RepB/Spo0J family partition protein | | afdb-uniprot50 | AF-A0A3D3Q403-F1-MODEL\_V4 | 1.0 | 1.066e-08 | 244 | 0.175 | 194 | 150 | 6 | 14 | 205 | 17 | 202 | Chromosome partitioning protein ParB | Chromosome partitioning protein ParB | | afdb-uniprot50 | AF-A0A2E8Y707-F1-MODEL\_V4 | 1.0 | 4.881e-10 | 244 | 0.198 | 277 | 192 | 11 | 19 | 284 | 21 | 278 | ParB domain-containing protein | ParB domain-containing protein | | afdb-uniprot50 | AF-A0A316RSR3-F1-MODEL\_V4 | 1.0 | 5.607e-11 | 244 | 0.193 | 316 | 195 | 15 | 1 | 284 | 5 | 292 | Stage 0 sporulation protein J | Stage 0 sporulation protein J | | afdb-uniprot50 | AF-A0A2M9YJ10-F1-MODEL\_V4 | 1.0 | 1.254e-08 | 244 | 0.171 | 268 | 185 | 16 | 1 | 246 | 32 | 284 | Chromophore lyase | Chromophore lyase | | afdb-uniprot50 | AF-A0A1I1EQZ5-F1-MODEL\_V4 | 1.0 | 1.324e-08 | 244 | 0.197 | 192 | 139 | 8 | 13 | 195 | 19 | 204 | ParB/RepB/Spo0J family partition protein | ParB/RepB/Spo0J family partition protein | | afdb-uniprot50 | AF-A0A0D5MBW0-F1-MODEL\_V4 | 1.0 | 6.552e-09 | 244 | 0.167 | 298 | 195 | 16 | 4 | 271 | 15 | 289 | ParB-like partition protein | ParB-like partition protein | | afdb-uniprot50 | AF-A0A7X6YTY0-F1-MODEL\_V4 | 1.0 | 3.637e-11 | 243 | 0.176 | 301 | 208 | 18 | 3 | 282 | 27 | 308 | ParB/RepB/Spo0J family partition protein | ParB/RepB/Spo0J family partition protein | | afdb-uniprot50 | AF-F7UI03-F1-MODEL\_V4 | 1.0 | 3.7e-08 | 243 | 0.15 | 305 | 211 | 16 | 1 | 291 | 75 | 345 | Chromosome partitioning protein | Chromosome partitioning protein | | afdb-uniprot50 | AF-A0A0C1N062-F1-MODEL\_V4 | 1.0 | 2.274e-08 | 242 | 0.13 | 292 | 185 | 16 | 13 | 287 | 26 | 265 | Probable chromosome-partitioning protein ParB | Probable chromosome-partitioning protein ParB | | afdb-uniprot50 | AF-A0A7C6ZXT2-F1-MODEL\_V4 | 1.0 | 1.644e-08 | 242 | 0.183 | 273 | 185 | 10 | 13 | 271 | 6 | 254 | ParB/RepB/Spo0J family partition protein | ParB/RepB/Spo0J family partition protein | | afdb-uniprot50 | AF-A0A2D7KGV3-F1-MODEL\_V4 | 1.0 | 6.753e-10 | 242 | 0.169 | 307 | 201 | 13 | 3 | 282 | 11 | 290 | Chromosome partitioning protein ParB | Chromosome partitioning protein ParB | | afdb-uniprot50 | AF-A0A066YW29-F1-MODEL\_V4 | 1.0 | 1.406e-10 | 242 | 0.211 | 312 | 202 | 17 | 1 | 290 | 21 | 310 | Plasmid partitioning protein ParB | Plasmid partitioning protein ParB | | afdb-uniprot50 | AF-A0A1Y3TUR7-F1-MODEL\_V4 | 1.0 | 1.431e-07 | 242 | 0.107 | 260 | 191 | 9 | 20 | 243 | 24 | 278 | ParB domain-containing protein | ParB domain-containing protein | | afdb-uniprot50 | AF-A0A7V4MZE4-F1-MODEL\_V4 | 1.0 | 8.851e-10 | 242 | 0.149 | 314 | 203 | 14 | 19 | 291 | 27 | 317 | ParB/RepB/Spo0J family partition protein | ParB/RepB/Spo0J family partition protein | | afdb-uniprot50 | AF-A0A5K1NR86-F1-MODEL\_V4 | 1.0 | 1.324e-08 | 242 | 0.156 | 307 | 198 | 13 | 1 | 273 | 6 | 285 | ParB domain-containing protein | ParB domain-containing protein | | afdb-uniprot50 | AF-A0A3E2TLN0-F1-MODEL\_V4 | 1.0 | 1.225e-09 | 241 | 0.161 | 298 | 207 | 12 | 4 | 283 | 38 | 310 | ParB/RepB/Spo0J family partition protein | ParB/RepB/Spo0J family partition protein | | afdb-uniprot50 | AF-A0A0K2SP45-F1-MODEL\_V4 | 1.0 | 4.486e-09 | 241 | 0.148 | 282 | 180 | 12 | 19 | 291 | 3 | 233 | Chromosome partitioning protein ParB | Chromosome partitioning protein ParB | | afdb-uniprot50 | AF-A0A3G9J1Q0-F1-MODEL\_V4 | 1.0 | 1.017e-10 | 241 | 0.204 | 289 | 192 | 13 | 19 | 291 | 75 | 341 | Chromosome partitioning protein ParB | Chromosome partitioning protein ParB | | afdb-uniprot50 | AF-A0A517ZB25-F1-MODEL\_V4 | 1.0 | 1.98e-07 | 241 | 0.209 | 196 | 123 | 12 | 19 | 206 | 4 | 175 | DNA methylase | DNA methylase | | afdb-uniprot50 | AF-A0A348VT13-F1-MODEL\_V4 | 1.0 | 4.25e-09 | 240 | 0.159 | 182 | 136 | 5 | 18 | 195 | 1 | 169 | Chromosome partitioning protein ParB | Chromosome partitioning protein ParB | | afdb-uniprot50 | AF-A0A6L7GCN8-F1-MODEL\_V4 | 1.0 | 6.552e-09 | 240 | 0.229 | 222 | 157 | 11 | 19 | 234 | 17 | 230 | ParB/RepB/Spo0J family partition protein | ParB/RepB/Spo0J family partition protein | | afdb-uniprot50 | AF-A0A0W0ZRH2-F1-MODEL\_V4 | 1.0 | 7.129e-10 | 240 | 0.182 | 285 | 198 | 12 | 13 | 282 | 13 | 277 | Probable chromosome-partitioning protein ParB | Probable chromosome-partitioning protein ParB | | afdb-uniprot50 | AF-A0A2M7R6K7-F1-MODEL\_V4 | 1.0 | 1.293e-09 | 240 | 0.197 | 284 | 197 | 13 | 13 | 284 | 24 | 288 | ParB domain-containing protein | ParB domain-containing protein | | afdb-uniprot50 | AF-A0A850MS07-F1-MODEL\_V4 | 1.0 | 1.683e-07 | 240 | 0.214 | 233 | 153 | 11 | 19 | 246 | 3 | 210 | ParB/RepB/Spo0J family partition protein | ParB/RepB/Spo0J family partition protein | | afdb-uniprot50 | AF-A0A1G9MR58-F1-MODEL\_V4 | 1.0 | 7.656e-07 | 240 | 0.115 | 217 | 165 | 11 | 2 | 204 | 26 | 229 | Chromosome partitioning protein, ParB family | Chromosome partitioning protein, ParB family | | afdb-uniprot50 | AF-A0A7R8P8J7-F1-MODEL\_V4 | 1.0 | 3.222e-07 | 240 | 0.175 | 228 | 140 | 13 | 13 | 205 | 3 | 217 | ParB family chromosome partitioning protein | ParB family chromosome partitioning protein | | afdb-uniprot50 | AF-A0A729SM52-F1-MODEL\_V4 | 1.0 | 2.757e-09 | 239 | 0.2 | 225 | 151 | 11 | 1 | 214 | 6 | 212 | ParB/RepB/Spo0J family partition protein | ParB/RepB/Spo0J family partition protein | | afdb-uniprot50 | AF-A0A536BIE7-F1-MODEL\_V4 | 1.0 | 1.254e-08 | 239 | 0.2 | 210 | 154 | 7 | 3 | 205 | 15 | 217 | ParB/RepB/Spo0J family partition protein | ParB/RepB/Spo0J family partition protein | | afdb-uniprot50 | AF-A0A377TSX5-F1-MODEL\_V4 | 1.0 | 1.284e-07 | 239 | 0.172 | 266 | 162 | 13 | 37 | 290 | 36 | 255 | PRTRC system ParB family protein | PRTRC system ParB family protein | | afdb-uniprot50 | AF-A0A0F5MQM1-F1-MODEL\_V4 | 1.0 | 7.892e-08 | 239 | 0.148 | 310 | 191 | 13 | 1 | 289 | 21 | 278 | Probable chromosome-partitioning protein ParB | Probable chromosome-partitioning protein ParB | | afdb-uniprot50 | AF-A0A6H3NI83-F1-MODEL\_V4 | 1.0 | 1.735e-08 | 239 | 0.164 | 267 | 188 | 12 | 7 | 246 | 13 | 271 | ParB/RepB/Spo0J family partition protein | ParB/RepB/Spo0J family partition protein | | afdb-uniprot50 | AF-A0A3A8HK35-F1-MODEL\_V4 | 1.0 | 4.352e-08 | 239 | 0.153 | 312 | 183 | 15 | 1 | 286 | 6 | 262 | ParB/RepB/Spo0J family partition protein | ParB/RepB/Spo0J family partition protein | | afdb-uniprot50 | AF-A0A1V6A6D7-F1-MODEL\_V4 | 1.0 | 2.235e-11 | 239 | 0.166 | 295 | 208 | 12 | 5 | 282 | 18 | 291 | Putative chromosome-partitioning protein ParB | Putative chromosome-partitioning protein ParB | | afdb-uniprot50 | AF-A0A2S9K0I3-F1-MODEL\_V4 | 1.0 | 1.217e-07 | 239 | 0.169 | 306 | 190 | 18 | 13 | 291 | 34 | 302 | Chromosome partitioning protein ParB | Chromosome partitioning protein ParB | | afdb-uniprot50 | AF-C2GFK6-F1-MODEL\_V4 | 1.0 | 4.624e-10 | 239 | 0.164 | 285 | 190 | 13 | 20 | 282 | 76 | 334 | ParB-like protein | ParB-like protein | | afdb-uniprot50 | AF-A0A2H9SV72-F1-MODEL\_V4 | 1.0 | 1.746e-10 | 238 | 0.188 | 302 | 197 | 15 | 1 | 282 | 1 | 274 | Chromosome partitioning protein | Chromosome partitioning protein | | afdb-uniprot50 | AF-A0A538PBW6-F1-MODEL\_V4 | 1.0 | 1.118e-06 | 238 | 0.171 | 221 | 144 | 10 | 12 | 201 | 7 | 219 | ParB/RepB/Spo0J family partition protein | ParB/RepB/Spo0J family partition protein | | afdb-uniprot50 | AF-A0A6L7U0D4-F1-MODEL\_V4 | 1.0 | 7.944e-10 | 238 | 0.183 | 321 | 206 | 15 | 1 | 289 | 11 | 307 | ParB/RepB/Spo0J family partition protein | ParB/RepB/Spo0J family partition protein | | afdb-uniprot50 | AF-A0A0R2KRI4-F1-MODEL\_V4 | 1.0 | 3.814e-09 | 238 | 0.2 | 290 | 177 | 16 | 13 | 271 | 15 | 280 | Chromosome partitioning protein, DNA-binding protein | Chromosome partitioning protein, DNA-binding protein | | afdb-uniprot50 | AF-A0A3D4JLM1-F1-MODEL\_V4 | 1.0 | 6.207e-09 | 238 | 0.151 | 311 | 194 | 11 | 1 | 246 | 1 | 306 | Chromosome partitioning protein ParB | Chromosome partitioning protein ParB | | afdb-uniprot50 | AF-A0A345J9F5-F1-MODEL\_V4 | 1.0 | 4.352e-08 | 238 | 0.15 | 280 | 188 | 15 | 7 | 273 | 65 | 307 | Partitioning protein, ParB family | Partitioning protein, ParB family | | afdb-uniprot50 | AF-A0A2N3GCV6-F1-MODEL\_V4 | 1.0 | 2.98e-08 | 238 | 0.186 | 193 | 145 | 8 | 14 | 199 | 190 | 377 | ParB domain-containing protein | ParB domain-containing protein | | afdb-uniprot50 | AF-A0A4Q3IEN1-F1-MODEL\_V4 | 1.0 | 2.534e-08 | 237 | 0.177 | 186 | 142 | 6 | 4 | 182 | 9 | 190 | ParB/RepB/Spo0J family partition protein | ParB/RepB/Spo0J family partition protein | | afdb-uniprot50 | AF-A0A1U7GP86-F1-MODEL\_V4 | 1.0 | 5.842e-07 | 237 | 0.159 | 257 | 162 | 13 | 23 | 246 | 2 | 237 | ParB domain-containing protein | ParB domain-containing protein | | afdb-uniprot50 | AF-A0A2H0NAZ6-F1-MODEL\_V4 | 1.0 | 4.85e-08 | 236 | 0.139 | 294 | 198 | 14 | 13 | 287 | 1 | 258 | ParB domain-containing protein | ParB domain-containing protein | | afdb-uniprot50 | AF-A0A535EWL3-F1-MODEL\_V4 | 1.0 | 1.356e-07 | 236 | 0.142 | 287 | 188 | 12 | 7 | 271 | 55 | 305 | ParB/RepB/Spo0J family partition protein | ParB/RepB/Spo0J family partition protein | | afdb-uniprot50 | AF-A0A523SL81-F1-MODEL\_V4 | 1.0 | 2.385e-06 | 236 | 0.167 | 191 | 143 | 10 | 19 | 202 | 4 | 185 | ParB/RepB/Spo0J family partition protein | ParB/RepB/Spo0J family partition protein | | afdb-uniprot50 | AF-A0A1W2CT08-F1-MODEL\_V4 | 1.0 | 3.423e-09 | 236 | 0.16 | 343 | 198 | 14 | 18 | 287 | 1 | 326 | Chromosome partitioning protein, ParB family | Chromosome partitioning protein, ParB family | | afdb-uniprot50 | AF-A0A7C7L2N7-F1-MODEL\_V4 | 1.0 | 1.921e-06 | 235 | 0.217 | 138 | 99 | 6 | 9 | 142 | 20 | 152 | ParB/RepB/Spo0J family partition protein | ParB/RepB/Spo0J family partition protein | | afdb-uniprot50 | AF-A0A3D1XDE0-F1-MODEL\_V4 | 1.0 | 9.124e-11 | 235 | 0.184 | 298 | 214 | 11 | 3 | 287 | 10 | 291 | ParB domain-containing protein | ParB domain-containing protein | | afdb-uniprot50 | AF-A0A6M3M0X8-F1-MODEL\_V4 | 1.0 | 3.505e-08 | 235 | 0.159 | 308 | 207 | 16 | 13 | 291 | 2 | 286 | KorB domain-containing protein | KorB domain-containing protein | | afdb-uniprot50 | AF-D7BJI1-F1-MODEL\_V4 | 1.0 | 9.006e-07 | 235 | 0.185 | 216 | 142 | 12 | 19 | 205 | 1 | 211 | ParB-like partition protein | ParB-like partition protein | | afdb-uniprot50 | AF-A0A3D4YD69-F1-MODEL\_V4 | 1.0 | 2.154e-08 | 234 | 0.125 | 294 | 187 | 17 | 13 | 287 | 30 | 272 | ParB domain-containing protein | ParB domain-containing protein | | afdb-uniprot50 | AF-A0A3C0QI21-F1-MODEL\_V4 | 1.0 | 3.166e-10 | 234 | 0.129 | 301 | 217 | 16 | 1 | 282 | 14 | 288 | Chromosome partitioning protein ParB | Chromosome partitioning protein ParB | | afdb-uniprot50 | AF-A0A2W1B217-F1-MODEL\_V4 | 1.0 | 3.505e-08 | 233 | 0.132 | 265 | 179 | 9 | 13 | 273 | 6 | 223 | Chromosome segregation protein Spo0J, contains ParB-like nuclease domain | Chromosome segregation protein Spo0J, contains ParB-like nuclease domain | | afdb-uniprot50 | AF-A0A848FHG5-F1-MODEL\_V4 | 1.0 | 3.906e-08 | 233 | 0.144 | 311 | 208 | 20 | 1 | 288 | 64 | 339 | ParB/RepB/Spo0J family partition protein | ParB/RepB/Spo0J family partition protein | | afdb-uniprot50 | AF-A0A7X6ZRM7-F1-MODEL\_V4 | 1.0 | 4.624e-10 | 233 | 0.182 | 290 | 197 | 15 | 13 | 282 | 82 | 351 | ParB/RepB/Spo0J family partition protein | ParB/RepB/Spo0J family partition protein | | afdb-uniprot50 | AF-A0A3D1WJI5-F1-MODEL\_V4 | 1.0 | 6.356e-08 | 232 | 0.178 | 269 | 171 | 16 | 19 | 270 | 3 | 238 | Chromosome partitioning protein ParB | Chromosome partitioning protein ParB | | afdb-uniprot50 | AF-A0A1C6C1D8-F1-MODEL\_V4 | 1.0 | 2.675e-08 | 232 | 0.134 | 335 | 218 | 15 | 1 | 291 | 1 | 307 | Probable chromosome-partitioning protein parB | Probable chromosome-partitioning protein parB | | afdb-uniprot50 | AF-A0A0E2D6A4-F1-MODEL\_V4 | 1.0 | 5.119e-08 | 232 | 0.147 | 271 | 200 | 11 | 1 | 247 | 29 | 292 | ParB-like protein | ParB-like protein | | afdb-uniprot50 | AF-B6WUQ8-F1-MODEL\_V4 | 1.0 | 9.343e-10 | 232 | 0.177 | 310 | 198 | 14 | 14 | 291 | 34 | 318 | ParB-like protein | ParB-like protein | | afdb-uniprot50 | AF-A0A523SL66-F1-MODEL\_V4 | 1.0 | 9.283e-08 | 232 | 0.172 | 267 | 172 | 10 | 21 | 262 | 1 | 243 | ParB/RepB/Spo0J family partition protein | ParB/RepB/Spo0J family partition protein | | afdb-uniprot50 | AF-A0A0C2AKN6-F1-MODEL\_V4 | 1.0 | 1.153e-07 | 232 | 0.165 | 308 | 196 | 13 | 1 | 272 | 4 | 286 | ParB domain-containing protein | ParB domain-containing protein | | afdb-uniprot50 | AF-A0A3D4YDN4-F1-MODEL\_V4 | 1.0 | 5.704e-08 | 231 | 0.133 | 306 | 171 | 14 | 1 | 275 | 13 | 255 | ParB domain-containing protein | ParB domain-containing protein | | afdb-uniprot50 | AF-A0A3C0WTF4-F1-MODEL\_V4 | 1.0 | 8.794e-08 | 231 | 0.159 | 251 | 177 | 12 | 21 | 246 | 1 | 242 | ParB domain-containing protein | ParB domain-containing protein | | afdb-uniprot50 | AF-A0A7C2RDB0-F1-MODEL\_V4 | 1.0 | 1.397e-08 | 231 | 0.151 | 284 | 195 | 11 | 15 | 287 | 40 | 288 | ParB/RepB/Spo0J family partition protein | ParB/RepB/Spo0J family partition protein | | afdb-uniprot50 | AF-A0A2K8WTV7-F1-MODEL\_V4 | 1.0 | 1.595e-07 | 231 | 0.157 | 229 | 149 | 10 | 10 | 200 | 16 | 238 | ParB-like partitioning protein | ParB-like partitioning protein | | afdb-uniprot50 | AF-A0A0F6IBY4-F1-MODEL\_V4 | 1.0 | 1.644e-08 | 231 | 0.128 | 295 | 204 | 15 | 7 | 287 | 38 | 293 | ParB-like protein | ParB-like protein | | afdb-uniprot50 | AF-A0A3P3DPP2-F1-MODEL\_V4 | 1.0 | 2.98e-08 | 231 | 0.148 | 297 | 196 | 14 | 19 | 287 | 52 | 319 | Plasmid partitioning protein RepB | Plasmid partitioning protein RepB | | afdb-uniprot50 | AF-A0A6P2FYS8-F1-MODEL\_V4 | 1.0 | 3.789e-07 | 231 | 0.137 | 283 | 200 | 16 | 20 | 282 | 66 | 324 | ParB/RepB/Spo0J family partition protein | ParB/RepB/Spo0J family partition protein | | afdb-uniprot50 | AF-A0A523VK37-F1-MODEL\_V4 | 1.0 | 1.465e-06 | 231 | 0.148 | 283 | 149 | 8 | 8 | 200 | 250 | 530 | MPN domain-containing protein | MPN domain-containing protein | | afdb-uniprot50 | AF-A0A7U9T1Z3-F1-MODEL\_V4 | 1.0 | 1.431e-07 | 230 | 0.17 | 240 | 152 | 9 | 13 | 214 | 23 | 253 | Chromosome-partitioning protein Spo0J | Chromosome-partitioning protein Spo0J | | afdb-uniprot50 | AF-A0A6H1Z970-F1-MODEL\_V4 | 1.0 | 3.401e-07 | 230 | 0.198 | 196 | 125 | 11 | 19 | 206 | 4 | 175 | Putative methyltransferase | Putative methyltransferase | | afdb-uniprot50 | AF-A0A8B2NJQ1-F1-MODEL\_V4 | 1.0 | 1.876e-07 | 230 | 0.193 | 181 | 142 | 4 | 18 | 196 | 1 | 179 | Chromosome partitioning protein ParB | Chromosome partitioning protein ParB | | afdb-uniprot50 | AF-X1DCG4-F1-MODEL\_V4 | 1.0 | 4.594e-08 | 229 | 0.149 | 174 | 140 | 5 | 13 | 182 | 21 | 190 | ParB domain-containing protein | ParB domain-containing protein | | afdb-uniprot50 | AF-A0A7X7UCM7-F1-MODEL\_V4 | 1.0 | 4.486e-09 | 229 | 0.18 | 321 | 191 | 16 | 1 | 282 | 5 | 292 | ParB/RepB/Spo0J family partition protein | ParB/RepB/Spo0J family partition protein | | afdb-uniprot50 | AF-A0A7I7Y4I0-F1-MODEL\_V4 | 1.0 | 6.753e-10 | 229 | 0.203 | 304 | 192 | 15 | 13 | 290 | 17 | 296 | Chromosome partitioning protein ParB | Chromosome partitioning protein ParB | | afdb-uniprot50 | AF-A0A4U9YID6-F1-MODEL\_V4 | 1.0 | 3.789e-07 | 229 | 0.123 | 234 | 165 | 7 | 4 | 202 | 13 | 241 | ParB-like nuclease protein | ParB-like nuclease protein | | afdb-uniprot50 | AF-A0A7W9PDS6-F1-MODEL\_V4 | 1.0 | 1.041e-09 | 229 | 0.183 | 299 | 188 | 16 | 14 | 282 | 57 | 329 | ParB family chromosome partitioning protein | ParB family chromosome partitioning protein | | afdb-uniprot50 | AF-A0A6I7P420-F1-MODEL\_V4 | 1.0 | 2.329e-07 | 229 | 0.164 | 323 | 202 | 16 | 1 | 286 | 2 | 293 | ParB/RepB/Spo0J family partition protein | ParB/RepB/Spo0J family partition protein | | afdb-uniprot50 | AF-A0A496Q7M5-F1-MODEL\_V4 | 1.0 | 2.028e-06 | 228 | 0.169 | 230 | 163 | 11 | 27 | 247 | 7 | 217 | ParB domain-containing protein | ParB domain-containing protein | | afdb-uniprot50 | AF-A0A4Q3UFB4-F1-MODEL\_V4 | 1.0 | 4.123e-08 | 228 | 0.157 | 305 | 199 | 18 | 13 | 290 | 18 | 291 | ParB/RepB/Spo0J family partition protein | ParB/RepB/Spo0J family partition protein | | afdb-uniprot50 | AF-A0A2N2TMN6-F1-MODEL\_V4 | 1.0 | 1.595e-07 | 228 | 0.155 | 309 | 199 | 18 | 13 | 291 | 20 | 296 | Chromosome partitioning protein ParB | Chromosome partitioning protein ParB | | afdb-uniprot50 | AF-A0A1Y4UN77-F1-MODEL\_V4 | 1.0 | 3.445e-11 | 227 | 0.169 | 306 | 195 | 14 | 1 | 282 | 1 | 271 | Chromosome partitioning protein ParB | Chromosome partitioning protein ParB | | afdb-uniprot50 | AF-A0A523VV34-F1-MODEL\_V4 | 1.0 | 3.321e-08 | 227 | 0.17 | 281 | 170 | 11 | 18 | 258 | 1 | 258 | ParB/RepB/Spo0J family partition protein | ParB/RepB/Spo0J family partition protein | | afdb-uniprot50 | AF-A0A6N7YJT5-F1-MODEL\_V4 | 1.0 | 3.166e-10 | 227 | 0.174 | 316 | 213 | 15 | 1 | 291 | 80 | 372 | ParB/RepB/Spo0J family partition protein | ParB/RepB/Spo0J family partition protein | | afdb-uniprot50 | AF-A0A326UQP0-F1-MODEL\_V4 | 1.0 | 6.356e-08 | 227 | 0.194 | 304 | 210 | 10 | 5 | 287 | 35 | 324 | ParB/RepB/Spo0J family partition protein | ParB/RepB/Spo0J family partition protein | | afdb-uniprot50 | AF-A0A3D8P5H7-F1-MODEL\_V4 | 1.0 | 2.154e-08 | 227 | 0.185 | 308 | 171 | 16 | 13 | 287 | 6 | 266 | ParB domain-containing protein | ParB domain-containing protein | | afdb-uniprot50 | AF-A0A1G1X644-F1-MODEL\_V4 | 1.0 | 2.344e-09 | 226 | 0.162 | 308 | 201 | 16 | 11 | 291 | 21 | 298 | ParB domain-containing protein | ParB domain-containing protein | | afdb-uniprot50 | AF-B4RIU1-F1-MODEL\_V4 | 1.0 | 4.123e-08 | 226 | 0.233 | 201 | 137 | 10 | 3 | 195 | 9 | 200 | ParB-like partition protein | ParB-like partition protein | | afdb-uniprot50 | AF-A0A1F5NRR0-F1-MODEL\_V4 | 1.0 | 1.993e-09 | 226 | 0.207 | 280 | 183 | 12 | 20 | 282 | 59 | 316 | ParB domain-containing protein | ParB domain-containing protein | | afdb-uniprot50 | AF-K6TXH8-F1-MODEL\_V4 | 1.0 | 4.123e-08 | 226 | 0.157 | 317 | 189 | 15 | 1 | 246 | 6 | 315 | Putative transcriptional regulator | Putative transcriptional regulator | | afdb-uniprot50 | AF-A0A5B8VFP4-F1-MODEL\_V4 | 1.0 | 1.324e-08 | 226 | 0.101 | 385 | 208 | 17 | 13 | 287 | 23 | 379 | ParB/RepB/Spo0J family partition protein | ParB/RepB/Spo0J family partition protein | | afdb-uniprot50 | AF-A0A1R3U4M9-F1-MODEL\_V4 | 1.0 | 4.705e-07 | 226 | 0.151 | 283 | 191 | 14 | 18 | 284 | 1 | 250 | Putative chromosome-partitioning protein ParB | Putative chromosome-partitioning protein ParB | | afdb-uniprot50 | AF-A0A1Q6TFJ3-F1-MODEL\_V4 | 1.0 | 3.146e-08 | 225 | 0.131 | 320 | 199 | 14 | 1 | 291 | 1 | 270 | ParB domain-containing protein | ParB domain-containing protein | | afdb-uniprot50 | AF-A0A1T1DHW5-F1-MODEL\_V4 | 1.0 | 2.961e-06 | 225 | 0.128 | 226 | 174 | 10 | 1 | 214 | 22 | 236 | Chromosome partitioning protein ParB | Chromosome partitioning protein ParB | | afdb-uniprot50 | AF-A0A1C6C4L8-F1-MODEL\_V4 | 1.0 | 3.401e-07 | 225 | 0.163 | 232 | 154 | 9 | 4 | 202 | 196 | 420 | Probable chromosome-partitioning protein parB | Probable chromosome-partitioning protein parB | | afdb-uniprot50 | AF-A0A212AB45-F1-MODEL\_V4 | 1.0 | 3.146e-08 | 224 | 0.217 | 216 | 144 | 8 | 13 | 224 | 7 | 201 | ParB domain-containing protein | ParB domain-containing protein | | afdb-uniprot50 | AF-A0A7Y7A5E4-F1-MODEL\_V4 | 1.0 | 5.704e-08 | 224 | 0.213 | 253 | 161 | 14 | 47 | 282 | 2 | 233 | ParB/RepB/Spo0J family partition protein | ParB/RepB/Spo0J family partition protein | | afdb-uniprot50 | AF-R5KEP9-F1-MODEL\_V4 | 1.0 | 2.154e-08 | 224 | 0.129 | 331 | 198 | 13 | 1 | 246 | 1 | 326 | ParB domain protein nuclease | ParB domain protein nuclease | | afdb-uniprot50 | AF-A0A1Q6QJG1-F1-MODEL\_V4 | 1.0 | 3.906e-08 | 223 | 0.158 | 322 | 195 | 14 | 13 | 287 | 26 | 318 | ParB domain-containing protein | ParB domain-containing protein | | afdb-uniprot50 | AF-A0A4V5NLE5-F1-MODEL\_V4 | 1.0 | 1.98e-07 | 223 | 0.131 | 312 | 224 | 15 | 1 | 286 | 32 | 322 | Plasmid partitioning protein RepB | Plasmid partitioning protein RepB | | afdb-uniprot50 | AF-A0A853IEB4-F1-MODEL\_V4 | 1.0 | 9.568e-09 | 223 | 0.155 | 327 | 209 | 19 | 1 | 291 | 1 | 296 | ParB/RepB/Spo0J family partition protein | ParB/RepB/Spo0J family partition protein | | afdb-uniprot50 | AF-A0A1Q3TEL5-F1-MODEL\_V4 | 1.0 | 1.475e-08 | 222 | 0.192 | 317 | 177 | 17 | 1 | 272 | 1 | 283 | ParB domain-containing protein | ParB domain-containing protein | | afdb-uniprot50 | AF-A0A1F5DHD6-F1-MODEL\_V4 | 1.0 | 1.092e-07 | 222 | 0.143 | 278 | 184 | 15 | 19 | 261 | 19 | 277 | ParB domain-containing protein | ParB domain-containing protein | | afdb-uniprot50 | AF-A0A0H5Q929-F1-MODEL\_V4 | 1.0 | 5.153e-10 | 221 | 0.166 | 301 | 211 | 14 | 1 | 282 | 6 | 285 | ParB domain-containing protein | ParB domain-containing protein | | afdb-uniprot50 | AF-A0A1F9VR61-F1-MODEL\_V4 | 1.0 | 1.225e-09 | 221 | 0.206 | 296 | 199 | 14 | 13 | 291 | 22 | 298 | ParB domain-containing protein | ParB domain-containing protein | | afdb-uniprot50 | AF-A0A127CRA7-F1-MODEL\_V4 | 1.0 | 8.794e-08 | 221 | 0.151 | 291 | 197 | 15 | 15 | 287 | 1 | 259 | ParB domain-containing protein | ParB domain-containing protein | | afdb-uniprot50 | AF-A0A535EHP5-F1-MODEL\_V4 | 1.0 | 2.274e-08 | 220 | 0.172 | 308 | 205 | 13 | 1 | 282 | 1 | 284 | ParB/RepB/Spo0J family partition protein | ParB/RepB/Spo0J family partition protein | | afdb-uniprot50 | AF-A0A0D0PKE9-F1-MODEL\_V4 | 1.0 | 3.423e-09 | 220 | 0.169 | 301 | 219 | 11 | 7 | 291 | 64 | 349 | Plasmid partitioning protein ParB | Plasmid partitioning protein ParB | | afdb-uniprot50 | AF-Q5U9L6-F1-MODEL\_V4 | 1.0 | 2.578e-05 | 220 | 0.168 | 148 | 110 | 8 | 56 | 196 | 1 | 142 | Putative ParB partitioning protein | Putative ParB partitioning protein | | afdb-uniprot50 | AF-A0A800CKI2-F1-MODEL\_V4 | 1.0 | 3.126e-06 | 220 | 0.145 | 233 | 145 | 9 | 19 | 199 | 1 | 231 | Uncharacterized protein | Uncharacterized protein | | afdb-uniprot50 | AF-A0A3L6ZQY3-F1-MODEL\_V4 | 1.0 | 2.041e-08 | 219 | 0.19 | 231 | 145 | 11 | 1 | 196 | 26 | 249 | ParB/RepB/Spo0J family partition protein | ParB/RepB/Spo0J family partition protein | | afdb-uniprot50 | AF-A0A259Q3N7-F1-MODEL\_V4 | 1.0 | 3.789e-07 | 219 | 0.148 | 277 | 198 | 12 | 1 | 265 | 15 | 265 | ParB domain-containing protein | ParB domain-containing protein | | afdb-uniprot50 | AF-E1M761-F1-MODEL\_V4 | 1.0 | 2.595e-07 | 218 | 0.19 | 231 | 164 | 10 | 56 | 282 | 1 | 212 | Stage 0 sporulation protein J | Stage 0 sporulation protein J | | afdb-uniprot50 | AF-A0A2R4G3E4-F1-MODEL\_V4 | 1.0 | 6.356e-08 | 218 | 0.142 | 316 | 184 | 16 | 13 | 286 | 15 | 285 | ParB domain-containing protein | ParB domain-containing protein | | afdb-uniprot50 | AF-A0A2N4X5C0-F1-MODEL\_V4 | 1.0 | 6.167e-07 | 218 | 0.188 | 244 | 162 | 12 | 23 | 260 | 2 | 215 | Chromosome partitioning protein ParB | Chromosome partitioning protein ParB | | afdb-uniprot50 | AF-A0A109E1J8-F1-MODEL\_V4 | 1.0 | 6.167e-07 | 218 | 0.145 | 302 | 209 | 17 | 13 | 291 | 65 | 340 | ParB domain-containing protein | ParB domain-containing protein | | afdb-uniprot50 | AF-A0A1G3BTE6-F1-MODEL\_V4 | 1.0 | 1.465e-06 | 217 | 0.111 | 288 | 205 | 12 | 15 | 290 | 1 | 249 | ParB domain-containing protein | ParB domain-containing protein | | afdb-uniprot50 | AF-A0A3A8ZTF9-F1-MODEL\_V4 | 1.0 | 3.052e-07 | 216 | 0.158 | 239 | 146 | 11 | 13 | 210 | 2 | 226 | Chromosome partitioning protein ParB | Chromosome partitioning protein ParB | | afdb-uniprot50 | AF-F0RQA6-F1-MODEL\_V4 | 1.0 | 7.253e-07 | 216 | 0.158 | 233 | 153 | 7 | 4 | 199 | 10 | 236 | ParB-like partition protein | ParB-like partition protein | | afdb-uniprot50 | AF-A0A125DMD0-F1-MODEL\_V4 | 1.0 | 3.321e-08 | 216 | 0.15 | 318 | 190 | 17 | 5 | 282 | 57 | 334 | Chromosome partitioning protein ParB | Chromosome partitioning protein ParB | | afdb-uniprot50 | AF-A0A7W9RWB0-F1-MODEL\_V4 | 1.0 | 4.736e-09 | 216 | 0.17 | 281 | 191 | 16 | 1 | 272 | 1 | 248 | ParB family chromosome partitioning protein | ParB family chromosome partitioning protein | | afdb-uniprot50 | AF-A0A1V6GT79-F1-MODEL\_V4 | 1.0 | 8.794e-08 | 216 | 0.168 | 296 | 170 | 16 | 19 | 282 | 3 | 254 | Methyltransferase | Methyltransferase | | afdb-uniprot50 | AF-A0A6C2U9R7-F1-MODEL\_V4 | 1.0 | 1.595e-07 | 215 | 0.141 | 276 | 188 | 10 | 18 | 287 | 12 | 244 | Chromosome-partitioning protein Spo0J | Chromosome-partitioning protein Spo0J | | afdb-uniprot50 | AF-A0A0R2QCK2-F1-MODEL\_V4 | 1.0 | 4.624e-10 | 215 | 0.165 | 314 | 190 | 14 | 13 | 284 | 34 | 317 | ParB domain-containing protein | ParB domain-containing protein | | afdb-uniprot50 | AF-W7X354-F1-MODEL\_V4 | 1.0 | 1.431e-07 | 215 | 0.149 | 314 | 194 | 20 | 13 | 290 | 57 | 333 | ParB/RepB/Spo0J family partition protein | ParB/RepB/Spo0J family partition protein | | afdb-uniprot50 | AF-A0A355VXV9-F1-MODEL\_V4 | 1.0 | 1.511e-07 | 214 | 0.126 | 300 | 190 | 15 | 1 | 287 | 24 | 264 | ParB domain-containing protein | ParB domain-containing protein | | afdb-uniprot50 | AF-A0A836VUS9-F1-MODEL\_V4 | 1.0 | 3.676e-06 | 213 | 0.182 | 170 | 126 | 5 | 2 | 169 | 14 | 172 | ParB/RepB/Spo0J family partition protein | ParB/RepB/Spo0J family partition protein | | afdb-uniprot50 | AF-A0A0F9FPY9-F1-MODEL\_V4 | 1.0 | 3.401e-07 | 213 | 0.157 | 280 | 178 | 13 | 16 | 287 | 10 | 239 | ParB domain-containing protein | ParB domain-containing protein | | afdb-uniprot50 | AF-A0A7X6X5M1-F1-MODEL\_V4 | 1.0 | 1.066e-08 | 213 | 0.194 | 262 | 182 | 10 | 3 | 246 | 23 | 273 | ParB/RepB/Spo0J family partition protein | ParB/RepB/Spo0J family partition protein | | afdb-uniprot50 | AF-A0A4R5TV04-F1-MODEL\_V4 | 1.0 | 2.91e-09 | 213 | 0.189 | 291 | 194 | 13 | 13 | 283 | 49 | 317 | ParB/RepB/Spo0J family partition protein | ParB/RepB/Spo0J family partition protein | | afdb-uniprot50 | AF-A0A2S0L073-F1-MODEL\_V4 | 1.0 | 3.881e-06 | 213 | 0.175 | 188 | 134 | 7 | 21 | 203 | 2 | 173 | ParB domain-containing protein | ParB domain-containing protein | | afdb-uniprot50 | AF-A0A660M752-F1-MODEL\_V4 | 1.0 | 8.331e-08 | 212 | 0.222 | 171 | 123 | 6 | 3 | 169 | 20 | 184 | ParB/RepB/Spo0J family partition protein | ParB/RepB/Spo0J family partition protein | | afdb-uniprot50 | AF-A0A378AKT6-F1-MODEL\_V4 | 1.0 | 5.086e-06 | 211 | 0.173 | 260 | 159 | 12 | 44 | 290 | 9 | 225 | PRTRC system ParB family protein | PRTRC system ParB family protein | | afdb-uniprot50 | AF-A0A2R7NXB0-F1-MODEL\_V4 | 1.0 | 2.739e-07 | 211 | 0.19 | 284 | 182 | 17 | 1 | 258 | 62 | 323 | Chromosome partitioning protein ParB | Chromosome partitioning protein ParB | | afdb-uniprot50 | AF-A0A246BKX2-F1-MODEL\_V4 | 1.0 | 1.034e-07 | 210 | 0.159 | 320 | 183 | 11 | 7 | 282 | 15 | 292 | ParB domain-containing protein | ParB domain-containing protein | | afdb-uniprot50 | AF-D6TUM3-F1-MODEL\_V4 | 1.0 | 2.274e-08 | 210 | 0.187 | 336 | 186 | 17 | 14 | 287 | 42 | 352 | ParB-like partition protein | ParB-like partition protein | | afdb-uniprot50 | AF-A0A160UJD6-F1-MODEL\_V4 | 1.0 | 6.509e-07 | 209 | 0.179 | 178 | 136 | 5 | 1 | 172 | 21 | 194 | Chromosome partitioning protein CDS | Chromosome partitioning protein CDS | | afdb-uniprot50 | AF-A0A5E4I9I8-F1-MODEL\_V4 | 1.0 | 6.509e-07 | 209 | 0.14 | 271 | 178 | 16 | 21 | 255 | 2 | 253 | Nucleoid occlusion protein | Nucleoid occlusion protein | | afdb-uniprot50 | AF-A0A257C4G3-F1-MODEL\_V4 | 1.0 | 4.352e-08 | 209 | 0.17 | 276 | 185 | 11 | 11 | 273 | 22 | 266 | ParB domain-containing protein | ParB domain-containing protein | | afdb-uniprot50 | AF-A0A0D6T7F5-F1-MODEL\_V4 | 1.0 | 8.082e-07 | 209 | 0.205 | 185 | 138 | 7 | 19 | 200 | 4 | 182 | ParB domain-containing protein | ParB domain-containing protein | | afdb-uniprot50 | AF-A0A0G1ITF5-F1-MODEL\_V4 | 1.0 | 4.705e-07 | 208 | 0.172 | 220 | 142 | 10 | 13 | 211 | 1 | 201 | ParB domain-containing protein | ParB domain-containing protein | | afdb-uniprot50 | AF-A0A2E3SMI7-F1-MODEL\_V4 | 1.0 | 3.126e-06 | 208 | 0.147 | 257 | 184 | 13 | 13 | 246 | 1 | 245 | ParB domain-containing protein | ParB domain-containing protein | | afdb-uniprot50 | AF-A0A845DAT5-F1-MODEL\_V4 | 1.0 | 2.757e-09 | 208 | 0.135 | 317 | 209 | 14 | 13 | 288 | 17 | 309 | ParB/RepB/Spo0J family partition protein | ParB/RepB/Spo0J family partition protein | | afdb-uniprot50 | AF-X1SKH0-F1-MODEL\_V4 | 1.0 | 9.283e-08 | 208 | 0.18 | 266 | 170 | 13 | 22 | 255 | 3 | 252 | Uncharacterized protein | Uncharacterized protein | | afdb-uniprot50 | AF-A0A1F5P5H6-F1-MODEL\_V4 | 1.0 | 1.324e-08 | 208 | 0.175 | 302 | 191 | 16 | 14 | 287 | 64 | 335 | ParB domain-containing protein | ParB domain-containing protein | | afdb-uniprot50 | AF-A0A2H0QRG0-F1-MODEL\_V4 | 1.0 | 5.404e-08 | 207 | 0.106 | 311 | 203 | 18 | 10 | 287 | 24 | 292 | ParB domain-containing protein | ParB domain-containing protein | | afdb-uniprot50 | AF-A0A443KCM7-F1-MODEL\_V4 | 1.0 | 3.59e-07 | 205 | 0.127 | 290 | 203 | 14 | 20 | 287 | 42 | 303 | Plasmid partitioning protein RepB | Plasmid partitioning protein RepB | | afdb-uniprot50 | AF-A0A2A2GEW1-F1-MODEL\_V4 | 1.0 | 2.458e-07 | 205 | 0.147 | 299 | 196 | 18 | 19 | 287 | 60 | 329 | Plasmid partitioning protein RepB | Plasmid partitioning protein RepB | | afdb-uniprot50 | AF-Q21QH4-F1-MODEL\_V4 | 1.0 | 2.329e-07 | 205 | 0.161 | 279 | 170 | 14 | 13 | 284 | 48 | 269 | ParB-like partition proteins | ParB-like partition proteins | | afdb-uniprot50 | AF-A0A6N8VJ22-F1-MODEL\_V4 | 1.0 | 2.739e-07 | 204 | 0.185 | 178 | 130 | 5 | 1 | 167 | 15 | 188 | ParB/RepB/Spo0J family partition protein | ParB/RepB/Spo0J family partition protein | | afdb-uniprot50 | AF-A0A2E7CGY7-F1-MODEL\_V4 | 1.0 | 1.98e-07 | 204 | 0.131 | 296 | 217 | 11 | 1 | 291 | 1 | 261 | HTH cro/C1-type domain-containing protein | HTH cro/C1-type domain-containing protein | | afdb-uniprot50 | AF-A0A258WWT9-F1-MODEL\_V4 | 1.0 | 7.892e-08 | 204 | 0.132 | 271 | 200 | 11 | 19 | 282 | 37 | 279 | ParB domain-containing protein | ParB domain-containing protein | | afdb-uniprot50 | AF-A0A539CYY2-F1-MODEL\_V4 | 1.0 | 6.167e-07 | 203 | 0.175 | 291 | 189 | 11 | 1 | 272 | 4 | 262 | ParB family protein | ParB family protein | | afdb-uniprot50 | AF-A0A2H0TB13-F1-MODEL\_V4 | 1.0 | 8.385e-10 | 203 | 0.166 | 289 | 203 | 11 | 9 | 282 | 17 | 282 | Chromosome partitioning protein ParB | Chromosome partitioning protein ParB | | afdb-uniprot50 | AF-A0A2D8EI10-F1-MODEL\_V4 | 1.0 | 9.006e-07 | 203 | 0.189 | 290 | 185 | 15 | 13 | 287 | 6 | 260 | ParB domain-containing protein | ParB domain-containing protein | | afdb-uniprot50 | AF-K1MKW0-F1-MODEL\_V4 | 1.0 | 3.032e-05 | 201 | 0.122 | 171 | 132 | 8 | 3 | 160 | 10 | 175 | ParB domain-containing protein | ParB domain-containing protein | | afdb-uniprot50 | AF-A0A2V9LAX5-F1-MODEL\_V4 | 1.0 | 0.0001052 | 200 | 0.192 | 114 | 87 | 3 | 1 | 114 | 5 | 113 | ParB domain-containing protein | ParB domain-containing protein | | afdb-uniprot50 | AF-A0A1B2YUD7-F1-MODEL\_V4 | 1.0 | 0.0001908 | 200 | 0.137 | 116 | 92 | 5 | 14 | 127 | 26 | 135 | Chromosome (Plasmid) partitioning protein ParB | Chromosome (Plasmid) partitioning protein ParB | | afdb-uniprot50 | AF-G5JF03-F1-MODEL\_V4 | 1.0 | 2.329e-07 | 200 | 0.146 | 287 | 170 | 15 | 12 | 243 | 36 | 302 | ParB-like protein-partition protein | ParB-like protein-partition protein | | afdb-uniprot50 | AF-C5B6N2-F1-MODEL\_V4 | 1.0 | 1.465e-06 | 200 | 0.219 | 187 | 135 | 8 | 13 | 193 | 24 | 205 | Putative plasmid stabilization protein | Putative plasmid stabilization protein | | afdb-uniprot50 | AF-A0A3R6DKU2-F1-MODEL\_V4 | 1.0 | 4.457e-07 | 199 | 0.113 | 317 | 200 | 14 | 12 | 278 | 18 | 303 | Chromosome partitioning protein ParB | Chromosome partitioning protein ParB | | afdb-uniprot50 | AF-A0A239PFQ7-F1-MODEL\_V4 | 1.0 | 1.059e-06 | 199 | 0.153 | 287 | 173 | 15 | 14 | 267 | 16 | 265 | Chromosome partitioning protein, ParB family | Chromosome partitioning protein, ParB family | | afdb-uniprot50 | AF-H4F7S0-F1-MODEL\_V4 | 1.0 | 3.401e-07 | 198 | 0.163 | 305 | 179 | 18 | 16 | 287 | 1 | 262 | Plasmid partitioning protein RepB | Plasmid partitioning protein RepB | | afdb-uniprot50 | AF-A0A0W7WJQ7-F1-MODEL\_V4 | 1.0 | 5.243e-07 | 196 | 0.174 | 310 | 199 | 18 | 7 | 291 | 44 | 321 | ParB domain-containing protein | ParB domain-containing protein | | afdb-uniprot50 | AF-A0A2E9QLE2-F1-MODEL\_V4 | 1.0 | 1.82e-06 | 195 | 0.108 | 304 | 209 | 14 | 1 | 282 | 26 | 289 | ParB domain-containing protein | ParB domain-containing protein | | afdb-uniprot50 | AF-A0A379PPU0-F1-MODEL\_V4 | 1.0 | 1.315e-06 | 195 | 0.126 | 300 | 204 | 18 | 13 | 287 | 61 | 327 | Chromosome partitioning protein ParB | Chromosome partitioning protein ParB | | afdb-uniprot50 | AF-A0A0N9UTQ1-F1-MODEL\_V4 | 1.0 | 1.388e-06 | 195 | 0.176 | 187 | 143 | 6 | 18 | 200 | 1 | 180 | ParB domain-containing protein | ParB domain-containing protein | | afdb-uniprot50 | AF-A0A2N2LVB4-F1-MODEL\_V4 | 1.0 | 1.595e-07 | 193 | 0.164 | 256 | 187 | 11 | 21 | 262 | 1 | 243 | ParB domain-containing protein | ParB domain-containing protein | | afdb-uniprot50 | AF-A0A842ZP40-F1-MODEL\_V4 | 1.0 | 4e-07 | 193 | 0.163 | 306 | 188 | 17 | 5 | 290 | 25 | 282 | Uncharacterized protein | Uncharacterized protein | | afdb-uniprot50 | AF-A0A1B6BH88-F1-MODEL\_V4 | 1.0 | 9.445e-05 | 192 | 0.202 | 143 | 99 | 6 | 13 | 144 | 24 | 162 | Plasmid partitioning protein ParB | Plasmid partitioning protein ParB | | afdb-uniprot50 | AF-A0A4R8HXN8-F1-MODEL\_V4 | 1.0 | 6.315e-06 | 192 | 0.218 | 160 | 119 | 3 | 19 | 175 | 5 | 161 | ParB family chromosome partitioning protein | ParB family chromosome partitioning protein | | afdb-uniprot50 | AF-A0A523SL68-F1-MODEL\_V4 | 1.0 | 1.724e-06 | 191 | 0.134 | 289 | 178 | 14 | 13 | 291 | 4 | 230 | ParB/RepB/Spo0J family partition protein | ParB/RepB/Spo0J family partition protein | | afdb-uniprot50 | AF-A0A519DTN3-F1-MODEL\_V4 | 1.0 | 9.006e-07 | 191 | 0.171 | 280 | 188 | 12 | 8 | 271 | 59 | 310 | ParB/RepB/Spo0J family partition protein | ParB/RepB/Spo0J family partition protein | | afdb-uniprot50 | AF-F7JID4-F1-MODEL\_V4 | 1.0 | 3.201e-05 | 189 | 0.219 | 155 | 104 | 8 | 7 | 148 | 15 | 165 | ParB domain-containing protein | ParB domain-containing protein | | afdb-uniprot50 | AF-L0A5U5-F1-MODEL\_V4 | 1.0 | 9.006e-07 | 189 | 0.163 | 306 | 193 | 16 | 14 | 287 | 52 | 326 | ParB-like partition protein | ParB-like partition protein | | afdb-uniprot50 | AF-B9L4Q1-F1-MODEL\_V4 | 1.0 | 1.993e-09 | 189 | 0.172 | 337 | 207 | 16 | 2 | 291 | 15 | 326 | Stage 0 sporulation protein J | Stage 0 sporulation protein J | | afdb-uniprot50 | AF-A0A0A3XRA4-F1-MODEL\_V4 | 1.0 | 2.274e-08 | 189 | 0.169 | 277 | 192 | 10 | 19 | 281 | 1 | 253 | Transcriptional regulator | Transcriptional regulator | | afdb-uniprot50 | AF-X1JNN1-F1-MODEL\_V4 | 1.0 | 0.000407 | 188 | 0.111 | 134 | 111 | 6 | 19 | 146 | 11 | 142 | ParB domain-containing protein | ParB domain-containing protein | | afdb-uniprot50 | AF-A0A350T1Y3-F1-MODEL\_V4 | 1.0 | 1.831e-08 | 188 | 0.154 | 297 | 214 | 13 | 1 | 287 | 10 | 279 | Stage 0 sporulation protein J | Stage 0 sporulation protein J | | afdb-uniprot50 | AF-A0A3E4UB54-F1-MODEL\_V4 | 1.0 | 1.118e-06 | 188 | 0.136 | 345 | 192 | 13 | 14 | 282 | 50 | 364 | Chromosome partitioning protein ParB | Chromosome partitioning protein ParB | | afdb-uniprot50 | AF-Q892Z6-F1-MODEL\_V4 | 1.0 | 1.276e-05 | 187 | 0.135 | 199 | 122 | 9 | 1 | 156 | 6 | 197 | Chromosome partitioning parB family protein | Chromosome partitioning parB family protein | | afdb-uniprot50 | AF-A0A1G0JK78-F1-MODEL\_V4 | 1.0 | 1.547e-06 | 186 | 0.137 | 204 | 156 | 9 | 19 | 214 | 1 | 192 | ParB domain-containing protein | ParB domain-containing protein | | afdb-uniprot50 | AF-A0A845UHA6-F1-MODEL\_V4 | 1.0 | 1.431e-07 | 185 | 0.126 | 365 | 216 | 17 | 1 | 287 | 38 | 377 | ParB/RepB/Spo0J family partition protein | ParB/RepB/Spo0J family partition protein | | afdb-uniprot50 | AF-A0A7V8X4E6-F1-MODEL\_V4 | 1.0 | 3.299e-06 | 184 | 0.16 | 168 | 123 | 6 | 9 | 163 | 50 | 212 | ParB/RepB/Spo0J family partition protein | ParB/RepB/Spo0J family partition protein | | afdb-uniprot50 | AF-A0A3A0AS42-F1-MODEL\_V4 | 1.0 | 1.066e-08 | 184 | 0.148 | 329 | 197 | 15 | 1 | 287 | 12 | 299 | Stage 0 sporulation protein J | Stage 0 sporulation protein J | | afdb-uniprot50 | AF-A0A2N0BFB6-F1-MODEL\_V4 | 1.0 | 0.0002787 | 183 | 0.15 | 113 | 86 | 3 | 13 | 119 | 20 | 128 | Chromosome partitioning protein ParB | Chromosome partitioning protein ParB | | afdb-uniprot50 | AF-A0A0F9K647-F1-MODEL\_V4 | 1.0 | 1.028e-05 | 183 | 0.114 | 315 | 210 | 13 | 27 | 291 | 26 | 321 | Uncharacterized protein | Uncharacterized protein | | afdb-uniprot50 | AF-A0A0B6RLI6-F1-MODEL\_V4 | 1.0 | 6.71e-08 | 182 | 0.177 | 310 | 162 | 14 | 3 | 291 | 1 | 238 | ParB-like nuclease | ParB-like nuclease | | afdb-uniprot50 | AF-A0A352R4S9-F1-MODEL\_V4 | 1.0 | 3.222e-07 | 182 | 0.161 | 285 | 185 | 11 | 14 | 288 | 60 | 300 | ParB domain-containing protein | ParB domain-containing protein | | afdb-uniprot50 | AF-W7B412-F1-MODEL\_V4 | 1.0 | 2.192e-05 | 181 | 0.172 | 162 | 111 | 9 | 1 | 156 | 1 | 145 | Putative Partition protein ParB-like protein | Putative Partition protein ParB-like protein | | afdb-uniprot50 | AF-A0A535YXC4-F1-MODEL\_V4 | 1.0 | 9.064e-09 | 181 | 0.177 | 298 | 186 | 13 | 13 | 280 | 23 | 291 | ParB/RepB/Spo0J family partition protein | ParB/RepB/Spo0J family partition protein | | afdb-uniprot50 | AF-A0A2D9IDC7-F1-MODEL\_V4 | 1.0 | 4.85e-08 | 179 | 0.166 | 294 | 191 | 17 | 18 | 291 | 1 | 260 | ParB domain-containing protein | ParB domain-containing protein | | afdb-uniprot50 | AF-A0A840SV32-F1-MODEL\_V4 | 1.0 | 7.083e-08 | 178 | 0.16 | 361 | 184 | 15 | 19 | 290 | 3 | 333 | ParB family chromosome partitioning protein | ParB family chromosome partitioning protein | | afdb-uniprot50 | AF-A0A373Q515-F1-MODEL\_V4 | 1.0 | 0.0001537 | 177 | 0.173 | 144 | 111 | 4 | 1 | 140 | 1 | 140 | ParB/RepB/Spo0J family partition protein | ParB/RepB/Spo0J family partition protein | | afdb-uniprot50 | AF-A0A3D9TAG4-F1-MODEL\_V4 | 1.0 | 8.082e-07 | 176 | 0.187 | 203 | 143 | 11 | 13 | 200 | 81 | 276 | ParB/RepB/Spo0J family partition protein | ParB/RepB/Spo0J family partition protein | | afdb-uniprot50 | AF-A0A1I3QL22-F1-MODEL\_V4 | 1.0 | 2.206e-07 | 176 | 0.198 | 272 | 181 | 11 | 19 | 280 | 4 | 248 | ParB family protein | ParB family protein | | afdb-uniprot50 | AF-A0A355VWD7-F1-MODEL\_V4 | 1.0 | 1.059e-06 | 175 | 0.123 | 283 | 178 | 14 | 19 | 287 | 34 | 260 | ParB domain-containing protein | ParB domain-containing protein | | afdb-uniprot50 | AF-A0A1U9XHD1-F1-MODEL\_V4 | 1.0 | 4.565e-06 | 175 | 0.156 | 281 | 182 | 11 | 60 | 291 | 2 | 276 | Uncharacterized protein | Uncharacterized protein | | afdb-uniprot50 | AF-A0A0N1HEN7-F1-MODEL\_V4 | 1.0 | 1.633e-06 | 173 | 0.169 | 213 | 144 | 10 | 19 | 206 | 5 | 209 | Uncharacterized protein | Uncharacterized protein | | afdb-uniprot50 | AF-Q1ZJV4-F1-MODEL\_V4 | 1.0 | 9.506e-07 | 173 | 0.168 | 303 | 188 | 18 | 20 | 287 | 76 | 349 | Partitioning protein B | Partitioning protein B | | afdb-uniprot50 | AF-A0A7V9N3J7-F1-MODEL\_V4 | 1.0 | 1.284e-07 | 173 | 0.173 | 328 | 200 | 14 | 9 | 290 | 24 | 326 | ParB/RepB/Spo0J family partition protein | ParB/RepB/Spo0J family partition protein | | afdb-uniprot50 | AF-A0A3B8MU94-F1-MODEL\_V4 | 1.0 | 0.0004296 | 170 | 0.194 | 113 | 78 | 7 | 1 | 106 | 1 | 107 | Chromosome partitioning protein ParB | Chromosome partitioning protein ParB | | afdb-uniprot50 | AF-A0A0Q8LGQ4-F1-MODEL\_V4 | 1.0 | 9.506e-07 | 169 | 0.176 | 215 | 154 | 8 | 4 | 199 | 8 | 218 | ParB domain-containing protein | ParB domain-containing protein | | afdb-uniprot50 | AF-A0A564Q3X4-F1-MODEL\_V4 | 1.0 | 1.863e-05 | 167 | 0.095 | 272 | 166 | 11 | 18 | 247 | 1 | 234 | Nucleoid occlusion protein | Nucleoid occlusion protein | | afdb-uniprot50 | AF-A0A527TY97-F1-MODEL\_V4 | 1.0 | 3.401e-07 | 167 | 0.172 | 284 | 192 | 13 | 13 | 282 | 1 | 255 | ParB/RepB/Spo0J family partition protein | ParB/RepB/Spo0J family partition protein | | afdb-uniprot50 | AF-A0A2A4MGC7-F1-MODEL\_V4 | 1.0 | 1.092e-07 | 166 | 0.132 | 354 | 207 | 15 | 13 | 287 | 3 | 335 | ParB domain-containing protein | ParB domain-containing protein | | afdb-uniprot50 | AF-E9SG08-F1-MODEL\_V4 | 1.0 | 5.209e-05 | 165 | 0.165 | 169 | 114 | 6 | 1 | 160 | 1 | 151 | ParB-like protein | ParB-like protein | | afdb-uniprot50 | AF-A0A5C7ERN6-F1-MODEL\_V4 | 1.0 | 2.873e-05 | 165 | 0.169 | 213 | 147 | 9 | 18 | 205 | 1 | 208 | ParB domain-containing protein | ParB domain-containing protein | | afdb-uniprot50 | AF-A0A7V9VJT7-F1-MODEL\_V4 | 1.0 | 1.876e-07 | 163 | 0.137 | 320 | 204 | 14 | 8 | 291 | 49 | 332 | ParB/RepB/Spo0J family partition protein | ParB/RepB/Spo0J family partition protein | | afdb-uniprot50 | AF-A0A2N1TR59-F1-MODEL\_V4 | 1.0 | 6.666e-06 | 163 | 0.164 | 291 | 173 | 12 | 12 | 271 | 74 | 325 | Uncharacterized protein | Uncharacterized protein | | afdb-uniprot50 | AF-A0A351AWR7-F1-MODEL\_V4 | 1.0 | 0.0005631 | 162 | 0.174 | 109 | 82 | 4 | 1 | 107 | 3 | 105 | Chromosome partitioning protein ParB | Chromosome partitioning protein ParB | | afdb-uniprot50 | AF-A0A1W9WV54-F1-MODEL\_V4 | 1.0 | 3.881e-06 | 160 | 0.141 | 283 | 163 | 17 | 8 | 282 | 15 | 225 | ParB domain-containing protein | ParB domain-containing protein | | afdb-uniprot50 | AF-F5YI81-F1-MODEL\_V4 | 1.0 | 6.71e-08 | 160 | 0.154 | 317 | 199 | 15 | 1 | 287 | 1 | 278 | ParB domain-containing protein | ParB domain-containing protein | | afdb-uniprot50 | AF-A0A2W5PVF2-F1-MODEL\_V4 | 1.0 | 8.082e-07 | 159 | 0.159 | 358 | 187 | 17 | 20 | 291 | 14 | 343 | ParB domain-containing protein | ParB domain-containing protein | | afdb-uniprot50 | AF-A0A2W6A9B4-F1-MODEL\_V4 | 1.0 | 1.82e-06 | 158 | 0.106 | 310 | 219 | 13 | 2 | 275 | 1 | 288 | ParB domain-containing protein | ParB domain-containing protein | | afdb-uniprot50 | AF-A0A7V8WTB0-F1-MODEL\_V4 | 1.0 | 2.329e-07 | 157 | 0.16 | 323 | 196 | 13 | 7 | 291 | 42 | 327 | ParB/RepB/Spo0J family partition protein | ParB/RepB/Spo0J family partition protein | | afdb-uniprot50 | AF-A0A8B5XDA0-F1-MODEL\_V4 | 1.0 | 3.401e-07 | 156 | 0.124 | 321 | 218 | 18 | 8 | 282 | 14 | 317 | ParB/RepB/Spo0J family partition protein | ParB/RepB/Spo0J family partition protein | | afdb-uniprot50 | AF-A0A1S8D0Y6-F1-MODEL\_V4 | 1.0 | 4.705e-07 | 156 | 0.14 | 291 | 200 | 17 | 18 | 291 | 1 | 258 | ParB domain-containing protein | ParB domain-containing protein | | afdb-uniprot50 | AF-A0A7Z1UWI7-F1-MODEL\_V4 | 1.0 | 3.222e-07 | 156 | 0.148 | 289 | 198 | 14 | 18 | 290 | 1 | 257 | ParB domain-containing protein | ParB domain-containing protein | | afdb-uniprot50 | AF-A0A1M5FTR8-F1-MODEL\_V4 | 1.0 | 3.881e-06 | 155 | 0.158 | 265 | 188 | 13 | 29 | 281 | 1 | 242 | ParB/RepB/Spo0J family partition protein | ParB/RepB/Spo0J family partition protein | | afdb-uniprot50 | AF-A0A5C7YCL1-F1-MODEL\_V4 | 1.0 | 2.314e-05 | 154 | 0.209 | 229 | 147 | 10 | 71 | 282 | 12 | 223 | ParB/RepB/Spo0J family partition protein | ParB/RepB/Spo0J family partition protein | | afdb-uniprot50 | AF-J7QGM7-F1-MODEL\_V4 | 1.0 | 1.388e-06 | 154 | 0.174 | 293 | 191 | 13 | 13 | 283 | 1 | 264 | ParB domain protein nuclease | ParB domain protein nuclease | | afdb-uniprot50 | AF-A0A370KZ30-F1-MODEL\_V4 | 1.0 | 4.705e-07 | 154 | 0.174 | 356 | 191 | 17 | 13 | 287 | 57 | 390 | ParB/RepB/Spo0J family partition protein | ParB/RepB/Spo0J family partition protein | | afdb-uniprot50 | AF-A0A1M4X5Z2-F1-MODEL\_V4 | 1.0 | 1.145e-05 | 152 | 0.147 | 278 | 179 | 10 | 19 | 273 | 20 | 262 | ParB-like nuclease domain-containing protein | ParB-like nuclease domain-containing protein | | afdb-uniprot50 | AF-A0A534ZDR6-F1-MODEL\_V4 | 1.0 | 0.001338 | 150 | 0.201 | 114 | 86 | 3 | 1 | 114 | 9 | 117 | ParB/RepB/Spo0J family partition protein | ParB/RepB/Spo0J family partition protein | | afdb-uniprot50 | AF-A0A7Y4NL96-F1-MODEL\_V4 | 1.0 | 0.002561 | 145 | 0.261 | 130 | 74 | 2 | 136 | 265 | 1 | 108 | Uncharacterized protein | Uncharacterized protein | | afdb-uniprot50 | AF-A0A6P1J9Y2-F1-MODEL\_V4 | 1.0 | 0.0002369 | 145 | 0.196 | 204 | 134 | 5 | 98 | 272 | 1 | 203 | Uncharacterized protein | Uncharacterized protein | | afdb-uniprot50 | AF-A0A246JU63-F1-MODEL\_V4 | 1.0 | 4.324e-06 | 145 | 0.172 | 279 | 182 | 15 | 17 | 280 | 76 | 320 | ParB domain-containing protein | ParB domain-containing protein | | afdb-uniprot50 | AF-A0A4Q2LMW1-F1-MODEL\_V4 | 1.0 | 9.445e-05 | 144 | 0.222 | 207 | 131 | 9 | 98 | 288 | 7 | 199 | ParB/RepB/Spo0J family partition protein | ParB/RepB/Spo0J family partition protein | | afdb-uniprot50 | AF-A0A2H9SQB1-F1-MODEL\_V4 | 1.0 | 2.259e-06 | 138 | 0.169 | 289 | 181 | 15 | 1 | 265 | 3 | 256 | Chromosome partitioning protein ParB | Chromosome partitioning protein ParB | | afdb-uniprot50 | AF-A0A5C8ILI9-F1-MODEL\_V4 | 1.0 | 7.841e-06 | 134 | 0.185 | 296 | 176 | 18 | 7 | 272 | 19 | 279 | ParB/RepB/Spo0J family partition protein | ParB/RepB/Spo0J family partition protein | | afdb-uniprot50 | AF-A0A2J0L660-F1-MODEL\_V4 | 1.0 | 0.0004535 | 131 | 0.175 | 233 | 170 | 10 | 60 | 282 | 1 | 221 | Uncharacterized protein | Uncharacterized protein | | afdb-uniprot50 | AF-A0A3B8V5W7-F1-MODEL\_V4 | 1.0 | 0.002854 | 126 | 0.179 | 184 | 132 | 5 | 107 | 282 | 18 | 190 | HTH\_ParB domain-containing protein | HTH\_ParB domain-containing protein | | afdb-uniprot50 | AF-A0A2T5GRZ6-F1-MODEL\_V4 | 1.0 | 0.0003278 | 119 | 0.132 | 257 | 181 | 11 | 37 | 287 | 464 | 684 | ParB-like nuclease family protein | ParB-like nuclease family protein | |
| Top keywords  (threshold 1.00e-02 (evalue)) | **ParB, partition, Spo0J, RepB, domain\_containing, partitioning, Chromosome, chromosome\_partitioning, ParB\_like, Probable** |
| Output files | ../../similar\_structures/28\_FANPEZAQ\_CDS\_0028\_afdb-proteome\_foldseek.tsv ../../similar\_structures/28\_FANPEZAQ\_CDS\_0028\_afdb-uniprot50\_foldseek.tsv ../../similar\_structures/28\_FANPEZAQ\_CDS\_0028\_merged.svg ../../similar\_structures/28\_FANPEZAQ\_CDS\_0028\_pdb\_foldseek.tsv |

  
  
  

Return to summary | Go to previous | Go to next

  


---

**Sequence/structure alignments coloring**  
Each object in the alignment figures is colored according to its E-value following this color coding:

1e-100
10

**References:**  
1) Steinegger M, Meier M, Mirdita M, Vöhringer H, Haunsberger S J, and Söding J (2019) HH-suite3 for fast remote homology detection and deep protein annotation, BMC Bioinformatics, 473. doi: 10.1186/s12859-019-3019-7  
2) Jumper J, Evans R, Pritzel A, ..., Hassabis D (2021) Highly accurate protein structure prediction with AlphaFold, Nature, 596. doi: 10.1038/s41586-021-03819-2  
3) van Kempen M, Kim S, Tumescheit C, Mirdita M, Lee J, Gilchrist CLM, Söding J, and Steinegger M (2023) Fast and accurate protein structure search with Foldseek. Nature Biotechnology. doi: 10.1038/s41587-023-01773-0
