# Supplementary material for: Completing the BASEL phage collection to unlock hidden diversity for systematic exploration of phage–host interactions
Source: PLoS Biol. 2025 Apr 7;23(4):e3003063. doi: 10.1371/journal.pbio.3003063 (PMC11990801; doi:10.1371/journal.pbio.3003063)
Supplement: S2 Data — (ZIP) [file pbio.3003063.s009.zip › entries/29.html]

FANPEZAQ\_CDS\_0029


Return to summary | Go to previous | Go to next

|  |  |
| --- | --- |
| FANPEZAQ\_CDS\_0029 Page creation date: 02 Sep 2024, 12:00  Project folder: n/a  Input sequences file: Escherichia\_virus\_HeidiAbel.gb | hypothetical domain\_containing phage periplasmic kow eef2k atpase duf2158 fragment p143733 vi\_10070 nc\_018850\_p11 mh020244\_p60 carboxypeptidase regulatory\_like nitrogen fixation nifz duf2171 snoal\_like prpf4b peptidylprolyl isomerase duf5641 stress\_induced\_phosphoprotein j |

### Sequence information

|  |  |
| --- | --- |
| Name | FANPEZAQ\_CDS\_0029  29\_FANPEZAQ\_CDS\_0029 (pipeline id) |
| Imported annotations | Escherichia\_virus\_HeidiAbel Bas97 |
| Protein sequence | MKGELKAGGLALVYGLRVRPEGNGKCVRLIELASYRQSELLAMALGEPVERWWRVYGLLP GNEVAQVRAQNLLPIDGDDQFLDSATGEGLNRLAEQFGFKPAELTA |
| Number of residues | 106 |
| Molecular weight (Da) | 11614.18 |
| Output files | ../../query\_sequences/29\_FANPEZAQ\_CDS\_0029.fasta |

### Putative domain architecture and protein family

#### Search results (HHblits)1

|  |  |
| --- | --- |
| Domain family databases searched | Pfam, Ncbi-cd, Cath, Phrogs |
| Results, scheme(s)  (Top layers only; threshold 1.00e-03 (evalue)) | xml version="1.0" encoding="utf-8" standalone="no"?       2024-09-02T21:08:18.544717 image/svg+xml   Matplotlib v3.7.2, https://matplotlib.org/ |
| Results, table  (E-value ≤ 1.00e-03 (evalue)) | | db | id | prob | evalue | pvalue | score | cols | query | query\_len | template | template\_len | name | description | | --- | --- | --- | --- | --- | --- | --- | --- | --- | --- | --- | --- | --- | | phrogs | 2529 | 99.9 | 4.2e-29 | 5.7e-33 | 170.3 | 82 | (1, 84) | 106 | (1, 93) | 101 | NA | NA; Category: unknown function; p143733 VI\_10070 | | phrogs | 28276 | 99.6 | 7.2e-20 | 8.2e-24 | 123.4 | 85 | (2, 91) | 106 | (4, 102) | 104 | NA | NA; Category: unknown function; NC\_018850\_p11 | | phrogs | 6513 | 94.3 | 0.00087 | 1e-07 | 40.3 | 52 | (1, 57) | 106 | (1, 53) | 63 | NA | NA; Category: unknown function; MH020244\_p60 | |
| Top keywords  (threshold 1.00e-03 (evalue)) | **p143733, VI\_10070, NC\_018850\_p11, MH020244\_p60** |
| Output files | ../../domain\_architecture/29\_FANPEZAQ\_CDS\_0029\_cath.hhr ../../domain\_architecture/29\_FANPEZAQ\_CDS\_0029\_merged.svg ../../domain\_architecture/29\_FANPEZAQ\_CDS\_0029\_ncbi-cd.hhr ../../domain\_architecture/29\_FANPEZAQ\_CDS\_0029\_pfam.hhr ../../domain\_architecture/29\_FANPEZAQ\_CDS\_0029\_phrogs.hhr |

### Identical protein sequences/structures

#### Search results

|  |  |
| --- | --- |
| Protein sequence databases searched | Pdb, Swissprot, Refseq |
| Identical proteins found | -- |
| Top keywords | -- |
| Output files | -- |

### Similar protein sequences/structures

#### Sequence similarity search results (HHblits)1

|  |  |
| --- | --- |
| Sequence databases searched | Uniclust, Pdb70 |
| Results, scheme(s)  (Top layers only, threshold 1.00e-03 (evalue)) | xml version="1.0" encoding="utf-8" standalone="no"?       2024-09-02T21:08:41.986776 image/svg+xml   Matplotlib v3.7.2, https://matplotlib.org/ |
| Results, table(s)  (threshold 1.00e-03 (evalue)) | | db | id | prob | evalue | pvalue | score | cols | query | query\_len | template | template\_len | name | description | | --- | --- | --- | --- | --- | --- | --- | --- | --- | --- | --- | --- | --- | | uniclust | UniRef100\_A0A0D9A4N1 | 100.0 | 1.1e-35 | 2.8e-41 | 206.2 | 94 | (1, 105) | 106 | (7, 112) | 117 | Uncharacterized protein | Uncharacterized protein | | uniclust | UniRef100\_A0A010SYJ8 | 100.0 | 5.5e-34 | 1.4e-39 | 197.5 | 94 | (1, 105) | 106 | (15, 114) | 114 | Uncharacterized protein | Uncharacterized protein | | uniclust | UniRef100\_A0A0Q8YUL3 | 99.9 | 3.6e-32 | 8.2e-38 | 184.2 | 94 | (1, 105) | 106 | (1, 101) | 101 | KOW domain-containing protein | KOW domain-containing protein | | uniclust | UniRef100\_A0A1C7BVV0 | 99.9 | 6e-31 | 1.3e-36 | 177.3 | 90 | (1, 103) | 106 | (3, 98) | 98 | Phage protein | Phage protein | | uniclust | UniRef100\_A0A1X7M6R0 | 99.9 | 4.5e-27 | 1.1e-32 | 161.4 | 91 | (1, 102) | 106 | (2, 104) | 104 | Carboxypeptidase regulatory-like domain-containing protein | Carboxypeptidase regulatory-like domain-containing protein | | uniclust | UniRef100\_A0A088FQM9 | 99.8 | 6.2e-23 | 1.4e-28 | 140.8 | 96 | (1, 106) | 106 | (1, 105) | 105 | ATPase | ATPase | | uniclust | UniRef100\_A0A3G7U449 | 99.8 | 3.7e-22 | 7.6e-28 | 136.4 | 90 | (1, 100) | 106 | (1, 102) | 107 | Phage protein | Phage protein | | uniclust | UniRef100\_A0A5E7ADD6 | 99.8 | 4e-22 | 8.1e-28 | 130.7 | 83 | (1, 103) | 106 | (1, 84) | 84 | Uncharacterized protein | Uncharacterized protein | | uniclust | UniRef100\_A0A1G8V272 | 99.8 | 8.4e-22 | 1.7e-27 | 131.4 | 89 | (1, 102) | 106 | (1, 91) | 91 | Uncharacterized protein | Uncharacterized protein | | uniclust | UniRef100\_A0A225TZ33 | 99.7 | 2.3e-21 | 4.8e-27 | 132.3 | 92 | (1, 102) | 106 | (1, 102) | 102 | Uncharacterized protein | Uncharacterized protein | | uniclust | UniRef100\_A0A022G1Y5 | 99.7 | 3.9e-21 | 9.9e-27 | 130.9 | 70 | (1, 81) | 106 | (1, 83) | 94 | Uncharacterized protein | Uncharacterized protein | | uniclust | UniRef100\_A0A2X3KFA9 | 99.7 | 1.9e-20 | 3.6e-26 | 133.3 | 91 | (2, 103) | 106 | (1, 106) | 149 | Uncharacterized protein | Uncharacterized protein | | uniclust | UniRef100\_A0A144RG41 | 99.7 | 2.5e-20 | 5.1e-26 | 126.6 | 90 | (2, 102) | 106 | (1, 102) | 102 | Uncharacterized protein | Uncharacterized protein | | uniclust | UniRef100\_A0A2Z5A9T9 | 99.7 | 8.9e-20 | 1.9e-25 | 125.7 | 91 | (1, 102) | 106 | (1, 93) | 108 | Uncharacterized protein | Uncharacterized protein | | uniclust | UniRef100\_A0A132DRQ2 | 99.6 | 2.8e-19 | 6.7e-25 | 124.4 | 82 | (1, 102) | 106 | (1, 95) | 108 | Uncharacterized protein | Uncharacterized protein | | uniclust | UniRef100\_A0A104MV53 | 99.6 | 1.7e-18 | 4.5e-24 | 126.5 | 75 | (1, 81) | 106 | (10, 97) | 145 | Uncharacterized protein | Uncharacterized protein | | uniclust | UniRef100\_A0A0A8RLE3 | 99.6 | 3e-18 | 5.6e-24 | 113.0 | 89 | (1, 103) | 106 | (1, 90) | 90 | Uncharacterized protein | Uncharacterized protein | | uniclust | UniRef100\_A0A071LWX4 | 99.6 | 5.5e-18 | 1.1e-23 | 121.6 | 87 | (2, 99) | 106 | (26, 125) | 146 | Periplasmic protein | Periplasmic protein | | uniclust | UniRef100\_A0A0N0JSM0 | 99.4 | 1.2e-15 | 2.9e-21 | 108.8 | 96 | (2, 104) | 106 | (9, 113) | 120 | Uncharacterized protein | Uncharacterized protein | | uniclust | UniRef100\_A0A483MTB8 | 99.4 | 2.9e-15 | 5.6e-21 | 102.1 | 90 | (2, 102) | 106 | (1, 104) | 104 | Phage protein | Phage protein | | uniclust | UniRef100\_A0A0J6LF42 | 99.3 | 9.5e-15 | 1.9e-20 | 101.5 | 81 | (1, 81) | 106 | (1, 91) | 114 | Uncharacterized protein | Uncharacterized protein | | uniclust | UniRef100\_UPI001C60F1CF | 99.3 | 1.6e-14 | 2.9e-20 | 97.7 | 86 | (2, 98) | 106 | (1, 97) | 102 | hypothetical protein | hypothetical protein | | uniclust | UniRef100\_A0A062ISY1 | 99.3 | 1.9e-14 | 4.5e-20 | 100.2 | 75 | (1, 81) | 106 | (1, 82) | 102 | Phage protein | Phage protein | | uniclust | UniRef100\_UPI0008FB2880 | 99.2 | 5.4e-14 | 1e-19 | 97.5 | 88 | (2, 104) | 106 | (1, 89) | 120 | hypothetical protein | hypothetical protein | | uniclust | UniRef100\_UPI001FC9648A | 99.2 | 8.3e-14 | 1.5e-19 | 88.3 | 66 | (23, 101) | 106 | (3, 68) | 68 | hypothetical protein | hypothetical protein | | uniclust | UniRef100\_UPI00200A17EF | 99.2 | 8.4e-14 | 1.5e-19 | 87.6 | 65 | (1, 103) | 106 | (1, 65) | 65 | hypothetical protein | hypothetical protein | | uniclust | UniRef100\_A0A0U3TCB6 | 99.2 | 1.1e-13 | 2e-19 | 95.3 | 89 | (1, 100) | 106 | (1, 106) | 108 | Uncharacterized protein | Uncharacterized protein | | uniclust | UniRef100\_A0A1C4DD36 | 99.2 | 1.7e-13 | 3.3e-19 | 99.6 | 79 | (2, 81) | 106 | (52, 143) | 152 | Periplasmic protein | Periplasmic protein | | uniclust | UniRef100\_A0A103BH94 | 99.2 | 1.5e-13 | 3.5e-19 | 99.1 | 64 | (3, 81) | 106 | (18, 94) | 126 | Nitrogen fixation protein NifZ | Nitrogen fixation protein NifZ | | uniclust | UniRef100\_A0A109EH43 | 99.1 | 4.8e-13 | 1.1e-18 | 94.8 | 64 | (4, 81) | 106 | (11, 87) | 114 | Uncharacterized protein | Uncharacterized protein | | uniclust | UniRef100\_A0A7Y9IXU9 | 99.0 | 2.3e-12 | 4.5e-18 | 89.3 | 63 | (3, 78) | 106 | (1, 76) | 106 | Uncharacterized protein | Uncharacterized protein | | uniclust | UniRef100\_UPI00209847FF | 99.0 | 2.6e-12 | 4.7e-18 | 85.3 | 77 | (2, 81) | 106 | (1, 82) | 87 | hypothetical protein | hypothetical protein | | uniclust | UniRef100\_A0A1B4LB38 | 98.8 | 3.7e-11 | 8.3e-17 | 84.6 | 84 | (3, 104) | 106 | (1, 94) | 104 | DUF2158 domain-containing protein | DUF2158 domain-containing protein | | uniclust | UniRef100\_A0A1E4FXU0 | 98.7 | 1.3e-10 | 2.9e-16 | 81.5 | 69 | (1, 78) | 106 | (14, 83) | 101 | Uncharacterized protein | Uncharacterized protein | | uniclust | UniRef100\_UPI0009AC47EE | 98.7 | 2e-10 | 3.8e-16 | 74.9 | 58 | (2, 60) | 106 | (1, 67) | 74 | hypothetical protein | hypothetical protein | | uniclust | UniRef100\_UPI0011B2021C | 98.7 | 2.3e-10 | 4.3e-16 | 80.4 | 81 | (1, 81) | 106 | (1, 96) | 121 | hypothetical protein | hypothetical protein | | uniclust | UniRef100\_UPI00059FEC15 | 98.6 | 3e-10 | 5.4e-16 | 76.8 | 71 | (2, 78) | 106 | (1, 78) | 93 | hypothetical protein | hypothetical protein | | uniclust | UniRef100\_UPI00203A5C56 | 98.6 | 4.7e-10 | 8.6e-16 | 73.9 | 45 | (49, 103) | 106 | (21, 79) | 79 | hypothetical protein | hypothetical protein | | uniclust | UniRef100\_A0A639VA07 | 98.5 | 8.1e-10 | 1.5e-15 | 69.7 | 55 | (1, 56) | 106 | (1, 58) | 61 | Uncharacterized protein | Uncharacterized protein | | uniclust | UniRef100\_A0A3G3H4G5 | 98.5 | 8.6e-10 | 1.7e-15 | 78.1 | 62 | (3, 78) | 106 | (5, 79) | 113 | Uncharacterized protein | Uncharacterized protein | | uniclust | UniRef100\_UPI0013D61B36 | 98.5 | 9.3e-10 | 1.7e-15 | 73.9 | 66 | (1, 81) | 106 | (1, 78) | 88 | hypothetical protein | hypothetical protein | | uniclust | UniRef100\_A0A839T1F5 | 98.5 | 9.4e-10 | 1.7e-15 | 74.7 | 75 | (1, 78) | 106 | (1, 78) | 94 | Uncharacterized protein | Uncharacterized protein | | uniclust | UniRef100\_A0A2W7MJX6 | 98.5 | 1.4e-09 | 2.6e-15 | 74.9 | 75 | (1, 78) | 106 | (1, 76) | 96 | Uncharacterized protein | Uncharacterized protein | | uniclust | UniRef100\_UPI0004890263 | 98.4 | 2.1e-09 | 4.2e-15 | 75.1 | 76 | (3, 78) | 106 | (1, 91) | 103 | hypothetical protein | hypothetical protein | | uniclust | UniRef100\_A0A068YI59 | 98.4 | 4.3e-09 | 8.7e-15 | 73.1 | 71 | (3, 81) | 106 | (1, 78) | 95 | Uncharacterized protein | Uncharacterized protein | | uniclust | UniRef100\_A0A859WEJ4 | 98.3 | 8.6e-09 | 1.6e-14 | 71.4 | 83 | (2, 101) | 106 | (1, 96) | 97 | Uncharacterized protein | Uncharacterized protein | | uniclust | UniRef100\_A0A7R8MJT1 | 98.3 | 1.3e-08 | 2.4e-14 | 71.3 | 77 | (2, 78) | 106 | (1, 88) | 111 | ATPase | ATPase | | uniclust | UniRef100\_UPI001556132D | 98.3 | 1.4e-08 | 2.6e-14 | 69.7 | 75 | (1, 81) | 106 | (1, 85) | 98 | hypothetical protein | hypothetical protein | | uniclust | UniRef100\_A0A1B4C0H0 | 98.2 | 1.4e-08 | 3.1e-14 | 73.0 | 62 | (4, 81) | 106 | (12, 87) | 110 | Uncharacterized protein | Uncharacterized protein | | uniclust | UniRef100\_A0A6D2G8R2 | 98.2 | 1.9e-08 | 3.5e-14 | 73.9 | 60 | (2, 62) | 106 | (1, 69) | 154 | Periplasmic protein | Periplasmic protein | | uniclust | UniRef100\_UPI00052A5558 | 98.2 | 2.4e-08 | 4.5e-14 | 69.7 | 65 | (3, 79) | 106 | (4, 83) | 103 | hypothetical protein | hypothetical protein | | uniclust | UniRef100\_A0A096FBG0 | 98.2 | 3.1e-08 | 5.8e-14 | 69.3 | 75 | (1, 78) | 106 | (1, 85) | 103 | DUF2171 domain-containing protein | DUF2171 domain-containing protein | | uniclust | UniRef100\_A0A2X3IPC5 | 98.1 | 3.6e-08 | 6.6e-14 | 65.7 | 60 | (22, 81) | 106 | (2, 71) | 80 | Periplasmic protein | Periplasmic protein | | uniclust | UniRef100\_A0A1Y0EPI5 | 98.1 | 4e-08 | 7.4e-14 | 71.7 | 77 | (1, 78) | 106 | (34, 120) | 144 | Uncharacterized protein | Uncharacterized protein | | uniclust | UniRef100\_UPI001C0BF18E | 98.1 | 4.3e-08 | 7.9e-14 | 68.1 | 72 | (3, 78) | 106 | (1, 85) | 103 | hypothetical protein | hypothetical protein | | uniclust | UniRef100\_A0A3Q9D7Q0 | 98.1 | 4.5e-08 | 8.3e-14 | 63.8 | 53 | (1, 57) | 106 | (1, 59) | 70 | Uncharacterized protein | Uncharacterized protein | | uniclust | UniRef100\_A0A7U0GBP7 | 98.1 | 4.9e-08 | 9.8e-14 | 72.7 | 79 | (2, 81) | 106 | (1, 115) | 146 | Uncharacterized protein | Uncharacterized protein | | uniclust | UniRef100\_A0A1H3ELA5 | 98.1 | 5.8e-08 | 1.1e-13 | 60.1 | 25 | (2, 27) | 106 | (4, 28) | 52 | Uncharacterized protein | Uncharacterized protein | | uniclust | UniRef100\_A0A7H4NPC2 | 98.0 | 1.1e-07 | 2e-13 | 61.7 | 47 | (46, 102) | 106 | (11, 67) | 67 | Uncharacterized protein | Uncharacterized protein | | uniclust | UniRef100\_A0A4U9HTB8 | 98.0 | 1.3e-07 | 2.4e-13 | 55.8 | 36 | (2, 38) | 106 | (1, 39) | 40 | Uncharacterized protein | Uncharacterized protein | | uniclust | UniRef100\_A0A0U4IIC9 | 98.0 | 1.3e-07 | 2.4e-13 | 47.6 | 18 | (73, 100) | 106 | (1, 18) | 19 | Uncharacterized protein | Uncharacterized protein | | uniclust | UniRef100\_A0A5P8D787 | 98.0 | 1.5e-07 | 2.9e-13 | 66.1 | 77 | (1, 78) | 106 | (1, 86) | 103 | Uncharacterized protein | Uncharacterized protein | | uniclust | UniRef100\_A0A2G9C6E2 | 97.9 | 3.4e-07 | 6.3e-13 | 63.5 | 77 | (3, 81) | 106 | (1, 83) | 99 | Uncharacterized protein | Uncharacterized protein | | uniclust | UniRef100\_A0A965ESD1 | 97.8 | 3.5e-07 | 6.7e-13 | 65.8 | 66 | (1, 78) | 106 | (13, 98) | 117 | Uncharacterized protein (Fragment) | Uncharacterized protein (Fragment) | | uniclust | UniRef100\_A0A142IE47 | 97.8 | 4.7e-07 | 9.2e-13 | 66.2 | 89 | (4, 103) | 106 | (15, 125) | 125 | Uncharacterized protein | Uncharacterized protein | | uniclust | UniRef100\_X1N3C4 | 97.8 | 5.5e-07 | 1e-12 | 55.2 | 26 | (67, 102) | 106 | (22, 47) | 48 | Uncharacterized protein (Fragment) | Uncharacterized protein (Fragment) | | uniclust | UniRef100\_UPI00115D64C9 | 97.8 | 6e-07 | 1.1e-12 | 59.5 | 33 | (2, 35) | 106 | (1, 33) | 72 | hypothetical protein | hypothetical protein | | uniclust | UniRef100\_A0A0K8P477 | 97.7 | 7.5e-07 | 1.5e-12 | 64.7 | 75 | (3, 81) | 106 | (10, 97) | 117 | Uncharacterized protein | Uncharacterized protein | | uniclust | UniRef100\_A0A519G502 | 97.7 | 8.1e-07 | 1.6e-12 | 62.7 | 70 | (1, 81) | 106 | (1, 82) | 97 | KOW domain-containing protein | KOW domain-containing protein | | uniclust | UniRef100\_A0A6J5LA11 | 97.7 | 8.9e-07 | 1.6e-12 | 60.1 | 74 | (5, 99) | 106 | (2, 83) | 85 | Uncharacterized protein | Uncharacterized protein | | uniclust | UniRef100\_A0A239N1G3 | 97.7 | 1.3e-06 | 2.4e-12 | 51.9 | 25 | (67, 101) | 106 | (15, 39) | 39 | Uncharacterized protein | Uncharacterized protein | | uniclust | UniRef100\_UPI0011BDCDA8 | 97.7 | 1.3e-06 | 2.5e-12 | 61.2 | 75 | (2, 77) | 106 | (1, 87) | 103 | hypothetical protein | hypothetical protein | | uniclust | UniRef100\_A0A516KWJ6 | 97.6 | 1.3e-06 | 2.8e-12 | 61.9 | 85 | (1, 102) | 106 | (2, 92) | 97 | Uncharacterized protein | Uncharacterized protein | | uniclust | UniRef100\_A0A6I6SLJ2 | 97.6 | 1.7e-06 | 3.3e-12 | 61.4 | 76 | (1, 81) | 106 | (1, 86) | 100 | Uncharacterized protein | Uncharacterized protein | | uniclust | UniRef100\_A0A244E847 | 97.6 | 1.7e-06 | 3.5e-12 | 62.2 | 65 | (1, 78) | 106 | (1, 72) | 108 | Uncharacterized protein | Uncharacterized protein | | uniclust | UniRef100\_UPI0009E75DDD | 97.6 | 2.3e-06 | 4.4e-12 | 63.8 | 60 | (3, 78) | 106 | (26, 98) | 141 | hypothetical protein | hypothetical protein | | uniclust | UniRef100\_A0A0K6IDV6 | 97.5 | 2.6e-06 | 5.1e-12 | 66.2 | 67 | (3, 78) | 106 | (1, 78) | 185 | Uncharacterized protein | Uncharacterized protein | | uniclust | UniRef100\_UPI0015E516B4 | 97.5 | 3e-06 | 5.6e-12 | 53.0 | 42 | (2, 44) | 106 | (1, 42) | 52 | hypothetical protein | hypothetical protein | | uniclust | UniRef100\_UPI0019579779 | 97.5 | 3.4e-06 | 6.3e-12 | 56.2 | 32 | (2, 34) | 106 | (1, 32) | 74 | hypothetical protein | hypothetical protein | | uniclust | UniRef100\_A0A158D246 | 97.5 | 3.7e-06 | 7.1e-12 | 58.3 | 64 | (3, 78) | 106 | (1, 79) | 88 | Uncharacterized protein | Uncharacterized protein | | uniclust | UniRef100\_A0A7D5XW54 | 97.5 | 3.8e-06 | 7.2e-12 | 62.8 | 79 | (1, 81) | 106 | (1, 91) | 149 | Uncharacterized protein | Uncharacterized protein | | uniclust | UniRef100\_UPI000E2FD569 | 97.5 | 4e-06 | 7.3e-12 | 55.2 | 48 | (3, 60) | 106 | (1, 48) | 69 | hypothetical protein | hypothetical protein | | uniclust | UniRef100\_A0A2M7ZW18 | 97.5 | 4.1e-06 | 7.6e-12 | 59.7 | 77 | (1, 78) | 106 | (1, 88) | 112 | Uncharacterized protein | Uncharacterized protein | | uniclust | UniRef100\_A0A0G3BHA1 | 97.5 | 4.2e-06 | 7.8e-12 | 60.5 | 69 | (3, 78) | 106 | (1, 81) | 123 | Uncharacterized protein | Uncharacterized protein | | uniclust | UniRef100\_A0A1W2AP14 | 97.5 | 4.3e-06 | 8.1e-12 | 63.3 | 97 | (3, 105) | 106 | (4, 128) | 163 | Uncharacterized protein | Uncharacterized protein | | uniclust | UniRef100\_UPI001C462278 | 97.5 | 4.5e-06 | 8.3e-12 | 61.2 | 67 | (1, 78) | 106 | (1, 86) | 136 | hypothetical protein | hypothetical protein | | uniclust | UniRef100\_UPI00187D4FC2 | 97.4 | 5.6e-06 | 1e-11 | 64.5 | 60 | (3, 78) | 106 | (4, 75) | 212 | hypothetical protein | hypothetical protein | | uniclust | UniRef100\_A0A1S5R1F1 | 97.4 | 6.8e-06 | 1.2e-11 | 58.8 | 86 | (3, 100) | 106 | (6, 105) | 113 | Uncharacterized protein | Uncharacterized protein | | uniclust | UniRef100\_UPI001F19C64C | 97.4 | 7.4e-06 | 1.4e-11 | 52.6 | 44 | (3, 60) | 106 | (9, 52) | 59 | hypothetical protein | hypothetical protein | | uniclust | UniRef100\_A0A1H5SET1 | 97.4 | 7.9e-06 | 1.4e-11 | 59.1 | 81 | (1, 81) | 106 | (1, 90) | 122 | Uncharacterized protein | Uncharacterized protein | | uniclust | UniRef100\_A0A2T7SZR4 | 97.3 | 8.9e-06 | 1.6e-11 | 59.0 | 70 | (3, 78) | 106 | (1, 80) | 123 | Uncharacterized protein | Uncharacterized protein | | uniclust | UniRef100\_UPI0016401755 | 97.3 | 9.9e-06 | 1.8e-11 | 56.7 | 65 | (3, 81) | 106 | (5, 83) | 96 | hypothetical protein | hypothetical protein | | uniclust | UniRef100\_UPI0006AC61BC | 97.3 | 1e-05 | 2e-11 | 56.4 | 65 | (3, 78) | 106 | (1, 80) | 92 | hypothetical protein | hypothetical protein | | uniclust | UniRef100\_A0A1T1ANT1 | 97.3 | 1.2e-05 | 2.2e-11 | 56.5 | 72 | (3, 81) | 106 | (1, 87) | 100 | Uncharacterized protein | Uncharacterized protein | | uniclust | UniRef100\_A0A873WNJ2 | 97.3 | 1.3e-05 | 2.4e-11 | 60.3 | 80 | (2, 81) | 106 | (1, 109) | 146 | Uncharacterized protein | Uncharacterized protein | | uniclust | UniRef100\_A0A257FV18 | 97.3 | 1.3e-05 | 2.5e-11 | 58.0 | 69 | (1, 78) | 106 | (12, 100) | 122 | KOW domain-containing protein | KOW domain-containing protein | | uniclust | UniRef100\_UPI0018CA9FA7 | 97.2 | 1.7e-05 | 3.1e-11 | 54.8 | 61 | (2, 78) | 106 | (1, 72) | 89 | hypothetical protein | hypothetical protein | | uniclust | UniRef100\_A0A965KUK1 | 97.2 | 1.9e-05 | 3.8e-11 | 57.9 | 69 | (3, 77) | 106 | (8, 91) | 115 | Uncharacterized protein | Uncharacterized protein | | uniclust | UniRef100\_UPI000A5B9EFB | 97.2 | 2.3e-05 | 4.2e-11 | 58.7 | 45 | (3, 59) | 106 | (1, 45) | 151 | hypothetical protein | hypothetical protein | | uniclust | UniRef100\_A0A0Q0XEJ4 | 97.2 | 2.2e-05 | 4.6e-11 | 57.4 | 70 | (1, 81) | 106 | (18, 96) | 109 | Uncharacterized protein | Uncharacterized protein | | uniclust | UniRef100\_UPI002150BB13 | 97.1 | 2.6e-05 | 4.9e-11 | 59.9 | 79 | (2, 104) | 106 | (3, 94) | 182 | hypothetical protein | hypothetical protein | | uniclust | UniRef100\_A0A060BAN8 | 97.1 | 2.5e-05 | 4.9e-11 | 55.2 | 70 | (1, 78) | 106 | (1, 71) | 91 | DUF2158 domain-containing protein | DUF2158 domain-containing protein | | uniclust | UniRef100\_A0A6G5Y4H1 | 97.1 | 3.9e-05 | 7.2e-11 | 52.2 | 66 | (1, 81) | 106 | (1, 69) | 80 | Uncharacterized protein | Uncharacterized protein | | uniclust | UniRef100\_A0A315DX93 | 97.0 | 5.8e-05 | 1.1e-10 | 53.0 | 78 | (3, 81) | 106 | (1, 87) | 95 | Uncharacterized protein | Uncharacterized protein | | uniclust | UniRef100\_A0A1W2BP56 | 97.0 | 5.5e-05 | 1.1e-10 | 56.4 | 88 | (2, 90) | 106 | (1, 101) | 127 | Uncharacterized protein | Uncharacterized protein | | uniclust | UniRef100\_UPI001E552E10 | 97.0 | 6.1e-05 | 1.1e-10 | 59.1 | 33 | (3, 36) | 106 | (121, 153) | 207 | hypothetical protein | hypothetical protein | | uniclust | UniRef100\_A0A924KGG3 | 97.0 | 6.2e-05 | 1.1e-10 | 53.7 | 80 | (1, 81) | 106 | (1, 88) | 105 | Uncharacterized protein | Uncharacterized protein | | uniclust | UniRef100\_A0A4R8FRD1 | 97.0 | 6.4e-05 | 1.2e-10 | 53.0 | 72 | (2, 78) | 106 | (3, 76) | 92 | Uncharacterized protein | Uncharacterized protein | | uniclust | UniRef100\_A0A653R688 | 96.9 | 9.4e-05 | 1.8e-10 | 53.4 | 71 | (2, 78) | 106 | (1, 88) | 106 | Uncharacterized protein | Uncharacterized protein | | uniclust | UniRef100\_A0A1M7R589 | 96.8 | 0.00011 | 2.1e-10 | 53.2 | 70 | (3, 78) | 106 | (1, 87) | 115 | Uncharacterized protein | Uncharacterized protein | | uniclust | UniRef100\_A0A7U4PBC5 | 96.8 | 0.00011 | 2.1e-10 | 53.3 | 33 | (3, 36) | 106 | (9, 41) | 107 | Uncharacterized protein | Uncharacterized protein | | uniclust | UniRef100\_A0A071MBJ7 | 96.8 | 0.00014 | 2.5e-10 | 51.6 | 65 | (3, 81) | 106 | (6, 83) | 99 | Uncharacterized protein | Uncharacterized protein | | uniclust | UniRef100\_UPI0011AE5093 | 96.8 | 0.00014 | 2.6e-10 | 52.7 | 31 | (1, 32) | 106 | (1, 33) | 115 | hypothetical protein | hypothetical protein | | uniclust | UniRef100\_A0A3D8K3M3 | 96.8 | 0.00014 | 2.9e-10 | 53.9 | 66 | (3, 78) | 106 | (1, 78) | 114 | Uncharacterized protein | Uncharacterized protein | | uniclust | UniRef100\_A0A828SWE6 | 96.8 | 0.00016 | 3e-10 | 50.0 | 71 | (2, 78) | 106 | (1, 79) | 83 | Uncharacterized protein | Uncharacterized protein | | uniclust | UniRef100\_UPI0012F7E7BF | 96.7 | 0.00021 | 3.9e-10 | 52.2 | 74 | (1, 77) | 106 | (1, 80) | 119 | hypothetical protein | hypothetical protein | | uniclust | UniRef100\_UPI0012AFD91F | 96.7 | 0.00023 | 4.2e-10 | 46.9 | 33 | (2, 35) | 106 | (1, 36) | 63 | hypothetical protein | hypothetical protein | | uniclust | UniRef100\_UPI002150BB13 | 96.7 | 0.00024 | 4.3e-10 | 55.2 | 65 | (3, 81) | 106 | (94, 171) | 182 | hypothetical protein | hypothetical protein | | uniclust | UniRef100\_UPI000667E3A6 | 96.6 | 0.00032 | 5.9e-10 | 47.5 | 44 | (3, 60) | 106 | (4, 47) | 73 | hypothetical protein | hypothetical protein | | uniclust | UniRef100\_A0A2T7SZN9 | 96.6 | 0.00033 | 6e-10 | 51.1 | 30 | (3, 33) | 106 | (21, 52) | 116 | Uncharacterized protein | Uncharacterized protein | | uniclust | UniRef100\_A0A1G3GYN4 | 96.5 | 0.00036 | 7.4e-10 | 50.8 | 33 | (2, 36) | 106 | (1, 33) | 100 | Uncharacterized protein | Uncharacterized protein | | uniclust | UniRef100\_A0A5P8D3D0 | 96.5 | 0.0004 | 7.4e-10 | 49.2 | 68 | (2, 78) | 106 | (1, 80) | 96 | Uncharacterized protein | Uncharacterized protein | | uniclust | UniRef100\_C5A7S6 | 96.5 | 0.00039 | 7.5e-10 | 52.0 | 70 | (3, 78) | 106 | (11, 83) | 127 | Uncharacterized protein | Uncharacterized protein | | uniclust | UniRef100\_UPI000539086C | 96.5 | 0.00042 | 8.2e-10 | 50.8 | 76 | (3, 79) | 106 | (8, 96) | 111 | hypothetical protein | hypothetical protein | | uniclust | UniRef100\_UPI0021F1A385 | 96.5 | 0.00048 | 8.8e-10 | 45.6 | 35 | (1, 37) | 106 | (1, 35) | 64 | hypothetical protein | hypothetical protein | | uniclust | UniRef100\_UPI0011CD8128 | 96.5 | 0.00048 | 8.9e-10 | 51.9 | 32 | (3, 35) | 106 | (52, 83) | 144 | hypothetical protein | hypothetical protein | | uniclust | UniRef100\_UPI001D17D500 | 96.5 | 0.0005 | 9.5e-10 | 48.0 | 83 | (2, 102) | 106 | (1, 85) | 85 | hypothetical protein | hypothetical protein | | uniclust | UniRef100\_A0A431L7L8 | 96.5 | 0.00054 | 1e-09 | 50.0 | 74 | (1, 78) | 106 | (1, 88) | 115 | Uncharacterized protein | Uncharacterized protein | | uniclust | UniRef100\_A0A4Y5TPK8 | 96.4 | 0.00059 | 1.1e-09 | 50.9 | 77 | (2, 81) | 106 | (1, 116) | 129 | Uncharacterized protein | Uncharacterized protein | | uniclust | UniRef100\_A0A315E1P7 | 96.4 | 0.00063 | 1.2e-09 | 55.7 | 31 | (3, 34) | 106 | (4, 34) | 272 | Uncharacterized protein | Uncharacterized protein | | uniclust | UniRef100\_A0A0F9SEI4 | 96.4 | 0.00065 | 1.2e-09 | 48.1 | 64 | (3, 79) | 106 | (1, 68) | 94 | SnoaL-like domain-containing protein | SnoaL-like domain-containing protein | | uniclust | UniRef100\_A0A6S7CP95 | 96.4 | 0.00067 | 1.2e-09 | 48.9 | 63 | (3, 81) | 106 | (1, 73) | 105 | Uncharacterized protein | Uncharacterized protein | | uniclust | UniRef100\_UPI0005CA7972 | 96.4 | 0.0007 | 1.3e-09 | 47.5 | 67 | (2, 78) | 106 | (1, 68) | 89 | hypothetical protein | hypothetical protein | | uniclust | UniRef100\_A0A1D2SK25 | 96.3 | 0.0008 | 1.5e-09 | 48.1 | 71 | (3, 77) | 106 | (1, 71) | 100 | Uncharacterized protein | Uncharacterized protein | | uniclust | UniRef100\_UPI0013FDE139 | 96.3 | 0.00082 | 1.5e-09 | 43.0 | 26 | (67, 102) | 106 | (27, 52) | 52 | hypothetical protein | hypothetical protein | |
| Top keywords  (threshold 1.00e-03 (evalue)) | **hypothetical, domain\_containing, Phage, Periplasmic, KOW, ATPase, DUF2158, Fragment, Carboxypeptidase, regulatory\_like** |
| Output files | ../../similar\_sequences/29\_FANPEZAQ\_CDS\_0029\_merged.svg ../../similar\_sequences/29\_FANPEZAQ\_CDS\_0029\_pdb70.a3m ../../similar\_sequences/29\_FANPEZAQ\_CDS\_0029\_pdb70.hhr ../../similar\_sequences/29\_FANPEZAQ\_CDS\_0029\_uniclust.a3m ../../similar\_sequences/29\_FANPEZAQ\_CDS\_0029\_uniclust.hhr |

#### Structure prediction (AlphaFold)2

|  |  |
| --- | --- |
| Stats | xml version="1.0" encoding="utf-8" standalone="no"?       2024-09-02T21:09:29.303587 image/svg+xml   Matplotlib v3.7.2, https://matplotlib.org/ |
| Predicted structure | **NGL Viewer Controls:**  - Center: *Left-Click* - Rotate: *Left-Click + Drag* - Translate: *Right-Click + Drag* - Zoom: *Shift + Left-Click + Drag* |
| Output files | ../../predicted\_structures/29\_FANPEZAQ\_CDS\_0029/features.pkl ../../predicted\_structures/29\_FANPEZAQ\_CDS\_0029/ranked\_0.pdb ../../predicted\_structures/29\_FANPEZAQ\_CDS\_0029/ranked\_0\_plots.svg ../../predicted\_structures/29\_FANPEZAQ\_CDS\_0029/result\_model\_1\_ptm\_pred\_0.pkl |

#### Structure similarity search results (Foldseek)3

|  |  |
| --- | --- |
| Structure databases searched | Pdb, Afdb-proteome, Afdb-uniprot50 |
| Results, scheme(s)  (Top layers only, threshold 1.00e-02 (evalue)) | xml version="1.0" encoding="utf-8" standalone="no"?       2024-09-02T21:11:01.797539 image/svg+xml   Matplotlib v3.7.2, https://matplotlib.org/ |
| Results, table  (threshold 1.00e-02 (evalue)) | | db | id | prob | evalue | bits | fident | alnlen | mismatch | gapopen | qstart | qend | tstart | tend | name | description | | --- | --- | --- | --- | --- | --- | --- | --- | --- | --- | --- | --- | --- | --- | --- | | afdb-uniprot50 | AF-A0A7S1FJM8-F1-MODEL\_V4 | 1.0 | 0.002565 | 147 | 0.269 | 78 | 41 | 3 | 1 | 78 | 35 | 96 | Hypothetical protein | Hypothetical protein | | afdb-uniprot50 | AF-A0A3M8RD56-F1-MODEL\_V4 | 1.0 | 0.0004608 | 144 | 0.235 | 89 | 53 | 3 | 1 | 86 | 1 | 77 | Uncharacterized protein | Uncharacterized protein | | afdb-uniprot50 | AF-A0A7S2FYS6-F1-MODEL\_V4 | 1.0 | 0.005353 | 139 | 0.256 | 78 | 43 | 3 | 3 | 80 | 8 | 70 | Hypothetical protein | Hypothetical protein | | afdb-uniprot50 | AF-A0A7S2C143-F1-MODEL\_V4 | 1.0 | 0.005691 | 139 | 0.194 | 77 | 47 | 2 | 1 | 77 | 42 | 103 | Hypothetical protein | Hypothetical protein | | afdb-uniprot50 | AF-A0A7S1FKI3-F1-MODEL\_V4 | 1.0 | 0.007273 | 139 | 0.233 | 77 | 47 | 2 | 1 | 77 | 89 | 153 | Hypothetical protein | Hypothetical protein | | afdb-uniprot50 | AF-A0A812UX06-F1-MODEL\_V4 | 1.0 | 0.009882 | 138 | 0.216 | 83 | 48 | 4 | 1 | 82 | 1 | 67 | Hypothetical protein | Hypothetical protein | | afdb-uniprot50 | AF-A0A812LB95-F1-MODEL\_V4 | 1.0 | 0.001087 | 138 | 0.257 | 97 | 55 | 5 | 2 | 96 | 196 | 277 | Hypothetical protein | Hypothetical protein | | afdb-uniprot50 | AF-A0A0G4HT18-F1-MODEL\_V4 | 1.0 | 0.003278 | 138 | 0.246 | 77 | 43 | 2 | 1 | 77 | 1 | 62 | Uncharacterized protein | Uncharacterized protein | | afdb-uniprot50 | AF-A0A7S2QCT9-F1-MODEL\_V4 | 1.0 | 0.001229 | 137 | 0.206 | 97 | 59 | 3 | 4 | 98 | 6 | 86 | Hypothetical protein | Hypothetical protein | | afdb-uniprot50 | AF-A0A7S1WPA2-F1-MODEL\_V4 | 1.0 | 0.008742 | 136 | 0.21 | 76 | 45 | 3 | 3 | 77 | 2 | 63 | Hypothetical protein | Hypothetical protein | | afdb-uniprot50 | AF-A0A7S1LFS6-F1-MODEL\_V4 | 1.0 | 0.001477 | 132 | 0.269 | 89 | 47 | 4 | 1 | 87 | 103 | 175 | Hypothetical protein | Hypothetical protein | | afdb-uniprot50 | AF-A0A0G4G8E2-F1-MODEL\_V4 | 1.0 | 0.006051 | 132 | 0.22 | 86 | 50 | 3 | 1 | 85 | 1 | 70 | Uncharacterized protein | Uncharacterized protein | | afdb-uniprot50 | AF-A0A813A0V9-F1-MODEL\_V4 | 1.0 | 0.0008 | 129 | 0.218 | 96 | 59 | 2 | 5 | 98 | 94 | 175 | Eef2k protein | Eef2k protein | | afdb-uniprot50 | AF-A0A7S4BX12-F1-MODEL\_V4 | 1.0 | 0.002727 | 129 | 0.238 | 88 | 49 | 4 | 1 | 85 | 214 | 286 | Hypothetical protein | Hypothetical protein | | afdb-uniprot50 | AF-A0A7S1B199-F1-MODEL\_V4 | 1.0 | 0.006051 | 128 | 0.244 | 86 | 50 | 3 | 2 | 87 | 130 | 200 | Hypothetical protein | Hypothetical protein | | afdb-uniprot50 | AF-A0A812ZDA0-F1-MODEL\_V4 | 1.0 | 0.001156 | 127 | 0.22 | 100 | 62 | 3 | 1 | 98 | 97 | 182 | Eef2k protein | Eef2k protein | | afdb-uniprot50 | AF-A0A3D9EXJ8-F1-MODEL\_V4 | 1.0 | 0.005353 | 126 | 0.247 | 89 | 43 | 3 | 3 | 78 | 2 | 79 | Uncharacterized protein | Uncharacterized protein | | afdb-uniprot50 | AF-A0A813JW72-F1-MODEL\_V4 | 1.0 | 0.002565 | 125 | 0.241 | 112 | 62 | 5 | 1 | 101 | 76 | 175 | Hypothetical protein | Hypothetical protein | | afdb-uniprot50 | AF-A0A813I3Y3-F1-MODEL\_V4 | 1.0 | 0.006051 | 125 | 0.302 | 86 | 41 | 4 | 2 | 87 | 62 | 128 | Hypothetical protein | Hypothetical protein | | afdb-uniprot50 | AF-A0A7S0BCM2-F1-MODEL\_V4 | 1.0 | 0.008742 | 125 | 0.255 | 86 | 49 | 2 | 2 | 87 | 17 | 87 | Hypothetical protein | Hypothetical protein | | afdb-uniprot50 | AF-A0A813CZE1-F1-MODEL\_V4 | 1.0 | 0.004735 | 124 | 0.257 | 97 | 55 | 5 | 2 | 96 | 269 | 350 | Hypothetical protein | Hypothetical protein | | afdb-uniprot50 | AF-A0A812TPH0-F1-MODEL\_V4 | 1.0 | 0.001775 | 123 | 0.226 | 97 | 59 | 2 | 4 | 98 | 64 | 146 | Eef2k protein | Eef2k protein | | afdb-uniprot50 | AF-A0A0M0JX70-F1-MODEL\_V4 | 1.0 | 0.001888 | 122 | 0.166 | 102 | 70 | 3 | 5 | 106 | 21 | 107 | Uncharacterized protein | Uncharacterized protein | | afdb-uniprot50 | AF-A0A0D9A4N1-F1-MODEL\_V4 | 1.0 | 0.005034 | 120 | 0.268 | 108 | 49 | 4 | 1 | 88 | 1 | 98 | Uncharacterized protein | Uncharacterized protein | | afdb-uniprot50 | AF-A0A7S3X5C1-F1-MODEL\_V4 | 1.0 | 0.0009044 | 120 | 0.225 | 111 | 57 | 5 | 3 | 98 | 6 | 102 | Hypothetical protein | Hypothetical protein | | afdb-uniprot50 | AF-A0A7S1J6S3-F1-MODEL\_V4 | 1.0 | 0.006434 | 120 | 0.188 | 85 | 53 | 3 | 3 | 87 | 10 | 78 | Hypothetical protein | Hypothetical protein | | afdb-uniprot50 | AF-A0A813JBM0-F1-MODEL\_V4 | 1.0 | 0.008222 | 120 | 0.235 | 85 | 48 | 4 | 3 | 86 | 284 | 352 | Hypothetical protein | Hypothetical protein | | afdb-uniprot50 | AF-A0A812RL46-F1-MODEL\_V4 | 1.0 | 0.007273 | 120 | 0.257 | 97 | 55 | 5 | 2 | 96 | 481 | 562 | Hypothetical protein | Hypothetical protein | | afdb-uniprot50 | AF-A0A7S0HQB5-F1-MODEL\_V4 | 1.0 | 0.006434 | 119 | 0.233 | 90 | 49 | 4 | 2 | 87 | 100 | 173 | Hypothetical protein | Hypothetical protein | | afdb-uniprot50 | AF-A0A812MRQ4-F1-MODEL\_V4 | 1.0 | 0.007733 | 119 | 0.22 | 86 | 53 | 2 | 1 | 86 | 186 | 257 | Hypothetical protein | Hypothetical protein | | afdb-uniprot50 | AF-A0A7S0FC98-F1-MODEL\_V4 | 1.0 | 0.009294 | 117 | 0.255 | 86 | 46 | 3 | 5 | 88 | 120 | 189 | Hypothetical protein | Hypothetical protein | | afdb-uniprot50 | AF-A0A0F9XCY2-F1-MODEL\_V4 | 1.0 | 0.003485 | 116 | 0.153 | 104 | 71 | 3 | 3 | 106 | 2 | 88 | Uncharacterized protein | Uncharacterized protein | | afdb-uniprot50 | AF-A0A7S2C3M0-F1-MODEL\_V4 | 1.0 | 0.003083 | 116 | 0.223 | 103 | 58 | 4 | 4 | 106 | 51 | 131 | Hypothetical protein | Hypothetical protein | | afdb-uniprot50 | AF-A0A7S3WU97-F1-MODEL\_V4 | 1.0 | 0.008742 | 116 | 0.206 | 87 | 53 | 3 | 1 | 87 | 1 | 71 | Hypothetical protein | Hypothetical protein | | afdb-uniprot50 | AF-A0A813GKT4-F1-MODEL\_V4 | 1.0 | 0.001229 | 114 | 0.225 | 102 | 55 | 4 | 2 | 103 | 74 | 151 | Hypothetical protein | Hypothetical protein | | afdb-uniprot50 | AF-A0A7S1WSB6-F1-MODEL\_V4 | 1.0 | 0.00684 | 111 | 0.209 | 105 | 63 | 5 | 4 | 106 | 1 | 87 | Hypothetical protein | Hypothetical protein | | afdb-uniprot50 | AF-A0A813E516-F1-MODEL\_V4 | 1.0 | 0.006051 | 111 | 0.295 | 105 | 52 | 5 | 2 | 106 | 59 | 141 | Hypothetical protein | Hypothetical protein | | afdb-uniprot50 | AF-R1DKV3-F1-MODEL\_V4 | 1.0 | 0.003705 | 111 | 0.217 | 101 | 57 | 4 | 5 | 98 | 66 | 151 | Uncharacterized protein | Uncharacterized protein | | afdb-uniprot50 | AF-A0A7S4HC21-F1-MODEL\_V4 | 1.0 | 0.006434 | 111 | 0.196 | 102 | 66 | 4 | 5 | 105 | 13 | 99 | Hypothetical protein | Hypothetical protein | | afdb-uniprot50 | AF-A0A7S2GV41-F1-MODEL\_V4 | 1.0 | 0.009882 | 110 | 0.145 | 103 | 59 | 3 | 4 | 106 | 3 | 76 | Hypothetical protein | Hypothetical protein | | afdb-uniprot50 | AF-A0A812IG67-F1-MODEL\_V4 | 1.0 | 0.009294 | 110 | 0.203 | 103 | 56 | 6 | 2 | 103 | 100 | 177 | Hypothetical protein | Hypothetical protein | | afdb-uniprot50 | AF-A0A7S0KF53-F1-MODEL\_V4 | 1.0 | 0.006051 | 110 | 0.15 | 106 | 68 | 5 | 1 | 106 | 10 | 93 | Hypothetical protein | Hypothetical protein | | afdb-uniprot50 | AF-A0A812NLF2-F1-MODEL\_V4 | 1.0 | 0.007733 | 110 | 0.242 | 103 | 59 | 4 | 1 | 102 | 260 | 344 | PRPF4B protein | PRPF4B protein | | afdb-uniprot50 | AF-A0A1Q9ET22-F1-MODEL\_V4 | 1.0 | 0.007733 | 110 | 0.215 | 102 | 64 | 2 | 1 | 102 | 560 | 645 | Peptidylprolyl isomerase | Peptidylprolyl isomerase | | afdb-uniprot50 | AF-A0A7S1EZX2-F1-MODEL\_V4 | 1.0 | 0.007273 | 109 | 0.247 | 113 | 52 | 5 | 1 | 98 | 43 | 137 | Hypothetical protein | Hypothetical protein | | afdb-uniprot50 | AF-A0A7S3BEY9-F1-MODEL\_V4 | 1.0 | 0.004735 | 108 | 0.266 | 105 | 59 | 4 | 2 | 106 | 9 | 95 | Hypothetical protein | Hypothetical protein | | afdb-uniprot50 | AF-A0A7S3TII7-F1-MODEL\_V4 | 1.0 | 0.008222 | 107 | 0.207 | 111 | 66 | 6 | 3 | 106 | 2 | 97 | Hypothetical protein | Hypothetical protein | | afdb-uniprot50 | AF-E9GT83-F1-MODEL\_V4 | 1.0 | 0.004453 | 106 | 0.2 | 90 | 59 | 4 | 1 | 88 | 339 | 417 | DUF5641 domain-containing protein | DUF5641 domain-containing protein | | afdb-uniprot50 | AF-A0A7S4VFS2-F1-MODEL\_V4 | 1.0 | 0.009294 | 104 | 0.231 | 95 | 59 | 4 | 4 | 98 | 361 | 441 | Hypothetical protein | Hypothetical protein | | afdb-uniprot50 | AF-A0A7S0FA90-F1-MODEL\_V4 | 1.0 | 0.007273 | 104 | 0.191 | 89 | 55 | 3 | 1 | 87 | 173 | 246 | Hypothetical protein | Hypothetical protein | | afdb-uniprot50 | AF-R1B7D7-F1-MODEL\_V4 | 1.0 | 0.005691 | 103 | 0.185 | 124 | 61 | 3 | 3 | 104 | 170 | 275 | Uncharacterized protein | Uncharacterized protein | | afdb-uniprot50 | AF-A0A2V4FDJ0-F1-MODEL\_V4 | 1.0 | 0.003278 | 102 | 0.247 | 121 | 57 | 9 | 1 | 102 | 1 | 106 | Uncharacterized protein | Uncharacterized protein | | afdb-uniprot50 | AF-A0A7S0F8T3-F1-MODEL\_V4 | 1.0 | 0.008742 | 100 | 0.166 | 114 | 63 | 6 | 4 | 101 | 16 | 113 | Hypothetical protein | Hypothetical protein | | afdb-uniprot50 | AF-A0A7S1TQQ4-F1-MODEL\_V4 | 0.999 | 0.008742 | 99 | 0.175 | 108 | 68 | 4 | 2 | 103 | 21 | 113 | Hypothetical protein | Hypothetical protein | | afdb-uniprot50 | AF-A0A1Q9EN27-F1-MODEL\_V4 | 0.999 | 0.009294 | 99 | 0.202 | 99 | 63 | 5 | 1 | 99 | 603 | 685 | Stress-induced-phosphoprotein 1 | Stress-induced-phosphoprotein 1 | | afdb-uniprot50 | AF-A0A7S4RQG6-F1-MODEL\_V4 | 0.999 | 0.00684 | 97 | 0.22 | 109 | 63 | 4 | 1 | 106 | 70 | 159 | Hypothetical protein | Hypothetical protein | | afdb-uniprot50 | AF-K8F462-F1-MODEL\_V4 | 0.998 | 0.00684 | 95 | 0.186 | 107 | 69 | 5 | 1 | 106 | 13 | 102 | Uncharacterized protein | Uncharacterized protein | | afdb-uniprot50 | AF-A0A812KFV9-F1-MODEL\_V4 | 0.998 | 0.009294 | 94 | 0.191 | 120 | 61 | 4 | 5 | 106 | 307 | 408 | Hypothetical protein | Hypothetical protein | | afdb-uniprot50 | AF-A0A6V7Y6U1-F1-MODEL\_V4 | 0.997 | 0.007733 | 91 | 0.166 | 108 | 75 | 5 | 2 | 106 | 49 | 144 | Hypothetical protein | Hypothetical protein | | afdb-uniprot50 | AF-K8EAH1-F1-MODEL\_V4 | 0.991 | 0.009294 | 84 | 0.168 | 113 | 73 | 3 | 3 | 106 | 2 | 102 | J domain-containing protein | J domain-containing protein | |
| Top keywords  (threshold 1.00e-02 (evalue)) | **Hypothetical, Eef2k, domain\_containing, PRPF4B, Peptidylprolyl, isomerase, DUF5641, Stress\_induced\_phosphoprotein, J** |
| Output files | ../../similar\_structures/29\_FANPEZAQ\_CDS\_0029\_afdb-proteome\_foldseek.tsv ../../similar\_structures/29\_FANPEZAQ\_CDS\_0029\_afdb-uniprot50\_foldseek.tsv ../../similar\_structures/29\_FANPEZAQ\_CDS\_0029\_merged.svg ../../similar\_structures/29\_FANPEZAQ\_CDS\_0029\_pdb\_foldseek.tsv |

  
  
  

Return to summary | Go to previous | Go to next

  


---

**Sequence/structure alignments coloring**  
Each object in the alignment figures is colored according to its E-value following this color coding:

1e-100
10

**References:**  
1) Steinegger M, Meier M, Mirdita M, Vöhringer H, Haunsberger S J, and Söding J (2019) HH-suite3 for fast remote homology detection and deep protein annotation, BMC Bioinformatics, 473. doi: 10.1186/s12859-019-3019-7  
2) Jumper J, Evans R, Pritzel A, ..., Hassabis D (2021) Highly accurate protein structure prediction with AlphaFold, Nature, 596. doi: 10.1038/s41586-021-03819-2  
3) van Kempen M, Kim S, Tumescheit C, Mirdita M, Lee J, Gilchrist CLM, Söding J, and Steinegger M (2023) Fast and accurate protein structure search with Foldseek. Nature Biotechnology. doi: 10.1038/s41587-023-01773-0
