# Supplementary material for: Completing the BASEL phage collection to unlock hidden diversity for systematic exploration of phage–host interactions
Source: PLoS Biol. 2025 Apr 7;23(4):e3003063. doi: 10.1371/journal.pbio.3003063 (PMC11990801; doi:10.1371/journal.pbio.3003063)
Supplement: S2 Data — (ZIP) [file pbio.3003063.s009.zip › entries/3.html]

FANPEZAQ\_CDS\_0003


Return to summary | Go to previous | Go to next

|  |  |
| --- | --- |
| FANPEZAQ\_CDS\_0003 Page creation date: 02 Sep 2024, 12:00  Project folder: n/a  Input sequences file: Escherichia\_virus\_HeidiAbel.gb | domain\_containing bppu\_n light phage receptor fab duf2479 hypothetical bppu dolichyl\_diphosphooligosaccharide\_\_protein glycosyltransferase n\_terminal immunoglobulin fibronectin motile sperm tail type repeat and ig\_like antibody heavy cell fragment copc sodium channel alpha leucine\_rich sequence putative region kda wall anchor type\_iii peptidylprolyl isomerase primosomal replication prib pric iii signal baseplate upper ig gamma transmembrane |

### Sequence information

|  |  |
| --- | --- |
| Name | FANPEZAQ\_CDS\_0003  03\_FANPEZAQ\_CDS\_0003 (pipeline id) |
| Imported annotations | Escherichia\_virus\_HeidiAbel Bas97 |
| Protein sequence | MKCSQLPQKITAGLTFESRLNLPDYPATEWAVRLLLRGPQSIDVPAVVDGKTSVIRAAAG VTGTWPAGEYWYSIRATRGSDVVEVGSGQITIMPDLAAAEAGYTAKTHAQRTLEAIEAVI EKRASMDQERYRINNRELYRTPIADLLKLRDLYRLEVAREQQAQRGCNPFGRKVRVSLR |
| Number of residues | 179 |
| Molecular weight (Da) | 19973.62 |
| Output files | ../../query\_sequences/03\_FANPEZAQ\_CDS\_0003.fasta |

### Putative domain architecture and protein family

#### Search results (HHblits)1

|  |  |
| --- | --- |
| Domain family databases searched | Pfam, Ncbi-cd, Cath, Phrogs |
| Results, scheme(s)  (Top layers only; threshold 1.00e-03 (evalue)) | xml version="1.0" encoding="utf-8" standalone="no"?       2024-09-02T21:08:12.337035 image/svg+xml   Matplotlib v3.7.2, https://matplotlib.org/ |
| Results, table  (E-value ≤ 1.00e-03 (evalue)) | | db | id | prob | evalue | pvalue | score | cols | query | query\_len | template | template\_len | name | description | | --- | --- | --- | --- | --- | --- | --- | --- | --- | --- | --- | --- | --- | | phrogs | 5774 | 100.0 | 2.4e-42 | 3e-46 | 271.9 | 171 | (5, 178) | 179 | (2, 173) | 176 | head-tail joining | head-tail joining; Category: connector; NC\_009016\_p5 | | phrogs | 1627 | 99.7 | 5.8e-23 | 7.5e-27 | 149.9 | 94 | (3, 96) | 179 | (1, 107) | 109 | NA | NA; Category: unknown function; NC\_024367\_p52 | | phrogs | 118 | 99.5 | 4.9e-19 | 6.4e-23 | 119.2 | 62 | (106, 169) | 179 | (3, 64) | 70 | head-tail adaptor | head-tail adaptor; Category: connector; p69560 VI\_02299 | | phrogs | 35772 | 97.3 | 1.9e-07 | 2.1e-11 | 66.1 | 56 | (106, 163) | 179 | (66, 121) | 136 | NA | NA; Category: unknown function; p130612 VI\_06329 | | phrogs | 20307 | 96.8 | 2.2e-06 | 2.5e-10 | 54.6 | 57 | (107, 163) | 179 | (2, 58) | 70 | NA | NA; Category: unknown function; p166236 VI\_12298 | |
| Top keywords  (threshold 1.00e-03 (evalue)) | **head\_tail, connector, joining, NC\_009016\_p5, NC\_024367\_p52, adaptor, p69560, VI\_02299, p130612, VI\_06329** |
| Output files | ../../domain\_architecture/03\_FANPEZAQ\_CDS\_0003\_cath.hhr ../../domain\_architecture/03\_FANPEZAQ\_CDS\_0003\_merged.svg ../../domain\_architecture/03\_FANPEZAQ\_CDS\_0003\_ncbi-cd.hhr ../../domain\_architecture/03\_FANPEZAQ\_CDS\_0003\_pfam.hhr ../../domain\_architecture/03\_FANPEZAQ\_CDS\_0003\_phrogs.hhr |

### Identical protein sequences/structures

#### Search results

|  |  |
| --- | --- |
| Protein sequence databases searched | Pdb, Swissprot, Refseq |
| Identical proteins found | -- |
| Top keywords | -- |
| Output files | -- |

### Similar protein sequences/structures

#### Sequence similarity search results (HHblits)1

|  |  |
| --- | --- |
| Sequence databases searched | Uniclust, Pdb70 |
| Results, scheme(s)  (Top layers only, threshold 1.00e-03 (evalue)) | xml version="1.0" encoding="utf-8" standalone="no"?       2024-09-02T21:08:28.900931 image/svg+xml   Matplotlib v3.7.2, https://matplotlib.org/ |
| Results, table(s)  (threshold 1.00e-03 (evalue)) | | db | id | prob | evalue | pvalue | score | cols | query | query\_len | template | template\_len | name | description | | --- | --- | --- | --- | --- | --- | --- | --- | --- | --- | --- | --- | --- | | uniclust | UniRef100\_A0A0A8TGY6 | 100.0 | 1.8e-51 | 4.3e-57 | 329.9 | 176 | (2, 179) | 179 | (24, 200) | 218 | Uncharacterized protein | Uncharacterized protein | | uniclust | UniRef100\_A0A165R292 | 100.0 | 1.3e-45 | 3e-51 | 290.3 | 175 | (2, 179) | 179 | (10, 195) | 206 | Uncharacterized protein | Uncharacterized protein | | uniclust | UniRef100\_A0A060H347 | 100.0 | 6.8e-45 | 1.4e-50 | 275.1 | 179 | (1, 179) | 179 | (5, 184) | 185 | Uncharacterized protein | Uncharacterized protein | | uniclust | UniRef100\_A0A0S8DP01 | 100.0 | 7.6e-44 | 1.6e-49 | 279.5 | 178 | (2, 179) | 179 | (8, 198) | 209 | Uncharacterized protein | Uncharacterized protein | | uniclust | UniRef100\_A0A2P1VF72 | 100.0 | 3e-38 | 6.2e-44 | 234.4 | 144 | (35, 179) | 179 | (4, 147) | 150 | Uncharacterized protein | Uncharacterized protein | | uniclust | UniRef100\_A0A1W6TB99 | 100.0 | 3e-34 | 5.6e-40 | 214.5 | 174 | (4, 179) | 179 | (1, 175) | 175 | Uncharacterized protein | Uncharacterized protein | | uniclust | UniRef100\_A0A0F9GVA5 | 99.9 | 2.8e-31 | 5.4e-37 | 201.5 | 162 | (1, 165) | 179 | (1, 164) | 184 | Uncharacterized protein (Fragment) | Uncharacterized protein (Fragment) | | uniclust | UniRef100\_A0A510E8K6 | 99.9 | 1.9e-30 | 3.7e-36 | 198.4 | 174 | (2, 179) | 179 | (1, 194) | 195 | Uncharacterized protein | Uncharacterized protein | | uniclust | UniRef100\_A0A1F9VFN4 | 99.9 | 6.2e-29 | 1.2e-34 | 192.3 | 170 | (5, 179) | 179 | (20, 191) | 197 | Phage tail protein | Phage tail protein | | uniclust | UniRef100\_A0A088FQK4 | 99.9 | 3.5e-28 | 7.1e-34 | 171.9 | 101 | (78, 179) | 179 | (2, 102) | 103 | Uncharacterized protein | Uncharacterized protein | | uniclust | UniRef100\_A0A2D5ZTJ7 | 99.9 | 1.4e-27 | 2.8e-33 | 181.9 | 170 | (4, 179) | 179 | (1, 171) | 171 | Uncharacterized protein | Uncharacterized protein | | uniclust | UniRef100\_A0A1J5SRA9 | 99.9 | 7.9e-27 | 1.5e-32 | 177.4 | 175 | (4, 179) | 179 | (1, 187) | 187 | Uncharacterized protein | Uncharacterized protein | | uniclust | UniRef100\_Q1ILS4 | 99.9 | 3.2e-25 | 5.8e-31 | 167.9 | 171 | (8, 179) | 179 | (10, 182) | 201 | Uncharacterized protein | Uncharacterized protein | | uniclust | UniRef100\_A0A510E7U2 | 99.9 | 3.9e-25 | 7.2e-31 | 163.9 | 166 | (4, 179) | 179 | (2, 168) | 172 | Uncharacterized protein | Uncharacterized protein | | uniclust | UniRef100\_A0A0N8FSS6 | 99.8 | 1.1e-22 | 2e-28 | 154.6 | 155 | (6, 161) | 179 | (12, 171) | 184 | DUF1254 domain-containing protein (Fragment) | DUF1254 domain-containing protein (Fragment) | | uniclust | UniRef100\_A0A090V164 | 99.8 | 2.7e-22 | 5.8e-28 | 158.6 | 161 | (1, 175) | 179 | (4, 168) | 185 | Uncharacterized protein | Uncharacterized protein | | uniclust | UniRef100\_A0A2E7ZMH9 | 99.7 | 3.5e-21 | 6.7e-27 | 138.7 | 109 | (69, 179) | 179 | (1, 114) | 125 | Uncharacterized protein (Fragment) | Uncharacterized protein (Fragment) | | uniclust | UniRef100\_A0A1V5CWH5 | 99.7 | 7.2e-21 | 1.3e-26 | 144.3 | 174 | (4, 179) | 179 | (1, 193) | 194 | Uncharacterized protein | Uncharacterized protein | | uniclust | UniRef100\_A0A3B9Q8J6 | 99.7 | 8.9e-21 | 1.8e-26 | 147.3 | 169 | (7, 179) | 179 | (2, 181) | 182 | Uncharacterized protein | Uncharacterized protein | | uniclust | UniRef100\_A0A958HLF9 | 99.7 | 2.7e-20 | 4.9e-26 | 144.8 | 170 | (5, 177) | 179 | (50, 230) | 233 | Uncharacterized protein | Uncharacterized protein | | uniclust | UniRef100\_UPI0016849037 | 99.7 | 3.6e-20 | 6.6e-26 | 139.5 | 170 | (3, 179) | 179 | (10, 182) | 182 | hypothetical protein | hypothetical protein | | uniclust | UniRef100\_A0A2D7GUN5 | 99.7 | 6.2e-20 | 1.1e-25 | 136.7 | 165 | (7, 178) | 179 | (3, 168) | 168 | Uncharacterized protein | Uncharacterized protein | | uniclust | UniRef100\_A0A969NUQ2 | 99.7 | 1.8e-19 | 3.3e-25 | 135.3 | 175 | (1, 179) | 179 | (1, 176) | 177 | Uncharacterized protein | Uncharacterized protein | | uniclust | UniRef100\_A0A286GPM6 | 99.7 | 2e-19 | 3.7e-25 | 133.3 | 146 | (9, 160) | 179 | (2, 147) | 161 | Uncharacterized protein | Uncharacterized protein | | uniclust | UniRef100\_A0A812JEV6 | 99.6 | 4.7e-19 | 8.6e-25 | 169.0 | 153 | (8, 160) | 179 | (960, 1116) | 4451 | Phage portal protein | Phage portal protein | | uniclust | UniRef100\_UPI00101302DA | 99.6 | 8.8e-19 | 1.6e-24 | 124.7 | 120 | (5, 126) | 179 | (1, 121) | 122 | hypothetical protein | hypothetical protein | | uniclust | UniRef100\_A0A071LUC0 | 99.6 | 1.7e-18 | 3.6e-24 | 133.3 | 139 | (4, 164) | 179 | (1, 141) | 161 | Uncharacterized protein | Uncharacterized protein | | uniclust | UniRef100\_UPI001982702E | 99.6 | 8.3e-18 | 1.5e-23 | 115.1 | 95 | (62, 157) | 179 | (1, 95) | 95 | hypothetical protein | hypothetical protein | | uniclust | UniRef100\_A0A934RPA6 | 99.6 | 8.9e-18 | 1.6e-23 | 125.6 | 145 | (9, 156) | 179 | (2, 153) | 168 | Uncharacterized protein | Uncharacterized protein | | uniclust | UniRef100\_A0A6H1ZNP5 | 99.5 | 1.5e-17 | 2.8e-23 | 126.3 | 167 | (6, 178) | 179 | (13, 182) | 186 | Uncharacterized protein | Uncharacterized protein | | uniclust | UniRef100\_A0A0Q7SMP9 | 99.5 | 5.6e-17 | 1.1e-22 | 124.8 | 176 | (2, 178) | 179 | (1, 187) | 187 | Uncharacterized protein | Uncharacterized protein | | uniclust | UniRef100\_A0A974WVA0 | 99.5 | 8.4e-17 | 1.5e-22 | 120.9 | 156 | (4, 161) | 179 | (2, 160) | 170 | Uncharacterized protein | Uncharacterized protein | | uniclust | UniRef100\_A0A2U1B226 | 99.5 | 1.4e-16 | 2.6e-22 | 120.1 | 134 | (23, 165) | 179 | (12, 148) | 159 | Uncharacterized protein | Uncharacterized protein | | uniclust | UniRef100\_A0A965HE76 | 99.4 | 1.6e-15 | 2.9e-21 | 108.3 | 107 | (71, 178) | 179 | (7, 119) | 119 | Uncharacterized protein | Uncharacterized protein | | uniclust | UniRef100\_A0A6J5RTA8 | 99.4 | 2e-15 | 3.7e-21 | 115.0 | 160 | (4, 167) | 179 | (7, 170) | 184 | Uncharacterized protein | Uncharacterized protein | | uniclust | UniRef100\_A0A968GWW4 | 99.3 | 4.5e-15 | 8.2e-21 | 111.7 | 150 | (6, 158) | 179 | (3, 155) | 167 | Uncharacterized protein | Uncharacterized protein | | uniclust | UniRef100\_A0A2D9MYC7 | 99.3 | 5.8e-15 | 1.1e-20 | 107.3 | 109 | (5, 114) | 179 | (8, 118) | 132 | Uncharacterized protein | Uncharacterized protein | | uniclust | UniRef100\_UPI0018F8B0B0 | 99.3 | 9.4e-15 | 1.7e-20 | 106.8 | 135 | (4, 139) | 179 | (1, 136) | 136 | hypothetical protein | hypothetical protein | | uniclust | UniRef100\_A0A3S0DLF1 | 99.3 | 2.9e-14 | 5.3e-20 | 105.7 | 128 | (33, 161) | 179 | (2, 136) | 148 | Uncharacterized protein | Uncharacterized protein | | uniclust | UniRef100\_A0A0F9JR02 | 99.2 | 2.7e-14 | 5.9e-20 | 109.7 | 103 | (1, 103) | 179 | (2, 118) | 134 | BppU N-terminal domain-containing protein | BppU N-terminal domain-containing protein | | uniclust | UniRef100\_A0A8S5N661 | 99.2 | 3.7e-14 | 7e-20 | 108.4 | 129 | (31, 163) | 179 | (18, 148) | 164 | Uncharacterized protein | Uncharacterized protein | | uniclust | UniRef100\_A0A0F9QC72 | 99.2 | 7.6e-14 | 1.4e-19 | 106.5 | 156 | (5, 161) | 179 | (10, 166) | 180 | Uncharacterized protein | Uncharacterized protein | | uniclust | UniRef100\_A0A377P1N6 | 99.1 | 2e-13 | 4.1e-19 | 106.6 | 155 | (3, 179) | 179 | (6, 166) | 170 | Uncharacterized protein | Uncharacterized protein | | uniclust | UniRef100\_UPI00035F2C0A | 99.1 | 3.8e-13 | 7.1e-19 | 101.8 | 125 | (35, 166) | 179 | (29, 154) | 167 | hypothetical protein | hypothetical protein | | uniclust | UniRef100\_A0A1Z8QIZ8 | 99.1 | 4.5e-13 | 8.6e-19 | 100.5 | 110 | (3, 115) | 179 | (16, 131) | 141 | Uncharacterized protein | Uncharacterized protein | | uniclust | UniRef100\_A0A2A4LXZ7 | 99.1 | 7e-13 | 1.3e-18 | 93.3 | 67 | (5, 71) | 179 | (8, 76) | 99 | Uncharacterized protein (Fragment) | Uncharacterized protein (Fragment) | | uniclust | UniRef100\_UPI0022F406F8 | 99.0 | 1.4e-12 | 2.6e-18 | 98.1 | 141 | (23, 163) | 179 | (2, 147) | 158 | hypothetical protein | hypothetical protein | | uniclust | UniRef100\_A0A061NTW5 | 99.0 | 1.2e-12 | 2.7e-18 | 94.4 | 57 | (104, 162) | 179 | (13, 69) | 88 | Phage protein | Phage protein | | uniclust | UniRef100\_A0A1B1IUI2 | 99.0 | 1.3e-12 | 2.8e-18 | 98.2 | 99 | (1, 99) | 179 | (2, 113) | 117 | Tail protein | Tail protein | | uniclust | UniRef100\_A0A2E7ZNQ6 | 99.0 | 2.3e-12 | 4.2e-18 | 94.6 | 118 | (4, 123) | 179 | (1, 132) | 133 | Uncharacterized protein | Uncharacterized protein | | uniclust | UniRef100\_A0A0F9ESU0 | 99.0 | 2.3e-12 | 5e-18 | 96.8 | 96 | (4, 99) | 179 | (6, 114) | 117 | Uncharacterized protein | Uncharacterized protein | | uniclust | UniRef100\_A0A9E5RBB4 | 99.0 | 3.4e-12 | 6.3e-18 | 97.3 | 153 | (2, 163) | 179 | (1, 154) | 172 | Uncharacterized protein | Uncharacterized protein | | uniclust | UniRef100\_A0A098F6N2 | 99.0 | 3.3e-12 | 7.2e-18 | 91.4 | 56 | (105, 161) | 179 | (13, 68) | 87 | Peptidylprolyl isomerase | Peptidylprolyl isomerase | | uniclust | UniRef100\_A0A0B0HXU3 | 99.0 | 3.3e-12 | 7.5e-18 | 92.8 | 57 | (105, 163) | 179 | (15, 71) | 92 | Uncharacterized protein | Uncharacterized protein | | uniclust | UniRef100\_A0A031G636 | 99.0 | 4.3e-12 | 9e-18 | 90.1 | 60 | (106, 166) | 179 | (9, 68) | 86 | Preprotein translocase subunit SecA | Preprotein translocase subunit SecA | | uniclust | UniRef100\_A0A2D3WPF3 | 98.9 | 5.7e-12 | 1.1e-17 | 87.0 | 76 | (104, 179) | 179 | (5, 83) | 84 | Uncharacterized protein | Uncharacterized protein | | uniclust | UniRef100\_A0A564WI89 | 98.9 | 6.7e-12 | 1.2e-17 | 94.8 | 99 | (63, 161) | 179 | (49, 147) | 160 | Uncharacterized protein | Uncharacterized protein | | uniclust | UniRef100\_UPI001E2E0B13 | 98.9 | 8.6e-12 | 1.6e-17 | 85.7 | 81 | (41, 122) | 179 | (3, 83) | 88 | hypothetical protein | hypothetical protein | | uniclust | UniRef100\_A0A8S0I2S7 | 98.9 | 9.8e-12 | 1.8e-17 | 90.1 | 108 | (4, 111) | 179 | (1, 120) | 121 | Uncharacterized protein | Uncharacterized protein | | uniclust | UniRef100\_A0A5Q0TJP6 | 98.9 | 1.1e-11 | 2.3e-17 | 86.9 | 73 | (104, 178) | 179 | (9, 81) | 81 | Phage tail protein | Phage tail protein | | uniclust | UniRef100\_A0A1C6BMD3 | 98.8 | 2.4e-11 | 5.1e-17 | 85.9 | 57 | (104, 161) | 179 | (5, 61) | 80 | Peptidylprolyl isomerase | Peptidylprolyl isomerase | | uniclust | UniRef100\_A0A1G1JJJ1 | 98.8 | 2.9e-11 | 5.6e-17 | 81.3 | 52 | (108, 159) | 179 | (5, 57) | 71 | Phage tail protein | Phage tail protein | | uniclust | UniRef100\_A0A073IS86 | 98.8 | 2.9e-11 | 5.8e-17 | 83.7 | 54 | (107, 161) | 179 | (5, 58) | 77 | Uncharacterized protein | Uncharacterized protein | | uniclust | UniRef100\_A0A081NV45 | 98.8 | 2.8e-11 | 6.2e-17 | 88.3 | 72 | (104, 178) | 179 | (18, 89) | 93 | Uncharacterized protein | Uncharacterized protein | | uniclust | UniRef100\_A0A3C0VX60 | 98.8 | 3.7e-11 | 6.9e-17 | 85.3 | 89 | (25, 114) | 179 | (1, 91) | 105 | Uncharacterized protein (Fragment) | Uncharacterized protein (Fragment) | | uniclust | UniRef100\_A0A0A8JM71 | 98.8 | 3.6e-11 | 7.6e-17 | 86.3 | 72 | (104, 178) | 179 | (16, 87) | 90 | Uncharacterized protein | Uncharacterized protein | | uniclust | UniRef100\_A0A193LN67 | 98.7 | 9e-11 | 1.7e-16 | 92.6 | 156 | (1, 178) | 179 | (24, 185) | 187 | Uncharacterized protein | Uncharacterized protein | | uniclust | UniRef100\_A0A0F9J4G4 | 98.7 | 1.1e-10 | 2.5e-16 | 91.4 | 97 | (3, 99) | 179 | (22, 137) | 140 | BppU N-terminal domain-containing protein | BppU N-terminal domain-containing protein | | uniclust | UniRef100\_A0A1B7HN81 | 98.6 | 3e-10 | 6.1e-16 | 89.7 | 160 | (2, 179) | 179 | (1, 166) | 168 | Uncharacterized protein | Uncharacterized protein | | uniclust | UniRef100\_A0A069I7I0 | 98.6 | 3.1e-10 | 6.6e-16 | 80.9 | 59 | (106, 166) | 179 | (14, 72) | 84 | Primosomal replication protein PriB/PriC domain protein | Primosomal replication protein PriB/PriC domain protein | | uniclust | UniRef100\_UPI00197DA380 | 98.6 | 6.1e-10 | 1.1e-15 | 77.7 | 89 | (88, 179) | 179 | (1, 92) | 92 | hypothetical protein | hypothetical protein | | uniclust | UniRef100\_A0A0K0KVG3 | 98.5 | 7.3e-10 | 1.6e-15 | 85.4 | 99 | (1, 99) | 179 | (13, 125) | 128 | Virion structural protein | Virion structural protein | | uniclust | UniRef100\_A0A0T2MKH9 | 98.4 | 2e-09 | 4.2e-15 | 78.7 | 58 | (105, 164) | 179 | (9, 66) | 95 | Uncharacterized protein | Uncharacterized protein | | uniclust | UniRef100\_A0A0Q4JQ41 | 98.4 | 2.3e-09 | 4.9e-15 | 77.4 | 58 | (104, 163) | 179 | (6, 63) | 87 | Uncharacterized protein | Uncharacterized protein | | uniclust | UniRef100\_UPI000D3BEE15 | 98.4 | 3.2e-09 | 5.8e-15 | 71.4 | 59 | (104, 162) | 179 | (5, 63) | 73 | hypothetical protein | hypothetical protein | | uniclust | UniRef100\_A0A3B9VER5 | 98.4 | 3.1e-09 | 6.3e-15 | 81.2 | 97 | (3, 99) | 179 | (5, 120) | 127 | BppU N-terminal domain-containing protein | BppU N-terminal domain-containing protein | | uniclust | UniRef100\_A0A101WGN2 | 98.4 | 3.5e-09 | 7.5e-15 | 77.3 | 63 | (105, 167) | 179 | (15, 79) | 92 | Chemotaxis protein | Chemotaxis protein | | uniclust | UniRef100\_A0A6J5QJF9 | 98.3 | 6.3e-09 | 1.3e-14 | 77.7 | 89 | (4, 93) | 179 | (10, 106) | 107 | Uncharacterized protein | Uncharacterized protein | | uniclust | UniRef100\_A0A1G1LJR7 | 98.3 | 7.4e-09 | 1.4e-14 | 69.2 | 53 | (109, 161) | 179 | (4, 57) | 70 | Phage tail protein | Phage tail protein | | uniclust | UniRef100\_A0A125WEA2 | 98.3 | 6.8e-09 | 1.4e-14 | 75.6 | 74 | (104, 179) | 179 | (17, 94) | 95 | Phage associated protein | Phage associated protein | | uniclust | UniRef100\_A0A0A2SJL1 | 98.2 | 1.7e-08 | 3.1e-14 | 69.0 | 54 | (106, 161) | 179 | (6, 59) | 76 | Phage protein | Phage protein | | uniclust | UniRef100\_A0A0A8TH18 | 98.2 | 1.4e-08 | 3.2e-14 | 81.7 | 97 | (3, 99) | 179 | (32, 146) | 152 | BppU N-terminal domain-containing protein | BppU N-terminal domain-containing protein | | uniclust | UniRef100\_A0A072T1F6 | 98.2 | 1.4e-08 | 3.4e-14 | 82.6 | 95 | (5, 99) | 179 | (23, 137) | 165 | Auto-transporter adhesin head GIN domain-containing protein | Auto-transporter adhesin head GIN domain-containing protein | | uniclust | UniRef100\_A0A142XVQ1 | 98.2 | 2.1e-08 | 4.3e-14 | 72.5 | 49 | (108, 157) | 179 | (5, 53) | 87 | Peptidylprolyl isomerase | Peptidylprolyl isomerase | | uniclust | UniRef100\_A0A0F9I9A9 | 98.2 | 2.3e-08 | 5e-14 | 77.1 | 98 | (1, 99) | 179 | (5, 119) | 123 | Uncharacterized protein | Uncharacterized protein | | uniclust | UniRef100\_A0A2D5YE29 | 98.2 | 2.9e-08 | 6.1e-14 | 75.7 | 98 | (2, 99) | 179 | (3, 113) | 116 | BppU N-terminal domain-containing protein | BppU N-terminal domain-containing protein | | uniclust | UniRef100\_A0A2D6E192 | 98.1 | 4.5e-08 | 9.4e-14 | 76.1 | 99 | (1, 99) | 179 | (9, 124) | 131 | Uncharacterized protein | Uncharacterized protein | | uniclust | UniRef100\_A0A432F9K8 | 98.1 | 5.4e-08 | 9.9e-14 | 65.4 | 54 | (5, 58) | 179 | (7, 62) | 70 | Uncharacterized protein | Uncharacterized protein | | uniclust | UniRef100\_UPI000E2A48E6 | 98.1 | 5.5e-08 | 1.1e-13 | 74.2 | 127 | (2, 145) | 179 | (1, 132) | 139 | hypothetical protein | hypothetical protein | | uniclust | UniRef100\_A0A1Q6KUC0 | 98.1 | 5.7e-08 | 1.1e-13 | 69.7 | 62 | (104, 166) | 179 | (14, 75) | 87 | Peptidylprolyl isomerase | Peptidylprolyl isomerase | | uniclust | UniRef100\_A0A2E9Y0J9 | 98.1 | 6.9e-08 | 1.3e-13 | 66.5 | 63 | (115, 178) | 179 | (15, 80) | 80 | Uncharacterized protein (Fragment) | Uncharacterized protein (Fragment) | | uniclust | UniRef100\_A0A023D777 | 98.0 | 7.6e-08 | 1.6e-13 | 74.2 | 99 | (1, 100) | 179 | (3, 113) | 120 | Uncharacterized protein | Uncharacterized protein | | uniclust | UniRef100\_A0A6G5Y298 | 98.0 | 8.6e-08 | 1.6e-13 | 67.2 | 57 | (104, 161) | 179 | (10, 66) | 80 | Phage protein | Phage protein | | uniclust | UniRef100\_UPI002251F4A5 | 98.0 | 9.9e-08 | 1.8e-13 | 67.5 | 87 | (4, 90) | 179 | (5, 93) | 93 | hypothetical protein | hypothetical protein | | uniclust | UniRef100\_A0A1J6Y2J5 | 98.0 | 9.4e-08 | 1.9e-13 | 73.4 | 97 | (3, 99) | 179 | (7, 116) | 127 | Uncharacterized protein | Uncharacterized protein | | uniclust | UniRef100\_A0A3R8K1E5 | 98.0 | 1.4e-07 | 2.6e-13 | 68.1 | 56 | (108, 163) | 179 | (33, 88) | 101 | Uncharacterized protein | Uncharacterized protein | | uniclust | UniRef100\_A0A075KCY2 | 98.0 | 1.4e-07 | 3e-13 | 66.2 | 54 | (107, 161) | 179 | (5, 58) | 72 | Uncharacterized protein | Uncharacterized protein | | uniclust | UniRef100\_A0A7X3ZJZ6 | 97.9 | 2.1e-07 | 3.9e-13 | 62.9 | 58 | (104, 161) | 179 | (4, 61) | 71 | Uncharacterized protein | Uncharacterized protein | | uniclust | UniRef100\_A0A3D2IPX1 | 97.9 | 2.4e-07 | 4.4e-13 | 64.9 | 49 | (109, 158) | 179 | (17, 65) | 86 | Peptidylprolyl isomerase | Peptidylprolyl isomerase | | uniclust | UniRef100\_UPI001F055D47 | 97.9 | 2.4e-07 | 4.5e-13 | 73.3 | 70 | (106, 178) | 179 | (35, 105) | 189 | DUF6148 family protein | DUF6148 family protein | | uniclust | UniRef100\_A0A0F9R4H3 | 97.9 | 2e-07 | 4.6e-13 | 72.4 | 98 | (1, 98) | 179 | (4, 119) | 120 | BppU N-terminal domain-containing protein | BppU N-terminal domain-containing protein | | uniclust | UniRef100\_A0A060H7N8 | 97.9 | 2.9e-07 | 5.8e-13 | 71.9 | 61 | (104, 166) | 179 | (52, 112) | 136 | Phage-related protein | Phage-related protein | | uniclust | UniRef100\_A0A974WXV3 | 97.8 | 3.9e-07 | 7.2e-13 | 66.0 | 88 | (71, 160) | 179 | (6, 93) | 104 | Uncharacterized protein | Uncharacterized protein | | uniclust | UniRef100\_A0A661I002 | 97.8 | 3.5e-07 | 7.2e-13 | 65.5 | 52 | (109, 161) | 179 | (8, 59) | 80 | Uncharacterized protein | Uncharacterized protein | | uniclust | UniRef100\_A0A1Q8QVH5 | 97.8 | 3.5e-07 | 7.4e-13 | 66.6 | 57 | (105, 163) | 179 | (8, 64) | 85 | PH domain-containing protein | PH domain-containing protein | | uniclust | UniRef100\_A0A177QGG6 | 97.8 | 4.5e-07 | 8.5e-13 | 67.9 | 96 | (2, 97) | 179 | (12, 115) | 116 | CBM2 domain-containing protein | CBM2 domain-containing protein | | uniclust | UniRef100\_A0A7V3MME9 | 97.8 | 5.1e-07 | 1e-12 | 68.6 | 97 | (1, 98) | 179 | (1, 109) | 113 | DUF2479 domain-containing protein | DUF2479 domain-containing protein | | uniclust | UniRef100\_A0A1Q6PWT8 | 97.8 | 5.8e-07 | 1.2e-12 | 64.7 | 62 | (105, 166) | 179 | (10, 71) | 82 | Uncharacterized protein | Uncharacterized protein | | uniclust | UniRef100\_A0A8J7TTR6 | 97.8 | 6.5e-07 | 1.2e-12 | 60.9 | 56 | (110, 166) | 179 | (5, 60) | 72 | Uncharacterized protein | Uncharacterized protein | | uniclust | UniRef100\_A0A022G2W7 | 97.8 | 5.9e-07 | 1.3e-12 | 68.1 | 60 | (105, 166) | 179 | (18, 77) | 106 | Primosomal replication protein PriB/PriC domain protein | Primosomal replication protein PriB/PriC domain protein | | uniclust | UniRef100\_A0A2A4ZZL1 | 97.7 | 7.6e-07 | 1.5e-12 | 61.8 | 59 | (107, 167) | 179 | (4, 62) | 73 | Primosomal replication protein PriB/PriC domain protein | Primosomal replication protein PriB/PriC domain protein | | uniclust | UniRef100\_L8M8S2 | 97.7 | 8.5e-07 | 1.6e-12 | 61.2 | 67 | (108, 178) | 179 | (8, 75) | 78 | Uncharacterized protein | Uncharacterized protein | | uniclust | UniRef100\_A0A090G6L9 | 97.7 | 7.2e-07 | 1.7e-12 | 69.6 | 96 | (1, 99) | 179 | (2, 116) | 119 | BppU N-terminal domain-containing protein | BppU N-terminal domain-containing protein | | uniclust | UniRef100\_K2K034 | 97.7 | 1.2e-06 | 2.2e-12 | 59.5 | 60 | (105, 166) | 179 | (4, 63) | 71 | Uncharacterized protein | Uncharacterized protein | | uniclust | UniRef100\_UPI001CCE3333 | 97.7 | 1.3e-06 | 2.3e-12 | 62.1 | 57 | (101, 158) | 179 | (19, 75) | 91 | hypothetical protein | hypothetical protein | | uniclust | UniRef100\_A0A346JU94 | 97.7 | 1.2e-06 | 2.3e-12 | 62.1 | 61 | (101, 163) | 179 | (8, 68) | 85 | Uncharacterized protein | Uncharacterized protein | | uniclust | UniRef100\_UPI001E29A3BE | 97.6 | 1.5e-06 | 2.8e-12 | 58.3 | 60 | (4, 63) | 179 | (6, 65) | 66 | hypothetical protein | hypothetical protein | | uniclust | UniRef100\_A0A1M6NZM2 | 97.6 | 1.5e-06 | 3.2e-12 | 66.1 | 92 | (6, 99) | 179 | (7, 105) | 107 | BppU N-terminal domain-containing protein | BppU N-terminal domain-containing protein | | uniclust | UniRef100\_A0A0K8PBJ4 | 97.6 | 1.6e-06 | 3.3e-12 | 66.7 | 91 | (7, 98) | 179 | (11, 116) | 118 | BppU N-terminal domain-containing protein | BppU N-terminal domain-containing protein | | uniclust | UniRef100\_A0A1Y1R2U1 | 97.6 | 1.9e-06 | 3.8e-12 | 67.7 | 91 | (9, 99) | 179 | (13, 117) | 134 | Uncharacterized protein | Uncharacterized protein | | uniclust | UniRef100\_A0A1Q8G4U9 | 97.6 | 2.1e-06 | 3.9e-12 | 58.8 | 59 | (107, 167) | 179 | (2, 60) | 69 | Primosomal replication protein PriB/PriC domain protein | Primosomal replication protein PriB/PriC domain protein | | uniclust | UniRef100\_A0A315ENZ3 | 97.6 | 2.2e-06 | 4.4e-12 | 67.3 | 103 | (1, 103) | 179 | (8, 123) | 135 | Uncharacterized protein | Uncharacterized protein | | uniclust | UniRef100\_UPI000A3F844A | 97.6 | 2.5e-06 | 4.6e-12 | 61.2 | 75 | (5, 79) | 179 | (12, 92) | 95 | hypothetical protein | hypothetical protein | | uniclust | UniRef100\_A0A1V4WKC7 | 97.5 | 4e-06 | 7.9e-12 | 59.3 | 51 | (109, 161) | 179 | (6, 56) | 76 | Uncharacterized protein | Uncharacterized protein | | uniclust | UniRef100\_A0A3S0P0G6 | 97.5 | 4.2e-06 | 8.3e-12 | 63.6 | 90 | (8, 97) | 179 | (15, 115) | 116 | Uncharacterized protein | Uncharacterized protein | | uniclust | UniRef100\_UPI001C940115 | 97.5 | 4.8e-06 | 8.8e-12 | 58.3 | 74 | (102, 176) | 179 | (7, 80) | 82 | hypothetical protein | hypothetical protein | | uniclust | UniRef100\_A0A023NI20 | 97.4 | 4.2e-06 | 8.9e-12 | 64.3 | 98 | (1, 98) | 179 | (1, 109) | 111 | BppU N-terminal domain-containing protein | BppU N-terminal domain-containing protein | | uniclust | UniRef100\_UPI001880E38C | 97.4 | 5.7e-06 | 1e-11 | 59.4 | 75 | (105, 179) | 179 | (13, 92) | 94 | hypothetical protein | hypothetical protein | | uniclust | UniRef100\_A0A268E9X3 | 97.4 | 5.2e-06 | 1.1e-11 | 58.8 | 54 | (106, 161) | 179 | (6, 59) | 73 | Uncharacterized protein | Uncharacterized protein | | uniclust | UniRef100\_UPI0023578956 | 97.4 | 6.2e-06 | 1.1e-11 | 59.0 | 59 | (106, 166) | 179 | (22, 80) | 92 | DUF6148 family protein | DUF6148 family protein | | uniclust | UniRef100\_UPI001FBB5195 | 97.4 | 6.6e-06 | 1.2e-11 | 53.2 | 34 | (128, 161) | 179 | (4, 37) | 53 | peptidylprolyl isomerase | peptidylprolyl isomerase | | uniclust | UniRef100\_A0A1F7S430 | 97.4 | 6.4e-06 | 1.2e-11 | 57.3 | 54 | (106, 161) | 179 | (6, 59) | 72 | Uncharacterized protein | Uncharacterized protein | | uniclust | UniRef100\_A0A2E5PQX4 | 97.4 | 6.6e-06 | 1.2e-11 | 59.8 | 78 | (3, 80) | 179 | (11, 99) | 101 | Uncharacterized protein | Uncharacterized protein | | uniclust | UniRef100\_A0A7C9BLN2 | 97.4 | 7.1e-06 | 1.4e-11 | 61.8 | 88 | (9, 97) | 179 | (14, 108) | 109 | Uncharacterized protein | Uncharacterized protein | | uniclust | UniRef100\_A0A2D6FIR8 | 97.4 | 6.9e-06 | 1.5e-11 | 65.5 | 98 | (2, 99) | 179 | (11, 126) | 133 | Cohesin domain-containing protein | Cohesin domain-containing protein | | uniclust | UniRef100\_A0A496ZSP7 | 97.3 | 8.9e-06 | 1.8e-11 | 62.2 | 94 | (5, 99) | 179 | (2, 111) | 114 | BppU N-terminal domain-containing protein | BppU N-terminal domain-containing protein | | uniclust | UniRef100\_A0A9D2L9Q1 | 97.3 | 1.1e-05 | 2.1e-11 | 58.4 | 50 | (110, 160) | 179 | (31, 80) | 98 | Peptidylprolyl isomerase | Peptidylprolyl isomerase | | uniclust | UniRef100\_A0A1M5RH09 | 97.3 | 1.2e-05 | 2.3e-11 | 59.1 | 60 | (105, 166) | 179 | (29, 88) | 98 | Uncharacterized protein | Uncharacterized protein | | uniclust | UniRef100\_A0A1V6E205 | 97.3 | 1.3e-05 | 2.4e-11 | 55.0 | 52 | (109, 160) | 179 | (4, 56) | 71 | Uncharacterized protein | Uncharacterized protein | | uniclust | UniRef100\_A0A094YXQ6 | 97.3 | 1.1e-05 | 2.4e-11 | 62.7 | 91 | (8, 99) | 179 | (8, 109) | 111 | YtkA-like domain-containing protein | YtkA-like domain-containing protein | | uniclust | UniRef100\_A0A948EQ09 | 97.3 | 1.4e-05 | 2.7e-11 | 55.4 | 61 | (105, 166) | 179 | (3, 63) | 71 | Uncharacterized protein | Uncharacterized protein | | uniclust | UniRef100\_A0A6J5NE21 | 97.2 | 1.4e-05 | 2.8e-11 | 62.6 | 95 | (5, 99) | 179 | (6, 121) | 125 | Uncharacterized protein | Uncharacterized protein | | uniclust | UniRef100\_A0A0B3S182 | 97.2 | 1.5e-05 | 2.9e-11 | 55.7 | 53 | (109, 161) | 179 | (6, 58) | 70 | Uncharacterized protein | Uncharacterized protein | | uniclust | UniRef100\_A0A064AM50 | 97.2 | 1.6e-05 | 3.2e-11 | 57.5 | 54 | (105, 160) | 179 | (8, 61) | 79 | Uncharacterized protein | Uncharacterized protein | | uniclust | UniRef100\_A0A3Q9BR07 | 97.2 | 1.9e-05 | 3.7e-11 | 55.7 | 58 | (107, 166) | 179 | (7, 64) | 75 | Uncharacterized protein | Uncharacterized protein | | uniclust | UniRef100\_A0A959C7P8 | 97.2 | 2e-05 | 3.8e-11 | 60.5 | 93 | (4, 97) | 179 | (14, 125) | 126 | Uncharacterized protein | Uncharacterized protein | | uniclust | UniRef100\_A0A2D7FMC3 | 97.2 | 2.1e-05 | 3.9e-11 | 57.2 | 69 | (3, 71) | 179 | (16, 90) | 99 | Uncharacterized protein | Uncharacterized protein | | uniclust | UniRef100\_A0A0R3MN91 | 97.2 | 1.9e-05 | 3.9e-11 | 56.9 | 52 | (109, 161) | 179 | (5, 56) | 79 | Uncharacterized protein | Uncharacterized protein | | uniclust | UniRef100\_A0A949G187 | 97.2 | 2e-05 | 4.1e-11 | 62.0 | 90 | (6, 95) | 179 | (13, 126) | 126 | Uncharacterized protein | Uncharacterized protein | | uniclust | UniRef100\_UPI001431CBAE | 97.2 | 2.3e-05 | 4.2e-11 | 58.6 | 107 | (64, 178) | 179 | (3, 114) | 116 | hypothetical protein | hypothetical protein | | uniclust | UniRef100\_A0A5B8C3L8 | 97.2 | 2.5e-05 | 4.6e-11 | 53.5 | 53 | (109, 161) | 179 | (6, 58) | 70 | Phage tail protein | Phage tail protein | | uniclust | UniRef100\_A0A7V2T445 | 97.1 | 2.9e-05 | 5.4e-11 | 56.6 | 55 | (108, 163) | 179 | (3, 57) | 93 | Primosomal replication protein PriB/PriC domain protein | Primosomal replication protein PriB/PriC domain protein | | uniclust | UniRef100\_A0A0F9BY64 | 97.1 | 3e-05 | 5.6e-11 | 53.8 | 63 | (3, 65) | 179 | (6, 75) | 75 | Uncharacterized protein (Fragment) | Uncharacterized protein (Fragment) | | uniclust | UniRef100\_A0A0F7L4W2 | 97.1 | 3.3e-05 | 6e-11 | 53.0 | 47 | (113, 161) | 179 | (9, 55) | 70 | Uncharacterized protein | Uncharacterized protein | | uniclust | UniRef100\_A0A6M3KMZ3 | 97.1 | 3.1e-05 | 6.2e-11 | 60.7 | 96 | (4, 99) | 179 | (8, 120) | 126 | Putative tail protein | Putative tail protein | | uniclust | UniRef100\_A0A850BA57 | 97.1 | 4e-05 | 7.3e-11 | 54.4 | 57 | (43, 99) | 179 | (22, 80) | 84 | MBG domain-containing protein | MBG domain-containing protein | | uniclust | UniRef100\_UPI000CE90355 | 97.0 | 4.5e-05 | 8.3e-11 | 52.9 | 59 | (107, 167) | 179 | (7, 65) | 74 | DUF6148 family protein | DUF6148 family protein | | uniclust | UniRef100\_E3HBM6 | 97.0 | 4.8e-05 | 8.9e-11 | 52.5 | 55 | (106, 161) | 179 | (5, 59) | 72 | Uncharacterized protein | Uncharacterized protein | | uniclust | UniRef100\_A0A956K140 | 97.0 | 5e-05 | 9.1e-11 | 53.5 | 62 | (104, 167) | 179 | (3, 68) | 80 | Uncharacterized protein | Uncharacterized protein | | uniclust | UniRef100\_A0A2W5LUH5 | 97.0 | 4.8e-05 | 9.2e-11 | 55.0 | 58 | (107, 166) | 179 | (13, 70) | 84 | Primosomal replication protein PriB/PriC domain protein | Primosomal replication protein PriB/PriC domain protein | | uniclust | UniRef100\_A0A0S2ZD68 | 97.0 | 4.8e-05 | 1e-10 | 60.6 | 52 | (108, 161) | 179 | (67, 118) | 135 | Phage protein | Phage protein | | uniclust | UniRef100\_A0A0F9B885 | 97.0 | 5.7e-05 | 1.1e-10 | 53.6 | 54 | (108, 161) | 179 | (7, 60) | 77 | Uncharacterized protein | Uncharacterized protein | | uniclust | UniRef100\_A0A0E3F1B5 | 97.0 | 5.8e-05 | 1.1e-10 | 62.4 | 94 | (6, 99) | 179 | (7, 115) | 191 | WW domain-containing protein | WW domain-containing protein | | uniclust | UniRef100\_A0A0A0YPM7 | 96.9 | 5.9e-05 | 1.4e-10 | 59.6 | 98 | (1, 99) | 179 | (1, 115) | 118 | Uncharacterized protein | Uncharacterized protein | | uniclust | UniRef100\_A0A1T5JQ31 | 96.9 | 8.4e-05 | 1.6e-10 | 57.0 | 89 | (6, 97) | 179 | (12, 117) | 118 | Uncharacterized protein | Uncharacterized protein | | uniclust | UniRef100\_UPI00063BFA67 | 96.9 | 9e-05 | 1.6e-10 | 59.3 | 103 | (53, 165) | 179 | (45, 148) | 175 | hypothetical protein | hypothetical protein | | uniclust | UniRef100\_A0A4R5VYM7 | 96.9 | 8.2e-05 | 1.7e-10 | 58.0 | 98 | (1, 98) | 179 | (1, 117) | 118 | BppU N-terminal domain-containing protein | BppU N-terminal domain-containing protein | | uniclust | UniRef100\_C6S6G3 | 96.9 | 9.7e-05 | 1.8e-10 | 56.2 | 73 | (104, 178) | 179 | (49, 125) | 125 | Putative phage associated protein | Putative phage associated protein | | uniclust | UniRef100\_A0A955V1P7 | 96.8 | 0.00011 | 2e-10 | 49.6 | 54 | (5, 58) | 179 | (7, 61) | 63 | Uncharacterized protein (Fragment) | Uncharacterized protein (Fragment) | | uniclust | UniRef100\_UPI0009772D17 | 96.8 | 0.0001 | 2e-10 | 56.7 | 91 | (4, 94) | 179 | (5, 107) | 111 | hypothetical protein | hypothetical protein | | uniclust | UniRef100\_A0A1G8EFZ3 | 96.8 | 0.00013 | 2.7e-10 | 57.4 | 96 | (1, 98) | 179 | (1, 115) | 118 | Uncharacterized protein | Uncharacterized protein | | uniclust | UniRef100\_A0A965I963 | 96.8 | 0.00015 | 2.9e-10 | 53.2 | 42 | (118, 160) | 179 | (26, 67) | 85 | Uncharacterized protein | Uncharacterized protein | | uniclust | UniRef100\_A0A6V8M2G4 | 96.8 | 0.00015 | 3e-10 | 56.0 | 97 | (1, 97) | 179 | (1, 110) | 111 | BppU N-terminal domain-containing protein | BppU N-terminal domain-containing protein | | uniclust | UniRef100\_A0A075KJY6 | 96.7 | 0.00017 | 3.4e-10 | 56.9 | 95 | (3, 97) | 179 | (12, 121) | 125 | BppU N-terminal domain-containing protein | BppU N-terminal domain-containing protein | | uniclust | UniRef100\_A0A6M3J343 | 96.7 | 0.0002 | 3.9e-10 | 50.8 | 50 | (110, 160) | 179 | (6, 55) | 71 | Uncharacterized protein | Uncharacterized protein | | uniclust | UniRef100\_A0A173W983 | 96.7 | 0.00022 | 4.6e-10 | 57.4 | 91 | (6, 96) | 179 | (32, 133) | 136 | Uncharacterized protein | Uncharacterized protein | | uniclust | UniRef100\_A0A9D8STH6 | 96.6 | 0.00033 | 6e-10 | 52.4 | 63 | (105, 172) | 179 | (37, 99) | 109 | Uncharacterized protein | Uncharacterized protein | | uniclust | UniRef100\_A0A1U9NPV2 | 96.6 | 0.00032 | 6.2e-10 | 50.0 | 52 | (108, 160) | 179 | (4, 55) | 72 | Uncharacterized protein | Uncharacterized protein | | uniclust | UniRef100\_A0A7J9RFL5 | 96.6 | 0.00034 | 6.3e-10 | 48.3 | 57 | (41, 97) | 179 | (9, 68) | 69 | Uncharacterized protein | Uncharacterized protein | | uniclust | UniRef100\_UPI00187511A5 | 96.6 | 0.00037 | 6.8e-10 | 50.3 | 72 | (62, 143) | 179 | (15, 87) | 88 | hypothetical protein | hypothetical protein | | uniclust | UniRef100\_A0A2D5MP89 | 96.5 | 0.00039 | 7.2e-10 | 52.7 | 93 | (4, 96) | 179 | (4, 110) | 111 | Uncharacterized protein | Uncharacterized protein | | uniclust | UniRef100\_A0A0J6HKU7 | 96.5 | 0.00036 | 7.4e-10 | 54.3 | 92 | (4, 96) | 179 | (3, 106) | 112 | Uncharacterized protein | Uncharacterized protein | | uniclust | UniRef100\_A0A0W1G8D0 | 96.5 | 0.00036 | 7.6e-10 | 55.2 | 78 | (2, 79) | 179 | (1, 97) | 119 | Uncharacterized protein | Uncharacterized protein | | uniclust | UniRef100\_A0A081MYL7 | 96.5 | 0.00045 | 8.7e-10 | 49.1 | 52 | (106, 158) | 179 | (5, 60) | 71 | Phage tail protein | Phage tail protein | | uniclust | UniRef100\_A0A7Y4VZ35 | 96.5 | 0.00046 | 8.8e-10 | 53.0 | 65 | (109, 178) | 179 | (46, 110) | 115 | Uncharacterized protein | Uncharacterized protein | | uniclust | UniRef100\_A0A2E5T0B5 | 96.5 | 0.00044 | 8.8e-10 | 55.4 | 96 | (4, 99) | 179 | (5, 125) | 136 | Uncharacterized protein | Uncharacterized protein | | uniclust | UniRef100\_A0A929FI38 | 96.5 | 0.00047 | 9.1e-10 | 53.4 | 92 | (7, 98) | 179 | (13, 117) | 119 | Uncharacterized protein | Uncharacterized protein | | uniclust | UniRef100\_UPI00201A6BDC | 96.5 | 0.00054 | 9.9e-10 | 47.8 | 63 | (115, 178) | 179 | (5, 72) | 72 | hypothetical protein | hypothetical protein | | uniclust | UniRef100\_A0A1V5IZW5 | 96.4 | 0.00055 | 1e-09 | 48.4 | 59 | (104, 163) | 179 | (2, 64) | 77 | GpW protein | GpW protein | | uniclust | UniRef100\_A0A2V2FWH8 | 96.4 | 0.00058 | 1.1e-09 | 50.3 | 64 | (104, 167) | 179 | (13, 76) | 89 | Uncharacterized protein | Uncharacterized protein | | uniclust | UniRef100\_A0A4U8YKJ0 | 96.4 | 0.00057 | 1.1e-09 | 49.2 | 55 | (105, 160) | 179 | (7, 61) | 75 | Uncharacterized protein | Uncharacterized protein | | uniclust | UniRef100\_A0A2U0TA70 | 96.4 | 0.00053 | 1.1e-09 | 54.3 | 94 | (3, 99) | 179 | (5, 115) | 118 | Nuclear transport factor 2 family protein | Nuclear transport factor 2 family protein | | uniclust | UniRef100\_A0A016XJH1 | 96.4 | 0.0005 | 1.1e-09 | 51.7 | 50 | (108, 161) | 179 | (16, 65) | 85 | Phage tail protein | Phage tail protein | | uniclust | UniRef100\_A0A381C5X6 | 96.4 | 0.00059 | 1.2e-09 | 49.2 | 52 | (108, 161) | 179 | (5, 56) | 72 | Uncharacterized protein | Uncharacterized protein | | uniclust | UniRef100\_A0A0F9H6E2 | 96.4 | 0.00062 | 1.2e-09 | 48.7 | 53 | (107, 161) | 179 | (5, 57) | 74 | Uncharacterized protein | Uncharacterized protein | | uniclust | UniRef100\_A0A350IBU3 | 96.4 | 0.00064 | 1.2e-09 | 52.6 | 91 | (9, 99) | 179 | (10, 120) | 122 | Uncharacterized protein | Uncharacterized protein | | uniclust | UniRef100\_A0A521CSU3 | 96.4 | 0.0007 | 1.3e-09 | 47.0 | 64 | (108, 179) | 179 | (5, 68) | 69 | GpW protein | GpW protein | | uniclust | UniRef100\_A0A2W4YA14 | 96.4 | 0.00068 | 1.3e-09 | 49.3 | 56 | (109, 166) | 179 | (6, 61) | 83 | Primosomal replication protein PriB/PriC domain protein | Primosomal replication protein PriB/PriC domain protein | | uniclust | UniRef100\_A0A009I3H9 | 96.3 | 0.00069 | 1.5e-09 | 53.9 | 94 | (2, 96) | 179 | (3, 117) | 120 | Uncharacterized protein | Uncharacterized protein | | uniclust | UniRef100\_A0A061Y8V6 | 96.3 | 0.00078 | 1.5e-09 | 50.9 | 59 | (107, 167) | 179 | (16, 74) | 100 | Prophage protein | Prophage protein | | uniclust | UniRef100\_A0A0F9D8Q2 | 96.3 | 0.00099 | 1.8e-09 | 50.0 | 93 | (5, 97) | 179 | (5, 107) | 108 | BppU N-terminal domain-containing protein | BppU N-terminal domain-containing protein | | uniclust | UniRef100\_A0A846QP15 | 96.3 | 0.00098 | 1.9e-09 | 46.0 | 48 | (112, 160) | 179 | (4, 51) | 63 | GpW protein | GpW protein | | uniclust | UniRef100\_A0A0J6WRI4 | 96.3 | 0.00084 | 1.9e-09 | 53.8 | 92 | (6, 98) | 179 | (11, 117) | 121 | BppU N-terminal domain-containing protein | BppU N-terminal domain-containing protein | |
| Top keywords  (threshold 1.00e-03 (evalue)) | **domain\_containing, hypothetical, BppU, N\_terminal, Phage, tail, Fragment, Peptidylprolyl, isomerase, Primosomal** |
| Output files | ../../similar\_sequences/03\_FANPEZAQ\_CDS\_0003\_merged.svg ../../similar\_sequences/03\_FANPEZAQ\_CDS\_0003\_pdb70.a3m ../../similar\_sequences/03\_FANPEZAQ\_CDS\_0003\_pdb70.hhr ../../similar\_sequences/03\_FANPEZAQ\_CDS\_0003\_uniclust.a3m ../../similar\_sequences/03\_FANPEZAQ\_CDS\_0003\_uniclust.hhr |

#### Structure prediction (AlphaFold)2

|  |  |
| --- | --- |
| Stats | xml version="1.0" encoding="utf-8" standalone="no"?       2024-09-02T21:09:00.613669 image/svg+xml   Matplotlib v3.7.2, https://matplotlib.org/ |
| Predicted structure | **NGL Viewer Controls:**  - Center: *Left-Click* - Rotate: *Left-Click + Drag* - Translate: *Right-Click + Drag* - Zoom: *Shift + Left-Click + Drag* |
| Output files | ../../predicted\_structures/03\_FANPEZAQ\_CDS\_0003/features.pkl ../../predicted\_structures/03\_FANPEZAQ\_CDS\_0003/ranked\_0.pdb ../../predicted\_structures/03\_FANPEZAQ\_CDS\_0003/ranked\_0\_plots.svg ../../predicted\_structures/03\_FANPEZAQ\_CDS\_0003/result\_model\_1\_ptm\_pred\_0.pkl |

#### Structure similarity search results (Foldseek)3

|  |  |
| --- | --- |
| Structure databases searched | Pdb, Afdb-proteome, Afdb-uniprot50 |
| Results, scheme(s)  (Top layers only, threshold 1.00e-02 (evalue)) | xml version="1.0" encoding="utf-8" standalone="no"?       2024-09-02T21:10:08.411433 image/svg+xml   Matplotlib v3.7.2, https://matplotlib.org/ |
| Results, table  (threshold 1.00e-02 (evalue)) | | db | id | prob | evalue | bits | fident | alnlen | mismatch | gapopen | qstart | qend | tstart | tend | name | description | | --- | --- | --- | --- | --- | --- | --- | --- | --- | --- | --- | --- | --- | --- | --- | | pdb | 8GTD\_M | 1.0 | 5.143e-14 | 410 | 0.303 | 181 | 107 | 8 | 7 | 178 | 8 | 178 | Head-to-tail joining protein | Head-to-tail joining protein | | pdb | 6SWS\_E | 1.0 | 0.0002162 | 131 | 0.159 | 94 | 67 | 5 | 7 | 97 | 6 | 90 | Phosphoinositide 3-kinase adapter protein 1 | Phosphoinositide 3-kinase adapter protein 1 | | pdb | 7KDD\_I | 1.0 | 0.0007933 | 103 | 0.12 | 108 | 69 | 5 | 7 | 94 | 7 | 108 | SM5-1 Fab antibody light chain | SM5-1 Fab antibody light chain | | pdb | 4V96\_AN | 1.0 | 0.0001921 | 103 | 0.103 | 126 | 102 | 5 | 9 | 124 | 21 | 145 | ORF48 | ORF48 | | pdb | 1OAR\_O | 0.999 | 0.00152 | 98 | 0.112 | 107 | 70 | 4 | 7 | 94 | 5 | 105 | IMMUNOGLOBULING E | IMMUNOGLOBULING E | | pdb | 7E5S\_Q | 0.998 | 0.003687 | 93 | 0.114 | 105 | 68 | 5 | 9 | 94 | 10 | 108 | FC05 light chain | FC05 light chain | | pdb | 7XW6\_L | 0.997 | 0.002438 | 92 | 0.081 | 111 | 71 | 6 | 7 | 94 | 7 | 109 | M22 antibody light chain | M22 antibody light chain | | pdb | 8DIM\_C | 0.997 | 0.0007933 | 91 | 0.099 | 111 | 70 | 7 | 7 | 94 | 5 | 108 | CR6261 Fab light chain | CR6261 Fab light chain | | pdb | 7JG2\_E | 0.997 | 0.00415 | 91 | 0.164 | 97 | 63 | 8 | 6 | 95 | 449 | 534 | Polymeric immunoglobulin receptor | Polymeric immunoglobulin receptor | | pdb | 1OAX\_O | 0.996 | 0.00415 | 90 | 0.112 | 107 | 70 | 4 | 7 | 94 | 6 | 106 | IMMUNOGLOBULIN E | IMMUNOGLOBULIN E | | pdb | 7UR6\_K | 0.996 | 0.002298 | 90 | 0.083 | 108 | 72 | 6 | 7 | 93 | 6 | 107 | Light Chain | Light Chain | | pdb | 6MID\_L | 0.996 | 0.003088 | 90 | 0.102 | 107 | 73 | 3 | 7 | 94 | 7 | 109 | monoclonal antibody ZIKV-195 light chain | monoclonal antibody ZIKV-195 light chain | | pdb | 8JJ5\_B | 0.996 | 0.0008416 | 90 | 0.134 | 134 | 96 | 8 | 3 | 124 | 7 | 132 | Uroplakin 2 | Uroplakin 2 | | pdb | 7UAR\_L | 0.996 | 0.00135 | 89 | 0.104 | 105 | 69 | 4 | 9 | 94 | 11 | 109 | C1717 Fab Light Chain | C1717 Fab Light Chain | | pdb | 7TGF\_B | 0.996 | 0.003476 | 89 | 0.075 | 119 | 71 | 5 | 9 | 93 | 10 | 123 | VHH camelid antibody | VHH camelid antibody | | pdb | 5YAX\_A | 0.996 | 0.005256 | 89 | 0.103 | 106 | 68 | 5 | 9 | 94 | 129 | 227 | scFv1 antibody | scFv1 antibody | | pdb | 1MH5\_H | 0.995 | 0.008433 | 88 | 0.108 | 120 | 70 | 6 | 9 | 100 | 10 | 120 | IMMUNOGLOBULIN MS6-164 | IMMUNOGLOBULIN MS6-164 | | pdb | 7T0W\_L | 0.994 | 0.006275 | 87 | 0.127 | 110 | 68 | 5 | 7 | 94 | 5 | 108 | Fab115 light chain, IgG1 | Fab115 light chain, IgG1 | | pdb | 4V96\_AK | 0.994 | 0.0006644 | 87 | 0.117 | 128 | 97 | 7 | 9 | 124 | 21 | 144 | ORF48 | ORF48 | | pdb | 7PA8\_KKK | 0.993 | 0.003687 | 86 | 0.117 | 111 | 67 | 8 | 7 | 94 | 6 | 108 | Fab 27C2 light chain | Fab 27C2 light chain | | pdb | 6UYN\_L | 0.993 | 0.00152 | 86 | 0.107 | 121 | 77 | 7 | 7 | 104 | 5 | 117 | CR6261 Fab light chain | CR6261 Fab light chain | | pdb | 5CUS\_O | 0.993 | 0.006658 | 86 | 0.116 | 103 | 70 | 2 | 9 | 94 | 10 | 108 | Fab LC region of KTN3379 | Fab LC region of KTN3379 | | pdb | 8TEA\_G | 0.991 | 0.003687 | 84 | 0.102 | 107 | 67 | 6 | 9 | 94 | 10 | 108 | CS2pt1p2\_A10L Fab light chain | CS2pt1p2\_A10L Fab light chain | | pdb | 7XW7\_B | 0.991 | 0.002911 | 84 | 0.09 | 110 | 71 | 5 | 7 | 94 | 6 | 108 | K1-70 scFv light chain | K1-70 scFv light chain | | pdb | 4GXU\_P | 0.991 | 0.001612 | 84 | 0.103 | 106 | 69 | 6 | 9 | 94 | 11 | 110 | Antibody 1F1, light chain | Antibody 1F1, light chain | | pdb | 6WIT\_L | 0.991 | 0.00415 | 84 | 0.107 | 112 | 68 | 7 | 7 | 94 | 6 | 109 | NHP GN1-SD7 Fab Light Chain | NHP GN1-SD7 Fab Light Chain | | pdb | 6WAS\_B | 0.991 | 0.006275 | 84 | 0.111 | 117 | 76 | 6 | 7 | 101 | 7 | 117 | GN1\_PA8 Fab Light chain | GN1\_PA8 Fab Light chain | | pdb | 6UUP\_B | 0.991 | 0.005576 | 84 | 0.115 | 104 | 67 | 5 | 9 | 94 | 128 | 224 | Anti-CD33 conditional scFv | Anti-CD33 conditional scFv | | pdb | 2A9N\_L | 0.99 | 0.005576 | 83 | 0.09 | 110 | 71 | 7 | 7 | 94 | 5 | 107 | fluorescein-scfv light chain | fluorescein-scfv light chain | | pdb | 3T0W\_B | 0.988 | 0.009491 | 82 | 0.091 | 109 | 72 | 5 | 7 | 94 | 7 | 109 | immunoglobulin variable lambda domain | immunoglobulin variable lambda domain | | pdb | 6OO0\_L | 0.988 | 0.007493 | 82 | 0.09 | 110 | 71 | 5 | 7 | 94 | 6 | 108 | NC-Cow1 light chain | NC-Cow1 light chain | | pdb | 7U9G\_D | 0.986 | 0.005576 | 81 | 0.082 | 109 | 73 | 5 | 7 | 94 | 6 | 108 | RVA122 Fab Light Chain | RVA122 Fab Light Chain | | pdb | 6U0L\_Q | 0.986 | 0.007493 | 81 | 0.116 | 112 | 66 | 6 | 7 | 94 | 7 | 109 | E51 Fab light chain | E51 Fab light chain | | pdb | 6QN7\_L | 0.986 | 0.008946 | 81 | 0.121 | 107 | 71 | 3 | 7 | 94 | 5 | 107 | Light chain of bovine anti-RSV B13 | Light chain of bovine anti-RSV B13 | | pdb | 6EQC\_D | 0.986 | 0.008433 | 81 | 0.09 | 111 | 72 | 5 | 9 | 95 | 127 | 232 | scFv of 9C12 antibody | scFv of 9C12 antibody | | pdb | 6CNV\_L | 0.984 | 0.007493 | 80 | 0.127 | 110 | 67 | 7 | 7 | 94 | 6 | 108 | CR9114 Light chain | CR9114 Light chain | | pdb | 6WIT\_B | 0.984 | 0.002298 | 80 | 0.117 | 136 | 81 | 9 | 7 | 115 | 5 | 128 | NHP GN1-SD7 Fab Light Chain | NHP GN1-SD7 Fab Light Chain | | pdb | 7FAU\_D | 0.981 | 0.008946 | 79 | 0.117 | 119 | 66 | 6 | 9 | 92 | 10 | 124 | NB\_1B11 | NB\_1B11 | | pdb | 7PA8\_III | 0.981 | 0.007493 | 79 | 0.117 | 111 | 67 | 7 | 7 | 94 | 6 | 108 | Fab 27C2 light chain | Fab 27C2 light chain | | pdb | 7Q9F\_J | 0.981 | 0.007493 | 79 | 0.067 | 118 | 78 | 5 | 9 | 100 | 9 | 120 | Beta-50 heavy chain | Beta-50 heavy chain | | pdb | 8DY5\_B | 0.981 | 0.009491 | 79 | 0.101 | 118 | 79 | 5 | 7 | 103 | 7 | 118 | spFv CAT2200 LH | spFv CAT2200 LH | | pdb | 7T3M\_J | 0.978 | 0.009491 | 78 | 0.1 | 109 | 72 | 6 | 7 | 94 | 6 | 109 | Antibody 2-7 scFv, variable light chain VL | Antibody 2-7 scFv, variable light chain VL | | pdb | 6IDI\_J | 0.978 | 0.008433 | 78 | 0.101 | 108 | 70 | 5 | 7 | 93 | 5 | 106 | Fab 1H10 light chain (V-region) | Fab 1H10 light chain (V-region) | | pdb | 8A67\_H | 0.978 | 0.00467 | 78 | 0.075 | 120 | 79 | 5 | 9 | 100 | 10 | 125 | Synthetic Nanobody NbSL3.3Q | Synthetic Nanobody NbSL3.3Q | | pdb | 6V8I\_BJ | 0.978 | 0.001131 | 78 | 0.139 | 158 | 98 | 10 | 2 | 128 | 15 | 165 | Fiber Lower, gp62 | Fiber Lower, gp62 | | pdb | 3MLT\_A | 0.978 | 0.007949 | 78 | 0.126 | 111 | 68 | 8 | 7 | 94 | 7 | 111 | Human monoclonal anti-HIV-1 gp120 V3 antibody 2557 Fab light chain | Human monoclonal anti-HIV-1 gp120 V3 antibody 2557 Fab light chain | | pdb | 7UP9\_H | 0.975 | 0.006658 | 77 | 0.081 | 111 | 74 | 5 | 7 | 92 | 9 | 116 | Fab 2D3 heavy chain | Fab 2D3 heavy chain | | pdb | 4HKB\_B | 0.975 | 0.007063 | 77 | 0.158 | 101 | 63 | 7 | 7 | 95 | 6 | 96 | CH67 light chain | CH67 light chain | | pdb | 5CUS\_L | 0.975 | 0.008433 | 77 | 0.114 | 105 | 68 | 4 | 9 | 94 | 10 | 108 | Fab LC region of KTN3379 | Fab LC region of KTN3379 | | pdb | 4Y5X\_B | 0.971 | 0.008433 | 76 | 0.117 | 111 | 67 | 7 | 7 | 94 | 7 | 109 | diabody 310 VH domain | diabody 310 VH domain | | pdb | 7S2S\_A | 0.971 | 0.005915 | 76 | 0.09 | 122 | 69 | 6 | 9 | 93 | 10 | 126 | IL2Rb-binding nanobody | IL2Rb-binding nanobody | | pdb | 7MLH\_E | 0.971 | 0.007063 | 76 | 0.076 | 117 | 79 | 4 | 8 | 100 | 10 | 121 | IgE Heavy chain | IgE Heavy chain | | pdb | 5OCK\_L | 0.971 | 0.005915 | 76 | 0.145 | 137 | 76 | 9 | 7 | 115 | 7 | 130 | Human ACPA E4 Fab fragment - Light chain | Human ACPA E4 Fab fragment - Light chain | | pdb | 7SJ0\_L | 0.967 | 0.004402 | 75 | 0.127 | 110 | 70 | 6 | 5 | 94 | 4 | 107 | A7V3 Fab light chain | A7V3 Fab light chain | | pdb | 8DD3\_J | 0.961 | 0.005576 | 74 | 0.061 | 113 | 74 | 6 | 9 | 95 | 10 | 116 | IgG2b Fab Heavy Chain | IgG2b Fab Heavy Chain | | pdb | 7AQG\_C | 0.961 | 0.007949 | 74 | 0.071 | 112 | 75 | 5 | 9 | 93 | 11 | 120 | VHH-2w-64 (Nb64) | VHH-2w-64 (Nb64) | | pdb | 1MH5\_B | 0.961 | 0.007493 | 74 | 0.093 | 118 | 74 | 6 | 9 | 100 | 10 | 120 | IMMUNOGLOBULIN MS6-164 | IMMUNOGLOBULIN MS6-164 | | pdb | 6U59\_H | 0.956 | 0.008433 | 73 | 0.059 | 117 | 74 | 8 | 9 | 95 | 8 | 118 | rabbit antibody 13B Fragment antigen binding heavy chain | rabbit antibody 13B Fragment antigen binding heavy chain | | pdb | 6ZG3\_J | 0.956 | 0.003687 | 73 | 0.058 | 120 | 73 | 5 | 9 | 93 | 10 | 124 | CA14381 nanobody | CA14381 nanobody | | pdb | 6LCS\_A | 0.956 | 0.007493 | 73 | 0.104 | 144 | 100 | 6 | 7 | 124 | 8 | 148 | VH-SARAH | VH-SARAH | | pdb | 5HBV\_D | 0.956 | 0.003687 | 73 | 0.11 | 136 | 80 | 6 | 7 | 107 | 9 | 138 | Fab35, Heavy Chain | Fab35, Heavy Chain | | pdb | 7RFC\_H | 0.956 | 0.009491 | 73 | 0.082 | 121 | 79 | 6 | 9 | 100 | 10 | 127 | mAb1382 Heavy Chain | mAb1382 Heavy Chain | | pdb | 7FEJ\_L | 0.949 | 0.00415 | 72 | 0.119 | 109 | 67 | 7 | 7 | 94 | 6 | 106 | IG LAMDA CHAIN VARIABLE REGION | IG LAMDA CHAIN VARIABLE REGION | | pdb | 7W9M\_B | 0.949 | 0.0004945 | 72 | 0.084 | 189 | 116 | 8 | 7 | 162 | 8 | 172 | Sodium channel subunit beta-1 | Sodium channel subunit beta-1 | | pdb | 6MEK\_E | 0.949 | 0.009491 | 72 | 0.117 | 136 | 83 | 8 | 7 | 115 | 6 | 131 | HEPC46 Light Chain | HEPC46 Light Chain | | pdb | 7N4M\_L | 0.949 | 0.008946 | 72 | 0.12 | 133 | 85 | 7 | 7 | 115 | 7 | 131 | WRAIR-2151 antibody Fab light chain | WRAIR-2151 antibody Fab light chain | | pdb | 7WQV\_N | 0.949 | 0.007063 | 72 | 0.126 | 111 | 67 | 7 | 7 | 94 | 129 | 232 | Ab08 | Ab08 | | pdb | 7M8J\_H | 0.941 | 0.004402 | 71 | 0.094 | 117 | 69 | 7 | 9 | 95 | 10 | 119 | CM25 Fab - Heavy Chain | CM25 Fab - Heavy Chain | | pdb | 7TJ9\_B | 0.941 | 0.001432 | 71 | 0.107 | 149 | 94 | 9 | 7 | 124 | 8 | 148 | Sodium channel subunit beta-3 | Sodium channel subunit beta-3 | | pdb | 6II4\_L | 0.941 | 0.009491 | 71 | 0.111 | 135 | 84 | 9 | 7 | 115 | 7 | 131 | Light chain of L4A-14 Fab | Light chain of L4A-14 Fab | | pdb | 7LFD\_H | 0.941 | 0.009491 | 71 | 0.106 | 122 | 76 | 5 | 7 | 100 | 9 | 125 | Fab 7D6 heavy chain | Fab 7D6 heavy chain | | pdb | 7M1H\_B | 0.933 | 0.007949 | 70 | 0.102 | 117 | 68 | 5 | 9 | 92 | 10 | 122 | JPU-D12 | JPU-D12 | | pdb | 6MWR\_C | 0.933 | 0.005576 | 70 | 0.101 | 138 | 87 | 8 | 9 | 115 | 10 | 141 | G7 Gamma chain T cell receptor | G7 Gamma chain T cell receptor | | pdb | 7JXD\_D | 0.923 | 0.009491 | 69 | 0.116 | 137 | 82 | 11 | 7 | 115 | 7 | 132 | S2A4 antigen-binding (Fab) fragment | S2A4 antigen-binding (Fab) fragment | | pdb | 5XCV\_B | 0.912 | 0.007063 | 68 | 0.112 | 142 | 83 | 9 | 7 | 124 | 7 | 129 | VL-SARAH(S37C) chimera,VL-SARAH(S37C) chimera | VL-SARAH(S37C) chimera,VL-SARAH(S37C) chimera | | pdb | 8F6O\_A | 0.9 | 0.008433 | 67 | 0.064 | 124 | 79 | 7 | 8 | 100 | 10 | 127 | h22B3 Fab heavy chain | h22B3 Fab heavy chain | | pdb | 7T0R\_H | 0.872 | 0.008433 | 65 | 0.071 | 126 | 79 | 6 | 7 | 101 | 9 | 127 | Ibalizumab Heavy Chain | Ibalizumab Heavy Chain | | pdb | 7K5Y\_N | 0.855 | 0.007063 | 64 | 0.073 | 122 | 69 | 6 | 7 | 95 | 117 | 227 | scFv20 | scFv20 | | pdb | 6DB6\_H | 0.837 | 0.008433 | 63 | 0.072 | 125 | 75 | 6 | 9 | 100 | 11 | 127 | Human monoclonal anti-HIV-1 gp120 V3 antibody 311-11D Fab heavy chain | Human monoclonal anti-HIV-1 gp120 V3 antibody 311-11D Fab heavy chain | | pdb | 5L7U\_B | 0.326 | 0.009491 | 45 | 0.104 | 143 | 70 | 12 | 8 | 96 | 50 | 188 | Glycoside hydrolase | Glycoside hydrolase | | afdb-proteome | AF-F1QIR0-F1-MODEL\_V4 | 1.0 | 0.0001196 | 116 | 0.112 | 133 | 84 | 9 | 7 | 128 | 201 | 310 | Phosphoinositide-3-kinase adaptor protein 1 | Phosphoinositide-3-kinase adaptor protein 1 | | afdb-proteome | AF-Q9EQ32-F1-MODEL\_V4 | 1.0 | 5.549e-05 | 114 | 0.137 | 138 | 80 | 9 | 7 | 118 | 184 | 308 | Phosphoinositide 3-kinase adapter protein 1 | Phosphoinositide 3-kinase adapter protein 1 | | afdb-proteome | AF-A0A044VIN6-F1-MODEL\_V4 | 1.0 | 0.00627 | 111 | 0.113 | 97 | 73 | 5 | 5 | 96 | 250 | 338 | Uncharacterized protein | Uncharacterized protein | | afdb-proteome | AF-Q32J71-F1-MODEL\_V4 | 1.0 | 0.004666 | 108 | 0.157 | 89 | 66 | 6 | 8 | 92 | 684 | 767 | Uncharacterized protein | Uncharacterized protein | | afdb-proteome | AF-Q6ZUJ8-F1-MODEL\_V4 | 1.0 | 7.029e-05 | 103 | 0.147 | 156 | 87 | 10 | 7 | 129 | 183 | 325 | Phosphoinositide 3-kinase adapter protein 1 | Phosphoinositide 3-kinase adapter protein 1 | | afdb-proteome | AF-Q32J72-F1-MODEL\_V4 | 1.0 | 0.004146 | 101 | 0.123 | 97 | 68 | 7 | 8 | 96 | 356 | 443 | Uncharacterized protein | Uncharacterized protein | | afdb-proteome | AF-G4V6G9-F1-MODEL\_V4 | 1.0 | 0.0002903 | 100 | 0.17 | 129 | 93 | 6 | 9 | 132 | 441 | 560 | Putative titin | Putative titin | | afdb-proteome | AF-Q2FX74-F1-MODEL\_V4 | 0.999 | 0.001004 | 96 | 0.125 | 192 | 121 | 11 | 9 | 179 | 24 | 189 | Phage tail fiber protein, putative | Phage tail fiber protein, putative | | afdb-proteome | AF-Q641Y0-F1-MODEL\_V4 | 0.999 | 0.0009463 | 96 | 0.095 | 147 | 102 | 9 | 7 | 134 | 307 | 441 | Dolichyl-diphosphooligosaccharide--protein glycosyltransferase 48 kDa subunit | Dolichyl-diphosphooligosaccharide--protein glycosyltransferase 48 kDa subunit | | afdb-proteome | AF-A0A1U7F3R9-F1-MODEL\_V4 | 0.999 | 0.008425 | 96 | 0.173 | 92 | 61 | 5 | 7 | 95 | 358 | 437 | Bm6990 | Bm6990 | | afdb-proteome | AF-O54734-F1-MODEL\_V4 | 0.998 | 0.002436 | 94 | 0.097 | 133 | 90 | 8 | 7 | 124 | 307 | 424 | Dolichyl-diphosphooligosaccharide--protein glycosyltransferase 48 kDa subunit | Dolichyl-diphosphooligosaccharide--protein glycosyltransferase 48 kDa subunit | | afdb-proteome | AF-A0A044QQS6-F1-MODEL\_V4 | 0.998 | 0.002908 | 93 | 0.131 | 145 | 103 | 12 | 7 | 136 | 305 | 441 | Dolichyl-diphosphooligosaccharide--protein glycosyltransferase 48 kDa subunit | Dolichyl-diphosphooligosaccharide--protein glycosyltransferase 48 kDa subunit | | afdb-proteome | AF-O95932-F1-MODEL\_V4 | 0.998 | 0.009482 | 93 | 0.139 | 129 | 84 | 8 | 9 | 113 | 31 | 156 | Protein-glutamine gamma-glutamyltransferase 6 | Protein-glutamine gamma-glutamyltransferase 6 | | afdb-proteome | AF-A0A077ZB36-F1-MODEL\_V4 | 0.998 | 0.005571 | 93 | 0.141 | 92 | 73 | 5 | 7 | 93 | 528 | 618 | Plexin A4 | Plexin A4 | | afdb-proteome | AF-A0A0H3GY50-F1-MODEL\_V4 | 0.997 | 0.0008409 | 92 | 0.117 | 179 | 112 | 12 | 6 | 145 | 3 | 174 | Uncharacterized protein | Uncharacterized protein | | afdb-proteome | AF-A0A0G2K7L7-F1-MODEL\_V4 | 0.997 | 0.0004138 | 91 | 0.096 | 155 | 115 | 9 | 6 | 142 | 249 | 396 | ANTXR-like | ANTXR-like | | afdb-proteome | AF-D3ZH36-F1-MODEL\_V4 | 0.997 | 0.000494 | 91 | 0.099 | 171 | 128 | 8 | 10 | 178 | 303 | 449 | Extracellular leucine-rich repeat and fibronectin type III domain-containing 2 | Extracellular leucine-rich repeat and fibronectin type III domain-containing 2 | | afdb-proteome | AF-A0A0G2JUH9-F1-MODEL\_V4 | 0.996 | 0.0006258 | 90 | 0.145 | 186 | 119 | 10 | 1 | 166 | 3 | 168 | SKICH domain-containing protein | SKICH domain-containing protein | | afdb-proteome | AF-A0A0K0DYW0-F1-MODEL\_V4 | 0.996 | 0.002164 | 90 | 0.11 | 154 | 104 | 9 | 7 | 140 | 308 | 448 | Dolichyl-diphosphooligosaccharide--protein glycosyltransferase 48 kDa subunit | Dolichyl-diphosphooligosaccharide--protein glycosyltransferase 48 kDa subunit | | afdb-proteome | AF-Q32J74-F1-MODEL\_V4 | 0.996 | 0.00591 | 90 | 0.177 | 96 | 62 | 7 | 8 | 95 | 1032 | 1118 | Uncharacterized protein | Uncharacterized protein | | afdb-proteome | AF-P39656-F1-MODEL\_V4 | 0.996 | 0.002164 | 89 | 0.088 | 147 | 103 | 9 | 7 | 134 | 322 | 456 | Dolichyl-diphosphooligosaccharide--protein glycosyltransferase 48 kDa subunit | Dolichyl-diphosphooligosaccharide--protein glycosyltransferase 48 kDa subunit | | afdb-proteome | AF-Q04864-F1-MODEL\_V4 | 0.995 | 0.002908 | 88 | 0.12 | 141 | 110 | 8 | 8 | 142 | 194 | 326 | Proto-oncogene c-Rel | Proto-oncogene c-Rel | | afdb-proteome | AF-A0A5P3FZ45-F1-MODEL\_V4 | 0.995 | 0.002296 | 88 | 0.129 | 177 | 111 | 12 | 4 | 164 | 13 | 162 | Uncharacterized protein | Uncharacterized protein | | afdb-proteome | AF-A0A3P7FSY3-F1-MODEL\_V4 | 0.994 | 0.003472 | 87 | 0.09 | 154 | 105 | 9 | 7 | 141 | 272 | 409 | Dolichyl-diphosphooligosaccharide--protein glycosyltransferase 48 kDa subunit | Dolichyl-diphosphooligosaccharide--protein glycosyltransferase 48 kDa subunit | | afdb-proteome | AF-A0A2R8QBU7-F1-MODEL\_V4 | 0.994 | 0.0004657 | 87 | 0.113 | 185 | 124 | 9 | 10 | 179 | 297 | 456 | LRRCT domain-containing protein | LRRCT domain-containing protein | | afdb-proteome | AF-Q9NZC2-F1-MODEL\_V4 | 0.993 | 0.0002903 | 86 | 0.14 | 213 | 116 | 12 | 9 | 179 | 25 | 212 | Triggering receptor expressed on myeloid cells 2 | Triggering receptor expressed on myeloid cells 2 | | afdb-proteome | AF-F1Q6X1-F1-MODEL\_V4 | 0.993 | 0.001272 | 86 | 0.096 | 156 | 103 | 9 | 7 | 142 | 225 | 362 | ANTXR cell adhesion molecule 1d | ANTXR cell adhesion molecule 1d | | afdb-proteome | AF-G5EFS1-F1-MODEL\_V4 | 0.993 | 0.001431 | 86 | 0.112 | 125 | 89 | 9 | 7 | 126 | 49 | 156 | ANK\_REP\_REGION domain-containing protein | ANK\_REP\_REGION domain-containing protein | | afdb-proteome | AF-A0A5K4F288-F1-MODEL\_V4 | 0.993 | 0.000892 | 86 | 0.164 | 170 | 102 | 10 | 9 | 163 | 708 | 852 | Kettin/titin-related protein | Kettin/titin-related protein | | afdb-proteome | AF-A0A0N4UPP3-F1-MODEL\_V4 | 0.993 | 0.002584 | 86 | 0.114 | 157 | 103 | 9 | 4 | 142 | 779 | 917 | Uncharacterized protein | Uncharacterized protein | | afdb-proteome | AF-P01740-F1-MODEL\_V4 | 0.992 | 0.008425 | 85 | 0.104 | 115 | 72 | 7 | 8 | 95 | 25 | 135 | T-cell receptor gamma chain V region V108A | T-cell receptor gamma chain V region V108A | | afdb-proteome | AF-C6T7K0-F1-MODEL\_V4 | 0.992 | 0.001065 | 85 | 0.12 | 150 | 96 | 12 | 1 | 126 | 70 | 207 | Signal sequence receptor subunit alpha | Signal sequence receptor subunit alpha | | afdb-proteome | AF-F1RDW2-F1-MODEL\_V4 | 0.992 | 0.00039 | 85 | 0.117 | 162 | 108 | 9 | 10 | 146 | 558 | 709 | Si:ch211-180f4.1 | Si:ch211-180f4.1 | | afdb-proteome | AF-Q68FM6-F1-MODEL\_V4 | 0.992 | 0.0005899 | 85 | 0.08 | 175 | 129 | 8 | 10 | 178 | 303 | 451 | Protein phosphatase 1 regulatory subunit 29 | Protein phosphatase 1 regulatory subunit 29 | | afdb-proteome | AF-Q9P7K7-F1-MODEL\_V4 | 0.991 | 0.001349 | 84 | 0.101 | 168 | 119 | 7 | 8 | 165 | 32 | 177 | Uncharacterized protein C21C3.17c | Uncharacterized protein C21C3.17c | | afdb-proteome | AF-A0A175VWL7-F1-MODEL\_V4 | 0.991 | 0.002436 | 84 | 0.094 | 201 | 141 | 10 | 8 | 179 | 126 | 314 | Uncharacterized protein | Uncharacterized protein | | afdb-proteome | AF-Q8BVM2-F1-MODEL\_V4 | 0.991 | 0.000892 | 84 | 0.084 | 154 | 112 | 10 | 7 | 142 | 260 | 402 | Anthrax toxin receptor-like | Anthrax toxin receptor-like | | afdb-proteome | AF-Q61809-F1-MODEL\_V4 | 0.991 | 0.001349 | 84 | 0.104 | 153 | 108 | 8 | 10 | 142 | 533 | 676 | Leucine-rich repeat neuronal protein 1 | Leucine-rich repeat neuronal protein 1 | | afdb-proteome | AF-Q8BHK2-F1-MODEL\_V4 | 0.99 | 0.00039 | 83 | 0.115 | 208 | 124 | 10 | 7 | 178 | 32 | 215 | Sodium channel subunit beta-3 | Sodium channel subunit beta-3 | | afdb-proteome | AF-Q6QX36-F1-MODEL\_V4 | 0.99 | 0.000892 | 83 | 0.108 | 212 | 129 | 13 | 3 | 179 | 22 | 208 | CRKD-binding protein | CRKD-binding protein | | afdb-proteome | AF-A6NF34-F1-MODEL\_V4 | 0.99 | 0.001349 | 83 | 0.14 | 149 | 103 | 7 | 7 | 142 | 259 | 395 | Anthrax toxin receptor-like | Anthrax toxin receptor-like | | afdb-proteome | AF-A0A5K4F3S2-F1-MODEL\_V4 | 0.99 | 0.001431 | 83 | 0.161 | 204 | 109 | 11 | 9 | 179 | 708 | 882 | Kettin/titin-related protein | Kettin/titin-related protein | | afdb-proteome | AF-A0A0N4UJG6-F1-MODEL\_V4 | 0.988 | 0.003085 | 82 | 0.083 | 156 | 105 | 11 | 7 | 142 | 305 | 442 | Dolichyl-diphosphooligosaccharide--protein glycosyltransferase 48 kDa subunit | Dolichyl-diphosphooligosaccharide--protein glycosyltransferase 48 kDa subunit | | afdb-proteome | AF-Q6AWS7-F1-MODEL\_V4 | 0.986 | 0.001065 | 81 | 0.129 | 154 | 89 | 11 | 1 | 126 | 65 | 201 | Signal sequence receptor subunit alpha | Signal sequence receptor subunit alpha | | afdb-proteome | AF-K0EVZ8-F1-MODEL\_V4 | 0.986 | 0.009482 | 81 | 0.125 | 120 | 87 | 6 | 7 | 122 | 543 | 648 | Non-specific serine/threonine protein kinase | Non-specific serine/threonine protein kinase | | afdb-proteome | AF-P0C7U0-F1-MODEL\_V4 | 0.986 | 0.001199 | 81 | 0.135 | 185 | 121 | 8 | 5 | 178 | 323 | 479 | Protein ELFN1 | Protein ELFN1 | | afdb-proteome | AF-Q9JK00-F1-MODEL\_V4 | 0.984 | 0.0006639 | 80 | 0.115 | 208 | 124 | 10 | 7 | 178 | 32 | 215 | Sodium channel subunit beta-3 | Sodium channel subunit beta-3 | | afdb-proteome | AF-Q8BG84-F1-MODEL\_V4 | 0.984 | 0.001813 | 80 | 0.13 | 176 | 118 | 8 | 9 | 179 | 38 | 183 | Leukocyte-associated immunoglobulin-like receptor 1 | Leukocyte-associated immunoglobulin-like receptor 1 | | afdb-proteome | AF-Q7TQN4-F1-MODEL\_V4 | 0.984 | 0.00204 | 80 | 0.137 | 145 | 104 | 9 | 8 | 142 | 202 | 335 | Nuclear factor kappaB subunit p65 | Nuclear factor kappaB subunit p65 | | afdb-proteome | AF-Q09147-F1-MODEL\_V4 | 0.984 | 0.0004657 | 80 | 0.131 | 175 | 105 | 9 | 8 | 164 | 495 | 640 | Fibroblast growth factor receptor homolog 2 | Fibroblast growth factor receptor homolog 2 | | afdb-proteome | AF-Q9NY72-F1-MODEL\_V4 | 0.981 | 0.0007926 | 79 | 0.096 | 217 | 128 | 11 | 3 | 179 | 26 | 214 | Sodium channel subunit beta-3 | Sodium channel subunit beta-3 | | afdb-proteome | AF-B4FE49-F1-MODEL\_V4 | 0.981 | 0.0009463 | 79 | 0.128 | 195 | 115 | 13 | 8 | 162 | 78 | 257 | Signal sequence receptor subunit alpha | Signal sequence receptor subunit alpha | | afdb-proteome | AF-Q6ZLK0-F1-MODEL\_V4 | 0.981 | 0.009482 | 79 | 0.111 | 135 | 87 | 9 | 7 | 124 | 310 | 428 | Dolichyl-diphosphooligosaccharide--protein glycosyltransferase 48 kDa subunit | Dolichyl-diphosphooligosaccharide--protein glycosyltransferase 48 kDa subunit | | afdb-proteome | AF-Q9ESY6-F1-MODEL\_V4 | 0.981 | 0.001431 | 79 | 0.088 | 147 | 106 | 8 | 10 | 142 | 531 | 663 | Leucine-rich repeat neuronal protein 3 | Leucine-rich repeat neuronal protein 3 | | afdb-proteome | AF-Q8C8T7-F1-MODEL\_V4 | 0.981 | 0.001611 | 79 | 0.13 | 184 | 122 | 8 | 5 | 178 | 323 | 478 | Protein ELFN1 | Protein ELFN1 | | afdb-proteome | AF-A0A2R8RRG5-F1-MODEL\_V4 | 0.978 | 0.001709 | 78 | 0.092 | 228 | 128 | 12 | 3 | 178 | 24 | 224 | Si:rp71-81e14.2 | Si:rp71-81e14.2 | | afdb-proteome | AF-P15307-F1-MODEL\_V4 | 0.978 | 0.00627 | 78 | 0.112 | 142 | 107 | 9 | 8 | 142 | 194 | 323 | Proto-oncogene c-Rel | Proto-oncogene c-Rel | | afdb-proteome | AF-Q93Z16-F1-MODEL\_V4 | 0.978 | 0.005571 | 78 | 0.115 | 182 | 122 | 13 | 9 | 168 | 455 | 619 | Dolichyl-diphosphooligosaccharide--protein glycosyltransferase subunit 2 | Dolichyl-diphosphooligosaccharide--protein glycosyltransferase subunit 2 | | afdb-proteome | AF-D3ZAV8-F1-MODEL\_V4 | 0.978 | 0.005571 | 78 | 0.112 | 142 | 109 | 6 | 10 | 142 | 535 | 668 | Leucine-rich repeat neuronal 2 | Leucine-rich repeat neuronal 2 | | afdb-proteome | AF-Q9P244-F1-MODEL\_V4 | 0.978 | 0.002741 | 78 | 0.14 | 157 | 99 | 8 | 12 | 142 | 434 | 580 | Leucine-rich repeat and fibronectin type III domain-containing protein 1 | Leucine-rich repeat and fibronectin type III domain-containing protein 1 | | afdb-proteome | AF-A9Q7H1-F1-MODEL\_V4 | 0.975 | 0.001272 | 77 | 0.114 | 192 | 111 | 10 | 3 | 163 | 22 | 185 | RIKEN cDNA 9830107B12 gene | RIKEN cDNA 9830107B12 gene | | afdb-proteome | AF-F8W4B3-F1-MODEL\_V4 | 0.975 | 0.001709 | 77 | 0.105 | 219 | 127 | 13 | 3 | 179 | 47 | 238 | Sodium channel subunit beta-3 | Sodium channel subunit beta-3 | | afdb-proteome | AF-O59866-F1-MODEL\_V4 | 0.975 | 0.002584 | 77 | 0.089 | 156 | 103 | 11 | 7 | 136 | 290 | 432 | Dolichyl-diphosphooligosaccharide--protein glycosyltransferase subunit wbp1 | Dolichyl-diphosphooligosaccharide--protein glycosyltransferase subunit wbp1 | | afdb-proteome | AF-A0A0H2UKN7-F1-MODEL\_V4 | 0.971 | 0.0004138 | 76 | 0.091 | 197 | 123 | 8 | 3 | 166 | 8 | 181 | Sodium channel, voltage-gated, type I, beta b | Sodium channel, voltage-gated, type I, beta b | | afdb-proteome | AF-Q07699-F1-MODEL\_V4 | 0.971 | 0.0006258 | 76 | 0.107 | 195 | 114 | 9 | 7 | 166 | 27 | 196 | Sodium channel subunit beta-1 | Sodium channel subunit beta-1 | | afdb-proteome | AF-F1QJZ8-F1-MODEL\_V4 | 0.971 | 0.002908 | 76 | 0.124 | 177 | 121 | 8 | 9 | 179 | 473 | 621 | Sema domain, immunoglobulin domain (Ig), transmembrane domain (TM) and short cytoplasmic domain, (semaphorin) 4Gb | Sema domain, immunoglobulin domain (Ig), transmembrane domain (TM) and short cytoplasmic domain, (semaphorin) 4Gb | | afdb-proteome | AF-F1Q7Y5-F1-MODEL\_V4 | 0.971 | 0.002164 | 76 | 0.096 | 177 | 130 | 7 | 12 | 179 | 330 | 485 | Extracellular leucine-rich repeat and fibronectin type III domain-containing 1b | Extracellular leucine-rich repeat and fibronectin type III domain-containing 1b | | afdb-proteome | AF-Q5R3F8-F1-MODEL\_V4 | 0.971 | 0.00204 | 76 | 0.093 | 181 | 127 | 9 | 10 | 179 | 303 | 457 | Protein phosphatase 1 regulatory subunit 29 | Protein phosphatase 1 regulatory subunit 29 | | afdb-proteome | AF-D3ZZ44-F1-MODEL\_V4 | 0.971 | 0.002164 | 76 | 0.129 | 186 | 120 | 9 | 5 | 178 | 323 | 478 | Extracellular leucine-rich repeat and fibronectin type III domain-containing 1 | Extracellular leucine-rich repeat and fibronectin type III domain-containing 1 | | afdb-proteome | AF-Q04207-F1-MODEL\_V4 | 0.967 | 0.003684 | 75 | 0.144 | 145 | 102 | 10 | 8 | 142 | 202 | 334 | Transcription factor p65 | Transcription factor p65 | | afdb-proteome | AF-A0A0D2DWU6-F1-MODEL\_V4 | 0.961 | 0.001518 | 74 | 0.151 | 172 | 99 | 11 | 9 | 142 | 57 | 219 | Signal sequence receptor subunit alpha | Signal sequence receptor subunit alpha | | afdb-proteome | AF-A0A1C1C8U1-F1-MODEL\_V4 | 0.961 | 0.001199 | 74 | 0.146 | 177 | 99 | 12 | 9 | 142 | 57 | 224 | Signal sequence receptor subunit alpha | Signal sequence receptor subunit alpha | | afdb-proteome | AF-Q8I3K4-F1-MODEL\_V4 | 0.961 | 0.005251 | 74 | 0.086 | 184 | 119 | 6 | 7 | 141 | 103 | 286 | Uncharacterized protein | Uncharacterized protein | | afdb-proteome | AF-A0A2I3BPJ2-F1-MODEL\_V4 | 0.961 | 0.003908 | 74 | 0.115 | 199 | 128 | 11 | 8 | 179 | 258 | 435 | Predicted gene, 30083 | Predicted gene, 30083 | | afdb-proteome | AF-M9MM94-F1-MODEL\_V4 | 0.961 | 0.001923 | 74 | 0.093 | 172 | 110 | 7 | 8 | 142 | 577 | 739 | Sema domain, immunoglobulin domain (Ig), short basic domain, secreted, (semaphorin) 3B | Sema domain, immunoglobulin domain (Ig), short basic domain, secreted, (semaphorin) 3B | | afdb-proteome | AF-F8W525-F1-MODEL\_V4 | 0.956 | 0.002296 | 73 | 0.115 | 182 | 115 | 9 | 9 | 168 | 33 | 190 | CD79a molecule, immunoglobulin-associated alpha | CD79a molecule, immunoglobulin-associated alpha | | afdb-proteome | AF-Q00954-F1-MODEL\_V4 | 0.956 | 0.0008409 | 73 | 0.106 | 198 | 111 | 11 | 7 | 166 | 27 | 196 | Sodium channel subunit beta-1 | Sodium channel subunit beta-1 | | afdb-proteome | AF-P97952-F1-MODEL\_V4 | 0.956 | 0.000892 | 73 | 0.1 | 199 | 111 | 11 | 7 | 166 | 27 | 196 | Sodium channel subunit beta-1 | Sodium channel subunit beta-1 | | afdb-proteome | AF-Q6DFX2-F1-MODEL\_V4 | 0.956 | 0.005251 | 73 | 0.121 | 156 | 100 | 8 | 7 | 142 | 226 | 364 | Anthrax toxin receptor 2 | Anthrax toxin receptor 2 | | afdb-proteome | AF-A2AS37-F1-MODEL\_V4 | 0.949 | 0.002164 | 72 | 0.12 | 191 | 111 | 14 | 7 | 153 | 140 | 317 | Expressed sequence AI182371 | Expressed sequence AI182371 | | afdb-proteome | AF-B0S5A5-F1-MODEL\_V4 | 0.949 | 0.005571 | 72 | 0.123 | 162 | 105 | 9 | 6 | 142 | 250 | 399 | Si:ch211-150o23.3 | Si:ch211-150o23.3 | | afdb-proteome | AF-A0A2R8S050-F1-MODEL\_V4 | 0.949 | 0.004398 | 72 | 0.116 | 154 | 95 | 10 | 8 | 142 | 231 | 362 | RHD domain-containing protein | RHD domain-containing protein | | afdb-proteome | AF-P48551-F1-MODEL\_V4 | 0.949 | 0.007942 | 72 | 0.101 | 157 | 108 | 9 | 10 | 142 | 142 | 289 | Interferon alpha/beta receptor 2 | Interferon alpha/beta receptor 2 | | afdb-proteome | AF-X1WEK5-F1-MODEL\_V4 | 0.949 | 0.003085 | 72 | 0.082 | 145 | 103 | 10 | 8 | 142 | 313 | 437 | V-rel avian reticuloendotheliosis viral oncogene homolog B | V-rel avian reticuloendotheliosis viral oncogene homolog B | | afdb-proteome | AF-P0C7J6-F1-MODEL\_V4 | 0.949 | 0.00591 | 72 | 0.132 | 158 | 100 | 8 | 12 | 142 | 434 | 581 | Leucine-rich repeat and fibronectin type III domain-containing protein 1 | Leucine-rich repeat and fibronectin type III domain-containing protein 1 | | afdb-proteome | AF-Q4LFA9-F1-MODEL\_V4 | 0.949 | 0.004146 | 72 | 0.145 | 192 | 118 | 11 | 4 | 179 | 585 | 746 | Semaphorin-3G | Semaphorin-3G | | afdb-proteome | AF-I1LIQ3-F1-MODEL\_V4 | 0.941 | 0.001611 | 71 | 0.145 | 158 | 85 | 12 | 8 | 128 | 80 | 224 | Signal sequence receptor subunit alpha | Signal sequence receptor subunit alpha | | afdb-proteome | AF-Q7Z6A9-F1-MODEL\_V4 | 0.941 | 0.00113 | 71 | 0.137 | 167 | 98 | 11 | 9 | 142 | 47 | 200 | B- and T-lymphocyte attenuator | B- and T-lymphocyte attenuator | | afdb-proteome | AF-Q9CZT5-F1-MODEL\_V4 | 0.941 | 0.003273 | 71 | 0.125 | 160 | 105 | 7 | 12 | 142 | 473 | 626 | Vasorin | Vasorin | | afdb-proteome | AF-A0A0D2DMU8-F1-MODEL\_V4 | 0.933 | 0.002584 | 70 | 0.112 | 240 | 129 | 12 | 8 | 179 | 44 | 267 | Unplaced genomic scaffold supercont1.5, whole genome shotgun sequence | Unplaced genomic scaffold supercont1.5, whole genome shotgun sequence | | afdb-proteome | AF-Q8NET5-F1-MODEL\_V4 | 0.923 | 0.003273 | 69 | 0.127 | 172 | 100 | 12 | 8 | 142 | 53 | 211 | NFAT activation molecule 1 | NFAT activation molecule 1 | | afdb-proteome | AF-Q63203-F1-MODEL\_V4 | 0.923 | 0.003472 | 69 | 0.12 | 149 | 102 | 6 | 7 | 142 | 127 | 259 | Low affinity immunoglobulin gamma Fc region receptor II | Low affinity immunoglobulin gamma Fc region receptor II | | afdb-proteome | AF-F7FEU1-F1-MODEL\_V4 | 0.923 | 0.001709 | 69 | 0.105 | 209 | 116 | 14 | 7 | 165 | 26 | 213 | V-set and immunoglobulin domain-containing 4 | V-set and immunoglobulin domain-containing 4 | | afdb-proteome | AF-T1NXB5-F1-MODEL\_V4 | 0.923 | 0.003273 | 69 | 0.103 | 213 | 124 | 13 | 9 | 178 | 35 | 223 | V-set and transmembrane domain-containing protein 4 | V-set and transmembrane domain-containing protein 4 | | afdb-proteome | AF-Q96PJ5-F1-MODEL\_V4 | 0.923 | 0.007057 | 69 | 0.108 | 148 | 107 | 9 | 7 | 142 | 297 | 431 | Fc receptor-like protein 4 | Fc receptor-like protein 4 | | afdb-proteome | AF-P97484-F1-MODEL\_V4 | 0.923 | 0.001611 | 69 | 0.118 | 203 | 120 | 11 | 5 | 179 | 529 | 700 | Leukocyte immunoglobulin-like receptor subfamily B member 3 | Leukocyte immunoglobulin-like receptor subfamily B member 3 | | afdb-proteome | AF-O75325-F1-MODEL\_V4 | 0.912 | 0.003684 | 68 | 0.108 | 147 | 102 | 7 | 10 | 142 | 535 | 666 | Leucine-rich repeat neuronal protein 2 | Leucine-rich repeat neuronal protein 2 | | afdb-proteome | AF-Q14517-F9-MODEL\_V4 | 0.912 | 0.003908 | 68 | 0.155 | 154 | 96 | 10 | 6 | 142 | 629 | 765 | Protocadherin Fat 1 | Protocadherin Fat 1 | | afdb-proteome | AF-F1LU58-F1-MODEL\_V4 | 0.9 | 0.003684 | 67 | 0.095 | 200 | 137 | 10 | 3 | 167 | 9 | 199 | Triggering receptor-expressed on myeloid cells-like 4 | Triggering receptor-expressed on myeloid cells-like 4 | | afdb-proteome | AF-Q5Z9P6-F1-MODEL\_V4 | 0.9 | 0.002584 | 67 | 0.121 | 181 | 107 | 14 | 1 | 145 | 71 | 235 | Signal sequence receptor subunit alpha | Signal sequence receptor subunit alpha | | afdb-proteome | AF-F6TUL9-F1-MODEL\_V4 | 0.9 | 0.001813 | 67 | 0.115 | 216 | 113 | 15 | 7 | 170 | 26 | 215 | V-set and immunoglobulin domain-containing 4 | V-set and immunoglobulin domain-containing 4 | | afdb-proteome | AF-Q8K558-F1-MODEL\_V4 | 0.9 | 0.00591 | 67 | 0.111 | 225 | 119 | 13 | 3 | 179 | 20 | 211 | Trem-like transcript 1 protein | Trem-like transcript 1 protein | | afdb-proteome | AF-K7MP75-F1-MODEL\_V4 | 0.9 | 0.004146 | 67 | 0.125 | 175 | 118 | 11 | 7 | 168 | 392 | 544 | Uncharacterized protein | Uncharacterized protein | | afdb-proteome | AF-Q9FYG2-F1-MODEL\_V4 | 0.9 | 0.00495 | 67 | 0.127 | 173 | 123 | 11 | 7 | 168 | 453 | 608 | Calmodulin-binding transcription activator 4 | Calmodulin-binding transcription activator 4 | | afdb-proteome | AF-A0A7I4KBK6-F1-MODEL\_V4 | 0.9 | 0.007486 | 67 | 0.107 | 158 | 106 | 8 | 8 | 142 | 703 | 848 | "BMA-FLN-2, isoform a | "BMA-FLN-2, isoform a | | afdb-proteome | AF-P01732-F1-MODEL\_V4 | 0.887 | 0.003908 | 66 | 0.158 | 221 | 123 | 13 | 6 | 179 | 29 | 233 | T-cell surface glycoprotein CD8 alpha chain | T-cell surface glycoprotein CD8 alpha chain | | afdb-proteome | AF-A0A0N4STN9-F1-MODEL\_V4 | 0.887 | 0.009482 | 66 | 0.082 | 195 | 121 | 11 | 6 | 179 | 144 | 301 | Novel immune-type receptor 1k | Novel immune-type receptor 1k | | afdb-proteome | AF-B8A473-F1-MODEL\_V4 | 0.887 | 0.00591 | 66 | 0.086 | 208 | 130 | 10 | 9 | 179 | 34 | 218 | V-set and transmembrane domain-containing 4a | V-set and transmembrane domain-containing 4a | | afdb-proteome | AF-P08101-F1-MODEL\_V4 | 0.887 | 0.00495 | 66 | 0.138 | 144 | 105 | 7 | 7 | 142 | 125 | 257 | Low affinity immunoglobulin gamma Fc region receptor II | Low affinity immunoglobulin gamma Fc region receptor II | | afdb-proteome | AF-Q0JDP3-F1-MODEL\_V4 | 0.887 | 0.004398 | 66 | 0.149 | 174 | 119 | 13 | 7 | 169 | 456 | 611 | Os04g0388500 protein | Os04g0388500 protein | | afdb-proteome | AF-Q8TBE3-F1-MODEL\_V4 | 0.872 | 0.005251 | 65 | 0.107 | 167 | 100 | 10 | 12 | 142 | 9 | 162 | Fibronectin type III domain-containing protein 9 | Fibronectin type III domain-containing protein 9 | | afdb-proteome | AF-Q57965-F1-MODEL\_V4 | 0.872 | 0.007057 | 65 | 0.13 | 153 | 108 | 11 | 7 | 143 | 34 | 177 | Uncharacterized protein MJ0545 | Uncharacterized protein MJ0545 | | afdb-proteome | AF-Q4QQR8-F1-MODEL\_V4 | 0.872 | 0.004398 | 65 | 0.09 | 211 | 129 | 11 | 9 | 178 | 35 | 223 | Uncharacterized protein RGD1310423 | Uncharacterized protein RGD1310423 | | afdb-proteome | AF-U7PRC0-F1-MODEL\_V4 | 0.872 | 0.009482 | 65 | 0.087 | 137 | 96 | 6 | 8 | 124 | 124 | 251 | Uncharacterized protein | Uncharacterized protein | | afdb-proteome | AF-Q7TNJ4-F1-MODEL\_V4 | 0.872 | 0.00591 | 65 | 0.126 | 150 | 106 | 9 | 8 | 142 | 297 | 436 | Amphoterin-induced protein 2 | Amphoterin-induced protein 2 | | afdb-proteome | AF-Q1LVE1-F1-MODEL\_V4 | 0.872 | 0.00591 | 65 | 0.103 | 193 | 126 | 11 | 8 | 179 | 573 | 739 | Sema domain, immunoglobulin domain (Ig), short basic domain, secreted, (semaphorin) 3H | Sema domain, immunoglobulin domain (Ig), short basic domain, secreted, (semaphorin) 3H | | afdb-proteome | AF-O75015-F1-MODEL\_V4 | 0.855 | 0.008938 | 64 | 0.12 | 124 | 89 | 6 | 7 | 125 | 115 | 223 | Low affinity immunoglobulin gamma Fc region receptor III-B | Low affinity immunoglobulin gamma Fc region receptor III-B | | afdb-proteome | AF-A0A1D6ECQ6-F1-MODEL\_V4 | 0.855 | 0.007486 | 64 | 0.119 | 176 | 119 | 12 | 7 | 168 | 427 | 580 | Calmodulin-binding transcription activator 4 | Calmodulin-binding transcription activator 4 | | afdb-proteome | AF-Q4DYJ9-F1-MODEL\_V4 | 0.855 | 0.008938 | 64 | 0.102 | 214 | 126 | 17 | 9 | 168 | 1080 | 1281 | Uncharacterized protein | Uncharacterized protein | | afdb-proteome | AF-Q9D659-F1-MODEL\_V4 | 0.837 | 0.00591 | 63 | 0.105 | 228 | 121 | 14 | 9 | 179 | 43 | 244 | V-type immunoglobulin domain-containing suppressor of T-cell activation | V-type immunoglobulin domain-containing suppressor of T-cell activation | | afdb-proteome | AF-Q8IW00-F1-MODEL\_V4 | 0.837 | 0.007942 | 63 | 0.109 | 220 | 119 | 14 | 9 | 179 | 35 | 226 | V-set and transmembrane domain-containing protein 4 | V-set and transmembrane domain-containing protein 4 | | afdb-proteome | AF-O75022-F1-MODEL\_V4 | 0.817 | 0.003908 | 62 | 0.104 | 192 | 119 | 11 | 8 | 179 | 333 | 491 | Leukocyte immunoglobulin-like receptor subfamily B member 3 | Leukocyte immunoglobulin-like receptor subfamily B member 3 | | afdb-proteome | AF-Q18438-F1-MODEL\_V4 | 0.795 | 0.001813 | 61 | 0.075 | 198 | 127 | 11 | 7 | 158 | 15 | 202 | Motile sperm domain-containing protein 1 | Motile sperm domain-containing protein 1 | | afdb-proteome | AF-P08637-F1-MODEL\_V4 | 0.795 | 0.005571 | 61 | 0.096 | 176 | 118 | 8 | 7 | 177 | 115 | 254 | Low affinity immunoglobulin gamma Fc region receptor III-A | Low affinity immunoglobulin gamma Fc region receptor III-A | | afdb-proteome | AF-O15041-F1-MODEL\_V4 | 0.772 | 0.007057 | 60 | 0.111 | 198 | 125 | 12 | 9 | 179 | 591 | 764 | Semaphorin-3E | Semaphorin-3E | | afdb-proteome | AF-Q5RJS6-F1-MODEL\_V4 | 0.747 | 0.005251 | 59 | 0.086 | 196 | 128 | 8 | 8 | 159 | 24 | 212 | Motile sperm domain-containing protein 1 | Motile sperm domain-containing protein 1 | | afdb-proteome | AF-F1R9R3-F1-MODEL\_V4 | 0.747 | 0.003684 | 59 | 0.107 | 205 | 123 | 11 | 7 | 159 | 22 | 218 | Motile sperm domain-containing protein 1 | Motile sperm domain-containing protein 1 | | afdb-proteome | AF-Q9D5K1-F1-MODEL\_V4 | 0.747 | 0.004398 | 59 | 0.144 | 201 | 110 | 17 | 9 | 178 | 90 | 259 | Transmembrane protein 81 | Transmembrane protein 81 | | afdb-proteome | AF-G4LVE3-F1-MODEL\_V4 | 0.747 | 0.001349 | 59 | 0.137 | 167 | 102 | 12 | 7 | 141 | 13 | 169 | MSP domain-containing protein | MSP domain-containing protein | | afdb-proteome | AF-Q9C0C4-F1-MODEL\_V4 | 0.747 | 0.007942 | 59 | 0.137 | 203 | 108 | 13 | 6 | 179 | 561 | 725 | Semaphorin-4C | Semaphorin-4C | | afdb-proteome | AF-Q59YF4-F1-MODEL\_V4 | 0.72 | 0.00627 | 58 | 0.106 | 187 | 100 | 12 | 6 | 142 | 43 | 212 | Increased recombination centers protein 22-1 | Increased recombination centers protein 22-1 | | afdb-proteome | AF-A0A0G2KSM2-F1-MODEL\_V4 | 0.72 | 0.008938 | 58 | 0.097 | 185 | 111 | 13 | 8 | 142 | 794 | 972 | Uncharacterized protein | Uncharacterized protein | | afdb-proteome | AF-Q59XW9-F1-MODEL\_V4 | 0.692 | 0.00627 | 57 | 0.112 | 187 | 99 | 12 | 6 | 142 | 43 | 212 | Increased recombination centers protein 22-2 | Increased recombination centers protein 22-2 | | afdb-proteome | AF-Q4CMW8-F1-MODEL\_V4 | 0.692 | 0.002164 | 57 | 0.114 | 244 | 131 | 13 | 9 | 178 | 679 | 911 | Uncharacterized protein | Uncharacterized protein | | afdb-proteome | AF-D3ZAE6-F1-MODEL\_V4 | 0.663 | 0.007057 | 56 | 0.139 | 186 | 100 | 8 | 12 | 178 | 473 | 617 | RCG49849 | RCG49849 | | afdb-proteome | AF-F6PZL4-F1-MODEL\_V4 | 0.663 | 0.008938 | 56 | 0.089 | 178 | 107 | 10 | 7 | 163 | 533 | 676 | Predicted gene 15448 | Predicted gene 15448 | | afdb-proteome | AF-F8VQ94-F1-MODEL\_V4 | 0.632 | 0.009482 | 55 | 0.099 | 181 | 106 | 11 | 5 | 163 | 529 | 674 | Paired-Ig-like receptor A2 | Paired-Ig-like receptor A2 | | afdb-proteome | AF-Q9UJG1-F1-MODEL\_V4 | 0.537 | 0.007942 | 52 | 0.094 | 201 | 121 | 11 | 8 | 159 | 24 | 212 | Motile sperm domain-containing protein 1 | Motile sperm domain-containing protein 1 | | afdb-proteome | AF-F1QU86-F1-MODEL\_V4 | 0.326 | 0.008425 | 45 | 0.126 | 197 | 114 | 10 | 9 | 179 | 600 | 764 | Sema domain, immunoglobulin domain (Ig), transmembrane domain (TM) and short cytoplasmic domain, (semaphorin) 4Bb | Sema domain, immunoglobulin domain (Ig), transmembrane domain (TM) and short cytoplasmic domain, (semaphorin) 4Bb | | afdb-uniprot50 | AF-E4PPT5-F1-MODEL\_V4 | 1.0 | 1.554e-22 | 848 | 0.514 | 171 | 83 | 0 | 4 | 174 | 2 | 172 | Uncharacterized protein | Uncharacterized protein | | afdb-uniprot50 | AF-A0A515BE33-F1-MODEL\_V4 | 1.0 | 5.702e-22 | 820 | 0.502 | 177 | 86 | 2 | 5 | 179 | 3 | 179 | Uncharacterized protein | Uncharacterized protein | | afdb-uniprot50 | AF-A0A291LZK4-F1-MODEL\_V4 | 1.0 | 3.35e-22 | 816 | 0.485 | 173 | 89 | 0 | 6 | 178 | 5 | 177 | Uncharacterized protein | Uncharacterized protein | | afdb-uniprot50 | AF-A0A143DE12-F1-MODEL\_V4 | 1.0 | 1.752e-21 | 799 | 0.439 | 173 | 97 | 0 | 6 | 178 | 4 | 176 | Uncharacterized protein | Uncharacterized protein | | afdb-uniprot50 | AF-A0A1H9YC26-F1-MODEL\_V4 | 1.0 | 2.228e-19 | 752 | 0.342 | 175 | 115 | 0 | 4 | 178 | 2 | 176 | Uncharacterized protein | Uncharacterized protein | | afdb-uniprot50 | AF-A0A4V1V5L7-F1-MODEL\_V4 | 1.0 | 6.443e-20 | 729 | 0.404 | 173 | 103 | 0 | 6 | 178 | 24 | 196 | Uncharacterized protein | Uncharacterized protein | | afdb-uniprot50 | AF-A0A846VV37-F1-MODEL\_V4 | 1.0 | 1.03e-21 | 705 | 0.477 | 174 | 91 | 0 | 5 | 178 | 4 | 177 | Uncharacterized protein | Uncharacterized protein | | afdb-uniprot50 | AF-A0A3A0FKV7-F1-MODEL\_V4 | 1.0 | 5.736e-19 | 698 | 0.407 | 179 | 102 | 2 | 4 | 178 | 5 | 183 | Uncharacterized protein | Uncharacterized protein | | afdb-uniprot50 | AF-A0A1L2B383-F1-MODEL\_V4 | 1.0 | 1.756e-20 | 686 | 0.408 | 181 | 95 | 2 | 3 | 178 | 7 | 180 | Uncharacterized protein | Uncharacterized protein | | afdb-uniprot50 | AF-A0A6A9VNP5-F1-MODEL\_V4 | 1.0 | 1.984e-18 | 681 | 0.314 | 175 | 117 | 3 | 6 | 179 | 3 | 175 | Uncharacterized protein | Uncharacterized protein | | afdb-uniprot50 | AF-A0A831XDR5-F1-MODEL\_V4 | 1.0 | 1.038e-17 | 677 | 0.307 | 179 | 118 | 4 | 5 | 178 | 3 | 180 | Uncharacterized protein | Uncharacterized protein | | afdb-uniprot50 | AF-A0A2N1AP69-F1-MODEL\_V4 | 1.0 | 1.036e-18 | 667 | 0.518 | 164 | 78 | 1 | 16 | 179 | 1 | 163 | Uncharacterized protein | Uncharacterized protein | | afdb-uniprot50 | AF-A0A0H3ZTY7-F1-MODEL\_V4 | 1.0 | 4.285e-17 | 664 | 0.345 | 159 | 103 | 1 | 5 | 163 | 2 | 159 | Uncharacterized protein | Uncharacterized protein | | afdb-uniprot50 | AF-A0A4Y3TFT0-F1-MODEL\_V4 | 1.0 | 3.189e-17 | 649 | 0.35 | 177 | 110 | 5 | 7 | 179 | 4 | 179 | Uncharacterized protein | Uncharacterized protein | | afdb-uniprot50 | AF-A0A359LUV7-F1-MODEL\_V4 | 1.0 | 3.189e-17 | 647 | 0.359 | 181 | 109 | 4 | 4 | 178 | 6 | 185 | Uncharacterized protein | Uncharacterized protein | | afdb-uniprot50 | AF-A0A3C0NSL4-F1-MODEL\_V4 | 1.0 | 3.383e-17 | 643 | 0.297 | 178 | 120 | 2 | 6 | 178 | 1 | 178 | Uncharacterized protein | Uncharacterized protein | | afdb-uniprot50 | AF-A0A5Y3B0U0-F1-MODEL\_V4 | 1.0 | 1.314e-17 | 638 | 0.503 | 159 | 79 | 0 | 21 | 179 | 4 | 162 | Uncharacterized protein | Uncharacterized protein | | afdb-uniprot50 | AF-A0A1G2ZKV4-F1-MODEL\_V4 | 1.0 | 1.038e-17 | 630 | 0.311 | 183 | 116 | 4 | 4 | 179 | 5 | 184 | Uncharacterized protein | Uncharacterized protein | | afdb-uniprot50 | AF-U5QDV1-F1-MODEL\_V4 | 1.0 | 7.294e-17 | 624 | 0.376 | 170 | 103 | 3 | 5 | 171 | 2 | 171 | Uncharacterized protein | Uncharacterized protein | | afdb-uniprot50 | AF-A0A369R582-F1-MODEL\_V4 | 1.0 | 1.241e-16 | 616 | 0.308 | 178 | 119 | 3 | 4 | 177 | 6 | 183 | Uncharacterized protein | Uncharacterized protein | | afdb-uniprot50 | AF-A0A0U5FTR8-F1-MODEL\_V4 | 1.0 | 8.209e-17 | 601 | 0.385 | 184 | 102 | 7 | 4 | 179 | 6 | 186 | Putative phage-related protein | Putative phage-related protein | | afdb-uniprot50 | AF-A0A7J0BYR9-F1-MODEL\_V4 | 1.0 | 1.482e-16 | 600 | 0.327 | 159 | 101 | 3 | 6 | 163 | 4 | 157 | Uncharacterized protein | Uncharacterized protein | | afdb-uniprot50 | AF-A0A7X2TJT6-F1-MODEL\_V4 | 1.0 | 1.763e-18 | 599 | 0.469 | 164 | 86 | 1 | 16 | 178 | 1 | 164 | Uncharacterized protein | Uncharacterized protein | | afdb-uniprot50 | AF-A0A542RRX4-F1-MODEL\_V4 | 1.0 | 1.241e-16 | 597 | 0.312 | 179 | 116 | 4 | 4 | 178 | 6 | 181 | Uncharacterized protein | Uncharacterized protein | | afdb-uniprot50 | AF-A0A7M1L5E3-F1-MODEL\_V4 | 1.0 | 3.012e-16 | 595 | 0.301 | 176 | 118 | 3 | 6 | 179 | 8 | 180 | Uncharacterized protein | Uncharacterized protein | | afdb-uniprot50 | AF-A0A679JLL1-F1-MODEL\_V4 | 1.0 | 5.439e-16 | 593 | 0.312 | 176 | 113 | 4 | 6 | 179 | 9 | 178 | Uncharacterized protein | Uncharacterized protein | | afdb-uniprot50 | AF-A0A6M3J2Y0-F1-MODEL\_V4 | 1.0 | 1.097e-19 | 591 | 0.386 | 176 | 104 | 4 | 6 | 179 | 2 | 175 | Uncharacterized protein | Uncharacterized protein | | afdb-uniprot50 | AF-A0A2G6D355-F1-MODEL\_V4 | 1.0 | 2.383e-15 | 591 | 0.263 | 167 | 117 | 4 | 5 | 166 | 5 | 170 | Uncharacterized protein | Uncharacterized protein | | afdb-uniprot50 | AF-A0A1F9B6L6-F1-MODEL\_V4 | 1.0 | 1.773e-15 | 589 | 0.282 | 177 | 122 | 3 | 6 | 178 | 8 | 183 | Uncharacterized protein | Uncharacterized protein | | afdb-uniprot50 | AF-A0A2A5ADZ7-F1-MODEL\_V4 | 1.0 | 5.439e-16 | 583 | 0.3 | 180 | 116 | 7 | 7 | 177 | 10 | 188 | Uncharacterized protein | Uncharacterized protein | | afdb-uniprot50 | AF-A0A3A4NQ75-F1-MODEL\_V4 | 1.0 | 1.32e-15 | 580 | 0.297 | 178 | 119 | 4 | 6 | 179 | 7 | 182 | Uncharacterized protein | Uncharacterized protein | | afdb-uniprot50 | AF-A0A7C8LXQ6-F1-MODEL\_V4 | 1.0 | 5.77e-16 | 578 | 0.298 | 174 | 111 | 4 | 6 | 178 | 5 | 168 | Uncharacterized protein | Uncharacterized protein | | afdb-uniprot50 | AF-A0A3B9NUU4-F1-MODEL\_V4 | 1.0 | 1.4e-15 | 574 | 0.306 | 183 | 113 | 7 | 6 | 178 | 8 | 186 | Uncharacterized protein | Uncharacterized protein | | afdb-uniprot50 | AF-A0A5N0TG52-F1-MODEL\_V4 | 1.0 | 4.294e-16 | 571 | 0.287 | 174 | 118 | 3 | 7 | 179 | 5 | 173 | Uncharacterized protein | Uncharacterized protein | | afdb-uniprot50 | AF-A0A1X7L1C8-F1-MODEL\_V4 | 1.0 | 1.77e-16 | 569 | 0.361 | 177 | 100 | 4 | 6 | 178 | 4 | 171 | Uncharacterized protein | Uncharacterized protein | | afdb-uniprot50 | AF-A0A1V6GEE1-F1-MODEL\_V4 | 1.0 | 3.012e-16 | 569 | 0.27 | 196 | 121 | 6 | 5 | 178 | 4 | 199 | Uncharacterized protein | Uncharacterized protein | | afdb-uniprot50 | AF-W0E3G9-F1-MODEL\_V4 | 1.0 | 4.048e-16 | 568 | 0.306 | 186 | 117 | 6 | 4 | 179 | 7 | 190 | Uncharacterized protein | Uncharacterized protein | | afdb-uniprot50 | AF-A0A254T8E3-F1-MODEL\_V4 | 1.0 | 2.383e-15 | 568 | 0.234 | 183 | 130 | 6 | 4 | 178 | 15 | 195 | Uncharacterized protein | Uncharacterized protein | | afdb-uniprot50 | AF-A0A6H2H8M8-F1-MODEL\_V4 | 1.0 | 1.576e-15 | 566 | 0.314 | 178 | 114 | 5 | 7 | 178 | 13 | 188 | Uncharacterized protein | Uncharacterized protein | | afdb-uniprot50 | AF-A0A2W5J2E3-F1-MODEL\_V4 | 1.0 | 1.996e-15 | 561 | 0.284 | 179 | 119 | 5 | 8 | 179 | 4 | 180 | Uncharacterized protein | Uncharacterized protein | | afdb-uniprot50 | AF-A0A3C1GA42-F1-MODEL\_V4 | 1.0 | 1.485e-15 | 557 | 0.275 | 185 | 122 | 6 | 6 | 179 | 7 | 190 | Uncharacterized protein | Uncharacterized protein | | afdb-uniprot50 | AF-A0A2E1P353-F1-MODEL\_V4 | 1.0 | 1.485e-15 | 552 | 0.297 | 188 | 116 | 7 | 4 | 178 | 12 | 196 | Uncharacterized protein | Uncharacterized protein | | afdb-uniprot50 | AF-A0A3S5CWM4-F1-MODEL\_V4 | 1.0 | 8.726e-16 | 551 | 0.267 | 176 | 121 | 5 | 5 | 179 | 2 | 170 | Uncharacterized protein | Uncharacterized protein | | afdb-uniprot50 | AF-Z9JLC1-F1-MODEL\_V4 | 1.0 | 2.523e-16 | 550 | 0.386 | 137 | 84 | 0 | 6 | 142 | 10 | 146 | Uncharacterized protein | Uncharacterized protein | | afdb-uniprot50 | AF-A0A2E2KNA8-F1-MODEL\_V4 | 1.0 | 3.808e-17 | 549 | 0.335 | 176 | 112 | 4 | 6 | 179 | 3 | 175 | Uncharacterized protein | Uncharacterized protein | | afdb-uniprot50 | AF-A0A843YIL2-F1-MODEL\_V4 | 1.0 | 5.439e-16 | 549 | 0.302 | 185 | 117 | 6 | 1 | 178 | 2 | 181 | Uncharacterized protein | Uncharacterized protein | | afdb-uniprot50 | AF-A0A1C2K2F5-F1-MODEL\_V4 | 1.0 | 1.101e-17 | 544 | 0.348 | 175 | 108 | 4 | 6 | 179 | 2 | 171 | Uncharacterized protein | Uncharacterized protein | | afdb-uniprot50 | AF-A0A4T2A8A7-F1-MODEL\_V4 | 1.0 | 1.675e-14 | 543 | 0.321 | 174 | 108 | 4 | 6 | 178 | 5 | 169 | Uncharacterized protein | Uncharacterized protein | | afdb-uniprot50 | AF-A0A1Y5TZC7-F1-MODEL\_V4 | 1.0 | 4.842e-15 | 540 | 0.316 | 183 | 116 | 5 | 4 | 179 | 6 | 186 | Uncharacterized protein | Uncharacterized protein | | afdb-uniprot50 | AF-A0A0S9N2Z7-F1-MODEL\_V4 | 1.0 | 5.137e-15 | 539 | 0.284 | 183 | 119 | 7 | 6 | 179 | 6 | 185 | Uncharacterized protein | Uncharacterized protein | | afdb-uniprot50 | AF-A0A423HS27-F1-MODEL\_V4 | 1.0 | 4.564e-15 | 536 | 0.287 | 174 | 118 | 4 | 6 | 178 | 2 | 170 | Uncharacterized protein | Uncharacterized protein | | afdb-uniprot50 | AF-A0A2D5ZTJ7-F1-MODEL\_V4 | 1.0 | 1.885e-14 | 531 | 0.225 | 173 | 131 | 3 | 6 | 177 | 3 | 173 | Uncharacterized protein | Uncharacterized protein | | afdb-uniprot50 | AF-A0A6L4BCL0-F1-MODEL\_V4 | 1.0 | 8.225e-16 | 530 | 0.302 | 175 | 112 | 3 | 5 | 178 | 2 | 167 | Uncharacterized protein | Uncharacterized protein | | afdb-uniprot50 | AF-A0A368L7T0-F1-MODEL\_V4 | 1.0 | 1.246e-14 | 524 | 0.247 | 186 | 128 | 6 | 6 | 179 | 7 | 192 | Uncharacterized protein | Uncharacterized protein | | afdb-uniprot50 | AF-Q1ILS4-F1-MODEL\_V4 | 1.0 | 6.481e-17 | 524 | 0.268 | 175 | 127 | 1 | 5 | 178 | 7 | 181 | Uncharacterized protein | Uncharacterized protein | | afdb-uniprot50 | AF-A0A661FDS9-F1-MODEL\_V4 | 1.0 | 3.83e-14 | 522 | 0.259 | 181 | 125 | 6 | 6 | 178 | 3 | 182 | Uncharacterized protein | Uncharacterized protein | | afdb-uniprot50 | AF-A0A1F9VFN4-F1-MODEL\_V4 | 1.0 | 4.833e-16 | 520 | 0.343 | 160 | 102 | 3 | 7 | 164 | 22 | 180 | Uncharacterized protein | Uncharacterized protein | | afdb-uniprot50 | AF-E7C7Y6-F1-MODEL\_V4 | 1.0 | 3.611e-14 | 518 | 0.254 | 185 | 124 | 6 | 4 | 176 | 12 | 194 | Uncharacterized protein | Uncharacterized protein | | afdb-uniprot50 | AF-A0A2V5PK36-F1-MODEL\_V4 | 1.0 | 1.877e-16 | 518 | 0.349 | 163 | 99 | 5 | 5 | 161 | 24 | 185 | Uncharacterized protein | Uncharacterized protein | | afdb-uniprot50 | AF-A0A2Z6UL02-F1-MODEL\_V4 | 1.0 | 2.004e-13 | 515 | 0.258 | 170 | 121 | 4 | 9 | 177 | 5 | 170 | Phage-related protein | Phage-related protein | | afdb-uniprot50 | AF-A0A0S8DP01-F1-MODEL\_V4 | 1.0 | 1.675e-14 | 513 | 0.225 | 186 | 132 | 5 | 6 | 179 | 3 | 188 | Uncharacterized protein | Uncharacterized protein | | afdb-uniprot50 | AF-A0A2A5BDD7-F1-MODEL\_V4 | 1.0 | 9.859e-14 | 511 | 0.26 | 165 | 116 | 4 | 6 | 166 | 7 | 169 | Uncharacterized protein | Uncharacterized protein | | afdb-uniprot50 | AF-A0A3B9NQ74-F1-MODEL\_V4 | 1.0 | 8.258e-14 | 511 | 0.26 | 184 | 121 | 6 | 6 | 178 | 14 | 193 | Uncharacterized protein | Uncharacterized protein | | afdb-uniprot50 | AF-A0A838M0T7-F1-MODEL\_V4 | 1.0 | 1.044e-14 | 509 | 0.241 | 182 | 124 | 6 | 7 | 179 | 4 | 180 | Uncharacterized protein | Uncharacterized protein | | afdb-uniprot50 | AF-A0A165R292-F1-MODEL\_V4 | 1.0 | 3.215e-13 | 504 | 0.26 | 184 | 126 | 5 | 4 | 179 | 11 | 192 | Uncharacterized protein | Uncharacterized protein | | afdb-uniprot50 | AF-A0A6A4RDA2-F1-MODEL\_V4 | 1.0 | 3.823e-15 | 503 | 0.36 | 147 | 91 | 3 | 7 | 150 | 3 | 149 | Uncharacterized protein | Uncharacterized protein | | afdb-uniprot50 | AF-A0A1T1IZJ5-F1-MODEL\_V4 | 1.0 | 7.769e-15 | 498 | 0.405 | 143 | 80 | 2 | 1 | 141 | 1 | 140 | Uncharacterized protein | Uncharacterized protein | | afdb-uniprot50 | AF-A0A2E0ENC4-F1-MODEL\_V4 | 1.0 | 2.121e-14 | 494 | 0.248 | 181 | 127 | 5 | 4 | 179 | 6 | 182 | Uncharacterized protein | Uncharacterized protein | | afdb-uniprot50 | AF-A0A7V8KQN1-F1-MODEL\_V4 | 1.0 | 3.403e-14 | 492 | 0.285 | 182 | 118 | 6 | 4 | 178 | 10 | 186 | Uncharacterized protein | Uncharacterized protein | | afdb-uniprot50 | AF-A0A1Z9Q622-F1-MODEL\_V4 | 1.0 | 4.311e-14 | 488 | 0.226 | 181 | 128 | 6 | 7 | 178 | 15 | 192 | Uncharacterized protein | Uncharacterized protein | | afdb-uniprot50 | AF-A0A2D7GUN5-F1-MODEL\_V4 | 1.0 | 1.678e-13 | 487 | 0.274 | 175 | 117 | 6 | 6 | 178 | 2 | 168 | Uncharacterized protein | Uncharacterized protein | | afdb-uniprot50 | AF-A0A7G8BY96-F1-MODEL\_V4 | 1.0 | 1.406e-13 | 487 | 0.245 | 179 | 129 | 4 | 6 | 179 | 21 | 198 | Uncharacterized protein | Uncharacterized protein | | afdb-uniprot50 | AF-A0A5P9IVF8-F1-MODEL\_V4 | 1.0 | 1.046e-13 | 484 | 0.264 | 178 | 125 | 5 | 1 | 175 | 1 | 175 | Uncharacterized protein | Uncharacterized protein | | afdb-uniprot50 | AF-A0A7X8AH15-F1-MODEL\_V4 | 1.0 | 6.519e-14 | 481 | 0.308 | 185 | 107 | 5 | 14 | 178 | 1 | 184 | Uncharacterized protein | Uncharacterized protein | | afdb-uniprot50 | AF-A0A653JDQ3-F1-MODEL\_V4 | 1.0 | 4.573e-14 | 479 | 0.274 | 182 | 119 | 6 | 4 | 178 | 6 | 181 | Uncharacterized protein | Uncharacterized protein | | afdb-uniprot50 | AF-A0A1M7FPB6-F1-MODEL\_V4 | 1.0 | 6.507e-15 | 477 | 0.219 | 182 | 134 | 3 | 6 | 179 | 3 | 184 | Uncharacterized protein | Uncharacterized protein | | afdb-uniprot50 | AF-A0A7T6ARA8-F1-MODEL\_V4 | 1.0 | 9.859e-14 | 472 | 0.237 | 177 | 125 | 6 | 6 | 179 | 8 | 177 | Uncharacterized protein | Uncharacterized protein | | afdb-uniprot50 | AF-A0A1J5SRA9-F1-MODEL\_V4 | 1.0 | 3.403e-14 | 471 | 0.196 | 183 | 138 | 4 | 6 | 179 | 3 | 185 | Uncharacterized protein | Uncharacterized protein | | afdb-uniprot50 | AF-A0A1B3WBU8-F1-MODEL\_V4 | 1.0 | 6.121e-16 | 466 | 0.341 | 173 | 106 | 5 | 7 | 178 | 6 | 171 | Uncharacterized protein | Uncharacterized protein | | afdb-uniprot50 | AF-A0A2A5DGI2-F1-MODEL\_V4 | 1.0 | 4.582e-13 | 466 | 0.24 | 166 | 118 | 5 | 7 | 166 | 4 | 167 | Uncharacterized protein | Uncharacterized protein | | afdb-uniprot50 | AF-A0A2E7WAD6-F1-MODEL\_V4 | 1.0 | 4.852e-14 | 466 | 0.225 | 186 | 131 | 5 | 7 | 179 | 4 | 189 | Uncharacterized protein | Uncharacterized protein | | afdb-uniprot50 | AF-A0A1M7U224-F1-MODEL\_V4 | 1.0 | 3.215e-13 | 465 | 0.272 | 180 | 118 | 6 | 6 | 179 | 11 | 183 | Uncharacterized protein | Uncharacterized protein | | afdb-uniprot50 | AF-A0A0Q7SMP9-F1-MODEL\_V4 | 1.0 | 1.78e-13 | 461 | 0.266 | 180 | 124 | 5 | 6 | 177 | 5 | 184 | Uncharacterized protein | Uncharacterized protein | | afdb-uniprot50 | AF-A0A1L3ZRN8-F1-MODEL\_V4 | 1.0 | 4.072e-13 | 461 | 0.306 | 189 | 110 | 8 | 6 | 178 | 12 | 195 | Uncharacterized protein | Uncharacterized protein | | afdb-uniprot50 | AF-A0A844IFC1-F1-MODEL\_V4 | 1.0 | 1.585e-12 | 458 | 0.214 | 177 | 120 | 5 | 6 | 166 | 5 | 178 | Uncharacterized protein | Uncharacterized protein | | afdb-uniprot50 | AF-A0A2M7YUE3-F1-MODEL\_V4 | 1.0 | 1.322e-14 | 456 | 0.303 | 178 | 116 | 5 | 4 | 178 | 6 | 178 | Uncharacterized protein | Uncharacterized protein | | afdb-uniprot50 | AF-A0A286GPM6-F1-MODEL\_V4 | 1.0 | 9.879e-13 | 438 | 0.263 | 171 | 115 | 3 | 9 | 179 | 2 | 161 | Uncharacterized protein | Uncharacterized protein | | afdb-uniprot50 | AF-A0A1G0RDT9-F1-MODEL\_V4 | 1.0 | 2.004e-13 | 436 | 0.265 | 177 | 119 | 7 | 7 | 179 | 6 | 175 | Uncharacterized protein | Uncharacterized protein | | afdb-uniprot50 | AF-A0A501WT91-F1-MODEL\_V4 | 1.0 | 3.625e-12 | 423 | 0.25 | 180 | 118 | 6 | 7 | 179 | 10 | 179 | Uncharacterized protein | Uncharacterized protein | | afdb-uniprot50 | AF-A0A6H1ZNP5-F1-MODEL\_V4 | 1.0 | 7.815e-12 | 407 | 0.229 | 174 | 127 | 4 | 9 | 179 | 16 | 185 | Uncharacterized protein | Uncharacterized protein | | afdb-uniprot50 | AF-F5R899-F1-MODEL\_V4 | 1.0 | 1.892e-12 | 407 | 0.272 | 180 | 122 | 6 | 6 | 179 | 8 | 184 | Phage-related protein | Phage-related protein | | afdb-uniprot50 | AF-A0A6M4Y9K2-F1-MODEL\_V4 | 1.0 | 8.795e-12 | 399 | 0.558 | 136 | 58 | 1 | 46 | 179 | 3 | 138 | Uncharacterized protein | Uncharacterized protein | | afdb-uniprot50 | AF-A0A7Y0NUN9-F1-MODEL\_V4 | 1.0 | 1.182e-11 | 393 | 0.144 | 159 | 130 | 4 | 6 | 160 | 8 | 164 | Uncharacterized protein | Uncharacterized protein | | afdb-uniprot50 | AF-A0A0F9IDA6-F1-MODEL\_V4 | 1.0 | 7.352e-13 | 389 | 0.278 | 187 | 124 | 5 | 1 | 179 | 1 | 184 | Uncharacterized protein | Uncharacterized protein | | afdb-uniprot50 | AF-A0A1V5CWH5-F1-MODEL\_V4 | 1.0 | 1.78e-13 | 389 | 0.211 | 194 | 130 | 7 | 5 | 179 | 2 | 191 | Uncharacterized protein | Uncharacterized protein | | afdb-uniprot50 | AF-A0A0F9JRZ2-F1-MODEL\_V4 | 1.0 | 5.167e-12 | 382 | 0.234 | 196 | 124 | 8 | 6 | 179 | 9 | 200 | Uncharacterized protein | Uncharacterized protein | | afdb-uniprot50 | AF-A0A358KKZ0-F1-MODEL\_V4 | 1.0 | 2.016e-10 | 381 | 0.232 | 125 | 93 | 2 | 6 | 128 | 9 | 132 | Uncharacterized protein | Uncharacterized protein | | afdb-uniprot50 | AF-A0A6L6JB30-F1-MODEL\_V4 | 1.0 | 8.795e-12 | 381 | 0.485 | 140 | 71 | 1 | 40 | 178 | 1 | 140 | Uncharacterized protein | Uncharacterized protein | | afdb-uniprot50 | AF-A0A0N8FSS6-F1-MODEL\_V4 | 1.0 | 2.402e-11 | 381 | 0.312 | 144 | 89 | 4 | 6 | 142 | 12 | 152 | Uncharacterized protein | Uncharacterized protein | | afdb-uniprot50 | AF-A0A3B9Q8J6-F1-MODEL\_V4 | 1.0 | 6.558e-11 | 364 | 0.209 | 181 | 131 | 6 | 6 | 178 | 1 | 177 | Uncharacterized protein | Uncharacterized protein | | afdb-uniprot50 | AF-A0A0F9QC72-F1-MODEL\_V4 | 1.0 | 4.591e-12 | 362 | 0.239 | 171 | 110 | 5 | 6 | 166 | 11 | 171 | Uncharacterized protein | Uncharacterized protein | | afdb-uniprot50 | AF-A0A848V5M5-F1-MODEL\_V4 | 1.0 | 1.259e-09 | 356 | 0.251 | 159 | 107 | 5 | 29 | 177 | 3 | 159 | Uncharacterized protein | Uncharacterized protein | | afdb-uniprot50 | AF-A0A2E7ZNQ6-F1-MODEL\_V4 | 1.0 | 1.691e-09 | 340 | 0.206 | 131 | 88 | 5 | 7 | 123 | 4 | 132 | Uncharacterized protein | Uncharacterized protein | | afdb-uniprot50 | AF-A0A3B9NUI5-F1-MODEL\_V4 | 1.0 | 1.333e-10 | 339 | 0.185 | 183 | 130 | 9 | 6 | 178 | 9 | 182 | Uncharacterized protein | Uncharacterized protein | | afdb-uniprot50 | AF-A0A1F9UT01-F1-MODEL\_V4 | 1.0 | 9.918e-11 | 317 | 0.275 | 189 | 119 | 9 | 6 | 179 | 8 | 193 | Uncharacterized protein | Uncharacterized protein | | afdb-uniprot50 | AF-A0A7V8FKH2-F1-MODEL\_V4 | 1.0 | 6.985e-09 | 313 | 0.518 | 108 | 52 | 0 | 57 | 164 | 4 | 111 | Uncharacterized protein | Uncharacterized protein | | afdb-uniprot50 | AF-A0A2P1VF72-F1-MODEL\_V4 | 1.0 | 3.444e-08 | 302 | 0.33 | 142 | 85 | 3 | 40 | 179 | 2 | 135 | Uncharacterized protein | Uncharacterized protein | | afdb-uniprot50 | AF-A0A564WI89-F1-MODEL\_V4 | 1.0 | 3.246e-08 | 299 | 0.21 | 171 | 121 | 5 | 7 | 176 | 3 | 160 | Uncharacterized protein | Uncharacterized protein | | afdb-uniprot50 | AF-A0A2V2GR66-F1-MODEL\_V4 | 1.0 | 1.338e-08 | 284 | 0.242 | 165 | 103 | 8 | 7 | 165 | 1 | 149 | Uncharacterized protein | Uncharacterized protein | | afdb-uniprot50 | AF-A0A432FDA3-F1-MODEL\_V4 | 1.0 | 9.423e-07 | 282 | 0.311 | 90 | 58 | 3 | 6 | 92 | 9 | 97 | Uncharacterized protein | Uncharacterized protein | | afdb-uniprot50 | AF-A0A0F9KXQ4-F1-MODEL\_V4 | 1.0 | 1.194e-06 | 264 | 0.091 | 98 | 84 | 4 | 9 | 102 | 5 | 101 | Uncharacterized protein | Uncharacterized protein | | afdb-uniprot50 | AF-A0A3G9FQ52-F1-MODEL\_V4 | 1.0 | 3.072e-06 | 260 | 0.553 | 94 | 42 | 0 | 86 | 179 | 5 | 98 | Uncharacterized protein | Uncharacterized protein | | afdb-uniprot50 | AF-A0A1F8QKN5-F1-MODEL\_V4 | 1.0 | 8.34e-09 | 260 | 0.287 | 167 | 109 | 4 | 22 | 179 | 1 | 166 | Uncharacterized protein | Uncharacterized protein | | afdb-uniprot50 | AF-A0A382W2M1-F1-MODEL\_V4 | 1.0 | 1.702e-06 | 247 | 0.21 | 100 | 67 | 4 | 9 | 96 | 9 | 108 | Uncharacterized protein | Uncharacterized protein | | afdb-uniprot50 | AF-A0A0A2Y143-F1-MODEL\_V4 | 1.0 | 1.343e-06 | 246 | 0.17 | 100 | 71 | 3 | 9 | 96 | 7 | 106 | Uncharacterized protein | Uncharacterized protein | | afdb-uniprot50 | AF-A0A0E3UJT2-F1-MODEL\_V4 | 1.0 | 1.915e-06 | 239 | 0.2 | 105 | 69 | 5 | 9 | 98 | 18 | 122 | Uncharacterized protein | Uncharacterized protein | | afdb-uniprot50 | AF-A0A2E3JRL0-F1-MODEL\_V4 | 1.0 | 3.668e-06 | 237 | 0.205 | 102 | 70 | 4 | 9 | 99 | 10 | 111 | Uncharacterized protein | Uncharacterized protein | | afdb-uniprot50 | AF-A0A2D6TNC0-F1-MODEL\_V4 | 1.0 | 1.915e-06 | 236 | 0.173 | 104 | 74 | 4 | 9 | 100 | 8 | 111 | Uncharacterized protein | Uncharacterized protein | | afdb-uniprot50 | AF-A0A2E5AWZ1-F1-MODEL\_V4 | 1.0 | 4.929e-06 | 233 | 0.198 | 101 | 68 | 4 | 9 | 96 | 9 | 109 | Uncharacterized protein | Uncharacterized protein | | afdb-uniprot50 | AF-A6FS73-F1-MODEL\_V4 | 1.0 | 2.286e-06 | 232 | 0.156 | 102 | 75 | 3 | 9 | 100 | 5 | 105 | Uncharacterized protein | Uncharacterized protein | | afdb-uniprot50 | AF-A0A0F9JR02-F1-MODEL\_V4 | 1.0 | 3.259e-06 | 231 | 0.247 | 105 | 65 | 6 | 6 | 96 | 5 | 109 | Uncharacterized protein | Uncharacterized protein | | afdb-uniprot50 | AF-A0A0F9D8Q2-F1-MODEL\_V4 | 1.0 | 7.027e-06 | 228 | 0.176 | 102 | 74 | 5 | 5 | 96 | 5 | 106 | Uncharacterized protein | Uncharacterized protein | | afdb-uniprot50 | AF-A0A2Z3H230-F1-MODEL\_V4 | 1.0 | 9.997e-07 | 228 | 0.174 | 109 | 72 | 4 | 10 | 100 | 1 | 109 | Uncharacterized protein | Uncharacterized protein | | afdb-uniprot50 | AF-A0A3C1DS41-F1-MODEL\_V4 | 1.0 | 3.892e-06 | 227 | 0.198 | 106 | 70 | 6 | 6 | 96 | 7 | 112 | Uncharacterized protein | Uncharacterized protein | | afdb-uniprot50 | AF-A0A3B0VIW3-F1-MODEL\_V4 | 1.0 | 8.883e-07 | 225 | 0.13 | 107 | 87 | 5 | 7 | 108 | 1 | 106 | Uncharacterized protein | Uncharacterized protein | | afdb-uniprot50 | AF-A0A3N5X8Y3-F1-MODEL\_V4 | 1.0 | 5.548e-06 | 224 | 0.155 | 103 | 72 | 6 | 9 | 96 | 10 | 112 | Uncharacterized protein | Uncharacterized protein | | afdb-uniprot50 | AF-A0A2E9KUT5-F1-MODEL\_V4 | 1.0 | 2.426e-06 | 223 | 0.203 | 113 | 75 | 5 | 9 | 106 | 8 | 120 | Uncharacterized protein | Uncharacterized protein | | afdb-uniprot50 | AF-A0A381YBA8-F1-MODEL\_V4 | 1.0 | 9.442e-06 | 220 | 0.2 | 100 | 68 | 5 | 9 | 96 | 11 | 110 | Uncharacterized protein | Uncharacterized protein | | afdb-uniprot50 | AF-A0A7X7UJS9-F1-MODEL\_V4 | 1.0 | 2.286e-06 | 219 | 0.152 | 105 | 80 | 6 | 9 | 104 | 8 | 112 | Uncharacterized protein | Uncharacterized protein | | afdb-uniprot50 | AF-A0A5M8IDW4-F1-MODEL\_V4 | 1.0 | 3.892e-06 | 214 | 0.175 | 108 | 74 | 6 | 6 | 98 | 9 | 116 | Uncharacterized protein | Uncharacterized protein | | afdb-uniprot50 | AF-A0A418M5Y9-F1-MODEL\_V4 | 1.0 | 3.465e-05 | 213 | 0.175 | 91 | 69 | 4 | 9 | 96 | 17 | 104 | Uncharacterized protein | Uncharacterized protein | | afdb-uniprot50 | AF-A0A2D6E192-F1-MODEL\_V4 | 1.0 | 8.9e-06 | 213 | 0.194 | 103 | 68 | 6 | 9 | 96 | 8 | 110 | Uncharacterized protein | Uncharacterized protein | | afdb-uniprot50 | AF-A0A382DI07-F1-MODEL\_V4 | 1.0 | 3.266e-05 | 212 | 0.13 | 100 | 77 | 4 | 9 | 98 | 21 | 120 | Uncharacterized protein | Uncharacterized protein | | afdb-uniprot50 | AF-A0A2I1XBL1-F1-MODEL\_V4 | 1.0 | 3.465e-05 | 209 | 0.161 | 99 | 74 | 3 | 9 | 98 | 8 | 106 | Uncharacterized protein | Uncharacterized protein | | afdb-uniprot50 | AF-A0A1H9GD31-F1-MODEL\_V4 | 1.0 | 6.243e-06 | 206 | 0.186 | 118 | 74 | 7 | 1 | 96 | 1 | 118 | Uncharacterized protein | Uncharacterized protein | | afdb-uniprot50 | AF-A0A378PHR1-F1-MODEL\_V4 | 1.0 | 4.137e-05 | 205 | 0.126 | 103 | 78 | 6 | 9 | 99 | 6 | 108 | Uncharacterized protein | Uncharacterized protein | | afdb-uniprot50 | AF-A0A1B7X5C5-F1-MODEL\_V4 | 1.0 | 2.43e-05 | 203 | 0.163 | 104 | 74 | 5 | 9 | 99 | 13 | 116 | Uncharacterized protein | Uncharacterized protein | | afdb-uniprot50 | AF-A0A0F9R4H3-F1-MODEL\_V4 | 1.0 | 2.578e-05 | 203 | 0.157 | 108 | 73 | 7 | 9 | 98 | 12 | 119 | Uncharacterized protein | Uncharacterized protein | | afdb-uniprot50 | AF-A0A849MUW1-F1-MODEL\_V4 | 1.0 | 6.243e-06 | 203 | 0.178 | 123 | 79 | 5 | 9 | 109 | 9 | 131 | Uncharacterized protein | Uncharacterized protein | | afdb-uniprot50 | AF-A0A1T0ACG6-F1-MODEL\_V4 | 1.0 | 2.735e-05 | 200 | 0.166 | 108 | 76 | 7 | 9 | 104 | 5 | 110 | Uncharacterized protein | Uncharacterized protein | | afdb-uniprot50 | AF-A0A3S0DMG9-F1-MODEL\_V4 | 1.0 | 7.469e-05 | 200 | 0.115 | 104 | 76 | 5 | 9 | 96 | 8 | 111 | Uncharacterized protein | Uncharacterized protein | | afdb-uniprot50 | AF-A0A5C7PP54-F1-MODEL\_V4 | 1.0 | 1.607e-05 | 199 | 0.168 | 101 | 72 | 3 | 9 | 97 | 4 | 104 | Uncharacterized protein | Uncharacterized protein | | afdb-uniprot50 | AF-A0A378UGU1-F1-MODEL\_V4 | 1.0 | 1.809e-05 | 199 | 0.183 | 109 | 80 | 4 | 9 | 108 | 7 | 115 | Uncharacterized protein | Uncharacterized protein | | afdb-uniprot50 | AF-A0A2N0B444-F1-MODEL\_V4 | 1.0 | 1.512e-06 | 198 | 0.136 | 139 | 98 | 7 | 5 | 130 | 4 | 133 | Uncharacterized protein | Uncharacterized protein | | afdb-uniprot50 | AF-A0A2N2D0B1-F1-MODEL\_V4 | 1.0 | 3.899e-05 | 197 | 0.149 | 107 | 76 | 5 | 9 | 107 | 2 | 101 | Uncharacterized protein | Uncharacterized protein | | afdb-uniprot50 | AF-A0A257NKB8-F1-MODEL\_V4 | 1.0 | 1.269e-05 | 197 | 0.178 | 112 | 77 | 8 | 9 | 107 | 6 | 115 | Uncharacterized protein | Uncharacterized protein | | afdb-uniprot50 | AF-A0A497T2U7-F1-MODEL\_V4 | 1.0 | 3.676e-05 | 197 | 0.138 | 108 | 73 | 6 | 9 | 96 | 9 | 116 | Uncharacterized protein | Uncharacterized protein | | afdb-uniprot50 | AF-A0A3N4MWR2-F1-MODEL\_V4 | 1.0 | 1.127e-05 | 196 | 0.214 | 112 | 74 | 6 | 3 | 100 | 2 | 113 | Uncharacterized protein | Uncharacterized protein | | afdb-uniprot50 | AF-A0A1Y3RG66-F1-MODEL\_V4 | 1.0 | 1.604e-06 | 196 | 0.177 | 124 | 87 | 7 | 7 | 124 | 13 | 127 | Uncharacterized protein | Uncharacterized protein | | afdb-uniprot50 | AF-A0A1S1CDS1-F1-MODEL\_V4 | 1.0 | 5.537e-07 | 196 | 0.168 | 166 | 106 | 3 | 9 | 142 | 9 | 174 | Uncharacterized protein | Uncharacterized protein | | afdb-uniprot50 | AF-A0A4Q4GW66-F1-MODEL\_V4 | 1.0 | 2.578e-05 | 195 | 0.13 | 107 | 78 | 7 | 6 | 98 | 1 | 106 | Uncharacterized protein | Uncharacterized protein | | afdb-uniprot50 | AF-A0A351GJN3-F1-MODEL\_V4 | 1.0 | 5.548e-06 | 194 | 0.166 | 114 | 69 | 7 | 9 | 96 | 12 | 125 | Uncharacterized protein | Uncharacterized protein | | afdb-uniprot50 | AF-A0A2D5QIA3-F1-MODEL\_V4 | 1.0 | 1.515e-05 | 194 | 0.151 | 119 | 72 | 5 | 9 | 98 | 9 | 127 | Uncharacterized protein | Uncharacterized protein | | afdb-uniprot50 | AF-A0A090MR43-F1-MODEL\_V4 | 1.0 | 9.461e-05 | 193 | 0.118 | 110 | 82 | 4 | 5 | 99 | 4 | 113 | Uncharacterized protein | Uncharacterized protein | | afdb-uniprot50 | AF-A0A838JK26-F1-MODEL\_V4 | 1.0 | 8.406e-05 | 191 | 0.095 | 105 | 78 | 5 | 9 | 96 | 4 | 108 | Uncharacterized protein | Uncharacterized protein | | afdb-uniprot50 | AF-A0A3E0R3L4-F1-MODEL\_V4 | 1.0 | 7.469e-05 | 189 | 0.16 | 106 | 71 | 4 | 9 | 96 | 8 | 113 | Uncharacterized protein | Uncharacterized protein | | afdb-uniprot50 | AF-A0A2D8B2N1-F1-MODEL\_V4 | 1.0 | 1.428e-05 | 189 | 0.136 | 117 | 81 | 5 | 4 | 100 | 12 | 128 | Uncharacterized protein | Uncharacterized protein | | afdb-uniprot50 | AF-A0A1Q5PL77-F1-MODEL\_V4 | 1.0 | 1.705e-05 | 188 | 0.123 | 105 | 81 | 6 | 8 | 107 | 47 | 145 | Uncharacterized protein | Uncharacterized protein | | afdb-uniprot50 | AF-A0A2L0W7Z5-F1-MODEL\_V4 | 1.0 | 0.0001518 | 185 | 0.123 | 105 | 75 | 5 | 9 | 96 | 8 | 112 | Uncharacterized protein | Uncharacterized protein | | afdb-uniprot50 | AF-A0A524LAF8-F1-MODEL\_V4 | 1.0 | 1.915e-06 | 182 | 0.153 | 143 | 93 | 6 | 9 | 142 | 8 | 131 | Uncharacterized protein | Uncharacterized protein | | afdb-uniprot50 | AF-A0A7C5R5E6-F1-MODEL\_V4 | 1.0 | 5.559e-05 | 181 | 0.154 | 110 | 75 | 5 | 9 | 100 | 10 | 119 | Uncharacterized protein | Uncharacterized protein | | afdb-uniprot50 | AF-A0A1Y3SHK2-F1-MODEL\_V4 | 1.0 | 2.896e-06 | 180 | 0.157 | 146 | 104 | 9 | 6 | 144 | 12 | 145 | Uncharacterized protein | Uncharacterized protein | | afdb-uniprot50 | AF-A0A397Q662-F1-MODEL\_V4 | 1.0 | 0.0001431 | 179 | 0.109 | 110 | 81 | 7 | 9 | 101 | 5 | 114 | Uncharacterized protein | Uncharacterized protein | | afdb-uniprot50 | AF-A0A0F9S9C7-F1-MODEL\_V4 | 1.0 | 8.918e-05 | 179 | 0.165 | 103 | 68 | 5 | 11 | 95 | 14 | 116 | Uncharacterized protein | Uncharacterized protein | | afdb-uniprot50 | AF-A0A495J3S7-F1-MODEL\_V4 | 1.0 | 2.159e-05 | 179 | 0.133 | 120 | 83 | 6 | 9 | 107 | 3 | 122 | Uncharacterized protein | Uncharacterized protein | | afdb-uniprot50 | AF-A0A3C0VX60-F1-MODEL\_V4 | 1.0 | 7.924e-05 | 178 | 0.21 | 119 | 78 | 3 | 25 | 141 | 1 | 105 | Uncharacterized protein | Uncharacterized protein | | afdb-uniprot50 | AF-A0A0F9HMU7-F1-MODEL\_V4 | 1.0 | 3.259e-06 | 178 | 0.136 | 146 | 101 | 7 | 3 | 142 | 2 | 128 | Uncharacterized protein | Uncharacterized protein | | afdb-uniprot50 | AF-A0A447CZS3-F1-MODEL\_V4 | 1.0 | 0.0001065 | 177 | 0.14 | 114 | 75 | 5 | 9 | 105 | 8 | 115 | Uncharacterized protein | Uncharacterized protein | | afdb-uniprot50 | AF-A0A1H4UM15-F1-MODEL\_V4 | 1.0 | 0.0001518 | 177 | 0.129 | 116 | 84 | 6 | 9 | 107 | 8 | 123 | Uncharacterized protein | Uncharacterized protein | | afdb-uniprot50 | AF-A0A662BHI3-F1-MODEL\_V4 | 1.0 | 2.286e-06 | 177 | 0.125 | 143 | 107 | 6 | 9 | 143 | 6 | 138 | Uncharacterized protein | Uncharacterized protein | | afdb-uniprot50 | AF-A0A1H6QBZ3-F1-MODEL\_V4 | 1.0 | 2.578e-05 | 176 | 0.169 | 118 | 71 | 7 | 6 | 101 | 12 | 124 | Uncharacterized protein | Uncharacterized protein | | afdb-uniprot50 | AF-F9GSL4-F1-MODEL\_V4 | 1.0 | 3.676e-05 | 176 | 0.129 | 116 | 83 | 7 | 9 | 108 | 10 | 123 | Uncharacterized protein | Uncharacterized protein | | afdb-uniprot50 | AF-A0A7C3YAM0-F1-MODEL\_V4 | 1.0 | 7.469e-05 | 176 | 0.168 | 95 | 65 | 6 | 8 | 95 | 66 | 153 | Uncharacterized protein | Uncharacterized protein | | afdb-uniprot50 | AF-M5V2Z7-F1-MODEL\_V4 | 1.0 | 2.291e-05 | 176 | 0.124 | 137 | 99 | 7 | 5 | 128 | 4 | 132 | Uncharacterized protein | Uncharacterized protein | | afdb-uniprot50 | AF-A0A432R0Z8-F1-MODEL\_V4 | 1.0 | 0.0001004 | 175 | 0.102 | 107 | 77 | 5 | 9 | 96 | 5 | 111 | Uncharacterized protein | Uncharacterized protein | | afdb-uniprot50 | AF-A0A7W5UGR6-F1-MODEL\_V4 | 1.0 | 0.0001708 | 175 | 0.13 | 115 | 76 | 7 | 9 | 106 | 8 | 115 | Uncharacterized protein | Uncharacterized protein | | afdb-uniprot50 | AF-A0A7V6LFQ4-F1-MODEL\_V4 | 1.0 | 3.458e-06 | 175 | 0.158 | 164 | 106 | 8 | 9 | 142 | 23 | 184 | Uncharacterized protein | Uncharacterized protein | | afdb-uniprot50 | AF-R6F1T1-F1-MODEL\_V4 | 1.0 | 4.939e-05 | 175 | 0.158 | 120 | 94 | 5 | 9 | 124 | 20 | 136 | Uncharacterized protein | Uncharacterized protein | | afdb-uniprot50 | AF-A0A418VEF3-F1-MODEL\_V4 | 1.0 | 4.939e-05 | 171 | 0.178 | 112 | 80 | 5 | 1 | 100 | 3 | 114 | Uncharacterized protein | Uncharacterized protein | | afdb-uniprot50 | AF-A0A4R8HZ05-F1-MODEL\_V4 | 1.0 | 4.388e-05 | 169 | 0.14 | 114 | 81 | 6 | 9 | 107 | 5 | 116 | Uncharacterized protein | Uncharacterized protein | | afdb-uniprot50 | AF-A0A417HAH9-F1-MODEL\_V4 | 1.0 | 3.266e-05 | 169 | 0.175 | 120 | 87 | 5 | 7 | 120 | 13 | 126 | Uncharacterized protein | Uncharacterized protein | | afdb-uniprot50 | AF-A0A658JVP5-F1-MODEL\_V4 | 1.0 | 8.9e-06 | 169 | 0.103 | 155 | 118 | 6 | 8 | 144 | 13 | 164 | Uncharacterized protein | Uncharacterized protein | | afdb-uniprot50 | AF-A0A1Z8QCH1-F1-MODEL\_V4 | 1.0 | 9.461e-05 | 168 | 0.14 | 121 | 75 | 5 | 9 | 100 | 21 | 141 | Uncharacterized protein | Uncharacterized protein | | afdb-uniprot50 | AF-A0A1B1UD36-F1-MODEL\_V4 | 1.0 | 0.0002583 | 167 | 0.122 | 106 | 74 | 5 | 9 | 95 | 5 | 110 | Uncharacterized protein | Uncharacterized protein | | afdb-uniprot50 | AF-A0A844AGF1-F1-MODEL\_V4 | 1.0 | 0.0001812 | 167 | 0.081 | 111 | 82 | 6 | 9 | 99 | 5 | 115 | Uncharacterized protein | Uncharacterized protein | | afdb-uniprot50 | AF-A0A5C8SNL3-F1-MODEL\_V4 | 1.0 | 0.000113 | 167 | 0.159 | 113 | 77 | 6 | 5 | 99 | 9 | 121 | Uncharacterized protein | Uncharacterized protein | | afdb-uniprot50 | AF-A0A382BVZ5-F1-MODEL\_V4 | 1.0 | 3.676e-05 | 167 | 0.152 | 138 | 72 | 6 | 9 | 104 | 13 | 147 | Uncharacterized protein | Uncharacterized protein | | afdb-uniprot50 | AF-A0A1Q4CYS2-F1-MODEL\_V4 | 1.0 | 0.000525 | 167 | 0.191 | 99 | 70 | 2 | 8 | 96 | 110 | 208 | Uncharacterized protein | Uncharacterized protein | | afdb-uniprot50 | AF-A0A1C5N9C8-F1-MODEL\_V4 | 1.0 | 4.646e-06 | 167 | 0.152 | 144 | 108 | 5 | 5 | 144 | 14 | 147 | Uncharacterized protein | Uncharacterized protein | | afdb-uniprot50 | AF-A0A5P3MUE5-F1-MODEL\_V4 | 1.0 | 5.229e-06 | 167 | 0.127 | 172 | 117 | 5 | 9 | 165 | 9 | 162 | Uncharacterized protein | Uncharacterized protein | | afdb-uniprot50 | AF-A0A1C5PNG8-F1-MODEL\_V4 | 1.0 | 0.0001431 | 166 | 0.11 | 109 | 86 | 8 | 7 | 106 | 1 | 107 | Uncharacterized protein | Uncharacterized protein | | afdb-uniprot50 | AF-A0A1Y3XMP8-F1-MODEL\_V4 | 1.0 | 1.428e-05 | 166 | 0.146 | 123 | 90 | 6 | 9 | 124 | 14 | 128 | Uncharacterized protein | Uncharacterized protein | | afdb-uniprot50 | AF-A0A651G663-F1-MODEL\_V4 | 1.0 | 0.0002295 | 165 | 0.145 | 117 | 76 | 8 | 9 | 104 | 5 | 118 | Uncharacterized protein | Uncharacterized protein | | afdb-uniprot50 | AF-A0A1I3T8Y1-F1-MODEL\_V4 | 1.0 | 0.000204 | 164 | 0.095 | 115 | 77 | 5 | 9 | 96 | 15 | 129 | Uncharacterized protein | Uncharacterized protein | | afdb-uniprot50 | AF-A0A5C7LQ67-F1-MODEL\_V4 | 1.0 | 5.874e-07 | 163 | 0.138 | 188 | 123 | 7 | 9 | 158 | 10 | 196 | Uncharacterized protein | Uncharacterized protein | | afdb-uniprot50 | AF-A0A1U7GKV9-F1-MODEL\_V4 | 1.0 | 2.291e-05 | 163 | 0.182 | 137 | 94 | 6 | 9 | 128 | 7 | 142 | Uncharacterized protein | Uncharacterized protein | | afdb-uniprot50 | AF-A0A7X5E0T7-F1-MODEL\_V4 | 1.0 | 3.892e-06 | 162 | 0.144 | 166 | 114 | 5 | 8 | 166 | 17 | 161 | Uncharacterized protein | Uncharacterized protein | | afdb-uniprot50 | AF-A0A7J3I1Y6-F1-MODEL\_V4 | 1.0 | 2.43e-05 | 160 | 0.128 | 132 | 104 | 7 | 1 | 124 | 1 | 129 | Uncharacterized protein | Uncharacterized protein | | afdb-uniprot50 | AF-A0A1Y3ZCV4-F1-MODEL\_V4 | 1.0 | 2.73e-06 | 160 | 0.152 | 170 | 114 | 8 | 6 | 166 | 10 | 158 | Uncharacterized protein | Uncharacterized protein | | afdb-uniprot50 | AF-A0A5F2B0I3-F1-MODEL\_V4 | 1.0 | 1.127e-05 | 160 | 0.188 | 143 | 82 | 9 | 9 | 129 | 7 | 137 | Uncharacterized protein | Uncharacterized protein | | afdb-uniprot50 | AF-A0A7M3JY29-F1-MODEL\_V4 | 1.0 | 4.929e-06 | 160 | 0.18 | 144 | 82 | 8 | 9 | 129 | 7 | 137 | Uncharacterized protein | Uncharacterized protein | | afdb-uniprot50 | AF-A0A662SJ11-F1-MODEL\_V4 | 1.0 | 1.428e-05 | 159 | 0.138 | 152 | 97 | 9 | 7 | 142 | 736 | 869 | SBP\_bac\_5 domain-containing protein | SBP\_bac\_5 domain-containing protein | | afdb-uniprot50 | AF-A0A443JEA3-F1-MODEL\_V4 | 1.0 | 0.0003683 | 158 | 0.188 | 106 | 74 | 4 | 3 | 96 | 108 | 213 | Uncharacterized protein | Uncharacterized protein | | afdb-uniprot50 | AF-A0A353H0K0-F1-MODEL\_V4 | 1.0 | 5.559e-05 | 158 | 0.169 | 124 | 71 | 6 | 9 | 101 | 197 | 319 | Ig-like domain-containing protein | Ig-like domain-containing protein | | afdb-uniprot50 | AF-J9UW90-F1-MODEL\_V4 | 1.0 | 0.0006268 | 158 | 0.128 | 101 | 71 | 4 | 7 | 93 | 718 | 815 | Solute-binding protein like protein | Solute-binding protein like protein | | afdb-uniprot50 | AF-A0A518G2U2-F1-MODEL\_V4 | 1.0 | 0.0003272 | 157 | 0.111 | 108 | 78 | 4 | 8 | 97 | 24 | 131 | Uncharacterized protein | Uncharacterized protein | | afdb-uniprot50 | AF-A0A1Y4LVL1-F1-MODEL\_V4 | 1.0 | 1.705e-05 | 157 | 0.164 | 146 | 103 | 8 | 7 | 145 | 16 | 149 | Uncharacterized protein | Uncharacterized protein | | afdb-uniprot50 | AF-A0A1Y4HDA8-F1-MODEL\_V4 | 1.0 | 4.137e-05 | 157 | 0.14 | 128 | 95 | 6 | 9 | 127 | 2 | 123 | Uncharacterized protein | Uncharacterized protein | | afdb-uniprot50 | AF-A0A243KKJ3-F1-MODEL\_V4 | 1.0 | 3.465e-05 | 156 | 0.106 | 150 | 117 | 6 | 6 | 142 | 1 | 146 | BppU\_N domain-containing protein | BppU\_N domain-containing protein | | afdb-uniprot50 | AF-A0A562NRU4-F1-MODEL\_V4 | 1.0 | 0.000525 | 155 | 0.181 | 99 | 70 | 4 | 8 | 96 | 116 | 213 | Uncharacterized protein | Uncharacterized protein | | afdb-uniprot50 | AF-A0A2R7Z2V5-F1-MODEL\_V4 | 1.0 | 6.256e-05 | 155 | 0.161 | 105 | 74 | 6 | 9 | 107 | 48 | 144 | ZnMc domain-containing protein | ZnMc domain-containing protein | | afdb-uniprot50 | AF-A0A1Y3ZUZ1-F1-MODEL\_V4 | 1.0 | 4.38e-06 | 154 | 0.186 | 161 | 96 | 9 | 7 | 142 | 12 | 162 | Uncharacterized protein | Uncharacterized protein | | afdb-uniprot50 | AF-A0A2N6C104-F1-MODEL\_V4 | 1.0 | 0.0006268 | 153 | 0.181 | 99 | 72 | 6 | 4 | 96 | 2 | 97 | Uncharacterized protein | Uncharacterized protein | | afdb-uniprot50 | AF-A0A1V5X591-F1-MODEL\_V4 | 1.0 | 0.0008423 | 153 | 0.204 | 83 | 54 | 4 | 26 | 96 | 111 | 193 | Uncharacterized protein | Uncharacterized protein | | afdb-uniprot50 | AF-A0A6M1VR84-F1-MODEL\_V4 | 1.0 | 5.24e-05 | 152 | 0.132 | 143 | 102 | 9 | 9 | 134 | 2 | 139 | BppU family phage baseplate upper protein | BppU family phage baseplate upper protein | | afdb-uniprot50 | AF-A0A448VHT3-F1-MODEL\_V4 | 1.0 | 1.919e-05 | 152 | 0.157 | 190 | 116 | 9 | 9 | 166 | 8 | 185 | Uncharacterized protein | Uncharacterized protein | | afdb-uniprot50 | AF-A0A6G7X1N8-F1-MODEL\_V4 | 1.0 | 7.454e-06 | 151 | 0.155 | 167 | 91 | 7 | 6 | 129 | 2 | 161 | Uncharacterized protein | Uncharacterized protein | | afdb-uniprot50 | AF-A0A2B0J102-F1-MODEL\_V4 | 1.0 | 4.388e-05 | 150 | 0.128 | 125 | 102 | 4 | 8 | 127 | 5 | 127 | BppU\_N domain-containing protein | BppU\_N domain-containing protein | | afdb-uniprot50 | AF-A0A1Y3ZD32-F1-MODEL\_V4 | 1.0 | 0.0001431 | 150 | 0.155 | 109 | 81 | 5 | 15 | 122 | 2 | 100 | Uncharacterized protein | Uncharacterized protein | | afdb-uniprot50 | AF-A0A1Q6JK56-F1-MODEL\_V4 | 1.0 | 1.269e-05 | 150 | 0.191 | 136 | 92 | 6 | 5 | 125 | 18 | 150 | Uncharacterized protein | Uncharacterized protein | | afdb-uniprot50 | AF-T3D9W5-F1-MODEL\_V4 | 1.0 | 9.442e-06 | 149 | 0.103 | 164 | 118 | 7 | 9 | 166 | 19 | 159 | Uncharacterized protein | Uncharacterized protein | | afdb-uniprot50 | AF-A0A1Y4EZM3-F1-MODEL\_V4 | 1.0 | 4.939e-05 | 149 | 0.144 | 152 | 105 | 7 | 6 | 136 | 17 | 164 | Uncharacterized protein | Uncharacterized protein | | afdb-uniprot50 | AF-A0A6C2CBK0-F1-MODEL\_V4 | 1.0 | 2.291e-05 | 149 | 0.146 | 150 | 107 | 9 | 8 | 142 | 22 | 165 | Collagen-like protein | Collagen-like protein | | afdb-uniprot50 | AF-A0A487D5S2-F1-MODEL\_V4 | 1.0 | 1.346e-05 | 149 | 0.13 | 168 | 115 | 8 | 9 | 166 | 19 | 165 | Phage pre-neck appendage-like protein | Phage pre-neck appendage-like protein | | afdb-uniprot50 | AF-G0J1Z2-F1-MODEL\_V4 | 1.0 | 0.0003085 | 148 | 0.118 | 110 | 79 | 6 | 9 | 100 | 12 | 121 | Uncharacterized protein | Uncharacterized protein | | afdb-uniprot50 | AF-A0A7C5VHK6-F1-MODEL\_V4 | 1.0 | 0.0003085 | 148 | 0.12 | 108 | 80 | 8 | 9 | 107 | 700 | 801 | SBP\_bac\_5 domain-containing protein | SBP\_bac\_5 domain-containing protein | | afdb-uniprot50 | AF-A0A3D6CSD6-F1-MODEL\_V4 | 1.0 | 0.0004145 | 147 | 0.204 | 98 | 65 | 4 | 6 | 94 | 1 | 94 | Uncharacterized protein | Uncharacterized protein | | afdb-uniprot50 | AF-A0A089QFE0-F1-MODEL\_V4 | 1.0 | 3.266e-05 | 147 | 0.09 | 154 | 116 | 9 | 7 | 142 | 8 | 155 | Phage-associated protein, putative structural protein | Phage-associated protein, putative structural protein | | afdb-uniprot50 | AF-A0A1Y3X1Y0-F1-MODEL\_V4 | 1.0 | 1.919e-05 | 147 | 0.142 | 169 | 117 | 7 | 6 | 166 | 10 | 158 | Uncharacterized protein | Uncharacterized protein | | afdb-uniprot50 | AF-A0A2E6W1A1-F1-MODEL\_V4 | 1.0 | 0.0008423 | 146 | 0.193 | 93 | 61 | 6 | 9 | 95 | 7 | 91 | Uncharacterized protein | Uncharacterized protein | | afdb-uniprot50 | AF-A0A4Q2YKC0-F1-MODEL\_V4 | 1.0 | 0.0001518 | 146 | 0.157 | 121 | 82 | 7 | 8 | 108 | 4 | 124 | Uncharacterized protein | Uncharacterized protein | | afdb-uniprot50 | AF-A0A843HUJ8-F1-MODEL\_V4 | 1.0 | 3.676e-05 | 146 | 0.154 | 123 | 85 | 6 | 6 | 109 | 1 | 123 | Uncharacterized protein | Uncharacterized protein | | afdb-uniprot50 | AF-A0A0Q8VPZ8-F1-MODEL\_V4 | 1.0 | 0.0001349 | 146 | 0.126 | 119 | 97 | 5 | 9 | 124 | 254 | 368 | Uncharacterized protein | Uncharacterized protein | | afdb-uniprot50 | AF-A0A137PPG3-F1-MODEL\_V4 | 1.0 | 0.000794 | 144 | 0.096 | 114 | 93 | 4 | 3 | 108 | 15 | 126 | BppU\_N domain-containing protein | BppU\_N domain-containing protein | | afdb-uniprot50 | AF-A0A4P9YNR0-F1-MODEL\_V4 | 1.0 | 2.291e-05 | 144 | 0.131 | 182 | 125 | 9 | 9 | 178 | 58 | 218 | Dolichyl-diphosphooligosaccharide--protein glycosyltransferase subunit 2 | Dolichyl-diphosphooligosaccharide--protein glycosyltransferase subunit 2 | | afdb-uniprot50 | AF-A0A3R6UUE4-F1-MODEL\_V4 | 1.0 | 7.454e-06 | 144 | 0.111 | 171 | 128 | 5 | 8 | 164 | 16 | 176 | Uncharacterized protein | Uncharacterized protein | | afdb-uniprot50 | AF-A0A3C0GD26-F1-MODEL\_V4 | 1.0 | 6.256e-05 | 143 | 0.107 | 140 | 104 | 6 | 7 | 125 | 1 | 140 | Uncharacterized protein | Uncharacterized protein | | afdb-uniprot50 | AF-Q6NTZ0-F1-MODEL\_V4 | 1.0 | 0.0002295 | 143 | 0.161 | 130 | 90 | 7 | 7 | 126 | 5 | 125 | MGC81410 protein | MGC81410 protein | | afdb-uniprot50 | AF-A0A1K2HBG8-F1-MODEL\_V4 | 1.0 | 7.469e-05 | 143 | 0.106 | 150 | 114 | 8 | 8 | 142 | 10 | 154 | Collagen triple helix repeat-containing protein | Collagen triple helix repeat-containing protein | | afdb-uniprot50 | AF-A0A3D2H3M5-F1-MODEL\_V4 | 1.0 | 0.000204 | 143 | 0.156 | 128 | 92 | 7 | 9 | 124 | 209 | 332 | Uncharacterized protein | Uncharacterized protein | | afdb-uniprot50 | AF-A0A2D6EZF0-F1-MODEL\_V4 | 1.0 | 0.0001923 | 142 | 0.085 | 140 | 104 | 6 | 9 | 142 | 5 | 126 | Uncharacterized protein | Uncharacterized protein | | afdb-uniprot50 | AF-A0A373N6D8-F1-MODEL\_V4 | 1.0 | 2.735e-05 | 142 | 0.115 | 147 | 106 | 9 | 8 | 141 | 11 | 146 | Uncharacterized protein | Uncharacterized protein | | afdb-uniprot50 | AF-A0A3R6XI26-F1-MODEL\_V4 | 1.0 | 6.256e-05 | 142 | 0.13 | 199 | 130 | 12 | 8 | 179 | 16 | 198 | Collagen-like protein | Collagen-like protein | | afdb-uniprot50 | AF-A0A7H9CMM2-F1-MODEL\_V4 | 1.0 | 9.461e-05 | 142 | 0.11 | 136 | 100 | 7 | 6 | 129 | 7 | 133 | Uncharacterized protein | Uncharacterized protein | | afdb-uniprot50 | AF-U2L7Y7-F1-MODEL\_V4 | 1.0 | 7.908e-06 | 142 | 0.158 | 158 | 110 | 8 | 14 | 163 | 47 | 189 | Uncharacterized protein | Uncharacterized protein | | afdb-uniprot50 | AF-D9SVZ5-F1-MODEL\_V4 | 1.0 | 0.0002435 | 142 | 0.1 | 130 | 98 | 7 | 8 | 126 | 18 | 139 | Uncharacterized protein | Uncharacterized protein | | afdb-uniprot50 | AF-A0A6I4LXN3-F1-MODEL\_V4 | 1.0 | 0.0002295 | 141 | 0.159 | 113 | 74 | 5 | 6 | 98 | 2 | 113 | Uncharacterized protein | Uncharacterized protein | | afdb-uniprot50 | AF-A0A3S0BU18-F1-MODEL\_V4 | 1.0 | 2.159e-05 | 141 | 0.144 | 152 | 101 | 6 | 5 | 142 | 27 | 163 | Uncharacterized protein | Uncharacterized protein | | afdb-uniprot50 | AF-A0A3N1XSC8-F1-MODEL\_V4 | 1.0 | 2.291e-05 | 141 | 0.167 | 131 | 87 | 10 | 9 | 127 | 11 | 131 | Uncharacterized protein | Uncharacterized protein | | afdb-uniprot50 | AF-A0A7K3NLH1-F1-MODEL\_V4 | 1.0 | 0.001712 | 140 | 0.141 | 99 | 79 | 4 | 6 | 101 | 1 | 96 | Uncharacterized protein | Uncharacterized protein | | afdb-uniprot50 | AF-A0A2E3J263-F1-MODEL\_V4 | 1.0 | 9.461e-05 | 140 | 0.158 | 126 | 93 | 7 | 8 | 125 | 550 | 670 | Uncharacterized protein | Uncharacterized protein | | afdb-uniprot50 | AF-D1AG96-F1-MODEL\_V4 | 1.0 | 0.0002295 | 139 | 0.102 | 136 | 110 | 6 | 9 | 136 | 21 | 152 | BppU\_N domain-containing protein | BppU\_N domain-containing protein | | afdb-uniprot50 | AF-A0A090Z5B2-F1-MODEL\_V4 | 1.0 | 2.578e-05 | 139 | 0.125 | 184 | 126 | 7 | 6 | 178 | 15 | 174 | BppU\_N domain-containing protein | BppU\_N domain-containing protein | | afdb-uniprot50 | AF-A0A2N5DZ13-F1-MODEL\_V4 | 1.0 | 0.002589 | 139 | 0.095 | 94 | 76 | 6 | 8 | 95 | 134 | 224 | Uncharacterized protein | Uncharacterized protein | | afdb-uniprot50 | AF-C9L790-F1-MODEL\_V4 | 1.0 | 3.078e-05 | 139 | 0.148 | 175 | 113 | 8 | 8 | 165 | 11 | 166 | Uncharacterized protein | Uncharacterized protein | | afdb-uniprot50 | AF-A0A417BVX3-F1-MODEL\_V4 | 1.0 | 1.919e-05 | 139 | 0.132 | 174 | 115 | 10 | 8 | 166 | 16 | 168 | Uncharacterized protein | Uncharacterized protein | | afdb-uniprot50 | AF-A0A844KFR1-F1-MODEL\_V4 | 1.0 | 9.461e-05 | 138 | 0.105 | 170 | 118 | 8 | 5 | 166 | 16 | 159 | Uncharacterized protein | Uncharacterized protein | | afdb-uniprot50 | AF-A0A3C1IA62-F1-MODEL\_V4 | 1.0 | 0.0001349 | 138 | 0.092 | 140 | 110 | 10 | 8 | 134 | 12 | 147 | Uncharacterized protein | Uncharacterized protein | | afdb-uniprot50 | AF-A0A1N6WK55-F1-MODEL\_V4 | 1.0 | 0.000113 | 138 | 0.139 | 165 | 116 | 6 | 9 | 165 | 23 | 169 | Lysophospholipase L1 | Lysophospholipase L1 | | afdb-uniprot50 | AF-A0A350VIV5-F1-MODEL\_V4 | 1.0 | 5.559e-05 | 137 | 0.163 | 171 | 125 | 8 | 5 | 166 | 16 | 177 | Uncharacterized protein | Uncharacterized protein | | afdb-uniprot50 | AF-A0A5P3XC75-F1-MODEL\_V4 | 1.0 | 2.902e-05 | 137 | 0.14 | 171 | 118 | 7 | 9 | 165 | 25 | 180 | DUF2479 domain-containing protein | DUF2479 domain-containing protein | | afdb-uniprot50 | AF-A0A7X9K8L6-F1-MODEL\_V4 | 1.0 | 0.0008423 | 137 | 0.148 | 101 | 81 | 3 | 9 | 107 | 408 | 505 | Peptidase\_C25 domain-containing protein | Peptidase\_C25 domain-containing protein | | afdb-uniprot50 | AF-A0A6N1ESY5-F1-MODEL\_V4 | 1.0 | 0.001006 | 137 | 0.121 | 132 | 104 | 7 | 7 | 128 | 9 | 138 | Pectate\_lyase\_3 domain-containing protein | Pectate\_lyase\_3 domain-containing protein | | afdb-uniprot50 | AF-A0A7C5W505-F1-MODEL\_V4 | 1.0 | 0.0003907 | 137 | 0.178 | 129 | 81 | 7 | 7 | 119 | 199 | 318 | Uncharacterized protein | Uncharacterized protein | | afdb-uniprot50 | AF-A0A174D4S5-F1-MODEL\_V4 | 1.0 | 6.256e-05 | 137 | 0.114 | 174 | 122 | 11 | 9 | 165 | 20 | 178 | Choline binding protein | Choline binding protein | | afdb-uniprot50 | AF-R9N9F0-F1-MODEL\_V4 | 1.0 | 0.0002908 | 137 | 0.169 | 124 | 95 | 5 | 9 | 128 | 21 | 140 | Uncharacterized protein | Uncharacterized protein | | afdb-uniprot50 | AF-A0A429GIY2-F1-MODEL\_V4 | 1.0 | 0.0003272 | 137 | 0.172 | 116 | 80 | 6 | 7 | 109 | 732 | 844 | CGP-CTERM sorting domain-containing protein | CGP-CTERM sorting domain-containing protein | | afdb-uniprot50 | AF-A0A496KGP7-F1-MODEL\_V4 | 1.0 | 0.001613 | 136 | 0.12 | 108 | 83 | 3 | 9 | 107 | 7 | 111 | Uncharacterized protein | Uncharacterized protein | | afdb-uniprot50 | AF-A0A7Y9PCR7-F1-MODEL\_V4 | 1.0 | 0.00244 | 136 | 0.193 | 88 | 59 | 4 | 26 | 101 | 123 | 210 | Uncharacterized protein | Uncharacterized protein | | afdb-uniprot50 | AF-R8RDA6-F1-MODEL\_V4 | 1.0 | 3.465e-05 | 136 | 0.13 | 153 | 115 | 6 | 6 | 142 | 15 | 165 | BppU\_N domain-containing protein | BppU\_N domain-containing protein | | afdb-uniprot50 | AF-A0A1H3XCM1-F1-MODEL\_V4 | 1.0 | 0.0001708 | 136 | 0.126 | 166 | 115 | 7 | 9 | 166 | 22 | 165 | BppU\_N domain-containing protein | BppU\_N domain-containing protein | | afdb-uniprot50 | AF-A0A2A8VJ41-F1-MODEL\_V4 | 1.0 | 6.637e-05 | 136 | 0.148 | 155 | 110 | 8 | 6 | 142 | 15 | 165 | BppU\_N domain-containing protein | BppU\_N domain-containing protein | | afdb-uniprot50 | AF-A0A2B5HPR6-F1-MODEL\_V4 | 1.0 | 7.041e-05 | 136 | 0.148 | 135 | 93 | 9 | 8 | 127 | 17 | 144 | Uncharacterized protein | Uncharacterized protein | | afdb-uniprot50 | AF-A0A1I3QIP6-F1-MODEL\_V4 | 1.0 | 8.39e-06 | 136 | 0.121 | 189 | 127 | 11 | 9 | 164 | 24 | 206 | Uncharacterized protein | Uncharacterized protein | | afdb-uniprot50 | AF-A0A2H3KVU9-F1-MODEL\_V4 | 1.0 | 0.00244 | 135 | 0.211 | 90 | 62 | 5 | 7 | 95 | 41 | 122 | Uncharacterized protein | Uncharacterized protein | | afdb-uniprot50 | AF-A0A1C5U595-F1-MODEL\_V4 | 1.0 | 0.0002295 | 135 | 0.139 | 151 | 111 | 10 | 9 | 148 | 22 | 164 | Uncharacterized protein | Uncharacterized protein | | afdb-uniprot50 | AF-A0A0P9E8S0-F1-MODEL\_V4 | 1.0 | 0.000113 | 135 | 0.155 | 174 | 117 | 11 | 8 | 167 | 38 | 195 | Uncharacterized protein | Uncharacterized protein | | afdb-uniprot50 | AF-A0A4P6ZYX7-F1-MODEL\_V4 | 1.0 | 0.0002295 | 135 | 0.149 | 161 | 105 | 9 | 9 | 158 | 23 | 162 | Uncharacterized protein | Uncharacterized protein | | afdb-uniprot50 | AF-A0A837IKU0-F1-MODEL\_V4 | 1.0 | 0.000161 | 134 | 0.126 | 134 | 105 | 5 | 7 | 135 | 31 | 157 | Peptidase, M23/M37 family | Peptidase, M23/M37 family | | afdb-uniprot50 | AF-A0A1C4ERV9-F1-MODEL\_V4 | 1.0 | 3.078e-05 | 134 | 0.141 | 156 | 110 | 10 | 6 | 142 | 1 | 151 | BppU\_N domain-containing protein | BppU\_N domain-containing protein | | afdb-uniprot50 | AF-A0A3F3S3N3-F1-MODEL\_V4 | 1.0 | 0.0002295 | 134 | 0.119 | 142 | 107 | 6 | 7 | 135 | 18 | 154 | Hep\_Hag | Hep\_Hag | | afdb-uniprot50 | AF-A0A0E9N1Z6-F1-MODEL\_V4 | 1.0 | 0.003478 | 133 | 0.134 | 104 | 83 | 5 | 7 | 106 | 10 | 110 | Uncharacterized protein | Uncharacterized protein | | afdb-uniprot50 | AF-A0A2H9QAH0-F1-MODEL\_V4 | 1.0 | 0.001201 | 133 | 0.162 | 111 | 78 | 8 | 5 | 108 | 275 | 377 | Uncharacterized protein | Uncharacterized protein | | afdb-uniprot50 | AF-A0A497L8C9-F1-MODEL\_V4 | 1.0 | 0.007069 | 132 | 0.094 | 95 | 80 | 3 | 6 | 96 | 1 | 93 | Uncharacterized protein | Uncharacterized protein | | afdb-uniprot50 | AF-A0A0W8G563-F1-MODEL\_V4 | 1.0 | 0.003091 | 132 | 0.139 | 93 | 71 | 6 | 9 | 96 | 12 | 100 | Uncharacterized protein | Uncharacterized protein | | afdb-uniprot50 | AF-A0A848RGW3-F1-MODEL\_V4 | 1.0 | 5.24e-05 | 132 | 0.165 | 139 | 87 | 9 | 9 | 125 | 17 | 148 | BppU family phage baseplate upper protein | BppU family phage baseplate upper protein | | afdb-uniprot50 | AF-A0A846M644-F1-MODEL\_V4 | 1.0 | 2.43e-05 | 132 | 0.12 | 182 | 107 | 10 | 8 | 156 | 45 | 206 | Uncharacterized protein | Uncharacterized protein | | afdb-uniprot50 | AF-A0A1W7LNG8-F1-MODEL\_V4 | 1.0 | 0.0002295 | 132 | 0.083 | 168 | 122 | 8 | 9 | 166 | 23 | 168 | Uncharacterized protein | Uncharacterized protein | | afdb-uniprot50 | AF-A0A2C4PYW6-F1-MODEL\_V4 | 1.0 | 8.918e-05 | 132 | 0.134 | 156 | 114 | 9 | 2 | 142 | 11 | 160 | DUF2479 domain-containing protein | DUF2479 domain-containing protein | | afdb-uniprot50 | AF-A0A7J4FW38-F1-MODEL\_V4 | 1.0 | 0.00369 | 132 | 0.148 | 94 | 73 | 4 | 7 | 94 | 38 | 130 | Carboxypeptidase regulatory-like domain-containing protein | Carboxypeptidase regulatory-like domain-containing protein | | afdb-uniprot50 | AF-A0A174X6D8-F1-MODEL\_V4 | 1.0 | 2.578e-05 | 132 | 0.137 | 175 | 116 | 10 | 8 | 166 | 16 | 171 | Uncharacterized protein | Uncharacterized protein | | afdb-uniprot50 | AF-A0A2X3L147-F1-MODEL\_V4 | 1.0 | 0.0005909 | 131 | 0.096 | 104 | 83 | 7 | 10 | 107 | 2 | 100 | Big\_13 domain-containing protein | Big\_13 domain-containing protein | | afdb-uniprot50 | AF-A0A3L7SAM3-F1-MODEL\_V4 | 1.0 | 0.0002295 | 131 | 0.161 | 136 | 74 | 9 | 3 | 103 | 287 | 417 | Uncharacterized protein | Uncharacterized protein | | afdb-uniprot50 | AF-A0A3M2A537-F1-MODEL\_V4 | 1.0 | 0.0007484 | 131 | 0.236 | 93 | 56 | 7 | 7 | 95 | 52 | 133 | Uncharacterized protein | Uncharacterized protein | | afdb-uniprot50 | AF-A0A538K330-F1-MODEL\_V4 | 1.0 | 0.003279 | 131 | 0.127 | 94 | 72 | 4 | 7 | 95 | 261 | 349 | Uncharacterized protein | Uncharacterized protein | | afdb-uniprot50 | AF-A0A6A8DIG3-F1-MODEL\_V4 | 1.0 | 7.041e-05 | 131 | 0.145 | 193 | 129 | 11 | 8 | 169 | 21 | 208 | Uncharacterized protein | Uncharacterized protein | | afdb-uniprot50 | AF-A0A2P9GEF6-F1-MODEL\_V4 | 1.0 | 9.442e-06 | 131 | 0.095 | 209 | 138 | 10 | 8 | 179 | 90 | 284 | Uncharacterized protein | Uncharacterized protein | | afdb-uniprot50 | AF-A0A1L6LN89-F1-MODEL\_V4 | 1.0 | 0.00244 | 130 | 0.126 | 111 | 83 | 6 | 6 | 107 | 6 | 111 | Uncharacterized protein | Uncharacterized protein | | afdb-uniprot50 | AF-A0A377PLH1-F1-MODEL\_V4 | 1.0 | 0.000113 | 130 | 0.134 | 141 | 95 | 8 | 8 | 141 | 7 | 127 | Big\_13 domain-containing protein | Big\_13 domain-containing protein | | afdb-uniprot50 | AF-E3CAQ5-F1-MODEL\_V4 | 1.0 | 0.0003471 | 130 | 0.098 | 163 | 121 | 6 | 8 | 148 | 12 | 170 | Uncharacterized protein | Uncharacterized protein | | afdb-uniprot50 | AF-A0A1G2S722-F1-MODEL\_V4 | 1.0 | 0.000948 | 130 | 0.159 | 88 | 68 | 4 | 9 | 95 | 430 | 512 | Uncharacterized protein | Uncharacterized protein | | afdb-uniprot50 | AF-A0A354KRM3-F1-MODEL\_V4 | 1.0 | 2.159e-05 | 130 | 0.114 | 183 | 131 | 9 | 9 | 165 | 24 | 201 | Uncharacterized protein | Uncharacterized protein | | afdb-uniprot50 | AF-A0A1I3P6U5-F1-MODEL\_V4 | 1.0 | 0.001351 | 129 | 0.153 | 98 | 70 | 4 | 9 | 96 | 6 | 100 | Uncharacterized protein | Uncharacterized protein | | afdb-uniprot50 | AF-A0A2D8ESG9-F1-MODEL\_V4 | 1.0 | 0.0004949 | 129 | 0.108 | 148 | 75 | 6 | 6 | 96 | 5 | 152 | Uncharacterized protein | Uncharacterized protein | | afdb-uniprot50 | AF-A0A3D5KYB9-F1-MODEL\_V4 | 1.0 | 2.735e-05 | 129 | 0.125 | 167 | 118 | 7 | 9 | 166 | 20 | 167 | BppU\_N domain-containing protein | BppU\_N domain-containing protein | | afdb-uniprot50 | AF-A0A4S3LGE2-F1-MODEL\_V4 | 1.0 | 0.006663 | 129 | 0.172 | 93 | 67 | 5 | 8 | 96 | 208 | 294 | Ig-like domain-containing protein | Ig-like domain-containing protein | | afdb-uniprot50 | AF-A0A3C1CTL9-F1-MODEL\_V4 | 1.0 | 0.0002164 | 129 | 0.119 | 142 | 100 | 5 | 9 | 127 | 19 | 158 | Uncharacterized protein | Uncharacterized protein | | afdb-uniprot50 | AF-A0A366ZBU9-F1-MODEL\_V4 | 1.0 | 0.0001812 | 129 | 0.153 | 130 | 94 | 9 | 1 | 124 | 323 | 442 | Uncharacterized protein | Uncharacterized protein | | afdb-uniprot50 | AF-A0A662KU90-F1-MODEL\_V4 | 1.0 | 5.897e-05 | 128 | 0.097 | 154 | 117 | 5 | 8 | 160 | 5 | 137 | Uncharacterized protein | Uncharacterized protein | | afdb-uniprot50 | AF-A0A2E5D4P0-F1-MODEL\_V4 | 1.0 | 0.0004397 | 128 | 0.134 | 104 | 75 | 5 | 8 | 100 | 50 | 149 | Uncharacterized protein | Uncharacterized protein | | afdb-uniprot50 | AF-F1TEH3-F1-MODEL\_V4 | 1.0 | 0.001926 | 128 | 0.125 | 120 | 84 | 8 | 2 | 108 | 13 | 124 | BppU\_N domain-containing protein | BppU\_N domain-containing protein | | afdb-uniprot50 | AF-X1CLZ4-F1-MODEL\_V4 | 1.0 | 0.002168 | 128 | 0.099 | 111 | 85 | 7 | 8 | 108 | 132 | 237 | Uncharacterized protein | Uncharacterized protein | | afdb-uniprot50 | AF-A0A7W1I8A5-F1-MODEL\_V4 | 1.0 | 8.406e-05 | 128 | 0.123 | 146 | 100 | 12 | 7 | 134 | 272 | 407 | Uncharacterized protein | Uncharacterized protein | | afdb-uniprot50 | AF-A0A2X0V1N7-F1-MODEL\_V4 | 1.0 | 0.0001004 | 128 | 0.15 | 173 | 110 | 10 | 9 | 164 | 25 | 177 | Uncharacterized protein | Uncharacterized protein | | afdb-uniprot50 | AF-A0A4P8S4K1-F1-MODEL\_V4 | 1.0 | 0.0001923 | 128 | 0.101 | 177 | 121 | 9 | 6 | 164 | 19 | 175 | Uncharacterized protein | Uncharacterized protein | | afdb-uniprot50 | AF-A0A4Q7PQS7-F1-MODEL\_V4 | 1.0 | 0.0008936 | 127 | 0.125 | 128 | 98 | 8 | 9 | 127 | 26 | 148 | Uncharacterized protein | Uncharacterized protein | | afdb-uniprot50 | AF-A5ZRU3-F1-MODEL\_V4 | 1.0 | 0.0001708 | 127 | 0.113 | 176 | 125 | 8 | 3 | 166 | 5 | 161 | BppU\_N domain-containing protein | BppU\_N domain-containing protein | | afdb-uniprot50 | AF-A0A3E2WI43-F1-MODEL\_V4 | 1.0 | 4.388e-05 | 127 | 0.136 | 176 | 122 | 6 | 8 | 166 | 16 | 178 | Uncharacterized protein | Uncharacterized protein | | afdb-uniprot50 | AF-A0A7X2MU43-F1-MODEL\_V4 | 1.0 | 0.0003683 | 126 | 0.095 | 105 | 84 | 7 | 9 | 108 | 2 | 100 | Big\_13 domain-containing protein | Big\_13 domain-containing protein | | afdb-uniprot50 | AF-A0A3C0G3Z4-F1-MODEL\_V4 | 1.0 | 0.000794 | 126 | 0.079 | 138 | 104 | 5 | 9 | 142 | 3 | 121 | Uncharacterized protein | Uncharacterized protein | | afdb-uniprot50 | AF-A0A2S4GM85-F1-MODEL\_V4 | 1.0 | 0.0002295 | 126 | 0.124 | 169 | 119 | 7 | 5 | 166 | 16 | 162 | Uncharacterized protein | Uncharacterized protein | | afdb-uniprot50 | AF-A0A810ZRD3-F1-MODEL\_V4 | 1.0 | 0.001521 | 126 | 0.118 | 110 | 78 | 7 | 8 | 107 | 141 | 241 | Uncharacterized protein | Uncharacterized protein | | afdb-uniprot50 | AF-R8E0D3-F1-MODEL\_V4 | 1.0 | 5.897e-05 | 126 | 0.125 | 176 | 128 | 5 | 6 | 170 | 15 | 175 | BppU\_N domain-containing protein | BppU\_N domain-containing protein | | afdb-uniprot50 | AF-B0PAR7-F1-MODEL\_V4 | 1.0 | 0.0001065 | 126 | 0.129 | 170 | 122 | 5 | 8 | 166 | 159 | 313 | Uncharacterized protein | Uncharacterized protein | | afdb-uniprot50 | AF-A0A349MVT2-F1-MODEL\_V4 | 1.0 | 0.00844 | 126 | 0.197 | 91 | 66 | 4 | 9 | 95 | 351 | 438 | Uncharacterized protein | Uncharacterized protein | | afdb-uniprot50 | AF-X8HS60-F1-MODEL\_V4 | 1.0 | 0.0004145 | 125 | 0.148 | 135 | 96 | 7 | 7 | 127 | 20 | 149 | Uncharacterized protein | Uncharacterized protein | | afdb-uniprot50 | AF-A0A417HW05-F1-MODEL\_V4 | 1.0 | 4.388e-05 | 125 | 0.189 | 137 | 76 | 8 | 15 | 124 | 32 | 160 | Uncharacterized protein | Uncharacterized protein | | afdb-uniprot50 | AF-A0A2B9TYA6-F1-MODEL\_V4 | 1.0 | 0.000113 | 125 | 0.117 | 153 | 117 | 6 | 6 | 142 | 15 | 165 | BppU\_N domain-containing protein | BppU\_N domain-containing protein | | afdb-uniprot50 | AF-A0A446IBB5-F1-MODEL\_V4 | 1.0 | 9.461e-05 | 125 | 0.085 | 188 | 137 | 10 | 8 | 179 | 16 | 184 | DUF2479 domain-containing protein | DUF2479 domain-containing protein | | afdb-uniprot50 | AF-A0A2T5V3S1-F1-MODEL\_V4 | 1.0 | 0.0001349 | 125 | 0.14 | 185 | 122 | 8 | 6 | 178 | 15 | 174 | Uncharacterized protein DUF2479 | Uncharacterized protein DUF2479 | | afdb-uniprot50 | AF-R7H217-F1-MODEL\_V4 | 1.0 | 7.469e-05 | 125 | 0.129 | 162 | 114 | 10 | 6 | 144 | 6 | 163 | Uncharacterized protein | Uncharacterized protein | | afdb-uniprot50 | AF-R9MUQ2-F1-MODEL\_V4 | 1.0 | 0.0001349 | 125 | 0.114 | 184 | 123 | 8 | 5 | 178 | 16 | 169 | Uncharacterized protein | Uncharacterized protein | | afdb-uniprot50 | AF-A0A1T4KGE4-F1-MODEL\_V4 | 1.0 | 0.004406 | 125 | 0.145 | 110 | 85 | 5 | 4 | 107 | 384 | 490 | Uncharacterized protein | Uncharacterized protein | | afdb-uniprot50 | AF-A0A1R7IK63-F1-MODEL\_V4 | 1.0 | 7.924e-05 | 125 | 0.091 | 186 | 127 | 6 | 3 | 165 | 11 | 177 | Phage pre-neck appendage-like protein | Phage pre-neck appendage-like protein | | afdb-uniprot50 | AF-A0A544TAC3-F1-MODEL\_V4 | 1.0 | 0.000113 | 125 | 0.116 | 172 | 115 | 11 | 8 | 166 | 21 | 168 | NodB homology domain-containing protein | NodB homology domain-containing protein | | afdb-uniprot50 | AF-A0A2B0JB62-F1-MODEL\_V4 | 1.0 | 7.041e-05 | 124 | 0.13 | 138 | 90 | 8 | 6 | 124 | 15 | 141 | BppU\_N domain-containing protein | BppU\_N domain-containing protein | | afdb-uniprot50 | AF-A0A7C9H3D3-F1-MODEL\_V4 | 1.0 | 0.0004145 | 124 | 0.092 | 108 | 85 | 6 | 21 | 124 | 46 | 144 | BppU\_N domain-containing protein | BppU\_N domain-containing protein | | afdb-uniprot50 | AF-A0A2A8VGJ7-F1-MODEL\_V4 | 1.0 | 0.0001349 | 124 | 0.135 | 184 | 117 | 8 | 6 | 178 | 15 | 167 | BppU\_N domain-containing protein | BppU\_N domain-containing protein | | afdb-uniprot50 | AF-A0A1B1KZP8-F1-MODEL\_V4 | 1.0 | 0.0001708 | 124 | 0.146 | 178 | 126 | 6 | 4 | 170 | 13 | 175 | Phage protein | Phage protein | | afdb-uniprot50 | AF-U2TW11-F1-MODEL\_V4 | 1.0 | 0.000665 | 124 | 0.154 | 136 | 99 | 7 | 4 | 125 | 17 | 150 | Collagen triple helix repeat protein | Collagen triple helix repeat protein | | afdb-uniprot50 | AF-A0A396KPQ0-F1-MODEL\_V4 | 1.0 | 3.899e-05 | 124 | 0.121 | 173 | 124 | 8 | 8 | 164 | 16 | 176 | Uncharacterized protein | Uncharacterized protein | | afdb-uniprot50 | AF-A0A3N2FNP4-F1-MODEL\_V4 | 1.0 | 0.0007484 | 124 | 0.18 | 122 | 90 | 8 | 8 | 126 | 602 | 716 | Glycine rich protein | Glycine rich protein | | afdb-uniprot50 | AF-A0A2S4GT96-F1-MODEL\_V4 | 1.0 | 0.0001271 | 123 | 0.132 | 166 | 110 | 10 | 9 | 165 | 19 | 159 | Uncharacterized protein | Uncharacterized protein | | afdb-uniprot50 | AF-A0A0K9FG35-F1-MODEL\_V4 | 1.0 | 0.0008936 | 123 | 0.157 | 127 | 84 | 9 | 9 | 124 | 22 | 136 | Uncharacterized protein | Uncharacterized protein | | afdb-uniprot50 | AF-A0A506QRY5-F1-MODEL\_V4 | 1.0 | 0.003478 | 123 | 0.087 | 91 | 72 | 6 | 8 | 93 | 206 | 290 | BapA prefix-like domain-containing protein | BapA prefix-like domain-containing protein | | afdb-uniprot50 | AF-A0A6L6H013-F1-MODEL\_V4 | 1.0 | 0.0001065 | 123 | 0.129 | 178 | 113 | 9 | 9 | 166 | 24 | 179 | Uncharacterized protein | Uncharacterized protein | | afdb-uniprot50 | AF-A0A3R6NAE3-F1-MODEL\_V4 | 1.0 | 7.924e-05 | 123 | 0.109 | 174 | 122 | 9 | 8 | 166 | 16 | 171 | H\_lectin domain-containing protein | H\_lectin domain-containing protein | | afdb-uniprot50 | AF-A0A658JWQ7-F1-MODEL\_V4 | 1.0 | 5.24e-05 | 123 | 0.149 | 167 | 100 | 12 | 8 | 142 | 16 | 172 | Uncharacterized protein | Uncharacterized protein | | afdb-uniprot50 | AF-A0A2W4NIF2-F1-MODEL\_V4 | 1.0 | 0.00526 | 123 | 0.122 | 114 | 84 | 6 | 6 | 108 | 128 | 236 | Uncharacterized protein | Uncharacterized protein | | afdb-uniprot50 | AF-A0A0L0AZV1-F1-MODEL\_V4 | 1.0 | 0.004406 | 123 | 0.139 | 115 | 89 | 3 | 3 | 107 | 155 | 269 | Uncharacterized protein | Uncharacterized protein | | afdb-uniprot50 | AF-A0A414S2W6-F1-MODEL\_V4 | 1.0 | 0.0003683 | 123 | 0.129 | 170 | 129 | 9 | 9 | 168 | 21 | 181 | Uncharacterized protein | Uncharacterized protein | | afdb-uniprot50 | AF-A0A3S4JY58-F1-MODEL\_V4 | 1.0 | 0.0004397 | 122 | 0.127 | 110 | 77 | 8 | 8 | 107 | 33 | 133 | Adhesin for cattle intestine colonization | Adhesin for cattle intestine colonization | | afdb-uniprot50 | AF-A0A1C5W6G3-F1-MODEL\_V4 | 1.0 | 0.0002908 | 122 | 0.093 | 107 | 89 | 4 | 30 | 132 | 47 | 149 | BppU\_N domain-containing protein | BppU\_N domain-containing protein | | afdb-uniprot50 | AF-A0A8B3A5B4-F1-MODEL\_V4 | 1.0 | 6.637e-05 | 122 | 0.157 | 178 | 116 | 9 | 8 | 166 | 16 | 178 | Uncharacterized protein | Uncharacterized protein | | afdb-uniprot50 | AF-A0A7Y5ZE53-F1-MODEL\_V4 | 1.0 | 0.0003085 | 122 | 0.136 | 132 | 96 | 8 | 6 | 128 | 380 | 502 | LPXTG cell wall anchor domain-containing protein | LPXTG cell wall anchor domain-containing protein | | afdb-uniprot50 | AF-A0A7U9RBF8-F1-MODEL\_V4 | 1.0 | 0.0008423 | 122 | 0.139 | 136 | 100 | 8 | 5 | 128 | 14 | 144 | BppU\_N domain-containing protein | BppU\_N domain-containing protein | | afdb-uniprot50 | AF-C0VY35-F1-MODEL\_V4 | 1.0 | 0.0023 | 122 | 0.071 | 140 | 103 | 6 | 7 | 142 | 594 | 710 | LPXTG-motif cell wall anchor domain protein | LPXTG-motif cell wall anchor domain protein | | afdb-uniprot50 | AF-A0A4R8VD28-F1-MODEL\_V4 | 1.0 | 0.00592 | 122 | 0.152 | 105 | 77 | 9 | 7 | 107 | 557 | 653 | Big\_6 domain-containing protein | Big\_6 domain-containing protein | | afdb-uniprot50 | AF-A0A2D6WYC0-F1-MODEL\_V4 | 1.0 | 0.0002295 | 121 | 0.104 | 153 | 72 | 7 | 6 | 96 | 1 | 150 | Uncharacterized protein | Uncharacterized protein | | afdb-uniprot50 | AF-A0A1C5KJI7-F1-MODEL\_V4 | 1.0 | 0.0004949 | 121 | 0.101 | 128 | 97 | 7 | 8 | 124 | 14 | 134 | Uncharacterized protein | Uncharacterized protein | | afdb-uniprot50 | AF-A0A4Q9HRS1-F1-MODEL\_V4 | 1.0 | 0.000525 | 121 | 0.1 | 139 | 92 | 6 | 7 | 142 | 53 | 161 | Uncharacterized protein | Uncharacterized protein | | afdb-uniprot50 | AF-A0A7X2SX63-F1-MODEL\_V4 | 1.0 | 0.0001065 | 121 | 0.151 | 152 | 102 | 10 | 8 | 142 | 60 | 201 | Big\_13 domain-containing protein | Big\_13 domain-containing protein | | afdb-uniprot50 | AF-A0A1Q6MGK8-F1-MODEL\_V4 | 1.0 | 5.24e-05 | 121 | 0.138 | 180 | 120 | 6 | 8 | 166 | 36 | 201 | Uncharacterized protein | Uncharacterized protein | | afdb-uniprot50 | AF-A0A1B1LED8-F1-MODEL\_V4 | 1.0 | 0.0001271 | 121 | 0.134 | 171 | 124 | 6 | 9 | 170 | 20 | 175 | Phage protein | Phage protein | | afdb-uniprot50 | AF-A0A143ZVL5-F1-MODEL\_V4 | 1.0 | 0.0003085 | 121 | 0.132 | 173 | 119 | 8 | 6 | 166 | 18 | 171 | Uncharacterized protein | Uncharacterized protein | | afdb-uniprot50 | AF-A0A7V7HH80-F1-MODEL\_V4 | 1.0 | 0.0004665 | 121 | 0.122 | 122 | 93 | 7 | 17 | 127 | 32 | 150 | DUF2479 domain-containing protein | DUF2479 domain-containing protein | | afdb-uniprot50 | AF-A0A437UJ27-F1-MODEL\_V4 | 1.0 | 0.000665 | 121 | 0.134 | 163 | 112 | 8 | 8 | 152 | 24 | 175 | BppU\_N domain-containing protein | BppU\_N domain-containing protein | | afdb-uniprot50 | AF-F7JWE5-F1-MODEL\_V4 | 1.0 | 3.899e-05 | 120 | 0.094 | 148 | 114 | 8 | 7 | 141 | 10 | 150 | Uncharacterized protein | Uncharacterized protein | | afdb-uniprot50 | AF-A0A1V9QR56-F1-MODEL\_V4 | 1.0 | 0.0004665 | 120 | 0.054 | 148 | 120 | 8 | 8 | 142 | 9 | 149 | Uncharacterized protein | Uncharacterized protein | | afdb-uniprot50 | AF-A0A3B9GR26-F1-MODEL\_V4 | 1.0 | 0.0002908 | 120 | 0.134 | 164 | 116 | 8 | 9 | 164 | 20 | 165 | Uncharacterized protein | Uncharacterized protein | | afdb-uniprot50 | AF-A0A1C5MR39-F1-MODEL\_V4 | 1.0 | 0.004674 | 120 | 0.125 | 104 | 78 | 7 | 7 | 106 | 139 | 233 | Uncharacterized protein | Uncharacterized protein | | afdb-uniprot50 | AF-R5RJJ3-F1-MODEL\_V4 | 1.0 | 0.0001708 | 120 | 0.126 | 174 | 112 | 10 | 8 | 166 | 16 | 164 | Uncharacterized protein | Uncharacterized protein | | afdb-uniprot50 | AF-A0A350H2I6-F1-MODEL\_V4 | 1.0 | 0.0003683 | 120 | 0.121 | 156 | 108 | 9 | 9 | 141 | 20 | 169 | BppU\_N domain-containing protein | BppU\_N domain-containing protein | | afdb-uniprot50 | AF-A0A6P1Q0A7-F1-MODEL\_V4 | 1.0 | 0.00369 | 120 | 0.112 | 98 | 77 | 7 | 8 | 100 | 62 | 154 | Uncharacterized protein | Uncharacterized protein | | afdb-uniprot50 | AF-A0A0F9PSE9-F1-MODEL\_V4 | 1.0 | 0.005581 | 119 | 0.166 | 84 | 59 | 4 | 22 | 96 | 104 | 185 | Uncharacterized protein | Uncharacterized protein | | afdb-uniprot50 | AF-A0A1C6KJ53-F1-MODEL\_V4 | 1.0 | 0.0001708 | 119 | 0.12 | 224 | 133 | 14 | 9 | 179 | 3 | 215 | Collagen triple helix repeat (20 copies) | Collagen triple helix repeat (20 copies) | | afdb-uniprot50 | AF-A0A2V2FAF0-F1-MODEL\_V4 | 1.0 | 6.637e-05 | 119 | 0.165 | 163 | 109 | 7 | 15 | 166 | 12 | 158 | Uncharacterized protein | Uncharacterized protein | | afdb-uniprot50 | AF-A0A534IQ49-F1-MODEL\_V4 | 1.0 | 0.0003683 | 119 | 0.12 | 149 | 105 | 11 | 7 | 142 | 390 | 525 | Uncharacterized protein | Uncharacterized protein | | afdb-uniprot50 | AF-R5C5C3-F1-MODEL\_V4 | 1.0 | 3.078e-05 | 119 | 0.132 | 188 | 118 | 9 | 6 | 165 | 15 | 185 | BppU\_N domain-containing protein | BppU\_N domain-containing protein | | afdb-uniprot50 | AF-A0A354KQE9-F1-MODEL\_V4 | 1.0 | 2.902e-05 | 119 | 0.108 | 203 | 137 | 10 | 1 | 163 | 1 | 199 | Uncharacterized protein | Uncharacterized protein | | afdb-uniprot50 | AF-A0A810YRC3-F1-MODEL\_V4 | 1.0 | 0.00526 | 119 | 0.138 | 94 | 72 | 6 | 8 | 97 | 410 | 498 | Uncharacterized protein | Uncharacterized protein | | afdb-uniprot50 | AF-A0A2N1W3X1-F1-MODEL\_V4 | 1.0 | 0.002168 | 119 | 0.094 | 95 | 66 | 3 | 12 | 95 | 896 | 981 | Glyco\_hydro\_44 domain-containing protein | Glyco\_hydro\_44 domain-containing protein | | afdb-uniprot50 | AF-A0A7J3TMW7-F1-MODEL\_V4 | 1.0 | 0.004153 | 118 | 0.112 | 124 | 86 | 7 | 7 | 108 | 48 | 169 | Uncharacterized protein | Uncharacterized protein | | afdb-uniprot50 | AF-A0A1G4VBC8-F1-MODEL\_V4 | 1.0 | 0.0002164 | 118 | 0.139 | 172 | 123 | 11 | 3 | 164 | 14 | 170 | BppU\_N domain-containing protein | BppU\_N domain-containing protein | | afdb-uniprot50 | AF-A0A4W6F9B7-F1-MODEL\_V4 | 1.0 | 6.256e-05 | 118 | 0.077 | 206 | 140 | 9 | 9 | 177 | 24 | 216 | Ig-like domain-containing protein | Ig-like domain-containing protein | | afdb-uniprot50 | AF-A0A174CIG2-F1-MODEL\_V4 | 1.0 | 0.0008423 | 118 | 0.14 | 171 | 117 | 8 | 5 | 166 | 16 | 165 | Uncharacterized protein | Uncharacterized protein | | afdb-uniprot50 | AF-A0A8B6H4W0-F1-MODEL\_V4 | 1.0 | 0.0004665 | 118 | 0.098 | 142 | 99 | 7 | 5 | 142 | 162 | 278 | Uncharacterized protein | Uncharacterized protein | | afdb-uniprot50 | AF-A0A6N2ZND8-F1-MODEL\_V4 | 1.0 | 0.0001923 | 118 | 0.124 | 177 | 114 | 9 | 6 | 166 | 20 | 171 | BppU\_N domain-containing protein | BppU\_N domain-containing protein | | afdb-uniprot50 | AF-A0A2H3PGQ7-F1-MODEL\_V4 | 1.0 | 0.0004145 | 118 | 0.142 | 161 | 114 | 9 | 2 | 142 | 11 | 167 | BppU\_N domain-containing protein | BppU\_N domain-containing protein | | afdb-uniprot50 | AF-R0AR67-F1-MODEL\_V4 | 1.0 | 0.0001198 | 118 | 0.157 | 178 | 116 | 9 | 8 | 166 | 16 | 178 | Uncharacterized protein | Uncharacterized protein | | afdb-uniprot50 | AF-R6HGZ8-F1-MODEL\_V4 | 1.0 | 0.0001198 | 118 | 0.129 | 177 | 110 | 7 | 6 | 166 | 16 | 164 | BppU\_N domain-containing protein | BppU\_N domain-containing protein | | afdb-uniprot50 | AF-A0A1T0AWF2-F1-MODEL\_V4 | 1.0 | 0.009498 | 118 | 0.104 | 96 | 74 | 5 | 8 | 96 | 241 | 331 | Uncharacterized protein | Uncharacterized protein | | afdb-uniprot50 | AF-A0A843BS13-F1-MODEL\_V4 | 1.0 | 0.0004145 | 118 | 0.123 | 138 | 100 | 10 | 5 | 127 | 441 | 572 | Uncharacterized protein | Uncharacterized protein | | afdb-uniprot50 | AF-A0A1C6DXN6-F1-MODEL\_V4 | 1.0 | 0.001132 | 117 | 0.096 | 124 | 95 | 6 | 6 | 122 | 15 | 128 | Uncharacterized protein | Uncharacterized protein | | afdb-uniprot50 | AF-A0A1Y4G926-F1-MODEL\_V4 | 1.0 | 0.002168 | 117 | 0.145 | 131 | 94 | 8 | 9 | 125 | 21 | 147 | BppU\_N domain-containing protein | BppU\_N domain-containing protein | | afdb-uniprot50 | AF-C2VRW8-F1-MODEL\_V4 | 1.0 | 0.0002295 | 117 | 0.133 | 150 | 97 | 10 | 9 | 142 | 98 | 230 | Uncharacterized protein | Uncharacterized protein | | afdb-uniprot50 | AF-R9JK16-F1-MODEL\_V4 | 1.0 | 0.0003085 | 117 | 0.156 | 179 | 118 | 9 | 1 | 167 | 18 | 175 | Uncharacterized protein | Uncharacterized protein | | afdb-uniprot50 | AF-A0A7X8ZBB4-F1-MODEL\_V4 | 1.0 | 4.388e-05 | 117 | 0.173 | 173 | 108 | 10 | 9 | 166 | 11 | 163 | Uncharacterized protein | Uncharacterized protein | | afdb-uniprot50 | AF-A0A0H5SGG3-F1-MODEL\_V4 | 1.0 | 0.0004145 | 117 | 0.14 | 171 | 109 | 8 | 9 | 166 | 21 | 166 | Uncharacterized protein | Uncharacterized protein | | afdb-uniprot50 | AF-A0A316M9Y2-F1-MODEL\_V4 | 1.0 | 0.001816 | 117 | 0.094 | 169 | 125 | 7 | 9 | 166 | 22 | 173 | Uncharacterized protein | Uncharacterized protein | | afdb-uniprot50 | AF-A0A411WGX7-F1-MODEL\_V4 | 1.0 | 0.002168 | 116 | 0.113 | 106 | 76 | 7 | 8 | 104 | 55 | 151 | Ig-like domain-containing protein | Ig-like domain-containing protein | | afdb-uniprot50 | AF-A0A0F6W690-F1-MODEL\_V4 | 1.0 | 7.469e-05 | 116 | 0.156 | 147 | 99 | 7 | 7 | 142 | 138 | 270 | Uncharacterized protein | Uncharacterized protein | | afdb-uniprot50 | AF-A0A6J7FJT6-F1-MODEL\_V4 | 1.0 | 0.0001923 | 116 | 0.178 | 146 | 101 | 9 | 9 | 142 | 154 | 292 | Unannotated protein | Unannotated protein | | afdb-uniprot50 | AF-A0A3E2VR12-F1-MODEL\_V4 | 1.0 | 0.0004145 | 116 | 0.148 | 175 | 121 | 9 | 8 | 166 | 16 | 178 | Uncharacterized protein | Uncharacterized protein | | afdb-uniprot50 | AF-A0A0Q9JHR9-F1-MODEL\_V4 | 1.0 | 0.001926 | 116 | 0.101 | 108 | 77 | 8 | 8 | 107 | 166 | 261 | Uncharacterized protein | Uncharacterized protein | | afdb-uniprot50 | AF-A0A1C6ET81-F1-MODEL\_V4 | 1.0 | 0.001132 | 116 | 0.117 | 179 | 131 | 9 | 9 | 179 | 22 | 181 | Uncharacterized protein | Uncharacterized protein | | afdb-uniprot50 | AF-A0A7G5MXH1-F1-MODEL\_V4 | 1.0 | 0.0002741 | 116 | 0.165 | 169 | 104 | 9 | 6 | 166 | 17 | 156 | Uncharacterized protein | Uncharacterized protein | | afdb-uniprot50 | AF-A0A653VSI4-F1-MODEL\_V4 | 1.0 | 0.006281 | 116 | 0.104 | 125 | 99 | 6 | 9 | 124 | 23 | 143 | Uncharacterized protein | Uncharacterized protein | | afdb-uniprot50 | AF-A0A7S0INU3-F1-MODEL\_V4 | 1.0 | 0.001067 | 115 | 0.122 | 122 | 80 | 6 | 7 | 107 | 78 | 193 | Hypothetical protein | Hypothetical protein | | afdb-uniprot50 | AF-A0A6I2FDN3-F1-MODEL\_V4 | 1.0 | 0.0003907 | 115 | 0.143 | 132 | 92 | 9 | 7 | 131 | 38 | 155 | Uncharacterized protein | Uncharacterized protein | | afdb-uniprot50 | AF-A0A843Z2H5-F1-MODEL\_V4 | 1.0 | 0.000794 | 115 | 0.133 | 120 | 87 | 9 | 17 | 124 | 35 | 149 | DUF2479 domain-containing protein | DUF2479 domain-containing protein | | afdb-uniprot50 | AF-F8WU49-F1-MODEL\_V4 | 1.0 | 0.0008423 | 115 | 0.095 | 168 | 128 | 5 | 9 | 169 | 22 | 172 | Uncharacterized protein | Uncharacterized protein | | afdb-uniprot50 | AF-A0A7K3XZ12-F1-MODEL\_V4 | 1.0 | 0.0001198 | 115 | 0.121 | 181 | 117 | 10 | 6 | 166 | 22 | 180 | Uncharacterized protein | Uncharacterized protein | | afdb-uniprot50 | AF-A0A2V2FF44-F1-MODEL\_V4 | 1.0 | 0.0004949 | 115 | 0.109 | 146 | 106 | 10 | 9 | 138 | 27 | 164 | Uncharacterized protein | Uncharacterized protein | | afdb-uniprot50 | AF-K1GH27-F1-MODEL\_V4 | 1.0 | 0.0002908 | 115 | 0.154 | 181 | 121 | 8 | 9 | 179 | 21 | 179 | Uncharacterized protein | Uncharacterized protein | | afdb-uniprot50 | AF-A0A854ZIF3-F1-MODEL\_V4 | 1.0 | 0.0001812 | 115 | 0.139 | 179 | 112 | 11 | 8 | 162 | 17 | 177 | Uncharacterized protein | Uncharacterized protein | | afdb-uniprot50 | AF-A0A0F5HNP9-F1-MODEL\_V4 | 1.0 | 0.001521 | 115 | 0.104 | 163 | 114 | 6 | 9 | 164 | 23 | 160 | Phage pre-neck appendage-like protein | Phage pre-neck appendage-like protein | | afdb-uniprot50 | AF-N2B7B9-F1-MODEL\_V4 | 1.0 | 0.0004397 | 114 | 0.101 | 158 | 110 | 9 | 9 | 142 | 24 | 173 | Uncharacterized protein | Uncharacterized protein | | afdb-uniprot50 | AF-W0U658-F1-MODEL\_V4 | 1.0 | 0.0007484 | 114 | 0.083 | 167 | 120 | 7 | 9 | 166 | 21 | 163 | BppU\_N domain-containing protein | BppU\_N domain-containing protein | | afdb-uniprot50 | AF-A0A416RMF7-F1-MODEL\_V4 | 1.0 | 0.0001518 | 114 | 0.129 | 177 | 110 | 11 | 9 | 166 | 23 | 174 | Uncharacterized protein | Uncharacterized protein | | afdb-uniprot50 | AF-A0A4S2EF76-F1-MODEL\_V4 | 1.0 | 0.000557 | 114 | 0.105 | 151 | 106 | 9 | 8 | 142 | 9 | 146 | Collagen-like protein | Collagen-like protein | | afdb-uniprot50 | AF-A0A1B1LIW2-F1-MODEL\_V4 | 1.0 | 0.0003085 | 114 | 0.124 | 177 | 119 | 7 | 6 | 166 | 15 | 171 | Phage protein | Phage protein | | afdb-uniprot50 | AF-A0A7G9G6Y2-F1-MODEL\_V4 | 1.0 | 0.0007484 | 114 | 0.13 | 169 | 111 | 8 | 9 | 168 | 21 | 162 | BppU family phage baseplate upper protein | BppU family phage baseplate upper protein | | afdb-uniprot50 | AF-A0A3A9FK46-F1-MODEL\_V4 | 1.0 | 0.0001518 | 114 | 0.124 | 177 | 116 | 8 | 9 | 166 | 20 | 176 | Uncharacterized protein | Uncharacterized protein | | afdb-uniprot50 | AF-A0A6N7SV12-F1-MODEL\_V4 | 1.0 | 0.0004665 | 114 | 0.081 | 171 | 122 | 8 | 9 | 164 | 24 | 174 | DUF2479 domain-containing protein | DUF2479 domain-containing protein | | afdb-uniprot50 | AF-A0A658JQ79-F1-MODEL\_V4 | 1.0 | 0.001816 | 114 | 0.132 | 174 | 116 | 9 | 4 | 166 | 18 | 167 | DUF2479 domain-containing protein | DUF2479 domain-containing protein | | afdb-uniprot50 | AF-A0A1Q5PL34-F1-MODEL\_V4 | 1.0 | 0.004153 | 114 | 0.131 | 99 | 80 | 4 | 10 | 107 | 587 | 680 | Fibronectin type-III domain-containing protein | Fibronectin type-III domain-containing protein | | afdb-uniprot50 | AF-A0A177L7H1-F1-MODEL\_V4 | 1.0 | 0.001926 | 114 | 0.096 | 165 | 122 | 5 | 9 | 166 | 23 | 167 | BppU\_N domain-containing protein | BppU\_N domain-containing protein | | afdb-uniprot50 | AF-X0SIW3-F1-MODEL\_V4 | 1.0 | 0.008953 | 113 | 0.106 | 103 | 84 | 6 | 9 | 107 | 2 | 100 | Uncharacterized protein | Uncharacterized protein | | afdb-uniprot50 | AF-A0A2L2BRS6-F1-MODEL\_V4 | 1.0 | 0.001351 | 113 | 0.09 | 121 | 96 | 8 | 9 | 124 | 22 | 133 | Uncharacterized protein | Uncharacterized protein | | afdb-uniprot50 | AF-A0A0M6WYK5-F1-MODEL\_V4 | 1.0 | 0.0002741 | 113 | 0.113 | 176 | 112 | 11 | 9 | 166 | 24 | 173 | BppU\_N domain-containing protein | BppU\_N domain-containing protein | | afdb-uniprot50 | AF-A0A2H9MF23-F1-MODEL\_V4 | 1.0 | 0.002168 | 113 | 0.147 | 102 | 73 | 7 | 8 | 104 | 269 | 361 | Peptidase\_S8 domain-containing protein | Peptidase\_S8 domain-containing protein | | afdb-uniprot50 | AF-A0A6P5KX56-F1-MODEL\_V4 | 1.0 | 0.0002164 | 113 | 0.129 | 154 | 104 | 8 | 7 | 142 | 225 | 366 | intercellular adhesion molecule 3 isoform X2 | intercellular adhesion molecule 3 isoform X2 | | afdb-uniprot50 | AF-A0A1Q9J512-F1-MODEL\_V4 | 1.0 | 0.0008423 | 113 | 0.11 | 136 | 95 | 8 | 9 | 127 | 22 | 148 | BppU\_N domain-containing protein | BppU\_N domain-containing protein | | afdb-uniprot50 | AF-A0A033V779-F1-MODEL\_V4 | 1.0 | 0.0003683 | 113 | 0.122 | 155 | 104 | 8 | 20 | 166 | 48 | 178 | BppU\_N domain-containing protein | BppU\_N domain-containing protein | | afdb-uniprot50 | AF-A0A6M5FW15-F1-MODEL\_V4 | 1.0 | 0.0005909 | 113 | 0.176 | 170 | 108 | 9 | 9 | 166 | 22 | 171 | Uncharacterized protein | Uncharacterized protein | | afdb-uniprot50 | AF-A0A060YWJ5-F1-MODEL\_V4 | 1.0 | 0.007069 | 112 | 0.162 | 111 | 75 | 9 | 7 | 104 | 5 | 110 | Uncharacterized protein | Uncharacterized protein | | afdb-uniprot50 | AF-A0A1C5W5W4-F1-MODEL\_V4 | 1.0 | 0.000525 | 112 | 0.074 | 147 | 105 | 8 | 7 | 128 | 11 | 151 | Uncharacterized protein | Uncharacterized protein | | afdb-uniprot50 | AF-G8SD22-F1-MODEL\_V4 | 1.0 | 0.0006268 | 112 | 0.112 | 125 | 95 | 9 | 6 | 124 | 55 | 169 | Fibronectin type-III domain-containing protein | Fibronectin type-III domain-containing protein | | afdb-uniprot50 | AF-V5GR29-F1-MODEL\_V4 | 1.0 | 0.0001923 | 112 | 0.12 | 175 | 123 | 9 | 3 | 168 | 6 | 158 | Dolichyl-diphosphooligosaccharide--protein glycosyltransferase subunit 2 | Dolichyl-diphosphooligosaccharide--protein glycosyltransferase subunit 2 | | afdb-uniprot50 | AF-W0FKU2-F1-MODEL\_V4 | 1.0 | 0.0003683 | 112 | 0.122 | 171 | 121 | 5 | 8 | 166 | 37 | 190 | Uncharacterized protein | Uncharacterized protein | | afdb-uniprot50 | AF-A0A412T7C3-F1-MODEL\_V4 | 1.0 | 0.0003907 | 112 | 0.134 | 171 | 113 | 12 | 9 | 164 | 25 | 175 | DUF2479 domain-containing protein | DUF2479 domain-containing protein | | afdb-uniprot50 | AF-A0A133KN76-F1-MODEL\_V4 | 1.0 | 0.0003085 | 112 | 0.176 | 136 | 88 | 11 | 9 | 124 | 28 | 159 | Uncharacterized protein | Uncharacterized protein | | afdb-uniprot50 | AF-A0A316QDJ6-F1-MODEL\_V4 | 1.0 | 0.000794 | 112 | 0.108 | 166 | 121 | 7 | 8 | 165 | 18 | 164 | Uncharacterized protein | Uncharacterized protein | | afdb-uniprot50 | AF-A0A348ZCH6-F1-MODEL\_V4 | 1.0 | 0.002913 | 112 | 0.09 | 132 | 90 | 9 | 9 | 124 | 22 | 139 | Uncharacterized protein | Uncharacterized protein | | afdb-uniprot50 | AF-A0A261QKZ1-F1-MODEL\_V4 | 1.0 | 0.004153 | 112 | 0.097 | 123 | 96 | 6 | 9 | 124 | 23 | 137 | Uncharacterized protein | Uncharacterized protein | | afdb-uniprot50 | AF-A0A4U9VLC3-F1-MODEL\_V4 | 1.0 | 0.005581 | 112 | 0.105 | 95 | 75 | 6 | 8 | 97 | 10 | 99 | HYR domain | HYR domain | | afdb-uniprot50 | AF-A0A379AD61-F1-MODEL\_V4 | 1.0 | 0.004674 | 112 | 0.15 | 93 | 65 | 8 | 8 | 93 | 52 | 137 | Uncharacterized protein | Uncharacterized protein | | afdb-uniprot50 | AF-A0A3D3UB27-F1-MODEL\_V4 | 1.0 | 0.0003471 | 112 | 0.142 | 190 | 119 | 12 | 6 | 179 | 16 | 177 | BppU\_N domain-containing protein | BppU\_N domain-containing protein | | afdb-uniprot50 | AF-A0A2N2P695-F1-MODEL\_V4 | 1.0 | 0.007955 | 112 | 0.134 | 97 | 70 | 8 | 8 | 94 | 1066 | 1158 | Uncharacterized protein | Uncharacterized protein | | afdb-uniprot50 | AF-A0A350V7B0-F1-MODEL\_V4 | 1.0 | 0.0004397 | 111 | 0.094 | 170 | 125 | 9 | 1 | 156 | 12 | 166 | Uncharacterized protein | Uncharacterized protein | | afdb-uniprot50 | AF-A0A417GRQ1-F1-MODEL\_V4 | 1.0 | 4.137e-05 | 111 | 0.162 | 185 | 90 | 11 | 15 | 142 | 32 | 208 | Uncharacterized protein | Uncharacterized protein | | afdb-uniprot50 | AF-T2RFS3-F1-MODEL\_V4 | 1.0 | 0.0006268 | 111 | 0.122 | 163 | 111 | 5 | 15 | 165 | 12 | 154 | BppU\_N domain-containing protein | BppU\_N domain-containing protein | | afdb-uniprot50 | AF-A0A7C7QDX2-F1-MODEL\_V4 | 1.0 | 0.00244 | 111 | 0.174 | 132 | 77 | 9 | 7 | 124 | 267 | 380 | Uncharacterized protein | Uncharacterized protein | | afdb-uniprot50 | AF-A0A242WLZ3-F1-MODEL\_V4 | 1.0 | 0.001274 | 111 | 0.12 | 133 | 97 | 9 | 9 | 127 | 26 | 152 | BppU\_N domain-containing protein | BppU\_N domain-containing protein | | afdb-uniprot50 | AF-A0A1F4WKG2-F1-MODEL\_V4 | 1.0 | 0.004153 | 111 | 0.138 | 94 | 78 | 2 | 3 | 96 | 513 | 603 | Uncharacterized protein | Uncharacterized protein | | afdb-uniprot50 | AF-A0A8B6H4W6-F1-MODEL\_V4 | 1.0 | 0.007955 | 111 | 0.094 | 95 | 72 | 5 | 7 | 94 | 373 | 460 | Uncharacterized protein | Uncharacterized protein | | afdb-uniprot50 | AF-A0A7U9T1A0-F1-MODEL\_V4 | 1.0 | 0.001521 | 111 | 0.143 | 167 | 117 | 6 | 6 | 166 | 15 | 161 | Uncharacterized protein | Uncharacterized protein | | afdb-uniprot50 | AF-A0A143Z024-F1-MODEL\_V4 | 1.0 | 0.00244 | 111 | 0.147 | 170 | 118 | 7 | 8 | 166 | 24 | 177 | Uncharacterized protein | Uncharacterized protein | | afdb-uniprot50 | AF-A0A380DMD7-F1-MODEL\_V4 | 1.0 | 0.0007055 | 110 | 0.117 | 188 | 127 | 10 | 9 | 179 | 24 | 189 | Phage tail fiber protein | Phage tail fiber protein | | afdb-uniprot50 | AF-A0A823HEB2-F1-MODEL\_V4 | 1.0 | 0.001816 | 110 | 0.082 | 169 | 119 | 8 | 9 | 158 | 28 | 179 | DUF2479 domain-containing protein | DUF2479 domain-containing protein | | afdb-uniprot50 | AF-A0A037Z2D6-F1-MODEL\_V4 | 1.0 | 5.897e-05 | 110 | 0.175 | 171 | 103 | 11 | 9 | 166 | 11 | 156 | Uncharacterized protein | Uncharacterized protein | | afdb-uniprot50 | AF-A0A2V2G0U0-F1-MODEL\_V4 | 1.0 | 0.0004145 | 110 | 0.127 | 172 | 117 | 5 | 7 | 166 | 20 | 170 | Uncharacterized protein | Uncharacterized protein | | afdb-uniprot50 | AF-A0A3C2EKY6-F1-MODEL\_V4 | 1.0 | 0.0001518 | 110 | 0.195 | 169 | 102 | 12 | 9 | 166 | 11 | 156 | Uncharacterized protein | Uncharacterized protein | | afdb-uniprot50 | AF-A0A1N7F4Z8-F1-MODEL\_V4 | 1.0 | 0.0001431 | 110 | 0.136 | 168 | 110 | 10 | 3 | 142 | 17 | 177 | BppU\_N domain-containing protein | BppU\_N domain-containing protein | | afdb-uniprot50 | AF-A0A7M3YFZ2-F1-MODEL\_V4 | 1.0 | 0.0004397 | 110 | 0.101 | 148 | 113 | 9 | 7 | 142 | 472 | 611 | Uncharacterized protein | Uncharacterized protein | | afdb-uniprot50 | AF-A0A523W2L9-F1-MODEL\_V4 | 1.0 | 0.000113 | 110 | 0.142 | 176 | 138 | 8 | 8 | 179 | 457 | 623 | Uncharacterized protein | Uncharacterized protein | | afdb-uniprot50 | AF-A0A4Y8UNF9-F1-MODEL\_V4 | 1.0 | 0.001816 | 110 | 0.174 | 126 | 89 | 9 | 9 | 126 | 892 | 1010 | Fibronectin type III domain-containing protein | Fibronectin type III domain-containing protein | | afdb-uniprot50 | AF-A0A2E1ACQ2-F1-MODEL\_V4 | 1.0 | 0.0008936 | 110 | 0.227 | 110 | 69 | 5 | 7 | 109 | 669 | 769 | Uncharacterized protein | Uncharacterized protein | | afdb-uniprot50 | AF-A0A3B8NXB8-F1-MODEL\_V4 | 1.0 | 0.001613 | 109 | 0.135 | 118 | 95 | 4 | 14 | 124 | 32 | 149 | Uncharacterized protein | Uncharacterized protein | | afdb-uniprot50 | AF-A0A5R9C6T5-F1-MODEL\_V4 | 1.0 | 0.000665 | 109 | 0.126 | 190 | 126 | 11 | 1 | 170 | 13 | 182 | Uncharacterized protein | Uncharacterized protein | | afdb-uniprot50 | AF-A0A429ZSE8-F1-MODEL\_V4 | 1.0 | 0.001006 | 109 | 0.112 | 169 | 120 | 9 | 9 | 166 | 21 | 170 | BppU\_N domain-containing protein | BppU\_N domain-containing protein | | afdb-uniprot50 | AF-A0A173Z244-F1-MODEL\_V4 | 1.0 | 0.0003683 | 109 | 0.142 | 182 | 116 | 10 | 5 | 166 | 23 | 184 | Uncharacterized protein | Uncharacterized protein | | afdb-uniprot50 | AF-A0A6Z6TAU9-F1-MODEL\_V4 | 1.0 | 0.001434 | 109 | 0.092 | 183 | 122 | 11 | 7 | 169 | 25 | 183 | DUF2479 domain-containing protein | DUF2479 domain-containing protein | | afdb-uniprot50 | AF-A0A8B5WFU7-F1-MODEL\_V4 | 1.0 | 0.002913 | 109 | 0.137 | 109 | 82 | 5 | 6 | 107 | 630 | 733 | Uncharacterized protein | Uncharacterized protein | | afdb-uniprot50 | AF-A0A0R2D990-F1-MODEL\_V4 | 1.0 | 0.001132 | 109 | 0.181 | 138 | 84 | 6 | 9 | 142 | 340 | 452 | Uncharacterized protein | Uncharacterized protein | | afdb-uniprot50 | AF-A0A1S8S9Z7-F1-MODEL\_V4 | 1.0 | 0.000557 | 108 | 0.089 | 168 | 124 | 5 | 9 | 166 | 20 | 168 | Uncharacterized protein | Uncharacterized protein | | afdb-uniprot50 | AF-R5FZM6-F1-MODEL\_V4 | 1.0 | 0.0001923 | 108 | 0.132 | 181 | 114 | 10 | 8 | 166 | 16 | 175 | Uncharacterized protein | Uncharacterized protein | | afdb-uniprot50 | AF-A0A1C6EHP8-F1-MODEL\_V4 | 1.0 | 0.0003272 | 108 | 0.082 | 182 | 126 | 8 | 7 | 157 | 18 | 189 | Uncharacterized protein | Uncharacterized protein | | afdb-uniprot50 | AF-G9QBI2-F1-MODEL\_V4 | 1.0 | 0.0008936 | 108 | 0.137 | 153 | 99 | 9 | 9 | 142 | 235 | 373 | Uncharacterized protein | Uncharacterized protein | | afdb-uniprot50 | AF-A0A1F7QTB1-F1-MODEL\_V4 | 1.0 | 0.003091 | 108 | 0.104 | 105 | 81 | 5 | 8 | 107 | 42 | 138 | Glucanase | Glucanase | | afdb-uniprot50 | AF-R5L6I5-F1-MODEL\_V4 | 1.0 | 0.0006268 | 108 | 0.121 | 156 | 109 | 9 | 8 | 142 | 15 | 163 | Uncharacterized protein | Uncharacterized protein | | afdb-uniprot50 | AF-R7NRH2-F1-MODEL\_V4 | 1.0 | 0.00369 | 108 | 0.083 | 132 | 91 | 9 | 9 | 124 | 22 | 139 | Uncharacterized protein | Uncharacterized protein | | afdb-uniprot50 | AF-A0A1G7MZS4-F1-MODEL\_V4 | 1.0 | 0.001067 | 108 | 0.109 | 128 | 98 | 9 | 8 | 128 | 438 | 556 | LPXTG-motif cell wall anchor domain-containing protein | LPXTG-motif cell wall anchor domain-containing protein | | afdb-uniprot50 | AF-A0A2H9MLH3-F1-MODEL\_V4 | 1.0 | 0.0003085 | 108 | 0.102 | 146 | 113 | 7 | 9 | 142 | 480 | 619 | Peptidase\_S8 domain-containing protein | Peptidase\_S8 domain-containing protein | | afdb-uniprot50 | AF-A0A3A1XKV0-F1-MODEL\_V4 | 1.0 | 0.0002435 | 108 | 0.145 | 179 | 109 | 13 | 6 | 165 | 20 | 173 | Methyl-accepting transducer domain-containing protein | Methyl-accepting transducer domain-containing protein | | afdb-uniprot50 | AF-A0A374JQW7-F1-MODEL\_V4 | 1.0 | 0.0008423 | 108 | 0.105 | 171 | 110 | 10 | 9 | 164 | 25 | 167 | DUF2479 domain-containing protein | DUF2479 domain-containing protein | | afdb-uniprot50 | AF-A0A1C6DME9-F1-MODEL\_V4 | 1.0 | 0.0004397 | 107 | 0.122 | 179 | 113 | 10 | 9 | 173 | 19 | 167 | Domain of uncharacterized function (DUF2479) | Domain of uncharacterized function (DUF2479) | | afdb-uniprot50 | AF-B4DA57-F1-MODEL\_V4 | 1.0 | 0.001712 | 107 | 0.144 | 138 | 97 | 5 | 10 | 128 | 22 | 157 | Uncharacterized protein | Uncharacterized protein | | afdb-uniprot50 | AF-A0A1C5T6I9-F1-MODEL\_V4 | 1.0 | 0.0002741 | 107 | 0.082 | 182 | 125 | 7 | 5 | 166 | 11 | 170 | BppU\_N domain-containing protein | BppU\_N domain-containing protein | | afdb-uniprot50 | AF-K8DBU7-F1-MODEL\_V4 | 1.0 | 0.0001518 | 107 | 0.15 | 146 | 91 | 9 | 8 | 142 | 190 | 313 | Large repetitive protein | Large repetitive protein | | afdb-uniprot50 | AF-A0A6N7YBU7-F1-MODEL\_V4 | 1.0 | 0.0001518 | 107 | 0.149 | 181 | 115 | 8 | 8 | 166 | 37 | 200 | Uncharacterized protein | Uncharacterized protein | | afdb-uniprot50 | AF-A0A2D6NE59-F1-MODEL\_V4 | 1.0 | 0.0004665 | 107 | 0.136 | 132 | 91 | 8 | 8 | 126 | 258 | 379 | Uncharacterized protein | Uncharacterized protein | | afdb-uniprot50 | AF-A0A1Q6J949-F1-MODEL\_V4 | 1.0 | 0.0001708 | 107 | 0.142 | 183 | 126 | 7 | 7 | 166 | 12 | 186 | Uncharacterized protein | Uncharacterized protein | | afdb-uniprot50 | AF-V5MHJ2-F1-MODEL\_V4 | 1.0 | 0.0008936 | 107 | 0.118 | 144 | 99 | 10 | 3 | 127 | 16 | 150 | BppU\_N domain-containing protein | BppU\_N domain-containing protein | | afdb-uniprot50 | AF-W4VP65-F1-MODEL\_V4 | 1.0 | 0.003478 | 107 | 0.114 | 114 | 83 | 5 | 5 | 108 | 608 | 713 | Uncharacterized protein | Uncharacterized protein | | afdb-uniprot50 | AF-R8SYT5-F1-MODEL\_V4 | 1.0 | 0.001816 | 107 | 0.106 | 169 | 124 | 10 | 9 | 166 | 23 | 175 | SGNH\_hydro domain-containing protein | SGNH\_hydro domain-containing protein | | afdb-uniprot50 | AF-A0A7X2RXU3-F1-MODEL\_V4 | 1.0 | 0.001613 | 106 | 0.106 | 113 | 82 | 6 | 28 | 134 | 49 | 148 | DUF2479 domain-containing protein | DUF2479 domain-containing protein | | afdb-uniprot50 | AF-A0A1M6TYC1-F1-MODEL\_V4 | 1.0 | 0.0008423 | 106 | 0.093 | 193 | 140 | 11 | 9 | 179 | 26 | 205 | BppU\_N domain-containing protein | BppU\_N domain-containing protein | | afdb-uniprot50 | AF-R7K9N5-F1-MODEL\_V4 | 1.0 | 0.0002435 | 106 | 0.115 | 173 | 117 | 9 | 7 | 164 | 14 | 165 | Putative antireceptor | Putative antireceptor | | afdb-uniprot50 | AF-A0A396ZWI6-F1-MODEL\_V4 | 1.0 | 0.0007484 | 106 | 0.11 | 172 | 131 | 7 | 9 | 168 | 128 | 289 | Dolichyl-diphosphooligosaccharide--protein glycosyltransferase subunit 2 | Dolichyl-diphosphooligosaccharide--protein glycosyltransferase subunit 2 | | afdb-uniprot50 | AF-A0A395YDS6-F1-MODEL\_V4 | 1.0 | 0.0001708 | 106 | 0.112 | 177 | 117 | 8 | 8 | 166 | 14 | 168 | Uncharacterized protein | Uncharacterized protein | | afdb-uniprot50 | AF-A0A6L8THQ1-F1-MODEL\_V4 | 1.0 | 0.001351 | 106 | 0.128 | 171 | 111 | 9 | 7 | 163 | 18 | 164 | Uncharacterized protein | Uncharacterized protein | | afdb-uniprot50 | AF-A0A2H1FCV0-F1-MODEL\_V4 | 1.0 | 0.001201 | 105 | 0.12 | 133 | 93 | 9 | 7 | 128 | 47 | 166 | Uncharacterized protein | Uncharacterized protein | | afdb-uniprot50 | AF-A0A376LJQ6-F1-MODEL\_V4 | 1.0 | 0.00592 | 105 | 0.092 | 108 | 81 | 7 | 8 | 107 | 119 | 217 | Adhesin for cattle intestine colonization | Adhesin for cattle intestine colonization | | afdb-uniprot50 | AF-A0A6B1VLF8-F1-MODEL\_V4 | 1.0 | 0.0003272 | 105 | 0.127 | 149 | 99 | 7 | 6 | 125 | 13 | 159 | Uncharacterized protein | Uncharacterized protein | | afdb-uniprot50 | AF-A0A3E3EGS6-F1-MODEL\_V4 | 1.0 | 0.0006268 | 105 | 0.116 | 172 | 119 | 7 | 7 | 166 | 17 | 167 | Uncharacterized protein | Uncharacterized protein | | afdb-uniprot50 | AF-A0A417VHC6-F1-MODEL\_V4 | 1.0 | 0.001201 | 105 | 0.145 | 172 | 106 | 12 | 9 | 166 | 23 | 167 | DUF2479 domain-containing protein | DUF2479 domain-containing protein | | afdb-uniprot50 | AF-A0A2D3PVV1-F1-MODEL\_V4 | 1.0 | 0.0006268 | 105 | 0.121 | 157 | 111 | 6 | 17 | 164 | 31 | 169 | Uncharacterized protein | Uncharacterized protein | | afdb-uniprot50 | AF-A0A2W5XP03-F1-MODEL\_V4 | 1.0 | 0.006663 | 105 | 0.184 | 130 | 81 | 4 | 13 | 124 | 60 | 182 | Peptidase\_MA\_2 domain-containing protein | Peptidase\_MA\_2 domain-containing protein | | afdb-uniprot50 | AF-A0A6J8CMK5-F1-MODEL\_V4 | 1.0 | 0.002589 | 105 | 0.15 | 133 | 100 | 6 | 15 | 136 | 169 | 299 | Opine dehydrogenase | Opine dehydrogenase | | afdb-uniprot50 | AF-A0A1G8VNZ3-F1-MODEL\_V4 | 1.0 | 0.002168 | 105 | 0.132 | 158 | 107 | 7 | 17 | 166 | 35 | 170 | BppU\_N domain-containing protein | BppU\_N domain-containing protein | | afdb-uniprot50 | AF-A0A6H0TDU9-F1-MODEL\_V4 | 1.0 | 0.001351 | 105 | 0.152 | 171 | 115 | 10 | 9 | 166 | 22 | 175 | BppU family phage baseplate upper protein | BppU family phage baseplate upper protein | | afdb-uniprot50 | AF-D4M5A2-F1-MODEL\_V4 | 1.0 | 0.0003272 | 105 | 0.14 | 178 | 115 | 9 | 5 | 166 | 16 | 171 | Uncharacterized protein | Uncharacterized protein | | afdb-uniprot50 | AF-A0A356CXZ6-F1-MODEL\_V4 | 1.0 | 0.001613 | 104 | 0.098 | 173 | 119 | 8 | 6 | 166 | 16 | 163 | BppU\_N domain-containing protein | BppU\_N domain-containing protein | | afdb-uniprot50 | AF-A0A6P5L4Q4-F1-MODEL\_V4 | 1.0 | 0.0004949 | 104 | 0.114 | 157 | 103 | 8 | 7 | 142 | 138 | 279 | intercellular adhesion molecule 3 isoform X3 | intercellular adhesion molecule 3 isoform X3 | | afdb-uniprot50 | AF-A0A7X5CGT2-F1-MODEL\_V4 | 1.0 | 0.001434 | 104 | 0.11 | 172 | 116 | 10 | 9 | 166 | 59 | 207 | DUF2479 domain-containing protein | DUF2479 domain-containing protein | | afdb-uniprot50 | AF-A0A376VLZ8-F1-MODEL\_V4 | 1.0 | 0.001006 | 103 | 0.097 | 143 | 98 | 8 | 8 | 142 | 47 | 166 | Adhesin for cattle intestine colonization | Adhesin for cattle intestine colonization | | afdb-uniprot50 | AF-A0A4P7U9X2-F1-MODEL\_V4 | 1.0 | 0.0003907 | 103 | 0.164 | 164 | 87 | 8 | 8 | 124 | 43 | 203 | CopC domain-containing protein | CopC domain-containing protein | | afdb-uniprot50 | AF-R9MX48-F1-MODEL\_V4 | 1.0 | 0.0003471 | 103 | 0.153 | 143 | 98 | 5 | 29 | 170 | 41 | 161 | Uncharacterized protein | Uncharacterized protein | | afdb-uniprot50 | AF-A0A356J3M0-F1-MODEL\_V4 | 1.0 | 0.0004949 | 103 | 0.145 | 172 | 112 | 11 | 7 | 166 | 1 | 149 | Uncharacterized protein | Uncharacterized protein | | afdb-uniprot50 | AF-A0A844QZW9-F1-MODEL\_V4 | 1.0 | 0.001351 | 103 | 0.138 | 159 | 112 | 8 | 27 | 179 | 50 | 189 | DUF2479 domain-containing protein | DUF2479 domain-containing protein | | afdb-uniprot50 | AF-A0A347WIJ3-F1-MODEL\_V4 | 1.0 | 0.001201 | 103 | 0.112 | 187 | 107 | 9 | 6 | 168 | 21 | 172 | BppU\_N domain-containing protein | BppU\_N domain-containing protein | | afdb-uniprot50 | AF-A0A822PFD8-F1-MODEL\_V4 | 1.0 | 0.001006 | 103 | 0.121 | 165 | 109 | 7 | 15 | 165 | 35 | 177 | Uncharacterized protein | Uncharacterized protein | | afdb-uniprot50 | AF-A0A1G6RQ90-F1-MODEL\_V4 | 1.0 | 0.002746 | 103 | 0.107 | 140 | 98 | 10 | 5 | 124 | 23 | 155 | Uncharacterized protein | Uncharacterized protein | | afdb-uniprot50 | AF-A0A1Y4LS28-F1-MODEL\_V4 | 1.0 | 0.001613 | 103 | 0.146 | 177 | 118 | 7 | 5 | 167 | 12 | 169 | Uncharacterized protein | Uncharacterized protein | | afdb-uniprot50 | AF-A0A7V7SAY6-F1-MODEL\_V4 | 1.0 | 0.001521 | 103 | 0.147 | 136 | 91 | 10 | 9 | 127 | 22 | 149 | DUF2479 domain-containing protein | DUF2479 domain-containing protein | | afdb-uniprot50 | AF-W7L1A8-F1-MODEL\_V4 | 1.0 | 0.00526 | 103 | 0.093 | 171 | 127 | 8 | 9 | 166 | 23 | 178 | Phage pre-neck appendage-like protein | Phage pre-neck appendage-like protein | | afdb-uniprot50 | AF-A0A7K3W8B9-F1-MODEL\_V4 | 1.0 | 0.0004145 | 103 | 0.153 | 130 | 87 | 10 | 6 | 124 | 386 | 503 | LPXTG cell wall anchor domain-containing protein | LPXTG cell wall anchor domain-containing protein | | afdb-uniprot50 | AF-A0A1W9QEJ3-F1-MODEL\_V4 | 1.0 | 0.006663 | 103 | 0.085 | 128 | 83 | 6 | 9 | 108 | 165 | 286 | Uncharacterized protein | Uncharacterized protein | | afdb-uniprot50 | AF-A0A1C5VG89-F1-MODEL\_V4 | 1.0 | 0.007069 | 103 | 0.089 | 167 | 126 | 9 | 9 | 166 | 22 | 171 | Uncharacterized protein | Uncharacterized protein | | afdb-uniprot50 | AF-W0RS98-F1-MODEL\_V4 | 1.0 | 0.0003471 | 103 | 0.161 | 198 | 119 | 14 | 6 | 179 | 741 | 915 | CHASE2 domain protein | CHASE2 domain protein | | afdb-uniprot50 | AF-A2DAW7-F1-MODEL\_V4 | 1.0 | 0.002746 | 102 | 0.07 | 99 | 80 | 7 | 8 | 100 | 49 | 141 | Uncharacterized protein | Uncharacterized protein | | afdb-uniprot50 | AF-A2GME7-F1-MODEL\_V4 | 1.0 | 0.000557 | 102 | 0.156 | 128 | 93 | 9 | 6 | 124 | 44 | 165 | Uncharacterized protein | Uncharacterized protein | | afdb-uniprot50 | AF-A0A1T4K5N9-F1-MODEL\_V4 | 1.0 | 0.001351 | 102 | 0.118 | 185 | 124 | 11 | 1 | 166 | 15 | 179 | BppU\_N domain-containing protein | BppU\_N domain-containing protein | | afdb-uniprot50 | AF-A0A1H3BKQ8-F1-MODEL\_V4 | 1.0 | 0.000794 | 102 | 0.11 | 190 | 119 | 11 | 4 | 145 | 21 | 208 | Uncharacterized protein | Uncharacterized protein | | afdb-uniprot50 | AF-A0A662SM31-F1-MODEL\_V4 | 1.0 | 0.003478 | 102 | 0.152 | 131 | 84 | 8 | 7 | 124 | 269 | 385 | Uncharacterized protein | Uncharacterized protein | | afdb-uniprot50 | AF-A0A1Y4DR79-F1-MODEL\_V4 | 1.0 | 0.007499 | 102 | 0.13 | 138 | 98 | 8 | 4 | 125 | 55 | 186 | BppU\_N domain-containing protein | BppU\_N domain-containing protein | | afdb-uniprot50 | AF-A0A1S9UV81-F1-MODEL\_V4 | 1.0 | 0.0004949 | 102 | 0.164 | 140 | 95 | 7 | 19 | 142 | 43 | 176 | BppU\_N domain-containing protein | BppU\_N domain-containing protein | | afdb-uniprot50 | AF-A0A534I7T6-F1-MODEL\_V4 | 1.0 | 0.009498 | 102 | 0.123 | 105 | 79 | 6 | 8 | 107 | 443 | 539 | Uncharacterized protein | Uncharacterized protein | | afdb-uniprot50 | AF-A0A3E4GMK1-F1-MODEL\_V4 | 1.0 | 0.000204 | 102 | 0.106 | 178 | 122 | 8 | 7 | 166 | 11 | 169 | Uncharacterized protein | Uncharacterized protein | | afdb-uniprot50 | AF-A0A1P8J5T2-F1-MODEL\_V4 | 1.0 | 0.0006268 | 102 | 0.129 | 177 | 117 | 8 | 6 | 165 | 20 | 176 | Methyl-accepting transducer domain-containing protein | Methyl-accepting transducer domain-containing protein | | afdb-uniprot50 | AF-A0A522C888-F1-MODEL\_V4 | 1.0 | 0.001351 | 102 | 0.143 | 146 | 107 | 6 | 7 | 142 | 535 | 672 | DUF1929 domain-containing protein | DUF1929 domain-containing protein | | afdb-uniprot50 | AF-A0A101XYV2-F1-MODEL\_V4 | 1.0 | 0.000948 | 102 | 0.144 | 118 | 82 | 6 | 9 | 107 | 671 | 788 | Uncharacterized protein | Uncharacterized protein | | afdb-uniprot50 | AF-A0A2P2BRD5-F1-MODEL\_V4 | 1.0 | 0.001067 | 102 | 0.157 | 152 | 100 | 5 | 28 | 168 | 51 | 185 | BppU\_N domain-containing protein | BppU\_N domain-containing protein | | afdb-uniprot50 | AF-A0A867I3J4-F1-MODEL\_V4 | 1.0 | 0.0003471 | 102 | 0.136 | 176 | 104 | 10 | 8 | 163 | 24 | 171 | SGNH/GDSL hydrolase family protein | SGNH/GDSL hydrolase family protein | | afdb-uniprot50 | AF-A0A843J091-F1-MODEL\_V4 | 1.0 | 0.001006 | 102 | 0.097 | 134 | 98 | 6 | 31 | 143 | 51 | 182 | Uncharacterized protein | Uncharacterized protein | | afdb-uniprot50 | AF-A0A2N6SD88-F1-MODEL\_V4 | 1.0 | 0.003279 | 101 | 0.098 | 142 | 101 | 6 | 9 | 128 | 24 | 160 | Uncharacterized protein | Uncharacterized protein | | afdb-uniprot50 | AF-A0A3C0CBN8-F1-MODEL\_V4 | 1.0 | 0.00526 | 101 | 0.092 | 130 | 99 | 7 | 14 | 135 | 13 | 131 | Uncharacterized protein | Uncharacterized protein | | afdb-uniprot50 | AF-A0A3L6US60-F1-MODEL\_V4 | 1.0 | 0.001274 | 101 | 0.133 | 172 | 121 | 11 | 9 | 164 | 2 | 161 | Dolichyl-diphosphooligosaccharide--protein glycosyltransferase subunit 2 | Dolichyl-diphosphooligosaccharide--protein glycosyltransferase subunit 2 | | afdb-uniprot50 | AF-A0A349YTI5-F1-MODEL\_V4 | 1.0 | 0.001067 | 101 | 0.116 | 171 | 115 | 10 | 9 | 166 | 19 | 166 | Uncharacterized protein | Uncharacterized protein | | afdb-uniprot50 | AF-R6QMQ6-F1-MODEL\_V4 | 1.0 | 0.00244 | 101 | 0.108 | 157 | 104 | 6 | 29 | 179 | 49 | 175 | Hep/Hag repeat protein | Hep/Hag repeat protein | | afdb-uniprot50 | AF-A0A3D2E0M6-F1-MODEL\_V4 | 1.0 | 0.0003683 | 101 | 0.12 | 158 | 110 | 10 | 9 | 142 | 18 | 170 | Uncharacterized protein | Uncharacterized protein | | afdb-uniprot50 | AF-A0A822PCT9-F1-MODEL\_V4 | 1.0 | 0.002913 | 101 | 0.075 | 173 | 120 | 7 | 9 | 166 | 20 | 167 | Choline binding protein | Choline binding protein | | afdb-uniprot50 | AF-A0A0H2PBG6-F1-MODEL\_V4 | 1.0 | 0.001351 | 101 | 0.173 | 138 | 88 | 11 | 9 | 124 | 26 | 159 | Tail fiber protein | Tail fiber protein | | afdb-uniprot50 | AF-A0A1I0BNH9-F1-MODEL\_V4 | 1.0 | 0.0004949 | 101 | 0.095 | 146 | 108 | 6 | 22 | 164 | 32 | 156 | Uncharacterized protein | Uncharacterized protein | | afdb-uniprot50 | AF-A0A385NWF3-F1-MODEL\_V4 | 1.0 | 0.006663 | 101 | 0.131 | 144 | 104 | 7 | 9 | 138 | 23 | 159 | Uncharacterized protein | Uncharacterized protein | | afdb-uniprot50 | AF-A0A4Z2IX58-F1-MODEL\_V4 | 1.0 | 0.004153 | 101 | 0.111 | 144 | 113 | 7 | 7 | 142 | 497 | 633 | Deleted in malignant brain tumors 1 protein | Deleted in malignant brain tumors 1 protein | | afdb-uniprot50 | AF-A0A0F9I0H5-F1-MODEL\_V4 | 1.0 | 0.008953 | 101 | 0.165 | 115 | 61 | 8 | 9 | 105 | 643 | 740 | Uncharacterized protein | Uncharacterized protein | | afdb-uniprot50 | AF-A0A1I1EW75-F1-MODEL\_V4 | 1.0 | 0.003091 | 101 | 0.129 | 170 | 111 | 9 | 11 | 166 | 24 | 170 | Uncharacterized protein | Uncharacterized protein | | afdb-uniprot50 | AF-A0A7U9X8V9-F1-MODEL\_V4 | 1.0 | 0.0005909 | 101 | 0.098 | 193 | 128 | 7 | 9 | 179 | 21 | 189 | Autotransporter adhesin EhaG | Autotransporter adhesin EhaG | | afdb-uniprot50 | AF-A0A3M2LFC0-F1-MODEL\_V4 | 1.0 | 0.00369 | 100 | 0.136 | 139 | 92 | 10 | 9 | 128 | 43 | 172 | Uncharacterized protein | Uncharacterized protein | | afdb-uniprot50 | AF-A0A3S2MRH1-F1-MODEL\_V4 | 1.0 | 0.0008936 | 100 | 0.106 | 141 | 103 | 6 | 9 | 135 | 31 | 162 | ig domain-containing protein | ig domain-containing protein | | afdb-uniprot50 | AF-A0A412EM07-F1-MODEL\_V4 | 1.0 | 0.0007484 | 100 | 0.08 | 174 | 129 | 6 | 8 | 166 | 38 | 195 | Uncharacterized protein | Uncharacterized protein | | afdb-uniprot50 | AF-A0A086ZDU3-F1-MODEL\_V4 | 1.0 | 0.001926 | 100 | 0.166 | 150 | 89 | 9 | 8 | 127 | 8 | 151 | Tail fiber protein | Tail fiber protein | | afdb-uniprot50 | AF-A0A3B3D8Y5-F1-MODEL\_V4 | 1.0 | 0.002044 | 100 | 0.107 | 121 | 90 | 6 | 9 | 125 | 407 | 513 | Uncharacterized protein | Uncharacterized protein | | afdb-uniprot50 | AF-A0A523YCW7-F1-MODEL\_V4 | 1.0 | 0.0008936 | 100 | 0.118 | 144 | 103 | 7 | 6 | 142 | 545 | 671 | Fibronectin type-III domain-containing protein | Fibronectin type-III domain-containing protein | | afdb-uniprot50 | AF-A0A6B1ZRS2-F1-MODEL\_V4 | 1.0 | 0.001434 | 100 | 0.142 | 175 | 112 | 9 | 6 | 165 | 22 | 173 | Uncharacterized protein | Uncharacterized protein | | afdb-uniprot50 | AF-A0A0N0C451-F1-MODEL\_V4 | 1.0 | 0.001351 | 100 | 0.119 | 117 | 85 | 6 | 9 | 107 | 854 | 970 | Uncharacterized protein | Uncharacterized protein | | afdb-uniprot50 | AF-A0A1X2IR94-F1-MODEL\_V4 | 1.0 | 0.000948 | 100 | 0.168 | 166 | 108 | 10 | 9 | 165 | 63 | 207 | Uncharacterized protein | Uncharacterized protein | | afdb-uniprot50 | AF-A0A3C0II44-F1-MODEL\_V4 | 0.999 | 0.004153 | 99 | 0.102 | 146 | 111 | 9 | 6 | 142 | 14 | 148 | Uncharacterized protein | Uncharacterized protein | | afdb-uniprot50 | AF-A0A7V9P6P7-F1-MODEL\_V4 | 0.999 | 0.0007484 | 99 | 0.165 | 157 | 96 | 12 | 6 | 142 | 47 | 188 | Uncharacterized protein | Uncharacterized protein | | afdb-uniprot50 | AF-A0A1D7ULP3-F1-MODEL\_V4 | 0.999 | 0.0008423 | 99 | 0.148 | 175 | 111 | 10 | 9 | 166 | 26 | 179 | Uncharacterized protein | Uncharacterized protein | | afdb-uniprot50 | AF-A0A843IDY4-F1-MODEL\_V4 | 0.999 | 0.001926 | 99 | 0.118 | 144 | 97 | 9 | 7 | 143 | 106 | 226 | Uncharacterized protein | Uncharacterized protein | | afdb-uniprot50 | AF-A0A6S5JXD1-F1-MODEL\_V4 | 0.999 | 0.00526 | 99 | 0.172 | 110 | 71 | 8 | 8 | 107 | 251 | 350 | Uncharacterized protein | Uncharacterized protein | | afdb-uniprot50 | AF-A0A3R6NWS5-F1-MODEL\_V4 | 0.999 | 0.001712 | 99 | 0.135 | 184 | 113 | 12 | 8 | 166 | 21 | 183 | Uncharacterized protein | Uncharacterized protein | | afdb-uniprot50 | AF-A0A415D505-F1-MODEL\_V4 | 0.999 | 0.001274 | 99 | 0.155 | 116 | 87 | 3 | 30 | 138 | 38 | 149 | Uncharacterized protein | Uncharacterized protein | | afdb-uniprot50 | AF-A0A348Z913-F1-MODEL\_V4 | 0.999 | 0.0008936 | 99 | 0.114 | 174 | 107 | 12 | 9 | 166 | 22 | 164 | BppU\_N domain-containing protein | BppU\_N domain-containing protein | | afdb-uniprot50 | AF-X1G6M4-F1-MODEL\_V4 | 0.999 | 0.0023 | 99 | 0.113 | 141 | 114 | 7 | 8 | 145 | 130 | 262 | NosD domain-containing protein | NosD domain-containing protein | | afdb-uniprot50 | AF-C4IGZ0-F1-MODEL\_V4 | 0.999 | 0.001006 | 99 | 0.137 | 175 | 120 | 8 | 6 | 166 | 14 | 171 | Uncharacterized protein | Uncharacterized protein | | afdb-uniprot50 | AF-A0A7D6VTN3-F1-MODEL\_V4 | 0.999 | 0.00526 | 99 | 0.109 | 146 | 100 | 8 | 8 | 128 | 21 | 161 | Uncharacterized protein | Uncharacterized protein | | afdb-uniprot50 | AF-A0A429ZSL4-F1-MODEL\_V4 | 0.999 | 0.001613 | 99 | 0.147 | 217 | 119 | 13 | 1 | 178 | 1 | 190 | GP-PDE domain-containing protein | GP-PDE domain-containing protein | | afdb-uniprot50 | AF-A0A6I4NWJ7-F1-MODEL\_V4 | 0.999 | 0.004406 | 99 | 0.137 | 145 | 90 | 11 | 9 | 142 | 606 | 726 | Uncharacterized protein | Uncharacterized protein | | afdb-uniprot50 | AF-A0A4V6APB1-F1-MODEL\_V4 | 0.999 | 0.002589 | 98 | 0.138 | 152 | 103 | 9 | 7 | 142 | 279 | 418 | Deleted in malignant brain tumors 1 protein Hensin | Deleted in malignant brain tumors 1 protein Hensin | | afdb-uniprot50 | AF-A0A3D2W3M9-F1-MODEL\_V4 | 0.999 | 0.001067 | 98 | 0.141 | 156 | 100 | 9 | 6 | 142 | 21 | 161 | BppU\_N domain-containing protein | BppU\_N domain-containing protein | | afdb-uniprot50 | AF-A0A1D2KT36-F1-MODEL\_V4 | 0.999 | 0.002589 | 98 | 0.105 | 142 | 107 | 5 | 27 | 163 | 52 | 178 | DUF2479 domain-containing protein | DUF2479 domain-containing protein | | afdb-uniprot50 | AF-T3DU14-F1-MODEL\_V4 | 0.999 | 0.001434 | 98 | 0.098 | 152 | 115 | 9 | 9 | 142 | 19 | 166 | PQQ-like domain protein | PQQ-like domain protein | | afdb-uniprot50 | AF-A0A396L304-F1-MODEL\_V4 | 0.999 | 0.00244 | 98 | 0.08 | 175 | 127 | 7 | 7 | 166 | 11 | 166 | Uncharacterized protein | Uncharacterized protein | | afdb-uniprot50 | AF-A0A162U122-F1-MODEL\_V4 | 0.999 | 0.001434 | 98 | 0.163 | 153 | 102 | 7 | 6 | 142 | 54 | 196 | Secreted sialophospho protein | Secreted sialophospho protein | | afdb-uniprot50 | AF-A0A3Q9S137-F1-MODEL\_V4 | 0.999 | 0.005581 | 98 | 0.161 | 105 | 64 | 9 | 9 | 98 | 785 | 880 | Uncharacterized protein | Uncharacterized protein | | afdb-uniprot50 | AF-A0A6J8ASX6-F1-MODEL\_V4 | 0.999 | 0.001351 | 97 | 0.12 | 150 | 110 | 7 | 10 | 142 | 9 | 153 | Fibronectin type-III domain-containing protein | Fibronectin type-III domain-containing protein | | afdb-uniprot50 | AF-A0A846PSR2-F1-MODEL\_V4 | 0.999 | 0.003478 | 97 | 0.149 | 114 | 81 | 7 | 7 | 107 | 141 | 251 | Uncharacterized protein | Uncharacterized protein | | afdb-uniprot50 | AF-A2HSA7-F1-MODEL\_V4 | 0.999 | 0.001067 | 97 | 0.179 | 128 | 90 | 9 | 6 | 124 | 141 | 262 | Uncharacterized protein | Uncharacterized protein | | afdb-uniprot50 | AF-A0A3M6TAQ9-F1-MODEL\_V4 | 0.999 | 0.001006 | 97 | 0.083 | 155 | 107 | 11 | 4 | 142 | 28 | 163 | Uncharacterized protein | Uncharacterized protein | | afdb-uniprot50 | AF-R0AP54-F1-MODEL\_V4 | 0.999 | 0.00526 | 97 | 0.135 | 170 | 112 | 10 | 9 | 166 | 23 | 169 | BppU\_N domain-containing protein | BppU\_N domain-containing protein | | afdb-uniprot50 | AF-A0A397BUT5-F1-MODEL\_V4 | 0.999 | 0.001926 | 97 | 0.114 | 184 | 136 | 10 | 9 | 178 | 128 | 298 | Dolichyl-diphosphooligosaccharide--protein glycosyltransferase subunit 2 | Dolichyl-diphosphooligosaccharide--protein glycosyltransferase subunit 2 | | afdb-uniprot50 | AF-R5KP20-F1-MODEL\_V4 | 0.999 | 0.001434 | 97 | 0.119 | 159 | 106 | 8 | 22 | 179 | 36 | 161 | Uncharacterized protein | Uncharacterized protein | | afdb-uniprot50 | AF-A0A1F1KZP2-F1-MODEL\_V4 | 0.999 | 0.001926 | 97 | 0.137 | 174 | 121 | 8 | 5 | 166 | 15 | 171 | Uncharacterized protein | Uncharacterized protein | | afdb-uniprot50 | AF-A0A669B4J7-F1-MODEL\_V4 | 0.999 | 0.001521 | 97 | 0.188 | 143 | 99 | 9 | 9 | 142 | 433 | 567 | Uncharacterized protein | Uncharacterized protein | | afdb-uniprot50 | AF-A0A535MAP0-F1-MODEL\_V4 | 0.999 | 0.0005909 | 97 | 0.175 | 177 | 109 | 13 | 7 | 166 | 34 | 190 | Copper resistance protein CopC | Copper resistance protein CopC | | afdb-uniprot50 | AF-M1LNW5-F1-MODEL\_V4 | 0.999 | 0.00526 | 97 | 0.13 | 168 | 116 | 8 | 9 | 166 | 22 | 169 | Uncharacterized protein | Uncharacterized protein | | afdb-uniprot50 | AF-A0A8B1YVX0-F1-MODEL\_V4 | 0.999 | 0.0004397 | 97 | 0.135 | 206 | 136 | 11 | 3 | 179 | 8 | 200 | Uncharacterized protein | Uncharacterized protein | | afdb-uniprot50 | AF-A0A848B9H4-F1-MODEL\_V4 | 0.999 | 0.00526 | 97 | 0.137 | 175 | 117 | 10 | 9 | 166 | 22 | 179 | Uncharacterized protein | Uncharacterized protein | | afdb-uniprot50 | AF-A0A6N3A7F2-F1-MODEL\_V4 | 0.999 | 0.0008936 | 97 | 0.116 | 171 | 119 | 7 | 7 | 164 | 34 | 185 | Uncharacterized protein | Uncharacterized protein | | afdb-uniprot50 | AF-A0A7S8E8U1-F1-MODEL\_V4 | 0.999 | 0.001521 | 97 | 0.16 | 143 | 84 | 7 | 7 | 142 | 594 | 707 | PPC domain-containing protein | PPC domain-containing protein | | afdb-uniprot50 | AF-A0A523K1L5-F1-MODEL\_V4 | 0.999 | 0.00526 | 96 | 0.107 | 102 | 70 | 10 | 8 | 100 | 73 | 162 | Uncharacterized protein | Uncharacterized protein | | afdb-uniprot50 | AF-A0A256BLC4-F1-MODEL\_V4 | 0.999 | 0.002589 | 96 | 0.104 | 181 | 119 | 10 | 9 | 165 | 30 | 191 | BppU\_N domain-containing protein | BppU\_N domain-containing protein | | afdb-uniprot50 | AF-A0A7G5NVU1-F1-MODEL\_V4 | 0.999 | 0.004959 | 96 | 0.114 | 175 | 125 | 8 | 5 | 166 | 15 | 172 | Uncharacterized protein | Uncharacterized protein | | afdb-uniprot50 | AF-C0EIT1-F1-MODEL\_V4 | 0.999 | 0.002044 | 96 | 0.172 | 145 | 91 | 7 | 30 | 166 | 56 | 179 | BppU\_N domain-containing protein | BppU\_N domain-containing protein | | afdb-uniprot50 | AF-A0A7J2R513-F1-MODEL\_V4 | 0.999 | 0.001201 | 96 | 0.126 | 166 | 113 | 8 | 15 | 160 | 398 | 551 | VWA domain-containing protein | VWA domain-containing protein | | afdb-uniprot50 | AF-A0A2V8GIF6-F1-MODEL\_V4 | 0.999 | 0.001132 | 96 | 0.138 | 252 | 123 | 14 | 6 | 179 | 606 | 841 | Uncharacterized protein | Uncharacterized protein | | afdb-uniprot50 | AF-A0A2G6E620-F1-MODEL\_V4 | 0.998 | 0.002913 | 95 | 0.119 | 109 | 80 | 6 | 7 | 108 | 55 | 154 | Uncharacterized protein | Uncharacterized protein | | afdb-uniprot50 | AF-A0A543HW98-F1-MODEL\_V4 | 0.998 | 0.0002295 | 95 | 0.151 | 172 | 117 | 9 | 7 | 166 | 38 | 192 | Uncharacterized protein | Uncharacterized protein | | afdb-uniprot50 | AF-A0A4R8ZQF4-F1-MODEL\_V4 | 0.998 | 0.0001431 | 95 | 0.165 | 187 | 124 | 11 | 9 | 179 | 47 | 217 | Copper resistance protein CopC | Copper resistance protein CopC | | afdb-uniprot50 | AF-A0A7U9XHU5-F1-MODEL\_V4 | 0.998 | 0.001201 | 95 | 0.137 | 138 | 94 | 5 | 29 | 166 | 41 | 153 | Uncharacterized protein | Uncharacterized protein | | afdb-uniprot50 | AF-A0A6L5YI01-F1-MODEL\_V4 | 0.998 | 0.001613 | 95 | 0.085 | 199 | 124 | 13 | 8 | 168 | 19 | 197 | Uncharacterized protein | Uncharacterized protein | | afdb-uniprot50 | AF-A0A7V9I5K7-F1-MODEL\_V4 | 0.998 | 0.0007484 | 95 | 0.138 | 195 | 126 | 12 | 7 | 179 | 34 | 208 | Uncharacterized protein | Uncharacterized protein | | afdb-uniprot50 | AF-A0A174XQW7-F1-MODEL\_V4 | 0.998 | 0.00592 | 95 | 0.154 | 194 | 133 | 13 | 9 | 179 | 21 | 206 | Collagen triple helix repeat-containing protein | Collagen triple helix repeat-containing protein | | afdb-uniprot50 | AF-A0A2M7DL93-F1-MODEL\_V4 | 0.998 | 0.0002908 | 95 | 0.154 | 149 | 101 | 9 | 7 | 136 | 179 | 321 | DUF11 domain-containing protein | DUF11 domain-containing protein | | afdb-uniprot50 | AF-W7C4F2-F1-MODEL\_V4 | 0.998 | 0.001132 | 95 | 0.147 | 149 | 93 | 10 | 28 | 165 | 54 | 179 | BppU\_N domain-containing protein | BppU\_N domain-containing protein | | afdb-uniprot50 | AF-A0A7Y1YUI9-F1-MODEL\_V4 | 0.998 | 0.008953 | 95 | 0.163 | 110 | 73 | 9 | 8 | 107 | 32 | 132 | Uncharacterized protein | Uncharacterized protein | | afdb-uniprot50 | AF-A0A0Q5WDI7-F1-MODEL\_V4 | 0.998 | 0.001926 | 95 | 0.157 | 127 | 90 | 10 | 8 | 126 | 401 | 518 | Uncharacterized protein | Uncharacterized protein | | afdb-uniprot50 | AF-A0A523YH80-F1-MODEL\_V4 | 0.998 | 0.0008936 | 95 | 0.121 | 140 | 97 | 6 | 8 | 136 | 550 | 674 | Fibronectin type-III domain-containing protein | Fibronectin type-III domain-containing protein | | afdb-uniprot50 | AF-A0A2K4ZNI6-F1-MODEL\_V4 | 0.998 | 0.001613 | 95 | 0.128 | 140 | 99 | 6 | 28 | 164 | 50 | 169 | Uncharacterized protein | Uncharacterized protein | | afdb-uniprot50 | AF-A0A845QX82-F1-MODEL\_V4 | 0.998 | 0.007069 | 95 | 0.076 | 171 | 123 | 11 | 9 | 166 | 22 | 170 | DUF2479 domain-containing protein | DUF2479 domain-containing protein | | afdb-uniprot50 | AF-S4B432-F1-MODEL\_V4 | 0.998 | 0.0023 | 95 | 0.125 | 199 | 125 | 11 | 6 | 178 | 22 | 197 | Peptidase\_M14 domain-containing protein | Peptidase\_M14 domain-containing protein | | afdb-uniprot50 | AF-A0A1Z5IVA8-F1-MODEL\_V4 | 0.998 | 0.004153 | 95 | 0.089 | 189 | 124 | 11 | 6 | 166 | 25 | 193 | GDSL-like lipase/acylhydrolase | GDSL-like lipase/acylhydrolase | | afdb-uniprot50 | AF-A0A0Q8PIW8-F1-MODEL\_V4 | 0.998 | 0.0001923 | 94 | 0.131 | 183 | 111 | 10 | 4 | 164 | 35 | 191 | CopC domain-containing protein | CopC domain-containing protein | | afdb-uniprot50 | AF-A0A5F0FGF2-F1-MODEL\_V4 | 0.998 | 0.0001349 | 94 | 0.12 | 174 | 133 | 7 | 15 | 179 | 45 | 207 | Copper resistance protein CopC | Copper resistance protein CopC | | afdb-uniprot50 | AF-A0A813XUR2-F1-MODEL\_V4 | 0.998 | 0.000665 | 94 | 0.097 | 205 | 151 | 12 | 3 | 179 | 38 | 236 | Hypothetical protein | Hypothetical protein | | afdb-uniprot50 | AF-A0A3M1FDJ3-F1-MODEL\_V4 | 0.998 | 0.001816 | 94 | 0.097 | 134 | 93 | 7 | 9 | 124 | 245 | 368 | Uncharacterized protein | Uncharacterized protein | | afdb-uniprot50 | AF-A0A2X0QIE9-F1-MODEL\_V4 | 0.998 | 0.002168 | 94 | 0.097 | 154 | 108 | 9 | 20 | 166 | 48 | 177 | BppU\_N domain-containing protein | BppU\_N domain-containing protein | | afdb-uniprot50 | AF-A0A4Q7PK69-F1-MODEL\_V4 | 0.998 | 0.0023 | 94 | 0.154 | 175 | 111 | 12 | 9 | 166 | 26 | 180 | Uncharacterized protein | Uncharacterized protein | | afdb-uniprot50 | AF-A0A847WF40-F1-MODEL\_V4 | 0.998 | 0.002746 | 94 | 0.129 | 186 | 127 | 9 | 9 | 178 | 20 | 186 | Uncharacterized protein | Uncharacterized protein | | afdb-uniprot50 | AF-A0A6J8BSE7-F1-MODEL\_V4 | 0.998 | 0.009498 | 94 | 0.104 | 143 | 112 | 6 | 11 | 142 | 669 | 806 | Uncharacterized protein | Uncharacterized protein | | afdb-uniprot50 | AF-A0A6P7P392-F1-MODEL\_V4 | 0.998 | 0.001274 | 94 | 0.127 | 141 | 107 | 7 | 9 | 142 | 940 | 1071 | scavenger receptor cysteine-rich type 1 protein M160-like isoform X2 | scavenger receptor cysteine-rich type 1 protein M160-like isoform X2 | | afdb-uniprot50 | AF-A0A328L2A5-F1-MODEL\_V4 | 0.998 | 0.001434 | 93 | 0.102 | 176 | 122 | 12 | 6 | 166 | 19 | 173 | BppU\_N domain-containing protein | BppU\_N domain-containing protein | | afdb-uniprot50 | AF-A0A3R7YHD4-F1-MODEL\_V4 | 0.998 | 0.001006 | 93 | 0.146 | 157 | 87 | 9 | 9 | 142 | 185 | 317 | Uncharacterized protein | Uncharacterized protein | | afdb-uniprot50 | AF-A0A1J1HAE1-F1-MODEL\_V4 | 0.998 | 0.002746 | 93 | 0.092 | 151 | 112 | 6 | 9 | 135 | 74 | 223 | Uncharacterized protein | Uncharacterized protein | | afdb-uniprot50 | AF-A2H226-F1-MODEL\_V4 | 0.998 | 0.003478 | 93 | 0.172 | 122 | 92 | 7 | 9 | 124 | 230 | 348 | Uncharacterized protein | Uncharacterized protein | | afdb-uniprot50 | AF-A0A4Q9XZW9-F1-MODEL\_V4 | 0.998 | 0.002168 | 93 | 0.127 | 180 | 114 | 14 | 5 | 164 | 16 | 172 | DUF2479 domain-containing protein | DUF2479 domain-containing protein | | afdb-uniprot50 | AF-A0A420YQM6-F1-MODEL\_V4 | 0.998 | 0.001274 | 93 | 0.146 | 218 | 115 | 13 | 6 | 162 | 22 | 229 | Uncharacterized protein | Uncharacterized protein | | afdb-uniprot50 | AF-A0A7J2ISC6-F1-MODEL\_V4 | 0.998 | 0.004406 | 93 | 0.089 | 146 | 117 | 5 | 7 | 142 | 306 | 445 | DUF1565 domain-containing protein | DUF1565 domain-containing protein | | afdb-uniprot50 | AF-A0A1V5IGQ3-F1-MODEL\_V4 | 0.998 | 0.00592 | 93 | 0.161 | 124 | 77 | 10 | 6 | 108 | 522 | 639 | DUF3821 domain-containing protein | DUF3821 domain-containing protein | | afdb-uniprot50 | AF-A0A0C9MY19-F1-MODEL\_V4 | 0.998 | 0.003091 | 93 | 0.127 | 141 | 103 | 6 | 9 | 142 | 62 | 189 | Uncharacterized protein | Uncharacterized protein | | afdb-uniprot50 | AF-A0A7U9X263-F1-MODEL\_V4 | 0.998 | 0.007069 | 93 | 0.109 | 128 | 91 | 6 | 9 | 124 | 20 | 136 | Uncharacterized protein | Uncharacterized protein | | afdb-uniprot50 | AF-A0A7T4G8C7-F1-MODEL\_V4 | 0.998 | 0.002746 | 93 | 0.109 | 174 | 115 | 9 | 6 | 158 | 22 | 176 | SGNH/GDSL hydrolase family protein | SGNH/GDSL hydrolase family protein | | afdb-uniprot50 | AF-A0A6I7EES4-F1-MODEL\_V4 | 0.998 | 0.00369 | 93 | 0.123 | 138 | 97 | 11 | 9 | 127 | 21 | 153 | BppU family phage baseplate upper protein | BppU family phage baseplate upper protein | | afdb-uniprot50 | AF-A0A532TIZ1-F1-MODEL\_V4 | 0.997 | 0.004406 | 92 | 0.093 | 128 | 98 | 4 | 8 | 128 | 47 | 163 | Uncharacterized protein | Uncharacterized protein | | afdb-uniprot50 | AF-A0A495WU82-F1-MODEL\_V4 | 0.997 | 0.001067 | 92 | 0.142 | 190 | 123 | 11 | 9 | 179 | 55 | 223 | CopC domain-containing protein | CopC domain-containing protein | | afdb-uniprot50 | AF-A0A846KDD2-F1-MODEL\_V4 | 0.997 | 0.000525 | 92 | 0.115 | 165 | 109 | 12 | 20 | 164 | 52 | 199 | BppU family phage baseplate upper protein | BppU family phage baseplate upper protein | | afdb-uniprot50 | AF-D4FN16-F1-MODEL\_V4 | 0.997 | 0.001132 | 92 | 0.172 | 168 | 107 | 7 | 20 | 179 | 47 | 190 | BppU\_N domain-containing protein | BppU\_N domain-containing protein | | afdb-uniprot50 | AF-A0A1V5ZC38-F1-MODEL\_V4 | 0.997 | 0.001006 | 92 | 0.118 | 160 | 91 | 11 | 6 | 142 | 227 | 359 | Uncharacterized protein | Uncharacterized protein | | afdb-uniprot50 | AF-A0A242WL41-F1-MODEL\_V4 | 0.997 | 0.00526 | 92 | 0.139 | 165 | 110 | 12 | 8 | 142 | 20 | 182 | BppU\_N domain-containing protein | BppU\_N domain-containing protein | | afdb-uniprot50 | AF-A0A523YCV8-F1-MODEL\_V4 | 0.997 | 0.000794 | 92 | 0.12 | 149 | 99 | 7 | 9 | 142 | 835 | 966 | Fibronectin type-III domain-containing protein | Fibronectin type-III domain-containing protein | | afdb-uniprot50 | AF-D6SR61-F1-MODEL\_V4 | 0.997 | 0.002746 | 92 | 0.179 | 145 | 97 | 9 | 7 | 142 | 230 | 361 | Lipoprotein | Lipoprotein | | afdb-uniprot50 | AF-A0A6I4P3U8-F1-MODEL\_V4 | 0.997 | 0.000557 | 91 | 0.147 | 190 | 121 | 10 | 9 | 178 | 44 | 212 | CopC domain-containing protein | CopC domain-containing protein | | afdb-uniprot50 | AF-A0A6J7GNW7-F1-MODEL\_V4 | 0.997 | 0.004674 | 91 | 0.093 | 129 | 99 | 10 | 9 | 128 | 291 | 410 | Unannotated protein | Unannotated protein | | afdb-uniprot50 | AF-A0A1C5M3U0-F1-MODEL\_V4 | 0.997 | 0.003915 | 91 | 0.155 | 148 | 93 | 8 | 28 | 166 | 56 | 180 | Uncharacterized protein | Uncharacterized protein | | afdb-uniprot50 | AF-A0A7C4V780-F1-MODEL\_V4 | 0.997 | 0.003915 | 91 | 0.149 | 167 | 111 | 14 | 9 | 158 | 549 | 701 | Peptidase\_S8 domain-containing protein | Peptidase\_S8 domain-containing protein | | afdb-uniprot50 | AF-A0A101XV12-F1-MODEL\_V4 | 0.997 | 0.001926 | 91 | 0.152 | 118 | 81 | 7 | 9 | 107 | 730 | 847 | Uncharacterized protein | Uncharacterized protein | | afdb-uniprot50 | AF-N6VQ37-F1-MODEL\_V4 | 0.997 | 0.001351 | 91 | 0.099 | 131 | 97 | 10 | 5 | 128 | 845 | 961 | Uncharacterized protein | Uncharacterized protein | | afdb-uniprot50 | AF-A0A521H572-F1-MODEL\_V4 | 0.997 | 0.00592 | 91 | 0.162 | 135 | 96 | 9 | 11 | 136 | 878 | 1004 | VWA domain-containing protein | VWA domain-containing protein | | afdb-uniprot50 | AF-A0A3E2BTS2-F1-MODEL\_V4 | 0.996 | 0.003091 | 90 | 0.117 | 178 | 115 | 9 | 6 | 166 | 20 | 172 | BppU\_N domain-containing protein | BppU\_N domain-containing protein | | afdb-uniprot50 | AF-A0A6A4RWM8-F1-MODEL\_V4 | 0.996 | 0.002589 | 90 | 0.113 | 159 | 104 | 7 | 6 | 142 | 71 | 214 | 40S ribosomal protein S12 | 40S ribosomal protein S12 | | afdb-uniprot50 | AF-A0A7V3ZT25-F1-MODEL\_V4 | 0.996 | 0.002913 | 90 | 0.139 | 151 | 99 | 10 | 6 | 142 | 334 | 467 | Uncharacterized protein | Uncharacterized protein | | afdb-uniprot50 | AF-A0A844FIK1-F1-MODEL\_V4 | 0.996 | 0.00592 | 90 | 0.139 | 186 | 109 | 10 | 6 | 166 | 22 | 181 | Uncharacterized protein | Uncharacterized protein | | afdb-uniprot50 | AF-A0A3S2MDZ9-F1-MODEL\_V4 | 0.996 | 0.001712 | 90 | 0.127 | 157 | 103 | 10 | 9 | 142 | 662 | 807 | Uncharacterized protein | Uncharacterized protein | | afdb-uniprot50 | AF-A0A4Y5ZNF9-F1-MODEL\_V4 | 0.996 | 0.003915 | 89 | 0.123 | 146 | 99 | 9 | 8 | 142 | 54 | 181 | Ig-like domain-containing protein | Ig-like domain-containing protein | | afdb-uniprot50 | AF-A0A3A9CCX0-F1-MODEL\_V4 | 0.996 | 0.0023 | 89 | 0.128 | 148 | 97 | 7 | 30 | 166 | 48 | 174 | DUF2479 domain-containing protein | DUF2479 domain-containing protein | | afdb-uniprot50 | AF-A0A7J5ZBY5-F1-MODEL\_V4 | 0.996 | 0.001816 | 89 | 0.136 | 169 | 118 | 11 | 3 | 163 | 32 | 180 | Uncharacterized protein | Uncharacterized protein | | afdb-uniprot50 | AF-L1NGG6-F1-MODEL\_V4 | 0.996 | 0.001006 | 89 | 0.137 | 203 | 101 | 13 | 9 | 149 | 32 | 222 | Uncharacterized protein | Uncharacterized protein | | afdb-uniprot50 | AF-Q4L3Y3-F1-MODEL\_V4 | 0.996 | 0.004153 | 89 | 0.126 | 158 | 110 | 7 | 7 | 143 | 16 | 166 | Uncharacterized protein | Uncharacterized protein | | afdb-uniprot50 | AF-A0A399UNF6-F1-MODEL\_V4 | 0.995 | 0.0003085 | 88 | 0.148 | 202 | 114 | 11 | 8 | 179 | 61 | 234 | CopC domain-containing protein | CopC domain-containing protein | | afdb-uniprot50 | AF-A0A151MA98-F1-MODEL\_V4 | 0.995 | 0.000948 | 88 | 0.107 | 186 | 110 | 12 | 5 | 142 | 72 | 249 | Ig-like domain-containing protein | Ig-like domain-containing protein | | afdb-uniprot50 | AF-A0A852YGZ0-F1-MODEL\_V4 | 0.995 | 0.001201 | 88 | 0.139 | 194 | 122 | 13 | 9 | 179 | 63 | 234 | Uncharacterized protein | Uncharacterized protein | | afdb-uniprot50 | AF-A0A3M6TAX9-F1-MODEL\_V4 | 0.995 | 0.001816 | 88 | 0.132 | 174 | 113 | 12 | 7 | 159 | 72 | 228 | Uncharacterized protein | Uncharacterized protein | | afdb-uniprot50 | AF-A0A380CHS6-F1-MODEL\_V4 | 0.995 | 0.007069 | 88 | 0.145 | 165 | 103 | 13 | 17 | 166 | 36 | 177 | Putative phage tail protein | Putative phage tail protein | | afdb-uniprot50 | AF-A0A143Z9K5-F1-MODEL\_V4 | 0.995 | 0.007499 | 88 | 0.113 | 176 | 118 | 9 | 9 | 164 | 27 | 184 | Uncharacterized protein | Uncharacterized protein | | afdb-uniprot50 | AF-A0A6G0I273-F1-MODEL\_V4 | 0.995 | 0.002746 | 88 | 0.198 | 146 | 91 | 8 | 9 | 142 | 926 | 1057 | Scavenger receptor cysteine-rich type 1 protein M130 Soluble CD163 | Scavenger receptor cysteine-rich type 1 protein M130 Soluble CD163 | | afdb-uniprot50 | AF-A0A524CJ65-F1-MODEL\_V4 | 0.994 | 0.004959 | 87 | 0.126 | 126 | 92 | 7 | 9 | 126 | 203 | 318 | NosD domain-containing protein | NosD domain-containing protein | | afdb-uniprot50 | AF-J8KZ84-F1-MODEL\_V4 | 0.994 | 0.004153 | 87 | 0.14 | 164 | 112 | 10 | 8 | 142 | 22 | 185 | BppU\_N domain-containing protein | BppU\_N domain-containing protein | | afdb-uniprot50 | AF-A0A3Q0GK91-F1-MODEL\_V4 | 0.994 | 0.0006268 | 87 | 0.106 | 188 | 112 | 14 | 4 | 142 | 142 | 322 | uncharacterized protein LOC112550110 isoform X1 | uncharacterized protein LOC112550110 isoform X1 | | afdb-uniprot50 | AF-A0A6A8SHN8-F1-MODEL\_V4 | 0.994 | 0.007955 | 87 | 0.119 | 151 | 103 | 9 | 25 | 166 | 46 | 175 | DUF2479 domain-containing protein | DUF2479 domain-containing protein | | afdb-uniprot50 | AF-A0A351F507-F1-MODEL\_V4 | 0.994 | 0.004674 | 87 | 0.11 | 163 | 120 | 7 | 9 | 166 | 20 | 162 | Uncharacterized protein | Uncharacterized protein | | afdb-uniprot50 | AF-A0A7M7KDJ7-F1-MODEL\_V4 | 0.994 | 0.008953 | 87 | 0.092 | 151 | 118 | 10 | 8 | 142 | 747 | 894 | Uncharacterized protein | Uncharacterized protein | | afdb-uniprot50 | AF-A0A3E3EHK7-F1-MODEL\_V4 | 0.993 | 0.007955 | 86 | 0.075 | 146 | 110 | 7 | 22 | 164 | 36 | 159 | Uncharacterized protein | Uncharacterized protein | | afdb-uniprot50 | AF-A0A379AD69-F1-MODEL\_V4 | 0.993 | 0.00592 | 86 | 0.139 | 143 | 100 | 9 | 8 | 142 | 144 | 271 | Big\_13 domain-containing protein | Big\_13 domain-containing protein | | afdb-uniprot50 | AF-A0A381QTK1-F1-MODEL\_V4 | 0.993 | 0.003279 | 86 | 0.109 | 146 | 94 | 8 | 6 | 135 | 170 | 295 | Uncharacterized protein | Uncharacterized protein | | afdb-uniprot50 | AF-R9AHF4-F1-MODEL\_V4 | 0.993 | 0.0004949 | 86 | 0.145 | 200 | 118 | 15 | 7 | 179 | 228 | 401 | Dolichyl-diphosphooligosaccharide--protein glycosyltransferase subunit WBP1 | Dolichyl-diphosphooligosaccharide--protein glycosyltransferase subunit WBP1 | | afdb-uniprot50 | AF-A0A0E0UW38-F1-MODEL\_V4 | 0.993 | 0.00592 | 86 | 0.107 | 168 | 121 | 11 | 28 | 179 | 53 | 207 | Gp19 | Gp19 | | afdb-uniprot50 | AF-A0A3B3E2G3-F1-MODEL\_V4 | 0.993 | 0.0023 | 86 | 0.098 | 153 | 105 | 7 | 9 | 142 | 289 | 427 | Uncharacterized protein | Uncharacterized protein | | afdb-uniprot50 | AF-A0A6P7LDW9-F1-MODEL\_V4 | 0.993 | 0.00369 | 86 | 0.15 | 146 | 100 | 7 | 9 | 142 | 444 | 577 | scavenger receptor cysteine-rich type 1 protein M130-like | scavenger receptor cysteine-rich type 1 protein M130-like | | afdb-uniprot50 | AF-A0A0B0EE87-F1-MODEL\_V4 | 0.993 | 0.003915 | 86 | 0.14 | 164 | 120 | 7 | 15 | 166 | 499 | 653 | VWFA domain-containing protein | VWFA domain-containing protein | | afdb-uniprot50 | AF-Q32J73-F1-MODEL\_V4 | 0.993 | 0.006663 | 86 | 0.115 | 139 | 96 | 9 | 8 | 136 | 649 | 770 | Uncharacterized protein | Uncharacterized protein | | afdb-uniprot50 | AF-A0A6H3J5S0-F1-MODEL\_V4 | 0.992 | 0.0005909 | 85 | 0.154 | 181 | 122 | 9 | 14 | 179 | 6 | 170 | Copper resistance protein CopC | Copper resistance protein CopC | | afdb-uniprot50 | AF-A0A1H8GB40-F1-MODEL\_V4 | 0.992 | 0.001067 | 85 | 0.114 | 157 | 105 | 10 | 7 | 136 | 11 | 160 | PASTA domain, binds beta-lactams | PASTA domain, binds beta-lactams | | afdb-uniprot50 | AF-A0A497HKX8-F1-MODEL\_V4 | 0.992 | 0.004153 | 85 | 0.097 | 134 | 98 | 9 | 8 | 128 | 293 | 416 | CARDB domain-containing protein | CARDB domain-containing protein | | afdb-uniprot50 | AF-A0A4Y5ZTI8-F1-MODEL\_V4 | 0.992 | 0.007955 | 85 | 0.136 | 146 | 103 | 10 | 8 | 142 | 251 | 384 | Ig-like domain-containing protein | Ig-like domain-containing protein | | afdb-uniprot50 | AF-A0A6L3ENR8-F1-MODEL\_V4 | 0.992 | 0.002589 | 85 | 0.161 | 142 | 102 | 10 | 9 | 142 | 315 | 447 | Uncharacterized protein | Uncharacterized protein | | afdb-uniprot50 | AF-A0A354M6W3-F1-MODEL\_V4 | 0.992 | 0.003091 | 85 | 0.1 | 160 | 114 | 9 | 8 | 142 | 10 | 164 | Uncharacterized protein | Uncharacterized protein | | afdb-uniprot50 | AF-A0A2B5I3B6-F1-MODEL\_V4 | 0.992 | 0.00369 | 85 | 0.115 | 164 | 106 | 11 | 9 | 142 | 23 | 177 | BppU\_N domain-containing protein | BppU\_N domain-containing protein | | afdb-uniprot50 | AF-A0A368W6S3-F1-MODEL\_V4 | 0.992 | 0.001926 | 85 | 0.114 | 148 | 95 | 7 | 7 | 125 | 460 | 600 | FecR family protein | FecR family protein | | afdb-uniprot50 | AF-A0A814IBB4-F1-MODEL\_V4 | 0.992 | 0.003478 | 85 | 0.125 | 144 | 112 | 8 | 7 | 142 | 213 | 350 | Hypothetical protein | Hypothetical protein | | afdb-uniprot50 | AF-A0A0A6ZMX3-F1-MODEL\_V4 | 0.992 | 0.009498 | 85 | 0.11 | 145 | 94 | 9 | 8 | 142 | 467 | 586 | Membrane glycoprotein | Membrane glycoprotein | | afdb-uniprot50 | AF-A2F0K9-F1-MODEL\_V4 | 0.991 | 0.0003085 | 84 | 0.088 | 169 | 110 | 9 | 7 | 142 | 48 | 205 | Uncharacterized protein | Uncharacterized protein | | afdb-uniprot50 | AF-A0A7T9Z8U9-F1-MODEL\_V4 | 0.991 | 0.00592 | 84 | 0.134 | 141 | 97 | 6 | 7 | 142 | 51 | 171 | Excalibur calcium-binding domain-containing protein | Excalibur calcium-binding domain-containing protein | | afdb-uniprot50 | AF-A0A2N6QKG2-F1-MODEL\_V4 | 0.991 | 0.007955 | 84 | 0.109 | 182 | 132 | 11 | 8 | 166 | 24 | 198 | BppU\_N domain-containing protein | BppU\_N domain-containing protein | | afdb-uniprot50 | AF-A0A6B0AW78-F1-MODEL\_V4 | 0.991 | 0.00244 | 84 | 0.143 | 153 | 98 | 9 | 27 | 165 | 56 | 189 | DUF2479 domain-containing protein | DUF2479 domain-containing protein | | afdb-uniprot50 | AF-S9PVW1-F1-MODEL\_V4 | 0.991 | 0.00844 | 84 | 0.125 | 151 | 103 | 11 | 7 | 136 | 291 | 433 | Dolichyl-diphosphooligosaccharide--protein glycosyltransferase subunit WBP1 | Dolichyl-diphosphooligosaccharide--protein glycosyltransferase subunit WBP1 | | afdb-uniprot50 | AF-A0A5N5MH13-F1-MODEL\_V4 | 0.991 | 0.005581 | 84 | 0.135 | 140 | 105 | 8 | 9 | 142 | 347 | 476 | Uncharacterized protein | Uncharacterized protein | | afdb-uniprot50 | AF-D2RBM3-F1-MODEL\_V4 | 0.991 | 0.005581 | 84 | 0.122 | 179 | 116 | 10 | 6 | 165 | 20 | 176 | Methyl-accepting transducer domain-containing protein | Methyl-accepting transducer domain-containing protein | | afdb-uniprot50 | AF-X1G5Z6-F1-MODEL\_V4 | 0.99 | 0.001434 | 83 | 0.129 | 154 | 101 | 9 | 5 | 142 | 27 | 163 | Uncharacterized protein | Uncharacterized protein | | afdb-uniprot50 | AF-A0A3B4YJ65-F1-MODEL\_V4 | 0.99 | 0.00592 | 83 | 0.128 | 156 | 101 | 7 | 9 | 160 | 129 | 253 | Uncharacterized protein | Uncharacterized protein | | afdb-uniprot50 | AF-A0A518GX12-F1-MODEL\_V4 | 0.99 | 0.003915 | 83 | 0.105 | 190 | 119 | 9 | 6 | 164 | 79 | 248 | Uncharacterized protein | Uncharacterized protein | | afdb-uniprot50 | AF-A0A7J3SM07-F1-MODEL\_V4 | 0.99 | 0.006663 | 83 | 0.104 | 162 | 115 | 10 | 7 | 158 | 110 | 251 | Uncharacterized protein | Uncharacterized protein | | afdb-uniprot50 | AF-E1RGZ0-F1-MODEL\_V4 | 0.99 | 0.006663 | 83 | 0.115 | 130 | 82 | 5 | 8 | 115 | 31 | 149 | Uncharacterized protein | Uncharacterized protein | | afdb-uniprot50 | AF-A0A0L0SSW8-F1-MODEL\_V4 | 0.99 | 0.002168 | 83 | 0.1 | 200 | 133 | 13 | 7 | 179 | 294 | 473 | Dolichyl-diphosphooligosaccharide--protein glycosyltransferase subunit WBP1 | Dolichyl-diphosphooligosaccharide--protein glycosyltransferase subunit WBP1 | | afdb-uniprot50 | AF-A0A5M9ZIC2-F1-MODEL\_V4 | 0.99 | 0.002589 | 83 | 0.154 | 142 | 82 | 12 | 9 | 126 | 24 | 151 | Uncharacterized protein | Uncharacterized protein | | afdb-uniprot50 | AF-A0A7J9PQ88-F1-MODEL\_V4 | 0.99 | 0.003091 | 83 | 0.134 | 134 | 97 | 8 | 9 | 129 | 519 | 646 | Uncharacterized protein | Uncharacterized protein | | afdb-uniprot50 | AF-A0A842BP15-F1-MODEL\_V4 | 0.988 | 0.003478 | 82 | 0.156 | 160 | 91 | 8 | 9 | 142 | 120 | 261 | LPXTG cell wall anchor domain-containing protein | LPXTG cell wall anchor domain-containing protein | | afdb-uniprot50 | AF-A0A7J9SFD5-F1-MODEL\_V4 | 0.988 | 0.002913 | 82 | 0.116 | 129 | 93 | 7 | 9 | 124 | 305 | 425 | Uncharacterized protein | Uncharacterized protein | | afdb-uniprot50 | AF-A0A6G0ITN7-F1-MODEL\_V4 | 0.988 | 0.00244 | 82 | 0.13 | 161 | 99 | 9 | 3 | 142 | 296 | 436 | Deleted in malignant brain tumors 1 protein Glycoprotein 340 | Deleted in malignant brain tumors 1 protein Glycoprotein 340 | | afdb-uniprot50 | AF-A0A843LRD6-F1-MODEL\_V4 | 0.988 | 0.003478 | 82 | 0.106 | 160 | 93 | 12 | 6 | 142 | 281 | 413 | PGF-CTERM sorting domain-containing protein | PGF-CTERM sorting domain-containing protein | | afdb-uniprot50 | AF-A0A5C1G595-F1-MODEL\_V4 | 0.988 | 0.005581 | 82 | 0.115 | 173 | 113 | 10 | 3 | 142 | 15 | 180 | DUF2479 domain-containing protein | DUF2479 domain-containing protein | | afdb-uniprot50 | AF-A0A2M7AA77-F1-MODEL\_V4 | 0.988 | 0.007955 | 82 | 0.102 | 156 | 91 | 12 | 7 | 122 | 277 | 423 | Carb-bd\_dom\_fam9 domain-containing protein | Carb-bd\_dom\_fam9 domain-containing protein | | afdb-uniprot50 | AF-A0A6P7K4J8-F1-MODEL\_V4 | 0.986 | 0.009498 | 81 | 0.144 | 138 | 96 | 8 | 9 | 138 | 109 | 232 | uncharacterized protein LOC114449958 | uncharacterized protein LOC114449958 | | afdb-uniprot50 | AF-A0A2N0G476-F1-MODEL\_V4 | 0.986 | 0.004959 | 81 | 0.109 | 155 | 106 | 10 | 8 | 142 | 5 | 147 | Uncharacterized protein | Uncharacterized protein | | afdb-uniprot50 | AF-A0A535C1Z2-F1-MODEL\_V4 | 0.986 | 0.003091 | 81 | 0.145 | 144 | 100 | 11 | 6 | 135 | 168 | 302 | Uncharacterized protein | Uncharacterized protein | | afdb-uniprot50 | AF-A0A1V4ZS71-F1-MODEL\_V4 | 0.986 | 0.006281 | 81 | 0.155 | 154 | 79 | 12 | 9 | 142 | 203 | 325 | Uncharacterized protein | Uncharacterized protein | | afdb-uniprot50 | AF-A0A3M2QGJ1-F1-MODEL\_V4 | 0.986 | 0.003915 | 81 | 0.127 | 188 | 113 | 14 | 9 | 166 | 26 | 192 | Phage baseplate upper protein | Phage baseplate upper protein | | afdb-uniprot50 | AF-A0A4V6KU45-F1-MODEL\_V4 | 0.986 | 0.00592 | 81 | 0.121 | 189 | 119 | 12 | 8 | 178 | 356 | 515 | Protein of uncharacterized function (DUF1533) | Protein of uncharacterized function (DUF1533) | | afdb-uniprot50 | AF-A0A842F260-F1-MODEL\_V4 | 0.986 | 0.009498 | 81 | 0.153 | 117 | 80 | 6 | 29 | 142 | 423 | 523 | LPXTG cell wall anchor domain-containing protein | LPXTG cell wall anchor domain-containing protein | | afdb-uniprot50 | AF-A0A452J3I1-F1-MODEL\_V4 | 0.984 | 0.003478 | 80 | 0.071 | 167 | 118 | 10 | 9 | 145 | 28 | 187 | Ig-like domain-containing protein | Ig-like domain-containing protein | | afdb-uniprot50 | AF-H1Z1T2-F1-MODEL\_V4 | 0.984 | 0.003091 | 80 | 0.135 | 155 | 87 | 11 | 8 | 142 | 193 | 320 | Uncharacterized protein | Uncharacterized protein | | afdb-uniprot50 | AF-A0A1V1P2D0-F1-MODEL\_V4 | 0.984 | 0.003091 | 80 | 0.09 | 177 | 106 | 11 | 8 | 157 | 296 | 444 | Uncharacterized protein | Uncharacterized protein | | afdb-uniprot50 | AF-A0A2R5GPP2-F1-MODEL\_V4 | 0.984 | 0.001434 | 80 | 0.141 | 198 | 124 | 13 | 15 | 179 | 480 | 664 | DnaJ protein ERDJ2A | DnaJ protein ERDJ2A | | afdb-uniprot50 | AF-A0A1Q5P452-F1-MODEL\_V4 | 0.984 | 0.006663 | 80 | 0.12 | 141 | 95 | 7 | 31 | 164 | 50 | 168 | NodB homology domain-containing protein | NodB homology domain-containing protein | | afdb-uniprot50 | AF-A0A3N2CWC2-F1-MODEL\_V4 | 0.981 | 0.007069 | 79 | 0.127 | 173 | 115 | 9 | 8 | 166 | 46 | 196 | CopC domain-containing protein | CopC domain-containing protein | | afdb-uniprot50 | AF-A0A674NHX4-F1-MODEL\_V4 | 0.981 | 0.000948 | 79 | 0.091 | 174 | 110 | 12 | 7 | 142 | 51 | 214 | IGv domain-containing protein | IGv domain-containing protein | | afdb-uniprot50 | AF-A0A556UXS5-F1-MODEL\_V4 | 0.981 | 0.001132 | 79 | 0.131 | 160 | 114 | 9 | 7 | 149 | 21 | 172 | Motile sperm domain-containing protein 1 | Motile sperm domain-containing protein 1 | | afdb-uniprot50 | AF-A0A7K4B3J4-F1-MODEL\_V4 | 0.981 | 0.002746 | 79 | 0.113 | 158 | 91 | 10 | 9 | 142 | 241 | 373 | PGF-CTERM sorting domain-containing protein | PGF-CTERM sorting domain-containing protein | | afdb-uniprot50 | AF-A0A7D8H6E0-F1-MODEL\_V4 | 0.981 | 0.007069 | 79 | 0.134 | 164 | 105 | 12 | 8 | 142 | 22 | 177 | Uncharacterized protein | Uncharacterized protein | | afdb-uniprot50 | AF-A0A7J5YJ57-F1-MODEL\_V4 | 0.981 | 0.007069 | 79 | 0.059 | 202 | 159 | 7 | 9 | 179 | 322 | 523 | ZP domain-containing protein | ZP domain-containing protein | | afdb-uniprot50 | AF-A0A7J5XR45-F1-MODEL\_V4 | 0.981 | 0.007499 | 79 | 0.132 | 151 | 101 | 8 | 9 | 142 | 764 | 901 | Ig-like domain-containing protein | Ig-like domain-containing protein | | afdb-uniprot50 | AF-A0A3M1PQZ9-F1-MODEL\_V4 | 0.981 | 0.002589 | 79 | 0.125 | 208 | 124 | 12 | 15 | 178 | 42 | 235 | Tetratricopeptide repeat protein | Tetratricopeptide repeat protein | | afdb-uniprot50 | AF-A0A7K1J0H1-F1-MODEL\_V4 | 0.978 | 0.007499 | 78 | 0.117 | 128 | 95 | 9 | 9 | 128 | 102 | 219 | Uncharacterized protein | Uncharacterized protein | | afdb-uniprot50 | AF-A0A3Q3ABB6-F1-MODEL\_V4 | 0.978 | 0.006663 | 78 | 0.117 | 179 | 111 | 8 | 5 | 179 | 304 | 439 | Scavenger receptor cysteine-rich type 1 protein M160-like | Scavenger receptor cysteine-rich type 1 protein M160-like | | afdb-uniprot50 | AF-A0A834FJH2-F1-MODEL\_V4 | 0.978 | 0.00369 | 78 | 0.153 | 196 | 113 | 11 | 3 | 178 | 681 | 843 | Uncharacterized protein | Uncharacterized protein | | afdb-uniprot50 | AF-A0A7J5XPK3-F1-MODEL\_V4 | 0.978 | 0.00526 | 78 | 0.14 | 149 | 97 | 10 | 9 | 142 | 839 | 971 | Uncharacterized protein | Uncharacterized protein | | afdb-uniprot50 | AF-B8MP86-F1-MODEL\_V4 | 0.975 | 0.008953 | 77 | 0.096 | 218 | 147 | 9 | 3 | 179 | 12 | 220 | Uncharacterized protein | Uncharacterized protein | | afdb-uniprot50 | AF-A0A841ZTY5-F1-MODEL\_V4 | 0.975 | 0.007955 | 77 | 0.126 | 150 | 92 | 9 | 14 | 142 | 286 | 417 | LPXTG cell wall anchor domain-containing protein | LPXTG cell wall anchor domain-containing protein | | afdb-uniprot50 | AF-A0A537DD91-F1-MODEL\_V4 | 0.975 | 0.007069 | 77 | 0.125 | 167 | 128 | 10 | 7 | 165 | 642 | 798 | PKD domain-containing protein | PKD domain-containing protein | | afdb-uniprot50 | AF-A0A1Y1JV51-F1-MODEL\_V4 | 0.975 | 0.007499 | 77 | 0.093 | 193 | 130 | 8 | 10 | 164 | 30 | 215 | Uncharacterized protein | Uncharacterized protein | | afdb-uniprot50 | AF-A0A6A5FI04-F1-MODEL\_V4 | 0.971 | 0.00844 | 76 | 0.156 | 147 | 96 | 10 | 9 | 142 | 14 | 145 | Ig-like domain-containing protein | Ig-like domain-containing protein | | afdb-uniprot50 | AF-A0A7J3DL36-F1-MODEL\_V4 | 0.971 | 0.0006268 | 76 | 0.125 | 200 | 134 | 11 | 1 | 166 | 17 | 209 | Uncharacterized protein | Uncharacterized protein | | afdb-uniprot50 | AF-A0A1A8Q8P6-F1-MODEL\_V4 | 0.971 | 0.003915 | 76 | 0.1 | 139 | 105 | 6 | 10 | 142 | 103 | 227 | Fibronectin type-III domain-containing protein | Fibronectin type-III domain-containing protein | | afdb-uniprot50 | AF-A0A3S1AFG5-F1-MODEL\_V4 | 0.971 | 0.006663 | 76 | 0.1 | 149 | 102 | 7 | 5 | 142 | 286 | 413 | MG3 domain-containing protein | MG3 domain-containing protein | | afdb-uniprot50 | AF-A0A3P8Y1B5-F1-MODEL\_V4 | 0.971 | 0.006281 | 76 | 0.147 | 149 | 99 | 9 | 9 | 142 | 338 | 473 | Uncharacterized protein | Uncharacterized protein | | afdb-uniprot50 | AF-A0A0F8AG26-F1-MODEL\_V4 | 0.971 | 0.003279 | 76 | 0.065 | 168 | 126 | 7 | 6 | 142 | 324 | 491 | Transforming growth factor beta receptor type 3 | Transforming growth factor beta receptor type 3 | | afdb-uniprot50 | AF-A0A484DDS8-F1-MODEL\_V4 | 0.971 | 0.009498 | 76 | 0.164 | 158 | 94 | 9 | 3 | 142 | 632 | 769 | Uncharacterized protein | Uncharacterized protein | | afdb-uniprot50 | AF-A0A3P9JQK7-F1-MODEL\_V4 | 0.967 | 0.007499 | 75 | 0.114 | 192 | 123 | 14 | 8 | 164 | 56 | 235 | Ig-like domain-containing protein | Ig-like domain-containing protein | | afdb-uniprot50 | AF-A0A517XKX4-F1-MODEL\_V4 | 0.967 | 0.001521 | 75 | 0.137 | 175 | 104 | 7 | 9 | 141 | 600 | 769 | Leupeptin-inactivating enzyme 1 | Leupeptin-inactivating enzyme 1 | | afdb-uniprot50 | AF-A0A7Y2HJU2-F1-MODEL\_V4 | 0.967 | 0.002913 | 75 | 0.158 | 145 | 97 | 7 | 8 | 142 | 568 | 697 | Uncharacterized protein | Uncharacterized protein | | afdb-uniprot50 | AF-A0A0M0BEQ4-F1-MODEL\_V4 | 0.967 | 0.004674 | 75 | 0.074 | 135 | 98 | 9 | 5 | 128 | 901 | 1019 | Uncharacterized protein | Uncharacterized protein | | afdb-uniprot50 | AF-A0A662BMB6-F1-MODEL\_V4 | 0.967 | 0.003915 | 75 | 0.095 | 199 | 127 | 11 | 7 | 166 | 957 | 1141 | Uncharacterized protein | Uncharacterized protein | | afdb-uniprot50 | AF-A0A094B7D5-F1-MODEL\_V4 | 0.961 | 0.007499 | 74 | 0.137 | 153 | 103 | 10 | 8 | 142 | 113 | 254 | Uncharacterized protein | Uncharacterized protein | | afdb-uniprot50 | AF-A0A7X0XKQ9-F1-MODEL\_V4 | 0.961 | 0.003279 | 74 | 0.125 | 168 | 95 | 10 | 9 | 142 | 51 | 200 | LPXTG cell wall anchor domain-containing protein | LPXTG cell wall anchor domain-containing protein | | afdb-uniprot50 | AF-A0A1W9LDA3-F1-MODEL\_V4 | 0.961 | 0.009498 | 74 | 0.164 | 158 | 88 | 9 | 6 | 129 | 317 | 464 | Uncharacterized protein | Uncharacterized protein | | afdb-uniprot50 | AF-A0A672HNP6-F1-MODEL\_V4 | 0.956 | 0.001434 | 73 | 0.112 | 169 | 119 | 9 | 7 | 155 | 20 | 177 | Motile sperm domain-containing protein 1 | Motile sperm domain-containing protein 1 | | afdb-uniprot50 | AF-A0A6I9Y835-F1-MODEL\_V4 | 0.956 | 0.008953 | 73 | 0.102 | 185 | 134 | 12 | 8 | 179 | 77 | 242 | semaphorin-3F-like | semaphorin-3F-like | | afdb-uniprot50 | AF-A0A803VYX2-F1-MODEL\_V4 | 0.956 | 0.007499 | 73 | 0.138 | 202 | 111 | 9 | 9 | 179 | 740 | 909 | Uncharacterized protein | Uncharacterized protein | | afdb-uniprot50 | AF-A0A4W5L7M6-F1-MODEL\_V4 | 0.949 | 0.001816 | 72 | 0.095 | 168 | 109 | 10 | 9 | 142 | 29 | 187 | Ig-like domain-containing protein | Ig-like domain-containing protein | | afdb-uniprot50 | AF-A0A7J5XLP4-F1-MODEL\_V4 | 0.949 | 0.002589 | 72 | 0.097 | 154 | 113 | 8 | 7 | 142 | 47 | 192 | Motile sperm domain-containing protein 1 | Motile sperm domain-containing protein 1 | | afdb-uniprot50 | AF-A0A832TUV5-F1-MODEL\_V4 | 0.949 | 0.008953 | 72 | 0.111 | 162 | 88 | 10 | 6 | 142 | 171 | 301 | Uncharacterized protein | Uncharacterized protein | | afdb-uniprot50 | AF-A0A2G9S9Q7-F1-MODEL\_V4 | 0.949 | 0.004959 | 72 | 0.152 | 151 | 103 | 9 | 2 | 142 | 82 | 217 | Ig-like domain-containing protein | Ig-like domain-containing protein | | afdb-uniprot50 | AF-A0A6G0ITN3-F1-MODEL\_V4 | 0.949 | 0.00844 | 72 | 0.128 | 164 | 99 | 10 | 3 | 142 | 403 | 546 | Scavenger receptor cysteine-rich type 1 protein M130 | Scavenger receptor cysteine-rich type 1 protein M130 | | afdb-uniprot50 | AF-A0A843LMU9-F1-MODEL\_V4 | 0.949 | 0.009498 | 72 | 0.113 | 159 | 90 | 11 | 9 | 142 | 436 | 568 | PGF-CTERM sorting domain-containing protein | PGF-CTERM sorting domain-containing protein | | afdb-uniprot50 | AF-A0A7J4HUN3-F1-MODEL\_V4 | 0.941 | 0.00526 | 71 | 0.112 | 178 | 124 | 9 | 7 | 164 | 304 | 467 | Uncharacterized protein | Uncharacterized protein | | afdb-uniprot50 | AF-S8F0H2-F1-MODEL\_V4 | 0.941 | 0.002589 | 71 | 0.147 | 197 | 119 | 13 | 15 | 179 | 400 | 579 | Uncharacterized protein | Uncharacterized protein | | afdb-uniprot50 | AF-A0A8B6DDG9-F1-MODEL\_V4 | 0.941 | 0.009498 | 71 | 0.13 | 123 | 95 | 3 | 32 | 142 | 159 | 281 | Uncharacterized protein | Uncharacterized protein | | afdb-uniprot50 | AF-A0A2V1A8H1-F1-MODEL\_V4 | 0.941 | 0.00526 | 71 | 0.113 | 158 | 102 | 10 | 9 | 142 | 53 | 196 | SAC domain-containing protein | SAC domain-containing protein | | afdb-uniprot50 | AF-A0A6G7Z4I0-F1-MODEL\_V4 | 0.933 | 0.003279 | 70 | 0.119 | 151 | 107 | 7 | 31 | 166 | 45 | 184 | Fibronectin type-III domain-containing protein | Fibronectin type-III domain-containing protein | | afdb-uniprot50 | AF-A0A7J6B8F1-F1-MODEL\_V4 | 0.923 | 0.0023 | 69 | 0.109 | 191 | 131 | 12 | 7 | 178 | 87 | 257 | Motile sperm domain-containing protein 1 | Motile sperm domain-containing protein 1 | | afdb-uniprot50 | AF-A0A847I2Q6-F1-MODEL\_V4 | 0.923 | 0.004674 | 69 | 0.168 | 172 | 101 | 13 | 17 | 163 | 298 | 452 | VWA domain-containing protein | VWA domain-containing protein | | afdb-uniprot50 | AF-A0A087TGG5-F1-MODEL\_V4 | 0.923 | 0.004153 | 69 | 0.148 | 148 | 95 | 9 | 9 | 142 | 585 | 715 | Receptor protein-tyrosine kinase | Receptor protein-tyrosine kinase | | afdb-uniprot50 | AF-A0A2M9C4S0-F1-MODEL\_V4 | 0.912 | 0.008953 | 68 | 0.128 | 194 | 97 | 11 | 9 | 142 | 46 | 227 | Methionine-rich copper-binding protein CopC | Methionine-rich copper-binding protein CopC | | afdb-uniprot50 | AF-A0A1J0U2V1-F1-MODEL\_V4 | 0.912 | 0.004959 | 68 | 0.154 | 188 | 119 | 13 | 9 | 179 | 342 | 506 | Cadherin\_5 domain-containing protein | Cadherin\_5 domain-containing protein | | afdb-uniprot50 | AF-A0A3R9QQN6-F1-MODEL\_V4 | 0.912 | 0.004959 | 68 | 0.119 | 151 | 90 | 9 | 7 | 142 | 412 | 534 | Uncharacterized protein | Uncharacterized protein | | afdb-uniprot50 | AF-A0A3Q1GFR9-F1-MODEL\_V4 | 0.9 | 0.004674 | 67 | 0.105 | 151 | 111 | 7 | 7 | 142 | 21 | 162 | Motile sperm domain-containing protein 1 | Motile sperm domain-containing protein 1 | | afdb-uniprot50 | AF-A0A3Q1J8F6-F1-MODEL\_V4 | 0.9 | 0.00526 | 67 | 0.113 | 167 | 121 | 8 | 7 | 155 | 112 | 269 | Motile sperm domain-containing protein 1 | Motile sperm domain-containing protein 1 | | afdb-uniprot50 | AF-A0A075B1J7-F1-MODEL\_V4 | 0.9 | 0.008953 | 67 | 0.101 | 207 | 135 | 12 | 8 | 178 | 226 | 417 | Uncharacterized protein | Uncharacterized protein | | afdb-uniprot50 | AF-A0A670KDQ6-F1-MODEL\_V4 | 0.887 | 0.003915 | 66 | 0.105 | 151 | 108 | 7 | 8 | 142 | 24 | 163 | Motile sperm domain-containing protein 1 | Motile sperm domain-containing protein 1 | | afdb-uniprot50 | AF-A0A3M6TAK4-F1-MODEL\_V4 | 0.887 | 0.009498 | 66 | 0.106 | 169 | 113 | 11 | 15 | 160 | 25 | 178 | Uncharacterized protein | Uncharacterized protein | | afdb-uniprot50 | AF-A0A0S7IV27-F1-MODEL\_V4 | 0.872 | 0.004959 | 65 | 0.126 | 166 | 116 | 8 | 8 | 155 | 54 | 208 | Motile sperm domain-containing protein 1 | Motile sperm domain-containing protein 1 | | afdb-uniprot50 | AF-A0A6F9ANK6-F1-MODEL\_V4 | 0.872 | 0.003478 | 65 | 0.101 | 187 | 133 | 9 | 8 | 178 | 58 | 225 | Motile sperm domain-containing protein 1 | Motile sperm domain-containing protein 1 | | afdb-uniprot50 | AF-A0A497MZN8-F1-MODEL\_V4 | 0.855 | 0.00844 | 64 | 0.164 | 158 | 94 | 13 | 7 | 142 | 126 | 267 | Uncharacterized protein | Uncharacterized protein | | afdb-uniprot50 | AF-A0A2Y9RF76-F1-MODEL\_V4 | 0.855 | 0.00592 | 64 | 0.163 | 165 | 94 | 9 | 7 | 142 | 121 | 270 | T-cell-interacting, activating receptor on myeloid cells protein 1 isoform X5 | T-cell-interacting, activating receptor on myeloid cells protein 1 isoform X5 | | afdb-uniprot50 | AF-A0A7M3ZE92-F1-MODEL\_V4 | 0.855 | 0.007499 | 64 | 0.098 | 172 | 112 | 8 | 8 | 142 | 110 | 275 | Uncharacterized protein | Uncharacterized protein | | afdb-uniprot50 | AF-A0A832Y8B9-F1-MODEL\_V4 | 0.855 | 0.001132 | 64 | 0.168 | 184 | 104 | 12 | 7 | 164 | 117 | 277 | Uncharacterized protein | Uncharacterized protein | | afdb-uniprot50 | AF-A0A351H4X9-F1-MODEL\_V4 | 0.837 | 0.004153 | 63 | 0.087 | 240 | 126 | 13 | 5 | 153 | 22 | 259 | Uncharacterized protein | Uncharacterized protein | | afdb-uniprot50 | AF-A0A3B5BAV1-F1-MODEL\_V4 | 0.817 | 0.007955 | 62 | 0.119 | 167 | 116 | 8 | 8 | 155 | 24 | 178 | Motile sperm domain-containing protein 1 | Motile sperm domain-containing protein 1 | | afdb-uniprot50 | AF-A0A3Q8ZFA0-F1-MODEL\_V4 | 0.817 | 0.00592 | 62 | 0.122 | 179 | 120 | 8 | 15 | 166 | 20 | 188 | Uncharacterized protein | Uncharacterized protein | | afdb-uniprot50 | AF-A0A7X0FNP4-F1-MODEL\_V4 | 0.817 | 0.008953 | 62 | 0.191 | 157 | 93 | 12 | 6 | 142 | 579 | 721 | Uncharacterized protein | Uncharacterized protein | | afdb-uniprot50 | AF-A0A2D5X0L3-F1-MODEL\_V4 | 0.795 | 0.00244 | 61 | 0.157 | 210 | 115 | 13 | 9 | 179 | 121 | 307 | Uncharacterized protein | Uncharacterized protein | | afdb-uniprot50 | AF-A0A672YNI3-F1-MODEL\_V4 | 0.747 | 0.009498 | 59 | 0.113 | 150 | 108 | 7 | 8 | 141 | 24 | 164 | Motile sperm domain-containing protein 1 | Motile sperm domain-containing protein 1 | | afdb-uniprot50 | AF-A0A534JZD8-F1-MODEL\_V4 | 0.747 | 0.0007484 | 59 | 0.151 | 172 | 96 | 9 | 9 | 142 | 49 | 208 | Spt5-NGN domain-containing protein | Spt5-NGN domain-containing protein | | afdb-uniprot50 | AF-A0A0M2LTA1-F1-MODEL\_V4 | 0.72 | 0.009498 | 58 | 0.142 | 161 | 90 | 13 | 7 | 142 | 541 | 678 | Uncharacterized protein | Uncharacterized protein | | afdb-uniprot50 | AF-A0A075HIB3-F1-MODEL\_V4 | 0.692 | 0.004674 | 57 | 0.094 | 179 | 108 | 11 | 8 | 142 | 17 | 185 | Uncharacterized protein | Uncharacterized protein | |
| Top keywords  (threshold 1.00e-02 (evalue)) | **domain\_containing, BppU\_N, light, receptor, Fab, DUF2479, Dolichyl\_diphosphooligosaccharide\_\_protein, glycosyltransferase, Phage, immunoglobulin** |
| Output files | ../../similar\_structures/03\_FANPEZAQ\_CDS\_0003\_afdb-proteome\_foldseek.tsv ../../similar\_structures/03\_FANPEZAQ\_CDS\_0003\_afdb-uniprot50\_foldseek.tsv ../../similar\_structures/03\_FANPEZAQ\_CDS\_0003\_merged.svg ../../similar\_structures/03\_FANPEZAQ\_CDS\_0003\_pdb\_foldseek.tsv |

  
  
  

Return to summary | Go to previous | Go to next

  


---

**Sequence/structure alignments coloring**  
Each object in the alignment figures is colored according to its E-value following this color coding:

1e-100
10

**References:**  
1) Steinegger M, Meier M, Mirdita M, Vöhringer H, Haunsberger S J, and Söding J (2019) HH-suite3 for fast remote homology detection and deep protein annotation, BMC Bioinformatics, 473. doi: 10.1186/s12859-019-3019-7  
2) Jumper J, Evans R, Pritzel A, ..., Hassabis D (2021) Highly accurate protein structure prediction with AlphaFold, Nature, 596. doi: 10.1038/s41586-021-03819-2  
3) van Kempen M, Kim S, Tumescheit C, Mirdita M, Lee J, Gilchrist CLM, Söding J, and Steinegger M (2023) Fast and accurate protein structure search with Foldseek. Nature Biotechnology. doi: 10.1038/s41587-023-01773-0
