# Supplementary material for: Completing the BASEL phage collection to unlock hidden diversity for systematic exploration of phage–host interactions
Source: PLoS Biol. 2025 Apr 7;23(4):e3003063. doi: 10.1371/journal.pbio.3003063 (PMC11990801; doi:10.1371/journal.pbio.3003063)
Supplement: S2 Data — (ZIP) [file pbio.3003063.s009.zip › entries/32.html]

FANPEZAQ\_CDS\_0032


Return to summary | Go to previous | Go to next

|  |  |
| --- | --- |
| FANPEZAQ\_CDS\_0032 Page creation date: 02 Sep 2024, 12:00  Project folder: n/a  Input sequences file: Escherichia\_virus\_HeidiAbel.gb | integrase recombinase domain\_containing tyr site\_specific phage duf4102 prophage tyrosine\_type tyrosine xerd putative dna cp4\_57 xerc catalytic dna\_binding complex a tp ap and fragment yes integration recombination c\_terminal int cps\_53 arm\_type engineered core\_binding cb inta site protein\_dna binding structural in excision alpha bundle bacteriophage transposase mainly arch orthogonal hpi genomics gp |

### Sequence information

|  |  |
| --- | --- |
| Name | FANPEZAQ\_CDS\_0032  32\_FANPEZAQ\_CDS\_0032 (pipeline id) |
| Imported annotations | Escherichia\_virus\_HeidiAbel Bas97 |
| Protein sequence | MLTERQIQAAMRAVTSETVLNDGAAGRGSGSLRLRIRAGAKGPNATWQAVWWNAGKQTSK ALGRYPDLSLADARRKYETEVRDVLTAGRDPNAVVAMAESPTIEKLFQAYIQYLRDKETA TTHTSEHLLLTGKYNAADLLGRNTIASTIEPADIANVLAKGVKRGARRTTDMQRTAMMAA FNWAMKSTHDYTQENRMDWGLKYNPVAAVPRDQAANRTLDRNLSAEEMKHVWDNAPEQSG DVMRLVMCTGQRVIEVMRIAGADVDLKARLWTMPARKTKGRKHTHMVPLTTQAVDILTTL IDLYGDGYLFPARAGAKGDIIGIPSVSRGASRMAGIKPFTPRDLRRTWKSRAGDAGIDRF TRDLIQQHAQTDTGSKHYDRFDYLPQMREAMNKWEIWLRNAIA |
| Number of residues | 403 |
| Molecular weight (Da) | 45042.87 |
| Output files | ../../query\_sequences/32\_FANPEZAQ\_CDS\_0032.fasta |

### Putative domain architecture and protein family

#### Search results (HHblits)1

|  |  |
| --- | --- |
| Domain family databases searched | Pfam, Ncbi-cd, Cath, Phrogs |
| Results, scheme(s)  (Top layers only; threshold 1.00e-03 (evalue)) | xml version="1.0" encoding="utf-8" standalone="no"?       2024-09-02T21:08:19.283941 image/svg+xml   Matplotlib v3.7.2, https://matplotlib.org/ |
| Results, table  (E-value ≤ 1.00e-03 (evalue)) | | db | id | prob | evalue | pvalue | score | cols | query | query\_len | template | template\_len | name | description | | --- | --- | --- | --- | --- | --- | --- | --- | --- | --- | --- | --- | --- | | pfam | PF00589 | 99.1 | 8.5e-15 | 2e-18 | 116.3 | 159 | (221, 383) | 403 | (1, 171) | 173 | Phage\_integrase | Phage integrase family | | pfam | PF16795 | 98.7 | 1.5e-12 | 3.5e-16 | 104.9 | 153 | (221, 396) | 403 | (2, 170) | 171 | Phage\_integr\_3 | Archaeal phage integrase | | pfam | PF12835 | 98.1 | 4.3e-10 | 1e-13 | 85.4 | 126 | (217, 350) | 403 | (8, 148) | 149 | Integrase\_1 | Integrase | | pfam | PF11917 | 97.5 | 2.9e-08 | 6.4e-12 | 92.1 | 68 | (315, 382) | 403 | (174, 243) | 418 | DUF3435 | Protein of unknown function (DUF3435) | | pfam | PF16787 | 96.8 | 9.7e-07 | 2.1e-10 | 78.3 | 142 | (238, 383) | 403 | (29, 222) | 313 | NDC10\_II | Centromere DNA-binding protein complex CBF3 subunit, domain 2 | | ncbi-cd | cd01193 | 99.1 | 2e-15 | 5e-19 | 120.1 | 158 | (219, 380) | 403 | (2, 175) | 176 | INT\_IntI\_C | cd01193 INT\_IntI\_C; Integron integrase and similar protiens, C-terminal catalytic domain. Integron integrases mediate site-specific DNA recombination between a proximal primary site (attI) and a secondary target site (attC) found within mobile gene cassettes encoding resistance or virulence factors. | | ncbi-cd | cd01197 | 99.1 | 2.5e-15 | 6e-19 | 121.1 | 163 | (220, 386) | 403 | (4, 178) | 181 | INT\_FimBE\_like | cd01197 INT\_FimBE\_like; FimB and FimE and related proteins, integrase/recombinases. This CD includes proteins similar to E. | | ncbi-cd | cd00797 | 99.0 | 9.1e-15 | 2.2e-18 | 120.0 | 163 | (223, 389) | 403 | (1, 194) | 198 | INT\_RitB\_C\_like | cd00797 INT\_RitB\_C\_like; C-terminal catalytic domain of recombinase RitB, a component of the recombinase trio. | | ncbi-cd | cd01191 | 99.0 | 9.8e-15 | 2.3e-18 | 119.1 | 164 | (222, 392) | 403 | (1, 172) | 176 | INT\_C\_like\_2 | cd01191 INT\_C\_like\_2; Uncharacterized site-specific tyrosine recombinase, C-terminal catalytic domain. | | ncbi-cd | cd01188 | 99.0 | 1.3e-14 | 3.2e-18 | 116.7 | 161 | (224, 388) | 403 | (1, 176) | 179 | INT\_RitA\_C\_like | cd01188 INT\_RitA\_C\_like; C-terminal catalytic domain of recombinase RitA, a component of the recombinase trio. | | ncbi-cd | cd00801 | 99.0 | 1.7e-14 | 4.1e-18 | 115.9 | 171 | (224, 397) | 403 | (1, 179) | 180 | INT\_P4\_C | cd00801 INT\_P4\_C; Bacteriophage P4 integrase, C-terminal catalytic domain. P4-like integrases are found in temperate bacteriophages, integrative plasmids, pathogenicity and symbiosis islands, and other mobile genetic elements. | | ncbi-cd | cd01192 | 99.0 | 1.7e-14 | 4.2e-18 | 115.9 | 163 | (220, 387) | 403 | (4, 176) | 178 | INT\_C\_like\_3 | cd01192 INT\_C\_like\_3; Uncharacterized site-specific tyrosine recombinase, C-terminal catalytic domain. | | ncbi-cd | cd00796 | 99.0 | 2.8e-14 | 6.7e-18 | 111.9 | 155 | (221, 380) | 403 | (3, 162) | 162 | INT\_Rci\_Hp1\_C | cd00796 INT\_Rci\_Hp1\_C; Shufflon-specific DNA recombinase Rci and Bacteriophage Hp1\_like integrase, C-terminal catalytic domain. | | ncbi-cd | cd01196 | 99.0 | 3.2e-14 | 7.7e-18 | 115.1 | 161 | (224, 387) | 403 | (1, 180) | 183 | INT\_C\_like\_6 | cd01196 INT\_C\_like\_6; Uncharacterized site-specific tyrosine recombinase, C-terminal catalytic domain. | | ncbi-cd | cd01182 | 99.0 | 3.4e-14 | 8.2e-18 | 113.7 | 162 | (223, 388) | 403 | (1, 181) | 186 | INT\_RitC\_C\_like | cd01182 INT\_RitC\_C\_like; C-terminal catalytic domain of recombinase RitC, a component of the recombinase trio. | | ncbi-cd | cd01195 | 99.0 | 4e-14 | 9.5e-18 | 113.2 | 154 | (224, 381) | 403 | (1, 168) | 170 | INT\_C\_like\_5 | cd01195 INT\_C\_like\_5; Uncharacterized site-specific tyrosine recombinase, C-terminal catalytic domain. | | ncbi-cd | cd01186 | 98.9 | 4.5e-14 | 1.1e-17 | 112.4 | 163 | (222, 387) | 403 | (1, 182) | 184 | INT\_tnpA\_C\_Tn554 | cd01186 INT\_tnpA\_C\_Tn554; Putative Transposase A from transposon Tn554, C-terminal catalytic domain. This family includes putative Transposase A from transposon Tn554. | | ncbi-cd | cd00798 | 98.8 | 2e-13 | 4.8e-17 | 107.2 | 155 | (225, 384) | 403 | (1, 170) | 172 | INT\_XerDC\_C | cd00798 INT\_XerDC\_C; XerD and XerC integrases, C-terminal catalytic domains. XerDC-like integrases are involved in the site-specific integration and excision of lysogenic bacteriophage genomes, transposition of conjugative transposons, termination of chromosomal replication, and stable plasmid inheritance. | | ncbi-cd | cd01194 | 98.8 | 2e-13 | 4.8e-17 | 108.1 | 155 | (223, 379) | 403 | (1, 173) | 174 | INT\_C\_like\_4 | cd01194 INT\_C\_like\_4; Uncharacterized site-specific tyrosine recombinase, C-terminal catalytic domain. | | ncbi-cd | cd00800 | 98.8 | 2.7e-13 | 6.5e-17 | 107.6 | 152 | (226, 384) | 403 | (1, 158) | 161 | INT\_Lambda\_C | cd00800 INT\_Lambda\_C; C-terminal catalytic domain of Lambda integrase, a tyrosine-based site-specific recombinase. | | ncbi-cd | cd00799 | 98.8 | 4e-13 | 9.7e-17 | 108.7 | 150 | (238, 389) | 403 | (17, 184) | 188 | INT\_Cre\_C | cd00799 INT\_Cre\_C; C-terminal catalytic domain of Cre recombinase (also called integrase). Cre-like recombinases are tyrosine based site specific recombinases. | | ncbi-cd | cd01185 | 98.7 | 7e-13 | 1.7e-16 | 104.8 | 152 | (226, 384) | 403 | (1, 159) | 161 | INTN1\_C\_like | cd01185 INTN1\_C\_like; Integrase IntN1 of Bacteroides mobilizable transposon NBU1 and similar proteins, C-terminal catalytic domain. | | ncbi-cd | cd01184 | 98.7 | 8.6e-13 | 2.1e-16 | 104.1 | 156 | (223, 379) | 403 | (1, 179) | 180 | INT\_C\_like\_1 | cd01184 INT\_C\_like\_1; Uncharacterized site-specific tyrosine recombinase, C-terminal catalytic domain. | | ncbi-cd | cd01190 | 98.7 | 1.1e-12 | 2.6e-16 | 101.9 | 146 | (224, 378) | 403 | (1, 149) | 150 | INT\_StrepXerD\_C\_like | cd01190 INT\_StrepXerD\_C\_like; Putative XerD in Streptococcus pneumonia and similar proteins, C-terminal catalytic domain. | | ncbi-cd | cd00397 | 98.6 | 5.1e-12 | 1.2e-15 | 98.2 | 139 | (238, 379) | 403 | (18, 167) | 167 | DNA\_BRE\_C | cd00397 DNA\_BRE\_C; DNA breaking-rejoining enzymes, C-terminal catalytic domain. The DNA breaking-rejoining enzyme superfamily includes type IB topoisomerases and tyrosine based site-specific recombinases (integrases) that share the same fold in their catalytic domain containing conserved active site residues. | | ncbi-cd | cd01189 | 97.9 | 1.6e-09 | 3.8e-13 | 82.7 | 131 | (225, 379) | 403 | (1, 146) | 147 | INT\_ICEBs1\_C\_like | cd01189 INT\_ICEBs1\_C\_like; C-terminal catalytic domain of integrases from bacterial phages and conjugate transposons. | | ncbi-cd | cd11602 | 97.8 | 3.3e-09 | 7.8e-13 | 96.9 | 163 | (220, 387) | 403 | (127, 338) | 413 | Ndc10 | cd11602 Ndc10; Ndc10 component of the yeast centromere-binding factor 3. Ndc10 is a multidomain protein conserved in Saccharomycotina that interacts with kinetochore components. | | ncbi-cd | cd01187 | 97.7 | 8.8e-09 | 2.1e-12 | 78.7 | 116 | (239, 379) | 403 | (15, 141) | 142 | INT\_tnpB\_C\_Tn554 | cd01187 INT\_tnpB\_C\_Tn554; Putative Transposase B from transposon Tn554, C-terminal catalytic domain. This family includes putative Transposase B from transposon Tn554. | | cath | 4a8eA02 | 99.2 | 5.9e-16 | 1.1e-19 | 128.4 | 179 | (216, 400) | 403 | (3, 187) | 197 | Probable tyrosine recombinase xerc-like | CATHCODE: 1.10.443.10 NAME: Probable tyrosine recombinase xerc-like. Chain: a. Synonym: xer a. Engineered: yes SOURCE: Pyrococcus abyssi. Organism\_taxid: 29292. Strain: ge5 / orsay. Expressed in: escherichia coli. Expression\_system\_taxid: 469008. CLASS: Mainly Alpha, ARCH: Orthogonal Bundle, TOPOL: hpI Integrase; Chain A, HOMOL: Intergrase catalytic core | | cath | 2a3vA02 | 99.2 | 8.2e-16 | 1.5e-19 | 127.4 | 166 | (215, 384) | 403 | (4, 214) | 217 | Dna (31-mer) | CATHCODE: 1.10.443.10 NAME: Dna (31-mer). Chain: e, g. Engineered: yes. Dna (34-mer). Chain: f, h.Engineered: yes. Site-specific recombinase inti4. Chain: a, b, c, d. Engineered: yes SOURCE: Yes. Other\_details: sythetic construct. Yes. Other\_details: sythetic construct. CLASS: Mainly Alpha, ARCH: Orthogonal Bundle, TOPOL: hpI Integrase; Chain A, HOMOL: Intergrase catalytic core | | cath | 3nkhA00 | 99.2 | 1.2e-15 | 2.1e-19 | 127.8 | 179 | (219, 398) | 403 | (2, 222) | 224 | Integrase | CATHCODE: 1.10.443.10 NAME: Integrase. Chain: a, b. Fragment: residues 187-406. Engineered: yes SOURCE: Staphylococcus aureus subsp. Aureus usa300\_tch1516. Organism\_taxid: 451516. Strain: strain usa300 / tch1516. Gene: usa300hou\_0850. Expressed in: escherichia coli. Expression\_system\_taxid: 511693. CLASS: Mainly Alpha, ARCH: Orthogonal Bundle, TOPOL: hpI Integrase; Chain A, HOMOL: Intergrase catalytic core | | cath | 1f44A01 | 99.1 | 9.3e-15 | 1.7e-18 | 119.7 | 164 | (219, 384) | 403 | (2, 199) | 202 | Dna (5'- d(\*tp\*ap\*tp\*ap\*ap\*cp\*tp\*tp\*cp\*gp\*tp\*ap\*tp\*ap\*gp\*c)-3') | CATHCODE: 1.10.443.10 NAME: Dna (5'- d(\*tp\*ap\*tp\*ap\*ap\*cp\*tp\*tp\*cp\*gp\*tp\*ap\*tp\*ap\*gp\*c)-3'). Chain: m. Engineered: yes. Dna (5'- d(\*ap\*tp\*ap\*tp\*gp\*cp\*tp\*ap\*tp\*ap\*cp\*gp\*ap\*ap\*gp\*tp\*tp\*ap\*t)-3'). Chain: n. Engineered: yes. Cre recombinase. Chain: a. Synonym: recombinase - phage p1. Engineered: yes. Mutation: yes SOURCE: Yes. Yes. CLASS: Mainly Alpha, ARCH: Orthogonal Bundle, TOPOL: hpI Integrase; Chain A, HOMOL: Intergrase catalytic core | | cath | 1ae9A00 | 99.0 | 1.4e-14 | 2.5e-18 | 118.8 | 158 | (220, 384) | 403 | (1, 172) | 179 | Lambda integrase | CATHCODE: 1.10.443.10 NAME: Lambda integrase. Chain: a, b. Fragment: catalytic domain. Engineered:yes. Mutation: yes SOURCE: Enterobacteria phage lambda. Organism\_taxid: 10710. Expressed in: escherichia coli. Expression\_system\_taxid: 562 CLASS: Mainly Alpha, ARCH: Orthogonal Bundle, TOPOL: hpI Integrase; Chain A, HOMOL: Intergrase catalytic core | | cath | 1aihA00 | 98.8 | 4.6e-13 | 8.2e-17 | 108.7 | 154 | (218, 393) | 403 | (2, 162) | 170 | Hp1 integrase | CATHCODE: 1.10.443.10 NAME: Hp1 integrase. Chain: a, b, c, d. Fragment: catalytic domain, residues168 - 337. Engineered: yes SOURCE: Haemophilus phage hp1. Organism\_taxid: 10690. Strain: hp1c1. Cell\_line: haemophilus influenzae l10. Gene: genbank u24159. Expressed in: escherichia coli. Expression\_system\_taxid: 562. CLASS: Mainly Alpha, ARCH: Orthogonal Bundle, TOPOL: hpI Integrase; Chain A, HOMOL: Intergrase catalytic core | | cath | 3uxuA00 | 98.3 | 1.1e-10 | 2e-14 | 93.7 | 140 | (219, 384) | 403 | (3, 148) | 169 | Probable integrase | CATHCODE: 1.10.443.10 NAME: Probable integrase. Chain: a. Fragment: catalytic domain (176-334). Engineered: yes. Mutation: yes SOURCE: Sulfolobus virus 1. Organism\_taxid: 244589. Strain: sulfolobus solfataricus. Gene: d335. Expressed in: escherichia coli. Expression\_system\_taxid: 469008. CLASS: Mainly Alpha, ARCH: Orthogonal Bundle, TOPOL: hpI Integrase; Chain A, HOMOL: Intergrase catalytic core | | cath | 4dwpA02 | 97.9 | 3.8e-09 | 6.1e-13 | 93.9 | 163 | (220, 384) | 403 | (25, 217) | 227 | Protelomerase | CATHCODE: 1.10.443.30 NAME: Protelomerase. Chain: a. Engineered: yes. Dna (5'-d(\*tp\*tp\*ap\*cp\*ap\*ap\*tp\*ap\*ap\*cp\*ap\*ap\*tp\*ap\*t)- 3'). Chain: c. Engineered: yes. Dna (5'- d(\*cp\*ap\*tp\*gp\*ap\*tp\*ap\*tp\*tp\*gp\*tp\*tp\*ap\*tp\*tp\*gp\*tp\*ap\*a)-3'). Chain: d. Engineered: yes SOURCE: Agrobacterium tumefaciens. Organism\_taxid: 176299. Strain: c58 / atcc 33970. Gene: tela, atu2523. Expressed in: escherichia coli. Expression\_system\_taxid: 562. Yes. CLASS: Mainly Alpha, ARCH: Orthogonal Bundle, TOPOL: hpI Integrase; Chain A, HOMOL: Telomere resolvase | | cath | 2v6eA03 | 97.8 | 5.2e-09 | 8.6e-13 | 92.1 | 152 | (225, 382) | 403 | (32, 222) | 225 | Protelemorase | CATHCODE: 1.10.443.30 NAME: Protelemorase. Chain: a, b. Fragment: c-terminally truncated active resolvase, residues 1-538. Synonym: protelomerase telk538. Engineered: yes. Telrl. Chain: c, e. Other\_details: target site for telk. Telrl. Chain: d, f. Other\_details: target site for telk SOURCE: Klebsiella phage phiko2. Organism\_taxid: 255431. Expressed in: escherichia coli. Expression\_system\_taxid: 511693. CLASS: Mainly Alpha, ARCH: Orthogonal Bundle, TOPOL: hpI Integrase; Chain A, HOMOL: Telomere resolvase | | phrogs | 7244 | 100.0 | 2.4e-35 | 2.8e-39 | 264.4 | 222 | (41, 295) | 403 | (17, 250) | 269 | integrase | integrase; Category: integration and excision; p76383 VI\_09148 | | phrogs | 3423 | 99.9 | 1.5e-29 | 1.7e-33 | 221.8 | 102 | (1, 113) | 403 | (1, 109) | 217 | NA | NA; Category: unknown function; p166161 VI\_03799 | | phrogs | 216 | 99.9 | 2.9e-28 | 3.9e-32 | 228.8 | 211 | (168, 395) | 403 | (2, 250) | 254 | integrase | integrase; Category: integration and excision; p381219 VI\_06799 | | phrogs | 1 | 99.8 | 1.7e-23 | 2.4e-27 | 206.0 | 265 | (99, 392) | 403 | (61, 373) | 379 | integrase | integrase; Category: integration and excision; p428256 VI\_04636 | | phrogs | 5127 | 99.4 | 1.6e-17 | 1.9e-21 | 156.4 | 207 | (147, 401) | 403 | (127, 339) | 340 | integrase | integrase; Category: integration and excision; NC\_005361\_p7 | | phrogs | 15424 | 99.4 | 1.8e-17 | 2.1e-21 | 136.5 | 131 | (42, 187) | 403 | (23, 169) | 179 | NA | NA; Category: unknown function; p337988 VI\_03719 | | phrogs | 7685 | 98.5 | 1.3e-11 | 1.6e-15 | 94.8 | 85 | (305, 395) | 403 | (5, 95) | 99 | integrase | integrase; Category: integration and excision; p430610 VI\_04687 | | phrogs | 7269 | 98.0 | 1.7e-09 | 1.9e-13 | 82.5 | 67 | (324, 393) | 403 | (17, 86) | 105 | integrase | integrase; Category: integration and excision; p209079 VI\_03929 | | phrogs | 17497 | 97.9 | 5.4e-09 | 6.2e-13 | 83.7 | 97 | (303, 402) | 403 | (12, 117) | 145 | NA | NA; Category: unknown function; p9119 VI\_09989 | | phrogs | 22184 | 97.6 | 4.2e-08 | 4.8e-12 | 83.9 | 161 | (97, 273) | 403 | (65, 243) | 256 | NA | NA; Category: unknown function; p65886 VI\_01893 | | phrogs | 23271 | 97.2 | 2.9e-07 | 3.3e-11 | 64.0 | 44 | (338, 382) | 403 | (8, 51) | 67 | NA | NA; Category: unknown function; p201349 VI\_04413 | | phrogs | 24775 | 97.0 | 8.4e-07 | 9.5e-11 | 65.7 | 65 | (337, 402) | 403 | (26, 90) | 101 | NA | NA; Category: unknown function; p271771 VI\_09564 | | phrogs | 35951 | 96.5 | 7.5e-06 | 8.3e-10 | 59.3 | 56 | (337, 394) | 403 | (19, 74) | 97 | NA | NA; Category: unknown function; p215618 VI\_07463 | | phrogs | 34557 | 96.5 | 7.7e-06 | 8.5e-10 | 63.9 | 72 | (306, 382) | 403 | (68, 142) | 158 | integrase | integrase; Category: integration and excision; p391360 VI\_04907 | | phrogs | 24633 | 96.0 | 3.6e-05 | 4e-09 | 66.8 | 140 | (237, 380) | 403 | (92, 258) | 367 | NA | NA; Category: unknown function; p434924 VI\_10927 | | phrogs | 37709 | 94.9 | 0.00039 | 4.3e-08 | 51.4 | 83 | (169, 265) | 403 | (20, 103) | 116 | NA | NA; Category: unknown function; p376656 VI\_08697 | | phrogs | 25190 | 94.7 | 0.00055 | 6.1e-08 | 50.0 | 50 | (337, 387) | 403 | (45, 94) | 109 | integrase | integrase; Category: integration and excision; p65883 VI\_01893 | | phrogs | 9208 | 94.4 | 0.00083 | 9.4e-08 | 56.2 | 124 | (238, 382) | 403 | (105, 230) | 241 | integrase | integrase; Category: integration and excision; KX607102\_p66 | |
| Top keywords  (threshold 1.00e-03 (evalue)) | **integrase, catalytic, a, tp, ap, and, recombinase, yes, C\_terminal, Engineered** |
| Output files | ../../domain\_architecture/32\_FANPEZAQ\_CDS\_0032\_cath.hhr ../../domain\_architecture/32\_FANPEZAQ\_CDS\_0032\_merged.svg ../../domain\_architecture/32\_FANPEZAQ\_CDS\_0032\_ncbi-cd.hhr ../../domain\_architecture/32\_FANPEZAQ\_CDS\_0032\_pfam.hhr ../../domain\_architecture/32\_FANPEZAQ\_CDS\_0032\_phrogs.hhr |

### Identical protein sequences/structures

#### Search results

|  |  |
| --- | --- |
| Protein sequence databases searched | Pdb, Swissprot, Refseq |
| Identical proteins found | -- |
| Top keywords | -- |
| Output files | -- |

### Similar protein sequences/structures

#### Sequence similarity search results (HHblits)1

|  |  |
| --- | --- |
| Sequence databases searched | Uniclust, Pdb70 |
| Results, scheme(s)  (Top layers only, threshold 1.00e-03 (evalue)) | xml version="1.0" encoding="utf-8" standalone="no"?       2024-09-02T21:08:42.912040 image/svg+xml   Matplotlib v3.7.2, https://matplotlib.org/ |
| Results, table(s)  (threshold 1.00e-03 (evalue)) | | db | id | prob | evalue | pvalue | score | cols | query | query\_len | template | template\_len | name | description | | --- | --- | --- | --- | --- | --- | --- | --- | --- | --- | --- | --- | --- | | uniclust | UniRef100\_A0A023WX11 | 100.0 | 2.1e-70 | 3.7e-76 | 482.4 | 373 | (1, 402) | 403 | (94, 487) | 563 | Integrase | Integrase | | uniclust | UniRef100\_A0A078KVJ2 | 100.0 | 6.9e-69 | 1.2e-74 | 450.5 | 372 | (1, 402) | 403 | (35, 422) | 486 | Prophage CP4-57 integrase | Prophage CP4-57 integrase | | uniclust | UniRef100\_A0A009HH03 | 100.0 | 1.9e-66 | 3.3e-72 | 450.0 | 374 | (1, 402) | 403 | (70, 458) | 525 | Phage integrase family protein | Phage integrase family protein | | uniclust | UniRef100\_A0A010RTI3 | 100.0 | 3e-66 | 5.2e-72 | 464.3 | 374 | (1, 402) | 403 | (92, 480) | 605 | Integrase | Integrase | | uniclust | UniRef100\_A0A095BCC0 | 100.0 | 1.1e-65 | 2e-71 | 429.1 | 373 | (1, 402) | 403 | (5, 401) | 420 | Tyr recombinase domain-containing protein | Tyr recombinase domain-containing protein | | uniclust | UniRef100\_A0A062VJ35 | 100.0 | 1.9e-65 | 3.3e-71 | 447.7 | 376 | (1, 402) | 403 | (72, 483) | 578 | Integrase family protein | Integrase family protein | | uniclust | UniRef100\_A0A023WM80 | 100.0 | 3.6e-65 | 6.3e-71 | 430.8 | 374 | (1, 402) | 403 | (25, 413) | 477 | Integrase | Integrase | | uniclust | UniRef100\_A0A009ITW1 | 100.0 | 8.3e-65 | 1.5e-70 | 447.3 | 374 | (1, 402) | 403 | (98, 489) | 595 | Phage integrase family protein | Phage integrase family protein | | uniclust | UniRef100\_A0A031JRT9 | 100.0 | 1e-64 | 1.7e-70 | 451.6 | 373 | (1, 402) | 403 | (69, 462) | 531 | Integrase family protein | Integrase family protein | | uniclust | UniRef100\_A0A0F7PI78 | 100.0 | 1.5e-64 | 2.6e-70 | 432.9 | 367 | (1, 402) | 403 | (64, 439) | 472 | Site-specific recombinase XerD | Site-specific recombinase XerD | | uniclust | UniRef100\_A0A009GCG0 | 100.0 | 2.1e-64 | 3.8e-70 | 449.4 | 372 | (1, 401) | 403 | (80, 466) | 655 | Phage integrase family protein | Phage integrase family protein | | uniclust | UniRef100\_A0A3N5UFD5 | 100.0 | 2.3e-64 | 4e-70 | 415.6 | 372 | (1, 402) | 403 | (31, 416) | 439 | DUF4102 domain-containing protein (Fragment) | DUF4102 domain-containing protein (Fragment) | | uniclust | UniRef100\_A0A060HVV6 | 100.0 | 3.9e-64 | 6.8e-70 | 429.9 | 372 | (1, 401) | 403 | (32, 439) | 507 | Phage integrase/recombinase family protein | Phage integrase/recombinase family protein | | uniclust | UniRef100\_A0A009Q4Z9 | 100.0 | 4.3e-64 | 7.5e-70 | 445.6 | 374 | (1, 402) | 403 | (102, 493) | 604 | Phage integrase family protein | Phage integrase family protein | | uniclust | UniRef100\_A0A0J1CXV2 | 100.0 | 8.6e-64 | 1.5e-69 | 412.4 | 373 | (1, 401) | 403 | (20, 409) | 435 | Integrase | Integrase | | uniclust | UniRef100\_A0A010T4C0 | 100.0 | 1.8e-63 | 3.1e-69 | 441.0 | 374 | (1, 402) | 403 | (80, 470) | 595 | Integrase | Integrase | | uniclust | UniRef100\_A0A1I4E0U7 | 100.0 | 2.5e-63 | 4.4e-69 | 425.7 | 377 | (1, 403) | 403 | (67, 460) | 493 | Phage integrase family protein | Phage integrase family protein | | uniclust | UniRef100\_A0A069F866 | 100.0 | 5.1e-63 | 9.1e-69 | 427.2 | 391 | (1, 402) | 403 | (68, 483) | 551 | DUF4102 domain-containing protein | DUF4102 domain-containing protein | | uniclust | UniRef100\_A0A011NNI0 | 100.0 | 6.1e-63 | 1.1e-68 | 426.9 | 374 | (1, 402) | 403 | (54, 448) | 530 | Prophage CP4-57 integrase | Prophage CP4-57 integrase | | uniclust | UniRef100\_A0A095S8W4 | 100.0 | 1.5e-62 | 2.7e-68 | 420.2 | 373 | (1, 401) | 403 | (42, 434) | 517 | DNA integration/recombination/inversion protein | DNA integration/recombination/inversion protein | | uniclust | UniRef100\_A0A011P849 | 100.0 | 2.2e-62 | 3.9e-68 | 427.6 | 366 | (1, 400) | 403 | (58, 437) | 542 | Tyrosine recombinase XerC | Tyrosine recombinase XerC | | uniclust | UniRef100\_A0A1Y6D1R1 | 100.0 | 8.4e-62 | 1.5e-67 | 396.6 | 375 | (1, 402) | 403 | (6, 404) | 422 | Integrase | Integrase | | uniclust | UniRef100\_A0A0A8WLC3 | 100.0 | 1.4e-61 | 2.5e-67 | 411.3 | 364 | (1, 402) | 403 | (53, 440) | 506 | Integrase | Integrase | | uniclust | UniRef100\_A0A095AQU7 | 100.0 | 1.5e-61 | 2.6e-67 | 427.9 | 377 | (1, 403) | 403 | (96, 497) | 559 | Phage integrase | Phage integrase | | uniclust | UniRef100\_A0A086ME39 | 100.0 | 1.7e-61 | 3e-67 | 424.3 | 371 | (1, 402) | 403 | (59, 443) | 524 | Tyr recombinase domain-containing protein | Tyr recombinase domain-containing protein | | uniclust | UniRef100\_A0A0F3QL50 | 100.0 | 1.8e-61 | 3.3e-67 | 405.8 | 363 | (1, 398) | 403 | (24, 396) | 469 | Phage integrase family protein | Phage integrase family protein | | uniclust | UniRef100\_A0A084TAT8 | 100.0 | 2.2e-61 | 4e-67 | 406.5 | 375 | (1, 401) | 403 | (34, 429) | 526 | Integrase | Integrase | | uniclust | UniRef100\_A0A0B0Q1I0 | 100.0 | 2.3e-61 | 4e-67 | 409.6 | 371 | (1, 402) | 403 | (22, 409) | 437 | Integrase | Integrase | | uniclust | UniRef100\_A0A011PLM2 | 100.0 | 5.6e-61 | 9.8e-67 | 413.0 | 365 | (1, 398) | 403 | (41, 415) | 471 | Putative prophage CPS-53 integrase | Putative prophage CPS-53 integrase | | uniclust | UniRef100\_A0A1E5SVX7 | 100.0 | 1.1e-60 | 1.9e-66 | 406.5 | 376 | (1, 402) | 403 | (42, 440) | 502 | Tyr recombinase domain-containing protein | Tyr recombinase domain-containing protein | | uniclust | UniRef100\_A0A0C3I7B0 | 100.0 | 1.8e-60 | 3.3e-66 | 399.4 | 374 | (1, 401) | 403 | (59, 459) | 532 | Tyr recombinase domain-containing protein | Tyr recombinase domain-containing protein | | uniclust | UniRef100\_A0A023WV33 | 100.0 | 1.9e-60 | 3.4e-66 | 408.8 | 388 | (1, 402) | 403 | (41, 441) | 489 | Integrase | Integrase | | uniclust | UniRef100\_A0A0D7NHD3 | 100.0 | 2.5e-60 | 4.5e-66 | 408.9 | 372 | (1, 402) | 403 | (72, 479) | 529 | Tyr recombinase domain-containing protein | Tyr recombinase domain-containing protein | | uniclust | UniRef100\_A0A077N6L9 | 100.0 | 4.2e-60 | 7.5e-66 | 403.6 | 372 | (1, 402) | 403 | (69, 455) | 487 | Phage integrase family protein | Phage integrase family protein | | uniclust | UniRef100\_A0A0A8TPH3 | 100.0 | 4.3e-60 | 7.5e-66 | 399.4 | 356 | (6, 399) | 403 | (26, 393) | 439 | Phage integrase family protein | Phage integrase family protein | | uniclust | UniRef100\_A0A060NVI1 | 100.0 | 4.7e-60 | 8.4e-66 | 407.2 | 374 | (1, 402) | 403 | (62, 453) | 528 | Integrase | Integrase | | uniclust | UniRef100\_A0A084EX67 | 100.0 | 5.2e-60 | 9.3e-66 | 399.5 | 374 | (1, 401) | 403 | (28, 420) | 482 | Prophage CP4-57 integrase | Prophage CP4-57 integrase | | uniclust | UniRef100\_A0A013SVQ0 | 100.0 | 6.7e-60 | 1.2e-65 | 411.2 | 374 | (1, 401) | 403 | (55, 458) | 529 | Phage integrase family protein | Phage integrase family protein | | uniclust | UniRef100\_A0A059DPB2 | 100.0 | 6.9e-60 | 1.2e-65 | 392.8 | 366 | (1, 398) | 403 | (11, 415) | 454 | Tyr recombinase domain-containing protein | Tyr recombinase domain-containing protein | | uniclust | UniRef100\_A0A011PKW5 | 100.0 | 7.2e-60 | 1.3e-65 | 424.5 | 364 | (1, 402) | 403 | (156, 536) | 651 | Tyrosine recombinase XerD | Tyrosine recombinase XerD | | uniclust | UniRef100\_A0A1H3N2F0 | 100.0 | 9.1e-60 | 1.6e-65 | 395.4 | 364 | (2, 398) | 403 | (6, 381) | 421 | Site-specific recombinase XerD | Site-specific recombinase XerD | | uniclust | UniRef100\_A0A0B1JQG0 | 100.0 | 1.1e-59 | 1.9e-65 | 386.0 | 366 | (1, 400) | 403 | (20, 405) | 421 | DUF4102 domain-containing protein | DUF4102 domain-containing protein | | uniclust | UniRef100\_A0A066PM44 | 100.0 | 1.5e-59 | 2.6e-65 | 398.4 | 374 | (1, 402) | 403 | (45, 433) | 450 | Integrase Int | Integrase Int | | uniclust | UniRef100\_A0A0N8TYL7 | 100.0 | 4e-59 | 7.2e-65 | 391.8 | 373 | (1, 399) | 403 | (11, 413) | 458 | Tyrosine recombinase XerD | Tyrosine recombinase XerD | | uniclust | UniRef100\_A0A076LR81 | 100.0 | 4.2e-59 | 7.4e-65 | 402.1 | 375 | (2, 402) | 403 | (90, 497) | 525 | Prophage integrase | Prophage integrase | | uniclust | UniRef100\_A0A009Z8S4 | 100.0 | 5e-59 | 8.9e-65 | 388.2 | 373 | (1, 401) | 403 | (6, 406) | 468 | Phage integrase family protein | Phage integrase family protein | | uniclust | UniRef100\_A0A0C1EFS8 | 100.0 | 6.2e-59 | 1.1e-64 | 393.8 | 360 | (1, 402) | 403 | (23, 389) | 460 | Integrase | Integrase | | uniclust | UniRef100\_A0A010YUZ4 | 100.0 | 6.4e-59 | 1.1e-64 | 412.0 | 364 | (1, 398) | 403 | (65, 469) | 525 | Site-specific recombinase XerD | Site-specific recombinase XerD | | uniclust | UniRef100\_A0A011U6N9 | 100.0 | 9.9e-59 | 1.7e-64 | 409.9 | 365 | (1, 401) | 403 | (116, 512) | 613 | Integrase | Integrase | | uniclust | UniRef100\_A0A0A2YVI4 | 100.0 | 1.8e-58 | 3.2e-64 | 377.5 | 373 | (1, 401) | 403 | (7, 396) | 436 | Preprotein translocase | Preprotein translocase | | uniclust | UniRef100\_A0A1T4VSE3 | 100.0 | 1.8e-58 | 3.2e-64 | 390.3 | 401 | (1, 402) | 403 | (35, 447) | 473 | Phage integrase family protein | Phage integrase family protein | | uniclust | UniRef100\_A0A084EN63 | 100.0 | 1.9e-58 | 3.3e-64 | 397.8 | 373 | (1, 402) | 403 | (65, 468) | 532 | Phage integrase | Phage integrase | | uniclust | UniRef100\_A0A090T816 | 100.0 | 2e-58 | 3.5e-64 | 385.7 | 373 | (1, 401) | 403 | (16, 405) | 431 | Phage integrase | Phage integrase | | uniclust | UniRef100\_A0A1Q8YBY4 | 100.0 | 2.4e-58 | 4.2e-64 | 387.3 | 372 | (1, 402) | 403 | (7, 420) | 468 | Phage integrase domain protein | Phage integrase domain protein | | uniclust | UniRef100\_A0A011PJQ8 | 100.0 | 3e-58 | 5.2e-64 | 407.5 | 373 | (1, 402) | 403 | (104, 525) | 621 | Prophage CP4-57 integrase | Prophage CP4-57 integrase | | uniclust | UniRef100\_A0A0C1H4X8 | 100.0 | 7.1e-58 | 1.3e-63 | 377.3 | 371 | (1, 402) | 403 | (2, 390) | 420 | Integrase | Integrase | | uniclust | UniRef100\_A0A0A2I197 | 100.0 | 8.8e-58 | 1.6e-63 | 370.8 | 373 | (1, 401) | 403 | (7, 395) | 421 | Integrase | Integrase | | uniclust | UniRef100\_A0A009HMB5 | 100.0 | 9.8e-58 | 1.7e-63 | 395.9 | 373 | (1, 401) | 403 | (99, 502) | 587 | Integrase | Integrase | | uniclust | UniRef100\_A0A094ZP55 | 100.0 | 1e-57 | 1.8e-63 | 383.9 | 370 | (1, 402) | 403 | (29, 414) | 444 | Phage integrase family protein | Phage integrase family protein | | uniclust | UniRef100\_A0A011Q0B5 | 100.0 | 1e-57 | 1.8e-63 | 385.6 | 371 | (1, 401) | 403 | (30, 425) | 479 | Prophage CP4-57 integrase | Prophage CP4-57 integrase | | uniclust | UniRef100\_A0A2E3D5G4 | 100.0 | 1.2e-57 | 2.1e-63 | 385.2 | 371 | (2, 402) | 403 | (52, 446) | 463 | Tyr recombinase domain-containing protein | Tyr recombinase domain-containing protein | | uniclust | UniRef100\_A0A062GL24 | 100.0 | 1.7e-57 | 3e-63 | 364.2 | 372 | (3, 402) | 403 | (50, 437) | 457 | Phage integrase family protein | Phage integrase family protein | | uniclust | UniRef100\_A0A075MIC5 | 100.0 | 1.9e-57 | 3.4e-63 | 386.4 | 362 | (1, 401) | 403 | (24, 408) | 451 | Integrase | Integrase | | uniclust | UniRef100\_A0A077NDE6 | 100.0 | 6e-57 | 1.1e-62 | 378.0 | 385 | (1, 402) | 403 | (29, 430) | 468 | Tyr recombinase domain-containing protein | Tyr recombinase domain-containing protein | | uniclust | UniRef100\_A0A0F9AQZ4 | 100.0 | 6.7e-57 | 1.2e-62 | 371.0 | 374 | (1, 402) | 403 | (40, 443) | 451 | Core-binding (CB) domain-containing protein (Fragment) | Core-binding (CB) domain-containing protein (Fragment) | | uniclust | UniRef100\_A0A081RJF2 | 100.0 | 8.8e-57 | 1.6e-62 | 384.3 | 361 | (1, 399) | 403 | (68, 438) | 515 | Phage integrase | Phage integrase | | uniclust | UniRef100\_A0A083USI6 | 100.0 | 1e-56 | 1.8e-62 | 375.7 | 374 | (1, 401) | 403 | (38, 440) | 567 | Integrase | Integrase | | uniclust | UniRef100\_A0A011URX8 | 100.0 | 1.5e-56 | 2.7e-62 | 388.3 | 367 | (1, 402) | 403 | (67, 473) | 582 | Integrase | Integrase | | uniclust | UniRef100\_A0A009GC36 | 100.0 | 1.9e-56 | 3.3e-62 | 386.4 | 372 | (1, 402) | 403 | (54, 451) | 508 | Phage integrase family protein | Phage integrase family protein | | uniclust | UniRef100\_A0A013XU66 | 100.0 | 2e-56 | 3.6e-62 | 381.5 | 365 | (1, 402) | 403 | (37, 414) | 468 | Tyr recombinase domain-containing protein | Tyr recombinase domain-containing protein | | uniclust | UniRef100\_A0A3B9NVS3 | 100.0 | 2.6e-56 | 4.5e-62 | 371.3 | 340 | (1, 402) | 403 | (21, 366) | 386 | Tyr recombinase domain-containing protein | Tyr recombinase domain-containing protein | | uniclust | UniRef100\_A0A1G4SA28 | 100.0 | 5.6e-56 | 1e-61 | 364.6 | 373 | (1, 401) | 403 | (7, 397) | 441 | Integrase | Integrase | | uniclust | UniRef100\_A0A085JZ21 | 100.0 | 5.7e-56 | 1e-61 | 372.4 | 372 | (1, 401) | 403 | (55, 443) | 470 | Integrase (Fragment) | Integrase (Fragment) | | uniclust | UniRef100\_A0A087M879 | 100.0 | 6.6e-56 | 1.2e-61 | 376.8 | 372 | (1, 402) | 403 | (53, 454) | 471 | Tyr recombinase domain-containing protein | Tyr recombinase domain-containing protein | | uniclust | UniRef100\_A0A017HRW7 | 100.0 | 1.3e-55 | 2.4e-61 | 381.8 | 365 | (1, 400) | 403 | (83, 461) | 525 | Site-specific recombinase, phage integrase family | Site-specific recombinase, phage integrase family | | uniclust | UniRef100\_A0A2H5VUU6 | 100.0 | 1.7e-55 | 3.1e-61 | 352.2 | 361 | (1, 396) | 403 | (13, 383) | 396 | Prophage integrase IntA | Prophage integrase IntA | | uniclust | UniRef100\_A0A162TJ54 | 100.0 | 2.4e-55 | 4.4e-61 | 357.2 | 366 | (1, 401) | 403 | (25, 401) | 424 | Integrase | Integrase | | uniclust | UniRef100\_A0A059UX83 | 100.0 | 4.8e-55 | 8.7e-61 | 364.6 | 370 | (1, 401) | 403 | (35, 427) | 476 | Integrase | Integrase | | uniclust | UniRef100\_A0A0B0EHE1 | 100.0 | 6e-55 | 1e-60 | 379.7 | 328 | (33, 398) | 403 | (60, 391) | 471 | Site-specific tyrosine recombinase | Site-specific tyrosine recombinase | | uniclust | UniRef100\_A0A010JLF0 | 100.0 | 6.2e-55 | 1.1e-60 | 385.4 | 368 | (1, 400) | 403 | (81, 470) | 577 | Phage integrase | Phage integrase | | uniclust | UniRef100\_A0A009QLU8 | 100.0 | 6.5e-55 | 1.1e-60 | 382.3 | 374 | (1, 402) | 403 | (93, 481) | 539 | Phage integrase family protein | Phage integrase family protein | | uniclust | UniRef100\_A0A034T1P2 | 100.0 | 1.5e-54 | 2.8e-60 | 347.1 | 373 | (1, 401) | 403 | (2, 389) | 401 | Integrase family protein | Integrase family protein | | uniclust | UniRef100\_A0A2N1AM94 | 100.0 | 1.6e-54 | 2.9e-60 | 347.6 | 371 | (1, 401) | 403 | (7, 395) | 420 | Integrase | Integrase | | uniclust | UniRef100\_A0A132DDL7 | 100.0 | 1.9e-54 | 3.4e-60 | 366.0 | 322 | (44, 396) | 403 | (48, 376) | 425 | Site-specific recombinase XerD | Site-specific recombinase XerD | | uniclust | UniRef100\_A0A011P551 | 100.0 | 3.1e-54 | 5.5e-60 | 381.8 | 367 | (1, 400) | 403 | (110, 499) | 587 | Prophage CP4-57 integrase | Prophage CP4-57 integrase | | uniclust | UniRef100\_A0A0J7XSS8 | 100.0 | 3.5e-54 | 6.2e-60 | 369.3 | 365 | (1, 400) | 403 | (55, 432) | 523 | Integrase | Integrase | | uniclust | UniRef100\_A0A1E5Q3Z9 | 100.0 | 4e-54 | 7e-60 | 363.7 | 357 | (1, 399) | 403 | (13, 380) | 413 | Integrase | Integrase | | uniclust | UniRef100\_A0A068R0Z1 | 100.0 | 4.4e-54 | 7.8e-60 | 361.8 | 369 | (1, 402) | 403 | (50, 440) | 475 | Putative prophage terminase, large subunit | Putative prophage terminase, large subunit | | uniclust | UniRef100\_A0A0L1KDE5 | 100.0 | 5.2e-54 | 9.3e-60 | 363.0 | 372 | (1, 402) | 403 | (35, 437) | 474 | Integrase family protein | Integrase family protein | | uniclust | UniRef100\_A0A0G3XIB2 | 100.0 | 6.9e-54 | 1.3e-59 | 342.1 | 373 | (1, 402) | 403 | (1, 395) | 428 | Integrase | Integrase | | uniclust | UniRef100\_A0A059E4G9 | 100.0 | 1.4e-53 | 2.4e-59 | 369.2 | 367 | (1, 399) | 403 | (74, 463) | 511 | Tyr recombinase domain-containing protein | Tyr recombinase domain-containing protein | | uniclust | UniRef100\_A0A0B4WVT1 | 100.0 | 1.8e-53 | 3.3e-59 | 348.4 | 372 | (1, 402) | 403 | (35, 428) | 433 | Integrase/recombinase domain-containing protein | Integrase/recombinase domain-containing protein | | uniclust | UniRef100\_A0A094YPM0 | 100.0 | 1.9e-53 | 3.4e-59 | 365.7 | 319 | (45, 398) | 403 | (94, 418) | 472 | Site-specific recombinase, phage integrase family | Site-specific recombinase, phage integrase family | | uniclust | UniRef100\_A0A0C1ZFP6 | 100.0 | 2e-53 | 3.5e-59 | 353.9 | 362 | (1, 400) | 403 | (3, 379) | 414 | Integrase | Integrase | | uniclust | UniRef100\_A0A6M0JVR3 | 100.0 | 2.4e-53 | 4.3e-59 | 346.3 | 382 | (1, 403) | 403 | (6, 427) | 462 | Integrase arm-type DNA-binding domain-containing protein | Integrase arm-type DNA-binding domain-containing protein | | uniclust | UniRef100\_A0A0F3GSB0 | 100.0 | 2.9e-53 | 5.2e-59 | 360.6 | 326 | (33, 395) | 403 | (47, 378) | 410 | Phage integrase family protein | Phage integrase family protein | | uniclust | UniRef100\_A0A010RDT3 | 100.0 | 5.9e-53 | 1.1e-58 | 358.6 | 368 | (1, 400) | 403 | (79, 474) | 516 | Integrase | Integrase | | uniclust | UniRef100\_A0A0T5ZW92 | 100.0 | 6.1e-53 | 1.1e-58 | 337.7 | 328 | (44, 398) | 403 | (16, 349) | 367 | Putative phage integrase | Putative phage integrase | | uniclust | UniRef100\_A0A060VRP1 | 100.0 | 6.7e-53 | 1.2e-58 | 351.8 | 373 | (1, 401) | 403 | (33, 436) | 473 | Phage integrase family site-specific recombinase | Phage integrase family site-specific recombinase | | uniclust | UniRef100\_A0A0M9TXC0 | 100.0 | 9e-53 | 1.6e-58 | 343.1 | 367 | (1, 402) | 403 | (5, 398) | 422 | Site-specific recombinase, phage integrase family | Site-specific recombinase, phage integrase family | | uniclust | UniRef100\_A0A037ZN86 | 100.0 | 9.6e-53 | 1.7e-58 | 354.4 | 371 | (1, 402) | 403 | (23, 404) | 462 | Integrase | Integrase | | uniclust | UniRef100\_A0A068QUH7 | 100.0 | 1.1e-52 | 2e-58 | 356.3 | 370 | (1, 402) | 403 | (78, 465) | 544 | Integrase family protein | Integrase family protein | | uniclust | UniRef100\_A0A0B5KFG7 | 100.0 | 1.6e-52 | 2.9e-58 | 342.9 | 369 | (1, 397) | 403 | (31, 416) | 433 | Integrase | Integrase | | uniclust | UniRef100\_A0A1G7GLR9 | 100.0 | 1.7e-52 | 3e-58 | 348.1 | 377 | (2, 402) | 403 | (16, 449) | 466 | Site-specific recombinase XerD | Site-specific recombinase XerD | | uniclust | UniRef100\_A0A078KST6 | 100.0 | 1.9e-52 | 3.3e-58 | 342.8 | 376 | (1, 402) | 403 | (6, 394) | 426 | Integrase | Integrase | | uniclust | UniRef100\_A0A0E4BJQ1 | 100.0 | 1.9e-52 | 3.4e-58 | 341.0 | 361 | (1, 402) | 403 | (18, 393) | 415 | Site-specific integrase/recombinase | Site-specific integrase/recombinase | | uniclust | UniRef100\_A0A017HLQ6 | 100.0 | 2.1e-52 | 3.7e-58 | 344.1 | 362 | (1, 402) | 403 | (39, 408) | 427 | Putative phage-like integrase | Putative phage-like integrase | | uniclust | UniRef100\_A0A0M4T696 | 100.0 | 2.6e-52 | 4.7e-58 | 356.1 | 376 | (2, 402) | 403 | (65, 465) | 510 | Integrase | Integrase | | uniclust | UniRef100\_A0A1V0GPP3 | 100.0 | 7.9e-52 | 1.4e-57 | 349.7 | 374 | (1, 401) | 403 | (7, 394) | 445 | DUF4102 domain-containing protein | DUF4102 domain-containing protein | | uniclust | UniRef100\_A0A061JXB1 | 100.0 | 8e-52 | 1.4e-57 | 349.6 | 371 | (1, 401) | 403 | (77, 474) | 511 | DUF4102 domain-containing protein | DUF4102 domain-containing protein | | uniclust | UniRef100\_A0A1H1GCP0 | 100.0 | 9.1e-52 | 1.6e-57 | 339.1 | 376 | (1, 401) | 403 | (38, 451) | 462 | Integrase | Integrase | | uniclust | UniRef100\_A0A010INU5 | 100.0 | 9.9e-52 | 1.8e-57 | 345.4 | 373 | (1, 402) | 403 | (37, 454) | 479 | Phage integrase family protein | Phage integrase family protein | | uniclust | UniRef100\_A0A010RYA1 | 100.0 | 1.3e-51 | 2.3e-57 | 355.7 | 375 | (1, 401) | 403 | (85, 492) | 744 | Integrase | Integrase | | uniclust | UniRef100\_A0A0Q3PQJ9 | 100.0 | 1.5e-51 | 2.7e-57 | 351.1 | 371 | (1, 402) | 403 | (57, 442) | 469 | Tyr recombinase domain-containing protein | Tyr recombinase domain-containing protein | | uniclust | UniRef100\_A0A1V4AHU6 | 100.0 | 1.7e-51 | 3e-57 | 343.4 | 371 | (1, 400) | 403 | (11, 414) | 433 | Integrase | Integrase | | uniclust | UniRef100\_A0A0F9J930 | 100.0 | 2e-51 | 3.5e-57 | 342.5 | 325 | (43, 398) | 403 | (38, 367) | 419 | Tyr recombinase domain-containing protein | Tyr recombinase domain-containing protein | | uniclust | UniRef100\_A0A0F9K6W3 | 100.0 | 2e-51 | 3.6e-57 | 345.3 | 322 | (46, 397) | 403 | (42, 382) | 415 | Tyr recombinase domain-containing protein | Tyr recombinase domain-containing protein | | uniclust | UniRef100\_A0A058ZJX6 | 100.0 | 2.3e-51 | 4.1e-57 | 337.5 | 352 | (18, 400) | 403 | (46, 420) | 456 | Integrase family protein | Integrase family protein | | uniclust | UniRef100\_A0A1L8CP99 | 100.0 | 2.4e-51 | 4.3e-57 | 351.0 | 365 | (1, 401) | 403 | (20, 398) | 490 | Uncharacterized protein | Uncharacterized protein | | uniclust | UniRef100\_A0A097EN29 | 100.0 | 2.8e-51 | 4.9e-57 | 341.0 | 369 | (1, 402) | 403 | (7, 403) | 459 | Tyr recombinase domain-containing protein | Tyr recombinase domain-containing protein | | uniclust | UniRef100\_A0A077NQI5 | 100.0 | 3.6e-51 | 6.5e-57 | 332.5 | 373 | (1, 402) | 403 | (2, 391) | 407 | Integrase family protein | Integrase family protein | | uniclust | UniRef100\_A0A0D0LPS2 | 100.0 | 4.4e-51 | 7.9e-57 | 336.9 | 360 | (2, 395) | 403 | (40, 406) | 451 | Tyr recombinase domain-containing protein | Tyr recombinase domain-containing protein | | uniclust | UniRef100\_A0A013XV28 | 100.0 | 4.8e-51 | 8.5e-57 | 344.5 | 379 | (1, 401) | 403 | (43, 442) | 473 | Integrase | Integrase | | uniclust | UniRef100\_A0A1I9YDF8 | 100.0 | 5.2e-51 | 9.2e-57 | 339.1 | 362 | (3, 395) | 403 | (9, 418) | 434 | Site-specific integrase | Site-specific integrase | | uniclust | UniRef100\_A0A101VET0 | 100.0 | 7.7e-51 | 1.4e-56 | 338.8 | 366 | (1, 402) | 403 | (12, 418) | 484 | Tyr recombinase domain-containing protein | Tyr recombinase domain-containing protein | | uniclust | UniRef100\_A0A084TFE9 | 100.0 | 7.9e-51 | 1.4e-56 | 333.3 | 367 | (1, 401) | 403 | (31, 411) | 425 | Tyr recombinase domain-containing protein | Tyr recombinase domain-containing protein | | uniclust | UniRef100\_A0A1Y5D4R3 | 100.0 | 8.4e-51 | 1.5e-56 | 338.6 | 369 | (1, 402) | 403 | (23, 410) | 470 | Tyr recombinase domain-containing protein | Tyr recombinase domain-containing protein | | uniclust | UniRef100\_A0A0F9DQS8 | 100.0 | 9.4e-51 | 1.6e-56 | 355.4 | 330 | (43, 398) | 403 | (40, 399) | 495 | Tyr recombinase domain-containing protein | Tyr recombinase domain-containing protein | | uniclust | UniRef100\_A0A090RAS3 | 100.0 | 9.6e-51 | 1.7e-56 | 329.9 | 371 | (2, 401) | 403 | (10, 409) | 454 | Tyr recombinase domain-containing protein | Tyr recombinase domain-containing protein | | uniclust | UniRef100\_A0A069PA44 | 100.0 | 1.1e-50 | 2e-56 | 349.1 | 378 | (1, 402) | 403 | (60, 516) | 572 | Integrase | Integrase | | uniclust | UniRef100\_A0A0R2SAA9 | 100.0 | 1.2e-50 | 2.2e-56 | 326.8 | 362 | (1, 394) | 403 | (5, 377) | 385 | Tyr recombinase domain-containing protein | Tyr recombinase domain-containing protein | | uniclust | UniRef100\_A0A1H8KG10 | 100.0 | 1.3e-50 | 2.4e-56 | 329.0 | 374 | (1, 401) | 403 | (4, 395) | 453 | Integrase | Integrase | | uniclust | UniRef100\_A0A1M5SXE7 | 100.0 | 1.5e-50 | 2.7e-56 | 318.8 | 376 | (2, 402) | 403 | (7, 406) | 422 | Site-specific recombinase XerD | Site-specific recombinase XerD | | uniclust | UniRef100\_A0A0H2V028 | 100.0 | 1.7e-50 | 3e-56 | 327.0 | 370 | (1, 402) | 403 | (7, 391) | 409 | Putative integrase of prophage CP-933C | Putative integrase of prophage CP-933C | | uniclust | UniRef100\_A0A011SXD2 | 100.0 | 2.5e-50 | 4.4e-56 | 348.2 | 363 | (1, 400) | 403 | (61, 440) | 517 | DUF4102 domain-containing protein | DUF4102 domain-containing protein | | uniclust | UniRef100\_A0A1R3WVC8 | 100.0 | 2.8e-50 | 5e-56 | 328.9 | 361 | (1, 398) | 403 | (17, 393) | 408 | Integrase | Integrase | | uniclust | UniRef100\_A0A0A2WI49 | 100.0 | 3.6e-50 | 6.3e-56 | 349.5 | 321 | (43, 395) | 403 | (38, 368) | 432 | Integrase | Integrase | | uniclust | UniRef100\_A0A0S7XRQ7 | 100.0 | 4e-50 | 7.2e-56 | 334.4 | 334 | (30, 396) | 403 | (100, 439) | 475 | Tyr recombinase domain-containing protein (Fragment) | Tyr recombinase domain-containing protein (Fragment) | | uniclust | UniRef100\_A0A068Z6C2 | 100.0 | 4.5e-50 | 8.1e-56 | 324.5 | 370 | (2, 402) | 403 | (3, 386) | 399 | Site-specific integrase | Site-specific integrase | | uniclust | UniRef100\_A0A060UZS8 | 100.0 | 4.8e-50 | 8.5e-56 | 347.0 | 322 | (43, 398) | 403 | (67, 406) | 493 | Integrase | Integrase | | uniclust | UniRef100\_A0A076HE32 | 100.0 | 5e-50 | 9e-56 | 329.7 | 378 | (1, 400) | 403 | (22, 437) | 445 | Tyr recombinase domain-containing protein | Tyr recombinase domain-containing protein | | uniclust | UniRef100\_A0A061P7F4 | 100.0 | 5.7e-50 | 9.9e-56 | 352.0 | 326 | (46, 399) | 403 | (51, 409) | 494 | Integrase | Integrase | | uniclust | UniRef100\_A0A0D7E4E3 | 100.0 | 6.4e-50 | 1.1e-55 | 339.2 | 367 | (1, 400) | 403 | (46, 435) | 451 | Integrase DNA-binding domain-containing protein (Fragment) | Integrase DNA-binding domain-containing protein (Fragment) | | uniclust | UniRef100\_A0A059G1M6 | 100.0 | 6.7e-50 | 1.2e-55 | 318.9 | 374 | (1, 402) | 403 | (21, 416) | 436 | Integrase family protein (Fragment) | Integrase family protein (Fragment) | | uniclust | UniRef100\_A0A2K4WBA5 | 100.0 | 7.4e-50 | 1.3e-55 | 316.7 | 367 | (1, 400) | 403 | (2, 398) | 401 | Integrase | Integrase | | uniclust | UniRef100\_A0A1E8FE80 | 100.0 | 8.8e-50 | 1.6e-55 | 329.7 | 364 | (1, 394) | 403 | (59, 441) | 483 | Integrase | Integrase | | uniclust | UniRef100\_A0A0H3LRV4 | 100.0 | 9.7e-50 | 1.7e-55 | 333.4 | 377 | (1, 401) | 403 | (27, 430) | 455 | Phage-related integrase | Phage-related integrase | | uniclust | UniRef100\_A0A0D1EAI8 | 100.0 | 1.4e-49 | 2.4e-55 | 334.0 | 352 | (19, 400) | 403 | (30, 403) | 434 | IntA protein | IntA protein | | uniclust | UniRef100\_A0A0Q5CV90 | 100.0 | 2e-49 | 3.6e-55 | 332.6 | 378 | (1, 402) | 403 | (30, 488) | 522 | Integrase | Integrase | | uniclust | UniRef100\_A0A2R3F0U9 | 100.0 | 2.3e-49 | 4.1e-55 | 320.7 | 390 | (1, 401) | 403 | (47, 465) | 480 | Integrase | Integrase | | uniclust | UniRef100\_A0A0B0EHF7 | 100.0 | 2.4e-49 | 4.2e-55 | 329.5 | 319 | (45, 395) | 403 | (11, 333) | 389 | Phage integrase | Phage integrase | | uniclust | UniRef100\_A0A009HKV8 | 100.0 | 2.5e-49 | 4.4e-55 | 323.9 | 370 | (1, 401) | 403 | (2, 398) | 417 | Phage integrase family protein | Phage integrase family protein | | uniclust | UniRef100\_A0A0P9N4F7 | 100.0 | 5.2e-49 | 9.5e-55 | 311.5 | 372 | (1, 401) | 403 | (21, 406) | 415 | Integrase (Fragment) | Integrase (Fragment) | | uniclust | UniRef100\_A0A0G0N6A5 | 100.0 | 5.5e-49 | 9.9e-55 | 309.9 | 322 | (45, 398) | 403 | (8, 338) | 351 | Site-specific tyrosine recombinase | Site-specific tyrosine recombinase | | uniclust | UniRef100\_A0A191UGE2 | 100.0 | 5.8e-49 | 1.1e-54 | 320.3 | 373 | (1, 400) | 403 | (36, 430) | 450 | Integrase | Integrase | | uniclust | UniRef100\_A0A0D6IMZ0 | 100.0 | 6.2e-49 | 1.1e-54 | 309.6 | 370 | (2, 401) | 403 | (10, 401) | 420 | Prophage CP4-57 integrase | Prophage CP4-57 integrase | | uniclust | UniRef100\_A0A0P1E854 | 100.0 | 7.1e-49 | 1.3e-54 | 332.6 | 379 | (2, 401) | 403 | (86, 477) | 495 | Prophage CP4-57 integrase | Prophage CP4-57 integrase | | uniclust | UniRef100\_A0A345RBQ0 | 100.0 | 7.2e-49 | 1.3e-54 | 326.2 | 367 | (2, 402) | 403 | (37, 425) | 441 | Tyr recombinase domain-containing protein | Tyr recombinase domain-containing protein | | uniclust | UniRef100\_A0A1M3BHB6 | 100.0 | 7.7e-49 | 1.4e-54 | 331.8 | 326 | (28, 382) | 403 | (66, 421) | 477 | Site-specific integrase | Site-specific integrase | | uniclust | UniRef100\_A0A0T6BPG3 | 100.0 | 8.8e-49 | 1.5e-54 | 335.5 | 329 | (44, 400) | 403 | (34, 402) | 434 | Integrase | Integrase | | uniclust | UniRef100\_A0A0B3S3P6 | 100.0 | 9.4e-49 | 1.7e-54 | 337.2 | 366 | (1, 400) | 403 | (57, 441) | 479 | Putative integrase | Putative integrase | | uniclust | UniRef100\_A0A016QR91 | 100.0 | 1.1e-48 | 1.9e-54 | 355.0 | 330 | (43, 400) | 403 | (111, 474) | 581 | Site-specific integrase | Site-specific integrase | | uniclust | UniRef100\_A0A3M1JS39 | 100.0 | 1.2e-48 | 2.1e-54 | 304.6 | 367 | (1, 402) | 403 | (11, 389) | 391 | Site-specific integrase | Site-specific integrase | | uniclust | UniRef100\_A0A0M2U8J5 | 100.0 | 1.4e-48 | 2.5e-54 | 330.8 | 328 | (43, 399) | 403 | (62, 424) | 465 | Integrase | Integrase | | uniclust | UniRef100\_A0A068YXB4 | 100.0 | 1.5e-48 | 2.7e-54 | 338.8 | 373 | (1, 401) | 403 | (76, 493) | 585 | Site-specific integrase | Site-specific integrase | | uniclust | UniRef100\_A0A0B5FFN2 | 100.0 | 1.6e-48 | 2.8e-54 | 340.1 | 344 | (30, 398) | 403 | (32, 411) | 452 | Tyr recombinase domain-containing protein | Tyr recombinase domain-containing protein | | uniclust | UniRef100\_A0A0K1UCX2 | 100.0 | 1.6e-48 | 2.8e-54 | 324.3 | 376 | (1, 402) | 403 | (8, 443) | 460 | Integrase | Integrase | | uniclust | UniRef100\_A0A0A1MDV4 | 100.0 | 1.8e-48 | 3.1e-54 | 328.9 | 327 | (44, 399) | 403 | (25, 388) | 416 | Transposase from transposon Tn916 | Transposase from transposon Tn916 | | uniclust | UniRef100\_A0A0N1C1K5 | 100.0 | 1.7e-48 | 3.1e-54 | 317.5 | 372 | (1, 402) | 403 | (19, 421) | 428 | Tyr recombinase domain-containing protein | Tyr recombinase domain-containing protein | | uniclust | UniRef100\_A0A0S8KDU2 | 100.0 | 2.1e-48 | 3.8e-54 | 326.8 | 319 | (46, 398) | 403 | (50, 383) | 410 | Integrase | Integrase | | uniclust | UniRef100\_A0A136KDK1 | 100.0 | 2.5e-48 | 4.5e-54 | 326.4 | 324 | (46, 398) | 403 | (47, 393) | 424 | Putative integrase | Putative integrase | | uniclust | UniRef100\_A0A0J6ZLF5 | 100.0 | 2.6e-48 | 4.5e-54 | 340.9 | 328 | (43, 400) | 403 | (57, 416) | 475 | Integrase | Integrase | | uniclust | UniRef100\_A0A0E9MN93 | 100.0 | 2.7e-48 | 4.8e-54 | 317.4 | 368 | (1, 402) | 403 | (3, 383) | 395 | Putative recombinase | Putative recombinase | | uniclust | UniRef100\_A0A179D3R0 | 100.0 | 3.3e-48 | 5.8e-54 | 317.9 | 307 | (43, 381) | 403 | (32, 348) | 385 | Phage integrase | Phage integrase | | uniclust | UniRef100\_A0A096BD50 | 100.0 | 4.1e-48 | 7.2e-54 | 338.7 | 326 | (44, 399) | 403 | (86, 453) | 498 | Tyr recombinase domain-containing protein | Tyr recombinase domain-containing protein | | uniclust | UniRef100\_A0A0F2QSG4 | 100.0 | 4.5e-48 | 7.9e-54 | 324.0 | 339 | (31, 397) | 403 | (24, 391) | 435 | Integrase | Integrase | | uniclust | UniRef100\_A0A0S4KXJ1 | 100.0 | 5.1e-48 | 9e-54 | 318.8 | 320 | (44, 400) | 403 | (31, 360) | 399 | Putative Integrase family protein | Putative Integrase family protein | | uniclust | UniRef100\_A0A0S2JJG5 | 100.0 | 5.5e-48 | 9.6e-54 | 324.1 | 355 | (2, 395) | 403 | (11, 379) | 398 | Integrase | Integrase | | uniclust | UniRef100\_A0A0F8XP09 | 100.0 | 6e-48 | 1.1e-53 | 333.6 | 331 | (43, 398) | 403 | (123, 480) | 515 | Tyr recombinase domain-containing protein (Fragment) | Tyr recombinase domain-containing protein (Fragment) | | uniclust | UniRef100\_A0A061NSW1 | 100.0 | 6.3e-48 | 1.1e-53 | 340.8 | 328 | (46, 401) | 403 | (65, 427) | 478 | Integrase | Integrase | | uniclust | UniRef100\_A0A0C2YL66 | 100.0 | 6.7e-48 | 1.2e-53 | 321.4 | 367 | (1, 401) | 403 | (5, 409) | 440 | Integrase | Integrase | | uniclust | UniRef100\_A0A2M6ZPB3 | 100.0 | 7.1e-48 | 1.3e-53 | 320.4 | 324 | (44, 399) | 403 | (60, 396) | 414 | Site-specific integrase | Site-specific integrase | | uniclust | UniRef100\_A0A285ZA31 | 100.0 | 7.6e-48 | 1.3e-53 | 313.4 | 323 | (61, 402) | 403 | (3, 341) | 359 | Integrase | Integrase | | uniclust | UniRef100\_A0A083UQ90 | 100.0 | 7.7e-48 | 1.4e-53 | 315.4 | 336 | (15, 393) | 403 | (24, 372) | 388 | Site-specific integrase | Site-specific integrase | | uniclust | UniRef100\_A0A0A1YGP1 | 100.0 | 8.1e-48 | 1.4e-53 | 323.4 | 362 | (1, 402) | 403 | (20, 397) | 434 | Integrase | Integrase | | uniclust | UniRef100\_A0A7X4ILM6 | 100.0 | 1e-47 | 1.8e-53 | 317.9 | 350 | (16, 401) | 403 | (59, 416) | 433 | Tyrosine-type recombinase/integrase (Fragment) | Tyrosine-type recombinase/integrase (Fragment) | | uniclust | UniRef100\_A0A1S1V944 | 100.0 | 1.8e-47 | 3.2e-53 | 310.8 | 328 | (44, 399) | 403 | (19, 374) | 377 | Transposase | Transposase | | uniclust | UniRef100\_A0A3C1G4V3 | 100.0 | 1.9e-47 | 3.4e-53 | 308.1 | 325 | (45, 399) | 403 | (15, 360) | 385 | Site-specific integrase | Site-specific integrase | | uniclust | UniRef100\_A0A0N0U8F3 | 100.0 | 2.5e-47 | 4.3e-53 | 326.9 | 327 | (43, 398) | 403 | (36, 394) | 420 | Putative prophage phiRv2 integrase | Putative prophage phiRv2 integrase | | uniclust | UniRef100\_A0A0Q4X9F3 | 100.0 | 2.5e-47 | 4.5e-53 | 308.1 | 362 | (1, 398) | 403 | (10, 410) | 428 | Integrase | Integrase | | uniclust | UniRef100\_A0A1C7HF54 | 100.0 | 3.5e-47 | 6.2e-53 | 327.5 | 372 | (2, 401) | 403 | (35, 466) | 486 | Core-binding (CB) domain-containing protein | Core-binding (CB) domain-containing protein | | uniclust | UniRef100\_A0A017TBT8 | 100.0 | 3.9e-47 | 6.8e-53 | 328.9 | 337 | (32, 395) | 403 | (41, 393) | 461 | Integrase | Integrase | | uniclust | UniRef100\_A0A098RMQ0 | 100.0 | 5e-47 | 8.8e-53 | 331.1 | 330 | (43, 400) | 403 | (91, 461) | 505 | Integrase | Integrase | | uniclust | UniRef100\_A0A066RIY1 | 100.0 | 5.2e-47 | 9.3e-53 | 311.4 | 368 | (1, 400) | 403 | (19, 412) | 435 | Tyr recombinase domain-containing protein | Tyr recombinase domain-containing protein | | uniclust | UniRef100\_A0A089WLN7 | 100.0 | 6.3e-47 | 1.1e-52 | 314.0 | 316 | (43, 397) | 403 | (19, 353) | 406 | Recombinase XerD | Recombinase XerD | | uniclust | UniRef100\_A0A1G7YW28 | 100.0 | 7.5e-47 | 1.3e-52 | 314.7 | 326 | (44, 399) | 403 | (19, 377) | 426 | Phage integrase family protein | Phage integrase family protein | | uniclust | UniRef100\_A0A0F5RAY4 | 100.0 | 8.2e-47 | 1.4e-52 | 320.1 | 323 | (45, 397) | 403 | (31, 386) | 418 | Integrase | Integrase | | uniclust | UniRef100\_A0A023X4M9 | 100.0 | 8.3e-47 | 1.4e-52 | 341.8 | 326 | (45, 399) | 403 | (90, 451) | 615 | Phage integrase family | Phage integrase family | | uniclust | UniRef100\_A0A095TK40 | 100.0 | 8.6e-47 | 1.5e-52 | 325.6 | 368 | (1, 397) | 403 | (24, 452) | 482 | Phage integrase | Phage integrase | | uniclust | UniRef100\_A0A072N9D5 | 100.0 | 8.6e-47 | 1.5e-52 | 321.0 | 333 | (35, 398) | 403 | (20, 382) | 430 | Integrase | Integrase | | uniclust | UniRef100\_A0A1F5UR10 | 100.0 | 9.7e-47 | 1.7e-52 | 309.9 | 329 | (45, 399) | 403 | (17, 361) | 367 | Tyr recombinase domain-containing protein | Tyr recombinase domain-containing protein | | uniclust | UniRef100\_A0A0S2W7S6 | 100.0 | 9.8e-47 | 1.7e-52 | 321.1 | 326 | (45, 399) | 403 | (76, 436) | 523 | Prophage LambdaBa04, site-specific recombinase, phage integrase family | Prophage LambdaBa04, site-specific recombinase, phage integrase family | | uniclust | UniRef100\_A0A382NU96 | 100.0 | 9.8e-47 | 1.8e-52 | 289.8 | 271 | (1, 297) | 403 | (5, 287) | 288 | Core-binding (CB) domain-containing protein (Fragment) | Core-binding (CB) domain-containing protein (Fragment) | | uniclust | UniRef100\_A0A2D7A771 | 100.0 | 1.1e-46 | 1.9e-52 | 306.7 | 288 | (1, 313) | 403 | (17, 311) | 332 | Integrase | Integrase | | uniclust | UniRef100\_A0A1C3E882 | 100.0 | 1.2e-46 | 2.1e-52 | 305.1 | 371 | (2, 401) | 403 | (9, 405) | 417 | Tyr recombinase domain-containing protein | Tyr recombinase domain-containing protein | | uniclust | UniRef100\_A0A6L4B4K9 | 100.0 | 1.2e-46 | 2.3e-52 | 306.6 | 335 | (31, 396) | 403 | (104, 450) | 469 | Tyrosine-type recombinase/integrase | Tyrosine-type recombinase/integrase | | uniclust | UniRef100\_A0A0A0FAJ7 | 100.0 | 1.4e-46 | 2.5e-52 | 304.4 | 349 | (2, 382) | 403 | (95, 458) | 479 | Uncharacterized protein | Uncharacterized protein | | uniclust | UniRef100\_A0A3S3C496 | 100.0 | 1.4e-46 | 2.5e-52 | 307.4 | 369 | (2, 402) | 403 | (35, 416) | 444 | Site-specific integrase | Site-specific integrase | | uniclust | UniRef100\_A0A024PA33 | 100.0 | 1.6e-46 | 2.8e-52 | 330.1 | 338 | (33, 400) | 403 | (52, 441) | 494 | Tyrosine recombinase XerC | Tyrosine recombinase XerC | | uniclust | UniRef100\_A0A064AD33 | 100.0 | 1.7e-46 | 2.9e-52 | 331.1 | 329 | (43, 400) | 403 | (78, 452) | 528 | Integrase | Integrase | | uniclust | UniRef100\_A0A096AZ84 | 100.0 | 1.7e-46 | 3e-52 | 313.0 | 324 | (44, 400) | 403 | (32, 382) | 414 | Tyr recombinase domain-containing protein | Tyr recombinase domain-containing protein | | uniclust | UniRef100\_A0A0J9BJZ1 | 100.0 | 2e-46 | 3.5e-52 | 327.0 | 327 | (44, 400) | 403 | (60, 429) | 465 | Tyr recombinase domain-containing protein | Tyr recombinase domain-containing protein | | uniclust | UniRef100\_A0A072NST7 | 100.0 | 2e-46 | 3.6e-52 | 322.6 | 343 | (26, 400) | 403 | (64, 458) | 482 | Site-specific recombinase XerD | Site-specific recombinase XerD | | uniclust | UniRef100\_A0A0J9D0K7 | 100.0 | 2.3e-46 | 4.1e-52 | 309.2 | 351 | (30, 403) | 403 | (38, 431) | 444 | Tyrosine-type recombinase/integrase | Tyrosine-type recombinase/integrase | | uniclust | UniRef100\_A0A1Q7BDQ6 | 100.0 | 2.3e-46 | 4.2e-52 | 296.9 | 282 | (100, 402) | 403 | (25, 313) | 332 | Tyr recombinase domain-containing protein | Tyr recombinase domain-containing protein | | uniclust | UniRef100\_A0A0D7QFG1 | 100.0 | 2.5e-46 | 4.4e-52 | 304.5 | 370 | (1, 402) | 403 | (2, 424) | 437 | Tyr recombinase domain-containing protein | Tyr recombinase domain-containing protein | | uniclust | UniRef100\_A0A0A0FSB0 | 100.0 | 2.6e-46 | 4.6e-52 | 304.3 | 342 | (2, 382) | 403 | (49, 398) | 440 | Uncharacterized protein | Uncharacterized protein | | uniclust | UniRef100\_A0A0F3M6B8 | 100.0 | 2.7e-46 | 4.9e-52 | 296.7 | 364 | (1, 398) | 403 | (9, 395) | 408 | Integrase DNA-binding domain-containing protein | Integrase DNA-binding domain-containing protein | | uniclust | UniRef100\_A0A0B5NC73 | 100.0 | 2.9e-46 | 5e-52 | 327.1 | 325 | (46, 399) | 403 | (78, 435) | 478 | Site-specific integrase | Site-specific integrase | | uniclust | UniRef100\_A0A098BVB0 | 100.0 | 2.9e-46 | 5.1e-52 | 322.9 | 326 | (43, 398) | 403 | (55, 402) | 470 | Putative Site-specific recombinase XerD | Putative Site-specific recombinase XerD | | uniclust | UniRef100\_A0A1F3SLV7 | 100.0 | 3e-46 | 5.3e-52 | 315.8 | 305 | (45, 382) | 403 | (49, 373) | 434 | Tyr recombinase domain-containing protein | Tyr recombinase domain-containing protein | | uniclust | UniRef100\_A0A0H4IW46 | 100.0 | 3.1e-46 | 5.5e-52 | 302.4 | 376 | (1, 400) | 403 | (6, 410) | 444 | Integrase | Integrase | | uniclust | UniRef100\_A0A087QF00 | 100.0 | 4.3e-46 | 7.5e-52 | 318.6 | 331 | (43, 400) | 403 | (55, 426) | 467 | Toxin-antitoxin system, toxin component, PIN family | Toxin-antitoxin system, toxin component, PIN family | | uniclust | UniRef100\_A0A0A2WPT7 | 100.0 | 5.1e-46 | 9e-52 | 310.5 | 313 | (56, 396) | 403 | (14, 354) | 408 | Integrase | Integrase | | uniclust | UniRef100\_A0A0B7IVU8 | 100.0 | 5.8e-46 | 1e-51 | 306.5 | 368 | (2, 402) | 403 | (21, 401) | 418 | Tyr recombinase domain-containing protein | Tyr recombinase domain-containing protein | | uniclust | UniRef100\_A0A142WZZ4 | 100.0 | 6.3e-46 | 1.1e-51 | 316.4 | 323 | (44, 398) | 403 | (44, 391) | 436 | Putative prophage phiRv2 integrase | Putative prophage phiRv2 integrase | | uniclust | UniRef100\_A0A0D8IVH4 | 100.0 | 6.6e-46 | 1.2e-51 | 313.0 | 325 | (44, 398) | 403 | (32, 392) | 442 | Integrase | Integrase | | uniclust | UniRef100\_A0A1V0DCV2 | 100.0 | 9.5e-46 | 1.7e-51 | 313.3 | 320 | (44, 391) | 403 | (31, 388) | 407 | Tyr recombinase domain-containing protein | Tyr recombinase domain-containing protein | | uniclust | UniRef100\_A0A1X7BYL9 | 100.0 | 1.1e-45 | 2e-51 | 295.1 | 316 | (58, 401) | 403 | (10, 340) | 364 | Tyrosine recombinase XerD | Tyrosine recombinase XerD | | uniclust | UniRef100\_A0A024EK23 | 100.0 | 1.6e-45 | 2.8e-51 | 294.3 | 373 | (1, 401) | 403 | (8, 410) | 439 | Tyr recombinase domain-containing protein | Tyr recombinase domain-containing protein | | uniclust | UniRef100\_A0A059NZ30 | 100.0 | 1.6e-45 | 2.8e-51 | 322.2 | 333 | (43, 402) | 403 | (81, 461) | 483 | Integrase | Integrase | | uniclust | UniRef100\_A0A049DW49 | 100.0 | 1.6e-45 | 2.9e-51 | 314.4 | 322 | (43, 394) | 403 | (43, 385) | 433 | Tyr recombinase domain-containing protein | Tyr recombinase domain-containing protein | | uniclust | UniRef100\_A0A084TFD8 | 100.0 | 1.7e-45 | 3.1e-51 | 307.0 | 357 | (1, 402) | 403 | (22, 394) | 525 | Site-specific recombinase | Site-specific recombinase | | uniclust | UniRef100\_A0A522WA23 | 100.0 | 2.1e-45 | 3.7e-51 | 291.7 | 281 | (100, 402) | 403 | (14, 313) | 328 | Tyrosine-type recombinase/integrase | Tyrosine-type recombinase/integrase | | uniclust | UniRef100\_A0A085U0R6 | 100.0 | 2.2e-45 | 3.8e-51 | 313.5 | 382 | (1, 401) | 403 | (26, 463) | 495 | Phage integrase | Phage integrase | | uniclust | UniRef100\_A0A3D1TX51 | 100.0 | 2.2e-45 | 3.9e-51 | 302.7 | 318 | (31, 381) | 403 | (19, 357) | 402 | Site-specific integrase | Site-specific integrase | | uniclust | UniRef100\_A0A096FMR1 | 100.0 | 3.2e-45 | 5.9e-51 | 291.8 | 375 | (1, 402) | 403 | (1, 419) | 443 | Integrase | Integrase | | uniclust | UniRef100\_A0A099T6K6 | 100.0 | 3.6e-45 | 6.4e-51 | 299.3 | 320 | (54, 398) | 403 | (28, 364) | 384 | Integrase | Integrase | | uniclust | UniRef100\_A0A0F5ERZ1 | 100.0 | 3.9e-45 | 7.1e-51 | 287.0 | 372 | (1, 402) | 403 | (6, 399) | 425 | Phage integrase family site-specific recombinase | Phage integrase family site-specific recombinase | | uniclust | UniRef100\_A0A1U9UUM4 | 100.0 | 4.1e-45 | 7.3e-51 | 294.3 | 352 | (2, 382) | 403 | (6, 381) | 401 | Site-specific integrase | Site-specific integrase | | uniclust | UniRef100\_A0A011PK30 | 100.0 | 5e-45 | 8.8e-51 | 323.0 | 387 | (1, 402) | 403 | (48, 478) | 514 | Phage integrase family protein | Phage integrase family protein | | uniclust | UniRef100\_A0A086P927 | 100.0 | 4.9e-45 | 8.9e-51 | 290.5 | 373 | (1, 402) | 403 | (1, 392) | 476 | Integrase | Integrase | | uniclust | UniRef100\_A0A0D8IYN9 | 100.0 | 5.2e-45 | 9.2e-51 | 310.7 | 317 | (45, 392) | 403 | (84, 431) | 468 | Tyr recombinase domain-containing protein | Tyr recombinase domain-containing protein | | uniclust | UniRef100\_A0A0P1EBU4 | 100.0 | 5.6e-45 | 1e-50 | 309.3 | 371 | (2, 402) | 403 | (31, 432) | 464 | Site-specific recombinase XerD | Site-specific recombinase XerD | | uniclust | UniRef100\_A0A059DPD4 | 100.0 | 6.3e-45 | 1.1e-50 | 300.5 | 379 | (2, 402) | 403 | (14, 413) | 433 | Tyr recombinase domain-containing protein | Tyr recombinase domain-containing protein | | uniclust | UniRef100\_A0A1G6SUD2 | 100.0 | 6.5e-45 | 1.1e-50 | 316.0 | 326 | (45, 399) | 403 | (47, 410) | 478 | Integrase | Integrase | | uniclust | UniRef100\_A0A3D6EJP8 | 100.0 | 6.4e-45 | 1.2e-50 | 293.6 | 372 | (2, 402) | 403 | (19, 430) | 455 | Tyr recombinase domain-containing protein | Tyr recombinase domain-containing protein | | uniclust | UniRef100\_A0A011QXA4 | 100.0 | 7.5e-45 | 1.3e-50 | 320.2 | 330 | (43, 401) | 403 | (60, 430) | 500 | Integrase | Integrase | | uniclust | UniRef100\_A0A0F8YR70 | 100.0 | 7.6e-45 | 1.3e-50 | 309.9 | 323 | (43, 398) | 403 | (28, 366) | 462 | Tyr recombinase domain-containing protein (Fragment) | Tyr recombinase domain-containing protein (Fragment) | | uniclust | UniRef100\_A0A061NW99 | 100.0 | 8.5e-45 | 1.5e-50 | 304.9 | 331 | (43, 402) | 403 | (21, 391) | 417 | Phage integrase | Phage integrase | | uniclust | UniRef100\_A0A399RGX3 | 100.0 | 8.6e-45 | 1.5e-50 | 293.0 | 333 | (31, 400) | 403 | (3, 346) | 386 | Site-specific integrase | Site-specific integrase | | uniclust | UniRef100\_A0A060DST5 | 100.0 | 1.1e-44 | 2e-50 | 304.6 | 314 | (45, 392) | 403 | (38, 357) | 398 | Site-specific integrase | Site-specific integrase | | uniclust | UniRef100\_A0A177PVT5 | 100.0 | 1.2e-44 | 2.1e-50 | 311.7 | 327 | (31, 383) | 403 | (53, 437) | 488 | Integrase | Integrase | | uniclust | UniRef100\_A0A2E8GAL2 | 100.0 | 1.3e-44 | 2.2e-50 | 297.2 | 335 | (31, 397) | 403 | (32, 371) | 422 | Site-specific integrase | Site-specific integrase | | uniclust | UniRef100\_A0A421K355 | 100.0 | 1.6e-44 | 2.9e-50 | 285.2 | 373 | (1, 402) | 403 | (8, 388) | 401 | Tyr recombinase domain-containing protein | Tyr recombinase domain-containing protein | | uniclust | UniRef100\_A0A1L7AEE4 | 100.0 | 1.6e-44 | 2.9e-50 | 303.6 | 376 | (2, 403) | 403 | (22, 437) | 480 | Tyr recombinase domain-containing protein | Tyr recombinase domain-containing protein | | uniclust | UniRef100\_A0A081K581 | 100.0 | 1.9e-44 | 3.5e-50 | 298.5 | 372 | (1, 401) | 403 | (10, 426) | 477 | Tyr recombinase domain-containing protein | Tyr recombinase domain-containing protein | | uniclust | UniRef100\_A0A1F6PNS7 | 100.0 | 2.1e-44 | 3.7e-50 | 297.8 | 332 | (32, 394) | 403 | (34, 387) | 407 | Tyr recombinase domain-containing protein | Tyr recombinase domain-containing protein | | uniclust | UniRef100\_A0A0C9PF50 | 100.0 | 2.7e-44 | 4.9e-50 | 281.6 | 243 | (136, 400) | 403 | (2, 247) | 286 | Site-specific recombinase | Site-specific recombinase | | uniclust | UniRef100\_A0A0K2JBH0 | 100.0 | 3e-44 | 5.2e-50 | 313.7 | 322 | (44, 398) | 403 | (31, 386) | 429 | Integrase | Integrase | | uniclust | UniRef100\_A0A062WVW9 | 100.0 | 3.2e-44 | 5.5e-50 | 318.4 | 321 | (44, 393) | 403 | (77, 419) | 493 | Site-specific recombinase XerD | Site-specific recombinase XerD | | uniclust | UniRef100\_A0A090IT75 | 100.0 | 4e-44 | 7.2e-50 | 297.4 | 373 | (1, 401) | 403 | (75, 471) | 502 | Putative phage integrase | Putative phage integrase | | uniclust | UniRef100\_A0A1M3P5Q7 | 100.0 | 4.1e-44 | 7.2e-50 | 292.7 | 324 | (1, 354) | 403 | (5, 346) | 367 | Tyr recombinase domain-containing protein | Tyr recombinase domain-containing protein | | uniclust | UniRef100\_A0A011V5Q7 | 100.0 | 4.2e-44 | 7.5e-50 | 297.2 | 330 | (45, 399) | 403 | (17, 393) | 436 | Recombinase | Recombinase | | uniclust | UniRef100\_A0A031LMT3 | 100.0 | 4.3e-44 | 7.7e-50 | 302.3 | 374 | (1, 400) | 403 | (20, 428) | 460 | Integrase | Integrase | | uniclust | UniRef100\_A0A101VH43 | 100.0 | 5e-44 | 8.8e-50 | 283.9 | 245 | (139, 402) | 403 | (19, 272) | 294 | Tyr recombinase domain-containing protein | Tyr recombinase domain-containing protein | | uniclust | UniRef100\_A0A024L7S6 | 100.0 | 5e-44 | 9e-50 | 285.6 | 373 | (1, 401) | 403 | (13, 413) | 447 | DUF4102 domain-containing protein | DUF4102 domain-containing protein | | uniclust | UniRef100\_A0A090P338 | 100.0 | 5.2e-44 | 9.1e-50 | 298.5 | 311 | (45, 394) | 403 | (16, 351) | 384 | Integrase | Integrase | | uniclust | UniRef100\_A0A0W1DLE8 | 100.0 | 5.3e-44 | 9.6e-50 | 292.7 | 371 | (2, 401) | 403 | (12, 429) | 459 | Integrase | Integrase | | uniclust | UniRef100\_A0A166RGF6 | 100.0 | 5.7e-44 | 1e-49 | 301.6 | 329 | (45, 399) | 403 | (51, 411) | 442 | Site-specific recombinase XerD | Site-specific recombinase XerD | | uniclust | UniRef100\_A0A0M2NN08 | 100.0 | 7e-44 | 1.2e-49 | 303.1 | 311 | (45, 382) | 403 | (32, 383) | 432 | Prophage LambdaBa04, site-specific recombinase, phage integrase family | Prophage LambdaBa04, site-specific recombinase, phage integrase family | | uniclust | UniRef100\_A0A015UQW6 | 100.0 | 7.9e-44 | 1.4e-49 | 314.6 | 324 | (44, 398) | 403 | (76, 430) | 492 | Phage integrase family protein | Phage integrase family protein | | uniclust | UniRef100\_A0A086PDB9 | 100.0 | 7.8e-44 | 1.4e-49 | 298.0 | 356 | (1, 399) | 403 | (26, 394) | 428 | Integrase family protein | Integrase family protein | | uniclust | UniRef100\_A0A086ZVN3 | 100.0 | 8.4e-44 | 1.5e-49 | 304.0 | 323 | (44, 396) | 403 | (89, 439) | 495 | Integrase/recombinase | Integrase/recombinase | | uniclust | UniRef100\_A0A1V5MLF3 | 100.0 | 9e-44 | 1.6e-49 | 288.9 | 319 | (44, 400) | 403 | (14, 341) | 345 | Tyrosine recombinase XerC | Tyrosine recombinase XerC | | uniclust | UniRef100\_A0A2V8JLK3 | 100.0 | 1.1e-43 | 1.9e-49 | 296.1 | 332 | (33, 398) | 403 | (36, 389) | 438 | Tyr recombinase domain-containing protein | Tyr recombinase domain-containing protein | | uniclust | UniRef100\_A0A1M3IUQ0 | 100.0 | 1.1e-43 | 2e-49 | 281.0 | 323 | (43, 399) | 403 | (12, 350) | 360 | Tyr recombinase domain-containing protein | Tyr recombinase domain-containing protein | | uniclust | UniRef100\_A0A0J8SS38 | 100.0 | 1.2e-43 | 2.1e-49 | 284.4 | 343 | (1, 381) | 403 | (28, 388) | 414 | Site-specific recombinase | Site-specific recombinase | | uniclust | UniRef100\_A0A024P888 | 100.0 | 1.2e-43 | 2.1e-49 | 310.4 | 329 | (43, 398) | 403 | (36, 421) | 487 | Integrase | Integrase | | uniclust | UniRef100\_A0A024JYX4 | 100.0 | 1.3e-43 | 2.3e-49 | 302.8 | 329 | (43, 400) | 403 | (28, 392) | 431 | Phage integrase family protein | Phage integrase family protein | | uniclust | UniRef100\_A0A061M0A6 | 100.0 | 1.4e-43 | 2.5e-49 | 302.7 | 307 | (44, 382) | 403 | (29, 368) | 458 | Integrase | Integrase | | uniclust | UniRef100\_A0A143X2R8 | 100.0 | 1.5e-43 | 2.7e-49 | 303.3 | 327 | (45, 400) | 403 | (44, 412) | 486 | Site-specific tyrosine recombinase XerC | Site-specific tyrosine recombinase XerC | | uniclust | UniRef100\_A0A069AE41 | 100.0 | 1.5e-43 | 2.7e-49 | 311.7 | 329 | (44, 399) | 403 | (78, 457) | 542 | Prophage lambdaba04, site-specific recombinase, phage integrase family | Prophage lambdaba04, site-specific recombinase, phage integrase family | | uniclust | UniRef100\_A0A147J3P5 | 100.0 | 1.7e-43 | 3e-49 | 294.5 | 346 | (31, 400) | 403 | (51, 409) | 436 | Integrase (Fragment) | Integrase (Fragment) | | uniclust | UniRef100\_A0A075KBY8 | 100.0 | 1.7e-43 | 3e-49 | 300.5 | 339 | (32, 400) | 403 | (8, 391) | 405 | Integrase family protein | Integrase family protein | | uniclust | UniRef100\_A0A096CRB0 | 100.0 | 1.9e-43 | 3.4e-49 | 291.8 | 287 | (98, 402) | 403 | (94, 411) | 490 | Tyr recombinase domain-containing protein | Tyr recombinase domain-containing protein | | uniclust | UniRef100\_A0A009S4D1 | 100.0 | 2.1e-43 | 3.8e-49 | 291.2 | 372 | (1, 400) | 403 | (5, 402) | 442 | Phage integrase family protein | Phage integrase family protein | | uniclust | UniRef100\_A0A1H8A220 | 100.0 | 2.2e-43 | 3.9e-49 | 300.3 | 315 | (45, 399) | 403 | (67, 403) | 426 | Site-specific recombinase XerD | Site-specific recombinase XerD | | uniclust | UniRef100\_A0A014L1K2 | 100.0 | 2.3e-43 | 4.2e-49 | 298.1 | 326 | (44, 399) | 403 | (80, 437) | 472 | Integrase | Integrase | | uniclust | UniRef100\_A0A928E6Y9 | 100.0 | 2.6e-43 | 4.7e-49 | 283.1 | 366 | (1, 398) | 403 | (11, 403) | 409 | Site-specific integrase | Site-specific integrase | | uniclust | UniRef100\_A0A1J0KSW7 | 100.0 | 2.8e-43 | 5e-49 | 285.9 | 286 | (99, 401) | 403 | (9, 308) | 321 | Phage integrase family protein | Phage integrase family protein | | uniclust | UniRef100\_A0A2D9L8W3 | 100.0 | 2.9e-43 | 5.2e-49 | 281.6 | 342 | (2, 382) | 403 | (12, 376) | 390 | Integrase | Integrase | | uniclust | UniRef100\_A0A0K2H1N5 | 100.0 | 3.7e-43 | 6.5e-49 | 290.3 | 323 | (43, 395) | 403 | (24, 368) | 403 | Integrase | Integrase | | uniclust | UniRef100\_A0A0X8VBK9 | 100.0 | 3.7e-43 | 6.6e-49 | 290.1 | 315 | (44, 395) | 403 | (7, 346) | 371 | Tyrosine recombinase XerD | Tyrosine recombinase XerD | | uniclust | UniRef100\_A0A098AZG8 | 100.0 | 4e-43 | 7.1e-49 | 295.4 | 319 | (54, 401) | 403 | (30, 380) | 418 | Integrase protein | Integrase protein | | uniclust | UniRef100\_A0A011U459 | 100.0 | 4.5e-43 | 7.9e-49 | 309.4 | 321 | (45, 395) | 403 | (102, 442) | 562 | Integrase | Integrase | | uniclust | UniRef100\_A0A059N5D1 | 100.0 | 4.5e-43 | 8e-49 | 300.6 | 332 | (43, 401) | 403 | (33, 403) | 443 | Site-specific recombinase, phage integrase family | Site-specific recombinase, phage integrase family | | uniclust | UniRef100\_A0A0M7K4N7 | 100.0 | 4.6e-43 | 8.2e-49 | 287.6 | 286 | (99, 402) | 403 | (49, 361) | 404 | Prophage CP4-57 integrase | Prophage CP4-57 integrase | | uniclust | UniRef100\_A0A0F3MLM1 | 100.0 | 4.8e-43 | 8.6e-49 | 279.9 | 286 | (84, 400) | 403 | (7, 299) | 325 | Phage integrase family protein | Phage integrase family protein | | uniclust | UniRef100\_A0A059KUY7 | 100.0 | 4.9e-43 | 8.6e-49 | 306.8 | 368 | (1, 400) | 403 | (43, 425) | 507 | Integrase | Integrase | | uniclust | UniRef100\_A0A0A8JYV4 | 100.0 | 4.9e-43 | 8.7e-49 | 299.7 | 325 | (43, 398) | 403 | (53, 421) | 460 | Integrase | Integrase | | uniclust | UniRef100\_A0A4D7DB28 | 100.0 | 5e-43 | 9.1e-49 | 280.8 | 372 | (2, 402) | 403 | (10, 396) | 415 | Tyr recombinase domain-containing protein | Tyr recombinase domain-containing protein | | uniclust | UniRef100\_A0A1D7YT86 | 100.0 | 5.2e-43 | 9.2e-49 | 279.9 | 247 | (125, 398) | 403 | (10, 264) | 289 | Tyr recombinase domain-containing protein | Tyr recombinase domain-containing protein | | uniclust | UniRef100\_A0A073IUB0 | 100.0 | 5.2e-43 | 9.3e-49 | 292.7 | 314 | (35, 382) | 403 | (16, 387) | 422 | Integrase | Integrase | | uniclust | UniRef100\_A0A0B1ZRK3 | 100.0 | 5.3e-43 | 9.5e-49 | 295.0 | 363 | (2, 401) | 403 | (60, 448) | 539 | Tyr recombinase domain-containing protein | Tyr recombinase domain-containing protein | | uniclust | UniRef100\_A0A095YNC6 | 100.0 | 5.8e-43 | 1e-48 | 298.1 | 326 | (31, 396) | 403 | (67, 422) | 454 | Tyr recombinase domain-containing protein | Tyr recombinase domain-containing protein | | uniclust | UniRef100\_A0A0F6AIS4 | 100.0 | 5.9e-43 | 1e-48 | 289.4 | 264 | (122, 402) | 403 | (33, 311) | 358 | Tyr recombinase domain-containing protein | Tyr recombinase domain-containing protein | | uniclust | UniRef100\_A0A1G7JFK2 | 100.0 | 6.3e-43 | 1.1e-48 | 292.2 | 322 | (31, 396) | 403 | (57, 422) | 440 | Site-specific recombinase XerD | Site-specific recombinase XerD | | uniclust | UniRef100\_A0A1V6AYS9 | 100.0 | 6.7e-43 | 1.2e-48 | 287.0 | 320 | (43, 398) | 403 | (52, 384) | 410 | Prophage phiRv2 integrase | Prophage phiRv2 integrase | | uniclust | UniRef100\_A0A096A971 | 100.0 | 7.5e-43 | 1.3e-48 | 296.1 | 317 | (32, 381) | 403 | (33, 398) | 438 | Tyr recombinase domain-containing protein | Tyr recombinase domain-containing protein | | uniclust | UniRef100\_A0A136KB30 | 100.0 | 7.6e-43 | 1.3e-48 | 294.8 | 304 | (44, 382) | 403 | (35, 369) | 416 | Phage integrase | Phage integrase | | uniclust | UniRef100\_A0A0M9ZP03 | 100.0 | 8.1e-43 | 1.4e-48 | 290.5 | 343 | (32, 399) | 403 | (18, 393) | 419 | Tyr recombinase domain-containing protein | Tyr recombinase domain-containing protein | | uniclust | UniRef100\_A0A062WVF1 | 100.0 | 8.6e-43 | 1.5e-48 | 297.9 | 326 | (44, 399) | 403 | (40, 425) | 528 | Site-specific recombinase XerD | Site-specific recombinase XerD | | uniclust | UniRef100\_A0A069CWW0 | 100.0 | 9.6e-43 | 1.7e-48 | 298.0 | 329 | (43, 399) | 403 | (30, 397) | 420 | Phage integrase family protein (Fragment) | Phage integrase family protein (Fragment) | | uniclust | UniRef100\_A0A089ICH8 | 100.0 | 9.5e-43 | 1.7e-48 | 292.1 | 336 | (35, 398) | 403 | (50, 438) | 461 | Integrase | Integrase | | uniclust | UniRef100\_A0A068F1P9 | 100.0 | 9.6e-43 | 1.7e-48 | 299.5 | 308 | (44, 381) | 403 | (130, 463) | 544 | Integrase | Integrase | | uniclust | UniRef100\_A0A126QRX5 | 100.0 | 1.1e-42 | 1.9e-48 | 288.9 | 328 | (43, 399) | 403 | (76, 416) | 475 | Tyr recombinase domain-containing protein | Tyr recombinase domain-containing protein | | uniclust | UniRef100\_A0A099RM23 | 100.0 | 1.2e-42 | 2.1e-48 | 286.4 | 317 | (34, 382) | 403 | (17, 359) | 385 | Tyr recombinase domain-containing protein | Tyr recombinase domain-containing protein | | uniclust | UniRef100\_A0A1J5I4Z9 | 100.0 | 1.2e-42 | 2.2e-48 | 288.9 | 324 | (46, 401) | 403 | (37, 381) | 408 | Tyr recombinase domain-containing protein | Tyr recombinase domain-containing protein | | uniclust | UniRef100\_A0A0L6J7P5 | 100.0 | 1.2e-42 | 2.2e-48 | 299.8 | 341 | (32, 400) | 403 | (40, 426) | 493 | Integrase | Integrase | | uniclust | UniRef100\_A0A095ZM98 | 100.0 | 1.2e-42 | 2.2e-48 | 296.1 | 316 | (47, 395) | 403 | (57, 403) | 445 | Tyr recombinase domain-containing protein | Tyr recombinase domain-containing protein | | uniclust | UniRef100\_A0A0M2S0L9 | 100.0 | 1.3e-42 | 2.2e-48 | 294.6 | 324 | (26, 382) | 403 | (18, 364) | 418 | Tyr recombinase domain-containing protein | Tyr recombinase domain-containing protein | | uniclust | UniRef100\_UPI001488B3D3 | 100.0 | 1.2e-42 | 2.2e-48 | 271.8 | 360 | (1, 401) | 403 | (1, 370) | 379 | tyrosine-type recombinase/integrase | tyrosine-type recombinase/integrase | | uniclust | UniRef100\_A0A2T5MEE6 | 100.0 | 1.3e-42 | 2.3e-48 | 283.4 | 369 | (6, 399) | 403 | (10, 396) | 437 | Tyr recombinase domain-containing protein | Tyr recombinase domain-containing protein | | uniclust | UniRef100\_A0A096B432 | 100.0 | 1.3e-42 | 2.3e-48 | 292.4 | 306 | (69, 397) | 403 | (61, 395) | 562 | Tyr recombinase domain-containing protein | Tyr recombinase domain-containing protein | | uniclust | UniRef100\_A0A0Q5I7C0 | 100.0 | 1.6e-42 | 2.8e-48 | 287.8 | 333 | (34, 397) | 403 | (19, 387) | 433 | Integrase | Integrase | | uniclust | UniRef100\_A0A059T666 | 100.0 | 1.6e-42 | 2.9e-48 | 298.3 | 328 | (43, 398) | 403 | (60, 431) | 464 | Integrase | Integrase | | uniclust | UniRef100\_A0A024H3U4 | 100.0 | 1.7e-42 | 2.9e-48 | 295.6 | 327 | (43, 397) | 403 | (37, 395) | 432 | Phage integrase family protein | Phage integrase family protein | | uniclust | UniRef100\_A0A0X8JIH3 | 100.0 | 1.8e-42 | 3.2e-48 | 283.5 | 278 | (100, 399) | 403 | (35, 318) | 386 | Integrase | Integrase | | uniclust | UniRef100\_A0A081RCZ7 | 100.0 | 2e-42 | 3.5e-48 | 282.5 | 281 | (100, 402) | 403 | (4, 300) | 328 | Phage integrase | Phage integrase | | uniclust | UniRef100\_A0A1E7ILK3 | 100.0 | 2e-42 | 3.6e-48 | 279.1 | 346 | (31, 402) | 403 | (35, 412) | 434 | Tyr recombinase domain-containing protein | Tyr recombinase domain-containing protein | | uniclust | UniRef100\_A0A0H5D4Z8 | 100.0 | 2.1e-42 | 3.8e-48 | 290.6 | 383 | (1, 402) | 403 | (12, 451) | 486 | Prophage CP4-57 integrase | Prophage CP4-57 integrase | | uniclust | UniRef100\_A0A0F2NFY3 | 100.0 | 2.2e-42 | 3.9e-48 | 283.5 | 324 | (44, 396) | 403 | (74, 410) | 459 | Integrase | Integrase | | uniclust | UniRef100\_A0A3M5BZX2 | 100.0 | 2.9e-42 | 5.2e-48 | 272.9 | 277 | (99, 395) | 403 | (20, 309) | 347 | Integrase protein | Integrase protein | | uniclust | UniRef100\_A0A4P5VS75 | 100.0 | 3.4e-42 | 6.1e-48 | 286.3 | 310 | (44, 382) | 403 | (24, 349) | 415 | Integrase | Integrase | | uniclust | UniRef100\_A0A0H2XPX9 | 100.0 | 3.3e-42 | 6.1e-48 | 271.4 | 372 | (1, 401) | 403 | (26, 415) | 431 | Phage integrase | Phage integrase | | uniclust | UniRef100\_A0A0D0JR95 | 100.0 | 3.5e-42 | 6.2e-48 | 282.0 | 286 | (100, 402) | 403 | (51, 347) | 368 | Integrase | Integrase | | uniclust | UniRef100\_A0A081RFF4 | 100.0 | 3.7e-42 | 6.7e-48 | 278.5 | 286 | (99, 401) | 403 | (18, 314) | 371 | Integrase | Integrase | | uniclust | UniRef100\_A0A7T4N282 | 100.0 | 3.8e-42 | 6.9e-48 | 279.7 | 374 | (1, 401) | 403 | (2, 399) | 584 | Integrase arm-type DNA-binding domain-containing protein | Integrase arm-type DNA-binding domain-containing protein | | uniclust | UniRef100\_A0A1V5M8V7 | 100.0 | 4.2e-42 | 7.5e-48 | 280.6 | 311 | (45, 381) | 403 | (35, 361) | 414 | Putative prophage phiRv2 integrase | Putative prophage phiRv2 integrase | | uniclust | UniRef100\_A0A174EDH2 | 100.0 | 4.4e-42 | 7.8e-48 | 284.9 | 326 | (43, 398) | 403 | (49, 409) | 420 | Integrase | Integrase | | uniclust | UniRef100\_A0A1C3XG07 | 100.0 | 4.3e-42 | 7.8e-48 | 281.4 | 373 | (2, 402) | 403 | (42, 438) | 454 | Integrase | Integrase | | uniclust | UniRef100\_A0A1H6ZZ71 | 100.0 | 4.3e-42 | 7.9e-48 | 279.5 | 372 | (2, 400) | 403 | (67, 466) | 506 | Integrase | Integrase | | uniclust | UniRef100\_A0A089HSZ3 | 100.0 | 4.5e-42 | 8e-48 | 294.8 | 319 | (45, 384) | 403 | (60, 422) | 490 | Integrase | Integrase | | uniclust | UniRef100\_A0A059N3T8 | 100.0 | 4.7e-42 | 8.2e-48 | 301.0 | 325 | (44, 397) | 403 | (93, 474) | 519 | Site-specific recombinase, phage integrase family | Site-specific recombinase, phage integrase family | | uniclust | UniRef100\_A0A537CBF8 | 100.0 | 4.8e-42 | 8.7e-48 | 283.4 | 389 | (1, 401) | 403 | (8, 437) | 443 | DUF4102 domain-containing protein (Fragment) | DUF4102 domain-containing protein (Fragment) | | uniclust | UniRef100\_A0A550JG93 | 100.0 | 4.9e-42 | 8.9e-48 | 275.3 | 334 | (44, 402) | 403 | (30, 419) | 434 | Site-specific integrase | Site-specific integrase | | uniclust | UniRef100\_UPI00187E1C08 | 100.0 | 5e-42 | 9.2e-48 | 275.4 | 366 | (1, 397) | 403 | (4, 381) | 571 | site-specific integrase | site-specific integrase | | uniclust | UniRef100\_A0A3N5J444 | 100.0 | 6e-42 | 1.1e-47 | 270.5 | 333 | (31, 394) | 403 | (3, 356) | 366 | Site-specific integrase | Site-specific integrase | | uniclust | UniRef100\_A0A1G6JWN1 | 100.0 | 6.5e-42 | 1.2e-47 | 281.8 | 369 | (1, 400) | 403 | (75, 468) | 473 | Integrase DNA-binding domain-containing protein | Integrase DNA-binding domain-containing protein | | uniclust | UniRef100\_A0A011NT00 | 100.0 | 7.4e-42 | 1.3e-47 | 295.5 | 373 | (1, 400) | 403 | (130, 537) | 594 | Site-specific tyrosine recombinase XerC | Site-specific tyrosine recombinase XerC | | uniclust | UniRef100\_A0A0Q5R873 | 100.0 | 7.8e-42 | 1.4e-47 | 279.6 | 366 | (3, 402) | 403 | (15, 418) | 435 | Core-binding (CB) domain-containing protein | Core-binding (CB) domain-containing protein | | uniclust | UniRef100\_A0A173G9H3 | 100.0 | 8.5e-42 | 1.5e-47 | 283.6 | 338 | (27, 400) | 403 | (15, 375) | 393 | Uncharacterized protein | Uncharacterized protein | | uniclust | UniRef100\_A0A1F2SPC9 | 100.0 | 9e-42 | 1.6e-47 | 285.6 | 323 | (45, 400) | 403 | (41, 372) | 424 | Tyr recombinase domain-containing protein | Tyr recombinase domain-containing protein | | uniclust | UniRef100\_A0A061NKM5 | 100.0 | 1.1e-41 | 1.9e-47 | 283.0 | 276 | (99, 395) | 403 | (50, 339) | 353 | Integrase | Integrase | | uniclust | UniRef100\_A0A3B7DI48 | 100.0 | 1.1e-41 | 1.9e-47 | 275.9 | 251 | (134, 402) | 403 | (9, 277) | 307 | Tyr recombinase domain-containing protein | Tyr recombinase domain-containing protein | | uniclust | UniRef100\_A0A096B2D4 | 100.0 | 1.1e-41 | 2e-47 | 301.3 | 333 | (45, 398) | 403 | (91, 471) | 570 | Tyr recombinase domain-containing protein | Tyr recombinase domain-containing protein | | uniclust | UniRef100\_A0A3D1VDB8 | 100.0 | 1.1e-41 | 2e-47 | 280.0 | 327 | (54, 399) | 403 | (30, 404) | 407 | Tyr recombinase domain-containing protein | Tyr recombinase domain-containing protein | | uniclust | UniRef100\_A0A1L9BA63 | 100.0 | 1.3e-41 | 2.3e-47 | 290.7 | 330 | (34, 395) | 403 | (41, 422) | 502 | Tyr recombinase domain-containing protein | Tyr recombinase domain-containing protein | | uniclust | UniRef100\_A0A024YX43 | 100.0 | 1.3e-41 | 2.3e-47 | 295.0 | 327 | (44, 399) | 403 | (58, 415) | 584 | Integrase | Integrase | | uniclust | UniRef100\_A0A0F9J7V5 | 100.0 | 1.3e-41 | 2.4e-47 | 287.3 | 332 | (33, 394) | 403 | (20, 372) | 410 | Tyr recombinase domain-containing protein | Tyr recombinase domain-containing protein | | uniclust | UniRef100\_A0A1D8AFA3 | 100.0 | 1.3e-41 | 2.4e-47 | 277.0 | 353 | (1, 398) | 403 | (44, 411) | 436 | Integrase | Integrase | | uniclust | UniRef100\_A0A1I4ZYE7 | 100.0 | 1.3e-41 | 2.4e-47 | 274.8 | 286 | (1, 313) | 403 | (30, 330) | 339 | Integrase DNA-binding domain-containing protein | Integrase DNA-binding domain-containing protein | | uniclust | UniRef100\_A0A059N0G3 | 100.0 | 1.4e-41 | 2.5e-47 | 286.9 | 336 | (43, 401) | 403 | (30, 421) | 454 | Site-specific recombinase, phage integrase family | Site-specific recombinase, phage integrase family | | uniclust | UniRef100\_A0A0C4WKA9 | 100.0 | 1.4e-41 | 2.6e-47 | 294.4 | 386 | (1, 402) | 403 | (66, 504) | 539 | Phage integrase family protein | Phage integrase family protein | | uniclust | UniRef100\_A0A081NWM4 | 100.0 | 1.5e-41 | 2.7e-47 | 288.2 | 308 | (45, 382) | 403 | (83, 421) | 466 | Integrase | Integrase | | uniclust | UniRef100\_A0A0N0K2E9 | 100.0 | 1.5e-41 | 2.8e-47 | 276.3 | 371 | (1, 400) | 403 | (2, 394) | 435 | Tyr recombinase domain-containing protein (Fragment) | Tyr recombinase domain-containing protein (Fragment) | | uniclust | UniRef100\_A0A023CQB7 | 100.0 | 1.7e-41 | 2.9e-47 | 294.5 | 325 | (45, 399) | 403 | (75, 439) | 467 | Prophage LambdaBa04, site-specific recombinase, phage integrase family protein | Prophage LambdaBa04, site-specific recombinase, phage integrase family protein | | uniclust | UniRef100\_A0A2V2FPB6 | 100.0 | 1.7e-41 | 3e-47 | 280.1 | 314 | (54, 398) | 403 | (23, 372) | 404 | Site-specific integrase | Site-specific integrase | | uniclust | UniRef100\_A0A059G3I2 | 100.0 | 1.9e-41 | 3.3e-47 | 283.9 | 376 | (1, 402) | 403 | (25, 441) | 460 | Phage integrase | Phage integrase | | uniclust | UniRef100\_A0A0U4EAX9 | 100.0 | 2e-41 | 3.4e-47 | 296.9 | 322 | (44, 396) | 403 | (42, 393) | 441 | Integrase | Integrase | | uniclust | UniRef100\_Q1K3L0 | 100.0 | 1.9e-41 | 3.4e-47 | 267.9 | 327 | (30, 382) | 403 | (39, 382) | 417 | Phage integrase | Phage integrase | | uniclust | UniRef100\_A0A0E8XLM2 | 100.0 | 1.9e-41 | 3.4e-47 | 267.4 | 374 | (1, 401) | 403 | (23, 424) | 445 | Phage integrase family site specific recombinase | Phage integrase family site specific recombinase | | uniclust | UniRef100\_A0A090GDZ2 | 100.0 | 1.9e-41 | 3.4e-47 | 276.4 | 347 | (2, 381) | 403 | (27, 388) | 428 | Putative Phage integrase | Putative Phage integrase | | uniclust | UniRef100\_A0A1M6M0L3 | 100.0 | 2e-41 | 3.5e-47 | 273.7 | 282 | (1, 312) | 403 | (16, 308) | 358 | Phage integrase family protein | Phage integrase family protein | | uniclust | UniRef100\_A0A069AU07 | 100.0 | 2.3e-41 | 4e-47 | 286.7 | 325 | (44, 398) | 403 | (42, 406) | 440 | Integrase | Integrase | | uniclust | UniRef100\_A0A0M2UWX7 | 100.0 | 2.6e-41 | 4.6e-47 | 271.6 | 243 | (136, 397) | 403 | (10, 257) | 313 | Site-specific tyrosine recombinase (Fragment) | Site-specific tyrosine recombinase (Fragment) | | uniclust | UniRef100\_A0A6L4AW27 | 100.0 | 2.8e-41 | 5.2e-47 | 274.5 | 340 | (26, 399) | 403 | (70, 421) | 470 | Site-specific integrase | Site-specific integrase | | uniclust | UniRef100\_A0A1Y3R4K1 | 100.0 | 2.9e-41 | 5.2e-47 | 276.7 | 281 | (100, 399) | 403 | (93, 402) | 411 | Tyr recombinase domain-containing protein | Tyr recombinase domain-containing protein | | uniclust | UniRef100\_A0A2W5XPE9 | 100.0 | 3.4e-41 | 6e-47 | 281.5 | 316 | (55, 398) | 403 | (23, 364) | 392 | Site-specific integrase | Site-specific integrase | | uniclust | UniRef100\_A0A5C8B1S2 | 100.0 | 3.3e-41 | 6e-47 | 271.6 | 364 | (2, 402) | 403 | (9, 389) | 408 | DUF4102 domain-containing protein | DUF4102 domain-containing protein | | uniclust | UniRef100\_A0A1H5C2S2 | 100.0 | 3.4e-41 | 6.2e-47 | 271.3 | 371 | (2, 402) | 403 | (30, 412) | 421 | Tyr recombinase domain-containing protein | Tyr recombinase domain-containing protein | | uniclust | UniRef100\_A0A0F9D313 | 100.0 | 3.6e-41 | 6.3e-47 | 288.1 | 313 | (55, 398) | 403 | (69, 407) | 454 | Tyr recombinase domain-containing protein (Fragment) | Tyr recombinase domain-containing protein (Fragment) | | uniclust | UniRef100\_A0A1C9V0P7 | 100.0 | 3.8e-41 | 6.7e-47 | 294.0 | 323 | (43, 400) | 403 | (118, 451) | 539 | Integrase family protein | Integrase family protein | | uniclust | UniRef100\_A0A093UD42 | 100.0 | 3.9e-41 | 7.1e-47 | 270.6 | 373 | (1, 401) | 403 | (30, 430) | 521 | Integrase | Integrase | | uniclust | UniRef100\_A0A1M6EU03 | 100.0 | 3.9e-41 | 7.1e-47 | 268.7 | 309 | (43, 394) | 403 | (14, 333) | 347 | Site-specific recombinase XerD | Site-specific recombinase XerD | | uniclust | UniRef100\_A0A0H3ZTY6 | 100.0 | 4.2e-41 | 7.6e-47 | 269.5 | 361 | (4, 401) | 403 | (26, 407) | 410 | Phage integrase | Phage integrase | | uniclust | UniRef100\_A0A080N279 | 100.0 | 4.3e-41 | 7.7e-47 | 290.8 | 323 | (44, 395) | 403 | (96, 459) | 540 | Phage integrase family protein | Phage integrase family protein | | uniclust | UniRef100\_A0A0J1B6R9 | 100.0 | 4.4e-41 | 7.8e-47 | 288.7 | 312 | (43, 393) | 403 | (60, 379) | 436 | Site-specific recombinase XerD | Site-specific recombinase XerD | | uniclust | UniRef100\_A0A1V6JU51 | 100.0 | 4.5e-41 | 8e-47 | 287.6 | 275 | (100, 395) | 403 | (113, 413) | 455 | Tyrosine recombinase XerC | Tyrosine recombinase XerC | | uniclust | UniRef100\_A0A1C7HEN5 | 100.0 | 4.5e-41 | 8.1e-47 | 280.8 | 369 | (2, 402) | 403 | (8, 441) | 453 | Site-specific recombinase | Site-specific recombinase | | uniclust | UniRef100\_A0A127Q2R3 | 100.0 | 4.5e-41 | 8.1e-47 | 263.6 | 365 | (1, 401) | 403 | (2, 377) | 388 | Phage integrase family protein | Phage integrase family protein | | uniclust | UniRef100\_A0A2V3TQD5 | 100.0 | 4.7e-41 | 8.4e-47 | 281.0 | 325 | (31, 382) | 403 | (33, 397) | 438 | Site-specific recombinase XerD | Site-specific recombinase XerD | | uniclust | UniRef100\_A0A1Q8AQL2 | 100.0 | 4.8e-41 | 8.6e-47 | 266.2 | 245 | (144, 402) | 403 | (31, 289) | 344 | Tyr recombinase domain-containing protein | Tyr recombinase domain-containing protein | | uniclust | UniRef100\_A0A2E0W7N1 | 100.0 | 5.6e-41 | 9.9e-47 | 279.7 | 319 | (44, 395) | 403 | (27, 364) | 379 | Tyr recombinase domain-containing protein | Tyr recombinase domain-containing protein | | uniclust | UniRef100\_A0A017H9M4 | 100.0 | 5.8e-41 | 1e-46 | 290.8 | 323 | (32, 382) | 403 | (52, 417) | 470 | Phage integrase | Phage integrase | | uniclust | UniRef100\_A0A0B0EHS4 | 100.0 | 5.7e-41 | 1e-46 | 267.4 | 322 | (44, 395) | 403 | (7, 339) | 364 | Putative phage integrase | Putative phage integrase | | uniclust | UniRef100\_A0A011Q867 | 100.0 | 6.2e-41 | 1.1e-46 | 286.1 | 350 | (29, 402) | 403 | (48, 445) | 505 | Phage integrase family protein | Phage integrase family protein | | uniclust | UniRef100\_A0A011UHL6 | 100.0 | 6.3e-41 | 1.1e-46 | 287.1 | 327 | (46, 399) | 403 | (82, 526) | 558 | Integrase | Integrase | | uniclust | UniRef100\_A0A679JWT2 | 100.0 | 6.2e-41 | 1.1e-46 | 271.2 | 355 | (28, 402) | 403 | (26, 414) | 429 | Prophage integrase IntA | Prophage integrase IntA | | uniclust | UniRef100\_A0A2N5X4M8 | 100.0 | 6.2e-41 | 1.1e-46 | 266.8 | 380 | (2, 400) | 403 | (10, 424) | 426 | Tyr recombinase domain-containing protein | Tyr recombinase domain-containing protein | | uniclust | UniRef100\_A0A1F2TFY9 | 100.0 | 6.4e-41 | 1.1e-46 | 273.9 | 286 | (99, 401) | 403 | (27, 324) | 400 | Tyr recombinase domain-containing protein | Tyr recombinase domain-containing protein | | uniclust | UniRef100\_A0A1A0XD97 | 100.0 | 6.7e-41 | 1.2e-46 | 278.6 | 327 | (45, 399) | 403 | (18, 380) | 436 | Tyr recombinase domain-containing protein | Tyr recombinase domain-containing protein | | uniclust | UniRef100\_A0A0F3M5Y8 | 100.0 | 6.6e-41 | 1.2e-46 | 265.4 | 352 | (1, 382) | 403 | (10, 396) | 447 | Phage integrase family protein | Phage integrase family protein | | uniclust | UniRef100\_A0A084JMC9 | 100.0 | 6.9e-41 | 1.2e-46 | 284.0 | 319 | (45, 394) | 403 | (53, 417) | 441 | Tyr recombinase domain-containing protein | Tyr recombinase domain-containing protein | | uniclust | UniRef100\_A0A1H3NLR1 | 100.0 | 8e-41 | 1.4e-46 | 283.9 | 339 | (1, 382) | 403 | (29, 389) | 476 | Site-specific recombinase XerD | Site-specific recombinase XerD | | uniclust | UniRef100\_A0A098Y3T2 | 100.0 | 8.3e-41 | 1.5e-46 | 281.3 | 313 | (43, 382) | 403 | (48, 394) | 440 | Integrase | Integrase | | uniclust | UniRef100\_A0A0G3V0I4 | 100.0 | 8.3e-41 | 1.5e-46 | 276.6 | 311 | (45, 382) | 403 | (21, 376) | 411 | Site-specific recombinase XerD | Site-specific recombinase XerD | | uniclust | UniRef100\_A0A0Q6W4T8 | 100.0 | 8.4e-41 | 1.5e-46 | 269.3 | 285 | (1, 313) | 403 | (15, 315) | 336 | Tyr recombinase domain-containing protein | Tyr recombinase domain-containing protein | | uniclust | UniRef100\_A0A060DJC5 | 100.0 | 9.3e-41 | 1.6e-46 | 296.3 | 351 | (6, 394) | 403 | (77, 468) | 520 | Tyr recombinase domain-containing protein | Tyr recombinase domain-containing protein | | uniclust | UniRef100\_A0A4P7CHC9 | 100.0 | 9e-41 | 1.6e-46 | 266.7 | 373 | (1, 401) | 403 | (8, 404) | 407 | DUF4102 domain-containing protein | DUF4102 domain-containing protein | | uniclust | UniRef100\_A0A6J4TB91 | 100.0 | 9.2e-41 | 1.7e-46 | 273.3 | 321 | (32, 382) | 403 | (9, 358) | 407 | Integrase | Integrase | | uniclust | UniRef100\_A0A133KEU4 | 100.0 | 9.6e-41 | 1.7e-46 | 276.4 | 314 | (44, 396) | 403 | (9, 348) | 367 | Site-specific recombinase, phage integrase family | Site-specific recombinase, phage integrase family | | uniclust | UniRef100\_A0A2E5C3J8 | 100.0 | 9.4e-41 | 1.7e-46 | 262.9 | 371 | (3, 402) | 403 | (6, 404) | 418 | Integrase | Integrase | | uniclust | UniRef100\_A0A0A8HUI6 | 100.0 | 1e-40 | 1.8e-46 | 279.0 | 370 | (1, 402) | 403 | (36, 442) | 454 | Site-specific recombinase, phage integrase family (DUF4102 domain) | Site-specific recombinase, phage integrase family (DUF4102 domain) | | uniclust | UniRef100\_A0A068F5B1 | 100.0 | 1e-40 | 1.8e-46 | 285.0 | 320 | (43, 398) | 403 | (124, 467) | 556 | Tyrosine integrase | Tyrosine integrase | | uniclust | UniRef100\_A0A1Y3UD86 | 100.0 | 1e-40 | 1.9e-46 | 281.8 | 332 | (45, 398) | 403 | (51, 439) | 506 | Site-specific integrase | Site-specific integrase | | uniclust | UniRef100\_A0A0B5BC66 | 100.0 | 1.1e-40 | 1.9e-46 | 280.4 | 322 | (32, 394) | 403 | (10, 342) | 381 | Integrase | Integrase | | uniclust | UniRef100\_A0A1Q6YTJ6 | 100.0 | 1.1e-40 | 2e-46 | 277.9 | 331 | (44, 399) | 403 | (84, 442) | 463 | Tyr recombinase domain-containing protein | Tyr recombinase domain-containing protein | | uniclust | UniRef100\_A0A017HIM5 | 100.0 | 1.2e-40 | 2.2e-46 | 271.7 | 349 | (30, 400) | 403 | (8, 387) | 416 | Putative P4-family integrase | Putative P4-family integrase | | uniclust | UniRef100\_A0A174NPS2 | 100.0 | 1.3e-40 | 2.2e-46 | 284.7 | 340 | (32, 399) | 403 | (34, 467) | 480 | Site-specific recombinase XerD | Site-specific recombinase XerD | | uniclust | UniRef100\_A0A011UCH0 | 100.0 | 1.3e-40 | 2.3e-46 | 287.6 | 365 | (1, 401) | 403 | (40, 428) | 534 | Integrase | Integrase | | uniclust | UniRef100\_A0A2D5PMG0 | 100.0 | 1.3e-40 | 2.4e-46 | 275.5 | 307 | (67, 402) | 403 | (41, 356) | 376 | Tyr recombinase domain-containing protein | Tyr recombinase domain-containing protein | | uniclust | UniRef100\_A0A0F9PLM6 | 100.0 | 1.4e-40 | 2.4e-46 | 269.1 | 311 | (44, 382) | 403 | (18, 340) | 355 | Tyr recombinase domain-containing protein | Tyr recombinase domain-containing protein | | uniclust | UniRef100\_A0A0C1GP24 | 100.0 | 1.4e-40 | 2.4e-46 | 271.5 | 318 | (31, 369) | 403 | (8, 342) | 360 | Integrase | Integrase | | uniclust | UniRef100\_A0A0P1HC62 | 100.0 | 1.4e-40 | 2.4e-46 | 266.1 | 365 | (1, 401) | 403 | (10, 390) | 400 | Putative prophage CPS-53 integrase | Putative prophage CPS-53 integrase | | uniclust | UniRef100\_A0A1M3BIR1 | 100.0 | 1.5e-40 | 2.6e-46 | 286.9 | 317 | (43, 381) | 403 | (30, 409) | 540 | Site-specific integrase | Site-specific integrase | | uniclust | UniRef100\_A0A3R8YPI9 | 100.0 | 1.5e-40 | 2.8e-46 | 265.1 | 373 | (2, 401) | 403 | (15, 423) | 443 | Site-specific integrase | Site-specific integrase | | uniclust | UniRef100\_A0A076MHG8 | 100.0 | 1.7e-40 | 3e-46 | 284.2 | 333 | (35, 399) | 403 | (63, 488) | 539 | Integrase family protein | Integrase family protein | | uniclust | UniRef100\_A0A0U5I7C2 | 100.0 | 1.7e-40 | 3e-46 | 262.9 | 369 | (1, 399) | 403 | (5, 395) | 407 | Putative phage integrase family protein | Putative phage integrase family protein | | uniclust | UniRef100\_A0A829Y7U8 | 100.0 | 1.7e-40 | 3.1e-46 | 271.3 | 349 | (17, 397) | 403 | (24, 380) | 549 | Tyr recombinase domain-containing protein | Tyr recombinase domain-containing protein | | uniclust | UniRef100\_A0A651GHW1 | 100.0 | 1.8e-40 | 3.2e-46 | 275.8 | 338 | (28, 397) | 403 | (32, 418) | 449 | Site-specific integrase | Site-specific integrase | | uniclust | UniRef100\_A0A1H7D2D6 | 100.0 | 1.8e-40 | 3.3e-46 | 274.2 | 336 | (43, 402) | 403 | (19, 407) | 424 | Site-specific recombinase XerD | Site-specific recombinase XerD | | uniclust | UniRef100\_A0A090YSV9 | 100.0 | 1.9e-40 | 3.3e-46 | 272.2 | 281 | (101, 400) | 403 | (1, 317) | 331 | Phage integrase family protein | Phage integrase family protein | | uniclust | UniRef100\_A0A1F9TZH4 | 100.0 | 1.9e-40 | 3.5e-46 | 261.1 | 315 | (44, 398) | 403 | (8, 328) | 365 | Tyr recombinase domain-containing protein | Tyr recombinase domain-containing protein | | uniclust | UniRef100\_A0A2V9I892 | 100.0 | 2e-40 | 3.5e-46 | 264.6 | 267 | (99, 383) | 403 | (16, 306) | 346 | Tyr recombinase domain-containing protein (Fragment) | Tyr recombinase domain-containing protein (Fragment) | | uniclust | UniRef100\_A0A016QKK8 | 100.0 | 2e-40 | 3.5e-46 | 279.7 | 326 | (46, 400) | 403 | (105, 491) | 523 | Phage integrase | Phage integrase | | uniclust | UniRef100\_A0A011P559 | 100.0 | 2.1e-40 | 3.6e-46 | 286.3 | 298 | (43, 382) | 403 | (81, 395) | 440 | Integrase | Integrase | | uniclust | UniRef100\_A0A0D6EVV0 | 100.0 | 2.1e-40 | 3.8e-46 | 274.2 | 346 | (31, 399) | 403 | (12, 394) | 417 | Putative Phage related integrase | Putative Phage related integrase | | uniclust | UniRef100\_A0A090KHJ9 | 100.0 | 2.1e-40 | 3.8e-46 | 281.4 | 332 | (43, 399) | 403 | (33, 407) | 428 | Uncultured bacterium genome assembly Metasoil\_fosmids\_resub | Uncultured bacterium genome assembly Metasoil\_fosmids\_resub | | uniclust | UniRef100\_A0A024QGB3 | 100.0 | 2.2e-40 | 3.8e-46 | 284.7 | 322 | (54, 401) | 403 | (51, 412) | 445 | Tyrosine recombinase XerC | Tyrosine recombinase XerC | | uniclust | UniRef100\_A0A0C1U0L8 | 100.0 | 2.2e-40 | 3.9e-46 | 273.5 | 369 | (1, 402) | 403 | (9, 411) | 430 | Tyr recombinase domain-containing protein | Tyr recombinase domain-containing protein | | uniclust | UniRef100\_A0A7H1RNH4 | 100.0 | 2.4e-40 | 4.4e-46 | 270.6 | 356 | (2, 382) | 403 | (33, 471) | 500 | Site-specific integrase | Site-specific integrase | | uniclust | UniRef100\_A0A069CWN5 | 100.0 | 2.7e-40 | 4.7e-46 | 282.3 | 320 | (32, 382) | 403 | (25, 377) | 438 | Phage integrase | Phage integrase | | uniclust | UniRef100\_A0A0N0UZK9 | 100.0 | 2.8e-40 | 5e-46 | 265.6 | 307 | (69, 401) | 403 | (43, 376) | 389 | Phage integrase | Phage integrase | | uniclust | UniRef100\_A0A061NY33 | 100.0 | 2.9e-40 | 5.1e-46 | 279.1 | 317 | (44, 396) | 403 | (51, 377) | 397 | DNA integration/recombination/invertion protein | DNA integration/recombination/invertion protein | | uniclust | UniRef100\_A0A238H9E1 | 100.0 | 3e-40 | 5.3e-46 | 265.5 | 357 | (19, 401) | 403 | (20, 391) | 413 | Phage-related integrase | Phage-related integrase | | uniclust | UniRef100\_A0A011V4P1 | 100.0 | 3e-40 | 5.4e-46 | 277.6 | 327 | (44, 400) | 403 | (41, 426) | 546 | Integrase | Integrase | | uniclust | UniRef100\_A0A023XKK8 | 100.0 | 3.1e-40 | 5.5e-46 | 276.5 | 373 | (2, 403) | 403 | (67, 499) | 542 | Site-specific integrase/recombinase | Site-specific integrase/recombinase | | uniclust | UniRef100\_A0A1I3LKL9 | 100.0 | 3.4e-40 | 6.1e-46 | 271.7 | 307 | (44, 382) | 403 | (77, 419) | 459 | Site-specific recombinase XerD | Site-specific recombinase XerD | | uniclust | UniRef100\_A0A0T6W442 | 100.0 | 3.6e-40 | 6.4e-46 | 282.8 | 379 | (2, 400) | 403 | (32, 452) | 492 | Integrase | Integrase | | uniclust | UniRef100\_A0A1I6MIL3 | 100.0 | 3.6e-40 | 6.5e-46 | 268.9 | 341 | (32, 398) | 403 | (18, 379) | 397 | Site-specific recombinase XerD | Site-specific recombinase XerD | | uniclust | UniRef100\_A0A1H6QXH7 | 100.0 | 3.9e-40 | 7e-46 | 268.1 | 299 | (1, 332) | 403 | (33, 343) | 353 | Integrase DNA-binding domain-containing protein | Integrase DNA-binding domain-containing protein | | uniclust | UniRef100\_A0A0B2JJ45 | 100.0 | 4e-40 | 7.1e-46 | 274.7 | 319 | (44, 394) | 403 | (39, 406) | 444 | Integrase | Integrase | | uniclust | UniRef100\_A0A073J3R0 | 100.0 | 4.1e-40 | 7.5e-46 | 259.0 | 357 | (1, 400) | 403 | (7, 384) | 400 | Tyr recombinase domain-containing protein | Tyr recombinase domain-containing protein | | uniclust | UniRef100\_A0A022PDC8 | 100.0 | 4.3e-40 | 7.6e-46 | 267.1 | 286 | (1, 313) | 403 | (28, 325) | 339 | Integrase DNA-binding domain-containing protein | Integrase DNA-binding domain-containing protein | | uniclust | UniRef100\_A0A024Q9W8 | 100.0 | 4.5e-40 | 7.8e-46 | 283.4 | 272 | (99, 393) | 403 | (65, 354) | 390 | Tyrosine recombinase XerD | Tyrosine recombinase XerD | | uniclust | UniRef100\_A0A1J5D474 | 100.0 | 4.6e-40 | 8.1e-46 | 284.0 | 327 | (43, 398) | 403 | (53, 414) | 449 | Tyr recombinase domain-containing protein | Tyr recombinase domain-containing protein | | uniclust | UniRef100\_A0A9D1WD27 | 100.0 | 4.5e-40 | 8.2e-46 | 262.1 | 364 | (1, 401) | 403 | (8, 382) | 392 | Tyrosine-type recombinase/integrase | Tyrosine-type recombinase/integrase | | uniclust | UniRef100\_A0A355B445 | 100.0 | 4.7e-40 | 8.3e-46 | 264.2 | 292 | (1, 313) | 403 | (9, 308) | 337 | Tyr recombinase domain-containing protein (Fragment) | Tyr recombinase domain-containing protein (Fragment) | | uniclust | UniRef100\_A0A0A0DDR9 | 100.0 | 4.7e-40 | 8.4e-46 | 267.1 | 314 | (45, 396) | 403 | (9, 331) | 368 | Tyr recombinase domain-containing protein | Tyr recombinase domain-containing protein | | uniclust | UniRef100\_A0A090KIS3 | 100.0 | 4.8e-40 | 8.5e-46 | 266.4 | 277 | (101, 401) | 403 | (1, 307) | 328 | Uncultured bacterium genome assembly Metasoil\_fosmids\_resub | Uncultured bacterium genome assembly Metasoil\_fosmids\_resub | | uniclust | UniRef100\_A0A022N8Z9 | 100.0 | 4.9e-40 | 8.6e-46 | 288.4 | 317 | (34, 381) | 403 | (80, 432) | 480 | Integrase | Integrase | | uniclust | UniRef100\_A0A0B8N5H0 | 100.0 | 4.8e-40 | 8.6e-46 | 267.2 | 310 | (43, 381) | 403 | (11, 331) | 386 | Integrase | Integrase | | uniclust | UniRef100\_A0A1C6BGX5 | 100.0 | 5e-40 | 9e-46 | 271.7 | 330 | (31, 395) | 403 | (22, 389) | 414 | Integrase | Integrase | | uniclust | UniRef100\_A0A021VV25 | 100.0 | 5.2e-40 | 9.3e-46 | 270.0 | 338 | (27, 396) | 403 | (15, 375) | 473 | Integrase | Integrase | | uniclust | UniRef100\_A0A286GPU9 | 100.0 | 5.9e-40 | 1.1e-45 | 270.4 | 373 | (1, 401) | 403 | (8, 411) | 441 | Tyr recombinase domain-containing protein | Tyr recombinase domain-containing protein | | uniclust | UniRef100\_A0A1E3WRL1 | 100.0 | 6e-40 | 1.1e-45 | 261.5 | 360 | (2, 396) | 403 | (9, 381) | 385 | Tyrosine recombinase XerC | Tyrosine recombinase XerC | | uniclust | UniRef100\_A0A021VW42 | 100.0 | 6.1e-40 | 1.1e-45 | 274.2 | 323 | (43, 393) | 403 | (55, 409) | 437 | Integrase | Integrase | | uniclust | UniRef100\_A0A075M047 | 100.0 | 6.4e-40 | 1.1e-45 | 274.5 | 304 | (44, 381) | 403 | (20, 336) | 397 | Integrase | Integrase | | uniclust | UniRef100\_D5V8K7 | 100.0 | 6.6e-40 | 1.2e-45 | 262.3 | 277 | (108, 402) | 403 | (3, 287) | 326 | Phage integrase | Phage integrase | | uniclust | UniRef100\_A0A087MIL0 | 100.0 | 6.7e-40 | 1.2e-45 | 280.1 | 382 | (1, 400) | 403 | (29, 448) | 507 | Tyr recombinase domain-containing protein | Tyr recombinase domain-containing protein | | uniclust | UniRef100\_A0A1W2A448 | 100.0 | 7e-40 | 1.3e-45 | 267.6 | 303 | (45, 382) | 403 | (33, 351) | 419 | Site-specific recombinase XerD | Site-specific recombinase XerD | | uniclust | UniRef100\_A0A1G3JV15 | 100.0 | 7.1e-40 | 1.3e-45 | 266.0 | 274 | (100, 401) | 403 | (5, 288) | 317 | Tyr recombinase domain-containing protein | Tyr recombinase domain-containing protein | | uniclust | UniRef100\_A0A023D6X8 | 100.0 | 7.3e-40 | 1.3e-45 | 283.9 | 310 | (45, 394) | 403 | (81, 398) | 459 | Prophage integrase | Prophage integrase | | uniclust | UniRef100\_A0A096D865 | 100.0 | 7.3e-40 | 1.3e-45 | 274.4 | 280 | (102, 400) | 403 | (2, 313) | 343 | Tyr recombinase domain-containing protein | Tyr recombinase domain-containing protein | | uniclust | UniRef100\_A0A141RCN1 | 100.0 | 7.2e-40 | 1.3e-45 | 259.8 | 285 | (100, 401) | 403 | (24, 318) | 335 | Phage integrase family (Fragment) | Phage integrase family (Fragment) | | uniclust | UniRef100\_A0A1Q6RVP7 | 100.0 | 7.8e-40 | 1.4e-45 | 270.5 | 278 | (99, 395) | 403 | (24, 307) | 340 | Tyr recombinase domain-containing protein | Tyr recombinase domain-containing protein | | uniclust | UniRef100\_A0A1Q3QU81 | 100.0 | 8.4e-40 | 1.5e-45 | 266.5 | 319 | (56, 399) | 403 | (39, 374) | 415 | Tyr recombinase domain-containing protein | Tyr recombinase domain-containing protein | | uniclust | UniRef100\_A0A073KMD6 | 100.0 | 8.9e-40 | 1.6e-45 | 276.0 | 280 | (101, 400) | 403 | (65, 372) | 394 | Integrase | Integrase | | uniclust | UniRef100\_A0A1F3ST71 | 100.0 | 9.1e-40 | 1.6e-45 | 272.8 | 330 | (31, 394) | 403 | (28, 377) | 403 | Tyr recombinase domain-containing protein | Tyr recombinase domain-containing protein | | uniclust | UniRef100\_A0A239MDX8 | 100.0 | 9.3e-40 | 1.6e-45 | 263.9 | 273 | (1, 300) | 403 | (12, 296) | 307 | Phage integrase family protein | Phage integrase family protein | | uniclust | UniRef100\_A0A1C6BYJ1 | 100.0 | 9.6e-40 | 1.7e-45 | 270.6 | 325 | (44, 398) | 403 | (60, 430) | 519 | Integrase | Integrase | | uniclust | UniRef100\_A0A095YZI0 | 100.0 | 1e-39 | 1.8e-45 | 278.7 | 330 | (44, 396) | 403 | (48, 422) | 454 | Tyr recombinase domain-containing protein | Tyr recombinase domain-containing protein | | uniclust | UniRef100\_A0A059WLJ1 | 100.0 | 1.1e-39 | 2e-45 | 282.0 | 333 | (31, 394) | 403 | (68, 421) | 449 | Integrase family site specific recombinase | Integrase family site specific recombinase | | uniclust | UniRef100\_A0A0A0HGU1 | 100.0 | 1.1e-39 | 2e-45 | 278.9 | 307 | (43, 383) | 403 | (55, 389) | 468 | Site-specific recombinase XerD | Site-specific recombinase XerD | | uniclust | UniRef100\_A0A0Q4J458 | 100.0 | 1.2e-39 | 2.1e-45 | 265.3 | 310 | (44, 394) | 403 | (43, 357) | 374 | Tyr recombinase domain-containing protein | Tyr recombinase domain-containing protein | | uniclust | UniRef100\_A0A023LBH6 | 100.0 | 1.2e-39 | 2.2e-45 | 280.2 | 313 | (44, 394) | 403 | (79, 403) | 480 | Integrase | Integrase | | uniclust | UniRef100\_A0A177RBV1 | 100.0 | 1.3e-39 | 2.3e-45 | 270.1 | 329 | (45, 395) | 403 | (72, 417) | 441 | Tyr recombinase domain-containing protein | Tyr recombinase domain-containing protein | | uniclust | UniRef100\_A0A021X9S7 | 100.0 | 1.4e-39 | 2.4e-45 | 283.0 | 302 | (45, 382) | 403 | (96, 407) | 515 | Site-specific recombinase XerD | Site-specific recombinase XerD | | uniclust | UniRef100\_A0A060QBM0 | 100.0 | 1.4e-39 | 2.5e-45 | 268.3 | 311 | (45, 395) | 403 | (18, 340) | 374 | Integrase | Integrase | | uniclust | UniRef100\_A0A059UQM9 | 100.0 | 1.4e-39 | 2.6e-45 | 275.1 | 366 | (1, 401) | 403 | (25, 408) | 473 | Phage integrase | Phage integrase | | uniclust | UniRef100\_A0A009G861 | 100.0 | 1.5e-39 | 2.6e-45 | 285.2 | 309 | (32, 382) | 403 | (76, 394) | 477 | Phage integrase family protein | Phage integrase family protein | | uniclust | UniRef100\_A0A1M5XD08 | 100.0 | 1.6e-39 | 2.9e-45 | 265.1 | 330 | (44, 399) | 403 | (8, 360) | 462 | Site-specific recombinase XerD | Site-specific recombinase XerD | | uniclust | UniRef100\_A0A2E8DB97 | 100.0 | 1.6e-39 | 2.9e-45 | 269.3 | 331 | (32, 397) | 403 | (59, 405) | 438 | Tyr recombinase domain-containing protein | Tyr recombinase domain-containing protein | | uniclust | UniRef100\_A0A068SP06 | 100.0 | 1.7e-39 | 2.9e-45 | 287.0 | 322 | (32, 398) | 403 | (25, 364) | 507 | Integrative genetic element Ppu40, integrase | Integrative genetic element Ppu40, integrase | | uniclust | UniRef100\_A0A0T9M937 | 100.0 | 1.6e-39 | 2.9e-45 | 254.2 | 311 | (54, 393) | 403 | (2, 328) | 345 | Prophage CP4-57 integrase | Prophage CP4-57 integrase | | uniclust | UniRef100\_A0A096CRU8 | 100.0 | 1.7e-39 | 3e-45 | 265.0 | 311 | (44, 394) | 403 | (32, 350) | 351 | Tyr recombinase domain-containing protein | Tyr recombinase domain-containing protein | | uniclust | UniRef100\_A0A085K0B1 | 100.0 | 1.7e-39 | 3.1e-45 | 260.8 | 285 | (99, 403) | 403 | (20, 317) | 342 | Tyr recombinase domain-containing protein | Tyr recombinase domain-containing protein | | uniclust | UniRef100\_A0A0D4CL96 | 100.0 | 1.7e-39 | 3.1e-45 | 274.0 | 337 | (32, 400) | 403 | (29, 391) | 413 | Integrase | Integrase | | pdb70 | 1Z1B\_A | 100.0 | 3.4e-34 | 2.4e-38 | 255.7 | 325 | (27, 388) | 403 | (11, 351) | 356 | PROTEIN/DNA Complex | 1Z1B\_A PROTEIN/DNA Complex PROTEIN-DNA COMPLEX, DNA BINDING PROTEIN-DNA HET: PTR | | pdb70 | 5J0N\_H | 99.9 | 1.1e-33 | 7.9e-38 | 252.4 | 308 | (49, 383) | 403 | (24, 347) | 356 | Integrase, Integration host factor subunit | 5J0N\_H Integrase, Integration host factor subunit bacteriophage lambda, excision, site-specific recombination | | pdb70 | 1Z1G\_C | 99.9 | 1.5e-33 | 1.1e-37 | 251.4 | 320 | (27, 382) | 403 | (11, 346) | 356 | PROTEIN/DNA Complex | 1Z1G\_C PROTEIN/DNA Complex Protein-DNA complex, DNA BINDING PROTEIN-DNA HET: MSE | | pdb70 | 1Z19\_B | 99.9 | 6.4e-29 | 4.5e-33 | 213.5 | 263 | (101, 388) | 403 | (1, 278) | 283 | Integrase/DNA Complex | 1Z19\_B Integrase/DNA Complex PROTEIN-DNA COMPLEX, DNA BINDING PROTEIN-DNA | | pdb70 | 1P7D\_A | 99.9 | 6.4e-29 | 4.5e-33 | 213.5 | 265 | (101, 390) | 403 | (1, 280) | 283 | Lambda Integrase/DNA Complex | 1P7D\_A Lambda Integrase/DNA Complex PROTEIN-DNA COMPLEX, DNA Binding Protein-DNA HET: PTR | | pdb70 | 5VFZ\_A | 99.9 | 3.3e-28 | 2.4e-32 | 213.1 | 273 | (98, 400) | 403 | (14, 290) | 318 | Gp33 | 5VFZ\_A Gp33 Bacteriophage, Brujita, DNA-binding, Integrase, DNA HET: ACT, GOL | | pdb70 | 1A0P\_A | 99.9 | 1.3e-27 | 9.2e-32 | 205.8 | 264 | (106, 390) | 403 | (5, 288) | 290 | SITE-SPECIFIC RECOMBINASE XERD | 1A0P\_A SITE-SPECIFIC RECOMBINASE XERD XERD, RECOMBINASE, DNA BINDING, DNA | | pdb70 | 6EN1\_B | 99.9 | 1.4e-27 | 1e-31 | 208.3 | 271 | (101, 394) | 403 | (2, 315) | 317 | Int protein/DNA Complex | 6EN1\_B Int protein/DNA Complex transposase protein - DNA complex HET: PEG | | pdb70 | 5HXY\_A | 99.9 | 1.6e-27 | 1.1e-31 | 208.7 | 269 | (98, 394) | 403 | (34, 313) | 317 | Site-specific integrase/recombinase XerD related protein | 5HXY\_A Site-specific integrase/recombinase XerD related protein recombinase, XerA, RECOMBINATION HET: PO4, MSE | | pdb70 | 6EMY\_A | 99.9 | 4.8e-27 | 3.4e-31 | 204.8 | 272 | (101, 394) | 403 | (2, 315) | 317 | Int protein/DNA Complex | 6EMY\_A Int protein/DNA Complex transposase protein-DNA complex, tyrosine recombinase | | pdb70 | 2A3V\_D | 99.9 | 5.2e-27 | 3.6e-31 | 205.1 | 267 | (102, 395) | 403 | (4, 318) | 320 | site-specific recombinase IntI4/DNA Complex | 2A3V\_D site-specific recombinase IntI4/DNA Complex Protein-DNA complex, RECOMBINATION | | pdb70 | 5C6K\_B | 99.9 | 1e-26 | 7.2e-31 | 200.9 | 262 | (98, 394) | 403 | (11, 274) | 292 | Integrase | 5C6K\_B Integrase Integrase, tyrosine recombinase, integration, site-specific | | pdb70 | 3MGV\_D | 99.9 | 1.1e-26 | 7.6e-31 | 204.9 | 282 | (98, 395) | 403 | (15, 340) | 343 | Recombinase cre | 3MGV\_D Recombinase cre CRE-LOXP, TRANSITION STATE, ISOMERASE-DNA complex | | pdb70 | 4A8E\_A | 99.8 | 2.6e-26 | 1.9e-30 | 198.3 | 263 | (100, 394) | 403 | (6, 276) | 292 | PROBABLE TYROSINE RECOMBINASE XERC-LIKE | 4A8E\_A PROBABLE TYROSINE RECOMBINASE XERC-LIKE CELL CYCLE, CHROMOSOME DIMER RESOLUTION HET: SO4 | | pdb70 | 1XO0\_A | 99.8 | 4.1e-26 | 2.9e-30 | 199.4 | 274 | (106, 395) | 403 | (4, 321) | 324 | RECOMBINASE CRE/DNA Complex | 1XO0\_A RECOMBINASE CRE/DNA Complex CRE RECOMBINASE, HOLLIDAY JUNCTION, RECOMBINATION | | pdb70 | 4CRX\_B | 99.8 | 5.6e-26 | 3.9e-30 | 198.3 | 273 | (106, 394) | 403 | (4, 320) | 322 | CRE RECOMBINASE/DNA COMPLEX | 4CRX\_B CRE RECOMBINASE/DNA COMPLEX CRE RECOMBINASE, DNA BENDING, RECOMBINATION | | pdb70 | 5JJV\_B | 99.8 | 1.3e-25 | 8.9e-30 | 200.0 | 271 | (105, 395) | 403 | (56, 361) | 363 | Tyrosine recombinase XerH/DNA Complex | 5JJV\_B Tyrosine recombinase XerH/DNA Complex Xer, tyrosine recombinase, site-specific recombinase HET: GOL | | pdb70 | 5JK0\_C | 99.8 | 1.3e-25 | 8.9e-30 | 200.0 | 271 | (105, 395) | 403 | (56, 361) | 363 | Tyrosine recombinase XerH/DNA Complex | 5JK0\_C Tyrosine recombinase XerH/DNA Complex Xer, tyrosine recombinase, site-specific recombinase HET: GOL, EDO | | pdb70 | 4E0P\_A | 99.7 | 4.3e-21 | 3e-25 | 175.7 | 223 | (150, 383) | 403 | (166, 430) | 462 | Protelomerase | 4E0P\_A Protelomerase PROTELEMORASE, DNA BINDING PROTEIN-DNA complex HET: DT, EDO, PTR | | pdb70 | 4E0Z\_A | 99.7 | 4.4e-21 | 3.1e-25 | 175.8 | 220 | (150, 383) | 403 | (166, 430) | 462 | Protelomerase | 4E0Z\_A Protelomerase PROTELEMORASE, DNA BINDING PROTEIN-DNA complex HET: GOL, PTR, DT | | pdb70 | 4DWP\_A | 99.7 | 5e-21 | 3.5e-25 | 175.3 | 223 | (150, 383) | 403 | (166, 430) | 462 | Protelomerase, DNA | 4DWP\_A Protelomerase, DNA PROTELEMORASE, DNA BINDING PROTEIN-DNA complex HET: TMP | | pdb70 | 4F41\_A | 99.6 | 5.4e-20 | 3.8e-24 | 160.5 | 244 | (121, 383) | 403 | (24, 309) | 320 | Protelomerase/DNA complex | 4F41\_A Protelomerase/DNA complex RECOMBINATION-DNA complex | | pdb70 | 4F43\_A | 99.6 | 5.4e-20 | 3.8e-24 | 160.5 | 244 | (121, 383) | 403 | (24, 309) | 320 | Protelomerase/DNA complex | 4F43\_A Protelomerase/DNA complex RECOMBINATION-DNA complex | | pdb70 | 5DCF\_A | 99.5 | 1.6e-18 | 1.1e-22 | 147.5 | 172 | (221, 398) | 403 | (2, 189) | 275 | Tyrosine recombinase XerD,DNA translocase FtsK | 5DCF\_A Tyrosine recombinase XerD,DNA translocase FtsK Recombination | | pdb70 | 1AE9\_A | 99.5 | 2.2e-18 | 1.6e-22 | 136.3 | 156 | (221, 383) | 403 | (2, 171) | 179 | LAMBDA INTEGRASE | 1AE9\_A LAMBDA INTEGRASE DNA RECOMBINATION, INTEGRASE, SITE-SPECIFIC RECOMBINATION | | pdb70 | 3NKH\_A | 99.5 | 2.4e-18 | 1.7e-22 | 143.6 | 177 | (218, 398) | 403 | (21, 242) | 244 | Integrase | 3NKH\_A Integrase alpha-fold, MRSA protein, Structural Genomics HET: MRD, SO4, MPD, MSE | | pdb70 | 3NKH\_B | 99.5 | 2.4e-18 | 1.7e-22 | 143.6 | 177 | (218, 398) | 403 | (21, 242) | 244 | Integrase | 3NKH\_B Integrase alpha-fold, MRSA protein, Structural Genomics HET: SO4, MRD, MPD | | pdb70 | 1AE9\_B | 99.5 | 2.5e-18 | 1.8e-22 | 135.9 | 156 | (221, 383) | 403 | (2, 171) | 179 | LAMBDA INTEGRASE | 1AE9\_B LAMBDA INTEGRASE DNA RECOMBINATION, INTEGRASE, SITE-SPECIFIC RECOMBINATION | | pdb70 | 5DOR\_A | 99.4 | 1.3e-17 | 9.2e-22 | 131.8 | 156 | (221, 393) | 403 | (2, 157) | 176 | Integrase | 5DOR\_A Integrase tyrosine recombinase, integrase, hydrolase | | pdb70 | 5DOR\_D | 99.4 | 1.3e-17 | 9.2e-22 | 131.8 | 156 | (221, 393) | 403 | (2, 157) | 176 | Integrase | 5DOR\_D Integrase tyrosine recombinase, integrase, hydrolase | | pdb70 | 1AIH\_A | 99.3 | 1.1e-16 | 7.7e-21 | 125.4 | 150 | (220, 391) | 403 | (4, 160) | 170 | HP1 INTEGRASE | 1AIH\_A HP1 INTEGRASE DNA INTEGRATION, RECOMBINATION HET: SO4 | | pdb70 | 1AIH\_C | 99.3 | 1.1e-16 | 7.7e-21 | 125.4 | 150 | (220, 391) | 403 | (4, 160) | 170 | HP1 INTEGRASE | 1AIH\_C HP1 INTEGRASE DNA INTEGRATION, RECOMBINATION HET: SO4 | | pdb70 | 3VCF\_A | 99.0 | 1.4e-13 | 9.6e-18 | 106.6 | 152 | (220, 398) | 403 | (4, 160) | 163 | Probable integrase | 3VCF\_A Probable integrase catalyzes site-specific integration, RECOMBINATION | | pdb70 | 6GSA\_E | 98.9 | 2.5e-13 | 1.8e-17 | 126.5 | 257 | (119, 392) | 403 | (39, 409) | 562 | Centromere DNA-binding protein complex CBF3 | 6GSA\_E Centromere DNA-binding protein complex CBF3 Centromere, CDEIII-binding, LRR domain, DNA | | pdb70 | 3UXU\_A | 98.9 | 4.6e-13 | 3.2e-17 | 104.3 | 131 | (220, 382) | 403 | (4, 146) | 169 | Probable integrase | 3UXU\_A Probable integrase SSV1, Archaea, Archaeal virus, hyperthermophilic | | pdb70 | 4ACO\_A | 98.8 | 8.3e-13 | 5.9e-17 | 128.0 | 261 | (119, 396) | 403 | (38, 412) | 956 | CENTROMERE DNA-BINDING PROTEIN COMPLEX CBF3 | 4ACO\_A CENTROMERE DNA-BINDING PROTEIN COMPLEX CBF3 DNA BINDING PROTEIN | | pdb70 | 6GYP\_E | 98.8 | 8.3e-13 | 5.9e-17 | 128.0 | 261 | (119, 396) | 403 | (38, 412) | 956 | Centromere DNA-binding protein complex CBF3 | 6GYP\_E Centromere DNA-binding protein complex CBF3 Complex, CELL CYCLE HET: ARG, PHE, THR | | pdb70 | 3JTZ\_A | 98.0 | 3.6e-09 | 2.6e-13 | 71.5 | 59 | (30, 92) | 403 | (24, 82) | 88 | Integrase | 3JTZ\_A Integrase four stranded beta-sheet, DNA BINDING | | pdb70 | 3JU0\_A | 97.9 | 4.8e-09 | 3.4e-13 | 74.0 | 81 | (29, 114) | 403 | (23, 103) | 108 | Phage integrase | 3JU0\_A Phage integrase four stranded beta-sheet, DNA BINDING | | pdb70 | 3T79\_A | 97.8 | 1.2e-08 | 8.7e-13 | 90.0 | 238 | (119, 383) | 403 | (12, 303) | 402 | PROTEIN/DNA Complex | 3T79\_A PROTEIN/DNA Complex DNA recombinase, DNA binding, DNA | | pdb70 | 3SQI\_A | 97.5 | 7.1e-08 | 5e-12 | 87.5 | 238 | (119, 383) | 403 | (12, 303) | 534 | KLLA0E03807p | 3SQI\_A KLLA0E03807p DNA recombinase, DNA binding, DNA | | pdb70 | 2OXO\_A | 97.3 | 2.3e-07 | 1.6e-11 | 63.7 | 85 | (102, 192) | 403 | (2, 87) | 103 | Integrase | 2OXO\_A Integrase DNA-binding protein, four-helix bundle, DNA | | pdb70 | 3LYS\_A | 97.3 | 2.5e-07 | 1.8e-11 | 65.1 | 88 | (99, 192) | 403 | (4, 92) | 112 | Prophage pi2 protein 01, integrase | 3LYS\_A Prophage pi2 protein 01, integrase helical N-terminal domain, Structural Genomics HET: MSE | | pdb70 | 3LYS\_D | 97.3 | 2.5e-07 | 1.8e-11 | 65.1 | 88 | (99, 192) | 403 | (4, 92) | 112 | Prophage pi2 protein 01, integrase | 3LYS\_D Prophage pi2 protein 01, integrase helical N-terminal domain, Structural Genomics | | pdb70 | 2KKV\_A | 97.3 | 2.9e-07 | 2.1e-11 | 65.7 | 94 | (99, 209) | 403 | (5, 100) | 121 | Integrase | 2KKV\_A Integrase Protein structure, PSI, NESGC, Structural | | pdb70 | 2KHQ\_A | 97.3 | 3.8e-07 | 2.7e-11 | 63.8 | 94 | (101, 209) | 403 | (2, 96) | 110 | Integrase | 2KHQ\_A Integrase all-alpha, Structural Genomics, PSI-2, Protein | | pdb70 | 2KEY\_A | 97.2 | 6.6e-07 | 4.6e-11 | 62.8 | 88 | (98, 192) | 403 | (5, 95) | 112 | Putative phage integrase | 2KEY\_A Putative phage integrase Protein structure, PSI, NESG, STRUCTURAL | | pdb70 | 2KJ8\_A | 97.1 | 7.1e-07 | 5e-11 | 63.5 | 95 | (99, 209) | 403 | (3, 99) | 118 | Putative prophage CPS-53 integrase | 2KJ8\_A Putative prophage CPS-53 integrase integrase, intS, intC, yfdB, CPS-53 | | pdb70 | 2KIW\_A | 97.1 | 8.8e-07 | 6.2e-11 | 61.9 | 92 | (102, 209) | 403 | (1, 93) | 111 | Int protein | 2KIW\_A Int protein alpha, Structural Genomics, PSI-2, Protein | | pdb70 | 2KOB\_A | 96.9 | 2e-06 | 1.4e-10 | 59.8 | 92 | (101, 209) | 403 | (2, 94) | 108 | Uncharacterized protein | 2KOB\_A Uncharacterized protein alpha beta, Structural Genomics, PSI-2 | | pdb70 | 2KKP\_A | 96.9 | 2.1e-06 | 1.5e-10 | 60.6 | 96 | (99, 209) | 403 | (5, 103) | 117 | Phage integrase | 2KKP\_A Phage integrase SAM-like domain, alpha-helical bundle, Structural | | pdb70 | 2KD1\_A | 96.9 | 2.4e-06 | 1.7e-10 | 60.3 | 97 | (99, 210) | 403 | (4, 102) | 118 | DNA integration/recombination/invertion protein | 2KD1\_A DNA integration/recombination/invertion protein PROTEIN STRUCTURE INITIATIVE, STRUCTURAL GENOMICS | | pdb70 | 3NRW\_A | 96.8 | 3.1e-06 | 2.2e-10 | 59.5 | 96 | (99, 209) | 403 | (5, 104) | 117 | Phage integrase/site-specific recombinase | 3NRW\_A Phage integrase/site-specific recombinase alpha-helical domain, Structural Genomics, PSI-2 | | pdb70 | 2KJ9\_A | 96.8 | 3.7e-06 | 2.6e-10 | 59.5 | 95 | (98, 209) | 403 | (8, 104) | 118 | Integrase | 2KJ9\_A Integrase integrase, DNA\_BRE\_C superfamily, bacteriophage P4 | | pdb70 | 2KJ5\_A | 96.7 | 4.9e-06 | 3.5e-10 | 58.5 | 97 | (99, 210) | 403 | (5, 101) | 116 | Phage integrase | 2KJ5\_A Phage integrase phage integrase, GFT NMR, PSI-2 | | pdb70 | 2KHV\_A | 96.6 | 9.7e-06 | 6.9e-10 | 55.8 | 84 | (102, 192) | 403 | (2, 87) | 106 | Phage integrase | 2KHV\_A Phage integrase solution structure, GFT, NESG, STRUCTURAL | |
| Top keywords  (threshold 1.00e-03 (evalue)) | **Integrase, recombinase, domain\_containing, Tyr, Site\_specific, Phage, DNA, XerD, complex, Prophage** |
| Output files | ../../similar\_sequences/32\_FANPEZAQ\_CDS\_0032\_merged.svg ../../similar\_sequences/32\_FANPEZAQ\_CDS\_0032\_pdb70.a3m ../../similar\_sequences/32\_FANPEZAQ\_CDS\_0032\_pdb70.hhr ../../similar\_sequences/32\_FANPEZAQ\_CDS\_0032\_uniclust.a3m ../../similar\_sequences/32\_FANPEZAQ\_CDS\_0032\_uniclust.hhr |

#### Structure prediction (AlphaFold)2

|  |  |
| --- | --- |
| Stats | xml version="1.0" encoding="utf-8" standalone="no"?       2024-09-02T21:09:31.882498 image/svg+xml   Matplotlib v3.7.2, https://matplotlib.org/ |
| Predicted structure | **NGL Viewer Controls:**  - Center: *Left-Click* - Rotate: *Left-Click + Drag* - Translate: *Right-Click + Drag* - Zoom: *Shift + Left-Click + Drag* |
| Output files | ../../predicted\_structures/32\_FANPEZAQ\_CDS\_0032/features.pkl ../../predicted\_structures/32\_FANPEZAQ\_CDS\_0032/ranked\_0.pdb ../../predicted\_structures/32\_FANPEZAQ\_CDS\_0032/ranked\_0\_plots.svg ../../predicted\_structures/32\_FANPEZAQ\_CDS\_0032/result\_model\_1\_ptm\_pred\_0.pkl |

#### Structure similarity search results (Foldseek)3

|  |  |
| --- | --- |
| Structure databases searched | Pdb, Afdb-proteome, Afdb-uniprot50 |
| Results, scheme(s)  (Top layers only, threshold 1.00e-02 (evalue)) | xml version="1.0" encoding="utf-8" standalone="no"?       2024-09-02T21:11:06.448704 image/svg+xml   Matplotlib v3.7.2, https://matplotlib.org/ |
| Results, table  (threshold 1.00e-02 (evalue)) | | db | id | prob | evalue | bits | fident | alnlen | mismatch | gapopen | qstart | qend | tstart | tend | name | description | | --- | --- | --- | --- | --- | --- | --- | --- | --- | --- | --- | --- | --- | --- | --- | | pdb | 1Z19\_A | 1.0 | 8.811e-08 | 288 | 0.16 | 305 | 199 | 17 | 101 | 382 | 1 | 271 | Integrase | Integrase | | pdb | 5VFZ\_A | 1.0 | 8.943e-09 | 277 | 0.189 | 316 | 199 | 17 | 107 | 403 | 9 | 286 | Gp33 | Gp33 | | pdb | 1Z1G\_A | 1.0 | 6.942e-08 | 256 | 0.165 | 362 | 240 | 19 | 49 | 382 | 17 | 344 | Integrase | Integrase | | pdb | 5J0N\_E | 1.0 | 1.538e-06 | 249 | 0.166 | 306 | 199 | 16 | 99 | 382 | 1 | 272 | Integrase | Integrase | | pdb | 6EMY\_A | 1.0 | 2.637e-07 | 248 | 0.14 | 341 | 215 | 18 | 101 | 392 | 1 | 312 | Int protein | Int protein | | pdb | 4A8E\_A | 1.0 | 2.765e-07 | 246 | 0.167 | 311 | 202 | 15 | 104 | 403 | 2 | 266 | PROBABLE TYROSINE RECOMBINASE XERC-LIKE | PROBABLE TYROSINE RECOMBINASE XERC-LIKE | | pdb | 1Z1B\_A | 1.0 | 7.281e-08 | 244 | 0.157 | 380 | 243 | 21 | 36 | 382 | 9 | 344 | Integrase | Integrase | | pdb | 6EN0\_A | 1.0 | 4.247e-07 | 238 | 0.154 | 331 | 212 | 17 | 100 | 389 | 1 | 304 | Int protein | Int protein | | pdb | 1Z19\_B | 1.0 | 1.952e-06 | 237 | 0.163 | 311 | 198 | 18 | 101 | 382 | 1 | 278 | Integrase | Integrase | | pdb | 2A3V\_A | 1.0 | 1.398e-06 | 234 | 0.15 | 333 | 209 | 15 | 105 | 386 | 3 | 312 | site-specific recombinase IntI4 | site-specific recombinase IntI4 | | pdb | 6EN2\_B | 1.0 | 5.928e-07 | 234 | 0.132 | 339 | 222 | 17 | 100 | 392 | 1 | 313 | Int protein | Int protein | | pdb | 5J0N\_F | 1.0 | 1.981e-07 | 232 | 0.156 | 390 | 236 | 23 | 36 | 391 | 16 | 346 | Integrase | Integrase | | pdb | 1Z1G\_B | 1.0 | 2.765e-07 | 231 | 0.151 | 363 | 236 | 16 | 49 | 382 | 15 | 334 | Integrase | Integrase | | pdb | 2A3V\_C | 1.0 | 2.857e-06 | 225 | 0.15 | 325 | 204 | 14 | 107 | 382 | 6 | 307 | site-specific recombinase IntI4 | site-specific recombinase IntI4 | | pdb | 6EN1\_B | 1.0 | 1.398e-06 | 223 | 0.149 | 341 | 215 | 18 | 100 | 392 | 1 | 314 | Int protein | Int protein | | pdb | 6EMY\_B | 1.0 | 8.276e-07 | 219 | 0.132 | 340 | 220 | 20 | 101 | 392 | 1 | 313 | Int protein | Int protein | | pdb | 1P7D\_B | 1.0 | 6.126e-06 | 218 | 0.164 | 310 | 199 | 16 | 101 | 382 | 1 | 278 | Integrase | Integrase | | pdb | 6EN0\_B | 1.0 | 1.05e-06 | 218 | 0.144 | 338 | 213 | 15 | 101 | 392 | 1 | 308 | Int protein | Int protein | | pdb | 5HXY\_E | 1.0 | 4.454e-07 | 209 | 0.154 | 318 | 201 | 16 | 103 | 400 | 2 | 271 | Tyrosine recombinase XerA | Tyrosine recombinase XerA | | pdb | 5HXY\_B | 1.0 | 5.389e-07 | 205 | 0.154 | 318 | 196 | 17 | 103 | 400 | 2 | 266 | Tyrosine recombinase XerA | Tyrosine recombinase XerA | | pdb | 5HXY\_D | 1.0 | 1.398e-06 | 203 | 0.182 | 301 | 196 | 16 | 105 | 389 | 2 | 268 | Tyrosine recombinase XerA | Tyrosine recombinase XerA | | pdb | 2A3V\_B | 1.0 | 8.969e-06 | 201 | 0.157 | 318 | 196 | 14 | 107 | 375 | 6 | 300 | site-specific recombinase IntI4 | site-specific recombinase IntI4 | | pdb | 5HXY\_C | 1.0 | 7.524e-07 | 195 | 0.176 | 317 | 188 | 18 | 105 | 400 | 4 | 268 | Tyrosine recombinase XerA | Tyrosine recombinase XerA | | pdb | 5HXY\_F | 1.0 | 3.297e-06 | 185 | 0.184 | 319 | 181 | 23 | 105 | 400 | 2 | 264 | Tyrosine recombinase XerA | Tyrosine recombinase XerA | | pdb | 5JJV\_A | 1.0 | 4.323e-05 | 171 | 0.175 | 279 | 187 | 17 | 143 | 395 | 91 | 352 | Tyrosine recombinase XerH | Tyrosine recombinase XerH | | pdb | 3RMP\_C | 1.0 | 0.005077 | 163 | 0.284 | 88 | 48 | 7 | 1 | 87 | 1 | 74 | CP4-like integrase | CP4-like integrase | | pdb | 5JK0\_D | 1.0 | 1.138e-05 | 163 | 0.158 | 322 | 203 | 20 | 89 | 370 | 36 | 329 | Tyrosine recombinase XerH | Tyrosine recombinase XerH | | pdb | 5JK0\_A | 1.0 | 4.755e-05 | 156 | 0.172 | 336 | 221 | 20 | 90 | 395 | 37 | 345 | Tyrosine recombinase XerH | Tyrosine recombinase XerH | | pdb | 5C6K\_A | 1.0 | 0.003637 | 154 | 0.169 | 153 | 108 | 6 | 220 | 370 | 1 | 136 | Integrase | Integrase | | pdb | 3RMP\_A | 1.0 | 0.005857 | 153 | 0.28 | 89 | 47 | 7 | 1 | 86 | 1 | 75 | CP4-like integrase | CP4-like integrase | | pdb | 3JU0\_A | 1.0 | 0.003306 | 153 | 0.322 | 93 | 52 | 5 | 1 | 93 | 1 | 82 | Phage integrase | Phage integrase | | pdb | 5C6K\_B | 1.0 | 0.006143 | 138 | 0.16 | 175 | 114 | 7 | 221 | 393 | 1 | 144 | Integrase | Integrase | | pdb | 5JJV\_B | 1.0 | 0.0001121 | 136 | 0.165 | 350 | 216 | 19 | 70 | 395 | 58 | 355 | Tyrosine recombinase XerH | Tyrosine recombinase XerH | | pdb | 3NKH\_A | 1.0 | 0.006143 | 105 | 0.122 | 220 | 136 | 13 | 222 | 400 | 5 | 208 | Integrase | Integrase | | pdb | 3NKH\_B | 1.0 | 0.008177 | 105 | 0.14 | 221 | 139 | 10 | 222 | 400 | 6 | 217 | Integrase | Integrase | | afdb-proteome | AF-A0A0H3GN77-F1-MODEL\_V4 | 1.0 | 3.998e-20 | 714 | 0.231 | 419 | 273 | 17 | 2 | 402 | 3 | 390 | Putative site specific integrase | Putative site specific integrase | | afdb-proteome | AF-Q32GN0-F1-MODEL\_V4 | 1.0 | 1.645e-18 | 657 | 0.225 | 421 | 276 | 18 | 1 | 402 | 15 | 404 | Integrase | Integrase | | afdb-proteome | AF-P37326-F1-MODEL\_V4 | 1.0 | 2.527e-18 | 643 | 0.218 | 417 | 280 | 18 | 1 | 403 | 1 | 385 | Prophage integrase IntS | Prophage integrase IntS | | afdb-proteome | AF-P32053-F1-MODEL\_V4 | 1.0 | 3.012e-17 | 610 | 0.195 | 420 | 289 | 17 | 2 | 402 | 8 | 397 | Prophage integrase IntA | Prophage integrase IntA | | afdb-proteome | AF-Q4AAY5-F1-MODEL\_V4 | 1.0 | 5.417e-18 | 603 | 0.187 | 432 | 284 | 22 | 2 | 403 | 3 | 397 | Gifsy-1 prophage protein | Gifsy-1 prophage protein | | afdb-proteome | AF-A0A0H3GY91-F1-MODEL\_V4 | 1.0 | 1.384e-16 | 568 | 0.191 | 424 | 282 | 21 | 2 | 402 | 8 | 393 | Integrase family protein | Integrase family protein | | afdb-proteome | AF-A0A0H3GWB4-F1-MODEL\_V4 | 1.0 | 3.591e-16 | 560 | 0.183 | 420 | 290 | 19 | 2 | 402 | 3 | 388 | Tyr recombinase domain-containing protein | Tyr recombinase domain-containing protein | | afdb-proteome | AF-P76542-F1-MODEL\_V4 | 1.0 | 1.384e-16 | 555 | 0.203 | 422 | 280 | 23 | 2 | 402 | 6 | 392 | Prophage integrase IntZ | Prophage integrase IntZ | | afdb-proteome | AF-A0A0H3GIF3-F1-MODEL\_V4 | 1.0 | 1.127e-15 | 546 | 0.183 | 437 | 289 | 21 | 2 | 403 | 3 | 406 | Putative P4-type integrase | Putative P4-type integrase | | afdb-proteome | AF-Q5F9D7-F1-MODEL\_V4 | 1.0 | 1.2e-16 | 540 | 0.177 | 429 | 283 | 22 | 2 | 402 | 8 | 394 | Integrase | Integrase | | afdb-proteome | AF-A0A0H3GS99-F1-MODEL\_V4 | 1.0 | 2.967e-16 | 526 | 0.181 | 436 | 288 | 25 | 2 | 402 | 3 | 404 | Integrase | Integrase | | afdb-proteome | AF-P39347-F1-MODEL\_V4 | 1.0 | 4.938e-15 | 496 | 0.192 | 400 | 266 | 18 | 36 | 402 | 3 | 378 | Putative protein IntB | Putative protein IntB | | afdb-proteome | AF-P71298-F1-MODEL\_V4 | 1.0 | 5.179e-15 | 490 | 0.192 | 451 | 286 | 24 | 2 | 402 | 33 | 455 | Prophage integrase IntF | Prophage integrase IntF | | afdb-proteome | AF-Q8ZMR4-F1-MODEL\_V4 | 1.0 | 6.792e-14 | 460 | 0.193 | 377 | 247 | 20 | 2 | 354 | 8 | 351 | Fels-2 prophage protein | Fels-2 prophage protein | | afdb-proteome | AF-A0A0H3H506-F1-MODEL\_V4 | 1.0 | 3.495e-11 | 460 | 0.184 | 320 | 226 | 12 | 100 | 402 | 39 | 340 | Integrase | Integrase | | afdb-proteome | AF-A0A0H4IW46-F1-MODEL\_V4 | 1.0 | 4.639e-14 | 420 | 0.224 | 366 | 227 | 19 | 2 | 341 | 7 | 341 | Integrase | Integrase | | afdb-proteome | AF-Q9HXQ6-F1-MODEL\_V4 | 1.0 | 7.382e-10 | 372 | 0.181 | 319 | 208 | 18 | 101 | 393 | 2 | 293 | Tyrosine recombinase XerD | Tyrosine recombinase XerD | | afdb-proteome | AF-Q32BV7-F1-MODEL\_V4 | 1.0 | 1.915e-09 | 369 | 0.159 | 319 | 211 | 20 | 103 | 393 | 4 | 293 | Tyrosine recombinase XerD | Tyrosine recombinase XerD | | afdb-proteome | AF-P0A8P8-F1-MODEL\_V4 | 1.0 | 2.549e-09 | 368 | 0.166 | 319 | 209 | 19 | 103 | 393 | 4 | 293 | Tyrosine recombinase XerD | Tyrosine recombinase XerD | | afdb-proteome | AF-Q9CBU0-F1-MODEL\_V4 | 1.0 | 9.368e-10 | 346 | 0.18 | 327 | 198 | 15 | 106 | 402 | 3 | 289 | Tyrosine recombinase XerC | Tyrosine recombinase XerC | | afdb-proteome | AF-Q2FWP2-F1-MODEL\_V4 | 1.0 | 2.985e-10 | 345 | 0.171 | 374 | 248 | 16 | 46 | 400 | 10 | 340 | Integrase | Integrase | | afdb-proteome | AF-P9WF35-F1-MODEL\_V4 | 1.0 | 1.509e-09 | 336 | 0.182 | 328 | 196 | 16 | 106 | 402 | 4 | 290 | Tyrosine recombinase XerC | Tyrosine recombinase XerC | | afdb-proteome | AF-P0A8P6-F1-MODEL\_V4 | 1.0 | 8e-09 | 335 | 0.18 | 310 | 212 | 15 | 101 | 390 | 2 | 289 | Tyrosine recombinase XerC | Tyrosine recombinase XerC | | afdb-proteome | AF-Q8ZQI9-F1-MODEL\_V4 | 1.0 | 1.266e-10 | 334 | 0.154 | 428 | 257 | 21 | 36 | 395 | 19 | 409 | Putative Fels-1 prophage integrase | Putative Fels-1 prophage integrase | | afdb-proteome | AF-Q329Y7-F1-MODEL\_V4 | 1.0 | 1.015e-08 | 331 | 0.183 | 310 | 211 | 15 | 101 | 390 | 2 | 289 | Tyrosine recombinase XerC | Tyrosine recombinase XerC | | afdb-proteome | AF-K0ETD7-F1-MODEL\_V4 | 1.0 | 1.288e-08 | 328 | 0.175 | 337 | 205 | 20 | 103 | 393 | 2 | 311 | Tyrosine recombinase XerD | Tyrosine recombinase XerD | | afdb-proteome | AF-P76056-F1-MODEL\_V4 | 1.0 | 2.587e-10 | 327 | 0.151 | 442 | 275 | 21 | 25 | 401 | 2 | 408 | Prophage integrase IntR | Prophage integrase IntR | | afdb-proteome | AF-A0A1Y3JR42-F1-MODEL\_V4 | 1.0 | 6.711e-10 | 320 | 0.133 | 412 | 267 | 20 | 34 | 402 | 3 | 367 | Site-specific integrase | Site-specific integrase | | afdb-proteome | AF-Q5F975-F1-MODEL\_V4 | 1.0 | 2.549e-09 | 316 | 0.155 | 387 | 231 | 21 | 34 | 395 | 2 | 317 | Integrase | Integrase | | afdb-proteome | AF-A0A132P7M2-F1-MODEL\_V4 | 1.0 | 1.247e-09 | 312 | 0.144 | 409 | 263 | 22 | 32 | 400 | 2 | 363 | Integrase | Integrase | | afdb-proteome | AF-P24218-F1-MODEL\_V4 | 1.0 | 1.826e-09 | 310 | 0.166 | 396 | 244 | 17 | 46 | 402 | 9 | 357 | Prophage integrase IntD | Prophage integrase IntD | | afdb-proteome | AF-K0F374-F1-MODEL\_V4 | 1.0 | 3.038e-08 | 309 | 0.165 | 332 | 205 | 16 | 103 | 402 | 8 | 299 | Tyrosine recombinase XerC | Tyrosine recombinase XerC | | afdb-proteome | AF-Q8ZQB0-F1-MODEL\_V4 | 1.0 | 8.932e-10 | 309 | 0.168 | 428 | 253 | 20 | 36 | 396 | 10 | 401 | Gifsy-2 prophage integrase | Gifsy-2 prophage integrase | | afdb-proteome | AF-P55888-F1-MODEL\_V4 | 1.0 | 3.505e-08 | 308 | 0.172 | 308 | 213 | 17 | 103 | 390 | 6 | 291 | Tyrosine recombinase XerC | Tyrosine recombinase XerC | | afdb-proteome | AF-A0A0H3GH57-F1-MODEL\_V4 | 1.0 | 4.893e-08 | 306 | 0.171 | 314 | 213 | 17 | 99 | 390 | 3 | 291 | Tyrosine recombinase XerC | Tyrosine recombinase XerC | | afdb-proteome | AF-Q2FZ30-F1-MODEL\_V4 | 1.0 | 3.342e-08 | 303 | 0.185 | 313 | 203 | 13 | 106 | 390 | 4 | 292 | Tyrosine recombinase XerC | Tyrosine recombinase XerC | | afdb-proteome | AF-P76168-F1-MODEL\_V4 | 1.0 | 5.73e-09 | 301 | 0.163 | 386 | 220 | 17 | 75 | 401 | 33 | 374 | Putative defective protein IntQ | Putative defective protein IntQ | | afdb-proteome | AF-P46495-F1-MODEL\_V4 | 1.0 | 2.43e-09 | 300 | 0.159 | 364 | 224 | 20 | 34 | 370 | 2 | 310 | Putative integrase/recombinase HI\_1572 | Putative integrase/recombinase HI\_1572 | | afdb-proteome | AF-A0A4U3SYR4-F1-MODEL\_V4 | 1.0 | 8.8e-09 | 290 | 0.155 | 405 | 261 | 29 | 36 | 393 | 7 | 377 | Site-specific integrase | Site-specific integrase | | afdb-proteome | AF-A0A0H3GP78-F1-MODEL\_V4 | 1.0 | 3.084e-09 | 288 | 0.156 | 410 | 248 | 18 | 36 | 402 | 3 | 357 | Site-specific recombinase, phage integrase family | Site-specific recombinase, phage integrase family | | afdb-proteome | AF-Q32DW2-F1-MODEL\_V4 | 1.0 | 8.8e-09 | 287 | 0.163 | 368 | 239 | 21 | 48 | 382 | 17 | 348 | Integrase | Integrase | | afdb-proteome | AF-X8FIQ7-F1-MODEL\_V4 | 1.0 | 2.804e-09 | 284 | 0.188 | 419 | 253 | 28 | 22 | 402 | 2 | 371 | Phage integrase family protein | Phage integrase family protein | | afdb-proteome | AF-Q8ZMR5-F1-MODEL\_V4 | 1.0 | 1.351e-08 | 283 | 0.167 | 389 | 226 | 23 | 36 | 397 | 3 | 320 | Fels-2 prophage protein | Fels-2 prophage protein | | afdb-proteome | AF-Q5FAI3-F1-MODEL\_V4 | 1.0 | 1.269e-07 | 277 | 0.174 | 321 | 203 | 16 | 103 | 389 | 2 | 294 | Tyrosine recombinase XerC | Tyrosine recombinase XerC | | afdb-proteome | AF-Q8ZNX5-F1-MODEL\_V4 | 1.0 | 2.854e-07 | 269 | 0.17 | 317 | 210 | 21 | 93 | 383 | 6 | 295 | Putative phage integrase protein | Putative phage integrase protein | | afdb-proteome | AF-A0A133CSM1-F1-MODEL\_V4 | 1.0 | 2.075e-08 | 264 | 0.15 | 411 | 261 | 22 | 36 | 401 | 7 | 374 | Site-specific integrase | Site-specific integrase | | afdb-proteome | AF-P75969-F1-MODEL\_V4 | 1.0 | 2.511e-08 | 262 | 0.179 | 384 | 243 | 22 | 34 | 379 | 15 | 364 | Prophage integrase IntE | Prophage integrase IntE | | afdb-proteome | AF-A0A132Z757-F1-MODEL\_V4 | 1.0 | 1.117e-08 | 262 | 0.159 | 415 | 242 | 22 | 36 | 394 | 17 | 380 | Tyrosine-type recombinase/integrase | Tyrosine-type recombinase/integrase | | afdb-proteome | AF-A0A132ZA79-F1-MODEL\_V4 | 1.0 | 5.645e-08 | 261 | 0.137 | 416 | 277 | 21 | 34 | 400 | 3 | 385 | Recombinase | Recombinase | | afdb-proteome | AF-Q2FY83-F1-MODEL\_V4 | 1.0 | 2.762e-08 | 257 | 0.107 | 426 | 271 | 21 | 34 | 396 | 3 | 382 | Bacteriophage integrase | Bacteriophage integrase | | afdb-proteome | AF-A0A133CJD6-F1-MODEL\_V4 | 1.0 | 2.897e-08 | 254 | 0.127 | 409 | 263 | 20 | 34 | 400 | 2 | 358 | Site-specific integrase | Site-specific integrase | | afdb-proteome | AF-A0A0H3GTY0-F1-MODEL\_V4 | 1.0 | 3.139e-07 | 249 | 0.143 | 390 | 236 | 25 | 36 | 397 | 3 | 322 | Gp27 | Gp27 | | afdb-proteome | AF-Q2FX17-F1-MODEL\_V4 | 1.0 | 1e-07 | 248 | 0.145 | 384 | 242 | 27 | 46 | 392 | 11 | 345 | Integrase, phage family, putative | Integrase, phage family, putative | | afdb-proteome | AF-P9WMB3-F1-MODEL\_V4 | 1.0 | 1.269e-07 | 248 | 0.142 | 385 | 261 | 17 | 34 | 391 | 13 | 355 | Putative prophage phiRv2 integrase | Putative prophage phiRv2 integrase | | afdb-proteome | AF-A0A5P3G0X5-F1-MODEL\_V4 | 1.0 | 1.049e-07 | 230 | 0.128 | 445 | 270 | 29 | 14 | 400 | 1 | 385 | Site-specific integrase | Site-specific integrase | | afdb-proteome | AF-A0A132PA96-F1-MODEL\_V4 | 1.0 | 4.383e-07 | 226 | 0.126 | 364 | 214 | 20 | 97 | 401 | 17 | 335 | Integrase | Integrase | | afdb-proteome | AF-A0A133CEM3-F1-MODEL\_V4 | 1.0 | 2.359e-07 | 225 | 0.148 | 391 | 247 | 22 | 56 | 400 | 23 | 373 | Site-specific integrase | Site-specific integrase | | afdb-proteome | AF-C0H5A8-F1-MODEL\_V4 | 1.0 | 2.812e-06 | 223 | 0.146 | 328 | 222 | 14 | 99 | 402 | 188 | 481 | Tyrosine recombinase | Tyrosine recombinase | | afdb-proteome | AF-A0A0H3GQP3-F1-MODEL\_V4 | 1.0 | 2.949e-06 | 222 | 0.155 | 340 | 223 | 21 | 66 | 386 | 8 | 302 | Integrase family protein | Integrase family protein | | afdb-proteome | AF-A0A077ZHS4-F1-MODEL\_V4 | 1.0 | 6.027e-06 | 216 | 0.18 | 266 | 150 | 12 | 175 | 401 | 2 | 238 | MAPEG and Phage integrase and DUF72 domain contai ning protein | MAPEG and Phage integrase and DUF72 domain contai ning protein | | afdb-proteome | AF-Q8ZMP1-F1-MODEL\_V4 | 1.0 | 9.708e-06 | 205 | 0.23 | 200 | 124 | 10 | 2 | 186 | 8 | 192 | Putative integrase | Putative integrase | | afdb-proteome | AF-Q57813-F1-MODEL\_V4 | 1.0 | 1.421e-05 | 195 | 0.113 | 334 | 212 | 14 | 103 | 400 | 28 | 313 | Probable integrase/recombinase protein MJ0367 | Probable integrase/recombinase protein MJ0367 | | afdb-proteome | AF-A0A0H3GZJ0-F1-MODEL\_V4 | 1.0 | 2.641e-05 | 185 | 0.131 | 349 | 212 | 13 | 99 | 399 | 12 | 317 | Class I integron integrase | Class I integron integrase | | afdb-proteome | AF-Q327R4-F1-MODEL\_V4 | 1.0 | 0.0003002 | 159 | 0.276 | 123 | 71 | 6 | 2 | 117 | 3 | 114 | Arm-DNA-bind\_3 domain-containing protein | Arm-DNA-bind\_3 domain-containing protein | | afdb-proteome | AF-A0A132Z1U1-F1-MODEL\_V4 | 1.0 | 0.001196 | 159 | 0.145 | 172 | 120 | 9 | 227 | 392 | 16 | 166 | Integrase | Integrase | | afdb-proteome | AF-O25386-F1-MODEL\_V4 | 1.0 | 2.641e-05 | 158 | 0.146 | 355 | 224 | 19 | 70 | 401 | 57 | 355 | Tyrosine recombinase XerH | Tyrosine recombinase XerH | | afdb-proteome | AF-A0A0H3GVE8-F1-MODEL\_V4 | 1.0 | 0.0002481 | 156 | 0.155 | 303 | 170 | 16 | 137 | 400 | 7 | 262 | Phage integrase | Phage integrase | | afdb-proteome | AF-P0ADH5-F1-MODEL\_V4 | 1.0 | 0.002118 | 147 | 0.185 | 199 | 127 | 12 | 210 | 381 | 1 | 191 | Type 1 fimbriae regulatory protein FimB | Type 1 fimbriae regulatory protein FimB | | afdb-proteome | AF-Q8ZK19-F1-MODEL\_V4 | 1.0 | 0.004541 | 140 | 0.245 | 106 | 60 | 7 | 2 | 101 | 3 | 94 | Putative integrase | Putative integrase | | afdb-proteome | AF-Q7CQX8-F1-MODEL\_V4 | 1.0 | 0.006974 | 132 | 0.162 | 191 | 126 | 10 | 219 | 381 | 1 | 185 | Putative phage integrase | Putative phage integrase | | afdb-uniprot50 | AF-A0A2Z3I9J6-F1-MODEL\_V4 | 1.0 | 5.152e-36 | 1388 | 0.495 | 414 | 183 | 10 | 1 | 402 | 1 | 400 | Uncharacterized protein | Uncharacterized protein | | afdb-uniprot50 | AF-A0A7W4YVC1-F1-MODEL\_V4 | 1.0 | 2.454e-31 | 1266 | 0.39 | 422 | 222 | 13 | 8 | 402 | 6 | 419 | Integrase | Integrase | | afdb-uniprot50 | AF-V4NZZ0-F1-MODEL\_V4 | 1.0 | 1.656e-27 | 1102 | 0.298 | 415 | 263 | 14 | 2 | 402 | 14 | 414 | Integrase | Integrase | | afdb-uniprot50 | AF-B0SV31-F1-MODEL\_V4 | 1.0 | 4.796e-28 | 1092 | 0.308 | 415 | 259 | 13 | 2 | 402 | 4 | 404 | Integrase family protein | Integrase family protein | | afdb-uniprot50 | AF-A0A1I3YI60-F1-MODEL\_V4 | 1.0 | 1.078e-27 | 1090 | 0.306 | 430 | 258 | 14 | 1 | 402 | 1 | 418 | Tyr recombinase domain-containing protein | Tyr recombinase domain-containing protein | | afdb-uniprot50 | AF-A0A4T2A6U0-F1-MODEL\_V4 | 1.0 | 7.255e-27 | 1066 | 0.289 | 428 | 268 | 11 | 1 | 402 | 2 | 419 | Site-specific integrase | Site-specific integrase | | afdb-uniprot50 | AF-A0A1Q8T9Y1-F1-MODEL\_V4 | 1.0 | 4.295e-27 | 1029 | 0.301 | 425 | 259 | 16 | 1 | 402 | 1 | 410 | Tyr recombinase domain-containing protein | Tyr recombinase domain-containing protein | | afdb-uniprot50 | AF-A0A4R8F7U9-F1-MODEL\_V4 | 1.0 | 2.101e-27 | 1023 | 0.296 | 425 | 261 | 15 | 1 | 403 | 1 | 409 | Phage integrase family protein | Phage integrase family protein | | afdb-uniprot50 | AF-A0A1Y6D6E5-F1-MODEL\_V4 | 1.0 | 2.277e-26 | 1017 | 0.301 | 418 | 259 | 17 | 2 | 402 | 4 | 405 | Integrase | Integrase | | afdb-uniprot50 | AF-A0A1T4VSE3-F1-MODEL\_V4 | 1.0 | 9.514e-26 | 996 | 0.275 | 414 | 264 | 9 | 15 | 402 | 1 | 404 | Phage integrase family protein | Phage integrase family protein | | afdb-uniprot50 | AF-Q6LTT6-F1-MODEL\_V4 | 1.0 | 9.514e-26 | 977 | 0.259 | 443 | 273 | 16 | 1 | 402 | 1 | 429 | Hypothetical integrase | Hypothetical integrase | | afdb-uniprot50 | AF-A0A4Q5KHX9-F1-MODEL\_V4 | 1.0 | 1.685e-25 | 966 | 0.283 | 437 | 264 | 17 | 1 | 402 | 1 | 423 | DUF4102 domain-containing protein | DUF4102 domain-containing protein | | afdb-uniprot50 | AF-C6BWF0-F1-MODEL\_V4 | 1.0 | 1.916e-24 | 952 | 0.278 | 410 | 261 | 14 | 17 | 403 | 2 | 399 | Integrase family protein | Integrase family protein | | afdb-uniprot50 | AF-A0A379FVR2-F1-MODEL\_V4 | 1.0 | 1.945e-25 | 949 | 0.264 | 434 | 272 | 16 | 2 | 402 | 3 | 422 | Prophage CP4-57 integrase | Prophage CP4-57 integrase | | afdb-uniprot50 | AF-A0A6J6YBA8-F1-MODEL\_V4 | 1.0 | 5.29e-25 | 948 | 0.289 | 407 | 264 | 13 | 2 | 401 | 3 | 391 | Unannotated protein | Unannotated protein | | afdb-uniprot50 | AF-A0A1T4XEC3-F1-MODEL\_V4 | 1.0 | 6.714e-25 | 947 | 0.272 | 433 | 266 | 16 | 2 | 402 | 4 | 419 | Phage integrase family protein | Phage integrase family protein | | afdb-uniprot50 | AF-W3VAI2-F1-MODEL\_V4 | 1.0 | 6.714e-25 | 922 | 0.255 | 435 | 271 | 17 | 2 | 400 | 5 | 422 | Integrase | Integrase | | afdb-uniprot50 | AF-E6VX36-F1-MODEL\_V4 | 1.0 | 4.969e-24 | 920 | 0.274 | 411 | 263 | 15 | 18 | 402 | 1 | 402 | Integrase family protein | Integrase family protein | | afdb-uniprot50 | AF-A0A7Y4C939-F1-MODEL\_V4 | 1.0 | 2.942e-24 | 915 | 0.287 | 432 | 262 | 15 | 1 | 400 | 1 | 418 | Tyrosine-type recombinase/integrase | Tyrosine-type recombinase/integrase | | afdb-uniprot50 | AF-A0A7U5HNH5-F1-MODEL\_V4 | 1.0 | 4.518e-24 | 905 | 0.286 | 423 | 269 | 16 | 1 | 403 | 1 | 410 | Putative phage integrase | Putative phage integrase | | afdb-uniprot50 | AF-I3TMM1-F1-MODEL\_V4 | 1.0 | 1.397e-22 | 895 | 0.31 | 370 | 221 | 14 | 49 | 402 | 2 | 353 | Integrase family protein | Integrase family protein | | afdb-uniprot50 | AF-A0A1I4Q7X5-F1-MODEL\_V4 | 1.0 | 1.001e-22 | 871 | 0.228 | 437 | 290 | 15 | 1 | 402 | 14 | 438 | Uncharacterized protein | Uncharacterized protein | | afdb-uniprot50 | AF-A0A369C3G4-F1-MODEL\_V4 | 1.0 | 4.244e-23 | 853 | 0.265 | 425 | 256 | 17 | 1 | 402 | 7 | 398 | Site-specific recombinase XerD | Site-specific recombinase XerD | | afdb-uniprot50 | AF-A0A2P6JM49-F1-MODEL\_V4 | 1.0 | 3.188e-23 | 853 | 0.268 | 444 | 269 | 15 | 1 | 402 | 3 | 432 | Integrase | Integrase | | afdb-uniprot50 | AF-A0A837NCG4-F1-MODEL\_V4 | 1.0 | 2.855e-22 | 846 | 0.231 | 428 | 288 | 17 | 1 | 401 | 5 | 418 | Uncharacterized protein | Uncharacterized protein | | afdb-uniprot50 | AF-A0A5C8S084-F1-MODEL\_V4 | 1.0 | 4.385e-22 | 843 | 0.238 | 415 | 272 | 16 | 1 | 402 | 5 | 388 | Tyrosine-type recombinase/integrase | Tyrosine-type recombinase/integrase | | afdb-uniprot50 | AF-Q15Y37-F1-MODEL\_V4 | 1.0 | 1.084e-21 | 828 | 0.232 | 421 | 294 | 17 | 1 | 403 | 1 | 410 | Phage integrase | Phage integrase | | afdb-uniprot50 | AF-Q0A9S4-F1-MODEL\_V4 | 1.0 | 7.407e-22 | 824 | 0.257 | 443 | 275 | 18 | 2 | 402 | 6 | 436 | Phage integrase family protein | Phage integrase family protein | | afdb-uniprot50 | AF-A0A3D1IYE7-F1-MODEL\_V4 | 1.0 | 8.57e-19 | 809 | 0.326 | 315 | 193 | 7 | 100 | 402 | 11 | 318 | Tyr recombinase domain-containing protein | Tyr recombinase domain-containing protein | | afdb-uniprot50 | AF-A0A142BFK5-F1-MODEL\_V4 | 1.0 | 3.245e-21 | 805 | 0.23 | 452 | 285 | 15 | 1 | 402 | 3 | 441 | Uncharacterized protein | Uncharacterized protein | | afdb-uniprot50 | AF-A0A848W2S4-F1-MODEL\_V4 | 1.0 | 6.42e-22 | 803 | 0.264 | 416 | 256 | 20 | 3 | 403 | 10 | 390 | Tyrosine-type recombinase/integrase | Tyrosine-type recombinase/integrase | | afdb-uniprot50 | AF-A0A7T4WGI3-F1-MODEL\_V4 | 1.0 | 1.121e-20 | 792 | 0.237 | 413 | 267 | 19 | 9 | 403 | 6 | 388 | Tyrosine-type recombinase/integrase | Tyrosine-type recombinase/integrase | | afdb-uniprot50 | AF-A0A7G3ZYV6-F1-MODEL\_V4 | 1.0 | 2.438e-21 | 788 | 0.248 | 426 | 268 | 19 | 1 | 402 | 2 | 399 | Site-specific integrase | Site-specific integrase | | afdb-uniprot50 | AF-A0A423PYC9-F1-MODEL\_V4 | 1.0 | 4.531e-21 | 783 | 0.229 | 423 | 271 | 16 | 2 | 402 | 6 | 395 | Uncharacterized protein | Uncharacterized protein | | afdb-uniprot50 | AF-A0A850S105-F1-MODEL\_V4 | 1.0 | 6.325e-21 | 782 | 0.233 | 433 | 267 | 21 | 1 | 403 | 7 | 404 | Tyrosine-type recombinase/integrase | Tyrosine-type recombinase/integrase | | afdb-uniprot50 | AF-A0A1M6HN29-F1-MODEL\_V4 | 1.0 | 3.744e-21 | 781 | 0.234 | 426 | 265 | 20 | 2 | 403 | 3 | 391 | Site-specific recombinase XerD | Site-specific recombinase XerD | | afdb-uniprot50 | AF-A0A537RYP4-F1-MODEL\_V4 | 1.0 | 1.514e-21 | 780 | 0.254 | 437 | 257 | 20 | 2 | 402 | 4 | 407 | DUF4102 domain-containing protein | DUF4102 domain-containing protein | | afdb-uniprot50 | AF-A0A059G9S3-F1-MODEL\_V4 | 1.0 | 3.049e-20 | 775 | 0.251 | 417 | 275 | 18 | 2 | 402 | 6 | 401 | Integrase family protein | Integrase family protein | | afdb-uniprot50 | AF-A0A4T0UP22-F1-MODEL\_V4 | 1.0 | 3.689e-20 | 774 | 0.221 | 415 | 280 | 19 | 2 | 402 | 8 | 393 | DUF4102 domain-containing protein | DUF4102 domain-containing protein | | afdb-uniprot50 | AF-A0A2D6KBJ9-F1-MODEL\_V4 | 1.0 | 2.642e-20 | 773 | 0.265 | 388 | 247 | 16 | 32 | 403 | 12 | 377 | Uncharacterized protein | Uncharacterized protein | | afdb-uniprot50 | AF-A0A4R2L208-F1-MODEL\_V4 | 1.0 | 2.438e-21 | 765 | 0.238 | 428 | 265 | 20 | 2 | 402 | 7 | 400 | Integrase | Integrase | | afdb-uniprot50 | AF-A0A2P9HI64-F1-MODEL\_V4 | 1.0 | 6.03e-21 | 762 | 0.243 | 419 | 268 | 20 | 2 | 403 | 36 | 422 | Site-specific recombinase, phage integrase family | Site-specific recombinase, phage integrase family | | afdb-uniprot50 | AF-A0A504KCG3-F1-MODEL\_V4 | 1.0 | 1.019e-20 | 761 | 0.234 | 426 | 274 | 19 | 2 | 402 | 4 | 402 | DUF4102 domain-containing protein | DUF4102 domain-containing protein | | afdb-uniprot50 | AF-Q0EWR9-F1-MODEL\_V4 | 1.0 | 1.491e-20 | 758 | 0.233 | 419 | 274 | 18 | 2 | 403 | 3 | 391 | Integrative genetic element Gsu32, integrase | Integrative genetic element Gsu32, integrase | | afdb-uniprot50 | AF-A0A495GPM7-F1-MODEL\_V4 | 1.0 | 9.26e-21 | 755 | 0.247 | 424 | 266 | 19 | 2 | 403 | 4 | 396 | Integrase | Integrase | | afdb-uniprot50 | AF-A0A5Q0M768-F1-MODEL\_V4 | 1.0 | 2.813e-21 | 753 | 0.243 | 436 | 263 | 21 | 1 | 403 | 3 | 404 | Tyrosine-type recombinase/integrase | Tyrosine-type recombinase/integrase | | afdb-uniprot50 | AF-A0A2E1XW16-F1-MODEL\_V4 | 1.0 | 2.184e-20 | 751 | 0.243 | 415 | 270 | 18 | 2 | 402 | 6 | 390 | Tyr recombinase domain-containing protein | Tyr recombinase domain-containing protein | | afdb-uniprot50 | AF-A0A0A1F5C1-F1-MODEL\_V4 | 1.0 | 2.519e-20 | 749 | 0.238 | 415 | 271 | 17 | 3 | 402 | 5 | 389 | Integrase | Integrase | | afdb-uniprot50 | AF-Q2SP99-F1-MODEL\_V4 | 1.0 | 1.158e-19 | 741 | 0.223 | 426 | 280 | 19 | 2 | 403 | 4 | 402 | Integrase | Integrase | | afdb-uniprot50 | AF-A0A0S8AFG9-F1-MODEL\_V4 | 1.0 | 3.689e-20 | 740 | 0.243 | 419 | 264 | 19 | 1 | 402 | 2 | 384 | Integrase | Integrase | | afdb-uniprot50 | AF-A0A7C3WGF3-F1-MODEL\_V4 | 1.0 | 2.519e-20 | 740 | 0.237 | 433 | 266 | 22 | 2 | 402 | 6 | 406 | Site-specific integrase | Site-specific integrase | | afdb-uniprot50 | AF-A0A7V2T5X5-F1-MODEL\_V4 | 1.0 | 1.865e-19 | 732 | 0.251 | 418 | 269 | 16 | 2 | 402 | 14 | 404 | Site-specific integrase | Site-specific integrase | | afdb-uniprot50 | AF-A0A2N7M5E9-F1-MODEL\_V4 | 1.0 | 8.699e-20 | 731 | 0.229 | 431 | 273 | 22 | 2 | 402 | 4 | 405 | Integrase | Integrase | | afdb-uniprot50 | AF-A7K117-F1-MODEL\_V4 | 1.0 | 3.15e-19 | 730 | 0.211 | 431 | 281 | 17 | 1 | 401 | 3 | 404 | Site-specific recombinase, phage integrase family protein | Site-specific recombinase, phage integrase family protein | | afdb-uniprot50 | AF-U2H964-F1-MODEL\_V4 | 1.0 | 4.463e-20 | 730 | 0.232 | 421 | 273 | 18 | 2 | 402 | 66 | 456 | Integrase | Integrase | | afdb-uniprot50 | AF-M9RL77-F1-MODEL\_V4 | 1.0 | 1.004e-19 | 729 | 0.226 | 410 | 272 | 18 | 2 | 402 | 9 | 382 | Putative phage integrase | Putative phage integrase | | afdb-uniprot50 | AF-A0A849TNM8-F1-MODEL\_V4 | 1.0 | 1.053e-19 | 729 | 0.22 | 408 | 276 | 18 | 7 | 402 | 12 | 389 | Tyrosine-type recombinase/integrase | Tyrosine-type recombinase/integrase | | afdb-uniprot50 | AF-A0A1I3R028-F1-MODEL\_V4 | 1.0 | 7.189e-20 | 728 | 0.245 | 423 | 262 | 23 | 1 | 403 | 1 | 386 | Uncharacterized protein | Uncharacterized protein | | afdb-uniprot50 | AF-A0A0W1A9G8-F1-MODEL\_V4 | 1.0 | 1.695e-19 | 727 | 0.199 | 417 | 291 | 14 | 1 | 402 | 1 | 389 | Integrase | Integrase | | afdb-uniprot50 | AF-A0A454JJL7-F1-MODEL\_V4 | 1.0 | 1.274e-19 | 727 | 0.241 | 419 | 269 | 17 | 1 | 402 | 1 | 387 | DUF4102 domain-containing protein | DUF4102 domain-containing protein | | afdb-uniprot50 | AF-A0A7W2BWR4-F1-MODEL\_V4 | 1.0 | 1.616e-19 | 725 | 0.233 | 424 | 269 | 22 | 2 | 402 | 3 | 393 | Tyrosine-type recombinase/integrase | Tyrosine-type recombinase/integrase | | afdb-uniprot50 | AF-A0A1G5PTY7-F1-MODEL\_V4 | 1.0 | 2.082e-20 | 725 | 0.22 | 454 | 282 | 19 | 1 | 398 | 3 | 440 | Phage integrase family protein | Phage integrase family protein | | afdb-uniprot50 | AF-A0A6I2IUJ7-F1-MODEL\_V4 | 1.0 | 1.336e-19 | 722 | 0.208 | 423 | 283 | 18 | 1 | 402 | 1 | 392 | Tyrosine-type recombinase/integrase | Tyrosine-type recombinase/integrase | | afdb-uniprot50 | AF-A0A523JSY9-F1-MODEL\_V4 | 1.0 | 1.469e-19 | 722 | 0.233 | 416 | 267 | 21 | 2 | 401 | 5 | 384 | Site-specific integrase | Site-specific integrase | | afdb-uniprot50 | AF-A0A7Y7NB12-F1-MODEL\_V4 | 1.0 | 2.151e-19 | 722 | 0.237 | 437 | 263 | 19 | 1 | 402 | 7 | 408 | Integrase arm-type DNA-binding domain-containing protein | Integrase arm-type DNA-binding domain-containing protein | | afdb-uniprot50 | AF-A0A2T5NNU0-F1-MODEL\_V4 | 1.0 | 8.699e-20 | 720 | 0.201 | 417 | 288 | 21 | 1 | 402 | 7 | 393 | Integrase | Integrase | | afdb-uniprot50 | AF-A0A2H4W5G4-F1-MODEL\_V4 | 1.0 | 5.941e-20 | 718 | 0.227 | 453 | 285 | 19 | 1 | 402 | 10 | 448 | Tyr recombinase domain-containing protein | Tyr recombinase domain-containing protein | | afdb-uniprot50 | AF-A0A5C7LRP0-F1-MODEL\_V4 | 1.0 | 2.864e-19 | 715 | 0.228 | 428 | 270 | 21 | 2 | 402 | 27 | 421 | DUF4102 domain-containing protein | DUF4102 domain-containing protein | | afdb-uniprot50 | AF-A0A1X7AJC6-F1-MODEL\_V4 | 1.0 | 2.256e-19 | 713 | 0.248 | 450 | 276 | 15 | 1 | 402 | 3 | 438 | Phage integrase family protein | Phage integrase family protein | | afdb-uniprot50 | AF-A0A0W0U9B0-F1-MODEL\_V4 | 1.0 | 2.367e-19 | 712 | 0.215 | 418 | 282 | 17 | 1 | 402 | 4 | 391 | Phage-related integrase | Phage-related integrase | | afdb-uniprot50 | AF-A0A7C3KWL2-F1-MODEL\_V4 | 1.0 | 7.082e-19 | 712 | 0.228 | 433 | 264 | 22 | 2 | 402 | 6 | 400 | DUF4102 domain-containing protein | DUF4102 domain-containing protein | | afdb-uniprot50 | AF-A0A7V2S709-F1-MODEL\_V4 | 1.0 | 4.397e-19 | 710 | 0.198 | 413 | 281 | 20 | 2 | 402 | 3 | 377 | DUF4102 domain-containing protein | DUF4102 domain-containing protein | | afdb-uniprot50 | AF-A0A850T1C9-F1-MODEL\_V4 | 1.0 | 7.791e-19 | 710 | 0.236 | 427 | 272 | 23 | 2 | 401 | 6 | 405 | Integrase family protein | Integrase family protein | | afdb-uniprot50 | AF-A0A2Z6G8D0-F1-MODEL\_V4 | 1.0 | 2.256e-19 | 710 | 0.215 | 418 | 279 | 18 | 2 | 402 | 33 | 418 | Prophage integrase IntA | Prophage integrase IntA | | afdb-uniprot50 | AF-A0A2S7FLE3-F1-MODEL\_V4 | 1.0 | 4.256e-20 | 708 | 0.199 | 421 | 281 | 20 | 2 | 401 | 3 | 388 | DUF4102 domain-containing protein | DUF4102 domain-containing protein | | afdb-uniprot50 | AF-A0A1Q3UZH6-F1-MODEL\_V4 | 1.0 | 3.634e-19 | 708 | 0.252 | 419 | 270 | 18 | 2 | 403 | 6 | 398 | Uncharacterized protein | Uncharacterized protein | | afdb-uniprot50 | AF-Q5P090-F1-MODEL\_V4 | 1.0 | 6.854e-20 | 708 | 0.235 | 421 | 271 | 18 | 1 | 402 | 2 | 390 | Integrase | Integrase | | afdb-uniprot50 | AF-A0A7C1UI09-F1-MODEL\_V4 | 1.0 | 2.256e-19 | 708 | 0.239 | 413 | 262 | 19 | 9 | 402 | 18 | 397 | Site-specific integrase | Site-specific integrase | | afdb-uniprot50 | AF-V6F355-F1-MODEL\_V4 | 1.0 | 2.151e-19 | 707 | 0.222 | 422 | 278 | 19 | 2 | 402 | 8 | 400 | Putative prophage CPZ-55 integrase | Putative prophage CPZ-55 integrase | | afdb-uniprot50 | AF-A0A2N6AWZ1-F1-MODEL\_V4 | 1.0 | 3.465e-19 | 706 | 0.243 | 410 | 269 | 18 | 2 | 400 | 6 | 385 | Integrase | Integrase | | afdb-uniprot50 | AF-A0A2S5M9V6-F1-MODEL\_V4 | 1.0 | 2.184e-20 | 706 | 0.255 | 411 | 263 | 15 | 8 | 402 | 8 | 391 | Integrase | Integrase | | afdb-uniprot50 | AF-A0A7C1NNI4-F1-MODEL\_V4 | 1.0 | 4.91e-20 | 706 | 0.226 | 460 | 280 | 19 | 1 | 402 | 13 | 454 | Integrase | Integrase | | afdb-uniprot50 | AF-A0A7X9IKM6-F1-MODEL\_V4 | 1.0 | 2.864e-19 | 702 | 0.22 | 409 | 276 | 20 | 8 | 402 | 8 | 387 | Tyrosine-type recombinase/integrase | Tyrosine-type recombinase/integrase | | afdb-uniprot50 | AF-A0A1T4XZ14-F1-MODEL\_V4 | 1.0 | 3.304e-19 | 701 | 0.234 | 413 | 271 | 16 | 2 | 402 | 3 | 382 | Integrase | Integrase | | afdb-uniprot50 | AF-A0A1E7D9T2-F1-MODEL\_V4 | 1.0 | 2.445e-18 | 701 | 0.202 | 430 | 288 | 14 | 1 | 403 | 3 | 404 | Phage integrase | Phage integrase | | afdb-uniprot50 | AF-A0A1Q3IXG3-F1-MODEL\_V4 | 1.0 | 3.465e-19 | 701 | 0.227 | 426 | 270 | 23 | 2 | 403 | 3 | 393 | Integrase | Integrase | | afdb-uniprot50 | AF-A0A4R1J419-F1-MODEL\_V4 | 1.0 | 1.469e-19 | 701 | 0.213 | 422 | 280 | 20 | 2 | 402 | 3 | 393 | Site-specific recombinase XerD | Site-specific recombinase XerD | | afdb-uniprot50 | AF-A0A4T2A4G2-F1-MODEL\_V4 | 1.0 | 2.331e-18 | 701 | 0.213 | 422 | 280 | 21 | 2 | 402 | 3 | 393 | DUF4102 domain-containing protein | DUF4102 domain-containing protein | | afdb-uniprot50 | AF-A0A1Q3NZN8-F1-MODEL\_V4 | 1.0 | 1.158e-19 | 701 | 0.262 | 426 | 257 | 20 | 2 | 403 | 6 | 398 | Tyr recombinase domain-containing protein | Tyr recombinase domain-containing protein | | afdb-uniprot50 | AF-A0A522J360-F1-MODEL\_V4 | 1.0 | 7.428e-19 | 700 | 0.228 | 433 | 272 | 22 | 3 | 402 | 19 | 422 | Site-specific integrase | Site-specific integrase | | afdb-uniprot50 | AF-A0A5M8FH85-F1-MODEL\_V4 | 1.0 | 4.91e-20 | 700 | 0.239 | 464 | 271 | 22 | 1 | 403 | 1 | 443 | Integrase family protein | Integrase family protein | | afdb-uniprot50 | AF-A0A5Y1WL89-F1-MODEL\_V4 | 1.0 | 8.171e-19 | 699 | 0.2 | 420 | 282 | 21 | 2 | 402 | 3 | 387 | Tyrosine-type recombinase/integrase | Tyrosine-type recombinase/integrase | | afdb-uniprot50 | AF-H8YW01-F1-MODEL\_V4 | 1.0 | 1.274e-19 | 699 | 0.222 | 462 | 277 | 21 | 1 | 401 | 1 | 441 | Phage integrase family protein | Phage integrase family protein | | afdb-uniprot50 | AF-A0A127F2R1-F1-MODEL\_V4 | 1.0 | 2.864e-19 | 698 | 0.222 | 423 | 276 | 19 | 1 | 402 | 1 | 391 | Symbiosis island integrase | Symbiosis island integrase | | afdb-uniprot50 | AF-A0A837V9C3-F1-MODEL\_V4 | 1.0 | 3.634e-19 | 697 | 0.219 | 419 | 280 | 17 | 2 | 402 | 3 | 392 | Integrase | Integrase | | afdb-uniprot50 | AF-E6W6Y4-F1-MODEL\_V4 | 1.0 | 1.38e-18 | 695 | 0.224 | 423 | 272 | 20 | 2 | 402 | 8 | 396 | Integrase family protein | Integrase family protein | | afdb-uniprot50 | AF-A0A7X0ALK7-F1-MODEL\_V4 | 1.0 | 5.401e-20 | 695 | 0.215 | 423 | 278 | 18 | 1 | 402 | 1 | 390 | Integrase | Integrase | | afdb-uniprot50 | AF-A0A3D2H5V9-F1-MODEL\_V4 | 1.0 | 1.865e-19 | 695 | 0.226 | 428 | 274 | 17 | 2 | 402 | 4 | 401 | Integrase | Integrase | | afdb-uniprot50 | AF-A0A378IX19-F1-MODEL\_V4 | 1.0 | 1.316e-18 | 695 | 0.198 | 418 | 289 | 17 | 1 | 402 | 4 | 391 | Phage-related integrase | Phage-related integrase | | afdb-uniprot50 | AF-A0A1I5DK39-F1-MODEL\_V4 | 1.0 | 1.274e-19 | 695 | 0.216 | 424 | 275 | 18 | 2 | 402 | 98 | 487 | Integrase | Integrase | | afdb-uniprot50 | AF-A0A529Y462-F1-MODEL\_V4 | 1.0 | 6.438e-19 | 694 | 0.294 | 302 | 192 | 9 | 2 | 293 | 4 | 294 | Site-specific integrase | Site-specific integrase | | afdb-uniprot50 | AF-A0A2S9KTH3-F1-MODEL\_V4 | 1.0 | 4.193e-19 | 693 | 0.216 | 420 | 280 | 19 | 2 | 402 | 3 | 392 | Integrase | Integrase | | afdb-uniprot50 | AF-A0A4V3WAS3-F1-MODEL\_V4 | 1.0 | 3.998e-19 | 693 | 0.212 | 432 | 281 | 20 | 1 | 402 | 6 | 408 | DUF4102 domain-containing protein | DUF4102 domain-containing protein | | afdb-uniprot50 | AF-A0A2N2FX08-F1-MODEL\_V4 | 1.0 | 8.988e-19 | 692 | 0.223 | 443 | 265 | 23 | 1 | 402 | 2 | 406 | Integrase | Integrase | | afdb-uniprot50 | AF-A0A0P7Z5I7-F1-MODEL\_V4 | 1.0 | 4.612e-19 | 690 | 0.232 | 425 | 274 | 20 | 2 | 402 | 6 | 402 | Integrase | Integrase | | afdb-uniprot50 | AF-A0A7C3PY40-F1-MODEL\_V4 | 1.0 | 2.331e-18 | 689 | 0.214 | 447 | 267 | 28 | 2 | 402 | 6 | 414 | DUF4102 domain-containing protein | DUF4102 domain-containing protein | | afdb-uniprot50 | AF-A0A395JJS3-F1-MODEL\_V4 | 1.0 | 1.837e-18 | 688 | 0.207 | 414 | 280 | 16 | 1 | 398 | 1 | 382 | Integrase | Integrase | | afdb-uniprot50 | AF-A0A1Z8Q0M2-F1-MODEL\_V4 | 1.0 | 1.448e-18 | 686 | 0.186 | 419 | 294 | 18 | 2 | 402 | 3 | 392 | Tyr recombinase domain-containing protein | Tyr recombinase domain-containing protein | | afdb-uniprot50 | AF-A0A377P7I5-F1-MODEL\_V4 | 1.0 | 3.465e-19 | 685 | 0.219 | 419 | 278 | 18 | 2 | 402 | 3 | 390 | Prophage CPS-53 integrase | Prophage CPS-53 integrase | | afdb-uniprot50 | AF-A0A2N4XL17-F1-MODEL\_V4 | 1.0 | 7.428e-19 | 685 | 0.214 | 420 | 280 | 17 | 2 | 402 | 3 | 391 | Integrase | Integrase | | afdb-uniprot50 | AF-A0A239PYP0-F1-MODEL\_V4 | 1.0 | 3.634e-19 | 685 | 0.258 | 425 | 265 | 20 | 1 | 402 | 1 | 398 | Integrase | Integrase | | afdb-uniprot50 | AF-A0A840V7L5-F1-MODEL\_V4 | 1.0 | 3.998e-19 | 685 | 0.213 | 427 | 280 | 18 | 1 | 402 | 31 | 426 | Integrase | Integrase | | afdb-uniprot50 | AF-A0A2V7TR44-F1-MODEL\_V4 | 1.0 | 4.193e-19 | 684 | 0.23 | 417 | 273 | 21 | 2 | 402 | 6 | 390 | Uncharacterized protein | Uncharacterized protein | | afdb-uniprot50 | AF-Q5P707-F1-MODEL\_V4 | 1.0 | 3.812e-19 | 684 | 0.235 | 420 | 271 | 21 | 1 | 402 | 1 | 388 | Phage-related integrase | Phage-related integrase | | afdb-uniprot50 | AF-A0A845M1N2-F1-MODEL\_V4 | 1.0 | 1.67e-18 | 683 | 0.212 | 418 | 280 | 19 | 2 | 402 | 3 | 388 | Tyrosine-type recombinase/integrase | Tyrosine-type recombinase/integrase | | afdb-uniprot50 | AF-A0A143PQ67-F1-MODEL\_V4 | 1.0 | 1.088e-18 | 681 | 0.258 | 441 | 249 | 23 | 2 | 402 | 5 | 407 | Prophage CP4-57 integrase | Prophage CP4-57 integrase | | afdb-uniprot50 | AF-S9TLS3-F1-MODEL\_V4 | 1.0 | 7.791e-19 | 680 | 0.261 | 421 | 261 | 19 | 2 | 403 | 8 | 397 | Integrase | Integrase | | afdb-uniprot50 | AF-A0A1Y2J7Q5-F1-MODEL\_V4 | 1.0 | 2.297e-17 | 680 | 0.191 | 423 | 287 | 21 | 2 | 402 | 9 | 398 | Tyr recombinase domain-containing protein | Tyr recombinase domain-containing protein | | afdb-uniprot50 | AF-A0A317EG89-F1-MODEL\_V4 | 1.0 | 4.397e-19 | 679 | 0.222 | 423 | 274 | 20 | 2 | 402 | 9 | 398 | Integrase | Integrase | | afdb-uniprot50 | AF-A0A1H5IA46-F1-MODEL\_V4 | 1.0 | 4.193e-19 | 679 | 0.217 | 428 | 279 | 20 | 2 | 402 | 5 | 403 | Site-specific recombinase XerD | Site-specific recombinase XerD | | afdb-uniprot50 | AF-A0A290T5I9-F1-MODEL\_V4 | 1.0 | 1.255e-18 | 678 | 0.211 | 420 | 284 | 16 | 1 | 401 | 1 | 392 | Tyr recombinase domain-containing protein | Tyr recombinase domain-containing protein | | afdb-uniprot50 | AF-A0A7V7N0U3-F1-MODEL\_V4 | 1.0 | 1.518e-18 | 678 | 0.196 | 412 | 285 | 16 | 9 | 402 | 6 | 389 | DUF4102 domain-containing protein | DUF4102 domain-containing protein | | afdb-uniprot50 | AF-A0A286IFF7-F1-MODEL\_V4 | 1.0 | 2.367e-19 | 678 | 0.227 | 436 | 268 | 19 | 2 | 403 | 6 | 406 | Site-specific recombinase XerD | Site-specific recombinase XerD | | afdb-uniprot50 | AF-A0A2E0QXG3-F1-MODEL\_V4 | 1.0 | 8.171e-19 | 678 | 0.22 | 418 | 278 | 19 | 2 | 401 | 23 | 410 | Integrase | Integrase | | afdb-uniprot50 | AF-A0A2D8HMA2-F1-MODEL\_V4 | 1.0 | 8.57e-19 | 676 | 0.232 | 421 | 279 | 17 | 1 | 402 | 9 | 404 | Integrase | Integrase | | afdb-uniprot50 | AF-A0A522KQT0-F1-MODEL\_V4 | 1.0 | 3.255e-18 | 676 | 0.216 | 435 | 278 | 19 | 1 | 402 | 13 | 417 | DUF4102 domain-containing protein | DUF4102 domain-containing protein | | afdb-uniprot50 | AF-A0A7C8HSY9-F1-MODEL\_V4 | 1.0 | 4.837e-19 | 675 | 0.212 | 419 | 279 | 19 | 2 | 403 | 3 | 387 | Prophage integrase IntA | Prophage integrase IntA | | afdb-uniprot50 | AF-A0A4P0Z7G3-F1-MODEL\_V4 | 1.0 | 6.653e-18 | 675 | 0.209 | 429 | 278 | 18 | 2 | 402 | 8 | 403 | Phage integrase | Phage integrase | | afdb-uniprot50 | AF-A0A365QGV4-F1-MODEL\_V4 | 1.0 | 3.304e-19 | 675 | 0.219 | 441 | 272 | 23 | 2 | 403 | 7 | 414 | Integrase | Integrase | | afdb-uniprot50 | AF-A0A3S0XF73-F1-MODEL\_V4 | 1.0 | 8.594e-16 | 674 | 0.276 | 329 | 200 | 12 | 92 | 402 | 6 | 314 | Site-specific integrase | Site-specific integrase | | afdb-uniprot50 | AF-A0A1G0ESB5-F1-MODEL\_V4 | 1.0 | 6.139e-19 | 674 | 0.24 | 428 | 264 | 20 | 2 | 402 | 3 | 396 | Integrase | Integrase | | afdb-uniprot50 | AF-A0A1M7YL42-F1-MODEL\_V4 | 1.0 | 1.196e-18 | 673 | 0.207 | 425 | 283 | 17 | 2 | 402 | 4 | 398 | Integrase | Integrase | | afdb-uniprot50 | AF-A0A235EH93-F1-MODEL\_V4 | 1.0 | 6.753e-19 | 672 | 0.216 | 421 | 278 | 19 | 2 | 403 | 3 | 390 | Integrase | Integrase | | afdb-uniprot50 | AF-A0A847R4Z8-F1-MODEL\_V4 | 1.0 | 1.837e-18 | 671 | 0.178 | 419 | 297 | 17 | 1 | 403 | 1 | 388 | Integrase arm-type DNA-binding domain-containing protein | Integrase arm-type DNA-binding domain-containing protein | | afdb-uniprot50 | AF-A0A1S8CFL3-F1-MODEL\_V4 | 1.0 | 1.518e-18 | 671 | 0.214 | 419 | 280 | 17 | 2 | 402 | 3 | 390 | Integrase | Integrase | | afdb-uniprot50 | AF-A0A2V8IYL9-F1-MODEL\_V4 | 1.0 | 6.343e-18 | 671 | 0.214 | 429 | 280 | 21 | 2 | 402 | 6 | 405 | Uncharacterized protein | Uncharacterized protein | | afdb-uniprot50 | AF-A0A4P7R7H7-F1-MODEL\_V4 | 1.0 | 1.752e-18 | 671 | 0.222 | 418 | 280 | 19 | 2 | 402 | 3 | 392 | DUF4102 domain-containing protein | DUF4102 domain-containing protein | | afdb-uniprot50 | AF-A0A1P8UJ48-F1-MODEL\_V4 | 1.0 | 3.103e-18 | 670 | 0.213 | 435 | 279 | 18 | 2 | 402 | 7 | 412 | Uncharacterized protein | Uncharacterized protein | | afdb-uniprot50 | AF-A0A3B0YJF4-F1-MODEL\_V4 | 1.0 | 1.67e-18 | 669 | 0.205 | 419 | 282 | 19 | 2 | 403 | 10 | 394 | Integrase | Integrase | | afdb-uniprot50 | AF-Q31UY1-F1-MODEL\_V4 | 1.0 | 3.255e-18 | 669 | 0.205 | 428 | 287 | 18 | 2 | 402 | 80 | 481 | Putative integrase | Putative integrase | | afdb-uniprot50 | AF-A0A329B445-F1-MODEL\_V4 | 1.0 | 1.448e-18 | 668 | 0.223 | 420 | 276 | 18 | 1 | 402 | 1 | 388 | Integrase | Integrase | | afdb-uniprot50 | AF-A0A2N6DE38-F1-MODEL\_V4 | 1.0 | 1.592e-18 | 668 | 0.201 | 422 | 284 | 20 | 2 | 402 | 3 | 392 | Integrase | Integrase | | afdb-uniprot50 | AF-A0A7Y6D5N6-F1-MODEL\_V4 | 1.0 | 9.887e-19 | 668 | 0.217 | 418 | 280 | 17 | 2 | 402 | 3 | 390 | Integrase arm-type DNA-binding domain-containing protein | Integrase arm-type DNA-binding domain-containing protein | | afdb-uniprot50 | AF-A0A1H4U7X0-F1-MODEL\_V4 | 1.0 | 6.753e-19 | 668 | 0.212 | 465 | 293 | 19 | 1 | 402 | 2 | 456 | Phage integrase family protein | Phage integrase family protein | | afdb-uniprot50 | AF-A0A1C3H4D2-F1-MODEL\_V4 | 1.0 | 1.67e-18 | 667 | 0.208 | 423 | 278 | 20 | 1 | 402 | 1 | 387 | Integrase | Integrase | | afdb-uniprot50 | AF-A0A2S5NSX3-F1-MODEL\_V4 | 1.0 | 2.821e-18 | 667 | 0.223 | 420 | 280 | 20 | 2 | 402 | 3 | 395 | Integrase | Integrase | | afdb-uniprot50 | AF-A6QCY9-F1-MODEL\_V4 | 1.0 | 1.124e-17 | 667 | 0.219 | 424 | 279 | 19 | 2 | 402 | 8 | 402 | Site-specific recombinase, phage integrase family | Site-specific recombinase, phage integrase family | | afdb-uniprot50 | AF-A0A1Q3RET7-F1-MODEL\_V4 | 1.0 | 3.103e-18 | 666 | 0.221 | 424 | 275 | 20 | 2 | 402 | 3 | 394 | Integrase | Integrase | | afdb-uniprot50 | AF-A0A2P5IL45-F1-MODEL\_V4 | 1.0 | 1.67e-18 | 666 | 0.218 | 425 | 275 | 20 | 2 | 403 | 3 | 393 | Integrase | Integrase | | afdb-uniprot50 | AF-W7WZL9-F1-MODEL\_V4 | 1.0 | 8.171e-19 | 666 | 0.227 | 413 | 264 | 18 | 20 | 402 | 50 | 437 | Prophage CP4-57 integrase | Prophage CP4-57 integrase | | afdb-uniprot50 | AF-A0A6N7GTQ0-F1-MODEL\_V4 | 1.0 | 5.766e-18 | 665 | 0.244 | 417 | 269 | 17 | 2 | 402 | 30 | 416 | Tyrosine-type recombinase/integrase | Tyrosine-type recombinase/integrase | | afdb-uniprot50 | AF-A0A443UGA5-F1-MODEL\_V4 | 1.0 | 7.082e-19 | 664 | 0.201 | 432 | 282 | 15 | 2 | 402 | 4 | 403 | Site-specific integrase | Site-specific integrase | | afdb-uniprot50 | AF-A0A0J1CJU1-F1-MODEL\_V4 | 1.0 | 2.959e-18 | 664 | 0.222 | 423 | 275 | 19 | 1 | 402 | 2 | 391 | Integrase | Integrase | | afdb-uniprot50 | AF-C0N6F7-F1-MODEL\_V4 | 1.0 | 2.565e-18 | 663 | 0.189 | 427 | 289 | 20 | 2 | 402 | 3 | 398 | Site-specific recombinase, phage integrase family protein | Site-specific recombinase, phage integrase family protein | | afdb-uniprot50 | AF-A0A2N9L337-F1-MODEL\_V4 | 1.0 | 9.887e-19 | 663 | 0.189 | 422 | 290 | 19 | 2 | 402 | 3 | 393 | Phage integrase | Phage integrase | | afdb-uniprot50 | AF-W7WT06-F1-MODEL\_V4 | 1.0 | 6.653e-18 | 662 | 0.207 | 380 | 257 | 16 | 44 | 403 | 6 | 361 | Prophage CP4-57 integrase | Prophage CP4-57 integrase | | afdb-uniprot50 | AF-A0A509IV90-F1-MODEL\_V4 | 1.0 | 2.297e-17 | 662 | 0.211 | 383 | 263 | 17 | 36 | 402 | 2 | 361 | Integrase | Integrase | | afdb-uniprot50 | AF-A0A550FGH2-F1-MODEL\_V4 | 1.0 | 2.223e-18 | 662 | 0.209 | 420 | 286 | 14 | 2 | 402 | 8 | 400 | DUF4102 domain-containing protein | DUF4102 domain-containing protein | | afdb-uniprot50 | AF-A0A5W0NCW6-F1-MODEL\_V4 | 1.0 | 9.74e-18 | 661 | 0.235 | 417 | 274 | 17 | 2 | 402 | 3 | 390 | Tyrosine-type recombinase/integrase | Tyrosine-type recombinase/integrase | | afdb-uniprot50 | AF-A0A1G3KD23-F1-MODEL\_V4 | 1.0 | 1.255e-18 | 661 | 0.217 | 427 | 275 | 23 | 2 | 402 | 3 | 396 | Integrase | Integrase | | afdb-uniprot50 | AF-A0A6C1P3F8-F1-MODEL\_V4 | 1.0 | 3.103e-18 | 661 | 0.227 | 409 | 277 | 16 | 9 | 403 | 9 | 392 | Site-specific integrase | Site-specific integrase | | afdb-uniprot50 | AF-A0A2E0A4M5-F1-MODEL\_V4 | 1.0 | 3.414e-18 | 660 | 0.232 | 418 | 269 | 18 | 2 | 402 | 3 | 385 | Integrase | Integrase | | afdb-uniprot50 | AF-A0A2N8S9G8-F1-MODEL\_V4 | 1.0 | 1.518e-18 | 660 | 0.23 | 451 | 272 | 21 | 11 | 402 | 2 | 436 | Integrase | Integrase | | afdb-uniprot50 | AF-A0A7W4PUN0-F1-MODEL\_V4 | 1.0 | 2.12e-18 | 659 | 0.22 | 421 | 275 | 18 | 2 | 402 | 3 | 390 | Tyrosine-type recombinase/integrase | Tyrosine-type recombinase/integrase | | afdb-uniprot50 | AF-A0A2D6TQU3-F1-MODEL\_V4 | 1.0 | 1.927e-18 | 659 | 0.207 | 425 | 281 | 19 | 2 | 403 | 3 | 394 | Integrase | Integrase | | afdb-uniprot50 | AF-A0A1T4YJI4-F1-MODEL\_V4 | 1.0 | 6.048e-18 | 659 | 0.222 | 423 | 277 | 19 | 2 | 402 | 3 | 395 | Integrase | Integrase | | afdb-uniprot50 | AF-A0A3G8GZZ2-F1-MODEL\_V4 | 1.0 | 3.938e-18 | 659 | 0.207 | 419 | 286 | 16 | 2 | 402 | 8 | 398 | DUF4102 domain-containing protein | DUF4102 domain-containing protein | | afdb-uniprot50 | AF-A0A256GC42-F1-MODEL\_V4 | 1.0 | 2.738e-16 | 658 | 0.243 | 349 | 228 | 15 | 69 | 403 | 2 | 328 | Phage integrase family protein | Phage integrase family protein | | afdb-uniprot50 | AF-A0A7C7W234-F1-MODEL\_V4 | 1.0 | 1.141e-18 | 658 | 0.239 | 410 | 250 | 19 | 1 | 379 | 2 | 380 | Site-specific integrase | Site-specific integrase | | afdb-uniprot50 | AF-A0A6M1TNW1-F1-MODEL\_V4 | 1.0 | 1.022e-17 | 657 | 0.224 | 436 | 276 | 23 | 2 | 402 | 6 | 414 | Tyrosine-type recombinase/integrase | Tyrosine-type recombinase/integrase | | afdb-uniprot50 | AF-A0A1B4YTI6-F1-MODEL\_V4 | 1.0 | 6.653e-18 | 657 | 0.21 | 423 | 276 | 20 | 2 | 402 | 8 | 394 | Symbiosis island integrase | Symbiosis island integrase | | afdb-uniprot50 | AF-A0A4Q6XBY6-F1-MODEL\_V4 | 1.0 | 9.887e-19 | 656 | 0.225 | 430 | 273 | 23 | 1 | 403 | 7 | 403 | DUF4102 domain-containing protein | DUF4102 domain-containing protein | | afdb-uniprot50 | AF-A0A396S6Q7-F1-MODEL\_V4 | 1.0 | 4.332e-18 | 656 | 0.209 | 454 | 291 | 18 | 1 | 402 | 3 | 440 | Integrase | Integrase | | afdb-uniprot50 | AF-A0A376BJV7-F1-MODEL\_V4 | 1.0 | 2.021e-18 | 655 | 0.222 | 422 | 275 | 17 | 1 | 402 | 1 | 389 | Prophage CPS-53 integrase | Prophage CPS-53 integrase | | afdb-uniprot50 | AF-A0A850H6I7-F1-MODEL\_V4 | 1.0 | 8.05e-18 | 654 | 0.207 | 415 | 274 | 19 | 1 | 402 | 1 | 373 | Tyrosine-type recombinase/integrase | Tyrosine-type recombinase/integrase | | afdb-uniprot50 | AF-A0A2G8BY28-F1-MODEL\_V4 | 1.0 | 4.332e-18 | 654 | 0.201 | 417 | 287 | 18 | 1 | 401 | 1 | 387 | Integrase | Integrase | | afdb-uniprot50 | AF-A0A3S4N3C8-F1-MODEL\_V4 | 1.0 | 8.05e-18 | 654 | 0.213 | 416 | 286 | 17 | 2 | 403 | 3 | 391 | Prophage CPS-53 integrase | Prophage CPS-53 integrase | | afdb-uniprot50 | AF-A0A3A6TZY1-F1-MODEL\_V4 | 1.0 | 3.255e-18 | 653 | 0.214 | 414 | 277 | 18 | 1 | 399 | 1 | 381 | DUF4102 domain-containing protein | DUF4102 domain-containing protein | | afdb-uniprot50 | AF-A0A827TY01-F1-MODEL\_V4 | 1.0 | 1.296e-17 | 653 | 0.212 | 418 | 281 | 17 | 1 | 403 | 1 | 385 | Prophage integrase IntS | Prophage integrase IntS | | afdb-uniprot50 | AF-A0A5U9KLB9-F1-MODEL\_V4 | 1.0 | 8.57e-19 | 653 | 0.208 | 423 | 281 | 20 | 1 | 403 | 1 | 389 | DUF4102 domain-containing protein | DUF4102 domain-containing protein | | afdb-uniprot50 | AF-A0A317T8S8-F1-MODEL\_V4 | 1.0 | 4.765e-18 | 653 | 0.211 | 420 | 284 | 20 | 2 | 403 | 8 | 398 | Integrase | Integrase | | afdb-uniprot50 | AF-A0A1V2BER2-F1-MODEL\_V4 | 1.0 | 4.544e-18 | 653 | 0.21 | 423 | 280 | 20 | 2 | 402 | 8 | 398 | Tyr recombinase domain-containing protein | Tyr recombinase domain-containing protein | | afdb-uniprot50 | AF-A0A345DDB6-F1-MODEL\_V4 | 1.0 | 8.855e-18 | 653 | 0.2 | 424 | 285 | 19 | 1 | 402 | 6 | 397 | Prophage integrase IntA | Prophage integrase IntA | | afdb-uniprot50 | AF-A0A3V2YQD8-F1-MODEL\_V4 | 1.0 | 2.565e-18 | 652 | 0.204 | 421 | 285 | 19 | 1 | 403 | 1 | 389 | DUF4102 domain-containing protein | DUF4102 domain-containing protein | | afdb-uniprot50 | AF-A0A6N0IKJ5-F1-MODEL\_V4 | 1.0 | 4.998e-18 | 652 | 0.207 | 420 | 282 | 17 | 2 | 402 | 3 | 390 | Tyrosine-type recombinase/integrase | Tyrosine-type recombinase/integrase | | afdb-uniprot50 | AF-A0A5U6SL20-F1-MODEL\_V4 | 1.0 | 1.088e-18 | 652 | 0.213 | 431 | 270 | 20 | 2 | 403 | 3 | 393 | DUF4102 domain-containing protein | DUF4102 domain-containing protein | | afdb-uniprot50 | AF-A0A1J5TKJ8-F1-MODEL\_V4 | 1.0 | 3.414e-18 | 652 | 0.21 | 428 | 279 | 19 | 1 | 402 | 8 | 402 | Prophage CP4-57 integrase | Prophage CP4-57 integrase | | afdb-uniprot50 | AF-A0A1M7DHR6-F1-MODEL\_V4 | 1.0 | 1.196e-18 | 652 | 0.205 | 423 | 283 | 18 | 2 | 402 | 28 | 419 | Integrase | Integrase | | afdb-uniprot50 | AF-A0A841JT67-F1-MODEL\_V4 | 1.0 | 1.022e-17 | 651 | 0.191 | 422 | 290 | 16 | 1 | 402 | 2 | 392 | Integrase | Integrase | | afdb-uniprot50 | AF-A0A839LID3-F1-MODEL\_V4 | 1.0 | 6.977e-18 | 650 | 0.221 | 424 | 275 | 19 | 1 | 402 | 7 | 397 | Tyrosine-type recombinase/integrase | Tyrosine-type recombinase/integrase | | afdb-uniprot50 | AF-A0A7S7UC04-F1-MODEL\_V4 | 1.0 | 9.74e-18 | 650 | 0.189 | 427 | 288 | 19 | 2 | 402 | 3 | 397 | Uncharacterized protein | Uncharacterized protein | | afdb-uniprot50 | AF-A0A851GY06-F1-MODEL\_V4 | 1.0 | 3.938e-18 | 650 | 0.224 | 427 | 280 | 17 | 2 | 402 | 50 | 451 | Tyrosine-type recombinase/integrase | Tyrosine-type recombinase/integrase | | afdb-uniprot50 | AF-A0A257G8F0-F1-MODEL\_V4 | 1.0 | 9.74e-18 | 649 | 0.208 | 426 | 277 | 21 | 2 | 402 | 3 | 393 | Integrase | Integrase | | afdb-uniprot50 | AF-A0A7C1MG73-F1-MODEL\_V4 | 1.0 | 2.223e-18 | 649 | 0.196 | 422 | 283 | 19 | 1 | 402 | 3 | 388 | Site-specific integrase | Site-specific integrase | | afdb-uniprot50 | AF-A0A1I2A527-F1-MODEL\_V4 | 1.0 | 2.821e-18 | 649 | 0.21 | 422 | 280 | 21 | 1 | 402 | 1 | 389 | Integrase | Integrase | | afdb-uniprot50 | AF-A0A158DHE5-F1-MODEL\_V4 | 1.0 | 4.998e-18 | 649 | 0.22 | 421 | 274 | 21 | 2 | 402 | 8 | 394 | Integrase | Integrase | | afdb-uniprot50 | AF-A0A059FPK9-F1-MODEL\_V4 | 1.0 | 1.898e-17 | 648 | 0.227 | 426 | 269 | 24 | 2 | 402 | 3 | 393 | Phage integrase family site specific recombinase | Phage integrase family site specific recombinase | | afdb-uniprot50 | AF-E5Y508-F1-MODEL\_V4 | 1.0 | 4.998e-18 | 648 | 0.199 | 422 | 280 | 19 | 9 | 403 | 6 | 396 | Uncharacterized protein | Uncharacterized protein | | afdb-uniprot50 | AF-A0A381EDH6-F1-MODEL\_V4 | 1.0 | 2.223e-18 | 648 | 0.224 | 419 | 275 | 15 | 1 | 402 | 1 | 386 | Prophage CPS-53 integrase | Prophage CPS-53 integrase | | afdb-uniprot50 | AF-A0A437QDT5-F1-MODEL\_V4 | 1.0 | 4.131e-18 | 648 | 0.224 | 419 | 276 | 19 | 2 | 402 | 3 | 390 | DUF4102 domain-containing protein | DUF4102 domain-containing protein | | afdb-uniprot50 | AF-A0A1I7I1V6-F1-MODEL\_V4 | 1.0 | 7.675e-18 | 648 | 0.215 | 441 | 279 | 21 | 15 | 402 | 1 | 427 | Core-binding (CB) domain-containing protein | Core-binding (CB) domain-containing protein | | afdb-uniprot50 | AF-A0A1L3ZVP2-F1-MODEL\_V4 | 1.0 | 4.544e-18 | 647 | 0.221 | 419 | 262 | 21 | 2 | 403 | 4 | 375 | Uncharacterized protein | Uncharacterized protein | | afdb-uniprot50 | AF-A0A0F2QKR9-F1-MODEL\_V4 | 1.0 | 4.998e-18 | 647 | 0.192 | 415 | 285 | 17 | 13 | 402 | 5 | 394 | Tyr recombinase domain-containing protein | Tyr recombinase domain-containing protein | | afdb-uniprot50 | AF-A0A1W1BZB0-F1-MODEL\_V4 | 1.0 | 3.58e-18 | 647 | 0.183 | 426 | 293 | 18 | 2 | 402 | 8 | 403 | Integrase | Integrase | | afdb-uniprot50 | AF-A0A212JFC8-F1-MODEL\_V4 | 1.0 | 7.675e-18 | 647 | 0.213 | 421 | 288 | 19 | 1 | 402 | 5 | 401 | Site-specific recombinase, phage integrase family | Site-specific recombinase, phage integrase family | | afdb-uniprot50 | AF-H1Y3L2-F1-MODEL\_V4 | 1.0 | 1.726e-17 | 647 | 0.209 | 415 | 286 | 17 | 2 | 402 | 3 | 389 | Integrase family protein | Integrase family protein | | afdb-uniprot50 | AF-A0A1F0DZT6-F1-MODEL\_V4 | 1.0 | 7.318e-18 | 646 | 0.219 | 419 | 272 | 20 | 1 | 400 | 1 | 383 | Tyr recombinase domain-containing protein | Tyr recombinase domain-containing protein | | afdb-uniprot50 | AF-A0A2D6EM10-F1-MODEL\_V4 | 1.0 | 1.36e-17 | 646 | 0.206 | 426 | 283 | 21 | 2 | 403 | 3 | 397 | Integrase | Integrase | | afdb-uniprot50 | AF-A0A2X1WMX6-F1-MODEL\_V4 | 1.0 | 1.81e-17 | 646 | 0.217 | 423 | 268 | 19 | 1 | 402 | 5 | 385 | Prophage CP4-57 integrase | Prophage CP4-57 integrase | | afdb-uniprot50 | AF-A0A1G0XRY8-F1-MODEL\_V4 | 1.0 | 1.7e-16 | 645 | 0.215 | 358 | 247 | 13 | 60 | 402 | 1 | 339 | Integrase | Integrase | | afdb-uniprot50 | AF-A0A846UIU9-F1-MODEL\_V4 | 1.0 | 1.36e-17 | 645 | 0.214 | 397 | 266 | 18 | 25 | 402 | 4 | 373 | Integrase | Integrase | | afdb-uniprot50 | AF-A0A353D6A0-F1-MODEL\_V4 | 1.0 | 5.242e-18 | 645 | 0.211 | 425 | 279 | 21 | 1 | 403 | 11 | 401 | Tyr recombinase domain-containing protein | Tyr recombinase domain-containing protein | | afdb-uniprot50 | AF-I2DN67-F1-MODEL\_V4 | 1.0 | 1.898e-17 | 644 | 0.213 | 421 | 279 | 21 | 2 | 403 | 3 | 390 | Integrase | Integrase | | afdb-uniprot50 | AF-A0A486XD46-F1-MODEL\_V4 | 1.0 | 1.296e-17 | 644 | 0.191 | 423 | 286 | 19 | 2 | 402 | 8 | 396 | Prophage CP4-57 integrase | Prophage CP4-57 integrase | | afdb-uniprot50 | AF-A0A507WKG0-F1-MODEL\_V4 | 1.0 | 4.765e-18 | 644 | 0.208 | 423 | 284 | 18 | 2 | 402 | 3 | 396 | DUF4102 domain-containing protein | DUF4102 domain-containing protein | | afdb-uniprot50 | AF-A0A845SLH3-F1-MODEL\_V4 | 1.0 | 8.855e-18 | 644 | 0.215 | 422 | 279 | 20 | 2 | 402 | 8 | 398 | Tyrosine-type recombinase/integrase | Tyrosine-type recombinase/integrase | | afdb-uniprot50 | AF-P37326-F1-MODEL\_V4 | 1.0 | 9.287e-18 | 643 | 0.218 | 417 | 280 | 18 | 1 | 403 | 1 | 385 | Prophage integrase IntS | Prophage integrase IntS | | afdb-uniprot50 | AF-A0A0K8NTH3-F1-MODEL\_V4 | 1.0 | 4.998e-18 | 643 | 0.208 | 393 | 263 | 17 | 36 | 403 | 3 | 372 | Phage integrase | Phage integrase | | afdb-uniprot50 | AF-A0A2D0JZY9-F1-MODEL\_V4 | 1.0 | 8.05e-18 | 643 | 0.207 | 429 | 273 | 23 | 2 | 402 | 3 | 392 | Integrase | Integrase | | afdb-uniprot50 | AF-A0A290XAM6-F1-MODEL\_V4 | 1.0 | 7.791e-19 | 643 | 0.226 | 429 | 269 | 20 | 1 | 402 | 1 | 393 | Integrase | Integrase | | afdb-uniprot50 | AF-A0A523L477-F1-MODEL\_V4 | 1.0 | 1.569e-17 | 643 | 0.174 | 419 | 293 | 21 | 2 | 402 | 6 | 389 | DUF4102 domain-containing protein | DUF4102 domain-containing protein | | afdb-uniprot50 | AF-A0A0J6KK27-F1-MODEL\_V4 | 1.0 | 2.021e-18 | 643 | 0.202 | 430 | 283 | 18 | 2 | 403 | 3 | 400 | Tyr recombinase domain-containing protein | Tyr recombinase domain-containing protein | | afdb-uniprot50 | AF-A0A3M9XVK5-F1-MODEL\_V4 | 1.0 | 3.812e-19 | 643 | 0.24 | 415 | 266 | 18 | 1 | 398 | 17 | 399 | Site-specific integrase | Site-specific integrase | | afdb-uniprot50 | AF-A0A5V2R899-F1-MODEL\_V4 | 1.0 | 1.236e-17 | 643 | 0.196 | 417 | 284 | 16 | 9 | 402 | 11 | 399 | Tyrosine-type recombinase/integrase | Tyrosine-type recombinase/integrase | | afdb-uniprot50 | AF-A0A2C8ZKJ8-F1-MODEL\_V4 | 1.0 | 1.022e-17 | 643 | 0.233 | 415 | 270 | 23 | 9 | 402 | 75 | 462 | Integrase | Integrase | | afdb-uniprot50 | AF-A0A389ML60-F1-MODEL\_V4 | 1.0 | 9.74e-18 | 642 | 0.221 | 415 | 270 | 22 | 2 | 401 | 13 | 389 | Integrase | Integrase | | afdb-uniprot50 | AF-B8J055-F1-MODEL\_V4 | 1.0 | 2.69e-18 | 641 | 0.204 | 421 | 281 | 20 | 2 | 402 | 7 | 393 | Integrase family protein | Integrase family protein | | afdb-uniprot50 | AF-A0A1F0DV58-F1-MODEL\_V4 | 1.0 | 3.255e-18 | 641 | 0.214 | 425 | 277 | 19 | 2 | 402 | 3 | 394 | Integrase | Integrase | | afdb-uniprot50 | AF-A0A1Q8SPE1-F1-MODEL\_V4 | 1.0 | 4.131e-18 | 640 | 0.212 | 418 | 281 | 18 | 2 | 401 | 3 | 390 | Tyr recombinase domain-containing protein | Tyr recombinase domain-containing protein | | afdb-uniprot50 | AF-A0A7R6PGN4-F1-MODEL\_V4 | 1.0 | 4.998e-18 | 640 | 0.191 | 423 | 290 | 20 | 1 | 402 | 1 | 392 | Phage integrase family protein | Phage integrase family protein | | afdb-uniprot50 | AF-A0A7W0GKX6-F1-MODEL\_V4 | 1.0 | 7.675e-18 | 640 | 0.208 | 422 | 283 | 18 | 2 | 402 | 6 | 397 | Tyrosine-type recombinase/integrase | Tyrosine-type recombinase/integrase | | afdb-uniprot50 | AF-A0A5P3MQF5-F1-MODEL\_V4 | 1.0 | 7.318e-18 | 640 | 0.232 | 421 | 271 | 19 | 2 | 402 | 3 | 391 | DUF4102 domain-containing protein | DUF4102 domain-containing protein | | afdb-uniprot50 | AF-A0A3N1JQV1-F1-MODEL\_V4 | 1.0 | 8.855e-18 | 640 | 0.185 | 420 | 295 | 18 | 1 | 402 | 50 | 440 | Integrase | Integrase | | afdb-uniprot50 | AF-A0A6N7EZ98-F1-MODEL\_V4 | 1.0 | 2.19e-17 | 639 | 0.185 | 415 | 292 | 19 | 2 | 402 | 3 | 385 | Tyrosine-type recombinase/integrase | Tyrosine-type recombinase/integrase | | afdb-uniprot50 | AF-A0A2E1TM79-F1-MODEL\_V4 | 1.0 | 1.898e-17 | 639 | 0.2 | 420 | 285 | 20 | 2 | 403 | 3 | 389 | Integrase | Integrase | | afdb-uniprot50 | AF-W2UIP2-F1-MODEL\_V4 | 1.0 | 4.131e-18 | 638 | 0.189 | 427 | 287 | 21 | 2 | 402 | 3 | 396 | Prophage CP4-57 integrase | Prophage CP4-57 integrase | | afdb-uniprot50 | AF-A0A5F1HJD6-F1-MODEL\_V4 | 1.0 | 3.88e-17 | 638 | 0.215 | 422 | 279 | 20 | 2 | 402 | 8 | 398 | Integrase | Integrase | | afdb-uniprot50 | AF-A0A1C3E882-F1-MODEL\_V4 | 1.0 | 1.726e-17 | 637 | 0.194 | 421 | 290 | 16 | 2 | 403 | 3 | 393 | Tyr recombinase domain-containing protein | Tyr recombinase domain-containing protein | | afdb-uniprot50 | AF-A0A6L3SXM2-F1-MODEL\_V4 | 1.0 | 1.569e-17 | 637 | 0.215 | 418 | 275 | 18 | 2 | 402 | 13 | 394 | Tyrosine-type recombinase/integrase | Tyrosine-type recombinase/integrase | | afdb-uniprot50 | AF-A0A5C7R8L9-F1-MODEL\_V4 | 1.0 | 1.426e-17 | 637 | 0.193 | 429 | 284 | 20 | 1 | 402 | 4 | 397 | DUF4102 domain-containing protein | DUF4102 domain-containing protein | | afdb-uniprot50 | AF-A0A4Q6X2P3-F1-MODEL\_V4 | 1.0 | 6.653e-18 | 637 | 0.215 | 432 | 278 | 20 | 2 | 403 | 12 | 412 | DUF4102 domain-containing protein | DUF4102 domain-containing protein | | afdb-uniprot50 | AF-A0A2T7U8C2-F1-MODEL\_V4 | 1.0 | 6.977e-18 | 636 | 0.213 | 421 | 278 | 20 | 2 | 402 | 3 | 390 | Integrase | Integrase | | afdb-uniprot50 | AF-A0A4C3NQ15-F1-MODEL\_V4 | 1.0 | 2.915e-17 | 636 | 0.213 | 422 | 280 | 19 | 2 | 402 | 8 | 398 | DUF4102 domain-containing protein | DUF4102 domain-containing protein | | afdb-uniprot50 | AF-A0A7Y0GZP7-F1-MODEL\_V4 | 1.0 | 1.071e-17 | 636 | 0.215 | 426 | 279 | 18 | 2 | 402 | 9 | 404 | Integrase arm-type DNA-binding domain-containing protein | Integrase arm-type DNA-binding domain-containing protein | | afdb-uniprot50 | AF-A0A5F0WL98-F1-MODEL\_V4 | 1.0 | 4.332e-18 | 635 | 0.216 | 420 | 276 | 18 | 2 | 402 | 3 | 388 | DUF4102 domain-containing protein | DUF4102 domain-containing protein | | afdb-uniprot50 | AF-A0A848HR69-F1-MODEL\_V4 | 1.0 | 2.223e-18 | 635 | 0.227 | 422 | 273 | 17 | 2 | 402 | 3 | 392 | Tyrosine-type recombinase/integrase | Tyrosine-type recombinase/integrase | | afdb-uniprot50 | AF-A0A5H7WS47-F1-MODEL\_V4 | 1.0 | 1.179e-17 | 635 | 0.207 | 419 | 283 | 17 | 2 | 402 | 3 | 390 | DUF4102 domain-containing protein | DUF4102 domain-containing protein | | afdb-uniprot50 | AF-A0A1I7NE20-F1-MODEL\_V4 | 1.0 | 1.496e-17 | 635 | 0.226 | 419 | 273 | 19 | 2 | 402 | 5 | 390 | Site-specific recombinase XerD | Site-specific recombinase XerD | | afdb-uniprot50 | AF-A0A3M5ZHM3-F1-MODEL\_V4 | 1.0 | 6.874e-17 | 635 | 0.21 | 422 | 282 | 19 | 2 | 403 | 81 | 471 | Integrase | Integrase | | afdb-uniprot50 | AF-A0A376D7E0-F1-MODEL\_V4 | 1.0 | 2.779e-17 | 635 | 0.206 | 422 | 283 | 18 | 2 | 402 | 8 | 398 | Integrase from prophage | Integrase from prophage | | afdb-uniprot50 | AF-A0A6H2DRV2-F1-MODEL\_V4 | 1.0 | 1.179e-17 | 634 | 0.238 | 411 | 266 | 17 | 2 | 403 | 6 | 378 | Tyrosine-type recombinase/integrase | Tyrosine-type recombinase/integrase | | afdb-uniprot50 | AF-A0A0M0T6P3-F1-MODEL\_V4 | 1.0 | 3.527e-17 | 634 | 0.183 | 425 | 288 | 21 | 2 | 402 | 3 | 392 | Uncharacterized protein | Uncharacterized protein | | afdb-uniprot50 | AF-A0A1B3M9C7-F1-MODEL\_V4 | 1.0 | 2.297e-17 | 634 | 0.209 | 424 | 279 | 18 | 2 | 402 | 3 | 393 | Phage integrase family protein | Phage integrase family protein | | afdb-uniprot50 | AF-A0A1N6DLZ0-F1-MODEL\_V4 | 1.0 | 2.565e-18 | 634 | 0.18 | 422 | 291 | 17 | 2 | 402 | 3 | 390 | Integrase | Integrase | | afdb-uniprot50 | AF-A0A2N2UYZ7-F1-MODEL\_V4 | 1.0 | 1.179e-17 | 634 | 0.216 | 425 | 275 | 21 | 2 | 402 | 8 | 398 | Integrase | Integrase | | afdb-uniprot50 | AF-A0A7D6SXA8-F1-MODEL\_V4 | 1.0 | 1.726e-17 | 634 | 0.192 | 420 | 290 | 18 | 2 | 402 | 8 | 397 | Tyrosine-type recombinase/integrase | Tyrosine-type recombinase/integrase | | afdb-uniprot50 | AF-A0A2R5EJF6-F1-MODEL\_V4 | 1.0 | 2.565e-18 | 634 | 0.224 | 446 | 263 | 23 | 1 | 402 | 1 | 407 | Integrase | Integrase | | afdb-uniprot50 | AF-A0A4Q6X281-F1-MODEL\_V4 | 1.0 | 1.569e-17 | 633 | 0.225 | 416 | 272 | 23 | 9 | 402 | 11 | 398 | DUF4102 domain-containing protein | DUF4102 domain-containing protein | | afdb-uniprot50 | AF-A0A822P193-F1-MODEL\_V4 | 1.0 | 5.242e-18 | 632 | 0.22 | 418 | 272 | 20 | 1 | 398 | 1 | 384 | Putative prophage CPS-53 integrase CPS-53 (KpLE1) prophage | Putative prophage CPS-53 integrase CPS-53 (KpLE1) prophage | | afdb-uniprot50 | AF-A0A4V4RAT5-F1-MODEL\_V4 | 1.0 | 2.489e-16 | 632 | 0.19 | 421 | 294 | 20 | 2 | 403 | 3 | 395 | DUF4102 domain-containing protein | DUF4102 domain-containing protein | | afdb-uniprot50 | AF-A0A3A9I0N3-F1-MODEL\_V4 | 1.0 | 6.977e-18 | 632 | 0.214 | 425 | 275 | 21 | 2 | 402 | 16 | 405 | DUF4102 domain-containing protein | DUF4102 domain-containing protein | | afdb-uniprot50 | AF-A0A6G6WPD0-F1-MODEL\_V4 | 1.0 | 6.977e-18 | 632 | 0.2 | 430 | 284 | 22 | 1 | 402 | 5 | 402 | Integrase arm-type DNA-binding domain-containing protein | Integrase arm-type DNA-binding domain-containing protein | | afdb-uniprot50 | AF-A0A5P2H4F2-F1-MODEL\_V4 | 1.0 | 5.498e-18 | 632 | 0.197 | 425 | 273 | 17 | 1 | 398 | 24 | 407 | Tyrosine-type recombinase/integrase | Tyrosine-type recombinase/integrase | | afdb-uniprot50 | AF-A0A2W0G8P2-F1-MODEL\_V4 | 1.0 | 3.699e-17 | 631 | 0.196 | 417 | 289 | 18 | 1 | 403 | 1 | 385 | DUF4102 domain-containing protein | DUF4102 domain-containing protein | | afdb-uniprot50 | AF-A0A2D8CKN1-F1-MODEL\_V4 | 1.0 | 3.414e-18 | 631 | 0.211 | 421 | 276 | 20 | 2 | 402 | 5 | 389 | Uncharacterized protein | Uncharacterized protein | | afdb-uniprot50 | AF-A0A534HSB6-F1-MODEL\_V4 | 1.0 | 3.938e-18 | 631 | 0.212 | 418 | 287 | 18 | 1 | 402 | 2 | 393 | DUF4102 domain-containing protein | DUF4102 domain-containing protein | | afdb-uniprot50 | AF-A0A5C7LA00-F1-MODEL\_V4 | 1.0 | 3.755e-18 | 631 | 0.206 | 432 | 278 | 18 | 1 | 402 | 1 | 397 | DUF4102 domain-containing protein | DUF4102 domain-containing protein | | afdb-uniprot50 | AF-A0A522LW18-F1-MODEL\_V4 | 1.0 | 1.022e-17 | 631 | 0.216 | 402 | 270 | 19 | 14 | 402 | 26 | 395 | Site-specific integrase | Site-specific integrase | | afdb-uniprot50 | AF-A0A4Q7G062-F1-MODEL\_V4 | 1.0 | 1.296e-17 | 631 | 0.236 | 431 | 264 | 21 | 2 | 403 | 5 | 399 | Tyr recombinase domain-containing protein | Tyr recombinase domain-containing protein | | afdb-uniprot50 | AF-A0A7I7D2M7-F1-MODEL\_V4 | 1.0 | 2.19e-17 | 630 | 0.203 | 417 | 286 | 18 | 1 | 403 | 1 | 385 | Prophage integrase IntS | Prophage integrase IntS | | afdb-uniprot50 | AF-A0A3R0Q2U4-F1-MODEL\_V4 | 1.0 | 4.131e-18 | 630 | 0.212 | 423 | 279 | 20 | 1 | 403 | 1 | 389 | DUF4102 domain-containing protein | DUF4102 domain-containing protein | | afdb-uniprot50 | AF-A0A0F2R7F3-F1-MODEL\_V4 | 1.0 | 6.653e-18 | 630 | 0.192 | 425 | 285 | 21 | 2 | 402 | 3 | 393 | Integrase | Integrase | | afdb-uniprot50 | AF-A0A2N1ZXX0-F1-MODEL\_V4 | 1.0 | 1.645e-17 | 630 | 0.194 | 417 | 287 | 18 | 2 | 402 | 3 | 386 | Integrase | Integrase | | afdb-uniprot50 | AF-A0A3G2N436-F1-MODEL\_V4 | 1.0 | 1.022e-17 | 630 | 0.238 | 411 | 257 | 20 | 14 | 402 | 81 | 457 | DUF4102 domain-containing protein | DUF4102 domain-containing protein | | afdb-uniprot50 | AF-A0A4R1IAG8-F1-MODEL\_V4 | 1.0 | 1.426e-17 | 629 | 0.209 | 425 | 278 | 19 | 2 | 402 | 3 | 393 | Integrase | Integrase | | afdb-uniprot50 | AF-A0A7D6P8E2-F1-MODEL\_V4 | 1.0 | 2.779e-17 | 629 | 0.183 | 420 | 294 | 18 | 2 | 402 | 8 | 397 | Integrase | Integrase | | afdb-uniprot50 | AF-U7GEQ5-F1-MODEL\_V4 | 1.0 | 7.675e-18 | 629 | 0.2 | 430 | 276 | 20 | 2 | 403 | 3 | 392 | Uncharacterized protein | Uncharacterized protein | | afdb-uniprot50 | AF-A0A7H1M906-F1-MODEL\_V4 | 1.0 | 2.19e-17 | 629 | 0.214 | 419 | 278 | 20 | 2 | 401 | 3 | 389 | Phage integrase family protein | Phage integrase family protein | | afdb-uniprot50 | AF-A0A2D4TBE4-F1-MODEL\_V4 | 1.0 | 2.69e-18 | 629 | 0.201 | 437 | 289 | 20 | 2 | 402 | 10 | 422 | Uncharacterized protein | Uncharacterized protein | | afdb-uniprot50 | AF-A0A6I2IYN0-F1-MODEL\_V4 | 1.0 | 9.15e-17 | 628 | 0.205 | 413 | 290 | 18 | 1 | 403 | 1 | 385 | Tyrosine-type recombinase/integrase | Tyrosine-type recombinase/integrase | | afdb-uniprot50 | AF-D3PB12-F1-MODEL\_V4 | 1.0 | 1.071e-17 | 628 | 0.208 | 418 | 275 | 22 | 2 | 402 | 4 | 382 | Phage integrase | Phage integrase | | afdb-uniprot50 | AF-A0A376M5B4-F1-MODEL\_V4 | 1.0 | 2.409e-17 | 628 | 0.208 | 422 | 282 | 18 | 2 | 402 | 8 | 398 | Integrase from prophage | Integrase from prophage | | afdb-uniprot50 | AF-A0A1H8LKS2-F1-MODEL\_V4 | 1.0 | 1.496e-17 | 627 | 0.194 | 427 | 282 | 21 | 2 | 402 | 3 | 393 | Integrase | Integrase | | afdb-uniprot50 | AF-A0A1A7PDT9-F1-MODEL\_V4 | 1.0 | 1.056e-16 | 627 | 0.19 | 420 | 291 | 17 | 1 | 401 | 7 | 396 | Tyr recombinase domain-containing protein | Tyr recombinase domain-containing protein | | afdb-uniprot50 | AF-A0A560K1J7-F1-MODEL\_V4 | 1.0 | 1.898e-17 | 627 | 0.209 | 420 | 281 | 20 | 2 | 402 | 3 | 390 | Integrase | Integrase | | afdb-uniprot50 | AF-A0A7U1D0Y1-F1-MODEL\_V4 | 1.0 | 1.296e-17 | 627 | 0.197 | 441 | 290 | 20 | 1 | 402 | 3 | 418 | Tyrosine-type recombinase/integrase | Tyrosine-type recombinase/integrase | | afdb-uniprot50 | AF-A0A830ZYW7-F1-MODEL\_V4 | 1.0 | 3.645e-16 | 626 | 0.223 | 381 | 249 | 15 | 50 | 402 | 3 | 364 | Integrase | Integrase | | afdb-uniprot50 | AF-A0A806JFH6-F1-MODEL\_V4 | 1.0 | 3.363e-17 | 626 | 0.206 | 427 | 282 | 20 | 2 | 402 | 8 | 403 | Integrase | Integrase | | afdb-uniprot50 | AF-A0A1E4KI51-F1-MODEL\_V4 | 1.0 | 2.915e-17 | 625 | 0.195 | 420 | 291 | 19 | 1 | 402 | 3 | 393 | Tyr recombinase domain-containing protein | Tyr recombinase domain-containing protein | | afdb-uniprot50 | AF-A0A811GGD4-F1-MODEL\_V4 | 1.0 | 2.19e-17 | 625 | 0.193 | 423 | 293 | 18 | 1 | 403 | 9 | 403 | Prophage CPS-53 integrase | Prophage CPS-53 integrase | | afdb-uniprot50 | AF-A0A0H4P8E5-F1-MODEL\_V4 | 1.0 | 4.625e-16 | 624 | 0.244 | 360 | 233 | 14 | 67 | 402 | 30 | 374 | Integrase family protein | Integrase family protein | | afdb-uniprot50 | AF-A0A6L5XMX1-F1-MODEL\_V4 | 1.0 | 2.527e-17 | 624 | 0.194 | 422 | 289 | 20 | 1 | 402 | 1 | 391 | DUF4102 domain-containing protein | DUF4102 domain-containing protein | | afdb-uniprot50 | AF-A0A257HDF3-F1-MODEL\_V4 | 1.0 | 2.19e-17 | 623 | 0.215 | 426 | 275 | 24 | 2 | 402 | 3 | 394 | Integrase | Integrase | | afdb-uniprot50 | AF-A0A5B0T1R7-F1-MODEL\_V4 | 1.0 | 5.416e-17 | 623 | 0.208 | 423 | 283 | 19 | 2 | 403 | 10 | 401 | Tyrosine-type recombinase/integrase | Tyrosine-type recombinase/integrase | | afdb-uniprot50 | AF-A0A7M1AWK8-F1-MODEL\_V4 | 1.0 | 1.898e-17 | 623 | 0.183 | 431 | 295 | 21 | 2 | 402 | 8 | 411 | DUF4102 domain-containing protein | DUF4102 domain-containing protein | | afdb-uniprot50 | AF-A0A657B792-F1-MODEL\_V4 | 1.0 | 8.724e-17 | 623 | 0.21 | 422 | 281 | 20 | 2 | 402 | 8 | 398 | Tyr recombinase domain-containing protein | Tyr recombinase domain-containing protein | | afdb-uniprot50 | AF-A0A356X5P7-F1-MODEL\_V4 | 1.0 | 1.36e-17 | 623 | 0.21 | 428 | 287 | 17 | 2 | 403 | 7 | 409 | Uncharacterized protein | Uncharacterized protein | | afdb-uniprot50 | AF-A0A7X4GIX6-F1-MODEL\_V4 | 1.0 | 1.569e-17 | 623 | 0.186 | 450 | 289 | 20 | 1 | 403 | 7 | 426 | Tyrosine-type recombinase/integrase | Tyrosine-type recombinase/integrase | | afdb-uniprot50 | AF-A0A0A2WLL9-F1-MODEL\_V4 | 1.0 | 5.164e-17 | 622 | 0.208 | 417 | 284 | 19 | 2 | 403 | 8 | 393 | Phage integrase | Phage integrase | | afdb-uniprot50 | AF-A0A2G2DS92-F1-MODEL\_V4 | 1.0 | 3.88e-17 | 622 | 0.218 | 434 | 277 | 23 | 2 | 398 | 8 | 416 | Integrase | Integrase | | afdb-uniprot50 | AF-A0A2V8UYS4-F1-MODEL\_V4 | 1.0 | 2.229e-15 | 621 | 0.244 | 348 | 215 | 16 | 73 | 402 | 2 | 319 | Integrase | Integrase | | afdb-uniprot50 | AF-A0A0M3TU49-F1-MODEL\_V4 | 1.0 | 6.554e-17 | 621 | 0.183 | 415 | 296 | 18 | 2 | 403 | 6 | 390 | Tyr recombinase domain-containing protein | Tyr recombinase domain-containing protein | | afdb-uniprot50 | AF-A0A2L0VMP1-F1-MODEL\_V4 | 1.0 | 2.779e-17 | 621 | 0.213 | 427 | 273 | 23 | 2 | 402 | 3 | 392 | Integrase | Integrase | | afdb-uniprot50 | AF-A0A175VIU2-F1-MODEL\_V4 | 1.0 | 4.924e-17 | 621 | 0.207 | 424 | 282 | 21 | 2 | 402 | 6 | 398 | Integrase | Integrase | | afdb-uniprot50 | AF-X5NZA3-F1-MODEL\_V4 | 1.0 | 1.296e-17 | 621 | 0.211 | 406 | 268 | 19 | 18 | 403 | 1 | 374 | Integrase | Integrase | | afdb-uniprot50 | AF-A0A1N6J5W7-F1-MODEL\_V4 | 1.0 | 1.277e-16 | 620 | 0.19 | 421 | 292 | 19 | 2 | 402 | 3 | 394 | Integrase | Integrase | | afdb-uniprot50 | AF-Q5E028-F1-MODEL\_V4 | 1.0 | 2.915e-17 | 620 | 0.24 | 424 | 265 | 23 | 2 | 401 | 8 | 398 | Phage family integrase | Phage family integrase | | afdb-uniprot50 | AF-A0A380QSD2-F1-MODEL\_V4 | 1.0 | 1.34e-16 | 620 | 0.196 | 423 | 283 | 18 | 2 | 398 | 8 | 399 | Putative prophage integrase | Putative prophage integrase | | afdb-uniprot50 | AF-A0A198XHQ6-F1-MODEL\_V4 | 1.0 | 4.924e-17 | 619 | 0.197 | 421 | 271 | 20 | 1 | 403 | 1 | 372 | Prophage CP4-like integrase | Prophage CP4-like integrase | | afdb-uniprot50 | AF-A3UDD1-F1-MODEL\_V4 | 1.0 | 1.81e-17 | 619 | 0.169 | 426 | 293 | 18 | 2 | 402 | 3 | 392 | Symbiosis island integrase | Symbiosis island integrase | | afdb-uniprot50 | AF-A0A2G1AS07-F1-MODEL\_V4 | 1.0 | 8.318e-17 | 619 | 0.226 | 420 | 276 | 19 | 2 | 402 | 8 | 397 | Integrase | Integrase | | afdb-uniprot50 | AF-A0A0D0PRG3-F1-MODEL\_V4 | 1.0 | 5.164e-17 | 619 | 0.182 | 427 | 297 | 15 | 4 | 402 | 8 | 410 | Contig\_52, whole genome shotgun sequence | Contig\_52, whole genome shotgun sequence | | afdb-uniprot50 | AF-A0A382GF79-F1-MODEL\_V4 | 1.0 | 3.057e-17 | 618 | 0.199 | 427 | 284 | 25 | 1 | 402 | 1 | 394 | Tyr recombinase domain-containing protein | Tyr recombinase domain-containing protein | | afdb-uniprot50 | AF-A0A328TT78-F1-MODEL\_V4 | 1.0 | 1.991e-17 | 617 | 0.212 | 419 | 280 | 18 | 1 | 403 | 1 | 385 | Putative prophage CPS-53 integrase | Putative prophage CPS-53 integrase | | afdb-uniprot50 | AF-A0A3D1LLU3-F1-MODEL\_V4 | 1.0 | 1.898e-17 | 617 | 0.195 | 424 | 287 | 19 | 2 | 402 | 8 | 400 | Integrase | Integrase | | afdb-uniprot50 | AF-A0A0Q0CJV1-F1-MODEL\_V4 | 1.0 | 1.474e-16 | 617 | 0.21 | 422 | 279 | 22 | 2 | 402 | 39 | 427 | Integrase | Integrase | | afdb-uniprot50 | AF-A0A2D8ER74-F1-MODEL\_V4 | 1.0 | 8.318e-17 | 616 | 0.204 | 426 | 280 | 21 | 2 | 402 | 3 | 394 | Integrase | Integrase | | afdb-uniprot50 | AF-A0A858BC33-F1-MODEL\_V4 | 1.0 | 1.161e-16 | 616 | 0.216 | 421 | 279 | 21 | 2 | 402 | 8 | 397 | DUF4102 domain-containing protein | DUF4102 domain-containing protein | | afdb-uniprot50 | AF-A0A246JIN4-F1-MODEL\_V4 | 1.0 | 8.443e-18 | 616 | 0.221 | 425 | 272 | 20 | 2 | 402 | 3 | 392 | Integrase | Integrase | | afdb-uniprot50 | AF-A0A7D7VIT3-F1-MODEL\_V4 | 1.0 | 6.249e-17 | 616 | 0.204 | 421 | 286 | 18 | 1 | 402 | 2 | 392 | Tyrosine-type recombinase/integrase | Tyrosine-type recombinase/integrase | | afdb-uniprot50 | AF-A0A3P1XZM4-F1-MODEL\_V4 | 1.0 | 3.699e-17 | 616 | 0.208 | 427 | 279 | 22 | 2 | 402 | 14 | 407 | DUF4102 domain-containing protein | DUF4102 domain-containing protein | | afdb-uniprot50 | AF-A0A520PEP9-F1-MODEL\_V4 | 1.0 | 1.296e-17 | 616 | 0.214 | 448 | 275 | 19 | 1 | 402 | 11 | 427 | DUF4102 domain-containing protein | DUF4102 domain-containing protein | | afdb-uniprot50 | AF-C9M6R5-F1-MODEL\_V4 | 1.0 | 1.991e-17 | 615 | 0.199 | 421 | 279 | 18 | 1 | 402 | 1 | 382 | Site-specific recombinase, phage integrase family | Site-specific recombinase, phage integrase family | | afdb-uniprot50 | AF-A0A345H8S3-F1-MODEL\_V4 | 1.0 | 1.961e-16 | 615 | 0.179 | 423 | 294 | 21 | 2 | 400 | 3 | 396 | DUF4102 domain-containing protein | DUF4102 domain-containing protein | | afdb-uniprot50 | AF-A0A6G4WGV4-F1-MODEL\_V4 | 1.0 | 2.65e-17 | 615 | 0.235 | 434 | 268 | 23 | 1 | 402 | 4 | 405 | Integrase family protein | Integrase family protein | | afdb-uniprot50 | AF-A0A426QL33-F1-MODEL\_V4 | 1.0 | 3.88e-17 | 614 | 0.185 | 421 | 295 | 16 | 2 | 402 | 14 | 406 | DUF4102 domain-containing protein | DUF4102 domain-containing protein | | afdb-uniprot50 | AF-A0A0X8JKZ6-F1-MODEL\_V4 | 1.0 | 1.726e-17 | 614 | 0.186 | 428 | 282 | 19 | 2 | 402 | 3 | 391 | Integrase | Integrase | | afdb-uniprot50 | AF-A0A8B4S2H0-F1-MODEL\_V4 | 1.0 | 3.057e-17 | 614 | 0.204 | 446 | 284 | 22 | 1 | 402 | 1 | 419 | Prophage CPS-53 integrase | Prophage CPS-53 integrase | | afdb-uniprot50 | AF-A0A1I7M1P0-F1-MODEL\_V4 | 1.0 | 9.287e-18 | 614 | 0.217 | 432 | 272 | 20 | 2 | 402 | 6 | 402 | Site-specific recombinase XerD | Site-specific recombinase XerD | | afdb-uniprot50 | AF-A0A3D3CKM6-F1-MODEL\_V4 | 1.0 | 9.15e-17 | 613 | 0.242 | 408 | 260 | 19 | 1 | 398 | 1 | 369 | Integrase | Integrase | | afdb-uniprot50 | AF-A0A4U2UK76-F1-MODEL\_V4 | 1.0 | 8.318e-17 | 613 | 0.202 | 420 | 286 | 18 | 2 | 402 | 8 | 397 | DUF4102 domain-containing protein | DUF4102 domain-containing protein | | afdb-uniprot50 | AF-A0A4R1VLQ5-F1-MODEL\_V4 | 1.0 | 9.287e-18 | 613 | 0.204 | 426 | 284 | 20 | 2 | 402 | 5 | 400 | Integrase | Integrase | | afdb-uniprot50 | AF-A0A2U2CQD6-F1-MODEL\_V4 | 1.0 | 1.36e-17 | 613 | 0.185 | 431 | 282 | 19 | 2 | 401 | 33 | 425 | Integrase | Integrase | | afdb-uniprot50 | AF-A0A254N809-F1-MODEL\_V4 | 1.0 | 2.331e-18 | 613 | 0.191 | 465 | 278 | 22 | 2 | 402 | 3 | 433 | Integrase | Integrase | | afdb-uniprot50 | AF-A0A6L5XJW2-F1-MODEL\_V4 | 1.0 | 3.88e-17 | 613 | 0.198 | 424 | 284 | 21 | 2 | 402 | 3 | 393 | DUF4102 domain-containing protein | DUF4102 domain-containing protein | | afdb-uniprot50 | AF-A0A3T7XM81-F1-MODEL\_V4 | 1.0 | 9.15e-17 | 612 | 0.197 | 420 | 286 | 18 | 2 | 401 | 8 | 396 | DUF4102 domain-containing protein | DUF4102 domain-containing protein | | afdb-uniprot50 | AF-A0A7C8AXU5-F1-MODEL\_V4 | 1.0 | 5.416e-17 | 612 | 0.227 | 427 | 270 | 20 | 2 | 402 | 8 | 400 | Integrase arm-type DNA-binding domain-containing protein | Integrase arm-type DNA-binding domain-containing protein | | afdb-uniprot50 | AF-A0A0X3TJA3-F1-MODEL\_V4 | 1.0 | 3.527e-17 | 612 | 0.184 | 444 | 290 | 17 | 2 | 402 | 3 | 417 | Uncharacterized protein | Uncharacterized protein | | afdb-uniprot50 | AF-A0A317CJS7-F1-MODEL\_V4 | 1.0 | 3.057e-17 | 611 | 0.193 | 424 | 286 | 19 | 2 | 402 | 5 | 395 | Integrase | Integrase | | afdb-uniprot50 | AF-A0A1B1NW11-F1-MODEL\_V4 | 1.0 | 1.87e-16 | 611 | 0.222 | 427 | 272 | 20 | 1 | 402 | 7 | 398 | Prophage CP4-57 integrase | Prophage CP4-57 integrase | | afdb-uniprot50 | AF-A0A2S5QMZ1-F1-MODEL\_V4 | 1.0 | 6.874e-17 | 610 | 0.199 | 416 | 280 | 21 | 1 | 403 | 1 | 376 | Integrase | Integrase | | afdb-uniprot50 | AF-A0A560D0T2-F1-MODEL\_V4 | 1.0 | 1.296e-17 | 610 | 0.22 | 439 | 265 | 23 | 1 | 401 | 5 | 404 | Site-specific recombinase XerD | Site-specific recombinase XerD | | afdb-uniprot50 | AF-A0A2U8P8P1-F1-MODEL\_V4 | 1.0 | 1.991e-17 | 610 | 0.211 | 434 | 278 | 24 | 2 | 403 | 5 | 406 | DUF4102 domain-containing protein | DUF4102 domain-containing protein | | afdb-uniprot50 | AF-A0A661G1N2-F1-MODEL\_V4 | 1.0 | 2.65e-17 | 610 | 0.188 | 451 | 284 | 25 | 2 | 402 | 3 | 421 | Integrase | Integrase | | afdb-uniprot50 | AF-A0A095S8W4-F1-MODEL\_V4 | 1.0 | 3.207e-17 | 609 | 0.228 | 421 | 274 | 20 | 2 | 402 | 6 | 395 | DNA integration/recombination/inversion protein | DNA integration/recombination/inversion protein | | afdb-uniprot50 | AF-A0A2U2CNI2-F1-MODEL\_V4 | 1.0 | 5.242e-18 | 609 | 0.237 | 426 | 267 | 23 | 2 | 403 | 11 | 402 | Uncharacterized protein | Uncharacterized protein | | afdb-uniprot50 | AF-A0A379HBU4-F1-MODEL\_V4 | 1.0 | 4.268e-17 | 609 | 0.205 | 423 | 287 | 17 | 2 | 402 | 3 | 398 | Prophage CP4-57 integrase | Prophage CP4-57 integrase | | afdb-uniprot50 | AF-A0A7X8YJ22-F1-MODEL\_V4 | 1.0 | 1.056e-16 | 609 | 0.216 | 425 | 274 | 21 | 2 | 402 | 8 | 397 | Tyrosine-type recombinase/integrase | Tyrosine-type recombinase/integrase | | afdb-uniprot50 | AF-A0A066UH65-F1-MODEL\_V4 | 1.0 | 5.958e-17 | 608 | 0.181 | 418 | 300 | 17 | 2 | 402 | 3 | 395 | Phage integrase | Phage integrase | | afdb-uniprot50 | AF-A0A7W9B908-F1-MODEL\_V4 | 1.0 | 3.363e-17 | 608 | 0.208 | 427 | 278 | 22 | 1 | 402 | 1 | 392 | Integrase | Integrase | | afdb-uniprot50 | AF-A0A2G0Q224-F1-MODEL\_V4 | 1.0 | 1.783e-16 | 607 | 0.178 | 426 | 289 | 22 | 2 | 402 | 3 | 392 | Integrase | Integrase | | afdb-uniprot50 | AF-A0A380TVV2-F1-MODEL\_V4 | 1.0 | 7.561e-17 | 607 | 0.203 | 427 | 282 | 22 | 2 | 401 | 8 | 403 | Prophage CP4-57 integrase | Prophage CP4-57 integrase | | afdb-uniprot50 | AF-W2UZJ4-F1-MODEL\_V4 | 1.0 | 2.779e-17 | 607 | 0.193 | 419 | 288 | 15 | 2 | 402 | 14 | 400 | p4-like integrase | p4-like integrase | | afdb-uniprot50 | AF-A0A1G9U9P8-F1-MODEL\_V4 | 1.0 | 1.991e-17 | 607 | 0.203 | 422 | 283 | 17 | 2 | 402 | 3 | 392 | Integrase | Integrase | | afdb-uniprot50 | AF-A0A1M7T7F7-F1-MODEL\_V4 | 1.0 | 6.554e-17 | 606 | 0.175 | 417 | 294 | 19 | 2 | 402 | 3 | 385 | Integrase | Integrase | | afdb-uniprot50 | AF-A0A3Q9JNA5-F1-MODEL\_V4 | 1.0 | 1.056e-16 | 606 | 0.211 | 407 | 277 | 19 | 2 | 394 | 8 | 384 | DUF4102 domain-containing protein | DUF4102 domain-containing protein | | afdb-uniprot50 | AF-A0A7Y8SU33-F1-MODEL\_V4 | 1.0 | 2.409e-17 | 606 | 0.218 | 426 | 276 | 19 | 2 | 402 | 1 | 394 | Tyrosine-type recombinase/integrase | Tyrosine-type recombinase/integrase | | afdb-uniprot50 | AF-A0A259IRK1-F1-MODEL\_V4 | 1.0 | 9.596e-17 | 606 | 0.185 | 431 | 291 | 21 | 2 | 402 | 3 | 403 | Tyr recombinase domain-containing protein | Tyr recombinase domain-containing protein | | afdb-uniprot50 | AF-A0A7H9BH93-F1-MODEL\_V4 | 1.0 | 4.069e-17 | 605 | 0.202 | 429 | 281 | 20 | 1 | 402 | 15 | 409 | Tyrosine-type recombinase/integrase | Tyrosine-type recombinase/integrase | | afdb-uniprot50 | AF-A0A2Z2HFA7-F1-MODEL\_V4 | 1.0 | 2.158e-16 | 605 | 0.186 | 423 | 288 | 21 | 2 | 401 | 43 | 432 | Tyr recombinase domain-containing protein | Tyr recombinase domain-containing protein | | afdb-uniprot50 | AF-A0A6G5R766-F1-MODEL\_V4 | 1.0 | 2.527e-17 | 604 | 0.178 | 436 | 293 | 18 | 1 | 403 | 4 | 407 | Site-specific recombinase, phage integrase family (DUF4102 domain) | Site-specific recombinase, phage integrase family (DUF4102 domain) | | afdb-uniprot50 | AF-A0A509YI24-F1-MODEL\_V4 | 1.0 | 1.006e-16 | 604 | 0.185 | 416 | 292 | 18 | 2 | 400 | 23 | 408 | Integrase | Integrase | | afdb-uniprot50 | AF-A0A4P7REU5-F1-MODEL\_V4 | 1.0 | 1.81e-17 | 604 | 0.221 | 429 | 271 | 19 | 1 | 402 | 7 | 399 | DUF4102 domain-containing protein | DUF4102 domain-containing protein | | afdb-uniprot50 | AF-A0A6M8UC91-F1-MODEL\_V4 | 1.0 | 5.681e-17 | 603 | 0.225 | 417 | 264 | 20 | 2 | 391 | 8 | 392 | Integrase | Integrase | | afdb-uniprot50 | AF-A0A363CZK6-F1-MODEL\_V4 | 1.0 | 2.872e-16 | 603 | 0.165 | 428 | 302 | 18 | 2 | 403 | 8 | 406 | Uncharacterized protein | Uncharacterized protein | | afdb-uniprot50 | AF-A0A0F2P2B9-F1-MODEL\_V4 | 1.0 | 4.924e-17 | 602 | 0.189 | 428 | 291 | 18 | 2 | 402 | 3 | 401 | Integrase | Integrase | | afdb-uniprot50 | AF-A0A1G3TPX5-F1-MODEL\_V4 | 1.0 | 8.724e-17 | 602 | 0.204 | 420 | 289 | 19 | 2 | 402 | 8 | 401 | Integrase | Integrase | | afdb-uniprot50 | AF-A0A1I3RK68-F1-MODEL\_V4 | 1.0 | 8.318e-17 | 602 | 0.196 | 427 | 281 | 21 | 2 | 402 | 3 | 393 | Integrase | Integrase | | afdb-uniprot50 | AF-A0A1X7JHB3-F1-MODEL\_V4 | 1.0 | 1.474e-16 | 601 | 0.186 | 419 | 281 | 24 | 2 | 402 | 1 | 377 | Integrase | Integrase | | afdb-uniprot50 | AF-A0A8B0UBD6-F1-MODEL\_V4 | 1.0 | 4.009e-16 | 601 | 0.198 | 414 | 283 | 19 | 2 | 402 | 3 | 380 | Tyrosine-type recombinase/integrase | Tyrosine-type recombinase/integrase | | afdb-uniprot50 | AF-A0A239LDZ8-F1-MODEL\_V4 | 1.0 | 1.36e-17 | 601 | 0.205 | 424 | 281 | 19 | 2 | 402 | 3 | 393 | Integrase | Integrase | | afdb-uniprot50 | AF-A0A7W4WUX0-F1-MODEL\_V4 | 1.0 | 4.476e-17 | 601 | 0.189 | 449 | 287 | 22 | 1 | 402 | 1 | 419 | Integrase | Integrase | | afdb-uniprot50 | AF-A0A2G4ASH1-F1-MODEL\_V4 | 1.0 | 1.161e-16 | 600 | 0.202 | 415 | 286 | 17 | 2 | 402 | 3 | 386 | Putative prophage CPS-53 integrase | Putative prophage CPS-53 integrase | | afdb-uniprot50 | AF-A0A5J5FS73-F1-MODEL\_V4 | 1.0 | 5.416e-17 | 600 | 0.195 | 425 | 286 | 18 | 1 | 402 | 7 | 398 | Tyrosine-type recombinase/integrase | Tyrosine-type recombinase/integrase | | afdb-uniprot50 | AF-A0A1S8D336-F1-MODEL\_V4 | 1.0 | 1.898e-17 | 600 | 0.209 | 434 | 273 | 22 | 2 | 403 | 1 | 396 | Uncharacterized protein | Uncharacterized protein | | afdb-uniprot50 | AF-A0A1E7WYI8-F1-MODEL\_V4 | 1.0 | 4.069e-17 | 600 | 0.203 | 456 | 280 | 21 | 2 | 403 | 4 | 430 | Prophage CP4-57 integrase | Prophage CP4-57 integrase | | afdb-uniprot50 | AF-A0A212LBW6-F1-MODEL\_V4 | 1.0 | 1.991e-17 | 600 | 0.192 | 442 | 290 | 17 | 2 | 402 | 4 | 419 | Putative Integrase family protein | Putative Integrase family protein | | afdb-uniprot50 | AF-A0A5C8TMJ0-F1-MODEL\_V4 | 1.0 | 5.681e-17 | 599 | 0.22 | 421 | 268 | 22 | 2 | 401 | 18 | 399 | Tyrosine-type recombinase/integrase | Tyrosine-type recombinase/integrase | | afdb-uniprot50 | AF-D0X1Z0-F1-MODEL\_V4 | 1.0 | 1.783e-16 | 599 | 0.213 | 422 | 279 | 21 | 2 | 402 | 4 | 393 | Tyr recombinase domain-containing protein | Tyr recombinase domain-containing protein | | afdb-uniprot50 | AF-A0A5B0GL81-F1-MODEL\_V4 | 1.0 | 2.65e-17 | 599 | 0.214 | 428 | 273 | 22 | 2 | 402 | 8 | 399 | Tyrosine-type recombinase/integrase | Tyrosine-type recombinase/integrase | | afdb-uniprot50 | AF-A0A7C1QT18-F1-MODEL\_V4 | 1.0 | 1.7e-16 | 599 | 0.195 | 461 | 288 | 22 | 1 | 402 | 1 | 437 | Tyr recombinase domain-containing protein | Tyr recombinase domain-containing protein | | afdb-uniprot50 | AF-A0A6L9MH98-F1-MODEL\_V4 | 1.0 | 6.874e-17 | 598 | 0.198 | 424 | 284 | 18 | 1 | 402 | 3 | 392 | Tyrosine-type recombinase/integrase | Tyrosine-type recombinase/integrase | | afdb-uniprot50 | AF-A0A2G6HRL6-F1-MODEL\_V4 | 1.0 | 1.107e-16 | 598 | 0.204 | 435 | 276 | 23 | 2 | 402 | 5 | 403 | Integrase | Integrase | | afdb-uniprot50 | AF-H3RBK3-F1-MODEL\_V4 | 1.0 | 1.34e-16 | 597 | 0.201 | 426 | 279 | 19 | 2 | 402 | 8 | 397 | CP4-57 family phage integrase | CP4-57 family phage integrase | | afdb-uniprot50 | AF-A0A3S0V2Q9-F1-MODEL\_V4 | 1.0 | 1.898e-17 | 597 | 0.209 | 439 | 276 | 19 | 2 | 402 | 6 | 411 | DUF4102 domain-containing protein | DUF4102 domain-containing protein | | afdb-uniprot50 | AF-A0A1Y0HJN4-F1-MODEL\_V4 | 1.0 | 2.738e-16 | 596 | 0.169 | 425 | 300 | 22 | 1 | 402 | 5 | 399 | Prophage integrase IntA | Prophage integrase IntA | | afdb-uniprot50 | AF-A0A0N0IHF8-F1-MODEL\_V4 | 1.0 | 1.071e-17 | 596 | 0.173 | 433 | 291 | 19 | 2 | 402 | 9 | 406 | Tyr recombinase domain-containing protein | Tyr recombinase domain-containing protein | | afdb-uniprot50 | AF-A0A432ZBV6-F1-MODEL\_V4 | 1.0 | 3.159e-16 | 596 | 0.208 | 422 | 281 | 19 | 2 | 402 | 16 | 405 | Integrase | Integrase | | afdb-uniprot50 | AF-A0A2T0XJR5-F1-MODEL\_V4 | 1.0 | 8.443e-18 | 596 | 0.201 | 431 | 277 | 21 | 1 | 402 | 1 | 393 | Integrase | Integrase | | afdb-uniprot50 | AF-H0Q156-F1-MODEL\_V4 | 1.0 | 4.924e-17 | 596 | 0.213 | 427 | 279 | 21 | 2 | 402 | 28 | 423 | Uncharacterized protein | Uncharacterized protein | | afdb-uniprot50 | AF-A0A2E6A368-F1-MODEL\_V4 | 1.0 | 2.611e-16 | 595 | 0.21 | 422 | 279 | 20 | 1 | 402 | 6 | 393 | Integrase | Integrase | | afdb-uniprot50 | AF-A0A7M3B6Q3-F1-MODEL\_V4 | 1.0 | 7.21e-17 | 595 | 0.208 | 426 | 275 | 21 | 2 | 402 | 11 | 399 | Integrase | Integrase | | afdb-uniprot50 | AF-A0A845UVE8-F1-MODEL\_V4 | 1.0 | 1.496e-17 | 595 | 0.222 | 450 | 280 | 21 | 1 | 403 | 10 | 436 | Uncharacterized protein | Uncharacterized protein | | afdb-uniprot50 | AF-A0A482ITC2-F1-MODEL\_V4 | 1.0 | 3.363e-17 | 593 | 0.217 | 469 | 263 | 25 | 2 | 402 | 4 | 436 | DUF4102 domain-containing protein | DUF4102 domain-containing protein | | afdb-uniprot50 | AF-A0A5S4SE16-F1-MODEL\_V4 | 1.0 | 7.931e-17 | 593 | 0.204 | 446 | 284 | 21 | 1 | 402 | 1 | 419 | Tyrosine-type recombinase/integrase | Tyrosine-type recombinase/integrase | | afdb-uniprot50 | AF-D6VB17-F1-MODEL\_V4 | 1.0 | 2.297e-17 | 593 | 0.211 | 421 | 271 | 17 | 2 | 401 | 64 | 444 | Integrase family protein | Integrase family protein | | afdb-uniprot50 | AF-A0A0K1QM45-F1-MODEL\_V4 | 1.0 | 5.088e-16 | 592 | 0.218 | 411 | 267 | 22 | 2 | 391 | 3 | 380 | Integrase | Integrase | | afdb-uniprot50 | AF-A0A560WXP0-F1-MODEL\_V4 | 1.0 | 1.056e-16 | 592 | 0.207 | 429 | 283 | 23 | 2 | 402 | 11 | 410 | Integrase | Integrase | | afdb-uniprot50 | AF-A0A1H6GE73-F1-MODEL\_V4 | 1.0 | 1.006e-16 | 592 | 0.224 | 414 | 262 | 20 | 2 | 391 | 3 | 381 | Integrase | Integrase | | afdb-uniprot50 | AF-A0A2U0T8C2-F1-MODEL\_V4 | 1.0 | 4.41e-16 | 591 | 0.198 | 424 | 288 | 20 | 2 | 402 | 8 | 402 | Integrase | Integrase | | afdb-uniprot50 | AF-A0A6S6TC66-F1-MODEL\_V4 | 1.0 | 1.546e-16 | 591 | 0.188 | 429 | 290 | 20 | 2 | 402 | 9 | 407 | Integrase family protein | Integrase family protein | | afdb-uniprot50 | AF-A0A4R6Y5X0-F1-MODEL\_V4 | 1.0 | 2.057e-16 | 591 | 0.179 | 429 | 289 | 20 | 1 | 402 | 1 | 393 | Integrase | Integrase | | afdb-uniprot50 | AF-A0A4R1HYV8-F1-MODEL\_V4 | 1.0 | 7.21e-17 | 590 | 0.219 | 433 | 273 | 20 | 2 | 403 | 3 | 401 | Site-specific recombinase XerD | Site-specific recombinase XerD | | afdb-uniprot50 | AF-A0A5U3D0P1-F1-MODEL\_V4 | 1.0 | 7.561e-17 | 590 | 0.213 | 449 | 275 | 25 | 2 | 403 | 10 | 427 | Tyrosine-type recombinase/integrase | Tyrosine-type recombinase/integrase | | afdb-uniprot50 | AF-A0A2U0ZIK5-F1-MODEL\_V4 | 1.0 | 1.546e-16 | 589 | 0.211 | 431 | 274 | 25 | 2 | 403 | 3 | 396 | Integrase | Integrase | | afdb-uniprot50 | AF-A0A836Q5F6-F1-MODEL\_V4 | 1.0 | 4.41e-16 | 589 | 0.178 | 421 | 297 | 21 | 2 | 402 | 4 | 395 | DUF4102 domain-containing protein | DUF4102 domain-containing protein | | afdb-uniprot50 | AF-A0A5E9PHW5-F1-MODEL\_V4 | 1.0 | 2.057e-16 | 589 | 0.179 | 423 | 300 | 18 | 1 | 403 | 1 | 396 | Site-specific integrase | Site-specific integrase | | afdb-uniprot50 | AF-A0A4R6FNZ7-F1-MODEL\_V4 | 1.0 | 1.34e-16 | 589 | 0.192 | 421 | 280 | 21 | 9 | 402 | 7 | 394 | Integrase | Integrase | | afdb-uniprot50 | AF-A0A5N0TDP5-F1-MODEL\_V4 | 1.0 | 1.161e-16 | 589 | 0.201 | 421 | 285 | 18 | 2 | 402 | 8 | 397 | Tyrosine-type recombinase/integrase | Tyrosine-type recombinase/integrase | | afdb-uniprot50 | AF-A0A7Z9HGD8-F1-MODEL\_V4 | 1.0 | 7.561e-17 | 589 | 0.2 | 430 | 284 | 20 | 2 | 402 | 9 | 407 | DUF4102 domain-containing protein | DUF4102 domain-containing protein | | afdb-uniprot50 | AF-A0A1G4SA28-F1-MODEL\_V4 | 1.0 | 4.851e-16 | 589 | 0.169 | 426 | 293 | 20 | 2 | 402 | 3 | 392 | Integrase | Integrase | | afdb-uniprot50 | AF-A0A839GCA6-F1-MODEL\_V4 | 1.0 | 8.318e-17 | 589 | 0.197 | 426 | 283 | 21 | 2 | 403 | 3 | 393 | Integrase | Integrase | | afdb-uniprot50 | AF-A0A2N2SIH5-F1-MODEL\_V4 | 1.0 | 1.165e-13 | 588 | 0.237 | 311 | 206 | 11 | 102 | 402 | 10 | 299 | Integrase | Integrase | | afdb-uniprot50 | AF-A0A562IZK1-F1-MODEL\_V4 | 1.0 | 4.779e-15 | 588 | 0.233 | 355 | 232 | 17 | 78 | 402 | 19 | 363 | Phage integrase family protein | Phage integrase family protein | | afdb-uniprot50 | AF-A0A4R5TYE0-F1-MODEL\_V4 | 1.0 | 8.318e-17 | 588 | 0.196 | 418 | 292 | 20 | 1 | 402 | 1 | 390 | DUF4102 domain-containing protein | DUF4102 domain-containing protein | | afdb-uniprot50 | AF-A0A4D7WTQ9-F1-MODEL\_V4 | 1.0 | 8.724e-17 | 588 | 0.207 | 428 | 277 | 19 | 2 | 403 | 6 | 397 | DUF4102 domain-containing protein | DUF4102 domain-containing protein | | afdb-uniprot50 | AF-A0A811B9I7-F1-MODEL\_V4 | 1.0 | 7.931e-17 | 588 | 0.177 | 423 | 294 | 18 | 2 | 402 | 5 | 395 | Integrase | Integrase | | afdb-uniprot50 | AF-A0A2S5TDL1-F1-MODEL\_V4 | 1.0 | 3.475e-16 | 588 | 0.21 | 423 | 281 | 19 | 2 | 402 | 7 | 398 | Integrase | Integrase | | afdb-uniprot50 | AF-A0A5S9PCI4-F1-MODEL\_V4 | 1.0 | 1.783e-16 | 587 | 0.172 | 423 | 297 | 18 | 1 | 402 | 1 | 391 | Prophage integrase IntA | Prophage integrase IntA | | afdb-uniprot50 | AF-A0A096FMR1-F1-MODEL\_V4 | 1.0 | 1.81e-17 | 586 | 0.189 | 454 | 281 | 21 | 1 | 402 | 1 | 419 | Integrase | Integrase | | afdb-uniprot50 | AF-A0A069E4V0-F1-MODEL\_V4 | 1.0 | 1.107e-16 | 585 | 0.216 | 412 | 271 | 17 | 9 | 400 | 16 | 395 | Phage integrase | Phage integrase | | afdb-uniprot50 | AF-A0A0M0FY12-F1-MODEL\_V4 | 1.0 | 2.452e-15 | 585 | 0.223 | 362 | 234 | 13 | 71 | 402 | 47 | 391 | Integrase | Integrase | | afdb-uniprot50 | AF-A0A1W6MW71-F1-MODEL\_V4 | 1.0 | 1.961e-16 | 585 | 0.197 | 436 | 282 | 21 | 2 | 402 | 10 | 412 | Uncharacterized protein | Uncharacterized protein | | afdb-uniprot50 | AF-A0A368V6W8-F1-MODEL\_V4 | 1.0 | 4.205e-16 | 585 | 0.193 | 423 | 287 | 22 | 2 | 402 | 11 | 401 | Integrase | Integrase | | afdb-uniprot50 | AF-A0A844WU91-F1-MODEL\_V4 | 1.0 | 7.813e-16 | 584 | 0.173 | 421 | 299 | 17 | 2 | 402 | 8 | 399 | Tyrosine-type recombinase/integrase | Tyrosine-type recombinase/integrase | | afdb-uniprot50 | AF-A0A441UN45-F1-MODEL\_V4 | 1.0 | 1.124e-17 | 584 | 0.206 | 432 | 284 | 17 | 2 | 402 | 6 | 409 | DUF4102 domain-containing protein | DUF4102 domain-containing protein | | afdb-uniprot50 | AF-A0A5E7JF77-F1-MODEL\_V4 | 1.0 | 9.15e-17 | 584 | 0.204 | 450 | 292 | 19 | 1 | 400 | 3 | 436 | Core-binding (CB) domain-containing protein | Core-binding (CB) domain-containing protein | | afdb-uniprot50 | AF-A0A0P1ELQ8-F1-MODEL\_V4 | 1.0 | 6.249e-17 | 584 | 0.215 | 436 | 279 | 23 | 2 | 401 | 52 | 460 | Prophage CP4-57 integrase | Prophage CP4-57 integrase | | afdb-uniprot50 | AF-A0A292AK98-F1-MODEL\_V4 | 1.0 | 5.681e-17 | 583 | 0.217 | 436 | 260 | 22 | 18 | 403 | 6 | 410 | Integrase | Integrase | | afdb-uniprot50 | AF-A0A5P9HCV6-F1-MODEL\_V4 | 1.0 | 2.738e-16 | 583 | 0.19 | 430 | 281 | 24 | 2 | 402 | 11 | 402 | Prophage CPS-53 integrase | Prophage CPS-53 integrase | | afdb-uniprot50 | AF-A0A1Q3K5N4-F1-MODEL\_V4 | 1.0 | 2.779e-17 | 582 | 0.23 | 407 | 265 | 17 | 2 | 402 | 3 | 367 | Tyr recombinase domain-containing protein | Tyr recombinase domain-containing protein | | afdb-uniprot50 | AF-K8PFU5-F1-MODEL\_V4 | 1.0 | 5.416e-17 | 582 | 0.218 | 425 | 280 | 19 | 2 | 403 | 14 | 409 | Tyr recombinase domain-containing protein | Tyr recombinase domain-containing protein | | afdb-uniprot50 | AF-A0A3B0W1B8-F1-MODEL\_V4 | 1.0 | 1.006e-16 | 582 | 0.186 | 440 | 288 | 23 | 1 | 401 | 3 | 411 | Phage integrase | Phage integrase | | afdb-uniprot50 | AF-A0A0P7AIM3-F1-MODEL\_V4 | 1.0 | 1.961e-16 | 581 | 0.185 | 415 | 289 | 20 | 2 | 403 | 9 | 387 | Tyr recombinase domain-containing protein | Tyr recombinase domain-containing protein | | afdb-uniprot50 | AF-S6CXQ8-F1-MODEL\_V4 | 1.0 | 1.056e-16 | 581 | 0.213 | 435 | 272 | 19 | 2 | 402 | 6 | 404 | Phage integrase family protein | Phage integrase family protein | | afdb-uniprot50 | AF-A0A4Q4B2S9-F1-MODEL\_V4 | 1.0 | 5.597e-16 | 581 | 0.224 | 428 | 271 | 22 | 2 | 402 | 3 | 396 | DUF4102 domain-containing protein | DUF4102 domain-containing protein | | afdb-uniprot50 | AF-A0A348UTP6-F1-MODEL\_V4 | 1.0 | 1.815e-14 | 580 | 0.236 | 326 | 213 | 13 | 106 | 402 | 3 | 321 | Integrase | Integrase | | afdb-uniprot50 | AF-A0A7H4NYI2-F1-MODEL\_V4 | 1.0 | 1.32e-15 | 580 | 0.195 | 388 | 270 | 15 | 36 | 403 | 39 | 404 | Phage integrase | Phage integrase | | afdb-uniprot50 | AF-A0A7W6S310-F1-MODEL\_V4 | 1.0 | 4.625e-16 | 578 | 0.222 | 432 | 270 | 20 | 2 | 401 | 7 | 404 | Integrase | Integrase | | afdb-uniprot50 | AF-A0A6S5KU75-F1-MODEL\_V4 | 1.0 | 7.449e-16 | 577 | 0.218 | 417 | 264 | 20 | 1 | 394 | 1 | 378 | Integrase | Integrase | | afdb-uniprot50 | AF-A0A345DAL3-F1-MODEL\_V4 | 1.0 | 6.457e-16 | 577 | 0.19 | 440 | 292 | 22 | 2 | 402 | 6 | 420 | Prophage integrase IntA | Prophage integrase IntA | | afdb-uniprot50 | AF-A0A1M6UHH4-F1-MODEL\_V4 | 1.0 | 1.961e-16 | 577 | 0.191 | 423 | 292 | 20 | 8 | 400 | 15 | 417 | Arm-DNA-bind\_3 domain-containing protein | Arm-DNA-bind\_3 domain-containing protein | | afdb-uniprot50 | AF-N9NA50-F1-MODEL\_V4 | 1.0 | 1.546e-16 | 576 | 0.194 | 432 | 285 | 23 | 2 | 403 | 6 | 404 | Tyr recombinase domain-containing protein | Tyr recombinase domain-containing protein | | afdb-uniprot50 | AF-C3X4R8-F1-MODEL\_V4 | 1.0 | 9.15e-17 | 576 | 0.175 | 432 | 293 | 20 | 2 | 401 | 8 | 408 | Tyr recombinase domain-containing protein | Tyr recombinase domain-containing protein | | afdb-uniprot50 | AF-E0M0I1-F1-MODEL\_V4 | 1.0 | 1.056e-16 | 575 | 0.194 | 416 | 289 | 20 | 2 | 402 | 3 | 387 | Integrase family protein | Integrase family protein | | afdb-uniprot50 | AF-A0A7U2PLX0-F1-MODEL\_V4 | 1.0 | 8.443e-18 | 575 | 0.193 | 435 | 273 | 20 | 2 | 403 | 3 | 392 | Tyrosine-type recombinase/integrase | Tyrosine-type recombinase/integrase | | afdb-uniprot50 | AF-A0A840MZH1-F1-MODEL\_V4 | 1.0 | 5.87e-16 | 575 | 0.183 | 424 | 286 | 18 | 2 | 398 | 10 | 400 | Integrase | Integrase | | afdb-uniprot50 | AF-A0A6G8CS84-F1-MODEL\_V4 | 1.0 | 4.625e-16 | 575 | 0.202 | 425 | 283 | 21 | 2 | 403 | 11 | 402 | Tyrosine-type recombinase/integrase | Tyrosine-type recombinase/integrase | | afdb-uniprot50 | AF-A0A5E7WTN4-F1-MODEL\_V4 | 1.0 | 5.179e-14 | 574 | 0.216 | 360 | 245 | 15 | 60 | 402 | 2 | 341 | Prophage integrase IntS | Prophage integrase IntS | | afdb-uniprot50 | AF-H3RIA1-F1-MODEL\_V4 | 1.0 | 2.967e-15 | 574 | 0.208 | 378 | 252 | 15 | 53 | 402 | 1 | 359 | Site-specific recombinase, phage integrase family | Site-specific recombinase, phage integrase family | | afdb-uniprot50 | AF-A0A2K8UAW1-F1-MODEL\_V4 | 1.0 | 9.014e-16 | 574 | 0.207 | 404 | 267 | 17 | 25 | 400 | 4 | 382 | Uncharacterized protein | Uncharacterized protein | | afdb-uniprot50 | AF-A0A2K8L2Y2-F1-MODEL\_V4 | 1.0 | 2.572e-15 | 574 | 0.167 | 424 | 295 | 22 | 2 | 402 | 3 | 391 | Integrase | Integrase | | afdb-uniprot50 | AF-A0A398BNA6-F1-MODEL\_V4 | 1.0 | 1.474e-16 | 574 | 0.232 | 425 | 261 | 23 | 1 | 398 | 1 | 387 | DUF4102 domain-containing protein | DUF4102 domain-containing protein | | afdb-uniprot50 | AF-A0A3G2HYM0-F1-MODEL\_V4 | 1.0 | 1.218e-16 | 574 | 0.187 | 443 | 290 | 23 | 2 | 402 | 7 | 421 | DUF4102 domain-containing protein | DUF4102 domain-containing protein | | afdb-uniprot50 | AF-A0A2E6KN51-F1-MODEL\_V4 | 1.0 | 4.851e-16 | 573 | 0.179 | 423 | 285 | 17 | 9 | 402 | 6 | 395 | Integrase | Integrase | | afdb-uniprot50 | AF-A0A1L3EZE4-F1-MODEL\_V4 | 1.0 | 1.161e-16 | 573 | 0.2 | 435 | 282 | 18 | 2 | 400 | 7 | 411 | Uncharacterized protein | Uncharacterized protein | | afdb-uniprot50 | AF-A0A7W5ZZ75-F1-MODEL\_V4 | 1.0 | 4.069e-17 | 573 | 0.18 | 432 | 283 | 21 | 2 | 402 | 58 | 449 | Integrase | Integrase | | afdb-uniprot50 | AF-A0A6N8EHY5-F1-MODEL\_V4 | 1.0 | 3.057e-17 | 572 | 0.229 | 406 | 243 | 21 | 2 | 367 | 6 | 381 | DUF4102 domain-containing protein | DUF4102 domain-containing protein | | afdb-uniprot50 | AF-A0A559NFV8-F1-MODEL\_V4 | 1.0 | 7.449e-16 | 571 | 0.189 | 422 | 278 | 20 | 1 | 400 | 2 | 381 | Site-specific recombinase XerD | Site-specific recombinase XerD | | afdb-uniprot50 | AF-A0A7V2M8D0-F1-MODEL\_V4 | 1.0 | 2.158e-16 | 571 | 0.216 | 439 | 273 | 22 | 1 | 400 | 5 | 411 | DUF4102 domain-containing protein | DUF4102 domain-containing protein | | afdb-uniprot50 | AF-A0A6F8T1B2-F1-MODEL\_V4 | 1.0 | 3.159e-16 | 570 | 0.19 | 414 | 296 | 18 | 1 | 403 | 25 | 410 | Integrase | Integrase | | afdb-uniprot50 | AF-A0A158IH21-F1-MODEL\_V4 | 1.0 | 3.313e-16 | 570 | 0.193 | 455 | 282 | 23 | 1 | 403 | 6 | 427 | Phage integrase family protein | Phage integrase family protein | | afdb-uniprot50 | AF-A0A257V1X1-F1-MODEL\_V4 | 1.0 | 7.103e-16 | 569 | 0.21 | 423 | 271 | 17 | 2 | 398 | 9 | 394 | Uncharacterized protein | Uncharacterized protein | | afdb-uniprot50 | AF-A0A4Y3HQF9-F1-MODEL\_V4 | 1.0 | 7.813e-16 | 568 | 0.215 | 417 | 279 | 19 | 2 | 403 | 8 | 391 | Integrase | Integrase | | afdb-uniprot50 | AF-A0A4R0NXE3-F1-MODEL\_V4 | 1.0 | 1.161e-16 | 568 | 0.219 | 491 | 264 | 25 | 2 | 402 | 6 | 467 | DUF4102 domain-containing protein | DUF4102 domain-containing protein | | afdb-uniprot50 | AF-A0A1Q7BDQ6-F1-MODEL\_V4 | 1.0 | 6.267e-14 | 567 | 0.227 | 352 | 225 | 11 | 58 | 402 | 2 | 313 | Tyr recombinase domain-containing protein | Tyr recombinase domain-containing protein | | afdb-uniprot50 | AF-D6ZZC9-F1-MODEL\_V4 | 1.0 | 2.158e-16 | 567 | 0.201 | 422 | 281 | 22 | 1 | 401 | 5 | 391 | Integrase family protein | Integrase family protein | | afdb-uniprot50 | AF-A0A440JSK6-F1-MODEL\_V4 | 1.0 | 1.81e-17 | 567 | 0.226 | 432 | 264 | 24 | 2 | 403 | 5 | 396 | DUF4102 domain-containing protein | DUF4102 domain-containing protein | | afdb-uniprot50 | AF-A0A3G8M944-F1-MODEL\_V4 | 1.0 | 4.851e-16 | 567 | 0.204 | 431 | 279 | 22 | 2 | 403 | 4 | 399 | Site-specific integrase | Site-specific integrase | | afdb-uniprot50 | AF-A0A269PHV3-F1-MODEL\_V4 | 1.0 | 5.336e-16 | 567 | 0.204 | 425 | 280 | 20 | 2 | 402 | 8 | 398 | Integrase | Integrase | | afdb-uniprot50 | AF-A0A7V2T2N7-F1-MODEL\_V4 | 1.0 | 1.523e-15 | 567 | 0.193 | 429 | 285 | 20 | 2 | 402 | 3 | 398 | DUF4102 domain-containing protein | DUF4102 domain-containing protein | | afdb-uniprot50 | AF-A0A3S9XF91-F1-MODEL\_V4 | 1.0 | 9.915e-16 | 567 | 0.172 | 422 | 294 | 20 | 2 | 402 | 11 | 398 | DUF4102 domain-containing protein | DUF4102 domain-containing protein | | afdb-uniprot50 | AF-A0A2E2ZXV7-F1-MODEL\_V4 | 1.0 | 4.779e-15 | 566 | 0.193 | 382 | 257 | 15 | 47 | 402 | 1 | 357 | Integrase | Integrase | | afdb-uniprot50 | AF-A0A5R1LZU8-F1-MODEL\_V4 | 1.0 | 1.87e-16 | 566 | 0.198 | 423 | 277 | 20 | 2 | 403 | 4 | 385 | Uncharacterized protein | Uncharacterized protein | | afdb-uniprot50 | AF-A0A1V0KP21-F1-MODEL\_V4 | 1.0 | 5.336e-16 | 566 | 0.192 | 427 | 283 | 21 | 2 | 402 | 8 | 398 | Integrase | Integrase | | afdb-uniprot50 | AF-G4QGW8-F1-MODEL\_V4 | 1.0 | 2.738e-16 | 566 | 0.172 | 424 | 301 | 19 | 2 | 402 | 3 | 399 | DNA integration/recombination/inversion protein | DNA integration/recombination/inversion protein | | afdb-uniprot50 | AF-A0A0Q6E4W7-F1-MODEL\_V4 | 1.0 | 3.475e-16 | 566 | 0.205 | 443 | 289 | 21 | 2 | 402 | 13 | 434 | Core-binding (CB) domain-containing protein | Core-binding (CB) domain-containing protein | | afdb-uniprot50 | AF-A0A2E0QAK3-F1-MODEL\_V4 | 1.0 | 7.103e-16 | 565 | 0.194 | 426 | 283 | 20 | 2 | 402 | 8 | 398 | Integrase | Integrase | | afdb-uniprot50 | AF-A0A1F9LW06-F1-MODEL\_V4 | 1.0 | 1.474e-16 | 565 | 0.204 | 425 | 277 | 21 | 2 | 402 | 20 | 407 | Uncharacterized protein | Uncharacterized protein | | afdb-uniprot50 | AF-A0A7C7GTQ2-F1-MODEL\_V4 | 1.0 | 5.87e-16 | 565 | 0.193 | 424 | 288 | 20 | 2 | 402 | 21 | 413 | DUF4102 domain-containing protein | DUF4102 domain-containing protein | | afdb-uniprot50 | AF-A0A0F9YIN6-F1-MODEL\_V4 | 1.0 | 1.546e-16 | 564 | 0.219 | 424 | 276 | 20 | 1 | 402 | 6 | 396 | Uncharacterized protein | Uncharacterized protein | | afdb-uniprot50 | AF-A0A2D5IDG5-F1-MODEL\_V4 | 1.0 | 1.384e-15 | 564 | 0.168 | 439 | 296 | 17 | 2 | 401 | 1 | 409 | Integrase | Integrase | | afdb-uniprot50 | AF-A0A6L7JZA5-F1-MODEL\_V4 | 1.0 | 5.597e-16 | 563 | 0.214 | 419 | 271 | 21 | 1 | 400 | 3 | 382 | DUF4102 domain-containing protein | DUF4102 domain-containing protein | | afdb-uniprot50 | AF-A0A657B0N1-F1-MODEL\_V4 | 1.0 | 1.258e-15 | 563 | 0.195 | 425 | 287 | 21 | 2 | 402 | 7 | 400 | Uncharacterized protein | Uncharacterized protein | | afdb-uniprot50 | AF-A0A5Y6ETH5-F1-MODEL\_V4 | 1.0 | 2.158e-16 | 563 | 0.217 | 450 | 268 | 26 | 2 | 401 | 10 | 425 | Tyrosine-type recombinase/integrase | Tyrosine-type recombinase/integrase | | afdb-uniprot50 | AF-A0A534HXP5-F1-MODEL\_V4 | 1.0 | 8.194e-16 | 563 | 0.195 | 410 | 275 | 19 | 1 | 391 | 10 | 383 | DUF4102 domain-containing protein | DUF4102 domain-containing protein | | afdb-uniprot50 | AF-A0A7X6JDQ3-F1-MODEL\_V4 | 1.0 | 3.012e-16 | 562 | 0.229 | 418 | 272 | 20 | 10 | 403 | 2 | 393 | Tyrosine-type recombinase/integrase | Tyrosine-type recombinase/integrase | | afdb-uniprot50 | AF-A0A550KFW2-F1-MODEL\_V4 | 1.0 | 7.449e-16 | 562 | 0.205 | 428 | 278 | 24 | 2 | 403 | 8 | 399 | DUF4102 domain-containing protein | DUF4102 domain-containing protein | | afdb-uniprot50 | AF-A0A850S1N2-F1-MODEL\_V4 | 1.0 | 9.454e-16 | 562 | 0.183 | 447 | 291 | 18 | 1 | 402 | 2 | 419 | Tyrosine-type recombinase/integrase | Tyrosine-type recombinase/integrase | | afdb-uniprot50 | AF-A0A242NUI6-F1-MODEL\_V4 | 1.0 | 3.012e-16 | 562 | 0.193 | 445 | 288 | 18 | 1 | 402 | 1 | 417 | Tyr recombinase domain-containing protein | Tyr recombinase domain-containing protein | | afdb-uniprot50 | AF-A0A523H6V4-F1-MODEL\_V4 | 1.0 | 1.405e-16 | 561 | 0.199 | 416 | 274 | 18 | 2 | 394 | 15 | 394 | DUF4102 domain-containing protein | DUF4102 domain-containing protein | | afdb-uniprot50 | AF-A0A3E0DQX6-F1-MODEL\_V4 | 1.0 | 8.594e-16 | 561 | 0.194 | 426 | 283 | 20 | 2 | 402 | 7 | 397 | Integrase | Integrase | | afdb-uniprot50 | AF-A0A2V3US64-F1-MODEL\_V4 | 1.0 | 7.561e-17 | 561 | 0.202 | 445 | 277 | 24 | 1 | 403 | 1 | 409 | Site-specific recombinase XerD | Site-specific recombinase XerD | | afdb-uniprot50 | AF-D2TBY5-F1-MODEL\_V4 | 1.0 | 3.834e-13 | 560 | 0.185 | 323 | 229 | 14 | 96 | 402 | 3 | 307 | Int protein | Int protein | | afdb-uniprot50 | AF-A0A842J5Y3-F1-MODEL\_V4 | 1.0 | 1.597e-15 | 560 | 0.183 | 420 | 289 | 23 | 2 | 400 | 8 | 394 | Tyrosine-type recombinase/integrase | Tyrosine-type recombinase/integrase | | afdb-uniprot50 | AF-Q083S5-F1-MODEL\_V4 | 1.0 | 3.012e-16 | 560 | 0.195 | 419 | 288 | 18 | 2 | 402 | 3 | 390 | Phage integrase family protein | Phage integrase family protein | | afdb-uniprot50 | AF-A0A7W5GR49-F1-MODEL\_V4 | 1.0 | 4.205e-16 | 560 | 0.203 | 422 | 281 | 20 | 14 | 398 | 1 | 404 | Integrase | Integrase | | afdb-uniprot50 | AF-A0A5P9HXM8-F1-MODEL\_V4 | 1.0 | 2.263e-16 | 560 | 0.216 | 411 | 282 | 19 | 2 | 400 | 6 | 388 | Prophage CP4-57 integrase | Prophage CP4-57 integrase | | afdb-uniprot50 | AF-A0A5S3UGE8-F1-MODEL\_V4 | 1.0 | 2.738e-16 | 559 | 0.183 | 419 | 295 | 19 | 2 | 398 | 3 | 396 | Tyr recombinase domain-containing protein | Tyr recombinase domain-containing protein | | afdb-uniprot50 | AF-A0A4R6Y425-F1-MODEL\_V4 | 1.0 | 3.313e-16 | 559 | 0.173 | 455 | 290 | 19 | 1 | 402 | 4 | 425 | Integrase | Integrase | | afdb-uniprot50 | AF-A4F5J8-F1-MODEL\_V4 | 1.0 | 2.263e-16 | 558 | 0.191 | 417 | 275 | 22 | 2 | 389 | 3 | 386 | Uncharacterized protein | Uncharacterized protein | | afdb-uniprot50 | AF-A0A4Y6I9E4-F1-MODEL\_V4 | 1.0 | 2.158e-16 | 558 | 0.17 | 427 | 298 | 20 | 2 | 403 | 3 | 398 | DUF4102 domain-containing protein | DUF4102 domain-containing protein | | afdb-uniprot50 | AF-A0A3S0MUP2-F1-MODEL\_V4 | 1.0 | 2.373e-16 | 558 | 0.192 | 411 | 291 | 18 | 2 | 402 | 7 | 386 | Site-specific integrase | Site-specific integrase | | afdb-uniprot50 | AF-A0A6C9E150-F1-MODEL\_V4 | 1.0 | 1.621e-16 | 558 | 0.193 | 434 | 284 | 21 | 2 | 402 | 3 | 403 | Tyrosine-type recombinase/integrase | Tyrosine-type recombinase/integrase | | afdb-uniprot50 | AF-A0A369TCW8-F1-MODEL\_V4 | 1.0 | 8.594e-16 | 558 | 0.23 | 425 | 259 | 18 | 13 | 402 | 24 | 415 | DUF4102 domain-containing protein | DUF4102 domain-containing protein | | afdb-uniprot50 | AF-A0A1S9PNP5-F1-MODEL\_V4 | 1.0 | 1.32e-15 | 558 | 0.19 | 425 | 285 | 20 | 2 | 402 | 3 | 392 | Tyr recombinase domain-containing protein | Tyr recombinase domain-containing protein | | afdb-uniprot50 | AF-A0A2W7KZ90-F1-MODEL\_V4 | 1.0 | 2.618e-13 | 557 | 0.2 | 315 | 223 | 12 | 99 | 402 | 12 | 308 | Integrase | Integrase | | afdb-uniprot50 | AF-A0A2D7FTU9-F1-MODEL\_V4 | 1.0 | 2.872e-16 | 557 | 0.176 | 425 | 290 | 18 | 1 | 401 | 5 | 393 | Integrase | Integrase | | afdb-uniprot50 | AF-A0A8A8H8X0-F1-MODEL\_V4 | 1.0 | 9.014e-16 | 557 | 0.189 | 422 | 285 | 21 | 1 | 402 | 1 | 385 | Tyrosine-type recombinase/integrase | Tyrosine-type recombinase/integrase | | afdb-uniprot50 | AF-A0A7H8PYI9-F1-MODEL\_V4 | 1.0 | 1.384e-15 | 557 | 0.198 | 433 | 279 | 22 | 2 | 402 | 8 | 404 | Integrase arm-type DNA-binding domain-containing protein | Integrase arm-type DNA-binding domain-containing protein | | afdb-uniprot50 | AF-A0A562ZNZ3-F1-MODEL\_V4 | 1.0 | 5.336e-16 | 557 | 0.241 | 434 | 275 | 22 | 2 | 403 | 17 | 428 | Integrase family protein | Integrase family protein | | afdb-uniprot50 | AF-A0A3R0ZT27-F1-MODEL\_V4 | 1.0 | 2.229e-15 | 556 | 0.188 | 408 | 268 | 19 | 2 | 380 | 26 | 399 | DUF4102 domain-containing protein | DUF4102 domain-containing protein | | afdb-uniprot50 | AF-A0A1M7UU15-F1-MODEL\_V4 | 1.0 | 1.523e-15 | 556 | 0.167 | 431 | 301 | 23 | 2 | 402 | 3 | 405 | Arm-DNA-bind\_3 domain-containing protein | Arm-DNA-bind\_3 domain-containing protein | | afdb-uniprot50 | AF-A0A2K4MK02-F1-MODEL\_V4 | 1.0 | 3.485e-13 | 555 | 0.192 | 328 | 227 | 13 | 93 | 403 | 3 | 309 | Integrase | Integrase | | afdb-uniprot50 | AF-A0A1J5IS85-F1-MODEL\_V4 | 1.0 | 2.452e-15 | 555 | 0.199 | 416 | 272 | 20 | 2 | 391 | 3 | 383 | Integrase | Integrase | | afdb-uniprot50 | AF-A0A4Q5VI67-F1-MODEL\_V4 | 1.0 | 1.091e-15 | 555 | 0.189 | 439 | 287 | 27 | 2 | 402 | 8 | 415 | DUF4102 domain-containing protein | DUF4102 domain-containing protein | | afdb-uniprot50 | AF-A0A0K0WKY4-F1-MODEL\_V4 | 1.0 | 3.645e-16 | 554 | 0.19 | 405 | 270 | 18 | 8 | 402 | 9 | 365 | Tyr recombinase domain-containing protein | Tyr recombinase domain-containing protein | | afdb-uniprot50 | AF-A0A6S6UED4-F1-MODEL\_V4 | 1.0 | 1.243e-11 | 552 | 0.334 | 203 | 121 | 5 | 209 | 403 | 2 | 198 | Phage integrase family protein | Phage integrase family protein | | afdb-uniprot50 | AF-A0A3B7L947-F1-MODEL\_V4 | 1.0 | 1.597e-15 | 552 | 0.199 | 426 | 285 | 18 | 1 | 402 | 3 | 396 | DUF4102 domain-containing protein | DUF4102 domain-containing protein | | afdb-uniprot50 | AF-A0A8B6QQ55-F1-MODEL\_V4 | 1.0 | 2.304e-14 | 551 | 0.21 | 361 | 244 | 15 | 63 | 402 | 2 | 342 | Tyrosine-type recombinase/integrase | Tyrosine-type recombinase/integrase | | afdb-uniprot50 | AF-A0A1Z8RU83-F1-MODEL\_V4 | 1.0 | 1.32e-15 | 551 | 0.194 | 427 | 274 | 19 | 2 | 398 | 4 | 390 | Uncharacterized protein | Uncharacterized protein | | afdb-uniprot50 | AF-A0A2W4RA98-F1-MODEL\_V4 | 1.0 | 2.452e-15 | 551 | 0.183 | 419 | 283 | 22 | 3 | 403 | 6 | 383 | Integrase | Integrase | | afdb-uniprot50 | AF-A0A2N4WW21-F1-MODEL\_V4 | 1.0 | 1.621e-16 | 551 | 0.22 | 450 | 271 | 24 | 1 | 403 | 1 | 417 | Integrase | Integrase | | afdb-uniprot50 | AF-A0A560K4G8-F1-MODEL\_V4 | 1.0 | 2.489e-16 | 550 | 0.215 | 417 | 267 | 20 | 2 | 401 | 6 | 379 | Site-specific recombinase XerD | Site-specific recombinase XerD | | afdb-uniprot50 | AF-A0A2A3LHG3-F1-MODEL\_V4 | 1.0 | 5.088e-16 | 550 | 0.201 | 426 | 278 | 21 | 2 | 392 | 6 | 404 | Uncharacterized protein | Uncharacterized protein | | afdb-uniprot50 | AF-A0A370K4W5-F1-MODEL\_V4 | 1.0 | 2.611e-16 | 550 | 0.224 | 445 | 279 | 23 | 2 | 398 | 3 | 429 | Uncharacterized protein | Uncharacterized protein | | afdb-uniprot50 | AF-A0A562R5J7-F1-MODEL\_V4 | 1.0 | 8.467e-15 | 549 | 0.22 | 376 | 250 | 17 | 48 | 402 | 2 | 355 | Integrase | Integrase | | afdb-uniprot50 | AF-A0A369IHL0-F1-MODEL\_V4 | 1.0 | 5.87e-16 | 549 | 0.178 | 426 | 287 | 22 | 2 | 401 | 3 | 391 | DUF4102 domain-containing protein | DUF4102 domain-containing protein | | afdb-uniprot50 | AF-A0A7D4SJ89-F1-MODEL\_V4 | 1.0 | 1.384e-15 | 549 | 0.189 | 422 | 290 | 21 | 1 | 402 | 1 | 390 | Tyrosine-type recombinase/integrase | Tyrosine-type recombinase/integrase | | afdb-uniprot50 | AF-A0A1T0CMB0-F1-MODEL\_V4 | 1.0 | 1.757e-15 | 549 | 0.166 | 426 | 302 | 19 | 1 | 402 | 1 | 397 | Tyr recombinase domain-containing protein | Tyr recombinase domain-containing protein | | afdb-uniprot50 | AF-A0A4P7BRM8-F1-MODEL\_V4 | 1.0 | 9.454e-16 | 548 | 0.179 | 445 | 294 | 22 | 2 | 403 | 14 | 430 | DUF4102 domain-containing protein | DUF4102 domain-containing protein | | afdb-uniprot50 | AF-A0A258KZS7-F1-MODEL\_V4 | 1.0 | 6.156e-16 | 547 | 0.214 | 411 | 252 | 15 | 46 | 402 | 2 | 395 | Uncharacterized protein | Uncharacterized protein | | afdb-uniprot50 | AF-A0A7M1LFV5-F1-MODEL\_V4 | 1.0 | 2.452e-15 | 546 | 0.185 | 426 | 284 | 21 | 2 | 402 | 3 | 390 | Tyrosine-type recombinase/integrase | Tyrosine-type recombinase/integrase | | afdb-uniprot50 | AF-A0A5C4PWQ3-F1-MODEL\_V4 | 1.0 | 1.757e-15 | 546 | 0.201 | 421 | 278 | 20 | 9 | 401 | 11 | 401 | DUF4102 domain-containing protein | DUF4102 domain-containing protein | | afdb-uniprot50 | AF-A0A840JLT4-F1-MODEL\_V4 | 1.0 | 9.454e-16 | 546 | 0.194 | 427 | 284 | 21 | 2 | 402 | 8 | 400 | Integrase | Integrase | | afdb-uniprot50 | AF-A0A2D9M000-F1-MODEL\_V4 | 1.0 | 3.012e-16 | 546 | 0.175 | 439 | 282 | 21 | 2 | 402 | 9 | 405 | Integrase | Integrase | | afdb-uniprot50 | AF-A0A430DFY4-F1-MODEL\_V4 | 1.0 | 4.205e-16 | 546 | 0.203 | 447 | 275 | 24 | 1 | 400 | 1 | 413 | DUF4102 domain-containing protein | DUF4102 domain-containing protein | | afdb-uniprot50 | AF-A0A2A2EZC5-F1-MODEL\_V4 | 1.0 | 3.423e-15 | 545 | 0.185 | 420 | 282 | 18 | 9 | 402 | 11 | 396 | Integrase | Integrase | | afdb-uniprot50 | AF-A0A0M4L5J0-F1-MODEL\_V4 | 1.0 | 5.012e-15 | 544 | 0.171 | 437 | 285 | 23 | 1 | 402 | 2 | 396 | Tyr recombinase domain-containing protein | Tyr recombinase domain-containing protein | | afdb-uniprot50 | AF-A0A1H8G0Y4-F1-MODEL\_V4 | 1.0 | 1.384e-15 | 544 | 0.196 | 418 | 280 | 22 | 2 | 403 | 8 | 385 | Integrase | Integrase | | afdb-uniprot50 | AF-Q31FR8-F1-MODEL\_V4 | 1.0 | 2.196e-14 | 543 | 0.211 | 425 | 275 | 21 | 2 | 402 | 5 | 393 | Tyrosine recombinase | Tyrosine recombinase | | afdb-uniprot50 | AF-A0A369ZF09-F1-MODEL\_V4 | 1.0 | 1.842e-15 | 543 | 0.174 | 424 | 290 | 16 | 2 | 402 | 8 | 394 | DUF4102 domain-containing protein | DUF4102 domain-containing protein | | afdb-uniprot50 | AF-A0A4P7RZE8-F1-MODEL\_V4 | 1.0 | 7.103e-16 | 543 | 0.179 | 428 | 283 | 21 | 2 | 401 | 7 | 394 | DUF4102 domain-containing protein | DUF4102 domain-containing protein | | afdb-uniprot50 | AF-A0A0C5WK64-F1-MODEL\_V4 | 1.0 | 9.915e-16 | 543 | 0.184 | 450 | 283 | 25 | 2 | 402 | 10 | 424 | Putative prophage integrase | Putative prophage integrase | | afdb-uniprot50 | AF-A0A4Z0BRC7-F1-MODEL\_V4 | 1.0 | 3.645e-16 | 543 | 0.198 | 439 | 289 | 21 | 1 | 398 | 2 | 418 | Integrase | Integrase | | afdb-uniprot50 | AF-R9AZ04-F1-MODEL\_V4 | 1.0 | 1.364e-14 | 542 | 0.201 | 372 | 244 | 18 | 50 | 402 | 3 | 340 | Uncharacterized protein | Uncharacterized protein | | afdb-uniprot50 | AF-F3WK33-F1-MODEL\_V4 | 1.0 | 1.452e-15 | 542 | 0.211 | 383 | 258 | 17 | 1 | 370 | 1 | 352 | Putative prophage CPS-53 integrase | Putative prophage CPS-53 integrase | | afdb-uniprot50 | AF-Q1N825-F1-MODEL\_V4 | 1.0 | 1.24e-14 | 542 | 0.217 | 382 | 244 | 17 | 50 | 402 | 2 | 357 | Symbiosis island integrase | Symbiosis island integrase | | afdb-uniprot50 | AF-G9EN62-F1-MODEL\_V4 | 1.0 | 3.591e-15 | 542 | 0.211 | 411 | 288 | 17 | 1 | 400 | 1 | 386 | Uncharacterized protein | Uncharacterized protein | | afdb-uniprot50 | AF-A0A1A5PTB8-F1-MODEL\_V4 | 1.0 | 1.7e-16 | 542 | 0.18 | 415 | 283 | 21 | 9 | 402 | 9 | 387 | Tyr recombinase domain-containing protein | Tyr recombinase domain-containing protein | | afdb-uniprot50 | AF-A0A5C5GCA0-F1-MODEL\_V4 | 1.0 | 2.338e-15 | 542 | 0.202 | 404 | 278 | 17 | 9 | 398 | 44 | 417 | DUF4102 domain-containing protein | DUF4102 domain-containing protein | | afdb-uniprot50 | AF-D5RQ66-F1-MODEL\_V4 | 1.0 | 4.009e-16 | 542 | 0.202 | 444 | 284 | 22 | 9 | 403 | 7 | 429 | Site-specific recombinase, phage integrase family | Site-specific recombinase, phage integrase family | | afdb-uniprot50 | AF-A0A845U6K0-F1-MODEL\_V4 | 1.0 | 3.159e-16 | 541 | 0.192 | 416 | 290 | 20 | 1 | 403 | 6 | 388 | Tyrosine-type recombinase/integrase | Tyrosine-type recombinase/integrase | | afdb-uniprot50 | AF-A0A503K063-F1-MODEL\_V4 | 1.0 | 1.32e-15 | 541 | 0.158 | 429 | 292 | 22 | 2 | 402 | 6 | 393 | DUF4102 domain-containing protein | DUF4102 domain-containing protein | | afdb-uniprot50 | AF-A0A7H5AGS0-F1-MODEL\_V4 | 1.0 | 2.38e-13 | 540 | 0.193 | 336 | 236 | 16 | 82 | 402 | 7 | 322 | Phage integrase | Phage integrase | | afdb-uniprot50 | AF-A0A381F1W7-F1-MODEL\_V4 | 1.0 | 3.475e-16 | 540 | 0.193 | 398 | 274 | 22 | 1 | 382 | 7 | 373 | Prophage CP4-57 integrase | Prophage CP4-57 integrase | | afdb-uniprot50 | AF-A0A2N2RRY9-F1-MODEL\_V4 | 1.0 | 1.32e-15 | 540 | 0.198 | 403 | 268 | 20 | 34 | 403 | 25 | 405 | Integrase | Integrase | | afdb-uniprot50 | AF-A0A7G5KXN3-F1-MODEL\_V4 | 1.0 | 3.766e-15 | 540 | 0.167 | 431 | 295 | 19 | 2 | 402 | 3 | 399 | Tyrosine-type recombinase/integrase | Tyrosine-type recombinase/integrase | | afdb-uniprot50 | AF-Q8EJS4-F1-MODEL\_V4 | 1.0 | 1.144e-15 | 540 | 0.18 | 437 | 290 | 22 | 2 | 402 | 7 | 411 | Integrase bacteriophage P4 family | Integrase bacteriophage P4 family | | afdb-uniprot50 | AF-A0A239M372-F1-MODEL\_V4 | 1.0 | 2.229e-15 | 540 | 0.2 | 429 | 284 | 23 | 2 | 402 | 49 | 446 | Integrase | Integrase | | afdb-uniprot50 | AF-A0A1E2UTE1-F1-MODEL\_V4 | 1.0 | 3.159e-16 | 539 | 0.201 | 421 | 286 | 19 | 1 | 400 | 1 | 392 | Integrase | Integrase | | afdb-uniprot50 | AF-A0A1I5H7Y3-F1-MODEL\_V4 | 1.0 | 1.04e-15 | 538 | 0.219 | 423 | 269 | 21 | 2 | 403 | 4 | 386 | Integrase | Integrase | | afdb-uniprot50 | AF-A0A7S9REC9-F1-MODEL\_V4 | 1.0 | 3.423e-15 | 538 | 0.194 | 421 | 283 | 21 | 2 | 400 | 8 | 394 | Tyrosine-type recombinase/integrase | Tyrosine-type recombinase/integrase | | afdb-uniprot50 | AF-A0A0D6MPY2-F1-MODEL\_V4 | 1.0 | 2.698e-15 | 538 | 0.224 | 428 | 264 | 21 | 2 | 399 | 9 | 398 | Phage integrase | Phage integrase | | afdb-uniprot50 | AF-A0A6I4Z264-F1-MODEL\_V4 | 1.0 | 2.829e-15 | 538 | 0.169 | 430 | 293 | 18 | 2 | 401 | 18 | 413 | Tyrosine-type recombinase/integrase | Tyrosine-type recombinase/integrase | | afdb-uniprot50 | AF-A0A3A1PIJ8-F1-MODEL\_V4 | 1.0 | 8.88e-15 | 538 | 0.171 | 415 | 293 | 22 | 9 | 401 | 23 | 408 | DUF4102 domain-containing protein | DUF4102 domain-containing protein | | afdb-uniprot50 | AF-A0A2W4X528-F1-MODEL\_V4 | 1.0 | 1.144e-15 | 537 | 0.205 | 400 | 252 | 20 | 2 | 373 | 3 | 364 | Integrase | Integrase | | afdb-uniprot50 | AF-A0A378NE62-F1-MODEL\_V4 | 1.0 | 4.779e-15 | 537 | 0.205 | 419 | 275 | 18 | 2 | 397 | 8 | 391 | Prophage CP4-57 integrase | Prophage CP4-57 integrase | | afdb-uniprot50 | AF-A0A4P5WYK2-F1-MODEL\_V4 | 1.0 | 3.645e-16 | 537 | 0.231 | 414 | 273 | 20 | 2 | 402 | 18 | 399 | Integrase | Integrase | | afdb-uniprot50 | AF-A0A2S5PFB0-F1-MODEL\_V4 | 1.0 | 5.87e-16 | 536 | 0.199 | 417 | 275 | 17 | 9 | 400 | 16 | 398 | Integrase | Integrase | | afdb-uniprot50 | AF-A0A1T0A3C6-F1-MODEL\_V4 | 1.0 | 1.24e-14 | 536 | 0.186 | 423 | 292 | 17 | 2 | 403 | 7 | 398 | Prophage CP4-57 integrase | Prophage CP4-57 integrase | | afdb-uniprot50 | AF-A0A1G8PZJ9-F1-MODEL\_V4 | 1.0 | 2.338e-15 | 536 | 0.195 | 424 | 284 | 17 | 2 | 402 | 8 | 397 | Integrase | Integrase | | afdb-uniprot50 | AF-A0A7Y0E2C5-F1-MODEL\_V4 | 1.0 | 5.336e-16 | 536 | 0.201 | 422 | 276 | 21 | 2 | 398 | 8 | 393 | Tyrosine-type recombinase/integrase | Tyrosine-type recombinase/integrase | | afdb-uniprot50 | AF-A0A431PNN7-F1-MODEL\_V4 | 1.0 | 7.813e-16 | 536 | 0.206 | 431 | 273 | 20 | 1 | 398 | 8 | 402 | Site-specific integrase | Site-specific integrase | | afdb-uniprot50 | AF-A0A349YDR9-F1-MODEL\_V4 | 1.0 | 7.339e-15 | 536 | 0.195 | 441 | 280 | 21 | 2 | 398 | 8 | 417 | Integrase | Integrase | | afdb-uniprot50 | AF-A4A7S2-F1-MODEL\_V4 | 1.0 | 6.997e-15 | 535 | 0.2 | 435 | 282 | 23 | 2 | 403 | 8 | 409 | Integrase | Integrase | | afdb-uniprot50 | AF-A0A0T6W442-F1-MODEL\_V4 | 1.0 | 3.766e-15 | 535 | 0.192 | 394 | 282 | 17 | 29 | 400 | 32 | 411 | Integrase | Integrase | | afdb-uniprot50 | AF-A0A5C7RHY4-F1-MODEL\_V4 | 1.0 | 1.409e-13 | 534 | 0.207 | 361 | 254 | 12 | 56 | 402 | 11 | 353 | DUF4102 domain-containing protein | DUF4102 domain-containing protein | | afdb-uniprot50 | AF-A0A2N4XR17-F1-MODEL\_V4 | 1.0 | 3.475e-16 | 534 | 0.212 | 423 | 270 | 18 | 2 | 398 | 7 | 392 | Integrase | Integrase | | afdb-uniprot50 | AF-A0A241WFB4-F1-MODEL\_V4 | 1.0 | 1.025e-14 | 534 | 0.201 | 422 | 275 | 20 | 9 | 402 | 14 | 401 | Integrase | Integrase | | afdb-uniprot50 | AF-G2LET3-F1-MODEL\_V4 | 1.0 | 4.345e-15 | 533 | 0.202 | 370 | 263 | 15 | 44 | 400 | 9 | 359 | Integrase | Integrase | | afdb-uniprot50 | AF-A0A345QRY7-F1-MODEL\_V4 | 1.0 | 1.258e-15 | 533 | 0.196 | 428 | 279 | 24 | 2 | 403 | 9 | 397 | Integrase | Integrase | | afdb-uniprot50 | AF-A0A841S252-F1-MODEL\_V4 | 1.0 | 8.073e-15 | 533 | 0.183 | 436 | 286 | 23 | 2 | 402 | 13 | 413 | Integrase | Integrase | | afdb-uniprot50 | AF-A0A6M4GUT6-F1-MODEL\_V4 | 1.0 | 1.384e-15 | 532 | 0.181 | 436 | 285 | 23 | 2 | 401 | 4 | 403 | Prophage integrase IntA | Prophage integrase IntA | | afdb-uniprot50 | AF-A0A2N1U9J3-F1-MODEL\_V4 | 1.0 | 2.027e-15 | 532 | 0.194 | 422 | 289 | 19 | 2 | 402 | 3 | 394 | Integrase | Integrase | | afdb-uniprot50 | AF-A0A1E4GTV1-F1-MODEL\_V4 | 1.0 | 2.338e-15 | 532 | 0.207 | 420 | 276 | 19 | 2 | 398 | 9 | 394 | Uncharacterized protein | Uncharacterized protein | | afdb-uniprot50 | AF-A0A430DCH7-F1-MODEL\_V4 | 1.0 | 1.04e-15 | 532 | 0.198 | 448 | 286 | 23 | 2 | 402 | 7 | 428 | Tyr recombinase domain-containing protein | Tyr recombinase domain-containing protein | | afdb-uniprot50 | AF-A0A1B8PU39-F1-MODEL\_V4 | 1.0 | 4.142e-15 | 531 | 0.198 | 424 | 284 | 22 | 2 | 401 | 8 | 399 | Tyr recombinase domain-containing protein | Tyr recombinase domain-containing protein | | afdb-uniprot50 | AF-A0A6I4V2B2-F1-MODEL\_V4 | 1.0 | 3.591e-15 | 531 | 0.184 | 423 | 285 | 19 | 2 | 401 | 8 | 393 | Tyrosine-type recombinase/integrase | Tyrosine-type recombinase/integrase | | afdb-uniprot50 | AF-A0A7S7ALF1-F1-MODEL\_V4 | 1.0 | 8.467e-15 | 531 | 0.152 | 426 | 301 | 20 | 8 | 402 | 3 | 399 | Tyrosine-type recombinase/integrase | Tyrosine-type recombinase/integrase | | afdb-uniprot50 | AF-A0A3S5DFZ5-F1-MODEL\_V4 | 1.0 | 1.731e-14 | 530 | 0.203 | 374 | 254 | 15 | 50 | 403 | 2 | 351 | Tyrosine recombinase xerD | Tyrosine recombinase xerD | | afdb-uniprot50 | AF-A0A3B0XZC6-F1-MODEL\_V4 | 1.0 | 3.264e-15 | 530 | 0.188 | 424 | 282 | 20 | 2 | 402 | 4 | 388 | Tyr recombinase domain-containing protein | Tyr recombinase domain-containing protein | | afdb-uniprot50 | AF-A0A7V8XVF4-F1-MODEL\_V4 | 1.0 | 6.691e-12 | 529 | 0.286 | 251 | 159 | 9 | 156 | 402 | 3 | 237 | Site-specific integrase | Site-specific integrase | | afdb-uniprot50 | AF-A0A833P2C7-F1-MODEL\_V4 | 1.0 | 1.523e-15 | 529 | 0.196 | 433 | 283 | 23 | 1 | 403 | 13 | 410 | Integrase family | Integrase family | | afdb-uniprot50 | AF-A0A2A6L6G2-F1-MODEL\_V4 | 1.0 | 1.65e-14 | 529 | 0.206 | 441 | 286 | 23 | 2 | 402 | 9 | 425 | Integrase | Integrase | | afdb-uniprot50 | AF-A0A6M7UIT5-F1-MODEL\_V4 | 1.0 | 4.009e-16 | 529 | 0.185 | 415 | 281 | 22 | 9 | 402 | 64 | 442 | DUF4102 domain-containing protein | DUF4102 domain-containing protein | | afdb-uniprot50 | AF-A0A831RFM1-F1-MODEL\_V4 | 1.0 | 5.257e-15 | 528 | 0.249 | 401 | 239 | 24 | 2 | 367 | 3 | 376 | DUF4102 domain-containing protein | DUF4102 domain-containing protein | | afdb-uniprot50 | AF-A0A7H9F2Z4-F1-MODEL\_V4 | 1.0 | 5.336e-16 | 528 | 0.225 | 417 | 269 | 19 | 2 | 402 | 3 | 381 | Tyrosine-type recombinase/integrase | Tyrosine-type recombinase/integrase | | afdb-uniprot50 | AF-A0A7W4FUN8-F1-MODEL\_V4 | 1.0 | 6.361e-15 | 528 | 0.169 | 437 | 292 | 18 | 2 | 402 | 3 | 404 | Site-specific integrase | Site-specific integrase | | afdb-uniprot50 | AF-A0A2A4Y533-F1-MODEL\_V4 | 1.0 | 2.698e-15 | 528 | 0.153 | 469 | 298 | 24 | 1 | 402 | 10 | 446 | Tyr recombinase domain-containing protein | Tyr recombinase domain-containing protein | | afdb-uniprot50 | AF-A0A528IPC3-F1-MODEL\_V4 | 1.0 | 3.891e-14 | 527 | 0.29 | 227 | 150 | 6 | 1 | 224 | 3 | 221 | Site-specific integrase | Site-specific integrase | | afdb-uniprot50 | AF-A0A258Q6T4-F1-MODEL\_V4 | 1.0 | 2.829e-15 | 527 | 0.224 | 375 | 238 | 19 | 1 | 354 | 1 | 343 | Integrase | Integrase | | afdb-uniprot50 | AF-A0A7S6S5Y9-F1-MODEL\_V4 | 1.0 | 1.075e-14 | 527 | 0.187 | 415 | 275 | 20 | 9 | 400 | 11 | 386 | Integrase arm-type DNA-binding domain-containing protein | Integrase arm-type DNA-binding domain-containing protein | | afdb-uniprot50 | AF-V8FUN5-F1-MODEL\_V4 | 1.0 | 1.091e-15 | 527 | 0.191 | 422 | 272 | 19 | 2 | 402 | 3 | 376 | Tyr recombinase domain-containing protein | Tyr recombinase domain-containing protein | | afdb-uniprot50 | AF-A0A3E0Q8B8-F1-MODEL\_V4 | 1.0 | 2.738e-16 | 527 | 0.231 | 406 | 265 | 18 | 9 | 401 | 8 | 379 | DUF4102 domain-containing protein | DUF4102 domain-containing protein | | afdb-uniprot50 | AF-A0A661F4U9-F1-MODEL\_V4 | 1.0 | 2.158e-16 | 526 | 0.211 | 411 | 273 | 17 | 1 | 392 | 10 | 388 | Tyr recombinase domain-containing protein | Tyr recombinase domain-containing protein | | afdb-uniprot50 | AF-A0A4P7WFD3-F1-MODEL\_V4 | 1.0 | 8.467e-15 | 526 | 0.189 | 422 | 296 | 18 | 2 | 403 | 9 | 404 | DUF4102 domain-containing protein | DUF4102 domain-containing protein | | afdb-uniprot50 | AF-A0A7X4K6Q4-F1-MODEL\_V4 | 1.0 | 6.457e-16 | 526 | 0.224 | 424 | 256 | 23 | 13 | 400 | 5 | 391 | Tyrosine-type recombinase/integrase | Tyrosine-type recombinase/integrase | | afdb-uniprot50 | AF-A0A800GIQ3-F1-MODEL\_V4 | 1.0 | 3.112e-15 | 526 | 0.153 | 416 | 308 | 21 | 2 | 398 | 1 | 391 | Site-specific integrase | Site-specific integrase | | afdb-uniprot50 | AF-A0A1D8UTH6-F1-MODEL\_V4 | 1.0 | 4.779e-15 | 525 | 0.187 | 426 | 270 | 20 | 15 | 400 | 1 | 390 | Tyr recombinase domain-containing protein | Tyr recombinase domain-containing protein | | afdb-uniprot50 | AF-A0A158AZQ9-F1-MODEL\_V4 | 1.0 | 2.452e-15 | 525 | 0.189 | 432 | 276 | 23 | 9 | 398 | 11 | 410 | Phage integrase | Phage integrase | | afdb-uniprot50 | AF-A0A1X1PI96-F1-MODEL\_V4 | 1.0 | 7.813e-16 | 525 | 0.2 | 443 | 281 | 19 | 2 | 398 | 3 | 418 | Prophage integrase IntS | Prophage integrase IntS | | afdb-uniprot50 | AF-A0A2W4K9L1-F1-MODEL\_V4 | 1.0 | 8.073e-15 | 525 | 0.187 | 427 | 293 | 20 | 2 | 402 | 14 | 412 | Integrase | Integrase | | afdb-uniprot50 | AF-A0A7W7E8P4-F1-MODEL\_V4 | 1.0 | 7.471e-13 | 524 | 0.186 | 349 | 249 | 15 | 69 | 402 | 3 | 331 | Integrase | Integrase | | afdb-uniprot50 | AF-A0A6B8KHW6-F1-MODEL\_V4 | 1.0 | 1.24e-14 | 524 | 0.204 | 410 | 276 | 22 | 2 | 403 | 8 | 375 | Tyrosine-type recombinase/integrase | Tyrosine-type recombinase/integrase | | afdb-uniprot50 | AF-A0A241VD59-F1-MODEL\_V4 | 1.0 | 1.675e-15 | 524 | 0.167 | 425 | 303 | 19 | 1 | 402 | 9 | 405 | Tyr recombinase domain-containing protein | Tyr recombinase domain-containing protein | | afdb-uniprot50 | AF-A0A1W9HT15-F1-MODEL\_V4 | 1.0 | 4.779e-15 | 524 | 0.164 | 419 | 291 | 20 | 2 | 398 | 13 | 394 | Core-binding (CB) domain-containing protein | Core-binding (CB) domain-containing protein | | afdb-uniprot50 | AF-A0A7Y2KR74-F1-MODEL\_V4 | 1.0 | 1.091e-15 | 524 | 0.206 | 427 | 280 | 19 | 2 | 398 | 8 | 405 | Tyrosine-type recombinase/integrase | Tyrosine-type recombinase/integrase | | afdb-uniprot50 | AF-A0A5S4W293-F1-MODEL\_V4 | 1.0 | 9.314e-15 | 524 | 0.198 | 428 | 285 | 23 | 2 | 402 | 19 | 415 | DUF4102 domain-containing protein | DUF4102 domain-containing protein | | afdb-uniprot50 | AF-A0A141RCN1-F1-MODEL\_V4 | 1.0 | 1.324e-12 | 523 | 0.178 | 313 | 225 | 10 | 102 | 402 | 6 | 298 | Phage integrase family | Phage integrase family | | afdb-uniprot50 | AF-A0A7Y9SXB0-F1-MODEL\_V4 | 1.0 | 9.176e-14 | 523 | 0.216 | 374 | 244 | 16 | 53 | 402 | 1 | 349 | Integrase | Integrase | | afdb-uniprot50 | AF-Q3SUE5-F1-MODEL\_V4 | 1.0 | 1.144e-15 | 523 | 0.211 | 420 | 270 | 23 | 2 | 400 | 5 | 384 | Phage integrase | Phage integrase | | afdb-uniprot50 | AF-B1YRM4-F1-MODEL\_V4 | 1.0 | 6.792e-13 | 522 | 0.237 | 278 | 186 | 9 | 136 | 402 | 6 | 268 | Integrase family protein | Integrase family protein | | afdb-uniprot50 | AF-A0A7Z1LPX5-F1-MODEL\_V4 | 1.0 | 4.779e-15 | 522 | 0.205 | 424 | 273 | 21 | 2 | 403 | 8 | 389 | Integrase | Integrase | | afdb-uniprot50 | AF-A0A2M8QRZ4-F1-MODEL\_V4 | 1.0 | 6.997e-15 | 522 | 0.206 | 430 | 275 | 24 | 2 | 403 | 4 | 395 | Tyr recombinase domain-containing protein | Tyr recombinase domain-containing protein | | afdb-uniprot50 | AF-A0A196QT34-F1-MODEL\_V4 | 1.0 | 6.457e-16 | 522 | 0.191 | 448 | 282 | 22 | 2 | 400 | 8 | 424 | Tyr recombinase domain-containing protein | Tyr recombinase domain-containing protein | | afdb-uniprot50 | AF-A0A836Q3M7-F1-MODEL\_V4 | 1.0 | 9.769e-15 | 522 | 0.182 | 423 | 292 | 20 | 2 | 402 | 94 | 484 | DUF4102 domain-containing protein | DUF4102 domain-containing protein | | afdb-uniprot50 | AF-B1FYI2-F1-MODEL\_V4 | 1.0 | 6.38e-12 | 521 | 0.21 | 314 | 222 | 15 | 99 | 403 | 4 | 300 | Integrase family protein | Integrase family protein | | afdb-uniprot50 | AF-A0A424MZS9-F1-MODEL\_V4 | 1.0 | 4.142e-15 | 521 | 0.186 | 428 | 290 | 23 | 1 | 403 | 1 | 395 | DUF4102 domain-containing protein | DUF4102 domain-containing protein | | afdb-uniprot50 | AF-A0A3Z6QG17-F1-MODEL\_V4 | 1.0 | 3.423e-15 | 521 | 0.165 | 434 | 297 | 23 | 2 | 402 | 3 | 404 | DUF4102 domain-containing protein | DUF4102 domain-containing protein | | afdb-uniprot50 | AF-A0A1G3CT50-F1-MODEL\_V4 | 1.0 | 2.126e-15 | 521 | 0.187 | 426 | 290 | 18 | 2 | 403 | 8 | 401 | Uncharacterized protein | Uncharacterized protein | | afdb-uniprot50 | AF-A0A258D734-F1-MODEL\_V4 | 1.0 | 1.997e-14 | 520 | 0.188 | 440 | 279 | 20 | 2 | 398 | 8 | 412 | Integrase | Integrase | | afdb-uniprot50 | AF-A0A7X5Y2R4-F1-MODEL\_V4 | 1.0 | 3.274e-12 | 519 | 0.196 | 301 | 198 | 15 | 116 | 402 | 6 | 276 | Integrase | Integrase | | afdb-uniprot50 | AF-A0A2T3P7B2-F1-MODEL\_V4 | 1.0 | 1.842e-15 | 519 | 0.194 | 411 | 282 | 18 | 2 | 398 | 32 | 407 | Tyr recombinase domain-containing protein | Tyr recombinase domain-containing protein | | afdb-uniprot50 | AF-A0A378IJ47-F1-MODEL\_V4 | 1.0 | 2.345e-12 | 518 | 0.223 | 269 | 183 | 9 | 146 | 402 | 3 | 257 | Integrase | Integrase | | afdb-uniprot50 | AF-K2EIJ5-F1-MODEL\_V4 | 1.0 | 1.091e-15 | 518 | 0.197 | 416 | 269 | 25 | 2 | 401 | 4 | 370 | Tyr recombinase domain-containing protein | Tyr recombinase domain-containing protein | | afdb-uniprot50 | AF-A0A522G4K5-F1-MODEL\_V4 | 1.0 | 1.573e-14 | 518 | 0.215 | 418 | 279 | 21 | 2 | 403 | 5 | 389 | Site-specific integrase | Site-specific integrase | | afdb-uniprot50 | AF-A0A1N7FGQ2-F1-MODEL\_V4 | 1.0 | 1.258e-15 | 518 | 0.195 | 419 | 285 | 19 | 1 | 402 | 5 | 388 | Phage integrase family protein | Phage integrase family protein | | afdb-uniprot50 | AF-A0A6P1RCR7-F1-MODEL\_V4 | 1.0 | 1.24e-14 | 517 | 0.192 | 421 | 281 | 20 | 9 | 403 | 12 | 399 | DUF4102 domain-containing protein | DUF4102 domain-containing protein | | afdb-uniprot50 | AF-A0A7Z8ZRH8-F1-MODEL\_V4 | 1.0 | 8.194e-16 | 517 | 0.183 | 435 | 271 | 22 | 2 | 402 | 3 | 387 | Phage integrase family site specific recombinase | Phage integrase family site specific recombinase | | afdb-uniprot50 | AF-A0A1Y1SIP4-F1-MODEL\_V4 | 1.0 | 3.264e-15 | 516 | 0.196 | 428 | 273 | 21 | 2 | 398 | 4 | 391 | Integrase family protein | Integrase family protein | | afdb-uniprot50 | AF-A0A7Z0APY4-F1-MODEL\_V4 | 1.0 | 1.523e-15 | 516 | 0.194 | 426 | 282 | 19 | 9 | 403 | 3 | 398 | Integrase | Integrase | | afdb-uniprot50 | AF-A0A6L4AZ65-F1-MODEL\_V4 | 1.0 | 3.264e-15 | 515 | 0.197 | 405 | 276 | 18 | 2 | 392 | 9 | 378 | Tyrosine-type recombinase/integrase | Tyrosine-type recombinase/integrase | | afdb-uniprot50 | AF-A0A271K6L1-F1-MODEL\_V4 | 1.0 | 8.467e-15 | 514 | 0.204 | 421 | 274 | 23 | 2 | 403 | 8 | 386 | Uncharacterized protein | Uncharacterized protein | | afdb-uniprot50 | AF-A0A6N7GUR2-F1-MODEL\_V4 | 1.0 | 2.967e-15 | 514 | 0.182 | 432 | 287 | 18 | 2 | 403 | 8 | 403 | DUF4102 domain-containing protein | DUF4102 domain-containing protein | | afdb-uniprot50 | AF-A0A1G8B2A4-F1-MODEL\_V4 | 1.0 | 2.967e-15 | 513 | 0.202 | 414 | 267 | 23 | 2 | 401 | 4 | 368 | Site-specific recombinase XerD | Site-specific recombinase XerD | | afdb-uniprot50 | AF-A0A7W9S1I0-F1-MODEL\_V4 | 1.0 | 2.787e-14 | 513 | 0.179 | 418 | 286 | 19 | 2 | 398 | 5 | 386 | Integrase | Integrase | | afdb-uniprot50 | AF-A0A0G9KC29-F1-MODEL\_V4 | 1.0 | 1.009e-13 | 513 | 0.165 | 436 | 300 | 18 | 1 | 403 | 5 | 409 | Integrase | Integrase | | afdb-uniprot50 | AF-A0A7L8RUW8-F1-MODEL\_V4 | 1.0 | 3.95e-15 | 513 | 0.193 | 435 | 286 | 22 | 1 | 402 | 5 | 407 | Tyrosine-type recombinase/integrase | Tyrosine-type recombinase/integrase | | afdb-uniprot50 | AF-A0A2P8FFM7-F1-MODEL\_V4 | 1.0 | 1.147e-12 | 512 | 0.201 | 317 | 215 | 13 | 102 | 402 | 7 | 301 | Integrase | Integrase | | afdb-uniprot50 | AF-A0A6N9BY28-F1-MODEL\_V4 | 1.0 | 1.815e-14 | 512 | 0.215 | 390 | 256 | 20 | 38 | 403 | 2 | 365 | Tyrosine-type recombinase/integrase | Tyrosine-type recombinase/integrase | | afdb-uniprot50 | AF-A0A178L9N4-F1-MODEL\_V4 | 1.0 | 5.87e-16 | 512 | 0.204 | 415 | 276 | 18 | 8 | 400 | 2 | 384 | Uncharacterized protein | Uncharacterized protein | | afdb-uniprot50 | AF-A0A4V1V182-F1-MODEL\_V4 | 1.0 | 1.573e-14 | 512 | 0.193 | 423 | 281 | 24 | 1 | 401 | 5 | 389 | Site-specific integrase | Site-specific integrase | | afdb-uniprot50 | AF-A0A0Q7VZE2-F1-MODEL\_V4 | 1.0 | 3.834e-13 | 511 | 0.213 | 333 | 216 | 15 | 92 | 402 | 7 | 315 | Uncharacterized protein | Uncharacterized protein | | afdb-uniprot50 | AF-A0A7Y9WLG8-F1-MODEL\_V4 | 1.0 | 1.815e-14 | 511 | 0.179 | 413 | 274 | 23 | 2 | 400 | 7 | 368 | Integrase | Integrase | | afdb-uniprot50 | AF-C6XJP3-F1-MODEL\_V4 | 1.0 | 8.467e-15 | 511 | 0.179 | 423 | 283 | 22 | 2 | 398 | 9 | 393 | Integrase family protein | Integrase family protein | | afdb-uniprot50 | AF-A0A7X8TU71-F1-MODEL\_V4 | 1.0 | 1.5e-14 | 511 | 0.174 | 425 | 292 | 21 | 2 | 403 | 3 | 391 | Site-specific integrase | Site-specific integrase | | afdb-uniprot50 | AF-A0A6H3SY94-F1-MODEL\_V4 | 1.0 | 4.489e-14 | 511 | 0.18 | 415 | 281 | 20 | 14 | 402 | 22 | 403 | Phage integrase | Phage integrase | | afdb-uniprot50 | AF-A0A7G8Q4D4-F1-MODEL\_V4 | 1.0 | 4.557e-15 | 511 | 0.206 | 459 | 260 | 25 | 8 | 402 | 8 | 426 | Site-specific integrase | Site-specific integrase | | afdb-uniprot50 | AF-A0A2A4AWM1-F1-MODEL\_V4 | 1.0 | 6.997e-15 | 510 | 0.162 | 430 | 292 | 20 | 2 | 402 | 11 | 401 | Integrase | Integrase | | afdb-uniprot50 | AF-A0A3B9UHJ3-F1-MODEL\_V4 | 1.0 | 3.121e-12 | 509 | 0.189 | 332 | 224 | 14 | 90 | 402 | 1 | 306 | Integrase | Integrase | | afdb-uniprot50 | AF-A0A077QQF8-F1-MODEL\_V4 | 1.0 | 3.121e-12 | 509 | 0.192 | 327 | 235 | 10 | 86 | 402 | 9 | 316 | Integrase | Integrase | | afdb-uniprot50 | AF-A0A4Q7BLR8-F1-MODEL\_V4 | 1.0 | 2.658e-14 | 509 | 0.182 | 416 | 289 | 20 | 2 | 401 | 12 | 392 | Site-specific integrase | Site-specific integrase | | afdb-uniprot50 | AF-A0A142W0M6-F1-MODEL\_V4 | 1.0 | 5.783e-15 | 509 | 0.179 | 435 | 280 | 23 | 8 | 400 | 7 | 406 | Uncharacterized protein | Uncharacterized protein | | afdb-uniprot50 | AF-A0A443L961-F1-MODEL\_V4 | 1.0 | 2.094e-14 | 509 | 0.188 | 419 | 283 | 20 | 1 | 400 | 3 | 383 | DUF4102 domain-containing protein | DUF4102 domain-containing protein | | afdb-uniprot50 | AF-A0A6L6YGQ9-F1-MODEL\_V4 | 1.0 | 3.066e-14 | 507 | 0.176 | 420 | 287 | 21 | 1 | 398 | 1 | 383 | Tyrosine-type recombinase/integrase | Tyrosine-type recombinase/integrase | | afdb-uniprot50 | AF-A0A2I8Q3E1-F1-MODEL\_V4 | 1.0 | 2.658e-14 | 507 | 0.209 | 430 | 281 | 21 | 1 | 403 | 3 | 400 | Integrase | Integrase | | afdb-uniprot50 | AF-A0A1W9JA93-F1-MODEL\_V4 | 1.0 | 1.3e-14 | 507 | 0.181 | 425 | 292 | 18 | 16 | 402 | 8 | 414 | Tyr recombinase domain-containing protein | Tyr recombinase domain-containing protein | | afdb-uniprot50 | AF-A0A6A4RG79-F1-MODEL\_V4 | 1.0 | 7.339e-15 | 506 | 0.198 | 439 | 273 | 23 | 2 | 402 | 8 | 405 | DUF4102 domain-containing protein | DUF4102 domain-containing protein | | afdb-uniprot50 | AF-A0A1H0NXL1-F1-MODEL\_V4 | 1.0 | 1.573e-14 | 506 | 0.226 | 419 | 245 | 18 | 36 | 398 | 2 | 397 | Uncharacterized protein | Uncharacterized protein | | afdb-uniprot50 | AF-A0A560FA05-F1-MODEL\_V4 | 1.0 | 5.783e-15 | 506 | 0.221 | 393 | 250 | 21 | 2 | 371 | 3 | 362 | Integrase | Integrase | | afdb-uniprot50 | AF-A0A1A7QBR9-F1-MODEL\_V4 | 1.0 | 1.904e-14 | 505 | 0.172 | 417 | 294 | 21 | 8 | 402 | 6 | 393 | Tyr recombinase domain-containing protein | Tyr recombinase domain-containing protein | | afdb-uniprot50 | AF-A0A4R0BFY7-F1-MODEL\_V4 | 1.0 | 4.938e-14 | 505 | 0.178 | 437 | 304 | 21 | 1 | 402 | 6 | 422 | DUF4102 domain-containing protein | DUF4102 domain-containing protein | | afdb-uniprot50 | AF-A0A7H9JPV7-F1-MODEL\_V4 | 1.0 | 1.075e-14 | 505 | 0.179 | 434 | 291 | 22 | 2 | 402 | 3 | 404 | Integrase arm-type DNA-binding domain-containing protein | Integrase arm-type DNA-binding domain-containing protein | | afdb-uniprot50 | AF-A0A4Q3ZIF4-F1-MODEL\_V4 | 1.0 | 6.38e-12 | 504 | 0.198 | 312 | 221 | 13 | 102 | 402 | 18 | 311 | Integrase | Integrase | | afdb-uniprot50 | AF-A0A6N3R5Y9-F1-MODEL\_V4 | 1.0 | 9.481e-13 | 504 | 0.207 | 361 | 248 | 14 | 60 | 402 | 2 | 342 | Prophage CP4-57 integrase | Prophage CP4-57 integrase | | afdb-uniprot50 | AF-A0A2X3G9U9-F1-MODEL\_V4 | 1.0 | 5.975e-14 | 502 | 0.198 | 377 | 247 | 21 | 2 | 358 | 3 | 344 | Putative phage integrase | Putative phage integrase | | afdb-uniprot50 | AF-A0A3S1ZPH6-F1-MODEL\_V4 | 1.0 | 4.28e-14 | 502 | 0.195 | 430 | 260 | 24 | 2 | 403 | 8 | 379 | Site-specific integrase | Site-specific integrase | | afdb-uniprot50 | AF-A0A3N0U659-F1-MODEL\_V4 | 1.0 | 9.944e-13 | 501 | 0.192 | 358 | 252 | 12 | 62 | 402 | 2 | 339 | DUF4102 domain-containing protein | DUF4102 domain-containing protein | | afdb-uniprot50 | AF-M9RKD3-F1-MODEL\_V4 | 1.0 | 7.583e-14 | 501 | 0.19 | 425 | 284 | 23 | 1 | 398 | 4 | 395 | p4 family integrase | p4 family integrase | | afdb-uniprot50 | AF-A0A0E2PF44-F1-MODEL\_V4 | 1.0 | 9.454e-16 | 501 | 0.245 | 412 | 249 | 22 | 2 | 393 | 22 | 391 | Uncharacterized protein | Uncharacterized protein | | afdb-uniprot50 | AF-A0A2T5I0T1-F1-MODEL\_V4 | 1.0 | 1.573e-14 | 501 | 0.184 | 422 | 288 | 17 | 2 | 402 | 8 | 394 | Integrase | Integrase | | afdb-uniprot50 | AF-A0A7W2BK59-F1-MODEL\_V4 | 1.0 | 1.675e-15 | 500 | 0.213 | 421 | 266 | 23 | 2 | 402 | 5 | 380 | Tyrosine-type recombinase/integrase | Tyrosine-type recombinase/integrase | | afdb-uniprot50 | AF-A0A4D7QHQ2-F1-MODEL\_V4 | 1.0 | 1.997e-14 | 500 | 0.182 | 432 | 283 | 21 | 2 | 403 | 8 | 399 | DUF4102 domain-containing protein | DUF4102 domain-containing protein | | afdb-uniprot50 | AF-A0A0Q4C992-F1-MODEL\_V4 | 1.0 | 1.24e-14 | 500 | 0.171 | 432 | 287 | 23 | 9 | 403 | 7 | 404 | Tyr recombinase domain-containing protein | Tyr recombinase domain-containing protein | | afdb-uniprot50 | AF-A0A2A2F2K7-F1-MODEL\_V4 | 1.0 | 1.009e-13 | 500 | 0.156 | 422 | 303 | 16 | 2 | 402 | 8 | 397 | Integrase | Integrase | | afdb-uniprot50 | AF-A0A4Q3Y2Z4-F1-MODEL\_V4 | 1.0 | 1.075e-14 | 500 | 0.193 | 435 | 278 | 23 | 2 | 400 | 68 | 465 | Site-specific integrase | Site-specific integrase | | afdb-uniprot50 | AF-A0A5C9APT4-F1-MODEL\_V4 | 1.0 | 7.719e-12 | 499 | 0.187 | 315 | 222 | 11 | 102 | 403 | 5 | 298 | Prophage integrase IntS | Prophage integrase IntS | | afdb-uniprot50 | AF-A0A517SMG1-F1-MODEL\_V4 | 1.0 | 1.452e-15 | 499 | 0.201 | 411 | 284 | 15 | 2 | 401 | 9 | 386 | Prophage CPS-53 integrase | Prophage CPS-53 integrase | | afdb-uniprot50 | AF-A0A380T874-F1-MODEL\_V4 | 1.0 | 1.364e-14 | 499 | 0.177 | 450 | 284 | 22 | 2 | 403 | 7 | 418 | Integrase | Integrase | | afdb-uniprot50 | AF-A0A0S3PXE2-F1-MODEL\_V4 | 1.0 | 3.216e-14 | 498 | 0.215 | 380 | 264 | 18 | 36 | 403 | 3 | 360 | Putative prophage CPS-53 integrase | Putative prophage CPS-53 integrase | | afdb-uniprot50 | AF-A0A1I7D5K5-F1-MODEL\_V4 | 1.0 | 2.618e-13 | 498 | 0.201 | 372 | 247 | 17 | 53 | 400 | 7 | 352 | Site-specific recombinase XerD | Site-specific recombinase XerD | | afdb-uniprot50 | AF-A0A2G6I7H5-F1-MODEL\_V4 | 1.0 | 3.264e-15 | 498 | 0.19 | 430 | 258 | 23 | 2 | 389 | 7 | 388 | Integrase | Integrase | | afdb-uniprot50 | AF-A0A7C4E6T6-F1-MODEL\_V4 | 1.0 | 1.43e-14 | 498 | 0.183 | 441 | 285 | 21 | 9 | 402 | 14 | 426 | Site-specific integrase | Site-specific integrase | | afdb-uniprot50 | AF-A0A0Q8QF83-F1-MODEL\_V4 | 1.0 | 7.471e-13 | 497 | 0.19 | 331 | 237 | 14 | 81 | 400 | 8 | 318 | Uncharacterized protein | Uncharacterized protein | | afdb-uniprot50 | AF-A0A1X6ZV10-F1-MODEL\_V4 | 1.0 | 2.967e-15 | 497 | 0.2 | 440 | 271 | 24 | 1 | 396 | 29 | 431 | Putative prophage CPS-53 integrase | Putative prophage CPS-53 integrase | | afdb-uniprot50 | AF-A0A826ZPT6-F1-MODEL\_V4 | 1.0 | 1.655e-11 | 496 | 0.238 | 264 | 177 | 8 | 145 | 399 | 5 | 253 | Site-specific integrase | Site-specific integrase | | afdb-uniprot50 | AF-A0A6G7ZKC8-F1-MODEL\_V4 | 1.0 | 4.081e-14 | 496 | 0.185 | 420 | 291 | 20 | 2 | 403 | 5 | 391 | Tyrosine-type recombinase/integrase | Tyrosine-type recombinase/integrase | | afdb-uniprot50 | AF-P39347-F1-MODEL\_V4 | 1.0 | 1.815e-14 | 496 | 0.192 | 400 | 266 | 18 | 36 | 402 | 3 | 378 | Putative protein IntB | Putative protein IntB | | afdb-uniprot50 | AF-A0A090MGL5-F1-MODEL\_V4 | 1.0 | 1.182e-14 | 496 | 0.177 | 440 | 282 | 24 | 2 | 402 | 6 | 404 | Uncultured bacterium genome assembly Metasoil\_fosmids\_resub | Uncultured bacterium genome assembly Metasoil\_fosmids\_resub | | afdb-uniprot50 | AF-A0A2D8M202-F1-MODEL\_V4 | 1.0 | 1.075e-14 | 496 | 0.181 | 424 | 286 | 21 | 2 | 401 | 7 | 393 | Uncharacterized protein | Uncharacterized protein | | afdb-uniprot50 | AF-V4NAD2-F1-MODEL\_V4 | 1.0 | 4.217e-13 | 495 | 0.182 | 367 | 251 | 17 | 60 | 402 | 4 | 345 | Integrase | Integrase | | afdb-uniprot50 | AF-A0A7H1NS61-F1-MODEL\_V4 | 1.0 | 3.71e-14 | 495 | 0.172 | 423 | 286 | 24 | 9 | 400 | 12 | 401 | Prophage integrase IntA | Prophage integrase IntA | | afdb-uniprot50 | AF-A0A7Z2J7L8-F1-MODEL\_V4 | 1.0 | 1.43e-14 | 495 | 0.176 | 431 | 281 | 20 | 2 | 402 | 7 | 393 | Tyrosine-type recombinase/integrase | Tyrosine-type recombinase/integrase | | afdb-uniprot50 | AF-A0A836V8A1-F1-MODEL\_V4 | 1.0 | 4.142e-15 | 494 | 0.181 | 418 | 275 | 19 | 2 | 402 | 7 | 374 | Site-specific integrase | Site-specific integrase | | afdb-uniprot50 | AF-A0A1N7RJQ7-F1-MODEL\_V4 | 1.0 | 2.416e-14 | 494 | 0.175 | 421 | 292 | 21 | 2 | 403 | 45 | 429 | Integrase family protein | Integrase family protein | | afdb-uniprot50 | AF-A0A354Q9H2-F1-MODEL\_V4 | 1.0 | 1.078e-11 | 492 | 0.166 | 325 | 237 | 12 | 92 | 402 | 3 | 307 | Integrase | Integrase | | afdb-uniprot50 | AF-A0A238H9E1-F1-MODEL\_V4 | 1.0 | 4.57e-12 | 492 | 0.196 | 330 | 233 | 14 | 85 | 402 | 8 | 317 | Phage-related integrase | Phage-related integrase | | afdb-uniprot50 | AF-A0A562Y0T8-F1-MODEL\_V4 | 1.0 | 1.788e-13 | 492 | 0.154 | 420 | 311 | 20 | 1 | 402 | 3 | 396 | Tyrosine-type recombinase/integrase | Tyrosine-type recombinase/integrase | | afdb-uniprot50 | AF-E2CJQ7-F1-MODEL\_V4 | 1.0 | 6.361e-15 | 491 | 0.196 | 418 | 274 | 26 | 7 | 403 | 2 | 378 | Integrase family protein | Integrase family protein | | afdb-uniprot50 | AF-R8Y101-F1-MODEL\_V4 | 1.0 | 1.343e-13 | 491 | 0.184 | 417 | 275 | 22 | 15 | 402 | 24 | 404 | Tyr recombinase domain-containing protein | Tyr recombinase domain-containing protein | | afdb-uniprot50 | AF-A0A2W7C4Z9-F1-MODEL\_V4 | 1.0 | 2.829e-15 | 491 | 0.202 | 440 | 270 | 23 | 2 | 398 | 27 | 428 | Tyr recombinase domain-containing protein | Tyr recombinase domain-containing protein | | afdb-uniprot50 | AF-A0A372BUY1-F1-MODEL\_V4 | 1.0 | 1.967e-13 | 490 | 0.218 | 371 | 242 | 15 | 68 | 403 | 9 | 366 | Integrase | Integrase | | afdb-uniprot50 | AF-A0A848EHP0-F1-MODEL\_V4 | 1.0 | 1.3e-14 | 489 | 0.213 | 426 | 274 | 22 | 2 | 399 | 4 | 396 | Integrase arm-type DNA-binding domain-containing protein | Integrase arm-type DNA-binding domain-containing protein | | afdb-uniprot50 | AF-A0A0X3TES6-F1-MODEL\_V4 | 1.0 | 6.267e-14 | 489 | 0.177 | 416 | 303 | 18 | 1 | 402 | 5 | 395 | Tyr recombinase domain-containing protein | Tyr recombinase domain-containing protein | | afdb-uniprot50 | AF-A0A0P9UR54-F1-MODEL\_V4 | 1.0 | 6.361e-15 | 488 | 0.231 | 401 | 250 | 21 | 1 | 377 | 1 | 367 | Site-specific recombinase, phage integrase family protein | Site-specific recombinase, phage integrase family protein | | afdb-uniprot50 | AF-A0A4D7QK21-F1-MODEL\_V4 | 1.0 | 6.361e-15 | 488 | 0.212 | 438 | 260 | 26 | 2 | 401 | 8 | 398 | DUF4102 domain-containing protein | DUF4102 domain-containing protein | | afdb-uniprot50 | AF-A0A6I2A9Q0-F1-MODEL\_V4 | 1.0 | 3.121e-12 | 487 | 0.198 | 322 | 223 | 14 | 99 | 403 | 2 | 305 | Prophage integrase | Prophage integrase | | afdb-uniprot50 | AF-K2JTR5-F1-MODEL\_V4 | 1.0 | 1.452e-15 | 487 | 0.229 | 423 | 260 | 22 | 2 | 401 | 5 | 384 | Integrase family protein | Integrase family protein | | afdb-uniprot50 | AF-A0A6D0Y5S2-F1-MODEL\_V4 | 1.0 | 9.769e-15 | 487 | 0.197 | 431 | 279 | 23 | 2 | 402 | 8 | 401 | Integrase arm-type DNA-binding domain-containing protein | Integrase arm-type DNA-binding domain-containing protein | | afdb-uniprot50 | AF-A0A1X9SW21-F1-MODEL\_V4 | 1.0 | 2.534e-14 | 486 | 0.165 | 422 | 285 | 26 | 2 | 402 | 6 | 381 | Site-specific recombinase, phage integrase family (DUF4102 domain) | Site-specific recombinase, phage integrase family (DUF4102 domain) | | afdb-uniprot50 | AF-A0A839UE18-F1-MODEL\_V4 | 1.0 | 2.304e-14 | 486 | 0.196 | 412 | 282 | 15 | 2 | 398 | 25 | 402 | Integrase | Integrase | | afdb-uniprot50 | AF-A0A6N4CPC3-F1-MODEL\_V4 | 1.0 | 2.002e-11 | 485 | 0.195 | 281 | 199 | 9 | 137 | 403 | 11 | 278 | Uncharacterized protein | Uncharacterized protein | | afdb-uniprot50 | AF-A0A350NYS9-F1-MODEL\_V4 | 1.0 | 2.203e-11 | 485 | 0.18 | 310 | 213 | 10 | 117 | 401 | 4 | 297 | Integrase | Integrase | | afdb-uniprot50 | AF-N8XS28-F1-MODEL\_V4 | 1.0 | 7.23e-14 | 485 | 0.153 | 403 | 290 | 22 | 14 | 400 | 6 | 373 | Tyr recombinase domain-containing protein | Tyr recombinase domain-containing protein | | afdb-uniprot50 | AF-A0A6I2IYY5-F1-MODEL\_V4 | 1.0 | 5.975e-14 | 485 | 0.184 | 412 | 271 | 22 | 9 | 400 | 12 | 378 | Tyrosine-type recombinase/integrase | Tyrosine-type recombinase/integrase | | afdb-uniprot50 | AF-A0A060HK82-F1-MODEL\_V4 | 1.0 | 2.196e-14 | 484 | 0.202 | 355 | 241 | 18 | 34 | 368 | 7 | 339 | Tyr recombinase domain-containing protein | Tyr recombinase domain-containing protein | | afdb-uniprot50 | AF-Q129B4-F1-MODEL\_V4 | 1.0 | 8.749e-14 | 484 | 0.215 | 413 | 281 | 20 | 2 | 403 | 7 | 387 | Phage integrase | Phage integrase | | afdb-uniprot50 | AF-K2BD66-F1-MODEL\_V4 | 1.0 | 1.43e-14 | 483 | 0.197 | 394 | 239 | 22 | 2 | 356 | 7 | 362 | Tyr recombinase domain-containing protein | Tyr recombinase domain-containing protein | | afdb-uniprot50 | AF-A0A6B8JJH7-F1-MODEL\_V4 | 1.0 | 2.196e-14 | 483 | 0.226 | 411 | 259 | 20 | 37 | 398 | 2 | 402 | Integrase | Integrase | | afdb-uniprot50 | AF-A0A522YGD1-F1-MODEL\_V4 | 1.0 | 6.792e-13 | 482 | 0.193 | 362 | 242 | 16 | 44 | 380 | 5 | 341 | DUF4102 domain-containing protein | DUF4102 domain-containing protein | | afdb-uniprot50 | AF-A0A560MBT4-F1-MODEL\_V4 | 1.0 | 2.416e-14 | 482 | 0.17 | 423 | 288 | 21 | 9 | 402 | 11 | 399 | Integrase | Integrase | | afdb-uniprot50 | AF-A0A433ZTM7-F1-MODEL\_V4 | 1.0 | 1.343e-13 | 481 | 0.206 | 353 | 235 | 16 | 1 | 335 | 1 | 326 | Integrase | Integrase | | afdb-uniprot50 | AF-A0A5C7QE83-F1-MODEL\_V4 | 1.0 | 1.938e-12 | 481 | 0.196 | 325 | 211 | 14 | 90 | 402 | 64 | 350 | Integrase | Integrase | | afdb-uniprot50 | AF-A0A2W7FQS8-F1-MODEL\_V4 | 1.0 | 9.944e-13 | 480 | 0.227 | 361 | 244 | 16 | 60 | 401 | 1 | 345 | Integrase | Integrase | | afdb-uniprot50 | AF-A0A127K4V2-F1-MODEL\_V4 | 1.0 | 1.904e-14 | 480 | 0.189 | 432 | 294 | 23 | 2 | 403 | 5 | 410 | Uncharacterized protein | Uncharacterized protein | | afdb-uniprot50 | AF-A0A2W5Q3A9-F1-MODEL\_V4 | 1.0 | 1.815e-14 | 480 | 0.205 | 453 | 272 | 27 | 1 | 402 | 7 | 422 | Integrase | Integrase | | afdb-uniprot50 | AF-A0A379NCF1-F1-MODEL\_V4 | 1.0 | 2.063e-13 | 480 | 0.2 | 474 | 278 | 27 | 2 | 398 | 8 | 457 | Prophage CP4-57 integrase | Prophage CP4-57 integrase | | afdb-uniprot50 | AF-A0A7Y9VNF8-F1-MODEL\_V4 | 1.0 | 8.88e-15 | 480 | 0.194 | 436 | 277 | 21 | 2 | 400 | 8 | 406 | Integrase | Integrase | | afdb-uniprot50 | AF-A0A3D1P0H0-F1-MODEL\_V4 | 1.0 | 5.432e-14 | 478 | 0.193 | 392 | 253 | 20 | 2 | 366 | 3 | 358 | Integrase | Integrase | | afdb-uniprot50 | AF-Q6ALK6-F1-MODEL\_V4 | 1.0 | 3.216e-14 | 478 | 0.174 | 418 | 282 | 19 | 2 | 402 | 8 | 379 | Related to integrase | Related to integrase | | afdb-uniprot50 | AF-A0A0H4IWA8-F1-MODEL\_V4 | 1.0 | 1.221e-13 | 478 | 0.161 | 452 | 293 | 25 | 2 | 402 | 3 | 419 | Core-binding (CB) domain-containing protein | Core-binding (CB) domain-containing protein | | afdb-uniprot50 | AF-A0A176ZCC3-F1-MODEL\_V4 | 1.0 | 2.164e-13 | 478 | 0.203 | 442 | 247 | 19 | 44 | 402 | 6 | 425 | Tyr recombinase domain-containing protein | Tyr recombinase domain-containing protein | | afdb-uniprot50 | AF-A0A6I1J6L9-F1-MODEL\_V4 | 1.0 | 3.548e-11 | 477 | 0.182 | 317 | 217 | 14 | 104 | 402 | 2 | 294 | Putative integrase | Putative integrase | | afdb-uniprot50 | AF-A0A4U1BG56-F1-MODEL\_V4 | 1.0 | 1.025e-14 | 477 | 0.2 | 429 | 282 | 21 | 3 | 400 | 13 | 411 | DUF4102 domain-containing protein | DUF4102 domain-containing protein | | afdb-uniprot50 | AF-A0A368KM35-F1-MODEL\_V4 | 1.0 | 4.639e-13 | 476 | 0.199 | 361 | 239 | 16 | 46 | 401 | 17 | 332 | Integrase | Integrase | | afdb-uniprot50 | AF-G1UQY9-F1-MODEL\_V4 | 1.0 | 1.523e-15 | 476 | 0.19 | 404 | 257 | 15 | 1 | 372 | 1 | 366 | Uncharacterized protein | Uncharacterized protein | | afdb-uniprot50 | AF-A0A5J4E109-F1-MODEL\_V4 | 1.0 | 3.373e-14 | 476 | 0.203 | 432 | 276 | 23 | 2 | 402 | 6 | 400 | Tyr recombinase domain-containing protein | Tyr recombinase domain-containing protein | | afdb-uniprot50 | AF-X0UCK1-F1-MODEL\_V4 | 1.0 | 2.345e-12 | 475 | 0.241 | 298 | 192 | 14 | 39 | 320 | 2 | 281 | Tyr recombinase domain-containing protein | Tyr recombinase domain-containing protein | | afdb-uniprot50 | AF-A0A3G9G2H1-F1-MODEL\_V4 | 1.0 | 6.38e-12 | 475 | 0.224 | 325 | 214 | 15 | 93 | 403 | 2 | 302 | Putative plasmid stabilization protein | Putative plasmid stabilization protein | | afdb-uniprot50 | AF-A0A7V8FSQ5-F1-MODEL\_V4 | 1.0 | 9.944e-13 | 474 | 0.219 | 387 | 242 | 19 | 50 | 402 | 3 | 363 | Prophage integrase IntA | Prophage integrase IntA | | afdb-uniprot50 | AF-A0A3N5Y2X2-F1-MODEL\_V4 | 1.0 | 3.71e-14 | 474 | 0.181 | 429 | 286 | 22 | 2 | 403 | 8 | 398 | DUF4102 domain-containing protein | DUF4102 domain-containing protein | | afdb-uniprot50 | AF-A0A009KR62-F1-MODEL\_V4 | 1.0 | 1.55e-13 | 474 | 0.16 | 411 | 289 | 21 | 14 | 402 | 22 | 398 | Phage integrase family protein | Phage integrase family protein | | afdb-uniprot50 | AF-A0A1I1WIT5-F1-MODEL\_V4 | 1.0 | 5.783e-15 | 474 | 0.199 | 467 | 278 | 25 | 2 | 403 | 10 | 445 | Integrase | Integrase | | afdb-uniprot50 | AF-A0A7Y9NF75-F1-MODEL\_V4 | 1.0 | 8.492e-12 | 473 | 0.22 | 327 | 227 | 14 | 93 | 398 | 2 | 321 | Integrase | Integrase | | afdb-uniprot50 | AF-A0A0F2NU29-F1-MODEL\_V4 | 1.0 | 7.583e-14 | 473 | 0.197 | 405 | 275 | 19 | 1 | 395 | 5 | 369 | Uncharacterized protein | Uncharacterized protein | | afdb-uniprot50 | AF-A0A3M4E1T2-F1-MODEL\_V4 | 1.0 | 1.009e-13 | 472 | 0.177 | 422 | 286 | 21 | 2 | 401 | 8 | 390 | Tyr recombinase domain-containing protein | Tyr recombinase domain-containing protein | | afdb-uniprot50 | AF-A0A1W1V7M5-F1-MODEL\_V4 | 1.0 | 5.194e-11 | 471 | 0.175 | 320 | 218 | 14 | 101 | 402 | 1 | 292 | Integrase | Integrase | | afdb-uniprot50 | AF-A0A657BBC9-F1-MODEL\_V4 | 1.0 | 3.075e-11 | 470 | 0.182 | 350 | 240 | 14 | 65 | 402 | 3 | 318 | Tyr recombinase domain-containing protein | Tyr recombinase domain-containing protein | | afdb-uniprot50 | AF-A0A316G5Z1-F1-MODEL\_V4 | 1.0 | 5.179e-14 | 470 | 0.182 | 399 | 278 | 19 | 20 | 400 | 2 | 370 | Integrase | Integrase | | afdb-uniprot50 | AF-A0A5C7UIE7-F1-MODEL\_V4 | 1.0 | 4.938e-14 | 469 | 0.157 | 431 | 290 | 17 | 2 | 398 | 8 | 399 | Site-specific integrase | Site-specific integrase | | afdb-uniprot50 | AF-A0A3E1BRB2-F1-MODEL\_V4 | 1.0 | 3.95e-15 | 469 | 0.193 | 449 | 271 | 22 | 1 | 398 | 7 | 415 | Tyr recombinase domain-containing protein | Tyr recombinase domain-containing protein | | afdb-uniprot50 | AF-A0A2E7GWJ5-F1-MODEL\_V4 | 1.0 | 1.705e-13 | 468 | 0.165 | 418 | 295 | 20 | 2 | 400 | 8 | 390 | Uncharacterized protein | Uncharacterized protein | | afdb-uniprot50 | AF-A0A292AEJ5-F1-MODEL\_V4 | 1.0 | 1.904e-14 | 468 | 0.193 | 434 | 273 | 27 | 1 | 402 | 1 | 389 | Tyr recombinase domain-containing protein | Tyr recombinase domain-containing protein | | afdb-uniprot50 | AF-A0A842HU53-F1-MODEL\_V4 | 1.0 | 2.38e-13 | 466 | 0.169 | 430 | 292 | 20 | 2 | 403 | 4 | 396 | Tyrosine-type recombinase/integrase | Tyrosine-type recombinase/integrase | | afdb-uniprot50 | AF-A0A1J5E277-F1-MODEL\_V4 | 1.0 | 3.891e-14 | 465 | 0.205 | 409 | 293 | 17 | 2 | 401 | 3 | 388 | Tyr recombinase domain-containing protein | Tyr recombinase domain-containing protein | | afdb-uniprot50 | AF-A0A2W4L7D4-F1-MODEL\_V4 | 1.0 | 8.749e-14 | 465 | 0.201 | 412 | 275 | 19 | 1 | 401 | 66 | 434 | Integrase | Integrase | | afdb-uniprot50 | AF-A0A1G6IJD1-F1-MODEL\_V4 | 1.0 | 3.323e-13 | 464 | 0.193 | 387 | 264 | 19 | 37 | 402 | 2 | 361 | Integrase | Integrase | | afdb-uniprot50 | AF-A0A844XW65-F1-MODEL\_V4 | 1.0 | 5.975e-14 | 464 | 0.178 | 432 | 283 | 22 | 1 | 402 | 1 | 390 | Integrase arm-type DNA-binding domain-containing protein | Integrase arm-type DNA-binding domain-containing protein | | afdb-uniprot50 | AF-A0A2P2EDV4-F1-MODEL\_V4 | 1.0 | 1.478e-13 | 464 | 0.171 | 456 | 283 | 23 | 2 | 400 | 8 | 425 | Tyrosine recombinase XerD | Tyrosine recombinase XerD | | afdb-uniprot50 | AF-A0A2E7GWC7-F1-MODEL\_V4 | 1.0 | 1.997e-14 | 464 | 0.213 | 436 | 275 | 26 | 2 | 401 | 8 | 411 | Tyr recombinase domain-containing protein | Tyr recombinase domain-containing protein | | afdb-uniprot50 | AF-H5WK80-F1-MODEL\_V4 | 1.0 | 3.537e-14 | 464 | 0.187 | 464 | 283 | 23 | 2 | 403 | 7 | 438 | Site-specific recombinase XerD | Site-specific recombinase XerD | | afdb-uniprot50 | AF-A0A382QZ10-F1-MODEL\_V4 | 1.0 | 5.194e-11 | 463 | 0.16 | 330 | 242 | 13 | 86 | 401 | 9 | 317 | Uncharacterized protein | Uncharacterized protein | | afdb-uniprot50 | AF-A0A7R7GHM2-F1-MODEL\_V4 | 1.0 | 7.492e-10 | 461 | 0.225 | 200 | 145 | 4 | 212 | 403 | 3 | 200 | Uncharacterized protein | Uncharacterized protein | | afdb-uniprot50 | AF-A0A198XBH7-F1-MODEL\_V4 | 1.0 | 6.914e-11 | 460 | 0.208 | 317 | 215 | 16 | 101 | 402 | 2 | 297 | Integrase | Integrase | | afdb-uniprot50 | AF-A0A0B8ZHW5-F1-MODEL\_V4 | 1.0 | 6.573e-14 | 460 | 0.172 | 465 | 275 | 23 | 2 | 398 | 9 | 431 | Integrase family protein | Integrase family protein | | afdb-uniprot50 | AF-A0A6N8K839-F1-MODEL\_V4 | 1.0 | 7.251e-11 | 458 | 0.19 | 294 | 198 | 11 | 123 | 402 | 2 | 269 | Tyrosine-type recombinase/integrase | Tyrosine-type recombinase/integrase | | afdb-uniprot50 | AF-A5UG79-F1-MODEL\_V4 | 1.0 | 4.793e-12 | 458 | 0.2 | 365 | 242 | 14 | 60 | 401 | 1 | 338 | Prophage integrase | Prophage integrase | | afdb-uniprot50 | AF-A0A4P6L4N5-F1-MODEL\_V4 | 1.0 | 2.618e-13 | 458 | 0.193 | 428 | 284 | 23 | 2 | 403 | 9 | 401 | Site-specific integrase | Site-specific integrase | | afdb-uniprot50 | AF-A0A165RXR2-F1-MODEL\_V4 | 1.0 | 7.339e-15 | 458 | 0.177 | 439 | 267 | 23 | 2 | 402 | 7 | 389 | Putative prophage CPS-53 integrase | Putative prophage CPS-53 integrase | | afdb-uniprot50 | AF-A0A7C2SE25-F1-MODEL\_V4 | 1.0 | 5.352e-13 | 457 | 0.179 | 456 | 288 | 23 | 2 | 398 | 10 | 438 | DUF4102 domain-containing protein | DUF4102 domain-containing protein | | afdb-uniprot50 | AF-A0A825BDC2-F1-MODEL\_V4 | 1.0 | 4.423e-13 | 456 | 0.179 | 413 | 268 | 22 | 7 | 403 | 3 | 360 | Uncharacterized protein | Uncharacterized protein | | afdb-uniprot50 | AF-A0A7Y3Y9M4-F1-MODEL\_V4 | 1.0 | 3.021e-13 | 456 | 0.18 | 422 | 286 | 22 | 2 | 403 | 8 | 389 | Tyrosine-type recombinase/integrase | Tyrosine-type recombinase/integrase | | afdb-uniprot50 | AF-A0A376VWA4-F1-MODEL\_V4 | 1.0 | 1.881e-10 | 455 | 0.203 | 260 | 179 | 8 | 158 | 402 | 2 | 248 | Integrase | Integrase | | afdb-uniprot50 | AF-A0A0N0WLQ3-F1-MODEL\_V4 | 1.0 | 1.736e-11 | 455 | 0.21 | 275 | 182 | 11 | 141 | 400 | 9 | 263 | Site-specific recombinase | Site-specific recombinase | | afdb-uniprot50 | AF-A0A859IFR7-F1-MODEL\_V4 | 1.0 | 4.217e-13 | 455 | 0.201 | 382 | 248 | 21 | 2 | 357 | 8 | 358 | Tyrosine-type recombinase/integrase | Tyrosine-type recombinase/integrase | | afdb-uniprot50 | AF-A0A1G6PQW0-F1-MODEL\_V4 | 1.0 | 1.554e-10 | 453 | 0.192 | 291 | 204 | 11 | 123 | 403 | 5 | 274 | Integrase | Integrase | | afdb-uniprot50 | AF-A0A2E7FT18-F1-MODEL\_V4 | 1.0 | 6.894e-14 | 453 | 0.189 | 380 | 250 | 16 | 2 | 354 | 8 | 356 | Integrase | Integrase | | afdb-uniprot50 | AF-A0A286E6R0-F1-MODEL\_V4 | 1.0 | 2.888e-10 | 452 | 0.22 | 268 | 185 | 8 | 145 | 403 | 11 | 263 | Phage integrase family protein | Phage integrase family protein | | afdb-uniprot50 | AF-A0A536TMG5-F1-MODEL\_V4 | 1.0 | 7.251e-11 | 452 | 0.207 | 270 | 186 | 8 | 145 | 402 | 5 | 258 | Site-specific integrase | Site-specific integrase | | afdb-uniprot50 | AF-A0A3D8K2G3-F1-MODEL\_V4 | 1.0 | 5.993e-11 | 452 | 0.172 | 290 | 207 | 12 | 131 | 403 | 34 | 307 | Integrase | Integrase | | afdb-uniprot50 | AF-A0A2R7RSS5-F1-MODEL\_V4 | 1.0 | 2.063e-13 | 452 | 0.199 | 426 | 271 | 22 | 2 | 402 | 8 | 388 | Integrase | Integrase | | afdb-uniprot50 | AF-A0A2D5FF62-F1-MODEL\_V4 | 1.0 | 1.059e-13 | 452 | 0.159 | 458 | 298 | 22 | 1 | 402 | 7 | 433 | Integrase | Integrase | | afdb-uniprot50 | AF-A0A1X3KZ03-F1-MODEL\_V4 | 1.0 | 4.093e-11 | 451 | 0.197 | 293 | 197 | 11 | 123 | 402 | 19 | 286 | Site-specific recombinase, phage integrase family | Site-specific recombinase, phage integrase family | | afdb-uniprot50 | AF-A0A2N7I5S9-F1-MODEL\_V4 | 1.0 | 3.601e-12 | 451 | 0.171 | 368 | 257 | 16 | 60 | 402 | 1 | 345 | Integrase | Integrase | | afdb-uniprot50 | AF-A0A7W8J9T4-F1-MODEL\_V4 | 1.0 | 1.225e-10 | 450 | 0.188 | 276 | 201 | 7 | 136 | 402 | 8 | 269 | Integrase | Integrase | | afdb-uniprot50 | AF-A0A1J0S1K6-F1-MODEL\_V4 | 1.0 | 5.613e-13 | 450 | 0.166 | 420 | 297 | 21 | 2 | 403 | 6 | 390 | Tyr recombinase domain-containing protein | Tyr recombinase domain-containing protein | | afdb-uniprot50 | AF-N8Q3U9-F1-MODEL\_V4 | 1.0 | 2.1e-11 | 449 | 0.187 | 362 | 254 | 16 | 60 | 403 | 1 | 340 | Tyr recombinase domain-containing protein | Tyr recombinase domain-containing protein | | afdb-uniprot50 | AF-A0A1G3UJM0-F1-MODEL\_V4 | 1.0 | 3.168e-13 | 448 | 0.21 | 346 | 229 | 16 | 2 | 330 | 8 | 326 | Integrase | Integrase | | afdb-uniprot50 | AF-A0A1D7NNW2-F1-MODEL\_V4 | 1.0 | 2.196e-14 | 447 | 0.216 | 416 | 239 | 24 | 1 | 377 | 1 | 368 | Arm-DNA-bind\_3 domain-containing protein | Arm-DNA-bind\_3 domain-containing protein | | afdb-uniprot50 | AF-A0A3N5UFD5-F1-MODEL\_V4 | 1.0 | 8.342e-14 | 446 | 0.246 | 357 | 211 | 20 | 1 | 340 | 29 | 344 | DUF4102 domain-containing protein | DUF4102 domain-containing protein | | afdb-uniprot50 | AF-A0A178MZZ2-F1-MODEL\_V4 | 1.0 | 1.876e-13 | 446 | 0.191 | 417 | 269 | 24 | 2 | 401 | 5 | 370 | Uncharacterized protein | Uncharacterized protein | | afdb-uniprot50 | AF-K7A9W5-F1-MODEL\_V4 | 1.0 | 4.021e-13 | 445 | 0.182 | 421 | 284 | 23 | 1 | 401 | 7 | 387 | Tyr recombinase domain-containing protein | Tyr recombinase domain-containing protein | | afdb-uniprot50 | AF-A0A653HY48-F1-MODEL\_V4 | 1.0 | 1.482e-10 | 443 | 0.155 | 321 | 227 | 14 | 101 | 402 | 1 | 296 | Prophage CPS-53 integrase | Prophage CPS-53 integrase | | afdb-uniprot50 | AF-A0A5F0WJ90-F1-MODEL\_V4 | 1.0 | 7.836e-13 | 442 | 0.176 | 424 | 286 | 22 | 1 | 401 | 7 | 390 | Site-specific integrase | Site-specific integrase | | afdb-uniprot50 | AF-A0A6J4EDZ9-F1-MODEL\_V4 | 1.0 | 1.262e-12 | 442 | 0.2 | 425 | 275 | 20 | 1 | 401 | 9 | 392 | Recombinase | Recombinase | | afdb-uniprot50 | AF-A0A833JLG8-F1-MODEL\_V4 | 1.0 | 1.967e-13 | 442 | 0.17 | 441 | 278 | 23 | 16 | 403 | 34 | 439 | Uncharacterized protein | Uncharacterized protein | | afdb-uniprot50 | AF-A0A534I5Z6-F1-MODEL\_V4 | 1.0 | 4.952e-11 | 440 | 0.216 | 300 | 198 | 11 | 119 | 403 | 12 | 289 | Site-specific integrase | Site-specific integrase | | afdb-uniprot50 | AF-A0A5A7MRW9-F1-MODEL\_V4 | 1.0 | 2.932e-11 | 440 | 0.223 | 367 | 242 | 17 | 60 | 403 | 1 | 347 | Phage integrase | Phage integrase | | afdb-uniprot50 | AF-A0A5J6R848-F1-MODEL\_V4 | 1.0 | 3.021e-13 | 439 | 0.174 | 430 | 279 | 21 | 1 | 401 | 7 | 389 | Site-specific integrase | Site-specific integrase | | afdb-uniprot50 | AF-A0A3B9VKD5-F1-MODEL\_V4 | 1.0 | 4.021e-13 | 439 | 0.174 | 492 | 272 | 27 | 1 | 392 | 5 | 462 | Tyr recombinase domain-containing protein | Tyr recombinase domain-containing protein | | afdb-uniprot50 | AF-A0A1L9GM55-F1-MODEL\_V4 | 1.0 | 5.8e-12 | 438 | 0.204 | 387 | 238 | 18 | 60 | 402 | 1 | 361 | Uncharacterized protein | Uncharacterized protein | | afdb-uniprot50 | AF-A0A3M3DIU2-F1-MODEL\_V4 | 1.0 | 4.021e-13 | 438 | 0.208 | 427 | 264 | 23 | 2 | 401 | 7 | 386 | Putative Phage integrase | Putative Phage integrase | | afdb-uniprot50 | AF-A0A2P7NYP5-F1-MODEL\_V4 | 1.0 | 1.243e-11 | 437 | 0.18 | 361 | 254 | 14 | 62 | 402 | 4 | 342 | Integrase | Integrase | | afdb-uniprot50 | AF-A0A523H760-F1-MODEL\_V4 | 1.0 | 3.323e-13 | 436 | 0.206 | 359 | 229 | 18 | 2 | 337 | 9 | 334 | DUF4102 domain-containing protein | DUF4102 domain-containing protein | | afdb-uniprot50 | AF-A0A2E7FR99-F1-MODEL\_V4 | 1.0 | 2.423e-11 | 436 | 0.151 | 369 | 247 | 16 | 74 | 402 | 14 | 356 | Tyr recombinase domain-containing protein | Tyr recombinase domain-containing protein | | afdb-uniprot50 | AF-A0A5I4RF45-F1-MODEL\_V4 | 1.0 | 3.021e-13 | 436 | 0.165 | 405 | 266 | 23 | 2 | 370 | 3 | 371 | DUF4102 domain-containing protein | DUF4102 domain-containing protein | | afdb-uniprot50 | AF-A0A5P9CRG7-F1-MODEL\_V4 | 1.0 | 2.236e-12 | 436 | 0.163 | 416 | 299 | 19 | 2 | 401 | 11 | 393 | Integrase | Integrase | | afdb-uniprot50 | AF-I2Q1B1-F1-MODEL\_V4 | 1.0 | 1.55e-13 | 436 | 0.192 | 411 | 263 | 22 | 17 | 391 | 2 | 379 | Site-specific recombinase XerD | Site-specific recombinase XerD | | afdb-uniprot50 | AF-A0A0A7EEY8-F1-MODEL\_V4 | 1.0 | 4.423e-13 | 436 | 0.181 | 425 | 284 | 22 | 2 | 402 | 7 | 391 | Tyr recombinase domain-containing protein | Tyr recombinase domain-containing protein | | afdb-uniprot50 | AF-A0A5C8NMZ4-F1-MODEL\_V4 | 1.0 | 6.476e-13 | 436 | 0.18 | 437 | 278 | 23 | 9 | 402 | 10 | 409 | DUF4102 domain-containing protein | DUF4102 domain-containing protein | | afdb-uniprot50 | AF-B8KHE9-F1-MODEL\_V4 | 1.0 | 4.639e-13 | 436 | 0.199 | 426 | 272 | 22 | 9 | 402 | 20 | 408 | Site-specific recombinase, phage integrase family | Site-specific recombinase, phage integrase family | | afdb-uniprot50 | AF-A0A6P3BW65-F1-MODEL\_V4 | 1.0 | 1.602e-12 | 433 | 0.206 | 344 | 225 | 15 | 2 | 326 | 3 | 317 | Integrase | Integrase | | afdb-uniprot50 | AF-A0A0W0U2X8-F1-MODEL\_V4 | 1.0 | 4.722e-11 | 433 | 0.176 | 329 | 242 | 13 | 82 | 398 | 4 | 315 | Integrase | Integrase | | afdb-uniprot50 | AF-A0A1I2F649-F1-MODEL\_V4 | 1.0 | 4.865e-13 | 433 | 0.161 | 470 | 290 | 22 | 2 | 402 | 7 | 441 | Tyr recombinase domain-containing protein | Tyr recombinase domain-containing protein | | afdb-uniprot50 | AF-A0A375BMV2-F1-MODEL\_V4 | 1.0 | 3.666e-10 | 431 | 0.195 | 292 | 204 | 13 | 102 | 381 | 16 | 288 | Phage integrase | Phage integrase | | afdb-uniprot50 | AF-A0A3D0RCF0-F1-MODEL\_V4 | 1.0 | 3.834e-13 | 431 | 0.201 | 388 | 261 | 22 | 2 | 372 | 19 | 374 | Integrase | Integrase | | afdb-uniprot50 | AF-A0A2N5Y8G1-F1-MODEL\_V4 | 1.0 | 2.496e-13 | 431 | 0.193 | 419 | 283 | 25 | 2 | 402 | 51 | 432 | Site-specific integrase | Site-specific integrase | | afdb-uniprot50 | AF-A0A7C4EE33-F1-MODEL\_V4 | 1.0 | 1.262e-12 | 431 | 0.151 | 450 | 299 | 23 | 2 | 402 | 25 | 440 | DUF4102 domain-containing protein | DUF4102 domain-containing protein | | afdb-uniprot50 | AF-A0A257SZ13-F1-MODEL\_V4 | 1.0 | 1.531e-09 | 430 | 0.26 | 192 | 133 | 7 | 216 | 402 | 6 | 193 | Tyr recombinase domain-containing protein | Tyr recombinase domain-containing protein | | afdb-uniprot50 | AF-A0A8B4ZLY0-F1-MODEL\_V4 | 1.0 | 1.388e-12 | 429 | 0.179 | 430 | 275 | 23 | 2 | 401 | 10 | 391 | Site-specific integrase | Site-specific integrase | | afdb-uniprot50 | AF-A0A1G4ZAT7-F1-MODEL\_V4 | 1.0 | 4.57e-12 | 428 | 0.167 | 364 | 239 | 14 | 52 | 402 | 19 | 331 | Integrase | Integrase | | afdb-uniprot50 | AF-A0A1U9SCV8-F1-MODEL\_V4 | 1.0 | 1.046e-09 | 427 | 0.196 | 259 | 182 | 8 | 155 | 403 | 2 | 244 | Integrase | Integrase | | afdb-uniprot50 | AF-A0A7J0BQK4-F1-MODEL\_V4 | 1.0 | 3.177e-10 | 427 | 0.157 | 312 | 225 | 13 | 107 | 402 | 2 | 291 | Tyr recombinase domain-containing protein | Tyr recombinase domain-containing protein | | afdb-uniprot50 | AF-A0A3B9B7X0-F1-MODEL\_V4 | 1.0 | 6.792e-13 | 427 | 0.21 | 342 | 216 | 17 | 2 | 321 | 3 | 312 | Integrase | Integrase | | afdb-uniprot50 | AF-A0A845MU45-F1-MODEL\_V4 | 1.0 | 7.449e-16 | 427 | 0.185 | 420 | 290 | 22 | 2 | 402 | 3 | 389 | DUF4102 domain-containing protein | DUF4102 domain-containing protein | | afdb-uniprot50 | AF-F4MXM0-F1-MODEL\_V4 | 1.0 | 8.644e-10 | 426 | 0.236 | 258 | 170 | 9 | 153 | 401 | 2 | 241 | Integrase | Integrase | | afdb-uniprot50 | AF-A0A6N7AFU2-F1-MODEL\_V4 | 1.0 | 9.04e-13 | 426 | 0.178 | 415 | 300 | 16 | 2 | 403 | 3 | 389 | Phage integrase family protein | Phage integrase family protein | | afdb-uniprot50 | AF-A0A518KCN4-F1-MODEL\_V4 | 1.0 | 1.078e-11 | 424 | 0.175 | 365 | 241 | 14 | 46 | 401 | 14 | 327 | Site-specific tyrosine recombinase XerC | Site-specific tyrosine recombinase XerC | | afdb-uniprot50 | AF-A0A5C7W994-F1-MODEL\_V4 | 1.0 | 3.845e-10 | 423 | 0.174 | 298 | 210 | 9 | 115 | 403 | 2 | 272 | Integrase | Integrase | | afdb-uniprot50 | AF-A0A212KHW4-F1-MODEL\_V4 | 1.0 | 2.138e-09 | 423 | 0.169 | 318 | 230 | 12 | 98 | 402 | 2 | 298 | Phage integrase family protein | Phage integrase family protein | | afdb-uniprot50 | AF-A0A090IF55-F1-MODEL\_V4 | 1.0 | 6.174e-13 | 422 | 0.182 | 427 | 268 | 26 | 2 | 400 | 8 | 381 | Putative phage integrase | Putative phage integrase | | afdb-uniprot50 | AF-A0A512AL48-F1-MODEL\_V4 | 1.0 | 7.492e-10 | 421 | 0.203 | 280 | 193 | 12 | 136 | 402 | 5 | 267 | Tyr recombinase domain-containing protein | Tyr recombinase domain-containing protein | | afdb-uniprot50 | AF-G4A9X4-F1-MODEL\_V4 | 1.0 | 9.973e-10 | 420 | 0.207 | 256 | 175 | 10 | 159 | 403 | 2 | 240 | Prophage CP4-57 integrase | Prophage CP4-57 integrase | | afdb-uniprot50 | AF-A0A1X0SVT1-F1-MODEL\_V4 | 1.0 | 5.993e-11 | 420 | 0.194 | 318 | 219 | 16 | 99 | 401 | 9 | 304 | Integrase family protein | Integrase family protein | | afdb-uniprot50 | AF-A0A0H4IW46-F1-MODEL\_V4 | 1.0 | 1.705e-13 | 420 | 0.224 | 366 | 227 | 19 | 2 | 341 | 7 | 341 | Integrase | Integrase | | afdb-uniprot50 | AF-A0A2X4XHJ1-F1-MODEL\_V4 | 1.0 | 5.53e-12 | 420 | 0.195 | 389 | 243 | 20 | 2 | 356 | 3 | 355 | Prophage CP4-57 integrase | Prophage CP4-57 integrase | | afdb-uniprot50 | AF-A0A1W7QVM2-F1-MODEL\_V4 | 1.0 | 1.043e-12 | 420 | 0.141 | 444 | 298 | 23 | 2 | 402 | 10 | 413 | Site-specific recombinase XerD | Site-specific recombinase XerD | | afdb-uniprot50 | AF-A0A259Q3Y0-F1-MODEL\_V4 | 1.0 | 6.192e-10 | 419 | 0.173 | 317 | 223 | 13 | 102 | 402 | 1 | 294 | Integrase | Integrase | | afdb-uniprot50 | AF-A0A6P4G483-F1-MODEL\_V4 | 1.0 | 4.435e-10 | 418 | 0.227 | 299 | 193 | 13 | 116 | 398 | 8 | 284 | prophage CP4-57 integrase-like | prophage CP4-57 integrase-like | | afdb-uniprot50 | AF-A0A0F7HAR5-F1-MODEL\_V4 | 1.0 | 1.281e-13 | 418 | 0.196 | 422 | 270 | 23 | 2 | 402 | 3 | 376 | Arm-DNA-bind\_3 domain-containing protein | Arm-DNA-bind\_3 domain-containing protein | | afdb-uniprot50 | AF-A0A5E4S4Y3-F1-MODEL\_V4 | 1.0 | 1.012e-10 | 417 | 0.199 | 371 | 247 | 16 | 60 | 403 | 1 | 348 | Integrase | Integrase | | afdb-uniprot50 | AF-A0A2N6Q9F2-F1-MODEL\_V4 | 1.0 | 1.046e-09 | 416 | 0.176 | 267 | 190 | 7 | 149 | 402 | 1 | 250 | Tyr recombinase domain-containing protein | Tyr recombinase domain-containing protein | | afdb-uniprot50 | AF-A0A2R7MYK0-F1-MODEL\_V4 | 1.0 | 2.352e-09 | 415 | 0.156 | 314 | 234 | 11 | 100 | 402 | 24 | 317 | Integrase | Integrase | | afdb-uniprot50 | AF-A0A5P3AFX6-F1-MODEL\_V4 | 1.0 | 9.066e-10 | 415 | 0.175 | 313 | 229 | 12 | 99 | 400 | 10 | 304 | Prophage CP4-57 integrase | Prophage CP4-57 integrase | | afdb-uniprot50 | AF-A0A4D9X583-F1-MODEL\_V4 | 1.0 | 3.323e-13 | 415 | 0.208 | 378 | 231 | 21 | 2 | 346 | 3 | 345 | DUF4102 domain-containing protein | DUF4102 domain-containing protein | | afdb-uniprot50 | AF-A0A2L2XGH8-F1-MODEL\_V4 | 1.0 | 2.846e-09 | 414 | 0.176 | 306 | 216 | 14 | 101 | 393 | 1 | 283 | Tyrosine recombinase xerD | Tyrosine recombinase xerD | | afdb-uniprot50 | AF-A0A2K8W1H0-F1-MODEL\_V4 | 1.0 | 2.88e-13 | 414 | 0.197 | 380 | 233 | 20 | 2 | 346 | 3 | 345 | Prophage P4 integrase | Prophage P4 integrase | | afdb-uniprot50 | AF-A0A350JMT7-F1-MODEL\_V4 | 1.0 | 3.601e-12 | 414 | 0.162 | 436 | 296 | 25 | 2 | 402 | 8 | 409 | Tyr recombinase domain-containing protein | Tyr recombinase domain-containing protein | | afdb-uniprot50 | AF-A0A4U8Z4V2-F1-MODEL\_V4 | 1.0 | 7.858e-10 | 413 | 0.222 | 265 | 177 | 9 | 149 | 402 | 1 | 247 | Tyr recombinase domain-containing protein | Tyr recombinase domain-containing protein | | afdb-uniprot50 | AF-A0A2E4NJY1-F1-MODEL\_V4 | 1.0 | 7.858e-10 | 413 | 0.188 | 276 | 197 | 9 | 137 | 402 | 6 | 264 | Integrase | Integrase | | afdb-uniprot50 | AF-A0A1W2A448-F1-MODEL\_V4 | 1.0 | 2.837e-12 | 413 | 0.194 | 375 | 236 | 20 | 45 | 402 | 33 | 358 | Site-specific recombinase XerD | Site-specific recombinase XerD | | afdb-uniprot50 | AF-A0A1H4W6T1-F1-MODEL\_V4 | 1.0 | 6.285e-11 | 412 | 0.201 | 343 | 233 | 16 | 80 | 403 | 2 | 322 | Integrase | Integrase | | afdb-uniprot50 | AF-A0A7G8BGC1-F1-MODEL\_V4 | 1.0 | 1.392e-09 | 411 | 0.188 | 265 | 188 | 12 | 101 | 356 | 12 | 258 | Tyrosine-type recombinase/integrase | Tyrosine-type recombinase/integrase | | afdb-uniprot50 | AF-A0A399RGX3-F1-MODEL\_V4 | 1.0 | 2.626e-10 | 411 | 0.205 | 312 | 204 | 15 | 99 | 401 | 22 | 298 | Site-specific integrase | Site-specific integrase | | afdb-uniprot50 | AF-A0A2D9RK50-F1-MODEL\_V4 | 1.0 | 6.592e-11 | 411 | 0.183 | 366 | 251 | 19 | 60 | 402 | 1 | 341 | Integrase | Integrase | | afdb-uniprot50 | AF-A0A5P9EZI4-F1-MODEL\_V4 | 1.0 | 1.602e-12 | 411 | 0.17 | 398 | 260 | 20 | 1 | 375 | 2 | 352 | Prophage CP4-57 integrase | Prophage CP4-57 integrase | | afdb-uniprot50 | AF-A0A523WE57-F1-MODEL\_V4 | 1.0 | 1.094e-12 | 411 | 0.181 | 424 | 291 | 21 | 2 | 402 | 70 | 460 | Uncharacterized protein | Uncharacterized protein | | afdb-uniprot50 | AF-A0A436CCZ6-F1-MODEL\_V4 | 1.0 | 2.466e-09 | 409 | 0.184 | 271 | 192 | 11 | 146 | 402 | 9 | 264 | Integrase | Integrase | | afdb-uniprot50 | AF-A0A8B3J0Y8-F1-MODEL\_V4 | 1.0 | 1.554e-10 | 409 | 0.198 | 318 | 219 | 12 | 99 | 402 | 4 | 299 | Integrase | Integrase | | afdb-uniprot50 | AF-B1JDT1-F1-MODEL\_V4 | 1.0 | 2.236e-12 | 409 | 0.169 | 396 | 264 | 18 | 34 | 401 | 37 | 395 | Integrase family protein | Integrase family protein | | afdb-uniprot50 | AF-A0A722ZTZ9-F1-MODEL\_V4 | 1.0 | 1.456e-12 | 407 | 0.18 | 387 | 254 | 21 | 2 | 357 | 3 | 357 | DUF4102 domain-containing protein | DUF4102 domain-containing protein | | afdb-uniprot50 | AF-A0A6L2ZPK9-F1-MODEL\_V4 | 1.0 | 6.811e-10 | 406 | 0.15 | 345 | 249 | 12 | 69 | 402 | 6 | 317 | Integrase | Integrase | | afdb-uniprot50 | AF-A0A7Y4U7C5-F1-MODEL\_V4 | 1.0 | 2.795e-11 | 406 | 0.168 | 373 | 255 | 15 | 36 | 401 | 3 | 327 | Site-specific integrase | Site-specific integrase | | afdb-uniprot50 | AF-A0A0T5ZW92-F1-MODEL\_V4 | 1.0 | 4.229e-10 | 405 | 0.214 | 308 | 217 | 13 | 97 | 400 | 35 | 321 | Putative phage integrase | Putative phage integrase | | afdb-uniprot50 | AF-A0A2J8HSE2-F1-MODEL\_V4 | 1.0 | 5.8e-12 | 405 | 0.161 | 422 | 295 | 20 | 2 | 402 | 44 | 427 | Tyr recombinase domain-containing protein | Tyr recombinase domain-containing protein | | afdb-uniprot50 | AF-A0A0Q7TMR2-F1-MODEL\_V4 | 1.0 | 2.276e-10 | 404 | 0.202 | 360 | 233 | 19 | 69 | 398 | 2 | 337 | Tyr recombinase domain-containing protein | Tyr recombinase domain-containing protein | | afdb-uniprot50 | AF-A0A7U0PNP7-F1-MODEL\_V4 | 1.0 | 1.078e-11 | 404 | 0.153 | 423 | 309 | 20 | 2 | 402 | 6 | 401 | Integrase family protein | Integrase family protein | | afdb-uniprot50 | AF-A0A2N8M7R9-F1-MODEL\_V4 | 1.0 | 1.848e-12 | 403 | 0.169 | 414 | 273 | 17 | 9 | 389 | 14 | 389 | Integrase | Integrase | | afdb-uniprot50 | AF-A0A090SWG2-F1-MODEL\_V4 | 1.0 | 1.046e-09 | 402 | 0.175 | 347 | 249 | 15 | 74 | 402 | 4 | 331 | Phage integrase | Phage integrase | | afdb-uniprot50 | AF-A0A537S885-F1-MODEL\_V4 | 1.0 | 3.121e-12 | 402 | 0.17 | 429 | 288 | 17 | 2 | 390 | 10 | 410 | Site-specific integrase | Site-specific integrase | | afdb-uniprot50 | AF-A0A225DKJ3-F1-MODEL\_V4 | 1.0 | 7.251e-11 | 401 | 0.188 | 366 | 239 | 16 | 49 | 401 | 22 | 342 | Mobile element protein | Mobile element protein | | afdb-uniprot50 | AF-A0A257LMN8-F1-MODEL\_V4 | 1.0 | 2.795e-11 | 401 | 0.204 | 377 | 234 | 20 | 69 | 403 | 17 | 369 | Integrase | Integrase | | afdb-uniprot50 | AF-A0A6G6J4R1-F1-MODEL\_V4 | 1.0 | 2.705e-12 | 401 | 0.168 | 438 | 272 | 24 | 1 | 401 | 9 | 391 | Site-specific integrase | Site-specific integrase | | afdb-uniprot50 | AF-A0A0M2V4F1-F1-MODEL\_V4 | 1.0 | 5.53e-12 | 400 | 0.166 | 414 | 281 | 20 | 34 | 402 | 9 | 403 | Tyr recombinase domain-containing protein | Tyr recombinase domain-containing protein | | afdb-uniprot50 | AF-L8MDS0-F1-MODEL\_V4 | 1.0 | 1.66e-08 | 399 | 0.278 | 187 | 125 | 6 | 223 | 402 | 1 | 184 | Prophage PSPPH01, site-specific recombinase, phage integrase family | Prophage PSPPH01, site-specific recombinase, phage integrase family | | afdb-uniprot50 | AF-A0A5B9QPY5-F1-MODEL\_V4 | 1.0 | 4.722e-11 | 399 | 0.183 | 366 | 235 | 18 | 46 | 401 | 16 | 327 | Tyrosine recombinase XerC | Tyrosine recombinase XerC | | afdb-uniprot50 | AF-A0A0W0U4L5-F1-MODEL\_V4 | 1.0 | 3.495e-10 | 398 | 0.206 | 296 | 206 | 10 | 112 | 400 | 10 | 283 | Integrase | Integrase | | afdb-uniprot50 | AF-A0A510U2Z7-F1-MODEL\_V4 | 1.0 | 1.909e-11 | 398 | 0.172 | 395 | 266 | 20 | 34 | 402 | 35 | 394 | Recombinase | Recombinase | | afdb-uniprot50 | AF-A0A656BVP0-F1-MODEL\_V4 | 1.0 | 3.283e-09 | 395 | 0.209 | 262 | 178 | 9 | 154 | 403 | 3 | 247 | Prophage Sf6-like integrase | Prophage Sf6-like integrase | | afdb-uniprot50 | AF-A0A248XRU4-F1-MODEL\_V4 | 1.0 | 1.392e-09 | 395 | 0.182 | 279 | 205 | 8 | 130 | 402 | 1 | 262 | Integrase | Integrase | | afdb-uniprot50 | AF-A0A853R082-F1-MODEL\_V4 | 1.0 | 1.909e-11 | 395 | 0.148 | 405 | 277 | 20 | 8 | 389 | 9 | 368 | Uncharacterized protein | Uncharacterized protein | | afdb-uniprot50 | AF-A0A2Z4R7D1-F1-MODEL\_V4 | 1.0 | 2.846e-09 | 394 | 0.163 | 281 | 207 | 11 | 133 | 401 | 9 | 273 | Tyr recombinase domain-containing protein | Tyr recombinase domain-containing protein | | afdb-uniprot50 | AF-A0A378MUR8-F1-MODEL\_V4 | 1.0 | 1.207e-09 | 392 | 0.197 | 269 | 186 | 10 | 146 | 402 | 3 | 253 | Prophage CP4-57 integrase | Prophage CP4-57 integrase | | afdb-uniprot50 | AF-A0A7W1APG2-F1-MODEL\_V4 | 1.0 | 2.673e-08 | 391 | 0.216 | 203 | 150 | 6 | 207 | 402 | 2 | 202 | Site-specific integrase | Site-specific integrase | | afdb-uniprot50 | AF-A0A192D4V7-F1-MODEL\_V4 | 1.0 | 1.685e-09 | 390 | 0.224 | 250 | 164 | 12 | 137 | 373 | 3 | 235 | Uncharacterized protein | Uncharacterized protein | | afdb-uniprot50 | AF-A0A2A6Q2D7-F1-MODEL\_V4 | 1.0 | 5.041e-09 | 389 | 0.224 | 236 | 157 | 10 | 177 | 402 | 2 | 221 | Integrase | Integrase | | afdb-uniprot50 | AF-A0A1H6ZMK8-F1-MODEL\_V4 | 1.0 | 6.38e-12 | 389 | 0.179 | 430 | 277 | 22 | 2 | 401 | 12 | 395 | Integrase | Integrase | | afdb-uniprot50 | AF-A0A7W0ZY73-F1-MODEL\_V4 | 1.0 | 4.093e-11 | 389 | 0.128 | 484 | 312 | 30 | 2 | 403 | 5 | 460 | Tyr recombinase domain-containing protein | Tyr recombinase domain-containing protein | | afdb-uniprot50 | AF-A0A7T9ZF29-F1-MODEL\_V4 | 1.0 | 2.985e-09 | 388 | 0.18 | 310 | 209 | 14 | 108 | 403 | 7 | 285 | Tyrosine-type recombinase/integrase | Tyrosine-type recombinase/integrase | | afdb-uniprot50 | AF-A0A1G2ZM93-F1-MODEL\_V4 | 1.0 | 1.973e-10 | 388 | 0.197 | 390 | 247 | 16 | 18 | 403 | 4 | 331 | Tyr recombinase domain-containing protein | Tyr recombinase domain-containing protein | | afdb-uniprot50 | AF-I4CE33-F1-MODEL\_V4 | 1.0 | 5.993e-11 | 387 | 0.154 | 394 | 276 | 19 | 34 | 393 | 2 | 372 | Site-specific recombinase XerD | Site-specific recombinase XerD | | afdb-uniprot50 | AF-A0A5E5BYC9-F1-MODEL\_V4 | 1.0 | 2.352e-09 | 386 | 0.242 | 231 | 154 | 8 | 182 | 401 | 3 | 223 | Integrase | Integrase | | afdb-uniprot50 | AF-A0A5N9VWT1-F1-MODEL\_V4 | 1.0 | 1.71e-10 | 385 | 0.176 | 306 | 213 | 18 | 2 | 296 | 9 | 286 | DUF4102 domain-containing protein | DUF4102 domain-containing protein | | afdb-uniprot50 | AF-A0A378AUB5-F1-MODEL\_V4 | 1.0 | 2.352e-09 | 384 | 0.176 | 295 | 215 | 10 | 64 | 347 | 11 | 288 | Integrase | Integrase | | afdb-uniprot50 | AF-A0A845AEC5-F1-MODEL\_V4 | 1.0 | 6.792e-13 | 383 | 0.187 | 411 | 216 | 17 | 2 | 401 | 3 | 306 | Integrase arm-type DNA-binding domain-containing protein | Integrase arm-type DNA-binding domain-containing protein | | afdb-uniprot50 | AF-A0A7W5E2G6-F1-MODEL\_V4 | 1.0 | 3.029e-10 | 382 | 0.173 | 357 | 239 | 16 | 46 | 393 | 15 | 324 | Integrase | Integrase | | afdb-uniprot50 | AF-A0A1F5UBR0-F1-MODEL\_V4 | 1.0 | 5.714e-11 | 382 | 0.158 | 392 | 261 | 16 | 18 | 400 | 2 | 333 | Uncharacterized protein | Uncharacterized protein | | afdb-uniprot50 | AF-A0A485GR16-F1-MODEL\_V4 | 1.0 | 1.853e-09 | 380 | 0.206 | 286 | 184 | 9 | 146 | 402 | 11 | 282 | Integrase family protein | Integrase family protein | | afdb-uniprot50 | AF-A0A1X9T2A8-F1-MODEL\_V4 | 1.0 | 3.283e-09 | 379 | 0.157 | 324 | 236 | 15 | 90 | 398 | 4 | 305 | Site-specific recombinase, phage integrase family | Site-specific recombinase, phage integrase family | | afdb-uniprot50 | AF-A0A1I4QUM7-F1-MODEL\_V4 | 1.0 | 4.722e-11 | 379 | 0.199 | 321 | 211 | 14 | 1 | 303 | 6 | 298 | Phage integrase family protein | Phage integrase family protein | | afdb-uniprot50 | AF-A0A564WGE9-F1-MODEL\_V4 | 1.0 | 2.665e-11 | 379 | 0.179 | 441 | 277 | 20 | 1 | 402 | 9 | 403 | Integrase family protein | Integrase family protein | | afdb-uniprot50 | AF-A0A560UXU6-F1-MODEL\_V4 | 1.0 | 6.934e-08 | 377 | 0.252 | 190 | 132 | 6 | 220 | 402 | 1 | 187 | Phage integrase family protein | Phage integrase family protein | | afdb-uniprot50 | AF-A0A257H209-F1-MODEL\_V4 | 1.0 | 6.811e-10 | 377 | 0.167 | 358 | 250 | 15 | 67 | 399 | 9 | 343 | Uncharacterized protein | Uncharacterized protein | | afdb-uniprot50 | AF-A0A6A4TZN2-F1-MODEL\_V4 | 1.0 | 1.685e-09 | 377 | 0.198 | 332 | 210 | 17 | 99 | 401 | 29 | 333 | DNA integration/recombination/inversion protein | DNA integration/recombination/inversion protein | | afdb-uniprot50 | AF-A0A655W7E3-F1-MODEL\_V4 | 1.0 | 8.39e-08 | 376 | 0.211 | 189 | 141 | 4 | 220 | 402 | 1 | 187 | Phage integrase | Phage integrase | | afdb-uniprot50 | AF-A0A4Q3Z742-F1-MODEL\_V4 | 1.0 | 2.317e-08 | 376 | 0.248 | 197 | 129 | 6 | 217 | 400 | 1 | 191 | Tyr recombinase domain-containing protein | Tyr recombinase domain-containing protein | | afdb-uniprot50 | AF-A0A5J6QNY1-F1-MODEL\_V4 | 1.0 | 3.788e-09 | 376 | 0.178 | 319 | 221 | 14 | 102 | 401 | 8 | 304 | Site-specific integrase | Site-specific integrase | | afdb-uniprot50 | AF-A0A5B9QG65-F1-MODEL\_V4 | 1.0 | 7.492e-10 | 376 | 0.169 | 348 | 248 | 12 | 46 | 389 | 12 | 322 | Tyrosine recombinase XerD | Tyrosine recombinase XerD | | afdb-uniprot50 | AF-A0A7W2L6W8-F1-MODEL\_V4 | 1.0 | 2.352e-09 | 376 | 0.19 | 326 | 218 | 14 | 99 | 402 | 13 | 314 | Site-specific integrase | Site-specific integrase | | afdb-uniprot50 | AF-A0A257RB84-F1-MODEL\_V4 | 1.0 | 4.093e-11 | 375 | 0.167 | 442 | 289 | 18 | 2 | 403 | 10 | 412 | Tyr recombinase domain-containing protein | Tyr recombinase domain-containing protein | | afdb-uniprot50 | AF-A0A3C1G9D8-F1-MODEL\_V4 | 1.0 | 3.972e-09 | 374 | 0.167 | 304 | 211 | 16 | 101 | 391 | 13 | 287 | Uncharacterized protein | Uncharacterized protein | | afdb-uniprot50 | AF-A0A1E3M116-F1-MODEL\_V4 | 1.0 | 1.151e-09 | 374 | 0.186 | 322 | 213 | 16 | 101 | 401 | 1 | 294 | Uncharacterized protein | Uncharacterized protein | | afdb-uniprot50 | AF-S5MWJ7-F1-MODEL\_V4 | 1.0 | 2.31e-11 | 372 | 0.226 | 335 | 215 | 17 | 1 | 322 | 7 | 310 | Integrase | Integrase | | afdb-uniprot50 | AF-A0A1B6VTD4-F1-MODEL\_V4 | 1.0 | 6.303e-08 | 371 | 0.22 | 200 | 142 | 5 | 216 | 402 | 18 | 216 | Tyr recombinase domain-containing protein | Tyr recombinase domain-containing protein | | afdb-uniprot50 | AF-A0A533QJ01-F1-MODEL\_V4 | 1.0 | 2.069e-10 | 371 | 0.19 | 400 | 250 | 21 | 34 | 403 | 4 | 359 | Putative defective protein IntQ | Putative defective protein IntQ | | afdb-uniprot50 | AF-A0A3C7VZS0-F1-MODEL\_V4 | 1.0 | 1.486e-07 | 370 | 0.23 | 182 | 133 | 5 | 225 | 403 | 21 | 198 | Tyr recombinase domain-containing protein | Tyr recombinase domain-containing protein | | afdb-uniprot50 | AF-A0A0S8GP47-F1-MODEL\_V4 | 1.0 | 6.494e-10 | 370 | 0.168 | 380 | 263 | 18 | 30 | 393 | 2 | 344 | Uncharacterized protein | Uncharacterized protein | | afdb-uniprot50 | AF-A0A7Z7RKM6-F1-MODEL\_V4 | 1.0 | 1.439e-08 | 369 | 0.195 | 245 | 166 | 8 | 174 | 402 | 2 | 231 | Prophage CP4-57 integrase | Prophage CP4-57 integrase | | afdb-uniprot50 | AF-A0A0Y0K400-F1-MODEL\_V4 | 1.0 | 8.218e-13 | 369 | 0.222 | 382 | 226 | 23 | 2 | 354 | 7 | 346 | Integrase family protein | Integrase family protein | | afdb-uniprot50 | AF-A0A5P3VU28-F1-MODEL\_V4 | 1.0 | 2.31e-11 | 369 | 0.176 | 413 | 271 | 21 | 9 | 396 | 13 | 381 | Site-specific integrase | Site-specific integrase | | afdb-uniprot50 | AF-A0A518HSZ1-F1-MODEL\_V4 | 1.0 | 7.605e-11 | 368 | 0.156 | 429 | 237 | 20 | 46 | 403 | 15 | 389 | Site-specific tyrosine recombinase XerC | Site-specific tyrosine recombinase XerC | | afdb-uniprot50 | AF-Q1NEP9-F1-MODEL\_V4 | 1.0 | 2.038e-09 | 367 | 0.17 | 310 | 205 | 12 | 108 | 402 | 6 | 278 | Symbiosis island integrase | Symbiosis island integrase | | afdb-uniprot50 | AF-A0A5E4XII2-F1-MODEL\_V4 | 1.0 | 5.714e-11 | 367 | 0.173 | 358 | 237 | 18 | 15 | 346 | 1 | 325 | Integrase | Integrase | | afdb-uniprot50 | AF-A0A655PDZ5-F1-MODEL\_V4 | 1.0 | 1.417e-07 | 366 | 0.211 | 189 | 141 | 4 | 221 | 403 | 11 | 197 | Phage integrase | Phage integrase | | afdb-uniprot50 | AF-A0A354XKQ8-F1-MODEL\_V4 | 1.0 | 8.799e-08 | 365 | 0.247 | 182 | 127 | 5 | 225 | 401 | 25 | 201 | Integrase | Integrase | | afdb-uniprot50 | AF-A0A7V8QD16-F1-MODEL\_V4 | 1.0 | 2.236e-12 | 365 | 0.198 | 358 | 217 | 17 | 2 | 321 | 3 | 328 | Integrase arm-type DNA-binding domain-containing protein | Integrase arm-type DNA-binding domain-containing protein | | afdb-uniprot50 | AF-A0A377DPL2-F1-MODEL\_V4 | 1.0 | 1.559e-07 | 364 | 0.196 | 188 | 142 | 6 | 222 | 402 | 3 | 188 | Phage integrase | Phage integrase | | afdb-uniprot50 | AF-A0A509BEV6-F1-MODEL\_V4 | 1.0 | 1.486e-07 | 364 | 0.181 | 193 | 150 | 5 | 216 | 402 | 4 | 194 | Integrase family protein | Integrase family protein | | afdb-uniprot50 | AF-D4C538-F1-MODEL\_V4 | 1.0 | 1.715e-07 | 364 | 0.186 | 193 | 149 | 5 | 216 | 402 | 26 | 216 | Putative prophage CP4-57 integrase | Putative prophage CP4-57 integrase | | afdb-uniprot50 | AF-A0A0M2DWI1-F1-MODEL\_V4 | 1.0 | 8.516e-09 | 364 | 0.226 | 234 | 158 | 9 | 178 | 402 | 2 | 221 | Prophage CP4-57 integrase | Prophage CP4-57 integrase | | afdb-uniprot50 | AF-A0A694V927-F1-MODEL\_V4 | 1.0 | 2.1e-11 | 364 | 0.169 | 324 | 230 | 12 | 1 | 310 | 3 | 301 | DUF4102 domain-containing protein | DUF4102 domain-containing protein | | afdb-uniprot50 | AF-A0A2W5DWS9-F1-MODEL\_V4 | 1.0 | 1.741e-08 | 363 | 0.171 | 263 | 190 | 10 | 152 | 402 | 2 | 248 | Integrase | Integrase | | afdb-uniprot50 | AF-A0A212I7A4-F1-MODEL\_V4 | 1.0 | 1.635e-07 | 362 | 0.196 | 193 | 147 | 5 | 216 | 402 | 26 | 216 | Integrase CP4-57 prophage | Integrase CP4-57 prophage | | afdb-uniprot50 | AF-A0A3M3AIK9-F1-MODEL\_V4 | 1.0 | 1.247e-08 | 362 | 0.194 | 262 | 182 | 9 | 153 | 402 | 2 | 246 | Site-specific recombinase, phage integrase protein | Site-specific recombinase, phage integrase protein | | afdb-uniprot50 | AF-A0A1G0VC22-F1-MODEL\_V4 | 1.0 | 3.666e-10 | 362 | 0.134 | 380 | 256 | 19 | 36 | 400 | 3 | 324 | Uncharacterized protein | Uncharacterized protein | | afdb-uniprot50 | AF-A0A848CG60-F1-MODEL\_V4 | 1.0 | 1.015e-07 | 361 | 0.179 | 206 | 156 | 8 | 207 | 403 | 3 | 204 | Site-specific integrase | Site-specific integrase | | afdb-uniprot50 | AF-A0A1Q3YLC3-F1-MODEL\_V4 | 1.0 | 6.914e-11 | 361 | 0.217 | 303 | 193 | 16 | 2 | 290 | 3 | 275 | Integrase | Integrase | | afdb-uniprot50 | AF-A0A518K3T6-F1-MODEL\_V4 | 1.0 | 8.242e-10 | 361 | 0.185 | 382 | 260 | 17 | 23 | 402 | 2 | 334 | Tyrosine recombinase XerD | Tyrosine recombinase XerD | | afdb-uniprot50 | AF-D1AHL5-F1-MODEL\_V4 | 1.0 | 7.144e-10 | 361 | 0.161 | 389 | 260 | 19 | 17 | 393 | 8 | 342 | Integrase family protein | Integrase family protein | | afdb-uniprot50 | AF-A0A397QTS5-F1-MODEL\_V4 | 1.0 | 1.63e-10 | 360 | 0.148 | 398 | 272 | 17 | 17 | 396 | 11 | 359 | Site-specific recombinase XerD | Site-specific recombinase XerD | | afdb-uniprot50 | AF-A0A1M6IDQ9-F1-MODEL\_V4 | 1.0 | 3.721e-11 | 360 | 0.161 | 408 | 279 | 19 | 7 | 392 | 8 | 374 | Site-specific recombinase XerD | Site-specific recombinase XerD | | afdb-uniprot50 | AF-D8FA13-F1-MODEL\_V4 | 1.0 | 1.881e-10 | 359 | 0.172 | 383 | 262 | 15 | 34 | 402 | 3 | 344 | Site-specific recombinase, phage integrase family | Site-specific recombinase, phage integrase family | | afdb-uniprot50 | AF-A0A432UDR0-F1-MODEL\_V4 | 1.0 | 2.17e-10 | 358 | 0.178 | 375 | 245 | 16 | 42 | 402 | 30 | 355 | Tyr recombinase domain-containing protein | Tyr recombinase domain-containing protein | | afdb-uniprot50 | AF-G0ERT2-F1-MODEL\_V4 | 1.0 | 2.283e-07 | 357 | 0.197 | 192 | 143 | 4 | 220 | 402 | 20 | 209 | Prophage CP4-57 integrase IntA | Prophage CP4-57 integrase IntA | | afdb-uniprot50 | AF-A0A5C9CD61-F1-MODEL\_V4 | 1.0 | 1.606e-09 | 357 | 0.178 | 359 | 240 | 18 | 46 | 393 | 15 | 329 | Uncharacterized protein | Uncharacterized protein | | afdb-uniprot50 | AF-A0A847DD10-F1-MODEL\_V4 | 1.0 | 1.114e-10 | 356 | 0.186 | 369 | 267 | 18 | 45 | 402 | 36 | 382 | Tyrosine-type recombinase/integrase | Tyrosine-type recombinase/integrase | | afdb-uniprot50 | AF-A0A6I6VWS2-F1-MODEL\_V4 | 1.0 | 6.192e-10 | 355 | 0.257 | 299 | 183 | 14 | 55 | 334 | 9 | 287 | Uncharacterized protein | Uncharacterized protein | | afdb-uniprot50 | AF-A0A2V8AVC8-F1-MODEL\_V4 | 1.0 | 4.652e-10 | 355 | 0.199 | 371 | 246 | 18 | 46 | 393 | 19 | 361 | Uncharacterized protein | Uncharacterized protein | | afdb-uniprot50 | AF-A0A3R9ZJ42-F1-MODEL\_V4 | 1.0 | 6.934e-08 | 354 | 0.169 | 248 | 180 | 8 | 164 | 401 | 2 | 233 | Integrase | Integrase | | afdb-uniprot50 | AF-A0A6P2CVZ9-F1-MODEL\_V4 | 1.0 | 2.008e-08 | 354 | 0.205 | 307 | 207 | 15 | 101 | 400 | 1 | 277 | Uncharacterized protein | Uncharacterized protein | | afdb-uniprot50 | AF-A0A5Y2E3L5-F1-MODEL\_V4 | 1.0 | 2.283e-07 | 353 | 0.223 | 192 | 133 | 8 | 221 | 402 | 1 | 186 | Integrase | Integrase | | afdb-uniprot50 | AF-A0A395JEX9-F1-MODEL\_V4 | 1.0 | 3.234e-08 | 353 | 0.207 | 241 | 165 | 8 | 172 | 402 | 11 | 235 | Integrase | Integrase | | afdb-uniprot50 | AF-A0A1Y6KVZ1-F1-MODEL\_V4 | 1.0 | 1.978e-07 | 351 | 0.244 | 196 | 137 | 6 | 216 | 402 | 25 | 218 | Prophage CP4-57 integrase | Prophage CP4-57 integrase | | afdb-uniprot50 | AF-A0A2D4WFI9-F1-MODEL\_V4 | 1.0 | 1.439e-08 | 351 | 0.177 | 322 | 218 | 14 | 102 | 401 | 3 | 299 | Integrase | Integrase | | afdb-uniprot50 | AF-A0A6C0TPK5-F1-MODEL\_V4 | 1.0 | 7.976e-11 | 351 | 0.211 | 336 | 212 | 15 | 1 | 297 | 8 | 329 | Integrase | Integrase | | afdb-uniprot50 | AF-A0A376RHC7-F1-MODEL\_V4 | 1.0 | 1.185e-11 | 351 | 0.186 | 375 | 238 | 22 | 1 | 341 | 2 | 343 | Integrase | Integrase | | afdb-uniprot50 | AF-A0A142WXX8-F1-MODEL\_V4 | 1.0 | 1.285e-10 | 351 | 0.16 | 400 | 281 | 16 | 1 | 393 | 17 | 368 | Site-specific tyrosine recombinase XerC | Site-specific tyrosine recombinase XerC | | afdb-uniprot50 | AF-A0A1F7FFN7-F1-MODEL\_V4 | 1.0 | 1.881e-10 | 350 | 0.172 | 383 | 247 | 20 | 34 | 395 | 2 | 335 | Tyr recombinase domain-containing protein | Tyr recombinase domain-containing protein | | afdb-uniprot50 | AF-A0A5C9C4Q8-F1-MODEL\_V4 | 1.0 | 5.041e-09 | 349 | 0.156 | 376 | 259 | 14 | 34 | 400 | 5 | 331 | Tyrosine recombinase XerC | Tyrosine recombinase XerC | | afdb-uniprot50 | AF-A0A2N2IB33-F1-MODEL\_V4 | 1.0 | 9.973e-10 | 349 | 0.165 | 374 | 276 | 15 | 34 | 401 | 2 | 345 | Tyr recombinase domain-containing protein | Tyr recombinase domain-containing protein | | afdb-uniprot50 | AF-A0A7U9F051-F1-MODEL\_V4 | 1.0 | 1.081e-08 | 348 | 0.204 | 289 | 185 | 15 | 137 | 402 | 12 | 278 | Tyr recombinase domain-containing protein | Tyr recombinase domain-containing protein | | afdb-uniprot50 | AF-A0A2G6DGF4-F1-MODEL\_V4 | 1.0 | 1.685e-09 | 348 | 0.143 | 356 | 269 | 16 | 46 | 396 | 12 | 336 | Tyr recombinase domain-containing protein | Tyr recombinase domain-containing protein | | afdb-uniprot50 | AF-A0A6C2YUQ7-F1-MODEL\_V4 | 1.0 | 1.531e-09 | 348 | 0.155 | 361 | 261 | 13 | 46 | 393 | 15 | 344 | Uncharacterized protein | Uncharacterized protein | | afdb-uniprot50 | AF-A0A6L7K450-F1-MODEL\_V4 | 1.0 | 3.505e-07 | 347 | 0.209 | 186 | 138 | 6 | 221 | 399 | 9 | 192 | Site-specific integrase | Site-specific integrase | | afdb-uniprot50 | AF-A0A261WKU5-F1-MODEL\_V4 | 1.0 | 4.044e-07 | 347 | 0.233 | 184 | 130 | 6 | 222 | 398 | 35 | 214 | Integrase | Integrase | | afdb-uniprot50 | AF-A0A5S9ISB9-F1-MODEL\_V4 | 1.0 | 2.888e-10 | 347 | 0.155 | 392 | 243 | 19 | 46 | 400 | 8 | 348 | Recombinase | Recombinase | | afdb-uniprot50 | AF-A0A7Y9UX65-F1-MODEL\_V4 | 1.0 | 5.904e-10 | 347 | 0.165 | 406 | 269 | 24 | 29 | 401 | 19 | 387 | Integrase | Integrase | | afdb-uniprot50 | AF-A0A2T4J5A4-F1-MODEL\_V4 | 1.0 | 2.352e-09 | 346 | 0.165 | 350 | 235 | 16 | 54 | 378 | 3 | 320 | Integrase | Integrase | | afdb-uniprot50 | AF-A0A2S8GSK0-F1-MODEL\_V4 | 1.0 | 7.492e-10 | 346 | 0.183 | 354 | 242 | 16 | 48 | 392 | 15 | 330 | Uncharacterized protein | Uncharacterized protein | | afdb-uniprot50 | AF-A0A142XGY2-F1-MODEL\_V4 | 1.0 | 1.767e-09 | 346 | 0.172 | 360 | 256 | 12 | 46 | 400 | 15 | 337 | Site-specific tyrosine recombinase XerC | Site-specific tyrosine recombinase XerC | | afdb-uniprot50 | AF-A0A7V9DZ64-F1-MODEL\_V4 | 1.0 | 1.826e-08 | 345 | 0.19 | 309 | 207 | 12 | 94 | 388 | 5 | 284 | Site-specific integrase | Site-specific integrase | | afdb-uniprot50 | AF-A0A1Y3TSY9-F1-MODEL\_V4 | 1.0 | 3.495e-10 | 345 | 0.157 | 399 | 252 | 24 | 27 | 393 | 5 | 351 | Site-specific integrase | Site-specific integrase | | afdb-uniprot50 | AF-A0A849MCQ5-F1-MODEL\_V4 | 1.0 | 1.606e-09 | 344 | 0.173 | 374 | 259 | 17 | 34 | 400 | 4 | 334 | Site-specific integrase | Site-specific integrase | | afdb-uniprot50 | AF-A0A839ZCX8-F1-MODEL\_V4 | 1.0 | 3.186e-07 | 344 | 0.216 | 180 | 130 | 5 | 225 | 398 | 134 | 308 | Uncharacterized protein | Uncharacterized protein | | afdb-uniprot50 | AF-A0A3E0NP80-F1-MODEL\_V4 | 1.0 | 5.993e-11 | 344 | 0.16 | 423 | 282 | 23 | 8 | 395 | 7 | 391 | Site-specific integrase | Site-specific integrase | | afdb-uniprot50 | AF-A0A1I2MLX2-F1-MODEL\_V4 | 1.0 | 6.611e-08 | 343 | 0.186 | 241 | 167 | 10 | 175 | 402 | 2 | 226 | Phage integrase family protein | Phage integrase family protein | | afdb-uniprot50 | AF-A0A7Z0MUD3-F1-MODEL\_V4 | 1.0 | 1.372e-08 | 343 | 0.187 | 331 | 227 | 14 | 99 | 402 | 14 | 329 | Tyrosine-type recombinase/integrase | Tyrosine-type recombinase/integrase | | afdb-uniprot50 | AF-A0A829YLK3-F1-MODEL\_V4 | 1.0 | 7.164e-07 | 342 | 0.207 | 193 | 143 | 7 | 218 | 402 | 29 | 219 | Uncharacterized protein | Uncharacterized protein | | afdb-uniprot50 | AF-A0A382MCM1-F1-MODEL\_V4 | 1.0 | 8.39e-08 | 342 | 0.176 | 250 | 181 | 9 | 164 | 401 | 2 | 238 | Tyr recombinase domain-containing protein | Tyr recombinase domain-containing protein | | afdb-uniprot50 | AF-A0A1I4UQB1-F1-MODEL\_V4 | 1.0 | 2.673e-08 | 342 | 0.183 | 261 | 186 | 10 | 154 | 402 | 2 | 247 | Integrase | Integrase | | afdb-uniprot50 | AF-A0A6L7CNV4-F1-MODEL\_V4 | 1.0 | 3.342e-07 | 341 | 0.222 | 189 | 138 | 5 | 222 | 403 | 8 | 194 | Prophage integrase IntS | Prophage integrase IntS | | afdb-uniprot50 | AF-A0A0F2IVU2-F1-MODEL\_V4 | 1.0 | 1.606e-09 | 341 | 0.155 | 360 | 256 | 16 | 46 | 391 | 9 | 334 | Phage integrase | Phage integrase | | afdb-uniprot50 | AF-A0A6N8JJ97-F1-MODEL\_V4 | 1.0 | 6.01e-08 | 340 | 0.206 | 242 | 163 | 9 | 174 | 402 | 11 | 236 | Tyrosine-type recombinase/integrase | Tyrosine-type recombinase/integrase | | afdb-uniprot50 | AF-A0A3A0B0M4-F1-MODEL\_V4 | 1.0 | 4.105e-08 | 340 | 0.174 | 315 | 211 | 15 | 102 | 401 | 7 | 287 | Uncharacterized protein | Uncharacterized protein | | afdb-uniprot50 | AF-A0A2N1RVN7-F1-MODEL\_V4 | 1.0 | 1.63e-10 | 338 | 0.157 | 380 | 250 | 19 | 44 | 392 | 16 | 356 | Tyr recombinase domain-containing protein | Tyr recombinase domain-containing protein | | afdb-uniprot50 | AF-A0A823MIL1-F1-MODEL\_V4 | 1.0 | 1.799e-07 | 337 | 0.209 | 205 | 146 | 8 | 208 | 401 | 2 | 201 | Site-specific integrase | Site-specific integrase | | afdb-uniprot50 | AF-A0A2U0ZBZ1-F1-MODEL\_V4 | 1.0 | 1.21e-06 | 336 | 0.235 | 187 | 135 | 6 | 222 | 402 | 3 | 187 | Phage integrase family protein | Phage integrase family protein | | afdb-uniprot50 | AF-A0A828G9E6-F1-MODEL\_V4 | 1.0 | 1.531e-09 | 336 | 0.148 | 404 | 281 | 18 | 36 | 393 | 9 | 395 | Site-specific integrase | Site-specific integrase | | afdb-uniprot50 | AF-A0A6F8PSE3-F1-MODEL\_V4 | 1.0 | 7.514e-07 | 335 | 0.18 | 210 | 161 | 6 | 202 | 402 | 5 | 212 | Tyr recombinase domain-containing protein | Tyr recombinase domain-containing protein | | afdb-uniprot50 | AF-A0A2Z3H9V6-F1-MODEL\_V4 | 1.0 | 1.767e-09 | 333 | 0.172 | 370 | 241 | 17 | 46 | 400 | 15 | 334 | Uncharacterized protein | Uncharacterized protein | | afdb-uniprot50 | AF-A0A517XN73-F1-MODEL\_V4 | 1.0 | 4.879e-10 | 333 | 0.16 | 411 | 252 | 19 | 46 | 403 | 15 | 385 | Site-specific tyrosine recombinase XerC | Site-specific tyrosine recombinase XerC | | afdb-uniprot50 | AF-A7MTK6-F1-MODEL\_V4 | 1.0 | 4.967e-08 | 332 | 0.158 | 302 | 215 | 13 | 115 | 402 | 3 | 279 | Uncharacterized protein | Uncharacterized protein | | afdb-uniprot50 | AF-A0A6G4KUC7-F1-MODEL\_V4 | 1.0 | 3.332e-10 | 332 | 0.205 | 311 | 188 | 17 | 44 | 321 | 5 | 289 | Integrase arm-type DNA-binding domain-containing protein | Integrase arm-type DNA-binding domain-containing protein | | afdb-uniprot50 | AF-A0A3C0FAP9-F1-MODEL\_V4 | 1.0 | 5.546e-09 | 332 | 0.186 | 316 | 206 | 14 | 107 | 396 | 10 | 300 | Uncharacterized protein | Uncharacterized protein | | afdb-uniprot50 | AF-A0A285R8I8-F1-MODEL\_V4 | 1.0 | 2.673e-08 | 331 | 0.161 | 316 | 212 | 17 | 101 | 394 | 3 | 287 | Integrase/recombinase XerD | Integrase/recombinase XerD | | afdb-uniprot50 | AF-A0A2Z6ETG2-F1-MODEL\_V4 | 1.0 | 3.845e-10 | 331 | 0.191 | 308 | 202 | 16 | 2 | 291 | 3 | 281 | Integrase | Integrase | | afdb-uniprot50 | AF-A0A139K4Z7-F1-MODEL\_V4 | 1.0 | 3.084e-08 | 331 | 0.184 | 347 | 242 | 16 | 65 | 398 | 3 | 321 | Site-specific recombinase, phage integrase family | Site-specific recombinase, phage integrase family | | afdb-uniprot50 | AF-A0A6L8HXX8-F1-MODEL\_V4 | 1.0 | 1.715e-07 | 330 | 0.16 | 317 | 221 | 17 | 105 | 403 | 2 | 291 | Tyrosine-type recombinase/integrase | Tyrosine-type recombinase/integrase | | afdb-uniprot50 | AF-A0A7V3D6C0-F1-MODEL\_V4 | 1.0 | 7.742e-09 | 330 | 0.157 | 363 | 248 | 12 | 46 | 402 | 34 | 344 | Tyr recombinase domain-containing protein | Tyr recombinase domain-containing protein | | afdb-uniprot50 | AF-A0A4Q3C5E2-F1-MODEL\_V4 | 1.0 | 6.934e-08 | 329 | 0.171 | 221 | 171 | 6 | 189 | 401 | 4 | 220 | Integrase | Integrase | | afdb-uniprot50 | AF-A0A522JXQ1-F1-MODEL\_V4 | 1.0 | 1.71e-10 | 329 | 0.186 | 348 | 219 | 18 | 1 | 313 | 1 | 319 | DUF4102 domain-containing protein | DUF4102 domain-containing protein | | afdb-uniprot50 | AF-A0A7W2QM99-F1-MODEL\_V4 | 1.0 | 1.015e-07 | 328 | 0.21 | 266 | 178 | 9 | 152 | 402 | 2 | 250 | Site-specific integrase | Site-specific integrase | | afdb-uniprot50 | AF-A0A737IDC5-F1-MODEL\_V4 | 1.0 | 1.881e-10 | 328 | 0.176 | 328 | 209 | 17 | 2 | 300 | 3 | 298 | DUF4102 domain-containing protein | DUF4102 domain-containing protein | | afdb-uniprot50 | AF-A0A7W1JY51-F1-MODEL\_V4 | 1.0 | 8.644e-10 | 328 | 0.172 | 401 | 264 | 18 | 30 | 403 | 2 | 361 | Tyrosine-type recombinase/integrase | Tyrosine-type recombinase/integrase | | afdb-uniprot50 | AF-A0A537X7F3-F1-MODEL\_V4 | 1.0 | 1.559e-07 | 327 | 0.183 | 240 | 170 | 8 | 174 | 402 | 7 | 231 | Site-specific integrase | Site-specific integrase | | afdb-uniprot50 | AF-A0A447KKV0-F1-MODEL\_V4 | 1.0 | 8.799e-08 | 327 | 0.173 | 265 | 185 | 11 | 154 | 402 | 3 | 249 | Site-specific tyrosine recombinase XerC | Site-specific tyrosine recombinase XerC | | afdb-uniprot50 | AF-A0A2G6C9G2-F1-MODEL\_V4 | 1.0 | 1.331e-06 | 325 | 0.222 | 166 | 123 | 4 | 238 | 400 | 5 | 167 | Integrase | Integrase | | afdb-uniprot50 | AF-A0A7C9MK69-F1-MODEL\_V4 | 1.0 | 9.973e-10 | 325 | 0.171 | 378 | 258 | 18 | 34 | 400 | 2 | 335 | Tyrosine-type recombinase/integrase | Tyrosine-type recombinase/integrase | | afdb-uniprot50 | AF-A0A533BAT2-F1-MODEL\_V4 | 1.0 | 5.816e-09 | 325 | 0.176 | 386 | 268 | 19 | 36 | 401 | 3 | 358 | Uncharacterized protein | Uncharacterized protein | | afdb-uniprot50 | AF-A0A7X9AF86-F1-MODEL\_V4 | 1.0 | 8.39e-08 | 324 | 0.16 | 318 | 216 | 15 | 103 | 393 | 14 | 307 | Tyrosine recombinase XerC | Tyrosine recombinase XerC | | afdb-uniprot50 | AF-A0A132HFJ0-F1-MODEL\_V4 | 1.0 | 7.251e-11 | 324 | 0.175 | 411 | 277 | 18 | 8 | 392 | 11 | 385 | Tyrosine recombinase XerC | Tyrosine recombinase XerC | | afdb-uniprot50 | AF-A0A285Z377-F1-MODEL\_V4 | 1.0 | 8.366e-11 | 324 | 0.185 | 443 | 283 | 25 | 1 | 395 | 1 | 413 | Phage integrase family protein | Phage integrase family protein | | afdb-uniprot50 | AF-A0A521UXZ2-F1-MODEL\_V4 | 1.0 | 1.372e-08 | 323 | 0.167 | 329 | 223 | 17 | 90 | 397 | 70 | 368 | Uncharacterized protein | Uncharacterized protein | | afdb-uniprot50 | AF-A0A3M1TIK8-F1-MODEL\_V4 | 1.0 | 3.332e-10 | 321 | 0.255 | 247 | 146 | 8 | 1 | 219 | 9 | 245 | DUF4102 domain-containing protein | DUF4102 domain-containing protein | | afdb-uniprot50 | AF-N9BVU9-F1-MODEL\_V4 | 1.0 | 2.283e-07 | 321 | 0.184 | 309 | 207 | 15 | 101 | 402 | 1 | 271 | Tyr recombinase domain-containing protein | Tyr recombinase domain-containing protein | | afdb-uniprot50 | AF-A0A807ZPL9-F1-MODEL\_V4 | 1.0 | 2.17e-10 | 320 | 0.155 | 353 | 231 | 18 | 2 | 319 | 37 | 357 | Uncharacterized protein | Uncharacterized protein | | afdb-uniprot50 | AF-A0A519K9Q9-F1-MODEL\_V4 | 1.0 | 5.117e-10 | 320 | 0.14 | 405 | 263 | 21 | 47 | 400 | 20 | 390 | Tyr recombinase domain-containing protein | Tyr recombinase domain-containing protein | | afdb-uniprot50 | AF-A0A2W5FLA2-F1-MODEL\_V4 | 1.0 | 2.511e-07 | 319 | 0.201 | 238 | 165 | 8 | 177 | 402 | 2 | 226 | Integrase | Integrase | | afdb-uniprot50 | AF-M9R7N3-F1-MODEL\_V4 | 1.0 | 3.234e-08 | 318 | 0.211 | 289 | 175 | 14 | 136 | 396 | 6 | 269 | DNA integration/recombination/inversion protein | DNA integration/recombination/inversion protein | | afdb-uniprot50 | AF-A0A7X5VWX1-F1-MODEL\_V4 | 1.0 | 2.038e-09 | 318 | 0.157 | 382 | 274 | 17 | 34 | 403 | 3 | 348 | Tyrosine-type recombinase/integrase | Tyrosine-type recombinase/integrase | | afdb-uniprot50 | AF-A0A3D2VB80-F1-MODEL\_V4 | 1.0 | 6.398e-09 | 318 | 0.183 | 392 | 248 | 19 | 17 | 386 | 10 | 351 | Tyr recombinase domain-containing protein | Tyr recombinase domain-containing protein | | afdb-uniprot50 | AF-A0A382I9H7-F1-MODEL\_V4 | 1.0 | 1.396e-06 | 317 | 0.204 | 191 | 140 | 8 | 221 | 402 | 1 | 188 | Tyr recombinase domain-containing protein | Tyr recombinase domain-containing protein | | afdb-uniprot50 | AF-A0A6L8LZX1-F1-MODEL\_V4 | 1.0 | 1.799e-07 | 317 | 0.158 | 278 | 202 | 10 | 137 | 401 | 3 | 261 | Tyrosine-type recombinase/integrase | Tyrosine-type recombinase/integrase | | afdb-uniprot50 | AF-A0A177R241-F1-MODEL\_V4 | 1.0 | 1.03e-08 | 317 | 0.185 | 366 | 236 | 15 | 46 | 403 | 15 | 326 | Uncharacterized protein | Uncharacterized protein | | afdb-uniprot50 | AF-U7GQ01-F1-MODEL\_V4 | 1.0 | 2.144e-06 | 316 | 0.234 | 162 | 120 | 4 | 241 | 400 | 3 | 162 | Tyr recombinase domain-containing protein | Tyr recombinase domain-containing protein | | afdb-uniprot50 | AF-A0A3D1EUH0-F1-MODEL\_V4 | 1.0 | 9.368e-09 | 316 | 0.158 | 384 | 256 | 20 | 32 | 401 | 14 | 344 | Uncharacterized protein | Uncharacterized protein | | afdb-uniprot50 | AF-A0A534XE27-F1-MODEL\_V4 | 1.0 | 6.398e-09 | 316 | 0.151 | 382 | 278 | 16 | 25 | 399 | 3 | 345 | Site-specific integrase | Site-specific integrase | | afdb-uniprot50 | AF-A0A526YPQ5-F1-MODEL\_V4 | 1.0 | 2.812e-05 | 315 | 0.308 | 94 | 61 | 2 | 2 | 92 | 4 | 96 | DUF4102 domain-containing protein | DUF4102 domain-containing protein | | afdb-uniprot50 | AF-A0A7K4GUA7-F1-MODEL\_V4 | 1.0 | 4.105e-08 | 314 | 0.147 | 319 | 240 | 11 | 99 | 403 | 2 | 302 | Tyrosine-type recombinase/integrase | Tyrosine-type recombinase/integrase | | afdb-uniprot50 | AF-A0A146GF21-F1-MODEL\_V4 | 1.0 | 7.742e-09 | 314 | 0.131 | 379 | 264 | 17 | 15 | 377 | 1 | 330 | Site-specific recombinase XerC | Site-specific recombinase XerC | | afdb-uniprot50 | AF-A0A0Q7Z9S4-F1-MODEL\_V4 | 1.0 | 8.644e-10 | 313 | 0.142 | 414 | 280 | 18 | 4 | 378 | 12 | 389 | Tyr recombinase domain-containing protein | Tyr recombinase domain-containing protein | | afdb-uniprot50 | AF-G2I0W8-F1-MODEL\_V4 | 1.0 | 1.046e-09 | 312 | 0.181 | 391 | 233 | 25 | 36 | 403 | 7 | 333 | Phage DNA recombinase | Phage DNA recombinase | | afdb-uniprot50 | AF-A0A179RXE5-F1-MODEL\_V4 | 1.0 | 1.826e-08 | 312 | 0.178 | 381 | 250 | 15 | 49 | 390 | 2 | 358 | Integrase | Integrase | | afdb-uniprot50 | AF-A0A2V3ZGJ6-F1-MODEL\_V4 | 1.0 | 6.285e-11 | 311 | 0.156 | 415 | 248 | 21 | 9 | 354 | 11 | 392 | Uncharacterized protein | Uncharacterized protein | | afdb-uniprot50 | AF-K0DW31-F1-MODEL\_V4 | 1.0 | 4.229e-10 | 311 | 0.167 | 419 | 276 | 23 | 1 | 396 | 70 | 438 | Phage integrase family protein | Phage integrase family protein | | afdb-uniprot50 | AF-A0A0S8DIS8-F1-MODEL\_V4 | 1.0 | 5.041e-09 | 309 | 0.15 | 365 | 254 | 16 | 46 | 400 | 9 | 327 | Uncharacterized protein | Uncharacterized protein | | afdb-uniprot50 | AF-A8T4I4-F1-MODEL\_V4 | 1.0 | 2.43e-08 | 305 | 0.193 | 351 | 186 | 12 | 62 | 402 | 1 | 264 | DNA integration/recombination/invertion protein | DNA integration/recombination/invertion protein | | afdb-uniprot50 | AF-N9FYI4-F1-MODEL\_V4 | 1.0 | 6.831e-07 | 304 | 0.178 | 235 | 172 | 9 | 174 | 401 | 9 | 229 | Tyr recombinase domain-containing protein | Tyr recombinase domain-containing protein | | afdb-uniprot50 | AF-A0A2I8S603-F1-MODEL\_V4 | 1.0 | 1.635e-07 | 304 | 0.166 | 253 | 168 | 13 | 171 | 402 | 10 | 240 | Tyr recombinase domain-containing protein | Tyr recombinase domain-containing protein | | afdb-uniprot50 | AF-A0A433WR77-F1-MODEL\_V4 | 1.0 | 1.288e-07 | 304 | 0.179 | 295 | 190 | 13 | 119 | 403 | 14 | 266 | Uncharacterized protein | Uncharacterized protein | | afdb-uniprot50 | AF-A0A3M2QIT5-F1-MODEL\_V4 | 1.0 | 1.03e-08 | 304 | 0.164 | 384 | 258 | 18 | 36 | 400 | 5 | 344 | Site-specific integrase | Site-specific integrase | | afdb-uniprot50 | AF-A0A285GAL8-F1-MODEL\_V4 | 1.0 | 1.308e-08 | 303 | 0.174 | 356 | 229 | 19 | 48 | 386 | 67 | 374 | Site-specific recombinase XerD | Site-specific recombinase XerD | | afdb-uniprot50 | AF-A0A7C6W9D4-F1-MODEL\_V4 | 1.0 | 2.008e-08 | 302 | 0.15 | 351 | 232 | 16 | 48 | 386 | 62 | 358 | Tyrosine-type recombinase/integrase | Tyrosine-type recombinase/integrase | | afdb-uniprot50 | AF-A0A223MSG3-F1-MODEL\_V4 | 1.0 | 1.582e-08 | 301 | 0.17 | 364 | 251 | 19 | 45 | 393 | 21 | 348 | Tyr recombinase domain-containing protein | Tyr recombinase domain-containing protein | | afdb-uniprot50 | AF-A0A1F6LJM3-F1-MODEL\_V4 | 1.0 | 2.713e-09 | 301 | 0.17 | 387 | 260 | 20 | 36 | 401 | 6 | 352 | Uncharacterized protein | Uncharacterized protein | | afdb-uniprot50 | AF-A0A7X6WJT0-F1-MODEL\_V4 | 1.0 | 1.66e-08 | 300 | 0.13 | 375 | 254 | 22 | 45 | 393 | 23 | 351 | Tyrosine-type recombinase/integrase | Tyrosine-type recombinase/integrase | | afdb-uniprot50 | AF-A0A382T058-F1-MODEL\_V4 | 1.0 | 2.176e-07 | 297 | 0.191 | 324 | 212 | 17 | 69 | 371 | 2 | 296 | Tyr recombinase domain-containing protein | Tyr recombinase domain-containing protein | | afdb-uniprot50 | AF-A0A7U8TD11-F1-MODEL\_V4 | 1.0 | 2.713e-09 | 296 | 0.208 | 316 | 188 | 16 | 2 | 288 | 3 | 285 | Integrase arm-type DNA-binding domain-containing protein | Integrase arm-type DNA-binding domain-containing protein | | afdb-uniprot50 | AF-A0A538PJK5-F1-MODEL\_V4 | 1.0 | 2.713e-09 | 296 | 0.144 | 423 | 259 | 25 | 14 | 402 | 1 | 354 | Uncharacterized protein | Uncharacterized protein | | afdb-uniprot50 | AF-A0A5B9Q349-F1-MODEL\_V4 | 1.0 | 3.084e-08 | 296 | 0.158 | 409 | 234 | 16 | 46 | 387 | 15 | 380 | Site-specific tyrosine recombinase XerC | Site-specific tyrosine recombinase XerC | | afdb-uniprot50 | AF-A0A518HJQ9-F1-MODEL\_V4 | 1.0 | 2.762e-07 | 294 | 0.162 | 301 | 214 | 17 | 96 | 386 | 2 | 274 | Tyrosine recombinase XerD | Tyrosine recombinase XerD | | afdb-uniprot50 | AF-A0A521U8H4-F1-MODEL\_V4 | 1.0 | 1.065e-07 | 294 | 0.172 | 336 | 223 | 20 | 90 | 403 | 24 | 326 | Uncharacterized protein | Uncharacterized protein | | afdb-uniprot50 | AF-A0A5Q4H1X5-F1-MODEL\_V4 | 1.0 | 1.065e-07 | 294 | 0.186 | 290 | 190 | 16 | 111 | 382 | 2 | 263 | Integrase | Integrase | | afdb-uniprot50 | AF-A0A6M4YDB8-F1-MODEL\_V4 | 1.0 | 5.303e-06 | 291 | 0.187 | 176 | 131 | 6 | 231 | 400 | 2 | 171 | Tyr recombinase domain-containing protein | Tyr recombinase domain-containing protein | | afdb-uniprot50 | AF-A0A2W1JGV9-F1-MODEL\_V4 | 1.0 | 1.21e-06 | 291 | 0.19 | 205 | 142 | 6 | 197 | 395 | 1 | 187 | Tyrosine recombinase XerC | Tyrosine recombinase XerC | | afdb-uniprot50 | AF-A0A359DB79-F1-MODEL\_V4 | 1.0 | 9.229e-08 | 291 | 0.208 | 249 | 161 | 10 | 169 | 402 | 45 | 272 | Tyr recombinase domain-containing protein | Tyr recombinase domain-containing protein | | afdb-uniprot50 | AF-K8ZZH4-F1-MODEL\_V4 | 1.0 | 7.403e-06 | 290 | 0.195 | 184 | 137 | 7 | 225 | 403 | 22 | 199 | Site-specific recombinase, phage integrase family | Site-specific recombinase, phage integrase family | | afdb-uniprot50 | AF-A0A518G3G5-F1-MODEL\_V4 | 1.0 | 2.283e-07 | 289 | 0.175 | 297 | 197 | 16 | 105 | 382 | 10 | 277 | Tyrosine recombinase XerD | Tyrosine recombinase XerD | | afdb-uniprot50 | AF-A0A4P7BT87-F1-MODEL\_V4 | 1.0 | 1.189e-08 | 288 | 0.18 | 271 | 175 | 13 | 2 | 256 | 3 | 242 | DUF4102 domain-containing protein | DUF4102 domain-containing protein | | afdb-uniprot50 | AF-A0A3S4GT40-F1-MODEL\_V4 | 1.0 | 1.308e-08 | 288 | 0.199 | 291 | 182 | 15 | 2 | 274 | 11 | 268 | Integrase | Integrase | | afdb-uniprot50 | AF-A0A3D5W661-F1-MODEL\_V4 | 1.0 | 9.679e-08 | 287 | 0.18 | 326 | 193 | 12 | 92 | 401 | 6 | 273 | Tyr recombinase domain-containing protein | Tyr recombinase domain-containing protein | | afdb-uniprot50 | AF-A0A375A8W5-F1-MODEL\_V4 | 1.0 | 2.713e-09 | 287 | 0.186 | 321 | 196 | 18 | 2 | 289 | 25 | 313 | Mobile element protein | Mobile element protein | | afdb-uniprot50 | AF-A0A3N5K3C0-F1-MODEL\_V4 | 1.0 | 1.081e-08 | 287 | 0.15 | 404 | 241 | 22 | 17 | 398 | 2 | 325 | Site-specific integrase | Site-specific integrase | | afdb-uniprot50 | AF-A0A0P1I3C9-F1-MODEL\_V4 | 1.0 | 1.853e-09 | 284 | 0.151 | 408 | 279 | 21 | 1 | 378 | 3 | 373 | Uncharacterized protein | Uncharacterized protein | | afdb-uniprot50 | AF-A0A7G7EQR9-F1-MODEL\_V4 | 1.0 | 3.342e-07 | 283 | 0.158 | 322 | 230 | 14 | 99 | 403 | 26 | 323 | Tyrosine-type recombinase/integrase | Tyrosine-type recombinase/integrase | | afdb-uniprot50 | AF-A0A1I4UQD3-F1-MODEL\_V4 | 1.0 | 4.178e-06 | 281 | 0.177 | 180 | 139 | 5 | 228 | 402 | 2 | 177 | Phage integrase family protein | Phage integrase family protein | | afdb-uniprot50 | AF-A0A6L9LYB1-F1-MODEL\_V4 | 1.0 | 1.331e-06 | 279 | 0.168 | 326 | 205 | 15 | 100 | 402 | 6 | 288 | Tyrosine-type recombinase/integrase | Tyrosine-type recombinase/integrase | | afdb-uniprot50 | AF-A0A1Y5TZF1-F1-MODEL\_V4 | 1.0 | 1.509e-08 | 279 | 0.183 | 386 | 255 | 20 | 46 | 401 | 17 | 372 | Tyrosine recombinase XerC | Tyrosine recombinase XerC | | afdb-uniprot50 | AF-A0A0F2Q7Y2-F1-MODEL\_V4 | 1.0 | 2.854e-06 | 278 | 0.165 | 236 | 166 | 8 | 169 | 394 | 10 | 224 | Tyr recombinase domain-containing protein | Tyr recombinase domain-containing protein | | afdb-uniprot50 | AF-A0A3B0WP27-F1-MODEL\_V4 | 1.0 | 3.443e-09 | 278 | 0.135 | 413 | 290 | 23 | 3 | 393 | 8 | 375 | Tyr recombinase domain-containing protein | Tyr recombinase domain-containing protein | | afdb-uniprot50 | AF-A0A5M6HN79-F1-MODEL\_V4 | 1.0 | 7.742e-09 | 277 | 0.147 | 460 | 273 | 22 | 36 | 402 | 19 | 452 | Tyrosine-type recombinase/integrase | Tyrosine-type recombinase/integrase | | afdb-uniprot50 | AF-A0A7I9NUR5-F1-MODEL\_V4 | 1.0 | 5.303e-06 | 274 | 0.205 | 190 | 132 | 7 | 231 | 403 | 3 | 190 | Tyrosine-type recombinase/integrase | Tyrosine-type recombinase/integrase | | afdb-uniprot50 | AF-A0A6G7ZA35-F1-MODEL\_V4 | 1.0 | 4.515e-08 | 274 | 0.143 | 370 | 252 | 25 | 36 | 387 | 3 | 325 | Site-specific integrase | Site-specific integrase | | afdb-uniprot50 | AF-A0A3C2D4R1-F1-MODEL\_V4 | 1.0 | 4.967e-08 | 273 | 0.124 | 427 | 277 | 20 | 14 | 402 | 8 | 375 | Uncharacterized protein | Uncharacterized protein | | afdb-uniprot50 | AF-A0A2J7Y8I7-F1-MODEL\_V4 | 1.0 | 1.587e-05 | 272 | 0.222 | 166 | 124 | 3 | 241 | 402 | 2 | 166 | Prophage CP4-57 integrase | Prophage CP4-57 integrase | | afdb-uniprot50 | AF-A0A523IFE9-F1-MODEL\_V4 | 1.0 | 1.858e-06 | 272 | 0.208 | 235 | 166 | 7 | 169 | 400 | 4 | 221 | Site-specific integrase | Site-specific integrase | | afdb-uniprot50 | AF-A0A7C4XZS9-F1-MODEL\_V4 | 1.0 | 2.144e-06 | 271 | 0.26 | 192 | 121 | 10 | 195 | 376 | 2 | 182 | Site-specific integrase | Site-specific integrase | | afdb-uniprot50 | AF-A0A2V7QVN5-F1-MODEL\_V4 | 1.0 | 3.622e-06 | 271 | 0.15 | 318 | 212 | 12 | 100 | 403 | 1 | 274 | Tyr recombinase domain-containing protein | Tyr recombinase domain-containing protein | | afdb-uniprot50 | AF-A0A2E9PKL6-F1-MODEL\_V4 | 1.0 | 2.594e-06 | 270 | 0.19 | 236 | 158 | 10 | 178 | 401 | 2 | 216 | Tyr recombinase domain-containing protein | Tyr recombinase domain-containing protein | | afdb-uniprot50 | AF-A0A520LCH2-F1-MODEL\_V4 | 1.0 | 3.731e-08 | 270 | 0.154 | 435 | 263 | 23 | 13 | 393 | 4 | 387 | Core-binding (CB) domain-containing protein | Core-binding (CB) domain-containing protein | | afdb-uniprot50 | AF-A0A0P7LNL0-F1-MODEL\_V4 | 1.0 | 2.897e-07 | 269 | 0.162 | 259 | 177 | 14 | 9 | 252 | 12 | 245 | Integrase | Integrase | | afdb-uniprot50 | AF-A0A2M6ZPB3-F1-MODEL\_V4 | 1.0 | 2.713e-09 | 269 | 0.158 | 397 | 251 | 26 | 37 | 402 | 29 | 373 | Site-specific integrase | Site-specific integrase | | afdb-uniprot50 | AF-A0A223MSP1-F1-MODEL\_V4 | 1.0 | 1.049e-06 | 268 | 0.161 | 266 | 177 | 13 | 146 | 398 | 10 | 242 | Tyr recombinase domain-containing protein | Tyr recombinase domain-containing protein | | afdb-uniprot50 | AF-A0A562IZE3-F1-MODEL\_V4 | 1.0 | 1.417e-07 | 261 | 0.205 | 268 | 170 | 12 | 1 | 237 | 8 | 263 | Uncharacterized protein | Uncharacterized protein | | afdb-uniprot50 | AF-A0A1Y4HE01-F1-MODEL\_V4 | 1.0 | 1.015e-07 | 261 | 0.153 | 411 | 259 | 20 | 1 | 393 | 52 | 391 | Tyr recombinase domain-containing protein | Tyr recombinase domain-containing protein | | afdb-uniprot50 | AF-A0A1E4X750-F1-MODEL\_V4 | 1.0 | 1.509e-08 | 261 | 0.148 | 430 | 263 | 25 | 46 | 403 | 10 | 408 | Tyr recombinase domain-containing protein | Tyr recombinase domain-containing protein | | afdb-uniprot50 | AF-A0A8B3HTI2-F1-MODEL\_V4 | 1.0 | 1.417e-07 | 259 | 0.221 | 235 | 139 | 12 | 2 | 224 | 3 | 205 | DUF4102 domain-containing protein | DUF4102 domain-containing protein | | afdb-uniprot50 | AF-A0A1G7SLZ0-F1-MODEL\_V4 | 1.0 | 9.092e-07 | 259 | 0.198 | 232 | 144 | 13 | 181 | 402 | 2 | 201 | Phage integrase family protein | Phage integrase family protein | | afdb-uniprot50 | AF-A0A259CZR1-F1-MODEL\_V4 | 1.0 | 5.383e-07 | 259 | 0.193 | 243 | 156 | 13 | 171 | 402 | 28 | 241 | Integrase | Integrase | | afdb-uniprot50 | AF-A0A156H2Y6-F1-MODEL\_V4 | 1.0 | 7.627e-08 | 258 | 0.204 | 274 | 175 | 13 | 2 | 261 | 3 | 247 | Phage integrase family protein | Phage integrase family protein | | afdb-uniprot50 | AF-W2TX06-F1-MODEL\_V4 | 1.0 | 3.139e-06 | 258 | 0.175 | 239 | 164 | 10 | 169 | 396 | 4 | 220 | Site-specific recombinase, phage integrase family | Site-specific recombinase, phage integrase family | | afdb-uniprot50 | AF-A0A0H3I6J4-F1-MODEL\_V4 | 1.0 | 6.303e-08 | 257 | 0.188 | 292 | 189 | 16 | 2 | 276 | 3 | 263 | Prophage P4 integrase | Prophage P4 integrase | | afdb-uniprot50 | AF-A0A8A3NBE6-F1-MODEL\_V4 | 1.0 | 2.317e-08 | 257 | 0.185 | 394 | 239 | 17 | 2 | 339 | 26 | 393 | Integrase family protein | Integrase family protein | | afdb-uniprot50 | AF-A0A150J908-F1-MODEL\_V4 | 1.0 | 3.292e-06 | 256 | 0.148 | 290 | 200 | 13 | 107 | 386 | 2 | 254 | Putative tyrosine recombinase XerC-like protein | Putative tyrosine recombinase XerC-like protein | | afdb-uniprot50 | AF-A0A836V0C8-F1-MODEL\_V4 | 1.0 | 5.383e-07 | 256 | 0.143 | 293 | 207 | 15 | 2 | 279 | 9 | 272 | DUF4102 domain-containing protein | DUF4102 domain-containing protein | | afdb-uniprot50 | AF-A0A828R5W3-F1-MODEL\_V4 | 1.0 | 3.038e-07 | 255 | 0.217 | 235 | 140 | 12 | 2 | 224 | 3 | 205 | Integrase domain protein | Integrase domain protein | | afdb-uniprot50 | AF-A0A5F1DSR0-F1-MODEL\_V4 | 1.0 | 5.73e-08 | 254 | 0.195 | 292 | 182 | 16 | 2 | 274 | 3 | 260 | Integrase | Integrase | | afdb-uniprot50 | AF-A0A0C1H784-F1-MODEL\_V4 | 1.0 | 3.402e-05 | 252 | 0.159 | 169 | 130 | 6 | 221 | 382 | 39 | 202 | Virulence plasmid integrase pGP8-D | Virulence plasmid integrase pGP8-D | | afdb-uniprot50 | AF-A0A4R7C7L2-F1-MODEL\_V4 | 1.0 | 7.272e-08 | 252 | 0.161 | 341 | 216 | 19 | 2 | 317 | 8 | 303 | Integrase | Integrase | | afdb-uniprot50 | AF-A0A7X1NZC6-F1-MODEL\_V4 | 1.0 | 3.676e-07 | 252 | 0.148 | 376 | 252 | 17 | 49 | 401 | 11 | 341 | Tyrosine-type recombinase/integrase | Tyrosine-type recombinase/integrase | | afdb-uniprot50 | AF-A0A2E1ZBG2-F1-MODEL\_V4 | 1.0 | 1.065e-07 | 252 | 0.145 | 448 | 281 | 21 | 18 | 400 | 2 | 412 | Tyr recombinase domain-containing protein | Tyr recombinase domain-containing protein | | afdb-uniprot50 | AF-A0A845H3L3-F1-MODEL\_V4 | 1.0 | 1.171e-07 | 251 | 0.207 | 227 | 145 | 11 | 19 | 235 | 23 | 224 | Integrase arm-type DNA-binding domain-containing protein | Integrase arm-type DNA-binding domain-containing protein | | afdb-uniprot50 | AF-A0A109BW84-F1-MODEL\_V4 | 1.0 | 5.562e-06 | 249 | 0.173 | 254 | 182 | 10 | 148 | 392 | 4 | 238 | Integrase | Integrase | | afdb-uniprot50 | AF-A0A518I3J3-F1-MODEL\_V4 | 1.0 | 1.171e-07 | 249 | 0.17 | 370 | 235 | 19 | 46 | 402 | 15 | 325 | Site-specific tyrosine recombinase XerC | Site-specific tyrosine recombinase XerC | | afdb-uniprot50 | AF-A0A2A3TB72-F1-MODEL\_V4 | 1.0 | 1.331e-06 | 248 | 0.115 | 345 | 244 | 19 | 74 | 398 | 4 | 307 | Tyr recombinase domain-containing protein | Tyr recombinase domain-containing protein | | afdb-uniprot50 | AF-V7IKX7-F1-MODEL\_V4 | 1.0 | 2.511e-07 | 244 | 0.181 | 281 | 181 | 14 | 2 | 265 | 3 | 251 | Site-specific recombinase, phage integrase family | Site-specific recombinase, phage integrase family | | afdb-uniprot50 | AF-A0A0F9MB95-F1-MODEL\_V4 | 1.0 | 5.747e-05 | 243 | 0.166 | 180 | 136 | 5 | 221 | 393 | 11 | 183 | Tyr recombinase domain-containing protein | Tyr recombinase domain-containing protein | | afdb-uniprot50 | AF-A0A376MQW7-F1-MODEL\_V4 | 1.0 | 1.065e-07 | 239 | 0.221 | 235 | 139 | 12 | 2 | 224 | 3 | 205 | Phage integrase family site specific recombinase | Phage integrase family site specific recombinase | | afdb-uniprot50 | AF-A0A327LWG9-F1-MODEL\_V4 | 1.0 | 2.215e-05 | 238 | 0.204 | 186 | 126 | 7 | 217 | 382 | 5 | 188 | Tyr recombinase domain-containing protein | Tyr recombinase domain-containing protein | | afdb-uniprot50 | AF-A0A7U9B7Q9-F1-MODEL\_V4 | 1.0 | 2.762e-07 | 237 | 0.206 | 233 | 141 | 13 | 2 | 224 | 3 | 201 | Int | Int | | afdb-uniprot50 | AF-A0A5C9B685-F1-MODEL\_V4 | 1.0 | 3.505e-07 | 237 | 0.174 | 343 | 216 | 17 | 46 | 386 | 15 | 292 | Core-binding (CB) domain-containing protein | Core-binding (CB) domain-containing protein | | afdb-uniprot50 | AF-A0A4R3UKN9-F1-MODEL\_V4 | 1.0 | 9.092e-07 | 236 | 0.182 | 236 | 155 | 13 | 2 | 227 | 8 | 215 | Uncharacterized protein DUF4102 | Uncharacterized protein DUF4102 | | afdb-uniprot50 | AF-A0A0M1I116-F1-MODEL\_V4 | 1.0 | 1.92e-05 | 234 | 0.2 | 260 | 156 | 12 | 149 | 402 | 9 | 222 | Tyr recombinase domain-containing protein | Tyr recombinase domain-containing protein | | afdb-uniprot50 | AF-A0A7U4R041-F1-MODEL\_V4 | 1.0 | 1.033e-05 | 234 | 0.168 | 273 | 187 | 10 | 144 | 403 | 10 | 255 | Tyr recombinase domain-containing protein | Tyr recombinase domain-containing protein | | afdb-uniprot50 | AF-A0A3E0ILK8-F1-MODEL\_V4 | 1.0 | 1.587e-05 | 234 | 0.141 | 282 | 205 | 10 | 138 | 403 | 9 | 269 | Site-specific integrase | Site-specific integrase | | afdb-uniprot50 | AF-A0A750P3X5-F1-MODEL\_V4 | 1.0 | 1.664e-05 | 233 | 0.191 | 193 | 130 | 11 | 2 | 186 | 3 | 177 | DUF4102 domain-containing protein | DUF4102 domain-containing protein | | afdb-uniprot50 | AF-A0A5M6ILP2-F1-MODEL\_V4 | 1.0 | 1.831e-05 | 232 | 0.227 | 180 | 128 | 6 | 223 | 397 | 85 | 258 | Site-specific integrase | Site-specific integrase | | afdb-uniprot50 | AF-A0A352UWS7-F1-MODEL\_V4 | 1.0 | 1.886e-07 | 231 | 0.217 | 230 | 142 | 11 | 2 | 224 | 1 | 199 | Core-binding (CB) domain-containing protein | Core-binding (CB) domain-containing protein | | afdb-uniprot50 | AF-A0A7C1YJJ2-F1-MODEL\_V4 | 1.0 | 3.676e-07 | 231 | 0.199 | 251 | 161 | 14 | 2 | 246 | 9 | 225 | DUF4102 domain-containing protein | DUF4102 domain-containing protein | | afdb-uniprot50 | AF-A0A4S5BUU1-F1-MODEL\_V4 | 1.0 | 2.283e-07 | 231 | 0.16 | 412 | 214 | 21 | 9 | 402 | 17 | 314 | Site-specific integrase | Site-specific integrase | | afdb-uniprot50 | AF-A0A4R3L278-F1-MODEL\_V4 | 1.0 | 6.831e-07 | 230 | 0.185 | 221 | 145 | 12 | 2 | 216 | 3 | 194 | Uncharacterized protein DUF4102 | Uncharacterized protein DUF4102 | | afdb-uniprot50 | AF-A0A7G1LGL0-F1-MODEL\_V4 | 1.0 | 1.978e-07 | 230 | 0.137 | 458 | 272 | 24 | 36 | 402 | 4 | 429 | Uncharacterized protein | Uncharacterized protein | | afdb-uniprot50 | AF-A0A7J4TFH4-F1-MODEL\_V4 | 1.0 | 3.292e-06 | 229 | 0.156 | 332 | 205 | 15 | 83 | 400 | 7 | 277 | Tyrosine-type recombinase/integrase | Tyrosine-type recombinase/integrase | | afdb-uniprot50 | AF-A0A0X3UBE4-F1-MODEL\_V4 | 1.0 | 1.92e-05 | 226 | 0.132 | 294 | 184 | 13 | 146 | 393 | 11 | 279 | Tyr recombinase domain-containing protein | Tyr recombinase domain-containing protein | | afdb-uniprot50 | AF-A0A3L5H774-F1-MODEL\_V4 | 1.0 | 7.272e-08 | 225 | 0.188 | 335 | 191 | 21 | 2 | 290 | 3 | 302 | ISAs1 family transposase | ISAs1 family transposase | | afdb-uniprot50 | AF-A0A376SEM2-F1-MODEL\_V4 | 1.0 | 3.292e-06 | 223 | 0.173 | 253 | 177 | 11 | 36 | 275 | 3 | 236 | Integrase | Integrase | | afdb-uniprot50 | AF-U3TY72-F1-MODEL\_V4 | 1.0 | 1.1e-06 | 221 | 0.179 | 273 | 175 | 15 | 2 | 256 | 3 | 244 | Integrase | Integrase | | afdb-uniprot50 | AF-A0A450YSF1-F1-MODEL\_V4 | 1.0 | 5.833e-06 | 220 | 0.152 | 361 | 222 | 18 | 60 | 395 | 3 | 304 | Site-specific recombinase XerD | Site-specific recombinase XerD | | afdb-uniprot50 | AF-A0A1X3L5U9-F1-MODEL\_V4 | 1.0 | 2.358e-06 | 217 | 0.18 | 199 | 133 | 9 | 34 | 224 | 2 | 178 | Integrase | Integrase | | afdb-uniprot50 | AF-A0A7W1CIH9-F1-MODEL\_V4 | 1.0 | 1.137e-05 | 215 | 0.147 | 339 | 226 | 17 | 80 | 382 | 3 | 314 | Site-specific integrase | Site-specific integrase | | afdb-uniprot50 | AF-A0A7X2D969-F1-MODEL\_V4 | 1.0 | 0.000164 | 214 | 0.203 | 192 | 144 | 7 | 207 | 392 | 10 | 198 | Tyrosine-type recombinase/integrase | Tyrosine-type recombinase/integrase | | afdb-uniprot50 | AF-U9Z089-F1-MODEL\_V4 | 1.0 | 5.303e-06 | 213 | 0.198 | 227 | 142 | 11 | 36 | 245 | 3 | 206 | Core-binding (CB) domain-containing protein | Core-binding (CB) domain-containing protein | | afdb-uniprot50 | AF-A0A267T8A9-F1-MODEL\_V4 | 1.0 | 0.0003196 | 211 | 0.19 | 173 | 128 | 7 | 207 | 373 | 18 | 184 | Recombinase XerC | Recombinase XerC | | afdb-uniprot50 | AF-A0A7X5NGS5-F1-MODEL\_V4 | 1.0 | 8.022e-05 | 210 | 0.151 | 244 | 174 | 11 | 174 | 403 | 14 | 238 | Tyr recombinase domain-containing protein | Tyr recombinase domain-containing protein | | afdb-uniprot50 | AF-A0A7X7MFU5-F1-MODEL\_V4 | 1.0 | 0.0001175 | 209 | 0.194 | 190 | 133 | 10 | 207 | 386 | 9 | 188 | Tyrosine-type recombinase/integrase | Tyrosine-type recombinase/integrase | | afdb-uniprot50 | AF-Q46428-F1-MODEL\_V4 | 1.0 | 2.437e-05 | 209 | 0.15 | 279 | 199 | 14 | 99 | 358 | 2 | 261 | Tyr recombinase domain-containing protein | Tyr recombinase domain-containing protein | | afdb-uniprot50 | AF-A0A377M8M8-F1-MODEL\_V4 | 1.0 | 4.382e-06 | 208 | 0.15 | 232 | 157 | 12 | 9 | 224 | 12 | 219 | Putative integrase | Putative integrase | | afdb-uniprot50 | AF-A6FQ69-F1-MODEL\_V4 | 1.0 | 6.21e-07 | 207 | 0.153 | 431 | 250 | 22 | 46 | 393 | 11 | 409 | Phage integrase | Phage integrase | | afdb-uniprot50 | AF-V0AM31-F1-MODEL\_V4 | 1.0 | 7.058e-06 | 205 | 0.168 | 231 | 151 | 12 | 34 | 246 | 2 | 209 | Core-binding (CB) domain-containing protein | Core-binding (CB) domain-containing protein | | afdb-uniprot50 | AF-A0A4U6M1J7-F1-MODEL\_V4 | 1.0 | 3.622e-06 | 203 | 0.158 | 234 | 151 | 14 | 2 | 224 | 3 | 201 | DUF4102 domain-containing protein | DUF4102 domain-containing protein | | afdb-uniprot50 | AF-A0A2J4P481-F1-MODEL\_V4 | 1.0 | 0.0001355 | 201 | 0.172 | 185 | 118 | 8 | 174 | 340 | 8 | 175 | Integrase | Integrase | | afdb-uniprot50 | AF-A0A7Z7L533-F1-MODEL\_V4 | 1.0 | 2.474e-06 | 201 | 0.216 | 194 | 126 | 9 | 2 | 187 | 3 | 178 | Phage integrase family site specific recombinase | Phage integrase family site specific recombinase | | afdb-uniprot50 | AF-A0A702LGL0-F1-MODEL\_V4 | 1.0 | 4.821e-06 | 200 | 0.182 | 236 | 154 | 15 | 2 | 224 | 3 | 212 | DUF4102 domain-containing protein | DUF4102 domain-containing protein | | afdb-uniprot50 | AF-A0A381GQ17-F1-MODEL\_V4 | 1.0 | 1.858e-06 | 198 | 0.193 | 238 | 147 | 14 | 2 | 224 | 7 | 214 | Integrase | Integrase | | afdb-uniprot50 | AF-A0A3R7DQE1-F1-MODEL\_V4 | 1.0 | 1.831e-05 | 197 | 0.138 | 303 | 195 | 11 | 106 | 394 | 34 | 284 | Site-specific integrase | Site-specific integrase | | afdb-uniprot50 | AF-P0CE20-F1-MODEL\_V4 | 1.0 | 2.324e-05 | 194 | 0.141 | 347 | 220 | 21 | 77 | 393 | 16 | 314 | Virulence plasmid integrase pGP8-D | Virulence plasmid integrase pGP8-D | | afdb-uniprot50 | AF-A0A7U0UV57-F1-MODEL\_V4 | 1.0 | 5.303e-06 | 191 | 0.119 | 377 | 224 | 20 | 48 | 393 | 54 | 353 | IS200/IS605 family transposase | IS200/IS605 family transposase | | afdb-uniprot50 | AF-A0A7T8FLC7-F1-MODEL\_V4 | 1.0 | 8.541e-06 | 189 | 0.165 | 230 | 154 | 12 | 2 | 224 | 24 | 222 | Integrase arm-type DNA-binding domain-containing protein | Integrase arm-type DNA-binding domain-containing protein | | afdb-uniprot50 | AF-I0QS20-F1-MODEL\_V4 | 1.0 | 9.536e-07 | 188 | 0.17 | 335 | 211 | 23 | 2 | 322 | 3 | 284 | Integrase family protein | Integrase family protein | | afdb-uniprot50 | AF-A0A1Y4HE69-F1-MODEL\_V4 | 1.0 | 0.00154 | 187 | 0.196 | 188 | 131 | 9 | 216 | 393 | 3 | 180 | Tyr recombinase domain-containing protein | Tyr recombinase domain-containing protein | | afdb-uniprot50 | AF-A0A1M6T5P9-F1-MODEL\_V4 | 1.0 | 0.001335 | 187 | 0.192 | 182 | 142 | 4 | 222 | 401 | 11 | 189 | Phage integrase family protein | Phage integrase family protein | | afdb-uniprot50 | AF-A0A2D2DAJ7-F1-MODEL\_V4 | 1.0 | 0.0001804 | 185 | 0.127 | 244 | 174 | 12 | 147 | 370 | 7 | 231 | Integrase | Integrase | | afdb-uniprot50 | AF-A0A509CWS0-F1-MODEL\_V4 | 1.0 | 7.293e-05 | 184 | 0.184 | 201 | 144 | 9 | 2 | 197 | 3 | 188 | Bacteriophage integrase | Bacteriophage integrase | | afdb-uniprot50 | AF-A0A2V8LGT1-F1-MODEL\_V4 | 1.0 | 0.0005398 | 182 | 0.166 | 228 | 165 | 8 | 177 | 396 | 2 | 212 | Tyr recombinase domain-containing protein | Tyr recombinase domain-containing protein | | afdb-uniprot50 | AF-A0A450Z0R5-F1-MODEL\_V4 | 1.0 | 0.0001804 | 177 | 0.139 | 286 | 195 | 11 | 125 | 395 | 2 | 251 | Site-specific recombinase XerD | Site-specific recombinase XerD | | afdb-uniprot50 | AF-A0A3N5YUU7-F1-MODEL\_V4 | 1.0 | 0.0001355 | 176 | 0.122 | 293 | 193 | 20 | 146 | 402 | 12 | 276 | Site-specific integrase | Site-specific integrase | | afdb-uniprot50 | AF-Q46257-F1-MODEL\_V4 | 1.0 | 0.0002401 | 173 | 0.113 | 300 | 209 | 15 | 101 | 371 | 14 | 285 | Virulence plasmid integrase pGP7-D | Virulence plasmid integrase pGP7-D | | afdb-uniprot50 | AF-A0A2D4THJ6-F1-MODEL\_V4 | 1.0 | 0.003463 | 168 | 0.207 | 169 | 128 | 5 | 221 | 386 | 12 | 177 | Tyr recombinase domain-containing protein | Tyr recombinase domain-containing protein | | afdb-uniprot50 | AF-A0A538PKB2-F1-MODEL\_V4 | 1.0 | 0.001955 | 165 | 0.202 | 202 | 137 | 10 | 190 | 382 | 3 | 189 | Tyr recombinase domain-containing protein | Tyr recombinase domain-containing protein | | afdb-uniprot50 | AF-A0A1F6ZB97-F1-MODEL\_V4 | 1.0 | 7.293e-05 | 164 | 0.101 | 363 | 233 | 24 | 104 | 400 | 8 | 343 | Tyr recombinase domain-containing protein | Tyr recombinase domain-containing protein | | afdb-uniprot50 | AF-A0A1T5DBV7-F1-MODEL\_V4 | 1.0 | 7.649e-05 | 157 | 0.151 | 331 | 219 | 17 | 74 | 389 | 9 | 292 | Integrase/recombinase XerC | Integrase/recombinase XerC | | afdb-uniprot50 | AF-A0A0E2IVB2-F1-MODEL\_V4 | 1.0 | 0.003463 | 153 | 0.127 | 172 | 127 | 6 | 228 | 382 | 3 | 168 | Phage integrase family protein | Phage integrase family protein | | afdb-uniprot50 | AF-A0A4Z0R2I2-F1-MODEL\_V4 | 1.0 | 0.00461 | 149 | 0.115 | 182 | 133 | 4 | 221 | 396 | 15 | 174 | Tyr recombinase domain-containing protein | Tyr recombinase domain-containing protein | | afdb-uniprot50 | AF-A0A2S9IKS5-F1-MODEL\_V4 | 1.0 | 0.003809 | 147 | 0.185 | 200 | 137 | 9 | 212 | 400 | 7 | 191 | Integrase | Integrase | | afdb-uniprot50 | AF-A0A0T9T9N2-F1-MODEL\_V4 | 1.0 | 0.004395 | 137 | 0.134 | 171 | 119 | 7 | 50 | 211 | 20 | 170 | Integrase | Integrase | |
| Top keywords  (threshold 1.00e-02 (evalue)) | **Integrase, recombinase, domain\_containing, Tyr, Site\_specific, DUF4102, Tyrosine\_type, Phage, Prophage, Tyrosine** |
| Output files | ../../similar\_structures/32\_FANPEZAQ\_CDS\_0032\_afdb-proteome\_foldseek.tsv ../../similar\_structures/32\_FANPEZAQ\_CDS\_0032\_afdb-uniprot50\_foldseek.tsv ../../similar\_structures/32\_FANPEZAQ\_CDS\_0032\_merged.svg ../../similar\_structures/32\_FANPEZAQ\_CDS\_0032\_pdb\_foldseek.tsv |

  
  
  

Return to summary | Go to previous | Go to next

  


---

**Sequence/structure alignments coloring**  
Each object in the alignment figures is colored according to its E-value following this color coding:

1e-100
10

**References:**  
1) Steinegger M, Meier M, Mirdita M, Vöhringer H, Haunsberger S J, and Söding J (2019) HH-suite3 for fast remote homology detection and deep protein annotation, BMC Bioinformatics, 473. doi: 10.1186/s12859-019-3019-7  
2) Jumper J, Evans R, Pritzel A, ..., Hassabis D (2021) Highly accurate protein structure prediction with AlphaFold, Nature, 596. doi: 10.1038/s41586-021-03819-2  
3) van Kempen M, Kim S, Tumescheit C, Mirdita M, Lee J, Gilchrist CLM, Söding J, and Steinegger M (2023) Fast and accurate protein structure search with Foldseek. Nature Biotechnology. doi: 10.1038/s41587-023-01773-0
