# Supplementary material for: Completing the BASEL phage collection to unlock hidden diversity for systematic exploration of phage–host interactions
Source: PLoS Biol. 2025 Apr 7;23(4):e3003063. doi: 10.1371/journal.pbio.3003063 (PMC11990801; doi:10.1371/journal.pbio.3003063)
Supplement: S2 Data — (ZIP) [file pbio.3003063.s009.zip › entries/34.html]

FANPEZAQ\_CDS\_0034


Return to summary | Go to previous | Go to next

|  |  |
| --- | --- |
| FANPEZAQ\_CDS\_0034 Page creation date: 02 Sep 2024, 12:00  Project folder: n/a  Input sequences file: Escherichia\_virus\_HeidiAbel.gb | sprt\_like domain\_containing fragment sprt acidic repeat\_containing metalloprotease transcription elongation hypothetical zinc\_dependent spartan putative protein\_like with at the n terminus zinc isoform protease hydrolase peptidase m48 metalloproteinase mpr hmg box\_containing metallopeptidase c19g7 minigluzincin capsid |

### Sequence information

|  |  |
| --- | --- |
| Name | FANPEZAQ\_CDS\_0034  34\_FANPEZAQ\_CDS\_0034 (pipeline id) |
| Imported annotations |  |
| Protein sequence | MDREGWLNALAAKFAPRFKEFGYEMPKFRVSVGFCSSGARSSTAAQCWHSKCSSDNTFEI FIMPDQVEPYLVANHLWHELTHATVGFDCGHKGAFATVCKAVGLNGPMTATTPGEKFKEY VKPFLDELGPMPHAKLTFDRGMSIKVPRLRIGLDEGGDDGEGEEIEVAPVGGASTAKPKQ TTRLKKCECQECGYTVRVTQKWLEVGPPHCPEHGAMTPEGDDESSDE |
| Number of residues | 227 |
| Molecular weight (Da) | 24902.92 |
| Output files | ../../query\_sequences/34\_FANPEZAQ\_CDS\_0034.fasta |

### Putative domain architecture and protein family

#### Search results (HHblits)1

|  |  |
| --- | --- |
| Domain family databases searched | Pfam, Ncbi-cd, Cath, Phrogs |
| Results, scheme(s)  (Top layers only; threshold 1.00e-03 (evalue)) | xml version="1.0" encoding="utf-8" standalone="no"?       2024-09-02T21:08:19.725489 image/svg+xml   Matplotlib v3.7.2, https://matplotlib.org/ |
| Results, table  (E-value ≤ 1.00e-03 (evalue)) | | db | id | prob | evalue | pvalue | score | cols | query | query\_len | template | template\_len | name | description | | --- | --- | --- | --- | --- | --- | --- | --- | --- | --- | --- | --- | --- | | phrogs | 6361 | 100.0 | 2.6e-52 | 3.4e-56 | 356.6 | 185 | (1, 220) | 227 | (17, 204) | 204 | NA | NA; Category: unknown function; MG670586\_p45 | | phrogs | 1006 | 99.8 | 1.5e-23 | 1.8e-27 | 168.7 | 75 | (29, 112) | 227 | (29, 106) | 154 | NA | NA; Category: unknown function; JQ512844\_p79 | | phrogs | 10756 | 99.7 | 2.5e-21 | 3e-25 | 166.4 | 109 | (2, 111) | 227 | (4, 122) | 258 | NA | NA; Category: unknown function; NC\_019538\_p220 | | phrogs | 3768 | 99.5 | 3.1e-19 | 3.8e-23 | 148.6 | 73 | (29, 111) | 227 | (33, 108) | 192 | NA | NA; Category: unknown function; NC\_029000\_p177 | | phrogs | 6445 | 99.4 | 4.2e-18 | 5e-22 | 140.2 | 82 | (29, 116) | 227 | (31, 120) | 191 | NA | NA; Category: unknown function; NC\_019401\_p436 | | phrogs | 9897 | 99.4 | 5.8e-18 | 7e-22 | 144.8 | 105 | (2, 111) | 227 | (34, 157) | 248 | NA | NA; Category: unknown function; NC\_029009\_p209 | | phrogs | 27319 | 99.1 | 4.5e-15 | 5e-19 | 121.9 | 108 | (2, 113) | 227 | (44, 159) | 220 | NA | NA; Category: unknown function; NC\_021327\_p65 | | phrogs | 10840 | 99.0 | 5.5e-14 | 6.3e-18 | 116.8 | 76 | (29, 112) | 227 | (40, 118) | 223 | NA | NA; Category: unknown function; NC\_028683\_p287 | | phrogs | 24857 | 98.3 | 1.4e-10 | 1.6e-14 | 92.2 | 72 | (31, 111) | 227 | (32, 108) | 171 | NA | NA; Category: unknown function; AP018399\_p45 | | phrogs | 32550 | 96.4 | 1.2e-05 | 1.3e-09 | 60.1 | 103 | (2, 107) | 227 | (5, 127) | 131 | NA | NA; Category: unknown function; NC\_015251\_p422 | | phrogs | 1233 | 95.0 | 0.00029 | 4e-08 | 51.5 | 41 | (40, 87) | 227 | (32, 72) | 108 | NA | NA; Category: unknown function; p367521 VI\_04587 | | phrogs | 37767 | 94.2 | 0.00097 | 1.1e-07 | 57.9 | 68 | (40, 107) | 227 | (117, 202) | 442 | NA | NA; Category: unknown function; NC\_017972\_p199 | |
| Top keywords  (threshold 1.00e-03 (evalue)) | **MG670586\_p45, JQ512844\_p79, NC\_019538\_p220, NC\_029000\_p177, NC\_019401\_p436, NC\_029009\_p209, NC\_021327\_p65, NC\_028683\_p287, AP018399\_p45, NC\_015251\_p422** |
| Output files | ../../domain\_architecture/34\_FANPEZAQ\_CDS\_0034\_cath.hhr ../../domain\_architecture/34\_FANPEZAQ\_CDS\_0034\_merged.svg ../../domain\_architecture/34\_FANPEZAQ\_CDS\_0034\_ncbi-cd.hhr ../../domain\_architecture/34\_FANPEZAQ\_CDS\_0034\_pfam.hhr ../../domain\_architecture/34\_FANPEZAQ\_CDS\_0034\_phrogs.hhr |

### Identical protein sequences/structures

#### Search results

|  |  |
| --- | --- |
| Protein sequence databases searched | Pdb, Swissprot, Refseq |
| Identical proteins found | Refseq  - QIG65622.1: Hypothetical protein (Salmonella phage PT1) |
| Top keywords | **Hypothetical** |
| Output files | ../../identical\_sequences/34\_FANPEZAQ\_CDS\_0034\_refseq.fasta |

### Similar protein sequences/structures

#### Sequence similarity search results (HHblits)1

|  |  |
| --- | --- |
| Sequence databases searched | Uniclust, Pdb70 |
| Results, scheme(s)  (Top layers only, threshold 1.00e-03 (evalue)) | xml version="1.0" encoding="utf-8" standalone="no"?       2024-09-02T21:08:43.710066 image/svg+xml   Matplotlib v3.7.2, https://matplotlib.org/ |
| Results, table(s)  (threshold 1.00e-03 (evalue)) | | db | id | prob | evalue | pvalue | score | cols | query | query\_len | template | template\_len | name | description | | --- | --- | --- | --- | --- | --- | --- | --- | --- | --- | --- | --- | --- | | uniclust | UniRef100\_A0A024GXC7 | 100.0 | 7.8e-50 | 1.8e-55 | 344.1 | 190 | (1, 225) | 227 | (40, 229) | 259 | Uncharacterized protein | Uncharacterized protein | | uniclust | UniRef100\_A0A031JAB2 | 100.0 | 4.7e-49 | 9.8e-55 | 337.6 | 193 | (1, 225) | 227 | (50, 251) | 293 | Transcription elongation protein SprT | Transcription elongation protein SprT | | uniclust | UniRef100\_A0A088FRV0 | 100.0 | 7.8e-49 | 1.7e-54 | 339.2 | 193 | (1, 226) | 227 | (54, 248) | 280 | SprT-like domain-containing protein | SprT-like domain-containing protein | | uniclust | UniRef100\_A0A2D0W8X1 | 100.0 | 3.8e-39 | 8e-45 | 269.1 | 189 | (1, 227) | 227 | (23, 214) | 218 | Putative transcription elongation protein | Putative transcription elongation protein | | uniclust | UniRef100\_UPI0011A7918A | 100.0 | 1.9e-37 | 3.7e-43 | 264.5 | 204 | (1, 214) | 227 | (93, 303) | 309 | SprT family zinc-dependent metalloprotease | SprT family zinc-dependent metalloprotease | | uniclust | UniRef100\_A0A1C4U4K3 | 100.0 | 7.5e-34 | 1.4e-39 | 250.2 | 187 | (2, 219) | 227 | (256, 446) | 448 | SprT-like family protein | SprT-like family protein | | uniclust | UniRef100\_A0A1C6ST94 | 100.0 | 6.4e-33 | 1.2e-38 | 241.8 | 186 | (1, 218) | 227 | (247, 436) | 439 | SprT-like family protein | SprT-like family protein | | uniclust | UniRef100\_A0A090Y2V6 | 99.9 | 4.1e-32 | 9.4e-38 | 241.0 | 189 | (6, 226) | 227 | (51, 269) | 298 | SprT-like family protein | SprT-like family protein | | uniclust | UniRef100\_A0A2C9WX41 | 99.9 | 7.9e-31 | 1.5e-36 | 219.1 | 217 | (1, 220) | 227 | (1, 219) | 288 | SprT domain-containing protein | SprT domain-containing protein | | uniclust | UniRef100\_A0A257FUX5 | 99.9 | 4.9e-30 | 9.4e-36 | 203.6 | 165 | (1, 201) | 227 | (5, 169) | 171 | Uncharacterized protein | Uncharacterized protein | | uniclust | UniRef100\_A0A498AIE9 | 99.9 | 9.7e-30 | 1.8e-35 | 216.8 | 186 | (1, 218) | 227 | (153, 340) | 342 | SprT-like family protein | SprT-like family protein | | uniclust | UniRef100\_A0A143ZU24 | 99.9 | 2.3e-29 | 4.9e-35 | 216.3 | 177 | (7, 222) | 227 | (22, 223) | 250 | SprT-like family protein | SprT-like family protein | | uniclust | UniRef100\_A0A158S5S8 | 99.9 | 9.9e-28 | 1.9e-33 | 192.0 | 132 | (1, 132) | 227 | (4, 141) | 173 | Transcription elongation protein SprT | Transcription elongation protein SprT | | uniclust | UniRef100\_A0A014MPW4 | 99.9 | 1.8e-27 | 4.1e-33 | 214.6 | 190 | (9, 224) | 227 | (77, 287) | 336 | Uncharacterized protein | Uncharacterized protein | | uniclust | UniRef100\_A0A973MLF9 | 99.9 | 2.4e-27 | 4.4e-33 | 203.5 | 211 | (1, 220) | 227 | (6, 222) | 356 | Uncharacterized protein | Uncharacterized protein | | uniclust | UniRef100\_A0A0F9EIW7 | 99.9 | 3.4e-27 | 7.1e-33 | 186.6 | 128 | (67, 226) | 227 | (11, 138) | 143 | SprT-like domain-containing protein (Fragment) | SprT-like domain-containing protein (Fragment) | | uniclust | UniRef100\_A0A0F9B2V9 | 99.9 | 7.2e-27 | 1.4e-32 | 187.4 | 188 | (1, 226) | 227 | (1, 191) | 192 | SprT-like domain-containing protein (Fragment) | SprT-like domain-containing protein (Fragment) | | uniclust | UniRef100\_A0A661MH10 | 99.9 | 2.1e-26 | 3.8e-32 | 190.7 | 188 | (1, 218) | 227 | (30, 239) | 256 | Uncharacterized protein | Uncharacterized protein | | uniclust | UniRef100\_A0A4Q2J0L1 | 99.9 | 4e-26 | 7.3e-32 | 192.2 | 187 | (1, 218) | 227 | (102, 292) | 295 | Uncharacterized protein | Uncharacterized protein | | uniclust | UniRef100\_UPI0015E46A98 | 99.9 | 4.4e-26 | 8.4e-32 | 181.2 | 139 | (1, 139) | 227 | (12, 150) | 169 | hypothetical protein | hypothetical protein | | uniclust | UniRef100\_A0A1F6D4Y3 | 99.9 | 9.9e-26 | 1.9e-31 | 184.5 | 181 | (1, 223) | 227 | (9, 198) | 207 | SprT-like domain-containing protein | SprT-like domain-containing protein | | uniclust | UniRef100\_UPI001F48BFF7 | 99.9 | 4.6e-25 | 8.4e-31 | 181.7 | 183 | (2, 220) | 227 | (56, 240) | 242 | hypothetical protein | hypothetical protein | | uniclust | UniRef100\_A0A0D8B6L3 | 99.8 | 8.5e-25 | 1.8e-30 | 195.1 | 195 | (8, 224) | 227 | (45, 271) | 326 | SprT-like family | SprT-like family | | uniclust | UniRef100\_A0A1E7JES4 | 99.8 | 1.1e-24 | 2.5e-30 | 193.3 | 171 | (5, 221) | 227 | (47, 227) | 288 | SprT-like domain-containing protein | SprT-like domain-containing protein | | uniclust | UniRef100\_A0A315DXF0 | 99.8 | 3.3e-24 | 6.9e-30 | 175.2 | 136 | (3, 138) | 227 | (21, 161) | 178 | Uncharacterized protein | Uncharacterized protein | | uniclust | UniRef100\_D4Z6N5 | 99.8 | 1.1e-23 | 2e-29 | 161.8 | 126 | (1, 126) | 227 | (4, 135) | 136 | Transcription elongation protein SprT | Transcription elongation protein SprT | | uniclust | UniRef100\_A0A920EZT5 | 99.8 | 8.9e-23 | 1.7e-28 | 155.3 | 108 | (2, 109) | 227 | (9, 116) | 123 | Uncharacterized protein | Uncharacterized protein | | uniclust | UniRef100\_A0A010RP43 | 99.8 | 1.1e-22 | 2.8e-28 | 188.1 | 201 | (7, 224) | 227 | (77, 318) | 356 | Zinc metalloprotease | Zinc metalloprotease | | uniclust | UniRef100\_A0A090SJ74 | 99.8 | 2.3e-22 | 4.9e-28 | 176.4 | 203 | (8, 223) | 227 | (16, 266) | 291 | Zinc metalloproteinase Mpr protein | Zinc metalloproteinase Mpr protein | | uniclust | UniRef100\_A0A6G4A0P7 | 99.8 | 3.8e-22 | 7.5e-28 | 168.8 | 191 | (9, 221) | 227 | (5, 225) | 233 | SprT-like domain-containing protein | SprT-like domain-containing protein | | uniclust | UniRef100\_A0A1C6IQ91 | 99.8 | 9.4e-22 | 1.8e-27 | 165.3 | 178 | (3, 222) | 227 | (3, 206) | 236 | SprT-like family | SprT-like family | | uniclust | UniRef100\_A0A1C5HCK4 | 99.7 | 6.2e-21 | 1.2e-26 | 171.0 | 172 | (15, 222) | 227 | (39, 235) | 382 | SprT-like family protein | SprT-like family protein | | uniclust | UniRef100\_A0A644ZG81 | 99.7 | 7.6e-21 | 1.6e-26 | 156.4 | 135 | (57, 223) | 227 | (11, 165) | 171 | Uncharacterized protein | Uncharacterized protein | | uniclust | UniRef100\_A0A0A0HYB9 | 99.7 | 1.2e-20 | 2.7e-26 | 165.5 | 178 | (7, 221) | 227 | (12, 212) | 241 | SprT-like domain-containing protein | SprT-like domain-containing protein | | uniclust | UniRef100\_A0A4R0JGF1 | 99.7 | 3.4e-20 | 6.6e-26 | 163.6 | 168 | (17, 222) | 227 | (163, 353) | 355 | SprT-like family protein | SprT-like family protein | | uniclust | UniRef100\_A0A0D7CF42 | 99.7 | 3.2e-20 | 7.2e-26 | 161.6 | 182 | (5, 224) | 227 | (28, 233) | 237 | SprT-like domain-containing protein | SprT-like domain-containing protein | | uniclust | UniRef100\_A0A401QRK3 | 99.7 | 7.5e-20 | 1.6e-25 | 160.8 | 213 | (9, 226) | 227 | (28, 288) | 290 | SprT-like domain-containing protein | SprT-like domain-containing protein | | uniclust | UniRef100\_A0A3N5NIA4 | 99.6 | 7.2e-19 | 1.3e-24 | 136.0 | 123 | (2, 126) | 227 | (17, 140) | 142 | Uncharacterized protein (Fragment) | Uncharacterized protein (Fragment) | | uniclust | UniRef100\_A0A259FRY7 | 99.6 | 8.8e-18 | 1.6e-23 | 121.8 | 86 | (2, 87) | 227 | (6, 91) | 93 | SprT domain-containing protein (Fragment) | SprT domain-containing protein (Fragment) | | uniclust | UniRef100\_UPI002354798D | 99.5 | 1.3e-17 | 2.4e-23 | 126.7 | 111 | (1, 113) | 227 | (2, 118) | 124 | SprT-like domain-containing protein | SprT-like domain-containing protein | | uniclust | UniRef100\_A0A9E2ZVF6 | 99.5 | 6.9e-17 | 1.3e-22 | 138.1 | 170 | (16, 220) | 227 | (102, 290) | 292 | J domain-containing protein | J domain-containing protein | | uniclust | UniRef100\_UPI002280EFAF | 99.5 | 8.9e-17 | 1.6e-22 | 139.4 | 187 | (2, 219) | 227 | (109, 329) | 331 | SprT-like domain-containing protein | SprT-like domain-containing protein | | uniclust | UniRef100\_D0ZHK0 | 99.5 | 1.5e-16 | 2.8e-22 | 138.7 | 98 | (12, 114) | 227 | (119, 233) | 347 | Putative zinc metalloproteinase Mpr protein | Putative zinc metalloproteinase Mpr protein | | uniclust | UniRef100\_A0A0F9G8P3 | 99.5 | 1.6e-16 | 3.3e-22 | 119.7 | 83 | (108, 222) | 227 | (1, 84) | 98 | Uncharacterized protein | Uncharacterized protein | | uniclust | UniRef100\_A0A0S3TZF6 | 99.4 | 3e-16 | 6.4e-22 | 134.7 | 169 | (7, 222) | 227 | (10, 200) | 211 | SprT-like domain-containing protein | SprT-like domain-containing protein | | uniclust | UniRef100\_A0A011SPG8 | 99.4 | 3.1e-16 | 6.6e-22 | 135.6 | 96 | (29, 139) | 227 | (52, 150) | 232 | SprT-like domain-containing protein | SprT-like domain-containing protein | | uniclust | UniRef100\_UPI002162A182 | 99.4 | 4.1e-16 | 7.6e-22 | 135.1 | 168 | (7, 212) | 227 | (5, 202) | 325 | SprT-like domain-containing protein | SprT-like domain-containing protein | | uniclust | UniRef100\_UPI0013579F19 | 99.4 | 8.2e-16 | 1.5e-21 | 141.5 | 187 | (7, 223) | 227 | (5, 214) | 593 | SprT-like domain-containing protein | SprT-like domain-containing protein | | uniclust | UniRef100\_A0A7X6P1E1 | 99.4 | 8.7e-16 | 1.7e-21 | 138.7 | 192 | (13, 221) | 227 | (25, 236) | 420 | SprT-like domain-containing protein | SprT-like domain-containing protein | | uniclust | UniRef100\_UPI002108714C | 99.4 | 1.2e-15 | 2.2e-21 | 130.2 | 169 | (7, 220) | 227 | (85, 278) | 279 | SprT-like domain-containing protein | SprT-like domain-containing protein | | uniclust | UniRef100\_A0A068F4D8 | 99.4 | 1.6e-15 | 3.7e-21 | 135.6 | 73 | (29, 110) | 227 | (79, 154) | 268 | SprT-like protease | SprT-like protease | | uniclust | UniRef100\_A0A970D5F1 | 99.4 | 1.9e-15 | 3.8e-21 | 126.8 | 177 | (7, 224) | 227 | (14, 214) | 215 | SprT-like domain-containing protein | SprT-like domain-containing protein | | uniclust | UniRef100\_A0A451CCI8 | 99.4 | 2.3e-15 | 4.6e-21 | 124.7 | 58 | (57, 114) | 227 | (18, 83) | 183 | SprT-like family protein | SprT-like family protein | | uniclust | UniRef100\_A0A3D0Z8I8 | 99.4 | 2.8e-15 | 5.2e-21 | 129.9 | 170 | (7, 220) | 227 | (118, 316) | 317 | SprT-like domain-containing protein | SprT-like domain-containing protein | | uniclust | UniRef100\_A0A6H9YBG4 | 99.4 | 3e-15 | 5.6e-21 | 129.4 | 100 | (10, 114) | 227 | (10, 126) | 311 | SprT family zinc-dependent metalloprotease | SprT family zinc-dependent metalloprotease | | uniclust | UniRef100\_A0A1G7EMH9 | 99.4 | 2.8e-15 | 5.9e-21 | 126.1 | 108 | (7, 122) | 227 | (23, 143) | 190 | SprT-like family protein | SprT-like family protein | | uniclust | UniRef100\_A0A0Q8UQ63 | 99.3 | 3.3e-15 | 6.1e-21 | 119.1 | 132 | (2, 136) | 227 | (19, 151) | 165 | SprT-like domain-containing protein | SprT-like domain-containing protein | | uniclust | UniRef100\_A0A292IIQ0 | 99.3 | 3.4e-15 | 6.8e-21 | 129.4 | 174 | (7, 222) | 227 | (17, 213) | 248 | SprT-like domain-containing protein | SprT-like domain-containing protein | | uniclust | UniRef100\_A0A0F0KVC3 | 99.3 | 4.6e-15 | 1e-20 | 133.2 | 160 | (41, 223) | 227 | (72, 266) | 292 | SprT-like family protein | SprT-like family protein | | uniclust | UniRef100\_A0A966KII7 | 99.3 | 6.4e-15 | 1.2e-20 | 118.7 | 114 | (72, 221) | 227 | (13, 126) | 157 | Uncharacterized protein | Uncharacterized protein | | uniclust | UniRef100\_A0A6P0HX66 | 99.3 | 6.8e-15 | 1.3e-20 | 122.7 | 161 | (1, 201) | 227 | (33, 209) | 228 | SprT family zinc-dependent metalloprotease | SprT family zinc-dependent metalloprotease | | uniclust | UniRef100\_A0A1H9XF20 | 99.3 | 7.8e-15 | 1.6e-20 | 120.5 | 114 | (7, 123) | 227 | (7, 147) | 169 | SprT-like family protein (Fragment) | SprT-like family protein (Fragment) | | uniclust | UniRef100\_A0A068NQF1 | 99.3 | 9.1e-15 | 2.1e-20 | 126.1 | 79 | (24, 112) | 227 | (41, 125) | 200 | Acidic repeat-containing protein | Acidic repeat-containing protein | | uniclust | UniRef100\_A0A0F9ATJ3 | 99.3 | 1.9e-14 | 3.5e-20 | 120.9 | 199 | (2, 220) | 227 | (4, 215) | 217 | SprT-like domain-containing protein (Fragment) | SprT-like domain-containing protein (Fragment) | | uniclust | UniRef100\_A5EQM9 | 99.3 | 2.2e-14 | 3.9e-20 | 123.7 | 169 | (22, 215) | 227 | (100, 281) | 297 | SprT-like domain-containing protein | SprT-like domain-containing protein | | uniclust | UniRef100\_A0A1F3SVB2 | 99.3 | 1.8e-14 | 4.3e-20 | 124.9 | 83 | (14, 105) | 227 | (28, 120) | 198 | SprT-like domain-containing protein | SprT-like domain-containing protein | | uniclust | UniRef100\_A0A095WTQ5 | 99.3 | 2.1e-14 | 4.8e-20 | 127.0 | 101 | (15, 134) | 227 | (50, 153) | 242 | Transcription elongation protein SprT | Transcription elongation protein SprT | | uniclust | UniRef100\_A0A0K2SH02 | 99.2 | 3.4e-14 | 7.5e-20 | 121.0 | 86 | (4, 102) | 227 | (17, 105) | 188 | Metal-dependent hydrolase | Metal-dependent hydrolase | | uniclust | UniRef100\_A0A073IWH6 | 99.2 | 3.8e-14 | 7.6e-20 | 124.2 | 185 | (7, 224) | 227 | (22, 221) | 267 | SprT-like domain-containing protein | SprT-like domain-containing protein | | uniclust | UniRef100\_A0A0K2B1L4 | 99.2 | 4.8e-14 | 9.7e-20 | 117.1 | 144 | (42, 220) | 227 | (11, 178) | 179 | Uncharacterized protein | Uncharacterized protein | | uniclust | UniRef100\_A0A4P7ZT04 | 99.2 | 5.4e-14 | 1.1e-19 | 127.5 | 167 | (5, 216) | 227 | (24, 212) | 368 | SprT-like domain-containing protein | SprT-like domain-containing protein | | uniclust | UniRef100\_A0A146GBG5 | 99.2 | 5.8e-14 | 1.3e-19 | 127.2 | 86 | (16, 109) | 227 | (114, 207) | 298 | SprT-like family protein | SprT-like family protein | | uniclust | UniRef100\_UPI0003B474FE | 99.2 | 9.9e-14 | 1.8e-19 | 114.8 | 58 | (57, 114) | 227 | (18, 84) | 209 | SprT-like domain-containing protein | SprT-like domain-containing protein | | uniclust | UniRef100\_A0A5Q2W9L6 | 99.2 | 9.3e-14 | 1.8e-19 | 118.7 | 192 | (1, 220) | 227 | (10, 221) | 224 | SprT-like family protein | SprT-like family protein | | uniclust | UniRef100\_A0A7Y5RTJ4 | 99.2 | 1e-13 | 1.9e-19 | 119.7 | 167 | (5, 219) | 227 | (102, 278) | 294 | SprT-like domain-containing protein | SprT-like domain-containing protein | | uniclust | UniRef100\_A0A2E4W4M1 | 99.2 | 9.8e-14 | 1.9e-19 | 123.4 | 94 | (15, 108) | 227 | (59, 165) | 326 | SprT-like domain-containing protein | SprT-like domain-containing protein | | uniclust | UniRef100\_A0A1V3BVK3 | 99.2 | 1e-13 | 1.9e-19 | 120.1 | 211 | (11, 223) | 227 | (28, 278) | 294 | SprT-like domain-containing protein | SprT-like domain-containing protein | | uniclust | UniRef100\_A0A2D5NF12 | 99.2 | 8.9e-14 | 2.2e-19 | 123.0 | 77 | (25, 109) | 227 | (55, 139) | 222 | SprT-like domain-containing protein | SprT-like domain-containing protein | | uniclust | UniRef100\_A0A0B5FXD9 | 99.2 | 9.8e-14 | 2.2e-19 | 131.7 | 118 | (10, 132) | 227 | (74, 229) | 421 | SprT-like domain-containing protein | SprT-like domain-containing protein | | uniclust | UniRef100\_A0A174V594 | 99.2 | 1.2e-13 | 2.8e-19 | 120.1 | 92 | (5, 103) | 227 | (32, 126) | 208 | SprT domain-containing protein | SprT domain-containing protein | | uniclust | UniRef100\_A0A7J9XW55 | 99.2 | 1.8e-13 | 3.2e-19 | 109.7 | 122 | (68, 223) | 227 | (6, 142) | 164 | SprT domain-containing protein | SprT domain-containing protein | | uniclust | UniRef100\_A0A318K5J9 | 99.1 | 3.6e-13 | 7.4e-19 | 114.5 | 120 | (10, 129) | 227 | (32, 170) | 197 | SprT-like family protein | SprT-like family protein | | uniclust | UniRef100\_A0A0F8YGA8 | 99.1 | 4e-13 | 7.7e-19 | 109.1 | 130 | (68, 216) | 227 | (8, 147) | 163 | SprT-like domain-containing protein (Fragment) | SprT-like domain-containing protein (Fragment) | | uniclust | UniRef100\_A0A358QWY1 | 99.1 | 4.3e-13 | 7.9e-19 | 110.2 | 162 | (45, 220) | 227 | (5, 195) | 196 | SprT-like domain-containing protein | SprT-like domain-containing protein | | uniclust | UniRef100\_A0A354EFJ4 | 99.1 | 4.2e-13 | 9.2e-19 | 119.6 | 98 | (2, 112) | 227 | (93, 193) | 259 | SprT-like domain-containing protein | SprT-like domain-containing protein | | uniclust | UniRef100\_A0A6B1ALR6 | 99.1 | 6.7e-13 | 1.4e-18 | 118.9 | 96 | (3, 111) | 227 | (98, 196) | 298 | SprT family zinc-dependent metalloprotease | SprT family zinc-dependent metalloprotease | | uniclust | UniRef100\_A0A1L3GQR5 | 99.1 | 6.5e-13 | 1.4e-18 | 122.2 | 94 | (6, 112) | 227 | (170, 266) | 324 | SprT-like domain-containing protein | SprT-like domain-containing protein | | uniclust | UniRef100\_A0A0A0J0D7 | 99.1 | 8.8e-13 | 1.9e-18 | 119.3 | 88 | (11, 111) | 227 | (39, 129) | 294 | SprT-like domain-containing protein | SprT-like domain-containing protein | | uniclust | UniRef100\_A0A2W4I2B1 | 99.1 | 9.9e-13 | 2e-18 | 110.8 | 78 | (24, 112) | 227 | (18, 98) | 183 | SprT domain-containing protein | SprT domain-containing protein | | uniclust | UniRef100\_A0A2N1P657 | 99.0 | 1.2e-12 | 2.7e-18 | 115.0 | 88 | (4, 107) | 227 | (61, 150) | 221 | SprT-like domain-containing protein | SprT-like domain-containing protein | | uniclust | UniRef100\_A0A516MH30 | 99.0 | 1.5e-12 | 2.8e-18 | 108.2 | 169 | (19, 223) | 227 | (20, 208) | 211 | SprT-like domain-containing protein | SprT-like domain-containing protein | | uniclust | UniRef100\_A0A8R1E689 | 99.0 | 2.1e-12 | 3.8e-18 | 116.0 | 99 | (11, 114) | 227 | (8, 123) | 408 | SprT-like domain-containing protein | SprT-like domain-containing protein | | uniclust | UniRef100\_A0A2D6AHS8 | 99.0 | 2.3e-12 | 4.7e-18 | 110.9 | 88 | (23, 122) | 227 | (72, 167) | 218 | SprT-like domain-containing protein | SprT-like domain-containing protein | | uniclust | UniRef100\_A0A140DWM3 | 99.0 | 2.2e-12 | 5e-18 | 111.8 | 89 | (7, 102) | 227 | (12, 105) | 199 | SprT-like domain-containing protein | SprT-like domain-containing protein | | uniclust | UniRef100\_A0A2A4WS63 | 99.0 | 2.4e-12 | 5.4e-18 | 112.7 | 94 | (10, 112) | 227 | (36, 136) | 211 | SprT-like domain-containing protein | SprT-like domain-containing protein | | uniclust | UniRef100\_A0A933N799 | 99.0 | 3e-12 | 5.6e-18 | 108.2 | 182 | (20, 218) | 227 | (30, 235) | 237 | SprT-like domain-containing protein | SprT-like domain-containing protein | | uniclust | UniRef100\_A0A060JGM0 | 99.0 | 3.3e-12 | 6.6e-18 | 110.4 | 96 | (28, 139) | 227 | (93, 192) | 232 | SprT-like family | SprT-like family | | uniclust | UniRef100\_A0A6A8MVU3 | 99.0 | 3.3e-12 | 6.8e-18 | 116.2 | 97 | (28, 139) | 227 | (203, 302) | 337 | SprT-like domain-containing protein | SprT-like domain-containing protein | | uniclust | UniRef100\_A0A1U7GSA4 | 99.0 | 3.2e-12 | 7e-18 | 112.6 | 76 | (28, 112) | 227 | (43, 121) | 236 | SprT-like domain-containing protein | SprT-like domain-containing protein | | uniclust | UniRef100\_A0A9D1JYK7 | 99.0 | 4e-12 | 8.2e-18 | 98.6 | 96 | (90, 220) | 227 | (5, 106) | 107 | Uncharacterized protein | Uncharacterized protein | | uniclust | UniRef100\_A0A955QNM8 | 98.9 | 5.8e-12 | 1.1e-17 | 98.5 | 107 | (23, 129) | 227 | (15, 130) | 135 | SprT-like domain-containing protein (Fragment) | SprT-like domain-containing protein (Fragment) | | uniclust | UniRef100\_A0A076YKR6 | 98.9 | 6.2e-12 | 1.3e-17 | 108.1 | 74 | (29, 110) | 227 | (48, 124) | 205 | SprT-like domain-containing protein | SprT-like domain-containing protein | | uniclust | UniRef100\_A0A0K2GJ75 | 98.9 | 7e-12 | 1.5e-17 | 113.3 | 150 | (6, 220) | 227 | (31, 199) | 282 | SprT-like domain-containing protein | SprT-like domain-containing protein | | uniclust | UniRef100\_A0A1W9W5N7 | 98.9 | 7.4e-12 | 1.5e-17 | 107.6 | 111 | (8, 126) | 227 | (16, 154) | 207 | SprT-like domain-containing protein | SprT-like domain-containing protein | | uniclust | UniRef100\_A0A2K9UZU6 | 98.9 | 1.1e-11 | 2.2e-17 | 102.4 | 80 | (23, 107) | 227 | (47, 139) | 153 | Zinc metalloproteinase Mpr protein | Zinc metalloproteinase Mpr protein | | uniclust | UniRef100\_A0A950FD64 | 98.9 | 1.3e-11 | 2.5e-17 | 94.8 | 98 | (86, 220) | 227 | (2, 109) | 119 | Uncharacterized protein | Uncharacterized protein | | uniclust | UniRef100\_A0A2U9QIZ2 | 98.9 | 1.6e-11 | 2.9e-17 | 102.0 | 109 | (8, 122) | 227 | (6, 134) | 197 | Zinc metalloproteinase Mpr protein | Zinc metalloproteinase Mpr protein | | uniclust | UniRef100\_A0A8R1E603 | 98.9 | 1.9e-11 | 3.6e-17 | 117.3 | 99 | (11, 114) | 227 | (8, 123) | 833 | SprT-like domain-containing protein | SprT-like domain-containing protein | | uniclust | UniRef100\_A0A0I9NBQ0 | 98.9 | 1.8e-11 | 3.8e-17 | 120.0 | 130 | (23, 220) | 227 | (371, 507) | 590 | Bm7449, isoform a | Bm7449, isoform a | | uniclust | UniRef100\_A0A139TQJ4 | 98.8 | 2.1e-11 | 4.3e-17 | 107.6 | 75 | (28, 111) | 227 | (93, 172) | 244 | SprT-like domain-containing protein | SprT-like domain-containing protein | | uniclust | UniRef100\_A0A1F6LM96 | 98.8 | 2.6e-11 | 4.7e-17 | 112.0 | 195 | (7, 219) | 227 | (295, 512) | 514 | SprT-like domain-containing protein | SprT-like domain-containing protein | | uniclust | UniRef100\_A0A232F4E3 | 98.8 | 2.8e-11 | 6.2e-17 | 125.2 | 151 | (2, 219) | 227 | (756, 917) | 995 | SprT-like domain-containing protein | SprT-like domain-containing protein | | uniclust | UniRef100\_A0A0B5A5G0 | 98.8 | 3.3e-11 | 6.6e-17 | 102.1 | 92 | (28, 136) | 227 | (66, 160) | 190 | SprT-like protease | SprT-like protease | | uniclust | UniRef100\_A0A372ZJH5 | 98.8 | 3.9e-11 | 7.3e-17 | 107.4 | 181 | (16, 222) | 227 | (22, 244) | 356 | Uncharacterized protein | Uncharacterized protein | | uniclust | UniRef100\_A0A6J8E1A8 | 98.8 | 3.9e-11 | 8.5e-17 | 124.0 | 136 | (23, 222) | 227 | (870, 1013) | 1074 | Acidic repeat-containing protein | Acidic repeat-containing protein | | uniclust | UniRef100\_A0A653DTS8 | 98.8 | 4.2e-11 | 8.9e-17 | 121.9 | 150 | (2, 219) | 227 | (721, 882) | 945 | SprT-like domain-containing protein | SprT-like domain-containing protein | | uniclust | UniRef100\_A0A2L1K6I3 | 98.8 | 5e-11 | 9.3e-17 | 101.5 | 101 | (7, 115) | 227 | (24, 138) | 230 | Zinc metalloproteinase Mpr protein | Zinc metalloproteinase Mpr protein | | uniclust | UniRef100\_A0A2D5Z0A3 | 98.8 | 4.1e-11 | 9.6e-17 | 106.5 | 93 | (7, 108) | 227 | (39, 143) | 221 | SprT-like domain-containing protein | SprT-like domain-containing protein | | uniclust | UniRef100\_A0A1B6C646 | 98.8 | 4.3e-11 | 9.9e-17 | 121.9 | 133 | (23, 220) | 227 | (526, 669) | 763 | SprT-like domain-containing protein | SprT-like domain-containing protein | | uniclust | UniRef100\_A0A0S7ZXW9 | 98.8 | 4.7e-11 | 1e-16 | 109.4 | 93 | (10, 108) | 227 | (181, 285) | 309 | SprT-like domain-containing protein | SprT-like domain-containing protein | | uniclust | UniRef100\_A0A026WDD5 | 98.8 | 5.1e-11 | 1.2e-16 | 123.7 | 132 | (23, 219) | 227 | (773, 915) | 1001 | Acidic repeat-containing protein | Acidic repeat-containing protein | | uniclust | UniRef100\_UPI00111BAF9D | 98.8 | 6.3e-11 | 1.2e-16 | 89.0 | 73 | (57, 129) | 227 | (12, 93) | 96 | SprT-like domain-containing protein | SprT-like domain-containing protein | | uniclust | UniRef100\_A0A2H8TPB7 | 98.8 | 6.1e-11 | 1.3e-16 | 118.9 | 133 | (23, 220) | 227 | (467, 610) | 690 | Acidic repeat-containing protein | Acidic repeat-containing protein | | uniclust | UniRef100\_A0A2D7KWL3 | 98.8 | 6.8e-11 | 1.4e-16 | 100.4 | 75 | (29, 112) | 227 | (62, 139) | 195 | SprT domain-containing protein | SprT domain-containing protein | | uniclust | UniRef100\_A0A016TL32 | 98.8 | 6.8e-11 | 1.4e-16 | 115.0 | 133 | (23, 222) | 227 | (363, 503) | 591 | SprT-like domain-containing protein | SprT-like domain-containing protein | | uniclust | UniRef100\_A0A517XQ89 | 98.7 | 8.8e-11 | 1.7e-16 | 100.7 | 79 | (28, 111) | 227 | (89, 170) | 225 | SprT-like family protein | SprT-like family protein | | uniclust | UniRef100\_A0A7M7RG74 | 98.7 | 8.7e-11 | 1.8e-16 | 118.5 | 81 | (23, 106) | 227 | (642, 729) | 841 | SprT-like domain-containing protein | SprT-like domain-containing protein | | uniclust | UniRef100\_A0A143BLS1 | 98.7 | 9.9e-11 | 2e-16 | 107.2 | 92 | (11, 108) | 227 | (180, 282) | 323 | SprT-like domain-containing protein | SprT-like domain-containing protein | | uniclust | UniRef100\_A0A0S9KEU5 | 98.7 | 1e-10 | 2.1e-16 | 103.9 | 79 | (25, 112) | 227 | (19, 109) | 275 | SprT-like domain-containing protein | SprT-like domain-containing protein | | uniclust | UniRef100\_UPI000C6CB064 | 98.7 | 1.1e-10 | 2.2e-16 | 111.9 | 133 | (23, 221) | 227 | (294, 433) | 494 | acidic repeat-containing protein-like isoform X8 | acidic repeat-containing protein-like isoform X8 | | uniclust | UniRef100\_A0A0F8YXQ5 | 98.7 | 1.7e-10 | 3.1e-16 | 85.7 | 92 | (95, 224) | 227 | (1, 92) | 94 | Uncharacterized protein (Fragment) | Uncharacterized protein (Fragment) | | uniclust | UniRef100\_A0A023W5I8 | 98.7 | 1.6e-10 | 3.1e-16 | 97.6 | 75 | (29, 112) | 227 | (32, 109) | 197 | SprT-like protease | SprT-like protease | | uniclust | UniRef100\_A0A2J7R5L2 | 98.7 | 1.9e-10 | 3.8e-16 | 110.1 | 132 | (23, 220) | 227 | (320, 461) | 531 | SprT-like domain-containing protein | SprT-like domain-containing protein | | uniclust | UniRef100\_A0A7V9QDL4 | 98.7 | 1.8e-10 | 3.9e-16 | 101.2 | 89 | (11, 109) | 227 | (119, 218) | 227 | SprT-like domain-containing protein | SprT-like domain-containing protein | | uniclust | UniRef100\_A0A158PCN7 | 98.7 | 2.2e-10 | 4.1e-16 | 94.5 | 128 | (23, 219) | 227 | (8, 143) | 186 | SprT-like domain-containing protein | SprT-like domain-containing protein | | uniclust | UniRef100\_A0A0X3PXD0 | 98.7 | 2.1e-10 | 4.2e-16 | 112.0 | 77 | (23, 102) | 227 | (355, 445) | 610 | Acidic repeat-containing protein | Acidic repeat-containing protein | | uniclust | UniRef100\_A0A315CPA0 | 98.7 | 2.3e-10 | 4.3e-16 | 102.2 | 132 | (3, 134) | 227 | (217, 352) | 357 | Bacteriophage T5 Orf172 DNA-binding domain-containing protein | Bacteriophage T5 Orf172 DNA-binding domain-containing protein | | uniclust | UniRef100\_A0A1I7TER3 | 98.7 | 2.2e-10 | 4.5e-16 | 109.7 | 92 | (2, 102) | 227 | (294, 392) | 539 | SprT-like domain-containing protein | SprT-like domain-containing protein | | uniclust | UniRef100\_A0A2W0DS13 | 98.7 | 2.2e-10 | 4.5e-16 | 89.0 | 70 | (29, 107) | 227 | (22, 94) | 102 | M48 family peptidase (Fragment) | M48 family peptidase (Fragment) | | uniclust | UniRef100\_A0A517SCM0 | 98.7 | 2.4e-10 | 4.5e-16 | 98.5 | 180 | (5, 224) | 227 | (6, 216) | 229 | SprT-like family protein | SprT-like family protein | | uniclust | UniRef100\_A0A374MXY9 | 98.7 | 2.5e-10 | 4.7e-16 | 88.5 | 95 | (5, 103) | 227 | (8, 113) | 113 | SprT-like family protein | SprT-like family protein | | uniclust | UniRef100\_A0A0K2UAN4 | 98.6 | 2.4e-10 | 4.8e-16 | 113.5 | 147 | (10, 220) | 227 | (571, 728) | 791 | Putative LOC100745142 [Bombus impatiens] | Putative LOC100745142 [Bombus impatiens] | | uniclust | UniRef100\_A0A087Y402 | 98.6 | 2.2e-10 | 4.8e-16 | 115.8 | 134 | (23, 220) | 227 | (470, 610) | 734 | Acidic repeat-containing protein-like | Acidic repeat-containing protein-like | | uniclust | UniRef100\_UPI001E52AEAC | 98.6 | 2.7e-10 | 5e-16 | 92.3 | 132 | (61, 219) | 227 | (2, 153) | 155 | hypothetical protein | hypothetical protein | | uniclust | UniRef100\_UPI001A9BFEBE | 98.6 | 2.6e-10 | 5e-16 | 105.5 | 100 | (10, 114) | 227 | (142, 258) | 398 | SprT-like domain-containing protein | SprT-like domain-containing protein | | uniclust | UniRef100\_A0A2A9EM23 | 98.6 | 2.9e-10 | 5.7e-16 | 105.1 | 74 | (29, 111) | 227 | (93, 169) | 383 | SprT-like family protein | SprT-like family protein | | uniclust | UniRef100\_A0A1S7NI76 | 98.6 | 3.2e-10 | 6.2e-16 | 92.5 | 125 | (80, 224) | 227 | (1, 131) | 153 | Uncharacterized protein | Uncharacterized protein | | uniclust | UniRef100\_A0A960IJM6 | 98.6 | 3.1e-10 | 6.2e-16 | 96.2 | 77 | (28, 112) | 227 | (50, 128) | 191 | SprT-like domain-containing protein | SprT-like domain-containing protein | | uniclust | UniRef100\_A0A2E7W4W3 | 98.6 | 4.5e-10 | 8.2e-16 | 96.2 | 139 | (42, 217) | 227 | (77, 232) | 244 | SprT-like domain-containing protein | SprT-like domain-containing protein | | uniclust | UniRef100\_A0A430JN74 | 98.6 | 4.7e-10 | 9.3e-16 | 90.7 | 82 | (14, 108) | 227 | (10, 94) | 136 | SprT domain-containing protein | SprT domain-containing protein | | uniclust | UniRef100\_A0A0D8XAM0 | 98.6 | 5.5e-10 | 1e-15 | 101.3 | 140 | (11, 219) | 227 | (214, 363) | 403 | SprT-like domain-containing protein | SprT-like domain-containing protein | | uniclust | UniRef100\_A0A076GXD3 | 98.6 | 4.9e-10 | 1e-15 | 98.1 | 90 | (14, 104) | 227 | (28, 133) | 224 | SprT-like domain-containing protein | SprT-like domain-containing protein | | uniclust | UniRef100\_A0A2D3TGT7 | 98.6 | 5.6e-10 | 1e-15 | 90.7 | 47 | (68, 114) | 227 | (10, 62) | 154 | SprT-like domain-containing protein | SprT-like domain-containing protein | | uniclust | UniRef100\_A0A0S7MAB5 | 98.6 | 4.7e-10 | 1.1e-15 | 107.4 | 97 | (2, 104) | 227 | (149, 254) | 404 | ACRC (Fragment) | ACRC (Fragment) | | uniclust | UniRef100\_A0A2E4EZM8 | 98.6 | 5.4e-10 | 1.2e-15 | 96.6 | 85 | (14, 111) | 227 | (20, 107) | 192 | SprT-like domain-containing protein | SprT-like domain-containing protein | | uniclust | UniRef100\_A0A1E4ZTF4 | 98.6 | 5.8e-10 | 1.2e-15 | 100.4 | 88 | (17, 110) | 227 | (13, 109) | 297 | SprT-like domain-containing protein | SprT-like domain-containing protein | | uniclust | UniRef100\_A0A3B8MXE4 | 98.6 | 7.3e-10 | 1.3e-15 | 85.0 | 74 | (29, 111) | 227 | (23, 99) | 112 | SprT domain-containing protein (Fragment) | SprT domain-containing protein (Fragment) | | uniclust | UniRef100\_A0A6L8C1X2 | 98.5 | 7.6e-10 | 1.5e-15 | 95.8 | 93 | (5, 110) | 227 | (135, 230) | 235 | SprT-like domain-containing protein | SprT-like domain-containing protein | | uniclust | UniRef100\_UPI0013AEF1FA | 98.5 | 9e-10 | 1.7e-15 | 85.0 | 66 | (42, 107) | 227 | (11, 88) | 116 | SprT-like domain-containing protein | SprT-like domain-containing protein | | uniclust | UniRef100\_K1PVW6 | 98.5 | 8.7e-10 | 1.7e-15 | 95.3 | 135 | (23, 221) | 227 | (27, 169) | 227 | Acidic repeat-containing protein | Acidic repeat-containing protein | | uniclust | UniRef100\_UPI00156DE97E | 98.5 | 1e-09 | 1.9e-15 | 77.6 | 63 | (2, 64) | 227 | (5, 67) | 70 | hypothetical protein | hypothetical protein | | uniclust | UniRef100\_A0A0C2QPB3 | 98.5 | 1.1e-09 | 2.2e-15 | 96.2 | 171 | (4, 224) | 227 | (30, 222) | 229 | SprT family zinc-dependent metalloprotease | SprT family zinc-dependent metalloprotease | | uniclust | UniRef100\_A0A410T805 | 98.5 | 1.2e-09 | 2.4e-15 | 83.8 | 68 | (28, 104) | 227 | (29, 99) | 103 | SprT-like domain-containing protein | SprT-like domain-containing protein | | uniclust | UniRef100\_A0A2E9YH35 | 98.5 | 1.4e-09 | 2.6e-15 | 84.5 | 85 | (17, 101) | 227 | (24, 121) | 121 | SprT-like domain-containing protein (Fragment) | SprT-like domain-containing protein (Fragment) | | uniclust | UniRef100\_A0A6I3DIT0 | 98.5 | 1.6e-09 | 2.9e-15 | 80.7 | 70 | (30, 108) | 227 | (10, 82) | 93 | M48 family peptidase (Fragment) | M48 family peptidase (Fragment) | | uniclust | UniRef100\_A0A0A0Q2I3 | 98.5 | 1.4e-09 | 2.9e-15 | 95.8 | 59 | (42, 107) | 227 | (58, 119) | 227 | SprT-like domain-containing protein | SprT-like domain-containing protein | | uniclust | UniRef100\_A0A010SRF5 | 98.5 | 1.3e-09 | 3.2e-15 | 100.1 | 79 | (23, 112) | 227 | (86, 173) | 266 | Protein SprT | Protein SprT | | uniclust | UniRef100\_UPI001F3D9D2C | 98.5 | 1.8e-09 | 3.3e-15 | 80.7 | 45 | (175, 219) | 227 | (50, 94) | 95 | hypothetical protein | hypothetical protein | | uniclust | UniRef100\_UPI00201D51DF | 98.5 | 1.8e-09 | 3.3e-15 | 83.9 | 91 | (7, 105) | 227 | (17, 120) | 120 | SprT-like domain-containing protein | SprT-like domain-containing protein | | uniclust | UniRef100\_A0A368G165 | 98.5 | 2e-09 | 3.6e-15 | 96.3 | 144 | (10, 220) | 227 | (115, 266) | 342 | SprT-like domain-containing protein | SprT-like domain-containing protein | | uniclust | UniRef100\_A0A096MLG6 | 98.4 | 2.1e-09 | 4.3e-15 | 107.8 | 99 | (2, 106) | 227 | (580, 687) | 810 | Germ cell nuclear acidic peptidase | Germ cell nuclear acidic peptidase | | uniclust | UniRef100\_A0A139AMY7 | 98.4 | 2.2e-09 | 4.6e-15 | 93.5 | 77 | (22, 101) | 227 | (28, 114) | 200 | SprT-like domain-containing protein (Fragment) | SprT-like domain-containing protein (Fragment) | | uniclust | UniRef100\_A0A1Q3UR33 | 98.4 | 2.9e-09 | 5.3e-15 | 91.1 | 186 | (4, 223) | 227 | (12, 211) | 234 | Uncharacterized protein | Uncharacterized protein | | uniclust | UniRef100\_A0A3C0P523 | 98.4 | 3e-09 | 5.6e-15 | 78.7 | 86 | (101, 222) | 227 | (1, 86) | 89 | Uncharacterized protein (Fragment) | Uncharacterized protein (Fragment) | | uniclust | UniRef100\_A0A0S7Z910 | 98.4 | 2.7e-09 | 5.8e-15 | 97.8 | 84 | (23, 110) | 227 | (146, 237) | 286 | SprT-like domain-containing protein | SprT-like domain-containing protein | | uniclust | UniRef100\_A7RFH1 | 98.4 | 3.8e-09 | 7.3e-15 | 89.9 | 79 | (24, 105) | 227 | (41, 123) | 206 | SprT-like domain-containing protein (Fragment) | SprT-like domain-containing protein (Fragment) | | uniclust | UniRef100\_A0A0K8SMS5 | 98.4 | 3.9e-09 | 7.8e-15 | 93.0 | 132 | (23, 220) | 227 | (11, 154) | 235 | SprT-like domain-containing protein | SprT-like domain-containing protein | | uniclust | UniRef100\_UPI001F36BFBF | 98.4 | 4.6e-09 | 8.5e-15 | 90.1 | 53 | (62, 114) | 227 | (2, 63) | 239 | SprT-like domain-containing protein | SprT-like domain-containing protein | | uniclust | UniRef100\_UPI001FE9CB55 | 98.4 | 4.7e-09 | 8.6e-15 | 87.6 | 84 | (29, 121) | 227 | (19, 105) | 193 | SprT-like domain-containing protein | SprT-like domain-containing protein | | uniclust | UniRef100\_A0A962Q7G0 | 98.4 | 4.8e-09 | 8.8e-15 | 88.9 | 131 | (56, 219) | 227 | (62, 214) | 217 | SprT-like domain-containing protein | SprT-like domain-containing protein | | uniclust | UniRef100\_UPI001364B730 | 98.4 | 5.4e-09 | 9.8e-15 | 86.4 | 140 | (43, 220) | 227 | (21, 177) | 179 | SprT family zinc-dependent metalloprotease | SprT family zinc-dependent metalloprotease | | uniclust | UniRef100\_A0A6N9B6I9 | 98.4 | 5.4e-09 | 9.9e-15 | 76.0 | 63 | (42, 110) | 227 | (11, 76) | 80 | SprT domain-containing protein (Fragment) | SprT domain-containing protein (Fragment) | | uniclust | UniRef100\_A0A0F9F6D4 | 98.4 | 5.7e-09 | 1e-14 | 87.1 | 130 | (2, 134) | 227 | (24, 163) | 192 | SprT-like domain-containing protein (Fragment) | SprT-like domain-containing protein (Fragment) | | uniclust | UniRef100\_UPI00055C0F9E | 98.4 | 5.7e-09 | 1.1e-14 | 78.7 | 65 | (1, 66) | 227 | (17, 81) | 99 | hypothetical protein | hypothetical protein | | uniclust | UniRef100\_A0A2B4RRR3 | 98.3 | 5.5e-09 | 1.1e-14 | 104.6 | 77 | (23, 102) | 227 | (691, 772) | 883 | HMG box-containing protein C19G7.04 | HMG box-containing protein C19G7.04 | | uniclust | UniRef100\_A0A225DJ34 | 98.3 | 5.8e-09 | 1.1e-14 | 92.9 | 163 | (23, 220) | 227 | (106, 287) | 288 | SprT-like domain-containing protein | SprT-like domain-containing protein | | uniclust | UniRef100\_UPI0006B0C5A3 | 98.3 | 6e-09 | 1.2e-14 | 104.6 | 134 | (22, 220) | 227 | (764, 908) | 972 | mucin-4-like | mucin-4-like | | uniclust | UniRef100\_A0A0F9MGL9 | 98.3 | 6.3e-09 | 1.2e-14 | 88.5 | 148 | (28, 219) | 227 | (50, 220) | 222 | SprT-like domain-containing protein | SprT-like domain-containing protein | | uniclust | UniRef100\_A0A369RWZ3 | 98.3 | 6.1e-09 | 1.2e-14 | 102.6 | 155 | (2, 223) | 227 | (495, 655) | 707 | Acidic repeat-containing protein | Acidic repeat-containing protein | | uniclust | UniRef100\_T2M7F9 | 98.3 | 6.2e-09 | 1.2e-14 | 102.3 | 95 | (3, 103) | 227 | (422, 522) | 643 | Acidic repeat-containing protein | Acidic repeat-containing protein | | uniclust | UniRef100\_A0A063ZKH3 | 98.3 | 5.7e-09 | 1.3e-14 | 94.1 | 97 | (3, 105) | 227 | (60, 164) | 238 | SprT-like domain-containing protein | SprT-like domain-containing protein | | uniclust | UniRef100\_A0A1H6LC21 | 98.3 | 6.6e-09 | 1.3e-14 | 89.6 | 74 | (28, 110) | 227 | (52, 134) | 207 | Sprt-like family | Sprt-like family | | uniclust | UniRef100\_A0A7M2WWZ9 | 98.3 | 6.9e-09 | 1.4e-14 | 89.2 | 75 | (28, 112) | 227 | (34, 111) | 193 | SprT-like domain-containing protein | SprT-like domain-containing protein | | uniclust | UniRef100\_A0A0N7ZDR3 | 98.3 | 6.7e-09 | 1.4e-14 | 109.9 | 134 | (23, 220) | 227 | (1151, 1292) | 1372 | SprT-like domain-containing protein | SprT-like domain-containing protein | | uniclust | UniRef100\_A0A8F4NUB2 | 98.3 | 7.4e-09 | 1.4e-14 | 83.9 | 56 | (68, 123) | 227 | (34, 95) | 141 | Zinc metalloproteinase Mpr protein | Zinc metalloproteinase Mpr protein | | uniclust | UniRef100\_A0A9D4QSU8 | 98.3 | 7.5e-09 | 1.5e-14 | 107.3 | 134 | (23, 220) | 227 | (1093, 1233) | 1297 | Uncharacterized protein | Uncharacterized protein | | uniclust | UniRef100\_UPI0011194AF0 | 98.3 | 8.3e-09 | 1.5e-14 | 78.7 | 76 | (41, 116) | 227 | (5, 93) | 103 | SprT-like domain-containing protein | SprT-like domain-containing protein | | uniclust | UniRef100\_A0A2E0ELX0 | 98.3 | 8.6e-09 | 1.7e-14 | 87.9 | 73 | (29, 110) | 227 | (46, 121) | 186 | SprT-like domain-containing protein | SprT-like domain-containing protein | | uniclust | UniRef100\_UPI001C69A0ED | 98.3 | 9.9e-09 | 1.8e-14 | 81.1 | 74 | (42, 115) | 227 | (15, 99) | 131 | SprT-like domain-containing protein | SprT-like domain-containing protein | | uniclust | UniRef100\_A0A386ZLW0 | 98.3 | 1e-08 | 2e-14 | 77.3 | 46 | (178, 224) | 227 | (38, 83) | 90 | Uncharacterized protein | Uncharacterized protein | | uniclust | UniRef100\_A0A6A7FXS4 | 98.3 | 1.1e-08 | 2.2e-14 | 105.0 | 152 | (2, 220) | 227 | (1148, 1308) | 1364 | Acidic repeat-containing protein-like | Acidic repeat-containing protein-like | | uniclust | UniRef100\_A0A3E4PBP9 | 98.3 | 1.1e-08 | 2.2e-14 | 88.7 | 123 | (55, 219) | 227 | (49, 187) | 193 | SprT-like family protein | SprT-like family protein | | uniclust | UniRef100\_A0A444UUE3 | 98.3 | 1.1e-08 | 2.2e-14 | 90.2 | 81 | (22, 105) | 227 | (25, 113) | 225 | Acidic repeat-containing protein | Acidic repeat-containing protein | | uniclust | UniRef100\_A0A942C240 | 98.3 | 1.3e-08 | 2.4e-14 | 88.2 | 73 | (29, 110) | 227 | (21, 96) | 254 | SprT-like domain-containing protein | SprT-like domain-containing protein | | uniclust | UniRef100\_A0A090L5F7 | 98.3 | 1.3e-08 | 2.5e-14 | 97.9 | 130 | (23, 219) | 227 | (328, 463) | 495 | SprT-like domain-containing protein | SprT-like domain-containing protein | | uniclust | UniRef100\_UPI0023581BAD | 98.3 | 1.4e-08 | 2.6e-14 | 84.4 | 58 | (57, 114) | 227 | (27, 93) | 183 | SprT-like domain-containing protein | SprT-like domain-containing protein | | uniclust | UniRef100\_A0A3D4EUY1 | 98.3 | 1.4e-08 | 2.6e-14 | 79.8 | 50 | (59, 108) | 227 | (6, 63) | 126 | SprT-like domain-containing protein (Fragment) | SprT-like domain-containing protein (Fragment) | | uniclust | UniRef100\_UPI00190F8D28 | 98.3 | 1.4e-08 | 2.6e-14 | 81.5 | 64 | (57, 120) | 227 | (5, 77) | 133 | SprT-like domain-containing protein | SprT-like domain-containing protein | | uniclust | UniRef100\_A0A0R3T453 | 98.3 | 1.4e-08 | 2.6e-14 | 102.6 | 153 | (1, 219) | 227 | (391, 557) | 1025 | Lipoyl synthase, mitochondrial | Lipoyl synthase, mitochondrial | | uniclust | UniRef100\_A0A1Y6FUX7 | 98.2 | 1.5e-08 | 2.7e-14 | 72.0 | 50 | (1, 50) | 227 | (4, 54) | 69 | Uncharacterized protein | Uncharacterized protein | | uniclust | UniRef100\_A0A7Y4SVY5 | 98.2 | 1.6e-08 | 2.9e-14 | 86.7 | 168 | (23, 222) | 227 | (29, 226) | 230 | SprT-like domain-containing protein | SprT-like domain-containing protein | | uniclust | UniRef100\_A0A2W6D4Y0 | 98.2 | 1.8e-08 | 3.2e-14 | 84.3 | 90 | (18, 107) | 227 | (40, 147) | 191 | SprT-like domain-containing protein (Fragment) | SprT-like domain-containing protein (Fragment) | | uniclust | UniRef100\_A0A011NB53 | 98.2 | 1.5e-08 | 3.5e-14 | 92.7 | 74 | (30, 112) | 227 | (98, 179) | 256 | Protein SprT | Protein SprT | | uniclust | UniRef100\_A0A523HVA6 | 98.2 | 1.9e-08 | 3.5e-14 | 89.6 | 87 | (23, 121) | 227 | (171, 265) | 317 | DUF45 domain-containing protein | DUF45 domain-containing protein | | uniclust | UniRef100\_A0A2A5DH80 | 98.2 | 1.7e-08 | 3.5e-14 | 85.5 | 81 | (28, 116) | 227 | (44, 131) | 160 | SprT-like domain-containing protein | SprT-like domain-containing protein | | uniclust | UniRef100\_A0A4Z0WHF5 | 98.2 | 2e-08 | 3.7e-14 | 84.0 | 78 | (24, 112) | 227 | (39, 119) | 191 | M48 family peptidase | M48 family peptidase | | uniclust | UniRef100\_A0A4Y7LNE8 | 98.2 | 1.8e-08 | 3.7e-14 | 99.8 | 94 | (2, 101) | 227 | (366, 469) | 593 | EOG090X0464 | EOG090X0464 | | uniclust | UniRef100\_A0A060YC73 | 98.2 | 1.9e-08 | 3.8e-14 | 98.0 | 80 | (23, 105) | 227 | (349, 435) | 540 | SprT-like domain-containing protein | SprT-like domain-containing protein | | uniclust | UniRef100\_A0A519H8W8 | 98.2 | 2.3e-08 | 4.2e-14 | 72.8 | 65 | (68, 138) | 227 | (12, 76) | 79 | SprT-like domain-containing protein | SprT-like domain-containing protein | | uniclust | UniRef100\_A0A091CV90 | 98.2 | 2.1e-08 | 4.3e-14 | 99.4 | 99 | (2, 106) | 227 | (397, 504) | 658 | Acidic repeat-containing protein | Acidic repeat-containing protein | | uniclust | UniRef100\_A0A0F9VUA0 | 98.2 | 2.3e-08 | 4.3e-14 | 91.3 | 145 | (37, 222) | 227 | (41, 208) | 396 | SprT-like domain-containing protein | SprT-like domain-containing protein | | uniclust | UniRef100\_A0A965PII6 | 98.2 | 2.5e-08 | 4.6e-14 | 85.4 | 97 | (10, 108) | 227 | (9, 121) | 228 | SprT-like domain-containing protein | SprT-like domain-containing protein | | uniclust | UniRef100\_A0A1B6FR93 | 98.2 | 2.4e-08 | 4.7e-14 | 89.8 | 132 | (23, 219) | 227 | (76, 217) | 292 | SprT-like domain-containing protein | SprT-like domain-containing protein | | uniclust | UniRef100\_A0A158QQ23 | 98.2 | 2.8e-08 | 5.2e-14 | 88.5 | 131 | (23, 220) | 227 | (90, 226) | 314 | SprT-like domain-containing protein | SprT-like domain-containing protein | | uniclust | UniRef100\_A0A0P7TZE5 | 98.2 | 2.6e-08 | 5.6e-14 | 100.2 | 98 | (2, 105) | 227 | (438, 543) | 664 | Acidic repeat containing-like (Fragment) | Acidic repeat containing-like (Fragment) | | uniclust | UniRef100\_A0A2W4S5I9 | 98.2 | 3.1e-08 | 5.7e-14 | 88.3 | 102 | (5, 111) | 227 | (120, 228) | 314 | SprT-like domain-containing protein | SprT-like domain-containing protein | | uniclust | UniRef100\_A0A0C9VRQ7 | 98.2 | 2.9e-08 | 5.9e-14 | 96.9 | 95 | (2, 102) | 227 | (290, 392) | 522 | SprT-like domain-containing protein | SprT-like domain-containing protein | | uniclust | UniRef100\_A0A1Y3SUR1 | 98.1 | 3.1e-08 | 6.7e-14 | 81.6 | 94 | (5, 102) | 227 | (10, 103) | 129 | Peptidase M48 domain-containing protein | Peptidase M48 domain-containing protein | | uniclust | UniRef100\_A0A1W9WRE7 | 98.1 | 3.2e-08 | 6.7e-14 | 84.0 | 94 | (6, 110) | 227 | (27, 129) | 159 | SprT-like domain-containing protein (Fragment) | SprT-like domain-containing protein (Fragment) | | uniclust | UniRef100\_A0A0S2W4E2 | 98.1 | 3.7e-08 | 6.7e-14 | 71.5 | 44 | (177, 224) | 227 | (21, 64) | 77 | Uncharacterized protein | Uncharacterized protein | | uniclust | UniRef100\_A0A8S1EWA4 | 98.1 | 3.9e-08 | 7.2e-14 | 90.3 | 75 | (23, 102) | 227 | (239, 319) | 411 | SprT-like domain-containing protein | SprT-like domain-containing protein | | uniclust | UniRef100\_A0A7V9QTE1 | 98.1 | 4.2e-08 | 7.7e-14 | 82.8 | 73 | (29, 110) | 227 | (8, 83) | 201 | SprT-like domain-containing protein | SprT-like domain-containing protein | | uniclust | UniRef100\_A0A815EWT2 | 98.1 | 4.1e-08 | 7.9e-14 | 91.4 | 144 | (10, 220) | 227 | (174, 326) | 398 | SprT-like domain-containing protein | SprT-like domain-containing protein | | uniclust | UniRef100\_A0A967HJD9 | 98.1 | 4.4e-08 | 8.1e-14 | 83.2 | 92 | (13, 108) | 227 | (97, 200) | 211 | SprT family zinc-dependent metalloprotease | SprT family zinc-dependent metalloprotease | | uniclust | UniRef100\_A0A017SUS6 | 98.1 | 3.5e-08 | 8.8e-14 | 97.8 | 96 | (4, 110) | 227 | (82, 186) | 456 | DUF2786 domain-containing protein | DUF2786 domain-containing protein | | uniclust | UniRef100\_A0A6I3FXI3 | 98.1 | 4.9e-08 | 9e-14 | 85.3 | 95 | (29, 138) | 227 | (136, 233) | 263 | SprT-like domain-containing protein | SprT-like domain-containing protein | | uniclust | UniRef100\_A0A4X2L320 | 98.1 | 4.7e-08 | 9.1e-14 | 91.4 | 82 | (22, 106) | 227 | (191, 281) | 401 | SprT-like domain-containing protein | SprT-like domain-containing protein | | uniclust | UniRef100\_UPI001BEB5868 | 98.1 | 4.9e-08 | 9.2e-14 | 78.7 | 79 | (7, 88) | 227 | (21, 111) | 136 | SprT-like domain-containing protein | SprT-like domain-containing protein | | uniclust | UniRef100\_A0A2V5M5G1 | 98.1 | 4.9e-08 | 9.3e-14 | 86.1 | 86 | (17, 110) | 227 | (85, 178) | 256 | SprT-like domain-containing protein | SprT-like domain-containing protein | | uniclust | UniRef100\_A0A084VEQ8 | 98.1 | 4.4e-08 | 9.7e-14 | 100.9 | 78 | (24, 105) | 227 | (139, 225) | 772 | Protein with SprT-like domain at the N terminus | Protein with SprT-like domain at the N terminus | | uniclust | UniRef100\_A0A061D4E3 | 98.1 | 4.8e-08 | 9.8e-14 | 87.6 | 86 | (14, 105) | 227 | (52, 151) | 248 | SPRT domain containg protein | SPRT domain containg protein | | uniclust | UniRef100\_A0A1Z8XXQ1 | 98.1 | 5.3e-08 | 1e-13 | 85.5 | 72 | (29, 108) | 227 | (89, 168) | 243 | SprT-like domain-containing protein | SprT-like domain-containing protein | | uniclust | UniRef100\_A0A813R6P1 | 98.1 | 5.4e-08 | 1.1e-13 | 99.1 | 131 | (23, 220) | 227 | (758, 895) | 965 | SprT-like domain-containing protein | SprT-like domain-containing protein | | uniclust | UniRef100\_A0A060S9M2 | 98.1 | 5.1e-08 | 1.1e-13 | 99.1 | 96 | (4, 102) | 227 | (390, 497) | 663 | SprT-like domain-containing protein | SprT-like domain-containing protein | | uniclust | UniRef100\_A0A0N4YL49 | 98.1 | 6.1e-08 | 1.2e-13 | 82.5 | 89 | (69, 220) | 227 | (9, 104) | 191 | Putative nuclear protein (inferred by orthology to a S. mansoni protein) | Putative nuclear protein (inferred by orthology to a S. mansoni protein) | | uniclust | UniRef100\_A0A932U3F1 | 98.1 | 6.5e-08 | 1.2e-13 | 80.1 | 64 | (42, 111) | 227 | (41, 110) | 174 | SprT-like domain-containing protein | SprT-like domain-containing protein | | uniclust | UniRef100\_A0A2E4W6R4 | 98.1 | 6.3e-08 | 1.2e-13 | 82.8 | 93 | (8, 111) | 227 | (12, 111) | 195 | SprT-like domain-containing protein | SprT-like domain-containing protein | | uniclust | UniRef100\_A0A564Y0D1 | 98.1 | 6.4e-08 | 1.2e-13 | 91.6 | 97 | (1, 102) | 227 | (240, 350) | 481 | SprT-like domain-containing protein | SprT-like domain-containing protein | | uniclust | UniRef100\_A0A8J3FY24 | 98.1 | 6.9e-08 | 1.3e-13 | 82.7 | 171 | (15, 221) | 227 | (18, 210) | 222 | SprT-like domain-containing protein | SprT-like domain-containing protein | | uniclust | UniRef100\_UPI0020C4C488 | 98.1 | 6.9e-08 | 1.3e-13 | 80.1 | 135 | (58, 223) | 227 | (15, 167) | 176 | hypothetical protein | hypothetical protein | | uniclust | UniRef100\_A0A6A0HDZ2 | 98.1 | 6.7e-08 | 1.3e-13 | 95.7 | 134 | (23, 221) | 227 | (520, 662) | 740 | SprT-like domain-containing protein | SprT-like domain-containing protein | | uniclust | UniRef100\_UPI0002659249 | 98.1 | 7.5e-08 | 1.4e-13 | 86.5 | 71 | (29, 101) | 227 | (140, 216) | 331 | acidic repeat-containing protein | acidic repeat-containing protein | | uniclust | UniRef100\_A0A147E8E1 | 98.1 | 7.4e-08 | 1.4e-13 | 73.3 | 63 | (26, 97) | 227 | (29, 94) | 95 | SprT-like domain-containing protein (Fragment) | SprT-like domain-containing protein (Fragment) | | uniclust | UniRef100\_A0A8R1U967 | 98.0 | 7.8e-08 | 1.4e-13 | 93.7 | 135 | (23, 223) | 227 | (518, 658) | 776 | SprT-like domain-containing protein | SprT-like domain-containing protein | | uniclust | UniRef100\_A0A067RTN9 | 98.0 | 6e-08 | 1.4e-13 | 100.5 | 77 | (23, 105) | 227 | (145, 230) | 721 | Protein with SprT-like domain at the N terminus | Protein with SprT-like domain at the N terminus | | uniclust | UniRef100\_A0A131Z5L9 | 98.0 | 6.4e-08 | 1.5e-13 | 98.6 | 91 | (8, 104) | 227 | (113, 217) | 646 | Protein with SprT-like domain at the N terminus | Protein with SprT-like domain at the N terminus | | uniclust | UniRef100\_A0A067R6Y3 | 98.0 | 8e-08 | 1.5e-13 | 95.5 | 134 | (23, 220) | 227 | (665, 808) | 875 | Acidic repeat-containing protein | Acidic repeat-containing protein | | uniclust | UniRef100\_UPI001D0C490B | 98.0 | 8.4e-08 | 1.5e-13 | 75.4 | 83 | (42, 124) | 227 | (9, 105) | 122 | SprT-like domain-containing protein | SprT-like domain-containing protein | | uniclust | UniRef100\_A0A3P6QM62 | 98.0 | 8.5e-08 | 1.6e-13 | 77.1 | 89 | (8, 102) | 227 | (14, 109) | 141 | SprT-like domain-containing protein | SprT-like domain-containing protein | | uniclust | UniRef100\_A0A2V2LDH4 | 98.0 | 8.6e-08 | 1.6e-13 | 92.6 | 101 | (10, 115) | 227 | (527, 644) | 698 | SprT-like domain-containing protein | SprT-like domain-containing protein | | uniclust | UniRef100\_A0A947HSE2 | 98.0 | 8.8e-08 | 1.6e-13 | 80.6 | 115 | (12, 130) | 227 | (14, 150) | 194 | Uncharacterized protein (Fragment) | Uncharacterized protein (Fragment) | | uniclust | UniRef100\_A0A660QXE7 | 98.0 | 8.5e-08 | 1.6e-13 | 73.3 | 43 | (69, 111) | 227 | (4, 46) | 95 | SprT domain-containing protein (Fragment) | SprT domain-containing protein (Fragment) | | uniclust | UniRef100\_A0A8B8D7V9 | 98.0 | 8.9e-08 | 1.6e-13 | 89.1 | 135 | (23, 221) | 227 | (259, 401) | 461 | HMG box-containing protein C19G7.04-like | HMG box-containing protein C19G7.04-like | | uniclust | UniRef100\_UPI00186B3928 | 98.0 | 9.2e-08 | 1.7e-13 | 85.6 | 83 | (22, 107) | 227 | (129, 216) | 318 | acidic repeat-containing protein | acidic repeat-containing protein | | uniclust | UniRef100\_A0A8R1DRL3 | 98.0 | 1e-07 | 1.9e-13 | 95.6 | 92 | (1, 101) | 227 | (927, 1025) | 1163 | Tr-type G domain-containing protein | Tr-type G domain-containing protein | | uniclust | UniRef100\_A0A250G0W3 | 98.0 | 1.1e-07 | 2e-13 | 76.9 | 59 | (42, 106) | 227 | (62, 126) | 132 | SprT-like domain-containing protein | SprT-like domain-containing protein | | uniclust | UniRef100\_A0A0N4U7B3 | 98.0 | 1.1e-07 | 2.1e-13 | 85.7 | 127 | (29, 221) | 227 | (104, 236) | 302 | SprT-like domain-containing protein | SprT-like domain-containing protein | | uniclust | UniRef100\_A0A1S3GEE3 | 98.0 | 1.1e-07 | 2.3e-13 | 87.9 | 96 | (5, 106) | 227 | (94, 198) | 311 | Acidic repeat-containing protein | Acidic repeat-containing protein | | uniclust | UniRef100\_A0A069QD30 | 98.0 | 9.6e-08 | 2.3e-13 | 88.9 | 90 | (12, 106) | 227 | (41, 147) | 272 | SprT-like domain-containing protein | SprT-like domain-containing protein | | uniclust | UniRef100\_A0A8T0DKP9 | 98.0 | 1.3e-07 | 2.3e-13 | 85.1 | 78 | (22, 102) | 227 | (74, 165) | 329 | SprT-like domain-containing protein | SprT-like domain-containing protein | | uniclust | UniRef100\_UPI0008F98D6C | 98.0 | 1.3e-07 | 2.4e-13 | 95.3 | 76 | (23, 101) | 227 | (1008, 1093) | 1216 | uncharacterized protein LOC109037607 | uncharacterized protein LOC109037607 | | uniclust | UniRef100\_A0A813SQV5 | 98.0 | 1.2e-07 | 2.4e-13 | 91.8 | 87 | (9, 100) | 227 | (341, 435) | 566 | SprT-like domain-containing protein | SprT-like domain-containing protein | | uniclust | UniRef100\_A0A210QVD4 | 98.0 | 1.1e-07 | 2.5e-13 | 97.4 | 86 | (13, 104) | 227 | (67, 166) | 721 | SprT-like domain-containing protein Spartan | SprT-like domain-containing protein Spartan | | uniclust | UniRef100\_A0A2V0P5I1 | 98.0 | 1.2e-07 | 2.6e-13 | 94.6 | 71 | (29, 103) | 227 | (118, 196) | 569 | Protein with SprT-like domain at the N terminus | Protein with SprT-like domain at the N terminus | | uniclust | UniRef100\_UPI001E46D32E | 98.0 | 1.4e-07 | 2.7e-13 | 71.9 | 73 | (7, 86) | 227 | (14, 93) | 94 | SprT-like domain-containing protein | SprT-like domain-containing protein | | uniclust | UniRef100\_UPI0001CBADE3 | 98.0 | 1.5e-07 | 2.8e-13 | 88.4 | 80 | (23, 105) | 227 | (299, 387) | 499 | acidic repeat-containing protein-like | acidic repeat-containing protein-like | | uniclust | UniRef100\_UPI001E3CB0E9 | 98.0 | 1.6e-07 | 2.9e-13 | 72.0 | 66 | (42, 107) | 227 | (8, 89) | 103 | SprT-like domain-containing protein | SprT-like domain-containing protein | | uniclust | UniRef100\_A0A6L8CSP2 | 98.0 | 1.6e-07 | 3e-13 | 85.1 | 92 | (4, 108) | 227 | (165, 259) | 350 | SprT-like domain-containing protein | SprT-like domain-containing protein | | uniclust | UniRef100\_A0A2A4JTQ0 | 97.9 | 1.6e-07 | 3.1e-13 | 92.7 | 103 | (57, 220) | 227 | (427, 535) | 608 | SprT-like domain-containing protein | SprT-like domain-containing protein | | uniclust | UniRef100\_A0A095C996 | 97.9 | 1.5e-07 | 3.1e-13 | 96.0 | 97 | (4, 102) | 227 | (522, 635) | 772 | SprT-like domain-containing protein | SprT-like domain-containing protein | | uniclust | UniRef100\_A0A800A1Z7 | 97.9 | 1.8e-07 | 3.3e-13 | 79.3 | 65 | (57, 121) | 227 | (116, 189) | 201 | DUF35 domain-containing protein (Fragment) | DUF35 domain-containing protein (Fragment) | | uniclust | UniRef100\_A0A059LKE0 | 97.9 | 1.6e-07 | 3.3e-13 | 91.7 | 76 | (23, 102) | 227 | (195, 283) | 458 | SprT-like family protein | SprT-like family protein | | uniclust | UniRef100\_UPI00145EA0DD | 97.9 | 1.9e-07 | 3.4e-13 | 74.4 | 71 | (30, 109) | 227 | (31, 107) | 130 | SprT-like domain-containing protein | SprT-like domain-containing protein | | uniclust | UniRef100\_UPI001D095371 | 97.9 | 2.1e-07 | 3.9e-13 | 94.6 | 134 | (23, 220) | 227 | (1174, 1317) | 1385 | dentin sialophosphoprotein-like | dentin sialophosphoprotein-like | | uniclust | UniRef100\_A0A2P6WC38 | 97.9 | 2.2e-07 | 4.1e-13 | 79.5 | 81 | (42, 134) | 227 | (93, 176) | 215 | SprT-like domain-containing protein | SprT-like domain-containing protein | | uniclust | UniRef100\_A0A2R8FEQ8 | 97.9 | 2.4e-07 | 4.5e-13 | 80.7 | 76 | (25, 110) | 227 | (27, 105) | 247 | Zinc metallopeptidase SprT family | Zinc metallopeptidase SprT family | | uniclust | UniRef100\_A0A4Q6ELT1 | 97.9 | 2.5e-07 | 4.8e-13 | 80.5 | 91 | (12, 106) | 227 | (6, 110) | 222 | M48 family peptidase | M48 family peptidase | | uniclust | UniRef100\_A0A0R2KSK1 | 97.9 | 2.3e-07 | 4.8e-13 | 81.1 | 102 | (8, 114) | 227 | (25, 134) | 190 | SprT-like domain-containing protein | SprT-like domain-containing protein | | uniclust | UniRef100\_A0A7Y3I8T8 | 97.9 | 2.6e-07 | 4.8e-13 | 71.9 | 68 | (41, 108) | 227 | (30, 104) | 112 | SprT-like domain-containing protein | SprT-like domain-containing protein | | uniclust | UniRef100\_A0A085M4A3 | 97.9 | 2.5e-07 | 4.8e-13 | 89.3 | 144 | (11, 219) | 227 | (83, 236) | 554 | Alpha-carbonic anhydrase domain-containing protein | Alpha-carbonic anhydrase domain-containing protein | | uniclust | UniRef100\_A0A068WCX1 | 97.9 | 2.6e-07 | 4.9e-13 | 90.6 | 97 | (1, 102) | 227 | (407, 517) | 650 | Acidic repeat-containing protein | Acidic repeat-containing protein | | uniclust | UniRef100\_A0A2C9KYW2 | 97.9 | 2.7e-07 | 4.9e-13 | 91.3 | 91 | (10, 103) | 227 | (684, 781) | 899 | SprT-like domain-containing protein | SprT-like domain-containing protein | | uniclust | UniRef100\_A0A1M5NTB3 | 97.9 | 2.2e-07 | 4.9e-13 | 77.3 | 88 | (6, 102) | 227 | (24, 120) | 132 | SprT-like family protein | SprT-like family protein | | uniclust | UniRef100\_Q0A8D8 | 97.9 | 2.4e-07 | 4.9e-13 | 81.7 | 64 | (41, 110) | 227 | (83, 154) | 216 | SprT-like domain-containing protein | SprT-like domain-containing protein | | uniclust | UniRef100\_A0A0C2N032 | 97.9 | 2.3e-07 | 4.9e-13 | 86.7 | 75 | (23, 103) | 227 | (87, 170) | 312 | SprT-like domain-containing protein Spartan | SprT-like domain-containing protein Spartan | | uniclust | UniRef100\_A0A0A1X4E5 | 97.9 | 2.4e-07 | 5.1e-13 | 98.2 | 89 | (10, 103) | 227 | (883, 977) | 1114 | Acidic repeat-containing protein | Acidic repeat-containing protein | | uniclust | UniRef100\_A0A1V9X7L4 | 97.9 | 2.7e-07 | 5.2e-13 | 90.6 | 122 | (30, 220) | 227 | (506, 637) | 644 | HMG box-containing protein C19G7.04-like | HMG box-containing protein C19G7.04-like | | uniclust | UniRef100\_A0A8J1XJQ0 | 97.9 | 2.9e-07 | 5.3e-13 | 95.3 | 134 | (23, 220) | 227 | (1687, 1828) | 1885 | Ofus.G033133 protein | Ofus.G033133 protein | | uniclust | UniRef100\_A0A4Y7MT59 | 97.9 | 2.9e-07 | 5.4e-13 | 93.3 | 94 | (2, 101) | 227 | (854, 957) | 1080 | EOG090X0464 | EOG090X0464 | | uniclust | UniRef100\_A0A6P3R813 | 97.9 | 2.7e-07 | 5.8e-13 | 94.2 | 81 | (23, 106) | 227 | (348, 436) | 702 | Acidic repeat-containing protein isoform X4 | Acidic repeat-containing protein isoform X4 | | uniclust | UniRef100\_A0A0D0DSM7 | 97.9 | 2.7e-07 | 5.8e-13 | 90.1 | 95 | (3, 103) | 227 | (222, 324) | 459 | SprT-like domain-containing protein | SprT-like domain-containing protein | | uniclust | UniRef100\_A0A095Y7F4 | 97.9 | 2.7e-07 | 6.2e-13 | 85.3 | 94 | (5, 105) | 227 | (36, 140) | 261 | SprT-like domain-containing protein | SprT-like domain-containing protein | | uniclust | UniRef100\_A0A0D2G9M8 | 97.9 | 3.3e-07 | 6.2e-13 | 75.8 | 91 | (10, 106) | 227 | (10, 108) | 168 | SprT-like domain-containing protein | SprT-like domain-containing protein | | uniclust | UniRef100\_A0A2V2CUH8 | 97.9 | 3e-07 | 6.2e-13 | 79.5 | 93 | (12, 110) | 227 | (7, 108) | 177 | SprT-like domain-containing protein | SprT-like domain-containing protein | | uniclust | UniRef100\_UPI001E63D35C | 97.9 | 3.4e-07 | 6.3e-13 | 71.0 | 44 | (176, 219) | 227 | (57, 100) | 110 | hypothetical protein | hypothetical protein | | uniclust | UniRef100\_A0A193QM59 | 97.8 | 3.2e-07 | 6.5e-13 | 78.9 | 64 | (42, 111) | 227 | (54, 125) | 180 | Protein SprT | Protein SprT | | uniclust | UniRef100\_A0A016TZM6 | 97.8 | 3e-07 | 6.5e-13 | 92.5 | 76 | (23, 104) | 227 | (91, 175) | 566 | SprT-like domain-containing protein | SprT-like domain-containing protein | | uniclust | UniRef100\_A0A3L7P4X0 | 97.8 | 3.5e-07 | 7e-13 | 79.4 | 76 | (31, 112) | 227 | (48, 132) | 192 | SprT family protein | SprT family protein | | uniclust | UniRef100\_A0A948QZG9 | 97.8 | 3.8e-07 | 7.2e-13 | 81.4 | 106 | (8, 121) | 227 | (119, 237) | 266 | SprT-like domain-containing protein | SprT-like domain-containing protein | | uniclust | UniRef100\_A0A7J6MCN6 | 97.8 | 3.8e-07 | 7.2e-13 | 86.6 | 72 | (23, 101) | 227 | (324, 401) | 463 | SprT-like domain-containing protein | SprT-like domain-containing protein | | uniclust | UniRef100\_A0A183PK73 | 97.8 | 4.1e-07 | 7.4e-13 | 79.4 | 135 | (22, 220) | 227 | (30, 178) | 247 | SprT-like domain-containing protein | SprT-like domain-containing protein | | uniclust | UniRef100\_UPI001CA819BC | 97.8 | 3.8e-07 | 7.6e-13 | 81.9 | 88 | (8, 105) | 227 | (72, 168) | 251 | DNA-dependent metalloprotease dvc-1-like isoform X1 | DNA-dependent metalloprotease dvc-1-like isoform X1 | | uniclust | UniRef100\_A0A515MIG7 | 97.8 | 4.1e-07 | 7.7e-13 | 81.1 | 113 | (14, 137) | 227 | (127, 242) | 271 | SprT-like domain-containing protein | SprT-like domain-containing protein | | uniclust | UniRef100\_A0A0C2X8A7 | 97.8 | 3.9e-07 | 8e-13 | 87.3 | 96 | (2, 102) | 227 | (163, 265) | 421 | SprT-like domain-containing protein | SprT-like domain-containing protein | | uniclust | UniRef100\_UPI00138FF197 | 97.8 | 4.7e-07 | 8.6e-13 | 89.6 | 80 | (23, 105) | 227 | (294, 378) | 877 | uncharacterized protein LOC5522005 isoform X1 | uncharacterized protein LOC5522005 isoform X1 | | uniclust | UniRef100\_A0A1G6XTM0 | 97.8 | 4.4e-07 | 8.7e-13 | 83.9 | 99 | (9, 109) | 227 | (7, 118) | 325 | SprT-like family protein | SprT-like family protein | | uniclust | UniRef100\_A0A2E7CJ87 | 97.8 | 4.8e-07 | 8.8e-13 | 75.5 | 93 | (14, 110) | 227 | (6, 107) | 176 | SprT-like domain-containing protein | SprT-like domain-containing protein | | uniclust | UniRef100\_A0A0B0EPM6 | 97.8 | 3.9e-07 | 9.5e-13 | 91.3 | 65 | (42, 112) | 227 | (121, 194) | 487 | SprT-like family protein | SprT-like family protein | | uniclust | UniRef100\_A0A090RVQ2 | 97.8 | 4.6e-07 | 9.7e-13 | 76.4 | 66 | (41, 112) | 227 | (6, 79) | 146 | Protein sprT | Protein sprT | | uniclust | UniRef100\_A0A852Z1F4 | 97.8 | 5.3e-07 | 9.7e-13 | 71.1 | 78 | (121, 213) | 227 | (38, 115) | 120 | C2H2-type domain-containing protein | C2H2-type domain-containing protein | | uniclust | UniRef100\_A0A1G8BVJ5 | 97.8 | 5.5e-07 | 1e-12 | 78.8 | 81 | (23, 113) | 227 | (20, 104) | 252 | SprT-like family protein | SprT-like family protein | | uniclust | UniRef100\_A0A938L5N4 | 97.8 | 5.5e-07 | 1e-12 | 73.9 | 76 | (22, 109) | 227 | (23, 105) | 156 | SprT family zinc-dependent metalloprotease (Fragment) | SprT family zinc-dependent metalloprotease (Fragment) | | uniclust | UniRef100\_A0A2V7WKY5 | 97.8 | 5.2e-07 | 1e-12 | 77.3 | 86 | (7, 108) | 227 | (29, 118) | 185 | SprT-like domain-containing protein | SprT-like domain-containing protein | | uniclust | UniRef100\_A0A166KJM6 | 97.8 | 4.9e-07 | 1e-12 | 88.4 | 93 | (3, 102) | 227 | (246, 347) | 464 | SprT-like domain-containing protein | SprT-like domain-containing protein | | uniclust | UniRef100\_UPI000C776800 | 97.8 | 5.9e-07 | 1.1e-12 | 91.1 | 77 | (23, 102) | 227 | (1034, 1120) | 1237 | uncharacterized protein LOC111695131 | uncharacterized protein LOC111695131 | | uniclust | UniRef100\_A0A096NXZ5 | 97.8 | 5.5e-07 | 1.1e-12 | 84.7 | 86 | (14, 105) | 227 | (88, 187) | 376 | SprT-like N-terminal domain | SprT-like N-terminal domain | | uniclust | UniRef100\_A0A163K0V2 | 97.8 | 5.3e-07 | 1.1e-12 | 87.0 | 86 | (13, 105) | 227 | (184, 283) | 411 | SprT-like domain-containing protein | SprT-like domain-containing protein | | uniclust | UniRef100\_A0A183LV10 | 97.8 | 6.1e-07 | 1.2e-12 | 87.8 | 77 | (23, 102) | 227 | (410, 500) | 627 | SprT-like domain-containing protein | SprT-like domain-containing protein | | uniclust | UniRef100\_A0A0S4TDI0 | 97.8 | 5.7e-07 | 1.2e-12 | 83.3 | 76 | (23, 105) | 227 | (85, 169) | 286 | SprT-like domain-containing protein (Fragment) | SprT-like domain-containing protein (Fragment) | | uniclust | UniRef100\_A0A384DU94 | 97.8 | 6.2e-07 | 1.2e-12 | 86.7 | 82 | (22, 106) | 227 | (298, 387) | 497 | Acidic repeat-containing protein | Acidic repeat-containing protein | | uniclust | UniRef100\_A0A857ZTK7 | 97.8 | 6.8e-07 | 1.2e-12 | 71.0 | 41 | (68, 108) | 227 | (21, 64) | 125 | SprT-like domain-containing protein | SprT-like domain-containing protein | | uniclust | UniRef100\_A0A4R9A316 | 97.8 | 7.1e-07 | 1.3e-12 | 71.9 | 83 | (25, 116) | 227 | (19, 104) | 137 | M48 family peptidase | M48 family peptidase | | uniclust | UniRef100\_UPI001E2C7D93 | 97.7 | 7.2e-07 | 1.3e-12 | 76.9 | 67 | (42, 108) | 227 | (11, 98) | 223 | hypothetical protein | hypothetical protein | | uniclust | UniRef100\_A0A1F3AAF6 | 97.7 | 5.6e-07 | 1.3e-12 | 74.3 | 86 | (7, 102) | 227 | (11, 105) | 117 | Uncharacterized protein | Uncharacterized protein | | uniclust | UniRef100\_A0A927AK43 | 97.7 | 7.8e-07 | 1.4e-12 | 76.4 | 66 | (42, 107) | 227 | (41, 120) | 215 | Uncharacterized protein | Uncharacterized protein | | uniclust | UniRef100\_A0A015LDH0 | 97.7 | 7.3e-07 | 1.4e-12 | 83.9 | 86 | (13, 105) | 227 | (69, 168) | 366 | SprT-like domain-containing protein | SprT-like domain-containing protein | | uniclust | UniRef100\_A0A8S1E164 | 97.7 | 7.9e-07 | 1.4e-12 | 88.2 | 73 | (28, 102) | 227 | (699, 781) | 886 | SprT-like domain-containing protein | SprT-like domain-containing protein | | uniclust | UniRef100\_A0A6A4WPN6 | 97.7 | 8.1e-07 | 1.5e-12 | 89.0 | 143 | (15, 221) | 227 | (784, 943) | 1016 | Acidic repeat-containing protein | Acidic repeat-containing protein | | uniclust | UniRef100\_A0A1I7SSN3 | 97.7 | 8e-07 | 1.5e-12 | 90.0 | 86 | (12, 102) | 227 | (792, 884) | 964 | SprT-like domain-containing protein | SprT-like domain-containing protein | | uniclust | UniRef100\_A0A023W584 | 97.7 | 8.3e-07 | 1.5e-12 | 76.5 | 60 | (42, 107) | 227 | (59, 137) | 221 | SprT-like domain-containing protein | SprT-like domain-containing protein | | uniclust | UniRef100\_A0A5R8KJF2 | 97.7 | 8.4e-07 | 1.5e-12 | 79.4 | 85 | (15, 107) | 227 | (142, 233) | 299 | SprT family zinc-dependent metalloprotease | SprT family zinc-dependent metalloprotease | | uniclust | UniRef100\_A0A7W1MVA6 | 97.7 | 9.1e-07 | 1.7e-12 | 77.5 | 72 | (29, 108) | 227 | (85, 164) | 235 | SprT-like domain-containing protein | SprT-like domain-containing protein | | uniclust | UniRef100\_A0A060WHD9 | 97.7 | 7.8e-07 | 1.7e-12 | 91.5 | 84 | (14, 103) | 227 | (165, 262) | 709 | DNA-dependent metalloprotease SPRTN | DNA-dependent metalloprotease SPRTN | | uniclust | UniRef100\_A0A1Y1LCH4 | 97.7 | 8e-07 | 1.7e-12 | 88.9 | 87 | (13, 105) | 227 | (138, 238) | 547 | Protein with SprT-like domain at the N terminus | Protein with SprT-like domain at the N terminus | | uniclust | UniRef100\_A0A522YTE6 | 97.7 | 9.4e-07 | 1.7e-12 | 70.9 | 66 | (59, 124) | 227 | (60, 127) | 133 | Uncharacterized protein | Uncharacterized protein | | uniclust | UniRef100\_A0A7J7JVI6 | 97.7 | 9.8e-07 | 1.9e-12 | 74.1 | 77 | (23, 105) | 227 | (36, 121) | 165 | SprT-like domain-containing protein | SprT-like domain-containing protein | | uniclust | UniRef100\_A0A026WP05 | 97.7 | 8.4e-07 | 1.9e-12 | 91.1 | 73 | (30, 106) | 227 | (96, 177) | 672 | Protein with SprT-like domain at the N terminus (Fragment) | Protein with SprT-like domain at the N terminus (Fragment) | | uniclust | UniRef100\_A0A8T6V780 | 97.7 | 1e-06 | 1.9e-12 | 68.9 | 80 | (24, 114) | 227 | (19, 104) | 113 | SprT-like domain-containing protein | SprT-like domain-containing protein | | uniclust | UniRef100\_A0A154NZ86 | 97.7 | 1e-06 | 2e-12 | 91.4 | 134 | (23, 220) | 227 | (1195, 1338) | 1410 | Neuropeptides capa receptor | Neuropeptides capa receptor | | uniclust | UniRef100\_A0A075A2A6 | 97.7 | 1.1e-06 | 2e-12 | 88.5 | 77 | (23, 102) | 227 | (836, 926) | 1080 | SprT-like domain-containing protein | SprT-like domain-containing protein | | uniclust | UniRef100\_A0A1X7VLW4 | 97.7 | 1.1e-06 | 2.1e-12 | 84.4 | 75 | (23, 100) | 227 | (453, 533) | 593 | SprT-like domain-containing protein | SprT-like domain-containing protein | | uniclust | UniRef100\_A0A1J1I8Q7 | 97.7 | 1.1e-06 | 2.1e-12 | 92.4 | 75 | (29, 105) | 227 | (945, 1023) | 1139 | CLUMA\_CG009862, isoform A | CLUMA\_CG009862, isoform A | | uniclust | UniRef100\_UPI0002CC5A87 | 97.7 | 1.2e-06 | 2.2e-12 | 72.0 | 101 | (9, 112) | 227 | (6, 125) | 155 | SprT-like domain-containing protein | SprT-like domain-containing protein | | uniclust | UniRef100\_A0A024U161 | 97.7 | 1.1e-06 | 2.2e-12 | 83.2 | 72 | (29, 105) | 227 | (87, 167) | 365 | SprT-like domain-containing protein | SprT-like domain-containing protein | | uniclust | UniRef100\_A0A6J1SGE2 | 97.7 | 1.1e-06 | 2.2e-12 | 89.0 | 133 | (23, 220) | 227 | (748, 890) | 956 | Germ cell nuclear acidic protein-like isoform X3 | Germ cell nuclear acidic protein-like isoform X3 | | uniclust | UniRef100\_A0A5A8CGL8 | 97.7 | 1.2e-06 | 2.2e-12 | 77.8 | 89 | (8, 106) | 227 | (38, 136) | 265 | SprT-like domain-containing protein | SprT-like domain-containing protein | | uniclust | UniRef100\_A0A7J6QCS3 | 97.7 | 1.3e-06 | 2.4e-12 | 74.7 | 84 | (8, 98) | 227 | (49, 143) | 205 | SprT-like domain-containing protein (Fragment) | SprT-like domain-containing protein (Fragment) | | uniclust | UniRef100\_A0A6N9DWF4 | 97.7 | 1.3e-06 | 2.4e-12 | 75.5 | 77 | (22, 102) | 227 | (29, 122) | 218 | SprT family zinc-dependent metalloprotease | SprT family zinc-dependent metalloprotease | | uniclust | UniRef100\_A0A1V5RWR0 | 97.7 | 1.3e-06 | 2.4e-12 | 73.5 | 63 | (41, 104) | 227 | (37, 106) | 183 | SprT-like family protein | SprT-like family protein | | uniclust | UniRef100\_A0A6P8HBJ2 | 97.6 | 1.4e-06 | 2.6e-12 | 83.9 | 82 | (23, 107) | 227 | (292, 378) | 495 | Acidic repeat-containing protein-like | Acidic repeat-containing protein-like | | uniclust | UniRef100\_A0A7J6LXX1 | 97.6 | 1.3e-06 | 2.6e-12 | 92.4 | 72 | (23, 101) | 227 | (982, 1059) | 1207 | JmjC domain-containing protein | JmjC domain-containing protein | | uniclust | UniRef100\_A0A8T1MBU3 | 97.6 | 1.5e-06 | 2.7e-12 | 85.5 | 77 | (23, 102) | 227 | (131, 221) | 770 | lipoyl synthase (Fragment) | lipoyl synthase (Fragment) | | uniclust | UniRef100\_A0A353P8A1 | 97.6 | 1.5e-06 | 2.8e-12 | 77.2 | 67 | (41, 107) | 227 | (112, 196) | 278 | SprT-like domain-containing protein | SprT-like domain-containing protein | | uniclust | UniRef100\_A0A0N5CJR3 | 97.6 | 1.5e-06 | 2.8e-12 | 75.8 | 64 | (33, 101) | 227 | (60, 129) | 240 | SprT-like domain-containing protein | SprT-like domain-containing protein | | uniclust | UniRef100\_A0A965RUJ9 | 97.6 | 1.6e-06 | 2.9e-12 | 67.9 | 72 | (23, 105) | 227 | (38, 112) | 113 | Uncharacterized protein | Uncharacterized protein | | uniclust | UniRef100\_A0A2W1U3I7 | 97.6 | 1.6e-06 | 3.1e-12 | 80.1 | 77 | (25, 111) | 227 | (18, 97) | 340 | SprT-like domain-containing protein | SprT-like domain-containing protein | | uniclust | UniRef100\_UPI001F5BC89C | 97.6 | 1.8e-06 | 3.2e-12 | 65.3 | 73 | (7, 81) | 227 | (4, 88) | 91 | hypothetical protein | hypothetical protein | | uniclust | UniRef100\_UPI000289E565 | 97.6 | 1.8e-06 | 3.3e-12 | 68.7 | 79 | (7, 87) | 227 | (4, 94) | 125 | SprT-like domain-containing protein | SprT-like domain-containing protein | | uniclust | UniRef100\_A0A162V0U9 | 97.6 | 1.7e-06 | 3.4e-12 | 80.9 | 72 | (29, 105) | 227 | (115, 195) | 325 | SprT-like domain-containing protein | SprT-like domain-containing protein | | uniclust | UniRef100\_A0A4Y2D086 | 97.6 | 1.8e-06 | 3.5e-12 | 78.8 | 99 | (3, 107) | 227 | (128, 234) | 292 | Acidic repeat-containing protein | Acidic repeat-containing protein | | uniclust | UniRef100\_A0A925ICF3 | 97.6 | 2.1e-06 | 3.9e-12 | 74.8 | 88 | (29, 134) | 227 | (109, 199) | 235 | SprT-like domain-containing protein | SprT-like domain-containing protein | | uniclust | UniRef100\_A0A1I8A746 | 97.6 | 2.2e-06 | 4e-12 | 85.4 | 77 | (23, 102) | 227 | (645, 727) | 894 | SprT-like domain-containing protein | SprT-like domain-containing protein | | uniclust | UniRef100\_A0A7U9DY43 | 97.6 | 2.2e-06 | 4e-12 | 56.3 | 35 | (186, 221) | 227 | (2, 36) | 43 | Uncharacterized protein | Uncharacterized protein | | uniclust | UniRef100\_UPI00201F22D7 | 97.6 | 2.2e-06 | 4.1e-12 | 88.2 | 132 | (23, 221) | 227 | (1274, 1410) | 1470 | dentin sialophosphoprotein-like | dentin sialophosphoprotein-like | | uniclust | UniRef100\_UPI00193CA4CB | 97.6 | 2.3e-06 | 4.2e-12 | 88.5 | 134 | (23, 220) | 227 | (1367, 1509) | 1604 | uncharacterized protein LOC111060193 isoform X2 | uncharacterized protein LOC111060193 isoform X2 | | uniclust | UniRef100\_A0A7S0X6E5 | 97.6 | 2.1e-06 | 4.2e-12 | 84.4 | 77 | (23, 102) | 227 | (319, 405) | 519 | SprT-like domain-containing protein (Fragment) | SprT-like domain-containing protein (Fragment) | | uniclust | UniRef100\_A0A0L0DN51 | 97.6 | 2.4e-06 | 4.4e-12 | 81.8 | 93 | (3, 101) | 227 | (346, 445) | 551 | HMG box-containing protein | HMG box-containing protein | | uniclust | UniRef100\_A0A2P6TE25 | 97.6 | 2.3e-06 | 4.6e-12 | 80.1 | 77 | (23, 105) | 227 | (32, 116) | 329 | SprT-like domain-containing Spartan | SprT-like domain-containing Spartan | | uniclust | UniRef100\_A0A034V6Q8 | 97.6 | 2.4e-06 | 4.6e-12 | 79.5 | 76 | (29, 106) | 227 | (106, 190) | 364 | SprT-like domain-containing protein Spartan (Fragment) | SprT-like domain-containing protein Spartan (Fragment) | | uniclust | UniRef100\_A0A0C3RSD2 | 97.6 | 2.3e-06 | 4.6e-12 | 78.2 | 94 | (3, 102) | 227 | (45, 146) | 263 | SprT-like domain-containing protein (Fragment) | SprT-like domain-containing protein (Fragment) | | uniclust | UniRef100\_A0A956X7Z6 | 97.5 | 2.6e-06 | 4.8e-12 | 72.9 | 124 | (68, 220) | 227 | (52, 191) | 205 | Uncharacterized protein | Uncharacterized protein | | uniclust | UniRef100\_A0A080K9R0 | 97.5 | 2.3e-06 | 4.9e-12 | 70.5 | 70 | (22, 102) | 227 | (34, 110) | 123 | SprT-like family (Fragment) | SprT-like family (Fragment) | | uniclust | UniRef100\_A0A6L7XKZ6 | 97.5 | 2.6e-06 | 4.9e-12 | 75.5 | 65 | (42, 112) | 227 | (98, 165) | 254 | SprT-like domain-containing protein | SprT-like domain-containing protein | | uniclust | UniRef100\_A0A164WWE9 | 97.5 | 2.5e-06 | 5e-12 | 85.1 | 94 | (2, 101) | 227 | (366, 471) | 595 | Acidic repeat-containing-like protein | Acidic repeat-containing-like protein | | uniclust | UniRef100\_A0A2S5R2G5 | 97.5 | 2.7e-06 | 5e-12 | 71.1 | 65 | (42, 112) | 227 | (45, 122) | 172 | SprT-like domain-containing protein | SprT-like domain-containing protein | | uniclust | UniRef100\_A0A0D4DB50 | 97.5 | 2.7e-06 | 5.2e-12 | 77.1 | 83 | (24, 115) | 227 | (25, 113) | 272 | SprT-like protein | SprT-like protein | | uniclust | UniRef100\_A0A822CXB0 | 97.5 | 2.9e-06 | 5.3e-12 | 68.6 | 68 | (31, 103) | 227 | (3, 79) | 137 | SprT-like domain-containing protein | SprT-like domain-containing protein | | uniclust | UniRef100\_A0A433C399 | 97.5 | 3e-06 | 5.6e-12 | 67.9 | 75 | (25, 108) | 227 | (18, 102) | 120 | Uncharacterized protein | Uncharacterized protein | | uniclust | UniRef100\_A0A1B6DXM7 | 97.5 | 3.2e-06 | 5.9e-12 | 80.6 | 80 | (23, 105) | 227 | (315, 404) | 524 | SprT-like domain-containing protein | SprT-like domain-containing protein | | uniclust | UniRef100\_A0A2V2RIV7 | 97.5 | 3.3e-06 | 6e-12 | 72.0 | 82 | (13, 107) | 227 | (47, 135) | 196 | SprT-like domain-containing protein | SprT-like domain-containing protein | | uniclust | UniRef100\_UPI001EEAE6CF | 97.5 | 3.3e-06 | 6.1e-12 | 85.7 | 80 | (23, 105) | 227 | (931, 1019) | 1144 | uncharacterized protein LOC124357666 | uncharacterized protein LOC124357666 | | uniclust | UniRef100\_A0A1C5Q9L4 | 97.5 | 3.3e-06 | 6.4e-12 | 75.1 | 91 | (5, 102) | 227 | (38, 131) | 247 | SprT-like family | SprT-like family | | uniclust | UniRef100\_A0A4Y8K0Q1 | 97.5 | 3.5e-06 | 6.5e-12 | 72.8 | 79 | (27, 114) | 227 | (8, 89) | 218 | Uncharacterized protein | Uncharacterized protein | | uniclust | UniRef100\_A0A233HV64 | 97.5 | 2.9e-06 | 6.7e-12 | 75.6 | 50 | (58, 107) | 227 | (58, 110) | 189 | SprT-like domain-containing protein | SprT-like domain-containing protein | | uniclust | UniRef100\_A0A843HBC1 | 97.5 | 3.7e-06 | 6.8e-12 | 75.5 | 48 | (60, 107) | 227 | (115, 170) | 293 | SprT-like domain-containing protein | SprT-like domain-containing protein | | uniclust | UniRef100\_A0A2D5VXK3 | 97.5 | 3.7e-06 | 6.9e-12 | 69.1 | 94 | (7, 108) | 227 | (30, 131) | 152 | SprT-like domain-containing protein | SprT-like domain-containing protein | | uniclust | UniRef100\_A0A2S8IY47 | 97.5 | 3.8e-06 | 7.1e-12 | 62.6 | 44 | (176, 219) | 227 | (30, 73) | 83 | Uncharacterized protein | Uncharacterized protein | | uniclust | UniRef100\_A0A6V7K544 | 97.5 | 3.8e-06 | 7.1e-12 | 65.2 | 71 | (30, 104) | 227 | (2, 81) | 101 | SprT-like domain-containing protein (Fragment) | SprT-like domain-containing protein (Fragment) | | uniclust | UniRef100\_A0A077Z859 | 97.5 | 3.6e-06 | 7.4e-12 | 81.1 | 78 | (23, 106) | 227 | (70, 156) | 394 | SprT-like domain containing protein | SprT-like domain containing protein | | uniclust | UniRef100\_UPI0012E23595 | 97.5 | 4.2e-06 | 7.7e-12 | 74.6 | 76 | (29, 112) | 227 | (70, 148) | 277 | hypothetical protein | hypothetical protein | | uniclust | UniRef100\_A0A4D6E533 | 97.5 | 4.3e-06 | 7.9e-12 | 73.6 | 77 | (25, 111) | 227 | (21, 100) | 250 | SprT-like domain-containing protein | SprT-like domain-containing protein | | uniclust | UniRef100\_A0A131XCL1 | 97.5 | 4.1e-06 | 7.9e-12 | 81.2 | 84 | (14, 103) | 227 | (52, 149) | 506 | SprT-like domain-containing protein (Fragment) | SprT-like domain-containing protein (Fragment) | | uniclust | UniRef100\_F2UXT2 | 97.5 | 4.3e-06 | 8e-12 | 72.9 | 70 | (30, 108) | 227 | (69, 141) | 231 | SprT-like domain-containing protein | SprT-like domain-containing protein | | uniclust | UniRef100\_A0A6J4F9I4 | 97.5 | 4.4e-06 | 8e-12 | 64.3 | 40 | (180, 219) | 227 | (8, 48) | 99 | Transposase | Transposase | | uniclust | UniRef100\_A0A8S1DZ30 | 97.5 | 4.4e-06 | 8.2e-12 | 87.2 | 73 | (28, 102) | 227 | (188, 270) | 1809 | DH domain-containing protein | DH domain-containing protein | | uniclust | UniRef100\_A0A2W5X165 | 97.5 | 4.6e-06 | 8.5e-12 | 76.0 | 93 | (4, 106) | 227 | (158, 255) | 333 | SprT-like domain-containing protein | SprT-like domain-containing protein | | uniclust | UniRef100\_A0A0P9DLN8 | 97.5 | 4.3e-06 | 8.8e-12 | 68.3 | 104 | (90, 222) | 227 | (2, 114) | 117 | Uncharacterized protein (Fragment) | Uncharacterized protein (Fragment) | | uniclust | UniRef100\_A0A1S3H5D5 | 97.4 | 4.9e-06 | 9.1e-12 | 84.4 | 80 | (23, 105) | 227 | (915, 1003) | 1114 | Uncharacterized protein LOC106152248 | Uncharacterized protein LOC106152248 | | uniclust | UniRef100\_A0A138ZXI7 | 97.4 | 4.6e-06 | 9.2e-12 | 80.2 | 72 | (29, 105) | 227 | (139, 219) | 425 | Protein with SprT-like domain at the N terminus | Protein with SprT-like domain at the N terminus | | uniclust | UniRef100\_Q8EWL3 | 97.4 | 5e-06 | 9.2e-12 | 73.5 | 116 | (68, 222) | 227 | (87, 217) | 256 | SprT-like domain-containing protein | SprT-like domain-containing protein | | uniclust | UniRef100\_UPI0018851632 | 97.4 | 5.3e-06 | 9.7e-12 | 77.4 | 135 | (22, 221) | 227 | (197, 341) | 410 | acidic repeat-containing protein-like | acidic repeat-containing protein-like | | uniclust | UniRef100\_A0A0C2BZ93 | 97.4 | 5.1e-06 | 9.9e-12 | 68.5 | 75 | (23, 103) | 227 | (33, 116) | 137 | SprT-like domain-containing protein | SprT-like domain-containing protein | | uniclust | UniRef100\_A0A1F5UZX3 | 97.4 | 5.7e-06 | 1e-11 | 79.0 | 86 | (12, 105) | 227 | (350, 448) | 516 | Phospho-N-acetylmuramoyl-pentapeptide-transferase (Fragment) | Phospho-N-acetylmuramoyl-pentapeptide-transferase (Fragment) | | uniclust | UniRef100\_W6U0M4 | 97.4 | 5.7e-06 | 1.1e-11 | 82.4 | 97 | (1, 102) | 227 | (406, 516) | 858 | SprT-like domain-containing protein | SprT-like domain-containing protein | | uniclust | UniRef100\_A0A3P6HE70 | 97.4 | 5.7e-06 | 1.1e-11 | 73.1 | 97 | (1, 103) | 227 | (6, 117) | 255 | SprT-like domain-containing protein | SprT-like domain-containing protein | | uniclust | UniRef100\_A0A226D0T9 | 97.4 | 5.8e-06 | 1.1e-11 | 76.4 | 71 | (29, 103) | 227 | (50, 131) | 376 | SprT-like domain-containing protein Spartan | SprT-like domain-containing protein Spartan | | uniclust | UniRef100\_A0A146HCT3 | 97.4 | 5.5e-06 | 1.1e-11 | 81.6 | 82 | (23, 106) | 227 | (301, 390) | 568 | SprT-like domain-containing protein | SprT-like domain-containing protein | | uniclust | UniRef100\_A0A219YCL2 | 97.4 | 5.2e-06 | 1.1e-11 | 73.3 | 71 | (30, 110) | 227 | (39, 112) | 197 | SprT-like domain-containing protein | SprT-like domain-containing protein | | uniclust | UniRef100\_A0A353B0T0 | 97.4 | 5.9e-06 | 1.1e-11 | 68.4 | 69 | (42, 116) | 227 | (49, 118) | 157 | SprT-like domain-containing protein | SprT-like domain-containing protein | | uniclust | UniRef100\_A0A7J6ZA20 | 97.4 | 5.8e-06 | 1.1e-11 | 86.5 | 133 | (23, 220) | 227 | (1254, 1397) | 1457 | SprT-like domain-containing protein | SprT-like domain-containing protein | | uniclust | UniRef100\_A0A0D6LND9 | 97.4 | 5.9e-06 | 1.1e-11 | 77.8 | 80 | (16, 101) | 227 | (264, 349) | 447 | SprT-like domain-containing protein | SprT-like domain-containing protein | | uniclust | UniRef100\_A0A160TLN6 | 97.4 | 6e-06 | 1.1e-11 | 56.1 | 29 | (184, 212) | 227 | (1, 30) | 50 | Uncharacterized protein | Uncharacterized protein | | uniclust | UniRef100\_A0A5S6QT35 | 97.4 | 6e-06 | 1.1e-11 | 71.8 | 92 | (9, 103) | 227 | (69, 169) | 224 | SprT-like domain-containing protein | SprT-like domain-containing protein | | uniclust | UniRef100\_UPI0015AA2B96 | 97.4 | 6.4e-06 | 1.2e-11 | 68.7 | 42 | (73, 114) | 227 | (2, 49) | 166 | hypothetical protein | hypothetical protein | | uniclust | UniRef100\_UPI000A922881 | 97.4 | 6.7e-06 | 1.2e-11 | 73.4 | 56 | (57, 112) | 227 | (106, 164) | 277 | SprT-like domain-containing protein | SprT-like domain-containing protein | | uniclust | UniRef100\_A0A147BGG7 | 97.4 | 6.9e-06 | 1.3e-11 | 68.0 | 66 | (29, 99) | 227 | (60, 130) | 157 | Putative sprt-like metalloprotease (Fragment) | Putative sprt-like metalloprotease (Fragment) | | uniclust | UniRef100\_UPI000D7345FA | 97.4 | 6.9e-06 | 1.3e-11 | 74.4 | 90 | (9, 100) | 227 | (65, 160) | 310 | HMG box-containing protein C19G7.04-like | HMG box-containing protein C19G7.04-like | | uniclust | UniRef100\_A0A2N1WAY1 | 97.4 | 7.1e-06 | 1.3e-11 | 67.6 | 66 | (42, 110) | 227 | (65, 137) | 152 | SprT-like domain-containing protein (Fragment) | SprT-like domain-containing protein (Fragment) | | uniclust | UniRef100\_A0A067TZ07 | 97.4 | 6.5e-06 | 1.3e-11 | 81.2 | 93 | (11, 106) | 227 | (254, 358) | 477 | SprT-like domain-containing protein | SprT-like domain-containing protein | | uniclust | UniRef100\_A0A956U136 | 97.4 | 7.5e-06 | 1.4e-11 | 64.8 | 65 | (42, 112) | 227 | (9, 79) | 116 | SprT-like domain-containing protein (Fragment) | SprT-like domain-containing protein (Fragment) | | uniclust | UniRef100\_A0A7Y1ZPK4 | 97.4 | 7.4e-06 | 1.4e-11 | 72.3 | 74 | (29, 110) | 227 | (70, 151) | 225 | SprT-like domain-containing protein | SprT-like domain-containing protein | | uniclust | UniRef100\_A0A915GBT6 | 97.4 | 7.6e-06 | 1.4e-11 | 71.9 | 144 | (16, 222) | 227 | (30, 182) | 242 | SprT-like domain-containing protein | SprT-like domain-containing protein | | uniclust | UniRef100\_A0A947MTM0 | 97.4 | 8e-06 | 1.5e-11 | 55.3 | 40 | (179, 219) | 227 | (8, 47) | 49 | Uncharacterized protein | Uncharacterized protein | | uniclust | UniRef100\_A0A7W1KMR8 | 97.4 | 7.8e-06 | 1.5e-11 | 67.2 | 83 | (23, 111) | 227 | (35, 124) | 139 | SprT-like domain-containing protein | SprT-like domain-containing protein | | uniclust | UniRef100\_A0A1G8CW72 | 97.4 | 8.2e-06 | 1.5e-11 | 70.9 | 62 | (42, 109) | 227 | (68, 132) | 222 | SprT-like family protein | SprT-like family protein | | uniclust | UniRef100\_A0A6L7STI3 | 97.4 | 8.3e-06 | 1.5e-11 | 55.9 | 32 | (68, 99) | 227 | (20, 51) | 52 | SprT family zinc-dependent metalloprotease | SprT family zinc-dependent metalloprotease | | uniclust | UniRef100\_A0A1D2MB96 | 97.4 | 7.8e-06 | 1.5e-11 | 78.2 | 75 | (23, 103) | 227 | (78, 161) | 400 | SprT-like domain-containing protein Spartan (Fragment) | SprT-like domain-containing protein Spartan (Fragment) | | uniclust | UniRef100\_F0F968 | 97.4 | 7.7e-06 | 1.5e-11 | 73.3 | 97 | (4, 108) | 227 | (7, 115) | 224 | SprT-like domain-containing protein | SprT-like domain-containing protein | | uniclust | UniRef100\_A0A067P3B9 | 97.4 | 8.2e-06 | 1.6e-11 | 72.7 | 50 | (57, 106) | 227 | (86, 141) | 242 | SprT-like domain-containing protein (Fragment) | SprT-like domain-containing protein (Fragment) | | uniclust | UniRef100\_A0A820RHD1 | 97.4 | 8.6e-06 | 1.6e-11 | 78.7 | 68 | (29, 100) | 227 | (376, 449) | 578 | SprT-like domain-containing protein | SprT-like domain-containing protein | | uniclust | UniRef100\_A0A5Q3L5Z6 | 97.3 | 8.7e-06 | 1.6e-11 | 69.5 | 87 | (23, 118) | 227 | (24, 118) | 193 | SprT domain-containing protein | SprT domain-containing protein | | uniclust | UniRef100\_A0A0L8I9K9 | 97.3 | 8.8e-06 | 1.6e-11 | 79.5 | 134 | (29, 224) | 227 | (457, 601) | 658 | SprT-like domain-containing protein | SprT-like domain-containing protein | | uniclust | UniRef100\_A0A182IL28 | 97.3 | 8.7e-06 | 1.7e-11 | 80.2 | 75 | (29, 105) | 227 | (95, 178) | 575 | Protein with SprT-like domain at the N terminus | Protein with SprT-like domain at the N terminus | | uniclust | UniRef100\_A0A484AUX1 | 97.3 | 8.7e-06 | 1.7e-11 | 77.8 | 74 | (28, 103) | 227 | (205, 283) | 412 | SprT-like domain-containing protein | SprT-like domain-containing protein | | uniclust | UniRef100\_UPI001CC56835 | 97.3 | 9.3e-06 | 1.7e-11 | 71.9 | 78 | (22, 102) | 227 | (46, 132) | 256 | acidic repeat-containing protein-like | acidic repeat-containing protein-like | | uniclust | UniRef100\_UPI001BB153D4 | 97.3 | 9.3e-06 | 1.8e-11 | 81.6 | 95 | (3, 103) | 227 | (513, 616) | 730 | acidic repeat-containing protein-like | acidic repeat-containing protein-like | | uniclust | UniRef100\_UPI0018D5A09C | 97.3 | 9.8e-06 | 1.8e-11 | 62.4 | 44 | (71, 114) | 227 | (2, 45) | 98 | SprT-like domain-containing protein | SprT-like domain-containing protein | | uniclust | UniRef100\_A0A7J8EKG4 | 97.3 | 9.6e-06 | 1.8e-11 | 75.6 | 81 | (23, 106) | 227 | (44, 132) | 367 | SprT-like domain-containing protein | SprT-like domain-containing protein | | uniclust | UniRef100\_A0A2V5RCE3 | 97.3 | 1e-05 | 1.8e-11 | 67.6 | 73 | (28, 108) | 227 | (57, 136) | 165 | SprT-like domain-containing protein | SprT-like domain-containing protein | | uniclust | UniRef100\_UPI000BF750D2 | 97.3 | 1e-05 | 1.8e-11 | 77.2 | 69 | (29, 99) | 227 | (358, 432) | 497 | acidic repeat-containing protein-like isoform X2 | acidic repeat-containing protein-like isoform X2 | | uniclust | UniRef100\_M9V2A0 | 97.3 | 1e-05 | 1.9e-11 | 60.8 | 57 | (29, 93) | 227 | (25, 84) | 85 | Putative SprT zinc metallopeptidase | Putative SprT zinc metallopeptidase | | uniclust | UniRef100\_A0A0P4W780 | 97.3 | 9.3e-06 | 1.9e-11 | 80.3 | 87 | (14, 104) | 227 | (38, 139) | 519 | Protein with SprT-like domain at the N terminus | Protein with SprT-like domain at the N terminus | | uniclust | UniRef100\_A0A090N044 | 97.3 | 9.9e-06 | 1.9e-11 | 77.0 | 76 | (23, 104) | 227 | (33, 117) | 375 | SprT-like domain-containing protein | SprT-like domain-containing protein | | uniclust | UniRef100\_A0A7S4L4D1 | 97.3 | 1e-05 | 2e-11 | 73.0 | 79 | (23, 107) | 227 | (30, 117) | 258 | SprT-like domain-containing protein (Fragment) | SprT-like domain-containing protein (Fragment) | | uniclust | UniRef100\_A0A068WV36 | 97.3 | 9.2e-06 | 2e-11 | 79.4 | 78 | (28, 107) | 227 | (90, 183) | 387 | Protein with SprT-like domain at the N terminus | Protein with SprT-like domain at the N terminus | | uniclust | UniRef100\_A0A497HZ04 | 97.3 | 9.8e-06 | 2e-11 | 66.1 | 46 | (56, 102) | 227 | (54, 100) | 114 | Uncharacterized protein | Uncharacterized protein | | uniclust | UniRef100\_A0A6I8TF76 | 97.3 | 1.1e-05 | 2.1e-11 | 78.3 | 71 | (28, 100) | 227 | (361, 435) | 557 | Uncharacterized protein | Uncharacterized protein | | uniclust | UniRef100\_A0A3M7QTT4 | 97.3 | 1.2e-05 | 2.1e-11 | 73.8 | 78 | (23, 105) | 227 | (109, 191) | 338 | Acidic repeat-containing (Fragment) | Acidic repeat-containing (Fragment) | | uniclust | UniRef100\_A0A803J7Z6 | 97.3 | 1.1e-05 | 2.1e-11 | 72.1 | 78 | (22, 105) | 227 | (82, 166) | 235 | SprT-like domain-containing protein | SprT-like domain-containing protein | | uniclust | UniRef100\_A0A965MWP3 | 97.3 | 1.2e-05 | 2.1e-11 | 64.2 | 53 | (59, 112) | 227 | (4, 59) | 121 | SprT-like domain-containing protein | SprT-like domain-containing protein | | uniclust | UniRef100\_A0A350I387 | 97.3 | 1.2e-05 | 2.1e-11 | 64.1 | 60 | (42, 107) | 227 | (47, 114) | 120 | SprT-like domain-containing protein | SprT-like domain-containing protein | | uniclust | UniRef100\_A0A2H9ZV33 | 97.3 | 1e-05 | 2.2e-11 | 80.2 | 71 | (32, 106) | 227 | (87, 166) | 459 | SprT-like domain-containing protein | SprT-like domain-containing protein | | uniclust | UniRef100\_A0A224XN60 | 97.3 | 1.1e-05 | 2.2e-11 | 68.8 | 83 | (14, 103) | 227 | (69, 165) | 168 | SprT-like domain-containing protein (Fragment) | SprT-like domain-containing protein (Fragment) | | uniclust | UniRef100\_A0A8J1IYR3 | 97.3 | 1e-05 | 2.2e-11 | 74.3 | 89 | (8, 104) | 227 | (56, 151) | 246 | SprT-like domain-containing protein Spartan | SprT-like domain-containing protein Spartan | | uniclust | UniRef100\_B4CZ15 | 97.3 | 1.2e-05 | 2.2e-11 | 77.8 | 73 | (28, 108) | 227 | (437, 517) | 582 | Aminotransferase class V | Aminotransferase class V | | uniclust | UniRef100\_A0A2R5LIW9 | 97.3 | 1.2e-05 | 2.2e-11 | 70.6 | 132 | (24, 222) | 227 | (98, 234) | 238 | Putative sprt-like metalloprotease (Fragment) | Putative sprt-like metalloprotease (Fragment) | | uniclust | UniRef100\_A0A0C3CV02 | 97.3 | 1.1e-05 | 2.3e-11 | 75.9 | 89 | (15, 106) | 227 | (114, 214) | 332 | SprT-like domain-containing protein | SprT-like domain-containing protein | | uniclust | UniRef100\_A0A2E2K3M5 | 97.3 | 1.3e-05 | 2.4e-11 | 69.7 | 63 | (42, 110) | 227 | (50, 119) | 198 | SprT-like domain-containing protein | SprT-like domain-containing protein | | uniclust | UniRef100\_UPI0003592C47 | 97.3 | 1.3e-05 | 2.4e-11 | 82.7 | 133 | (24, 220) | 227 | (1156, 1293) | 1358 | uncharacterized protein LOC101846459 | uncharacterized protein LOC101846459 | | uniclust | UniRef100\_A0A1Y4R9A6 | 97.3 | 1.2e-05 | 2.4e-11 | 70.3 | 95 | (4, 104) | 227 | (13, 111) | 186 | SprT-like domain-containing protein | SprT-like domain-containing protein | | uniclust | UniRef100\_A0A183A629 | 97.3 | 1.3e-05 | 2.5e-11 | 71.1 | 43 | (57, 99) | 227 | (35, 82) | 239 | SprT-like domain-containing protein | SprT-like domain-containing protein | | uniclust | UniRef100\_A0A3M7P3R5 | 97.3 | 1.3e-05 | 2.5e-11 | 74.8 | 91 | (9, 105) | 227 | (55, 159) | 348 | SprT-like domain-containing Spartan | SprT-like domain-containing Spartan | | uniclust | UniRef100\_A0A1Z8WTI9 | 97.3 | 1.4e-05 | 2.5e-11 | 66.0 | 83 | (16, 109) | 227 | (41, 142) | 151 | SprT-like domain-containing protein | SprT-like domain-containing protein | | uniclust | UniRef100\_R4TMW7 | 97.3 | 1.4e-05 | 2.6e-11 | 70.9 | 44 | (68, 111) | 227 | (128, 175) | 256 | SprT-like domain-containing protein | SprT-like domain-containing protein | | uniclust | UniRef100\_A0A010RHC0 | 97.3 | 1.2e-05 | 2.6e-11 | 83.9 | 73 | (29, 103) | 227 | (444, 536) | 687 | SprT-like domain-containing protein | SprT-like domain-containing protein | | uniclust | UniRef100\_A0A7V9Q7G0 | 97.3 | 1.4e-05 | 2.6e-11 | 68.6 | 93 | (7, 112) | 227 | (24, 123) | 176 | SprT-like domain-containing protein | SprT-like domain-containing protein | | uniclust | UniRef100\_UPI001AC329C2 | 97.3 | 1.4e-05 | 2.6e-11 | 71.0 | 39 | (68, 106) | 227 | (218, 256) | 262 | hypothetical protein | hypothetical protein | | uniclust | UniRef100\_A0A1E1XDU2 | 97.3 | 1.4e-05 | 2.7e-11 | 68.9 | 69 | (28, 101) | 227 | (91, 164) | 206 | Putative sprt-like metalloprotease (Fragment) | Putative sprt-like metalloprotease (Fragment) | | uniclust | UniRef100\_A0A7D9HBJ1 | 97.3 | 1.4e-05 | 2.7e-11 | 81.5 | 80 | (22, 105) | 227 | (708, 792) | 909 | SprT-like domain-containing protein | SprT-like domain-containing protein | | uniclust | UniRef100\_A0A6P7TLT7 | 97.3 | 1.5e-05 | 2.7e-11 | 80.4 | 135 | (28, 224) | 227 | (735, 880) | 937 | Acidic repeat-containing protein-like | Acidic repeat-containing protein-like | | uniclust | UniRef100\_A0A248SJR1 | 97.3 | 1.5e-05 | 2.7e-11 | 69.3 | 74 | (42, 121) | 227 | (60, 139) | 218 | SprT-like domain-containing protein | SprT-like domain-containing protein | | uniclust | UniRef100\_A0A2R5L845 | 97.3 | 1.4e-05 | 2.7e-11 | 80.4 | 94 | (7, 106) | 227 | (58, 165) | 605 | Protein with SprT-like domain at the N terminus (Fragment) | Protein with SprT-like domain at the N terminus (Fragment) | | uniclust | UniRef100\_A0A3C1Z6Q3 | 97.2 | 1.6e-05 | 2.8e-11 | 70.9 | 80 | (22, 105) | 227 | (78, 177) | 264 | SprT-like domain-containing protein | SprT-like domain-containing protein | | uniclust | UniRef100\_A0A3E0KDZ8 | 97.2 | 1.6e-05 | 2.9e-11 | 68.4 | 67 | (25, 102) | 227 | (71, 140) | 202 | SprT-like domain-containing protein | SprT-like domain-containing protein | | uniclust | UniRef100\_A0A1V4KYG6 | 97.2 | 1.6e-05 | 3.1e-11 | 84.5 | 97 | (3, 105) | 227 | (1349, 1454) | 1566 | UDP-N-acetylglucosamine--peptide N-acetylglucosaminyltransferase 110 kDa subunit | UDP-N-acetylglucosamine--peptide N-acetylglucosaminyltransferase 110 kDa subunit | | uniclust | UniRef100\_A0A521H7S3 | 97.2 | 1.7e-05 | 3.2e-11 | 69.1 | 92 | (17, 111) | 227 | (75, 173) | 222 | SprT-like domain-containing protein | SprT-like domain-containing protein | | uniclust | UniRef100\_UPI00167373B6 | 97.2 | 1.8e-05 | 3.2e-11 | 67.6 | 52 | (59, 110) | 227 | (3, 65) | 189 | hypothetical protein | hypothetical protein | | uniclust | UniRef100\_A0A7S0GYB2 | 97.2 | 1.7e-05 | 3.2e-11 | 72.0 | 79 | (29, 110) | 227 | (114, 195) | 254 | SprT-like domain-containing protein | SprT-like domain-containing protein | | uniclust | UniRef100\_A0A6P1LIC7 | 97.2 | 1.8e-05 | 3.3e-11 | 71.1 | 144 | (42, 220) | 227 | (85, 251) | 280 | SprT-like domain-containing protein | SprT-like domain-containing protein | | uniclust | UniRef100\_A0A0B2W3V2 | 97.2 | 1.8e-05 | 3.4e-11 | 66.1 | 39 | (185, 223) | 227 | (65, 103) | 163 | Acidic repeat-containing protein | Acidic repeat-containing protein | | uniclust | UniRef100\_A0A087R6W8 | 97.2 | 1.8e-05 | 3.4e-11 | 79.5 | 71 | (29, 103) | 227 | (192, 271) | 683 | SprT-like domain-containing protein Spartan (Fragment) | SprT-like domain-containing protein Spartan (Fragment) | | uniclust | UniRef100\_A0A0H2STH3 | 97.2 | 1.9e-05 | 3.5e-11 | 72.6 | 97 | (1, 102) | 227 | (81, 184) | 344 | SprT-like domain-containing protein | SprT-like domain-containing protein | | uniclust | UniRef100\_A0A0F9VPM0 | 97.2 | 1.8e-05 | 3.7e-11 | 68.6 | 68 | (29, 102) | 227 | (33, 108) | 175 | SprT-like domain-containing protein | SprT-like domain-containing protein | | uniclust | UniRef100\_A0A6M2D328 | 97.2 | 1.9e-05 | 3.7e-11 | 70.8 | 89 | (2, 99) | 227 | (128, 222) | 255 | Putative sprt-like metalloprotease ovary overexpressed | Putative sprt-like metalloprotease ovary overexpressed | | uniclust | UniRef100\_A0A1Q1N996 | 97.2 | 2e-05 | 3.9e-11 | 68.7 | 65 | (42, 112) | 227 | (44, 114) | 196 | SprT-like domain-containing protein | SprT-like domain-containing protein | | uniclust | UniRef100\_A0A5A8CG24 | 97.2 | 2.1e-05 | 3.9e-11 | 72.7 | 72 | (29, 106) | 227 | (204, 285) | 361 | DHFR domain-containing protein | DHFR domain-containing protein | | uniclust | UniRef100\_A0A822IAK2 | 97.2 | 2.1e-05 | 3.9e-11 | 67.2 | 73 | (23, 102) | 227 | (64, 142) | 174 | (mimic poison frog) hypothetical protein (Fragment) | (mimic poison frog) hypothetical protein (Fragment) | | uniclust | UniRef100\_A0A8D8EVU5 | 97.2 | 2.2e-05 | 4e-11 | 70.7 | 73 | (30, 104) | 227 | (46, 127) | 285 | SprT-like domain-containing protein Spartan (Fragment) | SprT-like domain-containing protein Spartan (Fragment) | | uniclust | UniRef100\_A0A5J4WFS3 | 97.2 | 2.1e-05 | 4.1e-11 | 73.5 | 96 | (2, 102) | 227 | (150, 257) | 317 | Putative sprt family metallopeptidase | Putative sprt family metallopeptidase | | uniclust | UniRef100\_A0A0C2NES1 | 97.2 | 1.9e-05 | 4.2e-11 | 75.0 | 84 | (22, 112) | 227 | (31, 127) | 311 | SprT-like domain-containing protein | SprT-like domain-containing protein | | pdb70 | 4QHF\_A | 98.8 | 6.1e-13 | 6.4e-17 | 98.9 | 89 | (1, 104) | 227 | (1, 93) | 110 | Uncharacterized protein MJ1213 | 4QHF\_A Uncharacterized protein MJ1213 Minigluzincin, Proteolytic enzyme, HYDROLASE | | pdb70 | 4QHJ\_B | 98.8 | 7.5e-13 | 7.9e-17 | 98.3 | 89 | (1, 104) | 227 | (1, 93) | 110 | Uncharacterized protein MJ1213 | 4QHJ\_B Uncharacterized protein MJ1213 Minigluzincin, Proteolytic enzyme, HYDROLASE HET: ACT | | pdb70 | 6MDX\_A | 98.5 | 2.5e-11 | 2.6e-15 | 97.7 | 93 | (3, 105) | 227 | (14, 115) | 185 | SprT-like domain-containing protein Spartan/DNA Complex | 6MDX\_A SprT-like domain-containing protein Spartan/DNA Complex DPC repair, protease, DNA BINDING HET: MSE, PGE, FLC, MLZ | | pdb70 | 6MDW\_A | 98.4 | 7.1e-11 | 7.3e-15 | 96.0 | 95 | (3, 105) | 227 | (21, 124) | 194 | SprT-like domain-containing protein Spartan | 6MDW\_A SprT-like domain-containing protein Spartan DPC repair protease, DNA BINDING HET: ADP, FLC, MLZ | | pdb70 | 4JIX\_A | 98.3 | 1.5e-10 | 1.6e-14 | 84.7 | 86 | (4, 105) | 227 | (9, 97) | 112 | Uncharacterized protein MJ0123 | 4JIX\_A Uncharacterized protein MJ0123 Hydrolase, Metallopeptidase Zymogen, Minigluzincin HET: GOL, SO4 | | pdb70 | 4JIU\_A | 98.1 | 1.2e-09 | 1.3e-13 | 79.4 | 82 | (7, 106) | 227 | (2, 87) | 105 | Putative uncharacterized protein | 4JIU\_A Putative uncharacterized protein Hydrolase, Metallopeptidase Zymogen, Minigluzincin | | pdb70 | 5XBV\_A | 97.9 | 4.2e-09 | 3.9e-13 | 83.6 | 56 | (41, 105) | 227 | (77, 139) | 149 | Wss1p | 5XBV\_A Wss1p protease, HYDROLASE | | pdb70 | 5XBN\_A | 97.9 | 4.4e-09 | 4.1e-13 | 83.4 | 56 | (41, 105) | 227 | (77, 139) | 148 | Wss1p | 5XBN\_A Wss1p protease, HYDROLASE HET: MSE | | pdb70 | 5JIG\_A | 97.5 | 5.8e-08 | 5.6e-12 | 74.5 | 55 | (42, 105) | 227 | (61, 121) | 127 | Ubiquitin and WLM domain-containing metalloprotease | 5JIG\_A Ubiquitin and WLM domain-containing metalloprotease Metalloprotease, DNA-Repair, Endoprotease, Regulation, hydrolase | | pdb70 | 3CQB\_A | 95.8 | 7.4e-05 | 8.3e-09 | 52.1 | 68 | (5, 85) | 227 | (26, 96) | 107 | Probable protease htpX homolog (E.C.3.4.24.-) | 3CQB\_A Probable protease htpX homolog (E.C.3.4.24.-) Heat shock protein HtpX domain HET: EDO | | pdb70 | 6NCL\_c2 | 95.3 | 0.00029 | 2.3e-08 | 57.0 | 46 | (60, 105) | 227 | (83, 136) | 181 | PBCV-1 capsid | 6NCL\_c2 PBCV-1 capsid tape-measure protein, minor capsid proteins | | pdb70 | 6NCL\_c3 | 95.3 | 0.00029 | 2.3e-08 | 57.0 | 46 | (60, 105) | 227 | (83, 136) | 181 | PBCV-1 capsid | 6NCL\_c3 PBCV-1 capsid tape-measure protein, minor capsid proteins | |
| Top keywords  (threshold 1.00e-03 (evalue)) | **SprT\_like, domain\_containing, Fragment, Acidic, SprT, repeat\_containing, metalloprotease, hypothetical, Spartan, Putative** |
| Output files | ../../similar\_sequences/34\_FANPEZAQ\_CDS\_0034\_merged.svg ../../similar\_sequences/34\_FANPEZAQ\_CDS\_0034\_pdb70.a3m ../../similar\_sequences/34\_FANPEZAQ\_CDS\_0034\_pdb70.hhr ../../similar\_sequences/34\_FANPEZAQ\_CDS\_0034\_uniclust.a3m ../../similar\_sequences/34\_FANPEZAQ\_CDS\_0034\_uniclust.hhr |

#### Structure prediction (AlphaFold)2

|  |  |
| --- | --- |
| Stats | xml version="1.0" encoding="utf-8" standalone="no"?       2024-09-02T21:09:33.674706 image/svg+xml   Matplotlib v3.7.2, https://matplotlib.org/ |
| Predicted structure | **NGL Viewer Controls:**  - Center: *Left-Click* - Rotate: *Left-Click + Drag* - Translate: *Right-Click + Drag* - Zoom: *Shift + Left-Click + Drag* |
| Output files | ../../predicted\_structures/34\_FANPEZAQ\_CDS\_0034/features.pkl ../../predicted\_structures/34\_FANPEZAQ\_CDS\_0034/ranked\_0.pdb ../../predicted\_structures/34\_FANPEZAQ\_CDS\_0034/ranked\_0\_plots.svg ../../predicted\_structures/34\_FANPEZAQ\_CDS\_0034/result\_model\_1\_ptm\_pred\_0.pkl |

#### Structure similarity search results (Foldseek)3

|  |  |
| --- | --- |
| Structure databases searched | Pdb, Afdb-proteome, Afdb-uniprot50 |
| Results, scheme(s)  (Top layers only, threshold 1.00e-02 (evalue)) | xml version="1.0" encoding="utf-8" standalone="no"?       2024-09-02T21:11:06.896100 image/svg+xml   Matplotlib v3.7.2, https://matplotlib.org/ |
| Results, table  (threshold 1.00e-02 (evalue)) | | db | id | prob | evalue | bits | fident | alnlen | mismatch | gapopen | qstart | qend | tstart | tend | name | description | | --- | --- | --- | --- | --- | --- | --- | --- | --- | --- | --- | --- | --- | --- | --- | | afdb-uniprot50 | AF-A0A2W5L353-F1-MODEL\_V4 | 1.0 | 1.243e-23 | 760 | 0.497 | 231 | 101 | 4 | 1 | 224 | 1 | 223 | Transcription elongation protein SprT | Transcription elongation protein SprT | | afdb-uniprot50 | AF-A0A3N8XMK1-F1-MODEL\_V4 | 1.0 | 2.833e-21 | 701 | 0.43 | 230 | 119 | 5 | 1 | 220 | 12 | 239 | Transcription elongation protein SprT | Transcription elongation protein SprT | | afdb-uniprot50 | AF-A0A149SP00-F1-MODEL\_V4 | 1.0 | 3.791e-20 | 674 | 0.432 | 208 | 104 | 4 | 15 | 218 | 2 | 199 | Uncharacterized protein | Uncharacterized protein | | afdb-uniprot50 | AF-S9SWB4-F1-MODEL\_V4 | 1.0 | 2.096e-21 | 670 | 0.431 | 234 | 105 | 6 | 1 | 226 | 36 | 249 | Transcription elongation protein SprT | Transcription elongation protein SprT | | afdb-uniprot50 | AF-A0A1B0Z2A4-F1-MODEL\_V4 | 1.0 | 4.776e-19 | 669 | 0.392 | 227 | 94 | 6 | 1 | 220 | 1 | 190 | Uncharacterized protein | Uncharacterized protein | | afdb-uniprot50 | AF-A0A1V2BSP3-F1-MODEL\_V4 | 1.0 | 2.46e-19 | 648 | 0.404 | 225 | 98 | 3 | 2 | 221 | 5 | 198 | SprT-like domain-containing protein | SprT-like domain-containing protein | | afdb-uniprot50 | AF-A0A1L3ZVD3-F1-MODEL\_V4 | 1.0 | 7.738e-19 | 641 | 0.375 | 224 | 102 | 5 | 1 | 218 | 5 | 196 | SprT-like domain-containing protein | SprT-like domain-containing protein | | afdb-uniprot50 | AF-A0A494WBN0-F1-MODEL\_V4 | 1.0 | 4.277e-20 | 614 | 0.42 | 240 | 93 | 8 | 2 | 227 | 21 | 228 | Transcription elongation protein SprT | Transcription elongation protein SprT | | afdb-uniprot50 | AF-A0A349QHC6-F1-MODEL\_V4 | 1.0 | 1.318e-17 | 594 | 0.325 | 221 | 111 | 5 | 1 | 219 | 14 | 198 | Transcription elongation protein SprT | Transcription elongation protein SprT | | afdb-uniprot50 | AF-A0A7G7MAJ5-F1-MODEL\_V4 | 1.0 | 2.292e-18 | 591 | 0.409 | 222 | 92 | 6 | 2 | 217 | 5 | 193 | SprT-like domain-containing protein | SprT-like domain-containing protein | | afdb-uniprot50 | AF-A0A537PHM0-F1-MODEL\_V4 | 1.0 | 6.079e-19 | 586 | 0.381 | 223 | 99 | 6 | 1 | 218 | 54 | 242 | Transcription elongation protein SprT | Transcription elongation protein SprT | | afdb-uniprot50 | AF-A0A6N6S9L2-F1-MODEL\_V4 | 1.0 | 2.113e-16 | 568 | 0.333 | 222 | 109 | 4 | 1 | 218 | 1 | 187 | Transcription elongation protein SprT | Transcription elongation protein SprT | | afdb-uniprot50 | AF-A0A7W4YW64-F1-MODEL\_V4 | 1.0 | 7.285e-19 | 549 | 0.392 | 237 | 109 | 6 | 8 | 227 | 2 | 220 | Uncharacterized protein | Uncharacterized protein | | afdb-uniprot50 | AF-A0A1Q8Y9H4-F1-MODEL\_V4 | 1.0 | 4.146e-17 | 546 | 0.373 | 225 | 122 | 7 | 15 | 227 | 2 | 219 | Uncharacterized protein | Uncharacterized protein | | afdb-uniprot50 | AF-A0A257NL89-F1-MODEL\_V4 | 1.0 | 1.596e-18 | 543 | 0.371 | 234 | 109 | 6 | 1 | 227 | 8 | 210 | Uncharacterized protein | Uncharacterized protein | | afdb-uniprot50 | AF-A0A7Y4QDV3-F1-MODEL\_V4 | 1.0 | 3.675e-17 | 537 | 0.326 | 239 | 113 | 6 | 1 | 227 | 1 | 203 | Uncharacterized protein | Uncharacterized protein | | afdb-uniprot50 | AF-A0A562K4X6-F1-MODEL\_V4 | 1.0 | 7.135e-17 | 537 | 0.347 | 239 | 109 | 9 | 3 | 227 | 12 | 217 | Uncharacterized protein | Uncharacterized protein | | afdb-uniprot50 | AF-A0A2M8QJE3-F1-MODEL\_V4 | 1.0 | 3.903e-17 | 535 | 0.39 | 223 | 95 | 6 | 14 | 227 | 6 | 196 | Transcription elongation protein SprT | Transcription elongation protein SprT | | afdb-uniprot50 | AF-A0A4V0X9F2-F1-MODEL\_V4 | 1.0 | 9.547e-16 | 534 | 0.318 | 220 | 109 | 6 | 1 | 216 | 4 | 186 | Uncharacterized protein | Uncharacterized protein | | afdb-uniprot50 | AF-A0A7X4GG86-F1-MODEL\_V4 | 1.0 | 2.01e-17 | 526 | 0.357 | 224 | 104 | 5 | 1 | 218 | 27 | 216 | Transcription elongation protein SprT | Transcription elongation protein SprT | | afdb-uniprot50 | AF-A0A0U4H3G1-F1-MODEL\_V4 | 1.0 | 3.863e-16 | 516 | 0.36 | 222 | 104 | 6 | 1 | 218 | 8 | 195 | Transcription elongation protein SprT | Transcription elongation protein SprT | | afdb-uniprot50 | AF-A0A419HBS0-F1-MODEL\_V4 | 1.0 | 3.637e-16 | 516 | 0.351 | 222 | 123 | 7 | 1 | 218 | 6 | 210 | Uncharacterized protein | Uncharacterized protein | | afdb-uniprot50 | AF-A0A382AGK6-F1-MODEL\_V4 | 1.0 | 7.967e-16 | 513 | 0.297 | 222 | 119 | 3 | 1 | 221 | 1 | 186 | SprT-like domain-containing protein | SprT-like domain-containing protein | | afdb-uniprot50 | AF-A0A158SQJ2-F1-MODEL\_V4 | 1.0 | 2.113e-16 | 507 | 0.331 | 241 | 114 | 9 | 1 | 227 | 28 | 235 | Uncharacterized protein | Uncharacterized protein | | afdb-uniprot50 | AF-A0A498AIE9-F1-MODEL\_V4 | 1.0 | 2.69e-16 | 502 | 0.343 | 221 | 110 | 5 | 1 | 218 | 153 | 341 | SprT-like family protein | SprT-like family protein | | afdb-uniprot50 | AF-A0A5B8DFB0-F1-MODEL\_V4 | 1.0 | 1.371e-15 | 490 | 0.324 | 222 | 108 | 6 | 1 | 218 | 5 | 188 | SprT family zinc-dependent metalloprotease | SprT family zinc-dependent metalloprotease | | afdb-uniprot50 | AF-I6APB7-F1-MODEL\_V4 | 1.0 | 1.643e-15 | 490 | 0.308 | 230 | 118 | 8 | 2 | 227 | 24 | 216 | SprT-like family | SprT-like family | | afdb-uniprot50 | AF-A0A7W7AN23-F1-MODEL\_V4 | 1.0 | 1.343e-13 | 488 | 0.422 | 161 | 83 | 4 | 1 | 153 | 4 | 162 | SprT-like domain-containing protein | SprT-like domain-containing protein | | afdb-uniprot50 | AF-A0A7K3ZFI4-F1-MODEL\_V4 | 1.0 | 6.579e-15 | 488 | 0.285 | 228 | 120 | 6 | 1 | 222 | 10 | 200 | PH domain-containing protein | PH domain-containing protein | | afdb-uniprot50 | AF-A0A7W7A6F7-F1-MODEL\_V4 | 1.0 | 2.245e-16 | 474 | 0.34 | 241 | 105 | 8 | 1 | 226 | 4 | 205 | Uncharacterized protein | Uncharacterized protein | | afdb-uniprot50 | AF-A0A315D311-F1-MODEL\_V4 | 1.0 | 1.132e-14 | 470 | 0.29 | 220 | 118 | 4 | 1 | 218 | 5 | 188 | SprT-like domain-containing protein | SprT-like domain-containing protein | | afdb-uniprot50 | AF-A0A3D5EPJ0-F1-MODEL\_V4 | 1.0 | 4.269e-14 | 464 | 0.301 | 222 | 113 | 6 | 2 | 218 | 19 | 203 | Transcription elongation protein SprT | Transcription elongation protein SprT | | afdb-uniprot50 | AF-A0A4P5RR10-F1-MODEL\_V4 | 1.0 | 2.36e-15 | 464 | 0.303 | 221 | 113 | 5 | 1 | 218 | 22 | 204 | SprT-like domain-containing protein | SprT-like domain-containing protein | | afdb-uniprot50 | AF-A0A1E1F840-F1-MODEL\_V4 | 1.0 | 3.223e-16 | 461 | 0.411 | 214 | 85 | 7 | 23 | 227 | 24 | 205 | Uncharacterized protein | Uncharacterized protein | | afdb-uniprot50 | AF-K9CK51-F1-MODEL\_V4 | 1.0 | 7.423e-15 | 453 | 0.434 | 205 | 75 | 7 | 32 | 227 | 3 | 175 | Uncharacterized protein | Uncharacterized protein | | afdb-uniprot50 | AF-A0A212SZK8-F1-MODEL\_V4 | 1.0 | 3.783e-14 | 451 | 0.271 | 228 | 129 | 3 | 1 | 227 | 10 | 201 | SprT-like family protein | SprT-like family protein | | afdb-uniprot50 | AF-A0A2D6X6V1-F1-MODEL\_V4 | 1.0 | 2.198e-14 | 445 | 0.293 | 232 | 123 | 5 | 1 | 227 | 2 | 197 | Uncharacterized protein | Uncharacterized protein | | afdb-uniprot50 | AF-A0A520VTD7-F1-MODEL\_V4 | 1.0 | 2.972e-14 | 445 | 0.275 | 225 | 124 | 3 | 1 | 220 | 29 | 219 | M48 family peptidase | M48 family peptidase | | afdb-uniprot50 | AF-A0A1C6ST94-F1-MODEL\_V4 | 1.0 | 4.866e-15 | 445 | 0.342 | 222 | 107 | 7 | 3 | 218 | 249 | 437 | SprT-like family protein | SprT-like family protein | | afdb-uniprot50 | AF-A0A3N7GVC1-F1-MODEL\_V4 | 1.0 | 6.511e-14 | 443 | 0.301 | 222 | 117 | 8 | 2 | 218 | 6 | 194 | Uncharacterized protein | Uncharacterized protein | | afdb-uniprot50 | AF-A0A257FUX5-F1-MODEL\_V4 | 1.0 | 1.778e-11 | 440 | 0.351 | 148 | 95 | 1 | 1 | 147 | 5 | 152 | SprT-like domain-containing protein | SprT-like domain-containing protein | | afdb-uniprot50 | AF-A0A5E7XVZ1-F1-MODEL\_V4 | 1.0 | 4.816e-14 | 435 | 0.355 | 194 | 110 | 5 | 2 | 184 | 5 | 194 | Transcription elongation protein SprT | Transcription elongation protein SprT | | afdb-uniprot50 | AF-A0A5J6UEE7-F1-MODEL\_V4 | 1.0 | 2.048e-13 | 432 | 0.279 | 236 | 114 | 7 | 1 | 219 | 3 | 199 | SprT family zinc-dependent metalloprotease | SprT family zinc-dependent metalloprotease | | afdb-uniprot50 | AF-A0A1C4U4K3-F1-MODEL\_V4 | 1.0 | 4.581e-15 | 432 | 0.36 | 222 | 103 | 7 | 3 | 218 | 182 | 370 | SprT-like family protein | SprT-like family protein | | afdb-uniprot50 | AF-A0A0F9KWU5-F1-MODEL\_V4 | 1.0 | 9.829e-13 | 431 | 0.27 | 222 | 120 | 5 | 1 | 218 | 1 | 184 | SprT-like domain-containing protein | SprT-like domain-containing protein | | afdb-uniprot50 | AF-D6TL66-F1-MODEL\_V4 | 1.0 | 1.515e-13 | 430 | 0.276 | 221 | 116 | 6 | 3 | 218 | 12 | 193 | Uncharacterized protein | Uncharacterized protein | | afdb-uniprot50 | AF-A0A4Q2J0L1-F1-MODEL\_V4 | 1.0 | 2.335e-14 | 430 | 0.357 | 221 | 106 | 5 | 3 | 218 | 104 | 293 | Uncharacterized protein | Uncharacterized protein | | afdb-uniprot50 | AF-A0A661CLR8-F1-MODEL\_V4 | 1.0 | 2.769e-13 | 427 | 0.263 | 228 | 120 | 8 | 2 | 218 | 6 | 196 | SprT-like domain-containing protein | SprT-like domain-containing protein | | afdb-uniprot50 | AF-A0A849MYV3-F1-MODEL\_V4 | 1.0 | 3.353e-14 | 427 | 0.292 | 239 | 129 | 9 | 1 | 226 | 13 | 224 | Uncharacterized protein | Uncharacterized protein | | afdb-uniprot50 | AF-A0A2W5Y178-F1-MODEL\_V4 | 1.0 | 4.816e-14 | 421 | 0.321 | 233 | 134 | 11 | 1 | 227 | 10 | 224 | SprT-like domain-containing protein | SprT-like domain-containing protein | | afdb-uniprot50 | AF-A0A661MH10-F1-MODEL\_V4 | 1.0 | 1.343e-13 | 417 | 0.276 | 257 | 124 | 12 | 1 | 227 | 30 | 254 | SprT-like domain-containing protein | SprT-like domain-containing protein | | afdb-uniprot50 | AF-A0A2W6B5M7-F1-MODEL\_V4 | 1.0 | 5.831e-15 | 410 | 0.336 | 223 | 113 | 7 | 11 | 227 | 2 | 195 | Transcription elongation protein SprT | Transcription elongation protein SprT | | afdb-uniprot50 | AF-A0A2D6XC71-F1-MODEL\_V4 | 1.0 | 3.667e-11 | 405 | 0.329 | 155 | 99 | 3 | 2 | 153 | 6 | 158 | Transcription elongation protein SprT | Transcription elongation protein SprT | | afdb-uniprot50 | AF-A0A3N5NIA4-F1-MODEL\_V4 | 1.0 | 6.634e-10 | 403 | 0.32 | 128 | 84 | 2 | 1 | 127 | 16 | 141 | Uncharacterized protein | Uncharacterized protein | | afdb-uniprot50 | AF-A0A0F9B2V9-F1-MODEL\_V4 | 1.0 | 5.594e-11 | 402 | 0.327 | 159 | 104 | 1 | 1 | 156 | 1 | 159 | Uncharacterized protein | Uncharacterized protein | | afdb-uniprot50 | AF-A0A2E4HFW3-F1-MODEL\_V4 | 1.0 | 2.429e-12 | 396 | 0.241 | 228 | 125 | 7 | 2 | 218 | 8 | 198 | Uncharacterized protein | Uncharacterized protein | | afdb-uniprot50 | AF-A0A0Q7CQI4-F1-MODEL\_V4 | 1.0 | 2.997e-09 | 353 | 0.318 | 138 | 86 | 5 | 1 | 135 | 18 | 150 | Uncharacterized protein | Uncharacterized protein | | afdb-uniprot50 | AF-A0A160TKS1-F1-MODEL\_V4 | 1.0 | 5.819e-09 | 305 | 0.355 | 163 | 76 | 7 | 2 | 153 | 5 | 149 | SprT-like domain-containing protein | SprT-like domain-containing protein | | afdb-uniprot50 | AF-A0A315CPA0-F1-MODEL\_V4 | 1.0 | 2.45e-07 | 296 | 0.241 | 141 | 103 | 2 | 2 | 138 | 216 | 356 | Uncharacterized protein | Uncharacterized protein | | afdb-uniprot50 | AF-A0A1F6D4Y3-F1-MODEL\_V4 | 1.0 | 4.856e-09 | 295 | 0.179 | 239 | 143 | 9 | 1 | 227 | 9 | 206 | Uncharacterized protein | Uncharacterized protein | | afdb-uniprot50 | AF-A0A315EGI5-F1-MODEL\_V4 | 1.0 | 1.945e-08 | 284 | 0.289 | 159 | 98 | 4 | 2 | 150 | 20 | 173 | SprT-like domain-containing protein | SprT-like domain-containing protein | | afdb-uniprot50 | AF-A0A144J432-F1-MODEL\_V4 | 1.0 | 7.95e-10 | 283 | 0.404 | 163 | 62 | 5 | 68 | 227 | 1 | 131 | Transcription elongation protein | Transcription elongation protein | | afdb-uniprot50 | AF-A0A315DXF0-F1-MODEL\_V4 | 1.0 | 2.475e-08 | 283 | 0.296 | 162 | 102 | 6 | 3 | 155 | 22 | 180 | SprT-like domain-containing protein | SprT-like domain-containing protein | | afdb-uniprot50 | AF-A0A126REC0-F1-MODEL\_V4 | 1.0 | 7.18e-06 | 243 | 0.421 | 95 | 49 | 2 | 2 | 90 | 5 | 99 | Uncharacterized protein | Uncharacterized protein | | afdb-uniprot50 | AF-A0A259FRY7-F1-MODEL\_V4 | 1.0 | 3.054e-05 | 223 | 0.395 | 81 | 49 | 0 | 3 | 83 | 7 | 87 | SprT domain-containing protein | SprT domain-containing protein | | afdb-uniprot50 | AF-A0A4S2U167-F1-MODEL\_V4 | 1.0 | 4.216e-07 | 178 | 0.211 | 251 | 151 | 15 | 3 | 217 | 32 | 271 | Uncharacterized protein | Uncharacterized protein | | afdb-uniprot50 | AF-A0A1F6M955-F1-MODEL\_V4 | 1.0 | 6.83e-07 | 170 | 0.175 | 245 | 151 | 17 | 1 | 217 | 289 | 510 | SprT-like domain-containing protein | SprT-like domain-containing protein | | afdb-uniprot50 | AF-A0A7Y6IZN4-F1-MODEL\_V4 | 1.0 | 1.326e-06 | 169 | 0.194 | 257 | 165 | 13 | 3 | 227 | 8 | 254 | Uncharacterized protein | Uncharacterized protein | | afdb-uniprot50 | AF-A0A7D4X9Y0-F1-MODEL\_V4 | 1.0 | 2.424e-06 | 163 | 0.172 | 266 | 168 | 15 | 4 | 227 | 28 | 283 | Uncharacterized protein | Uncharacterized protein | | afdb-uniprot50 | AF-A0A6G9F5T1-F1-MODEL\_V4 | 1.0 | 2.424e-06 | 153 | 0.175 | 262 | 158 | 18 | 1 | 227 | 2 | 240 | Uncharacterized protein | Uncharacterized protein | | afdb-uniprot50 | AF-A0A429FBE9-F1-MODEL\_V4 | 1.0 | 7.626e-06 | 149 | 0.159 | 269 | 167 | 17 | 3 | 227 | 12 | 265 | Uncharacterized protein | Uncharacterized protein | | afdb-uniprot50 | AF-A0A1I1XSQ6-F1-MODEL\_V4 | 1.0 | 3.445e-05 | 136 | 0.171 | 239 | 167 | 13 | 1 | 217 | 17 | 246 | Uncharacterized protein | Uncharacterized protein | | afdb-uniprot50 | AF-A0A2S8IY47-F1-MODEL\_V4 | 1.0 | 0.0007934 | 129 | 0.411 | 85 | 44 | 4 | 146 | 227 | 2 | 83 | Uncharacterized protein | Uncharacterized protein | | afdb-uniprot50 | AF-A0A2I1DJ04-F1-MODEL\_V4 | 1.0 | 0.0005868 | 111 | 0.126 | 301 | 167 | 17 | 4 | 218 | 8 | 298 | Uncharacterized protein | Uncharacterized protein | | afdb-uniprot50 | AF-A0A429A0F0-F1-MODEL\_V4 | 1.0 | 0.000321 | 108 | 0.191 | 230 | 135 | 13 | 31 | 226 | 2 | 214 | Uncharacterized protein | Uncharacterized protein | | afdb-uniprot50 | AF-A0A179BPY3-F1-MODEL\_V4 | 1.0 | 0.003807 | 107 | 0.12 | 315 | 173 | 13 | 5 | 227 | 26 | 328 | SprT-like domain-containing protein | SprT-like domain-containing protein | | afdb-uniprot50 | AF-Q9AEZ9-F1-MODEL\_V4 | 0.999 | 0.0001756 | 98 | 0.166 | 228 | 145 | 13 | 40 | 227 | 15 | 237 | Uncharacterized protein | Uncharacterized protein | | afdb-uniprot50 | AF-A0A2N8TS96-F1-MODEL\_V4 | 0.992 | 0.003807 | 85 | 0.184 | 228 | 140 | 14 | 34 | 222 | 1 | 221 | Uncharacterized protein | Uncharacterized protein | |
| Top keywords  (threshold 1.00e-02 (evalue)) | **SprT\_like, domain\_containing, SprT, Transcription, elongation, zinc\_dependent, metalloprotease, PH, M48, peptidase** |
| Output files | ../../similar\_structures/34\_FANPEZAQ\_CDS\_0034\_afdb-proteome\_foldseek.tsv ../../similar\_structures/34\_FANPEZAQ\_CDS\_0034\_afdb-uniprot50\_foldseek.tsv ../../similar\_structures/34\_FANPEZAQ\_CDS\_0034\_merged.svg ../../similar\_structures/34\_FANPEZAQ\_CDS\_0034\_pdb\_foldseek.tsv |

  
  
  

Return to summary | Go to previous | Go to next

  


---

**Sequence/structure alignments coloring**  
Each object in the alignment figures is colored according to its E-value following this color coding:

1e-100
10

**References:**  
1) Steinegger M, Meier M, Mirdita M, Vöhringer H, Haunsberger S J, and Söding J (2019) HH-suite3 for fast remote homology detection and deep protein annotation, BMC Bioinformatics, 473. doi: 10.1186/s12859-019-3019-7  
2) Jumper J, Evans R, Pritzel A, ..., Hassabis D (2021) Highly accurate protein structure prediction with AlphaFold, Nature, 596. doi: 10.1038/s41586-021-03819-2  
3) van Kempen M, Kim S, Tumescheit C, Mirdita M, Lee J, Gilchrist CLM, Söding J, and Steinegger M (2023) Fast and accurate protein structure search with Foldseek. Nature Biotechnology. doi: 10.1038/s41587-023-01773-0
