# Supplementary material for: Completing the BASEL phage collection to unlock hidden diversity for systematic exploration of phage–host interactions
Source: PLoS Biol. 2025 Apr 7;23(4):e3003063. doi: 10.1371/journal.pbio.3003063 (PMC11990801; doi:10.1371/journal.pbio.3003063)
Supplement: S2 Data — (ZIP) [file pbio.3003063.s009.zip › entries/35.html]

FANPEZAQ\_CDS\_0035


Return to summary | Go to previous | Go to next

|  |  |
| --- | --- |
| FANPEZAQ\_CDS\_0035 Page creation date: 02 Sep 2024, 12:00  Project folder: n/a  Input sequences file: Escherichia\_virus\_HeidiAbel.gb | single\_stranded dna\_binding binding dna single\_strand replication beta a primosomal n complex mitochondrial fold yes ob fragment single engineered rna nucleic acid\_binding proteins organism\_taxid barrel coli mainly arch dihydrolipoamide acetyltransferase e2p ssb escherichia expressed in expression\_system\_taxid editing b rim1 mp18 gene c rna\_editing hypothetical strand strain and antibody putative stranded synonym |

### Sequence information

|  |  |
| --- | --- |
| Name | FANPEZAQ\_CDS\_0035  35\_FANPEZAQ\_CDS\_0035 (pipeline id) |
| Imported annotations |  |
| Protein sequence | MAGSLNKVEIIGNLGNDPEFRTLPNGNGVAQLSIATSETWKDKQTGEKKEKTEWHRVVIF GKLAEIASQYLRKGSKVFIEGALQTRKWQDQAGVDRYTTEIVVGMNGRLLMLSAANDNQA GGQRQQQQPGGGQQFSGGGQQRPPAQGAGNEPPMDFDDDIPF |
| Number of residues | 162 |
| Molecular weight (Da) | 17644.45 |
| Output files | ../../query\_sequences/35\_FANPEZAQ\_CDS\_0035.fasta |

### Putative domain architecture and protein family

#### Search results (HHblits)1

|  |  |
| --- | --- |
| Domain family databases searched | Pfam, Ncbi-cd, Cath, Phrogs |
| Results, scheme(s)  (Top layers only; threshold 1.00e-03 (evalue)) | xml version="1.0" encoding="utf-8" standalone="no"?       2024-09-02T21:08:19.988308 image/svg+xml   Matplotlib v3.7.2, https://matplotlib.org/ |
| Results, table  (E-value ≤ 1.00e-03 (evalue)) | | db | id | prob | evalue | pvalue | score | cols | query | query\_len | template | template\_len | name | description | | --- | --- | --- | --- | --- | --- | --- | --- | --- | --- | --- | --- | --- | | pfam | PF00436 | 99.3 | 7.9e-17 | 1.6e-20 | 105.1 | 94 | (5, 105) | 162 | (1, 98) | 102 | SSB | Single-strand binding protein family | | pfam | PF11506 | 99.2 | 2.8e-16 | 5.7e-20 | 101.1 | 92 | (4, 108) | 162 | (1, 92) | 94 | DUF3217 | Protein of unknown function (DUF3217) | | ncbi-cd | cd04496 | 99.2 | 4.2e-16 | 9.4e-20 | 100.9 | 95 | (8, 104) | 162 | (1, 95) | 100 | SSB\_OBF | cd04496 SSB\_OBF; SSB\_OBF: A subfamily of OB folds similar to the OB fold of ssDNA-binding protein (SSB). | | cath | 2cwaA01 | 99.6 | 4.6e-20 | 6.9e-24 | 123.0 | 112 | (1, 114) | 162 | (1, 112) | 126 | Single-strand binding protein | CATHCODE: 2.40.50.140 NAME: Single-strand binding protein. Chain: a. Synonym: ssb, helix-destabilizing protein, single-stranded DNA binding protein. Engineered: yes SOURCE: Thermus thermophilus. Organism\_taxid: 300852. Strain: hb8. Expressed in: escherichia coli. Expression\_system\_taxid: 562. CLASS: Mainly Beta, ARCH: Beta Barrel, TOPOL: OB fold (Dihydrolipoamide Acetyltransferase, E2P), HOMOL: Nucleic acid-binding proteins | | cath | 3ulpD00 | 99.5 | 4.5e-19 | 6.8e-23 | 118.0 | 114 | (1, 114) | 162 | (2, 122) | 124 | Single-strand binding protein | CATHCODE: 2.40.50.140 NAME: Single-strand binding protein. Chain: a, b, c, d. Fragment: unp residues 77-200. Engineered: yes. Dna (35-mer). Chain: r, q. Engineered: yes SOURCE: Plasmodium falciparum. Organism\_taxid: 5833. Gene: pfe0435c. Expressed in: escherichia coli. Expression\_system\_taxid: 562. Expression\_system\_vector\_type: pet21a. CLASS: Mainly Beta, ARCH: Beta Barrel, TOPOL: OB fold (Dihydrolipoamide Acetyltransferase, E2P), HOMOL: Nucleic acid-binding proteins | | cath | 1se8A02 | 99.5 | 4.6e-19 | 7e-23 | 116.8 | 108 | (1, 110) | 162 | (9, 116) | 117 | Single-strand binding protein | CATHCODE: 2.40.50.140 NAME: Single-strand binding protein. Chain: a. Synonym: ssb, helix-destabilizing protein. Engineered: yes SOURCE: Deinococcus radiodurans. Organism\_taxid: 1299. Gene: ssb, dr0099. Expressed in: escherichia coli. Expression\_system\_taxid: 562. CLASS: Mainly Beta, ARCH: Beta Barrel, TOPOL: OB fold (Dihydrolipoamide Acetyltransferase, E2P), HOMOL: Nucleic acid-binding proteins | | cath | 1ue6D00 | 99.5 | 5e-19 | 7.5e-23 | 116.9 | 114 | (2, 115) | 162 | (1, 115) | 119 | Single-strand binding protein | CATHCODE: 2.40.50.140 NAME: Single-strand binding protein. Chain: a, b, c, d. Synonym: single-stranded DNA-binding protein. Engineered: yes SOURCE: Mycobacterium tuberculosis. Organism\_taxid: 1773. Expressed in: escherichia coli. Expression\_system\_taxid: 562. Expression\_system\_vector\_type: plasmid. CLASS: Mainly Beta, ARCH: Beta Barrel, TOPOL: OB fold (Dihydrolipoamide Acetyltransferase, E2P), HOMOL: Nucleic acid-binding proteins | | cath | 5odnC00 | 99.5 | 5.9e-19 | 8.9e-23 | 114.6 | 108 | (1, 109) | 162 | (3, 110) | 110 | Single-stranded DNA-binding protein | CATHCODE: 2.40.50.140 NAME: Single-stranded DNA-binding protein. Chain: a, b, c, d, e, f, g, h. Synonym: ssb. Engineered: yes. Dna (5'-d(p\*tp\*tp\*tp\*t)-3'). Chain: i, m,j, k, n, q. Engineered: yes. Dna (5'-d(\*tp\*tp\*t)-3'). Chain: p, l. Engineered: yes SOURCE: Salinibacter ruber (strain dsm 13855 / m31). Organism\_taxid: 309807. Gene: ssb, sru\_0523. Expressed in: escherichia coli. Expression\_system\_taxid: 562. Yes. Organism\_scientific: salinibacter ruber. Organism\_taxid: 146919. CLASS: Mainly Beta, ARCH: Beta Barrel, TOPOL: OB fold (Dihydrolipoamide Acetyltransferase, E2P), HOMOL: Nucleic acid-binding proteins | | cath | 3k7uC00 | 99.5 | 6.3e-19 | 9.5e-23 | 116.5 | 110 | (1, 112) | 162 | (2, 117) | 118 | Antibody | CATHCODE: 2.40.50.140 NAME: Antibody. Chain: a. Engineered: yes. Mp18 RNA editing complex protein.Chain: c. Engineered: yes SOURCE: Lama glama. Organism\_taxid: 9844. Expressed in: escherichia coli. Expression\_system\_taxid: 562. CLASS: Mainly Beta, ARCH: Beta Barrel, TOPOL: OB fold (Dihydrolipoamide Acetyltransferase, E2P), HOMOL: Nucleic acid-binding proteins | | cath | 4damC00 | 99.5 | 1.4e-18 | 2e-22 | 117.8 | 114 | (1, 116) | 162 | (11, 125) | 128 | Single-stranded DNA-binding protein 1 | CATHCODE: 2.40.50.140 NAME: Single-stranded DNA-binding protein 1. Chain: a, b, c, d, e, f, g, h, i, j, k, l. Fragment: unp residues 1-116. Synonym: ssb 1, helix-destabilizing protein 1. Engineered: yes. Mutation: yes SOURCE: Streptomyces coelicolor. Organism\_taxid: 1902. Strain: m145. Gene: scc61a.04c, sco2683, ssb1. Expressed in: escherichia coli. Expression\_system\_taxid: 562. CLASS: Mainly Beta, ARCH: Beta Barrel, TOPOL: OB fold (Dihydrolipoamide Acetyltransferase, E2P), HOMOL: Nucleic acid-binding proteins | | cath | 2vw9B00 | 99.5 | 2.2e-18 | 3.4e-22 | 110.9 | 105 | (4, 109) | 162 | (1, 105) | 106 | Single-stranded DNA binding protein | CATHCODE: 2.40.50.140 NAME: Single-stranded DNA binding protein. Chain: a, b. Fragment: residues 1-134. Synonym: ssb, helix-destabilizing protein. Engineered: yes. Poly-dt. Chain: c. Engineered: yes SOURCE: Helicobacter pylori. Organism\_taxid: 85962. Strain: 26695. Expressed in: escherichia coli. Expression\_system\_taxid: 562. CLASS: Mainly Beta, ARCH: Beta Barrel, TOPOL: OB fold (Dihydrolipoamide Acetyltransferase, E2P), HOMOL: Nucleic acid-binding proteins | | cath | 3kojA00 | 99.4 | 5.9e-18 | 8.8e-22 | 110.4 | 100 | (1, 107) | 162 | (7, 107) | 108 | Uncharacterized protein ycf41 | CATHCODE: 2.40.50.140 NAME: Uncharacterized protein ycf41. Chain: a, b. Fragment: ssb domain. Engineered: yes. Mutation: yes SOURCE: Synechococcus elongatus pcc 6301. Anacystis nidulans. Organism\_taxid: 269084. Strain: atcc 27144/pcc 6301 /saug 1402/1. Gene: syc1425\_d, ycf41. Expressed in: escherichia coli bl21(de3). Expression\_system\_taxid: 469008. CLASS: Mainly Beta, ARCH: Beta Barrel, TOPOL: OB fold (Dihydrolipoamide Acetyltransferase, E2P), HOMOL: Nucleic acid-binding proteins | | cath | 5yyuB00 | 99.4 | 1.1e-17 | 1.6e-21 | 109.8 | 110 | (4, 116) | 162 | (1, 110) | 112 | Single-stranded DNA-binding protein | CATHCODE: 2.40.50.140 NAME: Single-stranded DNA-binding protein. Chain: a, b, c, d. Synonym: ssb. Engineered: yes SOURCE: Staphylococcus aureus subsp. Aureus ed98. Organism\_taxid: 681288. Expressed in: escherichia coli bl21. Expression\_system\_taxid: 511693. CLASS: Mainly Beta, ARCH: Beta Barrel, TOPOL: OB fold (Dihydrolipoamide Acetyltransferase, E2P), HOMOL: Nucleic acid-binding proteins | | cath | 5gqoA00 | 99.4 | 1.4e-17 | 2.2e-21 | 109.6 | 110 | (5, 116) | 162 | (1, 111) | 117 | Single-stranded DNA-binding protein | CATHCODE: 2.40.50.140 NAME: Single-stranded DNA-binding protein. Chain: a, b. Engineered: yes SOURCE: Mycobacterium smegmatis (strain atcc 700084 / mc(2)155). Organism\_taxid: 246196. Strain: atcc 700084 / mc(2)155. Gene: msmeg\_4701. Expressed in: escherichia coli. Expression\_system\_taxid: 511693. CLASS: Mainly Beta, ARCH: Beta Barrel, TOPOL: OB fold (Dihydrolipoamide Acetyltransferase, E2P), HOMOL: Nucleic acid-binding proteins | | cath | 1txyA00 | 99.4 | 2.8e-17 | 4.2e-21 | 105.8 | 103 | (4, 112) | 162 | (1, 103) | 104 | Primosomal replication protein n | CATHCODE: 2.40.50.140 NAME: Primosomal replication protein n. Chain: a, b. Engineered: yes SOURCE: Escherichia coli. Organism\_taxid: 562. Gene: prib, b4201. Expressed in: escherichia coli. Expression\_system\_taxid: 562 CLASS: Mainly Beta, ARCH: Beta Barrel, TOPOL: OB fold (Dihydrolipoamide Acetyltransferase, E2P), HOMOL: Nucleic acid-binding proteins | | cath | 3k8aB00 | 99.3 | 4.9e-17 | 7.4e-21 | 104.4 | 100 | (1, 110) | 162 | (3, 102) | 103 | Putative primosomal replication protein | CATHCODE: 2.40.50.140 NAME: Putative primosomal replication protein. Chain: a, b. Engineered: yes SOURCE: Neisseria gonorrhoeae fa 1090. Organism\_taxid: 242231. Strain: fa1090.Gene: ngo0582, prib. Expressed in: escherichia coli. Expression\_system\_taxid: 562. CLASS: Mainly Beta, ARCH: Beta Barrel, TOPOL: OB fold (Dihydrolipoamide Acetyltransferase, E2P), HOMOL: Nucleic acid-binding proteins | | cath | 3ullB00 | 99.3 | 5.3e-17 | 8e-21 | 108.9 | 114 | (1, 114) | 162 | (10, 129) | 132 | Dna binding protein | CATHCODE: 2.40.50.140 NAME: Dna binding protein. Chain: a, b SOURCE: Homo sapiens. Human. Organism\_taxid: 9606. Organelle: mitochondria CLASS: Mainly Beta, ARCH: Beta Barrel, TOPOL: OB fold (Dihydrolipoamide Acetyltransferase, E2P), HOMOL: Nucleic acid-binding proteins | | cath | 4gs3A00 | 99.3 | 8.8e-17 | 1.3e-20 | 104.4 | 99 | (1, 110) | 162 | (7, 106) | 107 | Single-stranded DNA-binding protein | CATHCODE: 2.40.50.140 NAME: Single-stranded DNA-binding protein. Chain: a. Fragment: unp residues 1-104. Engineered: yes SOURCE: Thermoanaerobacter tengcongensis. Organism\_taxid: 273068. Strain: dsm 15242 / jcm 11007 / nbrc 100824 / mb4. Gene: ssb, tte0829. Expressed in: escherichia coli. Expression\_system\_taxid: 469008. CLASS: Mainly Beta, ARCH: Beta Barrel, TOPOL: OB fold (Dihydrolipoamide Acetyltransferase, E2P), HOMOL: Nucleic acid-binding proteins | | cath | 3dm4A00 | 99.2 | 3.9e-16 | 5.9e-20 | 99.2 | 97 | (5, 110) | 162 | (1, 97) | 98 | Primosomal replication protein n | CATHCODE: 2.40.50.140 NAME: Primosomal replication protein n. Chain: a, b. Engineered: yes SOURCE: Bordetella pertussis. Organism\_taxid: 520. Gene: prib, bp2795 CLASS: Mainly Beta, ARCH: Beta Barrel, TOPOL: OB fold (Dihydrolipoamide Acetyltransferase, E2P), HOMOL: Nucleic acid-binding proteins | | cath | 3en2A00 | 99.2 | 2.5e-15 | 3.6e-19 | 96.8 | 86 | (4, 92) | 162 | (1, 86) | 101 | Probable primosomal replication protein n | CATHCODE: 2.40.50.140 NAME: Probable primosomal replication protein n. Chain: a. Engineered: yes. Mutation: yes SOURCE: Ralstonia solanacearum. Pseudomonas solanacearum. Organism\_taxid: 305.Expressed in: escherichia coli. CLASS: Mainly Beta, ARCH: Beta Barrel, TOPOL: OB fold (Dihydrolipoamide Acetyltransferase, E2P), HOMOL: Nucleic acid-binding proteins | | cath | 4dkaC00 | 99.1 | 3.2e-15 | 4.7e-19 | 97.1 | 90 | (3, 102) | 162 | (1, 92) | 105 | Single domain antibody vhh | CATHCODE: 2.40.50.140 NAME: Single domain antibody vhh. Chain: a, b. Engineered: yes. Rna-editing complex protein mp81. Chain: c, d. Engineered: yes. Mutation: yes SOURCE: Lama glama. Llama. Organism\_taxid: 9844. Expressed in: escherichia coli. Expression\_system\_taxid: 469008. CLASS: Mainly Beta, ARCH: Beta Barrel, TOPOL: OB fold (Dihydrolipoamide Acetyltransferase, E2P), HOMOL: Nucleic acid-binding proteins | | cath | 2hqlA01 | 99.1 | 9.7e-15 | 1.5e-18 | 92.6 | 93 | (1, 106) | 162 | (4, 96) | 97 | Hypothetical protein mg376 homolog | CATHCODE: 2.40.50.140 NAME: Hypothetical protein mg376 homolog. Chain: a, b, c, d, e, f. Synonym: g12\_orf104, single-stranded DNA-binding protein. Engineered: yes SOURCE: Mycoplasma pneumoniae. Organism\_taxid: 2104. Gene: mpn554, mp288. Expressed in: escherichia coli. Expression\_system\_taxid: 562. CLASS: Mainly Beta, ARCH: Beta Barrel, TOPOL: OB fold (Dihydrolipoamide Acetyltransferase, E2P), HOMOL: Nucleic acid-binding proteins | | phrogs | 44 | 100.0 | 6.1e-35 | 8.9e-39 | 215.9 | 92 | (5, 106) | 162 | (1, 92) | 121 | single strand DNA binding protein | single strand DNA binding protein; Category: DNA, RNA and nucleotide metabolism; p256451 VI\_01358 | | phrogs | 18154 | 99.8 | 2e-23 | 2.4e-27 | 151.2 | 79 | (73, 162) | 162 | (28, 113) | 113 | single strand DNA binding protein | single strand DNA binding protein; Category: DNA, RNA and nucleotide metabolism; MG641885\_p29 | | phrogs | 25370 | 98.8 | 5e-13 | 5.7e-17 | 81.7 | 41 | (4, 44) | 162 | (1, 41) | 45 | NA | NA; Category: unknown function; p432599 VI\_04995 | | phrogs | 7364 | 98.6 | 4.3e-12 | 4.9e-16 | 94.8 | 25 | (138, 162) | 162 | (134, 158) | 158 | single strand DNA binding protein | single strand DNA binding protein; Category: DNA, RNA and nucleotide metabolism; NC\_005891\_p29 | | phrogs | 26861 | 98.1 | 1.1e-09 | 1.2e-13 | 85.9 | 78 | (5, 91) | 162 | (134, 211) | 242 | NA | NA; Category: unknown function; p359533 VI\_01260 | | phrogs | 12631 | 97.5 | 6e-08 | 6.7e-12 | 63.9 | 39 | (2, 40) | 162 | (27, 65) | 78 | single strand DNA binding protein | single strand DNA binding protein; Category: DNA, RNA and nucleotide metabolism; p232911 VI\_05461 | | phrogs | 27667 | 96.1 | 2.9e-05 | 3.2e-09 | 48.5 | 31 | (77, 107) | 162 | (2, 32) | 61 | single strand DNA binding protein | single strand DNA binding protein; Category: DNA, RNA and nucleotide metabolism; NC\_004584\_p19 | | phrogs | 4477 | 95.9 | 4.4e-05 | 5.2e-09 | 63.5 | 14 | (149, 162) | 162 | (395, 408) | 408 | NA | NA; Category: unknown function; NC\_020201\_p2 | |
| Top keywords  (threshold 1.00e-03 (evalue)) | **Beta, yes, Engineered, OB, fold, a, Organism\_taxid, Mainly, ARCH, Barrel** |
| Output files | ../../domain\_architecture/35\_FANPEZAQ\_CDS\_0035\_cath.hhr ../../domain\_architecture/35\_FANPEZAQ\_CDS\_0035\_merged.svg ../../domain\_architecture/35\_FANPEZAQ\_CDS\_0035\_ncbi-cd.hhr ../../domain\_architecture/35\_FANPEZAQ\_CDS\_0035\_pfam.hhr ../../domain\_architecture/35\_FANPEZAQ\_CDS\_0035\_phrogs.hhr |

### Identical protein sequences/structures

#### Search results

|  |  |
| --- | --- |
| Protein sequence databases searched | Pdb, Swissprot, Refseq |
| Identical proteins found | -- |
| Top keywords | -- |
| Output files | -- |

### Similar protein sequences/structures

#### Sequence similarity search results (HHblits)1

|  |  |
| --- | --- |
| Sequence databases searched | Uniclust, Pdb70 |
| Results, scheme(s)  (Top layers only, threshold 1.00e-03 (evalue)) | xml version="1.0" encoding="utf-8" standalone="no"?       2024-09-02T21:08:44.373315 image/svg+xml   Matplotlib v3.7.2, https://matplotlib.org/ |
| Results, table(s)  (threshold 1.00e-03 (evalue)) | | db | id | prob | evalue | pvalue | score | cols | query | query\_len | template | template\_len | name | description | | --- | --- | --- | --- | --- | --- | --- | --- | --- | --- | --- | --- | --- | | uniclust | UniRef100\_A0A098S7E1 | 100.0 | 5.3e-45 | 1e-50 | 241.9 | 111 | (1, 115) | 162 | (1, 111) | 157 | Single-stranded DNA-binding protein | Single-stranded DNA-binding protein | | uniclust | UniRef100\_A0A1E7IFR0 | 100.0 | 1.2e-43 | 2.2e-49 | 234.2 | 116 | (2, 117) | 162 | (3, 118) | 183 | Single-stranded DNA-binding protein | Single-stranded DNA-binding protein | | uniclust | UniRef100\_A0A0H4JBR5 | 100.0 | 2.4e-43 | 4.7e-49 | 234.4 | 111 | (1, 115) | 162 | (3, 113) | 163 | Single-stranded DNA-binding protein | Single-stranded DNA-binding protein | | uniclust | UniRef100\_A0A011NXA6 | 100.0 | 3.4e-43 | 6.4e-49 | 248.0 | 112 | (1, 115) | 162 | (41, 152) | 272 | Single-stranded DNA-binding protein | Single-stranded DNA-binding protein | | uniclust | UniRef100\_A0A0K1FF79 | 100.0 | 5.7e-43 | 1.1e-48 | 238.0 | 113 | (1, 117) | 162 | (13, 125) | 180 | Single-stranded DNA-binding protein | Single-stranded DNA-binding protein | | uniclust | UniRef100\_A0A017T184 | 100.0 | 1e-42 | 2e-48 | 241.2 | 111 | (1, 115) | 162 | (18, 128) | 209 | Single-stranded DNA-binding protein | Single-stranded DNA-binding protein | | uniclust | UniRef100\_A0A060R893 | 100.0 | 1.6e-42 | 3.1e-48 | 229.8 | 115 | (1, 118) | 162 | (1, 115) | 171 | Single-stranded DNA-binding protein | Single-stranded DNA-binding protein | | uniclust | UniRef100\_A0A136KBY6 | 100.0 | 1.9e-42 | 3.5e-48 | 228.4 | 112 | (1, 116) | 162 | (24, 135) | 174 | Single-stranded DNA-binding protein | Single-stranded DNA-binding protein | | uniclust | UniRef100\_A0A074LI87 | 100.0 | 2e-42 | 3.9e-48 | 231.5 | 114 | (1, 118) | 162 | (18, 131) | 172 | Single-stranded DNA-binding protein | Single-stranded DNA-binding protein | | uniclust | UniRef100\_A0A0F9YE88 | 100.0 | 4e-42 | 7.7e-48 | 234.1 | 110 | (1, 114) | 162 | (30, 139) | 198 | Single-stranded DNA-binding protein | Single-stranded DNA-binding protein | | uniclust | UniRef100\_A0A023WX09 | 100.0 | 4.7e-42 | 9.1e-48 | 238.5 | 112 | (1, 116) | 162 | (30, 141) | 215 | Single-stranded DNA-binding protein | Single-stranded DNA-binding protein | | uniclust | UniRef100\_A0A069DGL3 | 100.0 | 4.9e-42 | 9.6e-48 | 237.2 | 112 | (1, 116) | 162 | (19, 130) | 203 | Single-stranded DNA-binding protein | Single-stranded DNA-binding protein | | uniclust | UniRef100\_A0A011P4T6 | 100.0 | 5.3e-42 | 1e-47 | 238.6 | 115 | (1, 115) | 162 | (26, 140) | 225 | Single-stranded DNA-binding protein | Single-stranded DNA-binding protein | | uniclust | UniRef100\_A0A0C5J9Y9 | 100.0 | 1.3e-41 | 2.5e-47 | 233.2 | 113 | (1, 116) | 162 | (40, 152) | 238 | Single-stranded DNA-binding protein | Single-stranded DNA-binding protein | | uniclust | UniRef100\_A0A0G0LTD2 | 100.0 | 2e-41 | 3.8e-47 | 223.1 | 112 | (1, 116) | 162 | (5, 116) | 159 | Single-stranded DNA-binding protein | Single-stranded DNA-binding protein | | uniclust | UniRef100\_A0A2E1E3C2 | 100.0 | 2.4e-41 | 4.6e-47 | 226.5 | 113 | (1, 118) | 162 | (17, 129) | 172 | Single-stranded DNA-binding protein | Single-stranded DNA-binding protein | | uniclust | UniRef100\_A0A011MH48 | 100.0 | 3e-41 | 5.9e-47 | 237.6 | 112 | (1, 115) | 162 | (28, 139) | 213 | Single-stranded DNA-binding protein | Single-stranded DNA-binding protein | | uniclust | UniRef100\_A0A015RX92 | 100.0 | 3.3e-41 | 6.3e-47 | 227.7 | 111 | (1, 115) | 162 | (12, 123) | 186 | Single-stranded DNA-binding protein | Single-stranded DNA-binding protein | | uniclust | UniRef100\_A0A084SEV2 | 100.0 | 8.5e-41 | 1.7e-46 | 235.1 | 112 | (1, 116) | 162 | (36, 147) | 215 | Single-stranded DNA-binding protein | Single-stranded DNA-binding protein | | uniclust | UniRef100\_A0A0D8J7K4 | 100.0 | 9e-41 | 1.7e-46 | 217.4 | 117 | (1, 121) | 162 | (1, 117) | 154 | Single-stranded DNA-binding protein | Single-stranded DNA-binding protein | | uniclust | UniRef100\_A0A066TQG3 | 100.0 | 1.1e-40 | 2.2e-46 | 221.5 | 111 | (3, 117) | 162 | (5, 115) | 177 | Single-stranded DNA-binding protein | Single-stranded DNA-binding protein | | uniclust | UniRef100\_A0A023E151 | 100.0 | 1.5e-40 | 2.9e-46 | 226.1 | 114 | (1, 118) | 162 | (4, 117) | 195 | Single-stranded DNA-binding protein | Single-stranded DNA-binding protein | | uniclust | UniRef100\_A0A0W0U4V5 | 100.0 | 1.8e-40 | 3.4e-46 | 221.9 | 111 | (1, 115) | 162 | (14, 124) | 173 | Single-stranded DNA-binding protein | Single-stranded DNA-binding protein | | uniclust | UniRef100\_A0A060B5W2 | 100.0 | 2.4e-40 | 4.5e-46 | 229.2 | 116 | (1, 116) | 162 | (34, 149) | 240 | Single-stranded DNA-binding protein | Single-stranded DNA-binding protein | | uniclust | UniRef100\_A0A097EQ76 | 100.0 | 3.7e-40 | 7e-46 | 217.4 | 110 | (4, 116) | 162 | (5, 114) | 172 | Single-stranded DNA-binding protein | Single-stranded DNA-binding protein | | uniclust | UniRef100\_A0A087M3H5 | 100.0 | 4e-40 | 7.6e-46 | 226.6 | 113 | (1, 117) | 162 | (2, 114) | 222 | Single-stranded DNA-binding protein | Single-stranded DNA-binding protein | | uniclust | UniRef100\_A0A087MC81 | 100.0 | 6.1e-40 | 1.2e-45 | 229.6 | 112 | (1, 118) | 162 | (36, 147) | 211 | Single-stranded DNA-binding protein | Single-stranded DNA-binding protein | | uniclust | UniRef100\_A0A0N1KPD2 | 100.0 | 6.6e-40 | 1.3e-45 | 220.6 | 111 | (1, 115) | 162 | (23, 133) | 183 | Single-stranded DNA-binding protein (Fragment) | Single-stranded DNA-binding protein (Fragment) | | uniclust | UniRef100\_A0A1A9RXR2 | 100.0 | 7.7e-40 | 1.5e-45 | 215.5 | 114 | (1, 119) | 162 | (2, 115) | 156 | Single-stranded DNA-binding protein | Single-stranded DNA-binding protein | | uniclust | UniRef100\_A0A0G1DJJ4 | 100.0 | 1.2e-39 | 2.3e-45 | 216.4 | 116 | (1, 120) | 162 | (2, 117) | 168 | Single-stranded DNA-binding protein | Single-stranded DNA-binding protein | | uniclust | UniRef100\_A0A0G1KT99 | 100.0 | 1.3e-39 | 2.4e-45 | 225.1 | 110 | (1, 113) | 162 | (23, 132) | 207 | Single-stranded DNA-binding protein | Single-stranded DNA-binding protein | | uniclust | UniRef100\_A0A072Y7C9 | 100.0 | 1.5e-39 | 2.9e-45 | 224.6 | 111 | (1, 117) | 162 | (15, 125) | 197 | Single-stranded DNA-binding protein | Single-stranded DNA-binding protein | | uniclust | UniRef100\_A0A143XWD3 | 100.0 | 1.7e-39 | 3.3e-45 | 222.1 | 111 | (1, 118) | 162 | (1, 113) | 192 | Single-stranded DNA-binding protein | Single-stranded DNA-binding protein | | uniclust | UniRef100\_A0A023CPK7 | 100.0 | 1.8e-39 | 3.5e-45 | 221.0 | 108 | (2, 116) | 162 | (18, 125) | 175 | Single-stranded DNA-binding protein | Single-stranded DNA-binding protein | | uniclust | UniRef100\_A0A014P655 | 100.0 | 2.9e-39 | 5.5e-45 | 225.1 | 114 | (1, 117) | 162 | (41, 154) | 270 | Single-stranded DNA-binding protein | Single-stranded DNA-binding protein | | uniclust | UniRef100\_A0A062XTB0 | 100.0 | 3.1e-39 | 5.9e-45 | 222.6 | 112 | (1, 116) | 162 | (16, 127) | 203 | Single-stranded DNA-binding protein | Single-stranded DNA-binding protein | | uniclust | UniRef100\_A0A0D2JCW1 | 100.0 | 3.2e-39 | 6.1e-45 | 220.9 | 110 | (1, 114) | 162 | (6, 115) | 204 | Single-stranded DNA-binding protein | Single-stranded DNA-binding protein | | uniclust | UniRef100\_A0A1E4NJ61 | 100.0 | 3.7e-39 | 6.9e-45 | 209.7 | 116 | (1, 119) | 162 | (1, 116) | 166 | Single-stranded DNA-binding protein | Single-stranded DNA-binding protein | | uniclust | UniRef100\_A0A0N8NST2 | 100.0 | 4.2e-39 | 8e-45 | 212.8 | 108 | (4, 117) | 162 | (1, 108) | 163 | Single-stranded DNA-binding protein | Single-stranded DNA-binding protein | | uniclust | UniRef100\_A0A0C7NPU9 | 100.0 | 4.7e-39 | 9.1e-45 | 217.7 | 111 | (4, 118) | 162 | (40, 150) | 187 | Single-stranded DNA-binding protein | Single-stranded DNA-binding protein | | uniclust | UniRef100\_A0A023WZ22 | 100.0 | 4.8e-39 | 9.5e-45 | 228.6 | 111 | (1, 115) | 162 | (27, 137) | 224 | Single-stranded DNA-binding protein | Single-stranded DNA-binding protein | | uniclust | UniRef100\_A0A011TCV2 | 100.0 | 5.3e-39 | 1e-44 | 224.0 | 115 | (1, 115) | 162 | (34, 150) | 215 | Single-stranded DNA-binding protein | Single-stranded DNA-binding protein | | uniclust | UniRef100\_A0A011V2J4 | 100.0 | 5.7e-39 | 1.1e-44 | 223.9 | 110 | (2, 117) | 162 | (23, 132) | 213 | Single-stranded DNA-binding protein | Single-stranded DNA-binding protein | | uniclust | UniRef100\_A0A022FZQ1 | 100.0 | 6.2e-39 | 1.2e-44 | 223.6 | 112 | (1, 115) | 162 | (23, 134) | 254 | Single-stranded DNA-binding protein | Single-stranded DNA-binding protein | | uniclust | UniRef100\_A0A023BY02 | 100.0 | 7.1e-39 | 1.4e-44 | 223.6 | 114 | (1, 117) | 162 | (29, 142) | 217 | Single-stranded DNA-binding protein | Single-stranded DNA-binding protein | | uniclust | UniRef100\_A0A095BFI2 | 100.0 | 7.3e-39 | 1.4e-44 | 211.4 | 114 | (1, 117) | 162 | (6, 120) | 172 | Single-stranded DNA-binding protein | Single-stranded DNA-binding protein | | uniclust | UniRef100\_A0A1F7RRQ4 | 100.0 | 7.3e-39 | 1.4e-44 | 216.1 | 113 | (2, 118) | 162 | (29, 141) | 185 | Single-stranded DNA-binding protein | Single-stranded DNA-binding protein | | uniclust | UniRef100\_A0A0M5JTF8 | 100.0 | 9.9e-39 | 1.9e-44 | 213.8 | 116 | (3, 118) | 162 | (4, 119) | 192 | Single-stranded DNA-binding protein | Single-stranded DNA-binding protein | | uniclust | UniRef100\_A0A088T2F7 | 100.0 | 1.2e-38 | 2.4e-44 | 221.7 | 111 | (1, 115) | 162 | (51, 161) | 212 | Single-stranded DNA-binding protein | Single-stranded DNA-binding protein | | uniclust | UniRef100\_A0A1F3BMS9 | 100.0 | 1.3e-38 | 2.5e-44 | 208.2 | 112 | (1, 116) | 162 | (1, 112) | 150 | Single-stranded DNA-binding protein | Single-stranded DNA-binding protein | | uniclust | UniRef100\_A0A0A8B1Q7 | 100.0 | 1.3e-38 | 2.5e-44 | 212.7 | 112 | (1, 116) | 162 | (7, 118) | 175 | Single-stranded DNA-binding protein | Single-stranded DNA-binding protein | | uniclust | UniRef100\_A0A098R1J5 | 100.0 | 1.8e-38 | 3.4e-44 | 212.7 | 112 | (1, 118) | 162 | (2, 113) | 160 | Single-stranded DNA-binding protein | Single-stranded DNA-binding protein | | uniclust | UniRef100\_A0A014M3K2 | 100.0 | 1.9e-38 | 3.5e-44 | 209.9 | 115 | (3, 117) | 162 | (4, 118) | 193 | Single-stranded DNA-binding protein | Single-stranded DNA-binding protein | | uniclust | UniRef100\_A0A010YT66 | 100.0 | 1.8e-38 | 3.6e-44 | 217.6 | 110 | (1, 114) | 162 | (15, 125) | 177 | Single-stranded DNA-binding protein | Single-stranded DNA-binding protein | | uniclust | UniRef100\_A0A1B8PKK7 | 100.0 | 2.1e-38 | 4e-44 | 213.1 | 115 | (1, 119) | 162 | (1, 115) | 187 | Single-stranded DNA-binding protein | Single-stranded DNA-binding protein | | uniclust | UniRef100\_A0A015YND2 | 100.0 | 2.4e-38 | 4.6e-44 | 216.3 | 115 | (1, 118) | 162 | (29, 144) | 188 | Single-stranded DNA-binding protein | Single-stranded DNA-binding protein | | uniclust | UniRef100\_A0A064AII8 | 100.0 | 2.3e-38 | 4.6e-44 | 223.6 | 111 | (1, 117) | 162 | (27, 137) | 228 | Single-stranded DNA-binding protein | Single-stranded DNA-binding protein | | uniclust | UniRef100\_A0A0A0X2I0 | 100.0 | 2.8e-38 | 5.4e-44 | 214.4 | 110 | (1, 116) | 162 | (31, 140) | 187 | Single-stranded DNA-binding protein | Single-stranded DNA-binding protein | | uniclust | UniRef100\_A0A091AL92 | 100.0 | 2.8e-38 | 5.5e-44 | 216.9 | 113 | (1, 118) | 162 | (15, 127) | 181 | Single-stranded DNA-binding protein | Single-stranded DNA-binding protein | | uniclust | UniRef100\_A0A009PIV2 | 100.0 | 2.8e-38 | 5.5e-44 | 220.1 | 112 | (1, 115) | 162 | (26, 137) | 202 | Single-stranded DNA-binding protein | Single-stranded DNA-binding protein | | uniclust | UniRef100\_A0A094W5Q5 | 100.0 | 3.4e-38 | 6.5e-44 | 211.2 | 110 | (1, 115) | 162 | (19, 128) | 175 | Single-stranded DNA-binding protein | Single-stranded DNA-binding protein | | uniclust | UniRef100\_A0A0B0EP20 | 100.0 | 3.6e-38 | 7.2e-44 | 215.2 | 110 | (2, 117) | 162 | (22, 131) | 170 | Single-stranded DNA-binding protein | Single-stranded DNA-binding protein | | uniclust | UniRef100\_A0A016XFS3 | 100.0 | 3.9e-38 | 7.8e-44 | 222.2 | 112 | (1, 116) | 162 | (36, 147) | 217 | Single-stranded DNA-binding protein | Single-stranded DNA-binding protein | | uniclust | UniRef100\_A0A0A1DMG2 | 100.0 | 4.5e-38 | 8.8e-44 | 215.8 | 112 | (1, 116) | 162 | (11, 122) | 191 | Single-stranded DNA-binding protein | Single-stranded DNA-binding protein | | uniclust | UniRef100\_A0A068NVF8 | 100.0 | 6.6e-38 | 1.3e-43 | 205.5 | 112 | (2, 119) | 162 | (1, 112) | 146 | Single-stranded DNA-binding protein | Single-stranded DNA-binding protein | | uniclust | UniRef100\_A0A2D3R7S5 | 100.0 | 8.1e-38 | 1.5e-43 | 205.7 | 117 | (1, 121) | 162 | (1, 117) | 170 | Single-stranded DNA-binding protein | Single-stranded DNA-binding protein | | uniclust | UniRef100\_A0A0F3GPA7 | 100.0 | 9.3e-38 | 1.8e-43 | 211.8 | 109 | (1, 113) | 162 | (14, 122) | 170 | Single-stranded DNA-binding protein | Single-stranded DNA-binding protein | | uniclust | UniRef100\_A0A077MG65 | 100.0 | 1.2e-37 | 2.3e-43 | 208.7 | 112 | (2, 116) | 162 | (12, 123) | 195 | Single-stranded DNA-binding protein | Single-stranded DNA-binding protein | | uniclust | UniRef100\_A0A096B1L2 | 100.0 | 1.2e-37 | 2.3e-43 | 205.9 | 112 | (2, 118) | 162 | (11, 122) | 180 | Single-stranded DNA-binding protein | Single-stranded DNA-binding protein | | uniclust | UniRef100\_A0A072Y3A3 | 100.0 | 1.4e-37 | 2.7e-43 | 210.0 | 111 | (1, 117) | 162 | (7, 117) | 154 | Single-stranded DNA-binding protein | Single-stranded DNA-binding protein | | uniclust | UniRef100\_A0A0G0BWT4 | 100.0 | 1.5e-37 | 2.8e-43 | 208.2 | 109 | (2, 114) | 162 | (11, 119) | 177 | Single-stranded DNA-binding protein | Single-stranded DNA-binding protein | | uniclust | UniRef100\_A0A1B1IQN2 | 100.0 | 1.5e-37 | 2.9e-43 | 206.3 | 111 | (1, 116) | 162 | (7, 117) | 153 | Single-stranded DNA-binding protein | Single-stranded DNA-binding protein | | uniclust | UniRef100\_A0A2W0AHC9 | 100.0 | 2.3e-37 | 4.4e-43 | 201.9 | 115 | (1, 118) | 162 | (1, 115) | 162 | Single-stranded DNA-binding protein | Single-stranded DNA-binding protein | | uniclust | UniRef100\_A0A1Y5D6G7 | 100.0 | 2.8e-37 | 5.2e-43 | 207.3 | 114 | (2, 119) | 162 | (21, 134) | 207 | Single-stranded DNA-binding protein | Single-stranded DNA-binding protein | | uniclust | UniRef100\_A0A0S8KNV4 | 100.0 | 3e-37 | 5.9e-43 | 207.3 | 112 | (1, 116) | 162 | (24, 135) | 162 | Single-stranded DNA-binding protein | Single-stranded DNA-binding protein | | uniclust | UniRef100\_A0A0G2Q8L7 | 100.0 | 3.4e-37 | 6.5e-43 | 209.4 | 117 | (1, 117) | 162 | (33, 151) | 200 | Single-stranded DNA-binding protein | Single-stranded DNA-binding protein | | uniclust | UniRef100\_A0A0H5QQE0 | 100.0 | 3.3e-37 | 6.5e-43 | 208.0 | 110 | (1, 117) | 162 | (5, 114) | 170 | Single-stranded DNA-binding protein | Single-stranded DNA-binding protein | | uniclust | UniRef100\_A0A023BVC0 | 100.0 | 4.6e-37 | 9e-43 | 215.9 | 112 | (1, 116) | 162 | (32, 143) | 210 | Single-stranded DNA-binding protein | Single-stranded DNA-binding protein | | uniclust | UniRef100\_A0A011VB38 | 100.0 | 5.6e-37 | 1.1e-42 | 217.0 | 115 | (1, 115) | 162 | (49, 165) | 266 | Single-stranded DNA-binding protein | Single-stranded DNA-binding protein | | uniclust | UniRef100\_A0A0E3UIY2 | 100.0 | 6.3e-37 | 1.2e-42 | 205.1 | 110 | (2, 115) | 162 | (1, 111) | 183 | Single-stranded DNA-binding protein | Single-stranded DNA-binding protein | | uniclust | UniRef100\_A0A1F7X0M2 | 100.0 | 7.6e-37 | 1.4e-42 | 202.2 | 113 | (1, 117) | 162 | (1, 113) | 176 | Single-stranded DNA-binding protein | Single-stranded DNA-binding protein | | uniclust | UniRef100\_A0A084SGN0 | 100.0 | 1.1e-36 | 2.1e-42 | 193.4 | 111 | (1, 115) | 162 | (4, 114) | 127 | Single-stranded DNA-binding protein (Fragment) | Single-stranded DNA-binding protein (Fragment) | | uniclust | UniRef100\_A0A0A7P4Y4 | 100.0 | 1.4e-36 | 2.7e-42 | 208.3 | 110 | (1, 115) | 162 | (18, 127) | 191 | Single-stranded DNA-binding protein | Single-stranded DNA-binding protein | | uniclust | UniRef100\_A0A0W0WBB9 | 100.0 | 1.6e-36 | 3.1e-42 | 202.6 | 109 | (1, 114) | 162 | (5, 113) | 154 | Single-stranded DNA-binding protein | Single-stranded DNA-binding protein | | uniclust | UniRef100\_A0A084JIF5 | 100.0 | 1.7e-36 | 3.4e-42 | 208.4 | 109 | (1, 117) | 162 | (16, 127) | 183 | Single-stranded DNA-binding protein | Single-stranded DNA-binding protein | | uniclust | UniRef100\_A0A0S8EKW5 | 100.0 | 2.9e-36 | 5.6e-42 | 199.6 | 109 | (3, 115) | 162 | (7, 115) | 175 | Single-stranded DNA-binding protein | Single-stranded DNA-binding protein | | uniclust | UniRef100\_A0A0B6WUT2 | 100.0 | 3.2e-36 | 6.2e-42 | 201.7 | 114 | (2, 119) | 162 | (17, 130) | 172 | Single-stranded DNA-binding protein | Single-stranded DNA-binding protein | | uniclust | UniRef100\_A0A0A8WRT0 | 100.0 | 3.9e-36 | 7.5e-42 | 199.5 | 112 | (2, 118) | 162 | (25, 136) | 162 | Single-stranded DNA-binding protein | Single-stranded DNA-binding protein | | uniclust | UniRef100\_A0A011RQU6 | 100.0 | 4.9e-36 | 9.4e-42 | 209.2 | 112 | (1, 118) | 162 | (15, 126) | 241 | Single-stranded DNA-binding protein | Single-stranded DNA-binding protein | | uniclust | UniRef100\_A0A1V5HJ78 | 100.0 | 8.8e-36 | 1.7e-41 | 195.9 | 109 | (1, 113) | 162 | (13, 121) | 159 | Single-stranded DNA-binding protein | Single-stranded DNA-binding protein | | uniclust | UniRef100\_A0A0M8K5Q7 | 100.0 | 8.8e-36 | 1.7e-41 | 202.8 | 111 | (1, 115) | 162 | (29, 139) | 201 | Single-stranded DNA-binding protein | Single-stranded DNA-binding protein | | uniclust | UniRef100\_A0A0G1IEC5 | 100.0 | 1.1e-35 | 2.2e-41 | 206.5 | 111 | (1, 116) | 162 | (47, 157) | 200 | Single-stranded DNA-binding protein | Single-stranded DNA-binding protein | | uniclust | UniRef100\_A0A1T4V337 | 100.0 | 1.2e-35 | 2.3e-41 | 205.8 | 112 | (1, 115) | 162 | (1, 112) | 218 | Single-stranded DNA-binding protein | Single-stranded DNA-binding protein | | uniclust | UniRef100\_A0A081UYA0 | 100.0 | 2.3e-35 | 4.3e-41 | 204.8 | 114 | (3, 116) | 162 | (70, 183) | 281 | Single-stranded DNA-binding protein | Single-stranded DNA-binding protein | | uniclust | UniRef100\_A0A014MER4 | 100.0 | 2.7e-35 | 5.3e-41 | 194.9 | 104 | (3, 116) | 162 | (1, 104) | 150 | Single-stranded DNA-binding protein | Single-stranded DNA-binding protein | | uniclust | UniRef100\_A0A0P6Y529 | 100.0 | 3.8e-35 | 7.2e-41 | 194.6 | 112 | (1, 116) | 162 | (23, 134) | 160 | Single-stranded DNA-binding protein | Single-stranded DNA-binding protein | | uniclust | UniRef100\_A0A0A7LLS2 | 100.0 | 3.9e-35 | 7.3e-41 | 190.1 | 114 | (1, 118) | 162 | (6, 119) | 148 | Single-stranded DNA-binding protein | Single-stranded DNA-binding protein | | uniclust | UniRef100\_A0A014LL70 | 100.0 | 3.9e-35 | 7.6e-41 | 210.0 | 110 | (1, 113) | 162 | (44, 154) | 253 | Single-stranded DNA-binding protein | Single-stranded DNA-binding protein | | uniclust | UniRef100\_A0A010RG06 | 100.0 | 4.3e-35 | 8e-41 | 196.8 | 116 | (1, 117) | 162 | (7, 122) | 218 | Single-stranded DNA-binding protein | Single-stranded DNA-binding protein | | uniclust | UniRef100\_A0A1V5DNT6 | 100.0 | 4.5e-35 | 8.8e-41 | 194.3 | 113 | (1, 118) | 162 | (3, 115) | 152 | Single-stranded DNA-binding protein | Single-stranded DNA-binding protein | | uniclust | UniRef100\_A0A095Z4E9 | 100.0 | 4.8e-35 | 9.2e-41 | 193.0 | 116 | (2, 120) | 162 | (1, 120) | 170 | Single-stranded DNA-binding protein | Single-stranded DNA-binding protein | | uniclust | UniRef100\_A0A1G0XIK8 | 100.0 | 5.9e-35 | 1.1e-40 | 198.8 | 113 | (1, 118) | 162 | (1, 114) | 201 | Single-stranded DNA-binding protein | Single-stranded DNA-binding protein | | uniclust | UniRef100\_A0A101WK01 | 100.0 | 7e-35 | 1.4e-40 | 191.2 | 112 | (2, 119) | 162 | (1, 112) | 144 | Single-stranded DNA-binding protein | Single-stranded DNA-binding protein | | uniclust | UniRef100\_A0A0M4CSQ1 | 100.0 | 7.7e-35 | 1.5e-40 | 195.7 | 113 | (1, 115) | 162 | (12, 124) | 178 | Single-stranded DNA-binding protein | Single-stranded DNA-binding protein | | uniclust | UniRef100\_A0A0P9EXD2 | 100.0 | 7.9e-35 | 1.5e-40 | 182.0 | 108 | (1, 117) | 162 | (1, 108) | 118 | Single-stranded DNA-binding protein (Fragment) | Single-stranded DNA-binding protein (Fragment) | | uniclust | UniRef100\_A0A1Z9MN17 | 100.0 | 9.4e-35 | 1.8e-40 | 193.7 | 101 | (2, 104) | 162 | (1, 101) | 157 | Single-stranded DNA-binding protein | Single-stranded DNA-binding protein | | uniclust | UniRef100\_A0A161QJ15 | 100.0 | 1.4e-34 | 2.7e-40 | 191.8 | 109 | (2, 114) | 162 | (6, 114) | 171 | Single-stranded DNA-binding protein | Single-stranded DNA-binding protein | | uniclust | UniRef100\_A0A017RY15 | 100.0 | 1.5e-34 | 3e-40 | 197.5 | 107 | (1, 116) | 162 | (29, 135) | 179 | Single-stranded DNA-binding protein | Single-stranded DNA-binding protein | | uniclust | UniRef100\_A0A136KKK6 | 100.0 | 1.6e-34 | 3.1e-40 | 188.8 | 112 | (1, 117) | 162 | (2, 113) | 137 | Single-stranded DNA-binding protein | Single-stranded DNA-binding protein | | uniclust | UniRef100\_A0A0J1BEB9 | 100.0 | 1.9e-34 | 3.5e-40 | 193.4 | 113 | (1, 118) | 162 | (36, 148) | 198 | Single-stranded DNA-binding protein | Single-stranded DNA-binding protein | | uniclust | UniRef100\_A0A0F9YYB5 | 100.0 | 3.1e-34 | 6e-40 | 199.4 | 110 | (3, 116) | 162 | (30, 139) | 223 | Single-stranded DNA-binding protein | Single-stranded DNA-binding protein | | uniclust | UniRef100\_A0A0P0P3Y9 | 100.0 | 4.1e-34 | 7.8e-40 | 190.1 | 115 | (1, 115) | 162 | (2, 118) | 195 | Single-stranded DNA-binding protein | Single-stranded DNA-binding protein | | uniclust | UniRef100\_A0A1F5RVU2 | 100.0 | 4.4e-34 | 8.5e-40 | 190.2 | 108 | (3, 114) | 162 | (27, 134) | 169 | Single-stranded DNA-binding protein | Single-stranded DNA-binding protein | | uniclust | UniRef100\_A0A0A2E6F1 | 100.0 | 4.7e-34 | 9e-40 | 189.0 | 114 | (1, 118) | 162 | (16, 129) | 171 | Single-stranded DNA-binding protein | Single-stranded DNA-binding protein | | uniclust | UniRef100\_A0A1B0XUD7 | 100.0 | 7.2e-34 | 1.4e-39 | 188.1 | 113 | (2, 118) | 162 | (10, 122) | 145 | Single-stranded DNA-binding protein | Single-stranded DNA-binding protein | | uniclust | UniRef100\_A0A134CMY2 | 100.0 | 8.2e-34 | 1.5e-39 | 186.3 | 103 | (1, 104) | 162 | (1, 103) | 168 | Single-stranded DNA-binding protein | Single-stranded DNA-binding protein | | uniclust | UniRef100\_A0A173R6J3 | 100.0 | 8.6e-34 | 1.7e-39 | 183.0 | 106 | (1, 115) | 162 | (4, 109) | 127 | Single-stranded DNA-binding protein | Single-stranded DNA-binding protein | | uniclust | UniRef100\_A0A0M1LCB6 | 100.0 | 1.1e-33 | 2e-39 | 188.7 | 106 | (1, 115) | 162 | (1, 106) | 159 | Single-stranded DNA-binding protein | Single-stranded DNA-binding protein | | uniclust | UniRef100\_A0A133N082 | 100.0 | 1.1e-33 | 2.1e-39 | 188.2 | 111 | (4, 118) | 162 | (1, 115) | 172 | Single-stranded DNA-binding protein | Single-stranded DNA-binding protein | | uniclust | UniRef100\_A0A094P9Y5 | 100.0 | 1.3e-33 | 2.6e-39 | 192.2 | 107 | (1, 107) | 162 | (20, 126) | 185 | Single-stranded DNA-binding protein | Single-stranded DNA-binding protein | | uniclust | UniRef100\_A0A031WI19 | 100.0 | 1.5e-33 | 2.9e-39 | 182.7 | 110 | (2, 117) | 162 | (7, 117) | 151 | Single-stranded DNA-binding protein | Single-stranded DNA-binding protein | | uniclust | UniRef100\_A0A1Y3UI13 | 100.0 | 2.3e-33 | 4.5e-39 | 186.9 | 113 | (1, 118) | 162 | (1, 113) | 158 | Single-stranded DNA-binding protein | Single-stranded DNA-binding protein | | uniclust | UniRef100\_A0A1F3DCZ5 | 100.0 | 2.4e-33 | 4.5e-39 | 187.9 | 113 | (3, 119) | 162 | (1, 113) | 182 | Single-stranded DNA-binding protein | Single-stranded DNA-binding protein | | uniclust | UniRef100\_A0A059E0M9 | 100.0 | 2.4e-33 | 4.5e-39 | 184.3 | 113 | (1, 118) | 162 | (11, 123) | 158 | Single-stranded DNA-binding protein | Single-stranded DNA-binding protein | | uniclust | UniRef100\_A0A068EXU3 | 100.0 | 3.2e-33 | 6.1e-39 | 188.8 | 107 | (2, 116) | 162 | (18, 124) | 169 | Single-stranded DNA-binding protein | Single-stranded DNA-binding protein | | uniclust | UniRef100\_A0A0V8CZ75 | 100.0 | 3.4e-33 | 6.5e-39 | 186.5 | 108 | (3, 119) | 162 | (6, 113) | 162 | tRNA-binding protein | tRNA-binding protein | | uniclust | UniRef100\_A0A0G0XSM8 | 100.0 | 3.9e-33 | 7.2e-39 | 182.6 | 108 | (2, 114) | 162 | (1, 108) | 180 | Single-stranded DNA-binding protein | Single-stranded DNA-binding protein | | uniclust | UniRef100\_A0A075MAN7 | 100.0 | 4.1e-33 | 7.7e-39 | 184.6 | 116 | (3, 119) | 162 | (4, 119) | 189 | Single-stranded DNA-binding protein | Single-stranded DNA-binding protein | | uniclust | UniRef100\_A0A0F0CQF3 | 100.0 | 4.9e-33 | 9.3e-39 | 189.9 | 110 | (1, 114) | 162 | (30, 139) | 197 | Single-stranded DNA-binding protein | Single-stranded DNA-binding protein | | uniclust | UniRef100\_A0A077UH19 | 100.0 | 5e-33 | 9.7e-39 | 187.0 | 112 | (2, 119) | 162 | (10, 121) | 166 | Single-stranded DNA-binding protein | Single-stranded DNA-binding protein | | uniclust | UniRef100\_A0A6B1E277 | 100.0 | 5.4e-33 | 1e-38 | 181.2 | 112 | (2, 117) | 162 | (1, 112) | 164 | Single-stranded DNA-binding protein | Single-stranded DNA-binding protein | | uniclust | UniRef100\_A0A011RMD1 | 100.0 | 5.6e-33 | 1.1e-38 | 201.5 | 110 | (1, 116) | 162 | (46, 155) | 262 | Single-stranded DNA-binding protein | Single-stranded DNA-binding protein | | uniclust | UniRef100\_A0A0M2HGC3 | 100.0 | 6.8e-33 | 1.3e-38 | 189.1 | 111 | (2, 112) | 162 | (20, 133) | 177 | Single-stranded DNA-binding protein | Single-stranded DNA-binding protein | | uniclust | UniRef100\_A0A133Z2U9 | 100.0 | 7.9e-33 | 1.5e-38 | 185.0 | 110 | (3, 115) | 162 | (10, 120) | 165 | Single-stranded DNA-binding protein | Single-stranded DNA-binding protein | | uniclust | UniRef100\_A0A024C9G7 | 100.0 | 9.8e-33 | 1.9e-38 | 188.4 | 113 | (2, 118) | 162 | (1, 113) | 199 | Single-stranded DNA-binding protein | Single-stranded DNA-binding protein | | uniclust | UniRef100\_A0A0Q6NU73 | 100.0 | 1.3e-32 | 2.4e-38 | 179.8 | 103 | (1, 104) | 162 | (1, 104) | 160 | Single-stranded DNA-binding protein | Single-stranded DNA-binding protein | | uniclust | UniRef100\_A0A0P6WWD0 | 100.0 | 1.5e-32 | 3e-38 | 183.1 | 100 | (2, 115) | 162 | (4, 103) | 148 | Single-stranded DNA-binding protein | Single-stranded DNA-binding protein | | uniclust | UniRef100\_A0A013VT29 | 100.0 | 1.7e-32 | 3.1e-38 | 177.2 | 116 | (1, 116) | 162 | (3, 120) | 159 | Single-stranded DNA-binding protein (Fragment) | Single-stranded DNA-binding protein (Fragment) | | uniclust | UniRef100\_A0A0J8YFX1 | 100.0 | 1.6e-32 | 3.1e-38 | 183.0 | 101 | (2, 111) | 162 | (12, 112) | 174 | Single-stranded DNA-binding protein | Single-stranded DNA-binding protein | | uniclust | UniRef100\_A0A1I0ACB5 | 100.0 | 1.9e-32 | 3.6e-38 | 177.3 | 106 | (1, 118) | 162 | (1, 106) | 125 | Single-strand DNA-binding protein | Single-strand DNA-binding protein | | uniclust | UniRef100\_A0A2S5CMA6 | 100.0 | 2.1e-32 | 3.8e-38 | 182.7 | 113 | (2, 117) | 162 | (49, 161) | 216 | Single-stranded DNA-binding protein | Single-stranded DNA-binding protein | | uniclust | UniRef100\_A0A235BXR5 | 100.0 | 2.4e-32 | 4.6e-38 | 176.9 | 110 | (2, 114) | 162 | (6, 115) | 156 | Single-stranded DNA-binding protein | Single-stranded DNA-binding protein | | uniclust | UniRef100\_A0A1B9LQJ7 | 99.9 | 3.5e-32 | 6.7e-38 | 178.3 | 146 | (1, 162) | 162 | (1, 146) | 161 | Single-stranded DNA-binding protein | Single-stranded DNA-binding protein | | uniclust | UniRef100\_A0A091BEV2 | 99.9 | 4.1e-32 | 7.7e-38 | 177.6 | 111 | (2, 115) | 162 | (3, 113) | 175 | Single-stranded DNA-binding protein (Fragment) | Single-stranded DNA-binding protein (Fragment) | | uniclust | UniRef100\_A0A2E4F7T1 | 99.9 | 5.2e-32 | 9.6e-38 | 178.3 | 111 | (1, 115) | 162 | (1, 111) | 192 | Single-stranded DNA-binding protein | Single-stranded DNA-binding protein | | uniclust | UniRef100\_A0A136Q8P1 | 99.9 | 5.3e-32 | 1e-37 | 181.0 | 106 | (2, 114) | 162 | (30, 135) | 178 | Single-stranded DNA-binding protein | Single-stranded DNA-binding protein | | uniclust | UniRef100\_A0A2V2DG25 | 99.9 | 6.2e-32 | 1.2e-37 | 179.7 | 106 | (5, 120) | 162 | (1, 106) | 182 | Single-stranded DNA-binding protein | Single-stranded DNA-binding protein | | uniclust | UniRef100\_A0A061JJT5 | 99.9 | 6.1e-32 | 1.2e-37 | 186.7 | 111 | (2, 118) | 162 | (26, 137) | 205 | Single-stranded DNA-binding protein | Single-stranded DNA-binding protein | | uniclust | UniRef100\_A0A0A0X352 | 99.9 | 6.7e-32 | 1.3e-37 | 180.6 | 109 | (2, 116) | 162 | (17, 125) | 170 | Single-stranded DNA-binding protein | Single-stranded DNA-binding protein | | uniclust | UniRef100\_A0A072Z0E3 | 99.9 | 8.1e-32 | 1.5e-37 | 168.9 | 111 | (2, 118) | 162 | (1, 112) | 120 | Single-stranded DNA-binding protein | Single-stranded DNA-binding protein | | uniclust | UniRef100\_A0A1F5BJ96 | 99.9 | 8.9e-32 | 1.7e-37 | 171.7 | 110 | (2, 117) | 162 | (7, 116) | 131 | Single-stranded DNA-binding protein | Single-stranded DNA-binding protein | | uniclust | UniRef100\_A0A1B3AYF2 | 99.9 | 9e-32 | 1.8e-37 | 182.0 | 108 | (2, 112) | 162 | (1, 110) | 165 | SsDNA binding protein | SsDNA binding protein | | uniclust | UniRef100\_A0A1F3XSW4 | 99.9 | 9.3e-32 | 1.8e-37 | 176.2 | 113 | (2, 118) | 162 | (14, 126) | 149 | Single-stranded DNA-binding protein | Single-stranded DNA-binding protein | | uniclust | UniRef100\_A0A136L6G9 | 99.9 | 1.1e-31 | 2e-37 | 168.1 | 113 | (1, 117) | 162 | (1, 113) | 123 | Single-stranded DNA-binding protein | Single-stranded DNA-binding protein | | uniclust | UniRef100\_A0A068MX81 | 99.9 | 1.4e-31 | 2.7e-37 | 182.4 | 108 | (1, 117) | 162 | (27, 135) | 174 | Single-stranded DNA-binding protein | Single-stranded DNA-binding protein | | uniclust | UniRef100\_A0A7C3ZBM8 | 99.9 | 1.4e-31 | 2.7e-37 | 177.2 | 111 | (2, 116) | 162 | (3, 113) | 176 | Single-stranded DNA-binding protein | Single-stranded DNA-binding protein | | uniclust | UniRef100\_A0A0P0DYN1 | 99.9 | 1.6e-31 | 3.1e-37 | 181.7 | 106 | (2, 107) | 162 | (24, 130) | 187 | Single-stranded DNA-binding protein | Single-stranded DNA-binding protein | | uniclust | UniRef100\_A0A0E4FY13 | 99.9 | 1.7e-31 | 3.2e-37 | 180.5 | 110 | (1, 113) | 162 | (1, 111) | 215 | Single-stranded DNA-binding protein | Single-stranded DNA-binding protein | | uniclust | UniRef100\_A0A0A6PYF7 | 99.9 | 2.2e-31 | 4.2e-37 | 172.9 | 110 | (2, 117) | 162 | (12, 122) | 147 | Single-stranded DNA-binding protein | Single-stranded DNA-binding protein | | uniclust | UniRef100\_A0A086PBI0 | 99.9 | 2.2e-31 | 4.3e-37 | 179.0 | 116 | (1, 117) | 162 | (19, 136) | 161 | Single-stranded DNA-binding protein | Single-stranded DNA-binding protein | | uniclust | UniRef100\_A0A0D5M2L7 | 99.9 | 2.6e-31 | 4.9e-37 | 160.4 | 95 | (1, 95) | 162 | (2, 96) | 98 | Single-stranded DNA-binding protein | Single-stranded DNA-binding protein | | uniclust | UniRef100\_A0A0C5EQ65 | 99.9 | 3.1e-31 | 5.7e-37 | 178.8 | 117 | (4, 120) | 162 | (52, 168) | 227 | Single-stranded DNA-binding protein | Single-stranded DNA-binding protein | | uniclust | UniRef100\_A0A0F9ENP5 | 99.9 | 3.1e-31 | 6e-37 | 175.2 | 114 | (1, 116) | 162 | (12, 128) | 160 | Single-stranded DNA-binding protein | Single-stranded DNA-binding protein | | uniclust | UniRef100\_A0A063KS07 | 99.9 | 3.6e-31 | 6.8e-37 | 181.9 | 112 | (4, 116) | 162 | (7, 118) | 239 | Single-stranded DNA-binding protein | Single-stranded DNA-binding protein | | uniclust | UniRef100\_A0A8S5LK82 | 99.9 | 4.6e-31 | 8.8e-37 | 170.9 | 107 | (2, 112) | 162 | (1, 107) | 145 | Single strand binding protein | Single strand binding protein | | uniclust | UniRef100\_A0A0K2H331 | 99.9 | 5.1e-31 | 1e-36 | 176.2 | 111 | (2, 117) | 162 | (1, 111) | 149 | Single-stranded DNA-binding protein | Single-stranded DNA-binding protein | | uniclust | UniRef100\_A0A0D6IC91 | 99.9 | 6.5e-31 | 1.2e-36 | 174.1 | 114 | (2, 118) | 162 | (1, 114) | 191 | Single-stranded DNA-binding protein | Single-stranded DNA-binding protein | | uniclust | UniRef100\_A0A016QTA0 | 99.9 | 6.9e-31 | 1.3e-36 | 193.4 | 111 | (2, 117) | 162 | (147, 257) | 309 | Single-stranded DNA-binding protein | Single-stranded DNA-binding protein | | uniclust | UniRef100\_A0A1V5FBC2 | 99.9 | 7.3e-31 | 1.4e-36 | 176.1 | 112 | (2, 117) | 162 | (5, 117) | 191 | Single-stranded DNA-binding protein | Single-stranded DNA-binding protein | | uniclust | UniRef100\_A0A0K8MB88 | 99.9 | 8.7e-31 | 1.7e-36 | 175.3 | 113 | (1, 117) | 162 | (31, 146) | 176 | Single-stranded DNA-binding protein | Single-stranded DNA-binding protein | | uniclust | UniRef100\_A0A0B8NGF8 | 99.9 | 9.5e-31 | 1.8e-36 | 182.3 | 111 | (1, 111) | 162 | (23, 135) | 218 | Single-stranded DNA-binding protein | Single-stranded DNA-binding protein | | uniclust | UniRef100\_A0A2I7N694 | 99.9 | 1e-30 | 1.9e-36 | 169.2 | 111 | (1, 120) | 162 | (1, 111) | 148 | Single-stranded DNA-binding protein | Single-stranded DNA-binding protein | | uniclust | UniRef100\_A0A022L9F6 | 99.9 | 1e-30 | 2e-36 | 187.6 | 107 | (1, 107) | 162 | (52, 159) | 240 | Single-stranded DNA-binding protein | Single-stranded DNA-binding protein | | uniclust | UniRef100\_A0A2W5CU06 | 99.9 | 1.2e-30 | 2.3e-36 | 170.3 | 107 | (1, 107) | 162 | (1, 108) | 167 | Single-stranded DNA-binding protein | Single-stranded DNA-binding protein | | uniclust | UniRef100\_A0A0N0JSL7 | 99.9 | 1.3e-30 | 2.4e-36 | 176.0 | 106 | (2, 116) | 162 | (27, 133) | 164 | Single-stranded DNA-binding protein | Single-stranded DNA-binding protein | | uniclust | UniRef100\_A0A127SX29 | 99.9 | 1.4e-30 | 2.7e-36 | 167.5 | 111 | (3, 116) | 162 | (1, 111) | 133 | Single-stranded DNA-binding protein | Single-stranded DNA-binding protein | | uniclust | UniRef100\_A0A0A7G012 | 99.9 | 1.5e-30 | 3e-36 | 169.6 | 109 | (1, 118) | 162 | (2, 110) | 133 | Single-stranded DNA-binding protein | Single-stranded DNA-binding protein | | uniclust | UniRef100\_A0A133Y031 | 99.9 | 2.2e-30 | 4.2e-36 | 169.6 | 113 | (1, 117) | 162 | (20, 132) | 140 | Single-stranded DNA-binding protein | Single-stranded DNA-binding protein | | uniclust | UniRef100\_A0A081PBG3 | 99.9 | 2.2e-30 | 4.3e-36 | 173.3 | 114 | (1, 118) | 162 | (11, 124) | 155 | Single-stranded DNA-binding protein | Single-stranded DNA-binding protein | | uniclust | UniRef100\_A0A0F6X1X7 | 99.9 | 2.6e-30 | 4.9e-36 | 174.2 | 107 | (4, 118) | 162 | (1, 107) | 177 | Single-stranded DNA-binding protein | Single-stranded DNA-binding protein | | uniclust | UniRef100\_A0A011TE40 | 99.9 | 2.8e-30 | 5.3e-36 | 171.0 | 111 | (3, 117) | 162 | (26, 143) | 172 | Single-stranded DNA-binding protein | Single-stranded DNA-binding protein | | uniclust | UniRef100\_A0A0C2APC6 | 99.9 | 3.3e-30 | 6.2e-36 | 172.2 | 105 | (2, 107) | 162 | (28, 133) | 194 | Single-stranded DNA-binding protein | Single-stranded DNA-binding protein | | uniclust | UniRef100\_A0A0D5YK57 | 99.9 | 4e-30 | 7.6e-36 | 153.9 | 85 | (1, 86) | 162 | (2, 86) | 87 | Single-strand binding protein | Single-strand binding protein | | uniclust | UniRef100\_A0A0G4E5Q2 | 99.9 | 4.3e-30 | 7.8e-36 | 171.2 | 118 | (1, 119) | 162 | (61, 178) | 233 | Single-stranded DNA-binding protein | Single-stranded DNA-binding protein | | uniclust | UniRef100\_A0A1V5ICE4 | 99.9 | 4.2e-30 | 7.9e-36 | 160.3 | 105 | (4, 117) | 162 | (1, 112) | 129 | Single-stranded DNA-binding protein | Single-stranded DNA-binding protein | | uniclust | UniRef100\_A0A0F7L5K1 | 99.9 | 4.6e-30 | 8.7e-36 | 171.7 | 107 | (3, 119) | 162 | (1, 108) | 174 | Single-strand DNA-binding protein | Single-strand DNA-binding protein | | uniclust | UniRef100\_A0A1G3M634 | 99.9 | 4.9e-30 | 9.5e-36 | 170.8 | 110 | (1, 114) | 162 | (1, 110) | 158 | Single-stranded DNA-binding protein | Single-stranded DNA-binding protein | | uniclust | UniRef100\_A0A220SXH4 | 99.9 | 6.7e-30 | 1.2e-35 | 175.0 | 113 | (4, 117) | 162 | (18, 130) | 261 | Single-stranded DNA-binding protein | Single-stranded DNA-binding protein | | uniclust | UniRef100\_A0A2W1TN85 | 99.9 | 6.9e-30 | 1.3e-35 | 174.7 | 106 | (1, 106) | 162 | (1, 107) | 199 | Single-stranded DNA-binding protein | Single-stranded DNA-binding protein | | uniclust | UniRef100\_A0A2S8SU52 | 99.9 | 7e-30 | 1.3e-35 | 175.7 | 111 | (2, 118) | 162 | (4, 114) | 220 | Single-stranded DNA-binding protein | Single-stranded DNA-binding protein | | uniclust | UniRef100\_A0A1G9EW49 | 99.9 | 7.4e-30 | 1.4e-35 | 170.4 | 106 | (2, 107) | 162 | (1, 111) | 168 | Single-stranded DNA-binding protein | Single-stranded DNA-binding protein | | uniclust | UniRef100\_A0A078KHX1 | 99.9 | 8.6e-30 | 1.6e-35 | 165.3 | 112 | (1, 115) | 162 | (3, 114) | 144 | Single-stranded DNA-binding protein | Single-stranded DNA-binding protein | | uniclust | UniRef100\_A0A1B0XTI5 | 99.9 | 8.6e-30 | 1.7e-35 | 169.9 | 103 | (1, 104) | 162 | (1, 104) | 151 | Primosome PriB/single-strand DNA-binding protein | Primosome PriB/single-strand DNA-binding protein | | uniclust | UniRef100\_A0A0G0GYK1 | 99.9 | 9.5e-30 | 1.8e-35 | 169.9 | 112 | (3, 118) | 162 | (5, 116) | 176 | Single-stranded DNA-binding protein | Single-stranded DNA-binding protein | | uniclust | UniRef100\_A0A2E1Q3N5 | 99.9 | 1.2e-29 | 2.4e-35 | 166.2 | 112 | (2, 118) | 162 | (1, 112) | 145 | Single-stranded DNA-binding protein | Single-stranded DNA-binding protein | | uniclust | UniRef100\_A0A3D5Q9W8 | 99.9 | 1.3e-29 | 2.5e-35 | 153.7 | 89 | (2, 91) | 162 | (7, 95) | 98 | Single-stranded DNA-binding protein (Fragment) | Single-stranded DNA-binding protein (Fragment) | | uniclust | UniRef100\_A0A1D7QSF8 | 99.9 | 1.4e-29 | 2.6e-35 | 177.2 | 109 | (4, 118) | 162 | (1, 109) | 227 | Single-stranded DNA-binding protein | Single-stranded DNA-binding protein | | uniclust | UniRef100\_A0A142XSZ2 | 99.9 | 1.4e-29 | 2.7e-35 | 171.4 | 114 | (2, 118) | 162 | (39, 158) | 205 | Single-stranded DNA-binding protein | Single-stranded DNA-binding protein | | uniclust | UniRef100\_A0A199YTL7 | 99.9 | 1.5e-29 | 2.8e-35 | 153.0 | 91 | (3, 96) | 162 | (1, 91) | 98 | Single-stranded DNA-binding protein | Single-stranded DNA-binding protein | | uniclust | UniRef100\_A0A5Q2N011 | 99.9 | 2.1e-29 | 3.8e-35 | 166.4 | 110 | (4, 117) | 162 | (1, 113) | 193 | Single-stranded DNA-binding protein | Single-stranded DNA-binding protein | | uniclust | UniRef100\_A0A538CVG0 | 99.9 | 2.5e-29 | 4.6e-35 | 170.6 | 113 | (3, 118) | 162 | (75, 187) | 233 | Single-stranded DNA-binding protein | Single-stranded DNA-binding protein | | uniclust | UniRef100\_A0A285MVQ1 | 99.9 | 3e-29 | 5.7e-35 | 166.0 | 111 | (1, 115) | 162 | (1, 111) | 163 | Single-stranded DNA-binding protein | Single-stranded DNA-binding protein | | uniclust | UniRef100\_A0A1V5ZP08 | 99.9 | 3.1e-29 | 5.8e-35 | 150.5 | 87 | (1, 88) | 162 | (2, 88) | 92 | Single-stranded DNA-binding protein | Single-stranded DNA-binding protein | | uniclust | UniRef100\_A0A132HW80 | 99.9 | 3.5e-29 | 6.6e-35 | 161.1 | 111 | (2, 117) | 162 | (1, 111) | 150 | Single-stranded DNA-binding protein | Single-stranded DNA-binding protein | | uniclust | UniRef100\_A0A1H4XXA5 | 99.9 | 3.5e-29 | 6.7e-35 | 161.6 | 111 | (2, 118) | 162 | (1, 111) | 133 | Single-stranded DNA-binding protein | Single-stranded DNA-binding protein | | uniclust | UniRef100\_A0A2D9X095 | 99.9 | 4.7e-29 | 9e-35 | 162.0 | 109 | (1, 118) | 162 | (6, 116) | 141 | Single-stranded DNA-binding protein | Single-stranded DNA-binding protein | | uniclust | UniRef100\_A0A157SR46 | 99.9 | 6.4e-29 | 1.2e-34 | 162.0 | 110 | (1, 116) | 162 | (18, 127) | 148 | Single-stranded DNA-binding protein | Single-stranded DNA-binding protein | | uniclust | UniRef100\_A0A0C1NEJ2 | 99.9 | 6.4e-29 | 1.2e-34 | 168.2 | 106 | (1, 115) | 162 | (27, 133) | 190 | Single-stranded DNA-binding protein | Single-stranded DNA-binding protein | | uniclust | UniRef100\_A0A127EFR2 | 99.9 | 7.4e-29 | 1.4e-34 | 159.6 | 109 | (5, 119) | 162 | (1, 110) | 133 | Single-stranded DNA-binding protein | Single-stranded DNA-binding protein | | uniclust | UniRef100\_A0A432EQF9 | 99.9 | 7.8e-29 | 1.5e-34 | 165.5 | 110 | (2, 115) | 162 | (7, 117) | 186 | Single-stranded DNA-binding protein | Single-stranded DNA-binding protein | | uniclust | UniRef100\_A0A1G6BP95 | 99.9 | 8.4e-29 | 1.6e-34 | 159.2 | 107 | (4, 117) | 162 | (1, 107) | 139 | Single-stranded DNA-binding protein | Single-stranded DNA-binding protein | | uniclust | UniRef100\_A0A061K5I9 | 99.9 | 8.9e-29 | 1.7e-34 | 171.0 | 115 | (4, 119) | 162 | (44, 158) | 268 | Single-stranded DNA-binding protein | Single-stranded DNA-binding protein | | uniclust | UniRef100\_A0A0A7I9E0 | 99.9 | 9.5e-29 | 1.8e-34 | 164.2 | 102 | (2, 104) | 162 | (7, 109) | 165 | Single-stranded DNA-binding protein | Single-stranded DNA-binding protein | | uniclust | UniRef100\_A0A2P4V2X1 | 99.9 | 1.2e-28 | 2.2e-34 | 147.5 | 89 | (1, 89) | 162 | (2, 90) | 90 | Single-stranded DNA-binding protein (Fragment) | Single-stranded DNA-binding protein (Fragment) | | uniclust | UniRef100\_A0A0B8QWW0 | 99.9 | 1.3e-28 | 2.5e-34 | 163.6 | 106 | (5, 117) | 162 | (9, 120) | 169 | Single-stranded DNA-binding protein | Single-stranded DNA-binding protein | | uniclust | UniRef100\_A0A081UAD3 | 99.9 | 1.6e-28 | 3e-34 | 165.4 | 108 | (1, 115) | 162 | (33, 140) | 174 | Single-stranded DNA-binding protein | Single-stranded DNA-binding protein | | uniclust | UniRef100\_A0A968VPK9 | 99.9 | 1.8e-28 | 3.3e-34 | 161.8 | 113 | (1, 117) | 162 | (1, 114) | 214 | Single-stranded DNA-binding protein | Single-stranded DNA-binding protein | | uniclust | UniRef100\_A0A090CZ19 | 99.9 | 1.7e-28 | 3.4e-34 | 164.6 | 103 | (3, 114) | 162 | (12, 115) | 168 | Single-stranded DNA-binding protein | Single-stranded DNA-binding protein | | uniclust | UniRef100\_A0A1G9SMN4 | 99.9 | 1.9e-28 | 3.6e-34 | 164.9 | 108 | (4, 117) | 162 | (1, 108) | 177 | Single-stranded DNA-binding protein | Single-stranded DNA-binding protein | | uniclust | UniRef100\_A0A1S2MBF9 | 99.9 | 1.9e-28 | 3.7e-34 | 162.9 | 110 | (3, 118) | 162 | (20, 129) | 153 | Single-stranded DNA-binding protein | Single-stranded DNA-binding protein | | uniclust | UniRef100\_A0A086Z1J5 | 99.9 | 1.9e-28 | 3.8e-34 | 162.8 | 111 | (3, 117) | 162 | (1, 112) | 151 | Single-stranded DNA-binding protein | Single-stranded DNA-binding protein | | uniclust | UniRef100\_A0A0N1D9D7 | 99.9 | 2.1e-28 | 4.1e-34 | 165.3 | 113 | (2, 118) | 162 | (1, 115) | 161 | Single-stranded DNA-binding protein | Single-stranded DNA-binding protein | | uniclust | UniRef100\_A0A0G1W755 | 99.9 | 2.2e-28 | 4.2e-34 | 159.2 | 113 | (1, 117) | 162 | (40, 152) | 162 | Single-stranded DNA-binding protein | Single-stranded DNA-binding protein | | uniclust | UniRef100\_A0A1Q6UZU3 | 99.9 | 2.3e-28 | 4.5e-34 | 157.5 | 109 | (4, 118) | 162 | (2, 110) | 137 | Single-stranded DNA-binding protein | Single-stranded DNA-binding protein | | uniclust | UniRef100\_A0A1F9CN51 | 99.9 | 2.6e-28 | 4.9e-34 | 171.6 | 112 | (2, 117) | 162 | (127, 238) | 265 | Single-stranded DNA-binding protein | Single-stranded DNA-binding protein | | uniclust | UniRef100\_A0A022LTY2 | 99.9 | 2.6e-28 | 5e-34 | 172.6 | 113 | (1, 113) | 162 | (30, 144) | 272 | Single-stranded DNA-binding protein | Single-stranded DNA-binding protein | | uniclust | UniRef100\_A0A5C8BC14 | 99.9 | 2.8e-28 | 5.3e-34 | 157.9 | 110 | (2, 118) | 162 | (8, 117) | 135 | Single-stranded DNA-binding protein | Single-stranded DNA-binding protein | | uniclust | UniRef100\_A0A1B9F675 | 99.9 | 3.8e-28 | 7.1e-34 | 160.1 | 111 | (1, 116) | 162 | (81, 191) | 212 | Single-stranded DNA-binding protein | Single-stranded DNA-binding protein | | uniclust | UniRef100\_A0A2V7U681 | 99.9 | 4.3e-28 | 8e-34 | 158.3 | 102 | (2, 104) | 162 | (1, 102) | 166 | Single-stranded DNA-binding protein | Single-stranded DNA-binding protein | | uniclust | UniRef100\_A0A0N1KRF3 | 99.9 | 6.3e-28 | 1.2e-33 | 151.2 | 111 | (1, 114) | 162 | (4, 114) | 120 | Single-stranded DNA-binding protein (Fragment) | Single-stranded DNA-binding protein (Fragment) | | uniclust | UniRef100\_A0A2Z4R767 | 99.9 | 6.4e-28 | 1.2e-33 | 156.6 | 113 | (2, 118) | 162 | (3, 115) | 169 | Single-stranded DNA-binding protein | Single-stranded DNA-binding protein | | uniclust | UniRef100\_A0A095Y5H8 | 99.9 | 6.5e-28 | 1.3e-33 | 161.5 | 110 | (3, 118) | 162 | (1, 110) | 161 | Single-stranded DNA-binding protein | Single-stranded DNA-binding protein | | uniclust | UniRef100\_A0A1F2Q8N9 | 99.9 | 6.7e-28 | 1.3e-33 | 154.9 | 113 | (2, 117) | 162 | (7, 119) | 142 | Single-stranded DNA-binding protein | Single-stranded DNA-binding protein | | uniclust | UniRef100\_A0A022KT57 | 99.9 | 6.8e-28 | 1.3e-33 | 167.5 | 107 | (1, 107) | 162 | (15, 122) | 226 | Single-stranded DNA-binding protein | Single-stranded DNA-binding protein | | uniclust | UniRef100\_A0A0X8KMA9 | 99.9 | 7.3e-28 | 1.4e-33 | 162.7 | 112 | (4, 121) | 162 | (1, 112) | 193 | Single-stranded DNA-binding protein | Single-stranded DNA-binding protein | | uniclust | UniRef100\_A0A1B3X7C3 | 99.9 | 8.3e-28 | 1.6e-33 | 157.1 | 108 | (5, 118) | 162 | (1, 108) | 152 | Single-stranded DNA-binding protein | Single-stranded DNA-binding protein | | uniclust | UniRef100\_A0A1R4GZN9 | 99.9 | 8.7e-28 | 1.6e-33 | 153.4 | 106 | (4, 118) | 162 | (1, 107) | 147 | Single-stranded DNA-binding protein | Single-stranded DNA-binding protein | | uniclust | UniRef100\_A0A0B5D8I7 | 99.9 | 8.7e-28 | 1.7e-33 | 168.7 | 107 | (1, 107) | 162 | (46, 153) | 218 | Single-stranded DNA-binding protein | Single-stranded DNA-binding protein | | uniclust | UniRef100\_A0A1F9CN51 | 99.9 | 9.2e-28 | 1.7e-33 | 168.9 | 109 | (1, 113) | 162 | (1, 109) | 265 | Single-stranded DNA-binding protein | Single-stranded DNA-binding protein | | uniclust | UniRef100\_A0A0A2VXI7 | 99.9 | 1e-27 | 1.9e-33 | 172.1 | 159 | (3, 161) | 162 | (4, 175) | 425 | Single-stranded DNA-binding protein | Single-stranded DNA-binding protein | | uniclust | UniRef100\_A0A942N2T9 | 99.9 | 1.2e-27 | 2.1e-33 | 155.2 | 112 | (3, 118) | 162 | (52, 163) | 188 | Single-stranded DNA-binding protein | Single-stranded DNA-binding protein | | uniclust | UniRef100\_A0A1C6R7A7 | 99.9 | 1.6e-27 | 3e-33 | 157.1 | 105 | (3, 107) | 162 | (1, 106) | 155 | Single-stranded DNA-binding protein | Single-stranded DNA-binding protein | | uniclust | UniRef100\_A0A5D4T3F4 | 99.9 | 1.7e-27 | 3.1e-33 | 156.8 | 111 | (4, 120) | 162 | (1, 111) | 184 | Single-stranded DNA-binding protein | Single-stranded DNA-binding protein | | uniclust | UniRef100\_A0A0A0RT45 | 99.9 | 2e-27 | 3.7e-33 | 152.7 | 107 | (4, 117) | 162 | (1, 107) | 157 | Single-stranded DNA-binding protein | Single-stranded DNA-binding protein | | uniclust | UniRef100\_A0A077ELF4 | 99.9 | 2e-27 | 3.7e-33 | 151.0 | 115 | (1, 119) | 162 | (14, 128) | 138 | Single-stranded DNA-binding protein | Single-stranded DNA-binding protein | | uniclust | UniRef100\_A0A945T384 | 99.9 | 2e-27 | 3.8e-33 | 150.8 | 110 | (4, 117) | 162 | (1, 110) | 149 | Single-stranded DNA-binding protein | Single-stranded DNA-binding protein | | uniclust | UniRef100\_A0A1T4VV37 | 99.9 | 2.4e-27 | 4.5e-33 | 161.4 | 103 | (1, 104) | 162 | (1, 103) | 226 | Single-stranded DNA-binding protein | Single-stranded DNA-binding protein | | uniclust | UniRef100\_A0A1C6LRJ7 | 99.9 | 2.5e-27 | 4.7e-33 | 159.3 | 107 | (5, 115) | 162 | (1, 107) | 177 | Single-stranded DNA-binding protein | Single-stranded DNA-binding protein | | uniclust | UniRef100\_A0A8T6YXL0 | 99.9 | 2.6e-27 | 4.8e-33 | 146.7 | 110 | (3, 116) | 162 | (1, 110) | 125 | Single-stranded DNA-binding protein | Single-stranded DNA-binding protein | | uniclust | UniRef100\_A0A141RPF2 | 99.9 | 2.7e-27 | 5e-33 | 152.3 | 126 | (37, 162) | 162 | (2, 130) | 145 | Single-stranded DNA-binding protein | Single-stranded DNA-binding protein | | uniclust | UniRef100\_A0A1F2WYY6 | 99.9 | 2.7e-27 | 5e-33 | 151.7 | 104 | (3, 118) | 162 | (22, 125) | 145 | Single-stranded DNA-binding protein | Single-stranded DNA-binding protein | | uniclust | UniRef100\_A0A078KY52 | 99.9 | 3.3e-27 | 6.3e-33 | 151.5 | 110 | (1, 116) | 162 | (1, 110) | 141 | Single-stranded DNA-binding protein | Single-stranded DNA-binding protein | | uniclust | UniRef100\_A0A0K1HH46 | 99.9 | 4.3e-27 | 8e-33 | 155.1 | 112 | (3, 118) | 162 | (1, 112) | 174 | Single-stranded DNA-binding protein | Single-stranded DNA-binding protein | | uniclust | UniRef100\_A0A014MGY3 | 99.9 | 4.5e-27 | 8.9e-33 | 162.2 | 111 | (2, 118) | 162 | (3, 113) | 184 | Single-stranded DNA-binding protein | Single-stranded DNA-binding protein | | uniclust | UniRef100\_A0A259R8S6 | 99.9 | 5e-27 | 9.5e-33 | 152.3 | 73 | (42, 117) | 162 | (5, 77) | 143 | Single-stranded DNA-binding protein (Fragment) | Single-stranded DNA-binding protein (Fragment) | | uniclust | UniRef100\_A0A3D1TXE1 | 99.9 | 5.5e-27 | 1e-32 | 150.1 | 101 | (4, 117) | 162 | (28, 128) | 138 | Single-stranded DNA-binding protein | Single-stranded DNA-binding protein | | uniclust | UniRef100\_A0A9D7S067 | 99.9 | 5.7e-27 | 1e-32 | 164.4 | 112 | (1, 115) | 162 | (1, 112) | 342 | Single-stranded DNA-binding protein | Single-stranded DNA-binding protein | | uniclust | UniRef100\_A0A173TLD0 | 99.9 | 1.1e-26 | 2.1e-32 | 153.5 | 105 | (4, 117) | 162 | (1, 105) | 177 | Single-stranded DNA-binding protein | Single-stranded DNA-binding protein | | uniclust | UniRef100\_A0A0K2JFD7 | 99.9 | 1.1e-26 | 2.2e-32 | 152.4 | 106 | (3, 120) | 162 | (1, 106) | 164 | Single-stranded DNA-binding protein | Single-stranded DNA-binding protein | | uniclust | UniRef100\_A0A0C1I8N4 | 99.9 | 1.2e-26 | 2.3e-32 | 159.7 | 109 | (2, 116) | 162 | (24, 132) | 180 | Single-stranded DNA-binding protein | Single-stranded DNA-binding protein | | uniclust | UniRef100\_A0A077XQ49 | 99.9 | 1.3e-26 | 2.5e-32 | 145.1 | 111 | (1, 115) | 162 | (1, 111) | 131 | Single-stranded DNA-binding protein | Single-stranded DNA-binding protein | | uniclust | UniRef100\_A0A059XWD8 | 99.9 | 1.3e-26 | 2.6e-32 | 160.5 | 109 | (2, 118) | 162 | (3, 111) | 188 | Single-stranded DNA-binding protein | Single-stranded DNA-binding protein | | uniclust | UniRef100\_A0A1E9Q8Q7 | 99.9 | 1.7e-26 | 3.3e-32 | 157.2 | 106 | (1, 107) | 162 | (1, 112) | 186 | Single-stranded DNA-binding protein | Single-stranded DNA-binding protein | | uniclust | UniRef100\_A0A0R2CM34 | 99.9 | 1.8e-26 | 3.4e-32 | 146.3 | 111 | (4, 120) | 162 | (1, 111) | 149 | Single-stranded DNA-binding protein | Single-stranded DNA-binding protein | | uniclust | UniRef100\_A0A523YRH3 | 99.9 | 2.1e-26 | 3.8e-32 | 143.0 | 108 | (2, 115) | 162 | (1, 108) | 140 | Single-stranded DNA-binding protein | Single-stranded DNA-binding protein | | uniclust | UniRef100\_A0A1C7HIC5 | 99.9 | 2.1e-26 | 3.9e-32 | 157.5 | 112 | (2, 117) | 162 | (1, 112) | 226 | Single-stranded DNA-binding protein | Single-stranded DNA-binding protein | | uniclust | UniRef100\_A0A160LK02 | 99.9 | 2.1e-26 | 4e-32 | 154.9 | 101 | (4, 119) | 162 | (1, 101) | 182 | Single-stranded DNA-binding protein | Single-stranded DNA-binding protein | | uniclust | UniRef100\_A0A0K1F1H1 | 99.9 | 2.2e-26 | 4.1e-32 | 146.5 | 110 | (3, 115) | 162 | (1, 111) | 159 | Single-stranded DNA-binding protein | Single-stranded DNA-binding protein | | uniclust | UniRef100\_A0A2D3LPR9 | 99.9 | 2.4e-26 | 4.5e-32 | 147.9 | 107 | (5, 115) | 162 | (1, 107) | 129 | Single-stranded DNA-binding protein | Single-stranded DNA-binding protein | | uniclust | UniRef100\_A0A0U2Q367 | 99.9 | 2.5e-26 | 4.6e-32 | 163.3 | 114 | (4, 117) | 162 | (169, 282) | 344 | Single-stranded DNA-binding protein | Single-stranded DNA-binding protein | | uniclust | UniRef100\_A0A0A8X393 | 99.9 | 2.7e-26 | 4.9e-32 | 153.4 | 109 | (3, 117) | 162 | (69, 177) | 208 | Single-stranded DNA-binding protein | Single-stranded DNA-binding protein | | uniclust | UniRef100\_A0A238ZN22 | 99.9 | 2.9e-26 | 5.4e-32 | 138.9 | 102 | (1, 103) | 162 | (1, 103) | 105 | Single-stranded DNA-binding protein | Single-stranded DNA-binding protein | | uniclust | UniRef100\_A0A1V6FE91 | 99.9 | 2.9e-26 | 5.4e-32 | 158.5 | 111 | (2, 116) | 162 | (1, 112) | 249 | Single-stranded DNA-binding protein | Single-stranded DNA-binding protein | | uniclust | UniRef100\_A0A0S1SZJ6 | 99.9 | 2.9e-26 | 5.5e-32 | 150.4 | 109 | (1, 117) | 162 | (16, 126) | 141 | Single-stranded DNA-binding protein | Single-stranded DNA-binding protein | | uniclust | UniRef100\_A0A1Y4GBS3 | 99.9 | 3.1e-26 | 5.7e-32 | 148.9 | 113 | (3, 120) | 162 | (39, 151) | 189 | Single-stranded DNA-binding protein | Single-stranded DNA-binding protein | | uniclust | UniRef100\_A0A7V3PS15 | 99.9 | 3.2e-26 | 6.1e-32 | 145.2 | 103 | (5, 118) | 162 | (1, 103) | 122 | Single-stranded DNA-binding protein | Single-stranded DNA-binding protein | | uniclust | UniRef100\_A0A401FYS3 | 99.9 | 3.6e-26 | 6.7e-32 | 160.8 | 114 | (2, 118) | 162 | (1, 114) | 347 | Single-stranded DNA-binding protein | Single-stranded DNA-binding protein | | uniclust | UniRef100\_A0A1G6X935 | 99.9 | 3.6e-26 | 6.7e-32 | 151.7 | 113 | (1, 117) | 162 | (67, 179) | 184 | Single-stranded DNA-binding protein | Single-stranded DNA-binding protein | | uniclust | UniRef100\_A0A7Z9G7J1 | 99.9 | 3.8e-26 | 7.1e-32 | 160.2 | 114 | (3, 120) | 162 | (1, 114) | 339 | Single-stranded DNA-binding protein (Fragment) | Single-stranded DNA-binding protein (Fragment) | | uniclust | UniRef100\_A0A0G1VV93 | 99.9 | 3.8e-26 | 7.1e-32 | 153.4 | 111 | (2, 116) | 162 | (57, 167) | 194 | Single-stranded DNA-binding protein | Single-stranded DNA-binding protein | | uniclust | UniRef100\_A0A1G9Q5V4 | 99.9 | 4.1e-26 | 7.7e-32 | 146.9 | 99 | (3, 116) | 162 | (4, 102) | 145 | Single-stranded DNA-binding protein | Single-stranded DNA-binding protein | | uniclust | UniRef100\_A0A0M4QA89 | 99.9 | 4.2e-26 | 7.9e-32 | 156.3 | 104 | (3, 106) | 162 | (35, 139) | 210 | Single-stranded DNA-binding protein | Single-stranded DNA-binding protein | | uniclust | UniRef100\_A0A9D9DB79 | 99.9 | 4.3e-26 | 8.1e-32 | 159.8 | 112 | (1, 115) | 162 | (1, 112) | 268 | Single-stranded DNA-binding protein | Single-stranded DNA-binding protein | | uniclust | UniRef100\_A0A010SZ24 | 99.9 | 4.8e-26 | 9.4e-32 | 163.3 | 111 | (1, 115) | 162 | (31, 147) | 237 | Single-stranded DNA-binding protein | Single-stranded DNA-binding protein | | uniclust | UniRef100\_A0A132MZK2 | 99.9 | 5.6e-26 | 1.1e-31 | 148.4 | 112 | (1, 112) | 162 | (1, 114) | 159 | Single-stranded DNA-binding protein | Single-stranded DNA-binding protein | | uniclust | UniRef100\_UPI001CCB6140 | 99.9 | 6.8e-26 | 1.2e-31 | 144.4 | 113 | (2, 117) | 162 | (1, 113) | 165 | single-stranded DNA-binding protein | single-stranded DNA-binding protein | | uniclust | UniRef100\_A0A7C7W444 | 99.9 | 7.3e-26 | 1.3e-31 | 151.4 | 114 | (2, 118) | 162 | (1, 114) | 231 | Single-stranded DNA-binding protein | Single-stranded DNA-binding protein | | uniclust | UniRef100\_A0A920JQB4 | 99.9 | 1.1e-25 | 2.1e-31 | 147.6 | 114 | (3, 119) | 162 | (55, 168) | 200 | Single-stranded DNA-binding protein | Single-stranded DNA-binding protein | | uniclust | UniRef100\_A0A1M5RIB4 | 99.9 | 1.1e-25 | 2.1e-31 | 142.0 | 110 | (3, 119) | 162 | (1, 111) | 135 | Single-stranded DNA-binding protein | Single-stranded DNA-binding protein | | uniclust | UniRef100\_A0A1C6AGN4 | 99.9 | 1.2e-25 | 2.2e-31 | 149.9 | 106 | (5, 117) | 162 | (1, 106) | 180 | Single-stranded DNA-binding protein | Single-stranded DNA-binding protein | | uniclust | UniRef100\_A0A0F0LRE6 | 99.9 | 1.3e-25 | 2.5e-31 | 159.5 | 106 | (2, 108) | 162 | (35, 141) | 234 | Single-stranded DNA-binding protein | Single-stranded DNA-binding protein | | uniclust | UniRef100\_A0A2D6E675 | 99.9 | 1.5e-25 | 2.8e-31 | 145.4 | 100 | (4, 114) | 162 | (1, 100) | 140 | Single-stranded DNA-binding protein | Single-stranded DNA-binding protein | | uniclust | UniRef100\_A0A2N1TAT9 | 99.9 | 1.5e-25 | 2.9e-31 | 148.4 | 112 | (2, 117) | 162 | (1, 112) | 197 | Single-stranded DNA-binding protein | Single-stranded DNA-binding protein | | uniclust | UniRef100\_A0A1H9QB91 | 99.9 | 1.5e-25 | 2.9e-31 | 145.3 | 103 | (2, 104) | 162 | (12, 115) | 155 | Single-stranded DNA-binding protein (Fragment) | Single-stranded DNA-binding protein (Fragment) | | uniclust | UniRef100\_A0A8H9CAI2 | 99.9 | 1.6e-25 | 3e-31 | 141.1 | 117 | (1, 118) | 162 | (1, 118) | 153 | Single-stranded DNA-binding protein | Single-stranded DNA-binding protein | | uniclust | UniRef100\_A0A350T707 | 99.9 | 1.6e-25 | 3e-31 | 143.7 | 112 | (1, 116) | 162 | (1, 112) | 173 | Single-stranded DNA-binding protein (Fragment) | Single-stranded DNA-binding protein (Fragment) | | uniclust | UniRef100\_A0A077KLD4 | 99.9 | 1.6e-25 | 3.1e-31 | 152.3 | 100 | (9, 114) | 162 | (30, 129) | 173 | Single-stranded DNA-binding protein | Single-stranded DNA-binding protein | | uniclust | UniRef100\_A0A1C6B8W9 | 99.9 | 1.7e-25 | 3.2e-31 | 147.2 | 108 | (3, 115) | 162 | (64, 171) | 205 | Single-stranded DNA-binding protein | Single-stranded DNA-binding protein | | uniclust | UniRef100\_A0A931S483 | 99.9 | 1.9e-25 | 3.5e-31 | 141.2 | 110 | (2, 115) | 162 | (1, 110) | 146 | Single-stranded DNA-binding protein | Single-stranded DNA-binding protein | | uniclust | UniRef100\_A0A0D4C1Z6 | 99.9 | 2e-25 | 3.8e-31 | 148.4 | 100 | (3, 104) | 162 | (19, 119) | 167 | Single-stranded DNA-binding protein | Single-stranded DNA-binding protein | | uniclust | UniRef100\_A0A317ZLK3 | 99.9 | 2.1e-25 | 4e-31 | 144.6 | 105 | (2, 116) | 162 | (11, 116) | 140 | Single-stranded DNA-binding protein | Single-stranded DNA-binding protein | | uniclust | UniRef100\_A0A087D5W7 | 99.9 | 2.2e-25 | 4.2e-31 | 160.6 | 107 | (1, 107) | 162 | (14, 121) | 236 | Single-stranded DNA-binding protein | Single-stranded DNA-binding protein | | uniclust | UniRef100\_A0A5C7PTQ0 | 99.9 | 2.5e-25 | 4.7e-31 | 145.6 | 111 | (4, 118) | 162 | (1, 111) | 165 | Single-stranded DNA-binding protein | Single-stranded DNA-binding protein | | uniclust | UniRef100\_A0A379VN86 | 99.9 | 2.6e-25 | 4.7e-31 | 151.4 | 112 | (4, 115) | 162 | (5, 116) | 263 | Single-stranded DNA-binding protein | Single-stranded DNA-binding protein | | uniclust | UniRef100\_A0A3L7TTE1 | 99.9 | 3.3e-25 | 6e-31 | 148.0 | 113 | (2, 118) | 162 | (66, 178) | 227 | Single-stranded DNA-binding protein | Single-stranded DNA-binding protein | | uniclust | UniRef100\_A0A084F1M5 | 99.9 | 3.2e-25 | 6e-31 | 149.9 | 107 | (4, 118) | 162 | (1, 108) | 192 | Single-stranded DNA-binding protein | Single-stranded DNA-binding protein | | uniclust | UniRef100\_A0A2H0VXD5 | 99.9 | 3.7e-25 | 7e-31 | 147.1 | 106 | (1, 115) | 162 | (20, 126) | 185 | Single-stranded DNA-binding protein | Single-stranded DNA-binding protein | | uniclust | UniRef100\_A0A9D2B152 | 99.9 | 3.8e-25 | 7.1e-31 | 157.5 | 110 | (1, 113) | 162 | (17, 126) | 311 | Single-stranded DNA-binding protein | Single-stranded DNA-binding protein | | uniclust | UniRef100\_A0A1I7YM23 | 99.9 | 4.2e-25 | 7.7e-31 | 159.5 | 119 | (1, 120) | 162 | (271, 389) | 434 | Single-stranded DNA-binding protein | Single-stranded DNA-binding protein | | uniclust | UniRef100\_A0A6J5R9U9 | 99.9 | 4.3e-25 | 8.1e-31 | 144.5 | 109 | (3, 118) | 162 | (22, 130) | 153 | Single-stranded DNA-binding protein | Single-stranded DNA-binding protein | | uniclust | UniRef100\_A0A174F184 | 99.9 | 4.4e-25 | 8.4e-31 | 155.4 | 109 | (3, 118) | 162 | (19, 130) | 254 | Single-stranded DNA-binding protein | Single-stranded DNA-binding protein | | uniclust | UniRef100\_A0A011NB88 | 99.9 | 4.7e-25 | 8.8e-31 | 139.9 | 109 | (5, 116) | 162 | (1, 109) | 147 | Single-stranded DNA-binding protein | Single-stranded DNA-binding protein | | uniclust | UniRef100\_A0A0E3XA19 | 99.9 | 4.5e-25 | 8.8e-31 | 158.0 | 106 | (1, 107) | 162 | (32, 138) | 237 | DNA binding protein | DNA binding protein | | uniclust | UniRef100\_A0A087D6Y5 | 99.9 | 4.8e-25 | 9.2e-31 | 146.9 | 105 | (2, 107) | 162 | (1, 109) | 167 | Single-stranded DNA-binding protein | Single-stranded DNA-binding protein | | uniclust | UniRef100\_A0A0Q8L5D1 | 99.9 | 5.1e-25 | 9.7e-31 | 144.3 | 108 | (1, 116) | 162 | (2, 110) | 143 | Helix-destabilizing protein | Helix-destabilizing protein | | uniclust | UniRef100\_A0A061LY12 | 99.8 | 5.4e-25 | 1.1e-30 | 160.2 | 107 | (1, 108) | 162 | (40, 147) | 264 | Single-stranded DNA-binding protein | Single-stranded DNA-binding protein | | uniclust | UniRef100\_A0A140DWS2 | 99.8 | 5.5e-25 | 1.1e-30 | 144.6 | 111 | (1, 118) | 162 | (2, 112) | 123 | Single-stranded DNA-binding protein | Single-stranded DNA-binding protein | | uniclust | UniRef100\_A0A0L0HLE3 | 99.8 | 5.8e-25 | 1.1e-30 | 145.4 | 108 | (4, 115) | 162 | (32, 139) | 158 | Single-strand binding protein | Single-strand binding protein | | uniclust | UniRef100\_A0A1H7BM52 | 99.8 | 6.5e-25 | 1.2e-30 | 155.4 | 112 | (2, 113) | 162 | (131, 244) | 306 | Single-stranded DNA-binding protein | Single-stranded DNA-binding protein | | uniclust | UniRef100\_A0A4Q0D392 | 99.8 | 6.6e-25 | 1.2e-30 | 148.2 | 104 | (4, 113) | 162 | (1, 104) | 184 | Single-stranded DNA-binding protein | Single-stranded DNA-binding protein | | uniclust | UniRef100\_A0A075V2L9 | 99.8 | 6.5e-25 | 1.3e-30 | 149.2 | 106 | (1, 106) | 162 | (12, 118) | 170 | Single-stranded DNA-binding protein | Single-stranded DNA-binding protein | | uniclust | UniRef100\_A0A2N2PZR6 | 99.8 | 7.1e-25 | 1.3e-30 | 138.7 | 112 | (2, 117) | 162 | (37, 148) | 156 | Single-stranded DNA-binding protein | Single-stranded DNA-binding protein | | uniclust | UniRef100\_A0A0Q6VAK4 | 99.8 | 7.1e-25 | 1.4e-30 | 147.9 | 107 | (1, 107) | 162 | (1, 108) | 163 | Single-stranded DNA-binding protein | Single-stranded DNA-binding protein | | uniclust | UniRef100\_V7ZAW8 | 99.8 | 8.2e-25 | 1.5e-30 | 148.9 | 113 | (1, 117) | 162 | (87, 199) | 261 | Single-stranded DNA-binding protein | Single-stranded DNA-binding protein | | uniclust | UniRef100\_A0A7Z8Y919 | 99.8 | 8.8e-25 | 1.7e-30 | 147.4 | 102 | (3, 104) | 162 | (1, 104) | 183 | Single-stranded DNA-binding protein | Single-stranded DNA-binding protein | | uniclust | UniRef100\_A0A1N6NNQ4 | 99.8 | 9.4e-25 | 1.7e-30 | 144.0 | 113 | (1, 117) | 162 | (45, 157) | 207 | Single-stranded DNA-binding protein | Single-stranded DNA-binding protein | | uniclust | UniRef100\_A0A1V5QGS9 | 99.8 | 9.6e-25 | 1.8e-30 | 139.5 | 106 | (3, 117) | 162 | (1, 106) | 140 | Single-stranded DNA-binding protein | Single-stranded DNA-binding protein | | uniclust | UniRef100\_A0A2E8QTK5 | 99.8 | 1e-24 | 1.9e-30 | 146.1 | 110 | (1, 115) | 162 | (73, 182) | 215 | Single-stranded DNA-binding protein | Single-stranded DNA-binding protein | | uniclust | UniRef100\_A0A0M4MLW5 | 99.8 | 1e-24 | 1.9e-30 | 137.2 | 103 | (4, 107) | 162 | (1, 105) | 128 | Single-stranded DNA-binding protein | Single-stranded DNA-binding protein | | uniclust | UniRef100\_A0A1H0IKV7 | 99.8 | 1e-24 | 2e-30 | 144.6 | 106 | (2, 107) | 162 | (1, 107) | 154 | Single-stranded DNA-binding protein | Single-stranded DNA-binding protein | | uniclust | UniRef100\_A0A090VXM6 | 99.8 | 1.1e-24 | 2e-30 | 132.7 | 85 | (1, 86) | 162 | (1, 85) | 95 | Single-stranded DNA-binding protein | Single-stranded DNA-binding protein | | uniclust | UniRef100\_A0A8S5LHZ5 | 99.8 | 1.1e-24 | 2e-30 | 147.4 | 105 | (3, 118) | 162 | (100, 204) | 232 | Single strand binding protein | Single strand binding protein | | uniclust | UniRef100\_A0A1G7H7E7 | 99.8 | 1.1e-24 | 2e-30 | 145.3 | 106 | (5, 118) | 162 | (1, 106) | 188 | Single-stranded DNA-binding protein | Single-stranded DNA-binding protein | | uniclust | UniRef100\_A0A1T4KFM7 | 99.8 | 1.2e-24 | 2.3e-30 | 152.9 | 109 | (3, 119) | 162 | (1, 110) | 228 | Single-stranded DNA-binding protein | Single-stranded DNA-binding protein | | uniclust | UniRef100\_A0A086N2V0 | 99.8 | 1.3e-24 | 2.5e-30 | 154.1 | 107 | (1, 108) | 162 | (35, 142) | 224 | Single-stranded DNA-binding protein | Single-stranded DNA-binding protein | | uniclust | UniRef100\_A0A0D8L8J0 | 99.8 | 1.6e-24 | 3e-30 | 130.9 | 92 | (2, 93) | 162 | (3, 94) | 104 | Single-stranded DNA-binding protein (Fragment) | Single-stranded DNA-binding protein (Fragment) | | uniclust | UniRef100\_A4J7S4 | 99.8 | 1.7e-24 | 3.2e-30 | 146.0 | 106 | (4, 119) | 162 | (1, 107) | 225 | Single-stranded DNA-binding protein | Single-stranded DNA-binding protein | | uniclust | UniRef100\_A0A6L7UXF6 | 99.8 | 1.9e-24 | 3.5e-30 | 142.8 | 107 | (1, 115) | 162 | (59, 165) | 191 | Single-stranded DNA-binding protein | Single-stranded DNA-binding protein | | uniclust | UniRef100\_A0A3M1VW93 | 99.8 | 2e-24 | 3.7e-30 | 143.9 | 111 | (2, 116) | 162 | (89, 199) | 222 | Single-stranded DNA-binding protein | Single-stranded DNA-binding protein | | uniclust | UniRef100\_A0A2U8BT43 | 99.8 | 2.1e-24 | 3.9e-30 | 138.0 | 113 | (1, 113) | 162 | (1, 115) | 137 | Single-stranded DNA-binding protein | Single-stranded DNA-binding protein | | uniclust | UniRef100\_A0A0A3IX47 | 99.8 | 2.1e-24 | 4e-30 | 139.4 | 110 | (2, 117) | 162 | (9, 118) | 154 | Single-stranded DNA-binding protein | Single-stranded DNA-binding protein | | uniclust | UniRef100\_A0A060JLA2 | 99.8 | 2.3e-24 | 4.4e-30 | 147.3 | 106 | (1, 107) | 162 | (4, 110) | 177 | Single-stranded DNA-binding protein | Single-stranded DNA-binding protein | | uniclust | UniRef100\_A0A024LSH6 | 99.8 | 2.4e-24 | 4.5e-30 | 140.6 | 114 | (2, 115) | 162 | (34, 149) | 183 | Single-stranded DNA-binding protein | Single-stranded DNA-binding protein | | uniclust | UniRef100\_A0A2N5PKE3 | 99.8 | 2.8e-24 | 5.2e-30 | 135.4 | 105 | (5, 117) | 162 | (1, 106) | 136 | Single-stranded DNA-binding protein | Single-stranded DNA-binding protein | | uniclust | UniRef100\_A0A2E3Q240 | 99.8 | 2.9e-24 | 5.5e-30 | 138.0 | 89 | (20, 118) | 162 | (4, 93) | 132 | Single-stranded DNA-binding protein (Fragment) | Single-stranded DNA-binding protein (Fragment) | | uniclust | UniRef100\_UPI00232B6A13 | 99.8 | 3.1e-24 | 5.7e-30 | 148.3 | 111 | (2, 117) | 162 | (79, 189) | 292 | single-stranded DNA-binding protein | single-stranded DNA-binding protein | | uniclust | UniRef100\_A0A4Q4WRP0 | 99.8 | 3.1e-24 | 5.7e-30 | 144.6 | 110 | (2, 115) | 162 | (112, 221) | 241 | Single-stranded DNA-binding protein | Single-stranded DNA-binding protein | | uniclust | UniRef100\_A0A021VMQ7 | 99.8 | 3e-24 | 5.8e-30 | 157.0 | 107 | (1, 107) | 162 | (45, 152) | 278 | Single-stranded DNA-binding protein | Single-stranded DNA-binding protein | | uniclust | UniRef100\_UPI000AD85BCB | 99.8 | 3.3e-24 | 6.1e-30 | 150.8 | 103 | (5, 107) | 162 | (88, 191) | 255 | single-stranded DNA-binding protein | single-stranded DNA-binding protein | | uniclust | UniRef100\_A0A066YRZ6 | 99.8 | 3.2e-24 | 6.2e-30 | 156.2 | 106 | (1, 107) | 162 | (44, 150) | 269 | Single-stranded DNA-binding protein | Single-stranded DNA-binding protein | | uniclust | UniRef100\_A0A1G6H2Z0 | 99.8 | 3.5e-24 | 6.7e-30 | 150.1 | 104 | (2, 106) | 162 | (3, 108) | 213 | Single-stranded DNA-binding protein | Single-stranded DNA-binding protein | | uniclust | UniRef100\_A0A1H4CFD5 | 99.8 | 3.8e-24 | 7.2e-30 | 146.7 | 105 | (3, 107) | 162 | (1, 106) | 194 | Single-stranded DNA-binding protein | Single-stranded DNA-binding protein | | uniclust | UniRef100\_A0A077XMJ6 | 99.8 | 4.3e-24 | 8.2e-30 | 133.5 | 65 | (52, 119) | 162 | (2, 66) | 105 | Single-stranded DNA-binding protein | Single-stranded DNA-binding protein | | uniclust | UniRef100\_A0A3L7X4Y5 | 99.8 | 4.5e-24 | 8.2e-30 | 140.7 | 111 | (3, 117) | 162 | (68, 179) | 204 | Single-stranded DNA-binding protein | Single-stranded DNA-binding protein | | uniclust | UniRef100\_A0A516L280 | 99.8 | 4.5e-24 | 8.4e-30 | 135.5 | 101 | (2, 104) | 162 | (1, 101) | 140 | Putative single-stranded DNA-binding protein | Putative single-stranded DNA-binding protein | | uniclust | UniRef100\_A0A011SNI5 | 99.8 | 4.4e-24 | 8.7e-30 | 156.4 | 111 | (1, 112) | 162 | (41, 152) | 264 | Single-stranded DNA-binding protein | Single-stranded DNA-binding protein | | uniclust | UniRef100\_A0A1B8PQN4 | 99.8 | 5.2e-24 | 9.7e-30 | 139.9 | 107 | (2, 112) | 162 | (3, 109) | 179 | Single-stranded DNA-binding protein | Single-stranded DNA-binding protein | | uniclust | UniRef100\_A0A239FC50 | 99.8 | 5.5e-24 | 1e-29 | 137.1 | 104 | (1, 104) | 162 | (1, 105) | 138 | Single-stranded DNA-binding protein | Single-stranded DNA-binding protein | | uniclust | UniRef100\_A0A2W6AP70 | 99.8 | 5.5e-24 | 1e-29 | 142.8 | 109 | (3, 118) | 162 | (44, 152) | 175 | Single-stranded DNA-binding protein | Single-stranded DNA-binding protein | | uniclust | UniRef100\_A0A0G1ZRH2 | 99.8 | 5.5e-24 | 1.1e-29 | 137.0 | 80 | (31, 114) | 162 | (3, 82) | 128 | Single-stranded DNA-binding protein | Single-stranded DNA-binding protein | | uniclust | UniRef100\_A0A0I9UJK8 | 99.8 | 5.5e-24 | 1.1e-29 | 140.9 | 105 | (3, 117) | 162 | (12, 116) | 150 | Single-stranded DNA-binding protein | Single-stranded DNA-binding protein | | uniclust | UniRef100\_A0A0D5A1F4 | 99.8 | 6.4e-24 | 1.2e-29 | 137.2 | 117 | (1, 117) | 162 | (1, 120) | 178 | Single-stranded DNA-binding protein | Single-stranded DNA-binding protein | | uniclust | UniRef100\_A0A5K7ZGM1 | 99.8 | 7.4e-24 | 1.4e-29 | 147.5 | 104 | (4, 119) | 162 | (113, 216) | 239 | Single-stranded DNA-binding protein | Single-stranded DNA-binding protein | | uniclust | UniRef100\_A0A1Y4CWR4 | 99.8 | 8e-24 | 1.5e-29 | 135.0 | 111 | (4, 118) | 162 | (1, 111) | 163 | Single-stranded DNA-binding protein | Single-stranded DNA-binding protein | | uniclust | UniRef100\_UPI002022FF3E | 99.8 | 8.6e-24 | 1.6e-29 | 134.1 | 100 | (2, 104) | 162 | (1, 101) | 157 | single-stranded DNA-binding protein | single-stranded DNA-binding protein | | uniclust | UniRef100\_A0A174BNI7 | 99.8 | 9.6e-24 | 1.8e-29 | 144.2 | 109 | (3, 118) | 162 | (5, 114) | 222 | Single-stranded DNA-binding protein | Single-stranded DNA-binding protein | | uniclust | UniRef100\_A0A1R4KH11 | 99.8 | 9.5e-24 | 1.8e-29 | 147.0 | 109 | (3, 111) | 162 | (2, 113) | 230 | Single-stranded DNA-binding protein | Single-stranded DNA-binding protein | | uniclust | UniRef100\_A0A432GXJ5 | 99.8 | 1e-23 | 1.8e-29 | 141.7 | 113 | (2, 117) | 162 | (81, 194) | 234 | Single-stranded DNA-binding protein | Single-stranded DNA-binding protein | | uniclust | UniRef100\_A0A143ZUS2 | 99.8 | 9.5e-24 | 1.8e-29 | 142.3 | 107 | (3, 118) | 162 | (20, 127) | 159 | Single-stranded DNA-binding protein | Single-stranded DNA-binding protein | | uniclust | UniRef100\_A0A1M3LAQ8 | 99.8 | 9.7e-24 | 1.9e-29 | 139.2 | 111 | (2, 118) | 162 | (7, 117) | 141 | Single-stranded DNA-binding protein | Single-stranded DNA-binding protein | | uniclust | UniRef100\_A0A1E8Y6D8 | 99.8 | 1e-23 | 1.9e-29 | 138.6 | 108 | (5, 118) | 162 | (1, 108) | 145 | Single-stranded DNA-binding protein | Single-stranded DNA-binding protein | | uniclust | UniRef100\_A0A516LMM7 | 99.8 | 1.1e-23 | 2e-29 | 134.8 | 109 | (2, 114) | 162 | (1, 111) | 145 | Single-stranded DNA-binding protein | Single-stranded DNA-binding protein | | uniclust | UniRef100\_A0A173S9Z8 | 99.8 | 1.2e-23 | 2.2e-29 | 138.2 | 108 | (1, 115) | 162 | (27, 136) | 183 | Single-stranded DNA-binding protein | Single-stranded DNA-binding protein | | uniclust | UniRef100\_A0A069A419 | 99.8 | 1.2e-23 | 2.2e-29 | 133.5 | 106 | (5, 116) | 162 | (1, 107) | 136 | Putative phage single-strand DNA-binding protein | Putative phage single-strand DNA-binding protein | | uniclust | UniRef100\_A0A2N6T258 | 99.8 | 1.2e-23 | 2.3e-29 | 134.8 | 108 | (2, 113) | 162 | (1, 109) | 141 | Single-stranded DNA-binding protein | Single-stranded DNA-binding protein | | uniclust | UniRef100\_A0A1W0D608 | 99.8 | 1.3e-23 | 2.3e-29 | 136.2 | 103 | (5, 116) | 162 | (1, 103) | 170 | Single-stranded DNA-binding protein | Single-stranded DNA-binding protein | | uniclust | UniRef100\_A0A547EAB1 | 99.8 | 1.4e-23 | 2.5e-29 | 147.1 | 113 | (2, 117) | 162 | (1, 113) | 324 | Single-stranded DNA-binding protein | Single-stranded DNA-binding protein | | uniclust | UniRef100\_A0A6L6LUW6 | 99.8 | 1.5e-23 | 2.7e-29 | 142.4 | 108 | (4, 115) | 162 | (105, 213) | 253 | Single-stranded DNA-binding protein | Single-stranded DNA-binding protein | | uniclust | UniRef100\_A0A348YKY9 | 99.8 | 1.7e-23 | 3.1e-29 | 137.6 | 110 | (2, 115) | 162 | (1, 110) | 199 | Single-stranded DNA-binding protein | Single-stranded DNA-binding protein | | uniclust | UniRef100\_A0A0F2KWS5 | 99.8 | 1.7e-23 | 3.1e-29 | 139.6 | 104 | (4, 116) | 162 | (1, 104) | 180 | Single-stranded DNA-binding protein | Single-stranded DNA-binding protein | | uniclust | UniRef100\_A0A192A7M0 | 99.8 | 1.7e-23 | 3.2e-29 | 135.0 | 112 | (2, 117) | 162 | (1, 112) | 157 | Single-stranded DNA-binding protein | Single-stranded DNA-binding protein | | uniclust | UniRef100\_A0A2P6F917 | 99.8 | 1.9e-23 | 3.6e-29 | 123.1 | 80 | (4, 90) | 162 | (1, 81) | 83 | Single-stranded DNA-binding protein ssb | Single-stranded DNA-binding protein ssb | | uniclust | UniRef100\_A0A1I2DXE7 | 99.8 | 1.9e-23 | 3.7e-29 | 144.7 | 106 | (1, 107) | 162 | (1, 107) | 186 | Single-stranded DNA-binding protein | Single-stranded DNA-binding protein | | uniclust | UniRef100\_A0A1D3K7U2 | 99.8 | 2e-23 | 3.7e-29 | 141.1 | 111 | (4, 116) | 162 | (22, 133) | 204 | Single-stranded DNA-binding protein | Single-stranded DNA-binding protein | | uniclust | UniRef100\_UPI000CD26B27 | 99.8 | 2e-23 | 3.8e-29 | 133.5 | 115 | (1, 118) | 162 | (1, 115) | 148 | single-stranded DNA-binding protein | single-stranded DNA-binding protein | | uniclust | UniRef100\_A0A2E1ZIW0 | 99.8 | 2.2e-23 | 4.1e-29 | 130.2 | 66 | (49, 118) | 162 | (12, 77) | 107 | Single-stranded DNA-binding protein | Single-stranded DNA-binding protein | | uniclust | UniRef100\_A0A0F0GWP1 | 99.8 | 2.4e-23 | 4.6e-29 | 145.1 | 106 | (1, 107) | 162 | (15, 121) | 205 | Single-stranded DNA-binding protein | Single-stranded DNA-binding protein | | uniclust | UniRef100\_A0A088FAZ2 | 99.8 | 2.6e-23 | 4.9e-29 | 134.9 | 103 | (3, 116) | 162 | (1, 103) | 142 | Single-stranded DNA binding protein | Single-stranded DNA binding protein | | uniclust | UniRef100\_A0A2E5LYI8 | 99.8 | 2.7e-23 | 4.9e-29 | 144.9 | 114 | (2, 118) | 162 | (1, 114) | 310 | Multifunctional fusion protein | Multifunctional fusion protein | | uniclust | UniRef100\_A0A7J6YTU1 | 99.8 | 2.8e-23 | 5.2e-29 | 138.4 | 113 | (1, 116) | 162 | (1, 115) | 219 | BON domain-containing protein | BON domain-containing protein | | uniclust | UniRef100\_A0A968VRQ2 | 99.8 | 2.9e-23 | 5.4e-29 | 139.2 | 114 | (1, 118) | 162 | (1, 114) | 230 | Single-stranded DNA-binding protein (Fragment) | Single-stranded DNA-binding protein (Fragment) | | uniclust | UniRef100\_A6DQM0 | 99.8 | 3.2e-23 | 5.9e-29 | 138.3 | 111 | (2, 116) | 162 | (1, 112) | 208 | Single-stranded DNA-binding protein | Single-stranded DNA-binding protein | | uniclust | UniRef100\_A0A7V1C2I3 | 99.8 | 3.2e-23 | 6e-29 | 140.1 | 111 | (2, 116) | 162 | (99, 209) | 244 | Single-stranded DNA-binding protein | Single-stranded DNA-binding protein | | uniclust | UniRef100\_A0A0F4L723 | 99.8 | 3.2e-23 | 6e-29 | 130.1 | 105 | (2, 116) | 162 | (5, 110) | 122 | Single-stranded DNA-binding protein | Single-stranded DNA-binding protein | | uniclust | UniRef100\_A0A7X9LYH1 | 99.8 | 3.5e-23 | 6.5e-29 | 141.7 | 109 | (4, 118) | 162 | (1, 109) | 245 | Single-stranded DNA-binding protein | Single-stranded DNA-binding protein | | uniclust | UniRef100\_A0A535NDE5 | 99.8 | 3.6e-23 | 6.7e-29 | 133.3 | 112 | (3, 117) | 162 | (39, 151) | 157 | Single-stranded DNA-binding protein | Single-stranded DNA-binding protein | | uniclust | UniRef100\_A0A078MU66 | 99.8 | 3.5e-23 | 6.7e-29 | 147.6 | 111 | (2, 113) | 162 | (13, 124) | 227 | Single-stranded DNA-binding protein | Single-stranded DNA-binding protein | | uniclust | UniRef100\_A0A212Q4T3 | 99.8 | 3.7e-23 | 6.9e-29 | 133.4 | 98 | (3, 114) | 162 | (1, 98) | 136 | Single-stranded DNA-binding protein | Single-stranded DNA-binding protein | | uniclust | UniRef100\_A0A1F3Y0G0 | 99.8 | 3.7e-23 | 7.1e-29 | 136.8 | 112 | (2, 116) | 162 | (12, 124) | 149 | Single-stranded DNA-binding protein | Single-stranded DNA-binding protein | | uniclust | UniRef100\_A0A1R4G6W5 | 99.8 | 3.8e-23 | 7.4e-29 | 146.3 | 104 | (3, 107) | 162 | (21, 125) | 234 | Single-stranded DNA-binding protein | Single-stranded DNA-binding protein | | uniclust | UniRef100\_A0A031H3M6 | 99.8 | 4e-23 | 7.6e-29 | 141.8 | 103 | (4, 107) | 162 | (1, 104) | 203 | Single-stranded DNA-binding protein | Single-stranded DNA-binding protein | | uniclust | UniRef100\_A0A0Q2TWN2 | 99.8 | 4.1e-23 | 7.7e-29 | 129.8 | 111 | (3, 117) | 162 | (1, 112) | 126 | Single-stranded DNA-binding protein | Single-stranded DNA-binding protein | | uniclust | UniRef100\_A0A075MHM8 | 99.8 | 4.4e-23 | 8.1e-29 | 142.5 | 111 | (4, 115) | 162 | (82, 192) | 287 | Single-stranded DNA-binding protein | Single-stranded DNA-binding protein | | uniclust | UniRef100\_A0A241XSG0 | 99.8 | 4.5e-23 | 8.3e-29 | 133.2 | 112 | (1, 116) | 162 | (1, 112) | 162 | Single-stranded DNA-binding protein | Single-stranded DNA-binding protein | | uniclust | UniRef100\_A0A023EH54 | 99.8 | 4.4e-23 | 8.3e-29 | 140.4 | 109 | (3, 116) | 162 | (67, 176) | 194 | Putative single-stranded dna-binding protein (Fragment) | Putative single-stranded dna-binding protein (Fragment) | | uniclust | UniRef100\_A0A955A0C7 | 99.8 | 4.8e-23 | 8.9e-29 | 120.4 | 89 | (2, 91) | 162 | (1, 89) | 89 | Single-stranded DNA-binding protein (Fragment) | Single-stranded DNA-binding protein (Fragment) | | uniclust | UniRef100\_A0A193GL42 | 99.8 | 4.9e-23 | 9.1e-29 | 134.0 | 114 | (1, 117) | 162 | (1, 122) | 148 | Single-stranded DNA-binding protein | Single-stranded DNA-binding protein | | uniclust | UniRef100\_UPI001556A47A | 99.8 | 5.9e-23 | 1.1e-28 | 135.3 | 110 | (5, 118) | 162 | (1, 112) | 169 | single-stranded DNA-binding protein | single-stranded DNA-binding protein | | uniclust | UniRef100\_A0A6J5JVU9 | 99.8 | 6.4e-23 | 1.2e-28 | 133.3 | 113 | (1, 117) | 162 | (2, 114) | 152 | Single-stranded DNA-binding protein | Single-stranded DNA-binding protein | | uniclust | UniRef100\_A0A218QUP3 | 99.8 | 6.5e-23 | 1.2e-28 | 127.0 | 106 | (2, 117) | 162 | (3, 109) | 113 | Single-stranded DNA-binding protein | Single-stranded DNA-binding protein | | uniclust | UniRef100\_A0A0S4SVH8 | 99.8 | 6.4e-23 | 1.2e-28 | 131.1 | 102 | (4, 115) | 162 | (1, 102) | 126 | Single-stranded DNA-binding protein | Single-stranded DNA-binding protein | | uniclust | UniRef100\_A0A939ATV5 | 99.8 | 7.3e-23 | 1.3e-28 | 137.1 | 113 | (2, 118) | 162 | (107, 219) | 226 | Single-stranded DNA-binding protein | Single-stranded DNA-binding protein | | uniclust | UniRef100\_A0A0Q4S0Q6 | 99.8 | 7.5e-23 | 1.5e-28 | 139.1 | 100 | (9, 113) | 162 | (36, 135) | 165 | Single-stranded DNA-binding protein | Single-stranded DNA-binding protein | | uniclust | UniRef100\_A0A076NYD0 | 99.8 | 8.1e-23 | 1.5e-28 | 132.0 | 110 | (1, 114) | 162 | (52, 161) | 162 | Single-stranded DNA-binding protein | Single-stranded DNA-binding protein | | uniclust | UniRef100\_A0A256VI30 | 99.8 | 8.1e-23 | 1.5e-28 | 125.8 | 93 | (5, 104) | 162 | (1, 93) | 107 | Single-stranded DNA-binding protein | Single-stranded DNA-binding protein | | uniclust | UniRef100\_A0A0Q6GEB3 | 99.8 | 8.4e-23 | 1.6e-28 | 142.8 | 112 | (3, 114) | 162 | (38, 151) | 218 | Single-stranded DNA-binding protein | Single-stranded DNA-binding protein | | uniclust | UniRef100\_A0A662ZJD1 | 99.8 | 9.9e-23 | 1.8e-28 | 141.3 | 112 | (2, 117) | 162 | (3, 115) | 265 | Single-stranded DNA-binding protein | Single-stranded DNA-binding protein | | uniclust | UniRef100\_A0A2I6TCS3 | 99.8 | 9.9e-23 | 1.8e-28 | 126.1 | 112 | (2, 117) | 162 | (1, 112) | 124 | Single-stranded DNA-binding protein | Single-stranded DNA-binding protein | | uniclust | UniRef100\_A0A024H220 | 99.8 | 1e-22 | 1.9e-28 | 141.1 | 106 | (2, 108) | 162 | (5, 111) | 213 | Single-stranded DNA-binding protein | Single-stranded DNA-binding protein | | uniclust | UniRef100\_A0A6N7R1D9 | 99.8 | 1.1e-22 | 2.1e-28 | 147.5 | 100 | (3, 105) | 162 | (238, 337) | 395 | Single-stranded DNA-binding protein | Single-stranded DNA-binding protein | | uniclust | UniRef100\_A0A1G2BSC3 | 99.8 | 1.3e-22 | 2.3e-28 | 131.2 | 111 | (2, 115) | 162 | (39, 149) | 176 | Single-stranded DNA-binding protein | Single-stranded DNA-binding protein | | uniclust | UniRef100\_A0A1I0QCF5 | 99.8 | 1.2e-22 | 2.3e-28 | 135.1 | 107 | (5, 117) | 162 | (43, 149) | 175 | Single-stranded DNA-binding protein | Single-stranded DNA-binding protein | | uniclust | UniRef100\_A0A2D7A136 | 99.8 | 1.3e-22 | 2.5e-28 | 135.7 | 106 | (2, 115) | 162 | (1, 106) | 163 | Single-stranded DNA-binding protein | Single-stranded DNA-binding protein | | uniclust | UniRef100\_A0A1G6YCT7 | 99.8 | 1.4e-22 | 2.6e-28 | 141.0 | 101 | (1, 104) | 162 | (26, 128) | 202 | Single-strand DNA-binding protein | Single-strand DNA-binding protein | | uniclust | UniRef100\_A0A3D0NZB0 | 99.8 | 1.4e-22 | 2.7e-28 | 127.9 | 83 | (28, 117) | 162 | (2, 84) | 117 | Single-stranded DNA-binding protein (Fragment) | Single-stranded DNA-binding protein (Fragment) | | uniclust | UniRef100\_A0A7V8JJS2 | 99.8 | 1.5e-22 | 2.8e-28 | 134.4 | 112 | (1, 115) | 162 | (1, 112) | 212 | Single-stranded DNA-binding protein | Single-stranded DNA-binding protein | | uniclust | UniRef100\_A0A1C5RGB2 | 99.8 | 1.5e-22 | 2.8e-28 | 128.9 | 110 | (2, 118) | 162 | (7, 116) | 123 | Single-stranded DNA-binding protein | Single-stranded DNA-binding protein | | uniclust | UniRef100\_A0A016QTA0 | 99.8 | 1.5e-22 | 3e-28 | 150.6 | 109 | (1, 113) | 162 | (23, 131) | 309 | Single-stranded DNA-binding protein | Single-stranded DNA-binding protein | | uniclust | UniRef100\_A0A3D2SXS3 | 99.8 | 1.6e-22 | 3e-28 | 131.8 | 111 | (1, 115) | 162 | (43, 153) | 186 | Single-stranded DNA-binding protein | Single-stranded DNA-binding protein | | uniclust | UniRef100\_A0A2D8B5S5 | 99.8 | 1.8e-22 | 3.3e-28 | 124.3 | 105 | (4, 116) | 162 | (1, 105) | 118 | Single-stranded DNA-binding protein | Single-stranded DNA-binding protein | | uniclust | UniRef100\_L7W1E2 | 99.8 | 1.8e-22 | 3.4e-28 | 139.2 | 113 | (2, 118) | 162 | (144, 257) | 282 | Single-stranded DNA-binding protein | Single-stranded DNA-binding protein | | uniclust | UniRef100\_A0A1H3N9P1 | 99.8 | 2e-22 | 3.8e-28 | 133.6 | 112 | (1, 115) | 162 | (11, 122) | 174 | Single-stranded DNA-binding protein | Single-stranded DNA-binding protein | | uniclust | UniRef100\_A0A6N9JJD1 | 99.8 | 2.1e-22 | 3.8e-28 | 134.6 | 111 | (1, 118) | 162 | (1, 113) | 180 | Single-stranded DNA-binding protein | Single-stranded DNA-binding protein | | uniclust | UniRef100\_A0A0F5H0G2 | 99.8 | 2.1e-22 | 3.9e-28 | 139.7 | 108 | (3, 118) | 162 | (1, 108) | 210 | Single-stranded DNA-binding protein | Single-stranded DNA-binding protein | | uniclust | UniRef100\_A0A2T2WWQ9 | 99.8 | 2.5e-22 | 4.8e-28 | 124.3 | 83 | (30, 118) | 162 | (2, 84) | 111 | Single-stranded DNA-binding protein | Single-stranded DNA-binding protein | | uniclust | UniRef100\_A0A143XLA3 | 99.8 | 2.6e-22 | 4.9e-28 | 132.7 | 107 | (3, 118) | 162 | (8, 117) | 157 | Single-stranded DNA-binding protein | Single-stranded DNA-binding protein | | uniclust | UniRef100\_A0A6N6KQM9 | 99.8 | 2.8e-22 | 5.1e-28 | 130.8 | 111 | (3, 117) | 162 | (44, 161) | 186 | Single-stranded DNA-binding protein | Single-stranded DNA-binding protein | | uniclust | UniRef100\_A0A1N6N149 | 99.8 | 2.7e-22 | 5.1e-28 | 130.2 | 77 | (40, 119) | 162 | (1, 82) | 136 | Single-stranded DNA-binding protein | Single-stranded DNA-binding protein | | uniclust | UniRef100\_A0A070A9C6 | 99.8 | 2.8e-22 | 5.3e-28 | 131.5 | 115 | (1, 115) | 162 | (2, 119) | 157 | Single-stranded DNA-binding protein | Single-stranded DNA-binding protein | | uniclust | UniRef100\_A0A7X2TPA8 | 99.8 | 2.9e-22 | 5.5e-28 | 132.2 | 107 | (2, 112) | 162 | (1, 109) | 145 | Single-stranded DNA-binding protein | Single-stranded DNA-binding protein | | uniclust | UniRef100\_A0A2E4UVR0 | 99.8 | 3e-22 | 5.7e-28 | 129.5 | 113 | (2, 118) | 162 | (9, 121) | 154 | Single-stranded DNA-binding protein | Single-stranded DNA-binding protein | | uniclust | UniRef100\_A0A0F9MDI7 | 99.8 | 3.2e-22 | 5.9e-28 | 129.2 | 108 | (2, 113) | 162 | (66, 173) | 174 | Single-stranded DNA-binding protein | Single-stranded DNA-binding protein | | uniclust | UniRef100\_A0A0F9AA01 | 99.8 | 3.2e-22 | 6e-28 | 128.2 | 99 | (1, 110) | 162 | (1, 99) | 127 | Single-stranded DNA-binding protein | Single-stranded DNA-binding protein | | uniclust | UniRef100\_UPI0013D7D236 | 99.8 | 3.3e-22 | 6.2e-28 | 118.1 | 90 | (23, 115) | 162 | (2, 91) | 93 | single-stranded DNA-binding protein | single-stranded DNA-binding protein | | uniclust | UniRef100\_A0A966R2Y3 | 99.8 | 3.4e-22 | 6.3e-28 | 125.4 | 110 | (1, 115) | 162 | (31, 140) | 144 | Single-stranded DNA-binding protein | Single-stranded DNA-binding protein | | uniclust | UniRef100\_A0A2D9CDS7 | 99.8 | 4.2e-22 | 8e-28 | 129.6 | 106 | (4, 118) | 162 | (1, 107) | 138 | Single-stranded DNA-binding protein | Single-stranded DNA-binding protein | | uniclust | UniRef100\_A0A4R0XKR1 | 99.8 | 4.4e-22 | 8.2e-28 | 133.1 | 107 | (3, 119) | 162 | (15, 121) | 183 | Single-stranded DNA-binding protein | Single-stranded DNA-binding protein | | uniclust | UniRef100\_A0A0B7BQE9 | 99.8 | 4.7e-22 | 8.8e-28 | 132.3 | 110 | (2, 116) | 162 | (44, 154) | 165 | Single-stranded DNA-binding protein | Single-stranded DNA-binding protein | | uniclust | UniRef100\_A0A383B706 | 99.8 | 5.9e-22 | 1.1e-27 | 123.3 | 104 | (1, 104) | 162 | (29, 133) | 136 | Single-stranded DNA-binding protein (Fragment) | Single-stranded DNA-binding protein (Fragment) | | uniclust | UniRef100\_A0A1C9WY15 | 99.8 | 5.8e-22 | 1.1e-27 | 136.2 | 108 | (4, 111) | 162 | (6, 116) | 181 | Single-stranded DNA-binding protein | Single-stranded DNA-binding protein | | uniclust | UniRef100\_A0A1D8ETT1 | 99.8 | 6.2e-22 | 1.2e-27 | 115.2 | 84 | (4, 88) | 162 | (1, 85) | 85 | Single-stranded DNA binding domain protein | Single-stranded DNA binding domain protein | | uniclust | UniRef100\_A0A2E0R4C7 | 99.8 | 6.7e-22 | 1.2e-27 | 113.9 | 79 | (5, 89) | 162 | (1, 79) | 79 | Single-stranded DNA-binding protein (Fragment) | Single-stranded DNA-binding protein (Fragment) | | uniclust | UniRef100\_A0A7T0KD10 | 99.8 | 6.8e-22 | 1.3e-27 | 129.1 | 112 | (4, 118) | 162 | (1, 117) | 166 | Single-stranded DNA-binding protein | Single-stranded DNA-binding protein | | uniclust | UniRef100\_A0A4Q3ILA5 | 99.8 | 7.8e-22 | 1.5e-27 | 117.3 | 85 | (1, 85) | 162 | (1, 85) | 93 | Single-stranded DNA-binding protein (Fragment) | Single-stranded DNA-binding protein (Fragment) | | uniclust | UniRef100\_A0A022LPA2 | 99.8 | 7.6e-22 | 1.5e-27 | 137.4 | 107 | (1, 107) | 162 | (1, 108) | 204 | Single-stranded DNA-binding protein | Single-stranded DNA-binding protein | | uniclust | UniRef100\_A0A2S8PDV4 | 99.8 | 8.3e-22 | 1.5e-27 | 146.7 | 110 | (3, 118) | 162 | (400, 509) | 559 | Single-stranded DNA-binding protein | Single-stranded DNA-binding protein | | uniclust | UniRef100\_A0A6P1ZCH8 | 99.8 | 8.8e-22 | 1.6e-27 | 131.0 | 110 | (2, 115) | 162 | (75, 185) | 213 | Single-stranded DNA-binding protein | Single-stranded DNA-binding protein | | uniclust | UniRef100\_A0A3M4E9N4 | 99.8 | 8.8e-22 | 1.6e-27 | 126.1 | 97 | (1, 98) | 162 | (67, 163) | 164 | Single-stranded DNA-binding protein (Fragment) | Single-stranded DNA-binding protein (Fragment) | | uniclust | UniRef100\_A0A034W0Y3 | 99.8 | 9.5e-22 | 1.8e-27 | 135.7 | 108 | (3, 115) | 162 | (82, 190) | 205 | Single-stranded DNA-binding protein, mitochondrial | Single-stranded DNA-binding protein, mitochondrial | | uniclust | UniRef100\_A0A1M3GUQ3 | 99.8 | 9.6e-22 | 1.8e-27 | 133.0 | 113 | (4, 117) | 162 | (1, 115) | 186 | Single-stranded DNA-binding protein | Single-stranded DNA-binding protein | | uniclust | UniRef100\_A0A935RAW8 | 99.8 | 1e-21 | 1.9e-27 | 131.4 | 109 | (4, 115) | 162 | (1, 109) | 222 | Single-stranded DNA-binding protein | Single-stranded DNA-binding protein | | uniclust | UniRef100\_A0A292IIP5 | 99.8 | 1e-21 | 1.9e-27 | 134.4 | 104 | (5, 118) | 162 | (1, 104) | 187 | Single-stranded DNA-binding protein | Single-stranded DNA-binding protein | | uniclust | UniRef100\_A0A923AJ74 | 99.8 | 1.1e-21 | 2e-27 | 123.6 | 83 | (34, 120) | 162 | (1, 83) | 122 | Single-stranded DNA-binding protein | Single-stranded DNA-binding protein | | uniclust | UniRef100\_A0A026W545 | 99.8 | 1.1e-21 | 2e-27 | 129.9 | 108 | (3, 115) | 162 | (38, 146) | 163 | Single-stranded DNA-binding protein | Single-stranded DNA-binding protein | | uniclust | UniRef100\_A0A928CL58 | 99.8 | 1.1e-21 | 2.1e-27 | 126.9 | 106 | (2, 116) | 162 | (28, 133) | 175 | Single-stranded DNA-binding protein | Single-stranded DNA-binding protein | | uniclust | UniRef100\_A0A068SFW7 | 99.8 | 1.3e-21 | 2.4e-27 | 126.9 | 108 | (3, 115) | 162 | (27, 134) | 149 | Single-stranded dna-binding protein | Single-stranded dna-binding protein | | uniclust | UniRef100\_UPI000A9471B9 | 99.7 | 1.3e-21 | 2.5e-27 | 112.9 | 77 | (4, 83) | 162 | (1, 78) | 78 | single-stranded DNA-binding protein | single-stranded DNA-binding protein | | uniclust | UniRef100\_A0A1F0Z846 | 99.7 | 1.4e-21 | 2.6e-27 | 127.7 | 100 | (3, 104) | 162 | (1, 101) | 141 | Single-stranded DNA-binding protein | Single-stranded DNA-binding protein | | uniclust | UniRef100\_A0A845H8A1 | 99.7 | 1.4e-21 | 2.6e-27 | 131.9 | 105 | (11, 118) | 162 | (94, 199) | 236 | Single-stranded DNA-binding protein | Single-stranded DNA-binding protein | | uniclust | UniRef100\_A0A059VWJ1 | 99.7 | 1.4e-21 | 2.8e-27 | 146.5 | 107 | (1, 107) | 162 | (50, 158) | 336 | Single-stranded DNA-binding protein | Single-stranded DNA-binding protein | | uniclust | UniRef100\_A0A2H0QGF7 | 99.7 | 1.5e-21 | 2.8e-27 | 120.4 | 94 | (2, 104) | 162 | (1, 94) | 102 | Single-stranded DNA-binding protein | Single-stranded DNA-binding protein | | uniclust | UniRef100\_A0A137PND5 | 99.7 | 1.5e-21 | 2.8e-27 | 130.3 | 109 | (4, 119) | 162 | (16, 124) | 187 | Single-stranded DNA-binding protein | Single-stranded DNA-binding protein | | uniclust | UniRef100\_A0A8J4ZKB2 | 99.7 | 1.6e-21 | 2.9e-27 | 152.9 | 121 | (1, 121) | 162 | (1, 123) | 1036 | DNA topoisomerase (ATP-hydrolyzing) | DNA topoisomerase (ATP-hydrolyzing) | | uniclust | UniRef100\_A0A6J5KSU9 | 99.7 | 1.6e-21 | 3e-27 | 127.7 | 112 | (2, 114) | 162 | (1, 113) | 145 | Ssb Single-stranded DNA-binding protein | Ssb Single-stranded DNA-binding protein | | uniclust | UniRef100\_UPI001B42314A | 99.7 | 1.7e-21 | 3.1e-27 | 125.5 | 103 | (2, 104) | 162 | (4, 106) | 153 | single-stranded DNA-binding protein | single-stranded DNA-binding protein | | uniclust | UniRef100\_A0A7C3X101 | 99.7 | 1.7e-21 | 3.2e-27 | 125.4 | 108 | (4, 115) | 162 | (8, 116) | 169 | Single-stranded DNA-binding protein | Single-stranded DNA-binding protein | | uniclust | UniRef100\_A0A2U3LGW3 | 99.7 | 1.8e-21 | 3.3e-27 | 122.5 | 107 | (3, 117) | 162 | (6, 112) | 128 | Single-stranded DNA-binding protein | Single-stranded DNA-binding protein | | uniclust | UniRef100\_A0A1Q6YS66 | 99.7 | 1.9e-21 | 3.5e-27 | 129.0 | 108 | (4, 115) | 162 | (82, 189) | 207 | Single-stranded DNA-binding protein | Single-stranded DNA-binding protein | | uniclust | UniRef100\_A0A952LKT7 | 99.7 | 2e-21 | 3.6e-27 | 124.6 | 110 | (4, 117) | 162 | (1, 111) | 164 | Single-stranded DNA-binding protein | Single-stranded DNA-binding protein | | uniclust | UniRef100\_A0A0E3B7S8 | 99.7 | 2e-21 | 3.7e-27 | 125.2 | 110 | (1, 114) | 162 | (1, 111) | 143 | Single-stranded DNA-binding protein | Single-stranded DNA-binding protein | | uniclust | UniRef100\_A0A1G8T5L8 | 99.7 | 2e-21 | 3.7e-27 | 139.1 | 105 | (3, 107) | 162 | (42, 147) | 265 | Single-stranded DNA-binding protein | Single-stranded DNA-binding protein | | uniclust | UniRef100\_A0A086Z1D6 | 99.7 | 2.1e-21 | 3.9e-27 | 138.5 | 106 | (1, 107) | 162 | (39, 145) | 271 | Single-stranded DNA-binding protein | Single-stranded DNA-binding protein | | uniclust | UniRef100\_A0A0F0KXD3 | 99.7 | 2.1e-21 | 4e-27 | 130.4 | 101 | (3, 104) | 162 | (7, 108) | 166 | Single-stranded DNA-binding protein | Single-stranded DNA-binding protein | | uniclust | UniRef100\_A0A5J6Z6V0 | 99.7 | 2.2e-21 | 4.1e-27 | 131.1 | 103 | (3, 106) | 162 | (1, 103) | 188 | Single-stranded DNA-binding protein | Single-stranded DNA-binding protein | | uniclust | UniRef100\_A0A095YG51 | 99.7 | 2.4e-21 | 4.6e-27 | 138.7 | 106 | (1, 106) | 162 | (50, 156) | 241 | Single-stranded DNA-binding protein | Single-stranded DNA-binding protein | | uniclust | UniRef100\_A0A0K2GF50 | 99.7 | 2.5e-21 | 4.6e-27 | 124.9 | 107 | (4, 115) | 162 | (32, 138) | 144 | Single-stranded DNA-binding protein | Single-stranded DNA-binding protein | | uniclust | UniRef100\_G1DI79 | 99.7 | 2.5e-21 | 4.7e-27 | 128.0 | 98 | (7, 105) | 162 | (63, 161) | 203 | Single-stranded DNA binding protein | Single-stranded DNA binding protein | | uniclust | UniRef100\_A0A2S1PPN8 | 99.7 | 2.7e-21 | 5.1e-27 | 132.4 | 111 | (4, 115) | 162 | (5, 115) | 229 | Single-stranded DNA-binding protein | Single-stranded DNA-binding protein | | uniclust | UniRef100\_A0A443SA52 | 99.7 | 2.8e-21 | 5.3e-27 | 129.2 | 108 | (4, 117) | 162 | (40, 148) | 165 | Single-stranded DNA-binding protein-like isoform X2 | Single-stranded DNA-binding protein-like isoform X2 | | uniclust | UniRef100\_A0A074U9W4 | 99.7 | 2.9e-21 | 5.4e-27 | 133.2 | 116 | (1, 116) | 162 | (1, 118) | 278 | Single-stranded DNA-binding protein | Single-stranded DNA-binding protein | | uniclust | UniRef100\_UPI001E2AA1AE | 99.7 | 2.9e-21 | 5.4e-27 | 125.5 | 107 | (3, 118) | 162 | (41, 148) | 179 | single-stranded DNA-binding protein | single-stranded DNA-binding protein | | uniclust | UniRef100\_A0A126ZWG1 | 99.7 | 3.1e-21 | 5.8e-27 | 133.8 | 101 | (6, 106) | 162 | (95, 196) | 254 | Single-stranded DNA-binding protein | Single-stranded DNA-binding protein | | uniclust | UniRef100\_A0A0K2RXI4 | 99.7 | 3.2e-21 | 5.9e-27 | 130.8 | 104 | (4, 108) | 162 | (1, 105) | 191 | Single-stranded DNA-binding protein | Single-stranded DNA-binding protein | | uniclust | UniRef100\_A0A0B5D4L6 | 99.7 | 3.2e-21 | 6.1e-27 | 137.1 | 103 | (2, 104) | 162 | (31, 135) | 242 | Single-stranded DNA-binding protein | Single-stranded DNA-binding protein | | uniclust | UniRef100\_A0A1I4QA87 | 99.7 | 3.3e-21 | 6.1e-27 | 131.9 | 103 | (5, 117) | 162 | (1, 103) | 222 | Single-stranded DNA-binding protein | Single-stranded DNA-binding protein | | uniclust | UniRef100\_A0A0L7B6A5 | 99.7 | 3.3e-21 | 6.3e-27 | 130.7 | 103 | (2, 104) | 162 | (1, 106) | 192 | Single-stranded DNA-binding protein | Single-stranded DNA-binding protein | | uniclust | UniRef100\_A0A971KRS6 | 99.7 | 3.4e-21 | 6.3e-27 | 118.9 | 111 | (2, 116) | 162 | (1, 111) | 129 | Single-stranded DNA-binding protein | Single-stranded DNA-binding protein | | uniclust | UniRef100\_A0A7C6LEL5 | 99.7 | 3.6e-21 | 6.6e-27 | 135.2 | 107 | (4, 117) | 162 | (1, 107) | 321 | Single-stranded DNA-binding protein | Single-stranded DNA-binding protein | | uniclust | UniRef100\_A0A7Z9QKL3 | 99.7 | 3.6e-21 | 6.6e-27 | 123.0 | 101 | (3, 112) | 162 | (36, 136) | 160 | Single-stranded DNA-binding protein | Single-stranded DNA-binding protein | | uniclust | UniRef100\_A0A2D9WLM2 | 99.7 | 3.6e-21 | 6.6e-27 | 138.3 | 106 | (3, 113) | 162 | (210, 315) | 347 | Single-stranded DNA-binding protein (Fragment) | Single-stranded DNA-binding protein (Fragment) | | pdb70 | 1EQQ\_B | 100.0 | 5.5e-34 | 4.4e-38 | 201.5 | 160 | (2, 162) | 162 | (3, 178) | 178 | SINGLE STRANDED DNA BINDING PROTEIN/RNA | 1EQQ\_B SINGLE STRANDED DNA BINDING PROTEIN/RNA BETA BARREL, PROTEIN-DNA COMPLEX, REPLICATION-RNA | | pdb70 | 4MZ9\_D | 99.9 | 9e-34 | 7.2e-38 | 200.4 | 160 | (2, 162) | 162 | (3, 178) | 178 | Single-stranded DNA-binding protein | 4MZ9\_D Single-stranded DNA-binding protein Single strand DNA-binding domain, SSB | | pdb70 | 5ODN\_B | 99.9 | 1.8e-30 | 1.5e-34 | 185.8 | 117 | (1, 121) | 162 | (29, 145) | 196 | Single-stranded DNA-binding protein/DNA Complex | 5ODN\_B Single-stranded DNA-binding protein/DNA Complex Single-Strand Binding protein, DNA BINDING | | pdb70 | 5ODN\_F | 99.9 | 1.8e-30 | 1.5e-34 | 185.8 | 117 | (1, 121) | 162 | (29, 145) | 196 | Single-stranded DNA-binding protein/DNA Complex | 5ODN\_F Single-stranded DNA-binding protein/DNA Complex Single-Strand Binding protein, DNA BINDING | | pdb70 | 5ODP\_A | 99.9 | 3.6e-30 | 2.9e-34 | 184.2 | 118 | (1, 122) | 162 | (29, 146) | 196 | Single-stranded DNA-binding protein/DNA Complex | 5ODP\_A Single-stranded DNA-binding protein/DNA Complex Single-Strand Binding protein, DNA BINDING | | pdb70 | 3PGZ\_B | 99.9 | 6.3e-28 | 5e-32 | 172.1 | 118 | (1, 121) | 162 | (22, 144) | 193 | Single-stranded DNA-binding protein | 3PGZ\_B Single-stranded DNA-binding protein SSGCID, BARTONELLA HENSELAE, SINGLE-STRAND BINDING | | pdb70 | 5GQO\_A | 99.9 | 7.3e-28 | 5.9e-32 | 171.4 | 111 | (1, 116) | 162 | (21, 132) | 190 | Single-stranded DNA-binding protein | 5GQO\_A Single-stranded DNA-binding protein OB fold, inter-subunit clamp, Paralogous | | pdb70 | 5GQO\_B | 99.9 | 7.3e-28 | 5.9e-32 | 171.4 | 111 | (1, 116) | 162 | (21, 132) | 190 | Single-stranded DNA-binding protein | 5GQO\_B Single-stranded DNA-binding protein OB fold, inter-subunit clamp, Paralogous | | pdb70 | 1UE1\_B | 99.9 | 2.9e-27 | 2.3e-31 | 164.6 | 111 | (2, 115) | 162 | (1, 112) | 164 | Single-strand binding protein | 1UE1\_B Single-strand binding protein OLIGONUCLEOTIDE BINDING FOLD, DNA-BINDING PROTEIN | | pdb70 | 3TQY\_A | 99.9 | 7.7e-27 | 6.1e-31 | 161.5 | 118 | (1, 121) | 162 | (4, 121) | 158 | Single-stranded DNA-binding protein (E.C.2.7.7.6) | 3TQY\_A Single-stranded DNA-binding protein (E.C.2.7.7.6) DNA replication, TRANSFERASE HET: MSE | | pdb70 | 3TQY\_C | 99.9 | 7.7e-27 | 6.1e-31 | 161.5 | 118 | (1, 121) | 162 | (4, 121) | 158 | Single-stranded DNA-binding protein (E.C.2.7.7.6) | 3TQY\_C Single-stranded DNA-binding protein (E.C.2.7.7.6) DNA replication, TRANSFERASE HET: MSE | | pdb70 | 1Z9F\_A | 99.8 | 3.4e-26 | 2.7e-30 | 157.4 | 116 | (1, 120) | 162 | (12, 127) | 153 | Single-strand binding protein | 1Z9F\_A Single-strand binding protein TM0604, single stranded DNA-binding protein | | pdb70 | 3AFP\_A | 99.8 | 5.7e-26 | 4.6e-30 | 158.6 | 114 | (2, 118) | 162 | (1, 115) | 168 | Single-stranded DNA-binding protein | 3AFP\_A Single-stranded DNA-binding protein OB-fold, quaternary structure and stability | | pdb70 | 1QVC\_A | 99.8 | 6.6e-26 | 5.3e-30 | 154.5 | 122 | (1, 122) | 162 | (1, 122) | 145 | E.COLI SINGLE STRANDED DNA BINDING | 1QVC\_A E.COLI SINGLE STRANDED DNA BINDING BETA-BARREL, SINGLE STRANDED DNA BINDING | | pdb70 | 1QVC\_B | 99.8 | 6.6e-26 | 5.3e-30 | 154.5 | 122 | (1, 122) | 162 | (1, 122) | 145 | E.COLI SINGLE STRANDED DNA BINDING | 1QVC\_B E.COLI SINGLE STRANDED DNA BINDING BETA-BARREL, SINGLE STRANDED DNA BINDING | | pdb70 | 1SE8\_A | 99.8 | 7.3e-26 | 5.9e-30 | 170.8 | 115 | (1, 120) | 162 | (125, 239) | 301 | Single-strand binding protein | 1SE8\_A Single-strand binding protein Single-strand binding protein, DNA BINDING | | pdb70 | 3UDG\_C | 99.8 | 7.3e-26 | 5.9e-30 | 170.8 | 115 | (1, 120) | 162 | (125, 239) | 301 | Single-stranded DNA-binding protein/DNA complex | 3UDG\_C Single-stranded DNA-binding protein/DNA complex SSB, OB fold, beta-barrel, single-stranded HET: TMP | | pdb70 | 3EIV\_B | 99.8 | 9.3e-25 | 7.3e-29 | 155.8 | 114 | (2, 118) | 162 | (1, 115) | 199 | Crystal Structure of Single-stranded DNA-binding | 3EIV\_B Crystal Structure of Single-stranded DNA-binding Single-stranded DNA-binding protein, Streptomyces Coelicolor | | pdb70 | 1KAW\_A | 99.8 | 9.4e-25 | 7.6e-29 | 147.0 | 120 | (1, 120) | 162 | (1, 120) | 135 | SINGLE-STRANDED DNA BINDING PROTEIN | 1KAW\_A SINGLE-STRANDED DNA BINDING PROTEIN DNA-BINDING PROTEIN, SINGLE STRANDED DNA | | pdb70 | 6IRQ\_C | 99.8 | 2.6e-24 | 2.1e-28 | 142.1 | 112 | (1, 115) | 162 | (1, 112) | 121 | Single-stranded DNA-binding protein/DNA Complex | 6IRQ\_C Single-stranded DNA-binding protein/DNA Complex single-strand DNA binding protein, DNA | | pdb70 | 3LGJ\_B | 99.8 | 4.3e-24 | 3.4e-28 | 149.0 | 116 | (2, 120) | 162 | (20, 140) | 169 | Single-stranded DNA-binding protein | 3LGJ\_B Single-stranded DNA-binding protein NIAID, Cat Scratch Fever, Rochalimaea | | pdb70 | 2FXQ\_A | 99.8 | 1.1e-23 | 9.1e-28 | 155.8 | 111 | (2, 120) | 162 | (125, 235) | 264 | Single-strand binding protein | 2FXQ\_A Single-strand binding protein 5 strand beta-sheet extended loops | | pdb70 | 1EYG\_A | 99.8 | 1.2e-23 | 9.6e-28 | 137.8 | 113 | (1, 113) | 162 | (2, 114) | 116 | SINGLE-STRAND DNA-BINDING PROTEIN/DNA COMPLEX | 1EYG\_A SINGLE-STRAND DNA-BINDING PROTEIN/DNA COMPLEX PROTEIN-DNA COMPLEX OB fold | | pdb70 | 3ULP\_C | 99.8 | 2.6e-23 | 2.1e-27 | 137.7 | 116 | (1, 116) | 162 | (2, 121) | 124 | Single-strand binding protein/DNA complex | 3ULP\_C Single-strand binding protein/DNA complex OB-fold, DNA binding, single-stranded DNA | | pdb70 | 3ULP\_D | 99.8 | 2.6e-23 | 2.1e-27 | 137.7 | 116 | (1, 116) | 162 | (2, 121) | 124 | Single-strand binding protein/DNA complex | 3ULP\_D Single-strand binding protein/DNA complex OB-fold, DNA binding, single-stranded DNA | | pdb70 | 3A5U\_A | 99.8 | 2.7e-23 | 2.2e-27 | 138.7 | 114 | (2, 118) | 162 | (1, 115) | 130 | Single-stranded DNA-binding protein/DNA complex | 3A5U\_A Single-stranded DNA-binding protein/DNA complex DNA binding protein, DNA damage | | pdb70 | 3A5U\_B | 99.8 | 2.7e-23 | 2.2e-27 | 138.7 | 114 | (2, 118) | 162 | (1, 115) | 130 | Single-stranded DNA-binding protein/DNA complex | 3A5U\_B Single-stranded DNA-binding protein/DNA complex DNA binding protein, DNA damage | | pdb70 | 2VW9\_A | 99.7 | 4.6e-23 | 3.7e-27 | 138.4 | 113 | (4, 120) | 162 | (1, 113) | 134 | SINGLE-STRANDED DNA BINDING PROTEIN | 2VW9\_A SINGLE-STRANDED DNA BINDING PROTEIN DNA REPLICATION, SINGLE-STRANDED DNA, SINGLE-STRANDED | | pdb70 | 2VW9\_B | 99.7 | 4.6e-23 | 3.7e-27 | 138.4 | 113 | (4, 120) | 162 | (1, 113) | 134 | SINGLE-STRANDED DNA BINDING PROTEIN | 2VW9\_B SINGLE-STRANDED DNA BINDING PROTEIN DNA REPLICATION, SINGLE-STRANDED DNA, SINGLE-STRANDED | | pdb70 | 6BHW\_B | 99.7 | 4.6e-23 | 3.8e-27 | 135.6 | 113 | (1, 119) | 162 | (1, 113) | 119 | Single-stranded DNA-binding protein A | 6BHW\_B Single-stranded DNA-binding protein A Single-stranded DNA binding protein, DNA HET: EDO, PEG | | pdb70 | 6BHW\_C | 99.7 | 4.6e-23 | 3.8e-27 | 135.6 | 113 | (1, 119) | 162 | (1, 113) | 119 | Single-stranded DNA-binding protein A | 6BHW\_C Single-stranded DNA-binding protein A Single-stranded DNA binding protein, DNA HET: PEG, EDO | | pdb70 | 3VDY\_A | 99.7 | 1.1e-22 | 8.8e-27 | 132.9 | 113 | (1, 119) | 162 | (1, 113) | 116 | Single-stranded DNA-binding protein ssbB/DNA complex | 3VDY\_A Single-stranded DNA-binding protein ssbB/DNA complex OB fold, single-strand DNA binding | | pdb70 | 4DAM\_A | 99.7 | 1.6e-22 | 1.3e-26 | 134.8 | 111 | (1, 116) | 162 | (11, 122) | 128 | Single-stranded DNA-binding protein 1 | 4DAM\_A Single-stranded DNA-binding protein 1 OB-fold, DNA-binding, single-stranded DNA, DNA | | pdb70 | 4DAM\_F | 99.7 | 1.6e-22 | 1.3e-26 | 134.8 | 111 | (1, 116) | 162 | (11, 122) | 128 | Single-stranded DNA-binding protein 1 | 4DAM\_F Single-stranded DNA-binding protein 1 OB-fold, DNA-binding, single-stranded DNA, DNA | | pdb70 | 6RUP\_B | 99.7 | 1.8e-22 | 1.4e-26 | 137.0 | 116 | (1, 119) | 162 | (18, 139) | 142 | Single-stranded DNA-binding protein, mitochondrial, SER-SER-SER-SER | 6RUP\_B Single-stranded DNA-binding protein, mitochondrial, SER-SER-SER-SER Single-stranded DNA binding protein, mitochondria | | pdb70 | 1S3O\_A | 99.7 | 2.4e-22 | 2e-26 | 134.5 | 115 | (1, 118) | 162 | (10, 130) | 132 | Single-stranded DNA-binding protein, mitochondrial | 1S3O\_A Single-stranded DNA-binding protein, mitochondrial OB fold, DNA BINDING PROTEIN | | pdb70 | 5YYU\_A | 99.7 | 3.8e-22 | 3.1e-26 | 129.9 | 107 | (4, 116) | 162 | (1, 107) | 112 | Single-stranded DNA-binding protein | 5YYU\_A Single-stranded DNA-binding protein single-strand DNA binding protein, Staphylococcus | | pdb70 | 6CQO\_D | 99.7 | 6.5e-22 | 5.3e-26 | 129.8 | 107 | (3, 120) | 162 | (1, 108) | 119 | Single-stranded DNA-binding protein RIM1, mitochondrial | 6CQO\_D Single-stranded DNA-binding protein RIM1, mitochondrial Mitochondrial single-stranded DNA binding proteins HET: MSE | | pdb70 | 2CCZ\_A | 99.7 | 7.1e-22 | 5.8e-26 | 130.8 | 110 | (1, 119) | 162 | (4, 113) | 123 | PRIMOSOMAL REPLICATION PROTEIN N | 2CCZ\_A PRIMOSOMAL REPLICATION PROTEIN N DNA/REPLICATION, PRIMOSOME, PRIB, DNA REPLICATION | | pdb70 | 5XGT\_A | 99.7 | 8.5e-22 | 6.9e-26 | 128.0 | 105 | (4, 114) | 162 | (1, 105) | 111 | Single-stranded DNA-binding protein | 5XGT\_A Single-stranded DNA-binding protein single-strand DNA binding protein, SsbA | | pdb70 | 5WQV\_B | 99.7 | 1.9e-21 | 1.5e-25 | 124.5 | 103 | (4, 115) | 162 | (1, 103) | 104 | Primosomal replication protein N | 5WQV\_B Primosomal replication protein N DNA binding protein, Bacterial DNA | | pdb70 | 3KOJ\_A | 99.7 | 1.9e-21 | 1.5e-25 | 125.7 | 100 | (1, 110) | 162 | (7, 107) | 108 | uncharacterized protein ycf41 | 3KOJ\_A uncharacterized protein ycf41 single-strand binding protein family, PF00436 HET: MSE | | pdb70 | 3KOJ\_B | 99.7 | 1.9e-21 | 1.5e-25 | 125.7 | 100 | (1, 110) | 162 | (7, 107) | 108 | uncharacterized protein ycf41 | 3KOJ\_B uncharacterized protein ycf41 single-strand binding protein family, PF00436 | | pdb70 | 3K8A\_B | 99.7 | 2.7e-21 | 2.2e-25 | 123.7 | 96 | (1, 101) | 162 | (3, 98) | 103 | Putative primosomal replication protein | 3K8A\_B Putative primosomal replication protein beta-barrel, OB-fold, DNA binding protein | | pdb70 | 2IHF\_A | 99.7 | 2.9e-21 | 2.3e-25 | 140.7 | 110 | (1, 115) | 162 | (1, 110) | 239 | Single-stranded DNA-binding protein | 2IHF\_A Single-stranded DNA-binding protein Single-stranded DNA binding protein (SSB) | | pdb70 | 2FXQ\_A | 99.7 | 4.1e-21 | 3.3e-25 | 141.8 | 109 | (1, 114) | 162 | (1, 109) | 264 | Single-strand binding protein | 2FXQ\_A Single-strand binding protein 5 strand beta-sheet extended loops | | pdb70 | 3K81\_C | 99.7 | 4.5e-21 | 3.6e-25 | 132.9 | 117 | (1, 119) | 162 | (18, 137) | 164 | MP18 RNA editing complex protein | 3K81\_C MP18 RNA editing complex protein KREPA6, VHH, Single domain antibody | | pdb70 | 4APV\_A | 99.7 | 4.9e-21 | 4e-25 | 124.5 | 105 | (4, 117) | 162 | (1, 105) | 112 | PRIMOSOMAL REPLICATION PROTEIN N | 4APV\_A PRIMOSOMAL REPLICATION PROTEIN N REPLICATION, PRIB PRIMOSOME SSDNA BINDING | | pdb70 | 1V1Q\_B | 99.7 | 5.3e-21 | 4.2e-25 | 128.4 | 110 | (1, 119) | 162 | (15, 124) | 134 | PRIMOSOMAL REPLICATION PROTEIN N | 1V1Q\_B PRIMOSOMAL REPLICATION PROTEIN N PRIMOSOME, DNA REPLICATION, DNA BINDING HET: CYS | | pdb70 | 2IHF\_A | 99.6 | 6.7e-21 | 5.5e-25 | 138.7 | 103 | (2, 112) | 162 | (125, 227) | 239 | Single-stranded DNA-binding protein | 2IHF\_A Single-stranded DNA-binding protein Single-stranded DNA binding protein (SSB) | | pdb70 | 1SE8\_A | 99.6 | 7.7e-21 | 6.2e-25 | 143.0 | 110 | (1, 114) | 162 | (1, 111) | 301 | Single-strand binding protein | 1SE8\_A Single-strand binding protein Single-strand binding protein, DNA BINDING | | pdb70 | 3UDG\_C | 99.6 | 7.7e-21 | 6.2e-25 | 143.0 | 110 | (1, 114) | 162 | (1, 111) | 301 | Single-stranded DNA-binding protein/DNA complex | 3UDG\_C Single-stranded DNA-binding protein/DNA complex SSB, OB fold, beta-barrel, single-stranded HET: TMP | | pdb70 | 1WOC\_C | 99.6 | 8.7e-21 | 7.1e-25 | 121.0 | 102 | (5, 115) | 162 | (1, 102) | 103 | Primosomal replication protein n | 1WOC\_C Primosomal replication protein n Oligonucleotide Binding fold, DNA BINDING | | pdb70 | 3K7U\_C | 99.6 | 1.5e-20 | 1.2e-24 | 128.1 | 111 | (1, 117) | 162 | (2, 119) | 148 | PROTEIN | 3K7U\_C PROTEIN RNA-editing, OB-fold, RNA-editing proteins, kinetoplastids | | pdb70 | 3STB\_D | 99.6 | 2e-20 | 1.6e-24 | 126.9 | 114 | (3, 118) | 162 | (1, 117) | 145 | single domain antibody VHH, KREPA3 | 3STB\_D single domain antibody VHH, KREPA3 RNA EDITING, Editosome, Nanobody, Single | | pdb70 | 4DNI\_A | 99.6 | 4.6e-20 | 3.7e-24 | 136.1 | 102 | (1, 104) | 162 | (142, 245) | 257 | Fusion protein of RNA-editing complex | 4DNI\_A Fusion protein of RNA-editing complex KREPA3, KREPA6, Editosome, Protein/RNA binding | | pdb70 | 3STB\_C | 99.6 | 6.7e-20 | 5.3e-24 | 124.9 | 97 | (1, 103) | 162 | (40, 137) | 148 | single domain antibody VHH, KREPA3 | 3STB\_C single domain antibody VHH, KREPA3 RNA EDITING, Editosome, Nanobody, Single | | pdb70 | 4GS3\_A | 99.6 | 9.4e-20 | 7.7e-24 | 117.2 | 98 | (2, 113) | 162 | (8, 106) | 107 | Single-stranded DNA-binding protein | 4GS3\_A Single-stranded DNA-binding protein primosome, oligonucleotide/oligosaccharide binding domain, DNA | | pdb70 | 4DK6\_C | 99.6 | 1.3e-19 | 1e-23 | 116.3 | 91 | (3, 103) | 162 | (1, 93) | 105 | single domain antibody VHH, RNA-editing | 4DK6\_C single domain antibody VHH, RNA-editing KREPA1, VHH, Single domain antibody | | pdb70 | 4DKA\_D | 99.6 | 1.3e-19 | 1e-23 | 116.3 | 91 | (3, 103) | 162 | (1, 93) | 105 | single domain antibody VHH, RNA-editing | 4DKA\_D single domain antibody VHH, RNA-editing KREPA1, VHH, Single domain antibody | | pdb70 | 3KLW\_B | 99.6 | 1.6e-19 | 1.3e-23 | 114.1 | 97 | (5, 113) | 162 | (1, 97) | 98 | Primosomal replication protein n | 3KLW\_B Primosomal replication protein n STRUCTURAL GENOMICS, PSI-2, PROTEIN STRUCTURE HET: MSE | | pdb70 | 4FDB\_A | 99.6 | 2.5e-19 | 2.1e-23 | 114.3 | 85 | (4, 91) | 162 | (1, 85) | 101 | Probable primosomal replication protein n | 4FDB\_A Probable primosomal replication protein n Structural Genomics, PSI-Biology, Protein Structure | | pdb70 | 3FHW\_A | 99.5 | 3e-19 | 2.4e-23 | 116.5 | 103 | (5, 119) | 162 | (1, 103) | 115 | Primosomal replication protein n | 3FHW\_A Primosomal replication protein n priB BpR162 X-RAY NESG, Structural HET: MSE | | pdb70 | 3KLW\_A | 99.5 | 9.4e-19 | 7.7e-23 | 110.6 | 97 | (5, 113) | 162 | (1, 97) | 98 | Primosomal replication protein n | 3KLW\_A Primosomal replication protein n STRUCTURAL GENOMICS, PSI-2, PROTEIN STRUCTURE | | pdb70 | 2HQL\_B | 99.5 | 1.7e-18 | 1.3e-22 | 112.1 | 93 | (3, 111) | 162 | (6, 98) | 110 | Hypothetical protein MG376 homolog | 2HQL\_B Hypothetical protein MG376 homolog Structural genomics, conserved hypothetical protein | | pdb70 | 2HQL\_D | 99.5 | 1.7e-18 | 1.3e-22 | 112.1 | 93 | (3, 111) | 162 | (6, 98) | 110 | Hypothetical protein MG376 homolog | 2HQL\_D Hypothetical protein MG376 homolog Structural genomics, conserved hypothetical protein | | pdb70 | 4DNI\_A | 99.3 | 2e-16 | 1.6e-20 | 116.3 | 97 | (1, 103) | 162 | (26, 123) | 257 | Fusion protein of RNA-editing complex | 4DNI\_A Fusion protein of RNA-editing complex KREPA3, KREPA6, Editosome, Protein/RNA binding | |
| Top keywords  (threshold 1.00e-03 (evalue)) | **Single\_stranded, DNA\_binding, DNA, binding, Single\_strand, Fragment, Single, replication, complex, Primosomal** |
| Output files | ../../similar\_sequences/35\_FANPEZAQ\_CDS\_0035\_merged.svg ../../similar\_sequences/35\_FANPEZAQ\_CDS\_0035\_pdb70.a3m ../../similar\_sequences/35\_FANPEZAQ\_CDS\_0035\_pdb70.hhr ../../similar\_sequences/35\_FANPEZAQ\_CDS\_0035\_uniclust.a3m ../../similar\_sequences/35\_FANPEZAQ\_CDS\_0035\_uniclust.hhr |

#### Structure prediction (AlphaFold)2

|  |  |
| --- | --- |
| Stats | xml version="1.0" encoding="utf-8" standalone="no"?       2024-09-02T21:09:34.582616 image/svg+xml   Matplotlib v3.7.2, https://matplotlib.org/ |
| Predicted structure | **NGL Viewer Controls:**  - Center: *Left-Click* - Rotate: *Left-Click + Drag* - Translate: *Right-Click + Drag* - Zoom: *Shift + Left-Click + Drag* |
| Output files | ../../predicted\_structures/35\_FANPEZAQ\_CDS\_0035/features.pkl ../../predicted\_structures/35\_FANPEZAQ\_CDS\_0035/ranked\_0.pdb ../../predicted\_structures/35\_FANPEZAQ\_CDS\_0035/ranked\_0\_plots.svg ../../predicted\_structures/35\_FANPEZAQ\_CDS\_0035/result\_model\_1\_ptm\_pred\_0.pkl |

#### Structure similarity search results (Foldseek)3

|  |  |
| --- | --- |
| Structure databases searched | Pdb, Afdb-proteome, Afdb-uniprot50 |
| Results, scheme(s)  (Top layers only, threshold 1.00e-02 (evalue)) | xml version="1.0" encoding="utf-8" standalone="no"?       2024-09-02T21:11:11.159462 image/svg+xml   Matplotlib v3.7.2, https://matplotlib.org/ |
| Results, table  (threshold 1.00e-02 (evalue)) | | db | id | prob | evalue | bits | fident | alnlen | mismatch | gapopen | qstart | qend | tstart | tend | name | description | | --- | --- | --- | --- | --- | --- | --- | --- | --- | --- | --- | --- | --- | --- | --- | | pdb | 7F2N\_A | 1.0 | 5.8e-16 | 725 | 0.625 | 112 | 42 | 0 | 2 | 113 | 1 | 112 | Single-stranded DNA-binding protein | Single-stranded DNA-binding protein | | pdb | 4MZ9\_C | 1.0 | 8.448e-16 | 712 | 0.631 | 114 | 42 | 0 | 1 | 114 | 1 | 114 | Single-stranded DNA-binding protein | Single-stranded DNA-binding protein | | pdb | 3PGZ\_B | 1.0 | 2.906e-15 | 670 | 0.631 | 114 | 40 | 2 | 2 | 113 | 1 | 114 | Single-stranded DNA-binding protein | Single-stranded DNA-binding protein | | pdb | 3TQY\_C | 1.0 | 4.232e-15 | 664 | 0.618 | 110 | 39 | 1 | 4 | 113 | 2 | 108 | Single-stranded DNA-binding protein | Single-stranded DNA-binding protein | | pdb | 7F2N\_C | 1.0 | 9.994e-15 | 662 | 0.603 | 111 | 40 | 2 | 2 | 112 | 1 | 107 | Single-stranded DNA-binding protein | Single-stranded DNA-binding protein | | pdb | 1EYG\_D | 1.0 | 1.804e-14 | 639 | 0.616 | 112 | 40 | 1 | 1 | 112 | 1 | 109 | SINGLE-STRAND DNA-BINDING PROTEIN | SINGLE-STRAND DNA-BINDING PROTEIN | | pdb | 5YUO\_C | 1.0 | 7.693e-14 | 627 | 0.6 | 110 | 38 | 1 | 4 | 113 | 2 | 105 | Single-stranded DNA-binding protein | Single-stranded DNA-binding protein | | pdb | 1QVC\_D | 1.0 | 7.291e-14 | 619 | 0.571 | 112 | 47 | 1 | 1 | 112 | 1 | 111 | SINGLE STRANDED DNA BINDING PROTEIN MONOMER | SINGLE STRANDED DNA BINDING PROTEIN MONOMER | | pdb | 6JDG\_D | 1.0 | 5.574e-14 | 613 | 0.6 | 110 | 37 | 2 | 4 | 113 | 2 | 104 | Single-stranded DNA-binding protein | Single-stranded DNA-binding protein | | pdb | 6JDG\_A | 1.0 | 1.466e-13 | 611 | 0.572 | 110 | 37 | 1 | 4 | 113 | 2 | 101 | Single-stranded DNA-binding protein | Single-stranded DNA-binding protein | | pdb | 7F2N\_D | 1.0 | 1.917e-13 | 611 | 0.571 | 112 | 39 | 3 | 2 | 113 | 1 | 103 | Single-stranded DNA-binding protein | Single-stranded DNA-binding protein | | pdb | 5YUN\_B | 1.0 | 1.466e-13 | 609 | 0.576 | 111 | 39 | 1 | 4 | 114 | 1 | 103 | Single-stranded DNA-binding protein | Single-stranded DNA-binding protein | | pdb | 5YUN\_A | 1.0 | 2.252e-13 | 602 | 0.576 | 111 | 40 | 1 | 4 | 114 | 1 | 104 | Single-stranded DNA-binding protein | Single-stranded DNA-binding protein | | pdb | 7F2N\_B | 1.0 | 1.12e-13 | 600 | 0.543 | 116 | 43 | 2 | 2 | 117 | 1 | 106 | Single-stranded DNA-binding protein | Single-stranded DNA-binding protein | | pdb | 5YUO\_D | 1.0 | 2.646e-13 | 597 | 0.581 | 110 | 37 | 2 | 4 | 113 | 2 | 102 | Single-stranded DNA-binding protein | Single-stranded DNA-binding protein | | pdb | 1EYG\_B | 1.0 | 2.792e-13 | 596 | 0.587 | 114 | 36 | 3 | 1 | 113 | 1 | 104 | SINGLE-STRAND DNA-BINDING PROTEIN | SINGLE-STRAND DNA-BINDING PROTEIN | | pdb | 7F25\_B | 1.0 | 2.252e-13 | 596 | 0.575 | 113 | 40 | 3 | 2 | 113 | 1 | 106 | Single-stranded DNA-binding protein 1 | Single-stranded DNA-binding protein 1 | | pdb | 7VUM\_A | 1.0 | 4.066e-13 | 594 | 0.572 | 110 | 37 | 1 | 4 | 113 | 2 | 101 | Single-stranded DNA-binding protein | Single-stranded DNA-binding protein | | pdb | 5YUN\_D | 1.0 | 5.922e-13 | 594 | 0.585 | 111 | 37 | 1 | 2 | 112 | 1 | 102 | Single-stranded DNA-binding protein | Single-stranded DNA-binding protein | | pdb | 3PGZ\_A | 1.0 | 1.546e-13 | 594 | 0.614 | 114 | 37 | 3 | 2 | 113 | 1 | 109 | Single-stranded DNA-binding protein | Single-stranded DNA-binding protein | | pdb | 6IRQ\_C | 1.0 | 7.341e-13 | 575 | 0.581 | 110 | 39 | 2 | 4 | 113 | 2 | 104 | Single-stranded DNA-binding protein | Single-stranded DNA-binding protein | | pdb | 5YUO\_B | 1.0 | 9.101e-13 | 568 | 0.545 | 110 | 39 | 2 | 4 | 113 | 2 | 100 | Single-stranded DNA-binding protein | Single-stranded DNA-binding protein | | pdb | 5YUO\_A | 1.0 | 1.069e-12 | 563 | 0.558 | 111 | 38 | 2 | 4 | 114 | 1 | 100 | Single-stranded DNA-binding protein | Single-stranded DNA-binding protein | | pdb | 1SRU\_D | 1.0 | 1.734e-12 | 559 | 0.584 | 113 | 34 | 4 | 1 | 112 | 1 | 101 | Single-strand binding protein | Single-strand binding protein | | pdb | 1EQQ\_B | 1.0 | 5.612e-13 | 558 | 0.555 | 117 | 51 | 1 | 1 | 117 | 1 | 116 | SINGLE STRANDED DNA BINDING PROTEIN | SINGLE STRANDED DNA BINDING PROTEIN | | pdb | 3TQY\_D | 1.0 | 1.399e-12 | 548 | 0.545 | 110 | 37 | 3 | 4 | 113 | 2 | 98 | Single-stranded DNA-binding protein | Single-stranded DNA-binding protein | | pdb | 1SRU\_A | 1.0 | 4.559e-12 | 543 | 0.584 | 113 | 33 | 4 | 1 | 112 | 1 | 100 | Single-strand binding protein | Single-strand binding protein | | pdb | 1SRU\_B | 1.0 | 3.13e-12 | 541 | 0.553 | 112 | 35 | 3 | 1 | 112 | 1 | 97 | Single-strand binding protein | Single-strand binding protein | | pdb | 3ULP\_C | 1.0 | 2.967e-12 | 539 | 0.4 | 115 | 67 | 2 | 1 | 113 | 2 | 116 | Single-strand binding protein | Single-strand binding protein | | pdb | 3ULP\_A | 1.0 | 3.13e-12 | 534 | 0.4 | 115 | 65 | 3 | 1 | 113 | 2 | 114 | Single-strand binding protein | Single-strand binding protein | | pdb | 6JDG\_B | 1.0 | 7.006e-12 | 532 | 0.554 | 110 | 36 | 3 | 4 | 113 | 2 | 98 | Single-stranded DNA-binding protein | Single-stranded DNA-binding protein | | pdb | 1SRU\_C | 1.0 | 9.67e-12 | 529 | 0.553 | 112 | 34 | 3 | 1 | 112 | 1 | 96 | Single-strand binding protein | Single-strand binding protein | | pdb | 7VUM\_C | 1.0 | 1.408e-11 | 524 | 0.545 | 110 | 36 | 3 | 4 | 113 | 1 | 96 | Single-stranded DNA-binding protein | Single-stranded DNA-binding protein | | pdb | 5ODN\_C | 1.0 | 1.265e-11 | 524 | 0.446 | 112 | 50 | 2 | 1 | 112 | 3 | 102 | Single-stranded DNA-binding protein | Single-stranded DNA-binding protein | | pdb | 7VUM\_B | 1.0 | 1.944e-11 | 522 | 0.559 | 109 | 34 | 2 | 4 | 112 | 1 | 95 | Single-stranded DNA-binding protein | Single-stranded DNA-binding protein | | pdb | 5ODN\_H | 1.0 | 1.265e-11 | 512 | 0.428 | 112 | 50 | 3 | 1 | 112 | 2 | 99 | Single-stranded DNA-binding protein | Single-stranded DNA-binding protein | | pdb | 5ODN\_F | 1.0 | 6.292e-12 | 504 | 0.442 | 113 | 55 | 3 | 1 | 113 | 1 | 105 | Single-stranded DNA-binding protein | Single-stranded DNA-binding protein | | pdb | 1KAW\_A | 1.0 | 9.164e-12 | 503 | 0.55 | 109 | 35 | 4 | 4 | 112 | 2 | 96 | SINGLE-STRANDED DNA BINDING PROTEIN | SINGLE-STRANDED DNA BINDING PROTEIN | | pdb | 5ODN\_G | 1.0 | 1.335e-11 | 503 | 0.446 | 112 | 52 | 4 | 1 | 112 | 1 | 102 | Single-stranded DNA-binding protein | Single-stranded DNA-binding protein | | pdb | 5ODN\_E | 1.0 | 4.095e-12 | 503 | 0.472 | 110 | 52 | 3 | 1 | 110 | 1 | 104 | Single-stranded DNA-binding protein | Single-stranded DNA-binding protein | | pdb | 3ULP\_D | 1.0 | 1.568e-11 | 503 | 0.391 | 115 | 65 | 4 | 1 | 113 | 2 | 113 | Single-strand binding protein | Single-strand binding protein | | pdb | 3ULP\_B | 1.0 | 1.408e-11 | 502 | 0.4 | 115 | 65 | 4 | 1 | 113 | 2 | 114 | Single-strand binding protein | Single-strand binding protein | | pdb | 5ODN\_D | 1.0 | 9.67e-12 | 498 | 0.437 | 112 | 50 | 3 | 1 | 112 | 8 | 106 | Single-stranded DNA-binding protein | Single-stranded DNA-binding protein | | pdb | 5ODN\_B | 1.0 | 4.321e-12 | 497 | 0.463 | 110 | 54 | 3 | 1 | 110 | 1 | 105 | Single-stranded DNA-binding protein | Single-stranded DNA-binding protein | | pdb | 2VW9\_B | 1.0 | 2.41e-11 | 476 | 0.373 | 107 | 65 | 2 | 4 | 110 | 1 | 105 | SINGLE-STRANDED DNA BINDING PROTEIN | SINGLE-STRANDED DNA BINDING PROTEIN | | pdb | 5ODP\_A | 1.0 | 4.623e-10 | 450 | 0.405 | 111 | 52 | 3 | 4 | 114 | 2 | 98 | Single-stranded DNA-binding protein | Single-stranded DNA-binding protein | | pdb | 7F5Y\_B | 1.0 | 2.164e-11 | 446 | 0.313 | 118 | 78 | 3 | 1 | 117 | 1 | 116 | Single-stranded DNA-binding protein | Single-stranded DNA-binding protein | | pdb | 7YM1\_B | 1.0 | 1.274e-10 | 436 | 0.336 | 107 | 65 | 3 | 4 | 110 | 1 | 101 | Single-stranded DNA-binding protein | Single-stranded DNA-binding protein | | pdb | 1X3E\_B | 1.0 | 1.274e-10 | 419 | 0.284 | 116 | 78 | 3 | 6 | 117 | 4 | 118 | Single-strand binding protein | Single-strand binding protein | | pdb | 5ODP\_G | 1.0 | 3.174e-10 | 415 | 0.401 | 107 | 50 | 4 | 2 | 108 | 1 | 93 | Single-stranded DNA-binding protein | Single-stranded DNA-binding protein | | pdb | 3LGJ\_B | 1.0 | 1.152e-09 | 409 | 0.482 | 114 | 40 | 5 | 2 | 113 | 1 | 97 | Single-stranded DNA-binding protein | Single-stranded DNA-binding protein | | pdb | 6BHW\_G | 1.0 | 8.346e-10 | 409 | 0.333 | 105 | 63 | 3 | 4 | 108 | 1 | 98 | Single-stranded DNA-binding protein A | Single-stranded DNA-binding protein A | | pdb | 6RUP\_A | 1.0 | 1.353e-09 | 408 | 0.35 | 114 | 69 | 3 | 1 | 113 | 2 | 111 | Single-stranded DNA-binding protein, mitochondrial | Single-stranded DNA-binding protein, mitochondrial | | pdb | 3LGJ\_A | 1.0 | 1.971e-09 | 405 | 0.46 | 115 | 40 | 5 | 2 | 114 | 1 | 95 | Single-stranded DNA-binding protein | Single-stranded DNA-binding protein | | pdb | 7F5Z\_B | 1.0 | 3.008e-10 | 401 | 0.305 | 118 | 77 | 4 | 1 | 117 | 1 | 114 | Single-stranded DNA-binding protein | Single-stranded DNA-binding protein | | pdb | 7D8J\_A | 1.0 | 6.047e-10 | 393 | 0.338 | 118 | 72 | 3 | 4 | 121 | 1 | 112 | Single-stranded DNA-binding protein | Single-stranded DNA-binding protein | | pdb | 1S3O\_A | 1.0 | 2.443e-09 | 391 | 0.333 | 114 | 68 | 3 | 1 | 113 | 1 | 107 | Single-stranded DNA-binding protein, mitochondrial | Single-stranded DNA-binding protein, mitochondrial | | pdb | 5YYU\_B | 1.0 | 2.194e-09 | 387 | 0.361 | 105 | 63 | 3 | 4 | 108 | 1 | 101 | Single-stranded DNA-binding protein | Single-stranded DNA-binding protein | | pdb | 7F5Z\_A | 1.0 | 7.909e-10 | 386 | 0.305 | 118 | 75 | 4 | 1 | 117 | 1 | 112 | Single-stranded DNA-binding protein | Single-stranded DNA-binding protein | | pdb | 6BHX\_C | 1.0 | 3.196e-09 | 385 | 0.3 | 110 | 67 | 2 | 1 | 110 | 1 | 100 | Single-stranded DNA-binding protein A | Single-stranded DNA-binding protein A | | pdb | 3A5U\_A | 1.0 | 5.147e-10 | 383 | 0.305 | 118 | 78 | 4 | 1 | 117 | 1 | 115 | Single-stranded DNA-binding protein | Single-stranded DNA-binding protein | | pdb | 7DEP\_A | 1.0 | 6.089e-09 | 376 | 0.33 | 109 | 63 | 3 | 4 | 112 | 1 | 99 | Single-stranded DNA-binding protein | Single-stranded DNA-binding protein | | pdb | 6BHW\_E | 1.0 | 1.042e-08 | 374 | 0.308 | 107 | 65 | 2 | 4 | 110 | 2 | 99 | Single-stranded DNA-binding protein A | Single-stranded DNA-binding protein A | | pdb | 1SE8\_A | 1.0 | 8.867e-09 | 373 | 0.336 | 110 | 62 | 5 | 5 | 114 | 115 | 213 | Single-strand binding protein | Single-strand binding protein | | pdb | 6RUP\_B | 1.0 | 9.873e-09 | 371 | 0.333 | 114 | 63 | 3 | 1 | 113 | 8 | 109 | Single-stranded DNA-binding protein, mitochondrial | Single-stranded DNA-binding protein, mitochondrial | | pdb | 1X3F\_B | 1.0 | 3.558e-09 | 370 | 0.282 | 117 | 72 | 4 | 6 | 118 | 4 | 112 | Single-strand binding protein | Single-strand binding protein | | pdb | 2IHF\_A | 1.0 | 6.425e-09 | 370 | 0.286 | 115 | 77 | 3 | 1 | 115 | 1 | 110 | Single-stranded DNA-binding protein | Single-stranded DNA-binding protein | | pdb | 6BHX\_D | 1.0 | 1.042e-08 | 369 | 0.299 | 107 | 64 | 3 | 4 | 110 | 1 | 96 | Single-stranded DNA-binding protein A | Single-stranded DNA-binding protein A | | pdb | 6BHW\_B | 1.0 | 6.425e-09 | 369 | 0.327 | 107 | 63 | 3 | 4 | 110 | 2 | 99 | Single-stranded DNA-binding protein A | Single-stranded DNA-binding protein A | | pdb | 3VDY\_A | 1.0 | 6.089e-09 | 368 | 0.289 | 107 | 68 | 4 | 4 | 110 | 2 | 100 | Single-stranded DNA-binding protein ssbB | Single-stranded DNA-binding protein ssbB | | pdb | 6BHW\_F | 1.0 | 9.873e-09 | 365 | 0.295 | 105 | 63 | 3 | 4 | 108 | 2 | 95 | Single-stranded DNA-binding protein A | Single-stranded DNA-binding protein A | | pdb | 6BHW\_C | 1.0 | 1.099e-08 | 363 | 0.29 | 110 | 68 | 3 | 1 | 110 | 1 | 100 | Single-stranded DNA-binding protein A | Single-stranded DNA-binding protein A | | pdb | 5XGT\_A | 1.0 | 2.871e-09 | 362 | 0.342 | 105 | 65 | 3 | 4 | 108 | 1 | 101 | Single-stranded DNA-binding protein | Single-stranded DNA-binding protein | | pdb | 1X3G\_A | 1.0 | 2.871e-09 | 361 | 0.26 | 115 | 76 | 4 | 7 | 118 | 4 | 112 | Single-strand binding protein | Single-strand binding protein | | pdb | 3VDY\_B | 1.0 | 3.218e-08 | 360 | 0.27 | 111 | 67 | 5 | 4 | 114 | 1 | 97 | Single-stranded DNA-binding protein ssbB | Single-stranded DNA-binding protein ssbB | | pdb | 7DEP\_B | 1.0 | 2.871e-09 | 360 | 0.361 | 105 | 59 | 5 | 4 | 108 | 1 | 97 | Single-stranded DNA-binding protein | Single-stranded DNA-binding protein | | pdb | 3ULL\_A | 1.0 | 1.224e-08 | 360 | 0.289 | 114 | 72 | 4 | 1 | 113 | 1 | 106 | DNA BINDING PROTEIN | DNA BINDING PROTEIN | | pdb | 3AFQ\_B | 1.0 | 7.964e-09 | 358 | 0.247 | 121 | 76 | 5 | 3 | 117 | 1 | 112 | Single-stranded DNA-binding protein | Single-stranded DNA-binding protein | | pdb | 1X3G\_B | 1.0 | 9.357e-09 | 357 | 0.271 | 118 | 74 | 4 | 6 | 117 | 3 | 114 | Single-strand binding protein | Single-strand binding protein | | pdb | 3EIV\_C | 1.0 | 4.655e-09 | 355 | 0.277 | 108 | 67 | 4 | 2 | 108 | 1 | 98 | Single-stranded DNA-binding protein 2 | Single-stranded DNA-binding protein 2 | | pdb | 6BHW\_A | 1.0 | 2.21e-08 | 353 | 0.277 | 108 | 66 | 3 | 1 | 108 | 1 | 96 | Single-stranded DNA-binding protein A | Single-stranded DNA-binding protein A | | pdb | 1UE5\_B | 1.0 | 6.089e-09 | 353 | 0.271 | 118 | 68 | 6 | 6 | 117 | 3 | 108 | Single-strand binding protein | Single-strand binding protein | | pdb | 5YYU\_C | 1.0 | 7.153e-09 | 352 | 0.323 | 105 | 63 | 4 | 4 | 108 | 1 | 97 | Single-stranded DNA-binding protein | Single-stranded DNA-binding protein | | pdb | 3EIV\_B | 1.0 | 3.558e-09 | 352 | 0.282 | 117 | 74 | 4 | 1 | 117 | 1 | 107 | Single-stranded DNA-binding protein 2 | Single-stranded DNA-binding protein 2 | | pdb | 7D8J\_B | 1.0 | 8.867e-09 | 351 | 0.355 | 107 | 58 | 5 | 4 | 110 | 1 | 96 | Single-stranded DNA-binding protein | Single-stranded DNA-binding protein | | pdb | 1UE1\_B | 1.0 | 5.468e-09 | 342 | 0.277 | 119 | 77 | 6 | 4 | 117 | 1 | 115 | Single-strand binding protein | Single-strand binding protein | | pdb | 1UE1\_A | 1.0 | 4.411e-09 | 341 | 0.263 | 114 | 76 | 3 | 7 | 117 | 4 | 112 | Single-strand binding protein | Single-strand binding protein | | pdb | 6BHW\_D | 1.0 | 2.21e-08 | 338 | 0.299 | 107 | 61 | 4 | 4 | 110 | 2 | 94 | Single-stranded DNA-binding protein A | Single-stranded DNA-binding protein A | | pdb | 3A5U\_B | 1.0 | 8.867e-09 | 338 | 0.275 | 120 | 73 | 6 | 1 | 117 | 1 | 109 | Single-stranded DNA-binding protein | Single-stranded DNA-binding protein | | pdb | 6BHX\_B | 1.0 | 4.21e-08 | 336 | 0.29 | 110 | 66 | 4 | 1 | 110 | 1 | 98 | Single-stranded DNA-binding protein A | Single-stranded DNA-binding protein A | | pdb | 2CWA\_A | 1.0 | 8.929e-08 | 335 | 0.33 | 112 | 61 | 4 | 3 | 114 | 109 | 206 | Single-strand binding protein | Single-strand binding protein | | pdb | 2DUD\_B | 1.0 | 9.422e-08 | 331 | 0.281 | 110 | 59 | 6 | 4 | 112 | 2 | 92 | Single-stranded DNA-binding protein | Single-stranded DNA-binding protein | | pdb | 3AFP\_B | 1.0 | 2.094e-08 | 329 | 0.27 | 122 | 75 | 7 | 2 | 117 | 1 | 114 | Single-stranded DNA-binding protein | Single-stranded DNA-binding protein | | pdb | 7YM1\_A | 1.0 | 2.739e-08 | 324 | 0.323 | 105 | 62 | 3 | 4 | 108 | 1 | 96 | Single-stranded DNA-binding protein | Single-stranded DNA-binding protein | | pdb | 7F5Y\_A | 1.0 | 1.042e-08 | 324 | 0.25 | 120 | 74 | 6 | 1 | 117 | 3 | 109 | Single-stranded DNA-binding protein | Single-stranded DNA-binding protein | | pdb | 3ULL\_B | 1.0 | 2.225e-07 | 322 | 0.286 | 115 | 69 | 4 | 1 | 114 | 1 | 103 | DNA BINDING PROTEIN | DNA BINDING PROTEIN | | pdb | 1UE5\_A | 1.0 | 8.404e-09 | 321 | 0.252 | 115 | 79 | 4 | 6 | 117 | 4 | 114 | Single-strand binding protein | Single-strand binding protein | | pdb | 3UDG\_B | 1.0 | 9.422e-08 | 319 | 0.336 | 110 | 58 | 5 | 5 | 114 | 120 | 214 | Single-stranded DNA-binding protein | Single-stranded DNA-binding protein | | pdb | 6BHW\_H | 1.0 | 1.612e-07 | 318 | 0.296 | 108 | 66 | 4 | 4 | 110 | 2 | 100 | Single-stranded DNA-binding protein A | Single-stranded DNA-binding protein A | | pdb | 2FXQ\_A | 1.0 | 2.89e-08 | 318 | 0.29 | 117 | 65 | 6 | 1 | 114 | 96 | 197 | Single-strand binding protein | Single-strand binding protein | | pdb | 6BHX\_A | 1.0 | 9.942e-08 | 316 | 0.29 | 110 | 64 | 4 | 1 | 110 | 1 | 96 | Single-stranded DNA-binding protein A | Single-stranded DNA-binding protein A | | pdb | 2DUD\_A | 1.0 | 1.998e-07 | 315 | 0.296 | 108 | 61 | 5 | 4 | 110 | 2 | 95 | Single-stranded DNA-binding protein | Single-stranded DNA-binding protein | | pdb | 1X3E\_A | 1.0 | 1.689e-08 | 314 | 0.234 | 115 | 77 | 5 | 6 | 117 | 3 | 109 | Single-strand binding protein | Single-strand binding protein | | pdb | 3AFP\_A | 1.0 | 1.881e-08 | 311 | 0.252 | 111 | 74 | 4 | 7 | 117 | 4 | 105 | Single-stranded DNA-binding protein | Single-stranded DNA-binding protein | | pdb | 5YYU\_D | 1.0 | 4.473e-07 | 308 | 0.309 | 110 | 54 | 5 | 4 | 113 | 1 | 88 | Single-stranded DNA-binding protein | Single-stranded DNA-binding protein | | pdb | 3UDG\_A | 1.0 | 3.99e-08 | 306 | 0.336 | 107 | 64 | 4 | 5 | 110 | 3 | 103 | Single-stranded DNA-binding protein | Single-stranded DNA-binding protein | | pdb | 3UDG\_C | 1.0 | 6.826e-08 | 306 | 0.308 | 107 | 67 | 3 | 5 | 110 | 3 | 103 | Single-stranded DNA-binding protein | Single-stranded DNA-binding protein | | pdb | 3AFQ\_C | 1.0 | 1.049e-07 | 302 | 0.213 | 117 | 71 | 6 | 6 | 117 | 4 | 104 | Single-stranded DNA-binding protein | Single-stranded DNA-binding protein | | pdb | 1UE6\_C | 1.0 | 1.448e-07 | 302 | 0.267 | 112 | 69 | 3 | 6 | 117 | 3 | 101 | Single-strand binding protein | Single-strand binding protein | | pdb | 2IHE\_A | 1.0 | 5.81e-08 | 295 | 0.299 | 107 | 70 | 3 | 5 | 110 | 4 | 106 | Single-stranded DNA-binding protein | Single-stranded DNA-binding protein | | pdb | 4DNI\_A | 1.0 | 8.521e-07 | 291 | 0.208 | 120 | 87 | 6 | 1 | 114 | 139 | 256 | Fusion protein of RNA-editing complex proteins MP42 and MP18 | Fusion protein of RNA-editing complex proteins MP42 and MP18 | | pdb | 1UE6\_D | 1.0 | 5.545e-07 | 286 | 0.23 | 117 | 70 | 5 | 6 | 117 | 3 | 104 | Single-strand binding protein | Single-strand binding protein | | pdb | 1X3F\_A | 1.0 | 1.049e-07 | 286 | 0.263 | 114 | 70 | 4 | 7 | 117 | 4 | 106 | Single-strand binding protein | Single-strand binding protein | | pdb | 3AFQ\_D | 1.0 | 2.758e-07 | 280 | 0.217 | 115 | 75 | 6 | 6 | 117 | 4 | 106 | Single-stranded DNA-binding protein | Single-stranded DNA-binding protein | | pdb | 3AFQ\_A | 1.0 | 3.807e-07 | 276 | 0.228 | 118 | 74 | 5 | 3 | 117 | 1 | 104 | Single-stranded DNA-binding protein | Single-stranded DNA-binding protein | | pdb | 1WOC\_D | 1.0 | 3.608e-07 | 272 | 0.134 | 104 | 83 | 4 | 5 | 108 | 1 | 97 | Primosomal replication protein n | Primosomal replication protein n | | pdb | 4APV\_A | 1.0 | 3.608e-07 | 271 | 0.16 | 106 | 79 | 4 | 5 | 110 | 1 | 96 | PRIMOSOMAL REPLICATION PROTEIN N | PRIMOSOMAL REPLICATION PROTEIN N | | pdb | 3K80\_D | 1.0 | 2.364e-06 | 270 | 0.203 | 113 | 74 | 7 | 4 | 114 | 2 | 100 | MP18 RNA editing complex protein | MP18 RNA editing complex protein | | pdb | 3K81\_D | 1.0 | 1.907e-06 | 269 | 0.203 | 113 | 80 | 7 | 4 | 113 | 1 | 106 | MP18 RNA editing complex protein | MP18 RNA editing complex protein | | pdb | 3EIV\_A | 1.0 | 7.653e-07 | 268 | 0.23 | 113 | 68 | 5 | 5 | 117 | 1 | 94 | Single-stranded DNA-binding protein 2 | Single-stranded DNA-binding protein 2 | | pdb | 3K80\_C | 1.0 | 2.123e-06 | 266 | 0.203 | 113 | 77 | 7 | 4 | 114 | 1 | 102 | MP18 RNA editing complex protein | MP18 RNA editing complex protein | | pdb | 1UE6\_B | 1.0 | 4.239e-07 | 265 | 0.226 | 115 | 69 | 6 | 6 | 117 | 3 | 100 | Single-strand binding protein | Single-strand binding protein | | pdb | 1UE7\_C | 1.0 | 1.623e-06 | 263 | 0.25 | 120 | 64 | 6 | 2 | 117 | 1 | 98 | Single-strand binding protein | Single-strand binding protein | | pdb | 1UE6\_A | 1.0 | 3.071e-07 | 263 | 0.243 | 115 | 72 | 5 | 6 | 117 | 4 | 106 | Single-strand binding protein | Single-strand binding protein | | pdb | 2PNH\_A | 1.0 | 8.521e-07 | 262 | 0.121 | 107 | 83 | 4 | 4 | 110 | 1 | 96 | Primosomal replication protein n | Primosomal replication protein n | | pdb | 1UE7\_D | 1.0 | 2.364e-06 | 258 | 0.226 | 115 | 61 | 6 | 7 | 116 | 4 | 95 | Single-strand binding protein | Single-strand binding protein | | pdb | 4DAM\_B | 1.0 | 6.874e-07 | 258 | 0.232 | 116 | 76 | 5 | 7 | 121 | 5 | 108 | Single-stranded DNA-binding protein 1 | Single-stranded DNA-binding protein 1 | | pdb | 1Z9F\_A | 1.0 | 3.834e-06 | 256 | 0.3 | 110 | 55 | 6 | 1 | 110 | 1 | 88 | Single-strand binding protein | Single-strand binding protein | | pdb | 1UE7\_B | 1.0 | 2.241e-06 | 256 | 0.232 | 116 | 59 | 6 | 7 | 117 | 4 | 94 | Single-strand binding protein | Single-strand binding protein | | pdb | 5WQV\_A | 1.0 | 3.263e-06 | 255 | 0.111 | 108 | 82 | 4 | 5 | 112 | 1 | 94 | Primosomal replication protein N | Primosomal replication protein N | | pdb | 1TXY\_A | 1.0 | 2.012e-06 | 249 | 0.121 | 107 | 79 | 4 | 4 | 110 | 1 | 92 | Primosomal replication protein n | Primosomal replication protein n | | pdb | 2CCZ\_B | 1.0 | 1.115e-06 | 246 | 0.128 | 101 | 84 | 2 | 1 | 101 | 2 | 98 | PRIMOSOMAL REPLICATION PROTEIN N | PRIMOSOMAL REPLICATION PROTEIN N | | pdb | 2PNH\_B | 1.0 | 1.807e-06 | 243 | 0.121 | 107 | 81 | 4 | 4 | 110 | 1 | 94 | Primosomal replication protein n | Primosomal replication protein n | | pdb | 3K81\_C | 1.0 | 9.054e-06 | 241 | 0.201 | 114 | 78 | 7 | 4 | 114 | 2 | 105 | MP18 RNA editing complex protein | MP18 RNA editing complex protein | | pdb | 3STB\_D | 1.0 | 9.054e-06 | 240 | 0.21 | 114 | 80 | 8 | 4 | 114 | 2 | 108 | MP18 RNA editing complex protein | MP18 RNA editing complex protein | | pdb | 3KLW\_A | 1.0 | 1.391e-05 | 239 | 0.136 | 110 | 83 | 5 | 5 | 114 | 1 | 98 | Primosomal replication protein n | Primosomal replication protein n | | pdb | 3STB\_C | 1.0 | 1.008e-05 | 236 | 0.173 | 115 | 86 | 7 | 1 | 112 | 3 | 111 | RNA-editing complex protein MP42 | RNA-editing complex protein MP42 | | pdb | 3EIV\_D | 1.0 | 2.123e-06 | 232 | 0.216 | 111 | 68 | 6 | 7 | 117 | 5 | 96 | Single-stranded DNA-binding protein 2 | Single-stranded DNA-binding protein 2 | | pdb | 1V1Q\_A | 1.0 | 1.807e-06 | 232 | 0.127 | 102 | 81 | 4 | 2 | 102 | 3 | 97 | PRIMOSOMAL REPLICATION PROTEIN N | PRIMOSOMAL REPLICATION PROTEIN N | | pdb | 1UE7\_A | 1.0 | 5.583e-06 | 231 | 0.226 | 115 | 70 | 5 | 6 | 117 | 3 | 101 | Single-strand binding protein | Single-strand binding protein | | pdb | 6CQM\_D | 1.0 | 6.921e-06 | 229 | 0.207 | 111 | 74 | 5 | 4 | 113 | 2 | 99 | Single-stranded DNA-binding protein RIM1, mitochondrial | Single-stranded DNA-binding protein RIM1, mitochondrial | | pdb | 4DAM\_H | 1.0 | 5.255e-07 | 229 | 0.232 | 116 | 76 | 5 | 7 | 121 | 5 | 108 | Single-stranded DNA-binding protein 1 | Single-stranded DNA-binding protein 1 | | pdb | 3K8A\_A | 1.0 | 7.303e-06 | 228 | 0.151 | 112 | 82 | 5 | 3 | 114 | 2 | 100 | Putative primosomal replication protein | Putative primosomal replication protein | | pdb | 3KOJ\_A | 1.0 | 4.535e-05 | 221 | 0.163 | 110 | 68 | 6 | 1 | 109 | 1 | 87 | uncharacterized protein ycf41 | uncharacterized protein ycf41 | | pdb | 6CQO\_D | 1.0 | 1.391e-05 | 213 | 0.207 | 111 | 74 | 6 | 4 | 113 | 2 | 99 | Single-stranded DNA-binding protein RIM1, mitochondrial | Single-stranded DNA-binding protein RIM1, mitochondrial | | pdb | 5WQV\_B | 1.0 | 1.008e-05 | 211 | 0.122 | 98 | 73 | 3 | 5 | 102 | 1 | 85 | Primosomal replication protein N | Primosomal replication protein N | | pdb | 6CQM\_F | 1.0 | 7.706e-06 | 209 | 0.216 | 111 | 70 | 6 | 4 | 113 | 2 | 96 | Single-stranded DNA-binding protein RIM1, mitochondrial | Single-stranded DNA-binding protein RIM1, mitochondrial | | pdb | 3K7U\_C | 1.0 | 7.354e-05 | 207 | 0.191 | 115 | 74 | 7 | 4 | 116 | 1 | 98 | MP18 RNA editing complex protein | MP18 RNA editing complex protein | | pdb | 3EN2\_A | 1.0 | 6.97e-05 | 206 | 0.119 | 109 | 78 | 6 | 4 | 112 | 1 | 91 | Probable primosomal replication protein n | Probable primosomal replication protein n | | pdb | 3KOJ\_B | 1.0 | 8.188e-05 | 205 | 0.163 | 110 | 69 | 6 | 1 | 109 | 1 | 88 | uncharacterized protein ycf41 | uncharacterized protein ycf41 | | pdb | 4DAM\_E | 1.0 | 2.495e-06 | 205 | 0.243 | 123 | 78 | 6 | 4 | 122 | 1 | 112 | Single-stranded DNA-binding protein 1 | Single-stranded DNA-binding protein 1 | | pdb | 6CQM\_A | 1.0 | 1.635e-05 | 204 | 0.214 | 112 | 70 | 6 | 4 | 114 | 2 | 96 | Single-stranded DNA-binding protein RIM1, mitochondrial | Single-stranded DNA-binding protein RIM1, mitochondrial | | pdb | 6CQO\_F | 1.0 | 1.184e-05 | 203 | 0.216 | 111 | 69 | 7 | 4 | 113 | 2 | 95 | Single-stranded DNA-binding protein RIM1, mitochondrial | Single-stranded DNA-binding protein RIM1, mitochondrial | | pdb | 4FDB\_A | 1.0 | 6.26e-05 | 203 | 0.146 | 109 | 76 | 7 | 4 | 111 | 1 | 93 | Probable primosomal replication protein n | Probable primosomal replication protein n | | pdb | 8FAK\_A | 1.0 | 3.114e-05 | 196 | 0.12 | 108 | 84 | 4 | 5 | 112 | 1 | 97 | Primosomal replication protein N | Primosomal replication protein N | | pdb | 3FHW\_B | 1.0 | 3.86e-05 | 189 | 0.169 | 112 | 81 | 6 | 5 | 116 | 1 | 100 | Primosomal replication protein n | Primosomal replication protein n | | pdb | 6CQM\_B | 1.0 | 2.951e-05 | 188 | 0.196 | 112 | 69 | 6 | 4 | 114 | 1 | 92 | Single-stranded DNA-binding protein RIM1, mitochondrial | Single-stranded DNA-binding protein RIM1, mitochondrial | | pdb | 4DAM\_L | 1.0 | 2.651e-05 | 183 | 0.237 | 97 | 59 | 6 | 7 | 101 | 5 | 88 | Single-stranded DNA-binding protein 1 | Single-stranded DNA-binding protein 1 | | pdb | 3KLW\_B | 1.0 | 8.64e-05 | 182 | 0.16 | 106 | 73 | 7 | 5 | 110 | 1 | 90 | Primosomal replication protein n | Primosomal replication protein n | | pdb | 6CQM\_C | 1.0 | 9.117e-05 | 182 | 0.205 | 112 | 72 | 7 | 4 | 114 | 1 | 96 | Single-stranded DNA-binding protein RIM1, mitochondrial | Single-stranded DNA-binding protein RIM1, mitochondrial | | pdb | 4DAM\_J | 1.0 | 1.549e-05 | 181 | 0.228 | 118 | 74 | 6 | 7 | 122 | 5 | 107 | Single-stranded DNA-binding protein 1 | Single-stranded DNA-binding protein 1 | | pdb | 6CQO\_H | 1.0 | 9.117e-05 | 176 | 0.196 | 112 | 66 | 7 | 4 | 113 | 2 | 91 | Single-stranded DNA-binding protein RIM1, mitochondrial | Single-stranded DNA-binding protein RIM1, mitochondrial | | pdb | 6CQK\_A | 1.0 | 0.0001478 | 175 | 0.2 | 110 | 71 | 5 | 4 | 113 | 1 | 93 | SsDNA-binding protein essential for mitochondrial genome maintenance | SsDNA-binding protein essential for mitochondrial genome maintenance | | pdb | 1TXY\_B | 1.0 | 8.64e-05 | 174 | 0.135 | 81 | 62 | 3 | 4 | 84 | 1 | 73 | Primosomal replication protein n | Primosomal replication protein n | | pdb | 6CQK\_B | 1.0 | 0.0001478 | 166 | 0.214 | 112 | 67 | 7 | 4 | 113 | 1 | 93 | SsDNA-binding protein essential for mitochondrial genome maintenance | SsDNA-binding protein essential for mitochondrial genome maintenance | | pdb | 6CQO\_E | 1.0 | 0.0001071 | 166 | 0.203 | 113 | 68 | 7 | 4 | 114 | 2 | 94 | Single-stranded DNA-binding protein RIM1, mitochondrial | Single-stranded DNA-binding protein RIM1, mitochondrial | | pdb | 4DAM\_C | 1.0 | 5.622e-05 | 166 | 0.264 | 102 | 56 | 6 | 4 | 101 | 1 | 87 | Single-stranded DNA-binding protein 1 | Single-stranded DNA-binding protein 1 | | pdb | 5GQO\_B | 1.0 | 0.0001478 | 165 | 0.171 | 99 | 65 | 5 | 6 | 101 | 3 | 87 | Single-stranded DNA-binding protein | Single-stranded DNA-binding protein | | pdb | 3F2B\_A | 1.0 | 0.0002397 | 161 | 0.189 | 116 | 63 | 9 | 6 | 117 | 12 | 100 | DNA-directed DNA polymerase III alpha chain | DNA-directed DNA polymerase III alpha chain | | pdb | 4GS3\_A | 1.0 | 0.0004328 | 156 | 0.171 | 105 | 68 | 6 | 6 | 110 | 3 | 88 | Single-stranded DNA-binding protein | Single-stranded DNA-binding protein | | pdb | 4DAM\_G | 1.0 | 5.05e-05 | 155 | 0.267 | 101 | 56 | 6 | 4 | 101 | 1 | 86 | Single-stranded DNA-binding protein 1 | Single-stranded DNA-binding protein 1 | | pdb | 3F2D\_A | 1.0 | 0.0004567 | 151 | 0.198 | 116 | 62 | 10 | 6 | 117 | 12 | 100 | GEOBACILLUS KAUSTOPHILUS DNA POLC | GEOBACILLUS KAUSTOPHILUS DNA POLC | | pdb | 2HQL\_E | 1.0 | 5.932e-05 | 147 | 0.134 | 119 | 75 | 10 | 4 | 117 | 1 | 96 | Hypothetical protein MG376 homolog | Hypothetical protein MG376 homolog | | pdb | 5GQO\_A | 1.0 | 0.002288 | 141 | 0.158 | 120 | 72 | 5 | 5 | 124 | 1 | 91 | Single-stranded DNA-binding protein | Single-stranded DNA-binding protein | | pdb | 4DK3\_C | 1.0 | 0.006347 | 139 | 0.15 | 106 | 66 | 5 | 7 | 112 | 2 | 83 | RNA-editing complex protein MP81 | RNA-editing complex protein MP81 | | pdb | 2HQL\_C | 1.0 | 0.0002972 | 129 | 0.161 | 118 | 67 | 8 | 4 | 117 | 1 | 90 | Hypothetical protein MG376 homolog | Hypothetical protein MG376 homolog | | pdb | 2HQL\_F | 1.0 | 0.001845 | 122 | 0.144 | 118 | 67 | 10 | 4 | 117 | 1 | 88 | Hypothetical protein MG376 homolog | Hypothetical protein MG376 homolog | | pdb | 8C5Z\_A | 1.0 | 0.001489 | 119 | 0.166 | 108 | 65 | 6 | 8 | 111 | 73 | 159 | Replication factor A | Replication factor A | | pdb | 8OEL\_A | 1.0 | 0.006697 | 113 | 0.155 | 109 | 65 | 7 | 8 | 111 | 17 | 103 | Replication factor A | Replication factor A | | pdb | 8AAJ\_A | 1.0 | 0.006347 | 112 | 0.155 | 109 | 65 | 7 | 8 | 111 | 77 | 163 | Replication factor A | Replication factor A | | pdb | 8OEJ\_D | 1.0 | 0.006697 | 109 | 0.146 | 116 | 71 | 8 | 8 | 117 | 17 | 110 | Replication factor A | Replication factor A | | pdb | 4YWK\_B | 0.999 | 0.002688 | 98 | 0.117 | 102 | 71 | 5 | 1 | 101 | 106 | 189 | Cell division control protein 21 | Cell division control protein 21 | | pdb | 6RAX\_4 | 0.984 | 0.00413 | 80 | 0.123 | 162 | 81 | 9 | 2 | 110 | 124 | 277 | DNA replication licensing factor MCM4 | DNA replication licensing factor MCM4 | | afdb-proteome | AF-A0A0H3GL04-F1-MODEL\_V4 | 1.0 | 8.933e-21 | 770 | 0.583 | 173 | 61 | 2 | 1 | 162 | 2 | 174 | Single-stranded DNA-binding protein | Single-stranded DNA-binding protein | | afdb-proteome | AF-P0AGE0-F1-MODEL\_V4 | 1.0 | 2.349e-20 | 757 | 0.584 | 178 | 58 | 3 | 1 | 162 | 1 | 178 | Single-stranded DNA-binding protein | Single-stranded DNA-binding protein | | afdb-proteome | AF-P0A2F6-F1-MODEL\_V4 | 1.0 | 3.421e-20 | 750 | 0.596 | 176 | 57 | 5 | 1 | 162 | 1 | 176 | Single-stranded DNA-binding protein 1 | Single-stranded DNA-binding protein 1 | | afdb-proteome | AF-Q327X2-F1-MODEL\_V4 | 1.0 | 2.478e-20 | 739 | 0.587 | 177 | 58 | 3 | 1 | 162 | 2 | 178 | Single-stranded DNA-binding protein | Single-stranded DNA-binding protein | | afdb-proteome | AF-A0A0H3GZP4-F1-MODEL\_V4 | 1.0 | 9.058e-19 | 723 | 0.491 | 171 | 77 | 2 | 1 | 161 | 2 | 172 | Single-stranded DNA-binding protein | Single-stranded DNA-binding protein | | afdb-proteome | AF-P40947-F1-MODEL\_V4 | 1.0 | 5.017e-19 | 701 | 0.535 | 168 | 69 | 4 | 1 | 162 | 1 | 165 | Single-stranded DNA-binding protein | Single-stranded DNA-binding protein | | afdb-proteome | AF-Q93GP7-F1-MODEL\_V4 | 1.0 | 1.185e-18 | 695 | 0.508 | 177 | 66 | 4 | 1 | 162 | 2 | 172 | Single-stranded DNA-binding protein 2 | Single-stranded DNA-binding protein 2 | | afdb-proteome | AF-P44409-F1-MODEL\_V4 | 1.0 | 9.624e-18 | 628 | 0.537 | 173 | 64 | 7 | 1 | 162 | 1 | 168 | Single-stranded DNA-binding protein | Single-stranded DNA-binding protein | | afdb-proteome | AF-Q5F7Y0-F1-MODEL\_V4 | 1.0 | 1.03e-15 | 569 | 0.406 | 177 | 83 | 4 | 4 | 162 | 2 | 174 | Single-stranded DNA-binding protein | Single-stranded DNA-binding protein | | afdb-proteome | AF-O25841-F1-MODEL\_V4 | 1.0 | 5.823e-13 | 513 | 0.309 | 152 | 101 | 2 | 4 | 155 | 1 | 148 | Single-stranded DNA-binding protein | Single-stranded DNA-binding protein | | afdb-proteome | AF-Q8I415-F1-MODEL\_V4 | 1.0 | 1.786e-13 | 485 | 0.317 | 173 | 104 | 5 | 4 | 162 | 81 | 253 | Single-stranded DNA-binding protein | Single-stranded DNA-binding protein | | afdb-proteome | AF-P34496-F1-MODEL\_V4 | 1.0 | 9.574e-11 | 462 | 0.336 | 116 | 71 | 4 | 2 | 115 | 51 | 162 | Single-stranded DNA-binding protein, mitochondrial | Single-stranded DNA-binding protein, mitochondrial | | afdb-proteome | AF-O69302-F1-MODEL\_V4 | 1.0 | 6.483e-13 | 460 | 0.308 | 159 | 98 | 4 | 4 | 162 | 1 | 147 | Single-stranded DNA-binding protein | Single-stranded DNA-binding protein | | afdb-proteome | AF-B8A5I7-F1-MODEL\_V4 | 1.0 | 1.066e-10 | 458 | 0.344 | 122 | 71 | 3 | 1 | 116 | 25 | 143 | Single-stranded DNA-binding protein 1 | Single-stranded DNA-binding protein 1 | | afdb-proteome | AF-Q04837-F1-MODEL\_V4 | 1.0 | 1.125e-10 | 451 | 0.333 | 120 | 71 | 3 | 4 | 117 | 29 | 145 | Single-stranded DNA-binding protein, mitochondrial | Single-stranded DNA-binding protein, mitochondrial | | afdb-proteome | AF-Q9CYR0-F1-MODEL\_V4 | 1.0 | 1.187e-10 | 444 | 0.333 | 123 | 74 | 3 | 1 | 117 | 26 | 146 | Single-stranded DNA-binding protein, mitochondrial | Single-stranded DNA-binding protein, mitochondrial | | afdb-proteome | AF-A0A0K0DUQ6-F1-MODEL\_V4 | 1.0 | 7.37e-10 | 442 | 0.23 | 117 | 86 | 2 | 2 | 118 | 65 | 177 | Uncharacterized protein | Uncharacterized protein | | afdb-proteome | AF-P54622-F1-MODEL\_V4 | 1.0 | 3.475e-10 | 440 | 0.296 | 118 | 77 | 4 | 1 | 117 | 34 | 146 | Single-stranded DNA-binding protein, mitochondrial | Single-stranded DNA-binding protein, mitochondrial | | afdb-proteome | AF-P28042-F1-MODEL\_V4 | 1.0 | 1.01e-10 | 440 | 0.325 | 129 | 77 | 4 | 1 | 122 | 26 | 151 | Single-stranded DNA-binding protein, mitochondrial | Single-stranded DNA-binding protein, mitochondrial | | afdb-proteome | AF-Q8DNH4-F1-MODEL\_V4 | 1.0 | 1.729e-10 | 437 | 0.33 | 112 | 68 | 5 | 4 | 115 | 1 | 105 | Single-stranded DNA-binding protein | Single-stranded DNA-binding protein | | afdb-proteome | AF-Q2G112-F1-MODEL\_V4 | 1.0 | 3.248e-12 | 419 | 0.343 | 157 | 94 | 6 | 4 | 159 | 1 | 149 | Single-stranded DNA-binding protein | Single-stranded DNA-binding protein | | afdb-proteome | AF-A0A133CLV7-F1-MODEL\_V4 | 1.0 | 3.248e-12 | 414 | 0.306 | 160 | 97 | 7 | 4 | 159 | 1 | 150 | Single-stranded DNA-binding protein | Single-stranded DNA-binding protein | | afdb-proteome | AF-Q84J78-F1-MODEL\_V4 | 1.0 | 1.195e-09 | 410 | 0.266 | 120 | 80 | 5 | 4 | 117 | 70 | 187 | Single-stranded DNA-binding protein, mitochondrial | Single-stranded DNA-binding protein, mitochondrial | | afdb-proteome | AF-P9WGD5-F1-MODEL\_V4 | 1.0 | 2.483e-12 | 408 | 0.288 | 170 | 107 | 6 | 1 | 162 | 1 | 164 | Single-stranded DNA-binding protein | Single-stranded DNA-binding protein | | afdb-proteome | AF-X8FMG9-F1-MODEL\_V4 | 1.0 | 6.888e-12 | 408 | 0.254 | 169 | 117 | 4 | 1 | 162 | 1 | 167 | Single-stranded DNA-binding protein | Single-stranded DNA-binding protein | | afdb-proteome | AF-I1LB73-F1-MODEL\_V4 | 1.0 | 1.482e-09 | 402 | 0.273 | 117 | 77 | 5 | 3 | 113 | 85 | 199 | Uncharacterized protein | Uncharacterized protein | | afdb-proteome | AF-K0FER8-F1-MODEL\_V4 | 1.0 | 8.539e-12 | 398 | 0.294 | 163 | 107 | 5 | 1 | 159 | 1 | 159 | Single-stranded DNA-binding protein | Single-stranded DNA-binding protein | | afdb-proteome | AF-P46390-F1-MODEL\_V4 | 1.0 | 6.888e-12 | 396 | 0.264 | 170 | 115 | 5 | 1 | 162 | 1 | 168 | Single-stranded DNA-binding protein | Single-stranded DNA-binding protein | | afdb-proteome | AF-Q6L4W0-F1-MODEL\_V4 | 1.0 | 2.675e-09 | 388 | 0.237 | 122 | 87 | 4 | 4 | 120 | 75 | 195 | Os05g0509700 protein | Os05g0509700 protein | | afdb-proteome | AF-Q9LII1-F1-MODEL\_V4 | 1.0 | 1.837e-09 | 387 | 0.244 | 127 | 86 | 4 | 1 | 117 | 76 | 202 | Nucleic acid-binding, OB-fold-like protein | Nucleic acid-binding, OB-fold-like protein | | afdb-proteome | AF-P66855-F1-MODEL\_V4 | 1.0 | 6.574e-11 | 385 | 0.277 | 162 | 108 | 6 | 4 | 162 | 1 | 156 | Single-stranded DNA-binding protein | Single-stranded DNA-binding protein | | afdb-proteome | AF-K7N5B5-F1-MODEL\_V4 | 1.0 | 4.11e-09 | 381 | 0.246 | 130 | 90 | 4 | 4 | 129 | 85 | 210 | Uncharacterized protein | Uncharacterized protein | | afdb-proteome | AF-C4IYS0-F1-MODEL\_V4 | 1.0 | 3.896e-09 | 380 | 0.244 | 127 | 92 | 4 | 3 | 125 | 74 | 200 | Uncharacterized protein | Uncharacterized protein | | afdb-proteome | AF-K0F0D9-F1-MODEL\_V4 | 1.0 | 3.099e-11 | 379 | 0.236 | 165 | 114 | 6 | 4 | 162 | 2 | 160 | Single-stranded DNA-binding protein | Single-stranded DNA-binding protein | | afdb-proteome | AF-A0A132ZIN5-F1-MODEL\_V4 | 1.0 | 5.303e-11 | 370 | 0.272 | 169 | 109 | 4 | 4 | 162 | 1 | 165 | Single-stranded DNA-binding protein | Single-stranded DNA-binding protein | | afdb-proteome | AF-Q2FWF7-F1-MODEL\_V4 | 1.0 | 8.263e-09 | 369 | 0.286 | 115 | 74 | 5 | 4 | 118 | 1 | 107 | Single-stranded DNA-binding protein | Single-stranded DNA-binding protein | | afdb-proteome | AF-B4FRF4-F1-MODEL\_V4 | 1.0 | 5.096e-09 | 365 | 0.253 | 126 | 82 | 4 | 1 | 115 | 85 | 209 | Nucleic acid-binding OB-fold-like protein | Nucleic acid-binding OB-fold-like protein | | afdb-proteome | AF-Q5VP75-F1-MODEL\_V4 | 1.0 | 1.492e-08 | 359 | 0.28 | 125 | 80 | 3 | 1 | 115 | 85 | 209 | Os01g0642900 protein | Os01g0642900 protein | | afdb-proteome | AF-J9FJ05-F1-MODEL\_V4 | 1.0 | 3.164e-08 | 355 | 0.173 | 115 | 89 | 3 | 2 | 116 | 67 | 175 | Single-strand binding protein | Single-strand binding protein | | afdb-proteome | AF-I1K195-F1-MODEL\_V4 | 1.0 | 8.263e-09 | 353 | 0.244 | 135 | 92 | 4 | 1 | 125 | 65 | 199 | Uncharacterized protein | Uncharacterized protein | | afdb-proteome | AF-A0A0N4U7K8-F1-MODEL\_V4 | 1.0 | 9.64e-10 | 343 | 0.192 | 171 | 101 | 4 | 1 | 136 | 53 | 221 | Uncharacterized protein | Uncharacterized protein | | afdb-proteome | AF-A0A0K0JYI2-F1-MODEL\_V4 | 1.0 | 1.024e-08 | 336 | 0.187 | 139 | 102 | 4 | 2 | 136 | 67 | 198 | Bm9218 | Bm9218 | | afdb-proteome | AF-K0F5S9-F1-MODEL\_V4 | 1.0 | 9.64e-10 | 329 | 0.193 | 155 | 105 | 5 | 10 | 145 | 2 | 155 | Single-stranded DNA-binding protein | Single-stranded DNA-binding protein | | afdb-proteome | AF-A0A044UXX8-F1-MODEL\_V4 | 1.0 | 1.34e-08 | 321 | 0.137 | 153 | 121 | 4 | 2 | 154 | 67 | 208 | Uncharacterized protein | Uncharacterized protein | | afdb-proteome | AF-Q0JDX1-F1-MODEL\_V4 | 1.0 | 2.862e-07 | 317 | 0.271 | 114 | 73 | 6 | 8 | 114 | 1 | 111 | Os04g0363700 protein | Os04g0363700 protein | | afdb-proteome | AF-Q4CR03-F1-MODEL\_V4 | 1.0 | 7.885e-08 | 305 | 0.18 | 144 | 113 | 5 | 2 | 142 | 19 | 160 | RNA editing complex protein MP18, putative | RNA editing complex protein MP18, putative | | afdb-proteome | AF-Q75GK2-F1-MODEL\_V4 | 1.0 | 2.693e-08 | 298 | 0.229 | 157 | 103 | 4 | 1 | 157 | 74 | 212 | Os03g0633900 protein | Os03g0633900 protein | | afdb-proteome | AF-Q38B90-F1-MODEL\_V4 | 1.0 | 1.212e-07 | 297 | 0.195 | 148 | 113 | 6 | 2 | 145 | 19 | 164 | MP18 RNA editing complex protein | MP18 RNA editing complex protein | | afdb-proteome | AF-Q4CZ11-F1-MODEL\_V4 | 1.0 | 1.031e-07 | 296 | 0.164 | 146 | 113 | 7 | 2 | 141 | 19 | 161 | RNA editing complex protein MP18, putative | RNA editing complex protein MP18, putative | | afdb-proteome | AF-A4ICR4-F1-MODEL\_V4 | 1.0 | 8.779e-08 | 296 | 0.172 | 151 | 114 | 6 | 1 | 142 | 72 | 220 | MP18\_RNA\_editing\_complex\_protein\_-\_putative | MP18\_RNA\_editing\_complex\_protein\_-\_putative | | afdb-proteome | AF-A0A0R0I0B6-F1-MODEL\_V4 | 1.0 | 8.32e-08 | 295 | 0.213 | 145 | 96 | 4 | 1 | 145 | 122 | 248 | Uncharacterized protein | Uncharacterized protein | | afdb-proteome | AF-Q8ZK82-F1-MODEL\_V4 | 1.0 | 1.031e-07 | 290 | 0.131 | 114 | 89 | 5 | 4 | 117 | 1 | 104 | Primosomal replication protein N | Primosomal replication protein N | | afdb-proteome | AF-C0NDW9-F1-MODEL\_V4 | 1.0 | 5.414e-08 | 289 | 0.273 | 117 | 74 | 5 | 2 | 115 | 27 | 135 | Single-stranded DNA-binding protein | Single-stranded DNA-binding protein | | afdb-proteome | AF-I1JF51-F1-MODEL\_V4 | 1.0 | 1.031e-07 | 289 | 0.213 | 145 | 90 | 5 | 1 | 141 | 66 | 190 | Uncharacterized protein | Uncharacterized protein | | afdb-proteome | AF-Q328J8-F1-MODEL\_V4 | 1.0 | 1.031e-07 | 286 | 0.131 | 114 | 89 | 5 | 4 | 117 | 1 | 104 | Primosomal replication protein N | Primosomal replication protein N | | afdb-proteome | AF-A0A1C1D2M3-F1-MODEL\_V4 | 1.0 | 3.923e-08 | 284 | 0.225 | 124 | 83 | 6 | 1 | 120 | 27 | 141 | Single-stranded DNA-binding protein RIM1, mitochondrial | Single-stranded DNA-binding protein RIM1, mitochondrial | | afdb-proteome | AF-C4J9S9-F1-MODEL\_V4 | 1.0 | 7.473e-08 | 281 | 0.207 | 159 | 112 | 4 | 1 | 159 | 71 | 215 | Protein OSB2 chloroplastic | Protein OSB2 chloroplastic | | afdb-proteome | AF-U7PR10-F1-MODEL\_V4 | 1.0 | 1.502e-07 | 280 | 0.26 | 119 | 77 | 4 | 2 | 117 | 28 | 138 | Uncharacterized protein | Uncharacterized protein | | afdb-proteome | AF-A0A0D2GAT6-F1-MODEL\_V4 | 1.0 | 5.131e-08 | 279 | 0.216 | 125 | 85 | 6 | 1 | 121 | 27 | 142 | Unplaced genomic scaffold supercont1.7, whole genome shotgun sequence | Unplaced genomic scaffold supercont1.7, whole genome shotgun sequence | | afdb-proteome | AF-Q4DKA2-F1-MODEL\_V4 | 1.0 | 3.186e-07 | 278 | 0.181 | 138 | 89 | 6 | 4 | 120 | 13 | 147 | Uncharacterized protein | Uncharacterized protein | | afdb-proteome | AF-P07013-F1-MODEL\_V4 | 1.0 | 2.712e-07 | 276 | 0.131 | 114 | 89 | 5 | 4 | 117 | 1 | 104 | Primosomal replication protein N | Primosomal replication protein N | | afdb-proteome | AF-Q4D8F9-F1-MODEL\_V4 | 1.0 | 4.398e-07 | 270 | 0.169 | 136 | 89 | 6 | 6 | 120 | 38 | 170 | Uncharacterized protein | Uncharacterized protein | | afdb-proteome | AF-A0A175VYV7-F1-MODEL\_V4 | 1.0 | 8.779e-08 | 267 | 0.255 | 129 | 82 | 6 | 1 | 123 | 25 | 145 | Single-stranded DNA-binding protein | Single-stranded DNA-binding protein | | afdb-proteome | AF-K0ES78-F1-MODEL\_V4 | 1.0 | 4.863e-08 | 267 | 0.193 | 165 | 120 | 5 | 4 | 156 | 1 | 164 | Single-strand DNA binding protein (Modular protein) | Single-strand DNA binding protein (Modular protein) | | afdb-proteome | AF-O53205-F1-MODEL\_V4 | 1.0 | 8.32e-08 | 259 | 0.183 | 158 | 119 | 6 | 10 | 162 | 1 | 153 | Uncharacterized protein | Uncharacterized protein | | afdb-proteome | AF-Q57YG2-F1-MODEL\_V4 | 1.0 | 9.328e-07 | 252 | 0.185 | 140 | 83 | 7 | 4 | 115 | 13 | 149 | Uncharacterized protein Tb927.8.680, mitochondrial | Uncharacterized protein Tb927.8.680, mitochondrial | | afdb-proteome | AF-P44748-F1-MODEL\_V4 | 1.0 | 5.167e-07 | 251 | 0.157 | 114 | 85 | 4 | 1 | 110 | 1 | 107 | Primosomal replication protein N | Primosomal replication protein N | | afdb-proteome | AF-X8F2P4-F1-MODEL\_V4 | 1.0 | 2.436e-07 | 249 | 0.14 | 171 | 123 | 7 | 6 | 155 | 4 | 171 | Single-stranded DNA-binding protein | Single-stranded DNA-binding protein | | afdb-proteome | AF-A0A0K0JYI1-F1-MODEL\_V4 | 1.0 | 1.875e-06 | 249 | 0.181 | 127 | 90 | 5 | 2 | 127 | 55 | 168 | Bm9217 | Bm9217 | | afdb-proteome | AF-Q10GV8-F1-MODEL\_V4 | 1.0 | 6.805e-06 | 243 | 0.145 | 103 | 83 | 3 | 2 | 102 | 52 | 151 | Os03g0611700 protein | Os03g0611700 protein | | afdb-proteome | AF-A0A3Q0KRK1-F1-MODEL\_V4 | 1.0 | 9.328e-07 | 237 | 0.17 | 129 | 96 | 7 | 2 | 127 | 33 | 153 | Uncharacterized protein | Uncharacterized protein | | afdb-proteome | AF-Q5F924-F1-MODEL\_V4 | 1.0 | 7.181e-06 | 236 | 0.169 | 112 | 80 | 6 | 3 | 114 | 2 | 100 | Primosomal replication protein N | Primosomal replication protein N | | afdb-proteome | AF-P32445-F1-MODEL\_V4 | 1.0 | 3.572e-06 | 230 | 0.208 | 120 | 85 | 4 | 2 | 120 | 16 | 126 | Single-stranded DNA-binding protein RIM1, mitochondrial | Single-stranded DNA-binding protein RIM1, mitochondrial | | afdb-proteome | AF-A4ICD3-F1-MODEL\_V4 | 1.0 | 8.902e-06 | 229 | 0.184 | 114 | 78 | 6 | 2 | 113 | 526 | 626 | RNA editing comple protein MP63 | RNA editing comple protein MP63 | | afdb-proteome | AF-K7M5J7-F1-MODEL\_V4 | 1.0 | 4.197e-06 | 223 | 0.151 | 112 | 82 | 5 | 6 | 115 | 70 | 170 | Uncharacterized protein | Uncharacterized protein | | afdb-proteome | AF-Q38AE3-F1-MODEL\_V4 | 1.0 | 2.47e-05 | 223 | 0.166 | 114 | 80 | 7 | 2 | 113 | 487 | 587 | RNA editing complex protein MP63 | RNA editing complex protein MP63 | | afdb-proteome | AF-A0A3Q0KDA2-F1-MODEL\_V4 | 1.0 | 1.979e-06 | 221 | 0.103 | 135 | 101 | 4 | 1 | 117 | 49 | 181 | Uncharacterized protein | Uncharacterized protein | | afdb-proteome | AF-Q8GWJ4-F1-MODEL\_V4 | 1.0 | 1.359e-06 | 215 | 0.202 | 148 | 89 | 9 | 6 | 148 | 82 | 205 | Protein OSB3, chloroplastic/mitochondrial | Protein OSB3, chloroplastic/mitochondrial | | afdb-proteome | AF-A0A0R0GAG9-F1-MODEL\_V4 | 1.0 | 4.931e-06 | 212 | 0.168 | 113 | 83 | 4 | 6 | 116 | 76 | 179 | Uncharacterized protein | Uncharacterized protein | | afdb-proteome | AF-A0A1D6KXT6-F1-MODEL\_V4 | 1.0 | 2.341e-05 | 206 | 0.159 | 119 | 89 | 4 | 2 | 117 | 58 | 168 | Protein OSB1 mitochondrial | Protein OSB1 mitochondrial | | afdb-proteome | AF-Q4E153-F1-MODEL\_V4 | 1.0 | 3.796e-05 | 205 | 0.131 | 114 | 84 | 5 | 2 | 113 | 490 | 590 | RNA editing complex protein MP63, putative | RNA editing complex protein MP63, putative | | afdb-proteome | AF-Q8GXH3-F1-MODEL\_V4 | 1.0 | 9.911e-06 | 203 | 0.141 | 156 | 113 | 4 | 1 | 156 | 94 | 228 | Protein OSB2, chloroplastic | Protein OSB2, chloroplastic | | afdb-proteome | AF-O14087-F1-MODEL\_V4 | 1.0 | 6.112e-06 | 202 | 0.177 | 124 | 90 | 5 | 7 | 129 | 27 | 139 | Single-stranded DNA-binding protein rim1, mitochondrial | Single-stranded DNA-binding protein rim1, mitochondrial | | afdb-proteome | AF-C6T7T9-F1-MODEL\_V4 | 1.0 | 1.523e-05 | 190 | 0.197 | 152 | 98 | 7 | 6 | 150 | 37 | 171 | Uncharacterized protein | Uncharacterized protein | | afdb-proteome | AF-Q5JKX9-F1-MODEL\_V4 | 1.0 | 1.296e-05 | 188 | 0.165 | 157 | 105 | 7 | 6 | 156 | 83 | 219 | Os01g0949060 protein | Os01g0949060 protein | | afdb-proteome | AF-Q586L9-F1-MODEL\_V4 | 1.0 | 4.459e-05 | 188 | 0.161 | 142 | 85 | 9 | 2 | 113 | 625 | 762 | RNA-editing complex protein MP81 | RNA-editing complex protein MP81 | | afdb-proteome | AF-Q9FYJ2-F1-MODEL\_V4 | 1.0 | 1.104e-05 | 184 | 0.184 | 163 | 99 | 7 | 6 | 151 | 73 | 218 | Protein OSB4, chloroplastic | Protein OSB4, chloroplastic | | afdb-proteome | AF-Q9SX99-F1-MODEL\_V4 | 1.0 | 0.0001378 | 182 | 0.094 | 137 | 109 | 5 | 1 | 135 | 51 | 174 | Protein OSB1, mitochondrial | Protein OSB1, mitochondrial | | afdb-proteome | AF-A4HRK3-F1-MODEL\_V4 | 1.0 | 3.409e-05 | 168 | 0.128 | 202 | 130 | 10 | 2 | 162 | 242 | 438 | Hypothetical\_protein\_-\_conserved | Hypothetical\_protein\_-\_conserved | | afdb-proteome | AF-A0A1D6G4L8-F1-MODEL\_V4 | 1.0 | 6.155e-05 | 163 | 0.122 | 188 | 120 | 6 | 6 | 162 | 94 | 267 | Protein OSB2 chloroplastic | Protein OSB2 chloroplastic | | afdb-proteome | AF-Q9I5K8-F1-MODEL\_V4 | 1.0 | 0.0008554 | 112 | 0.142 | 154 | 116 | 7 | 1 | 149 | 2 | 144 | Helix destabilizing protein of bacteriophage Pf1 | Helix destabilizing protein of bacteriophage Pf1 | | afdb-proteome | AF-A0A1C1CYU6-F1-MODEL\_V4 | 1.0 | 0.00959 | 108 | 0.109 | 119 | 89 | 4 | 1 | 116 | 176 | 280 | Replication protein A subunit | Replication protein A subunit | | afdb-proteome | AF-A0A0R0FI03-F1-MODEL\_V4 | 0.997 | 0.007332 | 91 | 0.121 | 156 | 109 | 11 | 3 | 157 | 258 | 386 | Uncharacterized protein | Uncharacterized protein | | afdb-proteome | AF-A0A044UXX9-F1-MODEL\_V4 | 0.991 | 0.002504 | 84 | 0.155 | 135 | 86 | 6 | 12 | 137 | 65 | 180 | Uncharacterized protein | Uncharacterized protein | | afdb-uniprot50 | AF-A0A8A7QY70-F1-MODEL\_V4 | 1.0 | 4.882e-17 | 813 | 0.643 | 115 | 41 | 0 | 1 | 115 | 2 | 116 | Single-stranded DNA-binding protein | Single-stranded DNA-binding protein | | afdb-uniprot50 | AF-A0A5T2WJW7-F1-MODEL\_V4 | 1.0 | 1.318e-20 | 813 | 0.613 | 163 | 60 | 2 | 1 | 162 | 2 | 162 | Single-stranded DNA-binding protein | Single-stranded DNA-binding protein | | afdb-uniprot50 | AF-Q8D254-F1-MODEL\_V4 | 1.0 | 2.181e-17 | 810 | 0.652 | 118 | 40 | 1 | 1 | 117 | 1 | 118 | Single-stranded DNA-binding protein | Single-stranded DNA-binding protein | | afdb-uniprot50 | AF-A0A5T5YDS6-F1-MODEL\_V4 | 1.0 | 5.928e-20 | 791 | 0.586 | 162 | 65 | 1 | 1 | 162 | 2 | 161 | Single-stranded DNA-binding protein | Single-stranded DNA-binding protein | | afdb-uniprot50 | AF-Q9RHF4-F1-MODEL\_V4 | 1.0 | 5.618e-20 | 788 | 0.601 | 163 | 62 | 2 | 1 | 162 | 2 | 162 | Single-stranded DNA-binding protein 2 | Single-stranded DNA-binding protein 2 | | afdb-uniprot50 | AF-A0A8B4JY02-F1-MODEL\_V4 | 1.0 | 9.11e-20 | 787 | 0.607 | 163 | 59 | 3 | 1 | 162 | 20 | 178 | Single-strand binding protein | Single-strand binding protein | | afdb-uniprot50 | AF-A0A7X5QH72-F1-MODEL\_V4 | 1.0 | 8.633e-20 | 784 | 0.617 | 162 | 59 | 2 | 1 | 162 | 2 | 160 | Single-stranded DNA-binding protein | Single-stranded DNA-binding protein | | afdb-uniprot50 | AF-A0A2C6DMN1-F1-MODEL\_V4 | 1.0 | 5.928e-20 | 783 | 0.603 | 169 | 60 | 2 | 1 | 162 | 2 | 170 | Single-stranded DNA-binding protein | Single-stranded DNA-binding protein | | afdb-uniprot50 | AF-A0A759M963-F1-MODEL\_V4 | 1.0 | 2.949e-20 | 782 | 0.621 | 164 | 58 | 3 | 1 | 162 | 1 | 162 | Single-stranded DNA-binding protein | Single-stranded DNA-binding protein | | afdb-uniprot50 | AF-V5Z4Q9-F1-MODEL\_V4 | 1.0 | 5.618e-20 | 782 | 0.573 | 164 | 67 | 2 | 1 | 162 | 2 | 164 | Single-stranded DNA-binding protein | Single-stranded DNA-binding protein | | afdb-uniprot50 | AF-P28046-F1-MODEL\_V4 | 1.0 | 3.464e-20 | 778 | 0.612 | 173 | 56 | 2 | 1 | 162 | 2 | 174 | Single-stranded DNA-binding protein | Single-stranded DNA-binding protein | | afdb-uniprot50 | AF-A0A8A9F1R6-F1-MODEL\_V4 | 1.0 | 1.014e-19 | 777 | 0.573 | 164 | 68 | 2 | 1 | 162 | 2 | 165 | Single-stranded DNA-binding protein | Single-stranded DNA-binding protein | | afdb-uniprot50 | AF-A0A410X808-F1-MODEL\_V4 | 1.0 | 2.51e-20 | 772 | 0.622 | 175 | 53 | 4 | 1 | 162 | 2 | 176 | Single-stranded DNA-binding protein | Single-stranded DNA-binding protein | | afdb-uniprot50 | AF-A0A6I7D2R0-F1-MODEL\_V4 | 1.0 | 4.815e-19 | 770 | 0.588 | 163 | 62 | 3 | 1 | 162 | 2 | 160 | Single-stranded DNA-binding protein | Single-stranded DNA-binding protein | | afdb-uniprot50 | AF-A0A068YZG5-F1-MODEL\_V4 | 1.0 | 4.532e-20 | 768 | 0.581 | 172 | 62 | 2 | 1 | 162 | 2 | 173 | Single-stranded DNA-binding protein | Single-stranded DNA-binding protein | | afdb-uniprot50 | AF-C0ARM9-F1-MODEL\_V4 | 1.0 | 1.919e-20 | 767 | 0.622 | 172 | 54 | 3 | 2 | 162 | 39 | 210 | Single-stranded DNA-binding protein | Single-stranded DNA-binding protein | | afdb-uniprot50 | AF-E9CNB8-F1-MODEL\_V4 | 1.0 | 2.395e-19 | 765 | 0.576 | 163 | 66 | 2 | 1 | 162 | 2 | 162 | Single-stranded DNA-binding protein | Single-stranded DNA-binding protein | | afdb-uniprot50 | AF-A0A763N3M4-F1-MODEL\_V4 | 1.0 | 6.094e-16 | 764 | 0.601 | 113 | 44 | 1 | 1 | 113 | 2 | 113 | Single-stranded DNA-binding protein | Single-stranded DNA-binding protein | | afdb-uniprot50 | AF-A0A542C9S8-F1-MODEL\_V4 | 1.0 | 1.4e-19 | 764 | 0.586 | 162 | 65 | 1 | 1 | 162 | 2 | 161 | Single-stranded DNA-binding protein | Single-stranded DNA-binding protein | | afdb-uniprot50 | AF-A0A085GLM7-F1-MODEL\_V4 | 1.0 | 2.025e-20 | 764 | 0.598 | 177 | 56 | 3 | 1 | 162 | 2 | 178 | Single-stranded DNA-binding protein | Single-stranded DNA-binding protein | | afdb-uniprot50 | AF-A0A250B243-F1-MODEL\_V4 | 1.0 | 6.964e-20 | 764 | 0.589 | 178 | 57 | 2 | 1 | 162 | 2 | 179 | Single-stranded DNA-binding protein | Single-stranded DNA-binding protein | | afdb-uniprot50 | AF-A0A3N6SJW5-F1-MODEL\_V4 | 1.0 | 5.657e-19 | 763 | 0.561 | 162 | 71 | 0 | 1 | 162 | 2 | 163 | Single-stranded DNA-binding protein | Single-stranded DNA-binding protein | | afdb-uniprot50 | AF-A0A5V0FD94-F1-MODEL\_V4 | 1.0 | 2.039e-19 | 762 | 0.611 | 162 | 61 | 1 | 1 | 162 | 2 | 161 | Single-stranded DNA-binding protein | Single-stranded DNA-binding protein | | afdb-uniprot50 | AF-A0A1W6B523-F1-MODEL\_V4 | 1.0 | 5.046e-20 | 762 | 0.607 | 176 | 55 | 3 | 1 | 162 | 2 | 177 | Single-stranded DNA-binding protein | Single-stranded DNA-binding protein | | afdb-uniprot50 | AF-A0A090NBG8-F1-MODEL\_V4 | 1.0 | 6.6e-20 | 760 | 0.584 | 178 | 58 | 3 | 1 | 162 | 22 | 199 | Single-stranded DNA-binding protein | Single-stranded DNA-binding protein | | afdb-uniprot50 | AF-A0A068RC77-F1-MODEL\_V4 | 1.0 | 8.633e-20 | 759 | 0.581 | 172 | 62 | 2 | 1 | 162 | 2 | 173 | Single-stranded DNA-binding protein | Single-stranded DNA-binding protein | | afdb-uniprot50 | AF-A0A2W5S122-F1-MODEL\_V4 | 1.0 | 2.395e-19 | 759 | 0.521 | 182 | 66 | 2 | 1 | 162 | 2 | 182 | Single-stranded DNA-binding protein | Single-stranded DNA-binding protein | | afdb-uniprot50 | AF-A0A2P8VNA9-F1-MODEL\_V4 | 1.0 | 2.649e-20 | 758 | 0.596 | 181 | 54 | 3 | 1 | 162 | 2 | 182 | Single-stranded DNA-binding protein | Single-stranded DNA-binding protein | | afdb-uniprot50 | AF-A0A3A4CYF8-F1-MODEL\_V4 | 1.0 | 1.645e-19 | 757 | 0.586 | 162 | 64 | 1 | 1 | 162 | 2 | 160 | Single-stranded DNA-binding protein | Single-stranded DNA-binding protein | | afdb-uniprot50 | AF-A0A6B1YL18-F1-MODEL\_V4 | 1.0 | 1.043e-15 | 756 | 0.548 | 113 | 51 | 0 | 1 | 113 | 1 | 113 | Single-stranded DNA-binding protein | Single-stranded DNA-binding protein | | afdb-uniprot50 | AF-A0A6D2GFG8-F1-MODEL\_V4 | 1.0 | 8.182e-20 | 756 | 0.585 | 176 | 59 | 4 | 1 | 162 | 2 | 177 | Single-stranded DNA-binding protein | Single-stranded DNA-binding protein | | afdb-uniprot50 | AF-T0NVG8-F1-MODEL\_V4 | 1.0 | 3.681e-19 | 755 | 0.581 | 165 | 65 | 2 | 1 | 162 | 2 | 165 | Single-stranded DNA-binding protein | Single-stranded DNA-binding protein | | afdb-uniprot50 | AF-A0A7W3BKJ0-F1-MODEL\_V4 | 1.0 | 2.528e-19 | 755 | 0.545 | 165 | 69 | 2 | 1 | 162 | 2 | 163 | Single-stranded DNA-binding protein | Single-stranded DNA-binding protein | | afdb-uniprot50 | AF-A0A850L2I0-F1-MODEL\_V4 | 1.0 | 5.081e-19 | 754 | 0.564 | 163 | 60 | 3 | 1 | 162 | 1 | 153 | Single-stranded DNA-binding protein | Single-stranded DNA-binding protein | | afdb-uniprot50 | AF-A0A657IF21-F1-MODEL\_V4 | 1.0 | 4.815e-19 | 754 | 0.549 | 162 | 71 | 1 | 1 | 162 | 2 | 161 | Single-stranded DNA-binding protein | Single-stranded DNA-binding protein | | afdb-uniprot50 | AF-A0A637XTZ4-F1-MODEL\_V4 | 1.0 | 1.257e-19 | 754 | 0.591 | 174 | 59 | 4 | 1 | 162 | 2 | 175 | Single-stranded DNA-binding protein | Single-stranded DNA-binding protein | | afdb-uniprot50 | AF-C6CKS0-F1-MODEL\_V4 | 1.0 | 6.6e-20 | 754 | 0.582 | 182 | 56 | 5 | 1 | 162 | 1 | 182 | Single-stranded DNA-binding protein | Single-stranded DNA-binding protein | | afdb-uniprot50 | AF-D4I2A3-F1-MODEL\_V4 | 1.0 | 5.618e-20 | 752 | 0.587 | 182 | 55 | 4 | 1 | 162 | 2 | 183 | Single-stranded DNA-binding protein | Single-stranded DNA-binding protein | | afdb-uniprot50 | AF-A0A376WPU8-F1-MODEL\_V4 | 1.0 | 2.528e-19 | 751 | 0.6 | 165 | 60 | 3 | 1 | 162 | 1 | 162 | Single-stranded DNA-binding protein | Single-stranded DNA-binding protein | | afdb-uniprot50 | AF-A0A2J0PNB2-F1-MODEL\_V4 | 1.0 | 1.4e-19 | 751 | 0.577 | 168 | 61 | 3 | 1 | 162 | 1 | 164 | Single-stranded DNA-binding protein | Single-stranded DNA-binding protein | | afdb-uniprot50 | AF-A0A1Y0LD14-F1-MODEL\_V4 | 1.0 | 1.07e-19 | 751 | 0.576 | 177 | 60 | 3 | 1 | 162 | 2 | 178 | Single-stranded DNA-binding protein | Single-stranded DNA-binding protein | | afdb-uniprot50 | AF-A0A2Z5T340-F1-MODEL\_V4 | 1.0 | 3.966e-16 | 750 | 0.561 | 121 | 52 | 1 | 1 | 120 | 2 | 122 | Single-stranded DNA-binding protein | Single-stranded DNA-binding protein | | afdb-uniprot50 | AF-A0A4Q4A5A0-F1-MODEL\_V4 | 1.0 | 1.831e-19 | 750 | 0.584 | 171 | 55 | 4 | 1 | 162 | 2 | 165 | Single-stranded DNA-binding protein | Single-stranded DNA-binding protein | | afdb-uniprot50 | AF-A0A348DL17-F1-MODEL\_V4 | 1.0 | 6.6e-20 | 750 | 0.576 | 177 | 60 | 3 | 1 | 162 | 2 | 178 | Single-stranded DNA-binding protein | Single-stranded DNA-binding protein | | afdb-uniprot50 | AF-A0A0A2VXI7-F1-MODEL\_V4 | 1.0 | 5.618e-20 | 749 | 0.605 | 175 | 56 | 2 | 1 | 162 | 2 | 176 | Single-stranded DNA-binding protein | Single-stranded DNA-binding protein | | afdb-uniprot50 | AF-A0A419B249-F1-MODEL\_V4 | 1.0 | 1.831e-19 | 748 | 0.612 | 165 | 61 | 2 | 1 | 162 | 2 | 166 | Single-stranded DNA-binding protein | Single-stranded DNA-binding protein | | afdb-uniprot50 | AF-A0A5X6EUH9-F1-MODEL\_V4 | 1.0 | 1.327e-19 | 748 | 0.596 | 166 | 63 | 2 | 1 | 162 | 2 | 167 | Single-stranded DNA-binding protein | Single-stranded DNA-binding protein | | afdb-uniprot50 | AF-A0A710F8C2-F1-MODEL\_V4 | 1.0 | 6.646e-19 | 747 | 0.543 | 162 | 72 | 1 | 1 | 162 | 2 | 161 | Single-stranded DNA-binding protein | Single-stranded DNA-binding protein | | afdb-uniprot50 | AF-A0A5J6VYW7-F1-MODEL\_V4 | 1.0 | 3.133e-19 | 746 | 0.556 | 178 | 63 | 3 | 1 | 162 | 2 | 179 | Single-stranded DNA-binding protein | Single-stranded DNA-binding protein | | afdb-uniprot50 | AF-A0A7W2V7Q4-F1-MODEL\_V4 | 1.0 | 6.964e-20 | 745 | 0.571 | 175 | 62 | 2 | 1 | 162 | 2 | 176 | Single-stranded DNA-binding protein | Single-stranded DNA-binding protein | | afdb-uniprot50 | AF-A0A826P733-F1-MODEL\_V4 | 1.0 | 1.735e-19 | 744 | 0.6 | 165 | 60 | 3 | 1 | 162 | 1 | 162 | Single-stranded DNA-binding protein | Single-stranded DNA-binding protein | | afdb-uniprot50 | AF-A0A1W6C1A7-F1-MODEL\_V4 | 1.0 | 3.681e-19 | 743 | 0.558 | 170 | 64 | 3 | 1 | 162 | 2 | 168 | Single-stranded DNA-binding protein | Single-stranded DNA-binding protein | | afdb-uniprot50 | AF-A0A2V2SUM0-F1-MODEL\_V4 | 1.0 | 4.385e-17 | 742 | 0.551 | 136 | 59 | 1 | 1 | 136 | 1 | 134 | Single-stranded DNA-binding protein | Single-stranded DNA-binding protein | | afdb-uniprot50 | AF-A0A4Q9D8C0-F1-MODEL\_V4 | 1.0 | 2.667e-19 | 742 | 0.613 | 168 | 55 | 3 | 1 | 162 | 1 | 164 | Single-stranded DNA-binding protein | Single-stranded DNA-binding protein | | afdb-uniprot50 | AF-A0A0B6X7H2-F1-MODEL\_V4 | 1.0 | 8.693e-19 | 742 | 0.508 | 169 | 76 | 2 | 1 | 162 | 1 | 169 | Single-stranded DNA-binding protein | Single-stranded DNA-binding protein | | afdb-uniprot50 | AF-A0A5U5MJG5-F1-MODEL\_V4 | 1.0 | 7.862e-18 | 741 | 0.565 | 145 | 58 | 2 | 1 | 140 | 2 | 146 | Single-stranded DNA-binding protein | Single-stranded DNA-binding protein | | afdb-uniprot50 | AF-A0A5X2B0T4-F1-MODEL\_V4 | 1.0 | 6.298e-19 | 741 | 0.537 | 162 | 73 | 1 | 1 | 162 | 2 | 161 | Single-stranded DNA-binding protein | Single-stranded DNA-binding protein | | afdb-uniprot50 | AF-A0A4P9VSW3-F1-MODEL\_V4 | 1.0 | 7.4e-19 | 741 | 0.559 | 159 | 69 | 1 | 4 | 162 | 3 | 160 | Single-stranded DNA-binding protein | Single-stranded DNA-binding protein | | afdb-uniprot50 | AF-A0A3M4A4U1-F1-MODEL\_V4 | 1.0 | 2.096e-15 | 740 | 0.607 | 112 | 43 | 1 | 1 | 112 | 2 | 112 | Single-stranded DNA-binding protein | Single-stranded DNA-binding protein | | afdb-uniprot50 | AF-A0A703UWP0-F1-MODEL\_V4 | 1.0 | 1.668e-17 | 740 | 0.566 | 143 | 53 | 2 | 1 | 142 | 2 | 136 | Single-stranded DNA-binding protein | Single-stranded DNA-binding protein | | afdb-uniprot50 | AF-A0A0J9B2X7-F1-MODEL\_V4 | 1.0 | 5.081e-19 | 739 | 0.539 | 165 | 66 | 2 | 1 | 162 | 2 | 159 | Single-stranded DNA-binding protein | Single-stranded DNA-binding protein | | afdb-uniprot50 | AF-A0A7S4LY53-F1-MODEL\_V4 | 1.0 | 6.964e-20 | 739 | 0.589 | 178 | 57 | 4 | 1 | 162 | 2 | 179 | Single-stranded DNA-binding protein | Single-stranded DNA-binding protein | | afdb-uniprot50 | AF-A0A369WG84-F1-MODEL\_V4 | 1.0 | 3.489e-19 | 739 | 0.534 | 187 | 62 | 3 | 1 | 162 | 1 | 187 | Single-stranded DNA-binding protein | Single-stranded DNA-binding protein | | afdb-uniprot50 | AF-A0A411WM66-F1-MODEL\_V4 | 1.0 | 2.723e-16 | 737 | 0.496 | 129 | 60 | 1 | 1 | 129 | 2 | 125 | Single-stranded DNA-binding protein | Single-stranded DNA-binding protein | | afdb-uniprot50 | AF-A0A6G6PMY1-F1-MODEL\_V4 | 1.0 | 2.395e-19 | 735 | 0.603 | 169 | 60 | 3 | 1 | 162 | 1 | 169 | Single-stranded DNA-binding protein | Single-stranded DNA-binding protein | | afdb-uniprot50 | AF-A0A2P5IIN3-F1-MODEL\_V4 | 1.0 | 3.133e-19 | 734 | 0.595 | 168 | 62 | 3 | 1 | 162 | 2 | 169 | Single-stranded DNA-binding protein | Single-stranded DNA-binding protein | | afdb-uniprot50 | AF-A0A811DAC9-F1-MODEL\_V4 | 1.0 | 2.395e-19 | 734 | 0.572 | 180 | 59 | 4 | 1 | 162 | 1 | 180 | Single-stranded DNA-binding protein 1 | Single-stranded DNA-binding protein 1 | | afdb-uniprot50 | AF-C0Q532-F1-MODEL\_V4 | 1.0 | 2.814e-19 | 734 | 0.596 | 176 | 57 | 5 | 1 | 162 | 42 | 217 | Single-stranded DNA-binding protein | Single-stranded DNA-binding protein | | afdb-uniprot50 | AF-A0A6N8F438-F1-MODEL\_V4 | 1.0 | 1.973e-16 | 732 | 0.573 | 129 | 53 | 1 | 1 | 129 | 1 | 127 | Single-stranded DNA-binding protein | Single-stranded DNA-binding protein | | afdb-uniprot50 | AF-A0A1P8KIA2-F1-MODEL\_V4 | 1.0 | 1.487e-18 | 732 | 0.549 | 162 | 63 | 3 | 1 | 162 | 2 | 153 | Single-stranded DNA-binding protein | Single-stranded DNA-binding protein | | afdb-uniprot50 | AF-Q9KUW2-F1-MODEL\_V4 | 1.0 | 5.969e-19 | 732 | 0.564 | 179 | 59 | 5 | 1 | 162 | 1 | 177 | Single-stranded DNA-binding protein | Single-stranded DNA-binding protein | | afdb-uniprot50 | AF-A0A611K7F6-F1-MODEL\_V4 | 1.0 | 2.814e-19 | 732 | 0.568 | 174 | 60 | 4 | 1 | 162 | 1 | 171 | Single-stranded DNA-binding protein | Single-stranded DNA-binding protein | | afdb-uniprot50 | AF-A0A2S6IZE8-F1-MODEL\_V4 | 1.0 | 7.972e-16 | 731 | 0.605 | 119 | 44 | 2 | 1 | 117 | 1 | 118 | Single-stranded DNA-binding protein | Single-stranded DNA-binding protein | | afdb-uniprot50 | AF-A0A7G2IKD2-F1-MODEL\_V4 | 1.0 | 1.217e-16 | 731 | 0.601 | 128 | 42 | 2 | 1 | 127 | 15 | 134 | Single-stranded DNA-binding protein | Single-stranded DNA-binding protein | | afdb-uniprot50 | AF-A0A419ARW4-F1-MODEL\_V4 | 1.0 | 1.07e-19 | 730 | 0.586 | 184 | 54 | 3 | 1 | 162 | 2 | 185 | Single-stranded DNA-binding protein | Single-stranded DNA-binding protein | | afdb-uniprot50 | AF-A0A3D8ULM4-F1-MODEL\_V4 | 1.0 | 5.116e-18 | 729 | 0.524 | 162 | 69 | 2 | 1 | 162 | 2 | 155 | Single-stranded DNA-binding protein | Single-stranded DNA-binding protein | | afdb-uniprot50 | AF-A0A629K0S7-F1-MODEL\_V4 | 1.0 | 1.2e-18 | 728 | 0.526 | 167 | 67 | 3 | 1 | 155 | 1 | 167 | Single-stranded DNA-binding protein | Single-stranded DNA-binding protein | | afdb-uniprot50 | AF-V0AMD6-F1-MODEL\_V4 | 1.0 | 1.419e-17 | 726 | 0.575 | 146 | 52 | 3 | 1 | 144 | 1 | 138 | Single-stranded DNA-binding protein | Single-stranded DNA-binding protein | | afdb-uniprot50 | AF-A0A2Z3HU03-F1-MODEL\_V4 | 1.0 | 4.595e-18 | 726 | 0.524 | 164 | 65 | 2 | 1 | 162 | 1 | 153 | Single-stranded DNA-binding protein | Single-stranded DNA-binding protein | | afdb-uniprot50 | AF-A0A7H8I9U7-F1-MODEL\_V4 | 1.0 | 8.239e-19 | 726 | 0.577 | 168 | 65 | 3 | 1 | 162 | 1 | 168 | Single-stranded DNA-binding protein | Single-stranded DNA-binding protein | | afdb-uniprot50 | AF-A0A630JIS0-F1-MODEL\_V4 | 1.0 | 6.011e-18 | 725 | 0.573 | 150 | 52 | 4 | 1 | 138 | 1 | 150 | Single-stranded DNA-binding protein | Single-stranded DNA-binding protein | | afdb-uniprot50 | AF-A0A0C4WV47-F1-MODEL\_V4 | 1.0 | 3.707e-18 | 725 | 0.554 | 164 | 65 | 3 | 1 | 162 | 1 | 158 | Single-stranded DNA-binding protein | Single-stranded DNA-binding protein | | afdb-uniprot50 | AF-A0A0F5ARE6-F1-MODEL\_V4 | 1.0 | 1.946e-18 | 725 | 0.524 | 181 | 66 | 3 | 1 | 162 | 2 | 181 | Single-stranded DNA-binding protein | Single-stranded DNA-binding protein | | afdb-uniprot50 | AF-A0A2U8I3I1-F1-MODEL\_V4 | 1.0 | 2.166e-18 | 725 | 0.518 | 164 | 77 | 1 | 1 | 162 | 2 | 165 | Single-stranded DNA-binding protein | Single-stranded DNA-binding protein | | afdb-uniprot50 | AF-A0A2D5T2D5-F1-MODEL\_V4 | 1.0 | 1.078e-18 | 725 | 0.55 | 167 | 70 | 1 | 1 | 162 | 1 | 167 | Single-stranded DNA-binding protein | Single-stranded DNA-binding protein | | afdb-uniprot50 | AF-A0A139DMR2-F1-MODEL\_V4 | 1.0 | 5.474e-16 | 724 | 0.552 | 123 | 55 | 0 | 1 | 123 | 1 | 123 | Single-stranded DNA-binding protein | Single-stranded DNA-binding protein | | afdb-uniprot50 | AF-G5SJV1-F1-MODEL\_V4 | 1.0 | 1.208e-17 | 724 | 0.564 | 147 | 55 | 2 | 1 | 146 | 2 | 140 | Single-stranded DNA-binding protein | Single-stranded DNA-binding protein | | afdb-uniprot50 | AF-D3RW92-F1-MODEL\_V4 | 1.0 | 4.127e-18 | 724 | 0.518 | 162 | 70 | 2 | 1 | 162 | 1 | 154 | Single-stranded DNA-binding protein | Single-stranded DNA-binding protein | | afdb-uniprot50 | AF-A0A2R4NNX4-F1-MODEL\_V4 | 1.0 | 2.286e-18 | 723 | 0.537 | 162 | 66 | 2 | 1 | 162 | 1 | 153 | Single-stranded DNA-binding protein | Single-stranded DNA-binding protein | | afdb-uniprot50 | AF-A0A0H3GZP4-F1-MODEL\_V4 | 1.0 | 3.329e-18 | 723 | 0.491 | 171 | 77 | 2 | 1 | 161 | 2 | 172 | Single-stranded DNA-binding protein | Single-stranded DNA-binding protein | | afdb-uniprot50 | AF-A0A2Z3HV94-F1-MODEL\_V4 | 1.0 | 1.656e-18 | 723 | 0.519 | 181 | 67 | 4 | 1 | 162 | 2 | 181 | Single-stranded DNA-binding protein | Single-stranded DNA-binding protein | | afdb-uniprot50 | AF-A0A3D2B3S2-F1-MODEL\_V4 | 1.0 | 8.296e-18 | 722 | 0.567 | 148 | 55 | 2 | 1 | 139 | 2 | 149 | Single-stranded DNA-binding protein | Single-stranded DNA-binding protein | | afdb-uniprot50 | AF-A0A7Y2N5T2-F1-MODEL\_V4 | 1.0 | 4.127e-18 | 722 | 0.546 | 163 | 70 | 2 | 1 | 162 | 1 | 160 | Single-stranded DNA-binding protein | Single-stranded DNA-binding protein | | afdb-uniprot50 | AF-A0A4R7JHL5-F1-MODEL\_V4 | 1.0 | 1.487e-18 | 721 | 0.55 | 169 | 68 | 3 | 1 | 162 | 1 | 168 | Single-stranded DNA-binding protein | Single-stranded DNA-binding protein | | afdb-uniprot50 | AF-A0A1B9LCJ4-F1-MODEL\_V4 | 1.0 | 8.239e-19 | 721 | 0.531 | 173 | 70 | 4 | 1 | 162 | 1 | 173 | Single-stranded DNA-binding protein | Single-stranded DNA-binding protein | | afdb-uniprot50 | AF-A0A3B1E364-F1-MODEL\_V4 | 1.0 | 7.808e-19 | 721 | 0.529 | 172 | 71 | 3 | 1 | 162 | 2 | 173 | Single-stranded DNA-binding protein | Single-stranded DNA-binding protein | | afdb-uniprot50 | AF-A0A4D7J836-F1-MODEL\_V4 | 1.0 | 1.857e-17 | 720 | 0.493 | 162 | 69 | 1 | 1 | 162 | 2 | 150 | Single-stranded DNA-binding protein | Single-stranded DNA-binding protein | | afdb-uniprot50 | AF-A0A5K7YKA1-F1-MODEL\_V4 | 1.0 | 1.487e-18 | 720 | 0.544 | 167 | 67 | 4 | 1 | 162 | 1 | 163 | Single-stranded DNA-binding protein | Single-stranded DNA-binding protein | | afdb-uniprot50 | AF-A0A2E9T352-F1-MODEL\_V4 | 1.0 | 1.2e-18 | 720 | 0.495 | 200 | 63 | 4 | 1 | 162 | 1 | 200 | Single-stranded DNA-binding protein | Single-stranded DNA-binding protein | | afdb-uniprot50 | AF-A0A3S4HKI4-F1-MODEL\_V4 | 1.0 | 5.081e-19 | 720 | 0.558 | 179 | 62 | 4 | 1 | 162 | 1 | 179 | Single-stranded DNA-binding protein | Single-stranded DNA-binding protein | | afdb-uniprot50 | AF-A3JEM4-F1-MODEL\_V4 | 1.0 | 1.419e-17 | 719 | 0.541 | 155 | 70 | 1 | 1 | 155 | 29 | 182 | Single-stranded DNA-binding protein | Single-stranded DNA-binding protein | | afdb-uniprot50 | AF-A0A703J8T7-F1-MODEL\_V4 | 1.0 | 3.221e-15 | 718 | 0.647 | 105 | 37 | 0 | 1 | 105 | 2 | 106 | Single-stranded DNA-binding protein | Single-stranded DNA-binding protein | | afdb-uniprot50 | AF-A0A0H4A0G0-F1-MODEL\_V4 | 1.0 | 2.873e-16 | 718 | 0.576 | 130 | 51 | 2 | 1 | 129 | 2 | 128 | Single-stranded DNA-binding protein | Single-stranded DNA-binding protein | | afdb-uniprot50 | AF-A0A7U4TA53-F1-MODEL\_V4 | 1.0 | 5.361e-19 | 718 | 0.543 | 184 | 62 | 4 | 1 | 162 | 2 | 185 | Single-stranded DNA-binding protein | Single-stranded DNA-binding protein | | afdb-uniprot50 | AF-A0A4V3X5D4-F1-MODEL\_V4 | 1.0 | 7.061e-18 | 717 | 0.536 | 151 | 68 | 2 | 1 | 149 | 1 | 151 | Single-stranded DNA-binding protein | Single-stranded DNA-binding protein | | afdb-uniprot50 | AF-A0A709YGK6-F1-MODEL\_V4 | 1.0 | 3.329e-18 | 717 | 0.57 | 156 | 56 | 3 | 1 | 145 | 2 | 157 | Single-stranded DNA-binding protein | Single-stranded DNA-binding protein | | afdb-uniprot50 | AF-A0A316FAR0-F1-MODEL\_V4 | 1.0 | 9.173e-19 | 717 | 0.553 | 177 | 61 | 5 | 1 | 162 | 2 | 175 | Single-stranded DNA-binding protein | Single-stranded DNA-binding protein | | afdb-uniprot50 | AF-A0A5J6WRZ9-F1-MODEL\_V4 | 1.0 | 8.693e-19 | 717 | 0.573 | 169 | 61 | 3 | 1 | 162 | 2 | 166 | Single-stranded DNA-binding protein | Single-stranded DNA-binding protein | | afdb-uniprot50 | AF-A0A3M1FWU7-F1-MODEL\_V4 | 1.0 | 2.053e-18 | 716 | 0.514 | 169 | 74 | 2 | 1 | 162 | 1 | 168 | Single-stranded DNA-binding protein | Single-stranded DNA-binding protein | | afdb-uniprot50 | AF-A0A5V5DT66-F1-MODEL\_V4 | 1.0 | 3.993e-15 | 715 | 0.66 | 103 | 35 | 0 | 1 | 103 | 2 | 104 | Single-stranded DNA-binding protein | Single-stranded DNA-binding protein | | afdb-uniprot50 | AF-A0A3G2N7B1-F1-MODEL\_V4 | 1.0 | 3.911e-18 | 715 | 0.539 | 163 | 63 | 5 | 1 | 162 | 1 | 152 | Single-stranded DNA-binding protein | Single-stranded DNA-binding protein | | afdb-uniprot50 | AF-A0A1V0HPB3-F1-MODEL\_V4 | 1.0 | 2.286e-18 | 715 | 0.524 | 166 | 69 | 3 | 1 | 162 | 1 | 160 | Single-stranded DNA-binding protein | Single-stranded DNA-binding protein | | afdb-uniprot50 | AF-A0A1X3M0N9-F1-MODEL\_V4 | 1.0 | 8.296e-18 | 715 | 0.519 | 154 | 71 | 2 | 1 | 151 | 14 | 167 | Single-stranded DNA-binding protein | Single-stranded DNA-binding protein | | afdb-uniprot50 | AF-A0A379CU75-F1-MODEL\_V4 | 1.0 | 2.053e-18 | 715 | 0.548 | 164 | 67 | 1 | 1 | 157 | 2 | 165 | Single-stranded DNA-binding protein | Single-stranded DNA-binding protein | | afdb-uniprot50 | AF-A0A3B8TV62-F1-MODEL\_V4 | 1.0 | 4.627e-17 | 714 | 0.568 | 146 | 60 | 2 | 1 | 146 | 1 | 143 | Single-stranded DNA-binding protein | Single-stranded DNA-binding protein | | afdb-uniprot50 | AF-A0A2P5T016-F1-MODEL\_V4 | 1.0 | 2.545e-18 | 714 | 0.511 | 170 | 75 | 3 | 1 | 162 | 1 | 170 | Single-stranded DNA-binding protein | Single-stranded DNA-binding protein | | afdb-uniprot50 | AF-A0A659R992-F1-MODEL\_V4 | 1.0 | 7.451e-18 | 713 | 0.552 | 152 | 59 | 2 | 1 | 151 | 2 | 145 | Single-stranded DNA-binding protein | Single-stranded DNA-binding protein | | afdb-uniprot50 | AF-U7R096-F1-MODEL\_V4 | 1.0 | 8.754e-18 | 713 | 0.527 | 163 | 66 | 3 | 1 | 162 | 2 | 154 | Single-stranded DNA-binding protein | Single-stranded DNA-binding protein | | afdb-uniprot50 | AF-A0A7Y8YBZ3-F1-MODEL\_V4 | 1.0 | 4.127e-18 | 713 | 0.549 | 162 | 65 | 1 | 1 | 162 | 1 | 154 | Single-stranded DNA-binding protein | Single-stranded DNA-binding protein | | afdb-uniprot50 | AF-A0A707B4Y7-F1-MODEL\_V4 | 1.0 | 2.834e-18 | 713 | 0.524 | 162 | 68 | 2 | 1 | 161 | 2 | 155 | Single-stranded DNA-binding protein | Single-stranded DNA-binding protein | | afdb-uniprot50 | AF-A0A610WMJ1-F1-MODEL\_V4 | 1.0 | 4.355e-18 | 712 | 0.521 | 161 | 68 | 2 | 1 | 160 | 2 | 154 | Single-stranded DNA-binding protein | Single-stranded DNA-binding protein | | afdb-uniprot50 | AF-A0A6G2B591-F1-MODEL\_V4 | 1.0 | 5.398e-18 | 712 | 0.56 | 164 | 62 | 4 | 1 | 162 | 2 | 157 | Single-stranded DNA-binding protein | Single-stranded DNA-binding protein | | afdb-uniprot50 | AF-A0A6G9HLE5-F1-MODEL\_V4 | 1.0 | 3.011e-17 | 711 | 0.537 | 147 | 64 | 2 | 1 | 144 | 2 | 147 | Single-stranded DNA-binding protein | Single-stranded DNA-binding protein | | afdb-uniprot50 | AF-A0A6G7CNE3-F1-MODEL\_V4 | 1.0 | 7.451e-18 | 711 | 0.527 | 163 | 55 | 3 | 1 | 162 | 2 | 143 | Single-stranded DNA-binding protein | Single-stranded DNA-binding protein | | afdb-uniprot50 | AF-A0A6C8WF48-F1-MODEL\_V4 | 1.0 | 3.329e-18 | 711 | 0.563 | 158 | 58 | 3 | 1 | 147 | 2 | 159 | Single-stranded DNA-binding protein | Single-stranded DNA-binding protein | | afdb-uniprot50 | AF-A0A750BRM1-F1-MODEL\_V4 | 1.0 | 3.707e-18 | 711 | 0.481 | 162 | 81 | 1 | 1 | 162 | 2 | 160 | Single-stranded DNA-binding protein | Single-stranded DNA-binding protein | | afdb-uniprot50 | AF-A0A370S7B0-F1-MODEL\_V4 | 1.0 | 1.844e-18 | 711 | 0.51 | 186 | 66 | 4 | 1 | 162 | 1 | 185 | Single-stranded DNA-binding protein | Single-stranded DNA-binding protein | | afdb-uniprot50 | AF-A0A418YH34-F1-MODEL\_V4 | 1.0 | 1.487e-18 | 710 | 0.57 | 163 | 61 | 4 | 1 | 162 | 2 | 156 | Single-stranded DNA-binding protein | Single-stranded DNA-binding protein | | afdb-uniprot50 | AF-E0WRV0-F1-MODEL\_V4 | 1.0 | 1.487e-18 | 709 | 0.5 | 176 | 74 | 3 | 1 | 162 | 2 | 177 | Single-stranded DNA-binding protein | Single-stranded DNA-binding protein | | afdb-uniprot50 | AF-A0A383D6A9-F1-MODEL\_V4 | 1.0 | 1.987e-15 | 708 | 0.553 | 112 | 50 | 0 | 3 | 114 | 2 | 113 | Uncharacterized protein | Uncharacterized protein | | afdb-uniprot50 | AF-A0A5U8SVX5-F1-MODEL\_V4 | 1.0 | 5.116e-18 | 708 | 0.635 | 151 | 49 | 3 | 1 | 145 | 1 | 151 | Single-stranded DNA-binding protein | Single-stranded DNA-binding protein | | afdb-uniprot50 | AF-P28044-F1-MODEL\_V4 | 1.0 | 3.707e-18 | 707 | 0.502 | 175 | 73 | 4 | 1 | 162 | 2 | 175 | Plasmid-derived single-stranded DNA-binding protein | Plasmid-derived single-stranded DNA-binding protein | | afdb-uniprot50 | AF-A0A2S5JEG1-F1-MODEL\_V4 | 1.0 | 3.376e-16 | 706 | 0.576 | 130 | 53 | 2 | 1 | 129 | 1 | 129 | Single-stranded DNA-binding protein | Single-stranded DNA-binding protein | | afdb-uniprot50 | AF-A0A0H3ZW72-F1-MODEL\_V4 | 1.0 | 3.352e-17 | 706 | 0.533 | 163 | 54 | 5 | 1 | 162 | 2 | 143 | Single-stranded DNA-binding protein | Single-stranded DNA-binding protein | | afdb-uniprot50 | AF-A0A2V4ZPU8-F1-MODEL\_V4 | 1.0 | 5.116e-18 | 706 | 0.527 | 165 | 74 | 2 | 1 | 162 | 1 | 164 | Single-stranded DNA-binding protein | Single-stranded DNA-binding protein | | afdb-uniprot50 | AF-A0A2D0IKG4-F1-MODEL\_V4 | 1.0 | 6.692e-18 | 706 | 0.488 | 170 | 79 | 3 | 1 | 162 | 2 | 171 | Single-stranded DNA-binding protein | Single-stranded DNA-binding protein | | afdb-uniprot50 | AF-P18022-F1-MODEL\_V4 | 1.0 | 4.127e-18 | 706 | 0.482 | 176 | 76 | 4 | 1 | 162 | 1 | 175 | Plasmid-derived single-stranded DNA-binding protein | Plasmid-derived single-stranded DNA-binding protein | | afdb-uniprot50 | AF-A0A383C086-F1-MODEL\_V4 | 1.0 | 2.599e-15 | 705 | 0.616 | 112 | 41 | 2 | 1 | 111 | 1 | 111 | Uncharacterized protein | Uncharacterized protein | | afdb-uniprot50 | AF-A0A369F188-F1-MODEL\_V4 | 1.0 | 1.429e-16 | 705 | 0.559 | 143 | 55 | 3 | 1 | 143 | 2 | 136 | Single-stranded DNA-binding protein | Single-stranded DNA-binding protein | | afdb-uniprot50 | AF-A0A7W3G190-F1-MODEL\_V4 | 1.0 | 9.237e-18 | 705 | 0.53 | 162 | 64 | 3 | 1 | 162 | 2 | 151 | Single-stranded DNA-binding protein | Single-stranded DNA-binding protein | | afdb-uniprot50 | AF-A0A379X3G7-F1-MODEL\_V4 | 1.0 | 1.208e-17 | 705 | 0.478 | 163 | 82 | 2 | 1 | 162 | 2 | 162 | Single-stranded DNA-binding protein | Single-stranded DNA-binding protein | | afdb-uniprot50 | AF-A0A1I4SDU3-F1-MODEL\_V4 | 1.0 | 4.127e-18 | 704 | 0.533 | 163 | 75 | 1 | 1 | 162 | 14 | 176 | Single-stranded DNA-binding protein | Single-stranded DNA-binding protein | | afdb-uniprot50 | AF-T1CAF7-F1-MODEL\_V4 | 1.0 | 7.608e-15 | 702 | 0.539 | 113 | 49 | 1 | 1 | 113 | 1 | 110 | Single-stranded DNA-binding protein | Single-stranded DNA-binding protein | | afdb-uniprot50 | AF-A0A0F6TUU1-F1-MODEL\_V4 | 1.0 | 4.916e-16 | 702 | 0.492 | 130 | 66 | 0 | 1 | 130 | 2 | 131 | Single-stranded DNA-binding protein | Single-stranded DNA-binding protein | | afdb-uniprot50 | AF-A0A2X2BQR0-F1-MODEL\_V4 | 1.0 | 1.208e-17 | 702 | 0.559 | 161 | 47 | 3 | 2 | 162 | 3 | 139 | Single-stranded DNA-binding protein | Single-stranded DNA-binding protein | | afdb-uniprot50 | AF-A0A357KLJ1-F1-MODEL\_V4 | 1.0 | 4.355e-18 | 701 | 0.53 | 164 | 68 | 3 | 1 | 162 | 1 | 157 | Single-stranded DNA-binding protein | Single-stranded DNA-binding protein | | afdb-uniprot50 | AF-A0A850RHP0-F1-MODEL\_V4 | 1.0 | 1.208e-17 | 701 | 0.478 | 163 | 81 | 2 | 1 | 162 | 1 | 160 | Single-stranded DNA-binding protein | Single-stranded DNA-binding protein | | afdb-uniprot50 | AF-A0A5R9R0W8-F1-MODEL\_V4 | 1.0 | 1.208e-17 | 701 | 0.527 | 163 | 75 | 2 | 1 | 162 | 1 | 162 | Single-stranded DNA-binding protein | Single-stranded DNA-binding protein | | afdb-uniprot50 | AF-A0A1T1H873-F1-MODEL\_V4 | 1.0 | 8.296e-18 | 701 | 0.494 | 168 | 79 | 2 | 1 | 162 | 1 | 168 | Single-stranded DNA-binding protein | Single-stranded DNA-binding protein | | afdb-uniprot50 | AF-A0A7M2QME3-F1-MODEL\_V4 | 1.0 | 7.4e-19 | 700 | 0.568 | 169 | 60 | 3 | 1 | 162 | 2 | 164 | Single-stranded DNA-binding protein | Single-stranded DNA-binding protein | | afdb-uniprot50 | AF-A0A7Z9CPI0-F1-MODEL\_V4 | 1.0 | 8.754e-18 | 700 | 0.469 | 162 | 83 | 1 | 1 | 162 | 2 | 160 | Single-stranded DNA-binding protein | Single-stranded DNA-binding protein | | afdb-uniprot50 | AF-G5QBI0-F1-MODEL\_V4 | 1.0 | 3.155e-18 | 700 | 0.525 | 175 | 70 | 4 | 1 | 162 | 2 | 176 | Single-stranded DNA-binding protein | Single-stranded DNA-binding protein | | afdb-uniprot50 | AF-A0A485A5Z5-F1-MODEL\_V4 | 1.0 | 6.692e-18 | 699 | 0.525 | 160 | 72 | 2 | 1 | 156 | 2 | 161 | Single-stranded DNA-binding protein | Single-stranded DNA-binding protein | | afdb-uniprot50 | AF-A0A706BN23-F1-MODEL\_V4 | 1.0 | 7.061e-18 | 698 | 0.521 | 161 | 68 | 2 | 1 | 160 | 2 | 154 | Single-stranded DNA-binding protein | Single-stranded DNA-binding protein | | afdb-uniprot50 | AF-A0A1B3B8T4-F1-MODEL\_V4 | 1.0 | 8.296e-18 | 698 | 0.533 | 165 | 68 | 3 | 1 | 162 | 2 | 160 | Single-stranded DNA-binding protein | Single-stranded DNA-binding protein | | afdb-uniprot50 | AF-A0A1G5PGY1-F1-MODEL\_V4 | 1.0 | 3.329e-18 | 698 | 0.486 | 187 | 70 | 4 | 1 | 162 | 1 | 186 | Single-stranded DNA-binding protein | Single-stranded DNA-binding protein | | afdb-uniprot50 | AF-A0A2X2V858-F1-MODEL\_V4 | 1.0 | 4.849e-18 | 697 | 0.53 | 162 | 72 | 1 | 1 | 162 | 2 | 159 | Single-stranded DNA-binding protein | Single-stranded DNA-binding protein | | afdb-uniprot50 | AF-A0A2N3J8Y0-F1-MODEL\_V4 | 1.0 | 5.398e-18 | 697 | 0.551 | 165 | 67 | 4 | 1 | 162 | 1 | 161 | Single-stranded DNA-binding protein | Single-stranded DNA-binding protein | | afdb-uniprot50 | AF-A0A7V7RBU2-F1-MODEL\_V4 | 1.0 | 3.155e-18 | 696 | 0.555 | 162 | 60 | 3 | 1 | 162 | 1 | 150 | Single-stranded DNA-binding protein | Single-stranded DNA-binding protein | | afdb-uniprot50 | AF-A0A5R8ZPZ3-F1-MODEL\_V4 | 1.0 | 9.746e-18 | 695 | 0.496 | 165 | 69 | 3 | 1 | 162 | 1 | 154 | Single-stranded DNA-binding protein | Single-stranded DNA-binding protein | | afdb-uniprot50 | AF-A0A3Q8D6N7-F1-MODEL\_V4 | 1.0 | 6.342e-18 | 695 | 0.461 | 171 | 81 | 2 | 1 | 162 | 2 | 170 | Single-stranded DNA-binding protein | Single-stranded DNA-binding protein | | afdb-uniprot50 | AF-Q93GP7-F1-MODEL\_V4 | 1.0 | 4.355e-18 | 695 | 0.508 | 177 | 66 | 4 | 1 | 162 | 2 | 172 | Single-stranded DNA-binding protein 2 | Single-stranded DNA-binding protein 2 | | afdb-uniprot50 | AF-A0A659LI46-F1-MODEL\_V4 | 1.0 | 1.275e-17 | 695 | 0.481 | 162 | 77 | 1 | 1 | 162 | 2 | 156 | Single-stranded DNA-binding protein | Single-stranded DNA-binding protein | | afdb-uniprot50 | AF-A0A5P9CCU7-F1-MODEL\_V4 | 1.0 | 1.085e-17 | 694 | 0.53 | 164 | 74 | 3 | 1 | 162 | 1 | 163 | Single-stranded DNA-binding protein | Single-stranded DNA-binding protein | | afdb-uniprot50 | AF-U9Z7F7-F1-MODEL\_V4 | 1.0 | 7.451e-18 | 694 | 0.511 | 174 | 71 | 3 | 2 | 162 | 44 | 216 | Single-stranded DNA-binding protein | Single-stranded DNA-binding protein | | afdb-uniprot50 | AF-A0A2E1TLI2-F1-MODEL\_V4 | 1.0 | 7.21e-15 | 693 | 0.581 | 117 | 45 | 2 | 1 | 117 | 1 | 113 | Single-stranded DNA-binding protein | Single-stranded DNA-binding protein | | afdb-uniprot50 | AF-Q8K933-F1-MODEL\_V4 | 1.0 | 7.451e-18 | 693 | 0.518 | 164 | 75 | 3 | 1 | 162 | 1 | 162 | Single-stranded DNA-binding protein | Single-stranded DNA-binding protein | | afdb-uniprot50 | AF-A0A379WHK7-F1-MODEL\_V4 | 1.0 | 1.76e-17 | 693 | 0.478 | 161 | 83 | 1 | 1 | 160 | 2 | 162 | Single-stranded DNA-binding protein | Single-stranded DNA-binding protein | | afdb-uniprot50 | AF-A0A5Q0DJH1-F1-MODEL\_V4 | 1.0 | 7.608e-15 | 692 | 0.551 | 116 | 48 | 2 | 1 | 116 | 1 | 112 | Single-stranded DNA-binding protein | Single-stranded DNA-binding protein | | afdb-uniprot50 | AF-Q8L2D1-F1-MODEL\_V4 | 1.0 | 1.58e-17 | 692 | 0.509 | 157 | 71 | 3 | 1 | 154 | 1 | 154 | Single-stranded DNA-binding protein | Single-stranded DNA-binding protein | | afdb-uniprot50 | AF-A0A6G7VFM5-F1-MODEL\_V4 | 1.0 | 1.145e-17 | 692 | 0.512 | 162 | 74 | 3 | 1 | 162 | 2 | 158 | Single-stranded DNA-binding protein | Single-stranded DNA-binding protein | | afdb-uniprot50 | AF-A0A375H7Q9-F1-MODEL\_V4 | 1.0 | 4.849e-18 | 692 | 0.568 | 174 | 56 | 3 | 1 | 162 | 1 | 167 | Single-stranded DNA-binding protein | Single-stranded DNA-binding protein | | afdb-uniprot50 | AF-A0A1C4A5X5-F1-MODEL\_V4 | 1.0 | 4.156e-17 | 691 | 0.518 | 164 | 58 | 5 | 1 | 162 | 1 | 145 | Single-stranded DNA-binding protein | Single-stranded DNA-binding protein | | afdb-uniprot50 | AF-A0A3G2HPE7-F1-MODEL\_V4 | 1.0 | 1.498e-17 | 691 | 0.506 | 162 | 78 | 2 | 1 | 162 | 1 | 160 | Single-stranded DNA-binding protein | Single-stranded DNA-binding protein | | afdb-uniprot50 | AF-A0A661FAJ4-F1-MODEL\_V4 | 1.0 | 6.739e-17 | 690 | 0.515 | 159 | 59 | 3 | 4 | 162 | 1 | 141 | Single-stranded DNA-binding protein | Single-stranded DNA-binding protein | | afdb-uniprot50 | AF-A0A485D3V8-F1-MODEL\_V4 | 1.0 | 4.882e-17 | 690 | 0.487 | 154 | 76 | 1 | 1 | 154 | 2 | 152 | Single-stranded DNA-binding protein | Single-stranded DNA-binding protein | | afdb-uniprot50 | AF-A0A4Y3HW35-F1-MODEL\_V4 | 1.0 | 1.419e-17 | 690 | 0.509 | 163 | 72 | 3 | 1 | 162 | 2 | 157 | Single-stranded DNA-binding protein | Single-stranded DNA-binding protein | | afdb-uniprot50 | AF-A0A7X9ENL8-F1-MODEL\_V4 | 1.0 | 3.707e-18 | 690 | 0.588 | 158 | 50 | 3 | 1 | 143 | 1 | 158 | Single-stranded DNA-binding protein | Single-stranded DNA-binding protein | | afdb-uniprot50 | AF-A0A6B9GFR9-F1-MODEL\_V4 | 1.0 | 2.181e-17 | 690 | 0.469 | 164 | 84 | 2 | 1 | 162 | 2 | 164 | Single-stranded DNA-binding protein | Single-stranded DNA-binding protein | | afdb-uniprot50 | AF-A0A1V4T3M5-F1-MODEL\_V4 | 1.0 | 1.208e-17 | 690 | 0.496 | 163 | 79 | 2 | 1 | 162 | 2 | 162 | Single-stranded DNA-binding protein | Single-stranded DNA-binding protein | | afdb-uniprot50 | AF-A0A349ECJ1-F1-MODEL\_V4 | 1.0 | 5.512e-15 | 689 | 0.603 | 116 | 42 | 2 | 3 | 117 | 2 | 114 | Single-stranded DNA-binding protein | Single-stranded DNA-binding protein | | afdb-uniprot50 | AF-A0A5C8BHE1-F1-MODEL\_V4 | 1.0 | 7.21e-15 | 689 | 0.533 | 118 | 51 | 2 | 1 | 117 | 1 | 115 | Single-stranded DNA-binding protein | Single-stranded DNA-binding protein | | afdb-uniprot50 | AF-A0A5U2F1N9-F1-MODEL\_V4 | 1.0 | 3.177e-17 | 689 | 0.466 | 163 | 77 | 2 | 1 | 162 | 2 | 155 | Single-stranded DNA-binding protein | Single-stranded DNA-binding protein | | afdb-uniprot50 | AF-A0A3M5NM76-F1-MODEL\_V4 | 1.0 | 1.217e-16 | 688 | 0.527 | 146 | 62 | 3 | 1 | 146 | 2 | 140 | Single-stranded DNA-binding protein | Single-stranded DNA-binding protein | | afdb-uniprot50 | AF-A0A1I4SIB2-F1-MODEL\_V4 | 1.0 | 2.181e-17 | 688 | 0.527 | 163 | 72 | 3 | 1 | 162 | 1 | 159 | Single-stranded DNA-binding protein | Single-stranded DNA-binding protein | | afdb-uniprot50 | AF-A0A0S2SIW1-F1-MODEL\_V4 | 1.0 | 1.844e-18 | 688 | 0.526 | 186 | 64 | 5 | 1 | 162 | 1 | 186 | Single-stranded DNA-binding protein | Single-stranded DNA-binding protein | | afdb-uniprot50 | AF-C8CGN8-F1-MODEL\_V4 | 1.0 | 3.177e-17 | 688 | 0.487 | 162 | 82 | 1 | 1 | 162 | 2 | 162 | Single-stranded DNA-binding protein | Single-stranded DNA-binding protein | | afdb-uniprot50 | AF-A0A6G9I4I2-F1-MODEL\_V4 | 1.0 | 3.352e-17 | 688 | 0.478 | 161 | 83 | 1 | 1 | 161 | 2 | 161 | Single-stranded DNA-binding protein | Single-stranded DNA-binding protein | | afdb-uniprot50 | AF-A0A4V5J5Y1-F1-MODEL\_V4 | 1.0 | 5.187e-16 | 687 | 0.557 | 140 | 54 | 3 | 1 | 140 | 2 | 133 | Single-stranded DNA-binding protein | Single-stranded DNA-binding protein | | afdb-uniprot50 | AF-A0A2P0QEJ1-F1-MODEL\_V4 | 1.0 | 2.853e-17 | 687 | 0.506 | 162 | 75 | 2 | 1 | 162 | 1 | 157 | Single-stranded DNA-binding protein | Single-stranded DNA-binding protein | | afdb-uniprot50 | AF-A0A1S1HM16-F1-MODEL\_V4 | 1.0 | 5.398e-18 | 686 | 0.536 | 164 | 63 | 5 | 1 | 162 | 1 | 153 | Single-stranded DNA-binding protein | Single-stranded DNA-binding protein | | afdb-uniprot50 | AF-A0A177YDV3-F1-MODEL\_V4 | 1.0 | 1.345e-17 | 685 | 0.527 | 165 | 72 | 4 | 1 | 162 | 1 | 162 | Single-stranded DNA-binding protein | Single-stranded DNA-binding protein | | afdb-uniprot50 | AF-A0A6B1HYZ8-F1-MODEL\_V4 | 1.0 | 8.296e-18 | 685 | 0.517 | 174 | 70 | 4 | 1 | 162 | 2 | 173 | Single-stranded DNA-binding protein | Single-stranded DNA-binding protein | | afdb-uniprot50 | AF-A0A3T9QRY1-F1-MODEL\_V4 | 1.0 | 9.746e-18 | 685 | 0.562 | 169 | 67 | 5 | 1 | 162 | 2 | 170 | Single-stranded DNA-binding protein | Single-stranded DNA-binding protein | | afdb-uniprot50 | AF-A0A377BE37-F1-MODEL\_V4 | 1.0 | 1.028e-17 | 684 | 0.544 | 169 | 67 | 4 | 1 | 162 | 1 | 166 | Single-stranded DNA-binding protein | Single-stranded DNA-binding protein | | afdb-uniprot50 | AF-A0A2K8U2W3-F1-MODEL\_V4 | 1.0 | 1.145e-17 | 684 | 0.494 | 168 | 76 | 4 | 1 | 162 | 30 | 194 | Single-stranded DNA-binding protein | Single-stranded DNA-binding protein | | afdb-uniprot50 | AF-A0A377TIF1-F1-MODEL\_V4 | 1.0 | 6.692e-18 | 684 | 0.518 | 162 | 67 | 3 | 1 | 162 | 2 | 152 | Single-stranded DNA-binding protein | Single-stranded DNA-binding protein | | afdb-uniprot50 | AF-A0A3C0J9N0-F1-MODEL\_V4 | 1.0 | 1.896e-14 | 683 | 0.525 | 116 | 52 | 1 | 1 | 116 | 1 | 113 | Single-stranded DNA-binding protein | Single-stranded DNA-binding protein | | afdb-uniprot50 | AF-A0A7I6PS02-F1-MODEL\_V4 | 1.0 | 7.451e-18 | 683 | 0.541 | 168 | 64 | 5 | 1 | 162 | 2 | 162 | Single-stranded DNA-binding protein | Single-stranded DNA-binding protein | | afdb-uniprot50 | AF-U3U1Y0-F1-MODEL\_V4 | 1.0 | 3.911e-18 | 683 | 0.537 | 173 | 67 | 4 | 1 | 162 | 1 | 171 | Single-stranded DNA-binding protein | Single-stranded DNA-binding protein | | afdb-uniprot50 | AF-A0A7H4M7I9-F1-MODEL\_V4 | 1.0 | 1.374e-14 | 681 | 0.57 | 107 | 45 | 1 | 1 | 106 | 1 | 107 | Single-stranded DNA-binding protein | Single-stranded DNA-binding protein | | afdb-uniprot50 | AF-A0A827CVI6-F1-MODEL\_V4 | 1.0 | 6.431e-16 | 681 | 0.424 | 146 | 83 | 1 | 1 | 145 | 2 | 147 | Single-stranded DNA-binding protein | Single-stranded DNA-binding protein | | afdb-uniprot50 | AF-A0A850F6I9-F1-MODEL\_V4 | 1.0 | 9.746e-18 | 681 | 0.517 | 174 | 72 | 4 | 1 | 162 | 1 | 174 | Single-stranded DNA-binding protein | Single-stranded DNA-binding protein | | afdb-uniprot50 | AF-A0A128F9E4-F1-MODEL\_V4 | 1.0 | 5.696e-18 | 681 | 0.528 | 178 | 65 | 4 | 1 | 162 | 2 | 176 | Single-stranded DNA-binding protein | Single-stranded DNA-binding protein | | afdb-uniprot50 | AF-U1SCZ3-F1-MODEL\_V4 | 1.0 | 5.398e-18 | 681 | 0.542 | 166 | 68 | 4 | 1 | 162 | 25 | 186 | Single-stranded DNA-binding protein | Single-stranded DNA-binding protein | | afdb-uniprot50 | AF-A0A447LUG1-F1-MODEL\_V4 | 1.0 | 8.754e-18 | 680 | 0.509 | 163 | 71 | 2 | 1 | 162 | 2 | 156 | Single-stranded DNA-binding protein | Single-stranded DNA-binding protein | | afdb-uniprot50 | AF-A0A451D2Q2-F1-MODEL\_V4 | 1.0 | 2.302e-17 | 680 | 0.476 | 170 | 81 | 1 | 1 | 162 | 2 | 171 | Single-stranded DNA-binding protein | Single-stranded DNA-binding protein | | afdb-uniprot50 | AF-Q8L2A6-F1-MODEL\_V4 | 1.0 | 3.329e-18 | 680 | 0.553 | 179 | 63 | 5 | 1 | 162 | 1 | 179 | Single-stranded DNA-binding protein | Single-stranded DNA-binding protein | | afdb-uniprot50 | AF-A0A6F8PXD7-F1-MODEL\_V4 | 1.0 | 2.704e-17 | 680 | 0.468 | 160 | 84 | 1 | 4 | 162 | 3 | 162 | Single-stranded DNA-binding protein | Single-stranded DNA-binding protein | | afdb-uniprot50 | AF-A0A0G4E5Q2-F1-MODEL\_V4 | 1.0 | 2.853e-17 | 680 | 0.533 | 165 | 73 | 2 | 1 | 162 | 61 | 224 | Single-stranded DNA-binding protein | Single-stranded DNA-binding protein | | afdb-uniprot50 | AF-A7TUJ4-F1-MODEL\_V4 | 1.0 | 5.816e-15 | 679 | 0.631 | 114 | 36 | 3 | 1 | 113 | 1 | 109 | Single-stranded DNA-binding protein | Single-stranded DNA-binding protein | | afdb-uniprot50 | AF-A0A4R1NRS4-F1-MODEL\_V4 | 1.0 | 7.111e-17 | 679 | 0.506 | 162 | 71 | 4 | 1 | 162 | 1 | 153 | Single-stranded DNA-binding protein | Single-stranded DNA-binding protein | | afdb-uniprot50 | AF-A0A5Y2QF66-F1-MODEL\_V4 | 1.0 | 5.398e-18 | 679 | 0.565 | 168 | 54 | 4 | 5 | 162 | 1 | 159 | Single-stranded DNA-binding protein | Single-stranded DNA-binding protein | | afdb-uniprot50 | AF-A0A5N7ZQN8-F1-MODEL\_V4 | 1.0 | 5.512e-15 | 678 | 0.552 | 123 | 50 | 3 | 1 | 122 | 1 | 119 | Single-stranded DNA-binding protein | Single-stranded DNA-binding protein | | afdb-uniprot50 | AF-A0A3B8PC41-F1-MODEL\_V4 | 1.0 | 2.723e-16 | 678 | 0.542 | 142 | 60 | 2 | 1 | 142 | 1 | 137 | Single-stranded DNA-binding protein | Single-stranded DNA-binding protein | | afdb-uniprot50 | AF-A0A2N1WWE7-F1-MODEL\_V4 | 1.0 | 9.301e-17 | 678 | 0.503 | 151 | 74 | 1 | 1 | 151 | 1 | 150 | Single-stranded DNA-binding protein | Single-stranded DNA-binding protein | | afdb-uniprot50 | AF-X2JM87-F1-MODEL\_V4 | 1.0 | 1.959e-17 | 678 | 0.458 | 170 | 81 | 2 | 2 | 162 | 156 | 323 | Single-stranded DNA-binding protein | Single-stranded DNA-binding protein | | afdb-uniprot50 | AF-Q1XGN9-F1-MODEL\_V4 | 1.0 | 9.814e-17 | 677 | 0.532 | 154 | 67 | 2 | 1 | 154 | 1 | 149 | Single-stranded DNA-binding protein | Single-stranded DNA-binding protein | | afdb-uniprot50 | AF-A0A2D5QH01-F1-MODEL\_V4 | 1.0 | 3.938e-17 | 677 | 0.521 | 165 | 72 | 3 | 1 | 162 | 1 | 161 | Single-stranded DNA-binding protein | Single-stranded DNA-binding protein | | afdb-uniprot50 | AF-A0A7C9GLE3-F1-MODEL\_V4 | 1.0 | 4.627e-17 | 677 | 0.472 | 161 | 83 | 2 | 1 | 160 | 1 | 160 | Single-stranded DNA-binding protein | Single-stranded DNA-binding protein | | afdb-uniprot50 | AF-A0A2N7Y370-F1-MODEL\_V4 | 1.0 | 1.959e-17 | 675 | 0.539 | 165 | 65 | 4 | 1 | 162 | 1 | 157 | Single-stranded DNA-binding protein | Single-stranded DNA-binding protein | | afdb-uniprot50 | AF-A0A427EMB1-F1-MODEL\_V4 | 1.0 | 2.853e-17 | 675 | 0.503 | 165 | 74 | 3 | 1 | 162 | 1 | 160 | Single-stranded DNA-binding protein | Single-stranded DNA-binding protein | | afdb-uniprot50 | AF-A0A1H4ZXY2-F1-MODEL\_V4 | 1.0 | 4.882e-17 | 675 | 0.512 | 162 | 77 | 2 | 1 | 162 | 1 | 160 | Single-stranded DNA-binding protein | Single-stranded DNA-binding protein | | afdb-uniprot50 | AF-A0A377WAK0-F1-MODEL\_V4 | 1.0 | 8.754e-18 | 675 | 0.497 | 167 | 71 | 3 | 1 | 162 | 1 | 159 | Single-stranded DNA-binding protein | Single-stranded DNA-binding protein | | afdb-uniprot50 | AF-A0A5K1SDM4-F1-MODEL\_V4 | 1.0 | 6.052e-17 | 674 | 0.515 | 159 | 64 | 3 | 4 | 162 | 3 | 148 | Single-stranded DNA-binding protein | Single-stranded DNA-binding protein | | afdb-uniprot50 | AF-A0A437JJK4-F1-MODEL\_V4 | 1.0 | 6.386e-17 | 674 | 0.545 | 154 | 65 | 2 | 1 | 154 | 1 | 149 | Single-stranded DNA-binding protein | Single-stranded DNA-binding protein | | afdb-uniprot50 | AF-A0A346E026-F1-MODEL\_V4 | 1.0 | 9.814e-17 | 673 | 0.478 | 163 | 71 | 3 | 1 | 162 | 1 | 150 | Single-stranded DNA-binding protein | Single-stranded DNA-binding protein | | afdb-uniprot50 | AF-A0A378N1F6-F1-MODEL\_V4 | 1.0 | 1.797e-14 | 672 | 0.598 | 102 | 40 | 1 | 1 | 102 | 1 | 101 | Single-stranded DNA-binding protein | Single-stranded DNA-binding protein | | afdb-uniprot50 | AF-A0A5E5QEC3-F1-MODEL\_V4 | 1.0 | 9.951e-15 | 672 | 0.508 | 120 | 56 | 3 | 1 | 118 | 1 | 119 | Single-stranded DNA-binding protein | Single-stranded DNA-binding protein | | afdb-uniprot50 | AF-A0A7G7WKN4-F1-MODEL\_V4 | 1.0 | 9.366e-16 | 672 | 0.609 | 133 | 50 | 2 | 1 | 132 | 1 | 132 | Single-stranded DNA-binding protein | Single-stranded DNA-binding protein | | afdb-uniprot50 | AF-A0A7Z9WS61-F1-MODEL\_V4 | 1.0 | 1.76e-17 | 672 | 0.484 | 165 | 80 | 2 | 1 | 162 | 1 | 163 | Single-stranded DNA-binding protein | Single-stranded DNA-binding protein | | afdb-uniprot50 | AF-A0A3Q9MM08-F1-MODEL\_V4 | 1.0 | 1.58e-17 | 672 | 0.473 | 169 | 82 | 1 | 1 | 162 | 2 | 170 | Single-stranded DNA-binding protein | Single-stranded DNA-binding protein | | afdb-uniprot50 | AF-A0A844P9B2-F1-MODEL\_V4 | 1.0 | 4.021e-14 | 671 | 0.447 | 114 | 62 | 1 | 1 | 113 | 2 | 115 | Single-stranded DNA-binding protein | Single-stranded DNA-binding protein | | afdb-uniprot50 | AF-A0A2D8P5X7-F1-MODEL\_V4 | 1.0 | 2.853e-17 | 671 | 0.487 | 160 | 77 | 2 | 4 | 162 | 2 | 157 | Single-stranded DNA-binding protein | Single-stranded DNA-binding protein | | afdb-uniprot50 | AF-A0A4D6Y9S2-F1-MODEL\_V4 | 1.0 | 3.177e-17 | 671 | 0.45 | 171 | 79 | 2 | 1 | 162 | 1 | 165 | Single-stranded DNA-binding protein | Single-stranded DNA-binding protein | | afdb-uniprot50 | AF-A0A6G7A8E4-F1-MODEL\_V4 | 1.0 | 1.275e-17 | 670 | 0.538 | 169 | 70 | 4 | 1 | 162 | 1 | 168 | Single-stranded DNA-binding protein | Single-stranded DNA-binding protein | | afdb-uniprot50 | AF-A0A839HF94-F1-MODEL\_V4 | 1.0 | 4.627e-17 | 670 | 0.443 | 160 | 89 | 0 | 1 | 160 | 2 | 161 | Single-stranded DNA-binding protein | Single-stranded DNA-binding protein | | afdb-uniprot50 | AF-A0A2V1GX48-F1-MODEL\_V4 | 1.0 | 2.704e-17 | 670 | 0.49 | 163 | 78 | 3 | 1 | 162 | 2 | 160 | Single-stranded DNA-binding protein | Single-stranded DNA-binding protein | | afdb-uniprot50 | AF-A0A220SXH4-F1-MODEL\_V4 | 1.0 | 1.58e-17 | 670 | 0.483 | 180 | 69 | 4 | 1 | 162 | 2 | 175 | Single-stranded DNA-binding protein | Single-stranded DNA-binding protein | | afdb-uniprot50 | AF-A0A6S6RS03-F1-MODEL\_V4 | 1.0 | 2.227e-14 | 669 | 0.548 | 113 | 48 | 1 | 1 | 113 | 1 | 110 | Single-stranded DNA-binding protein | Single-stranded DNA-binding protein | | afdb-uniprot50 | AF-A0A6I7PW49-F1-MODEL\_V4 | 1.0 | 1.87e-16 | 669 | 0.435 | 163 | 75 | 3 | 1 | 162 | 2 | 148 | Single-stranded DNA-binding protein | Single-stranded DNA-binding protein | | afdb-uniprot50 | AF-Q0E842-F1-MODEL\_V4 | 1.0 | 1.345e-17 | 669 | 0.537 | 162 | 62 | 5 | 1 | 162 | 15 | 163 | Single-stranded DNA-binding protein | Single-stranded DNA-binding protein | | afdb-uniprot50 | AF-A0A023RQ42-F1-MODEL\_V4 | 1.0 | 1.498e-17 | 669 | 0.5 | 190 | 67 | 5 | 1 | 162 | 2 | 191 | Single-stranded DNA-binding protein | Single-stranded DNA-binding protein | | afdb-uniprot50 | AF-A0A6I4WDE5-F1-MODEL\_V4 | 1.0 | 1.419e-17 | 669 | 0.53 | 179 | 62 | 6 | 1 | 162 | 1 | 174 | Single-stranded DNA-binding protein | Single-stranded DNA-binding protein | | afdb-uniprot50 | AF-A0A3S9XDD4-F1-MODEL\_V4 | 1.0 | 2.096e-15 | 668 | 0.53 | 130 | 55 | 2 | 1 | 130 | 2 | 125 | Single-stranded DNA-binding protein | Single-stranded DNA-binding protein | | afdb-uniprot50 | AF-A0A495VCG3-F1-MODEL\_V4 | 1.0 | 6.386e-17 | 668 | 0.469 | 162 | 78 | 2 | 1 | 162 | 1 | 154 | Single-stranded DNA-binding protein | Single-stranded DNA-binding protein | | afdb-uniprot50 | AF-A0A1Z9NM89-F1-MODEL\_V4 | 1.0 | 3.032e-16 | 667 | 0.484 | 163 | 56 | 4 | 1 | 162 | 1 | 136 | Single-stranded DNA-binding protein | Single-stranded DNA-binding protein | | afdb-uniprot50 | AF-A0A4V1B5F1-F1-MODEL\_V4 | 1.0 | 2.082e-16 | 666 | 0.515 | 161 | 62 | 4 | 3 | 162 | 2 | 147 | Single-stranded DNA-binding protein | Single-stranded DNA-binding protein | | afdb-uniprot50 | AF-A0A443XDC5-F1-MODEL\_V4 | 1.0 | 5.736e-17 | 666 | 0.488 | 172 | 76 | 4 | 1 | 162 | 2 | 171 | Single-stranded DNA-binding protein | Single-stranded DNA-binding protein | | afdb-uniprot50 | AF-R1F8G1-F1-MODEL\_V4 | 1.0 | 1.76e-17 | 666 | 0.489 | 194 | 67 | 4 | 1 | 162 | 2 | 195 | Single-stranded DNA-binding protein | Single-stranded DNA-binding protein | | afdb-uniprot50 | AF-N6YXK7-F1-MODEL\_V4 | 1.0 | 5.776e-16 | 665 | 0.541 | 146 | 57 | 4 | 1 | 146 | 1 | 136 | Single-stranded DNA-binding protein | Single-stranded DNA-binding protein | | afdb-uniprot50 | AF-A0A1F0UKE2-F1-MODEL\_V4 | 1.0 | 2.181e-17 | 664 | 0.57 | 163 | 61 | 6 | 1 | 162 | 1 | 155 | Single-stranded DNA-binding protein | Single-stranded DNA-binding protein | | afdb-uniprot50 | AF-A0A5J5FSE3-F1-MODEL\_V4 | 1.0 | 1.519e-15 | 664 | 0.474 | 135 | 64 | 1 | 4 | 138 | 219 | 346 | Single-stranded DNA-binding protein | Single-stranded DNA-binding protein | | afdb-uniprot50 | AF-A0A2E1N8X2-F1-MODEL\_V4 | 1.0 | 6.739e-17 | 663 | 0.524 | 164 | 62 | 5 | 1 | 162 | 1 | 150 | Single-stranded DNA-binding protein | Single-stranded DNA-binding protein | | afdb-uniprot50 | AF-A0A2D5RVG7-F1-MODEL\_V4 | 1.0 | 4.882e-17 | 663 | 0.531 | 160 | 68 | 3 | 1 | 160 | 1 | 153 | Single-stranded DNA-binding protein | Single-stranded DNA-binding protein | | afdb-uniprot50 | AF-A0A3G9IRR3-F1-MODEL\_V4 | 1.0 | 1.085e-17 | 663 | 0.492 | 189 | 69 | 4 | 1 | 162 | 35 | 223 | Single-stranded DNA-binding protein | Single-stranded DNA-binding protein | | afdb-uniprot50 | AF-A0A7D5LTB4-F1-MODEL\_V4 | 1.0 | 1.161e-15 | 662 | 0.478 | 142 | 73 | 1 | 1 | 142 | 1 | 141 | Single-stranded DNA-binding protein | Single-stranded DNA-binding protein | | afdb-uniprot50 | AF-A0A4R3W6J2-F1-MODEL\_V4 | 1.0 | 2.563e-17 | 662 | 0.533 | 163 | 61 | 5 | 1 | 162 | 1 | 149 | Single-stranded DNA-binding protein | Single-stranded DNA-binding protein | | afdb-uniprot50 | AF-Q88QK5-F1-MODEL\_V4 | 1.0 | 2.302e-17 | 662 | 0.489 | 182 | 72 | 4 | 1 | 162 | 1 | 181 | Single-stranded DNA-binding protein | Single-stranded DNA-binding protein | | afdb-uniprot50 | AF-U4SJQ3-F1-MODEL\_V4 | 1.0 | 6.386e-17 | 661 | 0.533 | 163 | 59 | 5 | 1 | 162 | 1 | 147 | Single-stranded DNA-binding protein | Single-stranded DNA-binding protein | | afdb-uniprot50 | AF-A0A521H8Q0-F1-MODEL\_V4 | 1.0 | 1.093e-16 | 661 | 0.466 | 163 | 80 | 4 | 1 | 162 | 1 | 157 | Single-stranded DNA-binding protein | Single-stranded DNA-binding protein | | afdb-uniprot50 | AF-D4HQH8-F1-MODEL\_V4 | 1.0 | 2.181e-17 | 661 | 0.494 | 170 | 76 | 1 | 2 | 161 | 43 | 212 | Single-stranded DNA-binding protein | Single-stranded DNA-binding protein | | afdb-uniprot50 | AF-A0A7H9QSM4-F1-MODEL\_V4 | 1.0 | 2.893e-15 | 660 | 0.477 | 136 | 68 | 2 | 1 | 135 | 1 | 134 | Single-stranded DNA-binding protein | Single-stranded DNA-binding protein | | afdb-uniprot50 | AF-A0A0E1CS86-F1-MODEL\_V4 | 1.0 | 3.732e-17 | 660 | 0.472 | 180 | 74 | 4 | 2 | 162 | 87 | 264 | Single-stranded DNA-binding protein | Single-stranded DNA-binding protein | | afdb-uniprot50 | AF-A0A3S0XBA3-F1-MODEL\_V4 | 1.0 | 1.093e-16 | 659 | 0.524 | 162 | 64 | 3 | 1 | 162 | 1 | 149 | Single-stranded DNA-binding protein | Single-stranded DNA-binding protein | | afdb-uniprot50 | AF-A0A2N1DPM0-F1-MODEL\_V4 | 1.0 | 2.853e-17 | 659 | 0.488 | 168 | 77 | 3 | 1 | 162 | 2 | 166 | Single-stranded DNA-binding protein | Single-stranded DNA-binding protein | | afdb-uniprot50 | AF-A0A0B8Q6J5-F1-MODEL\_V4 | 1.0 | 2.318e-16 | 658 | 0.503 | 147 | 64 | 2 | 1 | 146 | 2 | 140 | Single-stranded DNA-binding protein | Single-stranded DNA-binding protein | | afdb-uniprot50 | AF-A0A7Y0HDL1-F1-MODEL\_V4 | 1.0 | 1.275e-17 | 658 | 0.486 | 181 | 73 | 5 | 1 | 162 | 1 | 180 | Single-stranded DNA-binding protein | Single-stranded DNA-binding protein | | afdb-uniprot50 | AF-A0A521VAV2-F1-MODEL\_V4 | 1.0 | 4.724e-14 | 657 | 0.558 | 102 | 44 | 1 | 1 | 102 | 1 | 101 | Single-stranded DNA-binding protein | Single-stranded DNA-binding protein | | afdb-uniprot50 | AF-A0A5C2HL42-F1-MODEL\_V4 | 1.0 | 1.153e-16 | 657 | 0.457 | 164 | 84 | 3 | 1 | 162 | 1 | 161 | Single-stranded DNA-binding protein | Single-stranded DNA-binding protein | | afdb-uniprot50 | AF-A0A379VN86-F1-MODEL\_V4 | 1.0 | 6.739e-17 | 657 | 0.506 | 164 | 67 | 4 | 1 | 162 | 1 | 152 | Single-stranded DNA-binding protein | Single-stranded DNA-binding protein | | afdb-uniprot50 | AF-Q0I5U0-F1-MODEL\_V4 | 1.0 | 8.815e-17 | 656 | 0.527 | 163 | 62 | 5 | 1 | 162 | 1 | 149 | Single-stranded DNA-binding protein | Single-stranded DNA-binding protein | | afdb-uniprot50 | AF-A0A6I4RC67-F1-MODEL\_V4 | 1.0 | 1.508e-16 | 656 | 0.49 | 163 | 72 | 5 | 1 | 162 | 1 | 153 | Single-stranded DNA-binding protein | Single-stranded DNA-binding protein | | afdb-uniprot50 | AF-A0A1A5XLL0-F1-MODEL\_V4 | 1.0 | 2.446e-16 | 656 | 0.423 | 196 | 75 | 4 | 1 | 162 | 1 | 192 | Single-stranded DNA-binding protein | Single-stranded DNA-binding protein | | afdb-uniprot50 | AF-A0A5J6Q657-F1-MODEL\_V4 | 1.0 | 7.16e-16 | 655 | 0.401 | 162 | 84 | 2 | 1 | 162 | 1 | 149 | Single-stranded DNA-binding protein | Single-stranded DNA-binding protein | | afdb-uniprot50 | AF-A0A841HPC4-F1-MODEL\_V4 | 1.0 | 1.498e-17 | 655 | 0.518 | 166 | 64 | 5 | 1 | 162 | 1 | 154 | Single-strand DNA-binding protein | Single-strand DNA-binding protein | | afdb-uniprot50 | AF-E1QMP1-F1-MODEL\_V4 | 1.0 | 6.386e-17 | 655 | 0.49 | 161 | 74 | 3 | 2 | 162 | 80 | 232 | Single-stranded DNA-binding protein | Single-stranded DNA-binding protein | | afdb-uniprot50 | AF-A0A2T6GBE3-F1-MODEL\_V4 | 1.0 | 9.814e-17 | 654 | 0.439 | 166 | 88 | 2 | 1 | 162 | 1 | 165 | Single-stranded DNA-binding protein | Single-stranded DNA-binding protein | | afdb-uniprot50 | AF-A0A432WBF7-F1-MODEL\_V4 | 1.0 | 6.386e-17 | 654 | 0.478 | 165 | 80 | 3 | 1 | 162 | 2 | 163 | Single-stranded DNA-binding protein | Single-stranded DNA-binding protein | | afdb-uniprot50 | AF-A0A0E2KWP8-F1-MODEL\_V4 | 1.0 | 5.736e-17 | 654 | 0.493 | 162 | 74 | 2 | 1 | 162 | 2 | 155 | Single-stranded DNA-binding protein | Single-stranded DNA-binding protein | | afdb-uniprot50 | AF-D9Z566-F1-MODEL\_V4 | 1.0 | 9.814e-17 | 654 | 0.494 | 174 | 74 | 3 | 2 | 162 | 38 | 210 | Single-stranded DNA-binding protein | Single-stranded DNA-binding protein | | afdb-uniprot50 | AF-A0A376YLQ2-F1-MODEL\_V4 | 1.0 | 4.882e-17 | 654 | 0.478 | 186 | 65 | 5 | 1 | 162 | 41 | 218 | Single-stranded DNA-binding protein | Single-stranded DNA-binding protein | | afdb-uniprot50 | AF-A0A382MFE2-F1-MODEL\_V4 | 1.0 | 3.612e-14 | 653 | 0.516 | 122 | 55 | 2 | 1 | 122 | 1 | 118 | Uncharacterized protein | Uncharacterized protein | | afdb-uniprot50 | AF-A0A4V6Y5D9-F1-MODEL\_V4 | 1.0 | 7.21e-15 | 653 | 0.488 | 127 | 62 | 2 | 1 | 124 | 2 | 128 | Single-stranded DNA-binding protein | Single-stranded DNA-binding protein | | afdb-uniprot50 | AF-A0A427E7X0-F1-MODEL\_V4 | 1.0 | 3.938e-17 | 653 | 0.517 | 168 | 72 | 5 | 1 | 162 | 1 | 165 | Single-stranded DNA-binding protein | Single-stranded DNA-binding protein | | afdb-uniprot50 | AF-A0A6S6WKZ6-F1-MODEL\_V4 | 1.0 | 1.58e-17 | 653 | 0.497 | 187 | 66 | 4 | 1 | 162 | 2 | 185 | Single-stranded DNA-binding protein | Single-stranded DNA-binding protein | | afdb-uniprot50 | AF-A0A6B0YI34-F1-MODEL\_V4 | 1.0 | 1.519e-15 | 652 | 0.521 | 140 | 58 | 4 | 1 | 138 | 1 | 133 | Single-stranded DNA-binding protein | Single-stranded DNA-binding protein | | afdb-uniprot50 | AF-A0A1Z8N4J9-F1-MODEL\_V4 | 1.0 | 5.856e-14 | 652 | 0.456 | 116 | 60 | 1 | 1 | 116 | 1 | 113 | Single-stranded DNA-binding protein | Single-stranded DNA-binding protein | | afdb-uniprot50 | AF-A0A6N8S0Z9-F1-MODEL\_V4 | 1.0 | 2.873e-16 | 652 | 0.509 | 157 | 69 | 4 | 1 | 156 | 1 | 150 | Single-stranded DNA-binding protein | Single-stranded DNA-binding protein | | afdb-uniprot50 | AF-A0A0R2U7J0-F1-MODEL\_V4 | 1.0 | 8.47e-15 | 651 | 0.536 | 138 | 52 | 2 | 1 | 138 | 1 | 126 | Single-stranded DNA-binding protein | Single-stranded DNA-binding protein | | afdb-uniprot50 | AF-A0A450WC46-F1-MODEL\_V4 | 1.0 | 1.093e-16 | 651 | 0.515 | 165 | 62 | 4 | 1 | 162 | 1 | 150 | Single-stranded DNA-binding protein | Single-stranded DNA-binding protein | | afdb-uniprot50 | AF-A0A1E7Q8I5-F1-MODEL\_V4 | 1.0 | 9.814e-17 | 651 | 0.515 | 157 | 70 | 2 | 1 | 157 | 2 | 152 | Single-stranded DNA-binding protein | Single-stranded DNA-binding protein | | afdb-uniprot50 | AF-A0A853I684-F1-MODEL\_V4 | 1.0 | 7.917e-17 | 651 | 0.512 | 162 | 74 | 4 | 1 | 162 | 1 | 157 | Single-stranded DNA-binding protein | Single-stranded DNA-binding protein | | afdb-uniprot50 | AF-A0A4R6P349-F1-MODEL\_V4 | 1.0 | 4.95e-15 | 650 | 0.542 | 118 | 52 | 2 | 1 | 117 | 1 | 117 | Single-stranded DNA-binding protein | Single-stranded DNA-binding protein | | afdb-uniprot50 | AF-A0A3A5L220-F1-MODEL\_V4 | 1.0 | 3.537e-17 | 650 | 0.537 | 162 | 71 | 3 | 1 | 162 | 1 | 158 | Single-stranded DNA-binding protein | Single-stranded DNA-binding protein | | afdb-uniprot50 | AF-A0A6M8T6X9-F1-MODEL\_V4 | 1.0 | 9.301e-17 | 650 | 0.542 | 164 | 58 | 5 | 1 | 162 | 1 | 149 | Single-stranded DNA-binding protein | Single-stranded DNA-binding protein | | afdb-uniprot50 | AF-A0A2G2KZ72-F1-MODEL\_V4 | 1.0 | 1.153e-16 | 650 | 0.461 | 182 | 78 | 4 | 1 | 162 | 2 | 183 | Single-stranded DNA-binding protein | Single-stranded DNA-binding protein | | afdb-uniprot50 | AF-S6GE41-F1-MODEL\_V4 | 1.0 | 7.111e-17 | 650 | 0.512 | 162 | 74 | 2 | 1 | 162 | 1 | 157 | Single-stranded DNA-binding protein | Single-stranded DNA-binding protein | | afdb-uniprot50 | AF-A0A1V3JSR9-F1-MODEL\_V4 | 1.0 | 1.987e-15 | 649 | 0.529 | 136 | 58 | 3 | 1 | 135 | 1 | 131 | Single-stranded DNA-binding protein | Single-stranded DNA-binding protein | | afdb-uniprot50 | AF-A0A635JC17-F1-MODEL\_V4 | 1.0 | 1.691e-15 | 649 | 0.425 | 162 | 68 | 3 | 1 | 162 | 1 | 137 | Single-stranded DNA-binding protein | Single-stranded DNA-binding protein | | afdb-uniprot50 | AF-A0A6N7E2V4-F1-MODEL\_V4 | 1.0 | 3.537e-17 | 649 | 0.507 | 189 | 61 | 6 | 4 | 162 | 3 | 189 | Single-stranded DNA-binding protein | Single-stranded DNA-binding protein | | afdb-uniprot50 | AF-A0A3M0A0X9-F1-MODEL\_V4 | 1.0 | 9.301e-17 | 649 | 0.481 | 164 | 80 | 2 | 1 | 162 | 1 | 161 | Single-stranded DNA-binding protein | Single-stranded DNA-binding protein | | afdb-uniprot50 | AF-H9ACF8-F1-MODEL\_V4 | 1.0 | 1.05e-14 | 648 | 0.564 | 117 | 44 | 1 | 1 | 117 | 2 | 111 | Single-stranded DNA-binding protein | Single-stranded DNA-binding protein | | afdb-uniprot50 | AF-A0A1C3H441-F1-MODEL\_V4 | 1.0 | 1.508e-16 | 648 | 0.537 | 162 | 56 | 7 | 1 | 162 | 1 | 143 | Single-stranded DNA-binding protein | Single-stranded DNA-binding protein | | afdb-uniprot50 | AF-G2FAV3-F1-MODEL\_V4 | 1.0 | 8.815e-17 | 648 | 0.5 | 162 | 67 | 4 | 1 | 162 | 1 | 148 | Single-stranded DNA-binding protein | Single-stranded DNA-binding protein | | afdb-uniprot50 | AF-A0A7Y2XRP8-F1-MODEL\_V4 | 1.0 | 8.815e-17 | 648 | 0.515 | 165 | 70 | 5 | 1 | 162 | 1 | 158 | Single-stranded DNA-binding protein | Single-stranded DNA-binding protein | | afdb-uniprot50 | AF-A0A1A9Z0W8-F1-MODEL\_V4 | 1.0 | 3.732e-17 | 648 | 0.464 | 170 | 83 | 4 | 1 | 162 | 1 | 170 | Uncharacterized protein | Uncharacterized protein | | afdb-uniprot50 | AF-A0A023RSX4-F1-MODEL\_V4 | 1.0 | 4.627e-17 | 648 | 0.5 | 172 | 74 | 5 | 2 | 162 | 24 | 194 | Single-stranded DNA-binding protein | Single-stranded DNA-binding protein | | afdb-uniprot50 | AF-A0A849HYH5-F1-MODEL\_V4 | 1.0 | 4.021e-14 | 647 | 0.504 | 117 | 52 | 3 | 1 | 115 | 1 | 113 | Single-stranded DNA-binding protein | Single-stranded DNA-binding protein | | afdb-uniprot50 | AF-A0A5N0TC18-F1-MODEL\_V4 | 1.0 | 2.446e-16 | 647 | 0.503 | 163 | 72 | 4 | 1 | 162 | 1 | 155 | Single-stranded DNA-binding protein | Single-stranded DNA-binding protein | | afdb-uniprot50 | AF-A0A4R3Y4S6-F1-MODEL\_V4 | 1.0 | 3.221e-15 | 646 | 0.541 | 133 | 54 | 3 | 1 | 133 | 1 | 126 | Single-stranded DNA-binding protein | Single-stranded DNA-binding protein | | afdb-uniprot50 | AF-W1J8L6-F1-MODEL\_V4 | 1.0 | 1.036e-16 | 646 | 0.452 | 170 | 81 | 4 | 1 | 162 | 1 | 166 | Single-stranded DNA-binding protein | Single-stranded DNA-binding protein | | afdb-uniprot50 | AF-A0A7C1ZZ21-F1-MODEL\_V4 | 1.0 | 4.243e-14 | 645 | 0.564 | 108 | 46 | 1 | 1 | 108 | 1 | 107 | Single-stranded DNA-binding protein | Single-stranded DNA-binding protein | | afdb-uniprot50 | AF-A0A7D5QRG0-F1-MODEL\_V4 | 1.0 | 1.036e-16 | 645 | 0.459 | 172 | 80 | 4 | 1 | 162 | 1 | 169 | Single-stranded DNA-binding protein | Single-stranded DNA-binding protein | | afdb-uniprot50 | AF-P59927-F1-MODEL\_V4 | 1.0 | 2.302e-17 | 645 | 0.526 | 173 | 67 | 5 | 1 | 162 | 1 | 169 | Single-stranded DNA-binding protein | Single-stranded DNA-binding protein | | afdb-uniprot50 | AF-N6YC46-F1-MODEL\_V4 | 1.0 | 6.739e-17 | 644 | 0.538 | 167 | 65 | 5 | 1 | 162 | 1 | 160 | Single-stranded DNA-binding protein | Single-stranded DNA-binding protein | | afdb-uniprot50 | AF-A0A455TMM6-F1-MODEL\_V4 | 1.0 | 6.052e-17 | 644 | 0.5 | 170 | 75 | 1 | 2 | 161 | 108 | 277 | Single-stranded DNA-binding protein | Single-stranded DNA-binding protein | | afdb-uniprot50 | AF-A0A0M3G1E7-F1-MODEL\_V4 | 1.0 | 1.87e-16 | 643 | 0.503 | 163 | 63 | 4 | 1 | 162 | 1 | 146 | Single-stranded DNA-binding protein | Single-stranded DNA-binding protein | | afdb-uniprot50 | AF-A0A524PZ59-F1-MODEL\_V4 | 1.0 | 2.873e-16 | 643 | 0.5 | 162 | 70 | 4 | 1 | 162 | 1 | 151 | Single-stranded DNA-binding protein | Single-stranded DNA-binding protein | | afdb-uniprot50 | AF-A0A1H2DVA7-F1-MODEL\_V4 | 1.0 | 2.318e-16 | 643 | 0.451 | 164 | 86 | 4 | 1 | 162 | 1 | 162 | Single-stranded DNA-binding protein | Single-stranded DNA-binding protein | | afdb-uniprot50 | AF-A0A0P7E436-F1-MODEL\_V4 | 1.0 | 3.938e-17 | 643 | 0.473 | 188 | 66 | 5 | 1 | 162 | 1 | 181 | Single-stranded DNA-binding protein | Single-stranded DNA-binding protein | | afdb-uniprot50 | AF-S7U9Q9-F1-MODEL\_V4 | 1.0 | 1.772e-16 | 642 | 0.473 | 167 | 81 | 3 | 1 | 162 | 1 | 165 | Single-stranded DNA-binding protein | Single-stranded DNA-binding protein | | afdb-uniprot50 | AF-A0A2D2CLI4-F1-MODEL\_V4 | 1.0 | 3.938e-17 | 642 | 0.492 | 189 | 69 | 4 | 1 | 162 | 67 | 255 | Single-stranded DNA-binding protein | Single-stranded DNA-binding protein | | afdb-uniprot50 | AF-A0A7V5VC36-F1-MODEL\_V4 | 1.0 | 3.376e-16 | 641 | 0.509 | 163 | 65 | 4 | 1 | 162 | 2 | 150 | Single-stranded DNA-binding protein | Single-stranded DNA-binding protein | | afdb-uniprot50 | AF-A0A3M3QJJ1-F1-MODEL\_V4 | 1.0 | 1.857e-17 | 641 | 0.486 | 187 | 70 | 5 | 1 | 162 | 7 | 192 | Single-stranded DNA-binding protein | Single-stranded DNA-binding protein | | afdb-uniprot50 | AF-A0A2N0WZ92-F1-MODEL\_V4 | 1.0 | 1.284e-16 | 640 | 0.49 | 165 | 75 | 5 | 1 | 162 | 2 | 160 | Single-stranded DNA-binding protein | Single-stranded DNA-binding protein | | afdb-uniprot50 | AF-A0A833GI75-F1-MODEL\_V4 | 1.0 | 2.599e-15 | 639 | 0.463 | 151 | 70 | 3 | 1 | 151 | 1 | 140 | Single-stranded DNA-binding protein | Single-stranded DNA-binding protein | | afdb-uniprot50 | AF-A0A1D8KD04-F1-MODEL\_V4 | 1.0 | 4.659e-16 | 639 | 0.468 | 160 | 75 | 3 | 3 | 162 | 2 | 151 | Single-stranded DNA-binding protein | Single-stranded DNA-binding protein | | afdb-uniprot50 | AF-A0A0N0IGT7-F1-MODEL\_V4 | 1.0 | 7.16e-16 | 638 | 0.454 | 154 | 71 | 3 | 1 | 154 | 1 | 141 | Single-stranded DNA-binding protein | Single-stranded DNA-binding protein | | afdb-uniprot50 | AF-A0A523MI06-F1-MODEL\_V4 | 1.0 | 3.199e-16 | 638 | 0.496 | 163 | 68 | 5 | 1 | 162 | 1 | 150 | Single-stranded DNA-binding protein | Single-stranded DNA-binding protein | | afdb-uniprot50 | AF-A0A482ZYE6-F1-MODEL\_V4 | 1.0 | 1.429e-16 | 638 | 0.527 | 163 | 68 | 4 | 1 | 162 | 1 | 155 | Single-stranded DNA-binding protein | Single-stranded DNA-binding protein | | afdb-uniprot50 | AF-A0A7C8ECW2-F1-MODEL\_V4 | 1.0 | 1.374e-14 | 638 | 0.472 | 125 | 60 | 2 | 3 | 127 | 27 | 145 | Single-stranded DNA-binding protein | Single-stranded DNA-binding protein | | afdb-uniprot50 | AF-A0A375IS87-F1-MODEL\_V4 | 1.0 | 1.093e-16 | 638 | 0.491 | 175 | 72 | 4 | 1 | 162 | 1 | 171 | Single-stranded DNA-binding protein | Single-stranded DNA-binding protein | | afdb-uniprot50 | AF-A0A1S2DF59-F1-MODEL\_V4 | 1.0 | 6.386e-17 | 638 | 0.488 | 176 | 73 | 4 | 1 | 162 | 1 | 173 | Single-stranded DNA-binding protein | Single-stranded DNA-binding protein | | afdb-uniprot50 | AF-A0A7C4XSU7-F1-MODEL\_V4 | 1.0 | 1.108e-14 | 637 | 0.589 | 117 | 45 | 3 | 1 | 117 | 1 | 114 | Single-stranded DNA-binding protein | Single-stranded DNA-binding protein | | afdb-uniprot50 | AF-A0A2X4DRV2-F1-MODEL\_V4 | 1.0 | 2.873e-16 | 637 | 0.533 | 163 | 53 | 5 | 1 | 162 | 1 | 141 | Single-stranded DNA-binding protein | Single-stranded DNA-binding protein | | afdb-uniprot50 | AF-A0A1V3K7V4-F1-MODEL\_V4 | 1.0 | 1.603e-15 | 637 | 0.492 | 138 | 63 | 4 | 1 | 137 | 1 | 132 | Single-stranded DNA-binding protein | Single-stranded DNA-binding protein | | afdb-uniprot50 | AF-A0A1J5S6P7-F1-MODEL\_V4 | 1.0 | 2.723e-16 | 637 | 0.512 | 162 | 70 | 5 | 1 | 162 | 1 | 153 | Single-stranded DNA-binding protein | Single-stranded DNA-binding protein | | afdb-uniprot50 | AF-A0A0F6L5J4-F1-MODEL\_V4 | 1.0 | 1.177e-13 | 636 | 0.526 | 112 | 49 | 2 | 4 | 115 | 1 | 108 | Single-stranded DNA-binding protein | Single-stranded DNA-binding protein | | afdb-uniprot50 | AF-A0A832N208-F1-MODEL\_V4 | 1.0 | 1.217e-16 | 636 | 0.533 | 165 | 56 | 7 | 1 | 162 | 1 | 147 | Single-stranded DNA-binding protein | Single-stranded DNA-binding protein | | afdb-uniprot50 | AF-A0A2H0DHP9-F1-MODEL\_V4 | 1.0 | 2.563e-17 | 636 | 0.511 | 180 | 66 | 5 | 1 | 162 | 1 | 176 | Single-stranded DNA-binding protein | Single-stranded DNA-binding protein | | afdb-uniprot50 | AF-A0A7C8AUH4-F1-MODEL\_V4 | 1.0 | 8.876e-16 | 635 | 0.521 | 163 | 57 | 5 | 1 | 162 | 1 | 143 | Single-stranded DNA-binding protein | Single-stranded DNA-binding protein | | afdb-uniprot50 | AF-A0A556UN06-F1-MODEL\_V4 | 1.0 | 7.972e-16 | 635 | 0.5 | 162 | 62 | 4 | 1 | 162 | 1 | 143 | Single-stranded DNA-binding protein | Single-stranded DNA-binding protein | | afdb-uniprot50 | AF-A0A5E4RMM5-F1-MODEL\_V4 | 1.0 | 1.429e-16 | 635 | 0.488 | 170 | 73 | 4 | 1 | 162 | 1 | 164 | Single-stranded DNA-binding protein | Single-stranded DNA-binding protein | | afdb-uniprot50 | AF-A0A165FGM2-F1-MODEL\_V4 | 1.0 | 2.082e-16 | 635 | 0.471 | 174 | 74 | 5 | 1 | 162 | 1 | 168 | Single-stranded DNA-binding protein | Single-stranded DNA-binding protein | | afdb-uniprot50 | AF-E1VGN3-F1-MODEL\_V4 | 1.0 | 7.917e-17 | 635 | 0.466 | 180 | 75 | 4 | 1 | 162 | 2 | 178 | Single-stranded DNA-binding protein | Single-stranded DNA-binding protein | | afdb-uniprot50 | AF-A0A832XC38-F1-MODEL\_V4 | 1.0 | 6.052e-17 | 635 | 0.429 | 198 | 72 | 6 | 1 | 162 | 1 | 193 | Single-stranded DNA-binding protein | Single-stranded DNA-binding protein | | afdb-uniprot50 | AF-A0A1F4BVL2-F1-MODEL\_V4 | 1.0 | 1.987e-15 | 634 | 0.545 | 132 | 58 | 2 | 1 | 132 | 1 | 130 | Single-stranded DNA-binding protein | Single-stranded DNA-binding protein | | afdb-uniprot50 | AF-A0A0Q8U118-F1-MODEL\_V4 | 1.0 | 7.503e-17 | 634 | 0.526 | 167 | 65 | 5 | 1 | 162 | 1 | 158 | Single-stranded DNA-binding protein | Single-stranded DNA-binding protein | | afdb-uniprot50 | AF-A0A3T0K3E2-F1-MODEL\_V4 | 1.0 | 1.284e-16 | 634 | 0.473 | 169 | 75 | 4 | 1 | 162 | 1 | 162 | Single-stranded DNA-binding protein | Single-stranded DNA-binding protein | | afdb-uniprot50 | AF-A0A3Q8WB38-F1-MODEL\_V4 | 1.0 | 1.153e-16 | 634 | 0.487 | 162 | 76 | 2 | 1 | 162 | 1 | 155 | Single-stranded DNA-binding protein | Single-stranded DNA-binding protein | | afdb-uniprot50 | AF-A0A651I5E3-F1-MODEL\_V4 | 1.0 | 7.16e-16 | 633 | 0.455 | 156 | 78 | 3 | 1 | 156 | 14 | 162 | Single-stranded DNA-binding protein | Single-stranded DNA-binding protein | | afdb-uniprot50 | AF-A0A353PC78-F1-MODEL\_V4 | 1.0 | 8.354e-17 | 633 | 0.477 | 178 | 74 | 4 | 1 | 162 | 2 | 176 | Single-stranded DNA-binding protein | Single-stranded DNA-binding protein | | afdb-uniprot50 | AF-A0A379ANG6-F1-MODEL\_V4 | 1.0 | 4.156e-17 | 633 | 0.508 | 183 | 64 | 5 | 1 | 162 | 1 | 178 | Single-stranded DNA-binding protein | Single-stranded DNA-binding protein | | afdb-uniprot50 | AF-A0A143WNF0-F1-MODEL\_V4 | 1.0 | 5.856e-14 | 632 | 0.527 | 110 | 51 | 1 | 1 | 110 | 1 | 109 | Single-stranded DNA-binding protein | Single-stranded DNA-binding protein | | afdb-uniprot50 | AF-A0A0R2PAL9-F1-MODEL\_V4 | 1.0 | 3.562e-16 | 632 | 0.503 | 163 | 62 | 5 | 1 | 162 | 1 | 145 | Single-stranded DNA-binding protein | Single-stranded DNA-binding protein | | afdb-uniprot50 | AF-A4VHR0-F1-MODEL\_V4 | 1.0 | 8.815e-17 | 632 | 0.518 | 164 | 68 | 5 | 5 | 162 | 1 | 159 | Single-stranded DNA-binding protein | Single-stranded DNA-binding protein | | afdb-uniprot50 | AF-A0A7J6YTU1-F1-MODEL\_V4 | 1.0 | 2.742e-15 | 632 | 0.506 | 144 | 65 | 4 | 1 | 142 | 1 | 140 | BON domain-containing protein | BON domain-containing protein | | afdb-uniprot50 | AF-B3PK66-F1-MODEL\_V4 | 1.0 | 1.093e-16 | 631 | 0.472 | 199 | 65 | 5 | 1 | 162 | 40 | 235 | Single-stranded DNA-binding protein | Single-stranded DNA-binding protein | | afdb-uniprot50 | AF-W7Q024-F1-MODEL\_V4 | 1.0 | 4.659e-16 | 630 | 0.487 | 162 | 69 | 3 | 1 | 162 | 1 | 148 | Single-stranded DNA-binding protein | Single-stranded DNA-binding protein | | afdb-uniprot50 | AF-G2JBS1-F1-MODEL\_V4 | 1.0 | 3.199e-16 | 630 | 0.521 | 165 | 65 | 5 | 1 | 162 | 1 | 154 | Single-stranded DNA-binding protein | Single-stranded DNA-binding protein | | afdb-uniprot50 | AF-G2DCM5-F1-MODEL\_V4 | 1.0 | 6.739e-17 | 630 | 0.5 | 162 | 67 | 3 | 1 | 162 | 9 | 156 | Single-stranded DNA-binding protein | Single-stranded DNA-binding protein | | afdb-uniprot50 | AF-A0A0J5P3F7-F1-MODEL\_V4 | 1.0 | 3.352e-17 | 630 | 0.524 | 185 | 57 | 7 | 1 | 162 | 1 | 177 | Single-stranded DNA-binding protein | Single-stranded DNA-binding protein | | afdb-uniprot50 | AF-A0A375HE91-F1-MODEL\_V4 | 1.0 | 4.446e-15 | 630 | 0.517 | 139 | 54 | 3 | 1 | 139 | 1 | 126 | Single-stranded DNA-binding protein | Single-stranded DNA-binding protein | | afdb-uniprot50 | AF-A0A3N2D590-F1-MODEL\_V4 | 1.0 | 2.082e-16 | 630 | 0.461 | 169 | 81 | 2 | 1 | 162 | 2 | 167 | Single-stranded DNA-binding protein | Single-stranded DNA-binding protein | | afdb-uniprot50 | AF-A0A4Q3R8Z3-F1-MODEL\_V4 | 1.0 | 3.053e-15 | 629 | 0.5 | 140 | 61 | 4 | 1 | 137 | 1 | 134 | Single-stranded DNA-binding protein | Single-stranded DNA-binding protein | | afdb-uniprot50 | AF-A0A1A7Q9A2-F1-MODEL\_V4 | 1.0 | 7.111e-17 | 629 | 0.52 | 175 | 66 | 5 | 1 | 162 | 1 | 170 | Single-stranded DNA-binding protein | Single-stranded DNA-binding protein | | afdb-uniprot50 | AF-A0A2D6NS79-F1-MODEL\_V4 | 1.0 | 2.082e-16 | 629 | 0.5 | 158 | 69 | 5 | 1 | 155 | 1 | 151 | Single-stranded DNA-binding protein | Single-stranded DNA-binding protein | | afdb-uniprot50 | AF-A0A849P2X6-F1-MODEL\_V4 | 1.0 | 3.399e-15 | 628 | 0.472 | 161 | 59 | 3 | 1 | 161 | 1 | 135 | Single-stranded DNA-binding protein | Single-stranded DNA-binding protein | | afdb-uniprot50 | AF-A0A2E7MNJ4-F1-MODEL\_V4 | 1.0 | 1.1e-15 | 628 | 0.472 | 161 | 68 | 3 | 2 | 162 | 3 | 146 | Single-stranded DNA-binding protein | Single-stranded DNA-binding protein | | afdb-uniprot50 | AF-A0A1F7FD92-F1-MODEL\_V4 | 1.0 | 3.562e-16 | 628 | 0.432 | 162 | 88 | 3 | 1 | 162 | 1 | 158 | Single-stranded DNA-binding protein | Single-stranded DNA-binding protein | | afdb-uniprot50 | AF-A0A0E3Y7R2-F1-MODEL\_V4 | 1.0 | 2.723e-16 | 628 | 0.436 | 174 | 74 | 3 | 1 | 162 | 2 | 163 | Single-stranded DNA-binding protein | Single-stranded DNA-binding protein | | afdb-uniprot50 | AF-A0A661EDJ0-F1-MODEL\_V4 | 1.0 | 6.094e-16 | 628 | 0.43 | 165 | 88 | 2 | 1 | 162 | 1 | 162 | Single-stranded DNA-binding protein | Single-stranded DNA-binding protein | | afdb-uniprot50 | AF-A0A380N429-F1-MODEL\_V4 | 1.0 | 5.187e-16 | 628 | 0.457 | 164 | 83 | 3 | 1 | 162 | 2 | 161 | Single-stranded DNA-binding protein | Single-stranded DNA-binding protein | | afdb-uniprot50 | AF-A0A154R4W2-F1-MODEL\_V4 | 1.0 | 4.185e-16 | 628 | 0.439 | 166 | 86 | 2 | 1 | 162 | 2 | 164 | Single-stranded DNA-binding protein | Single-stranded DNA-binding protein | | afdb-uniprot50 | AF-A0A561JT25-F1-MODEL\_V4 | 1.0 | 1.439e-15 | 627 | 0.493 | 162 | 66 | 3 | 1 | 162 | 1 | 146 | Single-stranded DNA-binding protein | Single-stranded DNA-binding protein | | afdb-uniprot50 | AF-A0A4U0YG63-F1-MODEL\_V4 | 1.0 | 7.16e-16 | 627 | 0.529 | 155 | 64 | 4 | 1 | 154 | 2 | 148 | Single-stranded DNA-binding protein | Single-stranded DNA-binding protein | | afdb-uniprot50 | AF-A0A381ECU1-F1-MODEL\_V4 | 1.0 | 1.87e-16 | 627 | 0.45 | 171 | 79 | 3 | 4 | 162 | 3 | 170 | Single-stranded DNA-binding protein | Single-stranded DNA-binding protein | | afdb-uniprot50 | AF-A0A3D1EPD5-F1-MODEL\_V4 | 1.0 | 1.311e-13 | 626 | 0.555 | 108 | 46 | 2 | 1 | 108 | 1 | 106 | Single-stranded DNA-binding protein | Single-stranded DNA-binding protein | | afdb-uniprot50 | AF-V8G2X8-F1-MODEL\_V4 | 1.0 | 1.05e-14 | 626 | 0.511 | 129 | 55 | 3 | 1 | 129 | 1 | 121 | Single-stranded DNA-binding protein | Single-stranded DNA-binding protein | | afdb-uniprot50 | AF-A0A7X7BUF0-F1-MODEL\_V4 | 1.0 | 1.603e-15 | 626 | 0.503 | 163 | 65 | 4 | 1 | 162 | 2 | 149 | Single-stranded DNA-binding protein | Single-stranded DNA-binding protein | | afdb-uniprot50 | AF-A0A5Q0EJT3-F1-MODEL\_V4 | 1.0 | 3.199e-16 | 626 | 0.506 | 166 | 64 | 4 | 2 | 162 | 3 | 155 | Single-stranded DNA-binding protein | Single-stranded DNA-binding protein | | afdb-uniprot50 | AF-A0A857TRT2-F1-MODEL\_V4 | 1.0 | 3.966e-16 | 626 | 0.442 | 165 | 85 | 4 | 1 | 162 | 2 | 162 | Single-stranded DNA-binding protein | Single-stranded DNA-binding protein | | afdb-uniprot50 | AF-A0A2E9KR01-F1-MODEL\_V4 | 1.0 | 1.1e-15 | 625 | 0.466 | 163 | 68 | 3 | 1 | 162 | 1 | 145 | Single-stranded DNA-binding protein | Single-stranded DNA-binding protein | | afdb-uniprot50 | AF-A0A1F9Y6H0-F1-MODEL\_V4 | 1.0 | 1.364e-15 | 625 | 0.401 | 162 | 91 | 3 | 1 | 162 | 1 | 156 | Single-stranded DNA-binding protein | Single-stranded DNA-binding protein | | afdb-uniprot50 | AF-A0A1B4G7F0-F1-MODEL\_V4 | 1.0 | 9.301e-17 | 625 | 0.471 | 174 | 76 | 5 | 1 | 162 | 1 | 170 | Single-stranded DNA-binding protein | Single-stranded DNA-binding protein | | afdb-uniprot50 | AF-G4MIH5-F1-MODEL\_V4 | 1.0 | 3.562e-16 | 625 | 0.443 | 176 | 81 | 3 | 1 | 162 | 28 | 200 | Single-stranded DNA-binding protein | Single-stranded DNA-binding protein | | afdb-uniprot50 | AF-Q9PHE7-F1-MODEL\_V4 | 1.0 | 6.475e-15 | 624 | 0.432 | 162 | 66 | 3 | 1 | 162 | 1 | 136 | Single-stranded DNA-binding protein 1 | Single-stranded DNA-binding protein 1 | | afdb-uniprot50 | AF-A0A261RM57-F1-MODEL\_V4 | 1.0 | 3.785e-15 | 624 | 0.447 | 163 | 63 | 5 | 1 | 162 | 1 | 137 | Single-stranded DNA-binding protein | Single-stranded DNA-binding protein | | afdb-uniprot50 | AF-G9ZJG1-F1-MODEL\_V4 | 1.0 | 3.785e-15 | 624 | 0.489 | 143 | 66 | 2 | 2 | 140 | 17 | 156 | Single-stranded DNA-binding protein | Single-stranded DNA-binding protein | | afdb-uniprot50 | AF-A0A516V4K4-F1-MODEL\_V4 | 1.0 | 4.416e-16 | 624 | 0.439 | 164 | 86 | 3 | 1 | 162 | 1 | 160 | Single-stranded DNA-binding protein | Single-stranded DNA-binding protein | | afdb-uniprot50 | AF-A0A451CMM4-F1-MODEL\_V4 | 1.0 | 3.199e-16 | 624 | 0.437 | 160 | 84 | 2 | 1 | 160 | 1 | 154 | Single-stranded DNA-binding protein | Single-stranded DNA-binding protein | | afdb-uniprot50 | AF-A0A348HBX9-F1-MODEL\_V4 | 1.0 | 1.679e-16 | 623 | 0.482 | 168 | 76 | 4 | 1 | 162 | 26 | 188 | Single-stranded DNA-binding protein | Single-stranded DNA-binding protein | | afdb-uniprot50 | AF-A0A522K774-F1-MODEL\_V4 | 1.0 | 3.266e-13 | 622 | 0.512 | 117 | 52 | 3 | 1 | 116 | 1 | 113 | Single-stranded DNA-binding protein | Single-stranded DNA-binding protein | | afdb-uniprot50 | AF-A6EM42-F1-MODEL\_V4 | 1.0 | 9.496e-14 | 622 | 0.434 | 122 | 66 | 1 | 1 | 122 | 1 | 119 | Single-stranded DNA-binding protein | Single-stranded DNA-binding protein | | afdb-uniprot50 | AF-A0A2G2QFS4-F1-MODEL\_V4 | 1.0 | 5.187e-16 | 622 | 0.471 | 159 | 75 | 3 | 4 | 162 | 2 | 151 | Single-stranded DNA-binding protein | Single-stranded DNA-binding protein | | afdb-uniprot50 | AF-A0A1M5YJ27-F1-MODEL\_V4 | 1.0 | 1.987e-15 | 622 | 0.453 | 163 | 77 | 6 | 1 | 162 | 1 | 152 | Single-stranded DNA-binding protein | Single-stranded DNA-binding protein | | afdb-uniprot50 | AF-A0A2M7SYI0-F1-MODEL\_V4 | 1.0 | 3.966e-16 | 622 | 0.469 | 164 | 76 | 4 | 1 | 162 | 1 | 155 | Single-stranded DNA-binding protein | Single-stranded DNA-binding protein | | afdb-uniprot50 | AF-A0A3D4KFN2-F1-MODEL\_V4 | 1.0 | 8.412e-16 | 622 | 0.49 | 159 | 77 | 2 | 4 | 162 | 3 | 157 | Single-stranded DNA-binding protein | Single-stranded DNA-binding protein | | afdb-uniprot50 | AF-A0A431KLH7-F1-MODEL\_V4 | 1.0 | 3.562e-16 | 622 | 0.475 | 166 | 78 | 5 | 1 | 162 | 1 | 161 | Single-stranded DNA-binding protein | Single-stranded DNA-binding protein | | afdb-uniprot50 | AF-A0A380MSC4-F1-MODEL\_V4 | 1.0 | 5.776e-16 | 621 | 0.503 | 163 | 64 | 5 | 1 | 162 | 1 | 147 | Single-stranded DNA-binding protein | Single-stranded DNA-binding protein | | afdb-uniprot50 | AF-A0A1E4ZSC0-F1-MODEL\_V4 | 1.0 | 4.416e-16 | 621 | 0.469 | 162 | 75 | 5 | 4 | 162 | 3 | 156 | Single-stranded DNA-binding protein | Single-stranded DNA-binding protein | | afdb-uniprot50 | AF-A0A6H9SJ48-F1-MODEL\_V4 | 1.0 | 7.16e-16 | 621 | 0.484 | 157 | 74 | 4 | 1 | 154 | 1 | 153 | Single-stranded DNA-binding protein | Single-stranded DNA-binding protein | | afdb-uniprot50 | AF-A0A2J9QVZ0-F1-MODEL\_V4 | 1.0 | 3.095e-13 | 619 | 0.457 | 118 | 61 | 3 | 1 | 117 | 1 | 116 | Single-stranded DNA-binding protein | Single-stranded DNA-binding protein | | afdb-uniprot50 | AF-A0A7C4FIT9-F1-MODEL\_V4 | 1.0 | 1.529e-14 | 619 | 0.472 | 127 | 61 | 3 | 1 | 127 | 1 | 121 | Single-stranded DNA-binding protein | Single-stranded DNA-binding protein | | afdb-uniprot50 | AF-A0A6L3NBL8-F1-MODEL\_V4 | 1.0 | 2.096e-15 | 619 | 0.487 | 154 | 68 | 4 | 1 | 154 | 1 | 143 | Single-stranded DNA-binding protein | Single-stranded DNA-binding protein | | afdb-uniprot50 | AF-A0A1F9LDU6-F1-MODEL\_V4 | 1.0 | 3.447e-13 | 618 | 0.485 | 107 | 52 | 2 | 8 | 114 | 1 | 104 | Single-stranded DNA-binding protein | Single-stranded DNA-binding protein | | afdb-uniprot50 | AF-A0A3D4GG74-F1-MODEL\_V4 | 1.0 | 1.459e-13 | 618 | 0.462 | 121 | 62 | 2 | 1 | 121 | 2 | 119 | Single-stranded DNA-binding protein | Single-stranded DNA-binding protein | | afdb-uniprot50 | AF-A0A2D7RFL5-F1-MODEL\_V4 | 1.0 | 2.599e-15 | 618 | 0.493 | 162 | 65 | 3 | 1 | 162 | 1 | 145 | Single-stranded DNA-binding protein | Single-stranded DNA-binding protein | | afdb-uniprot50 | AF-A0A4R7QAL6-F1-MODEL\_V4 | 1.0 | 1.909e-13 | 618 | 0.46 | 115 | 59 | 1 | 4 | 118 | 1 | 112 | Single-stranded DNA-binding protein | Single-stranded DNA-binding protein | | afdb-uniprot50 | AF-S7UTG7-F1-MODEL\_V4 | 1.0 | 3.032e-16 | 618 | 0.446 | 177 | 83 | 5 | 1 | 162 | 1 | 177 | Single-stranded DNA-binding protein | Single-stranded DNA-binding protein | | afdb-uniprot50 | AF-A0A7V8JJS2-F1-MODEL\_V4 | 1.0 | 1.987e-15 | 617 | 0.421 | 173 | 86 | 3 | 1 | 162 | 1 | 170 | Single-stranded DNA-binding protein | Single-stranded DNA-binding protein | | afdb-uniprot50 | AF-A0A2G6T2T9-F1-MODEL\_V4 | 1.0 | 3.032e-16 | 617 | 0.514 | 169 | 69 | 4 | 1 | 162 | 1 | 163 | Single-stranded DNA-binding protein | Single-stranded DNA-binding protein | | afdb-uniprot50 | AF-A0A3M8FH74-F1-MODEL\_V4 | 1.0 | 1.973e-16 | 617 | 0.444 | 162 | 85 | 3 | 1 | 162 | 1 | 157 | Single-stranded DNA-binding protein | Single-stranded DNA-binding protein | | afdb-uniprot50 | AF-A0A369ZFS8-F1-MODEL\_V4 | 1.0 | 8.938e-15 | 616 | 0.531 | 126 | 53 | 3 | 4 | 129 | 2 | 121 | Single-stranded DNA-binding protein | Single-stranded DNA-binding protein | | afdb-uniprot50 | AF-A0A1B8QYT8-F1-MODEL\_V4 | 1.0 | 7.972e-16 | 616 | 0.518 | 162 | 59 | 6 | 1 | 162 | 1 | 143 | Single-stranded DNA-binding protein | Single-stranded DNA-binding protein | | afdb-uniprot50 | AF-H0Q1C2-F1-MODEL\_V4 | 1.0 | 1.439e-15 | 616 | 0.487 | 162 | 72 | 4 | 1 | 162 | 1 | 151 | Single-stranded DNA-binding protein | Single-stranded DNA-binding protein | | afdb-uniprot50 | AF-A0A315BKE1-F1-MODEL\_V4 | 1.0 | 3.758e-16 | 616 | 0.509 | 161 | 72 | 4 | 4 | 162 | 1 | 156 | Single-stranded DNA-binding protein | Single-stranded DNA-binding protein | | afdb-uniprot50 | AF-A0A2N1I3V0-F1-MODEL\_V4 | 1.0 | 2.197e-16 | 616 | 0.48 | 179 | 72 | 6 | 1 | 162 | 2 | 176 | Single-stranded DNA-binding protein | Single-stranded DNA-binding protein | | afdb-uniprot50 | AF-A0A523Q0M1-F1-MODEL\_V4 | 1.0 | 8.139e-13 | 615 | 0.41 | 117 | 66 | 1 | 1 | 117 | 1 | 114 | Single-stranded DNA-binding protein | Single-stranded DNA-binding protein | | afdb-uniprot50 | AF-A0A2A4T3P5-F1-MODEL\_V4 | 1.0 | 3.447e-13 | 615 | 0.435 | 117 | 63 | 1 | 1 | 117 | 1 | 114 | Single-stranded DNA-binding protein | Single-stranded DNA-binding protein | | afdb-uniprot50 | AF-A0A418WRR6-F1-MODEL\_V4 | 1.0 | 5.187e-16 | 615 | 0.511 | 174 | 72 | 5 | 1 | 162 | 22 | 194 | Single-stranded DNA-binding protein | Single-stranded DNA-binding protein | | afdb-uniprot50 | AF-A0A318L053-F1-MODEL\_V4 | 1.0 | 3.562e-16 | 615 | 0.49 | 163 | 76 | 3 | 1 | 162 | 1 | 157 | Single-stranded DNA-binding protein | Single-stranded DNA-binding protein | | afdb-uniprot50 | AF-A4JUL0-F1-MODEL\_V4 | 1.0 | 4.916e-16 | 615 | 0.398 | 188 | 84 | 3 | 1 | 162 | 10 | 194 | Single-stranded DNA-binding protein | Single-stranded DNA-binding protein | | afdb-uniprot50 | AF-A0A6N4TA35-F1-MODEL\_V4 | 1.0 | 1.883e-15 | 614 | 0.407 | 162 | 79 | 4 | 1 | 162 | 1 | 145 | Single-stranded DNA-binding protein | Single-stranded DNA-binding protein | | afdb-uniprot50 | AF-A0A7W0C683-F1-MODEL\_V4 | 1.0 | 3.399e-15 | 614 | 0.456 | 162 | 73 | 4 | 1 | 162 | 1 | 147 | Single-stranded DNA-binding protein | Single-stranded DNA-binding protein | | afdb-uniprot50 | AF-A0A6N7CZF7-F1-MODEL\_V4 | 1.0 | 4.416e-16 | 614 | 0.487 | 162 | 65 | 4 | 1 | 162 | 1 | 144 | Single-stranded DNA-binding protein | Single-stranded DNA-binding protein | | afdb-uniprot50 | AF-A0A496NF27-F1-MODEL\_V4 | 1.0 | 2.581e-16 | 614 | 0.493 | 160 | 71 | 4 | 1 | 160 | 1 | 150 | Single-stranded DNA-binding protein | Single-stranded DNA-binding protein | | afdb-uniprot50 | AF-A0A3A5ANI7-F1-MODEL\_V4 | 1.0 | 1.116e-13 | 613 | 0.453 | 119 | 62 | 2 | 4 | 122 | 1 | 116 | Single-stranded DNA-binding protein | Single-stranded DNA-binding protein | | afdb-uniprot50 | AF-A0A369ZBV2-F1-MODEL\_V4 | 1.0 | 2.334e-15 | 613 | 0.484 | 163 | 58 | 6 | 1 | 162 | 1 | 138 | Single-stranded DNA-binding protein | Single-stranded DNA-binding protein | | afdb-uniprot50 | AF-A0A2G9C242-F1-MODEL\_V4 | 1.0 | 1.691e-15 | 613 | 0.452 | 157 | 76 | 4 | 1 | 157 | 1 | 147 | Single-stranded DNA-binding protein | Single-stranded DNA-binding protein | | afdb-uniprot50 | AF-A0A424WBH3-F1-MODEL\_V4 | 1.0 | 1.1e-15 | 613 | 0.453 | 163 | 75 | 4 | 1 | 162 | 1 | 150 | Single-stranded DNA-binding protein | Single-stranded DNA-binding protein | | afdb-uniprot50 | AF-A0A6N8BTZ9-F1-MODEL\_V4 | 1.0 | 7.16e-16 | 613 | 0.402 | 164 | 89 | 4 | 1 | 162 | 1 | 157 | Single-stranded DNA-binding protein | Single-stranded DNA-binding protein | | afdb-uniprot50 | AF-A0A109WX80-F1-MODEL\_V4 | 1.0 | 5.474e-16 | 613 | 0.435 | 179 | 77 | 4 | 1 | 162 | 1 | 172 | Single-stranded DNA-binding protein | Single-stranded DNA-binding protein | | afdb-uniprot50 | AF-A0A252ER09-F1-MODEL\_V4 | 1.0 | 1.161e-15 | 613 | 0.431 | 167 | 87 | 2 | 3 | 162 | 2 | 167 | Single-stranded DNA-binding protein | Single-stranded DNA-binding protein | | afdb-uniprot50 | AF-G0AGI2-F1-MODEL\_V4 | 1.0 | 4.416e-16 | 613 | 0.476 | 170 | 74 | 4 | 2 | 162 | 27 | 190 | Single-stranded DNA-binding protein | Single-stranded DNA-binding protein | | afdb-uniprot50 | AF-A0A2N5LXF7-F1-MODEL\_V4 | 1.0 | 4.916e-16 | 613 | 0.438 | 162 | 88 | 2 | 1 | 162 | 1 | 159 | Single-stranded DNA-binding protein | Single-stranded DNA-binding protein | | afdb-uniprot50 | AF-E6V3D0-F1-MODEL\_V4 | 1.0 | 3.562e-16 | 613 | 0.432 | 192 | 75 | 3 | 2 | 162 | 42 | 230 | Single-stranded DNA-binding protein | Single-stranded DNA-binding protein | | afdb-uniprot50 | AF-V2UYE3-F1-MODEL\_V4 | 1.0 | 1.169e-14 | 612 | 0.5 | 136 | 60 | 3 | 4 | 138 | 3 | 131 | Single-stranded DNA-binding protein | Single-stranded DNA-binding protein | | afdb-uniprot50 | AF-I2E0G5-F1-MODEL\_V4 | 1.0 | 8.027e-15 | 612 | 0.46 | 139 | 72 | 1 | 5 | 143 | 1 | 136 | Single-stranded DNA-binding protein | Single-stranded DNA-binding protein | | afdb-uniprot50 | AF-A0A543Q277-F1-MODEL\_V4 | 1.0 | 1.093e-16 | 612 | 0.505 | 170 | 70 | 7 | 1 | 162 | 1 | 164 | Single-stranded DNA-binding protein | Single-stranded DNA-binding protein | | afdb-uniprot50 | AF-A0A2T5MJC2-F1-MODEL\_V4 | 1.0 | 3.562e-16 | 612 | 0.502 | 175 | 68 | 6 | 1 | 162 | 1 | 169 | Single-stranded DNA-binding protein | Single-stranded DNA-binding protein | | afdb-uniprot50 | AF-A0A7T4USW3-F1-MODEL\_V4 | 1.0 | 1.87e-16 | 612 | 0.46 | 191 | 69 | 7 | 1 | 162 | 1 | 186 | Single-stranded DNA-binding protein | Single-stranded DNA-binding protein | | afdb-uniprot50 | AF-A0A661CUE1-F1-MODEL\_V4 | 1.0 | 1.364e-15 | 611 | 0.477 | 159 | 70 | 5 | 4 | 162 | 2 | 147 | Single-stranded DNA-binding protein | Single-stranded DNA-binding protein | | afdb-uniprot50 | AF-A0A524BBB9-F1-MODEL\_V4 | 1.0 | 9.883e-16 | 611 | 0.484 | 165 | 71 | 5 | 1 | 162 | 1 | 154 | Single-stranded DNA-binding protein | Single-stranded DNA-binding protein | | afdb-uniprot50 | AF-A0A259DP20-F1-MODEL\_V4 | 1.0 | 2.893e-15 | 610 | 0.427 | 159 | 74 | 4 | 4 | 162 | 2 | 143 | Single-stranded DNA-binding protein | Single-stranded DNA-binding protein | | afdb-uniprot50 | AF-A0A450Z8W8-F1-MODEL\_V4 | 1.0 | 6.094e-16 | 610 | 0.463 | 166 | 78 | 4 | 1 | 162 | 1 | 159 | Single-stranded DNA-binding protein | Single-stranded DNA-binding protein | | afdb-uniprot50 | AF-A0A7V8I1N6-F1-MODEL\_V4 | 1.0 | 4.185e-16 | 610 | 0.443 | 185 | 74 | 7 | 1 | 162 | 1 | 179 | Single-stranded DNA-binding protein | Single-stranded DNA-binding protein | | afdb-uniprot50 | AF-A0A654KG85-F1-MODEL\_V4 | 1.0 | 2.934e-13 | 609 | 0.487 | 121 | 58 | 2 | 1 | 121 | 1 | 117 | Single-stranded DNA-binding protein | Single-stranded DNA-binding protein | | afdb-uniprot50 | AF-A0A496W424-F1-MODEL\_V4 | 1.0 | 1.284e-16 | 609 | 0.487 | 164 | 76 | 6 | 1 | 162 | 1 | 158 | Single-stranded DNA-binding protein | Single-stranded DNA-binding protein | | afdb-uniprot50 | AF-A0A157KWT1-F1-MODEL\_V4 | 1.0 | 1.896e-14 | 608 | 0.42 | 157 | 70 | 5 | 1 | 156 | 1 | 137 | Single-stranded DNA-binding protein | Single-stranded DNA-binding protein | | afdb-uniprot50 | AF-A0A3D0DYT0-F1-MODEL\_V4 | 1.0 | 3.785e-15 | 608 | 0.457 | 153 | 70 | 4 | 1 | 153 | 1 | 140 | Single-stranded DNA-binding protein | Single-stranded DNA-binding protein | | afdb-uniprot50 | AF-V4N8N1-F1-MODEL\_V4 | 1.0 | 1.797e-14 | 608 | 0.47 | 136 | 64 | 3 | 1 | 136 | 1 | 128 | Single-stranded DNA-binding protein | Single-stranded DNA-binding protein | | afdb-uniprot50 | AF-A0A8B4QQV6-F1-MODEL\_V4 | 1.0 | 5.897e-13 | 608 | 0.407 | 113 | 64 | 1 | 1 | 113 | 1 | 110 | Helix-destabilizing protein | Helix-destabilizing protein | | afdb-uniprot50 | AF-A0A6S6RZC4-F1-MODEL\_V4 | 1.0 | 2.082e-16 | 608 | 0.419 | 162 | 93 | 1 | 1 | 162 | 1 | 161 | Single-stranded DNA-binding protein | Single-stranded DNA-binding protein | | afdb-uniprot50 | AF-A0A0F9C7M3-F1-MODEL\_V4 | 1.0 | 1.293e-15 | 608 | 0.397 | 156 | 85 | 3 | 1 | 156 | 1 | 147 | Uncharacterized protein | Uncharacterized protein | | afdb-uniprot50 | AF-A0A1H6F8Q9-F1-MODEL\_V4 | 1.0 | 2.48e-14 | 607 | 0.456 | 160 | 62 | 3 | 3 | 162 | 2 | 136 | Single-stranded DNA-binding protein | Single-stranded DNA-binding protein | | afdb-uniprot50 | AF-A0A2E1QNC3-F1-MODEL\_V4 | 1.0 | 1.108e-14 | 607 | 0.432 | 164 | 66 | 6 | 1 | 162 | 1 | 139 | Single-stranded DNA-binding protein | Single-stranded DNA-binding protein | | afdb-uniprot50 | AF-G2Z404-F1-MODEL\_V4 | 1.0 | 2.934e-13 | 607 | 0.408 | 120 | 68 | 1 | 1 | 120 | 1 | 117 | Single-stranded DNA-binding protein | Single-stranded DNA-binding protein | | afdb-uniprot50 | AF-A0A1V3KBP0-F1-MODEL\_V4 | 1.0 | 1.161e-15 | 607 | 0.49 | 163 | 63 | 6 | 1 | 162 | 1 | 144 | Single-stranded DNA-binding protein | Single-stranded DNA-binding protein | | afdb-uniprot50 | AF-A0A383D837-F1-MODEL\_V4 | 1.0 | 4.446e-15 | 607 | 0.429 | 163 | 74 | 4 | 1 | 162 | 2 | 146 | Uncharacterized protein | Uncharacterized protein | | afdb-uniprot50 | AF-A0A101GB98-F1-MODEL\_V4 | 1.0 | 2.096e-15 | 607 | 0.432 | 162 | 85 | 2 | 1 | 162 | 2 | 156 | Single-stranded DNA-binding protein | Single-stranded DNA-binding protein | | afdb-uniprot50 | AF-A0A1D9CYH9-F1-MODEL\_V4 | 1.0 | 1.883e-15 | 607 | 0.481 | 162 | 76 | 2 | 4 | 160 | 3 | 161 | Single-stranded DNA-binding protein | Single-stranded DNA-binding protein | | afdb-uniprot50 | AF-A0A151FIX3-F1-MODEL\_V4 | 1.0 | 7.972e-16 | 606 | 0.457 | 177 | 76 | 6 | 1 | 162 | 1 | 172 | Single-stranded DNA-binding protein | Single-stranded DNA-binding protein | | afdb-uniprot50 | AF-A0A0S8BBA7-F1-MODEL\_V4 | 1.0 | 9.366e-16 | 605 | 0.46 | 165 | 71 | 7 | 1 | 162 | 1 | 150 | Single-stranded DNA-binding protein | Single-stranded DNA-binding protein | | afdb-uniprot50 | AF-A0A345P7X1-F1-MODEL\_V4 | 1.0 | 7.16e-16 | 605 | 0.459 | 159 | 83 | 2 | 4 | 162 | 3 | 158 | Single-stranded DNA-binding protein | Single-stranded DNA-binding protein | | afdb-uniprot50 | AF-A0A5P2H9B6-F1-MODEL\_V4 | 1.0 | 9.301e-17 | 605 | 0.491 | 175 | 74 | 4 | 1 | 162 | 1 | 173 | Single-stranded DNA-binding protein | Single-stranded DNA-binding protein | | afdb-uniprot50 | AF-A0A2D9I903-F1-MODEL\_V4 | 1.0 | 3.447e-13 | 604 | 0.458 | 120 | 62 | 1 | 1 | 120 | 1 | 117 | Single-stranded DNA-binding protein | Single-stranded DNA-binding protein | | afdb-uniprot50 | AF-A0A2P5L9K4-F1-MODEL\_V4 | 1.0 | 7.21e-15 | 604 | 0.427 | 159 | 72 | 3 | 4 | 162 | 1 | 140 | Single-stranded DNA-binding protein | Single-stranded DNA-binding protein | | afdb-uniprot50 | AF-A0A5N3PH37-F1-MODEL\_V4 | 1.0 | 1.161e-15 | 604 | 0.485 | 173 | 77 | 5 | 1 | 162 | 1 | 172 | Single-stranded DNA-binding protein | Single-stranded DNA-binding protein | | afdb-uniprot50 | AF-A0A1R4EI52-F1-MODEL\_V4 | 1.0 | 6.094e-16 | 604 | 0.512 | 162 | 73 | 2 | 4 | 162 | 3 | 161 | Single-stranded DNA-binding protein | Single-stranded DNA-binding protein | | afdb-uniprot50 | AF-A0A7W3VKR4-F1-MODEL\_V4 | 1.0 | 8.589e-13 | 603 | 0.371 | 113 | 68 | 1 | 1 | 113 | 1 | 110 | Single-stranded DNA-binding protein | Single-stranded DNA-binding protein | | afdb-uniprot50 | AF-A0A011Q4A3-F1-MODEL\_V4 | 1.0 | 3.587e-15 | 603 | 0.45 | 151 | 78 | 2 | 4 | 154 | 3 | 148 | Single-stranded DNA-binding protein | Single-stranded DNA-binding protein | | afdb-uniprot50 | AF-A0A378T3Z6-F1-MODEL\_V4 | 1.0 | 2.446e-16 | 603 | 0.5 | 166 | 73 | 5 | 3 | 162 | 2 | 163 | Single-stranded DNA-binding protein | Single-stranded DNA-binding protein | | afdb-uniprot50 | AF-A0A3D0SLK3-F1-MODEL\_V4 | 1.0 | 4.185e-16 | 603 | 0.467 | 171 | 77 | 6 | 1 | 162 | 1 | 166 | Single-stranded DNA-binding protein | Single-stranded DNA-binding protein | | afdb-uniprot50 | AF-A0A0Q9PKG3-F1-MODEL\_V4 | 1.0 | 1.093e-16 | 602 | 0.466 | 178 | 78 | 4 | 1 | 162 | 1 | 177 | Single-stranded DNA-binding protein | Single-stranded DNA-binding protein | | afdb-uniprot50 | AF-A0A2D3R7S5-F1-MODEL\_V4 | 1.0 | 3.993e-15 | 602 | 0.452 | 157 | 78 | 3 | 1 | 157 | 1 | 149 | Single-stranded DNA-binding protein | Single-stranded DNA-binding protein | | afdb-uniprot50 | AF-K0MA94-F1-MODEL\_V4 | 1.0 | 8.412e-16 | 602 | 0.456 | 173 | 75 | 4 | 2 | 162 | 22 | 187 | Single-stranded DNA-binding protein | Single-stranded DNA-binding protein | | afdb-uniprot50 | AF-A0A2V8KTP6-F1-MODEL\_V4 | 1.0 | 4.692e-15 | 601 | 0.423 | 163 | 80 | 4 | 1 | 162 | 1 | 150 | Single-stranded DNA-binding protein | Single-stranded DNA-binding protein | | afdb-uniprot50 | AF-A0A522RZG9-F1-MODEL\_V4 | 1.0 | 1.161e-15 | 601 | 0.403 | 186 | 81 | 4 | 1 | 162 | 1 | 180 | Single-stranded DNA-binding protein | Single-stranded DNA-binding protein | | afdb-uniprot50 | AF-A0A523HF25-F1-MODEL\_V4 | 1.0 | 9.062e-13 | 600 | 0.431 | 116 | 61 | 2 | 3 | 117 | 2 | 113 | Single-stranded DNA-binding protein | Single-stranded DNA-binding protein | | afdb-uniprot50 | AF-A0A411SZB9-F1-MODEL\_V4 | 1.0 | 4.446e-15 | 600 | 0.447 | 163 | 77 | 4 | 1 | 162 | 1 | 151 | Single-stranded DNA-binding protein | Single-stranded DNA-binding protein | | afdb-uniprot50 | AF-A0A238H9F9-F1-MODEL\_V4 | 1.0 | 2.318e-16 | 600 | 0.45 | 173 | 81 | 4 | 2 | 162 | 24 | 194 | Single-stranded DNA-binding protein | Single-stranded DNA-binding protein | | afdb-uniprot50 | AF-A0A2S5CMA6-F1-MODEL\_V4 | 1.0 | 1.161e-15 | 600 | 0.45 | 173 | 78 | 3 | 2 | 162 | 46 | 213 | Single-stranded DNA-binding protein | Single-stranded DNA-binding protein | | afdb-uniprot50 | AF-A0A4R6QQT1-F1-MODEL\_V4 | 1.0 | 1.043e-15 | 600 | 0.425 | 188 | 75 | 4 | 1 | 162 | 35 | 215 | Single-stranded DNA-binding protein | Single-stranded DNA-binding protein | | afdb-uniprot50 | AF-A0A4R6DSH9-F1-MODEL\_V4 | 1.0 | 9.496e-14 | 599 | 0.457 | 129 | 63 | 3 | 1 | 129 | 1 | 122 | Single-stranded DNA-binding protein | Single-stranded DNA-binding protein | | afdb-uniprot50 | AF-A0A1V3L736-F1-MODEL\_V4 | 1.0 | 9.366e-16 | 599 | 0.476 | 170 | 64 | 6 | 1 | 162 | 1 | 153 | Single-stranded DNA-binding protein | Single-stranded DNA-binding protein | | afdb-uniprot50 | AF-A0A0G3ID38-F1-MODEL\_V4 | 1.0 | 7.555e-16 | 599 | 0.45 | 171 | 79 | 5 | 1 | 162 | 1 | 165 | Single-stranded DNA-binding protein | Single-stranded DNA-binding protein | | afdb-uniprot50 | AF-A0A3N9EZF1-F1-MODEL\_V4 | 1.0 | 8.412e-16 | 599 | 0.441 | 170 | 83 | 4 | 1 | 162 | 1 | 166 | Single-stranded DNA-binding protein | Single-stranded DNA-binding protein | | afdb-uniprot50 | AF-A0A833GPH7-F1-MODEL\_V4 | 1.0 | 1.87e-16 | 599 | 0.5 | 166 | 74 | 5 | 1 | 162 | 1 | 161 | Single-stranded DNA-binding protein | Single-stranded DNA-binding protein | | afdb-uniprot50 | AF-S7TLS7-F1-MODEL\_V4 | 1.0 | 2.197e-16 | 599 | 0.479 | 167 | 76 | 5 | 1 | 162 | 1 | 161 | Single-stranded DNA-binding protein | Single-stranded DNA-binding protein | | afdb-uniprot50 | AF-A0A5S4SIA5-F1-MODEL\_V4 | 1.0 | 2.35e-14 | 598 | 0.493 | 146 | 60 | 4 | 1 | 145 | 1 | 133 | Single-stranded DNA-binding protein | Single-stranded DNA-binding protein | | afdb-uniprot50 | AF-A0A2E5IAI4-F1-MODEL\_V4 | 1.0 | 5.224e-15 | 598 | 0.417 | 163 | 79 | 5 | 1 | 162 | 1 | 148 | Single-stranded DNA-binding protein | Single-stranded DNA-binding protein | | afdb-uniprot50 | AF-A0A2D9CGL3-F1-MODEL\_V4 | 1.0 | 2.873e-16 | 598 | 0.5 | 162 | 74 | 3 | 1 | 159 | 1 | 158 | Single-stranded DNA-binding protein | Single-stranded DNA-binding protein | | afdb-uniprot50 | AF-A0A2W5QBP7-F1-MODEL\_V4 | 1.0 | 1.32e-12 | 598 | 0.347 | 118 | 76 | 1 | 1 | 117 | 157 | 274 | Single-stranded DNA-binding protein | Single-stranded DNA-binding protein | | afdb-uniprot50 | AF-A0A0A1H6Y8-F1-MODEL\_V4 | 1.0 | 9.951e-15 | 597 | 0.447 | 161 | 65 | 5 | 3 | 162 | 2 | 139 | Single-stranded DNA-binding protein | Single-stranded DNA-binding protein | | afdb-uniprot50 | AF-A0A5C7NPC6-F1-MODEL\_V4 | 1.0 | 8.027e-15 | 597 | 0.425 | 160 | 71 | 4 | 3 | 162 | 2 | 140 | Single-stranded DNA-binding protein | Single-stranded DNA-binding protein | | afdb-uniprot50 | AF-A0A4Q7B5T0-F1-MODEL\_V4 | 1.0 | 4.049e-13 | 596 | 0.532 | 107 | 49 | 1 | 4 | 110 | 3 | 108 | Single-stranded DNA-binding protein | Single-stranded DNA-binding protein | | afdb-uniprot50 | AF-A0A2N2Y9Q4-F1-MODEL\_V4 | 1.0 | 9.062e-13 | 596 | 0.435 | 117 | 63 | 1 | 1 | 117 | 1 | 114 | Single-stranded DNA-binding protein | Single-stranded DNA-binding protein | | afdb-uniprot50 | AF-A0A318INE9-F1-MODEL\_V4 | 1.0 | 3.587e-15 | 596 | 0.445 | 164 | 68 | 6 | 1 | 162 | 1 | 143 | Single-stranded DNA-binding protein | Single-stranded DNA-binding protein | | afdb-uniprot50 | AF-A0A6S6M805-F1-MODEL\_V4 | 1.0 | 1.169e-14 | 596 | 0.415 | 159 | 89 | 2 | 4 | 162 | 1 | 155 | Single-stranded DNA-binding protein | Single-stranded DNA-binding protein | | afdb-uniprot50 | AF-A0A4Q4WRP0-F1-MODEL\_V4 | 1.0 | 4.477e-14 | 596 | 0.468 | 126 | 65 | 2 | 2 | 127 | 112 | 235 | Uncharacterized protein | Uncharacterized protein | | afdb-uniprot50 | AF-A0A3D3HIB1-F1-MODEL\_V4 | 1.0 | 5.296e-13 | 595 | 0.367 | 117 | 71 | 1 | 1 | 117 | 1 | 114 | Single-stranded DNA-binding protein | Single-stranded DNA-binding protein | | afdb-uniprot50 | AF-A0A1X7C379-F1-MODEL\_V4 | 1.0 | 7.661e-14 | 595 | 0.507 | 130 | 59 | 3 | 1 | 130 | 1 | 125 | Single-stranded DNA-binding protein | Single-stranded DNA-binding protein | | afdb-uniprot50 | AF-A0A653A015-F1-MODEL\_V4 | 1.0 | 3.587e-15 | 595 | 0.463 | 164 | 62 | 7 | 1 | 162 | 1 | 140 | Single-stranded DNA-binding protein | Single-stranded DNA-binding protein | | afdb-uniprot50 | AF-A0A2N0WIC8-F1-MODEL\_V4 | 1.0 | 4.416e-16 | 595 | 0.49 | 165 | 72 | 4 | 4 | 160 | 3 | 163 | Single-stranded DNA-binding protein | Single-stranded DNA-binding protein | | afdb-uniprot50 | AF-A0A1Z4C5J3-F1-MODEL\_V4 | 1.0 | 5.776e-16 | 595 | 0.467 | 169 | 77 | 3 | 4 | 162 | 1 | 166 | Single-stranded DNA-binding protein | Single-stranded DNA-binding protein | | afdb-uniprot50 | AF-A0A660N4U4-F1-MODEL\_V4 | 1.0 | 6.833e-15 | 594 | 0.443 | 142 | 74 | 3 | 4 | 143 | 2 | 140 | Single-stranded DNA-binding protein | Single-stranded DNA-binding protein | | afdb-uniprot50 | AF-A0A536S686-F1-MODEL\_V4 | 1.0 | 6.566e-13 | 594 | 0.442 | 113 | 60 | 1 | 2 | 114 | 36 | 145 | Single-stranded DNA-binding protein | Single-stranded DNA-binding protein | | afdb-uniprot50 | AF-A0A5J6PZX2-F1-MODEL\_V4 | 1.0 | 4.659e-16 | 594 | 0.444 | 171 | 81 | 4 | 3 | 162 | 2 | 169 | Single-stranded DNA-binding protein | Single-stranded DNA-binding protein | | afdb-uniprot50 | AF-G4E3E4-F1-MODEL\_V4 | 1.0 | 6.786e-16 | 594 | 0.423 | 170 | 86 | 4 | 1 | 162 | 1 | 166 | Single-stranded DNA-binding protein | Single-stranded DNA-binding protein | | afdb-uniprot50 | AF-A0A1V4GVS6-F1-MODEL\_V4 | 1.0 | 6.786e-16 | 593 | 0.494 | 170 | 72 | 5 | 1 | 160 | 1 | 166 | Single-stranded DNA-binding protein | Single-stranded DNA-binding protein | | afdb-uniprot50 | AF-A0A2D5RWR9-F1-MODEL\_V4 | 1.0 | 5.474e-16 | 592 | 0.469 | 162 | 79 | 3 | 1 | 158 | 1 | 159 | Single-stranded DNA-binding protein | Single-stranded DNA-binding protein | | afdb-uniprot50 | AF-A0A1V1X1A7-F1-MODEL\_V4 | 1.0 | 1.529e-14 | 591 | 0.421 | 159 | 73 | 4 | 4 | 162 | 1 | 140 | Single-stranded DNA-binding protein | Single-stranded DNA-binding protein | | afdb-uniprot50 | AF-A0A645DBS0-F1-MODEL\_V4 | 1.0 | 4.446e-15 | 591 | 0.395 | 162 | 88 | 3 | 1 | 162 | 2 | 153 | Single-stranded DNA-binding protein | Single-stranded DNA-binding protein | | afdb-uniprot50 | AF-F5T0V4-F1-MODEL\_V4 | 1.0 | 5.224e-15 | 590 | 0.479 | 148 | 70 | 3 | 4 | 148 | 2 | 145 | Single-stranded DNA-binding protein | Single-stranded DNA-binding protein | | afdb-uniprot50 | AF-A0A6B1AVA2-F1-MODEL\_V4 | 1.0 | 1.784e-15 | 590 | 0.447 | 161 | 81 | 4 | 1 | 160 | 1 | 154 | Single-stranded DNA-binding protein | Single-stranded DNA-binding protein | | afdb-uniprot50 | AF-A0A7W8HGC3-F1-MODEL\_V4 | 1.0 | 1.987e-15 | 590 | 0.437 | 167 | 84 | 5 | 1 | 162 | 1 | 162 | Single-stranded DNA-binding protein | Single-stranded DNA-binding protein | | afdb-uniprot50 | AF-A0A7X4KLH9-F1-MODEL\_V4 | 1.0 | 1.519e-15 | 589 | 0.43 | 179 | 72 | 4 | 1 | 162 | 1 | 166 | Single-stranded DNA-binding protein | Single-stranded DNA-binding protein | | afdb-uniprot50 | AF-A0A7V2HAB0-F1-MODEL\_V4 | 1.0 | 1.393e-12 | 588 | 0.376 | 117 | 70 | 1 | 4 | 120 | 2 | 115 | Single-stranded DNA-binding protein | Single-stranded DNA-binding protein | | afdb-uniprot50 | AF-A0A3N5PE79-F1-MODEL\_V4 | 1.0 | 4.021e-14 | 588 | 0.477 | 134 | 61 | 4 | 1 | 129 | 1 | 130 | Single-stranded DNA-binding protein | Single-stranded DNA-binding protein | | afdb-uniprot50 | AF-A0A1D2RY17-F1-MODEL\_V4 | 1.0 | 1.169e-14 | 588 | 0.413 | 162 | 78 | 3 | 1 | 162 | 1 | 145 | Single-stranded DNA-binding protein | Single-stranded DNA-binding protein | | afdb-uniprot50 | AF-A1VVC7-F1-MODEL\_V4 | 1.0 | 3.758e-16 | 588 | 0.479 | 167 | 80 | 3 | 1 | 162 | 1 | 165 | Single-stranded DNA-binding protein | Single-stranded DNA-binding protein | | afdb-uniprot50 | AF-E8LIW7-F1-MODEL\_V4 | 1.0 | 1.896e-14 | 588 | 0.4 | 155 | 89 | 2 | 1 | 154 | 1 | 152 | Single-stranded DNA-binding protein | Single-stranded DNA-binding protein | | afdb-uniprot50 | AF-A0A7Y6YA81-F1-MODEL\_V4 | 1.0 | 4.243e-14 | 587 | 0.456 | 162 | 61 | 4 | 1 | 162 | 1 | 135 | Single-stranded DNA-binding protein | Single-stranded DNA-binding protein | | afdb-uniprot50 | AF-A0A6A0IKH7-F1-MODEL\_V4 | 1.0 | 4.95e-15 | 587 | 0.429 | 163 | 69 | 6 | 1 | 162 | 1 | 140 | Single-stranded DNA-binding protein | Single-stranded DNA-binding protein | | afdb-uniprot50 | AF-A0A3A5L1K9-F1-MODEL\_V4 | 1.0 | 2.096e-15 | 587 | 0.456 | 171 | 79 | 6 | 1 | 162 | 1 | 166 | Single-stranded DNA-binding protein | Single-stranded DNA-binding protein | | afdb-uniprot50 | AF-A0A547EAB1-F1-MODEL\_V4 | 1.0 | 2.212e-15 | 587 | 0.443 | 158 | 71 | 4 | 1 | 157 | 1 | 142 | Single-stranded DNA-binding protein | Single-stranded DNA-binding protein | | afdb-uniprot50 | AF-A0A523H4Y8-F1-MODEL\_V4 | 1.0 | 3.266e-13 | 586 | 0.428 | 133 | 71 | 2 | 1 | 133 | 1 | 128 | Single-stranded DNA-binding protein | Single-stranded DNA-binding protein | | afdb-uniprot50 | AF-A0A1N7DQ25-F1-MODEL\_V4 | 1.0 | 1.883e-15 | 586 | 0.417 | 163 | 85 | 3 | 4 | 162 | 2 | 158 | Single-stranded DNA-binding protein | Single-stranded DNA-binding protein | | afdb-uniprot50 | AF-V4P5D9-F1-MODEL\_V4 | 1.0 | 4.446e-15 | 586 | 0.53 | 164 | 71 | 6 | 1 | 162 | 1 | 160 | Single-stranded DNA-binding protein | Single-stranded DNA-binding protein | | afdb-uniprot50 | AF-A0A179BNQ8-F1-MODEL\_V4 | 1.0 | 6.786e-16 | 586 | 0.491 | 169 | 73 | 6 | 1 | 162 | 1 | 163 | Single-stranded DNA-binding protein | Single-stranded DNA-binding protein | | afdb-uniprot50 | AF-A0A7V0XHS0-F1-MODEL\_V4 | 1.0 | 1.987e-15 | 586 | 0.414 | 169 | 87 | 4 | 2 | 162 | 3 | 167 | Single-stranded DNA-binding protein | Single-stranded DNA-binding protein | | afdb-uniprot50 | AF-V8G3X1-F1-MODEL\_V4 | 1.0 | 5.776e-16 | 586 | 0.456 | 162 | 78 | 5 | 1 | 162 | 1 | 152 | Single-stranded DNA-binding protein | Single-stranded DNA-binding protein | | afdb-uniprot50 | AF-A0A1S9ZKL9-F1-MODEL\_V4 | 1.0 | 4.659e-16 | 586 | 0.493 | 160 | 72 | 4 | 4 | 162 | 2 | 153 | Single-stranded DNA-binding protein | Single-stranded DNA-binding protein | | afdb-uniprot50 | AF-A0A3C0DYM7-F1-MODEL\_V4 | 1.0 | 6.88e-14 | 585 | 0.465 | 131 | 65 | 3 | 1 | 131 | 1 | 126 | Single-stranded DNA-binding protein | Single-stranded DNA-binding protein | | afdb-uniprot50 | AF-A0A519KFE2-F1-MODEL\_V4 | 1.0 | 2.954e-12 | 585 | 0.424 | 113 | 62 | 1 | 1 | 113 | 1 | 110 | Single-stranded DNA-binding protein | Single-stranded DNA-binding protein | | afdb-uniprot50 | AF-A0A7Y3IWH6-F1-MODEL\_V4 | 1.0 | 7.16e-16 | 585 | 0.47 | 185 | 62 | 7 | 4 | 162 | 3 | 177 | Single-stranded DNA-binding protein | Single-stranded DNA-binding protein | | afdb-uniprot50 | AF-A0A1G2ATN0-F1-MODEL\_V4 | 1.0 | 3.095e-13 | 584 | 0.401 | 117 | 67 | 2 | 4 | 120 | 2 | 115 | Single-stranded DNA-binding protein | Single-stranded DNA-binding protein | | afdb-uniprot50 | AF-A0A2S5QA54-F1-MODEL\_V4 | 1.0 | 4.692e-15 | 584 | 0.425 | 162 | 77 | 5 | 4 | 162 | 2 | 150 | Single-stranded DNA-binding protein | Single-stranded DNA-binding protein | | afdb-uniprot50 | AF-A0A6L7MV45-F1-MODEL\_V4 | 1.0 | 9.883e-16 | 584 | 0.482 | 168 | 73 | 5 | 1 | 162 | 1 | 160 | Single-stranded DNA-binding protein | Single-stranded DNA-binding protein | | afdb-uniprot50 | AF-A0A0T6W6Z2-F1-MODEL\_V4 | 1.0 | 5.512e-15 | 584 | 0.438 | 162 | 86 | 2 | 4 | 162 | 3 | 162 | Single-stranded DNA-binding protein | Single-stranded DNA-binding protein | | afdb-uniprot50 | AF-A0A1G2BSC3-F1-MODEL\_V4 | 1.0 | 6.18e-14 | 584 | 0.402 | 139 | 76 | 3 | 1 | 136 | 38 | 172 | Single-stranded DNA-binding protein | Single-stranded DNA-binding protein | | afdb-uniprot50 | AF-A0A7U3ZFE1-F1-MODEL\_V4 | 1.0 | 5.816e-15 | 584 | 0.404 | 163 | 91 | 4 | 2 | 162 | 15 | 173 | Single-stranded DNA-binding protein | Single-stranded DNA-binding protein | | afdb-uniprot50 | AF-A0A6M8T455-F1-MODEL\_V4 | 1.0 | 5.856e-14 | 583 | 0.496 | 125 | 59 | 2 | 3 | 127 | 2 | 122 | Single-stranded DNA-binding protein | Single-stranded DNA-binding protein | | afdb-uniprot50 | AF-A0A3M6R1S4-F1-MODEL\_V4 | 1.0 | 6.833e-15 | 583 | 0.481 | 162 | 60 | 5 | 1 | 162 | 1 | 138 | Single-stranded DNA-binding protein | Single-stranded DNA-binding protein | | afdb-uniprot50 | AF-A0A7C1KV95-F1-MODEL\_V4 | 1.0 | 2.599e-15 | 583 | 0.366 | 161 | 97 | 3 | 4 | 162 | 2 | 159 | Single-stranded DNA-binding protein | Single-stranded DNA-binding protein | | afdb-uniprot50 | AF-A0A6L4B9L0-F1-MODEL\_V4 | 1.0 | 9.366e-16 | 583 | 0.463 | 192 | 69 | 5 | 1 | 162 | 1 | 188 | Single-stranded DNA-binding protein | Single-stranded DNA-binding protein | | afdb-uniprot50 | AF-A0A2W4U1S0-F1-MODEL\_V4 | 1.0 | 6.137e-15 | 583 | 0.45 | 160 | 81 | 4 | 4 | 162 | 1 | 154 | Single-stranded DNA-binding protein | Single-stranded DNA-binding protein | | afdb-uniprot50 | AF-A0A1P8MPZ6-F1-MODEL\_V4 | 1.0 | 4.049e-13 | 582 | 0.42 | 119 | 62 | 3 | 1 | 112 | 2 | 120 | Single-stranded DNA-binding protein | Single-stranded DNA-binding protein | | afdb-uniprot50 | AF-A0A661T5P7-F1-MODEL\_V4 | 1.0 | 8.47e-15 | 582 | 0.429 | 163 | 69 | 6 | 1 | 162 | 1 | 140 | Single-stranded DNA-binding protein | Single-stranded DNA-binding protein | | afdb-uniprot50 | AF-A0A0M4SYI8-F1-MODEL\_V4 | 1.0 | 2.212e-15 | 582 | 0.452 | 159 | 79 | 2 | 4 | 162 | 3 | 153 | Single-stranded DNA-binding protein | Single-stranded DNA-binding protein | | afdb-uniprot50 | AF-A0A011NB88-F1-MODEL\_V4 | 1.0 | 1.614e-14 | 581 | 0.425 | 162 | 74 | 4 | 5 | 162 | 1 | 147 | Single-stranded DNA-binding protein | Single-stranded DNA-binding protein | | afdb-uniprot50 | AF-A0A1F3CA35-F1-MODEL\_V4 | 1.0 | 1.293e-15 | 581 | 0.425 | 160 | 82 | 4 | 4 | 162 | 1 | 151 | Single-stranded DNA-binding protein | Single-stranded DNA-binding protein | | afdb-uniprot50 | AF-A0A6I2H1I0-F1-MODEL\_V4 | 1.0 | 2.318e-16 | 581 | 0.468 | 173 | 76 | 6 | 1 | 162 | 1 | 168 | Single-stranded DNA-binding protein | Single-stranded DNA-binding protein | | afdb-uniprot50 | AF-A0A2E7E9P7-F1-MODEL\_V4 | 1.0 | 1.691e-15 | 581 | 0.442 | 174 | 81 | 5 | 1 | 162 | 1 | 170 | Single-stranded DNA-binding protein | Single-stranded DNA-binding protein | | afdb-uniprot50 | AF-A0A4V1J3T3-F1-MODEL\_V4 | 1.0 | 6.52e-14 | 580 | 0.472 | 129 | 65 | 3 | 1 | 129 | 1 | 126 | Single-stranded DNA-binding protein | Single-stranded DNA-binding protein | | afdb-uniprot50 | AF-A0A2G6LHZ8-F1-MODEL\_V4 | 1.0 | 8.412e-16 | 580 | 0.471 | 178 | 70 | 5 | 4 | 162 | 3 | 175 | Single-stranded DNA-binding protein | Single-stranded DNA-binding protein | | afdb-uniprot50 | AF-A0A3C0C8R5-F1-MODEL\_V4 | 1.0 | 4.95e-15 | 580 | 0.386 | 168 | 94 | 2 | 1 | 162 | 1 | 165 | Single-stranded DNA-binding protein | Single-stranded DNA-binding protein | | afdb-uniprot50 | AF-A0A349MDR0-F1-MODEL\_V4 | 1.0 | 6.52e-14 | 579 | 0.466 | 135 | 70 | 2 | 1 | 135 | 1 | 133 | Single-stranded DNA-binding protein | Single-stranded DNA-binding protein | | afdb-uniprot50 | AF-A0A2X1LM92-F1-MODEL\_V4 | 1.0 | 2.35e-14 | 579 | 0.471 | 142 | 65 | 3 | 1 | 142 | 2 | 133 | Single-stranded DNA-binding protein | Single-stranded DNA-binding protein | | afdb-uniprot50 | AF-A0A661IS78-F1-MODEL\_V4 | 1.0 | 8.47e-15 | 579 | 0.408 | 159 | 72 | 6 | 4 | 162 | 3 | 139 | Single-stranded DNA-binding protein | Single-stranded DNA-binding protein | | afdb-uniprot50 | AF-A0A845GCZ9-F1-MODEL\_V4 | 1.0 | 6.137e-15 | 579 | 0.418 | 160 | 88 | 4 | 1 | 160 | 1 | 155 | Single-stranded DNA-binding protein | Single-stranded DNA-binding protein | | afdb-uniprot50 | AF-A0A2D2LXU4-F1-MODEL\_V4 | 1.0 | 2.463e-15 | 579 | 0.456 | 160 | 78 | 3 | 4 | 162 | 3 | 154 | Single-stranded DNA-binding protein | Single-stranded DNA-binding protein | | afdb-uniprot50 | AF-A0A535GRY2-F1-MODEL\_V4 | 1.0 | 9.562e-13 | 578 | 0.401 | 117 | 67 | 1 | 4 | 120 | 1 | 114 | Single-stranded DNA-binding protein | Single-stranded DNA-binding protein | | afdb-uniprot50 | AF-A0A0M9TYI8-F1-MODEL\_V4 | 1.0 | 6.88e-14 | 578 | 0.427 | 159 | 68 | 3 | 4 | 162 | 1 | 136 | Single-stranded DNA-binding protein | Single-stranded DNA-binding protein | | afdb-uniprot50 | AF-A0A383C8L9-F1-MODEL\_V4 | 1.0 | 9.431e-15 | 578 | 0.457 | 164 | 69 | 7 | 1 | 162 | 1 | 146 | Uncharacterized protein | Uncharacterized protein | | afdb-uniprot50 | AF-A0A448TTN8-F1-MODEL\_V4 | 1.0 | 7.21e-15 | 578 | 0.472 | 163 | 69 | 6 | 1 | 162 | 1 | 147 | Single-stranded DNA-binding protein | Single-stranded DNA-binding protein | | afdb-uniprot50 | AF-A0LIN0-F1-MODEL\_V4 | 1.0 | 1.519e-15 | 578 | 0.433 | 173 | 79 | 6 | 1 | 162 | 20 | 184 | Single-stranded DNA-binding protein | Single-stranded DNA-binding protein | | afdb-uniprot50 | AF-A0A401FYS3-F1-MODEL\_V4 | 1.0 | 2e-14 | 578 | 0.515 | 132 | 51 | 3 | 1 | 132 | 1 | 119 | Single-stranded DNA-binding protein | Single-stranded DNA-binding protein | | afdb-uniprot50 | AF-Q5I719-F1-MODEL\_V4 | 1.0 | 2e-14 | 577 | 0.415 | 154 | 79 | 4 | 1 | 151 | 1 | 146 | Single-stranded DNA-binding protein | Single-stranded DNA-binding protein | | afdb-uniprot50 | AF-A0A2E8ZYW6-F1-MODEL\_V4 | 1.0 | 3.811e-14 | 576 | 0.383 | 159 | 78 | 3 | 4 | 162 | 2 | 140 | Single-stranded DNA-binding protein | Single-stranded DNA-binding protein | | afdb-uniprot50 | AF-A0A654E3W4-F1-MODEL\_V4 | 1.0 | 5.512e-15 | 576 | 0.46 | 163 | 75 | 6 | 1 | 162 | 1 | 151 | Single-stranded DNA-binding protein | Single-stranded DNA-binding protein | | afdb-uniprot50 | AF-A0A7X8GXX0-F1-MODEL\_V4 | 1.0 | 2.78e-13 | 576 | 0.448 | 125 | 63 | 3 | 1 | 125 | 1 | 119 | Single-stranded DNA-binding protein | Single-stranded DNA-binding protein | | afdb-uniprot50 | AF-A0A2A2T4T3-F1-MODEL\_V4 | 1.0 | 1.449e-14 | 574 | 0.472 | 161 | 62 | 6 | 3 | 162 | 2 | 140 | Single-stranded DNA-binding protein | Single-stranded DNA-binding protein | | afdb-uniprot50 | AF-B7J8U2-F1-MODEL\_V4 | 1.0 | 1.691e-15 | 574 | 0.466 | 163 | 73 | 6 | 1 | 162 | 1 | 150 | Single-stranded DNA-binding protein | Single-stranded DNA-binding protein | | afdb-uniprot50 | AF-A0A448CRE5-F1-MODEL\_V4 | 1.0 | 7.21e-15 | 574 | 0.418 | 160 | 85 | 3 | 1 | 157 | 1 | 155 | Single-stranded DNA-binding protein | Single-stranded DNA-binding protein | | afdb-uniprot50 | AF-A0A1A9X6E7-F1-MODEL\_V4 | 1.0 | 1.47e-12 | 574 | 0.637 | 102 | 37 | 0 | 16 | 117 | 433 | 534 | Replicative DNA helicase | Replicative DNA helicase | | afdb-uniprot50 | AF-A0A812ILX3-F1-MODEL\_V4 | 1.0 | 2.742e-15 | 573 | 0.451 | 177 | 71 | 6 | 1 | 162 | 1 | 166 | Ssb protein | Ssb protein | | afdb-uniprot50 | AF-A0A2U3KAX6-F1-MODEL\_V4 | 1.0 | 1.883e-15 | 573 | 0.419 | 174 | 83 | 6 | 1 | 162 | 1 | 168 | Single-stranded DNA-binding protein | Single-stranded DNA-binding protein | | afdb-uniprot50 | AF-H6REC4-F1-MODEL\_V4 | 1.0 | 4.508e-13 | 572 | 0.4 | 135 | 73 | 3 | 1 | 135 | 1 | 127 | Single-stranded DNA-binding protein | Single-stranded DNA-binding protein | | afdb-uniprot50 | AF-A0A424NW40-F1-MODEL\_V4 | 1.0 | 2.78e-13 | 572 | 0.351 | 162 | 76 | 3 | 1 | 162 | 1 | 133 | Single-stranded DNA-binding protein | Single-stranded DNA-binding protein | | afdb-uniprot50 | AF-H0A2S4-F1-MODEL\_V4 | 1.0 | 5.816e-15 | 572 | 0.469 | 164 | 81 | 5 | 1 | 162 | 1 | 160 | Single-stranded DNA-binding protein | Single-stranded DNA-binding protein | | afdb-uniprot50 | AF-A0A521PQ87-F1-MODEL\_V4 | 1.0 | 2.48e-14 | 572 | 0.383 | 146 | 86 | 2 | 2 | 145 | 16 | 159 | Single-stranded DNA-binding protein | Single-stranded DNA-binding protein | | afdb-uniprot50 | AF-A0A2T2UL83-F1-MODEL\_V4 | 1.0 | 3.221e-15 | 572 | 0.388 | 175 | 89 | 7 | 1 | 162 | 1 | 170 | Single-stranded DNA-binding protein | Single-stranded DNA-binding protein | | afdb-uniprot50 | AF-Q8Y2B4-F1-MODEL\_V4 | 1.0 | 3.587e-15 | 572 | 0.42 | 183 | 80 | 7 | 1 | 162 | 1 | 178 | Single-stranded DNA-binding protein | Single-stranded DNA-binding protein | | afdb-uniprot50 | AF-A0A1W1H8U1-F1-MODEL\_V4 | 1.0 | 1.614e-14 | 572 | 0.408 | 159 | 86 | 4 | 1 | 156 | 1 | 154 | Single-stranded DNA-binding protein | Single-stranded DNA-binding protein | | afdb-uniprot50 | AF-A0A662Z639-F1-MODEL\_V4 | 1.0 | 8.938e-15 | 572 | 0.406 | 172 | 89 | 3 | 1 | 160 | 1 | 171 | Single-stranded DNA-binding protein | Single-stranded DNA-binding protein | | afdb-uniprot50 | AF-A0A831ZMD0-F1-MODEL\_V4 | 1.0 | 1.822e-12 | 571 | 0.436 | 110 | 60 | 2 | 1 | 110 | 1 | 108 | Single-stranded DNA-binding protein | Single-stranded DNA-binding protein | | afdb-uniprot50 | AF-A0A496A9V7-F1-MODEL\_V4 | 1.0 | 1.449e-14 | 571 | 0.364 | 162 | 90 | 4 | 1 | 162 | 1 | 149 | Single-stranded DNA-binding protein | Single-stranded DNA-binding protein | | afdb-uniprot50 | AF-A0A419IWL4-F1-MODEL\_V4 | 1.0 | 6.137e-15 | 571 | 0.396 | 164 | 93 | 3 | 1 | 162 | 2 | 161 | Single-stranded DNA-binding protein | Single-stranded DNA-binding protein | | afdb-uniprot50 | AF-F0EYB2-F1-MODEL\_V4 | 1.0 | 7.714e-13 | 570 | 0.407 | 113 | 66 | 1 | 4 | 116 | 3 | 114 | Single-stranded DNA-binding protein | Single-stranded DNA-binding protein | | afdb-uniprot50 | AF-A0A7V4A6M8-F1-MODEL\_V4 | 1.0 | 2.111e-14 | 570 | 0.408 | 159 | 71 | 5 | 4 | 162 | 3 | 138 | Single-stranded DNA-binding protein | Single-stranded DNA-binding protein | | afdb-uniprot50 | AF-A0A0A2YFY3-F1-MODEL\_V4 | 1.0 | 4.95e-15 | 570 | 0.505 | 170 | 59 | 7 | 1 | 162 | 1 | 153 | Single-stranded DNA-binding protein | Single-stranded DNA-binding protein | | afdb-uniprot50 | AF-F9GVF4-F1-MODEL\_V4 | 1.0 | 8.412e-16 | 570 | 0.469 | 164 | 75 | 5 | 1 | 162 | 1 | 154 | Single-stranded DNA-binding protein | Single-stranded DNA-binding protein | | afdb-uniprot50 | AF-A0A0A6S9K9-F1-MODEL\_V4 | 1.0 | 1.691e-15 | 570 | 0.41 | 178 | 87 | 5 | 1 | 162 | 1 | 176 | Single-stranded DNA-binding protein | Single-stranded DNA-binding protein | | afdb-uniprot50 | AF-A0A356VXD0-F1-MODEL\_V4 | 1.0 | 3.864e-12 | 569 | 0.656 | 96 | 32 | 1 | 4 | 98 | 3 | 98 | Single-stranded DNA-binding protein | Single-stranded DNA-binding protein | | afdb-uniprot50 | AF-A0A7R9AGQ2-F1-MODEL\_V4 | 1.0 | 4.049e-13 | 569 | 0.466 | 120 | 63 | 1 | 3 | 122 | 2 | 120 | Hypothetical protein | Hypothetical protein | | afdb-uniprot50 | AF-A0A2S4N7Z1-F1-MODEL\_V4 | 1.0 | 2.028e-12 | 569 | 0.391 | 120 | 70 | 1 | 1 | 120 | 1 | 117 | Single-stranded DNA-binding protein | Single-stranded DNA-binding protein | | afdb-uniprot50 | AF-A0A831YQ24-F1-MODEL\_V4 | 1.0 | 4.692e-15 | 569 | 0.396 | 174 | 89 | 4 | 1 | 162 | 1 | 170 | Single-stranded DNA-binding protein | Single-stranded DNA-binding protein | | afdb-uniprot50 | AF-A0A335FS28-F1-MODEL\_V4 | 1.0 | 4.049e-13 | 568 | 0.486 | 115 | 57 | 2 | 4 | 118 | 3 | 115 | Single-stranded DNA-binding protein | Single-stranded DNA-binding protein | | afdb-uniprot50 | AF-A0A2V8QGU9-F1-MODEL\_V4 | 1.0 | 2.028e-12 | 568 | 0.439 | 107 | 59 | 1 | 4 | 110 | 2 | 107 | Single-stranded DNA-binding protein | Single-stranded DNA-binding protein | | afdb-uniprot50 | AF-A0A2C6UNF7-F1-MODEL\_V4 | 1.0 | 1.123e-12 | 568 | 0.378 | 132 | 74 | 2 | 1 | 132 | 1 | 124 | Single-stranded DNA-binding protein | Single-stranded DNA-binding protein | | afdb-uniprot50 | AF-A0A2W7INV0-F1-MODEL\_V4 | 1.0 | 4.214e-15 | 568 | 0.465 | 172 | 75 | 7 | 1 | 162 | 1 | 165 | Single-stranded DNA-binding protein | Single-stranded DNA-binding protein | | afdb-uniprot50 | AF-A0A2N9WUQ3-F1-MODEL\_V4 | 1.0 | 3.47e-12 | 567 | 0.418 | 117 | 64 | 2 | 1 | 117 | 1 | 113 | Single-stranded DNA-binding protein | Single-stranded DNA-binding protein | | afdb-uniprot50 | AF-A0A2D4XQA4-F1-MODEL\_V4 | 1.0 | 5.296e-13 | 567 | 0.408 | 125 | 66 | 2 | 5 | 129 | 1 | 117 | Single-stranded DNA-binding protein | Single-stranded DNA-binding protein | | afdb-uniprot50 | AF-A0A535DEE2-F1-MODEL\_V4 | 1.0 | 4.273e-13 | 567 | 0.458 | 120 | 61 | 3 | 1 | 120 | 1 | 116 | Single-stranded DNA-binding protein | Single-stranded DNA-binding protein | | afdb-uniprot50 | AF-A0A2E1JJ68-F1-MODEL\_V4 | 1.0 | 7.21e-15 | 567 | 0.453 | 163 | 71 | 4 | 1 | 162 | 1 | 146 | Single-stranded DNA-binding protein | Single-stranded DNA-binding protein | | afdb-uniprot50 | AF-A0A7S8EHJ2-F1-MODEL\_V4 | 1.0 | 3.074e-14 | 567 | 0.414 | 164 | 83 | 6 | 1 | 162 | 1 | 153 | Single-stranded DNA-binding protein | Single-stranded DNA-binding protein | | afdb-uniprot50 | AF-A0A2G4H6Y0-F1-MODEL\_V4 | 1.0 | 4.692e-15 | 567 | 0.425 | 167 | 79 | 6 | 4 | 162 | 3 | 160 | Single-stranded DNA-binding protein | Single-stranded DNA-binding protein | | afdb-uniprot50 | AF-A1WP86-F1-MODEL\_V4 | 1.0 | 7.972e-16 | 567 | 0.491 | 169 | 77 | 4 | 1 | 162 | 1 | 167 | Single-stranded DNA-binding protein | Single-stranded DNA-binding protein | | afdb-uniprot50 | AF-A0A7V4GHT9-F1-MODEL\_V4 | 1.0 | 3.587e-15 | 567 | 0.384 | 177 | 88 | 6 | 1 | 162 | 1 | 171 | Single-stranded DNA-binding protein | Single-stranded DNA-binding protein | | afdb-uniprot50 | AF-A0A2R3QD86-F1-MODEL\_V4 | 1.0 | 1.883e-15 | 567 | 0.475 | 162 | 73 | 3 | 1 | 162 | 1 | 150 | Single-stranded DNA-binding protein | Single-stranded DNA-binding protein | | afdb-uniprot50 | AF-A0A1D9HCR6-F1-MODEL\_V4 | 1.0 | 4.214e-15 | 567 | 0.434 | 198 | 72 | 7 | 1 | 162 | 1 | 194 | Single-stranded DNA-binding protein | Single-stranded DNA-binding protein | | afdb-uniprot50 | AF-A0A1W9TJB4-F1-MODEL\_V4 | 1.0 | 4.95e-15 | 566 | 0.462 | 162 | 67 | 6 | 1 | 162 | 1 | 142 | Single-stranded DNA-binding protein | Single-stranded DNA-binding protein | | afdb-uniprot50 | AF-A0A3D2TZ42-F1-MODEL\_V4 | 1.0 | 7.608e-15 | 566 | 0.386 | 163 | 88 | 5 | 1 | 162 | 1 | 152 | Single-stranded DNA-binding protein | Single-stranded DNA-binding protein | | afdb-uniprot50 | AF-A0A3M4E9N4-F1-MODEL\_V4 | 1.0 | 4.54e-12 | 566 | 0.642 | 98 | 34 | 1 | 1 | 98 | 67 | 163 | Single-stranded DNA-binding protein | Single-stranded DNA-binding protein | | afdb-uniprot50 | AF-A0A157QP91-F1-MODEL\_V4 | 1.0 | 4.95e-15 | 566 | 0.453 | 183 | 75 | 6 | 1 | 162 | 1 | 179 | Single-stranded DNA-binding protein | Single-stranded DNA-binding protein | | afdb-uniprot50 | AF-A0A7H9WP57-F1-MODEL\_V4 | 1.0 | 4.757e-13 | 565 | 0.39 | 128 | 72 | 3 | 4 | 131 | 2 | 123 | Single-stranded DNA-binding protein | Single-stranded DNA-binding protein | | afdb-uniprot50 | AF-A0A328B6D0-F1-MODEL\_V4 | 1.0 | 7.661e-14 | 565 | 0.414 | 140 | 79 | 2 | 4 | 142 | 1 | 138 | Single-stranded DNA-binding protein | Single-stranded DNA-binding protein | | afdb-uniprot50 | AF-A0A328BGG8-F1-MODEL\_V4 | 1.0 | 5.55e-14 | 565 | 0.386 | 150 | 81 | 4 | 4 | 150 | 1 | 142 | Single-stranded DNA-binding protein | Single-stranded DNA-binding protein | | afdb-uniprot50 | AF-A0A6J5FFK9-F1-MODEL\_V4 | 1.0 | 4.95e-15 | 565 | 0.447 | 163 | 67 | 4 | 4 | 162 | 2 | 145 | Single-stranded DNA-binding protein | Single-stranded DNA-binding protein | | afdb-uniprot50 | AF-A0A2E4DF34-F1-MODEL\_V4 | 1.0 | 5.512e-15 | 564 | 0.431 | 160 | 76 | 5 | 4 | 162 | 2 | 147 | Single-stranded DNA-binding protein | Single-stranded DNA-binding protein | | afdb-uniprot50 | AF-A7ILF7-F1-MODEL\_V4 | 1.0 | 2.48e-14 | 564 | 0.451 | 166 | 79 | 6 | 1 | 162 | 1 | 158 | Single-stranded DNA-binding protein | Single-stranded DNA-binding protein | | afdb-uniprot50 | AF-A0A7V6WJS5-F1-MODEL\_V4 | 1.0 | 6.137e-15 | 564 | 0.413 | 167 | 82 | 3 | 4 | 162 | 2 | 160 | Single-stranded DNA-binding protein | Single-stranded DNA-binding protein | | afdb-uniprot50 | AF-G4QAH2-F1-MODEL\_V4 | 1.0 | 7.608e-15 | 564 | 0.384 | 169 | 91 | 4 | 4 | 162 | 2 | 167 | Single-stranded DNA-binding protein | Single-stranded DNA-binding protein | | afdb-uniprot50 | AF-A0A1F6IFU5-F1-MODEL\_V4 | 1.0 | 6.475e-15 | 564 | 0.358 | 162 | 97 | 3 | 1 | 162 | 1 | 155 | Single-stranded DNA-binding protein | Single-stranded DNA-binding protein | | afdb-uniprot50 | AF-Q1N2F3-F1-MODEL\_V4 | 1.0 | 9.951e-15 | 564 | 0.46 | 163 | 79 | 5 | 1 | 162 | 1 | 155 | Single-stranded DNA-binding protein | Single-stranded DNA-binding protein | | afdb-uniprot50 | AF-A0A370U549-F1-MODEL\_V4 | 1.0 | 1.302e-14 | 564 | 0.408 | 164 | 85 | 4 | 1 | 160 | 1 | 156 | Single-stranded DNA-binding protein | Single-stranded DNA-binding protein | | afdb-uniprot50 | AF-A0A2E1MM44-F1-MODEL\_V4 | 1.0 | 2.913e-14 | 563 | 0.39 | 164 | 82 | 6 | 1 | 162 | 1 | 148 | Single-stranded DNA-binding protein | Single-stranded DNA-binding protein | | afdb-uniprot50 | AF-A0A383HK71-F1-MODEL\_V4 | 1.0 | 1.703e-14 | 563 | 0.431 | 183 | 77 | 4 | 6 | 162 | 7 | 188 | Single-stranded DNA-binding protein | Single-stranded DNA-binding protein | | afdb-uniprot50 | AF-R5ELR6-F1-MODEL\_V4 | 1.0 | 8.47e-15 | 563 | 0.381 | 173 | 93 | 4 | 1 | 162 | 1 | 170 | Single-stranded DNA-binding protein | Single-stranded DNA-binding protein | | afdb-uniprot50 | AF-A0A257GV23-F1-MODEL\_V4 | 1.0 | 5.333e-12 | 562 | 0.427 | 117 | 64 | 1 | 1 | 117 | 1 | 114 | Single-stranded DNA-binding protein | Single-stranded DNA-binding protein | | afdb-uniprot50 | AF-A0A2S0N5P2-F1-MODEL\_V4 | 1.0 | 5.55e-14 | 562 | 0.424 | 153 | 74 | 2 | 4 | 156 | 3 | 141 | Single-stranded DNA-binding protein | Single-stranded DNA-binding protein | | afdb-uniprot50 | AF-A0A2E2SU00-F1-MODEL\_V4 | 1.0 | 4.477e-14 | 562 | 0.368 | 163 | 84 | 4 | 1 | 162 | 1 | 145 | Single-stranded DNA-binding protein | Single-stranded DNA-binding protein | | afdb-uniprot50 | AF-A0A2G6HT26-F1-MODEL\_V4 | 1.0 | 4.214e-15 | 562 | 0.406 | 172 | 83 | 6 | 1 | 162 | 1 | 163 | Single-stranded DNA-binding protein | Single-stranded DNA-binding protein | | afdb-uniprot50 | AF-A0A6N9BWC5-F1-MODEL\_V4 | 1.0 | 2.893e-15 | 562 | 0.434 | 168 | 88 | 2 | 1 | 162 | 1 | 167 | Single-stranded DNA-binding protein | Single-stranded DNA-binding protein | | afdb-uniprot50 | AF-A0A7R8ZXC5-F1-MODEL\_V4 | 1.0 | 2.463e-15 | 562 | 0.421 | 171 | 80 | 4 | 5 | 159 | 1 | 168 | Hypothetical protein | Hypothetical protein | | afdb-uniprot50 | AF-A0A7X9IAE8-F1-MODEL\_V4 | 1.0 | 1.065e-12 | 561 | 0.346 | 127 | 78 | 2 | 1 | 127 | 1 | 122 | Single-stranded DNA-binding protein | Single-stranded DNA-binding protein | | afdb-uniprot50 | AF-A0A1B1Y8T8-F1-MODEL\_V4 | 1.0 | 2.243e-13 | 561 | 0.388 | 162 | 72 | 5 | 1 | 162 | 1 | 135 | Single-stranded DNA-binding protein | Single-stranded DNA-binding protein | | afdb-uniprot50 | AF-A0A1E4K5Q6-F1-MODEL\_V4 | 1.0 | 6.137e-15 | 561 | 0.463 | 179 | 75 | 5 | 1 | 162 | 1 | 175 | Single-stranded DNA-binding protein | Single-stranded DNA-binding protein | | afdb-uniprot50 | AF-L2F912-F1-MODEL\_V4 | 1.0 | 6.833e-15 | 561 | 0.407 | 167 | 88 | 3 | 4 | 162 | 3 | 166 | Single-stranded DNA-binding protein | Single-stranded DNA-binding protein | | afdb-uniprot50 | AF-A0A535N5L0-F1-MODEL\_V4 | 1.0 | 3.864e-12 | 561 | 0.4 | 115 | 66 | 1 | 3 | 117 | 199 | 310 | Single-stranded DNA-binding protein | Single-stranded DNA-binding protein | | afdb-uniprot50 | AF-A0A534RKS9-F1-MODEL\_V4 | 1.0 | 4.302e-12 | 560 | 0.401 | 117 | 67 | 1 | 1 | 117 | 115 | 228 | Single-stranded DNA-binding protein | Single-stranded DNA-binding protein | | afdb-uniprot50 | AF-F0IH13-F1-MODEL\_V4 | 1.0 | 1.185e-12 | 558 | 0.418 | 117 | 66 | 2 | 1 | 117 | 13 | 127 | Single-stranded DNA-binding protein | Single-stranded DNA-binding protein | | afdb-uniprot50 | AF-A0A4Q5VAN1-F1-MODEL\_V4 | 1.0 | 6.88e-14 | 558 | 0.459 | 135 | 65 | 2 | 1 | 135 | 1 | 127 | Single-stranded DNA-binding protein | Single-stranded DNA-binding protein | | afdb-uniprot50 | AF-A0A1A7QB56-F1-MODEL\_V4 | 1.0 | 5.26e-14 | 558 | 0.468 | 143 | 70 | 3 | 1 | 143 | 1 | 137 | Single-stranded DNA-binding protein | Single-stranded DNA-binding protein | | afdb-uniprot50 | AF-A0A2E9VE14-F1-MODEL\_V4 | 1.0 | 2.617e-14 | 558 | 0.444 | 162 | 70 | 4 | 1 | 162 | 2 | 143 | Single-stranded DNA-binding protein | Single-stranded DNA-binding protein | | afdb-uniprot50 | AF-A0A1F8TSK2-F1-MODEL\_V4 | 1.0 | 1.529e-14 | 558 | 0.516 | 151 | 68 | 2 | 12 | 162 | 2 | 147 | Single-stranded DNA-binding protein | Single-stranded DNA-binding protein | | afdb-uniprot50 | AF-E1YGH0-F1-MODEL\_V4 | 1.0 | 3.399e-15 | 558 | 0.425 | 160 | 77 | 4 | 3 | 162 | 21 | 165 | Single-stranded DNA-binding protein | Single-stranded DNA-binding protein | | afdb-uniprot50 | AF-A0A7Z9G7J1-F1-MODEL\_V4 | 1.0 | 6.137e-15 | 558 | 0.4 | 160 | 89 | 5 | 4 | 162 | 2 | 155 | Single-stranded DNA-binding protein | Single-stranded DNA-binding protein | | afdb-uniprot50 | AF-A0A661TMS4-F1-MODEL\_V4 | 1.0 | 6.137e-15 | 557 | 0.416 | 168 | 85 | 6 | 4 | 162 | 1 | 164 | Single-stranded DNA-binding protein | Single-stranded DNA-binding protein | | afdb-uniprot50 | AF-A0A7X8D708-F1-MODEL\_V4 | 1.0 | 1.225e-15 | 557 | 0.47 | 172 | 79 | 4 | 1 | 162 | 1 | 170 | Single-stranded DNA-binding protein | Single-stranded DNA-binding protein | | afdb-uniprot50 | AF-A0A3A1YSF5-F1-MODEL\_V4 | 1.0 | 1.043e-15 | 557 | 0.424 | 179 | 84 | 5 | 1 | 162 | 1 | 177 | Single-stranded DNA-binding protein | Single-stranded DNA-binding protein | | afdb-uniprot50 | AF-A0A1W9L078-F1-MODEL\_V4 | 1.0 | 1.374e-14 | 556 | 0.425 | 160 | 80 | 4 | 3 | 162 | 2 | 149 | Single-stranded DNA-binding protein | Single-stranded DNA-binding protein | | afdb-uniprot50 | AF-A0A662BHB8-F1-MODEL\_V4 | 1.0 | 6.928e-13 | 555 | 0.419 | 131 | 69 | 4 | 1 | 131 | 1 | 124 | Single-stranded DNA-binding protein | Single-stranded DNA-binding protein | | afdb-uniprot50 | AF-A0A7K1X431-F1-MODEL\_V4 | 1.0 | 1.726e-12 | 555 | 0.406 | 128 | 68 | 3 | 1 | 128 | 1 | 120 | Single-stranded DNA-binding protein | Single-stranded DNA-binding protein | | afdb-uniprot50 | AF-A0A1G2BK10-F1-MODEL\_V4 | 1.0 | 7.714e-13 | 555 | 0.429 | 128 | 65 | 3 | 4 | 130 | 2 | 122 | Single-stranded DNA-binding protein | Single-stranded DNA-binding protein | | afdb-uniprot50 | AF-A0A2B8A0F0-F1-MODEL\_V4 | 1.0 | 2.014e-13 | 555 | 0.441 | 145 | 71 | 5 | 1 | 144 | 1 | 136 | Single-stranded DNA-binding protein | Single-stranded DNA-binding protein | | afdb-uniprot50 | AF-G8R207-F1-MODEL\_V4 | 1.0 | 3.423e-14 | 555 | 0.45 | 162 | 73 | 3 | 1 | 162 | 1 | 146 | Single-stranded DNA-binding protein | Single-stranded DNA-binding protein | | afdb-uniprot50 | AF-A0A5C8ILK4-F1-MODEL\_V4 | 1.0 | 3.612e-14 | 555 | 0.388 | 162 | 84 | 5 | 1 | 162 | 1 | 147 | Single-stranded DNA-binding protein | Single-stranded DNA-binding protein | | afdb-uniprot50 | AF-A0A2H0L162-F1-MODEL\_V4 | 1.0 | 3.423e-14 | 555 | 0.422 | 154 | 82 | 4 | 1 | 154 | 1 | 147 | Single-stranded DNA-binding protein | Single-stranded DNA-binding protein | | afdb-uniprot50 | AF-A0A3D1BFD1-F1-MODEL\_V4 | 1.0 | 3.221e-15 | 555 | 0.436 | 165 | 85 | 5 | 1 | 162 | 1 | 160 | Single-stranded DNA-binding protein | Single-stranded DNA-binding protein | | afdb-uniprot50 | AF-A0A1F7BR67-F1-MODEL\_V4 | 1.0 | 1.529e-14 | 555 | 0.418 | 160 | 84 | 3 | 1 | 160 | 17 | 167 | Single-stranded DNA-binding protein | Single-stranded DNA-binding protein | | afdb-uniprot50 | AF-A0A5B5VMW8-F1-MODEL\_V4 | 1.0 | 9.562e-13 | 554 | 0.436 | 133 | 69 | 2 | 4 | 136 | 1 | 127 | Single-stranded DNA-binding protein | Single-stranded DNA-binding protein | | afdb-uniprot50 | AF-A0A1Z8MPJ0-F1-MODEL\_V4 | 1.0 | 1.909e-13 | 554 | 0.327 | 159 | 82 | 3 | 4 | 162 | 2 | 135 | Single-stranded DNA-binding protein | Single-stranded DNA-binding protein | | afdb-uniprot50 | AF-A0A1F9RAQ1-F1-MODEL\_V4 | 1.0 | 4.446e-15 | 554 | 0.419 | 162 | 86 | 5 | 1 | 162 | 1 | 154 | Single-stranded DNA-binding protein | Single-stranded DNA-binding protein | | afdb-uniprot50 | AF-A0A1F8B8P6-F1-MODEL\_V4 | 1.0 | 1.374e-14 | 554 | 0.37 | 162 | 96 | 3 | 1 | 162 | 1 | 156 | Single-stranded DNA-binding protein | Single-stranded DNA-binding protein | | afdb-uniprot50 | AF-A0A3B9MCP7-F1-MODEL\_V4 | 1.0 | 1.636e-12 | 553 | 0.423 | 118 | 61 | 3 | 1 | 114 | 14 | 128 | Single-stranded DNA-binding protein | Single-stranded DNA-binding protein | | afdb-uniprot50 | AF-A0A378XHW0-F1-MODEL\_V4 | 1.0 | 4.79e-12 | 553 | 0.347 | 118 | 72 | 2 | 1 | 117 | 1 | 114 | Single-stranded DNA-binding protein | Single-stranded DNA-binding protein | | afdb-uniprot50 | AF-A0A5C7JQL9-F1-MODEL\_V4 | 1.0 | 3.811e-14 | 553 | 0.385 | 174 | 73 | 6 | 1 | 162 | 1 | 152 | Single-stranded DNA-binding protein | Single-stranded DNA-binding protein | | afdb-uniprot50 | AF-A0A193KJZ2-F1-MODEL\_V4 | 1.0 | 2.35e-14 | 553 | 0.441 | 163 | 84 | 5 | 3 | 162 | 2 | 160 | Single-stranded DNA-binding protein | Single-stranded DNA-binding protein | | afdb-uniprot50 | AF-A0A352LLW6-F1-MODEL\_V4 | 1.0 | 1.302e-14 | 553 | 0.442 | 165 | 81 | 7 | 1 | 162 | 1 | 157 | Single-stranded DNA-binding protein | Single-stranded DNA-binding protein | | afdb-uniprot50 | AF-A0A0S8G146-F1-MODEL\_V4 | 1.0 | 1.715e-13 | 552 | 0.427 | 159 | 68 | 4 | 4 | 162 | 3 | 138 | Single-stranded DNA-binding protein | Single-stranded DNA-binding protein | | afdb-uniprot50 | AF-A0A1G4UBM1-F1-MODEL\_V4 | 1.0 | 2.366e-13 | 552 | 0.424 | 139 | 72 | 4 | 1 | 137 | 1 | 133 | Single-stranded DNA-binding protein | Single-stranded DNA-binding protein | | afdb-uniprot50 | AF-A0A7X3VRR7-F1-MODEL\_V4 | 1.0 | 2.497e-13 | 552 | 0.321 | 146 | 93 | 2 | 5 | 150 | 1 | 140 | Single-stranded DNA-binding protein | Single-stranded DNA-binding protein | | afdb-uniprot50 | AF-A0A2H0BUB7-F1-MODEL\_V4 | 1.0 | 1.374e-14 | 552 | 0.385 | 166 | 94 | 4 | 1 | 162 | 2 | 163 | Single-stranded DNA-binding protein | Single-stranded DNA-binding protein | | afdb-uniprot50 | AF-A0A4Y6J6J4-F1-MODEL\_V4 | 1.0 | 3.074e-14 | 552 | 0.341 | 170 | 96 | 4 | 1 | 160 | 2 | 165 | Single-stranded DNA-binding protein | Single-stranded DNA-binding protein | | afdb-uniprot50 | AF-A0A0G1V161-F1-MODEL\_V4 | 1.0 | 4.508e-13 | 551 | 0.388 | 126 | 74 | 2 | 4 | 127 | 2 | 126 | Single-stranded DNA-binding protein | Single-stranded DNA-binding protein | | afdb-uniprot50 | AF-A0A143XJT9-F1-MODEL\_V4 | 1.0 | 1.703e-14 | 551 | 0.392 | 191 | 81 | 4 | 4 | 162 | 1 | 188 | Single-stranded DNA-binding protein | Single-stranded DNA-binding protein | | afdb-uniprot50 | AF-A0A517VMC9-F1-MODEL\_V4 | 1.0 | 3.244e-14 | 550 | 0.377 | 167 | 84 | 7 | 1 | 162 | 1 | 152 | Single-stranded DNA-binding protein | Single-stranded DNA-binding protein | | afdb-uniprot50 | AF-A0A1Y1T5Q8-F1-MODEL\_V4 | 1.0 | 1.449e-14 | 550 | 0.325 | 175 | 102 | 3 | 1 | 162 | 1 | 172 | Single-stranded DNA-binding protein | Single-stranded DNA-binding protein | | afdb-uniprot50 | AF-A0A1F6RWV3-F1-MODEL\_V4 | 1.0 | 2.334e-15 | 550 | 0.427 | 166 | 82 | 4 | 4 | 162 | 2 | 161 | Single-stranded DNA-binding protein | Single-stranded DNA-binding protein | | afdb-uniprot50 | AF-A0A1W9NY84-F1-MODEL\_V4 | 1.0 | 5.333e-12 | 550 | 0.394 | 119 | 68 | 2 | 1 | 118 | 2 | 117 | Single-stranded DNA-binding protein | Single-stranded DNA-binding protein | | afdb-uniprot50 | AF-A0A4P5V4F4-F1-MODEL\_V4 | 1.0 | 2.617e-14 | 550 | 0.373 | 166 | 95 | 4 | 1 | 162 | 1 | 161 | Single-stranded DNA-binding protein | Single-stranded DNA-binding protein | | afdb-uniprot50 | AF-A0A2E9H291-F1-MODEL\_V4 | 1.0 | 6.52e-14 | 550 | 0.417 | 163 | 83 | 4 | 1 | 155 | 1 | 159 | Single-stranded DNA-binding protein | Single-stranded DNA-binding protein | | afdb-uniprot50 | AF-A0A2H0SKG1-F1-MODEL\_V4 | 1.0 | 7.26e-14 | 550 | 0.351 | 165 | 101 | 2 | 1 | 162 | 2 | 163 | Single-stranded DNA-binding protein | Single-stranded DNA-binding protein | | afdb-uniprot50 | AF-A0A554VRH5-F1-MODEL\_V4 | 1.0 | 1.726e-12 | 549 | 0.424 | 125 | 66 | 3 | 4 | 128 | 1 | 119 | Single-stranded DNA-binding protein | Single-stranded DNA-binding protein | | afdb-uniprot50 | AF-A0A258BZL2-F1-MODEL\_V4 | 1.0 | 3.244e-14 | 549 | 0.409 | 161 | 78 | 3 | 3 | 162 | 2 | 146 | Single-stranded DNA-binding protein | Single-stranded DNA-binding protein | | afdb-uniprot50 | AF-W7W8R5-F1-MODEL\_V4 | 1.0 | 2.334e-15 | 549 | 0.437 | 167 | 87 | 4 | 1 | 162 | 1 | 165 | Single-stranded DNA-binding protein | Single-stranded DNA-binding protein | | afdb-uniprot50 | AF-A0A563D9X7-F1-MODEL\_V4 | 1.0 | 3.47e-12 | 548 | 0.401 | 127 | 68 | 3 | 1 | 127 | 1 | 119 | Single-stranded DNA-binding protein | Single-stranded DNA-binding protein | | afdb-uniprot50 | AF-A0A2D6FJM0-F1-MODEL\_V4 | 1.0 | 8.529e-14 | 548 | 0.429 | 163 | 76 | 5 | 1 | 162 | 2 | 148 | Single-stranded DNA-binding protein | Single-stranded DNA-binding protein | | afdb-uniprot50 | AF-A0A257H2V1-F1-MODEL\_V4 | 1.0 | 9.431e-15 | 548 | 0.404 | 178 | 78 | 5 | 1 | 162 | 1 | 166 | Single-stranded DNA-binding protein | Single-stranded DNA-binding protein | | afdb-uniprot50 | AF-A0A5M4B7C0-F1-MODEL\_V4 | 1.0 | 1.636e-12 | 547 | 0.353 | 133 | 80 | 2 | 1 | 130 | 1 | 130 | Single-stranded DNA-binding protein | Single-stranded DNA-binding protein | | afdb-uniprot50 | AF-R6A491-F1-MODEL\_V4 | 1.0 | 3.423e-14 | 546 | 0.422 | 180 | 81 | 7 | 1 | 162 | 1 | 175 | Single-stranded DNA-binding protein | Single-stranded DNA-binding protein | | afdb-uniprot50 | AF-R5I4N3-F1-MODEL\_V4 | 1.0 | 6.137e-15 | 546 | 0.386 | 194 | 83 | 4 | 4 | 162 | 2 | 194 | Single-stranded DNA-binding protein | Single-stranded DNA-binding protein | | afdb-uniprot50 | AF-A0A7C4TH37-F1-MODEL\_V4 | 1.0 | 5.897e-13 | 545 | 0.355 | 135 | 81 | 3 | 1 | 134 | 5 | 134 | Single-stranded DNA-binding protein | Single-stranded DNA-binding protein | | afdb-uniprot50 | AF-A0A3N5P7G3-F1-MODEL\_V4 | 1.0 | 4.477e-14 | 545 | 0.419 | 162 | 72 | 5 | 1 | 162 | 1 | 140 | Single-stranded DNA-binding protein | Single-stranded DNA-binding protein | | afdb-uniprot50 | AF-A0A1W9QM69-F1-MODEL\_V4 | 1.0 | 3.074e-14 | 545 | 0.364 | 159 | 83 | 4 | 4 | 162 | 2 | 142 | Single-stranded DNA-binding protein | Single-stranded DNA-binding protein | | afdb-uniprot50 | AF-A0A6L7TFT4-F1-MODEL\_V4 | 1.0 | 3.423e-14 | 545 | 0.374 | 171 | 90 | 4 | 1 | 162 | 1 | 163 | Single-stranded DNA-binding protein | Single-stranded DNA-binding protein | | afdb-uniprot50 | AF-A0A2N7RSM6-F1-MODEL\_V4 | 1.0 | 5.816e-15 | 545 | 0.391 | 171 | 90 | 4 | 1 | 162 | 1 | 166 | Single-stranded DNA-binding protein | Single-stranded DNA-binding protein | | afdb-uniprot50 | AF-A0A535RBC2-F1-MODEL\_V4 | 1.0 | 4.077e-12 | 544 | 0.396 | 111 | 64 | 2 | 4 | 114 | 1 | 108 | Single-stranded DNA-binding protein | Single-stranded DNA-binding protein | | afdb-uniprot50 | AF-A0A7Z9YCJ8-F1-MODEL\_V4 | 1.0 | 2.497e-13 | 544 | 0.36 | 133 | 83 | 2 | 1 | 133 | 1 | 131 | Single-stranded DNA-binding protein | Single-stranded DNA-binding protein | | afdb-uniprot50 | AF-A0A351KW12-F1-MODEL\_V4 | 1.0 | 1.715e-13 | 544 | 0.381 | 160 | 78 | 5 | 4 | 162 | 3 | 142 | Single-stranded DNA-binding protein | Single-stranded DNA-binding protein | | afdb-uniprot50 | AF-A0A3M0YZZ3-F1-MODEL\_V4 | 1.0 | 1.26e-11 | 544 | 0.393 | 117 | 68 | 1 | 1 | 117 | 3 | 116 | Single-stranded DNA-binding protein | Single-stranded DNA-binding protein | | afdb-uniprot50 | AF-A0A3D4IIJ5-F1-MODEL\_V4 | 1.0 | 8.938e-15 | 544 | 0.402 | 174 | 83 | 6 | 1 | 162 | 2 | 166 | Single-stranded DNA-binding protein | Single-stranded DNA-binding protein | | afdb-uniprot50 | AF-A0A2W6YVK2-F1-MODEL\_V4 | 1.0 | 1.169e-14 | 544 | 0.454 | 176 | 80 | 6 | 1 | 162 | 1 | 174 | Single-stranded DNA-binding protein | Single-stranded DNA-binding protein | | afdb-uniprot50 | AF-A0A0F9SXG5-F1-MODEL\_V4 | 1.0 | 1.459e-13 | 543 | 0.401 | 162 | 78 | 6 | 1 | 162 | 1 | 143 | Uncharacterized protein | Uncharacterized protein | | afdb-uniprot50 | AF-A0A077DE37-F1-MODEL\_V4 | 1.0 | 3.423e-14 | 543 | 0.406 | 165 | 86 | 5 | 3 | 162 | 2 | 159 | Single-stranded DNA-binding protein | Single-stranded DNA-binding protein | | afdb-uniprot50 | AF-A0A1F7VHB8-F1-MODEL\_V4 | 1.0 | 1.374e-14 | 543 | 0.413 | 167 | 86 | 5 | 4 | 162 | 3 | 165 | Single-stranded DNA-binding protein | Single-stranded DNA-binding protein | | afdb-uniprot50 | AF-A0A350CXW3-F1-MODEL\_V4 | 1.0 | 1.459e-13 | 543 | 0.387 | 155 | 88 | 3 | 1 | 155 | 20 | 167 | Single-stranded DNA-binding protein | Single-stranded DNA-binding protein | | afdb-uniprot50 | AF-A0A4R3I482-F1-MODEL\_V4 | 1.0 | 2.334e-15 | 543 | 0.449 | 187 | 77 | 5 | 1 | 162 | 2 | 187 | Single-stranded DNA-binding protein | Single-stranded DNA-binding protein | | afdb-uniprot50 | AF-A0A2M7DG48-F1-MODEL\_V4 | 1.0 | 2.243e-13 | 542 | 0.327 | 162 | 97 | 2 | 1 | 162 | 1 | 150 | Single-stranded DNA-binding protein | Single-stranded DNA-binding protein | | afdb-uniprot50 | AF-A0A2M8EIY2-F1-MODEL\_V4 | 1.0 | 4.477e-14 | 542 | 0.367 | 155 | 92 | 3 | 1 | 155 | 1 | 149 | Single-stranded DNA-binding protein | Single-stranded DNA-binding protein | | afdb-uniprot50 | AF-A0A1F1C449-F1-MODEL\_V4 | 1.0 | 7.714e-13 | 540 | 0.341 | 117 | 71 | 2 | 4 | 120 | 3 | 113 | Single-stranded DNA-binding protein | Single-stranded DNA-binding protein | | afdb-uniprot50 | AF-R5E1D3-F1-MODEL\_V4 | 1.0 | 6.18e-14 | 540 | 0.389 | 167 | 88 | 5 | 1 | 161 | 1 | 159 | Single-stranded DNA-binding protein | Single-stranded DNA-binding protein | | afdb-uniprot50 | AF-A0A4P6FUU1-F1-MODEL\_V4 | 1.0 | 4.985e-14 | 540 | 0.355 | 163 | 98 | 6 | 4 | 162 | 2 | 161 | Single-stranded DNA-binding protein | Single-stranded DNA-binding protein | | afdb-uniprot50 | AF-S5RPZ6-F1-MODEL\_V4 | 1.0 | 6.137e-15 | 540 | 0.379 | 182 | 82 | 4 | 1 | 162 | 1 | 171 | Single-stranded DNA-binding protein | Single-stranded DNA-binding protein | | afdb-uniprot50 | AF-A0A2H1YK25-F1-MODEL\_V4 | 1.0 | 6.266e-12 | 539 | 0.377 | 127 | 75 | 2 | 1 | 127 | 1 | 123 | Single-stranded DNA-binding protein | Single-stranded DNA-binding protein | | afdb-uniprot50 | AF-A0A517YVP6-F1-MODEL\_V4 | 1.0 | 1.002e-13 | 539 | 0.368 | 160 | 89 | 4 | 4 | 162 | 3 | 151 | Single-stranded DNA-binding protein | Single-stranded DNA-binding protein | | afdb-uniprot50 | AF-A0A7C4ZBJ7-F1-MODEL\_V4 | 1.0 | 2.617e-14 | 539 | 0.358 | 159 | 94 | 3 | 4 | 162 | 3 | 153 | Single-stranded DNA-binding protein | Single-stranded DNA-binding protein | | afdb-uniprot50 | AF-A0A124FXW4-F1-MODEL\_V4 | 1.0 | 3.423e-14 | 539 | 0.337 | 169 | 96 | 5 | 1 | 162 | 2 | 161 | Single-stranded DNA-binding protein | Single-stranded DNA-binding protein | | afdb-uniprot50 | AF-A0A1G1CF15-F1-MODEL\_V4 | 1.0 | 2.48e-14 | 539 | 0.366 | 169 | 96 | 4 | 1 | 162 | 2 | 166 | Single-stranded DNA-binding protein | Single-stranded DNA-binding protein | | afdb-uniprot50 | AF-A0A4V0XBV8-F1-MODEL\_V4 | 1.0 | 9e-14 | 539 | 0.364 | 162 | 96 | 3 | 1 | 161 | 1 | 156 | Single-stranded DNA-binding protein | Single-stranded DNA-binding protein | | afdb-uniprot50 | AF-A0A662ZJD1-F1-MODEL\_V4 | 1.0 | 4.021e-14 | 539 | 0.386 | 163 | 92 | 4 | 1 | 159 | 2 | 160 | Single-stranded DNA-binding protein | Single-stranded DNA-binding protein | | afdb-uniprot50 | AF-X0YPY6-F1-MODEL\_V4 | 1.0 | 2.366e-13 | 538 | 0.363 | 146 | 90 | 1 | 5 | 150 | 1 | 143 | Uncharacterized protein | Uncharacterized protein | | afdb-uniprot50 | AF-A0A660MIK8-F1-MODEL\_V4 | 1.0 | 2.8e-12 | 537 | 0.343 | 128 | 81 | 1 | 1 | 128 | 1 | 125 | Single-stranded DNA-binding protein | Single-stranded DNA-binding protein | | afdb-uniprot50 | AF-A0A2D6STV9-F1-MODEL\_V4 | 1.0 | 6.88e-14 | 537 | 0.367 | 166 | 96 | 6 | 1 | 162 | 1 | 161 | Single-stranded DNA-binding protein | Single-stranded DNA-binding protein | | afdb-uniprot50 | AF-A0A6H9LAI1-F1-MODEL\_V4 | 1.0 | 8.027e-15 | 537 | 0.4 | 170 | 90 | 6 | 1 | 162 | 1 | 166 | Single-stranded DNA-binding protein | Single-stranded DNA-binding protein | | afdb-uniprot50 | AF-A0A0B7IME9-F1-MODEL\_V4 | 1.0 | 3.47e-12 | 536 | 0.375 | 128 | 74 | 2 | 2 | 129 | 3 | 124 | Single-stranded DNA-binding protein | Single-stranded DNA-binding protein | | afdb-uniprot50 | AF-U2C4X8-F1-MODEL\_V4 | 1.0 | 2.635e-13 | 536 | 0.381 | 139 | 81 | 2 | 1 | 139 | 1 | 134 | Single-stranded DNA-binding protein | Single-stranded DNA-binding protein | | afdb-uniprot50 | AF-A0A1F5BEK8-F1-MODEL\_V4 | 1.0 | 7.661e-14 | 536 | 0.376 | 162 | 88 | 5 | 2 | 162 | 4 | 153 | Single-stranded DNA-binding protein | Single-stranded DNA-binding protein | | afdb-uniprot50 | AF-T1D461-F1-MODEL\_V4 | 1.0 | 1.529e-14 | 535 | 0.445 | 164 | 83 | 4 | 4 | 162 | 3 | 163 | Single-strand DNA-binding protein | Single-strand DNA-binding protein | | afdb-uniprot50 | AF-A0A1Y1Q967-F1-MODEL\_V4 | 1.0 | 7.608e-15 | 535 | 0.396 | 174 | 91 | 4 | 1 | 162 | 1 | 172 | Single-stranded DNA-binding protein | Single-stranded DNA-binding protein | | afdb-uniprot50 | AF-A0A2A4U0H7-F1-MODEL\_V4 | 1.0 | 2.48e-14 | 535 | 0.367 | 185 | 93 | 2 | 1 | 162 | 2 | 185 | Single-stranded DNA-binding protein | Single-stranded DNA-binding protein | | afdb-uniprot50 | AF-A0A4Q7G2J8-F1-MODEL\_V4 | 1.0 | 1.311e-13 | 535 | 0.389 | 167 | 88 | 6 | 1 | 162 | 26 | 183 | Single-stranded DNA-binding protein | Single-stranded DNA-binding protein | | afdb-uniprot50 | AF-A0A315EK95-F1-MODEL\_V4 | 1.0 | 3.637e-13 | 534 | 0.404 | 146 | 73 | 3 | 1 | 146 | 1 | 132 | Single-stranded DNA-binding protein | Single-stranded DNA-binding protein | | afdb-uniprot50 | AF-A0A2M7CI87-F1-MODEL\_V4 | 1.0 | 3.074e-14 | 534 | 0.349 | 163 | 99 | 3 | 4 | 162 | 3 | 162 | Single-stranded DNA-binding protein | Single-stranded DNA-binding protein | | afdb-uniprot50 | AF-A0A7L4ZN54-F1-MODEL\_V4 | 1.0 | 5.26e-14 | 534 | 0.329 | 176 | 101 | 2 | 1 | 162 | 1 | 173 | Single-stranded DNA-binding protein | Single-stranded DNA-binding protein | | afdb-uniprot50 | AF-A0A7X6U5F3-F1-MODEL\_V4 | 1.0 | 4.823e-11 | 533 | 0.366 | 101 | 64 | 0 | 1 | 101 | 1 | 101 | Single-stranded DNA-binding protein | Single-stranded DNA-binding protein | | afdb-uniprot50 | AF-A0A644Y581-F1-MODEL\_V4 | 1.0 | 4.077e-12 | 533 | 0.393 | 127 | 67 | 3 | 4 | 129 | 2 | 119 | Single-stranded DNA-binding protein | Single-stranded DNA-binding protein | | afdb-uniprot50 | AF-A0A0B8QEM1-F1-MODEL\_V4 | 1.0 | 7.26e-14 | 533 | 0.457 | 153 | 75 | 4 | 12 | 162 | 2 | 148 | Single-stranded DNA-binding protein | Single-stranded DNA-binding protein | | afdb-uniprot50 | AF-A0A2D8XDB3-F1-MODEL\_V4 | 1.0 | 1.116e-13 | 533 | 0.347 | 161 | 92 | 5 | 4 | 162 | 1 | 150 | Single-stranded DNA-binding protein | Single-stranded DNA-binding protein | | afdb-uniprot50 | AF-A0A2E6JYQ6-F1-MODEL\_V4 | 1.0 | 3.244e-14 | 533 | 0.397 | 161 | 78 | 4 | 3 | 162 | 2 | 144 | Single-stranded DNA-binding protein | Single-stranded DNA-binding protein | | afdb-uniprot50 | AF-A0A6L7VM16-F1-MODEL\_V4 | 1.0 | 1.449e-14 | 533 | 0.436 | 181 | 76 | 9 | 1 | 162 | 1 | 174 | Single-stranded DNA-binding protein | Single-stranded DNA-binding protein | | afdb-uniprot50 | AF-A0A848FDV8-F1-MODEL\_V4 | 1.0 | 2.761e-14 | 533 | 0.371 | 191 | 87 | 5 | 1 | 162 | 1 | 187 | Single-stranded DNA-binding protein | Single-stranded DNA-binding protein | | afdb-uniprot50 | AF-A0A365P1J8-F1-MODEL\_V4 | 1.0 | 4.79e-12 | 532 | 0.392 | 130 | 76 | 1 | 1 | 130 | 1 | 127 | Single-stranded DNA-binding protein | Single-stranded DNA-binding protein | | afdb-uniprot50 | AF-A0A255ZVS7-F1-MODEL\_V4 | 1.0 | 1.636e-12 | 532 | 0.377 | 135 | 76 | 2 | 2 | 136 | 3 | 129 | Single-stranded DNA-binding protein | Single-stranded DNA-binding protein | | afdb-uniprot50 | AF-A0A1F2Q8N9-F1-MODEL\_V4 | 1.0 | 2.14e-12 | 532 | 0.338 | 139 | 83 | 3 | 4 | 138 | 2 | 135 | Single-stranded DNA-binding protein | Single-stranded DNA-binding protein | | afdb-uniprot50 | AF-A0A7Y4RCW9-F1-MODEL\_V4 | 1.0 | 9.496e-14 | 532 | 0.335 | 164 | 96 | 5 | 1 | 162 | 1 | 153 | Single-stranded DNA-binding protein | Single-stranded DNA-binding protein | | afdb-uniprot50 | AF-A0A7T9BXX1-F1-MODEL\_V4 | 1.0 | 9.562e-13 | 532 | 0.328 | 143 | 90 | 3 | 1 | 141 | 1 | 139 | Single-stranded DNA-binding protein | Single-stranded DNA-binding protein | | afdb-uniprot50 | AF-A0A081BZQ6-F1-MODEL\_V4 | 1.0 | 3.074e-14 | 531 | 0.376 | 162 | 97 | 2 | 1 | 162 | 2 | 159 | Single-stranded DNA-binding protein | Single-stranded DNA-binding protein | | afdb-uniprot50 | AF-A0A662ZPE4-F1-MODEL\_V4 | 1.0 | 1.311e-13 | 531 | 0.351 | 162 | 98 | 2 | 1 | 162 | 1 | 155 | Single-stranded DNA-binding protein | Single-stranded DNA-binding protein | | afdb-uniprot50 | AF-A0A1H9CG65-F1-MODEL\_V4 | 1.0 | 2.258e-12 | 530 | 0.345 | 136 | 85 | 2 | 1 | 136 | 1 | 132 | Single-stranded DNA-binding protein | Single-stranded DNA-binding protein | | afdb-uniprot50 | AF-A0A0G0IUI9-F1-MODEL\_V4 | 1.0 | 1.809e-13 | 530 | 0.408 | 159 | 84 | 3 | 4 | 162 | 2 | 150 | Single-stranded DNA-binding protein | Single-stranded DNA-binding protein | | afdb-uniprot50 | AF-K1ZMT3-F1-MODEL\_V4 | 1.0 | 8.529e-14 | 530 | 0.355 | 163 | 97 | 4 | 3 | 162 | 2 | 159 | Single-stranded DNA-binding protein | Single-stranded DNA-binding protein | | afdb-uniprot50 | AF-A0A139KGR1-F1-MODEL\_V4 | 1.0 | 2.913e-14 | 530 | 0.395 | 167 | 91 | 6 | 1 | 162 | 1 | 162 | Single-stranded DNA-binding protein | Single-stranded DNA-binding protein | | afdb-uniprot50 | AF-A0A4Q7V7A6-F1-MODEL\_V4 | 1.0 | 1.131e-11 | 528 | 0.394 | 119 | 68 | 2 | 4 | 122 | 1 | 115 | Single-stranded DNA-binding protein | Single-stranded DNA-binding protein | | afdb-uniprot50 | AF-A0A847B064-F1-MODEL\_V4 | 1.0 | 4.508e-13 | 527 | 0.327 | 162 | 95 | 4 | 1 | 162 | 1 | 148 | Single-stranded DNA-binding protein | Single-stranded DNA-binding protein | | afdb-uniprot50 | AF-A0A2H0VIG2-F1-MODEL\_V4 | 1.0 | 1.529e-14 | 527 | 0.359 | 181 | 93 | 5 | 1 | 162 | 2 | 178 | Single-stranded DNA-binding protein | Single-stranded DNA-binding protein | | afdb-uniprot50 | AF-A6DBN5-F1-MODEL\_V4 | 1.0 | 1.057e-13 | 526 | 0.356 | 160 | 89 | 5 | 4 | 162 | 1 | 147 | Single-stranded DNA-binding protein | Single-stranded DNA-binding protein | | afdb-uniprot50 | AF-A0A2H0YD16-F1-MODEL\_V4 | 1.0 | 3.423e-14 | 526 | 0.422 | 161 | 83 | 4 | 4 | 162 | 2 | 154 | Single-stranded DNA-binding protein | Single-stranded DNA-binding protein | | afdb-uniprot50 | AF-A0A1V5SBT0-F1-MODEL\_V4 | 1.0 | 4.985e-14 | 526 | 0.391 | 161 | 88 | 5 | 4 | 162 | 3 | 155 | Single-stranded DNA-binding protein | Single-stranded DNA-binding protein | | afdb-uniprot50 | AF-A0A660S5G9-F1-MODEL\_V4 | 1.0 | 1.402e-11 | 524 | 0.302 | 119 | 76 | 3 | 1 | 115 | 1 | 116 | Single-stranded DNA-binding protein | Single-stranded DNA-binding protein | | afdb-uniprot50 | AF-A0A1G2R9I5-F1-MODEL\_V4 | 1.0 | 4.021e-14 | 524 | 0.408 | 159 | 88 | 3 | 4 | 162 | 2 | 154 | Single-stranded DNA-binding protein | Single-stranded DNA-binding protein | | afdb-uniprot50 | AF-A0A2M6WFV0-F1-MODEL\_V4 | 1.0 | 3.074e-14 | 524 | 0.391 | 184 | 81 | 7 | 4 | 162 | 2 | 179 | Single-stranded DNA-binding protein | Single-stranded DNA-binding protein | | afdb-uniprot50 | AF-A0A2S9Y8A6-F1-MODEL\_V4 | 1.0 | 1.169e-14 | 524 | 0.391 | 166 | 95 | 3 | 1 | 162 | 1 | 164 | Single-stranded DNA-binding protein | Single-stranded DNA-binding protein | | afdb-uniprot50 | AF-A0A536IEW5-F1-MODEL\_V4 | 1.0 | 4.508e-13 | 523 | 0.413 | 138 | 75 | 4 | 1 | 136 | 1 | 134 | Single-stranded DNA-binding protein | Single-stranded DNA-binding protein | | afdb-uniprot50 | AF-A0A257K0X4-F1-MODEL\_V4 | 1.0 | 5.02e-13 | 523 | 0.337 | 154 | 89 | 5 | 1 | 154 | 1 | 141 | Single-stranded DNA-binding protein | Single-stranded DNA-binding protein | | afdb-uniprot50 | AF-A0A7V3EK99-F1-MODEL\_V4 | 1.0 | 5.26e-14 | 523 | 0.371 | 167 | 92 | 6 | 1 | 162 | 1 | 159 | Single-stranded DNA-binding protein | Single-stranded DNA-binding protein | | afdb-uniprot50 | AF-A0A4P6X7E8-F1-MODEL\_V4 | 1.0 | 1.551e-12 | 522 | 0.372 | 137 | 81 | 3 | 1 | 137 | 1 | 132 | Single-stranded DNA-binding protein | Single-stranded DNA-binding protein | | afdb-uniprot50 | AF-A0A7T9FFD8-F1-MODEL\_V4 | 1.0 | 1.116e-13 | 522 | 0.378 | 164 | 82 | 7 | 1 | 162 | 1 | 146 | Single-stranded DNA-binding protein | Single-stranded DNA-binding protein | | afdb-uniprot50 | AF-A0A7Z9KRQ6-F1-MODEL\_V4 | 1.0 | 3.891e-11 | 521 | 0.272 | 110 | 77 | 1 | 4 | 113 | 3 | 109 | Single-stranded DNA-binding protein | Single-stranded DNA-binding protein | | afdb-uniprot50 | AF-K1WZW4-F1-MODEL\_V4 | 1.0 | 1.48e-11 | 520 | 0.317 | 129 | 78 | 4 | 1 | 128 | 1 | 120 | Single-stranded DNA-binding protein | Single-stranded DNA-binding protein | | afdb-uniprot50 | AF-A0A4U0GGN8-F1-MODEL\_V4 | 1.0 | 1.922e-12 | 520 | 0.337 | 145 | 82 | 4 | 1 | 145 | 2 | 132 | Single-stranded DNA-binding protein | Single-stranded DNA-binding protein | | afdb-uniprot50 | AF-A0A2D5BSE2-F1-MODEL\_V4 | 1.0 | 4.243e-14 | 520 | 0.392 | 163 | 90 | 6 | 1 | 162 | 1 | 155 | Single-stranded DNA-binding protein | Single-stranded DNA-binding protein | | afdb-uniprot50 | AF-A0A6N9D2R3-F1-MODEL\_V4 | 1.0 | 1.625e-13 | 520 | 0.397 | 171 | 88 | 6 | 1 | 162 | 1 | 165 | Single-stranded DNA-binding protein | Single-stranded DNA-binding protein | | afdb-uniprot50 | AF-R4YT41-F1-MODEL\_V4 | 1.0 | 3.244e-14 | 520 | 0.419 | 181 | 84 | 5 | 1 | 162 | 1 | 179 | Single-stranded DNA-binding protein | Single-stranded DNA-binding protein | | afdb-uniprot50 | AF-A0A1E8VZ74-F1-MODEL\_V4 | 1.0 | 2.8e-12 | 519 | 0.404 | 126 | 63 | 2 | 4 | 129 | 2 | 115 | Single-stranded DNA-binding protein | Single-stranded DNA-binding protein | | afdb-uniprot50 | AF-A0A1V4QTT8-F1-MODEL\_V4 | 1.0 | 1.909e-13 | 519 | 0.381 | 160 | 91 | 3 | 1 | 160 | 1 | 152 | Single-stranded DNA-binding protein | Single-stranded DNA-binding protein | | afdb-uniprot50 | AF-A0A2D6Q1P2-F1-MODEL\_V4 | 1.0 | 3.811e-14 | 519 | 0.357 | 176 | 97 | 4 | 1 | 162 | 1 | 174 | Single-stranded DNA-binding protein | Single-stranded DNA-binding protein | | afdb-uniprot50 | AF-A0A1F3X205-F1-MODEL\_V4 | 1.0 | 4.302e-12 | 518 | 0.384 | 125 | 67 | 3 | 4 | 128 | 3 | 117 | Single-stranded DNA-binding protein | Single-stranded DNA-binding protein | | afdb-uniprot50 | AF-A0A7Y4XJK4-F1-MODEL\_V4 | 1.0 | 1.116e-13 | 518 | 0.397 | 166 | 88 | 4 | 3 | 162 | 2 | 161 | Single-stranded DNA-binding protein | Single-stranded DNA-binding protein | | afdb-uniprot50 | AF-A0A827P8J5-F1-MODEL\_V4 | 1.0 | 6.18e-14 | 517 | 0.611 | 144 | 51 | 2 | 21 | 162 | 1 | 141 | Single-stranded DNA-binding protein | Single-stranded DNA-binding protein | | afdb-uniprot50 | AF-A0A3B9CYY7-F1-MODEL\_V4 | 1.0 | 4.54e-12 | 516 | 0.365 | 134 | 78 | 4 | 1 | 132 | 1 | 129 | Single-stranded DNA-binding protein | Single-stranded DNA-binding protein | | afdb-uniprot50 | AF-A0A7X7RFW5-F1-MODEL\_V4 | 1.0 | 3.095e-13 | 516 | 0.31 | 164 | 101 | 5 | 1 | 162 | 1 | 154 | Single-stranded DNA-binding protein | Single-stranded DNA-binding protein | | afdb-uniprot50 | AF-A0A3L7QDN6-F1-MODEL\_V4 | 1.0 | 4.724e-14 | 516 | 0.329 | 173 | 104 | 4 | 1 | 162 | 1 | 172 | Single-stranded DNA-binding protein | Single-stranded DNA-binding protein | | afdb-uniprot50 | AF-A9AYW2-F1-MODEL\_V4 | 1.0 | 2.042e-11 | 515 | 0.375 | 120 | 69 | 4 | 1 | 118 | 3 | 118 | Single-stranded DNA-binding protein | Single-stranded DNA-binding protein | | afdb-uniprot50 | AF-A0A660NA14-F1-MODEL\_V4 | 1.0 | 1.47e-12 | 515 | 0.308 | 146 | 91 | 4 | 4 | 142 | 3 | 145 | Single-stranded DNA-binding protein | Single-stranded DNA-binding protein | | afdb-uniprot50 | AF-A0A7X6AFC1-F1-MODEL\_V4 | 1.0 | 2.366e-13 | 515 | 0.333 | 168 | 100 | 5 | 1 | 161 | 3 | 165 | Single-stranded DNA-binding protein | Single-stranded DNA-binding protein | | afdb-uniprot50 | AF-A0A7C0ZN74-F1-MODEL\_V4 | 1.0 | 4.021e-14 | 514 | 0.353 | 167 | 99 | 2 | 4 | 162 | 2 | 167 | Single-stranded DNA-binding protein | Single-stranded DNA-binding protein | | afdb-uniprot50 | AF-A0A432GXJ5-F1-MODEL\_V4 | 1.0 | 3.095e-13 | 514 | 0.3 | 163 | 105 | 4 | 1 | 162 | 75 | 229 | Single-stranded DNA-binding protein | Single-stranded DNA-binding protein | | afdb-uniprot50 | AF-A0A317HH03-F1-MODEL\_V4 | 1.0 | 2.383e-12 | 513 | 0.387 | 129 | 77 | 2 | 1 | 128 | 8 | 135 | Single-stranded DNA-binding protein | Single-stranded DNA-binding protein | | afdb-uniprot50 | AF-A0A2V2EFG8-F1-MODEL\_V4 | 1.0 | 6.566e-13 | 513 | 0.358 | 162 | 86 | 5 | 4 | 162 | 2 | 148 | Single-stranded DNA-binding protein | Single-stranded DNA-binding protein | | afdb-uniprot50 | AF-A0A2E8VMN4-F1-MODEL\_V4 | 1.0 | 4.757e-13 | 513 | 0.333 | 153 | 92 | 4 | 1 | 148 | 6 | 153 | Single-stranded DNA-binding protein | Single-stranded DNA-binding protein | | afdb-uniprot50 | AF-A0A7X8BKP9-F1-MODEL\_V4 | 1.0 | 2.028e-12 | 512 | 0.342 | 146 | 83 | 3 | 1 | 146 | 5 | 137 | Single-stranded DNA-binding protein | Single-stranded DNA-binding protein | | afdb-uniprot50 | AF-A0A2M6ZHX2-F1-MODEL\_V4 | 1.0 | 6.222e-13 | 512 | 0.318 | 157 | 98 | 4 | 1 | 156 | 1 | 149 | Single-stranded DNA-binding protein | Single-stranded DNA-binding protein | | afdb-uniprot50 | AF-F4T715-F1-MODEL\_V4 | 1.0 | 3.811e-14 | 512 | 0.506 | 158 | 67 | 2 | 16 | 162 | 1 | 158 | Single-stranded DNA-binding protein | Single-stranded DNA-binding protein | | afdb-uniprot50 | AF-A0A3E4Z701-F1-MODEL\_V4 | 1.0 | 9e-14 | 512 | 0.379 | 166 | 94 | 4 | 4 | 162 | 1 | 164 | Single-stranded DNA-binding protein | Single-stranded DNA-binding protein | | afdb-uniprot50 | AF-A0A1T0AXC1-F1-MODEL\_V4 | 1.0 | 6.657e-11 | 511 | 0.35 | 117 | 73 | 1 | 1 | 117 | 1 | 114 | Single-stranded DNA-binding protein | Single-stranded DNA-binding protein | | afdb-uniprot50 | AF-A0A2E6Z2B5-F1-MODEL\_V4 | 1.0 | 7.714e-13 | 511 | 0.308 | 159 | 96 | 3 | 4 | 162 | 1 | 145 | Single-stranded DNA-binding protein | Single-stranded DNA-binding protein | | afdb-uniprot50 | AF-A0A7C5KF93-F1-MODEL\_V4 | 1.0 | 1.177e-13 | 511 | 0.327 | 168 | 102 | 4 | 1 | 162 | 1 | 163 | Single-stranded DNA-binding protein | Single-stranded DNA-binding protein | | afdb-uniprot50 | AF-A0A7C3URN1-F1-MODEL\_V4 | 1.0 | 1.26e-11 | 510 | 0.391 | 115 | 69 | 1 | 3 | 117 | 7 | 120 | Single-stranded DNA-binding protein | Single-stranded DNA-binding protein | | afdb-uniprot50 | AF-A0A7W0J0Y0-F1-MODEL\_V4 | 1.0 | 1.123e-12 | 510 | 0.324 | 154 | 93 | 6 | 1 | 154 | 1 | 143 | Single-stranded DNA-binding protein | Single-stranded DNA-binding protein | | afdb-uniprot50 | AF-A0A1L5KLY0-F1-MODEL\_V4 | 1.0 | 2.227e-14 | 510 | 0.584 | 159 | 51 | 2 | 19 | 162 | 1 | 159 | Single-stranded DNA-binding protein | Single-stranded DNA-binding protein | | afdb-uniprot50 | AF-A0A1X4XY99-F1-MODEL\_V4 | 1.0 | 7.767e-12 | 509 | 0.325 | 126 | 81 | 2 | 4 | 129 | 2 | 123 | Single-stranded DNA-binding protein | Single-stranded DNA-binding protein | | afdb-uniprot50 | AF-A0A1G0AMD8-F1-MODEL\_V4 | 1.0 | 5.054e-12 | 509 | 0.314 | 143 | 91 | 2 | 1 | 143 | 1 | 136 | Single-stranded DNA-binding protein | Single-stranded DNA-binding protein | | afdb-uniprot50 | AF-A0A661XBR9-F1-MODEL\_V4 | 1.0 | 3.289e-12 | 509 | 0.343 | 128 | 78 | 3 | 1 | 128 | 1 | 122 | Single-stranded DNA-binding protein | Single-stranded DNA-binding protein | | afdb-uniprot50 | AF-A0A7C5UPK2-F1-MODEL\_V4 | 1.0 | 1.715e-13 | 509 | 0.364 | 159 | 90 | 3 | 1 | 156 | 2 | 152 | Single-stranded DNA-binding protein | Single-stranded DNA-binding protein | | afdb-uniprot50 | AF-A0A2E3HXA7-F1-MODEL\_V4 | 1.0 | 8.708e-11 | 508 | 0.343 | 99 | 65 | 0 | 4 | 102 | 3 | 101 | Single-stranded DNA-binding protein | Single-stranded DNA-binding protein | | afdb-uniprot50 | AF-A0A250FUA3-F1-MODEL\_V4 | 1.0 | 7.31e-13 | 508 | 0.333 | 156 | 94 | 3 | 1 | 156 | 1 | 146 | Single-stranded DNA-binding protein | Single-stranded DNA-binding protein | | afdb-uniprot50 | AF-A0A379MNE0-F1-MODEL\_V4 | 1.0 | 1.54e-13 | 508 | 0.4 | 160 | 88 | 4 | 4 | 162 | 1 | 153 | Single-stranded DNA-binding protein | Single-stranded DNA-binding protein | | afdb-uniprot50 | AF-A0A1D3K7U2-F1-MODEL\_V4 | 1.0 | 4.508e-13 | 508 | 0.335 | 173 | 102 | 6 | 1 | 162 | 2 | 172 | Single-stranded DNA-binding protein | Single-stranded DNA-binding protein | | afdb-uniprot50 | AF-A0A2E5LYI8-F1-MODEL\_V4 | 1.0 | 1.54e-13 | 508 | 0.356 | 160 | 95 | 3 | 3 | 162 | 2 | 153 | Multifunctional fusion protein | Multifunctional fusion protein | | afdb-uniprot50 | AF-A0A0P6Y529-F1-MODEL\_V4 | 1.0 | 3.312e-11 | 507 | 0.336 | 119 | 75 | 2 | 1 | 118 | 3 | 118 | Single-stranded DNA-binding protein | Single-stranded DNA-binding protein | | afdb-uniprot50 | AF-A0A7C4NQI9-F1-MODEL\_V4 | 1.0 | 1.561e-11 | 506 | 0.318 | 116 | 76 | 2 | 3 | 116 | 2 | 116 | Single-stranded DNA-binding protein | Single-stranded DNA-binding protein | | afdb-uniprot50 | AF-A0A2S1QWE8-F1-MODEL\_V4 | 1.0 | 1.251e-12 | 506 | 0.349 | 166 | 85 | 5 | 1 | 162 | 1 | 147 | Single-stranded DNA-binding protein | Single-stranded DNA-binding protein | | afdb-uniprot50 | AF-A0A653HVB8-F1-MODEL\_V4 | 1.0 | 6.222e-13 | 506 | 0.339 | 159 | 97 | 4 | 4 | 162 | 1 | 151 | Single-stranded DNA-binding protein | Single-stranded DNA-binding protein | | afdb-uniprot50 | AF-A0A2T3ITI4-F1-MODEL\_V4 | 1.0 | 2.78e-13 | 506 | 0.331 | 163 | 101 | 4 | 4 | 158 | 1 | 163 | Single-stranded DNA-binding protein | Single-stranded DNA-binding protein | | afdb-uniprot50 | AF-A0A0G0BK79-F1-MODEL\_V4 | 1.0 | 4.985e-14 | 506 | 0.329 | 170 | 105 | 3 | 1 | 162 | 2 | 170 | Single-stranded DNA-binding protein | Single-stranded DNA-binding protein | | afdb-uniprot50 | AF-A0A3M1FQV6-F1-MODEL\_V4 | 1.0 | 2.383e-12 | 505 | 0.47 | 136 | 67 | 2 | 10 | 145 | 1 | 131 | Single-stranded DNA-binding protein | Single-stranded DNA-binding protein | | afdb-uniprot50 | AF-A0A257LTW8-F1-MODEL\_V4 | 1.0 | 6.976e-12 | 505 | 0.338 | 136 | 86 | 2 | 1 | 136 | 5 | 136 | Single-stranded DNA-binding protein | Single-stranded DNA-binding protein | | afdb-uniprot50 | AF-A0A7X3UCI6-F1-MODEL\_V4 | 1.0 | 1.715e-13 | 505 | 0.393 | 160 | 76 | 4 | 3 | 162 | 2 | 140 | Single-stranded DNA-binding protein | Single-stranded DNA-binding protein | | afdb-uniprot50 | AF-A0A1J5HHA6-F1-MODEL\_V4 | 1.0 | 2.243e-13 | 505 | 0.312 | 160 | 101 | 5 | 4 | 162 | 3 | 154 | Single-stranded DNA-binding protein | Single-stranded DNA-binding protein | | afdb-uniprot50 | AF-A0A7C7W444-F1-MODEL\_V4 | 1.0 | 3.139e-11 | 505 | 0.304 | 115 | 79 | 1 | 3 | 117 | 2 | 115 | Single-stranded DNA-binding protein | Single-stranded DNA-binding protein | | afdb-uniprot50 | AF-A0A5C5X2Q6-F1-MODEL\_V4 | 1.0 | 1.32e-12 | 504 | 0.344 | 145 | 87 | 5 | 1 | 144 | 1 | 138 | Single-stranded DNA-binding protein | Single-stranded DNA-binding protein | | afdb-uniprot50 | AF-A0A2N3G5B3-F1-MODEL\_V4 | 1.0 | 4.508e-13 | 504 | 0.339 | 153 | 94 | 5 | 1 | 152 | 1 | 147 | Single-stranded DNA-binding protein | Single-stranded DNA-binding protein | | afdb-uniprot50 | AF-A0A661ED08-F1-MODEL\_V4 | 1.0 | 6.88e-14 | 504 | 0.382 | 170 | 89 | 6 | 4 | 162 | 3 | 167 | Single-stranded DNA-binding protein | Single-stranded DNA-binding protein | | afdb-uniprot50 | AF-A0A2E7GB44-F1-MODEL\_V4 | 1.0 | 2.399e-11 | 503 | 0.375 | 112 | 69 | 1 | 4 | 115 | 3 | 113 | Single-stranded DNA-binding protein | Single-stranded DNA-binding protein | | afdb-uniprot50 | AF-A0A060R893-F1-MODEL\_V4 | 1.0 | 2.8e-12 | 503 | 0.34 | 141 | 88 | 3 | 4 | 143 | 1 | 137 | Single-stranded DNA-binding protein | Single-stranded DNA-binding protein | | afdb-uniprot50 | AF-A0A7W1TQ49-F1-MODEL\_V4 | 1.0 | 7.025e-11 | 502 | 0.356 | 115 | 71 | 1 | 1 | 115 | 4 | 115 | Single-stranded DNA-binding protein | Single-stranded DNA-binding protein | | afdb-uniprot50 | AF-A0A6M8W133-F1-MODEL\_V4 | 1.0 | 9.062e-13 | 502 | 0.5 | 154 | 57 | 7 | 10 | 162 | 1 | 135 | Single-stranded DNA-binding protein | Single-stranded DNA-binding protein | | afdb-uniprot50 | AF-A0A538CVG0-F1-MODEL\_V4 | 1.0 | 2.78e-13 | 502 | 0.329 | 173 | 91 | 3 | 2 | 162 | 80 | 239 | Single-stranded DNA-binding protein | Single-stranded DNA-binding protein | | afdb-uniprot50 | AF-W0PFC5-F1-MODEL\_V4 | 1.0 | 3.495e-11 | 501 | 0.279 | 118 | 83 | 2 | 1 | 117 | 1 | 117 | Single-strand binding protein | Single-strand binding protein | | afdb-uniprot50 | AF-A0A838E1P8-F1-MODEL\_V4 | 1.0 | 1.551e-12 | 501 | 0.301 | 169 | 86 | 6 | 1 | 162 | 1 | 144 | Single-stranded DNA-binding protein | Single-stranded DNA-binding protein | | afdb-uniprot50 | AF-A0A7K2CRM9-F1-MODEL\_V4 | 1.0 | 3.837e-13 | 501 | 0.308 | 162 | 108 | 2 | 1 | 162 | 1 | 158 | Single-stranded DNA-binding protein | Single-stranded DNA-binding protein | | afdb-uniprot50 | AF-A0A3M0Z921-F1-MODEL\_V4 | 1.0 | 3.266e-13 | 500 | 0.37 | 178 | 91 | 6 | 1 | 162 | 1 | 173 | Single-stranded DNA-binding protein | Single-stranded DNA-binding protein | | afdb-uniprot50 | AF-A0A2H0DH32-F1-MODEL\_V4 | 1.0 | 5.628e-12 | 499 | 0.307 | 140 | 88 | 3 | 1 | 136 | 1 | 135 | Single-stranded DNA-binding protein | Single-stranded DNA-binding protein | | afdb-uniprot50 | AF-I4B8K2-F1-MODEL\_V4 | 1.0 | 2.653e-12 | 499 | 0.296 | 162 | 99 | 2 | 1 | 162 | 1 | 147 | Single-stranded DNA-binding protein | Single-stranded DNA-binding protein | | afdb-uniprot50 | AF-A0A1S9ZTE5-F1-MODEL\_V4 | 1.0 | 1.551e-12 | 499 | 0.293 | 167 | 91 | 4 | 4 | 162 | 2 | 149 | Single-stranded DNA-binding protein | Single-stranded DNA-binding protein | | afdb-uniprot50 | AF-A0A2M7TEI6-F1-MODEL\_V4 | 1.0 | 9.062e-13 | 499 | 0.337 | 163 | 95 | 5 | 1 | 162 | 2 | 152 | Single-stranded DNA-binding protein | Single-stranded DNA-binding protein | | afdb-uniprot50 | AF-A0A1H2FT79-F1-MODEL\_V4 | 1.0 | 1.809e-13 | 499 | 0.337 | 172 | 100 | 6 | 1 | 162 | 1 | 168 | Single-stranded DNA-binding protein | Single-stranded DNA-binding protein | | afdb-uniprot50 | AF-A0A2N1TAT9-F1-MODEL\_V4 | 1.0 | 4.477e-14 | 498 | 0.353 | 167 | 98 | 4 | 4 | 162 | 3 | 167 | Single-stranded DNA-binding protein | Single-stranded DNA-binding protein | | afdb-uniprot50 | AF-A0A836SQ28-F1-MODEL\_V4 | 1.0 | 5.054e-12 | 497 | 0.313 | 137 | 87 | 3 | 3 | 135 | 7 | 140 | Single-stranded DNA-binding protein | Single-stranded DNA-binding protein | | afdb-uniprot50 | AF-A0A7V3VUI2-F1-MODEL\_V4 | 1.0 | 1.636e-12 | 497 | 0.299 | 137 | 91 | 2 | 3 | 135 | 7 | 142 | Single-stranded DNA-binding protein | Single-stranded DNA-binding protein | | afdb-uniprot50 | AF-A0A7V3V0E3-F1-MODEL\_V4 | 1.0 | 6.611e-12 | 497 | 0.325 | 126 | 80 | 2 | 4 | 129 | 26 | 146 | Single-stranded DNA-binding protein | Single-stranded DNA-binding protein | | afdb-uniprot50 | AF-A0A090LE13-F1-MODEL\_V4 | 1.0 | 1.023e-10 | 497 | 0.245 | 118 | 86 | 1 | 1 | 118 | 62 | 176 | Primosome PriB/single-strand DNA-binding family and Single-strand DNA-binding family and Nucleic acid-binding, OB-fold domain-containing protein | Primosome PriB/single-strand DNA-binding family and Single-strand DNA-binding family and Nucleic acid-binding, OB-fold domain-containing protein | | afdb-uniprot50 | AF-A0A1Y4GFY9-F1-MODEL\_V4 | 1.0 | 6.222e-13 | 496 | 0.292 | 157 | 96 | 4 | 1 | 157 | 1 | 142 | Single-stranded DNA-binding protein | Single-stranded DNA-binding protein | | afdb-uniprot50 | AF-A0A4Q7V6Z3-F1-MODEL\_V4 | 1.0 | 7.31e-13 | 495 | 0.356 | 146 | 83 | 4 | 1 | 136 | 1 | 145 | Single-stranded DNA-binding protein | Single-stranded DNA-binding protein | | afdb-uniprot50 | AF-A0A523V6H0-F1-MODEL\_V4 | 1.0 | 1.822e-12 | 495 | 0.282 | 163 | 100 | 5 | 1 | 162 | 1 | 147 | Single-stranded DNA-binding protein | Single-stranded DNA-binding protein | | afdb-uniprot50 | AF-A0A2D5WAR7-F1-MODEL\_V4 | 1.0 | 8.589e-13 | 495 | 0.312 | 163 | 101 | 5 | 1 | 162 | 1 | 153 | Single-stranded DNA-binding protein | Single-stranded DNA-binding protein | | afdb-uniprot50 | AF-A0A381E7Q0-F1-MODEL\_V4 | 1.0 | 2.934e-13 | 495 | 0.309 | 168 | 105 | 6 | 1 | 161 | 2 | 165 | Single-stranded DNA-binding protein | Single-stranded DNA-binding protein | | afdb-uniprot50 | AF-A0A7C3YLT4-F1-MODEL\_V4 | 1.0 | 5.589e-13 | 494 | 0.339 | 162 | 88 | 5 | 1 | 162 | 1 | 143 | Single-stranded DNA-binding protein | Single-stranded DNA-binding protein | | afdb-uniprot50 | AF-A0A3M2CSC7-F1-MODEL\_V4 | 1.0 | 8.589e-13 | 494 | 0.304 | 161 | 106 | 3 | 4 | 162 | 3 | 159 | Single-stranded DNA-binding protein | Single-stranded DNA-binding protein | | afdb-uniprot50 | AF-A0A1V6GP20-F1-MODEL\_V4 | 1.0 | 9.562e-13 | 494 | 0.345 | 165 | 99 | 6 | 1 | 162 | 1 | 159 | Single-stranded DNA-binding protein | Single-stranded DNA-binding protein | | afdb-uniprot50 | AF-A0A2M8NDZ2-F1-MODEL\_V4 | 1.0 | 1.185e-12 | 494 | 0.297 | 168 | 107 | 6 | 1 | 162 | 2 | 164 | Single-stranded DNA-binding protein | Single-stranded DNA-binding protein | | afdb-uniprot50 | AF-A0A7C2DEI9-F1-MODEL\_V4 | 1.0 | 9.062e-13 | 493 | 0.359 | 164 | 94 | 5 | 1 | 162 | 1 | 155 | Single-stranded DNA-binding protein | Single-stranded DNA-binding protein | | afdb-uniprot50 | AF-A0A010YT66-F1-MODEL\_V4 | 1.0 | 1.123e-12 | 493 | 0.288 | 163 | 102 | 4 | 1 | 162 | 1 | 150 | Single-stranded DNA-binding protein | Single-stranded DNA-binding protein | | afdb-uniprot50 | AF-A0A7M3PPS8-F1-MODEL\_V4 | 1.0 | 7.26e-14 | 493 | 0.457 | 175 | 77 | 4 | 1 | 162 | 26 | 195 | Single-stranded DNA-binding protein | Single-stranded DNA-binding protein | | afdb-uniprot50 | AF-A0A523RKR1-F1-MODEL\_V4 | 1.0 | 4.508e-13 | 493 | 0.274 | 208 | 93 | 7 | 1 | 162 | 1 | 196 | Single-stranded DNA-binding protein | Single-stranded DNA-binding protein | | afdb-uniprot50 | AF-A0A557PGW7-F1-MODEL\_V4 | 1.0 | 2.819e-11 | 492 | 0.267 | 157 | 87 | 3 | 4 | 159 | 3 | 132 | Single-stranded DNA-binding protein | Single-stranded DNA-binding protein | | afdb-uniprot50 | AF-A0A7W1XXL3-F1-MODEL\_V4 | 1.0 | 1.393e-12 | 492 | 0.333 | 159 | 93 | 5 | 4 | 162 | 2 | 147 | Single-stranded DNA-binding protein | Single-stranded DNA-binding protein | | afdb-uniprot50 | AF-A0A1Y4DPV1-F1-MODEL\_V4 | 1.0 | 2.653e-12 | 491 | 0.314 | 159 | 105 | 2 | 4 | 162 | 2 | 156 | Single-stranded DNA-binding protein | Single-stranded DNA-binding protein | | afdb-uniprot50 | AF-A0A3M0WEQ2-F1-MODEL\_V4 | 1.0 | 7.767e-12 | 490 | 0.301 | 159 | 90 | 3 | 4 | 162 | 1 | 138 | Single-stranded DNA-binding protein | Single-stranded DNA-binding protein | | afdb-uniprot50 | AF-A0A6L3YVY1-F1-MODEL\_V4 | 1.0 | 1.251e-12 | 490 | 0.364 | 170 | 96 | 7 | 1 | 162 | 1 | 166 | Single-stranded DNA-binding protein | Single-stranded DNA-binding protein | | afdb-uniprot50 | AF-G2LEN2-F1-MODEL\_V4 | 1.0 | 1.185e-12 | 489 | 0.345 | 159 | 87 | 3 | 4 | 162 | 2 | 143 | Single-stranded DNA-binding protein | Single-stranded DNA-binding protein | | afdb-uniprot50 | AF-A0A2N6QFG8-F1-MODEL\_V4 | 1.0 | 1.393e-12 | 489 | 0.317 | 151 | 95 | 3 | 1 | 149 | 1 | 145 | Single-stranded DNA-binding protein | Single-stranded DNA-binding protein | | afdb-uniprot50 | AF-A0A1F9XKK2-F1-MODEL\_V4 | 1.0 | 2.042e-11 | 488 | 0.333 | 123 | 79 | 2 | 1 | 123 | 4 | 123 | Single-stranded DNA-binding protein | Single-stranded DNA-binding protein | | afdb-uniprot50 | AF-A0A7Y2SQU7-F1-MODEL\_V4 | 1.0 | 1.48e-11 | 488 | 0.287 | 132 | 88 | 3 | 1 | 129 | 4 | 132 | Single-stranded DNA-binding protein | Single-stranded DNA-binding protein | | afdb-uniprot50 | AF-A0A1H4XXA5-F1-MODEL\_V4 | 1.0 | 3.289e-12 | 488 | 0.32 | 159 | 85 | 5 | 4 | 162 | 1 | 136 | Single-stranded DNA-binding protein | Single-stranded DNA-binding protein | | afdb-uniprot50 | AF-A0A1Y3UKN9-F1-MODEL\_V4 | 1.0 | 8.648e-12 | 487 | 0.3 | 160 | 81 | 4 | 4 | 162 | 2 | 131 | Single-stranded DNA-binding protein | Single-stranded DNA-binding protein | | afdb-uniprot50 | AF-A0A7X6ICS6-F1-MODEL\_V4 | 1.0 | 2.042e-11 | 487 | 0.27 | 159 | 87 | 4 | 4 | 162 | 2 | 131 | Single-stranded DNA-binding protein | Single-stranded DNA-binding protein | | afdb-uniprot50 | AF-A0A2E0L7G6-F1-MODEL\_V4 | 1.0 | 4.54e-12 | 487 | 0.308 | 159 | 85 | 5 | 4 | 162 | 2 | 135 | Single-stranded DNA-binding protein | Single-stranded DNA-binding protein | | afdb-uniprot50 | AF-S3XEZ1-F1-MODEL\_V4 | 1.0 | 4.757e-13 | 487 | 0.358 | 159 | 91 | 4 | 4 | 162 | 1 | 148 | Single-stranded DNA-binding protein | Single-stranded DNA-binding protein | | afdb-uniprot50 | AF-A0A354CZB3-F1-MODEL\_V4 | 1.0 | 2.954e-12 | 486 | 0.337 | 154 | 92 | 4 | 1 | 154 | 1 | 144 | Single-stranded DNA-binding protein | Single-stranded DNA-binding protein | | afdb-uniprot50 | AF-A0A2E8DUA8-F1-MODEL\_V4 | 1.0 | 1.551e-12 | 486 | 0.246 | 162 | 100 | 3 | 1 | 162 | 16 | 155 | Single-stranded DNA-binding protein | Single-stranded DNA-binding protein | | afdb-uniprot50 | AF-A0A073IMY4-F1-MODEL\_V4 | 1.0 | 8.589e-13 | 486 | 0.303 | 181 | 97 | 6 | 1 | 162 | 1 | 171 | Single-stranded DNA-binding protein | Single-stranded DNA-binding protein | | afdb-uniprot50 | AF-A0A2M8NSD8-F1-MODEL\_V4 | 1.0 | 8.648e-12 | 485 | 0.385 | 114 | 68 | 2 | 1 | 114 | 1 | 112 | Single-stranded DNA-binding protein | Single-stranded DNA-binding protein | | afdb-uniprot50 | AF-A0A257B1D5-F1-MODEL\_V4 | 1.0 | 4.302e-12 | 485 | 0.337 | 151 | 86 | 2 | 4 | 154 | 2 | 138 | Single-stranded DNA-binding protein | Single-stranded DNA-binding protein | | afdb-uniprot50 | AF-A0A1F9VFL1-F1-MODEL\_V4 | 1.0 | 5.333e-12 | 485 | 0.341 | 161 | 89 | 4 | 3 | 162 | 2 | 146 | Single-stranded DNA-binding protein | Single-stranded DNA-binding protein | | afdb-uniprot50 | AF-A0A2D6HC40-F1-MODEL\_V4 | 1.0 | 2.78e-13 | 485 | 0.319 | 166 | 102 | 3 | 3 | 162 | 2 | 162 | Single-stranded DNA-binding protein | Single-stranded DNA-binding protein | | afdb-uniprot50 | AF-A0A495LT57-F1-MODEL\_V4 | 1.0 | 1.123e-12 | 485 | 0.333 | 162 | 92 | 4 | 1 | 162 | 21 | 166 | Single-stranded DNA-binding protein | Single-stranded DNA-binding protein | | afdb-uniprot50 | AF-A0A351JNY7-F1-MODEL\_V4 | 1.0 | 3.662e-12 | 484 | 0.28 | 146 | 98 | 2 | 3 | 148 | 2 | 140 | Single-stranded DNA-binding protein | Single-stranded DNA-binding protein | | afdb-uniprot50 | AF-A0A1F2X262-F1-MODEL\_V4 | 1.0 | 1.822e-12 | 484 | 0.287 | 167 | 97 | 6 | 1 | 162 | 1 | 150 | Single-stranded DNA-binding protein | Single-stranded DNA-binding protein | | afdb-uniprot50 | AF-A0A7X8AGS9-F1-MODEL\_V4 | 1.0 | 4.79e-12 | 483 | 0.339 | 159 | 89 | 4 | 4 | 162 | 1 | 143 | Single-stranded DNA-binding protein | Single-stranded DNA-binding protein | | afdb-uniprot50 | AF-A0A7C3RHW0-F1-MODEL\_V4 | 1.0 | 9.062e-13 | 482 | 0.341 | 155 | 95 | 4 | 1 | 154 | 3 | 151 | Single-stranded DNA-binding protein | Single-stranded DNA-binding protein | | afdb-uniprot50 | AF-W5YUV6-F1-MODEL\_V4 | 1.0 | 6.222e-13 | 482 | 0.447 | 161 | 80 | 3 | 4 | 162 | 1 | 154 | Single-stranded DNA-binding protein | Single-stranded DNA-binding protein | | afdb-uniprot50 | AF-A0A0K1F1N5-F1-MODEL\_V4 | 1.0 | 8.589e-13 | 482 | 0.331 | 160 | 97 | 3 | 4 | 162 | 2 | 152 | Single-stranded DNA-binding protein | Single-stranded DNA-binding protein | | afdb-uniprot50 | AF-A0A845H8A1-F1-MODEL\_V4 | 1.0 | 1.123e-12 | 481 | 0.453 | 152 | 72 | 4 | 12 | 162 | 95 | 236 | Single-stranded DNA-binding protein | Single-stranded DNA-binding protein | | afdb-uniprot50 | AF-A0A2A5A532-F1-MODEL\_V4 | 1.0 | 7.714e-13 | 480 | 0.293 | 160 | 101 | 3 | 4 | 162 | 3 | 151 | Single-stranded DNA-binding protein | Single-stranded DNA-binding protein | | afdb-uniprot50 | AF-H0E9H1-F1-MODEL\_V4 | 1.0 | 4.508e-13 | 480 | 0.327 | 162 | 99 | 4 | 4 | 162 | 2 | 156 | Single-stranded DNA-binding protein | Single-stranded DNA-binding protein | | afdb-uniprot50 | AF-A0A0S1SMJ0-F1-MODEL\_V4 | 1.0 | 1.123e-12 | 480 | 0.375 | 141 | 79 | 2 | 5 | 137 | 128 | 267 | Single-stranded DNA-binding protein | Single-stranded DNA-binding protein | | afdb-uniprot50 | AF-A0A437UU05-F1-MODEL\_V4 | 1.0 | 6.222e-13 | 479 | 0.349 | 169 | 97 | 5 | 4 | 162 | 2 | 167 | Single-stranded DNA-binding protein | Single-stranded DNA-binding protein | | afdb-uniprot50 | AF-A0A0F5PBD5-F1-MODEL\_V4 | 1.0 | 1.47e-12 | 476 | 0.355 | 163 | 97 | 4 | 1 | 162 | 1 | 156 | Single-stranded DNA-binding protein | Single-stranded DNA-binding protein | | afdb-uniprot50 | AF-A0A1S9ZZ07-F1-MODEL\_V4 | 1.0 | 1.625e-13 | 476 | 0.383 | 167 | 89 | 6 | 4 | 162 | 1 | 161 | Single-stranded DNA-binding protein | Single-stranded DNA-binding protein | | afdb-uniprot50 | AF-A0A3D0VSK9-F1-MODEL\_V4 | 1.0 | 2.839e-10 | 475 | 0.336 | 113 | 72 | 2 | 2 | 114 | 34 | 143 | Single-stranded DNA-binding protein | Single-stranded DNA-binding protein | | afdb-uniprot50 | AF-A0A0G0L6C8-F1-MODEL\_V4 | 1.0 | 4.049e-13 | 475 | 0.344 | 183 | 86 | 8 | 4 | 162 | 3 | 175 | Single-stranded DNA-binding protein | Single-stranded DNA-binding protein | | afdb-uniprot50 | AF-A0A1F3IVI4-F1-MODEL\_V4 | 1.0 | 3.918e-10 | 474 | 0.345 | 110 | 69 | 2 | 5 | 114 | 1 | 107 | Single-stranded DNA-binding protein | Single-stranded DNA-binding protein | | afdb-uniprot50 | AF-A0A348YKY9-F1-MODEL\_V4 | 1.0 | 6.566e-13 | 474 | 0.359 | 164 | 93 | 6 | 4 | 162 | 3 | 159 | Single-stranded DNA-binding protein | Single-stranded DNA-binding protein | | afdb-uniprot50 | AF-A0A1B0C486-F1-MODEL\_V4 | 1.0 | 4.273e-13 | 474 | 0.5 | 150 | 68 | 2 | 17 | 161 | 336 | 483 | Uncharacterized protein | Uncharacterized protein | | afdb-uniprot50 | AF-A0A0H3CMN1-F1-MODEL\_V4 | 1.0 | 3.662e-12 | 473 | 0.535 | 142 | 52 | 3 | 21 | 162 | 2 | 129 | Single-stranded DNA-binding protein | Single-stranded DNA-binding protein | | afdb-uniprot50 | AF-J0WGW8-F1-MODEL\_V4 | 1.0 | 4.54e-12 | 471 | 0.331 | 154 | 89 | 4 | 1 | 154 | 24 | 163 | Single-stranded DNA-binding protein | Single-stranded DNA-binding protein | | afdb-uniprot50 | AF-A0A7S4G7Z5-F1-MODEL\_V4 | 1.0 | 2.258e-12 | 471 | 0.269 | 167 | 112 | 4 | 2 | 162 | 187 | 349 | Hypothetical protein | Hypothetical protein | | afdb-uniprot50 | AF-A0A1F9TW11-F1-MODEL\_V4 | 1.0 | 5.938e-12 | 470 | 0.321 | 168 | 82 | 7 | 1 | 162 | 1 | 142 | Single-stranded DNA-binding protein | Single-stranded DNA-binding protein | | afdb-uniprot50 | AF-A0A6I2VGK0-F1-MODEL\_V4 | 1.0 | 1.009e-12 | 470 | 0.333 | 165 | 94 | 5 | 2 | 162 | 19 | 171 | Single-stranded DNA-binding protein | Single-stranded DNA-binding protein | | afdb-uniprot50 | AF-A0A1M3GUQ3-F1-MODEL\_V4 | 1.0 | 3.289e-12 | 470 | 0.308 | 162 | 106 | 5 | 4 | 162 | 1 | 159 | Single-stranded DNA-binding protein | Single-stranded DNA-binding protein | | afdb-uniprot50 | AF-A0A5B8U1B7-F1-MODEL\_V4 | 1.0 | 4.757e-13 | 467 | 0.339 | 171 | 91 | 4 | 2 | 162 | 45 | 203 | Single-stranded DNA-binding protein | Single-stranded DNA-binding protein | | afdb-uniprot50 | AF-A0A7J7BRE6-F1-MODEL\_V4 | 1.0 | 5.856e-14 | 467 | 0.443 | 187 | 69 | 3 | 10 | 162 | 543 | 728 | MFS domain-containing protein | MFS domain-containing protein | | afdb-uniprot50 | AF-W0FMQ2-F1-MODEL\_V4 | 1.0 | 1.726e-12 | 466 | 0.352 | 159 | 94 | 4 | 4 | 162 | 2 | 151 | Single-stranded DNA-binding protein | Single-stranded DNA-binding protein | | afdb-uniprot50 | AF-A0A354WRT5-F1-MODEL\_V4 | 1.0 | 3.837e-13 | 466 | 0.312 | 176 | 93 | 5 | 5 | 162 | 1 | 166 | Single-stranded DNA-binding protein | Single-stranded DNA-binding protein | | afdb-uniprot50 | AF-A0A7V4E1N2-F1-MODEL\_V4 | 1.0 | 1.936e-11 | 463 | 0.302 | 149 | 90 | 4 | 1 | 144 | 1 | 140 | Single-stranded DNA-binding protein | Single-stranded DNA-binding protein | | afdb-uniprot50 | AF-A0A1H1L2W3-F1-MODEL\_V4 | 1.0 | 1.26e-11 | 463 | 0.327 | 159 | 88 | 5 | 4 | 162 | 2 | 141 | Single-stranded DNA-binding protein | Single-stranded DNA-binding protein | | afdb-uniprot50 | AF-A0A7T5RMB9-F1-MODEL\_V4 | 1.0 | 1.922e-12 | 463 | 0.339 | 162 | 90 | 5 | 1 | 162 | 5 | 149 | Single-stranded DNA-binding protein | Single-stranded DNA-binding protein | | afdb-uniprot50 | AF-A0A7V2YYU3-F1-MODEL\_V4 | 1.0 | 3.289e-12 | 463 | 0.29 | 165 | 107 | 4 | 4 | 162 | 3 | 163 | Single-stranded DNA-binding protein | Single-stranded DNA-binding protein | | afdb-uniprot50 | AF-A0A4T9TD94-F1-MODEL\_V4 | 1.0 | 4.302e-12 | 463 | 0.301 | 159 | 104 | 3 | 4 | 162 | 2 | 153 | Single-stranded DNA-binding protein | Single-stranded DNA-binding protein | | afdb-uniprot50 | AF-A0A0F9VUL0-F1-MODEL\_V4 | 1.0 | 1.251e-12 | 461 | 0.279 | 168 | 107 | 6 | 1 | 162 | 18 | 177 | Uncharacterized protein | Uncharacterized protein | | afdb-uniprot50 | AF-A0A210VRK1-F1-MODEL\_V4 | 1.0 | 1.726e-12 | 460 | 0.349 | 166 | 98 | 6 | 1 | 160 | 1 | 162 | Single-stranded DNA-binding protein | Single-stranded DNA-binding protein | | afdb-uniprot50 | AF-A0A2E3VXK3-F1-MODEL\_V4 | 1.0 | 5.706e-10 | 459 | 0.327 | 113 | 73 | 1 | 4 | 116 | 2 | 111 | Single-stranded DNA-binding protein | Single-stranded DNA-binding protein | | afdb-uniprot50 | AF-A0A1M2URQ5-F1-MODEL\_V4 | 1.0 | 1.48e-11 | 458 | 0.372 | 161 | 83 | 4 | 4 | 162 | 1 | 145 | Single-stranded DNA-binding protein | Single-stranded DNA-binding protein | | afdb-uniprot50 | AF-A0A537YUJ5-F1-MODEL\_V4 | 1.0 | 1.123e-12 | 458 | 0.295 | 159 | 104 | 4 | 6 | 162 | 4 | 156 | Single-stranded DNA-binding protein | Single-stranded DNA-binding protein | | afdb-uniprot50 | AF-A0A2H0S6Z3-F1-MODEL\_V4 | 1.0 | 8.139e-13 | 458 | 0.352 | 156 | 99 | 2 | 5 | 160 | 223 | 376 | Single-stranded DNA-binding protein | Single-stranded DNA-binding protein | | afdb-uniprot50 | AF-A0A0P6YDB4-F1-MODEL\_V4 | 1.0 | 5.125e-10 | 456 | 0.3 | 110 | 75 | 2 | 1 | 109 | 1 | 109 | Single-stranded DNA-binding protein | Single-stranded DNA-binding protein | | afdb-uniprot50 | AF-A0A7W2H3Y3-F1-MODEL\_V4 | 1.0 | 4.54e-12 | 456 | 0.283 | 162 | 105 | 5 | 1 | 162 | 1 | 151 | Single-stranded DNA-binding protein | Single-stranded DNA-binding protein | | afdb-uniprot50 | AF-A0A1C5MNJ8-F1-MODEL\_V4 | 1.0 | 1.194e-11 | 456 | 0.283 | 159 | 107 | 3 | 4 | 162 | 2 | 153 | Single-stranded DNA-binding protein | Single-stranded DNA-binding protein | | afdb-uniprot50 | AF-A0A7V2CKI6-F1-MODEL\_V4 | 1.0 | 1.32e-12 | 456 | 0.284 | 172 | 109 | 6 | 1 | 162 | 1 | 168 | Single-stranded DNA-binding protein | Single-stranded DNA-binding protein | | afdb-uniprot50 | AF-A0A2N2XER4-F1-MODEL\_V4 | 1.0 | 5.37e-11 | 454 | 0.33 | 139 | 84 | 2 | 10 | 142 | 2 | 137 | Single-stranded DNA-binding protein | Single-stranded DNA-binding protein | | afdb-uniprot50 | AF-A0A639KX21-F1-MODEL\_V4 | 1.0 | 2.69e-10 | 453 | 0.353 | 113 | 70 | 3 | 4 | 115 | 3 | 113 | Single-stranded DNA-binding protein | Single-stranded DNA-binding protein | | afdb-uniprot50 | AF-A0A533RM11-F1-MODEL\_V4 | 1.0 | 3.687e-11 | 453 | 0.308 | 159 | 96 | 4 | 4 | 162 | 2 | 146 | Single-stranded DNA-binding protein | Single-stranded DNA-binding protein | | afdb-uniprot50 | AF-A0A1G1CN45-F1-MODEL\_V4 | 1.0 | 2.954e-12 | 453 | 0.367 | 158 | 92 | 3 | 10 | 162 | 2 | 156 | Single-stranded DNA-binding protein | Single-stranded DNA-binding protein | | afdb-uniprot50 | AF-A0A7X7NPE2-F1-MODEL\_V4 | 1.0 | 1.338e-10 | 450 | 0.313 | 115 | 76 | 3 | 4 | 117 | 2 | 114 | Single-stranded DNA-binding protein | Single-stranded DNA-binding protein | | afdb-uniprot50 | AF-A0A2N4Z5P7-F1-MODEL\_V4 | 1.0 | 5.667e-11 | 450 | 0.25 | 156 | 115 | 2 | 1 | 155 | 2 | 156 | Single-stranded DNA-binding protein | Single-stranded DNA-binding protein | | afdb-uniprot50 | AF-C4F8E6-F1-MODEL\_V4 | 1.0 | 1.268e-10 | 449 | 0.31 | 161 | 73 | 5 | 4 | 162 | 2 | 126 | Single-stranded DNA-binding protein | Single-stranded DNA-binding protein | | afdb-uniprot50 | AF-A0A1G6KJG1-F1-MODEL\_V4 | 1.0 | 3.139e-11 | 449 | 0.341 | 161 | 87 | 6 | 4 | 162 | 2 | 145 | Single-stranded DNA-binding protein | Single-stranded DNA-binding protein | | afdb-uniprot50 | AF-A0A7Y3KW57-F1-MODEL\_V4 | 1.0 | 1.072e-11 | 449 | 0.245 | 163 | 101 | 4 | 4 | 162 | 2 | 146 | Single-stranded DNA-binding protein | Single-stranded DNA-binding protein | | afdb-uniprot50 | AF-A0A6M0LP34-F1-MODEL\_V4 | 1.0 | 1.131e-11 | 449 | 0.259 | 162 | 106 | 6 | 4 | 160 | 3 | 155 | Single-stranded DNA-binding protein | Single-stranded DNA-binding protein | | afdb-uniprot50 | AF-A0A2D2LX13-F1-MODEL\_V4 | 1.0 | 4.54e-12 | 449 | 0.349 | 163 | 95 | 4 | 4 | 162 | 1 | 156 | Single-stranded DNA-binding protein | Single-stranded DNA-binding protein | | afdb-uniprot50 | AF-A0A7V5N906-F1-MODEL\_V4 | 1.0 | 8.648e-12 | 449 | 0.275 | 185 | 102 | 5 | 4 | 162 | 3 | 181 | Single-stranded DNA-binding protein | Single-stranded DNA-binding protein | | afdb-uniprot50 | AF-A0A349D2W1-F1-MODEL\_V4 | 1.0 | 1.922e-12 | 448 | 0.343 | 172 | 95 | 6 | 2 | 162 | 459 | 623 | Single-stranded DNA-binding protein | Single-stranded DNA-binding protein | | afdb-uniprot50 | AF-A0A7X7YU40-F1-MODEL\_V4 | 1.0 | 3.139e-11 | 446 | 0.254 | 165 | 97 | 5 | 1 | 162 | 1 | 142 | Single-stranded DNA-binding protein | Single-stranded DNA-binding protein | | afdb-uniprot50 | AF-L1NBJ9-F1-MODEL\_V4 | 1.0 | 2.057e-10 | 446 | 0.176 | 136 | 109 | 1 | 1 | 136 | 4 | 136 | Single-stranded DNA-binding protein | Single-stranded DNA-binding protein | | afdb-uniprot50 | AF-A0A7C3PY12-F1-MODEL\_V4 | 1.0 | 4.54e-12 | 446 | 0.251 | 175 | 113 | 5 | 3 | 162 | 2 | 173 | Single-stranded DNA-binding protein | Single-stranded DNA-binding protein | | afdb-uniprot50 | AF-A0A136NW82-F1-MODEL\_V4 | 1.0 | 6.657e-11 | 445 | 0.401 | 122 | 69 | 4 | 10 | 130 | 2 | 120 | Single-stranded DNA-binding protein | Single-stranded DNA-binding protein | | afdb-uniprot50 | AF-T1A6S3-F1-MODEL\_V4 | 1.0 | 1.834e-11 | 445 | 0.308 | 136 | 87 | 3 | 3 | 132 | 2 | 136 | Single-strand DNA-binding protein | Single-strand DNA-binding protein | | afdb-uniprot50 | AF-A0A7C1ENX2-F1-MODEL\_V4 | 1.0 | 7.361e-12 | 445 | 0.325 | 132 | 84 | 3 | 1 | 129 | 14 | 143 | Single-stranded DNA-binding protein | Single-stranded DNA-binding protein | | afdb-uniprot50 | AF-A0A6B1E277-F1-MODEL\_V4 | 1.0 | 2.497e-13 | 445 | 0.318 | 185 | 99 | 4 | 4 | 162 | 2 | 185 | Single-stranded DNA-binding protein | Single-stranded DNA-binding protein | | afdb-uniprot50 | AF-A0A6I2YPP7-F1-MODEL\_V4 | 1.0 | 1.338e-10 | 444 | 0.283 | 120 | 79 | 2 | 4 | 117 | 1 | 119 | Single-stranded DNA-binding protein | Single-stranded DNA-binding protein | | afdb-uniprot50 | AF-A0A6L9IS86-F1-MODEL\_V4 | 1.0 | 1.936e-11 | 444 | 0.279 | 129 | 86 | 3 | 1 | 129 | 1 | 122 | Single-stranded DNA-binding protein | Single-stranded DNA-binding protein | | afdb-uniprot50 | AF-A0A5C7U259-F1-MODEL\_V4 | 1.0 | 1.131e-11 | 444 | 0.288 | 163 | 103 | 6 | 1 | 161 | 23 | 174 | Single-stranded DNA-binding protein | Single-stranded DNA-binding protein | | afdb-uniprot50 | AF-A0A415JIS1-F1-MODEL\_V4 | 1.0 | 1.147e-09 | 443 | 0.263 | 114 | 80 | 2 | 4 | 117 | 2 | 111 | Single-stranded DNA-binding protein | Single-stranded DNA-binding protein | | afdb-uniprot50 | AF-A0A3M1DGA7-F1-MODEL\_V4 | 1.0 | 1.268e-10 | 443 | 0.22 | 159 | 101 | 5 | 5 | 162 | 1 | 137 | Single-stranded DNA-binding protein | Single-stranded DNA-binding protein | | afdb-uniprot50 | AF-A0A0K0DUQ6-F1-MODEL\_V4 | 1.0 | 2.709e-09 | 442 | 0.23 | 117 | 86 | 2 | 2 | 118 | 65 | 177 | Uncharacterized protein | Uncharacterized protein | | afdb-uniprot50 | AF-A0A1E3LMY7-F1-MODEL\_V4 | 1.0 | 5.706e-10 | 441 | 0.235 | 136 | 92 | 2 | 1 | 136 | 1 | 124 | Single-stranded DNA-binding protein | Single-stranded DNA-binding protein | | afdb-uniprot50 | AF-I3UGJ2-F1-MODEL\_V4 | 1.0 | 3.139e-11 | 441 | 0.236 | 152 | 111 | 3 | 1 | 151 | 1 | 148 | Single-stranded DNA-binding protein | Single-stranded DNA-binding protein | | afdb-uniprot50 | AF-A0A640Y9W9-F1-MODEL\_V4 | 1.0 | 7.767e-12 | 441 | 0.333 | 162 | 102 | 4 | 2 | 162 | 59 | 215 | Single-stranded DNA-binding protein | Single-stranded DNA-binding protein | | afdb-uniprot50 | AF-A0A6J5JVU9-F1-MODEL\_V4 | 1.0 | 1.572e-10 | 440 | 0.37 | 127 | 72 | 3 | 1 | 127 | 1 | 119 | Single-stranded DNA-binding protein | Single-stranded DNA-binding protein | | afdb-uniprot50 | AF-A0A7C3DLR0-F1-MODEL\_V4 | 1.0 | 4.302e-12 | 440 | 0.349 | 166 | 79 | 7 | 4 | 162 | 1 | 144 | Single-stranded DNA-binding protein | Single-stranded DNA-binding protein | | afdb-uniprot50 | AF-J5MVP4-F1-MODEL\_V4 | 1.0 | 1.834e-11 | 440 | 0.447 | 134 | 66 | 4 | 1 | 129 | 1 | 131 | Single-stranded DNA-binding protein | Single-stranded DNA-binding protein | | afdb-uniprot50 | AF-A0A0F9Z6S5-F1-MODEL\_V4 | 1.0 | 2.274e-11 | 438 | 0.333 | 159 | 91 | 6 | 4 | 160 | 3 | 148 | Single-stranded DNA-binding protein | Single-stranded DNA-binding protein | | afdb-uniprot50 | AF-A0A532UZN4-F1-MODEL\_V4 | 1.0 | 2.274e-11 | 437 | 0.303 | 165 | 105 | 6 | 1 | 160 | 1 | 160 | Single-stranded DNA-binding protein | Single-stranded DNA-binding protein | | afdb-uniprot50 | AF-A0A220QRB1-F1-MODEL\_V4 | 1.0 | 3.662e-12 | 436 | 0.367 | 174 | 89 | 3 | 2 | 155 | 156 | 328 | Single-stranded DNA-binding protein | Single-stranded DNA-binding protein | | afdb-uniprot50 | AF-A0A2W6BUT5-F1-MODEL\_V4 | 1.0 | 5.333e-12 | 435 | 0.317 | 173 | 84 | 6 | 4 | 162 | 2 | 154 | Single-stranded DNA-binding protein | Single-stranded DNA-binding protein | | afdb-uniprot50 | AF-S5DJE5-F1-MODEL\_V4 | 1.0 | 1.202e-10 | 434 | 0.267 | 131 | 87 | 4 | 4 | 129 | 2 | 128 | Single-stranded DNA-binding protein | Single-stranded DNA-binding protein | | afdb-uniprot50 | AF-A0A2H0WY68-F1-MODEL\_V4 | 1.0 | 3.864e-12 | 433 | 0.343 | 163 | 94 | 5 | 10 | 162 | 2 | 161 | Single-stranded DNA-binding protein | Single-stranded DNA-binding protein | | afdb-uniprot50 | AF-A0A0D8HFS3-F1-MODEL\_V4 | 1.0 | 7.767e-12 | 431 | 0.282 | 163 | 113 | 2 | 3 | 162 | 2 | 163 | Single-stranded DNA-binding protein | Single-stranded DNA-binding protein | | afdb-uniprot50 | AF-A0A133ZE68-F1-MODEL\_V4 | 1.0 | 2.042e-11 | 430 | 0.253 | 162 | 113 | 4 | 4 | 160 | 2 | 160 | Single-stranded DNA-binding protein | Single-stranded DNA-binding protein | | afdb-uniprot50 | AF-A0A7V1U542-F1-MODEL\_V4 | 1.0 | 4.332e-11 | 427 | 0.285 | 161 | 95 | 7 | 1 | 157 | 1 | 145 | Single-stranded DNA-binding protein | Single-stranded DNA-binding protein | | afdb-uniprot50 | AF-A0A6A8M953-F1-MODEL\_V4 | 1.0 | 2.042e-11 | 427 | 0.327 | 162 | 99 | 5 | 5 | 162 | 1 | 156 | Single-stranded DNA-binding protein | Single-stranded DNA-binding protein | | afdb-uniprot50 | AF-A0A6I1MPE9-F1-MODEL\_V4 | 1.0 | 1.03e-09 | 427 | 0.296 | 118 | 81 | 2 | 1 | 117 | 1 | 117 | Single-stranded DNA-binding protein | Single-stranded DNA-binding protein | | afdb-uniprot50 | AF-A0A2V2G4L0-F1-MODEL\_V4 | 1.0 | 7.025e-11 | 426 | 0.265 | 158 | 105 | 3 | 5 | 162 | 1 | 147 | Single-stranded DNA-binding protein | Single-stranded DNA-binding protein | | afdb-uniprot50 | AF-A0A3E2TPK5-F1-MODEL\_V4 | 1.0 | 4.362e-10 | 425 | 0.271 | 162 | 92 | 5 | 1 | 162 | 1 | 136 | Single-stranded DNA-binding protein | Single-stranded DNA-binding protein | | afdb-uniprot50 | AF-A0A4U5MFD9-F1-MODEL\_V4 | 1.0 | 1.583e-09 | 425 | 0.28 | 125 | 84 | 4 | 2 | 123 | 56 | 177 | Uncharacterized protein | Uncharacterized protein | | afdb-uniprot50 | AF-A0A4S2EXC4-F1-MODEL\_V4 | 1.0 | 1.072e-11 | 425 | 0.284 | 186 | 105 | 5 | 4 | 162 | 2 | 186 | Single-stranded DNA-binding protein | Single-stranded DNA-binding protein | | afdb-uniprot50 | AF-A0A3A1YFT4-F1-MODEL\_V4 | 1.0 | 9.125e-12 | 425 | 0.284 | 165 | 110 | 5 | 1 | 162 | 1 | 160 | Single-stranded DNA-binding protein | Single-stranded DNA-binding protein | | afdb-uniprot50 | AF-A0A1W6BYF7-F1-MODEL\_V4 | 1.0 | 4.332e-11 | 424 | 0.22 | 154 | 111 | 4 | 5 | 154 | 1 | 149 | Single-stranded DNA-binding protein | Single-stranded DNA-binding protein | | afdb-uniprot50 | AF-A0A1Y3SED9-F1-MODEL\_V4 | 1.0 | 4.332e-11 | 423 | 0.228 | 162 | 110 | 4 | 1 | 162 | 29 | 175 | Single-stranded DNA-binding protein | Single-stranded DNA-binding protein | | afdb-uniprot50 | AF-A0A356LBF3-F1-MODEL\_V4 | 1.0 | 1.023e-10 | 422 | 0.239 | 146 | 104 | 4 | 3 | 142 | 35 | 179 | Uncharacterized protein | Uncharacterized protein | | afdb-uniprot50 | AF-A0A1L8N1R4-F1-MODEL\_V4 | 1.0 | 5.125e-10 | 418 | 0.205 | 146 | 110 | 2 | 3 | 148 | 2 | 141 | Single-stranded DNA-binding protein | Single-stranded DNA-binding protein | | afdb-uniprot50 | AF-A0A5U6CUN8-F1-MODEL\_V4 | 1.0 | 3.312e-11 | 417 | 0.561 | 139 | 51 | 2 | 24 | 162 | 1 | 129 | Single-stranded DNA-binding protein | Single-stranded DNA-binding protein | | afdb-uniprot50 | AF-A0A4S2ER89-F1-MODEL\_V4 | 1.0 | 1.139e-10 | 417 | 0.258 | 147 | 107 | 2 | 1 | 146 | 6 | 151 | Single-stranded DNA-binding protein | Single-stranded DNA-binding protein | | afdb-uniprot50 | AF-A0A1F2YCG8-F1-MODEL\_V4 | 1.0 | 4.106e-11 | 414 | 0.281 | 160 | 93 | 6 | 1 | 155 | 1 | 143 | Single-stranded DNA-binding protein | Single-stranded DNA-binding protein | | afdb-uniprot50 | AF-A0A2N3QHM9-F1-MODEL\_V4 | 1.0 | 8.196e-12 | 414 | 0.325 | 160 | 104 | 4 | 4 | 162 | 2 | 158 | Single-stranded DNA-binding protein | Single-stranded DNA-binding protein | | afdb-uniprot50 | AF-A0A364VE05-F1-MODEL\_V4 | 1.0 | 3.139e-11 | 414 | 0.306 | 150 | 94 | 5 | 1 | 144 | 19 | 164 | Single-stranded DNA-binding protein | Single-stranded DNA-binding protein | | afdb-uniprot50 | AF-A0A3C1CGQ0-F1-MODEL\_V4 | 1.0 | 1.08e-10 | 413 | 0.229 | 144 | 105 | 2 | 4 | 142 | 1 | 143 | Single-stranded DNA-binding protein | Single-stranded DNA-binding protein | | afdb-uniprot50 | AF-A0A6G9HQU0-F1-MODEL\_V4 | 1.0 | 4.508e-13 | 413 | 0.448 | 165 | 80 | 3 | 1 | 154 | 1 | 165 | Single-stranded DNA-binding protein | Single-stranded DNA-binding protein | | afdb-uniprot50 | AF-U2KLY9-F1-MODEL\_V4 | 1.0 | 4.106e-11 | 412 | 0.227 | 158 | 115 | 3 | 3 | 160 | 2 | 152 | Single-stranded DNA-binding protein | Single-stranded DNA-binding protein | | afdb-uniprot50 | AF-A0A523Z5E7-F1-MODEL\_V4 | 1.0 | 5.979e-11 | 410 | 0.227 | 158 | 111 | 4 | 5 | 162 | 9 | 155 | Single-stranded DNA-binding protein | Single-stranded DNA-binding protein | | afdb-uniprot50 | AF-A0A7C4ZDT3-F1-MODEL\_V4 | 1.0 | 1.268e-10 | 409 | 0.309 | 152 | 101 | 2 | 4 | 155 | 3 | 150 | Single-stranded DNA-binding protein | Single-stranded DNA-binding protein | | afdb-uniprot50 | AF-A0A2H0Y1Q2-F1-MODEL\_V4 | 1.0 | 1.03e-09 | 408 | 0.259 | 135 | 95 | 3 | 1 | 135 | 21 | 150 | Single-stranded DNA-binding protein | Single-stranded DNA-binding protein | | afdb-uniprot50 | AF-A0A5N9VXX7-F1-MODEL\_V4 | 1.0 | 1.572e-10 | 407 | 0.576 | 125 | 48 | 2 | 29 | 151 | 3 | 124 | Single-stranded DNA-binding protein | Single-stranded DNA-binding protein | | afdb-uniprot50 | AF-A0A848J9W6-F1-MODEL\_V4 | 1.0 | 1.936e-11 | 407 | 0.273 | 161 | 101 | 6 | 4 | 154 | 2 | 156 | Single-stranded DNA-binding protein | Single-stranded DNA-binding protein | | afdb-uniprot50 | AF-A0A1W9S8T2-F1-MODEL\_V4 | 1.0 | 2.672e-11 | 407 | 0.298 | 184 | 102 | 6 | 1 | 162 | 4 | 182 | Single-stranded DNA-binding protein | Single-stranded DNA-binding protein | | afdb-uniprot50 | AF-A0A7V7XET6-F1-MODEL\_V4 | 1.0 | 3.495e-11 | 405 | 0.281 | 160 | 100 | 5 | 4 | 162 | 2 | 147 | Single-stranded DNA-binding protein | Single-stranded DNA-binding protein | | afdb-uniprot50 | AF-A0A556R9X6-F1-MODEL\_V4 | 1.0 | 5.09e-11 | 404 | 0.289 | 152 | 100 | 5 | 6 | 154 | 3 | 149 | Single-stranded DNA-binding protein | Single-stranded DNA-binding protein | | afdb-uniprot50 | AF-A0A7K3XWR7-F1-MODEL\_V4 | 1.0 | 1.847e-10 | 403 | 0.222 | 162 | 115 | 4 | 4 | 162 | 2 | 155 | Single-stranded DNA-binding protein | Single-stranded DNA-binding protein | | afdb-uniprot50 | AF-A0A1Y3S4L9-F1-MODEL\_V4 | 1.0 | 1.202e-10 | 402 | 0.238 | 159 | 102 | 5 | 2 | 160 | 76 | 215 | Single-stranded DNA-binding protein | Single-stranded DNA-binding protein | | afdb-uniprot50 | AF-X0UIT5-F1-MODEL\_V4 | 1.0 | 1.949e-10 | 399 | 0.269 | 156 | 98 | 4 | 8 | 162 | 1 | 141 | Uncharacterized protein | Uncharacterized protein | | afdb-uniprot50 | AF-A0A509MKJ1-F1-MODEL\_V4 | 1.0 | 1.936e-11 | 398 | 0.31 | 164 | 93 | 7 | 7 | 162 | 4 | 155 | Single-stranded DNA-binding protein | Single-stranded DNA-binding protein | | afdb-uniprot50 | AF-A0A7V9J1J1-F1-MODEL\_V4 | 1.0 | 3.312e-11 | 398 | 0.286 | 171 | 110 | 6 | 1 | 162 | 1 | 168 | Single-stranded DNA-binding protein | Single-stranded DNA-binding protein | | afdb-uniprot50 | AF-V8QYI1-F1-MODEL\_V4 | 1.0 | 8.769e-10 | 397 | 0.263 | 133 | 92 | 4 | 1 | 130 | 1 | 130 | Uncharacterized protein | Uncharacterized protein | | afdb-uniprot50 | AF-A0A2D7YMR2-F1-MODEL\_V4 | 1.0 | 1.422e-09 | 397 | 0.263 | 133 | 92 | 4 | 1 | 131 | 44 | 172 | Single-stranded DNA-binding protein | Single-stranded DNA-binding protein | | afdb-uniprot50 | AF-A0A447TPT0-F1-MODEL\_V4 | 1.0 | 4.106e-11 | 394 | 0.545 | 143 | 52 | 4 | 29 | 160 | 5 | 145 | Single-stranded DNA-binding protein | Single-stranded DNA-binding protein | | afdb-uniprot50 | AF-A0A5C7LUW6-F1-MODEL\_V4 | 1.0 | 7.821e-11 | 394 | 0.308 | 159 | 99 | 5 | 1 | 152 | 38 | 192 | Single-stranded DNA-binding protein | Single-stranded DNA-binding protein | | afdb-uniprot50 | AF-A0A7U0YZF2-F1-MODEL\_V4 | 1.0 | 1.268e-10 | 390 | 0.279 | 161 | 101 | 6 | 7 | 158 | 5 | 159 | Single-stranded DNA-binding protein | Single-stranded DNA-binding protein | | afdb-uniprot50 | AF-G5H4S1-F1-MODEL\_V4 | 1.0 | 6.353e-10 | 389 | 0.287 | 160 | 100 | 6 | 5 | 162 | 1 | 148 | Single-stranded DNA-binding protein | Single-stranded DNA-binding protein | | afdb-uniprot50 | AF-A0A6L6LUW6-F1-MODEL\_V4 | 1.0 | 1.49e-10 | 389 | 0.26 | 161 | 107 | 6 | 3 | 162 | 104 | 253 | Single-stranded DNA-binding protein | Single-stranded DNA-binding protein | | afdb-uniprot50 | AF-A0A415C335-F1-MODEL\_V4 | 1.0 | 2.399e-11 | 388 | 0.296 | 162 | 102 | 8 | 1 | 155 | 1 | 157 | Single-stranded DNA-binding protein | Single-stranded DNA-binding protein | | afdb-uniprot50 | AF-A0A0F9XHB2-F1-MODEL\_V4 | 1.0 | 2.071e-09 | 387 | 0.212 | 160 | 102 | 4 | 4 | 162 | 2 | 138 | Uncharacterized protein | Uncharacterized protein | | afdb-uniprot50 | AF-A0A6G2BJE3-F1-MODEL\_V4 | 1.0 | 2.399e-11 | 387 | 0.245 | 163 | 110 | 4 | 1 | 154 | 1 | 159 | Single-stranded DNA-binding protein | Single-stranded DNA-binding protein | | afdb-uniprot50 | AF-A0A6N9CAY7-F1-MODEL\_V4 | 1.0 | 1.422e-09 | 386 | 0.331 | 145 | 85 | 3 | 18 | 162 | 1 | 133 | Single-stranded DNA-binding protein | Single-stranded DNA-binding protein | | afdb-uniprot50 | AF-A0A1S2GGX7-F1-MODEL\_V4 | 1.0 | 2.819e-11 | 384 | 0.293 | 160 | 106 | 5 | 5 | 160 | 1 | 157 | Single-stranded DNA-binding protein | Single-stranded DNA-binding protein | | afdb-uniprot50 | AF-A0A2I2AAY6-F1-MODEL\_V4 | 1.0 | 7.464e-10 | 381 | 0.192 | 161 | 123 | 3 | 4 | 162 | 3 | 158 | Single-stranded DNA-binding protein | Single-stranded DNA-binding protein | | afdb-uniprot50 | AF-A0A3B9YJQ0-F1-MODEL\_V4 | 1.0 | 1.963e-09 | 380 | 0.26 | 146 | 94 | 5 | 1 | 142 | 4 | 139 | Single-stranded DNA-binding protein | Single-stranded DNA-binding protein | | afdb-uniprot50 | AF-G9YJ09-F1-MODEL\_V4 | 1.0 | 1.147e-09 | 378 | 0.309 | 152 | 86 | 4 | 12 | 162 | 2 | 135 | Single-stranded DNA-binding protein | Single-stranded DNA-binding protein | | afdb-uniprot50 | AF-A0A3G6ISV9-F1-MODEL\_V4 | 1.0 | 5.125e-10 | 378 | 0.256 | 152 | 103 | 5 | 5 | 154 | 1 | 144 | Single-stranded DNA-binding protein | Single-stranded DNA-binding protein | | afdb-uniprot50 | AF-A0A2I1Y967-F1-MODEL\_V4 | 1.0 | 1.49e-10 | 378 | 0.169 | 165 | 126 | 6 | 4 | 162 | 1 | 160 | Single-stranded DNA-binding protein | Single-stranded DNA-binding protein | | afdb-uniprot50 | AF-A0A7V9BRX3-F1-MODEL\_V4 | 1.0 | 3.945e-09 | 377 | 0.253 | 130 | 87 | 6 | 4 | 130 | 3 | 125 | Single-stranded DNA-binding protein | Single-stranded DNA-binding protein | | afdb-uniprot50 | AF-A0A1Y2MLA3-F1-MODEL\_V4 | 1.0 | 1.751e-10 | 377 | 0.277 | 155 | 99 | 7 | 4 | 154 | 2 | 147 | Single-stranded DNA-binding protein | Single-stranded DNA-binding protein | | afdb-uniprot50 | AF-A0A2D0IR08-F1-MODEL\_V4 | 1.0 | 2.839e-10 | 376 | 0.493 | 144 | 58 | 2 | 30 | 162 | 2 | 141 | Single-stranded DNA-binding protein | Single-stranded DNA-binding protein | | afdb-uniprot50 | AF-A0A2N6T258-F1-MODEL\_V4 | 1.0 | 5.706e-10 | 372 | 0.253 | 142 | 100 | 4 | 4 | 144 | 3 | 139 | Single-stranded DNA-binding protein | Single-stranded DNA-binding protein | | afdb-uniprot50 | AF-A0A2N6V0F4-F1-MODEL\_V4 | 1.0 | 9.696e-11 | 372 | 0.251 | 183 | 113 | 8 | 1 | 162 | 1 | 180 | Single-stranded DNA-binding protein | Single-stranded DNA-binding protein | | afdb-uniprot50 | AF-A0A7V6GLJ9-F1-MODEL\_V4 | 1.0 | 1.49e-10 | 371 | 0.267 | 168 | 95 | 7 | 4 | 162 | 2 | 150 | Single-stranded DNA-binding protein | Single-stranded DNA-binding protein | | afdb-uniprot50 | AF-A0A7Y0Y5A9-F1-MODEL\_V4 | 1.0 | 1.751e-10 | 370 | 0.288 | 163 | 106 | 6 | 4 | 162 | 2 | 158 | Single-stranded DNA-binding protein | Single-stranded DNA-binding protein | | afdb-uniprot50 | AF-A0A3N2P4R5-F1-MODEL\_V4 | 1.0 | 1.572e-10 | 370 | 0.239 | 171 | 115 | 7 | 4 | 162 | 2 | 169 | Single-stranded DNA-binding protein | Single-stranded DNA-binding protein | | afdb-uniprot50 | AF-A0A1T4P1L0-F1-MODEL\_V4 | 1.0 | 3.945e-09 | 369 | 0.33 | 127 | 76 | 5 | 5 | 129 | 1 | 120 | Single-stranded DNA-binding protein | Single-stranded DNA-binding protein | | afdb-uniprot50 | AF-A0A6J5CGD1-F1-MODEL\_V4 | 1.0 | 5.125e-10 | 369 | 0.276 | 163 | 106 | 5 | 4 | 162 | 3 | 157 | Single-stranded DNA-binding protein | Single-stranded DNA-binding protein | | afdb-uniprot50 | AF-A0A417WSE5-F1-MODEL\_V4 | 1.0 | 2.69e-10 | 366 | 0.283 | 159 | 108 | 4 | 5 | 161 | 1 | 155 | Single-stranded DNA-binding protein | Single-stranded DNA-binding protein | | afdb-uniprot50 | AF-S3A3R1-F1-MODEL\_V4 | 1.0 | 1.087e-09 | 365 | 0.306 | 137 | 89 | 5 | 5 | 139 | 1 | 133 | Single-stranded DNA-binding protein | Single-stranded DNA-binding protein | | afdb-uniprot50 | AF-A0A2N6TVJ7-F1-MODEL\_V4 | 1.0 | 1.5e-09 | 365 | 0.261 | 149 | 102 | 5 | 1 | 146 | 53 | 196 | Uncharacterized protein | Uncharacterized protein | | afdb-uniprot50 | AF-A0A1X3AD52-F1-MODEL\_V4 | 1.0 | 7.074e-10 | 364 | 0.269 | 156 | 103 | 5 | 1 | 152 | 1 | 149 | Single-stranded DNA-binding protein | Single-stranded DNA-binding protein | | afdb-uniprot50 | AF-A0A6L9SUC9-F1-MODEL\_V4 | 1.0 | 1.087e-09 | 361 | 0.243 | 164 | 114 | 4 | 1 | 158 | 1 | 160 | Single-stranded DNA-binding protein | Single-stranded DNA-binding protein | | afdb-uniprot50 | AF-A0A1F6QQY9-F1-MODEL\_V4 | 1.0 | 7.986e-08 | 360 | 0.196 | 112 | 87 | 2 | 4 | 115 | 2 | 110 | Uncharacterized protein | Uncharacterized protein | | afdb-uniprot50 | AF-A0A366INY2-F1-MODEL\_V4 | 1.0 | 3.713e-10 | 360 | 0.264 | 155 | 105 | 6 | 5 | 154 | 1 | 151 | Single-stranded DNA-binding protein | Single-stranded DNA-binding protein | | afdb-uniprot50 | AF-A0A4Y6QZ46-F1-MODEL\_V4 | 1.0 | 5.408e-10 | 359 | 0.246 | 162 | 112 | 5 | 5 | 162 | 1 | 156 | Single-stranded DNA-binding protein | Single-stranded DNA-binding protein | | afdb-uniprot50 | AF-A0A2S9Z3F5-F1-MODEL\_V4 | 1.0 | 1.087e-09 | 358 | 0.246 | 166 | 114 | 5 | 1 | 162 | 66 | 224 | Single-stranded DNA-binding protein | Single-stranded DNA-binding protein | | afdb-uniprot50 | AF-A0A7T0PDH1-F1-MODEL\_V4 | 1.0 | 1.202e-10 | 357 | 0.291 | 161 | 106 | 5 | 6 | 161 | 3 | 160 | Single-stranded DNA-binding protein | Single-stranded DNA-binding protein | | afdb-uniprot50 | AF-Q2MLT4-F1-MODEL\_V4 | 1.0 | 2.549e-10 | 357 | 0.248 | 173 | 116 | 6 | 1 | 162 | 1 | 170 | Single-stranded DNA-binding protein | Single-stranded DNA-binding protein | | afdb-uniprot50 | AF-A0A1I2DXE7-F1-MODEL\_V4 | 1.0 | 2.433e-09 | 357 | 0.233 | 133 | 89 | 6 | 5 | 129 | 1 | 128 | Single-stranded DNA-binding protein | Single-stranded DNA-binding protein | | afdb-uniprot50 | AF-B8DTM4-F1-MODEL\_V4 | 1.0 | 4.134e-10 | 357 | 0.244 | 168 | 117 | 5 | 1 | 162 | 1 | 164 | Single-stranded DNA-binding protein | Single-stranded DNA-binding protein | | afdb-uniprot50 | AF-A0A1V3G0K3-F1-MODEL\_V4 | 1.0 | 3.161e-10 | 356 | 0.235 | 153 | 110 | 6 | 5 | 154 | 1 | 149 | Single-stranded DNA-binding protein | Single-stranded DNA-binding protein | | afdb-uniprot50 | AF-A0A2W4PIC5-F1-MODEL\_V4 | 1.0 | 8.31e-10 | 355 | 0.238 | 155 | 102 | 6 | 7 | 154 | 4 | 149 | Single-stranded DNA-binding protein | Single-stranded DNA-binding protein | | afdb-uniprot50 | AF-A0A4R7L5A8-F1-MODEL\_V4 | 1.0 | 2.071e-09 | 354 | 0.254 | 161 | 104 | 7 | 4 | 162 | 2 | 148 | Single-stranded DNA-binding protein | Single-stranded DNA-binding protein | | afdb-uniprot50 | AF-A0A1E8CUC1-F1-MODEL\_V4 | 1.0 | 5.483e-08 | 354 | 0.31 | 103 | 68 | 3 | 7 | 108 | 5 | 105 | Single-stranded DNA-binding protein | Single-stranded DNA-binding protein | | afdb-uniprot50 | AF-A0A6N7WM32-F1-MODEL\_V4 | 1.0 | 2.306e-09 | 353 | 0.272 | 136 | 93 | 4 | 5 | 137 | 1 | 133 | Single-stranded DNA-binding protein | Single-stranded DNA-binding protein | | afdb-uniprot50 | AF-A0A0A1DMG2-F1-MODEL\_V4 | 1.0 | 2.071e-09 | 352 | 0.236 | 152 | 103 | 5 | 4 | 154 | 1 | 140 | Single-stranded DNA-binding protein | Single-stranded DNA-binding protein | | afdb-uniprot50 | AF-A0A2J8GAC6-F1-MODEL\_V4 | 1.0 | 6.021e-10 | 352 | 0.236 | 169 | 118 | 7 | 1 | 162 | 39 | 203 | Single-stranded DNA-binding protein | Single-stranded DNA-binding protein | | afdb-uniprot50 | AF-A0A2V4NL91-F1-MODEL\_V4 | 1.0 | 4.857e-10 | 351 | 0.254 | 161 | 109 | 5 | 2 | 154 | 28 | 185 | Single-stranded DNA-binding protein | Single-stranded DNA-binding protein | | afdb-uniprot50 | AF-A0A2A3F440-F1-MODEL\_V4 | 1.0 | 1.348e-09 | 350 | 0.2 | 165 | 122 | 6 | 4 | 162 | 1 | 161 | Uncharacterized protein | Uncharacterized protein | | afdb-uniprot50 | AF-A0A0G7ZN84-F1-MODEL\_V4 | 1.0 | 4.891e-09 | 348 | 0.225 | 164 | 115 | 2 | 4 | 158 | 1 | 161 | Single-stranded DNA-binding protein | Single-stranded DNA-binding protein | | afdb-uniprot50 | AF-A0A212UIC7-F1-MODEL\_V4 | 1.0 | 4.362e-10 | 347 | 0.262 | 164 | 105 | 7 | 4 | 154 | 2 | 162 | Single-stranded DNA-binding protein | Single-stranded DNA-binding protein | | afdb-uniprot50 | AF-A0A1S1D7V1-F1-MODEL\_V4 | 1.0 | 2.549e-10 | 346 | 0.239 | 163 | 115 | 5 | 6 | 162 | 3 | 162 | Single-stranded DNA-binding protein | Single-stranded DNA-binding protein | | afdb-uniprot50 | AF-A0A0N1D9D7-F1-MODEL\_V4 | 1.0 | 1.671e-09 | 345 | 0.236 | 161 | 107 | 6 | 4 | 162 | 2 | 148 | Single-stranded DNA-binding protein | Single-stranded DNA-binding protein | | afdb-uniprot50 | AF-M1UST2-F1-MODEL\_V4 | 1.0 | 1.422e-09 | 345 | 0.236 | 152 | 106 | 4 | 1 | 144 | 1 | 150 | Single-stranded DNA-binding protein | Single-stranded DNA-binding protein | | afdb-uniprot50 | AF-A0A4Z1E4B9-F1-MODEL\_V4 | 1.0 | 5.125e-10 | 345 | 0.215 | 167 | 121 | 5 | 4 | 162 | 1 | 165 | Single-stranded DNA-binding protein | Single-stranded DNA-binding protein | | afdb-uniprot50 | AF-A0A7W3IVP1-F1-MODEL\_V4 | 1.0 | 4.603e-10 | 344 | 0.245 | 167 | 114 | 5 | 6 | 162 | 3 | 167 | Single-stranded DNA-binding protein | Single-stranded DNA-binding protein | | afdb-uniprot50 | AF-C6R1H3-F1-MODEL\_V4 | 1.0 | 3.161e-10 | 342 | 0.236 | 165 | 116 | 5 | 6 | 162 | 3 | 165 | Single-stranded DNA-binding protein | Single-stranded DNA-binding protein | | afdb-uniprot50 | AF-A0A1F1ZNZ2-F1-MODEL\_V4 | 1.0 | 2.416e-10 | 341 | 0.275 | 160 | 104 | 6 | 5 | 155 | 1 | 157 | Single-stranded DNA-binding protein | Single-stranded DNA-binding protein | | afdb-uniprot50 | AF-A0A1G3ZU57-F1-MODEL\_V4 | 1.0 | 1.5e-09 | 341 | 0.232 | 176 | 117 | 6 | 2 | 162 | 3 | 175 | Uncharacterized protein | Uncharacterized protein | | afdb-uniprot50 | AF-A0A0K8Q012-F1-MODEL\_V4 | 1.0 | 9.253e-10 | 340 | 0.253 | 166 | 109 | 6 | 3 | 156 | 2 | 164 | Single-stranded DNA-binding protein | Single-stranded DNA-binding protein | | afdb-uniprot50 | AF-A0A657BBA4-F1-MODEL\_V4 | 1.0 | 3.335e-10 | 339 | 0.482 | 170 | 55 | 5 | 23 | 162 | 2 | 168 | Single-stranded DNA-binding protein | Single-stranded DNA-binding protein | | afdb-uniprot50 | AF-A0A6H9WNQ8-F1-MODEL\_V4 | 1.0 | 7.074e-10 | 339 | 0.206 | 174 | 123 | 6 | 1 | 162 | 50 | 220 | Single-stranded DNA-binding protein | Single-stranded DNA-binding protein | | afdb-uniprot50 | AF-L1ME36-F1-MODEL\_V4 | 1.0 | 2.085e-08 | 338 | 0.203 | 133 | 99 | 4 | 2 | 129 | 5 | 135 | Single-strand binding family protein | Single-strand binding family protein | | afdb-uniprot50 | AF-A0A3G6J8M0-F1-MODEL\_V4 | 1.0 | 5.445e-09 | 337 | 0.226 | 137 | 89 | 5 | 7 | 128 | 5 | 139 | Single-stranded DNA-binding protein | Single-stranded DNA-binding protein | | afdb-uniprot50 | AF-A0A1D9FGE8-F1-MODEL\_V4 | 1.0 | 6.75e-09 | 335 | 0.208 | 149 | 107 | 5 | 8 | 151 | 1 | 143 | Single-stranded DNA-binding protein | Single-stranded DNA-binding protein | | afdb-uniprot50 | AF-A0A449A2H2-F1-MODEL\_V4 | 1.0 | 1.357e-08 | 335 | 0.16 | 162 | 129 | 3 | 5 | 162 | 1 | 159 | Single-stranded DNA-binding protein | Single-stranded DNA-binding protein | | afdb-uniprot50 | AF-A0A7Z0VU71-F1-MODEL\_V4 | 1.0 | 1.422e-09 | 333 | 0.209 | 167 | 117 | 5 | 1 | 162 | 1 | 157 | Uncharacterized protein | Uncharacterized protein | | afdb-uniprot50 | AF-A0A3D5W1P2-F1-MODEL\_V4 | 1.0 | 1.348e-09 | 333 | 0.211 | 161 | 115 | 6 | 4 | 154 | 2 | 160 | Uncharacterized protein | Uncharacterized protein | | afdb-uniprot50 | AF-A0A0M2LZ75-F1-MODEL\_V4 | 1.0 | 2.216e-07 | 332 | 0.187 | 112 | 85 | 5 | 4 | 114 | 1 | 107 | Uncharacterized protein | Uncharacterized protein | | afdb-uniprot50 | AF-A0A6I7R8T6-F1-MODEL\_V4 | 1.0 | 3.568e-08 | 331 | 0.23 | 130 | 92 | 5 | 1 | 129 | 1 | 123 | Single-stranded DNA-binding protein | Single-stranded DNA-binding protein | | afdb-uniprot50 | AF-A0A2G2FL91-F1-MODEL\_V4 | 1.0 | 1.095e-08 | 329 | 0.208 | 139 | 105 | 3 | 3 | 138 | 25 | 161 | Single-stranded DNA-binding protein | Single-stranded DNA-binding protein | | afdb-uniprot50 | AF-A0A7M1SYA3-F1-MODEL\_V4 | 1.0 | 3.543e-09 | 329 | 0.189 | 190 | 123 | 7 | 1 | 162 | 1 | 187 | Single-stranded DNA-binding protein | Single-stranded DNA-binding protein | | afdb-uniprot50 | AF-A0A7C6GI43-F1-MODEL\_V4 | 1.0 | 2.185e-09 | 328 | 0.229 | 183 | 118 | 7 | 1 | 162 | 36 | 216 | Single-stranded DNA-binding protein | Single-stranded DNA-binding protein | | afdb-uniprot50 | AF-W6JX67-F1-MODEL\_V4 | 1.0 | 2.878e-08 | 327 | 0.269 | 126 | 88 | 4 | 4 | 127 | 1 | 124 | Single-stranded DNA-binding protein | Single-stranded DNA-binding protein | | afdb-uniprot50 | AF-A0A2I1I393-F1-MODEL\_V4 | 1.0 | 4.635e-09 | 327 | 0.188 | 175 | 127 | 5 | 1 | 162 | 7 | 179 | Single-stranded DNA-binding protein | Single-stranded DNA-binding protein | | afdb-uniprot50 | AF-A0A239QU82-F1-MODEL\_V4 | 1.0 | 3.358e-09 | 326 | 0.19 | 168 | 124 | 7 | 4 | 162 | 2 | 166 | Single-stranded DNA-binding protein | Single-stranded DNA-binding protein | | afdb-uniprot50 | AF-A0A560W8C9-F1-MODEL\_V4 | 1.0 | 1.976e-08 | 325 | 0.224 | 125 | 87 | 5 | 4 | 120 | 1 | 123 | Single-stranded DNA-binding protein | Single-stranded DNA-binding protein | | afdb-uniprot50 | AF-A0A2U1ZT47-F1-MODEL\_V4 | 1.0 | 3.016e-09 | 325 | 0.22 | 163 | 119 | 5 | 1 | 162 | 1 | 156 | Single-stranded DNA-binding protein | Single-stranded DNA-binding protein | | afdb-uniprot50 | AF-A0A5B8ARK9-F1-MODEL\_V4 | 1.0 | 2.306e-09 | 324 | 0.202 | 163 | 112 | 6 | 3 | 157 | 2 | 154 | Single-stranded DNA-binding protein | Single-stranded DNA-binding protein | | afdb-uniprot50 | AF-A0A5N5RH10-F1-MODEL\_V4 | 1.0 | 3.543e-09 | 324 | 0.226 | 168 | 118 | 5 | 1 | 159 | 1 | 165 | Single-stranded DNA-binding protein | Single-stranded DNA-binding protein | | afdb-uniprot50 | AF-A0A5C7PTQ0-F1-MODEL\_V4 | 1.0 | 2.567e-09 | 323 | 0.252 | 170 | 106 | 9 | 1 | 162 | 1 | 157 | Single-stranded DNA-binding protein | Single-stranded DNA-binding protein | | afdb-uniprot50 | AF-A0A1F0QJF3-F1-MODEL\_V4 | 1.0 | 3.205e-08 | 322 | 0.203 | 123 | 89 | 4 | 1 | 120 | 1 | 117 | Single-stranded DNA-binding protein | Single-stranded DNA-binding protein | | afdb-uniprot50 | AF-A0A498C414-F1-MODEL\_V4 | 1.0 | 2.433e-09 | 321 | 0.247 | 174 | 112 | 7 | 5 | 162 | 1 | 171 | Single-stranded DNA-binding protein | Single-stranded DNA-binding protein | | afdb-uniprot50 | AF-A0A7K1J6Z0-F1-MODEL\_V4 | 1.0 | 3.739e-09 | 321 | 0.229 | 196 | 115 | 8 | 1 | 162 | 1 | 194 | Single-stranded DNA-binding protein | Single-stranded DNA-binding protein | | afdb-uniprot50 | AF-A0A4Q5B4F8-F1-MODEL\_V4 | 1.0 | 3.543e-09 | 321 | 0.231 | 164 | 114 | 6 | 1 | 154 | 1 | 162 | Single-strand binding protein | Single-strand binding protein | | afdb-uniprot50 | AF-N6WBX6-F1-MODEL\_V4 | 1.0 | 3.358e-09 | 321 | 0.207 | 169 | 121 | 7 | 2 | 162 | 7 | 170 | Single-stranded DNA-binding protein | Single-stranded DNA-binding protein | | afdb-uniprot50 | AF-A0A6N9YPB8-F1-MODEL\_V4 | 1.0 | 5.746e-09 | 320 | 0.235 | 174 | 115 | 9 | 1 | 161 | 1 | 169 | Single-stranded DNA-binding protein | Single-stranded DNA-binding protein | | afdb-uniprot50 | AF-A0A0A7I9E0-F1-MODEL\_V4 | 1.0 | 1.037e-08 | 318 | 0.224 | 169 | 108 | 7 | 4 | 154 | 2 | 165 | Uncharacterized protein | Uncharacterized protein | | afdb-uniprot50 | AF-A0A6G7Z2E4-F1-MODEL\_V4 | 1.0 | 3.358e-09 | 318 | 0.203 | 167 | 118 | 6 | 5 | 162 | 1 | 161 | Single-stranded DNA-binding protein | Single-stranded DNA-binding protein | | afdb-uniprot50 | AF-A0A6A9V298-F1-MODEL\_V4 | 1.0 | 1.886e-07 | 318 | 0.213 | 122 | 86 | 3 | 2 | 122 | 56 | 168 | Single-stranded DNA-binding protein | Single-stranded DNA-binding protein | | afdb-uniprot50 | AF-A0A2W0CY63-F1-MODEL\_V4 | 1.0 | 3.543e-09 | 317 | 0.204 | 161 | 118 | 5 | 4 | 162 | 2 | 154 | Single-stranded DNA-binding protein | Single-stranded DNA-binding protein | | afdb-uniprot50 | AF-A0A3N4YNY9-F1-MODEL\_V4 | 1.0 | 1.422e-09 | 317 | 0.244 | 176 | 113 | 8 | 1 | 162 | 1 | 170 | Single-stranded DNA-binding protein | Single-stranded DNA-binding protein | | afdb-uniprot50 | AF-A0A6I1P9S7-F1-MODEL\_V4 | 1.0 | 1.095e-08 | 316 | 0.182 | 159 | 125 | 5 | 1 | 156 | 1 | 157 | Single-stranded DNA-binding protein | Single-stranded DNA-binding protein | | afdb-uniprot50 | AF-A0A7T0PDA0-F1-MODEL\_V4 | 1.0 | 7.123e-09 | 315 | 0.196 | 168 | 120 | 7 | 4 | 162 | 1 | 162 | Single-stranded DNA-binding protein | Single-stranded DNA-binding protein | | afdb-uniprot50 | AF-A0A7T0LMC8-F1-MODEL\_V4 | 1.0 | 3.016e-09 | 315 | 0.215 | 167 | 117 | 7 | 4 | 160 | 2 | 164 | Single-stranded DNA-binding protein | Single-stranded DNA-binding protein | | afdb-uniprot50 | AF-A0A6B8VYJ9-F1-MODEL\_V4 | 1.0 | 4.393e-09 | 314 | 0.211 | 189 | 115 | 7 | 6 | 162 | 4 | 190 | Single-stranded DNA-binding protein | Single-stranded DNA-binding protein | | afdb-uniprot50 | AF-A0A087AGL6-F1-MODEL\_V4 | 1.0 | 9.317e-09 | 314 | 0.223 | 170 | 120 | 7 | 1 | 162 | 1 | 166 | Single-stranded DNA-binding protein | Single-stranded DNA-binding protein | | afdb-uniprot50 | AF-A0A7K0K8X1-F1-MODEL\_V4 | 1.0 | 4.891e-09 | 314 | 0.182 | 175 | 126 | 5 | 4 | 162 | 2 | 175 | Single-stranded DNA-binding protein | Single-stranded DNA-binding protein | | afdb-uniprot50 | AF-A0A1G0GPX9-F1-MODEL\_V4 | 1.0 | 2.747e-07 | 313 | 0.183 | 120 | 95 | 1 | 1 | 120 | 4 | 120 | Uncharacterized protein | Uncharacterized protein | | afdb-uniprot50 | AF-A0A540R783-F1-MODEL\_V4 | 1.0 | 8.83e-09 | 312 | 0.254 | 169 | 110 | 8 | 5 | 162 | 1 | 164 | Single-stranded DNA-binding protein | Single-stranded DNA-binding protein | | afdb-uniprot50 | AF-A0A1H5DK09-F1-MODEL\_V4 | 1.0 | 7.931e-09 | 312 | 0.198 | 171 | 122 | 6 | 1 | 162 | 1 | 165 | Single-stranded DNA-binding protein | Single-stranded DNA-binding protein | | afdb-uniprot50 | AF-A0A852W0M8-F1-MODEL\_V4 | 1.0 | 5.786e-08 | 312 | 0.184 | 125 | 96 | 4 | 7 | 127 | 5 | 127 | Single-strand DNA-binding protein | Single-strand DNA-binding protein | | afdb-uniprot50 | AF-D1NUV4-F1-MODEL\_V4 | 1.0 | 1.873e-08 | 310 | 0.222 | 162 | 118 | 4 | 1 | 156 | 1 | 160 | Single-strand binding family protein | Single-strand binding family protein | | afdb-uniprot50 | AF-A0A6N9Z2N4-F1-MODEL\_V4 | 1.0 | 9.831e-09 | 310 | 0.21 | 166 | 119 | 7 | 1 | 159 | 1 | 161 | Single-stranded DNA-binding protein | Single-stranded DNA-binding protein | | afdb-uniprot50 | AF-A0A2S8ZS37-F1-MODEL\_V4 | 1.0 | 4.635e-09 | 308 | 0.248 | 165 | 113 | 7 | 6 | 162 | 3 | 164 | Single-stranded DNA-binding protein | Single-stranded DNA-binding protein | | afdb-uniprot50 | AF-A0A2N3QFT5-F1-MODEL\_V4 | 1.0 | 9.831e-09 | 308 | 0.225 | 200 | 115 | 6 | 1 | 162 | 1 | 198 | Single-stranded DNA-binding protein | Single-stranded DNA-binding protein | | afdb-uniprot50 | AF-A0A1H4WAE0-F1-MODEL\_V4 | 1.0 | 7.931e-09 | 307 | 0.22 | 159 | 117 | 5 | 5 | 162 | 1 | 153 | Single-stranded DNA-binding protein | Single-stranded DNA-binding protein | | afdb-uniprot50 | AF-A0A3D5VZ01-F1-MODEL\_V4 | 1.0 | 1.976e-08 | 307 | 0.181 | 171 | 125 | 7 | 1 | 162 | 1 | 165 | Single-stranded DNA-binding protein | Single-stranded DNA-binding protein | | afdb-uniprot50 | AF-A0A3A4KSQ4-F1-MODEL\_V4 | 1.0 | 2.45e-08 | 306 | 0.217 | 161 | 105 | 7 | 1 | 160 | 6 | 146 | Single-stranded DNA-binding protein | Single-stranded DNA-binding protein | | afdb-uniprot50 | AF-A0A1I6G343-F1-MODEL\_V4 | 1.0 | 1.286e-08 | 306 | 0.224 | 156 | 114 | 5 | 6 | 156 | 3 | 156 | Single-stranded DNA-binding protein | Single-stranded DNA-binding protein | | afdb-uniprot50 | AF-A0A3L6ZP49-F1-MODEL\_V4 | 1.0 | 7.931e-09 | 306 | 0.188 | 159 | 116 | 6 | 4 | 157 | 3 | 153 | Single-stranded DNA-binding protein | Single-stranded DNA-binding protein | | afdb-uniprot50 | AF-K6WX02-F1-MODEL\_V4 | 1.0 | 3.016e-09 | 304 | 0.218 | 169 | 115 | 8 | 4 | 160 | 1 | 164 | Single-stranded DNA-binding protein | Single-stranded DNA-binding protein | | afdb-uniprot50 | AF-A0A1R4FAL8-F1-MODEL\_V4 | 1.0 | 5.161e-09 | 304 | 0.215 | 181 | 114 | 9 | 1 | 162 | 1 | 172 | Uncharacterized protein | Uncharacterized protein | | afdb-uniprot50 | AF-A0A387BUL9-F1-MODEL\_V4 | 1.0 | 6.442e-08 | 303 | 0.223 | 134 | 98 | 5 | 1 | 131 | 1 | 131 | Single-stranded DNA-binding protein | Single-stranded DNA-binding protein | | afdb-uniprot50 | AF-A0A1F6RE83-F1-MODEL\_V4 | 1.0 | 1.682e-08 | 303 | 0.214 | 168 | 121 | 4 | 4 | 162 | 2 | 167 | Single-stranded DNA-binding protein | Single-stranded DNA-binding protein | | afdb-uniprot50 | AF-A0A7X8M9H6-F1-MODEL\_V4 | 1.0 | 8.426e-08 | 303 | 0.219 | 141 | 104 | 5 | 1 | 138 | 30 | 167 | Single-stranded DNA-binding protein | Single-stranded DNA-binding protein | | afdb-uniprot50 | AF-A0A5C8I7Q5-F1-MODEL\_V4 | 1.0 | 8.368e-09 | 303 | 0.244 | 172 | 112 | 7 | 6 | 162 | 3 | 171 | Single-stranded DNA-binding protein | Single-stranded DNA-binding protein | | afdb-uniprot50 | AF-A0A2A9D112-F1-MODEL\_V4 | 1.0 | 3.037e-08 | 303 | 0.203 | 177 | 115 | 9 | 1 | 160 | 17 | 184 | Single-strand DNA-binding protein | Single-strand DNA-binding protein | | afdb-uniprot50 | AF-A0A6P0HKI8-F1-MODEL\_V4 | 1.0 | 1.682e-08 | 302 | 0.196 | 173 | 119 | 6 | 4 | 162 | 2 | 168 | Single-stranded DNA-binding protein | Single-stranded DNA-binding protein | | afdb-uniprot50 | AF-A0A6I3JGH7-F1-MODEL\_V4 | 1.0 | 1.521e-07 | 301 | 0.155 | 129 | 101 | 5 | 1 | 124 | 1 | 126 | Single-stranded DNA-binding protein | Single-stranded DNA-binding protein | | afdb-uniprot50 | AF-B1VGP3-F1-MODEL\_V4 | 1.0 | 4.192e-08 | 301 | 0.195 | 169 | 126 | 6 | 1 | 161 | 49 | 215 | Single-stranded DNA-binding protein | Single-stranded DNA-binding protein | | afdb-uniprot50 | AF-A0A176QDX5-F1-MODEL\_V4 | 1.0 | 3.945e-09 | 300 | 0.235 | 170 | 112 | 8 | 4 | 157 | 1 | 168 | Single-stranded DNA-binding protein | Single-stranded DNA-binding protein | | afdb-uniprot50 | AF-A0A7X7K7I0-F1-MODEL\_V4 | 1.0 | 1.775e-08 | 299 | 0.173 | 161 | 106 | 5 | 1 | 136 | 1 | 159 | Single-stranded DNA-binding protein | Single-stranded DNA-binding protein | | afdb-uniprot50 | AF-A0A087BDX7-F1-MODEL\_V4 | 1.0 | 1.976e-08 | 299 | 0.205 | 170 | 123 | 5 | 1 | 160 | 1 | 168 | Single-stranded DNA-binding protein | Single-stranded DNA-binding protein | | afdb-uniprot50 | AF-A0A430FWQ9-F1-MODEL\_V4 | 1.0 | 6.75e-09 | 299 | 0.225 | 182 | 117 | 7 | 1 | 162 | 1 | 178 | Single-stranded DNA-binding protein | Single-stranded DNA-binding protein | | afdb-uniprot50 | AF-A0A2N6UPV5-F1-MODEL\_V4 | 1.0 | 1.286e-08 | 298 | 0.19 | 163 | 119 | 6 | 2 | 154 | 29 | 188 | Single-stranded DNA-binding protein | Single-stranded DNA-binding protein | | afdb-uniprot50 | AF-A0A4Q9KLV9-F1-MODEL\_V4 | 1.0 | 2.728e-08 | 297 | 0.19 | 173 | 123 | 8 | 5 | 162 | 1 | 171 | Single-stranded DNA-binding protein | Single-stranded DNA-binding protein | | afdb-uniprot50 | AF-A0A3G5T3X2-F1-MODEL\_V4 | 1.0 | 5.196e-08 | 296 | 0.174 | 166 | 125 | 7 | 1 | 162 | 1 | 158 | Single-stranded DNA-binding protein | Single-stranded DNA-binding protein | | afdb-uniprot50 | AF-A0A6L4WZV7-F1-MODEL\_V4 | 1.0 | 2.085e-08 | 296 | 0.211 | 170 | 123 | 7 | 1 | 162 | 1 | 167 | Single-stranded DNA-binding protein | Single-stranded DNA-binding protein | | afdb-uniprot50 | AF-A0A4Y8UKJ6-F1-MODEL\_V4 | 1.0 | 5.233e-07 | 294 | 0.21 | 119 | 86 | 4 | 3 | 117 | 2 | 116 | Uncharacterized protein | Uncharacterized protein | | afdb-uniprot50 | AF-A0A1R4HUB9-F1-MODEL\_V4 | 1.0 | 8.891e-08 | 294 | 0.179 | 156 | 117 | 6 | 1 | 151 | 1 | 150 | Single-strand binding protein/Primosomal replication protein n | Single-strand binding protein/Primosomal replication protein n | | afdb-uniprot50 | AF-A0A1X0E5M8-F1-MODEL\_V4 | 1.0 | 1.511e-08 | 294 | 0.201 | 169 | 117 | 7 | 6 | 162 | 4 | 166 | Single-stranded DNA-binding protein | Single-stranded DNA-binding protein | | afdb-uniprot50 | AF-A0A849PFU8-F1-MODEL\_V4 | 1.0 | 4.925e-08 | 294 | 0.155 | 180 | 128 | 8 | 1 | 162 | 1 | 174 | Single-stranded DNA-binding protein | Single-stranded DNA-binding protein | | afdb-uniprot50 | AF-A0A2P8EFA0-F1-MODEL\_V4 | 1.0 | 2.2e-08 | 294 | 0.223 | 170 | 117 | 7 | 4 | 162 | 1 | 166 | Single-stranded DNA-binding protein | Single-stranded DNA-binding protein | | afdb-uniprot50 | AF-C0W1B7-F1-MODEL\_V4 | 1.0 | 1.511e-08 | 294 | 0.207 | 178 | 121 | 8 | 1 | 162 | 34 | 207 | Single-stranded DNA-binding protein | Single-stranded DNA-binding protein | | afdb-uniprot50 | AF-A0A6I5N066-F1-MODEL\_V4 | 1.0 | 3.381e-08 | 292 | 0.226 | 168 | 119 | 7 | 1 | 162 | 1 | 163 | Single-stranded DNA-binding protein | Single-stranded DNA-binding protein | | afdb-uniprot50 | AF-A0A2W5B054-F1-MODEL\_V4 | 1.0 | 7.172e-08 | 291 | 0.207 | 169 | 124 | 6 | 1 | 161 | 15 | 181 | Single-stranded DNA-binding protein | Single-stranded DNA-binding protein | | afdb-uniprot50 | AF-X7ZHF1-F1-MODEL\_V4 | 1.0 | 3.765e-08 | 291 | 0.209 | 162 | 119 | 7 | 4 | 162 | 1 | 156 | Single-stranded DNA-binding protein | Single-stranded DNA-binding protein | | afdb-uniprot50 | AF-A0A852XD35-F1-MODEL\_V4 | 1.0 | 1.594e-08 | 290 | 0.218 | 169 | 117 | 6 | 7 | 162 | 5 | 171 | Single-strand DNA-binding protein | Single-strand DNA-binding protein | | afdb-uniprot50 | AF-A0A1A3DF40-F1-MODEL\_V4 | 1.0 | 2.878e-08 | 288 | 0.198 | 166 | 121 | 7 | 6 | 162 | 4 | 166 | Single-stranded DNA-binding protein | Single-stranded DNA-binding protein | | afdb-uniprot50 | AF-A0A6N2TFT1-F1-MODEL\_V4 | 1.0 | 3.381e-08 | 288 | 0.169 | 159 | 120 | 6 | 7 | 157 | 6 | 160 | Single-stranded DNA-binding protein | Single-stranded DNA-binding protein | | afdb-uniprot50 | AF-A0A1X3PBD3-F1-MODEL\_V4 | 1.0 | 3.973e-08 | 287 | 0.182 | 164 | 123 | 8 | 1 | 162 | 6 | 160 | Uncharacterized protein | Uncharacterized protein | | afdb-uniprot50 | AF-A0A1X0DBK5-F1-MODEL\_V4 | 1.0 | 4.192e-08 | 287 | 0.204 | 166 | 117 | 7 | 6 | 162 | 4 | 163 | Single-stranded DNA-binding protein | Single-stranded DNA-binding protein | | afdb-uniprot50 | AF-A0A087D7I9-F1-MODEL\_V4 | 1.0 | 2.585e-08 | 287 | 0.217 | 170 | 121 | 7 | 1 | 162 | 1 | 166 | Single-stranded DNA-binding protein | Single-stranded DNA-binding protein | | afdb-uniprot50 | AF-A0A1H1ZTF3-F1-MODEL\_V4 | 1.0 | 2.085e-08 | 286 | 0.214 | 177 | 106 | 10 | 4 | 154 | 1 | 170 | Single-stranded DNA-binding protein | Single-stranded DNA-binding protein | | afdb-uniprot50 | AF-A0A1Q4TRE2-F1-MODEL\_V4 | 1.0 | 2.585e-08 | 285 | 0.203 | 172 | 116 | 7 | 6 | 162 | 4 | 169 | Single-stranded DNA-binding protein | Single-stranded DNA-binding protein | | afdb-uniprot50 | AF-A0A2S9YZ45-F1-MODEL\_V4 | 1.0 | 2.45e-08 | 285 | 0.209 | 181 | 119 | 7 | 4 | 162 | 2 | 180 | Uncharacterized protein | Uncharacterized protein | | afdb-uniprot50 | AF-X8AMK4-F1-MODEL\_V4 | 1.0 | 8.891e-08 | 284 | 0.225 | 151 | 104 | 6 | 6 | 151 | 4 | 146 | Single-stranded DNA-binding protein | Single-stranded DNA-binding protein | | afdb-uniprot50 | AF-A0A4R4ZQF2-F1-MODEL\_V4 | 1.0 | 6.797e-08 | 284 | 0.195 | 164 | 108 | 5 | 4 | 162 | 2 | 146 | Single-stranded DNA-binding protein | Single-stranded DNA-binding protein | | afdb-uniprot50 | AF-A0A100XCE1-F1-MODEL\_V4 | 1.0 | 5.483e-08 | 282 | 0.191 | 162 | 121 | 5 | 6 | 160 | 4 | 162 | Single-stranded DNA-binding protein | Single-stranded DNA-binding protein | | afdb-uniprot50 | AF-A0A8A6ALU5-F1-MODEL\_V4 | 1.0 | 4.925e-08 | 281 | 0.18 | 166 | 123 | 7 | 4 | 162 | 2 | 161 | Single-stranded DNA-binding protein | Single-stranded DNA-binding protein | | afdb-uniprot50 | AF-A0A1W9ZUU9-F1-MODEL\_V4 | 1.0 | 4.925e-08 | 280 | 0.195 | 164 | 122 | 7 | 6 | 162 | 4 | 164 | Uncharacterized protein | Uncharacterized protein | | afdb-uniprot50 | AF-A0A430FPW2-F1-MODEL\_V4 | 1.0 | 1.227e-07 | 280 | 0.232 | 172 | 118 | 6 | 1 | 162 | 2 | 169 | Single-strand binding protein | Single-strand binding protein | | afdb-uniprot50 | AF-A0A3R6LGZ0-F1-MODEL\_V4 | 1.0 | 1.873e-08 | 278 | 0.194 | 149 | 105 | 5 | 2 | 148 | 16 | 151 | Single-stranded DNA-binding protein | Single-stranded DNA-binding protein | | afdb-uniprot50 | AF-A0A1X0IMP9-F1-MODEL\_V4 | 1.0 | 9.9e-08 | 278 | 0.185 | 156 | 116 | 6 | 6 | 156 | 4 | 153 | Single-stranded DNA-binding protein | Single-stranded DNA-binding protein | | afdb-uniprot50 | AF-A0A4U2YR72-F1-MODEL\_V4 | 1.0 | 1.694e-07 | 278 | 0.18 | 166 | 123 | 8 | 1 | 160 | 1 | 159 | Single-stranded DNA-binding protein | Single-stranded DNA-binding protein | | afdb-uniprot50 | AF-A0A1X1TEZ9-F1-MODEL\_V4 | 1.0 | 1.366e-07 | 277 | 0.171 | 157 | 121 | 6 | 4 | 154 | 1 | 154 | Single-stranded DNA-binding protein | Single-stranded DNA-binding protein | | afdb-uniprot50 | AF-A0A3A3Z1T3-F1-MODEL\_V4 | 1.0 | 4.192e-08 | 277 | 0.235 | 153 | 105 | 3 | 1 | 151 | 1 | 143 | Single-stranded DNA-binding protein | Single-stranded DNA-binding protein | | afdb-uniprot50 | AF-A0A7Z0J5J7-F1-MODEL\_V4 | 1.0 | 5.196e-08 | 277 | 0.202 | 163 | 118 | 7 | 6 | 162 | 3 | 159 | Single-strand DNA-binding protein | Single-strand DNA-binding protein | | afdb-uniprot50 | AF-C5BXK5-F1-MODEL\_V4 | 1.0 | 7.568e-08 | 276 | 0.18 | 166 | 125 | 6 | 4 | 162 | 2 | 163 | Single-stranded DNA-binding protein | Single-stranded DNA-binding protein | | afdb-uniprot50 | AF-A0A7G6Z5V3-F1-MODEL\_V4 | 1.0 | 7.986e-08 | 276 | 0.164 | 158 | 119 | 7 | 4 | 157 | 3 | 151 | Single-stranded DNA-binding protein | Single-stranded DNA-binding protein | | afdb-uniprot50 | AF-A0A101AME2-F1-MODEL\_V4 | 1.0 | 1.227e-07 | 274 | 0.175 | 160 | 122 | 6 | 6 | 162 | 4 | 156 | Single-stranded DNA-binding protein | Single-stranded DNA-binding protein | | afdb-uniprot50 | AF-A0A3P1SET8-F1-MODEL\_V4 | 1.0 | 4.667e-08 | 272 | 0.17 | 170 | 124 | 8 | 1 | 162 | 1 | 161 | Single-stranded DNA-binding protein | Single-stranded DNA-binding protein | | afdb-uniprot50 | AF-A0A0R3FMM0-F1-MODEL\_V4 | 1.0 | 1.163e-07 | 271 | 0.183 | 158 | 117 | 7 | 4 | 155 | 1 | 152 | Single-stranded DNA-binding protein | Single-stranded DNA-binding protein | | afdb-uniprot50 | AF-A0A3N4ZQ97-F1-MODEL\_V4 | 1.0 | 1.045e-07 | 270 | 0.172 | 185 | 123 | 6 | 3 | 162 | 2 | 181 | Single-stranded DNA-binding protein | Single-stranded DNA-binding protein | | afdb-uniprot50 | AF-A0A1Y0C1S9-F1-MODEL\_V4 | 1.0 | 9.9e-08 | 269 | 0.218 | 160 | 112 | 5 | 2 | 160 | 22 | 169 | Single-stranded DNA-binding protein | Single-stranded DNA-binding protein | | afdb-uniprot50 | AF-A0A7S8MY85-F1-MODEL\_V4 | 1.0 | 9.382e-08 | 268 | 0.173 | 161 | 122 | 7 | 1 | 154 | 1 | 157 | Single-stranded DNA-binding protein | Single-stranded DNA-binding protein | | afdb-uniprot50 | AF-A0A1Q4A4B6-F1-MODEL\_V4 | 1.0 | 1.787e-07 | 266 | 0.133 | 165 | 132 | 6 | 3 | 162 | 2 | 160 | Single-stranded DNA-binding protein | Single-stranded DNA-binding protein | | afdb-uniprot50 | AF-A0A061LX28-F1-MODEL\_V4 | 1.0 | 2.1e-07 | 266 | 0.18 | 166 | 127 | 6 | 1 | 160 | 1 | 163 | Single-stranded DNA-binding protein | Single-stranded DNA-binding protein | | afdb-uniprot50 | AF-A0A7T2TH27-F1-MODEL\_V4 | 1.0 | 3.791e-07 | 265 | 0.165 | 157 | 126 | 5 | 1 | 156 | 1 | 153 | Single-stranded DNA-binding protein | Single-stranded DNA-binding protein | | afdb-uniprot50 | AF-A0A086ZVX5-F1-MODEL\_V4 | 1.0 | 6.105e-08 | 265 | 0.217 | 179 | 116 | 9 | 1 | 159 | 1 | 175 | Single-strand binding protein | Single-strand binding protein | | afdb-uniprot50 | AF-A0A1E9HDT3-F1-MODEL\_V4 | 1.0 | 1.102e-07 | 263 | 0.215 | 167 | 117 | 8 | 1 | 162 | 5 | 162 | Uncharacterized protein | Uncharacterized protein | | afdb-uniprot50 | AF-A0A4Q7NSB1-F1-MODEL\_V4 | 1.0 | 1.99e-07 | 262 | 0.168 | 166 | 120 | 7 | 4 | 154 | 1 | 163 | Single-stranded DNA-binding protein | Single-stranded DNA-binding protein | | afdb-uniprot50 | AF-A0A7Y9YDV5-F1-MODEL\_V4 | 1.0 | 2.467e-07 | 260 | 0.14 | 164 | 129 | 7 | 1 | 156 | 1 | 160 | Single-stranded DNA-binding protein | Single-stranded DNA-binding protein | | afdb-uniprot50 | AF-A0A178LXY2-F1-MODEL\_V4 | 1.0 | 1.99e-07 | 258 | 0.175 | 160 | 117 | 5 | 6 | 160 | 4 | 153 | Single-stranded DNA-binding protein | Single-stranded DNA-binding protein | | afdb-uniprot50 | AF-A0A4P6MVX0-F1-MODEL\_V4 | 1.0 | 1.605e-07 | 258 | 0.171 | 181 | 116 | 8 | 5 | 154 | 1 | 178 | Single-stranded DNA-binding protein | Single-stranded DNA-binding protein | | afdb-uniprot50 | AF-A0A2S0KF83-F1-MODEL\_V4 | 1.0 | 2.603e-07 | 257 | 0.191 | 157 | 115 | 7 | 7 | 154 | 5 | 158 | Single-stranded DNA-binding protein | Single-stranded DNA-binding protein | | afdb-uniprot50 | AF-A0A7Z0EC79-F1-MODEL\_V4 | 1.0 | 6.845e-07 | 254 | 0.135 | 155 | 123 | 7 | 1 | 150 | 54 | 202 | Single-stranded DNA-binding protein | Single-stranded DNA-binding protein | | afdb-uniprot50 | AF-A0A6N4W4B9-F1-MODEL\_V4 | 1.0 | 4.959e-07 | 248 | 0.155 | 161 | 122 | 6 | 7 | 156 | 5 | 162 | Single-stranded DNA-binding protein | Single-stranded DNA-binding protein | | afdb-uniprot50 | AF-A0A2D9NY87-F1-MODEL\_V4 | 1.0 | 2.603e-07 | 245 | 0.173 | 167 | 125 | 6 | 1 | 162 | 14 | 172 | Uncharacterized protein | Uncharacterized protein | |
| Top keywords  (threshold 1.00e-02 (evalue)) | **DNA\_binding, Single\_stranded, Single\_strand, binding, replication, mitochondrial, Primosomal, n, A, complex** |
| Output files | ../../similar\_structures/35\_FANPEZAQ\_CDS\_0035\_afdb-proteome\_foldseek.tsv ../../similar\_structures/35\_FANPEZAQ\_CDS\_0035\_afdb-uniprot50\_foldseek.tsv ../../similar\_structures/35\_FANPEZAQ\_CDS\_0035\_merged.svg ../../similar\_structures/35\_FANPEZAQ\_CDS\_0035\_pdb\_foldseek.tsv |

  
  
  

Return to summary | Go to previous | Go to next

  


---

**Sequence/structure alignments coloring**  
Each object in the alignment figures is colored according to its E-value following this color coding:

1e-100
10

**References:**  
1) Steinegger M, Meier M, Mirdita M, Vöhringer H, Haunsberger S J, and Söding J (2019) HH-suite3 for fast remote homology detection and deep protein annotation, BMC Bioinformatics, 473. doi: 10.1186/s12859-019-3019-7  
2) Jumper J, Evans R, Pritzel A, ..., Hassabis D (2021) Highly accurate protein structure prediction with AlphaFold, Nature, 596. doi: 10.1038/s41586-021-03819-2  
3) van Kempen M, Kim S, Tumescheit C, Mirdita M, Lee J, Gilchrist CLM, Söding J, and Steinegger M (2023) Fast and accurate protein structure search with Foldseek. Nature Biotechnology. doi: 10.1038/s41587-023-01773-0
