# Supplementary material for: Completing the BASEL phage collection to unlock hidden diversity for systematic exploration of phage–host interactions
Source: PLoS Biol. 2025 Apr 7;23(4):e3003063. doi: 10.1371/journal.pbio.3003063 (PMC11990801; doi:10.1371/journal.pbio.3003063)
Supplement: S2 Data — (ZIP) [file pbio.3003063.s009.zip › entries/36.html]

FANPEZAQ\_CDS\_0036


Return to summary | Go to previous | Go to next

|  |  |
| --- | --- |
| FANPEZAQ\_CDS\_0036 Page creation date: 02 Sep 2024, 12:00  Project folder: n/a  Input sequences file: Escherichia\_virus\_HeidiAbel.gb | dksa c4\_type domain\_containing trar transcriptional regulator zinc finger dnak rna conjugal transfer polymerase\_binding transcription factor fragment suppressor molecular chaperone dna\_binding general stress 16o duf2116 zn\_ribbon putative phage polymerase plasmid c\_4 type hypothetical dna\_directed alpha structural zn |

### Sequence information

|  |  |
| --- | --- |
| Name | FANPEZAQ\_CDS\_0036  36\_FANPEZAQ\_CDS\_0036 (pipeline id) |
| Imported annotations |  |
| Protein sequence | MTTNSRTIAALFAVSRMVTTTKSARLHLVNSGCTMADDIDRACEREEQERAAMIAAHLQK APAVKAVARGYCLNCGEDFPEGSKKIYCDADCANDHAAHLKRK |
| Number of residues | 103 |
| Molecular weight (Da) | 11242.75 |
| Output files | ../../query\_sequences/36\_FANPEZAQ\_CDS\_0036.fasta |

### Putative domain architecture and protein family

#### Search results (HHblits)1

|  |  |
| --- | --- |
| Domain family databases searched | Pfam, Ncbi-cd, Cath, Phrogs |
| Results, scheme(s)  (Top layers only; threshold 1.00e-03 (evalue)) | xml version="1.0" encoding="utf-8" standalone="no"?       2024-09-02T21:08:20.209596 image/svg+xml   Matplotlib v3.7.2, https://matplotlib.org/ |
| Results, table  (E-value ≤ 1.00e-03 (evalue)) | | db | id | prob | evalue | pvalue | score | cols | query | query\_len | template | template\_len | name | description | | --- | --- | --- | --- | --- | --- | --- | --- | --- | --- | --- | --- | --- | | phrogs | 54 | 99.7 | 1.4e-21 | 2e-25 | 120.1 | 62 | (35, 98) | 103 | (1, 62) | 63 | DksA-like zinc-finger protein | DksA-like zinc-finger protein; Category: other; MF490239\_p53 | |
| Top keywords  (threshold 1.00e-03 (evalue)) | **DksA\_like, zinc\_finger, other, MF490239\_p53** |
| Output files | ../../domain\_architecture/36\_FANPEZAQ\_CDS\_0036\_cath.hhr ../../domain\_architecture/36\_FANPEZAQ\_CDS\_0036\_merged.svg ../../domain\_architecture/36\_FANPEZAQ\_CDS\_0036\_ncbi-cd.hhr ../../domain\_architecture/36\_FANPEZAQ\_CDS\_0036\_pfam.hhr ../../domain\_architecture/36\_FANPEZAQ\_CDS\_0036\_phrogs.hhr |

### Identical protein sequences/structures

#### Search results

|  |  |
| --- | --- |
| Protein sequence databases searched | Pdb, Swissprot, Refseq |
| Identical proteins found | -- |
| Top keywords | -- |
| Output files | -- |

### Similar protein sequences/structures

#### Sequence similarity search results (HHblits)1

|  |  |
| --- | --- |
| Sequence databases searched | Uniclust, Pdb70 |
| Results, scheme(s)  (Top layers only, threshold 1.00e-03 (evalue)) | xml version="1.0" encoding="utf-8" standalone="no"?       2024-09-02T21:08:45.027118 image/svg+xml   Matplotlib v3.7.2, https://matplotlib.org/ |
| Results, table(s)  (threshold 1.00e-03 (evalue)) | | db | id | prob | evalue | pvalue | score | cols | query | query\_len | template | template\_len | name | description | | --- | --- | --- | --- | --- | --- | --- | --- | --- | --- | --- | --- | --- | | uniclust | UniRef100\_A0A061JN07 | 99.5 | 1.1e-17 | 2.5e-23 | 113.6 | 90 | (6, 101) | 103 | (6, 100) | 109 | DksA C4-type domain-containing protein | DksA C4-type domain-containing protein | | uniclust | UniRef100\_A0A031IV80 | 99.5 | 1.4e-17 | 3.5e-23 | 108.5 | 67 | (33, 101) | 103 | (1, 71) | 82 | Prophage PSPPH06, DksA/TraR family C4-type zinc finger protein | Prophage PSPPH06, DksA/TraR family C4-type zinc finger protein | | uniclust | UniRef100\_A0A084Z0P9 | 99.5 | 2.7e-17 | 6.6e-23 | 110.6 | 72 | (29, 102) | 103 | (2, 78) | 98 | DksA C4-type domain-containing protein | DksA C4-type domain-containing protein | | uniclust | UniRef100\_A0A022PBT0 | 99.5 | 3.5e-17 | 8.4e-23 | 106.2 | 67 | (32, 102) | 103 | (1, 72) | 79 | Transcriptional regulator, TraR/DksA family | Transcriptional regulator, TraR/DksA family | | uniclust | UniRef100\_A0A059ZYI4 | 99.5 | 1.8e-16 | 4.3e-22 | 108.5 | 71 | (28, 101) | 103 | (11, 87) | 108 | DksA C4-type domain-containing protein | DksA C4-type domain-containing protein | | uniclust | UniRef100\_A0A009YU33 | 99.4 | 2.6e-16 | 6.4e-22 | 106.0 | 71 | (29, 102) | 103 | (2, 77) | 98 | Prokaryotic dksA/traR C4-type zinc finger family protein | Prokaryotic dksA/traR C4-type zinc finger family protein | | uniclust | UniRef100\_A0A011MGP2 | 99.4 | 4.7e-16 | 1.1e-21 | 107.7 | 68 | (28, 99) | 103 | (8, 80) | 117 | Molecular chaperone DnaK | Molecular chaperone DnaK | | uniclust | UniRef100\_A0A0F2KS34 | 99.4 | 8.5e-16 | 2e-21 | 102.0 | 68 | (30, 100) | 103 | (7, 79) | 89 | DksA C4-type domain-containing protein | DksA C4-type domain-containing protein | | uniclust | UniRef100\_A0A0A8RLH0 | 99.4 | 1.1e-15 | 2.6e-21 | 100.0 | 66 | (32, 102) | 103 | (6, 76) | 83 | Uncharacterized protein | Uncharacterized protein | | uniclust | UniRef100\_A0A086CZD8 | 99.4 | 2.2e-15 | 5.1e-21 | 100.1 | 74 | (27, 102) | 103 | (5, 83) | 90 | DksA C4-type domain-containing protein | DksA C4-type domain-containing protein | | uniclust | UniRef100\_A0A023WVJ6 | 99.3 | 2.6e-15 | 6e-21 | 106.3 | 71 | (27, 101) | 103 | (42, 117) | 136 | Conjugal transfer protein TraR | Conjugal transfer protein TraR | | uniclust | UniRef100\_A0A0H3PCF4 | 99.3 | 3.9e-15 | 8.2e-21 | 96.9 | 69 | (32, 102) | 103 | (2, 75) | 83 | DksA C4-type domain-containing protein | DksA C4-type domain-containing protein | | uniclust | UniRef100\_A0A0C5DZD0 | 99.3 | 9.9e-15 | 2.3e-20 | 93.9 | 65 | (35, 102) | 103 | (1, 70) | 74 | DksA C4-type domain-containing protein | DksA C4-type domain-containing protein | | uniclust | UniRef100\_A0A090IG19 | 99.3 | 1.4e-14 | 3.3e-20 | 94.9 | 65 | (35, 101) | 103 | (1, 74) | 82 | Uncharacterized phage protein | Uncharacterized phage protein | | uniclust | UniRef100\_A0A011NC41 | 99.2 | 2.9e-14 | 6.3e-20 | 95.6 | 71 | (30, 101) | 103 | (7, 85) | 98 | DksA-like zinc finger domain containing protein | DksA-like zinc finger domain containing protein | | uniclust | UniRef100\_A0A0C1EKQ1 | 99.2 | 3.3e-14 | 6.5e-20 | 94.4 | 70 | (27, 99) | 103 | (12, 86) | 97 | DksA C4-type domain-containing protein | DksA C4-type domain-containing protein | | uniclust | UniRef100\_A0A2A5G8T1 | 99.2 | 3.8e-14 | 8.9e-20 | 92.8 | 67 | (35, 102) | 103 | (1, 75) | 81 | Conjugal transfer protein TraR | Conjugal transfer protein TraR | | uniclust | UniRef100\_A0A140D8Z6 | 99.2 | 4.7e-14 | 1.1e-19 | 96.1 | 69 | (32, 102) | 103 | (1, 74) | 104 | Conjugal transfer protein TraR | Conjugal transfer protein TraR | | uniclust | UniRef100\_A0A0N0E3Y8 | 99.2 | 6e-14 | 1.3e-19 | 90.2 | 68 | (34, 102) | 103 | (1, 73) | 75 | Transcriptional regulator, TraR/DksA family | Transcriptional regulator, TraR/DksA family | | uniclust | UniRef100\_A0A0Q4N459 | 99.2 | 8.9e-14 | 2.1e-19 | 93.5 | 68 | (31, 102) | 103 | (5, 72) | 95 | DUF2116 family Zn-ribbon domain-containing protein | DUF2116 family Zn-ribbon domain-containing protein | | uniclust | UniRef100\_A0A0A2X9Z0 | 99.2 | 1.1e-13 | 2.4e-19 | 91.9 | 70 | (31, 101) | 103 | (4, 80) | 88 | DksA C4-type domain-containing protein | DksA C4-type domain-containing protein | | uniclust | UniRef100\_A0A3M1K5W8 | 99.1 | 2.2e-13 | 4.9e-19 | 90.1 | 69 | (32, 101) | 103 | (3, 80) | 87 | TraR/DksA family transcriptional regulator | TraR/DksA family transcriptional regulator | | uniclust | UniRef100\_A0A060H8P7 | 99.1 | 3.7e-13 | 8.5e-19 | 107.5 | 75 | (27, 102) | 103 | (233, 320) | 329 | RNA polymerase-binding transcription factor DksA | RNA polymerase-binding transcription factor DksA | | uniclust | UniRef100\_A0A317Q359 | 99.1 | 5e-13 | 1e-18 | 85.7 | 64 | (35, 102) | 103 | (3, 71) | 75 | Phage/conjugal plasmid C-4 type zinc finger TraR family protein (Fragment) | Phage/conjugal plasmid C-4 type zinc finger TraR family protein (Fragment) | | uniclust | UniRef100\_A0A010RU16 | 99.1 | 6.4e-13 | 1.4e-18 | 85.7 | 64 | (33, 101) | 103 | (1, 69) | 74 | Conjugal transfer protein TraR | Conjugal transfer protein TraR | | uniclust | UniRef100\_A0A193QIP3 | 99.1 | 6.3e-13 | 1.5e-18 | 90.0 | 69 | (28, 102) | 103 | (9, 77) | 97 | Phage protein | Phage protein | | uniclust | UniRef100\_A0A1W1XTU0 | 99.1 | 7.9e-13 | 1.8e-18 | 86.7 | 63 | (33, 101) | 103 | (5, 67) | 81 | DUF2116 family Zn-ribbon domain-containing protein | DUF2116 family Zn-ribbon domain-containing protein | | uniclust | UniRef100\_A0A177QP03 | 99.1 | 9e-13 | 2e-18 | 87.1 | 69 | (30, 101) | 103 | (4, 75) | 84 | DksA C4-type domain-containing protein | DksA C4-type domain-containing protein | | uniclust | UniRef100\_A0A034SXG4 | 99.0 | 1e-12 | 2.4e-18 | 89.6 | 75 | (15, 102) | 103 | (2, 76) | 101 | DUF2116 family Zn-ribbon domain-containing protein | DUF2116 family Zn-ribbon domain-containing protein | | uniclust | UniRef100\_A0A062GXM3 | 99.0 | 1.1e-12 | 2.4e-18 | 86.8 | 66 | (32, 102) | 103 | (1, 70) | 87 | Prokaryotic dksA/traR C4-type zinc finger family protein | Prokaryotic dksA/traR C4-type zinc finger family protein | | uniclust | UniRef100\_A0A0Q8QW33 | 99.0 | 1.5e-12 | 3.4e-18 | 86.9 | 67 | (31, 102) | 103 | (7, 73) | 89 | DUF2116 family Zn-ribbon domain-containing protein | DUF2116 family Zn-ribbon domain-containing protein | | uniclust | UniRef100\_A0A1U9ZAF7 | 99.0 | 2.2e-12 | 4.4e-18 | 81.2 | 63 | (35, 101) | 103 | (1, 63) | 67 | Uncharacterized protein | Uncharacterized protein | | uniclust | UniRef100\_A0A0J7J789 | 99.0 | 2.1e-12 | 4.5e-18 | 86.4 | 60 | (38, 101) | 103 | (21, 85) | 92 | Transcriptional regulator, TraR/DksA family | Transcriptional regulator, TraR/DksA family | | uniclust | UniRef100\_A0A091AIU5 | 99.0 | 1.8e-12 | 4.6e-18 | 96.1 | 65 | (35, 100) | 103 | (76, 153) | 174 | DksA C4-type domain-containing protein | DksA C4-type domain-containing protein | | uniclust | UniRef100\_A0A0H3A990 | 99.0 | 2.5e-12 | 5e-18 | 84.4 | 68 | (32, 100) | 103 | (3, 79) | 87 | Transcriptional regulator, TraR/DksA family | Transcriptional regulator, TraR/DksA family | | uniclust | UniRef100\_A0A1G3M3M1 | 99.0 | 4e-12 | 8e-18 | 80.8 | 62 | (36, 99) | 103 | (1, 67) | 72 | DksA C4-type domain-containing protein (Fragment) | DksA C4-type domain-containing protein (Fragment) | | uniclust | UniRef100\_A0A031GDH3 | 99.0 | 3.7e-12 | 9.6e-18 | 94.2 | 68 | (32, 100) | 103 | (65, 145) | 169 | C4-type zinc finger protein, DksA/TraR family | C4-type zinc finger protein, DksA/TraR family | | uniclust | UniRef100\_A0A1F2RT93 | 99.0 | 3.9e-12 | 9.7e-18 | 93.9 | 69 | (31, 100) | 103 | (61, 142) | 168 | DksA C4-type domain-containing protein | DksA C4-type domain-containing protein | | uniclust | UniRef100\_A0A0B0VDY3 | 98.9 | 5.4e-12 | 1.1e-17 | 84.7 | 70 | (31, 102) | 103 | (7, 81) | 96 | Conjugal transfer protein TraR | Conjugal transfer protein TraR | | uniclust | UniRef100\_A0A0H3JK08 | 98.9 | 6.6e-12 | 1.5e-17 | 84.4 | 64 | (33, 100) | 103 | (9, 78) | 91 | RNA polymerase-binding transcription factor | RNA polymerase-binding transcription factor | | uniclust | UniRef100\_A0A1B9JT42 | 98.9 | 8.2e-12 | 1.7e-17 | 80.4 | 64 | (35, 100) | 103 | (1, 69) | 74 | DksA C4-type domain-containing protein | DksA C4-type domain-containing protein | | uniclust | UniRef100\_A0A0A1VI61 | 98.9 | 7.6e-12 | 2e-17 | 92.0 | 67 | (34, 101) | 103 | (59, 138) | 161 | DnaK suppressor protein | DnaK suppressor protein | | uniclust | UniRef100\_A0A076FE71 | 98.9 | 7.9e-12 | 2e-17 | 93.6 | 70 | (32, 102) | 103 | (81, 163) | 184 | DnaK suppressor protein | DnaK suppressor protein | | uniclust | UniRef100\_A0A1G2UZM8 | 98.9 | 9.3e-12 | 2.2e-17 | 90.4 | 71 | (28, 99) | 103 | (48, 131) | 149 | DksA C4-type domain-containing protein | DksA C4-type domain-containing protein | | uniclust | UniRef100\_A0A0C2UA16 | 98.9 | 1.1e-11 | 2.4e-17 | 80.9 | 65 | (35, 102) | 103 | (1, 70) | 79 | DksA C4-type domain-containing protein | DksA C4-type domain-containing protein | | uniclust | UniRef100\_A0A059XN07 | 98.9 | 1e-11 | 2.8e-17 | 94.5 | 65 | (35, 100) | 103 | (74, 151) | 208 | Conjugal transfer protein TraR | Conjugal transfer protein TraR | | uniclust | UniRef100\_A0A1G8NB44 | 98.9 | 1.3e-11 | 2.8e-17 | 81.1 | 67 | (32, 102) | 103 | (4, 77) | 81 | DksA/traR C4-type zinc finger | DksA/traR C4-type zinc finger | | uniclust | UniRef100\_A0A1T4LZW9 | 98.9 | 1.5e-11 | 3.3e-17 | 94.3 | 70 | (31, 101) | 103 | (120, 202) | 226 | Transcriptional regulator, TraR/DksA family | Transcriptional regulator, TraR/DksA family | | uniclust | UniRef100\_A0A2E4HLP3 | 98.9 | 1.6e-11 | 3.4e-17 | 84.1 | 72 | (30, 102) | 103 | (10, 94) | 104 | DksA C4-type domain-containing protein | DksA C4-type domain-containing protein | | uniclust | UniRef100\_A0A0P0MAF7 | 98.9 | 1.6e-11 | 3.5e-17 | 101.7 | 72 | (29, 101) | 103 | (350, 434) | 444 | RNA polymerase-binding transcription factor DksA | RNA polymerase-binding transcription factor DksA | | uniclust | UniRef100\_A0A0K2G9N8 | 98.8 | 1.9e-11 | 4.6e-17 | 94.1 | 69 | (31, 100) | 103 | (129, 210) | 228 | DnaK suppressor protein (Modular protein) | DnaK suppressor protein (Modular protein) | | uniclust | UniRef100\_A0A1I3XGG3 | 98.8 | 3.2e-11 | 6.8e-17 | 87.3 | 77 | (23, 100) | 103 | (50, 139) | 155 | Transcriptional regulator, TraR/DksA family | Transcriptional regulator, TraR/DksA family | | uniclust | UniRef100\_A0A2H4TYU1 | 98.8 | 3.7e-11 | 7.2e-17 | 77.0 | 65 | (34, 102) | 103 | (1, 70) | 76 | Uncharacterized protein | Uncharacterized protein | | uniclust | UniRef100\_A0A0G0NEV6 | 98.8 | 4.1e-11 | 8.6e-17 | 85.9 | 68 | (35, 103) | 103 | (62, 142) | 144 | Transcriptional regulator, TraR/DksA family | Transcriptional regulator, TraR/DksA family | | uniclust | UniRef100\_A0A031GZT8 | 98.8 | 3.9e-11 | 8.6e-17 | 81.9 | 68 | (30, 101) | 103 | (9, 76) | 101 | DUF2116 family Zn-ribbon domain-containing protein | DUF2116 family Zn-ribbon domain-containing protein | | uniclust | UniRef100\_A0A1F5P3U3 | 98.8 | 5.4e-11 | 1.1e-16 | 85.0 | 67 | (34, 101) | 103 | (40, 119) | 139 | DksA C4-type domain-containing protein | DksA C4-type domain-containing protein | | uniclust | UniRef100\_A0A1F2RZ99 | 98.8 | 5e-11 | 1.1e-16 | 89.2 | 64 | (33, 97) | 103 | (81, 157) | 183 | DksA C4-type domain-containing protein | DksA C4-type domain-containing protein | | uniclust | UniRef100\_A0A109RVY0 | 98.8 | 5.6e-11 | 1.2e-16 | 77.4 | 66 | (35, 102) | 103 | (1, 66) | 78 | Uncharacterized protein | Uncharacterized protein | | uniclust | UniRef100\_A0A017TF53 | 98.8 | 4.8e-11 | 1.2e-16 | 90.8 | 67 | (34, 101) | 103 | (92, 171) | 202 | C4-type zinc finger protein, DksA/TraR family | C4-type zinc finger protein, DksA/TraR family | | uniclust | UniRef100\_A0A072T172 | 98.7 | 5.8e-11 | 1.5e-16 | 87.6 | 67 | (33, 100) | 103 | (61, 140) | 161 | TraR/DksA family transcriptional regulator | TraR/DksA family transcriptional regulator | | uniclust | UniRef100\_A0A1F9QKH6 | 98.7 | 6.5e-11 | 1.5e-16 | 88.5 | 71 | (29, 100) | 103 | (91, 174) | 179 | DksA C4-type domain-containing protein | DksA C4-type domain-containing protein | | uniclust | UniRef100\_A0A023D0P2 | 98.7 | 7.6e-11 | 1.6e-16 | 88.8 | 73 | (28, 101) | 103 | (112, 197) | 204 | RNA polymerase-binding transcription factor DksA | RNA polymerase-binding transcription factor DksA | | uniclust | UniRef100\_A0A099KCV7 | 98.7 | 7.7e-11 | 1.7e-16 | 84.8 | 68 | (32, 100) | 103 | (51, 131) | 143 | Transcriptional regulator, TraR/DksA family | Transcriptional regulator, TraR/DksA family | | uniclust | UniRef100\_A0A021XD72 | 98.7 | 6.4e-11 | 1.7e-16 | 90.5 | 63 | (38, 101) | 103 | (102, 177) | 209 | DnaK suppressor protein | DnaK suppressor protein | | uniclust | UniRef100\_A0A1F8GT99 | 98.7 | 7.4e-11 | 1.8e-16 | 86.6 | 70 | (31, 101) | 103 | (51, 133) | 156 | DksA C4-type domain-containing protein | DksA C4-type domain-containing protein | | uniclust | UniRef100\_A0A0M9UC17 | 98.7 | 8.3e-11 | 2e-16 | 87.3 | 70 | (32, 102) | 103 | (59, 141) | 169 | DksA C4-type domain-containing protein | DksA C4-type domain-containing protein | | uniclust | UniRef100\_A0A1F3SZI2 | 98.7 | 1.1e-10 | 2.1e-16 | 82.0 | 65 | (36, 101) | 103 | (43, 120) | 132 | DksA C4-type domain-containing protein | DksA C4-type domain-containing protein | | uniclust | UniRef100\_A0A124FE87 | 98.7 | 9.6e-11 | 2.2e-16 | 86.8 | 68 | (33, 101) | 103 | (64, 144) | 170 | Transcriptional regulator, TraR/DksA family | Transcriptional regulator, TraR/DksA family | | uniclust | UniRef100\_A0A0H3KWF3 | 98.7 | 1.3e-10 | 2.4e-16 | 79.3 | 92 | (5, 101) | 103 | (6, 105) | 121 | DksA C4-type domain-containing protein | DksA C4-type domain-containing protein | | uniclust | UniRef100\_A0A011SK02 | 98.7 | 9.5e-11 | 2.5e-16 | 87.3 | 64 | (36, 100) | 103 | (78, 154) | 173 | Molecular chaperone DnaK | Molecular chaperone DnaK | | uniclust | UniRef100\_A0A0D6QI25 | 98.7 | 1.1e-10 | 2.5e-16 | 85.4 | 65 | (32, 97) | 103 | (49, 126) | 157 | RNA polymerase-binding transcription factor DksA | RNA polymerase-binding transcription factor DksA | | uniclust | UniRef100\_A0A085GNS1 | 98.7 | 1.4e-10 | 2.9e-16 | 87.2 | 75 | (27, 102) | 103 | (93, 180) | 198 | RNA polymerase-binding transcription factor DksA | RNA polymerase-binding transcription factor DksA | | uniclust | UniRef100\_A0A010SR92 | 98.7 | 1.6e-10 | 3.3e-16 | 90.9 | 75 | (27, 102) | 103 | (190, 277) | 286 | RNA polymerase-binding transcription factor DksA | RNA polymerase-binding transcription factor DksA | | uniclust | UniRef100\_A0A1B1EDL0 | 98.7 | 1.9e-10 | 3.9e-16 | 74.7 | 66 | (35, 101) | 103 | (1, 74) | 78 | DksA C4-type domain-containing protein | DksA C4-type domain-containing protein | | uniclust | UniRef100\_A0A106QCK5 | 98.7 | 2e-10 | 4.2e-16 | 80.0 | 73 | (29, 102) | 103 | (22, 99) | 115 | DksA C4-type domain-containing protein | DksA C4-type domain-containing protein | | uniclust | UniRef100\_A0A1F9ALS5 | 98.7 | 2e-10 | 4.2e-16 | 82.6 | 69 | (31, 100) | 103 | (58, 139) | 145 | DksA C4-type domain-containing protein | DksA C4-type domain-containing protein | | uniclust | UniRef100\_A0A1V4XBY8 | 98.7 | 2.3e-10 | 4.7e-16 | 75.6 | 65 | (36, 101) | 103 | (1, 84) | 86 | RNA polymerase-binding transcription factor DksA | RNA polymerase-binding transcription factor DksA | | uniclust | UniRef100\_A0A1G1JYH2 | 98.6 | 2.2e-10 | 5e-16 | 87.9 | 65 | (36, 101) | 103 | (135, 212) | 218 | DksA C4-type domain-containing protein | DksA C4-type domain-containing protein | | uniclust | UniRef100\_A0A013XQT7 | 98.6 | 2.3e-10 | 5.8e-16 | 84.7 | 66 | (35, 101) | 103 | (74, 152) | 163 | DksA C4-type domain-containing protein | DksA C4-type domain-containing protein | | uniclust | UniRef100\_A0A1J4Z2A6 | 98.6 | 2.5e-10 | 6e-16 | 82.9 | 66 | (35, 101) | 103 | (58, 136) | 144 | DksA C4-type domain-containing protein | DksA C4-type domain-containing protein | | uniclust | UniRef100\_A0A523N3V7 | 98.6 | 3e-10 | 6e-16 | 78.0 | 65 | (34, 99) | 103 | (29, 106) | 109 | DksA C4-type domain-containing protein | DksA C4-type domain-containing protein | | uniclust | UniRef100\_A0A011U6K4 | 98.6 | 2.9e-10 | 6.5e-16 | 88.9 | 67 | (34, 101) | 103 | (125, 204) | 260 | RNA polymerase-binding transcription factor DksA | RNA polymerase-binding transcription factor DksA | | uniclust | UniRef100\_A0A656H975 | 98.6 | 3.7e-10 | 7.3e-16 | 74.3 | 67 | (35, 102) | 103 | (1, 73) | 85 | C4-type zinc finger DksA/TraR family protein | C4-type zinc finger DksA/TraR family protein | | uniclust | UniRef100\_A0A0S7Z4A0 | 98.6 | 3.5e-10 | 7.5e-16 | 85.2 | 70 | (32, 102) | 103 | (98, 180) | 198 | DksA C4-type domain-containing protein | DksA C4-type domain-containing protein | | uniclust | UniRef100\_A0A074M994 | 98.6 | 3.3e-10 | 7.8e-16 | 80.8 | 64 | (37, 101) | 103 | (43, 119) | 126 | DksA C4-type domain-containing protein | DksA C4-type domain-containing protein | | uniclust | UniRef100\_A0A1F3BDN4 | 98.6 | 3.5e-10 | 7.9e-16 | 88.2 | 67 | (34, 101) | 103 | (145, 224) | 246 | DksA C4-type domain-containing protein | DksA C4-type domain-containing protein | | uniclust | UniRef100\_A0A0U2WWH0 | 98.6 | 4.4e-10 | 9e-16 | 72.3 | 63 | (35, 102) | 103 | (1, 67) | 72 | DksA C4-type domain-containing protein | DksA C4-type domain-containing protein | | uniclust | UniRef100\_A0A1W9HQR9 | 98.6 | 4.3e-10 | 9.3e-16 | 82.8 | 66 | (35, 101) | 103 | (42, 120) | 164 | DksA C4-type domain-containing protein | DksA C4-type domain-containing protein | | uniclust | UniRef100\_A0A1J5DNA3 | 98.6 | 4.6e-10 | 9.9e-16 | 84.0 | 71 | (30, 101) | 103 | (42, 126) | 187 | DksA C4-type domain-containing protein | DksA C4-type domain-containing protein | | uniclust | UniRef100\_A0A0F9CJ95 | 98.6 | 3.8e-10 | 1e-15 | 84.9 | 65 | (36, 101) | 103 | (86, 163) | 183 | DksA C4-type domain-containing protein | DksA C4-type domain-containing protein | | uniclust | UniRef100\_A0A011NRS6 | 98.6 | 4.7e-10 | 1e-15 | 85.9 | 74 | (28, 102) | 103 | (108, 194) | 222 | RNA polymerase-binding transcription factor DksA | RNA polymerase-binding transcription factor DksA | | uniclust | UniRef100\_A0A2M8KEH6 | 98.6 | 4.6e-10 | 1e-15 | 74.0 | 38 | (63, 101) | 103 | (33, 75) | 79 | DksA C4-type domain-containing protein (Fragment) | DksA C4-type domain-containing protein (Fragment) | | uniclust | UniRef100\_A0A0A6UQS0 | 98.6 | 5.1e-10 | 1.1e-15 | 82.6 | 65 | (36, 101) | 103 | (90, 167) | 170 | Conjugal transfer protein TraR | Conjugal transfer protein TraR | | uniclust | UniRef100\_A0A1F2QVT2 | 98.6 | 4.9e-10 | 1.2e-15 | 83.1 | 69 | (32, 101) | 103 | (59, 140) | 164 | DksA C4-type domain-containing protein | DksA C4-type domain-containing protein | | uniclust | UniRef100\_A0A0B0EK80 | 98.6 | 5.4e-10 | 1.3e-15 | 83.7 | 70 | (30, 100) | 103 | (46, 128) | 176 | DNA-binding protein | DNA-binding protein | | uniclust | UniRef100\_A0A0S8CIM9 | 98.6 | 6e-10 | 1.4e-15 | 87.1 | 72 | (30, 102) | 103 | (90, 175) | 249 | DksA C4-type domain-containing protein | DksA C4-type domain-containing protein | | uniclust | UniRef100\_A0A1Q7CPT7 | 98.6 | 6.7e-10 | 1.4e-15 | 75.5 | 63 | (38, 101) | 103 | (19, 94) | 100 | DksA C4-type domain-containing protein | DksA C4-type domain-containing protein | | uniclust | UniRef100\_A0A1H9LGI8 | 98.6 | 6.8e-10 | 1.4e-15 | 71.5 | 64 | (33, 103) | 103 | (1, 66) | 71 | Phage/conjugal plasmid C-4 type zinc finger protein, TraR family | Phage/conjugal plasmid C-4 type zinc finger protein, TraR family | | uniclust | UniRef100\_A0A2E1M7Z0 | 98.5 | 7.4e-10 | 1.5e-15 | 88.1 | 69 | (30, 99) | 103 | (232, 313) | 318 | DksA C4-type domain-containing protein | DksA C4-type domain-containing protein | | uniclust | UniRef100\_A0A059KKD9 | 98.5 | 7e-10 | 1.6e-15 | 81.8 | 62 | (39, 101) | 103 | (73, 147) | 160 | Putative transcriptional regulator, TraR/DksA family | Putative transcriptional regulator, TraR/DksA family | | uniclust | UniRef100\_A0A0B0EI80 | 98.5 | 7.9e-10 | 1.7e-15 | 79.7 | 68 | (32, 100) | 103 | (52, 132) | 142 | Transcriptional regulator | Transcriptional regulator | | uniclust | UniRef100\_A0A0A2ZRM8 | 98.5 | 8.4e-10 | 1.7e-15 | 70.8 | 63 | (36, 101) | 103 | (1, 63) | 70 | DUF2116 family Zn-ribbon domain-containing protein | DUF2116 family Zn-ribbon domain-containing protein | | uniclust | UniRef100\_A0A0Q1A6I1 | 98.5 | 7.6e-10 | 1.8e-15 | 82.1 | 66 | (36, 102) | 103 | (81, 159) | 165 | DksA C4-type domain-containing protein | DksA C4-type domain-containing protein | | uniclust | UniRef100\_A0A0B5BBV4 | 98.5 | 7.8e-10 | 1.8e-15 | 82.1 | 63 | (37, 100) | 103 | (79, 154) | 166 | Molecular chaperone DnaK | Molecular chaperone DnaK | | uniclust | UniRef100\_A0A1F9CTD9 | 98.5 | 9.5e-10 | 1.9e-15 | 79.1 | 66 | (35, 101) | 103 | (48, 126) | 148 | DksA C4-type domain-containing protein | DksA C4-type domain-containing protein | | uniclust | UniRef100\_A0A0L8AA28 | 98.5 | 9.4e-10 | 2e-15 | 74.0 | 73 | (30, 102) | 103 | (8, 83) | 91 | Conjugal transfer protein TraR | Conjugal transfer protein TraR | | uniclust | UniRef100\_A0A017HA46 | 98.5 | 9.2e-10 | 2e-15 | 78.6 | 57 | (40, 99) | 103 | (37, 98) | 131 | Putative Zinc-finger | Putative Zinc-finger | | uniclust | UniRef100\_A0A1G2SET6 | 98.5 | 9.4e-10 | 2e-15 | 76.0 | 40 | (62, 102) | 103 | (56, 100) | 106 | DksA C4-type domain-containing protein | DksA C4-type domain-containing protein | | uniclust | UniRef100\_A0A161WT96 | 98.5 | 1.1e-09 | 2.1e-15 | 71.2 | 65 | (35, 103) | 103 | (1, 66) | 77 | DksA C4-type domain-containing protein | DksA C4-type domain-containing protein | | uniclust | UniRef100\_A0A2D8MXE8 | 98.5 | 1.1e-09 | 2.1e-15 | 82.9 | 68 | (34, 102) | 103 | (124, 204) | 214 | DksA C4-type domain-containing protein | DksA C4-type domain-containing protein | | uniclust | UniRef100\_A0A094X2I6 | 98.5 | 1e-09 | 2.4e-15 | 82.0 | 65 | (34, 99) | 103 | (56, 133) | 175 | Putative dnaK suppressor | Putative dnaK suppressor | | uniclust | UniRef100\_A0A0F9Z1F6 | 98.5 | 1.1e-09 | 2.6e-15 | 82.6 | 65 | (34, 99) | 103 | (70, 147) | 186 | Transcriptional regulator, TraR/DksA family | Transcriptional regulator, TraR/DksA family | | uniclust | UniRef100\_A0A0A8R9Y5 | 98.5 | 1.5e-09 | 2.8e-15 | 76.6 | 65 | (31, 99) | 103 | (79, 148) | 148 | DksA C4-type domain-containing protein | DksA C4-type domain-containing protein | | uniclust | UniRef100\_A0A0X8JHH5 | 98.5 | 1.7e-09 | 3.6e-15 | 79.9 | 67 | (31, 98) | 103 | (46, 125) | 170 | Transcriptional regulator | Transcriptional regulator | | uniclust | UniRef100\_A0A0G0JDQ4 | 98.5 | 1.6e-09 | 3.7e-15 | 76.5 | 66 | (33, 99) | 103 | (35, 113) | 118 | Transcriptional regulator, TraR/DksA family | Transcriptional regulator, TraR/DksA family | | uniclust | UniRef100\_A0A1F3SGG8 | 98.5 | 1.7e-09 | 3.7e-15 | 81.2 | 69 | (31, 100) | 103 | (69, 150) | 180 | DksA C4-type domain-containing protein | DksA C4-type domain-containing protein | | uniclust | UniRef100\_A0A060NLE4 | 98.4 | 1.7e-09 | 4.2e-15 | 81.0 | 61 | (40, 101) | 103 | (87, 160) | 173 | DnaK suppressor protein | DnaK suppressor protein | | uniclust | UniRef100\_A0A7C4ETC6 | 98.4 | 2.2e-09 | 4.3e-15 | 81.6 | 66 | (35, 101) | 103 | (137, 215) | 242 | TraR/DksA family transcriptional regulator | TraR/DksA family transcriptional regulator | | uniclust | UniRef100\_A0A2G6EIW2 | 98.4 | 2.4e-09 | 4.6e-15 | 70.6 | 71 | (30, 101) | 103 | (10, 86) | 87 | Conjugal transfer protein TraR | Conjugal transfer protein TraR | | uniclust | UniRef100\_A0A521SK58 | 98.4 | 2.4e-09 | 5e-15 | 75.2 | 65 | (35, 100) | 103 | (32, 109) | 120 | Transcriptional regulator, TraR/DksA family protein | Transcriptional regulator, TraR/DksA family protein | | uniclust | UniRef100\_A0A2A3LML0 | 98.4 | 2.4e-09 | 5.1e-15 | 70.3 | 63 | (35, 100) | 103 | (1, 68) | 78 | DksA C4-type domain-containing protein | DksA C4-type domain-containing protein | | uniclust | UniRef100\_UPI001CB70961 | 98.4 | 2.7e-09 | 5.2e-15 | 73.2 | 71 | (30, 101) | 103 | (33, 108) | 112 | TraR/DksA family transcriptional regulator | TraR/DksA family transcriptional regulator | | uniclust | UniRef100\_A0A2E2AXQ7 | 98.4 | 2.5e-09 | 5.2e-15 | 76.8 | 67 | (32, 99) | 103 | (48, 127) | 138 | DksA C4-type domain-containing protein | DksA C4-type domain-containing protein | | uniclust | UniRef100\_A0A1G3ZX42 | 98.4 | 2.3e-09 | 5.4e-15 | 86.3 | 64 | (36, 100) | 103 | (189, 265) | 306 | DksA C4-type domain-containing protein | DksA C4-type domain-containing protein | | uniclust | UniRef100\_A0A068VTU4 | 98.4 | 2.9e-09 | 6e-15 | 82.7 | 65 | (36, 101) | 103 | (161, 238) | 244 | Transcriptional regulators, TraR/DksA family | Transcriptional regulators, TraR/DksA family | | uniclust | UniRef100\_A0A0F2J1Q2 | 98.4 | 3e-09 | 6.4e-15 | 81.8 | 68 | (32, 100) | 103 | (126, 206) | 220 | RNA polymerase-binding, DksA | RNA polymerase-binding, DksA | | uniclust | UniRef100\_A0A024YPC6 | 98.4 | 2.8e-09 | 6.5e-15 | 89.9 | 65 | (37, 102) | 103 | (402, 479) | 481 | DNA-binding protein | DNA-binding protein | | uniclust | UniRef100\_A0A2A5B6B7 | 98.4 | 2.8e-09 | 6.6e-15 | 78.3 | 69 | (32, 101) | 103 | (64, 145) | 150 | DksA C4-type domain-containing protein | DksA C4-type domain-containing protein | | uniclust | UniRef100\_A0A059IV93 | 98.4 | 3.1e-09 | 7.3e-15 | 81.2 | 68 | (33, 101) | 103 | (94, 174) | 198 | Putative dnaK suppressor protein | Putative dnaK suppressor protein | | uniclust | UniRef100\_A0A1B4G123 | 98.4 | 4e-09 | 8.5e-15 | 68.5 | 63 | (35, 101) | 103 | (1, 64) | 73 | Conjugal transfer protein TraR | Conjugal transfer protein TraR | | uniclust | UniRef100\_A0A5T2HSX0 | 98.4 | 4.6e-09 | 8.7e-15 | 67.2 | 64 | (32, 99) | 103 | (2, 70) | 74 | TraR/DksA family transcriptional regulator | TraR/DksA family transcriptional regulator | | uniclust | UniRef100\_A0A2P5N6U8 | 98.3 | 5.3e-09 | 1.1e-14 | 70.6 | 67 | (35, 102) | 103 | (13, 84) | 92 | DksA C4-type domain-containing protein | DksA C4-type domain-containing protein | | uniclust | UniRef100\_A0A0S3AHW3 | 98.3 | 5.2e-09 | 1.1e-14 | 76.1 | 37 | (63, 100) | 103 | (95, 136) | 144 | TraR/DksA family transcriptional regulator | TraR/DksA family transcriptional regulator | | uniclust | UniRef100\_A0A081UWW5 | 98.3 | 4.7e-09 | 1.2e-14 | 79.8 | 60 | (40, 100) | 103 | (98, 170) | 189 | Molecular chaperone DnaK | Molecular chaperone DnaK | | uniclust | UniRef100\_A0A0F2NQD6 | 98.3 | 5.3e-09 | 1.2e-14 | 76.7 | 65 | (36, 101) | 103 | (56, 133) | 149 | DksA C4-type domain-containing protein | DksA C4-type domain-containing protein | | uniclust | UniRef100\_A0A0F9FJ65 | 98.3 | 5.7e-09 | 1.3e-14 | 81.0 | 67 | (33, 100) | 103 | (113, 192) | 231 | DksA C4-type domain-containing protein (Fragment) | DksA C4-type domain-containing protein (Fragment) | | uniclust | UniRef100\_A0A0B8WAV0 | 98.3 | 5.4e-09 | 1.3e-14 | 78.0 | 66 | (36, 102) | 103 | (62, 140) | 168 | Molecular chaperone DnaK | Molecular chaperone DnaK | | uniclust | UniRef100\_A0A0A0GV12 | 98.3 | 6.9e-09 | 1.3e-14 | 68.9 | 63 | (35, 101) | 103 | (2, 69) | 89 | Protein traR | Protein traR | | uniclust | UniRef100\_A0A171D6Y6 | 98.3 | 5.9e-09 | 1.4e-14 | 76.1 | 62 | (39, 101) | 103 | (61, 135) | 143 | Conjugal transfer protein TraR | Conjugal transfer protein TraR | | uniclust | UniRef100\_A0A061NPW7 | 98.3 | 6.6e-09 | 1.5e-14 | 74.8 | 37 | (62, 99) | 103 | (88, 129) | 131 | DnaK suppressor protein | DnaK suppressor protein | | uniclust | UniRef100\_A0A0N8QAU1 | 98.3 | 9.3e-09 | 1.7e-14 | 70.0 | 65 | (32, 99) | 103 | (40, 109) | 110 | TraR/DksA family transcriptional regulator | TraR/DksA family transcriptional regulator | | uniclust | UniRef100\_A0A0R2XJZ4 | 98.3 | 8.5e-09 | 1.8e-14 | 84.9 | 65 | (36, 101) | 103 | (279, 356) | 399 | DksA C4-type domain-containing protein | DksA C4-type domain-containing protein | | uniclust | UniRef100\_A0A1E4Q6M0 | 98.3 | 9.2e-09 | 1.8e-14 | 66.4 | 61 | (35, 101) | 103 | (1, 66) | 73 | DksA C4-type domain-containing protein | DksA C4-type domain-containing protein | | uniclust | UniRef100\_A0A0D0WTJ9 | 98.3 | 8.1e-09 | 1.9e-14 | 75.3 | 38 | (62, 100) | 103 | (89, 131) | 141 | Conjugal transfer protein TraR | Conjugal transfer protein TraR | | uniclust | UniRef100\_A0A3J9VMX4 | 98.3 | 1e-08 | 2e-14 | 66.2 | 64 | (36, 100) | 103 | (1, 70) | 75 | TraR/DksA family transcriptional regulator | TraR/DksA family transcriptional regulator | | uniclust | UniRef100\_A0A0T5ZLC1 | 98.3 | 8.8e-09 | 2e-14 | 78.6 | 68 | (32, 100) | 103 | (105, 185) | 197 | TraR/DksA family transcriptional regulator, DnaK suppressor protein | TraR/DksA family transcriptional regulator, DnaK suppressor protein | | uniclust | UniRef100\_A0A2D5I4F6 | 98.3 | 1e-08 | 2.1e-14 | 77.5 | 74 | (27, 101) | 103 | (117, 203) | 207 | RNA polymerase-binding transcription factor DksA | RNA polymerase-binding transcription factor DksA | | uniclust | UniRef100\_UPI00218380DB | 98.3 | 1.1e-08 | 2.1e-14 | 70.4 | 70 | (27, 100) | 103 | (43, 117) | 122 | TraR/DksA family transcriptional regulator | TraR/DksA family transcriptional regulator | | uniclust | UniRef100\_A0A106BVN3 | 98.3 | 1.1e-08 | 2.2e-14 | 66.4 | 68 | (34, 102) | 103 | (1, 68) | 73 | DUF2116 family Zn-ribbon domain-containing protein | DUF2116 family Zn-ribbon domain-containing protein | | uniclust | UniRef100\_A0A0Q8PNH8 | 98.3 | 9.2e-09 | 2.2e-14 | 78.5 | 64 | (36, 100) | 103 | (110, 186) | 194 | DksA C4-type domain-containing protein | DksA C4-type domain-containing protein | | uniclust | UniRef100\_A0A1M4XPA5 | 98.3 | 1.2e-08 | 2.2e-14 | 71.8 | 64 | (36, 100) | 103 | (26, 102) | 135 | Transcriptional regulator, TraR/DksA family | Transcriptional regulator, TraR/DksA family | | uniclust | UniRef100\_A0A1H6CVK7 | 98.3 | 1.1e-08 | 2.4e-14 | 71.7 | 35 | (63, 98) | 103 | (74, 113) | 116 | DksA/traR C4-type zinc finger | DksA/traR C4-type zinc finger | | uniclust | UniRef100\_A0A1Z9D4L2 | 98.3 | 1.1e-08 | 2.4e-14 | 80.9 | 62 | (37, 99) | 103 | (206, 280) | 282 | DksA C4-type domain-containing protein | DksA C4-type domain-containing protein | | uniclust | UniRef100\_A0A0G3V2G2 | 98.3 | 1.1e-08 | 2.5e-14 | 81.5 | 60 | (39, 99) | 103 | (212, 284) | 286 | DnaK suppressor protein | DnaK suppressor protein | | uniclust | UniRef100\_A0A0B6WW65 | 98.3 | 1.1e-08 | 2.5e-14 | 78.1 | 37 | (63, 100) | 103 | (134, 175) | 206 | Transcriptional regulator, TraR/DksA family | Transcriptional regulator, TraR/DksA family | | uniclust | UniRef100\_A0A1Z8V918 | 98.3 | 1.1e-08 | 2.5e-14 | 73.5 | 38 | (63, 101) | 103 | (84, 126) | 133 | DksA C4-type domain-containing protein | DksA C4-type domain-containing protein | | uniclust | UniRef100\_A0A1W9UTQ3 | 98.3 | 1.1e-08 | 2.5e-14 | 77.3 | 59 | (36, 95) | 103 | (105, 176) | 185 | DksA C4-type domain-containing protein | DksA C4-type domain-containing protein | | uniclust | UniRef100\_A0A1F8N3L7 | 98.3 | 1.2e-08 | 2.5e-14 | 74.8 | 68 | (34, 102) | 103 | (67, 147) | 149 | DksA C4-type domain-containing protein | DksA C4-type domain-containing protein | | uniclust | UniRef100\_A0A1G3XWY7 | 98.3 | 1.3e-08 | 2.6e-14 | 76.7 | 37 | (63, 100) | 103 | (138, 179) | 190 | DksA C4-type domain-containing protein | DksA C4-type domain-containing protein | | uniclust | UniRef100\_A0A096H0I5 | 98.2 | 1.1e-08 | 2.7e-14 | 75.8 | 38 | (63, 101) | 103 | (102, 144) | 155 | Molecular chaperone DnaK | Molecular chaperone DnaK | | uniclust | UniRef100\_A0A072N576 | 98.2 | 1.2e-08 | 2.7e-14 | 75.2 | 65 | (36, 101) | 103 | (56, 133) | 151 | Transcriptional regulator, TraR/DksA family | Transcriptional regulator, TraR/DksA family | | uniclust | UniRef100\_A0A2W6C1C1 | 98.2 | 1.2e-08 | 2.8e-14 | 76.3 | 69 | (31, 100) | 103 | (52, 133) | 172 | DksA C4-type domain-containing protein | DksA C4-type domain-containing protein | | uniclust | UniRef100\_A0A1F4QCC0 | 98.2 | 1.3e-08 | 2.8e-14 | 75.5 | 67 | (33, 100) | 103 | (55, 134) | 165 | DksA C4-type domain-containing protein | DksA C4-type domain-containing protein | | uniclust | UniRef100\_A0A1F9TZQ5 | 98.2 | 1.4e-08 | 2.9e-14 | 74.9 | 72 | (27, 99) | 103 | (77, 161) | 164 | DksA C4-type domain-containing protein | DksA C4-type domain-containing protein | | uniclust | UniRef100\_A0A1V8P4S1 | 98.2 | 1.5e-08 | 3e-14 | 68.8 | 68 | (31, 99) | 103 | (18, 91) | 97 | DksA C4-type domain-containing protein | DksA C4-type domain-containing protein | | uniclust | UniRef100\_A0A0Q7CUW1 | 98.2 | 1.4e-08 | 3e-14 | 72.1 | 38 | (63, 101) | 103 | (76, 118) | 123 | DksA C4-type domain-containing protein | DksA C4-type domain-containing protein | | uniclust | UniRef100\_A0A5Q0TJ53 | 98.2 | 1.4e-08 | 3e-14 | 65.0 | 35 | (67, 102) | 103 | (22, 61) | 66 | Transcriptional regulator | Transcriptional regulator | | uniclust | UniRef100\_A0A497AXA6 | 98.2 | 1.6e-08 | 3.2e-14 | 68.3 | 62 | (40, 102) | 103 | (11, 85) | 94 | DksA C4-type domain-containing protein | DksA C4-type domain-containing protein | | uniclust | UniRef100\_A0A1V6JBW9 | 98.2 | 1.4e-08 | 3.2e-14 | 82.3 | 63 | (39, 102) | 103 | (217, 292) | 319 | General stress protein 16O | General stress protein 16O | | uniclust | UniRef100\_A0A1F8N7W5 | 98.2 | 1.6e-08 | 3.3e-14 | 74.9 | 64 | (36, 100) | 103 | (54, 130) | 163 | DksA C4-type domain-containing protein | DksA C4-type domain-containing protein | | uniclust | UniRef100\_A0A522BF86 | 98.2 | 1.6e-08 | 3.4e-14 | 64.2 | 36 | (64, 100) | 103 | (18, 58) | 64 | DNA-binding protein (Fragment) | DNA-binding protein (Fragment) | | uniclust | UniRef100\_A0A021VUD3 | 98.2 | 1.6e-08 | 3.6e-14 | 77.2 | 64 | (36, 100) | 103 | (126, 202) | 203 | DNA-binding protein | DNA-binding protein | | uniclust | UniRef100\_A0A0H1AEC4 | 98.2 | 1.7e-08 | 3.7e-14 | 77.3 | 65 | (36, 101) | 103 | (125, 202) | 208 | DNA-binding protein | DNA-binding protein | | uniclust | UniRef100\_A0A2M9QWY4 | 98.2 | 1.8e-08 | 3.7e-14 | 66.4 | 64 | (35, 99) | 103 | (1, 71) | 78 | DksA C4-type domain-containing protein | DksA C4-type domain-containing protein | | uniclust | UniRef100\_A0A327X3U0 | 98.2 | 1.9e-08 | 3.8e-14 | 64.3 | 61 | (35, 101) | 103 | (1, 61) | 69 | Uncharacterized protein | Uncharacterized protein | | uniclust | UniRef100\_A0A022LFJ2 | 98.2 | 1.6e-08 | 3.9e-14 | 77.6 | 39 | (62, 101) | 103 | (139, 182) | 200 | Molecular chaperone DnaK | Molecular chaperone DnaK | | uniclust | UniRef100\_A0A014MPS1 | 98.2 | 1.8e-08 | 4.5e-14 | 75.0 | 39 | (62, 101) | 103 | (101, 144) | 160 | Molecular chaperone DnaK | Molecular chaperone DnaK | | uniclust | UniRef100\_A0A089YWC5 | 98.2 | 2e-08 | 4.5e-14 | 86.2 | 62 | (37, 99) | 103 | (494, 568) | 569 | DksA C4-type domain-containing protein | DksA C4-type domain-containing protein | | uniclust | UniRef100\_A0A139TQP8 | 98.2 | 2.1e-08 | 4.6e-14 | 81.6 | 65 | (36, 101) | 103 | (233, 310) | 349 | C4-type zinc finger protein, DksA/TraR family | C4-type zinc finger protein, DksA/TraR family | | uniclust | UniRef100\_A0A099TCY4 | 98.2 | 1.7e-08 | 4.6e-14 | 74.3 | 62 | (39, 101) | 103 | (61, 135) | 150 | Dimethylmenaquinone methyltransferase | Dimethylmenaquinone methyltransferase | | uniclust | UniRef100\_A0A1M6SXT8 | 98.2 | 2.3e-08 | 4.8e-14 | 72.8 | 64 | (35, 99) | 103 | (44, 120) | 151 | Transcriptional regulator, TraR/DksA family | Transcriptional regulator, TraR/DksA family | | uniclust | UniRef100\_A0A011U294 | 98.2 | 2.1e-08 | 4.8e-14 | 75.4 | 38 | (63, 101) | 103 | (118, 160) | 172 | Conjugal transfer protein TraR | Conjugal transfer protein TraR | | uniclust | UniRef100\_A0A1J4SK56 | 98.2 | 2.5e-08 | 4.9e-14 | 70.2 | 66 | (33, 99) | 103 | (39, 117) | 123 | DksA C4-type domain-containing protein | DksA C4-type domain-containing protein | | uniclust | UniRef100\_UPI00226C8043 | 98.2 | 2.8e-08 | 5.1e-14 | 68.3 | 64 | (36, 102) | 103 | (48, 116) | 118 | TraR/DksA C4-type zinc finger protein | TraR/DksA C4-type zinc finger protein | | uniclust | UniRef100\_A0A3A0CR10 | 98.2 | 2.6e-08 | 5.2e-14 | 77.4 | 78 | (23, 101) | 103 | (153, 243) | 260 | DksA C4-type domain-containing protein | DksA C4-type domain-containing protein | | uniclust | UniRef100\_A0A015MT88 | 98.2 | 2.1e-08 | 5.2e-14 | 82.2 | 60 | (37, 97) | 103 | (99, 171) | 336 | Molecular chaperone DnaK | Molecular chaperone DnaK | | uniclust | UniRef100\_A0A0K1JR25 | 98.2 | 2.6e-08 | 5.4e-14 | 81.3 | 63 | (37, 100) | 103 | (283, 358) | 359 | DksA C4-type domain-containing protein | DksA C4-type domain-containing protein | | uniclust | UniRef100\_A0A378VW00 | 98.2 | 3e-08 | 5.5e-14 | 68.9 | 60 | (35, 97) | 103 | (1, 65) | 127 | Phage associated protein | Phage associated protein | | uniclust | UniRef100\_A0A2E2EEP1 | 98.2 | 2.9e-08 | 5.6e-14 | 72.8 | 65 | (34, 99) | 103 | (45, 122) | 168 | DksA C4-type domain-containing protein | DksA C4-type domain-containing protein | | uniclust | UniRef100\_UPI0009DCB1FE | 98.2 | 3.2e-08 | 5.9e-14 | 66.5 | 65 | (34, 99) | 103 | (29, 101) | 102 | TraR/DksA C4-type zinc finger protein | TraR/DksA C4-type zinc finger protein | | uniclust | UniRef100\_A0A2V7YIT4 | 98.2 | 3e-08 | 6.1e-14 | 67.8 | 38 | (62, 100) | 103 | (51, 93) | 100 | DksA C4-type domain-containing protein | DksA C4-type domain-containing protein | | uniclust | UniRef100\_A0A024QBA7 | 98.1 | 2.8e-08 | 6.4e-14 | 73.0 | 56 | (38, 94) | 103 | (63, 131) | 146 | General stress protein 16O | General stress protein 16O | | uniclust | UniRef100\_A0A126SXD5 | 98.1 | 3.4e-08 | 6.9e-14 | 75.6 | 74 | (28, 102) | 103 | (129, 215) | 218 | RNA polymerase-binding transcription factor DksA | RNA polymerase-binding transcription factor DksA | | uniclust | UniRef100\_A0A6J5L1I0 | 98.1 | 3.9e-08 | 7.1e-14 | 61.0 | 57 | (35, 94) | 103 | (1, 62) | 63 | DksA-like zinc finger domain containing protein | DksA-like zinc finger domain containing protein | | uniclust | UniRef100\_A0A359KPZ9 | 98.1 | 3.5e-08 | 7.2e-14 | 71.4 | 37 | (63, 100) | 103 | (87, 128) | 142 | DksA C4-type domain-containing protein | DksA C4-type domain-containing protein | | uniclust | UniRef100\_A0A031FUW9 | 98.1 | 3.4e-08 | 7.3e-14 | 72.1 | 37 | (63, 100) | 103 | (93, 134) | 145 | DnaK suppressor protein | DnaK suppressor protein | | uniclust | UniRef100\_A0A099KYM7 | 98.1 | 3.1e-08 | 7.5e-14 | 72.4 | 66 | (35, 101) | 103 | (57, 135) | 140 | Transcriptional regulator, TraR/DksA family | Transcriptional regulator, TraR/DksA family | | uniclust | UniRef100\_A0A1F3T813 | 98.1 | 3.5e-08 | 7.5e-14 | 74.8 | 67 | (33, 100) | 103 | (56, 135) | 189 | DksA C4-type domain-containing protein | DksA C4-type domain-containing protein | | uniclust | UniRef100\_A0A2A4PPZ5 | 98.1 | 3.9e-08 | 7.7e-14 | 63.8 | 61 | (35, 101) | 103 | (1, 66) | 74 | DksA C4-type domain-containing protein | DksA C4-type domain-containing protein | | uniclust | UniRef100\_A0A085FRZ0 | 98.1 | 3.4e-08 | 7.8e-14 | 72.5 | 66 | (35, 101) | 103 | (51, 129) | 145 | C4-type zinc finger protein, DksA/TraR family | C4-type zinc finger protein, DksA/TraR family | | uniclust | UniRef100\_A0A084SHE3 | 98.1 | 3.4e-08 | 7.9e-14 | 71.4 | 63 | (36, 99) | 103 | (52, 127) | 131 | DksA C4-type domain-containing protein | DksA C4-type domain-containing protein | | uniclust | UniRef100\_A0A1I9LJW4 | 98.1 | 4.1e-08 | 8.2e-14 | 59.8 | 44 | (35, 81) | 103 | (1, 44) | 52 | TraR/DksA family transcriptional regulator | TraR/DksA family transcriptional regulator | | uniclust | UniRef100\_A0A7C5UR01 | 98.1 | 4.3e-08 | 8.3e-14 | 65.7 | 60 | (38, 98) | 103 | (20, 92) | 93 | TraR/DksA family transcriptional regulator | TraR/DksA family transcriptional regulator | | uniclust | UniRef100\_A0A0D8L7R9 | 98.1 | 4.2e-08 | 8.3e-14 | 63.8 | 63 | (35, 102) | 103 | (1, 63) | 74 | DUF2116 family Zn-ribbon domain-containing protein | DUF2116 family Zn-ribbon domain-containing protein | | uniclust | UniRef100\_A0A940WD45 | 98.1 | 4.7e-08 | 8.6e-14 | 65.5 | 65 | (36, 101) | 103 | (2, 79) | 99 | Molecular chaperone DnaK | Molecular chaperone DnaK | | uniclust | UniRef100\_A0A2A4WBF5 | 98.1 | 4.6e-08 | 9.1e-14 | 62.1 | 38 | (63, 101) | 103 | (19, 61) | 65 | DksA C4-type domain-containing protein | DksA C4-type domain-containing protein | | uniclust | UniRef100\_A0A3A0EJC5 | 98.1 | 4.7e-08 | 9.3e-14 | 73.5 | 70 | (30, 100) | 103 | (105, 187) | 194 | DksA C4-type domain-containing protein | DksA C4-type domain-containing protein | | uniclust | UniRef100\_UPI0004B56B7F | 98.1 | 5.2e-08 | 9.5e-14 | 58.2 | 36 | (63, 99) | 103 | (7, 47) | 51 | TraR/DksA family transcriptional regulator | TraR/DksA family transcriptional regulator | | uniclust | UniRef100\_A0A031IJ02 | 98.1 | 4e-08 | 9.5e-14 | 77.8 | 62 | (37, 99) | 103 | (59, 133) | 252 | Conjugal transfer protein TraR | Conjugal transfer protein TraR | | uniclust | UniRef100\_A0A8T5Z470 | 98.1 | 5.2e-08 | 9.6e-14 | 61.2 | 62 | (35, 98) | 103 | (1, 67) | 67 | Conjugal transfer protein TraR | Conjugal transfer protein TraR | | uniclust | UniRef100\_A0A063BFQ5 | 98.1 | 4.4e-08 | 9.8e-14 | 73.4 | 38 | (62, 100) | 103 | (119, 161) | 169 | Transcriptional regulator, TraR/DksA family | Transcriptional regulator, TraR/DksA family | | uniclust | UniRef100\_A0A059ZVK0 | 98.1 | 4.5e-08 | 9.8e-14 | 69.9 | 71 | (30, 101) | 103 | (5, 84) | 124 | DksA C4-type domain-containing protein | DksA C4-type domain-containing protein | | uniclust | UniRef100\_A0A7C5QEY6 | 98.1 | 4.9e-08 | 1e-13 | 68.8 | 69 | (33, 102) | 103 | (35, 116) | 120 | Conjugal transfer protein TraR | Conjugal transfer protein TraR | | uniclust | UniRef100\_A0A1G1F137 | 98.1 | 4.8e-08 | 1e-13 | 77.4 | 66 | (33, 99) | 103 | (179, 257) | 265 | DksA C4-type domain-containing protein | DksA C4-type domain-containing protein | | uniclust | UniRef100\_A0A1Q6YPU8 | 98.1 | 4.7e-08 | 1.1e-13 | 73.1 | 63 | (37, 100) | 103 | (74, 149) | 164 | DksA C4-type domain-containing protein | DksA C4-type domain-containing protein | | uniclust | UniRef100\_A0A1B1LRN6 | 98.1 | 4.9e-08 | 1.1e-13 | 71.1 | 71 | (30, 101) | 103 | (40, 125) | 140 | DksA C4-type domain-containing protein | DksA C4-type domain-containing protein | | uniclust | UniRef100\_A0A0D5NN62 | 98.1 | 4.9e-08 | 1.1e-13 | 76.4 | 57 | (37, 94) | 103 | (52, 121) | 230 | Conjugal transfer protein TraR | Conjugal transfer protein TraR | | uniclust | UniRef100\_A0A024P9L5 | 98.1 | 5.2e-08 | 1.2e-13 | 76.0 | 63 | (37, 100) | 103 | (65, 140) | 229 | General stress protein 16O | General stress protein 16O | | uniclust | UniRef100\_A0A936IL68 | 98.1 | 6.1e-08 | 1.2e-13 | 68.8 | 61 | (39, 100) | 103 | (48, 121) | 132 | TraR/DksA family transcriptional regulator | TraR/DksA family transcriptional regulator | | uniclust | UniRef100\_A0A095UJM2 | 98.1 | 5.2e-08 | 1.2e-13 | 70.4 | 40 | (62, 102) | 103 | (84, 128) | 131 | DnaK suppressor protein | DnaK suppressor protein | | uniclust | UniRef100\_A0A3C0KQS3 | 98.1 | 6.3e-08 | 1.2e-13 | 63.9 | 65 | (36, 101) | 103 | (5, 75) | 81 | DksA C4-type domain-containing protein | DksA C4-type domain-containing protein | | uniclust | UniRef100\_A0A0M7FD39 | 98.1 | 5.8e-08 | 1.3e-13 | 64.5 | 69 | (31, 101) | 103 | (6, 74) | 79 | Conjugal transfer protein TraR | Conjugal transfer protein TraR | | uniclust | UniRef100\_A0A011NM41 | 98.1 | 5.7e-08 | 1.3e-13 | 76.8 | 70 | (31, 101) | 103 | (151, 233) | 250 | RNA polymerase-binding transcription factor DksA | RNA polymerase-binding transcription factor DksA | | uniclust | UniRef100\_A0A0K2SKL3 | 98.1 | 6.2e-08 | 1.4e-13 | 77.9 | 65 | (37, 102) | 103 | (70, 147) | 296 | Transcriptional regulator | Transcriptional regulator | | uniclust | UniRef100\_A0A063KX61 | 98.1 | 6.8e-08 | 1.4e-13 | 72.9 | 74 | (27, 101) | 103 | (94, 180) | 197 | RNA polymerase-binding transcription factor DksA | RNA polymerase-binding transcription factor DksA | | uniclust | UniRef100\_A0A094JEJ5 | 98.0 | 6.9e-08 | 1.6e-13 | 70.6 | 39 | (63, 102) | 103 | (94, 137) | 141 | DksA C4-type domain-containing protein | DksA C4-type domain-containing protein | | uniclust | UniRef100\_A0A1G0XCN3 | 98.0 | 7.5e-08 | 1.6e-13 | 71.9 | 38 | (63, 101) | 103 | (113, 155) | 169 | DksA C4-type domain-containing protein | DksA C4-type domain-containing protein | | uniclust | UniRef100\_A0A084SZT3 | 98.0 | 7.7e-08 | 1.7e-13 | 71.6 | 34 | (65, 99) | 103 | (110, 148) | 163 | Conjugal transfer protein TraR | Conjugal transfer protein TraR | | uniclust | UniRef100\_A0A354P9W1 | 98.0 | 9.2e-08 | 1.7e-13 | 65.1 | 68 | (33, 101) | 103 | (10, 90) | 108 | DksA C4-type domain-containing protein (Fragment) | DksA C4-type domain-containing protein (Fragment) | | uniclust | UniRef100\_A0A1I5WIC7 | 98.0 | 8e-08 | 1.7e-13 | 64.9 | 66 | (35, 101) | 103 | (4, 82) | 88 | DnaK suppressor protein | DnaK suppressor protein | | uniclust | UniRef100\_A0A014N519 | 98.0 | 8e-08 | 1.7e-13 | 77.3 | 62 | (37, 99) | 103 | (229, 303) | 304 | DNA-binding protein | DNA-binding protein | | uniclust | UniRef100\_A0A1E7ZE78 | 98.0 | 8.2e-08 | 1.7e-13 | 63.4 | 66 | (36, 102) | 103 | (1, 75) | 77 | DksA C4-type domain-containing protein | DksA C4-type domain-containing protein | | uniclust | UniRef100\_A0A4P6WX91 | 98.0 | 9.5e-08 | 1.8e-13 | 64.2 | 61 | (37, 101) | 103 | (31, 96) | 100 | TraR/DksA family transcriptional regulator | TraR/DksA family transcriptional regulator | | uniclust | UniRef100\_A0A5M8P5R7 | 98.0 | 9.5e-08 | 1.8e-13 | 56.9 | 37 | (64, 101) | 103 | (7, 48) | 49 | DksA C4-type domain-containing protein (Fragment) | DksA C4-type domain-containing protein (Fragment) | | uniclust | UniRef100\_A0A250L6W6 | 98.0 | 8.6e-08 | 1.8e-13 | 72.0 | 36 | (63, 99) | 103 | (132, 172) | 181 | DksA C4-type domain-containing protein | DksA C4-type domain-containing protein | | uniclust | UniRef100\_A0A136KHD1 | 98.0 | 8.1e-08 | 1.8e-13 | 68.7 | 62 | (36, 98) | 103 | (48, 122) | 123 | General stress protein 16O | General stress protein 16O | | uniclust | UniRef100\_A0A3M0YYI6 | 98.0 | 8.6e-08 | 1.8e-13 | 70.0 | 36 | (63, 99) | 103 | (86, 126) | 146 | TraR/DksA family transcriptional regulator | TraR/DksA family transcriptional regulator | | uniclust | UniRef100\_UPI000A104C83 | 98.0 | 8.9e-08 | 1.8e-13 | 61.2 | 34 | (67, 101) | 103 | (26, 64) | 65 | TraR/DksA C4-type zinc finger protein | TraR/DksA C4-type zinc finger protein | | uniclust | UniRef100\_A0A1G2BEF3 | 98.0 | 8e-08 | 1.9e-13 | 72.0 | 37 | (62, 99) | 103 | (110, 151) | 164 | DksA C4-type domain-containing protein | DksA C4-type domain-containing protein | | uniclust | UniRef100\_A0A081FU55 | 98.0 | 1e-07 | 1.9e-13 | 63.8 | 39 | (63, 102) | 103 | (43, 86) | 91 | DksA C4-type domain-containing protein | DksA C4-type domain-containing protein | | uniclust | UniRef100\_A0A358PEC4 | 98.0 | 9.2e-08 | 2e-13 | 67.0 | 59 | (36, 95) | 103 | (37, 108) | 110 | Conjugal transfer protein TraR | Conjugal transfer protein TraR | | uniclust | UniRef100\_UPI000681A24E | 98.0 | 1e-07 | 2e-13 | 62.1 | 65 | (36, 101) | 103 | (1, 70) | 77 | TraR/DksA C4-type zinc finger protein | TraR/DksA C4-type zinc finger protein | | uniclust | UniRef100\_A0A521HPH1 | 98.0 | 1e-07 | 2.1e-13 | 62.6 | 32 | (67, 99) | 103 | (21, 57) | 75 | DksA C4-type domain-containing protein (Fragment) | DksA C4-type domain-containing protein (Fragment) | | uniclust | UniRef100\_A0A3N5HTP4 | 98.0 | 1e-07 | 2.2e-13 | 62.6 | 38 | (63, 101) | 103 | (29, 71) | 75 | DksA C4-type domain-containing protein (Fragment) | DksA C4-type domain-containing protein (Fragment) | | uniclust | UniRef100\_A0A6A7Y5C6 | 98.0 | 1.2e-07 | 2.2e-13 | 65.7 | 66 | (35, 102) | 103 | (50, 120) | 122 | DksA C4-type domain-containing protein | DksA C4-type domain-containing protein | | uniclust | UniRef100\_A0A1E7J167 | 98.0 | 1e-07 | 2.2e-13 | 76.5 | 37 | (63, 100) | 103 | (86, 127) | 282 | DksA C4-type domain-containing protein | DksA C4-type domain-containing protein | | uniclust | UniRef100\_A0A1F4UBE2 | 98.0 | 1.2e-07 | 2.3e-13 | 61.5 | 60 | (39, 99) | 103 | (2, 74) | 75 | DksA C4-type domain-containing protein | DksA C4-type domain-containing protein | | uniclust | UniRef100\_A0A0Q5ZHN8 | 98.0 | 1.1e-07 | 2.4e-13 | 69.4 | 63 | (33, 99) | 103 | (63, 130) | 139 | DksA C4-type domain-containing protein | DksA C4-type domain-containing protein | | uniclust | UniRef100\_A0A0F7GCS4 | 98.0 | 1.1e-07 | 2.4e-13 | 70.3 | 62 | (39, 101) | 103 | (64, 138) | 149 | Putative DNA-binding protein | Putative DNA-binding protein | | uniclust | UniRef100\_A0A2S5R2M0 | 98.0 | 1.2e-07 | 2.5e-13 | 63.3 | 34 | (67, 101) | 103 | (37, 75) | 83 | DksA C4-type domain-containing protein | DksA C4-type domain-containing protein | | uniclust | UniRef100\_A0A2T5CG19 | 98.0 | 1.4e-07 | 2.5e-13 | 68.0 | 64 | (35, 99) | 103 | (67, 143) | 152 | DksA C4-type domain-containing protein | DksA C4-type domain-containing protein | | uniclust | UniRef100\_A0A0M4DJJ5 | 98.0 | 1.2e-07 | 2.6e-13 | 82.0 | 62 | (37, 99) | 103 | (512, 586) | 587 | DNA-binding protein | DNA-binding protein | | uniclust | UniRef100\_A0A9E5MJY6 | 98.0 | 1.4e-07 | 2.7e-13 | 60.8 | 63 | (36, 99) | 103 | (1, 69) | 73 | Conjugal transfer protein TraR | Conjugal transfer protein TraR | | uniclust | UniRef100\_UPI001C11592D | 98.0 | 1.5e-07 | 2.8e-13 | 63.1 | 68 | (30, 101) | 103 | (12, 84) | 98 | TraR/DksA C4-type zinc finger protein | TraR/DksA C4-type zinc finger protein | | uniclust | UniRef100\_A0A2W4MHC5 | 98.0 | 1.3e-07 | 2.8e-13 | 68.4 | 38 | (63, 101) | 103 | (84, 126) | 129 | DksA C4-type domain-containing protein | DksA C4-type domain-containing protein | | uniclust | UniRef100\_A0A3N7CE60 | 98.0 | 1.5e-07 | 2.9e-13 | 62.5 | 65 | (35, 100) | 103 | (1, 80) | 88 | DksA C4-type domain-containing protein | DksA C4-type domain-containing protein | | uniclust | UniRef100\_A0A349E9S1 | 98.0 | 1.4e-07 | 2.9e-13 | 66.1 | 70 | (31, 101) | 103 | (29, 111) | 112 | DksA C4-type domain-containing protein | DksA C4-type domain-containing protein | | uniclust | UniRef100\_A0A2V6NZ32 | 98.0 | 1.4e-07 | 3e-13 | 72.0 | 63 | (34, 97) | 103 | (48, 123) | 194 | DksA C4-type domain-containing protein | DksA C4-type domain-containing protein | | uniclust | UniRef100\_A0A2G6DF11 | 98.0 | 1.6e-07 | 3e-13 | 63.2 | 68 | (35, 103) | 103 | (1, 81) | 100 | DksA C4-type domain-containing protein | DksA C4-type domain-containing protein | | uniclust | UniRef100\_A0A136NY37 | 98.0 | 1.4e-07 | 3e-13 | 67.2 | 63 | (36, 99) | 103 | (43, 118) | 121 | TraR/DksA family transcriptional regulator | TraR/DksA family transcriptional regulator | | uniclust | UniRef100\_A0A023WNM1 | 98.0 | 1.2e-07 | 3.1e-13 | 72.1 | 38 | (62, 100) | 103 | (123, 165) | 181 | Conjugal transfer protein TraR | Conjugal transfer protein TraR | | uniclust | UniRef100\_A0A2G4KCU6 | 97.9 | 1.7e-07 | 3.1e-13 | 65.9 | 69 | (30, 100) | 103 | (54, 127) | 133 | DksA C4-type domain-containing protein | DksA C4-type domain-containing protein | | uniclust | UniRef100\_A0A6F8XJD3 | 97.9 | 1.6e-07 | 3.2e-13 | 75.6 | 62 | (37, 99) | 103 | (247, 321) | 321 | DksA C4-type domain-containing protein | DksA C4-type domain-containing protein | | uniclust | UniRef100\_A0A1N7MQG6 | 97.9 | 1.7e-07 | 3.5e-13 | 61.8 | 51 | (46, 99) | 103 | (15, 70) | 78 | Transcriptional regulator, TraR/DksA family | Transcriptional regulator, TraR/DksA family | | uniclust | UniRef100\_A0A5Q0EGG8 | 97.9 | 1.9e-07 | 3.6e-13 | 59.6 | 37 | (63, 100) | 103 | (20, 61) | 68 | TraR/DksA C4-type zinc finger protein | TraR/DksA C4-type zinc finger protein | | uniclust | UniRef100\_A0A2D5B804 | 97.9 | 1.7e-07 | 3.6e-13 | 71.5 | 70 | (31, 101) | 103 | (110, 192) | 194 | DksA C4-type domain-containing protein | DksA C4-type domain-containing protein | | uniclust | UniRef100\_A0A1Q6VM36 | 97.9 | 1.9e-07 | 3.7e-13 | 67.5 | 70 | (31, 101) | 103 | (65, 147) | 150 | DksA C4-type domain-containing protein (Fragment) | DksA C4-type domain-containing protein (Fragment) | | uniclust | UniRef100\_A0A3M1Q0B7 | 97.9 | 2e-07 | 3.8e-13 | 69.9 | 64 | (35, 99) | 103 | (57, 133) | 198 | TraR/DksA family transcriptional regulator | TraR/DksA family transcriptional regulator | | uniclust | UniRef100\_UPI001D046260 | 97.9 | 2.1e-07 | 3.9e-13 | 62.5 | 66 | (31, 100) | 103 | (11, 81) | 98 | TraR/DksA C4-type zinc finger protein | TraR/DksA C4-type zinc finger protein | | uniclust | UniRef100\_A0A2S3QGI0 | 97.9 | 1.9e-07 | 4e-13 | 72.9 | 61 | (37, 98) | 103 | (50, 123) | 235 | Conjugal transfer protein TraR | Conjugal transfer protein TraR | | uniclust | UniRef100\_A0A101FKR2 | 97.9 | 1.8e-07 | 4e-13 | 69.9 | 63 | (37, 100) | 103 | (68, 145) | 163 | DNA binding protein, DksA/TraR family | DNA binding protein, DksA/TraR family | | uniclust | UniRef100\_A0A3D3CQ74 | 97.9 | 2.2e-07 | 4.2e-13 | 58.0 | 37 | (63, 100) | 103 | (10, 51) | 60 | Molecular chaperone DnaK (Fragment) | Molecular chaperone DnaK (Fragment) | | uniclust | UniRef100\_A0A7C6PEY8 | 97.9 | 2.4e-07 | 4.4e-13 | 71.1 | 68 | (31, 99) | 103 | (178, 250) | 257 | TraR/DksA family transcriptional regulator | TraR/DksA family transcriptional regulator | | uniclust | UniRef100\_A0A2E2XIR7 | 97.9 | 2.1e-07 | 4.7e-13 | 67.4 | 65 | (34, 99) | 103 | (47, 124) | 129 | Dimethylmenaquinone methyltransferase | Dimethylmenaquinone methyltransferase | | uniclust | UniRef100\_A0A254TH52 | 97.9 | 2.3e-07 | 4.8e-13 | 66.9 | 37 | (63, 100) | 103 | (85, 126) | 134 | DksA C4-type domain-containing protein | DksA C4-type domain-containing protein | | uniclust | UniRef100\_S9TQT2 | 97.9 | 2.5e-07 | 4.8e-13 | 60.4 | 65 | (35, 102) | 103 | (1, 70) | 77 | Conjugal transfer protein TraR | Conjugal transfer protein TraR | | uniclust | UniRef100\_A0A7X5VPI8 | 97.9 | 2.5e-07 | 5e-13 | 73.1 | 69 | (30, 99) | 103 | (142, 223) | 279 | DksA C4-type domain-containing protein | DksA C4-type domain-containing protein | | uniclust | UniRef100\_A0A096BHG0 | 97.9 | 2.2e-07 | 5e-13 | 74.2 | 62 | (37, 99) | 103 | (74, 148) | 259 | Molecular chaperone DnaK | Molecular chaperone DnaK | | uniclust | UniRef100\_A0A2N9MYM1 | 97.9 | 2.8e-07 | 5.1e-13 | 55.9 | 35 | (64, 99) | 103 | (9, 48) | 54 | DksA C4-type domain-containing protein | DksA C4-type domain-containing protein | | uniclust | UniRef100\_A0A258HF81 | 97.9 | 2.8e-07 | 5.2e-13 | 61.7 | 60 | (39, 99) | 103 | (16, 88) | 96 | DksA C4-type domain-containing protein | DksA C4-type domain-containing protein | | uniclust | UniRef100\_A0A2E3VVZ9 | 97.9 | 2.5e-07 | 5.2e-13 | 68.7 | 66 | (34, 100) | 103 | (78, 156) | 159 | Conjugal transfer protein TraR | Conjugal transfer protein TraR | | uniclust | UniRef100\_A0A3A1P1K3 | 97.9 | 2.7e-07 | 5.3e-13 | 57.1 | 32 | (68, 100) | 103 | (15, 51) | 55 | DksA C4-type domain-containing protein | DksA C4-type domain-containing protein | | uniclust | UniRef100\_A0A1G6Q4B8 | 97.9 | 2.5e-07 | 5.4e-13 | 65.4 | 66 | (30, 96) | 103 | (30, 108) | 113 | DksA/traR C4-type zinc finger | DksA/traR C4-type zinc finger | | uniclust | UniRef100\_A0A0P7RX32 | 97.9 | 2.6e-07 | 5.4e-13 | 61.8 | 66 | (32, 101) | 103 | (6, 76) | 83 | TraR/DksA family transcriptional regulator | TraR/DksA family transcriptional regulator | | uniclust | UniRef100\_A0A2V8LIK5 | 97.9 | 2.7e-07 | 5.4e-13 | 60.3 | 37 | (63, 100) | 103 | (22, 63) | 72 | DksA C4-type domain-containing protein | DksA C4-type domain-containing protein | | uniclust | UniRef100\_A0A2W4MDT4 | 97.9 | 2.9e-07 | 5.5e-13 | 66.7 | 62 | (37, 99) | 103 | (51, 125) | 150 | Conjugal transfer protein TraR (Fragment) | Conjugal transfer protein TraR (Fragment) | | uniclust | UniRef100\_A0A524JED7 | 97.9 | 2.8e-07 | 5.5e-13 | 64.6 | 55 | (39, 97) | 103 | (57, 116) | 118 | TraR/DksA family transcriptional regulator | TraR/DksA family transcriptional regulator | | uniclust | UniRef100\_A0A1F2RVJ7 | 97.9 | 2.8e-07 | 5.6e-13 | 66.6 | 38 | (62, 100) | 103 | (78, 120) | 136 | DksA C4-type domain-containing protein | DksA C4-type domain-containing protein | | uniclust | UniRef100\_A0A259NJV5 | 97.9 | 2.5e-07 | 5.7e-13 | 66.1 | 36 | (63, 99) | 103 | (77, 117) | 119 | DksA C4-type domain-containing protein | DksA C4-type domain-containing protein | | uniclust | UniRef100\_A0A519BE40 | 97.9 | 3e-07 | 5.8e-13 | 66.9 | 70 | (32, 102) | 103 | (50, 132) | 159 | TraR/DksA family transcriptional regulator | TraR/DksA family transcriptional regulator | | uniclust | UniRef100\_A0A2E9I554 | 97.9 | 2.8e-07 | 5.9e-13 | 61.6 | 34 | (63, 97) | 103 | (42, 80) | 82 | DksA C4-type domain-containing protein | DksA C4-type domain-containing protein | | uniclust | UniRef100\_UPI0009BB9C06 | 97.9 | 3.1e-07 | 5.9e-13 | 57.3 | 35 | (67, 102) | 103 | (13, 52) | 60 | TraR/DksA C4-type zinc finger protein | TraR/DksA C4-type zinc finger protein | | uniclust | UniRef100\_A0A521I243 | 97.9 | 2.9e-07 | 5.9e-13 | 66.4 | 66 | (34, 100) | 103 | (41, 119) | 132 | TraR/DksA family transcriptional regulator | TraR/DksA family transcriptional regulator | | uniclust | UniRef100\_A0A7C3RPK9 | 97.9 | 2.9e-07 | 5.9e-13 | 70.0 | 63 | (32, 95) | 103 | (43, 118) | 195 | TraR/DksA family transcriptional regulator | TraR/DksA family transcriptional regulator | | uniclust | UniRef100\_A0A1D9H525 | 97.9 | 2.9e-07 | 6.3e-13 | 71.3 | 64 | (36, 100) | 103 | (78, 154) | 210 | DksA C4-type domain-containing protein | DksA C4-type domain-containing protein | | uniclust | UniRef100\_A0A2D6ISF3 | 97.9 | 3.4e-07 | 6.4e-13 | 64.0 | 67 | (34, 101) | 103 | (44, 115) | 119 | DksA C4-type domain-containing protein | DksA C4-type domain-containing protein | | uniclust | UniRef100\_A0A379YCN4 | 97.8 | 3.5e-07 | 6.5e-13 | 61.4 | 61 | (35, 99) | 103 | (1, 66) | 97 | DnaK suppressor protein | DnaK suppressor protein | | uniclust | UniRef100\_A0A060NKP5 | 97.8 | 2.9e-07 | 6.6e-13 | 78.1 | 70 | (32, 102) | 103 | (361, 443) | 448 | RNA polymerase-binding transcription factor DksA | RNA polymerase-binding transcription factor DksA | | uniclust | UniRef100\_A0A2D8UDS4 | 97.8 | 3.3e-07 | 6.8e-13 | 69.9 | 64 | (36, 100) | 103 | (79, 159) | 196 | DksA C4-type domain-containing protein | DksA C4-type domain-containing protein | | uniclust | UniRef100\_A0A3D3KCQ9 | 97.8 | 3.7e-07 | 6.8e-13 | 60.1 | 65 | (29, 99) | 103 | (16, 85) | 86 | Molecular chaperone DnaK | Molecular chaperone DnaK | | uniclust | UniRef100\_A0A2V7CK04 | 97.8 | 3.8e-07 | 7.1e-13 | 67.2 | 64 | (35, 99) | 103 | (96, 172) | 174 | DksA C4-type domain-containing protein | DksA C4-type domain-containing protein | | uniclust | UniRef100\_A0A1Q7D0Z5 | 97.8 | 3.8e-07 | 7.1e-13 | 66.4 | 66 | (35, 101) | 103 | (76, 154) | 157 | DksA C4-type domain-containing protein | DksA C4-type domain-containing protein | | uniclust | UniRef100\_A0A014QDA9 | 97.8 | 3.5e-07 | 7.4e-13 | 74.4 | 70 | (31, 101) | 103 | (244, 326) | 331 | RNA polymerase-binding transcription factor DksA | RNA polymerase-binding transcription factor DksA | | uniclust | UniRef100\_A0A098S5Y4 | 97.8 | 3e-07 | 7.6e-13 | 68.3 | 38 | (63, 101) | 103 | (103, 145) | 151 | DksA C4-type domain-containing protein | DksA C4-type domain-containing protein | | uniclust | UniRef100\_A0A9D1R109 | 97.8 | 4e-07 | 7.6e-13 | 65.8 | 66 | (34, 100) | 103 | (32, 110) | 149 | TraR/DksA family transcriptional regulator | TraR/DksA family transcriptional regulator | | uniclust | UniRef100\_A0A6N6N474 | 97.8 | 4.3e-07 | 7.8e-13 | 66.6 | 67 | (31, 98) | 103 | (91, 170) | 176 | TraR/DksA family transcriptional regulator | TraR/DksA family transcriptional regulator | | uniclust | UniRef100\_A0A368U9L5 | 97.8 | 4.3e-07 | 8e-13 | 60.1 | 70 | (31, 103) | 103 | (11, 85) | 89 | TraR/DksA family transcriptional regulator | TraR/DksA family transcriptional regulator | | uniclust | UniRef100\_A0A520BYY6 | 97.8 | 4.2e-07 | 8.1e-13 | 62.7 | 36 | (63, 99) | 103 | (54, 94) | 105 | TraR/DksA family transcriptional regulator | TraR/DksA family transcriptional regulator | | uniclust | UniRef100\_A0A4Q7DA94 | 97.8 | 4.1e-07 | 8.2e-13 | 57.8 | 37 | (63, 100) | 103 | (21, 62) | 63 | RNA polymerase-binding transcription factor DksA | RNA polymerase-binding transcription factor DksA | | uniclust | UniRef100\_A0A0S8E9J3 | 97.8 | 4.2e-07 | 8.4e-13 | 68.7 | 62 | (37, 99) | 103 | (91, 165) | 187 | DksA C4-type domain-containing protein | DksA C4-type domain-containing protein | | uniclust | UniRef100\_A0A537JIW5 | 97.8 | 4.7e-07 | 8.6e-13 | 60.0 | 64 | (36, 100) | 103 | (9, 85) | 89 | DksA C4-type domain-containing protein | DksA C4-type domain-containing protein | | uniclust | UniRef100\_A0A2I8QNI8 | 97.8 | 4.8e-07 | 8.8e-13 | 62.0 | 66 | (32, 101) | 103 | (34, 104) | 110 | DksA C4-type domain-containing protein | DksA C4-type domain-containing protein | | uniclust | UniRef100\_A0A061A4Y2 | 97.8 | 4e-07 | 9.1e-13 | 67.6 | 36 | (63, 99) | 103 | (104, 144) | 152 | DNA-binding protein | DNA-binding protein | | uniclust | UniRef100\_A0A931US23 | 97.8 | 5e-07 | 9.2e-13 | 69.2 | 69 | (31, 100) | 103 | (36, 117) | 249 | TraR/DksA family transcriptional regulator | TraR/DksA family transcriptional regulator | | uniclust | UniRef100\_A0A0A6UJY1 | 97.8 | 4.7e-07 | 9.4e-13 | 68.2 | 63 | (37, 100) | 103 | (107, 182) | 184 | Conjugal transfer protein TraR | Conjugal transfer protein TraR | | uniclust | UniRef100\_A0A017SZY0 | 97.8 | 4.4e-07 | 9.8e-13 | 69.4 | 69 | (33, 102) | 103 | (94, 175) | 188 | C4-type zinc finger protein, DksA/TraR family | C4-type zinc finger protein, DksA/TraR family | | uniclust | UniRef100\_A0A059V4A5 | 97.8 | 5e-07 | 1e-12 | 61.0 | 65 | (35, 102) | 103 | (14, 83) | 89 | DksA C4-type domain-containing protein | DksA C4-type domain-containing protein | | uniclust | UniRef100\_I2NPZ6 | 97.8 | 5.5e-07 | 1e-12 | 66.8 | 86 | (15, 101) | 103 | (90, 188) | 192 | RNA polymerase-binding transcription factor DksA | RNA polymerase-binding transcription factor DksA | | uniclust | UniRef100\_A0A1Q7DI09 | 97.8 | 5.3e-07 | 1e-12 | 59.4 | 35 | (66, 101) | 103 | (37, 76) | 78 | DksA C4-type domain-containing protein | DksA C4-type domain-containing protein | | uniclust | UniRef100\_A0A0P0FQ45 | 97.8 | 5.2e-07 | 1.1e-12 | 71.9 | 65 | (36, 101) | 103 | (172, 249) | 282 | General stress protein 16O | General stress protein 16O | | uniclust | UniRef100\_A0A147EFJ8 | 97.8 | 4.9e-07 | 1.1e-12 | 60.4 | 36 | (63, 99) | 103 | (36, 76) | 79 | DksA C4-type domain-containing protein | DksA C4-type domain-containing protein | | uniclust | UniRef100\_A0A7Y8L3J6 | 97.8 | 5.9e-07 | 1.1e-12 | 54.4 | 34 | (64, 98) | 103 | (4, 42) | 53 | TraR/DksA C4-type zinc finger protein | TraR/DksA C4-type zinc finger protein | | uniclust | UniRef100\_A0A1M6JC87 | 97.8 | 5.8e-07 | 1.2e-12 | 68.0 | 60 | (38, 98) | 103 | (61, 133) | 189 | Transcriptional regulator, TraR/DksA family | Transcriptional regulator, TraR/DksA family | | uniclust | UniRef100\_A0A3C0SA11 | 97.8 | 6.1e-07 | 1.2e-12 | 52.0 | 33 | (66, 99) | 103 | (2, 39) | 40 | DksA C4-type domain-containing protein (Fragment) | DksA C4-type domain-containing protein (Fragment) | | uniclust | UniRef100\_A0A0J5GIB6 | 97.8 | 6.4e-07 | 1.2e-12 | 56.8 | 31 | (68, 99) | 103 | (31, 66) | 68 | DksA C4-type domain-containing protein | DksA C4-type domain-containing protein | | uniclust | UniRef100\_K2K8W1 | 97.8 | 6.4e-07 | 1.2e-12 | 56.9 | 63 | (35, 101) | 103 | (1, 64) | 69 | Uncharacterized protein | Uncharacterized protein | | uniclust | UniRef100\_A0A091ATI3 | 97.8 | 6.4e-07 | 1.2e-12 | 58.0 | 63 | (35, 102) | 103 | (1, 66) | 73 | DksA C4-type domain-containing protein | DksA C4-type domain-containing protein | | uniclust | UniRef100\_A0A0G4E598 | 97.8 | 5.7e-07 | 1.2e-12 | 63.6 | 73 | (28, 101) | 103 | (15, 100) | 112 | C4-type zinc finger protein, DksA/TraR family | C4-type zinc finger protein, DksA/TraR family | | uniclust | UniRef100\_A0A2V9DB68 | 97.8 | 6.3e-07 | 1.2e-12 | 59.6 | 36 | (63, 99) | 103 | (34, 74) | 82 | DksA C4-type domain-containing protein | DksA C4-type domain-containing protein | | uniclust | UniRef100\_A0A149QS87 | 97.8 | 6.7e-07 | 1.3e-12 | 58.6 | 63 | (36, 99) | 103 | (6, 76) | 78 | DksA C4-type domain-containing protein | DksA C4-type domain-containing protein | | uniclust | UniRef100\_A0A0S7ZBX2 | 97.8 | 6e-07 | 1.3e-12 | 68.1 | 62 | (38, 100) | 103 | (82, 158) | 180 | DksA C4-type domain-containing protein | DksA C4-type domain-containing protein | | uniclust | UniRef100\_A0A1Y3QGI6 | 97.7 | 6.8e-07 | 1.3e-12 | 67.5 | 61 | (37, 98) | 103 | (49, 122) | 189 | DksA C4-type domain-containing protein | DksA C4-type domain-containing protein | | uniclust | UniRef100\_A0A949QK11 | 97.7 | 7e-07 | 1.3e-12 | 54.4 | 38 | (63, 101) | 103 | (6, 48) | 52 | TraR/DksA C4-type zinc finger protein | TraR/DksA C4-type zinc finger protein | | uniclust | UniRef100\_A0A3L7RXF9 | 97.7 | 7.1e-07 | 1.4e-12 | 62.3 | 37 | (63, 100) | 103 | (41, 82) | 114 | TraR/DksA family transcriptional regulator (Fragment) | TraR/DksA family transcriptional regulator (Fragment) | | uniclust | UniRef100\_UPI000376C2D9 | 97.7 | 7.4e-07 | 1.4e-12 | 58.0 | 65 | (35, 100) | 103 | (1, 72) | 74 | hypothetical protein | hypothetical protein | | uniclust | UniRef100\_A0A0K6H4T7 | 97.7 | 7.7e-07 | 1.5e-12 | 59.6 | 35 | (63, 98) | 103 | (39, 78) | 84 | Transcriptional regulator, TraR/DksA family | Transcriptional regulator, TraR/DksA family | | uniclust | UniRef100\_UPI001D0B57A9 | 97.7 | 8.4e-07 | 1.6e-12 | 54.5 | 35 | (66, 101) | 103 | (11, 50) | 57 | TraR/DksA family transcriptional regulator | TraR/DksA family transcriptional regulator | | uniclust | UniRef100\_A0A1H1X644 | 97.7 | 8.1e-07 | 1.6e-12 | 71.3 | 62 | (37, 99) | 103 | (233, 307) | 307 | RNA polymerase-binding protein DksA | RNA polymerase-binding protein DksA | | uniclust | UniRef100\_A0A447JDU8 | 97.7 | 8.8e-07 | 1.6e-12 | 62.1 | 63 | (35, 101) | 103 | (1, 68) | 127 | site-specific DNA-methyltransferase (adenine-specific) | site-specific DNA-methyltransferase (adenine-specific) | | uniclust | UniRef100\_A0A2V9X0D4 | 97.7 | 8.6e-07 | 1.6e-12 | 53.9 | 34 | (64, 98) | 103 | (11, 49) | 51 | DksA C4-type domain-containing protein | DksA C4-type domain-containing protein | | uniclust | UniRef100\_A0A0K8QN02 | 97.7 | 8.6e-07 | 1.6e-12 | 59.3 | 65 | (35, 100) | 103 | (1, 76) | 87 | C4-type zinc finger DksA/TraR family protein | C4-type zinc finger DksA/TraR family protein | | uniclust | UniRef100\_A0A7C2IE66 | 97.7 | 9.5e-07 | 1.7e-12 | 60.9 | 39 | (63, 102) | 103 | (54, 97) | 113 | DksA C4-type domain-containing protein | DksA C4-type domain-containing protein | | uniclust | UniRef100\_A0A375H4V9 | 97.7 | 8e-07 | 1.7e-12 | 58.5 | 67 | (35, 102) | 103 | (1, 70) | 72 | Uncharacterized protein | Uncharacterized protein | | uniclust | UniRef100\_A0A090ARX1 | 97.7 | 8.7e-07 | 1.8e-12 | 59.0 | 60 | (36, 101) | 103 | (1, 61) | 80 | Conserved uncharacterized protein | Conserved uncharacterized protein | | uniclust | UniRef100\_A0A0A6CYW5 | 97.7 | 8.7e-07 | 1.8e-12 | 58.4 | 60 | (35, 99) | 103 | (1, 60) | 73 | Conjugal transfer protein TraR | Conjugal transfer protein TraR | | uniclust | UniRef100\_A0A7Y5VRL4 | 97.7 | 1e-06 | 1.9e-12 | 63.9 | 70 | (30, 100) | 103 | (63, 145) | 160 | TraR/DksA family transcriptional regulator | TraR/DksA family transcriptional regulator | | uniclust | UniRef100\_A0A177HY54 | 97.7 | 9.4e-07 | 1.9e-12 | 77.6 | 61 | (38, 99) | 103 | (686, 759) | 760 | RNA polymerase-binding transcription factor DksA | RNA polymerase-binding transcription factor DksA | | uniclust | UniRef100\_A0A142XHA1 | 97.7 | 9e-07 | 1.9e-12 | 64.0 | 66 | (33, 99) | 103 | (48, 126) | 129 | General stress protein 16O | General stress protein 16O | | uniclust | UniRef100\_A0A7Y6CRV9 | 97.7 | 1e-06 | 2e-12 | 66.5 | 62 | (36, 98) | 103 | (122, 196) | 197 | TraR/DksA family transcriptional regulator | TraR/DksA family transcriptional regulator | | uniclust | UniRef100\_A0A7V2X9Q0 | 97.7 | 1.1e-06 | 2.1e-12 | 63.3 | 67 | (32, 99) | 103 | (56, 135) | 152 | TraR/DksA family transcriptional regulator | TraR/DksA family transcriptional regulator | | uniclust | UniRef100\_A0A0H2KR58 | 97.7 | 9.8e-07 | 2.1e-12 | 65.7 | 36 | (63, 99) | 103 | (113, 153) | 158 | DksA C4-type domain-containing protein | DksA C4-type domain-containing protein | | uniclust | UniRef100\_A0A9E2N3C8 | 97.7 | 1.2e-06 | 2.1e-12 | 63.3 | 38 | (64, 102) | 103 | (89, 131) | 153 | TraR/DksA family transcriptional regulator | TraR/DksA family transcriptional regulator | | uniclust | UniRef100\_A0A9D6TUP0 | 97.7 | 1.2e-06 | 2.1e-12 | 58.3 | 39 | (63, 102) | 103 | (33, 76) | 89 | TraR/DksA C4-type zinc finger protein | TraR/DksA C4-type zinc finger protein | | uniclust | UniRef100\_A0A3D1TEN9 | 97.7 | 1e-06 | 2.1e-12 | 60.9 | 38 | (63, 101) | 103 | (47, 89) | 96 | DksA C4-type domain-containing protein (Fragment) | DksA C4-type domain-containing protein (Fragment) | | uniclust | UniRef100\_A0A929HNH4 | 97.7 | 1.2e-06 | 2.2e-12 | 67.6 | 70 | (31, 101) | 103 | (152, 234) | 260 | TraR/DksA family transcriptional regulator | TraR/DksA family transcriptional regulator | | uniclust | UniRef100\_A0A146G6K6 | 97.7 | 1.2e-06 | 2.2e-12 | 67.4 | 65 | (36, 101) | 103 | (105, 182) | 218 | RNA polymerase-binding transcription factor DksA | RNA polymerase-binding transcription factor DksA | | uniclust | UniRef100\_A0A536HB94 | 97.7 | 1.2e-06 | 2.3e-12 | 56.1 | 36 | (65, 101) | 103 | (29, 69) | 72 | DksA C4-type domain-containing protein | DksA C4-type domain-containing protein | | uniclust | UniRef100\_A0A1G8LDJ8 | 97.7 | 1.1e-06 | 2.3e-12 | 68.8 | 60 | (37, 97) | 103 | (75, 147) | 219 | RNA polymerase-binding protein DksA | RNA polymerase-binding protein DksA | | uniclust | UniRef100\_A0A7Y2DU12 | 97.7 | 1.2e-06 | 2.4e-12 | 63.2 | 62 | (37, 99) | 103 | (48, 122) | 131 | DksA C4-type domain-containing protein | DksA C4-type domain-containing protein | | uniclust | UniRef100\_A0A4Q3NJL8 | 97.7 | 1.2e-06 | 2.4e-12 | 66.4 | 36 | (63, 99) | 103 | (136, 176) | 193 | TraR/DksA family transcriptional regulator | TraR/DksA family transcriptional regulator | | uniclust | UniRef100\_A0A6B0XPH2 | 97.7 | 1.2e-06 | 2.4e-12 | 72.8 | 68 | (31, 99) | 103 | (377, 457) | 463 | TraR/DksA family transcriptional regulator | TraR/DksA family transcriptional regulator | | uniclust | UniRef100\_A0A7C5ZAZ5 | 97.7 | 1.2e-06 | 2.4e-12 | 71.3 | 68 | (31, 99) | 103 | (293, 373) | 375 | DksA C4-type domain-containing protein | DksA C4-type domain-containing protein | | uniclust | UniRef100\_A0A1V4XFM2 | 97.7 | 1.3e-06 | 2.4e-12 | 64.2 | 65 | (35, 100) | 103 | (58, 135) | 159 | RNA polymerase-binding transcription factor DksA | RNA polymerase-binding transcription factor DksA | | uniclust | UniRef100\_A0A1G3QAA7 | 97.7 | 1.2e-06 | 2.5e-12 | 65.0 | 66 | (34, 100) | 103 | (58, 136) | 158 | DksA C4-type domain-containing protein | DksA C4-type domain-containing protein | | uniclust | UniRef100\_UPI001FE60161 | 97.7 | 1.4e-06 | 2.5e-12 | 59.0 | 67 | (31, 101) | 103 | (20, 91) | 99 | TraR/DksA family transcriptional regulator | TraR/DksA family transcriptional regulator | | uniclust | UniRef100\_A0A889IPF0 | 97.6 | 1.3e-06 | 2.6e-12 | 58.4 | 59 | (40, 99) | 103 | (9, 74) | 87 | DksA C4-type domain-containing protein | DksA C4-type domain-containing protein | | uniclust | UniRef100\_A0A081K6D0 | 97.6 | 1.2e-06 | 2.6e-12 | 64.6 | 38 | (63, 101) | 103 | (97, 139) | 141 | Molecular chaperone DnaK | Molecular chaperone DnaK | | uniclust | UniRef100\_A0A0F7JX09 | 97.6 | 1.5e-06 | 2.8e-12 | 59.2 | 64 | (36, 100) | 103 | (1, 72) | 96 | DksA C4-type domain-containing protein | DksA C4-type domain-containing protein | | uniclust | UniRef100\_UPI001F35BB70 | 97.6 | 1.6e-06 | 2.9e-12 | 53.3 | 31 | (68, 99) | 103 | (15, 50) | 56 | TraR/DksA family transcriptional regulator | TraR/DksA family transcriptional regulator | | uniclust | UniRef100\_A0A336N326 | 97.6 | 1.5e-06 | 2.9e-12 | 56.5 | 66 | (35, 101) | 103 | (1, 72) | 73 | DksA C4-type domain-containing protein | DksA C4-type domain-containing protein | | uniclust | UniRef100\_A0A0K3R167 | 97.6 | 1.6e-06 | 2.9e-12 | 59.5 | 70 | (30, 101) | 103 | (10, 84) | 103 | Conjugal transfer protein TraR | Conjugal transfer protein TraR | | uniclust | UniRef100\_A0A7V3BK14 | 97.6 | 1.5e-06 | 2.9e-12 | 63.8 | 67 | (32, 99) | 103 | (74, 153) | 159 | TraR/DksA family transcriptional regulator | TraR/DksA family transcriptional regulator | | uniclust | UniRef100\_A0A846Q3K0 | 97.6 | 1.6e-06 | 3e-12 | 57.1 | 37 | (63, 100) | 103 | (33, 74) | 77 | DksA C4-type domain-containing protein (Fragment) | DksA C4-type domain-containing protein (Fragment) | | uniclust | UniRef100\_A0A8H9EJZ3 | 97.6 | 1.6e-06 | 3.1e-12 | 57.1 | 35 | (67, 102) | 103 | (33, 72) | 76 | TraR/DksA family transcriptional regulator | TraR/DksA family transcriptional regulator | | uniclust | UniRef100\_A0A3B9NYN9 | 97.6 | 1.7e-06 | 3.2e-12 | 53.3 | 34 | (68, 102) | 103 | (8, 48) | 54 | DksA C4-type domain-containing protein | DksA C4-type domain-containing protein | | uniclust | UniRef100\_UPI0002E76F17 | 97.6 | 1.7e-06 | 3.3e-12 | 55.8 | 62 | (34, 102) | 103 | (2, 63) | 70 | hypothetical protein | hypothetical protein | | uniclust | UniRef100\_UPI001D0D1C56 | 97.6 | 1.7e-06 | 3.3e-12 | 52.8 | 35 | (65, 100) | 103 | (12, 50) | 51 | TraR/DksA C4-type zinc finger protein | TraR/DksA C4-type zinc finger protein | | uniclust | UniRef100\_A0A1V4S0C0 | 97.6 | 1.8e-06 | 3.4e-12 | 66.0 | 68 | (31, 99) | 103 | (38, 119) | 238 | DksA C4-type domain-containing protein | DksA C4-type domain-containing protein | | uniclust | UniRef100\_B9BF14 | 97.6 | 1.8e-06 | 3.6e-12 | 67.9 | 64 | (36, 100) | 103 | (167, 243) | 252 | Transcriptional regulator, TraR/DksA family | Transcriptional regulator, TraR/DksA family | | uniclust | UniRef100\_A0A0G0PVY8 | 97.6 | 1.7e-06 | 3.7e-12 | 66.7 | 63 | (37, 100) | 103 | (100, 175) | 200 | DnaK suppressor protein (Modular protein) | DnaK suppressor protein (Modular protein) | | uniclust | UniRef100\_A0A3A4A6E0 | 97.6 | 1.9e-06 | 3.7e-12 | 54.7 | 35 | (63, 98) | 103 | (20, 59) | 61 | DksA C4-type domain-containing protein | DksA C4-type domain-containing protein | | uniclust | UniRef100\_A0A2D9HEW6 | 97.6 | 1.8e-06 | 3.8e-12 | 64.5 | 35 | (64, 99) | 103 | (116, 155) | 157 | DksA C4-type domain-containing protein | DksA C4-type domain-containing protein | | uniclust | UniRef100\_M4SM17 | 97.6 | 2e-06 | 3.8e-12 | 59.4 | 69 | (28, 99) | 103 | (14, 82) | 109 | Uncharacterized protein | Uncharacterized protein | | uniclust | UniRef100\_A0A2E4X1U5 | 97.6 | 1.8e-06 | 3.8e-12 | 63.9 | 64 | (36, 100) | 103 | (46, 122) | 148 | RNA polymerase-binding protein DksA | RNA polymerase-binding protein DksA | | uniclust | UniRef100\_A0A1F3SKA7 | 97.6 | 1.9e-06 | 3.9e-12 | 62.8 | 67 | (32, 99) | 103 | (39, 118) | 141 | DksA C4-type domain-containing protein | DksA C4-type domain-containing protein | | uniclust | UniRef100\_A0A1V5UVG4 | 97.6 | 2e-06 | 3.9e-12 | 67.7 | 62 | (37, 99) | 103 | (175, 249) | 257 | General stress protein 16O | General stress protein 16O | | uniclust | UniRef100\_A0A953US15 | 97.6 | 2.2e-06 | 4e-12 | 62.6 | 74 | (24, 98) | 103 | (24, 110) | 165 | TraR/DksA family transcriptional regulator | TraR/DksA family transcriptional regulator | | uniclust | UniRef100\_A0A7C4I3K4 | 97.6 | 2.1e-06 | 4e-12 | 52.2 | 38 | (63, 101) | 103 | (3, 45) | 49 | Transcriptional regulator, TraR/DksA family protein (Fragment) | Transcriptional regulator, TraR/DksA family protein (Fragment) | | uniclust | UniRef100\_UPI001FEB62E6 | 97.6 | 2.2e-06 | 4.1e-12 | 51.9 | 33 | (67, 100) | 103 | (4, 41) | 52 | TraR/DksA C4-type zinc finger protein | TraR/DksA C4-type zinc finger protein | | uniclust | UniRef100\_A0A5Q0M9K3 | 97.6 | 2.2e-06 | 4.1e-12 | 62.6 | 63 | (36, 99) | 103 | (67, 142) | 153 | TraR/DksA family transcriptional regulator | TraR/DksA family transcriptional regulator | | uniclust | UniRef100\_A0A258C2E0 | 97.6 | 2.2e-06 | 4.1e-12 | 52.6 | 33 | (67, 100) | 103 | (14, 51) | 52 | DksA C4-type domain-containing protein | DksA C4-type domain-containing protein | | uniclust | UniRef100\_A0A0J0V0N8 | 97.6 | 2e-06 | 4.2e-12 | 67.6 | 61 | (39, 100) | 103 | (150, 223) | 225 | Transcriptional regulator, TraR/DksA family | Transcriptional regulator, TraR/DksA family | | uniclust | UniRef100\_A0A0G0L1H4 | 97.6 | 1.8e-06 | 4.3e-12 | 64.6 | 62 | (36, 98) | 103 | (72, 146) | 153 | DksA C4-type domain-containing protein | DksA C4-type domain-containing protein | | uniclust | UniRef100\_A0A345GTX4 | 97.6 | 2.3e-06 | 4.4e-12 | 56.0 | 63 | (35, 100) | 103 | (4, 66) | 74 | DUF2116 family Zn-ribbon domain-containing protein | DUF2116 family Zn-ribbon domain-containing protein | | uniclust | UniRef100\_A0A9C8GJI6 | 97.6 | 2.4e-06 | 4.4e-12 | 61.6 | 36 | (63, 99) | 103 | (110, 150) | 151 | Molecular chaperone DnaK | Molecular chaperone DnaK | | uniclust | UniRef100\_A0A944V385 | 97.6 | 2.4e-06 | 4.5e-12 | 53.9 | 40 | (63, 103) | 103 | (20, 64) | 65 | DksA C4-type domain-containing protein | DksA C4-type domain-containing protein | | uniclust | UniRef100\_A0A250KX83 | 97.6 | 2.3e-06 | 4.5e-12 | 58.7 | 67 | (34, 101) | 103 | (2, 82) | 96 | Transcriptional regulator, TraR/DksA family | Transcriptional regulator, TraR/DksA family | | uniclust | UniRef100\_A0A3E0P096 | 97.6 | 2.2e-06 | 4.5e-12 | 65.3 | 66 | (33, 99) | 103 | (105, 183) | 189 | TraR/DksA family transcriptional regulator | TraR/DksA family transcriptional regulator | | uniclust | UniRef100\_A0A0J7LZ52 | 97.6 | 2.2e-06 | 4.7e-12 | 56.3 | 51 | (42, 95) | 103 | (11, 66) | 70 | DksA C4-type domain-containing protein | DksA C4-type domain-containing protein | | uniclust | UniRef100\_A0A010ZWH0 | 97.5 | 2.1e-06 | 4.9e-12 | 65.8 | 35 | (63, 98) | 103 | (123, 162) | 182 | DnaK suppressor protein | DnaK suppressor protein | | uniclust | UniRef100\_A0A2E3W236 | 97.5 | 2.8e-06 | 5.2e-12 | 63.8 | 65 | (36, 101) | 103 | (81, 158) | 202 | DksA C4-type domain-containing protein | DksA C4-type domain-containing protein | | uniclust | UniRef100\_A0A2V6NWY6 | 97.5 | 2.8e-06 | 5.2e-12 | 54.7 | 39 | (63, 102) | 103 | (15, 58) | 73 | DksA C4-type domain-containing protein | DksA C4-type domain-containing protein | | uniclust | UniRef100\_A0A142WZJ9 | 97.5 | 2.3e-06 | 5.2e-12 | 68.9 | 64 | (34, 98) | 103 | (49, 125) | 264 | General stress protein 16O | General stress protein 16O | | uniclust | UniRef100\_A0A086P6H6 | 97.5 | 2.3e-06 | 5.2e-12 | 65.3 | 35 | (62, 97) | 103 | (96, 135) | 177 | DnaK suppressor protein | DnaK suppressor protein | | uniclust | UniRef100\_A0A5Q2RKE7 | 97.5 | 2.6e-06 | 5.2e-12 | 60.7 | 35 | (63, 98) | 103 | (83, 122) | 123 | DksA C4-type domain-containing protein | DksA C4-type domain-containing protein | | uniclust | UniRef100\_A0A965KYD4 | 97.5 | 2.9e-06 | 5.2e-12 | 61.5 | 65 | (35, 100) | 103 | (80, 149) | 154 | DksA C4-type domain-containing protein | DksA C4-type domain-containing protein | | uniclust | UniRef100\_A0A3D1BL39 | 97.5 | 2.7e-06 | 5.3e-12 | 57.1 | 36 | (63, 99) | 103 | (30, 70) | 86 | Transcriptional regulator, TraR/DksA family protein (Fragment) | Transcriptional regulator, TraR/DksA family protein (Fragment) | | uniclust | UniRef100\_A0A1F3X404 | 97.5 | 2.9e-06 | 5.6e-12 | 55.4 | 37 | (63, 100) | 103 | (26, 67) | 75 | DksA C4-type domain-containing protein | DksA C4-type domain-containing protein | | uniclust | UniRef100\_A0A3D8HS68 | 97.5 | 3.3e-06 | 6e-12 | 61.0 | 66 | (35, 101) | 103 | (52, 130) | 150 | RNA polymerase-binding protein DksA | RNA polymerase-binding protein DksA | | uniclust | UniRef100\_A0A0G0G8U5 | 97.5 | 2.7e-06 | 6.1e-12 | 63.8 | 59 | (38, 97) | 103 | (80, 151) | 155 | Transcriptional regulator, TraR/DksA family | Transcriptional regulator, TraR/DksA family | | uniclust | UniRef100\_A0A0G0CFG9 | 97.5 | 2.6e-06 | 6.2e-12 | 62.8 | 64 | (33, 97) | 103 | (57, 133) | 138 | Transcriptional regulator, TraR/DksA family | Transcriptional regulator, TraR/DksA family | | uniclust | UniRef100\_UPI0003651E65 | 97.5 | 3.1e-06 | 6.2e-12 | 56.4 | 63 | (39, 102) | 103 | (8, 75) | 78 | hypothetical protein | hypothetical protein | | uniclust | UniRef100\_A0A2R4VRC7 | 97.5 | 3.3e-06 | 6.3e-12 | 52.3 | 33 | (68, 101) | 103 | (11, 48) | 54 | DksA C4-type domain-containing protein | DksA C4-type domain-containing protein | | uniclust | UniRef100\_A0A0F3Q901 | 97.5 | 3.1e-06 | 6.4e-12 | 62.9 | 71 | (30, 101) | 103 | (65, 148) | 153 | Putative dnaK suppressor-like protein | Putative dnaK suppressor-like protein | | uniclust | UniRef100\_A0A350NQT1 | 97.5 | 3.4e-06 | 7e-12 | 60.9 | 37 | (63, 100) | 103 | (27, 68) | 130 | DksA C4-type domain-containing protein | DksA C4-type domain-containing protein | | uniclust | UniRef100\_G5LKE7 | 97.5 | 3.6e-06 | 7.1e-12 | 52.9 | 34 | (67, 101) | 103 | (13, 51) | 58 | Putative Zinc-finger containing protein (Fragment) | Putative Zinc-finger containing protein (Fragment) | | uniclust | UniRef100\_A0A1H9NNW1 | 97.5 | 3.5e-06 | 7.3e-12 | 61.9 | 58 | (37, 95) | 103 | (45, 115) | 140 | Transcriptional regulator, TraR/DksA family | Transcriptional regulator, TraR/DksA family | | uniclust | UniRef100\_A0A518LQ83 | 97.5 | 3.4e-06 | 7.3e-12 | 64.8 | 64 | (36, 100) | 103 | (109, 185) | 189 | General stress protein 16O | General stress protein 16O | | uniclust | UniRef100\_A0A1E7HCG8 | 97.5 | 3.9e-06 | 7.5e-12 | 64.1 | 37 | (64, 101) | 103 | (75, 116) | 203 | DksA C4-type domain-containing protein | DksA C4-type domain-containing protein | | uniclust | UniRef100\_A0A0K1NCL4 | 97.5 | 3.8e-06 | 7.6e-12 | 62.1 | 38 | (63, 101) | 103 | (104, 146) | 152 | DksA C4-type domain-containing protein | DksA C4-type domain-containing protein | | uniclust | UniRef100\_A0A1Q7U7Y8 | 97.5 | 4.2e-06 | 7.7e-12 | 60.6 | 71 | (30, 101) | 103 | (24, 107) | 153 | DksA C4-type domain-containing protein | DksA C4-type domain-containing protein | | uniclust | UniRef100\_A0A0W8E1D5 | 97.5 | 3.7e-06 | 8e-12 | 63.6 | 61 | (38, 99) | 103 | (48, 121) | 167 | Dnak suppressor protein | Dnak suppressor protein | | uniclust | UniRef100\_A0A1Q7SQ33 | 97.5 | 4.1e-06 | 8.4e-12 | 63.7 | 38 | (63, 101) | 103 | (104, 146) | 181 | DksA C4-type domain-containing protein | DksA C4-type domain-containing protein | | uniclust | UniRef100\_A0A2E3P7D0 | 97.5 | 4.4e-06 | 8.5e-12 | 53.4 | 54 | (34, 93) | 103 | (2, 60) | 65 | Molecular chaperone DnaK | Molecular chaperone DnaK | | uniclust | UniRef100\_A0A223PH24 | 97.5 | 4.2e-06 | 8.7e-12 | 59.2 | 37 | (63, 100) | 103 | (68, 109) | 111 | Molecular chaperone DnaK | Molecular chaperone DnaK | | uniclust | UniRef100\_A0A1V1W784 | 97.4 | 4.4e-06 | 9e-12 | 59.9 | 35 | (63, 98) | 103 | (74, 113) | 122 | C4-type zinc finger protein, DksA/TraR family | C4-type zinc finger protein, DksA/TraR family | | uniclust | UniRef100\_A0A7Y8LK13 | 97.4 | 4.9e-06 | 9.4e-12 | 53.4 | 35 | (64, 99) | 103 | (14, 53) | 67 | TraR/DksA C4-type zinc finger protein | TraR/DksA C4-type zinc finger protein | | uniclust | UniRef100\_A0A0D8FU18 | 97.4 | 4.6e-06 | 9.6e-12 | 65.5 | 54 | (44, 98) | 103 | (148, 214) | 232 | General stress protein 16O | General stress protein 16O | | uniclust | UniRef100\_A0A0F7HEF4 | 97.4 | 4.9e-06 | 9.6e-12 | 58.8 | 52 | (46, 100) | 103 | (15, 71) | 117 | DksA C4-type domain-containing protein | DksA C4-type domain-containing protein | | uniclust | UniRef100\_A0A3N5HZ15 | 97.4 | 5.3e-06 | 9.8e-12 | 60.3 | 63 | (36, 99) | 103 | (79, 154) | 157 | TraR/DksA family transcriptional regulator | TraR/DksA family transcriptional regulator | | uniclust | UniRef100\_A0A925I2M1 | 97.4 | 5.1e-06 | 9.8e-12 | 57.4 | 38 | (63, 101) | 103 | (54, 96) | 103 | TraR/DksA family transcriptional regulator | TraR/DksA family transcriptional regulator | | uniclust | UniRef100\_A0A1G1XR92 | 97.4 | 5.2e-06 | 1e-11 | 60.2 | 39 | (63, 102) | 103 | (82, 125) | 140 | DksA C4-type domain-containing protein | DksA C4-type domain-containing protein | | uniclust | UniRef100\_A0A2H0RQV3 | 97.4 | 5.6e-06 | 1e-11 | 57.1 | 36 | (63, 99) | 103 | (50, 90) | 109 | DksA C4-type domain-containing protein | DksA C4-type domain-containing protein | | uniclust | UniRef100\_A0A0S4KQC6 | 97.4 | 5e-06 | 1.1e-11 | 63.1 | 69 | (31, 100) | 103 | (84, 165) | 176 | DksA C4-type domain-containing protein | DksA C4-type domain-containing protein | | uniclust | UniRef100\_A0A0G0A276 | 97.4 | 5.2e-06 | 1.1e-11 | 63.7 | 38 | (63, 101) | 103 | (123, 165) | 187 | DNA-binding protein | DNA-binding protein | | uniclust | UniRef100\_A0A0G1FXN7 | 97.4 | 4.6e-06 | 1.1e-11 | 61.4 | 36 | (62, 98) | 103 | (91, 131) | 135 | DksA C4-type domain-containing protein | DksA C4-type domain-containing protein | | uniclust | UniRef100\_A0A7C3SEN0 | 97.4 | 5.3e-06 | 1.1e-11 | 55.1 | 35 | (63, 98) | 103 | (35, 74) | 75 | DksA C4-type domain-containing protein | DksA C4-type domain-containing protein | | uniclust | UniRef100\_A0A067LRQ7 | 97.4 | 5.1e-06 | 1.1e-11 | 61.9 | 36 | (63, 99) | 103 | (103, 143) | 148 | Suppressor protein DnaK | Suppressor protein DnaK | | uniclust | UniRef100\_UPI0019149785 | 97.4 | 6.3e-06 | 1.2e-11 | 53.1 | 63 | (35, 101) | 103 | (1, 64) | 72 | hypothetical protein | hypothetical protein | | uniclust | UniRef100\_A0A1V3RQS2 | 97.4 | 5.8e-06 | 1.2e-11 | 56.6 | 64 | (33, 97) | 103 | (11, 82) | 92 | DksA C4-type domain-containing protein | DksA C4-type domain-containing protein | | uniclust | UniRef100\_A0A1G2XD98 | 97.4 | 5.6e-06 | 1.2e-11 | 64.9 | 67 | (32, 99) | 103 | (82, 161) | 222 | DksA C4-type domain-containing protein | DksA C4-type domain-containing protein | | uniclust | UniRef100\_UPI0009FAB1EC | 97.4 | 6.4e-06 | 1.2e-11 | 61.1 | 59 | (37, 96) | 103 | (49, 120) | 181 | TraR/DksA C4-type zinc finger protein | TraR/DksA C4-type zinc finger protein | | uniclust | UniRef100\_UPI001F4393EB | 97.4 | 6.5e-06 | 1.2e-11 | 52.9 | 32 | (67, 99) | 103 | (30, 66) | 71 | TraR/DksA C4-type zinc finger protein | TraR/DksA C4-type zinc finger protein | | uniclust | UniRef100\_A0A1U9NPL5 | 97.4 | 5.5e-06 | 1.2e-11 | 67.2 | 65 | (34, 99) | 103 | (141, 218) | 284 | General stress protein 16O | General stress protein 16O | | uniclust | UniRef100\_A0A411YA84 | 97.4 | 6.6e-06 | 1.2e-11 | 60.9 | 62 | (34, 96) | 103 | (99, 173) | 177 | TraR/DksA family transcriptional regulator | TraR/DksA family transcriptional regulator | | uniclust | UniRef100\_UPI002362D03C | 97.4 | 6.6e-06 | 1.2e-11 | 50.2 | 33 | (68, 101) | 103 | (11, 48) | 53 | TraR/DksA C4-type zinc finger protein | TraR/DksA C4-type zinc finger protein | | uniclust | UniRef100\_A0A0S4V0E8 | 97.4 | 6.7e-06 | 1.2e-11 | 54.4 | 36 | (63, 99) | 103 | (42, 82) | 84 | DnaK suppressor protein | DnaK suppressor protein | | uniclust | UniRef100\_A0A356T9N2 | 97.4 | 6.2e-06 | 1.3e-11 | 67.8 | 71 | (30, 101) | 103 | (251, 334) | 346 | RNA polymerase-binding protein DksA | RNA polymerase-binding protein DksA | | uniclust | UniRef100\_A0A7V5HPI1 | 97.4 | 6.3e-06 | 1.3e-11 | 51.6 | 34 | (63, 97) | 103 | (14, 52) | 55 | TraR/DksA family transcriptional regulator | TraR/DksA family transcriptional regulator | | uniclust | UniRef100\_A0A377TLM7 | 97.4 | 7.1e-06 | 1.3e-11 | 61.6 | 74 | (27, 101) | 103 | (42, 128) | 197 | RNA polymerase-binding transcription factor DksA | RNA polymerase-binding transcription factor DksA | | uniclust | UniRef100\_A0A7C3B7W3 | 97.4 | 6.8e-06 | 1.3e-11 | 47.9 | 31 | (64, 95) | 103 | (3, 38) | 40 | TraR/DksA family transcriptional regulator | TraR/DksA family transcriptional regulator | | uniclust | UniRef100\_A0A3N5XHU1 | 97.4 | 7.2e-06 | 1.3e-11 | 56.6 | 35 | (63, 98) | 103 | (60, 99) | 109 | DksA C4-type domain-containing protein (Fragment) | DksA C4-type domain-containing protein (Fragment) | | uniclust | UniRef100\_A0A1Z3LXK0 | 97.4 | 6.7e-06 | 1.3e-11 | 58.3 | 53 | (44, 99) | 103 | (43, 100) | 117 | DksA C4-type domain-containing protein | DksA C4-type domain-containing protein | | uniclust | UniRef100\_A0A2X1UWH1 | 97.4 | 6.9e-06 | 1.3e-11 | 55.0 | 67 | (33, 100) | 103 | (3, 77) | 83 | Phage/conjugal plasmid C-4 type zinc finger protein, TraR family | Phage/conjugal plasmid C-4 type zinc finger protein, TraR family | | uniclust | UniRef100\_A0A2V8PR07 | 97.4 | 7.5e-06 | 1.4e-11 | 55.2 | 62 | (36, 98) | 103 | (9, 83) | 94 | DksA C4-type domain-containing protein | DksA C4-type domain-containing protein | | uniclust | UniRef100\_A0A951CX43 | 97.4 | 7.7e-06 | 1.4e-11 | 52.5 | 36 | (63, 99) | 103 | (16, 56) | 70 | TraR/DksA C4-type zinc finger protein | TraR/DksA C4-type zinc finger protein | | uniclust | UniRef100\_A0A7V2MWM1 | 97.4 | 7.9e-06 | 1.5e-11 | 59.2 | 38 | (63, 101) | 103 | (100, 142) | 152 | TraR/DksA family transcriptional regulator | TraR/DksA family transcriptional regulator | | uniclust | UniRef100\_A0A1G3ZPZ8 | 97.4 | 7.2e-06 | 1.5e-11 | 69.7 | 65 | (36, 101) | 103 | (352, 429) | 477 | DksA C4-type domain-containing protein | DksA C4-type domain-containing protein | | uniclust | UniRef100\_A0A0J6YR85 | 97.4 | 7.3e-06 | 1.5e-11 | 64.3 | 68 | (31, 99) | 103 | (149, 229) | 231 | DksA C4-type domain-containing protein | DksA C4-type domain-containing protein | | uniclust | UniRef100\_A0A7C5J814 | 97.4 | 8.1e-06 | 1.5e-11 | 54.5 | 64 | (36, 100) | 103 | (1, 82) | 88 | DksA C4-type domain-containing protein | DksA C4-type domain-containing protein | | uniclust | UniRef100\_A0A2V8ELD6 | 97.4 | 7.6e-06 | 1.5e-11 | 63.1 | 37 | (63, 100) | 103 | (144, 185) | 209 | DksA C4-type domain-containing protein | DksA C4-type domain-containing protein | | uniclust | UniRef100\_A0A540V863 | 97.4 | 7.8e-06 | 1.5e-11 | 52.7 | 62 | (36, 102) | 103 | (1, 62) | 67 | DUF2116 family Zn-ribbon domain-containing protein | DUF2116 family Zn-ribbon domain-containing protein | | uniclust | UniRef100\_A0A2V7UG18 | 97.4 | 8.2e-06 | 1.5e-11 | 58.9 | 63 | (36, 99) | 103 | (71, 146) | 148 | DksA C4-type domain-containing protein | DksA C4-type domain-containing protein | | uniclust | UniRef100\_A0A0C1W9X3 | 97.4 | 7.4e-06 | 1.5e-11 | 60.4 | 67 | (35, 102) | 103 | (51, 130) | 142 | DksA C4-type domain-containing protein | DksA C4-type domain-containing protein | | uniclust | UniRef100\_A0A2N8GFW6 | 97.3 | 8.7e-06 | 1.6e-11 | 48.6 | 46 | (35, 81) | 103 | (1, 47) | 47 | Conjugal transfer protein TraR (Fragment) | Conjugal transfer protein TraR (Fragment) | | uniclust | UniRef100\_UPI0022357C3F | 97.3 | 8.9e-06 | 1.6e-11 | 53.2 | 35 | (63, 98) | 103 | (7, 46) | 78 | TraR/DksA family transcriptional regulator | TraR/DksA family transcriptional regulator | | uniclust | UniRef100\_A0A940R1G3 | 97.3 | 9e-06 | 1.6e-11 | 59.7 | 65 | (35, 100) | 103 | (39, 116) | 165 | C4-type zinc finger protein, DksA/TraR family | C4-type zinc finger protein, DksA/TraR family | | uniclust | UniRef100\_A0A2E4ZEP8 | 97.3 | 8.8e-06 | 1.7e-11 | 56.7 | 35 | (63, 98) | 103 | (65, 104) | 109 | DksA C4-type domain-containing protein | DksA C4-type domain-containing protein | | uniclust | UniRef100\_A0A2D6N8B5 | 97.3 | 9.1e-06 | 1.8e-11 | 58.2 | 36 | (63, 99) | 103 | (76, 116) | 125 | Conjugal transfer protein TraR | Conjugal transfer protein TraR | | uniclust | UniRef100\_A0A836SUD0 | 97.3 | 9.5e-06 | 1.8e-11 | 53.0 | 36 | (64, 100) | 103 | (10, 50) | 73 | DksA C4-type domain-containing protein | DksA C4-type domain-containing protein | | uniclust | UniRef100\_A0A1F9A4Z3 | 97.3 | 9.1e-06 | 1.8e-11 | 57.9 | 71 | (31, 102) | 103 | (35, 118) | 119 | DksA C4-type domain-containing protein | DksA C4-type domain-containing protein | | uniclust | UniRef100\_A0A2T2V596 | 97.3 | 9.1e-06 | 1.8e-11 | 56.8 | 64 | (37, 101) | 103 | (25, 101) | 104 | DksA C4-type domain-containing protein | DksA C4-type domain-containing protein | | uniclust | UniRef100\_A0A4R2CHW8 | 97.3 | 8.9e-06 | 1.8e-11 | 60.7 | 38 | (63, 101) | 103 | (112, 154) | 155 | TraR/DksA family transcriptional regulator | TraR/DksA family transcriptional regulator | | uniclust | UniRef100\_A0A0G0VR09 | 97.3 | 7.9e-06 | 1.8e-11 | 68.6 | 35 | (63, 98) | 103 | (310, 349) | 365 | DNA-binding protein | DNA-binding protein | | uniclust | UniRef100\_A0A497A8C1 | 97.3 | 9.7e-06 | 1.9e-11 | 54.4 | 36 | (63, 99) | 103 | (36, 76) | 84 | Molecular chaperone DnaK (Fragment) | Molecular chaperone DnaK (Fragment) | | uniclust | UniRef100\_A0A2E7BH60 | 97.3 | 1e-05 | 1.9e-11 | 58.2 | 68 | (31, 99) | 103 | (39, 119) | 143 | DksA C4-type domain-containing protein | DksA C4-type domain-containing protein | | uniclust | UniRef100\_A0A7Z9JQQ5 | 97.3 | 9.9e-06 | 1.9e-11 | 52.8 | 35 | (66, 101) | 103 | (31, 70) | 71 | RNA polymerase-binding protein DksA | RNA polymerase-binding protein DksA | | uniclust | UniRef100\_A0A1H7ZBE4 | 97.3 | 9.8e-06 | 1.9e-11 | 52.6 | 63 | (35, 99) | 103 | (1, 66) | 67 | Transcriptional regulator, TraR/DksA family | Transcriptional regulator, TraR/DksA family | | uniclust | UniRef100\_A0A0S2HZL1 | 97.3 | 8.8e-06 | 1.9e-11 | 60.1 | 38 | (63, 101) | 103 | (94, 136) | 138 | RNA polymerase-binding protein DksA | RNA polymerase-binding protein DksA | | uniclust | UniRef100\_A0A934NEW0 | 97.3 | 1.1e-05 | 2e-11 | 51.0 | 32 | (63, 95) | 103 | (23, 59) | 63 | TraR/DksA C4-type zinc finger protein | TraR/DksA C4-type zinc finger protein | | uniclust | UniRef100\_A0A1Z9AE00 | 97.3 | 9.9e-06 | 2e-11 | 64.7 | 65 | (36, 101) | 103 | (158, 235) | 264 | DksA C4-type domain-containing protein (Fragment) | DksA C4-type domain-containing protein (Fragment) | | uniclust | UniRef100\_A0A317E8Z2 | 97.3 | 1.1e-05 | 2.1e-11 | 51.5 | 60 | (36, 99) | 103 | (1, 67) | 68 | Conjugal transfer protein TraR | Conjugal transfer protein TraR | | uniclust | UniRef100\_A0A177RA40 | 97.3 | 1.2e-05 | 2.1e-11 | 58.8 | 62 | (36, 98) | 103 | (44, 118) | 158 | DksA C4-type domain-containing protein (Fragment) | DksA C4-type domain-containing protein (Fragment) | | uniclust | UniRef100\_A0A0B8P4P1 | 97.3 | 1.1e-05 | 2.2e-11 | 51.4 | 39 | (63, 102) | 103 | (17, 60) | 63 | C4-type zinc finger protein | C4-type zinc finger protein | | uniclust | UniRef100\_A0A084SYV1 | 97.3 | 1.1e-05 | 2.2e-11 | 58.7 | 37 | (63, 100) | 103 | (80, 121) | 133 | DksA | DksA | | uniclust | UniRef100\_A0A1G1E393 | 97.3 | 9.8e-06 | 2.2e-11 | 66.0 | 33 | (63, 96) | 103 | (89, 126) | 281 | DksA C4-type domain-containing protein | DksA C4-type domain-containing protein | | uniclust | UniRef100\_A0A496XS46 | 97.3 | 1.2e-05 | 2.2e-11 | 56.2 | 69 | (29, 98) | 103 | (32, 113) | 114 | DksA C4-type domain-containing protein | DksA C4-type domain-containing protein | | uniclust | UniRef100\_A0A4R4LN85 | 97.3 | 1.2e-05 | 2.2e-11 | 59.2 | 32 | (63, 95) | 103 | (130, 166) | 168 | Molecular chaperone DnaK | Molecular chaperone DnaK | | uniclust | UniRef100\_A0A2D5E8I5 | 97.3 | 1e-05 | 2.3e-11 | 68.1 | 64 | (36, 100) | 103 | (286, 362) | 375 | DksA C4-type domain-containing protein | DksA C4-type domain-containing protein | | uniclust | UniRef100\_A0A0U2YW82 | 97.3 | 1.2e-05 | 2.3e-11 | 54.5 | 34 | (63, 97) | 103 | (57, 95) | 97 | C4\_traR\_proteo: phage/conjugal plasmid C-4 type zinc finger protein, TraR | C4\_traR\_proteo: phage/conjugal plasmid C-4 type zinc finger protein, TraR | | uniclust | UniRef100\_A0A1A9UK84 | 97.3 | 1.3e-05 | 2.4e-11 | 56.8 | 65 | (27, 99) | 103 | (54, 118) | 128 | Ribosomal protein L9 domain-containing protein | Ribosomal protein L9 domain-containing protein | | uniclust | UniRef100\_A0A257H7G2 | 97.3 | 1.3e-05 | 2.4e-11 | 46.6 | 33 | (66, 99) | 103 | (3, 40) | 41 | DksA C4-type domain-containing protein | DksA C4-type domain-containing protein | | uniclust | UniRef100\_A0A357XRK3 | 97.3 | 1.3e-05 | 2.4e-11 | 64.6 | 64 | (35, 99) | 103 | (267, 343) | 356 | DksA C4-type domain-containing protein | DksA C4-type domain-containing protein | | uniclust | UniRef100\_A0A081NL60 | 97.3 | 1.3e-05 | 2.5e-11 | 52.8 | 65 | (36, 101) | 103 | (1, 71) | 75 | DksA C4-type domain-containing protein | DksA C4-type domain-containing protein | | uniclust | UniRef100\_A0A7Y6Z4L2 | 97.3 | 1.4e-05 | 2.5e-11 | 57.3 | 65 | (35, 101) | 103 | (62, 134) | 137 | TraR/DksA C4-type zinc finger protein | TraR/DksA C4-type zinc finger protein | | uniclust | UniRef100\_A0A257XAG9 | 97.3 | 1.2e-05 | 2.6e-11 | 59.5 | 65 | (35, 100) | 103 | (56, 133) | 144 | DksA C4-type domain-containing protein | DksA C4-type domain-containing protein | | uniclust | UniRef100\_Q31HW7 | 97.3 | 1.4e-05 | 2.7e-11 | 55.4 | 68 | (31, 102) | 103 | (42, 110) | 111 | Uncharacterized protein | Uncharacterized protein | | uniclust | UniRef100\_A0A845GB54 | 97.2 | 1.5e-05 | 2.8e-11 | 53.4 | 37 | (63, 100) | 103 | (47, 88) | 89 | TraR/DksA family transcriptional regulator | TraR/DksA family transcriptional regulator | | uniclust | UniRef100\_A0A968KN48 | 97.2 | 1.5e-05 | 2.8e-11 | 49.2 | 35 | (66, 101) | 103 | (4, 43) | 56 | YteA family sporulation protein | YteA family sporulation protein | | uniclust | UniRef100\_A0A535JRC0 | 97.2 | 1.5e-05 | 2.8e-11 | 54.8 | 33 | (65, 98) | 103 | (67, 104) | 105 | DksA C4-type domain-containing protein | DksA C4-type domain-containing protein | | pdb70 | 5W1S\_M | 99.4 | 7.3e-18 | 7.1e-22 | 106.1 | 66 | (35, 102) | 103 | (1, 71) | 79 | DNA-directed RNA polymerase subunit alpha | 5W1S\_M DNA-directed RNA polymerase subunit alpha RNA polymerase, TraR, TRANSFERASE | | pdb70 | 6PST\_N | 99.4 | 2e-17 | 2e-21 | 101.4 | 65 | (36, 102) | 103 | (1, 70) | 72 | DNA-directed RNA polymerase subunit alpha | 6PST\_N DNA-directed RNA polymerase subunit alpha TRANSCRIPTION, transcription-dna complex HET: 1N7 | | pdb70 | 6PSV\_N | 99.4 | 2e-17 | 2e-21 | 101.4 | 65 | (36, 102) | 103 | (1, 70) | 72 | DNA-directed RNA polymerase subunit alpha | 6PSV\_N DNA-directed RNA polymerase subunit alpha TRANSCRIPTION, transcription-dna complex HET: 1N7 | | pdb70 | 4IJJ\_B | 99.2 | 1e-15 | 1e-19 | 104.1 | 51 | (48, 102) | 103 | (77, 132) | 136 | Putative C4-type zinc finger protein | 4IJJ\_B Putative C4-type zinc finger protein DksA fold, transcription factor, RNA HET: SO4 | | pdb70 | 6PTG\_B | 99.2 | 1.7e-15 | 1.7e-19 | 102.7 | 50 | (48, 101) | 103 | (75, 129) | 132 | DnaK Suppressor | 6PTG\_B DnaK Suppressor SSGCID, Structural Genomics, Seattle Structural | | pdb70 | 1TJL\_B | 99.2 | 2.5e-15 | 2.4e-19 | 104.1 | 51 | (48, 102) | 103 | (93, 148) | 151 | DnaK suppressor protein | 1TJL\_B DnaK suppressor protein DksA, transcription factor, RNA polymerase HET: ZN | | pdb70 | 5VSW\_M | 99.2 | 2.5e-15 | 2.4e-19 | 104.1 | 51 | (48, 102) | 103 | (93, 148) | 151 | DNA-directed RNA polymerase subunit alpha | 5VSW\_M DNA-directed RNA polymerase subunit alpha RNA polymerase, DksA, ppGpp, transferase-dna HET: G4P | | pdb70 | 2KQ9\_A | 99.1 | 1.5e-14 | 1.5e-18 | 95.2 | 44 | (48, 95) | 103 | (63, 111) | 112 | DnaK suppressor protein | 2KQ9\_A DnaK suppressor protein Zinc binding protein, Structural Genomics HET: ZN | | pdb70 | 2KGO\_A | 99.0 | 3.6e-14 | 3.3e-18 | 94.7 | 60 | (40, 102) | 103 | (29, 93) | 108 | Uncharacterized protein ybiI | 2KGO\_A Uncharacterized protein ybiI Zn finger, partially disordered, structural HET: ZN | |
| Top keywords  (threshold 1.00e-03 (evalue)) | **DksA, C4\_type, domain\_containing, TraR, transcriptional, regulator, zinc, finger, DnaK, RNA** |
| Output files | ../../similar\_sequences/36\_FANPEZAQ\_CDS\_0036\_merged.svg ../../similar\_sequences/36\_FANPEZAQ\_CDS\_0036\_pdb70.a3m ../../similar\_sequences/36\_FANPEZAQ\_CDS\_0036\_pdb70.hhr ../../similar\_sequences/36\_FANPEZAQ\_CDS\_0036\_uniclust.a3m ../../similar\_sequences/36\_FANPEZAQ\_CDS\_0036\_uniclust.hhr |

#### Structure prediction (AlphaFold)2

|  |  |
| --- | --- |
| Stats | xml version="1.0" encoding="utf-8" standalone="no"?       2024-09-02T21:09:35.392415 image/svg+xml   Matplotlib v3.7.2, https://matplotlib.org/ |
| Predicted structure | **NGL Viewer Controls:**  - Center: *Left-Click* - Rotate: *Left-Click + Drag* - Translate: *Right-Click + Drag* - Zoom: *Shift + Left-Click + Drag* |
| Output files | ../../predicted\_structures/36\_FANPEZAQ\_CDS\_0036/features.pkl ../../predicted\_structures/36\_FANPEZAQ\_CDS\_0036/ranked\_0.pdb ../../predicted\_structures/36\_FANPEZAQ\_CDS\_0036/ranked\_0\_plots.svg ../../predicted\_structures/36\_FANPEZAQ\_CDS\_0036/result\_model\_1\_ptm\_pred\_0.pkl |

#### Structure similarity search results (Foldseek)3

|  |  |
| --- | --- |
| Structure databases searched | Pdb, Afdb-proteome, Afdb-uniprot50 |
| Results, scheme(s)  (Top layers only, threshold 1.00e-02 (evalue)) | xml version="1.0" encoding="utf-8" standalone="no"?       2024-09-02T21:11:11.331828 image/svg+xml   Matplotlib v3.7.2, https://matplotlib.org/ |
| Results, table  (threshold 1.00e-02 (evalue)) | | db | id | prob | evalue | bits | fident | alnlen | mismatch | gapopen | qstart | qend | tstart | tend | name | description | | --- | --- | --- | --- | --- | --- | --- | --- | --- | --- | --- | --- | --- | --- | --- | | afdb-uniprot50 | AF-Q31HW7-F1-MODEL\_V4 | 1.0 | 0.002348 | 108 | 0.281 | 103 | 63 | 5 | 1 | 103 | 18 | 109 | Uncharacterized protein | Uncharacterized protein | |
| Top keywords  (threshold 1.00e-02 (evalue)) | -- |
| Output files | ../../similar\_structures/36\_FANPEZAQ\_CDS\_0036\_afdb-proteome\_foldseek.tsv ../../similar\_structures/36\_FANPEZAQ\_CDS\_0036\_afdb-uniprot50\_foldseek.tsv ../../similar\_structures/36\_FANPEZAQ\_CDS\_0036\_merged.svg ../../similar\_structures/36\_FANPEZAQ\_CDS\_0036\_pdb\_foldseek.tsv |

  
  
  

Return to summary | Go to previous | Go to next

  


---

**Sequence/structure alignments coloring**  
Each object in the alignment figures is colored according to its E-value following this color coding:

1e-100
10

**References:**  
1) Steinegger M, Meier M, Mirdita M, Vöhringer H, Haunsberger S J, and Söding J (2019) HH-suite3 for fast remote homology detection and deep protein annotation, BMC Bioinformatics, 473. doi: 10.1186/s12859-019-3019-7  
2) Jumper J, Evans R, Pritzel A, ..., Hassabis D (2021) Highly accurate protein structure prediction with AlphaFold, Nature, 596. doi: 10.1038/s41586-021-03819-2  
3) van Kempen M, Kim S, Tumescheit C, Mirdita M, Lee J, Gilchrist CLM, Söding J, and Steinegger M (2023) Fast and accurate protein structure search with Foldseek. Nature Biotechnology. doi: 10.1038/s41587-023-01773-0
