# Supplementary material for: Completing the BASEL phage collection to unlock hidden diversity for systematic exploration of phage–host interactions
Source: PLoS Biol. 2025 Apr 7;23(4):e3003063. doi: 10.1371/journal.pbio.3003063 (PMC11990801; doi:10.1371/journal.pbio.3003063)
Supplement: S2 Data — (ZIP) [file pbio.3003063.s009.zip › entries/38.html]

FANPEZAQ\_CDS\_0038


Return to summary | Go to previous | Go to next

|  |  |
| --- | --- |
| FANPEZAQ\_CDS\_0038 Page creation date: 02 Sep 2024, 12:00  Project folder: n/a  Input sequences file: Escherichia\_virus\_HeidiAbel.gb | domain\_containing helix\_turn\_helix dna\_binding regulator hth\_17 transcriptional excisionase dna binding alpa merr phage a putative tp and regulatory ap yes cp gp multidrug\_efflux transporter engineered transcription complex escherichia hth coli organism\_taxid arch excision expressed in expression\_system\_taxid alpha integration gene packaging mainly orthogonal bundle b terminase activator hth\_type d xis \_3' c |

### Sequence information

|  |  |
| --- | --- |
| Name | FANPEZAQ\_CDS\_0038  38\_FANPEZAQ\_CDS\_0038 (pipeline id) |
| Imported annotations |  |
| Protein sequence | MAQPAQKQSTYLTPQELVLRWNGAVTTGTLANWRNKGKGPAYTKFGSRVRYSITSVEAYE AKHMIGANDNEEC |
| Number of residues | 73 |
| Molecular weight (Da) | 8136.05 |
| Output files | ../../query\_sequences/38\_FANPEZAQ\_CDS\_0038.fasta |

### Putative domain architecture and protein family

#### Search results (HHblits)1

|  |  |
| --- | --- |
| Domain family databases searched | Pfam, Ncbi-cd, Cath, Phrogs |
| Results, scheme(s)  (Top layers only; threshold 1.00e-03 (evalue)) | xml version="1.0" encoding="utf-8" standalone="no"?       2024-09-02T21:08:20.579766 image/svg+xml   Matplotlib v3.7.2, https://matplotlib.org/ |
| Results, table  (E-value ≤ 1.00e-03 (evalue)) | | db | id | prob | evalue | pvalue | score | cols | query | query\_len | template | template\_len | name | description | | --- | --- | --- | --- | --- | --- | --- | --- | --- | --- | --- | --- | --- | | pfam | PF09035 | 97.8 | 4.7e-09 | 7.7e-13 | 41.5 | 56 | (6, 63) | 73 | (10, 66) | 69 | Tn916-Xis | Excisionase from transposon Tn916 | | pfam | PF06806 | 97.8 | 7.2e-09 | 1.2e-12 | 41.3 | 57 | (5, 63) | 73 | (3, 69) | 70 | DUF1233 | Putative excisionase (DUF1233) | | pfam | PF10743 | 97.7 | 1e-08 | 1.7e-12 | 42.7 | 62 | (1, 64) | 73 | (1, 70) | 87 | Phage\_Cox | Regulatory phage protein cox | | pfam | PF04936 | 97.4 | 7e-08 | 1.2e-11 | 45.9 | 55 | (8, 64) | 73 | (11, 66) | 186 | DUF658 | Protein of unknown function (DUF658) | | pfam | PF04645 | 97.2 | 1.8e-07 | 2.9e-11 | 44.0 | 51 | (8, 63) | 73 | (16, 70) | 181 | DUF603 | Protein of unknown function, DUF603 | | pfam | PF07618 | 97.1 | 3.5e-07 | 5.7e-11 | 35.0 | 47 | (13, 62) | 73 | (1, 55) | 57 | DUF1580 | Protein of unknown function (DUF1580) | | pfam | PF04936 | 96.9 | 1.1e-06 | 1.8e-10 | 41.6 | 39 | (12, 52) | 73 | (89, 127) | 186 | DUF658 | Protein of unknown function (DUF658) | | pfam | PF12964 | 96.9 | 1.2e-06 | 2e-10 | 36.3 | 53 | (8, 62) | 73 | (42, 95) | 96 | DUF3853 | Protein of unknown function (DUF3853) | | pfam | PF12728 | 96.8 | 1.5e-06 | 2.4e-10 | 30.8 | 47 | (12, 62) | 73 | (2, 49) | 51 | HTH\_17 | Helix-turn-helix domain | | pfam | PF07278 | 96.8 | 1.5e-06 | 2.5e-10 | 39.1 | 50 | (12, 64) | 73 | (2, 51) | 147 | DUF1441 | Protein of unknown function (DUF1441) | | pfam | PF05930 | 96.8 | 1.8e-06 | 3e-10 | 30.5 | 48 | (10, 59) | 73 | (2, 51) | 52 | Phage\_AlpA | Prophage CP4-57 regulatory protein (AlpA) | | pfam | PF07037 | 96.7 | 2.5e-06 | 4.1e-10 | 36.4 | 50 | (12, 63) | 73 | (1, 55) | 113 | DUF1323 | Putative transcription regulator (DUF1323) | | pfam | PF13411 | 96.6 | 3.7e-06 | 6e-10 | 31.9 | 48 | (12, 61) | 73 | (1, 49) | 70 | MerR\_1 | MerR HTH family regulatory protein | | pfam | PF20038 | 96.5 | 4.3e-06 | 7.1e-10 | 31.2 | 54 | (8, 63) | 73 | (4, 64) | 66 | HTH\_59 | Helix-turn-helix domain | | pfam | PF07471 | 96.4 | 6.6e-06 | 1.1e-09 | 37.1 | 52 | (11, 64) | 73 | (2, 54) | 162 | Phage\_Nu1 | Phage DNA packaging protein Nu1 | | pfam | PF20063 | 96.4 | 7.9e-06 | 1.3e-09 | 28.2 | 48 | (10, 61) | 73 | (2, 49) | 51 | DUF6462 | Family of unknown function (DUF6462) | | pfam | PF13171 | 96.3 | 9.6e-06 | 1.6e-09 | 38.3 | 44 | (11, 56) | 73 | (1, 51) | 199 | DUF4004 | Protein of unknown function (DUF4004) | | pfam | PF07825 | 96.2 | 1.6e-05 | 2.6e-09 | 31.3 | 44 | (11, 56) | 73 | (2, 48) | 72 | Exc | Excisionase-like protein | | pfam | PF13591 | 95.3 | 0.00015 | 2.4e-08 | 28.3 | 46 | (12, 59) | 73 | (1, 47) | 86 | MerR\_2 | MerR HTH family regulatory protein | | pfam | PF11242 | 94.5 | 0.00046 | 7.7e-08 | 27.0 | 29 | (7, 37) | 73 | (10, 38) | 64 | DUF2774 | Protein of unknown function (DUF2774) | | pfam | PF17443 | 94.2 | 0.0007 | 1.1e-07 | 25.3 | 32 | (6, 39) | 73 | (3, 34) | 62 | pXO2-72 | Uncharacterized protein pXO2-72 | | pfam | PF00376 | 94.0 | 0.00083 | 1.4e-07 | 20.7 | 26 | (14, 41) | 73 | (2, 27) | 38 | MerR | MerR family regulatory protein | | pfam | PF08876 | 93.8 | 0.001 | 1.7e-07 | 28.5 | 32 | (10, 43) | 73 | (23, 54) | 109 | DUF1836 | Domain of unknown function (DUF1836) | | cath | 4lhfA00 | 98.2 | 2.7e-10 | 3.6e-14 | 55.0 | 61 | (1, 64) | 73 | (1, 61) | 91 | Regulatory protein cox | CATHCODE: 6.10.200.10 NAME: Regulatory protein cox. Chain: a. Engineered: yes SOURCE: Enterobacteria phage p2. Organism\_taxid: 10679. Gene: cox. Expressed in: escherichia coli. Expression\_system\_taxid: 469008. CLASS: Special, ARCH: Helix non-globular, TOPOL: Multidrug-efflux Transporter Regulator; Chain: A; Domain 2, HOMOL: Regulatory phage protein Cox | | cath | 2kvvA00 | 98.2 | 4.7e-10 | 6.3e-14 | 53.4 | 60 | (3, 65) | 73 | (2, 72) | 78 | Putative excisionase | CATHCODE: 1.10.1660.60 NAME: Putative excisionase. Chain: a. Engineered: yes SOURCE: Klebsiella pneumoniae. Organism\_taxid: 272620. Strain: atcc 700721 / mgh 78578. Gene: kpn78578\_13480, kpn\_01377. Expressed in: escherichia coli. Expression\_system\_taxid: 469008. CLASS: Mainly Alpha, ARCH: Orthogonal Bundle, TOPOL: Multidrug-efflux Transporter Regulator; Chain: A; Domain 2, HOMOL: Putative excisionased domain DUF1233 | | cath | 1y6uA01 | 97.4 | 1.1e-07 | 1.5e-11 | 38.5 | 47 | (10, 59) | 73 | (1, 49) | 49 | Excisionase from transposon tn916 | CATHCODE: 3.90.105.50 NAME: Excisionase from transposon tn916. Chain: a. Synonym: xis. Engineered:yes SOURCE: Enterococcus faecalis. Organism\_taxid: 1351. Gene: xis. Expressed in:escherichia coli. Expression\_system\_taxid: 562. CLASS: Alpha Beta, ARCH: Alpha-Beta Complex, TOPOL: Molybdopterin biosynthesis moea protein, domain 2, HOMOL: Molybdopterin biosynthesis moea protein, domain 2 | | cath | 1z4hA01 | 97.3 | 1.6e-07 | 2.2e-11 | 39.0 | 52 | (11, 65) | 73 | (2, 54) | 58 | Tor inhibition protein | CATHCODE: 1.10.238.160 NAME: Tor inhibition protein. Chain: a. Synonym: tori. Engineered: yes SOURCE: Escherichia coli. Organism\_taxid: 562. Expressed in: escherichia colibl21(de3). Expression\_system\_taxid: 469008. CLASS: Mainly Alpha, ARCH: Orthogonal Bundle, TOPOL: Recoverin; domain 1, HOMOL: Recoverin; domain 1 | | cath | 1j9iA00 | 96.0 | 2.9e-05 | 4.1e-09 | 32.6 | 52 | (11, 66) | 73 | (2, 56) | 68 | Terminase small subunit | CATHCODE: 1.10.10.10 NAME: Terminase small subunit. Chain: a, b. Fragment: DNA binding domain, residues 1-68. Synonym: DNA packaging protein nu1. Gpnu1 dbd. Engineered: yes SOURCE: Enterobacteria phage lambda. Organism\_taxid: 10710. Gene: nu1. Expressed in: escherichia coli. Expression\_system\_taxid: 562. Expression\_system\_vector\_type: plasmid CLASS: Mainly Alpha, ARCH: Orthogonal Bundle, TOPOL: Arc Repressor Mutant, subunit A, HOMOL: Winged helix-like DNA-binding domain superfamily/Winged helix DNA-binding domain | | cath | 1pm6A00 | 95.9 | 4.3e-05 | 5.9e-09 | 33.4 | 43 | (11, 56) | 73 | (2, 48) | 72 | Excisionase | CATHCODE: 1.10.1660.20 NAME: Excisionase. Chain: a. Engineered: yes. Mutation: yes SOURCE: Enterobacteria phage hk022. Organism\_taxid: 10742. Gene: xis. Expressed in: escherichia coli. Expression\_system\_taxid: 562. CLASS: Mainly Alpha, ARCH: Orthogonal Bundle, TOPOL: Multidrug-efflux Transporter Regulator; Chain: A; Domain 2, HOMOL: Multidrug-efflux Transporter Regulator; Chain: A; Domain 2 | | cath | 3gpvA00 | 95.6 | 7.9e-05 | 1.1e-08 | 35.6 | 55 | (5, 62) | 73 | (10, 66) | 128 | Transcriptional regulator, merr family | CATHCODE: 1.10.1660.10 NAME: Transcriptional regulator, merr family. Chain: a, b. Engineered: yes SOURCE: Bacillus thuringiensis serovar konkukian. Organism\_taxid: 180856. Gene: bt9727\_1615. Expressed in: escherichia coli. Expression\_system\_taxid: 562. CLASS: Mainly Alpha, ARCH: Orthogonal Bundle, TOPOL: Multidrug-efflux Transporter Regulator; Chain: A; Domain 2, HOMOL: Multidrug-efflux Transporter Regulator; Chain: A; Domain 2 | | cath | 4r4eB00 | 95.5 | 0.00011 | 1.6e-08 | 31.7 | 56 | (2, 60) | 73 | (2, 58) | 84 | Hth-type transcriptional regulator glnr | CATHCODE: 1.10.1660.10 NAME: Hth-type transcriptional regulator glnr. Chain: b, a. Engineered: yes.Dna (5'-d(\*ap\*tp\*tp\*cp\*tp\*gp\*ap\*cp\*a)-3'). Chain: d. Engineered: yes.Other\_details: cognate DNA. Dna (5'-d(\*tp\*gp\*tp\*cp\*ap\*gp\*tp\*a)-3'). Chain: e. Engineered: yes. Other\_details: cognate DNA (complement to 2) SOURCE: Bacillus subtilis subsp. Subtilis. Organism\_taxid: 224308. Strain: 168. Gene: glnr, bsu17450. Expressed in: escherichia coli. Expression\_system\_taxid: 562. Yes. Organism\_scientific: synthetic DNA. Organism\_taxid: 32630. CLASS: Mainly Alpha, ARCH: Orthogonal Bundle, TOPOL: Multidrug-efflux Transporter Regulator; Chain: A; Domain 2, HOMOL: Multidrug-efflux Transporter Regulator; Chain: A; Domain 2 | | cath | 1r8eA02 | 95.3 | 0.00017 | 2.4e-08 | 29.9 | 51 | (9, 62) | 73 | (3, 56) | 75 | 5'-d(\*gp\*ap\*cp\*cp\*cp\*tp\*cp\*cp\*cp\*cp\*tp\*tp\*ap\*gp\*gp\*gp\*gp\*ap \*gp\*gp\*gp\*tp\*c)-3' | CATHCODE: 1.10.1660.10 NAME: 5'-d(\*gp\*ap\*cp\*cp\*cp\*tp\*cp\*cp\*cp\*cp\*tp\*tp\*ap\*gp\*gp\*gp\*gp\*ap \*gp\*gp\*gp\*tp\*c)-3'. Chain: b. Engineered: yes. Multidrug-efflux transporter regulator. Chain: a. Engineered: yes SOURCE: Yes. CLASS: Mainly Alpha, ARCH: Orthogonal Bundle, TOPOL: Multidrug-efflux Transporter Regulator; Chain: A; Domain 2, HOMOL: Multidrug-efflux Transporter Regulator; Chain: A; Domain 2 | | cath | 5i41B00 | 95.1 | 0.00024 | 3.4e-08 | 28.6 | 50 | (10, 62) | 73 | (2, 52) | 69 | Chromosome-anchoring protein raca | CATHCODE: 1.10.1660.10 NAME: Chromosome-anchoring protein raca. Chain: b. Engineered: yes SOURCE: Bacillus subtilis. Organism\_taxid: 224308. Strain: 168. Gene: raca, ywkc, bsu37030. Expressed in: escherichia coli. Expression\_system\_taxid: 562 CLASS: Mainly Alpha, ARCH: Orthogonal Bundle, TOPOL: Multidrug-efflux Transporter Regulator; Chain: A; Domain 2, HOMOL: Multidrug-efflux Transporter Regulator; Chain: A; Domain 2 | | cath | 2zhgA00 | 95.0 | 0.00029 | 3.9e-08 | 34.0 | 55 | (3, 60) | 73 | (3, 58) | 135 | Redox-sensitive transcriptional activator soxr | CATHCODE: 1.10.1660.10 NAME: Redox-sensitive transcriptional activator soxr. Chain: a. Engineered: yes. Dna (5'- d(\*dgp\*dcp\*dcp\*dtp\*dcp\*dap\*dap\*dgp\*dtp\*dtp\*dap\*dap\*dcp\*dtp\*dtp\*dgp\*da p\*dgp\*dgp\*dc)-3'). Chain: b. Engineered: yes SOURCE: Escherichia coli. Organism\_taxid: 83333. Strain: k12. Gene: soxr. Expressed in: escherichia coli. Expression\_system\_taxid: 562. Expression\_system\_vector\_type: plasmid. CLASS: Mainly Alpha, ARCH: Orthogonal Bundle, TOPOL: Multidrug-efflux Transporter Regulator; Chain: A; Domain 2, HOMOL: Multidrug-efflux Transporter Regulator; Chain: A; Domain 2 | | cath | 3hh0A01 | 94.8 | 0.00036 | 5e-08 | 28.9 | 49 | (10, 61) | 73 | (3, 53) | 74 | Transcriptional regulator, merr family | CATHCODE: 1.10.1660.10 NAME: Transcriptional regulator, merr family. Chain: a, b, c, d. Engineered:yes SOURCE: Bacillus cereus atcc 14579. Organism\_taxid: 226900. Atcc: 14579. Gene:bc\_0953. Expressed in: escherichia coli. Expression\_system\_taxid: 562. CLASS: Mainly Alpha, ARCH: Orthogonal Bundle, TOPOL: Multidrug-efflux Transporter Regulator; Chain: A; Domain 2, HOMOL: Multidrug-efflux Transporter Regulator; Chain: A; Domain 2 | | cath | 2jmlA00 | 94.8 | 0.00037 | 5.1e-08 | 29.7 | 52 | (9, 62) | 73 | (3, 56) | 81 | Dna binding domain/transcriptional regulator | CATHCODE: 1.10.1660.10 NAME: Dna binding domain/transcriptional regulator. Chain: a. Fragment: n-terminal domain, residues 1-78. Engineered: yes SOURCE: Myxococcus xanthus. Organism\_taxid: 246197. Strain: dk 1622. Expressed in: escherichia coli bl21(de3). Expression\_system\_taxid: 469008. CLASS: Mainly Alpha, ARCH: Orthogonal Bundle, TOPOL: Multidrug-efflux Transporter Regulator; Chain: A; Domain 2, HOMOL: Multidrug-efflux Transporter Regulator; Chain: A; Domain 2 | | cath | 5d8cA00 | 94.7 | 0.00043 | 5.9e-08 | 33.3 | 49 | (10, 61) | 73 | (3, 53) | 137 | Merr family regulator protein | CATHCODE: 1.10.1660.10 NAME: Merr family regulator protein. Chain: a, b. Engineered: yes. Dna (5'- d(\*cp\*tp\*tp\*ap\*gp\*ap\*gp\*tp\*tp\*cp\*ap\*cp\*tp\*cp\*tp\*ap\*ap\*g)-3'). Chain: c. Engineered: yes. Other\_details: promoter sequence for adhc-estd operon and nmlr gene.. Dna (5'- d(\*cp\*tp\*tp\*ap\*gp\*ap\*gp\*tp\*gp\*ap\*ap\*cp\*tp\*cp\*tp\*ap\*ap\*g)-3'). Chain: d. Engineered: yes SOURCE: Haemophilus influenzae (strain atcc 51907 / dsm 11121 / kw20 / rd). Organism\_taxid: 71421. Strain: atcc 51907 / dsm 11121 / kw20 / rd. Gene:hi\_0186. Expressed in: escherichia coli. Expression\_system\_taxid: 469008. CLASS: Mainly Alpha, ARCH: Orthogonal Bundle, TOPOL: Multidrug-efflux Transporter Regulator; Chain: A; Domain 2, HOMOL: Multidrug-efflux Transporter Regulator; Chain: A; Domain 2 | | cath | 3vw4A02 | 94.1 | 0.00096 | 1.3e-07 | 24.4 | 27 | (9, 37) | 73 | (5, 31) | 33 | Rep | CATHCODE: 1.10.1220.10 NAME: Rep. Chain: a, b. Fragment: DNA-bindig domain. Synonym: replication initiator cole2-rep. Engineered: yes. Dna (5'- d(p\*ap\*ap\*tp\*gp\*ap\*gp\*ap\*cp\*cp\*ap\*gp\*ap\*tp\*ap\*ap\*gp\*cp\*cp\*tp\*tp\*ap\*tp \*c)-3'). Chain: c, e. Engineered: yes. Dna (5'- d(p\*gp\*ap\*tp\*ap\*ap\*gp\*gp\*cp\*tp\*tp\*ap\*tp\*cp\*tp\*gp\*gp\*tp\*cp\*tp\*cp\*ap\*tp \*t)-3'). Chain: d, f. Engineered: yes SOURCE: Escherichia coli. Organism\_taxid: 562. Gene: rep. Expressed in: escherichia coli. Expression\_system\_taxid: 562. CLASS: Mainly Alpha, ARCH: Orthogonal Bundle, TOPOL: Arc Repressor Mutant, HOMOL: Met repressor-like | | phrogs | 66 | 99.1 | 7e-15 | 8.7e-19 | 73.9 | 67 | (2, 70) | 73 | (8, 77) | 81 | excisionase and transcriptional regulator | excisionase and transcriptional regulator; Category: integration and excision; NC\_023692\_p35 | | phrogs | 6401 | 98.6 | 8.6e-12 | 9.9e-16 | 60.1 | 62 | (3, 66) | 73 | (3, 64) | 66 | DNA binding protein | DNA binding protein; Category: DNA, RNA and nucleotide metabolism; MG757155\_p60 | | phrogs | 10045 | 98.6 | 1.2e-11 | 1.4e-15 | 58.5 | 54 | (5, 61) | 73 | (3, 57) | 62 | HTH DNA binding protein | HTH DNA binding protein; Category: DNA, RNA and nucleotide metabolism; KU963258\_p53 | | phrogs | 15534 | 98.4 | 4e-11 | 4.6e-15 | 60.6 | 58 | (7, 68) | 73 | (32, 89) | 93 | HTH DNA binding protein | HTH DNA binding protein; Category: DNA, RNA and nucleotide metabolism; NC\_028930\_p36 | | phrogs | 7338 | 98.2 | 3e-10 | 3.5e-14 | 58.0 | 60 | (7, 69) | 73 | (34, 93) | 97 | NA | NA; Category: unknown function; p137519 VI\_04448 | | phrogs | 2907 | 98.2 | 3e-10 | 3.5e-14 | 59.0 | 59 | (5, 66) | 73 | (3, 65) | 103 | excisionase | excisionase; Category: integration and excision; p256170 VI\_09967 | | phrogs | 668 | 98.1 | 5.9e-10 | 7.3e-14 | 56.1 | 53 | (6, 63) | 73 | (31, 84) | 85 | HTH DNA binding protein | HTH DNA binding protein; Category: DNA, RNA and nucleotide metabolism; p164379 VI\_04615 | | phrogs | 2790 | 98.1 | 8.2e-10 | 9.3e-14 | 55.8 | 63 | (6, 70) | 73 | (20, 83) | 91 | excisionase and transcriptional regulator | excisionase and transcriptional regulator; Category: integration and excision; p43040 VI\_12007 | | phrogs | 2474 | 97.9 | 5.3e-09 | 6.1e-13 | 51.1 | 55 | (3, 59) | 73 | (7, 62) | 74 | MerR-like transcriptional regulator | MerR-like transcriptional regulator; Category: transcription regulation; NC\_026585\_p102 | | phrogs | 57 | 97.8 | 5.5e-09 | 6.8e-13 | 58.4 | 57 | (9, 67) | 73 | (2, 60) | 173 | terminase small subunit | terminase small subunit; Category: head and packaging; p277333 VI\_02593 | | phrogs | 6620 | 97.8 | 7e-09 | 8.1e-13 | 57.6 | 52 | (9, 62) | 73 | (3, 55) | 170 | transposase | transposase; Category: integration and excision; p336225 VI\_06314 | | phrogs | 4691 | 97.8 | 9.2e-09 | 1e-12 | 49.5 | 54 | (6, 61) | 73 | (13, 66) | 70 | excisionase | excisionase; Category: integration and excision; p211020 VI\_00698 | | phrogs | 4745 | 97.7 | 1.2e-08 | 1.4e-12 | 48.7 | 54 | (9, 64) | 73 | (2, 63) | 65 | DNA binding protein | DNA binding protein; Category: DNA, RNA and nucleotide metabolism; KR093652\_p84 | | phrogs | 25262 | 97.7 | 1.9e-08 | 2.1e-12 | 50.5 | 60 | (6, 68) | 73 | (21, 80) | 96 | NA | NA; Category: unknown function; p39263 VI\_01014 | | phrogs | 9834 | 97.5 | 5.2e-08 | 5.9e-12 | 46.5 | 58 | (8, 68) | 73 | (3, 62) | 67 | excisionase and transcriptional regulator | excisionase and transcriptional regulator; Category: integration and excision; p40253 VI\_09193 | | phrogs | 11671 | 97.5 | 6.7e-08 | 7.7e-12 | 51.1 | 57 | (4, 63) | 73 | (12, 69) | 122 | NA | NA; Category: unknown function; p36461 VI\_11179 | | phrogs | 2655 | 97.5 | 6.9e-08 | 8.1e-12 | 46.9 | 56 | (9, 66) | 73 | (3, 58) | 70 | excisionase | excisionase; Category: integration and excision; p346156 VI\_04054 | | phrogs | 575 | 97.4 | 1.1e-07 | 1.3e-11 | 45.9 | 33 | (7, 41) | 73 | (10, 42) | 67 | Cox-like excisionase and repressor | Cox-like excisionase and repressor; Category: integration and excision; p264496 VI\_02100 | | phrogs | 1969 | 97.2 | 2.6e-07 | 2.9e-11 | 47.4 | 52 | (7, 61) | 73 | (50, 101) | 103 | NA | NA; Category: unknown function; NC\_023689\_p106 | | phrogs | 1864 | 97.2 | 3.4e-07 | 3.9e-11 | 44.0 | 51 | (9, 63) | 73 | (18, 68) | 71 | NA | NA; Category: unknown function; p434791 VI\_03009 | | phrogs | 7735 | 97.1 | 4.6e-07 | 5.2e-11 | 43.8 | 54 | (6, 62) | 73 | (12, 66) | 74 | excisionase | excisionase; Category: integration and excision; p109736 VI\_12389 | | phrogs | 2620 | 97.0 | 1e-06 | 1.1e-10 | 48.5 | 60 | (6, 67) | 73 | (74, 139) | 187 | excisionase and transcriptional regulator | excisionase and transcriptional regulator; Category: integration and excision; p31239 VI\_02649 | | phrogs | 7506 | 96.8 | 2.4e-06 | 2.7e-10 | 41.6 | 56 | (1, 59) | 73 | (4, 59) | 85 | recombination directionality factor | recombination directionality factor; Category: integration and excision; NC\_012788\_p34 | | phrogs | 1729 | 96.7 | 3.2e-06 | 3.7e-10 | 45.5 | 53 | (9, 63) | 73 | (95, 148) | 152 | NA | NA; Category: unknown function; NC\_016435\_p45 | | phrogs | 5866 | 96.6 | 4.6e-06 | 5.1e-10 | 41.1 | 57 | (8, 66) | 73 | (36, 93) | 96 | NA | NA; Category: unknown function; JX409894\_p1 | | phrogs | 6355 | 96.3 | 1.4e-05 | 1.6e-09 | 39.7 | 57 | (8, 66) | 73 | (16, 78) | 89 | NA | NA; Category: unknown function; NC\_031098\_p35 | | phrogs | 2528 | 96.2 | 1.9e-05 | 2.2e-09 | 39.0 | 61 | (7, 69) | 73 | (24, 84) | 89 | NA | NA; Category: unknown function; p416682 VI\_10147 | | phrogs | 2538 | 96.2 | 2.3e-05 | 2.8e-09 | 40.8 | 30 | (9, 40) | 73 | (14, 43) | 113 | terminase small subunit | terminase small subunit; Category: head and packaging; p133796 VI\_05051 | | phrogs | 151 | 96.0 | 3.7e-05 | 4.6e-09 | 38.2 | 28 | (9, 38) | 73 | (20, 47) | 91 | transposase | transposase; Category: integration and excision; p268658 VI\_04551 | | phrogs | 10458 | 96.0 | 4.2e-05 | 4.8e-09 | 38.0 | 50 | (9, 61) | 73 | (39, 90) | 93 | NA | NA; Category: unknown function; p298856 VI\_05329 | | phrogs | 7676 | 95.9 | 4.4e-05 | 5e-09 | 36.4 | 31 | (6, 38) | 73 | (11, 41) | 75 | NA | NA; Category: unknown function; NC\_023688\_p227 | | phrogs | 24579 | 95.9 | 5e-05 | 5.7e-09 | 39.0 | 34 | (5, 40) | 73 | (25, 58) | 126 | terminase small subunit | terminase small subunit; Category: head and packaging; KP836356\_p30 | | phrogs | 28156 | 95.8 | 6.2e-05 | 6.9e-09 | 39.2 | 53 | (6, 61) | 73 | (4, 57) | 156 | NA | NA; Category: unknown function; JF937093\_p45 | | phrogs | 20793 | 95.8 | 7.4e-05 | 8.3e-09 | 36.1 | 52 | (9, 62) | 73 | (5, 57) | 86 | NA | NA; Category: unknown function; NC\_022918\_p114 | | phrogs | 629 | 95.7 | 8.4e-05 | 9.8e-09 | 36.8 | 45 | (11, 56) | 73 | (3, 50) | 86 | excisionase | excisionase; Category: integration and excision; p127807 VI\_03659 | | phrogs | 22784 | 95.7 | 9.3e-05 | 1e-08 | 34.9 | 54 | (9, 68) | 73 | (15, 68) | 76 | NA | NA; Category: unknown function; p30848 VI\_02556 | | phrogs | 17173 | 95.7 | 9.5e-05 | 1.1e-08 | 35.8 | 55 | (9, 67) | 73 | (41, 96) | 98 | NA | NA; Category: unknown function; p170583 VI\_00608 | | phrogs | 7950 | 95.6 | 0.00011 | 1.3e-08 | 33.2 | 27 | (8, 36) | 73 | (10, 36) | 53 | transcriptional repressor | transcriptional repressor; Category: transcription regulation; p141193 VI\_04895 | | phrogs | 2951 | 95.5 | 0.00012 | 1.3e-08 | 35.7 | 50 | (8, 60) | 73 | (23, 75) | 90 | NA | NA; Category: unknown function; p434966 VI\_08277 | | phrogs | 19880 | 95.5 | 0.00015 | 1.6e-08 | 38.4 | 47 | (9, 57) | 73 | (84, 130) | 151 | transcriptional regulator | transcriptional regulator; Category: transcription regulation; MG720309\_p38 | | phrogs | 33310 | 95.3 | 0.00021 | 2.4e-08 | 37.4 | 49 | (9, 61) | 73 | (99, 147) | 151 | NA | NA; Category: unknown function; p240132 VI\_01320 | | phrogs | 11967 | 95.2 | 0.00025 | 2.8e-08 | 34.3 | 46 | (8, 56) | 73 | (14, 62) | 87 | NA | NA; Category: unknown function; KX898400\_p53 | | phrogs | 534 | 95.1 | 0.00026 | 3e-08 | 34.2 | 27 | (9, 37) | 73 | (23, 49) | 87 | DNA binding protein | DNA binding protein; Category: DNA, RNA and nucleotide metabolism; p132807 VI\_04689 | | phrogs | 9099 | 94.9 | 0.00037 | 4.1e-08 | 34.8 | 59 | (6, 66) | 73 | (52, 111) | 115 | NA | NA; Category: unknown function; p72437 VI\_12339 | | phrogs | 35 | 94.9 | 0.00036 | 4.4e-08 | 32.8 | 27 | (12, 40) | 73 | (17, 43) | 75 | transcriptional repressor | transcriptional repressor; Category: transcription regulation; p323300 VI\_10199 | | phrogs | 4494 | 94.9 | 0.00041 | 4.7e-08 | 34.1 | 31 | (8, 40) | 73 | (14, 44) | 88 | NA | NA; Category: unknown function; p409661 VI\_06907 | | phrogs | 9848 | 94.7 | 0.00053 | 6e-08 | 33.8 | 49 | (11, 62) | 73 | (32, 81) | 89 | NA | NA; Category: unknown function; KC595516\_p66 | | phrogs | 12527 | 94.7 | 0.00057 | 6.3e-08 | 34.4 | 53 | (10, 65) | 73 | (28, 80) | 130 | excisionase | excisionase; Category: integration and excision; p35305 VI\_03424 | | phrogs | 17819 | 94.4 | 0.00081 | 9.2e-08 | 30.9 | 30 | (7, 38) | 73 | (18, 47) | 64 | NA | NA; Category: unknown function; p411564 VI\_01840 | | phrogs | 8013 | 94.3 | 0.00087 | 9.9e-08 | 38.4 | 50 | (8, 60) | 73 | (195, 244) | 259 | NA | NA; Category: unknown function; p437881 VI\_04144 | | phrogs | 5751 | 94.3 | 0.00092 | 1.1e-07 | 36.1 | 27 | (10, 38) | 73 | (23, 49) | 164 | terminase large subunit | terminase large subunit; Category: head and packaging; p273217 VI\_06917 | | phrogs | 309 | 94.2 | 0.00092 | 1.1e-07 | 35.9 | 26 | (10, 37) | 73 | (22, 47) | 153 | terminase small subunit | terminase small subunit; Category: head and packaging; p301521 VI\_00938 | | phrogs | 3218 | 94.2 | 0.00099 | 1.1e-07 | 35.2 | 56 | (10, 67) | 73 | (32, 93) | 152 | terminase small subunit | terminase small subunit; Category: head and packaging; p174882 VI\_05927 | |
| Top keywords  (threshold 1.00e-03 (evalue)) | **A, tp, Regulator, and, ap, DNA, yes, cp, gp, Engineered** |
| Output files | ../../domain\_architecture/38\_FANPEZAQ\_CDS\_0038\_cath.hhr ../../domain\_architecture/38\_FANPEZAQ\_CDS\_0038\_merged.svg ../../domain\_architecture/38\_FANPEZAQ\_CDS\_0038\_ncbi-cd.hhr ../../domain\_architecture/38\_FANPEZAQ\_CDS\_0038\_pfam.hhr ../../domain\_architecture/38\_FANPEZAQ\_CDS\_0038\_phrogs.hhr |

### Identical protein sequences/structures

#### Search results

|  |  |
| --- | --- |
| Protein sequence databases searched | Pdb, Swissprot, Refseq |
| Identical proteins found | -- |
| Top keywords | -- |
| Output files | -- |

### Similar protein sequences/structures

#### Sequence similarity search results (HHblits)1

|  |  |
| --- | --- |
| Sequence databases searched | Uniclust, Pdb70 |
| Results, scheme(s)  (Top layers only, threshold 1.00e-03 (evalue)) | xml version="1.0" encoding="utf-8" standalone="no"?       2024-09-02T21:08:45.791884 image/svg+xml   Matplotlib v3.7.2, https://matplotlib.org/ |
| Results, table(s)  (threshold 1.00e-03 (evalue)) | | db | id | prob | evalue | pvalue | score | cols | query | query\_len | template | template\_len | name | description | | --- | --- | --- | --- | --- | --- | --- | --- | --- | --- | --- | --- | --- | | uniclust | UniRef100\_A0A0N7LYK3 | 99.8 | 4.9e-24 | 9.7e-30 | 110.8 | 68 | (1, 70) | 73 | (8, 75) | 82 | Helix-turn-helix domain protein | Helix-turn-helix domain protein | | uniclust | UniRef100\_A0A0H5Q954 | 99.8 | 2.3e-23 | 4.6e-29 | 110.0 | 66 | (2, 69) | 73 | (5, 70) | 89 | Helix-turn-helix domain-containing protein | Helix-turn-helix domain-containing protein | | uniclust | UniRef100\_A0A0F2N5I2 | 99.8 | 3.4e-22 | 6.9e-28 | 106.0 | 63 | (4, 68) | 73 | (17, 79) | 90 | Helix-turn-helix domain-containing protein | Helix-turn-helix domain-containing protein | | uniclust | UniRef100\_A0A0B5I6H3 | 99.8 | 6.1e-22 | 1.2e-27 | 100.7 | 67 | (2, 70) | 73 | (1, 67) | 72 | Helix-turn-helix domain-containing protein | Helix-turn-helix domain-containing protein | | uniclust | UniRef100\_A0A1F8XFD9 | 99.8 | 8.2e-22 | 1.6e-27 | 103.9 | 61 | (6, 68) | 73 | (14, 74) | 88 | Helix-turn-helix domain-containing protein | Helix-turn-helix domain-containing protein | | uniclust | UniRef100\_A0A0M4QCQ6 | 99.8 | 8.9e-22 | 1.7e-27 | 105.1 | 63 | (4, 68) | 73 | (29, 91) | 99 | Excisionase | Excisionase | | uniclust | UniRef100\_A0A059G6X6 | 99.7 | 1.7e-21 | 3.3e-27 | 102.5 | 67 | (1, 69) | 73 | (15, 81) | 88 | Helix-turn-helix domain-containing protein | Helix-turn-helix domain-containing protein | | uniclust | UniRef100\_A0A0Q8DCD6 | 99.7 | 2.1e-21 | 4.1e-27 | 98.5 | 65 | (2, 68) | 73 | (1, 65) | 72 | Helix-turn-helix domain-containing protein | Helix-turn-helix domain-containing protein | | uniclust | UniRef100\_A0A072MSX1 | 99.7 | 2.1e-21 | 4.3e-27 | 103.1 | 62 | (6, 69) | 73 | (12, 77) | 89 | Helix-turn-helix domain-containing protein | Helix-turn-helix domain-containing protein | | uniclust | UniRef100\_A0A059G7C8 | 99.7 | 2.2e-21 | 4.5e-27 | 104.1 | 63 | (6, 70) | 73 | (11, 73) | 95 | Helix-turn-helix domain-containing protein | Helix-turn-helix domain-containing protein | | uniclust | UniRef100\_A0A087AQ78 | 99.7 | 2.4e-21 | 4.9e-27 | 106.2 | 64 | (4, 69) | 73 | (32, 95) | 110 | Helix-turn-helix domain-containing protein | Helix-turn-helix domain-containing protein | | uniclust | UniRef100\_A0A099FD25 | 99.7 | 2.8e-21 | 5.6e-27 | 105.0 | 66 | (2, 69) | 73 | (11, 76) | 104 | Helix-turn-helix domain-containing protein | Helix-turn-helix domain-containing protein | | uniclust | UniRef100\_A0A0C9PRY0 | 99.7 | 2.9e-21 | 5.9e-27 | 102.3 | 63 | (2, 67) | 73 | (7, 69) | 89 | Phosphotransferase system mannitol/fructose-specific IIA domain | Phosphotransferase system mannitol/fructose-specific IIA domain | | uniclust | UniRef100\_A0A0F5PY42 | 99.7 | 3.3e-21 | 6.7e-27 | 105.4 | 64 | (5, 70) | 73 | (21, 84) | 106 | Transcriptional regulator, AlpA family | Transcriptional regulator, AlpA family | | uniclust | UniRef100\_A0A0J5S0Y2 | 99.7 | 3.4e-21 | 6.9e-27 | 100.8 | 65 | (2, 68) | 73 | (8, 72) | 82 | Helix-turn-helix domain-containing protein | Helix-turn-helix domain-containing protein | | uniclust | UniRef100\_A0A0F9Z934 | 99.7 | 5.9e-21 | 1.2e-26 | 99.4 | 64 | (4, 70) | 73 | (10, 73) | 80 | Helix-turn-helix domain-containing protein | Helix-turn-helix domain-containing protein | | uniclust | UniRef100\_A0A031GGX7 | 99.7 | 6.2e-21 | 1.2e-26 | 99.1 | 62 | (5, 68) | 73 | (10, 71) | 80 | Helix-turn-helix domain protein | Helix-turn-helix domain protein | | uniclust | UniRef100\_A0A0K3BH05 | 99.7 | 8.9e-21 | 1.8e-26 | 99.2 | 65 | (3, 69) | 73 | (14, 78) | 84 | Helix-turn-helix domain-containing protein | Helix-turn-helix domain-containing protein | | uniclust | UniRef100\_A0A0H1R5M7 | 99.7 | 9.5e-21 | 1.9e-26 | 99.9 | 65 | (3, 69) | 73 | (11, 75) | 89 | Helix-turn-helix domain-containing protein | Helix-turn-helix domain-containing protein | | uniclust | UniRef100\_A0A0Q8Y888 | 99.7 | 1.1e-20 | 2.1e-26 | 99.7 | 59 | (6, 66) | 73 | (15, 73) | 89 | Helix-turn-helix domain-containing protein | Helix-turn-helix domain-containing protein | | uniclust | UniRef100\_A0A013WJ46 | 99.7 | 1.1e-20 | 2.2e-26 | 101.8 | 68 | (1, 70) | 73 | (12, 80) | 101 | Helix-turn-helix domain-containing protein | Helix-turn-helix domain-containing protein | | uniclust | UniRef100\_A0A1E7YNR3 | 99.7 | 1.5e-20 | 3e-26 | 99.6 | 63 | (2, 67) | 73 | (12, 74) | 93 | DNA-binding protein | DNA-binding protein | | uniclust | UniRef100\_A0A0S7XKK0 | 99.7 | 1.6e-20 | 3.1e-26 | 97.5 | 64 | (2, 68) | 73 | (1, 64) | 85 | Helix-turn-helix domain-containing protein | Helix-turn-helix domain-containing protein | | uniclust | UniRef100\_A0A0H1R6E4 | 99.7 | 1.8e-20 | 3.5e-26 | 99.1 | 62 | (7, 70) | 73 | (15, 76) | 91 | Helix-turn-helix domain-containing protein | Helix-turn-helix domain-containing protein | | uniclust | UniRef100\_A0A0W8GA41 | 99.7 | 1.8e-20 | 3.5e-26 | 99.2 | 66 | (3, 70) | 73 | (12, 77) | 91 | Helix-turn-helix domain-containing protein | Helix-turn-helix domain-containing protein | | uniclust | UniRef100\_A0A0S8E603 | 99.7 | 1.8e-20 | 3.7e-26 | 99.0 | 61 | (3, 66) | 73 | (9, 69) | 88 | Helix-turn-helix domain-containing protein | Helix-turn-helix domain-containing protein | | uniclust | UniRef100\_A0A7X6PMZ9 | 99.7 | 2.2e-20 | 4.2e-26 | 94.8 | 65 | (2, 68) | 73 | (5, 70) | 73 | Helix-turn-helix domain-containing protein | Helix-turn-helix domain-containing protein | | uniclust | UniRef100\_A0A1F1ED30 | 99.7 | 2.3e-20 | 4.6e-26 | 97.2 | 66 | (2, 69) | 73 | (10, 75) | 80 | Helix-turn-helix domain-containing protein | Helix-turn-helix domain-containing protein | | uniclust | UniRef100\_A0A0D8HFS2 | 99.7 | 2.8e-20 | 5.6e-26 | 99.7 | 64 | (2, 68) | 73 | (13, 76) | 95 | Helix-turn-helix domain protein | Helix-turn-helix domain protein | | uniclust | UniRef100\_A0A059G1X5 | 99.7 | 2.8e-20 | 5.7e-26 | 97.9 | 68 | (1, 70) | 73 | (5, 73) | 84 | Helix-turn-helix domain-containing protein | Helix-turn-helix domain-containing protein | | uniclust | UniRef100\_A0A0D6JKC0 | 99.7 | 3.1e-20 | 6.1e-26 | 98.4 | 60 | (9, 70) | 73 | (24, 84) | 91 | Helix-turn-helix domain-containing protein | Helix-turn-helix domain-containing protein | | uniclust | UniRef100\_A0A126NYS1 | 99.7 | 3.2e-20 | 6.5e-26 | 97.8 | 67 | (2, 70) | 73 | (7, 74) | 85 | Helix-turn-helix domain-containing protein | Helix-turn-helix domain-containing protein | | uniclust | UniRef100\_A0A077AYW7 | 99.7 | 3.4e-20 | 6.6e-26 | 95.4 | 65 | (3, 69) | 73 | (6, 70) | 76 | Helix-turn-helix domain-containing protein | Helix-turn-helix domain-containing protein | | uniclust | UniRef100\_A0A0K6HIE1 | 99.7 | 3.4e-20 | 6.9e-26 | 101.9 | 59 | (5, 65) | 73 | (24, 82) | 112 | Helix-turn-helix domain | Helix-turn-helix domain | | uniclust | UniRef100\_A0A094ZE53 | 99.7 | 4.2e-20 | 8.3e-26 | 97.4 | 63 | (6, 70) | 73 | (11, 74) | 87 | AlpA family phage regulatory protein | AlpA family phage regulatory protein | | uniclust | UniRef100\_A0A062WXZ1 | 99.7 | 4.2e-20 | 8.3e-26 | 101.0 | 62 | (6, 69) | 73 | (31, 92) | 110 | Transcriptional regulator, AlpA family | Transcriptional regulator, AlpA family | | uniclust | UniRef100\_A0A0A0BTB2 | 99.7 | 4.2e-20 | 8.4e-26 | 97.4 | 57 | (9, 67) | 73 | (18, 74) | 85 | Excisionase | Excisionase | | uniclust | UniRef100\_A0A059DTE2 | 99.7 | 5.1e-20 | 1.1e-25 | 100.5 | 61 | (3, 66) | 73 | (14, 74) | 103 | Helix-turn-helix domain-containing protein | Helix-turn-helix domain-containing protein | | uniclust | UniRef100\_A0A022MTG3 | 99.7 | 5.2e-20 | 1.1e-25 | 97.5 | 57 | (10, 68) | 73 | (18, 74) | 86 | DNA-binding protein | DNA-binding protein | | uniclust | UniRef100\_A0A0J6SUB7 | 99.7 | 5.6e-20 | 1.1e-25 | 99.1 | 61 | (8, 70) | 73 | (28, 88) | 99 | Helix-turn-helix domain-containing protein | Helix-turn-helix domain-containing protein | | uniclust | UniRef100\_A0A076JIU9 | 99.7 | 6.3e-20 | 1.2e-25 | 98.9 | 60 | (9, 70) | 73 | (26, 85) | 102 | Putative transcriptional regulator | Putative transcriptional regulator | | uniclust | UniRef100\_A0A0Q0HYW1 | 99.7 | 6.3e-20 | 1.3e-25 | 96.4 | 64 | (3, 68) | 73 | (5, 69) | 85 | Helix-turn-helix domain protein | Helix-turn-helix domain protein | | uniclust | UniRef100\_A0A0B5ERS0 | 99.7 | 6.6e-20 | 1.3e-25 | 98.2 | 59 | (8, 68) | 73 | (31, 89) | 98 | DNA-binding protein | DNA-binding protein | | uniclust | UniRef100\_A0A0G3GU35 | 99.7 | 6.9e-20 | 1.3e-25 | 92.4 | 65 | (3, 69) | 73 | (2, 66) | 70 | Transcriptional regulator, AlpA family | Transcriptional regulator, AlpA family | | uniclust | UniRef100\_A0A0K2GIG6 | 99.7 | 7.8e-20 | 1.5e-25 | 98.7 | 63 | (3, 68) | 73 | (12, 74) | 102 | Helix-turn-helix domain-containing protein | Helix-turn-helix domain-containing protein | | uniclust | UniRef100\_A0A099JLS6 | 99.7 | 8.7e-20 | 1.7e-25 | 97.3 | 60 | (8, 69) | 73 | (17, 77) | 92 | Prophage regulatory protein | Prophage regulatory protein | | uniclust | UniRef100\_A0A0A6UQH0 | 99.7 | 9.6e-20 | 1.9e-25 | 93.5 | 66 | (2, 69) | 73 | (10, 75) | 79 | Excisionase | Excisionase | | uniclust | UniRef100\_A0A081HUQ7 | 99.7 | 1.4e-19 | 2.8e-25 | 95.6 | 62 | (4, 67) | 73 | (12, 73) | 88 | Helix-turn-helix domain-containing protein | Helix-turn-helix domain-containing protein | | uniclust | UniRef100\_A0A094WFN5 | 99.7 | 1.4e-19 | 2.8e-25 | 98.3 | 64 | (5, 70) | 73 | (18, 81) | 106 | DNA-binding protein | DNA-binding protein | | uniclust | UniRef100\_A0A1L7AKC8 | 99.7 | 1.4e-19 | 2.8e-25 | 98.0 | 61 | (4, 66) | 73 | (21, 82) | 102 | Helix-turn-helix domain-containing protein | Helix-turn-helix domain-containing protein | | uniclust | UniRef100\_A0A1F5RZJ7 | 99.7 | 1.4e-19 | 2.9e-25 | 95.5 | 65 | (1, 68) | 73 | (5, 69) | 87 | Helix-turn-helix domain-containing protein | Helix-turn-helix domain-containing protein | | uniclust | UniRef100\_A0A3A5AND8 | 99.7 | 1.6e-19 | 3.1e-25 | 92.9 | 65 | (2, 69) | 73 | (2, 66) | 76 | DNA-binding protein | DNA-binding protein | | uniclust | UniRef100\_A0A1M4LEU1 | 99.7 | 1.7e-19 | 3.3e-25 | 94.6 | 62 | (6, 69) | 73 | (14, 75) | 86 | Helix-turn-helix domain-containing protein | Helix-turn-helix domain-containing protein | | uniclust | UniRef100\_A0A0L6JWJ1 | 99.7 | 1.7e-19 | 3.3e-25 | 94.8 | 65 | (1, 68) | 73 | (6, 70) | 85 | DNA binding domain protein, excisionase family | DNA binding domain protein, excisionase family | | uniclust | UniRef100\_A0A062VY92 | 99.7 | 2e-19 | 3.8e-25 | 92.5 | 59 | (9, 69) | 73 | (15, 73) | 79 | Helix-turn-helix domain-containing protein | Helix-turn-helix domain-containing protein | | uniclust | UniRef100\_A0A0F9NKP1 | 99.7 | 2e-19 | 4e-25 | 92.9 | 64 | (2, 68) | 73 | (3, 66) | 75 | Helix-turn-helix domain-containing protein | Helix-turn-helix domain-containing protein | | uniclust | UniRef100\_A0A0B9AT96 | 99.7 | 2.2e-19 | 4.3e-25 | 91.3 | 65 | (1, 68) | 73 | (1, 65) | 69 | Helix-turn-helix domain-containing protein | Helix-turn-helix domain-containing protein | | uniclust | UniRef100\_A0A090MQQ0 | 99.6 | 2.3e-19 | 4.6e-25 | 95.2 | 66 | (3, 70) | 73 | (9, 74) | 89 | Helix-turn-helix domain protein | Helix-turn-helix domain protein | | uniclust | UniRef100\_A0A1A9CVS8 | 99.6 | 2.4e-19 | 4.6e-25 | 93.8 | 66 | (1, 68) | 73 | (9, 74) | 85 | Helix-turn-helix domain-containing protein | Helix-turn-helix domain-containing protein | | uniclust | UniRef100\_A0A0A7I9V8 | 99.6 | 2.4e-19 | 4.8e-25 | 96.5 | 65 | (3, 69) | 73 | (14, 78) | 98 | Helix-turn-helix domain-containing protein | Helix-turn-helix domain-containing protein | | uniclust | UniRef100\_A0A0K0N7A4 | 99.6 | 2.6e-19 | 5.3e-25 | 95.6 | 61 | (7, 69) | 73 | (26, 86) | 94 | Helix-turn-helix domain-containing protein | Helix-turn-helix domain-containing protein | | uniclust | UniRef100\_A0A059E4I3 | 99.6 | 2.9e-19 | 5.7e-25 | 95.8 | 65 | (4, 70) | 73 | (21, 85) | 98 | Helix-turn-helix domain-containing protein | Helix-turn-helix domain-containing protein | | uniclust | UniRef100\_A0A0A1CWK4 | 99.6 | 2.9e-19 | 5.7e-25 | 91.0 | 64 | (1, 66) | 73 | (1, 64) | 72 | Helix-turn-helix domain-containing protein | Helix-turn-helix domain-containing protein | | uniclust | UniRef100\_A0A066PHX5 | 99.6 | 3e-19 | 5.9e-25 | 96.8 | 66 | (3, 70) | 73 | (18, 83) | 107 | Helix-turn-helix domain-containing protein | Helix-turn-helix domain-containing protein | | uniclust | UniRef100\_A0A089LM03 | 99.6 | 3.3e-19 | 6.6e-25 | 92.1 | 63 | (3, 68) | 73 | (1, 63) | 76 | Excisionase | Excisionase | | uniclust | UniRef100\_A0A1S9CZ14 | 99.6 | 3.5e-19 | 6.8e-25 | 89.9 | 63 | (5, 69) | 73 | (4, 66) | 70 | Helix-turn-helix domain-containing protein | Helix-turn-helix domain-containing protein | | uniclust | UniRef100\_A0A0M0BEZ1 | 99.6 | 3.5e-19 | 6.9e-25 | 93.2 | 60 | (1, 63) | 73 | (1, 60) | 82 | Helix-turn-helix domain-containing protein | Helix-turn-helix domain-containing protein | | uniclust | UniRef100\_A0A068F4K0 | 99.6 | 3.4e-19 | 6.9e-25 | 93.0 | 65 | (1, 67) | 73 | (4, 69) | 79 | Excise | Excise | | uniclust | UniRef100\_A0A1U7GLT6 | 99.6 | 3.6e-19 | 7.1e-25 | 92.4 | 62 | (5, 68) | 73 | (8, 69) | 80 | Helix-turn-helix domain-containing protein | Helix-turn-helix domain-containing protein | | uniclust | UniRef100\_A0A0M2HKS0 | 99.6 | 3.9e-19 | 7.5e-25 | 96.4 | 66 | (2, 69) | 73 | (7, 72) | 107 | Helix-turn-helix domain protein | Helix-turn-helix domain protein | | uniclust | UniRef100\_A0A077M4I0 | 99.6 | 4e-19 | 8e-25 | 97.0 | 57 | (9, 67) | 73 | (25, 81) | 107 | Helix-turn-helix domain-containing protein | Helix-turn-helix domain-containing protein | | uniclust | UniRef100\_A0A061LXH0 | 99.6 | 4.3e-19 | 8.6e-25 | 94.6 | 61 | (8, 70) | 73 | (22, 82) | 91 | Helix-turn-helix domain-containing protein | Helix-turn-helix domain-containing protein | | uniclust | UniRef100\_A0A1F6T9P4 | 99.6 | 4.4e-19 | 8.6e-25 | 94.3 | 61 | (8, 70) | 73 | (17, 77) | 91 | Helix-turn-helix domain-containing protein | Helix-turn-helix domain-containing protein | | uniclust | UniRef100\_A0A087BZX1 | 99.6 | 4.7e-19 | 9.3e-25 | 92.0 | 59 | (9, 69) | 73 | (16, 74) | 80 | DNA binding domain, excisionase family | DNA binding domain, excisionase family | | uniclust | UniRef100\_A0A068Z4E0 | 99.6 | 5.5e-19 | 1.1e-24 | 97.1 | 61 | (7, 69) | 73 | (33, 94) | 108 | Helix-turn-helix domain-containing protein | Helix-turn-helix domain-containing protein | | uniclust | UniRef100\_A0A133L1R7 | 99.6 | 5.7e-19 | 1.1e-24 | 92.7 | 62 | (6, 69) | 73 | (14, 76) | 83 | Transcriptional regulator | Transcriptional regulator | | uniclust | UniRef100\_A0A0H1RFW3 | 99.6 | 6e-19 | 1.2e-24 | 92.2 | 62 | (6, 69) | 73 | (14, 75) | 80 | Helix-turn-helix domain-containing protein | Helix-turn-helix domain-containing protein | | uniclust | UniRef100\_A0A1F4N4R1 | 99.6 | 6.3e-19 | 1.3e-24 | 92.2 | 63 | (3, 68) | 73 | (1, 63) | 80 | Helix-turn-helix domain-containing protein | Helix-turn-helix domain-containing protein | | uniclust | UniRef100\_A0A094S3N1 | 99.6 | 6.4e-19 | 1.3e-24 | 93.4 | 66 | (2, 69) | 73 | (8, 73) | 87 | AlpA family transcriptional regulator | AlpA family transcriptional regulator | | uniclust | UniRef100\_A0A061LYD6 | 99.6 | 6.9e-19 | 1.4e-24 | 95.5 | 64 | (4, 69) | 73 | (29, 92) | 106 | Ethanolamine utilization protein EutA | Ethanolamine utilization protein EutA | | uniclust | UniRef100\_A0A0B2Y500 | 99.6 | 7.1e-19 | 1.4e-24 | 92.2 | 68 | (1, 70) | 73 | (4, 71) | 85 | DNA-binding protein | DNA-binding protein | | uniclust | UniRef100\_A0A0K8PHS6 | 99.6 | 7.4e-19 | 1.5e-24 | 96.1 | 58 | (8, 67) | 73 | (28, 86) | 109 | Helix-turn-helix domain-containing protein | Helix-turn-helix domain-containing protein | | uniclust | UniRef100\_A0A0S4LPK6 | 99.6 | 8.7e-19 | 1.7e-24 | 94.4 | 62 | (3, 67) | 73 | (17, 78) | 101 | Putative DNA binding domain, excisionase family | Putative DNA binding domain, excisionase family | | uniclust | UniRef100\_A0A011UV86 | 99.6 | 8.9e-19 | 1.8e-24 | 97.1 | 61 | (7, 69) | 73 | (27, 87) | 116 | Transcriptional regulator | Transcriptional regulator | | uniclust | UniRef100\_A0A069E1R7 | 99.6 | 1e-18 | 2e-24 | 92.0 | 65 | (4, 70) | 73 | (14, 78) | 82 | Helix-turn-helix domain-containing protein | Helix-turn-helix domain-containing protein | | uniclust | UniRef100\_A0A1Y0BBP6 | 99.6 | 1.1e-18 | 2.1e-24 | 90.2 | 61 | (5, 68) | 73 | (2, 62) | 75 | B42 | B42 | | uniclust | UniRef100\_A0A1A3ELP5 | 99.6 | 1.2e-18 | 2.3e-24 | 90.9 | 65 | (4, 70) | 73 | (11, 75) | 82 | Helix-turn-helix domain-containing protein | Helix-turn-helix domain-containing protein | | uniclust | UniRef100\_A0A090REP0 | 99.6 | 1.2e-18 | 2.4e-24 | 89.2 | 63 | (5, 69) | 73 | (2, 65) | 72 | Transcriptional regulator | Transcriptional regulator | | uniclust | UniRef100\_A0A099KUC7 | 99.6 | 1.2e-18 | 2.4e-24 | 92.6 | 65 | (1, 67) | 73 | (2, 67) | 89 | Helix-turn-helix domain-containing protein | Helix-turn-helix domain-containing protein | | uniclust | UniRef100\_A0A0L1KD87 | 99.6 | 1.3e-18 | 2.6e-24 | 92.3 | 62 | (6, 69) | 73 | (18, 79) | 93 | Helix-turn-helix domain-containing protein | Helix-turn-helix domain-containing protein | | uniclust | UniRef100\_A0A0S8JD43 | 99.6 | 1.4e-18 | 2.8e-24 | 91.4 | 56 | (2, 60) | 73 | (6, 61) | 83 | Helix-turn-helix domain-containing protein | Helix-turn-helix domain-containing protein | | uniclust | UniRef100\_A0A1G9V0Q2 | 99.6 | 1.5e-18 | 2.9e-24 | 91.2 | 64 | (4, 69) | 73 | (8, 71) | 82 | Helix-turn-helix domain-containing protein | Helix-turn-helix domain-containing protein | | uniclust | UniRef100\_A0A444PYM9 | 99.6 | 1.5e-18 | 2.9e-24 | 89.1 | 63 | (2, 66) | 73 | (1, 63) | 72 | DNA-binding protein | DNA-binding protein | | uniclust | UniRef100\_A0A014NQA7 | 99.6 | 1.6e-18 | 3.1e-24 | 91.1 | 57 | (8, 66) | 73 | (18, 74) | 89 | Helix-turn-helix domain-containing protein | Helix-turn-helix domain-containing protein | | uniclust | UniRef100\_A0A0N8HY41 | 99.6 | 1.7e-18 | 3.4e-24 | 90.4 | 60 | (8, 69) | 73 | (18, 77) | 80 | Helix-turn-helix domain-containing protein | Helix-turn-helix domain-containing protein | | uniclust | UniRef100\_A0A0Q2LK60 | 99.6 | 1.8e-18 | 3.4e-24 | 88.6 | 64 | (5, 70) | 73 | (3, 66) | 74 | Helix-turn-helix domain-containing protein | Helix-turn-helix domain-containing protein | | uniclust | UniRef100\_A0A083XX69 | 99.6 | 1.8e-18 | 3.5e-24 | 91.5 | 61 | (7, 69) | 73 | (12, 72) | 88 | Helix-turn-helix domain-containing protein | Helix-turn-helix domain-containing protein | | uniclust | UniRef100\_A0A176YVV7 | 99.6 | 1.9e-18 | 3.7e-24 | 89.1 | 64 | (4, 69) | 73 | (6, 69) | 77 | HTH merR-type domain-containing protein | HTH merR-type domain-containing protein | | uniclust | UniRef100\_A0A1F9V6F5 | 99.6 | 1.9e-18 | 3.7e-24 | 93.0 | 60 | (6, 68) | 73 | (19, 78) | 98 | Helix-turn-helix domain-containing protein | Helix-turn-helix domain-containing protein | | uniclust | UniRef100\_A0A2T3IMR8 | 99.6 | 2.1e-18 | 4.1e-24 | 89.9 | 65 | (2, 68) | 73 | (9, 74) | 80 | AlpA family phage regulatory protein | AlpA family phage regulatory protein | | uniclust | UniRef100\_A0A059DSQ7 | 99.6 | 2.3e-18 | 4.5e-24 | 92.1 | 62 | (6, 69) | 73 | (17, 78) | 98 | Helix-turn-helix domain-containing protein | Helix-turn-helix domain-containing protein | | uniclust | UniRef100\_A0A022LRD1 | 99.6 | 2.3e-18 | 4.5e-24 | 92.4 | 63 | (4, 68) | 73 | (26, 88) | 97 | Helix-turn-helix domain-containing protein | Helix-turn-helix domain-containing protein | | uniclust | UniRef100\_A0A0S7XS04 | 99.6 | 2.4e-18 | 4.8e-24 | 88.3 | 65 | (3, 70) | 73 | (2, 66) | 73 | Helix-turn-helix domain-containing protein | Helix-turn-helix domain-containing protein | | uniclust | UniRef100\_A0A0A8B4D0 | 99.6 | 2.6e-18 | 5e-24 | 94.9 | 60 | (7, 68) | 73 | (35, 95) | 120 | Transcriptional regulator | Transcriptional regulator | | uniclust | UniRef100\_A0A0R0LNK5 | 99.6 | 2.6e-18 | 5.1e-24 | 90.8 | 67 | (1, 69) | 73 | (1, 67) | 87 | Helix-turn-helix domain-containing protein | Helix-turn-helix domain-containing protein | | uniclust | UniRef100\_A0A086PDB8 | 99.6 | 2.6e-18 | 5.2e-24 | 92.7 | 58 | (8, 67) | 73 | (27, 84) | 99 | Helix-turn-helix domain-containing protein | Helix-turn-helix domain-containing protein | | uniclust | UniRef100\_A0A099KYA7 | 99.6 | 2.6e-18 | 5.3e-24 | 91.2 | 65 | (2, 68) | 73 | (12, 77) | 86 | Phage transcriptional regulator, AlpA | Phage transcriptional regulator, AlpA | | uniclust | UniRef100\_A0A1H6A7S1 | 99.6 | 3.5e-18 | 6.8e-24 | 88.6 | 59 | (9, 69) | 73 | (16, 74) | 82 | Helix-turn-helix domain-containing protein | Helix-turn-helix domain-containing protein | | uniclust | UniRef100\_A0A078BLR7 | 99.6 | 3.7e-18 | 7.3e-24 | 90.5 | 63 | (5, 69) | 73 | (22, 84) | 88 | Putative Bacteriophage related protein | Putative Bacteriophage related protein | | uniclust | UniRef100\_A0A069S043 | 99.6 | 4.4e-18 | 8.7e-24 | 89.2 | 65 | (2, 69) | 73 | (16, 80) | 83 | MerR HTH regulatory family protein | MerR HTH regulatory family protein | | uniclust | UniRef100\_A0A0F9NA78 | 99.6 | 4.4e-18 | 8.8e-24 | 88.9 | 65 | (1, 68) | 73 | (2, 66) | 79 | Helix-turn-helix domain-containing protein | Helix-turn-helix domain-containing protein | | uniclust | UniRef100\_A0A081FVL7 | 99.6 | 4.5e-18 | 8.8e-24 | 91.3 | 62 | (3, 67) | 73 | (17, 78) | 96 | Putative phage protein | Putative phage protein | | uniclust | UniRef100\_A0A0B5FUY6 | 99.6 | 5.1e-18 | 9.8e-24 | 89.5 | 60 | (3, 65) | 73 | (7, 66) | 89 | DNA-binding protein | DNA-binding protein | | uniclust | UniRef100\_A0A011TBA6 | 99.6 | 5.2e-18 | 1e-23 | 91.7 | 65 | (3, 69) | 73 | (13, 77) | 102 | Helix-turn-helix domain-containing protein | Helix-turn-helix domain-containing protein | | uniclust | UniRef100\_A0A357ZGF1 | 99.6 | 5.3e-18 | 1e-23 | 87.2 | 62 | (7, 70) | 73 | (2, 64) | 76 | Helix-turn-helix domain-containing protein | Helix-turn-helix domain-containing protein | | uniclust | UniRef100\_A0A132H9Y5 | 99.6 | 5.8e-18 | 1.1e-23 | 91.7 | 62 | (3, 67) | 73 | (22, 83) | 110 | Helix-turn-helix domain-containing protein | Helix-turn-helix domain-containing protein | | uniclust | UniRef100\_A0A1G7WEL0 | 99.6 | 6.1e-18 | 1.2e-23 | 85.7 | 61 | (4, 66) | 73 | (6, 67) | 69 | Helix-turn-helix domain-containing protein | Helix-turn-helix domain-containing protein | | uniclust | UniRef100\_A0A0R3MKB8 | 99.6 | 6.2e-18 | 1.2e-23 | 90.2 | 60 | (9, 70) | 73 | (16, 75) | 94 | Helix-turn-helix domain-containing protein | Helix-turn-helix domain-containing protein | | uniclust | UniRef100\_A0A0F5MUR4 | 99.6 | 6.2e-18 | 1.2e-23 | 90.3 | 60 | (6, 67) | 73 | (21, 80) | 93 | DNA-binding protein | DNA-binding protein | | uniclust | UniRef100\_A0A0J1FJT4 | 99.6 | 6.2e-18 | 1.2e-23 | 85.0 | 56 | (9, 68) | 73 | (3, 58) | 64 | Helix-turn-helix domain protein | Helix-turn-helix domain protein | | uniclust | UniRef100\_A0A0C9PVN7 | 99.6 | 6.2e-18 | 1.3e-23 | 88.0 | 66 | (1, 69) | 73 | (3, 68) | 77 | Phosphotransferase system mannitol/fructose-specific IIA domain | Phosphotransferase system mannitol/fructose-specific IIA domain | | uniclust | UniRef100\_A0A1X0ZRW0 | 99.6 | 6.8e-18 | 1.3e-23 | 84.2 | 60 | (8, 69) | 73 | (3, 63) | 68 | Helix-turn-helix domain-containing protein | Helix-turn-helix domain-containing protein | | uniclust | UniRef100\_A0A0B3S532 | 99.6 | 6.7e-18 | 1.3e-23 | 91.6 | 62 | (7, 70) | 73 | (34, 96) | 102 | Phage regulator-like protein | Phage regulator-like protein | | uniclust | UniRef100\_A0A0J6YTM7 | 99.6 | 7e-18 | 1.4e-23 | 87.2 | 65 | (4, 70) | 73 | (1, 66) | 77 | Helix-turn-helix domain protein | Helix-turn-helix domain protein | | uniclust | UniRef100\_A0A075NWM2 | 99.6 | 7.1e-18 | 1.4e-23 | 93.8 | 62 | (5, 68) | 73 | (28, 95) | 123 | Helix-turn-helix domain-containing protein | Helix-turn-helix domain-containing protein | | uniclust | UniRef100\_A0A259S763 | 99.6 | 7.3e-18 | 1.4e-23 | 87.5 | 63 | (2, 67) | 73 | (6, 68) | 79 | Helix-turn-helix domain-containing protein | Helix-turn-helix domain-containing protein | | uniclust | UniRef100\_A0A380TD81 | 99.6 | 7.2e-18 | 1.4e-23 | 92.5 | 60 | (10, 71) | 73 | (34, 94) | 106 | Transcriptional regulator | Transcriptional regulator | | uniclust | UniRef100\_A0A060UPH2 | 99.6 | 7.4e-18 | 1.5e-23 | 91.6 | 60 | (9, 70) | 73 | (36, 96) | 103 | Helix-turn-helix domain-containing protein | Helix-turn-helix domain-containing protein | | uniclust | UniRef100\_A0A0H3LYR5 | 99.6 | 7.6e-18 | 1.5e-23 | 87.7 | 59 | (8, 68) | 73 | (18, 76) | 80 | Helix-turn-helix domain-containing protein | Helix-turn-helix domain-containing protein | | uniclust | UniRef100\_A0A087F9Y2 | 99.6 | 7.6e-18 | 1.5e-23 | 90.6 | 61 | (7, 69) | 73 | (26, 87) | 95 | Helix-turn-helix domain-containing protein | Helix-turn-helix domain-containing protein | | uniclust | UniRef100\_A0A0F7N9N1 | 99.6 | 8e-18 | 1.5e-23 | 90.9 | 58 | (8, 67) | 73 | (25, 82) | 104 | Helix-turn-helix domain-containing protein | Helix-turn-helix domain-containing protein | | uniclust | UniRef100\_A0A1E4NHF0 | 99.6 | 7.9e-18 | 1.6e-23 | 89.6 | 61 | (7, 69) | 73 | (26, 86) | 89 | Helix-turn-helix domain-containing protein | Helix-turn-helix domain-containing protein | | uniclust | UniRef100\_A0A0S7X7T0 | 99.6 | 8.1e-18 | 1.6e-23 | 87.4 | 62 | (4, 68) | 73 | (6, 67) | 78 | Helix-turn-helix domain-containing protein | Helix-turn-helix domain-containing protein | | uniclust | UniRef100\_A0A010YHV7 | 99.6 | 8.2e-18 | 1.6e-23 | 89.5 | 63 | (5, 69) | 73 | (24, 87) | 92 | DNA-binding protein, excisionase family | DNA-binding protein, excisionase family | | uniclust | UniRef100\_A0A0M9EFS7 | 99.6 | 8.8e-18 | 1.7e-23 | 89.1 | 61 | (8, 70) | 73 | (19, 80) | 93 | Helix-turn-helix domain protein | Helix-turn-helix domain protein | | uniclust | UniRef100\_A0A2A6RG20 | 99.6 | 8.7e-18 | 1.7e-23 | 90.2 | 62 | (4, 68) | 73 | (10, 71) | 98 | Helix-turn-helix domain-containing protein | Helix-turn-helix domain-containing protein | | uniclust | UniRef100\_A0A327KP88 | 99.6 | 8.9e-18 | 1.7e-23 | 87.8 | 62 | (6, 69) | 73 | (1, 62) | 86 | Helix-turn-helix domain-containing protein | Helix-turn-helix domain-containing protein | | uniclust | UniRef100\_A0A2H9N9A0 | 99.6 | 8.8e-18 | 1.7e-23 | 88.2 | 62 | (6, 69) | 73 | (13, 78) | 85 | Helix-turn-helix domain-containing protein | Helix-turn-helix domain-containing protein | | uniclust | UniRef100\_A0A097IEB9 | 99.6 | 9.6e-18 | 1.9e-23 | 88.7 | 65 | (4, 70) | 73 | (14, 78) | 87 | MerR family transcriptional regulator | MerR family transcriptional regulator | | uniclust | UniRef100\_A0A0G1M4Q2 | 99.6 | 1e-17 | 2e-23 | 85.2 | 64 | (4, 70) | 73 | (1, 64) | 67 | Excisionase/Xis, DNA-binding protein | Excisionase/Xis, DNA-binding protein | | uniclust | UniRef100\_A0A0Q5ZYK8 | 99.6 | 1.1e-17 | 2.1e-23 | 87.7 | 60 | (9, 70) | 73 | (18, 77) | 84 | Helix-turn-helix domain-containing protein | Helix-turn-helix domain-containing protein | | uniclust | UniRef100\_A0A061STS5 | 99.5 | 1.1e-17 | 2.3e-23 | 87.9 | 62 | (7, 70) | 73 | (12, 73) | 82 | AlpA family transcriptional regulator | AlpA family transcriptional regulator | | uniclust | UniRef100\_A0A2S5MS32 | 99.5 | 1.2e-17 | 2.4e-23 | 86.4 | 63 | (5, 69) | 73 | (13, 76) | 79 | DNA-binding protein | DNA-binding protein | | uniclust | UniRef100\_A0A0F6TRE5 | 99.5 | 1.2e-17 | 2.4e-23 | 85.9 | 62 | (5, 68) | 73 | (10, 72) | 75 | Helix-turn-helix domain-containing protein | Helix-turn-helix domain-containing protein | | uniclust | UniRef100\_A0A1X1MNM6 | 99.5 | 1.3e-17 | 2.4e-23 | 84.1 | 57 | (8, 66) | 73 | (15, 71) | 74 | Helix-turn-helix domain-containing protein | Helix-turn-helix domain-containing protein | | uniclust | UniRef100\_A0A1H8Y5U8 | 99.5 | 1.2e-17 | 2.4e-23 | 86.8 | 65 | (4, 70) | 73 | (6, 70) | 83 | Helix-turn-helix domain-containing protein | Helix-turn-helix domain-containing protein | | uniclust | UniRef100\_A0A2D5PMF7 | 99.5 | 1.3e-17 | 2.5e-23 | 86.8 | 63 | (3, 67) | 73 | (4, 66) | 79 | Terminase | Terminase | | uniclust | UniRef100\_A0A080N3X4 | 99.5 | 1.4e-17 | 2.7e-23 | 86.1 | 61 | (8, 70) | 73 | (16, 76) | 79 | DNA-binding protein | DNA-binding protein | | uniclust | UniRef100\_A0A062VFU0 | 99.5 | 1.4e-17 | 2.8e-23 | 91.0 | 62 | (3, 67) | 73 | (14, 75) | 106 | DNA-binding protein | DNA-binding protein | | uniclust | UniRef100\_A0A0F4QZF8 | 99.5 | 1.4e-17 | 2.8e-23 | 86.7 | 63 | (6, 70) | 73 | (11, 74) | 81 | Helix-turn-helix domain-containing protein | Helix-turn-helix domain-containing protein | | uniclust | UniRef100\_A0A0F2RWG3 | 99.5 | 1.5e-17 | 2.9e-23 | 86.7 | 62 | (5, 69) | 73 | (13, 74) | 78 | Helix-turn-helix domain-containing protein | Helix-turn-helix domain-containing protein | | uniclust | UniRef100\_A0A075MKU4 | 99.5 | 1.5e-17 | 3e-23 | 87.0 | 64 | (4, 69) | 73 | (2, 65) | 84 | Helix-turn-helix domain-containing protein | Helix-turn-helix domain-containing protein | | uniclust | UniRef100\_A0A2S8SD78 | 99.5 | 1.5e-17 | 3e-23 | 86.9 | 65 | (3, 69) | 73 | (7, 72) | 81 | Helix-turn-helix protein | Helix-turn-helix protein | | uniclust | UniRef100\_A0A0Q0XEN5 | 99.5 | 1.6e-17 | 3.1e-23 | 90.1 | 65 | (4, 70) | 73 | (24, 88) | 105 | Transcriptional regulator | Transcriptional regulator | | uniclust | UniRef100\_A0A061M0H8 | 99.5 | 1.7e-17 | 3.2e-23 | 89.7 | 57 | (8, 66) | 73 | (31, 87) | 102 | Excisionase | Excisionase | | uniclust | UniRef100\_A0A1J4YZ76 | 99.5 | 1.6e-17 | 3.3e-23 | 89.4 | 62 | (3, 67) | 73 | (9, 70) | 96 | Helix-turn-helix domain-containing protein | Helix-turn-helix domain-containing protein | | uniclust | UniRef100\_A0A071MIW0 | 99.5 | 1.6e-17 | 3.3e-23 | 88.9 | 61 | (6, 68) | 73 | (15, 76) | 92 | Helix-turn-helix domain-containing protein | Helix-turn-helix domain-containing protein | | uniclust | UniRef100\_A0A081N1X2 | 99.5 | 1.8e-17 | 3.5e-23 | 87.2 | 66 | (2, 69) | 73 | (13, 79) | 85 | AlpA family transcriptional regulator | AlpA family transcriptional regulator | | uniclust | UniRef100\_A0A0N8K6P2 | 99.5 | 1.8e-17 | 3.5e-23 | 84.8 | 59 | (6, 67) | 73 | (1, 59) | 69 | DNA binding domain, excisionase family | DNA binding domain, excisionase family | | uniclust | UniRef100\_A0A0S7XYB7 | 99.5 | 1.8e-17 | 3.6e-23 | 87.6 | 61 | (6, 68) | 73 | (21, 82) | 89 | Helix-turn-helix domain-containing protein | Helix-turn-helix domain-containing protein | | uniclust | UniRef100\_A0A098RX04 | 99.5 | 1.8e-17 | 3.6e-23 | 88.4 | 63 | (4, 68) | 73 | (18, 81) | 92 | AlpA family phage regulatory protein | AlpA family phage regulatory protein | | uniclust | UniRef100\_A0A0U3NKV4 | 99.5 | 1.9e-17 | 3.7e-23 | 88.0 | 62 | (7, 70) | 73 | (17, 78) | 91 | Helix-turn-helix domain-containing protein | Helix-turn-helix domain-containing protein | | uniclust | UniRef100\_A0A074TFL5 | 99.5 | 2e-17 | 3.9e-23 | 90.4 | 59 | (10, 70) | 73 | (33, 91) | 113 | Helix-turn-helix domain-containing protein | Helix-turn-helix domain-containing protein | | uniclust | UniRef100\_A0A096CY27 | 99.5 | 2.1e-17 | 4e-23 | 87.2 | 61 | (4, 67) | 73 | (20, 80) | 95 | AlpA family transcriptional regulator | AlpA family transcriptional regulator | | uniclust | UniRef100\_A0A0T5PC48 | 99.5 | 2.2e-17 | 4.3e-23 | 87.2 | 63 | (3, 67) | 73 | (1, 64) | 88 | Putative transcriptional regulator | Putative transcriptional regulator | | uniclust | UniRef100\_A0A0I9TFY0 | 99.5 | 2.3e-17 | 4.4e-23 | 82.8 | 62 | (6, 69) | 73 | (2, 63) | 67 | DNA-binding protein | DNA-binding protein | | uniclust | UniRef100\_A0A142XZZ0 | 99.5 | 2.3e-17 | 4.5e-23 | 89.0 | 57 | (6, 64) | 73 | (28, 84) | 100 | Prophage CP4-57 regulatory protein (AlpA) | Prophage CP4-57 regulatory protein (AlpA) | | uniclust | UniRef100\_A0A0F9IQP4 | 99.5 | 2.3e-17 | 4.5e-23 | 86.0 | 62 | (4, 68) | 73 | (12, 73) | 79 | Helix-turn-helix domain-containing protein | Helix-turn-helix domain-containing protein | | uniclust | UniRef100\_A0A1H1FCL2 | 99.5 | 2.3e-17 | 4.6e-23 | 88.5 | 63 | (5, 69) | 73 | (11, 74) | 91 | Helix-turn-helix domain-containing protein | Helix-turn-helix domain-containing protein | | uniclust | UniRef100\_A0A024EAZ7 | 99.5 | 2.4e-17 | 4.6e-23 | 89.6 | 62 | (3, 67) | 73 | (15, 76) | 106 | Phage transcriptional regulator, AlpA | Phage transcriptional regulator, AlpA | | uniclust | UniRef100\_A0A0F3IQQ3 | 99.5 | 2.3e-17 | 4.7e-23 | 88.3 | 66 | (1, 68) | 73 | (6, 72) | 90 | Transcriptional regulator | Transcriptional regulator | | uniclust | UniRef100\_A0A024P892 | 99.5 | 2.4e-17 | 4.8e-23 | 89.6 | 61 | (5, 68) | 73 | (18, 78) | 98 | DNA binding domain, excisionase family | DNA binding domain, excisionase family | | uniclust | UniRef100\_A0A1F7JH96 | 99.5 | 2.5e-17 | 4.9e-23 | 82.1 | 60 | (8, 70) | 73 | (1, 60) | 63 | Helix-turn-helix domain-containing protein | Helix-turn-helix domain-containing protein | | uniclust | UniRef100\_A0A011Q608 | 99.5 | 2.6e-17 | 5.1e-23 | 87.9 | 60 | (9, 70) | 73 | (27, 87) | 92 | Helix-turn-helix domain protein | Helix-turn-helix domain protein | | uniclust | UniRef100\_A0A151AMP3 | 99.5 | 2.5e-17 | 5.1e-23 | 88.0 | 62 | (3, 67) | 73 | (12, 73) | 90 | Helix-turn-helix domain protein | Helix-turn-helix domain protein | | uniclust | UniRef100\_A0A0D1MDJ0 | 99.5 | 2.9e-17 | 5.6e-23 | 86.6 | 62 | (3, 66) | 73 | (6, 67) | 90 | Helix-turn-helix domain-containing protein | Helix-turn-helix domain-containing protein | | uniclust | UniRef100\_A0A0K8JIR0 | 99.5 | 2.9e-17 | 5.6e-23 | 86.0 | 64 | (1, 67) | 73 | (1, 64) | 82 | Helix-turn-helix domain-containing protein | Helix-turn-helix domain-containing protein | | uniclust | UniRef100\_A0A087BB61 | 99.5 | 2.8e-17 | 5.6e-23 | 88.6 | 63 | (6, 70) | 73 | (24, 86) | 97 | Putative phage excisionase | Putative phage excisionase | | uniclust | UniRef100\_A0A060NMA2 | 99.5 | 2.9e-17 | 5.7e-23 | 86.0 | 66 | (2, 69) | 73 | (10, 75) | 85 | Helix-turn-helix domain-containing protein | Helix-turn-helix domain-containing protein | | uniclust | UniRef100\_A0A2U2MS54 | 99.5 | 3.1e-17 | 6.1e-23 | 85.9 | 64 | (5, 70) | 73 | (11, 74) | 81 | Excisionase | Excisionase | | uniclust | UniRef100\_A0A0Q6FGU9 | 99.5 | 3.4e-17 | 6.6e-23 | 87.9 | 58 | (7, 66) | 73 | (24, 81) | 96 | DNA-binding protein | DNA-binding protein | | uniclust | UniRef100\_A0A078BM66 | 99.5 | 3.3e-17 | 6.6e-23 | 85.3 | 63 | (1, 65) | 73 | (5, 67) | 78 | Putative Phage transcriptional regulator, AlpA | Putative Phage transcriptional regulator, AlpA | | uniclust | UniRef100\_A0A353H9N9 | 99.5 | 3.3e-17 | 6.6e-23 | 86.7 | 59 | (5, 66) | 73 | (6, 64) | 87 | DNA-binding protein | DNA-binding protein | | uniclust | UniRef100\_A0A0N1B023 | 99.5 | 3.3e-17 | 6.7e-23 | 86.0 | 64 | (4, 69) | 73 | (9, 72) | 80 | AlpA family transcriptional regulator | AlpA family transcriptional regulator | | uniclust | UniRef100\_A0A068YKC6 | 99.5 | 3.5e-17 | 6.9e-23 | 84.9 | 66 | (1, 68) | 73 | (1, 68) | 78 | Helix-turn-helix domain-containing protein | Helix-turn-helix domain-containing protein | | uniclust | UniRef100\_A0A090MSV2 | 99.5 | 3.8e-17 | 7.3e-23 | 88.0 | 65 | (4, 68) | 73 | (13, 77) | 102 | Helix-turn-helix domain-containing protein | Helix-turn-helix domain-containing protein | | uniclust | UniRef100\_A0A1V5E809 | 99.5 | 3.8e-17 | 7.3e-23 | 87.3 | 63 | (2, 67) | 73 | (11, 73) | 97 | Helix-turn-helix domain protein | Helix-turn-helix domain protein | | uniclust | UniRef100\_A0A2E3AS91 | 99.5 | 3.8e-17 | 7.4e-23 | 85.1 | 68 | (2, 71) | 73 | (8, 77) | 79 | DNA-binding protein | DNA-binding protein | | uniclust | UniRef100\_A0A0F8ZPA5 | 99.5 | 3.9e-17 | 7.6e-23 | 83.3 | 60 | (6, 68) | 73 | (8, 67) | 71 | Helix-turn-helix domain-containing protein (Fragment) | Helix-turn-helix domain-containing protein (Fragment) | | uniclust | UniRef100\_A0A066UD20 | 99.5 | 4e-17 | 7.9e-23 | 88.0 | 62 | (6, 69) | 73 | (18, 80) | 97 | Prophage CP4-57 regulatory protein AlpA | Prophage CP4-57 regulatory protein AlpA | | uniclust | UniRef100\_A0A0U2WJB7 | 99.5 | 4.1e-17 | 8e-23 | 85.7 | 62 | (5, 69) | 73 | (9, 70) | 87 | Excisionase family DNA-binding domain-containing protein | Excisionase family DNA-binding domain-containing protein | | uniclust | UniRef100\_A0A086PAC3 | 99.5 | 4.2e-17 | 8.1e-23 | 84.5 | 64 | (1, 66) | 73 | (1, 66) | 79 | Phage transcriptional regulator AlpA | Phage transcriptional regulator AlpA | | uniclust | UniRef100\_A0A1C0A4S8 | 99.5 | 4.2e-17 | 8.3e-23 | 85.2 | 61 | (5, 68) | 73 | (14, 74) | 79 | DNA-binding protein | DNA-binding protein | | uniclust | UniRef100\_A0A1G3X112 | 99.5 | 4.2e-17 | 8.4e-23 | 86.0 | 64 | (2, 68) | 73 | (9, 72) | 84 | Helix-turn-helix domain-containing protein | Helix-turn-helix domain-containing protein | | uniclust | UniRef100\_A0A1G5P793 | 99.5 | 4.6e-17 | 9e-23 | 85.6 | 63 | (4, 68) | 73 | (6, 69) | 86 | Transcriptional regulator, AlpA family | Transcriptional regulator, AlpA family | | uniclust | UniRef100\_A0A086MZX1 | 99.5 | 4.6e-17 | 9e-23 | 87.1 | 56 | (10, 67) | 73 | (35, 90) | 96 | Excisionase | Excisionase | | uniclust | UniRef100\_A0A1I4TIB0 | 99.5 | 4.6e-17 | 9.1e-23 | 88.0 | 60 | (10, 71) | 73 | (30, 89) | 99 | Helix-turn-helix domain-containing protein | Helix-turn-helix domain-containing protein | | uniclust | UniRef100\_A0A931WHN7 | 99.5 | 4.9e-17 | 9.3e-23 | 83.5 | 63 | (3, 68) | 73 | (3, 65) | 80 | Helix-turn-helix domain-containing protein | Helix-turn-helix domain-containing protein | | uniclust | UniRef100\_A0A2W4KKW8 | 99.5 | 5.3e-17 | 1e-22 | 85.3 | 58 | (9, 69) | 73 | (12, 69) | 84 | Excisionase | Excisionase | | uniclust | UniRef100\_A0A1G8RV34 | 99.5 | 5.4e-17 | 1.1e-22 | 84.4 | 61 | (3, 66) | 73 | (2, 62) | 80 | DNA binding domain-containing protein, excisionase family | DNA binding domain-containing protein, excisionase family | | uniclust | UniRef100\_A0A0C2YUN8 | 99.5 | 5.6e-17 | 1.1e-22 | 83.8 | 65 | (1, 67) | 73 | (1, 65) | 79 | Helix-turn-helix domain-containing protein | Helix-turn-helix domain-containing protein | | uniclust | UniRef100\_A0A2N9JAS9 | 99.5 | 5.5e-17 | 1.1e-22 | 83.8 | 61 | (6, 69) | 73 | (8, 68) | 75 | DNA binding domain-containing protein, excisionase family | DNA binding domain-containing protein, excisionase family | | uniclust | UniRef100\_A0A099GKU9 | 99.5 | 5.8e-17 | 1.1e-22 | 86.3 | 59 | (9, 69) | 73 | (22, 80) | 91 | Helix-turn-helix domain-containing protein | Helix-turn-helix domain-containing protein | | uniclust | UniRef100\_A0A0P6XBV6 | 99.5 | 6.1e-17 | 1.2e-22 | 86.9 | 60 | (4, 66) | 73 | (17, 76) | 95 | Helix-turn-helix domain-containing protein | Helix-turn-helix domain-containing protein | | uniclust | UniRef100\_A0A0F5ESX0 | 99.5 | 6.1e-17 | 1.2e-22 | 87.3 | 62 | (5, 68) | 73 | (16, 78) | 93 | AlpA family phage regulatory protein | AlpA family phage regulatory protein | | uniclust | UniRef100\_A0A164B9G5 | 99.5 | 6.5e-17 | 1.3e-22 | 86.0 | 61 | (7, 69) | 73 | (11, 71) | 94 | Helix-turn-helix domain-containing protein | Helix-turn-helix domain-containing protein | | uniclust | UniRef100\_A0A2E7GW86 | 99.5 | 6.5e-17 | 1.3e-22 | 83.0 | 60 | (7, 68) | 73 | (6, 65) | 75 | DNA-binding protein | DNA-binding protein | | uniclust | UniRef100\_A0A1F8ZYZ8 | 99.5 | 6.6e-17 | 1.3e-22 | 85.9 | 60 | (4, 66) | 73 | (8, 67) | 89 | Helix-turn-helix domain-containing protein | Helix-turn-helix domain-containing protein | | uniclust | UniRef100\_A0A0F7HAF8 | 99.5 | 6.6e-17 | 1.3e-22 | 84.4 | 59 | (7, 67) | 73 | (10, 68) | 78 | Response regulator inhibitor for tor operon | Response regulator inhibitor for tor operon | | uniclust | UniRef100\_A0A2A3YGA8 | 99.5 | 7.1e-17 | 1.3e-22 | 81.4 | 60 | (6, 67) | 73 | (11, 70) | 72 | Helix-turn-helix domain-containing protein | Helix-turn-helix domain-containing protein | | uniclust | UniRef100\_A0A1H6SP13 | 99.5 | 7e-17 | 1.4e-22 | 85.9 | 62 | (6, 69) | 73 | (25, 87) | 92 | Transcriptional regulator, AlpA family | Transcriptional regulator, AlpA family | | uniclust | UniRef100\_A0A0Q9SUY6 | 99.5 | 6.9e-17 | 1.4e-22 | 85.1 | 60 | (7, 68) | 73 | (23, 82) | 84 | Excisionase | Excisionase | | uniclust | UniRef100\_A0A0F9MAA1 | 99.5 | 7e-17 | 1.4e-22 | 84.9 | 58 | (7, 67) | 73 | (2, 59) | 85 | Helix-turn-helix domain-containing protein | Helix-turn-helix domain-containing protein | | uniclust | UniRef100\_A0A2S5CX75 | 99.5 | 8.2e-17 | 1.6e-22 | 82.6 | 60 | (3, 65) | 73 | (3, 62) | 73 | Helix-turn-helix domain-containing protein | Helix-turn-helix domain-containing protein | | uniclust | UniRef100\_A0A0R3E754 | 99.5 | 8.6e-17 | 1.7e-22 | 84.3 | 61 | (6, 69) | 73 | (18, 78) | 88 | Helix-turn-helix domain-containing protein | Helix-turn-helix domain-containing protein | | uniclust | UniRef100\_A0A2T6CQ99 | 99.5 | 8.5e-17 | 1.7e-22 | 83.3 | 64 | (3, 68) | 73 | (12, 75) | 78 | Helix-turn-helix domain-containing protein | Helix-turn-helix domain-containing protein | | uniclust | UniRef100\_A0A1A3DQH9 | 99.5 | 8.6e-17 | 1.7e-22 | 82.6 | 64 | (3, 68) | 73 | (6, 69) | 75 | Helix-turn-helix domain-containing protein | Helix-turn-helix domain-containing protein | | uniclust | UniRef100\_A0A255R1K7 | 99.5 | 8.6e-17 | 1.7e-22 | 86.2 | 55 | (8, 64) | 73 | (27, 81) | 94 | Helix-turn-helix domain-containing protein | Helix-turn-helix domain-containing protein | | uniclust | UniRef100\_A0A1F0QJQ0 | 99.5 | 8.8e-17 | 1.7e-22 | 83.2 | 57 | (11, 69) | 73 | (18, 74) | 79 | Helix-turn-helix domain-containing protein | Helix-turn-helix domain-containing protein | | uniclust | UniRef100\_A0A0C9PAJ7 | 99.5 | 8.7e-17 | 1.7e-22 | 84.8 | 58 | (9, 69) | 73 | (22, 79) | 85 | Helix-turn-helix domain-containing protein | Helix-turn-helix domain-containing protein | | uniclust | UniRef100\_A0A1G9DMJ4 | 99.5 | 8.8e-17 | 1.7e-22 | 83.4 | 61 | (7, 70) | 73 | (12, 72) | 76 | DNA binding domain-containing protein, excisionase family | DNA binding domain-containing protein, excisionase family | | uniclust | UniRef100\_A0A0G0M802 | 99.5 | 9.4e-17 | 1.8e-22 | 83.2 | 62 | (4, 68) | 73 | (4, 66) | 77 | Binding domain protein, excisionase family protein | Binding domain protein, excisionase family protein | | uniclust | UniRef100\_A0A017HNG0 | 99.5 | 9.5e-17 | 1.8e-22 | 86.6 | 61 | (6, 68) | 73 | (20, 81) | 101 | Phage transcriptional regulator, AlpA | Phage transcriptional regulator, AlpA | | uniclust | UniRef100\_A0A2H0DID7 | 99.5 | 1e-16 | 1.9e-22 | 81.4 | 62 | (3, 67) | 73 | (1, 63) | 70 | Helix-turn-helix domain-containing protein | Helix-turn-helix domain-containing protein | | uniclust | UniRef100\_A0A2V9PDW9 | 99.5 | 1e-16 | 1.9e-22 | 83.5 | 64 | (4, 68) | 73 | (11, 74) | 82 | DNA-binding protein | DNA-binding protein | | uniclust | UniRef100\_A0A0B8Q8F9 | 99.5 | 1e-16 | 2e-22 | 88.1 | 65 | (3, 69) | 73 | (24, 89) | 108 | Transcriptional regulator | Transcriptional regulator | | uniclust | UniRef100\_A0A2N1ZXV4 | 99.5 | 1.1e-16 | 2.1e-22 | 83.6 | 65 | (1, 67) | 73 | (1, 66) | 80 | Transcriptional regulator | Transcriptional regulator | | uniclust | UniRef100\_A0A1F2R0F2 | 99.5 | 1e-16 | 2.1e-22 | 84.8 | 57 | (8, 67) | 73 | (11, 67) | 82 | Helix-turn-helix domain-containing protein | Helix-turn-helix domain-containing protein | | uniclust | UniRef100\_A0A1X1PI46 | 99.5 | 1.1e-16 | 2.1e-22 | 82.3 | 61 | (6, 68) | 73 | (7, 67) | 75 | Helix-turn-helix domain-containing protein | Helix-turn-helix domain-containing protein | | uniclust | UniRef100\_A0A2V9WFU4 | 99.5 | 1.1e-16 | 2.2e-22 | 84.5 | 66 | (1, 68) | 73 | (1, 67) | 86 | Helix-turn-helix domain-containing protein | Helix-turn-helix domain-containing protein | | uniclust | UniRef100\_A0A017HTQ8 | 99.5 | 1.1e-16 | 2.2e-22 | 86.3 | 61 | (6, 68) | 73 | (21, 82) | 94 | DNA-binding protein, putative | DNA-binding protein, putative | | uniclust | UniRef100\_A0A0B4XAG0 | 99.5 | 1.1e-16 | 2.2e-22 | 87.2 | 60 | (6, 67) | 73 | (24, 83) | 108 | Helix-turn-helix domain-containing protein | Helix-turn-helix domain-containing protein | | uniclust | UniRef100\_A0A011QGD0 | 99.5 | 1.2e-16 | 2.3e-22 | 87.0 | 60 | (5, 67) | 73 | (30, 89) | 114 | DNA binding domain, excisionase family | DNA binding domain, excisionase family | | uniclust | UniRef100\_A0A109IMN0 | 99.5 | 1.2e-16 | 2.3e-22 | 81.5 | 60 | (7, 68) | 73 | (11, 70) | 78 | Transcriptional regulator, AlpA family | Transcriptional regulator, AlpA family | | uniclust | UniRef100\_A0A0P6X5W5 | 99.5 | 1.2e-16 | 2.3e-22 | 85.5 | 60 | (4, 66) | 73 | (9, 68) | 95 | DNA-binding protein | DNA-binding protein | | uniclust | UniRef100\_A0A077M1T7 | 99.5 | 1.2e-16 | 2.3e-22 | 84.3 | 63 | (4, 68) | 73 | (18, 80) | 86 | Helix-turn-helix domain-containing protein | Helix-turn-helix domain-containing protein | | uniclust | UniRef100\_A0A0F7PLT3 | 99.5 | 1.2e-16 | 2.4e-22 | 85.7 | 66 | (1, 68) | 73 | (3, 73) | 96 | Helix-turn-helix domain | Helix-turn-helix domain | | uniclust | UniRef100\_A0A0F5Q0G5 | 99.5 | 1.2e-16 | 2.4e-22 | 84.2 | 64 | (3, 69) | 73 | (1, 64) | 84 | DNA binding domain-containing protein, excisionase family | DNA binding domain-containing protein, excisionase family | | uniclust | UniRef100\_A0A136K2Y7 | 99.5 | 1.2e-16 | 2.4e-22 | 82.2 | 64 | (2, 68) | 73 | (1, 64) | 74 | Helix-turn-helix domain protein | Helix-turn-helix domain protein | | uniclust | UniRef100\_A0A022LBF6 | 99.5 | 1.3e-16 | 2.5e-22 | 83.4 | 61 | (7, 68) | 73 | (16, 76) | 84 | Helix-turn-helix domain-containing protein | Helix-turn-helix domain-containing protein | | uniclust | UniRef100\_A0A0K8MEP3 | 99.5 | 1.3e-16 | 2.5e-22 | 84.8 | 61 | (7, 69) | 73 | (24, 85) | 93 | Helix-turn-helix domain protein | Helix-turn-helix domain protein | | uniclust | UniRef100\_A0A258IUU4 | 99.5 | 1.4e-16 | 2.7e-22 | 83.3 | 67 | (2, 70) | 73 | (8, 74) | 82 | DNA-binding protein | DNA-binding protein | | uniclust | UniRef100\_A0A496RWF2 | 99.5 | 1.4e-16 | 2.7e-22 | 81.2 | 62 | (5, 68) | 73 | (3, 64) | 74 | Helix-turn-helix domain-containing protein | Helix-turn-helix domain-containing protein | | uniclust | UniRef100\_A0A077M848 | 99.5 | 1.5e-16 | 2.9e-22 | 86.6 | 62 | (2, 66) | 73 | (12, 73) | 110 | Phage transcriptional regulator, AlpA | Phage transcriptional regulator, AlpA | | uniclust | UniRef100\_A0A0T6APZ4 | 99.5 | 1.5e-16 | 2.9e-22 | 85.8 | 62 | (5, 69) | 73 | (25, 86) | 94 | Excisionase | Excisionase | | uniclust | UniRef100\_A0A066UG88 | 99.5 | 1.5e-16 | 3e-22 | 85.3 | 59 | (9, 69) | 73 | (32, 91) | 95 | Excisionase | Excisionase | | uniclust | UniRef100\_A0A0H4J0C0 | 99.5 | 1.6e-16 | 3.1e-22 | 87.1 | 61 | (6, 68) | 73 | (21, 82) | 106 | AlpA family transcriptional regulator | AlpA family transcriptional regulator | | uniclust | UniRef100\_A0A4R9BK06 | 99.5 | 1.6e-16 | 3.1e-22 | 86.2 | 60 | (10, 71) | 73 | (38, 97) | 101 | DNA-binding protein | DNA-binding protein | | uniclust | UniRef100\_A0A127K3W8 | 99.5 | 1.6e-16 | 3.2e-22 | 88.0 | 61 | (7, 69) | 73 | (45, 105) | 109 | Helix-turn-helix domain-containing protein | Helix-turn-helix domain-containing protein | | uniclust | UniRef100\_A0A066ZRH0 | 99.5 | 1.7e-16 | 3.3e-22 | 84.5 | 65 | (3, 69) | 73 | (7, 72) | 87 | AlpA family transcriptional regulator | AlpA family transcriptional regulator | | uniclust | UniRef100\_A0A0G3G4R0 | 99.5 | 1.7e-16 | 3.3e-22 | 83.3 | 64 | (3, 68) | 73 | (9, 73) | 82 | AlpA family transcriptional regulator | AlpA family transcriptional regulator | | uniclust | UniRef100\_A0A1V6CFG2 | 99.5 | 1.8e-16 | 3.4e-22 | 79.9 | 58 | (6, 66) | 73 | (2, 59) | 73 | Helix-turn-helix domain protein | Helix-turn-helix domain protein | | uniclust | UniRef100\_A0A0F8YFR4 | 99.5 | 1.8e-16 | 3.5e-22 | 82.8 | 62 | (3, 67) | 73 | (10, 71) | 84 | HTH merR-type domain-containing protein | HTH merR-type domain-containing protein | | uniclust | UniRef100\_A0A167I6B1 | 99.5 | 1.9e-16 | 3.8e-22 | 86.0 | 59 | (8, 68) | 73 | (25, 83) | 98 | Helix-turn-helix domain-containing protein | Helix-turn-helix domain-containing protein | | uniclust | UniRef100\_A0A086Y3N3 | 99.5 | 2e-16 | 3.9e-22 | 84.1 | 64 | (2, 67) | 73 | (14, 79) | 86 | AlpA family transcriptional regulator | AlpA family transcriptional regulator | | uniclust | UniRef100\_A0A0Q7G9F4 | 99.5 | 2.1e-16 | 4e-22 | 78.2 | 60 | (6, 67) | 73 | (1, 60) | 62 | Helix-turn-helix domain-containing protein | Helix-turn-helix domain-containing protein | | uniclust | UniRef100\_A0A098B1F2 | 99.5 | 2.1e-16 | 4e-22 | 80.8 | 64 | (2, 68) | 73 | (6, 69) | 78 | DNA binding domain, putative | DNA binding domain, putative | | uniclust | UniRef100\_A0A136KDH9 | 99.5 | 2e-16 | 4e-22 | 85.7 | 58 | (6, 66) | 73 | (28, 85) | 99 | Helix-turn-helix domain protein | Helix-turn-helix domain protein | | uniclust | UniRef100\_A0A261TDA7 | 99.5 | 2.1e-16 | 4.1e-22 | 86.8 | 58 | (5, 64) | 73 | (7, 65) | 110 | Helix-turn-helix domain-containing protein | Helix-turn-helix domain-containing protein | | uniclust | UniRef100\_A0A1I5LC99 | 99.5 | 2e-16 | 4.1e-22 | 84.3 | 64 | (3, 69) | 73 | (16, 79) | 88 | DNA binding domain-containing protein, excisionase family | DNA binding domain-containing protein, excisionase family | | uniclust | UniRef100\_A0A1Q7SV79 | 99.5 | 2e-16 | 4.1e-22 | 82.3 | 64 | (2, 68) | 73 | (2, 66) | 76 | Helix-turn-helix domain-containing protein | Helix-turn-helix domain-containing protein | | uniclust | UniRef100\_A0A0G0RVY6 | 99.5 | 2.2e-16 | 4.3e-22 | 83.2 | 62 | (3, 67) | 73 | (7, 68) | 86 | Binding domain protein, excisionase family protein | Binding domain protein, excisionase family protein | | uniclust | UniRef100\_A0A2N2NCM9 | 99.5 | 2.2e-16 | 4.3e-22 | 82.5 | 59 | (7, 68) | 73 | (13, 71) | 79 | DNA-binding protein | DNA-binding protein | | uniclust | UniRef100\_A0A086ZPG8 | 99.5 | 2.3e-16 | 4.4e-22 | 85.0 | 60 | (4, 66) | 73 | (12, 71) | 101 | DNA binding domain-containing protein, excisionase family | DNA binding domain-containing protein, excisionase family | | uniclust | UniRef100\_A0A085ADN8 | 99.4 | 2.3e-16 | 4.5e-22 | 82.4 | 64 | (3, 68) | 73 | (3, 66) | 79 | AlpA family phage regulatory protein | AlpA family phage regulatory protein | | uniclust | UniRef100\_A0A031JQZ3 | 99.4 | 2.3e-16 | 4.5e-22 | 87.0 | 59 | (9, 69) | 73 | (36, 94) | 107 | Helix-turn-helix domain-containing protein | Helix-turn-helix domain-containing protein | | uniclust | UniRef100\_A0A010YJ93 | 99.4 | 2.3e-16 | 4.6e-22 | 83.7 | 61 | (5, 67) | 73 | (13, 73) | 88 | DNA-binding protein, excisionase family | DNA-binding protein, excisionase family | | uniclust | UniRef100\_A0A0A3IXC5 | 99.4 | 2.4e-16 | 4.8e-22 | 83.7 | 62 | (4, 68) | 73 | (12, 73) | 89 | Excisionase | Excisionase | | uniclust | UniRef100\_A0A086MTU0 | 99.4 | 2.4e-16 | 4.8e-22 | 84.4 | 60 | (7, 68) | 73 | (29, 89) | 92 | Excisionase | Excisionase | | uniclust | UniRef100\_A0A0W0SED0 | 99.4 | 2.5e-16 | 4.9e-22 | 82.4 | 64 | (4, 69) | 73 | (1, 64) | 84 | DNA-binding protein | DNA-binding protein | | uniclust | UniRef100\_A0A059XS14 | 99.4 | 2.5e-16 | 5e-22 | 85.1 | 64 | (2, 67) | 73 | (11, 75) | 95 | AlpA family transcriptional regulator | AlpA family transcriptional regulator | | uniclust | UniRef100\_A0A1J5D8F8 | 99.4 | 2.5e-16 | 5.1e-22 | 82.4 | 63 | (2, 67) | 73 | (1, 63) | 79 | Helix-turn-helix domain-containing protein | Helix-turn-helix domain-containing protein | | uniclust | UniRef100\_A0A0F2RJT8 | 99.4 | 2.7e-16 | 5.3e-22 | 84.2 | 63 | (6, 70) | 73 | (23, 86) | 100 | Helix-turn-helix domain-containing protein | Helix-turn-helix domain-containing protein | | uniclust | UniRef100\_A0A3M0XFV3 | 99.4 | 2.8e-16 | 5.3e-22 | 83.1 | 59 | (5, 66) | 73 | (4, 62) | 93 | DNA-binding protein | DNA-binding protein | | uniclust | UniRef100\_A0A1V4VR13 | 99.4 | 2.8e-16 | 5.4e-22 | 81.1 | 65 | (2, 69) | 73 | (1, 65) | 75 | Helix-turn-helix domain protein | Helix-turn-helix domain protein | | uniclust | UniRef100\_A0A1N7PI06 | 99.4 | 2.8e-16 | 5.5e-22 | 84.9 | 62 | (2, 65) | 73 | (16, 78) | 101 | Prophage regulatory protein | Prophage regulatory protein | | uniclust | UniRef100\_A0A0F2R716 | 99.4 | 2.8e-16 | 5.6e-22 | 83.1 | 64 | (7, 72) | 73 | (18, 81) | 82 | Helix-turn-helix domain-containing protein | Helix-turn-helix domain-containing protein | | uniclust | UniRef100\_A0A1G2ZI97 | 99.4 | 2.9e-16 | 5.6e-22 | 83.7 | 53 | (8, 63) | 73 | (22, 74) | 91 | Helix-turn-helix domain-containing protein | Helix-turn-helix domain-containing protein | | uniclust | UniRef100\_A0A1F1E662 | 99.4 | 2.9e-16 | 5.7e-22 | 83.2 | 59 | (9, 69) | 73 | (19, 80) | 88 | Helix-turn-helix domain-containing protein | Helix-turn-helix domain-containing protein | | uniclust | UniRef100\_A0A0G3V2N5 | 99.4 | 2.9e-16 | 5.7e-22 | 84.9 | 58 | (8, 68) | 73 | (22, 79) | 97 | Transcriptional regulator, AlpA family | Transcriptional regulator, AlpA family | | uniclust | UniRef100\_A0A089IG40 | 99.4 | 3.2e-16 | 6.3e-22 | 79.7 | 65 | (1, 69) | 73 | (1, 65) | 69 | Helix-turn-helix domain-containing protein | Helix-turn-helix domain-containing protein | | uniclust | UniRef100\_A0A1F3TX82 | 99.4 | 3.3e-16 | 6.3e-22 | 81.8 | 61 | (4, 67) | 73 | (11, 71) | 84 | Excisionase | Excisionase | | uniclust | UniRef100\_A0A191WES9 | 99.4 | 3.4e-16 | 6.7e-22 | 83.6 | 58 | (5, 65) | 73 | (11, 68) | 90 | Helix-turn-helix domain-containing protein | Helix-turn-helix domain-containing protein | | uniclust | UniRef100\_A0A0C1RLB9 | 99.4 | 3.4e-16 | 6.8e-22 | 82.4 | 58 | (8, 68) | 73 | (19, 76) | 84 | Helix-turn-helix domain-containing protein | Helix-turn-helix domain-containing protein | | uniclust | UniRef100\_A0A0C1X3E4 | 99.4 | 3.6e-16 | 7e-22 | 82.9 | 65 | (4, 70) | 73 | (16, 84) | 93 | DNA-binding protein | DNA-binding protein | | uniclust | UniRef100\_A0A1G1K0V6 | 99.4 | 3.6e-16 | 7e-22 | 82.9 | 57 | (6, 65) | 73 | (9, 65) | 93 | Helix-turn-helix domain-containing protein | Helix-turn-helix domain-containing protein | | uniclust | UniRef100\_A0A067A1U4 | 99.4 | 3.5e-16 | 7e-22 | 86.4 | 65 | (2, 68) | 73 | (28, 93) | 109 | AlpA family transcriptional regulator | AlpA family transcriptional regulator | | uniclust | UniRef100\_A0A2E7A109 | 99.4 | 3.8e-16 | 7.2e-22 | 80.6 | 57 | (7, 65) | 73 | (22, 80) | 82 | Helix-turn-helix domain-containing protein | Helix-turn-helix domain-containing protein | | uniclust | UniRef100\_A0A060UYJ0 | 99.4 | 3.7e-16 | 7.2e-22 | 83.1 | 63 | (4, 68) | 73 | (15, 78) | 87 | AlpA family transcriptional regulator | AlpA family transcriptional regulator | | uniclust | UniRef100\_A0A060ZVZ6 | 99.4 | 4e-16 | 7.7e-22 | 86.2 | 60 | (8, 69) | 73 | (53, 112) | 120 | Helix-turn-helix domain-containing protein | Helix-turn-helix domain-containing protein | | uniclust | UniRef100\_A0A1F8XWQ5 | 99.4 | 3.9e-16 | 7.8e-22 | 88.8 | 52 | (10, 64) | 73 | (19, 70) | 132 | Helix-turn-helix domain-containing protein | Helix-turn-helix domain-containing protein | | uniclust | UniRef100\_A0A1U9USI9 | 99.4 | 4e-16 | 7.9e-22 | 80.6 | 62 | (3, 66) | 73 | (9, 70) | 76 | DNA-binding protein | DNA-binding protein | | uniclust | UniRef100\_A0A6P0HA59 | 99.4 | 4.2e-16 | 7.9e-22 | 81.7 | 60 | (7, 68) | 73 | (11, 70) | 93 | Helix-turn-helix domain-containing protein | Helix-turn-helix domain-containing protein | | uniclust | UniRef100\_A0A1Y0UY49 | 99.4 | 4.2e-16 | 8.3e-22 | 79.9 | 66 | (1, 68) | 73 | (1, 68) | 72 | Helix-turn-helix domain-containing protein | Helix-turn-helix domain-containing protein | | uniclust | UniRef100\_A0A3A5M606 | 99.4 | 4.4e-16 | 8.4e-22 | 80.8 | 57 | (8, 66) | 73 | (20, 76) | 80 | DNA-binding protein | DNA-binding protein | | uniclust | UniRef100\_A0A1V5FMB9 | 99.4 | 4.4e-16 | 8.5e-22 | 79.9 | 64 | (1, 66) | 73 | (1, 65) | 77 | Helix-turn-helix domain protein | Helix-turn-helix domain protein | | uniclust | UniRef100\_A0A1V5QDI4 | 99.4 | 4.2e-16 | 8.5e-22 | 81.2 | 64 | (3, 69) | 73 | (4, 67) | 75 | Helix-turn-helix domain protein | Helix-turn-helix domain protein | | uniclust | UniRef100\_A0A095XXS8 | 99.4 | 4.4e-16 | 8.7e-22 | 81.4 | 64 | (4, 68) | 73 | (8, 71) | 78 | HTH merR-type domain-containing protein | HTH merR-type domain-containing protein | | uniclust | UniRef100\_A0A5B0D528 | 99.4 | 4.5e-16 | 8.8e-22 | 77.9 | 59 | (5, 65) | 73 | (2, 60) | 63 | Helix-turn-helix domain-containing protein | Helix-turn-helix domain-containing protein | | uniclust | UniRef100\_A0A1I3XCD0 | 99.4 | 4.5e-16 | 8.9e-22 | 83.4 | 54 | (9, 64) | 73 | (20, 73) | 92 | Helix-turn-helix domain-containing protein | Helix-turn-helix domain-containing protein | | uniclust | UniRef100\_A0A1W9XLA3 | 99.4 | 4.5e-16 | 8.9e-22 | 85.1 | 63 | (6, 70) | 73 | (31, 94) | 103 | Helix-turn-helix domain-containing protein | Helix-turn-helix domain-containing protein | | uniclust | UniRef100\_A0A0Q8V938 | 99.4 | 4.7e-16 | 8.9e-22 | 84.0 | 58 | (9, 68) | 73 | (42, 99) | 108 | Helix-turn-helix domain-containing protein | Helix-turn-helix domain-containing protein | | uniclust | UniRef100\_A0A0F4ICJ3 | 99.4 | 4.8e-16 | 9.2e-22 | 82.8 | 60 | (9, 70) | 73 | (38, 97) | 100 | Helix-turn-helix domain-containing protein | Helix-turn-helix domain-containing protein | | uniclust | UniRef100\_A0A011U9H2 | 99.4 | 4.9e-16 | 9.5e-22 | 85.3 | 60 | (8, 69) | 73 | (21, 80) | 112 | Transcriptional regulator | Transcriptional regulator | | uniclust | UniRef100\_A0A0C1R836 | 99.4 | 5e-16 | 9.7e-22 | 85.7 | 59 | (7, 68) | 73 | (28, 86) | 120 | Helix-turn-helix domain-containing protein | Helix-turn-helix domain-containing protein | | uniclust | UniRef100\_A0A0D6ZBS2 | 99.4 | 5.1e-16 | 9.8e-22 | 79.7 | 60 | (6, 68) | 73 | (7, 66) | 75 | Helix-turn-helix domain-containing protein | Helix-turn-helix domain-containing protein | | uniclust | UniRef100\_A0A096BCL5 | 99.4 | 5.1e-16 | 1e-21 | 82.5 | 59 | (7, 68) | 73 | (18, 76) | 89 | Helix-turn-helix domain-containing protein | Helix-turn-helix domain-containing protein | | uniclust | UniRef100\_A0A2E3YEM9 | 99.4 | 5.3e-16 | 1e-21 | 82.4 | 59 | (5, 66) | 73 | (6, 64) | 96 | Helix-turn-helix domain-containing protein | Helix-turn-helix domain-containing protein | | uniclust | UniRef100\_A0A1H3K8W7 | 99.4 | 5.4e-16 | 1e-21 | 79.0 | 59 | (7, 67) | 73 | (9, 68) | 70 | Transcriptional regulator, AlpA family | Transcriptional regulator, AlpA family | | uniclust | UniRef100\_A0A0G1DFI7 | 99.4 | 5.4e-16 | 1.1e-21 | 83.8 | 62 | (3, 67) | 73 | (16, 82) | 95 | Helix-turn-helix domain-containing protein | Helix-turn-helix domain-containing protein | | uniclust | UniRef100\_A0A953LVT0 | 99.4 | 5.7e-16 | 1.1e-21 | 83.7 | 63 | (5, 69) | 73 | (32, 94) | 103 | Helix-turn-helix domain-containing protein | Helix-turn-helix domain-containing protein | | uniclust | UniRef100\_A0A136KUN2 | 99.4 | 6.2e-16 | 1.2e-21 | 96.0 | 65 | (1, 67) | 73 | (18, 82) | 272 | MerR family transcriptional regulator | MerR family transcriptional regulator | | uniclust | UniRef100\_A0A0J1BH83 | 99.4 | 6.2e-16 | 1.2e-21 | 82.8 | 55 | (9, 65) | 73 | (21, 75) | 88 | Helix-turn-helix domain-containing protein | Helix-turn-helix domain-containing protein | | uniclust | UniRef100\_A0A0F7GG26 | 99.4 | 6.4e-16 | 1.3e-21 | 95.5 | 61 | (1, 64) | 73 | (38, 98) | 256 | Nitrogen regulation protein NR(I) | Nitrogen regulation protein NR(I) | | uniclust | UniRef100\_A0A1V1PRD8 | 99.4 | 7e-16 | 1.4e-21 | 82.9 | 61 | (7, 69) | 73 | (26, 86) | 97 | Helix-turn-helix domain-containing protein | Helix-turn-helix domain-containing protein | | uniclust | UniRef100\_A0A023WP26 | 99.4 | 6.6e-16 | 1.4e-21 | 84.1 | 63 | (5, 69) | 73 | (19, 82) | 96 | Transcriptional regulator | Transcriptional regulator | | uniclust | UniRef100\_A0A062UK24 | 99.4 | 7.1e-16 | 1.4e-21 | 81.9 | 60 | (8, 69) | 73 | (16, 75) | 90 | Helix-turn-helix domain-containing protein | Helix-turn-helix domain-containing protein | | uniclust | UniRef100\_A0A7L8RVP0 | 99.4 | 7.5e-16 | 1.4e-21 | 81.6 | 62 | (6, 69) | 73 | (27, 88) | 95 | Helix-turn-helix domain-containing protein | Helix-turn-helix domain-containing protein | | uniclust | UniRef100\_A0A2E6LLT9 | 99.4 | 7.6e-16 | 1.4e-21 | 75.8 | 55 | (10, 66) | 73 | (2, 57) | 60 | AlpA family transcriptional regulator | AlpA family transcriptional regulator | | uniclust | UniRef100\_A0A0A0B9Q1 | 99.4 | 7.6e-16 | 1.5e-21 | 80.2 | 59 | (4, 64) | 73 | (19, 78) | 81 | Excisionase | Excisionase | | uniclust | UniRef100\_A0A0M0T6P6 | 99.4 | 7.7e-16 | 1.5e-21 | 81.1 | 63 | (4, 68) | 73 | (9, 71) | 85 | Uncharacterized protein | Uncharacterized protein | | uniclust | UniRef100\_A0A1Q6XGV3 | 99.4 | 8.2e-16 | 1.6e-21 | 80.8 | 64 | (3, 68) | 73 | (17, 80) | 87 | Helix-turn-helix domain-containing protein | Helix-turn-helix domain-containing protein | | uniclust | UniRef100\_A0A0G0MGN7 | 99.4 | 8.4e-16 | 1.6e-21 | 79.9 | 61 | (3, 67) | 73 | (8, 68) | 77 | Excise: DNA binding domain, excisionase fami | Excise: DNA binding domain, excisionase fami | | uniclust | UniRef100\_A0A1F6Q9F2 | 99.4 | 8.7e-16 | 1.7e-21 | 79.5 | 58 | (8, 68) | 73 | (20, 77) | 81 | Helix-turn-helix domain-containing protein | Helix-turn-helix domain-containing protein | | uniclust | UniRef100\_A0A0Q5WB56 | 99.4 | 8.5e-16 | 1.7e-21 | 81.8 | 57 | (9, 68) | 73 | (23, 79) | 88 | Helix-turn-helix domain-containing protein | Helix-turn-helix domain-containing protein | | uniclust | UniRef100\_A0A542ZXW8 | 99.4 | 8.9e-16 | 1.7e-21 | 77.0 | 61 | (6, 68) | 73 | (2, 63) | 66 | Helix-turn-helix protein | Helix-turn-helix protein | | uniclust | UniRef100\_A0A0Q9QUK2 | 99.4 | 9.4e-16 | 1.8e-21 | 80.3 | 57 | (9, 67) | 73 | (18, 74) | 85 | Helix-turn-helix domain-containing protein | Helix-turn-helix domain-containing protein | | uniclust | UniRef100\_A0A1Q6JKS2 | 99.4 | 9.9e-16 | 1.9e-21 | 78.1 | 61 | (5, 67) | 73 | (9, 70) | 75 | Helix-turn-helix domain-containing protein | Helix-turn-helix domain-containing protein | | uniclust | UniRef100\_A0A3B7DB92 | 99.4 | 1e-15 | 1.9e-21 | 77.1 | 58 | (8, 67) | 73 | (11, 68) | 71 | DNA-binding protein | DNA-binding protein | | uniclust | UniRef100\_A0A1C5D3R9 | 99.4 | 1e-15 | 2e-21 | 79.6 | 59 | (5, 66) | 73 | (13, 71) | 80 | DNA binding domain-containing protein, excisionase family | DNA binding domain-containing protein, excisionase family | | uniclust | UniRef100\_A0A068Z9N5 | 99.4 | 1e-15 | 2e-21 | 81.7 | 64 | (3, 68) | 73 | (6, 70) | 87 | AlpA family transcriptional regulator | AlpA family transcriptional regulator | | uniclust | UniRef100\_A0A1A9BV43 | 99.4 | 1.1e-15 | 2.1e-21 | 77.5 | 60 | (5, 66) | 73 | (2, 62) | 67 | Helix-turn-helix domain-containing protein | Helix-turn-helix domain-containing protein | | uniclust | UniRef100\_A0A2H1STW7 | 99.4 | 1.1e-15 | 2.1e-21 | 80.6 | 62 | (4, 68) | 73 | (29, 90) | 97 | Excisionase (Modular protein) | Excisionase (Modular protein) | | uniclust | UniRef100\_A0A0G0QHG5 | 99.4 | 1.1e-15 | 2.1e-21 | 79.8 | 61 | (5, 68) | 73 | (6, 70) | 78 | Binding domain protein, excisionase family protein | Binding domain protein, excisionase family protein | | uniclust | UniRef100\_A0A0B3SSZ9 | 99.4 | 1.1e-15 | 2.2e-21 | 79.5 | 59 | (6, 66) | 73 | (21, 79) | 82 | Transcriptional repressor, MerR family protein | Transcriptional repressor, MerR family protein | | uniclust | UniRef100\_A0A6I5VMA1 | 99.4 | 1.2e-15 | 2.2e-21 | 81.0 | 60 | (8, 69) | 73 | (25, 85) | 93 | Helix-turn-helix domain-containing protein | Helix-turn-helix domain-containing protein | | uniclust | UniRef100\_A0A1E3YDB6 | 99.4 | 1.2e-15 | 2.3e-21 | 91.7 | 63 | (4, 69) | 73 | (28, 90) | 229 | Response regulatory domain-containing protein | Response regulatory domain-containing protein | | uniclust | UniRef100\_A0A1H8HT30 | 99.4 | 1.2e-15 | 2.3e-21 | 80.1 | 62 | (1, 64) | 73 | (1, 63) | 81 | Transcriptional regulator, AlpA family | Transcriptional regulator, AlpA family | | uniclust | UniRef100\_A0A1H6INB4 | 99.4 | 1.2e-15 | 2.4e-21 | 81.9 | 61 | (5, 68) | 73 | (18, 78) | 90 | DNA binding domain-containing protein, excisionase family | DNA binding domain-containing protein, excisionase family | | uniclust | UniRef100\_A0A0F8ZBU4 | 99.4 | 1.3e-15 | 2.5e-21 | 78.3 | 58 | (7, 67) | 73 | (1, 58) | 76 | Helix-turn-helix domain-containing protein | Helix-turn-helix domain-containing protein | | uniclust | UniRef100\_A0A1J1EMG5 | 99.4 | 1.3e-15 | 2.5e-21 | 78.3 | 57 | (7, 66) | 73 | (5, 61) | 71 | DNA binding domain, excisionase family | DNA binding domain, excisionase family | | uniclust | UniRef100\_A0A353GQK3 | 99.4 | 1.3e-15 | 2.5e-21 | 77.4 | 62 | (6, 69) | 73 | (1, 65) | 70 | Helix-turn-helix domain-containing protein | Helix-turn-helix domain-containing protein | | uniclust | UniRef100\_A0A5N8YNY4 | 99.4 | 1.3e-15 | 2.5e-21 | 81.8 | 59 | (7, 67) | 73 | (23, 81) | 98 | Helix-turn-helix domain-containing protein | Helix-turn-helix domain-containing protein | | uniclust | UniRef100\_A0A1M3GUP1 | 99.4 | 1.4e-15 | 2.6e-21 | 80.3 | 59 | (6, 66) | 73 | (2, 60) | 88 | Helix-turn-helix domain-containing protein | Helix-turn-helix domain-containing protein | | uniclust | UniRef100\_A0A1C2G2R7 | 99.4 | 1.3e-15 | 2.6e-21 | 81.6 | 63 | (2, 66) | 73 | (5, 68) | 89 | Helix-turn-helix domain-containing protein | Helix-turn-helix domain-containing protein | | uniclust | UniRef100\_A0A023D901 | 99.4 | 1.5e-15 | 2.8e-21 | 81.1 | 60 | (8, 69) | 73 | (13, 72) | 98 | Helix-turn-helix domain-containing protein | Helix-turn-helix domain-containing protein | | uniclust | UniRef100\_A0A061YB04 | 99.4 | 1.4e-15 | 2.9e-21 | 81.6 | 65 | (3, 69) | 73 | (9, 75) | 91 | AlpA family phage regulatory protein | AlpA family phage regulatory protein | | uniclust | UniRef100\_A0A1I0GNV9 | 99.4 | 1.5e-15 | 2.9e-21 | 79.6 | 55 | (8, 64) | 73 | (15, 69) | 88 | Helix-turn-helix domain-containing protein | Helix-turn-helix domain-containing protein | | uniclust | UniRef100\_A0A4Q2YA13 | 99.4 | 1.5e-15 | 2.9e-21 | 79.7 | 59 | (8, 68) | 73 | (20, 78) | 83 | DNA-binding protein (Fragment) | DNA-binding protein (Fragment) | | uniclust | UniRef100\_A0A1M6UHM6 | 99.4 | 1.5e-15 | 2.9e-21 | 81.6 | 62 | (6, 69) | 73 | (24, 85) | 98 | Helix-turn-helix domain-containing protein | Helix-turn-helix domain-containing protein | | uniclust | UniRef100\_A0A0C1C693 | 99.4 | 1.5e-15 | 2.9e-21 | 79.9 | 63 | (4, 68) | 73 | (5, 68) | 79 | Uncharacterized protein ORF88 | Uncharacterized protein ORF88 | | uniclust | UniRef100\_A0A099F695 | 99.4 | 1.5e-15 | 3e-21 | 81.6 | 61 | (7, 69) | 73 | (18, 78) | 97 | Helix-turn-helix domain-containing protein | Helix-turn-helix domain-containing protein | | uniclust | UniRef100\_A0A0K1EPI1 | 99.4 | 1.6e-15 | 3e-21 | 81.0 | 62 | (4, 68) | 73 | (12, 73) | 98 | DNA-binding protein | DNA-binding protein | | uniclust | UniRef100\_A0A2D8NLB9 | 99.4 | 1.6e-15 | 3.2e-21 | 79.1 | 60 | (7, 68) | 73 | (12, 71) | 82 | Excisionase | Excisionase | | uniclust | UniRef100\_A0A1H3INP0 | 99.4 | 1.7e-15 | 3.2e-21 | 77.3 | 62 | (6, 69) | 73 | (2, 63) | 74 | Helix-turn-helix domain-containing protein | Helix-turn-helix domain-containing protein | | uniclust | UniRef100\_A0A2H9U2J9 | 99.4 | 1.7e-15 | 3.2e-21 | 75.0 | 59 | (8, 68) | 73 | (1, 59) | 63 | AlpA family transcriptional regulator | AlpA family transcriptional regulator | | uniclust | UniRef100\_A0A085DXN1 | 99.4 | 1.6e-15 | 3.2e-21 | 83.5 | 63 | (4, 68) | 73 | (17, 80) | 106 | AlpA family transcriptional regulator | AlpA family transcriptional regulator | | uniclust | UniRef100\_A0A0F2RL48 | 99.4 | 1.7e-15 | 3.3e-21 | 83.5 | 59 | (9, 69) | 73 | (39, 99) | 109 | Helix-turn-helix domain-containing protein | Helix-turn-helix domain-containing protein | | uniclust | UniRef100\_A0A0B2F8R1 | 99.4 | 1.7e-15 | 3.3e-21 | 79.8 | 60 | (5, 67) | 73 | (12, 71) | 83 | Helix-turn-helix domain-containing protein | Helix-turn-helix domain-containing protein | | uniclust | UniRef100\_A0A1H1ZMM5 | 99.4 | 1.8e-15 | 3.3e-21 | 78.6 | 60 | (8, 69) | 73 | (23, 82) | 85 | DNA binding domain-containing protein, excisionase family | DNA binding domain-containing protein, excisionase family | | uniclust | UniRef100\_A0A7W8EEW1 | 99.4 | 1.8e-15 | 3.4e-21 | 78.1 | 62 | (6, 69) | 73 | (14, 75) | 78 | Putative DNA-binding transcriptional regulator AlpA | Putative DNA-binding transcriptional regulator AlpA | | uniclust | UniRef100\_A0A1A0KLC7 | 99.4 | 1.8e-15 | 3.4e-21 | 79.6 | 58 | (9, 68) | 73 | (21, 78) | 87 | Helix-turn-helix domain-containing protein | Helix-turn-helix domain-containing protein | | uniclust | UniRef100\_A0A0M9CB32 | 99.4 | 1.8e-15 | 3.6e-21 | 82.0 | 60 | (6, 67) | 73 | (19, 79) | 100 | Helix-turn-helix domain-containing protein | Helix-turn-helix domain-containing protein | | uniclust | UniRef100\_A0A011NTG0 | 99.4 | 1.9e-15 | 3.6e-21 | 81.9 | 59 | (4, 65) | 73 | (25, 83) | 109 | DNA binding domain, excisionase family | DNA binding domain, excisionase family | | uniclust | UniRef100\_A0A2A4I9V0 | 99.4 | 1.9e-15 | 3.7e-21 | 78.7 | 62 | (7, 70) | 73 | (17, 78) | 83 | DNA-binding protein | DNA-binding protein | | uniclust | UniRef100\_A0A087DCF9 | 99.4 | 1.9e-15 | 3.7e-21 | 78.4 | 61 | (5, 67) | 73 | (9, 70) | 78 | Helix-turn-helix domain-containing protein | Helix-turn-helix domain-containing protein | | uniclust | UniRef100\_A0A1F9ABL8 | 99.4 | 1.9e-15 | 3.7e-21 | 78.8 | 58 | (7, 67) | 73 | (19, 76) | 86 | Helix-turn-helix domain-containing protein | Helix-turn-helix domain-containing protein | | uniclust | UniRef100\_A0A1C6IJV6 | 99.4 | 1.9e-15 | 3.7e-21 | 77.3 | 63 | (1, 66) | 73 | (1, 64) | 69 | DNA binding domain, excisionase family | DNA binding domain, excisionase family | | uniclust | UniRef100\_A0A1V5YRC9 | 99.4 | 2e-15 | 3.9e-21 | 78.5 | 58 | (5, 64) | 73 | (7, 64) | 80 | Helix-turn-helix domain protein | Helix-turn-helix domain protein | | uniclust | UniRef100\_A0A0K9ETV7 | 99.4 | 2e-15 | 3.9e-21 | 76.2 | 61 | (5, 68) | 73 | (2, 62) | 66 | Helix-turn-helix domain-containing protein | Helix-turn-helix domain-containing protein | | uniclust | UniRef100\_A0A011UIH5 | 99.4 | 2e-15 | 4.1e-21 | 78.8 | 63 | (2, 67) | 73 | (6, 68) | 77 | Excisionase | Excisionase | | uniclust | UniRef100\_A0A291ILY0 | 99.4 | 2e-15 | 4.1e-21 | 81.1 | 60 | (8, 69) | 73 | (21, 81) | 91 | Helix-turn-helix domain-containing protein | Helix-turn-helix domain-containing protein | | uniclust | UniRef100\_A0A081HYW1 | 99.4 | 2.1e-15 | 4.1e-21 | 78.9 | 63 | (2, 66) | 73 | (9, 71) | 81 | Helix-turn-helix domain-containing protein | Helix-turn-helix domain-containing protein | | uniclust | UniRef100\_A0A229VYW8 | 99.4 | 2.1e-15 | 4.2e-21 | 82.9 | 61 | (8, 70) | 73 | (28, 88) | 111 | Phage transcriptional regulator AlpA | Phage transcriptional regulator AlpA | | uniclust | UniRef100\_A0A0D6EU63 | 99.4 | 2.1e-15 | 4.2e-21 | 81.0 | 57 | (10, 68) | 73 | (22, 80) | 92 | Phage transcriptional regulator, AlpA | Phage transcriptional regulator, AlpA | | uniclust | UniRef100\_A0A2S8GGC5 | 99.4 | 2.2e-15 | 4.2e-21 | 78.0 | 57 | (7, 65) | 73 | (9, 65) | 76 | Excisionase | Excisionase | | uniclust | UniRef100\_A0A1F9CTC9 | 99.4 | 2.2e-15 | 4.3e-21 | 78.0 | 58 | (3, 63) | 73 | (1, 58) | 77 | Helix-turn-helix domain-containing protein | Helix-turn-helix domain-containing protein | | uniclust | UniRef100\_A0A1D2R863 | 99.4 | 2.2e-15 | 4.3e-21 | 78.6 | 59 | (8, 68) | 73 | (11, 70) | 84 | Helix-turn-helix domain-containing protein | Helix-turn-helix domain-containing protein | | uniclust | UniRef100\_A0A0K8QST4 | 99.4 | 2.2e-15 | 4.3e-21 | 79.2 | 58 | (5, 65) | 73 | (6, 63) | 83 | Transcriptional regulator, AlpA family | Transcriptional regulator, AlpA family | | uniclust | UniRef100\_A0A522NUE7 | 99.4 | 2.2e-15 | 4.4e-21 | 76.7 | 54 | (9, 65) | 73 | (10, 64) | 67 | DNA-binding protein | DNA-binding protein | | uniclust | UniRef100\_A0A135IGU9 | 99.4 | 2.3e-15 | 4.5e-21 | 75.2 | 56 | (12, 69) | 73 | (2, 57) | 66 | Helix-turn-helix domain-containing protein | Helix-turn-helix domain-containing protein | | uniclust | UniRef100\_A0A6N4ULT1 | 99.4 | 2.3e-15 | 4.5e-21 | 77.3 | 63 | (4, 68) | 73 | (2, 66) | 74 | Helix-turn-helix domain-containing protein | Helix-turn-helix domain-containing protein | | uniclust | UniRef100\_A0A024H216 | 99.4 | 2.3e-15 | 4.5e-21 | 80.9 | 59 | (9, 70) | 73 | (26, 84) | 94 | Uncharacterized domain protein | Uncharacterized domain protein | | uniclust | UniRef100\_A0A0F2JEK6 | 99.4 | 2.3e-15 | 4.6e-21 | 77.5 | 60 | (6, 68) | 73 | (2, 63) | 72 | Helix-turn-helix domain-containing protein | Helix-turn-helix domain-containing protein | | uniclust | UniRef100\_A0A1I4YVV8 | 99.4 | 2.4e-15 | 4.7e-21 | 82.1 | 64 | (4, 69) | 73 | (20, 83) | 102 | DNA binding domain-containing protein, excisionase family | DNA binding domain-containing protein, excisionase family | | uniclust | UniRef100\_A0A0K2ZQU6 | 99.4 | 2.4e-15 | 4.7e-21 | 81.7 | 62 | (5, 68) | 73 | (22, 85) | 99 | Uncharacterized protein | Uncharacterized protein | | uniclust | UniRef100\_A0A961Y0H3 | 99.4 | 2.5e-15 | 4.7e-21 | 78.7 | 67 | (1, 69) | 73 | (1, 71) | 88 | Helix-turn-helix domain-containing protein | Helix-turn-helix domain-containing protein | | uniclust | UniRef100\_A0A0K2SNP2 | 99.4 | 2.5e-15 | 4.8e-21 | 82.7 | 59 | (1, 62) | 73 | (4, 62) | 110 | Molybdenum-pterin binding protein | Molybdenum-pterin binding protein | | uniclust | UniRef100\_A0A1H8TNU4 | 99.4 | 2.6e-15 | 4.8e-21 | 77.7 | 59 | (8, 68) | 73 | (20, 78) | 83 | Transcriptional regulator, AlpA family | Transcriptional regulator, AlpA family | | uniclust | UniRef100\_A0A1F2WGB9 | 99.4 | 2.5e-15 | 4.8e-21 | 81.2 | 60 | (6, 68) | 73 | (19, 78) | 103 | Helix-turn-helix domain-containing protein (Fragment) | Helix-turn-helix domain-containing protein (Fragment) | | uniclust | UniRef100\_A0A0S7WQI0 | 99.4 | 2.5e-15 | 4.9e-21 | 80.7 | 57 | (7, 66) | 73 | (23, 79) | 94 | Helix-turn-helix domain-containing protein | Helix-turn-helix domain-containing protein | | uniclust | UniRef100\_A0A3E0Q7Z1 | 99.4 | 2.6e-15 | 5e-21 | 78.0 | 64 | (1, 67) | 73 | (1, 64) | 78 | DNA-binding protein | DNA-binding protein | | uniclust | UniRef100\_A0A0C2VMS3 | 99.4 | 2.6e-15 | 5.1e-21 | 77.1 | 61 | (6, 69) | 73 | (2, 62) | 72 | Helix-turn-helix domain-containing protein | Helix-turn-helix domain-containing protein | | uniclust | UniRef100\_A0A089N5B4 | 99.4 | 2.7e-15 | 5.2e-21 | 79.4 | 62 | (5, 69) | 73 | (14, 75) | 88 | Helix-turn-helix domain-containing protein | Helix-turn-helix domain-containing protein | | uniclust | UniRef100\_A0A2C8F565 | 99.4 | 2.8e-15 | 5.3e-21 | 79.6 | 59 | (7, 67) | 73 | (30, 88) | 92 | Helix-turn-helix domain-containing protein | Helix-turn-helix domain-containing protein | | uniclust | UniRef100\_A0A068NIB7 | 99.4 | 2.8e-15 | 5.4e-21 | 82.5 | 59 | (6, 67) | 73 | (32, 90) | 109 | Excisionase | Excisionase | | uniclust | UniRef100\_A0A0M6XPV5 | 99.4 | 2.9e-15 | 5.5e-21 | 74.5 | 54 | (7, 62) | 73 | (10, 63) | 66 | DNA binding domain, excisionase family | DNA binding domain, excisionase family | | uniclust | UniRef100\_A0A0G3BTD9 | 99.4 | 3e-15 | 5.8e-21 | 77.2 | 64 | (5, 70) | 73 | (4, 67) | 78 | Helix-turn-helix domain-containing protein | Helix-turn-helix domain-containing protein | | uniclust | UniRef100\_A0A085K4B9 | 99.4 | 3e-15 | 5.8e-21 | 83.4 | 61 | (7, 69) | 73 | (24, 84) | 121 | DNA-binding protein | DNA-binding protein | | uniclust | UniRef100\_A0A0C1AYG1 | 99.4 | 3e-15 | 5.8e-21 | 81.0 | 59 | (10, 70) | 73 | (37, 96) | 100 | Helix-turn-helix domain-containing protein | Helix-turn-helix domain-containing protein | | uniclust | UniRef100\_A0A326TZW7 | 99.3 | 3.2e-15 | 6.2e-21 | 81.6 | 59 | (6, 67) | 73 | (14, 72) | 104 | PTS system nitrogen regulatory IIA component | PTS system nitrogen regulatory IIA component | | uniclust | UniRef100\_A0A7T0C1C5 | 99.3 | 3.2e-15 | 6.2e-21 | 79.7 | 60 | (6, 68) | 73 | (1, 60) | 96 | Helix-turn-helix domain-containing protein | Helix-turn-helix domain-containing protein | | uniclust | UniRef100\_A0A1F5N9I8 | 99.3 | 3.2e-15 | 6.2e-21 | 79.3 | 62 | (3, 67) | 73 | (16, 77) | 88 | Helix-turn-helix domain-containing protein | Helix-turn-helix domain-containing protein | | uniclust | UniRef100\_A0A0A2WTF0 | 99.3 | 3.3e-15 | 6.3e-21 | 77.9 | 60 | (3, 65) | 73 | (7, 66) | 80 | Helix-turn-helix domain-containing protein | Helix-turn-helix domain-containing protein | | uniclust | UniRef100\_A0A0A5HUG6 | 99.3 | 3.2e-15 | 6.4e-21 | 80.8 | 62 | (6, 69) | 73 | (21, 83) | 95 | AlpA family transcriptional regulator | AlpA family transcriptional regulator | | uniclust | UniRef100\_L0J4B7 | 99.3 | 3.4e-15 | 6.5e-21 | 80.2 | 64 | (2, 67) | 73 | (1, 64) | 103 | Helix-turn-helix domain-containing protein | Helix-turn-helix domain-containing protein | | uniclust | UniRef100\_A0A2E2QBI3 | 99.3 | 3.4e-15 | 6.7e-21 | 78.7 | 61 | (3, 65) | 73 | (7, 72) | 86 | DNA-binding protein | DNA-binding protein | | uniclust | UniRef100\_A0A0G0BKN9 | 99.3 | 3.5e-15 | 6.8e-21 | 78.7 | 60 | (5, 67) | 73 | (17, 78) | 84 | Transcriptional regulator, MerR family | Transcriptional regulator, MerR family | | uniclust | UniRef100\_A0A0D5M2K3 | 99.3 | 3.4e-15 | 6.8e-21 | 80.5 | 56 | (8, 65) | 73 | (20, 76) | 92 | Phage transcriptional regulator, AlpA | Phage transcriptional regulator, AlpA | | uniclust | UniRef100\_A0A0A2XY55 | 99.3 | 3.5e-15 | 6.9e-21 | 80.3 | 61 | (6, 68) | 73 | (23, 84) | 94 | AlpA family phage regulatory protein | AlpA family phage regulatory protein | | uniclust | UniRef100\_A0A3D3UV23 | 99.3 | 3.6e-15 | 7e-21 | 79.4 | 60 | (2, 63) | 73 | (7, 66) | 90 | Helix-turn-helix domain-containing protein | Helix-turn-helix domain-containing protein | | uniclust | UniRef100\_A0A1A0M401 | 99.3 | 3.7e-15 | 7.1e-21 | 78.8 | 65 | (5, 70) | 73 | (9, 73) | 89 | Helix-turn-helix domain-containing protein | Helix-turn-helix domain-containing protein | | uniclust | UniRef100\_A0A143B7R0 | 99.3 | 3.7e-15 | 7.1e-21 | 80.3 | 60 | (5, 67) | 73 | (30, 89) | 99 | DNA-binding protein | DNA-binding protein | | uniclust | UniRef100\_A0A485B3H2 | 99.3 | 3.8e-15 | 7.1e-21 | 74.2 | 56 | (8, 66) | 73 | (2, 57) | 69 | Predicted transcriptional regulator | Predicted transcriptional regulator | | uniclust | UniRef100\_A0A0Q7JLQ8 | 99.3 | 3.7e-15 | 7.1e-21 | 76.0 | 65 | (1, 67) | 73 | (1, 66) | 70 | Helix-turn-helix domain-containing protein | Helix-turn-helix domain-containing protein | | uniclust | UniRef100\_A0A644TYX9 | 99.3 | 3.7e-15 | 7.4e-21 | 80.8 | 59 | (9, 70) | 73 | (38, 96) | 98 | Helix-turn-helix domain-containing protein | Helix-turn-helix domain-containing protein | | uniclust | UniRef100\_A0A0K8QVH0 | 99.3 | 3.9e-15 | 7.5e-21 | 80.2 | 58 | (5, 65) | 73 | (18, 75) | 106 | Protein containing DNA binding domain, excisionase family | Protein containing DNA binding domain, excisionase family | | uniclust | UniRef100\_A0A1G8QBS7 | 99.3 | 4e-15 | 7.7e-21 | 77.9 | 62 | (6, 69) | 73 | (13, 74) | 83 | Transcriptional regulator, AlpA family | Transcriptional regulator, AlpA family | | uniclust | UniRef100\_A0A2E8B578 | 99.3 | 4.2e-15 | 7.8e-21 | 79.4 | 58 | (7, 67) | 73 | (38, 95) | 104 | DNA-binding protein | DNA-binding protein | | uniclust | UniRef100\_A0A0F8YEC9 | 99.3 | 4e-15 | 8e-21 | 79.4 | 58 | (6, 65) | 73 | (14, 71) | 88 | Helix-turn-helix domain-containing protein | Helix-turn-helix domain-containing protein | | uniclust | UniRef100\_A0A0B0DF89 | 99.3 | 4.1e-15 | 8e-21 | 77.4 | 65 | (3, 69) | 73 | (11, 75) | 79 | Helix-turn-helix domain-containing protein | Helix-turn-helix domain-containing protein | | uniclust | UniRef100\_A0A177QRW9 | 99.3 | 4.2e-15 | 8.2e-21 | 83.8 | 59 | (6, 67) | 73 | (21, 79) | 131 | Helix-turn-helix domain-containing protein | Helix-turn-helix domain-containing protein | | uniclust | UniRef100\_A0A0T6A0K1 | 99.3 | 4.2e-15 | 8.3e-21 | 76.4 | 60 | (4, 66) | 73 | (3, 62) | 72 | Helix-turn-helix domain-containing protein | Helix-turn-helix domain-containing protein | | uniclust | UniRef100\_A0A1X1MGL1 | 99.3 | 4.6e-15 | 8.8e-21 | 77.0 | 59 | (5, 65) | 73 | (3, 61) | 84 | Helix-turn-helix domain-containing protein | Helix-turn-helix domain-containing protein | | uniclust | UniRef100\_A0A059E7V9 | 99.3 | 4.6e-15 | 8.9e-21 | 82.0 | 61 | (7, 69) | 73 | (42, 102) | 118 | DNA-binding protein | DNA-binding protein | | uniclust | UniRef100\_A0A109LZ39 | 99.3 | 4.6e-15 | 8.9e-21 | 75.9 | 57 | (7, 65) | 73 | (13, 70) | 73 | Helix-turn-helix domain-containing protein | Helix-turn-helix domain-containing protein | | uniclust | UniRef100\_A0A022GBV9 | 99.3 | 4.6e-15 | 8.9e-21 | 82.1 | 61 | (8, 70) | 73 | (38, 98) | 117 | Transcriptional regulator | Transcriptional regulator | | uniclust | UniRef100\_A0A087L5X3 | 99.3 | 4.5e-15 | 8.9e-21 | 79.8 | 64 | (3, 68) | 73 | (8, 73) | 92 | AlpA family transcriptional regulator | AlpA family transcriptional regulator | | uniclust | UniRef100\_A0A0M7B8D3 | 99.3 | 4.7e-15 | 9e-21 | 81.3 | 59 | (7, 67) | 73 | (2, 60) | 115 | Helix-turn-helix domain protein | Helix-turn-helix domain protein | | uniclust | UniRef100\_A0A094PUR9 | 99.3 | 4.7e-15 | 9e-21 | 78.0 | 61 | (5, 67) | 73 | (13, 73) | 88 | Helix-turn-helix domain-containing protein | Helix-turn-helix domain-containing protein | | uniclust | UniRef100\_A0A1N6NC24 | 99.3 | 4.6e-15 | 9e-21 | 77.7 | 59 | (8, 68) | 73 | (19, 77) | 83 | Helix-turn-helix domain-containing protein | Helix-turn-helix domain-containing protein | | uniclust | UniRef100\_A0A078LJ41 | 99.3 | 4.7e-15 | 9.4e-21 | 80.4 | 66 | (1, 68) | 73 | (5, 71) | 96 | Transcriptional regulator | Transcriptional regulator | | uniclust | UniRef100\_A0A366HET7 | 99.3 | 5e-15 | 9.5e-21 | 78.3 | 60 | (8, 69) | 73 | (18, 77) | 92 | Helix-turn-helix protein | Helix-turn-helix protein | | uniclust | UniRef100\_A0A1B4S5B3 | 99.3 | 5e-15 | 9.7e-21 | 85.1 | 65 | (2, 68) | 73 | (14, 78) | 146 | Helix-turn-helix domain-containing protein | Helix-turn-helix domain-containing protein | | uniclust | UniRef100\_A0A1H1RBV9 | 99.3 | 4.9e-15 | 9.7e-21 | 77.5 | 55 | (7, 64) | 73 | (14, 68) | 79 | DNA binding domain-containing protein, excisionase family | DNA binding domain-containing protein, excisionase family | | uniclust | UniRef100\_A0A2G2K1G3 | 99.3 | 5e-15 | 9.8e-21 | 80.2 | 60 | (6, 68) | 73 | (18, 77) | 97 | DNA-binding protein | DNA-binding protein | | uniclust | UniRef100\_A0A290ZH97 | 99.3 | 5.2e-15 | 9.9e-21 | 76.1 | 67 | (1, 69) | 73 | (1, 68) | 79 | Excisionase | Excisionase | | uniclust | UniRef100\_A0A382E9C4 | 99.3 | 5.4e-15 | 1.1e-20 | 78.2 | 64 | (5, 70) | 73 | (14, 77) | 87 | Helix-turn-helix domain-containing protein | Helix-turn-helix domain-containing protein | | uniclust | UniRef100\_A0A2G2GU36 | 99.3 | 5.5e-15 | 1.1e-20 | 78.1 | 62 | (7, 70) | 73 | (13, 79) | 86 | Helix-turn-helix domain-containing protein | Helix-turn-helix domain-containing protein | | uniclust | UniRef100\_A0A9E1W7L4 | 99.3 | 5.7e-15 | 1.1e-20 | 75.9 | 61 | (8, 70) | 73 | (14, 74) | 79 | Helix-turn-helix domain-containing protein | Helix-turn-helix domain-containing protein | | uniclust | UniRef100\_A0A011V604 | 99.3 | 5.5e-15 | 1.1e-20 | 79.2 | 61 | (6, 68) | 73 | (19, 79) | 93 | AlpA family transcriptional regulator | AlpA family transcriptional regulator | | uniclust | UniRef100\_A0A1H3REZ3 | 99.3 | 5.6e-15 | 1.1e-20 | 74.2 | 59 | (5, 66) | 73 | (4, 62) | 66 | Putative molybdopterin biosynthesis protein | Putative molybdopterin biosynthesis protein | | uniclust | UniRef100\_A0A4D7DJZ9 | 99.3 | 5.8e-15 | 1.1e-20 | 73.8 | 61 | (5, 67) | 73 | (3, 63) | 66 | Helix-turn-helix domain-containing protein | Helix-turn-helix domain-containing protein | | uniclust | UniRef100\_A0A4U1KY23 | 99.3 | 5.9e-15 | 1.1e-20 | 73.2 | 57 | (12, 70) | 73 | (1, 58) | 64 | Helix-turn-helix domain-containing protein | Helix-turn-helix domain-containing protein | | uniclust | UniRef100\_A0A1A9SZU8 | 99.3 | 5.8e-15 | 1.1e-20 | 77.5 | 59 | (8, 68) | 73 | (2, 61) | 81 | Helix-turn-helix domain-containing protein | Helix-turn-helix domain-containing protein | | uniclust | UniRef100\_A0A0K2GF18 | 99.3 | 5.9e-15 | 1.1e-20 | 77.2 | 58 | (7, 67) | 73 | (11, 68) | 81 | Helix-turn-helix domain-containing protein | Helix-turn-helix domain-containing protein | | uniclust | UniRef100\_A0A087MDQ3 | 99.3 | 6e-15 | 1.2e-20 | 77.2 | 64 | (2, 67) | 73 | (13, 76) | 81 | Helix-turn-helix domain-containing protein | Helix-turn-helix domain-containing protein | | uniclust | UniRef100\_A0A9D8HGG9 | 99.3 | 6.3e-15 | 1.2e-20 | 78.9 | 62 | (5, 68) | 73 | (5, 66) | 106 | Helix-turn-helix domain-containing protein | Helix-turn-helix domain-containing protein | | uniclust | UniRef100\_A0A399Y389 | 99.3 | 6.1e-15 | 1.2e-20 | 76.9 | 59 | (7, 68) | 73 | (1, 59) | 80 | AlpA family transcriptional regulator | AlpA family transcriptional regulator | | uniclust | UniRef100\_A0A097ELU5 | 99.3 | 6.1e-15 | 1.2e-20 | 78.9 | 58 | (8, 67) | 73 | (19, 76) | 93 | AlpA family transcriptional regulator | AlpA family transcriptional regulator | | uniclust | UniRef100\_A0A084EIT9 | 99.3 | 6.4e-15 | 1.2e-20 | 77.2 | 63 | (6, 68) | 73 | (20, 82) | 95 | Helix-turn-helix domain-containing protein | Helix-turn-helix domain-containing protein | | uniclust | UniRef100\_A0A356W9G0 | 99.3 | 6.2e-15 | 1.2e-20 | 77.9 | 60 | (8, 69) | 73 | (23, 83) | 89 | Helix-turn-helix domain-containing protein | Helix-turn-helix domain-containing protein | | uniclust | UniRef100\_A0A1M5BQS2 | 99.3 | 6.4e-15 | 1.2e-20 | 80.1 | 62 | (5, 68) | 73 | (22, 87) | 109 | Helix-turn-helix domain-containing protein | Helix-turn-helix domain-containing protein | | uniclust | UniRef100\_A0A0K2B6L2 | 99.3 | 6.4e-15 | 1.2e-20 | 78.8 | 64 | (3, 68) | 73 | (14, 78) | 95 | PCQ3\_68 | PCQ3\_68 | | uniclust | UniRef100\_A0A7W7ZCS8 | 99.3 | 6.5e-15 | 1.2e-20 | 77.8 | 56 | (9, 66) | 73 | (16, 71) | 90 | Putative DNA-binding transcriptional regulator AlpA | Putative DNA-binding transcriptional regulator AlpA | | uniclust | UniRef100\_A0A1Q5VLA1 | 99.3 | 6.5e-15 | 1.3e-20 | 78.2 | 64 | (3, 68) | 73 | (8, 72) | 86 | AlpA family transcriptional regulator | AlpA family transcriptional regulator | | uniclust | UniRef100\_A0A017HLU1 | 99.3 | 6.5e-15 | 1.3e-20 | 78.6 | 58 | (9, 68) | 73 | (23, 83) | 89 | Helix-turn-helix domain-containing protein | Helix-turn-helix domain-containing protein | | uniclust | UniRef100\_A0A0C1GY24 | 99.3 | 6.5e-15 | 1.3e-20 | 78.1 | 64 | (3, 68) | 73 | (6, 70) | 84 | DNA-binding protein | DNA-binding protein | | uniclust | UniRef100\_A0A1H1H733 | 99.3 | 6.8e-15 | 1.3e-20 | 76.0 | 59 | (7, 67) | 73 | (10, 68) | 78 | Helix-turn-helix domain-containing protein | Helix-turn-helix domain-containing protein | | uniclust | UniRef100\_A0A0P7CAH9 | 99.3 | 6.9e-15 | 1.4e-20 | 78.4 | 62 | (4, 68) | 73 | (6, 67) | 88 | Helix-turn-helix domain-containing protein | Helix-turn-helix domain-containing protein | | uniclust | UniRef100\_A0A0K1JJ15 | 99.3 | 6.8e-15 | 1.4e-20 | 80.0 | 62 | (5, 68) | 73 | (16, 77) | 97 | Helix-turn-helix domain-containing protein | Helix-turn-helix domain-containing protein | | uniclust | UniRef100\_A0A011QUG7 | 99.3 | 7e-15 | 1.4e-20 | 77.4 | 61 | (7, 69) | 73 | (15, 75) | 85 | Helix-turn-helix domain protein | Helix-turn-helix domain protein | | uniclust | UniRef100\_A0A0D4DU24 | 99.3 | 7.2e-15 | 1.4e-20 | 80.4 | 58 | (9, 68) | 73 | (21, 78) | 115 | DNA-binding protein | DNA-binding protein | | uniclust | UniRef100\_A0A2H0LJP8 | 99.3 | 7.2e-15 | 1.4e-20 | 76.8 | 56 | (8, 66) | 73 | (6, 61) | 84 | Helix-turn-helix domain-containing protein | Helix-turn-helix domain-containing protein | | uniclust | UniRef100\_A0A1A7QHD2 | 99.3 | 7.2e-15 | 1.4e-20 | 75.8 | 57 | (4, 62) | 73 | (10, 66) | 76 | Helix-turn-helix domain-containing protein | Helix-turn-helix domain-containing protein | | uniclust | UniRef100\_A0A2N3QTQ8 | 99.3 | 7.5e-15 | 1.4e-20 | 75.3 | 57 | (8, 66) | 73 | (14, 71) | 77 | Phage transcriptional regulator, AlpA | Phage transcriptional regulator, AlpA | | uniclust | UniRef100\_A0A1M6DCH5 | 99.3 | 7.6e-15 | 1.4e-20 | 74.7 | 62 | (6, 69) | 73 | (8, 69) | 74 | Helix-turn-helix domain-containing protein | Helix-turn-helix domain-containing protein | | uniclust | UniRef100\_A0A381XKI2 | 99.3 | 7.5e-15 | 1.5e-20 | 74.0 | 62 | (2, 66) | 73 | (2, 63) | 66 | Helix-turn-helix domain-containing protein | Helix-turn-helix domain-containing protein | | uniclust | UniRef100\_A0A1V4VK00 | 99.3 | 7.5e-15 | 1.5e-20 | 74.9 | 58 | (6, 66) | 73 | (5, 62) | 71 | Helix-turn-helix domain protein | Helix-turn-helix domain protein | | uniclust | UniRef100\_A0A060ZRS2 | 99.3 | 7.6e-15 | 1.5e-20 | 77.3 | 61 | (6, 69) | 73 | (16, 76) | 81 | DNA binding domain protein, excisionase family | DNA binding domain protein, excisionase family | | uniclust | UniRef100\_A0A0Q8DF19 | 99.3 | 7.7e-15 | 1.5e-20 | 79.3 | 56 | (9, 66) | 73 | (30, 85) | 98 | Helix-turn-helix domain-containing protein | Helix-turn-helix domain-containing protein | | uniclust | UniRef100\_A0A079YS49 | 99.3 | 7.7e-15 | 1.5e-20 | 80.9 | 56 | (7, 65) | 73 | (46, 101) | 104 | DNA-binding protein | DNA-binding protein | | uniclust | UniRef100\_A0A095UZ92 | 99.3 | 7.7e-15 | 1.6e-20 | 82.4 | 58 | (7, 67) | 73 | (52, 109) | 115 | HTH merR-type domain-containing protein | HTH merR-type domain-containing protein | | uniclust | UniRef100\_A0A1G9J2G5 | 99.3 | 8.3e-15 | 1.6e-20 | 73.3 | 58 | (6, 65) | 73 | (2, 59) | 65 | Helix-turn-helix domain-containing protein | Helix-turn-helix domain-containing protein | | uniclust | UniRef100\_A0A1H8Y2E5 | 99.3 | 8.7e-15 | 1.6e-20 | 77.6 | 56 | (9, 66) | 73 | (41, 96) | 98 | DNA binding domain-containing protein, excisionase family | DNA binding domain-containing protein, excisionase family | | uniclust | UniRef100\_A0A0P7YQL3 | 99.3 | 8.4e-15 | 1.7e-20 | 80.4 | 60 | (9, 70) | 73 | (42, 102) | 105 | Helix-turn-helix domain-containing protein | Helix-turn-helix domain-containing protein | | uniclust | UniRef100\_A0A8J2ZB08 | 99.3 | 8.8e-15 | 1.7e-20 | 74.3 | 67 | (1, 69) | 73 | (1, 67) | 77 | Helix-turn-helix domain-containing protein | Helix-turn-helix domain-containing protein | | uniclust | UniRef100\_A0A1F9Y074 | 99.3 | 9e-15 | 1.7e-20 | 76.1 | 62 | (6, 69) | 73 | (13, 76) | 81 | Helix-turn-helix domain-containing protein | Helix-turn-helix domain-containing protein | | uniclust | UniRef100\_A0A149UPN1 | 99.3 | 9e-15 | 1.7e-20 | 76.6 | 62 | (7, 70) | 73 | (15, 77) | 81 | AlpA family phage regulatory protein | AlpA family phage regulatory protein | | uniclust | UniRef100\_A0A1H0TUD0 | 99.3 | 9.6e-15 | 1.9e-20 | 75.1 | 64 | (4, 69) | 73 | (5, 69) | 73 | Transcriptional regulator, AlpA family | Transcriptional regulator, AlpA family | | uniclust | UniRef100\_A0A2W4KKL3 | 99.3 | 9.4e-15 | 1.9e-20 | 77.0 | 59 | (6, 67) | 73 | (8, 66) | 81 | DNA-binding protein | DNA-binding protein | | uniclust | UniRef100\_A0A371RLY0 | 99.3 | 1e-14 | 1.9e-20 | 75.2 | 61 | (6, 68) | 73 | (15, 76) | 78 | DNA-binding protein | DNA-binding protein | | uniclust | UniRef100\_A0A098U8C9 | 99.3 | 1e-14 | 2e-20 | 78.2 | 66 | (2, 69) | 73 | (14, 80) | 91 | AlpA family transcriptional regulator | AlpA family transcriptional regulator | | uniclust | UniRef100\_A0A839A0Y5 | 99.3 | 1e-14 | 2e-20 | 73.7 | 58 | (7, 66) | 73 | (1, 58) | 69 | Helix-turn-helix domain-containing protein | Helix-turn-helix domain-containing protein | | uniclust | UniRef100\_A0A2D6GE75 | 99.3 | 1e-14 | 2e-20 | 76.6 | 64 | (2, 67) | 73 | (7, 70) | 79 | DNA-binding protein | DNA-binding protein | | uniclust | UniRef100\_A0A0W1L165 | 99.3 | 1e-14 | 2e-20 | 78.5 | 57 | (6, 64) | 73 | (21, 78) | 92 | AlpA family transcriptional regulator | AlpA family transcriptional regulator | | uniclust | UniRef100\_A0A2N1W432 | 99.3 | 1e-14 | 2e-20 | 76.3 | 58 | (7, 67) | 73 | (1, 58) | 83 | DNA-binding protein | DNA-binding protein | | uniclust | UniRef100\_A0A139D2J3 | 99.3 | 1e-14 | 2e-20 | 73.9 | 57 | (9, 68) | 73 | (2, 58) | 65 | Helix-turn-helix domain-containing protein | Helix-turn-helix domain-containing protein | | uniclust | UniRef100\_A0A1I0F1X2 | 99.3 | 1.1e-14 | 2.1e-20 | 72.3 | 57 | (6, 65) | 73 | (2, 58) | 62 | DNA binding domain-containing protein, excisionase family | DNA binding domain-containing protein, excisionase family | | uniclust | UniRef100\_A0A1E7HLJ3 | 99.3 | 1.1e-14 | 2.1e-20 | 78.5 | 61 | (6, 68) | 73 | (27, 91) | 95 | Helix-turn-helix domain-containing protein | Helix-turn-helix domain-containing protein | | uniclust | UniRef100\_A0A1Q5RFL0 | 99.3 | 1.1e-14 | 2.1e-20 | 80.1 | 59 | (9, 69) | 73 | (27, 85) | 112 | Helix-turn-helix domain-containing protein | Helix-turn-helix domain-containing protein | | uniclust | UniRef100\_A0A5C5RVC0 | 99.3 | 1.2e-14 | 2.2e-20 | 77.5 | 59 | (8, 68) | 73 | (37, 95) | 98 | Helix-turn-helix domain-containing protein | Helix-turn-helix domain-containing protein | | uniclust | UniRef100\_A0A1P8XFB5 | 99.3 | 1.1e-14 | 2.2e-20 | 76.3 | 58 | (10, 69) | 73 | (11, 69) | 83 | DNA-binding protein | DNA-binding protein | | uniclust | UniRef100\_A0A085DXK9 | 99.3 | 1.1e-14 | 2.2e-20 | 81.1 | 62 | (5, 68) | 73 | (28, 90) | 113 | AlpA family transcriptional regulator | AlpA family transcriptional regulator | | uniclust | UniRef100\_A0A011NBN1 | 99.3 | 1.1e-14 | 2.3e-20 | 81.5 | 61 | (6, 68) | 73 | (25, 86) | 112 | DNA-binding protein | DNA-binding protein | | uniclust | UniRef100\_A0A0K9EV25 | 99.3 | 1.2e-14 | 2.3e-20 | 76.6 | 63 | (2, 66) | 73 | (1, 63) | 84 | Helix-turn-helix domain-containing protein | Helix-turn-helix domain-containing protein | | uniclust | UniRef100\_A0A085AAA5 | 99.3 | 1.1e-14 | 2.3e-20 | 79.1 | 64 | (3, 68) | 73 | (16, 80) | 97 | DNA-binding protein | DNA-binding protein | | uniclust | UniRef100\_A0A074TCW2 | 99.3 | 1.2e-14 | 2.3e-20 | 77.9 | 64 | (5, 70) | 73 | (14, 79) | 89 | Prophage CP4-57 regulatory | Prophage CP4-57 regulatory | | uniclust | UniRef100\_A0A142WZF1 | 99.3 | 1.2e-14 | 2.3e-20 | 78.0 | 61 | (4, 66) | 73 | (10, 71) | 94 | MerR family regulatory protein | MerR family regulatory protein | | uniclust | UniRef100\_A0A954DPY9 | 99.3 | 1.2e-14 | 2.3e-20 | 74.5 | 62 | (5, 68) | 73 | (2, 63) | 81 | Helix-turn-helix domain-containing protein | Helix-turn-helix domain-containing protein | | pdb70 | 1Z4H\_A | 98.6 | 9.9e-12 | 7.2e-16 | 53.1 | 62 | (3, 66) | 73 | (2, 63) | 66 | Tor inhibition protein | 1Z4H\_A Tor inhibition protein winged helix, reverse turn, PROTEIN | | pdb70 | 6AMA\_Y | 98.5 | 3.7e-11 | 2.7e-15 | 51.4 | 64 | (2, 67) | 73 | (5, 68) | 71 | DNA binding domain-containing protein, excisionase | 6AMA\_Y DNA binding domain-containing protein, excisionase BldC, S. coelicolor, developmental switch | | pdb70 | 6AMA\_A | 98.5 | 4.4e-11 | 3.2e-15 | 51.3 | 62 | (4, 67) | 73 | (7, 68) | 71 | DNA binding domain-containing protein, excisionase | 6AMA\_A DNA binding domain-containing protein, excisionase BldC, S. coelicolor, developmental switch | | pdb70 | 6AMK\_A | 98.4 | 5.9e-11 | 4.3e-15 | 51.1 | 60 | (6, 67) | 73 | (10, 69) | 72 | DNA binding domain-containing protein, excisionase | 6AMK\_A DNA binding domain-containing protein, excisionase BldC, Streptomyces, MerR-like, DNA BINDING HET: MSE | | pdb70 | 6HLK\_A | 98.4 | 1.2e-10 | 8.8e-15 | 57.0 | 58 | (6, 66) | 73 | (2, 60) | 153 | Redirecting phage packaging protein C | 6HLK\_A Redirecting phage packaging protein C Redirecting packaging protein, DNA Binding HET: MSE | | pdb70 | 6HN7\_A | 98.4 | 1.2e-10 | 8.8e-15 | 57.0 | 58 | (6, 66) | 73 | (2, 60) | 153 | Redirecting phage packaging protein C | 6HN7\_A Redirecting phage packaging protein C Redirecting packaging protein, DNA Binding | | pdb70 | 2ZHG\_A | 98.3 | 2.9e-10 | 2.1e-14 | 56.4 | 57 | (1, 59) | 73 | (1, 57) | 154 | Redox-sensitive transcriptional activator soxR/DNA Complex | 2ZHG\_A Redox-sensitive transcriptional activator soxR/DNA Complex oxidative stress, MerR family, Activator | | pdb70 | 2ZHH\_A | 98.3 | 2.9e-10 | 2.1e-14 | 56.4 | 57 | (1, 59) | 73 | (1, 57) | 154 | Redox-sensitive transcriptional activator soxR | 2ZHH\_A Redox-sensitive transcriptional activator soxR oxidative stress, MerR family, Activator | | pdb70 | 1Y6U\_A | 98.2 | 3.8e-10 | 2.8e-14 | 48.6 | 56 | (6, 64) | 73 | (11, 68) | 70 | Excisionase from transposon Tn916 | 1Y6U\_A Excisionase from transposon Tn916 DNA architectural protein, Tyrosine recombinase | | pdb70 | 4J2N\_B | 98.1 | 9.9e-10 | 7.2e-14 | 44.7 | 54 | (8, 63) | 73 | (3, 56) | 58 | Gp37 | 4J2N\_B Gp37 Winged-Helix, Doman Swap, Filament, VIRAL HET: SO4 | | pdb70 | 4J2N\_C | 98.1 | 9.9e-10 | 7.2e-14 | 44.7 | 54 | (8, 63) | 73 | (3, 56) | 58 | Gp37 | 4J2N\_C Gp37 Winged-Helix, Doman Swap, Filament, VIRAL HET: SO4 | | pdb70 | 3GPV\_B | 98.1 | 1.1e-09 | 7.9e-14 | 53.7 | 58 | (2, 61) | 73 | (7, 65) | 148 | Transcriptional regulator, MerR family | 3GPV\_B Transcriptional regulator, MerR family transcriptional regulator, MerR family, Protein | | pdb70 | 4LHF\_A | 98.1 | 1.9e-09 | 1.4e-13 | 49.3 | 57 | (5, 63) | 73 | (5, 69) | 91 | Regulatory protein cox | 4LHF\_A Regulatory protein cox helix-turn-helix, DNA binding, VIRAL PROTEIN | | pdb70 | 4R4E\_A | 98.0 | 2.2e-09 | 1.6e-13 | 47.2 | 57 | (3, 61) | 73 | (3, 59) | 84 | HTH-type transcriptional regulator GlnR | 4R4E\_A HTH-type transcriptional regulator GlnR TnrA/GlnR family, B. subtilis, nitrogen HET: CXS | | pdb70 | 5C8E\_G | 98.0 | 2.3e-09 | 1.6e-13 | 57.8 | 59 | (2, 62) | 73 | (17, 76) | 305 | Light-dependent transcriptional regulator CarH/DNA Complex | 5C8E\_G Light-dependent transcriptional regulator CarH/DNA Complex Transcription factor, light sensor, adenosylcobalamin-binding HET: B12, 5AD | | pdb70 | 5C8F\_A | 98.0 | 2.3e-09 | 1.6e-13 | 57.8 | 59 | (2, 62) | 73 | (17, 76) | 305 | Light-dependent transcriptional regulator CarH | 5C8F\_A Light-dependent transcriptional regulator CarH Transcription factor, light sensor, adenosylcobalamin-binding HET: B12 | | pdb70 | 6HN7\_B | 98.0 | 3.2e-09 | 2.3e-13 | 49.2 | 56 | (8, 66) | 73 | (2, 59) | 101 | Redirecting phage packaging protein C | 6HN7\_B Redirecting phage packaging protein C Redirecting packaging protein, DNA Binding | | pdb70 | 4R24\_B | 98.0 | 3.3e-09 | 2.4e-13 | 46.7 | 54 | (5, 60) | 73 | (2, 55) | 85 | HTH-type transcriptional regulator TnrA | 4R24\_B HTH-type transcriptional regulator TnrA TnrA, GS, B. subtilis, GlnK | | pdb70 | 3UCS\_B | 97.9 | 5.3e-09 | 3.9e-13 | 48.1 | 54 | (6, 61) | 73 | (3, 56) | 102 | Chaperone-modulator protein CbpM, Curved DNA-binding | 3UCS\_B Chaperone-modulator protein CbpM, Curved DNA-binding protein-protein complex, Structural Genomics, Montreal-Kingston | | pdb70 | 5CRL\_B | 97.9 | 6.2e-09 | 4.4e-13 | 50.1 | 54 | (6, 61) | 73 | (2, 56) | 134 | Mercuric resistance operon regulatory protein | 5CRL\_B Mercuric resistance operon regulatory protein transcription activator, MerR, mercury, P. | | pdb70 | 1J9I\_A | 97.8 | 1.2e-08 | 8.9e-13 | 43.2 | 54 | (11, 67) | 73 | (2, 57) | 68 | TERMINASE SMALL SUBUNIT | 1J9I\_A TERMINASE SMALL SUBUNIT DNA BINDING DOMAIN, HOMODIMER, VIRAL | | pdb70 | 4R22\_B | 97.8 | 1.7e-08 | 1.2e-12 | 43.8 | 54 | (6, 61) | 73 | (2, 55) | 82 | HTH-type transcriptional regulator TnrA | 4R22\_B HTH-type transcriptional regulator TnrA New family of transcription regulators | | pdb70 | 5XQL\_A | 97.7 | 2.3e-08 | 1.6e-12 | 52.8 | 57 | (2, 60) | 73 | (12, 70) | 291 | Multidrug-efflux transporter 1 regulator | 5XQL\_A Multidrug-efflux transporter 1 regulator Pseudomonas aeruginosa, transcriptional regulator, TRANSCRIPTION HET: C2E | | pdb70 | 5GPE\_C | 97.7 | 2.4e-08 | 1.7e-12 | 47.6 | 49 | (11, 61) | 73 | (1, 50) | 129 | Transcriptional regulator, MerR-family | 5GPE\_C Transcriptional regulator, MerR-family PbrR691, MerR family, Transcription activator | | pdb70 | 5GPE\_D | 97.7 | 2.4e-08 | 1.7e-12 | 47.6 | 49 | (11, 61) | 73 | (1, 50) | 129 | Transcriptional regulator, MerR-family | 5GPE\_D Transcriptional regulator, MerR-family PbrR691, MerR family, Transcription activator | | pdb70 | 4UA1\_A | 97.7 | 2.5e-08 | 1.8e-12 | 47.8 | 50 | (11, 62) | 73 | (2, 52) | 132 | Transcriptional regulator MerR | 4UA1\_A Transcriptional regulator MerR Metalloregulatory protein, DNA BINDING PROTEIN | | pdb70 | 4UA1\_B | 97.7 | 2.5e-08 | 1.8e-12 | 47.8 | 50 | (11, 62) | 73 | (2, 52) | 132 | Transcriptional regulator MerR | 4UA1\_B Transcriptional regulator MerR Metalloregulatory protein, DNA BINDING PROTEIN | | pdb70 | 4UA2\_C | 97.7 | 2.5e-08 | 1.8e-12 | 47.8 | 50 | (11, 62) | 73 | (2, 52) | 132 | Transcriptional regulator MerR | 4UA2\_C Transcriptional regulator MerR Metalloregulatory protein, DNA BINDING PROTEIN HET: MSE | | pdb70 | 3GP4\_A | 97.7 | 2.8e-08 | 2e-12 | 48.2 | 49 | (11, 61) | 73 | (2, 51) | 142 | Transcriptional regulator, MerR family | 3GP4\_A Transcriptional regulator, MerR family structural genomics, MerR, transcriptional regulator HET: GOL | | pdb70 | 3GP4\_B | 97.7 | 2.8e-08 | 2e-12 | 48.2 | 49 | (11, 61) | 73 | (2, 51) | 142 | Transcriptional regulator, MerR family | 3GP4\_B Transcriptional regulator, MerR family structural genomics, MerR, transcriptional regulator HET: GOL | | pdb70 | 3HH0\_A | 97.7 | 2.9e-08 | 2.1e-12 | 48.6 | 50 | (9, 60) | 73 | (2, 52) | 146 | Transcriptional regulator, MerR family | 3HH0\_A Transcriptional regulator, MerR family Transcriptional regulator, MerR, Protein Structure | | pdb70 | 3HH0\_B | 97.7 | 2.9e-08 | 2.1e-12 | 48.6 | 50 | (9, 60) | 73 | (2, 52) | 146 | Transcriptional regulator, MerR family | 3HH0\_B Transcriptional regulator, MerR family Transcriptional regulator, MerR, Protein Structure HET: MSE | | pdb70 | 6JGV\_A | 97.7 | 3.2e-08 | 2.3e-12 | 48.4 | 48 | (12, 61) | 73 | (1, 49) | 147 | CadR | 6JGV\_A CadR CadR, MerR family, cadmium regulator | | pdb70 | 6JGX\_A | 97.7 | 3.2e-08 | 2.3e-12 | 48.4 | 48 | (12, 61) | 73 | (1, 49) | 147 | CadR/DNA | 6JGX\_A CadR/DNA CadR, MerR family, cadmium regulator | | pdb70 | 6JNI\_A | 97.7 | 3.2e-08 | 2.3e-12 | 48.4 | 48 | (12, 61) | 73 | (1, 49) | 147 | CadR/DNA | 6JNI\_A CadR/DNA CadR, MerR family, cadmium regulator HET: ZN | | pdb70 | 3QAO\_A | 97.7 | 3.5e-08 | 2.5e-12 | 51.6 | 49 | (10, 60) | 73 | (2, 51) | 249 | MerR-like transcriptional regulator | 3QAO\_A MerR-like transcriptional regulator structural genomics, The Center for HET: GOL | | pdb70 | 5E01\_A | 97.6 | 4e-08 | 2.9e-12 | 46.7 | 50 | (9, 60) | 73 | (2, 52) | 128 | Uncharacterized HTH-type transcriptional regulator HI\_0186/DNA | 5E01\_A Uncharacterized HTH-type transcriptional regulator HI\_0186/DNA Transcription factor, MerR, thiol-based genetic | | pdb70 | 5E01\_B | 97.6 | 4e-08 | 2.9e-12 | 46.7 | 50 | (9, 60) | 73 | (2, 52) | 128 | Uncharacterized HTH-type transcriptional regulator HI\_0186/DNA | 5E01\_B Uncharacterized HTH-type transcriptional regulator HI\_0186/DNA Transcription factor, MerR, thiol-based genetic | | pdb70 | 1R8D\_A | 97.6 | 4.1e-08 | 2.9e-12 | 45.2 | 50 | (11, 62) | 73 | (2, 52) | 109 | transcription activator MtaN/DNA Complex | 1R8D\_A transcription activator MtaN/DNA Complex protein-DNA complex, Transcription-DNA COMPLEX HET: SO4 | | pdb70 | 1Q06\_B | 97.6 | 4.2e-08 | 3e-12 | 47.4 | 48 | (12, 61) | 73 | (1, 49) | 135 | Transcriptional regulator cueR | 1Q06\_B Transcriptional regulator cueR MerR family transcriptional regulator, copper | | pdb70 | 1Q07\_A | 97.6 | 4.2e-08 | 3e-12 | 47.4 | 48 | (12, 61) | 73 | (1, 49) | 135 | Transcriptional regulator cueR | 1Q07\_A Transcriptional regulator cueR MerR family transcriptional regulator, copper | | pdb70 | 4WLW\_A | 97.6 | 4.2e-08 | 3e-12 | 47.4 | 48 | (12, 61) | 73 | (1, 49) | 135 | TRANSCRIPTIONAL REGULATOR CUER/DNA Complex | 4WLW\_A TRANSCRIPTIONAL REGULATOR CUER/DNA Complex PROTEIN-DNA COMPLEX, MERR-FAMILY TRANSCRIPTION REGULATOR HET: MSE | | pdb70 | 6JGF\_A | 97.6 | 4.6e-08 | 3.4e-12 | 46.4 | 48 | (12, 61) | 73 | (1, 49) | 126 | CadR | 6JGF\_A CadR CadR, MerR family, cadmium regulator HET: MSE | | pdb70 | 1PM6\_A | 97.6 | 5e-08 | 3.6e-12 | 42.6 | 47 | (11, 57) | 73 | (2, 49) | 72 | Excisionase | 1PM6\_A Excisionase ANTIPARALLEL BETA-SHEET, WINGED-HELIX, CIS-TRANS-TRANS TRIPROLINE | | pdb70 | 5D8C\_A | 97.5 | 7.1e-08 | 5.1e-12 | 46.4 | 50 | (9, 60) | 73 | (2, 52) | 137 | Uncharacterized HTH-type transcriptional regulator HI\_0186/DNA | 5D8C\_A Uncharacterized HTH-type transcriptional regulator HI\_0186/DNA Transcription factor, MerR, thiol-based genetic | | pdb70 | 3Q2Y\_A | 97.5 | 7.9e-08 | 5.7e-12 | 50.6 | 51 | (8, 60) | 73 | (2, 54) | 284 | Multidrug-efflux transporter 1 regulator/DNA complex | 3Q2Y\_A Multidrug-efflux transporter 1 regulator/DNA complex Protein DNA complex, transcription regulator HET: GOL, ET | | pdb70 | 2OG0\_B | 97.5 | 8.7e-08 | 6.3e-12 | 38.8 | 46 | (11, 56) | 73 | (2, 48) | 52 | Excisionase/DNA Complex | 2OG0\_B Excisionase/DNA Complex PROTEIN-DNA COMPLEX, DNA ARCHITECTURAL PROTEIN | | pdb70 | 1R8E\_A | 97.5 | 9e-08 | 6.5e-12 | 49.9 | 51 | (8, 60) | 73 | (2, 54) | 278 | multidrug-efflux transporter regulator/DNA Complex | 1R8E\_A multidrug-efflux transporter regulator/DNA Complex PROTEIN-DNA COMPLEX, MERR-FAMILY TRANSCRIPTION ACTIVATOR HET: P4P, GOL, IMD | | pdb70 | 2JML\_A | 97.5 | 1e-07 | 7.3e-12 | 41.2 | 53 | (8, 62) | 73 | (2, 56) | 81 | DNA BINDING DOMAIN/TRANSCRIPTIONAL REGULATOR | 2JML\_A DNA BINDING DOMAIN/TRANSCRIPTIONAL REGULATOR anti-repressor, MerR, carotenogenesis, TRANSCRIPTION | | pdb70 | 3WHP\_A | 97.5 | 1.1e-07 | 8e-12 | 50.6 | 57 | (5, 63) | 73 | (5, 62) | 290 | Probable transcriptional regulator | 3WHP\_A Probable transcriptional regulator B12-binding domain, Rossmann fold, Four HET: B12 | | pdb70 | 2VZ4\_A | 97.5 | 1.2e-07 | 8.3e-12 | 43.6 | 49 | (11, 61) | 73 | (1, 50) | 108 | HTH-TYPE TRANSCRIPTIONAL ACTIVATOR TIPA | 2VZ4\_A HTH-TYPE TRANSCRIPTIONAL ACTIVATOR TIPA TRANSCRIPTION, RESISTANCE, ANTIBIOTIC, DNA-BINDING, STREPTOMYCES | | pdb70 | 2DG6\_A | 97.5 | 1.2e-07 | 8.7e-12 | 49.4 | 49 | (12, 62) | 73 | (1, 50) | 222 | putative transcriptional regulator | 2DG6\_A putative transcriptional regulator Winged-helix motif, MerR family, GENE | | pdb70 | 1RH6\_B | 97.4 | 2e-07 | 1.4e-11 | 38.2 | 47 | (11, 57) | 73 | (2, 49) | 55 | Excisionase/DNA Complex | 1RH6\_B Excisionase/DNA Complex Protein-DNA complex, DNA architectural protein | | pdb70 | 6P0U\_E | 97.4 | 2e-07 | 1.4e-11 | 38.2 | 47 | (11, 57) | 73 | (2, 49) | 55 | DNA-binding protein Fis, Excisionase/DNA Complex | 6P0U\_E DNA-binding protein Fis, Excisionase/DNA Complex Protein-DNA ternary complex, DNA shape | | pdb70 | 5I41\_B | 97.2 | 4.5e-07 | 3.3e-11 | 37.3 | 48 | (10, 59) | 73 | (2, 49) | 69 | Chromosome-anchoring protein RacA | 5I41\_B Chromosome-anchoring protein RacA RacA, axial filament, sporulation, B. | | pdb70 | 5I44\_A | 97.2 | 4.5e-07 | 3.3e-11 | 37.3 | 48 | (10, 59) | 73 | (2, 49) | 69 | Chromosome-anchoring protein RacA/DNA Complex | 5I44\_A Chromosome-anchoring protein RacA/DNA Complex RacA, B. subtilis, axial filament | | pdb70 | 5XBT\_B | 97.2 | 5.4e-07 | 3.9e-11 | 46.7 | 50 | (10, 61) | 73 | (2, 53) | 272 | Probable transcriptional regulator | 5XBT\_B Probable transcriptional regulator c-di-GMP, Receptor, HTH domain, DNA HET: GOL, PEG, IMD, C2E | | pdb70 | 2KFS\_A | 97.1 | 7.7e-07 | 5.6e-11 | 43.6 | 48 | (8, 58) | 73 | (28, 75) | 148 | Conserved hypothetical regulatory protein | 2KFS\_A Conserved hypothetical regulatory protein wHTH, DNA binding, Phosphorylation, DNA-binding | | pdb70 | 5YDC\_B | 96.9 | 2.5e-06 | 1.8e-10 | 41.4 | 49 | (5, 59) | 73 | (16, 64) | 142 | Uncharacterized HTH-type transcriptional regulator Rv1828 | 5YDC\_B Uncharacterized HTH-type transcriptional regulator Rv1828 DNA binding, HTH motif, promoter | | pdb70 | 5YDD\_B | 96.9 | 2.5e-06 | 1.8e-10 | 41.4 | 49 | (5, 59) | 73 | (16, 64) | 142 | Uncharacterized HTH-type transcriptional regulator Rv1828 | 5YDD\_B Uncharacterized HTH-type transcriptional regulator Rv1828 DNA binding, HTH motif, promoter HET: 8TU | | pdb70 | 5AF3\_B | 96.6 | 7.4e-06 | 5.3e-10 | 42.9 | 52 | (6, 59) | 73 | (12, 78) | 241 | VAPBC49 | 5AF3\_B VAPBC49 MYCOBACTERIUM TUBERCULOSIS, DNA BINDING, TA | | pdb70 | 6LND\_K | 96.4 | 1.5e-05 | 1.1e-09 | 44.1 | 62 | (8, 71) | 73 | (321, 389) | 396 | transposition protein TniQ | 6LND\_K transposition protein TniQ Type I-F CRISPR-Cas system: Csy HET: MSE | | pdb70 | 6PIG\_J | 96.3 | 2.4e-05 | 1.7e-09 | 42.9 | 60 | (9, 70) | 73 | (296, 362) | 369 | cas7 type I-F CRISPR-associated protein | 6PIG\_J cas7 type I-F CRISPR-associated protein CRISPR/Cas, Cascade, RNA BINDING PROTEIN | | pdb70 | 6V9P\_A | 96.3 | 2.6e-05 | 1.9e-09 | 43.1 | 60 | (9, 70) | 73 | (321, 387) | 395 | TniQ family protein | 6V9P\_A TniQ family protein Type I-F CRISPR-Cas system, transposition | |
| Top keywords  (threshold 1.00e-03 (evalue)) | **Helix\_turn\_helix, domain\_containing, regulator, transcriptional, DNA, excisionase, DNA\_binding, AlpA, binding, MerR** |
| Output files | ../../similar\_sequences/38\_FANPEZAQ\_CDS\_0038\_merged.svg ../../similar\_sequences/38\_FANPEZAQ\_CDS\_0038\_pdb70.a3m ../../similar\_sequences/38\_FANPEZAQ\_CDS\_0038\_pdb70.hhr ../../similar\_sequences/38\_FANPEZAQ\_CDS\_0038\_uniclust.a3m ../../similar\_sequences/38\_FANPEZAQ\_CDS\_0038\_uniclust.hhr |

#### Structure prediction (AlphaFold)2

|  |  |
| --- | --- |
| Stats | xml version="1.0" encoding="utf-8" standalone="no"?       2024-09-02T21:09:37.075729 image/svg+xml   Matplotlib v3.7.2, https://matplotlib.org/ |
| Predicted structure | **NGL Viewer Controls:**  - Center: *Left-Click* - Rotate: *Left-Click + Drag* - Translate: *Right-Click + Drag* - Zoom: *Shift + Left-Click + Drag* |
| Output files | ../../predicted\_structures/38\_FANPEZAQ\_CDS\_0038/features.pkl ../../predicted\_structures/38\_FANPEZAQ\_CDS\_0038/ranked\_0.pdb ../../predicted\_structures/38\_FANPEZAQ\_CDS\_0038/ranked\_0\_plots.svg ../../predicted\_structures/38\_FANPEZAQ\_CDS\_0038/result\_model\_1\_ptm\_pred\_0.pkl |

#### Structure similarity search results (Foldseek)3

|  |  |
| --- | --- |
| Structure databases searched | Pdb, Afdb-proteome, Afdb-uniprot50 |
| Results, scheme(s)  (Top layers only, threshold 1.00e-02 (evalue)) | xml version="1.0" encoding="utf-8" standalone="no"?       2024-09-02T21:11:13.160571 image/svg+xml   Matplotlib v3.7.2, https://matplotlib.org/ |
| Results, table  (threshold 1.00e-02 (evalue)) | | db | id | prob | evalue | bits | fident | alnlen | mismatch | gapopen | qstart | qend | tstart | tend | name | description | | --- | --- | --- | --- | --- | --- | --- | --- | --- | --- | --- | --- | --- | --- | --- | | pdb | 8DGL\_B | 1.0 | 0.008615 | 128 | 0.272 | 55 | 38 | 1 | 9 | 63 | 16 | 68 | Recombination Directionality Factor RdfS | Recombination Directionality Factor RdfS | | afdb-proteome | AF-O53399-F1-MODEL\_V4 | 1.0 | 0.002637 | 147 | 0.262 | 61 | 43 | 1 | 5 | 65 | 189 | 247 | HTH\_17 domain-containing protein | HTH\_17 domain-containing protein | | afdb-uniprot50 | AF-A0A2Z3IG45-F1-MODEL\_V4 | 1.0 | 5.132e-07 | 269 | 0.569 | 72 | 27 | 1 | 1 | 72 | 1 | 68 | Uncharacterized protein | Uncharacterized protein | | afdb-uniprot50 | AF-A0A158S0W0-F1-MODEL\_V4 | 1.0 | 1.744e-05 | 254 | 0.547 | 53 | 24 | 0 | 11 | 63 | 6 | 58 | Uncharacterized protein | Uncharacterized protein | | afdb-uniprot50 | AF-A0A375H718-F1-MODEL\_V4 | 1.0 | 6.265e-07 | 250 | 0.534 | 73 | 32 | 1 | 1 | 73 | 1 | 71 | Uncharacterized protein | Uncharacterized protein | | afdb-uniprot50 | AF-A0A5E7ZHS3-F1-MODEL\_V4 | 1.0 | 2.276e-05 | 235 | 0.39 | 64 | 38 | 1 | 1 | 64 | 1 | 63 | DNA-binding protein | DNA-binding protein | | afdb-uniprot50 | AF-A0A0S4UXE0-F1-MODEL\_V4 | 1.0 | 2.433e-05 | 230 | 0.365 | 63 | 40 | 0 | 11 | 73 | 5 | 67 | HTH\_17 domain-containing protein | HTH\_17 domain-containing protein | | afdb-uniprot50 | AF-A0A1Q4EAJ3-F1-MODEL\_V4 | 1.0 | 9.203e-05 | 228 | 0.396 | 58 | 35 | 0 | 7 | 64 | 2 | 59 | DNA-binding protein | DNA-binding protein | | afdb-uniprot50 | AF-A0A2T4HLL8-F1-MODEL\_V4 | 1.0 | 0.000179 | 220 | 0.368 | 57 | 36 | 0 | 7 | 63 | 2 | 58 | DNA-binding protein | DNA-binding protein | | afdb-uniprot50 | AF-A0A1C3NJQ6-F1-MODEL\_V4 | 1.0 | 1.527e-05 | 215 | 0.452 | 73 | 35 | 1 | 1 | 73 | 18 | 85 | Uncharacterized protein | Uncharacterized protein | | afdb-uniprot50 | AF-H4F9I4-F1-MODEL\_V4 | 1.0 | 1.864e-05 | 215 | 0.319 | 72 | 49 | 0 | 1 | 72 | 46 | 117 | Uncharacterized protein | Uncharacterized protein | | afdb-uniprot50 | AF-A0A841M6V1-F1-MODEL\_V4 | 1.0 | 5.057e-05 | 214 | 0.333 | 69 | 45 | 1 | 1 | 69 | 29 | 96 | Uncharacterized protein | Uncharacterized protein | | afdb-uniprot50 | AF-A0A5S4YCH6-F1-MODEL\_V4 | 1.0 | 9.203e-05 | 211 | 0.298 | 67 | 47 | 0 | 7 | 73 | 2 | 68 | Helix-turn-helix domain-containing protein | Helix-turn-helix domain-containing protein | | afdb-uniprot50 | AF-A0A227JR88-F1-MODEL\_V4 | 1.0 | 0.0002852 | 210 | 0.537 | 54 | 25 | 0 | 10 | 63 | 6 | 59 | HTH\_17 domain-containing protein | HTH\_17 domain-containing protein | | afdb-uniprot50 | AF-A0A1V1PTW5-F1-MODEL\_V4 | 1.0 | 7.538e-05 | 210 | 0.365 | 63 | 40 | 0 | 1 | 63 | 3 | 65 | Uncharacterized protein | Uncharacterized protein | | afdb-uniprot50 | AF-A0A2Z5UBR4-F1-MODEL\_V4 | 1.0 | 0.0003721 | 209 | 0.333 | 57 | 38 | 0 | 7 | 63 | 2 | 58 | Transcriptional regulator MerR family | Transcriptional regulator MerR family | | afdb-uniprot50 | AF-A0A145VQA6-F1-MODEL\_V4 | 1.0 | 6.174e-05 | 208 | 0.461 | 65 | 35 | 0 | 6 | 70 | 2 | 66 | Uncharacterized protein | Uncharacterized protein | | afdb-uniprot50 | AF-A0A356RFX9-F1-MODEL\_V4 | 1.0 | 0.0001466 | 208 | 0.344 | 58 | 36 | 2 | 7 | 63 | 1 | 57 | HTH\_17 domain-containing protein | HTH\_17 domain-containing protein | | afdb-uniprot50 | AF-A0A177IQV5-F1-MODEL\_V4 | 1.0 | 0.0001124 | 207 | 0.38 | 63 | 34 | 1 | 1 | 63 | 1 | 58 | HTH\_17 domain-containing protein | HTH\_17 domain-containing protein | | afdb-uniprot50 | AF-A0A6G4QV14-F1-MODEL\_V4 | 1.0 | 0.0001466 | 205 | 0.307 | 65 | 45 | 0 | 7 | 71 | 2 | 66 | Helix-turn-helix domain-containing protein | Helix-turn-helix domain-containing protein | | afdb-uniprot50 | AF-A0A504ERG4-F1-MODEL\_V4 | 1.0 | 9.836e-05 | 203 | 0.295 | 71 | 50 | 0 | 2 | 72 | 12 | 82 | Helix-turn-helix domain-containing protein | Helix-turn-helix domain-containing protein | | afdb-uniprot50 | AF-A0A2X0SMX0-F1-MODEL\_V4 | 1.0 | 0.0001372 | 202 | 0.38 | 63 | 37 | 1 | 1 | 63 | 17 | 77 | Uncharacterized protein | Uncharacterized protein | | afdb-uniprot50 | AF-A0A6L5FK90-F1-MODEL\_V4 | 1.0 | 0.000179 | 201 | 0.363 | 66 | 42 | 0 | 7 | 72 | 2 | 67 | DNA-binding protein | DNA-binding protein | | afdb-uniprot50 | AF-A0A1X7FPK0-F1-MODEL\_V4 | 1.0 | 0.0004543 | 200 | 0.363 | 55 | 35 | 0 | 7 | 61 | 2 | 56 | Uncharacterized protein | Uncharacterized protein | | afdb-uniprot50 | AF-A0A1M6DCH5-F1-MODEL\_V4 | 1.0 | 0.0001051 | 200 | 0.349 | 63 | 37 | 1 | 1 | 63 | 1 | 59 | Helix-turn-helix domain-containing protein | Helix-turn-helix domain-containing protein | | afdb-uniprot50 | AF-A0A1D2SFS9-F1-MODEL\_V4 | 1.0 | 3.875e-05 | 200 | 0.333 | 72 | 44 | 2 | 1 | 72 | 1 | 68 | Uncharacterized protein | Uncharacterized protein | | afdb-uniprot50 | AF-A0A0W8FLL0-F1-MODEL\_V4 | 1.0 | 0.0001372 | 199 | 0.333 | 66 | 43 | 1 | 7 | 72 | 3 | 67 | Uncharacterized protein | Uncharacterized protein | | afdb-uniprot50 | AF-A0A2S0XML1-F1-MODEL\_V4 | 1.0 | 0.0001124 | 198 | 0.367 | 68 | 38 | 1 | 1 | 63 | 23 | 90 | Uncharacterized protein | Uncharacterized protein | | afdb-uniprot50 | AF-T0GF19-F1-MODEL\_V4 | 1.0 | 0.000179 | 198 | 0.292 | 65 | 46 | 0 | 9 | 73 | 4 | 68 | Uncharacterized protein | Uncharacterized protein | | afdb-uniprot50 | AF-A0A0H3J1R4-F1-MODEL\_V4 | 1.0 | 0.0001124 | 197 | 0.333 | 69 | 41 | 1 | 1 | 69 | 1 | 64 | HTH transcriptional regulator | HTH transcriptional regulator | | afdb-uniprot50 | AF-A0A3B0N048-F1-MODEL\_V4 | 1.0 | 0.0002336 | 196 | 0.393 | 61 | 35 | 1 | 7 | 67 | 1 | 59 | HTH\_17 domain-containing protein | HTH\_17 domain-containing protein | | afdb-uniprot50 | AF-A0A222X839-F1-MODEL\_V4 | 1.0 | 0.0001567 | 196 | 0.409 | 61 | 34 | 1 | 11 | 71 | 5 | 63 | Uncharacterized protein | Uncharacterized protein | | afdb-uniprot50 | AF-A0A4P8HF99-F1-MODEL\_V4 | 1.0 | 0.0002668 | 194 | 0.333 | 66 | 42 | 2 | 1 | 66 | 1 | 64 | Helix-turn-helix domain-containing protein | Helix-turn-helix domain-containing protein | | afdb-uniprot50 | AF-A0A4Q2SXB6-F1-MODEL\_V4 | 1.0 | 0.0002852 | 194 | 0.328 | 67 | 45 | 0 | 1 | 67 | 24 | 90 | DNA-binding protein | DNA-binding protein | | afdb-uniprot50 | AF-A0A8B3L4A2-F1-MODEL\_V4 | 1.0 | 0.0003482 | 193 | 0.38 | 63 | 34 | 1 | 1 | 63 | 1 | 58 | DNA-binding protein | DNA-binding protein | | afdb-uniprot50 | AF-K2KUD9-F1-MODEL\_V4 | 1.0 | 0.0001372 | 192 | 0.396 | 63 | 36 | 1 | 11 | 73 | 5 | 65 | Uncharacterized protein | Uncharacterized protein | | afdb-uniprot50 | AF-A0A4Q3WF84-F1-MODEL\_V4 | 1.0 | 0.0002496 | 192 | 0.353 | 65 | 40 | 1 | 2 | 66 | 7 | 69 | DNA-binding protein | DNA-binding protein | | afdb-uniprot50 | AF-A0A7Y3WDS6-F1-MODEL\_V4 | 1.0 | 0.001153 | 192 | 0.381 | 55 | 34 | 0 | 9 | 63 | 4 | 58 | Helix-turn-helix domain-containing protein | Helix-turn-helix domain-containing protein | | afdb-uniprot50 | AF-A0A8A6KER8-F1-MODEL\_V4 | 1.0 | 0.0001675 | 191 | 0.417 | 67 | 34 | 1 | 12 | 73 | 1 | 67 | DNA-binding protein | DNA-binding protein | | afdb-uniprot50 | AF-A0A7Z8LR69-F1-MODEL\_V4 | 1.0 | 0.0002668 | 190 | 0.409 | 61 | 34 | 1 | 11 | 71 | 5 | 63 | Helix-turn-helix protein | Helix-turn-helix protein | | afdb-uniprot50 | AF-A0A099F695-F1-MODEL\_V4 | 1.0 | 0.0003048 | 189 | 0.328 | 67 | 43 | 1 | 7 | 73 | 1 | 65 | HTH\_17 domain-containing protein | HTH\_17 domain-containing protein | | afdb-uniprot50 | AF-A0A525JL77-F1-MODEL\_V4 | 1.0 | 0.0003977 | 189 | 0.349 | 63 | 39 | 1 | 1 | 63 | 1 | 61 | DNA-binding protein | DNA-binding protein | | afdb-uniprot50 | AF-A0A3A5K4U6-F1-MODEL\_V4 | 1.0 | 0.0001372 | 189 | 0.323 | 68 | 41 | 1 | 11 | 73 | 6 | 73 | DNA-binding protein | DNA-binding protein | | afdb-uniprot50 | AF-A0A2G5QHB5-F1-MODEL\_V4 | 1.0 | 0.0004251 | 189 | 0.311 | 61 | 42 | 0 | 11 | 71 | 4 | 64 | HTH\_17 domain-containing protein | HTH\_17 domain-containing protein | | afdb-uniprot50 | AF-A0A4R1R9W0-F1-MODEL\_V4 | 1.0 | 0.0008268 | 187 | 0.379 | 58 | 35 | 1 | 7 | 63 | 1 | 58 | Uncharacterized protein | Uncharacterized protein | | afdb-uniprot50 | AF-A0A1N6NC24-F1-MODEL\_V4 | 1.0 | 9.836e-05 | 187 | 0.378 | 74 | 40 | 3 | 1 | 73 | 6 | 74 | Uncharacterized protein | Uncharacterized protein | | afdb-uniprot50 | AF-R0EA79-F1-MODEL\_V4 | 1.0 | 0.0003977 | 187 | 0.333 | 66 | 42 | 1 | 1 | 66 | 16 | 79 | Uncharacterized protein | Uncharacterized protein | | afdb-uniprot50 | AF-A0A2N3AXX5-F1-MODEL\_V4 | 1.0 | 0.0002185 | 187 | 0.409 | 61 | 34 | 1 | 11 | 71 | 5 | 63 | HTH\_17 domain-containing protein | HTH\_17 domain-containing protein | | afdb-uniprot50 | AF-A0A1M3H0S1-F1-MODEL\_V4 | 1.0 | 0.000519 | 186 | 0.444 | 54 | 28 | 1 | 9 | 62 | 2 | 53 | Uncharacterized protein | Uncharacterized protein | | afdb-uniprot50 | AF-A0A7J5CN46-F1-MODEL\_V4 | 1.0 | 0.0002496 | 185 | 0.323 | 65 | 42 | 1 | 2 | 66 | 5 | 67 | Helix-turn-helix domain-containing protein | Helix-turn-helix domain-containing protein | | afdb-uniprot50 | AF-A0A1M3GUP1-F1-MODEL\_V4 | 1.0 | 0.0006772 | 185 | 0.444 | 54 | 28 | 1 | 10 | 63 | 4 | 55 | HTH\_17 domain-containing protein | HTH\_17 domain-containing protein | | afdb-uniprot50 | AF-A0A7Y4Y2M7-F1-MODEL\_V4 | 1.0 | 0.0001675 | 185 | 0.301 | 73 | 48 | 2 | 1 | 73 | 6 | 75 | DNA-binding protein | DNA-binding protein | | afdb-uniprot50 | AF-A0A2E2ZU57-F1-MODEL\_V4 | 1.0 | 0.0005928 | 184 | 0.333 | 63 | 37 | 1 | 1 | 63 | 1 | 58 | DNA-binding protein | DNA-binding protein | | afdb-uniprot50 | AF-A0A1H8ZIM8-F1-MODEL\_V4 | 1.0 | 0.0008836 | 184 | 0.389 | 59 | 34 | 1 | 7 | 65 | 3 | 59 | Uncharacterized protein | Uncharacterized protein | | afdb-uniprot50 | AF-A0A2E8PB88-F1-MODEL\_V4 | 1.0 | 0.0001466 | 184 | 0.367 | 68 | 40 | 2 | 2 | 68 | 8 | 73 | HTH\_17 domain-containing protein | HTH\_17 domain-containing protein | | afdb-uniprot50 | AF-A0A373FQ25-F1-MODEL\_V4 | 1.0 | 0.0004856 | 183 | 0.349 | 63 | 39 | 1 | 11 | 73 | 2 | 62 | DNA-binding protein | DNA-binding protein | | afdb-uniprot50 | AF-A0A4Q3YNY5-F1-MODEL\_V4 | 1.0 | 0.000519 | 183 | 0.359 | 64 | 39 | 1 | 10 | 73 | 4 | 65 | DNA-binding protein | DNA-binding protein | | afdb-uniprot50 | AF-A0A840STV4-F1-MODEL\_V4 | 1.0 | 0.0004543 | 183 | 0.403 | 62 | 35 | 1 | 10 | 71 | 4 | 63 | Putative site-specific integrase-resolvase | Putative site-specific integrase-resolvase | | afdb-uniprot50 | AF-A0A4R6RSK0-F1-MODEL\_V4 | 1.0 | 0.0001124 | 183 | 0.287 | 80 | 50 | 2 | 1 | 73 | 3 | 82 | Helix-turn-helix protein | Helix-turn-helix protein | | afdb-uniprot50 | AF-A0A554SP46-F1-MODEL\_V4 | 1.0 | 0.0008836 | 182 | 0.363 | 55 | 33 | 1 | 9 | 63 | 2 | 54 | Helix-turn-helix domain-containing protein | Helix-turn-helix domain-containing protein | | afdb-uniprot50 | AF-A0A5A7Y4Z2-F1-MODEL\_V4 | 1.0 | 0.0008836 | 182 | 0.403 | 57 | 32 | 1 | 11 | 67 | 8 | 62 | DNA-binding protein | DNA-binding protein | | afdb-uniprot50 | AF-A0A442F8A9-F1-MODEL\_V4 | 1.0 | 0.0003257 | 182 | 0.315 | 73 | 45 | 1 | 1 | 73 | 1 | 68 | DNA-binding protein | DNA-binding protein | | afdb-uniprot50 | AF-A0A2E8L6W0-F1-MODEL\_V4 | 1.0 | 0.0004543 | 182 | 0.384 | 65 | 38 | 1 | 9 | 73 | 14 | 76 | HTH\_17 domain-containing protein | HTH\_17 domain-containing protein | | afdb-uniprot50 | AF-A0A495BRN6-F1-MODEL\_V4 | 1.0 | 0.001009 | 182 | 0.392 | 51 | 29 | 1 | 11 | 61 | 5 | 53 | Uncharacterized protein | Uncharacterized protein | | afdb-uniprot50 | AF-A0A1L5BSX1-F1-MODEL\_V4 | 1.0 | 0.0005547 | 181 | 0.362 | 58 | 35 | 1 | 11 | 68 | 4 | 59 | DNA-binding protein | DNA-binding protein | | afdb-uniprot50 | AF-A0A1Y1R0V8-F1-MODEL\_V4 | 1.0 | 0.0003257 | 181 | 0.428 | 63 | 34 | 1 | 11 | 73 | 5 | 65 | HTH\_17 domain-containing protein | HTH\_17 domain-containing protein | | afdb-uniprot50 | AF-A0A4R6UD26-F1-MODEL\_V4 | 1.0 | 0.0003721 | 181 | 0.315 | 73 | 48 | 1 | 1 | 73 | 16 | 86 | Uncharacterized protein | Uncharacterized protein | | afdb-uniprot50 | AF-A0A5C7Y246-F1-MODEL\_V4 | 1.0 | 0.0005928 | 180 | 0.36 | 61 | 37 | 1 | 9 | 69 | 3 | 61 | DNA-binding protein | DNA-binding protein | | afdb-uniprot50 | AF-A0A1M3GVS8-F1-MODEL\_V4 | 1.0 | 0.0003257 | 180 | 0.388 | 67 | 38 | 2 | 7 | 73 | 1 | 64 | HTH\_17 domain-containing protein | HTH\_17 domain-containing protein | | afdb-uniprot50 | AF-A0A0G3IYW9-F1-MODEL\_V4 | 1.0 | 0.0002185 | 180 | 0.328 | 73 | 44 | 2 | 1 | 73 | 1 | 68 | Uncharacterized protein | Uncharacterized protein | | afdb-uniprot50 | AF-A0A2A2GKF9-F1-MODEL\_V4 | 1.0 | 0.0003257 | 180 | 0.264 | 68 | 48 | 1 | 2 | 69 | 6 | 71 | Terminase | Terminase | | afdb-uniprot50 | AF-A0A6V8MMJ1-F1-MODEL\_V4 | 1.0 | 0.0002336 | 180 | 0.285 | 70 | 48 | 1 | 1 | 70 | 7 | 74 | HTH\_17 domain-containing protein | HTH\_17 domain-containing protein | | afdb-uniprot50 | AF-A0A3M6RS62-F1-MODEL\_V4 | 1.0 | 0.0003977 | 180 | 0.328 | 64 | 39 | 2 | 1 | 62 | 14 | 75 | DNA-binding protein | DNA-binding protein | | afdb-uniprot50 | AF-A0A239NMV0-F1-MODEL\_V4 | 1.0 | 0.0002045 | 180 | 0.25 | 80 | 53 | 1 | 1 | 73 | 3 | 82 | HTH\_17 domain-containing protein | HTH\_17 domain-containing protein | | afdb-uniprot50 | AF-A0A7Y1SMM4-F1-MODEL\_V4 | 1.0 | 0.0004251 | 180 | 0.319 | 72 | 46 | 2 | 1 | 72 | 1 | 69 | Helix-turn-helix domain-containing protein | Helix-turn-helix domain-containing protein | | afdb-uniprot50 | AF-A0A347ULU5-F1-MODEL\_V4 | 1.0 | 0.0008268 | 179 | 0.393 | 61 | 35 | 1 | 11 | 71 | 5 | 63 | DNA-binding protein | DNA-binding protein | | afdb-uniprot50 | AF-A0A539DI59-F1-MODEL\_V4 | 1.0 | 0.0009444 | 179 | 0.317 | 63 | 43 | 0 | 1 | 63 | 7 | 69 | Uncharacterized protein | Uncharacterized protein | | afdb-uniprot50 | AF-A0A3D1ZSG9-F1-MODEL\_V4 | 1.0 | 0.0005547 | 178 | 0.383 | 60 | 35 | 1 | 11 | 70 | 5 | 62 | HTH\_17 domain-containing protein | HTH\_17 domain-containing protein | | afdb-uniprot50 | AF-A0A7J9VS78-F1-MODEL\_V4 | 1.0 | 0.0003257 | 178 | 0.357 | 70 | 42 | 2 | 4 | 73 | 2 | 68 | DNA-binding protein | DNA-binding protein | | afdb-uniprot50 | AF-A0A4P5VMN8-F1-MODEL\_V4 | 1.0 | 0.0004251 | 178 | 0.358 | 67 | 41 | 1 | 7 | 73 | 2 | 66 | Uncharacterized protein | Uncharacterized protein | | afdb-uniprot50 | AF-B1YV21-F1-MODEL\_V4 | 1.0 | 0.0003482 | 177 | 0.342 | 70 | 40 | 2 | 7 | 72 | 2 | 69 | HTH\_17 domain-containing protein | HTH\_17 domain-containing protein | | afdb-uniprot50 | AF-A0A653J2V4-F1-MODEL\_V4 | 1.0 | 0.0002852 | 177 | 0.318 | 66 | 41 | 2 | 2 | 65 | 3 | 66 | DNA-binding protein | DNA-binding protein | | afdb-uniprot50 | AF-A0A3A4KJX6-F1-MODEL\_V4 | 1.0 | 0.0006772 | 177 | 0.365 | 63 | 39 | 1 | 1 | 63 | 6 | 67 | DNA-binding protein | DNA-binding protein | | afdb-uniprot50 | AF-A0A807ZGS9-F1-MODEL\_V4 | 1.0 | 0.001505 | 177 | 0.327 | 58 | 37 | 1 | 8 | 65 | 43 | 98 | DNA-binding protein | DNA-binding protein | | afdb-uniprot50 | AF-E5Y5F8-F1-MODEL\_V4 | 1.0 | 0.0004543 | 177 | 0.277 | 72 | 50 | 1 | 1 | 72 | 33 | 102 | Uncharacterized protein | Uncharacterized protein | | afdb-uniprot50 | AF-A0A833D1M0-F1-MODEL\_V4 | 1.0 | 0.0005928 | 176 | 0.349 | 63 | 39 | 1 | 11 | 73 | 2 | 62 | Helix-turn-helix domain-containing protein | Helix-turn-helix domain-containing protein | | afdb-uniprot50 | AF-A0A2N6B1S7-F1-MODEL\_V4 | 1.0 | 0.0006336 | 176 | 0.333 | 63 | 40 | 1 | 11 | 73 | 5 | 65 | HTH\_17 domain-containing protein | HTH\_17 domain-containing protein | | afdb-uniprot50 | AF-A0A4V1RMP8-F1-MODEL\_V4 | 1.0 | 0.0009444 | 176 | 0.322 | 59 | 38 | 1 | 4 | 62 | 3 | 59 | DNA-binding protein | DNA-binding protein | | afdb-uniprot50 | AF-A0A383AGQ8-F1-MODEL\_V4 | 1.0 | 0.0005547 | 176 | 0.235 | 68 | 50 | 1 | 4 | 71 | 1 | 66 | Uncharacterized protein | Uncharacterized protein | | afdb-uniprot50 | AF-A0A433X345-F1-MODEL\_V4 | 1.0 | 0.0007736 | 176 | 0.318 | 66 | 43 | 1 | 8 | 73 | 4 | 67 | DNA-binding protein | DNA-binding protein | | afdb-uniprot50 | AF-A0A840XSZ3-F1-MODEL\_V4 | 1.0 | 0.0006772 | 176 | 0.365 | 63 | 38 | 1 | 11 | 73 | 5 | 65 | Putative site-specific integrase-resolvase | Putative site-specific integrase-resolvase | | afdb-uniprot50 | AF-A0A353PQN1-F1-MODEL\_V4 | 1.0 | 0.0003482 | 176 | 0.349 | 63 | 37 | 2 | 11 | 73 | 5 | 63 | HTH\_17 domain-containing protein | HTH\_17 domain-containing protein | | afdb-uniprot50 | AF-A0A2E7K219-F1-MODEL\_V4 | 1.0 | 0.0004543 | 176 | 0.343 | 67 | 42 | 1 | 7 | 73 | 2 | 66 | HTH\_17 domain-containing protein | HTH\_17 domain-containing protein | | afdb-uniprot50 | AF-A0A7L9SIQ9-F1-MODEL\_V4 | 1.0 | 0.0006336 | 175 | 0.269 | 63 | 44 | 1 | 11 | 73 | 5 | 65 | Helix-turn-helix domain-containing protein | Helix-turn-helix domain-containing protein | | afdb-uniprot50 | AF-A0A1M3IL58-F1-MODEL\_V4 | 1.0 | 0.001009 | 175 | 0.407 | 54 | 30 | 1 | 11 | 64 | 5 | 56 | Uncharacterized protein | Uncharacterized protein | | afdb-uniprot50 | AF-A0A2E3P4L9-F1-MODEL\_V4 | 1.0 | 0.0005928 | 175 | 0.419 | 62 | 33 | 2 | 11 | 72 | 5 | 63 | Helix-turn-helix domain-containing protein | Helix-turn-helix domain-containing protein | | afdb-uniprot50 | AF-A0A3D9U6K0-F1-MODEL\_V4 | 1.0 | 0.0002852 | 175 | 0.266 | 75 | 52 | 2 | 1 | 73 | 4 | 77 | Uncharacterized protein | Uncharacterized protein | | afdb-uniprot50 | AF-A0A329YBV5-F1-MODEL\_V4 | 1.0 | 0.0008836 | 175 | 0.306 | 62 | 43 | 0 | 11 | 72 | 6 | 67 | DNA-binding protein | DNA-binding protein | | afdb-uniprot50 | AF-A0A6C1KV97-F1-MODEL\_V4 | 1.0 | 0.0003721 | 175 | 0.315 | 73 | 45 | 1 | 1 | 73 | 1 | 68 | Helix-turn-helix domain-containing protein | Helix-turn-helix domain-containing protein | | afdb-uniprot50 | AF-A0A509Y4U3-F1-MODEL\_V4 | 1.0 | 0.0003048 | 175 | 0.367 | 68 | 41 | 1 | 2 | 69 | 17 | 82 | Helix-turn-helix domain-containing protein | Helix-turn-helix domain-containing protein | | afdb-uniprot50 | AF-A0A315ECV1-F1-MODEL\_V4 | 1.0 | 0.0001913 | 175 | 0.306 | 75 | 46 | 2 | 1 | 71 | 15 | 87 | HTH\_17 domain-containing protein | HTH\_17 domain-containing protein | | afdb-uniprot50 | AF-A0A6P0HA59-F1-MODEL\_V4 | 1.0 | 0.0004251 | 175 | 0.328 | 64 | 41 | 1 | 2 | 65 | 6 | 67 | Helix-turn-helix domain-containing protein | Helix-turn-helix domain-containing protein | | afdb-uniprot50 | AF-L7L686-F1-MODEL\_V4 | 1.0 | 0.001009 | 174 | 0.333 | 57 | 36 | 1 | 7 | 63 | 3 | 57 | Uncharacterized protein | Uncharacterized protein | | afdb-uniprot50 | AF-A0A5C7MH74-F1-MODEL\_V4 | 1.0 | 0.0003048 | 174 | 0.393 | 66 | 38 | 1 | 4 | 69 | 1 | 64 | DNA-binding protein | DNA-binding protein | | afdb-uniprot50 | AF-A0A2G2CSM9-F1-MODEL\_V4 | 1.0 | 0.0005547 | 174 | 0.235 | 68 | 50 | 1 | 1 | 68 | 1 | 66 | DNA-binding protein | DNA-binding protein | | afdb-uniprot50 | AF-A0A2G1YBU3-F1-MODEL\_V4 | 1.0 | 0.001153 | 174 | 0.317 | 63 | 41 | 1 | 4 | 66 | 2 | 62 | DNA-binding protein | DNA-binding protein | | afdb-uniprot50 | AF-A0A7G6WVR5-F1-MODEL\_V4 | 1.0 | 0.0002852 | 174 | 0.263 | 72 | 51 | 1 | 1 | 72 | 4 | 73 | Helix-turn-helix domain-containing protein | Helix-turn-helix domain-containing protein | | afdb-uniprot50 | AF-A0A7V7KBR4-F1-MODEL\_V4 | 1.0 | 0.0001567 | 174 | 0.378 | 74 | 42 | 3 | 1 | 73 | 3 | 73 | Helix-turn-helix domain-containing protein | Helix-turn-helix domain-containing protein | | afdb-uniprot50 | AF-A0A1F1WCH5-F1-MODEL\_V4 | 1.0 | 0.0003977 | 174 | 0.301 | 73 | 48 | 2 | 1 | 73 | 9 | 78 | HTH\_17 domain-containing protein | HTH\_17 domain-containing protein | | afdb-uniprot50 | AF-A0A1M6C0J0-F1-MODEL\_V4 | 1.0 | 0.0005928 | 173 | 0.313 | 67 | 44 | 1 | 7 | 73 | 2 | 66 | DNA binding domain-containing protein, excisionase family | DNA binding domain-containing protein, excisionase family | | afdb-uniprot50 | AF-A0A5B8IWQ0-F1-MODEL\_V4 | 1.0 | 0.0005928 | 173 | 0.343 | 64 | 39 | 2 | 1 | 63 | 3 | 64 | Helix-turn-helix domain-containing protein | Helix-turn-helix domain-containing protein | | afdb-uniprot50 | AF-A0A7V3JQH2-F1-MODEL\_V4 | 1.0 | 0.0004856 | 173 | 0.236 | 72 | 53 | 1 | 1 | 72 | 3 | 72 | DNA-binding protein | DNA-binding protein | | afdb-uniprot50 | AF-A0A4R8ZYI3-F1-MODEL\_V4 | 1.0 | 0.001009 | 173 | 0.41 | 56 | 30 | 2 | 11 | 66 | 9 | 61 | DNA-binding protein | DNA-binding protein | | afdb-uniprot50 | AF-A0A1I7L0T8-F1-MODEL\_V4 | 1.0 | 0.001079 | 173 | 0.303 | 66 | 44 | 1 | 8 | 73 | 23 | 86 | Uncharacterized protein | Uncharacterized protein | | afdb-uniprot50 | AF-A0A7W1M9X1-F1-MODEL\_V4 | 1.0 | 0.0001913 | 173 | 0.391 | 74 | 38 | 3 | 1 | 70 | 1 | 71 | Helix-turn-helix domain-containing protein | Helix-turn-helix domain-containing protein | | afdb-uniprot50 | AF-A0A521H311-F1-MODEL\_V4 | 1.0 | 0.0008836 | 172 | 0.327 | 61 | 39 | 1 | 11 | 71 | 6 | 64 | DNA-binding protein | DNA-binding protein | | afdb-uniprot50 | AF-A0A2D8GJA3-F1-MODEL\_V4 | 1.0 | 0.0008268 | 171 | 0.306 | 62 | 41 | 1 | 11 | 72 | 4 | 63 | DNA-binding protein | DNA-binding protein | | afdb-uniprot50 | AF-A0A317L7L9-F1-MODEL\_V4 | 1.0 | 0.0004543 | 171 | 0.378 | 66 | 36 | 2 | 11 | 73 | 5 | 68 | Helix-turn-helix domain-containing protein | Helix-turn-helix domain-containing protein | | afdb-uniprot50 | AF-A0A4S3M5G0-F1-MODEL\_V4 | 1.0 | 0.0006336 | 171 | 0.313 | 67 | 41 | 2 | 7 | 73 | 2 | 63 | DNA-binding protein | DNA-binding protein | | afdb-uniprot50 | AF-A0A2W7C0P0-F1-MODEL\_V4 | 1.0 | 0.004081 | 171 | 0.377 | 53 | 33 | 0 | 11 | 63 | 3 | 55 | DNA-binding protein | DNA-binding protein | | afdb-uniprot50 | AF-A0A1S2PGN3-F1-MODEL\_V4 | 1.0 | 0.001009 | 171 | 0.315 | 57 | 38 | 1 | 9 | 65 | 21 | 76 | DNA-binding protein | DNA-binding protein | | afdb-uniprot50 | AF-A0A0Q7BDA8-F1-MODEL\_V4 | 1.0 | 0.0004543 | 171 | 0.328 | 67 | 43 | 1 | 1 | 67 | 19 | 83 | HTH merR-type domain-containing protein | HTH merR-type domain-containing protein | | afdb-uniprot50 | AF-A0A4Y8PHP0-F1-MODEL\_V4 | 1.0 | 0.0004251 | 171 | 0.333 | 69 | 43 | 2 | 2 | 69 | 19 | 85 | DNA-binding protein | DNA-binding protein | | afdb-uniprot50 | AF-A0A2W7BSX5-F1-MODEL\_V4 | 1.0 | 0.001153 | 171 | 0.313 | 67 | 41 | 1 | 1 | 67 | 1 | 62 | Uncharacterized protein | Uncharacterized protein | | afdb-uniprot50 | AF-A0A2H5VV35-F1-MODEL\_V4 | 1.0 | 0.001009 | 170 | 0.322 | 62 | 38 | 2 | 11 | 72 | 4 | 61 | HTH\_17 domain-containing protein | HTH\_17 domain-containing protein | | afdb-uniprot50 | AF-G1V6J5-F1-MODEL\_V4 | 1.0 | 0.001153 | 170 | 0.303 | 66 | 42 | 2 | 1 | 66 | 1 | 62 | HTH\_17 domain-containing protein | HTH\_17 domain-containing protein | | afdb-uniprot50 | AF-A0A495A877-F1-MODEL\_V4 | 1.0 | 0.0005547 | 170 | 0.285 | 70 | 48 | 1 | 4 | 73 | 1 | 68 | DNA-binding protein | DNA-binding protein | | afdb-uniprot50 | AF-A0A6N7ZTL0-F1-MODEL\_V4 | 1.0 | 0.002243 | 170 | 0.285 | 56 | 38 | 1 | 8 | 63 | 9 | 62 | DNA-binding protein | DNA-binding protein | | afdb-uniprot50 | AF-A0A5B7QT10-F1-MODEL\_V4 | 1.0 | 0.0007736 | 170 | 0.333 | 60 | 38 | 1 | 3 | 62 | 6 | 63 | Helix-turn-helix domain-containing protein | Helix-turn-helix domain-containing protein | | afdb-uniprot50 | AF-A3WRZ7-F1-MODEL\_V4 | 1.0 | 0.0004251 | 170 | 0.337 | 83 | 40 | 2 | 1 | 73 | 1 | 78 | HTH\_17 domain-containing protein | HTH\_17 domain-containing protein | | afdb-uniprot50 | AF-M3AAY3-F1-MODEL\_V4 | 1.0 | 0.0006772 | 170 | 0.365 | 63 | 38 | 1 | 1 | 63 | 11 | 71 | HTH\_17 domain-containing protein | HTH\_17 domain-containing protein | | afdb-uniprot50 | AF-A3X0W1-F1-MODEL\_V4 | 1.0 | 0.001009 | 170 | 0.344 | 61 | 38 | 1 | 11 | 71 | 5 | 63 | Uncharacterized protein | Uncharacterized protein | | afdb-uniprot50 | AF-A0A7I7SIE3-F1-MODEL\_V4 | 1.0 | 0.0006772 | 170 | 0.291 | 72 | 49 | 1 | 1 | 72 | 10 | 79 | HTH\_17 domain-containing protein | HTH\_17 domain-containing protein | | afdb-uniprot50 | AF-A0A2D5PMF7-F1-MODEL\_V4 | 1.0 | 0.0005547 | 169 | 0.369 | 65 | 39 | 1 | 4 | 68 | 1 | 63 | Terminase | Terminase | | afdb-uniprot50 | AF-A0A562QVB6-F1-MODEL\_V4 | 1.0 | 0.001317 | 169 | 0.298 | 67 | 45 | 1 | 7 | 73 | 1 | 65 | Excisionase family DNA binding protein | Excisionase family DNA binding protein | | afdb-uniprot50 | AF-A0A5B8KXC5-F1-MODEL\_V4 | 1.0 | 0.0008836 | 169 | 0.396 | 63 | 36 | 1 | 11 | 73 | 5 | 65 | Helix-turn-helix domain-containing protein | Helix-turn-helix domain-containing protein | | afdb-uniprot50 | AF-H8YVZ3-F1-MODEL\_V4 | 1.0 | 0.0007736 | 169 | 0.278 | 61 | 41 | 2 | 4 | 63 | 3 | 61 | HTH\_17 domain-containing protein | HTH\_17 domain-containing protein | | afdb-uniprot50 | AF-A0A2S5P8W1-F1-MODEL\_V4 | 1.0 | 0.0004251 | 169 | 0.283 | 74 | 50 | 2 | 1 | 73 | 5 | 76 | HTH\_17 domain-containing protein | HTH\_17 domain-containing protein | | afdb-uniprot50 | AF-A0A1X1T3U5-F1-MODEL\_V4 | 1.0 | 0.001009 | 169 | 0.311 | 61 | 40 | 1 | 2 | 62 | 15 | 73 | Uncharacterized protein | Uncharacterized protein | | afdb-uniprot50 | AF-A0A7U7GDB5-F1-MODEL\_V4 | 1.0 | 0.002397 | 169 | 0.301 | 53 | 35 | 1 | 11 | 63 | 4 | 54 | HTH\_17 domain-containing protein | HTH\_17 domain-containing protein | | afdb-uniprot50 | AF-A0A6N1BNC1-F1-MODEL\_V4 | 1.0 | 0.0003257 | 169 | 0.311 | 77 | 46 | 2 | 1 | 72 | 26 | 100 | Helix-turn-helix domain-containing protein | Helix-turn-helix domain-containing protein | | afdb-uniprot50 | AF-A0A0J0YNG3-F1-MODEL\_V4 | 1.0 | 0.001317 | 168 | 0.349 | 63 | 38 | 2 | 7 | 69 | 3 | 62 | Transcriptional regulator, AlpA family | Transcriptional regulator, AlpA family | | afdb-uniprot50 | AF-A0A7M2X5E3-F1-MODEL\_V4 | 1.0 | 0.0007238 | 168 | 0.353 | 65 | 38 | 2 | 11 | 73 | 5 | 67 | Helix-turn-helix domain-containing protein | Helix-turn-helix domain-containing protein | | afdb-uniprot50 | AF-A0A7S7SJL7-F1-MODEL\_V4 | 1.0 | 0.0007736 | 168 | 0.301 | 73 | 47 | 2 | 1 | 73 | 1 | 69 | Helix-turn-helix domain-containing protein | Helix-turn-helix domain-containing protein | | afdb-uniprot50 | AF-A0A2D6TB11-F1-MODEL\_V4 | 1.0 | 0.0009444 | 168 | 0.349 | 63 | 39 | 1 | 11 | 73 | 5 | 65 | HTH\_17 domain-containing protein | HTH\_17 domain-containing protein | | afdb-uniprot50 | AF-A0A543I5H3-F1-MODEL\_V4 | 1.0 | 0.0006336 | 168 | 0.291 | 72 | 49 | 1 | 2 | 73 | 5 | 74 | Helix-turn-helix protein | Helix-turn-helix protein | | afdb-uniprot50 | AF-A0A7W1M9Q7-F1-MODEL\_V4 | 1.0 | 0.0007736 | 168 | 0.358 | 67 | 38 | 2 | 7 | 73 | 1 | 62 | Helix-turn-helix domain-containing protein | Helix-turn-helix domain-containing protein | | afdb-uniprot50 | AF-A0A3P1T187-F1-MODEL\_V4 | 1.0 | 0.001079 | 168 | 0.333 | 63 | 40 | 1 | 11 | 73 | 5 | 65 | DNA-binding protein | DNA-binding protein | | afdb-uniprot50 | AF-A0A7V2T8E4-F1-MODEL\_V4 | 1.0 | 0.0002668 | 168 | 0.257 | 70 | 49 | 2 | 4 | 73 | 2 | 68 | DNA-binding protein | DNA-binding protein | | afdb-uniprot50 | AF-A0A7K2THJ8-F1-MODEL\_V4 | 1.0 | 0.0009444 | 168 | 0.291 | 72 | 49 | 1 | 1 | 72 | 3 | 72 | DNA-binding protein | DNA-binding protein | | afdb-uniprot50 | AF-A0A2N8LFG3-F1-MODEL\_V4 | 1.0 | 0.002562 | 167 | 0.333 | 54 | 34 | 1 | 10 | 63 | 3 | 54 | DNA-binding protein | DNA-binding protein | | afdb-uniprot50 | AF-A0A2I1V145-F1-MODEL\_V4 | 1.0 | 0.001009 | 167 | 0.278 | 61 | 41 | 2 | 7 | 66 | 2 | 60 | DNA-binding protein | DNA-binding protein | | afdb-uniprot50 | AF-A0A5C7L536-F1-MODEL\_V4 | 1.0 | 0.0006772 | 167 | 0.26 | 73 | 49 | 2 | 1 | 73 | 1 | 68 | DNA-binding protein | DNA-binding protein | | afdb-uniprot50 | AF-A0A5P9H8R0-F1-MODEL\_V4 | 1.0 | 0.0005928 | 167 | 0.205 | 73 | 56 | 1 | 1 | 73 | 1 | 71 | Helix-turn-helix domain protein | Helix-turn-helix domain protein | | afdb-uniprot50 | AF-A0A5B2Z9L9-F1-MODEL\_V4 | 1.0 | 0.0003482 | 167 | 0.308 | 68 | 44 | 2 | 7 | 73 | 1 | 66 | Helix-turn-helix domain-containing protein | Helix-turn-helix domain-containing protein | | afdb-uniprot50 | AF-A0A850RC49-F1-MODEL\_V4 | 1.0 | 0.0007238 | 167 | 0.271 | 70 | 49 | 1 | 1 | 70 | 4 | 71 | Helix-turn-helix domain-containing protein | Helix-turn-helix domain-containing protein | | afdb-uniprot50 | AF-A0A2N3VFX6-F1-MODEL\_V4 | 1.0 | 0.001009 | 167 | 0.343 | 64 | 38 | 2 | 1 | 62 | 5 | 66 | Helix-turn-helix protein | Helix-turn-helix protein | | afdb-uniprot50 | AF-A0A1Q3UQ13-F1-MODEL\_V4 | 1.0 | 0.0006772 | 167 | 0.256 | 78 | 47 | 1 | 7 | 73 | 2 | 79 | DNA-binding protein | DNA-binding protein | | afdb-uniprot50 | AF-A0A2H6HB99-F1-MODEL\_V4 | 1.0 | 0.001009 | 167 | 0.241 | 62 | 45 | 1 | 11 | 72 | 7 | 66 | Helix-turn-helix domain protein | Helix-turn-helix domain protein | | afdb-uniprot50 | AF-A0A7W3P5A7-F1-MODEL\_V4 | 1.0 | 0.002927 | 166 | 0.32 | 53 | 34 | 1 | 11 | 63 | 6 | 56 | Excisionase family DNA binding protein | Excisionase family DNA binding protein | | afdb-uniprot50 | AF-A0A1A0UL62-F1-MODEL\_V4 | 1.0 | 0.001317 | 166 | 0.343 | 64 | 40 | 1 | 10 | 73 | 4 | 65 | HTH\_17 domain-containing protein | HTH\_17 domain-containing protein | | afdb-uniprot50 | AF-A0A6G9XNP3-F1-MODEL\_V4 | 1.0 | 0.001719 | 166 | 0.25 | 64 | 46 | 1 | 9 | 72 | 5 | 66 | Helix-turn-helix domain-containing protein | Helix-turn-helix domain-containing protein | | afdb-uniprot50 | AF-A0A167I6B1-F1-MODEL\_V4 | 1.0 | 0.002098 | 166 | 0.292 | 65 | 44 | 1 | 9 | 73 | 6 | 68 | HTH\_17 domain-containing protein | HTH\_17 domain-containing protein | | afdb-uniprot50 | AF-A0A3A4K0Q0-F1-MODEL\_V4 | 1.0 | 0.001837 | 166 | 0.246 | 65 | 47 | 1 | 9 | 73 | 6 | 68 | DNA-binding protein | DNA-binding protein | | afdb-uniprot50 | AF-A0A060NMA2-F1-MODEL\_V4 | 1.0 | 0.0003482 | 166 | 0.328 | 73 | 44 | 2 | 4 | 73 | 1 | 71 | Uncharacterized protein | Uncharacterized protein | | afdb-uniprot50 | AF-A0A1G7RCE4-F1-MODEL\_V4 | 1.0 | 0.000519 | 166 | 0.25 | 76 | 50 | 2 | 1 | 71 | 2 | 75 | Helix-turn-helix domain-containing protein | Helix-turn-helix domain-containing protein | | afdb-uniprot50 | AF-A0A2Z6GBU1-F1-MODEL\_V4 | 1.0 | 0.001079 | 166 | 0.306 | 62 | 41 | 1 | 11 | 72 | 5 | 64 | Uncharacterized protein | Uncharacterized protein | | afdb-uniprot50 | AF-A0A1G6T1N9-F1-MODEL\_V4 | 1.0 | 0.0008268 | 166 | 0.287 | 73 | 50 | 1 | 1 | 73 | 11 | 81 | Helix-turn-helix domain-containing protein | Helix-turn-helix domain-containing protein | | afdb-uniprot50 | AF-A0A5M8SNF4-F1-MODEL\_V4 | 1.0 | 0.0004856 | 166 | 0.275 | 80 | 49 | 3 | 1 | 73 | 13 | 90 | Helix-turn-helix domain-containing protein | Helix-turn-helix domain-containing protein | | afdb-uniprot50 | AF-A0A1E4MWD2-F1-MODEL\_V4 | 1.0 | 0.003343 | 165 | 0.321 | 56 | 36 | 1 | 11 | 66 | 2 | 55 | HTH\_17 domain-containing protein | HTH\_17 domain-containing protein | | afdb-uniprot50 | AF-A0A0H2KQU9-F1-MODEL\_V4 | 1.0 | 0.002397 | 165 | 0.288 | 52 | 35 | 1 | 11 | 62 | 4 | 53 | HTH\_17 domain-containing protein | HTH\_17 domain-containing protein | | afdb-uniprot50 | AF-A0A5C7YER4-F1-MODEL\_V4 | 1.0 | 0.000519 | 165 | 0.315 | 73 | 44 | 4 | 1 | 73 | 1 | 67 | DNA-binding protein | DNA-binding protein | | afdb-uniprot50 | AF-A0A2U1SGD4-F1-MODEL\_V4 | 1.0 | 0.0007736 | 165 | 0.315 | 73 | 48 | 1 | 1 | 73 | 2 | 72 | Excisionase | Excisionase | | afdb-uniprot50 | AF-A0A7R7GJG0-F1-MODEL\_V4 | 1.0 | 0.000519 | 165 | 0.365 | 63 | 34 | 2 | 11 | 73 | 5 | 61 | Uncharacterized protein | Uncharacterized protein | | afdb-uniprot50 | AF-A0A7Y9R7W6-F1-MODEL\_V4 | 1.0 | 0.0003977 | 165 | 0.328 | 67 | 39 | 2 | 1 | 63 | 3 | 67 | Uncharacterized protein | Uncharacterized protein | | afdb-uniprot50 | AF-A0A2E3RBG2-F1-MODEL\_V4 | 1.0 | 0.006084 | 165 | 0.346 | 52 | 34 | 0 | 11 | 62 | 4 | 55 | DNA-binding protein | DNA-binding protein | | afdb-uniprot50 | AF-A0A2X4RW04-F1-MODEL\_V4 | 1.0 | 0.001079 | 165 | 0.285 | 63 | 43 | 1 | 1 | 63 | 1 | 61 | Regulatory protein MerR | Regulatory protein MerR | | afdb-uniprot50 | AF-A0A1F9M8Y5-F1-MODEL\_V4 | 1.0 | 0.001153 | 165 | 0.285 | 63 | 43 | 1 | 1 | 63 | 6 | 66 | HTH\_17 domain-containing protein | HTH\_17 domain-containing protein | | afdb-uniprot50 | AF-A0A5J6ERQ1-F1-MODEL\_V4 | 1.0 | 0.0007238 | 165 | 0.287 | 66 | 46 | 1 | 2 | 67 | 13 | 77 | DNA-binding protein | DNA-binding protein | | afdb-uniprot50 | AF-A0A1E3Y8P5-F1-MODEL\_V4 | 1.0 | 0.0002336 | 165 | 0.217 | 78 | 54 | 3 | 1 | 73 | 1 | 76 | HTH\_17 domain-containing protein | HTH\_17 domain-containing protein | | afdb-uniprot50 | AF-A0A4P9SC28-F1-MODEL\_V4 | 1.0 | 0.0005547 | 165 | 0.26 | 73 | 51 | 2 | 1 | 72 | 11 | 81 | Helix-turn-helix domain-containing protein | Helix-turn-helix domain-containing protein | | afdb-uniprot50 | AF-A0A1M3GW08-F1-MODEL\_V4 | 1.0 | 0.000519 | 165 | 0.31 | 74 | 47 | 2 | 1 | 72 | 2 | 73 | Uncharacterized protein | Uncharacterized protein | | afdb-uniprot50 | AF-A0A7I7T0P9-F1-MODEL\_V4 | 1.0 | 0.0003482 | 165 | 0.283 | 74 | 49 | 2 | 1 | 72 | 48 | 119 | Uncharacterized protein | Uncharacterized protein | | afdb-uniprot50 | AF-A0A0M9VKJ4-F1-MODEL\_V4 | 1.0 | 0.0009444 | 164 | 0.412 | 63 | 35 | 1 | 1 | 63 | 1 | 61 | HTH\_17 domain-containing protein | HTH\_17 domain-containing protein | | afdb-uniprot50 | AF-A0A3T0DJV1-F1-MODEL\_V4 | 1.0 | 0.001837 | 164 | 0.266 | 60 | 41 | 2 | 7 | 65 | 2 | 59 | Excisionase | Excisionase | | afdb-uniprot50 | AF-A0A522CQA7-F1-MODEL\_V4 | 1.0 | 0.002562 | 164 | 0.271 | 59 | 41 | 1 | 11 | 69 | 5 | 61 | DNA-binding protein | DNA-binding protein | | afdb-uniprot50 | AF-A0A291RRP8-F1-MODEL\_V4 | 1.0 | 0.001317 | 164 | 0.272 | 66 | 45 | 2 | 1 | 65 | 1 | 64 | DNA-binding protein | DNA-binding protein | | afdb-uniprot50 | AF-A0A6I1KHJ9-F1-MODEL\_V4 | 1.0 | 0.0008268 | 164 | 0.314 | 70 | 46 | 1 | 4 | 73 | 1 | 68 | HTH\_17 domain-containing protein | HTH\_17 domain-containing protein | | afdb-uniprot50 | AF-A0A1J5V6Q7-F1-MODEL\_V4 | 1.0 | 0.0008268 | 164 | 0.313 | 67 | 43 | 2 | 1 | 66 | 1 | 65 | HTH\_17 domain-containing protein | HTH\_17 domain-containing protein | | afdb-uniprot50 | AF-A0A7Y6T9Q4-F1-MODEL\_V4 | 1.0 | 0.001317 | 164 | 0.276 | 65 | 43 | 2 | 1 | 63 | 1 | 63 | Helix-turn-helix domain-containing protein | Helix-turn-helix domain-containing protein | | afdb-uniprot50 | AF-A0A0N7LYK3-F1-MODEL\_V4 | 1.0 | 0.004081 | 164 | 0.309 | 55 | 36 | 1 | 9 | 63 | 5 | 57 | Helix-turn-helix domain protein | Helix-turn-helix domain protein | | afdb-uniprot50 | AF-F8AAE3-F1-MODEL\_V4 | 1.0 | 0.0007736 | 164 | 0.333 | 66 | 42 | 2 | 7 | 72 | 13 | 76 | HTH\_17 domain-containing protein | HTH\_17 domain-containing protein | | afdb-uniprot50 | AF-A0A0P7XPZ0-F1-MODEL\_V4 | 1.0 | 0.001009 | 164 | 0.276 | 65 | 45 | 1 | 2 | 66 | 13 | 75 | Putative site-specific integrase-resolvase | Putative site-specific integrase-resolvase | | afdb-uniprot50 | AF-A0A1M3H9S1-F1-MODEL\_V4 | 1.0 | 0.001009 | 164 | 0.349 | 63 | 36 | 2 | 11 | 73 | 5 | 62 | Uncharacterized protein | Uncharacterized protein | | afdb-uniprot50 | AF-A0A853UBA9-F1-MODEL\_V4 | 1.0 | 0.0002496 | 164 | 0.276 | 76 | 51 | 2 | 1 | 72 | 2 | 77 | Uncharacterized protein | Uncharacterized protein | | afdb-uniprot50 | AF-A0A843S3B5-F1-MODEL\_V4 | 1.0 | 0.0005547 | 164 | 0.253 | 75 | 52 | 2 | 1 | 73 | 1 | 73 | Helix-turn-helix domain-containing protein | Helix-turn-helix domain-containing protein | | afdb-uniprot50 | AF-A0A318WSP9-F1-MODEL\_V4 | 1.0 | 0.0006772 | 164 | 0.21 | 76 | 54 | 2 | 1 | 72 | 26 | 99 | AlpA family transcriptional regulator | AlpA family transcriptional regulator | | afdb-uniprot50 | AF-K2EJ62-F1-MODEL\_V4 | 1.0 | 0.0003721 | 164 | 0.315 | 73 | 44 | 3 | 1 | 73 | 58 | 124 | Uncharacterized protein | Uncharacterized protein | | afdb-uniprot50 | AF-D8F5B5-F1-MODEL\_V4 | 1.0 | 0.0001913 | 164 | 0.297 | 84 | 46 | 4 | 1 | 73 | 20 | 101 | Transcriptional regulator, MerR family | Transcriptional regulator, MerR family | | afdb-uniprot50 | AF-A0A1Y0CB63-F1-MODEL\_V4 | 1.0 | 0.001608 | 163 | 0.372 | 59 | 34 | 2 | 11 | 69 | 6 | 61 | DNA-binding protein | DNA-binding protein | | afdb-uniprot50 | AF-A0A7V0J3F2-F1-MODEL\_V4 | 1.0 | 0.003128 | 163 | 0.272 | 55 | 38 | 1 | 11 | 65 | 7 | 59 | DNA-binding protein | DNA-binding protein | | afdb-uniprot50 | AF-A0A387BVQ2-F1-MODEL\_V4 | 1.0 | 0.001719 | 163 | 0.295 | 61 | 41 | 1 | 3 | 63 | 2 | 60 | DNA-binding protein | DNA-binding protein | | afdb-uniprot50 | AF-A0A1X0H7C2-F1-MODEL\_V4 | 1.0 | 0.0005547 | 163 | 0.289 | 69 | 45 | 2 | 1 | 67 | 2 | 68 | DNA-binding protein | DNA-binding protein | | afdb-uniprot50 | AF-A0A347Q4W5-F1-MODEL\_V4 | 1.0 | 0.004662 | 163 | 0.327 | 55 | 35 | 1 | 9 | 63 | 7 | 59 | HTH\_17 domain-containing protein | HTH\_17 domain-containing protein | | afdb-uniprot50 | AF-A0A7Y9KJU0-F1-MODEL\_V4 | 1.0 | 0.0005928 | 163 | 0.32 | 78 | 47 | 2 | 1 | 73 | 1 | 77 | Putative DNA-binding transcriptional regulator AlpA | Putative DNA-binding transcriptional regulator AlpA | | afdb-uniprot50 | AF-A0A849C2U3-F1-MODEL\_V4 | 1.0 | 0.002397 | 163 | 0.265 | 64 | 45 | 1 | 10 | 73 | 7 | 68 | Helix-turn-helix domain-containing protein | Helix-turn-helix domain-containing protein | | afdb-uniprot50 | AF-A0A7Y6EM70-F1-MODEL\_V4 | 1.0 | 0.0004856 | 163 | 0.319 | 72 | 47 | 1 | 1 | 72 | 14 | 83 | Helix-turn-helix domain-containing protein | Helix-turn-helix domain-containing protein | | afdb-uniprot50 | AF-A0A316MH91-F1-MODEL\_V4 | 1.0 | 0.0005547 | 163 | 0.256 | 78 | 51 | 2 | 1 | 73 | 7 | 82 | DNA-binding protein | DNA-binding protein | | afdb-uniprot50 | AF-L0J4B7-F1-MODEL\_V4 | 1.0 | 0.001009 | 163 | 0.333 | 63 | 40 | 1 | 11 | 73 | 9 | 69 | Uncharacterized protein | Uncharacterized protein | | afdb-uniprot50 | AF-A0A3N6F8T7-F1-MODEL\_V4 | 1.0 | 0.002098 | 163 | 0.215 | 65 | 49 | 1 | 2 | 66 | 3 | 65 | Helix-turn-helix domain protein | Helix-turn-helix domain protein | | afdb-uniprot50 | AF-A0A6L5XLJ8-F1-MODEL\_V4 | 1.0 | 0.001317 | 162 | 0.36 | 61 | 37 | 1 | 12 | 72 | 2 | 60 | DNA-binding protein | DNA-binding protein | | afdb-uniprot50 | AF-A0A7C5CBG1-F1-MODEL\_V4 | 1.0 | 0.001408 | 162 | 0.344 | 61 | 38 | 1 | 12 | 72 | 1 | 59 | DNA-binding protein | DNA-binding protein | | afdb-uniprot50 | AF-A0A372MC99-F1-MODEL\_V4 | 1.0 | 0.002738 | 162 | 0.322 | 59 | 38 | 1 | 9 | 67 | 7 | 63 | DNA-binding protein | DNA-binding protein | | afdb-uniprot50 | AF-A0A4Q5Y382-F1-MODEL\_V4 | 1.0 | 0.001408 | 162 | 0.366 | 60 | 36 | 1 | 12 | 71 | 1 | 58 | DNA-binding protein | DNA-binding protein | | afdb-uniprot50 | AF-A0A0F2QGE5-F1-MODEL\_V4 | 1.0 | 0.001505 | 162 | 0.26 | 73 | 52 | 1 | 1 | 73 | 1 | 71 | HTH\_17 domain-containing protein | HTH\_17 domain-containing protein | | afdb-uniprot50 | AF-A0A853IUT5-F1-MODEL\_V4 | 1.0 | 0.0006772 | 162 | 0.305 | 72 | 47 | 2 | 1 | 72 | 1 | 69 | Helix-turn-helix domain-containing protein | Helix-turn-helix domain-containing protein | | afdb-uniprot50 | AF-A0A0S7CWQ4-F1-MODEL\_V4 | 1.0 | 0.001079 | 162 | 0.292 | 65 | 44 | 1 | 1 | 65 | 6 | 68 | HTH\_17 domain-containing protein | HTH\_17 domain-containing protein | | afdb-uniprot50 | AF-A0A321LRP0-F1-MODEL\_V4 | 1.0 | 0.0008268 | 162 | 0.3 | 70 | 47 | 1 | 4 | 73 | 2 | 69 | Excisionase | Excisionase | | afdb-uniprot50 | AF-A0A7X6SV09-F1-MODEL\_V4 | 1.0 | 0.0006336 | 162 | 0.226 | 75 | 54 | 3 | 1 | 73 | 2 | 74 | Helix-turn-helix domain-containing protein | Helix-turn-helix domain-containing protein | | afdb-uniprot50 | AF-A0A840A4S4-F1-MODEL\_V4 | 1.0 | 0.0008268 | 162 | 0.348 | 66 | 38 | 2 | 11 | 73 | 5 | 68 | Putative site-specific integrase-resolvase | Putative site-specific integrase-resolvase | | afdb-uniprot50 | AF-A0A7K1XH10-F1-MODEL\_V4 | 1.0 | 0.0009444 | 162 | 0.313 | 67 | 45 | 1 | 1 | 67 | 12 | 77 | Helix-turn-helix domain-containing protein | Helix-turn-helix domain-containing protein | | afdb-uniprot50 | AF-A0A259S496-F1-MODEL\_V4 | 1.0 | 0.0003257 | 162 | 0.297 | 74 | 49 | 2 | 1 | 73 | 1 | 72 | HTH\_17 domain-containing protein | HTH\_17 domain-containing protein | | afdb-uniprot50 | AF-A0A6V8MPT2-F1-MODEL\_V4 | 1.0 | 0.0008836 | 162 | 0.232 | 73 | 54 | 1 | 1 | 73 | 7 | 77 | HTH\_17 domain-containing protein | HTH\_17 domain-containing protein | | afdb-uniprot50 | AF-A0A7W1M9N3-F1-MODEL\_V4 | 1.0 | 0.001408 | 162 | 0.301 | 63 | 42 | 1 | 11 | 73 | 5 | 65 | DNA-binding protein | DNA-binding protein | | afdb-uniprot50 | AF-Q82S53-F1-MODEL\_V4 | 1.0 | 0.0005547 | 162 | 0.277 | 72 | 49 | 2 | 1 | 71 | 15 | 84 | Uncharacterized protein | Uncharacterized protein | | afdb-uniprot50 | AF-A0A4Q7G7Y9-F1-MODEL\_V4 | 1.0 | 0.001009 | 162 | 0.267 | 71 | 47 | 1 | 1 | 71 | 1 | 66 | DNA-binding protein | DNA-binding protein | | afdb-uniprot50 | AF-A0A315EIU2-F1-MODEL\_V4 | 1.0 | 0.0009444 | 162 | 0.348 | 66 | 38 | 3 | 1 | 63 | 15 | 78 | Uncharacterized protein | Uncharacterized protein | | afdb-uniprot50 | AF-A0A842HLN5-F1-MODEL\_V4 | 1.0 | 0.001505 | 161 | 0.301 | 63 | 42 | 1 | 11 | 73 | 5 | 65 | DNA-binding protein | DNA-binding protein | | afdb-uniprot50 | AF-A0A0Q4BJD0-F1-MODEL\_V4 | 1.0 | 0.002562 | 161 | 0.365 | 63 | 38 | 1 | 11 | 73 | 5 | 65 | HTH\_17 domain-containing protein | HTH\_17 domain-containing protein | | afdb-uniprot50 | AF-A0A4R2IPL2-F1-MODEL\_V4 | 1.0 | 0.001408 | 161 | 0.283 | 67 | 46 | 1 | 1 | 67 | 2 | 66 | Excisionase family DNA binding protein | Excisionase family DNA binding protein | | afdb-uniprot50 | AF-A0A1B8SCM6-F1-MODEL\_V4 | 1.0 | 0.001153 | 161 | 0.285 | 63 | 41 | 2 | 11 | 73 | 10 | 68 | HTH\_17 domain-containing protein | HTH\_17 domain-containing protein | | afdb-uniprot50 | AF-A0A7J9W6U6-F1-MODEL\_V4 | 1.0 | 0.003819 | 161 | 0.246 | 65 | 49 | 0 | 1 | 65 | 2 | 66 | Helix-turn-helix domain-containing protein | Helix-turn-helix domain-containing protein | | afdb-uniprot50 | AF-A0A2V8UL43-F1-MODEL\_V4 | 1.0 | 0.004081 | 161 | 0.333 | 51 | 32 | 1 | 11 | 61 | 4 | 52 | DNA-binding protein | DNA-binding protein | | afdb-uniprot50 | AF-A0A2H5F3C0-F1-MODEL\_V4 | 1.0 | 0.001317 | 161 | 0.191 | 73 | 57 | 1 | 1 | 73 | 1 | 71 | AlpA family transcriptional regulator | AlpA family transcriptional regulator | | afdb-uniprot50 | AF-A0A2N9N2Z5-F1-MODEL\_V4 | 1.0 | 0.001232 | 161 | 0.301 | 63 | 41 | 2 | 11 | 73 | 4 | 63 | HTH\_17 domain-containing protein | HTH\_17 domain-containing protein | | afdb-uniprot50 | AF-A0A315DB40-F1-MODEL\_V4 | 1.0 | 0.0009444 | 161 | 0.307 | 65 | 40 | 2 | 11 | 72 | 5 | 67 | Uncharacterized protein | Uncharacterized protein | | afdb-uniprot50 | AF-A0A7T1I020-F1-MODEL\_V4 | 1.0 | 0.001232 | 161 | 0.272 | 66 | 45 | 2 | 1 | 65 | 10 | 73 | Helix-turn-helix domain-containing protein | Helix-turn-helix domain-containing protein | | afdb-uniprot50 | AF-A0A0N1H048-F1-MODEL\_V4 | 1.0 | 0.0008836 | 161 | 0.298 | 67 | 46 | 1 | 2 | 68 | 13 | 78 | HTH\_17 domain-containing protein | HTH\_17 domain-containing protein | | afdb-uniprot50 | AF-A0A0Q8Q4S6-F1-MODEL\_V4 | 1.0 | 0.0005928 | 161 | 0.308 | 68 | 45 | 2 | 1 | 67 | 10 | 76 | HTH\_17 domain-containing protein | HTH\_17 domain-containing protein | | afdb-uniprot50 | AF-A3XET4-F1-MODEL\_V4 | 1.0 | 0.0008268 | 161 | 0.291 | 72 | 48 | 2 | 2 | 73 | 10 | 78 | Uncharacterized protein | Uncharacterized protein | | afdb-uniprot50 | AF-A0A2A4Z6F6-F1-MODEL\_V4 | 1.0 | 0.001079 | 161 | 0.26 | 73 | 49 | 2 | 1 | 73 | 1 | 68 | DNA-binding protein | DNA-binding protein | | afdb-uniprot50 | AF-A0A2X0SBJ5-F1-MODEL\_V4 | 1.0 | 0.000519 | 161 | 0.287 | 73 | 49 | 2 | 1 | 73 | 16 | 85 | Uncharacterized protein | Uncharacterized protein | | afdb-uniprot50 | AF-W0RB88-F1-MODEL\_V4 | 1.0 | 0.001232 | 161 | 0.253 | 71 | 51 | 1 | 1 | 71 | 12 | 80 | HTH\_17 domain-containing protein | HTH\_17 domain-containing protein | | afdb-uniprot50 | AF-A0A2V1NPH5-F1-MODEL\_V4 | 1.0 | 0.001608 | 161 | 0.311 | 61 | 41 | 1 | 9 | 69 | 30 | 89 | DNA-binding protein | DNA-binding protein | | afdb-uniprot50 | AF-A0A4S2B6Y8-F1-MODEL\_V4 | 1.0 | 0.0009444 | 161 | 0.343 | 64 | 38 | 2 | 2 | 63 | 10 | 71 | DNA-binding protein | DNA-binding protein | | afdb-uniprot50 | AF-A0A437M628-F1-MODEL\_V4 | 1.0 | 0.001009 | 161 | 0.243 | 74 | 53 | 2 | 1 | 73 | 16 | 87 | DNA-binding protein | DNA-binding protein | | afdb-uniprot50 | AF-S9ZET1-F1-MODEL\_V4 | 1.0 | 0.0006772 | 161 | 0.267 | 71 | 50 | 1 | 1 | 71 | 7 | 75 | Uncharacterized protein | Uncharacterized protein | | afdb-uniprot50 | AF-A0A7Z0JE43-F1-MODEL\_V4 | 1.0 | 0.002397 | 161 | 0.264 | 68 | 48 | 1 | 2 | 69 | 36 | 101 | HTH\_17 domain-containing protein | HTH\_17 domain-containing protein | | afdb-uniprot50 | AF-A0A1A2QLL9-F1-MODEL\_V4 | 1.0 | 0.001232 | 160 | 0.294 | 68 | 44 | 2 | 1 | 68 | 1 | 64 | HTH\_17 domain-containing protein | HTH\_17 domain-containing protein | | afdb-uniprot50 | AF-A0A4P8SWN0-F1-MODEL\_V4 | 1.0 | 0.001963 | 160 | 0.3 | 60 | 40 | 1 | 4 | 63 | 1 | 58 | Uncharacterized protein | Uncharacterized protein | | afdb-uniprot50 | AF-A0A3E0NRW3-F1-MODEL\_V4 | 1.0 | 0.001837 | 160 | 0.26 | 69 | 49 | 1 | 4 | 72 | 1 | 67 | DNA-binding protein | DNA-binding protein | | afdb-uniprot50 | AF-A0A7C6R8I4-F1-MODEL\_V4 | 1.0 | 0.001153 | 160 | 0.342 | 73 | 43 | 3 | 1 | 73 | 1 | 68 | Helix-turn-helix domain-containing protein | Helix-turn-helix domain-containing protein | | afdb-uniprot50 | AF-A0A850RHE4-F1-MODEL\_V4 | 1.0 | 0.001505 | 160 | 0.333 | 63 | 40 | 1 | 1 | 63 | 2 | 62 | Helix-turn-helix domain-containing protein | Helix-turn-helix domain-containing protein | | afdb-uniprot50 | AF-A0A2T7TRJ2-F1-MODEL\_V4 | 1.0 | 0.001505 | 160 | 0.328 | 67 | 40 | 2 | 7 | 73 | 2 | 63 | HTH\_17 domain-containing protein | HTH\_17 domain-containing protein | | afdb-uniprot50 | AF-A0A7C2GW26-F1-MODEL\_V4 | 1.0 | 0.001505 | 160 | 0.287 | 73 | 48 | 2 | 1 | 73 | 1 | 69 | Helix-turn-helix domain-containing protein | Helix-turn-helix domain-containing protein | | afdb-uniprot50 | AF-A0A6I3ZMZ5-F1-MODEL\_V4 | 1.0 | 0.001317 | 160 | 0.273 | 73 | 50 | 2 | 1 | 73 | 1 | 70 | Helix-turn-helix domain-containing protein | Helix-turn-helix domain-containing protein | | afdb-uniprot50 | AF-A0A4Q8QL74-F1-MODEL\_V4 | 1.0 | 0.001408 | 160 | 0.27 | 74 | 50 | 2 | 1 | 72 | 1 | 72 | DNA-binding protein | DNA-binding protein | | afdb-uniprot50 | AF-A0A1P8NCH9-F1-MODEL\_V4 | 1.0 | 0.001837 | 160 | 0.263 | 72 | 51 | 1 | 1 | 72 | 7 | 76 | HTH\_17 domain-containing protein | HTH\_17 domain-containing protein | | afdb-uniprot50 | AF-J0MNL2-F1-MODEL\_V4 | 1.0 | 0.003343 | 160 | 0.253 | 63 | 45 | 1 | 11 | 73 | 19 | 79 | Uncharacterized protein | Uncharacterized protein | | afdb-uniprot50 | AF-A0A1H1ZMM5-F1-MODEL\_V4 | 1.0 | 0.001232 | 160 | 0.263 | 72 | 51 | 1 | 1 | 72 | 18 | 87 | DNA binding domain-containing protein, excisionase family | DNA binding domain-containing protein, excisionase family | | afdb-uniprot50 | AF-K2DX72-F1-MODEL\_V4 | 1.0 | 0.002098 | 160 | 0.36 | 61 | 37 | 1 | 3 | 63 | 2 | 60 | Uncharacterized protein | Uncharacterized protein | | afdb-uniprot50 | AF-A0A437M765-F1-MODEL\_V4 | 1.0 | 0.002738 | 160 | 0.238 | 67 | 49 | 1 | 1 | 67 | 19 | 83 | Uncharacterized protein | Uncharacterized protein | | afdb-uniprot50 | AF-A0A2V9QRN0-F1-MODEL\_V4 | 1.0 | 0.001232 | 160 | 0.273 | 73 | 51 | 1 | 1 | 73 | 23 | 93 | HTH\_17 domain-containing protein | HTH\_17 domain-containing protein | | afdb-uniprot50 | AF-A0A238VQI5-F1-MODEL\_V4 | 1.0 | 0.002738 | 159 | 0.263 | 72 | 45 | 2 | 1 | 72 | 1 | 64 | Helix-turn-helix domain-containing protein | Helix-turn-helix domain-containing protein | | afdb-uniprot50 | AF-A0A7V9MYE0-F1-MODEL\_V4 | 1.0 | 0.002738 | 159 | 0.257 | 66 | 47 | 1 | 7 | 72 | 1 | 64 | Helix-turn-helix domain-containing protein | Helix-turn-helix domain-containing protein | | afdb-uniprot50 | AF-A0A1I1BCB6-F1-MODEL\_V4 | 1.0 | 0.001719 | 159 | 0.268 | 67 | 46 | 2 | 1 | 66 | 2 | 66 | Transcriptional regulator, AlpA family | Transcriptional regulator, AlpA family | | afdb-uniprot50 | AF-A0A1A2GHT1-F1-MODEL\_V4 | 1.0 | 0.004081 | 159 | 0.295 | 61 | 41 | 1 | 8 | 68 | 9 | 67 | HTH\_17 domain-containing protein | HTH\_17 domain-containing protein | | afdb-uniprot50 | AF-A0A4P9S7Y4-F1-MODEL\_V4 | 1.0 | 0.001317 | 159 | 0.363 | 66 | 39 | 2 | 7 | 71 | 2 | 65 | Helix-turn-helix domain-containing protein | Helix-turn-helix domain-containing protein | | afdb-uniprot50 | AF-I2ACQ1-F1-MODEL\_V4 | 1.0 | 0.001719 | 159 | 0.285 | 63 | 42 | 2 | 11 | 73 | 4 | 63 | HTH\_17 domain-containing protein | HTH\_17 domain-containing protein | | afdb-uniprot50 | AF-A0A7Y2W1R5-F1-MODEL\_V4 | 1.0 | 0.001963 | 159 | 0.306 | 62 | 41 | 1 | 1 | 62 | 1 | 60 | Helix-turn-helix domain-containing protein | Helix-turn-helix domain-containing protein | | afdb-uniprot50 | AF-A0A7L9BRT5-F1-MODEL\_V4 | 1.0 | 0.001232 | 159 | 0.328 | 70 | 45 | 1 | 4 | 73 | 1 | 68 | Helix-turn-helix domain-containing protein | Helix-turn-helix domain-containing protein | | afdb-uniprot50 | AF-A0A7M3MAM1-F1-MODEL\_V4 | 1.0 | 0.004983 | 159 | 0.24 | 54 | 41 | 0 | 10 | 63 | 17 | 70 | Uncharacterized protein | Uncharacterized protein | | afdb-uniprot50 | AF-A0A2G1QH89-F1-MODEL\_V4 | 1.0 | 0.0004543 | 159 | 0.269 | 78 | 50 | 3 | 1 | 73 | 1 | 76 | Transcriptional regulator | Transcriptional regulator | | afdb-uniprot50 | AF-A0A4Q7FM96-F1-MODEL\_V4 | 1.0 | 0.003343 | 159 | 0.311 | 61 | 40 | 1 | 5 | 65 | 13 | 71 | Transcriptional regulator | Transcriptional regulator | | afdb-uniprot50 | AF-A0A6N9BF07-F1-MODEL\_V4 | 1.0 | 0.0008268 | 159 | 0.266 | 75 | 51 | 2 | 1 | 73 | 1 | 73 | Helix-turn-helix domain-containing protein | Helix-turn-helix domain-containing protein | | afdb-uniprot50 | AF-A0A5N0E6B6-F1-MODEL\_V4 | 1.0 | 0.004662 | 159 | 0.28 | 57 | 39 | 1 | 9 | 65 | 5 | 59 | Helix-turn-helix domain-containing protein | Helix-turn-helix domain-containing protein | | afdb-uniprot50 | AF-A0A2U9NH93-F1-MODEL\_V4 | 1.0 | 0.0009444 | 159 | 0.292 | 65 | 42 | 2 | 1 | 63 | 9 | 71 | DNA-binding protein | DNA-binding protein | | afdb-uniprot50 | AF-A0A2E1IAE1-F1-MODEL\_V4 | 1.0 | 0.0005928 | 158 | 0.306 | 62 | 41 | 1 | 4 | 65 | 2 | 61 | DNA-binding protein | DNA-binding protein | | afdb-uniprot50 | AF-A0A366EM39-F1-MODEL\_V4 | 1.0 | 0.001719 | 158 | 0.349 | 63 | 39 | 1 | 11 | 73 | 7 | 67 | Helix-turn-helix protein | Helix-turn-helix protein | | afdb-uniprot50 | AF-A0A1H1LN62-F1-MODEL\_V4 | 1.0 | 0.001608 | 158 | 0.257 | 66 | 47 | 1 | 2 | 67 | 4 | 67 | Helix-turn-helix domain-containing protein | Helix-turn-helix domain-containing protein | | afdb-uniprot50 | AF-A0A2K8UDT2-F1-MODEL\_V4 | 1.0 | 0.0003721 | 158 | 0.301 | 73 | 43 | 3 | 1 | 73 | 1 | 65 | HTH\_17 domain-containing protein | HTH\_17 domain-containing protein | | afdb-uniprot50 | AF-T0HS16-F1-MODEL\_V4 | 1.0 | 0.001232 | 158 | 0.208 | 72 | 55 | 1 | 2 | 73 | 6 | 75 | Uncharacterized protein | Uncharacterized protein | | afdb-uniprot50 | AF-A0A6P0GKB9-F1-MODEL\_V4 | 1.0 | 0.002243 | 158 | 0.219 | 73 | 55 | 1 | 1 | 73 | 4 | 74 | Helix-turn-helix domain-containing protein | Helix-turn-helix domain-containing protein | | afdb-uniprot50 | AF-A0A286EZU0-F1-MODEL\_V4 | 1.0 | 0.001608 | 158 | 0.318 | 66 | 43 | 1 | 2 | 67 | 13 | 76 | Helix-turn-helix domain-containing protein | Helix-turn-helix domain-containing protein | | afdb-uniprot50 | AF-A0A7U3LS04-F1-MODEL\_V4 | 1.0 | 0.001837 | 158 | 0.287 | 66 | 43 | 2 | 10 | 73 | 4 | 67 | Uncharacterized protein | Uncharacterized protein | | afdb-uniprot50 | AF-A0A562C335-F1-MODEL\_V4 | 1.0 | 0.0008268 | 158 | 0.26 | 73 | 49 | 2 | 1 | 73 | 11 | 78 | AlpA family transcriptional regulator | AlpA family transcriptional regulator | | afdb-uniprot50 | AF-A0A4T0UKW9-F1-MODEL\_V4 | 1.0 | 0.0004251 | 158 | 0.309 | 71 | 44 | 3 | 1 | 67 | 8 | 77 | Helix-turn-helix domain-containing protein | Helix-turn-helix domain-containing protein | | afdb-uniprot50 | AF-A0A1M3IN55-F1-MODEL\_V4 | 1.0 | 0.001837 | 158 | 0.323 | 65 | 42 | 1 | 7 | 71 | 1 | 63 | HTH\_17 domain-containing protein | HTH\_17 domain-containing protein | | afdb-uniprot50 | AF-A0A1G7WPJ9-F1-MODEL\_V4 | 1.0 | 0.001505 | 158 | 0.26 | 73 | 51 | 2 | 1 | 73 | 13 | 82 | DNA binding domain-containing protein, excisionase family | DNA binding domain-containing protein, excisionase family | | afdb-uniprot50 | AF-A0A512T4M0-F1-MODEL\_V4 | 1.0 | 0.001408 | 158 | 0.26 | 69 | 49 | 1 | 4 | 72 | 1 | 67 | HTH\_17 domain-containing protein | HTH\_17 domain-containing protein | | afdb-uniprot50 | AF-A0A7Z0EDB2-F1-MODEL\_V4 | 1.0 | 0.001963 | 158 | 0.338 | 62 | 39 | 1 | 1 | 62 | 42 | 101 | Putative DNA-binding transcriptional regulator AlpA | Putative DNA-binding transcriptional regulator AlpA | | afdb-uniprot50 | AF-A0A7Y9SJU6-F1-MODEL\_V4 | 1.0 | 0.002738 | 157 | 0.338 | 62 | 39 | 1 | 1 | 62 | 1 | 60 | Excisionase family DNA binding protein | Excisionase family DNA binding protein | | afdb-uniprot50 | AF-A0A1B8SBD0-F1-MODEL\_V4 | 1.0 | 0.002243 | 157 | 0.285 | 70 | 44 | 2 | 4 | 73 | 1 | 64 | Excisionase | Excisionase | | afdb-uniprot50 | AF-A0A1V3NRC7-F1-MODEL\_V4 | 1.0 | 0.002243 | 157 | 0.206 | 63 | 48 | 1 | 11 | 73 | 6 | 66 | DNA-binding protein | DNA-binding protein | | afdb-uniprot50 | AF-Q5Z3A8-F1-MODEL\_V4 | 1.0 | 0.002243 | 157 | 0.317 | 63 | 40 | 2 | 1 | 63 | 1 | 60 | HTH\_17 domain-containing protein | HTH\_17 domain-containing protein | | afdb-uniprot50 | AF-A0A7G6U3R5-F1-MODEL\_V4 | 1.0 | 0.002562 | 157 | 0.222 | 63 | 47 | 1 | 1 | 63 | 3 | 63 | Helix-turn-helix domain-containing protein | Helix-turn-helix domain-containing protein | | afdb-uniprot50 | AF-A0A5N6S6V5-F1-MODEL\_V4 | 1.0 | 0.0006336 | 157 | 0.287 | 73 | 49 | 2 | 1 | 73 | 2 | 71 | DNA-binding protein | DNA-binding protein | | afdb-uniprot50 | AF-A0A4R5PE23-F1-MODEL\_V4 | 1.0 | 0.0009444 | 157 | 0.301 | 73 | 47 | 3 | 1 | 72 | 1 | 70 | DNA-binding protein | DNA-binding protein | | afdb-uniprot50 | AF-A0A2A3D3G3-F1-MODEL\_V4 | 1.0 | 0.001232 | 157 | 0.297 | 74 | 49 | 2 | 1 | 73 | 1 | 72 | Uncharacterized protein | Uncharacterized protein | | afdb-uniprot50 | AF-A0A4Y3UN51-F1-MODEL\_V4 | 1.0 | 0.0005547 | 157 | 0.306 | 75 | 48 | 2 | 1 | 73 | 1 | 73 | Uncharacterized protein | Uncharacterized protein | | afdb-uniprot50 | AF-A0A1I6Z5G2-F1-MODEL\_V4 | 1.0 | 0.001317 | 157 | 0.323 | 68 | 41 | 1 | 11 | 73 | 6 | 73 | Uncharacterized protein | Uncharacterized protein | | afdb-uniprot50 | AF-A0A3S0GSK5-F1-MODEL\_V4 | 1.0 | 0.002562 | 157 | 0.264 | 68 | 48 | 1 | 5 | 72 | 11 | 76 | DNA-binding protein | DNA-binding protein | | afdb-uniprot50 | AF-A0A7C3Q0U4-F1-MODEL\_V4 | 1.0 | 0.0007238 | 157 | 0.25 | 76 | 51 | 2 | 2 | 73 | 10 | 83 | DNA-binding protein | DNA-binding protein | | afdb-uniprot50 | AF-A0A1V4XWG2-F1-MODEL\_V4 | 1.0 | 0.002098 | 157 | 0.328 | 64 | 40 | 2 | 2 | 62 | 17 | 80 | Uncharacterized protein | Uncharacterized protein | | afdb-uniprot50 | AF-A0A7D5MY55-F1-MODEL\_V4 | 1.0 | 0.001963 | 157 | 0.303 | 66 | 43 | 2 | 9 | 73 | 2 | 65 | Helix-turn-helix domain-containing protein | Helix-turn-helix domain-containing protein | | afdb-uniprot50 | AF-A0A315BJ48-F1-MODEL\_V4 | 1.0 | 0.0003721 | 157 | 0.301 | 83 | 46 | 4 | 1 | 73 | 40 | 120 | Uncharacterized protein | Uncharacterized protein | | afdb-uniprot50 | AF-A0A1H3G4F0-F1-MODEL\_V4 | 1.0 | 0.003128 | 156 | 0.245 | 61 | 44 | 1 | 7 | 67 | 1 | 59 | Transcriptional regulator, AlpA family | Transcriptional regulator, AlpA family | | afdb-uniprot50 | AF-A0A1I6Y1L1-F1-MODEL\_V4 | 1.0 | 0.001009 | 156 | 0.338 | 62 | 37 | 2 | 11 | 72 | 6 | 63 | Transcriptional regulator, AlpA family | Transcriptional regulator, AlpA family | | afdb-uniprot50 | AF-S3AQA5-F1-MODEL\_V4 | 1.0 | 0.002927 | 156 | 0.253 | 63 | 45 | 1 | 1 | 63 | 3 | 63 | Excisionase family DNA binding domain-containing protein | Excisionase family DNA binding domain-containing protein | | afdb-uniprot50 | AF-A0A380MTT9-F1-MODEL\_V4 | 1.0 | 0.002243 | 156 | 0.292 | 65 | 42 | 2 | 1 | 65 | 2 | 62 | Excisionase or Xis | Excisionase or Xis | | afdb-uniprot50 | AF-A0A1Q8JCF0-F1-MODEL\_V4 | 1.0 | 0.0009444 | 156 | 0.268 | 67 | 46 | 2 | 1 | 66 | 1 | 65 | HTH\_17 domain-containing protein | HTH\_17 domain-containing protein | | afdb-uniprot50 | AF-A0A7Y0DBL9-F1-MODEL\_V4 | 1.0 | 0.001408 | 156 | 0.303 | 66 | 42 | 2 | 1 | 64 | 1 | 64 | Helix-turn-helix domain-containing protein | Helix-turn-helix domain-containing protein | | afdb-uniprot50 | AF-A0A150HHP1-F1-MODEL\_V4 | 1.0 | 0.001837 | 156 | 0.253 | 63 | 44 | 2 | 11 | 72 | 7 | 67 | Helix-turn-helix domain protein | Helix-turn-helix domain protein | | afdb-uniprot50 | AF-A0A494RQT9-F1-MODEL\_V4 | 1.0 | 0.001408 | 156 | 0.367 | 68 | 40 | 2 | 1 | 67 | 1 | 66 | DNA-binding protein | DNA-binding protein | | afdb-uniprot50 | AF-A0A2A4V5K8-F1-MODEL\_V4 | 1.0 | 0.001408 | 156 | 0.253 | 71 | 48 | 3 | 4 | 73 | 2 | 68 | HTH\_17 domain-containing protein | HTH\_17 domain-containing protein | | afdb-uniprot50 | AF-A0A3S4FDS9-F1-MODEL\_V4 | 1.0 | 0.002243 | 156 | 0.313 | 67 | 43 | 2 | 7 | 72 | 2 | 66 | HTH\_17 domain-containing protein | HTH\_17 domain-containing protein | | afdb-uniprot50 | AF-A0A5C7LIH8-F1-MODEL\_V4 | 1.0 | 0.001232 | 156 | 0.232 | 73 | 51 | 3 | 1 | 73 | 1 | 68 | DNA-binding protein | DNA-binding protein | | afdb-uniprot50 | AF-A0A6L5G2Y0-F1-MODEL\_V4 | 1.0 | 0.003819 | 156 | 0.3 | 60 | 39 | 2 | 7 | 65 | 2 | 59 | Helix-turn-helix domain-containing protein | Helix-turn-helix domain-containing protein | | afdb-uniprot50 | AF-A0A1A0KLC7-F1-MODEL\_V4 | 1.0 | 0.004662 | 156 | 0.222 | 63 | 47 | 1 | 11 | 73 | 7 | 67 | HTH\_17 domain-containing protein | HTH\_17 domain-containing protein | | afdb-uniprot50 | AF-A0A4U2Z9B1-F1-MODEL\_V4 | 1.0 | 0.002562 | 156 | 0.238 | 63 | 46 | 1 | 1 | 63 | 5 | 65 | Helix-turn-helix domain-containing protein | Helix-turn-helix domain-containing protein | | afdb-uniprot50 | AF-A0A4Y8QXZ4-F1-MODEL\_V4 | 1.0 | 0.001505 | 156 | 0.26 | 69 | 48 | 2 | 1 | 68 | 2 | 68 | DNA-binding protein | DNA-binding protein | | afdb-uniprot50 | AF-A0A2W7IHD9-F1-MODEL\_V4 | 1.0 | 0.0008836 | 156 | 0.315 | 76 | 43 | 2 | 1 | 69 | 1 | 74 | Helix-turn-helix protein | Helix-turn-helix protein | | afdb-uniprot50 | AF-A0A5B8RKC5-F1-MODEL\_V4 | 1.0 | 0.0007736 | 156 | 0.26 | 73 | 50 | 2 | 3 | 73 | 6 | 76 | HTH\_17 domain-containing protein | HTH\_17 domain-containing protein | | afdb-uniprot50 | AF-A0A4Q2SX89-F1-MODEL\_V4 | 1.0 | 0.001408 | 156 | 0.257 | 70 | 47 | 2 | 2 | 67 | 5 | 73 | DNA-binding protein | DNA-binding protein | | afdb-uniprot50 | AF-A0A7G7BH93-F1-MODEL\_V4 | 1.0 | 0.004362 | 156 | 0.305 | 59 | 39 | 1 | 9 | 67 | 21 | 77 | Helix-turn-helix domain-containing protein | Helix-turn-helix domain-containing protein | | afdb-uniprot50 | AF-A0A6M3XPC7-F1-MODEL\_V4 | 1.0 | 0.0009444 | 156 | 0.273 | 73 | 49 | 3 | 1 | 73 | 8 | 76 | Putative DNA binding, helix-turn-helix domain containing protein | Putative DNA binding, helix-turn-helix domain containing protein | | afdb-uniprot50 | AF-A0A1T4VUB8-F1-MODEL\_V4 | 1.0 | 0.004983 | 156 | 0.245 | 61 | 44 | 1 | 2 | 62 | 19 | 77 | Helix-turn-helix domain-containing protein | Helix-turn-helix domain-containing protein | | afdb-uniprot50 | AF-A0A4R9QI88-F1-MODEL\_V4 | 1.0 | 0.002098 | 156 | 0.275 | 69 | 48 | 1 | 5 | 73 | 11 | 77 | DNA-binding protein | DNA-binding protein | | afdb-uniprot50 | AF-A0A285VVA6-F1-MODEL\_V4 | 1.0 | 0.001963 | 156 | 0.253 | 67 | 47 | 2 | 1 | 67 | 28 | 91 | DNA binding domain-containing protein, excisionase family | DNA binding domain-containing protein, excisionase family | | afdb-uniprot50 | AF-A0A7X7D5U4-F1-MODEL\_V4 | 1.0 | 0.001079 | 156 | 0.293 | 75 | 49 | 2 | 1 | 73 | 3 | 75 | Helix-turn-helix domain-containing protein | Helix-turn-helix domain-containing protein | | afdb-uniprot50 | AF-A0A7X4A2Z2-F1-MODEL\_V4 | 1.0 | 0.0008268 | 156 | 0.256 | 74 | 52 | 2 | 1 | 73 | 34 | 105 | Helix-turn-helix domain-containing protein | Helix-turn-helix domain-containing protein | | afdb-uniprot50 | AF-A0A6N6VFE6-F1-MODEL\_V4 | 1.0 | 0.003343 | 155 | 0.285 | 63 | 43 | 1 | 11 | 73 | 4 | 64 | Helix-turn-helix domain-containing protein | Helix-turn-helix domain-containing protein | | afdb-uniprot50 | AF-A0A412KDG0-F1-MODEL\_V4 | 1.0 | 0.002098 | 155 | 0.333 | 63 | 38 | 2 | 12 | 72 | 2 | 62 | DNA-binding protein | DNA-binding protein | | afdb-uniprot50 | AF-A0A087BZX1-F1-MODEL\_V4 | 1.0 | 0.002397 | 155 | 0.318 | 66 | 43 | 1 | 7 | 72 | 2 | 65 | DNA binding domain, excisionase family | DNA binding domain, excisionase family | | afdb-uniprot50 | AF-A0A2U2MS54-F1-MODEL\_V4 | 1.0 | 0.003573 | 155 | 0.328 | 64 | 40 | 3 | 10 | 73 | 6 | 66 | Excisionase | Excisionase | | afdb-uniprot50 | AF-A0A1F1ZUF8-F1-MODEL\_V4 | 1.0 | 0.002397 | 155 | 0.246 | 65 | 46 | 2 | 10 | 73 | 4 | 66 | HTH\_17 domain-containing protein | HTH\_17 domain-containing protein | | afdb-uniprot50 | AF-F5XSJ0-F1-MODEL\_V4 | 1.0 | 0.001719 | 155 | 0.242 | 70 | 50 | 2 | 4 | 72 | 2 | 69 | HTH\_17 domain-containing protein | HTH\_17 domain-containing protein | | afdb-uniprot50 | AF-A0A4R6RE54-F1-MODEL\_V4 | 1.0 | 0.002927 | 155 | 0.262 | 61 | 43 | 1 | 11 | 71 | 5 | 63 | Helix-turn-helix protein | Helix-turn-helix protein | | afdb-uniprot50 | AF-A0A2G1YXW8-F1-MODEL\_V4 | 1.0 | 0.001408 | 155 | 0.318 | 69 | 41 | 2 | 9 | 73 | 2 | 68 | DNA-binding protein | DNA-binding protein | | afdb-uniprot50 | AF-A0A2V9L6Q6-F1-MODEL\_V4 | 1.0 | 0.001963 | 155 | 0.285 | 70 | 48 | 1 | 4 | 73 | 2 | 69 | HTH\_17 domain-containing protein | HTH\_17 domain-containing protein | | afdb-uniprot50 | AF-A0A0T1UKU9-F1-MODEL\_V4 | 1.0 | 0.001719 | 155 | 0.257 | 66 | 48 | 1 | 2 | 67 | 7 | 71 | DNA-binding protein | DNA-binding protein | | afdb-uniprot50 | AF-A0A526W0E5-F1-MODEL\_V4 | 1.0 | 0.001079 | 155 | 0.342 | 73 | 45 | 2 | 1 | 73 | 1 | 70 | Helix-turn-helix domain-containing protein | Helix-turn-helix domain-containing protein | | afdb-uniprot50 | AF-A0A6N0YFG5-F1-MODEL\_V4 | 1.0 | 0.003343 | 155 | 0.344 | 58 | 36 | 1 | 6 | 63 | 12 | 67 | DNA-binding protein | DNA-binding protein | | afdb-uniprot50 | AF-A0A4P6KDJ9-F1-MODEL\_V4 | 1.0 | 0.001153 | 155 | 0.205 | 73 | 55 | 2 | 1 | 73 | 4 | 73 | DNA-binding protein | DNA-binding protein | | afdb-uniprot50 | AF-A0A3G9G7L5-F1-MODEL\_V4 | 1.0 | 0.004081 | 155 | 0.258 | 62 | 42 | 2 | 9 | 70 | 15 | 72 | HTH\_17 domain-containing protein | HTH\_17 domain-containing protein | | afdb-uniprot50 | AF-A0A6A0QUD0-F1-MODEL\_V4 | 1.0 | 0.003128 | 155 | 0.25 | 64 | 46 | 1 | 2 | 65 | 15 | 76 | DNA-binding protein | DNA-binding protein | | afdb-uniprot50 | AF-A0A7Y0HTB1-F1-MODEL\_V4 | 1.0 | 0.003573 | 155 | 0.261 | 65 | 45 | 1 | 9 | 73 | 20 | 81 | Uncharacterized protein | Uncharacterized protein | | afdb-uniprot50 | AF-H6RNM7-F1-MODEL\_V4 | 1.0 | 0.002738 | 155 | 0.235 | 68 | 50 | 1 | 2 | 69 | 18 | 83 | Transcriptional regulator, MerR family | Transcriptional regulator, MerR family | | afdb-uniprot50 | AF-B7J9L7-F1-MODEL\_V4 | 1.0 | 0.001079 | 155 | 0.291 | 72 | 49 | 1 | 2 | 73 | 35 | 104 | Uncharacterized protein | Uncharacterized protein | | afdb-uniprot50 | AF-A0A2G4YWK8-F1-MODEL\_V4 | 1.0 | 0.003128 | 155 | 0.208 | 67 | 51 | 1 | 5 | 71 | 21 | 85 | HTH\_17 domain-containing protein | HTH\_17 domain-containing protein | | afdb-uniprot50 | AF-A0A3A5AUL8-F1-MODEL\_V4 | 1.0 | 0.003343 | 154 | 0.253 | 63 | 46 | 1 | 11 | 73 | 3 | 64 | Uncharacterized protein | Uncharacterized protein | | afdb-uniprot50 | AF-A0A1X1TZH7-F1-MODEL\_V4 | 1.0 | 0.002397 | 154 | 0.318 | 66 | 41 | 2 | 1 | 66 | 1 | 62 | Uncharacterized protein | Uncharacterized protein | | afdb-uniprot50 | AF-A0A100VZK0-F1-MODEL\_V4 | 1.0 | 0.001963 | 154 | 0.301 | 73 | 46 | 2 | 1 | 73 | 1 | 68 | HTH\_17 domain-containing protein | HTH\_17 domain-containing protein | | afdb-uniprot50 | AF-A0A2D6BJI6-F1-MODEL\_V4 | 1.0 | 0.001505 | 154 | 0.285 | 63 | 39 | 2 | 11 | 73 | 4 | 60 | HTH\_17 domain-containing protein | HTH\_17 domain-containing protein | | afdb-uniprot50 | AF-A0A517SMF5-F1-MODEL\_V4 | 1.0 | 0.0007736 | 154 | 0.243 | 74 | 53 | 2 | 1 | 73 | 1 | 72 | Helix-turn-helix domain protein | Helix-turn-helix domain protein | | afdb-uniprot50 | AF-A0A833FY91-F1-MODEL\_V4 | 1.0 | 0.002098 | 154 | 0.277 | 72 | 46 | 2 | 1 | 72 | 1 | 66 | Helix-turn-helix domain-containing protein | Helix-turn-helix domain-containing protein | | afdb-uniprot50 | AF-A0A849DSH8-F1-MODEL\_V4 | 1.0 | 0.0007736 | 154 | 0.232 | 73 | 54 | 2 | 1 | 73 | 4 | 74 | Helix-turn-helix domain-containing protein | Helix-turn-helix domain-containing protein | | afdb-uniprot50 | AF-A0A7X5U2M3-F1-MODEL\_V4 | 1.0 | 0.0006336 | 154 | 0.31 | 74 | 43 | 3 | 2 | 73 | 10 | 77 | Excisionase family DNA binding protein | Excisionase family DNA binding protein | | afdb-uniprot50 | AF-A0A4Q2KLM2-F1-MODEL\_V4 | 1.0 | 0.002397 | 154 | 0.311 | 77 | 45 | 3 | 1 | 72 | 1 | 74 | DNA-binding protein | DNA-binding protein | | afdb-uniprot50 | AF-A0A1G6EPZ3-F1-MODEL\_V4 | 1.0 | 0.0008268 | 154 | 0.258 | 85 | 49 | 3 | 1 | 73 | 1 | 83 | Uncharacterized protein | Uncharacterized protein | | afdb-uniprot50 | AF-A0A024H216-F1-MODEL\_V4 | 1.0 | 0.002562 | 154 | 0.265 | 64 | 45 | 1 | 10 | 73 | 23 | 84 | Uncharacterized domain protein | Uncharacterized domain protein | | afdb-uniprot50 | AF-A0A4R4DRZ7-F1-MODEL\_V4 | 1.0 | 0.001505 | 154 | 0.3 | 70 | 47 | 1 | 2 | 71 | 10 | 77 | DNA-binding protein | DNA-binding protein | | afdb-uniprot50 | AF-A0A2N3GRQ9-F1-MODEL\_V4 | 1.0 | 0.002243 | 154 | 0.26 | 69 | 48 | 2 | 5 | 73 | 5 | 70 | HTH\_17 domain-containing protein | HTH\_17 domain-containing protein | | afdb-uniprot50 | AF-A0A2A9DZ10-F1-MODEL\_V4 | 1.0 | 0.001079 | 154 | 0.191 | 73 | 56 | 2 | 1 | 73 | 28 | 97 | Excisionase family DNA binding protein | Excisionase family DNA binding protein | | afdb-uniprot50 | AF-A0A2L2Q153-F1-MODEL\_V4 | 1.0 | 0.001837 | 154 | 0.257 | 66 | 46 | 2 | 1 | 65 | 31 | 94 | DNA-binding protein | DNA-binding protein | | afdb-uniprot50 | AF-A0A2A4SJ14-F1-MODEL\_V4 | 1.0 | 0.001317 | 154 | 0.194 | 77 | 55 | 2 | 1 | 72 | 17 | 91 | HTH\_17 domain-containing protein | HTH\_17 domain-containing protein | | afdb-uniprot50 | AF-A0A2D6EPB2-F1-MODEL\_V4 | 1.0 | 0.004662 | 154 | 0.258 | 62 | 44 | 1 | 2 | 63 | 40 | 99 | HTH\_17 domain-containing protein | HTH\_17 domain-containing protein | | afdb-uniprot50 | AF-A0A7W9JAP3-F1-MODEL\_V4 | 1.0 | 0.002098 | 154 | 0.289 | 76 | 49 | 2 | 1 | 73 | 1 | 74 | Uncharacterized protein | Uncharacterized protein | | afdb-uniprot50 | AF-A0A4R4WWB4-F1-MODEL\_V4 | 1.0 | 0.005692 | 153 | 0.327 | 55 | 35 | 2 | 11 | 65 | 8 | 60 | DNA-binding protein | DNA-binding protein | | afdb-uniprot50 | AF-A0A0B2AB94-F1-MODEL\_V4 | 1.0 | 0.003343 | 153 | 0.317 | 63 | 41 | 2 | 12 | 73 | 1 | 62 | Uncharacterized protein | Uncharacterized protein | | afdb-uniprot50 | AF-A0A7S7TXP9-F1-MODEL\_V4 | 1.0 | 0.004983 | 153 | 0.285 | 63 | 42 | 2 | 11 | 73 | 4 | 63 | DNA-binding protein | DNA-binding protein | | afdb-uniprot50 | AF-A3VLE0-F1-MODEL\_V4 | 1.0 | 0.002243 | 153 | 0.242 | 70 | 48 | 3 | 4 | 73 | 2 | 66 | HTH\_17 domain-containing protein | HTH\_17 domain-containing protein | | afdb-uniprot50 | AF-A0A1X1RN46-F1-MODEL\_V4 | 1.0 | 0.001009 | 153 | 0.314 | 70 | 45 | 2 | 4 | 72 | 1 | 68 | HTH merR-type domain-containing protein | HTH merR-type domain-containing protein | | afdb-uniprot50 | AF-A0A7X6MSW7-F1-MODEL\_V4 | 1.0 | 0.005692 | 153 | 0.254 | 59 | 42 | 1 | 4 | 62 | 2 | 58 | Helix-turn-helix domain-containing protein | Helix-turn-helix domain-containing protein | | afdb-uniprot50 | AF-A0A6I0ENH1-F1-MODEL\_V4 | 1.0 | 0.003343 | 153 | 0.228 | 70 | 52 | 1 | 4 | 73 | 2 | 69 | Helix-turn-helix domain-containing protein | Helix-turn-helix domain-containing protein | | afdb-uniprot50 | AF-A0A1Q9W072-F1-MODEL\_V4 | 1.0 | 0.002738 | 153 | 0.189 | 74 | 55 | 3 | 1 | 73 | 1 | 70 | HTH\_17 domain-containing protein | HTH\_17 domain-containing protein | | afdb-uniprot50 | AF-A0A1H3DCW2-F1-MODEL\_V4 | 1.0 | 0.0007736 | 153 | 0.356 | 73 | 41 | 3 | 1 | 73 | 1 | 67 | Helix-turn-helix domain-containing protein | Helix-turn-helix domain-containing protein | | afdb-uniprot50 | AF-H5USK6-F1-MODEL\_V4 | 1.0 | 0.002397 | 153 | 0.222 | 63 | 46 | 2 | 1 | 63 | 12 | 71 | HTH\_17 domain-containing protein | HTH\_17 domain-containing protein | | afdb-uniprot50 | AF-A0A0N8HY41-F1-MODEL\_V4 | 1.0 | 0.001408 | 153 | 0.279 | 68 | 46 | 2 | 1 | 67 | 8 | 73 | Uncharacterized protein | Uncharacterized protein | | afdb-uniprot50 | AF-A0A2W7C802-F1-MODEL\_V4 | 1.0 | 0.0005928 | 153 | 0.283 | 74 | 49 | 2 | 1 | 72 | 4 | 75 | Uncharacterized protein | Uncharacterized protein | | afdb-uniprot50 | AF-A0A355T8Q2-F1-MODEL\_V4 | 1.0 | 0.001317 | 153 | 0.323 | 71 | 42 | 2 | 7 | 73 | 2 | 70 | DNA-binding protein | DNA-binding protein | | afdb-uniprot50 | AF-A0A366ZRD9-F1-MODEL\_V4 | 1.0 | 0.002397 | 153 | 0.235 | 68 | 49 | 2 | 1 | 67 | 6 | 71 | DNA-binding protein | DNA-binding protein | | afdb-uniprot50 | AF-A0A0Q8WXY6-F1-MODEL\_V4 | 1.0 | 0.002243 | 153 | 0.24 | 79 | 52 | 2 | 1 | 73 | 1 | 77 | HTH\_17 domain-containing protein | HTH\_17 domain-containing protein | | afdb-uniprot50 | AF-A0A2A3I5W9-F1-MODEL\_V4 | 1.0 | 0.003343 | 153 | 0.287 | 66 | 45 | 1 | 2 | 67 | 17 | 80 | Excisionase family DNA binding protein | Excisionase family DNA binding protein | | afdb-uniprot50 | AF-A0A2N8K0S6-F1-MODEL\_V4 | 1.0 | 0.001153 | 153 | 0.266 | 75 | 51 | 3 | 1 | 73 | 8 | 80 | Excisionase | Excisionase | | afdb-uniprot50 | AF-A0A521H308-F1-MODEL\_V4 | 1.0 | 0.001963 | 153 | 0.3 | 70 | 47 | 1 | 4 | 73 | 2 | 69 | DNA-binding protein | DNA-binding protein | | afdb-uniprot50 | AF-J1HLR3-F1-MODEL\_V4 | 1.0 | 0.002397 | 153 | 0.291 | 72 | 49 | 1 | 2 | 73 | 12 | 81 | DNA-binding helix-turn-helix protein | DNA-binding helix-turn-helix protein | | afdb-uniprot50 | AF-A0A0Q8Q995-F1-MODEL\_V4 | 1.0 | 0.007428 | 152 | 0.214 | 56 | 42 | 1 | 11 | 66 | 4 | 57 | HTH\_17 domain-containing protein | HTH\_17 domain-containing protein | | afdb-uniprot50 | AF-A0A1A2P8X3-F1-MODEL\_V4 | 1.0 | 0.002397 | 152 | 0.276 | 65 | 44 | 2 | 10 | 73 | 3 | 65 | Uncharacterized protein | Uncharacterized protein | | afdb-uniprot50 | AF-A0A2T4JGE1-F1-MODEL\_V4 | 1.0 | 0.001837 | 152 | 0.328 | 67 | 38 | 2 | 12 | 73 | 1 | 65 | Uncharacterized protein | Uncharacterized protein | | afdb-uniprot50 | AF-A0A355T7V6-F1-MODEL\_V4 | 1.0 | 0.002098 | 152 | 0.375 | 64 | 38 | 1 | 7 | 70 | 2 | 63 | DNA-binding protein | DNA-binding protein | | afdb-uniprot50 | AF-A0A5R8PA16-F1-MODEL\_V4 | 1.0 | 0.002243 | 152 | 0.26 | 73 | 50 | 3 | 1 | 72 | 1 | 70 | Helix-turn-helix domain-containing protein | Helix-turn-helix domain-containing protein | | afdb-uniprot50 | AF-A0A4Y4C815-F1-MODEL\_V4 | 1.0 | 0.0009444 | 152 | 0.297 | 74 | 45 | 3 | 1 | 73 | 2 | 69 | HTH\_17 domain-containing protein | HTH\_17 domain-containing protein | | afdb-uniprot50 | AF-A0A0C2YAS8-F1-MODEL\_V4 | 1.0 | 0.001608 | 152 | 0.301 | 73 | 47 | 2 | 1 | 73 | 1 | 69 | Uncharacterized protein | Uncharacterized protein | | afdb-uniprot50 | AF-A0A161VAV6-F1-MODEL\_V4 | 1.0 | 0.001963 | 152 | 0.205 | 73 | 56 | 1 | 1 | 73 | 2 | 72 | Helix-turn-helix domain protein | Helix-turn-helix domain protein | | afdb-uniprot50 | AF-A0A7W8EEW1-F1-MODEL\_V4 | 1.0 | 0.002243 | 152 | 0.243 | 74 | 52 | 2 | 1 | 72 | 3 | 74 | Putative DNA-binding transcriptional regulator AlpA | Putative DNA-binding transcriptional regulator AlpA | | afdb-uniprot50 | AF-A0A0M9CB32-F1-MODEL\_V4 | 1.0 | 0.003819 | 152 | 0.268 | 67 | 47 | 2 | 7 | 73 | 9 | 73 | HTH\_17 domain-containing protein | HTH\_17 domain-containing protein | | afdb-uniprot50 | AF-A0A542NQW3-F1-MODEL\_V4 | 1.0 | 0.004362 | 152 | 0.265 | 64 | 44 | 2 | 1 | 63 | 6 | 67 | Helix-turn-helix protein | Helix-turn-helix protein | | afdb-uniprot50 | AF-A0A7W0NGD1-F1-MODEL\_V4 | 1.0 | 0.002397 | 152 | 0.281 | 64 | 43 | 2 | 1 | 63 | 250 | 311 | Helix-turn-helix domain-containing protein | Helix-turn-helix domain-containing protein | | afdb-uniprot50 | AF-A0A0Q9KLL4-F1-MODEL\_V4 | 1.0 | 0.002098 | 151 | 0.272 | 66 | 42 | 3 | 1 | 65 | 1 | 61 | HTH\_17 domain-containing protein | HTH\_17 domain-containing protein | | afdb-uniprot50 | AF-A0A6G7PU95-F1-MODEL\_V4 | 1.0 | 0.002927 | 151 | 0.283 | 67 | 44 | 2 | 7 | 73 | 1 | 63 | Helix-turn-helix domain-containing protein | Helix-turn-helix domain-containing protein | | afdb-uniprot50 | AF-A0A7X6CXT2-F1-MODEL\_V4 | 1.0 | 0.003343 | 151 | 0.308 | 68 | 43 | 2 | 1 | 68 | 1 | 64 | Helix-turn-helix domain-containing protein | Helix-turn-helix domain-containing protein | | afdb-uniprot50 | AF-A0A059G3P2-F1-MODEL\_V4 | 1.0 | 0.004362 | 151 | 0.268 | 67 | 46 | 2 | 7 | 73 | 1 | 64 | HTH\_17 domain-containing protein | HTH\_17 domain-containing protein | | afdb-uniprot50 | AF-A0A661MGY5-F1-MODEL\_V4 | 1.0 | 0.003128 | 151 | 0.301 | 63 | 42 | 1 | 11 | 73 | 4 | 64 | DNA-binding protein | DNA-binding protein | | afdb-uniprot50 | AF-A0A1A2D498-F1-MODEL\_V4 | 1.0 | 0.003819 | 151 | 0.261 | 65 | 44 | 2 | 11 | 73 | 3 | 65 | Transcriptional regulator | Transcriptional regulator | | afdb-uniprot50 | AF-A0A1I4RPU8-F1-MODEL\_V4 | 1.0 | 0.004983 | 151 | 0.166 | 66 | 51 | 2 | 6 | 69 | 2 | 65 | Uncharacterized protein | Uncharacterized protein | | afdb-uniprot50 | AF-A0A2N6F548-F1-MODEL\_V4 | 1.0 | 0.003343 | 151 | 0.23 | 65 | 48 | 1 | 1 | 65 | 2 | 64 | HTH\_17 domain-containing protein | HTH\_17 domain-containing protein | | afdb-uniprot50 | AF-A0A258BKJ5-F1-MODEL\_V4 | 1.0 | 0.004081 | 151 | 0.285 | 63 | 43 | 2 | 11 | 73 | 4 | 64 | Uncharacterized protein | Uncharacterized protein | | afdb-uniprot50 | AF-A0A380MLV7-F1-MODEL\_V4 | 1.0 | 0.002738 | 151 | 0.287 | 66 | 44 | 2 | 1 | 65 | 2 | 65 | Excisionase or Xis | Excisionase or Xis | | afdb-uniprot50 | AF-A0A0N7M3V3-F1-MODEL\_V4 | 1.0 | 0.001153 | 151 | 0.357 | 70 | 42 | 2 | 4 | 72 | 2 | 69 | Helix-turn-helix domain protein | Helix-turn-helix domain protein | | afdb-uniprot50 | AF-A0A7C8G298-F1-MODEL\_V4 | 1.0 | 0.003343 | 151 | 0.287 | 73 | 49 | 2 | 1 | 73 | 2 | 71 | Helix-turn-helix domain-containing protein | Helix-turn-helix domain-containing protein | | afdb-uniprot50 | AF-A0A2G2M150-F1-MODEL\_V4 | 1.0 | 0.008485 | 151 | 0.254 | 55 | 39 | 1 | 10 | 64 | 4 | 56 | HTH\_17 domain-containing protein | HTH\_17 domain-containing protein | | afdb-uniprot50 | AF-A0A8A9YMU8-F1-MODEL\_V4 | 1.0 | 0.001079 | 151 | 0.205 | 73 | 55 | 2 | 2 | 73 | 6 | 76 | Helix-turn-helix domain-containing protein | Helix-turn-helix domain-containing protein | | afdb-uniprot50 | AF-A0A245ZQK2-F1-MODEL\_V4 | 1.0 | 0.001232 | 151 | 0.243 | 78 | 52 | 3 | 1 | 73 | 1 | 76 | Helix-turn-helix domain protein | Helix-turn-helix domain protein | | afdb-uniprot50 | AF-A0A1R1IPT8-F1-MODEL\_V4 | 1.0 | 0.003819 | 151 | 0.23 | 65 | 48 | 1 | 9 | 73 | 20 | 82 | DNA-binding protein | DNA-binding protein | | afdb-uniprot50 | AF-A0A429YW83-F1-MODEL\_V4 | 1.0 | 0.006084 | 151 | 0.301 | 63 | 39 | 1 | 1 | 63 | 1 | 58 | DNA-binding protein | DNA-binding protein | | afdb-uniprot50 | AF-A0A7W1M9M0-F1-MODEL\_V4 | 1.0 | 0.001837 | 151 | 0.308 | 68 | 44 | 2 | 6 | 73 | 2 | 66 | DNA-binding protein | DNA-binding protein | | afdb-uniprot50 | AF-A0A6N9RRQ5-F1-MODEL\_V4 | 1.0 | 0.003573 | 151 | 0.219 | 73 | 55 | 1 | 1 | 73 | 9 | 79 | Helix-turn-helix domain-containing protein | Helix-turn-helix domain-containing protein | | afdb-uniprot50 | AF-A0A6G4WQN4-F1-MODEL\_V4 | 1.0 | 0.0006772 | 151 | 0.246 | 77 | 52 | 2 | 1 | 73 | 14 | 88 | Helix-turn-helix domain-containing protein | Helix-turn-helix domain-containing protein | | afdb-uniprot50 | AF-A0A2G6Y1E4-F1-MODEL\_V4 | 1.0 | 0.003573 | 151 | 0.253 | 67 | 46 | 2 | 1 | 65 | 21 | 85 | Excisionase family DNA binding protein | Excisionase family DNA binding protein | | afdb-uniprot50 | AF-A0A2A3HXH1-F1-MODEL\_V4 | 1.0 | 0.002562 | 151 | 0.294 | 68 | 45 | 2 | 1 | 67 | 24 | 89 | Helix-turn-helix protein | Helix-turn-helix protein | | afdb-uniprot50 | AF-A0A386UE03-F1-MODEL\_V4 | 1.0 | 0.001232 | 151 | 0.333 | 63 | 35 | 2 | 11 | 73 | 110 | 165 | HTH\_17 domain-containing protein | HTH\_17 domain-containing protein | | afdb-uniprot50 | AF-A0A423UVZ0-F1-MODEL\_V4 | 1.0 | 0.003343 | 151 | 0.323 | 65 | 40 | 2 | 1 | 65 | 174 | 234 | DNA-binding protein | DNA-binding protein | | afdb-uniprot50 | AF-A0A2U9S8C5-F1-MODEL\_V4 | 1.0 | 0.002397 | 150 | 0.365 | 63 | 36 | 2 | 11 | 73 | 4 | 62 | DNA-binding protein | DNA-binding protein | | afdb-uniprot50 | AF-A0A2E7B4B3-F1-MODEL\_V4 | 1.0 | 0.005692 | 150 | 0.312 | 64 | 40 | 2 | 10 | 73 | 6 | 65 | DNA-binding protein | DNA-binding protein | | afdb-uniprot50 | AF-A0A1I1SH13-F1-MODEL\_V4 | 1.0 | 0.003343 | 150 | 0.232 | 73 | 49 | 3 | 1 | 73 | 1 | 66 | DNA binding domain-containing protein, excisionase family | DNA binding domain-containing protein, excisionase family | | afdb-uniprot50 | AF-A0A2D8ZUW2-F1-MODEL\_V4 | 1.0 | 0.001153 | 150 | 0.328 | 73 | 41 | 3 | 1 | 73 | 1 | 65 | DNA-binding protein | DNA-binding protein | | afdb-uniprot50 | AF-A0A6G2N519-F1-MODEL\_V4 | 1.0 | 0.0009444 | 150 | 0.253 | 67 | 48 | 2 | 1 | 66 | 2 | 67 | Helix-turn-helix domain-containing protein | Helix-turn-helix domain-containing protein | | afdb-uniprot50 | AF-A0A1X1RFI8-F1-MODEL\_V4 | 1.0 | 0.002098 | 150 | 0.205 | 73 | 53 | 2 | 1 | 73 | 1 | 68 | HTH\_17 domain-containing protein | HTH\_17 domain-containing protein | | afdb-uniprot50 | AF-A0A3N4Z623-F1-MODEL\_V4 | 1.0 | 0.002397 | 150 | 0.257 | 70 | 50 | 1 | 4 | 73 | 2 | 69 | Excisionase family DNA binding protein | Excisionase family DNA binding protein | | afdb-uniprot50 | AF-A0A846W105-F1-MODEL\_V4 | 1.0 | 0.004662 | 150 | 0.228 | 70 | 52 | 1 | 4 | 73 | 3 | 70 | Helix-turn-helix domain-containing protein | Helix-turn-helix domain-containing protein | | afdb-uniprot50 | AF-A0A809IK81-F1-MODEL\_V4 | 1.0 | 0.002562 | 150 | 0.285 | 63 | 43 | 1 | 1 | 63 | 7 | 67 | DNA-binding protein | DNA-binding protein | | afdb-uniprot50 | AF-A0A2E5BQF7-F1-MODEL\_V4 | 1.0 | 0.002397 | 150 | 0.271 | 70 | 48 | 2 | 2 | 70 | 10 | 77 | HTH\_17 domain-containing protein | HTH\_17 domain-containing protein | | afdb-uniprot50 | AF-A0A1C6LZ92-F1-MODEL\_V4 | 1.0 | 0.001963 | 150 | 0.216 | 74 | 52 | 3 | 1 | 73 | 10 | 78 | Helix-turn-helix domain-containing protein | Helix-turn-helix domain-containing protein | | afdb-uniprot50 | AF-A0A1G9CHM9-F1-MODEL\_V4 | 1.0 | 0.001963 | 150 | 0.243 | 78 | 54 | 2 | 1 | 73 | 1 | 78 | Uncharacterized protein | Uncharacterized protein | | afdb-uniprot50 | AF-A0A1G6EMA2-F1-MODEL\_V4 | 1.0 | 0.0006772 | 150 | 0.36 | 75 | 43 | 3 | 1 | 72 | 2 | 74 | Uncharacterized protein | Uncharacterized protein | | afdb-uniprot50 | AF-A0A2D8NLB9-F1-MODEL\_V4 | 1.0 | 0.002562 | 150 | 0.282 | 78 | 47 | 3 | 1 | 73 | 1 | 74 | Excisionase | Excisionase | | afdb-uniprot50 | AF-A0A4Y9QPR4-F1-MODEL\_V4 | 1.0 | 0.002738 | 150 | 0.25 | 76 | 52 | 3 | 1 | 73 | 2 | 75 | DNA-binding protein | DNA-binding protein | | afdb-uniprot50 | AF-A0A559VPY9-F1-MODEL\_V4 | 1.0 | 0.001505 | 150 | 0.287 | 73 | 47 | 2 | 1 | 73 | 16 | 83 | Excisionase family DNA binding protein | Excisionase family DNA binding protein | | afdb-uniprot50 | AF-A0A7W0L409-F1-MODEL\_V4 | 1.0 | 0.003819 | 150 | 0.232 | 73 | 54 | 1 | 1 | 73 | 16 | 86 | Helix-turn-helix domain-containing protein | Helix-turn-helix domain-containing protein | | afdb-uniprot50 | AF-A0A2E2QLK1-F1-MODEL\_V4 | 1.0 | 0.002738 | 150 | 0.228 | 70 | 51 | 2 | 1 | 70 | 18 | 84 | HTH\_17 domain-containing protein | HTH\_17 domain-containing protein | | afdb-uniprot50 | AF-A8LA07-F1-MODEL\_V4 | 1.0 | 0.001963 | 150 | 0.242 | 66 | 47 | 3 | 1 | 65 | 25 | 88 | Excision promoter, Xis | Excision promoter, Xis | | afdb-uniprot50 | AF-A0A0P1F1V2-F1-MODEL\_V4 | 1.0 | 0.004983 | 150 | 0.281 | 71 | 49 | 1 | 3 | 73 | 14 | 82 | Helix-turn-helix domain protein | Helix-turn-helix domain protein | | afdb-uniprot50 | AF-A0A2W1TE65-F1-MODEL\_V4 | 1.0 | 0.006084 | 149 | 0.295 | 61 | 41 | 1 | 12 | 72 | 1 | 59 | HTH\_17 domain-containing protein | HTH\_17 domain-containing protein | | afdb-uniprot50 | AF-A0A2W4LWT2-F1-MODEL\_V4 | 1.0 | 0.008485 | 149 | 0.298 | 57 | 38 | 1 | 11 | 67 | 4 | 58 | DNA-binding protein | DNA-binding protein | | afdb-uniprot50 | AF-A0A7C5TS90-F1-MODEL\_V4 | 1.0 | 0.004362 | 149 | 0.253 | 67 | 48 | 1 | 6 | 72 | 2 | 66 | DNA-binding protein | DNA-binding protein | | afdb-uniprot50 | AF-A0A7W7GUH3-F1-MODEL\_V4 | 1.0 | 0.004983 | 149 | 0.218 | 64 | 48 | 1 | 1 | 64 | 3 | 64 | Excisionase family DNA binding protein | Excisionase family DNA binding protein | | afdb-uniprot50 | AF-A0A2M8B589-F1-MODEL\_V4 | 1.0 | 0.009692 | 149 | 0.213 | 61 | 46 | 1 | 9 | 69 | 5 | 63 | Transcriptional regulator | Transcriptional regulator | | afdb-uniprot50 | AF-A0A7G2SRX6-F1-MODEL\_V4 | 1.0 | 0.003128 | 149 | 0.246 | 73 | 53 | 1 | 1 | 73 | 1 | 71 | HTH\_17 domain-containing protein | HTH\_17 domain-containing protein | | afdb-uniprot50 | AF-A0A2P8E071-F1-MODEL\_V4 | 1.0 | 0.002927 | 149 | 0.25 | 68 | 49 | 1 | 1 | 68 | 7 | 72 | Excisionase family DNA binding protein | Excisionase family DNA binding protein | | afdb-uniprot50 | AF-A0A2N3GRP0-F1-MODEL\_V4 | 1.0 | 0.003343 | 149 | 0.212 | 66 | 49 | 2 | 1 | 65 | 9 | 72 | HTH\_17 domain-containing protein | HTH\_17 domain-containing protein | | afdb-uniprot50 | AF-A0A7W0KKL6-F1-MODEL\_V4 | 1.0 | 0.002243 | 149 | 0.226 | 75 | 54 | 2 | 1 | 73 | 3 | 75 | Helix-turn-helix domain-containing protein | Helix-turn-helix domain-containing protein | | afdb-uniprot50 | AF-A0A3P3VTI3-F1-MODEL\_V4 | 1.0 | 0.001963 | 149 | 0.219 | 73 | 54 | 2 | 2 | 73 | 5 | 75 | DNA-binding protein | DNA-binding protein | | afdb-uniprot50 | AF-A0A399UEI2-F1-MODEL\_V4 | 1.0 | 0.003819 | 149 | 0.232 | 73 | 54 | 1 | 1 | 73 | 7 | 77 | DNA-binding protein | DNA-binding protein | | afdb-uniprot50 | AF-A0A7X5F027-F1-MODEL\_V4 | 1.0 | 0.004662 | 149 | 0.228 | 70 | 52 | 1 | 1 | 70 | 3 | 70 | Helix-turn-helix domain-containing protein | Helix-turn-helix domain-containing protein | | afdb-uniprot50 | AF-A0A0B5ERS0-F1-MODEL\_V4 | 1.0 | 0.001837 | 149 | 0.275 | 69 | 45 | 2 | 1 | 65 | 8 | 75 | DNA-binding protein | DNA-binding protein | | afdb-uniprot50 | AF-F6F0P7-F1-MODEL\_V4 | 1.0 | 0.005692 | 149 | 0.215 | 65 | 49 | 1 | 9 | 73 | 16 | 78 | Uncharacterized protein | Uncharacterized protein | | afdb-uniprot50 | AF-B6JJF5-F1-MODEL\_V4 | 1.0 | 0.002738 | 149 | 0.307 | 65 | 41 | 2 | 1 | 63 | 11 | 73 | HTH\_17 domain-containing protein | HTH\_17 domain-containing protein | | afdb-uniprot50 | AF-A0A2W5ETA6-F1-MODEL\_V4 | 1.0 | 0.002397 | 149 | 0.328 | 73 | 46 | 2 | 1 | 73 | 13 | 82 | DNA-binding protein | DNA-binding protein | | afdb-uniprot50 | AF-A0A1G6DPZ1-F1-MODEL\_V4 | 1.0 | 0.003819 | 149 | 0.333 | 63 | 40 | 1 | 11 | 73 | 14 | 74 | Uncharacterized protein | Uncharacterized protein | | afdb-uniprot50 | AF-A0A1M3Q4B2-F1-MODEL\_V4 | 1.0 | 0.005692 | 149 | 0.241 | 62 | 44 | 2 | 1 | 62 | 6 | 64 | DNA-binding protein | DNA-binding protein | | afdb-uniprot50 | AF-A0A316AUK9-F1-MODEL\_V4 | 1.0 | 0.006084 | 149 | 0.253 | 63 | 45 | 1 | 1 | 63 | 9 | 69 | Helix-turn-helix protein | Helix-turn-helix protein | | afdb-uniprot50 | AF-A0A7W3J5K9-F1-MODEL\_V4 | 1.0 | 0.002098 | 149 | 0.232 | 73 | 54 | 1 | 1 | 73 | 17 | 87 | Excisionase family DNA binding protein | Excisionase family DNA binding protein | | afdb-uniprot50 | AF-A0A435X2Z5-F1-MODEL\_V4 | 1.0 | 0.002243 | 149 | 0.253 | 75 | 51 | 2 | 2 | 73 | 32 | 104 | DNA-binding protein | DNA-binding protein | | afdb-uniprot50 | AF-A0A5A7WVK6-F1-MODEL\_V4 | 1.0 | 0.004983 | 148 | 0.205 | 68 | 52 | 1 | 6 | 73 | 2 | 67 | DNA-binding protein | DNA-binding protein | | afdb-uniprot50 | AF-A0A371P3H7-F1-MODEL\_V4 | 1.0 | 0.004662 | 148 | 0.235 | 68 | 49 | 2 | 1 | 68 | 1 | 65 | DNA-binding protein | DNA-binding protein | | afdb-uniprot50 | AF-A0A2H6H355-F1-MODEL\_V4 | 1.0 | 0.003573 | 148 | 0.279 | 68 | 47 | 1 | 6 | 73 | 2 | 67 | Helix-turn-helix domain protein | Helix-turn-helix domain protein | | afdb-uniprot50 | AF-A0A349HMZ8-F1-MODEL\_V4 | 1.0 | 0.001719 | 148 | 0.318 | 66 | 42 | 2 | 9 | 73 | 2 | 65 | DNA-binding protein | DNA-binding protein | | afdb-uniprot50 | AF-A0A4Q7ZEX0-F1-MODEL\_V4 | 1.0 | 0.001079 | 148 | 0.25 | 72 | 48 | 3 | 1 | 72 | 4 | 69 | AlpA family transcriptional regulator | AlpA family transcriptional regulator | | afdb-uniprot50 | AF-A0A7U3UU80-F1-MODEL\_V4 | 1.0 | 0.001608 | 148 | 0.279 | 68 | 44 | 2 | 1 | 65 | 1 | 66 | Uncharacterized protein | Uncharacterized protein | | afdb-uniprot50 | AF-A0A7T2WNU1-F1-MODEL\_V4 | 1.0 | 0.002927 | 148 | 0.273 | 73 | 47 | 3 | 1 | 70 | 1 | 70 | Helix-turn-helix domain-containing protein | Helix-turn-helix domain-containing protein | | afdb-uniprot50 | AF-A0A512JRF1-F1-MODEL\_V4 | 1.0 | 0.002243 | 148 | 0.239 | 71 | 52 | 1 | 2 | 72 | 3 | 71 | HTH\_17 domain-containing protein | HTH\_17 domain-containing protein | | afdb-uniprot50 | AF-A0A090MQQ0-F1-MODEL\_V4 | 1.0 | 0.004662 | 148 | 0.285 | 63 | 43 | 1 | 11 | 73 | 5 | 65 | Helix-turn-helix domain protein | Helix-turn-helix domain protein | | afdb-uniprot50 | AF-A0A229SBU2-F1-MODEL\_V4 | 1.0 | 0.002397 | 148 | 0.267 | 71 | 49 | 2 | 4 | 73 | 3 | 71 | HTH\_17 domain-containing protein | HTH\_17 domain-containing protein | | afdb-uniprot50 | AF-A0A4Q8TE58-F1-MODEL\_V4 | 1.0 | 0.003128 | 148 | 0.276 | 65 | 45 | 1 | 1 | 65 | 6 | 68 | DNA-binding protein | DNA-binding protein | | afdb-uniprot50 | AF-U5VTE3-F1-MODEL\_V4 | 1.0 | 0.003819 | 148 | 0.242 | 66 | 48 | 1 | 2 | 67 | 7 | 70 | Excisionase/Xis, DNA-binding protein | Excisionase/Xis, DNA-binding protein | | afdb-uniprot50 | AF-A0A1A2CNF7-F1-MODEL\_V4 | 1.0 | 0.002927 | 148 | 0.246 | 73 | 53 | 1 | 1 | 73 | 2 | 72 | HTH\_17 domain-containing protein | HTH\_17 domain-containing protein | | afdb-uniprot50 | AF-A0A238ZEI8-F1-MODEL\_V4 | 1.0 | 0.005326 | 148 | 0.265 | 64 | 45 | 1 | 10 | 73 | 15 | 76 | Uncharacterized protein | Uncharacterized protein | | afdb-uniprot50 | AF-A0A7C9M524-F1-MODEL\_V4 | 1.0 | 0.002562 | 148 | 0.328 | 73 | 47 | 2 | 1 | 73 | 1 | 71 | Helix-turn-helix domain-containing protein | Helix-turn-helix domain-containing protein | | afdb-uniprot50 | AF-A0A059GC80-F1-MODEL\_V4 | 1.0 | 0.004662 | 148 | 0.232 | 73 | 54 | 1 | 1 | 73 | 8 | 78 | HTH\_17 domain-containing protein | HTH\_17 domain-containing protein | | afdb-uniprot50 | AF-A0A349CXT0-F1-MODEL\_V4 | 1.0 | 0.001009 | 148 | 0.27 | 74 | 49 | 3 | 1 | 73 | 1 | 70 | HTH\_17 domain-containing protein | HTH\_17 domain-containing protein | | afdb-uniprot50 | AF-A0A2A4ZEI9-F1-MODEL\_V4 | 1.0 | 0.002098 | 148 | 0.256 | 74 | 50 | 2 | 1 | 71 | 13 | 84 | Uncharacterized protein | Uncharacterized protein | | afdb-uniprot50 | AF-E8RUQ5-F1-MODEL\_V4 | 1.0 | 0.004662 | 148 | 0.26 | 69 | 47 | 2 | 5 | 73 | 30 | 94 | HTH\_17 domain-containing protein | HTH\_17 domain-containing protein | | afdb-uniprot50 | AF-A0A6N8XP61-F1-MODEL\_V4 | 1.0 | 0.002738 | 148 | 0.24 | 75 | 53 | 2 | 1 | 73 | 26 | 98 | Helix-turn-helix domain-containing protein | Helix-turn-helix domain-containing protein | | afdb-uniprot50 | AF-A0A2N5C742-F1-MODEL\_V4 | 1.0 | 0.001608 | 148 | 0.287 | 73 | 47 | 2 | 1 | 70 | 13 | 83 | DNA-binding protein | DNA-binding protein | | afdb-uniprot50 | AF-A0A445MWH5-F1-MODEL\_V4 | 1.0 | 0.003573 | 147 | 0.294 | 68 | 46 | 1 | 6 | 73 | 2 | 67 | HTH\_17 domain-containing protein | HTH\_17 domain-containing protein | | afdb-uniprot50 | AF-A0A520X4P5-F1-MODEL\_V4 | 1.0 | 0.003573 | 147 | 0.246 | 73 | 49 | 3 | 1 | 73 | 1 | 67 | DNA-binding protein | DNA-binding protein | | afdb-uniprot50 | AF-A0A7X0FN29-F1-MODEL\_V4 | 1.0 | 0.003573 | 147 | 0.271 | 70 | 48 | 3 | 4 | 73 | 2 | 68 | Uncharacterized protein | Uncharacterized protein | | afdb-uniprot50 | AF-A0A4Z0KEZ5-F1-MODEL\_V4 | 1.0 | 0.002397 | 147 | 0.217 | 69 | 51 | 2 | 1 | 69 | 3 | 68 | DNA-binding protein | DNA-binding protein | | afdb-uniprot50 | AF-A0A243RM94-F1-MODEL\_V4 | 1.0 | 0.001963 | 147 | 0.25 | 72 | 52 | 1 | 1 | 72 | 1 | 70 | DNA-binding protein | DNA-binding protein | | afdb-uniprot50 | AF-A0A2A9K2C8-F1-MODEL\_V4 | 1.0 | 0.002098 | 147 | 0.253 | 71 | 49 | 2 | 1 | 69 | 1 | 69 | AlpA family transcriptional regulator | AlpA family transcriptional regulator | | afdb-uniprot50 | AF-A0A7J5BMM3-F1-MODEL\_V4 | 1.0 | 0.002243 | 147 | 0.238 | 67 | 47 | 2 | 1 | 65 | 2 | 66 | Helix-turn-helix domain-containing protein | Helix-turn-helix domain-containing protein | | afdb-uniprot50 | AF-A0A840EWM2-F1-MODEL\_V4 | 1.0 | 0.003819 | 147 | 0.219 | 73 | 53 | 3 | 1 | 73 | 1 | 69 | Uncharacterized protein | Uncharacterized protein | | afdb-uniprot50 | AF-A0A7Y7M2Z0-F1-MODEL\_V4 | 1.0 | 0.003343 | 147 | 0.223 | 67 | 48 | 2 | 1 | 65 | 6 | 70 | Helix-turn-helix domain-containing protein | Helix-turn-helix domain-containing protein | | afdb-uniprot50 | AF-A0A2K8Y4P9-F1-MODEL\_V4 | 1.0 | 0.005692 | 147 | 0.322 | 59 | 38 | 1 | 4 | 62 | 1 | 57 | Excisionase | Excisionase | | afdb-uniprot50 | AF-A0A2D6SS17-F1-MODEL\_V4 | 1.0 | 0.003128 | 147 | 0.257 | 70 | 50 | 1 | 4 | 73 | 1 | 68 | HTH\_17 domain-containing protein | HTH\_17 domain-containing protein | | afdb-uniprot50 | AF-A0A4Y9NPZ2-F1-MODEL\_V4 | 1.0 | 0.004662 | 147 | 0.222 | 63 | 47 | 1 | 11 | 73 | 13 | 73 | DNA-binding protein | DNA-binding protein | | afdb-uniprot50 | AF-A0A2V5JGJ3-F1-MODEL\_V4 | 1.0 | 0.003343 | 147 | 0.27 | 74 | 51 | 2 | 1 | 73 | 2 | 73 | DNA-binding protein | DNA-binding protein | | afdb-uniprot50 | AF-A0A522NW80-F1-MODEL\_V4 | 1.0 | 0.004983 | 147 | 0.25 | 72 | 52 | 1 | 2 | 73 | 8 | 77 | DNA-binding protein | DNA-binding protein | | afdb-uniprot50 | AF-A0A0D7CQX3-F1-MODEL\_V4 | 1.0 | 0.001317 | 147 | 0.295 | 71 | 42 | 2 | 2 | 65 | 7 | 76 | DNA-binding protein | DNA-binding protein | | afdb-uniprot50 | AF-A0A397LT87-F1-MODEL\_V4 | 1.0 | 0.002243 | 147 | 0.21 | 76 | 55 | 2 | 1 | 73 | 9 | 82 | AlpA family transcriptional regulator | AlpA family transcriptional regulator | | afdb-uniprot50 | AF-G2JA45-F1-MODEL\_V4 | 1.0 | 0.001963 | 147 | 0.315 | 73 | 45 | 3 | 1 | 73 | 1 | 68 | Uncharacterized protein | Uncharacterized protein | | afdb-uniprot50 | AF-A0A286GLX8-F1-MODEL\_V4 | 1.0 | 0.003343 | 147 | 0.208 | 72 | 55 | 1 | 1 | 72 | 16 | 85 | Helix-turn-helix domain-containing protein | Helix-turn-helix domain-containing protein | | afdb-uniprot50 | AF-A0A1V9K8R2-F1-MODEL\_V4 | 1.0 | 0.005692 | 147 | 0.271 | 59 | 41 | 1 | 9 | 67 | 30 | 86 | DNA-binding protein | DNA-binding protein | | afdb-uniprot50 | AF-A0A7Z0AIF4-F1-MODEL\_V4 | 1.0 | 0.001408 | 147 | 0.191 | 73 | 56 | 2 | 2 | 73 | 20 | 90 | Excisionase family DNA binding protein | Excisionase family DNA binding protein | | afdb-uniprot50 | AF-Q5P854-F1-MODEL\_V4 | 1.0 | 0.001153 | 147 | 0.32 | 75 | 46 | 3 | 1 | 72 | 17 | 89 | HTH\_17 domain-containing protein | HTH\_17 domain-containing protein | | afdb-uniprot50 | AF-A0A1G7ELU0-F1-MODEL\_V4 | 1.0 | 0.005692 | 147 | 0.38 | 63 | 36 | 2 | 11 | 73 | 41 | 100 | Helix-turn-helix domain-containing protein | Helix-turn-helix domain-containing protein | | afdb-uniprot50 | AF-A0A2W6S3C3-F1-MODEL\_V4 | 1.0 | 0.001408 | 147 | 0.273 | 73 | 50 | 2 | 1 | 72 | 19 | 89 | DNA-binding protein | DNA-binding protein | | afdb-uniprot50 | AF-M7A7X6-F1-MODEL\_V4 | 1.0 | 0.002927 | 146 | 0.312 | 64 | 39 | 3 | 1 | 62 | 1 | 61 | HTH\_17 domain-containing protein | HTH\_17 domain-containing protein | | afdb-uniprot50 | AF-A0A2K9DLX8-F1-MODEL\_V4 | 1.0 | 0.008485 | 146 | 0.312 | 64 | 41 | 2 | 1 | 63 | 2 | 63 | DNA-binding protein | DNA-binding protein | | afdb-uniprot50 | AF-A0A348G1J1-F1-MODEL\_V4 | 1.0 | 0.001408 | 146 | 0.231 | 69 | 46 | 2 | 4 | 72 | 2 | 63 | HTH\_17 domain-containing protein | HTH\_17 domain-containing protein | | afdb-uniprot50 | AF-A0A2S5X2A3-F1-MODEL\_V4 | 1.0 | 0.002927 | 146 | 0.271 | 70 | 45 | 2 | 1 | 66 | 1 | 68 | HTH\_17 domain-containing protein | HTH\_17 domain-containing protein | | afdb-uniprot50 | AF-A0A2U2AVN2-F1-MODEL\_V4 | 1.0 | 0.003819 | 146 | 0.295 | 61 | 39 | 2 | 2 | 62 | 9 | 65 | Excisionase | Excisionase | | afdb-uniprot50 | AF-A0A0B5HSA4-F1-MODEL\_V4 | 1.0 | 0.001963 | 146 | 0.257 | 70 | 47 | 2 | 1 | 67 | 3 | 70 | DNA-binding protein | DNA-binding protein | | afdb-uniprot50 | AF-A0A344WL72-F1-MODEL\_V4 | 1.0 | 0.002927 | 146 | 0.322 | 62 | 40 | 2 | 1 | 62 | 1 | 60 | Uncharacterized protein | Uncharacterized protein | | afdb-uniprot50 | AF-A0A6I7Y267-F1-MODEL\_V4 | 1.0 | 0.007428 | 146 | 0.265 | 64 | 45 | 1 | 1 | 64 | 8 | 69 | DNA-binding protein | DNA-binding protein | | afdb-uniprot50 | AF-A0A3A0G615-F1-MODEL\_V4 | 1.0 | 0.002927 | 146 | 0.246 | 73 | 51 | 2 | 1 | 73 | 2 | 70 | HTH\_17 domain-containing protein | HTH\_17 domain-containing protein | | afdb-uniprot50 | AF-A0A841CXJ6-F1-MODEL\_V4 | 1.0 | 0.001719 | 146 | 0.246 | 77 | 48 | 2 | 1 | 69 | 1 | 75 | Uncharacterized protein | Uncharacterized protein | | afdb-uniprot50 | AF-A0A6A7LAR2-F1-MODEL\_V4 | 1.0 | 0.003819 | 146 | 0.23 | 65 | 46 | 2 | 1 | 63 | 6 | 68 | Helix-turn-helix domain-containing protein | Helix-turn-helix domain-containing protein | | afdb-uniprot50 | AF-B0URF6-F1-MODEL\_V4 | 1.0 | 0.003128 | 146 | 0.242 | 70 | 51 | 1 | 1 | 70 | 2 | 69 | HTH\_17 domain-containing protein | HTH\_17 domain-containing protein | | afdb-uniprot50 | AF-A0A1H1VI02-F1-MODEL\_V4 | 1.0 | 0.001837 | 146 | 0.243 | 74 | 53 | 2 | 1 | 73 | 8 | 79 | DNA binding domain-containing protein, excisionase family | DNA binding domain-containing protein, excisionase family | | afdb-uniprot50 | AF-A0A3R8VZY2-F1-MODEL\_V4 | 1.0 | 0.001837 | 146 | 0.242 | 70 | 50 | 2 | 2 | 70 | 16 | 83 | DNA-binding protein | DNA-binding protein | | afdb-uniprot50 | AF-A0A846XD13-F1-MODEL\_V4 | 1.0 | 0.002098 | 146 | 0.275 | 69 | 46 | 3 | 2 | 68 | 21 | 87 | Helix-turn-helix domain-containing protein | Helix-turn-helix domain-containing protein | | afdb-uniprot50 | AF-A0A1C3RET6-F1-MODEL\_V4 | 1.0 | 0.002927 | 146 | 0.246 | 73 | 53 | 1 | 1 | 73 | 9 | 79 | Transcriptional regulator (Modular protein) | Transcriptional regulator (Modular protein) | | afdb-uniprot50 | AF-A0A2A2D798-F1-MODEL\_V4 | 1.0 | 0.003573 | 146 | 0.227 | 79 | 53 | 2 | 1 | 73 | 1 | 77 | Uncharacterized protein | Uncharacterized protein | | afdb-uniprot50 | AF-A0A6L7X0Z1-F1-MODEL\_V4 | 1.0 | 0.001608 | 146 | 0.246 | 73 | 52 | 2 | 1 | 72 | 45 | 115 | Helix-turn-helix domain-containing protein | Helix-turn-helix domain-containing protein | | afdb-uniprot50 | AF-A0A1W2FSX5-F1-MODEL\_V4 | 1.0 | 0.004983 | 145 | 0.25 | 64 | 45 | 2 | 1 | 63 | 2 | 63 | Helix-turn-helix domain-containing protein | Helix-turn-helix domain-containing protein | | afdb-uniprot50 | AF-A0A5B8C8T0-F1-MODEL\_V4 | 1.0 | 0.003573 | 145 | 0.277 | 72 | 46 | 3 | 1 | 72 | 1 | 66 | Helix-turn-helix domain-containing protein | Helix-turn-helix domain-containing protein | | afdb-uniprot50 | AF-A0A1V0KSN6-F1-MODEL\_V4 | 1.0 | 0.004081 | 145 | 0.268 | 67 | 45 | 3 | 7 | 72 | 2 | 65 | Excisionase | Excisionase | | afdb-uniprot50 | AF-A0A6L9SHQ0-F1-MODEL\_V4 | 1.0 | 0.007939 | 145 | 0.283 | 67 | 46 | 1 | 1 | 67 | 2 | 66 | Helix-turn-helix domain-containing protein | Helix-turn-helix domain-containing protein | | afdb-uniprot50 | AF-A0A7Y0CGF4-F1-MODEL\_V4 | 1.0 | 0.001153 | 145 | 0.287 | 73 | 45 | 3 | 1 | 73 | 3 | 68 | Helix-turn-helix domain-containing protein | Helix-turn-helix domain-containing protein | | afdb-uniprot50 | AF-A0A2W4I437-F1-MODEL\_V4 | 1.0 | 0.004983 | 145 | 0.257 | 66 | 46 | 2 | 1 | 65 | 3 | 66 | DNA-binding protein | DNA-binding protein | | afdb-uniprot50 | AF-A0A2W2MKA0-F1-MODEL\_V4 | 1.0 | 0.001963 | 145 | 0.246 | 73 | 52 | 2 | 1 | 73 | 2 | 71 | HTH\_17 domain-containing protein | HTH\_17 domain-containing protein | | afdb-uniprot50 | AF-A0A1P8X724-F1-MODEL\_V4 | 1.0 | 0.004081 | 145 | 0.232 | 73 | 52 | 2 | 1 | 73 | 2 | 70 | DNA-binding protein | DNA-binding protein | | afdb-uniprot50 | AF-A0A7W5FGQ6-F1-MODEL\_V4 | 1.0 | 0.003128 | 145 | 0.279 | 68 | 46 | 2 | 1 | 67 | 2 | 67 | Putative DNA-binding transcriptional regulator AlpA | Putative DNA-binding transcriptional regulator AlpA | | afdb-uniprot50 | AF-A0A7Y9GK84-F1-MODEL\_V4 | 1.0 | 0.003128 | 145 | 0.219 | 73 | 53 | 2 | 3 | 73 | 2 | 72 | Putative DNA-binding transcriptional regulator AlpA | Putative DNA-binding transcriptional regulator AlpA | | afdb-uniprot50 | AF-A0A645HPJ7-F1-MODEL\_V4 | 1.0 | 0.002738 | 145 | 0.219 | 73 | 55 | 1 | 1 | 73 | 1 | 71 | HTH\_17 domain-containing protein | HTH\_17 domain-containing protein | | afdb-uniprot50 | AF-A0A515KRJ5-F1-MODEL\_V4 | 1.0 | 0.003128 | 145 | 0.226 | 75 | 54 | 2 | 1 | 73 | 1 | 73 | Helix-turn-helix domain-containing protein | Helix-turn-helix domain-containing protein | | afdb-uniprot50 | AF-A0A2U3QJS9-F1-MODEL\_V4 | 1.0 | 0.003819 | 145 | 0.26 | 69 | 45 | 2 | 9 | 73 | 2 | 68 | Uncharacterized protein | Uncharacterized protein | | afdb-uniprot50 | AF-A0A511AET4-F1-MODEL\_V4 | 1.0 | 0.004662 | 145 | 0.26 | 73 | 53 | 1 | 1 | 73 | 6 | 77 | Uncharacterized protein | Uncharacterized protein | | afdb-uniprot50 | AF-A0A140GVH6-F1-MODEL\_V4 | 1.0 | 0.002927 | 145 | 0.177 | 79 | 57 | 3 | 1 | 73 | 1 | 77 | Phage transcriptional regulator AlpA | Phage transcriptional regulator AlpA | | afdb-uniprot50 | AF-A0A7X0VA92-F1-MODEL\_V4 | 1.0 | 0.002562 | 145 | 0.276 | 76 | 44 | 2 | 1 | 67 | 1 | 74 | Helix-turn-helix domain-containing protein | Helix-turn-helix domain-containing protein | | afdb-uniprot50 | AF-A0A7X3S6D7-F1-MODEL\_V4 | 1.0 | 0.004662 | 145 | 0.281 | 71 | 49 | 1 | 2 | 72 | 14 | 82 | Helix-turn-helix domain-containing protein | Helix-turn-helix domain-containing protein | | afdb-uniprot50 | AF-A0A1R1SIM1-F1-MODEL\_V4 | 1.0 | 0.007939 | 145 | 0.28 | 57 | 39 | 1 | 9 | 65 | 35 | 89 | DNA-binding protein | DNA-binding protein | | afdb-uniprot50 | AF-A0A2P2BX08-F1-MODEL\_V4 | 1.0 | 0.002243 | 145 | 0.305 | 72 | 45 | 3 | 2 | 71 | 4 | 72 | Transcriptional repressor, dcmR family (Modular protein) | Transcriptional repressor, dcmR family (Modular protein) | | afdb-uniprot50 | AF-A0A4R8WX82-F1-MODEL\_V4 | 1.0 | 0.005692 | 145 | 0.322 | 62 | 38 | 2 | 2 | 61 | 40 | 99 | Helix-turn-helix domain-containing protein | Helix-turn-helix domain-containing protein | | afdb-uniprot50 | AF-A0A2D9H295-F1-MODEL\_V4 | 1.0 | 0.0008268 | 145 | 0.276 | 76 | 46 | 4 | 1 | 73 | 44 | 113 | HTH\_17 domain-containing protein | HTH\_17 domain-containing protein | | afdb-uniprot50 | AF-D6KAR1-F1-MODEL\_V4 | 1.0 | 0.003573 | 145 | 0.214 | 70 | 48 | 2 | 1 | 65 | 44 | 111 | Excisionase family DNA-binding domain-containing protein | Excisionase family DNA-binding domain-containing protein | | afdb-uniprot50 | AF-A0A1Q7VQ48-F1-MODEL\_V4 | 1.0 | 0.005326 | 144 | 0.238 | 63 | 45 | 2 | 1 | 63 | 1 | 60 | HTH\_17 domain-containing protein | HTH\_17 domain-containing protein | | afdb-uniprot50 | AF-A0A562V6Z3-F1-MODEL\_V4 | 1.0 | 0.003819 | 144 | 0.25 | 68 | 46 | 3 | 1 | 68 | 1 | 63 | AlpA family transcriptional regulator | AlpA family transcriptional regulator | | afdb-uniprot50 | AF-A0A2N0TJM6-F1-MODEL\_V4 | 1.0 | 0.005326 | 144 | 0.257 | 66 | 45 | 2 | 9 | 73 | 6 | 68 | Uncharacterized protein | Uncharacterized protein | | afdb-uniprot50 | AF-A0A7Y4L6Z8-F1-MODEL\_V4 | 1.0 | 0.004662 | 144 | 0.185 | 70 | 54 | 2 | 1 | 70 | 1 | 67 | Helix-turn-helix domain-containing protein | Helix-turn-helix domain-containing protein | | afdb-uniprot50 | AF-A0A7I9Y7H1-F1-MODEL\_V4 | 1.0 | 0.005326 | 144 | 0.314 | 70 | 45 | 2 | 1 | 69 | 1 | 68 | HTH\_17 domain-containing protein | HTH\_17 domain-containing protein | | afdb-uniprot50 | AF-A0A3M2M828-F1-MODEL\_V4 | 1.0 | 0.004081 | 144 | 0.222 | 72 | 52 | 2 | 1 | 72 | 3 | 70 | Helix-turn-helix domain-containing protein | Helix-turn-helix domain-containing protein | | afdb-uniprot50 | AF-A0A1M5QRE5-F1-MODEL\_V4 | 1.0 | 0.004983 | 144 | 0.257 | 70 | 50 | 1 | 4 | 73 | 2 | 69 | Uncharacterized protein | Uncharacterized protein | | afdb-uniprot50 | AF-A0A445MRB1-F1-MODEL\_V4 | 1.0 | 0.002927 | 144 | 0.307 | 65 | 41 | 2 | 1 | 63 | 1 | 63 | HTH\_17 domain-containing protein | HTH\_17 domain-containing protein | | afdb-uniprot50 | AF-A0A077MB24-F1-MODEL\_V4 | 1.0 | 0.005326 | 144 | 0.238 | 67 | 49 | 1 | 1 | 67 | 6 | 70 | HTH\_17 domain-containing protein | HTH\_17 domain-containing protein | | afdb-uniprot50 | AF-B8EI03-F1-MODEL\_V4 | 1.0 | 0.001837 | 144 | 0.256 | 74 | 52 | 2 | 1 | 73 | 2 | 73 | Phage transcriptional regulator, AlpA | Phage transcriptional regulator, AlpA | | afdb-uniprot50 | AF-A0A0F3ILT4-F1-MODEL\_V4 | 1.0 | 0.004983 | 144 | 0.258 | 62 | 43 | 2 | 2 | 62 | 9 | 68 | Uncharacterized protein | Uncharacterized protein | | afdb-uniprot50 | AF-I9WCR2-F1-MODEL\_V4 | 1.0 | 0.006502 | 144 | 0.202 | 69 | 53 | 1 | 5 | 73 | 13 | 79 | HTH\_17 domain-containing protein | HTH\_17 domain-containing protein | | afdb-uniprot50 | AF-A0A3G7HH05-F1-MODEL\_V4 | 1.0 | 0.001963 | 144 | 0.256 | 74 | 52 | 2 | 1 | 73 | 9 | 80 | HTH\_17 domain-containing protein | HTH\_17 domain-containing protein | | afdb-uniprot50 | AF-A0A225SLN1-F1-MODEL\_V4 | 1.0 | 0.004983 | 144 | 0.303 | 66 | 44 | 1 | 8 | 73 | 13 | 76 | Uncharacterized protein | Uncharacterized protein | | afdb-uniprot50 | AF-A0A1H3I1Q4-F1-MODEL\_V4 | 1.0 | 0.001963 | 144 | 0.243 | 74 | 53 | 2 | 1 | 73 | 8 | 79 | DNA binding domain-containing protein, excisionase family | DNA binding domain-containing protein, excisionase family | | afdb-uniprot50 | AF-A0A2N0FF21-F1-MODEL\_V4 | 1.0 | 0.002243 | 144 | 0.253 | 71 | 47 | 2 | 1 | 66 | 12 | 81 | Helix-turn-helix protein | Helix-turn-helix protein | | afdb-uniprot50 | AF-A0A2T3GGH2-F1-MODEL\_V4 | 1.0 | 0.002562 | 144 | 0.241 | 87 | 49 | 3 | 1 | 73 | 2 | 85 | DNA-binding protein | DNA-binding protein | | afdb-uniprot50 | AF-A0A844B6Q9-F1-MODEL\_V4 | 1.0 | 0.005326 | 144 | 0.205 | 73 | 55 | 2 | 1 | 73 | 13 | 82 | Helix-turn-helix domain-containing protein | Helix-turn-helix domain-containing protein | | afdb-uniprot50 | AF-A0A5C8ULD5-F1-MODEL\_V4 | 1.0 | 0.004662 | 144 | 0.253 | 71 | 51 | 1 | 1 | 71 | 17 | 85 | DNA-binding protein | DNA-binding protein | | afdb-uniprot50 | AF-A0A4Y3VET6-F1-MODEL\_V4 | 1.0 | 0.004081 | 144 | 0.208 | 72 | 52 | 2 | 2 | 73 | 24 | 90 | HTH\_17 domain-containing protein | HTH\_17 domain-containing protein | | afdb-uniprot50 | AF-A0A846WWH9-F1-MODEL\_V4 | 1.0 | 0.002927 | 144 | 0.314 | 70 | 44 | 2 | 2 | 69 | 24 | 91 | Helix-turn-helix domain-containing protein | Helix-turn-helix domain-containing protein | | afdb-uniprot50 | AF-A0A8B5TVS5-F1-MODEL\_V4 | 1.0 | 0.003573 | 144 | 0.253 | 75 | 52 | 2 | 1 | 73 | 24 | 96 | Helix-turn-helix domain-containing protein | Helix-turn-helix domain-containing protein | | afdb-uniprot50 | AF-A0A7V8K6X7-F1-MODEL\_V4 | 1.0 | 0.008485 | 144 | 0.184 | 65 | 51 | 1 | 9 | 73 | 29 | 91 | Uncharacterized protein | Uncharacterized protein | | afdb-uniprot50 | AF-A0A503YCB6-F1-MODEL\_V4 | 1.0 | 0.001963 | 144 | 0.253 | 75 | 52 | 2 | 1 | 73 | 26 | 98 | Helix-turn-helix domain-containing protein | Helix-turn-helix domain-containing protein | | afdb-uniprot50 | AF-A0A2Z6AYS9-F1-MODEL\_V4 | 1.0 | 0.004362 | 144 | 0.267 | 71 | 49 | 2 | 3 | 73 | 40 | 107 | HTH\_17 domain-containing protein | HTH\_17 domain-containing protein | | afdb-uniprot50 | AF-D2S8Y0-F1-MODEL\_V4 | 1.0 | 0.002243 | 144 | 0.233 | 77 | 53 | 3 | 1 | 73 | 1 | 75 | Putative transcriptional regulator, MerR family | Putative transcriptional regulator, MerR family | | afdb-uniprot50 | AF-A0A6I6EB25-F1-MODEL\_V4 | 1.0 | 0.007939 | 143 | 0.269 | 63 | 44 | 1 | 1 | 63 | 1 | 61 | DNA-binding protein | DNA-binding protein | | afdb-uniprot50 | AF-A0A7W1TAK0-F1-MODEL\_V4 | 1.0 | 0.004081 | 143 | 0.317 | 63 | 41 | 2 | 1 | 63 | 1 | 61 | Helix-turn-helix domain-containing protein | Helix-turn-helix domain-containing protein | | afdb-uniprot50 | AF-A0A0F9SYS0-F1-MODEL\_V4 | 1.0 | 0.002927 | 143 | 0.25 | 72 | 48 | 3 | 1 | 69 | 1 | 69 | Uncharacterized protein | Uncharacterized protein | | afdb-uniprot50 | AF-A0A1C4T028-F1-MODEL\_V4 | 1.0 | 0.006502 | 143 | 0.261 | 65 | 46 | 1 | 1 | 65 | 6 | 68 | DNA binding domain-containing protein, excisionase family | DNA binding domain-containing protein, excisionase family | | afdb-uniprot50 | AF-A0A382E9C4-F1-MODEL\_V4 | 1.0 | 0.002243 | 143 | 0.297 | 74 | 48 | 2 | 1 | 72 | 1 | 72 | Uncharacterized protein | Uncharacterized protein | | afdb-uniprot50 | AF-A0A1P8XFC1-F1-MODEL\_V4 | 1.0 | 0.001153 | 143 | 0.333 | 75 | 41 | 3 | 1 | 73 | 2 | 69 | Uncharacterized protein | Uncharacterized protein | | afdb-uniprot50 | AF-A0A7W1GPM6-F1-MODEL\_V4 | 1.0 | 0.005692 | 143 | 0.285 | 63 | 42 | 2 | 11 | 73 | 4 | 63 | Helix-turn-helix domain-containing protein | Helix-turn-helix domain-containing protein | | afdb-uniprot50 | AF-A0A127ENA5-F1-MODEL\_V4 | 1.0 | 0.003573 | 143 | 0.256 | 74 | 52 | 2 | 1 | 73 | 1 | 72 | Excisionase family DNA binding domain-containing protein | Excisionase family DNA binding domain-containing protein | | afdb-uniprot50 | AF-A0A1C5D9B9-F1-MODEL\_V4 | 1.0 | 0.003343 | 143 | 0.246 | 73 | 47 | 2 | 1 | 67 | 1 | 71 | Helix-turn-helix domain-containing protein | Helix-turn-helix domain-containing protein | | afdb-uniprot50 | AF-A0A5H2Y9R4-F1-MODEL\_V4 | 1.0 | 0.004081 | 143 | 0.232 | 73 | 54 | 1 | 1 | 73 | 1 | 71 | HTH\_17 domain-containing protein | HTH\_17 domain-containing protein | | afdb-uniprot50 | AF-W6KAN3-F1-MODEL\_V4 | 1.0 | 0.002562 | 143 | 0.302 | 76 | 48 | 2 | 1 | 73 | 1 | 74 | Putative DNA binding domain protein, excisionase family | Putative DNA binding domain protein, excisionase family | | afdb-uniprot50 | AF-A0A512JRE3-F1-MODEL\_V4 | 1.0 | 0.002738 | 143 | 0.306 | 75 | 48 | 2 | 1 | 73 | 1 | 73 | HTH\_17 domain-containing protein | HTH\_17 domain-containing protein | | afdb-uniprot50 | AF-A0A4Z1CN04-F1-MODEL\_V4 | 1.0 | 0.002562 | 143 | 0.246 | 73 | 51 | 3 | 1 | 72 | 7 | 76 | DNA-binding protein | DNA-binding protein | | afdb-uniprot50 | AF-A0A6A8W264-F1-MODEL\_V4 | 1.0 | 0.00695 | 143 | 0.217 | 69 | 51 | 2 | 1 | 69 | 11 | 76 | AlpA family phage regulatory protein | AlpA family phage regulatory protein | | afdb-uniprot50 | AF-A0A6L7XK63-F1-MODEL\_V4 | 1.0 | 0.003343 | 143 | 0.285 | 77 | 49 | 2 | 1 | 73 | 1 | 75 | Helix-turn-helix domain-containing protein | Helix-turn-helix domain-containing protein | | afdb-uniprot50 | AF-A0A839UPM0-F1-MODEL\_V4 | 1.0 | 0.001719 | 143 | 0.222 | 72 | 53 | 2 | 3 | 73 | 12 | 81 | Putative DNA-binding transcriptional regulator AlpA | Putative DNA-binding transcriptional regulator AlpA | | afdb-uniprot50 | AF-A0A7I7VI49-F1-MODEL\_V4 | 1.0 | 0.006084 | 143 | 0.307 | 65 | 42 | 2 | 2 | 65 | 10 | 72 | HTH\_17 domain-containing protein | HTH\_17 domain-containing protein | | afdb-uniprot50 | AF-A0A0S3F4D4-F1-MODEL\_V4 | 1.0 | 0.003573 | 143 | 0.208 | 67 | 49 | 2 | 9 | 73 | 17 | 81 | Uncharacterized protein | Uncharacterized protein | | afdb-uniprot50 | AF-A0A2N3BAH4-F1-MODEL\_V4 | 1.0 | 0.003573 | 143 | 0.222 | 81 | 53 | 2 | 1 | 73 | 1 | 79 | Transcriptional regulator | Transcriptional regulator | | afdb-uniprot50 | AF-A0A062VUZ0-F1-MODEL\_V4 | 1.0 | 0.002098 | 143 | 0.229 | 74 | 54 | 2 | 1 | 73 | 7 | 78 | HTH\_17 domain-containing protein | HTH\_17 domain-containing protein | | afdb-uniprot50 | AF-A0A2A2ZNT2-F1-MODEL\_V4 | 1.0 | 0.003128 | 143 | 0.275 | 69 | 42 | 3 | 1 | 63 | 13 | 79 | DNA-binding protein | DNA-binding protein | | afdb-uniprot50 | AF-A0A420WJK2-F1-MODEL\_V4 | 1.0 | 0.004362 | 143 | 0.232 | 73 | 52 | 2 | 1 | 71 | 10 | 80 | Helix-turn-helix protein | Helix-turn-helix protein | | afdb-uniprot50 | AF-A0A367A4J1-F1-MODEL\_V4 | 1.0 | 0.006502 | 143 | 0.264 | 68 | 48 | 1 | 1 | 68 | 30 | 95 | HTH\_17 domain-containing protein | HTH\_17 domain-containing protein | | afdb-uniprot50 | AF-A0A1H8Y2E5-F1-MODEL\_V4 | 1.0 | 0.001608 | 143 | 0.253 | 75 | 44 | 2 | 1 | 65 | 24 | 96 | DNA binding domain-containing protein, excisionase family | DNA binding domain-containing protein, excisionase family | | afdb-uniprot50 | AF-A0A841NDL1-F1-MODEL\_V4 | 1.0 | 0.006502 | 143 | 0.261 | 65 | 44 | 2 | 2 | 64 | 39 | 101 | Excisionase family DNA binding protein | Excisionase family DNA binding protein | | afdb-uniprot50 | AF-A0A329LI23-F1-MODEL\_V4 | 1.0 | 0.004362 | 143 | 0.238 | 67 | 49 | 1 | 1 | 67 | 61 | 125 | HTH\_17 domain-containing protein | HTH\_17 domain-containing protein | | afdb-uniprot50 | AF-A0A2W4JHC9-F1-MODEL\_V4 | 1.0 | 0.006084 | 143 | 0.202 | 74 | 56 | 2 | 1 | 73 | 3 | 74 | HTH\_17 domain-containing protein | HTH\_17 domain-containing protein | | afdb-uniprot50 | AF-A0A2K4LH34-F1-MODEL\_V4 | 1.0 | 0.006084 | 142 | 0.269 | 63 | 44 | 1 | 11 | 73 | 3 | 63 | DNA-binding protein | DNA-binding protein | | afdb-uniprot50 | AF-A0A2H9N9A0-F1-MODEL\_V4 | 1.0 | 0.007939 | 142 | 0.285 | 63 | 42 | 2 | 11 | 72 | 4 | 64 | HTH\_17 domain-containing protein | HTH\_17 domain-containing protein | | afdb-uniprot50 | AF-A0A126UVT8-F1-MODEL\_V4 | 1.0 | 0.005326 | 142 | 0.236 | 72 | 51 | 3 | 1 | 72 | 1 | 68 | HTH\_17 domain-containing protein | HTH\_17 domain-containing protein | | afdb-uniprot50 | AF-A0A449CYP3-F1-MODEL\_V4 | 1.0 | 0.004081 | 142 | 0.231 | 69 | 49 | 2 | 1 | 67 | 1 | 67 | Uncharacterized protein | Uncharacterized protein | | afdb-uniprot50 | AF-A0A7Y0UIH7-F1-MODEL\_V4 | 1.0 | 0.006502 | 142 | 0.242 | 66 | 47 | 2 | 7 | 71 | 1 | 64 | Helix-turn-helix domain-containing protein | Helix-turn-helix domain-containing protein | | afdb-uniprot50 | AF-A0A7X5VIB6-F1-MODEL\_V4 | 1.0 | 0.004081 | 142 | 0.26 | 73 | 49 | 2 | 1 | 73 | 1 | 68 | Excisionase family DNA binding protein | Excisionase family DNA binding protein | | afdb-uniprot50 | AF-A0A7Z9V025-F1-MODEL\_V4 | 1.0 | 0.004983 | 142 | 0.274 | 62 | 43 | 1 | 2 | 63 | 4 | 63 | DNA-binding protein | DNA-binding protein | | afdb-uniprot50 | AF-A0A1B2A925-F1-MODEL\_V4 | 1.0 | 0.009692 | 142 | 0.234 | 64 | 46 | 2 | 10 | 73 | 8 | 68 | Helix-turn-helix domain protein | Helix-turn-helix domain protein | | afdb-uniprot50 | AF-A0A4Z1CGY7-F1-MODEL\_V4 | 1.0 | 0.003573 | 142 | 0.279 | 68 | 43 | 3 | 1 | 64 | 1 | 66 | DNA-binding protein | DNA-binding protein | | afdb-uniprot50 | AF-A0A6N6T802-F1-MODEL\_V4 | 1.0 | 0.001608 | 142 | 0.31 | 74 | 48 | 2 | 1 | 73 | 1 | 72 | Helix-turn-helix domain-containing protein | Helix-turn-helix domain-containing protein | | afdb-uniprot50 | AF-A0A382R345-F1-MODEL\_V4 | 1.0 | 0.004983 | 142 | 0.296 | 64 | 42 | 2 | 1 | 63 | 2 | 63 | HTH\_17 domain-containing protein | HTH\_17 domain-containing protein | | afdb-uniprot50 | AF-A0A1W6NWZ4-F1-MODEL\_V4 | 1.0 | 0.006084 | 142 | 0.186 | 75 | 57 | 2 | 1 | 73 | 3 | 75 | Phenylglyoxylate:acceptor oxidoreductase | Phenylglyoxylate:acceptor oxidoreductase | | afdb-uniprot50 | AF-A0A4S5ESI4-F1-MODEL\_V4 | 1.0 | 0.004662 | 142 | 0.218 | 64 | 47 | 2 | 1 | 63 | 13 | 74 | Helix-turn-helix domain-containing protein | Helix-turn-helix domain-containing protein | | afdb-uniprot50 | AF-A0A4V4RAK6-F1-MODEL\_V4 | 1.0 | 0.008485 | 142 | 0.246 | 65 | 44 | 1 | 2 | 66 | 20 | 79 | Uncharacterized protein | Uncharacterized protein | | afdb-uniprot50 | AF-A0A3N5ZHA9-F1-MODEL\_V4 | 1.0 | 0.003128 | 142 | 0.24 | 75 | 52 | 2 | 2 | 73 | 8 | 80 | DNA-binding protein | DNA-binding protein | | afdb-uniprot50 | AF-A0A7G8YVT3-F1-MODEL\_V4 | 1.0 | 0.001963 | 142 | 0.202 | 79 | 55 | 3 | 1 | 73 | 2 | 78 | Helix-turn-helix domain-containing protein | Helix-turn-helix domain-containing protein | | afdb-uniprot50 | AF-A0A7W1WKP4-F1-MODEL\_V4 | 1.0 | 0.002098 | 142 | 0.181 | 77 | 56 | 3 | 1 | 72 | 5 | 79 | Helix-turn-helix domain-containing protein | Helix-turn-helix domain-containing protein | | afdb-uniprot50 | AF-H5Y6A6-F1-MODEL\_V4 | 1.0 | 0.006084 | 142 | 0.154 | 71 | 57 | 2 | 1 | 71 | 15 | 82 | Uncharacterized protein | Uncharacterized protein | | afdb-uniprot50 | AF-A0A512NPT7-F1-MODEL\_V4 | 1.0 | 0.001719 | 142 | 0.256 | 74 | 48 | 3 | 1 | 72 | 17 | 85 | HTH\_17 domain-containing protein | HTH\_17 domain-containing protein | | afdb-uniprot50 | AF-A0A2V9IUE2-F1-MODEL\_V4 | 1.0 | 0.007939 | 142 | 0.222 | 63 | 47 | 1 | 11 | 73 | 3 | 63 | HTH\_17 domain-containing protein | HTH\_17 domain-containing protein | | afdb-uniprot50 | AF-A0A2S8CG40-F1-MODEL\_V4 | 1.0 | 0.004362 | 142 | 0.191 | 68 | 52 | 2 | 2 | 68 | 35 | 100 | HTH\_17 domain-containing protein | HTH\_17 domain-containing protein | | afdb-uniprot50 | AF-A0A328N1Y7-F1-MODEL\_V4 | 1.0 | 0.001719 | 142 | 0.281 | 71 | 45 | 3 | 2 | 72 | 43 | 107 | HTH\_17 domain-containing protein | HTH\_17 domain-containing protein | | afdb-uniprot50 | AF-A0A7Y9ZK00-F1-MODEL\_V4 | 1.0 | 0.009692 | 142 | 0.2 | 65 | 50 | 1 | 1 | 65 | 45 | 107 | Putative DNA-binding transcriptional regulator AlpA | Putative DNA-binding transcriptional regulator AlpA | | afdb-uniprot50 | AF-A0A327JJ67-F1-MODEL\_V4 | 1.0 | 0.003573 | 142 | 0.277 | 72 | 50 | 1 | 2 | 73 | 43 | 112 | HTH\_17 domain-containing protein | HTH\_17 domain-containing protein | | afdb-uniprot50 | AF-A0A2E8YX83-F1-MODEL\_V4 | 1.0 | 0.001719 | 142 | 0.263 | 76 | 51 | 3 | 1 | 73 | 44 | 117 | HTH\_17 domain-containing protein | HTH\_17 domain-containing protein | | afdb-uniprot50 | AF-A0A2G7CQS8-F1-MODEL\_V4 | 1.0 | 0.003819 | 142 | 0.228 | 70 | 49 | 2 | 1 | 67 | 62 | 129 | Helix-turn-helix protein | Helix-turn-helix protein | | afdb-uniprot50 | AF-A0A5B0V902-F1-MODEL\_V4 | 1.0 | 0.009692 | 141 | 0.222 | 63 | 47 | 1 | 11 | 73 | 4 | 64 | Helix-turn-helix domain-containing protein | Helix-turn-helix domain-containing protein | | afdb-uniprot50 | AF-A0A3N1AY40-F1-MODEL\_V4 | 1.0 | 0.007428 | 141 | 0.184 | 65 | 51 | 1 | 1 | 65 | 3 | 65 | Helix-turn-helix protein | Helix-turn-helix protein | | afdb-uniprot50 | AF-A0A1H8K0G4-F1-MODEL\_V4 | 1.0 | 0.003573 | 141 | 0.214 | 70 | 50 | 2 | 4 | 73 | 1 | 65 | DNA binding domain, excisionase family | DNA binding domain, excisionase family | | afdb-uniprot50 | AF-A0A1X0XIA3-F1-MODEL\_V4 | 1.0 | 0.003128 | 141 | 0.298 | 67 | 42 | 2 | 10 | 73 | 3 | 67 | Uncharacterized protein | Uncharacterized protein | | afdb-uniprot50 | AF-A0A2W6CXR4-F1-MODEL\_V4 | 1.0 | 0.004662 | 141 | 0.25 | 72 | 45 | 3 | 1 | 66 | 1 | 69 | HTH\_17 domain-containing protein | HTH\_17 domain-containing protein | | afdb-uniprot50 | AF-A0A7W0SNE2-F1-MODEL\_V4 | 1.0 | 0.006502 | 141 | 0.2 | 65 | 50 | 1 | 1 | 65 | 2 | 64 | Helix-turn-helix domain-containing protein | Helix-turn-helix domain-containing protein | | afdb-uniprot50 | AF-A0A2R4WUM3-F1-MODEL\_V4 | 1.0 | 0.001232 | 141 | 0.294 | 78 | 45 | 3 | 1 | 73 | 10 | 82 | DNA-binding protein | DNA-binding protein | | afdb-uniprot50 | AF-A0A386UJQ9-F1-MODEL\_V4 | 1.0 | 0.006502 | 141 | 0.171 | 76 | 58 | 2 | 1 | 73 | 6 | 79 | DNA-binding protein | DNA-binding protein | | afdb-uniprot50 | AF-A0A7Y0B853-F1-MODEL\_V4 | 1.0 | 0.002098 | 140 | 0.333 | 69 | 38 | 3 | 1 | 69 | 1 | 61 | Helix-turn-helix domain-containing protein | Helix-turn-helix domain-containing protein | | afdb-uniprot50 | AF-A0A1Q8KIY1-F1-MODEL\_V4 | 1.0 | 0.007939 | 140 | 0.276 | 65 | 42 | 2 | 3 | 67 | 2 | 61 | DNA binding domain, excisionase/Xis | DNA binding domain, excisionase/Xis | | afdb-uniprot50 | AF-A0A6G4X2R8-F1-MODEL\_V4 | 1.0 | 0.009068 | 140 | 0.268 | 67 | 45 | 2 | 1 | 67 | 5 | 67 | Helix-turn-helix domain-containing protein | Helix-turn-helix domain-containing protein | | afdb-uniprot50 | AF-K2AU10-F1-MODEL\_V4 | 1.0 | 0.006502 | 140 | 0.292 | 65 | 43 | 1 | 11 | 72 | 4 | 68 | Uncharacterized protein | Uncharacterized protein | | afdb-uniprot50 | AF-A0A543P9T2-F1-MODEL\_V4 | 1.0 | 0.003819 | 140 | 0.289 | 69 | 46 | 2 | 1 | 68 | 2 | 68 | Excisionase family DNA binding protein | Excisionase family DNA binding protein | | afdb-uniprot50 | AF-A0A2M8W6L7-F1-MODEL\_V4 | 1.0 | 0.009692 | 140 | 0.222 | 63 | 47 | 1 | 1 | 63 | 7 | 67 | AlpA family transcriptional regulator | AlpA family transcriptional regulator | | afdb-uniprot50 | AF-A0A0R3E397-F1-MODEL\_V4 | 1.0 | 0.004662 | 140 | 0.273 | 73 | 49 | 2 | 3 | 73 | 5 | 75 | Uncharacterized protein | Uncharacterized protein | | afdb-uniprot50 | AF-A0A1X1MGL1-F1-MODEL\_V4 | 1.0 | 0.005692 | 140 | 0.205 | 73 | 54 | 2 | 1 | 73 | 1 | 69 | Uncharacterized protein | Uncharacterized protein | | afdb-uniprot50 | AF-A0A2N1I4M7-F1-MODEL\_V4 | 1.0 | 0.004983 | 140 | 0.268 | 67 | 45 | 2 | 2 | 66 | 9 | 73 | Uncharacterized protein | Uncharacterized protein | | afdb-uniprot50 | AF-A0A516Q5F3-F1-MODEL\_V4 | 1.0 | 0.005326 | 140 | 0.202 | 69 | 51 | 2 | 1 | 67 | 1 | 67 | Helix-turn-helix domain-containing protein | Helix-turn-helix domain-containing protein | | afdb-uniprot50 | AF-A0A7J0BY77-F1-MODEL\_V4 | 1.0 | 0.001837 | 140 | 0.293 | 75 | 49 | 3 | 1 | 73 | 9 | 81 | HTH\_17 domain-containing protein | HTH\_17 domain-containing protein | | afdb-uniprot50 | AF-A0A530LMQ4-F1-MODEL\_V4 | 1.0 | 0.00695 | 140 | 0.246 | 69 | 50 | 1 | 5 | 73 | 11 | 77 | Helix-turn-helix domain-containing protein | Helix-turn-helix domain-containing protein | | afdb-uniprot50 | AF-A0A1E4PMA0-F1-MODEL\_V4 | 1.0 | 0.003819 | 140 | 0.279 | 68 | 42 | 2 | 1 | 63 | 4 | 69 | HTH\_17 domain-containing protein | HTH\_17 domain-containing protein | | afdb-uniprot50 | AF-A0A0V2FH72-F1-MODEL\_V4 | 1.0 | 0.001963 | 140 | 0.337 | 74 | 43 | 4 | 1 | 73 | 1 | 69 | Transcriptional regulator | Transcriptional regulator | | afdb-uniprot50 | AF-A5EBE1-F1-MODEL\_V4 | 1.0 | 0.002927 | 140 | 0.26 | 73 | 49 | 3 | 1 | 73 | 3 | 70 | Uncharacterized protein | Uncharacterized protein | | afdb-uniprot50 | AF-A0A7K0CZS0-F1-MODEL\_V4 | 1.0 | 0.005326 | 140 | 0.207 | 77 | 55 | 3 | 1 | 73 | 31 | 105 | HTH\_17 domain-containing protein | HTH\_17 domain-containing protein | | afdb-uniprot50 | AF-A0A653Z374-F1-MODEL\_V4 | 1.0 | 0.006084 | 140 | 0.285 | 63 | 42 | 2 | 1 | 62 | 33 | 93 | HTH\_17 domain-containing protein | HTH\_17 domain-containing protein | | afdb-uniprot50 | AF-A0A087KGM0-F1-MODEL\_V4 | 1.0 | 0.009068 | 139 | 0.225 | 62 | 46 | 1 | 6 | 67 | 2 | 61 | MerR family transcriptional regulator | MerR family transcriptional regulator | | afdb-uniprot50 | AF-A0A1Y4CKH7-F1-MODEL\_V4 | 1.0 | 0.006502 | 139 | 0.268 | 67 | 45 | 2 | 1 | 65 | 2 | 66 | HTH\_17 domain-containing protein | HTH\_17 domain-containing protein | | afdb-uniprot50 | AF-A0A7W8FGC6-F1-MODEL\_V4 | 1.0 | 0.00695 | 139 | 0.278 | 61 | 41 | 2 | 4 | 63 | 2 | 60 | Uncharacterized protein | Uncharacterized protein | | afdb-uniprot50 | AF-A0A0J6XLH8-F1-MODEL\_V4 | 1.0 | 0.006084 | 139 | 0.268 | 67 | 46 | 2 | 1 | 67 | 1 | 64 | DNA-binding protein | DNA-binding protein | | afdb-uniprot50 | AF-A0A0U3FN39-F1-MODEL\_V4 | 1.0 | 0.005326 | 139 | 0.236 | 72 | 49 | 2 | 1 | 72 | 5 | 70 | HTH\_17 domain-containing protein | HTH\_17 domain-containing protein | | afdb-uniprot50 | AF-A0A238Y6F1-F1-MODEL\_V4 | 1.0 | 0.008485 | 139 | 0.268 | 67 | 47 | 1 | 1 | 67 | 2 | 66 | DNA binding domain-containing protein, excisionase family | DNA binding domain-containing protein, excisionase family | | afdb-uniprot50 | AF-A0A7Y7M0V9-F1-MODEL\_V4 | 1.0 | 0.005326 | 139 | 0.253 | 63 | 44 | 2 | 1 | 62 | 6 | 66 | Helix-turn-helix domain-containing protein | Helix-turn-helix domain-containing protein | | afdb-uniprot50 | AF-A0A1H0BM39-F1-MODEL\_V4 | 1.0 | 0.003819 | 139 | 0.289 | 69 | 45 | 3 | 1 | 67 | 1 | 67 | DNA binding domain-containing protein, excisionase family | DNA binding domain-containing protein, excisionase family | | afdb-uniprot50 | AF-A0A4R8S6P8-F1-MODEL\_V4 | 1.0 | 0.003819 | 139 | 0.226 | 75 | 53 | 3 | 1 | 73 | 1 | 72 | Helix-turn-helix domain protein | Helix-turn-helix domain protein | | afdb-uniprot50 | AF-A0A1M5SRH2-F1-MODEL\_V4 | 1.0 | 0.005326 | 139 | 0.24 | 75 | 53 | 2 | 1 | 73 | 1 | 73 | Helix-turn-helix domain-containing protein | Helix-turn-helix domain-containing protein | | afdb-uniprot50 | AF-A0A4Q2RK28-F1-MODEL\_V4 | 1.0 | 0.008485 | 139 | 0.223 | 67 | 50 | 1 | 1 | 67 | 5 | 69 | DNA-binding protein | DNA-binding protein | | afdb-uniprot50 | AF-A0A200HEW4-F1-MODEL\_V4 | 1.0 | 0.004662 | 139 | 0.219 | 73 | 54 | 2 | 1 | 73 | 4 | 73 | HTH\_17 domain-containing protein | HTH\_17 domain-containing protein | | afdb-uniprot50 | AF-A0A2W6C6E5-F1-MODEL\_V4 | 1.0 | 0.004662 | 139 | 0.303 | 79 | 46 | 2 | 1 | 72 | 1 | 77 | DNA-binding protein | DNA-binding protein | | afdb-uniprot50 | AF-A0A1I1ZX04-F1-MODEL\_V4 | 1.0 | 0.003819 | 139 | 0.191 | 73 | 52 | 2 | 1 | 73 | 2 | 67 | DNA binding domain-containing protein, excisionase family | DNA binding domain-containing protein, excisionase family | | afdb-uniprot50 | AF-A0A1H9I537-F1-MODEL\_V4 | 1.0 | 0.003573 | 139 | 0.228 | 70 | 50 | 3 | 1 | 69 | 10 | 76 | Helix-turn-helix domain-containing protein | Helix-turn-helix domain-containing protein | | afdb-uniprot50 | AF-A0A7Z0J817-F1-MODEL\_V4 | 1.0 | 0.003819 | 139 | 0.26 | 73 | 50 | 3 | 1 | 73 | 11 | 79 | Putative DNA-binding transcriptional regulator AlpA | Putative DNA-binding transcriptional regulator AlpA | | afdb-uniprot50 | AF-A0A430F7K9-F1-MODEL\_V4 | 1.0 | 0.005692 | 139 | 0.211 | 71 | 51 | 3 | 4 | 71 | 2 | 70 | HTH\_17 domain-containing protein | HTH\_17 domain-containing protein | | afdb-uniprot50 | AF-A0A1I0Z939-F1-MODEL\_V4 | 1.0 | 0.003573 | 139 | 0.25 | 68 | 45 | 3 | 2 | 66 | 20 | 84 | Helix-turn-helix domain-containing protein | Helix-turn-helix domain-containing protein | | afdb-uniprot50 | AF-A0A4U7MSR7-F1-MODEL\_V4 | 1.0 | 0.007939 | 139 | 0.205 | 73 | 55 | 2 | 2 | 73 | 13 | 83 | Helix-turn-helix domain-containing protein | Helix-turn-helix domain-containing protein | | afdb-uniprot50 | AF-A0A5M9ZFW5-F1-MODEL\_V4 | 1.0 | 0.006502 | 139 | 0.206 | 63 | 48 | 1 | 1 | 63 | 25 | 85 | DNA-binding protein | DNA-binding protein | | afdb-uniprot50 | AF-A0A4R1K3A1-F1-MODEL\_V4 | 1.0 | 0.005692 | 139 | 0.238 | 67 | 47 | 2 | 6 | 72 | 42 | 104 | Uncharacterized protein | Uncharacterized protein | | afdb-uniprot50 | AF-A0A1I1T9U6-F1-MODEL\_V4 | 1.0 | 0.009692 | 139 | 0.257 | 66 | 46 | 2 | 2 | 67 | 41 | 103 | Helix-turn-helix domain-containing protein | Helix-turn-helix domain-containing protein | | afdb-uniprot50 | AF-A0A544Y1F3-F1-MODEL\_V4 | 1.0 | 0.00695 | 138 | 0.253 | 63 | 43 | 3 | 2 | 62 | 4 | 64 | Helix-turn-helix domain-containing protein | Helix-turn-helix domain-containing protein | | afdb-uniprot50 | AF-A0A7W3U6U0-F1-MODEL\_V4 | 1.0 | 0.006502 | 138 | 0.278 | 61 | 41 | 2 | 4 | 63 | 1 | 59 | Helix-turn-helix domain-containing protein | Helix-turn-helix domain-containing protein | | afdb-uniprot50 | AF-A0A4Y3WPK9-F1-MODEL\_V4 | 1.0 | 0.005692 | 138 | 0.238 | 67 | 45 | 2 | 11 | 73 | 5 | 69 | HTH\_17 domain-containing protein | HTH\_17 domain-containing protein | | afdb-uniprot50 | AF-A0A081HUQ7-F1-MODEL\_V4 | 1.0 | 0.005692 | 138 | 0.235 | 68 | 45 | 2 | 11 | 73 | 4 | 69 | HTH\_17 domain-containing protein | HTH\_17 domain-containing protein | | afdb-uniprot50 | AF-A0A2S5U1P4-F1-MODEL\_V4 | 1.0 | 0.001505 | 138 | 0.243 | 74 | 50 | 3 | 1 | 73 | 1 | 69 | Excisionase | Excisionase | | afdb-uniprot50 | AF-H6RJB0-F1-MODEL\_V4 | 1.0 | 0.003819 | 138 | 0.26 | 69 | 47 | 3 | 1 | 67 | 1 | 67 | Transcriptional regulator, MerR family | Transcriptional regulator, MerR family | | afdb-uniprot50 | AF-A0A0Q5J0X5-F1-MODEL\_V4 | 1.0 | 0.004362 | 138 | 0.24 | 75 | 50 | 3 | 1 | 73 | 6 | 75 | HTH\_17 domain-containing protein | HTH\_17 domain-containing protein | | afdb-uniprot50 | AF-A0A7I7MBG1-F1-MODEL\_V4 | 1.0 | 0.009692 | 138 | 0.223 | 67 | 51 | 1 | 1 | 67 | 8 | 73 | HTH\_17 domain-containing protein | HTH\_17 domain-containing protein | | afdb-uniprot50 | AF-A0A6D1I828-F1-MODEL\_V4 | 1.0 | 0.004983 | 138 | 0.222 | 72 | 54 | 2 | 2 | 72 | 6 | 76 | DNA-binding protein | DNA-binding protein | | afdb-uniprot50 | AF-A0A238JFU8-F1-MODEL\_V4 | 1.0 | 0.007939 | 138 | 0.218 | 64 | 47 | 2 | 1 | 63 | 12 | 73 | Uncharacterized protein | Uncharacterized protein | | afdb-uniprot50 | AF-A0A7Y9MMD5-F1-MODEL\_V4 | 1.0 | 0.002098 | 138 | 0.278 | 79 | 49 | 3 | 1 | 73 | 1 | 77 | Putative DNA-binding transcriptional regulator AlpA | Putative DNA-binding transcriptional regulator AlpA | | afdb-uniprot50 | AF-E9USC6-F1-MODEL\_V4 | 1.0 | 0.003573 | 138 | 0.246 | 73 | 51 | 3 | 1 | 72 | 12 | 81 | Conserved domain protein | Conserved domain protein | | afdb-uniprot50 | AF-A0A1V0KL81-F1-MODEL\_V4 | 1.0 | 0.004983 | 138 | 0.253 | 75 | 52 | 2 | 1 | 73 | 5 | 77 | HTH\_17 domain-containing protein | HTH\_17 domain-containing protein | | afdb-uniprot50 | AF-A0A7Y9J849-F1-MODEL\_V4 | 1.0 | 0.002738 | 138 | 0.194 | 72 | 52 | 2 | 2 | 73 | 18 | 83 | Putative DNA-binding transcriptional regulator AlpA | Putative DNA-binding transcriptional regulator AlpA | | afdb-uniprot50 | AF-A0A0F2QH64-F1-MODEL\_V4 | 1.0 | 0.007428 | 138 | 0.27 | 74 | 48 | 3 | 2 | 73 | 21 | 90 | HTH\_17 domain-containing protein | HTH\_17 domain-containing protein | | afdb-uniprot50 | AF-A0A2N5CW05-F1-MODEL\_V4 | 1.0 | 0.005326 | 138 | 0.26 | 73 | 50 | 4 | 1 | 73 | 11 | 79 | DNA-binding protein | DNA-binding protein | | afdb-uniprot50 | AF-A0A285KSA3-F1-MODEL\_V4 | 1.0 | 0.002243 | 138 | 0.266 | 75 | 50 | 2 | 1 | 72 | 24 | 96 | DNA binding domain-containing protein, excisionase family | DNA binding domain-containing protein, excisionase family | | afdb-uniprot50 | AF-A0A850Q143-F1-MODEL\_V4 | 1.0 | 0.004362 | 137 | 0.26 | 73 | 46 | 3 | 1 | 73 | 1 | 65 | Helix-turn-helix domain-containing protein | Helix-turn-helix domain-containing protein | | afdb-uniprot50 | AF-A0A1G7GIV7-F1-MODEL\_V4 | 1.0 | 0.009692 | 137 | 0.211 | 71 | 53 | 2 | 1 | 71 | 1 | 68 | Uncharacterized protein | Uncharacterized protein | | afdb-uniprot50 | AF-A0A7T8MJK6-F1-MODEL\_V4 | 1.0 | 0.005326 | 137 | 0.256 | 74 | 52 | 2 | 1 | 73 | 1 | 72 | Helix-turn-helix domain-containing protein | Helix-turn-helix domain-containing protein | | afdb-uniprot50 | AF-A0A101VLE6-F1-MODEL\_V4 | 1.0 | 0.006502 | 137 | 0.219 | 73 | 53 | 2 | 1 | 73 | 1 | 69 | HTH\_17 domain-containing protein | HTH\_17 domain-containing protein | | afdb-uniprot50 | AF-A0A1H0JZA4-F1-MODEL\_V4 | 1.0 | 0.005692 | 137 | 0.219 | 73 | 52 | 3 | 1 | 73 | 6 | 73 | DNA binding domain-containing protein, excisionase family | DNA binding domain-containing protein, excisionase family | | afdb-uniprot50 | AF-A0A543C838-F1-MODEL\_V4 | 1.0 | 0.004983 | 137 | 0.246 | 73 | 49 | 3 | 1 | 73 | 2 | 68 | Helix-turn-helix protein | Helix-turn-helix protein | | afdb-uniprot50 | AF-A0A3G2N4L3-F1-MODEL\_V4 | 1.0 | 0.003128 | 137 | 0.266 | 75 | 51 | 3 | 1 | 73 | 1 | 73 | DNA-binding protein | DNA-binding protein | | afdb-uniprot50 | AF-A0A101AJT1-F1-MODEL\_V4 | 1.0 | 0.008485 | 137 | 0.215 | 65 | 47 | 2 | 1 | 63 | 8 | 70 | HTH\_17 domain-containing protein | HTH\_17 domain-containing protein | | afdb-uniprot50 | AF-A0A846MX55-F1-MODEL\_V4 | 1.0 | 0.004662 | 137 | 0.207 | 77 | 45 | 2 | 1 | 63 | 1 | 75 | Uncharacterized protein | Uncharacterized protein | | afdb-uniprot50 | AF-A0A6N7ZMY0-F1-MODEL\_V4 | 1.0 | 0.004983 | 137 | 0.25 | 76 | 50 | 2 | 2 | 72 | 5 | 78 | Helix-turn-helix domain-containing protein | Helix-turn-helix domain-containing protein | | afdb-uniprot50 | AF-A0A0N1NIG7-F1-MODEL\_V4 | 1.0 | 0.004983 | 137 | 0.278 | 79 | 43 | 2 | 1 | 67 | 1 | 77 | HTH\_17 domain-containing protein | HTH\_17 domain-containing protein | | afdb-uniprot50 | AF-A0A2S9F4K8-F1-MODEL\_V4 | 1.0 | 0.007939 | 137 | 0.268 | 67 | 45 | 3 | 1 | 66 | 16 | 79 | DNA-binding protein | DNA-binding protein | | afdb-uniprot50 | AF-A0A7Z0BSY9-F1-MODEL\_V4 | 1.0 | 0.007939 | 137 | 0.226 | 75 | 54 | 2 | 1 | 73 | 10 | 82 | Putative DNA-binding transcriptional regulator AlpA | Putative DNA-binding transcriptional regulator AlpA | | afdb-uniprot50 | AF-A0A1G7SMF3-F1-MODEL\_V4 | 1.0 | 0.009068 | 137 | 0.178 | 73 | 57 | 2 | 1 | 72 | 9 | 79 | Uncharacterized protein | Uncharacterized protein | | afdb-uniprot50 | AF-A0A7W7ZCS8-F1-MODEL\_V4 | 1.0 | 0.006084 | 137 | 0.232 | 73 | 53 | 3 | 1 | 72 | 4 | 74 | Putative DNA-binding transcriptional regulator AlpA | Putative DNA-binding transcriptional regulator AlpA | | afdb-uniprot50 | AF-A0A2P2C867-F1-MODEL\_V4 | 1.0 | 0.003343 | 137 | 0.185 | 81 | 55 | 3 | 1 | 73 | 4 | 81 | Phage transcriptional regulator, AlpA | Phage transcriptional regulator, AlpA | | afdb-uniprot50 | AF-A0A0Q4WSX8-F1-MODEL\_V4 | 1.0 | 0.006502 | 137 | 0.24 | 79 | 52 | 2 | 3 | 73 | 17 | 95 | Uncharacterized protein | Uncharacterized protein | | afdb-uniprot50 | AF-A0A148N521-F1-MODEL\_V4 | 1.0 | 0.004662 | 136 | 0.279 | 68 | 45 | 3 | 7 | 73 | 2 | 66 | Excisionase | Excisionase | | afdb-uniprot50 | AF-A0A158A0S3-F1-MODEL\_V4 | 1.0 | 0.009068 | 136 | 0.23 | 65 | 48 | 1 | 9 | 73 | 2 | 64 | Uncharacterized protein | Uncharacterized protein | | afdb-uniprot50 | AF-A0A2P6WDG2-F1-MODEL\_V4 | 1.0 | 0.00695 | 136 | 0.194 | 67 | 51 | 2 | 7 | 73 | 3 | 66 | HTH\_17 domain-containing protein | HTH\_17 domain-containing protein | | afdb-uniprot50 | AF-A0A2G2QSP1-F1-MODEL\_V4 | 1.0 | 0.008485 | 136 | 0.257 | 66 | 46 | 2 | 11 | 73 | 3 | 68 | Excisionase | Excisionase | | afdb-uniprot50 | AF-A0A428YP86-F1-MODEL\_V4 | 1.0 | 0.005326 | 136 | 0.267 | 71 | 48 | 2 | 1 | 69 | 1 | 69 | HTH\_17 domain-containing protein | HTH\_17 domain-containing protein | | afdb-uniprot50 | AF-A0A3S3TAN4-F1-MODEL\_V4 | 1.0 | 0.009692 | 136 | 0.274 | 62 | 42 | 2 | 2 | 62 | 10 | 69 | DNA-binding protein | DNA-binding protein | | afdb-uniprot50 | AF-A0A1J0S147-F1-MODEL\_V4 | 1.0 | 0.003573 | 136 | 0.352 | 71 | 41 | 3 | 1 | 70 | 1 | 67 | DNA-binding protein | DNA-binding protein | | afdb-uniprot50 | AF-A0A165EL06-F1-MODEL\_V4 | 1.0 | 0.003573 | 136 | 0.25 | 76 | 51 | 4 | 1 | 73 | 1 | 73 | HTH\_17 domain-containing protein | HTH\_17 domain-containing protein | | afdb-uniprot50 | AF-A0A327KQE5-F1-MODEL\_V4 | 1.0 | 0.003128 | 136 | 0.24 | 79 | 52 | 3 | 1 | 73 | 1 | 77 | HTH\_17 domain-containing protein | HTH\_17 domain-containing protein | | afdb-uniprot50 | AF-A0A841L8L4-F1-MODEL\_V4 | 1.0 | 0.001719 | 136 | 0.24 | 75 | 53 | 3 | 1 | 73 | 6 | 78 | Uncharacterized protein | Uncharacterized protein | | afdb-uniprot50 | AF-A0A0L0LTJ3-F1-MODEL\_V4 | 1.0 | 0.004362 | 136 | 0.223 | 85 | 51 | 3 | 1 | 73 | 1 | 82 | Uncharacterized protein | Uncharacterized protein | | afdb-uniprot50 | AF-A0A1Q5AXY7-F1-MODEL\_V4 | 1.0 | 0.007428 | 136 | 0.208 | 67 | 50 | 2 | 1 | 67 | 15 | 78 | DNA-binding protein | DNA-binding protein | | afdb-uniprot50 | AF-A0A2P6WDT5-F1-MODEL\_V4 | 1.0 | 0.004362 | 136 | 0.272 | 77 | 49 | 3 | 1 | 73 | 8 | 81 | DNA-binding protein | DNA-binding protein | | afdb-uniprot50 | AF-A0A229VYW8-F1-MODEL\_V4 | 1.0 | 0.002243 | 136 | 0.252 | 87 | 48 | 4 | 1 | 72 | 1 | 85 | Phage transcriptional regulator AlpA | Phage transcriptional regulator AlpA | | afdb-uniprot50 | AF-A0A259MIW6-F1-MODEL\_V4 | 1.0 | 0.004362 | 136 | 0.253 | 71 | 49 | 1 | 1 | 67 | 29 | 99 | Uncharacterized protein | Uncharacterized protein | | afdb-uniprot50 | AF-A0A2T6M167-F1-MODEL\_V4 | 1.0 | 0.005692 | 136 | 0.298 | 67 | 43 | 2 | 2 | 66 | 89 | 153 | Uncharacterized protein | Uncharacterized protein | | afdb-uniprot50 | AF-A0A6S7F7W2-F1-MODEL\_V4 | 1.0 | 0.002562 | 136 | 0.253 | 75 | 52 | 3 | 1 | 73 | 63 | 135 | HTH\_17 domain-containing protein | HTH\_17 domain-containing protein | | afdb-uniprot50 | AF-A0A7V9RN05-F1-MODEL\_V4 | 1.0 | 0.007939 | 135 | 0.235 | 68 | 47 | 2 | 6 | 73 | 2 | 64 | Helix-turn-helix domain-containing protein | Helix-turn-helix domain-containing protein | | afdb-uniprot50 | AF-A0A7V1HES1-F1-MODEL\_V4 | 1.0 | 0.003819 | 135 | 0.239 | 71 | 51 | 2 | 4 | 73 | 1 | 69 | DNA-binding protein | DNA-binding protein | | afdb-uniprot50 | AF-A0A418KYP8-F1-MODEL\_V4 | 1.0 | 0.008485 | 135 | 0.257 | 66 | 44 | 2 | 1 | 63 | 5 | 68 | Uncharacterized protein | Uncharacterized protein | | afdb-uniprot50 | AF-A0A066PHX5-F1-MODEL\_V4 | 1.0 | 0.004362 | 135 | 0.225 | 71 | 52 | 2 | 4 | 73 | 1 | 69 | Helix-turn-helix domain-containing protein | Helix-turn-helix domain-containing protein | | afdb-uniprot50 | AF-A0A2J0TSF3-F1-MODEL\_V4 | 1.0 | 0.004983 | 135 | 0.26 | 73 | 48 | 3 | 1 | 73 | 8 | 74 | HTH\_17 domain-containing protein | HTH\_17 domain-containing protein | | afdb-uniprot50 | AF-A0A498B7D3-F1-MODEL\_V4 | 1.0 | 0.005326 | 135 | 0.298 | 67 | 44 | 2 | 2 | 67 | 12 | 76 | Excisionase family DNA binding protein | Excisionase family DNA binding protein | | afdb-uniprot50 | AF-A0A7V9PX81-F1-MODEL\_V4 | 1.0 | 0.009692 | 135 | 0.253 | 79 | 51 | 2 | 1 | 73 | 4 | 80 | Helix-turn-helix domain-containing protein | Helix-turn-helix domain-containing protein | | afdb-uniprot50 | AF-A0A2E2MPT9-F1-MODEL\_V4 | 1.0 | 0.004983 | 135 | 0.243 | 74 | 50 | 4 | 1 | 72 | 6 | 75 | DNA-binding protein | DNA-binding protein | | afdb-uniprot50 | AF-A0A3S0LBJ7-F1-MODEL\_V4 | 1.0 | 0.002927 | 135 | 0.263 | 76 | 50 | 3 | 2 | 73 | 17 | 90 | DNA-binding protein | DNA-binding protein | | afdb-uniprot50 | AF-A0A844ZUN2-F1-MODEL\_V4 | 1.0 | 0.00695 | 135 | 0.273 | 73 | 51 | 1 | 1 | 73 | 24 | 94 | Uncharacterized protein | Uncharacterized protein | | afdb-uniprot50 | AF-A0A6G6Z347-F1-MODEL\_V4 | 1.0 | 0.005692 | 135 | 0.202 | 74 | 56 | 3 | 1 | 73 | 3 | 74 | Helix-turn-helix domain-containing protein | Helix-turn-helix domain-containing protein | | afdb-uniprot50 | AF-A0A6H9WTP2-F1-MODEL\_V4 | 1.0 | 0.008485 | 135 | 0.268 | 67 | 47 | 1 | 1 | 67 | 40 | 104 | Helix-turn-helix domain-containing protein | Helix-turn-helix domain-containing protein | | afdb-uniprot50 | AF-A0A7W6IQJ9-F1-MODEL\_V4 | 1.0 | 0.00695 | 135 | 0.246 | 69 | 46 | 2 | 1 | 69 | 1 | 63 | Putative DNA-binding transcriptional regulator AlpA | Putative DNA-binding transcriptional regulator AlpA | | afdb-uniprot50 | AF-A0A2N2KGC4-F1-MODEL\_V4 | 1.0 | 0.004983 | 135 | 0.236 | 76 | 51 | 2 | 2 | 72 | 59 | 132 | Uncharacterized protein | Uncharacterized protein | | afdb-uniprot50 | AF-A0A401Z9H7-F1-MODEL\_V4 | 1.0 | 0.005692 | 134 | 0.2 | 75 | 51 | 3 | 1 | 73 | 1 | 68 | HTH\_17 domain-containing protein | HTH\_17 domain-containing protein | | afdb-uniprot50 | AF-A0A1H9VIH3-F1-MODEL\_V4 | 1.0 | 0.009068 | 134 | 0.26 | 69 | 49 | 2 | 1 | 69 | 1 | 67 | Prophage CP4-57 regulatory protein (AlpA) | Prophage CP4-57 regulatory protein (AlpA) | | afdb-uniprot50 | AF-A0A512DG42-F1-MODEL\_V4 | 1.0 | 0.007428 | 134 | 0.231 | 69 | 48 | 3 | 1 | 67 | 1 | 66 | HTH\_17 domain-containing protein | HTH\_17 domain-containing protein | | afdb-uniprot50 | AF-A0A0M2HKS0-F1-MODEL\_V4 | 1.0 | 0.005326 | 134 | 0.26 | 73 | 51 | 2 | 1 | 71 | 3 | 74 | Uncharacterized protein | Uncharacterized protein | | afdb-uniprot50 | AF-A0A525HJG6-F1-MODEL\_V4 | 1.0 | 0.004662 | 134 | 0.287 | 73 | 48 | 3 | 3 | 73 | 2 | 72 | DNA-binding protein | DNA-binding protein | | afdb-uniprot50 | AF-A0A1A2DCW1-F1-MODEL\_V4 | 1.0 | 0.005326 | 134 | 0.226 | 75 | 51 | 3 | 1 | 70 | 1 | 73 | Uncharacterized protein | Uncharacterized protein | | afdb-uniprot50 | AF-A0A1N7FDQ6-F1-MODEL\_V4 | 1.0 | 0.004983 | 134 | 0.231 | 69 | 45 | 2 | 1 | 63 | 3 | 69 | Uncharacterized protein | Uncharacterized protein | | afdb-uniprot50 | AF-A0A1F2UUZ3-F1-MODEL\_V4 | 1.0 | 0.004983 | 134 | 0.282 | 78 | 47 | 3 | 1 | 73 | 1 | 74 | HTH\_17 domain-containing protein | HTH\_17 domain-containing protein | | afdb-uniprot50 | AF-A0A1S6ERV9-F1-MODEL\_V4 | 1.0 | 0.008485 | 134 | 0.219 | 73 | 53 | 2 | 2 | 72 | 8 | 78 | HTH\_17 domain-containing protein | HTH\_17 domain-containing protein | | afdb-uniprot50 | AF-A0A8A3LV53-F1-MODEL\_V4 | 1.0 | 0.005326 | 134 | 0.21 | 76 | 57 | 2 | 1 | 73 | 5 | 80 | Helix-turn-helix domain-containing protein | Helix-turn-helix domain-containing protein | | afdb-uniprot50 | AF-A0A6B8MDK0-F1-MODEL\_V4 | 1.0 | 0.003573 | 134 | 0.27 | 74 | 50 | 3 | 1 | 72 | 10 | 81 | Helix-turn-helix domain-containing protein | Helix-turn-helix domain-containing protein | | afdb-uniprot50 | AF-A0A1W6ZN77-F1-MODEL\_V4 | 1.0 | 0.004983 | 134 | 0.205 | 78 | 54 | 2 | 2 | 73 | 4 | 79 | HTH\_17 domain-containing protein | HTH\_17 domain-containing protein | | afdb-uniprot50 | AF-A0A382CIH3-F1-MODEL\_V4 | 1.0 | 0.004662 | 134 | 0.213 | 75 | 54 | 3 | 1 | 73 | 4 | 75 | HTH\_17 domain-containing protein | HTH\_17 domain-containing protein | | afdb-uniprot50 | AF-A0A7Y7M2F2-F1-MODEL\_V4 | 1.0 | 0.004662 | 134 | 0.223 | 76 | 54 | 3 | 1 | 73 | 6 | 79 | Helix-turn-helix domain-containing protein | Helix-turn-helix domain-containing protein | | afdb-uniprot50 | AF-H6RRY8-F1-MODEL\_V4 | 1.0 | 0.00695 | 134 | 0.303 | 66 | 43 | 2 | 1 | 65 | 17 | 80 | Transcriptional regulator, MerR family | Transcriptional regulator, MerR family | | afdb-uniprot50 | AF-A0A7I7UXQ3-F1-MODEL\_V4 | 1.0 | 0.006502 | 134 | 0.205 | 73 | 55 | 3 | 1 | 73 | 21 | 90 | HTH\_17 domain-containing protein | HTH\_17 domain-containing protein | | afdb-uniprot50 | AF-A0A2P7ACK4-F1-MODEL\_V4 | 1.0 | 0.005326 | 133 | 0.202 | 79 | 53 | 3 | 1 | 73 | 1 | 75 | DNA-binding protein | DNA-binding protein | | afdb-uniprot50 | AF-A0A6V8MGW9-F1-MODEL\_V4 | 1.0 | 0.006084 | 133 | 0.26 | 73 | 51 | 2 | 1 | 73 | 7 | 76 | HTH\_17 domain-containing protein | HTH\_17 domain-containing protein | | afdb-uniprot50 | AF-A0A354W361-F1-MODEL\_V4 | 1.0 | 0.007428 | 133 | 0.212 | 66 | 47 | 3 | 1 | 63 | 2 | 65 | DNA-binding protein | DNA-binding protein | | afdb-uniprot50 | AF-A0A178HUC9-F1-MODEL\_V4 | 1.0 | 0.005326 | 133 | 0.285 | 77 | 47 | 3 | 1 | 73 | 8 | 80 | HTH\_17 domain-containing protein | HTH\_17 domain-containing protein | | afdb-uniprot50 | AF-A0A3A0FTT9-F1-MODEL\_V4 | 1.0 | 0.006502 | 133 | 0.236 | 72 | 49 | 2 | 2 | 73 | 16 | 81 | DNA-binding protein | DNA-binding protein | | afdb-uniprot50 | AF-A0A6L9W7M6-F1-MODEL\_V4 | 1.0 | 0.009692 | 133 | 0.205 | 73 | 56 | 1 | 1 | 73 | 2 | 72 | Helix-turn-helix domain-containing protein | Helix-turn-helix domain-containing protein | | afdb-uniprot50 | AF-A0A7Y7LZW5-F1-MODEL\_V4 | 1.0 | 0.005692 | 133 | 0.205 | 73 | 53 | 2 | 1 | 73 | 7 | 74 | Helix-turn-helix domain-containing protein | Helix-turn-helix domain-containing protein | | afdb-uniprot50 | AF-A0A6V8MQ78-F1-MODEL\_V4 | 1.0 | 0.004362 | 133 | 0.246 | 77 | 50 | 3 | 2 | 72 | 12 | 86 | HTH\_17 domain-containing protein | HTH\_17 domain-containing protein | | afdb-uniprot50 | AF-A0A4R0Z385-F1-MODEL\_V4 | 1.0 | 0.009068 | 133 | 0.25 | 72 | 52 | 1 | 2 | 73 | 29 | 98 | DNA-binding protein | DNA-binding protein | | afdb-uniprot50 | AF-A0A3M0HKQ5-F1-MODEL\_V4 | 1.0 | 0.009068 | 133 | 0.223 | 67 | 47 | 2 | 1 | 67 | 46 | 107 | AlpA family phage regulatory protein | AlpA family phage regulatory protein | | afdb-uniprot50 | AF-A0A0U5BBK8-F1-MODEL\_V4 | 1.0 | 0.005692 | 133 | 0.216 | 74 | 53 | 3 | 2 | 72 | 41 | 112 | Phage transcriptional regulator, AlpA | Phage transcriptional regulator, AlpA | | afdb-uniprot50 | AF-A0A7Y4MFA2-F1-MODEL\_V4 | 1.0 | 0.008485 | 133 | 0.238 | 67 | 48 | 2 | 2 | 67 | 5 | 69 | Helix-turn-helix domain-containing protein | Helix-turn-helix domain-containing protein | | afdb-uniprot50 | AF-A0A1Q8KWK1-F1-MODEL\_V4 | 1.0 | 0.003573 | 132 | 0.275 | 69 | 42 | 3 | 1 | 69 | 1 | 61 | HTH\_17 domain-containing protein | HTH\_17 domain-containing protein | | afdb-uniprot50 | AF-A0A7Y6NP98-F1-MODEL\_V4 | 1.0 | 0.007428 | 132 | 0.246 | 73 | 51 | 3 | 1 | 72 | 1 | 70 | Helix-turn-helix domain-containing protein | Helix-turn-helix domain-containing protein | | afdb-uniprot50 | AF-A0A7Y0VX78-F1-MODEL\_V4 | 1.0 | 0.006084 | 132 | 0.216 | 74 | 52 | 3 | 1 | 73 | 3 | 71 | Helix-turn-helix domain-containing protein | Helix-turn-helix domain-containing protein | | afdb-uniprot50 | AF-A0A2V5IYM0-F1-MODEL\_V4 | 1.0 | 0.009692 | 132 | 0.205 | 68 | 52 | 1 | 1 | 68 | 8 | 73 | Excisionase | Excisionase | | afdb-uniprot50 | AF-A0A7G9L3X4-F1-MODEL\_V4 | 1.0 | 0.009692 | 132 | 0.2 | 65 | 46 | 2 | 9 | 73 | 15 | 73 | Helix-turn-helix domain-containing protein | Helix-turn-helix domain-containing protein | | afdb-uniprot50 | AF-A0A2T0TPB9-F1-MODEL\_V4 | 1.0 | 0.00695 | 132 | 0.192 | 78 | 55 | 2 | 1 | 72 | 2 | 77 | Excisionase family DNA binding protein | Excisionase family DNA binding protein | | afdb-uniprot50 | AF-A0A1W1D8J7-F1-MODEL\_V4 | 1.0 | 0.003573 | 132 | 0.293 | 75 | 47 | 3 | 1 | 73 | 5 | 75 | HTH\_17 domain-containing protein | HTH\_17 domain-containing protein | | afdb-uniprot50 | AF-A0A7L5Z0X3-F1-MODEL\_V4 | 1.0 | 0.007939 | 132 | 0.232 | 73 | 53 | 2 | 2 | 73 | 5 | 75 | Helix-turn-helix domain-containing protein | Helix-turn-helix domain-containing protein | | afdb-uniprot50 | AF-A0A2V8IKF4-F1-MODEL\_V4 | 1.0 | 0.002562 | 132 | 0.209 | 86 | 53 | 3 | 1 | 73 | 38 | 121 | HTH\_17 domain-containing protein | HTH\_17 domain-containing protein | | afdb-uniprot50 | AF-A0A2E5FA21-F1-MODEL\_V4 | 1.0 | 0.006084 | 131 | 0.246 | 73 | 51 | 3 | 1 | 72 | 7 | 76 | Uncharacterized protein | Uncharacterized protein | | afdb-uniprot50 | AF-A0A365H3H9-F1-MODEL\_V4 | 1.0 | 0.006502 | 131 | 0.291 | 72 | 48 | 2 | 1 | 72 | 10 | 78 | DNA-binding protein | DNA-binding protein | | afdb-uniprot50 | AF-A0A1T4K0K2-F1-MODEL\_V4 | 1.0 | 0.004362 | 131 | 0.259 | 77 | 51 | 3 | 1 | 73 | 7 | 81 | Transcriptional regulator, AlpA family | Transcriptional regulator, AlpA family | | afdb-uniprot50 | AF-A0A7Z0GLK2-F1-MODEL\_V4 | 1.0 | 0.00695 | 131 | 0.285 | 77 | 49 | 5 | 1 | 73 | 9 | 83 | Uncharacterized protein | Uncharacterized protein | | afdb-uniprot50 | AF-A0A3S4WXW0-F1-MODEL\_V4 | 1.0 | 0.003819 | 131 | 0.294 | 78 | 48 | 3 | 1 | 73 | 1 | 76 | Predicted transcriptional regulator | Predicted transcriptional regulator | | afdb-uniprot50 | AF-A0A090MHP8-F1-MODEL\_V4 | 1.0 | 0.009068 | 131 | 0.18 | 72 | 57 | 1 | 2 | 73 | 27 | 96 | Uncultured bacterium genome assembly Metasoil\_fosmids\_resub | Uncultured bacterium genome assembly Metasoil\_fosmids\_resub | | afdb-uniprot50 | AF-A0A7Z7RKN0-F1-MODEL\_V4 | 1.0 | 0.009692 | 131 | 0.235 | 68 | 46 | 2 | 6 | 73 | 2 | 63 | Predicted transcriptional regulator | Predicted transcriptional regulator | | afdb-uniprot50 | AF-A0A239JAJ2-F1-MODEL\_V4 | 1.0 | 0.007428 | 130 | 0.246 | 69 | 44 | 2 | 11 | 73 | 5 | 71 | Uncharacterized protein | Uncharacterized protein | | afdb-uniprot50 | AF-A0A5C4W3D3-F1-MODEL\_V4 | 1.0 | 0.009068 | 130 | 0.25 | 64 | 44 | 2 | 1 | 62 | 4 | 65 | Helix-turn-helix domain-containing protein | Helix-turn-helix domain-containing protein | | afdb-uniprot50 | AF-A0A7V3NCW0-F1-MODEL\_V4 | 1.0 | 0.004983 | 130 | 0.227 | 79 | 53 | 3 | 1 | 73 | 51 | 127 | DNA-binding protein | DNA-binding protein | | afdb-uniprot50 | AF-A0A4Q2S2U5-F1-MODEL\_V4 | 1.0 | 0.006502 | 130 | 0.208 | 67 | 45 | 2 | 2 | 62 | 192 | 256 | Helix-turn-helix domain-containing protein | Helix-turn-helix domain-containing protein | | afdb-uniprot50 | AF-A0A1M6YKR5-F1-MODEL\_V4 | 1.0 | 0.008485 | 129 | 0.338 | 68 | 42 | 3 | 7 | 73 | 2 | 67 | Transcriptional regulator, AlpA family | Transcriptional regulator, AlpA family | | afdb-uniprot50 | AF-A0A355EX70-F1-MODEL\_V4 | 1.0 | 0.006084 | 129 | 0.297 | 74 | 48 | 3 | 1 | 72 | 1 | 72 | Uncharacterized protein | Uncharacterized protein | | afdb-uniprot50 | AF-A0A557SM49-F1-MODEL\_V4 | 1.0 | 0.009068 | 129 | 0.229 | 74 | 53 | 3 | 1 | 73 | 2 | 72 | Helix-turn-helix domain-containing protein | Helix-turn-helix domain-containing protein | | afdb-uniprot50 | AF-A0A0Q7K039-F1-MODEL\_V4 | 1.0 | 0.007428 | 129 | 0.205 | 78 | 53 | 3 | 1 | 72 | 2 | 76 | HTH\_17 domain-containing protein | HTH\_17 domain-containing protein | | afdb-uniprot50 | AF-I3ZJK5-F1-MODEL\_V4 | 1.0 | 0.00695 | 129 | 0.297 | 74 | 48 | 2 | 2 | 73 | 7 | 78 | HTH\_17 domain-containing protein | HTH\_17 domain-containing protein | | afdb-uniprot50 | AF-A0A522CQL4-F1-MODEL\_V4 | 1.0 | 0.007939 | 129 | 0.181 | 77 | 59 | 2 | 1 | 73 | 3 | 79 | DNA-binding protein | DNA-binding protein | | afdb-uniprot50 | AF-A0A3D9YWA9-F1-MODEL\_V4 | 1.0 | 0.009692 | 129 | 0.253 | 75 | 52 | 3 | 1 | 73 | 13 | 85 | Uncharacterized protein | Uncharacterized protein | | afdb-uniprot50 | AF-A0A3S8T1N0-F1-MODEL\_V4 | 1.0 | 0.007428 | 128 | 0.253 | 71 | 50 | 2 | 4 | 73 | 3 | 71 | DNA-binding protein | DNA-binding protein | | afdb-uniprot50 | AF-A0A840ARV2-F1-MODEL\_V4 | 1.0 | 0.006084 | 128 | 0.232 | 73 | 52 | 2 | 1 | 73 | 4 | 72 | Excisionase family DNA binding protein | Excisionase family DNA binding protein | | afdb-uniprot50 | AF-A0A382YY28-F1-MODEL\_V4 | 1.0 | 0.004362 | 128 | 0.23 | 78 | 54 | 3 | 1 | 73 | 1 | 77 | Uncharacterized protein | Uncharacterized protein | | afdb-uniprot50 | AF-A0A4R0IXT6-F1-MODEL\_V4 | 1.0 | 0.008485 | 128 | 0.228 | 70 | 50 | 2 | 4 | 73 | 2 | 67 | DNA-binding protein | DNA-binding protein | | afdb-uniprot50 | AF-A0A1M7TPV6-F1-MODEL\_V4 | 1.0 | 0.00695 | 128 | 0.236 | 72 | 52 | 2 | 2 | 72 | 9 | 78 | Uncharacterized protein | Uncharacterized protein | | afdb-uniprot50 | AF-F6EZQ7-F1-MODEL\_V4 | 1.0 | 0.009068 | 128 | 0.256 | 74 | 50 | 3 | 1 | 73 | 7 | 76 | Uncharacterized protein | Uncharacterized protein | | afdb-uniprot50 | AF-T0ZSB8-F1-MODEL\_V4 | 1.0 | 0.002243 | 128 | 0.272 | 77 | 46 | 4 | 4 | 72 | 3 | 77 | Uncharacterized protein | Uncharacterized protein | | afdb-uniprot50 | AF-A0A0K8MEP3-F1-MODEL\_V4 | 1.0 | 0.007939 | 127 | 0.213 | 75 | 54 | 3 | 1 | 72 | 1 | 73 | Helix-turn-helix domain protein | Helix-turn-helix domain protein | | afdb-uniprot50 | AF-A0A3S0AX38-F1-MODEL\_V4 | 1.0 | 0.008485 | 127 | 0.298 | 77 | 48 | 2 | 1 | 73 | 6 | 80 | DNA-binding protein | DNA-binding protein | | afdb-uniprot50 | AF-A0A2N2KEI7-F1-MODEL\_V4 | 1.0 | 0.009068 | 127 | 0.227 | 79 | 53 | 3 | 1 | 73 | 4 | 80 | DNA-binding protein | DNA-binding protein | | afdb-uniprot50 | AF-A0A8A7CSH7-F1-MODEL\_V4 | 1.0 | 0.009068 | 127 | 0.31 | 74 | 46 | 2 | 1 | 70 | 1 | 73 | Uncharacterized protein | Uncharacterized protein | | afdb-uniprot50 | AF-A0A1M3AAN7-F1-MODEL\_V4 | 1.0 | 0.005692 | 127 | 0.28 | 82 | 47 | 4 | 2 | 73 | 5 | 84 | Uncharacterized protein | Uncharacterized protein | | afdb-uniprot50 | AF-A0A1H3NPD0-F1-MODEL\_V4 | 1.0 | 0.008485 | 127 | 0.246 | 73 | 51 | 3 | 1 | 71 | 1 | 71 | DNA binding domain-containing protein, excisionase family | DNA binding domain-containing protein, excisionase family | | afdb-uniprot50 | AF-A0A2W4XEV9-F1-MODEL\_V4 | 1.0 | 0.008485 | 127 | 0.2 | 75 | 57 | 1 | 1 | 72 | 5 | 79 | Uncharacterized protein | Uncharacterized protein | | afdb-uniprot50 | AF-F4G8U6-F1-MODEL\_V4 | 1.0 | 0.007939 | 127 | 0.246 | 69 | 44 | 2 | 11 | 73 | 5 | 71 | Uncharacterized protein | Uncharacterized protein | | afdb-uniprot50 | AF-A0A3N0GII4-F1-MODEL\_V4 | 1.0 | 0.009692 | 126 | 0.21 | 76 | 55 | 3 | 1 | 73 | 1 | 74 | DNA-binding protein | DNA-binding protein | | afdb-uniprot50 | AF-A0A1V3R129-F1-MODEL\_V4 | 1.0 | 0.007428 | 126 | 0.26 | 73 | 48 | 3 | 1 | 73 | 3 | 69 | HTH\_17 domain-containing protein | HTH\_17 domain-containing protein | | afdb-uniprot50 | AF-A0A5S4WPV7-F1-MODEL\_V4 | 1.0 | 0.009692 | 126 | 0.259 | 77 | 51 | 3 | 1 | 72 | 1 | 76 | Uncharacterized protein | Uncharacterized protein | | afdb-uniprot50 | AF-A0A1M3DSQ0-F1-MODEL\_V4 | 1.0 | 0.007939 | 126 | 0.235 | 89 | 49 | 3 | 1 | 72 | 3 | 89 | HTH\_17 domain-containing protein | HTH\_17 domain-containing protein | | afdb-uniprot50 | AF-A0A7H4GTR2-F1-MODEL\_V4 | 1.0 | 0.008485 | 126 | 0.226 | 75 | 51 | 4 | 1 | 73 | 33 | 102 | Helix-turn-helix domain-containing protein | Helix-turn-helix domain-containing protein | | afdb-uniprot50 | AF-A0A2V8WJS3-F1-MODEL\_V4 | 1.0 | 0.006502 | 126 | 0.25 | 76 | 51 | 3 | 1 | 72 | 12 | 85 | HTH\_17 domain-containing protein | HTH\_17 domain-containing protein | | afdb-uniprot50 | AF-A0A1I7DBH4-F1-MODEL\_V4 | 1.0 | 0.009692 | 125 | 0.243 | 74 | 52 | 3 | 1 | 72 | 1 | 72 | Transcriptional regulator, AlpA family | Transcriptional regulator, AlpA family | | afdb-uniprot50 | AF-A0A516NU13-F1-MODEL\_V4 | 1.0 | 0.007428 | 125 | 0.277 | 72 | 44 | 3 | 1 | 72 | 1 | 64 | DNA-binding protein | DNA-binding protein | | afdb-uniprot50 | AF-A0A2W6VUQ2-F1-MODEL\_V4 | 1.0 | 0.009692 | 125 | 0.283 | 74 | 50 | 2 | 1 | 73 | 3 | 74 | HTH\_17 domain-containing protein | HTH\_17 domain-containing protein | | afdb-uniprot50 | AF-A0A3S0D818-F1-MODEL\_V4 | 1.0 | 0.006502 | 125 | 0.236 | 76 | 53 | 3 | 1 | 73 | 5 | 78 | DNA-binding protein | DNA-binding protein | | afdb-uniprot50 | AF-A0A5D4XRR2-F1-MODEL\_V4 | 1.0 | 0.00695 | 125 | 0.215 | 79 | 54 | 3 | 1 | 73 | 1 | 77 | Helix-turn-helix domain-containing protein | Helix-turn-helix domain-containing protein | | afdb-uniprot50 | AF-A0A2E7UH17-F1-MODEL\_V4 | 1.0 | 0.007428 | 125 | 0.283 | 81 | 48 | 3 | 1 | 73 | 1 | 79 | DNA-binding protein | DNA-binding protein | | afdb-uniprot50 | AF-A0A3F3GUT7-F1-MODEL\_V4 | 1.0 | 0.009692 | 125 | 0.213 | 75 | 55 | 3 | 1 | 73 | 2 | 74 | Excisionase DNA binding domain-containing protein | Excisionase DNA binding domain-containing protein | | afdb-uniprot50 | AF-A0A7C9KIR3-F1-MODEL\_V4 | 1.0 | 0.007939 | 124 | 0.246 | 77 | 50 | 3 | 1 | 73 | 6 | 78 | Helix-turn-helix domain-containing protein | Helix-turn-helix domain-containing protein | | afdb-uniprot50 | AF-A0A2V2R565-F1-MODEL\_V4 | 1.0 | 0.009692 | 124 | 0.256 | 82 | 50 | 4 | 1 | 73 | 2 | 81 | Uncharacterized protein | Uncharacterized protein | | afdb-uniprot50 | AF-E2S8B6-F1-MODEL\_V4 | 1.0 | 0.009692 | 124 | 0.226 | 75 | 54 | 2 | 1 | 73 | 18 | 90 | HTH\_17 domain-containing protein | HTH\_17 domain-containing protein | | afdb-uniprot50 | AF-A0A2T5JVL9-F1-MODEL\_V4 | 1.0 | 0.009692 | 123 | 0.243 | 74 | 53 | 2 | 1 | 73 | 8 | 79 | Helix-turn-helix protein | Helix-turn-helix protein | | afdb-uniprot50 | AF-A0A833LJH9-F1-MODEL\_V4 | 1.0 | 0.007939 | 122 | 0.232 | 73 | 53 | 2 | 1 | 73 | 6 | 75 | Helix-turn-helix domain-containing protein | Helix-turn-helix domain-containing protein | | afdb-uniprot50 | AF-A0A2E7ISN4-F1-MODEL\_V4 | 1.0 | 0.009692 | 121 | 0.214 | 70 | 51 | 3 | 1 | 68 | 1 | 68 | Transcriptional regulator | Transcriptional regulator | | afdb-uniprot50 | AF-A0A4R6WVG7-F1-MODEL\_V4 | 1.0 | 0.004662 | 121 | 0.217 | 78 | 54 | 3 | 1 | 73 | 1 | 76 | Uncharacterized protein | Uncharacterized protein | | afdb-uniprot50 | AF-A0A4P5WYP2-F1-MODEL\_V4 | 1.0 | 0.009692 | 119 | 0.23 | 78 | 51 | 5 | 1 | 73 | 1 | 74 | HTH\_17 domain-containing protein | HTH\_17 domain-containing protein | | afdb-uniprot50 | AF-F4CSF2-F1-MODEL\_V4 | 1.0 | 0.007428 | 118 | 0.277 | 72 | 41 | 3 | 1 | 68 | 22 | 86 | Prophage CP4-57 regulatory | Prophage CP4-57 regulatory | | afdb-uniprot50 | AF-A0A1Q4X2L2-F1-MODEL\_V4 | 1.0 | 0.003819 | 118 | 0.259 | 77 | 51 | 3 | 1 | 73 | 4 | 78 | HTH merR-type domain-containing protein | HTH merR-type domain-containing protein | | afdb-uniprot50 | AF-A0A087CGI1-F1-MODEL\_V4 | 1.0 | 0.008485 | 117 | 0.25 | 72 | 47 | 3 | 1 | 72 | 3 | 67 | Phage transcriptional regulator AlpA | Phage transcriptional regulator AlpA | |
| Top keywords  (threshold 1.00e-02 (evalue)) | **domain\_containing, DNA\_binding, Helix\_turn\_helix, HTH\_17, Excisionase, transcriptional, regulator, DNA, binding, AlpA** |
| Output files | ../../similar\_structures/38\_FANPEZAQ\_CDS\_0038\_afdb-proteome\_foldseek.tsv ../../similar\_structures/38\_FANPEZAQ\_CDS\_0038\_afdb-uniprot50\_foldseek.tsv ../../similar\_structures/38\_FANPEZAQ\_CDS\_0038\_merged.svg ../../similar\_structures/38\_FANPEZAQ\_CDS\_0038\_pdb\_foldseek.tsv |

  
  
  

Return to summary | Go to previous | Go to next

  


---

**Sequence/structure alignments coloring**  
Each object in the alignment figures is colored according to its E-value following this color coding:

1e-100
10

**References:**  
1) Steinegger M, Meier M, Mirdita M, Vöhringer H, Haunsberger S J, and Söding J (2019) HH-suite3 for fast remote homology detection and deep protein annotation, BMC Bioinformatics, 473. doi: 10.1186/s12859-019-3019-7  
2) Jumper J, Evans R, Pritzel A, ..., Hassabis D (2021) Highly accurate protein structure prediction with AlphaFold, Nature, 596. doi: 10.1038/s41586-021-03819-2  
3) van Kempen M, Kim S, Tumescheit C, Mirdita M, Lee J, Gilchrist CLM, Söding J, and Steinegger M (2023) Fast and accurate protein structure search with Foldseek. Nature Biotechnology. doi: 10.1038/s41587-023-01773-0
