# Supplementary material for: Completing the BASEL phage collection to unlock hidden diversity for systematic exploration of phage–host interactions
Source: PLoS Biol. 2025 Apr 7;23(4):e3003063. doi: 10.1371/journal.pbio.3003063 (PMC11990801; doi:10.1371/journal.pbio.3003063)
Supplement: S2 Data — (ZIP) [file pbio.3003063.s009.zip › entries/4.html]

FANPEZAQ\_CDS\_0004


Return to summary | Go to previous | Go to next

|  |  |
| --- | --- |
| FANPEZAQ\_CDS\_0004 Page creation date: 02 Sep 2024, 12:00  Project folder: n/a  Input sequences file: Escherichia\_virus\_HeidiAbel.gb | portal phage lambda fragment capsid putative duf935 head prophage and packaging domain\_containing bacteriophage minor terminase head\_tail phage\_related duf4055 spp1 preconnector hk97 gp6\_like parb protease phage\_mu\_f |

### Sequence information

|  |  |
| --- | --- |
| Name | FANPEZAQ\_CDS\_0004  04\_FANPEZAQ\_CDS\_0004 (pipeline id) |
| Imported annotations | Escherichia\_virus\_HeidiAbel Bas97 |
| Protein sequence | MQSIRAQSTGEPSLMKLWPFTRNKPAEQAQQPRTRRLPLNRLASRMFTGASVDRLGTAWG TQPLTADEVINKNQRILVARSREQAANNDYAKKFLRLCRNNIVGPKGVLLQAKSTDNTGK LDTLANEAIETAFDKWGHKSNCDVTGKLSWRAIQAACINSAAKDGEFFVRLVFGADAGPW GFALQLLDPQRCPVDMVEDRLNNGNFIRQGIEFNRYGRPVAYYFSTVDESDDNYRWSGRD FVRIPADEILHGFLDDMVGQKRGLPWMATSLFRMRHLGGMEEAALVNARAGANKMGFIEW DENSAGPEFEDDDELIIDSEPGEFQVLPQGARVKDWSPQYPNGEFAVFTKQMLRGMASGM GVPYNDLASDLEGVNFSSIRQGTLDSRENWKELQEWLIENLHQPVFEAWLPRALLAGRIT VKGRSLKPERIDRYSDIEWQPRRWAWIDPSSDVAAAERSKNNMLQSPGRIIREGGSDPDT VWKETASDVARMIEAYTQHGISREMAEKLVMQSMGMDKQLLMGQQNAEKPAE |
| Number of residues | 532 |
| Molecular weight (Da) | 60054.10 |
| Output files | ../../query\_sequences/04\_FANPEZAQ\_CDS\_0004.fasta |

### Putative domain architecture and protein family

#### Search results (HHblits)1

|  |  |
| --- | --- |
| Domain family databases searched | Pfam, Ncbi-cd, Cath, Phrogs |
| Results, scheme(s)  (Top layers only; threshold 1.00e-03 (evalue)) | xml version="1.0" encoding="utf-8" standalone="no"?       2024-09-02T21:08:12.560655 image/svg+xml   Matplotlib v3.7.2, https://matplotlib.org/ |
| Results, table  (E-value ≤ 1.00e-03 (evalue)) | | db | id | prob | evalue | pvalue | score | cols | query | query\_len | template | template\_len | name | description | | --- | --- | --- | --- | --- | --- | --- | --- | --- | --- | --- | --- | --- | | pfam | PF06074 | 99.8 | 2.8e-25 | 4.8e-29 | 224.8 | 351 | (56, 479) | 532 | (30, 391) | 504 | DUF935 | Protein of unknown function (DUF935) | | pfam | PF05136 | 99.7 | 2.8e-23 | 4.8e-27 | 198.4 | 330 | (53, 393) | 532 | (2, 333) | 334 | Phage\_portal\_2 | Phage portal protein, lambda family | | pfam | PF04860 | 99.7 | 1.3e-21 | 2.3e-25 | 185.4 | 324 | (89, 474) | 532 | (1, 332) | 332 | Phage\_portal | Phage portal protein | | pfam | PF06381 | 99.6 | 3e-20 | 5.3e-24 | 177.5 | 320 | (80, 463) | 532 | (2, 348) | 350 | DUF1073 | Protein of unknown function (DUF1073) | | pfam | PF07230 | 99.6 | 8.3e-20 | 1.5e-23 | 181.3 | 382 | (76, 486) | 532 | (3, 449) | 454 | Portal\_Gp20 | Bacteriophage T4-like portal protein (Gp20) | | pfam | PF05133 | 98.7 | 8.9e-13 | 1.6e-16 | 128.8 | 337 | (87, 475) | 532 | (54, 422) | 457 | Phage\_prot\_Gp6 | Phage portal protein, SPP1 Gp6-like | | phrogs | 21 | 100.0 | 3.1e-59 | 3.7e-63 | 486.4 | 447 | (40, 494) | 532 | (17, 502) | 533 | portal protein | portal protein; Category: head and packaging; p173499 VI\_06341 | | phrogs | 2662 | 99.9 | 6.5e-31 | 7.7e-35 | 272.9 | 344 | (81, 485) | 532 | (42, 391) | 685 | portal protein | portal protein; Category: head and packaging; NC\_023612\_p3 | | phrogs | 146 | 99.9 | 1.7e-29 | 2e-33 | 259.5 | 365 | (75, 492) | 532 | (60, 460) | 485 | portal protein | portal protein; Category: head and packaging; p353614 VI\_06430 | | phrogs | 12 | 99.9 | 3.4e-28 | 4.2e-32 | 248.4 | 344 | (76, 482) | 532 | (20, 369) | 434 | portal protein | portal protein; Category: head and packaging; p213127 VI\_06558 | | phrogs | 9232 | 99.6 | 5.7e-20 | 6.6e-24 | 173.7 | 365 | (79, 498) | 532 | (52, 425) | 479 | portal protein | portal protein; Category: head and packaging; NC\_004927\_p94 | | phrogs | 2271 | 99.4 | 6.4e-18 | 7.2e-22 | 152.4 | 394 | (57, 515) | 532 | (21, 418) | 620 | portal protein | portal protein; Category: head and packaging; p375194 VI\_08017 | | phrogs | 5929 | 99.4 | 9.3e-18 | 1.1e-21 | 145.0 | 150 | (310, 483) | 532 | (14, 167) | 231 | portal protein | portal protein; Category: head and packaging; p208881 VI\_08063 | | phrogs | 537 | 99.4 | 1.1e-17 | 1.3e-21 | 137.0 | 126 | (375, 504) | 532 | (2, 129) | 154 | portal protein | portal protein; Category: head and packaging; p386813 VI\_08621 | | phrogs | 12751 | 99.4 | 1.4e-17 | 1.6e-21 | 146.4 | 268 | (179, 476) | 532 | (12, 294) | 361 | NA | NA; Category: unknown function; p305174 VI\_10042 | | phrogs | 15528 | 99.4 | 3.8e-17 | 4.2e-21 | 153.8 | 361 | (82, 492) | 532 | (116, 516) | 698 | portal protein | portal protein; Category: head and packaging; NC\_005263\_p18 | | phrogs | 10963 | 99.1 | 5.1e-15 | 5.8e-19 | 139.4 | 369 | (54, 473) | 532 | (41, 435) | 509 | portal protein | portal protein; Category: head and packaging; KU160647\_p18 | | phrogs | 2739 | 99.0 | 4.3e-14 | 5e-18 | 139.1 | 364 | (73, 488) | 532 | (65, 446) | 494 | portal protein | portal protein; Category: head and packaging; MF547663\_p3 | | phrogs | 4660 | 99.0 | 7.8e-14 | 8.8e-18 | 132.1 | 364 | (80, 504) | 532 | (112, 501) | 765 | minor head protein | minor head protein; Category: head and packaging; LC102730\_p15 | | phrogs | 3753 | 98.7 | 1.6e-12 | 1.8e-16 | 124.3 | 351 | (77, 485) | 532 | (68, 448) | 490 | portal protein | portal protein; Category: head and packaging; MG596799\_p2 | | phrogs | 12037 | 98.2 | 2.7e-10 | 3e-14 | 90.8 | 176 | (43, 226) | 532 | (25, 205) | 223 | NA | NA; Category: unknown function; p423831 VI\_07821 | | phrogs | 3993 | 98.1 | 8e-10 | 8.9e-14 | 85.0 | 131 | (44, 175) | 532 | (18, 156) | 166 | NA | NA; Category: unknown function; p426125 VI\_07755 | | phrogs | 15615 | 97.7 | 1.7e-08 | 1.9e-12 | 81.4 | 141 | (74, 251) | 532 | (28, 179) | 180 | virion structural protein | virion structural protein; Category: head and packaging; p25678 VI\_08154 | | phrogs | 68 | 97.3 | 1.5e-07 | 1.8e-11 | 80.9 | 207 | (159, 409) | 532 | (2, 219) | 243 | portal protein | portal protein; Category: head and packaging; p356679 VI\_02767 | | phrogs | 11323 | 96.6 | 4.4e-06 | 5e-10 | 61.6 | 69 | (402, 479) | 532 | (5, 73) | 116 | NA | NA; Category: unknown function; p325568 VI\_04535 | | phrogs | 16240 | 96.4 | 1.1e-05 | 1.2e-09 | 76.2 | 393 | (73, 501) | 532 | (95, 515) | 635 | portal protein | portal protein; Category: head and packaging; NC\_010811\_p211 | | phrogs | 213 | 96.1 | 2.8e-05 | 3.2e-09 | 76.3 | 314 | (72, 406) | 532 | (46, 435) | 580 | portal protein | portal protein; Category: head and packaging; NC\_019401\_p248 | | phrogs | 33813 | 96.0 | 3.9e-05 | 4.4e-09 | 56.7 | 102 | (36, 138) | 532 | (22, 125) | 160 | NA | NA; Category: unknown function; p223265 VI\_05666 | | phrogs | 776 | 95.6 | 9.5e-05 | 1.1e-08 | 71.6 | 354 | (77, 485) | 532 | (29, 413) | 586 | portal protein | portal protein; Category: head and packaging; NC\_029005\_p52 | | phrogs | 27056 | 95.6 | 0.00012 | 1.3e-08 | 52.5 | 60 | (45, 104) | 532 | (22, 83) | 135 | NA | NA; Category: unknown function; p391015 VI\_06918 | | phrogs | 113 | 95.4 | 0.00015 | 1.8e-08 | 71.1 | 335 | (77, 481) | 532 | (52, 393) | 474 | portal protein | portal protein; Category: head and packaging; p68996 VI\_00707 | | phrogs | 11117 | 95.1 | 0.00027 | 3.1e-08 | 57.9 | 137 | (321, 478) | 532 | (29, 170) | 246 | portal protein | portal protein; Category: head and packaging; p100773 VI\_12396 | | phrogs | 11226 | 94.9 | 0.00041 | 4.6e-08 | 62.0 | 268 | (77, 389) | 532 | (33, 337) | 387 | portal protein | portal protein; Category: head and packaging; KC821625\_p80 | |
| Top keywords  (threshold 1.00e-03 (evalue)) | **portal, head, and, packaging, Phage, DUF935, lambda, DUF1073, Bacteriophage, T4\_like** |
| Output files | ../../domain\_architecture/04\_FANPEZAQ\_CDS\_0004\_cath.hhr ../../domain\_architecture/04\_FANPEZAQ\_CDS\_0004\_merged.svg ../../domain\_architecture/04\_FANPEZAQ\_CDS\_0004\_ncbi-cd.hhr ../../domain\_architecture/04\_FANPEZAQ\_CDS\_0004\_pfam.hhr ../../domain\_architecture/04\_FANPEZAQ\_CDS\_0004\_phrogs.hhr |

### Identical protein sequences/structures

#### Search results

|  |  |
| --- | --- |
| Protein sequence databases searched | Pdb, Swissprot, Refseq |
| Identical proteins found | -- |
| Top keywords | -- |
| Output files | -- |

### Similar protein sequences/structures

#### Sequence similarity search results (HHblits)1

|  |  |
| --- | --- |
| Sequence databases searched | Uniclust, Pdb70 |
| Results, scheme(s)  (Top layers only, threshold 1.00e-03 (evalue)) | xml version="1.0" encoding="utf-8" standalone="no"?       2024-09-02T21:08:29.764656 image/svg+xml   Matplotlib v3.7.2, https://matplotlib.org/ |
| Results, table(s)  (threshold 1.00e-03 (evalue)) | | db | id | prob | evalue | pvalue | score | cols | query | query\_len | template | template\_len | name | description | | --- | --- | --- | --- | --- | --- | --- | --- | --- | --- | --- | --- | --- | | uniclust | UniRef100\_A0A0S2KEQ2 | 100.0 | 4e-104 | 8e-110 | 812.0 | 482 | (7, 519) | 532 | (23, 512) | 559 | Phage portal protein, lambda | Phage portal protein, lambda | | uniclust | UniRef100\_A0A011VKJ3 | 100.0 | 2e-100 | 3e-106 | 810.0 | 476 | (12, 514) | 532 | (113, 598) | 677 | Portal protein | Portal protein | | uniclust | UniRef100\_A0A076G8T0 | 100.0 | 7.7e-97 | 2e-102 | 758.2 | 477 | (13, 516) | 532 | (74, 562) | 614 | Portal protein | Portal protein | | uniclust | UniRef100\_A0A060H8W7 | 100.0 | 1.1e-96 | 2e-102 | 783.4 | 455 | (43, 505) | 532 | (90, 547) | 640 | Portal protein | Portal protein | | uniclust | UniRef100\_A0A011QUJ3 | 100.0 | 1.7e-95 | 4e-101 | 763.6 | 450 | (44, 512) | 532 | (104, 561) | 683 | Phage portal protein, lambda family | Phage portal protein, lambda family | | uniclust | UniRef100\_A0A075KD33 | 100.0 | 2.6e-95 | 6e-101 | 770.2 | 477 | (17, 504) | 532 | (28, 528) | 606 | Phage portal protein, lambda family | Phage portal protein, lambda family | | uniclust | UniRef100\_A0A073ITU9 | 100.0 | 3.8e-95 | 8e-101 | 761.3 | 470 | (17, 504) | 532 | (29, 507) | 623 | Portal protein | Portal protein | | uniclust | UniRef100\_A0A014MMT4 | 100.0 | 1.6e-94 | 3e-100 | 765.0 | 475 | (15, 503) | 532 | (61, 567) | 698 | Portal protein | Portal protein | | uniclust | UniRef100\_A0A024LS66 | 100.0 | 1.6e-93 | 3.6e-99 | 745.4 | 447 | (45, 510) | 532 | (47, 502) | 545 | Phage portal protein | Phage portal protein | | uniclust | UniRef100\_A0A081NFB6 | 100.0 | 1.2e-86 | 2.5e-92 | 664.8 | 450 | (45, 512) | 532 | (55, 516) | 588 | Portal protein | Portal protein | | uniclust | UniRef100\_A0A024E891 | 100.0 | 1.9e-86 | 4.2e-92 | 692.0 | 446 | (44, 506) | 532 | (49, 507) | 598 | Portal protein lambda family | Portal protein lambda family | | uniclust | UniRef100\_A0A068Z022 | 100.0 | 3.1e-86 | 6.6e-92 | 702.1 | 453 | (46, 506) | 532 | (109, 600) | 711 | Phage portal protein | Phage portal protein | | uniclust | UniRef100\_A0A022G2U4 | 100.0 | 7.1e-84 | 1.5e-89 | 666.9 | 479 | (13, 511) | 532 | (121, 607) | 727 | Portal protein | Portal protein | | uniclust | UniRef100\_A0A0F9E9R9 | 100.0 | 8.5e-84 | 1.7e-89 | 643.4 | 472 | (17, 495) | 532 | (16, 512) | 539 | Phage portal protein (Fragment) | Phage portal protein (Fragment) | | uniclust | UniRef100\_A0A091FES5 | 100.0 | 4.7e-83 | 9.4e-89 | 644.0 | 469 | (19, 505) | 532 | (25, 503) | 601 | Phage portal protein | Phage portal protein | | uniclust | UniRef100\_A0A0F9KBE7 | 100.0 | 1.4e-82 | 2.9e-88 | 626.8 | 400 | (92, 508) | 532 | (4, 412) | 448 | Phage portal protein (Fragment) | Phage portal protein (Fragment) | | uniclust | UniRef100\_A0A073IWK7 | 100.0 | 1.5e-82 | 3e-88 | 631.9 | 462 | (9, 495) | 532 | (41, 510) | 556 | Phage portal protein | Phage portal protein | | uniclust | UniRef100\_A0A4Y1MVH7 | 100.0 | 5.7e-82 | 1.1e-87 | 636.3 | 480 | (11, 519) | 532 | (6, 503) | 651 | Portal protein | Portal protein | | uniclust | UniRef100\_A0A060H3H7 | 100.0 | 9.3e-82 | 1.9e-87 | 628.9 | 481 | (9, 508) | 532 | (40, 532) | 563 | Phage portal protein | Phage portal protein | | uniclust | UniRef100\_A0A0P1FA99 | 100.0 | 2.4e-81 | 4.9e-87 | 614.5 | 366 | (44, 419) | 532 | (34, 406) | 437 | Phage portal protein, lambda family | Phage portal protein, lambda family | | uniclust | UniRef100\_A0A0S9N407 | 100.0 | 2.8e-79 | 5.4e-85 | 595.9 | 457 | (44, 504) | 532 | (7, 469) | 499 | Portal protein | Portal protein | | uniclust | UniRef100\_A0A142Y555 | 100.0 | 2.9e-79 | 5.6e-85 | 600.1 | 492 | (12, 511) | 532 | (4, 512) | 609 | Phage portal protein, lambda family | Phage portal protein, lambda family | | uniclust | UniRef100\_A0A090N879 | 100.0 | 6.1e-79 | 1.1e-84 | 589.6 | 445 | (44, 505) | 532 | (28, 479) | 612 | Phage portal protein, lambda family | Phage portal protein, lambda family | | uniclust | UniRef100\_A0A1E3GXJ9 | 100.0 | 1.7e-78 | 3.1e-84 | 621.5 | 474 | (15, 516) | 532 | (2, 486) | 1194 | Phage portal protein, lambda family | Phage portal protein, lambda family | | uniclust | UniRef100\_A0A1D8IV87 | 100.0 | 3.3e-77 | 6.6e-83 | 603.2 | 442 | (44, 505) | 532 | (43, 503) | 669 | Phage portal protein | Phage portal protein | | uniclust | UniRef100\_A0A0Q6V110 | 100.0 | 6.7e-77 | 1.3e-82 | 595.0 | 477 | (12, 519) | 532 | (3, 496) | 568 | Portal protein | Portal protein | | uniclust | UniRef100\_A0A1A9VKE9 | 100.0 | 1e-75 | 1.9e-81 | 563.8 | 449 | (45, 514) | 532 | (233, 692) | 696 | Phage portal protein | Phage portal protein | | uniclust | UniRef100\_A0A510E8S9 | 100.0 | 1.3e-75 | 2.4e-81 | 584.0 | 453 | (43, 506) | 532 | (16, 474) | 962 | Phage portal protein | Phage portal protein | | uniclust | UniRef100\_A0A066T9U0 | 100.0 | 1.1e-74 | 2.2e-80 | 600.6 | 450 | (50, 509) | 532 | (102, 588) | 700 | Phage portal protein | Phage portal protein | | uniclust | UniRef100\_A0A437MJQ4 | 100.0 | 2.1e-73 | 4e-79 | 554.9 | 465 | (15, 511) | 532 | (4, 481) | 504 | Phage portal protein | Phage portal protein | | uniclust | UniRef100\_A0A0Q0CI78 | 100.0 | 1.1e-71 | 2.1e-77 | 534.6 | 346 | (154, 513) | 532 | (52, 406) | 433 | Histidinol phosphatase | Histidinol phosphatase | | uniclust | UniRef100\_A0A1J5EX01 | 100.0 | 3e-71 | 5.9e-77 | 545.1 | 448 | (47, 505) | 532 | (18, 492) | 525 | Phage portal protein | Phage portal protein | | uniclust | UniRef100\_A0A087M4C7 | 100.0 | 3.6e-71 | 7.6e-77 | 576.0 | 476 | (13, 505) | 532 | (21, 539) | 597 | Capsid protein | Capsid protein | | uniclust | UniRef100\_A0A0F9IDI9 | 100.0 | 4.8e-71 | 1e-76 | 584.0 | 431 | (43, 502) | 532 | (101, 536) | 652 | Portal protein | Portal protein | | uniclust | UniRef100\_A0A0F8ZRP6 | 100.0 | 3.8e-70 | 7.7e-76 | 532.2 | 363 | (132, 513) | 532 | (3, 374) | 405 | Phage portal protein (Fragment) | Phage portal protein (Fragment) | | uniclust | UniRef100\_A0A159Z5V5 | 100.0 | 2.6e-69 | 4.8e-75 | 535.4 | 446 | (45, 507) | 532 | (39, 492) | 873 | ATP-dependent Clp protease proteolytic subunit | ATP-dependent Clp protease proteolytic subunit | | uniclust | UniRef100\_A0A193LMF8 | 100.0 | 1.1e-68 | 2.2e-74 | 538.6 | 431 | (49, 493) | 532 | (49, 491) | 528 | Phage portal protein | Phage portal protein | | uniclust | UniRef100\_A0A6G1WEZ2 | 100.0 | 2.1e-68 | 4e-74 | 527.3 | 453 | (44, 504) | 532 | (35, 525) | 556 | Phage portal protein (Fragment) | Phage portal protein (Fragment) | | uniclust | UniRef100\_UPI00123DC76E | 100.0 | 3.5e-68 | 6.6e-74 | 523.9 | 472 | (18, 503) | 532 | (1, 512) | 705 | phage portal protein | phage portal protein | | uniclust | UniRef100\_A0A1R1LRL3 | 100.0 | 4e-67 | 7.8e-73 | 515.3 | 457 | (45, 514) | 532 | (28, 524) | 539 | Phage portal protein | Phage portal protein | | uniclust | UniRef100\_A0A071LT14 | 100.0 | 6.4e-67 | 1.3e-72 | 525.3 | 419 | (63, 493) | 532 | (61, 486) | 509 | Portal protein | Portal protein | | uniclust | UniRef100\_UPI002091CF7E | 100.0 | 7.2e-66 | 1.3e-71 | 506.0 | 480 | (16, 519) | 532 | (1, 500) | 884 | phage portal protein | phage portal protein | | uniclust | UniRef100\_A0A2D8T9H3 | 100.0 | 8.7e-66 | 1.7e-71 | 503.2 | 376 | (124, 506) | 532 | (2, 407) | 455 | Phage portal protein (Fragment) | Phage portal protein (Fragment) | | uniclust | UniRef100\_A0A1H6Z348 | 100.0 | 1.1e-64 | 2.2e-70 | 495.5 | 365 | (132, 504) | 532 | (1, 393) | 421 | Phage portal protein, lambda family (Fragment) | Phage portal protein, lambda family (Fragment) | | uniclust | UniRef100\_A0A0C2U5P6 | 100.0 | 2.1e-64 | 3.9e-70 | 470.4 | 441 | (44, 505) | 532 | (14, 459) | 463 | Phage portal protein | Phage portal protein | | uniclust | UniRef100\_A0A421BJ29 | 100.0 | 7.7e-64 | 1.4e-69 | 479.5 | 468 | (15, 508) | 532 | (150, 623) | 644 | Phage portal protein | Phage portal protein | | uniclust | UniRef100\_A0A0Q6MTA0 | 100.0 | 5.2e-63 | 1e-68 | 479.1 | 400 | (98, 517) | 532 | (13, 421) | 463 | Phage portal protein | Phage portal protein | | uniclust | UniRef100\_A0A2W4SHI4 | 100.0 | 7.9e-63 | 1.5e-68 | 475.8 | 392 | (16, 420) | 532 | (23, 423) | 445 | Phage portal protein (Fragment) | Phage portal protein (Fragment) | | uniclust | UniRef100\_A0A061KB43 | 100.0 | 1.5e-61 | 2.9e-67 | 479.2 | 456 | (47, 514) | 532 | (66, 559) | 608 | Phage portal protein | Phage portal protein | | uniclust | UniRef100\_A0A1Z9JFC0 | 100.0 | 2.9e-61 | 6e-67 | 496.5 | 421 | (44, 502) | 532 | (56, 490) | 578 | Phage portal protein | Phage portal protein | | uniclust | UniRef100\_A0A1G0ICK1 | 100.0 | 8.2e-61 | 1.6e-66 | 471.2 | 488 | (15, 516) | 532 | (52, 575) | 619 | Phage portal protein | Phage portal protein | | uniclust | UniRef100\_A0A175R9E3 | 100.0 | 1e-60 | 1.9e-66 | 451.3 | 435 | (43, 505) | 532 | (21, 460) | 472 | Portal protein | Portal protein | | uniclust | UniRef100\_A0A9E0NW25 | 100.0 | 1.4e-60 | 2.7e-66 | 462.8 | 400 | (92, 508) | 532 | (320, 728) | 741 | Phage portal protein | Phage portal protein | | uniclust | UniRef100\_A0A1B4C0H5 | 100.0 | 1.5e-60 | 2.7e-66 | 450.2 | 441 | (45, 505) | 532 | (22, 480) | 504 | Portal protein | Portal protein | | uniclust | UniRef100\_A0A443L3G4 | 100.0 | 2.9e-60 | 5.5e-66 | 444.8 | 373 | (16, 405) | 532 | (4, 383) | 384 | Phage portal protein (Fragment) | Phage portal protein (Fragment) | | uniclust | UniRef100\_A0A1V6HR06 | 100.0 | 4.6e-60 | 9.2e-66 | 482.5 | 461 | (16, 507) | 532 | (27, 500) | 662 | Phage portal protein, lambda family | Phage portal protein, lambda family | | uniclust | UniRef100\_A0A380AGT5 | 100.0 | 3.2e-59 | 6.2e-65 | 458.1 | 420 | (63, 495) | 532 | (61, 489) | 513 | Phage portal protein, lambda family | Phage portal protein, lambda family | | uniclust | UniRef100\_A0A353ZI73 | 100.0 | 3.8e-59 | 7.3e-65 | 457.6 | 448 | (45, 508) | 532 | (49, 523) | 552 | Phage portal protein | Phage portal protein | | uniclust | UniRef100\_A0A2M9P8F9 | 100.0 | 8.8e-59 | 1.7e-64 | 419.0 | 273 | (16, 300) | 532 | (2, 280) | 296 | Phage portal protein (Fragment) | Phage portal protein (Fragment) | | uniclust | UniRef100\_UPI002044A97D | 100.0 | 1.2e-58 | 2.2e-64 | 450.4 | 433 | (43, 504) | 532 | (20, 459) | 637 | phage portal protein | phage portal protein | | uniclust | UniRef100\_A0A1V5GTW5 | 100.0 | 1.3e-58 | 2.6e-64 | 441.0 | 292 | (209, 507) | 532 | (1, 295) | 328 | Phage portal protein, lambda family | Phage portal protein, lambda family | | uniclust | UniRef100\_A0A1S6TP94 | 100.0 | 2.6e-58 | 5.2e-64 | 459.4 | 397 | (66, 495) | 532 | (49, 453) | 486 | Phage portal protein, lambda family | Phage portal protein, lambda family | | uniclust | UniRef100\_A0A835Z6S7 | 100.0 | 3e-58 | 5.5e-64 | 444.6 | 468 | (16, 519) | 532 | (1, 487) | 695 | Phage portal protein | Phage portal protein | | uniclust | UniRef100\_A0A349EMI8 | 100.0 | 1.4e-57 | 2.9e-63 | 442.7 | 346 | (15, 365) | 532 | (11, 385) | 387 | Phage portal protein | Phage portal protein | | uniclust | UniRef100\_A0A3B8ZGJ9 | 100.0 | 1.6e-57 | 3e-63 | 442.3 | 437 | (43, 508) | 532 | (37, 479) | 609 | Phage portal protein | Phage portal protein | | uniclust | UniRef100\_A0A2N9AI26 | 100.0 | 1.8e-57 | 3.4e-63 | 458.4 | 447 | (45, 507) | 532 | (767, 1241) | 1265 | Terminase GpA (Modular protein) | Terminase GpA (Modular protein) | | uniclust | UniRef100\_A0A0S8GDJ6 | 100.0 | 1.9e-57 | 4e-63 | 473.7 | 428 | (44, 494) | 532 | (26, 478) | 605 | Phage portal protein | Phage portal protein | | uniclust | UniRef100\_A0A2E7BJT9 | 100.0 | 2e-57 | 4.1e-63 | 434.0 | 291 | (203, 513) | 532 | (12, 308) | 350 | Phage portal protein (Fragment) | Phage portal protein (Fragment) | | uniclust | UniRef100\_A0A0A2V4M4 | 100.0 | 2.8e-57 | 5.2e-63 | 470.7 | 455 | (46, 508) | 532 | (1183, 1679) | 1696 | Portal protein B | Portal protein B | | uniclust | UniRef100\_A0A1M4S7W9 | 100.0 | 4.2e-57 | 8.2e-63 | 425.6 | 300 | (44, 353) | 532 | (28, 342) | 342 | Phage portal protein, lambda family | Phage portal protein, lambda family | | uniclust | UniRef100\_A0A2X1LNZ6 | 100.0 | 6.6e-57 | 1.2e-62 | 428.1 | 430 | (63, 503) | 532 | (16, 468) | 484 | Lambda family phage portal protein | Lambda family phage portal protein | | uniclust | UniRef100\_A0A1G1LMU8 | 100.0 | 1.1e-56 | 2.1e-62 | 420.6 | 257 | (240, 503) | 532 | (7, 275) | 312 | Phage portal protein (Fragment) | Phage portal protein (Fragment) | | uniclust | UniRef100\_A0A1V1UMH2 | 100.0 | 1.3e-56 | 2.6e-62 | 447.9 | 434 | (63, 523) | 532 | (66, 532) | 547 | Phage portal protein, lambda family | Phage portal protein, lambda family | | uniclust | UniRef100\_A0A7X4AD15 | 100.0 | 4.5e-56 | 8.6e-62 | 428.6 | 404 | (43, 481) | 532 | (35, 441) | 472 | Phage portal protein | Phage portal protein | | uniclust | UniRef100\_A0A9E5UZN2 | 100.0 | 7.8e-56 | 1.4e-61 | 414.8 | 472 | (15, 506) | 532 | (9, 490) | 494 | Phage portal protein | Phage portal protein | | uniclust | UniRef100\_A0A011T786 | 100.0 | 9e-56 | 1.8e-61 | 450.9 | 429 | (61, 506) | 532 | (76, 550) | 597 | Capsid protein | Capsid protein | | uniclust | UniRef100\_A0A354UBL5 | 100.0 | 1.6e-54 | 3.1e-60 | 416.5 | 448 | (44, 505) | 532 | (22, 479) | 511 | Phage portal protein | Phage portal protein | | uniclust | UniRef100\_A0A0H4A1U0 | 100.0 | 1.7e-54 | 3.3e-60 | 426.6 | 464 | (14, 502) | 532 | (12, 482) | 512 | Phage portal protein | Phage portal protein | | uniclust | UniRef100\_A0A7G9NZU8 | 100.0 | 4.7e-54 | 8.7e-60 | 413.8 | 438 | (44, 487) | 532 | (53, 527) | 656 | Phage portal protein | Phage portal protein | | uniclust | UniRef100\_A0A0E2E9S4 | 100.0 | 6.1e-53 | 1.3e-58 | 434.5 | 409 | (71, 495) | 532 | (93, 543) | 590 | SPP1 family phage portal protein | SPP1 family phage portal protein | | uniclust | UniRef100\_A0A2D5SAY0 | 100.0 | 8.9e-53 | 1.8e-58 | 406.2 | 380 | (15, 416) | 532 | (11, 395) | 398 | Phage portal protein (Fragment) | Phage portal protein (Fragment) | | uniclust | UniRef100\_A0A1F4CAA0 | 100.0 | 2.1e-52 | 4e-58 | 380.4 | 288 | (208, 502) | 532 | (1, 312) | 338 | Phage portal protein (Fragment) | Phage portal protein (Fragment) | | uniclust | UniRef100\_A0A368TQV2 | 100.0 | 2.2e-52 | 4.1e-58 | 410.1 | 443 | (43, 505) | 532 | (183, 647) | 744 | Phage portal protein | Phage portal protein | | uniclust | UniRef100\_A0A3R8IU71 | 100.0 | 2.6e-52 | 4.9e-58 | 406.4 | 450 | (48, 505) | 532 | (57, 508) | 748 | Phage portal protein | Phage portal protein | | uniclust | UniRef100\_UPI00025BA5D3 | 100.0 | 2.8e-52 | 5.2e-58 | 409.3 | 444 | (45, 492) | 532 | (29, 516) | 823 | phage portal protein | phage portal protein | | uniclust | UniRef100\_A0A142WWE9 | 100.0 | 3.9e-52 | 8.1e-58 | 424.7 | 425 | (45, 506) | 532 | (29, 468) | 513 | Phage portal protein, lambda family | Phage portal protein, lambda family | | uniclust | UniRef100\_A0A062V9J7 | 100.0 | 4.8e-52 | 9.5e-58 | 394.2 | 317 | (44, 377) | 532 | (24, 344) | 344 | Phage portal protein, lambda family (Fragment) | Phage portal protein, lambda family (Fragment) | | uniclust | UniRef100\_A0A6L6JH12 | 100.0 | 1.1e-51 | 2.1e-57 | 395.2 | 390 | (7, 398) | 532 | (8, 414) | 425 | Phage portal protein (Fragment) | Phage portal protein (Fragment) | | uniclust | UniRef100\_W0IYG2 | 100.0 | 1.8e-51 | 3.3e-57 | 394.3 | 451 | (48, 505) | 532 | (43, 496) | 620 | Portal protein | Portal protein | | uniclust | UniRef100\_A0A0B8PGA6 | 100.0 | 8.5e-51 | 1.6e-56 | 388.4 | 433 | (64, 504) | 532 | (37, 477) | 501 | Phage portal protein | Phage portal protein | | uniclust | UniRef100\_A0A345DE62 | 100.0 | 2.9e-50 | 5.3e-56 | 379.0 | 430 | (46, 505) | 532 | (30, 471) | 509 | Phage portal protein | Phage portal protein | | uniclust | UniRef100\_A0A3L7RG21 | 100.0 | 5.4e-50 | 1.1e-55 | 379.8 | 335 | (155, 503) | 532 | (8, 344) | 372 | Phage portal protein | Phage portal protein | | uniclust | UniRef100\_A0A2S1XL37 | 100.0 | 1.1e-49 | 2.1e-55 | 359.2 | 253 | (16, 280) | 532 | (1, 260) | 261 | Phage portal protein | Phage portal protein | | uniclust | UniRef100\_A0A9E5G6J2 | 100.0 | 1.7e-49 | 3.1e-55 | 372.3 | 466 | (17, 505) | 532 | (5, 481) | 487 | Phage portal protein | Phage portal protein | | uniclust | UniRef100\_A0A524IJ31 | 100.0 | 1.7e-49 | 3.4e-55 | 392.4 | 431 | (44, 504) | 532 | (34, 479) | 542 | Phage portal protein (Fragment) | Phage portal protein (Fragment) | | uniclust | UniRef100\_UPI001F424B1F | 100.0 | 2.4e-49 | 4.4e-55 | 395.1 | 473 | (19, 510) | 532 | (512, 996) | 1019 | phage portal protein | phage portal protein | | uniclust | UniRef100\_A0A063ZY87 | 100.0 | 6e-49 | 1.1e-54 | 393.7 | 450 | (29, 493) | 532 | (574, 1056) | 1075 | Terminase | Terminase | | uniclust | UniRef100\_X0U7Z2 | 100.0 | 9e-49 | 1.7e-54 | 361.5 | 369 | (122, 505) | 532 | (10, 389) | 417 | Phage portal protein (Fragment) | Phage portal protein (Fragment) | | uniclust | UniRef100\_UPI0009FE0364 | 100.0 | 1e-48 | 1.9e-54 | 360.4 | 362 | (134, 507) | 532 | (6, 374) | 408 | phage portal protein | phage portal protein | | uniclust | UniRef100\_A0A072TET7 | 100.0 | 1.2e-48 | 2.1e-54 | 400.7 | 459 | (46, 512) | 532 | (803, 1302) | 1593 | Phage portal protein, lambda family protein (Fragment) | Phage portal protein, lambda family protein (Fragment) | | uniclust | UniRef100\_A0A2U3ERF1 | 100.0 | 1.1e-48 | 2.2e-54 | 382.3 | 332 | (46, 382) | 532 | (38, 413) | 420 | Phage portal protein (Fragment) | Phage portal protein (Fragment) | | uniclust | UniRef100\_A0A250DMY9 | 100.0 | 1.7e-48 | 3.3e-54 | 367.7 | 259 | (43, 301) | 532 | (21, 280) | 326 | Phage portal protein | Phage portal protein | | uniclust | UniRef100\_A0A376NRU5 | 100.0 | 2.1e-48 | 4.1e-54 | 370.9 | 345 | (15, 371) | 532 | (22, 374) | 391 | Phage portal protein, lambda family | Phage portal protein, lambda family | | uniclust | UniRef100\_A0A1T4WXT7 | 100.0 | 2.1e-48 | 4.3e-54 | 388.5 | 447 | (48, 504) | 532 | (29, 490) | 518 | Capsid protein | Capsid protein | | uniclust | UniRef100\_A0A661TNZ2 | 100.0 | 2.6e-48 | 4.8e-54 | 375.0 | 470 | (16, 495) | 532 | (94, 615) | 660 | Phage portal protein | Phage portal protein | | uniclust | UniRef100\_A0A3L7SGJ4 | 100.0 | 4.7e-48 | 8.6e-54 | 370.4 | 436 | (44, 507) | 532 | (33, 476) | 605 | Phage portal protein | Phage portal protein | | uniclust | UniRef100\_A0A177R1N7 | 100.0 | 6.5e-48 | 1.3e-53 | 392.2 | 402 | (57, 495) | 532 | (66, 484) | 537 | Phage portal protein | Phage portal protein | | uniclust | UniRef100\_A0A0S4TN73 | 100.0 | 7.9e-48 | 1.5e-53 | 355.7 | 258 | (16, 292) | 532 | (25, 287) | 303 | Phage portal protein (Fragment) | Phage portal protein (Fragment) | | uniclust | UniRef100\_A0A6G4KD87 | 100.0 | 1.1e-47 | 2e-53 | 359.1 | 349 | (87, 451) | 532 | (1, 358) | 363 | Phage portal protein (Fragment) | Phage portal protein (Fragment) | | uniclust | UniRef100\_A0A4P5XY08 | 100.0 | 1.1e-47 | 2.2e-53 | 378.9 | 421 | (44, 488) | 532 | (73, 498) | 616 | Phage portal protein | Phage portal protein | | uniclust | UniRef100\_UPI000A7FFD44 | 100.0 | 1.2e-47 | 2.2e-53 | 352.7 | 348 | (18, 371) | 532 | (1, 380) | 381 | phage portal protein | phage portal protein | | uniclust | UniRef100\_A0A0A1AA99 | 100.0 | 1.5e-47 | 2.8e-53 | 374.4 | 472 | (15, 509) | 532 | (22, 503) | 669 | Phage portal protein | Phage portal protein | | uniclust | UniRef100\_A0A812JEV6 | 100.0 | 3.2e-47 | 5.8e-53 | 404.0 | 419 | (65, 495) | 532 | (1153, 1576) | 4451 | Phage portal protein | Phage portal protein | | uniclust | UniRef100\_A0A0A0YPM0 | 100.0 | 3.8e-47 | 8e-53 | 393.2 | 413 | (66, 491) | 532 | (64, 527) | 555 | Portal protein | Portal protein | | uniclust | UniRef100\_A0A0F9AB75 | 100.0 | 6.2e-47 | 1.2e-52 | 351.4 | 258 | (243, 505) | 532 | (9, 278) | 300 | Phage portal protein (Fragment) | Phage portal protein (Fragment) | | uniclust | UniRef100\_A0A7J6YNN5 | 100.0 | 6.7e-47 | 1.2e-52 | 363.6 | 279 | (217, 507) | 532 | (333, 616) | 623 | DUF1016 domain-containing protein | DUF1016 domain-containing protein | | uniclust | UniRef100\_A0A2A2R1Z6 | 100.0 | 7.2e-47 | 1.5e-52 | 386.3 | 428 | (44, 503) | 532 | (35, 481) | 545 | Phage portal protein | Phage portal protein | | uniclust | UniRef100\_A0A954DEK2 | 100.0 | 8.4e-47 | 1.5e-52 | 359.4 | 435 | (45, 495) | 532 | (60, 514) | 560 | Phage portal protein | Phage portal protein | | uniclust | UniRef100\_A0A965PFI3 | 100.0 | 1.6e-46 | 2.9e-52 | 375.1 | 463 | (16, 510) | 532 | (4, 480) | 833 | Phage portal protein | Phage portal protein | | uniclust | UniRef100\_A0A973DZ03 | 100.0 | 1.9e-46 | 3.6e-52 | 360.0 | 445 | (61, 514) | 532 | (18, 500) | 522 | Phage portal protein | Phage portal protein | | uniclust | UniRef100\_A0A1G3M6A5 | 100.0 | 1.4e-45 | 2.6e-51 | 341.8 | 357 | (45, 419) | 532 | (58, 426) | 429 | Phage portal protein (Fragment) | Phage portal protein (Fragment) | | uniclust | UniRef100\_A0A2D6FNP9 | 100.0 | 1.6e-45 | 3.1e-51 | 355.4 | 428 | (44, 501) | 532 | (22, 460) | 501 | Phage portal protein | Phage portal protein | | uniclust | UniRef100\_A0A389MVE2 | 100.0 | 2.1e-45 | 4e-51 | 362.7 | 409 | (73, 505) | 532 | (89, 511) | 611 | Phage portal protein | Phage portal protein | | uniclust | UniRef100\_A0A1V5NFL2 | 100.0 | 4.9e-45 | 9e-51 | 367.0 | 436 | (43, 504) | 532 | (20, 482) | 1114 | Phage portal protein, lambda family | Phage portal protein, lambda family | | uniclust | UniRef100\_A0A7C8EMQ6 | 100.0 | 9.4e-45 | 1.7e-50 | 342.0 | 430 | (46, 505) | 532 | (51, 492) | 502 | Phage portal protein | Phage portal protein | | uniclust | UniRef100\_UPI0003A4D93C | 100.0 | 1.9e-44 | 3.6e-50 | 337.2 | 414 | (69, 509) | 532 | (42, 456) | 464 | phage portal protein | phage portal protein | | uniclust | UniRef100\_A0A2X2S8A3 | 100.0 | 2.9e-44 | 5.5e-50 | 333.3 | 317 | (153, 472) | 532 | (1, 348) | 349 | Phage portal protein | Phage portal protein | | uniclust | UniRef100\_A0A167I0Q7 | 100.0 | 3.9e-44 | 7.6e-50 | 342.9 | 355 | (141, 507) | 532 | (15, 400) | 418 | Plasmid partitioning protein ParB (Fragment) | Plasmid partitioning protein ParB (Fragment) | | uniclust | UniRef100\_A0A517Q5C2 | 100.0 | 4.3e-44 | 8.1e-50 | 343.0 | 462 | (15, 503) | 532 | (7, 481) | 504 | Phage portal protein, lambda family | Phage portal protein, lambda family | | uniclust | UniRef100\_A0A3M3BPQ1 | 100.0 | 4.3e-44 | 8.2e-50 | 326.2 | 292 | (126, 420) | 532 | (2, 307) | 314 | Portal protein (Fragment) | Portal protein (Fragment) | | uniclust | UniRef100\_A0A564WHL9 | 100.0 | 6.3e-44 | 1.2e-49 | 335.1 | 444 | (47, 506) | 532 | (4, 466) | 481 | Putative Phage portal protein | Putative Phage portal protein | | uniclust | UniRef100\_A0A120G5R1 | 100.0 | 6.5e-44 | 1.3e-49 | 334.2 | 250 | (248, 503) | 532 | (23, 290) | 322 | Phage portal protein, lambda family | Phage portal protein, lambda family | | uniclust | UniRef100\_A0A0F9MU33 | 100.0 | 1.1e-43 | 1.9e-49 | 337.2 | 459 | (46, 523) | 532 | (27, 523) | 537 | Phage portal protein, lambda family | Phage portal protein, lambda family | | uniclust | UniRef100\_UPI0021BB8F59 | 100.0 | 1.8e-43 | 3.2e-49 | 336.8 | 410 | (64, 501) | 532 | (62, 498) | 555 | phage portal protein | phage portal protein | | uniclust | UniRef100\_UPI000AC8D934 | 100.0 | 7.1e-43 | 1.3e-48 | 344.1 | 413 | (65, 492) | 532 | (70, 500) | 819 | phage portal protein | phage portal protein | | uniclust | UniRef100\_A0A965TTW4 | 100.0 | 8.2e-43 | 1.5e-48 | 337.0 | 467 | (17, 492) | 532 | (2, 507) | 522 | Phage portal protein | Phage portal protein | | uniclust | UniRef100\_A0A1G0RDT4 | 100.0 | 8.7e-43 | 1.6e-48 | 331.8 | 443 | (43, 491) | 532 | (32, 497) | 548 | Phage portal protein | Phage portal protein | | uniclust | UniRef100\_A0A1G3LXF9 | 100.0 | 1.3e-42 | 2.5e-48 | 320.2 | 355 | (146, 508) | 532 | (22, 389) | 403 | Phage portal protein | Phage portal protein | | uniclust | UniRef100\_A0A3B9NPV5 | 100.0 | 1.6e-42 | 3.2e-48 | 317.6 | 234 | (17, 254) | 532 | (1, 237) | 238 | Phage portal protein (Fragment) | Phage portal protein (Fragment) | | uniclust | UniRef100\_UPI0009B970C7 | 100.0 | 1.6e-42 | 3.3e-48 | 332.1 | 318 | (57, 381) | 532 | (46, 370) | 376 | phage portal protein | phage portal protein | | uniclust | UniRef100\_A0A0U1DBB2 | 100.0 | 2.6e-42 | 5.1e-48 | 311.7 | 229 | (271, 508) | 532 | (2, 238) | 261 | Putative phage portal protein | Putative phage portal protein | | uniclust | UniRef100\_A0A853IJZ4 | 100.0 | 4.2e-42 | 8.1e-48 | 307.5 | 226 | (248, 478) | 532 | (6, 240) | 240 | Phage portal protein (Fragment) | Phage portal protein (Fragment) | | uniclust | UniRef100\_A0A0F9HAI2 | 100.0 | 4.2e-42 | 8.4e-48 | 316.5 | 237 | (262, 507) | 532 | (2, 241) | 267 | Phage portal protein (Fragment) | Phage portal protein (Fragment) | | uniclust | UniRef100\_A0A0G4PZK6 | 100.0 | 4.7e-42 | 9.1e-48 | 319.4 | 283 | (216, 511) | 532 | (3, 288) | 313 | Phage portal protein, lambda family | Phage portal protein, lambda family | | uniclust | UniRef100\_A0A965PAQ9 | 100.0 | 7.4e-42 | 1.4e-47 | 333.5 | 406 | (45, 493) | 532 | (31, 451) | 644 | Phage portal protein | Phage portal protein | | uniclust | UniRef100\_UPI0003260BA1 | 100.0 | 9.4e-42 | 1.7e-47 | 322.2 | 347 | (15, 373) | 532 | (1, 359) | 503 | phage portal protein | phage portal protein | | uniclust | UniRef100\_A0A7X7YX50 | 100.0 | 9.8e-42 | 1.8e-47 | 321.4 | 450 | (20, 505) | 532 | (15, 476) | 494 | Phage portal protein | Phage portal protein | | uniclust | UniRef100\_A0A965P5L4 | 100.0 | 9.9e-42 | 1.8e-47 | 340.2 | 439 | (43, 518) | 532 | (32, 476) | 957 | Phage portal protein | Phage portal protein | | uniclust | UniRef100\_A0A166JYY8 | 100.0 | 1.5e-41 | 2.8e-47 | 327.7 | 406 | (73, 505) | 532 | (27, 438) | 463 | Portal protein | Portal protein | | uniclust | UniRef100\_A0A349JAV8 | 100.0 | 2e-41 | 3.6e-47 | 329.5 | 438 | (43, 506) | 532 | (34, 478) | 688 | Phage portal protein | Phage portal protein | | uniclust | UniRef100\_A0A2M9P8T1 | 100.0 | 3.4e-41 | 6.5e-47 | 305.5 | 253 | (249, 518) | 532 | (1, 258) | 281 | Phage portal protein (Fragment) | Phage portal protein (Fragment) | | uniclust | UniRef100\_A0A965I6N8 | 100.0 | 4.8e-41 | 8.9e-47 | 329.5 | 473 | (15, 514) | 532 | (1, 490) | 667 | Phage portal protein | Phage portal protein | | uniclust | UniRef100\_A0A3A1Y7Y1 | 100.0 | 6.3e-41 | 1.2e-46 | 330.9 | 430 | (45, 504) | 532 | (53, 485) | 534 | Phage portal protein | Phage portal protein | | uniclust | UniRef100\_A0A554XEQ4 | 100.0 | 6.9e-41 | 1.3e-46 | 316.2 | 315 | (43, 367) | 532 | (18, 336) | 500 | Putative esterase | Putative esterase | | uniclust | UniRef100\_A0A7X7H5B0 | 100.0 | 1.2e-40 | 2.2e-46 | 315.6 | 427 | (43, 491) | 532 | (25, 487) | 515 | Phage portal protein | Phage portal protein | | uniclust | UniRef100\_A0A7C4BYU0 | 100.0 | 1.2e-40 | 2.2e-46 | 310.9 | 313 | (46, 387) | 532 | (23, 336) | 446 | Phage portal protein (Fragment) | Phage portal protein (Fragment) | | uniclust | UniRef100\_C1AAN8 | 100.0 | 2.7e-40 | 5e-46 | 318.2 | 455 | (45, 509) | 532 | (41, 524) | 608 | Phage portal protein lambda family protein | Phage portal protein lambda family protein | | uniclust | UniRef100\_UPI000FCA5EF8 | 100.0 | 3.5e-40 | 6.5e-46 | 290.5 | 259 | (141, 402) | 532 | (5, 271) | 273 | phage portal protein | phage portal protein | | uniclust | UniRef100\_A0A062VDI3 | 100.0 | 3.9e-40 | 7.9e-46 | 299.3 | 183 | (317, 509) | 532 | (14, 199) | 229 | Lambda family phage portal protein | Lambda family phage portal protein | | uniclust | UniRef100\_A0A1H9QDY8 | 100.0 | 5e-40 | 9.1e-46 | 307.0 | 362 | (51, 416) | 532 | (31, 419) | 447 | Phage portal protein, lambda family | Phage portal protein, lambda family | | uniclust | UniRef100\_A0A0Q4ZHQ5 | 100.0 | 4.7e-40 | 9.4e-46 | 329.1 | 404 | (71, 502) | 532 | (65, 484) | 530 | Phage portal protein | Phage portal protein | | uniclust | UniRef100\_A0A929TYB1 | 100.0 | 6.2e-40 | 1.2e-45 | 298.2 | 280 | (16, 301) | 532 | (1, 281) | 305 | Phage portal protein (Fragment) | Phage portal protein (Fragment) | | uniclust | UniRef100\_A0A257VIH0 | 100.0 | 7.9e-40 | 1.5e-45 | 294.0 | 283 | (43, 341) | 532 | (10, 303) | 318 | Phage portal protein (Fragment) | Phage portal protein (Fragment) | | uniclust | UniRef100\_A0A812INR5 | 100.0 | 1.4e-39 | 2.5e-45 | 328.9 | 393 | (62, 467) | 532 | (53, 470) | 1131 | B protein | B protein | | uniclust | UniRef100\_A0A0S4U0W3 | 100.0 | 3.1e-39 | 5.7e-45 | 286.3 | 246 | (47, 301) | 532 | (8, 265) | 285 | Bacteriophage-like protein (Fragment) | Bacteriophage-like protein (Fragment) | | uniclust | UniRef100\_A0A0S8IF22 | 100.0 | 3.1e-39 | 6e-45 | 319.9 | 420 | (67, 494) | 532 | (63, 529) | 552 | Phage portal protein | Phage portal protein | | uniclust | UniRef100\_R9L6B1 | 100.0 | 4.5e-39 | 8.2e-45 | 295.7 | 366 | (16, 394) | 532 | (3, 382) | 383 | Lambda family phage portal protein | Lambda family phage portal protein | | uniclust | UniRef100\_A0A2W5C3N3 | 100.0 | 7e-39 | 1.3e-44 | 290.0 | 311 | (180, 503) | 532 | (2, 325) | 336 | Phage portal protein (Fragment) | Phage portal protein (Fragment) | | uniclust | UniRef100\_A0A0H2VTW3 | 100.0 | 8.5e-39 | 1.6e-44 | 297.5 | 339 | (165, 514) | 532 | (4, 372) | 388 | Head-tail preconnector gp5 | Head-tail preconnector gp5 | | uniclust | UniRef100\_UPI0020272EAC | 100.0 | 9.4e-39 | 1.7e-44 | 315.3 | 357 | (127, 490) | 532 | (3, 391) | 808 | phage portal protein | phage portal protein | | uniclust | UniRef100\_A0A0T7A5Q8 | 100.0 | 9.7e-39 | 1.8e-44 | 302.2 | 411 | (52, 472) | 532 | (18, 488) | 504 | Portal protein | Portal protein | | uniclust | UniRef100\_A0A376JWU1 | 100.0 | 9.8e-39 | 1.9e-44 | 302.3 | 345 | (63, 416) | 532 | (61, 414) | 415 | Phage portal protein, lambda family | Phage portal protein, lambda family | | uniclust | UniRef100\_A0A0S4TZ33 | 100.0 | 1.5e-38 | 3e-44 | 293.7 | 194 | (319, 516) | 532 | (11, 206) | 249 | Bacteriophage-related protein | Bacteriophage-related protein | | uniclust | UniRef100\_UPI0022F09839 | 100.0 | 3.5e-38 | 6.5e-44 | 292.5 | 348 | (126, 495) | 532 | (24, 378) | 415 | phage portal protein | phage portal protein | | uniclust | UniRef100\_A0A352V3P3 | 100.0 | 4.1e-38 | 7.6e-44 | 287.2 | 319 | (159, 495) | 532 | (7, 331) | 358 | Phage portal protein | Phage portal protein | | uniclust | UniRef100\_A0A6M3M268 | 100.0 | 5.4e-38 | 9.9e-44 | 298.2 | 471 | (4, 505) | 532 | (18, 500) | 520 | Putative portal protein | Putative portal protein | | uniclust | UniRef100\_A0A0F2PJU6 | 100.0 | 7e-38 | 1.3e-43 | 305.6 | 410 | (79, 505) | 532 | (66, 509) | 604 | Capsid protein | Capsid protein | | uniclust | UniRef100\_A0A165XDC1 | 100.0 | 8.7e-38 | 1.6e-43 | 306.5 | 428 | (75, 510) | 532 | (139, 727) | 742 | Phage portal protein, lambda family | Phage portal protein, lambda family | | uniclust | UniRef100\_A0A356GP31 | 100.0 | 1e-37 | 2e-43 | 287.0 | 229 | (13, 246) | 532 | (11, 247) | 260 | Phage portal protein (Fragment) | Phage portal protein (Fragment) | | uniclust | UniRef100\_A0A7X7KP38 | 100.0 | 1.3e-37 | 2.4e-43 | 295.9 | 422 | (45, 487) | 532 | (48, 491) | 524 | Phage portal protein | Phage portal protein | | uniclust | UniRef100\_A0A1V2N5C6 | 100.0 | 1.3e-37 | 2.5e-43 | 275.7 | 185 | (325, 519) | 532 | (2, 187) | 204 | Phage portal protein, lambda family | Phage portal protein, lambda family | | uniclust | UniRef100\_A0A354EMX8 | 100.0 | 1.4e-37 | 2.6e-43 | 286.6 | 291 | (180, 477) | 532 | (6, 302) | 389 | Phage portal protein | Phage portal protein | | uniclust | UniRef100\_A0A7W1G2Z3 | 100.0 | 1.4e-37 | 2.7e-43 | 301.8 | 447 | (46, 505) | 532 | (10, 475) | 519 | Phage portal protein | Phage portal protein | | uniclust | UniRef100\_A0A1X7MMD6 | 100.0 | 1.7e-37 | 3.1e-43 | 273.0 | 245 | (158, 405) | 532 | (2, 265) | 266 | Phage portal protein, lambda family | Phage portal protein, lambda family | | uniclust | UniRef100\_A0A376YBL2 | 100.0 | 2.6e-37 | 5e-43 | 286.1 | 307 | (78, 393) | 532 | (5, 347) | 348 | Capsid protein of prophage | Capsid protein of prophage | | uniclust | UniRef100\_A0A8X6LDJ2 | 100.0 | 3.3e-37 | 6e-43 | 265.3 | 205 | (163, 372) | 532 | (6, 216) | 226 | Phage portal protein | Phage portal protein | | uniclust | UniRef100\_A0A2V8RVC3 | 100.0 | 3.3e-37 | 6.4e-43 | 294.4 | 336 | (141, 506) | 532 | (1, 348) | 376 | Phage portal protein | Phage portal protein | | uniclust | UniRef100\_A0A0F9LPE5 | 100.0 | 4.6e-37 | 9e-43 | 302.2 | 382 | (56, 484) | 532 | (42, 443) | 478 | Portal protein | Portal protein | | uniclust | UniRef100\_A0A2W5DU94 | 100.0 | 6.5e-37 | 1.3e-42 | 280.5 | 216 | (283, 504) | 532 | (1, 220) | 242 | Phage portal protein (Fragment) | Phage portal protein (Fragment) | | uniclust | UniRef100\_A0A495YAC3 | 100.0 | 7.4e-37 | 1.5e-42 | 276.3 | 180 | (44, 233) | 532 | (24, 205) | 226 | Phage portal protein (Fragment) | Phage portal protein (Fragment) | | uniclust | UniRef100\_A0A0G4Q0C6 | 100.0 | 8.7e-37 | 1.7e-42 | 281.8 | 241 | (15, 267) | 532 | (22, 267) | 267 | Phage portal protein, lambda family | Phage portal protein, lambda family | | uniclust | UniRef100\_A0A6J5NU14 | 100.0 | 1e-36 | 2.1e-42 | 294.5 | 349 | (108, 488) | 532 | (2, 368) | 391 | Phage portal protein, lambda family | Phage portal protein, lambda family | | uniclust | UniRef100\_A0A060HCC5 | 100.0 | 1e-36 | 2.1e-42 | 275.7 | 181 | (318, 506) | 532 | (25, 205) | 233 | Portal protein | Portal protein | | uniclust | UniRef100\_A0A5C7Q7S1 | 100.0 | 1.2e-36 | 2.2e-42 | 288.9 | 424 | (44, 490) | 532 | (13, 459) | 512 | Phage portal protein | Phage portal protein | | uniclust | UniRef100\_A0A357ZWC2 | 100.0 | 2.1e-36 | 4.1e-42 | 305.7 | 425 | (48, 504) | 532 | (18, 465) | 581 | Phage portal protein | Phage portal protein | | uniclust | UniRef100\_A0A970C2J4 | 100.0 | 2.4e-36 | 4.5e-42 | 290.6 | 440 | (48, 495) | 532 | (59, 522) | 587 | Phage portal protein | Phage portal protein | | uniclust | UniRef100\_A0A9E0EC56 | 100.0 | 2.6e-36 | 4.7e-42 | 272.3 | 315 | (18, 361) | 532 | (1, 322) | 322 | Phage portal protein (Fragment) | Phage portal protein (Fragment) | | uniclust | UniRef100\_Q2CB91 | 100.0 | 2.7e-36 | 5.1e-42 | 266.8 | 234 | (249, 490) | 532 | (1, 237) | 245 | Probable bacteriophage-related protein | Probable bacteriophage-related protein | | uniclust | UniRef100\_UPI002012B078 | 100.0 | 3.1e-36 | 5.7e-42 | 300.5 | 334 | (21, 360) | 532 | (1, 353) | 907 | phage portal protein | phage portal protein | | uniclust | UniRef100\_UPI0002FED4F3 | 100.0 | 5.4e-36 | 1e-41 | 289.3 | 433 | (45, 503) | 532 | (37, 487) | 611 | phage portal protein | phage portal protein | | uniclust | UniRef100\_A0A2A2RL01 | 100.0 | 1.1e-35 | 2e-41 | 289.2 | 404 | (66, 504) | 532 | (56, 469) | 511 | Phage portal protein | Phage portal protein | | uniclust | UniRef100\_A0A2U1B6F7 | 100.0 | 1.1e-35 | 2e-41 | 273.0 | 338 | (154, 505) | 532 | (8, 350) | 373 | Lambda family phage portal protein | Lambda family phage portal protein | | uniclust | UniRef100\_UPI000DDF7C70 | 100.0 | 1.1e-35 | 2.2e-41 | 285.6 | 292 | (181, 487) | 532 | (33, 329) | 330 | phage portal protein | phage portal protein | | uniclust | UniRef100\_A0A8G2LY73 | 100.0 | 1.3e-35 | 2.4e-41 | 281.6 | 384 | (124, 518) | 532 | (3, 419) | 430 | Phage portal protein (Minor capsid protein) | Phage portal protein (Minor capsid protein) | | uniclust | UniRef100\_A0A0F9FZI9 | 100.0 | 1.6e-35 | 3.5e-41 | 315.0 | 396 | (72, 492) | 532 | (97, 516) | 607 | Phage portal protein | Phage portal protein | | uniclust | UniRef100\_A0A142Y7Z0 | 100.0 | 1.8e-35 | 3.6e-41 | 275.3 | 238 | (43, 299) | 532 | (38, 279) | 281 | Phage portal protein, lambda family | Phage portal protein, lambda family | | uniclust | UniRef100\_UPI000AC26F33 | 100.0 | 2.2e-35 | 4e-41 | 265.5 | 270 | (212, 490) | 532 | (35, 306) | 313 | phage portal protein | phage portal protein | | uniclust | UniRef100\_A0A813BI55 | 100.0 | 2.4e-35 | 4.4e-41 | 305.4 | 449 | (46, 505) | 532 | (85, 552) | 1651 | Peptidase S49 domain-containing protein | Peptidase S49 domain-containing protein | | uniclust | UniRef100\_UPI001BFF0A19 | 100.0 | 2.8e-35 | 5.1e-41 | 257.1 | 233 | (44, 285) | 532 | (11, 248) | 249 | phage portal protein | phage portal protein | | uniclust | UniRef100\_A0A0D8NGB3 | 100.0 | 2.8e-35 | 5.4e-41 | 275.2 | 252 | (46, 301) | 532 | (33, 309) | 360 | Capsid protein (Fragment) | Capsid protein (Fragment) | | uniclust | UniRef100\_A0A0S8GAH5 | 100.0 | 3.2e-35 | 6e-41 | 265.0 | 255 | (244, 505) | 532 | (1, 264) | 273 | Phage portal protein (Fragment) | Phage portal protein (Fragment) | | uniclust | UniRef100\_A0A0F9HR86 | 100.0 | 5.7e-35 | 1.1e-40 | 285.8 | 408 | (72, 495) | 532 | (80, 528) | 559 | Phage portal protein | Phage portal protein | | uniclust | UniRef100\_A0A953JK51 | 100.0 | 7.2e-35 | 1.3e-40 | 263.6 | 294 | (215, 523) | 532 | (3, 304) | 327 | Phage portal protein | Phage portal protein | | uniclust | UniRef100\_A0A2E3DCB1 | 100.0 | 7.9e-35 | 1.5e-40 | 279.7 | 448 | (43, 503) | 532 | (50, 545) | 571 | Phage portal protein | Phage portal protein | | uniclust | UniRef100\_A0A968GDE4 | 100.0 | 8.2e-35 | 1.5e-40 | 282.0 | 416 | (73, 505) | 532 | (57, 505) | 524 | Phage portal protein | Phage portal protein | | uniclust | UniRef100\_A0A948A818 | 100.0 | 8.8e-35 | 1.6e-40 | 258.6 | 259 | (30, 298) | 532 | (24, 285) | 285 | Phage portal protein (Fragment) | Phage portal protein (Fragment) | | uniclust | UniRef100\_UPI002041E7F5 | 100.0 | 1.1e-34 | 1.9e-40 | 292.9 | 381 | (122, 514) | 532 | (623, 1037) | 1052 | phage portal protein | phage portal protein | | uniclust | UniRef100\_A0A090G3P5 | 100.0 | 3.2e-34 | 6.2e-40 | 271.2 | 290 | (210, 507) | 532 | (4, 326) | 349 | Capsid protein of prophage | Capsid protein of prophage | | uniclust | UniRef100\_UPI000AD9D3B3 | 100.0 | 3.4e-34 | 6.3e-40 | 285.3 | 337 | (46, 391) | 532 | (18, 391) | 729 | phage portal protein | phage portal protein | | uniclust | UniRef100\_A0A0Q0BVW0 | 100.0 | 6e-34 | 1.2e-39 | 257.5 | 223 | (277, 508) | 532 | (1, 237) | 259 | Portal protein | Portal protein | | uniclust | UniRef100\_UPI0008FB6ED6 | 100.0 | 1e-33 | 1.8e-39 | 252.3 | 272 | (128, 464) | 532 | (2, 286) | 287 | phage portal protein | phage portal protein | | uniclust | UniRef100\_A0A1H2HHH0 | 100.0 | 1e-33 | 2e-39 | 275.4 | 431 | (45, 503) | 532 | (50, 496) | 523 | Capsid protein | Capsid protein | | uniclust | UniRef100\_UPI00207606F1 | 100.0 | 1.8e-33 | 3.3e-39 | 264.8 | 301 | (205, 513) | 532 | (113, 442) | 459 | phage portal protein | phage portal protein | | uniclust | UniRef100\_UPI00117A55C2 | 100.0 | 1.8e-33 | 3.3e-39 | 243.2 | 215 | (128, 348) | 532 | (5, 222) | 229 | phage portal protein | phage portal protein | | uniclust | UniRef100\_A0A9D0S255 | 100.0 | 2e-33 | 3.8e-39 | 244.4 | 185 | (318, 506) | 532 | (12, 196) | 208 | Phage portal protein | Phage portal protein | | uniclust | UniRef100\_A0A965PTT0 | 100.0 | 3.8e-33 | 7.2e-39 | 264.0 | 287 | (205, 505) | 532 | (15, 310) | 387 | Phage portal protein (Fragment) | Phage portal protein (Fragment) | | uniclust | UniRef100\_A0A062VID9 | 100.0 | 5.7e-33 | 1.1e-38 | 252.5 | 231 | (15, 284) | 532 | (1, 231) | 240 | Phage portal protein, lambda family (Fragment) | Phage portal protein, lambda family (Fragment) | | uniclust | UniRef100\_A0A955N1X2 | 100.0 | 7.7e-33 | 1.4e-38 | 254.4 | 310 | (186, 505) | 532 | (2, 327) | 369 | Phage portal protein (Fragment) | Phage portal protein (Fragment) | | uniclust | UniRef100\_A0A1Y1S1G3 | 100.0 | 8e-33 | 1.5e-38 | 264.0 | 415 | (72, 495) | 532 | (45, 498) | 522 | Phage portal protein | Phage portal protein | | uniclust | UniRef100\_A0A1E5NHB2 | 100.0 | 9.4e-33 | 1.8e-38 | 268.6 | 420 | (72, 505) | 532 | (64, 522) | 540 | Phage portal protein | Phage portal protein | | uniclust | UniRef100\_A0A5C7P2N1 | 100.0 | 1.4e-32 | 2.5e-38 | 263.0 | 494 | (17, 519) | 532 | (1, 512) | 535 | Phage portal protein | Phage portal protein | | uniclust | UniRef100\_A0A5C7PT24 | 100.0 | 1.6e-32 | 2.9e-38 | 263.9 | 480 | (20, 521) | 532 | (14, 532) | 563 | Phage portal protein | Phage portal protein | | uniclust | UniRef100\_UPI001C05E608 | 100.0 | 2.1e-32 | 3.8e-38 | 246.5 | 276 | (133, 412) | 532 | (2, 309) | 310 | phage portal protein | phage portal protein | | uniclust | UniRef100\_UPI001D09EB93 | 99.9 | 2.8e-32 | 5.2e-38 | 272.2 | 343 | (48, 397) | 532 | (26, 402) | 874 | phage portal protein | phage portal protein | | uniclust | UniRef100\_A0A3D5KD24 | 99.9 | 2.8e-32 | 5.2e-38 | 260.7 | 451 | (45, 516) | 532 | (51, 518) | 530 | Phage portal protein | Phage portal protein | | uniclust | UniRef100\_A0A257JFS0 | 99.9 | 5e-32 | 9.5e-38 | 230.8 | 168 | (16, 192) | 532 | (1, 173) | 173 | Portal protein (Fragment) | Portal protein (Fragment) | | uniclust | UniRef100\_A0A3C1GKI0 | 99.9 | 5.5e-32 | 1.1e-37 | 249.3 | 239 | (249, 494) | 532 | (1, 241) | 278 | Phage portal protein | Phage portal protein | | uniclust | UniRef100\_A0A6G1WRJ0 | 99.9 | 6.2e-32 | 1.2e-37 | 243.6 | 187 | (316, 506) | 532 | (37, 224) | 270 | Phage portal protein (Fragment) | Phage portal protein (Fragment) | | uniclust | UniRef100\_S2KXG2 | 99.9 | 1.2e-31 | 2.2e-37 | 240.1 | 242 | (253, 494) | 532 | (2, 255) | 295 | Lambda family phage portal protein | Lambda family phage portal protein | | uniclust | UniRef100\_UPI001FA956E3 | 99.9 | 1.2e-31 | 2.3e-37 | 243.0 | 315 | (18, 444) | 532 | (1, 324) | 325 | phage portal protein | phage portal protein | | uniclust | UniRef100\_A0A5I8XHP0 | 99.9 | 1.7e-31 | 3.1e-37 | 254.5 | 303 | (18, 331) | 532 | (1, 312) | 509 | Phage portal protein | Phage portal protein | | uniclust | UniRef100\_A0A0F8VDT1 | 99.9 | 2e-31 | 4.2e-37 | 247.0 | 213 | (11, 231) | 532 | (8, 225) | 234 | Uncharacterized protein (Fragment) | Uncharacterized protein (Fragment) | | uniclust | UniRef100\_A0A7W1T9L3 | 99.9 | 2.4e-31 | 4.5e-37 | 255.2 | 309 | (43, 371) | 532 | (29, 342) | 546 | Phage portal protein | Phage portal protein | | uniclust | UniRef100\_A0A357LHL5 | 99.9 | 3.3e-31 | 6e-37 | 252.6 | 414 | (78, 495) | 532 | (7, 462) | 509 | Phage portal protein | Phage portal protein | | uniclust | UniRef100\_A0A068SLY6 | 99.9 | 4.5e-31 | 8.3e-37 | 254.7 | 400 | (72, 495) | 532 | (63, 475) | 576 | Phage portal protein, lambda family | Phage portal protein, lambda family | | uniclust | UniRef100\_A0A0P7YIZ9 | 99.9 | 7e-31 | 1.3e-36 | 232.2 | 202 | (293, 505) | 532 | (1, 207) | 247 | Phage portal protein, lambda family | Phage portal protein, lambda family | | uniclust | UniRef100\_A0A0L6CQP6 | 99.9 | 7.2e-31 | 1.4e-36 | 229.5 | 167 | (336, 509) | 532 | (2, 169) | 209 | Phage portal protein, lambda family | Phage portal protein, lambda family | | uniclust | UniRef100\_A0A833G1E6 | 99.9 | 1.9e-30 | 3.6e-36 | 242.0 | 332 | (78, 423) | 532 | (29, 396) | 412 | Phage portal protein (Fragment) | Phage portal protein (Fragment) | | uniclust | UniRef100\_A0A0S9PWW8 | 99.9 | 1.8e-30 | 3.6e-36 | 242.6 | 233 | (45, 281) | 532 | (33, 281) | 283 | Phage portal protein | Phage portal protein | | uniclust | UniRef100\_UPI00117B6109 | 99.9 | 2.1e-30 | 3.9e-36 | 236.1 | 250 | (47, 301) | 532 | (44, 309) | 336 | phage portal protein | phage portal protein | | uniclust | UniRef100\_A0A7V1N7P6 | 99.9 | 3.9e-30 | 7.1e-36 | 238.5 | 310 | (49, 366) | 532 | (51, 388) | 389 | Phage portal protein (Fragment) | Phage portal protein (Fragment) | | uniclust | UniRef100\_A0A965PTZ3 | 99.9 | 4.1e-30 | 7.6e-36 | 234.8 | 336 | (82, 450) | 532 | (1, 341) | 342 | Phage portal protein (Fragment) | Phage portal protein (Fragment) | | uniclust | UniRef100\_A0A149VAP2 | 99.9 | 4.9e-30 | 9.1e-36 | 225.7 | 187 | (319, 509) | 532 | (26, 214) | 230 | Capsid protein (Fragment) | Capsid protein (Fragment) | | uniclust | UniRef100\_R7MYT1 | 99.9 | 1.4e-29 | 2.7e-35 | 219.6 | 165 | (338, 507) | 532 | (1, 166) | 179 | Phage portal protein | Phage portal protein | | uniclust | UniRef100\_A0A965PMJ0 | 99.9 | 1.5e-29 | 2.8e-35 | 238.4 | 304 | (207, 524) | 532 | (13, 319) | 448 | Phage portal protein | Phage portal protein | | uniclust | UniRef100\_UPI001E28734F | 99.9 | 1.7e-29 | 3.2e-35 | 236.2 | 266 | (221, 490) | 532 | (1, 295) | 417 | phage portal protein | phage portal protein | | uniclust | UniRef100\_A0A754E6Q2 | 99.9 | 2.6e-29 | 4.8e-35 | 252.3 | 320 | (28, 358) | 532 | (1, 329) | 907 | Phage portal protein | Phage portal protein | | uniclust | UniRef100\_A0A0H4TJT5 | 99.9 | 3e-29 | 5.7e-35 | 211.7 | 158 | (179, 341) | 532 | (4, 165) | 170 | Phage portal protein (Fragment) | Phage portal protein (Fragment) | | uniclust | UniRef100\_UPI0004956350 | 99.9 | 6.5e-29 | 1.2e-34 | 216.0 | 155 | (347, 505) | 532 | (1, 156) | 203 | phage portal protein | phage portal protein | | uniclust | UniRef100\_UPI001E588232 | 99.9 | 6.8e-29 | 1.3e-34 | 239.0 | 268 | (218, 489) | 532 | (1, 297) | 551 | phage portal protein | phage portal protein | | uniclust | UniRef100\_UPI00041184E5 | 99.9 | 7e-29 | 1.3e-34 | 226.4 | 306 | (74, 508) | 532 | (3, 322) | 335 | phage portal protein | phage portal protein | | uniclust | UniRef100\_UPI0002D45360 | 99.9 | 9.2e-29 | 1.7e-34 | 228.5 | 260 | (226, 495) | 532 | (11, 283) | 309 | phage portal protein | phage portal protein | | uniclust | UniRef100\_A0A2P7UVS4 | 99.9 | 1e-28 | 2e-34 | 223.8 | 200 | (178, 379) | 532 | (20, 256) | 256 | Uncharacterized protein | Uncharacterized protein | | uniclust | UniRef100\_A0A896Z248 | 99.9 | 1.4e-28 | 2.6e-34 | 219.6 | 206 | (44, 251) | 532 | (20, 229) | 280 | Portal protein | Portal protein | | uniclust | UniRef100\_A0A0F9H021 | 99.9 | 1.4e-28 | 2.6e-34 | 215.5 | 176 | (122, 301) | 532 | (11, 196) | 217 | Uncharacterized protein (Fragment) | Uncharacterized protein (Fragment) | | uniclust | UniRef100\_A0A2A4RG34 | 99.9 | 1.8e-28 | 3.4e-34 | 234.3 | 416 | (64, 504) | 532 | (46, 481) | 508 | Phage portal protein | Phage portal protein | | uniclust | UniRef100\_A0A081B6E1 | 99.9 | 1.7e-28 | 3.7e-34 | 258.3 | 439 | (12, 492) | 532 | (19, 486) | 532 | Conserved protein | Conserved protein | | uniclust | UniRef100\_UPI000B13FF44 | 99.9 | 2.1e-28 | 3.9e-34 | 238.0 | 278 | (122, 406) | 532 | (301, 609) | 611 | phage portal protein | phage portal protein | | uniclust | UniRef100\_A0A0F9Q1K6 | 99.9 | 2.2e-28 | 4.7e-34 | 257.5 | 380 | (55, 485) | 532 | (64, 466) | 510 | Portal protein | Portal protein | | uniclust | UniRef100\_A0A2T4JG48 | 99.9 | 2.8e-28 | 5.4e-34 | 229.4 | 291 | (46, 352) | 532 | (32, 359) | 360 | Phage portal protein | Phage portal protein | | uniclust | UniRef100\_A0A1V6E2F0 | 99.9 | 3e-28 | 5.5e-34 | 233.2 | 420 | (46, 489) | 532 | (17, 456) | 515 | Phage portal protein, lambda family | Phage portal protein, lambda family | | uniclust | UniRef100\_A0A812S2M1 | 99.9 | 3.5e-28 | 6.4e-34 | 244.8 | 441 | (45, 502) | 532 | (14, 478) | 922 | Gene 4 protein | Gene 4 protein | | uniclust | UniRef100\_A0A7W1J8B3 | 99.9 | 3.6e-28 | 6.7e-34 | 218.4 | 216 | (43, 275) | 532 | (33, 253) | 259 | Phage portal protein | Phage portal protein | | uniclust | UniRef100\_A0A827RQQ4 | 99.9 | 4.2e-28 | 8.1e-34 | 223.8 | 230 | (63, 299) | 532 | (58, 289) | 290 | Phage portal protein (Fragment) | Phage portal protein (Fragment) | | uniclust | UniRef100\_A0A966RAZ4 | 99.9 | 5.8e-28 | 1.1e-33 | 227.0 | 335 | (122, 505) | 532 | (3, 342) | 431 | Phage portal protein | Phage portal protein | | uniclust | UniRef100\_UPI0021C2EF29 | 99.9 | 5.9e-28 | 1.1e-33 | 207.5 | 170 | (323, 495) | 532 | (1, 170) | 191 | phage portal protein | phage portal protein | | uniclust | UniRef100\_UPI0018CC8607 | 99.9 | 7.2e-28 | 1.3e-33 | 218.4 | 254 | (43, 300) | 532 | (32, 312) | 315 | phage portal protein | phage portal protein | | uniclust | UniRef100\_UPI0002C9D6EA | 99.9 | 8.2e-28 | 1.6e-33 | 219.8 | 245 | (259, 514) | 532 | (8, 257) | 275 | phage portal protein | phage portal protein | | uniclust | UniRef100\_A0A0F9HAI8 | 99.9 | 8.5e-28 | 1.6e-33 | 224.7 | 316 | (183, 517) | 532 | (4, 341) | 349 | Phage portal protein (Fragment) | Phage portal protein (Fragment) | | uniclust | UniRef100\_A0A5M6I484 | 99.9 | 9.3e-28 | 1.7e-33 | 221.0 | 220 | (44, 267) | 532 | (19, 252) | 357 | Phage portal protein | Phage portal protein | | uniclust | UniRef100\_A0A2W5FLC9 | 99.9 | 9.8e-28 | 1.8e-33 | 216.4 | 245 | (255, 503) | 532 | (4, 275) | 302 | Phage portal protein (Fragment) | Phage portal protein (Fragment) | | uniclust | UniRef100\_A0A2X3JP92 | 99.9 | 1.2e-27 | 2.2e-33 | 219.2 | 271 | (240, 514) | 532 | (18, 318) | 333 | Phage portal protein (Minor capsid protein) | Phage portal protein (Minor capsid protein) | | uniclust | UniRef100\_A0A376JYF4 | 99.9 | 1.1e-27 | 2.3e-33 | 231.2 | 280 | (216, 506) | 532 | (29, 339) | 341 | Phage portal protein (Minor capsid protein) | Phage portal protein (Minor capsid protein) | | uniclust | UniRef100\_G1V7I5 | 99.9 | 1.4e-27 | 2.5e-33 | 211.1 | 239 | (253, 503) | 532 | (2, 244) | 258 | Phage portal protein (Fragment) | Phage portal protein (Fragment) | | uniclust | UniRef100\_UPI00226D29AD | 99.9 | 2e-27 | 3.7e-33 | 206.8 | 215 | (242, 469) | 532 | (4, 226) | 230 | phage portal protein | phage portal protein | | uniclust | UniRef100\_A0A142XEI8 | 99.9 | 2.1e-27 | 3.8e-33 | 229.9 | 382 | (72, 485) | 532 | (63, 464) | 498 | Phage portal protein, lambda family | Phage portal protein, lambda family | | uniclust | UniRef100\_UPI0022710B4D | 99.9 | 2.2e-27 | 4e-33 | 243.6 | 421 | (68, 506) | 532 | (721, 1192) | 1208 | phage terminase large subunit family protein | phage terminase large subunit family protein | | uniclust | UniRef100\_A0A1G0XUR8 | 99.9 | 2.4e-27 | 4.5e-33 | 234.4 | 398 | (73, 503) | 532 | (64, 498) | 544 | Phage portal protein | Phage portal protein | | uniclust | UniRef100\_A0A966R5B7 | 99.9 | 2.9e-27 | 5.4e-33 | 222.3 | 305 | (181, 503) | 532 | (29, 341) | 427 | Phage portal protein | Phage portal protein | | uniclust | UniRef100\_A0A0E3JS82 | 99.9 | 2.8e-27 | 5.4e-33 | 236.7 | 406 | (73, 511) | 532 | (61, 482) | 553 | Portal protein | Portal protein | | uniclust | UniRef100\_A0A5E8GT99 | 99.9 | 3.2e-27 | 5.8e-33 | 228.4 | 436 | (57, 502) | 532 | (30, 531) | 563 | Bacteriophage capsid protein | Bacteriophage capsid protein | | uniclust | UniRef100\_UPI001FEBC5EC | 99.9 | 3.3e-27 | 6e-33 | 211.8 | 188 | (261, 453) | 532 | (30, 225) | 288 | phage portal protein | phage portal protein | | uniclust | UniRef100\_A0A6M3IP50 | 99.9 | 3.7e-27 | 6.9e-33 | 213.2 | 261 | (247, 511) | 532 | (14, 281) | 307 | Putative portal protein (Fragment) | Putative portal protein (Fragment) | | uniclust | UniRef100\_A0A961H371 | 99.9 | 4.1e-27 | 7.6e-33 | 217.3 | 325 | (45, 400) | 532 | (23, 362) | 363 | Phage portal protein (Fragment) | Phage portal protein (Fragment) | | uniclust | UniRef100\_A0A450TW67 | 99.9 | 5.4e-27 | 9.8e-33 | 217.3 | 199 | (99, 301) | 532 | (11, 222) | 374 | Phage portal protein, lambda family | Phage portal protein, lambda family | | uniclust | UniRef100\_UPI0013B7ACB1 | 99.9 | 6.7e-27 | 1.2e-32 | 212.8 | 298 | (45, 354) | 532 | (14, 319) | 321 | phage portal protein | phage portal protein | | uniclust | UniRef100\_A0A9D8SJC5 | 99.9 | 6.9e-27 | 1.3e-32 | 222.0 | 424 | (46, 488) | 532 | (10, 452) | 468 | Phage portal protein | Phage portal protein | | uniclust | UniRef100\_A0A4Q6ELU1 | 99.9 | 6.9e-27 | 1.3e-32 | 202.0 | 170 | (329, 503) | 532 | (2, 172) | 192 | Phage portal protein | Phage portal protein | | uniclust | UniRef100\_A0A3C1YTA8 | 99.9 | 7.3e-27 | 1.4e-32 | 228.3 | 426 | (45, 493) | 532 | (19, 473) | 494 | Phage portal protein | Phage portal protein | | uniclust | UniRef100\_A0A6J5NYG1 | 99.9 | 8.1e-27 | 1.5e-32 | 231.0 | 385 | (71, 488) | 532 | (82, 490) | 622 | Portal\_lambda, phage portal protein, lambda family | Portal\_lambda, phage portal protein, lambda family | | uniclust | UniRef100\_A0A1H5Z513 | 99.9 | 1.1e-26 | 2.2e-32 | 198.8 | 145 | (362, 521) | 532 | (1, 146) | 167 | Phage portal protein, lambda family | Phage portal protein, lambda family | | uniclust | UniRef100\_X0TAA3 | 99.9 | 3e-26 | 5.5e-32 | 203.8 | 259 | (19, 300) | 532 | (4, 264) | 267 | Uncharacterized protein (Fragment) | Uncharacterized protein (Fragment) | | uniclust | UniRef100\_A0A2D0IJL4 | 99.9 | 3.3e-26 | 6.5e-32 | 197.4 | 150 | (356, 509) | 532 | (1, 152) | 166 | Capsid protein | Capsid protein | | uniclust | UniRef100\_UPI001EE63960 | 99.9 | 3.6e-26 | 6.6e-32 | 190.1 | 162 | (220, 381) | 532 | (1, 167) | 167 | phage portal protein | phage portal protein | | uniclust | UniRef100\_A0A9E3UTF1 | 99.9 | 4.4e-26 | 8.1e-32 | 219.2 | 387 | (73, 505) | 532 | (69, 480) | 524 | Phage portal protein | Phage portal protein | | uniclust | UniRef100\_A0A1Z4BY38 | 99.9 | 4.8e-26 | 8.8e-32 | 192.8 | 174 | (44, 226) | 532 | (11, 186) | 188 | Phage portal protein | Phage portal protein | | uniclust | UniRef100\_A0A257WRS9 | 99.9 | 5e-26 | 9.9e-32 | 218.6 | 263 | (207, 489) | 532 | (13, 290) | 327 | Phage portal protein | Phage portal protein | | uniclust | UniRef100\_A0A8J3DI50 | 99.9 | 6.2e-26 | 1.1e-31 | 218.3 | 414 | (45, 488) | 532 | (30, 472) | 527 | Phage portal protein | Phage portal protein | | uniclust | UniRef100\_A0A2T6DRH8 | 99.9 | 7e-26 | 1.3e-31 | 215.9 | 400 | (72, 505) | 532 | (32, 453) | 480 | Phage portal protein | Phage portal protein | | uniclust | UniRef100\_A0A1G8I036 | 99.9 | 7.7e-26 | 1.5e-31 | 217.5 | 247 | (46, 301) | 532 | (41, 303) | 400 | Phage portal protein, lambda family (Fragment) | Phage portal protein, lambda family (Fragment) | | uniclust | UniRef100\_A0A7V2ILH1 | 99.9 | 9.2e-26 | 1.7e-31 | 207.9 | 239 | (44, 301) | 532 | (19, 261) | 353 | Phage portal protein | Phage portal protein | | uniclust | UniRef100\_UPI001A7E5E67 | 99.9 | 1.6e-25 | 2.9e-31 | 224.0 | 201 | (319, 526) | 532 | (10, 215) | 808 | phage portal protein | phage portal protein | | uniclust | UniRef100\_UPI000A788E98 | 99.9 | 1.6e-25 | 2.9e-31 | 189.3 | 174 | (157, 332) | 532 | (1, 185) | 185 | phage portal protein | phage portal protein | | uniclust | UniRef100\_A0A2M7FBH1 | 99.9 | 1.8e-25 | 3.6e-31 | 193.0 | 156 | (15, 172) | 532 | (1, 158) | 159 | Phage portal protein (Fragment) | Phage portal protein (Fragment) | | uniclust | UniRef100\_A0A9E6UXJ6 | 99.9 | 2.6e-25 | 5e-31 | 185.4 | 120 | (351, 473) | 532 | (3, 123) | 145 | Phage portal protein | Phage portal protein | | uniclust | UniRef100\_A0A357XXP9 | 99.9 | 2.7e-25 | 5e-31 | 211.0 | 224 | (45, 290) | 532 | (166, 393) | 459 | Phage portal protein (Fragment) | Phage portal protein (Fragment) | | uniclust | UniRef100\_A0A2N8HBV6 | 99.9 | 3.1e-25 | 5.6e-31 | 211.0 | 388 | (73, 494) | 532 | (49, 445) | 466 | Phage portal protein | Phage portal protein | | uniclust | UniRef100\_A0A1G8HYN6 | 99.9 | 3.2e-25 | 6.1e-31 | 199.2 | 197 | (317, 517) | 532 | (26, 223) | 244 | Phage portal protein, lambda family (Fragment) | Phage portal protein, lambda family (Fragment) | | uniclust | UniRef100\_A0A167HQ01 | 99.9 | 3.3e-25 | 6.4e-31 | 206.3 | 240 | (45, 292) | 532 | (20, 274) | 296 | Phage portal protein (Fragment) | Phage portal protein (Fragment) | | uniclust | UniRef100\_UPI002265A757 | 99.9 | 3.6e-25 | 6.6e-31 | 198.8 | 238 | (250, 492) | 532 | (1, 248) | 284 | phage portal protein | phage portal protein | | uniclust | UniRef100\_UPI001FD9E691 | 99.9 | 3.7e-25 | 6.9e-31 | 186.5 | 148 | (176, 326) | 532 | (11, 163) | 165 | phage portal protein | phage portal protein | | uniclust | UniRef100\_A0A368KX58 | 99.9 | 4.1e-25 | 7.5e-31 | 205.1 | 248 | (99, 366) | 532 | (13, 262) | 373 | Phage portal protein | Phage portal protein | | uniclust | UniRef100\_A0A2G2K5M6 | 99.8 | 6.6e-25 | 1.3e-30 | 196.7 | 181 | (45, 229) | 532 | (30, 218) | 218 | Phage portal protein | Phage portal protein | | uniclust | UniRef100\_A0A3D4DT62 | 99.8 | 6.7e-25 | 1.4e-30 | 186.4 | 134 | (17, 173) | 532 | (1, 134) | 135 | Phage portal protein (Fragment) | Phage portal protein (Fragment) | | uniclust | UniRef100\_A0A954G1K1 | 99.8 | 7.5e-25 | 1.4e-30 | 201.4 | 286 | (206, 495) | 532 | (10, 316) | 343 | Phage portal protein (Fragment) | Phage portal protein (Fragment) | | uniclust | UniRef100\_A0A961U404 | 99.8 | 7.5e-25 | 1.4e-30 | 194.9 | 217 | (44, 269) | 532 | (47, 264) | 264 | Phage portal protein (Fragment) | Phage portal protein (Fragment) | | uniclust | UniRef100\_A0A661TPP3 | 99.8 | 1e-24 | 1.9e-30 | 193.8 | 181 | (319, 514) | 532 | (4, 185) | 225 | Phage portal protein (Fragment) | Phage portal protein (Fragment) | | uniclust | UniRef100\_A0A377DG37 | 99.8 | 1.2e-24 | 2.3e-30 | 196.1 | 233 | (57, 298) | 532 | (2, 248) | 250 | Phage portal protein (Minor capsid protein) | Phage portal protein (Minor capsid protein) | | uniclust | UniRef100\_A0A0A1DM99 | 99.8 | 1.5e-24 | 3.1e-30 | 230.9 | 416 | (54, 490) | 532 | (66, 518) | 626 | Phage portal protein | Phage portal protein | | uniclust | UniRef100\_UPI00227824CA | 99.8 | 1.7e-24 | 3.2e-30 | 192.6 | 217 | (148, 370) | 532 | (2, 226) | 263 | phage portal protein | phage portal protein | | uniclust | UniRef100\_A0A2E9VWF4 | 99.8 | 2.5e-24 | 4.7e-30 | 203.5 | 305 | (54, 385) | 532 | (53, 372) | 376 | Phage portal protein | Phage portal protein | | uniclust | UniRef100\_UPI000FD93CDA | 99.8 | 3.6e-24 | 6.6e-30 | 192.2 | 229 | (44, 276) | 532 | (35, 278) | 280 | phage portal protein | phage portal protein | | uniclust | UniRef100\_A0A2D6M8M0 | 99.8 | 3.8e-24 | 7e-30 | 208.9 | 427 | (57, 495) | 532 | (92, 556) | 599 | Phage portal protein | Phage portal protein | | uniclust | UniRef100\_A0A966SJ28 | 99.8 | 4.4e-24 | 8.1e-30 | 200.5 | 356 | (45, 419) | 532 | (23, 394) | 407 | Phage portal protein (Fragment) | Phage portal protein (Fragment) | | uniclust | UniRef100\_A0A9E5WIJ8 | 99.8 | 1.1e-23 | 2e-29 | 186.1 | 208 | (122, 340) | 532 | (18, 229) | 247 | Phage portal protein | Phage portal protein | | uniclust | UniRef100\_A0A954NMX5 | 99.8 | 1.2e-23 | 2.2e-29 | 177.0 | 172 | (209, 382) | 532 | (1, 173) | 176 | Phage portal protein (Fragment) | Phage portal protein (Fragment) | | uniclust | UniRef100\_A0A061P5J7 | 99.8 | 1.3e-23 | 2.8e-29 | 217.9 | 397 | (54, 488) | 532 | (51, 463) | 498 | Uncharacterized protein | Uncharacterized protein | | uniclust | UniRef100\_A0A3M1R6N2 | 99.8 | 1.6e-23 | 2.9e-29 | 187.7 | 201 | (44, 262) | 532 | (39, 243) | 243 | Phage portal protein (Fragment) | Phage portal protein (Fragment) | | uniclust | UniRef100\_A0A7X1E5Y1 | 99.8 | 1.9e-23 | 3.5e-29 | 199.9 | 398 | (73, 504) | 532 | (46, 470) | 480 | Phage portal protein | Phage portal protein | | uniclust | UniRef100\_A0A1V5U672 | 99.8 | 2e-23 | 3.9e-29 | 186.7 | 177 | (318, 509) | 532 | (5, 184) | 207 | Phage portal protein, lambda family | Phage portal protein, lambda family | | uniclust | UniRef100\_UPI001E4A37DB | 99.8 | 2.6e-23 | 4.7e-29 | 172.4 | 156 | (287, 457) | 532 | (2, 159) | 160 | phage portal protein | phage portal protein | | uniclust | UniRef100\_A0A376TZP2 | 99.8 | 2.7e-23 | 4.9e-29 | 194.3 | 234 | (277, 514) | 532 | (2, 258) | 387 | Capsid protein of prophage | Capsid protein of prophage | | uniclust | UniRef100\_A0A0B4ZPA5 | 99.8 | 2.7e-23 | 4.9e-29 | 210.0 | 349 | (46, 473) | 532 | (88, 451) | 881 | Serine protease | Serine protease | | uniclust | UniRef100\_A0A090G440 | 99.8 | 3.8e-23 | 7.1e-29 | 195.0 | 265 | (17, 300) | 532 | (5, 286) | 346 | Capsid protein of prophage | Capsid protein of prophage | | uniclust | UniRef100\_A0A376NS21 | 99.8 | 4.5e-23 | 8.3e-29 | 180.7 | 213 | (280, 502) | 532 | (1, 218) | 219 | Head-tail preconnector protein | Head-tail preconnector protein | | uniclust | UniRef100\_UPI002233EA83 | 99.8 | 4.9e-23 | 9e-29 | 190.8 | 198 | (46, 247) | 532 | (116, 332) | 357 | phage portal protein | phage portal protein | | uniclust | UniRef100\_A0A0J1CHN1 | 99.8 | 5.1e-23 | 1e-28 | 182.2 | 163 | (317, 479) | 532 | (7, 171) | 181 | Plasmid partitioning protein ParB (Fragment) | Plasmid partitioning protein ParB (Fragment) | | uniclust | UniRef100\_UPI001652C7CE | 99.8 | 5.9e-23 | 1.1e-28 | 180.2 | 225 | (69, 299) | 532 | (6, 233) | 233 | phage portal protein | phage portal protein | | uniclust | UniRef100\_A0A9E6BKT2 | 99.8 | 7.4e-23 | 1.4e-28 | 197.6 | 177 | (46, 232) | 532 | (21, 199) | 521 | Phage portal protein | Phage portal protein | | uniclust | UniRef100\_A0A2D9C8J7 | 99.8 | 7.4e-23 | 1.4e-28 | 185.4 | 231 | (263, 505) | 532 | (1, 244) | 271 | Phage portal protein (Fragment) | Phage portal protein (Fragment) | | uniclust | UniRef100\_A0A497KH68 | 99.8 | 7.7e-23 | 1.4e-28 | 194.3 | 321 | (72, 419) | 532 | (80, 419) | 444 | Phage portal protein (Fragment) | Phage portal protein (Fragment) | | uniclust | UniRef100\_UPI001CCED0AA | 99.8 | 8e-23 | 1.5e-28 | 181.6 | 239 | (26, 277) | 532 | (2, 245) | 255 | phage portal protein | phage portal protein | | uniclust | UniRef100\_A0A317F9M0 | 99.8 | 8.3e-23 | 1.6e-28 | 175.7 | 168 | (43, 226) | 532 | (15, 184) | 185 | Uncharacterized protein | Uncharacterized protein | | uniclust | UniRef100\_A0A7X6FP93 | 99.8 | 1.2e-22 | 2.2e-28 | 173.4 | 165 | (133, 300) | 532 | (3, 172) | 192 | Phage portal protein | Phage portal protein | | uniclust | UniRef100\_UPI000AF07CED | 99.8 | 1.4e-22 | 2.7e-28 | 175.3 | 149 | (348, 503) | 532 | (13, 162) | 211 | hypothetical protein | hypothetical protein | | uniclust | UniRef100\_UPI0004A0575F | 99.8 | 1.4e-22 | 2.7e-28 | 180.2 | 244 | (150, 401) | 532 | (3, 250) | 257 | phage portal protein | phage portal protein | | uniclust | UniRef100\_A0A2G1Z9F9 | 99.8 | 2e-22 | 3.7e-28 | 176.8 | 182 | (320, 505) | 532 | (30, 212) | 231 | Phage portal protein (Fragment) | Phage portal protein (Fragment) | | uniclust | UniRef100\_A0A2N2HIU1 | 99.8 | 2.5e-22 | 4.6e-28 | 176.6 | 179 | (318, 503) | 532 | (23, 201) | 235 | Phage portal protein (Fragment) | Phage portal protein (Fragment) | | uniclust | UniRef100\_A0A7W8DQU5 | 99.8 | 2.6e-22 | 4.8e-28 | 194.1 | 423 | (80, 524) | 532 | (59, 519) | 525 | Capsid protein | Capsid protein | | uniclust | UniRef100\_A0A6M3JYX7 | 99.8 | 3.6e-22 | 6.6e-28 | 197.9 | 381 | (54, 479) | 532 | (47, 439) | 678 | Putative portal protein | Putative portal protein | | uniclust | UniRef100\_A0A0S4U0U3 | 99.8 | 3.8e-22 | 7.1e-28 | 163.9 | 134 | (86, 229) | 532 | (2, 137) | 137 | Bacteriophage-related protein (Fragment) | Bacteriophage-related protein (Fragment) | | uniclust | UniRef100\_A0A250DLV1 | 99.8 | 3.9e-22 | 7.1e-28 | 179.5 | 224 | (280, 507) | 532 | (15, 244) | 279 | Phage portal protein | Phage portal protein | | uniclust | UniRef100\_UPI00209508A0 | 99.8 | 3.9e-22 | 7.2e-28 | 159.5 | 121 | (335, 455) | 532 | (3, 124) | 127 | phage portal protein | phage portal protein | | uniclust | UniRef100\_A0A965LHB1 | 99.8 | 4.7e-22 | 8.6e-28 | 179.7 | 267 | (15, 300) | 532 | (1, 276) | 288 | Phage portal protein (Fragment) | Phage portal protein (Fragment) | | uniclust | UniRef100\_UPI00054CCE67 | 99.8 | 4.7e-22 | 8.7e-28 | 172.5 | 179 | (240, 419) | 532 | (8, 193) | 196 | phage portal protein | phage portal protein | | uniclust | UniRef100\_Q9JMP2 | 99.8 | 7e-22 | 1.3e-27 | 161.7 | 127 | (293, 419) | 532 | (1, 132) | 145 | Orf4 protein | Orf4 protein | | uniclust | UniRef100\_A0A965PPH0 | 99.8 | 8.3e-22 | 1.5e-27 | 172.3 | 207 | (156, 374) | 532 | (3, 219) | 224 | Phage portal protein | Phage portal protein | | uniclust | UniRef100\_A0A6M3JUQ8 | 99.8 | 8.6e-22 | 1.6e-27 | 197.2 | 380 | (73, 492) | 532 | (112, 539) | 572 | Putative portal protein | Putative portal protein | | uniclust | UniRef100\_A0A827RRV0 | 99.8 | 9.6e-22 | 1.9e-27 | 179.3 | 169 | (319, 491) | 532 | (23, 191) | 217 | Phage portal protein | Phage portal protein | | uniclust | UniRef100\_A0A381KTT9 | 99.7 | 1.5e-21 | 2.8e-27 | 171.7 | 169 | (248, 416) | 532 | (22, 208) | 218 | Phage portal protein | Phage portal protein | | uniclust | UniRef100\_A0A357XSR0 | 99.7 | 1.8e-21 | 3.4e-27 | 188.2 | 456 | (16, 494) | 532 | (2, 494) | 519 | Phage portal protein | Phage portal protein | | uniclust | UniRef100\_A0A2W4N7H3 | 99.7 | 2e-21 | 3.6e-27 | 188.0 | 398 | (54, 490) | 532 | (41, 474) | 518 | Phage portal protein | Phage portal protein | | uniclust | UniRef100\_A0A376LQ97 | 99.7 | 2.1e-21 | 3.8e-27 | 181.4 | 232 | (46, 286) | 532 | (91, 338) | 375 | Phage portal protein (Minor capsid protein) | Phage portal protein (Minor capsid protein) | | uniclust | UniRef100\_A0A965N5Y9 | 99.7 | 2.4e-21 | 4.3e-27 | 158.7 | 122 | (317, 441) | 532 | (22, 144) | 145 | Phage portal protein (Fragment) | Phage portal protein (Fragment) | | uniclust | UniRef100\_A0A3L7VQ06 | 99.7 | 2.4e-21 | 4.4e-27 | 194.9 | 396 | (72, 493) | 532 | (76, 492) | 707 | Phage portal protein | Phage portal protein | | uniclust | UniRef100\_A0A6M3J4B5 | 99.7 | 2.7e-21 | 4.9e-27 | 181.0 | 339 | (161, 503) | 532 | (6, 362) | 380 | Putative portal protein | Putative portal protein | | uniclust | UniRef100\_A0A847I6D6 | 99.7 | 3.4e-21 | 6.3e-27 | 177.9 | 325 | (65, 417) | 532 | (4, 339) | 339 | Phage portal protein (Fragment) | Phage portal protein (Fragment) | | uniclust | UniRef100\_A0A9E1ZJH4 | 99.7 | 4e-21 | 7.3e-27 | 160.1 | 136 | (318, 458) | 532 | (2, 137) | 161 | Phage portal protein | Phage portal protein | | uniclust | UniRef100\_UPI0023560E02 | 99.7 | 4.7e-21 | 8.6e-27 | 185.1 | 408 | (74, 519) | 532 | (48, 481) | 506 | phage portal protein | phage portal protein | | uniclust | UniRef100\_A0A3C1GA38 | 99.7 | 5.2e-21 | 9.5e-27 | 177.4 | 179 | (45, 226) | 532 | (79, 259) | 349 | Phage portal protein | Phage portal protein | | uniclust | UniRef100\_J9FFD2 | 99.7 | 5.4e-21 | 9.9e-27 | 163.0 | 176 | (203, 382) | 532 | (6, 187) | 187 | Phage portal protein, lambda family (Fragment) | Phage portal protein, lambda family (Fragment) | | uniclust | UniRef100\_A0A1Q6JR94 | 99.7 | 5.8e-21 | 1.1e-26 | 166.8 | 160 | (14, 174) | 532 | (3, 172) | 178 | Phage portal protein | Phage portal protein | | uniclust | UniRef100\_A0A0F9FPS3 | 99.7 | 8.3e-21 | 1.5e-26 | 183.1 | 404 | (48, 493) | 532 | (45, 492) | 498 | Phage portal protein | Phage portal protein | | uniclust | UniRef100\_A0A2D7VYW9 | 99.7 | 1e-20 | 1.9e-26 | 160.4 | 133 | (366, 507) | 532 | (3, 137) | 161 | Phage portal protein (Fragment) | Phage portal protein (Fragment) | | uniclust | UniRef100\_A0A2W5HLR3 | 99.7 | 1.1e-20 | 2e-26 | 160.4 | 138 | (347, 495) | 532 | (2, 140) | 167 | Phage portal protein (Fragment) | Phage portal protein (Fragment) | | uniclust | UniRef100\_A0A970YYK5 | 99.7 | 1.3e-20 | 2.3e-26 | 177.6 | 331 | (45, 394) | 532 | (52, 394) | 397 | Phage portal protein (Fragment) | Phage portal protein (Fragment) | | uniclust | UniRef100\_A0A965PNA5 | 99.7 | 1.3e-20 | 2.4e-26 | 185.1 | 240 | (259, 507) | 532 | (2, 250) | 558 | Phage portal protein (Fragment) | Phage portal protein (Fragment) | | uniclust | UniRef100\_A0A074TJP9 | 99.7 | 1.2e-20 | 2.4e-26 | 174.8 | 189 | (317, 515) | 532 | (5, 195) | 236 | Capsid protein (Fragment) | Capsid protein (Fragment) | | uniclust | UniRef100\_A0A661KRT0 | 99.7 | 1.5e-20 | 2.8e-26 | 178.6 | 314 | (130, 478) | 532 | (2, 328) | 431 | Phage portal protein | Phage portal protein | | uniclust | UniRef100\_A0A7V3JP37 | 99.7 | 2.3e-20 | 4.3e-26 | 177.3 | 292 | (72, 393) | 532 | (60, 369) | 382 | Phage portal protein (Fragment) | Phage portal protein (Fragment) | | uniclust | UniRef100\_UPI002107534C | 99.7 | 2.9e-20 | 5.4e-26 | 150.2 | 116 | (327, 445) | 532 | (1, 116) | 132 | phage portal protein | phage portal protein | | uniclust | UniRef100\_A0A431I932 | 99.7 | 3.1e-20 | 5.8e-26 | 180.4 | 407 | (63, 494) | 532 | (79, 511) | 528 | Phage portal protein | Phage portal protein | | uniclust | UniRef100\_A0A9D8TK68 | 99.7 | 3.7e-20 | 6.8e-26 | 158.5 | 166 | (58, 227) | 532 | (7, 177) | 190 | Phage portal protein (Fragment) | Phage portal protein (Fragment) | | uniclust | UniRef100\_A0A7C3DW82 | 99.7 | 4.3e-20 | 7.9e-26 | 169.1 | 249 | (9, 301) | 532 | (25, 277) | 310 | Phage portal protein (Fragment) | Phage portal protein (Fragment) | | uniclust | UniRef100\_UPI00147320C4 | 99.7 | 4.3e-20 | 7.9e-26 | 187.8 | 180 | (318, 505) | 532 | (691, 872) | 886 | phage terminase large subunit family protein | phage terminase large subunit family protein | | uniclust | UniRef100\_UPI0022B34460 | 99.7 | 4.5e-20 | 8.3e-26 | 164.7 | 228 | (122, 373) | 532 | (7, 239) | 255 | phage portal protein | phage portal protein | | uniclust | UniRef100\_A0A358C6E0 | 99.7 | 4.8e-20 | 8.8e-26 | 163.7 | 184 | (316, 513) | 532 | (33, 227) | 245 | Phage portal protein (Fragment) | Phage portal protein (Fragment) | | uniclust | UniRef100\_A0A661H6X6 | 99.7 | 4.8e-20 | 8.9e-26 | 161.6 | 160 | (343, 506) | 532 | (14, 176) | 224 | Phage portal protein (Fragment) | Phage portal protein (Fragment) | | uniclust | UniRef100\_UPI001C83F08B | 99.7 | 5.6e-20 | 1e-25 | 160.7 | 181 | (203, 383) | 532 | (8, 218) | 219 | phage portal protein | phage portal protein | | uniclust | UniRef100\_A0A358B2C7 | 99.7 | 1e-19 | 1.8e-25 | 175.6 | 194 | (44, 254) | 532 | (16, 209) | 488 | LysR substrate-binding domain-containing protein | LysR substrate-binding domain-containing protein | | uniclust | UniRef100\_A0A0F9HE98 | 99.7 | 9.7e-20 | 1.9e-25 | 181.5 | 375 | (68, 495) | 532 | (42, 442) | 494 | Portal protein | Portal protein | | uniclust | UniRef100\_UPI0006A04A95 | 99.7 | 1.2e-19 | 2.2e-25 | 168.8 | 170 | (340, 513) | 532 | (162, 332) | 349 | phage portal protein | phage portal protein | | uniclust | UniRef100\_A0A2E9Y0J4 | 99.7 | 1.4e-19 | 2.6e-25 | 160.1 | 168 | (18, 197) | 532 | (1, 168) | 219 | Phage portal protein | Phage portal protein | | uniclust | UniRef100\_UPI0020A414D4 | 99.7 | 1.9e-19 | 3.5e-25 | 164.6 | 242 | (257, 503) | 532 | (8, 277) | 304 | phage portal protein | phage portal protein | | uniclust | UniRef100\_A0A376U7U2 | 99.7 | 1.9e-19 | 3.6e-25 | 153.3 | 148 | (319, 466) | 532 | (17, 165) | 165 | Capsid protein of prophage | Capsid protein of prophage | | uniclust | UniRef100\_A0A0F9F8Y8 | 99.7 | 1.9e-19 | 3.7e-25 | 159.5 | 152 | (15, 173) | 532 | (7, 173) | 186 | Uncharacterized protein | Uncharacterized protein | | uniclust | UniRef100\_A0A1Y5TZG3 | 99.7 | 2e-19 | 3.7e-25 | 157.1 | 174 | (209, 387) | 532 | (2, 177) | 216 | Phage portal protein, lambda family | Phage portal protein, lambda family | | uniclust | UniRef100\_UPI0012D43280 | 99.7 | 2.3e-19 | 4.2e-25 | 142.3 | 108 | (158, 266) | 532 | (4, 117) | 117 | phage portal protein | phage portal protein | | uniclust | UniRef100\_A0A4P9VMD7 | 99.6 | 2.5e-19 | 4.8e-25 | 161.3 | 184 | (54, 245) | 532 | (26, 216) | 216 | Phage portal protein | Phage portal protein | | uniclust | UniRef100\_A0A4Q8RPB7 | 99.6 | 3.3e-19 | 6.2e-25 | 155.4 | 168 | (219, 390) | 532 | (2, 197) | 197 | Phage portal protein (Fragment) | Phage portal protein (Fragment) | | uniclust | UniRef100\_A0A1F8QWT7 | 99.6 | 3.4e-19 | 6.7e-25 | 179.2 | 299 | (80, 417) | 532 | (7, 313) | 439 | Uncharacterized protein | Uncharacterized protein | | uniclust | UniRef100\_A0A941SHU7 | 99.6 | 3.8e-19 | 7.1e-25 | 172.6 | 434 | (45, 505) | 532 | (33, 484) | 513 | Phage portal protein | Phage portal protein | | uniclust | UniRef100\_A0A419ENK9 | 99.6 | 4.4e-19 | 8.1e-25 | 152.9 | 167 | (344, 519) | 532 | (11, 178) | 196 | Phage portal protein (Fragment) | Phage portal protein (Fragment) | | uniclust | UniRef100\_UPI00210FF08D | 99.6 | 4.9e-19 | 9e-25 | 157.6 | 168 | (274, 447) | 532 | (3, 175) | 246 | phage portal protein | phage portal protein | | uniclust | UniRef100\_A0A844M4D9 | 99.6 | 5.6e-19 | 1.1e-24 | 140.2 | 99 | (344, 453) | 532 | (2, 101) | 102 | Phage portal protein (Fragment) | Phage portal protein (Fragment) | | uniclust | UniRef100\_A0A142WUX4 | 99.6 | 6e-19 | 1.1e-24 | 170.8 | 402 | (46, 477) | 532 | (41, 456) | 497 | Phage portal protein, lambda family | Phage portal protein, lambda family | | uniclust | UniRef100\_A0A6P1B243 | 99.6 | 6.4e-19 | 1.2e-24 | 141.8 | 126 | (96, 222) | 532 | (2, 127) | 127 | Phage portal protein (Fragment) | Phage portal protein (Fragment) | | uniclust | UniRef100\_UPI000EB38119 | 99.6 | 9.4e-19 | 1.7e-24 | 153.4 | 168 | (17, 198) | 532 | (4, 174) | 219 | phage portal protein | phage portal protein | | uniclust | UniRef100\_A0A376SKF2 | 99.6 | 1.1e-18 | 2.1e-24 | 150.9 | 167 | (123, 300) | 532 | (3, 174) | 200 | Head-tail preconnector protein | Head-tail preconnector protein | | uniclust | UniRef100\_UPI0004A80172 | 99.6 | 2e-18 | 3.7e-24 | 155.0 | 177 | (319, 502) | 532 | (64, 240) | 259 | phage portal protein | phage portal protein | | uniclust | UniRef100\_UPI001FE83FA2 | 99.6 | 2.4e-18 | 4.5e-24 | 166.8 | 138 | (361, 505) | 532 | (322, 460) | 500 | ParB N-terminal domain-containing protein | ParB N-terminal domain-containing protein | | uniclust | UniRef100\_A0A431KHF4 | 99.6 | 2.6e-18 | 4.7e-24 | 173.7 | 387 | (56, 488) | 532 | (325, 729) | 784 | Phage portal protein | Phage portal protein | | uniclust | UniRef100\_A0A5C5X9Q3 | 99.6 | 3e-18 | 5.6e-24 | 172.4 | 398 | (74, 491) | 532 | (87, 525) | 559 | Phage portal protein, lambda family | Phage portal protein, lambda family | | uniclust | UniRef100\_A0A175RV87 | 99.6 | 3.2e-18 | 5.8e-24 | 162.4 | 318 | (78, 419) | 532 | (68, 398) | 406 | Portal protein | Portal protein | | uniclust | UniRef100\_X1FZP5 | 99.6 | 3.2e-18 | 5.9e-24 | 151.3 | 204 | (11, 218) | 532 | (16, 230) | 231 | Phage portal protein (Fragment) | Phage portal protein (Fragment) | | uniclust | UniRef100\_A0A3S4U6Y1 | 99.6 | 3.4e-18 | 6.2e-24 | 161.5 | 177 | (323, 509) | 532 | (1, 178) | 390 | ATP-dependent Clp protease proteolytic subunit | ATP-dependent Clp protease proteolytic subunit | | uniclust | UniRef100\_A0A934HCI9 | 99.6 | 4.3e-18 | 7.9e-24 | 164.7 | 367 | (73, 489) | 532 | (96, 481) | 484 | Uncharacterized protein | Uncharacterized protein | | uniclust | UniRef100\_UPI000A67C2A5 | 99.6 | 5e-18 | 9.4e-24 | 138.2 | 118 | (214, 335) | 532 | (6, 127) | 128 | phage portal protein | phage portal protein | | uniclust | UniRef100\_A0A0K0VKK6 | 99.6 | 5.7e-18 | 1e-23 | 153.8 | 187 | (317, 507) | 532 | (75, 263) | 281 | Plasmid partitioning protein ParB | Plasmid partitioning protein ParB | | uniclust | UniRef100\_UPI001F204F94 | 99.6 | 7.1e-18 | 1.3e-23 | 130.9 | 95 | (325, 419) | 532 | (2, 97) | 102 | phage portal protein | phage portal protein | | uniclust | UniRef100\_UPI002269D2B2 | 99.6 | 7.1e-18 | 1.3e-23 | 146.9 | 170 | (45, 214) | 532 | (31, 200) | 207 | phage portal protein | phage portal protein | | uniclust | UniRef100\_UPI000CEF4514 | 99.6 | 7.4e-18 | 1.4e-23 | 154.1 | 247 | (248, 495) | 532 | (5, 269) | 296 | phage portal protein | phage portal protein | | uniclust | UniRef100\_F3IS71 | 99.6 | 8.3e-18 | 1.5e-23 | 136.6 | 126 | (127, 255) | 532 | (3, 131) | 132 | Portal protein (Fragment) | Portal protein (Fragment) | | uniclust | UniRef100\_A0A1V5NFS7 | 99.6 | 9.9e-18 | 1.9e-23 | 145.0 | 145 | (357, 510) | 532 | (1, 145) | 168 | Phage portal protein, lambda family | Phage portal protein, lambda family | | uniclust | UniRef100\_A0A6J4FY55 | 99.6 | 1e-17 | 1.9e-23 | 150.7 | 165 | (44, 219) | 532 | (14, 178) | 261 | Phage portal protein | Phage portal protein | | uniclust | UniRef100\_A0A517YS81 | 99.5 | 1.1e-17 | 2.2e-23 | 149.9 | 165 | (43, 225) | 532 | (29, 197) | 212 | Phage portal protein, lambda family | Phage portal protein, lambda family | | uniclust | UniRef100\_A0A1U7AEE5 | 99.5 | 1.2e-17 | 2.2e-23 | 157.3 | 238 | (61, 301) | 532 | (56, 308) | 376 | Phage portal protein | Phage portal protein | | uniclust | UniRef100\_A0A7H4P585 | 99.5 | 1.6e-17 | 3.2e-23 | 155.5 | 178 | (45, 226) | 532 | (37, 223) | 253 | Phage portal protein, lambda family | Phage portal protein, lambda family | | uniclust | UniRef100\_A0A1H2PWF9 | 99.5 | 2.4e-17 | 4.4e-23 | 138.1 | 137 | (341, 488) | 532 | (5, 142) | 159 | Phage portal protein, lambda family (Fragment) | Phage portal protein, lambda family (Fragment) | | uniclust | UniRef100\_UPI00186BB8CC | 99.5 | 2.7e-17 | 4.9e-23 | 168.8 | 170 | (17, 198) | 532 | (700, 874) | 917 | phage terminase large subunit family protein | phage terminase large subunit family protein | | uniclust | UniRef100\_A0A1V5SIV4 | 99.5 | 2.7e-17 | 5e-23 | 134.9 | 104 | (393, 503) | 532 | (2, 105) | 139 | Phage portal protein, lambda family | Phage portal protein, lambda family | | uniclust | UniRef100\_UPI00052DD37F | 99.5 | 2.6e-17 | 5e-23 | 137.8 | 130 | (135, 275) | 532 | (2, 136) | 138 | phage portal protein | phage portal protein | | uniclust | UniRef100\_A0A659UH34 | 99.5 | 2.9e-17 | 5.3e-23 | 134.7 | 125 | (343, 467) | 532 | (9, 138) | 139 | Phage portal protein (Fragment) | Phage portal protein (Fragment) | | uniclust | UniRef100\_UPI0020205CB6 | 99.5 | 3.1e-17 | 5.7e-23 | 144.2 | 169 | (319, 492) | 532 | (32, 201) | 218 | phage portal protein | phage portal protein | | uniclust | UniRef100\_A0A662DL46 | 99.5 | 3.2e-17 | 5.8e-23 | 153.8 | 286 | (207, 495) | 532 | (22, 315) | 359 | Phage portal protein (Fragment) | Phage portal protein (Fragment) | | uniclust | UniRef100\_X0XGG6 | 99.5 | 3.3e-17 | 6e-23 | 146.1 | 168 | (319, 493) | 532 | (43, 213) | 241 | Phage portal protein (Fragment) | Phage portal protein (Fragment) | | uniclust | UniRef100\_A0A5R2MYS9 | 99.5 | 3.6e-17 | 7.3e-23 | 143.9 | 105 | (316, 420) | 532 | (36, 140) | 155 | Phage portal protein (Fragment) | Phage portal protein (Fragment) | | uniclust | UniRef100\_A0A3C0YQ03 | 99.5 | 4.9e-17 | 9.1e-23 | 151.1 | 240 | (249, 494) | 532 | (28, 279) | 331 | Phage portal protein | Phage portal protein | | uniclust | UniRef100\_A0A377MXP9 | 99.5 | 5.2e-17 | 9.5e-23 | 144.8 | 163 | (347, 514) | 532 | (60, 225) | 240 | Phage portal protein | Phage portal protein | | uniclust | UniRef100\_A0A268UA88 | 99.5 | 6e-17 | 1.1e-22 | 149.5 | 240 | (67, 403) | 532 | (50, 290) | 313 | Phage portal protein | Phage portal protein | | uniclust | UniRef100\_UPI001EEF44AD | 99.5 | 6.7e-17 | 1.2e-22 | 126.6 | 103 | (182, 285) | 532 | (1, 104) | 106 | phage portal protein | phage portal protein | | uniclust | UniRef100\_A0A2S1XDV8 | 99.5 | 7.9e-17 | 1.5e-22 | 146.7 | 187 | (32, 231) | 532 | (88, 280) | 281 | Phage portal protein | Phage portal protein | | uniclust | UniRef100\_UPI000500391C | 99.5 | 8.1e-17 | 1.5e-22 | 135.1 | 139 | (88, 231) | 532 | (4, 147) | 159 | phage portal protein | phage portal protein | | uniclust | UniRef100\_A0A966N5E4 | 99.5 | 8.2e-17 | 1.5e-22 | 152.4 | 222 | (66, 300) | 532 | (62, 302) | 320 | Phage portal protein | Phage portal protein | | uniclust | UniRef100\_UPI00163E7870 | 99.5 | 1.2e-16 | 2.2e-22 | 132.1 | 126 | (239, 364) | 532 | (8, 144) | 144 | phage portal protein | phage portal protein | | uniclust | UniRef100\_A0A660UHE4 | 99.5 | 1.2e-16 | 2.2e-22 | 140.8 | 178 | (45, 232) | 532 | (30, 212) | 219 | Phage portal protein (Fragment) | Phage portal protein (Fragment) | | uniclust | UniRef100\_A0A1H0DUF0 | 99.5 | 1.1e-16 | 2.2e-22 | 165.1 | 371 | (55, 469) | 532 | (40, 433) | 544 | Phage portal protein, SPP1 Gp6-like | Phage portal protein, SPP1 Gp6-like | | uniclust | UniRef100\_A0A142XP47 | 99.5 | 1.3e-16 | 2.5e-22 | 171.3 | 373 | (87, 490) | 532 | (4, 400) | 1882 | Phage portal protein, lambda family | Phage portal protein, lambda family | | uniclust | UniRef100\_A0A843Y6R3 | 99.5 | 1.7e-16 | 3e-22 | 146.2 | 238 | (73, 329) | 532 | (46, 289) | 304 | Phage portal protein | Phage portal protein | | uniclust | UniRef100\_UPI001CFD494D | 99.5 | 1.8e-16 | 3.3e-22 | 138.8 | 179 | (317, 505) | 532 | (9, 189) | 210 | phage portal protein | phage portal protein | | uniclust | UniRef100\_UPI0009670FCD | 99.5 | 1.9e-16 | 3.6e-22 | 135.8 | 148 | (317, 464) | 532 | (32, 181) | 182 | phage portal protein | phage portal protein | | uniclust | UniRef100\_UPI0013DF9E24 | 99.5 | 2e-16 | 3.6e-22 | 138.0 | 177 | (326, 506) | 532 | (1, 178) | 203 | phage portal protein | phage portal protein | | uniclust | UniRef100\_A0A961GYB0 | 99.5 | 2e-16 | 3.7e-22 | 135.7 | 158 | (45, 220) | 532 | (23, 182) | 182 | Phage portal protein (Fragment) | Phage portal protein (Fragment) | | uniclust | UniRef100\_A0A5C7PU34 | 99.5 | 2.1e-16 | 3.9e-22 | 148.0 | 209 | (269, 485) | 532 | (25, 234) | 349 | Phage portal protein | Phage portal protein | | uniclust | UniRef100\_A0A352V3P2 | 99.5 | 2.4e-16 | 4.3e-22 | 138.2 | 150 | (19, 175) | 532 | (6, 157) | 211 | Phage portal protein | Phage portal protein | | uniclust | UniRef100\_A0A379XPD9 | 99.5 | 2.3e-16 | 4.4e-22 | 137.1 | 143 | (356, 508) | 532 | (1, 144) | 178 | Phage portal protein, lambda family protein | Phage portal protein, lambda family protein | | uniclust | UniRef100\_A0A661I1H8 | 99.4 | 3e-16 | 5.6e-22 | 135.8 | 163 | (45, 229) | 532 | (15, 179) | 193 | Phage portal protein (Fragment) | Phage portal protein (Fragment) | | uniclust | UniRef100\_F2J6P5 | 99.4 | 3.1e-16 | 5.8e-22 | 136.9 | 128 | (240, 367) | 532 | (17, 148) | 205 | Portal protein GPB of phage lambda-like protein | Portal protein GPB of phage lambda-like protein | | uniclust | UniRef100\_A0A369RJ94 | 99.4 | 3.5e-16 | 6.6e-22 | 126.1 | 105 | (396, 508) | 532 | (6, 110) | 116 | Phage portal protein | Phage portal protein | | uniclust | UniRef100\_B4CWU6 | 99.4 | 3.8e-16 | 6.9e-22 | 140.5 | 177 | (99, 301) | 532 | (33, 209) | 252 | Bacteriophage capsid protein-like protein | Bacteriophage capsid protein-like protein | | uniclust | UniRef100\_A0A961K8S9 | 99.4 | 3.7e-16 | 7e-22 | 126.0 | 104 | (397, 507) | 532 | (2, 105) | 115 | Phage portal protein | Phage portal protein | | uniclust | UniRef100\_A0A1H5ZJX6 | 99.4 | 4e-16 | 7.7e-22 | 157.7 | 385 | (79, 531) | 532 | (65, 475) | 496 | Portal protein | Portal protein | | uniclust | UniRef100\_A0A076JPG9 | 99.4 | 3.7e-16 | 7.9e-22 | 167.5 | 275 | (148, 481) | 532 | (126, 404) | 513 | Phage portal protein | Phage portal protein | | uniclust | UniRef100\_A0A2X1JPZ6 | 99.4 | 4.4e-16 | 8.1e-22 | 133.3 | 151 | (357, 517) | 532 | (2, 153) | 168 | Portal protein (Head-tail preconnector protein) from prophage | Portal protein (Head-tail preconnector protein) from prophage | | uniclust | UniRef100\_A0A2X1K6P9 | 99.4 | 8e-16 | 1.5e-21 | 131.0 | 158 | (240, 398) | 532 | (6, 167) | 167 | Portal protein | Portal protein | | uniclust | UniRef100\_UPI0018CC948E | 99.4 | 8.3e-16 | 1.5e-21 | 141.4 | 166 | (327, 503) | 532 | (1, 167) | 298 | phage portal protein | phage portal protein | | uniclust | UniRef100\_A0A7W1KP99 | 99.4 | 1.2e-15 | 2.2e-21 | 149.8 | 396 | (54, 487) | 532 | (52, 477) | 524 | Phage portal protein | Phage portal protein | | uniclust | UniRef100\_A0A8J2Z721 | 99.4 | 1.4e-15 | 2.5e-21 | 129.3 | 138 | (43, 192) | 532 | (27, 164) | 169 | Uncharacterized protein | Uncharacterized protein | | uniclust | UniRef100\_UPI001FE2508B | 99.4 | 1.5e-15 | 2.7e-21 | 127.2 | 116 | (74, 198) | 532 | (6, 123) | 154 | phage portal protein | phage portal protein | | uniclust | UniRef100\_A0A3R5RY70 | 99.4 | 2.2e-15 | 4e-21 | 139.3 | 206 | (73, 300) | 532 | (49, 257) | 307 | Phage portal protein | Phage portal protein | | uniclust | UniRef100\_UPI0011BA491B | 99.4 | 2.2e-15 | 4.1e-21 | 129.0 | 160 | (240, 399) | 532 | (8, 174) | 177 | phage portal protein | phage portal protein | | uniclust | UniRef100\_UPI00227E5E2A | 99.4 | 2.6e-15 | 4.7e-21 | 124.1 | 112 | (48, 160) | 532 | (21, 137) | 141 | phage portal protein | phage portal protein | | uniclust | UniRef100\_B6Y8D8 | 99.4 | 2.6e-15 | 4.9e-21 | 123.2 | 92 | (44, 144) | 532 | (20, 111) | 114 | Putative phage portal protein | Putative phage portal protein | | uniclust | UniRef100\_A0A939WQF3 | 99.4 | 2.8e-15 | 5.2e-21 | 147.8 | 438 | (45, 506) | 532 | (24, 505) | 544 | Phage portal protein | Phage portal protein | | uniclust | UniRef100\_A0A0F8YUP6 | 99.4 | 2.8e-15 | 5.3e-21 | 119.2 | 96 | (16, 112) | 532 | (1, 99) | 101 | Uncharacterized protein (Fragment) | Uncharacterized protein (Fragment) | | uniclust | UniRef100\_UPI00210222F9 | 99.4 | 2.9e-15 | 5.3e-21 | 121.7 | 117 | (179, 300) | 532 | (2, 118) | 127 | phage portal protein | phage portal protein | | uniclust | UniRef100\_UPI00057803E2 | 99.4 | 3.1e-15 | 5.8e-21 | 115.5 | 80 | (151, 231) | 532 | (2, 83) | 86 | phage portal protein | phage portal protein | | uniclust | UniRef100\_A0A447U5T1 | 99.3 | 3.7e-15 | 6.7e-21 | 137.8 | 178 | (46, 227) | 532 | (87, 273) | 304 | Phage portal protein, lambda family | Phage portal protein, lambda family | | uniclust | UniRef100\_UPI001787C57E | 99.3 | 4.2e-15 | 7.7e-21 | 129.7 | 167 | (253, 419) | 532 | (1, 189) | 199 | phage portal protein | phage portal protein | | uniclust | UniRef100\_A0A8J7QZX4 | 99.3 | 4.9e-15 | 9e-21 | 124.0 | 125 | (369, 505) | 532 | (2, 126) | 152 | Phage portal protein, lambda family | Phage portal protein, lambda family | | uniclust | UniRef100\_UPI0022CDD105 | 99.3 | 5.5e-15 | 1e-20 | 140.9 | 288 | (73, 369) | 532 | (65, 390) | 392 | phage portal protein | phage portal protein | | uniclust | UniRef100\_A0A7X6J9Z5 | 99.3 | 5.7e-15 | 1e-20 | 120.8 | 127 | (319, 450) | 532 | (1, 130) | 132 | Phage portal protein | Phage portal protein | | uniclust | UniRef100\_UPI001111D749 | 99.3 | 5.7e-15 | 1.1e-20 | 115.7 | 95 | (323, 417) | 532 | (4, 98) | 98 | phage portal protein | phage portal protein | | uniclust | UniRef100\_A0A2G4JPU1 | 99.3 | 6.5e-15 | 1.2e-20 | 123.3 | 140 | (370, 514) | 532 | (2, 143) | 152 | Phage portal protein | Phage portal protein | | uniclust | UniRef100\_A0A2Z3H0N2 | 99.3 | 6.6e-15 | 1.3e-20 | 156.0 | 393 | (72, 495) | 532 | (89, 505) | 923 | Phage portal protein | Phage portal protein | | uniclust | UniRef100\_A0A0A7KFK9 | 99.3 | 7.1e-15 | 1.4e-20 | 151.9 | 365 | (72, 487) | 532 | (47, 443) | 476 | Phage portal protein | Phage portal protein | | uniclust | UniRef100\_UPI00191C467D | 99.3 | 8e-15 | 1.5e-20 | 124.9 | 123 | (18, 147) | 532 | (1, 126) | 169 | phage portal protein | phage portal protein | | uniclust | UniRef100\_A0A2M9FV85 | 99.3 | 8.9e-15 | 1.6e-20 | 124.3 | 131 | (374, 511) | 532 | (1, 132) | 166 | Uncharacterized protein | Uncharacterized protein | | uniclust | UniRef100\_A0A6N7AFZ5 | 99.3 | 1.6e-14 | 2.9e-20 | 118.8 | 97 | (93, 198) | 532 | (26, 124) | 135 | Lambda family phage portal protein (Fragment) | Lambda family phage portal protein (Fragment) | | uniclust | UniRef100\_A0A3C1GDR3 | 99.3 | 1.6e-14 | 3e-20 | 119.9 | 139 | (75, 230) | 532 | (3, 143) | 143 | Phage portal protein (Fragment) | Phage portal protein (Fragment) | |
| Top keywords  (threshold 1.00e-03 (evalue)) | **portal, Phage, Fragment, lambda, Capsid, Putative, prophage, terminase, Minor, domain\_containing** |
| Output files | ../../similar\_sequences/04\_FANPEZAQ\_CDS\_0004\_merged.svg ../../similar\_sequences/04\_FANPEZAQ\_CDS\_0004\_pdb70.a3m ../../similar\_sequences/04\_FANPEZAQ\_CDS\_0004\_pdb70.hhr ../../similar\_sequences/04\_FANPEZAQ\_CDS\_0004\_uniclust.a3m ../../similar\_sequences/04\_FANPEZAQ\_CDS\_0004\_uniclust.hhr |

#### Structure prediction (AlphaFold)2

|  |  |
| --- | --- |
| Stats | xml version="1.0" encoding="utf-8" standalone="no"?       2024-09-02T21:09:02.438651 image/svg+xml   Matplotlib v3.7.2, https://matplotlib.org/ |
| Predicted structure | **NGL Viewer Controls:**  - Center: *Left-Click* - Rotate: *Left-Click + Drag* - Translate: *Right-Click + Drag* - Zoom: *Shift + Left-Click + Drag* |
| Output files | ../../predicted\_structures/04\_FANPEZAQ\_CDS\_0004/features.pkl ../../predicted\_structures/04\_FANPEZAQ\_CDS\_0004/ranked\_0.pdb ../../predicted\_structures/04\_FANPEZAQ\_CDS\_0004/ranked\_0\_plots.svg ../../predicted\_structures/04\_FANPEZAQ\_CDS\_0004/result\_model\_1\_ptm\_pred\_0.pkl |

#### Structure similarity search results (Foldseek)3

|  |  |
| --- | --- |
| Structure databases searched | Pdb, Afdb-proteome, Afdb-uniprot50 |
| Results, scheme(s)  (Top layers only, threshold 1.00e-02 (evalue)) | xml version="1.0" encoding="utf-8" standalone="no"?       2024-09-02T21:10:12.685353 image/svg+xml   Matplotlib v3.7.2, https://matplotlib.org/ |
| Results, table  (threshold 1.00e-02 (evalue)) | | db | id | prob | evalue | bits | fident | alnlen | mismatch | gapopen | qstart | qend | tstart | tend | name | description | | --- | --- | --- | --- | --- | --- | --- | --- | --- | --- | --- | --- | --- | --- | --- | | pdb | 8GTD\_A | 1.0 | 1.243e-31 | 1160 | 0.328 | 456 | 277 | 10 | 47 | 495 | 1 | 434 | Portal protein | Portal protein | | pdb | 6QJT\_A | 1.0 | 5.678e-09 | 244 | 0.124 | 419 | 282 | 21 | 68 | 466 | 23 | 376 | Portal protein | Portal protein | | pdb | 4ZJN\_A | 1.0 | 3.578e-08 | 232 | 0.114 | 447 | 284 | 21 | 70 | 475 | 26 | 401 | Portal protein | Portal protein | | pdb | 8FQL\_A | 1.0 | 1.317e-08 | 231 | 0.13 | 420 | 261 | 26 | 83 | 478 | 4 | 343 | Portal protein | Portal protein | | pdb | 6TO8\_A | 1.0 | 4.906e-08 | 226 | 0.129 | 457 | 272 | 22 | 48 | 478 | 3 | 359 | Portal protein Rcc01684 | Portal protein Rcc01684 | | pdb | 5NGD\_A | 1.0 | 3.578e-08 | 222 | 0.122 | 482 | 289 | 26 | 70 | 517 | 21 | 402 | Portal protein | Portal protein | | pdb | 6UZC\_H | 1.0 | 2.447e-09 | 219 | 0.147 | 481 | 312 | 22 | 66 | 497 | 58 | 489 | Portal protein | Portal protein | | pdb | 6TOA\_B | 1.0 | 3.772e-08 | 211 | 0.142 | 464 | 264 | 26 | 48 | 478 | 3 | 365 | Portal protein Rcc01684 | Portal protein Rcc01684 | | pdb | 6TBA\_1A | 1.0 | 1.2e-07 | 206 | 0.13 | 459 | 269 | 25 | 48 | 478 | 3 | 359 | Portal protein Rcc01684 | Portal protein Rcc01684 | | pdb | 8CEZ\_A | 1.0 | 5.45e-08 | 202 | 0.125 | 423 | 260 | 24 | 83 | 478 | 24 | 363 | Portal protein | Portal protein | | pdb | 3JA7\_A | 1.0 | 6.055e-08 | 202 | 0.134 | 477 | 300 | 22 | 76 | 498 | 5 | 422 | Portal protein gp20 | Portal protein gp20 | | pdb | 6TUI\_A | 1.0 | 1.827e-07 | 200 | 0.141 | 465 | 267 | 29 | 48 | 478 | 3 | 369 | Portal protein Rcc01684 | Portal protein Rcc01684 | | pdb | 8FVH\_U | 1.0 | 4.709e-07 | 188 | 0.122 | 500 | 296 | 28 | 68 | 500 | 5 | 428 | E217 portal protein gp19 | E217 portal protein gp19 | | pdb | 6QWP\_A | 1.0 | 7.876e-08 | 185 | 0.122 | 539 | 303 | 31 | 68 | 519 | 16 | 471 | Portal protein | Portal protein | | pdb | 7BOU\_A | 1.0 | 8.301e-08 | 174 | 0.119 | 577 | 341 | 34 | 56 | 519 | 2 | 524 | Portal protein | Portal protein | | pdb | 2JES\_M | 1.0 | 3.906e-05 | 163 | 0.091 | 416 | 258 | 22 | 90 | 491 | 60 | 369 | PORTAL PROTEIN | PORTAL PROTEIN | | pdb | 7Z4W\_A | 1.0 | 4.52e-06 | 161 | 0.112 | 464 | 274 | 29 | 91 | 500 | 66 | 445 | Head completion protein gp15 | Head completion protein gp15 | | pdb | 6QX5\_I | 1.0 | 1.498e-06 | 156 | 0.125 | 533 | 315 | 30 | 68 | 525 | 14 | 470 | Portal protein | Portal protein | | pdb | 7EY6\_A | 1.0 | 1.849e-06 | 154 | 0.112 | 480 | 306 | 25 | 68 | 467 | 13 | 452 | Portal protein | Portal protein | | pdb | 7Y1C\_A | 1.0 | 4.709e-07 | 153 | 0.111 | 576 | 303 | 33 | 68 | 518 | 13 | 504 | phage connector protein | phage connector protein | | pdb | 8DGF\_H | 1.0 | 2.054e-06 | 143 | 0.109 | 477 | 287 | 27 | 68 | 464 | 15 | 433 | Portal protein | Portal protein | | pdb | 8CK0\_A | 1.0 | 3.663e-06 | 141 | 0.109 | 521 | 306 | 28 | 59 | 499 | 35 | 477 | Portal protein | Portal protein | | pdb | 6QXM\_A | 1.0 | 9.44e-06 | 140 | 0.117 | 409 | 260 | 24 | 68 | 416 | 6 | 373 | Portal protein | Portal protein | | pdb | 6R21\_A | 1.0 | 1.228e-05 | 137 | 0.118 | 481 | 287 | 27 | 68 | 458 | 16 | 449 | Tail tubular protein gp12 | Tail tubular protein gp12 | | pdb | 8PHO\_A | 1.0 | 1.037e-06 | 130 | 0.112 | 382 | 229 | 24 | 83 | 432 | 5 | 308 | Phage portal protein | Phage portal protein | | pdb | 8PHP\_A | 1.0 | 7.257e-06 | 127 | 0.093 | 437 | 244 | 23 | 83 | 487 | 5 | 321 | Phage portal protein | Phage portal protein | | pdb | 8PHU\_PF | 1.0 | 1.684e-05 | 125 | 0.097 | 449 | 238 | 26 | 83 | 487 | 5 | 330 | Portal protein | Portal protein | | pdb | 8PHU\_PG | 1.0 | 1.684e-05 | 125 | 0.103 | 446 | 243 | 26 | 83 | 487 | 5 | 334 | Portal protein | Portal protein | | pdb | 8PHU\_PH | 1.0 | 1.774e-05 | 124 | 0.098 | 455 | 240 | 26 | 83 | 487 | 5 | 339 | Portal protein | Portal protein | | pdb | 8PHU\_PI | 1.0 | 1.228e-05 | 121 | 0.108 | 441 | 242 | 24 | 83 | 487 | 5 | 330 | Portal protein | Portal protein | | pdb | 7Z44\_A | 1.0 | 6.27e-05 | 121 | 0.075 | 598 | 373 | 33 | 66 | 524 | 21 | 577 | Portal protein | Portal protein | | pdb | 7KLN\_A1 | 1.0 | 0.001131 | 119 | 0.082 | 461 | 271 | 26 | 52 | 473 | 10 | 357 | Portal protein | Portal protein | | pdb | 8PHU\_PA | 1.0 | 2.078e-05 | 116 | 0.098 | 448 | 238 | 24 | 83 | 487 | 5 | 329 | Portal protein | Portal protein | | pdb | 8PHU\_PD | 1.0 | 2.078e-05 | 116 | 0.1 | 449 | 238 | 24 | 83 | 487 | 5 | 331 | Portal protein | Portal protein | | pdb | 8PHU\_PE | 1.0 | 4.573e-05 | 116 | 0.096 | 464 | 240 | 26 | 83 | 487 | 5 | 348 | Portal protein | Portal protein | | pdb | 7Y22\_B | 1.0 | 0.0001118 | 113 | 0.109 | 422 | 227 | 24 | 68 | 418 | 8 | 351 | phage connector protein | phage connector protein | | pdb | 7Z4B\_ED | 1.0 | 0.0001455 | 108 | 0.086 | 610 | 373 | 34 | 66 | 527 | 21 | 593 | Portal protein | Portal protein | | pdb | 8PHU\_PC | 1.0 | 6.27e-05 | 107 | 0.095 | 460 | 238 | 24 | 83 | 487 | 5 | 341 | Portal protein | Portal protein | | pdb | 8PHU\_PJ | 1.0 | 6.966e-05 | 106 | 0.096 | 464 | 240 | 24 | 83 | 487 | 5 | 348 | Portal protein | Portal protein | | pdb | 8PHU\_PK | 1.0 | 0.0002216 | 103 | 0.094 | 468 | 238 | 26 | 83 | 487 | 5 | 349 | Portal protein | Portal protein | | pdb | 8PHU\_PB | 1.0 | 0.0001242 | 101 | 0.097 | 472 | 241 | 24 | 83 | 487 | 5 | 358 | Portal protein | Portal protein | | pdb | 7WMP\_A | 0.999 | 7.738e-05 | 98 | 0.108 | 560 | 334 | 30 | 66 | 515 | 27 | 530 | Nozzle protein gp25 | Nozzle protein gp25 | | pdb | 7Z4A\_K | 0.999 | 0.0002335 | 98 | 0.09 | 631 | 357 | 41 | 39 | 524 | 10 | 568 | Portal protein | Portal protein | | pdb | 8PHU\_PL | 0.998 | 0.0003375 | 93 | 0.093 | 479 | 240 | 24 | 83 | 487 | 5 | 363 | Portal protein | Portal protein | | pdb | 3KDR\_C | 0.991 | 0.006765 | 84 | 0.094 | 402 | 208 | 23 | 83 | 455 | 10 | 284 | HK97 Family Phage Portal Protein | HK97 Family Phage Portal Protein | | pdb | 1IJG\_A | 0.988 | 0.00249 | 82 | 0.132 | 257 | 161 | 15 | 155 | 400 | 41 | 246 | UPPER COLLAR PROTEIN | UPPER COLLAR PROTEIN | | pdb | 7SYA\_H | 0.986 | 0.0088 | 81 | 0.087 | 434 | 279 | 23 | 66 | 424 | 18 | 409 | Portal protein | Portal protein | | pdb | 7SZ6\_K | 0.984 | 0.004441 | 80 | 0.089 | 490 | 293 | 28 | 66 | 475 | 18 | 434 | Portal protein | Portal protein | | pdb | 1H5W\_C | 0.981 | 0.003239 | 79 | 0.115 | 260 | 163 | 18 | 155 | 399 | 34 | 241 | UPPER COLLAR PROTEIN | UPPER COLLAR PROTEIN | | pdb | 3KDR\_A | 0.981 | 0.009776 | 79 | 0.117 | 401 | 206 | 25 | 79 | 455 | 6 | 282 | HK97 Family Phage Portal Protein | HK97 Family Phage Portal Protein | | pdb | 7SFS\_A | 0.978 | 0.002242 | 78 | 0.122 | 627 | 310 | 40 | 67 | 522 | 17 | 573 | Gene 3 protein | Gene 3 protein | | pdb | 1H5W\_A | 0.967 | 0.005481 | 75 | 0.131 | 258 | 157 | 17 | 155 | 399 | 35 | 238 | UPPER COLLAR PROTEIN | UPPER COLLAR PROTEIN | | pdb | 1H5W\_B | 0.961 | 0.006765 | 74 | 0.128 | 257 | 164 | 15 | 155 | 399 | 36 | 244 | UPPER COLLAR PROTEIN | UPPER COLLAR PROTEIN | | pdb | 7SP4\_B | 0.933 | 0.002127 | 70 | 0.113 | 626 | 306 | 40 | 50 | 522 | 49 | 578 | Gene 5 protein | Gene 5 protein | | pdb | 4V4K\_N | 0.923 | 0.006418 | 69 | 0.11 | 615 | 317 | 40 | 67 | 524 | 17 | 558 | PACKAGED DNA STABILIZATION PROTEIN GP4 | PACKAGED DNA STABILIZATION PROTEIN GP4 | | pdb | 7QOI\_FB | 0.855 | 0.0052 | 64 | 0.101 | 631 | 324 | 33 | 90 | 523 | 74 | 658 | Portal protein gp20 | Portal protein gp20 | | pdb | 7QOI\_FC | 0.632 | 0.00713 | 55 | 0.093 | 654 | 326 | 36 | 71 | 523 | 77 | 664 | Portal protein gp20 | Portal protein gp20 | | afdb-proteome | AF-Q8ZQH0-F1-MODEL\_V4 | 1.0 | 1.11e-31 | 1140 | 0.206 | 500 | 369 | 12 | 39 | 532 | 21 | 498 | Fels-1 prophage protein | Fels-1 prophage protein | | afdb-proteome | AF-Q8ZQ95-F1-MODEL\_V4 | 1.0 | 1.017e-25 | 911 | 0.217 | 446 | 305 | 14 | 99 | 532 | 2 | 415 | Gifsy-2 prophage protein | Gifsy-2 prophage protein | | afdb-proteome | AF-Q8ZMZ5-F1-MODEL\_V4 | 1.0 | 4.039e-24 | 800 | 0.203 | 517 | 345 | 17 | 47 | 515 | 21 | 518 | Gifsy-1 prophage protein | Gifsy-1 prophage protein | | afdb-proteome | AF-Q2FYC7-F1-MODEL\_V4 | 1.0 | 3.655e-10 | 253 | 0.1 | 480 | 310 | 18 | 23 | 478 | 2 | 383 | Phage portal protein, HK97 family | Phage portal protein, HK97 family | | afdb-proteome | AF-A0A0H3GWD5-F1-MODEL\_V4 | 1.0 | 2.729e-08 | 220 | 0.114 | 490 | 304 | 26 | 14 | 478 | 1 | 385 | Phage portal protein, HK97 family | Phage portal protein, HK97 family | | afdb-proteome | AF-A0A0H3H1P8-F1-MODEL\_V4 | 1.0 | 6.22e-05 | 134 | 0.109 | 467 | 270 | 27 | 1 | 448 | 4 | 343 | Putative prophage presumed portal protein | Putative prophage presumed portal protein | | afdb-proteome | AF-A0A0H3GQE9-F1-MODEL\_V4 | 1.0 | 4.537e-05 | 127 | 0.108 | 478 | 249 | 27 | 2 | 446 | 8 | 341 | Putative phage portal protein | Putative phage portal protein | | afdb-proteome | AF-Q8ZMS9-F1-MODEL\_V4 | 1.0 | 0.0001053 | 124 | 0.119 | 427 | 247 | 21 | 1 | 397 | 4 | 331 | Fels-2 prophage protein | Fels-2 prophage protein | | afdb-proteome | AF-Q5F7S3-F1-MODEL\_V4 | 0.998 | 0.000459 | 94 | 0.082 | 695 | 359 | 36 | 49 | 516 | 2 | 644 | Uncharacterized protein | Uncharacterized protein | | afdb-uniprot50 | AF-A0A0D0QKS8-F1-MODEL\_V4 | 1.0 | 1.912e-69 | 2992 | 0.673 | 487 | 158 | 1 | 46 | 532 | 1 | 486 | Contig\_80, whole genome shotgun sequence | Contig\_80, whole genome shotgun sequence | | afdb-uniprot50 | AF-A0A0A8TLB7-F1-MODEL\_V4 | 1.0 | 2.05e-63 | 2629 | 0.568 | 512 | 214 | 3 | 13 | 518 | 3 | 513 | Phage-related portal protein | Phage-related portal protein | | afdb-uniprot50 | AF-A0A7Z1WDC6-F1-MODEL\_V4 | 1.0 | 2.698e-62 | 2544 | 0.537 | 530 | 231 | 6 | 4 | 532 | 1 | 517 | Phage portal protein | Phage portal protein | | afdb-uniprot50 | AF-E4PPT4-F1-MODEL\_V4 | 1.0 | 5.536e-59 | 2339 | 0.493 | 527 | 250 | 7 | 14 | 532 | 1 | 518 | Phage portal protein, lambda family | Phage portal protein, lambda family | | afdb-uniprot50 | AF-A0A1H9YBA9-F1-MODEL\_V4 | 1.0 | 1.369e-57 | 2293 | 0.454 | 537 | 264 | 7 | 1 | 532 | 13 | 525 | Phage portal protein, lambda family | Phage portal protein, lambda family | | afdb-uniprot50 | AF-A0A2S0MND2-F1-MODEL\_V4 | 1.0 | 5.601e-58 | 2274 | 0.471 | 539 | 263 | 8 | 1 | 532 | 1 | 524 | Phage portal protein | Phage portal protein | | afdb-uniprot50 | AF-A0A118DU69-F1-MODEL\_V4 | 1.0 | 5.733e-56 | 2256 | 0.498 | 495 | 219 | 8 | 46 | 532 | 1 | 474 | Portal protein | Portal protein | | afdb-uniprot50 | AF-A0A1X7L1E7-F1-MODEL\_V4 | 1.0 | 9.588e-57 | 2244 | 0.465 | 520 | 255 | 6 | 15 | 532 | 3 | 501 | Phage portal protein, lambda family | Phage portal protein, lambda family | | afdb-uniprot50 | AF-A0A837E1C6-F1-MODEL\_V4 | 1.0 | 3.613e-55 | 2203 | 0.463 | 516 | 239 | 6 | 27 | 532 | 2 | 489 | Portal protein | Portal protein | | afdb-uniprot50 | AF-A0A7H8DNI9-F1-MODEL\_V4 | 1.0 | 2.529e-54 | 2134 | 0.429 | 521 | 281 | 8 | 17 | 532 | 1 | 510 | Phage portal protein | Phage portal protein | | afdb-uniprot50 | AF-Q87CG8-F1-MODEL\_V4 | 1.0 | 6.257e-53 | 2082 | 0.449 | 530 | 259 | 9 | 1 | 523 | 3 | 506 | Phage-related portal protein | Phage-related portal protein | | afdb-uniprot50 | AF-A0A1F9UST4-F1-MODEL\_V4 | 1.0 | 2.002e-47 | 1861 | 0.404 | 495 | 279 | 6 | 13 | 502 | 1 | 484 | Phage portal protein | Phage portal protein | | afdb-uniprot50 | AF-A0A7J0BKD5-F1-MODEL\_V4 | 1.0 | 5.665e-48 | 1833 | 0.383 | 524 | 290 | 7 | 13 | 532 | 2 | 496 | Phage portal protein | Phage portal protein | | afdb-uniprot50 | AF-A0A7C8HWB8-F1-MODEL\_V4 | 1.0 | 6.991e-48 | 1817 | 0.362 | 533 | 317 | 7 | 2 | 532 | 12 | 523 | Uncharacterized protein | Uncharacterized protein | | afdb-uniprot50 | AF-A0A2G6D2V0-F1-MODEL\_V4 | 1.0 | 3.213e-47 | 1809 | 0.368 | 535 | 303 | 10 | 2 | 528 | 1 | 508 | Phage portal protein | Phage portal protein | | afdb-uniprot50 | AF-A0A3R8IU71-F1-MODEL\_V4 | 1.0 | 6.293e-48 | 1799 | 0.401 | 540 | 289 | 10 | 1 | 532 | 7 | 520 | Phage portal protein | Phage portal protein | | afdb-uniprot50 | AF-A0A2D7GUF0-F1-MODEL\_V4 | 1.0 | 1.921e-46 | 1791 | 0.33 | 526 | 318 | 8 | 15 | 532 | 1 | 500 | Phage portal protein | Phage portal protein | | afdb-uniprot50 | AF-A0A661FAN3-F1-MODEL\_V4 | 1.0 | 1.345e-45 | 1786 | 0.36 | 508 | 300 | 8 | 6 | 502 | 7 | 500 | Phage portal protein | Phage portal protein | | afdb-uniprot50 | AF-A0A1G2ZKX1-F1-MODEL\_V4 | 1.0 | 1.345e-45 | 1750 | 0.393 | 490 | 273 | 6 | 45 | 532 | 20 | 487 | Phage portal protein | Phage portal protein | | afdb-uniprot50 | AF-A0A0U5N3Z0-F1-MODEL\_V4 | 1.0 | 2.529e-45 | 1736 | 0.385 | 513 | 280 | 8 | 29 | 532 | 8 | 494 | Putative Phage portal protein, lambda family | Putative Phage portal protein, lambda family | | afdb-uniprot50 | AF-W0IYG2-F1-MODEL\_V4 | 1.0 | 4.698e-46 | 1718 | 0.381 | 532 | 293 | 7 | 4 | 532 | 10 | 508 | Portal protein | Portal protein | | afdb-uniprot50 | AF-A0A348SWC9-F1-MODEL\_V4 | 1.0 | 3.896e-44 | 1712 | 0.516 | 405 | 176 | 6 | 132 | 532 | 2 | 390 | Phage portal protein | Phage portal protein | | afdb-uniprot50 | AF-A0A542RSK1-F1-MODEL\_V4 | 1.0 | 1.791e-43 | 1701 | 0.358 | 491 | 289 | 6 | 26 | 496 | 13 | 497 | Lambda family phage portal protein | Lambda family phage portal protein | | afdb-uniprot50 | AF-A0A3A4NUK8-F1-MODEL\_V4 | 1.0 | 1.512e-44 | 1679 | 0.35 | 511 | 287 | 9 | 39 | 532 | 17 | 499 | Phage portal protein | Phage portal protein | | afdb-uniprot50 | AF-A0A0F9J630-F1-MODEL\_V4 | 1.0 | 1.548e-42 | 1673 | 0.372 | 454 | 270 | 7 | 46 | 495 | 14 | 456 | Uncharacterized protein | Uncharacterized protein | | afdb-uniprot50 | AF-A0A7X4CN61-F1-MODEL\_V4 | 1.0 | 2.558e-44 | 1671 | 0.373 | 533 | 312 | 7 | 2 | 532 | 23 | 535 | Phage portal protein | Phage portal protein | | afdb-uniprot50 | AF-A0A1Y0N464-F1-MODEL\_V4 | 1.0 | 7.41e-43 | 1658 | 0.341 | 495 | 302 | 9 | 44 | 532 | 26 | 502 | Uncharacterized protein | Uncharacterized protein | | afdb-uniprot50 | AF-A0A2X1U6Z4-F1-MODEL\_V4 | 1.0 | 8.935e-45 | 1658 | 0.36 | 530 | 295 | 11 | 23 | 532 | 2 | 507 | Putative phage portal protein | Putative phage portal protein | | afdb-uniprot50 | AF-A0A2A5BDL3-F1-MODEL\_V4 | 1.0 | 2.529e-45 | 1658 | 0.388 | 522 | 291 | 10 | 15 | 532 | 1 | 498 | Phage portal protein | Phage portal protein | | afdb-uniprot50 | AF-A0A3N2E0V0-F1-MODEL\_V4 | 1.0 | 8.329e-42 | 1653 | 0.318 | 502 | 291 | 10 | 46 | 532 | 1 | 466 | Lambda family phage portal protein | Lambda family phage portal protein | | afdb-uniprot50 | AF-A0A6M8STS1-F1-MODEL\_V4 | 1.0 | 2.097e-43 | 1651 | 0.382 | 494 | 267 | 9 | 43 | 531 | 19 | 479 | Phage portal protein | Phage portal protein | | afdb-uniprot50 | AF-A0A5Q0TIZ8-F1-MODEL\_V4 | 1.0 | 2.013e-42 | 1624 | 0.35 | 499 | 292 | 10 | 23 | 516 | 1 | 472 | Phage portal protein | Phage portal protein | | afdb-uniprot50 | AF-A0A1V5CVB9-F1-MODEL\_V4 | 1.0 | 5.935e-44 | 1622 | 0.319 | 536 | 322 | 7 | 15 | 532 | 1 | 511 | Phage portal protein, lambda family | Phage portal protein, lambda family | | afdb-uniprot50 | AF-A0A5N0TED7-F1-MODEL\_V4 | 1.0 | 1.791e-43 | 1621 | 0.348 | 522 | 297 | 12 | 27 | 531 | 3 | 498 | Phage portal protein | Phage portal protein | | afdb-uniprot50 | AF-A0A831XL27-F1-MODEL\_V4 | 1.0 | 4.379e-43 | 1617 | 0.34 | 505 | 294 | 8 | 21 | 502 | 1 | 489 | Phage portal protein | Phage portal protein | | afdb-uniprot50 | AF-A0A6M3KNK2-F1-MODEL\_V4 | 1.0 | 1.239e-43 | 1616 | 0.34 | 520 | 308 | 7 | 23 | 532 | 1 | 495 | Putative portal protein | Putative portal protein | | afdb-uniprot50 | AF-A0A653JFP4-F1-MODEL\_V4 | 1.0 | 1.129e-42 | 1612 | 0.333 | 531 | 314 | 6 | 15 | 532 | 1 | 504 | Phage portal protein, lambda family | Phage portal protein, lambda family | | afdb-uniprot50 | AF-A0A1Z9Q5E6-F1-MODEL\_V4 | 1.0 | 3.066e-42 | 1603 | 0.308 | 512 | 321 | 7 | 24 | 532 | 4 | 485 | Phage portal protein | Phage portal protein | | afdb-uniprot50 | AF-A0A6H1ZMX2-F1-MODEL\_V4 | 1.0 | 8.779e-42 | 1597 | 0.313 | 504 | 316 | 10 | 37 | 532 | 9 | 490 | Putative portal protein | Putative portal protein | | afdb-uniprot50 | AF-A0A2E7V4F4-F1-MODEL\_V4 | 1.0 | 3.27e-41 | 1594 | 0.315 | 514 | 323 | 10 | 23 | 532 | 2 | 490 | Phage portal protein | Phage portal protein | | afdb-uniprot50 | AF-A0A6L9M4Z4-F1-MODEL\_V4 | 1.0 | 8.677e-43 | 1593 | 0.355 | 531 | 307 | 11 | 8 | 531 | 1 | 503 | Phage portal protein | Phage portal protein | | afdb-uniprot50 | AF-A0A2D5VNS8-F1-MODEL\_V4 | 1.0 | 1.071e-42 | 1584 | 0.324 | 514 | 306 | 7 | 39 | 532 | 14 | 506 | Phage portal protein | Phage portal protein | | afdb-uniprot50 | AF-A0A1B3W9X7-F1-MODEL\_V4 | 1.0 | 2.978e-40 | 1578 | 0.331 | 479 | 288 | 7 | 26 | 502 | 30 | 478 | Phage portal protein | Phage portal protein | | afdb-uniprot50 | AF-A0A1Y5TZN6-F1-MODEL\_V4 | 1.0 | 2.013e-42 | 1578 | 0.335 | 531 | 317 | 11 | 15 | 532 | 1 | 508 | Phage portal protein, lambda family | Phage portal protein, lambda family | | afdb-uniprot50 | AF-A0A843YIQ3-F1-MODEL\_V4 | 1.0 | 2.147e-41 | 1569 | 0.287 | 528 | 352 | 8 | 5 | 532 | 1 | 504 | Phage portal protein | Phage portal protein | | afdb-uniprot50 | AF-A0A1E4UTF6-F1-MODEL\_V4 | 1.0 | 1.76e-40 | 1565 | 0.333 | 486 | 304 | 8 | 16 | 495 | 1 | 472 | Phage portal protein | Phage portal protein | | afdb-uniprot50 | AF-A0A7G3GB32-F1-MODEL\_V4 | 1.0 | 4.431e-42 | 1562 | 0.342 | 529 | 299 | 12 | 23 | 532 | 2 | 500 | Phage portal protein | Phage portal protein | | afdb-uniprot50 | AF-A0A1V6GE05-F1-MODEL\_V4 | 1.0 | 2.122e-42 | 1553 | 0.339 | 524 | 320 | 9 | 18 | 532 | 16 | 522 | Phage portal protein, lambda family | Phage portal protein, lambda family | | afdb-uniprot50 | AF-A0A1L3ZRM5-F1-MODEL\_V4 | 1.0 | 6.146e-41 | 1551 | 0.343 | 521 | 294 | 7 | 23 | 532 | 2 | 485 | Phage portal protein | Phage portal protein | | afdb-uniprot50 | AF-A0A7G8BY97-F1-MODEL\_V4 | 1.0 | 3.632e-41 | 1543 | 0.317 | 533 | 322 | 13 | 11 | 532 | 1 | 502 | Phage portal protein | Phage portal protein | | afdb-uniprot50 | AF-A0A7X8AGP4-F1-MODEL\_V4 | 1.0 | 2.172e-40 | 1543 | 0.36 | 475 | 282 | 9 | 39 | 502 | 6 | 469 | Phage portal protein | Phage portal protein | | afdb-uniprot50 | AF-A0A0F9FZF8-F1-MODEL\_V4 | 1.0 | 4.922e-42 | 1536 | 0.336 | 529 | 294 | 9 | 26 | 529 | 10 | 506 | Uncharacterized protein | Uncharacterized protein | | afdb-uniprot50 | AF-A0A4Y3TD15-F1-MODEL\_V4 | 1.0 | 1.584e-40 | 1535 | 0.364 | 475 | 282 | 7 | 29 | 502 | 13 | 468 | Phage portal protein | Phage portal protein | | afdb-uniprot50 | AF-A0A7Y4DYP6-F1-MODEL\_V4 | 1.0 | 1.426e-40 | 1535 | 0.355 | 475 | 278 | 6 | 58 | 532 | 16 | 462 | Phage portal protein | Phage portal protein | | afdb-uniprot50 | AF-W0E8V8-F1-MODEL\_V4 | 1.0 | 1.978e-39 | 1535 | 0.289 | 504 | 325 | 7 | 44 | 532 | 4 | 489 | Uncharacterized protein | Uncharacterized protein | | afdb-uniprot50 | AF-A0A6L4BC33-F1-MODEL\_V4 | 1.0 | 7.585e-41 | 1531 | 0.308 | 529 | 333 | 13 | 14 | 532 | 1 | 506 | Phage portal protein | Phage portal protein | | afdb-uniprot50 | AF-A0A1M7U252-F1-MODEL\_V4 | 1.0 | 1.409e-41 | 1528 | 0.328 | 536 | 316 | 10 | 1 | 531 | 7 | 503 | Phage portal protein, lambda family | Phage portal protein, lambda family | | afdb-uniprot50 | AF-A0A1F8QKN1-F1-MODEL\_V4 | 1.0 | 2.001e-38 | 1522 | 0.359 | 442 | 266 | 8 | 36 | 467 | 16 | 450 | Phage portal protein | Phage portal protein | | afdb-uniprot50 | AF-A0A1G0DQV6-F1-MODEL\_V4 | 1.0 | 1.353e-40 | 1521 | 0.3 | 503 | 309 | 11 | 44 | 532 | 29 | 502 | Phage portal protein | Phage portal protein | | afdb-uniprot50 | AF-A0A4Q0ZSC3-F1-MODEL\_V4 | 1.0 | 5.249e-41 | 1520 | 0.34 | 523 | 303 | 13 | 14 | 526 | 1 | 491 | Phage portal protein | Phage portal protein | | afdb-uniprot50 | AF-A0A7V8HP07-F1-MODEL\_V4 | 1.0 | 9.471e-40 | 1514 | 0.345 | 477 | 268 | 8 | 70 | 532 | 3 | 449 | Phage portal protein | Phage portal protein | | afdb-uniprot50 | AF-Q1ILS5-F1-MODEL\_V4 | 1.0 | 5.249e-41 | 1505 | 0.317 | 514 | 325 | 8 | 1 | 502 | 1 | 500 | Phage portal protein, lambda | Phage portal protein, lambda | | afdb-uniprot50 | AF-A0A844IP85-F1-MODEL\_V4 | 1.0 | 1.76e-40 | 1498 | 0.33 | 509 | 313 | 9 | 28 | 518 | 1 | 499 | Phage portal protein | Phage portal protein | | afdb-uniprot50 | AF-A0A848V4U3-F1-MODEL\_V4 | 1.0 | 9.982e-40 | 1496 | 0.314 | 487 | 312 | 10 | 24 | 502 | 1 | 473 | Phage portal protein | Phage portal protein | | afdb-uniprot50 | AF-A0A2Z6U1K4-F1-MODEL\_V4 | 1.0 | 6.828e-41 | 1496 | 0.295 | 541 | 350 | 10 | 2 | 532 | 1 | 520 | Phage-related protein | Phage-related protein | | afdb-uniprot50 | AF-A0A2D5ZTJ2-F1-MODEL\_V4 | 1.0 | 7.585e-41 | 1495 | 0.293 | 531 | 341 | 10 | 7 | 532 | 8 | 509 | Phage portal protein | Phage portal protein | | afdb-uniprot50 | AF-A0A227JR73-F1-MODEL\_V4 | 1.0 | 1.78e-39 | 1493 | 0.323 | 488 | 304 | 11 | 17 | 495 | 1 | 471 | Phage portal protein | Phage portal protein | | afdb-uniprot50 | AF-A0A369R324-F1-MODEL\_V4 | 1.0 | 6.146e-41 | 1489 | 0.319 | 539 | 310 | 11 | 1 | 531 | 15 | 504 | Phage portal protein | Phage portal protein | | afdb-uniprot50 | AF-A0A1M7FPC8-F1-MODEL\_V4 | 1.0 | 2.343e-38 | 1485 | 0.317 | 475 | 308 | 8 | 36 | 502 | 15 | 481 | Phage portal protein, lambda family | Phage portal protein, lambda family | | afdb-uniprot50 | AF-A0A844G1T9-F1-MODEL\_V4 | 1.0 | 3.308e-40 | 1466 | 0.308 | 534 | 319 | 11 | 20 | 532 | 9 | 512 | Phage portal protein | Phage portal protein | | afdb-uniprot50 | AF-A0A2A5DHS4-F1-MODEL\_V4 | 1.0 | 9.091e-39 | 1465 | 0.302 | 500 | 317 | 9 | 37 | 532 | 37 | 508 | Phage portal protein | Phage portal protein | | afdb-uniprot50 | AF-A0A3S0BB35-F1-MODEL\_V4 | 1.0 | 3.527e-39 | 1464 | 0.341 | 512 | 304 | 12 | 28 | 532 | 14 | 499 | Phage portal protein | Phage portal protein | | afdb-uniprot50 | AF-A0A3A0G2M7-F1-MODEL\_V4 | 1.0 | 8.183e-39 | 1452 | 0.287 | 498 | 319 | 8 | 45 | 532 | 3 | 474 | Phage portal protein | Phage portal protein | | afdb-uniprot50 | AF-A0A0Q7SMQ8-F1-MODEL\_V4 | 1.0 | 2.47e-38 | 1443 | 0.307 | 501 | 326 | 11 | 39 | 531 | 34 | 521 | Uncharacterized protein | Uncharacterized protein | | afdb-uniprot50 | AF-A0A286GNB7-F1-MODEL\_V4 | 1.0 | 8.986e-40 | 1440 | 0.318 | 518 | 316 | 15 | 15 | 519 | 1 | 494 | Phage portal protein, lambda family | Phage portal protein, lambda family | | afdb-uniprot50 | AF-A0A0S8DLM0-F1-MODEL\_V4 | 1.0 | 1.183e-38 | 1438 | 0.304 | 523 | 326 | 10 | 14 | 532 | 1 | 489 | Uncharacterized protein | Uncharacterized protein | | afdb-uniprot50 | AF-U5QE38-F1-MODEL\_V4 | 1.0 | 2.134e-37 | 1437 | 0.321 | 476 | 300 | 10 | 39 | 497 | 35 | 504 | Phage portal protein, lambda family | Phage portal protein, lambda family | | afdb-uniprot50 | AF-A0A0Q2WT08-F1-MODEL\_V4 | 1.0 | 2.223e-38 | 1434 | 0.47 | 393 | 194 | 8 | 1 | 386 | 3 | 388 | Portal protein | Portal protein | | afdb-uniprot50 | AF-A0A2A5AEH9-F1-MODEL\_V4 | 1.0 | 8.183e-39 | 1434 | 0.271 | 508 | 355 | 7 | 14 | 515 | 1 | 499 | Phage portal protein | Phage portal protein | | afdb-uniprot50 | AF-A0A7Y5N878-F1-MODEL\_V4 | 1.0 | 7.764e-39 | 1433 | 0.3 | 516 | 330 | 8 | 26 | 532 | 2 | 495 | Phage portal protein | Phage portal protein | | afdb-uniprot50 | AF-A0A355AHD4-F1-MODEL\_V4 | 1.0 | 1.898e-38 | 1426 | 0.295 | 508 | 334 | 8 | 30 | 532 | 14 | 502 | Phage portal protein | Phage portal protein | | afdb-uniprot50 | AF-A0A3B9Q8L9-F1-MODEL\_V4 | 1.0 | 2.47e-38 | 1415 | 0.3 | 515 | 319 | 12 | 35 | 532 | 12 | 502 | Phage portal protein | Phage portal protein | | afdb-uniprot50 | AF-A0A2V5PTT5-F1-MODEL\_V4 | 1.0 | 9.582e-39 | 1411 | 0.327 | 534 | 308 | 13 | 20 | 523 | 1 | 513 | Phage portal protein | Phage portal protein | | afdb-uniprot50 | AF-A0A2M7YUE4-F1-MODEL\_V4 | 1.0 | 1.749e-36 | 1410 | 0.353 | 456 | 274 | 7 | 29 | 475 | 1 | 444 | Phage portal protein | Phage portal protein | | afdb-uniprot50 | AF-A0A1F9VF65-F1-MODEL\_V4 | 1.0 | 1.689e-39 | 1405 | 0.292 | 541 | 325 | 10 | 15 | 531 | 33 | 539 | Phage portal protein | Phage portal protein | | afdb-uniprot50 | AF-A0A2E7WCF7-F1-MODEL\_V4 | 1.0 | 1.401e-37 | 1402 | 0.327 | 482 | 300 | 12 | 33 | 502 | 12 | 481 | Phage portal protein | Phage portal protein | | afdb-uniprot50 | AF-A0A166AHR0-F1-MODEL\_V4 | 1.0 | 4.642e-38 | 1396 | 0.302 | 545 | 315 | 10 | 15 | 532 | 1 | 507 | Phage portal protein, lambda family | Phage portal protein, lambda family | | afdb-uniprot50 | AF-A0A3B9NU50-F1-MODEL\_V4 | 1.0 | 5.499e-37 | 1393 | 0.296 | 495 | 308 | 9 | 44 | 532 | 25 | 485 | Phage portal protein | Phage portal protein | | afdb-uniprot50 | AF-A0A3S0CHF6-F1-MODEL\_V4 | 1.0 | 2.664e-36 | 1382 | 0.338 | 482 | 276 | 11 | 39 | 490 | 39 | 507 | Phage portal protein | Phage portal protein | | afdb-uniprot50 | AF-F5R8A0-F1-MODEL\_V4 | 1.0 | 7.855e-38 | 1379 | 0.317 | 514 | 295 | 10 | 22 | 490 | 6 | 508 | Phage-related protein | Phage-related protein | | afdb-uniprot50 | AF-A0A7Y5K1H3-F1-MODEL\_V4 | 1.0 | 7.071e-38 | 1373 | 0.282 | 534 | 335 | 9 | 20 | 532 | 2 | 508 | Phage portal protein | Phage portal protein | | afdb-uniprot50 | AF-F4BFR2-F1-MODEL\_V4 | 1.0 | 1.034e-36 | 1372 | 0.296 | 489 | 301 | 10 | 17 | 496 | 1 | 455 | Phage-related portal protein | Phage-related portal protein | | afdb-uniprot50 | AF-A0A3C0NVL9-F1-MODEL\_V4 | 1.0 | 9.415e-36 | 1362 | 0.339 | 439 | 263 | 10 | 85 | 502 | 1 | 433 | Phage portal protein | Phage portal protein | | afdb-uniprot50 | AF-A0A359LSW5-F1-MODEL\_V4 | 1.0 | 2.37e-37 | 1350 | 0.281 | 515 | 335 | 9 | 45 | 527 | 2 | 513 | Phage portal protein | Phage portal protein | | afdb-uniprot50 | AF-A0A0F9CUR0-F1-MODEL\_V4 | 1.0 | 2.995e-35 | 1321 | 0.252 | 507 | 346 | 10 | 36 | 532 | 31 | 514 | Uncharacterized protein | Uncharacterized protein | | afdb-uniprot50 | AF-A0A7Y0NUG5-F1-MODEL\_V4 | 1.0 | 2.696e-35 | 1310 | 0.282 | 492 | 323 | 11 | 23 | 502 | 12 | 485 | Lambda family phage portal protein | Lambda family phage portal protein | | afdb-uniprot50 | AF-A0A7T6AR04-F1-MODEL\_V4 | 1.0 | 8.475e-36 | 1310 | 0.295 | 501 | 312 | 15 | 39 | 517 | 26 | 507 | Phage portal protein | Phage portal protein | | afdb-uniprot50 | AF-A0A0B8PGA6-F1-MODEL\_V4 | 1.0 | 1.058e-34 | 1309 | 0.303 | 442 | 282 | 10 | 99 | 532 | 2 | 425 | Phage portal protein | Phage portal protein | | afdb-uniprot50 | AF-A0A7V3AD27-F1-MODEL\_V4 | 1.0 | 9.637e-34 | 1278 | 0.266 | 496 | 321 | 12 | 45 | 531 | 2 | 463 | Phage portal protein | Phage portal protein | | afdb-uniprot50 | AF-A0A6J4FT41-F1-MODEL\_V4 | 1.0 | 4.154e-34 | 1267 | 0.296 | 472 | 304 | 12 | 39 | 502 | 5 | 456 | Phage portal protein, lambda family | Phage portal protein, lambda family | | afdb-uniprot50 | AF-A0A4R2Z1J8-F1-MODEL\_V4 | 1.0 | 5.629e-35 | 1265 | 0.238 | 544 | 356 | 14 | 8 | 532 | 1 | 504 | Lambda family phage portal protein | Lambda family phage portal protein | | afdb-uniprot50 | AF-A0A108V769-F1-MODEL\_V4 | 1.0 | 3.03e-34 | 1256 | 0.265 | 505 | 320 | 11 | 45 | 530 | 3 | 475 | Portal protein | Portal protein | | afdb-uniprot50 | AF-A0A369T5A0-F1-MODEL\_V4 | 1.0 | 4.378e-34 | 1249 | 0.261 | 489 | 324 | 11 | 28 | 501 | 1 | 467 | Phage portal protein | Phage portal protein | | afdb-uniprot50 | AF-A0A7C2SUL5-F1-MODEL\_V4 | 1.0 | 4.669e-33 | 1246 | 0.272 | 470 | 311 | 12 | 47 | 502 | 23 | 475 | Phage portal protein | Phage portal protein | | afdb-uniprot50 | AF-M2ZLA5-F1-MODEL\_V4 | 1.0 | 5.762e-33 | 1245 | 0.276 | 474 | 288 | 12 | 67 | 532 | 5 | 431 | Lambda family phage portal protein | Lambda family phage portal protein | | afdb-uniprot50 | AF-A0A427D6X7-F1-MODEL\_V4 | 1.0 | 2.121e-33 | 1242 | 0.264 | 473 | 317 | 11 | 43 | 502 | 5 | 459 | Phage portal protein | Phage portal protein | | afdb-uniprot50 | AF-A0A6I6DRZ3-F1-MODEL\_V4 | 1.0 | 1.699e-34 | 1241 | 0.23 | 508 | 360 | 13 | 1 | 500 | 1 | 485 | Phage portal protein | Phage portal protein | | afdb-uniprot50 | AF-A0A6N9TLF9-F1-MODEL\_V4 | 1.0 | 1.631e-33 | 1238 | 0.248 | 491 | 336 | 14 | 39 | 515 | 28 | 499 | Phage portal protein | Phage portal protein | | afdb-uniprot50 | AF-B0KSY7-F1-MODEL\_V4 | 1.0 | 5.695e-34 | 1237 | 0.271 | 494 | 322 | 11 | 24 | 502 | 1 | 471 | Phage portal protein, lambda family | Phage portal protein, lambda family | | afdb-uniprot50 | AF-A0A1I1UG86-F1-MODEL\_V4 | 1.0 | 3.405e-33 | 1236 | 0.237 | 497 | 340 | 12 | 17 | 497 | 1 | 474 | Phage portal protein, lambda family | Phage portal protein, lambda family | | afdb-uniprot50 | AF-A0A2W6RMJ5-F1-MODEL\_V4 | 1.0 | 1.699e-34 | 1233 | 0.253 | 533 | 355 | 11 | 16 | 532 | 1 | 506 | Phage portal protein | Phage portal protein | | afdb-uniprot50 | AF-A0A380N598-F1-MODEL\_V4 | 1.0 | 3.231e-33 | 1227 | 0.243 | 513 | 342 | 15 | 34 | 532 | 12 | 492 | Phage portal protein, lambda family | Phage portal protein, lambda family | | afdb-uniprot50 | AF-A0A353UTN7-F1-MODEL\_V4 | 1.0 | 1.409e-32 | 1226 | 0.27 | 470 | 304 | 13 | 20 | 479 | 4 | 444 | Phage portal protein | Phage portal protein | | afdb-uniprot50 | AF-A0A564WHL9-F1-MODEL\_V4 | 1.0 | 7.495e-33 | 1226 | 0.251 | 504 | 326 | 12 | 46 | 531 | 3 | 473 | Putative Phage portal protein | Putative Phage portal protein | | afdb-uniprot50 | AF-F3Z2S4-F1-MODEL\_V4 | 1.0 | 6.668e-34 | 1226 | 0.256 | 503 | 339 | 11 | 39 | 532 | 30 | 506 | Phage portal protein, lambda family | Phage portal protein, lambda family | | afdb-uniprot50 | AF-A0A1U7MG31-F1-MODEL\_V4 | 1.0 | 1.887e-34 | 1223 | 0.282 | 509 | 319 | 12 | 39 | 532 | 21 | 498 | Phage portal protein, lambda family | Phage portal protein, lambda family | | afdb-uniprot50 | AF-A0A2R2IT64-F1-MODEL\_V4 | 1.0 | 5.467e-33 | 1222 | 0.252 | 495 | 327 | 10 | 46 | 529 | 20 | 482 | Portal protein | Portal protein | | afdb-uniprot50 | AF-A0A1A9VKE9-F1-MODEL\_V4 | 1.0 | 2.121e-33 | 1221 | 0.256 | 499 | 326 | 12 | 44 | 531 | 232 | 696 | Uncharacterized protein | Uncharacterized protein | | afdb-uniprot50 | AF-A0A0B6CWK4-F1-MODEL\_V4 | 1.0 | 8.326e-33 | 1219 | 0.242 | 512 | 327 | 14 | 20 | 515 | 2 | 468 | Phage portal protein, lambda family | Phage portal protein, lambda family | | afdb-uniprot50 | AF-A0A550EVA9-F1-MODEL\_V4 | 1.0 | 1.989e-34 | 1219 | 0.267 | 524 | 323 | 13 | 37 | 531 | 2 | 493 | Phage portal protein | Phage portal protein | | afdb-uniprot50 | AF-A0A255XT94-F1-MODEL\_V4 | 1.0 | 1.631e-33 | 1218 | 0.254 | 507 | 338 | 12 | 1 | 495 | 2 | 480 | Phage portal protein | Phage portal protein | | afdb-uniprot50 | AF-A0A2N5EXY9-F1-MODEL\_V4 | 1.0 | 1.393e-33 | 1216 | 0.272 | 488 | 313 | 14 | 39 | 502 | 8 | 477 | Phage portal protein | Phage portal protein | | afdb-uniprot50 | AF-A0A0F9M3A7-F1-MODEL\_V4 | 1.0 | 2.236e-33 | 1216 | 0.251 | 508 | 332 | 13 | 43 | 532 | 26 | 503 | Uncharacterized protein | Uncharacterized protein | | afdb-uniprot50 | AF-A0A3N1MH20-F1-MODEL\_V4 | 1.0 | 3.941e-34 | 1214 | 0.246 | 552 | 349 | 16 | 3 | 532 | 1 | 507 | Lambda family phage portal protein | Lambda family phage portal protein | | afdb-uniprot50 | AF-A0A2M9G2J3-F1-MODEL\_V4 | 1.0 | 1.547e-33 | 1211 | 0.258 | 499 | 337 | 13 | 15 | 502 | 1 | 477 | Phage portal protein | Phage portal protein | | afdb-uniprot50 | AF-A0A5E6YAG0-F1-MODEL\_V4 | 1.0 | 9.143e-34 | 1210 | 0.269 | 531 | 325 | 15 | 28 | 532 | 1 | 494 | Uncharacterized protein | Uncharacterized protein | | afdb-uniprot50 | AF-A0A024EJ49-F1-MODEL\_V4 | 1.0 | 1.083e-32 | 1207 | 0.267 | 475 | 313 | 12 | 45 | 502 | 8 | 464 | Portal protein lambda family | Portal protein lambda family | | afdb-uniprot50 | AF-A0A3N4ABJ5-F1-MODEL\_V4 | 1.0 | 1.565e-32 | 1206 | 0.262 | 476 | 320 | 11 | 42 | 502 | 10 | 469 | Phage portal protein | Phage portal protein | | afdb-uniprot50 | AF-A0A6I5YNG6-F1-MODEL\_V4 | 1.0 | 9.864e-32 | 1204 | 0.272 | 463 | 285 | 15 | 77 | 531 | 2 | 420 | Phage portal protein | Phage portal protein | | afdb-uniprot50 | AF-A0A0H3K144-F1-MODEL\_V4 | 1.0 | 2.791e-32 | 1204 | 0.285 | 470 | 304 | 11 | 36 | 502 | 24 | 464 | Uncharacterized protein | Uncharacterized protein | | afdb-uniprot50 | AF-A0A377P186-F1-MODEL\_V4 | 1.0 | 1.268e-32 | 1202 | 0.246 | 508 | 333 | 13 | 34 | 530 | 13 | 481 | Phage portal protein, lambda family | Phage portal protein, lambda family | | afdb-uniprot50 | AF-A0A6G6IPP9-F1-MODEL\_V4 | 1.0 | 9.25e-33 | 1200 | 0.251 | 508 | 329 | 12 | 41 | 530 | 11 | 485 | Phage portal protein | Phage portal protein | | afdb-uniprot50 | AF-A0A432ADL0-F1-MODEL\_V4 | 1.0 | 3.269e-32 | 1200 | 0.274 | 474 | 307 | 12 | 47 | 502 | 23 | 477 | Phage portal protein | Phage portal protein | | afdb-uniprot50 | AF-A0A285IZJ2-F1-MODEL\_V4 | 1.0 | 7.111e-33 | 1198 | 0.273 | 487 | 314 | 13 | 39 | 502 | 8 | 477 | Phage portal protein, lambda family | Phage portal protein, lambda family | | afdb-uniprot50 | AF-A0A7V4P0E4-F1-MODEL\_V4 | 1.0 | 3.269e-32 | 1197 | 0.291 | 442 | 285 | 9 | 72 | 502 | 2 | 426 | Phage portal protein | Phage portal protein | | afdb-uniprot50 | AF-A0A3M8C9W0-F1-MODEL\_V4 | 1.0 | 8.326e-33 | 1197 | 0.238 | 511 | 334 | 13 | 34 | 532 | 12 | 479 | Phage portal protein | Phage portal protein | | afdb-uniprot50 | AF-A0A285T1W8-F1-MODEL\_V4 | 1.0 | 3.445e-32 | 1193 | 0.248 | 471 | 327 | 13 | 35 | 502 | 6 | 452 | Phage portal protein, lambda family | Phage portal protein, lambda family | | afdb-uniprot50 | AF-A0A1G0RDT4-F1-MODEL\_V4 | 1.0 | 7.408e-34 | 1192 | 0.235 | 544 | 354 | 11 | 23 | 532 | 1 | 516 | Phage portal protein | Phage portal protein | | afdb-uniprot50 | AF-A0A0C6F7H6-F1-MODEL\_V4 | 1.0 | 6.144e-32 | 1189 | 0.266 | 462 | 313 | 13 | 45 | 502 | 15 | 454 | Portal protein | Portal protein | | afdb-uniprot50 | AF-A0A075KD33-F1-MODEL\_V4 | 1.0 | 1.337e-32 | 1187 | 0.248 | 504 | 334 | 11 | 39 | 532 | 24 | 492 | Phage portal protein, lambda family | Phage portal protein, lambda family | | afdb-uniprot50 | AF-A0A7W9SB80-F1-MODEL\_V4 | 1.0 | 8.879e-32 | 1185 | 0.224 | 495 | 353 | 10 | 23 | 504 | 2 | 478 | Lambda family phage portal protein | Lambda family phage portal protein | | afdb-uniprot50 | AF-A0A2S6ZDS2-F1-MODEL\_V4 | 1.0 | 2.648e-32 | 1185 | 0.251 | 474 | 321 | 12 | 45 | 502 | 6 | 461 | Phage portal protein | Phage portal protein | | afdb-uniprot50 | AF-A0A7T5UIB8-F1-MODEL\_V4 | 1.0 | 7.111e-33 | 1184 | 0.259 | 508 | 329 | 13 | 44 | 518 | 24 | 517 | Phage portal protein | Phage portal protein | | afdb-uniprot50 | AF-A0A0F9MU33-F1-MODEL\_V4 | 1.0 | 5.126e-34 | 1184 | 0.25 | 563 | 338 | 16 | 15 | 532 | 1 | 524 | Uncharacterized protein | Uncharacterized protein | | afdb-uniprot50 | AF-A0A2W0FY89-F1-MODEL\_V4 | 1.0 | 5.762e-33 | 1183 | 0.252 | 518 | 330 | 13 | 39 | 532 | 8 | 492 | Phage portal protein | Phage portal protein | | afdb-uniprot50 | AF-A0A1C3WPM1-F1-MODEL\_V4 | 1.0 | 1.189e-33 | 1183 | 0.25 | 531 | 342 | 14 | 13 | 532 | 4 | 489 | Phage portal protein, lambda family | Phage portal protein, lambda family | | afdb-uniprot50 | AF-A0A4U7J925-F1-MODEL\_V4 | 1.0 | 1.425e-31 | 1183 | 0.26 | 500 | 355 | 7 | 39 | 532 | 23 | 513 | Phage portal protein | Phage portal protein | | afdb-uniprot50 | AF-A0A4U6RHC0-F1-MODEL\_V4 | 1.0 | 4.978e-32 | 1181 | 0.247 | 509 | 345 | 12 | 28 | 529 | 1 | 478 | Phage portal protein | Phage portal protein | | afdb-uniprot50 | AF-D5EFB3-F1-MODEL\_V4 | 1.0 | 1.096e-31 | 1181 | 0.242 | 474 | 329 | 13 | 39 | 502 | 30 | 483 | Phage portal protein, lambda family | Phage portal protein, lambda family | | afdb-uniprot50 | AF-A0A3M1QZI3-F1-MODEL\_V4 | 1.0 | 2.648e-32 | 1181 | 0.229 | 501 | 356 | 12 | 39 | 532 | 27 | 504 | Phage portal protein | Phage portal protein | | afdb-uniprot50 | AF-A0A7S8C6A8-F1-MODEL\_V4 | 1.0 | 9.358e-32 | 1181 | 0.269 | 475 | 313 | 13 | 36 | 502 | 25 | 473 | Phage portal protein | Phage portal protein | | afdb-uniprot50 | AF-A0A2E7K4K1-F1-MODEL\_V4 | 1.0 | 2.06e-31 | 1181 | 0.243 | 480 | 332 | 13 | 35 | 502 | 20 | 480 | Phage portal protein | Phage portal protein | | afdb-uniprot50 | AF-A0A521H779-F1-MODEL\_V4 | 1.0 | 2.648e-32 | 1180 | 0.256 | 500 | 328 | 11 | 36 | 532 | 27 | 485 | Phage portal protein | Phage portal protein | | afdb-uniprot50 | AF-A0A2S4QSC7-F1-MODEL\_V4 | 1.0 | 2.908e-33 | 1180 | 0.227 | 541 | 363 | 14 | 8 | 532 | 1 | 502 | Phage portal protein | Phage portal protein | | afdb-uniprot50 | AF-A0A084XVZ6-F1-MODEL\_V4 | 1.0 | 2.384e-32 | 1179 | 0.238 | 477 | 330 | 11 | 39 | 502 | 8 | 464 | Phage portal protein, lambda family | Phage portal protein, lambda family | | afdb-uniprot50 | AF-A0A7G9NZU8-F1-MODEL\_V4 | 1.0 | 9.358e-32 | 1179 | 0.249 | 501 | 333 | 7 | 39 | 502 | 48 | 542 | Phage portal protein | Phage portal protein | | afdb-uniprot50 | AF-A0A516SAX6-F1-MODEL\_V4 | 1.0 | 1.932e-32 | 1178 | 0.266 | 492 | 331 | 12 | 23 | 502 | 1 | 474 | Phage portal protein | Phage portal protein | | afdb-uniprot50 | AF-A0A2M9EAZ3-F1-MODEL\_V4 | 1.0 | 3.445e-32 | 1177 | 0.268 | 470 | 291 | 14 | 62 | 502 | 5 | 450 | Phage portal protein | Phage portal protein | | afdb-uniprot50 | AF-A0A1E3G7C6-F1-MODEL\_V4 | 1.0 | 4.481e-32 | 1177 | 0.278 | 467 | 303 | 11 | 46 | 495 | 5 | 454 | Phage portal protein | Phage portal protein | | afdb-uniprot50 | AF-A0A073ITU9-F1-MODEL\_V4 | 1.0 | 3.589e-33 | 1176 | 0.241 | 518 | 354 | 12 | 34 | 532 | 17 | 514 | Uncharacterized protein | Uncharacterized protein | | afdb-uniprot50 | AF-A0A327Q828-F1-MODEL\_V4 | 1.0 | 7.495e-33 | 1174 | 0.264 | 510 | 334 | 15 | 36 | 531 | 23 | 505 | Lambda family phage portal protein | Lambda family phage portal protein | | afdb-uniprot50 | AF-A0A554UE06-F1-MODEL\_V4 | 1.0 | 1.954e-31 | 1174 | 0.261 | 486 | 334 | 11 | 23 | 502 | 3 | 469 | Phage portal protein | Phage portal protein | | afdb-uniprot50 | AF-A0A850IRK2-F1-MODEL\_V4 | 1.0 | 6.003e-34 | 1174 | 0.239 | 527 | 353 | 14 | 15 | 530 | 1 | 490 | Phage portal protein | Phage portal protein | | afdb-uniprot50 | AF-A0A7G6PYY7-F1-MODEL\_V4 | 1.0 | 1.217e-31 | 1172 | 0.255 | 501 | 320 | 17 | 24 | 502 | 1 | 470 | Phage portal protein | Phage portal protein | | afdb-uniprot50 | AF-F6AG24-F1-MODEL\_V4 | 1.0 | 3.827e-32 | 1172 | 0.234 | 524 | 348 | 11 | 23 | 532 | 1 | 485 | Phage portal protein, lambda family | Phage portal protein, lambda family | | afdb-uniprot50 | AF-A0A2A4ZZY4-F1-MODEL\_V4 | 1.0 | 1.565e-32 | 1172 | 0.242 | 511 | 352 | 14 | 34 | 532 | 12 | 499 | Phage portal protein | Phage portal protein | | afdb-uniprot50 | AF-A0A838Y2T1-F1-MODEL\_V4 | 1.0 | 2.679e-31 | 1170 | 0.272 | 452 | 289 | 12 | 71 | 519 | 2 | 416 | Phage portal protein | Phage portal protein | | afdb-uniprot50 | AF-Q24VH0-F1-MODEL\_V4 | 1.0 | 1.669e-31 | 1170 | 0.242 | 471 | 331 | 9 | 35 | 497 | 18 | 470 | Uncharacterized protein | Uncharacterized protein | | afdb-uniprot50 | AF-A0A6N7AIQ8-F1-MODEL\_V4 | 1.0 | 3.101e-32 | 1169 | 0.248 | 490 | 322 | 14 | 54 | 532 | 9 | 463 | Bacteriophage capsid protein | Bacteriophage capsid protein | | afdb-uniprot50 | AF-A0A0F2S3S0-F1-MODEL\_V4 | 1.0 | 1.352e-31 | 1169 | 0.274 | 459 | 304 | 12 | 54 | 502 | 4 | 443 | Phage portal protein | Phage portal protein | | afdb-uniprot50 | AF-A0A7C1RGC0-F1-MODEL\_V4 | 1.0 | 3.101e-32 | 1168 | 0.271 | 509 | 326 | 16 | 8 | 502 | 1 | 478 | Phage portal protein | Phage portal protein | | afdb-uniprot50 | AF-A0A3S0HXJ2-F1-MODEL\_V4 | 1.0 | 1.65e-32 | 1165 | 0.233 | 510 | 348 | 17 | 38 | 532 | 19 | 500 | Phage portal protein | Phage portal protein | | afdb-uniprot50 | AF-A0A839W5R8-F1-MODEL\_V4 | 1.0 | 2.513e-32 | 1165 | 0.227 | 496 | 350 | 13 | 14 | 488 | 1 | 484 | Lambda family phage portal protein | Lambda family phage portal protein | | afdb-uniprot50 | AF-A0A159Z5V5-F1-MODEL\_V4 | 1.0 | 4.669e-33 | 1164 | 0.258 | 523 | 340 | 17 | 20 | 532 | 4 | 488 | ATP-dependent Clp protease proteolytic subunit | ATP-dependent Clp protease proteolytic subunit | | afdb-uniprot50 | AF-A0A1I5C211-F1-MODEL\_V4 | 1.0 | 7.279e-31 | 1162 | 0.248 | 458 | 318 | 12 | 39 | 492 | 17 | 452 | Phage portal protein, lambda family | Phage portal protein, lambda family | | afdb-uniprot50 | AF-A0A846UIP3-F1-MODEL\_V4 | 1.0 | 1.232e-30 | 1162 | 0.212 | 471 | 344 | 12 | 39 | 496 | 14 | 470 | Lambda family phage portal protein | Lambda family phage portal protein | | afdb-uniprot50 | AF-A0A512H917-F1-MODEL\_V4 | 1.0 | 6.476e-32 | 1161 | 0.296 | 465 | 289 | 15 | 38 | 491 | 3 | 440 | Phage portal protein | Phage portal protein | | afdb-uniprot50 | AF-A0A431KJD1-F1-MODEL\_V4 | 1.0 | 3.101e-32 | 1161 | 0.234 | 499 | 342 | 13 | 39 | 517 | 21 | 499 | Phage portal protein | Phage portal protein | | afdb-uniprot50 | AF-A0A2N1QUE0-F1-MODEL\_V4 | 1.0 | 2.036e-32 | 1159 | 0.252 | 500 | 338 | 13 | 36 | 531 | 23 | 490 | Phage portal protein | Phage portal protein | | afdb-uniprot50 | AF-A0A6S5K3W5-F1-MODEL\_V4 | 1.0 | 1.65e-32 | 1157 | 0.234 | 490 | 339 | 13 | 17 | 488 | 1 | 472 | Uncharacterized protein | Uncharacterized protein | | afdb-uniprot50 | AF-A0A661TRQ9-F1-MODEL\_V4 | 1.0 | 4.302e-31 | 1157 | 0.241 | 464 | 322 | 14 | 36 | 486 | 19 | 465 | Phage portal protein | Phage portal protein | | afdb-uniprot50 | AF-V7HLM5-F1-MODEL\_V4 | 1.0 | 1.854e-31 | 1156 | 0.264 | 476 | 316 | 13 | 38 | 492 | 4 | 466 | Phage portal protein | Phage portal protein | | afdb-uniprot50 | AF-A0A1H0UU43-F1-MODEL\_V4 | 1.0 | 3.445e-32 | 1155 | 0.237 | 509 | 340 | 14 | 36 | 519 | 34 | 519 | Phage portal protein, lambda family | Phage portal protein, lambda family | | afdb-uniprot50 | AF-A0A6L7FGN1-F1-MODEL\_V4 | 1.0 | 3.101e-32 | 1154 | 0.221 | 547 | 365 | 18 | 1 | 532 | 2 | 502 | Phage portal protein | Phage portal protein | | afdb-uniprot50 | AF-R5BPP1-F1-MODEL\_V4 | 1.0 | 4.534e-31 | 1150 | 0.219 | 483 | 342 | 10 | 36 | 502 | 19 | 482 | Uncharacterized protein | Uncharacterized protein | | afdb-uniprot50 | AF-A0A354UBL5-F1-MODEL\_V4 | 1.0 | 2.412e-31 | 1149 | 0.259 | 494 | 324 | 13 | 15 | 502 | 1 | 458 | Phage portal protein | Phage portal protein | | afdb-uniprot50 | AF-A0A5E1A733-F1-MODEL\_V4 | 1.0 | 7.992e-32 | 1149 | 0.233 | 488 | 343 | 14 | 14 | 487 | 1 | 471 | Phage portal protein, lambda family | Phage portal protein, lambda family | | afdb-uniprot50 | AF-A0A7U3YJH6-F1-MODEL\_V4 | 1.0 | 4.978e-32 | 1148 | 0.225 | 514 | 353 | 16 | 39 | 532 | 29 | 517 | Phage portal protein, lambda family | Phage portal protein, lambda family | | afdb-uniprot50 | AF-A0A2A5EI97-F1-MODEL\_V4 | 1.0 | 7.194e-32 | 1147 | 0.252 | 492 | 335 | 12 | 57 | 532 | 2 | 476 | Phage portal protein | Phage portal protein | | afdb-uniprot50 | AF-A0A661RGN5-F1-MODEL\_V4 | 1.0 | 4.081e-31 | 1147 | 0.245 | 468 | 322 | 16 | 44 | 497 | 12 | 462 | Phage portal protein | Phage portal protein | | afdb-uniprot50 | AF-A0A654ALK5-F1-MODEL\_V4 | 1.0 | 1.669e-31 | 1147 | 0.225 | 502 | 345 | 12 | 39 | 532 | 21 | 486 | Portal protein | Portal protein | | afdb-uniprot50 | AF-A0A439TWD0-F1-MODEL\_V4 | 1.0 | 8.424e-32 | 1147 | 0.235 | 530 | 347 | 14 | 28 | 532 | 1 | 497 | Phage portal protein | Phage portal protein | | afdb-uniprot50 | AF-A0A1M7RIE4-F1-MODEL\_V4 | 1.0 | 8.879e-32 | 1146 | 0.244 | 498 | 329 | 13 | 39 | 532 | 38 | 492 | Phage portal protein, lambda family | Phage portal protein, lambda family | | afdb-uniprot50 | AF-A0A1Q8G4U8-F1-MODEL\_V4 | 1.0 | 2.036e-32 | 1145 | 0.22 | 513 | 350 | 14 | 34 | 532 | 12 | 488 | Uncharacterized protein | Uncharacterized protein | | afdb-uniprot50 | AF-A3WW86-F1-MODEL\_V4 | 1.0 | 3.674e-31 | 1144 | 0.27 | 470 | 311 | 13 | 36 | 502 | 5 | 445 | Probable bacteriophage-related protein | Probable bacteriophage-related protein | | afdb-uniprot50 | AF-A0A2R4BQC3-F1-MODEL\_V4 | 1.0 | 2.572e-30 | 1143 | 0.27 | 474 | 307 | 13 | 46 | 502 | 3 | 454 | Phage portal protein | Phage portal protein | | afdb-uniprot50 | AF-A0A1F3Y515-F1-MODEL\_V4 | 1.0 | 4.978e-32 | 1142 | 0.263 | 509 | 324 | 15 | 36 | 531 | 23 | 493 | Phage portal protein | Phage portal protein | | afdb-uniprot50 | AF-A0A6S6XWA1-F1-MODEL\_V4 | 1.0 | 6.144e-32 | 1141 | 0.253 | 508 | 334 | 14 | 39 | 532 | 4 | 480 | Phage portal protein | Phage portal protein | | afdb-uniprot50 | AF-D3NWR9-F1-MODEL\_V4 | 1.0 | 4.534e-31 | 1140 | 0.251 | 469 | 312 | 14 | 39 | 502 | 13 | 447 | Phage portal protein | Phage portal protein | | afdb-uniprot50 | AF-A0A413FZE1-F1-MODEL\_V4 | 1.0 | 2.44e-30 | 1140 | 0.237 | 475 | 333 | 7 | 39 | 500 | 22 | 480 | Phage portal protein | Phage portal protein | | afdb-uniprot50 | AF-H8NX02-F1-MODEL\_V4 | 1.0 | 1.602e-30 | 1138 | 0.218 | 490 | 336 | 11 | 53 | 531 | 1 | 454 | Uncharacterized protein | Uncharacterized protein | | afdb-uniprot50 | AF-B8IET8-F1-MODEL\_V4 | 1.0 | 1.502e-31 | 1138 | 0.233 | 510 | 336 | 12 | 49 | 532 | 20 | 500 | Phage portal protein, lambda family | Phage portal protein, lambda family | | afdb-uniprot50 | AF-A0A091FES5-F1-MODEL\_V4 | 1.0 | 4.534e-31 | 1137 | 0.224 | 507 | 351 | 16 | 37 | 532 | 16 | 491 | Uncharacterized protein | Uncharacterized protein | | afdb-uniprot50 | AF-A0A841S0J5-F1-MODEL\_V4 | 1.0 | 5.898e-31 | 1137 | 0.225 | 488 | 339 | 13 | 34 | 502 | 12 | 479 | Lambda family phage portal protein | Lambda family phage portal protein | | afdb-uniprot50 | AF-A0A7U0SE26-F1-MODEL\_V4 | 1.0 | 6.216e-31 | 1135 | 0.241 | 510 | 340 | 11 | 37 | 532 | 7 | 483 | Phage portal protein | Phage portal protein | | afdb-uniprot50 | AF-A0A3L8Q0N2-F1-MODEL\_V4 | 1.0 | 2.857e-30 | 1135 | 0.219 | 501 | 351 | 10 | 39 | 531 | 24 | 492 | Phage portal protein | Phage portal protein | | afdb-uniprot50 | AF-A0A2K4MTR6-F1-MODEL\_V4 | 1.0 | 5.829e-32 | 1135 | 0.247 | 513 | 326 | 14 | 35 | 532 | 18 | 485 | Phage portal protein | Phage portal protein | | afdb-uniprot50 | AF-A0A439EQB9-F1-MODEL\_V4 | 1.0 | 5.531e-32 | 1131 | 0.467 | 340 | 165 | 9 | 7 | 338 | 9 | 340 | Phage portal protein | Phage portal protein | | afdb-uniprot50 | AF-A0A1H2N8A6-F1-MODEL\_V4 | 1.0 | 2.679e-31 | 1131 | 0.208 | 518 | 376 | 13 | 32 | 532 | 10 | 510 | Phage portal protein | Phage portal protein | | afdb-uniprot50 | AF-A0A0T5PNS0-F1-MODEL\_V4 | 1.0 | 6.987e-30 | 1130 | 0.235 | 459 | 325 | 10 | 46 | 491 | 22 | 467 | Uncharacterized protein | Uncharacterized protein | | afdb-uniprot50 | AF-A0A810UW02-F1-MODEL\_V4 | 1.0 | 6.144e-32 | 1129 | 0.243 | 484 | 334 | 12 | 3 | 473 | 1 | 465 | Phage portal protein | Phage portal protein | | afdb-uniprot50 | AF-A0A379VWB5-F1-MODEL\_V4 | 1.0 | 2.824e-31 | 1129 | 0.239 | 493 | 329 | 14 | 54 | 532 | 6 | 466 | Phage portal protein | Phage portal protein | | afdb-uniprot50 | AF-A0A235H652-F1-MODEL\_V4 | 1.0 | 1.954e-31 | 1128 | 0.241 | 502 | 338 | 14 | 21 | 502 | 8 | 486 | Phage portal protein | Phage portal protein | | afdb-uniprot50 | AF-A0A081RFJ6-F1-MODEL\_V4 | 1.0 | 1.217e-31 | 1127 | 0.252 | 510 | 320 | 17 | 35 | 531 | 13 | 474 | Phage portal protein, lambda family | Phage portal protein, lambda family | | afdb-uniprot50 | AF-A0A843YX80-F1-MODEL\_V4 | 1.0 | 6.552e-31 | 1127 | 0.22 | 535 | 349 | 17 | 17 | 532 | 1 | 486 | Phage portal protein | Phage portal protein | | afdb-uniprot50 | AF-A0A7K0GNI4-F1-MODEL\_V4 | 1.0 | 5.967e-30 | 1127 | 0.238 | 462 | 327 | 11 | 46 | 497 | 23 | 469 | Phage portal protein | Phage portal protein | | afdb-uniprot50 | AF-A0A6G2BPX3-F1-MODEL\_V4 | 1.0 | 3.631e-32 | 1127 | 0.206 | 519 | 366 | 15 | 1 | 491 | 1 | 501 | Phage portal protein | Phage portal protein | | afdb-uniprot50 | AF-A0A3S0EHX2-F1-MODEL\_V4 | 1.0 | 6.476e-32 | 1126 | 0.242 | 527 | 347 | 16 | 36 | 532 | 39 | 543 | Phage portal protein | Phage portal protein | | afdb-uniprot50 | AF-A0A1Q4UUL1-F1-MODEL\_V4 | 1.0 | 1.876e-30 | 1125 | 0.373 | 410 | 219 | 8 | 130 | 532 | 2 | 380 | Uncharacterized protein | Uncharacterized protein | | afdb-uniprot50 | AF-A0A1V5WQS9-F1-MODEL\_V4 | 1.0 | 9.468e-31 | 1125 | 0.264 | 476 | 305 | 15 | 39 | 502 | 32 | 474 | Phage portal protein, lambda family | Phage portal protein, lambda family | | afdb-uniprot50 | AF-A0A7V1K4N8-F1-MODEL\_V4 | 1.0 | 8.326e-33 | 1125 | 0.218 | 527 | 358 | 15 | 1 | 495 | 10 | 514 | Phage portal protein | Phage portal protein | | afdb-uniprot50 | AF-A0A6J4FCQ3-F1-MODEL\_V4 | 1.0 | 6.552e-31 | 1123 | 0.234 | 507 | 334 | 14 | 39 | 531 | 5 | 471 | Bacteriophage capsid protein (Modular protein) | Bacteriophage capsid protein (Modular protein) | | afdb-uniprot50 | AF-A0A258LKW1-F1-MODEL\_V4 | 1.0 | 1.246e-29 | 1121 | 0.227 | 470 | 326 | 12 | 36 | 491 | 6 | 452 | Phage portal protein | Phage portal protein | | afdb-uniprot50 | AF-A0A560F1W8-F1-MODEL\_V4 | 1.0 | 5.829e-32 | 1120 | 0.244 | 557 | 350 | 15 | 1 | 532 | 6 | 516 | Lambda family phage portal protein | Lambda family phage portal protein | | afdb-uniprot50 | AF-A0A3S0V4X8-F1-MODEL\_V4 | 1.0 | 7.279e-31 | 1118 | 0.234 | 512 | 361 | 12 | 1 | 502 | 76 | 566 | Phage portal protein | Phage portal protein | | afdb-uniprot50 | AF-A0A4Y8MZU1-F1-MODEL\_V4 | 1.0 | 2.222e-29 | 1117 | 0.252 | 472 | 322 | 13 | 39 | 502 | 7 | 455 | Phage portal protein | Phage portal protein | | afdb-uniprot50 | AF-A0A1T5K0T3-F1-MODEL\_V4 | 1.0 | 1.689e-30 | 1117 | 0.227 | 475 | 341 | 14 | 36 | 502 | 13 | 469 | Phage portal protein, lambda family | Phage portal protein, lambda family | | afdb-uniprot50 | AF-A0A8B3PYM4-F1-MODEL\_V4 | 1.0 | 2.412e-31 | 1116 | 0.228 | 512 | 334 | 15 | 38 | 532 | 3 | 470 | Phage portal protein | Phage portal protein | | afdb-uniprot50 | AF-A0A2N9AI26-F1-MODEL\_V4 | 1.0 | 1.409e-32 | 1116 | 0.237 | 568 | 357 | 19 | 1 | 532 | 726 | 1253 | Terminase GpA (Modular protein) | Terminase GpA (Modular protein) | | afdb-uniprot50 | AF-A0A411X2P3-F1-MODEL\_V4 | 1.0 | 2.572e-30 | 1114 | 0.225 | 497 | 358 | 13 | 35 | 523 | 26 | 503 | Phage portal protein | Phage portal protein | | afdb-uniprot50 | AF-A0A363UL99-F1-MODEL\_V4 | 1.0 | 1.052e-30 | 1113 | 0.207 | 506 | 357 | 13 | 39 | 532 | 55 | 528 | Phage portal protein | Phage portal protein | | afdb-uniprot50 | AF-A0A142Y555-F1-MODEL\_V4 | 1.0 | 8.424e-32 | 1113 | 0.218 | 531 | 366 | 14 | 35 | 523 | 18 | 541 | Phage portal protein, lambda family | Phage portal protein, lambda family | | afdb-uniprot50 | AF-A0A1V5QSP9-F1-MODEL\_V4 | 1.0 | 1.854e-31 | 1113 | 0.254 | 535 | 340 | 15 | 15 | 524 | 1 | 501 | Phage portal protein, lambda family | Phage portal protein, lambda family | | afdb-uniprot50 | AF-A0A6L6JH12-F1-MODEL\_V4 | 1.0 | 1.298e-30 | 1112 | 0.48 | 312 | 154 | 4 | 2 | 309 | 1 | 308 | Phage portal protein | Phage portal protein | | afdb-uniprot50 | AF-A0A071LT14-F1-MODEL\_V4 | 1.0 | 1.442e-30 | 1110 | 0.227 | 488 | 349 | 11 | 17 | 498 | 1 | 466 | Uncharacterized protein | Uncharacterized protein | | afdb-uniprot50 | AF-A0A0Q0VFZ3-F1-MODEL\_V4 | 1.0 | 8.623e-30 | 1110 | 0.237 | 502 | 339 | 12 | 37 | 531 | 26 | 490 | Uncharacterized protein | Uncharacterized protein | | afdb-uniprot50 | AF-B3G2H0-F1-MODEL\_V4 | 1.0 | 8.424e-32 | 1110 | 0.222 | 543 | 378 | 14 | 1 | 517 | 92 | 616 | Phage portal protein | Phage portal protein | | afdb-uniprot50 | AF-A0A7Z0SJA8-F1-MODEL\_V4 | 1.0 | 1.854e-31 | 1109 | 0.228 | 529 | 345 | 16 | 23 | 530 | 2 | 488 | Phage portal protein | Phage portal protein | | afdb-uniprot50 | AF-A0A2M8G061-F1-MODEL\_V4 | 1.0 | 8.623e-30 | 1108 | 0.269 | 467 | 308 | 14 | 36 | 497 | 38 | 476 | Phage portal protein | Phage portal protein | | afdb-uniprot50 | AF-A0A1F4FSB3-F1-MODEL\_V4 | 1.0 | 3.526e-30 | 1107 | 0.245 | 473 | 311 | 13 | 39 | 502 | 28 | 463 | Phage portal protein | Phage portal protein | | afdb-uniprot50 | AF-A0A2D7VZC3-F1-MODEL\_V4 | 1.0 | 3.526e-30 | 1106 | 0.287 | 441 | 283 | 9 | 100 | 532 | 2 | 419 | Phage portal protein | Phage portal protein | | afdb-uniprot50 | AF-A0A2E5F847-F1-MODEL\_V4 | 1.0 | 4.081e-31 | 1106 | 0.24 | 503 | 331 | 15 | 46 | 531 | 25 | 493 | Phage portal protein | Phage portal protein | | afdb-uniprot50 | AF-A0A0G1R5P6-F1-MODEL\_V4 | 1.0 | 1.368e-30 | 1106 | 0.22 | 509 | 353 | 16 | 42 | 532 | 7 | 489 | Phage portal protein, lambda family | Phage portal protein, lambda family | | afdb-uniprot50 | AF-E0NXK2-F1-MODEL\_V4 | 1.0 | 1.368e-30 | 1106 | 0.219 | 505 | 361 | 11 | 39 | 532 | 22 | 504 | Phage portal protein, lambda family | Phage portal protein, lambda family | | afdb-uniprot50 | AF-A0A7W6WBD4-F1-MODEL\_V4 | 1.0 | 1.78e-30 | 1103 | 0.234 | 512 | 339 | 15 | 39 | 532 | 10 | 486 | Lambda family phage portal protein | Lambda family phage portal protein | | afdb-uniprot50 | AF-A0A2P7S021-F1-MODEL\_V4 | 1.0 | 2.469e-29 | 1100 | 0.257 | 462 | 309 | 13 | 46 | 502 | 2 | 434 | Phage portal protein | Phage portal protein | | afdb-uniprot50 | AF-A0A480BUD5-F1-MODEL\_V4 | 1.0 | 7.364e-30 | 1100 | 0.215 | 477 | 339 | 14 | 39 | 502 | 34 | 488 | Phage portal protein | Phage portal protein | | afdb-uniprot50 | AF-A0A2V4FUZ4-F1-MODEL\_V4 | 1.0 | 6.144e-32 | 1100 | 0.232 | 550 | 359 | 15 | 1 | 532 | 2 | 506 | Phage portal protein | Phage portal protein | | afdb-uniprot50 | AF-A0A858X372-F1-MODEL\_V4 | 1.0 | 1.689e-30 | 1099 | 0.271 | 461 | 298 | 13 | 44 | 502 | 20 | 444 | Phage portal protein | Phage portal protein | | afdb-uniprot50 | AF-C6BVY9-F1-MODEL\_V4 | 1.0 | 4.835e-30 | 1099 | 0.245 | 476 | 326 | 10 | 39 | 502 | 28 | 482 | Phage portal protein, lambda family | Phage portal protein, lambda family | | afdb-uniprot50 | AF-A0A3S2UY29-F1-MODEL\_V4 | 1.0 | 4.129e-30 | 1098 | 0.224 | 508 | 347 | 15 | 29 | 532 | 1 | 465 | Phage portal protein | Phage portal protein | | afdb-uniprot50 | AF-A0A4R6GV31-F1-MODEL\_V4 | 1.0 | 4.352e-30 | 1098 | 0.239 | 471 | 315 | 13 | 46 | 502 | 10 | 451 | Lambda family phage portal protein | Lambda family phage portal protein | | afdb-uniprot50 | AF-A0A3D2ITU5-F1-MODEL\_V4 | 1.0 | 4.129e-30 | 1098 | 0.23 | 494 | 344 | 12 | 35 | 519 | 18 | 484 | Phage portal protein | Phage portal protein | | afdb-uniprot50 | AF-A0A5P9H3D4-F1-MODEL\_V4 | 1.0 | 8.181e-30 | 1096 | 0.271 | 446 | 288 | 13 | 62 | 502 | 3 | 416 | Phage portal protein, lambda family | Phage portal protein, lambda family | | afdb-uniprot50 | AF-A0A1J5JBS4-F1-MODEL\_V4 | 1.0 | 6.906e-31 | 1096 | 0.272 | 466 | 289 | 13 | 83 | 532 | 2 | 433 | Phage portal protein | Phage portal protein | | afdb-uniprot50 | AF-F2K1G2-F1-MODEL\_V4 | 1.0 | 2.315e-30 | 1096 | 0.208 | 498 | 359 | 10 | 38 | 515 | 9 | 491 | Phage portal protein, lambda family | Phage portal protein, lambda family | | afdb-uniprot50 | AF-A0A348SU45-F1-MODEL\_V4 | 1.0 | 3.346e-30 | 1095 | 0.24 | 474 | 317 | 13 | 46 | 502 | 5 | 452 | Phage portal protein | Phage portal protein | | afdb-uniprot50 | AF-A0A6N6T1F2-F1-MODEL\_V4 | 1.0 | 7.762e-30 | 1095 | 0.24 | 495 | 331 | 12 | 26 | 502 | 2 | 469 | Phage portal protein | Phage portal protein | | afdb-uniprot50 | AF-A0A7C1N2Z9-F1-MODEL\_V4 | 1.0 | 2.572e-30 | 1093 | 0.219 | 505 | 349 | 14 | 39 | 531 | 39 | 510 | Phage portal protein | Phage portal protein | | afdb-uniprot50 | AF-A0A7C1Y793-F1-MODEL\_V4 | 1.0 | 1.459e-29 | 1093 | 0.236 | 478 | 326 | 15 | 42 | 496 | 3 | 464 | Phage portal protein | Phage portal protein | | afdb-uniprot50 | AF-A0A5P9JT86-F1-MODEL\_V4 | 1.0 | 1.109e-30 | 1092 | 0.247 | 513 | 345 | 15 | 1 | 502 | 4 | 486 | Phage portal protein | Phage portal protein | | afdb-uniprot50 | AF-A0A098ARS2-F1-MODEL\_V4 | 1.0 | 5.727e-29 | 1091 | 0.269 | 423 | 278 | 10 | 37 | 446 | 4 | 408 | Portal protein, lambda family,phage portal protein, lambda family,Phage portal protein, lambda family | Portal protein, lambda family,phage portal protein, lambda family,Phage portal protein, lambda family | | afdb-uniprot50 | AF-A0A1M7R7P3-F1-MODEL\_V4 | 1.0 | 2.084e-30 | 1091 | 0.225 | 509 | 339 | 15 | 39 | 532 | 29 | 497 | Phage portal protein, lambda family | Phage portal protein, lambda family | | afdb-uniprot50 | AF-A0A524RVZ8-F1-MODEL\_V4 | 1.0 | 1.096e-31 | 1091 | 0.227 | 589 | 351 | 17 | 1 | 502 | 19 | 590 | Phage portal protein | Phage portal protein | | afdb-uniprot50 | AF-A0A106BVU9-F1-MODEL\_V4 | 1.0 | 3.963e-29 | 1090 | 0.233 | 462 | 311 | 13 | 47 | 487 | 6 | 445 | Uncharacterized protein | Uncharacterized protein | | afdb-uniprot50 | AF-A0A6N9P4E6-F1-MODEL\_V4 | 1.0 | 3.346e-30 | 1088 | 0.232 | 500 | 351 | 11 | 39 | 532 | 24 | 496 | Phage portal protein | Phage portal protein | | afdb-uniprot50 | AF-A0A2G2DPB9-F1-MODEL\_V4 | 1.0 | 2.108e-29 | 1087 | 0.205 | 525 | 361 | 15 | 20 | 530 | 2 | 484 | Phage portal protein | Phage portal protein | | afdb-uniprot50 | AF-A0A0H4W134-F1-MODEL\_V4 | 1.0 | 2.146e-32 | 1085 | 0.256 | 558 | 319 | 15 | 39 | 524 | 4 | 537 | Phage portal protein, lambda family | Phage portal protein, lambda family | | afdb-uniprot50 | AF-A9C2C6-F1-MODEL\_V4 | 1.0 | 4.534e-31 | 1085 | 0.225 | 542 | 353 | 17 | 21 | 532 | 16 | 520 | Phage portal protein, lambda family | Phage portal protein, lambda family | | afdb-uniprot50 | AF-A0A0F2NL12-F1-MODEL\_V4 | 1.0 | 1.538e-29 | 1084 | 0.238 | 470 | 326 | 17 | 46 | 502 | 1 | 451 | Uncharacterized protein | Uncharacterized protein | | afdb-uniprot50 | AF-A0A4Q1AN27-F1-MODEL\_V4 | 1.0 | 5.434e-29 | 1084 | 0.201 | 501 | 359 | 10 | 30 | 508 | 1 | 482 | Phage portal protein | Phage portal protein | | afdb-uniprot50 | AF-A0A151FKE0-F1-MODEL\_V4 | 1.0 | 7.762e-30 | 1083 | 0.251 | 466 | 305 | 11 | 77 | 532 | 2 | 433 | Uncharacterized protein | Uncharacterized protein | | afdb-uniprot50 | AF-A0A7T5FFX4-F1-MODEL\_V4 | 1.0 | 6.289e-30 | 1082 | 0.224 | 500 | 332 | 15 | 46 | 532 | 23 | 479 | Phage portal protein | Phage portal protein | | afdb-uniprot50 | AF-A0A2N0WWU8-F1-MODEL\_V4 | 1.0 | 1.92e-28 | 1082 | 0.247 | 460 | 322 | 12 | 39 | 491 | 21 | 463 | Phage portal protein | Phage portal protein | | afdb-uniprot50 | AF-A0A1D8IV87-F1-MODEL\_V4 | 1.0 | 6.216e-31 | 1082 | 0.22 | 504 | 326 | 14 | 57 | 532 | 2 | 466 | Phage portal protein | Phage portal protein | | afdb-uniprot50 | AF-X6D259-F1-MODEL\_V4 | 1.0 | 3.917e-30 | 1080 | 0.262 | 483 | 314 | 12 | 36 | 502 | 25 | 481 | Uncharacterized protein | Uncharacterized protein | | afdb-uniprot50 | AF-A0A2E9SLJ8-F1-MODEL\_V4 | 1.0 | 2.572e-30 | 1078 | 0.25 | 496 | 309 | 17 | 14 | 495 | 1 | 447 | Phage portal protein | Phage portal protein | | afdb-uniprot50 | AF-A0A059KU09-F1-MODEL\_V4 | 1.0 | 1.442e-30 | 1078 | 0.209 | 520 | 359 | 15 | 32 | 519 | 10 | 509 | Capsid protein | Capsid protein | | afdb-uniprot50 | AF-A0A1E3G798-F1-MODEL\_V4 | 1.0 | 5.371e-30 | 1077 | 0.218 | 504 | 351 | 15 | 23 | 502 | 1 | 485 | Phage portal protein | Phage portal protein | | afdb-uniprot50 | AF-A0A0A8K3F6-F1-MODEL\_V4 | 1.0 | 3.211e-29 | 1076 | 0.247 | 464 | 316 | 16 | 29 | 487 | 1 | 436 | Uncharacterized protein | Uncharacterized protein | | afdb-uniprot50 | AF-A0A368TQV2-F1-MODEL\_V4 | 1.0 | 6.906e-31 | 1076 | 0.217 | 574 | 368 | 16 | 1 | 532 | 127 | 661 | Phage portal protein | Phage portal protein | | afdb-uniprot50 | AF-A4N155-F1-MODEL\_V4 | 1.0 | 1.476e-28 | 1075 | 0.227 | 436 | 312 | 12 | 39 | 464 | 21 | 441 | Bacteriophage capsid protein | Bacteriophage capsid protein | | afdb-uniprot50 | AF-A0A1T4W4N3-F1-MODEL\_V4 | 1.0 | 1.801e-29 | 1075 | 0.229 | 527 | 343 | 19 | 26 | 532 | 2 | 485 | Phage portal protein, lambda family | Phage portal protein, lambda family | | afdb-uniprot50 | AF-A0A5A7N0P2-F1-MODEL\_V4 | 1.0 | 4.835e-30 | 1075 | 0.264 | 476 | 305 | 15 | 35 | 491 | 15 | 464 | Phage portal protein | Phage portal protein | | afdb-uniprot50 | AF-A0A410P784-F1-MODEL\_V4 | 1.0 | 2.711e-30 | 1074 | 0.23 | 521 | 341 | 16 | 39 | 532 | 5 | 492 | Phage portal protein | Phage portal protein | | afdb-uniprot50 | AF-A0A765BM50-F1-MODEL\_V4 | 1.0 | 4.455e-28 | 1073 | 0.213 | 440 | 325 | 10 | 39 | 472 | 21 | 445 | Phage portal protein | Phage portal protein | | afdb-uniprot50 | AF-B6WS36-F1-MODEL\_V4 | 1.0 | 3.917e-30 | 1073 | 0.232 | 538 | 353 | 16 | 14 | 529 | 1 | 500 | Phage portal protein, lambda family | Phage portal protein, lambda family | | afdb-uniprot50 | AF-A0A521CT40-F1-MODEL\_V4 | 1.0 | 1.246e-29 | 1071 | 0.22 | 498 | 341 | 15 | 39 | 532 | 24 | 478 | Phage portal protein, lambda family | Phage portal protein, lambda family | | afdb-uniprot50 | AF-A0A6N3TG59-F1-MODEL\_V4 | 1.0 | 1.476e-28 | 1071 | 0.272 | 463 | 307 | 12 | 39 | 497 | 28 | 464 | Phage portal protein | Phage portal protein | | afdb-uniprot50 | AF-A0A0S9N407-F1-MODEL\_V4 | 1.0 | 6.289e-30 | 1070 | 0.35 | 428 | 238 | 12 | 125 | 532 | 2 | 409 | Uncharacterized protein | Uncharacterized protein | | afdb-uniprot50 | AF-A0A0A1W3J4-F1-MODEL\_V4 | 1.0 | 1.196e-28 | 1069 | 0.27 | 462 | 294 | 14 | 46 | 502 | 1 | 424 | Uncharacterized protein | Uncharacterized protein | | afdb-uniprot50 | AF-A0A562C7Q2-F1-MODEL\_V4 | 1.0 | 9.979e-31 | 1069 | 0.223 | 520 | 348 | 16 | 34 | 532 | 12 | 496 | Lambda family phage portal protein | Lambda family phage portal protein | | afdb-uniprot50 | AF-A0A1B1A8L3-F1-MODEL\_V4 | 1.0 | 1.077e-28 | 1068 | 0.235 | 463 | 321 | 12 | 36 | 490 | 25 | 462 | Uncharacterized protein | Uncharacterized protein | | afdb-uniprot50 | AF-A0A248JRM8-F1-MODEL\_V4 | 1.0 | 2.222e-29 | 1068 | 0.237 | 471 | 314 | 14 | 46 | 502 | 47 | 486 | Phage portal protein | Phage portal protein | | afdb-uniprot50 | AF-A0A3D1NR46-F1-MODEL\_V4 | 1.0 | 1.135e-28 | 1067 | 0.22 | 485 | 333 | 13 | 23 | 502 | 1 | 445 | Phage portal protein | Phage portal protein | | afdb-uniprot50 | AF-E2CJU7-F1-MODEL\_V4 | 1.0 | 9.691e-29 | 1066 | 0.228 | 472 | 327 | 10 | 44 | 497 | 12 | 464 | Phage portal protein, lambda family | Phage portal protein, lambda family | | afdb-uniprot50 | AF-A0A1M6Q0A4-F1-MODEL\_V4 | 1.0 | 1.232e-30 | 1064 | 0.247 | 533 | 348 | 15 | 2 | 530 | 1 | 484 | Phage portal protein, lambda family | Phage portal protein, lambda family | | afdb-uniprot50 | AF-A0A640W790-F1-MODEL\_V4 | 1.0 | 9.579e-30 | 1064 | 0.186 | 520 | 367 | 12 | 36 | 532 | 19 | 505 | Phage portal protein | Phage portal protein | | afdb-uniprot50 | AF-A0A1V5D6K6-F1-MODEL\_V4 | 1.0 | 1.52e-30 | 1064 | 0.232 | 520 | 349 | 14 | 39 | 532 | 33 | 528 | Phage portal protein, lambda family | Phage portal protein, lambda family | | afdb-uniprot50 | AF-E6X1P5-F1-MODEL\_V4 | 1.0 | 7.45e-29 | 1063 | 0.227 | 496 | 333 | 14 | 17 | 502 | 1 | 456 | Phage portal protein, lambda family | Phage portal protein, lambda family | | afdb-uniprot50 | AF-A0A6I6GTE6-F1-MODEL\_V4 | 1.0 | 7.762e-30 | 1061 | 0.214 | 523 | 371 | 16 | 32 | 532 | 10 | 514 | Phage portal protein | Phage portal protein | | afdb-uniprot50 | AF-A0A255YQI4-F1-MODEL\_V4 | 1.0 | 6.629e-30 | 1057 | 0.218 | 512 | 353 | 12 | 7 | 502 | 1 | 481 | Phage portal protein | Phage portal protein | | afdb-uniprot50 | AF-A0A5C7SHE2-F1-MODEL\_V4 | 1.0 | 3.917e-30 | 1056 | 0.22 | 536 | 362 | 17 | 20 | 532 | 27 | 529 | Phage portal protein | Phage portal protein | | afdb-uniprot50 | AF-A0A5C7LKW9-F1-MODEL\_V4 | 1.0 | 4.403e-29 | 1055 | 0.245 | 473 | 327 | 14 | 39 | 502 | 21 | 472 | Phage portal protein | Phage portal protein | | afdb-uniprot50 | AF-A0A085EXP1-F1-MODEL\_V4 | 1.0 | 6.363e-29 | 1055 | 0.234 | 481 | 324 | 14 | 36 | 502 | 28 | 478 | Putative Phage portal protein, lambda family | Putative Phage portal protein, lambda family | | afdb-uniprot50 | AF-A0A4R2P5J0-F1-MODEL\_V4 | 1.0 | 1.261e-28 | 1053 | 0.252 | 471 | 315 | 14 | 42 | 491 | 5 | 459 | Lambda family phage portal protein | Lambda family phage portal protein | | afdb-uniprot50 | AF-A0A524ITH3-F1-MODEL\_V4 | 1.0 | 1.801e-29 | 1053 | 0.233 | 509 | 334 | 16 | 35 | 532 | 22 | 485 | Phage portal protein | Phage portal protein | | afdb-uniprot50 | AF-A0A1F9BJ96-F1-MODEL\_V4 | 1.0 | 1.384e-29 | 1053 | 0.229 | 487 | 327 | 16 | 39 | 502 | 33 | 494 | Phage portal protein | Phage portal protein | | afdb-uniprot50 | AF-A0A1H2I371-F1-MODEL\_V4 | 1.0 | 2.775e-28 | 1052 | 0.25 | 472 | 320 | 11 | 39 | 498 | 19 | 468 | Phage portal protein, lambda family | Phage portal protein, lambda family | | afdb-uniprot50 | AF-A0A0S2KEQ2-F1-MODEL\_V4 | 1.0 | 9.195e-29 | 1048 | 0.241 | 501 | 333 | 13 | 36 | 532 | 24 | 481 | Phage portal protein, lambda | Phage portal protein, lambda | | afdb-uniprot50 | AF-A0A7R6ZZ48-F1-MODEL\_V4 | 1.0 | 6.108e-28 | 1047 | 0.194 | 488 | 333 | 13 | 42 | 519 | 5 | 442 | Uncharacterized protein | Uncharacterized protein | | afdb-uniprot50 | AF-A0A3B9ZLI4-F1-MODEL\_V4 | 1.0 | 2.469e-29 | 1047 | 0.25 | 491 | 329 | 15 | 36 | 502 | 34 | 509 | Phage portal protein | Phage portal protein | | afdb-uniprot50 | AF-B6IPY1-F1-MODEL\_V4 | 1.0 | 1.401e-28 | 1045 | 0.271 | 476 | 309 | 17 | 36 | 502 | 25 | 471 | Phage portal protein, lambda family | Phage portal protein, lambda family | | afdb-uniprot50 | AF-A0A345DE62-F1-MODEL\_V4 | 1.0 | 9.579e-30 | 1045 | 0.195 | 533 | 365 | 17 | 13 | 532 | 2 | 483 | Uncharacterized protein | Uncharacterized protein | | afdb-uniprot50 | AF-A0A7D7PEZ6-F1-MODEL\_V4 | 1.0 | 1.898e-29 | 1043 | 0.201 | 552 | 380 | 19 | 9 | 532 | 1 | 519 | Phage portal protein | Phage portal protein | | afdb-uniprot50 | AF-A0A4R2R7K8-F1-MODEL\_V4 | 1.0 | 7.538e-28 | 1042 | 0.263 | 433 | 294 | 11 | 74 | 502 | 2 | 413 | Lambda family phage portal protein | Lambda family phage portal protein | | afdb-uniprot50 | AF-A0A2E2DF85-F1-MODEL\_V4 | 1.0 | 1.92e-28 | 1042 | 0.239 | 472 | 321 | 15 | 36 | 502 | 23 | 461 | Phage portal protein | Phage portal protein | | afdb-uniprot50 | AF-A0A494X0D8-F1-MODEL\_V4 | 1.0 | 2.248e-28 | 1041 | 0.246 | 463 | 310 | 16 | 36 | 488 | 23 | 456 | Phage portal protein | Phage portal protein | | afdb-uniprot50 | AF-A0A2E3R5Z3-F1-MODEL\_V4 | 1.0 | 4.891e-29 | 1040 | 0.215 | 505 | 352 | 14 | 47 | 532 | 28 | 507 | Phage portal protein | Phage portal protein | | afdb-uniprot50 | AF-A0A3T0L2W9-F1-MODEL\_V4 | 1.0 | 2.498e-28 | 1038 | 0.226 | 477 | 334 | 15 | 39 | 497 | 29 | 488 | Phage portal protein | Phage portal protein | | afdb-uniprot50 | AF-A0A4R1Q774-F1-MODEL\_V4 | 1.0 | 6.289e-30 | 1038 | 0.228 | 521 | 332 | 12 | 37 | 532 | 31 | 506 | Lambda family phage portal protein | Lambda family phage portal protein | | afdb-uniprot50 | AF-E4LA53-F1-MODEL\_V4 | 1.0 | 2.342e-29 | 1038 | 0.221 | 501 | 352 | 11 | 39 | 532 | 28 | 497 | Phage portal protein, lambda family | Phage portal protein, lambda family | | afdb-uniprot50 | AF-A0A352L2C6-F1-MODEL\_V4 | 1.0 | 3.917e-30 | 1038 | 0.225 | 515 | 346 | 17 | 36 | 532 | 42 | 521 | Phage portal protein | Phage portal protein | | afdb-uniprot50 | AF-A0A844HP12-F1-MODEL\_V4 | 1.0 | 5.156e-29 | 1034 | 0.244 | 504 | 335 | 14 | 39 | 532 | 30 | 497 | Phage portal protein | Phage portal protein | | afdb-uniprot50 | AF-A0A5B9Y8G9-F1-MODEL\_V4 | 1.0 | 7.364e-30 | 1034 | 0.243 | 502 | 337 | 16 | 36 | 500 | 23 | 518 | Phage portal protein | Phage portal protein | | afdb-uniprot50 | AF-A0A2S6H5A5-F1-MODEL\_V4 | 1.0 | 9.691e-29 | 1033 | 0.246 | 466 | 306 | 15 | 45 | 502 | 9 | 437 | Lambda family phage portal protein | Lambda family phage portal protein | | afdb-uniprot50 | AF-A0A5C1B6Q3-F1-MODEL\_V4 | 1.0 | 3.249e-28 | 1033 | 0.222 | 476 | 333 | 13 | 36 | 502 | 27 | 474 | Phage portal protein | Phage portal protein | | afdb-uniprot50 | AF-A0A1V5E5F9-F1-MODEL\_V4 | 1.0 | 1.021e-28 | 1033 | 0.231 | 488 | 336 | 14 | 39 | 502 | 39 | 511 | Phage portal protein, lambda family | Phage portal protein, lambda family | | afdb-uniprot50 | AF-A0A268TNZ2-F1-MODEL\_V4 | 1.0 | 7.45e-29 | 1032 | 0.195 | 531 | 361 | 15 | 15 | 530 | 1 | 480 | Phage portal protein | Phage portal protein | | afdb-uniprot50 | AF-A0A4U8YLR7-F1-MODEL\_V4 | 1.0 | 1.077e-28 | 1030 | 0.222 | 481 | 323 | 12 | 45 | 502 | 44 | 496 | Phage portal protein lambda family | Phage portal protein lambda family | | afdb-uniprot50 | AF-A0A6P1EIH4-F1-MODEL\_V4 | 1.0 | 9.195e-29 | 1030 | 0.206 | 465 | 328 | 13 | 60 | 502 | 22 | 467 | Phage portal protein | Phage portal protein | | afdb-uniprot50 | AF-A0A7C5BTM8-F1-MODEL\_V4 | 1.0 | 2.024e-28 | 1029 | 0.247 | 488 | 316 | 17 | 18 | 502 | 4 | 443 | Phage portal protein | Phage portal protein | | afdb-uniprot50 | AF-A0A2E6KND4-F1-MODEL\_V4 | 1.0 | 1.476e-28 | 1029 | 0.241 | 505 | 343 | 14 | 39 | 532 | 25 | 500 | Phage portal protein | Phage portal protein | | afdb-uniprot50 | AF-A0A2A4V1I6-F1-MODEL\_V4 | 1.0 | 9.195e-29 | 1028 | 0.217 | 511 | 357 | 14 | 35 | 532 | 21 | 501 | Phage portal protein | Phage portal protein | | afdb-uniprot50 | AF-A0A037UQ03-F1-MODEL\_V4 | 1.0 | 6.363e-29 | 1028 | 0.236 | 482 | 323 | 16 | 37 | 502 | 28 | 480 | Portal protein | Portal protein | | afdb-uniprot50 | AF-A0A076LEP4-F1-MODEL\_V4 | 1.0 | 8.374e-28 | 1026 | 0.215 | 463 | 322 | 12 | 78 | 532 | 2 | 431 | Phage portal protein | Phage portal protein | | afdb-uniprot50 | AF-A0A3D1J2Q7-F1-MODEL\_V4 | 1.0 | 1.459e-29 | 1025 | 0.269 | 490 | 309 | 19 | 35 | 502 | 23 | 485 | Phage portal protein | Phage portal protein | | afdb-uniprot50 | AF-A0A4P7EGN2-F1-MODEL\_V4 | 1.0 | 1.261e-28 | 1024 | 0.237 | 501 | 339 | 16 | 36 | 523 | 25 | 495 | Phage portal protein | Phage portal protein | | afdb-uniprot50 | AF-A0A1L9BVE0-F1-MODEL\_V4 | 1.0 | 1.556e-28 | 1023 | 0.206 | 509 | 357 | 21 | 39 | 532 | 21 | 497 | Portal protein | Portal protein | | afdb-uniprot50 | AF-A0A1E3ZLT6-F1-MODEL\_V4 | 1.0 | 2.222e-29 | 1022 | 0.245 | 501 | 318 | 19 | 39 | 517 | 18 | 480 | Phage portal protein | Phage portal protein | | afdb-uniprot50 | AF-L7U545-F1-MODEL\_V4 | 1.0 | 9.195e-29 | 1022 | 0.225 | 519 | 344 | 15 | 34 | 532 | 13 | 493 | Phage portal protein | Phage portal protein | | afdb-uniprot50 | AF-G0ER26-F1-MODEL\_V4 | 1.0 | 8.724e-29 | 1021 | 0.253 | 477 | 311 | 15 | 36 | 502 | 18 | 459 | Phage portal protein lambda family | Phage portal protein lambda family | | afdb-uniprot50 | AF-A0A2W6W644-F1-MODEL\_V4 | 1.0 | 2.248e-28 | 1019 | 0.261 | 463 | 288 | 17 | 42 | 502 | 18 | 428 | Phage portal protein | Phage portal protein | | afdb-uniprot50 | AF-A0A2E5AE83-F1-MODEL\_V4 | 1.0 | 2.275e-27 | 1018 | 0.25 | 428 | 289 | 13 | 78 | 496 | 2 | 406 | Phage portal protein | Phage portal protein | | afdb-uniprot50 | AF-A0A2W5A5V5-F1-MODEL\_V4 | 1.0 | 1.92e-28 | 1017 | 0.208 | 494 | 350 | 15 | 23 | 491 | 3 | 480 | Phage portal protein | Phage portal protein | | afdb-uniprot50 | AF-W0DY98-F1-MODEL\_V4 | 1.0 | 6.865e-27 | 1016 | 0.269 | 430 | 274 | 13 | 78 | 502 | 2 | 396 | Portal protein | Portal protein | | afdb-uniprot50 | AF-A0A348FY34-F1-MODEL\_V4 | 1.0 | 9.805e-28 | 1014 | 0.251 | 474 | 315 | 15 | 30 | 488 | 1 | 449 | Phage portal protein | Phage portal protein | | afdb-uniprot50 | AF-A0A7C5PYI8-F1-MODEL\_V4 | 1.0 | 8.277e-29 | 1014 | 0.248 | 499 | 337 | 15 | 34 | 502 | 28 | 518 | Phage portal protein | Phage portal protein | | afdb-uniprot50 | AF-A0A1C3E9J0-F1-MODEL\_V4 | 1.0 | 4.276e-27 | 1013 | 0.211 | 453 | 326 | 12 | 79 | 523 | 2 | 431 | Phage portal protein | Phage portal protein | | afdb-uniprot50 | AF-A0A353ZI73-F1-MODEL\_V4 | 1.0 | 2e-29 | 1013 | 0.191 | 516 | 368 | 14 | 45 | 532 | 47 | 541 | Phage portal protein | Phage portal protein | | afdb-uniprot50 | AF-A0A497V0G5-F1-MODEL\_V4 | 1.0 | 7.151e-28 | 1012 | 0.202 | 498 | 358 | 15 | 35 | 530 | 25 | 485 | Lambda family phage portal protein | Lambda family phage portal protein | | afdb-uniprot50 | AF-A0A5C7T3J8-F1-MODEL\_V4 | 1.0 | 7.45e-29 | 1012 | 0.221 | 511 | 349 | 15 | 35 | 531 | 22 | 497 | Phage portal protein | Phage portal protein | | afdb-uniprot50 | AF-A0A841K9E4-F1-MODEL\_V4 | 1.0 | 7.853e-29 | 1012 | 0.238 | 512 | 337 | 15 | 35 | 532 | 18 | 490 | Lambda family phage portal protein | Lambda family phage portal protein | | afdb-uniprot50 | AF-A0A437MJQ4-F1-MODEL\_V4 | 1.0 | 2.024e-28 | 1011 | 0.224 | 482 | 326 | 16 | 36 | 502 | 29 | 477 | Phage portal protein | Phage portal protein | | afdb-uniprot50 | AF-A0A5C8P971-F1-MODEL\_V4 | 1.0 | 4.01e-28 | 1010 | 0.216 | 504 | 351 | 15 | 40 | 532 | 32 | 502 | Phage portal protein | Phage portal protein | | afdb-uniprot50 | AF-A0A166DQ97-F1-MODEL\_V4 | 1.0 | 4.403e-29 | 1009 | 0.226 | 507 | 337 | 15 | 21 | 502 | 1 | 477 | Phage portal protein, lambda family | Phage portal protein, lambda family | | afdb-uniprot50 | AF-A0A2E3QA51-F1-MODEL\_V4 | 1.0 | 3.249e-28 | 1009 | 0.231 | 483 | 326 | 16 | 36 | 502 | 32 | 485 | Phage portal protein | Phage portal protein | | afdb-uniprot50 | AF-A0A2D6BG92-F1-MODEL\_V4 | 1.0 | 1.196e-28 | 1008 | 0.245 | 465 | 316 | 12 | 42 | 502 | 17 | 450 | Phage portal protein | Phage portal protein | | afdb-uniprot50 | AF-A0A420DP32-F1-MODEL\_V4 | 1.0 | 2.633e-28 | 1008 | 0.247 | 497 | 318 | 15 | 23 | 502 | 2 | 459 | Lambda family phage portal protein | Lambda family phage portal protein | | afdb-uniprot50 | AF-A0A6N9P582-F1-MODEL\_V4 | 1.0 | 4.226e-28 | 1008 | 0.246 | 479 | 322 | 14 | 49 | 500 | 10 | 476 | Phage portal protein | Phage portal protein | | afdb-uniprot50 | AF-A0A6G5QFV0-F1-MODEL\_V4 | 1.0 | 7.235e-27 | 1007 | 0.239 | 505 | 322 | 17 | 15 | 500 | 1 | 462 | Phage portal protein, lambda family | Phage portal protein, lambda family | | afdb-uniprot50 | AF-A0A840JNA6-F1-MODEL\_V4 | 1.0 | 1.822e-28 | 1007 | 0.205 | 512 | 363 | 15 | 35 | 517 | 21 | 517 | Lambda family phage portal protein | Lambda family phage portal protein | | afdb-uniprot50 | AF-A0A518B2S3-F1-MODEL\_V4 | 1.0 | 3.425e-28 | 1005 | 0.203 | 516 | 375 | 12 | 39 | 529 | 9 | 513 | Phage portal protein, lambda family | Phage portal protein, lambda family | | afdb-uniprot50 | AF-A0A6I2J0E3-F1-MODEL\_V4 | 1.0 | 3.804e-28 | 1002 | 0.456 | 348 | 175 | 6 | 191 | 532 | 1 | 340 | Phage portal protein | Phage portal protein | | afdb-uniprot50 | AF-A0A1T4QCI5-F1-MODEL\_V4 | 1.0 | 1.033e-27 | 1002 | 0.211 | 511 | 355 | 14 | 17 | 501 | 1 | 489 | Phage portal protein, lambda family | Phage portal protein, lambda family | | afdb-uniprot50 | AF-A0A075MK45-F1-MODEL\_V4 | 1.0 | 1.417e-27 | 1001 | 0.215 | 506 | 338 | 16 | 46 | 517 | 6 | 486 | Phage portal protein, lambda family | Phage portal protein, lambda family | | afdb-uniprot50 | AF-A0A510IYQ6-F1-MODEL\_V4 | 1.0 | 5.007e-27 | 1000 | 0.228 | 416 | 296 | 12 | 35 | 439 | 18 | 419 | Phage portal protein | Phage portal protein | | afdb-uniprot50 | AF-A0A2Z6AZ44-F1-MODEL\_V4 | 1.0 | 1.92e-28 | 1000 | 0.216 | 512 | 357 | 15 | 21 | 500 | 1 | 500 | Lambda family phage portal protein | Lambda family phage portal protein | | afdb-uniprot50 | AF-A0A3P3D1Q4-F1-MODEL\_V4 | 1.0 | 1.033e-27 | 999 | 0.232 | 477 | 311 | 15 | 38 | 502 | 3 | 436 | Phage portal protein | Phage portal protein | | afdb-uniprot50 | AF-A0A7D7VGU6-F1-MODEL\_V4 | 1.0 | 2.807e-27 | 998 | 0.24 | 483 | 325 | 15 | 46 | 502 | 11 | 477 | Phage portal protein | Phage portal protein | | afdb-uniprot50 | AF-A0A6L6WAJ2-F1-MODEL\_V4 | 1.0 | 4.276e-27 | 998 | 0.202 | 475 | 341 | 13 | 36 | 495 | 20 | 471 | Phage portal protein | Phage portal protein | | afdb-uniprot50 | AF-K0NIH4-F1-MODEL\_V4 | 1.0 | 9.691e-29 | 998 | 0.209 | 550 | 368 | 16 | 8 | 532 | 1 | 508 | Phage portal protein, lambda family | Phage portal protein, lambda family | | afdb-uniprot50 | AF-A0A522VF55-F1-MODEL\_V4 | 1.0 | 2.602e-29 | 998 | 0.195 | 578 | 377 | 16 | 2 | 532 | 3 | 539 | Phage portal protein | Phage portal protein | | afdb-uniprot50 | AF-A0A225NFN7-F1-MODEL\_V4 | 1.0 | 2.959e-27 | 997 | 0.245 | 427 | 287 | 13 | 77 | 502 | 2 | 394 | Uncharacterized protein | Uncharacterized protein | | afdb-uniprot50 | AF-A0A2D8RC71-F1-MODEL\_V4 | 1.0 | 2.275e-27 | 996 | 0.248 | 466 | 313 | 15 | 39 | 502 | 28 | 458 | Phage portal protein | Phage portal protein | | afdb-uniprot50 | AF-A5V8D4-F1-MODEL\_V4 | 1.0 | 1.843e-27 | 996 | 0.242 | 474 | 319 | 17 | 36 | 496 | 27 | 473 | Phage portal protein, lambda family | Phage portal protein, lambda family | | afdb-uniprot50 | AF-A0A7G9GXH5-F1-MODEL\_V4 | 1.0 | 1.476e-28 | 995 | 0.23 | 508 | 332 | 15 | 39 | 525 | 35 | 504 | Phage portal protein | Phage portal protein | | afdb-uniprot50 | AF-A0A3P3DCQ1-F1-MODEL\_V4 | 1.0 | 1.033e-27 | 995 | 0.243 | 502 | 349 | 14 | 39 | 523 | 21 | 508 | Phage portal protein | Phage portal protein | | afdb-uniprot50 | AF-A0A1V2DSA5-F1-MODEL\_V4 | 1.0 | 2.664e-27 | 994 | 0.23 | 485 | 330 | 14 | 46 | 502 | 6 | 475 | Phage portal protein | Phage portal protein | | afdb-uniprot50 | AF-A0A7X9FN59-F1-MODEL\_V4 | 1.0 | 1.92e-28 | 994 | 0.226 | 547 | 363 | 19 | 15 | 532 | 1 | 516 | Phage portal protein | Phage portal protein | | afdb-uniprot50 | AF-A0A4V0XDV8-F1-MODEL\_V4 | 1.0 | 4.75e-27 | 994 | 0.214 | 485 | 339 | 13 | 34 | 502 | 12 | 470 | Phage portal protein | Phage portal protein | | afdb-uniprot50 | AF-A0A4U8YXU7-F1-MODEL\_V4 | 1.0 | 1.196e-28 | 994 | 0.233 | 506 | 344 | 15 | 48 | 523 | 23 | 514 | Phage portal protein lambda family | Phage portal protein lambda family | | afdb-uniprot50 | AF-A0A2W5SIJ8-F1-MODEL\_V4 | 1.0 | 7.853e-29 | 993 | 0.209 | 564 | 375 | 15 | 1 | 532 | 1 | 525 | Phage portal protein | Phage portal protein | | afdb-uniprot50 | AF-A0A316S9H5-F1-MODEL\_V4 | 1.0 | 3.652e-27 | 992 | 0.205 | 492 | 357 | 9 | 39 | 518 | 34 | 503 | Phage portal protein | Phage portal protein | | afdb-uniprot50 | AF-A0A225SMR3-F1-MODEL\_V4 | 1.0 | 3.119e-27 | 992 | 0.213 | 482 | 347 | 15 | 39 | 497 | 34 | 506 | Phage portal protein | Phage portal protein | | afdb-uniprot50 | AF-A0A0F9F3I2-F1-MODEL\_V4 | 1.0 | 1.21e-27 | 991 | 0.215 | 520 | 353 | 14 | 35 | 531 | 37 | 524 | Uncharacterized protein | Uncharacterized protein | | afdb-uniprot50 | AF-A0A0R3MA71-F1-MODEL\_V4 | 1.0 | 3.609e-28 | 990 | 0.201 | 536 | 377 | 16 | 2 | 502 | 1 | 520 | Uncharacterized protein | Uncharacterized protein | | afdb-uniprot50 | AF-A0A4U8S355-F1-MODEL\_V4 | 1.0 | 3.287e-27 | 989 | 0.204 | 503 | 329 | 15 | 15 | 499 | 1 | 450 | Phage portal protein | Phage portal protein | | afdb-uniprot50 | AF-A0A1I5HB10-F1-MODEL\_V4 | 1.0 | 1.401e-28 | 988 | 0.209 | 526 | 359 | 16 | 20 | 532 | 3 | 484 | Phage portal protein, lambda family | Phage portal protein, lambda family | | afdb-uniprot50 | AF-A0A1S1TN63-F1-MODEL\_V4 | 1.0 | 7.151e-28 | 988 | 0.225 | 488 | 341 | 18 | 35 | 498 | 19 | 493 | Phage portal protein | Phage portal protein | | afdb-uniprot50 | AF-A0A316NFS7-F1-MODEL\_V4 | 1.0 | 1.246e-29 | 988 | 0.216 | 532 | 344 | 14 | 40 | 532 | 36 | 533 | Phage portal protein | Phage portal protein | | afdb-uniprot50 | AF-A0A2N3BA45-F1-MODEL\_V4 | 1.0 | 6.179e-27 | 987 | 0.29 | 400 | 261 | 11 | 27 | 415 | 1 | 388 | Phage portal protein | Phage portal protein | | afdb-uniprot50 | AF-A0A7V2AKM7-F1-MODEL\_V4 | 1.0 | 1.344e-27 | 985 | 0.224 | 539 | 356 | 16 | 27 | 532 | 2 | 511 | Phage portal protein | Phage portal protein | | afdb-uniprot50 | AF-A0A7W6NJ63-F1-MODEL\_V4 | 1.0 | 2e-29 | 985 | 0.209 | 559 | 380 | 21 | 1 | 532 | 2 | 525 | Lambda family phage portal protein | Lambda family phage portal protein | | afdb-uniprot50 | AF-A0A7R7T6F2-F1-MODEL\_V4 | 1.0 | 4.276e-27 | 984 | 0.259 | 485 | 314 | 12 | 36 | 502 | 23 | 480 | Phage portal protein | Phage portal protein | | afdb-uniprot50 | AF-A0A4R3AAZ5-F1-MODEL\_V4 | 1.0 | 8.929e-27 | 983 | 0.252 | 468 | 307 | 12 | 44 | 496 | 2 | 441 | Lambda family phage portal protein | Lambda family phage portal protein | | afdb-uniprot50 | AF-A0A2E2Q7X0-F1-MODEL\_V4 | 1.0 | 5.498e-28 | 983 | 0.221 | 514 | 352 | 17 | 46 | 532 | 18 | 510 | Phage portal protein | Phage portal protein | | afdb-uniprot50 | AF-A0A4R3HW29-F1-MODEL\_V4 | 1.0 | 6.513e-27 | 983 | 0.229 | 480 | 341 | 16 | 40 | 497 | 35 | 507 | Lambda family phage portal protein | Lambda family phage portal protein | | afdb-uniprot50 | AF-A0A1G7C613-F1-MODEL\_V4 | 1.0 | 1.679e-26 | 982 | 0.26 | 433 | 286 | 9 | 62 | 491 | 1 | 402 | Phage portal protein, lambda family | Phage portal protein, lambda family | | afdb-uniprot50 | AF-A0A090KJ34-F1-MODEL\_V4 | 1.0 | 9.92e-27 | 982 | 0.178 | 504 | 369 | 13 | 36 | 532 | 27 | 492 | Phage portal protein | Phage portal protein | | afdb-uniprot50 | AF-H0T3Q1-F1-MODEL\_V4 | 1.0 | 1.135e-28 | 981 | 0.21 | 519 | 341 | 15 | 58 | 532 | 3 | 496 | Putative Phage portal protein, lambda | Putative Phage portal protein, lambda | | afdb-uniprot50 | AF-A0A7W0HL78-F1-MODEL\_V4 | 1.0 | 7.945e-28 | 981 | 0.24 | 520 | 351 | 17 | 1 | 498 | 28 | 525 | Lambda family phage portal protein | Lambda family phage portal protein | | afdb-uniprot50 | AF-A0A1T2XA84-F1-MODEL\_V4 | 1.0 | 9.195e-29 | 980 | 0.214 | 551 | 368 | 18 | 15 | 515 | 1 | 536 | Phage portal protein | Phage portal protein | | afdb-uniprot50 | AF-A0A016XIN5-F1-MODEL\_V4 | 1.0 | 1.843e-27 | 978 | 0.23 | 499 | 336 | 12 | 23 | 502 | 2 | 471 | Uncharacterized protein | Uncharacterized protein | | afdb-uniprot50 | AF-A0A117KHB7-F1-MODEL\_V4 | 1.0 | 4.507e-27 | 978 | 0.251 | 481 | 310 | 19 | 39 | 502 | 23 | 470 | Portal protein | Portal protein | | afdb-uniprot50 | AF-A0A0Q4S2Z1-F1-MODEL\_V4 | 1.0 | 1.556e-28 | 973 | 0.243 | 522 | 325 | 19 | 35 | 532 | 28 | 503 | Uncharacterized protein | Uncharacterized protein | | afdb-uniprot50 | AF-A0A363S2N1-F1-MODEL\_V4 | 1.0 | 4.949e-28 | 973 | 0.186 | 510 | 364 | 16 | 39 | 517 | 21 | 510 | Phage portal protein | Phage portal protein | | afdb-uniprot50 | AF-A0A3A9B6U4-F1-MODEL\_V4 | 1.0 | 2.807e-27 | 973 | 0.202 | 490 | 345 | 14 | 43 | 492 | 14 | 497 | Phage portal protein | Phage portal protein | | afdb-uniprot50 | AF-A0A6M3K3Y5-F1-MODEL\_V4 | 1.0 | 2.775e-28 | 973 | 0.201 | 531 | 372 | 16 | 39 | 530 | 52 | 569 | Putative portal protein | Putative portal protein | | afdb-uniprot50 | AF-A0A5C7Q6R0-F1-MODEL\_V4 | 1.0 | 6.513e-27 | 972 | 0.23 | 498 | 337 | 13 | 40 | 532 | 18 | 474 | Phage portal protein | Phage portal protein | | afdb-uniprot50 | AF-A0A4Y1MVH7-F1-MODEL\_V4 | 1.0 | 1.417e-27 | 972 | 0.224 | 531 | 331 | 17 | 39 | 532 | 27 | 513 | Portal protein | Portal protein | | afdb-uniprot50 | AF-A0A238KCR7-F1-MODEL\_V4 | 1.0 | 2.398e-27 | 971 | 0.233 | 492 | 334 | 17 | 35 | 517 | 18 | 475 | Phage portal protein, lambda family | Phage portal protein, lambda family | | afdb-uniprot50 | AF-A0A1V5N9M5-F1-MODEL\_V4 | 1.0 | 3.249e-28 | 971 | 0.213 | 533 | 357 | 15 | 17 | 532 | 1 | 488 | Phage portal protein, lambda family | Phage portal protein, lambda family | | afdb-uniprot50 | AF-A0A0M5L0Q6-F1-MODEL\_V4 | 1.0 | 3.804e-28 | 969 | 0.21 | 517 | 369 | 11 | 39 | 532 | 5 | 505 | Phage-related portal protein | Phage-related portal protein | | afdb-uniprot50 | AF-A0A643F376-F1-MODEL\_V4 | 1.0 | 3.505e-26 | 968 | 0.235 | 484 | 309 | 16 | 18 | 497 | 1 | 427 | Phage portal protein | Phage portal protein | | afdb-uniprot50 | AF-A0A557QLS5-F1-MODEL\_V4 | 1.0 | 1.511e-26 | 968 | 0.211 | 478 | 352 | 13 | 39 | 497 | 39 | 510 | Phage portal protein | Phage portal protein | | afdb-uniprot50 | AF-A0A1C1YRY8-F1-MODEL\_V4 | 1.0 | 2.891e-29 | 968 | 0.219 | 557 | 375 | 21 | 8 | 532 | 1 | 529 | Uncharacterized protein | Uncharacterized protein | | afdb-uniprot50 | AF-A0A5C5X2S3-F1-MODEL\_V4 | 1.0 | 2.275e-27 | 968 | 0.203 | 515 | 357 | 20 | 37 | 532 | 35 | 515 | Phage portal protein, lambda family | Phage portal protein, lambda family | | afdb-uniprot50 | AF-A0A1V6DZJ2-F1-MODEL\_V4 | 1.0 | 4.057e-27 | 967 | 0.2 | 505 | 355 | 15 | 39 | 521 | 18 | 495 | Phage portal protein, lambda family | Phage portal protein, lambda family | | afdb-uniprot50 | AF-A0A258A0N0-F1-MODEL\_V4 | 1.0 | 3.465e-27 | 967 | 0.205 | 526 | 346 | 14 | 39 | 532 | 20 | 505 | Phage portal protein | Phage portal protein | | afdb-uniprot50 | AF-A0A1V6E2A1-F1-MODEL\_V4 | 1.0 | 1.92e-28 | 967 | 0.246 | 528 | 330 | 18 | 33 | 530 | 36 | 525 | Phage portal protein, lambda family | Phage portal protein, lambda family | | afdb-uniprot50 | AF-A0A1P8QQ19-F1-MODEL\_V4 | 1.0 | 3.652e-27 | 967 | 0.227 | 484 | 340 | 16 | 36 | 496 | 31 | 503 | Phage portal protein | Phage portal protein | | afdb-uniprot50 | AF-A0A5M6HUJ4-F1-MODEL\_V4 | 1.0 | 5.498e-28 | 965 | 0.252 | 511 | 333 | 15 | 10 | 498 | 46 | 529 | Phage portal protein | Phage portal protein | | afdb-uniprot50 | AF-A0A0Q4HVV6-F1-MODEL\_V4 | 1.0 | 1.046e-26 | 964 | 0.255 | 443 | 288 | 14 | 75 | 502 | 2 | 417 | Portal protein | Portal protein | | afdb-uniprot50 | AF-A0A1Q6U834-F1-MODEL\_V4 | 1.0 | 3.465e-27 | 964 | 0.218 | 480 | 335 | 15 | 39 | 488 | 24 | 493 | Phage portal protein | Phage portal protein | | afdb-uniprot50 | AF-A0A2L1U4F6-F1-MODEL\_V4 | 1.0 | 1.196e-28 | 964 | 0.215 | 530 | 356 | 14 | 34 | 532 | 12 | 512 | Phage portal protein, lambda family | Phage portal protein, lambda family | | afdb-uniprot50 | AF-A0A259U583-F1-MODEL\_V4 | 1.0 | 1.728e-28 | 964 | 0.209 | 553 | 370 | 16 | 20 | 532 | 1 | 526 | Phage portal protein, lambda family | Phage portal protein, lambda family | | afdb-uniprot50 | AF-A0A4Q0SRN9-F1-MODEL\_V4 | 1.0 | 6.513e-27 | 963 | 0.229 | 471 | 323 | 16 | 29 | 484 | 1 | 446 | Uncharacterized protein | Uncharacterized protein | | afdb-uniprot50 | AF-A0A495PS99-F1-MODEL\_V4 | 1.0 | 2.527e-27 | 963 | 0.246 | 520 | 321 | 17 | 16 | 532 | 5 | 456 | Lambda family phage portal protein | Lambda family phage portal protein | | afdb-uniprot50 | AF-A0A1X9SVH6-F1-MODEL\_V4 | 1.0 | 7.715e-26 | 961 | 0.233 | 476 | 287 | 14 | 20 | 475 | 2 | 419 | Phage portal protein, lambda family | Phage portal protein, lambda family | | afdb-uniprot50 | AF-A0A1H9GFL6-F1-MODEL\_V4 | 1.0 | 6.179e-27 | 961 | 0.226 | 468 | 328 | 17 | 39 | 497 | 23 | 465 | Phage portal protein, lambda family | Phage portal protein, lambda family | | afdb-uniprot50 | AF-A0A1J5EX01-F1-MODEL\_V4 | 1.0 | 1.556e-28 | 961 | 0.198 | 520 | 353 | 19 | 46 | 532 | 12 | 500 | Phage portal protein | Phage portal protein | | afdb-uniprot50 | AF-A0A421BJ29-F1-MODEL\_V4 | 1.0 | 9.195e-29 | 961 | 0.219 | 573 | 356 | 19 | 1 | 532 | 111 | 632 | Phage portal protein | Phage portal protein | | afdb-uniprot50 | AF-A0A1E3GXJ9-F1-MODEL\_V4 | 1.0 | 2.072e-26 | 961 | 0.25 | 475 | 314 | 15 | 39 | 502 | 27 | 470 | Phage portal protein, lambda family | Phage portal protein, lambda family | | afdb-uniprot50 | AF-A0A2P9HNT3-F1-MODEL\_V4 | 1.0 | 3.849e-27 | 960 | 0.221 | 461 | 324 | 15 | 8 | 456 | 1 | 438 | Phage portal protein, lambda family | Phage portal protein, lambda family | | afdb-uniprot50 | AF-A0A2U2MXN7-F1-MODEL\_V4 | 1.0 | 6.108e-28 | 958 | 0.228 | 546 | 353 | 21 | 44 | 532 | 21 | 554 | Phage portal protein | Phage portal protein | | afdb-uniprot50 | AF-G1UXR1-F1-MODEL\_V4 | 1.0 | 9.303e-28 | 957 | 0.223 | 515 | 350 | 16 | 11 | 490 | 1 | 500 | Lambda family phage portal protein | Lambda family phage portal protein | | afdb-uniprot50 | AF-A0A2N8HQG3-F1-MODEL\_V4 | 1.0 | 2.807e-27 | 957 | 0.187 | 538 | 391 | 13 | 27 | 523 | 1 | 533 | Phage portal protein | Phage portal protein | | afdb-uniprot50 | AF-A0A1Y6CPS8-F1-MODEL\_V4 | 1.0 | 3.849e-27 | 952 | 0.207 | 492 | 340 | 17 | 52 | 516 | 11 | 479 | Phage portal protein, lambda family | Phage portal protein, lambda family | | afdb-uniprot50 | AF-A0A849WA89-F1-MODEL\_V4 | 1.0 | 7.235e-27 | 952 | 0.214 | 480 | 333 | 16 | 36 | 502 | 31 | 479 | Phage portal protein | Phage portal protein | | afdb-uniprot50 | AF-A0A1A8T9E5-F1-MODEL\_V4 | 1.0 | 1.943e-27 | 952 | 0.185 | 523 | 365 | 15 | 43 | 531 | 12 | 507 | Phage portal protein, lambda family | Phage portal protein, lambda family | | afdb-uniprot50 | AF-A0A367FXQ0-F1-MODEL\_V4 | 1.0 | 3.804e-28 | 952 | 0.237 | 523 | 352 | 16 | 36 | 518 | 23 | 538 | Phage portal protein | Phage portal protein | | afdb-uniprot50 | AF-A0A0P6VW69-F1-MODEL\_V4 | 1.0 | 1.769e-26 | 950 | 0.224 | 504 | 341 | 14 | 36 | 531 | 23 | 484 | Uncharacterized protein | Uncharacterized protein | | afdb-uniprot50 | AF-A0A212IX70-F1-MODEL\_V4 | 1.0 | 1.769e-26 | 950 | 0.223 | 488 | 336 | 16 | 44 | 495 | 15 | 495 | Lambda family phage portal protein | Lambda family phage portal protein | | afdb-uniprot50 | AF-A0A7X4AD15-F1-MODEL\_V4 | 1.0 | 3.652e-27 | 949 | 0.202 | 519 | 346 | 20 | 15 | 530 | 15 | 468 | Phage portal protein | Phage portal protein | | afdb-uniprot50 | AF-A0A7Z3TXU7-F1-MODEL\_V4 | 1.0 | 1.36e-26 | 949 | 0.221 | 497 | 327 | 18 | 20 | 490 | 3 | 465 | Phage portal protein | Phage portal protein | | afdb-uniprot50 | AF-A0A0K1NDB4-F1-MODEL\_V4 | 1.0 | 6.785e-28 | 949 | 0.211 | 562 | 342 | 13 | 17 | 532 | 8 | 514 | Portal protein | Portal protein | | afdb-uniprot50 | AF-A0A2A5A4N7-F1-MODEL\_V4 | 1.0 | 7.715e-26 | 948 | 0.21 | 471 | 333 | 13 | 36 | 497 | 30 | 470 | Phage portal protein | Phage portal protein | | afdb-uniprot50 | AF-A0A839H7H8-F1-MODEL\_V4 | 1.0 | 1.769e-26 | 947 | 0.22 | 495 | 330 | 15 | 46 | 532 | 31 | 477 | Phage portal protein | Phage portal protein | | afdb-uniprot50 | AF-A0A7C8HT84-F1-MODEL\_V4 | 1.0 | 1.224e-26 | 946 | 0.209 | 505 | 332 | 12 | 39 | 523 | 34 | 491 | Uncharacterized protein | Uncharacterized protein | | afdb-uniprot50 | AF-A0A1H5Z5E8-F1-MODEL\_V4 | 1.0 | 1.21e-27 | 946 | 0.222 | 543 | 337 | 16 | 5 | 502 | 1 | 503 | Phage portal protein | Phage portal protein | | afdb-uniprot50 | AF-A3VBF2-F1-MODEL\_V4 | 1.0 | 2.664e-27 | 945 | 0.226 | 538 | 335 | 20 | 20 | 532 | 1 | 482 | Uncharacterized protein | Uncharacterized protein | | afdb-uniprot50 | AF-E3H7C7-F1-MODEL\_V4 | 1.0 | 4.56e-26 | 945 | 0.195 | 486 | 349 | 12 | 39 | 494 | 29 | 502 | Phage portal protein, lambda family | Phage portal protein, lambda family | | afdb-uniprot50 | AF-A0A0G1Z1U3-F1-MODEL\_V4 | 1.0 | 1.511e-26 | 942 | 0.232 | 469 | 320 | 16 | 57 | 495 | 2 | 460 | Phage portal protein, lambda family | Phage portal protein, lambda family | | afdb-uniprot50 | AF-A0A134CAF6-F1-MODEL\_V4 | 1.0 | 1.21e-27 | 942 | 0.201 | 521 | 353 | 17 | 13 | 488 | 1 | 503 | Phage portal protein, lambda family | Phage portal protein, lambda family | | afdb-uniprot50 | AF-M2YEI3-F1-MODEL\_V4 | 1.0 | 1.659e-27 | 942 | 0.214 | 512 | 349 | 18 | 47 | 532 | 51 | 535 | Lambda family phage portal protein | Lambda family phage portal protein | | afdb-uniprot50 | AF-A0A2T5K540-F1-MODEL\_V4 | 1.0 | 3.652e-27 | 942 | 0.219 | 525 | 355 | 18 | 36 | 532 | 38 | 535 | Lambda family phage portal protein | Lambda family phage portal protein | | afdb-uniprot50 | AF-A0A546X7Y9-F1-MODEL\_V4 | 1.0 | 2.133e-28 | 939 | 0.194 | 541 | 382 | 20 | 1 | 515 | 6 | 518 | Phage portal protein | Phage portal protein | | afdb-uniprot50 | AF-A0A095YJQ5-F1-MODEL\_V4 | 1.0 | 5.562e-27 | 938 | 0.223 | 496 | 339 | 17 | 39 | 502 | 22 | 503 | Lambda family phage portal protein | Lambda family phage portal protein | | afdb-uniprot50 | AF-A0A369BH58-F1-MODEL\_V4 | 1.0 | 1.401e-28 | 938 | 0.233 | 530 | 347 | 20 | 39 | 523 | 31 | 545 | Lambda family phage portal protein | Lambda family phage portal protein | | afdb-uniprot50 | AF-A0A353GEE3-F1-MODEL\_V4 | 1.0 | 2.301e-26 | 937 | 0.219 | 515 | 360 | 16 | 23 | 532 | 1 | 478 | Uncharacterized protein | Uncharacterized protein | | afdb-uniprot50 | AF-A0A2U1WJ09-F1-MODEL\_V4 | 1.0 | 1.046e-26 | 937 | 0.202 | 500 | 337 | 16 | 46 | 502 | 3 | 483 | Uncharacterized protein | Uncharacterized protein | | afdb-uniprot50 | AF-A0A2V7CXU2-F1-MODEL\_V4 | 1.0 | 1.148e-27 | 936 | 0.207 | 554 | 351 | 16 | 9 | 532 | 1 | 496 | Phage portal protein | Phage portal protein | | afdb-uniprot50 | AF-A0A4V3ZNW4-F1-MODEL\_V4 | 1.0 | 1.344e-27 | 935 | 0.229 | 535 | 345 | 15 | 2 | 492 | 82 | 593 | Phage portal protein | Phage portal protein | | afdb-uniprot50 | AF-A0A7C3DQI9-F1-MODEL\_V4 | 1.0 | 7.715e-26 | 934 | 0.248 | 479 | 299 | 16 | 35 | 502 | 11 | 439 | Phage portal protein | Phage portal protein | | afdb-uniprot50 | AF-A0A5D0RSH2-F1-MODEL\_V4 | 1.0 | 3.155e-26 | 934 | 0.226 | 491 | 338 | 15 | 40 | 517 | 25 | 486 | Phage portal protein | Phage portal protein | | afdb-uniprot50 | AF-A0A840Z535-F1-MODEL\_V4 | 1.0 | 5.066e-26 | 934 | 0.189 | 476 | 342 | 16 | 39 | 502 | 27 | 470 | Lambda family phage portal protein | Lambda family phage portal protein | | afdb-uniprot50 | AF-D6KLF9-F1-MODEL\_V4 | 1.0 | 6.437e-28 | 934 | 0.191 | 564 | 380 | 16 | 13 | 532 | 1 | 532 | Phage portal protein, lambda family | Phage portal protein, lambda family | | afdb-uniprot50 | AF-A0A1Q6U9X6-F1-MODEL\_V4 | 1.0 | 1.224e-26 | 933 | 0.223 | 483 | 310 | 15 | 78 | 532 | 2 | 447 | Phage portal protein | Phage portal protein | | afdb-uniprot50 | AF-A0A7G6RHV5-F1-MODEL\_V4 | 1.0 | 1.004e-25 | 933 | 0.229 | 419 | 295 | 12 | 85 | 496 | 2 | 399 | Phage portal protein | Phage portal protein | | afdb-uniprot50 | AF-A0A1I3HJ62-F1-MODEL\_V4 | 1.0 | 3.465e-27 | 933 | 0.222 | 509 | 330 | 16 | 13 | 491 | 1 | 473 | Phage portal protein, lambda family | Phage portal protein, lambda family | | afdb-uniprot50 | AF-A0A101K665-F1-MODEL\_V4 | 1.0 | 4.695e-28 | 933 | 0.213 | 530 | 332 | 15 | 39 | 530 | 25 | 507 | Phage portal protein | Phage portal protein | | afdb-uniprot50 | AF-A0A385Q1G5-F1-MODEL\_V4 | 1.0 | 3.894e-26 | 932 | 0.224 | 494 | 341 | 15 | 36 | 519 | 23 | 484 | Phage portal protein | Phage portal protein | | afdb-uniprot50 | AF-A0A6I4TSN8-F1-MODEL\_V4 | 1.0 | 1.493e-27 | 932 | 0.181 | 588 | 411 | 19 | 1 | 532 | 2 | 574 | Phage portal protein | Phage portal protein | | afdb-uniprot50 | AF-A0A1E2RXZ6-F1-MODEL\_V4 | 1.0 | 6.945e-26 | 930 | 0.295 | 399 | 252 | 10 | 105 | 496 | 3 | 379 | Phage portal protein, lambda family | Phage portal protein, lambda family | | afdb-uniprot50 | AF-A0A5S4X1N8-F1-MODEL\_V4 | 1.0 | 1.058e-25 | 930 | 0.218 | 467 | 334 | 16 | 27 | 484 | 1 | 445 | Phage portal protein | Phage portal protein | | afdb-uniprot50 | AF-A0A4R3EC07-F1-MODEL\_V4 | 1.0 | 1.511e-26 | 930 | 0.22 | 480 | 324 | 17 | 35 | 492 | 18 | 469 | Lambda family phage portal protein | Lambda family phage portal protein | | afdb-uniprot50 | AF-A0A356C2B9-F1-MODEL\_V4 | 1.0 | 4.75e-27 | 928 | 0.21 | 538 | 356 | 12 | 39 | 531 | 29 | 542 | Phage portal protein | Phage portal protein | | afdb-uniprot50 | AF-A0A0Q7XTZ7-F1-MODEL\_V4 | 1.0 | 5.498e-28 | 928 | 0.197 | 547 | 355 | 20 | 20 | 532 | 1 | 497 | Uncharacterized protein | Uncharacterized protein | | afdb-uniprot50 | AF-Q72A26-F1-MODEL\_V4 | 1.0 | 5.277e-27 | 927 | 0.203 | 541 | 361 | 17 | 23 | 530 | 1 | 504 | Portal protein | Portal protein | | afdb-uniprot50 | AF-G1USE5-F1-MODEL\_V4 | 1.0 | 1.749e-27 | 926 | 0.209 | 529 | 348 | 20 | 39 | 532 | 31 | 524 | Lambda family phage portal protein | Lambda family phage portal protein | | afdb-uniprot50 | AF-A0A842IW31-F1-MODEL\_V4 | 1.0 | 1.769e-26 | 925 | 0.208 | 537 | 362 | 15 | 20 | 532 | 1 | 498 | Phage portal protein | Phage portal protein | | afdb-uniprot50 | AF-A0A1S2L7H0-F1-MODEL\_V4 | 1.0 | 3.287e-27 | 924 | 0.221 | 523 | 361 | 17 | 20 | 515 | 2 | 505 | Phage portal protein | Phage portal protein | | afdb-uniprot50 | AF-A0A835Z6S7-F1-MODEL\_V4 | 1.0 | 6.945e-26 | 924 | 0.228 | 482 | 316 | 18 | 39 | 502 | 27 | 470 | Phage portal protein | Phage portal protein | | afdb-uniprot50 | AF-A0A2P7V3N4-F1-MODEL\_V4 | 1.0 | 2.158e-27 | 923 | 0.212 | 513 | 343 | 17 | 36 | 495 | 23 | 527 | Phage portal protein | Phage portal protein | | afdb-uniprot50 | AF-A0A369XXM4-F1-MODEL\_V4 | 1.0 | 4.104e-26 | 922 | 0.226 | 468 | 323 | 14 | 49 | 497 | 40 | 487 | Phage portal protein | Phage portal protein | | afdb-uniprot50 | AF-A0A431PRZ4-F1-MODEL\_V4 | 1.0 | 7.715e-26 | 922 | 0.218 | 476 | 333 | 14 | 39 | 502 | 25 | 473 | Phage portal protein | Phage portal protein | | afdb-uniprot50 | AF-A3K1Z6-F1-MODEL\_V4 | 1.0 | 5.627e-26 | 921 | 0.204 | 488 | 334 | 15 | 35 | 490 | 21 | 486 | Phage portal protein, lambda | Phage portal protein, lambda | | afdb-uniprot50 | AF-A0A6L9LZ03-F1-MODEL\_V4 | 1.0 | 8.132e-26 | 921 | 0.214 | 514 | 356 | 16 | 15 | 497 | 1 | 497 | Phage portal protein | Phage portal protein | | afdb-uniprot50 | AF-A0A7X7KP38-F1-MODEL\_V4 | 1.0 | 3.287e-27 | 920 | 0.196 | 566 | 360 | 21 | 15 | 532 | 1 | 519 | Phage portal protein | Phage portal protein | | afdb-uniprot50 | AF-A0A259DE51-F1-MODEL\_V4 | 1.0 | 2.183e-26 | 918 | 0.233 | 441 | 294 | 13 | 98 | 532 | 3 | 405 | Phage portal protein | Phage portal protein | | afdb-uniprot50 | AF-A0A7X7H5B0-F1-MODEL\_V4 | 1.0 | 6.437e-28 | 917 | 0.21 | 538 | 339 | 18 | 36 | 521 | 10 | 513 | Phage portal protein | Phage portal protein | | afdb-uniprot50 | AF-A0A3L7Q8T8-F1-MODEL\_V4 | 1.0 | 2.301e-26 | 917 | 0.207 | 512 | 364 | 15 | 1 | 502 | 1 | 480 | Phage portal protein | Phage portal protein | | afdb-uniprot50 | AF-A0A509DVN1-F1-MODEL\_V4 | 1.0 | 2.587e-25 | 916 | 0.226 | 476 | 327 | 16 | 36 | 502 | 26 | 469 | Uncharacterized protein | Uncharacterized protein | | afdb-uniprot50 | AF-A0A5B9MAJ4-F1-MODEL\_V4 | 1.0 | 2.664e-27 | 916 | 0.206 | 581 | 383 | 17 | 12 | 532 | 3 | 565 | Phage portal protein, lambda family | Phage portal protein, lambda family | | afdb-uniprot50 | AF-A0A3D2ICL3-F1-MODEL\_V4 | 1.0 | 1.102e-26 | 915 | 0.205 | 521 | 360 | 14 | 35 | 515 | 21 | 527 | Phage portal protein | Phage portal protein | | afdb-uniprot50 | AF-A0A4Q7TER2-F1-MODEL\_V4 | 1.0 | 3.695e-26 | 914 | 0.222 | 476 | 307 | 11 | 41 | 467 | 12 | 473 | Lambda family phage portal protein | Lambda family phage portal protein | | afdb-uniprot50 | AF-A0A6H1ZM60-F1-MODEL\_V4 | 1.0 | 5.627e-26 | 913 | 0.211 | 530 | 353 | 18 | 39 | 532 | 33 | 533 | Putative portal protein | Putative portal protein | | afdb-uniprot50 | AF-A0A1M6LTT3-F1-MODEL\_V4 | 1.0 | 6.945e-26 | 912 | 0.234 | 498 | 335 | 13 | 39 | 500 | 17 | 504 | Phage portal protein, lambda family | Phage portal protein, lambda family | | afdb-uniprot50 | AF-A0A1L3GDR9-F1-MODEL\_V4 | 1.0 | 1.175e-25 | 912 | 0.207 | 482 | 346 | 15 | 35 | 488 | 25 | 498 | Phage portal protein | Phage portal protein | | afdb-uniprot50 | AF-A0A2U8WAV1-F1-MODEL\_V4 | 1.0 | 3.652e-27 | 912 | 0.186 | 563 | 385 | 21 | 13 | 517 | 1 | 548 | Phage portal protein | Phage portal protein | | afdb-uniprot50 | AF-E1JR91-F1-MODEL\_V4 | 1.0 | 9.412e-27 | 912 | 0.207 | 525 | 348 | 17 | 45 | 532 | 18 | 511 | Phage portal protein, lambda family | Phage portal protein, lambda family | | afdb-uniprot50 | AF-A0A369QQY9-F1-MODEL\_V4 | 1.0 | 1.511e-26 | 911 | 0.189 | 548 | 373 | 17 | 11 | 532 | 1 | 503 | Phage portal protein | Phage portal protein | | afdb-uniprot50 | AF-A0A353MHD9-F1-MODEL\_V4 | 1.0 | 8.929e-27 | 911 | 0.208 | 536 | 368 | 20 | 21 | 532 | 9 | 511 | Phage portal protein | Phage portal protein | | afdb-uniprot50 | AF-A0A854GYB8-F1-MODEL\_V4 | 1.0 | 2.183e-26 | 910 | 0.232 | 494 | 327 | 16 | 39 | 495 | 17 | 495 | Phage portal protein | Phage portal protein | | afdb-uniprot50 | AF-A0A1H1JS74-F1-MODEL\_V4 | 1.0 | 1.058e-25 | 910 | 0.211 | 491 | 335 | 13 | 45 | 496 | 28 | 505 | Phage portal protein, lambda family | Phage portal protein, lambda family | | afdb-uniprot50 | AF-E2CHI6-F1-MODEL\_V4 | 1.0 | 4.862e-25 | 909 | 0.215 | 478 | 333 | 13 | 39 | 502 | 23 | 472 | Phage portal protein, lambda family | Phage portal protein, lambda family | | afdb-uniprot50 | AF-A0A5C7P2N1-F1-MODEL\_V4 | 1.0 | 6.513e-27 | 909 | 0.171 | 549 | 393 | 18 | 23 | 532 | 10 | 535 | Phage portal protein | Phage portal protein | | afdb-uniprot50 | AF-A0A5E6U3P4-F1-MODEL\_V4 | 1.0 | 2.557e-26 | 909 | 0.217 | 501 | 339 | 18 | 39 | 503 | 33 | 516 | Uncharacterized protein | Uncharacterized protein | | afdb-uniprot50 | AF-K2IL77-F1-MODEL\_V4 | 1.0 | 4.507e-27 | 909 | 0.183 | 572 | 377 | 18 | 15 | 532 | 1 | 536 | Portal protein | Portal protein | | afdb-uniprot50 | AF-A0A349XPR7-F1-MODEL\_V4 | 1.0 | 9.634e-25 | 908 | 0.235 | 428 | 280 | 15 | 38 | 452 | 3 | 396 | Phage portal protein | Phage portal protein | | afdb-uniprot50 | AF-A0A2U0YK94-F1-MODEL\_V4 | 1.0 | 1.376e-25 | 908 | 0.226 | 451 | 307 | 12 | 98 | 532 | 2 | 426 | Lambda family phage portal protein | Lambda family phage portal protein | | afdb-uniprot50 | AF-A0A1H7TZE7-F1-MODEL\_V4 | 1.0 | 1.434e-26 | 908 | 0.187 | 571 | 373 | 21 | 20 | 532 | 9 | 546 | Phage portal protein, lambda family | Phage portal protein, lambda family | | afdb-uniprot50 | AF-A0A7Y4VYM6-F1-MODEL\_V4 | 1.0 | 2.301e-26 | 908 | 0.197 | 526 | 379 | 14 | 39 | 532 | 39 | 553 | Phage portal protein | Phage portal protein | | afdb-uniprot50 | AF-A0A2W4SHI4-F1-MODEL\_V4 | 1.0 | 2.356e-24 | 906 | 0.244 | 389 | 268 | 12 | 35 | 409 | 19 | 395 | Phage portal protein | Phage portal protein | | afdb-uniprot50 | AF-H1HCQ0-F1-MODEL\_V4 | 1.0 | 1.434e-26 | 904 | 0.206 | 509 | 348 | 15 | 39 | 517 | 29 | 511 | Lambda family phage portal protein | Lambda family phage portal protein | | afdb-uniprot50 | AF-A0A354U425-F1-MODEL\_V4 | 1.0 | 1.239e-25 | 903 | 0.216 | 521 | 337 | 15 | 20 | 532 | 2 | 459 | Uncharacterized protein | Uncharacterized protein | | afdb-uniprot50 | AF-A0A418VVD6-F1-MODEL\_V4 | 1.0 | 7.235e-27 | 903 | 0.207 | 525 | 349 | 17 | 45 | 519 | 18 | 525 | Phage portal protein | Phage portal protein | | afdb-uniprot50 | AF-A0A1Q6PWQ7-F1-MODEL\_V4 | 1.0 | 1.529e-25 | 903 | 0.212 | 484 | 342 | 17 | 49 | 500 | 51 | 527 | Phage portal protein | Phage portal protein | | afdb-uniprot50 | AF-A0A367V4E8-F1-MODEL\_V4 | 1.0 | 5.125e-25 | 902 | 0.235 | 442 | 294 | 15 | 75 | 502 | 2 | 413 | Uncharacterized protein | Uncharacterized protein | | afdb-uniprot50 | AF-A0A059KRD9-F1-MODEL\_V4 | 1.0 | 7.32e-26 | 901 | 0.204 | 508 | 350 | 19 | 39 | 532 | 22 | 489 | Uncharacterized protein | Uncharacterized protein | | afdb-uniprot50 | AF-A0A1H2EP65-F1-MODEL\_V4 | 1.0 | 1.046e-26 | 901 | 0.211 | 562 | 357 | 17 | 20 | 531 | 1 | 526 | Phage portal protein, lambda family | Phage portal protein, lambda family | | afdb-uniprot50 | AF-A0A521VMA3-F1-MODEL\_V4 | 1.0 | 2.84e-26 | 900 | 0.217 | 529 | 344 | 15 | 39 | 532 | 39 | 532 | Phage portal protein | Phage portal protein | | afdb-uniprot50 | AF-A0A1H5ZJ40-F1-MODEL\_V4 | 1.0 | 1.253e-24 | 899 | 0.253 | 442 | 290 | 15 | 39 | 466 | 23 | 438 | Phage portal protein, lambda family | Phage portal protein, lambda family | | afdb-uniprot50 | AF-A0A410NTY6-F1-MODEL\_V4 | 1.0 | 1.224e-26 | 899 | 0.217 | 537 | 358 | 20 | 33 | 524 | 25 | 544 | Phage portal protein | Phage portal protein | | afdb-uniprot50 | AF-G5NAD5-F1-MODEL\_V4 | 1.0 | 2.454e-25 | 898 | 0.241 | 460 | 301 | 15 | 73 | 502 | 1 | 442 | Phage portal protein | Phage portal protein | | afdb-uniprot50 | AF-A0A1W1BN33-F1-MODEL\_V4 | 1.0 | 1.698e-25 | 898 | 0.212 | 509 | 323 | 19 | 39 | 523 | 13 | 467 | Phage portal protein, lambda family | Phage portal protein, lambda family | | afdb-uniprot50 | AF-A0A5Q3L866-F1-MODEL\_V4 | 1.0 | 5.627e-26 | 898 | 0.213 | 535 | 361 | 19 | 31 | 532 | 5 | 512 | Phage portal protein, lambda family | Phage portal protein, lambda family | | afdb-uniprot50 | AF-A0A5A9EPH8-F1-MODEL\_V4 | 1.0 | 5.693e-25 | 898 | 0.215 | 479 | 327 | 16 | 39 | 497 | 28 | 477 | Phage portal protein | Phage portal protein | | afdb-uniprot50 | AF-A0A5C8BL97-F1-MODEL\_V4 | 1.0 | 6.252e-26 | 897 | 0.219 | 488 | 334 | 16 | 49 | 498 | 46 | 524 | Phage portal protein | Phage portal protein | | afdb-uniprot50 | AF-A0A1I5F286-F1-MODEL\_V4 | 1.0 | 8.929e-27 | 896 | 0.19 | 535 | 365 | 17 | 41 | 532 | 48 | 557 | Phage portal protein, lambda family | Phage portal protein, lambda family | | afdb-uniprot50 | AF-A0A517NLJ6-F1-MODEL\_V4 | 1.0 | 2.183e-26 | 895 | 0.189 | 550 | 374 | 20 | 9 | 531 | 1 | 505 | Phage portal protein, lambda family | Phage portal protein, lambda family | | afdb-uniprot50 | AF-A0A1V1UMH2-F1-MODEL\_V4 | 1.0 | 9.412e-27 | 895 | 0.191 | 554 | 374 | 20 | 7 | 523 | 1 | 517 | Phage portal protein, lambda family | Phage portal protein, lambda family | | afdb-uniprot50 | AF-G4KQ83-F1-MODEL\_V4 | 1.0 | 1.769e-26 | 895 | 0.195 | 548 | 362 | 17 | 39 | 532 | 35 | 557 | Putative portal protein | Putative portal protein | | afdb-uniprot50 | AF-A0A1B9VJM9-F1-MODEL\_V4 | 1.0 | 2.454e-25 | 895 | 0.212 | 508 | 333 | 16 | 38 | 495 | 13 | 503 | Phage portal protein, lambda family | Phage portal protein, lambda family | | afdb-uniprot50 | AF-A0A5B8BXE4-F1-MODEL\_V4 | 1.0 | 8.227e-25 | 893 | 0.179 | 491 | 366 | 15 | 39 | 502 | 34 | 514 | Phage portal protein | Phage portal protein | | afdb-uniprot50 | AF-A0A7Y4DJD1-F1-MODEL\_V4 | 1.0 | 6.325e-25 | 892 | 0.145 | 488 | 389 | 13 | 37 | 517 | 21 | 487 | Phage portal protein | Phage portal protein | | afdb-uniprot50 | AF-A0A2Z5ZE91-F1-MODEL\_V4 | 1.0 | 1.593e-26 | 891 | 0.194 | 585 | 362 | 20 | 5 | 532 | 15 | 546 | Phage portal protein | Phage portal protein | | afdb-uniprot50 | AF-A0A7C4ZHN2-F1-MODEL\_V4 | 1.0 | 1.434e-26 | 890 | 0.227 | 480 | 308 | 17 | 28 | 491 | 1 | 433 | Phage portal protein | Phage portal protein | | afdb-uniprot50 | AF-E3HBM5-F1-MODEL\_V4 | 1.0 | 2.209e-25 | 890 | 0.204 | 495 | 353 | 19 | 31 | 498 | 19 | 499 | Phage portal protein, lambda family | Phage portal protein, lambda family | | afdb-uniprot50 | AF-A0A349HLY6-F1-MODEL\_V4 | 1.0 | 8.571e-26 | 889 | 0.24 | 499 | 307 | 19 | 67 | 532 | 2 | 461 | Phage portal protein | Phage portal protein | | afdb-uniprot50 | AF-A0A841G3H8-F1-MODEL\_V4 | 1.0 | 1.865e-26 | 889 | 0.204 | 548 | 361 | 20 | 1 | 502 | 78 | 596 | Lambda family phage portal protein | Lambda family phage portal protein | | afdb-uniprot50 | AF-A0A1G5T351-F1-MODEL\_V4 | 1.0 | 2.183e-26 | 888 | 0.171 | 555 | 403 | 15 | 11 | 532 | 2 | 532 | Phage portal protein, lambda family | Phage portal protein, lambda family | | afdb-uniprot50 | AF-A0A6N1VH77-F1-MODEL\_V4 | 1.0 | 1.239e-25 | 886 | 0.221 | 515 | 337 | 18 | 35 | 532 | 24 | 491 | Phage portal protein | Phage portal protein | | afdb-uniprot50 | AF-A0A7J5WDZ0-F1-MODEL\_V4 | 1.0 | 1.887e-25 | 886 | 0.194 | 525 | 360 | 20 | 42 | 532 | 12 | 507 | Phage portal protein lambda | Phage portal protein lambda | | afdb-uniprot50 | AF-A0A268THM8-F1-MODEL\_V4 | 1.0 | 1.467e-24 | 885 | 0.212 | 480 | 312 | 18 | 38 | 500 | 19 | 449 | Uncharacterized protein | Uncharacterized protein | | afdb-uniprot50 | AF-A0A7C8EMQ6-F1-MODEL\_V4 | 1.0 | 1.004e-25 | 885 | 0.188 | 520 | 366 | 15 | 2 | 502 | 7 | 489 | Phage portal protein | Phage portal protein | | afdb-uniprot50 | AF-A0A068SLY6-F1-MODEL\_V4 | 1.0 | 5.277e-27 | 885 | 0.195 | 538 | 366 | 20 | 17 | 532 | 1 | 493 | Phage portal protein, lambda family | Phage portal protein, lambda family | | afdb-uniprot50 | AF-A0A1G2YTA2-F1-MODEL\_V4 | 1.0 | 4.377e-25 | 884 | 0.216 | 495 | 340 | 17 | 17 | 502 | 1 | 456 | Uncharacterized protein | Uncharacterized protein | | afdb-uniprot50 | AF-A0A7V3NBN7-F1-MODEL\_V4 | 1.0 | 1.79e-25 | 884 | 0.208 | 548 | 354 | 21 | 4 | 532 | 1 | 487 | Phage portal protein | Phage portal protein | | afdb-uniprot50 | AF-A0A2N5XX91-F1-MODEL\_V4 | 1.0 | 6.001e-25 | 884 | 0.228 | 490 | 320 | 16 | 36 | 502 | 23 | 477 | Phage portal protein | Phage portal protein | | afdb-uniprot50 | AF-A0A258VI72-F1-MODEL\_V4 | 1.0 | 5.066e-26 | 884 | 0.203 | 520 | 352 | 20 | 36 | 532 | 21 | 501 | Uncharacterized protein | Uncharacterized protein | | afdb-uniprot50 | AF-A0A389MVE2-F1-MODEL\_V4 | 1.0 | 4.75e-27 | 884 | 0.185 | 589 | 379 | 18 | 1 | 532 | 10 | 554 | Uncharacterized protein | Uncharacterized protein | | afdb-uniprot50 | AF-A0A063ZY87-F1-MODEL\_V4 | 1.0 | 7.626e-27 | 884 | 0.211 | 515 | 352 | 15 | 39 | 517 | 579 | 1075 | Uncharacterized protein | Uncharacterized protein | | afdb-uniprot50 | AF-A0A357BWI3-F1-MODEL\_V4 | 1.0 | 1.909e-24 | 883 | 0.23 | 521 | 337 | 17 | 17 | 531 | 1 | 463 | Uncharacterized protein | Uncharacterized protein | | afdb-uniprot50 | AF-A0A3C1F6B3-F1-MODEL\_V4 | 1.0 | 5.693e-25 | 883 | 0.193 | 490 | 346 | 16 | 40 | 492 | 41 | 518 | Phage portal protein | Phage portal protein | | afdb-uniprot50 | AF-A0A550KMN9-F1-MODEL\_V4 | 1.0 | 1.239e-25 | 882 | 0.192 | 571 | 386 | 17 | 6 | 532 | 1 | 540 | Phage portal protein | Phage portal protein | | afdb-uniprot50 | AF-A0A3D5KD24-F1-MODEL\_V4 | 1.0 | 2.557e-26 | 881 | 0.2 | 513 | 357 | 14 | 37 | 526 | 43 | 525 | Uncharacterized protein | Uncharacterized protein | | afdb-uniprot50 | AF-A0A4P8IPG7-F1-MODEL\_V4 | 1.0 | 5.339e-26 | 880 | 0.175 | 524 | 359 | 16 | 46 | 518 | 26 | 527 | Phage portal protein | Phage portal protein | | afdb-uniprot50 | AF-A0A786U7W6-F1-MODEL\_V4 | 1.0 | 1.63e-24 | 879 | 0.219 | 460 | 305 | 14 | 70 | 488 | 2 | 448 | Phage portal protein | Phage portal protein | | afdb-uniprot50 | AF-A0A796MCI7-F1-MODEL\_V4 | 1.0 | 4.104e-26 | 879 | 0.228 | 534 | 348 | 18 | 35 | 532 | 19 | 524 | Phage portal protein | Phage portal protein | | afdb-uniprot50 | AF-A0A530AI76-F1-MODEL\_V4 | 1.0 | 3.826e-23 | 878 | 0.262 | 362 | 236 | 10 | 70 | 417 | 2 | 346 | Phage portal protein | Phage portal protein | | afdb-uniprot50 | AF-A0A7X7K4M3-F1-MODEL\_V4 | 1.0 | 1.547e-24 | 878 | 0.217 | 493 | 323 | 15 | 45 | 532 | 4 | 438 | Phage portal protein | Phage portal protein | | afdb-uniprot50 | AF-A0A7Y5S0S4-F1-MODEL\_V4 | 1.0 | 3.894e-26 | 878 | 0.209 | 530 | 345 | 20 | 22 | 532 | 6 | 480 | Phage portal protein | Phage portal protein | | afdb-uniprot50 | AF-A0A6L8M286-F1-MODEL\_V4 | 1.0 | 1.058e-25 | 878 | 0.194 | 510 | 368 | 17 | 39 | 532 | 21 | 503 | Phage portal protein | Phage portal protein | | afdb-uniprot50 | AF-A0A497U413-F1-MODEL\_V4 | 1.0 | 7.406e-25 | 878 | 0.217 | 473 | 306 | 14 | 73 | 502 | 2 | 453 | Phage portal protein | Phage portal protein | | afdb-uniprot50 | AF-A0A706VWX3-F1-MODEL\_V4 | 1.0 | 1.679e-26 | 878 | 0.181 | 585 | 381 | 22 | 8 | 532 | 1 | 547 | Phage portal protein | Phage portal protein | | afdb-uniprot50 | AF-A0A239C998-F1-MODEL\_V4 | 1.0 | 9.034e-26 | 877 | 0.206 | 533 | 364 | 19 | 27 | 532 | 1 | 501 | Phage portal protein, lambda family | Phage portal protein, lambda family | | afdb-uniprot50 | AF-A0A661TNZ2-F1-MODEL\_V4 | 1.0 | 4.326e-26 | 877 | 0.18 | 571 | 376 | 17 | 6 | 497 | 59 | 616 | Phage portal protein | Phage portal protein | | afdb-uniprot50 | AF-A0A4Q3P809-F1-MODEL\_V4 | 1.0 | 2.209e-25 | 876 | 0.229 | 505 | 331 | 19 | 11 | 488 | 1 | 474 | Phage portal protein | Phage portal protein | | afdb-uniprot50 | AF-A0A5C7LHV8-F1-MODEL\_V4 | 1.0 | 1.058e-25 | 876 | 0.232 | 521 | 347 | 18 | 23 | 532 | 12 | 490 | Phage portal protein | Phage portal protein | | afdb-uniprot50 | AF-A0A2W6XAC1-F1-MODEL\_V4 | 1.0 | 2.096e-25 | 876 | 0.225 | 484 | 322 | 16 | 39 | 502 | 33 | 483 | Phage portal protein | Phage portal protein | | afdb-uniprot50 | AF-B6Y9F9-F1-MODEL\_V4 | 1.0 | 7.493e-24 | 875 | 0.295 | 369 | 239 | 8 | 135 | 496 | 4 | 358 | Putative phage portal protein | Putative phage portal protein | | afdb-uniprot50 | AF-A0A1M2YW43-F1-MODEL\_V4 | 1.0 | 6.325e-25 | 875 | 0.202 | 484 | 324 | 15 | 62 | 503 | 3 | 466 | Phage portal protein | Phage portal protein | | afdb-uniprot50 | AF-A0A2E2B1J6-F1-MODEL\_V4 | 1.0 | 6.666e-25 | 875 | 0.209 | 507 | 348 | 17 | 13 | 502 | 1 | 471 | Uncharacterized protein | Uncharacterized protein | | afdb-uniprot50 | AF-A0A1V5QVH4-F1-MODEL\_V4 | 1.0 | 1.376e-25 | 875 | 0.204 | 519 | 357 | 19 | 40 | 531 | 38 | 527 | Phage portal protein, lambda family | Phage portal protein, lambda family | | afdb-uniprot50 | AF-A0A5C5WDP0-F1-MODEL\_V4 | 1.0 | 1.529e-25 | 874 | 0.176 | 527 | 373 | 16 | 2 | 497 | 9 | 505 | Phage portal protein, lambda family | Phage portal protein, lambda family | | afdb-uniprot50 | AF-A0A1B1C3C9-F1-MODEL\_V4 | 1.0 | 3.94e-25 | 873 | 0.166 | 504 | 373 | 14 | 37 | 532 | 26 | 490 | Uncharacterized protein | Uncharacterized protein | | afdb-uniprot50 | AF-A0A2A5AG71-F1-MODEL\_V4 | 1.0 | 1.175e-25 | 872 | 0.185 | 571 | 370 | 18 | 20 | 532 | 1 | 534 | Uncharacterized protein | Uncharacterized protein | | afdb-uniprot50 | AF-A0A2W6T5F2-F1-MODEL\_V4 | 1.0 | 4.862e-25 | 871 | 0.182 | 509 | 336 | 16 | 53 | 503 | 13 | 499 | Phage portal protein | Phage portal protein | | afdb-uniprot50 | AF-A0A2S5TR06-F1-MODEL\_V4 | 1.0 | 6.001e-25 | 871 | 0.181 | 501 | 357 | 15 | 34 | 495 | 25 | 511 | Phage portal protein | Phage portal protein | | afdb-uniprot50 | AF-A0A6N1VGE5-F1-MODEL\_V4 | 1.0 | 4.326e-26 | 871 | 0.204 | 553 | 351 | 20 | 36 | 531 | 25 | 545 | Phage portal protein | Phage portal protein | | afdb-uniprot50 | AF-A0A1C7BGI4-F1-MODEL\_V4 | 1.0 | 4.613e-25 | 870 | 0.203 | 511 | 346 | 19 | 54 | 532 | 3 | 484 | Lambda family phage portal protein | Lambda family phage portal protein | | afdb-uniprot50 | AF-A0A3B8S5Y1-F1-MODEL\_V4 | 1.0 | 1.45e-25 | 870 | 0.21 | 512 | 351 | 18 | 34 | 499 | 12 | 516 | Phage portal protein | Phage portal protein | | afdb-uniprot50 | AF-A0A0F3IYT1-F1-MODEL\_V4 | 1.0 | 9.412e-27 | 869 | 0.243 | 484 | 310 | 16 | 36 | 500 | 23 | 469 | Uncharacterized protein | Uncharacterized protein | | afdb-uniprot50 | AF-A0A2P2E5U7-F1-MODEL\_V4 | 1.0 | 8.227e-25 | 869 | 0.204 | 490 | 341 | 18 | 39 | 502 | 30 | 496 | Uncharacterized protein | Uncharacterized protein | | afdb-uniprot50 | AF-A0A1I3H6N4-F1-MODEL\_V4 | 1.0 | 1.058e-25 | 869 | 0.191 | 527 | 360 | 18 | 39 | 532 | 27 | 520 | Phage portal protein, lambda family | Phage portal protein, lambda family | | afdb-uniprot50 | AF-A0A1G3M2B9-F1-MODEL\_V4 | 1.0 | 1.115e-25 | 868 | 0.198 | 544 | 352 | 22 | 24 | 532 | 1 | 495 | Phage portal protein | Phage portal protein | | afdb-uniprot50 | AF-A0A7C5LXR1-F1-MODEL\_V4 | 1.0 | 1.175e-25 | 868 | 0.198 | 524 | 362 | 17 | 22 | 532 | 28 | 506 | Phage portal protein | Phage portal protein | | afdb-uniprot50 | AF-B4CXQ8-F1-MODEL\_V4 | 1.0 | 3.738e-25 | 867 | 0.199 | 497 | 323 | 17 | 38 | 505 | 3 | 453 | Portal protein lambda | Portal protein lambda | | afdb-uniprot50 | AF-A0A6M3MCR2-F1-MODEL\_V4 | 1.0 | 3.505e-26 | 867 | 0.178 | 515 | 347 | 16 | 39 | 529 | 47 | 509 | Putative portal protein | Putative portal protein | | afdb-uniprot50 | AF-A0A843BDF6-F1-MODEL\_V4 | 1.0 | 4.104e-26 | 867 | 0.208 | 527 | 349 | 18 | 37 | 531 | 34 | 524 | Phage portal protein | Phage portal protein | | afdb-uniprot50 | AF-A0A2S8FUK2-F1-MODEL\_V4 | 1.0 | 3.029e-25 | 866 | 0.225 | 509 | 341 | 20 | 39 | 527 | 29 | 504 | Uncharacterized protein | Uncharacterized protein | | afdb-uniprot50 | AF-A0A1U9NQD9-F1-MODEL\_V4 | 1.0 | 4.326e-26 | 866 | 0.198 | 550 | 360 | 22 | 14 | 532 | 1 | 500 | Phage portal protein, lambda family | Phage portal protein, lambda family | | afdb-uniprot50 | AF-A0A2E3DCB1-F1-MODEL\_V4 | 1.0 | 7.32e-26 | 866 | 0.176 | 550 | 373 | 18 | 39 | 532 | 40 | 565 | Uncharacterized protein | Uncharacterized protein | | afdb-uniprot50 | AF-A0A1W9HA00-F1-MODEL\_V4 | 1.0 | 2.907e-24 | 865 | 0.246 | 486 | 306 | 18 | 24 | 502 | 1 | 433 | Uncharacterized protein | Uncharacterized protein | | afdb-uniprot50 | AF-A0A358PRF9-F1-MODEL\_V4 | 1.0 | 3.94e-25 | 865 | 0.221 | 483 | 323 | 15 | 85 | 527 | 3 | 472 | Phage portal protein | Phage portal protein | | afdb-uniprot50 | AF-A0A3A1YQG3-F1-MODEL\_V4 | 1.0 | 1.306e-25 | 865 | 0.186 | 542 | 376 | 19 | 4 | 532 | 1 | 490 | Uncharacterized protein | Uncharacterized protein | | afdb-uniprot50 | AF-S9S655-F1-MODEL\_V4 | 1.0 | 3.738e-25 | 863 | 0.19 | 556 | 367 | 22 | 27 | 532 | 13 | 535 | Phage portal protein, lambda family | Phage portal protein, lambda family | | afdb-uniprot50 | AF-A0A661I4X9-F1-MODEL\_V4 | 1.0 | 1.63e-24 | 862 | 0.176 | 493 | 344 | 16 | 39 | 521 | 6 | 446 | Phage portal protein | Phage portal protein | | afdb-uniprot50 | AF-A0A1M3AIF6-F1-MODEL\_V4 | 1.0 | 2.483e-24 | 862 | 0.176 | 533 | 348 | 18 | 52 | 532 | 5 | 498 | Phage portal protein | Phage portal protein | | afdb-uniprot50 | AF-A0A4V0I5D1-F1-MODEL\_V4 | 1.0 | 3.695e-26 | 862 | 0.206 | 561 | 371 | 21 | 1 | 532 | 1 | 516 | Uncharacterized protein | Uncharacterized protein | | afdb-uniprot50 | AF-A0A1Q3NUP4-F1-MODEL\_V4 | 1.0 | 6.666e-25 | 862 | 0.219 | 506 | 329 | 18 | 45 | 503 | 19 | 505 | Phage portal protein | Phage portal protein | | afdb-uniprot50 | AF-A0A4R3MIV3-F1-MODEL\_V4 | 1.0 | 1.253e-24 | 861 | 0.273 | 432 | 266 | 13 | 15 | 434 | 1 | 396 | Lambda family phage portal protein | Lambda family phage portal protein | | afdb-uniprot50 | AF-A0A2A5DIF0-F1-MODEL\_V4 | 1.0 | 4.428e-24 | 860 | 0.201 | 471 | 327 | 14 | 39 | 501 | 34 | 463 | Phage portal protein | Phage portal protein | | afdb-uniprot50 | AF-A0A0F9JDA0-F1-MODEL\_V4 | 1.0 | 1.529e-25 | 860 | 0.203 | 530 | 351 | 21 | 17 | 531 | 1 | 474 | Uncharacterized protein | Uncharacterized protein | | afdb-uniprot50 | AF-A0A2A4HXW4-F1-MODEL\_V4 | 1.0 | 5.465e-24 | 860 | 0.242 | 471 | 319 | 14 | 39 | 502 | 31 | 470 | Phage portal protein | Phage portal protein | | afdb-uniprot50 | AF-A0A317FAF6-F1-MODEL\_V4 | 1.0 | 9.634e-25 | 859 | 0.218 | 522 | 325 | 18 | 45 | 532 | 12 | 484 | Phage portal protein | Phage portal protein | | afdb-uniprot50 | AF-A0A5C7Q4H8-F1-MODEL\_V4 | 1.0 | 1.189e-24 | 858 | 0.217 | 511 | 334 | 20 | 27 | 502 | 3 | 482 | Phage portal protein | Phage portal protein | | afdb-uniprot50 | AF-A0A0Q0F5Y9-F1-MODEL\_V4 | 1.0 | 2.483e-24 | 857 | 0.225 | 453 | 305 | 17 | 77 | 496 | 2 | 441 | Portal protein, lambda family | Portal protein, lambda family | | afdb-uniprot50 | AF-A0A7X4AD17-F1-MODEL\_V4 | 1.0 | 3.782e-24 | 857 | 0.203 | 521 | 364 | 16 | 3 | 518 | 1 | 475 | Phage portal protein | Phage portal protein | | afdb-uniprot50 | AF-A0A3L7SGJ4-F1-MODEL\_V4 | 1.0 | 5.066e-26 | 857 | 0.215 | 544 | 346 | 22 | 1 | 532 | 4 | 478 | Phage portal protein | Phage portal protein | | afdb-uniprot50 | AF-A0A517SER7-F1-MODEL\_V4 | 1.0 | 1.07e-24 | 856 | 0.213 | 510 | 360 | 19 | 1 | 502 | 3 | 479 | Phage portal protein, lambda family | Phage portal protein, lambda family | | afdb-uniprot50 | AF-A0A1Y0FDI5-F1-MODEL\_V4 | 1.0 | 3.404e-24 | 855 | 0.27 | 403 | 258 | 14 | 68 | 451 | 2 | 387 | Uncharacterized protein | Uncharacterized protein | | afdb-uniprot50 | AF-A0A2T5K791-F1-MODEL\_V4 | 1.0 | 3.546e-25 | 854 | 0.232 | 504 | 317 | 21 | 15 | 502 | 1 | 450 | Lambda family phage portal protein | Lambda family phage portal protein | | afdb-uniprot50 | AF-A0A1W1Y8S5-F1-MODEL\_V4 | 1.0 | 6.945e-26 | 854 | 0.174 | 555 | 377 | 22 | 1 | 523 | 4 | 509 | Capsid protein | Capsid protein | | afdb-uniprot50 | AF-A0A7S6MI22-F1-MODEL\_V4 | 1.0 | 9.14e-25 | 853 | 0.192 | 524 | 353 | 15 | 42 | 515 | 23 | 526 | Phage portal protein | Phage portal protein | | afdb-uniprot50 | AF-A0A840JB72-F1-MODEL\_V4 | 1.0 | 2.035e-23 | 852 | 0.224 | 415 | 291 | 13 | 98 | 495 | 2 | 402 | Lambda family phage portal protein | Lambda family phage portal protein | | afdb-uniprot50 | AF-A0A3S0EMD5-F1-MODEL\_V4 | 1.0 | 2.235e-24 | 852 | 0.21 | 508 | 346 | 18 | 9 | 502 | 1 | 467 | Phage portal protein | Phage portal protein | | afdb-uniprot50 | AF-A0A8B2PKS7-F1-MODEL\_V4 | 1.0 | 3.738e-25 | 852 | 0.21 | 538 | 338 | 16 | 46 | 532 | 26 | 527 | Uncharacterized protein | Uncharacterized protein | | afdb-uniprot50 | AF-A0A3A1Y7Y1-F1-MODEL\_V4 | 1.0 | 1.015e-24 | 852 | 0.172 | 505 | 372 | 15 | 36 | 532 | 45 | 511 | Uncharacterized protein | Uncharacterized protein | | afdb-uniprot50 | AF-A0A2D2C211-F1-MODEL\_V4 | 1.0 | 7.806e-25 | 851 | 0.198 | 530 | 352 | 18 | 15 | 532 | 1 | 469 | Phage portal protein | Phage portal protein | | afdb-uniprot50 | AF-A0A7C1QAN0-F1-MODEL\_V4 | 1.0 | 8.227e-25 | 851 | 0.214 | 517 | 358 | 19 | 1 | 502 | 9 | 492 | Phage portal protein | Phage portal protein | | afdb-uniprot50 | AF-A0A1X0SWB6-F1-MODEL\_V4 | 1.0 | 4.862e-25 | 851 | 0.205 | 556 | 357 | 21 | 9 | 515 | 14 | 533 | Phage portal protein, lambda family | Phage portal protein, lambda family | | afdb-uniprot50 | AF-A0A6L6J9Q3-F1-MODEL\_V4 | 1.0 | 5.246e-23 | 850 | 0.516 | 269 | 119 | 4 | 266 | 532 | 41 | 300 | Phage portal protein | Phage portal protein | | afdb-uniprot50 | AF-A0A522WDL3-F1-MODEL\_V4 | 1.0 | 2.261e-23 | 850 | 0.256 | 393 | 265 | 10 | 122 | 502 | 5 | 382 | Phage portal protein | Phage portal protein | | afdb-uniprot50 | AF-A0A0F9KBE7-F1-MODEL\_V4 | 1.0 | 2.758e-24 | 850 | 0.274 | 422 | 262 | 9 | 127 | 532 | 7 | 400 | Uncharacterized protein | Uncharacterized protein | | afdb-uniprot50 | AF-A0A4P5XY08-F1-MODEL\_V4 | 1.0 | 3.986e-24 | 849 | 0.199 | 507 | 356 | 16 | 34 | 532 | 5 | 469 | Uncharacterized protein | Uncharacterized protein | | afdb-uniprot50 | AF-A0A496PDJ5-F1-MODEL\_V4 | 1.0 | 4.862e-25 | 848 | 0.202 | 500 | 325 | 17 | 81 | 532 | 2 | 475 | Phage portal protein | Phage portal protein | | afdb-uniprot50 | AF-A0A812S2M1-F1-MODEL\_V4 | 1.0 | 5.76e-24 | 847 | 0.19 | 483 | 352 | 19 | 43 | 502 | 12 | 478 | Gene 4 protein | Gene 4 protein | | afdb-uniprot50 | AF-A0A4Q6F5P2-F1-MODEL\_V4 | 1.0 | 1.336e-23 | 846 | 0.209 | 415 | 293 | 14 | 59 | 449 | 1 | 404 | Phage portal protein | Phage portal protein | | afdb-uniprot50 | AF-A0A4E0QSA5-F1-MODEL\_V4 | 1.0 | 9.247e-24 | 846 | 0.232 | 460 | 292 | 17 | 39 | 487 | 7 | 416 | Phage portal protein | Phage portal protein | | afdb-uniprot50 | AF-A0A6N8K7M5-F1-MODEL\_V4 | 1.0 | 5.125e-25 | 846 | 0.202 | 518 | 341 | 19 | 62 | 530 | 1 | 495 | Phage portal protein | Phage portal protein | | afdb-uniprot50 | AF-A0A2T6DLK8-F1-MODEL\_V4 | 1.0 | 3.23e-24 | 846 | 0.192 | 483 | 332 | 17 | 44 | 505 | 23 | 468 | Uncharacterized protein | Uncharacterized protein | | afdb-uniprot50 | AF-A0A518L4N8-F1-MODEL\_V4 | 1.0 | 6.666e-25 | 845 | 0.192 | 562 | 382 | 16 | 14 | 515 | 1 | 550 | Phage portal protein, lambda family | Phage portal protein, lambda family | | afdb-uniprot50 | AF-A0A443L3G4-F1-MODEL\_V4 | 1.0 | 6.474e-23 | 844 | 0.256 | 378 | 256 | 11 | 36 | 405 | 28 | 388 | Phage portal protein | Phage portal protein | | afdb-uniprot50 | AF-A0A2D8S0N3-F1-MODEL\_V4 | 1.0 | 4.377e-25 | 844 | 0.167 | 538 | 372 | 17 | 7 | 502 | 1 | 504 | Phage portal protein | Phage portal protein | | afdb-uniprot50 | AF-A0A085TW01-F1-MODEL\_V4 | 1.0 | 2.383e-23 | 843 | 0.255 | 391 | 267 | 11 | 105 | 491 | 3 | 373 | Phage portal protein, lambda family | Phage portal protein, lambda family | | afdb-uniprot50 | AF-A0A353B4R9-F1-MODEL\_V4 | 1.0 | 1.408e-23 | 843 | 0.214 | 508 | 329 | 19 | 19 | 519 | 11 | 455 | Uncharacterized protein | Uncharacterized protein | | afdb-uniprot50 | AF-U1YH26-F1-MODEL\_V4 | 1.0 | 1.027e-23 | 842 | 0.228 | 420 | 283 | 12 | 127 | 532 | 3 | 395 | Uncharacterized protein | Uncharacterized protein | | afdb-uniprot50 | AF-A0A1C7GVL9-F1-MODEL\_V4 | 1.0 | 5.76e-24 | 842 | 0.216 | 435 | 288 | 10 | 39 | 468 | 21 | 407 | Uncharacterized protein | Uncharacterized protein | | afdb-uniprot50 | AF-U7GCW3-F1-MODEL\_V4 | 1.0 | 2.328e-25 | 842 | 0.193 | 589 | 382 | 21 | 1 | 530 | 3 | 557 | Portal protein | Portal protein | | afdb-uniprot50 | AF-M5RV16-F1-MODEL\_V4 | 1.0 | 1.811e-24 | 841 | 0.201 | 545 | 377 | 20 | 1 | 531 | 14 | 514 | Phage portal protein, lambda family | Phage portal protein, lambda family | | afdb-uniprot50 | AF-A0A1E4N739-F1-MODEL\_V4 | 1.0 | 1.79e-25 | 841 | 0.172 | 568 | 377 | 21 | 21 | 532 | 32 | 562 | Phage portal protein | Phage portal protein | | afdb-uniprot50 | AF-A0A290MQ25-F1-MODEL\_V4 | 1.0 | 6.666e-25 | 840 | 0.189 | 550 | 359 | 17 | 39 | 532 | 21 | 539 | Phage portal protein | Phage portal protein | | afdb-uniprot50 | AF-A0A2D5FQ67-F1-MODEL\_V4 | 1.0 | 1.07e-24 | 838 | 0.18 | 560 | 382 | 21 | 2 | 502 | 6 | 547 | Uncharacterized protein | Uncharacterized protein | | afdb-uniprot50 | AF-A0A376ZFL5-F1-MODEL\_V4 | 1.0 | 4.377e-25 | 838 | 0.196 | 576 | 385 | 21 | 11 | 532 | 45 | 596 | Capsid protein of prophage | Capsid protein of prophage | | afdb-uniprot50 | AF-A0A078MHH5-F1-MODEL\_V4 | 1.0 | 1.887e-25 | 836 | 0.194 | 565 | 365 | 19 | 20 | 532 | 1 | 527 | Phage portal protein, lambda family | Phage portal protein, lambda family | | afdb-uniprot50 | AF-A0A653RBF9-F1-MODEL\_V4 | 1.0 | 3.738e-25 | 835 | 0.187 | 556 | 383 | 21 | 20 | 532 | 2 | 531 | Phage portal protein | Phage portal protein | | afdb-uniprot50 | AF-A0A087M4C7-F1-MODEL\_V4 | 1.0 | 5.402e-25 | 835 | 0.172 | 558 | 400 | 18 | 15 | 532 | 1 | 536 | Uncharacterized protein | Uncharacterized protein | | afdb-uniprot50 | AF-A0A4Z1R295-F1-MODEL\_V4 | 1.0 | 6.071e-24 | 833 | 0.179 | 536 | 352 | 21 | 49 | 532 | 4 | 503 | Phage portal protein | Phage portal protein | | afdb-uniprot50 | AF-A0A4Y7RX04-F1-MODEL\_V4 | 1.0 | 4.153e-25 | 833 | 0.213 | 548 | 346 | 19 | 39 | 532 | 17 | 533 | Uncharacterized protein | Uncharacterized protein | | afdb-uniprot50 | AF-A0A840FJN1-F1-MODEL\_V4 | 1.0 | 1.811e-24 | 833 | 0.177 | 512 | 366 | 15 | 39 | 502 | 29 | 533 | Lambda family phage portal protein | Lambda family phage portal protein | | afdb-uniprot50 | AF-A0A5C6C1F2-F1-MODEL\_V4 | 1.0 | 2.758e-24 | 832 | 0.198 | 530 | 350 | 19 | 27 | 532 | 8 | 486 | Phage portal protein, lambda family | Phage portal protein, lambda family | | afdb-uniprot50 | AF-A0A353BG30-F1-MODEL\_V4 | 1.0 | 2.012e-24 | 832 | 0.172 | 528 | 371 | 21 | 37 | 532 | 8 | 501 | Uncharacterized protein | Uncharacterized protein | | afdb-uniprot50 | AF-A0A1Z9JFC0-F1-MODEL\_V4 | 1.0 | 2.035e-23 | 831 | 0.169 | 520 | 364 | 19 | 27 | 530 | 11 | 478 | Uncharacterized protein | Uncharacterized protein | | afdb-uniprot50 | AF-A0A1Q3Z0R9-F1-MODEL\_V4 | 1.0 | 4.201e-24 | 831 | 0.211 | 486 | 329 | 14 | 58 | 502 | 3 | 475 | Phage portal protein | Phage portal protein | | afdb-uniprot50 | AF-A0A2E2KT33-F1-MODEL\_V4 | 1.0 | 2.035e-23 | 831 | 0.194 | 484 | 341 | 14 | 46 | 490 | 5 | 478 | Phage portal protein | Phage portal protein | | afdb-uniprot50 | AF-A0A7Y9FKF2-F1-MODEL\_V4 | 1.0 | 3.23e-24 | 830 | 0.231 | 513 | 322 | 17 | 35 | 531 | 24 | 480 | Lambda family phage portal protein | Lambda family phage portal protein | | afdb-uniprot50 | AF-A0A285YSW8-F1-MODEL\_V4 | 1.0 | 1.738e-23 | 830 | 0.169 | 495 | 337 | 15 | 62 | 502 | 5 | 479 | Phage portal protein, lambda family | Phage portal protein, lambda family | | afdb-uniprot50 | AF-D8JWB8-F1-MODEL\_V4 | 1.0 | 1.321e-24 | 830 | 0.194 | 535 | 365 | 15 | 18 | 502 | 1 | 519 | Phage portal protein, lambda family | Phage portal protein, lambda family | | afdb-uniprot50 | AF-A0A270BET9-F1-MODEL\_V4 | 1.0 | 6.666e-25 | 830 | 0.182 | 597 | 382 | 22 | 9 | 532 | 10 | 573 | Phage portal protein | Phage portal protein | | afdb-uniprot50 | AF-A0A7X3I6F0-F1-MODEL\_V4 | 1.0 | 6.666e-25 | 830 | 0.195 | 572 | 379 | 16 | 1 | 532 | 1 | 531 | Phage portal protein | Phage portal protein | | afdb-uniprot50 | AF-A0A2E7ZPP6-F1-MODEL\_V4 | 1.0 | 6.745e-24 | 829 | 0.163 | 515 | 364 | 17 | 39 | 532 | 5 | 473 | Uncharacterized protein | Uncharacterized protein | | afdb-uniprot50 | AF-A0A812INR5-F1-MODEL\_V4 | 1.0 | 1.083e-23 | 829 | 0.226 | 451 | 295 | 18 | 54 | 473 | 49 | 476 | B protein | B protein | | afdb-uniprot50 | AF-A0A357XSR0-F1-MODEL\_V4 | 1.0 | 3.588e-24 | 828 | 0.169 | 560 | 395 | 18 | 1 | 531 | 1 | 519 | Uncharacterized protein | Uncharacterized protein | | afdb-uniprot50 | AF-A0A259MIN2-F1-MODEL\_V4 | 1.0 | 1.07e-24 | 828 | 0.168 | 557 | 400 | 16 | 1 | 532 | 58 | 576 | Phage portal protein | Phage portal protein | | afdb-uniprot50 | AF-A0A1I3ZDY8-F1-MODEL\_V4 | 1.0 | 2.145e-23 | 826 | 0.201 | 486 | 335 | 18 | 35 | 502 | 20 | 470 | Phage portal protein, lambda family | Phage portal protein, lambda family | | afdb-uniprot50 | AF-A0A351VMH6-F1-MODEL\_V4 | 1.0 | 6.325e-25 | 826 | 0.182 | 548 | 373 | 18 | 15 | 497 | 1 | 538 | Phage portal protein | Phage portal protein | | afdb-uniprot50 | AF-A0A6I1IVV5-F1-MODEL\_V4 | 1.0 | 7.493e-24 | 825 | 0.198 | 515 | 339 | 17 | 49 | 522 | 7 | 488 | Phage portal protein | Phage portal protein | | afdb-uniprot50 | AF-A0A4Q3GP67-F1-MODEL\_V4 | 1.0 | 4.428e-24 | 825 | 0.189 | 506 | 354 | 16 | 23 | 488 | 1 | 490 | Phage portal protein | Phage portal protein | | afdb-uniprot50 | AF-A0A258L750-F1-MODEL\_V4 | 1.0 | 1.189e-24 | 825 | 0.19 | 530 | 366 | 19 | 23 | 502 | 1 | 517 | Phage portal protein | Phage portal protein | | afdb-uniprot50 | AF-A0A2U1SE48-F1-MODEL\_V4 | 1.0 | 2.209e-25 | 825 | 0.197 | 572 | 374 | 19 | 2 | 525 | 1 | 535 | Uncharacterized protein | Uncharacterized protein | | afdb-uniprot50 | AF-A0A7X8YIN0-F1-MODEL\_V4 | 1.0 | 2.012e-24 | 824 | 0.182 | 515 | 363 | 15 | 39 | 532 | 20 | 497 | Phage portal protein | Phage portal protein | | afdb-uniprot50 | AF-A0A518GNB7-F1-MODEL\_V4 | 1.0 | 1.811e-24 | 824 | 0.205 | 526 | 346 | 22 | 29 | 532 | 12 | 487 | Phage portal protein, lambda family | Phage portal protein, lambda family | | afdb-uniprot50 | AF-A0A4P7EGH2-F1-MODEL\_V4 | 1.0 | 7.109e-24 | 821 | 0.194 | 504 | 334 | 18 | 51 | 503 | 28 | 510 | Phage portal protein | Phage portal protein | | afdb-uniprot50 | AF-A0A420WDI6-F1-MODEL\_V4 | 1.0 | 2.454e-25 | 821 | 0.201 | 562 | 361 | 27 | 1 | 502 | 2 | 535 | Lambda family phage portal protein | Lambda family phage portal protein | | afdb-uniprot50 | AF-A0A5E8P2L6-F1-MODEL\_V4 | 1.0 | 6.214e-22 | 820 | 0.222 | 369 | 258 | 13 | 93 | 450 | 2 | 352 | Phage portal protein | Phage portal protein | | afdb-uniprot50 | AF-A0A349JAV8-F1-MODEL\_V4 | 1.0 | 3.63e-23 | 820 | 0.213 | 478 | 330 | 17 | 38 | 502 | 30 | 474 | Uncharacterized protein | Uncharacterized protein | | afdb-uniprot50 | AF-A0A7W1L746-F1-MODEL\_V4 | 1.0 | 3.1e-23 | 819 | 0.209 | 472 | 325 | 17 | 10 | 473 | 14 | 445 | Phage portal protein | Phage portal protein | | afdb-uniprot50 | AF-A0A530QV48-F1-MODEL\_V4 | 1.0 | 9.861e-23 | 818 | 0.303 | 356 | 231 | 7 | 148 | 492 | 2 | 351 | Phage portal protein | Phage portal protein | | afdb-uniprot50 | AF-A0A3E0N0Y2-F1-MODEL\_V4 | 1.0 | 1.909e-24 | 818 | 0.195 | 486 | 326 | 18 | 62 | 519 | 1 | 449 | Phage portal protein | Phage portal protein | | afdb-uniprot50 | AF-A0A7Z6W8D4-F1-MODEL\_V4 | 1.0 | 4.667e-24 | 818 | 0.18 | 525 | 368 | 14 | 11 | 497 | 1 | 501 | Phage portal protein | Phage portal protein | | afdb-uniprot50 | AF-A0A766GFH2-F1-MODEL\_V4 | 1.0 | 9.747e-24 | 817 | 0.176 | 522 | 346 | 18 | 59 | 523 | 1 | 495 | Phage portal protein | Phage portal protein | | afdb-uniprot50 | AF-A0A0F9R9A4-F1-MODEL\_V4 | 1.0 | 3.588e-24 | 817 | 0.183 | 540 | 367 | 20 | 23 | 532 | 1 | 496 | Uncharacterized protein | Uncharacterized protein | | afdb-uniprot50 | AF-A0A518CTW5-F1-MODEL\_V4 | 1.0 | 2.907e-24 | 817 | 0.167 | 543 | 383 | 19 | 27 | 532 | 2 | 512 | Phage portal protein, lambda family | Phage portal protein, lambda family | | afdb-uniprot50 | AF-A0A2D5TT87-F1-MODEL\_V4 | 1.0 | 3.986e-24 | 817 | 0.187 | 540 | 371 | 22 | 36 | 532 | 19 | 533 | Uncharacterized protein | Uncharacterized protein | | afdb-uniprot50 | AF-A0A1F9C0U9-F1-MODEL\_V4 | 1.0 | 7.493e-24 | 816 | 0.222 | 458 | 303 | 14 | 94 | 532 | 2 | 425 | Phage portal protein | Phage portal protein | | afdb-uniprot50 | AF-A0A518G2U8-F1-MODEL\_V4 | 1.0 | 1.931e-23 | 816 | 0.188 | 540 | 390 | 19 | 1 | 531 | 11 | 511 | Phage portal protein, lambda family | Phage portal protein, lambda family | | afdb-uniprot50 | AF-A0A517VMM7-F1-MODEL\_V4 | 1.0 | 1.811e-24 | 816 | 0.198 | 543 | 370 | 17 | 13 | 531 | 1 | 502 | Phage portal protein, lambda family | Phage portal protein, lambda family | | afdb-uniprot50 | AF-A0A5C5XSQ0-F1-MODEL\_V4 | 1.0 | 7.897e-24 | 815 | 0.218 | 517 | 334 | 20 | 28 | 532 | 14 | 472 | Phage portal protein, lambda family | Phage portal protein, lambda family | | afdb-uniprot50 | AF-A0A398BXH2-F1-MODEL\_V4 | 1.0 | 2.12e-24 | 815 | 0.192 | 583 | 392 | 23 | 2 | 532 | 17 | 572 | Phage portal protein | Phage portal protein | | afdb-uniprot50 | AF-A0A430EDN5-F1-MODEL\_V4 | 1.0 | 1.909e-24 | 814 | 0.242 | 495 | 312 | 18 | 39 | 517 | 32 | 479 | Phage portal protein | Phage portal protein | | afdb-uniprot50 | AF-A0A1C0SI27-F1-MODEL\_V4 | 1.0 | 2.791e-23 | 814 | 0.169 | 530 | 361 | 15 | 43 | 532 | 7 | 497 | Phage portal protein | Phage portal protein | | afdb-uniprot50 | AF-A0A7C7PZ16-F1-MODEL\_V4 | 1.0 | 8.672e-25 | 814 | 0.178 | 533 | 374 | 19 | 35 | 532 | 25 | 528 | Phage portal protein | Phage portal protein | | afdb-uniprot50 | AF-A0A1G7NEZ5-F1-MODEL\_V4 | 1.0 | 2.356e-24 | 814 | 0.192 | 550 | 358 | 25 | 15 | 501 | 1 | 527 | Phage portal protein, lambda family | Phage portal protein, lambda family | | afdb-uniprot50 | AF-A0A5N3PHC4-F1-MODEL\_V4 | 1.0 | 6.399e-24 | 814 | 0.169 | 550 | 382 | 20 | 24 | 532 | 12 | 527 | Phage portal protein | Phage portal protein | | afdb-uniprot50 | AF-A0A1N7SSK9-F1-MODEL\_V4 | 1.0 | 1.268e-23 | 813 | 0.168 | 533 | 351 | 17 | 57 | 532 | 2 | 499 | Phage portal | Phage portal | | afdb-uniprot50 | AF-A0A1M7D8F9-F1-MODEL\_V4 | 1.0 | 1.547e-24 | 813 | 0.188 | 541 | 351 | 20 | 7 | 501 | 1 | 499 | Phage portal protein, lambda family | Phage portal protein, lambda family | | afdb-uniprot50 | AF-A0A4P7WG88-F1-MODEL\_V4 | 1.0 | 1.467e-24 | 812 | 0.193 | 547 | 344 | 25 | 47 | 517 | 11 | 536 | Phage portal protein | Phage portal protein | | afdb-uniprot50 | AF-A0A3D4D8W6-F1-MODEL\_V4 | 1.0 | 9.522e-26 | 812 | 0.217 | 537 | 357 | 17 | 35 | 523 | 18 | 539 | Phage portal protein | Phage portal protein | | afdb-uniprot50 | AF-A0A2W5S548-F1-MODEL\_V4 | 1.0 | 2.012e-24 | 812 | 0.183 | 549 | 363 | 19 | 23 | 502 | 2 | 533 | Phage portal protein | Phage portal protein | | afdb-uniprot50 | AF-A0A1V5NFL2-F1-MODEL\_V4 | 1.0 | 2.758e-24 | 812 | 0.213 | 543 | 351 | 18 | 17 | 532 | 1 | 494 | Phage portal protein, lambda family | Phage portal protein, lambda family | | afdb-uniprot50 | AF-A0A522VYK9-F1-MODEL\_V4 | 1.0 | 6.904e-22 | 810 | 0.241 | 372 | 254 | 10 | 39 | 395 | 2 | 360 | Phage portal protein | Phage portal protein | | afdb-uniprot50 | AF-A0A7K1B1S8-F1-MODEL\_V4 | 1.0 | 3.404e-24 | 810 | 0.18 | 550 | 376 | 19 | 6 | 532 | 1 | 498 | Phage portal protein | Phage portal protein | | afdb-uniprot50 | AF-A0A7V9JT41-F1-MODEL\_V4 | 1.0 | 6.142e-23 | 808 | 0.202 | 480 | 324 | 18 | 60 | 532 | 1 | 428 | Phage portal protein | Phage portal protein | | afdb-uniprot50 | AF-A0A285M8B5-F1-MODEL\_V4 | 1.0 | 5.185e-24 | 808 | 0.179 | 562 | 375 | 21 | 30 | 532 | 14 | 548 | Phage portal protein, lambda family | Phage portal protein, lambda family | | afdb-uniprot50 | AF-A0A369U8Z9-F1-MODEL\_V4 | 1.0 | 7.806e-25 | 808 | 0.172 | 580 | 364 | 26 | 30 | 532 | 12 | 552 | Phage portal protein | Phage portal protein | | afdb-uniprot50 | AF-A0A7W6C1D9-F1-MODEL\_V4 | 1.0 | 3.192e-25 | 807 | 0.214 | 514 | 335 | 17 | 39 | 523 | 33 | 506 | Lambda family phage portal protein | Lambda family phage portal protein | | afdb-uniprot50 | AF-A0A420DHA7-F1-MODEL\_V4 | 1.0 | 6.399e-24 | 807 | 0.182 | 553 | 367 | 23 | 2 | 518 | 1 | 504 | Capsid protein | Capsid protein | | afdb-uniprot50 | AF-A0A1T4WXT7-F1-MODEL\_V4 | 1.0 | 6.142e-23 | 805 | 0.168 | 499 | 359 | 19 | 13 | 491 | 1 | 463 | Capsid protein | Capsid protein | | afdb-uniprot50 | AF-A0A0B1MGE1-F1-MODEL\_V4 | 1.0 | 3.484e-22 | 804 | 0.205 | 365 | 260 | 11 | 39 | 390 | 21 | 368 | Capsid protein | Capsid protein | | afdb-uniprot50 | AF-A0A6M1U479-F1-MODEL\_V4 | 1.0 | 1.268e-23 | 804 | 0.286 | 409 | 253 | 12 | 136 | 532 | 1 | 382 | Phage portal protein | Phage portal protein | | afdb-uniprot50 | AF-A0A5C7Q7S1-F1-MODEL\_V4 | 1.0 | 8.876e-23 | 803 | 0.187 | 497 | 341 | 17 | 47 | 515 | 16 | 477 | Phage portal protein | Phage portal protein | | afdb-uniprot50 | AF-A0A444KUH4-F1-MODEL\_V4 | 1.0 | 2.12e-24 | 803 | 0.209 | 553 | 340 | 20 | 46 | 532 | 10 | 531 | Phage portal protein | Phage portal protein | | afdb-uniprot50 | AF-A0A4Z0ITW1-F1-MODEL\_V4 | 1.0 | 1.189e-24 | 803 | 0.197 | 561 | 358 | 19 | 18 | 532 | 1 | 515 | Phage portal protein | Phage portal protein | | afdb-uniprot50 | AF-A0A1I3N5J6-F1-MODEL\_V4 | 1.0 | 2.356e-24 | 802 | 0.268 | 402 | 255 | 12 | 131 | 516 | 12 | 390 | Phage portal protein, lambda family | Phage portal protein, lambda family | | afdb-uniprot50 | AF-A0A3G8M317-F1-MODEL\_V4 | 1.0 | 3.986e-24 | 802 | 0.181 | 569 | 392 | 21 | 1 | 515 | 12 | 560 | Phage portal protein | Phage portal protein | | afdb-uniprot50 | AF-R9L6B1-F1-MODEL\_V4 | 1.0 | 9.861e-23 | 799 | 0.232 | 383 | 260 | 9 | 32 | 394 | 14 | 382 | Lambda family phage portal protein | Lambda family phage portal protein | | afdb-uniprot50 | AF-A0A1V6HR06-F1-MODEL\_V4 | 1.0 | 3.444e-23 | 798 | 0.16 | 556 | 389 | 19 | 20 | 531 | 1 | 522 | Phage portal protein, lambda family | Phage portal protein, lambda family | | afdb-uniprot50 | AF-A0A3M1LU04-F1-MODEL\_V4 | 1.0 | 1.039e-22 | 797 | 0.271 | 394 | 248 | 11 | 39 | 421 | 2 | 367 | Phage portal protein | Phage portal protein | | afdb-uniprot50 | AF-A0A3L4XBL8-F1-MODEL\_V4 | 1.0 | 1.141e-23 | 797 | 0.221 | 488 | 319 | 17 | 86 | 532 | 2 | 469 | Phage portal protein | Phage portal protein | | afdb-uniprot50 | AF-A0A2E5AGP1-F1-MODEL\_V4 | 1.0 | 4.667e-24 | 797 | 0.168 | 599 | 393 | 20 | 5 | 532 | 1 | 565 | Uncharacterized protein | Uncharacterized protein | | afdb-uniprot50 | AF-I6APC7-F1-MODEL\_V4 | 1.0 | 1.738e-23 | 796 | 0.16 | 492 | 348 | 16 | 62 | 532 | 62 | 509 | Bacteriophage capsid protein | Bacteriophage capsid protein | | afdb-uniprot50 | AF-A0A5S4WJH0-F1-MODEL\_V4 | 1.0 | 6.325e-25 | 795 | 0.179 | 568 | 356 | 22 | 40 | 532 | 16 | 548 | Phage portal protein | Phage portal protein | | afdb-uniprot50 | AF-C1AAN8-F1-MODEL\_V4 | 1.0 | 1.738e-23 | 795 | 0.151 | 548 | 387 | 17 | 20 | 532 | 24 | 528 | Phage portal protein lambda family protein | Phage portal protein lambda family protein | | afdb-uniprot50 | AF-I6B1B8-F1-MODEL\_V4 | 1.0 | 3.826e-23 | 794 | 0.195 | 523 | 354 | 18 | 35 | 532 | 25 | 505 | Bacteriophage capsid protein | Bacteriophage capsid protein | | afdb-uniprot50 | AF-A0A848VGF3-F1-MODEL\_V4 | 1.0 | 9.634e-25 | 793 | 0.194 | 541 | 365 | 20 | 24 | 532 | 10 | 511 | Phage portal protein | Phage portal protein | | afdb-uniprot50 | AF-A0A442I655-F1-MODEL\_V4 | 1.0 | 2.383e-23 | 793 | 0.172 | 556 | 395 | 16 | 14 | 532 | 1 | 528 | Phage portal protein | Phage portal protein | | afdb-uniprot50 | AF-A0A0P7Y6W9-F1-MODEL\_V4 | 1.0 | 1.875e-21 | 792 | 0.266 | 398 | 252 | 14 | 74 | 459 | 3 | 372 | Phage portal protein, lambda family | Phage portal protein, lambda family | | afdb-uniprot50 | AF-A0A1M7ZLP8-F1-MODEL\_V4 | 1.0 | 1.718e-24 | 792 | 0.202 | 579 | 353 | 29 | 11 | 532 | 1 | 527 | Phage portal protein, lambda family | Phage portal protein, lambda family | | afdb-uniprot50 | AF-A0A1G3M6A5-F1-MODEL\_V4 | 1.0 | 1.669e-22 | 789 | 0.242 | 421 | 285 | 14 | 11 | 418 | 26 | 425 | Phage portal protein | Phage portal protein | | afdb-uniprot50 | AF-A0A285D5Q3-F1-MODEL\_V4 | 1.0 | 3.94e-25 | 789 | 0.243 | 539 | 330 | 17 | 16 | 532 | 1 | 483 | Lambda family phage portal protein | Lambda family phage portal protein | | afdb-uniprot50 | AF-T0GB94-F1-MODEL\_V4 | 1.0 | 1.649e-23 | 789 | 0.211 | 544 | 365 | 21 | 27 | 531 | 11 | 529 | Uncharacterized protein | Uncharacterized protein | | afdb-uniprot50 | AF-A0A0F9E9R9-F1-MODEL\_V4 | 1.0 | 3.63e-23 | 786 | 0.214 | 439 | 293 | 16 | 39 | 439 | 24 | 448 | Uncharacterized protein | Uncharacterized protein | | afdb-uniprot50 | AF-A0A236MRM7-F1-MODEL\_V4 | 1.0 | 4.667e-24 | 786 | 0.183 | 546 | 347 | 20 | 39 | 523 | 159 | 666 | Phage portal protein (Minor capsid protein) | Phage portal protein (Minor capsid protein) | | afdb-uniprot50 | AF-A0A7U9F7S2-F1-MODEL\_V4 | 1.0 | 1.502e-22 | 785 | 0.269 | 419 | 259 | 14 | 127 | 532 | 2 | 386 | Uncharacterized protein | Uncharacterized protein | | afdb-uniprot50 | AF-A0A5C7PCJ8-F1-MODEL\_V4 | 1.0 | 2.758e-24 | 785 | 0.186 | 537 | 361 | 23 | 39 | 530 | 19 | 524 | Phage portal protein | Phage portal protein | | afdb-uniprot50 | AF-A0A662JPW1-F1-MODEL\_V4 | 1.0 | 8.421e-23 | 783 | 0.215 | 464 | 308 | 16 | 46 | 496 | 14 | 434 | Uncharacterized protein | Uncharacterized protein | | afdb-uniprot50 | AF-A0A3N9N4D9-F1-MODEL\_V4 | 1.0 | 1.039e-22 | 783 | 0.183 | 534 | 370 | 20 | 9 | 517 | 11 | 503 | Phage portal protein | Phage portal protein | | afdb-uniprot50 | AF-A0A7X9G897-F1-MODEL\_V4 | 1.0 | 6.142e-23 | 783 | 0.184 | 559 | 358 | 19 | 13 | 532 | 1 | 500 | Phage portal protein | Phage portal protein | | afdb-uniprot50 | AF-A0A1G3A9B5-F1-MODEL\_V4 | 1.0 | 1.141e-23 | 782 | 0.196 | 530 | 352 | 19 | 21 | 532 | 13 | 486 | Uncharacterized protein | Uncharacterized protein | | afdb-uniprot50 | AF-A0A2E5UE44-F1-MODEL\_V4 | 1.0 | 7.192e-23 | 782 | 0.165 | 539 | 369 | 16 | 42 | 532 | 15 | 520 | Phage portal protein | Phage portal protein | | afdb-uniprot50 | AF-A0A0Q4ZHQ5-F1-MODEL\_V4 | 1.0 | 1.485e-23 | 781 | 0.157 | 559 | 392 | 21 | 17 | 532 | 25 | 547 | Uncharacterized protein | Uncharacterized protein | | afdb-uniprot50 | AF-F7X6P0-F1-MODEL\_V4 | 1.0 | 1.141e-23 | 781 | 0.205 | 594 | 373 | 24 | 1 | 532 | 65 | 621 | Lambda family phage portal protein | Lambda family phage portal protein | | afdb-uniprot50 | AF-A0A518G4B4-F1-MODEL\_V4 | 1.0 | 9.356e-23 | 780 | 0.192 | 505 | 361 | 18 | 11 | 495 | 2 | 479 | Phage portal protein, lambda family | Phage portal protein, lambda family | | afdb-uniprot50 | AF-A0A496YY11-F1-MODEL\_V4 | 1.0 | 9.356e-23 | 779 | 0.18 | 525 | 367 | 19 | 20 | 532 | 1 | 474 | Uncharacterized protein | Uncharacterized protein | | afdb-uniprot50 | AF-A0A354U4Q5-F1-MODEL\_V4 | 1.0 | 5.529e-23 | 779 | 0.209 | 530 | 347 | 17 | 23 | 532 | 2 | 479 | Uncharacterized protein | Uncharacterized protein | | afdb-uniprot50 | AF-A0A259CCE8-F1-MODEL\_V4 | 1.0 | 1.009e-20 | 778 | 0.266 | 368 | 247 | 13 | 44 | 402 | 3 | 356 | Phage portal protein | Phage portal protein | | afdb-uniprot50 | AF-A0A3A0D2G7-F1-MODEL\_V4 | 1.0 | 1.779e-21 | 778 | 0.244 | 401 | 267 | 16 | 23 | 415 | 11 | 383 | Uncharacterized protein | Uncharacterized protein | | afdb-uniprot50 | AF-A0A1H2HHH0-F1-MODEL\_V4 | 1.0 | 9.861e-23 | 777 | 0.16 | 553 | 388 | 18 | 13 | 532 | 2 | 510 | Capsid protein | Capsid protein | | afdb-uniprot50 | AF-A0A0F9QX35-F1-MODEL\_V4 | 1.0 | 1.217e-22 | 777 | 0.216 | 490 | 340 | 18 | 1 | 475 | 9 | 469 | Uncharacterized protein | Uncharacterized protein | | afdb-uniprot50 | AF-A0A6N2ZN93-F1-MODEL\_V4 | 1.0 | 6.071e-24 | 775 | 0.206 | 513 | 351 | 19 | 27 | 495 | 1 | 501 | Phage portal protein, lambda family | Phage portal protein, lambda family | | afdb-uniprot50 | AF-A0A377PFS5-F1-MODEL\_V4 | 1.0 | 1.602e-21 | 774 | 0.236 | 422 | 280 | 14 | 122 | 532 | 4 | 394 | Phage portal protein, lambda family | Phage portal protein, lambda family | | afdb-uniprot50 | AF-A0A4D7QII8-F1-MODEL\_V4 | 1.0 | 1.649e-23 | 774 | 0.157 | 585 | 406 | 20 | 1 | 531 | 7 | 558 | Phage portal protein | Phage portal protein | | afdb-uniprot50 | AF-A0A480BUG0-F1-MODEL\_V4 | 1.0 | 9.576e-21 | 773 | 0.221 | 357 | 260 | 8 | 39 | 385 | 28 | 376 | Phage portal protein | Phage portal protein | | afdb-uniprot50 | AF-A0A3M9XN02-F1-MODEL\_V4 | 1.0 | 4.428e-24 | 772 | 0.18 | 575 | 382 | 25 | 17 | 532 | 1 | 545 | Phage portal protein | Phage portal protein | | afdb-uniprot50 | AF-Q727I3-F1-MODEL\_V4 | 1.0 | 1.268e-23 | 772 | 0.197 | 582 | 366 | 23 | 1 | 532 | 8 | 538 | Phage portal protein, lambda family | Phage portal protein, lambda family | | afdb-uniprot50 | AF-A0A1Z5H699-F1-MODEL\_V4 | 1.0 | 4.128e-21 | 770 | 0.232 | 404 | 268 | 13 | 46 | 418 | 18 | 410 | Uncharacterized protein | Uncharacterized protein | | afdb-uniprot50 | AF-A0A2E1P339-F1-MODEL\_V4 | 1.0 | 1.537e-20 | 769 | 0.316 | 275 | 181 | 2 | 31 | 305 | 12 | 279 | Phage portal protein | Phage portal protein | | afdb-uniprot50 | AF-A0A7W7NWS1-F1-MODEL\_V4 | 1.0 | 6.071e-24 | 769 | 0.179 | 557 | 363 | 21 | 39 | 532 | 7 | 532 | Lambda family phage portal protein | Lambda family phage portal protein | | afdb-uniprot50 | AF-A0A3A1YCY1-F1-MODEL\_V4 | 1.0 | 4.033e-23 | 768 | 0.18 | 542 | 376 | 21 | 16 | 532 | 12 | 510 | Uncharacterized protein | Uncharacterized protein | | afdb-uniprot50 | AF-A0A0E3C0X6-F1-MODEL\_V4 | 1.0 | 1.425e-22 | 767 | 0.218 | 421 | 296 | 12 | 105 | 517 | 2 | 397 | Capsid protein | Capsid protein | | afdb-uniprot50 | AF-A0A439EQ92-F1-MODEL\_V4 | 1.0 | 1.875e-21 | 765 | 0.218 | 389 | 278 | 12 | 122 | 502 | 1 | 371 | Phage portal protein | Phage portal protein | | afdb-uniprot50 | AF-A0A7V3AHA7-F1-MODEL\_V4 | 1.0 | 1.537e-20 | 764 | 0.274 | 331 | 215 | 9 | 46 | 366 | 3 | 318 | Phage portal protein | Phage portal protein | | afdb-uniprot50 | AF-A0A0H3X0U6-F1-MODEL\_V4 | 1.0 | 3.137e-22 | 764 | 0.178 | 548 | 361 | 25 | 44 | 532 | 6 | 523 | Phage portal protein | Phage portal protein | | afdb-uniprot50 | AF-A0A855TGI4-F1-MODEL\_V4 | 1.0 | 1.217e-22 | 763 | 0.174 | 482 | 350 | 15 | 58 | 531 | 12 | 453 | Uncharacterized protein | Uncharacterized protein | | afdb-uniprot50 | AF-A0A2D6XDS2-F1-MODEL\_V4 | 1.0 | 3.871e-22 | 763 | 0.187 | 507 | 345 | 18 | 39 | 520 | 19 | 483 | Uncharacterized protein | Uncharacterized protein | | afdb-uniprot50 | AF-A0A6P2LTT1-F1-MODEL\_V4 | 1.0 | 4.722e-23 | 763 | 0.236 | 452 | 270 | 17 | 24 | 459 | 1 | 393 | Portal protein | Portal protein | | afdb-uniprot50 | AF-A0A366G0P7-F1-MODEL\_V4 | 1.0 | 1.502e-22 | 762 | 0.191 | 518 | 324 | 20 | 74 | 532 | 3 | 484 | Lambda family phage portal protein | Lambda family phage portal protein | | afdb-uniprot50 | AF-A0A0E2L6R3-F1-MODEL\_V4 | 1.0 | 6.214e-22 | 761 | 0.236 | 431 | 277 | 14 | 49 | 439 | 25 | 443 | Portal protein | Portal protein | | afdb-uniprot50 | AF-A0A1Y1S1G3-F1-MODEL\_V4 | 1.0 | 1.832e-23 | 760 | 0.178 | 504 | 338 | 17 | 73 | 532 | 46 | 517 | Uncharacterized protein | Uncharacterized protein | | afdb-uniprot50 | AF-A0A1M4WC61-F1-MODEL\_V4 | 1.0 | 2.17e-22 | 759 | 0.179 | 536 | 356 | 21 | 20 | 502 | 12 | 516 | Phage portal protein, lambda family | Phage portal protein, lambda family | | afdb-uniprot50 | AF-A0A420Z232-F1-MODEL\_V4 | 1.0 | 9.861e-23 | 758 | 0.176 | 561 | 384 | 17 | 23 | 532 | 2 | 535 | Phage portal protein | Phage portal protein | | afdb-uniprot50 | AF-A0A7W1G457-F1-MODEL\_V4 | 1.0 | 2.261e-23 | 757 | 0.183 | 522 | 352 | 26 | 39 | 531 | 20 | 496 | Phage portal protein | Phage portal protein | | afdb-uniprot50 | AF-A0A1Z8KYX4-F1-MODEL\_V4 | 1.0 | 3.444e-23 | 756 | 0.194 | 529 | 364 | 21 | 39 | 532 | 32 | 533 | Phage portal protein | Phage portal protein | | afdb-uniprot50 | AF-A0A0T7A5Q8-F1-MODEL\_V4 | 1.0 | 1.352e-22 | 755 | 0.194 | 504 | 318 | 17 | 40 | 472 | 2 | 488 | Uncharacterized protein | Uncharacterized protein | | afdb-uniprot50 | AF-A0A154LMF9-F1-MODEL\_V4 | 1.0 | 2.261e-23 | 755 | 0.212 | 518 | 337 | 19 | 28 | 502 | 15 | 504 | Uncharacterized protein | Uncharacterized protein | | afdb-uniprot50 | AF-A0A1A6FM45-F1-MODEL\_V4 | 1.0 | 3.444e-23 | 752 | 0.18 | 538 | 354 | 23 | 51 | 532 | 1 | 507 | Phage portal protein | Phage portal protein | | afdb-uniprot50 | AF-A0A4R8FFK2-F1-MODEL\_V4 | 1.0 | 3.1e-23 | 752 | 0.157 | 583 | 381 | 24 | 13 | 532 | 2 | 537 | Lambda family phage portal protein | Lambda family phage portal protein | | afdb-uniprot50 | AF-A0A3C1QZ48-F1-MODEL\_V4 | 1.0 | 3.345e-21 | 750 | 0.231 | 437 | 299 | 15 | 28 | 456 | 10 | 417 | Uncharacterized protein | Uncharacterized protein | | afdb-uniprot50 | AF-A0A439RCC1-F1-MODEL\_V4 | 1.0 | 2.035e-23 | 749 | 0.173 | 588 | 374 | 24 | 2 | 532 | 7 | 539 | Phage portal protein | Phage portal protein | | afdb-uniprot50 | AF-A0A4R1PSV5-F1-MODEL\_V4 | 1.0 | 1.231e-21 | 747 | 0.301 | 358 | 225 | 11 | 161 | 502 | 2 | 350 | Lambda family phage portal protein | Lambda family phage portal protein | | afdb-uniprot50 | AF-A0A7W1IQN8-F1-MODEL\_V4 | 1.0 | 2.541e-22 | 747 | 0.192 | 498 | 326 | 15 | 46 | 502 | 2 | 464 | Phage portal protein | Phage portal protein | | afdb-uniprot50 | AF-A0A5C5VQX8-F1-MODEL\_V4 | 1.0 | 4.25e-23 | 747 | 0.179 | 556 | 362 | 22 | 29 | 532 | 15 | 528 | Phage portal protein, lambda family | Phage portal protein, lambda family | | afdb-uniprot50 | AF-A0A3C1YZ33-F1-MODEL\_V4 | 1.0 | 1.583e-22 | 744 | 0.167 | 548 | 370 | 17 | 37 | 532 | 8 | 521 | Uncharacterized protein | Uncharacterized protein | | afdb-uniprot50 | AF-A0A286IFC3-F1-MODEL\_V4 | 1.0 | 7.192e-23 | 744 | 0.17 | 521 | 350 | 22 | 26 | 503 | 24 | 505 | Lambda family phage portal protein | Lambda family phage portal protein | | afdb-uniprot50 | AF-A0A5C6BLH5-F1-MODEL\_V4 | 1.0 | 2.571e-21 | 743 | 0.184 | 482 | 332 | 18 | 39 | 495 | 10 | 455 | Phage portal protein, lambda family | Phage portal protein, lambda family | | afdb-uniprot50 | AF-D5SMQ0-F1-MODEL\_V4 | 1.0 | 6.214e-22 | 741 | 0.18 | 531 | 366 | 21 | 24 | 532 | 1 | 484 | Bacteriophage capsid protein-like protein | Bacteriophage capsid protein-like protein | | afdb-uniprot50 | AF-A0A537MC68-F1-MODEL\_V4 | 1.0 | 4.08e-22 | 741 | 0.16 | 561 | 394 | 19 | 1 | 532 | 4 | 516 | Phage portal protein | Phage portal protein | | afdb-uniprot50 | AF-A0A5C8A356-F1-MODEL\_V4 | 1.0 | 1.352e-22 | 737 | 0.202 | 558 | 374 | 20 | 2 | 532 | 23 | 536 | Phage portal protein | Phage portal protein | | afdb-uniprot50 | AF-A0A4Z0F5W2-F1-MODEL\_V4 | 1.0 | 3.444e-23 | 736 | 0.204 | 544 | 332 | 27 | 47 | 532 | 25 | 525 | Phage portal protein | Phage portal protein | | afdb-uniprot50 | AF-A0A354TJG1-F1-MODEL\_V4 | 1.0 | 1.052e-21 | 734 | 0.17 | 539 | 365 | 20 | 20 | 532 | 1 | 483 | Uncharacterized protein | Uncharacterized protein | | afdb-uniprot50 | AF-A0A4Q2YKT3-F1-MODEL\_V4 | 1.0 | 1.039e-22 | 733 | 0.203 | 575 | 371 | 23 | 5 | 532 | 3 | 537 | Phage portal protein | Phage portal protein | | afdb-uniprot50 | AF-A0A2T6DRH8-F1-MODEL\_V4 | 1.0 | 3.525e-21 | 730 | 0.183 | 517 | 351 | 21 | 46 | 532 | 4 | 479 | Uncharacterized protein | Uncharacterized protein | | afdb-uniprot50 | AF-A0A5C5Y9N3-F1-MODEL\_V4 | 1.0 | 1.854e-22 | 723 | 0.206 | 504 | 338 | 21 | 4 | 487 | 1 | 462 | Phage portal protein, lambda family | Phage portal protein, lambda family | | afdb-uniprot50 | AF-A0A3N5X2X0-F1-MODEL\_V4 | 1.0 | 4.833e-21 | 721 | 0.175 | 513 | 340 | 20 | 44 | 531 | 18 | 472 | Phage portal protein | Phage portal protein | | afdb-uniprot50 | AF-A0A1E5NHB2-F1-MODEL\_V4 | 1.0 | 1.583e-22 | 717 | 0.16 | 556 | 383 | 21 | 23 | 532 | 21 | 538 | Uncharacterized protein | Uncharacterized protein | | afdb-uniprot50 | AF-A0A127CHD1-F1-MODEL\_V4 | 1.0 | 1.095e-22 | 717 | 0.195 | 572 | 361 | 25 | 35 | 532 | 27 | 573 | Portal protein | Portal protein | | afdb-uniprot50 | AF-A0A5C7PT24-F1-MODEL\_V4 | 1.0 | 2.083e-21 | 715 | 0.177 | 534 | 378 | 21 | 22 | 532 | 48 | 543 | Phage portal protein | Phage portal protein | | afdb-uniprot50 | AF-A0A517R7D1-F1-MODEL\_V4 | 1.0 | 7.362e-21 | 714 | 0.177 | 523 | 350 | 23 | 36 | 532 | 3 | 471 | Phage portal protein, lambda family | Phage portal protein, lambda family | | afdb-uniprot50 | AF-A0A1W2ACJ6-F1-MODEL\_V4 | 1.0 | 7.277e-22 | 714 | 0.154 | 549 | 389 | 21 | 22 | 532 | 10 | 521 | Capsid protein | Capsid protein | | afdb-uniprot50 | AF-A0A2E9RXI4-F1-MODEL\_V4 | 1.0 | 2.571e-21 | 712 | 0.166 | 521 | 354 | 21 | 39 | 532 | 4 | 470 | Uncharacterized protein | Uncharacterized protein | | afdb-uniprot50 | AF-A0A2U2C460-F1-MODEL\_V4 | 1.0 | 1.298e-21 | 712 | 0.193 | 502 | 310 | 20 | 51 | 492 | 1 | 467 | Phage portal protein | Phage portal protein | | afdb-uniprot50 | AF-A0A5C7PZR6-F1-MODEL\_V4 | 1.0 | 1.009e-20 | 711 | 0.172 | 529 | 359 | 20 | 36 | 532 | 6 | 487 | Phage portal protein | Phage portal protein | | afdb-uniprot50 | AF-A0A258L7H2-F1-MODEL\_V4 | 1.0 | 1.779e-21 | 710 | 0.205 | 447 | 278 | 17 | 39 | 426 | 7 | 435 | Phage portal protein | Phage portal protein | | afdb-uniprot50 | AF-A0A846QDJ1-F1-MODEL\_V4 | 1.0 | 6.627e-21 | 706 | 0.179 | 513 | 352 | 17 | 15 | 502 | 1 | 469 | Capsid protein | Capsid protein | | afdb-uniprot50 | AF-A0A7X7IJJ8-F1-MODEL\_V4 | 1.0 | 3.567e-20 | 704 | 0.202 | 444 | 298 | 14 | 92 | 532 | 2 | 392 | Phage portal protein | Phage portal protein | | afdb-uniprot50 | AF-A0A6M3IJ20-F1-MODEL\_V4 | 1.0 | 4.833e-21 | 704 | 0.163 | 509 | 365 | 18 | 47 | 526 | 7 | 483 | Putative portal protein | Putative portal protein | | afdb-uniprot50 | AF-A0A2U1B6F7-F1-MODEL\_V4 | 1.0 | 5.154e-20 | 703 | 0.3 | 340 | 219 | 6 | 168 | 502 | 2 | 327 | Lambda family phage portal protein | Lambda family phage portal protein | | afdb-uniprot50 | AF-A0A7W1RRH0-F1-MODEL\_V4 | 1.0 | 7.448e-20 | 703 | 0.157 | 444 | 325 | 14 | 75 | 495 | 36 | 453 | Phage portal protein | Phage portal protein | | afdb-uniprot50 | AF-A0A7C5WID2-F1-MODEL\_V4 | 1.0 | 4.586e-21 | 703 | 0.155 | 539 | 391 | 18 | 19 | 532 | 12 | 511 | Phage portal protein | Phage portal protein | | afdb-uniprot50 | AF-A0A1V5QJV4-F1-MODEL\_V4 | 1.0 | 1.708e-20 | 701 | 0.207 | 457 | 307 | 14 | 99 | 532 | 2 | 426 | Phage portal protein, lambda family | Phage portal protein, lambda family | | afdb-uniprot50 | AF-A0A2Z6AIM3-F1-MODEL\_V4 | 1.0 | 2.083e-21 | 701 | 0.186 | 520 | 344 | 24 | 63 | 532 | 1 | 491 | Uncharacterized protein | Uncharacterized protein | | afdb-uniprot50 | AF-A0A5C5WMX5-F1-MODEL\_V4 | 1.0 | 2.742e-20 | 699 | 0.182 | 461 | 324 | 16 | 58 | 502 | 3 | 426 | Phage portal protein, lambda family | Phage portal protein, lambda family | | afdb-uniprot50 | AF-A0A5C5VCI3-F1-MODEL\_V4 | 1.0 | 1.688e-21 | 698 | 0.168 | 565 | 386 | 21 | 24 | 531 | 1 | 538 | Phage portal protein, lambda family | Phage portal protein, lambda family | | afdb-uniprot50 | AF-A0A8B4NDG1-F1-MODEL\_V4 | 1.0 | 4.639e-20 | 697 | 0.236 | 418 | 266 | 14 | 49 | 426 | 25 | 429 | Capsid protein of prophage | Capsid protein of prophage | | afdb-uniprot50 | AF-A0A3C1GAJ4-F1-MODEL\_V4 | 1.0 | 6.106e-19 | 693 | 0.383 | 295 | 165 | 4 | 234 | 515 | 4 | 294 | Phage portal protein | Phage portal protein | | afdb-uniprot50 | AF-A0A165U8M4-F1-MODEL\_V4 | 1.0 | 5.369e-21 | 691 | 0.154 | 589 | 371 | 23 | 15 | 532 | 1 | 533 | Phage portal protein, lambda family | Phage portal protein, lambda family | | afdb-uniprot50 | AF-A0A442NM56-F1-MODEL\_V4 | 1.0 | 1.121e-20 | 690 | 0.17 | 492 | 331 | 16 | 63 | 503 | 1 | 466 | Phage portal protein | Phage portal protein | | afdb-uniprot50 | AF-A0A840XZF9-F1-MODEL\_V4 | 1.0 | 6.035e-20 | 689 | 0.25 | 376 | 236 | 13 | 75 | 428 | 4 | 355 | Lambda family phage portal protein | Lambda family phage portal protein | | afdb-uniprot50 | AF-A0A0F9IQ30-F1-MODEL\_V4 | 1.0 | 2e-20 | 687 | 0.231 | 401 | 258 | 14 | 34 | 396 | 20 | 408 | Uncharacterized protein | Uncharacterized protein | | afdb-uniprot50 | AF-G9PUH7-F1-MODEL\_V4 | 1.0 | 1.246e-20 | 686 | 0.239 | 392 | 268 | 8 | 157 | 530 | 2 | 381 | Lambda family phage portal protein | Lambda family phage portal protein | | afdb-uniprot50 | AF-A0A833G1E6-F1-MODEL\_V4 | 1.0 | 1.196e-19 | 682 | 0.185 | 409 | 274 | 16 | 71 | 438 | 22 | 412 | Phage portal protein | Phage portal protein | | afdb-uniprot50 | AF-A0A3D2GU98-F1-MODEL\_V4 | 1.0 | 1.182e-20 | 682 | 0.208 | 455 | 308 | 19 | 39 | 456 | 32 | 471 | Uncharacterized protein | Uncharacterized protein | | afdb-uniprot50 | AF-A0A285MF00-F1-MODEL\_V4 | 1.0 | 1.52e-21 | 680 | 0.169 | 566 | 355 | 22 | 1 | 498 | 3 | 521 | Phage portal protein, lambda family | Phage portal protein, lambda family | | afdb-uniprot50 | AF-X0U7Z2-F1-MODEL\_V4 | 1.0 | 7.448e-20 | 679 | 0.208 | 437 | 291 | 13 | 113 | 532 | 3 | 401 | Uncharacterized protein | Uncharacterized protein | | afdb-uniprot50 | AF-A0A376UD89-F1-MODEL\_V4 | 1.0 | 1.639e-19 | 676 | 0.213 | 399 | 270 | 13 | 143 | 517 | 4 | 382 | Head-tail preconnector protein | Head-tail preconnector protein | | afdb-uniprot50 | AF-A0A1V1X572-F1-MODEL\_V4 | 1.0 | 3.608e-19 | 675 | 0.169 | 489 | 347 | 18 | 37 | 502 | 11 | 463 | Uncharacterized protein | Uncharacterized protein | | afdb-uniprot50 | AF-A0A0F9FJZ9-F1-MODEL\_V4 | 1.0 | 2.342e-20 | 675 | 0.185 | 555 | 362 | 21 | 22 | 532 | 43 | 551 | Uncharacterized protein | Uncharacterized protein | | afdb-uniprot50 | AF-A0A1H9QDY8-F1-MODEL\_V4 | 1.0 | 3.046e-20 | 672 | 0.196 | 453 | 301 | 16 | 28 | 433 | 1 | 437 | Phage portal protein, lambda family | Phage portal protein, lambda family | | afdb-uniprot50 | AF-A0A517QH17-F1-MODEL\_V4 | 1.0 | 1.196e-19 | 670 | 0.145 | 496 | 366 | 16 | 23 | 502 | 1 | 454 | Phage portal protein, lambda family | Phage portal protein, lambda family | | afdb-uniprot50 | AF-A0A1G3LXF9-F1-MODEL\_V4 | 1.0 | 1.328e-19 | 669 | 0.207 | 420 | 287 | 12 | 130 | 532 | 2 | 392 | Phage portal protein | Phage portal protein | | afdb-uniprot50 | AF-A0A6C2U4J9-F1-MODEL\_V4 | 1.0 | 1.384e-20 | 667 | 0.186 | 493 | 326 | 18 | 44 | 497 | 6 | 462 | Uncharacterized protein | Uncharacterized protein | | afdb-uniprot50 | AF-A0A2E8E056-F1-MODEL\_V4 | 1.0 | 4.586e-21 | 666 | 0.157 | 533 | 361 | 19 | 27 | 517 | 3 | 489 | Uncharacterized protein | Uncharacterized protein | | afdb-uniprot50 | AF-A0A5C5WP67-F1-MODEL\_V4 | 1.0 | 6.627e-21 | 663 | 0.15 | 537 | 377 | 21 | 13 | 515 | 2 | 493 | Phage portal protein, lambda family | Phage portal protein, lambda family | | afdb-uniprot50 | AF-A0A1V6CPT6-F1-MODEL\_V4 | 1.0 | 6.035e-20 | 653 | 0.171 | 513 | 339 | 25 | 39 | 503 | 19 | 493 | Phage portal protein, lambda family | Phage portal protein, lambda family | | afdb-uniprot50 | AF-A0A7G8VCB0-F1-MODEL\_V4 | 1.0 | 6.361e-20 | 649 | 0.174 | 517 | 311 | 20 | 89 | 530 | 6 | 481 | Phage portal protein | Phage portal protein | | afdb-uniprot50 | AF-A0A2X1LNZ6-F1-MODEL\_V4 | 1.0 | 1.843e-18 | 642 | 0.23 | 394 | 275 | 11 | 127 | 499 | 11 | 397 | Lambda family phage portal protein | Lambda family phage portal protein | | afdb-uniprot50 | AF-A0A2J6HXS8-F1-MODEL\_V4 | 1.0 | 8.569e-17 | 638 | 0.389 | 244 | 136 | 4 | 263 | 502 | 1 | 235 | Phage portal protein | Phage portal protein | | afdb-uniprot50 | AF-A0A844HAF1-F1-MODEL\_V4 | 1.0 | 3.848e-18 | 637 | 0.229 | 357 | 226 | 15 | 23 | 375 | 9 | 320 | Phage portal protein | Phage portal protein | | afdb-uniprot50 | AF-A0A410S8D1-F1-MODEL\_V4 | 1.0 | 8.721e-20 | 637 | 0.15 | 544 | 385 | 22 | 11 | 532 | 2 | 490 | Phage portal protein | Phage portal protein | | afdb-uniprot50 | AF-A0A518CCF6-F1-MODEL\_V4 | 1.0 | 6.783e-19 | 636 | 0.223 | 385 | 269 | 13 | 17 | 395 | 1 | 361 | Phage portal protein, lambda family | Phage portal protein, lambda family | | afdb-uniprot50 | AF-A0A3M3BPQ1-F1-MODEL\_V4 | 1.0 | 7.624e-18 | 635 | 0.323 | 278 | 172 | 6 | 160 | 422 | 4 | 280 | Portal protein | Portal protein | | afdb-uniprot50 | AF-A0A447JGS9-F1-MODEL\_V4 | 1.0 | 1.275e-18 | 634 | 0.211 | 436 | 278 | 17 | 47 | 433 | 34 | 452 | Phage portal protein, lambda family | Phage portal protein, lambda family | | afdb-uniprot50 | AF-A0A1H5ZK26-F1-MODEL\_V4 | 1.0 | 1.115e-16 | 633 | 0.259 | 301 | 208 | 7 | 122 | 413 | 13 | 307 | Phage portal protein, lambda family | Phage portal protein, lambda family | | afdb-uniprot50 | AF-A0A7W1G2Z3-F1-MODEL\_V4 | 1.0 | 9.689e-20 | 630 | 0.165 | 525 | 346 | 19 | 46 | 532 | 10 | 480 | Phage portal protein | Phage portal protein | | afdb-uniprot50 | AF-A0A827GZ56-F1-MODEL\_V4 | 1.0 | 3.286e-18 | 624 | 0.228 | 398 | 253 | 14 | 99 | 455 | 3 | 387 | Phage portal protein | Phage portal protein | | afdb-uniprot50 | AF-A0A177R1N7-F1-MODEL\_V4 | 1.0 | 8.823e-19 | 622 | 0.198 | 478 | 314 | 22 | 35 | 491 | 19 | 448 | Uncharacterized protein | Uncharacterized protein | | afdb-uniprot50 | AF-A0A3B9NPV5-F1-MODEL\_V4 | 1.0 | 2.071e-17 | 621 | 0.37 | 243 | 140 | 6 | 14 | 253 | 1 | 233 | Uncharacterized protein | Uncharacterized protein | | afdb-uniprot50 | AF-A0A257JLH4-F1-MODEL\_V4 | 1.0 | 5.276e-18 | 619 | 0.301 | 322 | 202 | 9 | 185 | 502 | 6 | 308 | Phage portal protein | Phage portal protein | | afdb-uniprot50 | AF-A0A853YYM9-F1-MODEL\_V4 | 1.0 | 8.469e-18 | 614 | 0.318 | 336 | 194 | 7 | 204 | 532 | 3 | 310 | Portal protein | Portal protein | | afdb-uniprot50 | AF-A0A0S8GDJ6-F1-MODEL\_V4 | 1.0 | 4.402e-20 | 614 | 0.168 | 582 | 352 | 23 | 26 | 532 | 2 | 526 | Uncharacterized protein | Uncharacterized protein | | afdb-uniprot50 | AF-A0A8B5P8M5-F1-MODEL\_V4 | 1.0 | 5.861e-18 | 611 | 0.19 | 442 | 283 | 15 | 23 | 413 | 2 | 419 | Phage portal protein | Phage portal protein | | afdb-uniprot50 | AF-A0A439F4D3-F1-MODEL\_V4 | 1.0 | 1.089e-18 | 608 | 0.229 | 393 | 253 | 14 | 160 | 532 | 4 | 366 | Uncharacterized protein | Uncharacterized protein | | afdb-uniprot50 | AF-A0A357LHL5-F1-MODEL\_V4 | 1.0 | 4.009e-19 | 607 | 0.167 | 519 | 352 | 24 | 73 | 532 | 2 | 499 | Phage portal protein | Phage portal protein | | afdb-uniprot50 | AF-A0A6G4KD87-F1-MODEL\_V4 | 1.0 | 4.558e-17 | 606 | 0.215 | 306 | 211 | 11 | 93 | 385 | 2 | 291 | Phage portal protein | Phage portal protein | | afdb-uniprot50 | AF-A0A0E3YYG8-F1-MODEL\_V4 | 1.0 | 1.678e-17 | 604 | 0.14 | 483 | 355 | 15 | 45 | 504 | 17 | 462 | Uncharacterized protein | Uncharacterized protein | | afdb-uniprot50 | AF-A0A1M7U6E7-F1-MODEL\_V4 | 1.0 | 5.626e-17 | 603 | 0.284 | 316 | 214 | 5 | 191 | 502 | 11 | 318 | Phage portal protein, lambda family | Phage portal protein, lambda family | | afdb-uniprot50 | AF-A0A554XEQ4-F1-MODEL\_V4 | 1.0 | 7.149e-19 | 601 | 0.246 | 397 | 266 | 13 | 36 | 416 | 8 | 387 | Putative esterase | Putative esterase | | afdb-uniprot50 | AF-A0A5U4DB71-F1-MODEL\_V4 | 1.0 | 1.115e-16 | 600 | 0.227 | 325 | 224 | 11 | 39 | 348 | 7 | 319 | Phage portal protein | Phage portal protein | | afdb-uniprot50 | AF-A0A380AGT5-F1-MODEL\_V4 | 1.0 | 1.592e-17 | 591 | 0.242 | 355 | 233 | 8 | 187 | 532 | 6 | 333 | Phage portal protein, lambda family | Phage portal protein, lambda family | | afdb-uniprot50 | AF-A0A357ZWC2-F1-MODEL\_V4 | 1.0 | 6.511e-18 | 588 | 0.147 | 507 | 365 | 16 | 51 | 532 | 12 | 476 | Uncharacterized protein | Uncharacterized protein | | afdb-uniprot50 | AF-A0A1L5KK25-F1-MODEL\_V4 | 1.0 | 4.749e-18 | 585 | 0.186 | 476 | 304 | 13 | 127 | 531 | 3 | 466 | Phage portal protein, lambda family | Phage portal protein, lambda family | | afdb-uniprot50 | AF-A0A3W5Y8G0-F1-MODEL\_V4 | 1.0 | 5.123e-16 | 584 | 0.225 | 319 | 222 | 9 | 165 | 474 | 4 | 306 | Phage portal protein | Phage portal protein | | afdb-uniprot50 | AF-A0A2U8HXA8-F1-MODEL\_V4 | 1.0 | 2.993e-17 | 583 | 0.268 | 339 | 220 | 9 | 207 | 532 | 1 | 324 | Phage portal protein | Phage portal protein | | afdb-uniprot50 | AF-A0A3L7RG21-F1-MODEL\_V4 | 1.0 | 7.713e-17 | 583 | 0.217 | 377 | 255 | 11 | 160 | 532 | 4 | 344 | Phage portal protein | Phage portal protein | | afdb-uniprot50 | AF-A0A847I6D6-F1-MODEL\_V4 | 1.0 | 1.988e-16 | 577 | 0.194 | 371 | 252 | 16 | 62 | 417 | 1 | 339 | Phage portal protein | Phage portal protein | | afdb-uniprot50 | AF-A0A7C4BYU0-F1-MODEL\_V4 | 1.0 | 2.497e-19 | 575 | 0.243 | 443 | 258 | 16 | 37 | 459 | 2 | 387 | Phage portal protein | Phage portal protein | | afdb-uniprot50 | AF-A0A1U7D0P3-F1-MODEL\_V4 | 1.0 | 5.93e-17 | 572 | 0.211 | 402 | 264 | 14 | 131 | 492 | 2 | 390 | Phage portal protein, lambda family | Phage portal protein, lambda family | | afdb-uniprot50 | AF-A0A3C1YTA8-F1-MODEL\_V4 | 1.0 | 3.694e-17 | 572 | 0.133 | 532 | 385 | 18 | 20 | 517 | 2 | 491 | Uncharacterized protein | Uncharacterized protein | | afdb-uniprot50 | AF-A0A1Y2XGJ0-F1-MODEL\_V4 | 1.0 | 4.558e-17 | 569 | 0.195 | 409 | 254 | 16 | 39 | 396 | 18 | 402 | Phage portal protein | Phage portal protein | | afdb-uniprot50 | AF-A0A166Z3X1-F1-MODEL\_V4 | 1.0 | 2.839e-17 | 568 | 0.194 | 427 | 285 | 13 | 135 | 519 | 2 | 411 | Phage portal protein, lambda family | Phage portal protein, lambda family | | afdb-uniprot50 | AF-B3CPN2-F1-MODEL\_V4 | 1.0 | 7.804e-16 | 566 | 0.292 | 291 | 193 | 4 | 211 | 496 | 112 | 394 | Putative phage portal protein | Putative phage portal protein | | afdb-uniprot50 | AF-A0A5C7PV87-F1-MODEL\_V4 | 1.0 | 1.698e-16 | 563 | 0.24 | 358 | 237 | 7 | 186 | 532 | 17 | 350 | Phage portal protein | Phage portal protein | | afdb-uniprot50 | AF-A0A0F9LPE5-F1-MODEL\_V4 | 1.0 | 6.863e-18 | 555 | 0.174 | 516 | 335 | 21 | 37 | 531 | 26 | 471 | Uncharacterized protein | Uncharacterized protein | | afdb-uniprot50 | AF-A0A3D1IXD3-F1-MODEL\_V4 | 1.0 | 2.556e-17 | 550 | 0.127 | 548 | 372 | 24 | 51 | 532 | 26 | 533 | Uncharacterized protein | Uncharacterized protein | | afdb-uniprot50 | AF-A0A5C7Q617-F1-MODEL\_V4 | 1.0 | 2.234e-15 | 548 | 0.245 | 310 | 215 | 8 | 187 | 487 | 14 | 313 | Phage portal protein | Phage portal protein | | afdb-uniprot50 | AF-A0A379XPD6-F1-MODEL\_V4 | 1.0 | 2.453e-16 | 548 | 0.187 | 347 | 253 | 10 | 39 | 374 | 21 | 349 | Phage portal protein, lambda family protein | Phage portal protein, lambda family protein | | afdb-uniprot50 | AF-A0A3S4M9N1-F1-MODEL\_V4 | 1.0 | 1.546e-15 | 542 | 0.225 | 341 | 228 | 8 | 196 | 532 | 1 | 309 | Phage portal protein, lambda family | Phage portal protein, lambda family | | afdb-uniprot50 | AF-A0A1V5GTW5-F1-MODEL\_V4 | 1.0 | 2.011e-15 | 540 | 0.286 | 304 | 191 | 5 | 233 | 532 | 6 | 287 | Phage portal protein, lambda family | Phage portal protein, lambda family | | afdb-uniprot50 | AF-A0A0P1FA99-F1-MODEL\_V4 | 1.0 | 1.305e-16 | 534 | 0.263 | 319 | 205 | 11 | 15 | 328 | 1 | 294 | Phage portal protein, lambda family | Phage portal protein, lambda family | | afdb-uniprot50 | AF-A0A250DMY9-F1-MODEL\_V4 | 1.0 | 7.713e-17 | 531 | 0.3 | 273 | 176 | 6 | 67 | 328 | 2 | 270 | Uncharacterized protein | Uncharacterized protein | | afdb-uniprot50 | AF-A0A484WP22-F1-MODEL\_V4 | 1.0 | 1.988e-16 | 530 | 0.206 | 411 | 269 | 15 | 122 | 490 | 3 | 398 | Capsid protein of prophage | Capsid protein of prophage | | afdb-uniprot50 | AF-A0A532UBS6-F1-MODEL\_V4 | 1.0 | 9.917e-18 | 530 | 0.156 | 497 | 311 | 19 | 39 | 490 | 18 | 451 | Uncharacterized protein | Uncharacterized protein | | afdb-uniprot50 | AF-A0A3M6EEB9-F1-MODEL\_V4 | 1.0 | 1.484e-14 | 528 | 0.291 | 257 | 169 | 6 | 58 | 311 | 3 | 249 | Portal protein | Portal protein | | afdb-uniprot50 | AF-A0A3C1XMB6-F1-MODEL\_V4 | 1.0 | 7.713e-17 | 526 | 0.207 | 371 | 235 | 13 | 39 | 357 | 15 | 378 | Phage portal protein | Phage portal protein | | afdb-uniprot50 | AF-A0A7W1T9L3-F1-MODEL\_V4 | 1.0 | 4.375e-16 | 522 | 0.221 | 374 | 246 | 13 | 21 | 381 | 15 | 356 | Phage portal protein | Phage portal protein | | afdb-uniprot50 | AF-A0A7X7KMY8-F1-MODEL\_V4 | 1.0 | 3.587e-15 | 519 | 0.237 | 341 | 221 | 14 | 20 | 352 | 9 | 318 | Phage portal protein | Phage portal protein | | afdb-uniprot50 | AF-A0A448MNG2-F1-MODEL\_V4 | 1.0 | 5.463e-15 | 515 | 0.22 | 295 | 208 | 9 | 39 | 328 | 21 | 298 | Bacteriophage capsid protein | Bacteriophage capsid protein | | afdb-uniprot50 | AF-B2I820-F1-MODEL\_V4 | 1.0 | 3.825e-14 | 513 | 0.435 | 216 | 115 | 2 | 317 | 532 | 1 | 209 | Putative phage related protein | Putative phage related protein | | afdb-uniprot50 | AF-A0A6C2YPG2-F1-MODEL\_V4 | 1.0 | 3.545e-16 | 513 | 0.144 | 486 | 349 | 23 | 39 | 491 | 58 | 509 | Uncharacterized protein | Uncharacterized protein | | afdb-uniprot50 | AF-A0A827BFZ7-F1-MODEL\_V4 | 1.0 | 1.07e-15 | 510 | 0.175 | 428 | 284 | 12 | 143 | 523 | 6 | 411 | Phage portal protein | Phage portal protein | | afdb-uniprot50 | AF-A0A7W8GZX7-F1-MODEL\_V4 | 1.0 | 3.229e-15 | 504 | 0.192 | 400 | 254 | 11 | 154 | 502 | 2 | 383 | Lambda family phage portal protein | Lambda family phage portal protein | | afdb-uniprot50 | AF-A0A2M9P8F9-F1-MODEL\_V4 | 1.0 | 3.671e-13 | 496 | 0.267 | 258 | 172 | 9 | 39 | 292 | 9 | 253 | Phage portal protein | Phage portal protein | | afdb-uniprot50 | AF-A0A497KH68-F1-MODEL\_V4 | 1.0 | 8.225e-16 | 493 | 0.146 | 452 | 302 | 20 | 39 | 456 | 42 | 443 | Uncharacterized protein | Uncharacterized protein | | afdb-uniprot50 | AF-A0A7J6YNN5-F1-MODEL\_V4 | 1.0 | 2.906e-15 | 491 | 0.268 | 316 | 198 | 8 | 220 | 530 | 336 | 623 | Uncharacterized protein | Uncharacterized protein | | afdb-uniprot50 | AF-J0PPA8-F1-MODEL\_V4 | 1.0 | 2.383e-14 | 488 | 0.232 | 314 | 221 | 6 | 201 | 502 | 2 | 307 | Lambda family phage portal protein | Lambda family phage portal protein | | afdb-uniprot50 | AF-F0SNL3-F1-MODEL\_V4 | 1.0 | 6.25e-17 | 486 | 0.148 | 564 | 364 | 25 | 24 | 517 | 35 | 551 | Uncharacterized protein | Uncharacterized protein | | afdb-uniprot50 | AF-A0A4C3GAR0-F1-MODEL\_V4 | 1.0 | 3.063e-15 | 484 | 0.175 | 415 | 285 | 14 | 150 | 523 | 376 | 774 | Phage terminase large subunit | Phage terminase large subunit | | afdb-uniprot50 | AF-A0A354EMX8-F1-MODEL\_V4 | 1.0 | 4.079e-13 | 482 | 0.266 | 300 | 208 | 8 | 179 | 473 | 5 | 297 | Phage portal protein | Phage portal protein | | afdb-uniprot50 | AF-A0A2E1P380-F1-MODEL\_V4 | 1.0 | 5.592e-13 | 479 | 0.297 | 262 | 162 | 6 | 275 | 532 | 2 | 245 | Phage portal protein | Phage portal protein | | afdb-uniprot50 | AF-A0A7I8Z9X4-F1-MODEL\_V4 | 1.0 | 3.671e-13 | 478 | 0.271 | 280 | 188 | 6 | 205 | 476 | 4 | 275 | Hypothetical protein | Hypothetical protein | | afdb-uniprot50 | AF-M1P158-F1-MODEL\_V4 | 1.0 | 5.758e-15 | 478 | 0.126 | 500 | 350 | 20 | 10 | 500 | 8 | 429 | Portal protein lambda | Portal protein lambda | | afdb-uniprot50 | AF-A0A827GZ82-F1-MODEL\_V4 | 1.0 | 2.647e-14 | 477 | 0.215 | 344 | 232 | 10 | 195 | 517 | 13 | 339 | Phage portal protein | Phage portal protein | | afdb-uniprot50 | AF-A0A6P1B243-F1-MODEL\_V4 | 1.0 | 1.601e-12 | 476 | 0.582 | 127 | 52 | 1 | 96 | 222 | 2 | 127 | Phage portal protein | Phage portal protein | | afdb-uniprot50 | AF-A0A353UWW7-F1-MODEL\_V4 | 1.0 | 5.592e-13 | 469 | 0.266 | 274 | 181 | 10 | 39 | 306 | 27 | 286 | Phage portal protein | Phage portal protein | | afdb-uniprot50 | AF-A0A484X983-F1-MODEL\_V4 | 1.0 | 1.095e-13 | 464 | 0.246 | 341 | 216 | 10 | 140 | 448 | 3 | 334 | Capsid protein of prophage | Capsid protein of prophage | | afdb-uniprot50 | AF-L8M8B8-F1-MODEL\_V4 | 1.0 | 3.305e-13 | 462 | 0.28 | 239 | 150 | 7 | 23 | 255 | 12 | 234 | Bacteriophage capsid protein | Bacteriophage capsid protein | | afdb-uniprot50 | AF-A0A0K3YI17-F1-MODEL\_V4 | 1.0 | 1.875e-12 | 459 | 0.234 | 277 | 199 | 8 | 214 | 486 | 4 | 271 | Portal protein (Head-tail preconnector protein) from prophage | Portal protein (Head-tail preconnector protein) from prophage | | afdb-uniprot50 | AF-A0A250DLV1-F1-MODEL\_V4 | 1.0 | 1.601e-12 | 457 | 0.319 | 263 | 152 | 4 | 277 | 532 | 12 | 254 | Uncharacterized protein | Uncharacterized protein | | afdb-uniprot50 | AF-A0A291A6H8-F1-MODEL\_V4 | 1.0 | 1.519e-12 | 457 | 0.275 | 272 | 176 | 5 | 244 | 502 | 3 | 266 | Phage portal protein | Phage portal protein | | afdb-uniprot50 | AF-A0A6I2J5G2-F1-MODEL\_V4 | 1.0 | 3.136e-13 | 453 | 0.442 | 190 | 99 | 4 | 1 | 189 | 3 | 186 | Phage portal protein | Phage portal protein | | afdb-uniprot50 | AF-A0A1G1LMU8-F1-MODEL\_V4 | 1.0 | 1.688e-12 | 452 | 0.288 | 253 | 170 | 4 | 253 | 502 | 1 | 246 | Phage portal protein | Phage portal protein | | afdb-uniprot50 | AF-A0A2V8RVC3-F1-MODEL\_V4 | 1.0 | 1.039e-13 | 447 | 0.152 | 387 | 273 | 14 | 160 | 532 | 5 | 350 | Uncharacterized protein | Uncharacterized protein | | afdb-uniprot50 | AF-A0A352V3P3-F1-MODEL\_V4 | 1.0 | 2.541e-13 | 444 | 0.225 | 337 | 209 | 10 | 210 | 531 | 3 | 302 | Uncharacterized protein | Uncharacterized protein | | afdb-uniprot50 | AF-A0A3S4CSS4-F1-MODEL\_V4 | 1.0 | 5.894e-13 | 442 | 0.218 | 316 | 214 | 8 | 218 | 532 | 5 | 288 | Phage portal protein, lambda family | Phage portal protein, lambda family | | afdb-uniprot50 | AF-A0A3C0I3F4-F1-MODEL\_V4 | 1.0 | 2.439e-12 | 441 | 0.244 | 245 | 162 | 9 | 34 | 264 | 13 | 248 | Uncharacterized protein | Uncharacterized protein | | afdb-uniprot50 | AF-A0A7V6YCH4-F1-MODEL\_V4 | 1.0 | 1.688e-12 | 441 | 0.252 | 317 | 216 | 6 | 221 | 532 | 1 | 301 | Phage portal protein | Phage portal protein | | afdb-uniprot50 | AF-A0A3M2YA61-F1-MODEL\_V4 | 1.0 | 1.367e-12 | 440 | 0.268 | 305 | 185 | 5 | 242 | 530 | 3 | 285 | Uncharacterized protein | Uncharacterized protein | | afdb-uniprot50 | AF-A0A258REZ0-F1-MODEL\_V4 | 1.0 | 7.274e-13 | 435 | 0.273 | 311 | 193 | 7 | 234 | 532 | 1 | 290 | Phage portal protein | Phage portal protein | | afdb-uniprot50 | AF-A0A754E6Q2-F1-MODEL\_V4 | 1.0 | 2.556e-17 | 435 | 0.157 | 595 | 347 | 25 | 39 | 532 | 11 | 551 | Phage portal protein | Phage portal protein | | afdb-uniprot50 | AF-A0A1F4CAA0-F1-MODEL\_V4 | 1.0 | 3.483e-13 | 430 | 0.247 | 360 | 209 | 12 | 208 | 532 | 1 | 333 | Phage portal protein | Phage portal protein | | afdb-uniprot50 | AF-A0A7C2ZHD3-F1-MODEL\_V4 | 1.0 | 2.975e-13 | 429 | 0.133 | 493 | 297 | 25 | 39 | 515 | 19 | 397 | Uncharacterized protein | Uncharacterized protein | | afdb-uniprot50 | AF-A0A7X7IZ38-F1-MODEL\_V4 | 1.0 | 1.168e-12 | 428 | 0.216 | 323 | 217 | 11 | 24 | 342 | 12 | 302 | Phage portal protein | Phage portal protein | | afdb-uniprot50 | AF-A0A497SVU5-F1-MODEL\_V4 | 1.0 | 5.244e-14 | 425 | 0.134 | 467 | 298 | 23 | 23 | 470 | 6 | 385 | Uncharacterized protein | Uncharacterized protein | | afdb-uniprot50 | AF-A0A378BW42-F1-MODEL\_V4 | 1.0 | 9.973e-13 | 423 | 0.233 | 325 | 212 | 14 | 217 | 532 | 4 | 300 | Phage portal protein, lambda family | Phage portal protein, lambda family | | afdb-uniprot50 | AF-A0A326U088-F1-MODEL\_V4 | 1.0 | 3.305e-13 | 419 | 0.141 | 509 | 314 | 24 | 27 | 523 | 3 | 400 | Uncharacterized protein | Uncharacterized protein | | afdb-uniprot50 | AF-A0A376JWU1-F1-MODEL\_V4 | 1.0 | 3.715e-12 | 418 | 0.214 | 261 | 179 | 10 | 20 | 264 | 5 | 255 | Phage portal protein, lambda family | Phage portal protein, lambda family | | afdb-uniprot50 | AF-A0A793I648-F1-MODEL\_V4 | 1.0 | 1.897e-11 | 412 | 0.217 | 276 | 201 | 6 | 195 | 466 | 13 | 277 | Phage portal protein | Phage portal protein | | afdb-uniprot50 | AF-A0A2H0LJS2-F1-MODEL\_V4 | 1.0 | 1.537e-11 | 412 | 0.209 | 282 | 204 | 6 | 254 | 532 | 9 | 274 | Phage portal protein | Phage portal protein | | afdb-uniprot50 | AF-A0A2W5H3L8-F1-MODEL\_V4 | 1.0 | 3.344e-12 | 411 | 0.211 | 307 | 208 | 11 | 36 | 330 | 23 | 307 | Phage portal protein | Phage portal protein | | afdb-uniprot50 | AF-A0A2D8T9H3-F1-MODEL\_V4 | 1.0 | 2.287e-13 | 411 | 0.185 | 372 | 245 | 11 | 192 | 532 | 22 | 366 | Phage portal protein | Phage portal protein | | afdb-uniprot50 | AF-K2GKL9-F1-MODEL\_V4 | 1.0 | 1.779e-12 | 408 | 0.221 | 334 | 203 | 11 | 39 | 328 | 27 | 347 | Uncharacterized protein | Uncharacterized protein | | afdb-uniprot50 | AF-H5UYW8-F1-MODEL\_V4 | 1.0 | 2.107e-11 | 406 | 0.216 | 310 | 209 | 8 | 226 | 532 | 2 | 280 | Putative portal protein | Putative portal protein | | afdb-uniprot50 | AF-A0A3D5T0J9-F1-MODEL\_V4 | 1.0 | 1.245e-11 | 405 | 0.238 | 310 | 206 | 6 | 226 | 532 | 1 | 283 | Phage portal protein | Phage portal protein | | afdb-uniprot50 | AF-A0A0F9HAI2-F1-MODEL\_V4 | 1.0 | 5.093e-12 | 396 | 0.364 | 233 | 135 | 4 | 303 | 524 | 1 | 231 | Uncharacterized protein | Uncharacterized protein | | afdb-uniprot50 | AF-A0A455T535-F1-MODEL\_V4 | 1.0 | 8.419e-14 | 396 | 0.139 | 587 | 348 | 29 | 3 | 531 | 6 | 493 | Uncharacterized protein | Uncharacterized protein | | afdb-uniprot50 | AF-M5JSY7-F1-MODEL\_V4 | 1.0 | 4.832e-12 | 395 | 0.2 | 374 | 236 | 13 | 168 | 502 | 2 | 351 | Phage portal protein, lambda | Phage portal protein, lambda | | afdb-uniprot50 | AF-A0A7C5MJ10-F1-MODEL\_V4 | 1.0 | 2.41e-13 | 394 | 0.113 | 527 | 335 | 25 | 39 | 532 | 7 | 434 | Phage portal protein | Phage portal protein | | afdb-uniprot50 | AF-A0A3A4ZEQ3-F1-MODEL\_V4 | 1.0 | 4.249e-14 | 394 | 0.131 | 556 | 358 | 26 | 37 | 518 | 107 | 611 | Phage portal protein | Phage portal protein | | afdb-uniprot50 | AF-A0A6M3XSW5-F1-MODEL\_V4 | 1.0 | 4.349e-12 | 392 | 0.166 | 396 | 273 | 15 | 160 | 518 | 5 | 380 | Putative portal protein | Putative portal protein | | afdb-uniprot50 | AF-A0A7V4ZD36-F1-MODEL\_V4 | 1.0 | 1.875e-12 | 392 | 0.139 | 493 | 286 | 31 | 15 | 477 | 1 | 385 | Uncharacterized protein | Uncharacterized protein | | afdb-uniprot50 | AF-A0A403NK32-F1-MODEL\_V4 | 1.0 | 4.127e-12 | 391 | 0.214 | 345 | 223 | 9 | 207 | 515 | 6 | 338 | Phage portal protein | Phage portal protein | | afdb-uniprot50 | AF-A0A367D8L0-F1-MODEL\_V4 | 1.0 | 1.821e-10 | 390 | 0.258 | 236 | 165 | 8 | 252 | 483 | 3 | 232 | Phage portal protein | Phage portal protein | | afdb-uniprot50 | AF-A0A6L9HC99-F1-MODEL\_V4 | 1.0 | 3.422e-10 | 387 | 0.233 | 253 | 182 | 3 | 244 | 487 | 1 | 250 | Phage portal protein | Phage portal protein | | afdb-uniprot50 | AF-A0A6M3IP50-F1-MODEL\_V4 | 1.0 | 3.21e-11 | 384 | 0.197 | 294 | 212 | 4 | 254 | 530 | 21 | 307 | Putative portal protein | Putative portal protein | | afdb-uniprot50 | AF-W8YFG7-F1-MODEL\_V4 | 1.0 | 4.584e-12 | 384 | 0.246 | 329 | 205 | 9 | 210 | 502 | 51 | 372 | Uncharacterized protein | Uncharacterized protein | | afdb-uniprot50 | AF-A0A402A2E6-F1-MODEL\_V4 | 1.0 | 2.57e-12 | 384 | 0.129 | 534 | 353 | 24 | 13 | 523 | 2 | 446 | Uncharacterized protein | Uncharacterized protein | | afdb-uniprot50 | AF-A0A0F9MZW4-F1-MODEL\_V4 | 1.0 | 2.383e-14 | 384 | 0.157 | 579 | 356 | 24 | 24 | 532 | 1 | 517 | Uncharacterized protein | Uncharacterized protein | | afdb-uniprot50 | AF-A0A5S5MFU0-F1-MODEL\_V4 | 1.0 | 4.946e-10 | 377 | 0.244 | 262 | 177 | 7 | 252 | 497 | 14 | 270 | Phage portal protein | Phage portal protein | | afdb-uniprot50 | AF-A0A368KX58-F1-MODEL\_V4 | 1.0 | 2.601e-11 | 377 | 0.23 | 286 | 186 | 10 | 99 | 379 | 13 | 269 | Phage portal protein | Phage portal protein | | afdb-uniprot50 | AF-A0A6M3LXD3-F1-MODEL\_V4 | 1.0 | 2.822e-13 | 377 | 0.141 | 515 | 333 | 30 | 39 | 525 | 19 | 452 | Putative portal protein | Putative portal protein | | afdb-uniprot50 | AF-A0A3V0YA20-F1-MODEL\_V4 | 1.0 | 3.422e-10 | 375 | 0.189 | 275 | 199 | 6 | 258 | 530 | 1 | 253 | Phage portal protein | Phage portal protein | | afdb-uniprot50 | AF-A0A0S7BFT1-F1-MODEL\_V4 | 1.0 | 7.274e-13 | 373 | 0.158 | 497 | 302 | 26 | 28 | 486 | 1 | 419 | Phage portal protein, lambda family | Phage portal protein, lambda family | | afdb-uniprot50 | AF-A0A402ARA1-F1-MODEL\_V4 | 1.0 | 1.121e-11 | 372 | 0.131 | 465 | 295 | 23 | 38 | 459 | 7 | 405 | Uncharacterized protein | Uncharacterized protein | | afdb-uniprot50 | AF-A0A0F9HE98-F1-MODEL\_V4 | 1.0 | 6.212e-13 | 372 | 0.138 | 535 | 345 | 23 | 39 | 523 | 11 | 479 | Uncharacterized protein | Uncharacterized protein | | afdb-uniprot50 | AF-A0A4P9VL88-F1-MODEL\_V4 | 1.0 | 1.009e-11 | 371 | 0.172 | 353 | 238 | 10 | 187 | 502 | 1 | 336 | Phage portal protein | Phage portal protein | | afdb-uniprot50 | AF-A0A7C4Z3X6-F1-MODEL\_V4 | 1.0 | 9.083e-12 | 370 | 0.151 | 529 | 317 | 25 | 39 | 532 | 4 | 435 | Phage portal protein | Phage portal protein | | afdb-uniprot50 | AF-A0A633NAI0-F1-MODEL\_V4 | 1.0 | 6.104e-10 | 368 | 0.22 | 209 | 146 | 5 | 157 | 357 | 2 | 201 | Phage portal protein | Phage portal protein | | afdb-uniprot50 | AF-A0A357YBI7-F1-MODEL\_V4 | 1.0 | 3.566e-11 | 368 | 0.193 | 315 | 220 | 8 | 211 | 497 | 1 | 309 | Uncharacterized protein | Uncharacterized protein | | afdb-uniprot50 | AF-A0A6J4K1M5-F1-MODEL\_V4 | 1.0 | 1.976e-12 | 367 | 0.145 | 510 | 312 | 25 | 35 | 488 | 7 | 448 | Uncharacterized protein | Uncharacterized protein | | afdb-uniprot50 | AF-A0A7V2ILH1-F1-MODEL\_V4 | 1.0 | 1.707e-11 | 364 | 0.216 | 323 | 215 | 11 | 17 | 328 | 1 | 296 | Phage portal protein | Phage portal protein | | afdb-uniprot50 | AF-A0A7C5LMG7-F1-MODEL\_V4 | 1.0 | 1.064e-11 | 364 | 0.163 | 435 | 259 | 24 | 39 | 439 | 4 | 367 | Uncharacterized protein | Uncharacterized protein | | afdb-uniprot50 | AF-A0A2N2NPH9-F1-MODEL\_V4 | 1.0 | 6.548e-13 | 357 | 0.14 | 497 | 312 | 28 | 37 | 488 | 7 | 433 | Uncharacterized protein | Uncharacterized protein | | afdb-uniprot50 | AF-A0A1R1LRL3-F1-MODEL\_V4 | 1.0 | 3.045e-11 | 356 | 0.187 | 383 | 233 | 16 | 186 | 519 | 7 | 360 | Phage portal protein | Phage portal protein | | afdb-uniprot50 | AF-A0A0F8ZDI4-F1-MODEL\_V4 | 1.0 | 7.848e-11 | 353 | 0.133 | 427 | 275 | 17 | 10 | 413 | 1 | 355 | Uncharacterized protein | Uncharacterized protein | | afdb-uniprot50 | AF-A0A120G5R1-F1-MODEL\_V4 | 1.0 | 3.422e-10 | 349 | 0.215 | 301 | 197 | 7 | 250 | 532 | 3 | 282 | Phage portal protein, lambda family | Phage portal protein, lambda family | | afdb-uniprot50 | AF-A0A7A7BND2-F1-MODEL\_V4 | 1.0 | 1.021e-10 | 348 | 0.169 | 353 | 220 | 12 | 187 | 491 | 8 | 335 | Phage portal protein | Phage portal protein | | afdb-uniprot50 | AF-A0A2T3BVF4-F1-MODEL\_V4 | 1.0 | 1.601e-12 | 346 | 0.144 | 589 | 340 | 29 | 17 | 508 | 1 | 522 | Uncharacterized protein | Uncharacterized protein | | afdb-uniprot50 | AF-A0A383EWE0-F1-MODEL\_V4 | 1.0 | 1.209e-09 | 340 | 0.307 | 228 | 140 | 5 | 305 | 532 | 1 | 210 | Uncharacterized protein | Uncharacterized protein | | afdb-uniprot50 | AF-A0A6M3KLI4-F1-MODEL\_V4 | 1.0 | 1.779e-12 | 340 | 0.15 | 505 | 326 | 27 | 54 | 532 | 21 | 448 | Putative portal protein | Putative portal protein | | afdb-uniprot50 | AF-A0A662DL46-F1-MODEL\_V4 | 1.0 | 3.081e-10 | 338 | 0.168 | 356 | 267 | 13 | 191 | 532 | 4 | 344 | Uncharacterized protein | Uncharacterized protein | | afdb-uniprot50 | AF-A0A377ECV1-F1-MODEL\_V4 | 1.0 | 7.94e-10 | 336 | 0.201 | 307 | 211 | 6 | 250 | 532 | 2 | 298 | Capsid protein of prophage | Capsid protein of prophage | | afdb-uniprot50 | AF-A0A0F9EPK0-F1-MODEL\_V4 | 1.0 | 1.62e-11 | 336 | 0.121 | 568 | 355 | 27 | 7 | 532 | 1 | 466 | Uncharacterized protein | Uncharacterized protein | | afdb-uniprot50 | AF-A0A0F8ZAV5-F1-MODEL\_V4 | 1.0 | 2.107e-11 | 335 | 0.148 | 396 | 263 | 23 | 39 | 412 | 21 | 364 | Uncharacterized protein | Uncharacterized protein | | afdb-uniprot50 | AF-A0A765BQZ8-F1-MODEL\_V4 | 1.0 | 3.607e-10 | 333 | 0.204 | 327 | 212 | 10 | 233 | 521 | 1 | 317 | Phage portal protein | Phage portal protein | | afdb-uniprot50 | AF-A0A662KLX8-F1-MODEL\_V4 | 1.0 | 1.121e-11 | 330 | 0.155 | 528 | 318 | 27 | 39 | 523 | 6 | 448 | Uncharacterized protein | Uncharacterized protein | | afdb-uniprot50 | AF-A0A810UT01-F1-MODEL\_V4 | 1.0 | 7.231e-09 | 329 | 0.232 | 232 | 164 | 5 | 244 | 465 | 1 | 228 | Uncharacterized protein | Uncharacterized protein | | afdb-uniprot50 | AF-A0A849R2Z3-F1-MODEL\_V4 | 1.0 | 1.707e-11 | 324 | 0.141 | 495 | 308 | 25 | 1 | 469 | 5 | 408 | Uncharacterized protein | Uncharacterized protein | | afdb-uniprot50 | AF-A0A842MGS5-F1-MODEL\_V4 | 1.0 | 1.021e-10 | 321 | 0.132 | 483 | 308 | 25 | 54 | 518 | 23 | 412 | DUF1073 domain-containing protein | DUF1073 domain-containing protein | | afdb-uniprot50 | AF-A0A1F9ALP8-F1-MODEL\_V4 | 1.0 | 1.475e-10 | 319 | 0.111 | 512 | 338 | 23 | 39 | 531 | 21 | 434 | Uncharacterized protein | Uncharacterized protein | | afdb-uniprot50 | AF-A0A497GNK8-F1-MODEL\_V4 | 1.0 | 4.452e-10 | 318 | 0.126 | 450 | 283 | 22 | 78 | 491 | 31 | 406 | Uncharacterized protein | Uncharacterized protein | | afdb-uniprot50 | AF-A0A0S8GAH5-F1-MODEL\_V4 | 1.0 | 6.509e-09 | 316 | 0.213 | 267 | 178 | 7 | 273 | 530 | 2 | 245 | Uncharacterized protein | Uncharacterized protein | | afdb-uniprot50 | AF-A0A774PAJ4-F1-MODEL\_V4 | 1.0 | 2.806e-09 | 313 | 0.167 | 310 | 207 | 7 | 252 | 523 | 26 | 322 | Phage portal protein | Phage portal protein | | afdb-uniprot50 | AF-A0A845CKT9-F1-MODEL\_V4 | 1.0 | 2.247e-10 | 310 | 0.14 | 506 | 300 | 30 | 50 | 515 | 3 | 413 | Uncharacterized protein | Uncharacterized protein | | afdb-uniprot50 | AF-A0A2T0YIX7-F1-MODEL\_V4 | 1.0 | 8.617e-12 | 308 | 0.139 | 546 | 335 | 33 | 39 | 506 | 29 | 517 | Uncharacterized protein | Uncharacterized protein | | afdb-uniprot50 | AF-A0A0A7G0D7-F1-MODEL\_V4 | 1.0 | 2.889e-11 | 307 | 0.136 | 520 | 325 | 26 | 37 | 500 | 7 | 458 | Portal domain protein | Portal domain protein | | afdb-uniprot50 | AF-A0A6D0Y5S4-F1-MODEL\_V4 | 1.0 | 8.467e-09 | 303 | 0.18 | 310 | 198 | 10 | 220 | 488 | 2 | 296 | Phage portal protein | Phage portal protein | | afdb-uniprot50 | AF-A0A497BG15-F1-MODEL\_V4 | 1.0 | 3.081e-10 | 303 | 0.151 | 495 | 291 | 27 | 40 | 517 | 17 | 399 | Uncharacterized protein | Uncharacterized protein | | afdb-uniprot50 | AF-A0A520NQA9-F1-MODEL\_V4 | 1.0 | 1.057e-07 | 300 | 0.342 | 143 | 89 | 1 | 45 | 187 | 15 | 152 | Phage portal protein | Phage portal protein | | afdb-uniprot50 | AF-A0A5C7PU34-F1-MODEL\_V4 | 1.0 | 9.406e-09 | 296 | 0.2 | 284 | 202 | 6 | 252 | 529 | 5 | 269 | Phage portal protein | Phage portal protein | | afdb-uniprot50 | AF-A0A7M3IAY5-F1-MODEL\_V4 | 1.0 | 8.127e-08 | 294 | 0.24 | 191 | 134 | 5 | 195 | 381 | 13 | 196 | Phage portal protein | Phage portal protein | | afdb-uniprot50 | AF-A0A496S006-F1-MODEL\_V4 | 1.0 | 4.452e-10 | 291 | 0.121 | 484 | 312 | 24 | 13 | 474 | 2 | 394 | Uncharacterized protein | Uncharacterized protein | | afdb-uniprot50 | AF-A0A830FYU9-F1-MODEL\_V4 | 1.0 | 3.247e-10 | 289 | 0.128 | 513 | 326 | 27 | 15 | 479 | 1 | 440 | Uncharacterized protein | Uncharacterized protein | | afdb-uniprot50 | AF-A0A1M4S7X1-F1-MODEL\_V4 | 1.0 | 4.102e-08 | 287 | 0.28 | 207 | 132 | 5 | 295 | 490 | 3 | 203 | Phage portal protein, lambda family | Phage portal protein, lambda family | | afdb-uniprot50 | AF-A0A256WIT2-F1-MODEL\_V4 | 1.0 | 5.431e-11 | 284 | 0.119 | 553 | 348 | 28 | 15 | 532 | 1 | 449 | Uncharacterized protein | Uncharacterized protein | | afdb-uniprot50 | AF-A0A1V5X4F2-F1-MODEL\_V4 | 1.0 | 5.004e-09 | 277 | 0.119 | 486 | 288 | 21 | 54 | 497 | 8 | 395 | Uncharacterized protein | Uncharacterized protein | | afdb-uniprot50 | AF-A0A6L7X1R6-F1-MODEL\_V4 | 1.0 | 2.526e-09 | 272 | 0.145 | 592 | 362 | 32 | 24 | 501 | 1 | 562 | Uncharacterized protein | Uncharacterized protein | | afdb-uniprot50 | AF-A0A7X7FQM4-F1-MODEL\_V4 | 1.0 | 1.697e-07 | 271 | 0.164 | 262 | 174 | 6 | 285 | 532 | 2 | 232 | Phage portal protein | Phage portal protein | | afdb-uniprot50 | AF-A0A0F9M865-F1-MODEL\_V4 | 1.0 | 7.94e-10 | 268 | 0.107 | 492 | 325 | 25 | 50 | 502 | 42 | 458 | Uncharacterized protein | Uncharacterized protein | | afdb-uniprot50 | AF-A0A2E9Y0J4-F1-MODEL\_V4 | 1.0 | 9.914e-09 | 264 | 0.247 | 222 | 137 | 5 | 45 | 264 | 17 | 210 | Uncharacterized protein | Uncharacterized protein | | afdb-uniprot50 | AF-A0A2V4ZQX8-F1-MODEL\_V4 | 1.0 | 4.557e-08 | 264 | 0.194 | 304 | 186 | 8 | 271 | 532 | 2 | 288 | Lambda family phage portal protein | Lambda family phage portal protein | | afdb-uniprot50 | AF-A0A1V5V193-F1-MODEL\_V4 | 1.0 | 8.467e-09 | 263 | 0.114 | 499 | 317 | 27 | 5 | 478 | 36 | 434 | Uncharacterized protein | Uncharacterized protein | | afdb-uniprot50 | AF-A0A7C6TUL3-F1-MODEL\_V4 | 1.0 | 8.127e-08 | 262 | 0.141 | 368 | 219 | 18 | 94 | 442 | 2 | 291 | Uncharacterized protein | Uncharacterized protein | | afdb-uniprot50 | AF-A0A258DZ30-F1-MODEL\_V4 | 1.0 | 1.789e-07 | 262 | 0.208 | 292 | 184 | 5 | 255 | 503 | 4 | 291 | Uncharacterized protein | Uncharacterized protein | | afdb-uniprot50 | AF-A0A1V5ZM23-F1-MODEL\_V4 | 1.0 | 1.224e-08 | 261 | 0.132 | 460 | 278 | 27 | 51 | 477 | 10 | 381 | Uncharacterized protein | Uncharacterized protein | | afdb-uniprot50 | AF-A0A2I1SLT0-F1-MODEL\_V4 | 1.0 | 1.033e-09 | 260 | 0.095 | 461 | 305 | 21 | 39 | 478 | 7 | 376 | Phage portal protein | Phage portal protein | | afdb-uniprot50 | AF-I9B6E5-F1-MODEL\_V4 | 1.0 | 2.023e-10 | 260 | 0.132 | 535 | 305 | 32 | 67 | 517 | 13 | 472 | Bacteriophage portal protein, SPP1 Gp6-like protein | Bacteriophage portal protein, SPP1 Gp6-like protein | | afdb-uniprot50 | AF-A0A7W3YMY9-F1-MODEL\_V4 | 1.0 | 4.747e-09 | 259 | 0.095 | 448 | 277 | 20 | 81 | 488 | 7 | 366 | Phage portal protein | Phage portal protein | | afdb-uniprot50 | AF-A0A8B1ASQ7-F1-MODEL\_V4 | 1.0 | 1.101e-08 | 258 | 0.109 | 448 | 279 | 21 | 51 | 478 | 13 | 360 | Phage portal protein | Phage portal protein | | afdb-uniprot50 | AF-A0A661CM99-F1-MODEL\_V4 | 1.0 | 3.463e-09 | 256 | 0.113 | 510 | 321 | 26 | 18 | 479 | 1 | 427 | Uncharacterized protein | Uncharacterized protein | | afdb-uniprot50 | AF-A0A143HAD2-F1-MODEL\_V4 | 1.0 | 2.957e-09 | 255 | 0.105 | 464 | 285 | 21 | 39 | 478 | 10 | 367 | Portal protein | Portal protein | | afdb-uniprot50 | AF-Q2FYC7-F1-MODEL\_V4 | 1.0 | 1.344e-09 | 253 | 0.1 | 480 | 310 | 18 | 23 | 478 | 2 | 383 | Phage portal protein, HK97 family | Phage portal protein, HK97 family | | afdb-uniprot50 | AF-A0A822IYQ1-F1-MODEL\_V4 | 1.0 | 9.914e-09 | 252 | 0.087 | 512 | 356 | 24 | 28 | 494 | 1 | 446 | Uncharacterized protein | Uncharacterized protein | | afdb-uniprot50 | AF-A0A7I8EK12-F1-MODEL\_V4 | 1.0 | 7.711e-08 | 251 | 0.128 | 389 | 262 | 15 | 14 | 372 | 1 | 342 | Uncharacterized protein | Uncharacterized protein | | afdb-uniprot50 | AF-A0A4Q6DI35-F1-MODEL\_V4 | 1.0 | 3.027e-07 | 249 | 0.207 | 222 | 161 | 5 | 292 | 502 | 3 | 220 | Phage portal protein | Phage portal protein | | afdb-uniprot50 | AF-A0A5T2E4A2-F1-MODEL\_V4 | 1.0 | 3.779e-06 | 248 | 0.246 | 166 | 119 | 5 | 270 | 431 | 2 | 165 | Phage portal protein | Phage portal protein | | afdb-uniprot50 | AF-A0A2R6MRI0-F1-MODEL\_V4 | 1.0 | 4.803e-08 | 248 | 0.118 | 480 | 296 | 28 | 27 | 467 | 1 | 392 | Uncharacterized protein | Uncharacterized protein | | afdb-uniprot50 | AF-A0A0F9NZU5-F1-MODEL\_V4 | 1.0 | 1.147e-09 | 248 | 0.131 | 600 | 340 | 33 | 67 | 532 | 24 | 576 | Uncharacterized protein | Uncharacterized protein | | afdb-uniprot50 | AF-A0A432QN30-F1-MODEL\_V4 | 1.0 | 1.101e-08 | 246 | 0.105 | 579 | 380 | 29 | 49 | 532 | 10 | 545 | Uncharacterized protein | Uncharacterized protein | | afdb-uniprot50 | AF-A0A7V1WC45-F1-MODEL\_V4 | 1.0 | 9.406e-09 | 245 | 0.125 | 496 | 316 | 26 | 11 | 473 | 1 | 411 | DUF935 family protein | DUF935 family protein | | afdb-uniprot50 | AF-A0A6S7F1S0-F1-MODEL\_V4 | 1.0 | 1.209e-09 | 245 | 0.13 | 512 | 309 | 24 | 18 | 464 | 6 | 446 | Uncharacterized protein | Uncharacterized protein | | afdb-uniprot50 | AF-A0A6B5FEG4-F1-MODEL\_V4 | 1.0 | 1.045e-08 | 244 | 0.105 | 419 | 270 | 19 | 83 | 478 | 4 | 340 | Phage portal protein | Phage portal protein | | afdb-uniprot50 | AF-W2E7Z9-F1-MODEL\_V4 | 1.0 | 9.406e-09 | 244 | 0.12 | 414 | 256 | 19 | 85 | 478 | 41 | 366 | ATP-dependent Clp protease proteolytic subunit | ATP-dependent Clp protease proteolytic subunit | | afdb-uniprot50 | AF-A0A7X7LEA5-F1-MODEL\_V4 | 1.0 | 5.494e-10 | 243 | 0.109 | 586 | 362 | 32 | 28 | 532 | 1 | 507 | Uncharacterized protein | Uncharacterized protein | | afdb-uniprot50 | AF-A0A3A4QJC1-F1-MODEL\_V4 | 1.0 | 3.847e-09 | 241 | 0.121 | 479 | 285 | 27 | 13 | 434 | 2 | 401 | DUF935 family protein | DUF935 family protein | | afdb-uniprot50 | AF-A0A843ANU4-F1-MODEL\_V4 | 1.0 | 1.864e-08 | 240 | 0.117 | 527 | 313 | 32 | 39 | 532 | 19 | 426 | DUF935 family protein | DUF935 family protein | | afdb-uniprot50 | AF-A0A6G1WRJ0-F1-MODEL\_V4 | 1.0 | 8.667e-07 | 239 | 0.256 | 218 | 138 | 5 | 316 | 532 | 18 | 212 | Phage portal protein | Phage portal protein | | afdb-uniprot50 | AF-A0A7Y9DWQ4-F1-MODEL\_V4 | 1.0 | 1.209e-09 | 239 | 0.158 | 481 | 300 | 26 | 59 | 495 | 46 | 465 | Uncharacterized protein | Uncharacterized protein | | afdb-uniprot50 | AF-A0A2E0IZX8-F1-MODEL\_V4 | 1.0 | 8.467e-09 | 238 | 0.126 | 484 | 297 | 20 | 15 | 478 | 1 | 378 | Phage portal protein | Phage portal protein | | afdb-uniprot50 | AF-A0A7V4AU95-F1-MODEL\_V4 | 1.0 | 5.062e-08 | 237 | 0.112 | 427 | 262 | 29 | 39 | 426 | 6 | 354 | DUF935 family protein | DUF935 family protein | | afdb-uniprot50 | AF-A0A7V5S056-F1-MODEL\_V4 | 1.0 | 9.135e-07 | 236 | 0.106 | 405 | 263 | 22 | 24 | 401 | 1 | 333 | DUF935 family protein | DUF935 family protein | | afdb-uniprot50 | AF-A0A662IQ42-F1-MODEL\_V4 | 1.0 | 1.864e-08 | 236 | 0.106 | 509 | 313 | 26 | 1 | 474 | 19 | 420 | Phage\_Mu\_F domain-containing protein | Phage\_Mu\_F domain-containing protein | | afdb-uniprot50 | AF-A0A1Q9KYC6-F1-MODEL\_V4 | 1.0 | 1.32e-06 | 235 | 0.16 | 292 | 194 | 7 | 270 | 523 | 5 | 283 | Phage portal protein | Phage portal protein | | afdb-uniprot50 | AF-A0A2I7SNJ0-F1-MODEL\_V4 | 1.0 | 1.592e-08 | 235 | 0.1 | 586 | 348 | 31 | 58 | 532 | 3 | 520 | Uncharacterized protein | Uncharacterized protein | | afdb-uniprot50 | AF-A0A1W9LYD6-F1-MODEL\_V4 | 1.0 | 1.359e-08 | 235 | 0.13 | 537 | 321 | 27 | 39 | 532 | 8 | 441 | Topoisom\_I domain-containing protein | Topoisom\_I domain-containing protein | | afdb-uniprot50 | AF-A0A2T0YNG5-F1-MODEL\_V4 | 1.0 | 4.803e-08 | 234 | 0.11 | 508 | 324 | 29 | 88 | 532 | 16 | 458 | SPP1 Gp6-like portal protein | SPP1 Gp6-like portal protein | | afdb-uniprot50 | AF-A0A662KXA9-F1-MODEL\_V4 | 1.0 | 3.19e-07 | 232 | 0.11 | 435 | 292 | 20 | 26 | 432 | 2 | 369 | Uncharacterized protein | Uncharacterized protein | | afdb-uniprot50 | AF-A0A4U3A7B6-F1-MODEL\_V4 | 1.0 | 3.324e-08 | 231 | 0.107 | 427 | 256 | 18 | 38 | 439 | 3 | 329 | Phage portal protein | Phage portal protein | | afdb-uniprot50 | AF-A0A7C3TS71-F1-MODEL\_V4 | 1.0 | 2.585e-07 | 229 | 0.13 | 414 | 273 | 21 | 125 | 523 | 5 | 346 | Phage portal protein | Phage portal protein | | afdb-uniprot50 | AF-A0A2K3ZHX0-F1-MODEL\_V4 | 1.0 | 1.964e-08 | 229 | 0.116 | 506 | 334 | 23 | 87 | 531 | 77 | 530 | Phage portal protein | Phage portal protein | | afdb-uniprot50 | AF-A0A376TZP2-F1-MODEL\_V4 | 1.0 | 3.19e-07 | 228 | 0.181 | 298 | 198 | 8 | 273 | 532 | 2 | 291 | Capsid protein of prophage | Capsid protein of prophage | | afdb-uniprot50 | AF-A0A0F9AB75-F1-MODEL\_V4 | 1.0 | 3.402e-06 | 227 | 0.205 | 229 | 166 | 4 | 285 | 502 | 2 | 225 | Uncharacterized protein | Uncharacterized protein | | afdb-uniprot50 | AF-A0A2A9EYD0-F1-MODEL\_V4 | 1.0 | 7.231e-09 | 227 | 0.139 | 501 | 305 | 30 | 72 | 532 | 48 | 462 | SPP1 Gp6-like portal protein | SPP1 Gp6-like portal protein | | afdb-uniprot50 | AF-A0A2H5WZA7-F1-MODEL\_V4 | 1.0 | 1.885e-07 | 226 | 0.102 | 431 | 272 | 29 | 39 | 439 | 11 | 356 | Uncharacterized protein | Uncharacterized protein | | afdb-uniprot50 | AF-A0A2A3U1G4-F1-MODEL\_V4 | 1.0 | 3.503e-08 | 226 | 0.095 | 419 | 267 | 19 | 83 | 478 | 32 | 361 | Phage portal protein | Phage portal protein | | afdb-uniprot50 | AF-A0A662UJK4-F1-MODEL\_V4 | 1.0 | 1.678e-08 | 226 | 0.155 | 496 | 304 | 29 | 66 | 517 | 20 | 444 | Uncharacterized protein | Uncharacterized protein | | afdb-uniprot50 | AF-A0A7V5ZGN1-F1-MODEL\_V4 | 1.0 | 3.228e-06 | 225 | 0.107 | 391 | 243 | 24 | 85 | 448 | 2 | 313 | DUF935 family protein | DUF935 family protein | | afdb-uniprot50 | AF-M2VUE3-F1-MODEL\_V4 | 1.0 | 6.175e-09 | 224 | 0.144 | 506 | 302 | 30 | 62 | 502 | 1 | 440 | Bacteriophage protein | Bacteriophage protein | | afdb-uniprot50 | AF-A0A0F9JGC3-F1-MODEL\_V4 | 1.0 | 2.327e-07 | 224 | 0.113 | 465 | 317 | 24 | 40 | 478 | 19 | 414 | Uncharacterized protein | Uncharacterized protein | | afdb-uniprot50 | AF-A0A167I0Q7-F1-MODEL\_V4 | 1.0 | 6.395e-06 | 223 | 0.234 | 239 | 147 | 5 | 252 | 462 | 4 | 234 | Plasmid partitioning protein ParB | Plasmid partitioning protein ParB | | afdb-uniprot50 | AF-A0A1G6LT02-F1-MODEL\_V4 | 1.0 | 5.928e-08 | 223 | 0.1 | 505 | 321 | 25 | 20 | 477 | 1 | 419 | Uncharacterized protein | Uncharacterized protein | | afdb-uniprot50 | AF-A0A2M8Q5N2-F1-MODEL\_V4 | 1.0 | 1.678e-08 | 222 | 0.121 | 592 | 354 | 32 | 43 | 532 | 6 | 533 | Uncharacterized protein | Uncharacterized protein | | afdb-uniprot50 | AF-S2KXG2-F1-MODEL\_V4 | 1.0 | 9.135e-07 | 221 | 0.216 | 236 | 158 | 5 | 301 | 532 | 5 | 217 | Lambda family phage portal protein | Lambda family phage portal protein | | afdb-uniprot50 | AF-A0A5C7P6L9-F1-MODEL\_V4 | 1.0 | 1.252e-06 | 220 | 0.168 | 315 | 215 | 11 | 244 | 531 | 4 | 298 | Phage portal protein | Phage portal protein | | afdb-uniprot50 | AF-A0A844QMZ9-F1-MODEL\_V4 | 1.0 | 8.127e-08 | 220 | 0.106 | 440 | 276 | 18 | 23 | 445 | 2 | 341 | Phage portal protein | Phage portal protein | | afdb-uniprot50 | AF-A0A099YCQ5-F1-MODEL\_V4 | 1.0 | 9.406e-09 | 218 | 0.119 | 469 | 277 | 25 | 39 | 478 | 6 | 367 | Portal protein | Portal protein | | afdb-uniprot50 | AF-A0A7K0ZTW4-F1-MODEL\_V4 | 1.0 | 7.316e-08 | 218 | 0.114 | 516 | 342 | 26 | 1 | 473 | 95 | 538 | DUF935 family protein | DUF935 family protein | | afdb-uniprot50 | AF-A0A0F9NYT3-F1-MODEL\_V4 | 1.0 | 1.29e-08 | 217 | 0.106 | 547 | 354 | 24 | 22 | 532 | 30 | 477 | Uncharacterized protein | Uncharacterized protein | | afdb-uniprot50 | AF-A0A0F9J430-F1-MODEL\_V4 | 1.0 | 6.585e-08 | 216 | 0.103 | 522 | 320 | 26 | 15 | 467 | 1 | 443 | Uncharacterized protein | Uncharacterized protein | | afdb-uniprot50 | AF-A0A6M3MEN7-F1-MODEL\_V4 | 1.0 | 4.374e-07 | 214 | 0.101 | 423 | 281 | 23 | 78 | 478 | 41 | 386 | Uncharacterized protein | Uncharacterized protein | | afdb-uniprot50 | AF-A0A1V6GNZ4-F1-MODEL\_V4 | 1.0 | 2.872e-07 | 212 | 0.125 | 501 | 315 | 28 | 11 | 476 | 1 | 413 | Phage Mu protein F like protein | Phage Mu protein F like protein | | afdb-uniprot50 | AF-A0A399WWF0-F1-MODEL\_V4 | 1.0 | 2.234e-06 | 210 | 0.095 | 408 | 255 | 19 | 39 | 407 | 6 | 338 | DUF935 family protein | DUF935 family protein | | afdb-uniprot50 | AF-C5R881-F1-MODEL\_V4 | 1.0 | 9.516e-08 | 210 | 0.105 | 473 | 282 | 23 | 23 | 476 | 19 | 369 | Phage portal protein, HK97 family | Phage portal protein, HK97 family | | afdb-uniprot50 | AF-A0A4P0YCK2-F1-MODEL\_V4 | 1.0 | 1.564e-05 | 209 | 0.201 | 213 | 155 | 3 | 321 | 532 | 1 | 199 | Capsid protein of prophage | Capsid protein of prophage | | afdb-uniprot50 | AF-A0A4D9WRN2-F1-MODEL\_V4 | 1.0 | 6.585e-08 | 209 | 0.1 | 498 | 318 | 23 | 51 | 515 | 32 | 432 | DUF935 family protein | DUF935 family protein | | afdb-uniprot50 | AF-A0A2H0A011-F1-MODEL\_V4 | 1.0 | 1.375e-07 | 209 | 0.105 | 492 | 329 | 27 | 27 | 485 | 3 | 416 | Uncharacterized protein | Uncharacterized protein | | afdb-uniprot50 | AF-A0A0R2G1M7-F1-MODEL\_V4 | 1.0 | 7.316e-08 | 208 | 0.122 | 458 | 286 | 26 | 92 | 499 | 34 | 425 | Uncharacterized protein | Uncharacterized protein | | afdb-uniprot50 | AF-A0A7U6KQJ3-F1-MODEL\_V4 | 1.0 | 1.003e-07 | 207 | 0.131 | 473 | 266 | 26 | 39 | 467 | 6 | 377 | Uncharacterized protein | Uncharacterized protein | | afdb-uniprot50 | AF-A0A1F6GLB2-F1-MODEL\_V4 | 1.0 | 2.453e-07 | 207 | 0.102 | 448 | 276 | 23 | 58 | 471 | 33 | 388 | Uncharacterized protein | Uncharacterized protein | | afdb-uniprot50 | AF-A0A1C3SC05-F1-MODEL\_V4 | 1.0 | 9.628e-07 | 207 | 0.106 | 507 | 342 | 31 | 67 | 495 | 9 | 482 | Phage protein | Phage protein | | afdb-uniprot50 | AF-A0A7C6ADP8-F1-MODEL\_V4 | 1.0 | 1.61e-07 | 207 | 0.095 | 554 | 337 | 32 | 29 | 532 | 1 | 440 | DUF935 family protein | DUF935 family protein | | afdb-uniprot50 | AF-A0A660E253-F1-MODEL\_V4 | 1.0 | 5.928e-08 | 205 | 0.095 | 471 | 293 | 21 | 38 | 478 | 3 | 370 | Phage portal protein [Lactobacillus brevis] | Phage portal protein [Lactobacillus brevis] | | afdb-uniprot50 | AF-A0A0F9MCG4-F1-MODEL\_V4 | 1.0 | 5.624e-08 | 205 | 0.108 | 600 | 360 | 31 | 6 | 530 | 1 | 500 | Uncharacterized protein | Uncharacterized protein | | afdb-uniprot50 | AF-A0A3C1G6A3-F1-MODEL\_V4 | 1.0 | 5.336e-08 | 205 | 0.114 | 488 | 324 | 30 | 17 | 476 | 1 | 408 | Uncharacterized protein | Uncharacterized protein | | afdb-uniprot50 | AF-A0A7V9U6J0-F1-MODEL\_V4 | 1.0 | 1.885e-07 | 202 | 0.108 | 535 | 330 | 29 | 66 | 532 | 9 | 464 | Phage portal protein | Phage portal protein | | afdb-uniprot50 | AF-A0A2G1Z9F9-F1-MODEL\_V4 | 1.0 | 6.741e-06 | 200 | 0.198 | 262 | 170 | 8 | 278 | 531 | 2 | 231 | Uncharacterized protein | Uncharacterized protein | | afdb-uniprot50 | AF-A0A377Q9Y2-F1-MODEL\_V4 | 1.0 | 7.801e-07 | 200 | 0.125 | 479 | 288 | 24 | 24 | 473 | 9 | 385 | Mu-like prophage protein gp29 | Mu-like prophage protein gp29 | | afdb-uniprot50 | AF-A0A353GK31-F1-MODEL\_V4 | 1.0 | 3.544e-07 | 200 | 0.106 | 534 | 323 | 27 | 4 | 477 | 1 | 440 | Uncharacterized protein | Uncharacterized protein | | afdb-uniprot50 | AF-A0A3D0N1B7-F1-MODEL\_V4 | 1.0 | 3.153e-08 | 199 | 0.107 | 504 | 321 | 25 | 9 | 478 | 32 | 440 | Uncharacterized protein | Uncharacterized protein | | afdb-uniprot50 | AF-A0A1U7GKP2-F1-MODEL\_V4 | 1.0 | 4.61e-07 | 198 | 0.106 | 547 | 364 | 29 | 51 | 532 | 18 | 504 | Uncharacterized protein | Uncharacterized protein | | afdb-uniprot50 | AF-A0A3N0ZNV0-F1-MODEL\_V4 | 1.0 | 2.327e-07 | 197 | 0.096 | 593 | 364 | 30 | 15 | 531 | 1 | 497 | DUF935 family protein | DUF935 family protein | | afdb-uniprot50 | AF-A0A6M3L929-F1-MODEL\_V4 | 1.0 | 3.027e-07 | 196 | 0.104 | 441 | 289 | 25 | 92 | 491 | 74 | 449 | Putative portal protein | Putative portal protein | | afdb-uniprot50 | AF-A0A4R7QR18-F1-MODEL\_V4 | 1.0 | 3.062e-06 | 194 | 0.101 | 435 | 295 | 22 | 69 | 473 | 34 | 402 | Uncharacterized protein DUF935 | Uncharacterized protein DUF935 | | afdb-uniprot50 | AF-A0A496UFM6-F1-MODEL\_V4 | 1.0 | 1.908e-06 | 194 | 0.075 | 492 | 295 | 25 | 77 | 488 | 8 | 419 | Uncharacterized protein | Uncharacterized protein | | afdb-uniprot50 | AF-A0A3N0ZBD8-F1-MODEL\_V4 | 1.0 | 3.19e-07 | 194 | 0.091 | 549 | 327 | 27 | 51 | 523 | 2 | 454 | DUF935 family protein | DUF935 family protein | | afdb-uniprot50 | AF-A0A5Y3MP01-F1-MODEL\_V4 | 1.0 | 1.987e-07 | 191 | 0.102 | 584 | 335 | 30 | 24 | 521 | 8 | 488 | DUF935 family protein | DUF935 family protein | | afdb-uniprot50 | AF-A0A0F9MWD7-F1-MODEL\_V4 | 1.0 | 7.022e-07 | 191 | 0.117 | 586 | 373 | 33 | 45 | 532 | 10 | 549 | Uncharacterized protein | Uncharacterized protein | | afdb-uniprot50 | AF-T1HRW6-F1-MODEL\_V4 | 1.0 | 4.374e-07 | 190 | 0.112 | 567 | 342 | 27 | 24 | 532 | 8 | 471 | Phage\_Mu\_F domain-containing protein | Phage\_Mu\_F domain-containing protein | | afdb-uniprot50 | AF-A0A640WJG5-F1-MODEL\_V4 | 1.0 | 4.859e-07 | 189 | 0.096 | 572 | 372 | 29 | 67 | 531 | 21 | 554 | Phage tail protein | Phage tail protein | | afdb-uniprot50 | AF-A0A843GSM3-F1-MODEL\_V4 | 1.0 | 1.127e-06 | 188 | 0.106 | 546 | 347 | 32 | 62 | 532 | 35 | 514 | Phage portal protein | Phage portal protein | | afdb-uniprot50 | AF-A0A7U5HK87-F1-MODEL\_V4 | 1.0 | 1.885e-07 | 187 | 0.127 | 432 | 270 | 26 | 108 | 498 | 2 | 367 | Phage portal protein | Phage portal protein | | afdb-uniprot50 | AF-A0A0Q4JBM3-F1-MODEL\_V4 | 1.0 | 1.188e-06 | 185 | 0.097 | 481 | 298 | 22 | 39 | 478 | 30 | 415 | Uncharacterized protein | Uncharacterized protein | | afdb-uniprot50 | AF-A0A432QRG5-F1-MODEL\_V4 | 1.0 | 3.363e-07 | 185 | 0.108 | 500 | 311 | 31 | 87 | 529 | 68 | 489 | DUF4055 domain-containing protein | DUF4055 domain-containing protein | | afdb-uniprot50 | AF-B8F5B1-F1-MODEL\_V4 | 1.0 | 7.022e-07 | 183 | 0.123 | 544 | 310 | 33 | 39 | 518 | 4 | 444 | Putative phage-related protein | Putative phage-related protein | | afdb-uniprot50 | AF-D8A1C1-F1-MODEL\_V4 | 1.0 | 6.741e-06 | 182 | 0.103 | 475 | 293 | 22 | 63 | 500 | 1 | 379 | Uncharacterized protein | Uncharacterized protein | | afdb-uniprot50 | AF-A0A7X7ERQ9-F1-MODEL\_V4 | 1.0 | 2.615e-06 | 181 | 0.09 | 473 | 316 | 25 | 28 | 473 | 1 | 386 | DUF935 family protein | DUF935 family protein | | afdb-uniprot50 | AF-A0A090G3P5-F1-MODEL\_V4 | 1.0 | 1.336e-05 | 180 | 0.183 | 261 | 182 | 8 | 274 | 532 | 24 | 255 | Capsid protein of prophage | Capsid protein of prophage | | afdb-uniprot50 | AF-Q30VX9-F1-MODEL\_V4 | 1.0 | 3.402e-06 | 179 | 0.104 | 469 | 308 | 20 | 38 | 479 | 19 | 402 | Uncharacterized protein | Uncharacterized protein | | afdb-uniprot50 | AF-E9CQ96-F1-MODEL\_V4 | 1.0 | 6.82e-05 | 178 | 0.189 | 201 | 134 | 6 | 254 | 429 | 27 | 223 | Putative phage portal protein | Putative phage portal protein | | afdb-uniprot50 | AF-A0A7X9E3K7-F1-MODEL\_V4 | 1.0 | 4.477e-05 | 177 | 0.083 | 431 | 287 | 23 | 67 | 475 | 2 | 346 | DUF935 family protein | DUF935 family protein | | afdb-uniprot50 | AF-A0A1Y1QSY6-F1-MODEL\_V4 | 1.0 | 7.801e-07 | 177 | 0.117 | 560 | 305 | 29 | 51 | 523 | 16 | 473 | Uncharacterized protein | Uncharacterized protein | | afdb-uniprot50 | AF-A0A1A9WW55-F1-MODEL\_V4 | 1.0 | 1.629e-06 | 176 | 0.101 | 560 | 339 | 25 | 24 | 517 | 8 | 469 | Phage\_Mu\_F domain-containing protein | Phage\_Mu\_F domain-containing protein | | afdb-uniprot50 | AF-A0A450Z5H8-F1-MODEL\_V4 | 1.0 | 7.105e-06 | 174 | 0.103 | 453 | 267 | 24 | 45 | 461 | 8 | 357 | Uncharacterized protein | Uncharacterized protein | | afdb-uniprot50 | AF-A0A3M0XMA1-F1-MODEL\_V4 | 1.0 | 1.07e-06 | 173 | 0.088 | 461 | 290 | 23 | 51 | 477 | 13 | 377 | DUF935 family protein | DUF935 family protein | | afdb-uniprot50 | AF-U4T7Q4-F1-MODEL\_V4 | 1.0 | 1.908e-06 | 173 | 0.117 | 469 | 292 | 22 | 39 | 474 | 4 | 383 | Mu-like prophage FluMu protein gp29 | Mu-like prophage FluMu protein gp29 | | afdb-uniprot50 | AF-A0A149TMR3-F1-MODEL\_V4 | 1.0 | 4.425e-06 | 173 | 0.092 | 455 | 296 | 21 | 58 | 474 | 39 | 414 | Uncharacterized protein | Uncharacterized protein | | afdb-uniprot50 | AF-A0A7W1RW72-F1-MODEL\_V4 | 1.0 | 2.872e-07 | 172 | 0.121 | 592 | 341 | 33 | 51 | 532 | 2 | 524 | DUF4055 domain-containing protein | DUF4055 domain-containing protein | | afdb-uniprot50 | AF-A0A2A2A6L3-F1-MODEL\_V4 | 1.0 | 1.32e-06 | 172 | 0.124 | 483 | 289 | 28 | 40 | 486 | 21 | 405 | Uncharacterized protein | Uncharacterized protein | | afdb-uniprot50 | AF-A0A840S794-F1-MODEL\_V4 | 1.0 | 6.395e-06 | 171 | 0.095 | 472 | 311 | 22 | 24 | 472 | 10 | 388 | Phage gp29-like protein | Phage gp29-like protein | | afdb-uniprot50 | AF-A0A2V3UB21-F1-MODEL\_V4 | 1.0 | 1.07e-06 | 170 | 0.113 | 511 | 305 | 32 | 63 | 487 | 8 | 456 | Uncharacterized protein DUF4055 | Uncharacterized protein DUF4055 | | afdb-uniprot50 | AF-A0A8B3NDI1-F1-MODEL\_V4 | 1.0 | 1.188e-06 | 170 | 0.103 | 524 | 298 | 31 | 63 | 488 | 10 | 459 | DUF4055 domain-containing protein | DUF4055 domain-containing protein | | afdb-uniprot50 | AF-A0A6G8AZX7-F1-MODEL\_V4 | 1.0 | 7.401e-07 | 170 | 0.11 | 433 | 265 | 21 | 39 | 454 | 4 | 333 | Phage portal protein | Phage portal protein | | afdb-uniprot50 | AF-A0A7Y5LKE7-F1-MODEL\_V4 | 1.0 | 3.586e-06 | 167 | 0.11 | 425 | 267 | 22 | 39 | 439 | 3 | 340 | DUF935 family protein | DUF935 family protein | | afdb-uniprot50 | AF-A0A849PHX0-F1-MODEL\_V4 | 1.0 | 8.416e-05 | 167 | 0.105 | 407 | 267 | 21 | 85 | 467 | 2 | 335 | DUF935 family protein | DUF935 family protein | | afdb-uniprot50 | AF-A0A254RDH1-F1-MODEL\_V4 | 1.0 | 5.462e-06 | 167 | 0.1 | 570 | 353 | 25 | 20 | 523 | 1 | 476 | Uncharacterized protein | Uncharacterized protein | | afdb-uniprot50 | AF-A0A7V5SQD7-F1-MODEL\_V4 | 1.0 | 2.51e-05 | 166 | 0.074 | 415 | 269 | 19 | 40 | 420 | 8 | 341 | DUF935 family protein | DUF935 family protein | | afdb-uniprot50 | AF-A0A353QZH6-F1-MODEL\_V4 | 1.0 | 1.127e-06 | 164 | 0.094 | 579 | 333 | 29 | 90 | 524 | 117 | 647 | Uncharacterized protein | Uncharacterized protein | | afdb-uniprot50 | AF-A0A4Y9PSX4-F1-MODEL\_V4 | 1.0 | 0.0001039 | 163 | 0.1 | 498 | 329 | 27 | 2 | 467 | 5 | 415 | Uncharacterized protein | Uncharacterized protein | | afdb-uniprot50 | AF-A0A838BWC4-F1-MODEL\_V4 | 1.0 | 3.228e-06 | 163 | 0.097 | 625 | 374 | 35 | 18 | 516 | 1 | 561 | Head-tail connector protein | Head-tail connector protein | | afdb-uniprot50 | AF-A0A7J4ZRG6-F1-MODEL\_V4 | 1.0 | 7.801e-07 | 163 | 0.129 | 570 | 337 | 30 | 63 | 523 | 3 | 522 | Phage tail protein | Phage tail protein | | afdb-uniprot50 | AF-A0A6M3IKJ8-F1-MODEL\_V4 | 1.0 | 4.664e-06 | 163 | 0.094 | 655 | 358 | 29 | 66 | 532 | 18 | 625 | Uncharacterized protein | Uncharacterized protein | | afdb-uniprot50 | AF-A0A357LVL4-F1-MODEL\_V4 | 1.0 | 2.481e-06 | 163 | 0.124 | 596 | 336 | 32 | 24 | 532 | 8 | 504 | Phage\_Mu\_F domain-containing protein | Phage\_Mu\_F domain-containing protein | | afdb-uniprot50 | AF-A0A329VRT9-F1-MODEL\_V4 | 1.0 | 1.202e-05 | 162 | 0.094 | 443 | 267 | 23 | 51 | 464 | 7 | 344 | Uncharacterized protein | Uncharacterized protein | | afdb-uniprot50 | AF-A0A2K2ZXI3-F1-MODEL\_V4 | 1.0 | 1.831e-05 | 162 | 0.095 | 430 | 284 | 26 | 90 | 473 | 86 | 456 | DUF4055 domain-containing protein | DUF4055 domain-containing protein | | afdb-uniprot50 | AF-A0A0F9HG25-F1-MODEL\_V4 | 1.0 | 8.319e-06 | 161 | 0.123 | 518 | 284 | 27 | 58 | 532 | 14 | 404 | Uncharacterized protein | Uncharacterized protein | | afdb-uniprot50 | AF-A0A840RLG2-F1-MODEL\_V4 | 1.0 | 2.905e-06 | 160 | 0.101 | 559 | 325 | 28 | 39 | 523 | 24 | 478 | Phage gp29-like protein | Phage gp29-like protein | | afdb-uniprot50 | AF-A0A1W1XTP3-F1-MODEL\_V4 | 1.0 | 7.488e-06 | 160 | 0.091 | 699 | 401 | 35 | 7 | 532 | 3 | 640 | Uncharacterized protein | Uncharacterized protein | | afdb-uniprot50 | AF-A0A3A8TWS2-F1-MODEL\_V4 | 1.0 | 1.717e-06 | 158 | 0.087 | 493 | 318 | 31 | 67 | 488 | 30 | 461 | DUF4055 domain-containing protein | DUF4055 domain-containing protein | | afdb-uniprot50 | AF-A0A6L5BK53-F1-MODEL\_V4 | 1.0 | 0.0001039 | 157 | 0.106 | 377 | 238 | 17 | 49 | 402 | 33 | 333 | Uncharacterized protein | Uncharacterized protein | | afdb-uniprot50 | AF-A0A450ZFK4-F1-MODEL\_V4 | 1.0 | 6.741e-06 | 156 | 0.119 | 568 | 304 | 30 | 27 | 518 | 2 | 449 | Mu-like prophage protein gp29 | Mu-like prophage protein gp29 | | afdb-uniprot50 | AF-A0A352Z9P4-F1-MODEL\_V4 | 1.0 | 1.141e-05 | 155 | 0.096 | 547 | 316 | 30 | 51 | 523 | 32 | 474 | DUF935 domain-containing protein | DUF935 domain-containing protein | | afdb-uniprot50 | AF-A0A3M8QA88-F1-MODEL\_V4 | 1.0 | 6.741e-06 | 155 | 0.08 | 635 | 380 | 33 | 66 | 532 | 31 | 629 | Uncharacterized protein | Uncharacterized protein | | afdb-uniprot50 | AF-A0A3N6V6C1-F1-MODEL\_V4 | 1.0 | 5.462e-06 | 155 | 0.118 | 624 | 344 | 33 | 68 | 525 | 39 | 622 | Uncharacterized protein | Uncharacterized protein | | afdb-uniprot50 | AF-A0A3B1CWW8-F1-MODEL\_V4 | 1.0 | 0.0001095 | 153 | 0.088 | 465 | 282 | 27 | 58 | 473 | 19 | 390 | Uncharacterized protein | Uncharacterized protein | | afdb-uniprot50 | AF-A0A3D9PWD8-F1-MODEL\_V4 | 1.0 | 2.94e-05 | 152 | 0.09 | 583 | 345 | 29 | 23 | 523 | 3 | 481 | Phage gp29-like protein | Phage gp29-like protein | | afdb-uniprot50 | AF-A0A6L7L088-F1-MODEL\_V4 | 1.0 | 7.985e-05 | 152 | 0.1 | 458 | 288 | 20 | 52 | 474 | 35 | 403 | DUF935 family protein | DUF935 family protein | | afdb-uniprot50 | AF-A0A0F9DSH9-F1-MODEL\_V4 | 1.0 | 8.416e-05 | 151 | 0.108 | 398 | 247 | 18 | 125 | 478 | 8 | 341 | Uncharacterized protein | Uncharacterized protein | | afdb-uniprot50 | AF-A0A515ERN2-F1-MODEL\_V4 | 1.0 | 7.985e-05 | 150 | 0.105 | 456 | 278 | 26 | 56 | 478 | 15 | 373 | DUF935 family protein | DUF935 family protein | | afdb-uniprot50 | AF-A0A7X7NIS7-F1-MODEL\_V4 | 1.0 | 1.027e-05 | 148 | 0.101 | 564 | 316 | 28 | 48 | 523 | 20 | 480 | DUF935 family protein | DUF935 family protein | | afdb-uniprot50 | AF-A0A2A2ARI2-F1-MODEL\_V4 | 1.0 | 0.0002286 | 147 | 0.091 | 470 | 314 | 21 | 36 | 475 | 23 | 409 | Uncharacterized protein | Uncharacterized protein | | afdb-uniprot50 | AF-A0A1V5KBQ0-F1-MODEL\_V4 | 1.0 | 6.395e-06 | 146 | 0.117 | 586 | 308 | 35 | 92 | 532 | 80 | 601 | Uncharacterized protein | Uncharacterized protein | | afdb-uniprot50 | AF-A0A6M8VBB9-F1-MODEL\_V4 | 1.0 | 0.0001501 | 145 | 0.103 | 387 | 245 | 19 | 38 | 398 | 2 | 312 | DUF935 family protein | DUF935 family protein | | afdb-uniprot50 | AF-A0A1V5V157-F1-MODEL\_V4 | 1.0 | 2.789e-05 | 145 | 0.094 | 614 | 354 | 30 | 24 | 532 | 1 | 517 | Phage Mu protein F like protein | Phage Mu protein F like protein | | afdb-uniprot50 | AF-X1C5I8-F1-MODEL\_V4 | 1.0 | 0.0002822 | 144 | 0.135 | 309 | 213 | 14 | 171 | 464 | 2 | 271 | Uncharacterized protein | Uncharacterized protein | | afdb-uniprot50 | AF-A0A2E7E6N9-F1-MODEL\_V4 | 1.0 | 9.35e-05 | 143 | 0.11 | 453 | 274 | 25 | 58 | 480 | 37 | 390 | Uncharacterized protein | Uncharacterized protein | | afdb-uniprot50 | AF-T2GD94-F1-MODEL\_V4 | 1.0 | 9.242e-06 | 142 | 0.099 | 575 | 331 | 28 | 54 | 523 | 30 | 522 | Uncharacterized protein | Uncharacterized protein | | afdb-uniprot50 | AF-A0A7C5UJ90-F1-MODEL\_V4 | 1.0 | 0.0002169 | 141 | 0.103 | 318 | 199 | 15 | 152 | 446 | 10 | 264 | DUF935 family protein | DUF935 family protein | | afdb-uniprot50 | AF-A0A6M3KQJ0-F1-MODEL\_V4 | 1.0 | 3.098e-05 | 141 | 0.1 | 546 | 342 | 30 | 38 | 523 | 18 | 474 | Phage\_Mu\_F domain-containing protein | Phage\_Mu\_F domain-containing protein | | afdb-uniprot50 | AF-A0A2S0MD11-F1-MODEL\_V4 | 1.0 | 4.248e-05 | 138 | 0.104 | 576 | 326 | 28 | 40 | 523 | 17 | 494 | DUF935 domain-containing protein | DUF935 domain-containing protein | | afdb-uniprot50 | AF-A0A2D6X058-F1-MODEL\_V4 | 1.0 | 4.248e-05 | 138 | 0.101 | 649 | 364 | 40 | 66 | 532 | 20 | 631 | Uncharacterized protein | Uncharacterized protein | | afdb-uniprot50 | AF-A0A0F9K8Y7-F1-MODEL\_V4 | 1.0 | 4.477e-05 | 137 | 0.11 | 609 | 308 | 34 | 66 | 468 | 18 | 598 | Uncharacterized protein | Uncharacterized protein | | afdb-uniprot50 | AF-A0A2E7DC09-F1-MODEL\_V4 | 1.0 | 2.144e-05 | 136 | 0.09 | 629 | 353 | 35 | 53 | 532 | 49 | 607 | Uncharacterized protein | Uncharacterized protein | | afdb-uniprot50 | AF-A0A2H0FN30-F1-MODEL\_V4 | 1.0 | 0.0001216 | 135 | 0.075 | 464 | 283 | 24 | 64 | 468 | 24 | 400 | Uncharacterized protein | Uncharacterized protein | | afdb-uniprot50 | AF-A0A0W8G0T3-F1-MODEL\_V4 | 1.0 | 0.000559 | 134 | 0.081 | 393 | 264 | 20 | 39 | 407 | 6 | 325 | Uncharacterized protein | Uncharacterized protein | | afdb-uniprot50 | AF-A0A847DLT8-F1-MODEL\_V4 | 1.0 | 5.824e-05 | 134 | 0.108 | 509 | 320 | 35 | 15 | 473 | 1 | 425 | DUF935 family protein | DUF935 family protein | | afdb-uniprot50 | AF-A0A2R4BP72-F1-MODEL\_V4 | 1.0 | 0.0001039 | 134 | 0.081 | 654 | 358 | 30 | 58 | 531 | 7 | 597 | Phage protein | Phage protein | | afdb-uniprot50 | AF-A0A357XXK9-F1-MODEL\_V4 | 1.0 | 0.002313 | 131 | 0.211 | 142 | 108 | 2 | 288 | 427 | 2 | 141 | Uncharacterized protein | Uncharacterized protein | | afdb-uniprot50 | AF-A0A0F8YUF6-F1-MODEL\_V4 | 1.0 | 0.0007665 | 130 | 0.12 | 341 | 210 | 14 | 207 | 519 | 1 | 279 | Uncharacterized protein | Uncharacterized protein | | afdb-uniprot50 | AF-A0A2H0Z0M0-F1-MODEL\_V4 | 1.0 | 0.0008975 | 130 | 0.084 | 377 | 228 | 17 | 62 | 407 | 25 | 315 | Uncharacterized protein | Uncharacterized protein | | afdb-uniprot50 | AF-A0A2E0VI79-F1-MODEL\_V4 | 1.0 | 9.855e-05 | 127 | 0.09 | 694 | 365 | 42 | 66 | 532 | 22 | 675 | Uncharacterized protein | Uncharacterized protein | | afdb-uniprot50 | AF-A0A521YNN5-F1-MODEL\_V4 | 1.0 | 0.0003304 | 127 | 0.082 | 667 | 375 | 32 | 40 | 532 | 6 | 609 | Uncharacterized protein | Uncharacterized protein | | afdb-uniprot50 | AF-A0A4Q6ACK7-F1-MODEL\_V4 | 1.0 | 0.0001582 | 126 | 0.103 | 414 | 257 | 25 | 62 | 397 | 9 | 386 | DUF4055 domain-containing protein | DUF4055 domain-containing protein | | afdb-uniprot50 | AF-A0A0F9CUA3-F1-MODEL\_V4 | 1.0 | 0.001367 | 120 | 0.148 | 304 | 218 | 12 | 252 | 532 | 17 | 302 | Uncharacterized protein | Uncharacterized protein | | afdb-uniprot50 | AF-A0A3N2M4B1-F1-MODEL\_V4 | 1.0 | 3.628e-05 | 120 | 0.105 | 453 | 264 | 23 | 70 | 407 | 3 | 429 | Uncharacterized protein | Uncharacterized protein | | afdb-uniprot50 | AF-A0A1Y1QPD1-F1-MODEL\_V4 | 1.0 | 0.0004298 | 117 | 0.097 | 731 | 395 | 40 | 21 | 532 | 1 | 685 | Uncharacterized protein | Uncharacterized protein | | afdb-uniprot50 | AF-A0A7J0AIQ2-F1-MODEL\_V4 | 1.0 | 0.0001501 | 116 | 0.114 | 456 | 256 | 23 | 70 | 408 | 2 | 426 | Uncharacterized protein | Uncharacterized protein | | afdb-uniprot50 | AF-A0A378VE46-F1-MODEL\_V4 | 1.0 | 0.0007665 | 113 | 0.101 | 533 | 340 | 25 | 43 | 523 | 21 | 466 | Phage protein | Phage protein | | afdb-uniprot50 | AF-A0A2D7XHA7-F1-MODEL\_V4 | 1.0 | 0.0006546 | 113 | 0.077 | 630 | 324 | 32 | 13 | 449 | 2 | 567 | Phage portal protein | Phage portal protein | | afdb-uniprot50 | AF-A0A4Q7AJZ6-F1-MODEL\_V4 | 1.0 | 0.0006546 | 112 | 0.101 | 599 | 353 | 33 | 66 | 515 | 35 | 596 | Portal protein p19 | Portal protein p19 | | afdb-uniprot50 | AF-A0A7Y5LIV9-F1-MODEL\_V4 | 1.0 | 0.002082 | 110 | 0.074 | 441 | 250 | 25 | 45 | 427 | 19 | 359 | DUF935 family protein | DUF935 family protein | | afdb-uniprot50 | AF-A0A0F9BIB7-F1-MODEL\_V4 | 1.0 | 0.001519 | 110 | 0.106 | 468 | 241 | 23 | 72 | 409 | 7 | 427 | Uncharacterized protein | Uncharacterized protein | | afdb-uniprot50 | AF-A0A4S2E2Z1-F1-MODEL\_V4 | 1.0 | 0.001874 | 101 | 0.12 | 475 | 255 | 23 | 109 | 521 | 19 | 392 | DUF935 family protein | DUF935 family protein | | afdb-uniprot50 | AF-R7J256-F1-MODEL\_V4 | 0.994 | 0.000997 | 87 | 0.1 | 456 | 266 | 26 | 71 | 404 | 3 | 436 | Uncharacterized protein | Uncharacterized protein | |
| Top keywords  (threshold 1.00e-02 (evalue)) | **portal, Phage, lambda, DUF935, Putative, Capsid, prophage, domain\_containing, Bacteriophage, Phage\_related** |
| Output files | ../../similar\_structures/04\_FANPEZAQ\_CDS\_0004\_afdb-proteome\_foldseek.tsv ../../similar\_structures/04\_FANPEZAQ\_CDS\_0004\_afdb-uniprot50\_foldseek.tsv ../../similar\_structures/04\_FANPEZAQ\_CDS\_0004\_merged.svg ../../similar\_structures/04\_FANPEZAQ\_CDS\_0004\_pdb\_foldseek.tsv |

  
  
  

Return to summary | Go to previous | Go to next

  


---

**Sequence/structure alignments coloring**  
Each object in the alignment figures is colored according to its E-value following this color coding:

1e-100
10

**References:**  
1) Steinegger M, Meier M, Mirdita M, Vöhringer H, Haunsberger S J, and Söding J (2019) HH-suite3 for fast remote homology detection and deep protein annotation, BMC Bioinformatics, 473. doi: 10.1186/s12859-019-3019-7  
2) Jumper J, Evans R, Pritzel A, ..., Hassabis D (2021) Highly accurate protein structure prediction with AlphaFold, Nature, 596. doi: 10.1038/s41586-021-03819-2  
3) van Kempen M, Kim S, Tumescheit C, Mirdita M, Lee J, Gilchrist CLM, Söding J, and Steinegger M (2023) Fast and accurate protein structure search with Foldseek. Nature Biotechnology. doi: 10.1038/s41587-023-01773-0
