# Supplementary material for: Completing the BASEL phage collection to unlock hidden diversity for systematic exploration of phage–host interactions
Source: PLoS Biol. 2025 Apr 7;23(4):e3003063. doi: 10.1371/journal.pbio.3003063 (PMC11990801; doi:10.1371/journal.pbio.3003063)
Supplement: S2 Data — (ZIP) [file pbio.3003063.s009.zip › entries/41.html]

FANPEZAQ\_CDS\_0041


Return to summary | Go to previous | Go to next

|  |  |
| --- | --- |
| FANPEZAQ\_CDS\_0041 Page creation date: 02 Sep 2024, 12:00  Project folder: n/a  Input sequences file: Escherichia\_virus\_HeidiAbel.gb | domain\_containing duf3799 exodeoxyribonuclease putative pddexk\_like e d pd\_ xk dna helicase nuclease\_like viii exonuclease pddexk\_1 nuclease fragment breaking\_rejoining 3'\_5' endonuclease\_like a superfamily atp\_dependent rece \_ cas4 crispr\_associated complex csa1 recb hypothetical phage type c containing i\_a yes lambda uvrd sap beta anp rep mitochondrial genome maintenance engineered escherichia coli gp60 |

### Sequence information

|  |  |
| --- | --- |
| Name | FANPEZAQ\_CDS\_0041  41\_FANPEZAQ\_CDS\_0041 (pipeline id) |
| Imported annotations |  |
| Protein sequence | MKPGVYEGIPNAEYHGGPGISKSGLDLVHRSPMHYHAVVTAANDRTPTAAQELGTAAHAL ILEPDVFADTYCLALRRSDVPDAIDDREVLVEMVNKLNEGRLAKLPTSGNKNELIARIME FTGWTGETSELEAMKGAELKAIIEKANESRTGLLPTSGSRHDLAYLLRSNGVKVTLWSDV LAEWTENNPGRMVLTPETWDQLHAMAAAVHAHPAAGSLLTSCPGEAEKSVYWIDATTGVL CRCRPDWWRDDNVIVDLKTTEDASPEGFAKSIANWRYDVQAAYYLDGVQQATGKRPKAFV FIAVEKKPPYGVGVYVLDSESVDLGRAQYQHDLRVYAECVRTGVWPGYGDKIQTISLPGW HANKNQHLLGAV |
| Number of residues | 372 |
| Molecular weight (Da) | 40833.65 |
| Output files | ../../query\_sequences/41\_FANPEZAQ\_CDS\_0041.fasta |

### Putative domain architecture and protein family

#### Search results (HHblits)1

|  |  |
| --- | --- |
| Domain family databases searched | Pfam, Ncbi-cd, Cath, Phrogs |
| Results, scheme(s)  (Top layers only; threshold 1.00e-03 (evalue)) | xml version="1.0" encoding="utf-8" standalone="no"?       2024-09-02T21:08:21.329101 image/svg+xml   Matplotlib v3.7.2, https://matplotlib.org/ |
| Results, table  (E-value ≤ 1.00e-03 (evalue)) | | db | id | prob | evalue | pvalue | score | cols | query | query\_len | template | template\_len | name | description | | --- | --- | --- | --- | --- | --- | --- | --- | --- | --- | --- | --- | --- | | pfam | PF12684 | 97.5 | 2.2e-08 | 5.4e-12 | 79.8 | 122 | (225, 348) | 372 | (95, 225) | 235 | DUF3799 | PDDEXK-like domain of unknown function (DUF3799) | | pfam | PF10926 | 96.8 | 1.1e-06 | 2.5e-10 | 78.1 | 91 | (238, 339) | 372 | (121, 218) | 406 | DUF2800 | Protein of unknown function (DUF2800) | | pfam | PF06023 | 96.5 | 3e-06 | 7.1e-10 | 68.9 | 105 | (226, 348) | 372 | (157, 266) | 281 | Csa1 | CRISPR-associated exonuclease Csa1 | | pfam | PF12705 | 95.5 | 6.7e-05 | 1.6e-08 | 59.4 | 63 | (238, 304) | 372 | (136, 202) | 272 | PDDEXK\_1 | PD-(D/E)XK nuclease superfamily | | pfam | PF01930 | 93.7 | 0.00077 | 1.8e-07 | 48.9 | 86 | (243, 347) | 372 | (57, 144) | 162 | Cas\_Cas4 | Domain of unknown function DUF83 | | cath | 3h4rA00 | 99.7 | 1.3e-21 | 2.1e-25 | 170.9 | 225 | (8, 365) | 372 | (1, 229) | 231 | Exodeoxyribonuclease 8 | CATHCODE: 3.90.320.10 NAME: Exodeoxyribonuclease 8. Chain: a. Fragment: c-terminal domain: unp residues 606-866. Synonym: exodeoxyribonuclease viii, exo viii. Engineered: yes. Mutation: yes SOURCE: Escherichia coli. Organism\_taxid: 83333. Strain: k-12. Gene: b1350, jw1344, rece. Expressed in: escherichia coli. Expression\_system\_taxid: 562. CLASS: Alpha Beta, ARCH: Alpha-Beta Complex, TOPOL: Lambda Exonuclease; Chain A, HOMOL: Lambda Exonuclease; Chain A | | cath | 3l0aA00 | 99.2 | 5.9e-16 | 9.4e-20 | 137.0 | 221 | (7, 348) | 372 | (2, 240) | 266 | Putative exonuclease | CATHCODE: 3.90.320.10 NAME: Putative exonuclease. Chain: a. Engineered: yes SOURCE: Eubacterium rectale. Organism\_taxid: 515619. Strain: atcc 33656 / vpi 0990. Gene: eubrec\_2131, rer070207002219. Expressed in: escherichia coli. Expression\_system\_taxid: 562. CLASS: Alpha Beta, ARCH: Alpha-Beta Complex, TOPOL: Lambda Exonuclease; Chain A, HOMOL: Lambda Exonuclease; Chain A | | cath | 3u4qA06 | 97.6 | 2.5e-08 | 4e-12 | 85.8 | 58 | (238, 296) | 372 | (163, 228) | 244 | Atp-dependent helicase/nuclease subunit a | CATHCODE: 3.90.320.10 NAME: Atp-dependent helicase/nuclease subunit a. Chain: a. Synonym: atp-dependent helicase/nuclease adda. Ec: 3.1.-.-, 3.6.4.12. Engineered: yes. Mutation: yes. Atp-dependent helicase/deoxyribonuclease subunit b. Chain: b. Synonym: atp-dependent helicase/nuclease addb. Ec: 3.1.-.-, 3.6.4.12. Engineered: yes. Mutation: yes. Dna (27-mer). Chain: x. Engineered: yes SOURCE: Bacillus subtilis. Organism\_taxid: 1423. Gene: adda, bsu10630. Expressed in: escherichia coli. Expression\_system\_taxid: 562. Bacillus subtilis. Organism\_taxid: 1423. Gene: addb, bsu10620. Expressed in: escherichia coli. Expression\_system\_taxid: 562. CLASS: Alpha Beta, ARCH: Alpha-Beta Complex, TOPOL: Lambda Exonuclease; Chain A, HOMOL: Lambda Exonuclease; Chain A | | cath | 4r5qA00 | 96.6 | 4.3e-06 | 6.9e-10 | 68.8 | 48 | (16, 63) | 372 | (52, 99) | 216 | Crispr-associated exonuclease, cas4 family | CATHCODE: 3.90.320.10 NAME: Crispr-associated exonuclease, cas4 family. Chain: a. Engineered: yes SOURCE: Pyrobaculum calidifontis jcm 11548. Organism\_taxid: 410359. Strain: jcm 11548 / va1. Gene: pcal\_0546. Expressed in: escherichia coli. Expression\_system\_taxid: 469008. CLASS: Alpha Beta, ARCH: Alpha-Beta Complex, TOPOL: Lambda Exonuclease; Chain A, HOMOL: Lambda Exonuclease; Chain A | | cath | 4ic1A00 | 96.2 | 1.4e-05 | 2.3e-09 | 63.7 | 49 | (237, 291) | 372 | (94, 146) | 206 | Uncharacterized protein | CATHCODE: 3.90.320.10 NAME: Uncharacterized protein. Chain: a, b, c, d, f, g, h, i, j, k. Engineered: yes SOURCE: Sulfolobus solfataricus. Organism\_taxid: 273057. Strain: atcc 35092 / dsm 1617 / jcm 11322 / p2. Gene: sso0001. Expressed in: escherichia coli. Expression\_system\_taxid: 469008. CLASS: Alpha Beta, ARCH: Alpha-Beta Complex, TOPOL: Lambda Exonuclease; Chain A, HOMOL: Lambda Exonuclease; Chain A | | phrogs | 412 | 100.0 | 1.1e-63 | 1.4e-67 | 473.5 | 248 | (2, 353) | 372 | (4, 265) | 267 | exonuclease VIII | exonuclease VIII; Category: DNA, RNA and nucleotide metabolism; p123465 VI\_04287 | | phrogs | 28222 | 99.9 | 1.5e-29 | 1.6e-33 | 250.5 | 276 | (4, 348) | 372 | (639, 924) | 941 | exonuclease | exonuclease; Category: DNA, RNA and nucleotide metabolism; NC\_020850\_p8 | | phrogs | 1227 | 99.9 | 6.3e-27 | 8.1e-31 | 218.9 | 150 | (195, 347) | 372 | (77, 246) | 268 | exonuclease | exonuclease; Category: DNA, RNA and nucleotide metabolism; KX456208\_p10 | |
| Top keywords  (threshold 1.00e-03 (evalue)) | **Exonuclease, A, yes, Lambda, Engineered, escherichia, coli, Organism\_taxid, Gene, Expressed** |
| Output files | ../../domain\_architecture/41\_FANPEZAQ\_CDS\_0041\_cath.hhr ../../domain\_architecture/41\_FANPEZAQ\_CDS\_0041\_merged.svg ../../domain\_architecture/41\_FANPEZAQ\_CDS\_0041\_ncbi-cd.hhr ../../domain\_architecture/41\_FANPEZAQ\_CDS\_0041\_pfam.hhr ../../domain\_architecture/41\_FANPEZAQ\_CDS\_0041\_phrogs.hhr |

### Identical protein sequences/structures

#### Search results

|  |  |
| --- | --- |
| Protein sequence databases searched | Pdb, Swissprot, Refseq |
| Identical proteins found | -- |
| Top keywords | -- |
| Output files | -- |

### Similar protein sequences/structures

#### Sequence similarity search results (HHblits)1

|  |  |
| --- | --- |
| Sequence databases searched | Uniclust, Pdb70 |
| Results, scheme(s)  (Top layers only, threshold 1.00e-03 (evalue)) | xml version="1.0" encoding="utf-8" standalone="no"?       2024-09-02T21:08:47.281446 image/svg+xml   Matplotlib v3.7.2, https://matplotlib.org/ |
| Results, table(s)  (threshold 1.00e-03 (evalue)) | | db | id | prob | evalue | pvalue | score | cols | query | query\_len | template | template\_len | name | description | | --- | --- | --- | --- | --- | --- | --- | --- | --- | --- | --- | --- | --- | | uniclust | UniRef100\_A0A088FVF0 | 100.0 | 3.3e-70 | 6.9e-76 | 514.8 | 357 | (1, 371) | 372 | (95, 456) | 476 | Putative exodeoxyribonuclease 8 PDDEXK-like domain-containing protein | Putative exodeoxyribonuclease 8 PDDEXK-like domain-containing protein | | uniclust | UniRef100\_A0A0F9QDF5 | 100.0 | 1.4e-63 | 3.1e-69 | 467.4 | 271 | (2, 368) | 372 | (73, 344) | 358 | Putative exodeoxyribonuclease 8 PDDEXK-like domain-containing protein | Putative exodeoxyribonuclease 8 PDDEXK-like domain-containing protein | | uniclust | UniRef100\_A0A0F6SEZ4 | 100.0 | 3.8e-63 | 8.2e-69 | 454.4 | 263 | (6, 365) | 372 | (34, 296) | 318 | Exodeoxyribonuclease VIII | Exodeoxyribonuclease VIII | | uniclust | UniRef100\_A0A0F5JU14 | 100.0 | 7.1e-63 | 1.5e-68 | 442.3 | 286 | (2, 370) | 372 | (23, 314) | 325 | Exonuclease VIII | Exonuclease VIII | | uniclust | UniRef100\_A0A0F9TR59 | 100.0 | 4.9e-60 | 1.1e-65 | 435.5 | 279 | (1, 368) | 372 | (25, 304) | 321 | Putative exodeoxyribonuclease 8 PDDEXK-like domain-containing protein | Putative exodeoxyribonuclease 8 PDDEXK-like domain-containing protein | | uniclust | UniRef100\_A0A0K6IY38 | 100.0 | 1.8e-59 | 3.8e-65 | 424.5 | 273 | (2, 369) | 372 | (61, 335) | 343 | Putative exodeoxyribonuclease 8 PDDEXK-like domain-containing protein | Putative exodeoxyribonuclease 8 PDDEXK-like domain-containing protein | | uniclust | UniRef100\_A0A023W6M4 | 100.0 | 9.6e-59 | 2e-64 | 432.0 | 269 | (1, 370) | 372 | (84, 358) | 384 | RecB-like exonuclease/helicase | RecB-like exonuclease/helicase | | uniclust | UniRef100\_A0A009G867 | 100.0 | 2.4e-58 | 4.9e-64 | 426.0 | 266 | (1, 368) | 372 | (87, 359) | 378 | Putative exodeoxyribonuclease 8 PDDEXK-like domain-containing protein | Putative exodeoxyribonuclease 8 PDDEXK-like domain-containing protein | | uniclust | UniRef100\_A0A011UJY5 | 100.0 | 1.4e-56 | 2.9e-62 | 430.1 | 266 | (2, 370) | 372 | (148, 419) | 504 | Putative exodeoxyribonuclease 8 PDDEXK-like domain-containing protein | Putative exodeoxyribonuclease 8 PDDEXK-like domain-containing protein | | uniclust | UniRef100\_A0A3D2A6G1 | 100.0 | 3.4e-54 | 6.5e-60 | 367.5 | 265 | (2, 361) | 372 | (3, 275) | 275 | Putative exodeoxyribonuclease 8 PDDEXK-like domain-containing protein | Putative exodeoxyribonuclease 8 PDDEXK-like domain-containing protein | | uniclust | UniRef100\_A0A011VDN1 | 100.0 | 1.2e-53 | 2.6e-59 | 395.3 | 280 | (2, 370) | 372 | (47, 339) | 361 | Putative exodeoxyribonuclease 8 PDDEXK-like domain-containing protein | Putative exodeoxyribonuclease 8 PDDEXK-like domain-containing protein | | uniclust | UniRef100\_A0A0F9EGN9 | 100.0 | 1.5e-53 | 3e-59 | 379.5 | 263 | (1, 364) | 372 | (8, 272) | 295 | Putative exodeoxyribonuclease 8 PDDEXK-like domain-containing protein (Fragment) | Putative exodeoxyribonuclease 8 PDDEXK-like domain-containing protein (Fragment) | | uniclust | UniRef100\_A0A1K1LD38 | 100.0 | 2.5e-51 | 5.1e-57 | 365.4 | 270 | (1, 368) | 372 | (9, 283) | 312 | Exodeoxyribonuclease VIII | Exodeoxyribonuclease VIII | | uniclust | UniRef100\_A0A318CJ92 | 100.0 | 3.9e-51 | 7.7e-57 | 361.4 | 265 | (1, 367) | 372 | (48, 312) | 317 | Putative exodeoxyribonuclease 8 PDDEXK-like domain-containing protein | Putative exodeoxyribonuclease 8 PDDEXK-like domain-containing protein | | uniclust | UniRef100\_A0A011MMW1 | 100.0 | 7.7e-51 | 1.4e-56 | 345.0 | 265 | (3, 367) | 372 | (6, 272) | 296 | Exodeoxyribonuclease 8 | Exodeoxyribonuclease 8 | | uniclust | UniRef100\_UPI0018DF9E2C | 100.0 | 3.4e-50 | 6.3e-56 | 351.4 | 369 | (2, 371) | 372 | (12, 454) | 459 | PD-(D/E)XK nuclease-like domain-containing protein | PD-(D/E)XK nuclease-like domain-containing protein | | uniclust | UniRef100\_A0A023ZU70 | 100.0 | 4.8e-48 | 1e-53 | 355.6 | 302 | (7, 370) | 372 | (50, 359) | 365 | Putative exodeoxyribonuclease VIII | Putative exodeoxyribonuclease VIII | | uniclust | UniRef100\_A0A2U9P084 | 100.0 | 2.4e-47 | 4.6e-53 | 351.3 | 268 | (2, 369) | 372 | (214, 491) | 499 | Putative exodeoxyribonuclease 8 PDDEXK-like domain-containing protein | Putative exodeoxyribonuclease 8 PDDEXK-like domain-containing protein | | uniclust | UniRef100\_A0A0F6B083 | 100.0 | 2.8e-47 | 5.6e-53 | 376.5 | 273 | (1, 372) | 372 | (610, 886) | 889 | Exodeoxyribonuclease 8 | Exodeoxyribonuclease 8 | | uniclust | UniRef100\_A0A1Z7ZMG2 | 100.0 | 4.8e-47 | 9.4e-53 | 330.7 | 261 | (2, 370) | 372 | (21, 285) | 296 | Putative exodeoxyribonuclease 8 PDDEXK-like domain-containing protein | Putative exodeoxyribonuclease 8 PDDEXK-like domain-containing protein | | uniclust | UniRef100\_A0A1E4WVT7 | 100.0 | 8e-47 | 1.5e-52 | 344.3 | 357 | (2, 368) | 372 | (100, 522) | 562 | Putative exodeoxyribonuclease 8 PDDEXK-like domain-containing protein | Putative exodeoxyribonuclease 8 PDDEXK-like domain-containing protein | | uniclust | UniRef100\_A0A076LUB6 | 100.0 | 1.1e-46 | 2.3e-52 | 385.2 | 354 | (2, 369) | 372 | (689, 1110) | 1223 | Exodeoxyribonuclease VIII | Exodeoxyribonuclease VIII | | uniclust | UniRef100\_A0A2W1SGM9 | 100.0 | 4.7e-46 | 9e-52 | 337.8 | 271 | (2, 368) | 372 | (166, 442) | 444 | Putative exodeoxyribonuclease 8 PDDEXK-like domain-containing protein | Putative exodeoxyribonuclease 8 PDDEXK-like domain-containing protein | | uniclust | UniRef100\_A0A6J5P3Q0 | 100.0 | 1.7e-45 | 3.5e-51 | 328.7 | 257 | (9, 365) | 372 | (33, 290) | 296 | Exodeoxyribonuclease 8, PDDEXK-like domain containing protein | Exodeoxyribonuclease 8, PDDEXK-like domain containing protein | | uniclust | UniRef100\_A0A061SV84 | 100.0 | 2e-45 | 4.1e-51 | 343.5 | 264 | (1, 368) | 372 | (91, 367) | 404 | Putative exodeoxyribonuclease 8 PDDEXK-like domain-containing protein | Putative exodeoxyribonuclease 8 PDDEXK-like domain-containing protein | | uniclust | UniRef100\_A0A0A0Q3N4 | 100.0 | 3.5e-45 | 6.7e-51 | 319.9 | 278 | (3, 371) | 372 | (78, 360) | 368 | PDDEXK-like domain | PDDEXK-like domain | | uniclust | UniRef100\_A0A0D8L788 | 100.0 | 8.2e-45 | 1.6e-50 | 335.4 | 352 | (2, 368) | 372 | (150, 569) | 577 | DNA breaking-rejoining protein | DNA breaking-rejoining protein | | uniclust | UniRef100\_A0A0F9G9B6 | 100.0 | 8.3e-45 | 1.7e-50 | 329.1 | 265 | (2, 368) | 372 | (15, 295) | 330 | Putative exodeoxyribonuclease 8 PDDEXK-like domain-containing protein | Putative exodeoxyribonuclease 8 PDDEXK-like domain-containing protein | | uniclust | UniRef100\_A0A0J6S4G7 | 100.0 | 3.7e-44 | 7.7e-50 | 330.6 | 295 | (1, 365) | 372 | (37, 368) | 373 | Putative exodeoxyribonuclease 8 PDDEXK-like domain-containing protein | Putative exodeoxyribonuclease 8 PDDEXK-like domain-containing protein | | uniclust | UniRef100\_A0A523QSH7 | 100.0 | 3.1e-43 | 5.9e-49 | 300.7 | 264 | (1, 364) | 372 | (21, 292) | 297 | Putative exodeoxyribonuclease 8 PDDEXK-like domain-containing protein | Putative exodeoxyribonuclease 8 PDDEXK-like domain-containing protein | | uniclust | UniRef100\_A0A0F9RJV3 | 100.0 | 8.3e-43 | 1.6e-48 | 299.9 | 266 | (3, 365) | 372 | (7, 273) | 291 | Putative exodeoxyribonuclease 8 PDDEXK-like domain-containing protein | Putative exodeoxyribonuclease 8 PDDEXK-like domain-containing protein | | uniclust | UniRef100\_A0A0F7L8T5 | 100.0 | 1.2e-42 | 2.4e-48 | 309.4 | 234 | (9, 349) | 372 | (39, 274) | 293 | Putative exodeoxyribonuclease 8 PDDEXK-like domain-containing protein | Putative exodeoxyribonuclease 8 PDDEXK-like domain-containing protein | | uniclust | UniRef100\_A0A7X5Z2D5 | 100.0 | 2.8e-42 | 5.5e-48 | 312.4 | 266 | (2, 369) | 372 | (124, 394) | 407 | Putative exodeoxyribonuclease 8 PDDEXK-like domain-containing protein | Putative exodeoxyribonuclease 8 PDDEXK-like domain-containing protein | | uniclust | UniRef100\_A0A501Y166 | 100.0 | 3.5e-42 | 6.8e-48 | 316.2 | 336 | (1, 357) | 372 | (86, 430) | 441 | Putative exodeoxyribonuclease 8 PDDEXK-like domain-containing protein | Putative exodeoxyribonuclease 8 PDDEXK-like domain-containing protein | | uniclust | UniRef100\_A0A4R4A4G8 | 100.0 | 6.9e-42 | 1.3e-47 | 285.8 | 255 | (3, 359) | 372 | (12, 270) | 273 | Exodeoxyribonuclease VIII | Exodeoxyribonuclease VIII | | uniclust | UniRef100\_A0A2D7NC52 | 100.0 | 8e-42 | 1.6e-47 | 297.1 | 260 | (3, 367) | 372 | (16, 277) | 289 | Putative exodeoxyribonuclease 8 PDDEXK-like domain-containing protein | Putative exodeoxyribonuclease 8 PDDEXK-like domain-containing protein | | uniclust | UniRef100\_A0A192A857 | 100.0 | 1.2e-41 | 2.5e-47 | 308.8 | 260 | (5, 366) | 372 | (110, 375) | 392 | Putative exodeoxyribonuclease 8 PDDEXK-like domain-containing protein | Putative exodeoxyribonuclease 8 PDDEXK-like domain-containing protein | | uniclust | UniRef100\_A0A254QH28 | 100.0 | 2.3e-41 | 4.5e-47 | 282.1 | 206 | (1, 306) | 372 | (6, 211) | 211 | Putative exodeoxyribonuclease 8 PDDEXK-like domain-containing protein (Fragment) | Putative exodeoxyribonuclease 8 PDDEXK-like domain-containing protein (Fragment) | | uniclust | UniRef100\_A0A661RLU8 | 100.0 | 7.1e-41 | 1.4e-46 | 289.6 | 272 | (1, 366) | 372 | (15, 296) | 306 | Putative exodeoxyribonuclease 8 PDDEXK-like domain-containing protein | Putative exodeoxyribonuclease 8 PDDEXK-like domain-containing protein | | uniclust | UniRef100\_A0A1X0YIJ6 | 100.0 | 1.2e-40 | 2.3e-46 | 300.8 | 353 | (2, 368) | 372 | (49, 469) | 481 | Exonuclease VIII (Fragment) | Exonuclease VIII (Fragment) | | uniclust | UniRef100\_A0A378FZ40 | 100.0 | 1.7e-40 | 3.3e-46 | 322.1 | 359 | (2, 369) | 372 | (579, 1000) | 1005 | Putative exodeoxyribonuclease VIII | Putative exodeoxyribonuclease VIII | | uniclust | UniRef100\_A0A2I7RCC9 | 100.0 | 2.1e-40 | 4.1e-46 | 282.9 | 208 | (1, 306) | 372 | (36, 247) | 253 | Uncharacterized protein | Uncharacterized protein | | uniclust | UniRef100\_A0A139BNH5 | 100.0 | 4.1e-40 | 8.1e-46 | 274.2 | 187 | (4, 288) | 372 | (15, 201) | 202 | Putative exodeoxyribonuclease 8 PDDEXK-like domain-containing protein (Fragment) | Putative exodeoxyribonuclease 8 PDDEXK-like domain-containing protein (Fragment) | | uniclust | UniRef100\_A0A090EAA3 | 100.0 | 5e-40 | 1e-45 | 303.5 | 307 | (1, 365) | 372 | (63, 407) | 415 | Putative exodeoxyribonuclease 8 PDDEXK-like domain-containing protein | Putative exodeoxyribonuclease 8 PDDEXK-like domain-containing protein | | uniclust | UniRef100\_A0A142XW82 | 100.0 | 1.8e-39 | 3.4e-45 | 276.2 | 258 | (10, 363) | 372 | (5, 268) | 285 | Exodeoxyribonuclease 8 | Exodeoxyribonuclease 8 | | uniclust | UniRef100\_UPI0005E1294F | 100.0 | 2e-39 | 3.6e-45 | 279.6 | 261 | (3, 366) | 372 | (94, 361) | 384 | PD-(D/E)XK nuclease-like domain-containing protein | PD-(D/E)XK nuclease-like domain-containing protein | | uniclust | UniRef100\_A0A0F6B562 | 100.0 | 3e-39 | 5.7e-45 | 314.3 | 353 | (2, 368) | 372 | (558, 978) | 987 | Exodeoxyribonuclease VIII-like protein | Exodeoxyribonuclease VIII-like protein | | uniclust | UniRef100\_E6QW59 | 100.0 | 3.8e-39 | 6.9e-45 | 281.2 | 354 | (5, 365) | 372 | (4, 424) | 427 | Putative exodeoxyribonuclease 8 PDDEXK-like domain-containing protein | Putative exodeoxyribonuclease 8 PDDEXK-like domain-containing protein | | uniclust | UniRef100\_A0A071LVK8 | 100.0 | 7.3e-39 | 1.4e-44 | 301.8 | 353 | (2, 368) | 372 | (324, 744) | 769 | Exodeoxyribonuclease VIII | Exodeoxyribonuclease VIII | | uniclust | UniRef100\_A0A370CF63 | 100.0 | 1e-38 | 1.9e-44 | 285.5 | 263 | (1, 362) | 372 | (11, 275) | 541 | Putative exodeoxyribonuclease 8 PDDEXK-like domain-containing protein | Putative exodeoxyribonuclease 8 PDDEXK-like domain-containing protein | | uniclust | UniRef100\_A0A076JDP6 | 100.0 | 9.3e-39 | 1.9e-44 | 285.6 | 261 | (6, 371) | 372 | (17, 296) | 299 | Phage-related exonuclease, PD-(D/E)XK superfamily | Phage-related exonuclease, PD-(D/E)XK superfamily | | uniclust | UniRef100\_A0A0S8J9S9 | 100.0 | 9.6e-39 | 1.9e-44 | 287.6 | 257 | (6, 359) | 372 | (69, 328) | 340 | Putative exodeoxyribonuclease 8 PDDEXK-like domain-containing protein | Putative exodeoxyribonuclease 8 PDDEXK-like domain-containing protein | | uniclust | UniRef100\_A0A448S1F5 | 100.0 | 1.7e-38 | 3.2e-44 | 278.8 | 267 | (1, 368) | 372 | (1, 273) | 451 | Exodeoxyribonuclease 8 | Exodeoxyribonuclease 8 | | uniclust | UniRef100\_A0A505CFK2 | 100.0 | 3.8e-38 | 7e-44 | 288.1 | 265 | (1, 364) | 372 | (414, 682) | 685 | Exodeoxyribonuclease 8 | Exodeoxyribonuclease 8 | | uniclust | UniRef100\_A0A1B3AZZ2 | 100.0 | 4.9e-38 | 9.1e-44 | 277.9 | 259 | (3, 370) | 372 | (168, 429) | 440 | Exonuclease | Exonuclease | | uniclust | UniRef100\_A0A2D8XAW4 | 100.0 | 6.3e-38 | 1.2e-43 | 268.5 | 261 | (3, 364) | 372 | (13, 283) | 309 | Putative exodeoxyribonuclease 8 PDDEXK-like domain-containing protein | Putative exodeoxyribonuclease 8 PDDEXK-like domain-containing protein | | uniclust | UniRef100\_A0A077ND06 | 100.0 | 1.6e-37 | 3.1e-43 | 264.0 | 261 | (1, 364) | 372 | (4, 268) | 276 | Phage-related exonuclease | Phage-related exonuclease | | uniclust | UniRef100\_A0A376LPM8 | 100.0 | 2e-37 | 3.8e-43 | 298.1 | 264 | (1, 363) | 372 | (505, 772) | 836 | Exonuclease VIII | Exonuclease VIII | | uniclust | UniRef100\_A0A1F9UXV9 | 100.0 | 2.8e-37 | 5.6e-43 | 269.3 | 253 | (8, 369) | 372 | (9, 265) | 273 | Uncharacterized protein | Uncharacterized protein | | uniclust | UniRef100\_A0A1Z9H6K7 | 100.0 | 3e-37 | 5.9e-43 | 266.5 | 239 | (4, 345) | 372 | (36, 276) | 280 | Putative exodeoxyribonuclease 8 PDDEXK-like domain-containing protein | Putative exodeoxyribonuclease 8 PDDEXK-like domain-containing protein | | uniclust | UniRef100\_A0A0P0IK53 | 100.0 | 6.1e-37 | 1.2e-42 | 268.2 | 307 | (7, 369) | 372 | (19, 342) | 349 | Exonuclease | Exonuclease | | uniclust | UniRef100\_A0A3B8UEV6 | 100.0 | 6.7e-37 | 1.2e-42 | 266.4 | 273 | (2, 370) | 372 | (111, 396) | 412 | Putative exodeoxyribonuclease 8 PDDEXK-like domain-containing protein | Putative exodeoxyribonuclease 8 PDDEXK-like domain-containing protein | | uniclust | UniRef100\_Q11ZS8 | 100.0 | 9.6e-37 | 1.8e-42 | 266.7 | 253 | (8, 360) | 372 | (82, 340) | 358 | Putative exodeoxyribonuclease 8 PDDEXK-like domain-containing protein | Putative exodeoxyribonuclease 8 PDDEXK-like domain-containing protein | | uniclust | UniRef100\_A0A379GM57 | 100.0 | 1.7e-36 | 3.1e-42 | 276.0 | 352 | (2, 368) | 372 | (150, 570) | 575 | Exodeoxyribonuclease 8 | Exodeoxyribonuclease 8 | | uniclust | UniRef100\_A0A0J9D3R0 | 100.0 | 2.7e-36 | 5.1e-42 | 268.8 | 275 | (2, 369) | 372 | (121, 432) | 442 | Putative exodeoxyribonuclease 8 PDDEXK-like domain-containing protein | Putative exodeoxyribonuclease 8 PDDEXK-like domain-containing protein | | uniclust | UniRef100\_UPI001C312083 | 100.0 | 5.7e-36 | 1e-41 | 256.7 | 262 | (2, 362) | 372 | (91, 358) | 359 | PD-(D/E)XK nuclease-like domain-containing protein | PD-(D/E)XK nuclease-like domain-containing protein | | uniclust | UniRef100\_A0A2X7FM56 | 100.0 | 5.8e-36 | 1.1e-41 | 281.6 | 355 | (2, 368) | 372 | (353, 773) | 776 | Putative phage exodeoxyribonuclease | Putative phage exodeoxyribonuclease | | uniclust | UniRef100\_A0A0F9KSW2 | 100.0 | 5.7e-36 | 1.1e-41 | 257.3 | 260 | (8, 365) | 372 | (10, 272) | 277 | Putative exodeoxyribonuclease 8 PDDEXK-like domain-containing protein | Putative exodeoxyribonuclease 8 PDDEXK-like domain-containing protein | | uniclust | UniRef100\_UPI001961519C | 100.0 | 1.5e-35 | 2.8e-41 | 253.2 | 261 | (8, 369) | 372 | (78, 347) | 349 | PD-(D/E)XK nuclease-like domain-containing protein | PD-(D/E)XK nuclease-like domain-containing protein | | uniclust | UniRef100\_A0A3N0CLS5 | 100.0 | 1.8e-35 | 3.5e-41 | 256.6 | 263 | (2, 362) | 372 | (12, 280) | 294 | Putative exodeoxyribonuclease 8 PDDEXK-like domain-containing protein | Putative exodeoxyribonuclease 8 PDDEXK-like domain-containing protein | | uniclust | UniRef100\_A0A2R5F5H8 | 100.0 | 1.9e-35 | 3.6e-41 | 254.4 | 256 | (7, 362) | 372 | (31, 289) | 292 | Coproporphyrinogen III oxidase | Coproporphyrinogen III oxidase | | uniclust | UniRef100\_H8FP23 | 100.0 | 2.4e-35 | 4.4e-41 | 254.4 | 267 | (2, 365) | 372 | (64, 350) | 378 | Putative exodeoxyribonuclease 8 PDDEXK-like domain-containing protein | Putative exodeoxyribonuclease 8 PDDEXK-like domain-containing protein | | uniclust | UniRef100\_A0A1V5GYE4 | 100.0 | 2.4e-35 | 4.7e-41 | 257.5 | 259 | (1, 365) | 372 | (9, 272) | 283 | Exodeoxyribonuclease 8 | Exodeoxyribonuclease 8 | | uniclust | UniRef100\_UPI001C47B09F | 100.0 | 3.7e-35 | 6.8e-41 | 254.1 | 258 | (7, 362) | 372 | (100, 378) | 389 | PD-(D/E)XK nuclease-like domain-containing protein | PD-(D/E)XK nuclease-like domain-containing protein | | uniclust | UniRef100\_A0A6J7VNU5 | 100.0 | 4.8e-35 | 9.2e-41 | 251.4 | 261 | (3, 365) | 372 | (9, 270) | 276 | Exodeoxyribonuclease 8, PDDEXK-like domain containing protein | Exodeoxyribonuclease 8, PDDEXK-like domain containing protein | | uniclust | UniRef100\_A0A0F8VZZ2 | 100.0 | 4.9e-35 | 9.4e-41 | 241.7 | 178 | (187, 367) | 372 | (33, 214) | 215 | Putative exodeoxyribonuclease 8 PDDEXK-like domain-containing protein (Fragment) | Putative exodeoxyribonuclease 8 PDDEXK-like domain-containing protein (Fragment) | | uniclust | UniRef100\_A0A2G2G2Z2 | 100.0 | 7.8e-35 | 1.5e-40 | 253.2 | 249 | (2, 348) | 372 | (5, 264) | 289 | Putative exodeoxyribonuclease 8 PDDEXK-like domain-containing protein | Putative exodeoxyribonuclease 8 PDDEXK-like domain-containing protein | | uniclust | UniRef100\_A0A437M7Z1 | 100.0 | 9.6e-35 | 1.8e-40 | 259.8 | 304 | (2, 363) | 372 | (45, 384) | 387 | Exodeoxyribonuclease 8 PDDEXK-like domain-containing protein | Exodeoxyribonuclease 8 PDDEXK-like domain-containing protein | | uniclust | UniRef100\_A0A377Q5C7 | 100.0 | 1.3e-34 | 2.3e-40 | 245.1 | 288 | (2, 353) | 372 | (10, 301) | 319 | Exodeoxyribonuclease 8 | Exodeoxyribonuclease 8 | | uniclust | UniRef100\_A0A086A3E8 | 100.0 | 1.5e-34 | 3.1e-40 | 268.8 | 308 | (3, 365) | 372 | (83, 405) | 416 | Uncharacterized protein | Uncharacterized protein | | uniclust | UniRef100\_A0A2I5T572 | 100.0 | 6e-34 | 1.2e-39 | 281.6 | 359 | (2, 369) | 372 | (552, 973) | 980 | Exodeoxyribonuclease VIII | Exodeoxyribonuclease VIII | | uniclust | UniRef100\_A0A7K0GP64 | 100.0 | 6.7e-34 | 1.4e-39 | 231.7 | 142 | (225, 366) | 372 | (9, 151) | 156 | Exodeoxyribonuclease VIII | Exodeoxyribonuclease VIII | | uniclust | UniRef100\_UPI001438A939 | 100.0 | 7.4e-34 | 1.4e-39 | 244.3 | 260 | (2, 365) | 372 | (49, 312) | 318 | PD-(D/E)XK nuclease-like domain-containing protein | PD-(D/E)XK nuclease-like domain-containing protein | | uniclust | UniRef100\_UPI0009ACE8C2 | 100.0 | 9e-34 | 1.7e-39 | 235.8 | 211 | (1, 306) | 372 | (41, 252) | 260 | PD-(D/E)XK nuclease-like domain-containing protein | PD-(D/E)XK nuclease-like domain-containing protein | | uniclust | UniRef100\_A0A433FYR1 | 100.0 | 9.2e-34 | 1.7e-39 | 245.3 | 281 | (4, 369) | 372 | (93, 379) | 384 | Putative exodeoxyribonuclease 8 PDDEXK-like domain-containing protein | Putative exodeoxyribonuclease 8 PDDEXK-like domain-containing protein | | uniclust | UniRef100\_A0A7Y6TYS2 | 100.0 | 1.6e-33 | 2.9e-39 | 243.2 | 260 | (4, 361) | 372 | (112, 374) | 374 | PD-(D/E)XK nuclease-like domain-containing protein | PD-(D/E)XK nuclease-like domain-containing protein | | uniclust | UniRef100\_A0A2D7BYZ1 | 100.0 | 1.6e-33 | 3e-39 | 240.9 | 248 | (2, 356) | 372 | (30, 279) | 284 | Putative exodeoxyribonuclease 8 PDDEXK-like domain-containing protein | Putative exodeoxyribonuclease 8 PDDEXK-like domain-containing protein | | uniclust | UniRef100\_A0A2S6AMK5 | 100.0 | 2.1e-33 | 3.8e-39 | 240.8 | 257 | (2, 361) | 372 | (81, 347) | 353 | RecE | RecE | | uniclust | UniRef100\_A0A759M5V4 | 100.0 | 3.3e-33 | 6.2e-39 | 248.9 | 309 | (3, 368) | 372 | (140, 461) | 474 | Putative exodeoxyribonuclease 8 PDDEXK-like domain-containing protein | Putative exodeoxyribonuclease 8 PDDEXK-like domain-containing protein | | uniclust | UniRef100\_A0A3S0UCX3 | 100.0 | 1.5e-32 | 2.8e-38 | 235.1 | 234 | (9, 349) | 372 | (1, 238) | 272 | Putative exodeoxyribonuclease 8 PDDEXK-like domain-containing protein | Putative exodeoxyribonuclease 8 PDDEXK-like domain-containing protein | | uniclust | UniRef100\_A0A0K8Q1C6 | 100.0 | 1.5e-32 | 3.1e-38 | 226.3 | 137 | (229, 366) | 372 | (1, 138) | 159 | Exodeoxyribonuclease 8 | Exodeoxyribonuclease 8 | | uniclust | UniRef100\_A0A6P1CUJ9 | 99.9 | 3.3e-32 | 6.1e-38 | 240.9 | 257 | (2, 363) | 372 | (6, 269) | 464 | Putative exodeoxyribonuclease 8 PDDEXK-like domain-containing protein | Putative exodeoxyribonuclease 8 PDDEXK-like domain-containing protein | | uniclust | UniRef100\_A0A5Q4ZYH7 | 99.9 | 3.1e-32 | 6.1e-38 | 266.1 | 369 | (2, 371) | 372 | (57, 623) | 683 | Uncharacterized protein | Uncharacterized protein | | uniclust | UniRef100\_A0A6H1ZNC6 | 99.9 | 3.6e-32 | 6.8e-38 | 235.8 | 287 | (2, 368) | 372 | (29, 324) | 325 | Putative exodeoxyribonuclease 8 PDDEXK-like domain-containing protein | Putative exodeoxyribonuclease 8 PDDEXK-like domain-containing protein | | uniclust | UniRef100\_A0A936Z580 | 99.9 | 4.8e-32 | 8.7e-38 | 228.7 | 264 | (5, 365) | 372 | (8, 301) | 307 | PD-(D/E)XK nuclease-like domain-containing protein | PD-(D/E)XK nuclease-like domain-containing protein | | uniclust | UniRef100\_A0A196PBF4 | 99.9 | 5.1e-32 | 1e-37 | 242.1 | 282 | (2, 365) | 372 | (20, 315) | 326 | Putative exodeoxyribonuclease 8 PDDEXK-like domain-containing protein | Putative exodeoxyribonuclease 8 PDDEXK-like domain-containing protein | | uniclust | UniRef100\_A0A9C7CW69 | 99.9 | 8.7e-32 | 1.6e-37 | 225.0 | 261 | (6, 362) | 372 | (17, 283) | 284 | Exodeoxyribonuclease 8 | Exodeoxyribonuclease 8 | | uniclust | UniRef100\_A0A0S8DSQ9 | 99.9 | 1.1e-31 | 2e-37 | 223.1 | 262 | (2, 364) | 372 | (3, 268) | 272 | Putative exodeoxyribonuclease 8 PDDEXK-like domain-containing protein | Putative exodeoxyribonuclease 8 PDDEXK-like domain-containing protein | | uniclust | UniRef100\_UPI0006FF5E4D | 99.9 | 1.8e-31 | 3.4e-37 | 230.2 | 179 | (186, 368) | 372 | (46, 231) | 256 | PD-(D/E)XK nuclease-like domain-containing protein | PD-(D/E)XK nuclease-like domain-containing protein | | uniclust | UniRef100\_A0A146GG86 | 99.9 | 2.1e-31 | 3.8e-37 | 224.2 | 269 | (1, 368) | 372 | (15, 293) | 298 | Putative exodeoxyribonuclease 8 PDDEXK-like domain-containing protein | Putative exodeoxyribonuclease 8 PDDEXK-like domain-containing protein | | uniclust | UniRef100\_A0A7C5SKK8 | 99.9 | 4.2e-31 | 7.9e-37 | 213.7 | 162 | (207, 370) | 372 | (5, 169) | 182 | Putative exodeoxyribonuclease 8 PDDEXK-like domain-containing protein | Putative exodeoxyribonuclease 8 PDDEXK-like domain-containing protein | | uniclust | UniRef100\_UPI001C485F61 | 99.9 | 8.2e-31 | 1.5e-36 | 225.9 | 280 | (2, 288) | 372 | (24, 362) | 362 | PD-(D/E)XK nuclease-like domain-containing protein | PD-(D/E)XK nuclease-like domain-containing protein | | uniclust | UniRef100\_I6APA3 | 99.9 | 9e-31 | 1.7e-36 | 223.8 | 285 | (4, 371) | 372 | (13, 317) | 320 | Putative exodeoxyribonuclease 8 PDDEXK-like domain-containing protein | Putative exodeoxyribonuclease 8 PDDEXK-like domain-containing protein | | uniclust | UniRef100\_A0A965K260 | 99.9 | 1.2e-30 | 2.2e-36 | 221.4 | 279 | (2, 363) | 372 | (10, 314) | 317 | Putative exodeoxyribonuclease 8 PDDEXK-like domain-containing protein | Putative exodeoxyribonuclease 8 PDDEXK-like domain-containing protein | | uniclust | UniRef100\_UPI00190851CF | 99.9 | 1.9e-30 | 3.4e-36 | 220.0 | 292 | (2, 363) | 372 | (14, 314) | 314 | PD-(D/E)XK nuclease-like domain-containing protein | PD-(D/E)XK nuclease-like domain-containing protein | | uniclust | UniRef100\_A0A0C1ZBA7 | 99.9 | 3.3e-30 | 6.5e-36 | 237.0 | 308 | (2, 366) | 372 | (62, 381) | 385 | Putative exodeoxyribonuclease 8 PDDEXK-like domain-containing protein | Putative exodeoxyribonuclease 8 PDDEXK-like domain-containing protein | | uniclust | UniRef100\_A0A2E7BLG2 | 99.9 | 3.8e-30 | 6.9e-36 | 217.6 | 257 | (7, 367) | 372 | (42, 304) | 307 | Putative exodeoxyribonuclease 8 PDDEXK-like domain-containing protein | Putative exodeoxyribonuclease 8 PDDEXK-like domain-containing protein | | uniclust | UniRef100\_A0A0K1H0Q4 | 99.9 | 5.9e-30 | 1.1e-35 | 241.8 | 263 | (5, 368) | 372 | (423, 692) | 700 | Exodeoxyribonuclease VIII | Exodeoxyribonuclease VIII | | uniclust | UniRef100\_UPI0015E1F6CA | 99.9 | 8e-30 | 1.7e-35 | 219.2 | 172 | (3, 265) | 372 | (7, 179) | 202 | PD-(D/E)XK nuclease-like domain-containing protein | PD-(D/E)XK nuclease-like domain-containing protein | | uniclust | UniRef100\_A0A2E5C063 | 99.9 | 9e-30 | 1.7e-35 | 211.3 | 231 | (3, 336) | 372 | (7, 239) | 243 | Putative exodeoxyribonuclease 8 PDDEXK-like domain-containing protein | Putative exodeoxyribonuclease 8 PDDEXK-like domain-containing protein | | uniclust | UniRef100\_A0A177W5I7 | 99.9 | 1e-29 | 1.9e-35 | 218.3 | 300 | (4, 365) | 372 | (15, 320) | 324 | Exonuclease VIII | Exonuclease VIII | | uniclust | UniRef100\_A0A0F8ZM74 | 99.9 | 1.3e-29 | 2.4e-35 | 208.2 | 180 | (184, 368) | 372 | (26, 205) | 215 | Putative exodeoxyribonuclease 8 PDDEXK-like domain-containing protein (Fragment) | Putative exodeoxyribonuclease 8 PDDEXK-like domain-containing protein (Fragment) | | uniclust | UniRef100\_UPI001E48B583 | 99.9 | 1.4e-29 | 2.5e-35 | 200.3 | 146 | (225, 370) | 372 | (7, 162) | 167 | PD-(D/E)XK nuclease-like domain-containing protein | PD-(D/E)XK nuclease-like domain-containing protein | | uniclust | UniRef100\_A0A2A2HMK2 | 99.9 | 1.8e-29 | 3.5e-35 | 232.7 | 262 | (5, 370) | 372 | (254, 522) | 531 | Putative exodeoxyribonuclease 8 PDDEXK-like domain-containing protein | Putative exodeoxyribonuclease 8 PDDEXK-like domain-containing protein | | uniclust | UniRef100\_A0A3M2CPY5 | 99.9 | 2.2e-29 | 4e-35 | 212.8 | 267 | (3, 362) | 372 | (16, 284) | 303 | Putative exodeoxyribonuclease 8 PDDEXK-like domain-containing protein | Putative exodeoxyribonuclease 8 PDDEXK-like domain-containing protein | | uniclust | UniRef100\_UPI0021473028 | 99.9 | 2.5e-29 | 4.6e-35 | 205.0 | 175 | (188, 367) | 372 | (56, 230) | 232 | PD-(D/E)XK nuclease-like domain-containing protein | PD-(D/E)XK nuclease-like domain-containing protein | | uniclust | UniRef100\_UPI00226FB7C9 | 99.9 | 3.3e-29 | 6.1e-35 | 221.4 | 268 | (7, 370) | 372 | (156, 440) | 445 | PD-(D/E)XK nuclease-like domain-containing protein | PD-(D/E)XK nuclease-like domain-containing protein | | uniclust | UniRef100\_A0A0B8T7Q5 | 99.9 | 4.8e-29 | 9.2e-35 | 217.8 | 243 | (19, 361) | 372 | (37, 286) | 288 | Exonuclease VIII, 5'-3' specific dsDNA exonuclease | Exonuclease VIII, 5'-3' specific dsDNA exonuclease | | uniclust | UniRef100\_A0A6H2A032 | 99.9 | 4.9e-29 | 9.3e-35 | 211.5 | 249 | (9, 363) | 372 | (2, 252) | 267 | Putative exodeoxyribonuclease 8 PDDEXK-like domain-containing protein | Putative exodeoxyribonuclease 8 PDDEXK-like domain-containing protein | | uniclust | UniRef100\_A0A3A1WJX1 | 99.9 | 7e-29 | 1.3e-34 | 217.9 | 272 | (2, 369) | 372 | (120, 408) | 418 | Putative exodeoxyribonuclease 8 PDDEXK-like domain-containing protein | Putative exodeoxyribonuclease 8 PDDEXK-like domain-containing protein | | uniclust | UniRef100\_A0A966V4M2 | 99.9 | 8.8e-29 | 1.6e-34 | 210.6 | 259 | (9, 363) | 372 | (1, 263) | 279 | Putative exodeoxyribonuclease 8 PDDEXK-like domain-containing protein | Putative exodeoxyribonuclease 8 PDDEXK-like domain-containing protein | | uniclust | UniRef100\_UPI0005A261FB | 99.9 | 9.2e-29 | 1.7e-34 | 203.6 | 215 | (2, 315) | 372 | (16, 241) | 248 | PD-(D/E)XK nuclease-like domain-containing protein | PD-(D/E)XK nuclease-like domain-containing protein | | uniclust | UniRef100\_A0A1B1IP21 | 99.9 | 1e-28 | 2.1e-34 | 223.1 | 211 | (11, 340) | 372 | (34, 247) | 269 | Putative exodeoxyribonuclease 8 PDDEXK-like domain-containing protein | Putative exodeoxyribonuclease 8 PDDEXK-like domain-containing protein | | uniclust | UniRef100\_A0A076HWB5 | 99.9 | 1.1e-28 | 2.3e-34 | 223.6 | 141 | (195, 341) | 372 | (97, 244) | 283 | Putative exodeoxyribonuclease 8 PDDEXK-like domain-containing protein | Putative exodeoxyribonuclease 8 PDDEXK-like domain-containing protein | | uniclust | UniRef100\_A0A2D9QDN0 | 99.9 | 1.4e-28 | 2.6e-34 | 202.5 | 238 | (19, 362) | 372 | (7, 248) | 248 | Putative exodeoxyribonuclease 8 PDDEXK-like domain-containing protein | Putative exodeoxyribonuclease 8 PDDEXK-like domain-containing protein | | uniclust | UniRef100\_A0A2Z3HVS0 | 99.9 | 1.5e-28 | 2.8e-34 | 199.4 | 168 | (189, 364) | 372 | (4, 175) | 180 | Putative exodeoxyribonuclease 8 PDDEXK-like domain-containing protein | Putative exodeoxyribonuclease 8 PDDEXK-like domain-containing protein | | uniclust | UniRef100\_A0A354CHA7 | 99.9 | 2.2e-28 | 4.2e-34 | 218.0 | 270 | (7, 369) | 372 | (53, 333) | 343 | Putative exodeoxyribonuclease 8 PDDEXK-like domain-containing protein | Putative exodeoxyribonuclease 8 PDDEXK-like domain-containing protein | | uniclust | UniRef100\_A0A497PDQ5 | 99.9 | 3.3e-28 | 6.1e-34 | 208.0 | 257 | (3, 362) | 372 | (9, 265) | 330 | Putative exodeoxyribonuclease 8 PDDEXK-like domain-containing protein | Putative exodeoxyribonuclease 8 PDDEXK-like domain-containing protein | | uniclust | UniRef100\_A0A8G2BHZ2 | 99.9 | 4.2e-28 | 7.7e-34 | 206.4 | 273 | (1, 370) | 372 | (3, 296) | 318 | Putative exodeoxyribonuclease 8 PDDEXK-like domain-containing protein | Putative exodeoxyribonuclease 8 PDDEXK-like domain-containing protein | | uniclust | UniRef100\_A0A6J5MSZ1 | 99.9 | 5.3e-28 | 9.7e-34 | 205.7 | 273 | (8, 364) | 372 | (30, 309) | 316 | Exodeoxyribonuclease 8, PDDEXK-like domain containing protein | Exodeoxyribonuclease 8, PDDEXK-like domain containing protein | | uniclust | UniRef100\_A0A7C1PFD3 | 99.9 | 6.2e-28 | 1.2e-33 | 213.2 | 255 | (8, 351) | 372 | (18, 291) | 344 | Putative exodeoxyribonuclease 8 PDDEXK-like domain-containing protein | Putative exodeoxyribonuclease 8 PDDEXK-like domain-containing protein | | uniclust | UniRef100\_A0A7T5UDE4 | 99.9 | 6.8e-28 | 1.3e-33 | 205.2 | 249 | (7, 353) | 372 | (57, 312) | 319 | PD-(D/E)XK nuclease-like domain-containing protein | PD-(D/E)XK nuclease-like domain-containing protein | | uniclust | UniRef100\_A0A0F9TVJ5 | 99.9 | 7e-28 | 1.3e-33 | 200.9 | 251 | (2, 353) | 372 | (15, 266) | 270 | Putative exodeoxyribonuclease 8 PDDEXK-like domain-containing protein | Putative exodeoxyribonuclease 8 PDDEXK-like domain-containing protein | | uniclust | UniRef100\_A0A2L0VPK0 | 99.9 | 1.2e-27 | 2.2e-33 | 207.6 | 263 | (2, 356) | 372 | (31, 316) | 344 | Putative exodeoxyribonuclease 8 PDDEXK-like domain-containing protein | Putative exodeoxyribonuclease 8 PDDEXK-like domain-containing protein | | uniclust | UniRef100\_A0A843HS31 | 99.9 | 1.4e-27 | 2.6e-33 | 207.1 | 237 | (8, 341) | 372 | (4, 249) | 289 | PD-(D/E)XK nuclease-like domain-containing protein | PD-(D/E)XK nuclease-like domain-containing protein | | uniclust | UniRef100\_A0A1G3FSC3 | 99.9 | 2e-27 | 3.8e-33 | 202.7 | 267 | (2, 364) | 372 | (9, 297) | 300 | Putative exodeoxyribonuclease 8 PDDEXK-like domain-containing protein | Putative exodeoxyribonuclease 8 PDDEXK-like domain-containing protein | | uniclust | UniRef100\_A0A0F9FLX8 | 99.9 | 2.5e-27 | 4.5e-33 | 197.8 | 260 | (1, 363) | 372 | (1, 267) | 271 | Putative exodeoxyribonuclease 8 PDDEXK-like domain-containing protein | Putative exodeoxyribonuclease 8 PDDEXK-like domain-containing protein | | uniclust | UniRef100\_UPI0015869DB5 | 99.9 | 2.6e-27 | 4.7e-33 | 194.4 | 182 | (175, 362) | 372 | (55, 237) | 239 | PD-(D/E)XK nuclease-like domain-containing protein | PD-(D/E)XK nuclease-like domain-containing protein | | uniclust | UniRef100\_A0A2D7PF36 | 99.9 | 2.6e-27 | 4.9e-33 | 204.2 | 235 | (6, 347) | 372 | (22, 274) | 294 | Putative exodeoxyribonuclease 8 PDDEXK-like domain-containing protein | Putative exodeoxyribonuclease 8 PDDEXK-like domain-containing protein | | uniclust | UniRef100\_A0A3M1GEU5 | 99.9 | 3.7e-27 | 6.9e-33 | 195.4 | 214 | (4, 310) | 372 | (24, 243) | 257 | Putative exodeoxyribonuclease 8 PDDEXK-like domain-containing protein (Fragment) | Putative exodeoxyribonuclease 8 PDDEXK-like domain-containing protein (Fragment) | | uniclust | UniRef100\_A0A3R8K6A3 | 99.9 | 3.6e-27 | 6.9e-33 | 203.1 | 265 | (3, 369) | 372 | (11, 293) | 296 | Putative exodeoxyribonuclease 8 PDDEXK-like domain-containing protein | Putative exodeoxyribonuclease 8 PDDEXK-like domain-containing protein | | uniclust | UniRef100\_A0A2D7GWI6 | 99.9 | 5.1e-27 | 9.3e-33 | 205.3 | 321 | (2, 350) | 372 | (35, 362) | 376 | SAP domain-containing protein | SAP domain-containing protein | | uniclust | UniRef100\_A0A2E5F7Y4 | 99.9 | 9.7e-27 | 1.8e-32 | 198.2 | 271 | (3, 364) | 372 | (24, 308) | 316 | Putative exodeoxyribonuclease 8 PDDEXK-like domain-containing protein | Putative exodeoxyribonuclease 8 PDDEXK-like domain-containing protein | | uniclust | UniRef100\_A0A6H1ZNA7 | 99.9 | 1.3e-26 | 2.4e-32 | 195.4 | 271 | (2, 363) | 372 | (9, 287) | 291 | Putative exodeoxyribonuclease 8 PDDEXK-like domain-containing protein | Putative exodeoxyribonuclease 8 PDDEXK-like domain-containing protein | | uniclust | UniRef100\_A0A524RVX3 | 99.9 | 1.8e-26 | 3.3e-32 | 204.2 | 296 | (6, 371) | 372 | (107, 430) | 437 | Putative exodeoxyribonuclease 8 PDDEXK-like domain-containing protein | Putative exodeoxyribonuclease 8 PDDEXK-like domain-containing protein | | uniclust | UniRef100\_A0A2N2TMM3 | 99.9 | 2.8e-26 | 5.1e-32 | 198.6 | 258 | (6, 365) | 372 | (89, 352) | 360 | Putative exodeoxyribonuclease 8 PDDEXK-like domain-containing protein | Putative exodeoxyribonuclease 8 PDDEXK-like domain-containing protein | | uniclust | UniRef100\_A0A3A0DGM8 | 99.9 | 2.8e-26 | 5.3e-32 | 209.5 | 249 | (8, 353) | 372 | (233, 485) | 490 | Putative exodeoxyribonuclease 8 PDDEXK-like domain-containing protein | Putative exodeoxyribonuclease 8 PDDEXK-like domain-containing protein | | uniclust | UniRef100\_UPI001CF244C9 | 99.9 | 3.5e-26 | 6.4e-32 | 187.1 | 187 | (181, 369) | 372 | (39, 229) | 232 | PD-(D/E)XK nuclease-like domain-containing protein | PD-(D/E)XK nuclease-like domain-containing protein | | uniclust | UniRef100\_UPI001675557B | 99.9 | 5.8e-26 | 1.1e-31 | 199.9 | 266 | (1, 363) | 372 | (92, 369) | 415 | PD-(D/E)XK nuclease-like domain-containing protein | PD-(D/E)XK nuclease-like domain-containing protein | | uniclust | UniRef100\_A0A496WM50 | 99.9 | 8.2e-26 | 1.5e-31 | 190.0 | 264 | (1, 353) | 372 | (1, 264) | 283 | Putative exodeoxyribonuclease 8 PDDEXK-like domain-containing protein | Putative exodeoxyribonuclease 8 PDDEXK-like domain-containing protein | | uniclust | UniRef100\_UPI001FE141F0 | 99.9 | 8.9e-26 | 1.6e-31 | 186.6 | 178 | (187, 367) | 372 | (52, 233) | 249 | PD-(D/E)XK nuclease-like domain-containing protein | PD-(D/E)XK nuclease-like domain-containing protein | | uniclust | UniRef100\_A0A966W4D5 | 99.9 | 9.8e-26 | 1.8e-31 | 183.1 | 176 | (186, 364) | 372 | (33, 211) | 219 | Putative exodeoxyribonuclease 8 PDDEXK-like domain-containing protein | Putative exodeoxyribonuclease 8 PDDEXK-like domain-containing protein | | uniclust | UniRef100\_A0A2E2D771 | 99.9 | 9.6e-26 | 1.9e-31 | 202.0 | 282 | (17, 364) | 372 | (40, 326) | 336 | Putative exodeoxyribonuclease 8 PDDEXK-like domain-containing protein | Putative exodeoxyribonuclease 8 PDDEXK-like domain-containing protein | | uniclust | UniRef100\_A0A1H5JK49 | 99.9 | 1e-25 | 1.9e-31 | 194.5 | 263 | (2, 366) | 372 | (5, 288) | 306 | Putative exodeoxyribonuclease 8 PDDEXK-like domain-containing protein | Putative exodeoxyribonuclease 8 PDDEXK-like domain-containing protein | | uniclust | UniRef100\_A0A518EYZ7 | 99.9 | 1.3e-25 | 2.4e-31 | 190.8 | 267 | (2, 366) | 372 | (11, 285) | 306 | Exodeoxyribonuclease 8 | Exodeoxyribonuclease 8 | | uniclust | UniRef100\_A0A1H6WGJ5 | 99.9 | 1.4e-25 | 2.6e-31 | 199.9 | 252 | (2, 351) | 372 | (32, 294) | 462 | Putative exodeoxyribonuclease 8 PDDEXK-like domain-containing protein | Putative exodeoxyribonuclease 8 PDDEXK-like domain-containing protein | | uniclust | UniRef100\_A0A2E8RC03 | 99.9 | 1.6e-25 | 2.9e-31 | 190.8 | 252 | (7, 349) | 372 | (5, 263) | 277 | Putative exodeoxyribonuclease 8 PDDEXK-like domain-containing protein | Putative exodeoxyribonuclease 8 PDDEXK-like domain-containing protein | | uniclust | UniRef100\_A0A0F9N5B6 | 99.9 | 1.9e-25 | 3.8e-31 | 199.7 | 271 | (7, 367) | 372 | (14, 320) | 327 | Putative exodeoxyribonuclease 8 PDDEXK-like domain-containing protein | Putative exodeoxyribonuclease 8 PDDEXK-like domain-containing protein | | uniclust | UniRef100\_A0A022N7N2 | 99.9 | 1.9e-25 | 4.1e-31 | 211.0 | 230 | (7, 348) | 372 | (60, 302) | 368 | Putative exodeoxyribonuclease 8 PDDEXK-like domain-containing protein | Putative exodeoxyribonuclease 8 PDDEXK-like domain-containing protein | | uniclust | UniRef100\_UPI001BB88D46 | 99.9 | 2.9e-25 | 5.4e-31 | 193.0 | 323 | (2, 351) | 372 | (21, 344) | 369 | PD-(D/E)XK nuclease-like domain-containing protein | PD-(D/E)XK nuclease-like domain-containing protein | | uniclust | UniRef100\_A0A2D7FJB4 | 99.9 | 4.5e-25 | 8.2e-31 | 185.9 | 256 | (5, 359) | 372 | (18, 277) | 285 | Putative exodeoxyribonuclease 8 PDDEXK-like domain-containing protein | Putative exodeoxyribonuclease 8 PDDEXK-like domain-containing protein | | uniclust | UniRef100\_A0A433CKI3 | 99.9 | 4.3e-25 | 8.4e-31 | 194.0 | 228 | (8, 343) | 372 | (24, 256) | 277 | Putative exodeoxyribonuclease 8 PDDEXK-like domain-containing protein | Putative exodeoxyribonuclease 8 PDDEXK-like domain-containing protein | | uniclust | UniRef100\_A0A0K1LMW8 | 99.9 | 4.7e-25 | 8.6e-31 | 196.0 | 230 | (2, 291) | 372 | (31, 269) | 447 | Nucleic acid-binding protein | Nucleic acid-binding protein | | uniclust | UniRef100\_A0A5M6B4U3 | 99.9 | 4.8e-25 | 9e-31 | 192.0 | 268 | (10, 364) | 372 | (21, 307) | 314 | Putative exodeoxyribonuclease 8 PDDEXK-like domain-containing protein | Putative exodeoxyribonuclease 8 PDDEXK-like domain-containing protein | | uniclust | UniRef100\_A0A516LLF0 | 99.9 | 5.2e-25 | 9.6e-31 | 184.6 | 265 | (2, 364) | 372 | (5, 271) | 274 | Putative exodeoxyribonuclease 8 | Putative exodeoxyribonuclease 8 | | uniclust | UniRef100\_A0A4Q7G2P0 | 99.8 | 7.4e-25 | 1.4e-30 | 195.0 | 301 | (2, 362) | 372 | (114, 449) | 453 | Putative exodeoxyribonuclease 8 PDDEXK-like domain-containing protein | Putative exodeoxyribonuclease 8 PDDEXK-like domain-containing protein | | uniclust | UniRef100\_A0A7X6FQD1 | 99.8 | 1.5e-24 | 2.8e-30 | 183.7 | 235 | (1, 291) | 372 | (16, 264) | 296 | Putative exodeoxyribonuclease 8 PDDEXK-like domain-containing protein | Putative exodeoxyribonuclease 8 PDDEXK-like domain-containing protein | | uniclust | UniRef100\_A0A385DTB7 | 99.8 | 2.7e-24 | 5.6e-30 | 201.6 | 294 | (7, 347) | 372 | (23, 340) | 365 | Exonuclease, PD-(D/E)XK superfamily | Exonuclease, PD-(D/E)XK superfamily | | uniclust | UniRef100\_A0A2D6XBJ2 | 99.8 | 3.1e-24 | 5.6e-30 | 179.7 | 262 | (7, 364) | 372 | (3, 267) | 269 | Putative exodeoxyribonuclease 8 PDDEXK-like domain-containing protein | Putative exodeoxyribonuclease 8 PDDEXK-like domain-containing protein | | uniclust | UniRef100\_A0A0F9U718 | 99.8 | 3.1e-24 | 5.6e-30 | 179.5 | 258 | (2, 356) | 372 | (4, 262) | 267 | Putative exodeoxyribonuclease 8 PDDEXK-like domain-containing protein | Putative exodeoxyribonuclease 8 PDDEXK-like domain-containing protein | | uniclust | UniRef100\_A0A0J6BHA2 | 99.8 | 4.2e-24 | 8e-30 | 183.6 | 248 | (7, 350) | 372 | (2, 256) | 282 | Putative exodeoxyribonuclease 8 PDDEXK-like domain-containing protein | Putative exodeoxyribonuclease 8 PDDEXK-like domain-containing protein | | uniclust | UniRef100\_UPI001E36E344 | 99.8 | 4.6e-24 | 8.5e-30 | 183.5 | 267 | (81, 368) | 372 | (15, 293) | 332 | PD-(D/E)XK nuclease-like domain-containing protein | PD-(D/E)XK nuclease-like domain-containing protein | | uniclust | UniRef100\_A0A1Q6ECM9 | 99.8 | 5.8e-24 | 1.1e-29 | 188.4 | 278 | (7, 365) | 372 | (37, 346) | 356 | Putative exodeoxyribonuclease 8 PDDEXK-like domain-containing protein | Putative exodeoxyribonuclease 8 PDDEXK-like domain-containing protein | | uniclust | UniRef100\_A0A2M9PH52 | 99.8 | 6.4e-24 | 1.2e-29 | 189.1 | 267 | (4, 367) | 372 | (9, 290) | 450 | Putative exodeoxyribonuclease 8 PDDEXK-like domain-containing protein | Putative exodeoxyribonuclease 8 PDDEXK-like domain-containing protein | | uniclust | UniRef100\_A0A1P8VVD3 | 99.8 | 7.3e-24 | 1.4e-29 | 194.4 | 232 | (2, 291) | 372 | (172, 413) | 550 | Nucleic acid-binding protein | Nucleic acid-binding protein | | uniclust | UniRef100\_A0A1F6GLK6 | 99.8 | 9.7e-24 | 1.8e-29 | 183.5 | 317 | (2, 364) | 372 | (17, 354) | 363 | Putative exodeoxyribonuclease 8 PDDEXK-like domain-containing protein | Putative exodeoxyribonuclease 8 PDDEXK-like domain-containing protein | | uniclust | UniRef100\_A0A7M1RQF0 | 99.8 | 9.7e-24 | 1.9e-29 | 191.6 | 291 | (8, 347) | 372 | (13, 324) | 347 | Exonuclease | Exonuclease | | uniclust | UniRef100\_A0A5C7PQS1 | 99.8 | 1.1e-23 | 2e-29 | 176.6 | 256 | (9, 362) | 372 | (1, 269) | 271 | Putative exodeoxyribonuclease 8 PDDEXK-like domain-containing protein | Putative exodeoxyribonuclease 8 PDDEXK-like domain-containing protein | | uniclust | UniRef100\_UPI001BC86F65 | 99.8 | 1.1e-23 | 2.1e-29 | 176.3 | 154 | (1, 246) | 372 | (115, 268) | 268 | PD-(D/E)XK nuclease-like domain-containing protein | PD-(D/E)XK nuclease-like domain-containing protein | | uniclust | UniRef100\_A0A5C7P550 | 99.8 | 1.3e-23 | 2.4e-29 | 180.3 | 230 | (19, 355) | 372 | (71, 301) | 324 | Putative exodeoxyribonuclease 8 PDDEXK-like domain-containing protein | Putative exodeoxyribonuclease 8 PDDEXK-like domain-containing protein | | uniclust | UniRef100\_UPI0007C549EE | 99.8 | 1.5e-23 | 2.8e-29 | 181.6 | 257 | (5, 365) | 372 | (80, 344) | 350 | PD-(D/E)XK nuclease-like domain-containing protein | PD-(D/E)XK nuclease-like domain-containing protein | | uniclust | UniRef100\_A0A1G3LP96 | 99.8 | 2e-23 | 3.8e-29 | 181.4 | 251 | (6, 362) | 372 | (4, 264) | 266 | Putative exodeoxyribonuclease 8 PDDEXK-like domain-containing protein | Putative exodeoxyribonuclease 8 PDDEXK-like domain-containing protein | | uniclust | UniRef100\_A0A2A2GZ94 | 99.8 | 2.6e-23 | 4.8e-29 | 174.3 | 167 | (191, 370) | 372 | (66, 238) | 247 | Putative exodeoxyribonuclease 8 PDDEXK-like domain-containing protein | Putative exodeoxyribonuclease 8 PDDEXK-like domain-containing protein | | uniclust | UniRef100\_A0A150AJT1 | 99.8 | 2.6e-23 | 5e-29 | 180.2 | 137 | (195, 337) | 372 | (93, 231) | 250 | Putative exodeoxyribonuclease 8 PDDEXK-like domain-containing protein | Putative exodeoxyribonuclease 8 PDDEXK-like domain-containing protein | | uniclust | UniRef100\_A0A966PZB7 | 99.8 | 2.7e-23 | 5e-29 | 174.5 | 248 | (19, 367) | 372 | (17, 268) | 273 | Putative exodeoxyribonuclease 8 PDDEXK-like domain-containing protein | Putative exodeoxyribonuclease 8 PDDEXK-like domain-containing protein | | uniclust | UniRef100\_A0A2D5TTD4 | 99.8 | 2.9e-23 | 5.4e-29 | 186.2 | 229 | (1, 283) | 372 | (7, 244) | 391 | Exodeoxyribonuclease 8 PDDEXK-like domain-containing protein | Exodeoxyribonuclease 8 PDDEXK-like domain-containing protein | | uniclust | UniRef100\_A0A3B8U8D5 | 99.8 | 3.1e-23 | 5.7e-29 | 177.8 | 269 | (19, 349) | 372 | (16, 287) | 320 | SAP domain-containing protein | SAP domain-containing protein | | uniclust | UniRef100\_A0A357XXN4 | 99.8 | 3.6e-23 | 6.7e-29 | 175.7 | 247 | (5, 348) | 372 | (8, 257) | 267 | Putative exodeoxyribonuclease 8 PDDEXK-like domain-containing protein | Putative exodeoxyribonuclease 8 PDDEXK-like domain-containing protein | | uniclust | UniRef100\_M3JBV3 | 99.8 | 3.9e-23 | 7.3e-29 | 174.5 | 224 | (8, 365) | 372 | (24, 249) | 253 | Bacteriophage protein | Bacteriophage protein | | uniclust | UniRef100\_A0A6J5S1P5 | 99.8 | 4.6e-23 | 8.4e-29 | 181.7 | 238 | (6, 347) | 372 | (5, 246) | 405 | Exodeoxyribonuclease 8, PDDEXK-like domain containing protein | Exodeoxyribonuclease 8, PDDEXK-like domain containing protein | | uniclust | UniRef100\_A0A2E7BUE9 | 99.8 | 4.6e-23 | 8.4e-29 | 177.1 | 263 | (6, 362) | 372 | (36, 313) | 325 | Putative exodeoxyribonuclease 8 PDDEXK-like domain-containing protein | Putative exodeoxyribonuclease 8 PDDEXK-like domain-containing protein | | uniclust | UniRef100\_A0A350PI72 | 99.8 | 5e-23 | 9.1e-29 | 168.1 | 178 | (10, 290) | 372 | (38, 216) | 221 | Putative exodeoxyribonuclease 8 PDDEXK-like domain-containing protein (Fragment) | Putative exodeoxyribonuclease 8 PDDEXK-like domain-containing protein (Fragment) | | uniclust | UniRef100\_A0A0F9ABF3 | 99.8 | 5.5e-23 | 1e-28 | 173.1 | 179 | (10, 291) | 372 | (97, 275) | 277 | Putative exodeoxyribonuclease 8 PDDEXK-like domain-containing protein (Fragment) | Putative exodeoxyribonuclease 8 PDDEXK-like domain-containing protein (Fragment) | | uniclust | UniRef100\_UPI000AEFFF0F | 99.8 | 5.5e-23 | 1e-28 | 168.9 | 179 | (187, 369) | 372 | (14, 196) | 205 | PD-(D/E)XK nuclease-like domain-containing protein | PD-(D/E)XK nuclease-like domain-containing protein | | uniclust | UniRef100\_A0A8T3QJZ4 | 99.8 | 6.3e-23 | 1.2e-28 | 179.6 | 263 | (5, 367) | 372 | (35, 312) | 320 | PD-(D/E)XK nuclease-like domain-containing protein | PD-(D/E)XK nuclease-like domain-containing protein | | uniclust | UniRef100\_UPI0013CB2100 | 99.8 | 8.3e-23 | 1.5e-28 | 172.7 | 211 | (154, 368) | 372 | (4, 224) | 263 | PD-(D/E)XK nuclease-like domain-containing protein | PD-(D/E)XK nuclease-like domain-containing protein | | uniclust | UniRef100\_A0A3C0EHM3 | 99.8 | 8.1e-23 | 1.6e-28 | 166.9 | 163 | (186, 355) | 372 | (12, 174) | 177 | Putative exodeoxyribonuclease 8 PDDEXK-like domain-containing protein | Putative exodeoxyribonuclease 8 PDDEXK-like domain-containing protein | | uniclust | UniRef100\_A0A2D6PI96 | 99.8 | 7.9e-23 | 1.6e-28 | 188.8 | 287 | (11, 347) | 372 | (8, 323) | 355 | PD-(D/E)XK endonuclease-like domain-containing protein | PD-(D/E)XK endonuclease-like domain-containing protein | | uniclust | UniRef100\_A0A661I8B6 | 99.8 | 1.4e-22 | 2.6e-28 | 171.5 | 242 | (3, 348) | 372 | (23, 271) | 286 | Putative exodeoxyribonuclease 8 PDDEXK-like domain-containing protein | Putative exodeoxyribonuclease 8 PDDEXK-like domain-containing protein | | uniclust | UniRef100\_A0A395XFE4 | 99.8 | 1.5e-22 | 2.8e-28 | 178.3 | 261 | (5, 366) | 372 | (95, 396) | 400 | Nuclease | Nuclease | | uniclust | UniRef100\_A0A516LE23 | 99.8 | 1.7e-22 | 3e-28 | 170.1 | 242 | (5, 343) | 372 | (9, 252) | 274 | Putative exodeoxyribonuclease 8 | Putative exodeoxyribonuclease 8 | | uniclust | UniRef100\_A0A966VAZ2 | 99.8 | 2e-22 | 3.6e-28 | 172.0 | 259 | (5, 359) | 372 | (35, 298) | 305 | Putative exodeoxyribonuclease 8 PDDEXK-like domain-containing protein | Putative exodeoxyribonuclease 8 PDDEXK-like domain-containing protein | | uniclust | UniRef100\_A0A7X8XER7 | 99.8 | 2.2e-22 | 4.1e-28 | 168.3 | 198 | (2, 295) | 372 | (20, 220) | 235 | Putative exodeoxyribonuclease 8 PDDEXK-like domain-containing protein (Fragment) | Putative exodeoxyribonuclease 8 PDDEXK-like domain-containing protein (Fragment) | | uniclust | UniRef100\_A0A6J5LX80 | 99.8 | 3.2e-22 | 6e-28 | 169.6 | 246 | (3, 350) | 372 | (5, 255) | 268 | Exodeoxyribonuclease 8, PDDEXK-like domain containing protein | Exodeoxyribonuclease 8, PDDEXK-like domain containing protein | | uniclust | UniRef100\_A0A968CQ55 | 99.8 | 3.6e-22 | 6.6e-28 | 165.5 | 227 | (2, 354) | 372 | (5, 243) | 244 | Putative exodeoxyribonuclease 8 PDDEXK-like domain-containing protein | Putative exodeoxyribonuclease 8 PDDEXK-like domain-containing protein | | uniclust | UniRef100\_A0A6I0ESC0 | 99.8 | 3.7e-22 | 6.8e-28 | 176.6 | 245 | (10, 351) | 372 | (158, 407) | 414 | Putative exodeoxyribonuclease 8 PDDEXK-like domain-containing protein | Putative exodeoxyribonuclease 8 PDDEXK-like domain-containing protein | | uniclust | UniRef100\_A0A2D7YTB8 | 99.8 | 4.1e-22 | 7.6e-28 | 168.2 | 258 | (4, 364) | 372 | (7, 278) | 280 | Putative exodeoxyribonuclease 8 PDDEXK-like domain-containing protein | Putative exodeoxyribonuclease 8 PDDEXK-like domain-containing protein | | uniclust | UniRef100\_A0A5C5X348 | 99.8 | 5.6e-22 | 1e-27 | 167.6 | 257 | (5, 365) | 372 | (12, 273) | 282 | Putative exodeoxyribonuclease 8 PDDEXK-like domain-containing protein | Putative exodeoxyribonuclease 8 PDDEXK-like domain-containing protein | | uniclust | UniRef100\_A0A2E8IY15 | 99.8 | 6.6e-22 | 1.2e-27 | 166.4 | 177 | (187, 365) | 372 | (93, 270) | 272 | Putative exodeoxyribonuclease 8 PDDEXK-like domain-containing protein | Putative exodeoxyribonuclease 8 PDDEXK-like domain-containing protein | | uniclust | UniRef100\_A0A935XAD7 | 99.8 | 7.9e-22 | 1.4e-27 | 164.8 | 146 | (188, 338) | 372 | (93, 240) | 258 | PD-(D/E)XK nuclease-like domain-containing protein | PD-(D/E)XK nuclease-like domain-containing protein | | uniclust | UniRef100\_A0A485A5W0 | 99.8 | 8.6e-22 | 1.6e-27 | 176.9 | 245 | (2, 249) | 372 | (163, 460) | 470 | Exodeoxyribonuclease 8 | Exodeoxyribonuclease 8 | | uniclust | UniRef100\_A0A2E4HLS4 | 99.8 | 1.1e-21 | 2.1e-27 | 178.2 | 282 | (11, 335) | 372 | (20, 319) | 343 | PD-(D/E)XK endonuclease-like domain-containing protein | PD-(D/E)XK endonuclease-like domain-containing protein | | uniclust | UniRef100\_A0A0G9KAG8 | 99.7 | 1.9e-21 | 3.6e-27 | 167.7 | 235 | (2, 341) | 372 | (20, 262) | 284 | Putative exodeoxyribonuclease 8 PDDEXK-like domain-containing protein | Putative exodeoxyribonuclease 8 PDDEXK-like domain-containing protein | | uniclust | UniRef100\_A0A6B9SPH0 | 99.7 | 2.7e-21 | 5e-27 | 161.0 | 225 | (7, 291) | 372 | (9, 241) | 249 | Putative exonuclease | Putative exonuclease | | uniclust | UniRef100\_UPI000C7CBD65 | 99.7 | 2.9e-21 | 5.3e-27 | 190.0 | 334 | (6, 348) | 372 | (868, 1312) | 1341 | hypothetical protein | hypothetical protein | | uniclust | UniRef100\_A0A5C7PJN5 | 99.7 | 3.1e-21 | 5.8e-27 | 163.0 | 262 | (7, 362) | 372 | (6, 274) | 278 | Putative exodeoxyribonuclease 8 PDDEXK-like domain-containing protein | Putative exodeoxyribonuclease 8 PDDEXK-like domain-containing protein | | uniclust | UniRef100\_A0A966RAL2 | 99.7 | 3.3e-21 | 6.1e-27 | 164.2 | 269 | (1, 370) | 372 | (2, 293) | 297 | Putative exodeoxyribonuclease 8 PDDEXK-like domain-containing protein | Putative exodeoxyribonuclease 8 PDDEXK-like domain-containing protein | | uniclust | UniRef100\_A0A948KE74 | 99.7 | 3.8e-21 | 7e-27 | 160.8 | 175 | (187, 368) | 372 | (57, 236) | 257 | PD-(D/E)XK nuclease-like domain-containing protein | PD-(D/E)XK nuclease-like domain-containing protein | | uniclust | UniRef100\_A0A076HWD4 | 99.7 | 3.7e-21 | 7e-27 | 169.2 | 126 | (205, 338) | 372 | (139, 268) | 309 | Putative exodeoxyribonuclease 8 PDDEXK-like domain-containing protein | Putative exodeoxyribonuclease 8 PDDEXK-like domain-containing protein | | uniclust | UniRef100\_A0A970WNN9 | 99.7 | 4.7e-21 | 8.7e-27 | 151.6 | 160 | (205, 366) | 372 | (1, 165) | 175 | Putative exodeoxyribonuclease 8 PDDEXK-like domain-containing protein | Putative exodeoxyribonuclease 8 PDDEXK-like domain-containing protein | | uniclust | UniRef100\_A0A956M5I9 | 99.7 | 5.4e-21 | 1e-26 | 161.9 | 247 | (2, 350) | 372 | (9, 275) | 283 | PD-(D/E)XK nuclease-like domain-containing protein (Fragment) | PD-(D/E)XK nuclease-like domain-containing protein (Fragment) | | uniclust | UniRef100\_A0A5T5VGN4 | 99.7 | 7.1e-21 | 1.3e-26 | 148.4 | 137 | (1, 233) | 372 | (1, 140) | 140 | Exodeoxyribonuclease 8 (Fragment) | Exodeoxyribonuclease 8 (Fragment) | | uniclust | UniRef100\_A0A0Q5QGX0 | 99.7 | 6.9e-21 | 1.3e-26 | 172.6 | 286 | (19, 367) | 372 | (46, 334) | 350 | Putative exodeoxyribonuclease 8 PDDEXK-like domain-containing protein | Putative exodeoxyribonuclease 8 PDDEXK-like domain-containing protein | | uniclust | UniRef100\_A0A2D8XKX9 | 99.7 | 7.9e-21 | 1.5e-26 | 165.1 | 242 | (17, 345) | 372 | (24, 267) | 278 | Putative exodeoxyribonuclease 8 PDDEXK-like domain-containing protein | Putative exodeoxyribonuclease 8 PDDEXK-like domain-containing protein | | uniclust | UniRef100\_A0A097ENH1 | 99.7 | 9.2e-21 | 1.7e-26 | 159.1 | 246 | (13, 364) | 372 | (2, 255) | 263 | Putative exodeoxyribonuclease 8 PDDEXK-like domain-containing protein | Putative exodeoxyribonuclease 8 PDDEXK-like domain-containing protein | | uniclust | UniRef100\_UPI0005F02F46 | 99.7 | 9.6e-21 | 1.8e-26 | 185.4 | 330 | (6, 349) | 372 | (540, 946) | 988 | hypothetical protein | hypothetical protein | | uniclust | UniRef100\_A0A965MNM5 | 99.7 | 9.8e-21 | 1.9e-26 | 166.6 | 265 | (6, 369) | 372 | (29, 302) | 304 | Putative exodeoxyribonuclease 8 PDDEXK-like domain-containing protein | Putative exodeoxyribonuclease 8 PDDEXK-like domain-containing protein | | uniclust | UniRef100\_A0A0F3IMN9 | 99.7 | 2.6e-20 | 4.7e-26 | 159.3 | 235 | (8, 343) | 372 | (47, 287) | 301 | Putative exodeoxyribonuclease 8 PDDEXK-like domain-containing protein | Putative exodeoxyribonuclease 8 PDDEXK-like domain-containing protein | | uniclust | UniRef100\_A0A936DK79 | 99.7 | 2.6e-20 | 4.8e-26 | 158.4 | 267 | (10, 364) | 372 | (2, 273) | 289 | PD-(D/E)XK nuclease-like domain-containing protein | PD-(D/E)XK nuclease-like domain-containing protein | | uniclust | UniRef100\_A0A3M1NIT7 | 99.7 | 3.4e-20 | 6.2e-26 | 156.9 | 258 | (8, 366) | 372 | (4, 264) | 277 | Putative exodeoxyribonuclease 8 PDDEXK-like domain-containing protein | Putative exodeoxyribonuclease 8 PDDEXK-like domain-containing protein | | uniclust | UniRef100\_A0A0F9AK76 | 99.7 | 3.5e-20 | 6.5e-26 | 154.8 | 176 | (184, 364) | 372 | (67, 247) | 252 | Putative exodeoxyribonuclease 8 PDDEXK-like domain-containing protein (Fragment) | Putative exodeoxyribonuclease 8 PDDEXK-like domain-containing protein (Fragment) | | uniclust | UniRef100\_A0A7Y6MP93 | 99.7 | 3.6e-20 | 6.7e-26 | 177.9 | 283 | (7, 348) | 372 | (641, 935) | 970 | PD-(D/E)XK nuclease-like domain-containing protein | PD-(D/E)XK nuclease-like domain-containing protein | | uniclust | UniRef100\_A0A517YVS6 | 99.7 | 3.9e-20 | 7.1e-26 | 158.0 | 260 | (3, 363) | 372 | (23, 294) | 298 | Exodeoxyribonuclease 8 | Exodeoxyribonuclease 8 | | uniclust | UniRef100\_A0A4V0Z515 | 99.7 | 4e-20 | 7.4e-26 | 148.1 | 171 | (191, 362) | 372 | (4, 183) | 188 | Putative exodeoxyribonuclease 8 PDDEXK-like domain-containing protein | Putative exodeoxyribonuclease 8 PDDEXK-like domain-containing protein | | uniclust | UniRef100\_A0A962Q8Y1 | 99.7 | 4.4e-20 | 8.1e-26 | 156.6 | 260 | (6, 365) | 372 | (4, 273) | 282 | PD-(D/E)XK nuclease-like domain-containing protein | PD-(D/E)XK nuclease-like domain-containing protein | | uniclust | UniRef100\_A0A6J5PGR2 | 99.7 | 5.3e-20 | 9.8e-26 | 155.9 | 263 | (3, 368) | 372 | (9, 276) | 279 | Exodeoxyribonuclease 8, PDDEXK-like domain containing protein | Exodeoxyribonuclease 8, PDDEXK-like domain containing protein | | uniclust | UniRef100\_A0A485AXQ3 | 99.7 | 5.9e-20 | 1.1e-25 | 142.1 | 78 | (1, 78) | 372 | (4, 81) | 136 | Exodeoxyribonuclease 8 | Exodeoxyribonuclease 8 | | uniclust | UniRef100\_A0A3S9D4G6 | 99.7 | 5.9e-20 | 1.1e-25 | 149.4 | 148 | (3, 261) | 372 | (21, 177) | 185 | Uncharacterized protein | Uncharacterized protein | | uniclust | UniRef100\_A0A955HL80 | 99.7 | 5.9e-20 | 1.1e-25 | 159.9 | 230 | (18, 348) | 372 | (26, 262) | 277 | PD-(D/E)XK nuclease-like domain-containing protein | PD-(D/E)XK nuclease-like domain-containing protein | | uniclust | UniRef100\_A0A1E4ZGR8 | 99.7 | 6.4e-20 | 1.2e-25 | 156.9 | 269 | (2, 364) | 372 | (7, 296) | 301 | Putative exodeoxyribonuclease 8 PDDEXK-like domain-containing protein | Putative exodeoxyribonuclease 8 PDDEXK-like domain-containing protein | | uniclust | UniRef100\_A0A511B8A3 | 99.7 | 7.2e-20 | 1.4e-25 | 147.1 | 147 | (215, 367) | 372 | (7, 156) | 168 | Putative exodeoxyribonuclease 8 PDDEXK-like domain-containing protein | Putative exodeoxyribonuclease 8 PDDEXK-like domain-containing protein | | uniclust | UniRef100\_A0A914CHP8 | 99.7 | 7.7e-20 | 1.4e-25 | 184.4 | 215 | (3, 313) | 372 | (6, 224) | 2182 | Putative exodeoxyribonuclease 8 PDDEXK-like domain-containing protein | Putative exodeoxyribonuclease 8 PDDEXK-like domain-containing protein | | uniclust | UniRef100\_A0A0P0DG67 | 99.7 | 8e-20 | 1.5e-25 | 161.4 | 278 | (1, 365) | 372 | (6, 324) | 337 | Putative exodeoxyribonuclease 8 PDDEXK-like domain-containing protein | Putative exodeoxyribonuclease 8 PDDEXK-like domain-containing protein | | uniclust | UniRef100\_A0A5C7PU20 | 99.7 | 8.2e-20 | 1.5e-25 | 154.7 | 254 | (13, 361) | 372 | (19, 277) | 278 | Putative exodeoxyribonuclease 8 PDDEXK-like domain-containing protein | Putative exodeoxyribonuclease 8 PDDEXK-like domain-containing protein | | uniclust | UniRef100\_Q6LJU7 | 99.7 | 1e-19 | 1.8e-25 | 177.1 | 335 | (1, 349) | 372 | (661, 1116) | 1148 | Uncharacterized protein | Uncharacterized protein | | uniclust | UniRef100\_A0A447XY29 | 99.7 | 1.1e-19 | 2.1e-25 | 156.2 | 210 | (153, 368) | 372 | (31, 250) | 254 | Putative phage exodeoxyribonuclease | Putative phage exodeoxyribonuclease | | uniclust | UniRef100\_A0A976KWG8 | 99.7 | 1.3e-19 | 2.4e-25 | 155.8 | 249 | (6, 338) | 372 | (9, 266) | 274 | Putative exodeoxyribonuclease 8 PDDEXK-like domain-containing protein | Putative exodeoxyribonuclease 8 PDDEXK-like domain-containing protein | | uniclust | UniRef100\_A0A2E1MZJ8 | 99.7 | 1.4e-19 | 2.7e-25 | 156.4 | 287 | (9, 347) | 372 | (1, 316) | 326 | Putative exodeoxyribonuclease 8 PDDEXK-like domain-containing protein | Putative exodeoxyribonuclease 8 PDDEXK-like domain-containing protein | | uniclust | UniRef100\_UPI0014383DDD | 99.7 | 1.8e-19 | 3.4e-25 | 152.0 | 186 | (5, 286) | 372 | (75, 264) | 269 | PD-(D/E)XK nuclease-like domain-containing protein | PD-(D/E)XK nuclease-like domain-containing protein | | uniclust | UniRef100\_A0A1I5SZ79 | 99.7 | 2.2e-19 | 4e-25 | 153.0 | 271 | (2, 362) | 372 | (4, 288) | 289 | Putative exodeoxyribonuclease 8 PDDEXK-like domain-containing protein | Putative exodeoxyribonuclease 8 PDDEXK-like domain-containing protein | | uniclust | UniRef100\_A0A7V6NHJ0 | 99.6 | 2.6e-19 | 5e-25 | 153.9 | 197 | (6, 315) | 372 | (8, 217) | 220 | Putative exodeoxyribonuclease 8 PDDEXK-like domain-containing protein | Putative exodeoxyribonuclease 8 PDDEXK-like domain-containing protein | | uniclust | UniRef100\_A0A963CJ35 | 99.6 | 2.8e-19 | 5.2e-25 | 140.8 | 135 | (2, 233) | 372 | (23, 158) | 166 | PD-(D/E)XK nuclease-like domain-containing protein (Fragment) | PD-(D/E)XK nuclease-like domain-containing protein (Fragment) | | uniclust | UniRef100\_A0A843GNI8 | 99.6 | 3.2e-19 | 5.8e-25 | 150.6 | 252 | (7, 361) | 372 | (15, 268) | 269 | PD-(D/E)XK nuclease-like domain-containing protein | PD-(D/E)XK nuclease-like domain-containing protein | | uniclust | UniRef100\_A0A0M4SKY7 | 99.6 | 3.1e-19 | 5.9e-25 | 155.6 | 233 | (6, 344) | 372 | (26, 261) | 294 | Putative exodeoxyribonuclease 8 PDDEXK-like domain-containing protein | Putative exodeoxyribonuclease 8 PDDEXK-like domain-containing protein | | uniclust | UniRef100\_UPI00158604F7 | 99.6 | 3.3e-19 | 6.4e-25 | 133.8 | 97 | (268, 365) | 372 | (2, 99) | 104 | PD-(D/E)XK nuclease-like domain-containing protein | PD-(D/E)XK nuclease-like domain-containing protein | | uniclust | UniRef100\_A0A3D1XDL4 | 99.6 | 3.7e-19 | 6.8e-25 | 145.3 | 161 | (187, 348) | 372 | (31, 193) | 211 | Putative exodeoxyribonuclease 8 PDDEXK-like domain-containing protein (Fragment) | Putative exodeoxyribonuclease 8 PDDEXK-like domain-containing protein (Fragment) | | uniclust | UniRef100\_A0A0F9I7X6 | 99.6 | 4.3e-19 | 7.9e-25 | 152.7 | 262 | (4, 362) | 372 | (18, 284) | 311 | Putative exodeoxyribonuclease 8 PDDEXK-like domain-containing protein | Putative exodeoxyribonuclease 8 PDDEXK-like domain-containing protein | | uniclust | UniRef100\_A0A2I7RBQ8 | 99.6 | 4.6e-19 | 8.4e-25 | 167.3 | 279 | (5, 349) | 372 | (445, 732) | 750 | Coil containing protein | Coil containing protein | | uniclust | UniRef100\_A0A3C0F448 | 99.6 | 4.5e-19 | 8.5e-25 | 147.3 | 167 | (11, 288) | 372 | (18, 187) | 194 | Putative exodeoxyribonuclease 8 PDDEXK-like domain-containing protein | Putative exodeoxyribonuclease 8 PDDEXK-like domain-containing protein | | uniclust | UniRef100\_UPI0005B4C7AB | 99.6 | 5e-19 | 9.2e-25 | 157.2 | 144 | (1, 236) | 372 | (244, 387) | 406 | PD-(D/E)XK nuclease-like domain-containing protein | PD-(D/E)XK nuclease-like domain-containing protein | | uniclust | UniRef100\_A0A967T6U3 | 99.6 | 6.1e-19 | 1.1e-24 | 140.8 | 165 | (197, 369) | 372 | (2, 179) | 181 | Putative exodeoxyribonuclease 8 PDDEXK-like domain-containing protein | Putative exodeoxyribonuclease 8 PDDEXK-like domain-containing protein | | uniclust | UniRef100\_A0A516LFV1 | 99.6 | 8.6e-19 | 1.6e-24 | 152.6 | 295 | (18, 347) | 372 | (4, 310) | 340 | Putative exodeoxyribonuclease 8 | Putative exodeoxyribonuclease 8 | | uniclust | UniRef100\_A0A482N4I2 | 99.6 | 3e-18 | 5.5e-24 | 135.5 | 160 | (206, 367) | 372 | (2, 167) | 168 | Putative exodeoxyribonuclease 8 PDDEXK-like domain-containing protein | Putative exodeoxyribonuclease 8 PDDEXK-like domain-containing protein | | uniclust | UniRef100\_R1HTJ0 | 99.6 | 3.9e-18 | 7.1e-24 | 131.9 | 128 | (242, 370) | 372 | (1, 134) | 146 | Gp60 protein (Fragment) | Gp60 protein (Fragment) | | uniclust | UniRef100\_A0A2H5BFV1 | 99.6 | 4e-18 | 7.3e-24 | 165.0 | 275 | (5, 348) | 372 | (635, 919) | 936 | Exodeoxyribonuclease type VIII | Exodeoxyribonuclease type VIII | | uniclust | UniRef100\_A0A098RLH1 | 99.6 | 4.2e-18 | 7.7e-24 | 139.4 | 189 | (2, 291) | 372 | (8, 204) | 211 | Putative exodeoxyribonuclease 8 PDDEXK-like domain-containing protein (Fragment) | Putative exodeoxyribonuclease 8 PDDEXK-like domain-containing protein (Fragment) | | uniclust | UniRef100\_A0A827N2G5 | 99.6 | 4.9e-18 | 8.9e-24 | 148.1 | 138 | (1, 232) | 372 | (1, 140) | 339 | Adenine methylase | Adenine methylase | | uniclust | UniRef100\_A0A6A6K1N9 | 99.6 | 7e-18 | 1.3e-23 | 153.3 | 124 | (225, 350) | 372 | (200, 323) | 490 | Putative exodeoxyribonuclease 8 PDDEXK-like domain-containing protein | Putative exodeoxyribonuclease 8 PDDEXK-like domain-containing protein | | uniclust | UniRef100\_A0A351GFU4 | 99.6 | 8.3e-18 | 1.6e-23 | 149.8 | 293 | (10, 346) | 372 | (2, 322) | 337 | Putative exodeoxyribonuclease 8 PDDEXK-like domain-containing protein | Putative exodeoxyribonuclease 8 PDDEXK-like domain-containing protein | | uniclust | UniRef100\_UPI00189A7229 | 99.6 | 8.7e-18 | 1.6e-23 | 136.6 | 170 | (193, 363) | 372 | (9, 181) | 190 | PD-(D/E)XK nuclease-like domain-containing protein | PD-(D/E)XK nuclease-like domain-containing protein | | uniclust | UniRef100\_UPI000C27CD9A | 99.6 | 1e-17 | 1.9e-23 | 131.5 | 145 | (201, 348) | 372 | (2, 148) | 159 | PD-(D/E)XK nuclease-like domain-containing protein | PD-(D/E)XK nuclease-like domain-containing protein | | uniclust | UniRef100\_A0A5P8D2N7 | 99.5 | 1.2e-17 | 2.3e-23 | 151.3 | 179 | (187, 368) | 372 | (210, 394) | 396 | Exonuclease | Exonuclease | | uniclust | UniRef100\_A0A345LVI2 | 99.5 | 1.3e-17 | 2.3e-23 | 131.8 | 148 | (1, 250) | 372 | (1, 148) | 155 | PD-(D/E)XK nuclease-like domain-containing protein | PD-(D/E)XK nuclease-like domain-containing protein | | uniclust | UniRef100\_A0A150JKQ2 | 99.5 | 1.3e-17 | 2.5e-23 | 153.0 | 274 | (8, 330) | 372 | (22, 313) | 382 | Uncharacterized protein | Uncharacterized protein | | uniclust | UniRef100\_A0A1T4X5K6 | 99.5 | 1.4e-17 | 2.6e-23 | 147.3 | 270 | (7, 317) | 372 | (23, 309) | 380 | Putative exodeoxyribonuclease 8 PDDEXK-like domain-containing protein | Putative exodeoxyribonuclease 8 PDDEXK-like domain-containing protein | | uniclust | UniRef100\_A0A3D2IK29 | 99.5 | 1.5e-17 | 2.7e-23 | 134.4 | 165 | (188, 357) | 372 | (14, 187) | 192 | Putative exodeoxyribonuclease 8 PDDEXK-like domain-containing protein (Fragment) | Putative exodeoxyribonuclease 8 PDDEXK-like domain-containing protein (Fragment) | | uniclust | UniRef100\_UPI001BAEF683 | 99.5 | 2.1e-17 | 3.8e-23 | 135.0 | 167 | (190, 359) | 372 | (32, 202) | 206 | PD-(D/E)XK nuclease-like domain-containing protein | PD-(D/E)XK nuclease-like domain-containing protein | | uniclust | UniRef100\_UPI00102DF95B | 99.5 | 2.8e-17 | 5.1e-23 | 142.3 | 237 | (2, 341) | 372 | (20, 272) | 315 | PD-(D/E)XK nuclease-like domain-containing protein | PD-(D/E)XK nuclease-like domain-containing protein | | uniclust | UniRef100\_UPI001EE8661C | 99.5 | 2.8e-17 | 5.2e-23 | 140.0 | 241 | (19, 347) | 372 | (22, 263) | 278 | PD-(D/E)XK nuclease-like domain-containing protein | PD-(D/E)XK nuclease-like domain-containing protein | | uniclust | UniRef100\_A0A4U9HTL9 | 99.5 | 5.2e-17 | 9.8e-23 | 160.2 | 227 | (2, 231) | 372 | (699, 978) | 980 | Exodeoxyribonuclease 8 | Exodeoxyribonuclease 8 | | uniclust | UniRef100\_A0A0E3B9I3 | 99.5 | 6.5e-17 | 1.2e-22 | 134.4 | 176 | (184, 363) | 372 | (31, 218) | 230 | Putative exodeoxyribonuclease 8 PDDEXK-like domain-containing protein | Putative exodeoxyribonuclease 8 PDDEXK-like domain-containing protein | | uniclust | UniRef100\_A0A4Q3BZQ0 | 99.5 | 8.5e-17 | 1.6e-22 | 139.9 | 207 | (2, 312) | 372 | (45, 266) | 276 | PD-(D/E)XK endonuclease-like domain-containing protein | PD-(D/E)XK endonuclease-like domain-containing protein | | uniclust | UniRef100\_A0A160CAK3 | 99.5 | 9.8e-17 | 1.8e-22 | 135.5 | 181 | (185, 367) | 372 | (45, 256) | 258 | Exonuclease | Exonuclease | | uniclust | UniRef100\_A0A6S6PGI9 | 99.5 | 1e-16 | 1.8e-22 | 127.2 | 142 | (1, 235) | 372 | (4, 151) | 168 | Putative exodeoxyribonuclease 8 PDDEXK-like domain-containing protein | Putative exodeoxyribonuclease 8 PDDEXK-like domain-containing protein | | uniclust | UniRef100\_A0A955A2S3 | 99.5 | 1.2e-16 | 2.2e-22 | 141.5 | 303 | (2, 364) | 372 | (27, 361) | 378 | PD-(D/E)XK nuclease-like domain-containing protein | PD-(D/E)XK nuclease-like domain-containing protein | | uniclust | UniRef100\_A0A5C7PD05 | 99.5 | 1.4e-16 | 2.5e-22 | 139.4 | 260 | (1, 365) | 372 | (51, 324) | 339 | Putative exodeoxyribonuclease 8 PDDEXK-like domain-containing protein | Putative exodeoxyribonuclease 8 PDDEXK-like domain-containing protein | | uniclust | UniRef100\_A0A5C8B335 | 99.5 | 1.7e-16 | 3.1e-22 | 134.6 | 251 | (9, 366) | 372 | (1, 254) | 265 | Putative exodeoxyribonuclease 8 PDDEXK-like domain-containing protein | Putative exodeoxyribonuclease 8 PDDEXK-like domain-containing protein | | uniclust | UniRef100\_A0A0K8Q145 | 99.5 | 1.7e-16 | 3.1e-22 | 126.2 | 138 | (2, 242) | 372 | (4, 142) | 170 | Exodeoxyribonuclease 8 | Exodeoxyribonuclease 8 | | uniclust | UniRef100\_UPI001E46E591 | 99.5 | 1.8e-16 | 3.4e-22 | 132.2 | 167 | (45, 262) | 372 | (14, 185) | 235 | PD-(D/E)XK nuclease-like domain-containing protein | PD-(D/E)XK nuclease-like domain-containing protein | | uniclust | UniRef100\_UPI001FEF4C2F | 99.5 | 2e-16 | 3.6e-22 | 129.7 | 168 | (192, 362) | 372 | (24, 196) | 207 | PD-(D/E)XK nuclease-like domain-containing protein | PD-(D/E)XK nuclease-like domain-containing protein | | uniclust | UniRef100\_A0A965PNL8 | 99.5 | 2.1e-16 | 3.9e-22 | 127.0 | 148 | (215, 364) | 372 | (4, 162) | 182 | Putative exodeoxyribonuclease 8 PDDEXK-like domain-containing protein | Putative exodeoxyribonuclease 8 PDDEXK-like domain-containing protein | | uniclust | UniRef100\_A0A218KSW6 | 99.4 | 2.5e-16 | 4.7e-22 | 136.3 | 130 | (209, 341) | 372 | (102, 233) | 261 | Putative exonuclease | Putative exonuclease | | uniclust | UniRef100\_UPI0018805882 | 99.4 | 2.8e-16 | 5.1e-22 | 121.0 | 78 | (1, 78) | 372 | (40, 117) | 138 | PD-(D/E)XK nuclease-like domain-containing protein | PD-(D/E)XK nuclease-like domain-containing protein | | uniclust | UniRef100\_UPI001354BAA8 | 99.4 | 2.9e-16 | 5.4e-22 | 122.8 | 101 | (186, 289) | 372 | (34, 135) | 152 | PD-(D/E)XK nuclease-like domain-containing protein | PD-(D/E)XK nuclease-like domain-containing protein | | uniclust | UniRef100\_A0A379GH20 | 99.4 | 3.1e-16 | 5.7e-22 | 135.9 | 131 | (2, 163) | 372 | (149, 280) | 312 | Exodeoxyribonuclease 8 | Exodeoxyribonuclease 8 | | uniclust | UniRef100\_A0A3U7CCZ6 | 99.4 | 3.1e-16 | 5.7e-22 | 128.7 | 78 | (1, 78) | 372 | (78, 155) | 208 | Exodeoxyribonuclease 8 (Fragment) | Exodeoxyribonuclease 8 (Fragment) | | uniclust | UniRef100\_A0A6I1JKT6 | 99.4 | 3.3e-16 | 6.1e-22 | 130.4 | 165 | (186, 366) | 372 | (45, 213) | 231 | Phage protein | Phage protein | | uniclust | UniRef100\_G5S9W1 | 99.4 | 3.8e-16 | 7e-22 | 131.5 | 78 | (1, 78) | 372 | (113, 190) | 243 | Exodeoxyribonuclease 8 | Exodeoxyribonuclease 8 | | uniclust | UniRef100\_UPI0021E8A40C | 99.4 | 3.9e-16 | 7.1e-22 | 139.1 | 169 | (190, 359) | 372 | (192, 383) | 391 | PD-(D/E)XK nuclease-like domain-containing protein | PD-(D/E)XK nuclease-like domain-containing protein | | uniclust | UniRef100\_A0A2E3D7E6 | 99.4 | 4.1e-16 | 7.6e-22 | 132.9 | 242 | (7, 358) | 372 | (3, 248) | 273 | Uncharacterized protein | Uncharacterized protein | | uniclust | UniRef100\_A0A3B9ZLY2 | 99.4 | 4.6e-16 | 8.5e-22 | 138.1 | 297 | (8, 350) | 372 | (15, 336) | 378 | Putative exodeoxyribonuclease 8 PDDEXK-like domain-containing protein | Putative exodeoxyribonuclease 8 PDDEXK-like domain-containing protein | | uniclust | UniRef100\_A0A3S4K5S1 | 99.4 | 4.7e-16 | 8.7e-22 | 126.9 | 130 | (2, 162) | 372 | (7, 137) | 179 | Putative phage exodeoxyribonuclease | Putative phage exodeoxyribonuclease | | uniclust | UniRef100\_UPI0004091708 | 99.4 | 5.1e-16 | 9.3e-22 | 128.5 | 130 | (1, 139) | 372 | (78, 213) | 220 | SAP domain-containing protein | SAP domain-containing protein | | uniclust | UniRef100\_A0A946HKP6 | 99.4 | 6.2e-16 | 1.1e-21 | 123.6 | 161 | (199, 364) | 372 | (4, 169) | 174 | Putative exodeoxyribonuclease 8 PDDEXK-like domain-containing protein | Putative exodeoxyribonuclease 8 PDDEXK-like domain-containing protein | | uniclust | UniRef100\_A0A359MF53 | 99.4 | 6.9e-16 | 1.3e-21 | 127.9 | 145 | (187, 343) | 372 | (36, 182) | 198 | Putative exodeoxyribonuclease 8 PDDEXK-like domain-containing protein (Fragment) | Putative exodeoxyribonuclease 8 PDDEXK-like domain-containing protein (Fragment) | | uniclust | UniRef100\_A0A942R8U6 | 99.4 | 7.1e-16 | 1.3e-21 | 122.4 | 127 | (1, 136) | 372 | (14, 141) | 166 | Uncharacterized protein (Fragment) | Uncharacterized protein (Fragment) | | uniclust | UniRef100\_M7RB49 | 99.4 | 8.2e-16 | 1.5e-21 | 124.4 | 78 | (1, 78) | 372 | (96, 173) | 188 | Uncharacterized protein (Fragment) | Uncharacterized protein (Fragment) | | uniclust | UniRef100\_UPI001CC2243F | 99.4 | 9.9e-16 | 1.9e-21 | 123.0 | 125 | (205, 337) | 372 | (1, 129) | 158 | PD-(D/E)XK nuclease-like domain-containing protein | PD-(D/E)XK nuclease-like domain-containing protein | | uniclust | UniRef100\_A0A1L3I482 | 99.4 | 1.6e-15 | 3e-21 | 116.4 | 103 | (205, 307) | 372 | (1, 110) | 134 | Exodeoxyribonuclease 8 | Exodeoxyribonuclease 8 | | uniclust | UniRef100\_A0A853IND3 | 99.4 | 1.6e-15 | 3e-21 | 125.1 | 119 | (187, 307) | 372 | (48, 166) | 189 | PD-(D/E)XK nuclease-like domain-containing protein (Fragment) | PD-(D/E)XK nuclease-like domain-containing protein (Fragment) | | uniclust | UniRef100\_A0A2A6DEY2 | 99.4 | 1.6e-15 | 3e-21 | 136.5 | 78 | (1, 78) | 372 | (293, 370) | 423 | Exodeoxyribonuclease 8 | Exodeoxyribonuclease 8 | | uniclust | UniRef100\_A0A139TR47 | 99.4 | 1.8e-15 | 3.7e-21 | 140.9 | 145 | (198, 349) | 372 | (142, 286) | 314 | PD-(D/E)XK endonuclease-like domain-containing protein | PD-(D/E)XK endonuclease-like domain-containing protein | | uniclust | UniRef100\_A0A377K6Z1 | 99.4 | 2.1e-15 | 3.9e-21 | 119.2 | 72 | (2, 73) | 372 | (46, 118) | 146 | Putative phage exodeoxyribonuclease | Putative phage exodeoxyribonuclease | | uniclust | UniRef100\_A0A1I7N9A7 | 99.4 | 2e-15 | 4e-21 | 153.7 | 149 | (188, 338) | 372 | (512, 674) | 717 | DNA 3'-5' helicase | DNA 3'-5' helicase | | uniclust | UniRef100\_UPI001CECFBF9 | 99.4 | 2.2e-15 | 4e-21 | 141.2 | 161 | (1, 169) | 372 | (415, 602) | 626 | DNA breaking-rejoining protein | DNA breaking-rejoining protein | | uniclust | UniRef100\_K1U9P6 | 99.4 | 2.5e-15 | 4.5e-21 | 141.0 | 291 | (13, 347) | 372 | (307, 614) | 628 | Protein containing Metallophosphoesterase domain protein | Protein containing Metallophosphoesterase domain protein | | uniclust | UniRef100\_UPI001A94C8F2 | 99.4 | 2.5e-15 | 4.7e-21 | 131.8 | 78 | (1, 78) | 372 | (213, 290) | 309 | PD-(D/E)XK nuclease-like domain-containing protein | PD-(D/E)XK nuclease-like domain-containing protein | | uniclust | UniRef100\_A0A021VU21 | 99.4 | 2.6e-15 | 5.3e-21 | 149.0 | 116 | (225, 350) | 372 | (272, 391) | 503 | Exodeoxyribonuclease V subunit beta | Exodeoxyribonuclease V subunit beta | | uniclust | UniRef100\_A0A0R1M3A8 | 99.3 | 3.1e-15 | 6e-21 | 129.2 | 148 | (195, 347) | 372 | (19, 175) | 203 | Putative exodeoxyribonuclease 8 PDDEXK-like domain-containing protein | Putative exodeoxyribonuclease 8 PDDEXK-like domain-containing protein | | uniclust | UniRef100\_UPI00210133BA | 99.3 | 4.6e-15 | 8.4e-21 | 115.3 | 127 | (187, 315) | 372 | (4, 139) | 143 | PD-(D/E)XK nuclease-like domain-containing protein | PD-(D/E)XK nuclease-like domain-containing protein | | uniclust | UniRef100\_A0A524P3G8 | 99.3 | 5e-15 | 9.2e-21 | 117.9 | 115 | (235, 351) | 372 | (6, 122) | 167 | Putative exodeoxyribonuclease 8 PDDEXK-like domain-containing protein (Fragment) | Putative exodeoxyribonuclease 8 PDDEXK-like domain-containing protein (Fragment) | | uniclust | UniRef100\_X1D8E3 | 99.3 | 5.4e-15 | 9.9e-21 | 100.9 | 65 | (2, 66) | 372 | (4, 70) | 70 | Uncharacterized protein (Fragment) | Uncharacterized protein (Fragment) | | uniclust | UniRef100\_A0A0R2JVU7 | 99.3 | 5.1e-15 | 1e-20 | 126.2 | 148 | (195, 348) | 372 | (11, 166) | 191 | Putative exodeoxyribonuclease 8 PDDEXK-like domain-containing protein | Putative exodeoxyribonuclease 8 PDDEXK-like domain-containing protein | | uniclust | UniRef100\_UPI000E170CDF | 99.3 | 5.9e-15 | 1.1e-20 | 108.4 | 74 | (5, 78) | 372 | (13, 87) | 103 | PD-(D/E)XK nuclease-like domain-containing protein | PD-(D/E)XK nuclease-like domain-containing protein | | uniclust | UniRef100\_A0A7X7QZ66 | 99.3 | 6.1e-15 | 1.1e-20 | 106.5 | 89 | (274, 362) | 372 | (3, 91) | 94 | Putative exodeoxyribonuclease 8 PDDEXK-like domain-containing protein | Putative exodeoxyribonuclease 8 PDDEXK-like domain-containing protein | | uniclust | UniRef100\_A0A238X2A8 | 99.3 | 6.8e-15 | 1.2e-20 | 126.5 | 140 | (199, 346) | 372 | (71, 215) | 285 | Putative exodeoxyribonuclease 8 PDDEXK-like domain-containing protein | Putative exodeoxyribonuclease 8 PDDEXK-like domain-containing protein | | uniclust | UniRef100\_A0A098LCC7 | 99.3 | 6.6e-15 | 1.4e-20 | 160.5 | 149 | (188, 338) | 372 | (891, 1053) | 1107 | DNA 3'-5' helicase | DNA 3'-5' helicase | | uniclust | UniRef100\_A0A0S7WJM6 | 99.3 | 6.9e-15 | 1.4e-20 | 139.4 | 212 | (19, 349) | 372 | (54, 272) | 348 | PD-(D/E)XK endonuclease-like domain-containing protein | PD-(D/E)XK endonuclease-like domain-containing protein | | uniclust | UniRef100\_A0A2D6B2Q9 | 99.3 | 8.7e-15 | 1.6e-20 | 126.1 | 252 | (3, 348) | 372 | (2, 277) | 288 | Putative exodeoxyribonuclease 8 PDDEXK-like domain-containing protein | Putative exodeoxyribonuclease 8 PDDEXK-like domain-containing protein | | uniclust | UniRef100\_A0A3D2IFL8 | 99.3 | 1e-14 | 1.9e-20 | 109.3 | 71 | (187, 260) | 372 | (44, 114) | 115 | Putative exodeoxyribonuclease 8 PDDEXK-like domain-containing protein (Fragment) | Putative exodeoxyribonuclease 8 PDDEXK-like domain-containing protein (Fragment) | | uniclust | UniRef100\_A0A0V8BEL4 | 99.3 | 1e-14 | 1.9e-20 | 128.7 | 227 | (5, 348) | 372 | (22, 270) | 293 | Prophage Lp1 protein 19 | Prophage Lp1 protein 19 | | uniclust | UniRef100\_UPI0022659236 | 99.3 | 1.4e-14 | 2.6e-20 | 118.8 | 175 | (188, 363) | 372 | (12, 193) | 201 | PD-(D/E)XK nuclease-like domain-containing protein | PD-(D/E)XK nuclease-like domain-containing protein | | uniclust | UniRef100\_A0A8S0I2N3 | 99.3 | 1.5e-14 | 2.8e-20 | 106.2 | 82 | (225, 308) | 372 | (14, 96) | 102 | Putative exodeoxyribonuclease 8 PDDEXK-like domain-containing protein | Putative exodeoxyribonuclease 8 PDDEXK-like domain-containing protein | | uniclust | UniRef100\_A0A1M5H3U8 | 99.3 | 1.5e-14 | 2.9e-20 | 142.6 | 149 | (188, 338) | 372 | (457, 619) | 658 | PD-(D/E)XK nuclease superfamily protein | PD-(D/E)XK nuclease superfamily protein | | uniclust | UniRef100\_A0A447JGP7 | 99.3 | 1.7e-14 | 3.2e-20 | 105.3 | 93 | (194, 289) | 372 | (2, 95) | 99 | Exonuclease VIII | Exonuclease VIII | | uniclust | UniRef100\_A0A368JHD6 | 99.3 | 1.7e-14 | 3.3e-20 | 120.9 | 133 | (201, 340) | 372 | (59, 194) | 215 | Putative exodeoxyribonuclease 8 PDDEXK-like domain-containing protein | Putative exodeoxyribonuclease 8 PDDEXK-like domain-containing protein | | uniclust | UniRef100\_A0A2A4RDJ8 | 99.3 | 2e-14 | 4.1e-20 | 153.9 | 224 | (19, 349) | 372 | (880, 1114) | 1152 | DNA 3'-5' helicase | DNA 3'-5' helicase | | uniclust | UniRef100\_A0A081PKL5 | 99.3 | 2.1e-14 | 4.3e-20 | 128.9 | 140 | (191, 337) | 372 | (76, 223) | 249 | Uncharacterized protein | Uncharacterized protein | | uniclust | UniRef100\_A0A3D8IYQ2 | 99.3 | 2.4e-14 | 4.3e-20 | 121.9 | 217 | (9, 335) | 372 | (1, 226) | 260 | Putative exodeoxyribonuclease 8 PDDEXK-like domain-containing protein | Putative exodeoxyribonuclease 8 PDDEXK-like domain-containing protein | | uniclust | UniRef100\_A0A1F9B6U3 | 99.3 | 2.3e-14 | 4.9e-20 | 137.7 | 140 | (199, 349) | 372 | (173, 321) | 362 | PD-(D/E)XK endonuclease-like domain-containing protein (Fragment) | PD-(D/E)XK endonuclease-like domain-containing protein (Fragment) | | uniclust | UniRef100\_A0A0C6EI61 | 99.3 | 2.5e-14 | 5e-20 | 140.9 | 114 | (225, 352) | 372 | (200, 317) | 491 | PD-(D/E)XK endonuclease-like domain-containing protein | PD-(D/E)XK endonuclease-like domain-containing protein | | uniclust | UniRef100\_A0A2X3JIG8 | 99.3 | 2.7e-14 | 5e-20 | 121.1 | 152 | (2, 160) | 372 | (67, 244) | 253 | Putative phage exodeoxyribonuclease | Putative phage exodeoxyribonuclease | | uniclust | UniRef100\_A0A350BS60 | 99.2 | 3e-14 | 5.6e-20 | 121.0 | 163 | (184, 348) | 372 | (57, 247) | 256 | Putative exodeoxyribonuclease 8 PDDEXK-like domain-containing protein | Putative exodeoxyribonuclease 8 PDDEXK-like domain-containing protein | | uniclust | UniRef100\_UPI001B805189 | 99.2 | 3.1e-14 | 5.6e-20 | 100.7 | 80 | (205, 285) | 372 | (1, 83) | 83 | PD-(D/E)XK nuclease-like domain-containing protein | PD-(D/E)XK nuclease-like domain-containing protein | | uniclust | UniRef100\_A0A1V5NKN1 | 99.2 | 3.1e-14 | 6.3e-20 | 142.0 | 232 | (8, 339) | 372 | (244, 493) | 538 | ATP-dependent helicase/deoxyribonuclease subunit B | ATP-dependent helicase/deoxyribonuclease subunit B | | uniclust | UniRef100\_A0A379CLP5 | 99.2 | 3.6e-14 | 6.5e-20 | 120.4 | 158 | (2, 165) | 372 | (24, 205) | 253 | Exodeoxyribonuclease 8 | Exodeoxyribonuclease 8 | | uniclust | UniRef100\_M4QBX2 | 99.2 | 4.2e-14 | 7.7e-20 | 125.6 | 176 | (31, 260) | 372 | (3, 182) | 361 | Putative exodeoxyribonuclease 8 PDDEXK-like domain-containing protein | Putative exodeoxyribonuclease 8 PDDEXK-like domain-containing protein | | uniclust | UniRef100\_A0A962Z9F4 | 99.2 | 4.4e-14 | 8e-20 | 116.4 | 134 | (191, 326) | 372 | (65, 205) | 205 | PD-(D/E)XK nuclease family protein (Fragment) | PD-(D/E)XK nuclease family protein (Fragment) | | uniclust | UniRef100\_A0A7C1JUS1 | 99.2 | 4.6e-14 | 8.4e-20 | 117.2 | 138 | (205, 349) | 372 | (1, 138) | 216 | Putative exodeoxyribonuclease 8 PDDEXK-like domain-containing protein | Putative exodeoxyribonuclease 8 PDDEXK-like domain-containing protein | | uniclust | UniRef100\_A0A098SBQ5 | 99.2 | 4.1e-14 | 9e-20 | 156.2 | 217 | (18, 338) | 372 | (824, 1056) | 1105 | DNA 3'-5' helicase | DNA 3'-5' helicase | | uniclust | UniRef100\_A0A059XC20 | 99.2 | 5.5e-14 | 1.1e-19 | 134.2 | 110 | (225, 350) | 372 | (199, 311) | 344 | PD-(D/E)XK nuclease superfamily (Fragment) | PD-(D/E)XK nuclease superfamily (Fragment) | | uniclust | UniRef100\_A0A0G0LEM3 | 99.2 | 6.2e-14 | 1.3e-19 | 136.1 | 231 | (14, 364) | 372 | (58, 303) | 429 | PD-(D/E)XK endonuclease-like domain-containing protein | PD-(D/E)XK endonuclease-like domain-containing protein | | uniclust | UniRef100\_A0A1T5CLE9 | 99.2 | 7.4e-14 | 1.4e-19 | 115.7 | 171 | (20, 306) | 372 | (1, 179) | 182 | Putative exodeoxyribonuclease 8 PDDEXK-like domain-containing protein | Putative exodeoxyribonuclease 8 PDDEXK-like domain-containing protein | | uniclust | UniRef100\_X7ZHD3 | 99.2 | 7.8e-14 | 1.4e-19 | 109.5 | 94 | (253, 346) | 372 | (9, 102) | 149 | Gp60 domain protein | Gp60 domain protein | | uniclust | UniRef100\_A0A6J5Q956 | 99.2 | 8.4e-14 | 1.5e-19 | 115.9 | 163 | (184, 350) | 372 | (15, 190) | 219 | Exodeoxyribonuclease 8, PDDEXK-like domain containing protein | Exodeoxyribonuclease 8, PDDEXK-like domain containing protein | | uniclust | UniRef100\_A0A6M3ITI9 | 99.2 | 8.4e-14 | 1.5e-19 | 122.0 | 259 | (8, 348) | 372 | (5, 290) | 321 | Putative exodeoxyribonuclease 8 PDDEXK-like domain-containing protein | Putative exodeoxyribonuclease 8 PDDEXK-like domain-containing protein | | uniclust | UniRef100\_A0A949QL58 | 99.2 | 8.6e-14 | 1.6e-19 | 104.4 | 109 | (254, 362) | 372 | (2, 113) | 114 | PD-(D/E)XK nuclease-like domain-containing protein | PD-(D/E)XK nuclease-like domain-containing protein | | uniclust | UniRef100\_UPI0015868581 | 99.2 | 8.6e-14 | 1.6e-19 | 98.5 | 66 | (1, 67) | 372 | (18, 83) | 83 | PD-(D/E)XK nuclease-like domain-containing protein | PD-(D/E)XK nuclease-like domain-containing protein | | uniclust | UniRef100\_UPI00215A86D0 | 99.2 | 9.2e-14 | 1.7e-19 | 99.5 | 75 | (229, 306) | 372 | (1, 76) | 88 | PD-(D/E)XK nuclease-like domain-containing protein | PD-(D/E)XK nuclease-like domain-containing protein | | uniclust | UniRef100\_A0A376LMM8 | 99.2 | 1.1e-13 | 2.1e-19 | 133.4 | 140 | (1, 232) | 372 | (613, 752) | 752 | Exonuclease VIII | Exonuclease VIII | | uniclust | UniRef100\_A0A958WPB8 | 99.2 | 1.2e-13 | 2.2e-19 | 119.0 | 137 | (194, 339) | 372 | (134, 276) | 280 | PD-(D/E)XK nuclease-like domain-containing protein | PD-(D/E)XK nuclease-like domain-containing protein | | uniclust | UniRef100\_UPI001FEDDF68 | 99.2 | 1.2e-13 | 2.3e-19 | 107.6 | 117 | (192, 310) | 372 | (24, 142) | 142 | PD-(D/E)XK nuclease-like domain-containing protein | PD-(D/E)XK nuclease-like domain-containing protein | | uniclust | UniRef100\_A0A5C7JQK3 | 99.2 | 1.3e-13 | 2.4e-19 | 120.3 | 249 | (15, 328) | 372 | (11, 265) | 310 | Putative exodeoxyribonuclease 8 PDDEXK-like domain-containing protein | Putative exodeoxyribonuclease 8 PDDEXK-like domain-containing protein | | uniclust | UniRef100\_UPI000DFC6B13 | 99.2 | 1.3e-13 | 2.5e-19 | 113.9 | 132 | (2, 142) | 372 | (37, 174) | 207 | SAP domain-containing protein | SAP domain-containing protein | | uniclust | UniRef100\_A0A1V1PGN8 | 99.2 | 1.6e-13 | 3.1e-19 | 122.0 | 255 | (7, 338) | 372 | (2, 277) | 300 | Exodeoxyribonuclease 8 PDDEXK-like domain-containing protein | Exodeoxyribonuclease 8 PDDEXK-like domain-containing protein | | uniclust | UniRef100\_A0A0F9EWN7 | 99.2 | 1.5e-13 | 3.2e-19 | 129.6 | 137 | (200, 352) | 372 | (133, 271) | 296 | PD-(D/E)XK endonuclease-like domain-containing protein (Fragment) | PD-(D/E)XK endonuclease-like domain-containing protein (Fragment) | | uniclust | UniRef100\_A0A2E9HYF9 | 99.2 | 1.8e-13 | 3.3e-19 | 121.6 | 177 | (184, 365) | 372 | (159, 346) | 357 | Putative exodeoxyribonuclease 8 PDDEXK-like domain-containing protein | Putative exodeoxyribonuclease 8 PDDEXK-like domain-containing protein | | uniclust | UniRef100\_A0A0S7ZE36 | 99.2 | 1.7e-13 | 3.4e-19 | 135.0 | 109 | (225, 349) | 372 | (323, 434) | 473 | DNA 3'-5' helicase (Fragment) | DNA 3'-5' helicase (Fragment) | | uniclust | UniRef100\_A0A8S5NP51 | 99.2 | 1.9e-13 | 3.5e-19 | 106.6 | 107 | (255, 362) | 372 | (33, 140) | 142 | Exodeoxyribonuclease 8 | Exodeoxyribonuclease 8 | | uniclust | UniRef100\_UPI001FDECBD7 | 99.2 | 2e-13 | 3.6e-19 | 109.1 | 118 | (245, 362) | 372 | (1, 127) | 165 | PD-(D/E)XK nuclease-like domain-containing protein | PD-(D/E)XK nuclease-like domain-containing protein | | uniclust | UniRef100\_A0A0G0NCS9 | 99.1 | 2.9e-13 | 5.8e-19 | 130.6 | 231 | (9, 348) | 372 | (143, 391) | 414 | DNA 3'-5' helicase (Fragment) | DNA 3'-5' helicase (Fragment) | | uniclust | UniRef100\_A0A0G1A9X7 | 99.1 | 3.6e-13 | 7.4e-19 | 141.2 | 218 | (18, 338) | 372 | (497, 731) | 777 | DNA 3'-5' helicase | DNA 3'-5' helicase | | uniclust | UniRef100\_A0A447U5S1 | 99.1 | 4.2e-13 | 7.8e-19 | 113.4 | 201 | (84, 308) | 372 | (22, 231) | 239 | Exodeoxyribonuclease VIII | Exodeoxyribonuclease VIII | | uniclust | UniRef100\_A0A139BNJ2 | 99.1 | 4.2e-13 | 8.2e-19 | 99.9 | 79 | (291, 370) | 372 | (4, 82) | 86 | Putative exodeoxyribonuclease 8 PDDEXK-like domain-containing protein | Putative exodeoxyribonuclease 8 PDDEXK-like domain-containing protein | | uniclust | UniRef100\_UPI001E519A63 | 99.1 | 4.6e-13 | 8.5e-19 | 109.4 | 150 | (190, 347) | 372 | (6, 161) | 189 | PD-(D/E)XK nuclease-like domain-containing protein | PD-(D/E)XK nuclease-like domain-containing protein | | uniclust | UniRef100\_A0A843FLM9 | 99.1 | 5.1e-13 | 9.9e-19 | 124.5 | 286 | (7, 343) | 372 | (23, 346) | 361 | PD-(D/E)XK nuclease-like domain-containing protein | PD-(D/E)XK nuclease-like domain-containing protein | | uniclust | UniRef100\_A0A0D5A873 | 99.1 | 5.2e-13 | 1e-18 | 125.7 | 105 | (236, 349) | 372 | (213, 321) | 391 | RecB family exonuclease | RecB family exonuclease | | uniclust | UniRef100\_UPI001F4ADC0F | 99.1 | 5.4e-13 | 1e-18 | 114.4 | 149 | (195, 348) | 372 | (49, 205) | 229 | PD-(D/E)XK nuclease-like domain-containing protein | PD-(D/E)XK nuclease-like domain-containing protein | | uniclust | UniRef100\_B6YS92 | 99.1 | 5.9e-13 | 1.1e-18 | 119.3 | 226 | (8, 292) | 372 | (15, 250) | 327 | Putative exodeoxyribonuclease 8 PDDEXK-like domain-containing protein | Putative exodeoxyribonuclease 8 PDDEXK-like domain-containing protein | | uniclust | UniRef100\_A0A160VHU9 | 99.1 | 5.8e-13 | 1.2e-18 | 124.0 | 120 | (225, 362) | 372 | (190, 324) | 327 | RecB family exonuclease | RecB family exonuclease | | uniclust | UniRef100\_A0A8E0IFE8 | 99.1 | 6.9e-13 | 1.3e-18 | 123.8 | 147 | (195, 347) | 372 | (370, 524) | 549 | Putative exodeoxyribonuclease 8 PDDEXK-like domain-containing protein | Putative exodeoxyribonuclease 8 PDDEXK-like domain-containing protein | | uniclust | UniRef100\_UPI0018CF9690 | 99.1 | 7.6e-13 | 1.4e-18 | 103.9 | 83 | (2, 84) | 372 | (54, 140) | 146 | hypothetical protein | hypothetical protein | | uniclust | UniRef100\_A0A3C0G174 | 99.1 | 8.2e-13 | 1.5e-18 | 99.3 | 93 | (253, 347) | 372 | (2, 94) | 113 | Putative exodeoxyribonuclease 8 PDDEXK-like domain-containing protein | Putative exodeoxyribonuclease 8 PDDEXK-like domain-containing protein | | uniclust | UniRef100\_UPI0009718900 | 99.1 | 8.2e-13 | 1.5e-18 | 107.1 | 163 | (201, 370) | 372 | (3, 170) | 179 | PD-(D/E)XK nuclease-like domain-containing protein | PD-(D/E)XK nuclease-like domain-containing protein | | uniclust | UniRef100\_A0A024YLP4 | 99.1 | 9.1e-13 | 1.7e-18 | 123.8 | 105 | (236, 349) | 372 | (253, 361) | 430 | Recombinase RecB | Recombinase RecB | | uniclust | UniRef100\_A0A4P0Y455 | 99.1 | 9.4e-13 | 1.8e-18 | 113.1 | 99 | (2, 102) | 372 | (130, 229) | 235 | Putative exodeoxyribonuclease VIII | Putative exodeoxyribonuclease VIII | | uniclust | UniRef100\_A0A177Q060 | 99.0 | 1e-12 | 2.1e-18 | 123.0 | 153 | (200, 364) | 372 | (125, 289) | 300 | PD-(D/E)XK endonuclease-like domain-containing protein | PD-(D/E)XK endonuclease-like domain-containing protein | | uniclust | UniRef100\_A0A066U5F6 | 99.0 | 1e-12 | 2.2e-18 | 128.5 | 126 | (199, 339) | 372 | (142, 270) | 367 | Recombinase RecB | Recombinase RecB | | uniclust | UniRef100\_A0A060HEP5 | 99.0 | 1.1e-12 | 2.2e-18 | 141.9 | 138 | (196, 349) | 372 | (926, 1067) | 1094 | DNA 3'-5' helicase | DNA 3'-5' helicase | | uniclust | UniRef100\_A0A0G0B930 | 99.0 | 1.1e-12 | 2.3e-18 | 129.6 | 110 | (225, 350) | 372 | (357, 470) | 489 | DNA 3'-5' helicase (Fragment) | DNA 3'-5' helicase (Fragment) | | uniclust | UniRef100\_A0A257LV29 | 99.0 | 1.3e-12 | 2.7e-18 | 119.6 | 113 | (225, 349) | 372 | (112, 227) | 258 | PD-(D/E)XK endonuclease-like domain-containing protein (Fragment) | PD-(D/E)XK endonuclease-like domain-containing protein (Fragment) | | uniclust | UniRef100\_A0A4R3AZ53 | 99.0 | 1.7e-12 | 3.2e-18 | 124.4 | 141 | (196, 349) | 372 | (340, 484) | 528 | RecB family exonuclease | RecB family exonuclease | | uniclust | UniRef100\_A0A0F9QNT3 | 99.0 | 1.6e-12 | 3.3e-18 | 142.8 | 110 | (225, 350) | 372 | (932, 1045) | 1088 | DNA 3'-5' helicase | DNA 3'-5' helicase | | uniclust | UniRef100\_A0A0F9YKX6 | 99.0 | 1.8e-12 | 3.6e-18 | 123.5 | 134 | (199, 347) | 372 | (140, 278) | 340 | PD-(D/E)XK endonuclease-like domain-containing protein | PD-(D/E)XK endonuclease-like domain-containing protein | | uniclust | UniRef100\_A0A0G1WC88 | 99.0 | 1.8e-12 | 3.7e-18 | 123.3 | 110 | (225, 349) | 372 | (190, 303) | 357 | UvrD/REP helicase (Fragment) | UvrD/REP helicase (Fragment) | | uniclust | UniRef100\_A0A6M3LWC3 | 99.0 | 2.3e-12 | 4.1e-18 | 113.4 | 280 | (19, 346) | 372 | (3, 296) | 317 | Putative exodeoxyribonuclease 8 PDDEXK-like domain-containing protein | Putative exodeoxyribonuclease 8 PDDEXK-like domain-containing protein | | uniclust | UniRef100\_UPI000E016246 | 99.0 | 2.3e-12 | 4.3e-18 | 109.7 | 134 | (2, 231) | 372 | (19, 162) | 248 | hypothetical protein | hypothetical protein | | uniclust | UniRef100\_A0A5V7WNI2 | 99.0 | 2.2e-12 | 4.4e-18 | 128.6 | 101 | (2, 104) | 372 | (329, 430) | 550 | Exodeoxyribonuclease (Fragment) | Exodeoxyribonuclease (Fragment) | | uniclust | UniRef100\_A0A327J2B3 | 99.0 | 2.3e-12 | 4.5e-18 | 136.4 | 110 | (225, 338) | 372 | (893, 1006) | 1032 | DNA 3'-5' helicase | DNA 3'-5' helicase | | uniclust | UniRef100\_A0A0F9YNH9 | 99.0 | 2.3e-12 | 4.7e-18 | 139.7 | 225 | (9, 337) | 372 | (814, 1056) | 1108 | DNA 3'-5' helicase | DNA 3'-5' helicase | | uniclust | UniRef100\_A0A117IC06 | 99.0 | 2.5e-12 | 4.7e-18 | 93.3 | 78 | (292, 369) | 372 | (5, 82) | 84 | RecE (Fragment) | RecE (Fragment) | | uniclust | UniRef100\_A0A0A0B9Q3 | 99.0 | 2.5e-12 | 4.8e-18 | 123.7 | 116 | (225, 350) | 372 | (185, 304) | 477 | Recombinase RecB | Recombinase RecB | | uniclust | UniRef100\_A0A9D7FBP7 | 99.0 | 2.7e-12 | 4.9e-18 | 96.1 | 70 | (7, 77) | 372 | (25, 95) | 109 | PD-(D/E)XK nuclease-like domain-containing protein | PD-(D/E)XK nuclease-like domain-containing protein | | uniclust | UniRef100\_A0A963IY46 | 99.0 | 2.7e-12 | 5e-18 | 98.4 | 106 | (253, 360) | 372 | (2, 108) | 125 | Uncharacterized protein | Uncharacterized protein | | uniclust | UniRef100\_A0A965VWR8 | 99.0 | 2.9e-12 | 5.4e-18 | 111.0 | 239 | (11, 290) | 372 | (16, 271) | 282 | PD-(D/E)XK endonuclease-like domain-containing protein (Fragment) | PD-(D/E)XK endonuclease-like domain-containing protein (Fragment) | | uniclust | UniRef100\_UPI001E64B484 | 99.0 | 3e-12 | 5.4e-18 | 102.9 | 96 | (2, 100) | 372 | (24, 119) | 166 | hypothetical protein | hypothetical protein | | uniclust | UniRef100\_A0A2E7DJB3 | 99.0 | 2.8e-12 | 5.5e-18 | 117.7 | 121 | (235, 363) | 372 | (174, 304) | 306 | PD-(D/E)XK endonuclease-like domain-containing protein | PD-(D/E)XK endonuclease-like domain-containing protein | | uniclust | UniRef100\_A0A4Q3PZX2 | 99.0 | 3.2e-12 | 6e-18 | 105.2 | 114 | (225, 338) | 372 | (36, 161) | 176 | Putative exodeoxyribonuclease 8 PDDEXK-like domain-containing protein (Fragment) | Putative exodeoxyribonuclease 8 PDDEXK-like domain-containing protein (Fragment) | | uniclust | UniRef100\_A0A349YBV5 | 99.0 | 3.5e-12 | 6.5e-18 | 109.6 | 138 | (195, 337) | 372 | (92, 231) | 242 | Putative exodeoxyribonuclease 8 PDDEXK-like domain-containing protein | Putative exodeoxyribonuclease 8 PDDEXK-like domain-containing protein | | uniclust | UniRef100\_A0A1I0PE57 | 99.0 | 3.5e-12 | 6.7e-18 | 120.9 | 149 | (188, 338) | 372 | (273, 438) | 468 | PD-(D/E)XK nuclease superfamily protein | PD-(D/E)XK nuclease superfamily protein | | uniclust | UniRef100\_A0A081S3H6 | 99.0 | 3.5e-12 | 7.1e-18 | 131.1 | 112 | (225, 349) | 372 | (585, 700) | 722 | DNA 3'-5' helicase (Fragment) | DNA 3'-5' helicase (Fragment) | | uniclust | UniRef100\_UPI000EC7A980 | 99.0 | 4.1e-12 | 7.5e-18 | 88.1 | 64 | (225, 288) | 372 | (5, 72) | 72 | PD-(D/E)XK nuclease-like domain-containing protein | PD-(D/E)XK nuclease-like domain-containing protein | | uniclust | UniRef100\_A0A5C7PXG6 | 99.0 | 4.3e-12 | 8e-18 | 94.7 | 70 | (3, 74) | 372 | (30, 100) | 107 | Putative exodeoxyribonuclease 8 PDDEXK-like domain-containing protein (Fragment) | Putative exodeoxyribonuclease 8 PDDEXK-like domain-containing protein (Fragment) | | uniclust | UniRef100\_A0A0G0K286 | 99.0 | 4.5e-12 | 8.9e-18 | 124.3 | 230 | (11, 347) | 372 | (239, 490) | 510 | DNA 3'-5' helicase | DNA 3'-5' helicase | | uniclust | UniRef100\_A0A968V6E1 | 99.0 | 5.1e-12 | 9.4e-18 | 111.8 | 262 | (7, 349) | 372 | (3, 293) | 329 | Uncharacterized protein | Uncharacterized protein | | uniclust | UniRef100\_A0A6M3J0N3 | 98.9 | 5.4e-12 | 9.9e-18 | 104.5 | 166 | (2, 262) | 372 | (8, 193) | 202 | Uncharacterized protein (Fragment) | Uncharacterized protein (Fragment) | | uniclust | UniRef100\_A0A0G0EV96 | 98.9 | 4.9e-12 | 1e-17 | 132.5 | 109 | (225, 349) | 372 | (606, 718) | 741 | DNA 3'-5' helicase | DNA 3'-5' helicase | | uniclust | UniRef100\_UPI002165B33C | 98.9 | 5.8e-12 | 1.1e-17 | 108.8 | 146 | (216, 363) | 372 | (104, 261) | 272 | PD-(D/E)XK nuclease-like domain-containing protein | PD-(D/E)XK nuclease-like domain-containing protein | | uniclust | UniRef100\_UPI000B1595CC | 98.9 | 5.9e-12 | 1.1e-17 | 111.9 | 211 | (4, 318) | 372 | (37, 262) | 341 | PD-(D/E)XK nuclease-like domain-containing protein | PD-(D/E)XK nuclease-like domain-containing protein | | uniclust | UniRef100\_A0A0F9D9H6 | 98.9 | 5.6e-12 | 1.2e-17 | 120.5 | 148 | (190, 351) | 372 | (132, 281) | 324 | PD-(D/E)XK endonuclease-like domain-containing protein | PD-(D/E)XK endonuclease-like domain-containing protein | | uniclust | UniRef100\_A0A1M7D7C6 | 98.9 | 6.5e-12 | 1.2e-17 | 109.0 | 138 | (192, 337) | 372 | (129, 273) | 282 | Putative exodeoxyribonuclease 8 PDDEXK-like domain-containing protein | Putative exodeoxyribonuclease 8 PDDEXK-like domain-containing protein | | uniclust | UniRef100\_A0A1F2WZJ7 | 98.9 | 6.2e-12 | 1.2e-17 | 117.0 | 162 | (187, 364) | 372 | (174, 347) | 353 | PD-(D/E)XK endonuclease-like domain-containing protein | PD-(D/E)XK endonuclease-like domain-containing protein | | uniclust | UniRef100\_A0A485BQR4 | 98.9 | 6.7e-12 | 1.2e-17 | 96.7 | 90 | (2, 93) | 372 | (36, 126) | 128 | Exodeoxyribonuclease 8 | Exodeoxyribonuclease 8 | | uniclust | UniRef100\_A0A0F9EXF6 | 98.9 | 6.7e-12 | 1.3e-17 | 106.4 | 166 | (2, 269) | 372 | (15, 199) | 211 | Exodeoxyribonuclease 8 PDDEXK-like domain-containing protein (Fragment) | Exodeoxyribonuclease 8 PDDEXK-like domain-containing protein (Fragment) | | uniclust | UniRef100\_A0A920Q8L2 | 98.9 | 6.8e-12 | 1.3e-17 | 108.9 | 126 | (8, 233) | 372 | (138, 263) | 283 | Putative exodeoxyribonuclease 8 PDDEXK-like domain-containing protein | Putative exodeoxyribonuclease 8 PDDEXK-like domain-containing protein | | uniclust | UniRef100\_UPI0022DE3438 | 98.9 | 6.9e-12 | 1.3e-17 | 104.9 | 133 | (199, 339) | 372 | (47, 186) | 215 | PD-(D/E)XK nuclease-like domain-containing protein | PD-(D/E)XK nuclease-like domain-containing protein | | uniclust | UniRef100\_A0A095Y853 | 98.9 | 7.2e-12 | 1.5e-17 | 135.1 | 137 | (195, 350) | 372 | (906, 1047) | 1071 | DNA 3'-5' helicase | DNA 3'-5' helicase | | uniclust | UniRef100\_A0A136KRS2 | 98.9 | 8.1e-12 | 1.5e-17 | 113.6 | 148 | (191, 339) | 372 | (128, 291) | 316 | ATP-dependent helicase/deoxyribonuclease subunit B | ATP-dependent helicase/deoxyribonuclease subunit B | | uniclust | UniRef100\_A0A0G3WJV3 | 98.9 | 8.4e-12 | 1.7e-17 | 117.7 | 132 | (199, 348) | 372 | (141, 276) | 309 | PD-(D/E)XK endonuclease-like domain-containing protein | PD-(D/E)XK endonuclease-like domain-containing protein | | uniclust | UniRef100\_A0A0F9YF59 | 98.9 | 8.5e-12 | 1.7e-17 | 124.1 | 127 | (225, 362) | 372 | (176, 316) | 482 | PD-(D/E)XK endonuclease-like domain-containing protein | PD-(D/E)XK endonuclease-like domain-containing protein | | uniclust | UniRef100\_A0A379YD88 | 98.9 | 9.3e-12 | 1.7e-17 | 101.7 | 153 | (210, 368) | 372 | (2, 164) | 168 | Exonuclease VIII | Exonuclease VIII | | uniclust | UniRef100\_A0A094PIS3 | 98.9 | 9.1e-12 | 1.8e-17 | 116.1 | 106 | (236, 350) | 372 | (161, 268) | 329 | PD-(D/E)XK endonuclease-like domain-containing protein | PD-(D/E)XK endonuclease-like domain-containing protein | | uniclust | UniRef100\_UPI0020C55E59 | 98.9 | 1e-11 | 1.9e-17 | 94.1 | 90 | (187, 279) | 372 | (23, 113) | 113 | PD-(D/E)XK nuclease-like domain-containing protein | PD-(D/E)XK nuclease-like domain-containing protein | | uniclust | UniRef100\_A0A1J4YR24 | 98.9 | 1.1e-11 | 2.1e-17 | 85.0 | 62 | (5, 68) | 372 | (5, 66) | 68 | Uncharacterized protein | Uncharacterized protein | | uniclust | UniRef100\_A0A0G0RCS7 | 98.9 | 1.1e-11 | 2.2e-17 | 123.0 | 112 | (225, 348) | 372 | (387, 501) | 531 | DNA 3'-5' helicase | DNA 3'-5' helicase | | uniclust | UniRef100\_A0A1G0YSF3 | 98.9 | 1.3e-11 | 2.7e-17 | 117.1 | 229 | (8, 349) | 372 | (6, 249) | 300 | PD-(D/E)XK endonuclease-like domain-containing protein | PD-(D/E)XK endonuclease-like domain-containing protein | | uniclust | UniRef100\_A0A5S9M3W7 | 98.9 | 1.6e-11 | 3.1e-17 | 101.2 | 148 | (2, 259) | 372 | (2, 155) | 155 | Putative exodeoxyribonuclease 8 PDDEXK-like domain-containing protein | Putative exodeoxyribonuclease 8 PDDEXK-like domain-containing protein | | uniclust | UniRef100\_UPI0017820ABF | 98.9 | 1.9e-11 | 3.5e-17 | 106.1 | 176 | (183, 361) | 372 | (63, 274) | 277 | PD-(D/E)XK nuclease-like domain-containing protein | PD-(D/E)XK nuclease-like domain-containing protein | | uniclust | UniRef100\_A0A024JZZ9 | 98.9 | 1.9e-11 | 3.5e-17 | 113.3 | 147 | (194, 349) | 372 | (124, 277) | 356 | Recombinase B | Recombinase B | | uniclust | UniRef100\_A0A5C5X7Z0 | 98.8 | 2.2e-11 | 4.1e-17 | 108.2 | 241 | (8, 351) | 372 | (13, 323) | 333 | Putative exodeoxyribonuclease 8 PDDEXK-like domain-containing protein | Putative exodeoxyribonuclease 8 PDDEXK-like domain-containing protein | | uniclust | UniRef100\_A0A350BX12 | 98.8 | 2.2e-11 | 4.3e-17 | 111.0 | 148 | (189, 338) | 372 | (53, 214) | 260 | DNA helicase UvrD (Fragment) | DNA helicase UvrD (Fragment) | | uniclust | UniRef100\_X0YC96 | 98.8 | 2.4e-11 | 4.4e-17 | 96.0 | 74 | (2, 78) | 372 | (6, 80) | 146 | Uncharacterized protein (Fragment) | Uncharacterized protein (Fragment) | | uniclust | UniRef100\_A0A1G6MCA1 | 98.8 | 2.4e-11 | 4.9e-17 | 130.7 | 141 | (197, 350) | 372 | (961, 1108) | 1131 | DNA 3'-5' helicase | DNA 3'-5' helicase | | uniclust | UniRef100\_A0A068NW26 | 98.8 | 2.6e-11 | 5.2e-17 | 114.6 | 213 | (17, 347) | 372 | (45, 268) | 323 | RecB family exonuclease | RecB family exonuclease | | uniclust | UniRef100\_A0A377E4A8 | 98.8 | 3.1e-11 | 5.7e-17 | 97.2 | 64 | (1, 64) | 372 | (94, 157) | 165 | Exonuclease VIII | Exonuclease VIII | | uniclust | UniRef100\_A0A962VA62 | 98.8 | 3.2e-11 | 5.9e-17 | 89.6 | 69 | (4, 77) | 372 | (22, 90) | 102 | Uncharacterized protein (Fragment) | Uncharacterized protein (Fragment) | | uniclust | UniRef100\_UPI000F6DF811 | 98.8 | 3.4e-11 | 6.2e-17 | 90.9 | 70 | (1, 75) | 372 | (1, 70) | 111 | hypothetical protein | hypothetical protein | | uniclust | UniRef100\_A0A2U3ERY8 | 98.8 | 3.8e-11 | 6.9e-17 | 109.5 | 166 | (188, 363) | 372 | (223, 394) | 413 | Exodeoxyribonuclease 8 PDDEXK-like domain-containing protein | Exodeoxyribonuclease 8 PDDEXK-like domain-containing protein | | uniclust | UniRef100\_A0A1G3BLT7 | 98.8 | 3.5e-11 | 6.9e-17 | 129.0 | 145 | (191, 348) | 372 | (963, 1112) | 1169 | DNA 3'-5' helicase (Fragment) | DNA 3'-5' helicase (Fragment) | | uniclust | UniRef100\_A0A0S8E471 | 98.8 | 3.6e-11 | 7.3e-17 | 129.3 | 112 | (225, 350) | 372 | (879, 994) | 1014 | DNA 3'-5' helicase | DNA 3'-5' helicase | | uniclust | UniRef100\_UPI0020CB8203 | 98.8 | 4.4e-11 | 8.1e-17 | 99.6 | 189 | (34, 290) | 372 | (3, 193) | 204 | PD-(D/E)XK nuclease-like domain-containing protein | PD-(D/E)XK nuclease-like domain-containing protein | | uniclust | UniRef100\_UPI0011161D0A | 98.8 | 4.5e-11 | 8.2e-17 | 88.7 | 77 | (2, 78) | 372 | (15, 92) | 101 | hypothetical protein | hypothetical protein | | uniclust | UniRef100\_UPI0007C58163 | 98.8 | 4.5e-11 | 8.3e-17 | 94.2 | 95 | (188, 291) | 372 | (10, 107) | 143 | PD-(D/E)XK nuclease-like domain-containing protein | PD-(D/E)XK nuclease-like domain-containing protein | | uniclust | UniRef100\_A0A2D9CKI8 | 98.8 | 4.6e-11 | 8.5e-17 | 108.6 | 138 | (198, 335) | 372 | (186, 348) | 399 | Putative exodeoxyribonuclease 8 PDDEXK-like domain-containing protein | Putative exodeoxyribonuclease 8 PDDEXK-like domain-containing protein | | uniclust | UniRef100\_UPI0020A48B88 | 98.8 | 5.3e-11 | 9.8e-17 | 102.2 | 95 | (231, 326) | 372 | (132, 230) | 254 | PD-(D/E)XK nuclease-like domain-containing protein | PD-(D/E)XK nuclease-like domain-containing protein | | uniclust | UniRef100\_A0A1V1FV31 | 98.8 | 5.3e-11 | 9.8e-17 | 105.1 | 152 | (188, 342) | 372 | (120, 290) | 314 | Exonucleaase VIII | Exonucleaase VIII | | uniclust | UniRef100\_A0A0F9G4G2 | 98.8 | 5.5e-11 | 1e-16 | 89.9 | 91 | (272, 363) | 372 | (4, 96) | 112 | Putative exodeoxyribonuclease 8 PDDEXK-like domain-containing protein (Fragment) | Putative exodeoxyribonuclease 8 PDDEXK-like domain-containing protein (Fragment) | | uniclust | UniRef100\_A0A3D2SYI2 | 98.8 | 5.8e-11 | 1.1e-16 | 101.9 | 70 | (236, 307) | 372 | (147, 216) | 252 | Putative exodeoxyribonuclease 8 PDDEXK-like domain-containing protein | Putative exodeoxyribonuclease 8 PDDEXK-like domain-containing protein | | uniclust | UniRef100\_A0A3N5V3F8 | 98.8 | 6e-11 | 1.1e-16 | 95.2 | 74 | (2, 77) | 372 | (28, 102) | 160 | Uncharacterized protein (Fragment) | Uncharacterized protein (Fragment) | | uniclust | UniRef100\_A0A1F2QKG7 | 98.8 | 5.5e-11 | 1.1e-16 | 113.5 | 101 | (236, 346) | 372 | (158, 264) | 320 | PD-(D/E)XK endonuclease-like domain-containing protein | PD-(D/E)XK endonuclease-like domain-containing protein | | uniclust | UniRef100\_A0A1M3BII6 | 98.8 | 5.5e-11 | 1.1e-16 | 133.0 | 145 | (193, 348) | 372 | (1236, 1383) | 1448 | DNA 3'-5' helicase (Fragment) | DNA 3'-5' helicase (Fragment) | | uniclust | UniRef100\_A0A1F9WEL2 | 98.8 | 6.9e-11 | 1.3e-16 | 111.8 | 103 | (198, 312) | 372 | (105, 211) | 399 | PD-(D/E)XK endonuclease-like domain-containing protein (Fragment) | PD-(D/E)XK endonuclease-like domain-containing protein (Fragment) | | uniclust | UniRef100\_A0A377TQV8 | 98.8 | 7.6e-11 | 1.4e-16 | 105.4 | 100 | (2, 103) | 372 | (239, 339) | 344 | Putative exodeoxyribonuclease VIII | Putative exodeoxyribonuclease VIII | | uniclust | UniRef100\_UPI001F44166F | 98.7 | 8e-11 | 1.5e-16 | 92.9 | 117 | (253, 370) | 372 | (12, 140) | 143 | PD-(D/E)XK nuclease-like domain-containing protein | PD-(D/E)XK nuclease-like domain-containing protein | | uniclust | UniRef100\_A0A1Z8ZBW6 | 98.7 | 7.7e-11 | 1.6e-16 | 128.0 | 121 | (225, 362) | 372 | (881, 1015) | 1018 | DNA 3'-5' helicase | DNA 3'-5' helicase | | uniclust | UniRef100\_UPI00208F1201 | 98.7 | 8.9e-11 | 1.6e-16 | 85.6 | 64 | (1, 69) | 372 | (1, 64) | 91 | hypothetical protein | hypothetical protein | | uniclust | UniRef100\_A0A0G2BII5 | 98.7 | 8.3e-11 | 1.6e-16 | 108.7 | 101 | (225, 337) | 372 | (143, 246) | 285 | UvrD/REP helicase (Fragment) | UvrD/REP helicase (Fragment) | | uniclust | UniRef100\_A0A3N0GKK1 | 98.7 | 8.7e-11 | 1.7e-16 | 110.1 | 103 | (235, 348) | 372 | (159, 264) | 332 | PD-(D/E)XK endonuclease-like domain-containing protein | PD-(D/E)XK endonuclease-like domain-containing protein | | uniclust | UniRef100\_UPI000B0585E9 | 98.7 | 1.1e-10 | 2e-16 | 96.0 | 73 | (188, 262) | 372 | (101, 175) | 185 | PD-(D/E)XK nuclease-like domain-containing protein | PD-(D/E)XK nuclease-like domain-containing protein | | uniclust | UniRef100\_A0A1Q7DWU8 | 98.7 | 1e-10 | 2.1e-16 | 119.7 | 110 | (225, 350) | 372 | (606, 719) | 738 | DNA 3'-5' helicase | DNA 3'-5' helicase | | uniclust | UniRef100\_A0A0G1VV66 | 98.7 | 1.1e-10 | 2.1e-16 | 121.3 | 146 | (190, 337) | 372 | (617, 778) | 814 | DNA 3'-5' helicase (Fragment) | DNA 3'-5' helicase (Fragment) | | uniclust | UniRef100\_A0A0P6X7G0 | 98.7 | 1.3e-10 | 2.6e-16 | 108.0 | 104 | (236, 350) | 372 | (155, 262) | 288 | PD-(D/E)XK endonuclease-like domain-containing protein | PD-(D/E)XK endonuclease-like domain-containing protein | | uniclust | UniRef100\_UPI00214D1424 | 98.7 | 1.4e-10 | 2.6e-16 | 86.1 | 69 | (298, 366) | 372 | (2, 70) | 100 | PD-(D/E)XK nuclease-like domain-containing protein | PD-(D/E)XK nuclease-like domain-containing protein | | uniclust | UniRef100\_UPI000DFB18C6 | 98.7 | 1.5e-10 | 2.7e-16 | 88.4 | 105 | (263, 368) | 372 | (3, 111) | 117 | PD-(D/E)XK nuclease-like domain-containing protein | PD-(D/E)XK nuclease-like domain-containing protein | | uniclust | UniRef100\_A0A537TML3 | 98.7 | 1.5e-10 | 2.8e-16 | 116.8 | 146 | (193, 351) | 372 | (508, 658) | 692 | DNA 3'-5' helicase (Fragment) | DNA 3'-5' helicase (Fragment) | | uniclust | UniRef100\_A0A0F9KKT2 | 98.7 | 1.4e-10 | 3e-16 | 107.8 | 117 | (237, 366) | 372 | (134, 260) | 265 | PD-(D/E)XK endonuclease-like domain-containing protein | PD-(D/E)XK endonuclease-like domain-containing protein | | uniclust | UniRef100\_UPI001E3ED308 | 98.7 | 1.7e-10 | 3.1e-16 | 95.0 | 164 | (7, 230) | 372 | (12, 185) | 185 | hypothetical protein | hypothetical protein | | uniclust | UniRef100\_A0A2V9BQB0 | 98.7 | 1.7e-10 | 3.3e-16 | 119.3 | 110 | (225, 350) | 372 | (632, 745) | 778 | DNA 3'-5' helicase | DNA 3'-5' helicase | | uniclust | UniRef100\_A0A1Q7DKN2 | 98.7 | 1.7e-10 | 3.4e-16 | 122.4 | 110 | (225, 350) | 372 | (855, 968) | 1013 | DNA 3'-5' helicase | DNA 3'-5' helicase | | uniclust | UniRef100\_A0A0C1UKG6 | 98.7 | 1.6e-10 | 3.4e-16 | 130.1 | 157 | (188, 350) | 372 | (859, 1034) | 1119 | PD-(D/E)XK endonuclease-like domain-containing protein | PD-(D/E)XK endonuclease-like domain-containing protein | | uniclust | UniRef100\_A0A973F465 | 98.7 | 1.9e-10 | 3.5e-16 | 100.2 | 228 | (9, 339) | 372 | (4, 241) | 277 | Putative exodeoxyribonuclease 8 PDDEXK-like domain-containing protein | Putative exodeoxyribonuclease 8 PDDEXK-like domain-containing protein | | uniclust | UniRef100\_UPI0020BD75F5 | 98.7 | 1.9e-10 | 3.5e-16 | 96.9 | 146 | (195, 346) | 372 | (32, 193) | 217 | PD-(D/E)XK nuclease-like domain-containing protein | PD-(D/E)XK nuclease-like domain-containing protein | | uniclust | UniRef100\_A0A1V5YSQ9 | 98.7 | 1.8e-10 | 3.8e-16 | 127.5 | 130 | (196, 337) | 372 | (1038, 1167) | 1194 | DNA 3'-5' helicase | DNA 3'-5' helicase | | uniclust | UniRef100\_A0A0B3AHC3 | 98.7 | 2e-10 | 4e-16 | 111.8 | 105 | (225, 349) | 372 | (193, 300) | 375 | PD-(D/E)XK endonuclease-like domain-containing protein | PD-(D/E)XK endonuclease-like domain-containing protein | | uniclust | UniRef100\_A0A101HUX6 | 98.7 | 2e-10 | 4.1e-16 | 121.5 | 112 | (226, 349) | 372 | (626, 740) | 766 | DNA 3'-5' helicase (Fragment) | DNA 3'-5' helicase (Fragment) | | uniclust | UniRef100\_UPI0007C48233 | 98.7 | 2.3e-10 | 4.2e-16 | 104.6 | 134 | (5, 231) | 372 | (254, 389) | 392 | hypothetical protein | hypothetical protein | | uniclust | UniRef100\_A0A1G1TB27 | 98.7 | 2.1e-10 | 4.2e-16 | 123.7 | 146 | (191, 338) | 372 | (865, 1040) | 1092 | DNA 3'-5' helicase | DNA 3'-5' helicase | | uniclust | UniRef100\_A0A074LL59 | 98.7 | 2e-10 | 4.5e-16 | 130.0 | 146 | (196, 349) | 372 | (1035, 1188) | 1220 | DNA 3'-5' helicase | DNA 3'-5' helicase | | uniclust | UniRef100\_A0A3Q8YTG7 | 98.7 | 2.5e-10 | 4.7e-16 | 89.9 | 106 | (253, 359) | 372 | (14, 124) | 140 | Putative exodeoxyribonuclease 8 PDDEXK-like domain-containing protein | Putative exodeoxyribonuclease 8 PDDEXK-like domain-containing protein | | uniclust | UniRef100\_A0A0G0MTT2 | 98.6 | 2.4e-10 | 4.9e-16 | 108.8 | 135 | (197, 350) | 372 | (118, 264) | 302 | RecB family exonuclease | RecB family exonuclease | | uniclust | UniRef100\_A0A1F2X2I5 | 98.6 | 2.5e-10 | 5.3e-16 | 126.8 | 159 | (188, 350) | 372 | (888, 1062) | 1142 | PD-(D/E)XK endonuclease-like domain-containing protein | PD-(D/E)XK endonuclease-like domain-containing protein | | uniclust | UniRef100\_A0A2W4MB63 | 98.6 | 2.8e-10 | 5.4e-16 | 112.3 | 158 | (190, 364) | 372 | (390, 558) | 563 | DNA 3'-5' helicase | DNA 3'-5' helicase | | uniclust | UniRef100\_A0A199YTG5 | 98.6 | 2.9e-10 | 5.6e-16 | 120.0 | 140 | (195, 338) | 372 | (827, 994) | 1330 | DNA 3'-5' helicase | DNA 3'-5' helicase | | uniclust | UniRef100\_A0A0S7XKG7 | 98.6 | 2.8e-10 | 6.1e-16 | 126.2 | 141 | (198, 344) | 372 | (917, 1061) | 1088 | DNA 3'-5' helicase (Fragment) | DNA 3'-5' helicase (Fragment) | | uniclust | UniRef100\_UPI001E3DABD6 | 98.6 | 3.7e-10 | 6.8e-16 | 84.6 | 86 | (252, 338) | 372 | (7, 94) | 104 | PD-(D/E)XK nuclease-like domain-containing protein | PD-(D/E)XK nuclease-like domain-containing protein | | uniclust | UniRef100\_A0A2E4L226 | 98.6 | 3.6e-10 | 6.8e-16 | 114.3 | 121 | (225, 361) | 372 | (654, 791) | 793 | DNA 3'-5' helicase | DNA 3'-5' helicase | | uniclust | UniRef100\_A0A0G1JH46 | 98.6 | 3.4e-10 | 6.9e-16 | 124.8 | 104 | (225, 338) | 372 | (1253, 1360) | 1396 | DNA 3'-5' helicase | DNA 3'-5' helicase | | uniclust | UniRef100\_A0A6N8NPZ1 | 98.6 | 4.2e-10 | 7.7e-16 | 93.6 | 112 | (136, 247) | 372 | (47, 189) | 190 | Exodeoxyribonuclease VIII (Fragment) | Exodeoxyribonuclease VIII (Fragment) | | uniclust | UniRef100\_A0A0B3ADA3 | 98.6 | 4.1e-10 | 8.4e-16 | 110.0 | 113 | (226, 350) | 372 | (170, 288) | 374 | PD-(D/E)XK endonuclease-like domain-containing protein | PD-(D/E)XK endonuclease-like domain-containing protein | | uniclust | UniRef100\_UPI000AE98C0E | 98.6 | 4.4e-10 | 8.4e-16 | 94.6 | 87 | (196, 287) | 372 | (71, 165) | 175 | PD-(D/E)XK nuclease-like domain-containing protein | PD-(D/E)XK nuclease-like domain-containing protein | | pdb70 | 3H4R\_A | 99.2 | 2.6e-15 | 3.1e-19 | 131.8 | 133 | (225, 364) | 372 | (126, 262) | 265 | Exodeoxyribonuclease 8 (E.C.3.1.11.-) | 3H4R\_A Exodeoxyribonuclease 8 (E.C.3.1.11.-) Exonuclease, Recombination, Hydrolase, Nuclease | | pdb70 | 5ZYU\_A | 97.7 | 1.5e-08 | 1.7e-12 | 89.6 | 116 | (224, 348) | 372 | (130, 250) | 254 | Mitochondrial genome maintenance exonuclease 1/DNA | 5ZYU\_A Mitochondrial genome maintenance exonuclease 1/DNA huamnMGME1, DNA complex, DNA exonuclease | | pdb70 | 5ZYW\_A | 97.7 | 1.5e-08 | 1.7e-12 | 89.6 | 116 | (224, 348) | 372 | (130, 250) | 254 | Mitochondrial genome maintenance exonuclease 1 | 5ZYW\_A Mitochondrial genome maintenance exonuclease 1 huamnMGME1, DNA complex, DNA exonuclease HET: TLA | | pdb70 | 5ZYT\_D | 97.6 | 3.2e-08 | 3.4e-12 | 92.3 | 115 | (224, 347) | 372 | (200, 319) | 324 | Mitochondrial genome maintenance exonuclease 1/DNA | 5ZYT\_D Mitochondrial genome maintenance exonuclease 1/DNA human MGME1, DNA complex, DNA | | pdb70 | 3L0A\_A | 97.4 | 1.2e-07 | 1.4e-11 | 83.5 | 119 | (225, 349) | 372 | (114, 240) | 266 | Putative exonuclease | 3L0A\_A Putative exonuclease RER070207002219, Putative exonuclease, Structural Genomics HET: PE4 | | pdb70 | 6PPJ\_A | 96.9 | 1.2e-06 | 1.5e-10 | 88.3 | 103 | (237, 349) | 372 | (911, 1023) | 1045 | UvrD/REP helicase (E.C.3.6.4.12), ATP-dependent DNA | 6PPJ\_A UvrD/REP helicase (E.C.3.6.4.12), ATP-dependent DNA DNA BINDING PROTEIN HET: ANP | | pdb70 | 6PPR\_A | 96.9 | 1.2e-06 | 1.5e-10 | 88.3 | 103 | (237, 349) | 372 | (911, 1023) | 1045 | UvrD/REP helicase, ATP-dependent DNA helicase | 6PPR\_A UvrD/REP helicase, ATP-dependent DNA helicase DNA, DNA BINDING PROTEIN, DNA HET: ANP | | pdb70 | 6PPU\_A | 96.9 | 1.3e-06 | 1.6e-10 | 85.3 | 103 | (237, 349) | 372 | (590, 702) | 724 | UvrD/REP helicase, ATP-dependent DNA helicase | 6PPU\_A UvrD/REP helicase, ATP-dependent DNA helicase DNA, DNA BINDING PROTEIN, DNA | | pdb70 | 6PPR\_B | 96.6 | 4e-06 | 4.9e-10 | 85.4 | 104 | (223, 341) | 372 | (979, 1088) | 1095 | UvrD/REP helicase, ATP-dependent DNA helicase | 6PPR\_B UvrD/REP helicase, ATP-dependent DNA helicase DNA, DNA BINDING PROTEIN, DNA HET: ANP | | pdb70 | 6PPJ\_B | 96.6 | 3.9e-06 | 4.9e-10 | 85.1 | 77 | (224, 307) | 372 | (980, 1062) | 1095 | UvrD/REP helicase (E.C.3.6.4.12), ATP-dependent DNA | 6PPJ\_B UvrD/REP helicase (E.C.3.6.4.12), ATP-dependent DNA DNA BINDING PROTEIN HET: ANP | | pdb70 | 3U4Q\_B | 96.3 | 1.5e-05 | 1.9e-09 | 81.3 | 50 | (238, 291) | 372 | (936, 992) | 1166 | ATP-dependent helicase/nuclease subunit A (E.C.3.1.-.- | 3U4Q\_B ATP-dependent helicase/nuclease subunit A (E.C.3.1.-.- Helicase, nuclease, Double strand DNA | | pdb70 | 4CEI\_B | 96.3 | 1.5e-05 | 1.9e-09 | 81.3 | 50 | (238, 291) | 372 | (936, 992) | 1166 | ATP-DEPENDENT HELICASE/NUCLEASE SUBUNIT A (E.C.3.1.-.- | 4CEI\_B ATP-DEPENDENT HELICASE/NUCLEASE SUBUNIT A (E.C.3.1.-.- HYDROLASE-DNA COMPLEX, HELICASE-NUCLEASE, BACTERIAL PROTEINS HET: ANP | | pdb70 | 4CEJ\_B | 96.3 | 1.5e-05 | 1.9e-09 | 81.3 | 50 | (238, 291) | 372 | (936, 992) | 1166 | ATP-DEPENDENT HELICASE/NUCLEASE SUBUNIT A (E.C.3.1.-.- | 4CEJ\_B ATP-DEPENDENT HELICASE/NUCLEASE SUBUNIT A (E.C.3.1.-.- HYDROLASE-DNA COMPLEX, HELICASE-NUCLEASE, DNA BREAKS HET: ANP | | pdb70 | 4IC1\_D | 95.9 | 4.3e-05 | 5.2e-09 | 62.7 | 58 | (238, 307) | 372 | (95, 156) | 206 | Uncharacterized protein | 4IC1\_D Uncharacterized protein Cas4, CRISPR, MCSG, Exonuclease, PSI-Biology HET: MN, SF4 | | pdb70 | 4IC1\_I | 95.9 | 4.3e-05 | 5.2e-09 | 62.7 | 58 | (238, 307) | 372 | (95, 156) | 206 | Uncharacterized protein | 4IC1\_I Uncharacterized protein Cas4, CRISPR, MCSG, Exonuclease, PSI-Biology HET: SF4, MN, MSE | | pdb70 | 3U4Q\_A | 95.8 | 6.8e-05 | 8.1e-09 | 77.9 | 83 | (222, 306) | 372 | (1124, 1226) | 1232 | ATP-dependent helicase/nuclease subunit A (E.C.3.1.-.- | 3U4Q\_A ATP-dependent helicase/nuclease subunit A (E.C.3.1.-.- Helicase, nuclease, Double strand DNA | | pdb70 | 4CEJ\_A | 95.8 | 6.8e-05 | 8.1e-09 | 77.9 | 83 | (222, 306) | 372 | (1124, 1226) | 1232 | ATP-DEPENDENT HELICASE/NUCLEASE SUBUNIT A (E.C.3.1.-.- | 4CEJ\_A ATP-DEPENDENT HELICASE/NUCLEASE SUBUNIT A (E.C.3.1.-.- HYDROLASE-DNA COMPLEX, HELICASE-NUCLEASE, DNA BREAKS HET: ANP | | pdb70 | 4CEI\_A | 95.8 | 7e-05 | 8.3e-09 | 77.8 | 83 | (222, 306) | 372 | (1124, 1226) | 1232 | ATP-DEPENDENT HELICASE/NUCLEASE SUBUNIT A (E.C.3.1.-.- | 4CEI\_A ATP-DEPENDENT HELICASE/NUCLEASE SUBUNIT A (E.C.3.1.-.- HYDROLASE-DNA COMPLEX, HELICASE-NUCLEASE, BACTERIAL PROTEINS HET: ANP | | pdb70 | 5EAN\_A | 95.6 | 0.0001 | 1.2e-08 | 76.4 | 71 | (223, 307) | 372 | (262, 343) | 1059 | DNA replication ATP-dependent helicase/nuclease DNA2/DNA | 5EAN\_A DNA replication ATP-dependent helicase/nuclease DNA2/DNA DNA binding protein, Hydrolase-DNA complex HET: ADP | | pdb70 | 1W36\_B | 95.2 | 0.00021 | 2.5e-08 | 74.0 | 54 | (238, 293) | 372 | (1059, 1123) | 1180 | EXODEOXYRIBONUCLEASE V BETA CHAIN (E.C.3.1.11.5) | 1W36\_B EXODEOXYRIBONUCLEASE V BETA CHAIN (E.C.3.1.11.5) RECOMBINATION, HELICASE, NUCLEASE, HYDROLASE, DNA | |
| Top keywords  (threshold 1.00e-03 (evalue)) | **domain\_containing, exodeoxyribonuclease, Putative, PDDEXK\_like, E, PD\_, D, XK, nuclease\_like, DNA** |
| Output files | ../../similar\_sequences/41\_FANPEZAQ\_CDS\_0041\_merged.svg ../../similar\_sequences/41\_FANPEZAQ\_CDS\_0041\_pdb70.a3m ../../similar\_sequences/41\_FANPEZAQ\_CDS\_0041\_pdb70.hhr ../../similar\_sequences/41\_FANPEZAQ\_CDS\_0041\_uniclust.a3m ../../similar\_sequences/41\_FANPEZAQ\_CDS\_0041\_uniclust.hhr |

#### Structure prediction (AlphaFold)2

|  |  |
| --- | --- |
| Stats | xml version="1.0" encoding="utf-8" standalone="no"?       2024-09-02T21:09:40.359380 image/svg+xml   Matplotlib v3.7.2, https://matplotlib.org/ |
| Predicted structure | **NGL Viewer Controls:**  - Center: *Left-Click* - Rotate: *Left-Click + Drag* - Translate: *Right-Click + Drag* - Zoom: *Shift + Left-Click + Drag* |
| Output files | ../../predicted\_structures/41\_FANPEZAQ\_CDS\_0041/features.pkl ../../predicted\_structures/41\_FANPEZAQ\_CDS\_0041/ranked\_0.pdb ../../predicted\_structures/41\_FANPEZAQ\_CDS\_0041/ranked\_0\_plots.svg ../../predicted\_structures/41\_FANPEZAQ\_CDS\_0041/result\_model\_1\_ptm\_pred\_0.pkl |

#### Structure similarity search results (Foldseek)3

|  |  |
| --- | --- |
| Structure databases searched | Pdb, Afdb-proteome, Afdb-uniprot50 |
| Results, scheme(s)  (Top layers only, threshold 1.00e-02 (evalue)) | xml version="1.0" encoding="utf-8" standalone="no"?       2024-09-02T21:11:17.549784 image/svg+xml   Matplotlib v3.7.2, https://matplotlib.org/ |
| Results, table  (threshold 1.00e-02 (evalue)) | | db | id | prob | evalue | bits | fident | alnlen | mismatch | gapopen | qstart | qend | tstart | tend | name | description | | --- | --- | --- | --- | --- | --- | --- | --- | --- | --- | --- | --- | --- | --- | --- | | pdb | 3H4R\_A | 1.0 | 9.159e-15 | 534 | 0.236 | 347 | 128 | 8 | 18 | 360 | 1 | 214 | Exodeoxyribonuclease 8 | Exodeoxyribonuclease 8 | | pdb | 3L0A\_A | 1.0 | 1.235e-07 | 191 | 0.161 | 241 | 154 | 13 | 142 | 356 | 40 | 258 | Putative exonuclease | Putative exonuclease | | pdb | 5ZYU\_B | 0.998 | 0.001179 | 93 | 0.14 | 264 | 163 | 17 | 98 | 344 | 7 | 223 | Mitochondrial genome maintenance exonuclease 1 | Mitochondrial genome maintenance exonuclease 1 | | pdb | 5ZYT\_A | 0.997 | 0.001054 | 92 | 0.123 | 252 | 164 | 16 | 97 | 342 | 19 | 219 | Mitochondrial genome maintenance exonuclease 1 | Mitochondrial genome maintenance exonuclease 1 | | pdb | 4IC1\_C | 0.991 | 0.008437 | 84 | 0.133 | 142 | 99 | 8 | 199 | 331 | 51 | 177 | Uncharacterized protein | Uncharacterized protein | | pdb | 3SZ5\_A | 0.933 | 0.004809 | 70 | 0.125 | 200 | 132 | 12 | 155 | 343 | 22 | 189 | Exonuclease | Exonuclease | | pdb | 4CEH\_A | 0.837 | 0.004809 | 63 | 0.123 | 323 | 149 | 19 | 6 | 315 | 953 | 1154 | ATP-DEPENDENT HELICASE/NUCLEASE SUBUNIT A | ATP-DEPENDENT HELICASE/NUCLEASE SUBUNIT A | | afdb-proteome | AF-Q8XF26-F1-MODEL\_V4 | 1.0 | 1.427e-24 | 759 | 0.222 | 436 | 265 | 12 | 1 | 372 | 536 | 961 | Gifsy-1 prophage protein | Gifsy-1 prophage protein | | afdb-proteome | AF-P15032-F1-MODEL\_V4 | 1.0 | 5.85e-22 | 668 | 0.274 | 368 | 164 | 9 | 1 | 364 | 595 | 863 | Exodeoxyribonuclease 8 | Exodeoxyribonuclease 8 | | afdb-proteome | AF-Q7AQ72-F1-MODEL\_V4 | 1.0 | 7.175e-08 | 194 | 0.119 | 351 | 202 | 15 | 7 | 351 | 4 | 253 | PDDEXK\_1 domain-containing protein | PDDEXK\_1 domain-containing protein | | afdb-proteome | AF-O33254-F1-MODEL\_V4 | 1.0 | 1.764e-07 | 188 | 0.141 | 353 | 180 | 17 | 9 | 351 | 9 | 248 | PDDEXK\_1 domain-containing protein | PDDEXK\_1 domain-containing protein | | afdb-proteome | AF-A0A077ZMK2-F1-MODEL\_V4 | 1.0 | 3.29e-05 | 127 | 0.181 | 248 | 164 | 13 | 101 | 322 | 878 | 1112 | DUF4494 and SNF2 N domain containing protein | DUF4494 and SNF2 N domain containing protein | | afdb-proteome | AF-K0F1Q7-F1-MODEL\_V4 | 1.0 | 2.484e-05 | 114 | 0.129 | 286 | 185 | 14 | 86 | 364 | 2 | 230 | Putative RecB family exonuclease | Putative RecB family exonuclease | | afdb-proteome | AF-A0A3Q0KJI8-F1-MODEL\_V4 | 0.999 | 0.000437 | 99 | 0.154 | 253 | 159 | 20 | 108 | 342 | 41 | 256 | Mitochondrial genome maintenance exonuclease 1 | Mitochondrial genome maintenance exonuclease 1 | | afdb-proteome | AF-Q55EN1-F1-MODEL\_V4 | 0.999 | 6.834e-05 | 98 | 0.127 | 360 | 228 | 20 | 11 | 360 | 72 | 355 | PDDEXK\_1 domain-containing protein | PDDEXK\_1 domain-containing protein | | afdb-proteome | AF-A9QY30-F1-MODEL\_V4 | 0.998 | 0.0005472 | 95 | 0.136 | 292 | 168 | 19 | 99 | 352 | 39 | 284 | Mitochondrial genome maintenance exonuclease 1 | Mitochondrial genome maintenance exonuclease 1 | | afdb-proteome | AF-Q5PPI6-F1-MODEL\_V4 | 0.975 | 0.002955 | 77 | 0.135 | 281 | 154 | 18 | 102 | 353 | 118 | 338 | Mitochondrial genome maintenance exonuclease 1 | Mitochondrial genome maintenance exonuclease 1 | | afdb-uniprot50 | AF-A0A158E8Q1-F1-MODEL\_V4 | 1.0 | 8.188e-48 | 1795 | 0.577 | 372 | 149 | 5 | 1 | 369 | 1 | 367 | Exonuclease VIII | Exonuclease VIII | | afdb-uniprot50 | AF-A0A8A6KNI9-F1-MODEL\_V4 | 1.0 | 2.488e-30 | 1192 | 0.36 | 374 | 136 | 6 | 1 | 366 | 4 | 282 | PD-(D/E)XK nuclease-like domain-containing protein | PD-(D/E)XK nuclease-like domain-containing protein | | afdb-uniprot50 | AF-A0A1V1UMV8-F1-MODEL\_V4 | 1.0 | 1.496e-31 | 1136 | 0.355 | 385 | 179 | 7 | 1 | 372 | 7 | 335 | Exodeoxyribonuclease 8 | Exodeoxyribonuclease 8 | | afdb-uniprot50 | AF-A0A4R2KU57-F1-MODEL\_V4 | 1.0 | 5.141e-32 | 1115 | 0.388 | 381 | 165 | 7 | 1 | 371 | 7 | 329 | PDDEXK-like uncharacterized protein DUF3799 | PDDEXK-like uncharacterized protein DUF3799 | | afdb-uniprot50 | AF-A0A2M7C2P3-F1-MODEL\_V4 | 1.0 | 8.184e-26 | 994 | 0.313 | 361 | 147 | 7 | 5 | 363 | 4 | 265 | Exodeoxyribonuclease VIII | Exodeoxyribonuclease VIII | | afdb-uniprot50 | AF-R1H4T8-F1-MODEL\_V4 | 1.0 | 2.112e-28 | 993 | 0.291 | 436 | 229 | 11 | 2 | 369 | 100 | 523 | DUF3799 domain-containing protein | DUF3799 domain-containing protein | | afdb-uniprot50 | AF-A0A7D5NWS8-F1-MODEL\_V4 | 1.0 | 3.131e-28 | 992 | 0.264 | 435 | 244 | 10 | 1 | 369 | 21 | 445 | PD-(D/E)XK nuclease-like domain-containing protein | PD-(D/E)XK nuclease-like domain-containing protein | | afdb-uniprot50 | AF-A0A1D2X562-F1-MODEL\_V4 | 1.0 | 1.691e-27 | 973 | 0.297 | 363 | 193 | 10 | 1 | 360 | 1 | 304 | DUF3799 domain-containing protein | DUF3799 domain-containing protein | | afdb-uniprot50 | AF-A0A349EF37-F1-MODEL\_V4 | 1.0 | 2.252e-25 | 954 | 0.327 | 363 | 142 | 6 | 4 | 364 | 7 | 269 | Exodeoxyribonuclease VIII | Exodeoxyribonuclease VIII | | afdb-uniprot50 | AF-D0XHM1-F1-MODEL\_V4 | 1.0 | 2.826e-24 | 946 | 0.308 | 357 | 144 | 7 | 8 | 363 | 2 | 256 | DUF3799 domain-containing protein | DUF3799 domain-containing protein | | afdb-uniprot50 | AF-A0A1A7Q996-F1-MODEL\_V4 | 1.0 | 7.332e-25 | 945 | 0.355 | 352 | 125 | 6 | 9 | 360 | 1 | 250 | Exodeoxyribonuclease VIII | Exodeoxyribonuclease VIII | | afdb-uniprot50 | AF-A0A1H0UVZ5-F1-MODEL\_V4 | 1.0 | 5.233e-25 | 920 | 0.327 | 372 | 148 | 10 | 1 | 366 | 4 | 279 | Exodeoxyribonuclease VIII | Exodeoxyribonuclease VIII | | afdb-uniprot50 | AF-A0A6J4XAC9-F1-MODEL\_V4 | 1.0 | 1.611e-24 | 917 | 0.297 | 360 | 156 | 6 | 1 | 360 | 5 | 267 | DUF3799 domain-containing protein | DUF3799 domain-containing protein | | afdb-uniprot50 | AF-A0A4Q6DHW1-F1-MODEL\_V4 | 1.0 | 6.569e-24 | 914 | 0.234 | 371 | 184 | 6 | 1 | 370 | 1 | 272 | DUF3799 domain-containing protein | DUF3799 domain-containing protein | | afdb-uniprot50 | AF-A0A227JR50-F1-MODEL\_V4 | 1.0 | 4.653e-27 | 906 | 0.341 | 372 | 158 | 12 | 5 | 370 | 46 | 336 | DUF3799 domain-containing protein | DUF3799 domain-containing protein | | afdb-uniprot50 | AF-A0A4D8QPW1-F1-MODEL\_V4 | 1.0 | 3.163e-24 | 903 | 0.287 | 362 | 154 | 9 | 1 | 361 | 9 | 267 | DUF3799 domain-containing protein | DUF3799 domain-containing protein | | afdb-uniprot50 | AF-A0A0K1QBP3-F1-MODEL\_V4 | 1.0 | 1.44e-24 | 903 | 0.308 | 363 | 152 | 10 | 1 | 360 | 19 | 285 | Exodeoxyribonuclease VIII | Exodeoxyribonuclease VIII | | afdb-uniprot50 | AF-A0A800EYB9-F1-MODEL\_V4 | 1.0 | 3.971e-23 | 897 | 0.252 | 372 | 178 | 7 | 1 | 369 | 3 | 277 | DUF3799 domain-containing protein | DUF3799 domain-containing protein | | afdb-uniprot50 | AF-A0A6M3KVF3-F1-MODEL\_V4 | 1.0 | 2.022e-23 | 896 | 0.281 | 373 | 167 | 10 | 1 | 371 | 6 | 279 | DUF3799 domain-containing protein | DUF3799 domain-containing protein | | afdb-uniprot50 | AF-A0A158DVS7-F1-MODEL\_V4 | 1.0 | 1.523e-24 | 891 | 0.267 | 366 | 164 | 10 | 2 | 361 | 8 | 275 | Exonuclease VIII | Exonuclease VIII | | afdb-uniprot50 | AF-A0A2M7J5F5-F1-MODEL\_V4 | 1.0 | 1.607e-25 | 889 | 0.313 | 361 | 147 | 7 | 5 | 363 | 65 | 326 | Exodeoxyribonuclease VIII | Exodeoxyribonuclease VIII | | afdb-uniprot50 | AF-A0A3B7N4J6-F1-MODEL\_V4 | 1.0 | 6.194e-25 | 887 | 0.301 | 371 | 156 | 11 | 1 | 367 | 1 | 272 | Exodeoxyribonuclease VIII | Exodeoxyribonuclease VIII | | afdb-uniprot50 | AF-A0A7W8M817-F1-MODEL\_V4 | 1.0 | 4.959e-24 | 887 | 0.276 | 373 | 165 | 9 | 1 | 367 | 9 | 282 | Exodeoxyribonuclease VIII | Exodeoxyribonuclease VIII | | afdb-uniprot50 | AF-A0A7C5FTZ9-F1-MODEL\_V4 | 1.0 | 2.394e-23 | 883 | 0.298 | 362 | 155 | 8 | 5 | 366 | 2 | 264 | Exodeoxyribonuclease VIII | Exodeoxyribonuclease VIII | | afdb-uniprot50 | AF-C5A7V4-F1-MODEL\_V4 | 1.0 | 4.2e-23 | 883 | 0.282 | 364 | 152 | 7 | 5 | 360 | 2 | 264 | Exonuclease VIII, 5'-3' specific dsDNA exonuclease | Exonuclease VIII, 5'-3' specific dsDNA exonuclease | | afdb-uniprot50 | AF-A0A5C5DDI0-F1-MODEL\_V4 | 1.0 | 8.679e-25 | 874 | 0.239 | 389 | 265 | 10 | 1 | 372 | 118 | 492 | DUF3799 domain-containing protein | DUF3799 domain-containing protein | | afdb-uniprot50 | AF-A0A838BVQ7-F1-MODEL\_V4 | 1.0 | 4.179e-25 | 872 | 0.328 | 362 | 138 | 8 | 1 | 360 | 1 | 259 | PD-(D/E)XK nuclease-like domain-containing protein | PD-(D/E)XK nuclease-like domain-containing protein | | afdb-uniprot50 | AF-A0A2G2QFT7-F1-MODEL\_V4 | 1.0 | 5.246e-24 | 871 | 0.291 | 371 | 161 | 6 | 1 | 371 | 14 | 282 | DUF3799 domain-containing protein | DUF3799 domain-containing protein | | afdb-uniprot50 | AF-A0A838LRU1-F1-MODEL\_V4 | 1.0 | 7.737e-26 | 869 | 0.317 | 369 | 183 | 15 | 2 | 363 | 5 | 311 | Uncharacterized protein | Uncharacterized protein | | afdb-uniprot50 | AF-D0Z7N0-F1-MODEL\_V4 | 1.0 | 4.168e-26 | 869 | 0.224 | 436 | 260 | 11 | 1 | 370 | 401 | 824 | Gifsy-1 prophage protein | Gifsy-1 prophage protein | | afdb-uniprot50 | AF-R7GUS0-F1-MODEL\_V4 | 1.0 | 2.532e-23 | 867 | 0.317 | 356 | 146 | 10 | 9 | 364 | 1 | 259 | DUF3799 domain-containing protein | DUF3799 domain-containing protein | | afdb-uniprot50 | AF-A0A2D7CSN2-F1-MODEL\_V4 | 1.0 | 9.205e-24 | 865 | 0.327 | 373 | 143 | 8 | 4 | 372 | 18 | 286 | Exodeoxyribonuclease VIII | Exodeoxyribonuclease VIII | | afdb-uniprot50 | AF-A0A6M3JML4-F1-MODEL\_V4 | 1.0 | 1.293e-22 | 864 | 0.267 | 367 | 162 | 9 | 1 | 364 | 2 | 264 | DUF3799 domain-containing protein | DUF3799 domain-containing protein | | afdb-uniprot50 | AF-A0A2E1R914-F1-MODEL\_V4 | 1.0 | 3.548e-23 | 864 | 0.304 | 364 | 149 | 9 | 2 | 360 | 8 | 272 | Exodeoxyribonuclease VIII | Exodeoxyribonuclease VIII | | afdb-uniprot50 | AF-A0A762GQR2-F1-MODEL\_V4 | 1.0 | 7.314e-26 | 862 | 0.249 | 433 | 250 | 15 | 1 | 369 | 687 | 1108 | Exodeoxyribonuclease | Exodeoxyribonuclease | | afdb-uniprot50 | AF-A0A149U469-F1-MODEL\_V4 | 1.0 | 2.819e-25 | 857 | 0.316 | 373 | 177 | 16 | 1 | 364 | 10 | 313 | DUF3799 domain-containing protein | DUF3799 domain-containing protein | | afdb-uniprot50 | AF-A0A612C5H5-F1-MODEL\_V4 | 1.0 | 1.519e-25 | 853 | 0.236 | 439 | 254 | 13 | 1 | 372 | 546 | 970 | Exodeoxyribonuclease | Exodeoxyribonuclease | | afdb-uniprot50 | AF-A0A5J0RYS5-F1-MODEL\_V4 | 1.0 | 2.382e-25 | 848 | 0.23 | 439 | 257 | 13 | 1 | 372 | 260 | 684 | Exodeoxyribonuclease | Exodeoxyribonuclease | | afdb-uniprot50 | AF-A0A0F9KCK9-F1-MODEL\_V4 | 1.0 | 1.527e-23 | 847 | 0.254 | 366 | 171 | 10 | 1 | 361 | 1 | 269 | DUF3799 domain-containing protein | DUF3799 domain-containing protein | | afdb-uniprot50 | AF-A0A0F9QDF5-F1-MODEL\_V4 | 1.0 | 2.841e-22 | 845 | 0.279 | 354 | 150 | 10 | 9 | 361 | 1 | 250 | DUF3799 domain-containing protein | DUF3799 domain-containing protein | | afdb-uniprot50 | AF-A0A1F9B5U7-F1-MODEL\_V4 | 1.0 | 1.092e-22 | 845 | 0.268 | 365 | 162 | 8 | 1 | 361 | 1 | 264 | DUF3799 domain-containing protein | DUF3799 domain-containing protein | | afdb-uniprot50 | AF-B7UGT8-F1-MODEL\_V4 | 1.0 | 1.615e-23 | 841 | 0.285 | 371 | 162 | 10 | 1 | 367 | 1 | 272 | Predicted exonuclease VIII/RecE-like protein | Predicted exonuclease VIII/RecE-like protein | | afdb-uniprot50 | AF-A0A2G2PPI1-F1-MODEL\_V4 | 1.0 | 1.09e-23 | 841 | 0.273 | 373 | 188 | 9 | 1 | 370 | 1 | 293 | DUF3799 domain-containing protein | DUF3799 domain-containing protein | | afdb-uniprot50 | AF-A0A2G2KSA4-F1-MODEL\_V4 | 1.0 | 3.548e-23 | 834 | 0.288 | 367 | 161 | 7 | 5 | 371 | 20 | 286 | DUF3799 domain-containing protein | DUF3799 domain-containing protein | | afdb-uniprot50 | AF-A0A5J2KB08-F1-MODEL\_V4 | 1.0 | 2.834e-23 | 831 | 0.285 | 371 | 162 | 9 | 1 | 367 | 1 | 272 | Exodeoxyribonuclease VIII | Exodeoxyribonuclease VIII | | afdb-uniprot50 | AF-A0A1Y6CQ16-F1-MODEL\_V4 | 1.0 | 6.967e-23 | 831 | 0.231 | 372 | 183 | 7 | 1 | 371 | 22 | 291 | Exodeoxyribonuclease VIII | Exodeoxyribonuclease VIII | | afdb-uniprot50 | AF-A0A6H0ITG2-F1-MODEL\_V4 | 1.0 | 4.7e-23 | 830 | 0.302 | 360 | 150 | 8 | 4 | 361 | 14 | 274 | DUF3799 domain-containing protein | DUF3799 domain-containing protein | | afdb-uniprot50 | AF-A0A1W9QRF2-F1-MODEL\_V4 | 1.0 | 1.225e-21 | 829 | 0.248 | 366 | 171 | 8 | 5 | 367 | 2 | 266 | DUF3799 domain-containing protein | DUF3799 domain-containing protein | | afdb-uniprot50 | AF-A0A4P5VJR3-F1-MODEL\_V4 | 1.0 | 9.228e-23 | 827 | 0.299 | 364 | 150 | 10 | 3 | 360 | 2 | 266 | DUF3799 domain-containing protein | DUF3799 domain-containing protein | | afdb-uniprot50 | AF-A0A1G2ZKS6-F1-MODEL\_V4 | 1.0 | 2.4e-22 | 827 | 0.255 | 364 | 166 | 10 | 1 | 360 | 4 | 266 | DUF3799 domain-containing protein | DUF3799 domain-containing protein | | afdb-uniprot50 | AF-A0A348HFN7-F1-MODEL\_V4 | 1.0 | 6.586e-23 | 827 | 0.283 | 370 | 153 | 10 | 1 | 362 | 6 | 271 | Dehydrogenases | Dehydrogenases | | afdb-uniprot50 | AF-A0A7U1K146-F1-MODEL\_V4 | 1.0 | 1.364e-23 | 825 | 0.281 | 376 | 157 | 14 | 1 | 367 | 1 | 272 | PD-(D/E)XK nuclease-like domain-containing protein | PD-(D/E)XK nuclease-like domain-containing protein | | afdb-uniprot50 | AF-A0A1J8PMA9-F1-MODEL\_V4 | 1.0 | 9.762e-23 | 825 | 0.28 | 363 | 160 | 12 | 1 | 361 | 11 | 274 | DUF3799 domain-containing protein | DUF3799 domain-containing protein | | afdb-uniprot50 | AF-A0A6G6JMC2-F1-MODEL\_V4 | 1.0 | 2.519e-25 | 825 | 0.238 | 436 | 250 | 13 | 1 | 368 | 480 | 901 | Exodeoxyribonuclease | Exodeoxyribonuclease | | afdb-uniprot50 | AF-E6QW59-F1-MODEL\_V4 | 1.0 | 3.512e-27 | 824 | 0.293 | 447 | 204 | 16 | 3 | 363 | 2 | 422 | Uncharacterized protein | Uncharacterized protein | | afdb-uniprot50 | AF-A0A4V2X429-F1-MODEL\_V4 | 1.0 | 6.586e-23 | 823 | 0.264 | 374 | 163 | 12 | 1 | 365 | 1 | 271 | Exodeoxyribonuclease VIII | Exodeoxyribonuclease VIII | | afdb-uniprot50 | AF-A0A1H3GZQ2-F1-MODEL\_V4 | 1.0 | 5.885e-23 | 823 | 0.267 | 367 | 181 | 10 | 1 | 361 | 3 | 287 | DUF3799 domain-containing protein | DUF3799 domain-containing protein | | afdb-uniprot50 | AF-A0A6M3LLV5-F1-MODEL\_V4 | 1.0 | 4.984e-22 | 820 | 0.27 | 377 | 162 | 10 | 1 | 369 | 1 | 272 | DUF3799 domain-containing protein | DUF3799 domain-containing protein | | afdb-uniprot50 | AF-A0A7T8DU22-F1-MODEL\_V4 | 1.0 | 1.027e-24 | 820 | 0.236 | 435 | 253 | 13 | 1 | 369 | 139 | 560 | PD-(D/E)XK nuclease-like domain-containing protein | PD-(D/E)XK nuclease-like domain-containing protein | | afdb-uniprot50 | AF-A0A7G8SH51-F1-MODEL\_V4 | 1.0 | 1.907e-24 | 818 | 0.216 | 438 | 259 | 14 | 1 | 369 | 498 | 920 | PD-(D/E)XK nuclease-like domain-containing protein | PD-(D/E)XK nuclease-like domain-containing protein | | afdb-uniprot50 | AF-A0A5E4PG85-F1-MODEL\_V4 | 1.0 | 3.354e-23 | 817 | 0.287 | 362 | 165 | 9 | 1 | 360 | 3 | 273 | Exodeoxyribonuclease 8 | Exodeoxyribonuclease 8 | | afdb-uniprot50 | AF-A0A4P7C0W9-F1-MODEL\_V4 | 1.0 | 1.158e-21 | 813 | 0.297 | 343 | 139 | 8 | 1 | 342 | 30 | 271 | Exodeoxyribonuclease VIII | Exodeoxyribonuclease VIII | | afdb-uniprot50 | AF-A0A2D7YR06-F1-MODEL\_V4 | 1.0 | 1.451e-21 | 809 | 0.298 | 355 | 137 | 8 | 9 | 361 | 1 | 245 | DUF3799 domain-containing protein | DUF3799 domain-containing protein | | afdb-uniprot50 | AF-A0A6M3IX22-F1-MODEL\_V4 | 1.0 | 1.156e-22 | 808 | 0.239 | 372 | 184 | 10 | 1 | 370 | 4 | 278 | DUF3799 domain-containing protein | DUF3799 domain-containing protein | | afdb-uniprot50 | AF-A0A663BKX6-F1-MODEL\_V4 | 1.0 | 7.332e-25 | 807 | 0.225 | 443 | 255 | 14 | 1 | 372 | 493 | 918 | Exodeoxyribonuclease 8 | Exodeoxyribonuclease 8 | | afdb-uniprot50 | AF-A0A4Q6DHU3-F1-MODEL\_V4 | 1.0 | 3.179e-22 | 806 | 0.206 | 377 | 194 | 9 | 1 | 371 | 5 | 282 | DUF3799 domain-containing protein | DUF3799 domain-containing protein | | afdb-uniprot50 | AF-A0A149SNY1-F1-MODEL\_V4 | 1.0 | 8.746e-22 | 805 | 0.254 | 369 | 161 | 11 | 9 | 368 | 1 | 264 | DUF3799 domain-containing protein | DUF3799 domain-containing protein | | afdb-uniprot50 | AF-A0A1C6FHM2-F1-MODEL\_V4 | 1.0 | 4.984e-22 | 804 | 0.28 | 364 | 156 | 8 | 7 | 364 | 3 | 266 | Exodeoxyribonuclease 8 | Exodeoxyribonuclease 8 | | afdb-uniprot50 | AF-A0A656QBR2-F1-MODEL\_V4 | 1.0 | 1.035e-21 | 804 | 0.248 | 358 | 171 | 7 | 3 | 360 | 10 | 269 | DUF3799 domain-containing protein | DUF3799 domain-containing protein | | afdb-uniprot50 | AF-A0A5C9BVQ4-F1-MODEL\_V4 | 1.0 | 6.241e-22 | 803 | 0.279 | 365 | 156 | 9 | 4 | 362 | 31 | 294 | DUF3799 domain-containing protein | DUF3799 domain-containing protein | | afdb-uniprot50 | AF-A0A2H9TC24-F1-MODEL\_V4 | 1.0 | 3.548e-23 | 803 | 0.252 | 372 | 213 | 12 | 1 | 368 | 15 | 325 | Exodeoxyribonuclease 8 | Exodeoxyribonuclease 8 | | afdb-uniprot50 | AF-A0A5M7LBW2-F1-MODEL\_V4 | 1.0 | 2.257e-24 | 802 | 0.231 | 436 | 253 | 13 | 1 | 368 | 529 | 950 | Exodeoxyribonuclease | Exodeoxyribonuclease | | afdb-uniprot50 | AF-A0A7S9S4Y9-F1-MODEL\_V4 | 1.0 | 1.451e-21 | 801 | 0.28 | 364 | 154 | 10 | 8 | 363 | 1 | 264 | Exodeoxyribonuclease VIII | Exodeoxyribonuclease VIII | | afdb-uniprot50 | AF-A0A1H2DRY6-F1-MODEL\_V4 | 1.0 | 1.033e-22 | 801 | 0.274 | 361 | 178 | 9 | 1 | 361 | 1 | 277 | Exodeoxyribonuclease VIII | Exodeoxyribonuclease VIII | | afdb-uniprot50 | AF-A0A2N3KY65-F1-MODEL\_V4 | 1.0 | 9.228e-23 | 801 | 0.228 | 380 | 191 | 10 | 1 | 370 | 3 | 290 | DUF3799 domain-containing protein | DUF3799 domain-containing protein | | afdb-uniprot50 | AF-A0A767X0T4-F1-MODEL\_V4 | 1.0 | 6.931e-25 | 801 | 0.227 | 440 | 258 | 12 | 1 | 372 | 1 | 426 | DNA breaking-rejoining protein | DNA breaking-rejoining protein | | afdb-uniprot50 | AF-A0A2Z3HXT5-F1-MODEL\_V4 | 1.0 | 4.984e-22 | 800 | 0.273 | 365 | 162 | 10 | 1 | 361 | 1 | 266 | Exodeoxyribonuclease VIII | Exodeoxyribonuclease VIII | | afdb-uniprot50 | AF-A0A5M6ITC2-F1-MODEL\_V4 | 1.0 | 5.9e-22 | 800 | 0.286 | 360 | 154 | 7 | 1 | 358 | 3 | 261 | DUF3799 domain-containing protein | DUF3799 domain-containing protein | | afdb-uniprot50 | AF-A0A742LIF7-F1-MODEL\_V4 | 1.0 | 8.679e-25 | 798 | 0.207 | 438 | 270 | 11 | 1 | 372 | 462 | 888 | DNA breaking-rejoining protein | DNA breaking-rejoining protein | | afdb-uniprot50 | AF-A0A2D6JHQ6-F1-MODEL\_V4 | 1.0 | 2.545e-21 | 797 | 0.243 | 361 | 165 | 9 | 9 | 363 | 5 | 263 | Exodeoxyribonuclease VIII | Exodeoxyribonuclease VIII | | afdb-uniprot50 | AF-A0A7Z1AHF7-F1-MODEL\_V4 | 1.0 | 2.145e-22 | 796 | 0.278 | 373 | 165 | 11 | 1 | 370 | 11 | 282 | DUF3799 domain-containing protein | DUF3799 domain-containing protein | | afdb-uniprot50 | AF-A0A741YHL3-F1-MODEL\_V4 | 1.0 | 6.552e-25 | 796 | 0.226 | 438 | 261 | 12 | 1 | 372 | 358 | 783 | Exodeoxyribonuclease | Exodeoxyribonuclease | | afdb-uniprot50 | AF-Q9AKZ1-F1-MODEL\_V4 | 1.0 | 5.578e-22 | 795 | 0.226 | 371 | 185 | 10 | 1 | 369 | 6 | 276 | DUF3799 domain-containing protein | DUF3799 domain-containing protein | | afdb-uniprot50 | AF-A0A212KLV9-F1-MODEL\_V4 | 1.0 | 3.971e-23 | 795 | 0.236 | 381 | 184 | 11 | 1 | 371 | 5 | 288 | DUF3799 domain-containing protein | DUF3799 domain-containing protein | | afdb-uniprot50 | AF-A0A1E4HKX6-F1-MODEL\_V4 | 1.0 | 7.815e-22 | 794 | 0.28 | 342 | 149 | 9 | 23 | 363 | 13 | 258 | DUF3799 domain-containing protein | DUF3799 domain-containing protein | | afdb-uniprot50 | AF-A0A1V2RAV6-F1-MODEL\_V4 | 1.0 | 3.557e-22 | 793 | 0.266 | 379 | 166 | 12 | 1 | 370 | 6 | 281 | Exodeoxyribonuclease 8 | Exodeoxyribonuclease 8 | | afdb-uniprot50 | AF-A0A2E2DCA2-F1-MODEL\_V4 | 1.0 | 1.921e-21 | 793 | 0.257 | 365 | 170 | 8 | 5 | 369 | 19 | 282 | DUF3799 domain-containing protein | DUF3799 domain-containing protein | | afdb-uniprot50 | AF-A0A250BLY8-F1-MODEL\_V4 | 1.0 | 1.216e-24 | 793 | 0.231 | 437 | 256 | 13 | 1 | 370 | 511 | 934 | Exodeoxyribonuclease | Exodeoxyribonuclease | | afdb-uniprot50 | AF-A0A6H0K5E3-F1-MODEL\_V4 | 1.0 | 2.012e-25 | 793 | 0.26 | 438 | 245 | 14 | 1 | 372 | 570 | 994 | Exodeoxyribonuclease | Exodeoxyribonuclease | | afdb-uniprot50 | AF-A0A3S0A9S4-F1-MODEL\_V4 | 1.0 | 1.713e-22 | 792 | 0.274 | 379 | 160 | 11 | 1 | 366 | 6 | 282 | DUF3799 domain-containing protein | DUF3799 domain-containing protein | | afdb-uniprot50 | AF-A0A346DXG2-F1-MODEL\_V4 | 1.0 | 6.586e-23 | 792 | 0.241 | 385 | 178 | 14 | 1 | 371 | 4 | 288 | DUF3799 domain-containing protein | DUF3799 domain-containing protein | | afdb-uniprot50 | AF-A0A5X6IFM5-F1-MODEL\_V4 | 1.0 | 2.819e-25 | 791 | 0.224 | 436 | 264 | 12 | 1 | 372 | 54 | 479 | Exodeoxyribonuclease | Exodeoxyribonuclease | | afdb-uniprot50 | AF-A0A6P1K602-F1-MODEL\_V4 | 1.0 | 7.332e-25 | 790 | 0.217 | 437 | 260 | 15 | 1 | 369 | 600 | 1022 | Exodeoxyribonuclease VIII | Exodeoxyribonuclease VIII | | afdb-uniprot50 | AF-A0A3D0NKI8-F1-MODEL\_V4 | 1.0 | 1.153e-23 | 789 | 0.277 | 367 | 192 | 10 | 1 | 359 | 9 | 310 | DUF3799 domain-containing protein | DUF3799 domain-containing protein | | afdb-uniprot50 | AF-G5S9R5-F1-MODEL\_V4 | 1.0 | 3.337e-25 | 789 | 0.221 | 438 | 263 | 13 | 1 | 372 | 177 | 602 | Exodeoxyribonuclease 8 | Exodeoxyribonuclease 8 | | afdb-uniprot50 | AF-A0A1Y2SD06-F1-MODEL\_V4 | 1.0 | 7.388e-22 | 788 | 0.266 | 368 | 167 | 9 | 1 | 364 | 1 | 269 | Exodeoxyribonuclease VIII | Exodeoxyribonuclease VIII | | afdb-uniprot50 | AF-A0A5W2M3A0-F1-MODEL\_V4 | 1.0 | 4.421e-25 | 787 | 0.224 | 436 | 264 | 12 | 1 | 372 | 143 | 568 | DNA breaking-rejoining protein | DNA breaking-rejoining protein | | afdb-uniprot50 | AF-A0A0F9LUR6-F1-MODEL\_V4 | 1.0 | 4.2e-23 | 786 | 0.271 | 364 | 177 | 9 | 1 | 364 | 25 | 300 | DUF3799 domain-containing protein | DUF3799 domain-containing protein | | afdb-uniprot50 | AF-A0A767X2R0-F1-MODEL\_V4 | 1.0 | 5.233e-25 | 786 | 0.224 | 436 | 264 | 12 | 1 | 372 | 99 | 524 | Exodeoxyribonuclease | Exodeoxyribonuclease | | afdb-uniprot50 | AF-A0A5V9PFN1-F1-MODEL\_V4 | 1.0 | 4.947e-25 | 785 | 0.224 | 436 | 264 | 12 | 1 | 372 | 474 | 899 | Exodeoxyribonuclease | Exodeoxyribonuclease | | afdb-uniprot50 | AF-A0A5C6BNI7-F1-MODEL\_V4 | 1.0 | 2.139e-23 | 784 | 0.254 | 377 | 183 | 11 | 2 | 366 | 20 | 310 | Exodeoxyribonuclease 8 | Exodeoxyribonuclease 8 | | afdb-uniprot50 | AF-A0A754MTK6-F1-MODEL\_V4 | 1.0 | 3.53e-25 | 784 | 0.228 | 438 | 260 | 13 | 1 | 372 | 142 | 567 | Exodeoxyribonuclease | Exodeoxyribonuclease | | afdb-uniprot50 | AF-A0A515CTF2-F1-MODEL\_V4 | 1.0 | 9.712e-25 | 784 | 0.215 | 437 | 261 | 13 | 1 | 369 | 612 | 1034 | DUF3799 domain-containing protein | DUF3799 domain-containing protein | | afdb-uniprot50 | AF-A0A0T9L9Z5-F1-MODEL\_V4 | 1.0 | 9.712e-25 | 783 | 0.228 | 438 | 254 | 14 | 1 | 369 | 159 | 581 | Exodeoxyribonuclease 8 | Exodeoxyribonuclease 8 | | afdb-uniprot50 | AF-A0A5V3EY84-F1-MODEL\_V4 | 1.0 | 3.53e-25 | 782 | 0.228 | 438 | 260 | 13 | 1 | 372 | 347 | 772 | Exodeoxyribonuclease | Exodeoxyribonuclease | | afdb-uniprot50 | AF-A0A4P8C6B6-F1-MODEL\_V4 | 1.0 | 1.907e-24 | 782 | 0.224 | 437 | 257 | 15 | 1 | 369 | 618 | 1040 | Exodeoxyribonuclease VIII | Exodeoxyribonuclease VIII | | afdb-uniprot50 | AF-A0A5Z8QTS3-F1-MODEL\_V4 | 1.0 | 6.552e-25 | 781 | 0.224 | 436 | 264 | 12 | 1 | 372 | 142 | 567 | Exodeoxyribonuclease | Exodeoxyribonuclease | | afdb-uniprot50 | AF-A0A5V8U3D0-F1-MODEL\_V4 | 1.0 | 1.087e-24 | 781 | 0.209 | 439 | 268 | 13 | 1 | 372 | 246 | 672 | DNA breaking-rejoining protein | DNA breaking-rejoining protein | | afdb-uniprot50 | AF-A0A2S4QN94-F1-MODEL\_V4 | 1.0 | 2.145e-22 | 781 | 0.218 | 385 | 231 | 10 | 1 | 372 | 749 | 1076 | Exodeoxyribonuclease | Exodeoxyribonuclease | | afdb-uniprot50 | AF-A0A2E0Z4I9-F1-MODEL\_V4 | 1.0 | 5.273e-22 | 780 | 0.258 | 367 | 173 | 10 | 3 | 364 | 8 | 280 | DUF3799 domain-containing protein | DUF3799 domain-containing protein | | afdb-uniprot50 | AF-A0A826KR83-F1-MODEL\_V4 | 1.0 | 2.99e-24 | 780 | 0.226 | 437 | 256 | 15 | 1 | 369 | 107 | 529 | Exonuclease VIII | Exonuclease VIII | | afdb-uniprot50 | AF-A0A726YLN1-F1-MODEL\_V4 | 1.0 | 4.421e-25 | 778 | 0.225 | 434 | 266 | 14 | 1 | 372 | 546 | 971 | Exodeoxyribonuclease | Exodeoxyribonuclease | | afdb-uniprot50 | AF-W1I0H3-F1-MODEL\_V4 | 1.0 | 5.233e-25 | 777 | 0.226 | 438 | 261 | 12 | 1 | 372 | 115 | 540 | Exodeoxyribonuclease VIII | Exodeoxyribonuclease VIII | | afdb-uniprot50 | AF-A0A378FZ40-F1-MODEL\_V4 | 1.0 | 3.961e-24 | 777 | 0.22 | 440 | 261 | 12 | 1 | 372 | 195 | 620 | Putative exodeoxyribonuclease VIII | Putative exodeoxyribonuclease VIII | | afdb-uniprot50 | AF-A0A723W028-F1-MODEL\_V4 | 1.0 | 5.535e-25 | 777 | 0.223 | 434 | 267 | 14 | 1 | 372 | 550 | 975 | Exodeoxyribonuclease | Exodeoxyribonuclease | | afdb-uniprot50 | AF-A0A839L3J4-F1-MODEL\_V4 | 1.0 | 6.586e-23 | 776 | 0.272 | 370 | 166 | 9 | 1 | 366 | 36 | 306 | PD-(D/E)XK nuclease-like domain-containing protein | PD-(D/E)XK nuclease-like domain-containing protein | | afdb-uniprot50 | AF-A0A808IEU1-F1-MODEL\_V4 | 1.0 | 9.181e-25 | 775 | 0.22 | 439 | 262 | 14 | 1 | 372 | 481 | 906 | Exodeoxyribonuclease VIII | Exodeoxyribonuclease VIII | | afdb-uniprot50 | AF-A0A759BTV9-F1-MODEL\_V4 | 1.0 | 1.286e-24 | 774 | 0.222 | 436 | 265 | 12 | 1 | 372 | 129 | 554 | Exodeoxyribonuclease | Exodeoxyribonuclease | | afdb-uniprot50 | AF-A0A5W6KM85-F1-MODEL\_V4 | 1.0 | 5.535e-25 | 773 | 0.21 | 438 | 269 | 15 | 1 | 372 | 166 | 592 | DNA breaking-rejoining protein | DNA breaking-rejoining protein | | afdb-uniprot50 | AF-A0A1G4AE34-F1-MODEL\_V4 | 1.0 | 9.275e-21 | 772 | 0.249 | 365 | 167 | 10 | 1 | 361 | 1 | 262 | DUF3799 domain-containing protein | DUF3799 domain-containing protein | | afdb-uniprot50 | AF-A0A7Z9CR49-F1-MODEL\_V4 | 1.0 | 1.027e-24 | 772 | 0.221 | 438 | 263 | 12 | 1 | 372 | 100 | 525 | Putative exodeoxyribonuclease VIII | Putative exodeoxyribonuclease VIII | | afdb-uniprot50 | AF-A0A839HGV9-F1-MODEL\_V4 | 1.0 | 2.274e-21 | 771 | 0.26 | 357 | 163 | 11 | 5 | 360 | 11 | 267 | PD-(D/E)XK nuclease-like domain-containing protein | PD-(D/E)XK nuclease-like domain-containing protein | | afdb-uniprot50 | AF-A0A764YUZ2-F1-MODEL\_V4 | 1.0 | 1.704e-24 | 770 | 0.222 | 436 | 265 | 12 | 1 | 372 | 271 | 696 | Exodeoxyribonuclease | Exodeoxyribonuclease | | afdb-uniprot50 | AF-A0A7I6ZDW9-F1-MODEL\_V4 | 1.0 | 2.841e-22 | 770 | 0.23 | 382 | 224 | 10 | 1 | 369 | 506 | 830 | Uncharacterized protein | Uncharacterized protein | | afdb-uniprot50 | AF-A0A0F9TR59-F1-MODEL\_V4 | 1.0 | 1.296e-21 | 769 | 0.246 | 369 | 186 | 10 | 1 | 367 | 11 | 289 | DUF3799 domain-containing protein | DUF3799 domain-containing protein | | afdb-uniprot50 | AF-A0A609CCL8-F1-MODEL\_V4 | 1.0 | 6.931e-25 | 768 | 0.212 | 438 | 268 | 15 | 1 | 372 | 487 | 913 | Exodeoxyribonuclease | Exodeoxyribonuclease | | afdb-uniprot50 | AF-A0A430DP05-F1-MODEL\_V4 | 1.0 | 4.984e-22 | 767 | 0.241 | 397 | 182 | 10 | 2 | 371 | 7 | 311 | DUF3799 domain-containing protein | DUF3799 domain-containing protein | | afdb-uniprot50 | AF-A0A3D0NII1-F1-MODEL\_V4 | 1.0 | 3.548e-23 | 765 | 0.311 | 360 | 145 | 11 | 5 | 360 | 51 | 311 | DUF3799 domain-containing protein | DUF3799 domain-containing protein | | afdb-uniprot50 | AF-A0A221DNX1-F1-MODEL\_V4 | 1.0 | 9.712e-25 | 765 | 0.216 | 435 | 262 | 13 | 1 | 369 | 456 | 877 | DUF3799 domain-containing protein | DUF3799 domain-containing protein | | afdb-uniprot50 | AF-A0A5V7BL52-F1-MODEL\_V4 | 1.0 | 5.564e-23 | 764 | 0.261 | 375 | 164 | 11 | 1 | 366 | 34 | 304 | Exonuclease VIII | Exonuclease VIII | | afdb-uniprot50 | AF-A0A7X4MGN6-F1-MODEL\_V4 | 1.0 | 4.19e-24 | 764 | 0.218 | 440 | 262 | 14 | 1 | 372 | 166 | 591 | DUF3799 domain-containing protein | DUF3799 domain-containing protein | | afdb-uniprot50 | AF-A0A6Y1QS66-F1-MODEL\_V4 | 1.0 | 1.216e-24 | 764 | 0.222 | 440 | 260 | 14 | 1 | 372 | 546 | 971 | Exodeoxyribonuclease | Exodeoxyribonuclease | | afdb-uniprot50 | AF-A0A377Q5C7-F1-MODEL\_V4 | 1.0 | 1.619e-22 | 763 | 0.252 | 368 | 203 | 14 | 1 | 363 | 9 | 309 | Exodeoxyribonuclease 8 | Exodeoxyribonuclease 8 | | afdb-uniprot50 | AF-A0A0P8LGK8-F1-MODEL\_V4 | 1.0 | 3.539e-24 | 762 | 0.217 | 436 | 267 | 12 | 1 | 372 | 656 | 1081 | Exodeoxyribonuclease | Exodeoxyribonuclease | | afdb-uniprot50 | AF-A0A0M8PNS2-F1-MODEL\_V4 | 1.0 | 2.15e-21 | 760 | 0.282 | 375 | 160 | 11 | 2 | 369 | 7 | 279 | RecE | RecE | | afdb-uniprot50 | AF-A0A4R0ER10-F1-MODEL\_V4 | 1.0 | 1.161e-20 | 760 | 0.208 | 375 | 195 | 8 | 2 | 368 | 9 | 289 | DUF3799 domain-containing protein | DUF3799 domain-containing protein | | afdb-uniprot50 | AF-Q8XF26-F1-MODEL\_V4 | 1.0 | 5.246e-24 | 759 | 0.222 | 436 | 265 | 12 | 1 | 372 | 536 | 961 | Gifsy-1 prophage protein | Gifsy-1 prophage protein | | afdb-uniprot50 | AF-A0A523QSH7-F1-MODEL\_V4 | 1.0 | 2.274e-21 | 758 | 0.239 | 371 | 171 | 10 | 1 | 363 | 21 | 288 | DUF3799 domain-containing protein | DUF3799 domain-containing protein | | afdb-uniprot50 | AF-A0A7V5Q1K7-F1-MODEL\_V4 | 1.0 | 2.406e-21 | 758 | 0.234 | 367 | 194 | 12 | 1 | 367 | 5 | 284 | DUF3799 domain-containing protein | DUF3799 domain-containing protein | | afdb-uniprot50 | AF-A0A2I8NWI0-F1-MODEL\_V4 | 1.0 | 5.246e-24 | 758 | 0.222 | 436 | 265 | 11 | 1 | 372 | 571 | 996 | Exodeoxyribonuclease VIII | Exodeoxyribonuclease VIII | | afdb-uniprot50 | AF-A0A318FG45-F1-MODEL\_V4 | 1.0 | 2.526e-24 | 758 | 0.227 | 440 | 258 | 13 | 1 | 372 | 594 | 1019 | Exodeoxyribonuclease VIII | Exodeoxyribonuclease VIII | | afdb-uniprot50 | AF-A0A6C7DAJ4-F1-MODEL\_V4 | 1.0 | 1.44e-24 | 757 | 0.224 | 437 | 263 | 14 | 1 | 372 | 542 | 967 | Exodeoxyribonuclease | Exodeoxyribonuclease | | afdb-uniprot50 | AF-A0A420S4T2-F1-MODEL\_V4 | 1.0 | 1.721e-20 | 756 | 0.241 | 373 | 175 | 10 | 8 | 372 | 10 | 282 | DUF3799 domain-containing protein | DUF3799 domain-containing protein | | afdb-uniprot50 | AF-D6PKU1-F1-MODEL\_V4 | 1.0 | 3.575e-20 | 755 | 0.249 | 365 | 163 | 10 | 1 | 360 | 1 | 259 | Exonuclease VIII 5 > 3 specific dsDNA exonuclease | Exonuclease VIII 5 > 3 specific dsDNA exonuclease | | afdb-uniprot50 | AF-A0A6N7BWK4-F1-MODEL\_V4 | 1.0 | 4.466e-21 | 755 | 0.254 | 370 | 171 | 9 | 1 | 360 | 1 | 275 | DUF3799 domain-containing protein | DUF3799 domain-containing protein | | afdb-uniprot50 | AF-A0A1I3YJV0-F1-MODEL\_V4 | 1.0 | 4.466e-21 | 755 | 0.246 | 373 | 175 | 11 | 1 | 371 | 5 | 273 | DUF3799 domain-containing protein | DUF3799 domain-containing protein | | afdb-uniprot50 | AF-A0A564TYU2-F1-MODEL\_V4 | 1.0 | 6.21e-24 | 752 | 0.221 | 433 | 263 | 13 | 1 | 369 | 203 | 625 | Exodeoxyribonuclease 8 | Exodeoxyribonuclease 8 | | afdb-uniprot50 | AF-A0A5E9SPN3-F1-MODEL\_V4 | 1.0 | 1.527e-23 | 752 | 0.217 | 436 | 259 | 15 | 1 | 368 | 427 | 848 | Exonuclease VIII | Exonuclease VIII | | afdb-uniprot50 | AF-A0A2D9CGT1-F1-MODEL\_V4 | 1.0 | 1.161e-20 | 750 | 0.262 | 358 | 158 | 10 | 5 | 360 | 3 | 256 | DUF3799 domain-containing protein | DUF3799 domain-containing protein | | afdb-uniprot50 | AF-A0A0P0EB10-F1-MODEL\_V4 | 1.0 | 1.821e-20 | 750 | 0.252 | 372 | 174 | 9 | 3 | 371 | 2 | 272 | DUF3799 domain-containing protein | DUF3799 domain-containing protein | | afdb-uniprot50 | AF-A0A756I411-F1-MODEL\_V4 | 1.0 | 2.388e-24 | 749 | 0.223 | 438 | 262 | 12 | 1 | 372 | 54 | 479 | DNA breaking-rejoining protein | DNA breaking-rejoining protein | | afdb-uniprot50 | AF-A0A2S9I8G0-F1-MODEL\_V4 | 1.0 | 9.712e-25 | 749 | 0.232 | 439 | 255 | 14 | 1 | 371 | 430 | 854 | Exodeoxyribonuclease VIII | Exodeoxyribonuclease VIII | | afdb-uniprot50 | AF-A0A754B5G1-F1-MODEL\_V4 | 1.0 | 3.013e-21 | 749 | 0.233 | 385 | 225 | 10 | 1 | 372 | 569 | 896 | Exodeoxyribonuclease VIII | Exodeoxyribonuclease VIII | | afdb-uniprot50 | AF-A0A480BQL0-F1-MODEL\_V4 | 1.0 | 2.552e-20 | 747 | 0.229 | 357 | 173 | 6 | 9 | 364 | 1 | 256 | DUF3799 domain-containing protein | DUF3799 domain-containing protein | | afdb-uniprot50 | AF-A0A0Q9C5I5-F1-MODEL\_V4 | 1.0 | 7.002e-21 | 746 | 0.27 | 374 | 166 | 11 | 1 | 372 | 2 | 270 | DUF3799 domain-containing protein | DUF3799 domain-containing protein | | afdb-uniprot50 | AF-A0A7T8PWF9-F1-MODEL\_V4 | 1.0 | 3.991e-21 | 746 | 0.265 | 343 | 170 | 9 | 20 | 361 | 2 | 263 | PD-(D/E)XK nuclease-like domain-containing protein | PD-(D/E)XK nuclease-like domain-containing protein | | afdb-uniprot50 | AF-A0A2L1VE72-F1-MODEL\_V4 | 1.0 | 7.407e-21 | 746 | 0.238 | 378 | 180 | 11 | 1 | 368 | 2 | 281 | Nuclease | Nuclease | | afdb-uniprot50 | AF-A0A090V4Q6-F1-MODEL\_V4 | 1.0 | 4.432e-24 | 746 | 0.231 | 436 | 261 | 12 | 1 | 372 | 814 | 1239 | Putative exodeoxyribonuclease VIII | Putative exodeoxyribonuclease VIII | | afdb-uniprot50 | AF-A0A0F9EGN9-F1-MODEL\_V4 | 1.0 | 2.848e-21 | 745 | 0.258 | 363 | 165 | 9 | 1 | 360 | 4 | 265 | DUF3799 domain-containing protein | DUF3799 domain-containing protein | | afdb-uniprot50 | AF-A0A3C1SW29-F1-MODEL\_V4 | 1.0 | 9.786e-22 | 745 | 0.28 | 363 | 153 | 10 | 1 | 363 | 44 | 298 | DUF3799 domain-containing protein | DUF3799 domain-containing protein | | afdb-uniprot50 | AF-A0A7W3KNY4-F1-MODEL\_V4 | 1.0 | 4.466e-21 | 744 | 0.286 | 366 | 154 | 10 | 1 | 360 | 11 | 275 | Exodeoxyribonuclease VIII | Exodeoxyribonuclease VIII | | afdb-uniprot50 | AF-A0A376RV81-F1-MODEL\_V4 | 1.0 | 6.967e-23 | 744 | 0.219 | 438 | 258 | 15 | 1 | 369 | 136 | 558 | Putative phage exodeoxyribonuclease | Putative phage exodeoxyribonuclease | | afdb-uniprot50 | AF-A0A6M3KS37-F1-MODEL\_V4 | 1.0 | 4.232e-20 | 743 | 0.26 | 361 | 163 | 10 | 3 | 360 | 2 | 261 | DUF3799 domain-containing protein | DUF3799 domain-containing protein | | afdb-uniprot50 | AF-A0A7W3F3C1-F1-MODEL\_V4 | 1.0 | 1.619e-22 | 743 | 0.227 | 382 | 225 | 11 | 1 | 369 | 664 | 988 | PD-(D/E)XK nuclease-like domain-containing protein | PD-(D/E)XK nuclease-like domain-containing protein | | afdb-uniprot50 | AF-A0A6B1U9G5-F1-MODEL\_V4 | 1.0 | 2.257e-24 | 743 | 0.217 | 437 | 266 | 15 | 1 | 372 | 680 | 1105 | Exodeoxyribonuclease | Exodeoxyribonuclease | | afdb-uniprot50 | AF-A0A0Q5C9J8-F1-MODEL\_V4 | 1.0 | 2.156e-20 | 742 | 0.24 | 370 | 183 | 9 | 1 | 369 | 1 | 273 | DUF3799 domain-containing protein | DUF3799 domain-containing protein | | afdb-uniprot50 | AF-A0A7W3A5W8-F1-MODEL\_V4 | 1.0 | 2.545e-21 | 742 | 0.214 | 382 | 228 | 10 | 1 | 368 | 535 | 858 | PD-(D/E)XK nuclease-like domain-containing protein | PD-(D/E)XK nuclease-like domain-containing protein | | afdb-uniprot50 | AF-A0A5W0BDJ2-F1-MODEL\_V4 | 1.0 | 1.03e-23 | 742 | 0.24 | 440 | 252 | 15 | 1 | 371 | 561 | 987 | Exonuclease VIII | Exonuclease VIII | | afdb-uniprot50 | AF-A0A448S1F5-F1-MODEL\_V4 | 1.0 | 9.205e-24 | 741 | 0.282 | 375 | 166 | 9 | 1 | 371 | 1 | 276 | Exodeoxyribonuclease 8 | Exodeoxyribonuclease 8 | | afdb-uniprot50 | AF-A0A3S2UWS8-F1-MODEL\_V4 | 1.0 | 3.005e-22 | 740 | 0.284 | 394 | 148 | 12 | 2 | 361 | 24 | 317 | Uncharacterized protein | Uncharacterized protein | | afdb-uniprot50 | AF-A0A7Y7QAL0-F1-MODEL\_V4 | 1.0 | 2.263e-23 | 740 | 0.264 | 367 | 207 | 7 | 1 | 359 | 97 | 408 | PD-(D/E)XK nuclease-like domain-containing protein | PD-(D/E)XK nuclease-like domain-containing protein | | afdb-uniprot50 | AF-A0A4Q3L0R6-F1-MODEL\_V4 | 1.0 | 7.835e-21 | 739 | 0.239 | 364 | 192 | 11 | 6 | 369 | 1 | 279 | DUF3799 domain-containing protein | DUF3799 domain-containing protein | | afdb-uniprot50 | AF-A0A2S4QV97-F1-MODEL\_V4 | 1.0 | 2.139e-23 | 737 | 0.217 | 436 | 267 | 11 | 1 | 372 | 658 | 1083 | Exodeoxyribonuclease | Exodeoxyribonuclease | | afdb-uniprot50 | AF-A0A0F9TVJ5-F1-MODEL\_V4 | 1.0 | 6.636e-20 | 736 | 0.215 | 358 | 179 | 7 | 1 | 357 | 14 | 270 | DUF3799 domain-containing protein | DUF3799 domain-containing protein | | afdb-uniprot50 | AF-A0A7Y9J8Y8-F1-MODEL\_V4 | 1.0 | 1.717e-21 | 735 | 0.256 | 374 | 171 | 12 | 1 | 372 | 33 | 301 | DUF3799 domain-containing protein | DUF3799 domain-containing protein | | afdb-uniprot50 | AF-A0A5U5MI79-F1-MODEL\_V4 | 1.0 | 4.688e-24 | 734 | 0.229 | 418 | 248 | 12 | 1 | 354 | 142 | 549 | Exodeoxyribonuclease | Exodeoxyribonuclease | | afdb-uniprot50 | AF-A0A3L9YEK8-F1-MODEL\_V4 | 1.0 | 8.768e-21 | 733 | 0.247 | 371 | 171 | 9 | 8 | 368 | 36 | 308 | PDDEXK-like uncharacterized protein DUF3799 | PDDEXK-like uncharacterized protein DUF3799 | | afdb-uniprot50 | AF-A0A542ZT56-F1-MODEL\_V4 | 1.0 | 2.699e-20 | 732 | 0.222 | 377 | 179 | 13 | 1 | 369 | 4 | 274 | PDDEXK-like uncharacterized protein DUF3799 | PDDEXK-like uncharacterized protein DUF3799 | | afdb-uniprot50 | AF-A0A3M1FF26-F1-MODEL\_V4 | 1.0 | 3.195e-20 | 730 | 0.25 | 363 | 159 | 12 | 1 | 361 | 1 | 252 | DUF3799 domain-containing protein | DUF3799 domain-containing protein | | afdb-uniprot50 | AF-A0A1E4IEB0-F1-MODEL\_V4 | 1.0 | 7.002e-21 | 730 | 0.249 | 361 | 184 | 10 | 3 | 360 | 7 | 283 | DUF3799 domain-containing protein | DUF3799 domain-containing protein | | afdb-uniprot50 | AF-A0A1X0DYE3-F1-MODEL\_V4 | 1.0 | 3.991e-21 | 729 | 0.257 | 376 | 171 | 14 | 1 | 365 | 4 | 282 | DUF3799 domain-containing protein | DUF3799 domain-containing protein | | afdb-uniprot50 | AF-M3VCA4-F1-MODEL\_V4 | 1.0 | 9.275e-21 | 728 | 0.281 | 362 | 154 | 13 | 1 | 360 | 6 | 263 | DUF3799 domain-containing protein | DUF3799 domain-containing protein | | afdb-uniprot50 | AF-A0A828EP49-F1-MODEL\_V4 | 1.0 | 3.354e-23 | 727 | 0.229 | 436 | 256 | 16 | 1 | 369 | 258 | 680 | Exonuclease VIII | Exonuclease VIII | | afdb-uniprot50 | AF-B8FNJ4-F1-MODEL\_V4 | 1.0 | 5.93e-20 | 726 | 0.212 | 357 | 177 | 7 | 5 | 360 | 4 | 257 | Putative bacteriophage protein | Putative bacteriophage protein | | afdb-uniprot50 | AF-A0A2W1PQ23-F1-MODEL\_V4 | 1.0 | 1.375e-20 | 726 | 0.252 | 372 | 177 | 13 | 1 | 367 | 1 | 276 | RecE | RecE | | afdb-uniprot50 | AF-A0A100Y6B0-F1-MODEL\_V4 | 1.0 | 7.407e-21 | 724 | 0.279 | 368 | 157 | 12 | 9 | 368 | 1 | 268 | DUF3799 domain-containing protein | DUF3799 domain-containing protein | | afdb-uniprot50 | AF-A0A2J6NWE3-F1-MODEL\_V4 | 1.0 | 8.768e-21 | 724 | 0.256 | 382 | 187 | 14 | 1 | 371 | 2 | 297 | DUF3799 domain-containing protein | DUF3799 domain-containing protein | | afdb-uniprot50 | AF-A0A6H1ZNA7-F1-MODEL\_V4 | 1.0 | 1.038e-20 | 723 | 0.221 | 366 | 186 | 11 | 3 | 360 | 10 | 284 | DUF3799 domain-containing protein | DUF3799 domain-containing protein | | afdb-uniprot50 | AF-A0A2E3N1L6-F1-MODEL\_V4 | 1.0 | 1.826e-19 | 722 | 0.245 | 366 | 168 | 10 | 9 | 361 | 3 | 273 | DUF3799 domain-containing protein | DUF3799 domain-containing protein | | afdb-uniprot50 | AF-A0A6M3JA96-F1-MODEL\_V4 | 1.0 | 5.915e-21 | 721 | 0.247 | 371 | 199 | 10 | 1 | 368 | 3 | 296 | DUF3799 domain-containing protein | DUF3799 domain-containing protein | | afdb-uniprot50 | AF-A0A0F9U718-F1-MODEL\_V4 | 1.0 | 1.931e-19 | 720 | 0.202 | 360 | 189 | 7 | 1 | 359 | 3 | 265 | DUF3799 domain-containing protein | DUF3799 domain-containing protein | | afdb-uniprot50 | AF-A0A6M3Y0Y1-F1-MODEL\_V4 | 1.0 | 2.863e-19 | 720 | 0.237 | 367 | 174 | 8 | 1 | 360 | 2 | 269 | DUF3799 domain-containing protein | DUF3799 domain-containing protein | | afdb-uniprot50 | AF-A0A653VM09-F1-MODEL\_V4 | 1.0 | 2.848e-21 | 719 | 0.232 | 370 | 184 | 11 | 5 | 370 | 7 | 280 | DUF3799 domain-containing protein | DUF3799 domain-containing protein | | afdb-uniprot50 | AF-A0A7W6CSA3-F1-MODEL\_V4 | 1.0 | 6.257e-21 | 719 | 0.244 | 385 | 181 | 10 | 2 | 368 | 6 | 298 | DUF3799 domain-containing protein | DUF3799 domain-containing protein | | afdb-uniprot50 | AF-A0A654C1H5-F1-MODEL\_V4 | 1.0 | 1.807e-23 | 719 | 0.221 | 437 | 258 | 14 | 1 | 369 | 603 | 1025 | DUF3799 domain-containing protein | DUF3799 domain-containing protein | | afdb-uniprot50 | AF-A0A2T3BX21-F1-MODEL\_V4 | 1.0 | 1.101e-19 | 718 | 0.246 | 365 | 167 | 14 | 1 | 364 | 3 | 260 | DUF3799 domain-containing protein | DUF3799 domain-containing protein | | afdb-uniprot50 | AF-A0A4P6GRI8-F1-MODEL\_V4 | 1.0 | 2.4e-22 | 718 | 0.261 | 363 | 181 | 8 | 1 | 360 | 60 | 338 | DUF3799 domain-containing protein | DUF3799 domain-containing protein | | afdb-uniprot50 | AF-D4LDT1-F1-MODEL\_V4 | 1.0 | 1.378e-19 | 717 | 0.243 | 362 | 163 | 9 | 9 | 361 | 1 | 260 | DUF3799 domain-containing protein | DUF3799 domain-containing protein | | afdb-uniprot50 | AF-A0A5B9W8V5-F1-MODEL\_V4 | 1.0 | 8.79e-20 | 717 | 0.247 | 367 | 172 | 9 | 5 | 369 | 2 | 266 | Exodeoxyribonuclease 8 | Exodeoxyribonuclease 8 | | afdb-uniprot50 | AF-A0A370CF63-F1-MODEL\_V4 | 1.0 | 4.7e-23 | 717 | 0.253 | 386 | 174 | 13 | 1 | 371 | 11 | 297 | DUF3799 domain-containing protein | DUF3799 domain-containing protein | | afdb-uniprot50 | AF-A0A6B3LMA0-F1-MODEL\_V4 | 1.0 | 4.477e-20 | 716 | 0.254 | 365 | 169 | 14 | 5 | 360 | 14 | 284 | DUF3799 domain-containing protein | DUF3799 domain-containing protein | | afdb-uniprot50 | AF-A0A5C8A0A4-F1-MODEL\_V4 | 1.0 | 3.773e-21 | 716 | 0.245 | 374 | 175 | 10 | 1 | 370 | 45 | 315 | DUF3799 domain-containing protein | DUF3799 domain-containing protein | | afdb-uniprot50 | AF-A0A1Z9X1Y0-F1-MODEL\_V4 | 1.0 | 5.273e-22 | 714 | 0.214 | 382 | 218 | 8 | 1 | 358 | 39 | 362 | DUF3799 domain-containing protein | DUF3799 domain-containing protein | | afdb-uniprot50 | AF-A0A2E8DZF3-F1-MODEL\_V4 | 1.0 | 3.38e-20 | 712 | 0.271 | 364 | 147 | 11 | 7 | 360 | 10 | 265 | DUF3799 domain-containing protein | DUF3799 domain-containing protein | | afdb-uniprot50 | AF-A0A759M5V4-F1-MODEL\_V4 | 1.0 | 3.763e-22 | 712 | 0.237 | 383 | 226 | 12 | 1 | 372 | 116 | 443 | DUF3799 domain-containing protein | DUF3799 domain-containing protein | | afdb-uniprot50 | AF-A0A0F9K1Z8-F1-MODEL\_V4 | 1.0 | 4.736e-20 | 711 | 0.23 | 368 | 182 | 9 | 1 | 361 | 22 | 295 | DUF3799 domain-containing protein | DUF3799 domain-containing protein | | afdb-uniprot50 | AF-A0A2D4TU41-F1-MODEL\_V4 | 1.0 | 9.228e-23 | 711 | 0.238 | 382 | 248 | 12 | 1 | 358 | 41 | 403 | Uncharacterized protein | Uncharacterized protein | | afdb-uniprot50 | AF-B6XEX5-F1-MODEL\_V4 | 1.0 | 4.984e-22 | 709 | 0.252 | 384 | 219 | 13 | 1 | 372 | 148 | 475 | DUF3799 domain-containing protein | DUF3799 domain-containing protein | | afdb-uniprot50 | AF-A0A7Z1T1G3-F1-MODEL\_V4 | 1.0 | 2.027e-22 | 708 | 0.283 | 371 | 163 | 9 | 1 | 367 | 567 | 838 | Exonuclease VIII | Exonuclease VIII | | afdb-uniprot50 | AF-A0A0Q6ECD3-F1-MODEL\_V4 | 1.0 | 1.375e-20 | 707 | 0.232 | 375 | 188 | 11 | 1 | 371 | 4 | 282 | DUF3799 domain-containing protein | DUF3799 domain-containing protein | | afdb-uniprot50 | AF-A0A722GKL2-F1-MODEL\_V4 | 1.0 | 7.37e-23 | 707 | 0.211 | 420 | 253 | 11 | 19 | 372 | 2 | 409 | DNA breaking-rejoining protein | DNA breaking-rejoining protein | | afdb-uniprot50 | AF-A0A1C6GFL6-F1-MODEL\_V4 | 1.0 | 3.028e-19 | 706 | 0.242 | 363 | 169 | 10 | 9 | 364 | 1 | 264 | Exodeoxyribonuclease 8 | Exodeoxyribonuclease 8 | | afdb-uniprot50 | AF-E6WYE1-F1-MODEL\_V4 | 1.0 | 5.93e-20 | 701 | 0.273 | 355 | 154 | 10 | 9 | 361 | 1 | 253 | DUF3799 domain-containing protein | DUF3799 domain-containing protein | | afdb-uniprot50 | AF-C7R228-F1-MODEL\_V4 | 1.0 | 1.931e-19 | 701 | 0.248 | 382 | 170 | 12 | 1 | 369 | 3 | 280 | DUF3799 domain-containing protein | DUF3799 domain-containing protein | | afdb-uniprot50 | AF-A0A845GHL0-F1-MODEL\_V4 | 1.0 | 1.232e-19 | 699 | 0.236 | 363 | 172 | 12 | 2 | 360 | 16 | 277 | Uncharacterized protein | Uncharacterized protein | | afdb-uniprot50 | AF-A0A0S8DSQ9-F1-MODEL\_V4 | 1.0 | 6.653e-19 | 698 | 0.205 | 365 | 185 | 7 | 1 | 361 | 2 | 265 | DUF3799 domain-containing protein | DUF3799 domain-containing protein | | afdb-uniprot50 | AF-A0A0C1AUR5-F1-MODEL\_V4 | 1.0 | 2.706e-19 | 697 | 0.237 | 379 | 174 | 14 | 1 | 370 | 4 | 276 | DUF3799 domain-containing protein | DUF3799 domain-containing protein | | afdb-uniprot50 | AF-A0A7S9HDT4-F1-MODEL\_V4 | 1.0 | 4.011e-19 | 697 | 0.241 | 377 | 177 | 12 | 1 | 372 | 20 | 292 | PD-(D/E)XK nuclease-like domain-containing protein | PD-(D/E)XK nuclease-like domain-containing protein | | afdb-uniprot50 | AF-A0A1V5Z6N5-F1-MODEL\_V4 | 1.0 | 1.627e-20 | 696 | 0.276 | 383 | 159 | 14 | 1 | 370 | 10 | 287 | Exodeoxyribonuclease 8 | Exodeoxyribonuclease 8 | | afdb-uniprot50 | AF-A0A742LDT8-F1-MODEL\_V4 | 1.0 | 2.145e-22 | 696 | 0.265 | 369 | 159 | 13 | 1 | 360 | 196 | 461 | DUF3799 domain-containing protein | DUF3799 domain-containing protein | | afdb-uniprot50 | AF-A0A6G7XGW4-F1-MODEL\_V4 | 1.0 | 7.875e-19 | 695 | 0.231 | 372 | 181 | 11 | 1 | 370 | 3 | 271 | DUF3799 domain-containing protein | DUF3799 domain-containing protein | | afdb-uniprot50 | AF-A0A366DC85-F1-MODEL\_V4 | 1.0 | 8.309e-20 | 695 | 0.238 | 378 | 175 | 15 | 1 | 368 | 1 | 275 | PDDEXK-like uncharacterized protein DUF3799 | PDDEXK-like uncharacterized protein DUF3799 | | afdb-uniprot50 | AF-A0A0J8U4U2-F1-MODEL\_V4 | 1.0 | 1.458e-19 | 694 | 0.243 | 374 | 178 | 12 | 2 | 371 | 7 | 279 | DUF3799 domain-containing protein | DUF3799 domain-containing protein | | afdb-uniprot50 | AF-A0A3D3U8P6-F1-MODEL\_V4 | 1.0 | 4.748e-19 | 693 | 0.235 | 361 | 164 | 8 | 9 | 358 | 1 | 260 | DUF3799 domain-containing protein | DUF3799 domain-containing protein | | afdb-uniprot50 | AF-A0A606Y4Y5-F1-MODEL\_V4 | 1.0 | 1.531e-22 | 693 | 0.27 | 370 | 167 | 10 | 1 | 366 | 541 | 811 | Exonuclease VIII | Exonuclease VIII | | afdb-uniprot50 | AF-A0A0F9FLX8-F1-MODEL\_V4 | 1.0 | 7.445e-19 | 690 | 0.198 | 373 | 189 | 10 | 1 | 366 | 1 | 270 | DUF3799 domain-containing protein | DUF3799 domain-containing protein | | afdb-uniprot50 | AF-A0A6Y5LE31-F1-MODEL\_V4 | 1.0 | 3.179e-22 | 690 | 0.262 | 369 | 160 | 13 | 1 | 360 | 501 | 766 | DUF1391 domain-containing protein | DUF1391 domain-containing protein | | afdb-uniprot50 | AF-A0A7V9ZES2-F1-MODEL\_V4 | 1.0 | 1.636e-18 | 689 | 0.174 | 361 | 195 | 7 | 1 | 360 | 7 | 265 | PD-(D/E)XK nuclease-like domain-containing protein | PD-(D/E)XK nuclease-like domain-containing protein | | afdb-uniprot50 | AF-A0A2E7ZS37-F1-MODEL\_V4 | 1.0 | 1.717e-21 | 689 | 0.266 | 372 | 188 | 12 | 5 | 364 | 10 | 308 | DUF3799 domain-containing protein | DUF3799 domain-containing protein | | afdb-uniprot50 | AF-A0A2I1I759-F1-MODEL\_V4 | 1.0 | 2.286e-19 | 688 | 0.223 | 372 | 172 | 13 | 1 | 363 | 6 | 269 | DUF3799 domain-containing protein | DUF3799 domain-containing protein | | afdb-uniprot50 | AF-A0A5C7NLS0-F1-MODEL\_V4 | 1.0 | 5.01e-20 | 688 | 0.242 | 383 | 183 | 14 | 1 | 371 | 11 | 298 | DUF3799 domain-containing protein | DUF3799 domain-containing protein | | afdb-uniprot50 | AF-A0A2E5HBV2-F1-MODEL\_V4 | 1.0 | 5.62e-19 | 686 | 0.261 | 367 | 165 | 10 | 1 | 364 | 2 | 265 | DUF3799 domain-containing protein | DUF3799 domain-containing protein | | afdb-uniprot50 | AF-A0A0W7W9N2-F1-MODEL\_V4 | 1.0 | 5.01e-20 | 685 | 0.228 | 380 | 189 | 10 | 2 | 371 | 7 | 292 | DUF3799 domain-containing protein | DUF3799 domain-containing protein | | afdb-uniprot50 | AF-A0A6M3JG38-F1-MODEL\_V4 | 1.0 | 1.382e-18 | 683 | 0.243 | 365 | 170 | 12 | 1 | 360 | 4 | 267 | DUF3799 domain-containing protein | DUF3799 domain-containing protein | | afdb-uniprot50 | AF-A0A6I4WMS0-F1-MODEL\_V4 | 1.0 | 1.615e-23 | 683 | 0.226 | 398 | 252 | 9 | 3 | 358 | 34 | 417 | PD-(D/E)XK nuclease-like domain-containing protein | PD-(D/E)XK nuclease-like domain-containing protein | | afdb-uniprot50 | AF-A0A0K6IY38-F1-MODEL\_V4 | 1.0 | 5.62e-19 | 682 | 0.299 | 321 | 120 | 9 | 52 | 363 | 2 | 226 | DUF3799 domain-containing protein | DUF3799 domain-containing protein | | afdb-uniprot50 | AF-A0A8B5X8J1-F1-MODEL\_V4 | 1.0 | 6.653e-19 | 682 | 0.224 | 356 | 175 | 6 | 8 | 359 | 1 | 259 | Uncharacterized protein | Uncharacterized protein | | afdb-uniprot50 | AF-A0A1Q5A5E3-F1-MODEL\_V4 | 1.0 | 1.826e-19 | 682 | 0.237 | 375 | 179 | 10 | 1 | 368 | 16 | 290 | DUF3799 domain-containing protein | DUF3799 domain-containing protein | | afdb-uniprot50 | AF-A0A5X3VD53-F1-MODEL\_V4 | 1.0 | 1.447e-22 | 682 | 0.216 | 424 | 250 | 12 | 17 | 372 | 1 | 410 | Exodeoxyribonuclease | Exodeoxyribonuclease | | afdb-uniprot50 | AF-A0A418GY69-F1-MODEL\_V4 | 1.0 | 4.454e-22 | 682 | 0.267 | 370 | 168 | 9 | 1 | 366 | 277 | 547 | Exodeoxyribonuclease 8 | Exodeoxyribonuclease 8 | | afdb-uniprot50 | AF-A0A7U5YQG6-F1-MODEL\_V4 | 1.0 | 1.619e-22 | 682 | 0.262 | 369 | 160 | 11 | 1 | 360 | 567 | 832 | Exonuclease VIII | Exonuclease VIII | | afdb-uniprot50 | AF-A0A494WBY5-F1-MODEL\_V4 | 1.0 | 7.02e-20 | 681 | 0.244 | 384 | 183 | 10 | 2 | 371 | 17 | 307 | DUF3799 domain-containing protein | DUF3799 domain-containing protein | | afdb-uniprot50 | AF-A0A2D6F114-F1-MODEL\_V4 | 1.0 | 1.038e-20 | 681 | 0.251 | 366 | 201 | 13 | 5 | 360 | 8 | 310 | DUF3799 domain-containing protein | DUF3799 domain-containing protein | | afdb-uniprot50 | AF-A0A827ZGB3-F1-MODEL\_V4 | 1.0 | 1.917e-22 | 681 | 0.265 | 373 | 165 | 11 | 1 | 366 | 501 | 771 | Exodeoxyribonuclease VIII | Exodeoxyribonuclease VIII | | afdb-uniprot50 | AF-A0A7U0UP56-F1-MODEL\_V4 | 1.0 | 2.418e-19 | 680 | 0.231 | 371 | 173 | 14 | 2 | 367 | 5 | 268 | PD-(D/E)XK nuclease-like domain-containing protein | PD-(D/E)XK nuclease-like domain-containing protein | | afdb-uniprot50 | AF-A0A7C5WUZ4-F1-MODEL\_V4 | 1.0 | 3.584e-19 | 680 | 0.254 | 365 | 161 | 12 | 6 | 364 | 11 | 270 | DUF3799 domain-containing protein | DUF3799 domain-containing protein | | afdb-uniprot50 | AF-A0A5C1APE4-F1-MODEL\_V4 | 1.0 | 1.3e-20 | 679 | 0.209 | 411 | 218 | 15 | 1 | 367 | 9 | 356 | Exodeoxyribonuclease 8 | Exodeoxyribonuclease 8 | | afdb-uniprot50 | AF-A0A1K1LD38-F1-MODEL\_V4 | 1.0 | 2.706e-19 | 678 | 0.232 | 375 | 178 | 14 | 1 | 366 | 1 | 274 | Exodeoxyribonuclease VIII | Exodeoxyribonuclease VIII | | afdb-uniprot50 | AF-A0A730CAU1-F1-MODEL\_V4 | 1.0 | 1.534e-21 | 678 | 0.205 | 418 | 254 | 11 | 21 | 372 | 1 | 406 | Exodeoxyribonuclease | Exodeoxyribonuclease | | afdb-uniprot50 | AF-A0A6M3J3Y5-F1-MODEL\_V4 | 1.0 | 1.041e-19 | 677 | 0.238 | 360 | 169 | 10 | 7 | 361 | 3 | 262 | DUF3799 domain-containing protein | DUF3799 domain-containing protein | | afdb-uniprot50 | AF-A0A829DNE5-F1-MODEL\_V4 | 1.0 | 3.005e-22 | 677 | 0.265 | 373 | 165 | 11 | 1 | 366 | 616 | 886 | Uncharacterized protein | Uncharacterized protein | | afdb-uniprot50 | AF-A0A731HUQ6-F1-MODEL\_V4 | 1.0 | 2.027e-22 | 676 | 0.214 | 424 | 251 | 12 | 17 | 372 | 1 | 410 | Exodeoxyribonuclease | Exodeoxyribonuclease | | afdb-uniprot50 | AF-A0A2A7UMV7-F1-MODEL\_V4 | 1.0 | 5.62e-19 | 675 | 0.22 | 376 | 181 | 12 | 1 | 370 | 6 | 275 | RecE | RecE | | afdb-uniprot50 | AF-A0A0A1DF13-F1-MODEL\_V4 | 1.0 | 1.232e-19 | 675 | 0.25 | 376 | 178 | 14 | 1 | 372 | 18 | 293 | Phage exonuclease | Phage exonuclease | | afdb-uniprot50 | AF-A0A849A8M1-F1-MODEL\_V4 | 1.0 | 1.458e-19 | 673 | 0.272 | 370 | 169 | 10 | 1 | 365 | 1 | 275 | Uncharacterized protein | Uncharacterized protein | | afdb-uniprot50 | AF-A0A2N3VGV8-F1-MODEL\_V4 | 1.0 | 3.584e-19 | 673 | 0.238 | 382 | 175 | 13 | 2 | 368 | 6 | 286 | PDDEXK-like uncharacterized protein DUF3799 | PDDEXK-like uncharacterized protein DUF3799 | | afdb-uniprot50 | AF-A0A6X8AL85-F1-MODEL\_V4 | 1.0 | 6.984e-22 | 673 | 0.264 | 374 | 164 | 11 | 1 | 366 | 237 | 507 | Exodeoxyribonuclease 8 | Exodeoxyribonuclease 8 | | afdb-uniprot50 | AF-A0A7H9CND5-F1-MODEL\_V4 | 1.0 | 4.748e-19 | 672 | 0.225 | 360 | 169 | 11 | 9 | 358 | 1 | 260 | Putative exonuclease VIII | Putative exonuclease VIII | | afdb-uniprot50 | AF-A0A2D9C7V6-F1-MODEL\_V4 | 1.0 | 5.326e-18 | 671 | 0.222 | 359 | 172 | 12 | 4 | 360 | 2 | 255 | DUF3799 domain-containing protein | DUF3799 domain-containing protein | | afdb-uniprot50 | AF-A0A2D5F9Z8-F1-MODEL\_V4 | 1.0 | 2.699e-20 | 671 | 0.253 | 355 | 169 | 9 | 5 | 357 | 2 | 262 | DUF3799 domain-containing protein | DUF3799 domain-containing protein | | afdb-uniprot50 | AF-A0A730GR09-F1-MODEL\_V4 | 1.0 | 8.746e-22 | 671 | 0.21 | 418 | 252 | 11 | 21 | 372 | 1 | 406 | DNA breaking-rejoining protein | DNA breaking-rejoining protein | | afdb-uniprot50 | AF-A0A378Y5Z6-F1-MODEL\_V4 | 1.0 | 8.79e-20 | 670 | 0.249 | 373 | 170 | 13 | 1 | 367 | 1 | 269 | Exodeoxyribonuclease 8 | Exodeoxyribonuclease 8 | | afdb-uniprot50 | AF-A0A3M9MM27-F1-MODEL\_V4 | 1.0 | 3.028e-19 | 669 | 0.255 | 368 | 169 | 15 | 2 | 360 | 10 | 281 | DUF3799 domain-containing protein | DUF3799 domain-containing protein | | afdb-uniprot50 | AF-A0A3A5MMF7-F1-MODEL\_V4 | 1.0 | 1.041e-19 | 669 | 0.237 | 371 | 183 | 11 | 2 | 367 | 36 | 311 | DUF3799 domain-containing protein | DUF3799 domain-containing protein | | afdb-uniprot50 | AF-A0A638M5J8-F1-MODEL\_V4 | 1.0 | 3.005e-22 | 669 | 0.264 | 375 | 163 | 12 | 1 | 366 | 314 | 584 | Exodeoxyribonuclease 8 | Exodeoxyribonuclease 8 | | afdb-uniprot50 | AF-A0A854GB88-F1-MODEL\_V4 | 1.0 | 7.855e-20 | 668 | 0.229 | 370 | 174 | 12 | 8 | 370 | 34 | 299 | Uncharacterized protein | Uncharacterized protein | | afdb-uniprot50 | AF-P15032-F1-MODEL\_V4 | 1.0 | 2.15e-21 | 668 | 0.274 | 368 | 164 | 9 | 1 | 364 | 595 | 863 | Exodeoxyribonuclease 8 | Exodeoxyribonuclease 8 | | afdb-uniprot50 | AF-A0A0N0A2F3-F1-MODEL\_V4 | 1.0 | 4.477e-20 | 667 | 0.257 | 381 | 169 | 14 | 1 | 369 | 23 | 301 | DUF3799 domain-containing protein | DUF3799 domain-containing protein | | afdb-uniprot50 | AF-A0A3L7AHV8-F1-MODEL\_V4 | 1.0 | 8.309e-20 | 667 | 0.224 | 383 | 187 | 12 | 2 | 372 | 18 | 302 | DUF3799 domain-containing protein | DUF3799 domain-containing protein | | afdb-uniprot50 | AF-A0A7Z0J579-F1-MODEL\_V4 | 1.0 | 1.726e-19 | 666 | 0.263 | 376 | 171 | 13 | 1 | 368 | 5 | 282 | DUF3799 domain-containing protein | DUF3799 domain-containing protein | | afdb-uniprot50 | AF-Q6LJU7-F1-MODEL\_V4 | 1.0 | 1.296e-21 | 666 | 0.185 | 501 | 266 | 17 | 1 | 372 | 661 | 1148 | DUF3799 domain-containing protein | DUF3799 domain-containing protein | | afdb-uniprot50 | AF-A0A709X174-F1-MODEL\_V4 | 1.0 | 6.619e-21 | 665 | 0.206 | 411 | 248 | 11 | 28 | 372 | 2 | 400 | DUF3799 domain-containing protein | DUF3799 domain-containing protein | | afdb-uniprot50 | AF-A0A2U9P084-F1-MODEL\_V4 | 1.0 | 1.296e-21 | 665 | 0.273 | 377 | 164 | 11 | 2 | 368 | 163 | 439 | DUF3799 domain-containing protein | DUF3799 domain-containing protein | | afdb-uniprot50 | AF-A0A1X3IAK9-F1-MODEL\_V4 | 1.0 | 9.251e-22 | 665 | 0.268 | 373 | 164 | 11 | 1 | 366 | 319 | 589 | Exodeoxyribonuclease 8 (Exodeoxyribonuclease VIII) (EXOVIII) | Exodeoxyribonuclease 8 (Exodeoxyribonuclease VIII) (EXOVIII) | | afdb-uniprot50 | AF-A0A536UDP7-F1-MODEL\_V4 | 1.0 | 5.62e-19 | 664 | 0.23 | 373 | 164 | 11 | 20 | 370 | 1 | 272 | DUF3799 domain-containing protein | DUF3799 domain-containing protein | | afdb-uniprot50 | AF-A0A1Z9Q5Q2-F1-MODEL\_V4 | 1.0 | 1.306e-18 | 663 | 0.199 | 366 | 186 | 10 | 1 | 361 | 5 | 268 | DUF3799 domain-containing protein | DUF3799 domain-containing protein | | afdb-uniprot50 | AF-A0A1G3M6I0-F1-MODEL\_V4 | 1.0 | 1.826e-19 | 663 | 0.254 | 370 | 181 | 13 | 1 | 360 | 22 | 306 | DUF3799 domain-containing protein | DUF3799 domain-containing protein | | afdb-uniprot50 | AF-A0A735S3T4-F1-MODEL\_V4 | 1.0 | 2.692e-21 | 663 | 0.272 | 370 | 166 | 9 | 1 | 366 | 121 | 391 | Exonuclease VIII | Exonuclease VIII | | afdb-uniprot50 | AF-A0A2W6R8J9-F1-MODEL\_V4 | 1.0 | 2.558e-19 | 662 | 0.235 | 373 | 174 | 13 | 2 | 371 | 11 | 275 | RecE | RecE | | afdb-uniprot50 | AF-A0A2W1QND0-F1-MODEL\_V4 | 1.0 | 1.164e-19 | 661 | 0.262 | 373 | 176 | 11 | 1 | 370 | 24 | 300 | DUF3799 domain-containing protein | DUF3799 domain-containing protein | | afdb-uniprot50 | AF-A0A1M5I3B0-F1-MODEL\_V4 | 1.0 | 2.043e-19 | 661 | 0.24 | 366 | 171 | 9 | 1 | 360 | 53 | 317 | Exodeoxyribonuclease VIII | Exodeoxyribonuclease VIII | | afdb-uniprot50 | AF-A0A7Z7VL18-F1-MODEL\_V4 | 1.0 | 2.841e-22 | 661 | 0.259 | 374 | 232 | 11 | 1 | 359 | 77 | 420 | SAP domain-containing protein | SAP domain-containing protein | | afdb-uniprot50 | AF-A0A0L6CK16-F1-MODEL\_V4 | 1.0 | 7.445e-19 | 660 | 0.241 | 368 | 166 | 14 | 5 | 362 | 9 | 273 | DUF3799 domain-containing protein | DUF3799 domain-containing protein | | afdb-uniprot50 | AF-A0A4Q8AK80-F1-MODEL\_V4 | 1.0 | 1.103e-18 | 659 | 0.236 | 363 | 179 | 11 | 1 | 360 | 2 | 269 | PDDEXK-like uncharacterized protein DUF3799 | PDDEXK-like uncharacterized protein DUF3799 | | afdb-uniprot50 | AF-A0A759U666-F1-MODEL\_V4 | 1.0 | 5.592e-21 | 659 | 0.203 | 417 | 254 | 11 | 22 | 372 | 1 | 405 | Exodeoxyribonuclease | Exodeoxyribonuclease | | afdb-uniprot50 | AF-A0A5T3EKQ1-F1-MODEL\_V4 | 1.0 | 3.557e-22 | 659 | 0.231 | 411 | 234 | 14 | 1 | 343 | 550 | 946 | Exodeoxyribonuclease | Exodeoxyribonuclease | | afdb-uniprot50 | AF-L7VX35-F1-MODEL\_V4 | 1.0 | 4.243e-19 | 658 | 0.211 | 355 | 176 | 9 | 9 | 361 | 1 | 253 | Exodeoxyribonuclease VIII | Exodeoxyribonuclease VIII | | afdb-uniprot50 | AF-A0A1H1XHG2-F1-MODEL\_V4 | 1.0 | 1.232e-19 | 658 | 0.231 | 372 | 187 | 14 | 1 | 368 | 14 | 290 | DUF3799 domain-containing protein | DUF3799 domain-containing protein | | afdb-uniprot50 | AF-A0A4V2NXX9-F1-MODEL\_V4 | 1.0 | 1.926e-20 | 658 | 0.269 | 371 | 165 | 11 | 2 | 370 | 61 | 327 | DUF3799 domain-containing protein | DUF3799 domain-containing protein | | afdb-uniprot50 | AF-A0A191WF22-F1-MODEL\_V4 | 1.0 | 5.035e-18 | 657 | 0.237 | 370 | 174 | 14 | 1 | 369 | 4 | 266 | DUF3799 domain-containing protein | DUF3799 domain-containing protein | | afdb-uniprot50 | AF-A0A367HZT2-F1-MODEL\_V4 | 1.0 | 4.748e-19 | 657 | 0.247 | 372 | 167 | 12 | 9 | 368 | 1 | 271 | DUF3799 domain-containing protein | DUF3799 domain-containing protein | | afdb-uniprot50 | AF-A0A2G8BG73-F1-MODEL\_V4 | 1.0 | 2.424e-18 | 656 | 0.247 | 367 | 167 | 13 | 1 | 361 | 4 | 267 | DUF3799 domain-containing protein | DUF3799 domain-containing protein | | afdb-uniprot50 | AF-A0A7D7WG90-F1-MODEL\_V4 | 1.0 | 4.243e-19 | 656 | 0.252 | 364 | 171 | 11 | 4 | 366 | 2 | 265 | DUF3799 domain-containing protein | DUF3799 domain-containing protein | | afdb-uniprot50 | AF-A0A497R8U6-F1-MODEL\_V4 | 1.0 | 2.043e-19 | 655 | 0.242 | 371 | 170 | 12 | 1 | 365 | 45 | 310 | DUF3799 domain-containing protein | DUF3799 domain-containing protein | | afdb-uniprot50 | AF-A0A7W4YZB4-F1-MODEL\_V4 | 1.0 | 6.653e-19 | 654 | 0.224 | 379 | 187 | 9 | 2 | 370 | 4 | 285 | DUF3799 domain-containing protein | DUF3799 domain-containing protein | | afdb-uniprot50 | AF-A0A4Y3NBY8-F1-MODEL\_V4 | 1.0 | 1.164e-19 | 654 | 0.252 | 376 | 175 | 14 | 2 | 372 | 53 | 327 | DUF3799 domain-containing protein | DUF3799 domain-containing protein | | afdb-uniprot50 | AF-A0A634SIY9-F1-MODEL\_V4 | 1.0 | 8.288e-21 | 654 | 0.27 | 374 | 162 | 12 | 1 | 366 | 85 | 355 | Exonuclease VIII | Exonuclease VIII | | afdb-uniprot50 | AF-A0A1T4USH8-F1-MODEL\_V4 | 1.0 | 5.286e-21 | 653 | 0.242 | 375 | 209 | 12 | 1 | 361 | 44 | 357 | Exodeoxyribonuclease 8 | Exodeoxyribonuclease 8 | | afdb-uniprot50 | AF-A0A7S7FKE8-F1-MODEL\_V4 | 1.0 | 4.243e-19 | 652 | 0.255 | 376 | 160 | 15 | 1 | 360 | 5 | 276 | PD-(D/E)XK nuclease-like domain-containing protein | PD-(D/E)XK nuclease-like domain-containing protein | | afdb-uniprot50 | AF-A0A0M2HG29-F1-MODEL\_V4 | 1.0 | 1.926e-20 | 652 | 0.258 | 372 | 175 | 13 | 4 | 371 | 37 | 311 | Exodeoxyribonuclease 8 | Exodeoxyribonuclease 8 | | afdb-uniprot50 | AF-A0A142XVC8-F1-MODEL\_V4 | 1.0 | 7.875e-19 | 651 | 0.216 | 360 | 178 | 12 | 10 | 363 | 3 | 264 | Exodeoxyribonuclease 8 | Exodeoxyribonuclease 8 | | afdb-uniprot50 | AF-A0A843H5C9-F1-MODEL\_V4 | 1.0 | 6.653e-19 | 651 | 0.239 | 355 | 198 | 12 | 1 | 351 | 1 | 287 | PD-(D/E)XK nuclease-like domain-containing protein | PD-(D/E)XK nuclease-like domain-containing protein | | afdb-uniprot50 | AF-A0A6I5FGL7-F1-MODEL\_V4 | 1.0 | 1.167e-18 | 650 | 0.228 | 377 | 182 | 12 | 1 | 368 | 13 | 289 | DUF3799 domain-containing protein | DUF3799 domain-containing protein | | afdb-uniprot50 | AF-A0A0F9G9B6-F1-MODEL\_V4 | 1.0 | 3.388e-19 | 650 | 0.211 | 388 | 192 | 14 | 1 | 372 | 5 | 294 | DUF3799 domain-containing protein | DUF3799 domain-containing protein | | afdb-uniprot50 | AF-A0A2W5NKZ8-F1-MODEL\_V4 | 1.0 | 1.926e-20 | 650 | 0.249 | 373 | 188 | 9 | 5 | 371 | 15 | 301 | DUF3799 domain-containing protein | DUF3799 domain-containing protein | | afdb-uniprot50 | AF-H8FP23-F1-MODEL\_V4 | 1.0 | 9.811e-21 | 650 | 0.248 | 391 | 177 | 13 | 1 | 371 | 63 | 356 | DUF3799 domain-containing protein | DUF3799 domain-containing protein | | afdb-uniprot50 | AF-A0A7Y5UCT3-F1-MODEL\_V4 | 1.0 | 1.462e-18 | 649 | 0.243 | 365 | 167 | 13 | 1 | 361 | 3 | 262 | DUF3799 domain-containing protein | DUF3799 domain-containing protein | | afdb-uniprot50 | AF-A0A661DE24-F1-MODEL\_V4 | 1.0 | 1.31e-17 | 649 | 0.224 | 361 | 172 | 8 | 5 | 360 | 2 | 259 | DUF3799 domain-containing protein | DUF3799 domain-containing protein | | afdb-uniprot50 | AF-A0A2E9H4D8-F1-MODEL\_V4 | 1.0 | 2.848e-21 | 649 | 0.238 | 382 | 213 | 14 | 1 | 359 | 47 | 373 | SAP domain-containing protein | SAP domain-containing protein | | afdb-uniprot50 | AF-A0A524KUU5-F1-MODEL\_V4 | 1.0 | 2.877e-17 | 648 | 0.242 | 342 | 153 | 9 | 1 | 336 | 1 | 242 | DUF3799 domain-containing protein | DUF3799 domain-containing protein | | afdb-uniprot50 | AF-A0A848KZI4-F1-MODEL\_V4 | 1.0 | 1.936e-18 | 648 | 0.236 | 380 | 171 | 14 | 1 | 369 | 12 | 283 | Uncharacterized protein | Uncharacterized protein | | afdb-uniprot50 | AF-A0A5C7Q151-F1-MODEL\_V4 | 1.0 | 5.313e-19 | 648 | 0.246 | 369 | 196 | 12 | 2 | 366 | 7 | 297 | DUF3799 domain-containing protein | DUF3799 domain-containing protein | | afdb-uniprot50 | AF-A0A447PEW2-F1-MODEL\_V4 | 1.0 | 1.816e-21 | 648 | 0.265 | 376 | 162 | 12 | 1 | 367 | 391 | 661 | Exonuclease VIII | Exonuclease VIII | | afdb-uniprot50 | AF-A0A354UC05-F1-MODEL\_V4 | 1.0 | 4.021e-18 | 647 | 0.212 | 353 | 180 | 8 | 9 | 357 | 13 | 271 | DUF3799 domain-containing protein | DUF3799 domain-containing protein | | afdb-uniprot50 | AF-A0A5C2FIG8-F1-MODEL\_V4 | 1.0 | 2.713e-18 | 647 | 0.219 | 374 | 179 | 15 | 4 | 369 | 3 | 271 | DUF3799 domain-containing protein | DUF3799 domain-containing protein | | afdb-uniprot50 | AF-A0A5C7QK33-F1-MODEL\_V4 | 1.0 | 1.826e-19 | 646 | 0.232 | 375 | 193 | 12 | 1 | 364 | 6 | 296 | DUF3799 domain-containing protein | DUF3799 domain-containing protein | | afdb-uniprot50 | AF-A0A7X0NGZ1-F1-MODEL\_V4 | 1.0 | 5.606e-20 | 646 | 0.209 | 397 | 216 | 8 | 1 | 357 | 34 | 372 | Exodeoxyribonuclease VIII | Exodeoxyribonuclease VIII | | afdb-uniprot50 | AF-A0A164JW40-F1-MODEL\_V4 | 1.0 | 5.945e-19 | 645 | 0.238 | 369 | 169 | 12 | 9 | 368 | 1 | 266 | DUF3799 domain-containing protein | DUF3799 domain-containing protein | | afdb-uniprot50 | AF-A0A7Y5JXA5-F1-MODEL\_V4 | 1.0 | 8.331e-19 | 645 | 0.237 | 362 | 173 | 13 | 1 | 359 | 5 | 266 | RecE | RecE | | afdb-uniprot50 | AF-A0A0J9D3R0-F1-MODEL\_V4 | 1.0 | 2.552e-20 | 645 | 0.226 | 406 | 186 | 11 | 2 | 371 | 118 | 431 | DUF3799 domain-containing protein | DUF3799 domain-containing protein | | afdb-uniprot50 | AF-A0A7X9ENY2-F1-MODEL\_V4 | 1.0 | 3.397e-18 | 644 | 0.241 | 365 | 165 | 13 | 3 | 359 | 2 | 262 | DUF3799 domain-containing protein | DUF3799 domain-containing protein | | afdb-uniprot50 | AF-A0A1S2WIB5-F1-MODEL\_V4 | 1.0 | 5.035e-18 | 644 | 0.229 | 374 | 181 | 12 | 2 | 369 | 8 | 280 | RecE | RecE | | afdb-uniprot50 | AF-A0A7X7ZLD9-F1-MODEL\_V4 | 1.0 | 2.167e-18 | 644 | 0.24 | 362 | 172 | 8 | 1 | 361 | 11 | 270 | DUF3799 domain-containing protein | DUF3799 domain-containing protein | | afdb-uniprot50 | AF-A0A022LJE0-F1-MODEL\_V4 | 1.0 | 4.748e-19 | 644 | 0.231 | 384 | 193 | 15 | 1 | 372 | 3 | 296 | DUF3799 domain-containing protein | DUF3799 domain-containing protein | | afdb-uniprot50 | AF-A0A2D7NC52-F1-MODEL\_V4 | 1.0 | 1.17e-17 | 643 | 0.203 | 358 | 177 | 10 | 3 | 357 | 5 | 257 | DUF3799 domain-containing protein | DUF3799 domain-containing protein | | afdb-uniprot50 | AF-A0A1N2I403-F1-MODEL\_V4 | 1.0 | 4.243e-19 | 643 | 0.219 | 369 | 185 | 15 | 1 | 360 | 5 | 279 | RecE | RecE | | afdb-uniprot50 | AF-A0A753B4R6-F1-MODEL\_V4 | 1.0 | 2.545e-21 | 643 | 0.268 | 376 | 161 | 12 | 1 | 367 | 621 | 891 | Exodeoxyribonuclease VIII | Exodeoxyribonuclease VIII | | afdb-uniprot50 | AF-A0A0F6SEZ4-F1-MODEL\_V4 | 1.0 | 9.346e-18 | 642 | 0.197 | 354 | 186 | 8 | 9 | 360 | 1 | 258 | Exodeoxyribonuclease VIII | Exodeoxyribonuclease VIII | | afdb-uniprot50 | AF-A0A2T3IFT8-F1-MODEL\_V4 | 1.0 | 8.768e-21 | 640 | 0.242 | 375 | 209 | 12 | 1 | 361 | 89 | 402 | DUF3799 domain-containing protein | DUF3799 domain-containing protein | | afdb-uniprot50 | AF-D2U156-F1-MODEL\_V4 | 1.0 | 8.352e-18 | 639 | 0.193 | 366 | 190 | 9 | 1 | 363 | 1 | 264 | Exodeoxyribonuclease 8 | Exodeoxyribonuclease 8 | | afdb-uniprot50 | AF-N6YA75-F1-MODEL\_V4 | 1.0 | 5.313e-19 | 639 | 0.243 | 358 | 159 | 9 | 8 | 360 | 43 | 293 | DUF3799 domain-containing protein | DUF3799 domain-containing protein | | afdb-uniprot50 | AF-A0A2D8W508-F1-MODEL\_V4 | 1.0 | 1.73e-18 | 639 | 0.225 | 381 | 186 | 11 | 2 | 372 | 15 | 296 | DUF3799 domain-containing protein | DUF3799 domain-containing protein | | afdb-uniprot50 | AF-A0A646KLD2-F1-MODEL\_V4 | 1.0 | 1.101e-19 | 639 | 0.259 | 381 | 166 | 14 | 1 | 368 | 45 | 322 | DUF3799 domain-containing protein | DUF3799 domain-containing protein | | afdb-uniprot50 | AF-A0A7Y6TYS2-F1-MODEL\_V4 | 1.0 | 5.01e-20 | 639 | 0.23 | 365 | 176 | 9 | 1 | 360 | 109 | 373 | PD-(D/E)XK nuclease-like domain-containing protein | PD-(D/E)XK nuclease-like domain-containing protein | | afdb-uniprot50 | AF-A0A7Y5TGE9-F1-MODEL\_V4 | 1.0 | 3.036e-18 | 638 | 0.223 | 363 | 171 | 13 | 1 | 357 | 3 | 260 | DUF3799 domain-containing protein | DUF3799 domain-containing protein | | afdb-uniprot50 | AF-A0A518EYZ7-F1-MODEL\_V4 | 1.0 | 1.382e-18 | 637 | 0.184 | 373 | 198 | 12 | 2 | 366 | 11 | 285 | Exodeoxyribonuclease 8 | Exodeoxyribonuclease 8 | | afdb-uniprot50 | AF-A0A560CFR0-F1-MODEL\_V4 | 1.0 | 2.424e-18 | 637 | 0.232 | 378 | 180 | 11 | 1 | 371 | 7 | 281 | PDDEXK-like uncharacterized protein DUF3799 | PDDEXK-like uncharacterized protein DUF3799 | | afdb-uniprot50 | AF-A0A2D7BYZ1-F1-MODEL\_V4 | 1.0 | 2.048e-18 | 636 | 0.218 | 361 | 171 | 10 | 1 | 357 | 16 | 269 | DUF3799 domain-containing protein | DUF3799 domain-containing protein | | afdb-uniprot50 | AF-A0A3M8FHL9-F1-MODEL\_V4 | 1.0 | 5.606e-20 | 636 | 0.231 | 371 | 214 | 15 | 5 | 368 | 13 | 319 | DUF3799 domain-containing protein | DUF3799 domain-containing protein | | afdb-uniprot50 | AF-A0A2W5UA26-F1-MODEL\_V4 | 1.0 | 2.706e-19 | 636 | 0.192 | 384 | 242 | 12 | 1 | 372 | 21 | 348 | DUF3799 domain-containing protein | DUF3799 domain-containing protein | | afdb-uniprot50 | AF-A0A8B2U0W5-F1-MODEL\_V4 | 1.0 | 9.251e-22 | 635 | 0.241 | 397 | 247 | 13 | 3 | 358 | 99 | 482 | Uncharacterized protein | Uncharacterized protein | | afdb-uniprot50 | AF-A0A2W6WBB9-F1-MODEL\_V4 | 1.0 | 2.855e-20 | 634 | 0.246 | 385 | 184 | 11 | 1 | 371 | 78 | 370 | DUF3799 domain-containing protein | DUF3799 domain-containing protein | | afdb-uniprot50 | AF-Q6LHC4-F1-MODEL\_V4 | 1.0 | 7.895e-18 | 633 | 0.377 | 196 | 111 | 3 | 178 | 370 | 26 | 213 | DUF3799 domain-containing protein | DUF3799 domain-containing protein | | afdb-uniprot50 | AF-A0A7W4B1R6-F1-MODEL\_V4 | 1.0 | 3.773e-21 | 633 | 0.262 | 381 | 231 | 11 | 1 | 357 | 34 | 388 | PD-(D/E)XK nuclease-like domain-containing protein | PD-(D/E)XK nuclease-like domain-containing protein | | afdb-uniprot50 | AF-A0A100WID5-F1-MODEL\_V4 | 1.0 | 7.463e-18 | 632 | 0.237 | 375 | 174 | 17 | 2 | 366 | 7 | 279 | DUF3799 domain-containing protein | DUF3799 domain-containing protein | | afdb-uniprot50 | AF-A0A6M3LIW3-F1-MODEL\_V4 | 1.0 | 1.83e-18 | 632 | 0.22 | 381 | 188 | 13 | 1 | 370 | 3 | 285 | DUF3799 domain-containing protein | DUF3799 domain-containing protein | | afdb-uniprot50 | AF-A0A3S9D344-F1-MODEL\_V4 | 1.0 | 1.382e-18 | 632 | 0.212 | 382 | 200 | 11 | 1 | 371 | 15 | 306 | DUF3799 domain-containing protein | DUF3799 domain-containing protein | | afdb-uniprot50 | AF-A0A2X1UAB7-F1-MODEL\_V4 | 1.0 | 5.313e-19 | 632 | 0.225 | 377 | 182 | 13 | 1 | 370 | 57 | 330 | Gp60 protein | Gp60 protein | | afdb-uniprot50 | AF-A0A1G4X2S6-F1-MODEL\_V4 | 1.0 | 7.426e-20 | 632 | 0.257 | 376 | 170 | 13 | 2 | 371 | 73 | 345 | PD-(D/E)XK nuclease superfamily protein | PD-(D/E)XK nuclease superfamily protein | | afdb-uniprot50 | AF-A0A6G7XI10-F1-MODEL\_V4 | 1.0 | 7.895e-18 | 630 | 0.232 | 375 | 187 | 11 | 2 | 371 | 7 | 285 | DUF3799 domain-containing protein | DUF3799 domain-containing protein | | afdb-uniprot50 | AF-A0A5R8NB53-F1-MODEL\_V4 | 1.0 | 9.322e-19 | 629 | 0.223 | 367 | 173 | 13 | 1 | 360 | 5 | 266 | DUF3799 domain-containing protein | DUF3799 domain-containing protein | | afdb-uniprot50 | AF-A0A3N5PIH7-F1-MODEL\_V4 | 1.0 | 5.945e-19 | 629 | 0.222 | 391 | 183 | 17 | 1 | 372 | 6 | 294 | DUF3799 domain-containing protein | DUF3799 domain-containing protein | | afdb-uniprot50 | AF-A0A7Y9DWL9-F1-MODEL\_V4 | 1.0 | 5.3e-20 | 629 | 0.217 | 400 | 182 | 17 | 1 | 371 | 1 | 298 | DUF3799 domain-containing protein | DUF3799 domain-containing protein | | afdb-uniprot50 | AF-N1MBF6-F1-MODEL\_V4 | 1.0 | 3.036e-18 | 628 | 0.4 | 205 | 114 | 4 | 172 | 369 | 19 | 221 | DUF3799 domain-containing protein | DUF3799 domain-containing protein | | afdb-uniprot50 | AF-A0A7W7XTG8-F1-MODEL\_V4 | 1.0 | 2.87e-18 | 628 | 0.248 | 370 | 168 | 13 | 1 | 364 | 11 | 276 | DUF3799 domain-containing protein | DUF3799 domain-containing protein | | afdb-uniprot50 | AF-A0A142XW82-F1-MODEL\_V4 | 1.0 | 7.915e-17 | 628 | 0.206 | 368 | 189 | 11 | 10 | 371 | 5 | 275 | Exodeoxyribonuclease 8 | Exodeoxyribonuclease 8 | | afdb-uniprot50 | AF-A0A0F9IPA0-F1-MODEL\_V4 | 1.0 | 5.035e-18 | 628 | 0.198 | 377 | 195 | 10 | 1 | 367 | 10 | 289 | DUF3799 domain-containing protein | DUF3799 domain-containing protein | | afdb-uniprot50 | AF-A0A7J5DV78-F1-MODEL\_V4 | 1.0 | 1.103e-18 | 628 | 0.255 | 379 | 180 | 12 | 1 | 369 | 7 | 293 | RecE | RecE | | afdb-uniprot50 | AF-A0A2D8XAW4-F1-MODEL\_V4 | 1.0 | 4.243e-19 | 628 | 0.269 | 368 | 152 | 12 | 11 | 365 | 7 | 270 | DUF3799 domain-containing protein | DUF3799 domain-containing protein | | afdb-uniprot50 | AF-W7WYY6-F1-MODEL\_V4 | 1.0 | 3.594e-18 | 628 | 0.185 | 372 | 188 | 12 | 1 | 360 | 13 | 281 | Exodeoxyribonuclease 8 | Exodeoxyribonuclease 8 | | afdb-uniprot50 | AF-A0A4D8R4H4-F1-MODEL\_V4 | 1.0 | 1.726e-19 | 628 | 0.217 | 390 | 194 | 15 | 1 | 371 | 7 | 304 | DUF3799 domain-containing protein | DUF3799 domain-containing protein | | afdb-uniprot50 | AF-A0A7G8J5Q1-F1-MODEL\_V4 | 1.0 | 1.826e-19 | 626 | 0.25 | 371 | 192 | 12 | 2 | 360 | 10 | 306 | DUF3799 domain-containing protein | DUF3799 domain-containing protein | | afdb-uniprot50 | AF-A0A291RDB1-F1-MODEL\_V4 | 1.0 | 2.706e-19 | 625 | 0.246 | 373 | 174 | 11 | 3 | 370 | 46 | 316 | RecE | RecE | | afdb-uniprot50 | AF-A0A269PKN2-F1-MODEL\_V4 | 1.0 | 3.792e-19 | 625 | 0.198 | 387 | 223 | 12 | 1 | 360 | 1 | 327 | DUF3799 domain-containing protein | DUF3799 domain-containing protein | | afdb-uniprot50 | AF-A0A6G2NMW0-F1-MODEL\_V4 | 1.0 | 3.38e-20 | 625 | 0.27 | 377 | 165 | 14 | 1 | 368 | 160 | 435 | DUF3799 domain-containing protein | DUF3799 domain-containing protein | | afdb-uniprot50 | AF-A0A069T421-F1-MODEL\_V4 | 1.0 | 1.64e-17 | 624 | 0.213 | 366 | 177 | 15 | 1 | 361 | 3 | 262 | DUF3799 domain-containing protein | DUF3799 domain-containing protein | | afdb-uniprot50 | AF-A0A2A9CPI8-F1-MODEL\_V4 | 1.0 | 3.02e-20 | 623 | 0.264 | 390 | 161 | 18 | 2 | 372 | 17 | 299 | PDDEXK-like uncharacterized protein DUF3799 | PDDEXK-like uncharacterized protein DUF3799 | | afdb-uniprot50 | AF-A0A5C8H0D8-F1-MODEL\_V4 | 1.0 | 7.426e-20 | 623 | 0.265 | 377 | 168 | 12 | 1 | 368 | 231 | 507 | DUF3799 domain-containing protein | DUF3799 domain-containing protein | | afdb-uniprot50 | AF-A0A852R0H0-F1-MODEL\_V4 | 1.0 | 7.463e-18 | 622 | 0.248 | 354 | 163 | 12 | 1 | 347 | 5 | 262 | Uncharacterized protein | Uncharacterized protein | | afdb-uniprot50 | AF-A0A3D6C748-F1-MODEL\_V4 | 1.0 | 2.424e-18 | 622 | 0.211 | 369 | 176 | 13 | 3 | 361 | 7 | 270 | Exonuclease VIII | Exonuclease VIII | | afdb-uniprot50 | AF-A0A178MMD6-F1-MODEL\_V4 | 1.0 | 3.594e-18 | 619 | 0.223 | 362 | 171 | 12 | 18 | 369 | 12 | 273 | DUF3799 domain-containing protein | DUF3799 domain-containing protein | | afdb-uniprot50 | AF-W5TN31-F1-MODEL\_V4 | 1.0 | 9.861e-19 | 619 | 0.228 | 368 | 172 | 12 | 1 | 360 | 36 | 299 | DUF3799 domain-containing protein | DUF3799 domain-containing protein | | afdb-uniprot50 | AF-A0A1F8JB47-F1-MODEL\_V4 | 1.0 | 7.875e-19 | 618 | 0.22 | 381 | 179 | 16 | 1 | 367 | 3 | 279 | DUF3799 domain-containing protein | DUF3799 domain-containing protein | | afdb-uniprot50 | AF-A0A3M2CPY5-F1-MODEL\_V4 | 1.0 | 1.735e-17 | 618 | 0.194 | 370 | 203 | 9 | 1 | 368 | 14 | 290 | DUF3799 domain-containing protein | DUF3799 domain-containing protein | | afdb-uniprot50 | AF-A0A635X7L7-F1-MODEL\_V4 | 1.0 | 5.286e-21 | 618 | 0.208 | 412 | 248 | 11 | 27 | 372 | 3 | 402 | DNA breaking-rejoining protein | DNA breaking-rejoining protein | | afdb-uniprot50 | AF-A0A7U6J3P3-F1-MODEL\_V4 | 1.0 | 5.93e-20 | 618 | 0.146 | 545 | 286 | 19 | 1 | 371 | 56 | 595 | DUF3799 domain-containing protein | DUF3799 domain-containing protein | | afdb-uniprot50 | AF-A0A7X5U491-F1-MODEL\_V4 | 1.0 | 8.352e-18 | 617 | 0.22 | 372 | 192 | 12 | 9 | 372 | 1 | 282 | DUF3799 domain-containing protein | DUF3799 domain-containing protein | | afdb-uniprot50 | AF-A0A5B8R4A6-F1-MODEL\_V4 | 1.0 | 1.926e-20 | 617 | 0.241 | 372 | 244 | 13 | 1 | 359 | 64 | 410 | DUF3799 domain-containing protein | DUF3799 domain-containing protein | | afdb-uniprot50 | AF-A0A0F9N017-F1-MODEL\_V4 | 1.0 | 9.346e-18 | 615 | 0.218 | 379 | 187 | 17 | 2 | 369 | 5 | 285 | DUF3799 domain-containing protein | DUF3799 domain-containing protein | | afdb-uniprot50 | AF-A0A7U2VEE6-F1-MODEL\_V4 | 1.0 | 1.926e-20 | 615 | 0.242 | 380 | 183 | 12 | 1 | 369 | 196 | 481 | DUF3799 domain-containing protein | DUF3799 domain-containing protein | | afdb-uniprot50 | AF-A0A7L5Z1X1-F1-MODEL\_V4 | 1.0 | 3.036e-18 | 614 | 0.251 | 362 | 168 | 14 | 2 | 361 | 5 | 265 | PD-(D/E)XK nuclease-like domain-containing protein | PD-(D/E)XK nuclease-like domain-containing protein | | afdb-uniprot50 | AF-A0A6M6JHN5-F1-MODEL\_V4 | 1.0 | 3.801e-18 | 614 | 0.214 | 391 | 191 | 14 | 1 | 371 | 7 | 301 | DUF3799 domain-containing protein | DUF3799 domain-containing protein | | afdb-uniprot50 | AF-A0A5M8H4X0-F1-MODEL\_V4 | 1.0 | 3.792e-19 | 614 | 0.251 | 378 | 170 | 13 | 1 | 367 | 57 | 332 | DUF3799 domain-containing protein | DUF3799 domain-containing protein | | afdb-uniprot50 | AF-A0A2D7FJB4-F1-MODEL\_V4 | 1.0 | 4.265e-17 | 612 | 0.165 | 368 | 208 | 10 | 1 | 366 | 14 | 284 | DUF3799 domain-containing protein | DUF3799 domain-containing protein | | afdb-uniprot50 | AF-A0A0P0YX84-F1-MODEL\_V4 | 1.0 | 3.801e-18 | 612 | 0.221 | 384 | 190 | 13 | 2 | 371 | 16 | 304 | DUF3799 domain-containing protein | DUF3799 domain-containing protein | | afdb-uniprot50 | AF-A0A7M1R1P4-F1-MODEL\_V4 | 1.0 | 2.713e-18 | 611 | 0.218 | 380 | 187 | 15 | 1 | 371 | 5 | 283 | PD-(D/E)XK nuclease-like domain-containing protein | PD-(D/E)XK nuclease-like domain-containing protein | | afdb-uniprot50 | AF-A0A6H2A4F4-F1-MODEL\_V4 | 1.0 | 1.936e-18 | 611 | 0.217 | 372 | 191 | 14 | 2 | 360 | 8 | 292 | DUF3799 domain-containing protein | DUF3799 domain-containing protein | | afdb-uniprot50 | AF-A0A2E7BLG2-F1-MODEL\_V4 | 1.0 | 1.106e-17 | 611 | 0.23 | 373 | 173 | 12 | 5 | 369 | 40 | 306 | DUF3799 domain-containing protein | DUF3799 domain-containing protein | | afdb-uniprot50 | AF-A0A4Q6UDT3-F1-MODEL\_V4 | 1.0 | 1.462e-18 | 611 | 0.264 | 375 | 166 | 13 | 3 | 368 | 49 | 322 | DUF3799 domain-containing protein | DUF3799 domain-containing protein | | afdb-uniprot50 | AF-A0A7K1LBD0-F1-MODEL\_V4 | 1.0 | 5.023e-19 | 611 | 0.242 | 379 | 175 | 12 | 1 | 369 | 54 | 330 | DUF3799 domain-containing protein | DUF3799 domain-containing protein | | afdb-uniprot50 | AF-A0A7W5AMD2-F1-MODEL\_V4 | 1.0 | 1.462e-18 | 610 | 0.237 | 383 | 179 | 17 | 1 | 369 | 11 | 294 | DUF3799 domain-containing protein | DUF3799 domain-containing protein | | afdb-uniprot50 | AF-A0A3D1IV21-F1-MODEL\_V4 | 1.0 | 1.167e-18 | 610 | 0.238 | 381 | 184 | 14 | 2 | 369 | 7 | 294 | DUF3799 domain-containing protein | DUF3799 domain-containing protein | | afdb-uniprot50 | AF-A0A3B9WYA0-F1-MODEL\_V4 | 1.0 | 1.644e-16 | 609 | 0.193 | 357 | 179 | 9 | 2 | 357 | 4 | 252 | DUF3799 domain-containing protein | DUF3799 domain-containing protein | | afdb-uniprot50 | AF-A0A1V5V1L8-F1-MODEL\_V4 | 1.0 | 4.511e-17 | 607 | 0.233 | 359 | 172 | 13 | 5 | 357 | 6 | 267 | Exonuclease VIII | Exonuclease VIII | | afdb-uniprot50 | AF-A0A4Q3G6R8-F1-MODEL\_V4 | 1.0 | 4.265e-17 | 607 | 0.22 | 385 | 184 | 14 | 1 | 372 | 5 | 286 | DUF3799 domain-containing protein | DUF3799 domain-containing protein | | afdb-uniprot50 | AF-A0A3C0M8N0-F1-MODEL\_V4 | 1.0 | 3.036e-18 | 607 | 0.214 | 382 | 186 | 10 | 2 | 371 | 14 | 293 | DUF3799 domain-containing protein | DUF3799 domain-containing protein | | afdb-uniprot50 | AF-A0A7G8A5U5-F1-MODEL\_V4 | 1.0 | 1.631e-19 | 607 | 0.24 | 375 | 187 | 15 | 3 | 371 | 73 | 355 | DUF3799 domain-containing protein | DUF3799 domain-containing protein | | afdb-uniprot50 | AF-A0A2E7BUE9-F1-MODEL\_V4 | 1.0 | 1.306e-18 | 605 | 0.215 | 376 | 186 | 12 | 5 | 365 | 35 | 316 | DUF3799 domain-containing protein | DUF3799 domain-containing protein | | afdb-uniprot50 | AF-A0A0F9KSW2-F1-MODEL\_V4 | 1.0 | 2.298e-17 | 604 | 0.21 | 371 | 190 | 11 | 1 | 366 | 1 | 273 | DUF3799 domain-containing protein | DUF3799 domain-containing protein | | afdb-uniprot50 | AF-A0A231HCK3-F1-MODEL\_V4 | 1.0 | 2.292e-18 | 604 | 0.23 | 368 | 175 | 14 | 1 | 361 | 30 | 296 | Exodeoxyribonuclease 8 | Exodeoxyribonuclease 8 | | afdb-uniprot50 | AF-A0A1M5MMI5-F1-MODEL\_V4 | 1.0 | 3.397e-18 | 604 | 0.222 | 382 | 190 | 12 | 2 | 371 | 14 | 300 | DUF3799 domain-containing protein | DUF3799 domain-containing protein | | afdb-uniprot50 | AF-A0A6L5VLS5-F1-MODEL\_V4 | 1.0 | 7.895e-18 | 603 | 0.247 | 340 | 153 | 9 | 31 | 366 | 4 | 244 | DUF3799 domain-containing protein | DUF3799 domain-containing protein | | afdb-uniprot50 | AF-A0A3B8U8D5-F1-MODEL\_V4 | 1.0 | 2.161e-19 | 603 | 0.208 | 365 | 221 | 12 | 5 | 364 | 3 | 304 | SAP domain-containing protein | SAP domain-containing protein | | afdb-uniprot50 | AF-A0A350P1N0-F1-MODEL\_V4 | 1.0 | 1.941e-17 | 600 | 0.238 | 369 | 162 | 13 | 5 | 361 | 4 | 265 | DUF3799 domain-containing protein | DUF3799 domain-containing protein | | afdb-uniprot50 | AF-N6UG32-F1-MODEL\_V4 | 1.0 | 2.713e-18 | 600 | 0.244 | 377 | 177 | 8 | 1 | 366 | 42 | 321 | DUF3799 domain-containing protein | DUF3799 domain-containing protein | | afdb-uniprot50 | AF-A0A4P6HS96-F1-MODEL\_V4 | 1.0 | 2.713e-18 | 600 | 0.231 | 376 | 189 | 10 | 1 | 366 | 38 | 323 | DUF3799 domain-containing protein | DUF3799 domain-containing protein | | afdb-uniprot50 | AF-A0A7Y0U3A1-F1-MODEL\_V4 | 1.0 | 2.431e-17 | 599 | 0.221 | 370 | 178 | 13 | 1 | 361 | 8 | 276 | DUF3799 domain-containing protein | DUF3799 domain-containing protein | | afdb-uniprot50 | AF-A0A705UGF7-F1-MODEL\_V4 | 1.0 | 2.855e-20 | 599 | 0.209 | 406 | 243 | 11 | 33 | 372 | 2 | 395 | DNA breaking-rejoining protein | DNA breaking-rejoining protein | | afdb-uniprot50 | AF-A0A3V4IUN9-F1-MODEL\_V4 | 1.0 | 4.997e-21 | 599 | 0.217 | 410 | 243 | 14 | 25 | 368 | 2 | 399 | DUF3799 domain-containing protein | DUF3799 domain-containing protein | | afdb-uniprot50 | AF-A0A3D6BSW6-F1-MODEL\_V4 | 1.0 | 7.073e-17 | 598 | 0.206 | 358 | 170 | 10 | 3 | 348 | 10 | 265 | DUF3799 domain-containing protein | DUF3799 domain-containing protein | | afdb-uniprot50 | AF-A0A0F9RJV3-F1-MODEL\_V4 | 1.0 | 7.073e-17 | 598 | 0.192 | 363 | 184 | 8 | 1 | 361 | 5 | 260 | DUF3799 domain-containing protein | DUF3799 domain-containing protein | | afdb-uniprot50 | AF-A0A524RVX3-F1-MODEL\_V4 | 1.0 | 2.558e-19 | 598 | 0.228 | 399 | 210 | 15 | 1 | 371 | 102 | 430 | DUF3799 domain-containing protein | DUF3799 domain-containing protein | | afdb-uniprot50 | AF-A0A2T6LPT7-F1-MODEL\_V4 | 1.0 | 3.792e-19 | 597 | 0.25 | 387 | 178 | 14 | 2 | 371 | 60 | 351 | PDDEXK-like uncharacterized protein DUF3799 | PDDEXK-like uncharacterized protein DUF3799 | | afdb-uniprot50 | AF-A0A5C7P0Q3-F1-MODEL\_V4 | 1.0 | 7.915e-17 | 596 | 0.204 | 376 | 175 | 13 | 1 | 358 | 2 | 271 | DUF3799 domain-containing protein | DUF3799 domain-containing protein | | afdb-uniprot50 | AF-A0A2E5F7Y4-F1-MODEL\_V4 | 1.0 | 2.87e-18 | 596 | 0.231 | 385 | 188 | 17 | 1 | 370 | 22 | 313 | DUF3799 domain-containing protein | DUF3799 domain-containing protein | | afdb-uniprot50 | AF-A0A7K3LW35-F1-MODEL\_V4 | 1.0 | 9.322e-19 | 596 | 0.223 | 371 | 180 | 12 | 2 | 371 | 58 | 321 | DUF3799 domain-containing protein | DUF3799 domain-containing protein | | afdb-uniprot50 | AF-A0A100W6Y0-F1-MODEL\_V4 | 1.0 | 3.028e-19 | 595 | 0.22 | 363 | 191 | 13 | 1 | 360 | 53 | 326 | Gp60 | Gp60 | | afdb-uniprot50 | AF-A0A757V7J2-F1-MODEL\_V4 | 1.0 | 2.699e-20 | 595 | 0.204 | 410 | 250 | 13 | 27 | 372 | 2 | 399 | DNA breaking-rejoining protein | DNA breaking-rejoining protein | | afdb-uniprot50 | AF-A0A7Y8WZA1-F1-MODEL\_V4 | 1.0 | 4.031e-17 | 593 | 0.226 | 371 | 174 | 14 | 1 | 364 | 6 | 270 | PD-(D/E)XK nuclease-like domain-containing protein | PD-(D/E)XK nuclease-like domain-containing protein | | afdb-uniprot50 | AF-A0A496WM50-F1-MODEL\_V4 | 1.0 | 1.469e-16 | 593 | 0.205 | 370 | 203 | 11 | 1 | 370 | 1 | 279 | DUF3799 domain-containing protein | DUF3799 domain-containing protein | | afdb-uniprot50 | AF-A0A7Y6MP93-F1-MODEL\_V4 | 1.0 | 5.62e-19 | 593 | 0.174 | 384 | 240 | 12 | 5 | 369 | 639 | 964 | PD-(D/E)XK nuclease-like domain-containing protein | PD-(D/E)XK nuclease-like domain-containing protein | | afdb-uniprot50 | AF-A0A2D5TXR6-F1-MODEL\_V4 | 1.0 | 6.321e-17 | 591 | 0.219 | 351 | 161 | 9 | 1 | 344 | 12 | 256 | DUF3799 domain-containing protein | DUF3799 domain-containing protein | | afdb-uniprot50 | AF-G2KMZ2-F1-MODEL\_V4 | 1.0 | 1.238e-17 | 590 | 0.203 | 402 | 220 | 15 | 1 | 363 | 20 | 360 | SAP domain-containing protein | SAP domain-containing protein | | afdb-uniprot50 | AF-A0A6M3IKZ8-F1-MODEL\_V4 | 1.0 | 4.265e-17 | 589 | 0.228 | 367 | 174 | 12 | 2 | 361 | 9 | 273 | DUF3799 domain-containing protein | DUF3799 domain-containing protein | | afdb-uniprot50 | AF-A0A5R8P622-F1-MODEL\_V4 | 1.0 | 6.289e-19 | 589 | 0.218 | 375 | 181 | 16 | 1 | 368 | 77 | 346 | DUF3799 domain-containing protein | DUF3799 domain-containing protein | | afdb-uniprot50 | AF-A0A7K2J285-F1-MODEL\_V4 | 1.0 | 4.031e-17 | 588 | 0.371 | 202 | 116 | 6 | 177 | 369 | 61 | 260 | DUF3799 domain-containing protein | DUF3799 domain-containing protein | | afdb-uniprot50 | AF-A0A6H1Z9N2-F1-MODEL\_V4 | 1.0 | 2.87e-18 | 588 | 0.229 | 362 | 210 | 13 | 9 | 362 | 1 | 301 | DUF3799 domain-containing protein | DUF3799 domain-containing protein | | afdb-uniprot50 | AF-A0A1F6GLK6-F1-MODEL\_V4 | 1.0 | 1.378e-19 | 587 | 0.21 | 408 | 225 | 17 | 1 | 372 | 16 | 362 | DUF3799 domain-containing protein | DUF3799 domain-containing protein | | afdb-uniprot50 | AF-A0A359JZU9-F1-MODEL\_V4 | 1.0 | 8.309e-20 | 587 | 0.218 | 380 | 230 | 14 | 3 | 359 | 35 | 370 | SAP domain-containing protein | SAP domain-containing protein | | afdb-uniprot50 | AF-A0A2E1XRD1-F1-MODEL\_V4 | 1.0 | 3.811e-17 | 586 | 0.193 | 377 | 189 | 13 | 2 | 367 | 5 | 277 | DUF3799 domain-containing protein | DUF3799 domain-containing protein | | afdb-uniprot50 | AF-A0A161XAN9-F1-MODEL\_V4 | 1.0 | 5.023e-19 | 586 | 0.235 | 391 | 190 | 12 | 2 | 371 | 13 | 315 | Exodeoxyribonuclease 8 | Exodeoxyribonuclease 8 | | afdb-uniprot50 | AF-A0A8B3WJL6-F1-MODEL\_V4 | 1.0 | 1.389e-16 | 585 | 0.2 | 374 | 190 | 13 | 1 | 365 | 5 | 278 | RecE | RecE | | afdb-uniprot50 | AF-A0A853KWC9-F1-MODEL\_V4 | 1.0 | 1.936e-18 | 585 | 0.206 | 397 | 219 | 14 | 1 | 361 | 39 | 375 | Uncharacterized protein | Uncharacterized protein | | afdb-uniprot50 | AF-A0A2W1SGM9-F1-MODEL\_V4 | 1.0 | 5.62e-19 | 585 | 0.247 | 372 | 180 | 11 | 1 | 367 | 110 | 386 | DUF3799 domain-containing protein | DUF3799 domain-containing protein | | afdb-uniprot50 | AF-A0A5Y7B1G5-F1-MODEL\_V4 | 1.0 | 1.926e-20 | 585 | 0.21 | 408 | 244 | 13 | 31 | 372 | 1 | 396 | Exodeoxyribonuclease | Exodeoxyribonuclease | | afdb-uniprot50 | AF-A0A661RLU8-F1-MODEL\_V4 | 1.0 | 9.886e-18 | 584 | 0.217 | 381 | 184 | 13 | 1 | 366 | 8 | 289 | DUF3799 domain-containing protein | DUF3799 domain-containing protein | | afdb-uniprot50 | AF-A0A2D4XKL8-F1-MODEL\_V4 | 1.0 | 3.228e-16 | 583 | 0.2 | 354 | 169 | 12 | 1 | 352 | 1 | 242 | DUF3799 domain-containing protein | DUF3799 domain-containing protein | | afdb-uniprot50 | AF-A0A7T4IB75-F1-MODEL\_V4 | 1.0 | 4.265e-17 | 583 | 0.232 | 365 | 174 | 7 | 12 | 366 | 2 | 270 | PD-(D/E)XK nuclease-like domain-containing protein | PD-(D/E)XK nuclease-like domain-containing protein | | afdb-uniprot50 | AF-A0A762RJE4-F1-MODEL\_V4 | 1.0 | 4.001e-20 | 582 | 0.232 | 391 | 218 | 12 | 1 | 323 | 12 | 388 | Exodeoxyribonuclease | Exodeoxyribonuclease | | afdb-uniprot50 | AF-A0A4Y8YA29-F1-MODEL\_V4 | 1.0 | 3.584e-19 | 582 | 0.263 | 380 | 166 | 16 | 1 | 368 | 235 | 512 | DUF3799 domain-containing protein | DUF3799 domain-containing protein | | afdb-uniprot50 | AF-A0A146GG86-F1-MODEL\_V4 | 1.0 | 1.385e-17 | 581 | 0.194 | 380 | 197 | 11 | 1 | 370 | 15 | 295 | DUF3799 domain-containing protein | DUF3799 domain-containing protein | | afdb-uniprot50 | AF-A0A2T0JIJ1-F1-MODEL\_V4 | 1.0 | 3.211e-18 | 581 | 0.225 | 385 | 183 | 17 | 1 | 372 | 17 | 299 | PDDEXK-like uncharacterized protein DUF3799 | PDDEXK-like uncharacterized protein DUF3799 | | afdb-uniprot50 | AF-I6APA3-F1-MODEL\_V4 | 1.0 | 2.571e-17 | 581 | 0.206 | 387 | 204 | 16 | 1 | 366 | 8 | 312 | DUF3799 domain-containing protein | DUF3799 domain-containing protein | | afdb-uniprot50 | AF-A0A6P1IM96-F1-MODEL\_V4 | 1.0 | 2.571e-17 | 580 | 0.198 | 363 | 183 | 11 | 5 | 361 | 33 | 293 | DUF3799 domain-containing protein | DUF3799 domain-containing protein | | afdb-uniprot50 | AF-A0A2E0GW99-F1-MODEL\_V4 | 1.0 | 1.469e-16 | 578 | 0.206 | 382 | 184 | 15 | 1 | 368 | 1 | 277 | DUF3799 domain-containing protein | DUF3799 domain-containing protein | | afdb-uniprot50 | AF-A0A3B8UEV6-F1-MODEL\_V4 | 1.0 | 4.488e-19 | 578 | 0.238 | 386 | 181 | 15 | 1 | 371 | 110 | 397 | DUF3799 domain-containing protein | DUF3799 domain-containing protein | | afdb-uniprot50 | AF-A0A0F9QMY9-F1-MODEL\_V4 | 1.0 | 1.109e-16 | 577 | 0.211 | 364 | 180 | 13 | 9 | 360 | 12 | 280 | DUF3799 domain-containing protein | DUF3799 domain-containing protein | | afdb-uniprot50 | AF-A0A7X5Z2D5-F1-MODEL\_V4 | 1.0 | 8.812e-19 | 576 | 0.239 | 376 | 178 | 11 | 1 | 371 | 124 | 396 | DUF3799 domain-containing protein | DUF3799 domain-containing protein | | afdb-uniprot50 | AF-Q11ZS8-F1-MODEL\_V4 | 1.0 | 2.172e-17 | 575 | 0.196 | 381 | 192 | 9 | 2 | 368 | 41 | 321 | DUF3799 domain-containing protein | DUF3799 domain-containing protein | | afdb-uniprot50 | AF-A0A844B7Y8-F1-MODEL\_V4 | 1.0 | 1.167e-18 | 574 | 0.191 | 382 | 192 | 12 | 3 | 367 | 82 | 363 | Uncharacterized protein | Uncharacterized protein | | afdb-uniprot50 | AF-A0A2D6XBJ2-F1-MODEL\_V4 | 1.0 | 8.88e-16 | 573 | 0.198 | 362 | 191 | 13 | 6 | 364 | 2 | 267 | DUF3799 domain-containing protein | DUF3799 domain-containing protein | | afdb-uniprot50 | AF-A0A7X6NUU3-F1-MODEL\_V4 | 1.0 | 2.172e-17 | 573 | 0.255 | 384 | 169 | 15 | 1 | 371 | 13 | 292 | DUF3799 domain-containing protein | DUF3799 domain-containing protein | | afdb-uniprot50 | AF-A0A2U1AQD0-F1-MODEL\_V4 | 1.0 | 5.991e-16 | 572 | 0.2 | 359 | 185 | 12 | 5 | 358 | 8 | 269 | PDDEXK-like uncharacterized protein DUF3799 | PDDEXK-like uncharacterized protein DUF3799 | | afdb-uniprot50 | AF-T0HIE6-F1-MODEL\_V4 | 1.0 | 8.373e-17 | 572 | 0.189 | 386 | 196 | 15 | 1 | 371 | 19 | 302 | DUF3799 domain-containing protein | DUF3799 domain-containing protein | | afdb-uniprot50 | AF-A0A5V6MQJ5-F1-MODEL\_V4 | 1.0 | 1.631e-19 | 571 | 0.212 | 404 | 240 | 11 | 35 | 372 | 2 | 393 | Exodeoxyribonuclease | Exodeoxyribonuclease | | afdb-uniprot50 | AF-J9SAI7-F1-MODEL\_V4 | 1.0 | 5.945e-19 | 570 | 0.259 | 370 | 167 | 13 | 1 | 369 | 192 | 455 | DUF3799 domain-containing protein | DUF3799 domain-containing protein | | afdb-uniprot50 | AF-A0A559PZG8-F1-MODEL\_V4 | 1.0 | 1.17e-17 | 569 | 0.232 | 387 | 204 | 14 | 2 | 372 | 14 | 323 | PDDEXK-like uncharacterized protein DUF3799 | PDDEXK-like uncharacterized protein DUF3799 | | afdb-uniprot50 | AF-A0A2Z5YJC4-F1-MODEL\_V4 | 1.0 | 4.511e-17 | 569 | 0.216 | 375 | 187 | 12 | 2 | 369 | 55 | 329 | DUF3799 domain-containing protein | DUF3799 domain-containing protein | | afdb-uniprot50 | AF-A0A433FYR1-F1-MODEL\_V4 | 1.0 | 9.861e-19 | 569 | 0.238 | 381 | 190 | 15 | 3 | 372 | 92 | 383 | DUF3799 domain-containing protein | DUF3799 domain-containing protein | | afdb-uniprot50 | AF-A0A3N0V111-F1-MODEL\_V4 | 1.0 | 1.554e-16 | 568 | 0.203 | 349 | 174 | 11 | 1 | 346 | 13 | 260 | DUF3799 domain-containing protein | DUF3799 domain-containing protein | | afdb-uniprot50 | AF-A0A7Z8LAH0-F1-MODEL\_V4 | 1.0 | 2.584e-15 | 568 | 0.198 | 352 | 177 | 8 | 9 | 360 | 8 | 254 | DUF3799 domain-containing protein | DUF3799 domain-containing protein | | afdb-uniprot50 | AF-A0A2S6AMK5-F1-MODEL\_V4 | 1.0 | 1.462e-18 | 568 | 0.247 | 368 | 164 | 13 | 1 | 358 | 80 | 344 | RecE | RecE | | afdb-uniprot50 | AF-A0A5R8NEL4-F1-MODEL\_V4 | 1.0 | 1.167e-18 | 568 | 0.205 | 374 | 187 | 14 | 1 | 368 | 163 | 432 | DUF3799 domain-containing protein | DUF3799 domain-containing protein | | afdb-uniprot50 | AF-A0A6H2A032-F1-MODEL\_V4 | 1.0 | 7.501e-16 | 567 | 0.203 | 358 | 170 | 13 | 9 | 361 | 1 | 248 | DUF3799 domain-containing protein | DUF3799 domain-containing protein | | afdb-uniprot50 | AF-A0A4Y9QDI6-F1-MODEL\_V4 | 1.0 | 1.554e-16 | 566 | 0.222 | 342 | 165 | 9 | 32 | 366 | 8 | 255 | DUF3799 domain-containing protein | DUF3799 domain-containing protein | | afdb-uniprot50 | AF-A0A2E1MYG3-F1-MODEL\_V4 | 1.0 | 1.558e-15 | 566 | 0.204 | 366 | 176 | 14 | 1 | 360 | 1 | 257 | DUF3799 domain-containing protein | DUF3799 domain-containing protein | | afdb-uniprot50 | AF-A0A5C5X348-F1-MODEL\_V4 | 1.0 | 2.584e-15 | 566 | 0.18 | 376 | 199 | 9 | 1 | 371 | 8 | 279 | DUF3799 domain-containing protein | DUF3799 domain-containing protein | | afdb-uniprot50 | AF-A0A2N2TMM3-F1-MODEL\_V4 | 1.0 | 6.305e-18 | 566 | 0.197 | 365 | 183 | 11 | 5 | 362 | 88 | 349 | DUF3799 domain-containing protein | DUF3799 domain-containing protein | | afdb-uniprot50 | AF-W0E2G2-F1-MODEL\_V4 | 1.0 | 1.558e-15 | 565 | 0.382 | 188 | 106 | 6 | 178 | 361 | 22 | 203 | Exonuclease VIII | Exonuclease VIII | | afdb-uniprot50 | AF-A0A5W6TLU2-F1-MODEL\_V4 | 1.0 | 1.84e-16 | 565 | 0.247 | 340 | 153 | 10 | 31 | 366 | 1 | 241 | Exonuclease VIII | Exonuclease VIII | | afdb-uniprot50 | AF-H0A060-F1-MODEL\_V4 | 1.0 | 5.649e-17 | 565 | 0.21 | 389 | 188 | 16 | 1 | 371 | 1 | 288 | DUF3799 domain-containing protein | DUF3799 domain-containing protein | | afdb-uniprot50 | AF-A0A7W7MM07-F1-MODEL\_V4 | 1.0 | 1.64e-17 | 563 | 0.243 | 399 | 170 | 17 | 1 | 372 | 6 | 299 | DUF3799 domain-containing protein | DUF3799 domain-containing protein | | afdb-uniprot50 | AF-A0A0F8VZZ2-F1-MODEL\_V4 | 1.0 | 3.83e-15 | 562 | 0.396 | 179 | 103 | 4 | 189 | 364 | 15 | 191 | DUF3799 domain-containing protein | DUF3799 domain-containing protein | | afdb-uniprot50 | AF-A0A2S4KG23-F1-MODEL\_V4 | 1.0 | 3.603e-17 | 562 | 0.197 | 369 | 179 | 12 | 5 | 360 | 11 | 275 | DUF3799 domain-containing protein | DUF3799 domain-containing protein | | afdb-uniprot50 | AF-Q21QE7-F1-MODEL\_V4 | 1.0 | 7.895e-18 | 562 | 0.213 | 379 | 178 | 16 | 5 | 364 | 53 | 330 | DUF3799 domain-containing protein | DUF3799 domain-containing protein | | afdb-uniprot50 | AF-A0A3D4T735-F1-MODEL\_V4 | 1.0 | 3.821e-16 | 561 | 0.254 | 318 | 134 | 8 | 53 | 364 | 4 | 224 | DUF3799 domain-containing protein | DUF3799 domain-containing protein | | afdb-uniprot50 | AF-A0A705Y2E6-F1-MODEL\_V4 | 1.0 | 4.477e-20 | 559 | 0.224 | 387 | 226 | 12 | 1 | 323 | 80 | 456 | Exodeoxyribonuclease | Exodeoxyribonuclease | | afdb-uniprot50 | AF-A0A3C0TWV8-F1-MODEL\_V4 | 1.0 | 1.31e-17 | 558 | 0.203 | 383 | 189 | 17 | 1 | 368 | 24 | 305 | DUF3799 domain-containing protein | DUF3799 domain-containing protein | | afdb-uniprot50 | AF-A0A759Y1M9-F1-MODEL\_V4 | 1.0 | 3.575e-20 | 557 | 0.226 | 389 | 223 | 13 | 1 | 323 | 80 | 456 | DNA breaking-rejoining protein | DNA breaking-rejoining protein | | afdb-uniprot50 | AF-A0A509MJM3-F1-MODEL\_V4 | 1.0 | 1.946e-16 | 556 | 0.203 | 369 | 185 | 11 | 1 | 360 | 6 | 274 | RecE | RecE | | afdb-uniprot50 | AF-A0A845UWG1-F1-MODEL\_V4 | 1.0 | 1.941e-17 | 556 | 0.227 | 383 | 186 | 16 | 1 | 371 | 5 | 289 | Uncharacterized protein | Uncharacterized protein | | afdb-uniprot50 | AF-A0A316RHU8-F1-MODEL\_V4 | 1.0 | 4.011e-19 | 555 | 0.218 | 380 | 212 | 13 | 1 | 364 | 57 | 367 | DUF3799 domain-containing protein | DUF3799 domain-containing protein | | afdb-uniprot50 | AF-A0A7W4CX72-F1-MODEL\_V4 | 1.0 | 8.857e-17 | 554 | 0.223 | 380 | 184 | 17 | 1 | 370 | 11 | 289 | PD-(D/E)XK nuclease-like domain-containing protein | PD-(D/E)XK nuclease-like domain-containing protein | | afdb-uniprot50 | AF-A0A761RJR1-F1-MODEL\_V4 | 1.0 | 2.855e-20 | 554 | 0.22 | 385 | 230 | 14 | 1 | 323 | 191 | 567 | Exodeoxyribonuclease | Exodeoxyribonuclease | | afdb-uniprot50 | AF-A0A316RSE2-F1-MODEL\_V4 | 1.0 | 1.244e-15 | 553 | 0.2 | 359 | 186 | 12 | 5 | 358 | 8 | 270 | DUF3799 domain-containing protein | DUF3799 domain-containing protein | | afdb-uniprot50 | AF-A0A7C5FX24-F1-MODEL\_V4 | 1.0 | 3.821e-16 | 553 | 0.189 | 354 | 188 | 11 | 5 | 356 | 45 | 301 | DUF3799 domain-containing protein | DUF3799 domain-containing protein | | afdb-uniprot50 | AF-A0A0F9I7X6-F1-MODEL\_V4 | 1.0 | 1.109e-16 | 553 | 0.185 | 388 | 199 | 14 | 4 | 371 | 17 | 307 | DUF3799 domain-containing protein | DUF3799 domain-containing protein | | afdb-uniprot50 | AF-A0A3R8K6A3-F1-MODEL\_V4 | 1.0 | 3.414e-16 | 552 | 0.194 | 391 | 193 | 15 | 1 | 372 | 9 | 296 | DUF3799 domain-containing protein | DUF3799 domain-containing protein | | afdb-uniprot50 | AF-A0A2E2S9B9-F1-MODEL\_V4 | 1.0 | 8.394e-16 | 552 | 0.196 | 382 | 185 | 11 | 1 | 364 | 7 | 284 | DUF3799 domain-containing protein | DUF3799 domain-containing protein | | afdb-uniprot50 | AF-E0MRN4-F1-MODEL\_V4 | 1.0 | 3.821e-16 | 552 | 0.17 | 388 | 207 | 11 | 2 | 371 | 15 | 305 | DUF3799 domain-containing protein | DUF3799 domain-containing protein | | afdb-uniprot50 | AF-A0A7J5DTI8-F1-MODEL\_V4 | 1.0 | 2.713e-18 | 552 | 0.246 | 378 | 183 | 10 | 1 | 368 | 84 | 369 | RecE | RecE | | afdb-uniprot50 | AF-A0A2D9QDN0-F1-MODEL\_V4 | 1.0 | 4.796e-15 | 551 | 0.21 | 346 | 161 | 10 | 20 | 360 | 8 | 246 | DUF3799 domain-containing protein | DUF3799 domain-containing protein | | afdb-uniprot50 | AF-A0A2V3UAZ7-F1-MODEL\_V4 | 1.0 | 5.354e-16 | 551 | 0.187 | 399 | 226 | 14 | 1 | 362 | 8 | 345 | PDDEXK-like uncharacterized protein DUF3799 | PDDEXK-like uncharacterized protein DUF3799 | | afdb-uniprot50 | AF-A0A7V9TIF0-F1-MODEL\_V4 | 1.0 | 1.739e-16 | 550 | 0.214 | 341 | 163 | 10 | 18 | 358 | 27 | 262 | PD-(D/E)XK nuclease-like domain-containing protein | PD-(D/E)XK nuclease-like domain-containing protein | | afdb-uniprot50 | AF-A0A163TGH6-F1-MODEL\_V4 | 1.0 | 6.321e-17 | 550 | 0.217 | 386 | 196 | 14 | 2 | 370 | 14 | 310 | DUF3799 domain-containing protein | DUF3799 domain-containing protein | | afdb-uniprot50 | AF-A0A2A7UDS5-F1-MODEL\_V4 | 1.0 | 5.635e-18 | 550 | 0.219 | 365 | 174 | 15 | 1 | 358 | 149 | 409 | DUF3799 domain-containing protein | DUF3799 domain-containing protein | | afdb-uniprot50 | AF-A0A2D7VYX6-F1-MODEL\_V4 | 1.0 | 1.392e-15 | 548 | 0.213 | 356 | 171 | 11 | 14 | 361 | 3 | 257 | DUF3799 domain-containing protein | DUF3799 domain-containing protein | | afdb-uniprot50 | AF-A0A2N3FUY9-F1-MODEL\_V4 | 1.0 | 1.55e-17 | 548 | 0.205 | 380 | 189 | 13 | 1 | 368 | 87 | 365 | DUF3799 domain-containing protein | DUF3799 domain-containing protein | | afdb-uniprot50 | AF-A0A757U2L4-F1-MODEL\_V4 | 1.0 | 1.101e-19 | 547 | 0.226 | 389 | 223 | 13 | 1 | 323 | 119 | 495 | Exodeoxyribonuclease | Exodeoxyribonuclease | | afdb-uniprot50 | AF-A0A5C7P550-F1-MODEL\_V4 | 1.0 | 4.772e-17 | 545 | 0.206 | 348 | 168 | 8 | 19 | 365 | 71 | 311 | DUF3799 domain-containing protein | DUF3799 domain-containing protein | | afdb-uniprot50 | AF-A0A1E4ZGR8-F1-MODEL\_V4 | 1.0 | 1.946e-16 | 544 | 0.19 | 388 | 201 | 13 | 1 | 368 | 6 | 300 | DUF3799 domain-containing protein | DUF3799 domain-containing protein | | afdb-uniprot50 | AF-A0A0Q6ZRJ8-F1-MODEL\_V4 | 1.0 | 2.178e-16 | 544 | 0.209 | 386 | 196 | 13 | 2 | 371 | 16 | 308 | DUF3799 domain-containing protein | DUF3799 domain-containing protein | | afdb-uniprot50 | AF-A0A3A1WJX1-F1-MODEL\_V4 | 1.0 | 8.352e-18 | 544 | 0.196 | 387 | 198 | 10 | 2 | 371 | 120 | 410 | DUF3799 domain-containing protein | DUF3799 domain-containing protein | | afdb-uniprot50 | AF-A0A2D6XD80-F1-MODEL\_V4 | 1.0 | 2.298e-17 | 543 | 0.211 | 355 | 184 | 9 | 8 | 359 | 4 | 265 | DUF3799 domain-containing protein | DUF3799 domain-containing protein | | afdb-uniprot50 | AF-A0A7W4AYG5-F1-MODEL\_V4 | 1.0 | 1.936e-18 | 543 | 0.212 | 372 | 252 | 14 | 1 | 357 | 89 | 434 | PD-(D/E)XK nuclease-like domain-containing protein | PD-(D/E)XK nuclease-like domain-containing protein | | afdb-uniprot50 | AF-A0A5Q4ZYH7-F1-MODEL\_V4 | 1.0 | 1.546e-18 | 543 | 0.145 | 548 | 282 | 21 | 8 | 371 | 30 | 574 | DUF3799 domain-containing protein | DUF3799 domain-containing protein | | afdb-uniprot50 | AF-A0A5C7PJN5-F1-MODEL\_V4 | 1.0 | 6.704e-16 | 542 | 0.21 | 366 | 186 | 13 | 5 | 362 | 4 | 274 | DUF3799 domain-containing protein | DUF3799 domain-containing protein | | afdb-uniprot50 | AF-A0A1I5SZ79-F1-MODEL\_V4 | 1.0 | 5.991e-16 | 540 | 0.172 | 376 | 203 | 15 | 1 | 360 | 3 | 286 | DUF3799 domain-containing protein | DUF3799 domain-containing protein | | afdb-uniprot50 | AF-A0A6L3A789-F1-MODEL\_V4 | 1.0 | 3.612e-16 | 536 | 0.198 | 363 | 184 | 13 | 8 | 358 | 40 | 307 | DUF3799 domain-containing protein | DUF3799 domain-containing protein | | afdb-uniprot50 | AF-A0A562ZN70-F1-MODEL\_V4 | 1.0 | 2.172e-17 | 536 | 0.201 | 363 | 180 | 13 | 5 | 360 | 104 | 363 | DUF3799 domain-containing protein | DUF3799 domain-containing protein | | afdb-uniprot50 | AF-A0A833IUC5-F1-MODEL\_V4 | 1.0 | 1.473e-15 | 535 | 0.333 | 225 | 138 | 6 | 154 | 369 | 4 | 225 | Uncharacterized protein | Uncharacterized protein | | afdb-uniprot50 | AF-A0A846T0U2-F1-MODEL\_V4 | 1.0 | 7.935e-16 | 534 | 0.177 | 366 | 192 | 9 | 9 | 371 | 1 | 260 | Uncharacterized protein | Uncharacterized protein | | afdb-uniprot50 | AF-A0A356J474-F1-MODEL\_V4 | 1.0 | 1.051e-15 | 533 | 0.213 | 360 | 181 | 12 | 9 | 362 | 30 | 293 | DUF3799 domain-containing protein | DUF3799 domain-containing protein | | afdb-uniprot50 | AF-A0A846LX61-F1-MODEL\_V4 | 1.0 | 5.991e-16 | 533 | 0.195 | 379 | 191 | 15 | 1 | 371 | 30 | 302 | Uncharacterized protein | Uncharacterized protein | | afdb-uniprot50 | AF-A0A7C1PFD3-F1-MODEL\_V4 | 1.0 | 3.228e-16 | 532 | 0.181 | 358 | 185 | 14 | 20 | 360 | 1 | 267 | DUF3799 domain-containing protein | DUF3799 domain-containing protein | | afdb-uniprot50 | AF-A0A0G1R8M5-F1-MODEL\_V4 | 1.0 | 1.956e-14 | 531 | 0.341 | 193 | 122 | 4 | 178 | 370 | 20 | 207 | GP60 protein | GP60 protein | | afdb-uniprot50 | AF-A0A2E5C063-F1-MODEL\_V4 | 1.0 | 3.83e-15 | 531 | 0.21 | 342 | 161 | 10 | 1 | 338 | 5 | 241 | DUF3799 domain-containing protein | DUF3799 domain-containing protein | | afdb-uniprot50 | AF-A0A429MIW0-F1-MODEL\_V4 | 1.0 | 2.892e-15 | 530 | 0.293 | 201 | 127 | 5 | 178 | 368 | 22 | 217 | Nuclease | Nuclease | | afdb-uniprot50 | AF-A0A7Y6AGS1-F1-MODEL\_V4 | 1.0 | 1.648e-15 | 530 | 0.302 | 218 | 127 | 3 | 157 | 368 | 37 | 235 | RecE | RecE | | afdb-uniprot50 | AF-A0A355H9R2-F1-MODEL\_V4 | 1.0 | 1.844e-15 | 529 | 0.21 | 342 | 165 | 10 | 1 | 340 | 5 | 243 | DUF3799 domain-containing protein | DUF3799 domain-containing protein | | afdb-uniprot50 | AF-A0A6M3J5F8-F1-MODEL\_V4 | 1.0 | 2.727e-16 | 529 | 0.209 | 387 | 192 | 18 | 7 | 367 | 3 | 301 | DUF3799 domain-containing protein | DUF3799 domain-containing protein | | afdb-uniprot50 | AF-A0A7V1BWR4-F1-MODEL\_V4 | 1.0 | 1.739e-16 | 529 | 0.184 | 385 | 193 | 15 | 9 | 361 | 12 | 307 | DUF3799 domain-containing protein | DUF3799 domain-containing protein | | afdb-uniprot50 | AF-A0A752A1T4-F1-MODEL\_V4 | 1.0 | 3.811e-17 | 529 | 0.199 | 407 | 219 | 15 | 5 | 367 | 22 | 365 | Exodeoxyribonuclease VIII | Exodeoxyribonuclease VIII | | afdb-uniprot50 | AF-A0A318CJ92-F1-MODEL\_V4 | 1.0 | 8.415e-15 | 528 | 0.239 | 359 | 151 | 12 | 1 | 357 | 5 | 243 | DUF3799 domain-containing protein | DUF3799 domain-containing protein | | afdb-uniprot50 | AF-A0A7D8UL27-F1-MODEL\_V4 | 1.0 | 4.042e-16 | 528 | 0.201 | 357 | 181 | 13 | 8 | 358 | 60 | 318 | DUF3799 domain-containing protein | DUF3799 domain-containing protein | | afdb-uniprot50 | AF-A0A3B9NQ75-F1-MODEL\_V4 | 1.0 | 2.178e-16 | 527 | 0.192 | 380 | 189 | 13 | 8 | 371 | 11 | 288 | DUF3799 domain-containing protein | DUF3799 domain-containing protein | | afdb-uniprot50 | AF-A0A7K1SKS8-F1-MODEL\_V4 | 1.0 | 1.306e-18 | 527 | 0.204 | 382 | 223 | 14 | 1 | 361 | 55 | 376 | DUF3799 domain-containing protein | DUF3799 domain-containing protein | | afdb-uniprot50 | AF-A0A497PDQ5-F1-MODEL\_V4 | 1.0 | 1.469e-16 | 526 | 0.171 | 367 | 196 | 13 | 1 | 363 | 4 | 266 | DUF3799 domain-containing protein | DUF3799 domain-containing protein | | afdb-uniprot50 | AF-A0A2E8IY15-F1-MODEL\_V4 | 1.0 | 3.236e-15 | 524 | 0.179 | 367 | 185 | 13 | 5 | 363 | 10 | 268 | DUF3799 domain-containing protein | DUF3799 domain-containing protein | | afdb-uniprot50 | AF-A0A7R7HDE4-F1-MODEL\_V4 | 1.0 | 4.275e-16 | 524 | 0.19 | 398 | 228 | 15 | 1 | 361 | 7 | 347 | Uncharacterized protein | Uncharacterized protein | | afdb-uniprot50 | AF-A0A3E5H9L3-F1-MODEL\_V4 | 1.0 | 9.346e-18 | 524 | 0.175 | 381 | 229 | 13 | 1 | 361 | 53 | 368 | DUF3799 domain-containing protein | DUF3799 domain-containing protein | | afdb-uniprot50 | AF-A0A4U8SHN3-F1-MODEL\_V4 | 1.0 | 1.316e-15 | 523 | 0.198 | 362 | 168 | 14 | 9 | 363 | 4 | 250 | DUF3799 domain-containing protein | DUF3799 domain-containing protein | | afdb-uniprot50 | AF-A0A6P1CUJ9-F1-MODEL\_V4 | 1.0 | 5.96e-18 | 523 | 0.217 | 368 | 176 | 13 | 1 | 361 | 5 | 267 | DUF3799 domain-containing protein | DUF3799 domain-containing protein | | afdb-uniprot50 | AF-A0A501WTJ5-F1-MODEL\_V4 | 1.0 | 1.389e-16 | 522 | 0.209 | 387 | 182 | 17 | 1 | 368 | 4 | 285 | DUF3799 domain-containing protein | DUF3799 domain-containing protein | | afdb-uniprot50 | AF-A0A0Q6C361-F1-MODEL\_V4 | 1.0 | 8.88e-16 | 522 | 0.174 | 390 | 216 | 13 | 2 | 371 | 14 | 317 | DUF3799 domain-containing protein | DUF3799 domain-containing protein | | afdb-uniprot50 | AF-A0A2D7EEB4-F1-MODEL\_V4 | 1.0 | 1.051e-15 | 522 | 0.17 | 386 | 224 | 16 | 1 | 371 | 13 | 317 | DUF3799 domain-containing protein | DUF3799 domain-containing protein | | afdb-uniprot50 | AF-A0A521STB6-F1-MODEL\_V4 | 1.0 | 3.236e-15 | 521 | 0.18 | 354 | 180 | 10 | 9 | 359 | 1 | 247 | DUF3799 domain-containing protein | DUF3799 domain-containing protein | | afdb-uniprot50 | AF-A0A6M3JLX9-F1-MODEL\_V4 | 1.0 | 7.091e-16 | 521 | 0.191 | 386 | 194 | 13 | 3 | 366 | 7 | 296 | DUF3799 domain-containing protein | DUF3799 domain-containing protein | | afdb-uniprot50 | AF-A0A5M6INY5-F1-MODEL\_V4 | 1.0 | 3.051e-16 | 520 | 0.181 | 386 | 195 | 17 | 2 | 369 | 7 | 289 | DUF3799 domain-containing protein | DUF3799 domain-containing protein | | afdb-uniprot50 | AF-A0A0Q6PBB0-F1-MODEL\_V4 | 1.0 | 2.178e-16 | 518 | 0.177 | 400 | 210 | 16 | 1 | 371 | 21 | 330 | DUF3799 domain-containing protein | DUF3799 domain-containing protein | | afdb-uniprot50 | AF-A0A354GE15-F1-MODEL\_V4 | 1.0 | 4.052e-15 | 517 | 0.193 | 372 | 175 | 15 | 3 | 360 | 2 | 262 | DUF3799 domain-containing protein | DUF3799 domain-containing protein | | afdb-uniprot50 | AF-A0A2W6TFB9-F1-MODEL\_V4 | 1.0 | 9.911e-17 | 517 | 0.206 | 388 | 206 | 15 | 2 | 371 | 45 | 348 | DUF3799 domain-containing protein | DUF3799 domain-containing protein | | afdb-uniprot50 | AF-A0A261QAX9-F1-MODEL\_V4 | 1.0 | 4.021e-18 | 517 | 0.212 | 381 | 220 | 14 | 1 | 361 | 62 | 382 | DUF3799 domain-containing protein | DUF3799 domain-containing protein | | afdb-uniprot50 | AF-A0A7K0K0E5-F1-MODEL\_V4 | 1.0 | 2.183e-15 | 516 | 0.247 | 371 | 164 | 18 | 1 | 360 | 16 | 282 | DUF3799 domain-containing protein | DUF3799 domain-containing protein | | afdb-uniprot50 | AF-F4SH48-F1-MODEL\_V4 | 1.0 | 7.875e-19 | 516 | 0.204 | 382 | 231 | 11 | 54 | 372 | 2 | 373 | Exodeoxyribonuclease VIII | Exodeoxyribonuclease VIII | | afdb-uniprot50 | AF-A0A8B5WVL2-F1-MODEL\_V4 | 1.0 | 4.772e-17 | 515 | 0.225 | 408 | 173 | 20 | 1 | 371 | 7 | 308 | Uncharacterized protein | Uncharacterized protein | | afdb-uniprot50 | AF-A0A447CPW6-F1-MODEL\_V4 | 1.0 | 5.663e-16 | 514 | 0.169 | 383 | 202 | 15 | 1 | 367 | 5 | 287 | DUF3799 domain-containing protein | DUF3799 domain-containing protein | | afdb-uniprot50 | AF-A0A850CSY3-F1-MODEL\_V4 | 1.0 | 2.315e-14 | 511 | 0.319 | 188 | 118 | 6 | 179 | 361 | 24 | 206 | Uncharacterized protein | Uncharacterized protein | | afdb-uniprot50 | AF-A0A844H0G1-F1-MODEL\_V4 | 1.0 | 6.337e-16 | 510 | 0.209 | 386 | 192 | 18 | 1 | 371 | 5 | 292 | Uncharacterized protein | Uncharacterized protein | | afdb-uniprot50 | AF-A0A6M3XHK2-F1-MODEL\_V4 | 1.0 | 4.275e-16 | 509 | 0.202 | 381 | 203 | 15 | 1 | 366 | 28 | 322 | DUF3799 domain-containing protein | DUF3799 domain-containing protein | | afdb-uniprot50 | AF-A0A3R6CYF7-F1-MODEL\_V4 | 1.0 | 6.305e-18 | 509 | 0.17 | 392 | 230 | 14 | 1 | 361 | 63 | 390 | DUF3799 domain-containing protein | DUF3799 domain-containing protein | | afdb-uniprot50 | AF-A0A843GNI8-F1-MODEL\_V4 | 1.0 | 2.183e-15 | 508 | 0.143 | 363 | 205 | 14 | 1 | 361 | 10 | 268 | PD-(D/E)XK nuclease-like domain-containing protein | PD-(D/E)XK nuclease-like domain-containing protein | | afdb-uniprot50 | AF-A0A5X8YNM2-F1-MODEL\_V4 | 1.0 | 6.006e-15 | 507 | 0.247 | 315 | 133 | 11 | 57 | 367 | 2 | 216 | Exonuclease VIII | Exonuclease VIII | | afdb-uniprot50 | AF-A0A5M6IB30-F1-MODEL\_V4 | 1.0 | 3.414e-16 | 507 | 0.206 | 382 | 188 | 13 | 2 | 367 | 18 | 300 | DUF3799 domain-containing protein | DUF3799 domain-containing protein | | afdb-uniprot50 | AF-A0A369QT25-F1-MODEL\_V4 | 1.0 | 5.991e-16 | 507 | 0.226 | 384 | 186 | 17 | 1 | 371 | 47 | 332 | DUF3799 domain-containing protein | DUF3799 domain-containing protein | | afdb-uniprot50 | AF-A0A2D7PF36-F1-MODEL\_V4 | 1.0 | 4.534e-15 | 506 | 0.207 | 376 | 168 | 15 | 5 | 359 | 20 | 286 | DUF3799 domain-containing protein | DUF3799 domain-containing protein | | afdb-uniprot50 | AF-A0A744K618-F1-MODEL\_V4 | 1.0 | 9.417e-15 | 505 | 0.242 | 313 | 134 | 9 | 58 | 366 | 2 | 215 | Exonuclease VIII | Exonuclease VIII | | afdb-uniprot50 | AF-A0A7C7TML4-F1-MODEL\_V4 | 1.0 | 6.738e-14 | 505 | 0.161 | 341 | 166 | 10 | 1 | 338 | 8 | 231 | DUF3799 domain-containing protein | DUF3799 domain-containing protein | | afdb-uniprot50 | AF-A0A1G0HWF1-F1-MODEL\_V4 | 1.0 | 1.84e-16 | 505 | 0.209 | 372 | 190 | 17 | 12 | 368 | 2 | 284 | DUF3799 domain-containing protein | DUF3799 domain-containing protein | | afdb-uniprot50 | AF-A0A317H245-F1-MODEL\_V4 | 1.0 | 1.051e-15 | 505 | 0.213 | 393 | 196 | 16 | 2 | 371 | 18 | 320 | DUF3799 domain-containing protein | DUF3799 domain-containing protein | | afdb-uniprot50 | AF-A0A7W6J8M0-F1-MODEL\_V4 | 1.0 | 3.414e-16 | 504 | 0.197 | 395 | 190 | 16 | 1 | 367 | 11 | 306 | DUF3799 domain-containing protein | DUF3799 domain-containing protein | | afdb-uniprot50 | AF-A0A8B3HGI6-F1-MODEL\_V4 | 1.0 | 8.437e-14 | 503 | 0.337 | 181 | 108 | 6 | 190 | 366 | 1 | 173 | Uncharacterized protein | Uncharacterized protein | | afdb-uniprot50 | AF-A0A1Z7ZMG2-F1-MODEL\_V4 | 1.0 | 1.247e-14 | 502 | 0.194 | 376 | 188 | 13 | 1 | 371 | 13 | 278 | DUF3799 domain-containing protein | DUF3799 domain-containing protein | | afdb-uniprot50 | AF-A0A0S9D1Q2-F1-MODEL\_V4 | 1.0 | 1.247e-14 | 501 | 0.279 | 326 | 129 | 12 | 43 | 364 | 2 | 225 | DUF3799 domain-containing protein | DUF3799 domain-containing protein | | afdb-uniprot50 | AF-A0A3A0DGM8-F1-MODEL\_V4 | 1.0 | 1.051e-15 | 500 | 0.205 | 355 | 181 | 12 | 8 | 358 | 233 | 490 | Uncharacterized protein | Uncharacterized protein | | afdb-uniprot50 | AF-S2W335-F1-MODEL\_V4 | 1.0 | 5.074e-15 | 498 | 0.191 | 361 | 192 | 13 | 2 | 361 | 3 | 264 | DUF3799 domain-containing protein | DUF3799 domain-containing protein | | afdb-uniprot50 | AF-A0A6N6ZVV7-F1-MODEL\_V4 | 1.0 | 1.316e-15 | 498 | 0.192 | 395 | 193 | 15 | 2 | 369 | 4 | 299 | DUF3799 domain-containing protein | DUF3799 domain-containing protein | | afdb-uniprot50 | AF-A0A3N0CLS5-F1-MODEL\_V4 | 1.0 | 3.83e-15 | 497 | 0.201 | 368 | 190 | 15 | 2 | 363 | 12 | 281 | DUF3799 domain-containing protein | DUF3799 domain-containing protein | | afdb-uniprot50 | AF-A0A7U2KNN2-F1-MODEL\_V4 | 1.0 | 1.179e-14 | 497 | 0.177 | 384 | 222 | 12 | 1 | 371 | 19 | 321 | PD-(D/E)XK nuclease-like domain-containing protein | PD-(D/E)XK nuclease-like domain-containing protein | | afdb-uniprot50 | AF-A0A2D7EZG3-F1-MODEL\_V4 | 1.0 | 2.315e-14 | 496 | 0.236 | 355 | 159 | 13 | 5 | 354 | 6 | 253 | DUF3799 domain-containing protein | DUF3799 domain-containing protein | | afdb-uniprot50 | AF-A0A1H5JK49-F1-MODEL\_V4 | 1.0 | 3.236e-15 | 496 | 0.204 | 387 | 189 | 18 | 1 | 371 | 4 | 287 | DUF3799 domain-containing protein | DUF3799 domain-containing protein | | afdb-uniprot50 | AF-A0A7V4U9Y0-F1-MODEL\_V4 | 1.0 | 1.844e-15 | 496 | 0.202 | 356 | 182 | 12 | 8 | 358 | 78 | 336 | DUF3799 domain-containing protein | DUF3799 domain-containing protein | | afdb-uniprot50 | AF-A0A373FBR7-F1-MODEL\_V4 | 1.0 | 4.052e-15 | 495 | 0.162 | 375 | 187 | 13 | 5 | 360 | 7 | 273 | DUF3799 domain-containing protein | DUF3799 domain-containing protein | | afdb-uniprot50 | AF-A0A5C0AA35-F1-MODEL\_V4 | 1.0 | 2.309e-15 | 495 | 0.179 | 385 | 195 | 17 | 1 | 369 | 6 | 285 | DUF3799 domain-containing protein | DUF3799 domain-containing protein | | afdb-uniprot50 | AF-A0A7Y0EXA6-F1-MODEL\_V4 | 1.0 | 4.796e-15 | 493 | 0.201 | 383 | 182 | 15 | 5 | 371 | 4 | 278 | DUF3799 domain-containing protein | DUF3799 domain-containing protein | | afdb-uniprot50 | AF-A0A4Q8QN88-F1-MODEL\_V4 | 1.0 | 2.069e-14 | 493 | 0.189 | 385 | 201 | 17 | 1 | 369 | 12 | 301 | DUF3799 domain-containing protein | DUF3799 domain-containing protein | | afdb-uniprot50 | AF-A0A509JFD6-F1-MODEL\_V4 | 1.0 | 2.178e-16 | 492 | 0.166 | 390 | 205 | 11 | 1 | 366 | 1 | 294 | Uncharacterized protein | Uncharacterized protein | | afdb-uniprot50 | AF-A0A5C7PU20-F1-MODEL\_V4 | 1.0 | 1.32e-14 | 491 | 0.188 | 361 | 193 | 13 | 6 | 361 | 12 | 277 | DUF3799 domain-containing protein | DUF3799 domain-containing protein | | afdb-uniprot50 | AF-A0A2D7YTB8-F1-MODEL\_V4 | 1.0 | 1.752e-13 | 491 | 0.176 | 375 | 192 | 15 | 1 | 361 | 4 | 275 | DUF3799 domain-containing protein | DUF3799 domain-containing protein | | afdb-uniprot50 | AF-A0A097ENH1-F1-MODEL\_V4 | 1.0 | 4.297e-14 | 488 | 0.196 | 366 | 178 | 15 | 14 | 370 | 3 | 261 | DUF3799 domain-containing protein | DUF3799 domain-containing protein | | afdb-uniprot50 | AF-A0A7C1JNN6-F1-MODEL\_V4 | 1.0 | 1.854e-13 | 487 | 0.192 | 333 | 170 | 11 | 25 | 356 | 2 | 236 | DUF3799 domain-containing protein | DUF3799 domain-containing protein | | afdb-uniprot50 | AF-Q4LBT7-F1-MODEL\_V4 | 1.0 | 4.275e-16 | 487 | 0.24 | 375 | 174 | 14 | 5 | 371 | 292 | 563 | RecE protein | RecE protein | | afdb-uniprot50 | AF-A0A3S0D0M8-F1-MODEL\_V4 | 1.0 | 1.056e-13 | 482 | 0.149 | 402 | 201 | 14 | 1 | 369 | 6 | 299 | Uncharacterized protein | Uncharacterized protein | | afdb-uniprot50 | AF-A0A5B8CGE1-F1-MODEL\_V4 | 1.0 | 1.562e-14 | 482 | 0.201 | 382 | 192 | 15 | 1 | 369 | 38 | 319 | DUF3799 domain-containing protein | DUF3799 domain-containing protein | | afdb-uniprot50 | AF-A0A077NJB7-F1-MODEL\_V4 | 1.0 | 1.059e-12 | 480 | 0.264 | 174 | 118 | 4 | 194 | 361 | 10 | 179 | Phage-related exonuclease | Phage-related exonuclease | | afdb-uniprot50 | AF-A0A318MLD0-F1-MODEL\_V4 | 1.0 | 2.069e-14 | 480 | 0.189 | 386 | 186 | 15 | 1 | 369 | 1 | 276 | DUF3799 domain-containing protein | DUF3799 domain-containing protein | | afdb-uniprot50 | AF-A0A6L9STJ4-F1-MODEL\_V4 | 1.0 | 3.83e-15 | 479 | 0.161 | 378 | 188 | 15 | 1 | 361 | 1 | 266 | DUF3799 domain-containing protein | DUF3799 domain-containing protein | | afdb-uniprot50 | AF-A0A3M1NIT7-F1-MODEL\_V4 | 1.0 | 1.251e-13 | 478 | 0.172 | 359 | 194 | 11 | 5 | 361 | 2 | 259 | DUF3799 domain-containing protein | DUF3799 domain-containing protein | | afdb-uniprot50 | AF-A0A437M7Z1-F1-MODEL\_V4 | 1.0 | 6.353e-15 | 478 | 0.162 | 413 | 256 | 14 | 1 | 370 | 44 | 409 | Uncharacterized protein | Uncharacterized protein | | afdb-uniprot50 | AF-A0A3G2V3T1-F1-MODEL\_V4 | 1.0 | 1.389e-16 | 476 | 0.188 | 408 | 219 | 22 | 1 | 361 | 15 | 357 | DUF3799 domain-containing protein | DUF3799 domain-containing protein | | afdb-uniprot50 | AF-A0A517YVS6-F1-MODEL\_V4 | 1.0 | 2.069e-14 | 475 | 0.17 | 382 | 192 | 13 | 1 | 364 | 21 | 295 | Exodeoxyribonuclease 8 | Exodeoxyribonuclease 8 | | afdb-uniprot50 | AF-A0A8A5F1F3-F1-MODEL\_V4 | 1.0 | 4.052e-15 | 475 | 0.196 | 387 | 199 | 18 | 1 | 364 | 10 | 307 | PD-(D/E)XK nuclease-like domain-containing protein | PD-(D/E)XK nuclease-like domain-containing protein | | afdb-uniprot50 | AF-A0A843HS31-F1-MODEL\_V4 | 1.0 | 7.127e-14 | 474 | 0.156 | 377 | 200 | 14 | 5 | 366 | 2 | 275 | PD-(D/E)XK nuclease-like domain-containing protein | PD-(D/E)XK nuclease-like domain-containing protein | | afdb-uniprot50 | AF-A0A5Y1VD64-F1-MODEL\_V4 | 1.0 | 1.12e-12 | 473 | 0.339 | 171 | 101 | 6 | 200 | 366 | 2 | 164 | Exonuclease VIII | Exonuclease VIII | | afdb-uniprot50 | AF-A0A2A5D9T2-F1-MODEL\_V4 | 1.0 | 2.747e-13 | 473 | 0.182 | 368 | 187 | 16 | 1 | 358 | 10 | 273 | DUF3799 domain-containing protein | DUF3799 domain-containing protein | | afdb-uniprot50 | AF-A0A358N4E2-F1-MODEL\_V4 | 1.0 | 2.189e-14 | 473 | 0.181 | 363 | 191 | 14 | 10 | 360 | 3 | 271 | DUF3799 domain-containing protein | DUF3799 domain-containing protein | | afdb-uniprot50 | AF-A0A1V5IXR4-F1-MODEL\_V4 | 1.0 | 8.925e-14 | 473 | 0.193 | 372 | 181 | 14 | 6 | 355 | 21 | 295 | Exonuclease VIII | Exonuclease VIII | | afdb-uniprot50 | AF-A0A6I0ESC0-F1-MODEL\_V4 | 1.0 | 2.584e-15 | 473 | 0.213 | 356 | 178 | 12 | 8 | 358 | 156 | 414 | DUF3799 domain-containing protein | DUF3799 domain-containing protein | | afdb-uniprot50 | AF-R5BQV7-F1-MODEL\_V4 | 1.0 | 3.228e-16 | 472 | 0.167 | 381 | 235 | 19 | 6 | 355 | 2 | 331 | DUF3799 domain-containing protein | DUF3799 domain-containing protein | | afdb-uniprot50 | AF-A0A1H4NL54-F1-MODEL\_V4 | 1.0 | 2.892e-15 | 471 | 0.16 | 411 | 235 | 16 | 1 | 360 | 16 | 367 | DUF3799 domain-containing protein | DUF3799 domain-containing protein | | afdb-uniprot50 | AF-A0A5N9VVT2-F1-MODEL\_V4 | 1.0 | 1.484e-12 | 469 | 0.349 | 169 | 98 | 6 | 194 | 358 | 1 | 161 | DUF3799 domain-containing protein | DUF3799 domain-containing protein | | afdb-uniprot50 | AF-A0A0Q6CAS4-F1-MODEL\_V4 | 1.0 | 3.059e-15 | 469 | 0.178 | 387 | 195 | 19 | 1 | 367 | 14 | 297 | DUF3799 domain-containing protein | DUF3799 domain-containing protein | | afdb-uniprot50 | AF-A0A0K1H0Q4-F1-MODEL\_V4 | 1.0 | 3.228e-16 | 469 | 0.206 | 377 | 185 | 13 | 5 | 371 | 416 | 688 | Exodeoxyribonuclease VIII | Exodeoxyribonuclease VIII | | afdb-uniprot50 | AF-A0A5V0VKT4-F1-MODEL\_V4 | 1.0 | 2.315e-14 | 468 | 0.232 | 293 | 191 | 7 | 90 | 372 | 2 | 270 | DUF3799 domain-containing protein | DUF3799 domain-containing protein | | afdb-uniprot50 | AF-A0A5C7PQS1-F1-MODEL\_V4 | 1.0 | 4.808e-14 | 468 | 0.184 | 364 | 188 | 14 | 9 | 360 | 1 | 267 | DUF3799 domain-containing protein | DUF3799 domain-containing protein | | afdb-uniprot50 | AF-A0A1F9UXV9-F1-MODEL\_V4 | 1.0 | 6.369e-14 | 468 | 0.157 | 363 | 176 | 11 | 17 | 359 | 8 | 260 | DUF3799 domain-containing protein | DUF3799 domain-containing protein | | afdb-uniprot50 | AF-A0A381SAS3-F1-MODEL\_V4 | 1.0 | 1.326e-12 | 465 | 0.21 | 290 | 132 | 8 | 52 | 340 | 2 | 195 | DUF3799 domain-containing protein | DUF3799 domain-containing protein | | afdb-uniprot50 | AF-A0A2U1TII1-F1-MODEL\_V4 | 1.0 | 8.902e-15 | 465 | 0.222 | 288 | 180 | 7 | 98 | 370 | 2 | 260 | Exodeoxyribonuclease VIII | Exodeoxyribonuclease VIII | | afdb-uniprot50 | AF-A0A0Q4WZ62-F1-MODEL\_V4 | 1.0 | 6.721e-15 | 465 | 0.181 | 386 | 193 | 20 | 2 | 367 | 13 | 295 | DUF3799 domain-containing protein | DUF3799 domain-containing protein | | afdb-uniprot50 | AF-A0A7R6V9M0-F1-MODEL\_V4 | 1.0 | 1.323e-13 | 463 | 0.172 | 354 | 185 | 13 | 9 | 358 | 1 | 250 | Exodeoxyribonuclease VIII | Exodeoxyribonuclease VIII | | afdb-uniprot50 | AF-A0A4P7L2A5-F1-MODEL\_V4 | 1.0 | 3.236e-15 | 463 | 0.213 | 374 | 183 | 13 | 5 | 371 | 254 | 523 | Exodeoxyribonuclease 8 | Exodeoxyribonuclease 8 | | afdb-uniprot50 | AF-A0A0Q8VDF8-F1-MODEL\_V4 | 1.0 | 4.083e-12 | 462 | 0.445 | 146 | 75 | 2 | 229 | 370 | 2 | 145 | DUF3799 domain-containing protein | DUF3799 domain-containing protein | | afdb-uniprot50 | AF-A0A6P1PXM4-F1-MODEL\_V4 | 1.0 | 6.353e-15 | 462 | 0.232 | 378 | 164 | 15 | 1 | 354 | 1 | 276 | Exodeoxyribonuclease 8 | Exodeoxyribonuclease 8 | | afdb-uniprot50 | AF-A0A8B3TCB0-F1-MODEL\_V4 | 1.0 | 1.477e-14 | 461 | 0.157 | 393 | 207 | 14 | 1 | 371 | 19 | 309 | Uncharacterized protein | Uncharacterized protein | | afdb-uniprot50 | AF-A0A0F9N5B6-F1-MODEL\_V4 | 1.0 | 8.902e-15 | 459 | 0.183 | 403 | 203 | 19 | 3 | 369 | 4 | 316 | DUF3799 domain-containing protein | DUF3799 domain-containing protein | | afdb-uniprot50 | AF-A0A841AAK3-F1-MODEL\_V4 | 1.0 | 4.796e-15 | 459 | 0.203 | 388 | 186 | 21 | 2 | 367 | 13 | 299 | Uncharacterized protein | Uncharacterized protein | | afdb-uniprot50 | AF-A0A165XES0-F1-MODEL\_V4 | 1.0 | 1.396e-14 | 459 | 0.149 | 401 | 243 | 17 | 1 | 363 | 10 | 350 | Exonuclease VIII | Exonuclease VIII | | afdb-uniprot50 | AF-A0A2Z5YI50-F1-MODEL\_V4 | 1.0 | 9.987e-14 | 458 | 0.206 | 368 | 188 | 16 | 1 | 361 | 5 | 275 | DUF3799 domain-containing protein | DUF3799 domain-containing protein | | afdb-uniprot50 | AF-A0A0F3IMN9-F1-MODEL\_V4 | 1.0 | 1.656e-13 | 457 | 0.181 | 353 | 181 | 11 | 9 | 356 | 48 | 297 | DUF3799 domain-containing protein | DUF3799 domain-containing protein | | afdb-uniprot50 | AF-A0A2Z3HVS0-F1-MODEL\_V4 | 1.0 | 3.449e-12 | 455 | 0.341 | 164 | 97 | 5 | 204 | 361 | 2 | 160 | DUF3799 domain-containing protein | DUF3799 domain-containing protein | | afdb-uniprot50 | AF-A0A354CHA7-F1-MODEL\_V4 | 1.0 | 8.415e-15 | 453 | 0.203 | 363 | 189 | 12 | 7 | 360 | 53 | 324 | DUF3799 domain-containing protein | DUF3799 domain-containing protein | | afdb-uniprot50 | AF-A0A832HXX1-F1-MODEL\_V4 | 1.0 | 1.051e-15 | 453 | 0.184 | 402 | 204 | 19 | 1 | 371 | 10 | 318 | Uncharacterized protein | Uncharacterized protein | | afdb-uniprot50 | AF-A0A2E8RC03-F1-MODEL\_V4 | 1.0 | 1.182e-13 | 452 | 0.207 | 367 | 179 | 14 | 8 | 363 | 5 | 270 | DUF3799 domain-containing protein | DUF3799 domain-containing protein | | afdb-uniprot50 | AF-X0VAD8-F1-MODEL\_V4 | 1.0 | 1.406e-11 | 451 | 0.417 | 134 | 77 | 1 | 232 | 364 | 2 | 135 | DUF3799 domain-containing protein | DUF3799 domain-containing protein | | afdb-uniprot50 | AF-A0A2A6NRI3-F1-MODEL\_V4 | 1.0 | 1.396e-14 | 448 | 0.164 | 407 | 235 | 16 | 1 | 361 | 64 | 411 | DUF3799 domain-containing protein | DUF3799 domain-containing protein | | afdb-uniprot50 | AF-A0A0F9AK76-F1-MODEL\_V4 | 1.0 | 1.966e-12 | 447 | 0.287 | 188 | 124 | 7 | 181 | 363 | 64 | 246 | DUF3799 domain-containing protein | DUF3799 domain-containing protein | | afdb-uniprot50 | AF-A0A5C7PD05-F1-MODEL\_V4 | 1.0 | 1.652e-14 | 444 | 0.162 | 381 | 200 | 15 | 1 | 367 | 51 | 326 | DUF3799 domain-containing protein | DUF3799 domain-containing protein | | afdb-uniprot50 | AF-A0A244CWF3-F1-MODEL\_V4 | 1.0 | 8.88e-16 | 444 | 0.156 | 409 | 242 | 16 | 1 | 372 | 54 | 396 | DUF3799 domain-containing protein | DUF3799 domain-containing protein | | afdb-uniprot50 | AF-A0A2D6B2Q9-F1-MODEL\_V4 | 1.0 | 9.987e-14 | 443 | 0.185 | 382 | 191 | 15 | 1 | 356 | 1 | 288 | DUF3799 domain-containing protein | DUF3799 domain-containing protein | | afdb-uniprot50 | AF-A0A3D1XDL4-F1-MODEL\_V4 | 1.0 | 2.194e-13 | 442 | 0.242 | 210 | 136 | 5 | 155 | 361 | 17 | 206 | DUF3799 domain-containing protein | DUF3799 domain-containing protein | | afdb-uniprot50 | AF-A0A7V9TKI5-F1-MODEL\_V4 | 1.0 | 3.44e-13 | 440 | 0.173 | 381 | 197 | 16 | 9 | 371 | 1 | 281 | Uncharacterized protein | Uncharacterized protein | | afdb-uniprot50 | AF-A0A2E9HYF9-F1-MODEL\_V4 | 1.0 | 1.112e-15 | 440 | 0.172 | 388 | 212 | 17 | 2 | 371 | 56 | 352 | DUF3799 domain-containing protein | DUF3799 domain-containing protein | | afdb-uniprot50 | AF-A0A4P7L155-F1-MODEL\_V4 | 1.0 | 1.656e-13 | 436 | 0.212 | 325 | 146 | 12 | 53 | 371 | 2 | 222 | Exodeoxyribonuclease 8 | Exodeoxyribonuclease 8 | | afdb-uniprot50 | AF-A0A709TYJ6-F1-MODEL\_V4 | 1.0 | 5.354e-16 | 434 | 0.223 | 335 | 205 | 8 | 83 | 372 | 4 | 328 | DNA breaking-rejoining protein | DNA breaking-rejoining protein | | afdb-uniprot50 | AF-A0A626E748-F1-MODEL\_V4 | 1.0 | 6.337e-16 | 434 | 0.223 | 335 | 205 | 8 | 83 | 372 | 7 | 331 | DNA breaking-rejoining protein | DNA breaking-rejoining protein | | afdb-uniprot50 | AF-M3JBV3-F1-MODEL\_V4 | 1.0 | 2.604e-12 | 433 | 0.164 | 359 | 166 | 9 | 9 | 366 | 25 | 250 | Bacteriophage protein | Bacteriophage protein | | afdb-uniprot50 | AF-A0A825MRR2-F1-MODEL\_V4 | 1.0 | 2.906e-13 | 432 | 0.193 | 331 | 157 | 12 | 40 | 361 | 5 | 234 | Uncharacterized protein | Uncharacterized protein | | afdb-uniprot50 | AF-A0A348FZE1-F1-MODEL\_V4 | 1.0 | 3.432e-14 | 432 | 0.196 | 391 | 190 | 19 | 2 | 371 | 15 | 302 | DUF3799 domain-containing protein | DUF3799 domain-containing protein | | afdb-uniprot50 | AF-A0A705SAZ7-F1-MODEL\_V4 | 1.0 | 3.821e-16 | 432 | 0.223 | 335 | 205 | 8 | 83 | 372 | 5 | 329 | DNA breaking-rejoining protein | DNA breaking-rejoining protein | | afdb-uniprot50 | AF-A0A741MSY8-F1-MODEL\_V4 | 1.0 | 3.821e-16 | 431 | 0.223 | 335 | 205 | 9 | 83 | 372 | 3 | 327 | DNA breaking-rejoining protein | DNA breaking-rejoining protein | | afdb-uniprot50 | AF-A0A3N2N6Q1-F1-MODEL\_V4 | 1.0 | 3.423e-15 | 430 | 0.181 | 392 | 214 | 19 | 5 | 367 | 35 | 348 | DUF3799 domain-containing protein | DUF3799 domain-containing protein | | afdb-uniprot50 | AF-A0A5U6R0D4-F1-MODEL\_V4 | 1.0 | 3.821e-16 | 429 | 0.22 | 336 | 205 | 9 | 83 | 372 | 4 | 328 | Exodeoxyribonuclease | Exodeoxyribonuclease | | afdb-uniprot50 | AF-A0A5X2NJW9-F1-MODEL\_V4 | 1.0 | 6.337e-16 | 429 | 0.223 | 335 | 205 | 8 | 83 | 372 | 5 | 329 | DNA breaking-rejoining protein | DNA breaking-rejoining protein | | afdb-uniprot50 | AF-A0A706H7T9-F1-MODEL\_V4 | 1.0 | 5.991e-16 | 428 | 0.222 | 337 | 203 | 9 | 83 | 372 | 6 | 330 | DNA breaking-rejoining protein | DNA breaking-rejoining protein | | afdb-uniprot50 | AF-A0A722TRM3-F1-MODEL\_V4 | 1.0 | 5.991e-16 | 427 | 0.223 | 335 | 205 | 9 | 83 | 372 | 3 | 327 | DNA breaking-rejoining protein | DNA breaking-rejoining protein | | afdb-uniprot50 | AF-A0A710PYI5-F1-MODEL\_V4 | 1.0 | 5.991e-16 | 426 | 0.223 | 335 | 205 | 8 | 83 | 372 | 5 | 329 | DNA breaking-rejoining protein | DNA breaking-rejoining protein | | afdb-uniprot50 | AF-A0A5C7M3T7-F1-MODEL\_V4 | 1.0 | 5.394e-13 | 425 | 0.186 | 359 | 188 | 17 | 9 | 362 | 1 | 260 | DUF3799 domain-containing protein | DUF3799 domain-containing protein | | afdb-uniprot50 | AF-A0A704YVF3-F1-MODEL\_V4 | 1.0 | 1.473e-15 | 425 | 0.22 | 335 | 206 | 8 | 83 | 372 | 7 | 331 | DNA breaking-rejoining protein | DNA breaking-rejoining protein | | afdb-uniprot50 | AF-A0A710ZUB8-F1-MODEL\_V4 | 1.0 | 5.061e-16 | 424 | 0.22 | 336 | 205 | 9 | 83 | 372 | 4 | 328 | Exodeoxyribonuclease | Exodeoxyribonuclease | | afdb-uniprot50 | AF-A0A705GFT1-F1-MODEL\_V4 | 1.0 | 1.176e-15 | 424 | 0.22 | 336 | 205 | 9 | 83 | 372 | 7 | 331 | Exodeoxyribonuclease | Exodeoxyribonuclease | | afdb-uniprot50 | AF-A0A705K906-F1-MODEL\_V4 | 1.0 | 6.337e-16 | 424 | 0.223 | 335 | 205 | 8 | 83 | 372 | 11 | 335 | DNA breaking-rejoining protein | DNA breaking-rejoining protein | | afdb-uniprot50 | AF-A0A6W0EZW0-F1-MODEL\_V4 | 1.0 | 1.316e-15 | 422 | 0.22 | 335 | 206 | 9 | 83 | 372 | 3 | 327 | Exodeoxyribonuclease | Exodeoxyribonuclease | | afdb-uniprot50 | AF-A0A5X0DDY5-F1-MODEL\_V4 | 1.0 | 5.991e-16 | 422 | 0.22 | 336 | 205 | 9 | 83 | 372 | 7 | 331 | Exodeoxyribonuclease | Exodeoxyribonuclease | | afdb-uniprot50 | AF-A0A7K0GP64-F1-MODEL\_V4 | 1.0 | 1.333e-10 | 421 | 0.52 | 121 | 58 | 0 | 241 | 361 | 1 | 121 | Exodeoxyribonuclease VIII | Exodeoxyribonuclease VIII | | afdb-uniprot50 | AF-A0A661I8B6-F1-MODEL\_V4 | 1.0 | 3.261e-12 | 421 | 0.179 | 357 | 182 | 13 | 9 | 358 | 29 | 281 | DUF3799 domain-containing protein | DUF3799 domain-containing protein | | afdb-uniprot50 | AF-A0A3T5P7X6-F1-MODEL\_V4 | 1.0 | 8.88e-16 | 421 | 0.223 | 335 | 205 | 8 | 83 | 372 | 7 | 331 | DNA breaking-rejoining protein | DNA breaking-rejoining protein | | afdb-uniprot50 | AF-A0A2U1SSX7-F1-MODEL\_V4 | 1.0 | 1.118e-13 | 421 | 0.195 | 405 | 203 | 17 | 1 | 371 | 14 | 329 | DUF3799 domain-containing protein | DUF3799 domain-containing protein | | afdb-uniprot50 | AF-A0A5W4VQX4-F1-MODEL\_V4 | 1.0 | 7.091e-16 | 421 | 0.219 | 337 | 204 | 9 | 83 | 372 | 18 | 342 | DNA breaking-rejoining protein | DNA breaking-rejoining protein | | afdb-uniprot50 | AF-B2KDD5-F1-MODEL\_V4 | 1.0 | 2.597e-13 | 420 | 0.144 | 387 | 206 | 15 | 6 | 367 | 1 | 287 | DUF3799 domain-containing protein | DUF3799 domain-containing protein | | afdb-uniprot50 | AF-A0A721STD1-F1-MODEL\_V4 | 1.0 | 1.244e-15 | 420 | 0.223 | 335 | 205 | 8 | 83 | 372 | 6 | 330 | DNA breaking-rejoining protein | DNA breaking-rejoining protein | | afdb-uniprot50 | AF-A0A630QVP1-F1-MODEL\_V4 | 1.0 | 4.319e-12 | 419 | 0.261 | 226 | 151 | 5 | 157 | 372 | 1 | 220 | DUF3799 domain-containing protein | DUF3799 domain-containing protein | | afdb-uniprot50 | AF-A0A5C8B335-F1-MODEL\_V4 | 1.0 | 1.966e-12 | 419 | 0.147 | 379 | 190 | 16 | 9 | 370 | 1 | 263 | DUF3799 domain-containing protein | DUF3799 domain-containing protein | | afdb-uniprot50 | AF-A0A722NJL0-F1-MODEL\_V4 | 1.0 | 1.051e-15 | 419 | 0.22 | 335 | 206 | 9 | 83 | 372 | 3 | 327 | Exodeoxyribonuclease | Exodeoxyribonuclease | | afdb-uniprot50 | AF-A0A3B9ZLY2-F1-MODEL\_V4 | 1.0 | 5.663e-16 | 419 | 0.193 | 387 | 241 | 21 | 7 | 368 | 13 | 353 | DUF3799 domain-containing protein | DUF3799 domain-containing protein | | afdb-uniprot50 | AF-A0A3U7DT70-F1-MODEL\_V4 | 1.0 | 2.085e-11 | 418 | 0.354 | 155 | 89 | 5 | 216 | 366 | 2 | 149 | Exonuclease VIII | Exonuclease VIII | | afdb-uniprot50 | AF-A0A2E1MZJ8-F1-MODEL\_V4 | 1.0 | 2.899e-14 | 418 | 0.183 | 382 | 220 | 18 | 9 | 354 | 1 | 326 | DUF3799 domain-containing protein | DUF3799 domain-containing protein | | afdb-uniprot50 | AF-A0A709BXU1-F1-MODEL\_V4 | 1.0 | 2.443e-15 | 418 | 0.22 | 336 | 205 | 9 | 83 | 372 | 3 | 327 | Exodeoxyribonuclease | Exodeoxyribonuclease | | afdb-uniprot50 | AF-A0A722CY88-F1-MODEL\_V4 | 1.0 | 8.394e-16 | 417 | 0.22 | 336 | 205 | 9 | 83 | 372 | 5 | 329 | Exodeoxyribonuclease | Exodeoxyribonuclease | | afdb-uniprot50 | AF-A0A6Y0XSK0-F1-MODEL\_V4 | 1.0 | 8.394e-16 | 417 | 0.22 | 336 | 205 | 9 | 83 | 372 | 5 | 329 | Exodeoxyribonuclease | Exodeoxyribonuclease | | afdb-uniprot50 | AF-A0A087B4Z0-F1-MODEL\_V4 | 1.0 | 4.557e-13 | 416 | 0.173 | 381 | 188 | 20 | 5 | 368 | 2 | 272 | Phage protein | Phage protein | | afdb-uniprot50 | AF-A0A6L6J4B9-F1-MODEL\_V4 | 1.0 | 2.194e-13 | 416 | 0.19 | 373 | 176 | 18 | 1 | 359 | 8 | 268 | DUF3799 domain-containing protein | DUF3799 domain-containing protein | | afdb-uniprot50 | AF-X8AH60-F1-MODEL\_V4 | 1.0 | 5.422e-11 | 413 | 0.345 | 168 | 102 | 6 | 207 | 369 | 3 | 167 | DUF3799 domain-containing protein | DUF3799 domain-containing protein | | afdb-uniprot50 | AF-A0A705X8M6-F1-MODEL\_V4 | 1.0 | 7.935e-16 | 413 | 0.215 | 338 | 204 | 10 | 83 | 372 | 11 | 335 | Exodeoxyribonuclease | Exodeoxyribonuclease | | afdb-uniprot50 | AF-A0A2E2D771-F1-MODEL\_V4 | 1.0 | 1.956e-14 | 409 | 0.175 | 353 | 214 | 16 | 19 | 359 | 24 | 311 | DUF3799 domain-containing protein | DUF3799 domain-containing protein | | afdb-uniprot50 | AF-A0A3S5DAA5-F1-MODEL\_V4 | 1.0 | 3.658e-11 | 407 | 0.299 | 147 | 100 | 2 | 221 | 364 | 10 | 156 | Exodeoxyribonuclease 8 | Exodeoxyribonuclease 8 | | afdb-uniprot50 | AF-A0A6N8TQN8-F1-MODEL\_V4 | 1.0 | 3.84e-14 | 407 | 0.126 | 331 | 225 | 12 | 15 | 343 | 43 | 311 | Uncharacterized protein | Uncharacterized protein | | afdb-uniprot50 | AF-A0A5W8UZN8-F1-MODEL\_V4 | 1.0 | 7.109e-15 | 404 | 0.23 | 321 | 192 | 9 | 97 | 372 | 2 | 312 | Exodeoxyribonuclease | Exodeoxyribonuclease | | afdb-uniprot50 | AF-A0A2E3D7E6-F1-MODEL\_V4 | 1.0 | 2.327e-12 | 402 | 0.174 | 373 | 191 | 14 | 8 | 372 | 4 | 267 | DUF3799 domain-containing protein | DUF3799 domain-containing protein | | afdb-uniprot50 | AF-A0A1M6GAE2-F1-MODEL\_V4 | 1.0 | 5.381e-14 | 397 | 0.163 | 355 | 228 | 13 | 19 | 370 | 49 | 337 | DUF3799 domain-containing protein | DUF3799 domain-containing protein | | afdb-uniprot50 | AF-A0A1H6WGJ5-F1-MODEL\_V4 | 1.0 | 3.84e-14 | 396 | 0.243 | 373 | 167 | 18 | 1 | 359 | 31 | 302 | DUF3799 domain-containing protein | DUF3799 domain-containing protein | | afdb-uniprot50 | AF-A0A5W2I5P1-F1-MODEL\_V4 | 1.0 | 3.63e-14 | 395 | 0.23 | 321 | 192 | 9 | 97 | 372 | 2 | 312 | Exodeoxyribonuclease | Exodeoxyribonuclease | | afdb-uniprot50 | AF-A0A495SNW1-F1-MODEL\_V4 | 1.0 | 4.297e-14 | 391 | 0.151 | 364 | 233 | 15 | 6 | 360 | 22 | 318 | PDDEXK-like uncharacterized protein DUF3799 | PDDEXK-like uncharacterized protein DUF3799 | | afdb-uniprot50 | AF-A0A318TK26-F1-MODEL\_V4 | 1.0 | 1.059e-12 | 390 | 0.185 | 400 | 220 | 23 | 3 | 361 | 10 | 344 | PDDEXK-like uncharacterized protein DUF3799 | PDDEXK-like uncharacterized protein DUF3799 | | afdb-uniprot50 | AF-A0A4Q2UH62-F1-MODEL\_V4 | 1.0 | 1.188e-11 | 388 | 0.189 | 364 | 144 | 10 | 9 | 359 | 3 | 228 | DUF3799 domain-containing protein | DUF3799 domain-containing protein | | afdb-uniprot50 | AF-A0A5V8R8Y3-F1-MODEL\_V4 | 1.0 | 3.63e-14 | 388 | 0.231 | 315 | 187 | 9 | 103 | 372 | 3 | 307 | DNA breaking-rejoining protein | DNA breaking-rejoining protein | | afdb-uniprot50 | AF-A0A369XIG4-F1-MODEL\_V4 | 1.0 | 8.057e-10 | 386 | 0.453 | 128 | 66 | 3 | 238 | 363 | 2 | 127 | DUF3799 domain-containing protein | DUF3799 domain-containing protein | | afdb-uniprot50 | AF-A0A3Z7BBV9-F1-MODEL\_V4 | 1.0 | 8.925e-14 | 386 | 0.231 | 315 | 187 | 9 | 103 | 372 | 1 | 305 | Exodeoxyribonuclease | Exodeoxyribonuclease | | afdb-uniprot50 | AF-A0A553GCW7-F1-MODEL\_V4 | 1.0 | 2.205e-11 | 385 | 0.161 | 341 | 150 | 8 | 1 | 338 | 2 | 209 | DUF3799 domain-containing protein | DUF3799 domain-containing protein | | afdb-uniprot50 | AF-A0A379GHA6-F1-MODEL\_V4 | 1.0 | 1.484e-12 | 385 | 0.242 | 264 | 166 | 9 | 105 | 358 | 5 | 244 | Phage-related exonuclease | Phage-related exonuclease | | afdb-uniprot50 | AF-A0A6M3ITI9-F1-MODEL\_V4 | 1.0 | 1.001e-12 | 385 | 0.182 | 390 | 203 | 15 | 5 | 358 | 2 | 311 | DUF3799 domain-containing protein | DUF3799 domain-containing protein | | afdb-uniprot50 | AF-A0A7U4E4Q7-F1-MODEL\_V4 | 1.0 | 7.597e-11 | 384 | 0.179 | 334 | 139 | 10 | 7 | 338 | 9 | 209 | DUF3799 domain-containing protein | DUF3799 domain-containing protein | | afdb-uniprot50 | AF-A0A662AYQ9-F1-MODEL\_V4 | 1.0 | 4.569e-12 | 384 | 0.153 | 391 | 194 | 20 | 1 | 356 | 1 | 289 | DUF3799 domain-containing protein | DUF3799 domain-containing protein | | afdb-uniprot50 | AF-A0A2K8Z643-F1-MODEL\_V4 | 1.0 | 2.211e-10 | 379 | 0.175 | 331 | 131 | 12 | 14 | 338 | 4 | 198 | DUF3799 domain-containing protein | DUF3799 domain-containing protein | | afdb-uniprot50 | AF-A0A2N7BEU4-F1-MODEL\_V4 | 1.0 | 1.578e-10 | 378 | 0.182 | 334 | 137 | 9 | 7 | 340 | 3 | 200 | DUF3799 domain-containing protein | DUF3799 domain-containing protein | | afdb-uniprot50 | AF-A0A2D8XKX9-F1-MODEL\_V4 | 1.0 | 9.489e-12 | 378 | 0.15 | 340 | 192 | 13 | 19 | 355 | 16 | 261 | DUF3799 domain-containing protein | DUF3799 domain-containing protein | | afdb-uniprot50 | AF-A0A857VCF5-F1-MODEL\_V4 | 1.0 | 1.863e-11 | 376 | 0.162 | 358 | 186 | 16 | 9 | 359 | 1 | 251 | Uncharacterized protein | Uncharacterized protein | | afdb-uniprot50 | AF-A0A0E3B9I3-F1-MODEL\_V4 | 1.0 | 1.574e-11 | 375 | 0.244 | 196 | 132 | 7 | 177 | 360 | 24 | 215 | DUF3799 domain-containing protein | DUF3799 domain-containing protein | | afdb-uniprot50 | AF-A0A433CKI3-F1-MODEL\_V4 | 1.0 | 4.58e-11 | 375 | 0.159 | 327 | 160 | 12 | 17 | 341 | 3 | 216 | DUF3799 domain-containing protein | DUF3799 domain-containing protein | | afdb-uniprot50 | AF-A0A0F8ZM74-F1-MODEL\_V4 | 1.0 | 2.211e-10 | 374 | 0.302 | 192 | 123 | 8 | 183 | 372 | 2 | 184 | DUF3799 domain-containing protein | DUF3799 domain-containing protein | | afdb-uniprot50 | AF-A0A721W8G7-F1-MODEL\_V4 | 1.0 | 6.082e-10 | 373 | 0.259 | 189 | 124 | 5 | 194 | 372 | 3 | 185 | DUF3799 domain-containing protein | DUF3799 domain-containing protein | | afdb-uniprot50 | AF-A0A7J5TSP0-F1-MODEL\_V4 | 1.0 | 3.869e-11 | 373 | 0.183 | 371 | 150 | 10 | 6 | 361 | 1 | 233 | DUF3799 domain-containing protein | DUF3799 domain-containing protein | | afdb-uniprot50 | AF-A0A2D9CGG8-F1-MODEL\_V4 | 1.0 | 1.123e-11 | 373 | 0.15 | 338 | 195 | 13 | 19 | 356 | 15 | 260 | DUF3799 domain-containing protein | DUF3799 domain-containing protein | | afdb-uniprot50 | AF-A0A1B9YEN4-F1-MODEL\_V4 | 1.0 | 7.145e-13 | 373 | 0.151 | 370 | 233 | 13 | 6 | 360 | 12 | 315 | DUF3799 domain-containing protein | DUF3799 domain-containing protein | | afdb-uniprot50 | AF-A0A7X9RXT8-F1-MODEL\_V4 | 1.0 | 4.845e-11 | 372 | 0.173 | 345 | 147 | 7 | 1 | 341 | 27 | 237 | DUF3799 domain-containing protein | DUF3799 domain-containing protein | | afdb-uniprot50 | AF-A0A7C2PXI9-F1-MODEL\_V4 | 1.0 | 1.26e-10 | 370 | 0.278 | 187 | 125 | 6 | 174 | 358 | 105 | 283 | DUF3799 domain-containing protein | DUF3799 domain-containing protein | | afdb-uniprot50 | AF-A0A7Z9YY02-F1-MODEL\_V4 | 1.0 | 3.475e-09 | 369 | 0.49 | 108 | 55 | 0 | 254 | 361 | 1 | 108 | Exodeoxyribonuclease VIII | Exodeoxyribonuclease VIII | | afdb-uniprot50 | AF-A0A352REG3-F1-MODEL\_V4 | 1.0 | 2.217e-09 | 368 | 0.294 | 139 | 93 | 4 | 203 | 340 | 2 | 136 | DUF3799 domain-containing protein | DUF3799 domain-containing protein | | afdb-uniprot50 | AF-A0A519U1A8-F1-MODEL\_V4 | 1.0 | 1.976e-10 | 367 | 0.159 | 377 | 157 | 10 | 1 | 371 | 10 | 232 | DUF3799 domain-containing protein | DUF3799 domain-containing protein | | afdb-uniprot50 | AF-I8ANZ2-F1-MODEL\_V4 | 1.0 | 1.574e-11 | 367 | 0.17 | 386 | 197 | 12 | 6 | 372 | 1 | 282 | Phage protein | Phage protein | | afdb-uniprot50 | AF-A0A4V0Z515-F1-MODEL\_V4 | 1.0 | 6.082e-10 | 366 | 0.287 | 181 | 119 | 4 | 189 | 360 | 2 | 181 | DUF3799 domain-containing protein | DUF3799 domain-containing protein | | afdb-uniprot50 | AF-A0A6X8JIS0-F1-MODEL\_V4 | 1.0 | 1.182e-13 | 364 | 0.228 | 306 | 174 | 7 | 1 | 250 | 457 | 756 | Exodeoxyribonuclease | Exodeoxyribonuclease | | afdb-uniprot50 | AF-A0A6Y3UI71-F1-MODEL\_V4 | 1.0 | 5.087e-14 | 363 | 0.219 | 328 | 201 | 10 | 1 | 273 | 219 | 546 | Exodeoxyribonuclease | Exodeoxyribonuclease | | afdb-uniprot50 | AF-A0A3D2IK29-F1-MODEL\_V4 | 1.0 | 1.766e-10 | 360 | 0.29 | 196 | 119 | 10 | 177 | 359 | 1 | 189 | DUF3799 domain-containing protein | DUF3799 domain-containing protein | | afdb-uniprot50 | AF-A0A485BQ07-F1-MODEL\_V4 | 1.0 | 2.455e-13 | 359 | 0.227 | 312 | 188 | 10 | 100 | 368 | 28 | 329 | Exonuclease VIII | Exonuclease VIII | | afdb-uniprot50 | AF-X0ST36-F1-MODEL\_V4 | 1.0 | 2.095e-09 | 357 | 0.357 | 137 | 87 | 1 | 234 | 369 | 5 | 141 | DUF3799 domain-containing protein | DUF3799 domain-containing protein | | afdb-uniprot50 | AF-A0A1W6ECB7-F1-MODEL\_V4 | 1.0 | 3.098e-10 | 356 | 0.158 | 359 | 156 | 11 | 8 | 358 | 2 | 222 | DUF3799 domain-containing protein | DUF3799 domain-containing protein | | afdb-uniprot50 | AF-A0A238X2A8-F1-MODEL\_V4 | 1.0 | 2.468e-11 | 355 | 0.196 | 357 | 140 | 12 | 1 | 349 | 1 | 218 | DUF3799 domain-containing protein | DUF3799 domain-containing protein | | afdb-uniprot50 | AF-A0A705E0Z0-F1-MODEL\_V4 | 1.0 | 1.182e-13 | 354 | 0.216 | 300 | 185 | 8 | 1 | 250 | 63 | 362 | Exodeoxyribonuclease | Exodeoxyribonuclease | | afdb-uniprot50 | AF-A0A758PAR8-F1-MODEL\_V4 | 1.0 | 2.074e-13 | 350 | 0.208 | 321 | 177 | 10 | 68 | 323 | 2 | 310 | DNA breaking-rejoining protein | DNA breaking-rejoining protein | | afdb-uniprot50 | AF-A0A7Y0FMZ2-F1-MODEL\_V4 | 1.0 | 2.211e-10 | 347 | 0.17 | 375 | 161 | 14 | 1 | 358 | 1 | 242 | DUF3799 domain-containing protein | DUF3799 domain-containing protein | | afdb-uniprot50 | AF-A0A7W4DRQ1-F1-MODEL\_V4 | 1.0 | 3.667e-10 | 343 | 0.153 | 359 | 182 | 15 | 9 | 355 | 1 | 249 | PD-(D/E)XK nuclease-like domain-containing protein | PD-(D/E)XK nuclease-like domain-containing protein | | afdb-uniprot50 | AF-A0A1G2Z6Y7-F1-MODEL\_V4 | 1.0 | 2.474e-10 | 341 | 0.22 | 200 | 143 | 7 | 164 | 359 | 25 | 215 | DUF3799 domain-containing protein | DUF3799 domain-containing protein | | afdb-uniprot50 | AF-A0A447XY29-F1-MODEL\_V4 | 1.0 | 4.093e-11 | 340 | 0.214 | 280 | 170 | 9 | 105 | 372 | 1 | 242 | Putative phage exodeoxyribonuclease | Putative phage exodeoxyribonuclease | | afdb-uniprot50 | AF-A0A1B1G3H1-F1-MODEL\_V4 | 1.0 | 1.067e-09 | 340 | 0.158 | 341 | 149 | 8 | 2 | 338 | 25 | 231 | DUF3799 domain-containing protein | DUF3799 domain-containing protein | | afdb-uniprot50 | AF-A0A5V5KL18-F1-MODEL\_V4 | 1.0 | 2.597e-13 | 340 | 0.222 | 328 | 200 | 10 | 1 | 273 | 39 | 366 | Exodeoxyribonuclease | Exodeoxyribonuclease | | afdb-uniprot50 | AF-A0A4S3LPF3-F1-MODEL\_V4 | 1.0 | 1.254e-12 | 339 | 0.214 | 308 | 176 | 8 | 1 | 250 | 485 | 784 | Uncharacterized protein | Uncharacterized protein | | afdb-uniprot50 | AF-A0A4Q2UMS3-F1-MODEL\_V4 | 1.0 | 6.082e-10 | 338 | 0.184 | 347 | 140 | 11 | 14 | 355 | 3 | 211 | DUF3799 domain-containing protein | DUF3799 domain-containing protein | | afdb-uniprot50 | AF-A0A1G3PFN4-F1-MODEL\_V4 | 1.0 | 1.333e-10 | 337 | 0.146 | 369 | 187 | 18 | 5 | 359 | 4 | 258 | DUF3799 domain-containing protein | DUF3799 domain-containing protein | | afdb-uniprot50 | AF-A0A485A5W0-F1-MODEL\_V4 | 1.0 | 1.757e-12 | 337 | 0.214 | 308 | 176 | 9 | 1 | 250 | 162 | 461 | Exodeoxyribonuclease 8 | Exodeoxyribonuclease 8 | | afdb-uniprot50 | AF-A0A5C5X7Z0-F1-MODEL\_V4 | 1.0 | 3.869e-11 | 336 | 0.154 | 415 | 191 | 18 | 6 | 356 | 11 | 329 | DUF3799 domain-containing protein | DUF3799 domain-containing protein | | afdb-uniprot50 | AF-A0A2U3ERY8-F1-MODEL\_V4 | 1.0 | 3.269e-11 | 335 | 0.159 | 376 | 176 | 12 | 5 | 369 | 154 | 400 | Uncharacterized protein | Uncharacterized protein | | afdb-uniprot50 | AF-A0A5X5VH27-F1-MODEL\_V4 | 1.0 | 1.326e-12 | 334 | 0.223 | 304 | 178 | 7 | 1 | 250 | 634 | 933 | DNA breaking-rejoining protein | DNA breaking-rejoining protein | | afdb-uniprot50 | AF-A0A7H1NU02-F1-MODEL\_V4 | 1.0 | 1.197e-08 | 333 | 0.261 | 149 | 103 | 6 | 224 | 369 | 8 | 152 | DUF3799 domain-containing protein | DUF3799 domain-containing protein | | afdb-uniprot50 | AF-A0A2E4HLS4-F1-MODEL\_V4 | 1.0 | 4.319e-12 | 332 | 0.135 | 361 | 241 | 17 | 8 | 345 | 2 | 314 | PDDEXK\_1 domain-containing protein | PDDEXK\_1 domain-containing protein | | afdb-uniprot50 | AF-A0A6Y4WD11-F1-MODEL\_V4 | 1.0 | 1.059e-12 | 330 | 0.218 | 297 | 182 | 8 | 1 | 247 | 110 | 406 | Exodeoxyribonuclease | Exodeoxyribonuclease | | afdb-uniprot50 | AF-A0A2D8BQ94-F1-MODEL\_V4 | 1.0 | 2.217e-09 | 319 | 0.13 | 353 | 180 | 14 | 11 | 357 | 10 | 241 | DUF3799 domain-containing protein | DUF3799 domain-containing protein | | afdb-uniprot50 | AF-A0A060RHE4-F1-MODEL\_V4 | 1.0 | 2.624e-09 | 318 | 0.133 | 381 | 194 | 15 | 9 | 363 | 1 | 271 | DUF3799 domain-containing protein | DUF3799 domain-containing protein | | afdb-uniprot50 | AF-A0A447MUU1-F1-MODEL\_V4 | 1.0 | 2.747e-13 | 318 | 0.179 | 340 | 213 | 11 | 33 | 311 | 9 | 343 | Exodeoxyribonuclease VIII | Exodeoxyribonuclease VIII | | afdb-uniprot50 | AF-A0A4Q2UGG0-F1-MODEL\_V4 | 1.0 | 1.126e-10 | 314 | 0.248 | 205 | 134 | 8 | 167 | 358 | 19 | 216 | DUF3799 domain-containing protein | DUF3799 domain-containing protein | | afdb-uniprot50 | AF-A0A0K8Q1C6-F1-MODEL\_V4 | 1.0 | 2.222e-08 | 309 | 0.431 | 116 | 64 | 2 | 256 | 371 | 2 | 115 | Exodeoxyribonuclease 8 | Exodeoxyribonuclease 8 | | afdb-uniprot50 | AF-A0A6G4MZF1-F1-MODEL\_V4 | 1.0 | 2.48e-09 | 306 | 0.125 | 391 | 200 | 15 | 6 | 370 | 1 | 275 | Nuclease | Nuclease | | afdb-uniprot50 | AF-A0A3M1KL91-F1-MODEL\_V4 | 1.0 | 5.164e-08 | 302 | 0.288 | 142 | 93 | 5 | 217 | 356 | 4 | 139 | DUF3799 domain-containing protein | DUF3799 domain-containing protein | | afdb-uniprot50 | AF-A0A7C1JUS1-F1-MODEL\_V4 | 1.0 | 9.585e-08 | 302 | 0.267 | 153 | 105 | 4 | 207 | 359 | 3 | 148 | DUF3799 domain-containing protein | DUF3799 domain-containing protein | | afdb-uniprot50 | AF-A0A5Y2EHS0-F1-MODEL\_V4 | 1.0 | 2.929e-10 | 302 | 0.254 | 283 | 106 | 8 | 1 | 277 | 49 | 232 | Exonuclease VIII | Exonuclease VIII | | afdb-uniprot50 | AF-A0A511B8A3-F1-MODEL\_V4 | 1.0 | 2.351e-08 | 297 | 0.26 | 161 | 105 | 7 | 222 | 372 | 12 | 168 | DUF3799 domain-containing protein | DUF3799 domain-containing protein | | afdb-uniprot50 | AF-A0A5M6A0E3-F1-MODEL\_V4 | 1.0 | 1.263e-09 | 297 | 0.218 | 215 | 133 | 10 | 177 | 364 | 21 | 227 | DUF3799 domain-containing protein | DUF3799 domain-containing protein | | afdb-uniprot50 | AF-A0A5X5NRN7-F1-MODEL\_V4 | 1.0 | 6.806e-10 | 295 | 0.244 | 229 | 139 | 7 | 108 | 326 | 2 | 206 | DUF3799 domain-containing protein | DUF3799 domain-containing protein | | afdb-uniprot50 | AF-A0A377TT21-F1-MODEL\_V4 | 1.0 | 1.971e-11 | 295 | 0.22 | 286 | 185 | 8 | 84 | 359 | 15 | 272 | Putative exodeoxyribonuclease VIII | Putative exodeoxyribonuclease VIII | | afdb-uniprot50 | AF-A0A350BS60-F1-MODEL\_V4 | 1.0 | 3.098e-10 | 291 | 0.218 | 275 | 159 | 13 | 114 | 354 | 4 | 256 | DUF3799 domain-containing protein | DUF3799 domain-containing protein | | afdb-uniprot50 | AF-A0A349YBV5-F1-MODEL\_V4 | 1.0 | 2.351e-08 | 288 | 0.226 | 159 | 116 | 5 | 187 | 343 | 84 | 237 | DUF3799 domain-containing protein | DUF3799 domain-containing protein | | afdb-uniprot50 | AF-A0A3R7HPJ5-F1-MODEL\_V4 | 1.0 | 2.211e-10 | 286 | 0.176 | 375 | 200 | 21 | 9 | 358 | 1 | 291 | DUF3799 domain-containing protein | DUF3799 domain-containing protein | | afdb-uniprot50 | AF-A0A2S8GSL0-F1-MODEL\_V4 | 1.0 | 3.475e-09 | 286 | 0.129 | 354 | 181 | 14 | 6 | 346 | 29 | 268 | Uncharacterized protein | Uncharacterized protein | | afdb-uniprot50 | AF-A0A7C5SKK8-F1-MODEL\_V4 | 1.0 | 3.493e-07 | 285 | 0.315 | 130 | 88 | 1 | 232 | 360 | 1 | 130 | DUF3799 domain-containing protein | DUF3799 domain-containing protein | | afdb-uniprot50 | AF-A0A352NZ68-F1-MODEL\_V4 | 1.0 | 1.986e-08 | 279 | 0.123 | 381 | 185 | 16 | 8 | 361 | 7 | 265 | DUF3799 domain-containing protein | DUF3799 domain-containing protein | | afdb-uniprot50 | AF-A0A1L3I482-F1-MODEL\_V4 | 1.0 | 2.487e-08 | 274 | 0.451 | 135 | 60 | 2 | 207 | 334 | 3 | 130 | Exodeoxyribonuclease 8 | Exodeoxyribonuclease 8 | | afdb-uniprot50 | AF-A0A706A9B5-F1-MODEL\_V4 | 1.0 | 7.2e-10 | 271 | 0.227 | 251 | 160 | 7 | 83 | 323 | 12 | 238 | Exodeoxyribonuclease | Exodeoxyribonuclease | | afdb-uniprot50 | AF-A0A1S8PH38-F1-MODEL\_V4 | 1.0 | 5.463e-08 | 270 | 0.137 | 370 | 179 | 17 | 8 | 357 | 2 | 251 | PD-(D/E)XK nuclease superfamily protein | PD-(D/E)XK nuclease superfamily protein | | afdb-uniprot50 | AF-Z9JM14-F1-MODEL\_V4 | 1.0 | 1.273e-06 | 265 | 0.394 | 109 | 64 | 2 | 255 | 363 | 8 | 114 | DUF3799 domain-containing protein | DUF3799 domain-containing protein | | afdb-uniprot50 | AF-A0A069CWL0-F1-MODEL\_V4 | 1.0 | 1.503e-07 | 264 | 0.113 | 360 | 184 | 12 | 6 | 344 | 1 | 246 | Phage-related protein | Phage-related protein | | afdb-uniprot50 | AF-A0A1V1PGN8-F1-MODEL\_V4 | 1.0 | 5.151e-09 | 261 | 0.158 | 379 | 211 | 19 | 6 | 358 | 1 | 297 | Uncharacterized protein | Uncharacterized protein | | afdb-uniprot50 | AF-A0A853IND3-F1-MODEL\_V4 | 1.0 | 1.014e-07 | 260 | 0.251 | 159 | 110 | 5 | 155 | 307 | 11 | 166 | PD-(D/E)XK nuclease-like domain-containing protein | PD-(D/E)XK nuclease-like domain-containing protein | | afdb-uniprot50 | AF-A0A8A3QEV9-F1-MODEL\_V4 | 1.0 | 8.566e-08 | 260 | 0.122 | 360 | 173 | 16 | 20 | 357 | 1 | 239 | PD-(D/E)XK nuclease-like domain-containing protein | PD-(D/E)XK nuclease-like domain-containing protein | | afdb-uniprot50 | AF-A0A6M3IM01-F1-MODEL\_V4 | 1.0 | 1.981e-09 | 260 | 0.133 | 352 | 183 | 15 | 6 | 348 | 4 | 242 | Putative PD-(D/E)XK nuclease superfamily protein | Putative PD-(D/E)XK nuclease superfamily protein | | afdb-uniprot50 | AF-A0A524P3G8-F1-MODEL\_V4 | 1.0 | 1.779e-07 | 258 | 0.29 | 148 | 97 | 4 | 230 | 371 | 1 | 146 | DUF3799 domain-containing protein | DUF3799 domain-containing protein | | afdb-uniprot50 | AF-A0A6G9Q4G4-F1-MODEL\_V4 | 1.0 | 3.484e-08 | 257 | 0.143 | 384 | 178 | 18 | 7 | 358 | 3 | 267 | DUF3799 domain-containing protein | DUF3799 domain-containing protein | | afdb-uniprot50 | AF-A0A0R1R1I5-F1-MODEL\_V4 | 1.0 | 1.503e-07 | 254 | 0.113 | 360 | 188 | 14 | 20 | 361 | 1 | 247 | DUF3799 domain-containing protein | DUF3799 domain-containing protein | | afdb-uniprot50 | AF-A0A2N5WAQ2-F1-MODEL\_V4 | 1.0 | 2.493e-07 | 252 | 0.121 | 369 | 176 | 12 | 18 | 356 | 3 | 253 | PD-(D/E)XK nuclease superfamily protein | PD-(D/E)XK nuclease superfamily protein | | afdb-uniprot50 | AF-A0A512PLA9-F1-MODEL\_V4 | 1.0 | 3.114e-08 | 252 | 0.124 | 379 | 188 | 18 | 7 | 359 | 41 | 301 | HTH cro/C1-type domain-containing protein | HTH cro/C1-type domain-containing protein | | afdb-uniprot50 | AF-F5VG10-F1-MODEL\_V4 | 1.0 | 3.106e-09 | 250 | 0.14 | 398 | 219 | 21 | 11 | 360 | 4 | 326 | DUF3799 domain-containing protein | DUF3799 domain-containing protein | | afdb-uniprot50 | AF-A0A3Q8YTG7-F1-MODEL\_V4 | 1.0 | 2.958e-06 | 246 | 0.306 | 111 | 73 | 2 | 253 | 359 | 14 | 124 | DUF3799 domain-containing protein | DUF3799 domain-containing protein | | afdb-uniprot50 | AF-X0TZX0-F1-MODEL\_V4 | 1.0 | 1.686e-06 | 246 | 0.176 | 249 | 110 | 4 | 1 | 249 | 6 | 159 | DUF3799 domain-containing protein | DUF3799 domain-containing protein | | afdb-uniprot50 | AF-A0A3C0G174-F1-MODEL\_V4 | 1.0 | 1.273e-06 | 243 | 0.339 | 106 | 68 | 2 | 253 | 358 | 2 | 105 | DUF3799 domain-containing protein | DUF3799 domain-containing protein | | afdb-uniprot50 | AF-R1HTJ0-F1-MODEL\_V4 | 1.0 | 7.674e-07 | 243 | 0.274 | 142 | 89 | 3 | 231 | 371 | 7 | 135 | Gp60 protein | Gp60 protein | | afdb-uniprot50 | AF-A0A1E3KRK4-F1-MODEL\_V4 | 1.0 | 6.858e-07 | 240 | 0.119 | 360 | 178 | 15 | 20 | 357 | 1 | 243 | DUF3799 domain-containing protein | DUF3799 domain-containing protein | | afdb-uniprot50 | AF-A0A842HJB6-F1-MODEL\_V4 | 1.0 | 1.877e-08 | 240 | 0.117 | 367 | 203 | 17 | 1 | 350 | 2 | 264 | PD-(D/E)XK nuclease family protein | PD-(D/E)XK nuclease family protein | | afdb-uniprot50 | AF-A0A379YD88-F1-MODEL\_V4 | 1.0 | 3.502e-06 | 239 | 0.225 | 151 | 101 | 5 | 229 | 369 | 2 | 146 | Exonuclease VIII | Exonuclease VIII | | afdb-uniprot50 | AF-A0A6I1JKT6-F1-MODEL\_V4 | 1.0 | 1.266e-08 | 239 | 0.278 | 201 | 117 | 9 | 178 | 371 | 39 | 218 | Phage protein | Phage protein | | afdb-uniprot50 | AF-A0A7C6QXB3-F1-MODEL\_V4 | 1.0 | 4.124e-08 | 237 | 0.123 | 340 | 177 | 12 | 16 | 347 | 3 | 229 | PD-(D/E)XK nuclease family protein | PD-(D/E)XK nuclease family protein | | afdb-uniprot50 | AF-A0A7X6AC15-F1-MODEL\_V4 | 1.0 | 9.585e-08 | 236 | 0.128 | 343 | 183 | 15 | 15 | 350 | 5 | 238 | PDDEXK\_1 domain-containing protein | PDDEXK\_1 domain-containing protein | | afdb-uniprot50 | AF-A0A3Z0FCG3-F1-MODEL\_V4 | 1.0 | 1.067e-09 | 234 | 0.215 | 279 | 157 | 8 | 53 | 273 | 5 | 279 | Exodeoxyribonuclease | Exodeoxyribonuclease | | afdb-uniprot50 | AF-A0A645IKQ3-F1-MODEL\_V4 | 1.0 | 6.876e-06 | 232 | 0.324 | 114 | 71 | 5 | 246 | 357 | 2 | 111 | DUF3799 domain-containing protein | DUF3799 domain-containing protein | | afdb-uniprot50 | AF-A0A8A7W4S7-F1-MODEL\_V4 | 1.0 | 1.27e-07 | 232 | 0.134 | 364 | 195 | 16 | 17 | 371 | 2 | 254 | PD-(D/E)XK nuclease family protein | PD-(D/E)XK nuclease family protein | | afdb-uniprot50 | AF-X0YVD2-F1-MODEL\_V4 | 1.0 | 8.077e-09 | 230 | 0.175 | 280 | 171 | 16 | 90 | 354 | 3 | 237 | DUF3799 domain-containing protein | DUF3799 domain-containing protein | | afdb-uniprot50 | AF-A0A139TR47-F1-MODEL\_V4 | 1.0 | 8.098e-08 | 230 | 0.131 | 374 | 211 | 12 | 1 | 371 | 1 | 263 | PDDEXK\_1 domain-containing protein | PDDEXK\_1 domain-containing protein | | afdb-uniprot50 | AF-A0A6B2LYD2-F1-MODEL\_V4 | 1.0 | 7.655e-08 | 230 | 0.117 | 332 | 186 | 13 | 17 | 343 | 19 | 248 | PD-(D/E)XK nuclease family protein | PD-(D/E)XK nuclease family protein | | afdb-uniprot50 | AF-X0SG33-F1-MODEL\_V4 | 1.0 | 2.624e-09 | 228 | 0.164 | 352 | 200 | 18 | 43 | 368 | 33 | 316 | Uncharacterized protein | Uncharacterized protein | | afdb-uniprot50 | AF-A0A1F9X0L3-F1-MODEL\_V4 | 1.0 | 1.343e-07 | 223 | 0.12 | 340 | 183 | 11 | 18 | 350 | 4 | 234 | PDDEXK\_1 domain-containing protein | PDDEXK\_1 domain-containing protein | | afdb-uniprot50 | AF-A0A1M3CJF3-F1-MODEL\_V4 | 1.0 | 2.493e-07 | 221 | 0.125 | 343 | 180 | 16 | 18 | 348 | 10 | 244 | PDDEXK\_1 domain-containing protein | PDDEXK\_1 domain-containing protein | | afdb-uniprot50 | AF-A0A661TMP3-F1-MODEL\_V4 | 1.0 | 2.943e-08 | 221 | 0.109 | 348 | 191 | 13 | 18 | 351 | 11 | 253 | PDDEXK\_1 domain-containing protein | PDDEXK\_1 domain-containing protein | | afdb-uniprot50 | AF-A0A2T6D714-F1-MODEL\_V4 | 1.0 | 1.2e-07 | 220 | 0.133 | 351 | 176 | 16 | 14 | 348 | 6 | 244 | PD-(D/E)XK nuclease family protein | PD-(D/E)XK nuclease family protein | | afdb-uniprot50 | AF-A0A517QQP5-F1-MODEL\_V4 | 1.0 | 1.503e-07 | 219 | 0.125 | 360 | 203 | 15 | 1 | 350 | 3 | 260 | PD-(D/E)XK nuclease superfamily protein | PD-(D/E)XK nuclease superfamily protein | | afdb-uniprot50 | AF-A0A1M6GGK6-F1-MODEL\_V4 | 1.0 | 6.841e-08 | 218 | 0.144 | 380 | 202 | 18 | 1 | 372 | 4 | 268 | Putative RecB family exonuclease | Putative RecB family exonuclease | | afdb-uniprot50 | AF-A0A831P8V5-F1-MODEL\_V4 | 1.0 | 2.351e-08 | 218 | 0.135 | 361 | 190 | 12 | 1 | 350 | 8 | 257 | PD-(D/E)XK nuclease family protein | PD-(D/E)XK nuclease family protein | | afdb-uniprot50 | AF-A0A1V9R6K6-F1-MODEL\_V4 | 1.0 | 4.374e-07 | 218 | 0.121 | 386 | 192 | 17 | 11 | 367 | 8 | 275 | DUF3799 domain-containing protein | DUF3799 domain-containing protein | | afdb-uniprot50 | AF-A0A722BMP5-F1-MODEL\_V4 | 1.0 | 2.369e-05 | 217 | 0.26 | 150 | 95 | 5 | 233 | 372 | 1 | 144 | DNA breaking-rejoining protein | DNA breaking-rejoining protein | | afdb-uniprot50 | AF-A0A0R2B3T1-F1-MODEL\_V4 | 1.0 | 2.357e-07 | 217 | 0.162 | 222 | 142 | 11 | 157 | 357 | 18 | 216 | DUF3799 domain-containing protein | DUF3799 domain-containing protein | | afdb-uniprot50 | AF-A0A2H0X8D0-F1-MODEL\_V4 | 1.0 | 1.779e-07 | 217 | 0.102 | 333 | 189 | 13 | 19 | 348 | 11 | 236 | PDDEXK\_1 domain-containing protein | PDDEXK\_1 domain-containing protein | | afdb-uniprot50 | AF-A0A3N9MVW9-F1-MODEL\_V4 | 1.0 | 9.061e-08 | 217 | 0.115 | 345 | 185 | 14 | 17 | 350 | 2 | 237 | PD-(D/E)XK nuclease family protein | PD-(D/E)XK nuclease family protein | | afdb-uniprot50 | AF-A0A5V0PBZ2-F1-MODEL\_V4 | 1.0 | 9.585e-08 | 216 | 0.195 | 235 | 128 | 5 | 71 | 250 | 2 | 230 | Uncharacterized protein | Uncharacterized protein | | afdb-uniprot50 | AF-X0YAJ3-F1-MODEL\_V4 | 1.0 | 3.909e-07 | 215 | 0.129 | 341 | 173 | 17 | 19 | 343 | 2 | 234 | PDDEXK\_1 domain-containing protein | PDDEXK\_1 domain-containing protein | | afdb-uniprot50 | AF-A0A6A7VXV8-F1-MODEL\_V4 | 1.0 | 2.797e-06 | 214 | 0.137 | 348 | 164 | 13 | 6 | 340 | 1 | 225 | Uncharacterized protein | Uncharacterized protein | | afdb-uniprot50 | AF-A0A2A2QKQ5-F1-MODEL\_V4 | 1.0 | 1.882e-07 | 214 | 0.132 | 378 | 210 | 16 | 1 | 372 | 9 | 274 | PDDEXK\_1 domain-containing protein | PDDEXK\_1 domain-containing protein | | afdb-uniprot50 | AF-A0A7X7QZ66-F1-MODEL\_V4 | 1.0 | 2.966e-05 | 213 | 0.348 | 89 | 58 | 0 | 273 | 361 | 2 | 90 | DUF3799 domain-containing protein | DUF3799 domain-containing protein | | afdb-uniprot50 | AF-A0A5W5XL97-F1-MODEL\_V4 | 1.0 | 2.369e-05 | 211 | 0.253 | 146 | 93 | 5 | 237 | 372 | 3 | 142 | DNA breaking-rejoining protein | DNA breaking-rejoining protein | | afdb-uniprot50 | AF-A0A0F9F1T3-F1-MODEL\_V4 | 1.0 | 4.65e-05 | 211 | 0.228 | 127 | 90 | 4 | 222 | 343 | 4 | 127 | DUF3799 domain-containing protein | DUF3799 domain-containing protein | | afdb-uniprot50 | AF-A0A496LXW8-F1-MODEL\_V4 | 1.0 | 7.674e-07 | 211 | 0.118 | 380 | 191 | 19 | 16 | 372 | 3 | 261 | DUF3799 domain-containing protein | DUF3799 domain-containing protein | | afdb-uniprot50 | AF-A0A3C0NN37-F1-MODEL\_V4 | 1.0 | 8.118e-07 | 210 | 0.131 | 379 | 173 | 17 | 6 | 361 | 1 | 246 | DUF3799 domain-containing protein | DUF3799 domain-containing protein | | afdb-uniprot50 | AF-A0A1M5WQQ9-F1-MODEL\_V4 | 1.0 | 2.637e-07 | 206 | 0.139 | 374 | 211 | 20 | 19 | 357 | 2 | 299 | Uncharacterized protein | Uncharacterized protein | | afdb-uniprot50 | AF-A0A6H2A5D7-F1-MODEL\_V4 | 1.0 | 1.073e-07 | 204 | 0.121 | 353 | 194 | 16 | 6 | 348 | 1 | 247 | Putative PD-(D/E)XK nuclease superfamily protein | Putative PD-(D/E)XK nuclease superfamily protein | | afdb-uniprot50 | AF-A0A2E0QSB0-F1-MODEL\_V4 | 1.0 | 3.493e-07 | 203 | 0.143 | 349 | 188 | 15 | 8 | 348 | 20 | 265 | PDDEXK\_1 domain-containing protein | PDDEXK\_1 domain-containing protein | | afdb-uniprot50 | AF-H6SML8-F1-MODEL\_V4 | 1.0 | 0.0002006 | 202 | 0.289 | 69 | 49 | 0 | 250 | 318 | 10 | 78 | DUF3799 domain-containing protein | DUF3799 domain-containing protein | | afdb-uniprot50 | AF-A0A512M8Q3-F1-MODEL\_V4 | 1.0 | 1.273e-06 | 201 | 0.117 | 340 | 186 | 15 | 18 | 350 | 24 | 256 | PDDEXK\_1 domain-containing protein | PDDEXK\_1 domain-containing protein | | afdb-uniprot50 | AF-A0A7C3TTB3-F1-MODEL\_V4 | 1.0 | 0.0001209 | 200 | 0.257 | 105 | 76 | 1 | 254 | 358 | 14 | 116 | DUF3799 domain-containing protein | DUF3799 domain-containing protein | | afdb-uniprot50 | AF-A0A7L6WNN6-F1-MODEL\_V4 | 1.0 | 4.894e-07 | 200 | 0.148 | 250 | 171 | 13 | 137 | 360 | 17 | 250 | PD-(D/E)XK nuclease-like domain-containing protein | PD-(D/E)XK nuclease-like domain-containing protein | | afdb-uniprot50 | AF-A0A1Z8QGD4-F1-MODEL\_V4 | 1.0 | 8.588e-07 | 200 | 0.101 | 345 | 183 | 13 | 18 | 347 | 5 | 237 | PDDEXK\_1 domain-containing protein | PDDEXK\_1 domain-containing protein | | afdb-uniprot50 | AF-A0A851GBJ6-F1-MODEL\_V4 | 1.0 | 1.27e-07 | 198 | 0.158 | 373 | 183 | 23 | 7 | 352 | 20 | 288 | PD-(D/E)XK nuclease family protein | PD-(D/E)XK nuclease family protein | | afdb-uniprot50 | AF-A0A556QJB4-F1-MODEL\_V4 | 1.0 | 1.347e-06 | 197 | 0.149 | 362 | 189 | 16 | 1 | 348 | 7 | 263 | PD-(D/E)XK nuclease family protein | PD-(D/E)XK nuclease family protein | | afdb-uniprot50 | AF-A0A0F9HJD9-F1-MODEL\_V4 | 1.0 | 1.014e-07 | 196 | 0.142 | 344 | 186 | 15 | 19 | 348 | 14 | 262 | PDDEXK\_1 domain-containing protein | PDDEXK\_1 domain-containing protein | | afdb-uniprot50 | AF-A0A758AID2-F1-MODEL\_V4 | 1.0 | 8.159e-05 | 195 | 0.236 | 127 | 84 | 4 | 253 | 372 | 20 | 140 | DNA breaking-rejoining protein | DNA breaking-rejoining protein | | afdb-uniprot50 | AF-A0A6A6K1N9-F1-MODEL\_V4 | 1.0 | 1.678e-08 | 195 | 0.179 | 318 | 152 | 9 | 43 | 359 | 123 | 332 | DUF3799 domain-containing protein | DUF3799 domain-containing protein | | afdb-uniprot50 | AF-A0A0R2JVU7-F1-MODEL\_V4 | 1.0 | 5.491e-06 | 194 | 0.185 | 183 | 118 | 10 | 197 | 357 | 3 | 176 | DUF3799 domain-containing protein | DUF3799 domain-containing protein | | afdb-uniprot50 | AF-A0A3A4UKW7-F1-MODEL\_V4 | 1.0 | 5.794e-07 | 193 | 0.131 | 373 | 187 | 20 | 17 | 359 | 4 | 269 | PD-(D/E)XK nuclease family protein | PD-(D/E)XK nuclease family protein | | afdb-uniprot50 | AF-A0A3A4NCG6-F1-MODEL\_V4 | 1.0 | 2.493e-07 | 193 | 0.112 | 355 | 206 | 12 | 1 | 351 | 15 | 264 | PD-(D/E)XK nuclease family protein | PD-(D/E)XK nuclease family protein | | afdb-uniprot50 | AF-A0A3R8KJC4-F1-MODEL\_V4 | 1.0 | 8.118e-07 | 193 | 0.127 | 352 | 198 | 15 | 7 | 351 | 47 | 296 | PD-(D/E)XK nuclease family protein | PD-(D/E)XK nuclease family protein | | afdb-uniprot50 | AF-A0A5T2DQK0-F1-MODEL\_V4 | 1.0 | 0.0001022 | 190 | 0.226 | 128 | 86 | 4 | 252 | 372 | 6 | 127 | DNA breaking-rejoining protein | DNA breaking-rejoining protein | | afdb-uniprot50 | AF-A0A1B1UKD2-F1-MODEL\_V4 | 1.0 | 0.000818 | 188 | 0.252 | 91 | 67 | 1 | 252 | 342 | 6 | 95 | Uncharacterized protein | Uncharacterized protein | | afdb-uniprot50 | AF-A0A6C2U3G2-F1-MODEL\_V4 | 1.0 | 3.302e-07 | 187 | 0.127 | 353 | 183 | 18 | 12 | 348 | 6 | 249 | PDDEXK\_1 domain-containing protein | PDDEXK\_1 domain-containing protein | | afdb-uniprot50 | AF-A0A5Y3WNM2-F1-MODEL\_V4 | 1.0 | 0.0001209 | 186 | 0.234 | 128 | 85 | 4 | 252 | 372 | 3 | 124 | DNA breaking-rejoining protein | DNA breaking-rejoining protein | | afdb-uniprot50 | AF-A0A4R1S4K0-F1-MODEL\_V4 | 1.0 | 1.996e-06 | 186 | 0.128 | 350 | 181 | 15 | 6 | 347 | 17 | 250 | RecB family exonuclease | RecB family exonuclease | | afdb-uniprot50 | AF-A0A5W2H069-F1-MODEL\_V4 | 1.0 | 9.061e-08 | 185 | 0.199 | 261 | 151 | 7 | 14 | 220 | 2 | 258 | Uncharacterized protein | Uncharacterized protein | | afdb-uniprot50 | AF-A0A5Y0QKR5-F1-MODEL\_V4 | 1.0 | 2.637e-07 | 185 | 0.19 | 257 | 150 | 7 | 1 | 203 | 21 | 273 | Exodeoxyribonuclease | Exodeoxyribonuclease | | afdb-uniprot50 | AF-A0A610YZ68-F1-MODEL\_V4 | 1.0 | 9.659e-05 | 184 | 0.228 | 127 | 85 | 4 | 253 | 372 | 18 | 138 | DUF3799 domain-containing protein | DUF3799 domain-containing protein | | afdb-uniprot50 | AF-A0A5P0ZGJ0-F1-MODEL\_V4 | 1.0 | 7.236e-08 | 184 | 0.15 | 346 | 177 | 20 | 43 | 357 | 4 | 263 | DUF3799 domain-containing protein | DUF3799 domain-containing protein | | afdb-uniprot50 | AF-A0A410SCK6-F1-MODEL\_V4 | 1.0 | 6.5e-06 | 180 | 0.168 | 196 | 142 | 5 | 178 | 369 | 6 | 184 | PD-(D/E)XK nuclease family protein | PD-(D/E)XK nuclease family protein | | afdb-uniprot50 | AF-A0A3A4A5A1-F1-MODEL\_V4 | 1.0 | 1.594e-06 | 176 | 0.134 | 358 | 203 | 16 | 1 | 350 | 1 | 259 | PD-(D/E)XK nuclease family protein | PD-(D/E)XK nuclease family protein | | afdb-uniprot50 | AF-A0A853QFX1-F1-MODEL\_V4 | 1.0 | 2.363e-06 | 175 | 0.152 | 275 | 149 | 14 | 116 | 356 | 2 | 226 | Uncharacterized protein | Uncharacterized protein | | afdb-uniprot50 | AF-A0A6M3IPF6-F1-MODEL\_V4 | 1.0 | 6.483e-07 | 174 | 0.109 | 346 | 182 | 15 | 8 | 342 | 1 | 231 | Putative PD-(D/E)XK nuclease superfamily protein | Putative PD-(D/E)XK nuclease superfamily protein | | afdb-uniprot50 | AF-A0A5S9M6A0-F1-MODEL\_V4 | 1.0 | 0.0001279 | 172 | 0.187 | 144 | 99 | 8 | 222 | 353 | 4 | 141 | DUF3799 domain-containing protein | DUF3799 domain-containing protein | | afdb-uniprot50 | AF-A0A7J9WA11-F1-MODEL\_V4 | 1.0 | 4.135e-07 | 170 | 0.122 | 342 | 185 | 17 | 18 | 351 | 164 | 398 | PDDEXK\_1 domain-containing protein | PDDEXK\_1 domain-containing protein | | afdb-uniprot50 | AF-A0A1V5IYR0-F1-MODEL\_V4 | 1.0 | 6.144e-06 | 169 | 0.109 | 338 | 187 | 16 | 18 | 347 | 5 | 236 | PD-(D/E)XK nuclease superfamily protein | PD-(D/E)XK nuclease superfamily protein | | afdb-uniprot50 | AF-A0A6M3LHK4-F1-MODEL\_V4 | 1.0 | 1.276e-05 | 168 | 0.142 | 218 | 135 | 13 | 177 | 357 | 13 | 215 | DUF3799 domain-containing protein | DUF3799 domain-containing protein | | afdb-uniprot50 | AF-A0A1I4S4P9-F1-MODEL\_V4 | 1.0 | 9.084e-07 | 168 | 0.101 | 345 | 191 | 17 | 7 | 343 | 158 | 391 | DNA helicase | DNA helicase | | afdb-uniprot50 | AF-X7ZHD3-F1-MODEL\_V4 | 1.0 | 0.0003939 | 167 | 0.258 | 124 | 75 | 1 | 253 | 359 | 9 | 132 | Gp60 domain protein | Gp60 domain protein | | afdb-uniprot50 | AF-A0A2N2ISB4-F1-MODEL\_V4 | 1.0 | 4.907e-06 | 166 | 0.121 | 361 | 179 | 17 | 1 | 341 | 1 | 243 | PDDEXK\_1 domain-containing protein | PDDEXK\_1 domain-containing protein | | afdb-uniprot50 | AF-A0A3N5JX30-F1-MODEL\_V4 | 1.0 | 0.0002811 | 165 | 0.19 | 147 | 103 | 6 | 196 | 338 | 2 | 136 | PDDEXK\_1 domain-containing protein | PDDEXK\_1 domain-containing protein | | afdb-uniprot50 | AF-A0A4Q3AU83-F1-MODEL\_V4 | 1.0 | 6.16e-05 | 163 | 0.15 | 179 | 136 | 10 | 178 | 349 | 6 | 175 | PD-(D/E)XK nuclease family protein | PD-(D/E)XK nuclease family protein | | afdb-uniprot50 | AF-A0A7V3L4A6-F1-MODEL\_V4 | 1.0 | 1.887e-06 | 163 | 0.121 | 361 | 205 | 19 | 19 | 350 | 10 | 287 | PDDEXK\_1 domain-containing protein | PDDEXK\_1 domain-containing protein | | afdb-uniprot50 | AF-A0A7C3FI44-F1-MODEL\_V4 | 1.0 | 1.511e-05 | 162 | 0.141 | 226 | 134 | 9 | 105 | 319 | 5 | 181 | PDDEXK\_1 domain-containing protein | PDDEXK\_1 domain-containing protein | | afdb-uniprot50 | AF-A0LJW0-F1-MODEL\_V4 | 1.0 | 3.511e-05 | 161 | 0.138 | 159 | 108 | 9 | 223 | 372 | 1 | 139 | PDDEXK\_1 domain-containing protein | PDDEXK\_1 domain-containing protein | | afdb-uniprot50 | AF-A0A842HHQ9-F1-MODEL\_V4 | 1.0 | 8.159e-05 | 160 | 0.17 | 182 | 124 | 12 | 182 | 348 | 5 | 174 | PD-(D/E)XK nuclease family protein | PD-(D/E)XK nuclease family protein | | afdb-uniprot50 | AF-A0A0F9T4J1-F1-MODEL\_V4 | 1.0 | 9.634e-06 | 160 | 0.111 | 270 | 177 | 11 | 111 | 370 | 8 | 224 | Uncharacterized protein | Uncharacterized protein | | afdb-uniprot50 | AF-A0A3C0ZKX1-F1-MODEL\_V4 | 1.0 | 2.111e-06 | 160 | 0.11 | 336 | 183 | 16 | 16 | 343 | 360 | 587 | DNA helicase | DNA helicase | | afdb-uniprot50 | AF-A0A6M3INL5-F1-MODEL\_V4 | 1.0 | 1.69e-05 | 159 | 0.106 | 244 | 169 | 8 | 111 | 349 | 8 | 207 | Putative PD-(D/E)XK nuclease superfamily protein | Putative PD-(D/E)XK nuclease superfamily protein | | afdb-uniprot50 | AF-A0A6J7E3Q5-F1-MODEL\_V4 | 1.0 | 3.129e-06 | 158 | 0.112 | 373 | 222 | 20 | 2 | 350 | 10 | 297 | Unannotated protein | Unannotated protein | | afdb-uniprot50 | AF-A0A5C7RJB2-F1-MODEL\_V4 | 1.0 | 1.347e-06 | 157 | 0.084 | 343 | 200 | 15 | 8 | 343 | 68 | 303 | DNA helicase | DNA helicase | | afdb-uniprot50 | AF-A0A6G2KJL8-F1-MODEL\_V4 | 1.0 | 7.292e-05 | 157 | 0.174 | 183 | 117 | 8 | 159 | 337 | 954 | 1106 | DNA helicase | DNA helicase | | afdb-uniprot50 | AF-A0A0F9G4G2-F1-MODEL\_V4 | 1.0 | 0.0008653 | 155 | 0.329 | 97 | 56 | 4 | 270 | 361 | 2 | 94 | DUF3799 domain-containing protein | DUF3799 domain-containing protein | | afdb-uniprot50 | AF-A0A7X9KIB3-F1-MODEL\_V4 | 1.0 | 0.0001695 | 155 | 0.167 | 137 | 102 | 7 | 201 | 333 | 7 | 135 | PDDEXK\_1 domain-containing protein | PDDEXK\_1 domain-containing protein | | afdb-uniprot50 | AF-A0A6M3IV43-F1-MODEL\_V4 | 1.0 | 1.276e-05 | 154 | 0.143 | 265 | 165 | 12 | 111 | 368 | 9 | 218 | Putative PD-(D/E)XK nuclease superfamily protein | Putative PD-(D/E)XK nuclease superfamily protein | | afdb-uniprot50 | AF-A0A3N5PX14-F1-MODEL\_V4 | 1.0 | 2.499e-06 | 154 | 0.113 | 352 | 175 | 17 | 17 | 346 | 337 | 573 | DNA helicase | DNA helicase | | afdb-uniprot50 | AF-A0A7W0FIM0-F1-MODEL\_V4 | 1.0 | 5.491e-06 | 150 | 0.11 | 335 | 180 | 16 | 16 | 338 | 568 | 796 | DNA helicase | DNA helicase | | afdb-uniprot50 | AF-A0A6L2R4U5-F1-MODEL\_V4 | 1.0 | 6.5e-06 | 150 | 0.093 | 353 | 201 | 18 | 7 | 351 | 702 | 943 | DNA helicase | DNA helicase | | afdb-uniprot50 | AF-R6ZK78-F1-MODEL\_V4 | 1.0 | 8.139e-06 | 149 | 0.118 | 355 | 190 | 12 | 6 | 350 | 80 | 321 | PDDEXK\_1 domain-containing protein | PDDEXK\_1 domain-containing protein | | afdb-uniprot50 | AF-A0A7W9ZL67-F1-MODEL\_V4 | 1.0 | 6.5e-06 | 149 | 0.098 | 376 | 213 | 18 | 6 | 370 | 115 | 375 | DNA helicase | DNA helicase | | afdb-uniprot50 | AF-A0A2H5VL30-F1-MODEL\_V4 | 1.0 | 4.135e-07 | 149 | 0.158 | 353 | 200 | 20 | 7 | 350 | 940 | 1204 | DNA helicase | DNA helicase | | afdb-uniprot50 | AF-A0A537ZYU4-F1-MODEL\_V4 | 1.0 | 4.894e-07 | 148 | 0.128 | 350 | 217 | 16 | 6 | 354 | 871 | 1133 | DNA helicase | DNA helicase | | afdb-uniprot50 | AF-A0A0R1TRL8-F1-MODEL\_V4 | 1.0 | 0.0004932 | 147 | 0.14 | 164 | 114 | 9 | 225 | 368 | 2 | 158 | DUF3799 domain-containing protein | DUF3799 domain-containing protein | | afdb-uniprot50 | AF-A0A706XGY7-F1-MODEL\_V4 | 1.0 | 4.639e-06 | 147 | 0.225 | 204 | 117 | 4 | 83 | 250 | 6 | 204 | Uncharacterized protein | Uncharacterized protein | | afdb-uniprot50 | AF-A0A7W1BLJ0-F1-MODEL\_V4 | 1.0 | 1.594e-06 | 147 | 0.129 | 348 | 193 | 21 | 7 | 338 | 637 | 890 | DNA helicase | DNA helicase | | afdb-uniprot50 | AF-A0A6G0A6A2-F1-MODEL\_V4 | 1.0 | 0.0001279 | 147 | 0.213 | 183 | 102 | 9 | 162 | 336 | 991 | 1139 | DNA helicase | DNA helicase | | afdb-uniprot50 | AF-A0A2H0LI12-F1-MODEL\_V4 | 1.0 | 0.0002512 | 146 | 0.156 | 147 | 107 | 5 | 208 | 351 | 2 | 134 | PDDEXK\_1 domain-containing protein | PDDEXK\_1 domain-containing protein | | afdb-uniprot50 | AF-A0A5Y3U2L9-F1-MODEL\_V4 | 1.0 | 6.144e-06 | 146 | 0.226 | 203 | 118 | 3 | 83 | 250 | 5 | 203 | DNA breaking-rejoining protein | DNA breaking-rejoining protein | | afdb-uniprot50 | AF-A0A2E7SPB8-F1-MODEL\_V4 | 1.0 | 1.69e-05 | 146 | 0.106 | 367 | 198 | 20 | 10 | 347 | 14 | 279 | PDDEXK\_1 domain-containing protein | PDDEXK\_1 domain-containing protein | | afdb-uniprot50 | AF-A0A843GQU3-F1-MODEL\_V4 | 1.0 | 0.003153 | 145 | 0.19 | 100 | 76 | 4 | 254 | 351 | 1 | 97 | PD-(D/E)XK nuclease-like domain-containing protein | PD-(D/E)XK nuclease-like domain-containing protein | | afdb-uniprot50 | AF-A0A3D5RXV7-F1-MODEL\_V4 | 1.0 | 0.0003327 | 145 | 0.174 | 149 | 102 | 9 | 201 | 344 | 7 | 139 | PDDEXK\_1 domain-containing protein | PDDEXK\_1 domain-containing protein | | afdb-uniprot50 | AF-A0A1V5U7X3-F1-MODEL\_V4 | 1.0 | 0.0001353 | 145 | 0.137 | 204 | 139 | 9 | 154 | 342 | 1 | 182 | PDDEXK\_1 domain-containing protein | PDDEXK\_1 domain-containing protein | | afdb-uniprot50 | AF-A0A6Y1UZP9-F1-MODEL\_V4 | 1.0 | 3.919e-06 | 145 | 0.226 | 203 | 118 | 4 | 83 | 250 | 3 | 201 | DNA breaking-rejoining protein | DNA breaking-rejoining protein | | afdb-uniprot50 | AF-A0A709G0R9-F1-MODEL\_V4 | 1.0 | 6.144e-06 | 145 | 0.226 | 203 | 118 | 3 | 83 | 250 | 6 | 204 | DNA breaking-rejoining protein | DNA breaking-rejoining protein | | afdb-uniprot50 | AF-U2QAP5-F1-MODEL\_V4 | 1.0 | 1.35e-05 | 145 | 0.074 | 334 | 197 | 15 | 16 | 343 | 109 | 336 | DNA helicase | DNA helicase | | afdb-uniprot50 | AF-A0A708WCW9-F1-MODEL\_V4 | 1.0 | 6.876e-06 | 144 | 0.226 | 203 | 118 | 3 | 83 | 250 | 4 | 202 | DNA breaking-rejoining protein | DNA breaking-rejoining protein | | afdb-uniprot50 | AF-A0A726Q5Q9-F1-MODEL\_V4 | 1.0 | 6.876e-06 | 144 | 0.226 | 203 | 118 | 3 | 83 | 250 | 4 | 202 | DNA breaking-rejoining protein | DNA breaking-rejoining protein | | afdb-uniprot50 | AF-A0A702ARL0-F1-MODEL\_V4 | 1.0 | 4.385e-06 | 144 | 0.225 | 204 | 117 | 4 | 83 | 250 | 5 | 203 | Uncharacterized protein | Uncharacterized protein | | afdb-uniprot50 | AF-A0A3M1HHZ9-F1-MODEL\_V4 | 1.0 | 8.61e-06 | 144 | 0.103 | 338 | 195 | 15 | 17 | 346 | 94 | 331 | PD-(D/E)XK nuclease family protein | PD-(D/E)XK nuclease family protein | | afdb-uniprot50 | AF-A0A3A2J0B7-F1-MODEL\_V4 | 1.0 | 1.598e-05 | 144 | 0.117 | 356 | 197 | 16 | 6 | 353 | 414 | 660 | DNA helicase | DNA helicase | | afdb-uniprot50 | AF-A0A7X8LCA3-F1-MODEL\_V4 | 1.0 | 1.019e-05 | 144 | 0.095 | 344 | 195 | 17 | 16 | 351 | 502 | 737 | DNA helicase | DNA helicase | | afdb-uniprot50 | AF-D8FG64-F1-MODEL\_V4 | 1.0 | 3.31e-06 | 143 | 0.122 | 336 | 188 | 18 | 16 | 335 | 8 | 252 | PDDEXK\_1 domain-containing protein | PDDEXK\_1 domain-containing protein | | afdb-uniprot50 | AF-A0A359LS55-F1-MODEL\_V4 | 1.0 | 9.107e-06 | 143 | 0.119 | 352 | 184 | 15 | 6 | 342 | 579 | 819 | PDDEXK\_1 domain-containing protein | PDDEXK\_1 domain-containing protein | | afdb-uniprot50 | AF-A0A327X879-F1-MODEL\_V4 | 1.0 | 3.929e-05 | 143 | 0.113 | 345 | 191 | 17 | 18 | 352 | 707 | 946 | DNA helicase | DNA helicase | | afdb-uniprot50 | AF-A0A722ZM29-F1-MODEL\_V4 | 1.0 | 6.876e-06 | 142 | 0.226 | 203 | 118 | 4 | 83 | 250 | 2 | 200 | DNA breaking-rejoining protein | DNA breaking-rejoining protein | | afdb-uniprot50 | AF-A0A607LSL1-F1-MODEL\_V4 | 1.0 | 8.61e-06 | 142 | 0.221 | 203 | 119 | 4 | 83 | 250 | 3 | 201 | Uncharacterized protein | Uncharacterized protein | | afdb-uniprot50 | AF-A0A614G6N4-F1-MODEL\_V4 | 1.0 | 8.61e-06 | 142 | 0.225 | 204 | 117 | 4 | 83 | 250 | 4 | 202 | Uncharacterized protein | Uncharacterized protein | | afdb-uniprot50 | AF-A0A611B465-F1-MODEL\_V4 | 1.0 | 9.107e-06 | 142 | 0.226 | 203 | 118 | 3 | 83 | 250 | 7 | 205 | DNA breaking-rejoining protein | DNA breaking-rejoining protein | | afdb-uniprot50 | AF-A0A1E5IMV9-F1-MODEL\_V4 | 1.0 | 9.634e-06 | 142 | 0.125 | 344 | 204 | 16 | 6 | 341 | 31 | 285 | PDDEXK\_1 domain-containing protein | PDDEXK\_1 domain-containing protein | | afdb-uniprot50 | AF-A0A538KGZ7-F1-MODEL\_V4 | 1.0 | 4.385e-06 | 142 | 0.116 | 359 | 181 | 14 | 6 | 341 | 912 | 1157 | DNA helicase | DNA helicase | | afdb-uniprot50 | AF-A0A708ZUJ8-F1-MODEL\_V4 | 1.0 | 9.634e-06 | 141 | 0.226 | 203 | 118 | 4 | 83 | 250 | 3 | 201 | DNA breaking-rejoining protein | DNA breaking-rejoining protein | | afdb-uniprot50 | AF-A0A704U7J8-F1-MODEL\_V4 | 1.0 | 4.907e-06 | 141 | 0.226 | 203 | 118 | 3 | 83 | 250 | 7 | 205 | DNA breaking-rejoining protein | DNA breaking-rejoining protein | | afdb-uniprot50 | AF-A0A704ZNC4-F1-MODEL\_V4 | 1.0 | 9.634e-06 | 141 | 0.225 | 204 | 117 | 4 | 83 | 250 | 7 | 205 | Uncharacterized protein | Uncharacterized protein | | afdb-uniprot50 | AF-A0A317GV55-F1-MODEL\_V4 | 1.0 | 7.694e-06 | 141 | 0.116 | 360 | 212 | 21 | 9 | 343 | 1 | 279 | PDDEXK\_1 domain-containing protein | PDDEXK\_1 domain-containing protein | | afdb-uniprot50 | AF-A0A3Z3JMT0-F1-MODEL\_V4 | 1.0 | 4.639e-06 | 140 | 0.225 | 204 | 117 | 4 | 83 | 250 | 7 | 205 | Uncharacterized protein | Uncharacterized protein | | afdb-uniprot50 | AF-I4EFM0-F1-MODEL\_V4 | 1.0 | 5.505e-05 | 140 | 0.167 | 173 | 122 | 10 | 193 | 353 | 32 | 194 | PDDEXK\_1 domain-containing protein | PDDEXK\_1 domain-containing protein | | afdb-uniprot50 | AF-R7JEN0-F1-MODEL\_V4 | 1.0 | 3.137e-05 | 140 | 0.119 | 351 | 188 | 16 | 11 | 351 | 336 | 575 | DNA helicase | DNA helicase | | afdb-uniprot50 | AF-A0A523W971-F1-MODEL\_V4 | 1.0 | 3.319e-05 | 140 | 0.128 | 359 | 187 | 19 | 7 | 350 | 659 | 906 | Dna2/Cas4 domain-containing protein | Dna2/Cas4 domain-containing protein | | afdb-uniprot50 | AF-A0A625UR15-F1-MODEL\_V4 | 1.0 | 1.14e-05 | 139 | 0.225 | 204 | 117 | 4 | 83 | 250 | 7 | 205 | Uncharacterized protein | Uncharacterized protein | | afdb-uniprot50 | AF-A0A0B5GGD9-F1-MODEL\_V4 | 1.0 | 2.001e-05 | 139 | 0.074 | 336 | 195 | 18 | 16 | 343 | 206 | 433 | DNA helicase | DNA helicase | | afdb-uniprot50 | AF-A0A254S109-F1-MODEL\_V4 | 1.0 | 1.14e-05 | 138 | 0.111 | 342 | 187 | 17 | 7 | 338 | 712 | 946 | DNA helicase | DNA helicase | | afdb-uniprot50 | AF-A0A538LVJ5-F1-MODEL\_V4 | 1.0 | 6.5e-06 | 138 | 0.151 | 330 | 175 | 14 | 17 | 338 | 854 | 1086 | DNA helicase | DNA helicase | | afdb-uniprot50 | AF-A0A2H0RW27-F1-MODEL\_V4 | 1.0 | 0.0001695 | 137 | 0.131 | 343 | 173 | 17 | 17 | 342 | 2 | 236 | PDDEXK\_1 domain-containing protein | PDDEXK\_1 domain-containing protein | | afdb-uniprot50 | AF-A0A7K0RIH0-F1-MODEL\_V4 | 1.0 | 9.107e-06 | 137 | 0.145 | 357 | 182 | 17 | 6 | 347 | 212 | 460 | Uncharacterized protein | Uncharacterized protein | | afdb-uniprot50 | AF-A0A1I6KGK5-F1-MODEL\_V4 | 1.0 | 4.65e-05 | 137 | 0.084 | 330 | 191 | 14 | 18 | 342 | 705 | 928 | DNA helicase | DNA helicase | | afdb-uniprot50 | AF-A0A1E5J1P3-F1-MODEL\_V4 | 1.0 | 4.65e-05 | 137 | 0.099 | 333 | 185 | 16 | 18 | 343 | 702 | 926 | DNA helicase | DNA helicase | | afdb-uniprot50 | AF-A0A2W5Y867-F1-MODEL\_V4 | 1.0 | 1.35e-05 | 137 | 0.142 | 364 | 167 | 16 | 9 | 338 | 902 | 1154 | DNA helicase | DNA helicase | | afdb-uniprot50 | AF-A0A3D0QKZ3-F1-MODEL\_V4 | 1.0 | 1.428e-05 | 136 | 0.106 | 338 | 206 | 15 | 6 | 334 | 1 | 251 | PDDEXK\_1 domain-containing protein | PDDEXK\_1 domain-containing protein | | afdb-uniprot50 | AF-A0A7S7SGN9-F1-MODEL\_V4 | 1.0 | 9.634e-06 | 136 | 0.091 | 382 | 204 | 16 | 9 | 371 | 609 | 866 | PD-(D/E)XK nuclease family protein | PD-(D/E)XK nuclease family protein | | afdb-uniprot50 | AF-R5VS20-F1-MODEL\_V4 | 1.0 | 7.292e-05 | 135 | 0.075 | 343 | 200 | 16 | 18 | 352 | 710 | 943 | DNA helicase | DNA helicase | | afdb-uniprot50 | AF-A0A538TBY8-F1-MODEL\_V4 | 1.0 | 0.001699 | 134 | 0.206 | 126 | 85 | 6 | 220 | 341 | 5 | 119 | PDDEXK\_1 domain-containing protein | PDDEXK\_1 domain-containing protein | | afdb-uniprot50 | AF-B1GYT7-F1-MODEL\_V4 | 1.0 | 1.511e-05 | 134 | 0.09 | 342 | 209 | 16 | 6 | 334 | 1 | 253 | PD-(D/E)XK nuclease family protein | PD-(D/E)XK nuclease family protein | | afdb-uniprot50 | AF-A0A7V4SV88-F1-MODEL\_V4 | 1.0 | 9.107e-06 | 134 | 0.136 | 345 | 216 | 16 | 17 | 350 | 5 | 278 | PD-(D/E)XK nuclease family protein | PD-(D/E)XK nuclease family protein | | afdb-uniprot50 | AF-A0A1G9NLF5-F1-MODEL\_V4 | 1.0 | 1.276e-05 | 134 | 0.126 | 378 | 188 | 19 | 20 | 358 | 3 | 277 | PD-(D/E)XK nuclease superfamily protein | PD-(D/E)XK nuclease superfamily protein | | afdb-uniprot50 | AF-A0A2G0E7V9-F1-MODEL\_V4 | 1.0 | 4.65e-05 | 134 | 0.077 | 335 | 194 | 17 | 18 | 345 | 360 | 586 | DNA helicase | DNA helicase | | afdb-uniprot50 | AF-A0A5F0K2G5-F1-MODEL\_V4 | 1.0 | 2.001e-05 | 134 | 0.098 | 344 | 189 | 16 | 8 | 342 | 665 | 896 | DNA helicase | DNA helicase | | afdb-uniprot50 | AF-R7LXF4-F1-MODEL\_V4 | 1.0 | 1.276e-05 | 134 | 0.129 | 370 | 197 | 19 | 11 | 371 | 762 | 1015 | DNA helicase | DNA helicase | | afdb-uniprot50 | AF-A0A383DLI3-F1-MODEL\_V4 | 1.0 | 0.0008653 | 133 | 0.179 | 167 | 108 | 9 | 182 | 343 | 3 | 145 | PDDEXK\_1 domain-containing protein | PDDEXK\_1 domain-containing protein | | afdb-uniprot50 | AF-K4ZR42-F1-MODEL\_V4 | 1.0 | 0.001084 | 133 | 0.175 | 148 | 96 | 8 | 238 | 363 | 7 | 150 | DUF3799 domain-containing protein | DUF3799 domain-containing protein | | afdb-uniprot50 | AF-A0A5C7U316-F1-MODEL\_V4 | 1.0 | 7.292e-05 | 133 | 0.1 | 377 | 199 | 20 | 18 | 370 | 3 | 263 | PD-(D/E)XK nuclease family protein | PD-(D/E)XK nuclease family protein | | afdb-uniprot50 | AF-A0A0S4SUN1-F1-MODEL\_V4 | 1.0 | 7.713e-05 | 133 | 0.117 | 410 | 182 | 15 | 9 | 344 | 6 | 309 | PD-(D/E)XK nuclease superfamily | PD-(D/E)XK nuclease superfamily | | afdb-uniprot50 | AF-A0A2E6AAK1-F1-MODEL\_V4 | 1.0 | 0.001797 | 132 | 0.137 | 153 | 102 | 10 | 202 | 344 | 98 | 230 | Uncharacterized protein | Uncharacterized protein | | afdb-uniprot50 | AF-A0A1W1IJA3-F1-MODEL\_V4 | 1.0 | 5.505e-05 | 132 | 0.093 | 332 | 188 | 15 | 18 | 343 | 705 | 929 | DNA helicase | DNA helicase | | afdb-uniprot50 | AF-A0A7Y1VN02-F1-MODEL\_V4 | 1.0 | 8.159e-05 | 131 | 0.158 | 246 | 152 | 13 | 115 | 351 | 18 | 217 | Uncharacterized protein | Uncharacterized protein | | afdb-uniprot50 | AF-A0A011A8K6-F1-MODEL\_V4 | 1.0 | 6.16e-05 | 131 | 0.149 | 207 | 134 | 12 | 161 | 343 | 44 | 232 | PDDEXK\_1 domain-containing protein | PDDEXK\_1 domain-containing protein | | afdb-uniprot50 | AF-A0A7C6NF67-F1-MODEL\_V4 | 1.0 | 5.505e-05 | 131 | 0.12 | 332 | 159 | 13 | 16 | 338 | 6 | 213 | PD-(D/E)XK nuclease family protein | PD-(D/E)XK nuclease family protein | | afdb-uniprot50 | AF-A0A7C1SR97-F1-MODEL\_V4 | 1.0 | 2.117e-05 | 131 | 0.093 | 375 | 226 | 21 | 19 | 372 | 14 | 295 | PD-(D/E)XK nuclease family protein | PD-(D/E)XK nuclease family protein | | afdb-uniprot50 | AF-A0A609LX09-F1-MODEL\_V4 | 1.0 | 0.001213 | 130 | 0.25 | 148 | 93 | 2 | 103 | 250 | 3 | 132 | Uncharacterized protein | Uncharacterized protein | | afdb-uniprot50 | AF-A0A5X8LAM5-F1-MODEL\_V4 | 1.0 | 0.0004407 | 130 | 0.231 | 190 | 123 | 4 | 88 | 272 | 6 | 177 | Uncharacterized protein | Uncharacterized protein | | afdb-uniprot50 | AF-A0A6N8NPZ1-F1-MODEL\_V4 | 1.0 | 3.929e-05 | 130 | 0.221 | 194 | 108 | 4 | 91 | 247 | 2 | 189 | Exodeoxyribonuclease VIII | Exodeoxyribonuclease VIII | | afdb-uniprot50 | AF-A0A0S8KUQ7-F1-MODEL\_V4 | 1.0 | 0.0001432 | 130 | 0.111 | 207 | 143 | 11 | 157 | 343 | 34 | 219 | Uncharacterized protein | Uncharacterized protein | | afdb-uniprot50 | AF-A0A6G0AGT9-F1-MODEL\_V4 | 1.0 | 2.966e-05 | 130 | 0.133 | 337 | 202 | 20 | 17 | 347 | 398 | 650 | DNA helicase | DNA helicase | | afdb-uniprot50 | AF-A0A2M7MLQ6-F1-MODEL\_V4 | 1.0 | 9.634e-06 | 130 | 0.107 | 343 | 209 | 16 | 18 | 347 | 920 | 1178 | DNA helicase | DNA helicase | | afdb-uniprot50 | AF-A0A3C0F7Z2-F1-MODEL\_V4 | 1.0 | 0.0004932 | 129 | 0.127 | 204 | 157 | 8 | 178 | 370 | 24 | 217 | Uncharacterized protein | Uncharacterized protein | | afdb-uniprot50 | AF-A0A832GQ63-F1-MODEL\_V4 | 1.0 | 9.634e-06 | 129 | 0.129 | 354 | 195 | 19 | 16 | 351 | 44 | 302 | Type I-A CRISPR-associated protein Cas4/Csa1 | Type I-A CRISPR-associated protein Cas4/Csa1 | | afdb-uniprot50 | AF-A0A0F8WAD0-F1-MODEL\_V4 | 1.0 | 0.004177 | 128 | 0.304 | 92 | 59 | 1 | 284 | 370 | 2 | 93 | Uncharacterized protein | Uncharacterized protein | | afdb-uniprot50 | AF-A0A382D5K0-F1-MODEL\_V4 | 1.0 | 0.0002657 | 128 | 0.176 | 187 | 106 | 12 | 164 | 343 | 34 | 179 | PDDEXK\_1 domain-containing protein | PDDEXK\_1 domain-containing protein | | afdb-uniprot50 | AF-K1UR72-F1-MODEL\_V4 | 1.0 | 0.0002122 | 128 | 0.106 | 254 | 169 | 12 | 114 | 364 | 5 | 203 | UvrD/REP helicase | UvrD/REP helicase | | afdb-uniprot50 | AF-A0A2M7X4F5-F1-MODEL\_V4 | 1.0 | 2.804e-05 | 127 | 0.12 | 348 | 188 | 18 | 7 | 342 | 749 | 990 | DNA helicase | DNA helicase | | afdb-uniprot50 | AF-A0A660LK76-F1-MODEL\_V4 | 1.0 | 3.319e-05 | 127 | 0.126 | 341 | 182 | 16 | 17 | 347 | 878 | 1112 | DNA helicase | DNA helicase | | afdb-uniprot50 | AF-A0A077ZMK2-F1-MODEL\_V4 | 1.0 | 0.0001209 | 127 | 0.181 | 248 | 164 | 13 | 101 | 322 | 878 | 1112 | DUF4494 and SNF2 N domain containing protein | DUF4494 and SNF2 N domain containing protein | | afdb-uniprot50 | AF-A0A7C4ZXI5-F1-MODEL\_V4 | 1.0 | 2.111e-06 | 126 | 0.131 | 364 | 196 | 24 | 22 | 370 | 59 | 317 | Type I-A CRISPR-associated protein Cas4/Csa1 | Type I-A CRISPR-associated protein Cas4/Csa1 | | afdb-uniprot50 | AF-A0A0F9SQ52-F1-MODEL\_V4 | 1.0 | 4.396e-05 | 126 | 0.122 | 384 | 193 | 18 | 9 | 370 | 945 | 1206 | DNA helicase | DNA helicase | | afdb-uniprot50 | AF-A0A6S6Y1F3-F1-MODEL\_V4 | 1.0 | 0.000818 | 125 | 0.307 | 127 | 71 | 4 | 254 | 372 | 43 | 160 | DUF3799 domain-containing protein | DUF3799 domain-containing protein | | afdb-uniprot50 | AF-A0A3B9YZQ0-F1-MODEL\_V4 | 1.0 | 3.929e-05 | 125 | 0.132 | 256 | 154 | 15 | 99 | 333 | 423 | 631 | DNA helicase | DNA helicase | | afdb-uniprot50 | AF-A0A6J6ESD5-F1-MODEL\_V4 | 1.0 | 7.713e-05 | 125 | 0.121 | 353 | 167 | 16 | 18 | 351 | 775 | 1003 | DNA helicase | DNA helicase | | afdb-uniprot50 | AF-A0A7V9VQS8-F1-MODEL\_V4 | 1.0 | 0.001084 | 125 | 0.167 | 143 | 112 | 4 | 196 | 335 | 958 | 1096 | DNA helicase | DNA helicase | | afdb-uniprot50 | AF-A0A2D6SUJ8-F1-MODEL\_V4 | 1.0 | 2.369e-05 | 124 | 0.122 | 367 | 177 | 19 | 6 | 343 | 287 | 537 | DNA helicase | DNA helicase | | afdb-uniprot50 | AF-A0A7W1NG24-F1-MODEL\_V4 | 1.0 | 0.001357 | 124 | 0.167 | 143 | 112 | 4 | 196 | 335 | 758 | 896 | DNA helicase | DNA helicase | | afdb-uniprot50 | AF-A0A506XSD7-F1-MODEL\_V4 | 1.0 | 1.35e-05 | 124 | 0.121 | 380 | 229 | 22 | 3 | 365 | 683 | 974 | PD-(D/E)XK nuclease family protein | PD-(D/E)XK nuclease family protein | | afdb-uniprot50 | AF-A0A7Y2X9K5-F1-MODEL\_V4 | 1.0 | 2.369e-05 | 124 | 0.085 | 397 | 224 | 18 | 6 | 358 | 903 | 1204 | DNA helicase | DNA helicase | | afdb-uniprot50 | AF-A0A7X7UZ96-F1-MODEL\_V4 | 1.0 | 2.966e-05 | 124 | 0.099 | 352 | 230 | 17 | 6 | 347 | 925 | 1199 | DNA helicase | DNA helicase | | afdb-uniprot50 | AF-A0A5U2XD06-F1-MODEL\_V4 | 1.0 | 0.004674 | 123 | 0.281 | 110 | 79 | 0 | 141 | 250 | 2 | 111 | Uncharacterized protein | Uncharacterized protein | | afdb-uniprot50 | AF-F3KJA7-F1-MODEL\_V4 | 1.0 | 0.0006533 | 123 | 0.128 | 164 | 122 | 9 | 182 | 343 | 35 | 179 | PDDEXK\_1 domain-containing protein | PDDEXK\_1 domain-containing protein | | afdb-uniprot50 | AF-A0A1Z9PHH6-F1-MODEL\_V4 | 1.0 | 0.0002973 | 122 | 0.123 | 227 | 132 | 11 | 178 | 343 | 38 | 258 | Uncharacterized protein | Uncharacterized protein | | afdb-uniprot50 | AF-A0A350P152-F1-MODEL\_V4 | 1.0 | 0.0005838 | 122 | 0.149 | 174 | 122 | 9 | 217 | 371 | 76 | 242 | Uncharacterized protein | Uncharacterized protein | | afdb-uniprot50 | AF-A0A1W9VNN7-F1-MODEL\_V4 | 1.0 | 6.893e-05 | 122 | 0.105 | 332 | 204 | 18 | 16 | 338 | 11 | 258 | PDDEXK\_1 domain-containing protein | PDDEXK\_1 domain-containing protein | | afdb-uniprot50 | AF-A0A7W0AR45-F1-MODEL\_V4 | 1.0 | 3.511e-05 | 122 | 0.127 | 352 | 193 | 16 | 7 | 347 | 565 | 813 | DNA helicase | DNA helicase | | afdb-uniprot50 | AF-A0A350UHQ6-F1-MODEL\_V4 | 1.0 | 4.919e-05 | 122 | 0.111 | 360 | 199 | 21 | 6 | 349 | 583 | 837 | PDDEXK\_1 domain-containing protein | PDDEXK\_1 domain-containing protein | | afdb-uniprot50 | AF-A0A3D3R7C6-F1-MODEL\_V4 | 1.0 | 7.292e-05 | 122 | 0.122 | 270 | 175 | 11 | 87 | 343 | 948 | 1168 | DNA helicase | DNA helicase | | afdb-uniprot50 | AF-A0A3M2E2F5-F1-MODEL\_V4 | 1.0 | 0.000731 | 121 | 0.131 | 183 | 137 | 10 | 196 | 367 | 6 | 177 | Double-strand break repair protein AddB | Double-strand break repair protein AddB | | afdb-uniprot50 | AF-A0A6N8BXU9-F1-MODEL\_V4 | 1.0 | 0.001699 | 121 | 0.1 | 149 | 113 | 7 | 200 | 338 | 80 | 217 | PD-(D/E)XK nuclease family protein | PD-(D/E)XK nuclease family protein | | afdb-uniprot50 | AF-A0A1Q4FIU6-F1-MODEL\_V4 | 1.0 | 0.0002006 | 121 | 0.2 | 200 | 129 | 13 | 167 | 346 | 31 | 219 | PDDEXK\_1 domain-containing protein | PDDEXK\_1 domain-containing protein | | afdb-uniprot50 | AF-A0A497SU83-F1-MODEL\_V4 | 1.0 | 2.506e-05 | 121 | 0.112 | 348 | 208 | 18 | 18 | 353 | 41 | 299 | Type I-A CRISPR-associated protein Cas4/Csa1 | Type I-A CRISPR-associated protein Cas4/Csa1 | | afdb-uniprot50 | AF-A0A7V7XH75-F1-MODEL\_V4 | 1.0 | 0.0001022 | 121 | 0.114 | 350 | 205 | 17 | 19 | 353 | 787 | 1046 | DNA helicase | DNA helicase | | afdb-uniprot50 | AF-A0A3E0QRG2-F1-MODEL\_V4 | 1.0 | 0.001283 | 120 | 0.14 | 206 | 129 | 10 | 179 | 343 | 14 | 212 | Uncharacterized protein | Uncharacterized protein | | afdb-uniprot50 | AF-A0A662HTM3-F1-MODEL\_V4 | 1.0 | 0.0001209 | 120 | 0.108 | 342 | 191 | 17 | 19 | 353 | 40 | 274 | Type I-A CRISPR-associated protein Cas4/Csa1 | Type I-A CRISPR-associated protein Cas4/Csa1 | | afdb-uniprot50 | AF-A0A7C4GM66-F1-MODEL\_V4 | 1.0 | 8.631e-05 | 120 | 0.12 | 349 | 201 | 18 | 14 | 351 | 42 | 295 | Type I-A CRISPR-associated protein Cas4/Csa1 | Type I-A CRISPR-associated protein Cas4/Csa1 | | afdb-uniprot50 | AF-A0A497Q0N2-F1-MODEL\_V4 | 1.0 | 4.919e-05 | 120 | 0.091 | 337 | 199 | 14 | 18 | 336 | 45 | 292 | Type I-A CRISPR-associated protein Cas4/Csa1 | Type I-A CRISPR-associated protein Cas4/Csa1 | | afdb-uniprot50 | AF-A0A7W1M4Q3-F1-MODEL\_V4 | 1.0 | 4.156e-05 | 120 | 0.123 | 348 | 179 | 14 | 7 | 339 | 250 | 486 | DNA helicase | DNA helicase | | afdb-uniprot50 | AF-A0A7C3DWA4-F1-MODEL\_V4 | 1.0 | 2.65e-05 | 120 | 0.101 | 366 | 207 | 17 | 11 | 368 | 390 | 641 | PD-(D/E)XK nuclease family protein | PD-(D/E)XK nuclease family protein | | afdb-uniprot50 | AF-A0A3L7WDI4-F1-MODEL\_V4 | 1.0 | 0.0001353 | 120 | 0.129 | 254 | 155 | 13 | 99 | 334 | 1033 | 1238 | DNA helicase | DNA helicase | | afdb-uniprot50 | AF-A0A136PHC7-F1-MODEL\_V4 | 1.0 | 0.001146 | 119 | 0.12 | 166 | 130 | 8 | 203 | 364 | 2 | 155 | Exonuclease V subunit beta | Exonuclease V subunit beta | | afdb-uniprot50 | AF-A0A7V9FD96-F1-MODEL\_V4 | 1.0 | 8.631e-05 | 119 | 0.122 | 383 | 173 | 18 | 18 | 369 | 7 | 257 | PD-(D/E)XK nuclease family protein | PD-(D/E)XK nuclease family protein | | afdb-uniprot50 | AF-A0A2P5LHJ5-F1-MODEL\_V4 | 1.0 | 0.0002375 | 119 | 0.111 | 394 | 205 | 22 | 1 | 368 | 4 | 278 | Double-strand break repair protein AddB | Double-strand break repair protein AddB | | afdb-uniprot50 | AF-A0A1F8NK16-F1-MODEL\_V4 | 1.0 | 3.714e-05 | 119 | 0.124 | 353 | 200 | 17 | 1 | 338 | 1 | 259 | PDDEXK\_1 domain-containing protein | PDDEXK\_1 domain-containing protein | | afdb-uniprot50 | AF-A0A0F9C255-F1-MODEL\_V4 | 1.0 | 5.204e-05 | 119 | 0.117 | 390 | 221 | 23 | 6 | 371 | 9 | 299 | PDDEXK\_1 domain-containing protein | PDDEXK\_1 domain-containing protein | | afdb-uniprot50 | AF-A0A3B8MFY8-F1-MODEL\_V4 | 1.0 | 8.631e-05 | 119 | 0.131 | 289 | 168 | 15 | 76 | 337 | 595 | 827 | DNA helicase | DNA helicase | | afdb-uniprot50 | AF-A0A357CMJ9-F1-MODEL\_V4 | 1.0 | 6.16e-05 | 119 | 0.126 | 372 | 183 | 17 | 20 | 372 | 895 | 1143 | DNA helicase | DNA helicase | | afdb-uniprot50 | AF-X0RWR8-F1-MODEL\_V4 | 1.0 | 0.0002006 | 118 | 0.14 | 227 | 140 | 13 | 157 | 364 | 7 | 197 | PDDEXK\_1 domain-containing protein | PDDEXK\_1 domain-containing protein | | afdb-uniprot50 | AF-E3HAC0-F1-MODEL\_V4 | 1.0 | 0.0005217 | 118 | 0.103 | 376 | 180 | 18 | 9 | 369 | 1 | 234 | PDDEXK\_1 domain-containing protein | PDDEXK\_1 domain-containing protein | | afdb-uniprot50 | AF-A0A1B8SLC9-F1-MODEL\_V4 | 1.0 | 0.0001896 | 118 | 0.155 | 186 | 133 | 11 | 180 | 354 | 67 | 239 | Uncharacterized protein | Uncharacterized protein | | afdb-uniprot50 | AF-A0A7C5JZ99-F1-MODEL\_V4 | 1.0 | 0.0004662 | 118 | 0.1 | 340 | 178 | 15 | 19 | 350 | 8 | 227 | PD-(D/E)XK nuclease family protein | PD-(D/E)XK nuclease family protein | | afdb-uniprot50 | AF-A0A1W9UX36-F1-MODEL\_V4 | 1.0 | 0.001901 | 118 | 0.176 | 159 | 109 | 9 | 200 | 346 | 94 | 242 | PDDEXK\_1 domain-containing protein | PDDEXK\_1 domain-containing protein | | afdb-uniprot50 | AF-A0A843DE36-F1-MODEL\_V4 | 1.0 | 0.0001793 | 118 | 0.107 | 354 | 198 | 14 | 6 | 349 | 728 | 973 | ATP-dependent helicase | ATP-dependent helicase | | afdb-uniprot50 | AF-A0A662I0G3-F1-MODEL\_V4 | 1.0 | 9.131e-05 | 117 | 0.128 | 351 | 203 | 19 | 17 | 355 | 44 | 303 | Type I-A CRISPR-associated protein Cas4/Csa1 | Type I-A CRISPR-associated protein Cas4/Csa1 | | afdb-uniprot50 | AF-A0A7V9KBP8-F1-MODEL\_V4 | 1.0 | 0.0001081 | 117 | 0.13 | 361 | 182 | 20 | 10 | 349 | 461 | 710 | DNA helicase | DNA helicase | | afdb-uniprot50 | AF-A0A1M3KGY8-F1-MODEL\_V4 | 1.0 | 0.0003939 | 116 | 0.091 | 337 | 184 | 15 | 19 | 350 | 8 | 227 | PDDEXK\_1 domain-containing protein | PDDEXK\_1 domain-containing protein | | afdb-uniprot50 | AF-A0A431HDA1-F1-MODEL\_V4 | 1.0 | 0.0001022 | 116 | 0.09 | 387 | 215 | 19 | 1 | 372 | 626 | 890 | PDDEXK\_1 domain-containing protein | PDDEXK\_1 domain-containing protein | | afdb-uniprot50 | AF-A0A351EJF5-F1-MODEL\_V4 | 1.0 | 3.929e-05 | 116 | 0.136 | 322 | 181 | 17 | 45 | 346 | 687 | 931 | DNA helicase | DNA helicase | | afdb-uniprot50 | AF-A0A6M8UY77-F1-MODEL\_V4 | 1.0 | 4.156e-05 | 116 | 0.123 | 355 | 205 | 20 | 6 | 347 | 907 | 1168 | DNA helicase | DNA helicase | | afdb-uniprot50 | AF-A0A7V4J4G6-F1-MODEL\_V4 | 1.0 | 0.0008653 | 115 | 0.158 | 196 | 124 | 12 | 196 | 370 | 60 | 235 | PDDEXK\_1 domain-containing protein | PDDEXK\_1 domain-containing protein | | afdb-uniprot50 | AF-A0A7V3LX21-F1-MODEL\_V4 | 1.0 | 0.0001793 | 115 | 0.138 | 239 | 173 | 11 | 111 | 338 | 44 | 260 | PDDEXK\_1 domain-containing protein | PDDEXK\_1 domain-containing protein | | afdb-uniprot50 | AF-A0A3M1GPK9-F1-MODEL\_V4 | 1.0 | 5.823e-05 | 115 | 0.117 | 332 | 174 | 16 | 20 | 337 | 938 | 1164 | DNA helicase | DNA helicase | | afdb-uniprot50 | AF-A0A4Q6FWM5-F1-MODEL\_V4 | 1.0 | 0.002011 | 114 | 0.202 | 153 | 103 | 9 | 205 | 343 | 28 | 175 | ATP-dependent DNA helicase | ATP-dependent DNA helicase | | afdb-uniprot50 | AF-A0A846AL31-F1-MODEL\_V4 | 1.0 | 0.002011 | 114 | 0.1 | 368 | 187 | 17 | 16 | 369 | 2 | 239 | PD-(D/E)XK nuclease family protein | PD-(D/E)XK nuclease family protein | | afdb-uniprot50 | AF-A0A0L8EQ63-F1-MODEL\_V4 | 1.0 | 0.0002512 | 114 | 0.088 | 326 | 203 | 15 | 43 | 354 | 10 | 255 | PDDEXK\_1 domain-containing protein | PDDEXK\_1 domain-containing protein | | afdb-uniprot50 | AF-A0A0F8WPB7-F1-MODEL\_V4 | 1.0 | 0.0002122 | 114 | 0.075 | 369 | 195 | 19 | 9 | 347 | 1 | 253 | Uncharacterized protein | Uncharacterized protein | | afdb-uniprot50 | AF-E0STX3-F1-MODEL\_V4 | 1.0 | 0.0001209 | 114 | 0.106 | 356 | 177 | 15 | 6 | 356 | 70 | 289 | CRISPR-associated protein, Csa1 family | CRISPR-associated protein, Csa1 family | | afdb-uniprot50 | AF-A0A2D5TWL9-F1-MODEL\_V4 | 1.0 | 0.0002512 | 113 | 0.141 | 233 | 139 | 10 | 111 | 338 | 51 | 227 | PDDEXK\_1 domain-containing protein | PDDEXK\_1 domain-containing protein | | afdb-uniprot50 | AF-A0A841KPM6-F1-MODEL\_V4 | 1.0 | 0.0009154 | 113 | 0.106 | 365 | 175 | 18 | 19 | 354 | 10 | 252 | CRISPR/Cas system-associated exonuclease Cas4 (RecB family) | CRISPR/Cas system-associated exonuclease Cas4 (RecB family) | | afdb-uniprot50 | AF-A0A7C0UD41-F1-MODEL\_V4 | 1.0 | 0.0001209 | 113 | 0.13 | 368 | 211 | 22 | 1 | 351 | 5 | 280 | Type I-A CRISPR-associated protein Cas4/Csa1 | Type I-A CRISPR-associated protein Cas4/Csa1 | | afdb-uniprot50 | AF-A0A7Y2DTL4-F1-MODEL\_V4 | 1.0 | 0.0002245 | 113 | 0.107 | 345 | 187 | 17 | 19 | 338 | 12 | 260 | PDDEXK\_1 domain-containing protein | PDDEXK\_1 domain-containing protein | | afdb-uniprot50 | AF-A0A850I7L6-F1-MODEL\_V4 | 1.0 | 0.0003145 | 113 | 0.131 | 205 | 139 | 12 | 180 | 371 | 81 | 259 | Uncharacterized protein | Uncharacterized protein | | afdb-uniprot50 | AF-R5VWW7-F1-MODEL\_V4 | 1.0 | 0.0006533 | 113 | 0.093 | 330 | 189 | 15 | 16 | 338 | 510 | 736 | DNA helicase | DNA helicase | | afdb-uniprot50 | AF-A0A554JBM9-F1-MODEL\_V4 | 1.0 | 0.0002006 | 113 | 0.11 | 380 | 211 | 23 | 12 | 369 | 758 | 1032 | DNA helicase | DNA helicase | | afdb-uniprot50 | AF-A0A2D4YUG9-F1-MODEL\_V4 | 1.0 | 0.0001896 | 112 | 0.119 | 251 | 159 | 16 | 104 | 344 | 26 | 224 | Exonuclease | Exonuclease | | afdb-uniprot50 | AF-A0A4P5V7J0-F1-MODEL\_V4 | 1.0 | 3.714e-05 | 112 | 0.125 | 310 | 174 | 19 | 63 | 333 | 240 | 491 | DNA helicase | DNA helicase | | afdb-uniprot50 | AF-A0A7W2B9I7-F1-MODEL\_V4 | 1.0 | 0.0001143 | 112 | 0.109 | 384 | 207 | 22 | 1 | 366 | 665 | 931 | PD-(D/E)XK nuclease family protein | PD-(D/E)XK nuclease family protein | | afdb-uniprot50 | AF-A0A497EM85-F1-MODEL\_V4 | 1.0 | 0.0006176 | 111 | 0.111 | 341 | 188 | 16 | 19 | 354 | 65 | 295 | Type I-A CRISPR-associated protein Cas4/Csa1 | Type I-A CRISPR-associated protein Cas4/Csa1 | | afdb-uniprot50 | AF-A0A2E5G5K7-F1-MODEL\_V4 | 1.0 | 0.0002811 | 111 | 0.123 | 371 | 205 | 20 | 17 | 370 | 677 | 944 | PDDEXK\_1 domain-containing protein | PDDEXK\_1 domain-containing protein | | afdb-uniprot50 | AF-A0A5E4HM91-F1-MODEL\_V4 | 1.0 | 0.002381 | 110 | 0.161 | 198 | 121 | 12 | 164 | 353 | 65 | 225 | Uncharacterized protein | Uncharacterized protein | | afdb-uniprot50 | AF-A0A496U7R8-F1-MODEL\_V4 | 1.0 | 0.0006911 | 109 | 0.101 | 353 | 202 | 17 | 17 | 363 | 627 | 870 | PDDEXK\_1 domain-containing protein | PDDEXK\_1 domain-containing protein | | afdb-uniprot50 | AF-A0A7C5FEZ8-F1-MODEL\_V4 | 1.0 | 0.007329 | 108 | 0.096 | 176 | 123 | 8 | 220 | 372 | 7 | 169 | PDDEXK\_1 domain-containing protein | PDDEXK\_1 domain-containing protein | | afdb-uniprot50 | AF-A0A1E5IMZ6-F1-MODEL\_V4 | 1.0 | 0.0004662 | 108 | 0.104 | 307 | 189 | 16 | 81 | 368 | 14 | 253 | PDDEXK\_1 domain-containing protein | PDDEXK\_1 domain-containing protein | | afdb-uniprot50 | AF-A0A7C4A432-F1-MODEL\_V4 | 1.0 | 0.0001602 | 108 | 0.11 | 345 | 205 | 21 | 17 | 341 | 6 | 268 | PDDEXK\_1 domain-containing protein | PDDEXK\_1 domain-containing protein | | afdb-uniprot50 | AF-A0A2N8HCS8-F1-MODEL\_V4 | 1.0 | 0.005853 | 107 | 0.188 | 106 | 72 | 4 | 249 | 348 | 32 | 129 | PDDEXK\_1 domain-containing protein | PDDEXK\_1 domain-containing protein | | afdb-uniprot50 | AF-A0A2E9QA28-F1-MODEL\_V4 | 1.0 | 0.0002122 | 107 | 0.117 | 282 | 166 | 19 | 86 | 346 | 49 | 268 | Uncharacterized protein | Uncharacterized protein | | afdb-uniprot50 | AF-A0A3M2AIE2-F1-MODEL\_V4 | 1.0 | 0.0001793 | 107 | 0.137 | 393 | 194 | 23 | 6 | 366 | 486 | 765 | PD-(D/E)XK nuclease family protein | PD-(D/E)XK nuclease family protein | | afdb-uniprot50 | AF-A0A1U9K8J1-F1-MODEL\_V4 | 1.0 | 3.137e-05 | 107 | 0.127 | 393 | 217 | 20 | 1 | 351 | 875 | 1183 | DNA helicase | DNA helicase | | afdb-uniprot50 | AF-A0A2M7TJF4-F1-MODEL\_V4 | 1.0 | 0.001518 | 106 | 0.1 | 319 | 167 | 17 | 31 | 341 | 3 | 209 | PDDEXK\_1 domain-containing protein | PDDEXK\_1 domain-containing protein | | afdb-uniprot50 | AF-A0A2M6ZV13-F1-MODEL\_V4 | 1.0 | 0.0006533 | 106 | 0.1 | 300 | 188 | 14 | 87 | 370 | 11 | 244 | PDDEXK\_1 domain-containing protein | PDDEXK\_1 domain-containing protein | | afdb-uniprot50 | AF-A0A7T8V446-F1-MODEL\_V4 | 1.0 | 0.002011 | 106 | 0.075 | 330 | 173 | 13 | 19 | 338 | 7 | 214 | PD-(D/E)XK nuclease family protein | PD-(D/E)XK nuclease family protein | | afdb-uniprot50 | AF-A0A7C7QVR4-F1-MODEL\_V4 | 1.0 | 0.0002657 | 106 | 0.125 | 366 | 190 | 22 | 18 | 366 | 39 | 291 | PDDEXK\_1 domain-containing protein | PDDEXK\_1 domain-containing protein | | afdb-uniprot50 | AF-A0A3L7YM13-F1-MODEL\_V4 | 1.0 | 0.0001793 | 106 | 0.117 | 247 | 151 | 16 | 111 | 352 | 870 | 1054 | DNA helicase | DNA helicase | | afdb-uniprot50 | AF-A0A382UHU8-F1-MODEL\_V4 | 1.0 | 0.001797 | 105 | 0.142 | 140 | 99 | 7 | 225 | 355 | 2 | 129 | PDDEXK\_1 domain-containing protein | PDDEXK\_1 domain-containing protein | | afdb-uniprot50 | AF-A0A2E5ASM7-F1-MODEL\_V4 | 1.0 | 0.0007733 | 105 | 0.15 | 226 | 136 | 15 | 137 | 346 | 44 | 229 | PDDEXK\_1 domain-containing protein | PDDEXK\_1 domain-containing protein | | afdb-uniprot50 | AF-A0A523LAM6-F1-MODEL\_V4 | 1.0 | 0.0004662 | 105 | 0.128 | 280 | 133 | 13 | 44 | 316 | 63 | 238 | PDDEXK\_1 domain-containing protein | PDDEXK\_1 domain-containing protein | | afdb-uniprot50 | AF-A0A160T011-F1-MODEL\_V4 | 1.0 | 0.0002122 | 105 | 0.149 | 287 | 165 | 12 | 83 | 350 | 6 | 232 | Uncharacterized protein | Uncharacterized protein | | afdb-uniprot50 | AF-A0A1V4IB94-F1-MODEL\_V4 | 1.0 | 0.004674 | 104 | 0.095 | 199 | 147 | 8 | 183 | 370 | 62 | 238 | PD-(D/E)XK nuclease superfamily protein | PD-(D/E)XK nuclease superfamily protein | | afdb-uniprot50 | AF-A0A7X9J9S1-F1-MODEL\_V4 | 1.0 | 0.000731 | 104 | 0.11 | 325 | 163 | 15 | 19 | 338 | 8 | 211 | PD-(D/E)XK nuclease family protein | PD-(D/E)XK nuclease family protein | | afdb-uniprot50 | AF-A0A3B9QJZ0-F1-MODEL\_V4 | 1.0 | 0.0003145 | 104 | 0.133 | 285 | 165 | 16 | 82 | 346 | 285 | 507 | DNA helicase | DNA helicase | | afdb-uniprot50 | AF-A0A7V7XI94-F1-MODEL\_V4 | 1.0 | 0.001797 | 103 | 0.093 | 342 | 169 | 17 | 16 | 346 | 16 | 227 | PD-(D/E)XK nuclease family protein | PD-(D/E)XK nuclease family protein | | afdb-uniprot50 | AF-A0A2E0ZUF9-F1-MODEL\_V4 | 1.0 | 0.0001896 | 103 | 0.147 | 338 | 178 | 16 | 6 | 332 | 232 | 470 | DNA helicase | DNA helicase | | afdb-uniprot50 | AF-A0A1Z8S8D1-F1-MODEL\_V4 | 1.0 | 0.001283 | 102 | 0.142 | 245 | 138 | 14 | 134 | 352 | 1 | 199 | PDDEXK\_1 domain-containing protein | PDDEXK\_1 domain-containing protein | | afdb-uniprot50 | AF-A0A2D9L0E1-F1-MODEL\_V4 | 1.0 | 0.0006176 | 102 | 0.136 | 234 | 153 | 14 | 168 | 372 | 52 | 265 | PDDEXK\_1 domain-containing protein | PDDEXK\_1 domain-containing protein | | afdb-uniprot50 | AF-A0A381XZY0-F1-MODEL\_V4 | 1.0 | 0.001901 | 101 | 0.162 | 209 | 119 | 10 | 197 | 369 | 15 | 203 | PDDEXK\_1 domain-containing protein | PDDEXK\_1 domain-containing protein | | afdb-uniprot50 | AF-A0A0F9FF23-F1-MODEL\_V4 | 1.0 | 0.006549 | 101 | 0.13 | 192 | 122 | 14 | 179 | 359 | 58 | 215 | Uncharacterized protein | Uncharacterized protein | | afdb-uniprot50 | AF-H0E406-F1-MODEL\_V4 | 1.0 | 0.0001353 | 101 | 0.14 | 300 | 159 | 16 | 82 | 338 | 691 | 934 | DNA helicase | DNA helicase | | afdb-uniprot50 | AF-A0A2P5NMI1-F1-MODEL\_V4 | 1.0 | 0.001901 | 101 | 0.111 | 384 | 224 | 17 | 1 | 371 | 744 | 1023 | Double-strand break repair protein AddB | Double-strand break repair protein AddB | | afdb-uniprot50 | AF-A0A292YCD1-F1-MODEL\_V4 | 1.0 | 0.0002122 | 101 | 0.142 | 379 | 177 | 21 | 7 | 350 | 952 | 1217 | DNA helicase | DNA helicase | | afdb-uniprot50 | AF-A0A705R219-F1-MODEL\_V4 | 1.0 | 0.004419 | 100 | 0.201 | 174 | 98 | 4 | 83 | 220 | 7 | 175 | Uncharacterized protein | Uncharacterized protein | | afdb-uniprot50 | AF-A0A7W1EQU2-F1-MODEL\_V4 | 0.999 | 0.0004166 | 99 | 0.123 | 299 | 167 | 20 | 84 | 356 | 38 | 267 | Uncharacterized protein | Uncharacterized protein | | afdb-uniprot50 | AF-A0A840DK55-F1-MODEL\_V4 | 0.999 | 0.0004662 | 99 | 0.135 | 400 | 189 | 24 | 9 | 368 | 663 | 945 | RecB family exonuclease | RecB family exonuclease | | afdb-uniprot50 | AF-A0A832FQV0-F1-MODEL\_V4 | 0.999 | 0.001283 | 98 | 0.169 | 206 | 119 | 13 | 161 | 362 | 14 | 171 | DUF911 domain-containing protein | DUF911 domain-containing protein | | afdb-uniprot50 | AF-A0A537W8D4-F1-MODEL\_V4 | 0.999 | 0.002127 | 98 | 0.122 | 204 | 140 | 12 | 154 | 351 | 8 | 178 | PDDEXK\_1 domain-containing protein | PDDEXK\_1 domain-containing protein | | afdb-uniprot50 | AF-A0A162LB28-F1-MODEL\_V4 | 0.999 | 0.003336 | 98 | 0.077 | 322 | 168 | 16 | 19 | 334 | 5 | 203 | PD-(D/E)XK nuclease superfamily protein | PD-(D/E)XK nuclease superfamily protein | | afdb-uniprot50 | AF-A0A1U9NQE7-F1-MODEL\_V4 | 0.999 | 0.004419 | 98 | 0.125 | 207 | 131 | 10 | 197 | 369 | 44 | 234 | PD-(D/E)XK nuclease superfamily protein | PD-(D/E)XK nuclease superfamily protein | | afdb-uniprot50 | AF-A0A350REJ8-F1-MODEL\_V4 | 0.999 | 0.0004407 | 98 | 0.16 | 331 | 194 | 17 | 32 | 334 | 73 | 347 | PDDEXK\_1 domain-containing protein | PDDEXK\_1 domain-containing protein | | afdb-uniprot50 | AF-A0A4P5SGY1-F1-MODEL\_V4 | 0.999 | 0.001084 | 98 | 0.13 | 284 | 197 | 14 | 104 | 372 | 670 | 918 | PDDEXK\_1 domain-containing protein | PDDEXK\_1 domain-containing protein | | afdb-uniprot50 | AF-U6B6L1-F1-MODEL\_V4 | 0.999 | 0.0006911 | 98 | 0.093 | 376 | 215 | 22 | 15 | 368 | 758 | 1029 | Inactivated superfamily I helicase | Inactivated superfamily I helicase | | afdb-uniprot50 | AF-A0A2S5MP45-F1-MODEL\_V4 | 0.999 | 0.00523 | 97 | 0.14 | 185 | 132 | 11 | 199 | 369 | 3 | 174 | Double-strand break repair protein AddB | Double-strand break repair protein AddB | | afdb-uniprot50 | AF-A0A662HJV4-F1-MODEL\_V4 | 0.999 | 0.003153 | 97 | 0.109 | 339 | 177 | 18 | 19 | 351 | 47 | 266 | Type I-A CRISPR-associated protein Cas4/Csa1 | Type I-A CRISPR-associated protein Cas4/Csa1 | | afdb-uniprot50 | AF-A0A1W9S0B8-F1-MODEL\_V4 | 0.999 | 0.0002245 | 97 | 0.16 | 286 | 166 | 13 | 38 | 319 | 557 | 772 | DNA helicase | DNA helicase | | afdb-uniprot50 | AF-A0A1Q3MWE1-F1-MODEL\_V4 | 0.999 | 0.003153 | 97 | 0.126 | 348 | 179 | 21 | 17 | 351 | 624 | 859 | PDDEXK\_1 domain-containing protein | PDDEXK\_1 domain-containing protein | | afdb-uniprot50 | AF-A0A7V4APT1-F1-MODEL\_V4 | 0.999 | 0.0004407 | 96 | 0.099 | 352 | 215 | 20 | 6 | 335 | 1 | 272 | PD-(D/E)XK nuclease family protein | PD-(D/E)XK nuclease family protein | | afdb-uniprot50 | AF-D9PF80-F1-MODEL\_V4 | 0.998 | 0.0005519 | 95 | 0.16 | 237 | 128 | 15 | 100 | 316 | 52 | 237 | PDDEXK\_1 domain-containing protein | PDDEXK\_1 domain-containing protein | | afdb-uniprot50 | AF-A0A6N8W5D8-F1-MODEL\_V4 | 0.998 | 0.0004932 | 95 | 0.121 | 355 | 162 | 17 | 18 | 354 | 358 | 580 | DNA helicase | DNA helicase | | afdb-uniprot50 | AF-A0A661U7B8-F1-MODEL\_V4 | 0.998 | 0.001146 | 95 | 0.144 | 242 | 145 | 10 | 116 | 319 | 862 | 1079 | DNA helicase | DNA helicase | | afdb-uniprot50 | AF-A0A3M1Y1D5-F1-MODEL\_V4 | 0.998 | 0.0004166 | 94 | 0.109 | 293 | 184 | 16 | 66 | 334 | 219 | 458 | DNA helicase | DNA helicase | | afdb-uniprot50 | AF-A0A356N865-F1-MODEL\_V4 | 0.998 | 0.002381 | 93 | 0.125 | 352 | 175 | 22 | 19 | 351 | 798 | 1035 | DNA helicase | DNA helicase | | afdb-uniprot50 | AF-A0A2M6YXS7-F1-MODEL\_V4 | 0.997 | 0.001901 | 92 | 0.128 | 367 | 173 | 15 | 17 | 349 | 956 | 1209 | DNA helicase | DNA helicase | | afdb-uniprot50 | AF-A0A7C1B943-F1-MODEL\_V4 | 0.997 | 0.0005217 | 91 | 0.168 | 285 | 155 | 14 | 43 | 319 | 691 | 901 | DNA helicase | DNA helicase | | afdb-uniprot50 | AF-A0A6I1QTZ0-F1-MODEL\_V4 | 0.997 | 0.001901 | 91 | 0.138 | 368 | 199 | 21 | 12 | 353 | 724 | 999 | UvrD-like helicase C-terminal domain-containing protein | UvrD-like helicase C-terminal domain-containing protein | | afdb-uniprot50 | AF-A0A0F8YJ77-F1-MODEL\_V4 | 0.996 | 0.008201 | 90 | 0.152 | 190 | 137 | 9 | 194 | 369 | 10 | 189 | Uncharacterized protein | Uncharacterized protein | | afdb-uniprot50 | AF-A0A2E5NP53-F1-MODEL\_V4 | 0.996 | 0.002818 | 90 | 0.098 | 283 | 144 | 12 | 43 | 318 | 927 | 1105 | DNA helicase | DNA helicase | | afdb-uniprot50 | AF-A0A5J4EAI4-F1-MODEL\_V4 | 0.995 | 0.002011 | 88 | 0.096 | 291 | 143 | 16 | 43 | 318 | 332 | 517 | DNA helicase | DNA helicase | | afdb-uniprot50 | AF-A0A2T4UF80-F1-MODEL\_V4 | 0.995 | 0.0006533 | 88 | 0.127 | 415 | 192 | 23 | 6 | 359 | 905 | 1210 | DNA helicase | DNA helicase | | afdb-uniprot50 | AF-A0A7C5FTP4-F1-MODEL\_V4 | 0.994 | 0.005853 | 87 | 0.135 | 229 | 144 | 13 | 182 | 372 | 11 | 223 | PDDEXK\_1 domain-containing protein | PDDEXK\_1 domain-containing protein | | afdb-uniprot50 | AF-A0A256Z4F0-F1-MODEL\_V4 | 0.993 | 0.001213 | 86 | 0.107 | 280 | 180 | 15 | 104 | 351 | 240 | 481 | Cas\_Cas4 domain-containing protein | Cas\_Cas4 domain-containing protein | | afdb-uniprot50 | AF-A0A1F8V201-F1-MODEL\_V4 | 0.991 | 0.004944 | 84 | 0.116 | 223 | 151 | 15 | 109 | 318 | 910 | 1099 | DNA helicase | DNA helicase | | afdb-uniprot50 | AF-A0A662G4I6-F1-MODEL\_V4 | 0.99 | 0.0003939 | 83 | 0.125 | 288 | 174 | 14 | 88 | 364 | 2 | 222 | Type I-A CRISPR-associated protein Cas4/Csa1 | Type I-A CRISPR-associated protein Cas4/Csa1 | | afdb-uniprot50 | AF-A0A7C5H0W5-F1-MODEL\_V4 | 0.99 | 0.0009683 | 83 | 0.121 | 347 | 230 | 18 | 23 | 351 | 13 | 302 | Dna2/Cas4 domain-containing protein | Dna2/Cas4 domain-containing protein | | afdb-uniprot50 | AF-A0A0S8BQY4-F1-MODEL\_V4 | 0.99 | 0.001797 | 83 | 0.122 | 237 | 130 | 14 | 87 | 316 | 270 | 435 | DNA helicase | DNA helicase | | afdb-uniprot50 | AF-A0A7C1YNJ4-F1-MODEL\_V4 | 0.99 | 0.003949 | 83 | 0.101 | 247 | 146 | 15 | 82 | 319 | 963 | 1142 | DNA helicase | DNA helicase | | afdb-uniprot50 | AF-A0A3C1LBA3-F1-MODEL\_V4 | 0.988 | 0.001606 | 82 | 0.145 | 337 | 183 | 20 | 35 | 333 | 353 | 622 | DNA helicase | DNA helicase | | afdb-uniprot50 | AF-A0A2N6N5L1-F1-MODEL\_V4 | 0.988 | 0.006928 | 82 | 0.104 | 286 | 140 | 14 | 45 | 318 | 596 | 777 | DNA helicase | DNA helicase | | afdb-uniprot50 | AF-A0A849E0V5-F1-MODEL\_V4 | 0.986 | 0.004674 | 81 | 0.095 | 240 | 142 | 15 | 87 | 319 | 223 | 394 | Uncharacterized protein | Uncharacterized protein | | afdb-uniprot50 | AF-Q83C98-F1-MODEL\_V4 | 0.986 | 0.006928 | 81 | 0.091 | 284 | 147 | 13 | 43 | 319 | 923 | 1102 | DNA helicase | DNA helicase | | afdb-uniprot50 | AF-A0A0F9E004-F1-MODEL\_V4 | 0.984 | 0.003949 | 80 | 0.112 | 213 | 130 | 10 | 159 | 343 | 13 | 194 | Uncharacterized protein | Uncharacterized protein | | afdb-uniprot50 | AF-A0A812L764-F1-MODEL\_V4 | 0.984 | 0.001024 | 80 | 0.15 | 378 | 222 | 22 | 42 | 359 | 27 | 365 | SWA2 protein | SWA2 protein | | afdb-uniprot50 | AF-A0A3C1TRC6-F1-MODEL\_V4 | 0.984 | 0.004944 | 80 | 0.126 | 252 | 130 | 15 | 82 | 316 | 584 | 762 | DNA helicase | DNA helicase | | afdb-uniprot50 | AF-A0A1G1K328-F1-MODEL\_V4 | 0.984 | 0.004674 | 80 | 0.128 | 357 | 199 | 19 | 7 | 338 | 860 | 1129 | DNA helicase | DNA helicase | | afdb-uniprot50 | AF-A0A1F8RNI5-F1-MODEL\_V4 | 0.981 | 0.005533 | 79 | 0.128 | 280 | 147 | 15 | 43 | 318 | 838 | 1024 | DNA helicase | DNA helicase | | afdb-uniprot50 | AF-A0A6B1AB89-F1-MODEL\_V4 | 0.981 | 0.001283 | 79 | 0.155 | 329 | 179 | 21 | 44 | 351 | 847 | 1097 | DNA helicase | DNA helicase | | afdb-uniprot50 | AF-A0A0F9TAF0-F1-MODEL\_V4 | 0.978 | 0.007329 | 78 | 0.134 | 276 | 157 | 14 | 105 | 359 | 17 | 231 | YqaJ domain-containing protein | YqaJ domain-containing protein | | afdb-uniprot50 | AF-A0A521I6S1-F1-MODEL\_V4 | 0.978 | 0.0009683 | 78 | 0.135 | 287 | 189 | 14 | 43 | 318 | 181 | 419 | DNA helicase | DNA helicase | | afdb-uniprot50 | AF-A0A5C7V023-F1-MODEL\_V4 | 0.975 | 0.007329 | 77 | 0.113 | 281 | 142 | 15 | 43 | 315 | 486 | 667 | DNA helicase | DNA helicase | | afdb-uniprot50 | AF-A0A812ZGY8-F1-MODEL\_V4 | 0.975 | 0.00225 | 77 | 0.123 | 308 | 166 | 18 | 100 | 372 | 280 | 518 | Hypothetical protein | Hypothetical protein | | afdb-uniprot50 | AF-A0A6M3JLX4-F1-MODEL\_V4 | 0.971 | 0.003733 | 76 | 0.118 | 185 | 136 | 11 | 170 | 343 | 34 | 202 | Putative exonuclease | Putative exonuclease | | afdb-uniprot50 | AF-A0A2D9JQL8-F1-MODEL\_V4 | 0.971 | 0.008675 | 76 | 0.114 | 287 | 144 | 16 | 42 | 319 | 951 | 1136 | DNA helicase | DNA helicase | | afdb-uniprot50 | AF-A0A1W9K967-F1-MODEL\_V4 | 0.933 | 0.009708 | 70 | 0.105 | 304 | 182 | 16 | 35 | 316 | 585 | 820 | DNA helicase | DNA helicase | | afdb-uniprot50 | AF-A0A812XXC2-F1-MODEL\_V4 | 0.933 | 0.003733 | 70 | 0.129 | 317 | 191 | 23 | 81 | 359 | 15 | 284 | Hypothetical protein | Hypothetical protein | | afdb-uniprot50 | AF-A0A2P4PVM3-F1-MODEL\_V4 | 0.772 | 0.00523 | 60 | 0.142 | 338 | 202 | 21 | 75 | 370 | 1 | 292 | Uncharacterized protein | Uncharacterized protein | |
| Top keywords  (threshold 1.00e-02 (evalue)) | **domain\_containing, DUF3799, Exodeoxyribonuclease, DNA, helicase, PD\_, D, E, XK, VIII** |
| Output files | ../../similar\_structures/41\_FANPEZAQ\_CDS\_0041\_afdb-proteome\_foldseek.tsv ../../similar\_structures/41\_FANPEZAQ\_CDS\_0041\_afdb-uniprot50\_foldseek.tsv ../../similar\_structures/41\_FANPEZAQ\_CDS\_0041\_merged.svg ../../similar\_structures/41\_FANPEZAQ\_CDS\_0041\_pdb\_foldseek.tsv |

  
  
  

Return to summary | Go to previous | Go to next

  


---

**Sequence/structure alignments coloring**  
Each object in the alignment figures is colored according to its E-value following this color coding:

1e-100
10

**References:**  
1) Steinegger M, Meier M, Mirdita M, Vöhringer H, Haunsberger S J, and Söding J (2019) HH-suite3 for fast remote homology detection and deep protein annotation, BMC Bioinformatics, 473. doi: 10.1186/s12859-019-3019-7  
2) Jumper J, Evans R, Pritzel A, ..., Hassabis D (2021) Highly accurate protein structure prediction with AlphaFold, Nature, 596. doi: 10.1038/s41586-021-03819-2  
3) van Kempen M, Kim S, Tumescheit C, Mirdita M, Lee J, Gilchrist CLM, Söding J, and Steinegger M (2023) Fast and accurate protein structure search with Foldseek. Nature Biotechnology. doi: 10.1038/s41587-023-01773-0
