# Supplementary material for: Completing the BASEL phage collection to unlock hidden diversity for systematic exploration of phage–host interactions
Source: PLoS Biol. 2025 Apr 7;23(4):e3003063. doi: 10.1371/journal.pbio.3003063 (PMC11990801; doi:10.1371/journal.pbio.3003063)
Supplement: S2 Data — (ZIP) [file pbio.3003063.s009.zip › entries/42.html]

FANPEZAQ\_CDS\_0042


Return to summary | Go to previous | Go to next

|  |  |
| --- | --- |
| FANPEZAQ\_CDS\_0042 Page creation date: 02 Sep 2024, 12:00  Project folder: n/a  Input sequences file: Escherichia\_virus\_HeidiAbel.gb | transcriptional domain\_containing regulator repressor hth cro c1\_type peptidase helix\_turn\_helix s24 phage putative c peptidase\_s24 and lexa peptisase transcription s24\_like dna hth\_type signal ci contains s26a s26b xre domains dna\_binding complex with regulation s26c structural binding |

### Sequence information

|  |  |
| --- | --- |
| Name | FANPEZAQ\_CDS\_0042  42\_FANPEZAQ\_CDS\_0042 (pipeline id) |
| Imported annotations |  |
| Protein sequence | MPNRIRERRQAAGMTLQDVAEKLGTTAVTVSRWEREPQRVTLPILDRLADAIGCRKEELL SATAVTANALFFNDGVMSTMSAFYGLPAESLAVVKVVTDSMEPTLLNGDSCVIDKSISWI DNAGIYAIAMNGEARMVRCQRRIDGNVRVLCDNELYKFDEVCTDDDLEVIGKVIGLNRKI |
| Number of residues | 180 |
| Molecular weight (Da) | 19913.70 |
| Output files | ../../query\_sequences/42\_FANPEZAQ\_CDS\_0042.fasta |

### Putative domain architecture and protein family

#### Search results (HHblits)1

|  |  |
| --- | --- |
| Domain family databases searched | Pfam, Ncbi-cd, Cath, Phrogs |
| Results, scheme(s)  (Top layers only; threshold 1.00e-03 (evalue)) | xml version="1.0" encoding="utf-8" standalone="no"?       2024-09-02T21:08:21.681344 image/svg+xml   Matplotlib v3.7.2, https://matplotlib.org/ |
| Results, table  (E-value ≤ 1.00e-03 (evalue)) | | db | id | prob | evalue | pvalue | score | cols | query | query\_len | template | template\_len | name | description | | --- | --- | --- | --- | --- | --- | --- | --- | --- | --- | --- | --- | --- | | pfam | PF00717 | 98.2 | 3e-10 | 4.8e-14 | 64.0 | 84 | (89, 174) | 180 | (32, 116) | 116 | Peptidase\_S24 | Peptidase S24-like | | pfam | PF08667 | 98.1 | 6.6e-10 | 1.1e-13 | 68.0 | 121 | (4, 140) | 180 | (9, 137) | 174 | BetR | BetR domain | | pfam | PF16452 | 97.4 | 8.5e-08 | 1.4e-11 | 52.6 | 66 | (105, 175) | 180 | (36, 101) | 101 | Phage\_CI\_C | Bacteriophage CI repressor C-terminal domain | | ncbi-cd | cd06529 | 97.0 | 4.8e-07 | 9.1e-11 | 49.3 | 79 | (94, 174) | 180 | (3, 81) | 81 | S24\_LexA-like | cd06529 S24\_LexA-like; Peptidase S24 LexA-like proteins are involved in the SOS response leading to the repair of single-stranded DNA within the bacterial cell. | | ncbi-cd | cd06462 | 96.2 | 1.1e-05 | 2.1e-09 | 43.9 | 64 | (94, 157) | 180 | (3, 68) | 84 | Peptidase\_S24\_S26 | cd06462 Peptidase\_S24\_S26; The S24, S26 LexA/signal peptidase superfamily contains LexA-related and type I signal peptidase families. | | ncbi-cd | cd06530 | 94.9 | 0.00023 | 4.3e-08 | 38.4 | 55 | (94, 153) | 180 | (3, 63) | 85 | S26\_SPase\_I | cd06530 S26\_SPase\_I; The S26 Type I signal peptidase (SPase; LepB; leader peptidase B; leader peptidase I. | | cath | 1jheA00 | 98.5 | 2.7e-11 | 3.2e-15 | 70.0 | 89 | (88, 179) | 180 | (40, 131) | 135 | Lexa repressor | CATHCODE: 2.10.109.10 NAME: Lexa repressor. Chain: a, b. Fragment: c-terminus, residues 68-202. Engineered: yes. Mutation: yes SOURCE: Escherichia coli. Organism\_taxid: 562. Gene: lexa. Expressed in: escherichia coli. Expression\_system\_taxid: 562 CLASS: Mainly Beta, ARCH: Ribbon, TOPOL: Umud Fragment, subunit A, HOMOL: Umud Fragment, subunit A | | cath | 1ay9A00 | 98.4 | 7.2e-11 | 8.5e-15 | 65.6 | 90 | (86, 179) | 180 | (15, 106) | 108 | Umud protein | CATHCODE: 2.10.109.10 NAME: Umud protein. Chain: a, b. SOURCE: Escherichia coli. Organism\_taxid: 562 CLASS: Mainly Beta, ARCH: Ribbon, TOPOL: Umud Fragment, subunit A, HOMOL: Umud Fragment, subunit A | | cath | 3k2zA02 | 98.4 | 9e-11 | 1.1e-14 | 65.9 | 87 | (90, 179) | 180 | (31, 118) | 119 | Lexa repressor | CATHCODE: 2.10.109.10 NAME: Lexa repressor. Chain: a, b. Engineered: yes. Mutation: yes SOURCE: Thermotoga maritima. Organism\_taxid: 2336. Gene: lexa, tm\_1082. Expressed in: escherichia coli. Expression\_system\_taxid: 562. CLASS: Mainly Beta, ARCH: Ribbon, TOPOL: Umud Fragment, subunit A, HOMOL: Umud Fragment, subunit A | | cath | 1f39A00 | 98.2 | 3.7e-10 | 4.3e-14 | 61.7 | 86 | (89, 178) | 180 | (3, 95) | 101 | Repressor protein ci | CATHCODE: 2.10.109.10 NAME: Repressor protein ci. Chain: a, b. Fragment: c-terminal domain. Engineered: yes SOURCE: Enterobacteria phage lambda. Organism\_taxid: 10710. Expressed in: escherichia coli. Expression\_system\_taxid: 562. Expression\_system\_vector\_type: plasmid. CLASS: Mainly Beta, ARCH: Ribbon, TOPOL: Umud Fragment, subunit A, HOMOL: Umud Fragment, subunit A | | cath | 1b12C01 | 97.9 | 5.4e-09 | 6.4e-13 | 60.7 | 89 | (90, 179) | 180 | (5, 118) | 137 | Signal peptidase i | CATHCODE: 2.10.109.10 NAME: Signal peptidase i. Chain: a, b, c, d. Fragment: catalytic domain. Synonym: spase i, leader peptidase i. Engineered: yes SOURCE: Escherichia coli. Organism\_taxid: 469008. Strain: bl21(de3). Cellular\_location: periplasm. Gene: lepb. Expressed in: escherichia coli bl21(de3). Expression\_system\_taxid: 469008. CLASS: Mainly Beta, ARCH: Ribbon, TOPOL: Umud Fragment, subunit A, HOMOL: Umud Fragment, subunit A | | cath | 2fjrA02 | 97.2 | 3.4e-07 | 4.1e-11 | 50.8 | 67 | (106, 179) | 180 | (44, 111) | 113 | Repressor protein ci | CATHCODE: 2.10.109.10 NAME: Repressor protein ci. Chain: a, b. Engineered: yes. Mutation: yes SOURCE: Enterobacteria phage 186. Organism\_taxid: 29252. Gene: ci. Expressed in: escherichia coli. Expression\_system\_taxid: 562 CLASS: Mainly Beta, ARCH: Ribbon, TOPOL: Umud Fragment, subunit A, HOMOL: Umud Fragment, subunit A | | cath | 4k8wA00 | 94.7 | 0.00054 | 6.4e-08 | 37.7 | 44 | (98, 141) | 180 | (2, 45) | 129 | Lepa | CATHCODE: 2.10.109.10 NAME: Lepa. Chain: a. Fragment: unp residues 45-173. Engineered: yes SOURCE: Streptococcus pyogenes. Organism\_taxid: 1314. Strain: 90/306s. Gene: lepa, sipa. Expressed in: escherichia coli. Expression\_system\_taxid: 469008. CLASS: Mainly Beta, ARCH: Ribbon, TOPOL: Umud Fragment, subunit A, HOMOL: Umud Fragment, subunit A | | phrogs | 4 | 99.7 | 4.3e-23 | 5.2e-27 | 148.6 | 160 | (14, 179) | 180 | (22, 208) | 209 | transcriptional repressor | transcriptional repressor; Category: transcription regulation; p259758 VI\_06270 | | phrogs | 811 | 99.7 | 2.2e-21 | 2.5e-25 | 131.1 | 92 | (85, 179) | 180 | (68, 161) | 165 | UmuD-like protein | UmuD-like protein; Category: DNA, RNA and nucleotide metabolism; p6911 VI\_09232 | | phrogs | 6201 | 99.7 | 2.6e-21 | 3e-25 | 125.7 | 92 | (85, 178) | 180 | (38, 132) | 139 | CI-like repressor | CI-like repressor; Category: transcription regulation; p89843 VI\_01594 | | phrogs | 10470 | 99.0 | 6.3e-14 | 7e-18 | 79.2 | 73 | (103, 179) | 180 | (4, 77) | 79 | UmuD-like protein | UmuD-like protein; Category: DNA, RNA and nucleotide metabolism; p434438 VI\_09939 | | phrogs | 2328 | 98.7 | 1.9e-12 | 2.2e-16 | 83.0 | 63 | (1, 64) | 180 | (19, 82) | 123 | transcriptional repressor | transcriptional repressor; Category: transcription regulation; MG099936\_p34 | | phrogs | 8558 | 98.7 | 3.8e-12 | 4.3e-16 | 74.6 | 73 | (102, 179) | 180 | (8, 82) | 87 | NA | NA; Category: unknown function; p316397 VI\_07324 | | phrogs | 1373 | 98.5 | 1.6e-11 | 1.9e-15 | 75.6 | 57 | (2, 59) | 180 | (35, 92) | 95 | transcriptional regulator | transcriptional regulator; Category: transcription regulation; NC\_005857\_p48 | | phrogs | 2967 | 98.4 | 7.4e-11 | 8.6e-15 | 79.5 | 62 | (1, 63) | 180 | (7, 69) | 167 | transcriptional regulator | transcriptional regulator; Category: transcription regulation; p123578 VI\_00485 | | phrogs | 7241 | 98.4 | 8.2e-11 | 9.3e-15 | 73.6 | 57 | (5, 62) | 180 | (66, 123) | 128 | transcriptional regulator | transcriptional regulator; Category: transcription regulation; p63620 VI\_05511 | | phrogs | 4583 | 98.2 | 4.8e-10 | 5.5e-14 | 65.3 | 62 | (2, 64) | 180 | (12, 74) | 77 | transcriptional repressor | transcriptional repressor; Category: transcription regulation; NC\_031243\_p35 | | phrogs | 1381 | 98.0 | 1.7e-09 | 1.9e-13 | 66.4 | 60 | (3, 65) | 180 | (11, 72) | 99 | plasmid antitoxin with HTH domain | plasmid antitoxin with HTH domain; Category: moron, auxiliary metabolic gene and host takeover; p79485 VI\_11488 | | phrogs | 4836 | 98.0 | 1.8e-09 | 2e-13 | 73.0 | 65 | (2, 67) | 180 | (84, 149) | 233 | CII-like regulator | CII-like regulator; Category: transcription regulation; p13677 VI\_07483 | | phrogs | 2439 | 97.8 | 6.2e-09 | 7.2e-13 | 78.7 | 61 | (2, 63) | 180 | (9, 70) | 385 | transcriptional regulator | transcriptional regulator; Category: transcription regulation; p298790 VI\_12393 | | phrogs | 147 | 97.8 | 8.4e-09 | 9.7e-13 | 64.3 | 67 | (1, 68) | 180 | (13, 82) | 112 | transcriptional regulator | transcriptional regulator; Category: transcription regulation; p315289 VI\_07968 | | phrogs | 2104 | 97.8 | 1e-08 | 1.2e-12 | 61.5 | 39 | (3, 42) | 180 | (33, 71) | 92 | NA | NA; Category: unknown function; p72070 VI\_03178 | | phrogs | 1468 | 97.6 | 2.5e-08 | 2.9e-12 | 56.5 | 56 | (2, 58) | 180 | (12, 67) | 69 | transcriptional repressor | transcriptional repressor; Category: transcription regulation; p307668 VI\_06058 | | phrogs | 4779 | 97.6 | 3.3e-08 | 3.8e-12 | 57.6 | 35 | (2, 36) | 180 | (3, 37) | 78 | transcriptional regulator | transcriptional regulator; Category: transcription regulation; p225361 VI\_06249 | | phrogs | 5823 | 97.6 | 3.8e-08 | 4.4e-12 | 74.0 | 63 | (1, 64) | 180 | (9, 74) | 410 | transcriptional regulator | transcriptional regulator; Category: transcription regulation; NC\_028952\_p26 | | phrogs | 2339 | 97.5 | 5.4e-08 | 6.3e-12 | 58.5 | 53 | (4, 56) | 180 | (32, 85) | 93 | HTH DNA binding protein | HTH DNA binding protein; Category: DNA, RNA and nucleotide metabolism; p425338 VI\_07226 | | phrogs | 6831 | 97.4 | 7.8e-08 | 8.9e-12 | 53.3 | 43 | (8, 50) | 180 | (10, 54) | 66 | transcriptional regulator | transcriptional regulator; Category: transcription regulation; p431926 VI\_12195 | | phrogs | 769 | 97.4 | 8e-08 | 9.2e-12 | 57.4 | 63 | (3, 67) | 180 | (4, 67) | 90 | transcriptional repressor | transcriptional repressor; Category: transcription regulation; p166433 VI\_07600 | | phrogs | 1337 | 97.4 | 1.1e-07 | 1.2e-11 | 52.8 | 52 | (2, 53) | 180 | (4, 57) | 61 | DNA binding protein | DNA binding protein; Category: DNA, RNA and nucleotide metabolism; p177899 VI\_00920 | | phrogs | 638 | 97.4 | 1.1e-07 | 1.3e-11 | 62.5 | 62 | (3, 65) | 180 | (5, 68) | 148 | transcriptional regulator | transcriptional regulator; Category: transcription regulation; p438490 VI\_02648 | | phrogs | 34939 | 97.2 | 3.3e-07 | 3.7e-11 | 61.7 | 66 | (3, 68) | 180 | (12, 78) | 276 | NA | NA; Category: unknown function; p54066 VI\_01167 | | phrogs | 3037 | 97.0 | 8.4e-07 | 9.6e-11 | 60.8 | 54 | (13, 67) | 180 | (29, 83) | 215 | transcriptional repressor | transcriptional repressor; Category: transcription regulation; p321370 VI\_07179 | | phrogs | 13326 | 97.0 | 8.6e-07 | 9.6e-11 | 55.8 | 64 | (3, 67) | 180 | (5, 71) | 161 | NA | NA; Category: unknown function; p201792 VI\_05516 | | phrogs | 4441 | 97.0 | 1e-06 | 1.2e-10 | 53.9 | 52 | (11, 63) | 180 | (14, 67) | 109 | HTH DNA binding protein | HTH DNA binding protein; Category: DNA, RNA and nucleotide metabolism; p152087 VI\_05530 | | phrogs | 9103 | 96.9 | 1.2e-06 | 1.3e-10 | 50.0 | 60 | (5, 65) | 180 | (13, 74) | 78 | transcriptional repressor | transcriptional repressor; Category: transcription regulation; KY624611\_p11 | | phrogs | 3642 | 96.9 | 1.3e-06 | 1.5e-10 | 51.5 | 51 | (4, 55) | 180 | (9, 60) | 90 | HTH DNA binding protein | HTH DNA binding protein; Category: DNA, RNA and nucleotide metabolism; p184909 VI\_05451 | | phrogs | 4739 | 96.9 | 1.4e-06 | 1.6e-10 | 57.0 | 64 | (3, 67) | 180 | (71, 135) | 216 | NA | NA; Category: unknown function; p402726 VI\_08646 | | phrogs | 35 | 96.8 | 1.7e-06 | 2.1e-10 | 49.9 | 57 | (4, 62) | 180 | (8, 67) | 75 | transcriptional repressor | transcriptional repressor; Category: transcription regulation; p323300 VI\_10199 | | phrogs | 3557 | 96.8 | 1.9e-06 | 2.1e-10 | 53.9 | 62 | (2, 65) | 180 | (15, 77) | 148 | transcriptional regulator | transcriptional regulator; Category: transcription regulation; p437537 VI\_09606 | | phrogs | 7950 | 96.8 | 2.6e-06 | 2.9e-10 | 45.0 | 39 | (2, 41) | 180 | (2, 40) | 53 | transcriptional repressor | transcriptional repressor; Category: transcription regulation; p141193 VI\_04895 | | phrogs | 18342 | 96.7 | 2.9e-06 | 3.2e-10 | 51.7 | 59 | (5, 63) | 180 | (23, 83) | 123 | transcriptional repressor | transcriptional repressor; Category: transcription regulation; p158151 VI\_11365 | | phrogs | 1702 | 96.7 | 2.9e-06 | 3.3e-10 | 51.6 | 63 | (2, 65) | 180 | (30, 96) | 112 | transcriptional repressor | transcriptional repressor; Category: transcription regulation; NC\_022052\_p39 | | phrogs | 6463 | 96.7 | 3.8e-06 | 4.3e-10 | 47.4 | 57 | (5, 62) | 180 | (3, 66) | 77 | HTH DNA binding protein | HTH DNA binding protein; Category: DNA, RNA and nucleotide metabolism; NC\_004683\_p47 | | phrogs | 5736 | 96.6 | 4.8e-06 | 5.3e-10 | 51.8 | 60 | (5, 65) | 180 | (11, 71) | 151 | transcriptional regulator | transcriptional regulator; Category: transcription regulation; p339615 VI\_06353 | | phrogs | 7126 | 96.6 | 4.9e-06 | 5.6e-10 | 51.6 | 52 | (13, 64) | 180 | (31, 83) | 123 | HTH DNA binding protein | HTH DNA binding protein; Category: DNA, RNA and nucleotide metabolism; NC\_031107\_p106 | | phrogs | 925 | 96.6 | 5.3e-06 | 6.1e-10 | 47.7 | 56 | (2, 58) | 180 | (15, 71) | 76 | HTH DNA binding protein | HTH DNA binding protein; Category: DNA, RNA and nucleotide metabolism; p18585 VI\_03080 | | phrogs | 1994 | 96.5 | 7.1e-06 | 7.9e-10 | 52.5 | 61 | (3, 64) | 180 | (2, 63) | 170 | DNA binding protein | DNA binding protein; Category: DNA, RNA and nucleotide metabolism; NC\_031023\_p45 | | phrogs | 18104 | 96.4 | 1.3e-05 | 1.4e-09 | 42.0 | 48 | (3, 53) | 180 | (5, 53) | 56 | transcriptional regulator | transcriptional regulator; Category: transcription regulation; NC\_025471\_p63 | | phrogs | 3949 | 96.3 | 1.4e-05 | 1.6e-09 | 51.5 | 58 | (5, 63) | 180 | (5, 64) | 163 | transcriptional repressor | transcriptional repressor; Category: transcription regulation; JQ300538\_p45 | | phrogs | 6223 | 96.3 | 1.7e-05 | 1.9e-09 | 44.9 | 33 | (10, 42) | 180 | (14, 46) | 77 | transcriptional repressor | transcriptional repressor; Category: transcription regulation; p192116 VI\_09192 | | phrogs | 955 | 96.1 | 3.2e-05 | 3.6e-09 | 50.2 | 65 | (1, 65) | 180 | (56, 123) | 174 | HTH DNA binding protein | HTH DNA binding protein; Category: DNA, RNA and nucleotide metabolism; JN699010\_p46 | | phrogs | 4739 | 96.1 | 3.3e-05 | 3.7e-09 | 50.3 | 64 | (1, 65) | 180 | (149, 213) | 216 | NA | NA; Category: unknown function; p402726 VI\_08646 | | phrogs | 7396 | 96.0 | 3.5e-05 | 4e-09 | 52.1 | 66 | (1, 67) | 180 | (13, 81) | 233 | NA | NA; Category: unknown function; p13562 VI\_08594 | | phrogs | 4268 | 96.0 | 3.7e-05 | 4.2e-09 | 45.4 | 50 | (4, 56) | 180 | (39, 89) | 94 | transcriptional repressor | transcriptional repressor; Category: transcription regulation; p79532 VI\_11488 | | phrogs | 8 | 96.0 | 3.8e-05 | 4.5e-09 | 46.1 | 41 | (14, 55) | 180 | (1, 42) | 95 | transcriptional regulator | transcriptional regulator; Category: transcription regulation; p309030 VI\_07697 | | phrogs | 18318 | 95.9 | 5.1e-05 | 5.8e-09 | 41.8 | 54 | (4, 57) | 180 | (12, 66) | 76 | HTH DNA binding protein | HTH DNA binding protein; Category: DNA, RNA and nucleotide metabolism; KU574722\_p511 | | phrogs | 38027 | 95.9 | 5.3e-05 | 5.9e-09 | 44.7 | 58 | (4, 62) | 180 | (61, 119) | 132 | transcriptional repressor | transcriptional repressor; Category: transcription regulation; NC\_030931\_p18 | | phrogs | 18068 | 95.9 | 5.4e-05 | 6e-09 | 46.1 | 60 | (5, 67) | 180 | (6, 66) | 139 | NA | NA; Category: unknown function; KR093642\_p39 | | phrogs | 14076 | 95.8 | 6.5e-05 | 7.3e-09 | 42.4 | 34 | (3, 36) | 180 | (17, 55) | 81 | transcriptional regulator | transcriptional regulator; Category: transcription regulation; p109717 VI\_00077 | | phrogs | 1529 | 95.8 | 6.5e-05 | 7.4e-09 | 41.3 | 38 | (2, 41) | 180 | (2, 39) | 65 | NA | NA; Category: unknown function; p439749 VI\_04529 | | phrogs | 32768 | 95.8 | 7.3e-05 | 8.1e-09 | 53.0 | 62 | (1, 62) | 180 | (10, 72) | 446 | NA | NA; Category: unknown function; KT151957\_p71 | | phrogs | 5301 | 95.6 | 9.9e-05 | 1.1e-08 | 45.8 | 52 | (10, 62) | 180 | (21, 73) | 143 | transcriptional repressor | transcriptional repressor; Category: transcription regulation; p166315 VI\_04146 | | phrogs | 38278 | 95.6 | 0.00011 | 1.2e-08 | 46.3 | 53 | (1, 55) | 180 | (18, 71) | 180 | NA | NA; Category: unknown function; p17072 VI\_01301 | | phrogs | 296 | 95.6 | 0.00011 | 1.3e-08 | 53.5 | 35 | (2, 36) | 180 | (7, 41) | 312 | DNA transposition protein | DNA transposition protein; Category: integration and excision; p199288 VI\_08191 | | phrogs | 517 | 95.5 | 0.00013 | 1.5e-08 | 43.9 | 31 | (5, 36) | 180 | (73, 103) | 107 | HTH DNA binding protein | HTH DNA binding protein; Category: DNA, RNA and nucleotide metabolism; MF668278\_p45 | | phrogs | 6962 | 95.5 | 0.00014 | 1.6e-08 | 38.4 | 33 | (4, 36) | 180 | (5, 37) | 59 | NA | NA; Category: unknown function; p75562 VI\_02384 | | phrogs | 725 | 95.4 | 0.00016 | 1.8e-08 | 43.7 | 41 | (12, 55) | 180 | (28, 68) | 106 | CII-like regulator | CII-like regulator; Category: transcription regulation; p72240 VI\_06449 | | phrogs | 3 | 95.3 | 0.00018 | 2.2e-08 | 41.3 | 50 | (2, 54) | 180 | (7, 59) | 80 | transcriptional repressor | transcriptional repressor; Category: transcription regulation; p29899 VI\_00998 | | phrogs | 4031 | 95.2 | 0.00022 | 2.5e-08 | 54.5 | 55 | (3, 58) | 180 | (518, 574) | 622 | NA | NA; Category: unknown function; p115970 VI\_05096 | | phrogs | 6499 | 95.1 | 0.00026 | 3e-08 | 45.7 | 51 | (5, 56) | 180 | (13, 70) | 157 | transcriptional regulator | transcriptional regulator; Category: transcription regulation; p14253 VI\_01278 | | phrogs | 4151 | 95.1 | 0.00028 | 3.2e-08 | 46.2 | 27 | (10, 36) | 180 | (17, 43) | 168 | DNA binding protein | DNA binding protein; Category: DNA, RNA and nucleotide metabolism; p284557 VI\_05952 | | phrogs | 34341 | 95.0 | 0.00032 | 3.6e-08 | 40.6 | 34 | (3, 36) | 180 | (74, 107) | 122 | transcriptional regulator | transcriptional regulator; Category: transcription regulation; MF360957\_p141 | | phrogs | 24325 | 95.0 | 0.00033 | 3.6e-08 | 41.2 | 61 | (2, 62) | 180 | (54, 115) | 133 | transcriptional regulator | transcriptional regulator; Category: transcription regulation; p318984 VI\_12108 | | phrogs | 2538 | 94.8 | 0.00043 | 5e-08 | 42.5 | 28 | (9, 36) | 180 | (12, 39) | 113 | terminase small subunit | terminase small subunit; Category: head and packaging; p133796 VI\_05051 | | phrogs | 17822 | 94.8 | 0.00047 | 5.3e-08 | 46.3 | 63 | (3, 67) | 180 | (10, 73) | 267 | NA | NA; Category: unknown function; p205501 VI\_03162 | | phrogs | 15562 | 94.8 | 0.00048 | 5.5e-08 | 44.4 | 49 | (94, 145) | 180 | (31, 80) | 185 | NA | NA; Category: unknown function; p342664 VI\_05383 | | phrogs | 1124 | 94.7 | 0.00051 | 5.9e-08 | 45.7 | 39 | (12, 52) | 180 | (16, 55) | 182 | transcriptional regulator | transcriptional regulator; Category: transcription regulation; p275302 VI\_05197 | | phrogs | 409 | 94.7 | 0.00053 | 6.2e-08 | 38.6 | 28 | (7, 36) | 180 | (5, 32) | 72 | transcriptional regulator | transcriptional regulator; Category: transcription regulation; p88852 VI\_07592 | | phrogs | 19579 | 94.6 | 0.00059 | 6.6e-08 | 44.7 | 51 | (12, 63) | 180 | (14, 65) | 225 | transcriptional regulator | transcriptional regulator; Category: transcription regulation; p68909 VI\_04167 | | phrogs | 7676 | 94.4 | 0.0008 | 9e-08 | 36.9 | 26 | (11, 36) | 180 | (14, 39) | 75 | NA | NA; Category: unknown function; NC\_023688\_p227 | |
| Top keywords  (threshold 1.00e-03 (evalue)) | **transcription, regulation, transcriptional, DNA, repressor, regulator, A, and, Fragment, Umud** |
| Output files | ../../domain\_architecture/42\_FANPEZAQ\_CDS\_0042\_cath.hhr ../../domain\_architecture/42\_FANPEZAQ\_CDS\_0042\_merged.svg ../../domain\_architecture/42\_FANPEZAQ\_CDS\_0042\_ncbi-cd.hhr ../../domain\_architecture/42\_FANPEZAQ\_CDS\_0042\_pfam.hhr ../../domain\_architecture/42\_FANPEZAQ\_CDS\_0042\_phrogs.hhr |

### Identical protein sequences/structures

#### Search results

|  |  |
| --- | --- |
| Protein sequence databases searched | Pdb, Swissprot, Refseq |
| Identical proteins found | -- |
| Top keywords | -- |
| Output files | -- |

### Similar protein sequences/structures

#### Sequence similarity search results (HHblits)1

|  |  |
| --- | --- |
| Sequence databases searched | Uniclust, Pdb70 |
| Results, scheme(s)  (Top layers only, threshold 1.00e-03 (evalue)) | xml version="1.0" encoding="utf-8" standalone="no"?       2024-09-02T21:08:48.043791 image/svg+xml   Matplotlib v3.7.2, https://matplotlib.org/ |
| Results, table(s)  (threshold 1.00e-03 (evalue)) | | db | id | prob | evalue | pvalue | score | cols | query | query\_len | template | template\_len | name | description | | --- | --- | --- | --- | --- | --- | --- | --- | --- | --- | --- | --- | --- | | uniclust | UniRef100\_A0A078LJD5 | 100.0 | 3.8e-37 | 7.1e-43 | 225.0 | 179 | (1, 180) | 180 | (78, 299) | 306 | Phage repressor protein | Phage repressor protein | | uniclust | UniRef100\_A0A0C1R042 | 100.0 | 2.3e-35 | 4.3e-41 | 203.2 | 177 | (1, 180) | 180 | (27, 225) | 228 | Cro/Cl family transcriptional regulator | Cro/Cl family transcriptional regulator | | uniclust | UniRef100\_A0A0F9H182 | 100.0 | 5.1e-35 | 9.5e-41 | 208.8 | 179 | (1, 180) | 180 | (37, 251) | 253 | HTH cro/C1-type domain-containing protein | HTH cro/C1-type domain-containing protein | | uniclust | UniRef100\_A0A521VUF4 | 100.0 | 4.6e-34 | 8.6e-40 | 194.6 | 178 | (1, 180) | 180 | (24, 234) | 236 | Peptidase S24 (Fragment) | Peptidase S24 (Fragment) | | uniclust | UniRef100\_A0A1M7T7T0 | 100.0 | 5.7e-34 | 1.1e-39 | 196.7 | 178 | (1, 179) | 180 | (30, 265) | 267 | Phage repressor protein C, contains Cro/C1-type HTH and peptisase s24 domains | Phage repressor protein C, contains Cro/C1-type HTH and peptisase s24 domains | | uniclust | UniRef100\_A0A078BMD6 | 100.0 | 7.9e-34 | 1.5e-39 | 205.2 | 177 | (1, 180) | 180 | (42, 256) | 280 | Putative phage repressor | Putative phage repressor | | uniclust | UniRef100\_A0A2G2CXL5 | 100.0 | 1.1e-33 | 2.1e-39 | 197.9 | 178 | (1, 180) | 180 | (33, 250) | 253 | HTH cro/C1-type domain-containing protein | HTH cro/C1-type domain-containing protein | | uniclust | UniRef100\_A0A075WVE9 | 100.0 | 1.8e-33 | 3.4e-39 | 196.5 | 178 | (1, 179) | 180 | (37, 241) | 244 | HTH cro/C1-type domain-containing protein | HTH cro/C1-type domain-containing protein | | uniclust | UniRef100\_A0A1W0D753 | 100.0 | 3.1e-33 | 5.7e-39 | 191.2 | 178 | (1, 179) | 180 | (29, 248) | 258 | HTH cro/C1-type domain-containing protein | HTH cro/C1-type domain-containing protein | | uniclust | UniRef100\_A0A139KEG7 | 100.0 | 3.5e-33 | 6.4e-39 | 188.4 | 176 | (2, 177) | 180 | (11, 222) | 227 | Peptidase S24-like protein | Peptidase S24-like protein | | uniclust | UniRef100\_A0A1V4GWD0 | 100.0 | 7.8e-33 | 1.5e-38 | 194.8 | 177 | (1, 179) | 180 | (23, 230) | 241 | HTH cro/C1-type domain-containing protein | HTH cro/C1-type domain-containing protein | | uniclust | UniRef100\_A0A066TBR7 | 100.0 | 9.6e-33 | 1.8e-38 | 197.0 | 178 | (1, 180) | 180 | (68, 299) | 315 | Putative transcriptional regulator | Putative transcriptional regulator | | uniclust | UniRef100\_A0A099K7Y6 | 100.0 | 1e-32 | 1.9e-38 | 204.6 | 177 | (1, 180) | 180 | (85, 301) | 327 | Phage C1 repressor | Phage C1 repressor | | uniclust | UniRef100\_A0A0C1W593 | 100.0 | 1.3e-32 | 2.4e-38 | 190.8 | 176 | (1, 177) | 180 | (16, 218) | 233 | Phage repressor protein | Phage repressor protein | | uniclust | UniRef100\_A0A022N9X9 | 100.0 | 2.3e-32 | 4.2e-38 | 196.3 | 178 | (1, 180) | 180 | (40, 271) | 275 | Peptidase S24 | Peptidase S24 | | uniclust | UniRef100\_A0A075CI58 | 99.9 | 3.7e-32 | 6.9e-38 | 192.3 | 165 | (15, 180) | 180 | (55, 256) | 275 | C repressor | C repressor | | uniclust | UniRef100\_A0A1H9B3Z2 | 99.9 | 7.6e-32 | 1.4e-37 | 184.9 | 178 | (1, 180) | 180 | (20, 228) | 230 | Helix-turn-helix | Helix-turn-helix | | uniclust | UniRef100\_A0A0S4SC53 | 99.9 | 8.1e-32 | 1.5e-37 | 190.9 | 178 | (1, 179) | 180 | (10, 233) | 253 | DNA-binding protein | DNA-binding protein | | uniclust | UniRef100\_A0A0J5GKK0 | 99.9 | 8.7e-32 | 1.6e-37 | 189.0 | 178 | (1, 178) | 180 | (29, 260) | 263 | HTH cro/C1-type domain-containing protein | HTH cro/C1-type domain-containing protein | | uniclust | UniRef100\_A0A0J7Y5V7 | 99.9 | 1.1e-31 | 2.1e-37 | 188.1 | 177 | (2, 180) | 180 | (37, 252) | 255 | HTH cro/C1-type domain-containing protein | HTH cro/C1-type domain-containing protein | | uniclust | UniRef100\_A0A011TVH5 | 99.9 | 1.3e-31 | 2.5e-37 | 187.6 | 179 | (1, 179) | 180 | (40, 259) | 264 | Transcriptional regulator | Transcriptional regulator | | uniclust | UniRef100\_A0A086WQD3 | 99.9 | 1.4e-31 | 2.6e-37 | 188.4 | 177 | (2, 179) | 180 | (26, 245) | 254 | Prophage transcriptional regulator | Prophage transcriptional regulator | | uniclust | UniRef100\_A0A013SC04 | 99.9 | 1.7e-31 | 3.2e-37 | 194.6 | 178 | (1, 178) | 180 | (49, 275) | 309 | Peptidase S24-like family protein | Peptidase S24-like family protein | | uniclust | UniRef100\_A0A060H7F8 | 99.9 | 3.1e-31 | 5.7e-37 | 190.7 | 173 | (1, 176) | 180 | (42, 269) | 281 | Peptidase S24 | Peptidase S24 | | uniclust | UniRef100\_A0A0G3CSZ6 | 99.9 | 3.6e-31 | 6.7e-37 | 182.3 | 176 | (1, 179) | 180 | (31, 243) | 246 | HTH cro/C1-type domain-containing protein | HTH cro/C1-type domain-containing protein | | uniclust | UniRef100\_A0A090IB00 | 99.9 | 4.4e-31 | 8.1e-37 | 187.4 | 178 | (1, 179) | 180 | (36, 251) | 254 | Uncharacterized phage protein, peptidase S24 family | Uncharacterized phage protein, peptidase S24 family | | uniclust | UniRef100\_A0A081NQ11 | 99.9 | 4.8e-31 | 8.9e-37 | 186.7 | 177 | (1, 179) | 180 | (55, 257) | 264 | Helix-turn-helix domain-containing protein | Helix-turn-helix domain-containing protein | | uniclust | UniRef100\_A0A095YT12 | 99.9 | 5.2e-31 | 9.7e-37 | 185.9 | 176 | (1, 179) | 180 | (23, 226) | 236 | HTH cro/C1-type domain-containing protein | HTH cro/C1-type domain-containing protein | | uniclust | UniRef100\_A0A0B1TYP4 | 99.9 | 6.3e-31 | 1.2e-36 | 188.3 | 176 | (3, 180) | 180 | (48, 270) | 284 | Putative transcriptional regulator | Putative transcriptional regulator | | uniclust | UniRef100\_A0A011VKK5 | 99.9 | 7.3e-31 | 1.3e-36 | 189.8 | 178 | (2, 180) | 180 | (69, 294) | 295 | Transcriptional regulator | Transcriptional regulator | | uniclust | UniRef100\_A0A098ELK1 | 99.9 | 8.1e-31 | 1.5e-36 | 188.5 | 178 | (1, 179) | 180 | (42, 245) | 255 | LexA repressor | LexA repressor | | uniclust | UniRef100\_A0A0K6IU92 | 99.9 | 9.8e-31 | 1.8e-36 | 188.8 | 177 | (2, 179) | 180 | (23, 313) | 316 | Peptidase S24-like/Helix-turn-helix | Peptidase S24-like/Helix-turn-helix | | uniclust | UniRef100\_A0A074V8P9 | 99.9 | 1e-30 | 1.9e-36 | 193.1 | 177 | (1, 179) | 180 | (54, 275) | 304 | Putative transcriptional regulator | Putative transcriptional regulator | | uniclust | UniRef100\_A0A0A2XT17 | 99.9 | 1.1e-30 | 2e-36 | 183.7 | 174 | (1, 176) | 180 | (22, 234) | 245 | Transcriptional regulator | Transcriptional regulator | | uniclust | UniRef100\_A0A1Y6CX45 | 99.9 | 1.1e-30 | 2e-36 | 180.5 | 176 | (1, 180) | 180 | (26, 240) | 242 | Phage repressor protein C, contains Cro/C1-type HTH and peptisase s24 domains | Phage repressor protein C, contains Cro/C1-type HTH and peptisase s24 domains | | uniclust | UniRef100\_A0A096ALY3 | 99.9 | 1.4e-30 | 2.6e-36 | 184.2 | 177 | (1, 180) | 180 | (34, 239) | 248 | HTH cro/C1-type domain-containing protein | HTH cro/C1-type domain-containing protein | | uniclust | UniRef100\_A0A094YT33 | 99.9 | 2e-30 | 3.8e-36 | 181.5 | 178 | (2, 180) | 180 | (28, 253) | 254 | Putative transcriptional regulator | Putative transcriptional regulator | | uniclust | UniRef100\_A0A101ETK8 | 99.9 | 2.2e-30 | 4e-36 | 173.5 | 176 | (1, 178) | 180 | (11, 213) | 230 | Putative prophage repressor | Putative prophage repressor | | uniclust | UniRef100\_A0A132DZT8 | 99.9 | 2.1e-30 | 4e-36 | 185.2 | 175 | (5, 180) | 180 | (47, 261) | 276 | Uncharacterized protein | Uncharacterized protein | | uniclust | UniRef100\_A0A1C5ZA08 | 99.9 | 2.2e-30 | 4.1e-36 | 176.4 | 178 | (1, 180) | 180 | (12, 219) | 222 | LexA repressor | LexA repressor | | uniclust | UniRef100\_A0A0H2MAD8 | 99.9 | 2.7e-30 | 5e-36 | 181.9 | 178 | (1, 180) | 180 | (37, 253) | 255 | Putative HTH-type transcriptional regulator | Putative HTH-type transcriptional regulator | | uniclust | UniRef100\_A0A084EVF3 | 99.9 | 2.9e-30 | 5.4e-36 | 182.7 | 178 | (2, 180) | 180 | (90, 312) | 313 | Putative HTH-type transcriptional regulator | Putative HTH-type transcriptional regulator | | uniclust | UniRef100\_A0A142BB31 | 99.9 | 3.6e-30 | 6.6e-36 | 174.1 | 175 | (1, 179) | 180 | (12, 200) | 203 | S24 family peptidase | S24 family peptidase | | uniclust | UniRef100\_A0A0Q2XP48 | 99.9 | 3.7e-30 | 6.9e-36 | 180.4 | 174 | (3, 180) | 180 | (35, 244) | 246 | HTH cro/C1-type domain-containing protein | HTH cro/C1-type domain-containing protein | | uniclust | UniRef100\_A0A1C3RIU3 | 99.9 | 4e-30 | 7.3e-36 | 175.5 | 177 | (1, 180) | 180 | (22, 235) | 237 | Putative Transcriptional regulator | Putative Transcriptional regulator | | uniclust | UniRef100\_A0A0A2XGJ6 | 99.9 | 4.3e-30 | 8e-36 | 178.9 | 177 | (1, 178) | 180 | (23, 241) | 247 | Transcriptional regulator | Transcriptional regulator | | uniclust | UniRef100\_A0A011VZ09 | 99.9 | 5.1e-30 | 9.4e-36 | 185.3 | 178 | (1, 180) | 180 | (56, 264) | 272 | LexA family transcriptional regulator | LexA family transcriptional regulator | | uniclust | UniRef100\_A0A173YIP3 | 99.9 | 5.4e-30 | 9.9e-36 | 176.0 | 177 | (1, 180) | 180 | (19, 227) | 230 | LexA repressor | LexA repressor | | uniclust | UniRef100\_A0A0Q7I1K8 | 99.9 | 5.9e-30 | 1.1e-35 | 179.1 | 177 | (4, 180) | 180 | (44, 261) | 262 | HTH cro/C1-type domain-containing protein | HTH cro/C1-type domain-containing protein | | uniclust | UniRef100\_A0A074VC69 | 99.9 | 6.1e-30 | 1.1e-35 | 183.0 | 175 | (1, 176) | 180 | (25, 243) | 280 | Putative transcriptional regulator | Putative transcriptional regulator | | uniclust | UniRef100\_A0A086PBI9 | 99.9 | 6.9e-30 | 1.3e-35 | 185.1 | 177 | (2, 180) | 180 | (83, 301) | 302 | Putative phage repressor | Putative phage repressor | | uniclust | UniRef100\_A0A248LHK2 | 99.9 | 7.4e-30 | 1.4e-35 | 172.7 | 178 | (2, 180) | 180 | (37, 260) | 261 | Repressor | Repressor | | uniclust | UniRef100\_A0A089HUS8 | 99.9 | 9.4e-30 | 1.7e-35 | 182.6 | 175 | (1, 179) | 180 | (61, 259) | 289 | HTH cro/C1-type domain-containing protein | HTH cro/C1-type domain-containing protein | | uniclust | UniRef100\_A0A0D8J1P7 | 99.9 | 9.5e-30 | 1.7e-35 | 171.1 | 174 | (1, 177) | 180 | (12, 213) | 219 | Helix-turn-helix domain-containing protein | Helix-turn-helix domain-containing protein | | uniclust | UniRef100\_A0A193GZ18 | 99.9 | 1e-29 | 1.8e-35 | 165.0 | 179 | (2, 180) | 180 | (11, 189) | 193 | Putative DNA binding helix-turn helix protein | Putative DNA binding helix-turn helix protein | | uniclust | UniRef100\_A0A017H4C1 | 99.9 | 1e-29 | 1.9e-35 | 181.7 | 177 | (1, 179) | 180 | (22, 231) | 271 | Peptidase S24 | Peptidase S24 | | uniclust | UniRef100\_A0A2E3AY80 | 99.9 | 1.2e-29 | 2.2e-35 | 169.9 | 174 | (2, 176) | 180 | (7, 216) | 222 | Peptidase S24 | Peptidase S24 | | uniclust | UniRef100\_A0A099GKA4 | 99.9 | 1.2e-29 | 2.2e-35 | 179.6 | 178 | (1, 179) | 180 | (46, 269) | 288 | Peptidase S24/S26A/S26B/S26C domain-containing protein | Peptidase S24/S26A/S26B/S26C domain-containing protein | | uniclust | UniRef100\_A0A1G0SGA6 | 99.9 | 1.3e-29 | 2.4e-35 | 169.7 | 177 | (1, 180) | 180 | (15, 226) | 227 | HTH cro/C1-type domain-containing protein | HTH cro/C1-type domain-containing protein | | uniclust | UniRef100\_A0A0Q1A481 | 99.9 | 1.6e-29 | 2.9e-35 | 173.5 | 180 | (1, 180) | 180 | (7, 221) | 226 | Uncharacterized protein | Uncharacterized protein | | uniclust | UniRef100\_A0A352NLU9 | 99.9 | 3.5e-29 | 6.5e-35 | 172.2 | 176 | (1, 179) | 180 | (59, 264) | 265 | LexA family transcriptional regulator | LexA family transcriptional regulator | | uniclust | UniRef100\_A0A073IPP6 | 99.9 | 3.6e-29 | 6.6e-35 | 175.6 | 176 | (1, 178) | 180 | (22, 244) | 252 | HTH cro/C1-type domain-containing protein | HTH cro/C1-type domain-containing protein | | uniclust | UniRef100\_A0A059L2Y0 | 99.9 | 5e-29 | 9.3e-35 | 184.3 | 175 | (2, 177) | 180 | (90, 320) | 370 | XRE family transcriptional regulator | XRE family transcriptional regulator | | uniclust | UniRef100\_A0A1G6D4T4 | 99.9 | 5.4e-29 | 1e-34 | 179.9 | 172 | (1, 178) | 180 | (75, 309) | 353 | Peptidase S24-like | Peptidase S24-like | | uniclust | UniRef100\_A0A080NM98 | 99.9 | 5.5e-29 | 1e-34 | 173.7 | 176 | (3, 180) | 180 | (53, 262) | 265 | LexA family transcriptional regulator | LexA family transcriptional regulator | | uniclust | UniRef100\_A0A062XFL7 | 99.9 | 5.7e-29 | 1e-34 | 178.3 | 176 | (1, 178) | 180 | (37, 267) | 275 | HTH cro/C1-type domain-containing protein | HTH cro/C1-type domain-containing protein | | uniclust | UniRef100\_A0A0Q8U4Q3 | 99.9 | 6.1e-29 | 1.1e-34 | 180.4 | 176 | (2, 179) | 180 | (33, 248) | 309 | Phage repressor protein | Phage repressor protein | | uniclust | UniRef100\_A0A009Z9C0 | 99.9 | 7.8e-29 | 1.4e-34 | 180.8 | 176 | (1, 176) | 180 | (55, 287) | 308 | Repressor protein CI | Repressor protein CI | | uniclust | UniRef100\_A0A062H0E0 | 99.9 | 9.4e-29 | 1.7e-34 | 167.0 | 178 | (1, 179) | 180 | (23, 235) | 238 | Helix-turn-helix family protein | Helix-turn-helix family protein | | uniclust | UniRef100\_A0A143B3W3 | 99.9 | 1e-28 | 1.9e-34 | 176.4 | 171 | (10, 180) | 180 | (58, 273) | 276 | LexA-related transcriptional regulator | LexA-related transcriptional regulator | | uniclust | UniRef100\_A0A3C1VZE1 | 99.9 | 1.4e-28 | 2.6e-34 | 168.7 | 174 | (1, 175) | 180 | (10, 225) | 233 | HTH cro/C1-type domain-containing protein | HTH cro/C1-type domain-containing protein | | uniclust | UniRef100\_A0A009FWP1 | 99.9 | 1.5e-28 | 2.7e-34 | 172.2 | 176 | (1, 177) | 180 | (16, 240) | 245 | Helix-turn-helix family protein | Helix-turn-helix family protein | | uniclust | UniRef100\_A0A009GET6 | 99.9 | 1.5e-28 | 2.7e-34 | 171.0 | 174 | (2, 176) | 180 | (43, 271) | 278 | Peptidase S24-like family protein | Peptidase S24-like family protein | | uniclust | UniRef100\_A0A060HCU5 | 99.9 | 1.6e-28 | 3e-34 | 175.5 | 176 | (1, 178) | 180 | (48, 264) | 271 | HTH cro/C1-type domain-containing protein | HTH cro/C1-type domain-containing protein | | uniclust | UniRef100\_A0A023D7Y1 | 99.9 | 2e-28 | 3.6e-34 | 172.6 | 175 | (2, 178) | 180 | (31, 251) | 256 | Transcriptional regulator phage repressor | Transcriptional regulator phage repressor | | uniclust | UniRef100\_A0A0K1F1X2 | 99.9 | 2.2e-28 | 4.1e-34 | 170.3 | 175 | (1, 177) | 180 | (23, 231) | 240 | HTH cro/C1-type domain-containing protein | HTH cro/C1-type domain-containing protein | | uniclust | UniRef100\_A0A023KYN1 | 99.9 | 2.3e-28 | 4.3e-34 | 178.6 | 175 | (1, 177) | 180 | (65, 282) | 314 | Helix-turn-helix transcriptional regulator | Helix-turn-helix transcriptional regulator | | uniclust | UniRef100\_A0A124G6Q3 | 99.9 | 2.5e-28 | 4.5e-34 | 167.5 | 178 | (1, 179) | 180 | (9, 243) | 245 | XRE family transcriptional regulator | XRE family transcriptional regulator | | uniclust | UniRef100\_A0A068A299 | 99.9 | 2.7e-28 | 5e-34 | 176.4 | 177 | (1, 179) | 180 | (34, 271) | 275 | Repressor | Repressor | | uniclust | UniRef100\_A0A013WWL1 | 99.9 | 3e-28 | 5.5e-34 | 175.5 | 177 | (3, 180) | 180 | (53, 262) | 275 | Peptidase S24 | Peptidase S24 | | uniclust | UniRef100\_A0A1F8XTB5 | 99.9 | 3e-28 | 5.6e-34 | 170.2 | 178 | (1, 180) | 180 | (46, 262) | 263 | HTH cro/C1-type domain-containing protein | HTH cro/C1-type domain-containing protein | | uniclust | UniRef100\_A0A1Y5FD13 | 99.9 | 4.3e-28 | 8e-34 | 163.6 | 174 | (4, 180) | 180 | (14, 217) | 218 | Peptidase S24/S26A/S26B/S26C domain-containing protein | Peptidase S24/S26A/S26B/S26C domain-containing protein | | uniclust | UniRef100\_A0A0F2J2G4 | 99.9 | 4.3e-28 | 8e-34 | 170.3 | 175 | (1, 178) | 180 | (44, 244) | 276 | Repressor protein | Repressor protein | | uniclust | UniRef100\_A0A0G4C027 | 99.9 | 4.5e-28 | 8.3e-34 | 172.1 | 177 | (1, 178) | 180 | (18, 249) | 255 | Peptidase S24-like protein | Peptidase S24-like protein | | uniclust | UniRef100\_A0A0B5QLH2 | 99.9 | 5.4e-28 | 9.9e-34 | 169.1 | 177 | (1, 179) | 180 | (21, 236) | 241 | HTH cro/C1-type domain-containing protein | HTH cro/C1-type domain-containing protein | | uniclust | UniRef100\_A0A073INQ6 | 99.9 | 6.6e-28 | 1.2e-33 | 162.9 | 176 | (1, 179) | 180 | (5, 228) | 238 | HTH cro/C1-type domain-containing protein | HTH cro/C1-type domain-containing protein | | uniclust | UniRef100\_A0A0R1Q5L8 | 99.9 | 7.1e-28 | 1.3e-33 | 163.9 | 174 | (2, 177) | 180 | (14, 211) | 218 | Peptidase s24-like protein | Peptidase s24-like protein | | uniclust | UniRef100\_A0A011VZ04 | 99.9 | 7.1e-28 | 1.3e-33 | 170.9 | 178 | (1, 179) | 180 | (14, 227) | 234 | DNA-binding protein | DNA-binding protein | | uniclust | UniRef100\_A0A011PI42 | 99.9 | 8.1e-28 | 1.5e-33 | 175.2 | 173 | (2, 176) | 180 | (86, 309) | 320 | Pyocin repressor protein | Pyocin repressor protein | | uniclust | UniRef100\_A0A1V5P064 | 99.9 | 8.3e-28 | 1.5e-33 | 169.0 | 179 | (1, 179) | 180 | (39, 248) | 265 | LexA repressor | LexA repressor | | uniclust | UniRef100\_A0A085ERU0 | 99.9 | 8.9e-28 | 1.7e-33 | 171.9 | 177 | (2, 179) | 180 | (36, 253) | 261 | Putative transcriptional regulator | Putative transcriptional regulator | | uniclust | UniRef100\_A0A134A0B4 | 99.9 | 9.9e-28 | 1.8e-33 | 172.0 | 176 | (1, 179) | 180 | (17, 260) | 267 | Peptidase S24-like protein | Peptidase S24-like protein | | uniclust | UniRef100\_A0A0M9GHV2 | 99.9 | 1.5e-27 | 2.8e-33 | 159.9 | 176 | (2, 179) | 180 | (27, 237) | 239 | Putative transcriptional regulator | Putative transcriptional regulator | | uniclust | UniRef100\_A0A1R1MJE6 | 99.9 | 1.6e-27 | 3e-33 | 162.4 | 177 | (1, 179) | 180 | (21, 213) | 216 | HTH cro/C1-type domain-containing protein | HTH cro/C1-type domain-containing protein | | uniclust | UniRef100\_A0A1F9FXD4 | 99.9 | 1.8e-27 | 3.4e-33 | 162.8 | 176 | (1, 179) | 180 | (26, 230) | 232 | HTH cro/C1-type domain-containing protein | HTH cro/C1-type domain-containing protein | | uniclust | UniRef100\_A0A074VB08 | 99.9 | 2.2e-27 | 4.1e-33 | 161.2 | 176 | (1, 178) | 180 | (24, 241) | 245 | HTH cro/C1-type domain-containing protein | HTH cro/C1-type domain-containing protein | | uniclust | UniRef100\_A0A084J761 | 99.9 | 2.2e-27 | 4.1e-33 | 174.8 | 175 | (1, 177) | 180 | (52, 283) | 335 | HTH cro/C1-type domain-containing protein | HTH cro/C1-type domain-containing protein | | uniclust | UniRef100\_A0A7C2V4D0 | 99.9 | 2.3e-27 | 4.2e-33 | 159.4 | 174 | (1, 177) | 180 | (19, 215) | 224 | Helix-turn-helix domain-containing protein | Helix-turn-helix domain-containing protein | | uniclust | UniRef100\_A0A081NDK8 | 99.9 | 3.1e-27 | 5.7e-33 | 158.6 | 166 | (14, 180) | 180 | (33, 231) | 232 | Repressor | Repressor | | uniclust | UniRef100\_A0A031IUW7 | 99.9 | 3.7e-27 | 6.9e-33 | 172.0 | 173 | (2, 176) | 180 | (78, 308) | 321 | Putative phage repressor | Putative phage repressor | | uniclust | UniRef100\_A0A926HWW3 | 99.9 | 3.8e-27 | 7e-33 | 153.6 | 176 | (2, 180) | 180 | (3, 185) | 186 | Helix-turn-helix domain-containing protein | Helix-turn-helix domain-containing protein | | uniclust | UniRef100\_A0A0Q8TCT1 | 99.9 | 4.6e-27 | 8.5e-33 | 163.0 | 175 | (5, 180) | 180 | (38, 241) | 242 | Peptidase S24/S26A/S26B/S26C domain-containing protein | Peptidase S24/S26A/S26B/S26C domain-containing protein | | uniclust | UniRef100\_A0A072NUH5 | 99.9 | 5e-27 | 9.3e-33 | 169.4 | 175 | (1, 178) | 180 | (15, 280) | 298 | SOS response transcriptional repressor, RecA-mediated autopeptidase | SOS response transcriptional repressor, RecA-mediated autopeptidase | | uniclust | UniRef100\_A0A075SG92 | 99.9 | 5.4e-27 | 1e-32 | 173.0 | 177 | (1, 179) | 180 | (76, 319) | 337 | Repressor | Repressor | | uniclust | UniRef100\_A0A0R1VT03 | 99.9 | 5.9e-27 | 1.1e-32 | 162.0 | 173 | (1, 175) | 180 | (21, 252) | 253 | Phage repressor like XRE family transcriptional regulator | Phage repressor like XRE family transcriptional regulator | | uniclust | UniRef100\_A0A060H2Y3 | 99.9 | 6.3e-27 | 1.2e-32 | 168.8 | 174 | (1, 175) | 180 | (82, 314) | 358 | Peptidase S24, S26A and S26B | Peptidase S24, S26A and S26B | | uniclust | UniRef100\_A0A0R1LCU9 | 99.9 | 6.4e-27 | 1.2e-32 | 163.8 | 172 | (1, 175) | 180 | (37, 269) | 286 | Peptidase S24-like protein | Peptidase S24-like protein | | uniclust | UniRef100\_A0A1B3JHZ4 | 99.9 | 6.7e-27 | 1.2e-32 | 159.8 | 175 | (1, 176) | 180 | (14, 238) | 247 | Phage repressor | Phage repressor | | uniclust | UniRef100\_A0A1C6GZH6 | 99.9 | 6.8e-27 | 1.3e-32 | 166.7 | 168 | (1, 176) | 180 | (22, 249) | 306 | LexA repressor | LexA repressor | | uniclust | UniRef100\_A0A0J6S859 | 99.9 | 7.4e-27 | 1.4e-32 | 166.6 | 171 | (1, 174) | 180 | (31, 275) | 283 | HTH cro/C1-type domain-containing protein | HTH cro/C1-type domain-containing protein | | uniclust | UniRef100\_A0A1W1Y2G5 | 99.9 | 8.4e-27 | 1.6e-32 | 165.7 | 177 | (1, 179) | 180 | (23, 247) | 265 | Phage repressor protein C, contains Cro/C1-type HTH and peptisase s24 domains | Phage repressor protein C, contains Cro/C1-type HTH and peptisase s24 domains | | uniclust | UniRef100\_A0A212QLE3 | 99.9 | 8.8e-27 | 1.6e-32 | 163.4 | 178 | (1, 179) | 180 | (38, 276) | 277 | Phage repressor protein C, contains Cro/C1-type HTH and peptisase s24 domains | Phage repressor protein C, contains Cro/C1-type HTH and peptisase s24 domains | | uniclust | UniRef100\_A0A024L3W0 | 99.9 | 9.1e-27 | 1.7e-32 | 171.1 | 175 | (1, 179) | 180 | (69, 281) | 312 | Helix-turn-helix domain-containing protein | Helix-turn-helix domain-containing protein | | uniclust | UniRef100\_A0A015NCW0 | 99.9 | 9.8e-27 | 1.8e-32 | 171.3 | 177 | (2, 178) | 180 | (62, 292) | 297 | HTH cro/C1-type domain-containing protein | HTH cro/C1-type domain-containing protein | | uniclust | UniRef100\_A0A1G7D0E1 | 99.9 | 1.3e-26 | 2.4e-32 | 154.8 | 164 | (16, 179) | 180 | (35, 224) | 226 | Peptidase S24-like | Peptidase S24-like | | uniclust | UniRef100\_A0A968IL67 | 99.9 | 1.7e-26 | 3.1e-32 | 151.1 | 175 | (3, 180) | 180 | (28, 235) | 237 | Helix-turn-helix domain-containing protein | Helix-turn-helix domain-containing protein | | uniclust | UniRef100\_A0A009PV59 | 99.9 | 1.7e-26 | 3.2e-32 | 162.4 | 178 | (1, 179) | 180 | (47, 256) | 271 | Helix-turn-helix family protein | Helix-turn-helix family protein | | uniclust | UniRef100\_A0A0D0RL49 | 99.9 | 1.9e-26 | 3.6e-32 | 162.7 | 175 | (1, 177) | 180 | (19, 242) | 251 | CI-like repressor, phage associated | CI-like repressor, phage associated | | uniclust | UniRef100\_A0A1V6DM68 | 99.9 | 2.4e-26 | 4.4e-32 | 159.0 | 180 | (1, 180) | 180 | (2, 241) | 244 | LexA repressor | LexA repressor | | uniclust | UniRef100\_A0A099I651 | 99.9 | 2.6e-26 | 4.9e-32 | 169.4 | 170 | (1, 176) | 180 | (53, 295) | 312 | HTH cro/C1-type domain-containing protein | HTH cro/C1-type domain-containing protein | | uniclust | UniRef100\_A0A0Q4FWG9 | 99.9 | 2.7e-26 | 5e-32 | 161.9 | 177 | (3, 180) | 180 | (57, 272) | 272 | HTH cro/C1-type domain-containing protein | HTH cro/C1-type domain-containing protein | | uniclust | UniRef100\_A0A1G3BMS2 | 99.9 | 2.7e-26 | 5e-32 | 158.4 | 176 | (1, 176) | 180 | (60, 259) | 265 | HTH cro/C1-type domain-containing protein | HTH cro/C1-type domain-containing protein | | uniclust | UniRef100\_A0A1G3L741 | 99.9 | 2.9e-26 | 5.3e-32 | 161.9 | 175 | (1, 177) | 180 | (10, 244) | 249 | HTH cro/C1-type domain-containing protein | HTH cro/C1-type domain-containing protein | | uniclust | UniRef100\_A0A090V113 | 99.9 | 3.1e-26 | 5.7e-32 | 163.8 | 175 | (1, 179) | 180 | (31, 239) | 253 | LexA repressor | LexA repressor | | uniclust | UniRef100\_UPI000FD88B00 | 99.9 | 3.3e-26 | 6.1e-32 | 155.2 | 174 | (2, 177) | 180 | (19, 237) | 241 | S24 family peptidase | S24 family peptidase | | uniclust | UniRef100\_A0A2S5N385 | 99.9 | 4.4e-26 | 8.2e-32 | 161.2 | 177 | (1, 179) | 180 | (66, 291) | 292 | HTH cro/C1-type domain-containing protein | HTH cro/C1-type domain-containing protein | | uniclust | UniRef100\_A0A0U3VIS1 | 99.9 | 4.6e-26 | 8.5e-32 | 156.4 | 176 | (1, 178) | 180 | (9, 211) | 226 | LexA family transcriptional regulator | LexA family transcriptional regulator | | uniclust | UniRef100\_A0A0S4R308 | 99.9 | 5.3e-26 | 9.8e-32 | 154.4 | 174 | (2, 177) | 180 | (10, 222) | 230 | DNA-binding protein | DNA-binding protein | | uniclust | UniRef100\_A0A023CWD3 | 99.9 | 5.4e-26 | 1e-31 | 153.2 | 176 | (2, 179) | 180 | (18, 215) | 225 | Xre family DNA-binding protein | Xre family DNA-binding protein | | uniclust | UniRef100\_A0A0Q2Y0V6 | 99.9 | 5.8e-26 | 1.1e-31 | 157.2 | 165 | (14, 179) | 180 | (1, 216) | 236 | Peptidase S24/S26A/S26B/S26C domain-containing protein | Peptidase S24/S26A/S26B/S26C domain-containing protein | | uniclust | UniRef100\_A0A068RDG1 | 99.9 | 5.8e-26 | 1.1e-31 | 156.8 | 174 | (1, 176) | 180 | (23, 222) | 232 | Helix-turn-helix/Peptidase S24-like domain-containing protein | Helix-turn-helix/Peptidase S24-like domain-containing protein | | uniclust | UniRef100\_A0A0A5HTQ8 | 99.9 | 6.4e-26 | 1.2e-31 | 157.4 | 162 | (15, 180) | 180 | (60, 243) | 250 | Transcriptional regulator | Transcriptional regulator | | uniclust | UniRef100\_A0A024E9Q4 | 99.9 | 7.9e-26 | 1.5e-31 | 164.5 | 176 | (3, 178) | 180 | (47, 277) | 284 | Phage repressor | Phage repressor | | uniclust | UniRef100\_A0A0Q3EP03 | 99.9 | 8.8e-26 | 1.6e-31 | 162.3 | 175 | (2, 176) | 180 | (52, 265) | 276 | Helix-turn-helix domain-containing protein | Helix-turn-helix domain-containing protein | | uniclust | UniRef100\_A0A377PS33 | 99.9 | 9e-26 | 1.7e-31 | 153.2 | 175 | (1, 180) | 180 | (4, 247) | 249 | LexA repressor | LexA repressor | | uniclust | UniRef100\_A0A369T885 | 99.9 | 9.5e-26 | 1.7e-31 | 153.6 | 176 | (1, 180) | 180 | (38, 255) | 257 | XRE family transcriptional regulator | XRE family transcriptional regulator | | uniclust | UniRef100\_A0A0C9MTW4 | 99.9 | 1.1e-25 | 2e-31 | 158.3 | 174 | (2, 179) | 180 | (36, 244) | 251 | DNA, contig: SP630 | DNA, contig: SP630 | | uniclust | UniRef100\_A0A098RK60 | 99.9 | 1.1e-25 | 2e-31 | 160.0 | 176 | (2, 178) | 180 | (23, 277) | 279 | Repressor | Repressor | | uniclust | UniRef100\_A0A081MYG4 | 99.9 | 1.1e-25 | 2.1e-31 | 158.0 | 178 | (1, 179) | 180 | (15, 219) | 225 | HTH cro/C1-type domain-containing protein | HTH cro/C1-type domain-containing protein | | uniclust | UniRef100\_A0A1F4HU16 | 99.9 | 1.2e-25 | 2.2e-31 | 158.4 | 175 | (1, 177) | 180 | (63, 281) | 286 | Repressor | Repressor | | uniclust | UniRef100\_A0A0V8LX08 | 99.9 | 1.2e-25 | 2.2e-31 | 154.7 | 172 | (1, 180) | 180 | (13, 216) | 228 | Transcriptional regulator | Transcriptional regulator | | uniclust | UniRef100\_A0A0K1HH28 | 99.9 | 1.2e-25 | 2.3e-31 | 155.0 | 174 | (2, 175) | 180 | (8, 237) | 256 | Putative transcriptional regulator, XRE family (Peptidase S24 LexA-like domain) | Putative transcriptional regulator, XRE family (Peptidase S24 LexA-like domain) | | uniclust | UniRef100\_A0A0C2YQB8 | 99.9 | 1.3e-25 | 2.3e-31 | 161.2 | 177 | (3, 180) | 180 | (54, 273) | 278 | Peptidase | Peptidase | | uniclust | UniRef100\_A0A0C3RMF8 | 99.9 | 1.3e-25 | 2.4e-31 | 151.3 | 101 | (79, 180) | 180 | (70, 172) | 175 | Peptidase S24/S26A/S26B/S26C domain-containing protein | Peptidase S24/S26A/S26B/S26C domain-containing protein | | uniclust | UniRef100\_A0A0D6H1B7 | 99.9 | 1.3e-25 | 2.4e-31 | 158.5 | 177 | (1, 177) | 180 | (21, 233) | 243 | LexA family transcriptional regulator | LexA family transcriptional regulator | | uniclust | UniRef100\_A0A090HXE6 | 99.9 | 1.3e-25 | 2.5e-31 | 158.1 | 176 | (1, 176) | 180 | (23, 235) | 242 | LexA protein | LexA protein | | uniclust | UniRef100\_A0A937YQE5 | 99.9 | 1.5e-25 | 2.7e-31 | 147.1 | 178 | (2, 180) | 180 | (35, 246) | 246 | Helix-turn-helix transcriptional regulator | Helix-turn-helix transcriptional regulator | | uniclust | UniRef100\_A0A1A9RLP6 | 99.9 | 1.5e-25 | 2.8e-31 | 156.4 | 175 | (2, 177) | 180 | (35, 253) | 264 | Peptidase S24/S26A/S26B/S26C domain-containing protein | Peptidase S24/S26A/S26B/S26C domain-containing protein | | uniclust | UniRef100\_A0A060RFY0 | 99.9 | 1.6e-25 | 3e-31 | 162.5 | 177 | (1, 179) | 180 | (29, 262) | 272 | Pleiotropic regulator of exopolysaccharide synthe sis, competence and biofilm formation Ftr, XRE family | Pleiotropic regulator of exopolysaccharide synthe sis, competence and biofilm formation Ftr, XRE family | | uniclust | UniRef100\_A0A1Q6T3X1 | 99.9 | 2.1e-25 | 3.9e-31 | 153.2 | 175 | (4, 179) | 180 | (10, 222) | 226 | Peptidase S24/S26A/S26B/S26C domain-containing protein | Peptidase S24/S26A/S26B/S26C domain-containing protein | | uniclust | UniRef100\_A0A061QGB9 | 99.9 | 2.2e-25 | 4e-31 | 160.0 | 176 | (2, 179) | 180 | (63, 252) | 265 | LexA repressor | LexA repressor | | uniclust | UniRef100\_A0A212IUA3 | 99.9 | 2.2e-25 | 4.1e-31 | 146.0 | 175 | (1, 179) | 180 | (3, 217) | 218 | Putative Phage repressor | Putative Phage repressor | | uniclust | UniRef100\_A0A0U4BEI9 | 99.9 | 2.3e-25 | 4.3e-31 | 159.2 | 175 | (1, 177) | 180 | (33, 256) | 266 | HTH cro/C1-type domain-containing protein | HTH cro/C1-type domain-containing protein | | uniclust | UniRef100\_A0A066SSH7 | 99.9 | 2.3e-25 | 4.3e-31 | 157.7 | 175 | (1, 179) | 180 | (37, 248) | 254 | Helix-turn-helix domain-containing protein | Helix-turn-helix domain-containing protein | | uniclust | UniRef100\_A0A0B1TSB9 | 99.9 | 2.9e-25 | 5.4e-31 | 156.1 | 177 | (2, 180) | 180 | (35, 281) | 282 | Putative transcriptional regulator | Putative transcriptional regulator | | uniclust | UniRef100\_A0A1I1VLL5 | 99.9 | 3e-25 | 5.5e-31 | 152.4 | 177 | (2, 179) | 180 | (21, 252) | 256 | Phage repressor protein C, contains Cro/C1-type HTH and peptisase s24 domains | Phage repressor protein C, contains Cro/C1-type HTH and peptisase s24 domains | | uniclust | UniRef100\_A0A077PXM9 | 99.9 | 3e-25 | 5.5e-31 | 157.3 | 172 | (2, 176) | 180 | (89, 319) | 324 | Repressor protein CI (Modular protein) | Repressor protein CI (Modular protein) | | uniclust | UniRef100\_A0A0F8XX58 | 99.9 | 3.7e-25 | 6.8e-31 | 157.5 | 177 | (2, 178) | 180 | (31, 267) | 277 | HTH cro/C1-type domain-containing protein (Fragment) | HTH cro/C1-type domain-containing protein (Fragment) | | uniclust | UniRef100\_A0A1C5L5I0 | 99.9 | 3.9e-25 | 7.3e-31 | 150.8 | 177 | (1, 178) | 180 | (12, 212) | 219 | LexA repressor | LexA repressor | | uniclust | UniRef100\_A0A0J1F8E5 | 99.9 | 4e-25 | 7.3e-31 | 158.2 | 176 | (1, 179) | 180 | (21, 226) | 291 | HTH-type transcriptional regulator Xre | HTH-type transcriptional regulator Xre | | uniclust | UniRef100\_A0A149SNV7 | 99.9 | 4.9e-25 | 9e-31 | 152.7 | 172 | (2, 175) | 180 | (18, 233) | 238 | HTH cro/C1-type domain-containing protein | HTH cro/C1-type domain-containing protein | | uniclust | UniRef100\_A0A352MTY7 | 99.9 | 4.9e-25 | 9.1e-31 | 147.7 | 175 | (1, 177) | 180 | (5, 193) | 195 | HTH cro/C1-type domain-containing protein | HTH cro/C1-type domain-containing protein | | uniclust | UniRef100\_A0A061QF65 | 99.9 | 5e-25 | 9.1e-31 | 150.3 | 169 | (12, 180) | 180 | (23, 226) | 227 | Repressor protein CI | Repressor protein CI | | uniclust | UniRef100\_A0A1V5PB98 | 99.9 | 5.2e-25 | 9.7e-31 | 148.8 | 172 | (3, 177) | 180 | (23, 226) | 230 | LexA repressor | LexA repressor | | uniclust | UniRef100\_A0A5C1MF53 | 99.8 | 5.5e-25 | 1e-30 | 150.4 | 175 | (2, 180) | 180 | (45, 263) | 263 | Helix-turn-helix transcriptional regulator | Helix-turn-helix transcriptional regulator | | uniclust | UniRef100\_A0A0Y7KSX8 | 99.8 | 5.6e-25 | 1e-30 | 148.9 | 179 | (1, 180) | 180 | (30, 256) | 256 | Helix-turn-helix domain-containing protein | Helix-turn-helix domain-containing protein | | uniclust | UniRef100\_A0A0K6I8B4 | 99.8 | 6.3e-25 | 1.2e-30 | 155.1 | 173 | (1, 175) | 180 | (62, 282) | 290 | Peptidase S24-like | Peptidase S24-like | | uniclust | UniRef100\_A0A419V0E4 | 99.8 | 6.4e-25 | 1.2e-30 | 146.3 | 175 | (1, 177) | 180 | (7, 206) | 210 | SOS-response transcriptional repressor LexA | SOS-response transcriptional repressor LexA | | uniclust | UniRef100\_A0A1M5MNV0 | 99.8 | 7.7e-25 | 1.4e-30 | 153.5 | 173 | (2, 175) | 180 | (29, 258) | 266 | Peptidase S24-like | Peptidase S24-like | | uniclust | UniRef100\_A0A133N482 | 99.8 | 8.6e-25 | 1.6e-30 | 154.2 | 176 | (1, 178) | 180 | (88, 282) | 286 | Repressor LexA | Repressor LexA | | uniclust | UniRef100\_A0A014MMR0 | 99.8 | 1.1e-24 | 2e-30 | 147.5 | 178 | (2, 179) | 180 | (4, 216) | 219 | Peptidase S24/S26A/S26B/S26C domain-containing protein | Peptidase S24/S26A/S26B/S26C domain-containing protein | | uniclust | UniRef100\_A0A099WLJ4 | 99.8 | 1.1e-24 | 2.1e-30 | 154.6 | 177 | (1, 177) | 180 | (60, 271) | 284 | HTH cro/C1-type domain-containing protein | HTH cro/C1-type domain-containing protein | | uniclust | UniRef100\_A0A0V8LXT6 | 99.8 | 1.2e-24 | 2.2e-30 | 148.7 | 175 | (1, 180) | 180 | (16, 231) | 233 | HTH cro/C1-type domain-containing protein | HTH cro/C1-type domain-containing protein | | uniclust | UniRef100\_A0A023D3P8 | 99.8 | 1.2e-24 | 2.2e-30 | 147.1 | 174 | (2, 178) | 180 | (36, 234) | 238 | Phage repressor | Phage repressor | | uniclust | UniRef100\_A0A071M1S3 | 99.8 | 1.6e-24 | 2.9e-30 | 151.0 | 175 | (1, 179) | 180 | (30, 239) | 241 | XRE family transcriptional regulator | XRE family transcriptional regulator | | uniclust | UniRef100\_A0A091C634 | 99.8 | 1.6e-24 | 2.9e-30 | 148.3 | 171 | (2, 174) | 180 | (8, 231) | 232 | Phage repressor | Phage repressor | | uniclust | UniRef100\_A0A1B1PWH0 | 99.8 | 2.1e-24 | 3.9e-30 | 142.6 | 177 | (2, 178) | 180 | (5, 227) | 229 | HTH cro/C1-type domain-containing protein | HTH cro/C1-type domain-containing protein | | uniclust | UniRef100\_A0A098F7G8 | 99.8 | 2.2e-24 | 4e-30 | 157.6 | 175 | (1, 177) | 180 | (14, 322) | 340 | SOS-response transcriptional repressor | SOS-response transcriptional repressor | | uniclust | UniRef100\_A0A015NHE4 | 99.8 | 2.3e-24 | 4.3e-30 | 153.3 | 177 | (1, 177) | 180 | (24, 242) | 256 | HTH cro/C1-type domain-containing protein | HTH cro/C1-type domain-containing protein | | uniclust | UniRef100\_F6DTI3 | 99.8 | 3.3e-24 | 6.1e-30 | 150.4 | 174 | (1, 177) | 180 | (8, 277) | 294 | LexA repressor | LexA repressor | | uniclust | UniRef100\_A0A2C9D6H6 | 99.8 | 3.6e-24 | 6.6e-30 | 142.6 | 176 | (2, 179) | 180 | (8, 207) | 209 | Pyocin repressor protein | Pyocin repressor protein | | uniclust | UniRef100\_A0A087MCA7 | 99.8 | 3.7e-24 | 6.8e-30 | 152.1 | 165 | (13, 179) | 180 | (53, 246) | 288 | Phage repressor protein | Phage repressor protein | | uniclust | UniRef100\_A0A523J2I9 | 99.8 | 4.2e-24 | 7.7e-30 | 149.3 | 176 | (3, 179) | 180 | (35, 245) | 246 | Helix-turn-helix domain-containing protein | Helix-turn-helix domain-containing protein | | uniclust | UniRef100\_A0A084UBN0 | 99.8 | 4.2e-24 | 7.8e-30 | 155.5 | 173 | (2, 179) | 180 | (44, 295) | 297 | Putative transcriptional regulator | Putative transcriptional regulator | | uniclust | UniRef100\_A0A1V5GIA7 | 99.8 | 5e-24 | 9.1e-30 | 153.7 | 172 | (1, 178) | 180 | (65, 296) | 330 | HTH-type transcriptional regulator ImmR | HTH-type transcriptional regulator ImmR | | uniclust | UniRef100\_A0A143Z9V0 | 99.8 | 5.3e-24 | 9.7e-30 | 144.9 | 177 | (1, 179) | 180 | (9, 256) | 261 | Phage repressor protein C, contains Cro/C1-type HTH and peptisase s24 domains | Phage repressor protein C, contains Cro/C1-type HTH and peptisase s24 domains | | uniclust | UniRef100\_A0A353R1K5 | 99.8 | 5.3e-24 | 9.7e-30 | 144.4 | 175 | (1, 180) | 180 | (27, 230) | 231 | HTH cro/C1-type domain-containing protein | HTH cro/C1-type domain-containing protein | | uniclust | UniRef100\_A0A3A4K5N6 | 99.8 | 5.5e-24 | 1e-29 | 141.3 | 175 | (1, 178) | 180 | (10, 203) | 205 | LexA family transcriptional regulator | LexA family transcriptional regulator | | uniclust | UniRef100\_A0A1H4AEP1 | 99.8 | 6.5e-24 | 1.2e-29 | 140.4 | 175 | (1, 180) | 180 | (5, 193) | 195 | SOS-response transcriptional repressor LexA (RecA-mediated autopeptidase) | SOS-response transcriptional repressor LexA (RecA-mediated autopeptidase) | | uniclust | UniRef100\_A0A2U0TA89 | 99.8 | 6.7e-24 | 1.2e-29 | 141.1 | 174 | (3, 179) | 180 | (28, 215) | 225 | Helix-turn-helix protein | Helix-turn-helix protein | | uniclust | UniRef100\_A0A2U3L084 | 99.8 | 7.8e-24 | 1.4e-29 | 144.9 | 180 | (1, 180) | 180 | (16, 246) | 247 | Putative phage repressor | Putative phage repressor | | uniclust | UniRef100\_A0A0K2GBJ9 | 99.8 | 1.5e-23 | 2.7e-29 | 149.1 | 176 | (2, 180) | 180 | (43, 231) | 256 | LexA repressor | LexA repressor | | uniclust | UniRef100\_A0A0S4W647 | 99.8 | 1.5e-23 | 2.8e-29 | 145.8 | 172 | (3, 176) | 180 | (12, 254) | 260 | Peptidase S24/S26A/S26B/S26C domain-containing protein | Peptidase S24/S26A/S26B/S26C domain-containing protein | | uniclust | UniRef100\_A0A101VVJ0 | 99.8 | 1.6e-23 | 3e-29 | 144.4 | 176 | (1, 179) | 180 | (18, 221) | 245 | Peptidase S24/S26A/S26B/S26C domain-containing protein | Peptidase S24/S26A/S26B/S26C domain-containing protein | | uniclust | UniRef100\_A0A1B7XER7 | 99.8 | 1.7e-23 | 3.1e-29 | 147.9 | 164 | (15, 180) | 180 | (37, 262) | 265 | Transcriptional regulator | Transcriptional regulator | | uniclust | UniRef100\_A0A098G5U5 | 99.8 | 1.9e-23 | 3.5e-29 | 148.2 | 178 | (1, 179) | 180 | (47, 262) | 274 | HTH cro/C1-type domain-containing protein | HTH cro/C1-type domain-containing protein | | uniclust | UniRef100\_A0A0Q5M1T3 | 99.8 | 1.9e-23 | 3.6e-29 | 142.6 | 179 | (1, 180) | 180 | (22, 248) | 248 | Peptidase S24 | Peptidase S24 | | uniclust | UniRef100\_A0A523U297 | 99.8 | 1.9e-23 | 3.6e-29 | 144.5 | 175 | (1, 177) | 180 | (23, 244) | 257 | Helix-turn-helix domain-containing protein | Helix-turn-helix domain-containing protein | | uniclust | UniRef100\_A0A072CT56 | 99.8 | 1.9e-23 | 3.6e-29 | 154.4 | 177 | (1, 179) | 180 | (61, 276) | 319 | Repressor | Repressor | | uniclust | UniRef100\_A0A084INR4 | 99.8 | 1.9e-23 | 3.6e-29 | 149.1 | 171 | (2, 178) | 180 | (41, 245) | 250 | Repressor protein c2 | Repressor protein c2 | | uniclust | UniRef100\_A0A1Q6U9K6 | 99.8 | 2e-23 | 3.7e-29 | 138.2 | 176 | (3, 178) | 180 | (7, 222) | 225 | HTH cro/C1-type domain-containing protein | HTH cro/C1-type domain-containing protein | | uniclust | UniRef100\_A0A3D4YWI1 | 99.8 | 2.2e-23 | 4.1e-29 | 141.5 | 172 | (1, 174) | 180 | (7, 213) | 233 | HTH cro/C1-type domain-containing protein | HTH cro/C1-type domain-containing protein | | uniclust | UniRef100\_A0A098EGV2 | 99.8 | 2.2e-23 | 4.1e-29 | 139.4 | 178 | (2, 180) | 180 | (12, 213) | 214 | DNA-binding protein | DNA-binding protein | | uniclust | UniRef100\_A0A1B8QAD9 | 99.8 | 2.3e-23 | 4.2e-29 | 143.1 | 178 | (1, 179) | 180 | (16, 224) | 230 | HTH cro/C1-type domain-containing protein | HTH cro/C1-type domain-containing protein | | uniclust | UniRef100\_A0A1G5MEX5 | 99.8 | 2.5e-23 | 4.6e-29 | 144.1 | 177 | (1, 179) | 180 | (39, 274) | 275 | Phage repressor protein C, contains Cro/C1-type HTH and peptisase s24 domains | Phage repressor protein C, contains Cro/C1-type HTH and peptisase s24 domains | | uniclust | UniRef100\_A0A023BWP2 | 99.8 | 2.5e-23 | 4.7e-29 | 152.5 | 164 | (2, 166) | 180 | (38, 241) | 302 | DNA-binding protein | DNA-binding protein | | uniclust | UniRef100\_A0A133XV83 | 99.8 | 2.6e-23 | 4.8e-29 | 143.4 | 175 | (1, 177) | 180 | (29, 234) | 250 | DNA-binding helix-turn-helix protein | DNA-binding helix-turn-helix protein | | uniclust | UniRef100\_A0A1F1DBG0 | 99.8 | 2.7e-23 | 5e-29 | 143.2 | 176 | (2, 179) | 180 | (23, 263) | 273 | Repressor | Repressor | | uniclust | UniRef100\_A0A014B1G1 | 99.8 | 2.7e-23 | 5e-29 | 147.4 | 179 | (1, 179) | 180 | (13, 234) | 243 | Helix-turn-helix family protein | Helix-turn-helix family protein | | uniclust | UniRef100\_A0A2S7JR70 | 99.8 | 2.9e-23 | 5.4e-29 | 146.1 | 178 | (2, 180) | 180 | (25, 299) | 299 | Peptidase S24/S26A/S26B/S26C domain-containing protein | Peptidase S24/S26A/S26B/S26C domain-containing protein | | uniclust | UniRef100\_A0A2X0V8H6 | 99.8 | 3e-23 | 5.6e-29 | 134.5 | 177 | (1, 180) | 180 | (15, 202) | 203 | LexA repressor | LexA repressor | | uniclust | UniRef100\_A0A0E3BVD5 | 99.8 | 4.3e-23 | 7.9e-29 | 142.2 | 175 | (2, 177) | 180 | (10, 231) | 236 | Repressor | Repressor | | uniclust | UniRef100\_A0A2N7Q1N3 | 99.8 | 4.4e-23 | 8.1e-29 | 136.9 | 175 | (1, 176) | 180 | (16, 210) | 220 | Phage repressor protein | Phage repressor protein | | uniclust | UniRef100\_A0A118HZG0 | 99.8 | 4.6e-23 | 8.5e-29 | 149.3 | 159 | (20, 180) | 180 | (172, 377) | 377 | HTH cro/C1-type domain-containing protein | HTH cro/C1-type domain-containing protein | | uniclust | UniRef100\_A0A077LIM9 | 99.8 | 4.7e-23 | 8.8e-29 | 147.2 | 173 | (2, 177) | 180 | (59, 271) | 277 | Repressor protein cI | Repressor protein cI | | uniclust | UniRef100\_A0A0D0JL38 | 99.8 | 6.4e-23 | 1.2e-28 | 142.6 | 172 | (2, 175) | 180 | (42, 254) | 264 | Phage-related repressor protein | Phage-related repressor protein | | uniclust | UniRef100\_A0A2D3WLJ9 | 99.8 | 6.7e-23 | 1.2e-28 | 142.4 | 175 | (1, 176) | 180 | (28, 252) | 262 | HTH cro/C1-type domain-containing protein | HTH cro/C1-type domain-containing protein | | uniclust | UniRef100\_A0A066TCP8 | 99.8 | 6.9e-23 | 1.3e-28 | 140.2 | 168 | (12, 180) | 180 | (39, 237) | 238 | HTH cro/C1-type domain-containing protein | HTH cro/C1-type domain-containing protein | | uniclust | UniRef100\_A0A0H4A1L4 | 99.8 | 7.1e-23 | 1.3e-28 | 137.4 | 173 | (1, 178) | 180 | (6, 202) | 211 | Phage repressor | Phage repressor | | uniclust | UniRef100\_A0A059V0W4 | 99.8 | 8.2e-23 | 1.5e-28 | 149.6 | 175 | (1, 177) | 180 | (60, 329) | 334 | Repressor | Repressor | | uniclust | UniRef100\_A0A0Q5CIB1 | 99.8 | 8.5e-23 | 1.6e-28 | 144.5 | 169 | (5, 176) | 180 | (48, 280) | 289 | Peptidase S24/S26A/S26B/S26C domain-containing protein | Peptidase S24/S26A/S26B/S26C domain-containing protein | | uniclust | UniRef100\_A0A162BGE3 | 99.8 | 1e-22 | 1.9e-28 | 137.7 | 172 | (1, 178) | 180 | (3, 214) | 225 | Repressor | Repressor | | uniclust | UniRef100\_A0A1G8JBR8 | 99.8 | 1.1e-22 | 2e-28 | 147.3 | 175 | (2, 176) | 180 | (101, 335) | 347 | Phage repressor protein C, contains Cro/C1-type HTH and peptisase s24 domains | Phage repressor protein C, contains Cro/C1-type HTH and peptisase s24 domains | | uniclust | UniRef100\_A0A0M2DVU2 | 99.8 | 1.1e-22 | 2e-28 | 141.7 | 176 | (4, 179) | 180 | (17, 250) | 254 | Peptidase S24/S26A/S26B/S26C domain-containing protein | Peptidase S24/S26A/S26B/S26C domain-containing protein | | uniclust | UniRef100\_A0A172WXK4 | 99.8 | 1.3e-22 | 2.5e-28 | 140.6 | 173 | (2, 176) | 180 | (23, 252) | 260 | XRE family transcriptional regulator | XRE family transcriptional regulator | | uniclust | UniRef100\_A0A1L8R4K9 | 99.8 | 1.4e-22 | 2.6e-28 | 138.7 | 174 | (1, 176) | 180 | (10, 240) | 244 | Multidrug transporter | Multidrug transporter | | uniclust | UniRef100\_A0A2M9FSW7 | 99.8 | 1.7e-22 | 3.1e-28 | 139.2 | 169 | (1, 174) | 180 | (13, 230) | 231 | HTH cro/C1-type domain-containing protein | HTH cro/C1-type domain-containing protein | | uniclust | UniRef100\_A0A085JF28 | 99.8 | 1.8e-22 | 3.3e-28 | 143.8 | 175 | (1, 178) | 180 | (47, 255) | 266 | Phage repressor protein C2 | Phage repressor protein C2 | | uniclust | UniRef100\_A0A2A2HM07 | 99.8 | 2e-22 | 3.6e-28 | 138.0 | 175 | (1, 177) | 180 | (17, 240) | 248 | Phage repressor protein | Phage repressor protein | | uniclust | UniRef100\_A0A0T9T2W8 | 99.8 | 2e-22 | 3.6e-28 | 138.2 | 173 | (2, 178) | 180 | (29, 219) | 236 | Uncharacterized HTH-type transcriptional regulator HI\_1476 | Uncharacterized HTH-type transcriptional regulator HI\_1476 | | uniclust | UniRef100\_A0A191HUA0 | 99.8 | 2e-22 | 3.7e-28 | 136.3 | 178 | (1, 180) | 180 | (35, 236) | 237 | HTH cro/C1-type domain-containing protein | HTH cro/C1-type domain-containing protein | | uniclust | UniRef100\_A0A0N8P2N3 | 99.8 | 2.3e-22 | 4.2e-28 | 121.7 | 92 | (89, 180) | 180 | (4, 97) | 101 | Peptidase S24/S26A/S26B/S26C domain-containing protein (Fragment) | Peptidase S24/S26A/S26B/S26C domain-containing protein (Fragment) | | uniclust | UniRef100\_A0A7X5Y7A7 | 99.8 | 2.4e-22 | 4.4e-28 | 136.3 | 177 | (2, 180) | 180 | (89, 306) | 306 | Phage repressor protein C with HTH and peptisase S24 domain | Phage repressor protein C with HTH and peptisase S24 domain | | uniclust | UniRef100\_UPI002147CCE2 | 99.8 | 2.8e-22 | 5.1e-28 | 136.2 | 175 | (2, 176) | 180 | (70, 295) | 309 | S24 family peptidase | S24 family peptidase | | uniclust | UniRef100\_A0A1E4G2P2 | 99.8 | 2.8e-22 | 5.1e-28 | 140.2 | 174 | (1, 178) | 180 | (34, 236) | 245 | HTH cro/C1-type domain-containing protein | HTH cro/C1-type domain-containing protein | | uniclust | UniRef100\_A0A1R1LYN7 | 99.8 | 2.9e-22 | 5.3e-28 | 133.8 | 174 | (1, 176) | 180 | (3, 221) | 227 | HTH cro/C1-type domain-containing protein | HTH cro/C1-type domain-containing protein | | uniclust | UniRef100\_A0A1C2DEK8 | 99.8 | 3.3e-22 | 6.1e-28 | 135.3 | 98 | (83, 180) | 180 | (86, 185) | 196 | Peptidase S24/S26A/S26B/S26C domain-containing protein | Peptidase S24/S26A/S26B/S26C domain-containing protein | | uniclust | UniRef100\_A0A2A2R811 | 99.8 | 3.4e-22 | 6.3e-28 | 140.0 | 176 | (1, 180) | 180 | (26, 241) | 262 | Peptidase S24/S26A/S26B/S26C domain-containing protein | Peptidase S24/S26A/S26B/S26C domain-containing protein | | uniclust | UniRef100\_A0A1D9GLV6 | 99.8 | 3.5e-22 | 6.4e-28 | 147.1 | 174 | (2, 176) | 180 | (65, 357) | 369 | Peptidase S24/S26A/S26B/S26C domain-containing protein | Peptidase S24/S26A/S26B/S26C domain-containing protein | | uniclust | UniRef100\_A0A4Q2J692 | 99.8 | 3.5e-22 | 6.5e-28 | 135.0 | 174 | (1, 178) | 180 | (4, 245) | 256 | LexA family transcriptional regulator | LexA family transcriptional regulator | | uniclust | UniRef100\_A0A0W8E3K7 | 99.8 | 3.6e-22 | 6.6e-28 | 147.5 | 176 | (1, 179) | 180 | (29, 274) | 333 | Sos-response repressor and protease lexa | Sos-response repressor and protease lexa | | uniclust | UniRef100\_A0A1H0VW04 | 99.8 | 3.8e-22 | 6.9e-28 | 137.9 | 172 | (4, 178) | 180 | (20, 235) | 240 | SOS-response transcriptional repressor LexA (RecA-mediated autopeptidase) | SOS-response transcriptional repressor LexA (RecA-mediated autopeptidase) | | uniclust | UniRef100\_A0A0K4HVA9 | 99.8 | 4e-22 | 7.4e-28 | 140.5 | 174 | (2, 176) | 180 | (48, 244) | 264 | Helix-turn-helix domain-containing protein | Helix-turn-helix domain-containing protein | | uniclust | UniRef100\_A0A1H6C042 | 99.8 | 4.2e-22 | 7.8e-28 | 143.4 | 176 | (2, 178) | 180 | (42, 282) | 309 | Helix-turn-helix | Helix-turn-helix | | uniclust | UniRef100\_A0A1D2UGZ7 | 99.8 | 5e-22 | 9.2e-28 | 143.3 | 174 | (1, 175) | 180 | (101, 327) | 333 | HTH cro/C1-type domain-containing protein | HTH cro/C1-type domain-containing protein | | uniclust | UniRef100\_A0A023WV23 | 99.8 | 5.8e-22 | 1.1e-27 | 143.6 | 173 | (1, 175) | 180 | (43, 247) | 260 | Cro/Cl family transcriptional regulator | Cro/Cl family transcriptional regulator | | uniclust | UniRef100\_A0A013SVQ8 | 99.8 | 6e-22 | 1.1e-27 | 148.7 | 174 | (1, 178) | 180 | (88, 300) | 332 | Helix-turn-helix family protein | Helix-turn-helix family protein | | uniclust | UniRef100\_A0A2W4N736 | 99.8 | 6.1e-22 | 1.1e-27 | 138.0 | 174 | (1, 176) | 180 | (68, 277) | 340 | HTH cro/C1-type domain-containing protein | HTH cro/C1-type domain-containing protein | | uniclust | UniRef100\_A0A196NVN3 | 99.8 | 7.3e-22 | 1.4e-27 | 132.9 | 176 | (4, 180) | 180 | (20, 235) | 236 | Peptidase S24/S26A/S26B/S26C domain-containing protein | Peptidase S24/S26A/S26B/S26C domain-containing protein | | uniclust | UniRef100\_A0A1R0YXM3 | 99.8 | 8.1e-22 | 1.5e-27 | 129.2 | 174 | (1, 179) | 180 | (4, 192) | 198 | HTH cro/C1-type domain-containing protein | HTH cro/C1-type domain-containing protein | | uniclust | UniRef100\_A0A292QUL5 | 99.8 | 8.9e-22 | 1.6e-27 | 133.4 | 163 | (12, 179) | 180 | (17, 201) | 210 | Peptidase S24/S26A/S26B/S26C domain-containing protein | Peptidase S24/S26A/S26B/S26C domain-containing protein | | uniclust | UniRef100\_A0A0E2BPP9 | 99.8 | 8.9e-22 | 1.6e-27 | 138.2 | 174 | (1, 176) | 180 | (38, 252) | 259 | SOS-response repressor/protease | SOS-response repressor/protease | | uniclust | UniRef100\_A0A077QL17 | 99.8 | 9.8e-22 | 1.8e-27 | 140.3 | 173 | (1, 177) | 180 | (14, 216) | 281 | Helix-turn-helix/peptidase S24-like domain protein (Modular protein) | Helix-turn-helix/peptidase S24-like domain protein (Modular protein) | | uniclust | UniRef100\_A0A268THE3 | 99.8 | 1.1e-21 | 2.1e-27 | 135.3 | 88 | (87, 177) | 180 | (175, 262) | 267 | HTH cro/C1-type domain-containing protein | HTH cro/C1-type domain-containing protein | | uniclust | UniRef100\_A0A0Q5GW81 | 99.7 | 1.4e-21 | 2.5e-27 | 127.8 | 97 | (84, 180) | 180 | (62, 160) | 161 | Peptidase S24/S26A/S26B/S26C domain-containing protein | Peptidase S24/S26A/S26B/S26C domain-containing protein | | uniclust | UniRef100\_A0A0A8GVX7 | 99.7 | 1.4e-21 | 2.6e-27 | 134.9 | 159 | (4, 177) | 180 | (30, 225) | 232 | Peptidase S24 LexA-like protein | Peptidase S24 LexA-like protein | | uniclust | UniRef100\_A0A084A6X1 | 99.7 | 1.4e-21 | 2.6e-27 | 137.8 | 176 | (1, 177) | 180 | (13, 254) | 297 | Putative transcriptional regulator | Putative transcriptional regulator | | uniclust | UniRef100\_A0A0U3FB36 | 99.7 | 1.5e-21 | 2.7e-27 | 132.0 | 178 | (1, 179) | 180 | (12, 219) | 227 | HTH/peptidase S24 family domain-containing protein | HTH/peptidase S24 family domain-containing protein | | uniclust | UniRef100\_A0A1G3FIK6 | 99.7 | 1.5e-21 | 2.7e-27 | 131.1 | 178 | (1, 179) | 180 | (16, 242) | 243 | Transcriptional regulator | Transcriptional regulator | | uniclust | UniRef100\_A0A1F9A3V9 | 99.7 | 1.5e-21 | 2.8e-27 | 126.2 | 93 | (87, 180) | 180 | (62, 156) | 158 | Peptidase S24/S26A/S26B/S26C domain-containing protein | Peptidase S24/S26A/S26B/S26C domain-containing protein | | uniclust | UniRef100\_A0A2E9H220 | 99.7 | 1.6e-21 | 2.9e-27 | 136.4 | 174 | (1, 175) | 180 | (39, 260) | 275 | HTH cro/C1-type domain-containing protein | HTH cro/C1-type domain-containing protein | | uniclust | UniRef100\_A0A2I0GID1 | 99.7 | 1.8e-21 | 3.3e-27 | 139.3 | 173 | (1, 174) | 180 | (11, 288) | 298 | HTH cro/C1-type domain-containing protein | HTH cro/C1-type domain-containing protein | | uniclust | UniRef100\_A0A4U0PYZ7 | 99.7 | 1.9e-21 | 3.5e-27 | 136.3 | 176 | (2, 178) | 180 | (96, 319) | 322 | Helix-turn-helix transcriptional regulator | Helix-turn-helix transcriptional regulator | | uniclust | UniRef100\_A0A1Q6I010 | 99.7 | 2e-21 | 3.6e-27 | 137.6 | 165 | (2, 168) | 180 | (54, 258) | 274 | HTH cro/C1-type domain-containing protein | HTH cro/C1-type domain-containing protein | | uniclust | UniRef100\_B6AS86 | 99.7 | 2e-21 | 3.6e-27 | 128.7 | 176 | (2, 179) | 180 | (7, 228) | 229 | Putative phage repressor | Putative phage repressor | | uniclust | UniRef100\_A0A1Y2K654 | 99.7 | 2e-21 | 3.6e-27 | 122.6 | 96 | (84, 180) | 180 | (30, 127) | 129 | Putative Cro/CI family transcriptional regulator | Putative Cro/CI family transcriptional regulator | | uniclust | UniRef100\_A0A016QME0 | 99.7 | 2e-21 | 3.7e-27 | 134.9 | 171 | (2, 176) | 180 | (30, 243) | 251 | LexA repressor | LexA repressor | | uniclust | UniRef100\_A0A640WCV9 | 99.7 | 2.1e-21 | 3.9e-27 | 129.2 | 173 | (2, 174) | 180 | (5, 234) | 242 | Helix-turn-helix domain-containing protein | Helix-turn-helix domain-containing protein | | uniclust | UniRef100\_UPI0009857A7F | 99.7 | 2.4e-21 | 4.4e-27 | 129.7 | 168 | (1, 176) | 180 | (10, 219) | 228 | XRE family transcriptional regulator | XRE family transcriptional regulator | | uniclust | UniRef100\_A0A071LTP5 | 99.7 | 2.4e-21 | 4.5e-27 | 140.9 | 100 | (80, 180) | 180 | (171, 272) | 279 | HTH Mu-type domain-containing protein | HTH Mu-type domain-containing protein | | uniclust | UniRef100\_A0A1E7YT01 | 99.7 | 2.8e-21 | 5.1e-27 | 137.7 | 171 | (5, 176) | 180 | (70, 292) | 305 | Peptidase S24/S26A/S26B/S26C domain-containing protein | Peptidase S24/S26A/S26B/S26C domain-containing protein | | uniclust | UniRef100\_A0A356M362 | 99.7 | 2.8e-21 | 5.1e-27 | 131.7 | 177 | (1, 179) | 180 | (12, 222) | 229 | HTH cro/C1-type domain-containing protein | HTH cro/C1-type domain-containing protein | | uniclust | UniRef100\_A0A081RAB9 | 99.7 | 2.9e-21 | 5.3e-27 | 120.2 | 99 | (80, 180) | 180 | (23, 122) | 123 | Putative phage repressor | Putative phage repressor | | uniclust | UniRef100\_A0A519BE94 | 99.7 | 2.9e-21 | 5.4e-27 | 130.3 | 178 | (1, 180) | 180 | (14, 234) | 234 | Helix-turn-helix domain-containing protein | Helix-turn-helix domain-containing protein | | uniclust | UniRef100\_A0A946HKU8 | 99.7 | 3.7e-21 | 6.8e-27 | 122.5 | 176 | (4, 180) | 180 | (2, 189) | 190 | Helix-turn-helix domain-containing protein | Helix-turn-helix domain-containing protein | | uniclust | UniRef100\_A0A011NA96 | 99.7 | 3.8e-21 | 7e-27 | 131.5 | 156 | (19, 176) | 180 | (1, 200) | 210 | Transcriptional regulator | Transcriptional regulator | | uniclust | UniRef100\_UPI001432A832 | 99.7 | 4e-21 | 7.4e-27 | 124.7 | 180 | (1, 180) | 180 | (3, 207) | 207 | helix-turn-helix domain-containing protein | helix-turn-helix domain-containing protein | | uniclust | UniRef100\_A0A971PC19 | 99.7 | 4.2e-21 | 7.7e-27 | 131.8 | 173 | (1, 176) | 180 | (3, 253) | 255 | LexA family transcriptional regulator | LexA family transcriptional regulator | | uniclust | UniRef100\_UPI0004923115 | 99.7 | 4.8e-21 | 8.9e-27 | 130.7 | 177 | (1, 179) | 180 | (10, 249) | 250 | helix-turn-helix domain-containing protein | helix-turn-helix domain-containing protein | | uniclust | UniRef100\_A0A0K2XET6 | 99.7 | 5e-21 | 9.3e-27 | 131.2 | 175 | (3, 178) | 180 | (38, 242) | 246 | Transcriptional regulator | Transcriptional regulator | | uniclust | UniRef100\_A0A9D8S324 | 99.7 | 5.7e-21 | 1.1e-26 | 123.9 | 174 | (2, 176) | 180 | (2, 185) | 192 | LexA family transcriptional regulator | LexA family transcriptional regulator | | uniclust | UniRef100\_A0A4Q2X6Y6 | 99.7 | 5.7e-21 | 1.1e-26 | 130.6 | 175 | (1, 178) | 180 | (12, 220) | 221 | LexA family transcriptional regulator | LexA family transcriptional regulator | | uniclust | UniRef100\_A0A0H0MNU5 | 99.7 | 5.7e-21 | 1.1e-26 | 131.8 | 171 | (2, 176) | 180 | (27, 232) | 246 | Helix-turn-helix domain-containing protein | Helix-turn-helix domain-containing protein | | uniclust | UniRef100\_A0A158HTA6 | 99.7 | 6.2e-21 | 1.1e-26 | 130.0 | 178 | (1, 179) | 180 | (8, 245) | 249 | LexA repressor | LexA repressor | | uniclust | UniRef100\_A0A239PSQ2 | 99.7 | 6.2e-21 | 1.1e-26 | 134.6 | 176 | (1, 179) | 180 | (59, 262) | 265 | Transcriptional regulator, contains XRE-family HTH domain | Transcriptional regulator, contains XRE-family HTH domain | | uniclust | UniRef100\_A0A136P7M0 | 99.7 | 7.2e-21 | 1.3e-26 | 132.0 | 179 | (1, 180) | 180 | (15, 221) | 222 | LexA repressor | LexA repressor | | uniclust | UniRef100\_A0A0J6YUX8 | 99.7 | 7.3e-21 | 1.3e-26 | 136.2 | 177 | (1, 179) | 180 | (95, 313) | 314 | HTH cro/C1-type domain-containing protein | HTH cro/C1-type domain-containing protein | | uniclust | UniRef100\_A0A068QRI4 | 99.7 | 9.3e-21 | 1.7e-26 | 137.6 | 173 | (1, 177) | 180 | (49, 261) | 270 | Repressor protein C2 | Repressor protein C2 | | uniclust | UniRef100\_A0A258GFY3 | 99.7 | 9.9e-21 | 1.8e-26 | 128.8 | 176 | (2, 177) | 180 | (6, 243) | 246 | HTH cro/C1-type domain-containing protein | HTH cro/C1-type domain-containing protein | | uniclust | UniRef100\_A0A367LYV8 | 99.7 | 1e-20 | 1.8e-26 | 114.4 | 96 | (84, 180) | 180 | (15, 113) | 114 | S24 family peptidase (Fragment) | S24 family peptidase (Fragment) | | uniclust | UniRef100\_A0A174MQZ3 | 99.7 | 1.1e-20 | 2.1e-26 | 129.4 | 175 | (1, 177) | 180 | (7, 215) | 226 | Antitoxin PezA | Antitoxin PezA | | uniclust | UniRef100\_A0A2P1S2C8 | 99.7 | 1.2e-20 | 2.2e-26 | 130.6 | 177 | (1, 179) | 180 | (7, 220) | 259 | HTH cro/C1-type domain-containing protein | HTH cro/C1-type domain-containing protein | | uniclust | UniRef100\_A0A0F2RP23 | 99.7 | 1.2e-20 | 2.2e-26 | 124.3 | 102 | (78, 180) | 180 | (58, 160) | 161 | Peptidase S24/S26A/S26B/S26C domain-containing protein | Peptidase S24/S26A/S26B/S26C domain-containing protein | | uniclust | UniRef100\_A0A258AUA7 | 99.7 | 1.2e-20 | 2.2e-26 | 137.8 | 175 | (3, 179) | 180 | (90, 307) | 313 | HTH cro/C1-type domain-containing protein | HTH cro/C1-type domain-containing protein | | uniclust | UniRef100\_UPI0013D78761 | 99.7 | 1.2e-20 | 2.3e-26 | 134.7 | 178 | (1, 180) | 180 | (4, 323) | 324 | S24 family peptidase | S24 family peptidase | | uniclust | UniRef100\_A0A3G9GCF8 | 99.7 | 1.3e-20 | 2.4e-26 | 117.3 | 100 | (80, 180) | 180 | (36, 138) | 138 | Probable transcription regulator | Probable transcription regulator | | uniclust | UniRef100\_A0A0N0MC17 | 99.7 | 1.5e-20 | 2.8e-26 | 126.0 | 98 | (82, 180) | 180 | (80, 180) | 180 | Peptidase S24/S26A/S26B/S26C domain-containing protein | Peptidase S24/S26A/S26B/S26C domain-containing protein | | uniclust | UniRef100\_A0A193FV67 | 99.7 | 1.5e-20 | 2.8e-26 | 140.5 | 174 | (3, 176) | 180 | (139, 384) | 392 | HTH cro/C1-type domain-containing protein | HTH cro/C1-type domain-containing protein | | uniclust | UniRef100\_A0A2P5MV31 | 99.7 | 1.7e-20 | 3e-26 | 131.1 | 177 | (1, 179) | 180 | (17, 322) | 323 | HTH cro/C1-type domain-containing protein | HTH cro/C1-type domain-containing protein | | uniclust | UniRef100\_A0A1B2I1E8 | 99.7 | 1.7e-20 | 3e-26 | 131.0 | 176 | (1, 179) | 180 | (32, 258) | 265 | HTH cro/C1-type domain-containing protein | HTH cro/C1-type domain-containing protein | | uniclust | UniRef100\_A0A0B1U779 | 99.7 | 1.8e-20 | 3.3e-26 | 134.8 | 177 | (3, 180) | 180 | (48, 269) | 273 | Putative DNA-binding protein | Putative DNA-binding protein | | uniclust | UniRef100\_A0A8S5PML4 | 99.7 | 1.9e-20 | 3.5e-26 | 134.1 | 174 | (2, 176) | 180 | (129, 345) | 355 | Helix-turn-helix domain protein | Helix-turn-helix domain protein | | uniclust | UniRef100\_A0A010SKQ2 | 99.7 | 2.1e-20 | 3.9e-26 | 137.2 | 174 | (1, 178) | 180 | (151, 361) | 368 | Sulfurtransferase | Sulfurtransferase | | uniclust | UniRef100\_A0A971C5L3 | 99.7 | 2.1e-20 | 3.9e-26 | 128.7 | 174 | (1, 176) | 180 | (10, 286) | 291 | Helix-turn-helix domain-containing protein | Helix-turn-helix domain-containing protein | | uniclust | UniRef100\_A0A6B1CMI7 | 99.7 | 2.1e-20 | 3.9e-26 | 131.9 | 173 | (4, 180) | 180 | (27, 261) | 278 | Helix-turn-helix transcriptional regulator | Helix-turn-helix transcriptional regulator | | uniclust | UniRef100\_A0A069PBS4 | 99.7 | 2.2e-20 | 4.1e-26 | 137.5 | 169 | (2, 174) | 180 | (62, 279) | 290 | Phage repressor protein | Phage repressor protein | | uniclust | UniRef100\_A0A087NEV6 | 99.7 | 2.2e-20 | 4.1e-26 | 114.0 | 93 | (87, 180) | 180 | (13, 108) | 109 | Peptidase S24/S26A/S26B/S26C domain-containing protein | Peptidase S24/S26A/S26B/S26C domain-containing protein | | uniclust | UniRef100\_A0A292SMX3 | 99.7 | 2.3e-20 | 4.3e-26 | 125.5 | 166 | (12, 179) | 180 | (27, 212) | 222 | Peptidase S24/S26A/S26B/S26C domain-containing protein | Peptidase S24/S26A/S26B/S26C domain-containing protein | | uniclust | UniRef100\_A0A1E7ZE28 | 99.7 | 2.5e-20 | 4.6e-26 | 129.4 | 175 | (1, 177) | 180 | (13, 202) | 206 | Peptidase S24/S26A/S26B/S26C domain-containing protein | Peptidase S24/S26A/S26B/S26C domain-containing protein | | uniclust | UniRef100\_A0A1C5MIN5 | 99.7 | 2.9e-20 | 5.4e-26 | 123.9 | 167 | (1, 179) | 180 | (7, 178) | 190 | Bifunctional HTH-domain containing protein/aminotransferase | Bifunctional HTH-domain containing protein/aminotransferase | | uniclust | UniRef100\_A0A9E5G3I3 | 99.7 | 2.9e-20 | 5.4e-26 | 126.7 | 179 | (1, 180) | 180 | (17, 246) | 251 | LexA family transcriptional regulator | LexA family transcriptional regulator | | uniclust | UniRef100\_A4U201 | 99.7 | 3.1e-20 | 5.7e-26 | 121.2 | 178 | (2, 180) | 180 | (6, 222) | 222 | Transcriptional regulator | Transcriptional regulator | | uniclust | UniRef100\_A0A3S0D8L6 | 99.7 | 3.7e-20 | 6.8e-26 | 125.9 | 166 | (12, 177) | 180 | (25, 219) | 224 | LexA family transcriptional regulator | LexA family transcriptional regulator | | uniclust | UniRef100\_A0A0E2NFI1 | 99.7 | 3.8e-20 | 7.1e-26 | 135.3 | 173 | (2, 176) | 180 | (46, 267) | 274 | Repressor | Repressor | | uniclust | UniRef100\_A0A2D5TJQ4 | 99.7 | 4e-20 | 7.3e-26 | 124.7 | 172 | (1, 176) | 180 | (12, 217) | 223 | HTH cro/C1-type domain-containing protein | HTH cro/C1-type domain-containing protein | | uniclust | UniRef100\_A0A1C3H508 | 99.7 | 4.4e-20 | 8.1e-26 | 126.6 | 91 | (86, 178) | 180 | (191, 281) | 284 | Phage repressor | Phage repressor | | uniclust | UniRef100\_A0A1F9LJ24 | 99.7 | 4.4e-20 | 8.2e-26 | 130.8 | 177 | (1, 177) | 180 | (36, 274) | 283 | HTH cro/C1-type domain-containing protein | HTH cro/C1-type domain-containing protein | | uniclust | UniRef100\_A0A1H5VJF6 | 99.7 | 4.6e-20 | 8.5e-26 | 127.8 | 174 | (2, 179) | 180 | (5, 274) | 290 | Peptidase S24-like | Peptidase S24-like | | uniclust | UniRef100\_A0A1G3PJ90 | 99.7 | 5.2e-20 | 9.6e-26 | 122.0 | 175 | (2, 178) | 180 | (4, 216) | 219 | HTH cro/C1-type domain-containing protein | HTH cro/C1-type domain-containing protein | | uniclust | UniRef100\_A0A7R6VQ36 | 99.7 | 5.3e-20 | 9.8e-26 | 123.8 | 177 | (1, 177) | 180 | (4, 229) | 239 | Phage repressor | Phage repressor | | uniclust | UniRef100\_A0A0T2MLF1 | 99.7 | 5.7e-20 | 1e-25 | 134.4 | 178 | (1, 178) | 180 | (68, 280) | 339 | Phage repressor | Phage repressor | | uniclust | UniRef100\_A0A3P1Y7B9 | 99.7 | 6.6e-20 | 1.2e-25 | 122.6 | 174 | (1, 175) | 180 | (13, 226) | 237 | LexA family transcriptional regulator | LexA family transcriptional regulator | | uniclust | UniRef100\_A0A1I2BPM5 | 99.7 | 6.8e-20 | 1.3e-25 | 131.6 | 175 | (1, 176) | 180 | (20, 249) | 299 | Phage repressor protein C, contains Cro/C1-type HTH and peptisase s24 domains | Phage repressor protein C, contains Cro/C1-type HTH and peptisase s24 domains | | uniclust | UniRef100\_A0A010RVE5 | 99.7 | 7.5e-20 | 1.4e-25 | 134.3 | 174 | (2, 178) | 180 | (85, 294) | 339 | Cro/Cl family transcriptional regulator | Cro/Cl family transcriptional regulator | | uniclust | UniRef100\_A0A7C6H9D8 | 99.7 | 7.7e-20 | 1.4e-25 | 122.7 | 174 | (4, 180) | 180 | (3, 214) | 215 | Helix-turn-helix transcriptional regulator | Helix-turn-helix transcriptional regulator | | uniclust | UniRef100\_A0A009PCU9 | 99.7 | 8.2e-20 | 1.5e-25 | 130.7 | 177 | (2, 179) | 180 | (50, 261) | 281 | Peptidase S24-like family protein | Peptidase S24-like family protein | | uniclust | UniRef100\_A0A2Z4PPI4 | 99.7 | 8.4e-20 | 1.6e-25 | 109.6 | 90 | (90, 180) | 180 | (8, 101) | 102 | Peptidase S24/S26A/S26B/S26C domain-containing protein | Peptidase S24/S26A/S26B/S26C domain-containing protein | | uniclust | UniRef100\_A0A2P8KL91 | 99.7 | 8.5e-20 | 1.6e-25 | 126.9 | 170 | (3, 177) | 180 | (35, 249) | 254 | Uncharacterized protein | Uncharacterized protein | | uniclust | UniRef100\_A0A1I1N4A7 | 99.7 | 1.1e-19 | 1.9e-25 | 122.0 | 178 | (1, 179) | 180 | (17, 232) | 235 | Phage repressor protein C, contains Cro/C1-type HTH and peptisase s24 domains | Phage repressor protein C, contains Cro/C1-type HTH and peptisase s24 domains | | uniclust | UniRef100\_A0A1B2Z1M4 | 99.7 | 1.1e-19 | 2e-25 | 128.9 | 176 | (1, 176) | 180 | (43, 248) | 291 | HTH cro/C1-type domain-containing protein | HTH cro/C1-type domain-containing protein | | uniclust | UniRef100\_A0A1Q3MNN2 | 99.7 | 1.1e-19 | 2.1e-25 | 124.3 | 170 | (2, 178) | 180 | (14, 202) | 222 | Peptidase S24/S26A/S26B/S26C domain-containing protein | Peptidase S24/S26A/S26B/S26C domain-containing protein | | uniclust | UniRef100\_A0A1U9LFW6 | 99.7 | 1.2e-19 | 2.2e-25 | 117.3 | 100 | (80, 180) | 180 | (61, 163) | 163 | Peptidase S24/S26A/S26B/S26C domain-containing protein | Peptidase S24/S26A/S26B/S26C domain-containing protein | | uniclust | UniRef100\_A0A847BTK1 | 99.7 | 1.3e-19 | 2.3e-25 | 106.4 | 83 | (95, 178) | 180 | (2, 84) | 88 | S24 family peptidase | S24 family peptidase | | uniclust | UniRef100\_A0A081J3X7 | 99.7 | 1.3e-19 | 2.3e-25 | 128.4 | 168 | (11, 179) | 180 | (66, 282) | 285 | Transcriptional regulator | Transcriptional regulator | | uniclust | UniRef100\_A0A942GU57 | 99.7 | 1.3e-19 | 2.3e-25 | 124.8 | 174 | (2, 178) | 180 | (23, 233) | 262 | Helix-turn-helix domain-containing protein | Helix-turn-helix domain-containing protein | | uniclust | UniRef100\_A0A0D6I1W4 | 99.7 | 1.3e-19 | 2.4e-25 | 110.6 | 93 | (87, 180) | 180 | (13, 108) | 110 | Repressor LexA | Repressor LexA | | uniclust | UniRef100\_A0A173T592 | 99.7 | 1.3e-19 | 2.4e-25 | 128.4 | 174 | (1, 175) | 180 | (36, 281) | 284 | Peptidase S24-like | Peptidase S24-like | | uniclust | UniRef100\_A0A1V5IGK5 | 99.7 | 1.5e-19 | 2.8e-25 | 126.5 | 172 | (1, 174) | 180 | (18, 248) | 259 | LexA repressor | LexA repressor | | uniclust | UniRef100\_A0A0W0R9T4 | 99.7 | 1.6e-19 | 3e-25 | 124.5 | 173 | (3, 177) | 180 | (10, 209) | 215 | HTH-type transcriptional regulator | HTH-type transcriptional regulator | | uniclust | UniRef100\_A0A0A1AFM4 | 99.7 | 1.6e-19 | 3e-25 | 120.8 | 177 | (1, 178) | 180 | (12, 216) | 218 | Helix-turn-helix domain-containing protein | Helix-turn-helix domain-containing protein | | uniclust | UniRef100\_A0A1F7T9S1 | 99.7 | 1.6e-19 | 3e-25 | 125.1 | 177 | (2, 179) | 180 | (26, 245) | 282 | HTH cro/C1-type domain-containing protein | HTH cro/C1-type domain-containing protein | | uniclust | UniRef100\_A0A0U2IWK9 | 99.7 | 1.7e-19 | 3e-25 | 115.2 | 98 | (82, 180) | 180 | (34, 134) | 134 | Putative peptidase S24 | Putative peptidase S24 | | uniclust | UniRef100\_A0A1C6E8P6 | 99.7 | 1.8e-19 | 3.4e-25 | 128.0 | 169 | (2, 177) | 180 | (8, 325) | 329 | Peptidase S24-like | Peptidase S24-like | | uniclust | UniRef100\_A0A0R6PHW4 | 99.7 | 1.9e-19 | 3.6e-25 | 128.2 | 175 | (3, 179) | 180 | (40, 258) | 278 | HTH cro/C1-type domain-containing protein | HTH cro/C1-type domain-containing protein | | uniclust | UniRef100\_A0A220S122 | 99.7 | 2e-19 | 3.7e-25 | 122.1 | 173 | (1, 174) | 180 | (19, 233) | 243 | HTH cro/C1-type domain-containing protein | HTH cro/C1-type domain-containing protein | | uniclust | UniRef100\_A0A1I5LCX6 | 99.7 | 2.1e-19 | 3.8e-25 | 131.5 | 176 | (1, 179) | 180 | (126, 369) | 374 | Peptidase S24-like | Peptidase S24-like | | uniclust | UniRef100\_A0A432UMV4 | 99.7 | 2.1e-19 | 3.9e-25 | 125.2 | 164 | (13, 177) | 180 | (50, 275) | 281 | Peptidase S24/S26A/S26B/S26C domain-containing protein | Peptidase S24/S26A/S26B/S26C domain-containing protein | | uniclust | UniRef100\_A0A848G1C2 | 99.7 | 2.2e-19 | 4e-25 | 119.7 | 176 | (1, 178) | 180 | (2, 222) | 224 | LexA family transcriptional regulator | LexA family transcriptional regulator | | uniclust | UniRef100\_A0A963CV82 | 99.7 | 2.2e-19 | 4e-25 | 116.1 | 176 | (1, 178) | 180 | (4, 203) | 206 | LexA family transcriptional regulator | LexA family transcriptional regulator | | uniclust | UniRef100\_A0A8T3RZ60 | 99.7 | 2.2e-19 | 4.1e-25 | 123.2 | 164 | (15, 179) | 180 | (27, 280) | 282 | LexA family transcriptional regulator | LexA family transcriptional regulator | | uniclust | UniRef100\_A0A2E3MGU9 | 99.6 | 2.7e-19 | 5e-25 | 119.3 | 176 | (2, 178) | 180 | (9, 222) | 226 | HTH cro/C1-type domain-containing protein | HTH cro/C1-type domain-containing protein | | uniclust | UniRef100\_A0A0B0SD21 | 99.6 | 3e-19 | 5.5e-25 | 121.9 | 173 | (3, 179) | 180 | (13, 220) | 231 | Peptidase S24 | Peptidase S24 | | uniclust | UniRef100\_A0A0A1H208 | 99.6 | 3e-19 | 5.6e-25 | 122.6 | 174 | (1, 176) | 180 | (14, 228) | 243 | Putative phage repressor | Putative phage repressor | | uniclust | UniRef100\_A0A085FSU8 | 99.6 | 3.1e-19 | 5.7e-25 | 129.2 | 171 | (1, 174) | 180 | (45, 262) | 271 | Putative phage repressor | Putative phage repressor | | uniclust | UniRef100\_A0A3D5VC65 | 99.6 | 3.2e-19 | 5.9e-25 | 122.0 | 178 | (3, 180) | 180 | (25, 262) | 262 | HTH cro/C1-type domain-containing protein | HTH cro/C1-type domain-containing protein | | uniclust | UniRef100\_A0A011UQX8 | 99.6 | 3.4e-19 | 6.3e-25 | 132.3 | 177 | (2, 179) | 180 | (96, 315) | 329 | Peptidase S24 | Peptidase S24 | | uniclust | UniRef100\_A0A1M7HEK4 | 99.6 | 3.6e-19 | 6.6e-25 | 124.6 | 175 | (2, 179) | 180 | (13, 259) | 271 | Peptidase S24-like | Peptidase S24-like | | uniclust | UniRef100\_A0A0K9N6J2 | 99.6 | 3.9e-19 | 7.3e-25 | 127.0 | 169 | (1, 177) | 180 | (12, 255) | 319 | Putative repressor | Putative repressor | | uniclust | UniRef100\_A0A0Y7I845 | 99.6 | 4.3e-19 | 7.9e-25 | 121.8 | 165 | (9, 174) | 180 | (28, 243) | 253 | Peptidase S24-like protein | Peptidase S24-like protein | | uniclust | UniRef100\_A0A095FKS9 | 99.6 | 4.3e-19 | 7.9e-25 | 120.5 | 176 | (1, 179) | 180 | (5, 229) | 232 | Transcriptional regulator | Transcriptional regulator | | uniclust | UniRef100\_G0EP36 | 99.6 | 5.2e-19 | 9.7e-25 | 121.3 | 174 | (1, 177) | 180 | (19, 254) | 262 | Putative phage repressor | Putative phage repressor | | uniclust | UniRef100\_A0A9D1SLB7 | 99.6 | 5.4e-19 | 1e-24 | 119.5 | 176 | (1, 180) | 180 | (8, 209) | 211 | LexA family transcriptional regulator | LexA family transcriptional regulator | | uniclust | UniRef100\_A0A0S6X5H7 | 99.6 | 6.2e-19 | 1.1e-24 | 106.3 | 95 | (83, 179) | 180 | (20, 114) | 115 | Peptidase S24/S26A/S26B/S26C domain-containing protein | Peptidase S24/S26A/S26B/S26C domain-containing protein | | uniclust | UniRef100\_A0A6S7CQU9 | 99.6 | 7.2e-19 | 1.3e-24 | 123.2 | 164 | (16, 180) | 180 | (114, 340) | 340 | Peptidase S24/S26A/S26B/S26C domain-containing protein | Peptidase S24/S26A/S26B/S26C domain-containing protein | | uniclust | UniRef100\_A0A0C6FN11 | 99.6 | 7.1e-19 | 1.3e-24 | 128.1 | 171 | (1, 174) | 180 | (41, 253) | 261 | Putative phage repressor | Putative phage repressor | | uniclust | UniRef100\_A0A1F3L8R9 | 99.6 | 7.6e-19 | 1.4e-24 | 121.2 | 158 | (1, 159) | 180 | (10, 210) | 238 | HTH cro/C1-type domain-containing protein | HTH cro/C1-type domain-containing protein | | uniclust | UniRef100\_A0A1F4NGE6 | 99.6 | 7.9e-19 | 1.5e-24 | 122.9 | 172 | (3, 174) | 180 | (35, 256) | 264 | HTH cro/C1-type domain-containing protein | HTH cro/C1-type domain-containing protein | | uniclust | UniRef100\_A0A1U9JWC3 | 99.6 | 8.1e-19 | 1.5e-24 | 123.5 | 170 | (1, 174) | 180 | (49, 265) | 271 | Putative DNA-binding protein | Putative DNA-binding protein | | uniclust | UniRef100\_A0A1F5AT30 | 99.6 | 9.3e-19 | 1.7e-24 | 120.3 | 178 | (2, 179) | 180 | (7, 240) | 251 | HTH cro/C1-type domain-containing protein | HTH cro/C1-type domain-containing protein | | uniclust | UniRef100\_UPI0009838998 | 99.6 | 9.6e-19 | 1.8e-24 | 116.9 | 169 | (3, 179) | 180 | (13, 214) | 218 | S24 family peptidase | S24 family peptidase | | uniclust | UniRef100\_A0A352SWL9 | 99.6 | 1.1e-18 | 2e-24 | 119.0 | 172 | (1, 175) | 180 | (5, 248) | 259 | HTH cro/C1-type domain-containing protein | HTH cro/C1-type domain-containing protein | | uniclust | UniRef100\_A0A1J5NH46 | 99.6 | 1.1e-18 | 2e-24 | 110.4 | 92 | (87, 179) | 180 | (45, 140) | 142 | Putative HTH-type transcriptional regulator | Putative HTH-type transcriptional regulator | | uniclust | UniRef100\_A0A1A7C1A4 | 99.6 | 1.2e-18 | 2.2e-24 | 123.9 | 159 | (15, 176) | 180 | (64, 257) | 266 | Peptidase S24-like protein | Peptidase S24-like protein | | uniclust | UniRef100\_A0A0F9HCG5 | 99.6 | 1.3e-18 | 2.4e-24 | 124.9 | 177 | (2, 178) | 180 | (67, 278) | 284 | HTH cro/C1-type domain-containing protein | HTH cro/C1-type domain-containing protein | | uniclust | UniRef100\_A0A1X0W151 | 99.6 | 1.7e-18 | 3.2e-24 | 122.7 | 172 | (3, 177) | 180 | (24, 227) | 247 | HTH cro/C1-type domain-containing protein | HTH cro/C1-type domain-containing protein | | uniclust | UniRef100\_A0A5B9DX04 | 99.6 | 1.8e-18 | 3.3e-24 | 118.6 | 175 | (2, 177) | 180 | (34, 251) | 262 | Helix-turn-helix transcriptional regulator | Helix-turn-helix transcriptional regulator | | uniclust | UniRef100\_A0A060I420 | 99.6 | 1.8e-18 | 3.3e-24 | 126.7 | 174 | (2, 177) | 180 | (79, 279) | 292 | Peptidase S24/S26 family protein | Peptidase S24/S26 family protein | | uniclust | UniRef100\_A0A077N0G1 | 99.6 | 1.8e-18 | 3.3e-24 | 125.2 | 161 | (1, 162) | 180 | (44, 235) | 295 | Helix-turn-helix/peptidase S24-like domain protein | Helix-turn-helix/peptidase S24-like domain protein | | uniclust | UniRef100\_A0A329BE56 | 99.6 | 2e-18 | 3.6e-24 | 121.1 | 170 | (1, 174) | 180 | (102, 306) | 316 | SOS-response transcriptional repressor LexA | SOS-response transcriptional repressor LexA | | uniclust | UniRef100\_A0A2A4NKI2 | 99.6 | 2.1e-18 | 3.8e-24 | 125.3 | 164 | (2, 166) | 180 | (52, 273) | 339 | Peptidase S24/S26A/S26B/S26C domain-containing protein | Peptidase S24/S26A/S26B/S26C domain-containing protein | | uniclust | UniRef100\_A0A085JGV9 | 99.6 | 2.2e-18 | 4.1e-24 | 122.6 | 170 | (2, 176) | 180 | (38, 239) | 307 | Phage repressor | Phage repressor | | uniclust | UniRef100\_A0A068SNY7 | 99.6 | 2.3e-18 | 4.2e-24 | 122.2 | 163 | (16, 178) | 180 | (90, 298) | 300 | Prophage MuSo2, transcriptional regulator, Cro/CI family | Prophage MuSo2, transcriptional regulator, Cro/CI family | | uniclust | UniRef100\_A0A1Y5TZI9 | 99.6 | 2.3e-18 | 4.2e-24 | 111.4 | 100 | (80, 180) | 180 | (61, 163) | 164 | Peptidase S24-like protein | Peptidase S24-like protein | | uniclust | UniRef100\_A0A098CTP1 | 99.6 | 2.4e-18 | 4.4e-24 | 120.3 | 173 | (1, 176) | 180 | (23, 266) | 277 | Transcriptional repressor DicA | Transcriptional repressor DicA | | uniclust | UniRef100\_A0A258L743 | 99.6 | 2.5e-18 | 4.6e-24 | 119.8 | 172 | (2, 177) | 180 | (26, 209) | 221 | HTH cro/C1-type domain-containing protein | HTH cro/C1-type domain-containing protein | | uniclust | UniRef100\_A0A2N1S0V3 | 99.6 | 2.5e-18 | 4.6e-24 | 117.3 | 172 | (2, 175) | 180 | (7, 227) | 236 | HTH cro/C1-type domain-containing protein | HTH cro/C1-type domain-containing protein | | uniclust | UniRef100\_A0A959R3J2 | 99.6 | 2.7e-18 | 4.9e-24 | 114.2 | 177 | (1, 179) | 180 | (4, 215) | 218 | Helix-turn-helix domain-containing protein | Helix-turn-helix domain-containing protein | | uniclust | UniRef100\_A0A0Q6W6Y0 | 99.6 | 2.7e-18 | 4.9e-24 | 123.5 | 160 | (17, 177) | 180 | (71, 251) | 267 | Peptidase S24/S26A/S26B/S26C domain-containing protein | Peptidase S24/S26A/S26B/S26C domain-containing protein | | uniclust | UniRef100\_A0A1I5T8W6 | 99.6 | 2.8e-18 | 5.2e-24 | 120.4 | 156 | (2, 157) | 180 | (25, 213) | 237 | Helix-turn-helix | Helix-turn-helix | | uniclust | UniRef100\_UPI0004669D61 | 99.6 | 3e-18 | 5.4e-24 | 111.5 | 130 | (1, 132) | 180 | (4, 167) | 171 | S24 family peptidase | S24 family peptidase | | uniclust | UniRef100\_A0A1F7T224 | 99.6 | 3.1e-18 | 5.6e-24 | 117.6 | 177 | (2, 179) | 180 | (39, 265) | 267 | HTH cro/C1-type domain-containing protein | HTH cro/C1-type domain-containing protein | | uniclust | UniRef100\_A0A940P805 | 99.6 | 3.3e-18 | 6e-24 | 111.8 | 143 | (1, 144) | 180 | (4, 172) | 176 | Helix-turn-helix transcriptional regulator (Fragment) | Helix-turn-helix transcriptional regulator (Fragment) | | uniclust | UniRef100\_A0A126NZ97 | 99.6 | 3.5e-18 | 6.4e-24 | 118.5 | 177 | (1, 178) | 180 | (12, 228) | 252 | HTH cro/C1-type domain-containing protein | HTH cro/C1-type domain-containing protein | | uniclust | UniRef100\_A0A3C0EH31 | 99.6 | 3.6e-18 | 6.7e-24 | 115.3 | 165 | (2, 167) | 180 | (11, 208) | 221 | HTH cro/C1-type domain-containing protein | HTH cro/C1-type domain-containing protein | | uniclust | UniRef100\_A0A1C3K7V4 | 99.6 | 3.7e-18 | 6.8e-24 | 124.9 | 178 | (1, 178) | 180 | (15, 343) | 357 | Putative phage repressor protein | Putative phage repressor protein | | uniclust | UniRef100\_A0A223PBQ1 | 99.6 | 3.7e-18 | 6.8e-24 | 120.0 | 163 | (13, 177) | 180 | (24, 233) | 241 | Transcriptional regulator | Transcriptional regulator | | uniclust | UniRef100\_A0A0C1MWF1 | 99.6 | 3.7e-18 | 6.8e-24 | 105.7 | 91 | (87, 179) | 180 | (8, 99) | 110 | Peptidase S24/S26A/S26B/S26C domain-containing protein | Peptidase S24/S26A/S26B/S26C domain-containing protein | | uniclust | UniRef100\_A0A2E4Q3Q2 | 99.6 | 3.9e-18 | 7.2e-24 | 116.5 | 174 | (1, 176) | 180 | (69, 298) | 302 | XRE family transcriptional regulator | XRE family transcriptional regulator | | uniclust | UniRef100\_A0A0S4SRP9 | 99.6 | 4.2e-18 | 7.7e-24 | 119.8 | 174 | (2, 177) | 180 | (7, 233) | 254 | Signal peptidase I | Signal peptidase I | | uniclust | UniRef100\_A0A352PSP8 | 99.6 | 5e-18 | 9.2e-24 | 112.9 | 100 | (80, 179) | 180 | (66, 169) | 171 | Peptidase S24/S26A/S26B/S26C domain-containing protein | Peptidase S24/S26A/S26B/S26C domain-containing protein | | uniclust | UniRef100\_A0A1M7RMF5 | 99.6 | 5.2e-18 | 9.6e-24 | 114.8 | 92 | (87, 180) | 180 | (81, 173) | 177 | SOS regulatory protein LexA | SOS regulatory protein LexA | | uniclust | UniRef100\_A0A0S2EQH0 | 99.6 | 5.6e-18 | 1e-23 | 120.5 | 173 | (2, 177) | 180 | (64, 256) | 268 | HTH cro/C1-type domain-containing protein | HTH cro/C1-type domain-containing protein | | uniclust | UniRef100\_A0A011Q9M4 | 99.6 | 6.7e-18 | 1.2e-23 | 115.2 | 174 | (2, 178) | 180 | (21, 217) | 222 | Putative HTH-type transcriptional regulator | Putative HTH-type transcriptional regulator | | uniclust | UniRef100\_UPI001403D558 | 99.6 | 6.9e-18 | 1.3e-23 | 120.4 | 176 | (1, 177) | 180 | (130, 390) | 394 | helix-turn-helix transcriptional regulator | helix-turn-helix transcriptional regulator | | uniclust | UniRef100\_A0A2P9HMT6 | 99.6 | 6.9e-18 | 1.3e-23 | 117.1 | 173 | (3, 176) | 180 | (8, 227) | 231 | HTH cro/C1-type domain-containing protein | HTH cro/C1-type domain-containing protein | | uniclust | UniRef100\_A0A2V2G387 | 99.6 | 7e-18 | 1.3e-23 | 120.8 | 167 | (12, 180) | 180 | (54, 251) | 251 | HTH cro/C1-type domain-containing protein | HTH cro/C1-type domain-containing protein | | uniclust | UniRef100\_A0A084A767 | 99.6 | 7.8e-18 | 1.4e-23 | 117.6 | 174 | (2, 177) | 180 | (20, 292) | 295 | Phage repressor | Phage repressor | | uniclust | UniRef100\_A0A927Y316 | 99.6 | 7.8e-18 | 1.4e-23 | 120.1 | 176 | (1, 178) | 180 | (3, 307) | 309 | HTH cro/C1-type domain-containing protein | HTH cro/C1-type domain-containing protein | | uniclust | UniRef100\_A0A0U3G7P0 | 99.6 | 7.9e-18 | 1.5e-23 | 111.7 | 174 | (3, 178) | 180 | (2, 195) | 198 | Helix-turn-helix domain-containing protein | Helix-turn-helix domain-containing protein | | uniclust | UniRef100\_A0A845JXB3 | 99.6 | 8e-18 | 1.5e-23 | 111.2 | 100 | (80, 179) | 180 | (129, 228) | 229 | Transcriptional regulator | Transcriptional regulator | | uniclust | UniRef100\_A0A1Q6G157 | 99.6 | 8.3e-18 | 1.5e-23 | 117.7 | 175 | (3, 179) | 180 | (25, 233) | 237 | Peptidase S24/S26A/S26B/S26C domain-containing protein | Peptidase S24/S26A/S26B/S26C domain-containing protein | | uniclust | UniRef100\_A0A5S3XEB8 | 99.6 | 8.4e-18 | 1.6e-23 | 114.3 | 176 | (2, 178) | 180 | (25, 211) | 217 | HTH cro/C1-type domain-containing protein | HTH cro/C1-type domain-containing protein | | uniclust | UniRef100\_A0A8I2F569 | 99.6 | 9e-18 | 1.7e-23 | 114.9 | 174 | (1, 175) | 180 | (17, 216) | 229 | Helix-turn-helix domain-containing protein | Helix-turn-helix domain-containing protein | | uniclust | UniRef100\_A0A1I7BNY7 | 99.6 | 1e-17 | 1.9e-23 | 115.1 | 93 | (85, 177) | 180 | (124, 219) | 223 | Peptidase S24-like | Peptidase S24-like | | uniclust | UniRef100\_A0A0S2SLA0 | 99.6 | 1.1e-17 | 2e-23 | 110.0 | 100 | (80, 179) | 180 | (53, 156) | 158 | Peptidase S24/S26A/S26B/S26C domain-containing protein | Peptidase S24/S26A/S26B/S26C domain-containing protein | | uniclust | UniRef100\_A0A172U149 | 99.6 | 1.1e-17 | 2e-23 | 118.1 | 165 | (3, 167) | 180 | (21, 235) | 260 | HTH cro/C1-type domain-containing protein | HTH cro/C1-type domain-containing protein | | uniclust | UniRef100\_A0A1F9G5D9 | 99.5 | 1.2e-17 | 2.3e-23 | 118.9 | 174 | (2, 176) | 180 | (31, 258) | 276 | HTH cro/C1-type domain-containing protein | HTH cro/C1-type domain-containing protein | | uniclust | UniRef100\_A0A2P7TC32 | 99.5 | 1.3e-17 | 2.4e-23 | 111.7 | 175 | (1, 178) | 180 | (10, 222) | 224 | Helix-turn-helix domain-containing protein | Helix-turn-helix domain-containing protein | | uniclust | UniRef100\_A0A0C2R644 | 99.5 | 1.3e-17 | 2.4e-23 | 122.6 | 87 | (88, 177) | 180 | (234, 322) | 330 | HTH cro/C1-type domain-containing protein | HTH cro/C1-type domain-containing protein | | uniclust | UniRef100\_A0A1I6T486 | 99.5 | 1.4e-17 | 2.6e-23 | 117.8 | 173 | (3, 178) | 180 | (5, 236) | 242 | Peptidase S24-like | Peptidase S24-like | | uniclust | UniRef100\_A0A381KSX4 | 99.5 | 1.5e-17 | 2.8e-23 | 112.8 | 179 | (1, 179) | 180 | (15, 215) | 216 | Helix-turn-helix | Helix-turn-helix | | uniclust | UniRef100\_A0A1F3SQC4 | 99.5 | 1.5e-17 | 2.8e-23 | 109.6 | 94 | (85, 180) | 180 | (55, 148) | 152 | Peptidase S24/S26A/S26B/S26C domain-containing protein | Peptidase S24/S26A/S26B/S26C domain-containing protein | | uniclust | UniRef100\_A0A031G478 | 99.5 | 1.6e-17 | 2.9e-23 | 118.3 | 177 | (3, 179) | 180 | (46, 251) | 254 | Peptidase s24-like domain protein | Peptidase s24-like domain protein | | uniclust | UniRef100\_A0A136LGU8 | 99.5 | 1.6e-17 | 3e-23 | 122.4 | 151 | (15, 166) | 180 | (36, 232) | 305 | Transcriptional regulator | Transcriptional regulator | | uniclust | UniRef100\_A0A066ZT37 | 99.5 | 1.7e-17 | 3.1e-23 | 120.0 | 99 | (80, 180) | 180 | (140, 239) | 258 | DNA polymerase V | DNA polymerase V | | uniclust | UniRef100\_A0A085EU53 | 99.5 | 1.7e-17 | 3.2e-23 | 120.8 | 179 | (1, 180) | 180 | (39, 263) | 278 | Helix-turn-helix protein | Helix-turn-helix protein | | uniclust | UniRef100\_A0A0E4A064 | 99.5 | 1.8e-17 | 3.3e-23 | 118.1 | 175 | (2, 177) | 180 | (38, 254) | 261 | Repressor | Repressor | | uniclust | UniRef100\_A0A554XCA7 | 99.5 | 1.9e-17 | 3.5e-23 | 116.5 | 88 | (89, 176) | 180 | (227, 316) | 325 | Peptidase S24-like protein | Peptidase S24-like protein | | uniclust | UniRef100\_A0A031INB5 | 99.5 | 1.9e-17 | 3.5e-23 | 120.6 | 165 | (11, 177) | 180 | (48, 302) | 307 | Prophage PSPPH03, Cro/CI family transcriptional regulator | Prophage PSPPH03, Cro/CI family transcriptional regulator | | uniclust | UniRef100\_A0A1Q3QX90 | 99.5 | 1.9e-17 | 3.6e-23 | 115.8 | 175 | (2, 177) | 180 | (49, 268) | 276 | HTH cro/C1-type domain-containing protein | HTH cro/C1-type domain-containing protein | | uniclust | UniRef100\_A0A1Q3VUE3 | 99.5 | 2e-17 | 3.6e-23 | 116.1 | 160 | (16, 175) | 180 | (47, 231) | 241 | HTH cro/C1-type domain-containing protein | HTH cro/C1-type domain-containing protein | | uniclust | UniRef100\_A0A6I4YRF4 | 99.5 | 2e-17 | 3.7e-23 | 118.2 | 160 | (15, 175) | 180 | (151, 358) | 370 | Helix-turn-helix domain-containing protein | Helix-turn-helix domain-containing protein | | uniclust | UniRef100\_A0A0B6CXM0 | 99.5 | 2.1e-17 | 3.8e-23 | 113.5 | 173 | (4, 177) | 180 | (11, 216) | 228 | Helix-turn-helix family protein | Helix-turn-helix family protein | | uniclust | UniRef100\_A0A0K8JF70 | 99.5 | 2.4e-17 | 4.4e-23 | 114.8 | 175 | (1, 177) | 180 | (10, 295) | 298 | HTH cro/C1-type domain-containing protein | HTH cro/C1-type domain-containing protein | | uniclust | UniRef100\_A0A940CVJ9 | 99.5 | 2.4e-17 | 4.4e-23 | 111.5 | 175 | (1, 177) | 180 | (3, 229) | 244 | LexA family transcriptional regulator | LexA family transcriptional regulator | | uniclust | UniRef100\_A0A315CMB6 | 99.5 | 2.4e-17 | 4.5e-23 | 110.6 | 174 | (1, 178) | 180 | (5, 201) | 206 | HTH cro/C1-type domain-containing protein | HTH cro/C1-type domain-containing protein | | uniclust | UniRef100\_A0A380LI00 | 99.5 | 2.6e-17 | 4.7e-23 | 109.7 | 169 | (9, 179) | 180 | (15, 198) | 201 | CI-like repressor, S. pneumoniae bacteriophage EJ-1 | CI-like repressor, S. pneumoniae bacteriophage EJ-1 | | uniclust | UniRef100\_A0A844PAU3 | 99.5 | 2.7e-17 | 5e-23 | 109.7 | 171 | (6, 180) | 180 | (28, 214) | 214 | Transcriptional regulator | Transcriptional regulator | | uniclust | UniRef100\_A0A7W2GRI4 | 99.5 | 3e-17 | 5.5e-23 | 114.1 | 162 | (16, 178) | 180 | (56, 265) | 272 | Helix-turn-helix domain-containing protein | Helix-turn-helix domain-containing protein | | uniclust | UniRef100\_A0A0A8HUB8 | 99.5 | 3e-17 | 5.6e-23 | 113.6 | 171 | (4, 175) | 180 | (18, 225) | 243 | Peptidase S24 LexA-like protein | Peptidase S24 LexA-like protein | | uniclust | UniRef100\_A0A8J8BJY7 | 99.5 | 3e-17 | 5.6e-23 | 112.4 | 173 | (4, 179) | 180 | (9, 211) | 245 | LexA family transcriptional regulator | LexA family transcriptional regulator | | uniclust | UniRef100\_A0A101VSQ7 | 99.5 | 3.1e-17 | 5.7e-23 | 114.7 | 172 | (1, 176) | 180 | (11, 213) | 228 | HTH cro/C1-type domain-containing protein | HTH cro/C1-type domain-containing protein | | uniclust | UniRef100\_A0A2D5Y4P2 | 99.5 | 3.2e-17 | 6e-23 | 112.3 | 164 | (13, 177) | 180 | (15, 197) | 200 | Peptidase S24/S26A/S26B/S26C domain-containing protein | Peptidase S24/S26A/S26B/S26C domain-containing protein | | uniclust | UniRef100\_A0A2S0UMT0 | 99.5 | 3.3e-17 | 6.1e-23 | 95.5 | 79 | (101, 179) | 180 | (1, 82) | 85 | Peptidase S24/S26A/S26B/S26C domain-containing protein | Peptidase S24/S26A/S26B/S26C domain-containing protein | | uniclust | UniRef100\_E5BEW3 | 99.5 | 3.6e-17 | 6.6e-23 | 116.0 | 173 | (4, 179) | 180 | (9, 310) | 345 | Putative phage head-tail adaptor | Putative phage head-tail adaptor | | uniclust | UniRef100\_A0A2A4XVH8 | 99.5 | 3.7e-17 | 6.9e-23 | 113.6 | 171 | (1, 174) | 180 | (4, 223) | 231 | HTH cro/C1-type domain-containing protein | HTH cro/C1-type domain-containing protein | | uniclust | UniRef100\_A0A0K2Y4A7 | 99.5 | 4.6e-17 | 8.5e-23 | 119.7 | 176 | (1, 177) | 180 | (10, 317) | 339 | LexA-family transcriptional regulator | LexA-family transcriptional regulator | | uniclust | UniRef100\_A0A2E9YV30 | 99.5 | 4.7e-17 | 8.6e-23 | 112.1 | 176 | (1, 176) | 180 | (10, 222) | 230 | HTH cro/C1-type domain-containing protein | HTH cro/C1-type domain-containing protein | | uniclust | UniRef100\_A0A0U5N406 | 99.5 | 4.8e-17 | 8.9e-23 | 117.6 | 156 | (2, 157) | 180 | (61, 281) | 303 | Putative Repressor protein C | Putative Repressor protein C | | uniclust | UniRef100\_A0A011VG60 | 99.5 | 5.2e-17 | 9.6e-23 | 117.5 | 168 | (3, 177) | 180 | (46, 234) | 281 | Peptidase S24 | Peptidase S24 | | uniclust | UniRef100\_A0A1A0FJR0 | 99.5 | 5.5e-17 | 1e-22 | 107.3 | 94 | (84, 177) | 180 | (82, 178) | 181 | Peptidase S24/S26A/S26B/S26C domain-containing protein | Peptidase S24/S26A/S26B/S26C domain-containing protein | | uniclust | UniRef100\_A0A2A4N3F3 | 99.5 | 5.6e-17 | 1e-22 | 114.9 | 176 | (2, 179) | 180 | (27, 281) | 293 | Peptidase S24/S26A/S26B/S26C domain-containing protein | Peptidase S24/S26A/S26B/S26C domain-containing protein | | uniclust | UniRef100\_A0A0A1VGJ5 | 99.5 | 5.6e-17 | 1e-22 | 106.4 | 99 | (80, 180) | 180 | (44, 143) | 153 | SOS-response transcriptional repressor | SOS-response transcriptional repressor | | uniclust | UniRef100\_A0A017RV89 | 99.5 | 5.6e-17 | 1e-22 | 117.1 | 176 | (1, 178) | 180 | (25, 243) | 268 | HTH cro/C1-type domain-containing protein | HTH cro/C1-type domain-containing protein | | uniclust | UniRef100\_A0A0N0ZPS4 | 99.5 | 5.9e-17 | 1.1e-22 | 114.6 | 176 | (2, 179) | 180 | (29, 241) | 259 | HTH cro/C1-type domain-containing protein | HTH cro/C1-type domain-containing protein | | uniclust | UniRef100\_A0A1F3XYF0 | 99.5 | 6.4e-17 | 1.2e-22 | 113.5 | 177 | (1, 177) | 180 | (20, 252) | 258 | HTH cro/C1-type domain-containing protein | HTH cro/C1-type domain-containing protein | | uniclust | UniRef100\_UPI0018DFAD8D | 99.5 | 6.5e-17 | 1.2e-22 | 101.6 | 159 | (2, 177) | 180 | (3, 162) | 164 | LexA family transcriptional regulator | LexA family transcriptional regulator | | uniclust | UniRef100\_A0A1A5RXV5 | 99.5 | 6.7e-17 | 1.2e-22 | 112.7 | 172 | (2, 174) | 180 | (76, 266) | 272 | HTH cro/C1-type domain-containing protein | HTH cro/C1-type domain-containing protein | | uniclust | UniRef100\_A0A0T2QE85 | 99.5 | 6.9e-17 | 1.3e-22 | 112.3 | 172 | (2, 179) | 180 | (31, 224) | 235 | HTH cro/C1-type domain-containing protein | HTH cro/C1-type domain-containing protein | | uniclust | UniRef100\_A0A7W1G0U4 | 99.5 | 7.5e-17 | 1.4e-22 | 108.9 | 160 | (6, 166) | 180 | (13, 200) | 238 | LexA family transcriptional regulator | LexA family transcriptional regulator | | uniclust | UniRef100\_A0A085EXQ7 | 99.5 | 8.2e-17 | 1.5e-22 | 118.5 | 171 | (2, 175) | 180 | (52, 269) | 279 | Putative phage repressor | Putative phage repressor | | uniclust | UniRef100\_A0A4Q3HVL5 | 99.5 | 8.3e-17 | 1.5e-22 | 97.5 | 99 | (80, 180) | 180 | (10, 109) | 111 | Error-prone repair protein UmuD (Fragment) | Error-prone repair protein UmuD (Fragment) | | uniclust | UniRef100\_A0A133MBD3 | 99.5 | 8.3e-17 | 1.5e-22 | 120.6 | 175 | (1, 178) | 180 | (25, 365) | 375 | DNA-binding helix-turn-helix protein | DNA-binding helix-turn-helix protein | | uniclust | UniRef100\_A0A7Z9U9M0 | 99.5 | 8.4e-17 | 1.6e-22 | 107.3 | 95 | (85, 180) | 180 | (76, 175) | 175 | Peptidase S24/S26A/S26B/S26C domain-containing protein | Peptidase S24/S26A/S26B/S26C domain-containing protein | | uniclust | UniRef100\_A0A7V3HNI8 | 99.5 | 8.6e-17 | 1.6e-22 | 108.2 | 174 | (1, 177) | 180 | (19, 257) | 262 | Helix-turn-helix domain-containing protein | Helix-turn-helix domain-containing protein | | uniclust | UniRef100\_A0A090CZE3 | 99.5 | 8.8e-17 | 1.6e-22 | 110.4 | 99 | (80, 180) | 180 | (96, 195) | 204 | Protein UmuD | Protein UmuD | | uniclust | UniRef100\_A0A124BRC6 | 99.5 | 9.7e-17 | 1.8e-22 | 112.8 | 172 | (2, 177) | 180 | (24, 223) | 233 | Helix-turn-helix domain protein | Helix-turn-helix domain protein | | uniclust | UniRef100\_A0A1B7JZC6 | 99.5 | 1e-16 | 1.9e-22 | 106.4 | 91 | (85, 176) | 180 | (68, 160) | 169 | Phage repressor | Phage repressor | | uniclust | UniRef100\_A0A2N9XWK5 | 99.5 | 1.1e-16 | 1.9e-22 | 106.9 | 91 | (85, 176) | 180 | (76, 167) | 174 | Peptidase S24/S26A/S26B/S26C domain-containing protein | Peptidase S24/S26A/S26B/S26C domain-containing protein | | uniclust | UniRef100\_A0A143XNR7 | 99.5 | 1.1e-16 | 2.1e-22 | 114.8 | 173 | (2, 178) | 180 | (20, 234) | 270 | Peptidase S24/S26A/S26B/S26C domain-containing protein | Peptidase S24/S26A/S26B/S26C domain-containing protein | | uniclust | UniRef100\_A0A1C3EE82 | 99.5 | 1.2e-16 | 2.2e-22 | 110.2 | 175 | (2, 176) | 180 | (89, 312) | 318 | HTH cro/C1-type domain-containing protein | HTH cro/C1-type domain-containing protein | | uniclust | UniRef100\_A0A1C2K2G3 | 99.5 | 1.3e-16 | 2.3e-22 | 110.9 | 174 | (1, 178) | 180 | (15, 221) | 229 | HTH cro/C1-type domain-containing protein | HTH cro/C1-type domain-containing protein | | uniclust | UniRef100\_A0A494X9D3 | 99.5 | 1.3e-16 | 2.4e-22 | 109.0 | 177 | (1, 177) | 180 | (13, 249) | 254 | LexA family transcriptional regulator | LexA family transcriptional regulator | | uniclust | UniRef100\_A0A351J0K7 | 99.5 | 1.3e-16 | 2.4e-22 | 107.4 | 171 | (2, 174) | 180 | (4, 237) | 238 | HTH cro/C1-type domain-containing protein | HTH cro/C1-type domain-containing protein | | uniclust | UniRef100\_A0A060BDL7 | 99.5 | 1.4e-16 | 2.6e-22 | 112.4 | 175 | (1, 179) | 180 | (17, 207) | 245 | XRE family transcriptional regulator | XRE family transcriptional regulator | | uniclust | UniRef100\_A0A1R1BA90 | 99.5 | 1.4e-16 | 2.6e-22 | 117.5 | 87 | (88, 177) | 180 | (279, 367) | 370 | HTH cro/C1-type domain-containing protein | HTH cro/C1-type domain-containing protein | | uniclust | UniRef100\_A0A123TEK7 | 99.5 | 1.4e-16 | 2.6e-22 | 107.2 | 91 | (87, 179) | 180 | (76, 166) | 174 | Putative phage repressor | Putative phage repressor | | uniclust | UniRef100\_A0A0U5F099 | 99.5 | 1.5e-16 | 2.8e-22 | 115.1 | 89 | (87, 176) | 180 | (204, 296) | 300 | Putative phage repressor | Putative phage repressor | | uniclust | UniRef100\_A0A0G0TYJ6 | 99.5 | 1.5e-16 | 2.8e-22 | 110.7 | 175 | (1, 179) | 180 | (11, 247) | 248 | LexA repressor | LexA repressor | | uniclust | UniRef100\_UPI001F49D1FF | 99.5 | 1.6e-16 | 2.9e-22 | 91.1 | 86 | (95, 180) | 180 | (3, 90) | 90 | S24 family peptidase | S24 family peptidase | | uniclust | UniRef100\_A0A098GCJ7 | 99.5 | 1.6e-16 | 2.9e-22 | 116.8 | 175 | (2, 178) | 180 | (41, 244) | 276 | Putative repressor protein from phage origin | Putative repressor protein from phage origin | | uniclust | UniRef100\_UPI0013CFBFF4 | 99.5 | 1.6e-16 | 2.9e-22 | 105.6 | 177 | (1, 179) | 180 | (18, 210) | 211 | LexA family transcriptional regulator | LexA family transcriptional regulator | | uniclust | UniRef100\_A0A031HR16 | 99.5 | 1.6e-16 | 2.9e-22 | 109.5 | 155 | (25, 180) | 180 | (62, 242) | 249 | Peptidase S24/S26A/S26B/S26C domain-containing protein | Peptidase S24/S26A/S26B/S26C domain-containing protein | | uniclust | UniRef100\_A0A6L7WV89 | 99.5 | 1.6e-16 | 3e-22 | 109.9 | 177 | (1, 180) | 180 | (29, 288) | 288 | LexA family transcriptional regulator | LexA family transcriptional regulator | | uniclust | UniRef100\_A0A179BUG2 | 99.5 | 1.7e-16 | 3.1e-22 | 115.2 | 173 | (1, 176) | 180 | (32, 250) | 257 | HTH cro/C1-type domain-containing protein | HTH cro/C1-type domain-containing protein | | uniclust | UniRef100\_UPI001E65DB02 | 99.5 | 1.8e-16 | 3.2e-22 | 105.7 | 173 | (1, 175) | 180 | (7, 228) | 235 | LexA family transcriptional regulator | LexA family transcriptional regulator | | uniclust | UniRef100\_A0A1H0WB76 | 99.5 | 1.8e-16 | 3.4e-22 | 98.5 | 90 | (88, 177) | 180 | (20, 114) | 122 | Peptidase S24-like | Peptidase S24-like | | uniclust | UniRef100\_A0A437MC40 | 99.5 | 1.8e-16 | 3.4e-22 | 113.2 | 154 | (2, 155) | 180 | (49, 254) | 279 | Helix-turn-helix transcriptional regulator | Helix-turn-helix transcriptional regulator | | uniclust | UniRef100\_A0A0Q4YH48 | 99.5 | 1.9e-16 | 3.5e-22 | 109.3 | 159 | (17, 175) | 180 | (57, 228) | 238 | Peptidase S24 | Peptidase S24 | | uniclust | UniRef100\_A0A0L6J7F1 | 99.5 | 1.9e-16 | 3.6e-22 | 115.4 | 169 | (2, 177) | 180 | (61, 243) | 266 | Xre family transcriptional regulator | Xre family transcriptional regulator | | uniclust | UniRef100\_A0A1I4Z2S5 | 99.5 | 2e-16 | 3.6e-22 | 99.1 | 98 | (80, 179) | 180 | (28, 126) | 132 | DNA polymerase V | DNA polymerase V | | uniclust | UniRef100\_A0A3C1EDA2 | 99.5 | 2e-16 | 3.7e-22 | 103.0 | 173 | (1, 180) | 180 | (3, 179) | 180 | HTH cro/C1-type domain-containing protein | HTH cro/C1-type domain-containing protein | | uniclust | UniRef100\_A0A191HVY2 | 99.5 | 2.1e-16 | 3.8e-22 | 99.6 | 92 | (87, 180) | 180 | (19, 115) | 127 | Peptidase S24/S26A/S26B/S26C domain-containing protein | Peptidase S24/S26A/S26B/S26C domain-containing protein | | uniclust | UniRef100\_A0A4R7DWV3 | 99.5 | 2.2e-16 | 4e-22 | 110.5 | 173 | (1, 177) | 180 | (16, 286) | 292 | Helix-turn-helix protein | Helix-turn-helix protein | | uniclust | UniRef100\_A0A0S4S9Z8 | 99.5 | 2.2e-16 | 4.1e-22 | 107.8 | 163 | (13, 180) | 180 | (21, 210) | 213 | Phage repressor protein | Phage repressor protein | | uniclust | UniRef100\_A0A0H3ZMC9 | 99.5 | 2.4e-16 | 4.4e-22 | 112.7 | 96 | (81, 176) | 180 | (141, 240) | 280 | HTH Mu-type domain-containing protein | HTH Mu-type domain-containing protein | | uniclust | UniRef100\_A0A0R6PGT9 | 99.5 | 2.4e-16 | 4.5e-22 | 106.0 | 173 | (6, 180) | 180 | (13, 196) | 197 | Peptidase S24/S26A/S26B/S26C domain-containing protein | Peptidase S24/S26A/S26B/S26C domain-containing protein | | uniclust | UniRef100\_A0A8X8TGA2 | 99.4 | 2.5e-16 | 4.7e-22 | 109.9 | 171 | (2, 178) | 180 | (7, 203) | 220 | Helix-turn-helix transcriptional regulator | Helix-turn-helix transcriptional regulator | | uniclust | UniRef100\_A0A6L7U9Q8 | 99.4 | 2.6e-16 | 4.8e-22 | 106.1 | 93 | (85, 179) | 180 | (146, 238) | 239 | LexA family transcriptional regulator | LexA family transcriptional regulator | | uniclust | UniRef100\_UPI000688D37D | 99.4 | 2.7e-16 | 4.9e-22 | 101.7 | 84 | (90, 176) | 180 | (67, 151) | 158 | S24/S26 family peptidase | S24/S26 family peptidase | | pdb70 | 3BDN\_A | 99.7 | 2.1e-22 | 1.4e-26 | 132.0 | 173 | (3, 179) | 180 | (16, 231) | 236 | PROTEIN/DNA Complex | 3BDN\_A PROTEIN/DNA Complex lambda, repressor, allostery, cooperativity, DNA | | pdb70 | 2FJR\_A | 99.6 | 1.1e-19 | 7.3e-24 | 115.0 | 167 | (2, 179) | 180 | (8, 187) | 189 | Repressor protein CI | 2FJR\_A Repressor protein CI genetic switch, regulation, repressor, cooperativity | | pdb70 | 1JHC\_A | 99.0 | 3.9e-14 | 2.6e-18 | 84.6 | 88 | (89, 179) | 180 | (41, 131) | 137 | LEXA REPRESSOR (E.C.3.4.21.88) | 1JHC\_A LEXA REPRESSOR (E.C.3.4.21.88) LexA SOS repressor, HYDROLASE | | pdb70 | 6A2Q\_A | 99.0 | 6.1e-14 | 4.1e-18 | 81.6 | 86 | (89, 179) | 180 | (31, 117) | 118 | LexA repressor (E.C.3.4.21.88) | 6A2Q\_A LexA repressor (E.C.3.4.21.88) Mycobacterium tuberculosis, LexA, SOS response HET: CME | | pdb70 | 6A2S\_C | 99.0 | 7.3e-14 | 4.9e-18 | 80.3 | 86 | (89, 179) | 180 | (24, 110) | 111 | LexA repressor (E.C.3.4.21.88) | 6A2S\_C LexA repressor (E.C.3.4.21.88) Mycobacterium tuberculosis, LexA, SOS response HET: CME, PEG, P6G | | pdb70 | 1F39\_A | 99.0 | 8e-14 | 5.4e-18 | 78.8 | 87 | (89, 179) | 180 | (3, 96) | 101 | REPRESSOR PROTEIN CI | 1F39\_A REPRESSOR PROTEIN CI COOPERATIVE OPERATOR BINDING, RECA-MEDIATED SELF-CLEAVAGE | | pdb70 | 4Y42\_I | 99.0 | 1.3e-13 | 8.5e-18 | 84.2 | 126 | (3, 138) | 180 | (16, 155) | 156 | Cyanase (E.C.4.2.1.104) | 4Y42\_I Cyanase (E.C.4.2.1.104) CynS, cyanase, lyase HET: GOL | | pdb70 | 6B6M\_A | 99.0 | 1.3e-13 | 8.5e-18 | 84.2 | 126 | (3, 138) | 180 | (16, 155) | 156 | Cyanate hydratase (E.C.4.2.1.104) | 6B6M\_A Cyanate hydratase (E.C.4.2.1.104) Cyanase, LYASE | | pdb70 | 3K2Z\_B | 98.9 | 1.8e-13 | 1.2e-17 | 86.4 | 88 | (89, 179) | 180 | (107, 195) | 196 | LexA repressor (E.C.3.4.21.88) | 3K2Z\_B LexA repressor (E.C.3.4.21.88) winged helix-turn-helix, repressor, LexA, SOS | | pdb70 | 1AY9\_B | 98.9 | 1.9e-13 | 1.3e-17 | 78.2 | 87 | (89, 179) | 180 | (18, 106) | 108 | UMUD PROTEIN | 1AY9\_B UMUD PROTEIN MUTAGENESIS PROTEIN, DNA REPAIR, HYDROLASE | | pdb70 | 1UMU\_B | 98.9 | 4.4e-13 | 2.9e-17 | 77.7 | 87 | (89, 179) | 180 | (25, 113) | 116 | UMUD' | 1UMU\_B UMUD' INDUCED MUTAGENESIS, SOS MUTAGENESIS, DNA HET: MSE | | pdb70 | 1JHF\_A | 98.8 | 6.4e-13 | 4.3e-17 | 84.3 | 159 | (12, 179) | 180 | (24, 198) | 202 | LEXA REPRESSOR (E.C.3.4.21.88) | 1JHF\_A LEXA REPRESSOR (E.C.3.4.21.88) LexA SOS repressor, HYDROLASE | | pdb70 | 2HNF\_A | 98.8 | 1.2e-12 | 8.1e-17 | 77.6 | 84 | (89, 176) | 180 | (42, 132) | 133 | Phage lambda repressor protein CI | 2HNF\_A Phage lambda repressor protein CI VIRAL PROTEIN | | pdb70 | 4N31\_B | 98.5 | 3.8e-11 | 2.6e-15 | 73.5 | 60 | (91, 151) | 180 | (29, 89) | 163 | SipA | 4N31\_B SipA Streptococcus pyogenes, pilus polymerisation, Signal HET: PO4, PTY | | pdb70 | 4N31\_A | 98.5 | 4.8e-11 | 3.2e-15 | 73.0 | 60 | (91, 151) | 180 | (29, 89) | 163 | SipA | 4N31\_A SipA Streptococcus pyogenes, pilus polymerisation, Signal HET: PO4, PTY | | pdb70 | 4ME8\_A | 98.2 | 7.1e-10 | 4.8e-14 | 66.8 | 57 | (94, 151) | 180 | (2, 60) | 151 | Signal peptidase I (E.C.3.4.21.89) | 4ME8\_A Signal peptidase I (E.C.3.4.21.89) PF00717 family, Peptidase\_S24, Structural Genomics | | pdb70 | 4NV4\_A | 98.1 | 2e-09 | 1.3e-13 | 66.5 | 59 | (91, 150) | 180 | (20, 83) | 176 | Signal peptidase I (E.C.3.4.21.89) | 4NV4\_A Signal peptidase I (E.C.3.4.21.89) Structural Genomics, NIAID, National Institute HET: PEG, PG4 | | pdb70 | 4NV4\_B | 98.0 | 2.2e-09 | 1.5e-13 | 66.3 | 59 | (91, 150) | 180 | (20, 83) | 176 | Signal peptidase I (E.C.3.4.21.89) | 4NV4\_B Signal peptidase I (E.C.3.4.21.89) Structural Genomics, NIAID, National Institute HET: PG4, PEG | | pdb70 | 1B12\_D | 98.0 | 2.5e-09 | 1.7e-13 | 69.6 | 60 | (92, 152) | 180 | (7, 85) | 248 | SIGNAL PEPTIDASE I (E.C.3.9.21.89) | 1B12\_D SIGNAL PEPTIDASE I (E.C.3.9.21.89) SERINE PROTEINASE, SERINE-DEPENDANT HYDROLASE, SIGNAL HET: 1PN | | pdb70 | 4K8W\_A | 98.0 | 3.1e-09 | 2.1e-13 | 62.1 | 55 | (97, 152) | 180 | (1, 56) | 129 | LepA | 4K8W\_A LepA Predominately beta-strand, S26A signal peptidase | | pdb70 | 2FKD\_F | 98.0 | 3.3e-09 | 2.2e-13 | 60.3 | 69 | (105, 179) | 180 | (40, 108) | 110 | Repressor protein CI | 2FKD\_F Repressor protein CI genetic switch, regulation, cooperativity, repressor | | pdb70 | 6B88\_B | 97.9 | 7.5e-09 | 5.1e-13 | 67.2 | 59 | (92, 151) | 180 | (6, 83) | 247 | Signal peptidase I (E.C.3.4.21.89), ILE-THR-LEU-LEU | 6B88\_B Signal peptidase I (E.C.3.4.21.89), ILE-THR-LEU-LEU Signal peptidase, SBDD, antibiotic, HYDROLASE HET: 1PE, CZD | | pdb70 | 2K9Q\_B | 97.7 | 2.2e-08 | 1.4e-12 | 52.9 | 62 | (2, 63) | 180 | (4, 65) | 77 | uncharacterized protein | 2K9Q\_B uncharacterized protein all helix, helix-turn-helix, Plasmid, Structural | | pdb70 | 4WVI\_A | 97.7 | 2.2e-08 | 1.5e-12 | 71.9 | 58 | (93, 151) | 180 | (380, 442) | 533 | Maltose binding protein - | 4WVI\_A Maltose binding protein - SpsB Type-I signal peptidase, Peptide HET: MAL | | pdb70 | 5J2Y\_A | 97.7 | 2.3e-08 | 1.5e-12 | 53.3 | 61 | (2, 62) | 180 | (13, 74) | 80 | Regulatory protein/DNA Complex | 5J2Y\_A Regulatory protein/DNA Complex Quorum-sensing repressor, gene regulation, RsaL-DNA HET: MSE | | pdb70 | 5J2Y\_B | 97.7 | 2.3e-08 | 1.5e-12 | 53.3 | 61 | (2, 62) | 180 | (13, 74) | 80 | Regulatory protein/DNA Complex | 5J2Y\_B Regulatory protein/DNA Complex Quorum-sensing repressor, gene regulation, RsaL-DNA HET: MSE | | pdb70 | 2KPJ\_A | 97.7 | 3.5e-08 | 2.4e-12 | 54.2 | 61 | (3, 63) | 180 | (12, 72) | 94 | SOS-response transcriptional repressor, LexA | 2KPJ\_A SOS-response transcriptional repressor, LexA NESG, GFT, Structural Genomics, PSI-2 | | pdb70 | 1LLI\_B | 97.6 | 3.8e-08 | 2.6e-12 | 53.9 | 53 | (10, 62) | 180 | (27, 79) | 92 | LAMBDA REPRESSOR (TRIPLE MUTANT)/DNA COMPLEX | 1LLI\_B LAMBDA REPRESSOR (TRIPLE MUTANT)/DNA COMPLEX PROTEIN-DNA COMPLEX, DOUBLE HELIX, TRANSCRIPTION-DNA | | pdb70 | 3KZ3\_B | 97.6 | 7e-08 | 4.7e-12 | 51.1 | 60 | (4, 63) | 180 | (16, 75) | 80 | Repressor protein CI | 3KZ3\_B Repressor protein CI Five helix bundle, DNA-binding, Repressor | | pdb70 | 4I6U\_A | 97.6 | 7.1e-08 | 4.8e-12 | 51.3 | 61 | (2, 62) | 180 | (13, 73) | 82 | Regulatory protein | 4I6U\_A Regulatory protein Restriction-modification, helix-turn-helix, transcriptional regulato, DNA HET: GOL, PEG, ACT | | pdb70 | 4I6R\_B | 97.5 | 9.9e-08 | 6.6e-12 | 50.7 | 62 | (2, 63) | 180 | (13, 74) | 82 | Regulatory protein | 4I6R\_B Regulatory protein Restriction-modification, helix-turn-helix, transcriptional regulator, DNA HET: GOL, SO4 | | pdb70 | 6B9R\_C | 97.4 | 1.4e-07 | 9.3e-12 | 66.5 | 63 | (1, 63) | 180 | (17, 79) | 450 | Methylphosphonate synthase | 6B9R\_C Methylphosphonate synthase Phosphonate, Hydroxymethylphosphonate, Iron, OXIDOREDUCTASE HET: 2HE, GOL | | pdb70 | 6B9R\_D | 97.4 | 1.4e-07 | 9.3e-12 | 66.5 | 63 | (1, 63) | 180 | (17, 79) | 450 | Methylphosphonate synthase | 6B9R\_D Methylphosphonate synthase Phosphonate, Hydroxymethylphosphonate, Iron, OXIDOREDUCTASE HET: GOL, 2HE | | pdb70 | 1Y9Q\_A | 97.4 | 1.4e-07 | 9.7e-12 | 58.6 | 62 | (2, 63) | 180 | (13, 74) | 192 | transcriptional regulator, HTH\_3 family | 1Y9Q\_A transcriptional regulator, HTH\_3 family HTH\_3 family, Transcriptional Regulaator, Strucutral | | pdb70 | 2EF8\_A | 97.3 | 2.7e-07 | 1.8e-11 | 49.3 | 62 | (2, 63) | 180 | (12, 77) | 84 | Putative transcription factor | 2EF8\_A Putative transcription factor helix-turn-helix, DNA binding protein, TRANSCRIPTION | | pdb70 | 3B7H\_A | 97.3 | 2.8e-07 | 1.9e-11 | 48.2 | 61 | (3, 63) | 180 | (10, 71) | 78 | Prophage Lp1 protein 11 | 3B7H\_A Prophage Lp1 protein 11 prophage Lp1 protein 11, structural | | pdb70 | 2P5T\_E | 97.3 | 2.9e-07 | 1.9e-11 | 55.5 | 62 | (2, 63) | 180 | (3, 64) | 158 | fragment of PezA helix-turn-helix motif | 2P5T\_E fragment of PezA helix-turn-helix motif postsegregational killing system, phosphoryltransferase, helix-turn-helix | | pdb70 | 2WIU\_B | 97.3 | 3e-07 | 2e-11 | 49.4 | 61 | (3, 63) | 180 | (15, 75) | 88 | PROTEIN HIPA (E.C.2.7.11.1), HTH-TYPE TRANSCRIPTIONAL | 2WIU\_B PROTEIN HIPA (E.C.2.7.11.1), HTH-TYPE TRANSCRIPTIONAL TRANSFERASE TRANSCRIPTION COMPLEX, SERINE KINASE HET: CL | | pdb70 | 5D4Z\_M | 97.3 | 3.2e-07 | 2.2e-11 | 51.5 | 61 | (2, 62) | 180 | (6, 68) | 107 | Repressor | 5D4Z\_M Repressor Repressor, DNA BINDING PROTEIN | | pdb70 | 2XI8\_B | 97.3 | 3.3e-07 | 2.2e-11 | 45.9 | 61 | (2, 62) | 180 | (3, 63) | 66 | PUTATIVE TRANSCRIPTION REGULATOR | 2XI8\_B PUTATIVE TRANSCRIPTION REGULATOR TRANSCRIPTION, HTH DNA-BINDING MOTIF HET: GOL | | pdb70 | 4MCT\_C | 97.3 | 3.5e-07 | 2.4e-11 | 52.8 | 62 | (2, 63) | 180 | (10, 71) | 125 | Antidote protein, Killer protein | 4MCT\_C Antidote protein, Killer protein bacterial toxins, biofilms, cell metabolism | | pdb70 | 3OMT\_A | 97.3 | 3.9e-07 | 2.6e-11 | 46.7 | 60 | (3, 62) | 180 | (11, 70) | 73 | uncharacterized protein | 3OMT\_A uncharacterized protein structural genomics, PSI-2, Protein Structure | | pdb70 | 1Y7Y\_A | 97.3 | 4.1e-07 | 2.7e-11 | 46.8 | 58 | (3, 60) | 180 | (16, 73) | 74 | C.AhdI | 1Y7Y\_A C.AhdI HELIX-TURN-HELIX, DNA-BINDING PROTEIN, TRANSCRIPTIONAL REGULATOR | | pdb70 | 3OMT\_B | 97.3 | 4.1e-07 | 2.7e-11 | 46.7 | 60 | (3, 62) | 180 | (11, 70) | 73 | uncharacterized protein | 3OMT\_B uncharacterized protein structural genomics, PSI-2, Protein Structure | | pdb70 | 6B9T\_D | 97.2 | 4.2e-07 | 2.8e-11 | 64.1 | 64 | (1, 64) | 180 | (16, 79) | 457 | Methylphosphonate synthase (E.C.1.13.11.73) | 6B9T\_D Methylphosphonate synthase (E.C.1.13.11.73) Phosphonate, Methylphosphonate, Iron, OXIDOREDUCTASE HET: 2HE, FMT | | pdb70 | 6B9T\_C | 97.2 | 4.5e-07 | 3e-11 | 64.0 | 64 | (1, 64) | 180 | (16, 79) | 457 | Methylphosphonate synthase (E.C.1.13.11.73) | 6B9T\_C Methylphosphonate synthase (E.C.1.13.11.73) Phosphonate, Methylphosphonate, Iron, OXIDOREDUCTASE HET: FMT, 2HE | | pdb70 | 3F6W\_C | 97.2 | 4.6e-07 | 3.1e-11 | 48.0 | 61 | (3, 63) | 180 | (17, 77) | 83 | XRE-family like protein | 3F6W\_C XRE-family like protein helix-turn-helix DNA binding protein HET: BTB, MSE | | pdb70 | 5UK3\_G | 97.2 | 4.8e-07 | 3.2e-11 | 55.5 | 62 | (2, 63) | 180 | (21, 82) | 176 | Uncharacterized protein | 5UK3\_G Uncharacterized protein Tetranychus urticae, cyanase, agricultural pest | | pdb70 | 5UK3\_I | 97.2 | 4.8e-07 | 3.2e-11 | 55.5 | 62 | (2, 63) | 180 | (21, 82) | 176 | Uncharacterized protein | 5UK3\_I Uncharacterized protein Tetranychus urticae, cyanase, agricultural pest | | pdb70 | 1ADR\_A | 97.2 | 5.9e-07 | 3.9e-11 | 46.6 | 61 | (3, 63) | 180 | (8, 68) | 76 | P22 C2 REPRESSOR (AMINO-TERMINAL DNA-BINDING | 1ADR\_A P22 C2 REPRESSOR (AMINO-TERMINAL DNA-BINDING TRANSCRIPTION REGULATION | | pdb70 | 6RNZ\_B | 97.2 | 6.5e-07 | 4.3e-11 | 44.7 | 59 | (3, 61) | 180 | (5, 63) | 66 | HTH-type transcriptional regulator DdrOC | 6RNZ\_B HTH-type transcriptional regulator DdrOC ----, DNA BINDING PROTEIN HET: GOL | | pdb70 | 3JXC\_L | 97.2 | 7e-07 | 4.6e-11 | 44.8 | 59 | (3, 61) | 180 | (7, 65) | 67 | Repressor protein C2/DNA Complex, THALLIUM | 3JXC\_L Repressor protein C2/DNA Complex, THALLIUM protein-DNA complex, DNA-binding, Repressor, Transcription | | pdb70 | 4RYK\_A | 97.2 | 7e-07 | 4.7e-11 | 59.0 | 62 | (1, 63) | 180 | (10, 71) | 306 | Lmo0325 protein | 4RYK\_A Lmo0325 protein Structural Genomics, PSI-Biology, Midwest Center HET: TLA, SUC | | pdb70 | 6JQ1\_B | 97.2 | 7e-07 | 4.7e-11 | 51.8 | 62 | (2, 63) | 180 | (7, 68) | 132 | Transcriptional regulator, XRE family | 6JQ1\_B Transcriptional regulator, XRE family Transcription factor, Xre, HTH, Dimerization | | pdb70 | 6QER\_C | 97.1 | 7.6e-07 | 5e-11 | 58.3 | 61 | (1, 61) | 180 | (7, 68) | 310 | Transcriptional regulator | 6QER\_C Transcriptional regulator Quorum sensing, Competence, TPR domain | | pdb70 | 5JUF\_A | 97.1 | 8.2e-07 | 5.4e-11 | 58.2 | 61 | (1, 61) | 180 | (7, 68) | 310 | Transcriptional regulator | 5JUF\_A Transcriptional regulator RNPP, Transcriptional activator, TPR, HTH HET: SO4 | | pdb70 | 3JXB\_C | 97.1 | 8.7e-07 | 5.7e-11 | 44.4 | 60 | (2, 61) | 180 | (6, 65) | 67 | Repressor protein C2/DNA Complex | 3JXB\_C Repressor protein C2/DNA Complex protein-DNA complex, DNA-binding, Repressor, Transcription | | pdb70 | 2R1J\_R | 97.1 | 8.9e-07 | 5.9e-11 | 44.5 | 59 | (3, 61) | 180 | (8, 66) | 68 | Repressor protein C2/DNA Complex | 2R1J\_R Repressor protein C2/DNA Complex Protein-DNA complex, Helix-turn-helix, DNA-binding, Repressor | | pdb70 | 2XIU\_A | 97.1 | 9.3e-07 | 6.1e-11 | 44.0 | 61 | (2, 62) | 180 | (3, 63) | 66 | CYLR2 | 2XIU\_A CYLR2 DNA BINDING PROTEIN, HTH-DNA BINDING HET: MTN | | pdb70 | 6HU8\_A | 97.1 | 1e-06 | 6.6e-11 | 58.0 | 62 | (1, 62) | 180 | (7, 69) | 310 | Uncharacterized protein | 6HU8\_A Uncharacterized protein RNPP family TPR domain HTH | | pdb70 | 6H49\_A | 97.1 | 9.9e-07 | 6.7e-11 | 53.0 | 62 | (2, 63) | 180 | (16, 77) | 157 | Orf20 | 6H49\_A Orf20 SaPI, Repressor, STRUCTURAL PROTEIN HET: SO4 | | pdb70 | 6H4C\_D | 97.1 | 9.9e-07 | 6.7e-11 | 53.0 | 62 | (2, 63) | 180 | (16, 77) | 157 | dUTPase, Orf20 | 6H4C\_D dUTPase, Orf20 SaPI, Repressor, Complex, STRUCTURAL PROTEIN HET: PEG | | pdb70 | 5W4M\_B | 97.1 | 1.1e-06 | 7.1e-11 | 57.0 | 61 | (1, 62) | 180 | (5, 65) | 280 | Transcriptional regulator | 5W4M\_B Transcriptional regulator DNA BINDING, PHEROMONE BINDING, REPEAT HET: GOL, DMS | | pdb70 | 4Z5H\_A | 97.1 | 1.1e-06 | 7.5e-11 | 44.8 | 58 | (3, 60) | 180 | (13, 70) | 72 | E.coli transcription repressor, HipB subunit | 4Z5H\_A E.coli transcription repressor, HipB subunit HTH motif, Transcription repressor, HipA | | pdb70 | 2B5A\_C | 97.1 | 1.2e-06 | 7.7e-11 | 45.4 | 60 | (3, 62) | 180 | (13, 72) | 77 | C.BclI | 2B5A\_C C.BclI HELIX-TURN-HELIX motif, GENE REGULATION | | pdb70 | 3BS3\_A | 97.0 | 1.3e-06 | 9e-11 | 44.9 | 60 | (3, 62) | 180 | (13, 72) | 76 | Putative DNA-binding protein | 3BS3\_A Putative DNA-binding protein DNA-binding, XRE-family, Structural Genomics, PSI-2 | | pdb70 | 5WOQ\_B | 97.0 | 1.4e-06 | 9.4e-11 | 49.8 | 62 | (2, 63) | 180 | (18, 79) | 120 | Transcriptional regulator ClgR | 5WOQ\_B Transcriptional regulator ClgR NIAID, structural genomics, transcription factor | | pdb70 | 5WOQ\_C | 97.0 | 1.4e-06 | 9.4e-11 | 49.8 | 62 | (2, 63) | 180 | (18, 79) | 120 | Transcriptional regulator ClgR | 5WOQ\_C Transcriptional regulator ClgR NIAID, structural genomics, transcription factor | | pdb70 | 4YG7\_C | 97.0 | 1.4e-06 | 9.4e-11 | 44.4 | 57 | (3, 59) | 180 | (12, 68) | 71 | HipB, HipA/DNA Complex | 4YG7\_C HipB, HipA/DNA Complex persistence, multidrug tolerance, autorepression, promoter | | pdb70 | 4Z59\_A | 97.0 | 1.4e-06 | 9.4e-11 | 44.4 | 57 | (3, 59) | 180 | (12, 68) | 71 | E.coli transcription repressor, HipB/DNA Complex | 4Z59\_A E.coli transcription repressor, HipB/DNA Complex HTH motif, Transcription repressor, HipA | | pdb70 | 2GRM\_A | 97.0 | 1.5e-06 | 9.9e-11 | 57.9 | 61 | (1, 62) | 180 | (4, 64) | 317 | PrgX, peptide | 2GRM\_A PrgX, peptide receptor, inhibitor, TRANSCRIPTION | | pdb70 | 5Y6A\_A | 97.0 | 1.5e-06 | 1e-10 | 51.8 | 62 | (2, 63) | 180 | (5, 67) | 151 | chain A and B | 5Y6A\_A chain A and B Unknown function, VIRAL PROTEIN | | pdb70 | 2BNM\_B | 97.0 | 1.5e-06 | 1e-10 | 54.1 | 62 | (2, 63) | 180 | (12, 74) | 198 | EPOXIDASE | 2BNM\_B EPOXIDASE OXIDOREDUCTASE, EPOXIDASE, CUPIN, HTH, CATION-DEPENDANT HET: SO4 | | pdb70 | 4J1X\_C | 97.0 | 1.7e-06 | 1.2e-10 | 53.8 | 62 | (2, 63) | 180 | (11, 73) | 197 | Epoxidase | 4J1X\_C Epoxidase keto product, Hydroxypropylphosphonic acid epoxidase HET: 1JJ | | pdb70 | 1B0N\_A | 97.0 | 1.8e-06 | 1.2e-10 | 48.4 | 62 | (2, 63) | 180 | (3, 65) | 111 | PROTEIN (SINR PROTEIN) | 1B0N\_A PROTEIN (SINR PROTEIN) TRANSCRIPTION REGULATOR, ANTAGONIST, SPORULATION | | pdb70 | 3KXA\_A | 97.0 | 1.8e-06 | 1.2e-10 | 50.9 | 61 | (3, 63) | 180 | (71, 131) | 141 | Putative uncharacterized protein | 3KXA\_A Putative uncharacterized protein NGO0477, Neisseria gonorrhoeae, New protein HET: CL, ASN, UNX, MSE | | pdb70 | 4YV9\_D | 97.0 | 1.8e-06 | 1.2e-10 | 56.1 | 61 | (2, 63) | 180 | (6, 66) | 284 | Transcriptional regulator, Cyclosporin A | 4YV9\_D Transcriptional regulator, Cyclosporin A DNA binding, pheromone binding, repeat HET: DAL, SO4, MVA, SAR, MSE, ABA, BMT, MLE | | pdb70 | 5TN0\_A | 97.0 | 1.9e-06 | 1.2e-10 | 43.3 | 62 | (2, 63) | 180 | (3, 65) | 69 | HTH-type transcriptional regulator SinR | 5TN0\_A HTH-type transcriptional regulator SinR biofilm formation, TRANSCRIPTION | | pdb70 | 2IUO\_B | 96.9 | 1.9e-06 | 1.3e-10 | 51.7 | 61 | (2, 62) | 180 | (15, 75) | 156 | CYANATE HYDRATASE (E.C.4.2.1.104) | 2IUO\_B CYANATE HYDRATASE (E.C.4.2.1.104) LYASE, CYANATE DEGRADATION HET: CL, SO4, BR | | pdb70 | 1R63\_A | 96.9 | 2.1e-06 | 1.4e-10 | 42.3 | 59 | (2, 61) | 180 | (3, 61) | 63 | REPRESSOR PROTEIN FROM BACTERIOPHAGE 434 | 1R63\_A REPRESSOR PROTEIN FROM BACTERIOPHAGE 434 GENE REGULATING PROTEIN, PHAGE 434 | | pdb70 | 1DW9\_C | 96.9 | 2.1e-06 | 1.4e-10 | 51.5 | 61 | (2, 62) | 180 | (15, 75) | 156 | CYANATE LYASE (E.C.4.3.99.1) | 1DW9\_C CYANATE LYASE (E.C.4.3.99.1) LYASE, CYANATE DEGRADATION, STRUCTURAL GENOMICS HET: CL, SO4, MSE | | pdb70 | 1DWK\_J | 96.9 | 2.1e-06 | 1.4e-10 | 51.5 | 61 | (2, 62) | 180 | (15, 75) | 156 | CYANATE HYDRATASE (E.C.4.2.1.104) | 1DWK\_J CYANATE HYDRATASE (E.C.4.2.1.104) LYASE, CYANATE DEGRADATION, PSI, PROTEIN HET: OXL, SO4, MSE | | pdb70 | 3OP9\_A | 96.9 | 2.2e-06 | 1.5e-10 | 48.1 | 61 | (3, 63) | 180 | (12, 72) | 114 | Pli0006 protein | 3OP9\_A Pli0006 protein Structural Genomics, PSI-2, Protein Structure | | pdb70 | 6F8H\_B | 96.9 | 2.4e-06 | 1.6e-10 | 47.6 | 62 | (2, 63) | 180 | (18, 80) | 105 | XRE family transcriptional regulator | 6F8H\_B XRE family transcriptional regulator GraA, HigA, antitoxin | | pdb70 | 3MLF\_A | 96.9 | 2.5e-06 | 1.7e-10 | 48.0 | 61 | (3, 63) | 180 | (26, 86) | 111 | transcriptional regulator | 3MLF\_A transcriptional regulator structural genomics, Helix-turn-helix XRE-family like | | pdb70 | 3MLF\_D | 96.9 | 2.5e-06 | 1.7e-10 | 48.0 | 61 | (3, 63) | 180 | (26, 86) | 111 | transcriptional regulator | 3MLF\_D transcriptional regulator structural genomics, Helix-turn-helix XRE-family like HET: MSE | | pdb70 | 1RIO\_B | 96.9 | 2.6e-06 | 1.8e-10 | 46.6 | 55 | (9, 63) | 180 | (27, 81) | 98 | sigma factor SigA/Repressor protein CI/DNA | 1RIO\_B sigma factor SigA/Repressor protein CI/DNA HELIX-TURN-HELIX, TRANSCRIPTION ACTIVATION, transcription-DNA COMPLEX HET: MSE | | pdb70 | 2ICT\_A | 96.9 | 2.7e-06 | 1.8e-10 | 46.2 | 61 | (3, 63) | 180 | (11, 71) | 94 | Putative HTH-type transcriptional regulator yddM | 2ICT\_A Putative HTH-type transcriptional regulator yddM helix-turn-helix, Structural Genomics, PSI-2, Protein | | pdb70 | 1R69\_A | 96.9 | 2.7e-06 | 1.8e-10 | 42.8 | 61 | (2, 63) | 180 | (3, 63) | 69 | 434 REPRESSOR (AMINO-TERMINAL DOMAIN) (R1-69) | 1R69\_A 434 REPRESSOR (AMINO-TERMINAL DOMAIN) (R1-69) GENE REGULATING PROTEIN | | pdb70 | 5D50\_A | 96.9 | 2.9e-06 | 1.9e-10 | 52.8 | 63 | (2, 64) | 180 | (10, 73) | 199 | Repressor, Anti-repressor protein | 5D50\_A Repressor, Anti-repressor protein Repressor, Anti-repressor, complex, DNA BINDING | | pdb70 | 5D50\_B | 96.9 | 2.9e-06 | 1.9e-10 | 52.8 | 63 | (2, 64) | 180 | (10, 73) | 199 | Repressor, Anti-repressor protein | 5D50\_B Repressor, Anti-repressor protein Repressor, Anti-repressor, complex, DNA BINDING | | pdb70 | 3IVP\_D | 96.9 | 2.9e-06 | 2e-10 | 48.6 | 61 | (3, 63) | 180 | (15, 75) | 126 | Putative transposon-related DNA-binding protein | 3IVP\_D Putative transposon-related DNA-binding protein APC62618, transposon-related DNA-binding, Clostridium difficile HET: MSE, PG4 | | pdb70 | 3FYA\_A | 96.9 | 2.9e-06 | 2e-10 | 46.5 | 61 | (3, 63) | 180 | (31, 91) | 99 | Regulatory protein | 3FYA\_A Regulatory protein transcriptional regulator, helix-turn-helix, Restriction-modification, TRANSCRIPTION | | pdb70 | 2MEZ\_A | 96.8 | 3e-06 | 2e-10 | 47.2 | 61 | (3, 63) | 180 | (13, 73) | 106 | Multiprotein Bridging Factor (MBP-like) | 2MEZ\_A Multiprotein Bridging Factor (MBP-like) Multiple Binding Factor MBF1, Helix-turn-Helix | | pdb70 | 2EWT\_A | 96.8 | 3.2e-06 | 2.1e-10 | 42.7 | 57 | (4, 60) | 180 | (12, 70) | 71 | putative DNA-binding protein | 2EWT\_A putative DNA-binding protein the DNA-binding domain of BldD HET: SO4 | | pdb70 | 4O8B\_A | 96.8 | 3.4e-06 | 2.3e-10 | 48.3 | 62 | (2, 63) | 180 | (3, 68) | 122 | Uncharacterized protein | 4O8B\_A Uncharacterized protein HTH motif, transcriptional activator, promoter | | pdb70 | 1X57\_A | 96.8 | 3.5e-06 | 2.3e-10 | 45.2 | 61 | (3, 63) | 180 | (16, 76) | 91 | Endothelial differentiation-related factor 1 | 1X57\_A Endothelial differentiation-related factor 1 EDF1, HMBF1alpha, helix-turn-helix, Structural Genomics | | pdb70 | 3O9X\_A | 96.8 | 4.1e-06 | 2.7e-10 | 48.4 | 57 | (3, 60) | 180 | (74, 130) | 133 | Uncharacterized HTH-type transcriptional regulator ygiT/DNA | 3O9X\_A Uncharacterized HTH-type transcriptional regulator ygiT/DNA HTH-XRE DNA binding motif, Transcriptional HET: GOL | | pdb70 | 5FD4\_B | 96.8 | 4.1e-06 | 2.7e-10 | 55.8 | 61 | (2, 62) | 180 | (32, 93) | 324 | ComR | 5FD4\_B ComR Streptococcus, Competence, Quorum sensing, ComR | | pdb70 | 3FYM\_A | 96.8 | 4.5e-06 | 3e-10 | 48.3 | 62 | (2, 63) | 180 | (5, 72) | 130 | Putative uncharacterized protein | 3FYM\_A Putative uncharacterized protein HTH DNA binding, DNA BINDING | | pdb70 | 1ZUG\_A | 96.7 | 4.8e-06 | 3.2e-10 | 42.2 | 60 | (3, 63) | 180 | (6, 65) | 71 | PHAGE 434 CRO PROTEIN | 1ZUG\_A PHAGE 434 CRO PROTEIN GENE REGULATING PROTEIN, TRANSCRIPTION REGULATION | | pdb70 | 2CRO\_A | 96.7 | 4.8e-06 | 3.2e-10 | 42.2 | 60 | (3, 63) | 180 | (6, 65) | 71 | 434 CRO PROTEIN | 2CRO\_A 434 CRO PROTEIN GENE REGULATING PROTEIN | | pdb70 | 5Y69\_A | 96.7 | 5.4e-06 | 3.6e-10 | 49.2 | 62 | (2, 63) | 180 | (5, 67) | 151 | chain A | 5Y69\_A chain A UNKNOWN FUCTION, VIRAL PROTEIN HET: MSE | | pdb70 | 3CEC\_A | 96.7 | 5.8e-06 | 3.9e-10 | 45.8 | 61 | (3, 63) | 180 | (21, 81) | 104 | Putative antidote protein of plasmid | 3CEC\_A Putative antidote protein of plasmid Structural genomics, Joint Center for | | pdb70 | 1S4K\_B | 96.7 | 6.3e-06 | 4.2e-10 | 46.9 | 59 | (2, 60) | 180 | (5, 65) | 120 | putative cytoplasmic protein ydil | 1S4K\_B putative cytoplasmic protein ydil structural genomics, MCSG, Salmonella typhimurium HET: MSE | | pdb70 | 3G5G\_G | 96.7 | 6.4e-06 | 4.3e-10 | 45.1 | 60 | (3, 62) | 180 | (31, 90) | 99 | Regulatory protein | 3G5G\_G Regulatory protein transcriptional regulator, helix-turn-helix, Restriction-modification, TRANSCRIPTION | | pdb70 | 3G5G\_H | 96.7 | 6.4e-06 | 4.3e-10 | 45.1 | 60 | (3, 62) | 180 | (31, 90) | 99 | Regulatory protein | 3G5G\_H Regulatory protein transcriptional regulator, helix-turn-helix, Restriction-modification, TRANSCRIPTION | | pdb70 | 1LMB\_4 | 96.7 | 6.6e-06 | 4.4e-10 | 44.0 | 55 | (9, 63) | 180 | (26, 80) | 92 | LAMBDA REPRESSOR/DNA COMPLEX | 1LMB\_4 LAMBDA REPRESSOR/DNA COMPLEX PROTEIN-DNA COMPLEX, DOUBLE HELIX, TRANSCRIPTION-DNA | | pdb70 | 5IFG\_B | 96.7 | 6.5e-06 | 4.4e-10 | 48.2 | 56 | (3, 60) | 180 | (82, 137) | 138 | mRNA interferase HigB (E.C.3.1.-.-), Antitoxin | 5IFG\_B mRNA interferase HigB (E.C.3.1.-.-), Antitoxin mRNA interferase, HYDROLASE-ANTITOXIN complex HET: MSE | | pdb70 | 6IRP\_A | 96.7 | 6.5e-06 | 4.4e-10 | 48.2 | 56 | (3, 60) | 180 | (82, 137) | 138 | Antitoxin HigA | 6IRP\_A Antitoxin HigA Antoxin, ANTITOXIN HET: MSE | | pdb70 | 6JQ4\_A | 96.7 | 6.5e-06 | 4.4e-10 | 48.2 | 56 | (3, 60) | 180 | (82, 137) | 138 | Antitoxin HigA | 6JQ4\_A Antitoxin HigA Helix-Turn-Helix Domain, ANTITOXIN | | pdb70 | 5V5T\_A | 96.7 | 6.8e-06 | 4.6e-10 | 54.6 | 61 | (2, 63) | 180 | (11, 71) | 306 | Conserved domain protein | 5V5T\_A Conserved domain protein TRANSCRIPTION REGULATOR, TRANSCRIPTION HET: MSE | | pdb70 | 5V5T\_D | 96.7 | 6.8e-06 | 4.6e-10 | 54.6 | 61 | (2, 63) | 180 | (11, 71) | 306 | Conserved domain protein | 5V5T\_D Conserved domain protein TRANSCRIPTION REGULATOR, TRANSCRIPTION HET: MSE | | pdb70 | 5V5U\_B | 96.7 | 6.8e-06 | 4.6e-10 | 54.6 | 61 | (2, 63) | 180 | (11, 71) | 306 | Conserved domain protein | 5V5U\_B Conserved domain protein TRANSCRIPTION REGULATOR, TRANSCRIPTION | | pdb70 | 3ZHI\_A | 96.6 | 8e-06 | 5.4e-10 | 43.3 | 60 | (3, 62) | 180 | (8, 76) | 83 | CI | 3ZHI\_A CI TRANSCRIPTION, TRANSCRIPTION REGULATION | | pdb70 | 3F51\_E | 96.6 | 8.4e-06 | 5.6e-10 | 46.0 | 61 | (3, 63) | 180 | (31, 91) | 117 | clp gene regulator (ClgR) | 3F51\_E clp gene regulator (ClgR) gene regulator, helix-turn-helix, transcriptional activator HET: MPD | | pdb70 | 3F52\_E | 96.6 | 8.4e-06 | 5.6e-10 | 46.0 | 61 | (3, 63) | 180 | (31, 91) | 117 | clp gene regulator (ClgR) | 3F52\_E clp gene regulator (ClgR) gene regulator, helix-turn-helix motif, transcriptional HET: GOL | | pdb70 | 4MCX\_C | 96.6 | 8.4e-06 | 5.6e-10 | 44.9 | 61 | (3, 63) | 180 | (11, 71) | 104 | Host inhibition of growth A | 4MCX\_C Host inhibition of growth A bacterial toxins, biofilms, cell metabolism | | pdb70 | 3PXP\_A | 96.6 | 8.9e-06 | 6e-10 | 53.8 | 62 | (2, 63) | 180 | (8, 79) | 292 | Helix-turn-helix domain protein | 3PXP\_A Helix-turn-helix domain protein DNA-BINDING, BASIC HELIX-LOOP-HELIX MOTIF, BHLH HET: MYR, EDO | | pdb70 | 3R1F\_N | 96.6 | 9.7e-06 | 6.5e-10 | 47.2 | 60 | (3, 62) | 180 | (10, 81) | 135 | ESX-1 secretion-associated regulator EspR | 3R1F\_N ESX-1 secretion-associated regulator EspR helix-turn-helix, transcription factor, helix-turn-helix transcription HET: MSE | | pdb70 | 2L49\_A | 96.6 | 9.8e-06 | 6.6e-10 | 44.1 | 56 | (3, 58) | 180 | (7, 62) | 99 | C protein | 2L49\_A C protein P2 bacteriophage, P2 C, direct | | pdb70 | 2XCJ\_B | 96.6 | 9.8e-06 | 6.6e-10 | 44.1 | 56 | (3, 58) | 180 | (7, 62) | 99 | C PROTEIN | 2XCJ\_B C PROTEIN DIRECT REPEATS, REPRESSOR, HELIX-TURN-HELIX, VIRAL HET: GOL | | pdb70 | 3VK0\_A | 96.6 | 1e-05 | 6.7e-10 | 45.6 | 62 | (3, 64) | 180 | (24, 85) | 114 | Transcriptional regulator | 3VK0\_A Transcriptional regulator HTH motif, XRE transcription factor | | pdb70 | 4MCX\_A | 96.6 | 1e-05 | 6.7e-10 | 44.5 | 61 | (3, 63) | 180 | (11, 71) | 104 | Host inhibition of growth A | 4MCX\_A Host inhibition of growth A bacterial toxins, biofilms, cell metabolism | | pdb70 | 3TYS\_A | 96.5 | 1.1e-05 | 7.1e-10 | 43.2 | 58 | (4, 62) | 180 | (28, 85) | 88 | Predicted transcriptional regulator | 3TYS\_A Predicted transcriptional regulator Structural Genomics, Center for Structural | | pdb70 | 2JVL\_A | 96.5 | 1.1e-05 | 7.3e-10 | 44.9 | 61 | (3, 63) | 180 | (37, 99) | 107 | TrMBF1 | 2JVL\_A TrMBF1 MBF1, Coactivator, Trichoderma reesei, helix-turn-helix | | pdb70 | 6AF3\_D | 96.5 | 1.1e-05 | 7.6e-10 | 43.8 | 59 | (4, 62) | 180 | (35, 93) | 97 | HigB toxin, HigA antitoxin | 6AF3\_D HigB toxin, HigA antitoxin Toxin-Antitoxin complex, TOXIN HET: MSE | | pdb70 | 6AF4\_B | 96.5 | 1.1e-05 | 7.6e-10 | 43.8 | 59 | (4, 62) | 180 | (35, 93) | 97 | HigB, HigA | 6AF4\_B HigB, HigA Toxin-Antitoxin complex, TOXIN | | pdb70 | 3QYX\_B | 96.5 | 1.1e-05 | 7.6e-10 | 46.8 | 61 | (2, 62) | 180 | (7, 79) | 133 | ESX-1 secretion-associated regulator EspR/DNA complex | 3QYX\_B ESX-1 secretion-associated regulator EspR/DNA complex N-terminal HTH motif, C-terminal dimerization | | pdb70 | 4NDW\_B | 96.5 | 1.2e-05 | 8.5e-10 | 47.9 | 60 | (3, 62) | 180 | (28, 99) | 153 | Nucleoid-associated protein EspR | 4NDW\_B Nucleoid-associated protein EspR ALL HELICAL, HELIX-TURN-HELIX MOTIF, TRANSACTIONAL | | pdb70 | 6CF1\_B | 96.5 | 1.3e-05 | 8.5e-10 | 47.0 | 61 | (3, 63) | 180 | (45, 105) | 138 | Antitoxin HigA | 6CF1\_B Antitoxin HigA Helix-turn-helix motif, transcriptional regulator, toxin-antitoxin | | pdb70 | 3PXP\_B | 96.5 | 1.3e-05 | 9e-10 | 52.9 | 62 | (2, 63) | 180 | (8, 79) | 292 | Helix-turn-helix domain protein | 3PXP\_B Helix-turn-helix domain protein DNA-BINDING, BASIC HELIX-LOOP-HELIX MOTIF, BHLH HET: MSE, EDO, MYR | | pdb70 | 3FMY\_A | 96.5 | 1.4e-05 | 9.3e-10 | 40.5 | 57 | (3, 60) | 180 | (14, 70) | 73 | Uncharacterized HTH-type transcriptional regulator ygiT | 3FMY\_A Uncharacterized HTH-type transcriptional regulator ygiT helix-turn-helix, DNA-binding, Transcription, Transcription regulation | | pdb70 | 2AW6\_B | 96.5 | 1.5e-05 | 9.8e-10 | 53.1 | 60 | (2, 62) | 180 | (5, 64) | 317 | PrgX | 2AW6\_B PrgX Repressor, pheromone, DNA binding, regulatory | | pdb70 | 5A7L\_A | 96.4 | 1.6e-05 | 1e-09 | 41.3 | 61 | (3, 63) | 180 | (7, 76) | 80 | CI | 5A7L\_A CI TRANSCRIPTION | | pdb70 | 3QWG\_A | 96.4 | 1.6e-05 | 1.1e-09 | 45.5 | 62 | (2, 63) | 180 | (7, 80) | 123 | ESX-1 secretion-associated regulator EspR | 3QWG\_A ESX-1 secretion-associated regulator EspR N-terminal helix-turn-helix motif, Transcription factor | | pdb70 | 2KZ8\_A | 96.4 | 1.7e-05 | 1.2e-09 | 45.5 | 58 | (3, 61) | 180 | (72, 129) | 131 | Uncharacterized HTH-type transcriptional regulator ygiT | 2KZ8\_A Uncharacterized HTH-type transcriptional regulator ygiT Zinc finger, Helix turn helix | | pdb70 | 2H8R\_B | 96.4 | 1.8e-05 | 1.2e-09 | 50.2 | 61 | (2, 62) | 180 | (33, 93) | 221 | Hepatocyte nuclear factor 1-beta/DNA Complex | 2H8R\_B Hepatocyte nuclear factor 1-beta/DNA Complex trasncription factor, POU, homeo, protein-DNA | | pdb70 | 2EBY\_A | 96.4 | 2e-05 | 1.4e-09 | 44.0 | 58 | (6, 63) | 180 | (17, 74) | 113 | Putative HTH-type transcriptional regulator ybaQ | 2EBY\_A Putative HTH-type transcriptional regulator ybaQ Hypothetical protein, JW0472, Structural Genomics HET: SO4 | | pdb70 | 6HPC\_A | 96.4 | 2.2e-05 | 1.4e-09 | 45.2 | 55 | (4, 58) | 180 | (81, 135) | 138 | Antitoxin HicB | 6HPC\_A Antitoxin HicB dna binding, helix-turn-helix, HTH, ANTITOXIN HET: MSE | | pdb70 | 1NEQ\_A | 96.3 | 2.3e-05 | 1.5e-09 | 39.8 | 58 | (3, 63) | 180 | (12, 69) | 74 | DNA-BINDING PROTEIN NER | 1NEQ\_A DNA-BINDING PROTEIN NER DNA-BINDING PROTEIN | | pdb70 | 2AUW\_B | 96.3 | 2.3e-05 | 1.5e-09 | 47.5 | 59 | (3, 62) | 180 | (93, 151) | 170 | hypothetical protein NE0471 | 2AUW\_B hypothetical protein NE0471 alpha-beta structure, Structural Genomics, PSI HET: GOL | | pdb70 | 3QQ6\_A | 96.3 | 2.4e-05 | 1.6e-09 | 40.4 | 59 | (4, 62) | 180 | (14, 73) | 78 | HTH-type transcriptional regulator sinR | 3QQ6\_A HTH-type transcriptional regulator sinR Helix-Turn-Helix motif, biofilm, repressor, transcriptional | | pdb70 | 4ICH\_A | 96.3 | 2.5e-05 | 1.7e-09 | 51.7 | 62 | (3, 64) | 180 | (33, 94) | 311 | Transcriptional regulator | 4ICH\_A Transcriptional regulator Structural Genomics, PSI-Biology, Midwest Center HET: MSE, B3P | | pdb70 | 6CHV\_B | 96.3 | 2.5e-05 | 1.7e-09 | 44.5 | 60 | (3, 62) | 180 | (28, 87) | 121 | Antitoxin HigA/DNA Complex | 6CHV\_B Antitoxin HigA/DNA Complex Helix-turn-helix DNA binding protein, DNA-protein | | pdb70 | 4PU7\_B | 96.3 | 2.6e-05 | 1.7e-09 | 44.3 | 59 | (4, 63) | 180 | (53, 111) | 118 | Toxin-antitoxin system antidote transcriptional repressor | 4PU7\_B Toxin-antitoxin system antidote transcriptional repressor Toxin Antitoxin System, TOXIN | | pdb70 | 3EUS\_A | 96.3 | 2.6e-05 | 1.7e-09 | 40.9 | 59 | (3, 61) | 180 | (17, 76) | 86 | DNA-binding protein | 3EUS\_A DNA-binding protein DNA binding protein, STRUCTURAL GENOMICS | | pdb70 | 2A6C\_A | 96.3 | 2.7e-05 | 1.8e-09 | 40.8 | 58 | (4, 61) | 180 | (22, 80) | 83 | Helix-turn-helix motif | 2A6C\_A Helix-turn-helix motif PUTATIVE TRANSCRIPTIONAL REGULATOR, STRUCTURAL GENOMICS HET: GOL, CIT, MSE | | pdb70 | 4PU4\_D | 96.3 | 2.8e-05 | 1.9e-09 | 44.1 | 59 | (4, 63) | 180 | (53, 111) | 118 | Toxin-antitoxin system toxin HipA family | 4PU4\_D Toxin-antitoxin system toxin HipA family Toxin Antitoxin System, TOXIN-ANTITOXIN-DNA complex | | pdb70 | 4GQM\_A | 96.3 | 2.8e-05 | 1.9e-09 | 44.0 | 62 | (2, 63) | 180 | (15, 82) | 118 | CT009 | 4GQM\_A CT009 helix-turn-helix, UNKNOWN FUNCTION | | pdb70 | 2WUS\_R | 96.3 | 2.9e-05 | 2e-09 | 43.5 | 61 | (3, 63) | 180 | (10, 76) | 112 | ROD SHAPE-DETERMINING PROTEIN MREB, PUTATIVE | 2WUS\_R ROD SHAPE-DETERMINING PROTEIN MREB, PUTATIVE STRUCTURAL PROTEIN, CELL WALL MORPHOGENESIS | | pdb70 | 2WUS\_S | 96.3 | 2.9e-05 | 2e-09 | 43.5 | 61 | (3, 63) | 180 | (10, 76) | 112 | ROD SHAPE-DETERMINING PROTEIN MREB, PUTATIVE | 2WUS\_S ROD SHAPE-DETERMINING PROTEIN MREB, PUTATIVE STRUCTURAL PROTEIN, CELL WALL MORPHOGENESIS | | pdb70 | 6U0I\_B | 96.3 | 3e-05 | 2e-09 | 45.5 | 54 | (4, 57) | 180 | (91, 144) | 148 | Antitoxin HicB | 6U0I\_B Antitoxin HicB structural genomics, Center for Structural HET: MSE | | pdb70 | 6F8S\_C | 96.2 | 3e-05 | 2e-09 | 42.1 | 60 | (4, 63) | 180 | (14, 74) | 99 | XRE family transcriptional regulator, Putative | 6F8S\_C XRE family transcriptional regulator, Putative GraA, GraT, HigA, HigB, GraTA HET: PEG, SO4 | | pdb70 | 2O38\_A | 96.2 | 3.3e-05 | 2.2e-09 | 43.8 | 60 | (4, 63) | 180 | (44, 104) | 120 | Hypothetical protein | 2O38\_A Hypothetical protein alpha-beta, helix-turn-helix, Structural Genomics, PSI-2 HET: ACY, MSE | | pdb70 | 2O38\_B | 96.2 | 3.3e-05 | 2.2e-09 | 43.8 | 60 | (4, 63) | 180 | (44, 104) | 120 | Hypothetical protein | 2O38\_B Hypothetical protein alpha-beta, helix-turn-helix, Structural Genomics, PSI-2 HET: ACY | | pdb70 | 4YBA\_B | 96.2 | 3.6e-05 | 2.4e-09 | 41.8 | 53 | (3, 55) | 180 | (12, 64) | 99 | C.kpn2I controller protein | 4YBA\_B C.kpn2I controller protein DNA-protein interactions, helix-turn-helix, restriction-modification, controller | | pdb70 | 6CHV\_D | 96.2 | 3.6e-05 | 2.4e-09 | 43.8 | 60 | (3, 62) | 180 | (28, 87) | 121 | Antitoxin HigA/DNA Complex | 6CHV\_D Antitoxin HigA/DNA Complex Helix-turn-helix DNA binding protein, DNA-protein | | pdb70 | 4JCY\_A | 96.2 | 4e-05 | 2.6e-09 | 41.3 | 62 | (2, 63) | 180 | (3, 68) | 98 | Csp231I C protein | 4JCY\_A Csp231I C protein helix-turn-helix, C controller protein, restriction-modification | | pdb70 | 4JQD\_E | 96.2 | 4.1e-05 | 2.7e-09 | 41.2 | 62 | (2, 63) | 180 | (3, 68) | 98 | Csp231I C protein | 4JQD\_E Csp231I C protein helix-turn-helix, C controller protein, restriction-modification | | pdb70 | 2PPX\_A | 96.1 | 4.9e-05 | 3.3e-09 | 41.4 | 57 | (4, 61) | 180 | (34, 90) | 99 | Uncharacterized protein Atu1735 | 2PPX\_A Uncharacterized protein Atu1735 Agrobacterium tumefaciens, HTH-motif, XRE-family, structural HET: GOL, SO4 | | pdb70 | 2AWI\_F | 96.1 | 4.9e-05 | 3.3e-09 | 50.4 | 60 | (2, 62) | 180 | (5, 64) | 317 | PrgX | 2AWI\_F PrgX Repressor, pheromone, DNA binding, regulatory HET: MSE | | pdb70 | 4GHJ\_A | 96.1 | 5e-05 | 3.4e-09 | 41.7 | 57 | (3, 61) | 180 | (39, 95) | 101 | Probable transcriptional regulator | 4GHJ\_A Probable transcriptional regulator Structural Genomics, NIAID, National Institute HET: MSE | | pdb70 | 4GHJ\_B | 96.1 | 5e-05 | 3.4e-09 | 41.7 | 57 | (3, 61) | 180 | (39, 95) | 101 | Probable transcriptional regulator | 4GHJ\_B Probable transcriptional regulator Structural Genomics, NIAID, National Institute | | pdb70 | 6B9R\_D | 96.0 | 5.7e-05 | 3.8e-09 | 53.0 | 63 | (2, 64) | 180 | (239, 301) | 450 | Methylphosphonate synthase | 6B9R\_D Methylphosphonate synthase Phosphonate, Hydroxymethylphosphonate, Iron, OXIDOREDUCTASE HET: GOL, 2HE | | pdb70 | 6B9R\_C | 96.0 | 5.7e-05 | 3.8e-09 | 53.0 | 63 | (2, 64) | 180 | (239, 301) | 450 | Methylphosphonate synthase | 6B9R\_C Methylphosphonate synthase Phosphonate, Hydroxymethylphosphonate, Iron, OXIDOREDUCTASE HET: 2HE, GOL | | pdb70 | 2HIN\_A | 96.0 | 5.7e-05 | 3.9e-09 | 38.5 | 54 | (5, 60) | 180 | (3, 58) | 71 | Repressor protein | 2HIN\_A Repressor protein TRANSCRIPTION FACTOR, DIMER INTERFACE, HELIX-TURN-HELIX HET: SO4 | | pdb70 | 2HIN\_B | 96.0 | 5.7e-05 | 3.9e-09 | 38.5 | 54 | (5, 60) | 180 | (3, 58) | 71 | Repressor protein | 2HIN\_B Repressor protein TRANSCRIPTION FACTOR, DIMER INTERFACE, HELIX-TURN-HELIX HET: SO4 | | pdb70 | 3BD1\_A | 96.0 | 5.8e-05 | 3.9e-09 | 39.1 | 58 | (4, 63) | 180 | (3, 62) | 79 | Cro protein | 3BD1\_A Cro protein transcription factor, helix-turn-helix, prophage, structural HET: GOL, SO4 | | pdb70 | 2OFY\_A | 96.0 | 6.2e-05 | 4.1e-09 | 39.5 | 50 | (13, 62) | 180 | (27, 77) | 86 | Putative XRE-family transcriptional regulator | 2OFY\_A Putative XRE-family transcriptional regulator TRANSCRIPTION REGULATOR, XRE-FAMILY, STRUCTURAL GENOMICS | | pdb70 | 2OFY\_B | 96.0 | 6.3e-05 | 4.2e-09 | 39.4 | 50 | (13, 62) | 180 | (27, 77) | 86 | Putative XRE-family transcriptional regulator | 2OFY\_B Putative XRE-family transcriptional regulator TRANSCRIPTION REGULATOR, XRE-FAMILY, STRUCTURAL GENOMICS HET: MSE | | pdb70 | 5Y24\_B | 96.0 | 6.6e-05 | 4.4e-09 | 51.4 | 56 | (1, 56) | 180 | (4, 62) | 396 | AimR transcriptional regulator, GLY-MET-PRO-ARG-GLY-ALA | 5Y24\_B AimR transcriptional regulator, GLY-MET-PRO-ARG-GLY-ALA DNA binding protein, peptide binding HET: MSE | | pdb70 | 5D50\_A | 96.0 | 7.1e-05 | 4.7e-09 | 46.2 | 60 | (5, 64) | 180 | (101, 162) | 199 | Repressor, Anti-repressor protein | 5D50\_A Repressor, Anti-repressor protein Repressor, Anti-repressor, complex, DNA BINDING | | pdb70 | 5D50\_B | 96.0 | 7.1e-05 | 4.7e-09 | 46.2 | 60 | (5, 64) | 180 | (101, 162) | 199 | Repressor, Anti-repressor protein | 5D50\_B Repressor, Anti-repressor protein Repressor, Anti-repressor, complex, DNA BINDING | | pdb70 | 3TRB\_B | 95.9 | 7.5e-05 | 5e-09 | 41.0 | 59 | (5, 63) | 180 | (18, 77) | 104 | Virulence-associated protein I | 3TRB\_B Virulence-associated protein I Mobile and extrachromosomal element functions HET: MSE | | pdb70 | 2OX6\_C | 95.9 | 7.4e-05 | 5e-09 | 45.0 | 51 | (3, 53) | 180 | (10, 60) | 166 | Hypothetical protein | 2OX6\_C Hypothetical protein Shewanella oneidensis, Structural genomics, PSI-2 HET: MG | | pdb70 | 1IC8\_B | 95.9 | 8e-05 | 5.4e-09 | 46.0 | 59 | (2, 60) | 180 | (32, 90) | 194 | HEPATOCYTE NUCLEAR FACTOR 1-ALPHA/DNA Complex | 1IC8\_B HEPATOCYTE NUCLEAR FACTOR 1-ALPHA/DNA Complex Transcription regulation DNA-binding | | pdb70 | 6FKG\_C | 95.9 | 8.2e-05 | 5.5e-09 | 41.5 | 57 | (3, 59) | 180 | (10, 69) | 115 | Uncharacterized protein Rv1989c, Uncharacterized protein | 6FKG\_C Uncharacterized protein Rv1989c, Uncharacterized protein Toxin-Antitoxin system Phosphorylase NAD+-binding, TOXIN HET: GOL | | pdb70 | 2OX6\_A | 95.9 | 8.4e-05 | 5.7e-09 | 44.8 | 51 | (3, 53) | 180 | (10, 60) | 166 | Hypothetical protein | 2OX6\_A Hypothetical protein Shewanella oneidensis, Structural genomics, PSI-2 HET: MG | | pdb70 | 5W8Y\_A | 95.9 | 8.5e-05 | 5.7e-09 | 37.7 | 56 | (4, 61) | 180 | (3, 60) | 73 | protein XPH1 | 5W8Y\_A protein XPH1 helix-turn-helix, structural evolution, conformational switch | | pdb70 | 3VWB\_A | 95.9 | 8.4e-05 | 5.8e-09 | 43.6 | 34 | (3, 37) | 180 | (32, 65) | 143 | Virulence regulon transcriptional activator virB/DNA | 3VWB\_A Virulence regulon transcriptional activator virB/DNA HTH DNA binding motif, dsDNA | | pdb70 | 3W2A\_A | 95.9 | 8.4e-05 | 5.8e-09 | 43.6 | 34 | (3, 37) | 180 | (32, 65) | 143 | Virulence regulon transcriptional activator VirB/DNA | 3W2A\_A Virulence regulon transcriptional activator VirB/DNA ParS like DNA binding sites | | pdb70 | 4YAR\_A | 95.9 | 8.5e-05 | 5.8e-09 | 51.9 | 61 | (3, 63) | 180 | (6, 67) | 443 | 2-Hydroxyethyl-phosphonate Dioxygenase (HEPD) | 4YAR\_A 2-Hydroxyethyl-phosphonate Dioxygenase (HEPD) Dioxygenase, OXIDOREDUCTASE HET: CD | | pdb70 | 5ZW6\_B | 95.8 | 0.0001 | 6.7e-09 | 50.3 | 56 | (1, 56) | 180 | (4, 62) | 391 | AimR transcriptional regulator, GLY-MET-PRO-ARG-GLY-ALA | 5ZW6\_B AimR transcriptional regulator, GLY-MET-PRO-ARG-GLY-ALA dimer, phage SPbeta, TRANSCRIPTION REGULATOR | | pdb70 | 2LCV\_A | 95.8 | 0.00011 | 7.2e-09 | 36.1 | 54 | (7, 60) | 180 | (3, 59) | 67 | HTH-type transcriptional repressor CytR | 2LCV\_A HTH-type transcriptional repressor CytR BACTERIAL GENE REPRESSOR, HELIX TURN | | pdb70 | 2MQK\_A | 95.7 | 0.00014 | 9.1e-09 | 35.6 | 37 | (3, 39) | 180 | (10, 46) | 65 | ATP-dependent target DNA activator B | 2MQK\_A ATP-dependent target DNA activator B HYDROLASE | | pdb70 | 1NR3\_A | 95.7 | 0.00014 | 9.2e-09 | 41.3 | 52 | (11, 62) | 180 | (3, 56) | 122 | DNA-BINDING PROTEIN TFX | 1NR3\_A DNA-BINDING PROTEIN TFX NORTHEAST STRUCTURAL GENOMICS CONSORTIUM, REDUCED-DIMENSIONALITY | | pdb70 | 1CJG\_A | 95.7 | 0.00014 | 9.2e-09 | 35.5 | 46 | (12, 57) | 180 | (2, 50) | 62 | LAC REPRESSOR HP62/DNA COMPLEX | 1CJG\_A LAC REPRESSOR HP62/DNA COMPLEX TRANSCRIPTION REGULATION, LAC OPERON, LAC | | pdb70 | 4YAR\_A | 95.7 | 0.00015 | 1.1e-08 | 50.6 | 62 | (2, 63) | 180 | (232, 294) | 443 | 2-Hydroxyethyl-phosphonate Dioxygenase (HEPD) | 4YAR\_A 2-Hydroxyethyl-phosphonate Dioxygenase (HEPD) Dioxygenase, OXIDOREDUCTASE HET: CD | | pdb70 | 2L1P\_A | 95.6 | 0.00017 | 1.1e-08 | 38.0 | 42 | (11, 54) | 180 | (30, 71) | 83 | DNA-binding protein SATB1 | 2L1P\_A DNA-binding protein SATB1 PSI-Biology, NESG, Structural Genomics, Protein | | pdb70 | 3NZL\_A | 95.6 | 0.00017 | 1.1e-08 | 38.0 | 42 | (11, 54) | 180 | (30, 71) | 83 | DNA-binding protein SATB1 | 3NZL\_A DNA-binding protein SATB1 alpha-helical domain, Structural Genomics, PSI-Biology | | pdb70 | 3ME5\_A | 95.6 | 0.00017 | 1.2e-08 | 51.0 | 40 | (2, 41) | 180 | (18, 63) | 482 | Cytosine-specific methyltransferase (E.C.2.1.1.37) | 3ME5\_A Cytosine-specific methyltransferase (E.C.2.1.1.37) STRUCTURAL GENOMICS, PROTEIN STRUCTURE INITIATIVE | | pdb70 | 6B9T\_C | 95.6 | 0.00018 | 1.2e-08 | 50.5 | 62 | (2, 63) | 180 | (244, 305) | 457 | Methylphosphonate synthase (E.C.1.13.11.73) | 6B9T\_C Methylphosphonate synthase (E.C.1.13.11.73) Phosphonate, Methylphosphonate, Iron, OXIDOREDUCTASE HET: FMT, 2HE | | pdb70 | 6B9T\_D | 95.6 | 0.00018 | 1.2e-08 | 50.4 | 61 | (3, 63) | 180 | (245, 305) | 457 | Methylphosphonate synthase (E.C.1.13.11.73) | 6B9T\_D Methylphosphonate synthase (E.C.1.13.11.73) Phosphonate, Methylphosphonate, Iron, OXIDOREDUCTASE HET: 2HE, FMT | | pdb70 | 6JG8\_A | 95.5 | 0.00021 | 1.4e-08 | 48.8 | 55 | (2, 56) | 180 | (6, 63) | 395 | AimR transcriptional regulator/DNA Complex | 6JG8\_A AimR transcriptional regulator/DNA Complex AimR, Apo, HTH, PEPTIDE BINDING | | pdb70 | 5J9I\_D | 95.5 | 0.00021 | 1.4e-08 | 39.1 | 58 | (3, 61) | 180 | (43, 100) | 104 | Antitoxin igA-2 | 5J9I\_D Antitoxin igA-2 toxin-antitoxin system, antitoxin | | pdb70 | 2QFC\_A | 95.4 | 0.0003 | 2e-08 | 45.0 | 58 | (2, 60) | 180 | (7, 64) | 293 | PlcR protein, C-terminus pentapeptide from | 2QFC\_A PlcR protein, C-terminus pentapeptide from TPR HTH, TRANSCRIPTION REGULATION HET: PEG | | pdb70 | 3U3W\_B | 95.4 | 0.0003 | 2e-08 | 45.0 | 58 | (2, 60) | 180 | (7, 64) | 293 | Transcriptional activator PlcR protein, C-terminus | 3U3W\_B Transcriptional activator PlcR protein, C-terminus ternary complex, PlcR-PAPR7-DNA, HTH DNA-binding | | pdb70 | 3D1N\_I | 95.3 | 0.00035 | 2.3e-08 | 40.6 | 50 | (2, 53) | 180 | (10, 65) | 151 | POU domain, class 6, transcription | 3D1N\_I POU domain, class 6, transcription Protein-DNA complex, Helix-turn-helix (HTH), DNA-binding HET: MSE | | pdb70 | 5ZVV\_B | 95.2 | 0.00037 | 2.5e-08 | 46.8 | 55 | (2, 56) | 180 | (3, 60) | 382 | AimR transcriptional regulator | 5ZVV\_B AimR transcriptional regulator dimer, phage phi3T, TRANSCRIPTION REGULATOR HET: GOL, MSE | | pdb70 | 1RZS\_A | 95.2 | 0.00044 | 2.9e-08 | 33.0 | 45 | (5, 50) | 180 | (3, 47) | 61 | Regulatory protein cro | 1RZS\_A Regulatory protein cro HELIX-TURN-HELIX, DNA-BINDING PROTEIN, STRUCTURAL EVOLUTION | | pdb70 | 5JAA\_A | 95.1 | 0.00044 | 2.9e-08 | 37.7 | 57 | (3, 60) | 180 | (42, 98) | 103 | Antitoxin igA-2, Toxin HigB-2 | 5JAA\_A Antitoxin igA-2, Toxin HigB-2 toxin-antitoxin system, toxin HET: MSE | | pdb70 | 3D1N\_N | 95.1 | 0.00046 | 3e-08 | 40.1 | 51 | (2, 54) | 180 | (10, 66) | 151 | POU domain, class 6, transcription | 3D1N\_N POU domain, class 6, transcription Protein-DNA complex, Helix-turn-helix (HTH), DNA-binding HET: MSE | | pdb70 | 1GT0\_C | 94.6 | 0.001 | 7e-08 | 39.0 | 49 | (2, 52) | 180 | (13, 67) | 159 | OCTAMER-BINDING TRANSCRIPTION FACTOR 1, TRANSCRIPTION | 1GT0\_C OCTAMER-BINDING TRANSCRIPTION FACTOR 1, TRANSCRIPTION TRANSCRIPTION FACTOR, POU FACTORS, SOX | |
| Top keywords  (threshold 1.00e-03 (evalue)) | **domain\_containing, repressor, transcriptional, regulator, HTH, cro, C1\_type, Peptidase, Helix\_turn\_helix, S24** |
| Output files | ../../similar\_sequences/42\_FANPEZAQ\_CDS\_0042\_merged.svg ../../similar\_sequences/42\_FANPEZAQ\_CDS\_0042\_pdb70.a3m ../../similar\_sequences/42\_FANPEZAQ\_CDS\_0042\_pdb70.hhr ../../similar\_sequences/42\_FANPEZAQ\_CDS\_0042\_uniclust.a3m ../../similar\_sequences/42\_FANPEZAQ\_CDS\_0042\_uniclust.hhr |

#### Structure prediction (AlphaFold)2

|  |  |
| --- | --- |
| Stats | xml version="1.0" encoding="utf-8" standalone="no"?       2024-09-02T21:09:41.312135 image/svg+xml   Matplotlib v3.7.2, https://matplotlib.org/ |
| Predicted structure | **NGL Viewer Controls:**  - Center: *Left-Click* - Rotate: *Left-Click + Drag* - Translate: *Right-Click + Drag* - Zoom: *Shift + Left-Click + Drag* |
| Output files | ../../predicted\_structures/42\_FANPEZAQ\_CDS\_0042/features.pkl ../../predicted\_structures/42\_FANPEZAQ\_CDS\_0042/ranked\_0.pdb ../../predicted\_structures/42\_FANPEZAQ\_CDS\_0042/ranked\_0\_plots.svg ../../predicted\_structures/42\_FANPEZAQ\_CDS\_0042/result\_model\_1\_ptm\_pred\_0.pkl |

#### Structure similarity search results (Foldseek)3

|  |  |
| --- | --- |
| Structure databases searched | Pdb, Afdb-proteome, Afdb-uniprot50 |
| Results, scheme(s)  (Top layers only, threshold 1.00e-02 (evalue)) | xml version="1.0" encoding="utf-8" standalone="no"?       2024-09-02T21:11:21.799040 image/svg+xml   Matplotlib v3.7.2, https://matplotlib.org/ |
| Results, table  (threshold 1.00e-02 (evalue)) | | db | id | prob | evalue | bits | fident | alnlen | mismatch | gapopen | qstart | qend | tstart | tend | name | description | | --- | --- | --- | --- | --- | --- | --- | --- | --- | --- | --- | --- | --- | --- | --- | | pdb | 3K2Z\_A | 1.0 | 8.188e-06 | 226 | 0.252 | 91 | 64 | 4 | 91 | 180 | 105 | 192 | LexA repressor | LexA repressor | | pdb | 6A2R\_A | 1.0 | 1.14e-06 | 222 | 0.3 | 103 | 61 | 5 | 84 | 180 | 2 | 99 | LexA repressor | LexA repressor | | pdb | 6A2S\_A | 1.0 | 2.157e-06 | 220 | 0.297 | 101 | 60 | 5 | 86 | 180 | 5 | 100 | LexA repressor | LexA repressor | | pdb | 3K2Z\_B | 1.0 | 5.782e-06 | 217 | 0.235 | 102 | 68 | 5 | 86 | 180 | 97 | 195 | LexA repressor | LexA repressor | | pdb | 6A2T\_A | 1.0 | 1.229e-05 | 194 | 0.307 | 91 | 57 | 4 | 91 | 180 | 9 | 94 | LexA repressor | LexA repressor | | pdb | 1UMU\_B | 1.0 | 6.232e-05 | 189 | 0.235 | 102 | 72 | 5 | 80 | 179 | 9 | 106 | UMUD' | UMUD' | | pdb | 8GMT\_A | 1.0 | 7.859e-05 | 184 | 0.237 | 101 | 71 | 5 | 80 | 178 | 2 | 98 | DNA polymerase V subunit UmuD | DNA polymerase V subunit UmuD | | pdb | 1JHH\_B | 1.0 | 8.825e-05 | 182 | 0.177 | 90 | 68 | 5 | 93 | 179 | 32 | 118 | LEXA REPRESSOR | LEXA REPRESSOR | | pdb | 1JHE\_B | 1.0 | 0.000105 | 180 | 0.166 | 90 | 69 | 5 | 93 | 179 | 38 | 124 | LEXA REPRESSOR | LEXA REPRESSOR | | pdb | 7ZRA\_B | 1.0 | 0.0001179 | 179 | 0.188 | 90 | 67 | 5 | 93 | 179 | 38 | 124 | LexA repressor | LexA repressor | | pdb | 1JHF\_B | 1.0 | 0.0001179 | 178 | 0.188 | 90 | 67 | 5 | 93 | 179 | 24 | 110 | LEXA REPRESSOR | LEXA REPRESSOR | | pdb | 8GMS\_B | 1.0 | 0.000105 | 175 | 0.188 | 90 | 67 | 5 | 93 | 179 | 27 | 113 | LexA repressor | LexA repressor | | pdb | 1KCA\_A | 1.0 | 9.911e-05 | 173 | 0.197 | 96 | 66 | 6 | 89 | 177 | 3 | 94 | REPRESSOR PROTEIN CI | REPRESSOR PROTEIN CI | | pdb | 2HO0\_A | 1.0 | 0.000177 | 165 | 0.217 | 92 | 65 | 7 | 89 | 177 | 41 | 128 | Repressor protein cI101-229DM-K192A | Repressor protein cI101-229DM-K192A | | pdb | 1JHH\_A | 1.0 | 0.0002815 | 165 | 0.177 | 90 | 68 | 5 | 93 | 179 | 111 | 197 | LEXA REPRESSOR | LEXA REPRESSOR | | pdb | 1F39\_B | 1.0 | 0.0001988 | 164 | 0.202 | 94 | 64 | 6 | 90 | 176 | 4 | 93 | REPRESSOR PROTEIN CI | REPRESSOR PROTEIN CI | | pdb | 8GMS\_A | 1.0 | 0.0002983 | 163 | 0.188 | 90 | 67 | 5 | 93 | 179 | 37 | 123 | LexA repressor | LexA repressor | | pdb | 8GMU\_A | 1.0 | 0.0001876 | 159 | 0.202 | 94 | 64 | 6 | 90 | 176 | 39 | 128 | Repressor protein cI | Repressor protein cI | | pdb | 3K3R\_F | 1.0 | 0.0002815 | 159 | 0.191 | 89 | 66 | 5 | 93 | 178 | 86 | 171 | LexA repressor | LexA repressor | | pdb | 2HNF\_A | 1.0 | 0.0002365 | 158 | 0.204 | 93 | 66 | 7 | 89 | 177 | 42 | 130 | Repressor protein cI101-229DM-K192A | Repressor protein cI101-229DM-K192A | | pdb | 3OMT\_B | 1.0 | 0.004296 | 157 | 0.278 | 61 | 44 | 0 | 3 | 63 | 6 | 66 | uncharacterized protein | uncharacterized protein | | pdb | 7JVT\_C | 1.0 | 4.777e-07 | 154 | 0.155 | 193 | 130 | 6 | 6 | 180 | 20 | 197 | Repressor protein CI | Repressor protein CI | | pdb | 3BS3\_A | 1.0 | 0.006084 | 153 | 0.254 | 59 | 44 | 0 | 3 | 61 | 4 | 62 | Putative DNA-binding protein | Putative DNA-binding protein | | pdb | 3JXC\_L | 1.0 | 0.003407 | 149 | 0.293 | 58 | 41 | 0 | 4 | 61 | 7 | 64 | Repressor protein C2 | Repressor protein C2 | | pdb | 1ADR\_A | 1.0 | 0.001132 | 148 | 0.264 | 68 | 50 | 0 | 4 | 71 | 9 | 76 | P22 C2 REPRESSOR | P22 C2 REPRESSOR | | pdb | 3JSO\_A | 1.0 | 1.16e-05 | 144 | 0.164 | 195 | 122 | 13 | 5 | 179 | 14 | 187 | LexA repressor | LexA repressor | | pdb | 3JSO\_B | 1.0 | 8.677e-06 | 140 | 0.168 | 196 | 118 | 12 | 5 | 179 | 14 | 185 | LexA repressor | LexA repressor | | pdb | 2FKD\_B | 1.0 | 0.002406 | 139 | 0.202 | 74 | 53 | 2 | 107 | 180 | 42 | 109 | Repressor protein CI | Repressor protein CI | | pdb | 3K3R\_E | 1.0 | 0.0012 | 138 | 0.179 | 89 | 67 | 5 | 93 | 178 | 87 | 172 | LexA repressor | LexA repressor | | pdb | 1ADR\_A | 1.0 | 0.002863 | 134 | 0.264 | 68 | 50 | 0 | 4 | 71 | 9 | 76 | P22 C2 REPRESSOR | P22 C2 REPRESSOR | | pdb | 1R63\_A | 1.0 | 0.007672 | 132 | 0.275 | 58 | 41 | 1 | 4 | 61 | 5 | 61 | REPRESSOR PROTEIN FROM BACTERIOPHAGE 434 | REPRESSOR PROTEIN FROM BACTERIOPHAGE 434 | | pdb | 1ADR\_A | 1.0 | 0.008616 | 124 | 0.264 | 68 | 50 | 0 | 4 | 71 | 9 | 76 | P22 C2 REPRESSOR | P22 C2 REPRESSOR | | pdb | 3JSP\_B | 1.0 | 8.328e-05 | 122 | 0.166 | 198 | 119 | 13 | 5 | 179 | 14 | 188 | LexA repressor | LexA repressor | | pdb | 4N31\_A | 1.0 | 0.0008976 | 121 | 0.118 | 135 | 72 | 5 | 90 | 179 | 7 | 139 | SipA | SipA | | pdb | 1ADR\_A | 1.0 | 0.008616 | 120 | 0.264 | 68 | 50 | 0 | 4 | 71 | 9 | 76 | P22 C2 REPRESSOR | P22 C2 REPRESSOR | | pdb | 3JSP\_A | 1.0 | 8.825e-05 | 119 | 0.172 | 197 | 124 | 13 | 5 | 179 | 14 | 193 | LexA repressor | LexA repressor | | pdb | 6JQ1\_A | 1.0 | 0.004296 | 112 | 0.264 | 87 | 63 | 1 | 4 | 89 | 7 | 93 | Transcriptional regulator, XRE family | Transcriptional regulator, XRE family | | pdb | 2FJR\_B | 1.0 | 4.153e-05 | 112 | 0.133 | 202 | 126 | 10 | 5 | 180 | 10 | 188 | Repressor protein CI | Repressor protein CI | | pdb | 3BDN\_A | 1.0 | 0.000167 | 112 | 0.14 | 235 | 131 | 10 | 2 | 180 | 11 | 230 | Lambda Repressor | Lambda Repressor | | pdb | 4N31\_B | 1.0 | 0.00227 | 104 | 0.133 | 135 | 68 | 5 | 91 | 180 | 3 | 133 | SipA | SipA | | pdb | 1ZZ6\_A | 0.999 | 7.859e-05 | 99 | 0.134 | 208 | 110 | 13 | 5 | 178 | 10 | 181 | Hydroxypropylphosphonic Acid Epoxidase | Hydroxypropylphosphonic Acid Epoxidase | | pdb | 1ZZ7\_B | 0.999 | 0.000167 | 96 | 0.134 | 201 | 112 | 13 | 5 | 178 | 9 | 174 | Hydroxyprophylphosphonic Acid Epoxidase | Hydroxyprophylphosphonic Acid Epoxidase | | pdb | 3OP9\_A | 0.998 | 0.00813 | 93 | 0.15 | 93 | 71 | 2 | 1 | 89 | 1 | 89 | Pli0006 protein | Pli0006 protein | | pdb | 1ZZ7\_A | 0.998 | 0.0002232 | 93 | 0.122 | 187 | 119 | 9 | 5 | 169 | 9 | 172 | Hydroxyprophylphosphonic Acid Epoxidase | Hydroxyprophylphosphonic Acid Epoxidase | | pdb | 2BNO\_A | 0.998 | 0.0002506 | 93 | 0.128 | 202 | 112 | 10 | 5 | 175 | 12 | 180 | EPOXIDASE | EPOXIDASE | | pdb | 7P2P\_A | 0.997 | 0.003407 | 91 | 0.184 | 119 | 81 | 6 | 76 | 178 | 17 | 135 | Signal peptidase complex catalytic subunit SEC11A | Signal peptidase complex catalytic subunit SEC11A | | pdb | 1ZZ6\_B | 0.997 | 0.0002106 | 91 | 0.134 | 193 | 114 | 11 | 5 | 169 | 10 | 177 | Hydroxypropylphosphonic Acid Epoxidase | Hydroxypropylphosphonic Acid Epoxidase | | pdb | 2BNN\_B | 0.997 | 0.0002106 | 91 | 0.129 | 208 | 114 | 12 | 5 | 174 | 11 | 189 | EPOXIDASE | EPOXIDASE | | pdb | 1ZZ9\_C | 0.997 | 0.0002656 | 91 | 0.125 | 200 | 115 | 11 | 5 | 169 | 10 | 184 | Hydroxypropylphosphonic Acid Epoxidase | Hydroxypropylphosphonic Acid Epoxidase | | pdb | 1ZZC\_A | 0.996 | 0.0004224 | 90 | 0.134 | 193 | 112 | 11 | 5 | 173 | 11 | 172 | hydroxypropylphosphonic acid epoxidase | hydroxypropylphosphonic acid epoxidase | | pdb | 1ZZ9\_A | 0.996 | 0.0004224 | 89 | 0.133 | 203 | 112 | 12 | 5 | 169 | 10 | 186 | Hydroxypropylphosphonic Acid Epoxidase | Hydroxypropylphosphonic Acid Epoxidase | | pdb | 1ZZ8\_C | 0.995 | 0.0003549 | 88 | 0.129 | 208 | 118 | 13 | 5 | 174 | 10 | 192 | Hydroxypropylphosphonic Acid Epoxidase | Hydroxypropylphosphonic Acid Epoxidase | | pdb | 1ZZB\_A | 0.994 | 0.0005327 | 87 | 0.131 | 197 | 113 | 11 | 5 | 176 | 10 | 173 | Hydroxypropylphosphonic Acid Epoxidase | Hydroxypropylphosphonic Acid Epoxidase | | pdb | 4J1X\_C | 0.994 | 0.0005982 | 87 | 0.121 | 206 | 112 | 10 | 5 | 169 | 12 | 189 | Epoxidase | Epoxidase | | pdb | 6B9R\_C | 0.949 | 0.003407 | 72 | 0.153 | 202 | 107 | 13 | 5 | 173 | 238 | 408 | Hydroxyethylphosphonate dioxygenase | Hydroxyethylphosphonate dioxygenase | | pdb | 3GBF\_A | 0.933 | 0.0005645 | 70 | 0.121 | 214 | 113 | 15 | 4 | 173 | 7 | 189 | PhpD | PhpD | | pdb | 3RZZ\_A | 0.923 | 0.001132 | 69 | 0.105 | 208 | 117 | 14 | 4 | 173 | 7 | 183 | Hydroxyethylphoshphonate Dioxygenase (PhpD) | Hydroxyethylphoshphonate Dioxygenase (PhpD) | | pdb | 1Y9Q\_A | 0.872 | 0.006832 | 65 | 0.117 | 187 | 115 | 12 | 4 | 169 | 12 | 169 | transcriptional regulator, HTH\_3 family | transcriptional regulator, HTH\_3 family | | pdb | 3G7D\_A | 0.772 | 0.00361 | 60 | 0.119 | 210 | 111 | 14 | 4 | 173 | 7 | 182 | PhpD | PhpD | | pdb | 4YAR\_A | 0.772 | 0.004296 | 60 | 0.109 | 220 | 118 | 14 | 4 | 173 | 5 | 196 | 2-hydroxyethylphosphonate dioxygenase | 2-hydroxyethylphosphonate dioxygenase | | afdb-proteome | AF-Q8ZQI4-F1-MODEL\_V4 | 1.0 | 4.604e-13 | 350 | 0.23 | 221 | 124 | 5 | 4 | 180 | 8 | 226 | Putative Fels-1 prophage transcriptional regulator | Putative Fels-1 prophage transcriptional regulator | | afdb-proteome | AF-Q5F9C0-F1-MODEL\_V4 | 1.0 | 6.006e-11 | 302 | 0.2 | 210 | 132 | 8 | 5 | 180 | 29 | 236 | Cro/Cl family transcriptional regulator | Cro/Cl family transcriptional regulator | | afdb-proteome | AF-Q9I551-F1-MODEL\_V4 | 1.0 | 1.915e-10 | 283 | 0.206 | 233 | 128 | 5 | 4 | 180 | 6 | 237 | Probable transcriptional regulator | Probable transcriptional regulator | | afdb-proteome | AF-A0A0H3GP86-F1-MODEL\_V4 | 1.0 | 1.005e-07 | 281 | 0.298 | 114 | 74 | 3 | 70 | 180 | 7 | 117 | Regulatory protein CI bacteriophage origin | Regulatory protein CI bacteriophage origin | | afdb-proteome | AF-P44207-F1-MODEL\_V4 | 1.0 | 2.874e-10 | 272 | 0.195 | 230 | 129 | 8 | 4 | 180 | 12 | 238 | Uncharacterized HTH-type transcriptional regulator HI\_1476 | Uncharacterized HTH-type transcriptional regulator HI\_1476 | | afdb-proteome | AF-Q5F7Q4-F1-MODEL\_V4 | 1.0 | 9.167e-10 | 270 | 0.147 | 231 | 142 | 5 | 4 | 180 | 7 | 236 | Repressor | Repressor | | afdb-proteome | AF-Q5F6C9-F1-MODEL\_V4 | 1.0 | 2.03e-10 | 263 | 0.168 | 225 | 135 | 7 | 4 | 176 | 19 | 243 | Repressor | Repressor | | afdb-proteome | AF-Q06553-F1-MODEL\_V4 | 1.0 | 1.458e-09 | 256 | 0.149 | 234 | 141 | 7 | 4 | 180 | 15 | 247 | HTH-type transcriptional regulator PrtR | HTH-type transcriptional regulator PrtR | | afdb-proteome | AF-Q5F6W7-F1-MODEL\_V4 | 1.0 | 2.759e-09 | 245 | 0.177 | 231 | 136 | 9 | 1 | 180 | 1 | 228 | Repressor | Repressor | | afdb-proteome | AF-P22493-F1-MODEL\_V4 | 1.0 | 1.237e-05 | 216 | 0.262 | 103 | 70 | 5 | 80 | 180 | 40 | 138 | Protein UmuD | Protein UmuD | | afdb-proteome | AF-Q2FX22-F1-MODEL\_V4 | 1.0 | 6.977e-09 | 211 | 0.174 | 229 | 128 | 9 | 4 | 174 | 13 | 238 | Phage repressor protein, putative | Phage repressor protein, putative | | afdb-proteome | AF-Q32H33-F1-MODEL\_V4 | 1.0 | 3.316e-05 | 204 | 0.233 | 103 | 73 | 5 | 80 | 180 | 40 | 138 | UmuD | UmuD | | afdb-proteome | AF-Q32F74-F1-MODEL\_V4 | 1.0 | 4.211e-08 | 204 | 0.151 | 218 | 132 | 10 | 6 | 179 | 24 | 232 | Repressor protein CI | Repressor protein CI | | afdb-proteome | AF-Q2FY89-F1-MODEL\_V4 | 1.0 | 2.648e-08 | 200 | 0.161 | 229 | 128 | 9 | 5 | 174 | 13 | 236 | Helix-turn-helix domain protein | Helix-turn-helix domain protein | | afdb-proteome | AF-P0AG11-F1-MODEL\_V4 | 1.0 | 5.588e-05 | 198 | 0.233 | 103 | 73 | 5 | 80 | 180 | 40 | 138 | Protein UmuD | Protein UmuD | | afdb-proteome | AF-P23831-F1-MODEL\_V4 | 1.0 | 4.976e-05 | 189 | 0.227 | 101 | 71 | 4 | 80 | 179 | 41 | 135 | Protein SamA | Protein SamA | | afdb-proteome | AF-A0A0H3H1L6-F1-MODEL\_V4 | 1.0 | 0.0001334 | 184 | 0.178 | 101 | 78 | 4 | 80 | 179 | 42 | 138 | Protein impA | Protein impA | | afdb-proteome | AF-P75974-F1-MODEL\_V4 | 1.0 | 5.401e-07 | 181 | 0.127 | 211 | 145 | 11 | 4 | 179 | 11 | 217 | Prophage repressor CohE | Prophage repressor CohE | | afdb-proteome | AF-P0A273-F1-MODEL\_V4 | 1.0 | 0.0001782 | 178 | 0.177 | 90 | 68 | 5 | 93 | 179 | 112 | 198 | LexA repressor | LexA repressor | | afdb-proteome | AF-A0A0H3GQ57-F1-MODEL\_V4 | 1.0 | 0.0002121 | 176 | 0.188 | 90 | 67 | 5 | 93 | 179 | 112 | 198 | LexA repressor | LexA repressor | | afdb-proteome | AF-P0A7C2-F1-MODEL\_V4 | 1.0 | 0.0002524 | 173 | 0.188 | 90 | 67 | 5 | 93 | 179 | 112 | 198 | LexA repressor | LexA repressor | | afdb-proteome | AF-Q327V0-F1-MODEL\_V4 | 1.0 | 0.0003182 | 171 | 0.188 | 90 | 67 | 5 | 93 | 179 | 112 | 198 | LexA repressor | LexA repressor | | afdb-proteome | AF-A0A0H3GN19-F1-MODEL\_V4 | 1.0 | 0.0004013 | 170 | 0.203 | 103 | 76 | 5 | 80 | 180 | 40 | 138 | DNA polymerase V subunit UmuD | DNA polymerase V subunit UmuD | | afdb-proteome | AF-P37452-F1-MODEL\_V4 | 1.0 | 0.0007167 | 167 | 0.188 | 90 | 67 | 5 | 93 | 179 | 118 | 204 | LexA repressor | LexA repressor | | afdb-proteome | AF-A0A2S7MUS3-F1-MODEL\_V4 | 1.0 | 3.661e-06 | 156 | 0.186 | 214 | 112 | 16 | 5 | 180 | 19 | 208 | LexA repressor | LexA repressor | | afdb-proteome | AF-Q9HWV1-F1-MODEL\_V4 | 1.0 | 0.002286 | 153 | 0.287 | 66 | 47 | 0 | 1 | 66 | 1 | 66 | Probable transcriptional regulator | Probable transcriptional regulator | | afdb-proteome | AF-A0A0H3H1N6-F1-MODEL\_V4 | 1.0 | 9.646e-07 | 142 | 0.185 | 199 | 126 | 11 | 5 | 179 | 10 | 196 | CI repressor | CI repressor | | afdb-proteome | AF-A0A133CLZ3-F1-MODEL\_V4 | 1.0 | 0.0009038 | 140 | 0.264 | 87 | 57 | 1 | 5 | 84 | 10 | 96 | Helix-turn-helix transcriptional regulator | Helix-turn-helix transcriptional regulator | | afdb-proteome | AF-Q57720-F1-MODEL\_V4 | 1.0 | 0.009193 | 139 | 0.23 | 65 | 50 | 0 | 1 | 65 | 1 | 65 | Uncharacterized HTH-type transcriptional regulator MJ0272 | Uncharacterized HTH-type transcriptional regulator MJ0272 | | afdb-proteome | AF-Q2FYU1-F1-MODEL\_V4 | 1.0 | 3.514e-05 | 138 | 0.189 | 216 | 112 | 15 | 5 | 180 | 15 | 207 | LexA repressor | LexA repressor | | afdb-proteome | AF-Q2G1V7-F1-MODEL\_V4 | 1.0 | 0.008186 | 137 | 0.212 | 66 | 52 | 0 | 1 | 66 | 1 | 66 | HTH cro/C1-type domain-containing protein | HTH cro/C1-type domain-containing protein | | afdb-proteome | AF-A0A132ZE74-F1-MODEL\_V4 | 1.0 | 0.006491 | 137 | 0.205 | 68 | 54 | 0 | 1 | 68 | 1 | 68 | Helix-turn-helix transcriptional regulator | Helix-turn-helix transcriptional regulator | | afdb-proteome | AF-Q4AAY3-F1-MODEL\_V4 | 1.0 | 8.244e-06 | 125 | 0.162 | 209 | 128 | 11 | 5 | 180 | 16 | 210 | Fels-2 prophage protein | Fels-2 prophage protein | | afdb-proteome | AF-Q8ZNJ5-F1-MODEL\_V4 | 1.0 | 0.0007167 | 121 | 0.136 | 125 | 90 | 4 | 61 | 179 | 43 | 155 | Putative peptidase | Putative peptidase | | afdb-proteome | AF-K7M8T7-F1-MODEL\_V4 | 1.0 | 0.0005683 | 120 | 0.196 | 117 | 74 | 7 | 76 | 177 | 9 | 120 | Signal peptidase I | Signal peptidase I | | afdb-proteome | AF-Q2FX38-F1-MODEL\_V4 | 1.0 | 0.006491 | 119 | 0.246 | 77 | 54 | 2 | 5 | 80 | 11 | 84 | Helix-turn-helix DNA binding protein | Helix-turn-helix DNA binding protein | | afdb-proteome | AF-I1KFJ2-F1-MODEL\_V4 | 1.0 | 0.0009038 | 116 | 0.196 | 117 | 74 | 7 | 76 | 177 | 36 | 147 | Signal peptidase I | Signal peptidase I | | afdb-proteome | AF-Q0DJD8-F1-MODEL\_V4 | 1.0 | 0.0007167 | 115 | 0.185 | 124 | 77 | 7 | 74 | 177 | 28 | 147 | Signal peptidase I | Signal peptidase I | | afdb-proteome | AF-P23873-F1-MODEL\_V4 | 1.0 | 0.009742 | 113 | 0.217 | 69 | 49 | 1 | 5 | 73 | 17 | 80 | Antitoxin HipB | Antitoxin HipB | | afdb-proteome | AF-Q9R0P6-F1-MODEL\_V4 | 1.0 | 0.0001682 | 113 | 0.209 | 153 | 91 | 8 | 45 | 177 | 4 | 146 | Signal peptidase complex catalytic subunit SEC11A | Signal peptidase complex catalytic subunit SEC11A | | afdb-proteome | AF-A0A0H3GXD8-F1-MODEL\_V4 | 1.0 | 0.009742 | 112 | 0.275 | 69 | 49 | 1 | 5 | 73 | 16 | 83 | DNA-binding transcriptional regulator HipB | DNA-binding transcriptional regulator HipB | | afdb-proteome | AF-A0A044QWB3-F1-MODEL\_V4 | 1.0 | 0.001813 | 111 | 0.17 | 117 | 77 | 7 | 76 | 177 | 2 | 113 | Signal peptidase complex catalytic subunit SEC11 | Signal peptidase complex catalytic subunit SEC11 | | afdb-proteome | AF-Q5A869-F1-MODEL\_V4 | 1.0 | 0.002422 | 111 | 0.162 | 117 | 81 | 6 | 74 | 177 | 21 | 133 | Signal peptidase complex catalytic subunit SEC11 | Signal peptidase complex catalytic subunit SEC11 | | afdb-proteome | AF-P06966-F1-MODEL\_V4 | 1.0 | 0.008186 | 110 | 0.255 | 90 | 61 | 1 | 4 | 87 | 11 | 100 | HTH-type transcriptional regulator DicA | HTH-type transcriptional regulator DicA | | afdb-proteome | AF-P15367-F1-MODEL\_V4 | 1.0 | 0.001921 | 106 | 0.158 | 120 | 79 | 9 | 74 | 177 | 21 | 134 | Signal peptidase complex catalytic subunit SEC11 | Signal peptidase complex catalytic subunit SEC11 | | afdb-proteome | AF-P42667-F1-MODEL\_V4 | 1.0 | 0.0001334 | 106 | 0.181 | 160 | 93 | 12 | 35 | 177 | 8 | 146 | Signal peptidase complex catalytic subunit SEC11A | Signal peptidase complex catalytic subunit SEC11A | | afdb-proteome | AF-K0F7R0-F1-MODEL\_V4 | 1.0 | 0.007725 | 105 | 0.215 | 93 | 61 | 3 | 5 | 85 | 40 | 132 | Putative DNA-binding protein | Putative DNA-binding protein | | afdb-proteome | AF-O97066-F1-MODEL\_V4 | 1.0 | 0.0001057 | 104 | 0.149 | 167 | 104 | 12 | 28 | 177 | 7 | 152 | Signal peptidase complex catalytic subunit SEC11 | Signal peptidase complex catalytic subunit SEC11 | | afdb-proteome | AF-A0A0D2F1V0-F1-MODEL\_V4 | 1.0 | 0.001921 | 104 | 0.161 | 130 | 79 | 7 | 74 | 177 | 30 | 155 | Signal peptidase complex catalytic subunit SEC11 | Signal peptidase complex catalytic subunit SEC11 | | afdb-proteome | AF-I1KAD4-F1-MODEL\_V4 | 1.0 | 0.001614 | 104 | 0.128 | 171 | 92 | 8 | 64 | 180 | 24 | 191 | Peptidase\_S26 domain-containing protein | Peptidase\_S26 domain-containing protein | | afdb-proteome | AF-P67812-F1-MODEL\_V4 | 1.0 | 0.0002834 | 103 | 0.219 | 155 | 87 | 11 | 45 | 177 | 4 | 146 | Signal peptidase complex catalytic subunit SEC11A | Signal peptidase complex catalytic subunit SEC11A | | afdb-proteome | AF-B8A1P4-F1-MODEL\_V4 | 1.0 | 0.0003787 | 102 | 0.159 | 163 | 103 | 9 | 30 | 177 | 4 | 147 | Signal peptidase I | Signal peptidase I | | afdb-proteome | AF-Q9BY50-F1-MODEL\_V4 | 1.0 | 0.0002001 | 102 | 0.168 | 160 | 95 | 12 | 35 | 177 | 20 | 158 | Signal peptidase complex catalytic subunit SEC11C | Signal peptidase complex catalytic subunit SEC11C | | afdb-proteome | AF-Q1LVK8-F1-MODEL\_V4 | 1.0 | 0.0001587 | 100 | 0.164 | 164 | 99 | 12 | 31 | 177 | 4 | 146 | Signal peptidase complex catalytic subunit SEC11 | Signal peptidase complex catalytic subunit SEC11 | | afdb-proteome | AF-Q9I6R3-F1-MODEL\_V4 | 0.999 | 0.0002247 | 98 | 0.136 | 198 | 113 | 8 | 1 | 169 | 1 | 169 | Probable transcriptional regulator | Probable transcriptional regulator | | afdb-proteome | AF-A0A132P6Q0-F1-MODEL\_V4 | 0.999 | 0.00729 | 97 | 0.123 | 113 | 84 | 7 | 74 | 177 | 17 | 123 | Signal peptidase | Signal peptidase | | afdb-proteome | AF-Q9N3D0-F1-MODEL\_V4 | 0.999 | 0.0009038 | 97 | 0.164 | 158 | 98 | 9 | 35 | 177 | 11 | 149 | Signal peptidase complex catalytic subunit SEC11 | Signal peptidase complex catalytic subunit SEC11 | | afdb-proteome | AF-B4FU77-F1-MODEL\_V4 | 0.999 | 0.001356 | 97 | 0.13 | 168 | 89 | 7 | 64 | 177 | 32 | 196 | Thylakoidal processing peptidase 1 chloroplastic | Thylakoidal processing peptidase 1 chloroplastic | | afdb-proteome | AF-Q9D8V7-F1-MODEL\_V4 | 0.999 | 0.0003003 | 96 | 0.168 | 160 | 95 | 12 | 35 | 177 | 20 | 158 | Signal peptidase complex catalytic subunit SEC11C | Signal peptidase complex catalytic subunit SEC11C | | afdb-proteome | AF-Q8ZP82-F1-MODEL\_V4 | 0.999 | 0.0002834 | 96 | 0.124 | 193 | 109 | 8 | 4 | 170 | 25 | 183 | Putative transcriptional regulator | Putative transcriptional regulator | | afdb-proteome | AF-Q54RP1-F1-MODEL\_V4 | 0.999 | 0.006491 | 96 | 0.176 | 125 | 75 | 6 | 74 | 173 | 151 | 272 | Uncharacterized protein | Uncharacterized protein | | afdb-proteome | AF-A0A077ZFD1-F1-MODEL\_V4 | 0.999 | 0.0002001 | 96 | 0.154 | 220 | 129 | 14 | 8 | 177 | 154 | 366 | Signal peptidase complex catalytic subunit SEC11 | Signal peptidase complex catalytic subunit SEC11 | | afdb-proteome | AF-Q5VN14-F1-MODEL\_V4 | 0.998 | 0.0009038 | 95 | 0.141 | 163 | 106 | 9 | 30 | 177 | 4 | 147 | Signal peptidase I | Signal peptidase I | | afdb-proteome | AF-I1MSH9-F1-MODEL\_V4 | 0.998 | 0.0006023 | 95 | 0.166 | 186 | 110 | 9 | 7 | 177 | 3 | 158 | Signal peptidase I | Signal peptidase I | | afdb-proteome | AF-Q86JD4-F1-MODEL\_V4 | 0.998 | 0.0008529 | 94 | 0.144 | 166 | 108 | 9 | 26 | 177 | 1 | 146 | Signal peptidase complex catalytic subunit sec11 | Signal peptidase complex catalytic subunit sec11 | | afdb-proteome | AF-Q9WTR7-F1-MODEL\_V4 | 0.998 | 0.0004253 | 94 | 0.168 | 160 | 95 | 12 | 35 | 177 | 20 | 158 | Signal peptidase complex catalytic subunit SEC11C | Signal peptidase complex catalytic subunit SEC11C | | afdb-proteome | AF-K0EK64-F1-MODEL\_V4 | 0.998 | 0.0002524 | 94 | 0.13 | 207 | 118 | 10 | 4 | 180 | 16 | 190 | XRE family transcriptional regulator | XRE family transcriptional regulator | | afdb-proteome | AF-A0A1D6QAA1-F1-MODEL\_V4 | 0.998 | 0.001208 | 94 | 0.135 | 170 | 103 | 8 | 28 | 177 | 2 | 147 | Signal peptidase I | Signal peptidase I | | afdb-proteome | AF-B4F8H6-F1-MODEL\_V4 | 0.998 | 0.00114 | 93 | 0.165 | 163 | 102 | 9 | 30 | 177 | 4 | 147 | Signal peptidase I | Signal peptidase I | | afdb-proteome | AF-Q9HVS2-F1-MODEL\_V4 | 0.997 | 0.0002001 | 92 | 0.165 | 200 | 110 | 12 | 4 | 175 | 9 | 179 | Probable transcriptional regulator | Probable transcriptional regulator | | afdb-proteome | AF-A0A1C1CFT4-F1-MODEL\_V4 | 0.997 | 0.0008048 | 92 | 0.157 | 178 | 101 | 10 | 26 | 177 | 1 | 155 | Signal peptidase complex catalytic subunit SEC11 | Signal peptidase complex catalytic subunit SEC11 | | afdb-proteome | AF-K0EMM4-F1-MODEL\_V4 | 0.997 | 0.0003574 | 92 | 0.141 | 191 | 117 | 7 | 4 | 175 | 17 | 179 | XRE family transcriptional regulator | XRE family transcriptional regulator | | afdb-proteome | AF-Q9SSR2-F1-MODEL\_V4 | 0.996 | 0.001614 | 90 | 0.165 | 163 | 102 | 9 | 30 | 177 | 4 | 147 | Signal peptidase I | Signal peptidase I | | afdb-proteome | AF-Q4DIR9-F1-MODEL\_V4 | 0.996 | 0.0006023 | 90 | 0.176 | 181 | 102 | 10 | 27 | 177 | 10 | 173 | Signal peptidase I | Signal peptidase I | | afdb-proteome | AF-A0A1D6L610-F1-MODEL\_V4 | 0.996 | 0.00729 | 90 | 0.141 | 162 | 74 | 8 | 72 | 173 | 278 | 434 | Peptidase/ serine-type peptidase | Peptidase/ serine-type peptidase | | afdb-proteome | AF-Q4DSS4-F1-MODEL\_V4 | 0.996 | 0.0008048 | 89 | 0.197 | 177 | 103 | 11 | 27 | 177 | 10 | 173 | Signal peptidase I | Signal peptidase I | | afdb-proteome | AF-Q9LW08-F1-MODEL\_V4 | 0.995 | 0.002286 | 88 | 0.153 | 163 | 104 | 9 | 30 | 177 | 4 | 147 | Signal peptidase I | Signal peptidase I | | afdb-proteome | AF-A0A0H3H3B8-F1-MODEL\_V4 | 0.995 | 0.001208 | 88 | 0.144 | 166 | 85 | 6 | 4 | 137 | 25 | 165 | Helix-turn-helix motif-containing protein | Helix-turn-helix motif-containing protein | | afdb-proteome | AF-A0A133CSK7-F1-MODEL\_V4 | 0.994 | 0.0008048 | 87 | 0.1 | 199 | 115 | 9 | 4 | 173 | 6 | 169 | Cro/Cl family transcriptional regulator | Cro/Cl family transcriptional regulator | | afdb-proteome | AF-C0NKT8-F1-MODEL\_V4 | 0.994 | 0.0008048 | 87 | 0.172 | 180 | 95 | 13 | 26 | 177 | 1 | 154 | Signal peptidase complex catalytic subunit SEC11 | Signal peptidase complex catalytic subunit SEC11 | | afdb-proteome | AF-A0A0H3GK51-F1-MODEL\_V4 | 0.994 | 0.001921 | 87 | 0.144 | 152 | 96 | 8 | 5 | 136 | 30 | 167 | Putative helix-turn-helix regulatory protein | Putative helix-turn-helix regulatory protein | | afdb-proteome | AF-Q57Z71-F1-MODEL\_V4 | 0.994 | 0.0009038 | 87 | 0.156 | 192 | 118 | 11 | 9 | 177 | 5 | 175 | Signal peptidase I | Signal peptidase I | | afdb-proteome | AF-K0F3Z4-F1-MODEL\_V4 | 0.993 | 0.00114 | 86 | 0.133 | 202 | 100 | 10 | 4 | 173 | 20 | 178 | DNA binding regulatory protein | DNA binding regulatory protein | | afdb-proteome | AF-Q9HUH8-F1-MODEL\_V4 | 0.993 | 0.0007595 | 86 | 0.142 | 197 | 108 | 7 | 4 | 169 | 23 | 189 | Probable transcriptional regulator | Probable transcriptional regulator | | afdb-proteome | AF-Q7Y0D0-F1-MODEL\_V4 | 0.993 | 0.008186 | 86 | 0.153 | 163 | 73 | 9 | 72 | 174 | 300 | 457 | Os03g0765200 protein | Os03g0765200 protein | | afdb-proteome | AF-A0A1D6GGF0-F1-MODEL\_V4 | 0.993 | 0.007725 | 86 | 0.148 | 162 | 75 | 8 | 72 | 174 | 293 | 450 | Thylakoidal processing peptidase 1 chloroplastic | Thylakoidal processing peptidase 1 chloroplastic | | afdb-proteome | AF-P77626-F1-MODEL\_V4 | 0.992 | 0.002035 | 85 | 0.14 | 150 | 100 | 5 | 5 | 140 | 12 | 146 | HTH-type transcriptional regulator SutR | HTH-type transcriptional regulator SutR | | afdb-proteome | AF-O04348-F1-MODEL\_V4 | 0.992 | 0.005781 | 85 | 0.16 | 175 | 82 | 9 | 57 | 174 | 147 | 313 | Thylakoidal processing peptidase 1, chloroplastic | Thylakoidal processing peptidase 1, chloroplastic | | afdb-proteome | AF-I1JTG1-F1-MODEL\_V4 | 0.992 | 0.002567 | 85 | 0.133 | 202 | 91 | 10 | 26 | 173 | 162 | 333 | Peptidase\_S26 domain-containing protein | Peptidase\_S26 domain-containing protein | | afdb-proteome | AF-X8FMT1-F1-MODEL\_V4 | 0.99 | 0.0008529 | 83 | 0.128 | 202 | 131 | 12 | 1 | 179 | 1 | 180 | Helix-turn-helix family protein | Helix-turn-helix family protein | | afdb-proteome | AF-Q32FP6-F1-MODEL\_V4 | 0.986 | 0.00343 | 81 | 0.14 | 150 | 100 | 5 | 5 | 140 | 12 | 146 | HTH cro/C1-type domain-containing protein | HTH cro/C1-type domain-containing protein | | afdb-proteome | AF-C1GU90-F1-MODEL\_V4 | 0.986 | 0.005148 | 81 | 0.155 | 187 | 100 | 10 | 26 | 177 | 1 | 164 | Signal peptidase complex catalytic subunit SEC11 | Signal peptidase complex catalytic subunit SEC11 | | afdb-proteome | AF-Q9I601-F1-MODEL\_V4 | 0.984 | 0.004584 | 80 | 0.162 | 154 | 91 | 6 | 5 | 136 | 15 | 152 | Probable transcriptional regulator | Probable transcriptional regulator | | afdb-proteome | AF-Q9I1G3-F1-MODEL\_V4 | 0.984 | 0.003237 | 80 | 0.101 | 158 | 97 | 7 | 5 | 136 | 17 | 155 | Probable transcriptional regulator | Probable transcriptional regulator | | afdb-proteome | AF-K0ES92-F1-MODEL\_V4 | 0.975 | 0.001523 | 77 | 0.163 | 190 | 109 | 11 | 5 | 172 | 12 | 173 | DNA-binding protein | DNA-binding protein | | afdb-proteome | AF-Q9I3Y5-F1-MODEL\_V4 | 0.975 | 0.003055 | 77 | 0.147 | 190 | 104 | 10 | 5 | 173 | 49 | 201 | Probable transcriptional regulator | Probable transcriptional regulator | | afdb-proteome | AF-A0A5K4EAL6-F1-MODEL\_V4 | 0.971 | 0.009193 | 76 | 0.165 | 139 | 79 | 8 | 68 | 173 | 7 | 141 | Mitochondrial signal peptidase (S26 family) | Mitochondrial signal peptidase (S26 family) | | afdb-proteome | AF-Q2G2A6-F1-MODEL\_V4 | 0.967 | 0.005455 | 75 | 0.125 | 176 | 111 | 9 | 4 | 153 | 6 | 164 | HTH cro/C1-type domain-containing protein | HTH cro/C1-type domain-containing protein | | afdb-proteome | AF-Q9I2L3-F1-MODEL\_V4 | 0.967 | 0.003055 | 75 | 0.16 | 200 | 109 | 11 | 4 | 171 | 10 | 182 | Probable transcriptional regulator | Probable transcriptional regulator | | afdb-proteome | AF-K0EYC2-F1-MODEL\_V4 | 0.967 | 0.002422 | 75 | 0.158 | 202 | 118 | 10 | 5 | 179 | 19 | 195 | Regulatory protein | Regulatory protein | | afdb-proteome | AF-A0A0H3GYK3-F1-MODEL\_V4 | 0.961 | 0.002157 | 74 | 0.162 | 197 | 113 | 11 | 4 | 173 | 15 | 186 | Putative regulator | Putative regulator | | afdb-proteome | AF-A0A0H3GP19-F1-MODEL\_V4 | 0.956 | 0.004082 | 73 | 0.135 | 184 | 113 | 8 | 5 | 169 | 11 | 167 | Putative regulator | Putative regulator | | afdb-proteome | AF-Q8ZPC6-F1-MODEL\_V4 | 0.949 | 0.004584 | 72 | 0.121 | 197 | 121 | 11 | 5 | 179 | 12 | 178 | Putative oxidoreductase | Putative oxidoreductase | | afdb-proteome | AF-Q32F57-F1-MODEL\_V4 | 0.949 | 0.005455 | 72 | 0.13 | 192 | 119 | 10 | 4 | 176 | 11 | 173 | HTH cro/C1-type domain-containing protein | HTH cro/C1-type domain-containing protein | | afdb-proteome | AF-Q8H0W1-F1-MODEL\_V4 | 0.933 | 0.003237 | 70 | 0.14 | 178 | 86 | 10 | 61 | 174 | 94 | 268 | Chloroplast processing peptidase | Chloroplast processing peptidase | | afdb-proteome | AF-Q8ZKS3-F1-MODEL\_V4 | 0.9 | 0.006879 | 67 | 0.11 | 199 | 104 | 8 | 5 | 172 | 18 | 174 | HTH cro/C1-type domain-containing protein | HTH cro/C1-type domain-containing protein | | afdb-proteome | AF-A0A133CSL8-F1-MODEL\_V4 | 0.872 | 0.008675 | 65 | 0.116 | 248 | 115 | 14 | 4 | 166 | 11 | 239 | Helix-turn-helix domain-containing protein | Helix-turn-helix domain-containing protein | | afdb-uniprot50 | AF-A0A139JVH4-F1-MODEL\_V4 | 1.0 | 3.747e-13 | 391 | 0.215 | 190 | 135 | 5 | 4 | 180 | 12 | 200 | DNA-binding helix-turn-helix protein | DNA-binding helix-turn-helix protein | | afdb-uniprot50 | AF-A0A316C0P5-F1-MODEL\_V4 | 1.0 | 5.306e-13 | 371 | 0.2 | 200 | 137 | 3 | 1 | 180 | 1 | 197 | DNA-binding XRE family transcriptional regulator | DNA-binding XRE family transcriptional regulator | | afdb-uniprot50 | AF-A0A1C7W9W0-F1-MODEL\_V4 | 1.0 | 5.622e-13 | 366 | 0.25 | 216 | 119 | 6 | 4 | 180 | 6 | 217 | HTH cro/C1-type domain-containing protein | HTH cro/C1-type domain-containing protein | | afdb-uniprot50 | AF-A0A661FLS5-F1-MODEL\_V4 | 1.0 | 4.458e-13 | 365 | 0.207 | 222 | 129 | 7 | 4 | 180 | 24 | 243 | HTH cro/C1-type domain-containing protein | HTH cro/C1-type domain-containing protein | | afdb-uniprot50 | AF-A0A2E3AY80-F1-MODEL\_V4 | 1.0 | 1.9e-12 | 362 | 0.276 | 210 | 117 | 7 | 4 | 180 | 9 | 216 | Peptidase S24 | Peptidase S24 | | afdb-uniprot50 | AF-A0A1S8YLB7-F1-MODEL\_V4 | 1.0 | 3.148e-13 | 360 | 0.243 | 222 | 121 | 7 | 4 | 180 | 6 | 225 | Transcriptional regulator | Transcriptional regulator | | afdb-uniprot50 | AF-A0A1Q6SZL5-F1-MODEL\_V4 | 1.0 | 1.422e-12 | 359 | 0.255 | 180 | 122 | 3 | 1 | 180 | 1 | 168 | HTH cro/C1-type domain-containing protein | HTH cro/C1-type domain-containing protein | | afdb-uniprot50 | AF-A0A7U4Q650-F1-MODEL\_V4 | 1.0 | 8.941e-13 | 359 | 0.226 | 221 | 125 | 6 | 4 | 180 | 6 | 224 | Helix-turn-helix transcriptional regulator | Helix-turn-helix transcriptional regulator | | afdb-uniprot50 | AF-Q57QA9-F1-MODEL\_V4 | 1.0 | 3.97e-13 | 359 | 0.27 | 222 | 110 | 7 | 4 | 177 | 6 | 223 | Regulatory protein | Regulatory protein | | afdb-uniprot50 | AF-A0A327JZK4-F1-MODEL\_V4 | 1.0 | 1.355e-08 | 350 | 0.349 | 106 | 65 | 2 | 78 | 180 | 49 | 153 | Peptidase\_S24 domain-containing protein | Peptidase\_S24 domain-containing protein | | afdb-uniprot50 | AF-A0A7V8G8N6-F1-MODEL\_V4 | 1.0 | 3.022e-12 | 348 | 0.215 | 213 | 130 | 6 | 4 | 180 | 28 | 239 | Putative HTH-type transcriptional regulator | Putative HTH-type transcriptional regulator | | afdb-uniprot50 | AF-A0A6F8VBR1-F1-MODEL\_V4 | 1.0 | 4.038e-12 | 348 | 0.213 | 239 | 124 | 6 | 4 | 180 | 10 | 246 | Repressor | Repressor | | afdb-uniprot50 | AF-A0A1H0M9N5-F1-MODEL\_V4 | 1.0 | 2.691e-12 | 348 | 0.222 | 216 | 128 | 6 | 4 | 180 | 51 | 265 | Phage repressor protein C, contains Cro/C1-type HTH and peptisase s24 domains | Phage repressor protein C, contains Cro/C1-type HTH and peptisase s24 domains | | afdb-uniprot50 | AF-A0LD08-F1-MODEL\_V4 | 1.0 | 4.279e-12 | 346 | 0.252 | 214 | 117 | 7 | 4 | 180 | 8 | 215 | Putative phage repressor | Putative phage repressor | | afdb-uniprot50 | AF-A0A2A4XTD6-F1-MODEL\_V4 | 1.0 | 3.393e-12 | 345 | 0.226 | 212 | 127 | 6 | 4 | 180 | 13 | 222 | HTH cro/C1-type domain-containing protein | HTH cro/C1-type domain-containing protein | | afdb-uniprot50 | AF-K2J5W2-F1-MODEL\_V4 | 1.0 | 6.757e-09 | 344 | 0.318 | 113 | 69 | 3 | 71 | 180 | 50 | 157 | Phage repressor | Phage repressor | | afdb-uniprot50 | AF-K2JV89-F1-MODEL\_V4 | 1.0 | 1.597e-12 | 343 | 0.252 | 206 | 121 | 6 | 4 | 180 | 25 | 226 | Putative phage repressor | Putative phage repressor | | afdb-uniprot50 | AF-A0A2X1U723-F1-MODEL\_V4 | 1.0 | 4.279e-12 | 343 | 0.21 | 219 | 131 | 7 | 2 | 180 | 230 | 446 | Putative regulatory protein | Putative regulatory protein | | afdb-uniprot50 | AF-A0A1I4Q661-F1-MODEL\_V4 | 1.0 | 7.642e-12 | 341 | 0.211 | 222 | 125 | 6 | 4 | 180 | 8 | 224 | HTH cro/C1-type domain-containing protein | HTH cro/C1-type domain-containing protein | | afdb-uniprot50 | AF-D4BUI1-F1-MODEL\_V4 | 1.0 | 7.212e-12 | 341 | 0.201 | 223 | 131 | 4 | 4 | 180 | 7 | 228 | DNA-binding helix-turn-helix protein | DNA-binding helix-turn-helix protein | | afdb-uniprot50 | AF-A0A315BKI3-F1-MODEL\_V4 | 1.0 | 5.143e-08 | 339 | 0.294 | 95 | 66 | 1 | 87 | 180 | 80 | 174 | Peptidase\_S24 domain-containing protein | Peptidase\_S24 domain-containing protein | | afdb-uniprot50 | AF-A0A495RIU6-F1-MODEL\_V4 | 1.0 | 3.202e-12 | 339 | 0.207 | 212 | 130 | 7 | 4 | 180 | 6 | 214 | Phage repressor protein C with HTH and peptisase S24 domain | Phage repressor protein C with HTH and peptisase S24 domain | | afdb-uniprot50 | AF-A0A2D5PK86-F1-MODEL\_V4 | 1.0 | 3.022e-12 | 339 | 0.213 | 225 | 129 | 5 | 4 | 180 | 6 | 230 | HTH cro/C1-type domain-containing protein | HTH cro/C1-type domain-containing protein | | afdb-uniprot50 | AF-U0ZRD1-F1-MODEL\_V4 | 1.0 | 5.719e-12 | 337 | 0.201 | 208 | 128 | 5 | 4 | 178 | 7 | 209 | HTH cro/C1-type domain-containing protein | HTH cro/C1-type domain-containing protein | | afdb-uniprot50 | AF-A0A2Z4R558-F1-MODEL\_V4 | 1.0 | 6.422e-12 | 337 | 0.192 | 213 | 135 | 5 | 4 | 180 | 9 | 220 | HTH cro/C1-type domain-containing protein | HTH cro/C1-type domain-containing protein | | afdb-uniprot50 | AF-A0A2R3QQH4-F1-MODEL\_V4 | 1.0 | 5.719e-12 | 337 | 0.206 | 208 | 131 | 6 | 4 | 180 | 28 | 232 | HTH cro/C1-type domain-containing protein | HTH cro/C1-type domain-containing protein | | afdb-uniprot50 | AF-A0A844BRP4-F1-MODEL\_V4 | 1.0 | 7.642e-12 | 335 | 0.228 | 197 | 128 | 4 | 4 | 180 | 9 | 201 | Helix-turn-helix domain-containing protein | Helix-turn-helix domain-containing protein | | afdb-uniprot50 | AF-A0A4U0PX94-F1-MODEL\_V4 | 1.0 | 8.582e-12 | 335 | 0.208 | 230 | 128 | 4 | 4 | 180 | 7 | 235 | Helix-turn-helix domain-containing protein | Helix-turn-helix domain-containing protein | | afdb-uniprot50 | AF-A0A836WXJ3-F1-MODEL\_V4 | 1.0 | 1.288e-11 | 334 | 0.221 | 226 | 126 | 5 | 1 | 180 | 1 | 222 | Helix-turn-helix transcriptional regulator | Helix-turn-helix transcriptional regulator | | afdb-uniprot50 | AF-A0A7T9LJB7-F1-MODEL\_V4 | 1.0 | 1.365e-11 | 333 | 0.207 | 222 | 131 | 6 | 2 | 180 | 5 | 224 | Helix-turn-helix transcriptional regulator | Helix-turn-helix transcriptional regulator | | afdb-uniprot50 | AF-A0A2P5N6V1-F1-MODEL\_V4 | 1.0 | 4.279e-12 | 333 | 0.19 | 242 | 129 | 6 | 5 | 180 | 41 | 281 | HTH cro/C1-type domain-containing protein | HTH cro/C1-type domain-containing protein | | afdb-uniprot50 | AF-A0A6N7BSU4-F1-MODEL\_V4 | 1.0 | 1.082e-11 | 332 | 0.218 | 197 | 127 | 4 | 4 | 180 | 8 | 197 | HTH cro/C1-type domain-containing protein | HTH cro/C1-type domain-containing protein | | afdb-uniprot50 | AF-A0A6F8VD58-F1-MODEL\_V4 | 1.0 | 7.642e-12 | 332 | 0.235 | 212 | 125 | 6 | 5 | 180 | 7 | 217 | HTH cro/C1-type domain-containing protein | HTH cro/C1-type domain-containing protein | | afdb-uniprot50 | AF-A0A2N9XPR0-F1-MODEL\_V4 | 1.0 | 5.45e-08 | 331 | 0.284 | 95 | 64 | 2 | 89 | 180 | 6 | 99 | Peptidase\_S24 domain-containing protein | Peptidase\_S24 domain-containing protein | | afdb-uniprot50 | AF-A0A2N9XWM8-F1-MODEL\_V4 | 1.0 | 2.565e-08 | 331 | 0.273 | 106 | 73 | 2 | 78 | 180 | 7 | 111 | Peptidase\_S24 domain-containing protein | Peptidase\_S24 domain-containing protein | | afdb-uniprot50 | AF-A0A6N7AJK2-F1-MODEL\_V4 | 1.0 | 1.824e-11 | 331 | 0.231 | 207 | 124 | 7 | 5 | 180 | 20 | 222 | Prophage MuMc02 S24 family peptidase | Prophage MuMc02 S24 family peptidase | | afdb-uniprot50 | AF-A0A1G0CQ54-F1-MODEL\_V4 | 1.0 | 5.45e-08 | 330 | 0.345 | 107 | 66 | 2 | 77 | 180 | 69 | 174 | Peptidase\_S24 domain-containing protein | Peptidase\_S24 domain-containing protein | | afdb-uniprot50 | AF-A0A139KEG7-F1-MODEL\_V4 | 1.0 | 1.365e-11 | 330 | 0.197 | 213 | 134 | 5 | 4 | 180 | 6 | 217 | Peptidase S24-like protein | Peptidase S24-like protein | | afdb-uniprot50 | AF-A0A2U0YHI6-F1-MODEL\_V4 | 1.0 | 1.933e-11 | 329 | 0.248 | 209 | 122 | 8 | 4 | 180 | 20 | 225 | Phage repressor protein C with HTH and peptisase S24 domain | Phage repressor protein C with HTH and peptisase S24 domain | | afdb-uniprot50 | AF-A0A1H7XMA1-F1-MODEL\_V4 | 1.0 | 5.092e-12 | 329 | 0.2 | 220 | 130 | 8 | 4 | 180 | 7 | 223 | Phage repressor protein C, contains Cro/C1-type HTH and peptisase s24 domains | Phage repressor protein C, contains Cro/C1-type HTH and peptisase s24 domains | | afdb-uniprot50 | AF-A0A7Y5VHZ7-F1-MODEL\_V4 | 1.0 | 1.215e-11 | 329 | 0.188 | 228 | 132 | 5 | 4 | 179 | 7 | 233 | Helix-turn-helix domain-containing protein | Helix-turn-helix domain-containing protein | | afdb-uniprot50 | AF-A0A2N9Y298-F1-MODEL\_V4 | 1.0 | 1.933e-11 | 328 | 0.197 | 192 | 137 | 3 | 4 | 180 | 6 | 195 | HTH cro/C1-type domain-containing protein | HTH cro/C1-type domain-containing protein | | afdb-uniprot50 | AF-A0A6S5X474-F1-MODEL\_V4 | 1.0 | 3.876e-11 | 328 | 0.2 | 209 | 131 | 4 | 4 | 180 | 11 | 215 | HTH cro/C1-type domain-containing protein | HTH cro/C1-type domain-containing protein | | afdb-uniprot50 | AF-A0A7Z6VI80-F1-MODEL\_V4 | 1.0 | 2.048e-11 | 328 | 0.2 | 215 | 130 | 6 | 4 | 180 | 8 | 218 | Helix-turn-helix transcriptional regulator | Helix-turn-helix transcriptional regulator | | afdb-uniprot50 | AF-B9Z333-F1-MODEL\_V4 | 1.0 | 8.582e-12 | 328 | 0.2 | 224 | 130 | 6 | 4 | 180 | 6 | 227 | Putative phage repressor | Putative phage repressor | | afdb-uniprot50 | AF-G8NRH6-F1-MODEL\_V4 | 1.0 | 8.582e-12 | 328 | 0.251 | 235 | 116 | 9 | 1 | 176 | 1 | 234 | Putative phage repressor | Putative phage repressor | | afdb-uniprot50 | AF-A0A7W5EVC8-F1-MODEL\_V4 | 1.0 | 2.88e-08 | 327 | 0.304 | 105 | 68 | 3 | 79 | 180 | 36 | 138 | Phage repressor protein C with HTH and peptisase S24 domain | Phage repressor protein C with HTH and peptisase S24 domain | | afdb-uniprot50 | AF-A0A1C4CK44-F1-MODEL\_V4 | 1.0 | 9.637e-12 | 327 | 0.226 | 216 | 123 | 6 | 5 | 180 | 9 | 220 | Peptidase S24-like | Peptidase S24-like | | afdb-uniprot50 | AF-G2ZXI3-F1-MODEL\_V4 | 1.0 | 2.17e-11 | 327 | 0.209 | 224 | 125 | 7 | 4 | 180 | 7 | 225 | Putative phage repressor | Putative phage repressor | | afdb-uniprot50 | AF-A0A3S2YW43-F1-MODEL\_V4 | 1.0 | 1.365e-11 | 327 | 0.205 | 238 | 127 | 7 | 4 | 180 | 22 | 258 | Peptidase\_S24 domain-containing protein | Peptidase\_S24 domain-containing protein | | afdb-uniprot50 | AF-A0A1H5XPH2-F1-MODEL\_V4 | 1.0 | 5.092e-12 | 327 | 0.212 | 231 | 126 | 8 | 5 | 180 | 33 | 262 | Phage repressor protein C, contains Cro/C1-type HTH and peptisase s24 domains | Phage repressor protein C, contains Cro/C1-type HTH and peptisase s24 domains | | afdb-uniprot50 | AF-D5RM48-F1-MODEL\_V4 | 1.0 | 1.082e-11 | 327 | 0.201 | 228 | 129 | 5 | 4 | 180 | 39 | 264 | Peptidase S24-like protein | Peptidase S24-like protein | | afdb-uniprot50 | AF-X2JS37-F1-MODEL\_V4 | 1.0 | 3.876e-11 | 327 | 0.218 | 220 | 127 | 7 | 4 | 180 | 72 | 289 | HTH cro/C1-type domain-containing protein | HTH cro/C1-type domain-containing protein | | afdb-uniprot50 | AF-A0A0Q5YYS1-F1-MODEL\_V4 | 1.0 | 9.637e-12 | 326 | 0.219 | 210 | 131 | 7 | 4 | 180 | 7 | 216 | HTH cro/C1-type domain-containing protein | HTH cro/C1-type domain-containing protein | | afdb-uniprot50 | AF-F3BKE6-F1-MODEL\_V4 | 1.0 | 9.094e-12 | 325 | 0.255 | 192 | 124 | 5 | 4 | 180 | 19 | 206 | HTH cro/C1-type domain-containing protein | HTH cro/C1-type domain-containing protein | | afdb-uniprot50 | AF-A0A1H2HQC4-F1-MODEL\_V4 | 1.0 | 5.775e-08 | 325 | 0.307 | 104 | 68 | 2 | 80 | 180 | 126 | 228 | Peptidase S24-like | Peptidase S24-like | | afdb-uniprot50 | AF-A0A3M2H9H5-F1-MODEL\_V4 | 1.0 | 1.082e-11 | 325 | 0.202 | 207 | 134 | 6 | 4 | 180 | 25 | 230 | XRE family transcriptional regulator | XRE family transcriptional regulator | | afdb-uniprot50 | AF-A0A1I3KW04-F1-MODEL\_V4 | 1.0 | 9.637e-12 | 325 | 0.196 | 224 | 133 | 4 | 4 | 180 | 7 | 230 | Peptidase S24-like | Peptidase S24-like | | afdb-uniprot50 | AF-A0A348HFP4-F1-MODEL\_V4 | 1.0 | 1.824e-11 | 325 | 0.221 | 221 | 127 | 7 | 4 | 180 | 12 | 231 | Predicted transcriptional regulator | Predicted transcriptional regulator | | afdb-uniprot50 | AF-A0A2I6S9H6-F1-MODEL\_V4 | 1.0 | 3.658e-11 | 325 | 0.189 | 232 | 131 | 6 | 4 | 180 | 6 | 235 | HTH cro/C1-type domain-containing protein | HTH cro/C1-type domain-containing protein | | afdb-uniprot50 | AF-A0A2G5FSI2-F1-MODEL\_V4 | 1.0 | 1.288e-11 | 325 | 0.209 | 215 | 127 | 8 | 4 | 180 | 37 | 246 | C repressor | C repressor | | afdb-uniprot50 | AF-A5EUX6-F1-MODEL\_V4 | 1.0 | 1.824e-11 | 325 | 0.188 | 228 | 131 | 7 | 5 | 180 | 80 | 305 | Regulatory protein RegA2 | Regulatory protein RegA2 | | afdb-uniprot50 | AF-A0A1Q6UEY7-F1-MODEL\_V4 | 1.0 | 6.922e-11 | 324 | 0.2 | 215 | 133 | 7 | 1 | 180 | 1 | 211 | HTH cro/C1-type domain-containing protein | HTH cro/C1-type domain-containing protein | | afdb-uniprot50 | AF-A0A2D9F6P6-F1-MODEL\_V4 | 1.0 | 2.048e-11 | 324 | 0.222 | 220 | 126 | 7 | 2 | 180 | 8 | 223 | HTH cro/C1-type domain-containing protein | HTH cro/C1-type domain-containing protein | | afdb-uniprot50 | AF-A0A1Y2S5X0-F1-MODEL\_V4 | 1.0 | 1.288e-11 | 324 | 0.19 | 221 | 129 | 9 | 4 | 178 | 9 | 225 | Transcriptional regulator | Transcriptional regulator | | afdb-uniprot50 | AF-A0A7I6ZJ48-F1-MODEL\_V4 | 1.0 | 6.12e-08 | 324 | 0.245 | 106 | 76 | 2 | 78 | 180 | 133 | 237 | Uncharacterized protein | Uncharacterized protein | | afdb-uniprot50 | AF-A0A7W5EKL1-F1-MODEL\_V4 | 1.0 | 2.437e-11 | 324 | 0.229 | 231 | 122 | 6 | 4 | 179 | 30 | 259 | Phage repressor protein C with HTH and peptisase S24 domain | Phage repressor protein C with HTH and peptisase S24 domain | | afdb-uniprot50 | AF-A0A0K6IU92-F1-MODEL\_V4 | 1.0 | 1.533e-11 | 324 | 0.192 | 265 | 124 | 5 | 4 | 180 | 23 | 285 | Peptidase S24-like/Helix-turn-helix | Peptidase S24-like/Helix-turn-helix | | afdb-uniprot50 | AF-J6DSP2-F1-MODEL\_V4 | 1.0 | 1.093e-07 | 323 | 0.304 | 105 | 69 | 2 | 79 | 180 | 36 | 139 | Prophage MuSo1, transcriptional regulator, Cro/CI family protein | Prophage MuSo1, transcriptional regulator, Cro/CI family protein | | afdb-uniprot50 | AF-A0A0F2RP23-F1-MODEL\_V4 | 1.0 | 1.227e-07 | 323 | 0.361 | 105 | 64 | 3 | 78 | 180 | 40 | 143 | Peptidase\_S24 domain-containing protein | Peptidase\_S24 domain-containing protein | | afdb-uniprot50 | AF-A0A1S6HW15-F1-MODEL\_V4 | 1.0 | 1.824e-11 | 323 | 0.2 | 205 | 134 | 6 | 4 | 180 | 16 | 218 | Helix-turn-helix domain | Helix-turn-helix domain | | afdb-uniprot50 | AF-A0A378NHI5-F1-MODEL\_V4 | 1.0 | 1.365e-11 | 323 | 0.21 | 214 | 129 | 8 | 4 | 180 | 7 | 217 | Uncharacterized HTH-type transcriptional regulator HI\_1476 | Uncharacterized HTH-type transcriptional regulator HI\_1476 | | afdb-uniprot50 | AF-A0A3B7PQJ3-F1-MODEL\_V4 | 1.0 | 1.533e-11 | 323 | 0.19 | 220 | 131 | 6 | 4 | 178 | 6 | 223 | Helix-turn-helix transcriptional regulator | Helix-turn-helix transcriptional regulator | | afdb-uniprot50 | AF-H8W5A1-F1-MODEL\_V4 | 1.0 | 5.45e-08 | 322 | 0.301 | 106 | 70 | 2 | 78 | 180 | 70 | 174 | Putative transcriptional regulator, Cro/CI family | Putative transcriptional regulator, Cro/CI family | | afdb-uniprot50 | AF-H8L1T9-F1-MODEL\_V4 | 1.0 | 2.9e-11 | 322 | 0.228 | 219 | 126 | 6 | 4 | 180 | 8 | 225 | Putative transcriptional regulator | Putative transcriptional regulator | | afdb-uniprot50 | AF-A0A443VFS7-F1-MODEL\_V4 | 1.0 | 2.17e-11 | 322 | 0.232 | 219 | 124 | 8 | 4 | 180 | 8 | 224 | HTH cro/C1-type domain-containing protein | HTH cro/C1-type domain-containing protein | | afdb-uniprot50 | AF-A0A2D6GSD7-F1-MODEL\_V4 | 1.0 | 9.637e-12 | 322 | 0.235 | 217 | 125 | 6 | 4 | 180 | 18 | 233 | Phage repressor protein C | Phage repressor protein C | | afdb-uniprot50 | AF-A0A367LYV8-F1-MODEL\_V4 | 1.0 | 1.031e-07 | 321 | 0.292 | 99 | 66 | 2 | 85 | 180 | 5 | 102 | S24 family peptidase | S24 family peptidase | | afdb-uniprot50 | AF-A0A5B9EHR9-F1-MODEL\_V4 | 1.0 | 7.283e-08 | 321 | 0.257 | 105 | 74 | 2 | 78 | 179 | 51 | 154 | S24 family peptidase | S24 family peptidase | | afdb-uniprot50 | AF-A0A1R1LYN7-F1-MODEL\_V4 | 1.0 | 1.446e-11 | 321 | 0.22 | 222 | 122 | 7 | 4 | 180 | 6 | 221 | HTH cro/C1-type domain-containing protein | HTH cro/C1-type domain-containing protein | | afdb-uniprot50 | AF-A0A561H5B3-F1-MODEL\_V4 | 1.0 | 1.824e-11 | 321 | 0.233 | 201 | 129 | 7 | 4 | 180 | 92 | 291 | Phage repressor protein C with HTH and peptisase S24 domain | Phage repressor protein C with HTH and peptisase S24 domain | | afdb-uniprot50 | AF-A0A192IPT4-F1-MODEL\_V4 | 1.0 | 3.234e-08 | 320 | 0.291 | 120 | 81 | 2 | 64 | 180 | 17 | 135 | Peptidase\_S24 domain-containing protein | Peptidase\_S24 domain-containing protein | | afdb-uniprot50 | AF-A0A0Q5GW81-F1-MODEL\_V4 | 1.0 | 6.12e-08 | 320 | 0.263 | 114 | 77 | 2 | 70 | 180 | 28 | 137 | Peptidase\_S24 domain-containing protein | Peptidase\_S24 domain-containing protein | | afdb-uniprot50 | AF-A0A317EGV6-F1-MODEL\_V4 | 1.0 | 1.721e-11 | 320 | 0.237 | 223 | 125 | 7 | 1 | 180 | 1 | 221 | HTH cro/C1-type domain-containing protein | HTH cro/C1-type domain-containing protein | | afdb-uniprot50 | AF-A0A4R2GU03-F1-MODEL\_V4 | 1.0 | 1.624e-11 | 320 | 0.202 | 217 | 130 | 5 | 3 | 180 | 11 | 223 | Helix-turn-helix protein | Helix-turn-helix protein | | afdb-uniprot50 | AF-G2IX61-F1-MODEL\_V4 | 1.0 | 1.933e-11 | 320 | 0.191 | 219 | 133 | 4 | 4 | 178 | 7 | 225 | Putative phage repressor | Putative phage repressor | | afdb-uniprot50 | AF-C6XCG7-F1-MODEL\_V4 | 1.0 | 1.031e-07 | 320 | 0.35 | 100 | 61 | 2 | 85 | 180 | 131 | 230 | Putative phage repressor | Putative phage repressor | | afdb-uniprot50 | AF-A0A3M1A2A7-F1-MODEL\_V4 | 1.0 | 1.64e-07 | 319 | 0.257 | 105 | 74 | 2 | 79 | 180 | 70 | 173 | Helix-turn-helix transcriptional regulator | Helix-turn-helix transcriptional regulator | | afdb-uniprot50 | AF-A0A7C3DGY8-F1-MODEL\_V4 | 1.0 | 1.824e-11 | 319 | 0.205 | 219 | 130 | 6 | 4 | 180 | 10 | 226 | Helix-turn-helix transcriptional regulator | Helix-turn-helix transcriptional regulator | | afdb-uniprot50 | AF-A0A439P541-F1-MODEL\_V4 | 1.0 | 7.335e-11 | 319 | 0.204 | 205 | 131 | 6 | 1 | 180 | 39 | 236 | Helix-turn-helix domain-containing protein | Helix-turn-helix domain-containing protein | | afdb-uniprot50 | AF-A0A2W6V6P1-F1-MODEL\_V4 | 1.0 | 1.933e-11 | 319 | 0.237 | 215 | 124 | 5 | 4 | 180 | 30 | 242 | Helix-turn-helix transcriptional regulator | Helix-turn-helix transcriptional regulator | | afdb-uniprot50 | AF-A0A1S1NYL5-F1-MODEL\_V4 | 1.0 | 1.933e-11 | 319 | 0.221 | 239 | 121 | 5 | 4 | 178 | 8 | 245 | Helix-turn-helix transcriptional regulator | Helix-turn-helix transcriptional regulator | | afdb-uniprot50 | AF-A0A5B8R9J4-F1-MODEL\_V4 | 1.0 | 4.888e-11 | 318 | 0.247 | 202 | 125 | 6 | 4 | 180 | 28 | 227 | Putative HTH-type transcriptional regulator | Putative HTH-type transcriptional regulator | | afdb-uniprot50 | AF-A0A5P1R9H6-F1-MODEL\_V4 | 1.0 | 2.737e-11 | 318 | 0.228 | 223 | 124 | 8 | 4 | 180 | 6 | 226 | Helix-turn-helix domain-containing protein | Helix-turn-helix domain-containing protein | | afdb-uniprot50 | AF-A0A077F5X8-F1-MODEL\_V4 | 1.0 | 2.437e-11 | 318 | 0.167 | 233 | 138 | 3 | 4 | 180 | 7 | 239 | Phage transcriptional regulator | Phage transcriptional regulator | | afdb-uniprot50 | AF-A0A1S6HSY5-F1-MODEL\_V4 | 1.0 | 1.365e-11 | 318 | 0.188 | 228 | 133 | 5 | 4 | 180 | 17 | 243 | Putative transcriptional regulator | Putative transcriptional regulator | | afdb-uniprot50 | AF-A0A5C1E747-F1-MODEL\_V4 | 1.0 | 2.737e-11 | 318 | 0.2 | 240 | 129 | 8 | 4 | 180 | 19 | 258 | HTH cro/C1-type domain-containing protein | HTH cro/C1-type domain-containing protein | | afdb-uniprot50 | AF-A0A2D2D3J4-F1-MODEL\_V4 | 1.0 | 2.3e-11 | 317 | 0.237 | 215 | 124 | 6 | 4 | 180 | 18 | 230 | Phage repressor protein | Phage repressor protein | | afdb-uniprot50 | AF-P71159-F1-MODEL\_V4 | 1.0 | 4.612e-11 | 317 | 0.192 | 228 | 130 | 5 | 5 | 180 | 7 | 232 | Regulatory protein | Regulatory protein | | afdb-uniprot50 | AF-A0A345Y6T3-F1-MODEL\_V4 | 1.0 | 2.583e-11 | 317 | 0.191 | 230 | 131 | 6 | 5 | 180 | 13 | 241 | Helix-turn-helix transcriptional regulator | Helix-turn-helix transcriptional regulator | | afdb-uniprot50 | AF-A0A0C1Q293-F1-MODEL\_V4 | 1.0 | 2.437e-11 | 317 | 0.209 | 220 | 128 | 7 | 5 | 180 | 26 | 243 | Repressor | Repressor | | afdb-uniprot50 | AF-A0A7Z8ZKU1-F1-MODEL\_V4 | 1.0 | 2.9e-11 | 317 | 0.186 | 220 | 134 | 7 | 4 | 180 | 32 | 249 | DNA-binding/peptidase S24 domain protein | DNA-binding/peptidase S24 domain protein | | afdb-uniprot50 | AF-A0A545SG22-F1-MODEL\_V4 | 1.0 | 3.257e-11 | 317 | 0.224 | 223 | 127 | 7 | 1 | 179 | 37 | 257 | Helix-turn-helix transcriptional regulator | Helix-turn-helix transcriptional regulator | | afdb-uniprot50 | AF-A0A1Q3UZ38-F1-MODEL\_V4 | 1.0 | 7.718e-08 | 316 | 0.292 | 113 | 72 | 3 | 71 | 180 | 3 | 110 | Peptidase\_S24 domain-containing protein | Peptidase\_S24 domain-containing protein | | afdb-uniprot50 | AF-A0A2G6PXF8-F1-MODEL\_V4 | 1.0 | 3.658e-11 | 316 | 0.248 | 209 | 122 | 8 | 4 | 180 | 7 | 212 | HTH cro/C1-type domain-containing protein | HTH cro/C1-type domain-containing protein | | afdb-uniprot50 | AF-F3QJB3-F1-MODEL\_V4 | 1.0 | 3.257e-11 | 316 | 0.203 | 221 | 129 | 6 | 4 | 180 | 7 | 224 | Peptidase S24-like protein | Peptidase S24-like protein | | afdb-uniprot50 | AF-A0A4Q9D5B2-F1-MODEL\_V4 | 1.0 | 1.721e-11 | 316 | 0.217 | 221 | 128 | 6 | 4 | 180 | 6 | 225 | Helix-turn-helix transcriptional regulator | Helix-turn-helix transcriptional regulator | | afdb-uniprot50 | AF-A0A7T0G1L7-F1-MODEL\_V4 | 1.0 | 3.658e-11 | 316 | 0.189 | 216 | 136 | 5 | 4 | 180 | 15 | 230 | Helix-turn-helix transcriptional regulator | Helix-turn-helix transcriptional regulator | | afdb-uniprot50 | AF-A0A516SAZ0-F1-MODEL\_V4 | 1.0 | 4.612e-11 | 316 | 0.213 | 234 | 126 | 7 | 4 | 180 | 7 | 239 | Helix-turn-helix domain-containing protein | Helix-turn-helix domain-containing protein | | afdb-uniprot50 | AF-A0A5C1MF53-F1-MODEL\_V4 | 1.0 | 4.612e-11 | 316 | 0.212 | 216 | 129 | 8 | 4 | 179 | 30 | 244 | Helix-turn-helix transcriptional regulator | Helix-turn-helix transcriptional regulator | | afdb-uniprot50 | AF-A4N1A7-F1-MODEL\_V4 | 1.0 | 6.486e-08 | 315 | 0.322 | 96 | 61 | 2 | 88 | 180 | 93 | 187 | Putative transcriptional regulator | Putative transcriptional regulator | | afdb-uniprot50 | AF-A0A562C8X5-F1-MODEL\_V4 | 1.0 | 2.737e-11 | 315 | 0.221 | 212 | 124 | 7 | 4 | 180 | 9 | 214 | Phage repressor protein C with HTH and peptisase S24 domain | Phage repressor protein C with HTH and peptisase S24 domain | | afdb-uniprot50 | AF-A0A318MXH2-F1-MODEL\_V4 | 1.0 | 3.257e-11 | 315 | 0.195 | 215 | 130 | 7 | 5 | 180 | 9 | 219 | Transcriptional regulator | Transcriptional regulator | | afdb-uniprot50 | AF-A0A7R6SVB1-F1-MODEL\_V4 | 1.0 | 1.039e-10 | 315 | 0.236 | 220 | 123 | 7 | 4 | 180 | 8 | 225 | Peptidase S24 | Peptidase S24 | | afdb-uniprot50 | AF-A0A1B9KJY8-F1-MODEL\_V4 | 1.0 | 3.876e-11 | 315 | 0.185 | 226 | 133 | 6 | 4 | 180 | 7 | 230 | HTH cro/C1-type domain-containing protein | HTH cro/C1-type domain-containing protein | | afdb-uniprot50 | AF-A0A3R8T613-F1-MODEL\_V4 | 1.0 | 1.147e-11 | 315 | 0.202 | 232 | 133 | 10 | 1 | 180 | 1 | 232 | Helix-turn-helix transcriptional regulator | Helix-turn-helix transcriptional regulator | | afdb-uniprot50 | AF-A0A1B3E902-F1-MODEL\_V4 | 1.0 | 3.451e-11 | 315 | 0.169 | 224 | 137 | 5 | 4 | 179 | 19 | 241 | Cro/Cl family transcriptional regulator | Cro/Cl family transcriptional regulator | | afdb-uniprot50 | AF-A0A358RV08-F1-MODEL\_V4 | 1.0 | 4.322e-08 | 314 | 0.271 | 114 | 75 | 3 | 70 | 180 | 5 | 113 | Peptidase\_S24 domain-containing protein | Peptidase\_S24 domain-containing protein | | afdb-uniprot50 | AF-A0A423DG37-F1-MODEL\_V4 | 1.0 | 1.166e-10 | 314 | 0.213 | 206 | 128 | 5 | 6 | 180 | 2 | 204 | HTH cro/C1-type domain-containing protein | HTH cro/C1-type domain-containing protein | | afdb-uniprot50 | AF-A0A5E4YQN1-F1-MODEL\_V4 | 1.0 | 6.532e-11 | 314 | 0.2 | 205 | 136 | 4 | 4 | 180 | 19 | 223 | Putative HTH-type transcriptional regulator | Putative HTH-type transcriptional regulator | | afdb-uniprot50 | AF-A0A6G6Q5W5-F1-MODEL\_V4 | 1.0 | 3.451e-11 | 314 | 0.177 | 225 | 136 | 5 | 4 | 180 | 6 | 229 | Helix-turn-helix transcriptional regulator | Helix-turn-helix transcriptional regulator | | afdb-uniprot50 | AF-A0A3D5BE89-F1-MODEL\_V4 | 1.0 | 3.074e-11 | 314 | 0.2 | 234 | 130 | 5 | 4 | 180 | 6 | 239 | XRE family transcriptional regulator | XRE family transcriptional regulator | | afdb-uniprot50 | AF-A0A2M8RY32-F1-MODEL\_V4 | 1.0 | 3.632e-08 | 313 | 0.232 | 116 | 82 | 4 | 70 | 180 | 9 | 122 | Peptidase\_S24 domain-containing protein | Peptidase\_S24 domain-containing protein | | afdb-uniprot50 | AF-U7P2I5-F1-MODEL\_V4 | 1.0 | 1.461e-07 | 313 | 0.285 | 105 | 71 | 2 | 79 | 180 | 49 | 152 | Peptidase\_S24 domain-containing protein | Peptidase\_S24 domain-containing protein | | afdb-uniprot50 | AF-A0A1L9CZL4-F1-MODEL\_V4 | 1.0 | 2.034e-08 | 313 | 0.251 | 127 | 84 | 4 | 60 | 180 | 75 | 196 | Peptidase\_S24 domain-containing protein | Peptidase\_S24 domain-containing protein | | afdb-uniprot50 | AF-E6W350-F1-MODEL\_V4 | 1.0 | 3.257e-11 | 313 | 0.235 | 212 | 125 | 6 | 4 | 180 | 6 | 215 | Peptidase S24/S26A/S26B, conserved region | Peptidase S24/S26A/S26B, conserved region | | afdb-uniprot50 | AF-A0A7Y6VAX6-F1-MODEL\_V4 | 1.0 | 1.824e-11 | 313 | 0.208 | 225 | 130 | 8 | 1 | 180 | 3 | 224 | Helix-turn-helix transcriptional regulator | Helix-turn-helix transcriptional regulator | | afdb-uniprot50 | AF-A0A1C0A0X4-F1-MODEL\_V4 | 1.0 | 6.532e-11 | 313 | 0.172 | 226 | 136 | 5 | 4 | 180 | 7 | 230 | HTH cro/C1-type domain-containing protein | HTH cro/C1-type domain-containing protein | | afdb-uniprot50 | AF-A0A2R3QPF1-F1-MODEL\_V4 | 1.0 | 3.451e-11 | 313 | 0.209 | 205 | 131 | 6 | 4 | 180 | 29 | 230 | C repressor | C repressor | | afdb-uniprot50 | AF-A0A1L3JAI5-F1-MODEL\_V4 | 1.0 | 3.658e-11 | 313 | 0.192 | 223 | 131 | 6 | 6 | 180 | 11 | 232 | Peptidase\_S24 domain-containing protein | Peptidase\_S24 domain-containing protein | | afdb-uniprot50 | AF-A0A2W6GVH0-F1-MODEL\_V4 | 1.0 | 4.612e-11 | 313 | 0.222 | 225 | 124 | 7 | 4 | 179 | 10 | 232 | Peptidase\_S24 domain-containing protein | Peptidase\_S24 domain-containing protein | | afdb-uniprot50 | AF-A0A318DG58-F1-MODEL\_V4 | 1.0 | 3.257e-11 | 313 | 0.214 | 233 | 123 | 6 | 5 | 178 | 8 | 239 | Phage repressor protein C with HTH and peptisase S24 domain | Phage repressor protein C with HTH and peptisase S24 domain | | afdb-uniprot50 | AF-A0A1C3H797-F1-MODEL\_V4 | 1.0 | 5.489e-11 | 313 | 0.218 | 215 | 129 | 6 | 4 | 180 | 28 | 241 | Putative transcriptional regulator | Putative transcriptional regulator | | afdb-uniprot50 | AF-A0A060H678-F1-MODEL\_V4 | 1.0 | 5.18e-11 | 313 | 0.176 | 243 | 131 | 7 | 5 | 179 | 7 | 248 | Peptidase S24 | Peptidase S24 | | afdb-uniprot50 | AF-A0A7T8EGD1-F1-MODEL\_V4 | 1.0 | 1.031e-07 | 312 | 0.292 | 106 | 70 | 3 | 78 | 180 | 76 | 179 | S24 family peptidase | S24 family peptidase | | afdb-uniprot50 | AF-A9L6K9-F1-MODEL\_V4 | 1.0 | 3.451e-11 | 312 | 0.197 | 208 | 134 | 5 | 4 | 180 | 7 | 212 | Putative phage repressor | Putative phage repressor | | afdb-uniprot50 | AF-A0A7X6EK24-F1-MODEL\_V4 | 1.0 | 9.25e-11 | 312 | 0.195 | 215 | 130 | 7 | 4 | 180 | 11 | 220 | Helix-turn-helix domain-containing protein | Helix-turn-helix domain-containing protein | | afdb-uniprot50 | AF-R5YMJ3-F1-MODEL\_V4 | 1.0 | 4.888e-11 | 312 | 0.164 | 213 | 139 | 4 | 4 | 178 | 10 | 221 | Putative transcriptional regulator repressor | Putative transcriptional regulator repressor | | afdb-uniprot50 | AF-A0A249DYA3-F1-MODEL\_V4 | 1.0 | 3.451e-11 | 312 | 0.194 | 226 | 133 | 4 | 4 | 180 | 6 | 231 | XRE family transcriptional regulator | XRE family transcriptional regulator | | afdb-uniprot50 | AF-A0A2W6WL33-F1-MODEL\_V4 | 1.0 | 6.532e-11 | 312 | 0.169 | 213 | 137 | 6 | 4 | 180 | 27 | 235 | HTH cro/C1-type domain-containing protein | HTH cro/C1-type domain-containing protein | | afdb-uniprot50 | AF-A0A1I1Q734-F1-MODEL\_V4 | 1.0 | 4.612e-11 | 312 | 0.211 | 227 | 126 | 7 | 5 | 180 | 31 | 255 | Phage repressor protein C, contains Cro/C1-type HTH and peptisase s24 domains | Phage repressor protein C, contains Cro/C1-type HTH and peptisase s24 domains | | afdb-uniprot50 | AF-A0A0F7KKY2-F1-MODEL\_V4 | 1.0 | 2.323e-07 | 311 | 0.317 | 107 | 69 | 2 | 77 | 180 | 35 | 140 | Peptidase\_S24 domain-containing protein | Peptidase\_S24 domain-containing protein | | afdb-uniprot50 | AF-A0A855FN50-F1-MODEL\_V4 | 1.0 | 4.58e-08 | 311 | 0.254 | 114 | 78 | 4 | 70 | 180 | 83 | 192 | Uncharacterized protein | Uncharacterized protein | | afdb-uniprot50 | AF-A0A7H1NQ78-F1-MODEL\_V4 | 1.0 | 3.658e-11 | 311 | 0.199 | 221 | 131 | 5 | 4 | 180 | 8 | 226 | HTH-type transcriptional regulator PrtR | HTH-type transcriptional regulator PrtR | | afdb-uniprot50 | AF-A0A437QDQ5-F1-MODEL\_V4 | 1.0 | 4.612e-11 | 311 | 0.235 | 225 | 123 | 7 | 4 | 180 | 8 | 231 | Helix-turn-helix transcriptional regulator | Helix-turn-helix transcriptional regulator | | afdb-uniprot50 | AF-A0A847S398-F1-MODEL\_V4 | 1.0 | 4.612e-11 | 311 | 0.243 | 230 | 119 | 8 | 4 | 180 | 7 | 234 | Helix-turn-helix transcriptional regulator | Helix-turn-helix transcriptional regulator | | afdb-uniprot50 | AF-A0A0D5N774-F1-MODEL\_V4 | 1.0 | 1.471e-10 | 310 | 0.215 | 200 | 128 | 8 | 5 | 180 | 8 | 202 | Peptidase\_S24 domain-containing protein | Peptidase\_S24 domain-containing protein | | afdb-uniprot50 | AF-A0A3D5BAV1-F1-MODEL\_V4 | 1.0 | 8.729e-11 | 310 | 0.193 | 202 | 134 | 4 | 5 | 180 | 10 | 208 | Peptidase\_S24 domain-containing protein | Peptidase\_S24 domain-containing protein | | afdb-uniprot50 | AF-A0A252A0T3-F1-MODEL\_V4 | 1.0 | 4.888e-11 | 310 | 0.21 | 204 | 127 | 7 | 4 | 180 | 14 | 210 | Repressor | Repressor | | afdb-uniprot50 | AF-A0A246HR45-F1-MODEL\_V4 | 1.0 | 2.048e-11 | 310 | 0.199 | 221 | 130 | 9 | 4 | 180 | 6 | 223 | Transcriptional regulator | Transcriptional regulator | | afdb-uniprot50 | AF-A0A3D3SH38-F1-MODEL\_V4 | 1.0 | 5.18e-11 | 310 | 0.2 | 219 | 131 | 7 | 4 | 180 | 7 | 223 | HTH cro/C1-type domain-containing protein | HTH cro/C1-type domain-containing protein | | afdb-uniprot50 | AF-A0A379CA04-F1-MODEL\_V4 | 1.0 | 4.612e-11 | 310 | 0.184 | 217 | 134 | 8 | 4 | 180 | 7 | 220 | Uncharacterized HTH-type transcriptional regulator HI\_1476 | Uncharacterized HTH-type transcriptional regulator HI\_1476 | | afdb-uniprot50 | AF-A0A1J0D5X4-F1-MODEL\_V4 | 1.0 | 6.922e-11 | 310 | 0.181 | 220 | 131 | 7 | 4 | 179 | 10 | 224 | HTH cro/C1-type domain-containing protein | HTH cro/C1-type domain-containing protein | | afdb-uniprot50 | AF-A0A495RI07-F1-MODEL\_V4 | 1.0 | 8.237e-11 | 310 | 0.195 | 225 | 131 | 4 | 4 | 180 | 5 | 227 | Phage repressor protein C with HTH and peptisase S24 domain | Phage repressor protein C with HTH and peptisase S24 domain | | afdb-uniprot50 | AF-A0A2E2KKV0-F1-MODEL\_V4 | 1.0 | 4.612e-11 | 310 | 0.251 | 215 | 118 | 5 | 4 | 180 | 19 | 228 | HTH cro/C1-type domain-containing protein | HTH cro/C1-type domain-containing protein | | afdb-uniprot50 | AF-D0IHH2-F1-MODEL\_V4 | 1.0 | 3.451e-11 | 310 | 0.205 | 229 | 130 | 5 | 4 | 180 | 9 | 237 | Predicted transcriptional regulator | Predicted transcriptional regulator | | afdb-uniprot50 | AF-A0A011NA96-F1-MODEL\_V4 | 1.0 | 9.733e-08 | 309 | 0.226 | 106 | 77 | 3 | 78 | 180 | 31 | 134 | Transcriptional regulator | Transcriptional regulator | | afdb-uniprot50 | AF-A0A1H2PS65-F1-MODEL\_V4 | 1.0 | 1.039e-10 | 309 | 0.201 | 218 | 128 | 8 | 4 | 180 | 6 | 218 | Phage repressor protein C, contains Cro/C1-type HTH and peptisase s24 domains | Phage repressor protein C, contains Cro/C1-type HTH and peptisase s24 domains | | afdb-uniprot50 | AF-A0A2J6JA73-F1-MODEL\_V4 | 1.0 | 6.164e-11 | 309 | 0.185 | 221 | 134 | 6 | 5 | 180 | 7 | 226 | Transcriptional regulator | Transcriptional regulator | | afdb-uniprot50 | AF-A0A346JU79-F1-MODEL\_V4 | 1.0 | 3.451e-11 | 309 | 0.223 | 224 | 120 | 9 | 4 | 177 | 7 | 226 | Helix-turn-helix transcriptional regulator | Helix-turn-helix transcriptional regulator | | afdb-uniprot50 | AF-A0A2U1TLL0-F1-MODEL\_V4 | 1.0 | 1.039e-10 | 308 | 0.206 | 208 | 132 | 6 | 4 | 180 | 12 | 217 | HTH cro/C1-type domain-containing protein | HTH cro/C1-type domain-containing protein | | afdb-uniprot50 | AF-A0A522BFE5-F1-MODEL\_V4 | 1.0 | 1.855e-10 | 308 | 0.22 | 213 | 127 | 6 | 4 | 180 | 14 | 223 | Helix-turn-helix transcriptional regulator | Helix-turn-helix transcriptional regulator | | afdb-uniprot50 | AF-A0A1G5CCK6-F1-MODEL\_V4 | 1.0 | 4.612e-11 | 308 | 0.235 | 212 | 127 | 8 | 2 | 180 | 17 | 226 | Phage repressor protein C, contains Cro/C1-type HTH and peptisase s24 domains | Phage repressor protein C, contains Cro/C1-type HTH and peptisase s24 domains | | afdb-uniprot50 | AF-A0A222FGK0-F1-MODEL\_V4 | 1.0 | 7.335e-11 | 308 | 0.204 | 215 | 131 | 6 | 4 | 180 | 18 | 230 | Transcriptional regulator | Transcriptional regulator | | afdb-uniprot50 | AF-A0A1N7DPA7-F1-MODEL\_V4 | 1.0 | 4.107e-11 | 308 | 0.193 | 227 | 129 | 6 | 4 | 178 | 6 | 230 | Phage repressor protein C, contains Cro/C1-type HTH and peptisase s24 domains | Phage repressor protein C, contains Cro/C1-type HTH and peptisase s24 domains | | afdb-uniprot50 | AF-A0A3T0ZVY7-F1-MODEL\_V4 | 1.0 | 3.658e-11 | 308 | 0.201 | 218 | 130 | 5 | 4 | 179 | 31 | 246 | Transcriptional regulator | Transcriptional regulator | | afdb-uniprot50 | AF-A0A149QP86-F1-MODEL\_V4 | 1.0 | 1.548e-07 | 307 | 0.301 | 93 | 61 | 2 | 88 | 177 | 46 | 137 | Repressor | Repressor | | afdb-uniprot50 | AF-A0A546XR11-F1-MODEL\_V4 | 1.0 | 5.775e-08 | 307 | 0.26 | 123 | 80 | 4 | 64 | 180 | 48 | 165 | Helix-turn-helix transcriptional regulator | Helix-turn-helix transcriptional regulator | | afdb-uniprot50 | AF-A0A2D9DW47-F1-MODEL\_V4 | 1.0 | 4.353e-11 | 307 | 0.188 | 207 | 134 | 8 | 5 | 180 | 10 | 213 | Peptidase\_S24 domain-containing protein | Peptidase\_S24 domain-containing protein | | afdb-uniprot50 | AF-A0A7X5QU28-F1-MODEL\_V4 | 1.0 | 8.729e-11 | 307 | 0.177 | 214 | 138 | 6 | 4 | 180 | 4 | 216 | Helix-turn-helix transcriptional regulator | Helix-turn-helix transcriptional regulator | | afdb-uniprot50 | AF-F3QJB4-F1-MODEL\_V4 | 1.0 | 1.236e-10 | 307 | 0.201 | 213 | 129 | 6 | 4 | 178 | 13 | 222 | Peptidase S24-like protein | Peptidase S24-like protein | | afdb-uniprot50 | AF-A0A657K4X9-F1-MODEL\_V4 | 1.0 | 7.335e-11 | 307 | 0.193 | 232 | 129 | 6 | 4 | 180 | 7 | 235 | HTH cro/C1-type domain-containing protein | HTH cro/C1-type domain-containing protein | | afdb-uniprot50 | AF-W6W8I6-F1-MODEL\_V4 | 1.0 | 1.166e-10 | 307 | 0.201 | 238 | 128 | 6 | 4 | 180 | 9 | 245 | Putative phage repressor | Putative phage repressor | | afdb-uniprot50 | AF-A0A8B3TEF1-F1-MODEL\_V4 | 1.0 | 5.18e-11 | 307 | 0.18 | 249 | 134 | 8 | 1 | 180 | 1 | 248 | Helix-turn-helix domain-containing protein | Helix-turn-helix domain-containing protein | | afdb-uniprot50 | AF-A0A7T5A214-F1-MODEL\_V4 | 1.0 | 4.612e-11 | 307 | 0.232 | 219 | 122 | 7 | 4 | 180 | 37 | 251 | LexA family transcriptional regulator | LexA family transcriptional regulator | | afdb-uniprot50 | AF-A0A286C7A4-F1-MODEL\_V4 | 1.0 | 6.12e-08 | 307 | 0.318 | 113 | 69 | 3 | 71 | 180 | 154 | 261 | Mu DNA-binding domain-containing protein | Mu DNA-binding domain-containing protein | | afdb-uniprot50 | AF-A0A743J265-F1-MODEL\_V4 | 1.0 | 4.853e-08 | 306 | 0.309 | 113 | 70 | 4 | 71 | 180 | 56 | 163 | Helix-turn-helix transcriptional regulator | Helix-turn-helix transcriptional regulator | | afdb-uniprot50 | AF-A0A2P1VM30-F1-MODEL\_V4 | 1.0 | 1.227e-07 | 306 | 0.245 | 106 | 75 | 3 | 78 | 180 | 104 | 207 | Transcriptional regulator | Transcriptional regulator | | afdb-uniprot50 | AF-A0A4R2GXK3-F1-MODEL\_V4 | 1.0 | 1.855e-10 | 306 | 0.212 | 212 | 127 | 9 | 4 | 180 | 18 | 224 | Phage repressor protein C with HTH and peptisase S24 domain | Phage repressor protein C with HTH and peptisase S24 domain | | afdb-uniprot50 | AF-A0A366FEU9-F1-MODEL\_V4 | 1.0 | 2.583e-11 | 306 | 0.222 | 220 | 123 | 8 | 4 | 180 | 6 | 220 | Phage repressor protein C with HTH and peptisase S24 domain | Phage repressor protein C with HTH and peptisase S24 domain | | afdb-uniprot50 | AF-A0A2J9DWQ2-F1-MODEL\_V4 | 1.0 | 7.335e-11 | 306 | 0.2 | 220 | 131 | 6 | 4 | 180 | 9 | 226 | Helix-turn-helix transcriptional regulator | Helix-turn-helix transcriptional regulator | | afdb-uniprot50 | AF-A0A7X2GXD4-F1-MODEL\_V4 | 1.0 | 4.107e-11 | 306 | 0.194 | 226 | 134 | 8 | 1 | 180 | 1 | 224 | Helix-turn-helix domain-containing protein | Helix-turn-helix domain-containing protein | | afdb-uniprot50 | AF-A0A852PQB2-F1-MODEL\_V4 | 1.0 | 3.451e-11 | 306 | 0.174 | 223 | 136 | 7 | 4 | 180 | 7 | 227 | Helix-turn-helix transcriptional regulator | Helix-turn-helix transcriptional regulator | | afdb-uniprot50 | AF-A0A7W6G492-F1-MODEL\_V4 | 1.0 | 4.178e-10 | 306 | 0.172 | 238 | 129 | 7 | 5 | 180 | 7 | 238 | Phage repressor protein C with HTH and peptisase S24 domain | Phage repressor protein C with HTH and peptisase S24 domain | | afdb-uniprot50 | AF-A0A495YMK1-F1-MODEL\_V4 | 1.0 | 1.166e-10 | 306 | 0.164 | 237 | 134 | 7 | 4 | 180 | 7 | 239 | Phage repressor protein | Phage repressor protein | | afdb-uniprot50 | AF-A0A1Y2K654-F1-MODEL\_V4 | 1.0 | 7.718e-08 | 305 | 0.211 | 118 | 84 | 4 | 66 | 180 | 1 | 112 | Putative Cro/CI family transcriptional regulator | Putative Cro/CI family transcriptional regulator | | afdb-uniprot50 | AF-A0A2W6YVV3-F1-MODEL\_V4 | 1.0 | 1.75e-10 | 305 | 0.198 | 212 | 133 | 6 | 4 | 180 | 4 | 213 | HTH cro/C1-type domain-containing protein | HTH cro/C1-type domain-containing protein | | afdb-uniprot50 | AF-A0A1X0TH41-F1-MODEL\_V4 | 1.0 | 1.559e-10 | 305 | 0.17 | 211 | 139 | 6 | 4 | 180 | 7 | 215 | HTH cro/C1-type domain-containing protein | HTH cro/C1-type domain-containing protein | | afdb-uniprot50 | AF-A0A4Q7BGU0-F1-MODEL\_V4 | 1.0 | 1.31e-10 | 305 | 0.213 | 220 | 123 | 5 | 4 | 180 | 6 | 218 | Peptidase S24 | Peptidase S24 | | afdb-uniprot50 | AF-A0A212KKI8-F1-MODEL\_V4 | 1.0 | 8.237e-11 | 305 | 0.22 | 209 | 129 | 6 | 5 | 180 | 14 | 221 | Putative Repressor protein CI | Putative Repressor protein CI | | afdb-uniprot50 | AF-A0A6N9SY62-F1-MODEL\_V4 | 1.0 | 1.471e-10 | 305 | 0.213 | 220 | 129 | 5 | 4 | 180 | 12 | 230 | Helix-turn-helix domain-containing protein | Helix-turn-helix domain-containing protein | | afdb-uniprot50 | AF-A0A7Y2QFS9-F1-MODEL\_V4 | 1.0 | 1.166e-10 | 305 | 0.171 | 228 | 132 | 6 | 4 | 178 | 8 | 231 | Helix-turn-helix transcriptional regulator | Helix-turn-helix transcriptional regulator | | afdb-uniprot50 | AF-A0A516SD59-F1-MODEL\_V4 | 1.0 | 5.489e-11 | 305 | 0.231 | 233 | 122 | 9 | 4 | 180 | 6 | 237 | Helix-turn-helix transcriptional regulator | Helix-turn-helix transcriptional regulator | | afdb-uniprot50 | AF-I7C2G6-F1-MODEL\_V4 | 1.0 | 2.737e-11 | 305 | 0.184 | 260 | 128 | 7 | 4 | 180 | 8 | 266 | Putative phage repressor | Putative phage repressor | | afdb-uniprot50 | AF-C4GFS1-F1-MODEL\_V4 | 1.0 | 1.236e-10 | 305 | 0.161 | 229 | 138 | 7 | 4 | 180 | 125 | 351 | DNA-binding helix-turn-helix protein | DNA-binding helix-turn-helix protein | | afdb-uniprot50 | AF-A0A554RHB1-F1-MODEL\_V4 | 1.0 | 1.952e-07 | 304 | 0.245 | 106 | 75 | 3 | 79 | 180 | 65 | 169 | Helix-turn-helix transcriptional regulator | Helix-turn-helix transcriptional regulator | | afdb-uniprot50 | AF-A0A345DBG2-F1-MODEL\_V4 | 1.0 | 1.471e-10 | 304 | 0.195 | 200 | 135 | 4 | 5 | 179 | 7 | 205 | HTH-type transcriptional regulator PrtR | HTH-type transcriptional regulator PrtR | | afdb-uniprot50 | AF-A0A2X1U757-F1-MODEL\_V4 | 1.0 | 8.729e-11 | 304 | 0.206 | 213 | 130 | 9 | 4 | 180 | 7 | 216 | Phage repressor protein, phage associated protein | Phage repressor protein, phage associated protein | | afdb-uniprot50 | AF-V2QBR0-F1-MODEL\_V4 | 1.0 | 5.489e-11 | 304 | 0.2 | 215 | 130 | 7 | 6 | 180 | 12 | 224 | HTH cro/C1-type domain-containing protein | HTH cro/C1-type domain-containing protein | | afdb-uniprot50 | AF-A0A376BT84-F1-MODEL\_V4 | 1.0 | 6.532e-11 | 304 | 0.233 | 214 | 125 | 7 | 4 | 180 | 16 | 227 | Uncharacterized HTH-type transcriptional regulator HI\_1476 | Uncharacterized HTH-type transcriptional regulator HI\_1476 | | afdb-uniprot50 | AF-A0A369YGT9-F1-MODEL\_V4 | 1.0 | 1.75e-10 | 304 | 0.185 | 221 | 133 | 7 | 4 | 180 | 7 | 224 | Helix-turn-helix transcriptional regulator | Helix-turn-helix transcriptional regulator | | afdb-uniprot50 | AF-A0A369T885-F1-MODEL\_V4 | 1.0 | 9.802e-11 | 304 | 0.169 | 218 | 136 | 8 | 4 | 180 | 41 | 254 | XRE family transcriptional regulator | XRE family transcriptional regulator | | afdb-uniprot50 | AF-A0A349XYC5-F1-MODEL\_V4 | 1.0 | 1.64e-07 | 303 | 0.271 | 114 | 75 | 3 | 70 | 180 | 9 | 117 | Helix-turn-helix transcriptional regulator | Helix-turn-helix transcriptional regulator | | afdb-uniprot50 | AF-A0A828SCT0-F1-MODEL\_V4 | 1.0 | 1.548e-07 | 303 | 0.287 | 108 | 72 | 3 | 77 | 180 | 45 | 151 | Peptidase S24-like family protein | Peptidase S24-like family protein | | afdb-uniprot50 | AF-U3U086-F1-MODEL\_V4 | 1.0 | 1.158e-07 | 303 | 0.28 | 107 | 71 | 4 | 78 | 180 | 60 | 164 | Repressor protein CI | Repressor protein CI | | afdb-uniprot50 | AF-A0A718TEP2-F1-MODEL\_V4 | 1.0 | 6.12e-08 | 303 | 0.309 | 113 | 70 | 4 | 71 | 180 | 63 | 170 | Helix-turn-helix transcriptional regulator | Helix-turn-helix transcriptional regulator | | afdb-uniprot50 | AF-A0A515D4V4-F1-MODEL\_V4 | 1.0 | 1.158e-07 | 303 | 0.303 | 112 | 71 | 4 | 72 | 180 | 80 | 187 | Helix-turn-helix transcriptional regulator | Helix-turn-helix transcriptional regulator | | afdb-uniprot50 | AF-C0N8P1-F1-MODEL\_V4 | 1.0 | 2.764e-07 | 303 | 0.256 | 109 | 75 | 2 | 77 | 180 | 102 | 209 | Peptidase S24-like domain protein | Peptidase S24-like domain protein | | afdb-uniprot50 | AF-A0A2L2BE93-F1-MODEL\_V4 | 1.0 | 9.802e-11 | 303 | 0.199 | 216 | 131 | 4 | 4 | 178 | 7 | 221 | HTH cro/C1-type domain-containing protein | HTH cro/C1-type domain-containing protein | | afdb-uniprot50 | AF-A0A8B5S1H8-F1-MODEL\_V4 | 1.0 | 6.164e-11 | 303 | 0.177 | 225 | 136 | 6 | 4 | 180 | 6 | 229 | Helix-turn-helix transcriptional regulator | Helix-turn-helix transcriptional regulator | | afdb-uniprot50 | AF-A0A0F4R425-F1-MODEL\_V4 | 1.0 | 5.489e-11 | 303 | 0.202 | 237 | 128 | 6 | 4 | 180 | 6 | 241 | HTH cro/C1-type domain-containing protein | HTH cro/C1-type domain-containing protein | | afdb-uniprot50 | AF-A0A3Z8HGE4-F1-MODEL\_V4 | 1.0 | 2.929e-07 | 302 | 0.34 | 100 | 61 | 3 | 83 | 179 | 3 | 100 | Helix-turn-helix transcriptional regulator | Helix-turn-helix transcriptional regulator | | afdb-uniprot50 | AF-A0A6L3ERI8-F1-MODEL\_V4 | 1.0 | 1.207e-08 | 302 | 0.236 | 127 | 87 | 3 | 60 | 178 | 1 | 125 | S24 family peptidase | S24 family peptidase | | afdb-uniprot50 | AF-A0A160BZ99-F1-MODEL\_V4 | 1.0 | 1.842e-07 | 302 | 0.224 | 107 | 78 | 3 | 79 | 180 | 49 | 155 | Peptidase\_S24 domain-containing protein | Peptidase\_S24 domain-containing protein | | afdb-uniprot50 | AF-A0A1C3EAH7-F1-MODEL\_V4 | 1.0 | 1.75e-10 | 302 | 0.242 | 194 | 127 | 4 | 4 | 180 | 18 | 208 | HTH cro/C1-type domain-containing protein | HTH cro/C1-type domain-containing protein | | afdb-uniprot50 | AF-K9RVQ1-F1-MODEL\_V4 | 1.0 | 8.237e-11 | 302 | 0.21 | 209 | 130 | 7 | 3 | 180 | 11 | 215 | Putative transcriptional regulator | Putative transcriptional regulator | | afdb-uniprot50 | AF-A0A2T1AI87-F1-MODEL\_V4 | 1.0 | 8.729e-11 | 302 | 0.221 | 212 | 129 | 4 | 4 | 180 | 9 | 219 | Helix-turn-helix protein | Helix-turn-helix protein | | afdb-uniprot50 | AF-B4RKJ1-F1-MODEL\_V4 | 1.0 | 8.729e-11 | 302 | 0.219 | 210 | 128 | 6 | 5 | 180 | 11 | 218 | Putative lambda repressor protein cI, putative phage associated protein | Putative lambda repressor protein cI, putative phage associated protein | | afdb-uniprot50 | AF-A0A1N6R0A9-F1-MODEL\_V4 | 1.0 | 1.652e-10 | 302 | 0.218 | 220 | 123 | 6 | 5 | 180 | 8 | 222 | Phage repressor protein C, contains Cro/C1-type HTH and peptisase s24 domains | Phage repressor protein C, contains Cro/C1-type HTH and peptisase s24 domains | | afdb-uniprot50 | AF-A0A0Q4U5S7-F1-MODEL\_V4 | 1.0 | 1.166e-10 | 302 | 0.184 | 211 | 137 | 5 | 4 | 180 | 15 | 224 | HTH cro/C1-type domain-containing protein | HTH cro/C1-type domain-containing protein | | afdb-uniprot50 | AF-A0A380TXN7-F1-MODEL\_V4 | 1.0 | 4.353e-11 | 302 | 0.189 | 216 | 134 | 7 | 4 | 180 | 12 | 225 | Putative phage repressor | Putative phage repressor | | afdb-uniprot50 | AF-A0A1R3U4A3-F1-MODEL\_V4 | 1.0 | 4.107e-11 | 302 | 0.213 | 225 | 127 | 7 | 4 | 180 | 12 | 234 | Putative HTH-type transcriptional regulator | Putative HTH-type transcriptional regulator | | afdb-uniprot50 | AF-A0A7G5DM43-F1-MODEL\_V4 | 1.0 | 6.532e-11 | 302 | 0.18 | 227 | 138 | 6 | 1 | 180 | 16 | 241 | Helix-turn-helix transcriptional regulator | Helix-turn-helix transcriptional regulator | | afdb-uniprot50 | AF-U1I7S2-F1-MODEL\_V4 | 1.0 | 3.074e-11 | 302 | 0.171 | 222 | 135 | 6 | 6 | 180 | 26 | 245 | Transcriptional regulator | Transcriptional regulator | | afdb-uniprot50 | AF-A0A154U565-F1-MODEL\_V4 | 1.0 | 2.608e-07 | 301 | 0.285 | 105 | 71 | 2 | 79 | 180 | 62 | 165 | Transcriptional regulator | Transcriptional regulator | | afdb-uniprot50 | AF-Q0FYV8-F1-MODEL\_V4 | 1.0 | 2.608e-07 | 301 | 0.273 | 106 | 74 | 1 | 78 | 180 | 78 | 183 | Prophage MuSo1, transcriptional regulator, Cro/CI family protein | Prophage MuSo1, transcriptional regulator, Cro/CI family protein | | afdb-uniprot50 | AF-A0A6G8C4E6-F1-MODEL\_V4 | 1.0 | 3.104e-07 | 301 | 0.237 | 97 | 71 | 2 | 87 | 180 | 95 | 191 | Helix-turn-helix transcriptional regulator | Helix-turn-helix transcriptional regulator | | afdb-uniprot50 | AF-A0A212J1Z6-F1-MODEL\_V4 | 1.0 | 8.237e-11 | 301 | 0.213 | 201 | 128 | 9 | 5 | 180 | 7 | 202 | Putative Phage repressor | Putative Phage repressor | | afdb-uniprot50 | AF-A0A7Y8SNM8-F1-MODEL\_V4 | 1.0 | 1.236e-10 | 301 | 0.199 | 216 | 123 | 8 | 6 | 180 | 2 | 208 | Helix-turn-helix transcriptional regulator | Helix-turn-helix transcriptional regulator | | afdb-uniprot50 | AF-A0A1G0HXS8-F1-MODEL\_V4 | 1.0 | 1.652e-10 | 301 | 0.209 | 210 | 129 | 6 | 7 | 180 | 3 | 211 | HTH cro/C1-type domain-containing protein | HTH cro/C1-type domain-containing protein | | afdb-uniprot50 | AF-A0A0M3G0U6-F1-MODEL\_V4 | 1.0 | 1.236e-10 | 301 | 0.211 | 217 | 130 | 6 | 4 | 180 | 7 | 222 | HTH cro/C1-type domain-containing protein | HTH cro/C1-type domain-containing protein | | afdb-uniprot50 | AF-G9ZDL4-F1-MODEL\_V4 | 1.0 | 1.559e-10 | 301 | 0.209 | 215 | 129 | 6 | 4 | 180 | 22 | 233 | DNA-binding helix-turn-helix protein | DNA-binding helix-turn-helix protein | | afdb-uniprot50 | AF-A0A1S7MQB7-F1-MODEL\_V4 | 1.0 | 1.388e-10 | 301 | 0.211 | 218 | 129 | 7 | 4 | 180 | 26 | 241 | Putative transcriptional regulator | Putative transcriptional regulator | | afdb-uniprot50 | AF-A0A7V8F8X1-F1-MODEL\_V4 | 1.0 | 2.479e-10 | 301 | 0.191 | 225 | 132 | 6 | 5 | 180 | 28 | 251 | HTH cro/C1-type domain-containing protein | HTH cro/C1-type domain-containing protein | | afdb-uniprot50 | AF-A0A077LIF9-F1-MODEL\_V4 | 1.0 | 4.888e-11 | 301 | 0.151 | 271 | 135 | 6 | 4 | 180 | 8 | 277 | Prophage PSPPH03, Cro/CI family transcriptional regulator | Prophage PSPPH03, Cro/CI family transcriptional regulator | | afdb-uniprot50 | AF-A0A808X786-F1-MODEL\_V4 | 1.0 | 2.764e-07 | 300 | 0.257 | 105 | 73 | 3 | 79 | 180 | 62 | 164 | Helix-turn-helix transcriptional regulator | Helix-turn-helix transcriptional regulator | | afdb-uniprot50 | AF-A0A380RBK7-F1-MODEL\_V4 | 1.0 | 3.914e-07 | 300 | 0.288 | 104 | 72 | 2 | 78 | 180 | 71 | 173 | Peptidase S24-like | Peptidase S24-like | | afdb-uniprot50 | AF-A0A4V1P805-F1-MODEL\_V4 | 1.0 | 1.471e-10 | 300 | 0.219 | 219 | 130 | 6 | 1 | 180 | 1 | 217 | Peptidase\_S24 domain-containing protein | Peptidase\_S24 domain-containing protein | | afdb-uniprot50 | AF-A0A5A7NJR8-F1-MODEL\_V4 | 1.0 | 2.339e-10 | 300 | 0.179 | 223 | 137 | 8 | 1 | 180 | 1 | 220 | Peptidase\_S24 domain-containing protein | Peptidase\_S24 domain-containing protein | | afdb-uniprot50 | AF-A0A377QCZ4-F1-MODEL\_V4 | 1.0 | 8.729e-11 | 300 | 0.19 | 215 | 131 | 8 | 8 | 180 | 10 | 223 | Uncharacterized HTH-type transcriptional regulator HI\_1476 | Uncharacterized HTH-type transcriptional regulator HI\_1476 | | afdb-uniprot50 | AF-A0A256C1Y2-F1-MODEL\_V4 | 1.0 | 2.784e-10 | 300 | 0.156 | 223 | 137 | 6 | 4 | 180 | 6 | 223 | HTH cro/C1-type domain-containing protein | HTH cro/C1-type domain-containing protein | | afdb-uniprot50 | AF-A0A256CUE4-F1-MODEL\_V4 | 1.0 | 2.083e-10 | 300 | 0.172 | 232 | 135 | 5 | 4 | 180 | 5 | 234 | HTH cro/C1-type domain-containing protein | HTH cro/C1-type domain-containing protein | | afdb-uniprot50 | AF-A0A7W2R0E4-F1-MODEL\_V4 | 1.0 | 1.236e-10 | 300 | 0.187 | 235 | 130 | 4 | 4 | 180 | 7 | 238 | Helix-turn-helix transcriptional regulator | Helix-turn-helix transcriptional regulator | | afdb-uniprot50 | AF-B7J7H3-F1-MODEL\_V4 | 1.0 | 1.039e-10 | 300 | 0.231 | 229 | 123 | 5 | 4 | 180 | 6 | 233 | Transcriptional regulator, putative | Transcriptional regulator, putative | | afdb-uniprot50 | AF-A0A6B0DRA6-F1-MODEL\_V4 | 1.0 | 1.855e-10 | 300 | 0.221 | 217 | 126 | 7 | 4 | 180 | 49 | 262 | Helix-turn-helix domain-containing protein | Helix-turn-helix domain-containing protein | | afdb-uniprot50 | AF-A0A376PCJ4-F1-MODEL\_V4 | 1.0 | 2.929e-07 | 299 | 0.252 | 107 | 74 | 4 | 78 | 180 | 46 | 150 | Phage repressor protein CI | Phage repressor protein CI | | afdb-uniprot50 | AF-A0A4R1YUP2-F1-MODEL\_V4 | 1.0 | 1.952e-07 | 299 | 0.266 | 105 | 73 | 2 | 79 | 180 | 59 | 162 | Peptidase S24-like protein | Peptidase S24-like protein | | afdb-uniprot50 | AF-Q9CNL1-F1-MODEL\_V4 | 1.0 | 2.461e-07 | 299 | 0.285 | 105 | 69 | 4 | 80 | 180 | 113 | 215 | HTH Mu-type domain-containing protein | HTH Mu-type domain-containing protein | | afdb-uniprot50 | AF-A0A556UNU0-F1-MODEL\_V4 | 1.0 | 9.25e-11 | 299 | 0.229 | 196 | 112 | 6 | 19 | 180 | 28 | 218 | Helix-turn-helix transcriptional regulator | Helix-turn-helix transcriptional regulator | | afdb-uniprot50 | AF-A0A3G3L9K2-F1-MODEL\_V4 | 1.0 | 1.559e-10 | 299 | 0.184 | 222 | 132 | 6 | 4 | 178 | 7 | 226 | Helix-turn-helix transcriptional regulator | Helix-turn-helix transcriptional regulator | | afdb-uniprot50 | AF-A0A2V1H275-F1-MODEL\_V4 | 1.0 | 1.966e-10 | 299 | 0.196 | 229 | 131 | 4 | 4 | 180 | 6 | 233 | Phage repressor protein | Phage repressor protein | | afdb-uniprot50 | AF-A0A2G2PID2-F1-MODEL\_V4 | 1.0 | 8.237e-11 | 299 | 0.203 | 226 | 130 | 5 | 5 | 180 | 8 | 233 | XRE family transcriptional regulator | XRE family transcriptional regulator | | afdb-uniprot50 | AF-C4LBA5-F1-MODEL\_V4 | 1.0 | 1.039e-10 | 299 | 0.198 | 227 | 130 | 6 | 4 | 180 | 31 | 255 | Putative phage repressor | Putative phage repressor | | afdb-uniprot50 | AF-A0A7X4W2Y3-F1-MODEL\_V4 | 1.0 | 8.237e-11 | 299 | 0.206 | 242 | 124 | 8 | 4 | 180 | 23 | 261 | Helix-turn-helix domain-containing protein | Helix-turn-helix domain-containing protein | | afdb-uniprot50 | AF-A0A2W1MLG5-F1-MODEL\_V4 | 1.0 | 3.451e-11 | 299 | 0.19 | 278 | 123 | 7 | 4 | 180 | 6 | 282 | Transcriptional regulator | Transcriptional regulator | | afdb-uniprot50 | AF-A0A7H5FDH5-F1-MODEL\_V4 | 1.0 | 2.608e-07 | 298 | 0.254 | 106 | 75 | 2 | 78 | 180 | 9 | 113 | Cro/Cl family transcriptional regulator | Cro/Cl family transcriptional regulator | | afdb-uniprot50 | AF-A0A1N7AVQ2-F1-MODEL\_V4 | 1.0 | 2.764e-07 | 298 | 0.245 | 102 | 75 | 2 | 79 | 180 | 53 | 152 | Peptidase S24-like | Peptidase S24-like | | afdb-uniprot50 | AF-A0A7S7LXX0-F1-MODEL\_V4 | 1.0 | 2.207e-10 | 298 | 0.187 | 213 | 134 | 6 | 5 | 180 | 8 | 218 | LexA family transcriptional regulator | LexA family transcriptional regulator | | afdb-uniprot50 | AF-A0A031LXB0-F1-MODEL\_V4 | 1.0 | 1.855e-10 | 298 | 0.19 | 221 | 135 | 5 | 4 | 180 | 7 | 227 | Peptidase S24 | Peptidase S24 | | afdb-uniprot50 | AF-A0A380MRE3-F1-MODEL\_V4 | 1.0 | 1.855e-10 | 298 | 0.195 | 230 | 131 | 6 | 4 | 180 | 6 | 234 | Uncharacterized HTH-type transcriptional regulator HI\_1476 | Uncharacterized HTH-type transcriptional regulator HI\_1476 | | afdb-uniprot50 | AF-A0A625Y966-F1-MODEL\_V4 | 1.0 | 1.75e-10 | 298 | 0.2 | 220 | 131 | 8 | 4 | 180 | 13 | 230 | Helix-turn-helix transcriptional regulator | Helix-turn-helix transcriptional regulator | | afdb-uniprot50 | AF-A0LBS2-F1-MODEL\_V4 | 1.0 | 2.784e-10 | 298 | 0.181 | 232 | 132 | 8 | 4 | 180 | 36 | 264 | Putative phage repressor | Putative phage repressor | | afdb-uniprot50 | AF-Q82XF6-F1-MODEL\_V4 | 1.0 | 2.323e-07 | 298 | 0.261 | 107 | 75 | 2 | 78 | 180 | 157 | 263 | Peptidase\_S24 domain-containing protein | Peptidase\_S24 domain-containing protein | | afdb-uniprot50 | AF-A0A514BU83-F1-MODEL\_V4 | 1.0 | 7.773e-11 | 298 | 0.173 | 231 | 136 | 11 | 4 | 180 | 38 | 267 | Helix-turn-helix transcriptional regulator | Helix-turn-helix transcriptional regulator | | afdb-uniprot50 | AF-A0A5Y2E1G9-F1-MODEL\_V4 | 1.0 | 7.718e-08 | 297 | 0.31 | 116 | 73 | 4 | 68 | 180 | 5 | 116 | Helix-turn-helix transcriptional regulator | Helix-turn-helix transcriptional regulator | | afdb-uniprot50 | AF-A0A556SZ41-F1-MODEL\_V4 | 1.0 | 1.031e-07 | 297 | 0.279 | 111 | 72 | 4 | 71 | 178 | 14 | 119 | Helix-turn-helix transcriptional regulator | Helix-turn-helix transcriptional regulator | | afdb-uniprot50 | AF-A0A1W9QTR7-F1-MODEL\_V4 | 1.0 | 1.301e-07 | 297 | 0.3 | 103 | 71 | 1 | 78 | 180 | 51 | 152 | Phage repressor protein | Phage repressor protein | | afdb-uniprot50 | AF-A3NLY9-F1-MODEL\_V4 | 1.0 | 1.31e-10 | 297 | 0.211 | 217 | 127 | 8 | 4 | 179 | 7 | 220 | Helix-turn-helix motif:Peptidase S24, S26A and S26B | Helix-turn-helix motif:Peptidase S24, S26A and S26B | | afdb-uniprot50 | AF-E6W293-F1-MODEL\_V4 | 1.0 | 2.339e-10 | 297 | 0.2 | 220 | 130 | 7 | 5 | 180 | 7 | 224 | CI repressor | CI repressor | | afdb-uniprot50 | AF-A0A0D5LT23-F1-MODEL\_V4 | 1.0 | 3.313e-10 | 297 | 0.187 | 219 | 129 | 5 | 5 | 180 | 12 | 224 | Peptidase\_S24 domain-containing protein | Peptidase\_S24 domain-containing protein | | afdb-uniprot50 | AF-I3YGS2-F1-MODEL\_V4 | 1.0 | 1.166e-10 | 297 | 0.238 | 218 | 123 | 6 | 4 | 180 | 12 | 227 | Putative transcriptional regulator | Putative transcriptional regulator | | afdb-uniprot50 | AF-A0A6G9IAJ9-F1-MODEL\_V4 | 1.0 | 2.479e-10 | 297 | 0.178 | 207 | 137 | 7 | 5 | 180 | 28 | 232 | Helix-turn-helix transcriptional regulator | Helix-turn-helix transcriptional regulator | | afdb-uniprot50 | AF-A0A1V0B9I0-F1-MODEL\_V4 | 1.0 | 4.107e-11 | 297 | 0.19 | 231 | 132 | 7 | 4 | 180 | 14 | 243 | HTH cro/C1-type domain-containing protein | HTH cro/C1-type domain-containing protein | | afdb-uniprot50 | AF-A0A3N9U3X2-F1-MODEL\_V4 | 1.0 | 1.039e-10 | 297 | 0.224 | 227 | 125 | 8 | 4 | 180 | 22 | 247 | Helix-turn-helix domain-containing protein | Helix-turn-helix domain-containing protein | | afdb-uniprot50 | AF-A0A2M9G0Q7-F1-MODEL\_V4 | 1.0 | 1.236e-10 | 297 | 0.209 | 243 | 121 | 7 | 4 | 180 | 21 | 258 | HTH cro/C1-type domain-containing protein | HTH cro/C1-type domain-containing protein | | afdb-uniprot50 | AF-A0A1B6W082-F1-MODEL\_V4 | 1.0 | 4.396e-07 | 296 | 0.254 | 106 | 75 | 2 | 78 | 180 | 22 | 126 | Peptidase\_S24 domain-containing protein | Peptidase\_S24 domain-containing protein | | afdb-uniprot50 | AF-A0A560TXL0-F1-MODEL\_V4 | 1.0 | 8.179e-08 | 296 | 0.26 | 115 | 77 | 3 | 70 | 180 | 28 | 138 | Peptidase S24-like protein | Peptidase S24-like protein | | afdb-uniprot50 | AF-A0A212KJY0-F1-MODEL\_V4 | 1.0 | 3.486e-07 | 296 | 0.273 | 106 | 72 | 3 | 78 | 180 | 49 | 152 | Phage repressor | Phage repressor | | afdb-uniprot50 | AF-A0A1F9A3V9-F1-MODEL\_V4 | 1.0 | 4.396e-07 | 296 | 0.291 | 103 | 70 | 2 | 80 | 180 | 55 | 156 | Peptidase\_S24 domain-containing protein | Peptidase\_S24 domain-containing protein | | afdb-uniprot50 | AF-N8ZST7-F1-MODEL\_V4 | 1.0 | 1.559e-10 | 296 | 0.188 | 196 | 139 | 4 | 4 | 180 | 7 | 201 | HTH cro/C1-type domain-containing protein | HTH cro/C1-type domain-containing protein | | afdb-uniprot50 | AF-A0A377R622-F1-MODEL\_V4 | 1.0 | 1.039e-10 | 296 | 0.188 | 217 | 135 | 6 | 3 | 179 | 2 | 217 | Uncharacterized HTH-type transcriptional regulator HI\_1476 | Uncharacterized HTH-type transcriptional regulator HI\_1476 | | afdb-uniprot50 | AF-A0A839LDZ9-F1-MODEL\_V4 | 1.0 | 1.75e-10 | 296 | 0.217 | 207 | 130 | 5 | 4 | 180 | 18 | 222 | Helix-turn-helix domain-containing protein | Helix-turn-helix domain-containing protein | | afdb-uniprot50 | AF-U5DI73-F1-MODEL\_V4 | 1.0 | 6.269e-10 | 296 | 0.202 | 207 | 132 | 5 | 4 | 180 | 18 | 221 | Putative transcriptional regulator | Putative transcriptional regulator | | afdb-uniprot50 | AF-A0A7X4HWJ8-F1-MODEL\_V4 | 1.0 | 2.784e-10 | 296 | 0.179 | 228 | 134 | 5 | 4 | 180 | 7 | 232 | Helix-turn-helix domain-containing protein | Helix-turn-helix domain-containing protein | | afdb-uniprot50 | AF-A0A7W6EDZ3-F1-MODEL\_V4 | 1.0 | 3.126e-10 | 296 | 0.224 | 209 | 126 | 8 | 4 | 180 | 30 | 234 | Phage repressor protein C with HTH and peptisase S24 domain | Phage repressor protein C with HTH and peptisase S24 domain | | afdb-uniprot50 | AF-A0A1H5YB65-F1-MODEL\_V4 | 1.0 | 2.929e-07 | 295 | 0.261 | 107 | 74 | 2 | 79 | 180 | 29 | 135 | Peptidase S24-like | Peptidase S24-like | | afdb-uniprot50 | AF-A0A1I7CAN9-F1-MODEL\_V4 | 1.0 | 2.068e-07 | 295 | 0.28 | 114 | 74 | 3 | 70 | 180 | 28 | 136 | Peptidase S24-like | Peptidase S24-like | | afdb-uniprot50 | AF-A0A0D9LFD7-F1-MODEL\_V4 | 1.0 | 1.966e-10 | 295 | 0.207 | 212 | 131 | 7 | 4 | 180 | 7 | 216 | Peptidase S24-like protein | Peptidase S24-like protein | | afdb-uniprot50 | AF-R1F2E5-F1-MODEL\_V4 | 1.0 | 1.31e-10 | 295 | 0.22 | 218 | 126 | 7 | 4 | 179 | 7 | 222 | Prophage MuSo1, transcriptional regulator, Cro/CI family protein | Prophage MuSo1, transcriptional regulator, Cro/CI family protein | | afdb-uniprot50 | AF-A0A0Q6T7L7-F1-MODEL\_V4 | 1.0 | 1.471e-10 | 295 | 0.184 | 222 | 133 | 7 | 4 | 179 | 6 | 225 | HTH cro/C1-type domain-containing protein | HTH cro/C1-type domain-containing protein | | afdb-uniprot50 | AF-A0A680P814-F1-MODEL\_V4 | 1.0 | 2.784e-10 | 295 | 0.207 | 222 | 121 | 8 | 4 | 176 | 6 | 221 | Helix-turn-helix transcriptional regulator | Helix-turn-helix transcriptional regulator | | afdb-uniprot50 | AF-A0A7Y7MC10-F1-MODEL\_V4 | 1.0 | 2.339e-10 | 295 | 0.247 | 226 | 121 | 6 | 4 | 180 | 6 | 231 | Helix-turn-helix transcriptional regulator | Helix-turn-helix transcriptional regulator | | afdb-uniprot50 | AF-A0A1Q3VUE3-F1-MODEL\_V4 | 1.0 | 7.335e-11 | 295 | 0.195 | 210 | 126 | 7 | 5 | 177 | 28 | 231 | HTH cro/C1-type domain-containing protein | HTH cro/C1-type domain-containing protein | | afdb-uniprot50 | AF-A0A202B2B8-F1-MODEL\_V4 | 1.0 | 1.966e-10 | 295 | 0.214 | 219 | 126 | 7 | 4 | 178 | 20 | 236 | HTH cro/C1-type domain-containing protein | HTH cro/C1-type domain-containing protein | | afdb-uniprot50 | AF-F9RZN7-F1-MODEL\_V4 | 1.0 | 2.784e-10 | 295 | 0.159 | 264 | 133 | 6 | 4 | 180 | 7 | 268 | Prophage MuSo1, Cro/CI family transcriptional regulator | Prophage MuSo1, Cro/CI family transcriptional regulator | | afdb-uniprot50 | AF-A0A5P1R1X3-F1-MODEL\_V4 | 1.0 | 1.652e-10 | 295 | 0.197 | 233 | 128 | 6 | 4 | 180 | 53 | 282 | Helix-turn-helix domain-containing protein | Helix-turn-helix domain-containing protein | | afdb-uniprot50 | AF-A0A1H9EDS2-F1-MODEL\_V4 | 1.0 | 2.207e-10 | 295 | 0.155 | 296 | 129 | 6 | 4 | 180 | 6 | 299 | Phage repressor protein C, contains Cro/C1-type HTH and peptisase s24 domains | Phage repressor protein C, contains Cro/C1-type HTH and peptisase s24 domains | | afdb-uniprot50 | AF-A0A1V3RQU3-F1-MODEL\_V4 | 1.0 | 8.179e-08 | 294 | 0.274 | 124 | 82 | 4 | 60 | 180 | 2 | 120 | Peptidase\_S24 domain-containing protein | Peptidase\_S24 domain-containing protein | | afdb-uniprot50 | AF-A0A257JTL0-F1-MODEL\_V4 | 1.0 | 9.342e-07 | 294 | 0.245 | 106 | 76 | 2 | 78 | 180 | 66 | 170 | Transcriptional regulator | Transcriptional regulator | | afdb-uniprot50 | AF-A0A7L9WS46-F1-MODEL\_V4 | 1.0 | 3.289e-07 | 294 | 0.269 | 104 | 72 | 2 | 80 | 180 | 99 | 201 | Uncharacterized protein | Uncharacterized protein | | afdb-uniprot50 | AF-A0A256C4D7-F1-MODEL\_V4 | 1.0 | 2.339e-10 | 294 | 0.186 | 215 | 138 | 7 | 1 | 180 | 1 | 213 | Transcriptional regulator | Transcriptional regulator | | afdb-uniprot50 | AF-A0A2L0I076-F1-MODEL\_V4 | 1.0 | 2.627e-10 | 294 | 0.218 | 211 | 125 | 7 | 4 | 179 | 8 | 213 | Peptidase S24 | Peptidase S24 | | afdb-uniprot50 | AF-A0A832USA2-F1-MODEL\_V4 | 1.0 | 4.427e-10 | 294 | 0.165 | 218 | 136 | 6 | 4 | 180 | 9 | 221 | Helix-turn-helix transcriptional regulator | Helix-turn-helix transcriptional regulator | | afdb-uniprot50 | AF-A0A1I0CD38-F1-MODEL\_V4 | 1.0 | 3.126e-10 | 294 | 0.193 | 217 | 133 | 6 | 4 | 180 | 9 | 223 | Phage repressor protein C, contains Cro/C1-type HTH and peptisase s24 domains | Phage repressor protein C, contains Cro/C1-type HTH and peptisase s24 domains | | afdb-uniprot50 | AF-A0A2G6CHA8-F1-MODEL\_V4 | 1.0 | 2.339e-10 | 294 | 0.214 | 219 | 129 | 5 | 4 | 180 | 6 | 223 | Transcriptional regulator | Transcriptional regulator | | afdb-uniprot50 | AF-A0A425B209-F1-MODEL\_V4 | 1.0 | 2.339e-10 | 294 | 0.2 | 224 | 128 | 6 | 5 | 180 | 3 | 223 | XRE family transcriptional regulator | XRE family transcriptional regulator | | afdb-uniprot50 | AF-A0A1A7PW04-F1-MODEL\_V4 | 1.0 | 5.489e-11 | 294 | 0.173 | 225 | 136 | 8 | 4 | 180 | 7 | 229 | Repressor | Repressor | | afdb-uniprot50 | AF-A0A2T5R3U0-F1-MODEL\_V4 | 1.0 | 5.489e-11 | 294 | 0.238 | 226 | 116 | 9 | 5 | 180 | 15 | 234 | Phage repressor protein C with HTH and peptisase S24 domain | Phage repressor protein C with HTH and peptisase S24 domain | | afdb-uniprot50 | AF-A0A139SW86-F1-MODEL\_V4 | 1.0 | 2.207e-10 | 294 | 0.172 | 232 | 137 | 5 | 4 | 180 | 7 | 238 | HTH cro/C1-type domain-containing protein | HTH cro/C1-type domain-containing protein | | afdb-uniprot50 | AF-A0A246GMR8-F1-MODEL\_V4 | 1.0 | 1.855e-10 | 294 | 0.193 | 238 | 128 | 7 | 4 | 180 | 8 | 242 | XRE family transcriptional regulator | XRE family transcriptional regulator | | afdb-uniprot50 | AF-A0A5E9N793-F1-MODEL\_V4 | 1.0 | 1.75e-10 | 294 | 0.193 | 248 | 128 | 6 | 5 | 180 | 8 | 255 | Helix-turn-helix domain-containing protein | Helix-turn-helix domain-containing protein | | afdb-uniprot50 | AF-A0A486QK96-F1-MODEL\_V4 | 1.0 | 1.093e-07 | 293 | 0.268 | 123 | 84 | 3 | 61 | 180 | 4 | 123 | Putative phage repressor protein CI | Putative phage repressor protein CI | | afdb-uniprot50 | AF-A0A653B0F6-F1-MODEL\_V4 | 1.0 | 2.068e-07 | 293 | 0.2 | 105 | 80 | 2 | 79 | 180 | 49 | 152 | Phage repressor protein C, contains Cro/C1-type HTH and peptisase s24 domains | Phage repressor protein C, contains Cro/C1-type HTH and peptisase s24 domains | | afdb-uniprot50 | AF-A0A5C4SB52-F1-MODEL\_V4 | 1.0 | 4.148e-07 | 293 | 0.276 | 105 | 72 | 3 | 79 | 180 | 56 | 159 | S24 family peptidase | S24 family peptidase | | afdb-uniprot50 | AF-A0A2J4SU95-F1-MODEL\_V4 | 1.0 | 1.64e-07 | 293 | 0.283 | 113 | 73 | 4 | 71 | 180 | 68 | 175 | Phage repressor protein | Phage repressor protein | | afdb-uniprot50 | AF-A0A5D3WFX3-F1-MODEL\_V4 | 1.0 | 2.339e-10 | 293 | 0.255 | 215 | 115 | 10 | 4 | 180 | 7 | 214 | Phage repressor protein C with HTH and peptisase S24 domain | Phage repressor protein C with HTH and peptisase S24 domain | | afdb-uniprot50 | AF-A0A2M7FQV6-F1-MODEL\_V4 | 1.0 | 8.237e-11 | 293 | 0.199 | 226 | 128 | 5 | 1 | 178 | 3 | 223 | HTH cro/C1-type domain-containing protein | HTH cro/C1-type domain-containing protein | | afdb-uniprot50 | AF-A0A1Y2Q9C5-F1-MODEL\_V4 | 1.0 | 1.652e-10 | 293 | 0.185 | 216 | 133 | 5 | 4 | 178 | 7 | 220 | HTH cro/C1-type domain-containing protein | HTH cro/C1-type domain-containing protein | | afdb-uniprot50 | AF-E6W0K3-F1-MODEL\_V4 | 1.0 | 2.784e-10 | 293 | 0.208 | 221 | 130 | 5 | 4 | 180 | 5 | 224 | Helix-turn-helix domain protein | Helix-turn-helix domain protein | | afdb-uniprot50 | AF-A0A1V3JA05-F1-MODEL\_V4 | 1.0 | 2.784e-10 | 293 | 0.178 | 219 | 135 | 8 | 4 | 180 | 7 | 222 | Transcriptional regulator | Transcriptional regulator | | afdb-uniprot50 | AF-A0A357I973-F1-MODEL\_V4 | 1.0 | 1.75e-10 | 293 | 0.183 | 218 | 133 | 6 | 5 | 178 | 11 | 227 | HTH cro/C1-type domain-containing protein | HTH cro/C1-type domain-containing protein | | afdb-uniprot50 | AF-A0A6P1WJJ3-F1-MODEL\_V4 | 1.0 | 6.269e-10 | 293 | 0.196 | 224 | 130 | 6 | 5 | 180 | 6 | 227 | Helix-turn-helix transcriptional regulator | Helix-turn-helix transcriptional regulator | | afdb-uniprot50 | AF-A0A2G6CCY5-F1-MODEL\_V4 | 1.0 | 3.126e-10 | 293 | 0.218 | 229 | 122 | 7 | 4 | 180 | 10 | 233 | Transcriptional regulator | Transcriptional regulator | | afdb-uniprot50 | AF-A0A1H1QPG0-F1-MODEL\_V4 | 1.0 | 1.559e-10 | 293 | 0.161 | 223 | 140 | 6 | 4 | 180 | 19 | 240 | Phage repressor protein C, contains Cro/C1-type HTH and peptisase s24 domains | Phage repressor protein C, contains Cro/C1-type HTH and peptisase s24 domains | | afdb-uniprot50 | AF-A0A4R1MGX5-F1-MODEL\_V4 | 1.0 | 2.784e-10 | 293 | 0.185 | 242 | 129 | 5 | 4 | 178 | 7 | 247 | Phage repressor protein C with HTH and peptisase S24 domain | Phage repressor protein C with HTH and peptisase S24 domain | | afdb-uniprot50 | AF-A0A5D3YA70-F1-MODEL\_V4 | 1.0 | 6.12e-08 | 293 | 0.276 | 130 | 78 | 4 | 63 | 180 | 123 | 248 | Phage repressor protein C with HTH and peptisase S24 domain | Phage repressor protein C with HTH and peptisase S24 domain | | afdb-uniprot50 | AF-A0A1F0H5F4-F1-MODEL\_V4 | 1.0 | 3.876e-11 | 293 | 0.21 | 209 | 128 | 7 | 5 | 176 | 37 | 245 | HTH cro/C1-type domain-containing protein | HTH cro/C1-type domain-containing protein | | afdb-uniprot50 | AF-A0A516WA19-F1-MODEL\_V4 | 1.0 | 1.388e-10 | 293 | 0.177 | 225 | 128 | 6 | 5 | 179 | 50 | 267 | Helix-turn-helix transcriptional regulator | Helix-turn-helix transcriptional regulator | | afdb-uniprot50 | AF-A0A2C9D6H6-F1-MODEL\_V4 | 1.0 | 2.192e-07 | 292 | 0.272 | 110 | 73 | 4 | 77 | 180 | 75 | 183 | Pyocin repressor protein | Pyocin repressor protein | | afdb-uniprot50 | AF-A0A5B8KTY6-F1-MODEL\_V4 | 1.0 | 5.583e-10 | 292 | 0.207 | 217 | 132 | 6 | 1 | 180 | 1 | 214 | Helix-turn-helix transcriptional regulator | Helix-turn-helix transcriptional regulator | | afdb-uniprot50 | AF-B9JYW3-F1-MODEL\_V4 | 1.0 | 1.652e-10 | 292 | 0.187 | 219 | 139 | 6 | 1 | 180 | 1 | 219 | Transcriptional regulator | Transcriptional regulator | | afdb-uniprot50 | AF-A0A1B8PUD1-F1-MODEL\_V4 | 1.0 | 1.559e-10 | 292 | 0.165 | 218 | 135 | 6 | 6 | 178 | 9 | 224 | HTH cro/C1-type domain-containing protein | HTH cro/C1-type domain-containing protein | | afdb-uniprot50 | AF-A0A844RUM5-F1-MODEL\_V4 | 1.0 | 3.511e-10 | 292 | 0.221 | 217 | 126 | 6 | 4 | 180 | 12 | 225 | Helix-turn-helix domain-containing protein | Helix-turn-helix domain-containing protein | | afdb-uniprot50 | AF-A0A2Z4RAG6-F1-MODEL\_V4 | 1.0 | 1.471e-10 | 292 | 0.191 | 219 | 133 | 7 | 4 | 180 | 9 | 225 | Peptidase\_S24 domain-containing protein | Peptidase\_S24 domain-containing protein | | afdb-uniprot50 | AF-A0A6M4XFP4-F1-MODEL\_V4 | 1.0 | 2.95e-10 | 292 | 0.197 | 223 | 126 | 7 | 5 | 180 | 16 | 232 | Helix-turn-helix transcriptional regulator | Helix-turn-helix transcriptional regulator | | afdb-uniprot50 | AF-A0A6V8N692-F1-MODEL\_V4 | 1.0 | 3.126e-10 | 292 | 0.222 | 234 | 119 | 7 | 5 | 179 | 3 | 232 | Transcriptional regulator | Transcriptional regulator | | afdb-uniprot50 | AF-A0A653HZL2-F1-MODEL\_V4 | 1.0 | 8.878e-10 | 292 | 0.165 | 223 | 137 | 5 | 4 | 178 | 13 | 234 | HTH cro/C1-type domain-containing protein | HTH cro/C1-type domain-containing protein | | afdb-uniprot50 | AF-A0A354IYV1-F1-MODEL\_V4 | 1.0 | 2.339e-10 | 292 | 0.168 | 231 | 137 | 6 | 4 | 180 | 7 | 236 | Peptidase | Peptidase | | afdb-uniprot50 | AF-A1W671-F1-MODEL\_V4 | 1.0 | 2.437e-11 | 292 | 0.247 | 202 | 119 | 7 | 4 | 180 | 44 | 237 | Putative phage repressor | Putative phage repressor | | afdb-uniprot50 | AF-A0A7W4FX54-F1-MODEL\_V4 | 1.0 | 2.083e-10 | 292 | 0.234 | 230 | 123 | 5 | 4 | 180 | 8 | 237 | Helix-turn-helix transcriptional regulator | Helix-turn-helix transcriptional regulator | | afdb-uniprot50 | AF-A0A7Y7YNG6-F1-MODEL\_V4 | 1.0 | 4.178e-10 | 292 | 0.179 | 234 | 133 | 8 | 4 | 180 | 14 | 245 | Helix-turn-helix transcriptional regulator | Helix-turn-helix transcriptional regulator | | afdb-uniprot50 | AF-A0A0J5QMV2-F1-MODEL\_V4 | 1.0 | 8.729e-11 | 292 | 0.206 | 247 | 124 | 6 | 4 | 180 | 7 | 251 | Putative transcriptional regulator | Putative transcriptional regulator | | afdb-uniprot50 | AF-Q2RVM0-F1-MODEL\_V4 | 1.0 | 1.388e-10 | 292 | 0.21 | 237 | 127 | 8 | 2 | 180 | 31 | 265 | Phage repressor | Phage repressor | | afdb-uniprot50 | AF-E1SGK7-F1-MODEL\_V4 | 1.0 | 2.929e-07 | 291 | 0.263 | 114 | 77 | 4 | 70 | 180 | 30 | 139 | 26 kDa repressor protein (Regulatory protein CI) | 26 kDa repressor protein (Regulatory protein CI) | | afdb-uniprot50 | AF-A0A1G7D0E1-F1-MODEL\_V4 | 1.0 | 2.339e-10 | 291 | 0.271 | 199 | 116 | 6 | 4 | 180 | 6 | 197 | Peptidase S24-like | Peptidase S24-like | | afdb-uniprot50 | AF-A0A2D5S2B6-F1-MODEL\_V4 | 1.0 | 2.627e-10 | 291 | 0.204 | 205 | 131 | 6 | 4 | 180 | 12 | 212 | HTH cro/C1-type domain-containing protein | HTH cro/C1-type domain-containing protein | | afdb-uniprot50 | AF-A0A829P4G5-F1-MODEL\_V4 | 1.0 | 7.46e-10 | 291 | 0.201 | 208 | 133 | 3 | 6 | 180 | 10 | 217 | Peptidase S24 | Peptidase S24 | | afdb-uniprot50 | AF-A0A1Q8ZSD3-F1-MODEL\_V4 | 1.0 | 1.855e-10 | 291 | 0.191 | 219 | 138 | 6 | 1 | 180 | 1 | 219 | Transcriptional regulator | Transcriptional regulator | | afdb-uniprot50 | AF-A0A368ALS6-F1-MODEL\_V4 | 1.0 | 4.691e-10 | 291 | 0.192 | 213 | 132 | 6 | 4 | 180 | 11 | 219 | HTH cro/C1-type domain-containing protein | HTH cro/C1-type domain-containing protein | | afdb-uniprot50 | AF-A0A329E845-F1-MODEL\_V4 | 1.0 | 3.72e-10 | 291 | 0.2 | 215 | 133 | 4 | 4 | 180 | 7 | 220 | Phage repressor protein C with HTH and peptisase S24 domain | Phage repressor protein C with HTH and peptisase S24 domain | | afdb-uniprot50 | AF-A0A1Q5TGM7-F1-MODEL\_V4 | 1.0 | 2.95e-10 | 291 | 0.163 | 220 | 138 | 8 | 4 | 180 | 9 | 225 | Transcriptional regulator | Transcriptional regulator | | afdb-uniprot50 | AF-A0A0C5S4V4-F1-MODEL\_V4 | 1.0 | 2.784e-10 | 291 | 0.184 | 233 | 131 | 5 | 4 | 180 | 7 | 236 | XRE family transcriptional regulator | XRE family transcriptional regulator | | afdb-uniprot50 | AF-A0A2D5RVH7-F1-MODEL\_V4 | 1.0 | 2.339e-10 | 291 | 0.214 | 233 | 127 | 5 | 4 | 180 | 8 | 240 | XRE family transcriptional regulator | XRE family transcriptional regulator | | afdb-uniprot50 | AF-A0A829TAF0-F1-MODEL\_V4 | 1.0 | 2.083e-10 | 291 | 0.185 | 232 | 134 | 5 | 4 | 180 | 14 | 245 | Predicted transcriptional regulator | Predicted transcriptional regulator | | afdb-uniprot50 | AF-A0A7T1GX48-F1-MODEL\_V4 | 1.0 | 2.627e-10 | 291 | 0.192 | 229 | 132 | 8 | 4 | 180 | 18 | 245 | Helix-turn-helix domain-containing protein | Helix-turn-helix domain-containing protein | | afdb-uniprot50 | AF-A0A1H6KDH3-F1-MODEL\_V4 | 1.0 | 4.396e-07 | 291 | 0.235 | 106 | 76 | 3 | 78 | 180 | 152 | 255 | Peptidase S24-like | Peptidase S24-like | | afdb-uniprot50 | AF-A0A015NHE4-F1-MODEL\_V4 | 1.0 | 4.691e-10 | 291 | 0.176 | 249 | 132 | 7 | 4 | 180 | 7 | 254 | HTH cro/C1-type domain-containing protein | HTH cro/C1-type domain-containing protein | | afdb-uniprot50 | AF-A0A0J1HD31-F1-MODEL\_V4 | 1.0 | 3.72e-10 | 291 | 0.17 | 252 | 131 | 8 | 4 | 180 | 26 | 274 | Peptidase\_S24 domain-containing protein | Peptidase\_S24 domain-containing protein | | afdb-uniprot50 | AF-A0A1Y1IYP3-F1-MODEL\_V4 | 1.0 | 1.652e-10 | 291 | 0.193 | 222 | 129 | 10 | 2 | 180 | 106 | 320 | Prophage transcriptional regulator | Prophage transcriptional regulator | | afdb-uniprot50 | AF-A0A377R2D0-F1-MODEL\_V4 | 1.0 | 1.461e-07 | 290 | 0.268 | 123 | 85 | 3 | 60 | 179 | 5 | 125 | Uncharacterized HTH-type transcriptional regulator HI\_1476 | Uncharacterized HTH-type transcriptional regulator HI\_1476 | | afdb-uniprot50 | AF-A0A2N7RSW1-F1-MODEL\_V4 | 1.0 | 4.936e-07 | 290 | 0.235 | 102 | 76 | 2 | 79 | 180 | 30 | 129 | LexA repressor | LexA repressor | | afdb-uniprot50 | AF-A0A6B8QMJ2-F1-MODEL\_V4 | 1.0 | 6.597e-07 | 290 | 0.285 | 105 | 71 | 2 | 79 | 180 | 71 | 174 | S24 family peptidase | S24 family peptidase | | afdb-uniprot50 | AF-A0A7W0C9Y1-F1-MODEL\_V4 | 1.0 | 1.668e-06 | 290 | 0.234 | 94 | 72 | 0 | 87 | 180 | 82 | 175 | Phage repressor protein C with HTH and peptisase S24 domain | Phage repressor protein C with HTH and peptisase S24 domain | | afdb-uniprot50 | AF-A0A6C1MWJ7-F1-MODEL\_V4 | 1.0 | 5.583e-10 | 290 | 0.184 | 211 | 136 | 6 | 4 | 180 | 7 | 215 | LexA family transcriptional regulator | LexA family transcriptional regulator | | afdb-uniprot50 | AF-A0A2N9XVJ2-F1-MODEL\_V4 | 1.0 | 3.313e-10 | 290 | 0.188 | 218 | 134 | 5 | 4 | 180 | 5 | 220 | HTH cro/C1-type domain-containing protein | HTH cro/C1-type domain-containing protein | | afdb-uniprot50 | AF-A0A6F8QDC6-F1-MODEL\_V4 | 1.0 | 4.971e-10 | 290 | 0.165 | 224 | 138 | 7 | 6 | 180 | 2 | 225 | HTH cro/C1-type domain-containing protein | HTH cro/C1-type domain-containing protein | | afdb-uniprot50 | AF-A0A6N9DJF1-F1-MODEL\_V4 | 1.0 | 7.46e-10 | 290 | 0.174 | 212 | 137 | 6 | 4 | 180 | 25 | 233 | Helix-turn-helix domain-containing protein | Helix-turn-helix domain-containing protein | | afdb-uniprot50 | AF-A0A3A4NEQ1-F1-MODEL\_V4 | 1.0 | 2.784e-10 | 290 | 0.174 | 223 | 136 | 5 | 4 | 180 | 17 | 237 | Helix-turn-helix transcriptional regulator | Helix-turn-helix transcriptional regulator | | afdb-uniprot50 | AF-A0A3M3NJT7-F1-MODEL\_V4 | 1.0 | 2.083e-10 | 290 | 0.188 | 249 | 128 | 5 | 5 | 180 | 8 | 255 | HTH cro/C1-type domain-containing protein | HTH cro/C1-type domain-containing protein | | afdb-uniprot50 | AF-A0A8B5ZRH6-F1-MODEL\_V4 | 1.0 | 7.04e-10 | 290 | 0.157 | 228 | 140 | 5 | 4 | 180 | 30 | 256 | Helix-turn-helix transcriptional regulator | Helix-turn-helix transcriptional regulator | | afdb-uniprot50 | AF-A0A6M4XRT2-F1-MODEL\_V4 | 1.0 | 1.952e-07 | 289 | 0.28 | 114 | 74 | 3 | 70 | 180 | 1 | 109 | S24 family peptidase | S24 family peptidase | | afdb-uniprot50 | AF-A0A376TJ78-F1-MODEL\_V4 | 1.0 | 2.068e-07 | 289 | 0.283 | 113 | 74 | 3 | 71 | 180 | 48 | 156 | Regulatory protein CI from bacteriophage origin | Regulatory protein CI from bacteriophage origin | | afdb-uniprot50 | AF-A0A1E3WK34-F1-MODEL\_V4 | 1.0 | 3.511e-10 | 289 | 0.202 | 212 | 131 | 7 | 4 | 180 | 10 | 218 | HTH cro/C1-type domain-containing protein | HTH cro/C1-type domain-containing protein | | afdb-uniprot50 | AF-A0A127MKS3-F1-MODEL\_V4 | 1.0 | 4.971e-10 | 289 | 0.184 | 217 | 131 | 7 | 4 | 180 | 8 | 218 | Putative HTH-type transcriptional regulator | Putative HTH-type transcriptional regulator | | afdb-uniprot50 | AF-A0A496L3E2-F1-MODEL\_V4 | 1.0 | 4.691e-10 | 289 | 0.21 | 223 | 129 | 4 | 4 | 180 | 7 | 228 | Helix-turn-helix transcriptional regulator | Helix-turn-helix transcriptional regulator | | afdb-uniprot50 | AF-A0A261R781-F1-MODEL\_V4 | 1.0 | 3.72e-10 | 289 | 0.187 | 224 | 133 | 6 | 4 | 179 | 6 | 228 | HTH cro/C1-type domain-containing protein | HTH cro/C1-type domain-containing protein | | afdb-uniprot50 | AF-A0A443J1K1-F1-MODEL\_V4 | 1.0 | 2.339e-10 | 289 | 0.186 | 225 | 134 | 6 | 4 | 180 | 10 | 233 | LexA family transcriptional regulator | LexA family transcriptional regulator | | afdb-uniprot50 | AF-A0A198XTH8-F1-MODEL\_V4 | 1.0 | 3.313e-10 | 289 | 0.201 | 228 | 130 | 7 | 4 | 180 | 6 | 232 | Transcriptional regulator | Transcriptional regulator | | afdb-uniprot50 | AF-A0A7X3VZG0-F1-MODEL\_V4 | 1.0 | 1.855e-10 | 289 | 0.214 | 238 | 117 | 8 | 5 | 180 | 10 | 239 | Helix-turn-helix transcriptional regulator | Helix-turn-helix transcriptional regulator | | afdb-uniprot50 | AF-A0A3L0WWP8-F1-MODEL\_V4 | 1.0 | 1.855e-10 | 289 | 0.199 | 241 | 126 | 5 | 4 | 180 | 17 | 254 | LexA family transcriptional regulator | LexA family transcriptional regulator | | afdb-uniprot50 | AF-A0A6I6QEU7-F1-MODEL\_V4 | 1.0 | 1.166e-10 | 289 | 0.18 | 271 | 127 | 5 | 4 | 180 | 6 | 275 | Helix-turn-helix domain-containing protein | Helix-turn-helix domain-containing protein | | afdb-uniprot50 | AF-A0A5E9U8S2-F1-MODEL\_V4 | 1.0 | 4.971e-10 | 289 | 0.149 | 267 | 136 | 4 | 4 | 180 | 16 | 281 | Helix-turn-helix domain-containing protein | Helix-turn-helix domain-containing protein | | afdb-uniprot50 | AF-A0A251ZV98-F1-MODEL\_V4 | 1.0 | 5.874e-07 | 288 | 0.311 | 93 | 60 | 2 | 87 | 176 | 5 | 96 | Repressor | Repressor | | afdb-uniprot50 | AF-A0A0Q4PTP6-F1-MODEL\_V4 | 1.0 | 2.461e-07 | 288 | 0.22 | 118 | 85 | 3 | 65 | 180 | 2 | 114 | Peptidase\_S24 domain-containing protein | Peptidase\_S24 domain-containing protein | | afdb-uniprot50 | AF-K1JKB5-F1-MODEL\_V4 | 1.0 | 1.227e-07 | 288 | 0.243 | 123 | 84 | 4 | 61 | 180 | 3 | 119 | Peptidase\_S24 domain-containing protein | Peptidase\_S24 domain-containing protein | | afdb-uniprot50 | AF-A0A7H4P4T5-F1-MODEL\_V4 | 1.0 | 2.323e-07 | 288 | 0.283 | 113 | 73 | 4 | 71 | 180 | 33 | 140 | Phage repressor | Phage repressor | | afdb-uniprot50 | AF-A0A1E4EGW2-F1-MODEL\_V4 | 1.0 | 6.225e-07 | 288 | 0.266 | 105 | 73 | 2 | 79 | 180 | 43 | 146 | Peptidase\_S24 domain-containing protein | Peptidase\_S24 domain-containing protein | | afdb-uniprot50 | AF-A0A515EKI5-F1-MODEL\_V4 | 1.0 | 5.231e-07 | 288 | 0.219 | 105 | 78 | 2 | 79 | 180 | 50 | 153 | Helix-turn-helix transcriptional regulator | Helix-turn-helix transcriptional regulator | | afdb-uniprot50 | AF-A0A5K1I4S0-F1-MODEL\_V4 | 1.0 | 4.658e-07 | 288 | 0.262 | 103 | 73 | 1 | 78 | 177 | 60 | 162 | HTH-type transcriptional regulator PrtR | HTH-type transcriptional regulator PrtR | | afdb-uniprot50 | AF-A0A7H5FFX3-F1-MODEL\_V4 | 1.0 | 6.99e-07 | 288 | 0.235 | 102 | 78 | 0 | 79 | 180 | 79 | 180 | Transcriptional regulator | Transcriptional regulator | | afdb-uniprot50 | AF-N9N570-F1-MODEL\_V4 | 1.0 | 3.511e-10 | 288 | 0.198 | 191 | 134 | 3 | 4 | 180 | 7 | 192 | HTH cro/C1-type domain-containing protein | HTH cro/C1-type domain-containing protein | | afdb-uniprot50 | AF-A0A285TSU5-F1-MODEL\_V4 | 1.0 | 4.691e-10 | 288 | 0.171 | 216 | 137 | 7 | 5 | 180 | 7 | 220 | Phage repressor protein C, contains Cro/C1-type HTH and peptisase s24 domains | Phage repressor protein C, contains Cro/C1-type HTH and peptisase s24 domains | | afdb-uniprot50 | AF-A0A1X0TJB0-F1-MODEL\_V4 | 1.0 | 1.388e-10 | 288 | 0.204 | 220 | 123 | 5 | 5 | 180 | 9 | 220 | HTH cro/C1-type domain-containing protein | HTH cro/C1-type domain-containing protein | | afdb-uniprot50 | AF-A0A2N9XSA8-F1-MODEL\_V4 | 1.0 | 5.583e-10 | 288 | 0.14 | 228 | 142 | 5 | 6 | 180 | 3 | 229 | HTH cro/C1-type domain-containing protein | HTH cro/C1-type domain-containing protein | | afdb-uniprot50 | AF-A0A376I3L8-F1-MODEL\_V4 | 1.0 | 4.691e-10 | 288 | 0.17 | 211 | 140 | 6 | 4 | 180 | 22 | 231 | DNA-binding/peptidase S24 domain protein | DNA-binding/peptidase S24 domain protein | | afdb-uniprot50 | AF-A0A3B0MMP0-F1-MODEL\_V4 | 1.0 | 3.313e-10 | 288 | 0.149 | 228 | 142 | 4 | 4 | 180 | 20 | 246 | HTH-type transcriptional regulator PrtR | HTH-type transcriptional regulator PrtR | | afdb-uniprot50 | AF-A0A5C8AJZ0-F1-MODEL\_V4 | 1.0 | 3.511e-10 | 288 | 0.194 | 252 | 124 | 6 | 4 | 180 | 7 | 254 | XRE family transcriptional regulator | XRE family transcriptional regulator | | afdb-uniprot50 | AF-A0A6L4AN93-F1-MODEL\_V4 | 1.0 | 8.878e-10 | 288 | 0.18 | 210 | 136 | 4 | 4 | 180 | 68 | 274 | Helix-turn-helix transcriptional regulator | Helix-turn-helix transcriptional regulator | | afdb-uniprot50 | AF-A0A510U6Q2-F1-MODEL\_V4 | 1.0 | 2.479e-10 | 287 | 0.213 | 183 | 135 | 4 | 5 | 180 | 15 | 195 | HTH cro/C1-type domain-containing protein | HTH cro/C1-type domain-containing protein | | afdb-uniprot50 | AF-F9T596-F1-MODEL\_V4 | 1.0 | 3.511e-10 | 287 | 0.211 | 208 | 126 | 4 | 4 | 180 | 7 | 207 | Repressor | Repressor | | afdb-uniprot50 | AF-A0A640W7B7-F1-MODEL\_V4 | 1.0 | 4.178e-10 | 287 | 0.166 | 210 | 136 | 7 | 4 | 180 | 11 | 214 | Helix-turn-helix domain-containing protein | Helix-turn-helix domain-containing protein | | afdb-uniprot50 | AF-A0A099KPN4-F1-MODEL\_V4 | 1.0 | 2.479e-10 | 287 | 0.211 | 227 | 122 | 9 | 1 | 180 | 1 | 217 | Putative phage repressor | Putative phage repressor | | afdb-uniprot50 | AF-A0A418WU97-F1-MODEL\_V4 | 1.0 | 3.126e-10 | 287 | 0.196 | 214 | 133 | 4 | 4 | 180 | 6 | 217 | XRE family transcriptional regulator | XRE family transcriptional regulator | | afdb-uniprot50 | AF-A0A7V2F687-F1-MODEL\_V4 | 1.0 | 2.784e-10 | 287 | 0.218 | 224 | 125 | 6 | 4 | 180 | 8 | 228 | Helix-turn-helix transcriptional regulator | Helix-turn-helix transcriptional regulator | | afdb-uniprot50 | AF-A0A2N2R9N5-F1-MODEL\_V4 | 1.0 | 4.612e-11 | 287 | 0.26 | 196 | 124 | 7 | 4 | 180 | 34 | 227 | Peptidase S24 | Peptidase S24 | | afdb-uniprot50 | AF-A0A7W2VIL3-F1-MODEL\_V4 | 1.0 | 8.378e-10 | 287 | 0.223 | 219 | 127 | 6 | 4 | 180 | 18 | 235 | Helix-turn-helix transcriptional regulator | Helix-turn-helix transcriptional regulator | | afdb-uniprot50 | AF-A0A2D6RTW2-F1-MODEL\_V4 | 1.0 | 2.95e-10 | 287 | 0.175 | 240 | 134 | 8 | 5 | 180 | 4 | 243 | Phage repressor protein | Phage repressor protein | | afdb-uniprot50 | AF-A0A1C3H9I3-F1-MODEL\_V4 | 1.0 | 5.916e-10 | 287 | 0.197 | 228 | 131 | 4 | 5 | 180 | 19 | 246 | HTH-type transcriptional regulator PrtR | HTH-type transcriptional regulator PrtR | | afdb-uniprot50 | AF-A0A2E7Q619-F1-MODEL\_V4 | 1.0 | 8.378e-10 | 287 | 0.213 | 230 | 123 | 9 | 5 | 180 | 40 | 265 | HTH cro/C1-type domain-containing protein | HTH cro/C1-type domain-containing protein | | afdb-uniprot50 | AF-A0A2I2MFF4-F1-MODEL\_V4 | 1.0 | 2.784e-10 | 287 | 0.193 | 233 | 130 | 8 | 4 | 180 | 106 | 336 | Helix-turn-helix protein | Helix-turn-helix protein | | afdb-uniprot50 | AF-A0A377M491-F1-MODEL\_V4 | 1.0 | 3.694e-07 | 286 | 0.271 | 114 | 76 | 4 | 70 | 180 | 10 | 119 | Putative repressor protein | Putative repressor protein | | afdb-uniprot50 | AF-A0A0Q4G0B2-F1-MODEL\_V4 | 1.0 | 6.99e-07 | 286 | 0.264 | 106 | 73 | 2 | 79 | 179 | 54 | 159 | Peptidase\_S24 domain-containing protein | Peptidase\_S24 domain-containing protein | | afdb-uniprot50 | AF-A0A5C7FWN9-F1-MODEL\_V4 | 1.0 | 5.231e-07 | 286 | 0.266 | 105 | 72 | 3 | 79 | 180 | 64 | 166 | S24 family peptidase | S24 family peptidase | | afdb-uniprot50 | AF-A0A1C3H412-F1-MODEL\_V4 | 1.0 | 4.148e-07 | 286 | 0.245 | 106 | 72 | 3 | 79 | 178 | 70 | 173 | Phage repressor | Phage repressor | | afdb-uniprot50 | AF-A0A264VM17-F1-MODEL\_V4 | 1.0 | 3.486e-07 | 286 | 0.223 | 112 | 80 | 4 | 72 | 180 | 77 | 184 | Peptidase\_S24 domain-containing protein | Peptidase\_S24 domain-containing protein | | afdb-uniprot50 | AF-A0A660NC27-F1-MODEL\_V4 | 1.0 | 5.583e-10 | 286 | 0.183 | 218 | 134 | 7 | 4 | 180 | 6 | 220 | S24 family peptidase | S24 family peptidase | | afdb-uniprot50 | AF-Q72BW5-F1-MODEL\_V4 | 1.0 | 1.559e-10 | 286 | 0.205 | 209 | 127 | 8 | 5 | 180 | 22 | 224 | Transcriptional regulator, putative | Transcriptional regulator, putative | | afdb-uniprot50 | AF-A0A220S1V6-F1-MODEL\_V4 | 1.0 | 2.479e-10 | 286 | 0.171 | 228 | 137 | 6 | 4 | 180 | 3 | 229 | Chromophore lyase | Chromophore lyase | | afdb-uniprot50 | AF-A0A5E4V8H1-F1-MODEL\_V4 | 1.0 | 4.971e-10 | 286 | 0.181 | 215 | 128 | 7 | 4 | 180 | 31 | 235 | Putative HTH-type transcriptional regulator | Putative HTH-type transcriptional regulator | | afdb-uniprot50 | AF-A0A7L5XVV2-F1-MODEL\_V4 | 1.0 | 4.691e-10 | 286 | 0.211 | 213 | 131 | 5 | 4 | 180 | 33 | 244 | Helix-turn-helix transcriptional regulator | Helix-turn-helix transcriptional regulator | | afdb-uniprot50 | AF-A0A1V3NCJ2-F1-MODEL\_V4 | 1.0 | 1.257e-09 | 286 | 0.202 | 222 | 129 | 6 | 5 | 180 | 29 | 248 | Peptidase\_S24 domain-containing protein | Peptidase\_S24 domain-containing protein | | afdb-uniprot50 | AF-A0A7T7Q6W5-F1-MODEL\_V4 | 1.0 | 3.72e-10 | 286 | 0.175 | 239 | 134 | 6 | 5 | 180 | 10 | 248 | Helix-turn-helix transcriptional regulator | Helix-turn-helix transcriptional regulator | | afdb-uniprot50 | AF-A0A0P7CEB0-F1-MODEL\_V4 | 1.0 | 3.72e-10 | 286 | 0.194 | 252 | 124 | 6 | 4 | 180 | 7 | 254 | XRE family transcriptional regulator | XRE family transcriptional regulator | | afdb-uniprot50 | AF-A0A7R7BN55-F1-MODEL\_V4 | 1.0 | 4.178e-10 | 285 | 0.198 | 202 | 136 | 4 | 1 | 180 | 1 | 198 | Uncharacterized protein | Uncharacterized protein | | afdb-uniprot50 | AF-A0A1V4T976-F1-MODEL\_V4 | 1.0 | 6.269e-10 | 285 | 0.2 | 215 | 132 | 6 | 4 | 180 | 5 | 217 | Peptidase\_S24 domain-containing protein | Peptidase\_S24 domain-containing protein | | afdb-uniprot50 | AF-A0A3S8YQ18-F1-MODEL\_V4 | 1.0 | 7.906e-10 | 285 | 0.223 | 210 | 125 | 9 | 4 | 180 | 13 | 217 | S24 family peptidase | S24 family peptidase | | afdb-uniprot50 | AF-A0A6G8YM25-F1-MODEL\_V4 | 1.0 | 1.471e-10 | 285 | 0.202 | 217 | 127 | 10 | 5 | 180 | 11 | 222 | Peptidase\_S24 domain-containing protein | Peptidase\_S24 domain-containing protein | | afdb-uniprot50 | AF-A0A7X2KYD5-F1-MODEL\_V4 | 1.0 | 1.323e-06 | 285 | 0.186 | 102 | 83 | 0 | 79 | 180 | 136 | 237 | DUF723 domain-containing protein | DUF723 domain-containing protein | | afdb-uniprot50 | AF-A0A7D7NB06-F1-MODEL\_V4 | 1.0 | 3.126e-10 | 285 | 0.149 | 234 | 141 | 5 | 4 | 180 | 5 | 237 | Chromophore lyase | Chromophore lyase | | afdb-uniprot50 | AF-A0A1B3JHZ4-F1-MODEL\_V4 | 1.0 | 2.784e-10 | 285 | 0.152 | 242 | 139 | 9 | 4 | 180 | 7 | 247 | Phage repressor | Phage repressor | | afdb-uniprot50 | AF-A0A4Q0YMX5-F1-MODEL\_V4 | 1.0 | 4.427e-10 | 285 | 0.202 | 227 | 130 | 7 | 4 | 180 | 91 | 316 | Uncharacterized protein | Uncharacterized protein | | afdb-uniprot50 | AF-A0A2N0WL24-F1-MODEL\_V4 | 1.0 | 1.64e-07 | 284 | 0.278 | 104 | 72 | 3 | 79 | 180 | 5 | 107 | Transcriptional regulator | Transcriptional regulator | | afdb-uniprot50 | AF-A0A1Z4C411-F1-MODEL\_V4 | 1.0 | 1.093e-07 | 284 | 0.316 | 117 | 71 | 4 | 65 | 177 | 4 | 115 | Peptidase\_S24 domain-containing protein | Peptidase\_S24 domain-containing protein | | afdb-uniprot50 | AF-W1ED85-F1-MODEL\_V4 | 1.0 | 3.289e-07 | 284 | 0.298 | 114 | 74 | 3 | 70 | 180 | 7 | 117 | COG2932: Predicted transcriptional regulator | COG2932: Predicted transcriptional regulator | | afdb-uniprot50 | AF-X1P3M5-F1-MODEL\_V4 | 1.0 | 4.148e-07 | 284 | 0.247 | 113 | 79 | 3 | 71 | 180 | 27 | 136 | Peptidase\_S24 domain-containing protein | Peptidase\_S24 domain-containing protein | | afdb-uniprot50 | AF-A0A0H3EGA9-F1-MODEL\_V4 | 1.0 | 3.313e-10 | 284 | 0.224 | 209 | 118 | 7 | 14 | 180 | 1 | 207 | Regulatory protein CI from bacteriophage origin | Regulatory protein CI from bacteriophage origin | | afdb-uniprot50 | AF-U7H6U4-F1-MODEL\_V4 | 1.0 | 2.207e-10 | 284 | 0.228 | 219 | 121 | 6 | 6 | 180 | 3 | 217 | HTH cro/C1-type domain-containing protein | HTH cro/C1-type domain-containing protein | | afdb-uniprot50 | AF-A0A7W7Y2E7-F1-MODEL\_V4 | 1.0 | 9.408e-10 | 284 | 0.208 | 216 | 128 | 8 | 5 | 180 | 7 | 219 | Phage repressor protein C with HTH and peptisase S24 domain | Phage repressor protein C with HTH and peptisase S24 domain | | afdb-uniprot50 | AF-A0A2X4G6V1-F1-MODEL\_V4 | 1.0 | 5.874e-07 | 284 | 0.252 | 107 | 74 | 6 | 78 | 180 | 113 | 217 | Peptidase S24-like protein | Peptidase S24-like protein | | afdb-uniprot50 | AF-A0A7W4B885-F1-MODEL\_V4 | 1.0 | 3.72e-10 | 284 | 0.201 | 233 | 130 | 7 | 1 | 180 | 1 | 230 | LexA family transcriptional regulator | LexA family transcriptional regulator | | afdb-uniprot50 | AF-A0A0H2MCK7-F1-MODEL\_V4 | 1.0 | 4.178e-10 | 284 | 0.186 | 215 | 136 | 6 | 4 | 180 | 18 | 231 | Putative HTH-type transcriptional regulator | Putative HTH-type transcriptional regulator | | afdb-uniprot50 | AF-A0A147GPB6-F1-MODEL\_V4 | 1.0 | 5.916e-10 | 284 | 0.168 | 225 | 139 | 8 | 4 | 180 | 8 | 232 | HTH cro/C1-type domain-containing protein | HTH cro/C1-type domain-containing protein | | afdb-uniprot50 | AF-A0A4Q5YJ50-F1-MODEL\_V4 | 1.0 | 2.627e-10 | 284 | 0.216 | 231 | 127 | 5 | 4 | 180 | 6 | 236 | Helix-turn-helix transcriptional regulator | Helix-turn-helix transcriptional regulator | | afdb-uniprot50 | AF-A4JD17-F1-MODEL\_V4 | 1.0 | 3.942e-10 | 284 | 0.222 | 225 | 126 | 10 | 4 | 180 | 15 | 238 | Putative phage repressor | Putative phage repressor | | afdb-uniprot50 | AF-N9M239-F1-MODEL\_V4 | 1.0 | 4.691e-10 | 284 | 0.193 | 233 | 129 | 6 | 4 | 178 | 7 | 238 | HTH cro/C1-type domain-containing protein | HTH cro/C1-type domain-containing protein | | afdb-uniprot50 | AF-A0A3S0MVQ0-F1-MODEL\_V4 | 1.0 | 4.971e-10 | 284 | 0.213 | 229 | 127 | 6 | 5 | 180 | 14 | 242 | XRE family transcriptional regulator | XRE family transcriptional regulator | | afdb-uniprot50 | AF-A0A6P2FI33-F1-MODEL\_V4 | 1.0 | 4.178e-10 | 284 | 0.172 | 232 | 133 | 9 | 5 | 179 | 22 | 251 | Pyocin repressor protein | Pyocin repressor protein | | afdb-uniprot50 | AF-A0A060H7F8-F1-MODEL\_V4 | 1.0 | 3.72e-10 | 284 | 0.205 | 229 | 125 | 9 | 5 | 178 | 29 | 255 | Peptidase S24 | Peptidase S24 | | afdb-uniprot50 | AF-A0A2S6NDQ8-F1-MODEL\_V4 | 1.0 | 5.916e-10 | 284 | 0.165 | 224 | 138 | 5 | 5 | 180 | 44 | 266 | HTH cro/C1-type domain-containing protein | HTH cro/C1-type domain-containing protein | | afdb-uniprot50 | AF-A0A6M3M5L7-F1-MODEL\_V4 | 1.0 | 1.559e-10 | 284 | 0.166 | 265 | 132 | 6 | 4 | 180 | 6 | 269 | Putative peptidase | Putative peptidase | | afdb-uniprot50 | AF-A0A1Y0G6S2-F1-MODEL\_V4 | 1.0 | 6.597e-07 | 284 | 0.31 | 100 | 65 | 2 | 78 | 173 | 201 | 300 | Peptidase\_S24 domain-containing protein | Peptidase\_S24 domain-containing protein | | afdb-uniprot50 | AF-A0A6B1DL16-F1-MODEL\_V4 | 1.0 | 2.339e-10 | 284 | 0.204 | 230 | 130 | 4 | 4 | 180 | 163 | 392 | Helix-turn-helix domain-containing protein | Helix-turn-helix domain-containing protein | | afdb-uniprot50 | AF-A0A429MKG1-F1-MODEL\_V4 | 1.0 | 5.231e-07 | 283 | 0.214 | 112 | 80 | 3 | 70 | 178 | 28 | 134 | S24 family peptidase | S24 family peptidase | | afdb-uniprot50 | AF-A0A839VM87-F1-MODEL\_V4 | 1.0 | 5.874e-07 | 283 | 0.247 | 105 | 74 | 3 | 79 | 180 | 100 | 202 | Uncharacterized protein | Uncharacterized protein | | afdb-uniprot50 | AF-A0A379LIF7-F1-MODEL\_V4 | 1.0 | 8.878e-10 | 283 | 0.159 | 226 | 135 | 8 | 5 | 178 | 10 | 232 | Uncharacterized HTH-type transcriptional regulator HI\_1476 | Uncharacterized HTH-type transcriptional regulator HI\_1476 | | afdb-uniprot50 | AF-A0A3D0LWT4-F1-MODEL\_V4 | 1.0 | 9.408e-10 | 283 | 0.195 | 230 | 130 | 8 | 4 | 180 | 6 | 233 | Peptidase\_S24 domain-containing protein | Peptidase\_S24 domain-containing protein | | afdb-uniprot50 | AF-A0A7G2S2Y5-F1-MODEL\_V4 | 1.0 | 2.627e-10 | 283 | 0.189 | 227 | 133 | 6 | 5 | 180 | 10 | 236 | XRE family transcriptional regulator | XRE family transcriptional regulator | | afdb-uniprot50 | AF-Q9I551-F1-MODEL\_V4 | 1.0 | 7.04e-10 | 283 | 0.206 | 233 | 128 | 5 | 4 | 180 | 6 | 237 | Probable transcriptional regulator | Probable transcriptional regulator | | afdb-uniprot50 | AF-A0A443LNA1-F1-MODEL\_V4 | 1.0 | 5.268e-10 | 283 | 0.2 | 225 | 131 | 7 | 4 | 180 | 15 | 238 | LexA family transcriptional regulator | LexA family transcriptional regulator | | afdb-uniprot50 | AF-A0A5D9DBC8-F1-MODEL\_V4 | 1.0 | 6.269e-10 | 283 | 0.195 | 235 | 125 | 6 | 4 | 177 | 68 | 299 | Helix-turn-helix transcriptional regulator | Helix-turn-helix transcriptional regulator | | afdb-uniprot50 | AF-V9H5H0-F1-MODEL\_V4 | 1.0 | 1.057e-09 | 282 | 0.205 | 200 | 135 | 8 | 1 | 180 | 1 | 196 | Peptidase\_S24 domain-containing protein | Peptidase\_S24 domain-containing protein | | afdb-uniprot50 | AF-A0A7T0BVD4-F1-MODEL\_V4 | 1.0 | 5.583e-10 | 282 | 0.19 | 221 | 132 | 7 | 5 | 180 | 3 | 221 | Helix-turn-helix transcriptional regulator | Helix-turn-helix transcriptional regulator | | afdb-uniprot50 | AF-A0A3B0IWS9-F1-MODEL\_V4 | 1.0 | 4.178e-10 | 282 | 0.157 | 228 | 139 | 5 | 4 | 180 | 6 | 231 | HTH-type transcriptional regulator PrtR | HTH-type transcriptional regulator PrtR | | afdb-uniprot50 | AF-A0A0N9VWL4-F1-MODEL\_V4 | 1.0 | 1.652e-10 | 282 | 0.173 | 236 | 133 | 7 | 6 | 180 | 6 | 240 | HTH cro/C1-type domain-containing protein | HTH cro/C1-type domain-containing protein | | afdb-uniprot50 | AF-A0A6V8IAU7-F1-MODEL\_V4 | 1.0 | 2.95e-10 | 282 | 0.206 | 232 | 118 | 8 | 4 | 180 | 19 | 239 | Putative HTH-type transcriptional regulator | Putative HTH-type transcriptional regulator | | afdb-uniprot50 | AF-A0A5C8WGV8-F1-MODEL\_V4 | 1.0 | 9.408e-10 | 282 | 0.193 | 232 | 126 | 6 | 4 | 180 | 20 | 245 | LexA family transcriptional regulator | LexA family transcriptional regulator | | afdb-uniprot50 | AF-C0N2Z7-F1-MODEL\_V4 | 1.0 | 1.057e-09 | 282 | 0.181 | 265 | 127 | 6 | 4 | 180 | 10 | 272 | Peptidase S24-like domain protein | Peptidase S24-like domain protein | | afdb-uniprot50 | AF-Q3JTQ6-F1-MODEL\_V4 | 1.0 | 4.178e-10 | 282 | 0.211 | 208 | 130 | 6 | 5 | 180 | 84 | 289 | Repressor protein, putative | Repressor protein, putative | | afdb-uniprot50 | AF-A0A511FR38-F1-MODEL\_V4 | 1.0 | 3.72e-10 | 281 | 0.217 | 198 | 125 | 7 | 4 | 175 | 15 | 208 | Repressor | Repressor | | afdb-uniprot50 | AF-A0A255ZI18-F1-MODEL\_V4 | 1.0 | 5.583e-10 | 281 | 0.179 | 217 | 129 | 5 | 5 | 179 | 7 | 216 | HTH cro/C1-type domain-containing protein | HTH cro/C1-type domain-containing protein | | afdb-uniprot50 | AF-A0A0S4TX81-F1-MODEL\_V4 | 1.0 | 2.627e-10 | 281 | 0.215 | 218 | 127 | 7 | 5 | 180 | 6 | 221 | Peptidase\_S24 domain-containing protein | Peptidase\_S24 domain-containing protein | | afdb-uniprot50 | AF-A0A7C4YFR3-F1-MODEL\_V4 | 1.0 | 1.559e-10 | 281 | 0.222 | 225 | 128 | 9 | 1 | 180 | 3 | 225 | Helix-turn-helix transcriptional regulator | Helix-turn-helix transcriptional regulator | | afdb-uniprot50 | AF-A0A1H0LEQ9-F1-MODEL\_V4 | 1.0 | 4.178e-10 | 281 | 0.229 | 227 | 126 | 7 | 1 | 180 | 1 | 225 | Phage repressor protein C, contains Cro/C1-type HTH and peptisase s24 domains | Phage repressor protein C, contains Cro/C1-type HTH and peptisase s24 domains | | afdb-uniprot50 | AF-A3JLF3-F1-MODEL\_V4 | 1.0 | 2.245e-09 | 281 | 0.163 | 226 | 139 | 5 | 4 | 180 | 13 | 237 | Probable transcriptional regulator | Probable transcriptional regulator | | afdb-uniprot50 | AF-A0A1C3ELF7-F1-MODEL\_V4 | 1.0 | 5.916e-10 | 281 | 0.167 | 233 | 137 | 6 | 4 | 180 | 7 | 238 | XRE family transcriptional regulator | XRE family transcriptional regulator | | afdb-uniprot50 | AF-A0A7Z0UXS2-F1-MODEL\_V4 | 1.0 | 7.46e-10 | 281 | 0.132 | 242 | 145 | 5 | 4 | 180 | 10 | 251 | Transcriptional regulator | Transcriptional regulator | | afdb-uniprot50 | AF-A0A7U2YMF5-F1-MODEL\_V4 | 1.0 | 1.057e-09 | 281 | 0.18 | 238 | 132 | 8 | 5 | 180 | 19 | 255 | Peptidase\_S24 domain-containing protein | Peptidase\_S24 domain-containing protein | | afdb-uniprot50 | AF-A0A554U561-F1-MODEL\_V4 | 1.0 | 1.057e-09 | 281 | 0.164 | 255 | 133 | 8 | 4 | 180 | 5 | 257 | LexA family transcriptional regulator | LexA family transcriptional regulator | | afdb-uniprot50 | AF-A0A1Q9R2D7-F1-MODEL\_V4 | 1.0 | 3.511e-10 | 281 | 0.158 | 240 | 137 | 5 | 6 | 180 | 22 | 261 | HTH-type transcriptional regulator PrtR | HTH-type transcriptional regulator PrtR | | afdb-uniprot50 | AF-B2U8I8-F1-MODEL\_V4 | 1.0 | 1.75e-10 | 281 | 0.186 | 241 | 130 | 7 | 4 | 180 | 62 | 300 | Putative phage repressor | Putative phage repressor | | afdb-uniprot50 | AF-T5KQ06-F1-MODEL\_V4 | 1.0 | 2.764e-07 | 280 | 0.276 | 112 | 74 | 6 | 73 | 180 | 1 | 109 | Peptidase\_S24 domain-containing protein | Peptidase\_S24 domain-containing protein | | afdb-uniprot50 | AF-A0A2U2BY69-F1-MODEL\_V4 | 1.0 | 6.225e-07 | 280 | 0.271 | 114 | 79 | 2 | 70 | 180 | 6 | 118 | Phage repressor protein | Phage repressor protein | | afdb-uniprot50 | AF-A0A127QZI9-F1-MODEL\_V4 | 1.0 | 2.764e-07 | 280 | 0.258 | 120 | 80 | 4 | 64 | 180 | 16 | 129 | Peptidase S24-like family protein | Peptidase S24-like family protein | | afdb-uniprot50 | AF-A0A6B2D5R2-F1-MODEL\_V4 | 1.0 | 1.12e-09 | 280 | 0.206 | 199 | 132 | 6 | 7 | 180 | 3 | 200 | HTH cro/C1-type domain-containing protein | HTH cro/C1-type domain-containing protein | | afdb-uniprot50 | AF-A0A3S4Z798-F1-MODEL\_V4 | 1.0 | 1.585e-09 | 280 | 0.18 | 211 | 138 | 4 | 4 | 180 | 6 | 215 | Transcriptional regulator | Transcriptional regulator | | afdb-uniprot50 | AF-A0A1Q8SUI1-F1-MODEL\_V4 | 1.0 | 6.644e-10 | 280 | 0.196 | 219 | 128 | 7 | 4 | 180 | 7 | 219 | HTH cro/C1-type domain-containing protein | HTH cro/C1-type domain-containing protein | | afdb-uniprot50 | AF-A0A7S7P6L2-F1-MODEL\_V4 | 1.0 | 5.916e-10 | 280 | 0.2 | 220 | 133 | 5 | 1 | 178 | 1 | 219 | Helix-turn-helix transcriptional regulator | Helix-turn-helix transcriptional regulator | | afdb-uniprot50 | AF-A0A3R9FTS3-F1-MODEL\_V4 | 1.0 | 9.97e-10 | 280 | 0.16 | 230 | 139 | 5 | 4 | 180 | 7 | 235 | XRE family transcriptional regulator | XRE family transcriptional regulator | | afdb-uniprot50 | AF-A0A806J3Z0-F1-MODEL\_V4 | 1.0 | 4.971e-10 | 280 | 0.165 | 230 | 135 | 9 | 5 | 180 | 12 | 238 | Bacteriophage transcriptional regulator | Bacteriophage transcriptional regulator | | afdb-uniprot50 | AF-A0A0C1K7W0-F1-MODEL\_V4 | 1.0 | 1.768e-06 | 279 | 0.217 | 92 | 71 | 1 | 89 | 180 | 12 | 102 | Repressor | Repressor | | afdb-uniprot50 | AF-A0A6G6J700-F1-MODEL\_V4 | 1.0 | 1.402e-06 | 279 | 0.274 | 102 | 74 | 0 | 79 | 180 | 82 | 183 | Helix-turn-helix transcriptional regulator | Helix-turn-helix transcriptional regulator | | afdb-uniprot50 | AF-A0A1I4RVT8-F1-MODEL\_V4 | 1.0 | 5.268e-10 | 279 | 0.211 | 227 | 127 | 7 | 4 | 180 | 6 | 230 | Peptidase S24-like | Peptidase S24-like | | afdb-uniprot50 | AF-A0A4D7AWP4-F1-MODEL\_V4 | 1.0 | 9.97e-10 | 279 | 0.204 | 220 | 130 | 7 | 4 | 180 | 16 | 233 | Helix-turn-helix transcriptional regulator | Helix-turn-helix transcriptional regulator | | afdb-uniprot50 | AF-A0A6G6JTQ1-F1-MODEL\_V4 | 1.0 | 4.971e-10 | 279 | 0.193 | 227 | 131 | 9 | 4 | 180 | 14 | 238 | Chromophore lyase | Chromophore lyase | | afdb-uniprot50 | AF-A0A2T6F9B1-F1-MODEL\_V4 | 1.0 | 4.691e-10 | 279 | 0.196 | 214 | 132 | 8 | 5 | 180 | 34 | 245 | HTH cro/C1-type domain-containing protein | HTH cro/C1-type domain-containing protein | | afdb-uniprot50 | AF-A0A3D4KIJ4-F1-MODEL\_V4 | 1.0 | 1.999e-09 | 279 | 0.152 | 243 | 133 | 8 | 4 | 178 | 14 | 251 | HTH cro/C1-type domain-containing protein | HTH cro/C1-type domain-containing protein | | afdb-uniprot50 | AF-A0A2L0WYT4-F1-MODEL\_V4 | 1.0 | 6.644e-10 | 279 | 0.211 | 232 | 127 | 5 | 4 | 180 | 20 | 250 | HTH cro/C1-type domain-containing protein | HTH cro/C1-type domain-containing protein | | afdb-uniprot50 | AF-A0A3E0WQD8-F1-MODEL\_V4 | 1.0 | 5.916e-10 | 279 | 0.224 | 241 | 121 | 9 | 5 | 180 | 15 | 254 | HTH cro/C1-type domain-containing protein | HTH cro/C1-type domain-containing protein | | afdb-uniprot50 | AF-A0A2D5F736-F1-MODEL\_V4 | 1.0 | 4.427e-10 | 279 | 0.223 | 224 | 126 | 7 | 4 | 180 | 38 | 260 | Peptidase | Peptidase | | afdb-uniprot50 | AF-A0A4S2Q5B1-F1-MODEL\_V4 | 1.0 | 7.04e-10 | 279 | 0.166 | 222 | 138 | 8 | 4 | 180 | 65 | 284 | XRE family transcriptional regulator | XRE family transcriptional regulator | | afdb-uniprot50 | AF-A0A2J4NYR4-F1-MODEL\_V4 | 1.0 | 3.486e-07 | 278 | 0.272 | 125 | 83 | 5 | 60 | 180 | 1 | 121 | Phage repressor protein | Phage repressor protein | | afdb-uniprot50 | AF-A0A1Y1QLB8-F1-MODEL\_V4 | 1.0 | 1.68e-09 | 278 | 0.183 | 202 | 138 | 4 | 5 | 180 | 10 | 210 | HTH cro/C1-type domain-containing protein | HTH cro/C1-type domain-containing protein | | afdb-uniprot50 | AF-A0A2S5TZG8-F1-MODEL\_V4 | 1.0 | 6.644e-10 | 278 | 0.221 | 212 | 114 | 6 | 15 | 180 | 5 | 211 | Helix-turn-helix transcriptional regulator | Helix-turn-helix transcriptional regulator | | afdb-uniprot50 | AF-A0A6G8AHU4-F1-MODEL\_V4 | 1.0 | 7.04e-10 | 278 | 0.195 | 225 | 134 | 8 | 1 | 180 | 1 | 223 | Helix-turn-helix transcriptional regulator | Helix-turn-helix transcriptional regulator | | afdb-uniprot50 | AF-A0A2L0PMA1-F1-MODEL\_V4 | 1.0 | 6.269e-10 | 278 | 0.209 | 210 | 128 | 9 | 5 | 177 | 15 | 223 | Putative transcriptional repressor | Putative transcriptional repressor | | afdb-uniprot50 | AF-A0A1N6NSV1-F1-MODEL\_V4 | 1.0 | 8.378e-10 | 278 | 0.188 | 223 | 126 | 5 | 4 | 180 | 20 | 233 | Phage repressor protein C, contains Cro/C1-type HTH and peptisase s24 domains | Phage repressor protein C, contains Cro/C1-type HTH and peptisase s24 domains | | afdb-uniprot50 | AF-A0A3C0XA60-F1-MODEL\_V4 | 1.0 | 7.906e-10 | 278 | 0.194 | 226 | 131 | 10 | 4 | 180 | 8 | 231 | Peptidase\_S24 domain-containing protein | Peptidase\_S24 domain-containing protein | | afdb-uniprot50 | AF-A0A1D9BCV5-F1-MODEL\_V4 | 1.0 | 4.691e-10 | 278 | 0.2 | 224 | 129 | 8 | 4 | 178 | 7 | 229 | Peptidase\_S24 domain-containing protein | Peptidase\_S24 domain-containing protein | | afdb-uniprot50 | AF-A0A1N6XE22-F1-MODEL\_V4 | 1.0 | 1.966e-10 | 278 | 0.19 | 231 | 129 | 8 | 5 | 180 | 12 | 239 | Phage repressor protein C, contains Cro/C1-type HTH and peptisase s24 domains | Phage repressor protein C, contains Cro/C1-type HTH and peptisase s24 domains | | afdb-uniprot50 | AF-D7N153-F1-MODEL\_V4 | 1.0 | 5.583e-10 | 278 | 0.183 | 212 | 135 | 9 | 5 | 180 | 36 | 245 | Peptidase\_S24 domain-containing protein | Peptidase\_S24 domain-containing protein | | afdb-uniprot50 | AF-A0A1G9CRS8-F1-MODEL\_V4 | 1.0 | 9.185e-08 | 278 | 0.291 | 127 | 81 | 4 | 61 | 180 | 127 | 251 | Peptidase S24-like | Peptidase S24-like | | afdb-uniprot50 | AF-A0A2N8C2G3-F1-MODEL\_V4 | 1.0 | 1.966e-10 | 278 | 0.177 | 236 | 132 | 7 | 6 | 180 | 22 | 256 | Helix-turn-helix domain-containing protein | Helix-turn-helix domain-containing protein | | afdb-uniprot50 | AF-A0A2E8IH19-F1-MODEL\_V4 | 1.0 | 8.878e-10 | 278 | 0.184 | 228 | 134 | 7 | 4 | 180 | 37 | 263 | Transcriptional regulator | Transcriptional regulator | | afdb-uniprot50 | AF-A0A1Y3KYL7-F1-MODEL\_V4 | 1.0 | 8.378e-10 | 278 | 0.193 | 243 | 129 | 8 | 4 | 180 | 44 | 285 | HTH cro/C1-type domain-containing protein | HTH cro/C1-type domain-containing protein | | afdb-uniprot50 | AF-A0A7C2AYL5-F1-MODEL\_V4 | 1.0 | 1.75e-10 | 278 | 0.156 | 274 | 133 | 6 | 4 | 180 | 14 | 286 | LexA family transcriptional regulator | LexA family transcriptional regulator | | afdb-uniprot50 | AF-A0A1N7DHM0-F1-MODEL\_V4 | 1.0 | 5.583e-10 | 277 | 0.195 | 215 | 129 | 7 | 4 | 180 | 7 | 215 | Phage repressor protein C, contains Cro/C1-type HTH and peptisase s24 domains | Phage repressor protein C, contains Cro/C1-type HTH and peptisase s24 domains | | afdb-uniprot50 | AF-Q1JZ74-F1-MODEL\_V4 | 1.0 | 1.257e-09 | 277 | 0.183 | 207 | 136 | 5 | 4 | 180 | 27 | 230 | Putative phage repressor | Putative phage repressor | | afdb-uniprot50 | AF-Q1H1H4-F1-MODEL\_V4 | 1.0 | 9.899e-07 | 277 | 0.309 | 97 | 64 | 1 | 87 | 180 | 140 | 236 | Putative phage repressor | Putative phage repressor | | afdb-uniprot50 | AF-A0A2N2QYU2-F1-MODEL\_V4 | 1.0 | 1.31e-10 | 277 | 0.275 | 196 | 121 | 8 | 4 | 180 | 42 | 235 | Peptidase S24 | Peptidase S24 | | afdb-uniprot50 | AF-A0A561MAZ7-F1-MODEL\_V4 | 1.0 | 1.585e-09 | 277 | 0.164 | 237 | 130 | 7 | 5 | 179 | 7 | 237 | Phage repressor protein C with HTH and peptisase S24 domain | Phage repressor protein C with HTH and peptisase S24 domain | | afdb-uniprot50 | AF-A0A6M8SNY5-F1-MODEL\_V4 | 1.0 | 5.268e-10 | 277 | 0.19 | 236 | 131 | 10 | 4 | 180 | 10 | 244 | Helix-turn-helix transcriptional regulator | Helix-turn-helix transcriptional regulator | | afdb-uniprot50 | AF-A0A510XCN0-F1-MODEL\_V4 | 1.0 | 1.186e-09 | 277 | 0.222 | 238 | 124 | 6 | 4 | 180 | 9 | 246 | HTH cro/C1-type domain-containing protein | HTH cro/C1-type domain-containing protein | | afdb-uniprot50 | AF-A0A843YNI7-F1-MODEL\_V4 | 1.0 | 2.784e-10 | 277 | 0.166 | 240 | 135 | 7 | 4 | 180 | 12 | 249 | Uncharacterized protein | Uncharacterized protein | | afdb-uniprot50 | AF-A0A6P2YEJ5-F1-MODEL\_V4 | 1.0 | 1.496e-09 | 277 | 0.168 | 232 | 137 | 5 | 4 | 179 | 17 | 248 | Putative phage repressor | Putative phage repressor | | afdb-uniprot50 | AF-A0A562RKT0-F1-MODEL\_V4 | 1.0 | 8.878e-10 | 277 | 0.175 | 256 | 130 | 6 | 4 | 179 | 4 | 258 | Peptidase S24-like protein | Peptidase S24-like protein | | afdb-uniprot50 | AF-A0A285VR71-F1-MODEL\_V4 | 1.0 | 1.412e-09 | 277 | 0.213 | 225 | 127 | 6 | 4 | 180 | 49 | 271 | Phage repressor protein C, contains Cro/C1-type HTH and peptisase s24 domains | Phage repressor protein C, contains Cro/C1-type HTH and peptisase s24 domains | | afdb-uniprot50 | AF-A0A846VQU4-F1-MODEL\_V4 | 1.0 | 4.148e-07 | 276 | 0.285 | 119 | 77 | 4 | 65 | 180 | 19 | 132 | S24 family peptidase | S24 family peptidase | | afdb-uniprot50 | AF-H0KGR1-F1-MODEL\_V4 | 1.0 | 2.831e-09 | 276 | 0.159 | 194 | 138 | 7 | 5 | 180 | 10 | 196 | DNA-binding protein RDGA | DNA-binding protein RDGA | | afdb-uniprot50 | AF-A0A848G1C2-F1-MODEL\_V4 | 1.0 | 2.119e-09 | 276 | 0.185 | 226 | 127 | 8 | 5 | 180 | 6 | 224 | LexA family transcriptional regulator | LexA family transcriptional regulator | | afdb-uniprot50 | AF-A0A2G6EVX0-F1-MODEL\_V4 | 1.0 | 4.971e-10 | 276 | 0.198 | 212 | 128 | 6 | 4 | 174 | 12 | 222 | HTH cro/C1-type domain-containing protein | HTH cro/C1-type domain-containing protein | | afdb-uniprot50 | AF-A0A1X0TFD1-F1-MODEL\_V4 | 1.0 | 6.644e-10 | 276 | 0.156 | 223 | 136 | 8 | 4 | 178 | 7 | 225 | HTH cro/C1-type domain-containing protein | HTH cro/C1-type domain-containing protein | | afdb-uniprot50 | AF-B0C586-F1-MODEL\_V4 | 1.0 | 1.332e-09 | 276 | 0.168 | 214 | 138 | 6 | 4 | 180 | 17 | 227 | Peptidase, S24 family | Peptidase, S24 family | | afdb-uniprot50 | AF-A0A1Z4BY84-F1-MODEL\_V4 | 1.0 | 1.057e-09 | 276 | 0.191 | 224 | 132 | 7 | 5 | 180 | 8 | 230 | XRE family transcriptional regulator | XRE family transcriptional regulator | | afdb-uniprot50 | AF-A0A4R0EQW7-F1-MODEL\_V4 | 1.0 | 1.257e-09 | 276 | 0.158 | 227 | 141 | 3 | 4 | 180 | 6 | 232 | Helix-turn-helix transcriptional regulator | Helix-turn-helix transcriptional regulator | | afdb-uniprot50 | AF-A0A7U9F4N3-F1-MODEL\_V4 | 1.0 | 9.97e-10 | 276 | 0.174 | 229 | 135 | 4 | 5 | 180 | 8 | 235 | Transcriptional regulator | Transcriptional regulator | | afdb-uniprot50 | AF-A0A7D5D6C4-F1-MODEL\_V4 | 1.0 | 6.269e-10 | 276 | 0.194 | 206 | 135 | 6 | 4 | 180 | 34 | 237 | Helix-turn-helix domain-containing protein | Helix-turn-helix domain-containing protein | | afdb-uniprot50 | AF-A0A0D6Q481-F1-MODEL\_V4 | 1.0 | 4.691e-10 | 276 | 0.206 | 232 | 125 | 7 | 4 | 179 | 22 | 250 | Transcriptional regulator phage repressor | Transcriptional regulator phage repressor | | afdb-uniprot50 | AF-A0A1G8G7E8-F1-MODEL\_V4 | 1.0 | 1.585e-09 | 276 | 0.16 | 250 | 134 | 7 | 5 | 180 | 11 | 258 | Phage repressor protein C, contains Cro/C1-type HTH and peptisase s24 domains | Phage repressor protein C, contains Cro/C1-type HTH and peptisase s24 domains | | afdb-uniprot50 | AF-A0A4U8YGI0-F1-MODEL\_V4 | 1.0 | 1.887e-09 | 276 | 0.205 | 234 | 128 | 4 | 4 | 180 | 35 | 267 | Peptidase s24/s26a/s26b/s26c | Peptidase s24/s26a/s26b/s26c | | afdb-uniprot50 | AF-B8L9T0-F1-MODEL\_V4 | 1.0 | 5.916e-10 | 276 | 0.194 | 226 | 131 | 9 | 4 | 180 | 74 | 297 | Phage-related repressor protein | Phage-related repressor protein | | afdb-uniprot50 | AF-A0A354J173-F1-MODEL\_V4 | 1.0 | 1.574e-06 | 275 | 0.2 | 105 | 79 | 2 | 79 | 179 | 25 | 128 | Helix-turn-helix transcriptional regulator | Helix-turn-helix transcriptional regulator | | afdb-uniprot50 | AF-B9BQH7-F1-MODEL\_V4 | 1.0 | 7.408e-07 | 275 | 0.259 | 108 | 74 | 4 | 78 | 180 | 38 | 144 | Prophage transcriptional regulator | Prophage transcriptional regulator | | afdb-uniprot50 | AF-A0A8B3VSP7-F1-MODEL\_V4 | 1.0 | 9.899e-07 | 275 | 0.23 | 104 | 74 | 4 | 79 | 179 | 51 | 151 | Putative phage repressor | Putative phage repressor | | afdb-uniprot50 | AF-C8N745-F1-MODEL\_V4 | 1.0 | 6.644e-10 | 275 | 0.218 | 211 | 124 | 9 | 4 | 180 | 6 | 209 | Peptidase S24-like protein | Peptidase S24-like protein | | afdb-uniprot50 | AF-A0A0Q3EYM7-F1-MODEL\_V4 | 1.0 | 9.408e-10 | 275 | 0.181 | 215 | 137 | 5 | 4 | 180 | 6 | 219 | Peptidase\_S24 domain-containing protein | Peptidase\_S24 domain-containing protein | | afdb-uniprot50 | AF-A0A6G9RL62-F1-MODEL\_V4 | 1.0 | 4.178e-10 | 275 | 0.221 | 217 | 123 | 12 | 4 | 180 | 13 | 223 | Helix-turn-helix transcriptional regulator | Helix-turn-helix transcriptional regulator | | afdb-uniprot50 | AF-A0A1X0TKP2-F1-MODEL\_V4 | 1.0 | 1.332e-09 | 275 | 0.169 | 224 | 131 | 8 | 4 | 178 | 7 | 224 | HTH cro/C1-type domain-containing protein | HTH cro/C1-type domain-containing protein | | afdb-uniprot50 | AF-A0A3S5GHX0-F1-MODEL\_V4 | 1.0 | 1.471e-10 | 275 | 0.19 | 210 | 127 | 7 | 4 | 172 | 7 | 214 | Transcriptional regulator | Transcriptional regulator | | afdb-uniprot50 | AF-A0A077NT47-F1-MODEL\_V4 | 1.0 | 5.916e-10 | 275 | 0.162 | 221 | 139 | 9 | 4 | 180 | 11 | 229 | 26 kDa repressor protein | 26 kDa repressor protein | | afdb-uniprot50 | AF-A0A522KYU9-F1-MODEL\_V4 | 1.0 | 1.412e-09 | 275 | 0.145 | 233 | 140 | 7 | 4 | 178 | 6 | 237 | LexA family transcriptional regulator | LexA family transcriptional regulator | | afdb-uniprot50 | AF-A0A846UWQ4-F1-MODEL\_V4 | 1.0 | 8.378e-10 | 275 | 0.184 | 228 | 127 | 8 | 5 | 179 | 14 | 235 | Phage repressor protein C with HTH and peptisase S24 domain | Phage repressor protein C with HTH and peptisase S24 domain | | afdb-uniprot50 | AF-A0A432UMV4-F1-MODEL\_V4 | 1.0 | 4.971e-10 | 275 | 0.163 | 233 | 133 | 7 | 7 | 178 | 12 | 243 | Peptidase\_S24 domain-containing protein | Peptidase\_S24 domain-containing protein | | afdb-uniprot50 | AF-A0A104MVH2-F1-MODEL\_V4 | 1.0 | 2.379e-09 | 275 | 0.178 | 230 | 133 | 6 | 4 | 178 | 20 | 248 | HTH cro/C1-type domain-containing protein | HTH cro/C1-type domain-containing protein | | afdb-uniprot50 | AF-A0A364H0J3-F1-MODEL\_V4 | 1.0 | 1.68e-09 | 275 | 0.159 | 232 | 139 | 5 | 4 | 179 | 29 | 260 | Peptidase S24-like protein | Peptidase S24-like protein | | afdb-uniprot50 | AF-A0A2E4Q3Q2-F1-MODEL\_V4 | 1.0 | 1.496e-09 | 275 | 0.215 | 232 | 126 | 6 | 4 | 180 | 72 | 302 | XRE family transcriptional regulator | XRE family transcriptional regulator | | afdb-uniprot50 | AF-A0A1H6SGR5-F1-MODEL\_V4 | 1.0 | 1.75e-10 | 275 | 0.153 | 273 | 133 | 8 | 5 | 180 | 35 | 306 | Peptidase S24-like | Peptidase S24-like | | afdb-uniprot50 | AF-A0A060H3F4-F1-MODEL\_V4 | 1.0 | 1.332e-09 | 275 | 0.169 | 248 | 132 | 6 | 4 | 178 | 69 | 315 | Uncharacterized protein | Uncharacterized protein | | afdb-uniprot50 | AF-A0A7U4R302-F1-MODEL\_V4 | 1.0 | 5.543e-07 | 274 | 0.258 | 112 | 75 | 5 | 70 | 179 | 25 | 130 | Peptidase\_S24 domain-containing protein | Peptidase\_S24 domain-containing protein | | afdb-uniprot50 | AF-A0A0S2SFV9-F1-MODEL\_V4 | 1.0 | 6.644e-10 | 274 | 0.175 | 216 | 131 | 7 | 10 | 180 | 2 | 215 | Transcriptional regulator | Transcriptional regulator | | afdb-uniprot50 | AF-A0A4Q4AGJ7-F1-MODEL\_V4 | 1.0 | 7.46e-10 | 274 | 0.207 | 217 | 131 | 7 | 4 | 180 | 12 | 227 | Helix-turn-helix transcriptional regulator | Helix-turn-helix transcriptional regulator | | afdb-uniprot50 | AF-A0A2U8I4J9-F1-MODEL\_V4 | 1.0 | 1.999e-09 | 274 | 0.154 | 226 | 138 | 5 | 4 | 180 | 8 | 229 | HTH cro/C1-type domain-containing protein | HTH cro/C1-type domain-containing protein | | afdb-uniprot50 | AF-A0A352Z9M4-F1-MODEL\_V4 | 1.0 | 1.412e-09 | 274 | 0.199 | 221 | 130 | 8 | 4 | 180 | 13 | 230 | HTH cro/C1-type domain-containing protein | HTH cro/C1-type domain-containing protein | | afdb-uniprot50 | AF-A0A2A7U2T6-F1-MODEL\_V4 | 1.0 | 1.585e-09 | 274 | 0.152 | 230 | 141 | 5 | 4 | 180 | 8 | 236 | Phage repressor protein C | Phage repressor protein C | | afdb-uniprot50 | AF-A0A346MWA8-F1-MODEL\_V4 | 1.0 | 1.257e-09 | 274 | 0.207 | 222 | 124 | 7 | 6 | 180 | 29 | 245 | Helix-turn-helix domain-containing protein | Helix-turn-helix domain-containing protein | | afdb-uniprot50 | AF-A0A069PBS4-F1-MODEL\_V4 | 1.0 | 6.644e-10 | 274 | 0.159 | 232 | 137 | 7 | 4 | 180 | 17 | 245 | Phage repressor protein | Phage repressor protein | | afdb-uniprot50 | AF-A0A7T4WC91-F1-MODEL\_V4 | 1.0 | 5.583e-10 | 274 | 0.168 | 238 | 134 | 8 | 5 | 180 | 25 | 260 | S24 family peptidase | S24 family peptidase | | afdb-uniprot50 | AF-A0A7Z0ACD2-F1-MODEL\_V4 | 1.0 | 6.644e-10 | 274 | 0.189 | 243 | 130 | 8 | 5 | 180 | 19 | 261 | Phage repressor protein C with HTH and peptisase S24 domain | Phage repressor protein C with HTH and peptisase S24 domain | | afdb-uniprot50 | AF-A0A2N9XWK5-F1-MODEL\_V4 | 1.0 | 3.486e-07 | 273 | 0.192 | 125 | 94 | 2 | 62 | 180 | 27 | 150 | Peptidase\_S24 domain-containing protein | Peptidase\_S24 domain-containing protein | | afdb-uniprot50 | AF-A0A806J765-F1-MODEL\_V4 | 1.0 | 7.85e-07 | 273 | 0.278 | 104 | 70 | 3 | 77 | 176 | 62 | 164 | LexA family repressor/S24 family protease | LexA family repressor/S24 family protease | | afdb-uniprot50 | AF-A0A1I4GAU7-F1-MODEL\_V4 | 1.0 | 2.245e-09 | 273 | 0.176 | 215 | 133 | 7 | 4 | 180 | 11 | 219 | HTH cro/C1-type domain-containing protein | HTH cro/C1-type domain-containing protein | | afdb-uniprot50 | AF-A0A261U5A9-F1-MODEL\_V4 | 1.0 | 1.12e-09 | 273 | 0.199 | 221 | 131 | 6 | 4 | 180 | 7 | 225 | HTH cro/C1-type domain-containing protein | HTH cro/C1-type domain-containing protein | | afdb-uniprot50 | AF-A0A1X0TEC1-F1-MODEL\_V4 | 1.0 | 8.878e-10 | 273 | 0.161 | 217 | 140 | 8 | 4 | 180 | 11 | 225 | Peptidase\_S24 domain-containing protein | Peptidase\_S24 domain-containing protein | | afdb-uniprot50 | AF-A0A425B2V4-F1-MODEL\_V4 | 1.0 | 1.057e-09 | 273 | 0.163 | 232 | 139 | 5 | 3 | 180 | 2 | 232 | Chromophore lyase | Chromophore lyase | | afdb-uniprot50 | AF-C3X398-F1-MODEL\_V4 | 1.0 | 6.269e-10 | 273 | 0.16 | 225 | 139 | 9 | 4 | 180 | 7 | 229 | HTH cro/C1-type domain-containing protein | HTH cro/C1-type domain-containing protein | | afdb-uniprot50 | AF-A0A3D5UZY1-F1-MODEL\_V4 | 1.0 | 1.412e-09 | 273 | 0.182 | 235 | 133 | 6 | 4 | 180 | 6 | 239 | HTH cro/C1-type domain-containing protein | HTH cro/C1-type domain-containing protein | | afdb-uniprot50 | AF-A0A2S5QN69-F1-MODEL\_V4 | 1.0 | 4.691e-10 | 273 | 0.184 | 233 | 132 | 9 | 5 | 180 | 11 | 242 | Phage repressor protein | Phage repressor protein | | afdb-uniprot50 | AF-A0A7Z1UV24-F1-MODEL\_V4 | 1.0 | 1.68e-09 | 273 | 0.171 | 227 | 136 | 7 | 5 | 180 | 27 | 252 | HTH cro/C1-type domain-containing protein | HTH cro/C1-type domain-containing protein | | afdb-uniprot50 | AF-A0A1I1VLZ3-F1-MODEL\_V4 | 1.0 | 4.936e-07 | 273 | 0.336 | 113 | 67 | 3 | 71 | 180 | 161 | 268 | Peptidase S24-like | Peptidase S24-like | | afdb-uniprot50 | AF-A0A4R7VY96-F1-MODEL\_V4 | 1.0 | 4.178e-10 | 273 | 0.151 | 258 | 134 | 6 | 6 | 180 | 22 | 277 | Phage repressor protein C with HTH and peptisase S24 domain | Phage repressor protein C with HTH and peptisase S24 domain | | afdb-uniprot50 | AF-A0A1G8LL79-F1-MODEL\_V4 | 1.0 | 2.95e-10 | 273 | 0.148 | 269 | 135 | 7 | 5 | 180 | 22 | 289 | Phage repressor protein C, contains Cro/C1-type HTH and peptisase s24 domains | Phage repressor protein C, contains Cro/C1-type HTH and peptisase s24 domains | | afdb-uniprot50 | AF-A0A2J0UCK8-F1-MODEL\_V4 | 1.0 | 4.738e-06 | 272 | 0.23 | 91 | 69 | 1 | 90 | 180 | 18 | 107 | Uncharacterized protein | Uncharacterized protein | | afdb-uniprot50 | AF-A0A1F1UL81-F1-MODEL\_V4 | 1.0 | 5.874e-07 | 272 | 0.247 | 109 | 77 | 3 | 77 | 180 | 45 | 153 | Peptidase\_S24 domain-containing protein | Peptidase\_S24 domain-containing protein | | afdb-uniprot50 | AF-A0A5E7AN99-F1-MODEL\_V4 | 1.0 | 3.914e-07 | 272 | 0.3 | 110 | 68 | 4 | 79 | 180 | 65 | 173 | Peptidase\_S24 domain-containing protein | Peptidase\_S24 domain-containing protein | | afdb-uniprot50 | AF-A0A1B1A083-F1-MODEL\_V4 | 1.0 | 1.842e-07 | 272 | 0.253 | 126 | 83 | 5 | 61 | 180 | 54 | 174 | Transcriptional regulator | Transcriptional regulator | | afdb-uniprot50 | AF-A0A661J126-F1-MODEL\_V4 | 1.0 | 1.496e-09 | 272 | 0.203 | 211 | 127 | 6 | 4 | 180 | 8 | 211 | Peptidase S24 | Peptidase S24 | | afdb-uniprot50 | AF-A0A0Q6LRH4-F1-MODEL\_V4 | 1.0 | 7.46e-10 | 272 | 0.185 | 226 | 128 | 8 | 5 | 179 | 7 | 227 | HTH cro/C1-type domain-containing protein | HTH cro/C1-type domain-containing protein | | afdb-uniprot50 | AF-A0A1G0MXC6-F1-MODEL\_V4 | 1.0 | 6.269e-10 | 272 | 0.219 | 219 | 123 | 8 | 5 | 180 | 17 | 230 | Repressor | Repressor | | afdb-uniprot50 | AF-A0A2A4V065-F1-MODEL\_V4 | 1.0 | 1.186e-09 | 272 | 0.163 | 238 | 131 | 8 | 4 | 180 | 9 | 239 | Peptidase S24 | Peptidase S24 | | afdb-uniprot50 | AF-A0A2J7TQM8-F1-MODEL\_V4 | 1.0 | 1.585e-09 | 272 | 0.185 | 248 | 130 | 4 | 5 | 180 | 8 | 255 | XRE family transcriptional regulator | XRE family transcriptional regulator | | afdb-uniprot50 | AF-A0A2E1HCN9-F1-MODEL\_V4 | 1.0 | 2.672e-09 | 272 | 0.17 | 241 | 134 | 6 | 4 | 180 | 43 | 281 | HTH cro/C1-type domain-containing protein | HTH cro/C1-type domain-containing protein | | afdb-uniprot50 | AF-A0A1D3JVS7-F1-MODEL\_V4 | 1.0 | 5.268e-10 | 272 | 0.121 | 264 | 141 | 5 | 7 | 180 | 24 | 286 | Propanediol utilization protein | Propanediol utilization protein | | afdb-uniprot50 | AF-A0A6I6SSB5-F1-MODEL\_V4 | 1.0 | 1.186e-09 | 272 | 0.204 | 235 | 125 | 6 | 4 | 177 | 58 | 291 | Helix-turn-helix transcriptional regulator | Helix-turn-helix transcriptional regulator | | afdb-uniprot50 | AF-A0A1B5D1A6-F1-MODEL\_V4 | 1.0 | 2.363e-06 | 271 | 0.254 | 106 | 75 | 2 | 78 | 180 | 66 | 170 | Putative HTH-type transcriptional regulator | Putative HTH-type transcriptional regulator | | afdb-uniprot50 | AF-A0A173HMY6-F1-MODEL\_V4 | 1.0 | 7.85e-07 | 271 | 0.22 | 109 | 78 | 3 | 78 | 180 | 100 | 207 | CI repressor protein | CI repressor protein | | afdb-uniprot50 | AF-A0A1C0A407-F1-MODEL\_V4 | 1.0 | 1.12e-09 | 271 | 0.184 | 206 | 127 | 7 | 14 | 180 | 6 | 209 | HTH cro/C1-type domain-containing protein | HTH cro/C1-type domain-containing protein | | afdb-uniprot50 | AF-A0A4R4G357-F1-MODEL\_V4 | 1.0 | 2.521e-09 | 271 | 0.198 | 196 | 121 | 5 | 17 | 180 | 27 | 218 | Phage repressor protein | Phage repressor protein | | afdb-uniprot50 | AF-A0A6I1ZNW8-F1-MODEL\_V4 | 1.0 | 2.245e-09 | 271 | 0.192 | 213 | 135 | 4 | 4 | 180 | 19 | 230 | Peptidase\_S24 domain-containing protein | Peptidase\_S24 domain-containing protein | | afdb-uniprot50 | AF-A0A742DF20-F1-MODEL\_V4 | 1.0 | 3.18e-09 | 271 | 0.159 | 226 | 140 | 6 | 4 | 180 | 8 | 232 | Helix-turn-helix transcriptional regulator | Helix-turn-helix transcriptional regulator | | afdb-uniprot50 | AF-A0A1S9ZSJ1-F1-MODEL\_V4 | 1.0 | 8.378e-10 | 271 | 0.167 | 233 | 136 | 7 | 4 | 180 | 7 | 237 | Uncharacterized HTH-type transcriptional regulator HI\_1476 | Uncharacterized HTH-type transcriptional regulator HI\_1476 | | afdb-uniprot50 | AF-A0A6I5AJN6-F1-MODEL\_V4 | 1.0 | 2.245e-09 | 271 | 0.213 | 220 | 124 | 9 | 4 | 178 | 13 | 228 | Helix-turn-helix domain-containing protein | Helix-turn-helix domain-containing protein | | afdb-uniprot50 | AF-A0A4P0TEV4-F1-MODEL\_V4 | 1.0 | 1.402e-06 | 270 | 0.254 | 106 | 75 | 1 | 79 | 180 | 67 | 172 | Transcriptional regulator | Transcriptional regulator | | afdb-uniprot50 | AF-A0A011TVH5-F1-MODEL\_V4 | 1.0 | 1.057e-09 | 270 | 0.206 | 203 | 125 | 6 | 14 | 180 | 1 | 203 | Transcriptional regulator | Transcriptional regulator | | afdb-uniprot50 | AF-A0A7D0IFV9-F1-MODEL\_V4 | 1.0 | 1.186e-09 | 270 | 0.19 | 226 | 132 | 9 | 4 | 180 | 6 | 229 | XRE family transcriptional regulator | XRE family transcriptional regulator | | afdb-uniprot50 | AF-A0A1I1ETS9-F1-MODEL\_V4 | 1.0 | 1.412e-09 | 270 | 0.208 | 225 | 128 | 7 | 4 | 180 | 6 | 228 | Phage repressor protein C, contains Cro/C1-type HTH and peptisase s24 domains | Phage repressor protein C, contains Cro/C1-type HTH and peptisase s24 domains | | afdb-uniprot50 | AF-A0A1M5I2V5-F1-MODEL\_V4 | 1.0 | 7.906e-10 | 270 | 0.194 | 221 | 122 | 5 | 15 | 180 | 14 | 233 | Phage repressor protein C, contains Cro/C1-type HTH and peptisase s24 domains | Phage repressor protein C, contains Cro/C1-type HTH and peptisase s24 domains | | afdb-uniprot50 | AF-A0A1N7UFW9-F1-MODEL\_V4 | 1.0 | 4.971e-10 | 270 | 0.132 | 272 | 140 | 6 | 4 | 180 | 14 | 284 | Propanediol utilization protein | Propanediol utilization protein | | afdb-uniprot50 | AF-A0A822VGQ0-F1-MODEL\_V4 | 1.0 | 1.049e-06 | 269 | 0.247 | 105 | 74 | 5 | 77 | 178 | 55 | 157 | Peptidase S24-like protein | Peptidase S24-like protein | | afdb-uniprot50 | AF-A0A1H3E809-F1-MODEL\_V4 | 1.0 | 1.887e-09 | 269 | 0.192 | 213 | 126 | 8 | 12 | 180 | 14 | 224 | Phage repressor protein C, contains Cro/C1-type HTH and peptisase s24 domains | Phage repressor protein C, contains Cro/C1-type HTH and peptisase s24 domains | | afdb-uniprot50 | AF-A0A5B9DY71-F1-MODEL\_V4 | 1.0 | 9.408e-10 | 269 | 0.214 | 224 | 126 | 9 | 5 | 180 | 4 | 225 | Helix-turn-helix transcriptional regulator | Helix-turn-helix transcriptional regulator | | afdb-uniprot50 | AF-A0A4R8FYR2-F1-MODEL\_V4 | 1.0 | 1.412e-09 | 269 | 0.199 | 226 | 131 | 6 | 4 | 180 | 8 | 232 | Phage repressor protein C with HTH and peptisase S24 domain | Phage repressor protein C with HTH and peptisase S24 domain | | afdb-uniprot50 | AF-A0A1L2B3E0-F1-MODEL\_V4 | 1.0 | 1.496e-09 | 269 | 0.19 | 247 | 128 | 6 | 4 | 178 | 6 | 252 | Helix-turn-helix transcriptional regulator | Helix-turn-helix transcriptional regulator | | afdb-uniprot50 | AF-A0A3M2VAP0-F1-MODEL\_V4 | 1.0 | 1.257e-09 | 269 | 0.178 | 224 | 136 | 5 | 5 | 180 | 31 | 254 | Putative prophage PSSB64-02, repressor protein | Putative prophage PSSB64-02, repressor protein | | afdb-uniprot50 | AF-A0A2E1XTS7-F1-MODEL\_V4 | 1.0 | 2.831e-09 | 269 | 0.175 | 211 | 138 | 6 | 5 | 180 | 78 | 287 | HTH cro/C1-type domain-containing protein | HTH cro/C1-type domain-containing protein | | afdb-uniprot50 | AF-A0A1I3KCB5-F1-MODEL\_V4 | 1.0 | 8.378e-10 | 269 | 0.139 | 266 | 138 | 4 | 5 | 180 | 22 | 286 | Helix-turn-helix | Helix-turn-helix | | afdb-uniprot50 | AF-A0A379GG64-F1-MODEL\_V4 | 1.0 | 2.229e-06 | 268 | 0.218 | 110 | 82 | 1 | 70 | 179 | 1 | 106 | Phage repressor protein | Phage repressor protein | | afdb-uniprot50 | AF-K9RXU0-F1-MODEL\_V4 | 1.0 | 4.396e-07 | 268 | 0.266 | 120 | 82 | 3 | 64 | 180 | 8 | 124 | Putative transcriptional regulator | Putative transcriptional regulator | | afdb-uniprot50 | AF-A0A839A2N0-F1-MODEL\_V4 | 1.0 | 6.017e-09 | 268 | 0.177 | 220 | 134 | 7 | 4 | 179 | 7 | 223 | Helix-turn-helix domain-containing protein | Helix-turn-helix domain-containing protein | | afdb-uniprot50 | AF-A0A0A0FDV9-F1-MODEL\_V4 | 1.0 | 4.503e-09 | 268 | 0.151 | 225 | 142 | 7 | 4 | 180 | 7 | 230 | Putative transcriptional regulator | Putative transcriptional regulator | | afdb-uniprot50 | AF-A0A7U0N6L3-F1-MODEL\_V4 | 1.0 | 1.412e-09 | 268 | 0.188 | 223 | 130 | 10 | 4 | 180 | 13 | 230 | Helix-turn-helix transcriptional regulator | Helix-turn-helix transcriptional regulator | | afdb-uniprot50 | AF-A0A1B8PKT6-F1-MODEL\_V4 | 1.0 | 2.521e-09 | 268 | 0.178 | 230 | 135 | 5 | 4 | 180 | 7 | 235 | HTH cro/C1-type domain-containing protein | HTH cro/C1-type domain-containing protein | | afdb-uniprot50 | AF-A0A1B1EDQ4-F1-MODEL\_V4 | 1.0 | 2.521e-09 | 268 | 0.186 | 225 | 132 | 8 | 5 | 180 | 18 | 240 | HTH cro/C1-type domain-containing protein | HTH cro/C1-type domain-containing protein | | afdb-uniprot50 | AF-A0A3N0V596-F1-MODEL\_V4 | 1.0 | 1.057e-09 | 268 | 0.182 | 230 | 134 | 9 | 4 | 180 | 16 | 244 | XRE family transcriptional regulator | XRE family transcriptional regulator | | afdb-uniprot50 | AF-A0A348DDK2-F1-MODEL\_V4 | 1.0 | 9.408e-10 | 268 | 0.2 | 224 | 130 | 7 | 4 | 180 | 35 | 256 | Repressor | Repressor | | afdb-uniprot50 | AF-A0A7Y7J3L6-F1-MODEL\_V4 | 1.0 | 2.379e-09 | 268 | 0.204 | 230 | 130 | 6 | 4 | 180 | 41 | 270 | Helix-turn-helix transcriptional regulator | Helix-turn-helix transcriptional regulator | | afdb-uniprot50 | AF-A0A2S9AU56-F1-MODEL\_V4 | 1.0 | 1.496e-09 | 268 | 0.125 | 278 | 141 | 7 | 4 | 180 | 14 | 290 | Propanediol utilization protein | Propanediol utilization protein | | afdb-uniprot50 | AF-A0A2D0KAP2-F1-MODEL\_V4 | 1.0 | 2.831e-09 | 268 | 0.157 | 235 | 139 | 5 | 4 | 180 | 97 | 330 | Repressor | Repressor | | afdb-uniprot50 | AF-A0A2W6VYN7-F1-MODEL\_V4 | 1.0 | 1.668e-06 | 267 | 0.26 | 100 | 67 | 4 | 87 | 180 | 5 | 103 | Uncharacterized protein | Uncharacterized protein | | afdb-uniprot50 | AF-A0A7G2K0K2-F1-MODEL\_V4 | 1.0 | 2.229e-06 | 267 | 0.252 | 111 | 79 | 1 | 70 | 180 | 1 | 107 | Prophage LambdaSo, transcriptional regulator, Cro/CI family | Prophage LambdaSo, transcriptional regulator, Cro/CI family | | afdb-uniprot50 | AF-A0A4Q8L9Y8-F1-MODEL\_V4 | 1.0 | 5.32e-06 | 267 | 0.191 | 94 | 74 | 2 | 87 | 180 | 20 | 111 | Uncharacterized protein | Uncharacterized protein | | afdb-uniprot50 | AF-A0A7W4SU23-F1-MODEL\_V4 | 1.0 | 1.496e-09 | 267 | 0.181 | 198 | 131 | 7 | 4 | 180 | 10 | 197 | Transcriptional regulator with XRE-family HTH domain | Transcriptional regulator with XRE-family HTH domain | | afdb-uniprot50 | AF-A0A349EQG1-F1-MODEL\_V4 | 1.0 | 4.249e-09 | 267 | 0.209 | 210 | 130 | 5 | 4 | 180 | 6 | 212 | HTH cro/C1-type domain-containing protein | HTH cro/C1-type domain-containing protein | | afdb-uniprot50 | AF-A0A7T7HH85-F1-MODEL\_V4 | 1.0 | 4.772e-09 | 267 | 0.18 | 211 | 138 | 6 | 4 | 180 | 15 | 224 | Helix-turn-helix domain-containing protein | Helix-turn-helix domain-containing protein | | afdb-uniprot50 | AF-A0A4R1J3T7-F1-MODEL\_V4 | 1.0 | 4.249e-09 | 267 | 0.206 | 228 | 122 | 8 | 5 | 178 | 7 | 229 | Peptidase S24-like protein | Peptidase S24-like protein | | afdb-uniprot50 | AF-A0A3N4NGB5-F1-MODEL\_V4 | 1.0 | 6.269e-10 | 267 | 0.208 | 221 | 125 | 7 | 4 | 176 | 7 | 225 | XRE family transcriptional regulator | XRE family transcriptional regulator | | afdb-uniprot50 | AF-A0A2A2B7J3-F1-MODEL\_V4 | 1.0 | 5.678e-09 | 267 | 0.135 | 228 | 135 | 6 | 5 | 178 | 16 | 235 | Peptidase\_S24 domain-containing protein | Peptidase\_S24 domain-containing protein | | afdb-uniprot50 | AF-A0A4U9HM21-F1-MODEL\_V4 | 1.0 | 2.653e-06 | 266 | 0.245 | 106 | 76 | 1 | 79 | 180 | 73 | 178 | Peptidase S24-like | Peptidase S24-like | | afdb-uniprot50 | AF-A0A377ICP7-F1-MODEL\_V4 | 1.0 | 5.143e-08 | 266 | 0.248 | 141 | 90 | 5 | 48 | 180 | 48 | 180 | LexA family repressor/S24 family protease | LexA family repressor/S24 family protease | | afdb-uniprot50 | AF-A0A7Y9BX19-F1-MODEL\_V4 | 1.0 | 6.757e-09 | 266 | 0.152 | 230 | 138 | 6 | 4 | 180 | 9 | 234 | Helix-turn-helix transcriptional regulator | Helix-turn-helix transcriptional regulator | | afdb-uniprot50 | AF-A0A1Y5STX6-F1-MODEL\_V4 | 1.0 | 1.496e-09 | 266 | 0.163 | 226 | 138 | 9 | 4 | 180 | 14 | 237 | HTH-type transcriptional regulator PrtR | HTH-type transcriptional regulator PrtR | | afdb-uniprot50 | AF-A0A7S9VBU4-F1-MODEL\_V4 | 1.0 | 3.257e-11 | 266 | 0.21 | 238 | 124 | 8 | 4 | 179 | 7 | 242 | Helix-turn-helix domain-containing protein | Helix-turn-helix domain-containing protein | | afdb-uniprot50 | AF-A0A369TDZ1-F1-MODEL\_V4 | 1.0 | 5.056e-09 | 266 | 0.181 | 242 | 135 | 6 | 1 | 180 | 8 | 248 | LexA family transcriptional regulator | LexA family transcriptional regulator | | afdb-uniprot50 | AF-A0A1W6K966-F1-MODEL\_V4 | 1.0 | 3.784e-09 | 266 | 0.192 | 250 | 128 | 5 | 4 | 180 | 6 | 254 | HTH-type transcriptional regulator PrtR | HTH-type transcriptional regulator PrtR | | afdb-uniprot50 | AF-A0A2G2PKM8-F1-MODEL\_V4 | 1.0 | 3e-09 | 266 | 0.188 | 239 | 129 | 6 | 4 | 180 | 30 | 265 | Cro/Cl family transcriptional regulator | Cro/Cl family transcriptional regulator | | afdb-uniprot50 | AF-A0A7G2K0R3-F1-MODEL\_V4 | 1.0 | 7.11e-06 | 265 | 0.271 | 92 | 67 | 0 | 89 | 180 | 5 | 96 | Prophage LambdaSo, transcriptional regulator, Cro/CI family | Prophage LambdaSo, transcriptional regulator, Cro/CI family | | afdb-uniprot50 | AF-A0A0E1W9H6-F1-MODEL\_V4 | 1.0 | 1.68e-09 | 265 | 0.217 | 207 | 127 | 6 | 6 | 180 | 11 | 214 | Putative repressor protein | Putative repressor protein | | afdb-uniprot50 | AF-A0A7M1S497-F1-MODEL\_V4 | 1.0 | 1.057e-09 | 265 | 0.182 | 208 | 136 | 7 | 5 | 180 | 12 | 217 | LexA family transcriptional regulator | LexA family transcriptional regulator | | afdb-uniprot50 | AF-E7H635-F1-MODEL\_V4 | 1.0 | 8.878e-10 | 265 | 0.219 | 214 | 128 | 7 | 4 | 180 | 7 | 218 | HTH cro/C1-type domain-containing protein | HTH cro/C1-type domain-containing protein | | afdb-uniprot50 | AF-A0A286U3Z1-F1-MODEL\_V4 | 1.0 | 2.379e-09 | 265 | 0.169 | 212 | 135 | 5 | 9 | 180 | 11 | 221 | Peptidase S24-like domain protein | Peptidase S24-like domain protein | | afdb-uniprot50 | AF-X2H633-F1-MODEL\_V4 | 1.0 | 1.999e-09 | 265 | 0.151 | 231 | 143 | 4 | 3 | 180 | 2 | 232 | Putative transcriptional regulator | Putative transcriptional regulator | | afdb-uniprot50 | AF-A0A212QJ24-F1-MODEL\_V4 | 1.0 | 2.672e-09 | 265 | 0.191 | 225 | 128 | 7 | 5 | 180 | 14 | 233 | Phage repressor protein C, contains Cro/C1-type HTH and peptisase s24 domains | Phage repressor protein C, contains Cro/C1-type HTH and peptisase s24 domains | | afdb-uniprot50 | AF-A0A2S5KUI7-F1-MODEL\_V4 | 1.0 | 1.887e-09 | 265 | 0.22 | 231 | 125 | 9 | 4 | 180 | 10 | 239 | Peptidase\_S24 domain-containing protein | Peptidase\_S24 domain-containing protein | | afdb-uniprot50 | AF-A0A2T5PGI5-F1-MODEL\_V4 | 1.0 | 2.379e-09 | 265 | 0.184 | 239 | 131 | 5 | 4 | 180 | 10 | 246 | Peptidase | Peptidase | | afdb-uniprot50 | AF-A0A1Q9R370-F1-MODEL\_V4 | 1.0 | 1.412e-09 | 265 | 0.14 | 277 | 137 | 9 | 4 | 180 | 6 | 281 | HTH cro/C1-type domain-containing protein | HTH cro/C1-type domain-containing protein | | afdb-uniprot50 | AF-A0A0W0N0W8-F1-MODEL\_V4 | 1.0 | 6.269e-10 | 265 | 0.14 | 270 | 138 | 6 | 4 | 180 | 14 | 282 | Propanediol utilization protein | Propanediol utilization protein | | afdb-uniprot50 | AF-A0A379D1W1-F1-MODEL\_V4 | 1.0 | 3.914e-07 | 264 | 0.248 | 125 | 85 | 5 | 60 | 180 | 2 | 121 | Uncharacterized HTH-type transcriptional regulator HI\_1476 | Uncharacterized HTH-type transcriptional regulator HI\_1476 | | afdb-uniprot50 | AF-A0A5E7J7C6-F1-MODEL\_V4 | 1.0 | 6.99e-07 | 264 | 0.213 | 122 | 91 | 3 | 63 | 180 | 6 | 126 | Peptidase\_S24 domain-containing protein | Peptidase\_S24 domain-containing protein | | afdb-uniprot50 | AF-A0A317DV49-F1-MODEL\_V4 | 1.0 | 2.521e-09 | 264 | 0.226 | 199 | 114 | 6 | 20 | 180 | 2 | 198 | HTH cro/C1-type domain-containing protein | HTH cro/C1-type domain-containing protein | | afdb-uniprot50 | AF-A0A3A8EIV5-F1-MODEL\_V4 | 1.0 | 1.186e-09 | 264 | 0.231 | 203 | 123 | 7 | 4 | 174 | 6 | 207 | Peptidase S24 | Peptidase S24 | | afdb-uniprot50 | AF-A0A850JR98-F1-MODEL\_V4 | 1.0 | 3.369e-09 | 264 | 0.207 | 212 | 129 | 7 | 4 | 180 | 10 | 217 | Helix-turn-helix transcriptional regulator | Helix-turn-helix transcriptional regulator | | afdb-uniprot50 | AF-J9Z8Z9-F1-MODEL\_V4 | 1.0 | 2.521e-09 | 264 | 0.216 | 217 | 122 | 10 | 5 | 178 | 8 | 219 | Repressor protein | Repressor protein | | afdb-uniprot50 | AF-A0A2P5LAB6-F1-MODEL\_V4 | 1.0 | 2.521e-09 | 264 | 0.191 | 219 | 133 | 7 | 4 | 180 | 7 | 223 | HTH cro/C1-type domain-containing protein | HTH cro/C1-type domain-containing protein | | afdb-uniprot50 | AF-A0A840M8M5-F1-MODEL\_V4 | 1.0 | 2.831e-09 | 264 | 0.188 | 218 | 136 | 6 | 4 | 180 | 15 | 232 | Phage repressor protein C with HTH and peptisase S24 domain | Phage repressor protein C with HTH and peptisase S24 domain | | afdb-uniprot50 | AF-A0A0U2B2W6-F1-MODEL\_V4 | 1.0 | 1.257e-09 | 264 | 0.19 | 236 | 131 | 8 | 1 | 180 | 3 | 234 | Peptidase\_S24 domain-containing protein | Peptidase\_S24 domain-containing protein | | afdb-uniprot50 | AF-A0A2N5ZG77-F1-MODEL\_V4 | 1.0 | 3.571e-09 | 264 | 0.173 | 213 | 138 | 6 | 4 | 180 | 36 | 246 | HTH cro/C1-type domain-containing protein | HTH cro/C1-type domain-containing protein | | afdb-uniprot50 | AF-A0A0S4IE42-F1-MODEL\_V4 | 1.0 | 1.496e-09 | 264 | 0.146 | 273 | 135 | 6 | 5 | 180 | 9 | 280 | LexA repressor | LexA repressor | | afdb-uniprot50 | AF-A0A0B5K5I8-F1-MODEL\_V4 | 1.0 | 1.12e-09 | 264 | 0.141 | 275 | 136 | 7 | 5 | 180 | 22 | 295 | Cro/Cl family transcriptional regulator | Cro/Cl family transcriptional regulator | | afdb-uniprot50 | AF-A0A7C9KFV0-F1-MODEL\_V4 | 1.0 | 1.186e-09 | 264 | 0.157 | 292 | 126 | 9 | 4 | 179 | 27 | 314 | Helix-turn-helix domain-containing protein | Helix-turn-helix domain-containing protein | | afdb-uniprot50 | AF-A0A1Q4D1Y9-F1-MODEL\_V4 | 1.0 | 8.319e-07 | 263 | 0.258 | 116 | 76 | 4 | 70 | 180 | 28 | 138 | Peptidase\_S24 domain-containing protein | Peptidase\_S24 domain-containing protein | | afdb-uniprot50 | AF-W7PA95-F1-MODEL\_V4 | 1.0 | 7.535e-06 | 263 | 0.196 | 102 | 82 | 0 | 79 | 180 | 87 | 188 | HTH-type transcriptional regulator RdgA | HTH-type transcriptional regulator RdgA | | afdb-uniprot50 | AF-A0A7V8JTY9-F1-MODEL\_V4 | 1.0 | 2.245e-09 | 263 | 0.212 | 202 | 122 | 6 | 5 | 173 | 15 | 212 | Putative HTH-type transcriptional regulator | Putative HTH-type transcriptional regulator | | afdb-uniprot50 | AF-A0A2D4RVR8-F1-MODEL\_V4 | 1.0 | 7.588e-09 | 263 | 0.196 | 214 | 134 | 5 | 4 | 180 | 7 | 219 | HTH cro/C1-type domain-containing protein | HTH cro/C1-type domain-containing protein | | afdb-uniprot50 | AF-A0A154WGA2-F1-MODEL\_V4 | 1.0 | 4.249e-09 | 263 | 0.161 | 223 | 140 | 11 | 1 | 180 | 4 | 222 | Peptidase S24 | Peptidase S24 | | afdb-uniprot50 | AF-A0A836MN97-F1-MODEL\_V4 | 1.0 | 8.878e-10 | 263 | 0.193 | 238 | 130 | 10 | 1 | 180 | 1 | 234 | Putative transcriptional regulator | Putative transcriptional regulator | | afdb-uniprot50 | AF-Q5F6C9-F1-MODEL\_V4 | 1.0 | 7.46e-10 | 263 | 0.168 | 225 | 135 | 7 | 4 | 176 | 19 | 243 | Repressor | Repressor | | afdb-uniprot50 | AF-A0A849P416-F1-MODEL\_V4 | 1.0 | 3.571e-09 | 263 | 0.203 | 221 | 127 | 7 | 5 | 180 | 38 | 254 | S24 family peptidase | S24 family peptidase | | afdb-uniprot50 | AF-A0A845BW56-F1-MODEL\_V4 | 1.0 | 3.571e-09 | 263 | 0.201 | 238 | 129 | 5 | 4 | 180 | 27 | 264 | Helix-turn-helix domain-containing protein | Helix-turn-helix domain-containing protein | | afdb-uniprot50 | AF-A0A5M8F983-F1-MODEL\_V4 | 1.0 | 4.427e-10 | 263 | 0.146 | 266 | 133 | 7 | 4 | 176 | 14 | 278 | Helix-turn-helix domain-containing protein | Helix-turn-helix domain-containing protein | | afdb-uniprot50 | AF-A0A1H9EPH6-F1-MODEL\_V4 | 1.0 | 3.313e-10 | 263 | 0.161 | 273 | 129 | 7 | 7 | 180 | 24 | 295 | Phage repressor protein C, contains Cro/C1-type HTH and peptisase s24 domains | Phage repressor protein C, contains Cro/C1-type HTH and peptisase s24 domains | | afdb-uniprot50 | AF-A0A239LDH9-F1-MODEL\_V4 | 1.0 | 2.379e-09 | 263 | 0.189 | 232 | 129 | 7 | 5 | 179 | 85 | 314 | Phage repressor protein C, contains Cro/C1-type HTH and peptisase s24 domains | Phage repressor protein C, contains Cro/C1-type HTH and peptisase s24 domains | | afdb-uniprot50 | AF-Q4QKX1-F1-MODEL\_V4 | 1.0 | 2.672e-09 | 263 | 0.138 | 246 | 142 | 6 | 4 | 180 | 74 | 318 | Uncharacterized protein | Uncharacterized protein | | afdb-uniprot50 | AF-A0A549SHD6-F1-MODEL\_V4 | 1.0 | 3.545e-06 | 262 | 0.204 | 98 | 71 | 3 | 89 | 180 | 13 | 109 | S24 family peptidase | S24 family peptidase | | afdb-uniprot50 | AF-A0A6G9VXL7-F1-MODEL\_V4 | 1.0 | 2.229e-06 | 262 | 0.254 | 106 | 75 | 2 | 78 | 180 | 40 | 144 | S24 family peptidase | S24 family peptidase | | afdb-uniprot50 | AF-A0A166UAP7-F1-MODEL\_V4 | 1.0 | 6.709e-06 | 262 | 0.288 | 104 | 72 | 2 | 78 | 180 | 70 | 172 | Peptidase\_S24 domain-containing protein | Peptidase\_S24 domain-containing protein | | afdb-uniprot50 | AF-A0A3B9IP20-F1-MODEL\_V4 | 1.0 | 1.999e-09 | 262 | 0.198 | 196 | 121 | 5 | 20 | 180 | 2 | 196 | Transcriptional regulator | Transcriptional regulator | | afdb-uniprot50 | AF-A0A554GDH4-F1-MODEL\_V4 | 1.0 | 1.768e-06 | 262 | 0.271 | 107 | 73 | 1 | 79 | 180 | 90 | 196 | Helix-turn-helix transcriptional regulator | Helix-turn-helix transcriptional regulator | | afdb-uniprot50 | AF-A0A5K1S6Z7-F1-MODEL\_V4 | 1.0 | 1.985e-06 | 262 | 0.257 | 105 | 75 | 1 | 79 | 180 | 98 | 202 | Helix-turn-helix transcriptional regulator | Helix-turn-helix transcriptional regulator | | afdb-uniprot50 | AF-A0A317EHX9-F1-MODEL\_V4 | 1.0 | 2.831e-09 | 262 | 0.19 | 215 | 134 | 6 | 4 | 180 | 7 | 219 | HTH cro/C1-type domain-containing protein | HTH cro/C1-type domain-containing protein | | afdb-uniprot50 | AF-A0A7H8UUY2-F1-MODEL\_V4 | 1.0 | 2.521e-09 | 262 | 0.19 | 215 | 129 | 8 | 6 | 180 | 14 | 223 | Helix-turn-helix transcriptional regulator | Helix-turn-helix transcriptional regulator | | afdb-uniprot50 | AF-A0A1I2E7J1-F1-MODEL\_V4 | 1.0 | 2.245e-09 | 262 | 0.172 | 237 | 135 | 8 | 1 | 180 | 1 | 233 | Peptidase S24-like | Peptidase S24-like | | afdb-uniprot50 | AF-A0A515DE75-F1-MODEL\_V4 | 1.0 | 8.378e-10 | 262 | 0.248 | 197 | 126 | 9 | 4 | 180 | 52 | 246 | S24 family peptidase | S24 family peptidase | | afdb-uniprot50 | AF-A0A7M3AWF2-F1-MODEL\_V4 | 1.0 | 2.672e-09 | 262 | 0.165 | 241 | 136 | 7 | 4 | 180 | 18 | 257 | LexA family transcriptional regulator | LexA family transcriptional regulator | | afdb-uniprot50 | AF-A0A5E4YYK6-F1-MODEL\_V4 | 1.0 | 3.369e-09 | 262 | 0.138 | 259 | 140 | 7 | 4 | 180 | 19 | 276 | Putative phage repressor | Putative phage repressor | | afdb-uniprot50 | AF-A0A266Q680-F1-MODEL\_V4 | 1.0 | 2.521e-09 | 262 | 0.191 | 267 | 121 | 11 | 4 | 180 | 19 | 280 | Peptidase\_S24 domain-containing protein | Peptidase\_S24 domain-containing protein | | afdb-uniprot50 | AF-A0A6D0P928-F1-MODEL\_V4 | 1.0 | 1.112e-06 | 261 | 0.241 | 116 | 81 | 4 | 68 | 180 | 5 | 116 | Peptidase\_S24 domain-containing protein | Peptidase\_S24 domain-containing protein | | afdb-uniprot50 | AF-A1VX46-F1-MODEL\_V4 | 1.0 | 7.161e-09 | 261 | 0.172 | 203 | 126 | 5 | 19 | 180 | 3 | 204 | Putative phage repressor | Putative phage repressor | | afdb-uniprot50 | AF-A0A2S9TGE6-F1-MODEL\_V4 | 1.0 | 5.056e-09 | 261 | 0.183 | 218 | 134 | 6 | 5 | 180 | 7 | 222 | Phage repressor protein | Phage repressor protein | | afdb-uniprot50 | AF-A0A2E5FYH6-F1-MODEL\_V4 | 1.0 | 8.041e-09 | 261 | 0.162 | 240 | 137 | 6 | 4 | 180 | 13 | 251 | HTH cro/C1-type domain-containing protein | HTH cro/C1-type domain-containing protein | | afdb-uniprot50 | AF-A0A317EB58-F1-MODEL\_V4 | 1.0 | 2.672e-09 | 261 | 0.191 | 225 | 128 | 10 | 5 | 180 | 34 | 253 | Peptidase\_S24 domain-containing protein | Peptidase\_S24 domain-containing protein | | afdb-uniprot50 | AF-A0A2S4J7K4-F1-MODEL\_V4 | 1.0 | 3e-09 | 261 | 0.176 | 255 | 131 | 7 | 4 | 180 | 6 | 259 | XRE family transcriptional regulator | XRE family transcriptional regulator | | afdb-uniprot50 | AF-A0A2N0ZNY9-F1-MODEL\_V4 | 1.0 | 7.161e-09 | 261 | 0.128 | 257 | 143 | 4 | 4 | 180 | 12 | 267 | HTH cro/C1-type domain-containing protein | HTH cro/C1-type domain-containing protein | | afdb-uniprot50 | AF-A0A3B8ZF36-F1-MODEL\_V4 | 1.0 | 1.412e-09 | 261 | 0.188 | 239 | 127 | 8 | 8 | 180 | 42 | 279 | Peptidase\_S24 domain-containing protein | Peptidase\_S24 domain-containing protein | | afdb-uniprot50 | AF-A0A0U4VN79-F1-MODEL\_V4 | 1.0 | 2.672e-09 | 261 | 0.149 | 274 | 133 | 7 | 5 | 180 | 9 | 280 | Repressor | Repressor | | afdb-uniprot50 | AF-A0A062GNF5-F1-MODEL\_V4 | 1.0 | 5.975e-06 | 260 | 0.247 | 89 | 63 | 2 | 93 | 178 | 3 | 90 | Peptidase S24-like family protein | Peptidase S24-like family protein | | afdb-uniprot50 | AF-A0A7T8S055-F1-MODEL\_V4 | 1.0 | 3.289e-07 | 260 | 0.195 | 128 | 93 | 5 | 61 | 180 | 41 | 166 | Helix-turn-helix transcriptional regulator | Helix-turn-helix transcriptional regulator | | afdb-uniprot50 | AF-A0A7U9HK71-F1-MODEL\_V4 | 1.0 | 2.521e-09 | 260 | 0.164 | 219 | 138 | 9 | 4 | 180 | 20 | 235 | Peptidase\_S24 domain-containing protein | Peptidase\_S24 domain-containing protein | | afdb-uniprot50 | AF-A0A7X5UB38-F1-MODEL\_V4 | 1.0 | 2.831e-09 | 260 | 0.157 | 229 | 136 | 8 | 5 | 180 | 15 | 239 | Peptidase\_S24 domain-containing protein | Peptidase\_S24 domain-containing protein | | afdb-uniprot50 | AF-A0A031LX11-F1-MODEL\_V4 | 1.0 | 3.571e-09 | 260 | 0.141 | 241 | 138 | 7 | 5 | 178 | 4 | 242 | Alkaline phosphatase | Alkaline phosphatase | | afdb-uniprot50 | AF-A0A258UIF6-F1-MODEL\_V4 | 1.0 | 7.161e-09 | 260 | 0.211 | 217 | 129 | 8 | 5 | 180 | 36 | 251 | HTH cro/C1-type domain-containing protein | HTH cro/C1-type domain-containing protein | | afdb-uniprot50 | AF-A0A6J4EE08-F1-MODEL\_V4 | 1.0 | 3.18e-09 | 260 | 0.163 | 238 | 138 | 5 | 4 | 180 | 16 | 253 | HTH-type transcriptional regulator PrtR | HTH-type transcriptional regulator PrtR | | afdb-uniprot50 | AF-A0A0P9ZT73-F1-MODEL\_V4 | 1.0 | 1.585e-09 | 260 | 0.169 | 266 | 130 | 7 | 5 | 180 | 3 | 267 | Cro/Cl family transcriptional regulator | Cro/Cl family transcriptional regulator | | afdb-uniprot50 | AF-A0A1L7NES5-F1-MODEL\_V4 | 1.0 | 2.504e-06 | 259 | 0.233 | 107 | 77 | 1 | 79 | 180 | 90 | 196 | Transcriptional regulator | Transcriptional regulator | | afdb-uniprot50 | AF-A0A081MYG4-F1-MODEL\_V4 | 1.0 | 1.014e-08 | 259 | 0.195 | 200 | 124 | 6 | 13 | 180 | 4 | 198 | HTH cro/C1-type domain-containing protein | HTH cro/C1-type domain-containing protein | | afdb-uniprot50 | AF-A0A419U229-F1-MODEL\_V4 | 1.0 | 1.257e-09 | 259 | 0.213 | 215 | 111 | 9 | 19 | 178 | 4 | 215 | Phage repressor protein C with HTH and peptisase S24 domain | Phage repressor protein C with HTH and peptisase S24 domain | | afdb-uniprot50 | AF-A0A2G2Q498-F1-MODEL\_V4 | 1.0 | 2.245e-09 | 259 | 0.195 | 225 | 128 | 8 | 1 | 180 | 5 | 221 | Transcriptional regulator | Transcriptional regulator | | afdb-uniprot50 | AF-A0A7Y1GCX0-F1-MODEL\_V4 | 1.0 | 2.104e-06 | 259 | 0.242 | 107 | 76 | 1 | 79 | 180 | 130 | 236 | Helix-turn-helix transcriptional regulator | Helix-turn-helix transcriptional regulator | | afdb-uniprot50 | AF-A0A094SR02-F1-MODEL\_V4 | 1.0 | 3.784e-09 | 259 | 0.21 | 228 | 125 | 11 | 4 | 180 | 13 | 236 | Peptidase\_S24 domain-containing protein | Peptidase\_S24 domain-containing protein | | afdb-uniprot50 | AF-A0A4Q3MRY5-F1-MODEL\_V4 | 1.0 | 3.369e-09 | 259 | 0.191 | 245 | 131 | 9 | 1 | 180 | 1 | 243 | LexA family transcriptional repressor | LexA family transcriptional repressor | | afdb-uniprot50 | AF-A0A7H1NPH5-F1-MODEL\_V4 | 1.0 | 6.757e-09 | 259 | 0.162 | 234 | 136 | 8 | 4 | 180 | 28 | 258 | HTH-type transcriptional regulator PrtR | HTH-type transcriptional regulator PrtR | | afdb-uniprot50 | AF-A0A1H8PP99-F1-MODEL\_V4 | 1.0 | 1.496e-09 | 258 | 0.2 | 215 | 119 | 6 | 14 | 180 | 1 | 210 | Helix-turn-helix | Helix-turn-helix | | afdb-uniprot50 | AF-A0A1I5S1T1-F1-MODEL\_V4 | 1.0 | 4.503e-09 | 258 | 0.205 | 224 | 130 | 9 | 1 | 180 | 1 | 220 | Phage repressor protein C, contains Cro/C1-type HTH and peptisase s24 domains | Phage repressor protein C, contains Cro/C1-type HTH and peptisase s24 domains | | afdb-uniprot50 | AF-A0A165VS19-F1-MODEL\_V4 | 1.0 | 2.504e-06 | 258 | 0.235 | 106 | 77 | 1 | 79 | 180 | 107 | 212 | Peptidase\_S24 domain-containing protein | Peptidase\_S24 domain-containing protein | | afdb-uniprot50 | AF-A0A378PYP5-F1-MODEL\_V4 | 1.0 | 5.678e-09 | 258 | 0.163 | 233 | 139 | 6 | 1 | 180 | 3 | 232 | Uncharacterized HTH-type transcriptional regulator HI\_1476 | Uncharacterized HTH-type transcriptional regulator HI\_1476 | | afdb-uniprot50 | AF-A0A386DUQ7-F1-MODEL\_V4 | 1.0 | 9.03e-09 | 258 | 0.206 | 213 | 128 | 4 | 4 | 176 | 39 | 250 | Putative phage repressor | Putative phage repressor | | afdb-uniprot50 | AF-A0A0P9KR81-F1-MODEL\_V4 | 1.0 | 2.831e-09 | 258 | 0.166 | 258 | 134 | 9 | 1 | 180 | 2 | 256 | HTH cro/C1-type domain-containing protein | HTH cro/C1-type domain-containing protein | | afdb-uniprot50 | AF-A0A7D5QHE9-F1-MODEL\_V4 | 1.0 | 4.249e-09 | 258 | 0.148 | 249 | 138 | 3 | 6 | 180 | 22 | 270 | Helix-turn-helix transcriptional regulator | Helix-turn-helix transcriptional regulator | | afdb-uniprot50 | AF-A0A8B6UYU7-F1-MODEL\_V4 | 1.0 | 1.057e-09 | 258 | 0.145 | 274 | 136 | 8 | 4 | 180 | 14 | 286 | Helix-turn-helix domain-containing protein | Helix-turn-helix domain-containing protein | | afdb-uniprot50 | AF-A0A4U1KS30-F1-MODEL\_V4 | 1.0 | 1.78e-09 | 258 | 0.15 | 285 | 132 | 7 | 4 | 180 | 6 | 288 | LexA family transcriptional regulator | LexA family transcriptional regulator | | afdb-uniprot50 | AF-A0A285Q3X1-F1-MODEL\_V4 | 1.0 | 4.249e-09 | 258 | 0.138 | 289 | 136 | 5 | 4 | 180 | 7 | 294 | Phage repressor protein C, contains Cro/C1-type HTH and peptisase s24 domains | Phage repressor protein C, contains Cro/C1-type HTH and peptisase s24 domains | | afdb-uniprot50 | AF-A0A3D4Z7E1-F1-MODEL\_V4 | 1.0 | 4.738e-06 | 257 | 0.27 | 96 | 66 | 4 | 87 | 180 | 17 | 110 | Peptidase\_S24 domain-containing protein | Peptidase\_S24 domain-containing protein | | afdb-uniprot50 | AF-A0A0R0C0L3-F1-MODEL\_V4 | 1.0 | 2.504e-06 | 257 | 0.257 | 101 | 66 | 5 | 87 | 180 | 22 | 120 | Uncharacterized protein | Uncharacterized protein | | afdb-uniprot50 | AF-F4G9T8-F1-MODEL\_V4 | 1.0 | 4.691e-10 | 257 | 0.256 | 187 | 110 | 7 | 15 | 180 | 8 | 186 | Peptidase S24/S26A/S26B, conserved region | Peptidase S24/S26A/S26B, conserved region | | afdb-uniprot50 | AF-A0A4P6X1R6-F1-MODEL\_V4 | 1.0 | 6.644e-10 | 257 | 0.251 | 187 | 111 | 7 | 15 | 180 | 8 | 186 | Peptidase S24-like protein | Peptidase S24-like protein | | afdb-uniprot50 | AF-A0A1G7S5X8-F1-MODEL\_V4 | 1.0 | 3.784e-09 | 257 | 0.227 | 202 | 121 | 7 | 13 | 180 | 2 | 202 | Helix-turn-helix domain-containing protein | Helix-turn-helix domain-containing protein | | afdb-uniprot50 | AF-A0A4R1V102-F1-MODEL\_V4 | 1.0 | 1.075e-08 | 257 | 0.168 | 202 | 141 | 4 | 5 | 180 | 36 | 236 | Phage repressor protein C with HTH and peptisase S24 domain | Phage repressor protein C with HTH and peptisase S24 domain | | afdb-uniprot50 | AF-A0A1A9RFF4-F1-MODEL\_V4 | 1.0 | 6.377e-09 | 257 | 0.181 | 231 | 135 | 5 | 4 | 180 | 7 | 237 | HTH cro/C1-type domain-containing protein | HTH cro/C1-type domain-containing protein | | afdb-uniprot50 | AF-A0A848FZX3-F1-MODEL\_V4 | 1.0 | 3.571e-09 | 257 | 0.188 | 239 | 131 | 7 | 4 | 180 | 7 | 244 | Helix-turn-helix transcriptional regulator | Helix-turn-helix transcriptional regulator | | afdb-uniprot50 | AF-A0A2E8IKW7-F1-MODEL\_V4 | 1.0 | 4.322e-08 | 257 | 0.141 | 254 | 139 | 5 | 4 | 180 | 9 | 260 | XRE family transcriptional regulator | XRE family transcriptional regulator | | afdb-uniprot50 | AF-A0A3M4BSU0-F1-MODEL\_V4 | 1.0 | 3e-09 | 257 | 0.173 | 236 | 131 | 6 | 6 | 180 | 35 | 267 | HTH cro/C1-type domain-containing protein | HTH cro/C1-type domain-containing protein | | afdb-uniprot50 | AF-A0A2Z4RT63-F1-MODEL\_V4 | 1.0 | 2.521e-09 | 257 | 0.138 | 267 | 136 | 7 | 7 | 180 | 24 | 289 | Helix-turn-helix domain-containing protein | Helix-turn-helix domain-containing protein | | afdb-uniprot50 | AF-A0A1E8PLB2-F1-MODEL\_V4 | 1.0 | 1.323e-06 | 256 | 0.228 | 118 | 83 | 4 | 60 | 174 | 2 | 114 | Peptidase\_S24 domain-containing protein | Peptidase\_S24 domain-containing protein | | afdb-uniprot50 | AF-A0A2S9K153-F1-MODEL\_V4 | 1.0 | 1.697e-05 | 256 | 0.2 | 100 | 75 | 2 | 86 | 180 | 31 | 130 | Peptidase\_S24 domain-containing protein | Peptidase\_S24 domain-containing protein | | afdb-uniprot50 | AF-A0A5C8BLB1-F1-MODEL\_V4 | 1.0 | 8.461e-06 | 256 | 0.21 | 100 | 77 | 2 | 78 | 176 | 51 | 149 | S24 family peptidase | S24 family peptidase | | afdb-uniprot50 | AF-A0A0F4NKC0-F1-MODEL\_V4 | 1.0 | 3.571e-09 | 256 | 0.193 | 207 | 131 | 7 | 4 | 179 | 5 | 206 | HTH cro/C1-type domain-containing protein | HTH cro/C1-type domain-containing protein | | afdb-uniprot50 | AF-R6P6E9-F1-MODEL\_V4 | 1.0 | 6.017e-09 | 256 | 0.186 | 214 | 127 | 8 | 5 | 180 | 7 | 211 | Phage transcriptional regulator | Phage transcriptional regulator | | afdb-uniprot50 | AF-D4XHG0-F1-MODEL\_V4 | 1.0 | 6.017e-09 | 256 | 0.168 | 220 | 136 | 9 | 4 | 180 | 8 | 223 | DNA-binding helix-turn-helix protein | DNA-binding helix-turn-helix protein | | afdb-uniprot50 | AF-A0A6N4T9B0-F1-MODEL\_V4 | 1.0 | 4.01e-09 | 256 | 0.168 | 225 | 136 | 8 | 5 | 179 | 7 | 230 | HTH cro/C1-type domain-containing protein | HTH cro/C1-type domain-containing protein | | afdb-uniprot50 | AF-A0A318DXJ5-F1-MODEL\_V4 | 1.0 | 8.521e-09 | 256 | 0.159 | 219 | 139 | 8 | 6 | 180 | 14 | 231 | HTH cro/C1-type domain-containing protein | HTH cro/C1-type domain-containing protein | | afdb-uniprot50 | AF-F2JZ54-F1-MODEL\_V4 | 1.0 | 1.436e-08 | 256 | 0.173 | 236 | 131 | 6 | 4 | 180 | 6 | 236 | Putative phage repressor | Putative phage repressor | | afdb-uniprot50 | AF-A0A2N6F112-F1-MODEL\_V4 | 1.0 | 2.521e-09 | 256 | 0.216 | 236 | 122 | 8 | 6 | 180 | 14 | 247 | Peptidase\_S24 domain-containing protein | Peptidase\_S24 domain-containing protein | | afdb-uniprot50 | AF-Q06553-F1-MODEL\_V4 | 1.0 | 5.358e-09 | 256 | 0.149 | 234 | 141 | 7 | 4 | 180 | 15 | 247 | HTH-type transcriptional regulator PrtR | HTH-type transcriptional regulator PrtR | | afdb-uniprot50 | AF-A0A7Z2ZDG1-F1-MODEL\_V4 | 1.0 | 1.999e-09 | 256 | 0.186 | 247 | 129 | 8 | 5 | 180 | 10 | 255 | Cro/Cl family transcriptional regulator | Cro/Cl family transcriptional regulator | | afdb-uniprot50 | AF-A0A3M3D8N0-F1-MODEL\_V4 | 1.0 | 5.678e-09 | 256 | 0.169 | 236 | 137 | 6 | 2 | 180 | 41 | 274 | Cro/CI family transcriptional regulator | Cro/CI family transcriptional regulator | | afdb-uniprot50 | AF-A0A6S7B3R8-F1-MODEL\_V4 | 1.0 | 4.772e-09 | 256 | 0.166 | 265 | 131 | 6 | 4 | 178 | 16 | 280 | HTH-type transcriptional regulator PrtR | HTH-type transcriptional regulator PrtR | | afdb-uniprot50 | AF-A0A3D3HTG8-F1-MODEL\_V4 | 1.0 | 6.757e-09 | 256 | 0.17 | 229 | 136 | 7 | 4 | 179 | 62 | 289 | Phage repressor protein | Phage repressor protein | | afdb-uniprot50 | AF-A0A7X3HDH1-F1-MODEL\_V4 | 1.0 | 7.588e-09 | 256 | 0.165 | 229 | 137 | 5 | 5 | 180 | 163 | 390 | Helix-turn-helix domain-containing protein | Helix-turn-helix domain-containing protein | | afdb-uniprot50 | AF-A0A6A7Z0J1-F1-MODEL\_V4 | 1.0 | 4.219e-06 | 255 | 0.245 | 106 | 76 | 1 | 79 | 180 | 65 | 170 | Peptidase\_S24 domain-containing protein | Peptidase\_S24 domain-containing protein | | afdb-uniprot50 | AF-A0A346N4Q6-F1-MODEL\_V4 | 1.0 | 5.678e-09 | 255 | 0.181 | 220 | 130 | 7 | 4 | 178 | 6 | 220 | Helix-turn-helix transcriptional regulator | Helix-turn-helix transcriptional regulator | | afdb-uniprot50 | AF-A0A1H2PQK0-F1-MODEL\_V4 | 1.0 | 3.18e-09 | 255 | 0.172 | 226 | 136 | 8 | 4 | 180 | 5 | 228 | Phage repressor protein C, contains Cro/C1-type HTH and peptisase s24 domains | Phage repressor protein C, contains Cro/C1-type HTH and peptisase s24 domains | | afdb-uniprot50 | AF-A0A2L0S440-F1-MODEL\_V4 | 1.0 | 1.279e-08 | 255 | 0.163 | 220 | 143 | 5 | 1 | 180 | 10 | 228 | HTH cro/C1-type domain-containing protein | HTH cro/C1-type domain-containing protein | | afdb-uniprot50 | AF-A0A4R3NNU1-F1-MODEL\_V4 | 1.0 | 1.811e-08 | 255 | 0.165 | 217 | 141 | 5 | 4 | 180 | 14 | 230 | Phage repressor protein C with HTH and peptisase S24 domain | Phage repressor protein C with HTH and peptisase S24 domain | | afdb-uniprot50 | AF-A0A7Y1BXH5-F1-MODEL\_V4 | 1.0 | 8.521e-09 | 255 | 0.157 | 242 | 139 | 6 | 4 | 180 | 14 | 255 | LexA family transcriptional regulator | LexA family transcriptional regulator | | afdb-uniprot50 | AF-W6W2S2-F1-MODEL\_V4 | 1.0 | 2.521e-09 | 255 | 0.143 | 265 | 135 | 7 | 7 | 180 | 24 | 287 | Putative phage repressor | Putative phage repressor | | afdb-uniprot50 | AF-A0A4U0PYZ7-F1-MODEL\_V4 | 1.0 | 4.249e-09 | 255 | 0.171 | 239 | 135 | 7 | 5 | 180 | 88 | 326 | Helix-turn-helix transcriptional regulator | Helix-turn-helix transcriptional regulator | | afdb-uniprot50 | AF-A0A2T5S3K6-F1-MODEL\_V4 | 1.0 | 1.112e-06 | 254 | 0.228 | 118 | 85 | 3 | 68 | 180 | 17 | 133 | Peptidase S24-like protein | Peptidase S24-like protein | | afdb-uniprot50 | AF-A0A845JXB3-F1-MODEL\_V4 | 1.0 | 5.678e-09 | 254 | 0.183 | 224 | 133 | 7 | 5 | 180 | 8 | 229 | Transcriptional regulator | Transcriptional regulator | | afdb-uniprot50 | AF-B5JYF6-F1-MODEL\_V4 | 1.0 | 1.139e-08 | 254 | 0.18 | 216 | 135 | 7 | 4 | 180 | 18 | 230 | Prophage MuMc02, peptidase, family S24 | Prophage MuMc02, peptidase, family S24 | | afdb-uniprot50 | AF-A0A370QQ69-F1-MODEL\_V4 | 1.0 | 5.056e-09 | 254 | 0.157 | 228 | 141 | 6 | 4 | 180 | 12 | 239 | Helix-turn-helix protein | Helix-turn-helix protein | | afdb-uniprot50 | AF-A0A1G5MEX5-F1-MODEL\_V4 | 1.0 | 6.757e-09 | 254 | 0.19 | 236 | 129 | 8 | 4 | 180 | 10 | 242 | Phage repressor protein C, contains Cro/C1-type HTH and peptisase s24 domains | Phage repressor protein C, contains Cro/C1-type HTH and peptisase s24 domains | | afdb-uniprot50 | AF-A0A3M5WRJ3-F1-MODEL\_V4 | 1.0 | 4.503e-09 | 254 | 0.166 | 246 | 134 | 6 | 6 | 180 | 99 | 344 | Putative transcriptional regulator | Putative transcriptional regulator | | afdb-uniprot50 | AF-A0A096AWJ2-F1-MODEL\_V4 | 1.0 | 1.402e-06 | 253 | 0.237 | 118 | 82 | 5 | 70 | 180 | 1 | 117 | Peptidase\_S24 domain-containing protein | Peptidase\_S24 domain-containing protein | | afdb-uniprot50 | AF-A0A8B3GBH4-F1-MODEL\_V4 | 1.0 | 3.757e-06 | 253 | 0.198 | 106 | 80 | 1 | 80 | 180 | 47 | 152 | Uncharacterized protein | Uncharacterized protein | | afdb-uniprot50 | AF-A0A4V1KJS4-F1-MODEL\_V4 | 1.0 | 1.522e-08 | 253 | 0.236 | 173 | 96 | 6 | 43 | 180 | 5 | 176 | Helix-turn-helix transcriptional regulator | Helix-turn-helix transcriptional regulator | | afdb-uniprot50 | AF-A0A1N7Q2M4-F1-MODEL\_V4 | 1.0 | 6.597e-07 | 253 | 0.236 | 127 | 86 | 4 | 61 | 180 | 75 | 197 | Peptidase S24-like | Peptidase S24-like | | afdb-uniprot50 | AF-A0A173JAB6-F1-MODEL\_V4 | 1.0 | 4.738e-06 | 253 | 0.177 | 107 | 83 | 1 | 78 | 179 | 83 | 189 | Transcriptional regulator | Transcriptional regulator | | afdb-uniprot50 | AF-A0A7X8V275-F1-MODEL\_V4 | 1.0 | 1.355e-08 | 253 | 0.219 | 210 | 123 | 7 | 4 | 176 | 6 | 211 | Helix-turn-helix domain-containing protein | Helix-turn-helix domain-containing protein | | afdb-uniprot50 | AF-A0A5C7QA30-F1-MODEL\_V4 | 1.0 | 8.521e-09 | 253 | 0.184 | 206 | 133 | 7 | 5 | 180 | 18 | 218 | Helix-turn-helix transcriptional regulator | Helix-turn-helix transcriptional regulator | | afdb-uniprot50 | AF-A0A449IEK0-F1-MODEL\_V4 | 1.0 | 6.377e-09 | 253 | 0.174 | 229 | 134 | 6 | 4 | 180 | 14 | 239 | Peptidase S24, S26A and S26B | Peptidase S24, S26A and S26B | | afdb-uniprot50 | AF-A0A7Y4UN23-F1-MODEL\_V4 | 1.0 | 2.245e-09 | 253 | 0.242 | 198 | 121 | 6 | 4 | 180 | 78 | 267 | S24 family peptidase | S24 family peptidase | | afdb-uniprot50 | AF-A0A0P7CW07-F1-MODEL\_V4 | 1.0 | 9.03e-09 | 253 | 0.163 | 239 | 137 | 6 | 5 | 180 | 29 | 267 | Transcriptional regulator | Transcriptional regulator | | afdb-uniprot50 | AF-A0A4R6DX28-F1-MODEL\_V4 | 1.0 | 4.503e-09 | 253 | 0.153 | 260 | 134 | 8 | 4 | 178 | 19 | 277 | Peptidase S24-like protein | Peptidase S24-like protein | | afdb-uniprot50 | AF-A0A1H2N8M0-F1-MODEL\_V4 | 1.0 | 2.672e-09 | 253 | 0.136 | 264 | 138 | 5 | 6 | 180 | 23 | 285 | Helix-turn-helix | Helix-turn-helix | | afdb-uniprot50 | AF-A0A3S4MVN3-F1-MODEL\_V4 | 1.0 | 4.01e-09 | 253 | 0.209 | 244 | 124 | 10 | 4 | 180 | 66 | 307 | CI repressor protein | CI repressor protein | | afdb-uniprot50 | AF-S6WAL0-F1-MODEL\_V4 | 1.0 | 2.104e-06 | 252 | 0.21 | 114 | 81 | 2 | 72 | 180 | 1 | 110 | Transcriptional regulator | Transcriptional regulator | | afdb-uniprot50 | AF-A0A1X0T0I3-F1-MODEL\_V4 | 1.0 | 1.112e-06 | 252 | 0.225 | 124 | 88 | 3 | 61 | 180 | 13 | 132 | Phage repressor | Phage repressor | | afdb-uniprot50 | AF-A0A5R9NXX0-F1-MODEL\_V4 | 1.0 | 8.521e-09 | 252 | 0.18 | 183 | 131 | 7 | 13 | 180 | 3 | 181 | XRE family transcriptional regulator | XRE family transcriptional regulator | | afdb-uniprot50 | AF-A0A839ILY4-F1-MODEL\_V4 | 1.0 | 1.139e-08 | 252 | 0.184 | 228 | 133 | 6 | 4 | 180 | 11 | 236 | Helix-turn-helix transcriptional regulator | Helix-turn-helix transcriptional regulator | | afdb-uniprot50 | AF-A0A2N9XC71-F1-MODEL\_V4 | 1.0 | 6.017e-09 | 252 | 0.167 | 233 | 137 | 8 | 4 | 180 | 8 | 239 | Chromophore lyase | Chromophore lyase | | afdb-uniprot50 | AF-A0A2Z6EVF8-F1-MODEL\_V4 | 1.0 | 4.249e-09 | 252 | 0.178 | 219 | 132 | 9 | 4 | 179 | 28 | 241 | Peptidase S24, S26A and S26B with Helix-turn-helix motif | Peptidase S24, S26A and S26B with Helix-turn-helix motif | | afdb-uniprot50 | AF-A0A1G4ZS22-F1-MODEL\_V4 | 1.0 | 1.279e-08 | 252 | 0.148 | 229 | 141 | 4 | 4 | 180 | 9 | 235 | Phage repressor protein C, contains Cro/C1-type HTH and peptisase s24 domains | Phage repressor protein C, contains Cro/C1-type HTH and peptisase s24 domains | | afdb-uniprot50 | AF-A0A7W8HG37-F1-MODEL\_V4 | 1.0 | 2.672e-09 | 252 | 0.168 | 249 | 133 | 8 | 5 | 180 | 12 | 259 | Phage repressor protein C with HTH and peptisase S24 domain | Phage repressor protein C with HTH and peptisase S24 domain | | afdb-uniprot50 | AF-W6WJM4-F1-MODEL\_V4 | 1.0 | 5.056e-09 | 252 | 0.165 | 224 | 139 | 5 | 4 | 180 | 37 | 259 | Putative phage repressor | Putative phage repressor | | afdb-uniprot50 | AF-K2C6Z1-F1-MODEL\_V4 | 1.0 | 2.155e-08 | 252 | 0.173 | 231 | 134 | 6 | 4 | 180 | 31 | 258 | HTH cro/C1-type domain-containing protein | HTH cro/C1-type domain-containing protein | | afdb-uniprot50 | AF-A0A0M7LNV7-F1-MODEL\_V4 | 1.0 | 5.678e-09 | 252 | 0.187 | 219 | 135 | 7 | 4 | 180 | 54 | 271 | DNA polymerase V subunit UmuD | DNA polymerase V subunit UmuD | | afdb-uniprot50 | AF-A0A2S8Y6M7-F1-MODEL\_V4 | 1.0 | 1.78e-09 | 252 | 0.138 | 267 | 136 | 7 | 7 | 180 | 24 | 289 | Propanediol utilization protein | Propanediol utilization protein | | afdb-uniprot50 | AF-A0A833N995-F1-MODEL\_V4 | 1.0 | 8.041e-09 | 252 | 0.146 | 314 | 134 | 6 | 1 | 180 | 61 | 374 | Phage cI repressor (ACLAME 5) | Phage cI repressor (ACLAME 5) | | afdb-uniprot50 | AF-A0A1K1W354-F1-MODEL\_V4 | 1.0 | 8.521e-09 | 251 | 0.23 | 182 | 93 | 5 | 41 | 180 | 3 | 179 | Phage repressor protein C, contains Cro/C1-type HTH and peptisase s24 domains | Phage repressor protein C, contains Cro/C1-type HTH and peptisase s24 domains | | afdb-uniprot50 | AF-U1ZQ79-F1-MODEL\_V4 | 1.0 | 8.461e-06 | 251 | 0.25 | 100 | 68 | 3 | 87 | 179 | 91 | 190 | Peptidase\_S24 domain-containing protein | Peptidase\_S24 domain-containing protein | | afdb-uniprot50 | AF-A0A7S9UK10-F1-MODEL\_V4 | 1.0 | 6.709e-06 | 251 | 0.223 | 103 | 78 | 2 | 79 | 180 | 98 | 199 | S24 family peptidase | S24 family peptidase | | afdb-uniprot50 | AF-A0A0A7S0B9-F1-MODEL\_V4 | 1.0 | 8.521e-09 | 251 | 0.202 | 222 | 124 | 9 | 8 | 180 | 3 | 220 | Putative transcriptional regulator | Putative transcriptional regulator | | afdb-uniprot50 | AF-A0A2E6QFD6-F1-MODEL\_V4 | 1.0 | 1.014e-08 | 251 | 0.186 | 215 | 131 | 7 | 4 | 180 | 16 | 224 | HTH cro/C1-type domain-containing protein | HTH cro/C1-type domain-containing protein | | afdb-uniprot50 | AF-A0A171KSF2-F1-MODEL\_V4 | 1.0 | 5.056e-09 | 251 | 0.173 | 219 | 131 | 9 | 7 | 180 | 6 | 219 | HTH cro/C1-type domain-containing protein | HTH cro/C1-type domain-containing protein | | afdb-uniprot50 | AF-A0A3R8XKJ5-F1-MODEL\_V4 | 1.0 | 6.377e-09 | 251 | 0.159 | 232 | 129 | 6 | 10 | 180 | 2 | 228 | XRE family transcriptional regulator | XRE family transcriptional regulator | | afdb-uniprot50 | AF-A0A3S0YRJ1-F1-MODEL\_V4 | 1.0 | 8.041e-09 | 251 | 0.194 | 226 | 130 | 8 | 4 | 180 | 9 | 231 | LexA family transcriptional regulator | LexA family transcriptional regulator | | afdb-uniprot50 | AF-A0A1G6NPZ1-F1-MODEL\_V4 | 1.0 | 6.377e-09 | 251 | 0.159 | 245 | 134 | 6 | 5 | 179 | 12 | 254 | Phage repressor protein C, contains Cro/C1-type HTH and peptisase s24 domains | Phage repressor protein C, contains Cro/C1-type HTH and peptisase s24 domains | | afdb-uniprot50 | AF-A0A2G4E5F7-F1-MODEL\_V4 | 1.0 | 9.97e-10 | 251 | 0.174 | 269 | 128 | 8 | 5 | 180 | 3 | 270 | Cro/Cl family transcriptional regulator | Cro/Cl family transcriptional regulator | | afdb-uniprot50 | AF-A0A8A8PHC1-F1-MODEL\_V4 | 1.0 | 1.919e-08 | 251 | 0.186 | 263 | 127 | 7 | 4 | 180 | 29 | 290 | Helix-turn-helix transcriptional regulator | Helix-turn-helix transcriptional regulator | | afdb-uniprot50 | AF-A0A8B4I5H6-F1-MODEL\_V4 | 1.0 | 8.521e-09 | 251 | 0.168 | 237 | 135 | 6 | 4 | 180 | 92 | 326 | Putative phage repressor | Putative phage repressor | | afdb-uniprot50 | AF-A0A2N9Y4B0-F1-MODEL\_V4 | 1.0 | 7.85e-07 | 250 | 0.184 | 125 | 93 | 4 | 57 | 178 | 8 | 126 | Peptidase\_S24 domain-containing protein | Peptidase\_S24 domain-containing protein | | afdb-uniprot50 | AF-A0A0V8T744-F1-MODEL\_V4 | 1.0 | 4.219e-06 | 250 | 0.152 | 105 | 85 | 2 | 79 | 180 | 82 | 185 | Cro/Cl family transcriptional regulator | Cro/Cl family transcriptional regulator | | afdb-uniprot50 | AF-C0EP73-F1-MODEL\_V4 | 1.0 | 4.772e-09 | 250 | 0.17 | 211 | 131 | 7 | 10 | 180 | 5 | 211 | Peptidase S24-like protein | Peptidase S24-like protein | | afdb-uniprot50 | AF-A0A1G5V1P4-F1-MODEL\_V4 | 1.0 | 1.075e-08 | 250 | 0.181 | 226 | 137 | 7 | 1 | 180 | 2 | 225 | Phage repressor protein C, contains Cro/C1-type HTH and peptisase s24 domains | Phage repressor protein C, contains Cro/C1-type HTH and peptisase s24 domains | | afdb-uniprot50 | AF-A0A1D2QRN9-F1-MODEL\_V4 | 1.0 | 4.772e-09 | 250 | 0.214 | 224 | 124 | 8 | 5 | 179 | 7 | 227 | Peptidase\_S24 domain-containing protein | Peptidase\_S24 domain-containing protein | | afdb-uniprot50 | AF-A0A6N8AT90-F1-MODEL\_V4 | 1.0 | 4.01e-09 | 250 | 0.196 | 229 | 124 | 10 | 5 | 180 | 11 | 232 | Helix-turn-helix transcriptional regulator | Helix-turn-helix transcriptional regulator | | afdb-uniprot50 | AF-A0A530R6J1-F1-MODEL\_V4 | 1.0 | 1.522e-08 | 250 | 0.185 | 221 | 133 | 8 | 5 | 180 | 26 | 244 | Helix-turn-helix domain-containing protein | Helix-turn-helix domain-containing protein | | afdb-uniprot50 | AF-A0A1B8Q949-F1-MODEL\_V4 | 1.0 | 4.249e-09 | 250 | 0.146 | 245 | 136 | 7 | 5 | 178 | 5 | 247 | HTH cro/C1-type domain-containing protein | HTH cro/C1-type domain-containing protein | | afdb-uniprot50 | AF-A0A7U3YNF5-F1-MODEL\_V4 | 1.0 | 1.207e-08 | 250 | 0.16 | 243 | 135 | 6 | 4 | 180 | 6 | 245 | Putative phage repressor | Putative phage repressor | | afdb-uniprot50 | AF-A0A433C752-F1-MODEL\_V4 | 1.0 | 2.245e-09 | 250 | 0.188 | 202 | 137 | 8 | 4 | 180 | 47 | 246 | S24 family peptidase | S24 family peptidase | | afdb-uniprot50 | AF-A0A2S7E1D1-F1-MODEL\_V4 | 1.0 | 4.01e-09 | 250 | 0.182 | 241 | 132 | 5 | 5 | 180 | 35 | 275 | HTH cro/C1-type domain-containing protein | HTH cro/C1-type domain-containing protein | | afdb-uniprot50 | AF-A0A495IUG7-F1-MODEL\_V4 | 1.0 | 2.155e-08 | 250 | 0.139 | 323 | 132 | 5 | 4 | 180 | 22 | 344 | Phage repressor protein C with HTH and peptisase S24 domain | Phage repressor protein C with HTH and peptisase S24 domain | | afdb-uniprot50 | AF-A0A837MMU5-F1-MODEL\_V4 | 1.0 | 5.975e-06 | 249 | 0.194 | 108 | 82 | 1 | 78 | 180 | 90 | 197 | Transcriptional regulator | Transcriptional regulator | | afdb-uniprot50 | AF-A0A2D5S0G5-F1-MODEL\_V4 | 1.0 | 5.358e-09 | 249 | 0.199 | 231 | 128 | 6 | 5 | 180 | 9 | 237 | Transcriptional regulator | Transcriptional regulator | | afdb-uniprot50 | AF-A0A6N8RY26-F1-MODEL\_V4 | 1.0 | 4.58e-08 | 249 | 0.17 | 234 | 136 | 7 | 4 | 180 | 18 | 250 | Helix-turn-helix domain-containing protein | Helix-turn-helix domain-containing protein | | afdb-uniprot50 | AF-A0A7T8M381-F1-MODEL\_V4 | 1.0 | 1.049e-06 | 248 | 0.258 | 120 | 73 | 4 | 64 | 180 | 6 | 112 | S24 family peptidase | S24 family peptidase | | afdb-uniprot50 | AF-A0A4S1MQ12-F1-MODEL\_V4 | 1.0 | 2.019e-05 | 248 | 0.184 | 92 | 73 | 2 | 89 | 180 | 27 | 116 | Uncharacterized protein | Uncharacterized protein | | afdb-uniprot50 | AF-A0A4R7VYY6-F1-MODEL\_V4 | 1.0 | 3.981e-06 | 248 | 0.224 | 116 | 81 | 2 | 70 | 180 | 26 | 137 | Peptidase S24-like protein | Peptidase S24-like protein | | afdb-uniprot50 | AF-A0A7G2JK01-F1-MODEL\_V4 | 1.0 | 1.355e-08 | 248 | 0.199 | 211 | 120 | 9 | 15 | 180 | 3 | 209 | Putative HTH-type transcriptional regulator | Putative HTH-type transcriptional regulator | | afdb-uniprot50 | AF-A0A2X1U872-F1-MODEL\_V4 | 1.0 | 4.772e-09 | 248 | 0.173 | 213 | 133 | 8 | 5 | 176 | 11 | 221 | Phage repressor protein, phage associated protein | Phage repressor protein, phage associated protein | | afdb-uniprot50 | AF-A0A3B7M1G8-F1-MODEL\_V4 | 1.0 | 1.075e-08 | 248 | 0.179 | 223 | 134 | 5 | 5 | 180 | 11 | 231 | Helix-turn-helix transcriptional regulator | Helix-turn-helix transcriptional regulator | | afdb-uniprot50 | AF-N8XK06-F1-MODEL\_V4 | 1.0 | 1.014e-08 | 248 | 0.145 | 227 | 144 | 5 | 1 | 180 | 14 | 237 | HTH cro/C1-type domain-containing protein | HTH cro/C1-type domain-containing protein | | afdb-uniprot50 | AF-A0A258NSS6-F1-MODEL\_V4 | 1.0 | 1.355e-08 | 248 | 0.222 | 225 | 129 | 6 | 2 | 180 | 58 | 282 | XRE family transcriptional regulator | XRE family transcriptional regulator | | afdb-uniprot50 | AF-A0A844RU21-F1-MODEL\_V4 | 1.0 | 6.017e-09 | 248 | 0.211 | 208 | 121 | 7 | 4 | 172 | 49 | 252 | Helix-turn-helix domain-containing protein | Helix-turn-helix domain-containing protein | | afdb-uniprot50 | AF-A0A2P5MV31-F1-MODEL\_V4 | 1.0 | 1.139e-08 | 248 | 0.158 | 290 | 129 | 8 | 4 | 180 | 6 | 293 | HTH cro/C1-type domain-containing protein | HTH cro/C1-type domain-containing protein | | afdb-uniprot50 | AF-A0A178J3C6-F1-MODEL\_V4 | 1.0 | 4.503e-09 | 247 | 0.187 | 197 | 121 | 8 | 14 | 180 | 3 | 190 | HTH cro/C1-type domain-containing protein | HTH cro/C1-type domain-containing protein | | afdb-uniprot50 | AF-A0A833L7F1-F1-MODEL\_V4 | 1.0 | 1.522e-08 | 247 | 0.184 | 206 | 133 | 6 | 4 | 180 | 9 | 208 | Uncharacterized protein | Uncharacterized protein | | afdb-uniprot50 | AF-A0A1H8RCZ9-F1-MODEL\_V4 | 1.0 | 5.358e-09 | 247 | 0.175 | 233 | 139 | 7 | 1 | 180 | 2 | 234 | Peptidase S24-like | Peptidase S24-like | | afdb-uniprot50 | AF-A0A7W6RC19-F1-MODEL\_V4 | 1.0 | 2.155e-08 | 247 | 0.186 | 241 | 121 | 6 | 14 | 180 | 1 | 240 | Phage repressor protein C with HTH and peptisase S24 domain | Phage repressor protein C with HTH and peptisase S24 domain | | afdb-uniprot50 | AF-B2I5H5-F1-MODEL\_V4 | 1.0 | 6.377e-09 | 247 | 0.172 | 243 | 132 | 9 | 4 | 178 | 7 | 248 | Putative phage repressor | Putative phage repressor | | afdb-uniprot50 | AF-A0A6G6IUW8-F1-MODEL\_V4 | 1.0 | 1.279e-08 | 247 | 0.159 | 245 | 137 | 5 | 5 | 180 | 10 | 254 | Helix-turn-helix domain-containing protein | Helix-turn-helix domain-containing protein | | afdb-uniprot50 | AF-A0A2E6JYY0-F1-MODEL\_V4 | 1.0 | 1.355e-08 | 247 | 0.146 | 226 | 142 | 4 | 5 | 180 | 34 | 258 | HTH cro/C1-type domain-containing protein | HTH cro/C1-type domain-containing protein | | afdb-uniprot50 | AF-A0A7Y4W621-F1-MODEL\_V4 | 1.0 | 5.358e-09 | 247 | 0.182 | 257 | 121 | 10 | 12 | 180 | 11 | 266 | Helix-turn-helix transcriptional regulator | Helix-turn-helix transcriptional regulator | | afdb-uniprot50 | AF-A0A143ZKM8-F1-MODEL\_V4 | 1.0 | 1.014e-08 | 246 | 0.2 | 204 | 124 | 5 | 16 | 180 | 6 | 209 | Phage repressor protein C, contains Cro/C1-type HTH and peptisase s24 domains | Phage repressor protein C, contains Cro/C1-type HTH and peptisase s24 domains | | afdb-uniprot50 | AF-A0A2D3WEV3-F1-MODEL\_V4 | 1.0 | 6.757e-09 | 246 | 0.187 | 208 | 133 | 5 | 5 | 180 | 15 | 218 | Phage repressor protein | Phage repressor protein | | afdb-uniprot50 | AF-C3X7V2-F1-MODEL\_V4 | 1.0 | 1.613e-08 | 246 | 0.208 | 216 | 130 | 6 | 4 | 180 | 6 | 219 | Peptidase S24-like protein | Peptidase S24-like protein | | afdb-uniprot50 | AF-A0A126Z9R7-F1-MODEL\_V4 | 1.0 | 5.056e-09 | 246 | 0.158 | 221 | 139 | 4 | 5 | 180 | 14 | 232 | Peptidase\_S24 domain-containing protein | Peptidase\_S24 domain-containing protein | | afdb-uniprot50 | AF-A0A1U9JXL2-F1-MODEL\_V4 | 1.0 | 5.45e-08 | 246 | 0.156 | 224 | 142 | 7 | 4 | 180 | 7 | 230 | Peptidase\_S24 domain-containing protein | Peptidase\_S24 domain-containing protein | | afdb-uniprot50 | AF-A0A832UN00-F1-MODEL\_V4 | 1.0 | 2.565e-08 | 246 | 0.2 | 235 | 124 | 6 | 5 | 180 | 10 | 239 | Uncharacterized protein | Uncharacterized protein | | afdb-uniprot50 | AF-A0A068T867-F1-MODEL\_V4 | 1.0 | 2.42e-08 | 245 | 0.191 | 204 | 121 | 6 | 16 | 179 | 5 | 204 | Putative transcriptional regulator, repressor | Putative transcriptional regulator, repressor | | afdb-uniprot50 | AF-A0A0Q9EUS2-F1-MODEL\_V4 | 1.0 | 1.522e-08 | 245 | 0.142 | 231 | 142 | 8 | 5 | 179 | 5 | 235 | HTH cro/C1-type domain-containing protein | HTH cro/C1-type domain-containing protein | | afdb-uniprot50 | AF-A0A1H3KSL9-F1-MODEL\_V4 | 1.0 | 2.88e-08 | 245 | 0.137 | 261 | 137 | 6 | 4 | 180 | 8 | 264 | Peptidase S24-like | Peptidase S24-like | | afdb-uniprot50 | AF-A0A2N2KCH0-F1-MODEL\_V4 | 1.0 | 8.041e-09 | 245 | 0.148 | 263 | 137 | 5 | 5 | 180 | 13 | 275 | Peptidase\_S24 domain-containing protein | Peptidase\_S24 domain-containing protein | | afdb-uniprot50 | AF-A0A327JDI9-F1-MODEL\_V4 | 1.0 | 2.718e-08 | 244 | 0.139 | 208 | 140 | 7 | 4 | 180 | 5 | 204 | Peptidase\_S24 domain-containing protein | Peptidase\_S24 domain-containing protein | | afdb-uniprot50 | AF-A0A4Z0BX65-F1-MODEL\_V4 | 1.0 | 1.613e-08 | 244 | 0.152 | 210 | 143 | 3 | 4 | 180 | 19 | 226 | Helix-turn-helix transcriptional regulator | Helix-turn-helix transcriptional regulator | | afdb-uniprot50 | AF-A0A1H1UI98-F1-MODEL\_V4 | 1.0 | 2.88e-08 | 244 | 0.165 | 242 | 132 | 7 | 4 | 180 | 12 | 248 | Peptidase\_S24 domain-containing protein | Peptidase\_S24 domain-containing protein | | afdb-uniprot50 | AF-J2XH41-F1-MODEL\_V4 | 1.0 | 1.709e-08 | 244 | 0.155 | 232 | 138 | 4 | 4 | 179 | 76 | 305 | Putative transcriptional regulator | Putative transcriptional regulator | | afdb-uniprot50 | AF-A0A1M7LJF6-F1-MODEL\_V4 | 1.0 | 7.588e-09 | 244 | 0.129 | 254 | 131 | 5 | 16 | 180 | 116 | 368 | Peptidase S24-like | Peptidase S24-like | | afdb-uniprot50 | AF-A0A0G9JXZ6-F1-MODEL\_V4 | 1.0 | 2.034e-08 | 243 | 0.197 | 197 | 121 | 6 | 19 | 180 | 3 | 197 | Phage repressor protein | Phage repressor protein | | afdb-uniprot50 | AF-A0A1Q4CNZ7-F1-MODEL\_V4 | 1.0 | 2.034e-08 | 243 | 0.172 | 214 | 120 | 8 | 6 | 180 | 2 | 197 | HTH cro/C1-type domain-containing protein | HTH cro/C1-type domain-containing protein | | afdb-uniprot50 | AF-A0A378CDN0-F1-MODEL\_V4 | 1.0 | 1.436e-08 | 243 | 0.233 | 193 | 109 | 6 | 26 | 180 | 4 | 195 | Putative phage repressor protein CI | Putative phage repressor protein CI | | afdb-uniprot50 | AF-A0A1B9LJP2-F1-MODEL\_V4 | 1.0 | 3.052e-08 | 243 | 0.177 | 220 | 131 | 5 | 4 | 180 | 10 | 222 | Transcriptional regulator | Transcriptional regulator | | afdb-uniprot50 | AF-A0A7U5AQA5-F1-MODEL\_V4 | 1.0 | 1.436e-08 | 243 | 0.181 | 209 | 136 | 8 | 5 | 180 | 28 | 234 | Peptidase S24-like family protein | Peptidase S24-like family protein | | afdb-uniprot50 | AF-A0A1F0J3A6-F1-MODEL\_V4 | 1.0 | 1.279e-08 | 243 | 0.169 | 230 | 133 | 7 | 9 | 180 | 14 | 243 | Transcriptional regulator | Transcriptional regulator | | afdb-uniprot50 | AF-A0A2R7RTB9-F1-MODEL\_V4 | 1.0 | 2.284e-08 | 243 | 0.15 | 232 | 139 | 5 | 4 | 179 | 9 | 238 | Transcriptional regulator | Transcriptional regulator | | afdb-uniprot50 | AF-A0A855KF29-F1-MODEL\_V4 | 1.0 | 1.075e-08 | 243 | 0.18 | 255 | 130 | 6 | 4 | 180 | 6 | 259 | Phage repressor protein C | Phage repressor protein C | | afdb-uniprot50 | AF-A0A015NCW0-F1-MODEL\_V4 | 1.0 | 7.161e-09 | 242 | 0.211 | 232 | 128 | 10 | 3 | 180 | 8 | 238 | HTH cro/C1-type domain-containing protein | HTH cro/C1-type domain-containing protein | | afdb-uniprot50 | AF-A0A2V4FY31-F1-MODEL\_V4 | 1.0 | 3.052e-08 | 242 | 0.182 | 235 | 134 | 5 | 4 | 180 | 14 | 248 | Helix-turn-helix transcriptional regulator | Helix-turn-helix transcriptional regulator | | afdb-uniprot50 | AF-A0A1S8DCK6-F1-MODEL\_V4 | 1.0 | 4.503e-09 | 242 | 0.207 | 246 | 124 | 9 | 5 | 180 | 3 | 247 | Peptidase\_S24 domain-containing protein | Peptidase\_S24 domain-containing protein | | afdb-uniprot50 | AF-A0A6J4E657-F1-MODEL\_V4 | 1.0 | 7.588e-09 | 242 | 0.167 | 245 | 123 | 7 | 16 | 180 | 24 | 267 | HTH cro/C1-type domain-containing protein | HTH cro/C1-type domain-containing protein | | afdb-uniprot50 | AF-A0A7X1TFZ1-F1-MODEL\_V4 | 1.0 | 8.041e-09 | 242 | 0.16 | 281 | 131 | 11 | 4 | 180 | 6 | 285 | Peptidase\_S24 domain-containing protein | Peptidase\_S24 domain-containing protein | | afdb-uniprot50 | AF-A0A4Q7D2I1-F1-MODEL\_V4 | 1.0 | 3.632e-08 | 242 | 0.167 | 215 | 139 | 6 | 5 | 180 | 103 | 316 | Helix-turn-helix domain-containing protein | Helix-turn-helix domain-containing protein | | afdb-uniprot50 | AF-A0A081B6G2-F1-MODEL\_V4 | 1.0 | 3.234e-08 | 241 | 0.202 | 202 | 121 | 7 | 16 | 180 | 7 | 205 | Putative phage repressor | Putative phage repressor | | afdb-uniprot50 | AF-A0A388SDE2-F1-MODEL\_V4 | 1.0 | 1.436e-08 | 241 | 0.18 | 194 | 140 | 4 | 4 | 180 | 14 | 205 | HTH cro/C1-type domain-containing protein | HTH cro/C1-type domain-containing protein | | afdb-uniprot50 | AF-A0A1C3GJA4-F1-MODEL\_V4 | 1.0 | 1.131e-05 | 241 | 0.205 | 107 | 80 | 1 | 79 | 180 | 83 | 189 | Pyocin repressor protein | Pyocin repressor protein | | afdb-uniprot50 | AF-A0A1E7QYY0-F1-MODEL\_V4 | 1.0 | 9.569e-09 | 241 | 0.174 | 241 | 133 | 7 | 5 | 180 | 8 | 247 | Helix-turn-helix transcriptional regulator | Helix-turn-helix transcriptional regulator | | afdb-uniprot50 | AF-A0A6S5P8N1-F1-MODEL\_V4 | 1.0 | 7.588e-09 | 241 | 0.156 | 237 | 138 | 7 | 5 | 180 | 15 | 250 | Transcriptional regulator | Transcriptional regulator | | afdb-uniprot50 | AF-A0A7Y0VS83-F1-MODEL\_V4 | 1.0 | 2.284e-08 | 241 | 0.172 | 232 | 137 | 6 | 1 | 179 | 1 | 230 | Helix-turn-helix transcriptional regulator | Helix-turn-helix transcriptional regulator | | afdb-uniprot50 | AF-A0A1F8VMR4-F1-MODEL\_V4 | 1.0 | 3.052e-08 | 240 | 0.211 | 199 | 120 | 6 | 13 | 180 | 4 | 196 | Peptidase\_S24 domain-containing protein | Peptidase\_S24 domain-containing protein | | afdb-uniprot50 | AF-A5UES4-F1-MODEL\_V4 | 1.0 | 4.078e-08 | 240 | 0.179 | 212 | 125 | 4 | 18 | 180 | 2 | 213 | Predicted transcriptional regulator | Predicted transcriptional regulator | | afdb-uniprot50 | AF-A0A1F0F535-F1-MODEL\_V4 | 1.0 | 1.919e-08 | 240 | 0.154 | 233 | 139 | 7 | 4 | 180 | 3 | 233 | Repressor | Repressor | | afdb-uniprot50 | AF-A0A2T5HFF1-F1-MODEL\_V4 | 1.0 | 1.207e-08 | 240 | 0.206 | 237 | 127 | 5 | 5 | 180 | 3 | 239 | Phage repressor protein C with HTH and peptisase S24 domain | Phage repressor protein C with HTH and peptisase S24 domain | | afdb-uniprot50 | AF-A0A809N3S2-F1-MODEL\_V4 | 1.0 | 1.014e-08 | 240 | 0.159 | 232 | 137 | 10 | 5 | 180 | 24 | 253 | Uncharacterized protein | Uncharacterized protein | | afdb-uniprot50 | AF-A0A853GEI4-F1-MODEL\_V4 | 1.0 | 8.179e-08 | 240 | 0.151 | 238 | 141 | 8 | 4 | 180 | 18 | 255 | Helix-turn-helix transcriptional regulator | Helix-turn-helix transcriptional regulator | | afdb-uniprot50 | AF-A0A246GY71-F1-MODEL\_V4 | 1.0 | 6.017e-09 | 240 | 0.132 | 272 | 139 | 8 | 5 | 180 | 3 | 273 | Phage repressor protein | Phage repressor protein | | afdb-uniprot50 | AF-A0A1H8V9H3-F1-MODEL\_V4 | 1.0 | 1.279e-08 | 240 | 0.165 | 247 | 135 | 8 | 5 | 180 | 32 | 278 | Phage repressor protein C, contains Cro/C1-type HTH and peptisase s24 domains | Phage repressor protein C, contains Cro/C1-type HTH and peptisase s24 domains | | afdb-uniprot50 | AF-A0A653E6P4-F1-MODEL\_V4 | 1.0 | 8.521e-09 | 240 | 0.155 | 244 | 138 | 7 | 5 | 180 | 94 | 337 | HTH-type transcriptional regulator PrtR (Modular protein) | HTH-type transcriptional regulator PrtR (Modular protein) | | afdb-uniprot50 | AF-A0A543HLB3-F1-MODEL\_V4 | 1.0 | 1.709e-08 | 239 | 0.168 | 232 | 134 | 9 | 4 | 180 | 7 | 234 | Phage repressor protein C with HTH and peptisase S24 domain | Phage repressor protein C with HTH and peptisase S24 domain | | afdb-uniprot50 | AF-A0A6M3M6C4-F1-MODEL\_V4 | 1.0 | 2.284e-08 | 239 | 0.181 | 237 | 134 | 6 | 4 | 180 | 5 | 241 | Putative peptidase | Putative peptidase | | afdb-uniprot50 | AF-E6QCB5-F1-MODEL\_V4 | 1.0 | 1.613e-08 | 239 | 0.182 | 235 | 128 | 7 | 4 | 180 | 14 | 242 | Putative 26 kDa repressor protein (Regulatory protein CI) | Putative 26 kDa repressor protein (Regulatory protein CI) | | afdb-uniprot50 | AF-A0A1M4WAA2-F1-MODEL\_V4 | 1.0 | 1.436e-08 | 239 | 0.181 | 220 | 124 | 6 | 16 | 180 | 25 | 243 | Peptidase S24-like | Peptidase S24-like | | afdb-uniprot50 | AF-A0A329VHA2-F1-MODEL\_V4 | 1.0 | 1.709e-08 | 239 | 0.176 | 232 | 131 | 8 | 7 | 180 | 16 | 245 | Phage repressor protein | Phage repressor protein | | afdb-uniprot50 | AF-A0A653E7Y4-F1-MODEL\_V4 | 1.0 | 1.198e-05 | 238 | 0.229 | 109 | 77 | 2 | 79 | 180 | 52 | 160 | HTH-type transcriptional regulator PrtR | HTH-type transcriptional regulator PrtR | | afdb-uniprot50 | AF-A0A5A7N7S5-F1-MODEL\_V4 | 1.0 | 1.548e-07 | 238 | 0.189 | 179 | 102 | 8 | 41 | 180 | 2 | 176 | Peptidase\_S24 domain-containing protein | Peptidase\_S24 domain-containing protein | | afdb-uniprot50 | AF-A0A6I1NN78-F1-MODEL\_V4 | 1.0 | 1.426e-05 | 238 | 0.214 | 107 | 79 | 1 | 79 | 180 | 94 | 200 | Transcriptional regulator | Transcriptional regulator | | afdb-uniprot50 | AF-A0A359LPK0-F1-MODEL\_V4 | 1.0 | 3.234e-08 | 238 | 0.171 | 204 | 139 | 7 | 4 | 180 | 9 | 209 | Peptidase\_S24 domain-containing protein | Peptidase\_S24 domain-containing protein | | afdb-uniprot50 | AF-A0A222FJU3-F1-MODEL\_V4 | 1.0 | 3.571e-09 | 238 | 0.194 | 211 | 132 | 7 | 6 | 180 | 9 | 217 | LexA family transcriptional repressor | LexA family transcriptional repressor | | afdb-uniprot50 | AF-G4QCW3-F1-MODEL\_V4 | 1.0 | 2.565e-08 | 238 | 0.153 | 222 | 139 | 8 | 5 | 180 | 4 | 222 | Putative phage repressor | Putative phage repressor | | afdb-uniprot50 | AF-A0A7U3BSK1-F1-MODEL\_V4 | 1.0 | 2.565e-08 | 238 | 0.186 | 220 | 136 | 7 | 4 | 180 | 19 | 238 | Transcriptional regulator | Transcriptional regulator | | afdb-uniprot50 | AF-A0A7D5DA53-F1-MODEL\_V4 | 1.0 | 3.427e-08 | 238 | 0.134 | 230 | 144 | 4 | 4 | 180 | 7 | 234 | Helix-turn-helix transcriptional regulator | Helix-turn-helix transcriptional regulator | | afdb-uniprot50 | AF-A0A7S9VER3-F1-MODEL\_V4 | 1.0 | 1.709e-08 | 238 | 0.173 | 242 | 131 | 5 | 5 | 177 | 4 | 245 | Helix-turn-helix transcriptional regulator | Helix-turn-helix transcriptional regulator | | afdb-uniprot50 | AF-A0A2W5EWM8-F1-MODEL\_V4 | 1.0 | 1.522e-08 | 238 | 0.176 | 250 | 133 | 8 | 4 | 180 | 14 | 263 | XRE family transcriptional regulator | XRE family transcriptional regulator | | afdb-uniprot50 | AF-A0A285N7N5-F1-MODEL\_V4 | 1.0 | 4.772e-09 | 238 | 0.172 | 267 | 132 | 10 | 1 | 180 | 1 | 265 | Phage repressor protein C, contains Cro/C1-type HTH and peptisase s24 domains | Phage repressor protein C, contains Cro/C1-type HTH and peptisase s24 domains | | afdb-uniprot50 | AF-B9TGR6-F1-MODEL\_V4 | 1.0 | 1.139e-08 | 238 | 0.19 | 221 | 125 | 8 | 5 | 174 | 21 | 238 | HTH cro/C1-type domain-containing protein | HTH cro/C1-type domain-containing protein | | afdb-uniprot50 | AF-A0A6I4YRF4-F1-MODEL\_V4 | 1.0 | 1.139e-08 | 238 | 0.207 | 226 | 127 | 10 | 4 | 180 | 133 | 355 | Helix-turn-helix domain-containing protein | Helix-turn-helix domain-containing protein | | afdb-uniprot50 | AF-A0A2R8CKN3-F1-MODEL\_V4 | 1.0 | 1.139e-08 | 237 | 0.173 | 207 | 134 | 9 | 4 | 180 | 7 | 206 | HTH cro/C1-type domain-containing protein | HTH cro/C1-type domain-containing protein | | afdb-uniprot50 | AF-A0A292SJV4-F1-MODEL\_V4 | 1.0 | 2.42e-08 | 237 | 0.186 | 209 | 127 | 5 | 4 | 179 | 7 | 205 | Helix-turn-helix domain-containing protein | Helix-turn-helix domain-containing protein | | afdb-uniprot50 | AF-A0A345PBQ8-F1-MODEL\_V4 | 1.0 | 2.718e-08 | 237 | 0.144 | 214 | 137 | 4 | 13 | 180 | 5 | 218 | Helix-turn-helix transcriptional regulator | Helix-turn-helix transcriptional regulator | | afdb-uniprot50 | AF-A0A427G804-F1-MODEL\_V4 | 1.0 | 2.718e-08 | 237 | 0.159 | 232 | 129 | 6 | 10 | 180 | 2 | 228 | XRE family transcriptional regulator | XRE family transcriptional regulator | | afdb-uniprot50 | AF-A0A853SDR3-F1-MODEL\_V4 | 1.0 | 3.052e-08 | 237 | 0.161 | 204 | 140 | 7 | 4 | 180 | 29 | 228 | Uncharacterized protein | Uncharacterized protein | | afdb-uniprot50 | AF-A0A241W2E3-F1-MODEL\_V4 | 1.0 | 1.014e-08 | 237 | 0.161 | 223 | 132 | 9 | 4 | 180 | 53 | 266 | HTH cro/C1-type domain-containing protein | HTH cro/C1-type domain-containing protein | | afdb-uniprot50 | AF-A0A2S9API7-F1-MODEL\_V4 | 1.0 | 2.565e-08 | 237 | 0.126 | 276 | 141 | 8 | 4 | 180 | 6 | 280 | HTH cro/C1-type domain-containing protein | HTH cro/C1-type domain-containing protein | | afdb-uniprot50 | AF-A0A5E6XVR5-F1-MODEL\_V4 | 1.0 | 2.42e-08 | 237 | 0.16 | 230 | 135 | 5 | 4 | 177 | 9 | 236 | HTH-type transcriptional regulator PrtR | HTH-type transcriptional regulator PrtR | | afdb-uniprot50 | AF-A0A2W0ES33-F1-MODEL\_V4 | 1.0 | 9.03e-09 | 237 | 0.141 | 275 | 136 | 8 | 5 | 180 | 11 | 284 | Cro/Cl family transcriptional regulator | Cro/Cl family transcriptional regulator | | afdb-uniprot50 | AF-A0A212IUA3-F1-MODEL\_V4 | 1.0 | 5.143e-08 | 236 | 0.206 | 199 | 111 | 7 | 24 | 180 | 3 | 196 | Putative Phage repressor | Putative Phage repressor | | afdb-uniprot50 | AF-A0A4R1PLM3-F1-MODEL\_V4 | 1.0 | 8.521e-09 | 236 | 0.148 | 216 | 125 | 7 | 7 | 180 | 24 | 222 | Phage repressor protein C with HTH and peptisase S24 domain | Phage repressor protein C with HTH and peptisase S24 domain | | afdb-uniprot50 | AF-A0A2Z4PXZ2-F1-MODEL\_V4 | 1.0 | 6.757e-09 | 236 | 0.183 | 224 | 124 | 10 | 14 | 180 | 4 | 225 | Cro/Cl family transcriptional regulator | Cro/Cl family transcriptional regulator | | afdb-uniprot50 | AF-A0A080NQX9-F1-MODEL\_V4 | 1.0 | 6.486e-08 | 236 | 0.175 | 217 | 138 | 7 | 5 | 180 | 19 | 235 | Peptidase S24-like family protein | Peptidase S24-like family protein | | afdb-uniprot50 | AF-A0A286BZZ3-F1-MODEL\_V4 | 1.0 | 6.873e-08 | 236 | 0.156 | 236 | 136 | 6 | 4 | 180 | 6 | 237 | Phage repressor protein C, contains Cro/C1-type HTH and peptisase s24 domains | Phage repressor protein C, contains Cro/C1-type HTH and peptisase s24 domains | | afdb-uniprot50 | AF-A0A1I7KDD2-F1-MODEL\_V4 | 1.0 | 3.052e-08 | 236 | 0.14 | 250 | 138 | 7 | 5 | 180 | 5 | 251 | Peptidase S24-like | Peptidase S24-like | | afdb-uniprot50 | AF-A0A3D2LZR0-F1-MODEL\_V4 | 1.0 | 1.873e-06 | 235 | 0.153 | 124 | 100 | 2 | 62 | 180 | 2 | 125 | Peptidase\_S24 domain-containing protein | Peptidase\_S24 domain-containing protein | | afdb-uniprot50 | AF-A0A3G6YKD9-F1-MODEL\_V4 | 1.0 | 3.346e-06 | 235 | 0.195 | 128 | 90 | 4 | 61 | 180 | 4 | 126 | Helix-turn-helix transcriptional regulator | Helix-turn-helix transcriptional regulator | | afdb-uniprot50 | AF-A0A837E4W0-F1-MODEL\_V4 | 1.0 | 1.574e-06 | 235 | 0.186 | 129 | 95 | 3 | 62 | 180 | 38 | 166 | Uncharacterized protein | Uncharacterized protein | | afdb-uniprot50 | AF-A9H6C0-F1-MODEL\_V4 | 1.0 | 1.279e-08 | 235 | 0.188 | 217 | 112 | 7 | 16 | 179 | 5 | 210 | Putative peptidase | Putative peptidase | | afdb-uniprot50 | AF-A0A072T9Z9-F1-MODEL\_V4 | 1.0 | 1.093e-07 | 235 | 0.159 | 219 | 131 | 8 | 14 | 180 | 1 | 218 | HTH cro/C1-type domain-containing protein | HTH cro/C1-type domain-containing protein | | afdb-uniprot50 | AF-A0A5M9J6V1-F1-MODEL\_V4 | 1.0 | 1.613e-08 | 235 | 0.176 | 227 | 127 | 7 | 10 | 180 | 2 | 224 | HTH-type transcriptional regulator PrtR | HTH-type transcriptional regulator PrtR | | afdb-uniprot50 | AF-A0A519E7W6-F1-MODEL\_V4 | 1.0 | 1.075e-08 | 235 | 0.192 | 218 | 133 | 9 | 4 | 180 | 55 | 270 | S24 family peptidase | S24 family peptidase | | afdb-uniprot50 | AF-A0A3R9Z5S5-F1-MODEL\_V4 | 1.0 | 1.031e-07 | 235 | 0.179 | 239 | 132 | 6 | 4 | 180 | 53 | 289 | Helix-turn-helix transcriptional regulator | Helix-turn-helix transcriptional regulator | | afdb-uniprot50 | AF-V8RAQ2-F1-MODEL\_V4 | 1.0 | 9.569e-09 | 235 | 0.121 | 271 | 140 | 6 | 7 | 180 | 24 | 293 | Propanediol utilization protein | Propanediol utilization protein | | afdb-uniprot50 | AF-A0A157S832-F1-MODEL\_V4 | 1.0 | 7.984e-06 | 234 | 0.221 | 122 | 92 | 3 | 60 | 180 | 8 | 127 | Transcriptional regulator | Transcriptional regulator | | afdb-uniprot50 | AF-A0A1E4G2P2-F1-MODEL\_V4 | 1.0 | 4.58e-08 | 234 | 0.161 | 211 | 137 | 7 | 4 | 180 | 26 | 230 | HTH cro/C1-type domain-containing protein | HTH cro/C1-type domain-containing protein | | afdb-uniprot50 | AF-A0A2N3B9K3-F1-MODEL\_V4 | 1.0 | 2.88e-08 | 234 | 0.173 | 231 | 135 | 6 | 4 | 180 | 13 | 241 | HTH cro/C1-type domain-containing protein | HTH cro/C1-type domain-containing protein | | afdb-uniprot50 | AF-A0A2E3KX44-F1-MODEL\_V4 | 1.0 | 2.155e-08 | 234 | 0.166 | 252 | 137 | 8 | 1 | 180 | 2 | 252 | Transcriptional regulator | Transcriptional regulator | | afdb-uniprot50 | AF-J8RZK5-F1-MODEL\_V4 | 1.0 | 1.697e-05 | 233 | 0.166 | 108 | 85 | 1 | 78 | 180 | 25 | 132 | Transcriptional regulator | Transcriptional regulator | | afdb-uniprot50 | AF-A0A7Y6QLG8-F1-MODEL\_V4 | 1.0 | 2.284e-08 | 233 | 0.18 | 210 | 132 | 9 | 4 | 180 | 29 | 231 | Peptidase\_S24 domain-containing protein | Peptidase\_S24 domain-containing protein | | afdb-uniprot50 | AF-A0A542RP09-F1-MODEL\_V4 | 1.0 | 2.42e-08 | 233 | 0.166 | 228 | 136 | 7 | 6 | 180 | 11 | 237 | Phage repressor protein C with HTH and peptisase S24 domain | Phage repressor protein C with HTH and peptisase S24 domain | | afdb-uniprot50 | AF-A0A109Q3B0-F1-MODEL\_V4 | 1.0 | 7.283e-08 | 233 | 0.163 | 232 | 140 | 6 | 1 | 180 | 1 | 230 | Peptidase\_S24 domain-containing protein | Peptidase\_S24 domain-containing protein | | afdb-uniprot50 | AF-A0A1I1CFP1-F1-MODEL\_V4 | 1.0 | 5.45e-08 | 233 | 0.161 | 229 | 139 | 8 | 5 | 180 | 11 | 239 | Peptidase S24-like | Peptidase S24-like | | afdb-uniprot50 | AF-A0A2D2DKA6-F1-MODEL\_V4 | 1.0 | 3.052e-08 | 233 | 0.131 | 235 | 142 | 4 | 7 | 180 | 26 | 259 | HTH cro/C1-type domain-containing protein | HTH cro/C1-type domain-containing protein | | afdb-uniprot50 | AF-A0A367M7U5-F1-MODEL\_V4 | 1.0 | 2.155e-08 | 233 | 0.132 | 271 | 139 | 8 | 5 | 180 | 11 | 280 | Phage repressor protein | Phage repressor protein | | afdb-uniprot50 | AF-A0A443K7M6-F1-MODEL\_V4 | 1.0 | 9.733e-08 | 233 | 0.149 | 294 | 130 | 8 | 4 | 179 | 7 | 298 | HTH cro/C1-type domain-containing protein | HTH cro/C1-type domain-containing protein | | afdb-uniprot50 | AF-A0A1Z5YTF2-F1-MODEL\_V4 | 1.0 | 4.853e-08 | 232 | 0.166 | 216 | 120 | 6 | 20 | 180 | 2 | 212 | HTH cro/C1-type domain-containing protein | HTH cro/C1-type domain-containing protein | | afdb-uniprot50 | AF-A0A1Q6U9K6-F1-MODEL\_V4 | 1.0 | 5.143e-08 | 232 | 0.171 | 216 | 134 | 8 | 6 | 180 | 9 | 220 | HTH cro/C1-type domain-containing protein | HTH cro/C1-type domain-containing protein | | afdb-uniprot50 | AF-A0A5C7JG03-F1-MODEL\_V4 | 1.0 | 3.234e-08 | 232 | 0.206 | 194 | 132 | 7 | 4 | 179 | 45 | 234 | Peptidase\_S24 domain-containing protein | Peptidase\_S24 domain-containing protein | | afdb-uniprot50 | AF-D5CU95-F1-MODEL\_V4 | 1.0 | 3.427e-08 | 232 | 0.226 | 225 | 114 | 8 | 4 | 174 | 15 | 233 | Putative phage repressor | Putative phage repressor | | afdb-uniprot50 | AF-A0A6B0DM06-F1-MODEL\_V4 | 1.0 | 9.569e-09 | 232 | 0.235 | 195 | 111 | 4 | 4 | 163 | 12 | 203 | Helix-turn-helix domain-containing protein | Helix-turn-helix domain-containing protein | | afdb-uniprot50 | AF-A0A5B8CV78-F1-MODEL\_V4 | 1.0 | 1.709e-08 | 231 | 0.198 | 181 | 106 | 4 | 33 | 174 | 4 | 184 | Helix-turn-helix transcriptional regulator | Helix-turn-helix transcriptional regulator | | afdb-uniprot50 | AF-A0A1Y5SGE5-F1-MODEL\_V4 | 1.0 | 2.88e-08 | 231 | 0.219 | 228 | 111 | 10 | 14 | 180 | 1 | 222 | Putative HTH-type transcriptional regulator | Putative HTH-type transcriptional regulator | | afdb-uniprot50 | AF-A0A6I1H3E1-F1-MODEL\_V4 | 1.0 | 7.283e-08 | 231 | 0.169 | 206 | 138 | 7 | 4 | 180 | 28 | 229 | Peptidase\_S24 domain-containing protein | Peptidase\_S24 domain-containing protein | | afdb-uniprot50 | AF-A0A845GQS4-F1-MODEL\_V4 | 1.0 | 4.322e-08 | 231 | 0.175 | 211 | 130 | 8 | 4 | 180 | 30 | 230 | Uncharacterized protein | Uncharacterized protein | | afdb-uniprot50 | AF-A0A2S7JR70-F1-MODEL\_V4 | 1.0 | 2.88e-08 | 231 | 0.191 | 282 | 121 | 10 | 4 | 180 | 5 | 284 | Uncharacterized protein | Uncharacterized protein | | afdb-uniprot50 | AF-A0A7Y5EC04-F1-MODEL\_V4 | 1.0 | 2.14e-05 | 230 | 0.166 | 108 | 85 | 1 | 78 | 180 | 51 | 158 | Transcriptional regulator | Transcriptional regulator | | afdb-uniprot50 | AF-A0A7U3GDM4-F1-MODEL\_V4 | 1.0 | 6.873e-08 | 230 | 0.125 | 215 | 150 | 5 | 4 | 180 | 8 | 222 | LexA family transcriptional regulator | LexA family transcriptional regulator | | afdb-uniprot50 | AF-Q1IFI8-F1-MODEL\_V4 | 1.0 | 6.486e-08 | 230 | 0.141 | 226 | 140 | 6 | 4 | 180 | 7 | 227 | Putative transcriptional regulator | Putative transcriptional regulator | | afdb-uniprot50 | AF-A0A1F7FIP4-F1-MODEL\_V4 | 1.0 | 4.078e-08 | 230 | 0.129 | 232 | 145 | 7 | 5 | 180 | 22 | 252 | HTH cro/C1-type domain-containing protein | HTH cro/C1-type domain-containing protein | | afdb-uniprot50 | AF-A0A679GE06-F1-MODEL\_V4 | 1.0 | 2.155e-08 | 230 | 0.132 | 271 | 139 | 7 | 5 | 180 | 6 | 275 | Peptidase\_S24 domain-containing protein | Peptidase\_S24 domain-containing protein | | afdb-uniprot50 | AF-A0A1F4NAY1-F1-MODEL\_V4 | 1.0 | 2.546e-05 | 229 | 0.221 | 104 | 77 | 4 | 79 | 180 | 78 | 179 | Peptidase\_S24 domain-containing protein | Peptidase\_S24 domain-containing protein | | afdb-uniprot50 | AF-A0A0M7MMT4-F1-MODEL\_V4 | 1.0 | 1.798e-05 | 229 | 0.257 | 97 | 66 | 2 | 87 | 180 | 90 | 183 | DNA polymerase V subunit UmuD | DNA polymerase V subunit UmuD | | afdb-uniprot50 | AF-A0A1P8END7-F1-MODEL\_V4 | 1.0 | 3.234e-08 | 229 | 0.168 | 219 | 139 | 6 | 4 | 180 | 11 | 228 | HTH cro/C1-type domain-containing protein | HTH cro/C1-type domain-containing protein | | afdb-uniprot50 | AF-A0A3D0L6H5-F1-MODEL\_V4 | 1.0 | 7.283e-08 | 229 | 0.17 | 217 | 133 | 7 | 8 | 180 | 15 | 228 | Peptidase\_S24 domain-containing protein | Peptidase\_S24 domain-containing protein | | afdb-uniprot50 | AF-A0A2X1WHQ7-F1-MODEL\_V4 | 1.0 | 2.42e-08 | 229 | 0.178 | 235 | 131 | 7 | 5 | 180 | 4 | 235 | Uncharacterized HTH-type transcriptional regulator HI\_1476 | Uncharacterized HTH-type transcriptional regulator HI\_1476 | | afdb-uniprot50 | AF-A0A3A5J7R3-F1-MODEL\_V4 | 1.0 | 6.873e-08 | 229 | 0.152 | 229 | 136 | 6 | 4 | 180 | 16 | 238 | LexA family transcriptional regulator | LexA family transcriptional regulator | | afdb-uniprot50 | AF-A0A358AXE2-F1-MODEL\_V4 | 1.0 | 8.667e-08 | 229 | 0.16 | 225 | 141 | 6 | 4 | 180 | 19 | 243 | Transcriptional regulator | Transcriptional regulator | | afdb-uniprot50 | AF-A0A074V4S2-F1-MODEL\_V4 | 1.0 | 9.185e-08 | 229 | 0.183 | 240 | 131 | 8 | 4 | 180 | 11 | 248 | Peptidase S24-like | Peptidase S24-like | | afdb-uniprot50 | AF-A0A6L7X9K4-F1-MODEL\_V4 | 1.0 | 2.284e-08 | 229 | 0.122 | 253 | 139 | 6 | 5 | 180 | 4 | 250 | S24 family peptidase | S24 family peptidase | | afdb-uniprot50 | AF-A0A6G6IVE1-F1-MODEL\_V4 | 1.0 | 4.58e-08 | 229 | 0.158 | 234 | 135 | 6 | 5 | 177 | 44 | 276 | Helix-turn-helix domain-containing protein | Helix-turn-helix domain-containing protein | | afdb-uniprot50 | AF-A0A348SFI4-F1-MODEL\_V4 | 1.0 | 5.45e-08 | 229 | 0.142 | 246 | 139 | 5 | 5 | 178 | 34 | 279 | HTH cro/C1-type domain-containing protein | HTH cro/C1-type domain-containing protein | | afdb-uniprot50 | AF-A0A7Z8D4G4-F1-MODEL\_V4 | 1.0 | 6.12e-08 | 228 | 0.159 | 207 | 121 | 7 | 26 | 180 | 1 | 206 | XRE family transcriptional regulator | XRE family transcriptional regulator | | afdb-uniprot50 | AF-A0A4D7B2Y1-F1-MODEL\_V4 | 1.0 | 3.052e-08 | 228 | 0.2 | 214 | 130 | 9 | 4 | 180 | 15 | 224 | HTH cro/C1-type domain-containing protein | HTH cro/C1-type domain-containing protein | | afdb-uniprot50 | AF-A0A7W7NEM4-F1-MODEL\_V4 | 1.0 | 4.078e-08 | 228 | 0.133 | 263 | 141 | 8 | 4 | 180 | 7 | 268 | Phage repressor protein C with HTH and peptisase S24 domain | Phage repressor protein C with HTH and peptisase S24 domain | | afdb-uniprot50 | AF-A0A1Y0ER74-F1-MODEL\_V4 | 1.0 | 3.234e-08 | 227 | 0.186 | 220 | 128 | 14 | 4 | 178 | 7 | 220 | Peptidase\_S24 domain-containing protein | Peptidase\_S24 domain-containing protein | | afdb-uniprot50 | AF-A0A1V5QRV5-F1-MODEL\_V4 | 1.0 | 4.58e-08 | 227 | 0.207 | 231 | 116 | 9 | 15 | 180 | 9 | 237 | HTH-type transcriptional regulator PrtR | HTH-type transcriptional regulator PrtR | | afdb-uniprot50 | AF-A0A0Q5KKZ7-F1-MODEL\_V4 | 1.0 | 4.322e-08 | 227 | 0.137 | 232 | 144 | 8 | 4 | 180 | 13 | 243 | Peptidase\_S24 domain-containing protein | Peptidase\_S24 domain-containing protein | | afdb-uniprot50 | AF-A0A220RCN3-F1-MODEL\_V4 | 1.0 | 7.718e-08 | 227 | 0.19 | 241 | 126 | 7 | 4 | 177 | 7 | 245 | Peptidase\_S24 domain-containing protein | Peptidase\_S24 domain-containing protein | | afdb-uniprot50 | AF-A0A1E7YT01-F1-MODEL\_V4 | 1.0 | 4.58e-08 | 227 | 0.163 | 238 | 137 | 6 | 4 | 180 | 8 | 244 | Peptidase\_S24 domain-containing protein | Peptidase\_S24 domain-containing protein | | afdb-uniprot50 | AF-A0A854CXB7-F1-MODEL\_V4 | 1.0 | 2.718e-08 | 227 | 0.175 | 245 | 130 | 9 | 2 | 178 | 5 | 245 | Transcriptional regulator | Transcriptional regulator | | afdb-uniprot50 | AF-A0A1H5XW02-F1-MODEL\_V4 | 1.0 | 6.12e-08 | 227 | 0.159 | 251 | 135 | 6 | 6 | 180 | 12 | 262 | Helix-turn-helix | Helix-turn-helix | | afdb-uniprot50 | AF-A0A239BRE0-F1-MODEL\_V4 | 1.0 | 2.034e-08 | 227 | 0.126 | 261 | 143 | 8 | 4 | 180 | 7 | 266 | Peptidase S24-like | Peptidase S24-like | | afdb-uniprot50 | AF-A0A1N7HEF4-F1-MODEL\_V4 | 1.0 | 3.234e-08 | 227 | 0.137 | 262 | 140 | 8 | 4 | 180 | 22 | 282 | Phage repressor protein C, contains Cro/C1-type HTH and peptisase s24 domains | Phage repressor protein C, contains Cro/C1-type HTH and peptisase s24 domains | | afdb-uniprot50 | AF-A0A1E7PYC2-F1-MODEL\_V4 | 1.0 | 7.283e-08 | 227 | 0.141 | 269 | 141 | 6 | 1 | 180 | 26 | 293 | HTH cro/C1-type domain-containing protein | HTH cro/C1-type domain-containing protein | | afdb-uniprot50 | AF-A0A1X0ZUR4-F1-MODEL\_V4 | 1.0 | 2.88e-08 | 226 | 0.179 | 223 | 126 | 6 | 4 | 179 | 7 | 219 | Transcriptional regulator | Transcriptional regulator | | afdb-uniprot50 | AF-A0A521EXD5-F1-MODEL\_V4 | 1.0 | 1.031e-07 | 226 | 0.157 | 222 | 141 | 7 | 4 | 180 | 10 | 230 | Peptidase S24-like | Peptidase S24-like | | afdb-uniprot50 | AF-A0A7C7ACW4-F1-MODEL\_V4 | 1.0 | 1.811e-08 | 226 | 0.156 | 230 | 140 | 5 | 4 | 180 | 7 | 235 | Helix-turn-helix transcriptional regulator | Helix-turn-helix transcriptional regulator | | afdb-uniprot50 | AF-A0A4R1XGS3-F1-MODEL\_V4 | 1.0 | 8.667e-08 | 226 | 0.181 | 232 | 132 | 10 | 5 | 180 | 7 | 236 | Phage repressor protein C with HTH and peptisase S24 domain | Phage repressor protein C with HTH and peptisase S24 domain | | afdb-uniprot50 | AF-A0A379JE71-F1-MODEL\_V4 | 1.0 | 7.283e-08 | 226 | 0.164 | 225 | 130 | 6 | 8 | 179 | 2 | 221 | Putative regulatory protein | Putative regulatory protein | | afdb-uniprot50 | AF-A0A2S2F875-F1-MODEL\_V4 | 1.0 | 9.733e-08 | 226 | 0.146 | 218 | 139 | 5 | 4 | 180 | 34 | 245 | Helix-turn-helix domain-containing protein | Helix-turn-helix domain-containing protein | | afdb-uniprot50 | AF-A0A1F4NGE6-F1-MODEL\_V4 | 1.0 | 2.192e-07 | 226 | 0.131 | 235 | 145 | 8 | 4 | 180 | 17 | 250 | HTH cro/C1-type domain-containing protein | HTH cro/C1-type domain-containing protein | | afdb-uniprot50 | AF-A0A7U9I271-F1-MODEL\_V4 | 1.0 | 4.322e-08 | 226 | 0.132 | 265 | 141 | 8 | 4 | 180 | 7 | 270 | Peptidase\_S24 domain-containing protein | Peptidase\_S24 domain-containing protein | | afdb-uniprot50 | AF-A0A7W8WGP6-F1-MODEL\_V4 | 1.0 | 5.143e-08 | 226 | 0.16 | 275 | 114 | 11 | 16 | 180 | 8 | 275 | Phage repressor protein C with HTH and peptisase S24 domain | Phage repressor protein C with HTH and peptisase S24 domain | | afdb-uniprot50 | AF-F2K1S3-F1-MODEL\_V4 | 1.0 | 6.486e-08 | 225 | 0.196 | 204 | 119 | 4 | 20 | 179 | 2 | 204 | Putative phage repressor | Putative phage repressor | | afdb-uniprot50 | AF-A0A6S7DEF1-F1-MODEL\_V4 | 1.0 | 3.234e-08 | 225 | 0.187 | 229 | 130 | 8 | 5 | 180 | 7 | 232 | HTH cro/C1-type domain-containing protein | HTH cro/C1-type domain-containing protein | | afdb-uniprot50 | AF-A0A7U5XY97-F1-MODEL\_V4 | 1.0 | 2.565e-08 | 225 | 0.214 | 210 | 123 | 10 | 4 | 180 | 37 | 237 | Peptidase\_S24 domain-containing protein | Peptidase\_S24 domain-containing protein | | afdb-uniprot50 | AF-A0A3G7V6R7-F1-MODEL\_V4 | 1.0 | 8.667e-08 | 225 | 0.163 | 239 | 136 | 5 | 4 | 180 | 14 | 250 | Phage cI repressor | Phage cI repressor | | afdb-uniprot50 | AF-A0A080NQD6-F1-MODEL\_V4 | 1.0 | 5.45e-08 | 225 | 0.153 | 247 | 137 | 11 | 4 | 180 | 8 | 252 | Peptidase S24-like family protein | Peptidase S24-like family protein | | afdb-uniprot50 | AF-A0A6M9CND3-F1-MODEL\_V4 | 1.0 | 7.718e-08 | 225 | 0.148 | 249 | 137 | 12 | 5 | 180 | 13 | 259 | Peptidase\_S24 domain-containing protein | Peptidase\_S24 domain-containing protein | | afdb-uniprot50 | AF-D8IV29-F1-MODEL\_V4 | 1.0 | 3.849e-08 | 225 | 0.157 | 266 | 132 | 8 | 4 | 180 | 6 | 268 | Peptidase S24 LexA-like protein | Peptidase S24 LexA-like protein | | afdb-uniprot50 | AF-A0A6B1CR96-F1-MODEL\_V4 | 1.0 | 1.031e-07 | 225 | 0.146 | 267 | 134 | 8 | 5 | 180 | 7 | 270 | S24 family peptidase | S24 family peptidase | | afdb-uniprot50 | AF-A0A1W2AAM9-F1-MODEL\_V4 | 1.0 | 1.919e-08 | 225 | 0.142 | 274 | 132 | 8 | 5 | 180 | 11 | 279 | Phage repressor protein C, contains Cro/C1-type HTH and peptisase s24 domains | Phage repressor protein C, contains Cro/C1-type HTH and peptisase s24 domains | | afdb-uniprot50 | AF-A0A4R5UKI9-F1-MODEL\_V4 | 1.0 | 1.093e-07 | 225 | 0.16 | 230 | 139 | 5 | 4 | 180 | 79 | 307 | LexA family transcriptional regulator | LexA family transcriptional regulator | | afdb-uniprot50 | AF-A0A7G2K0G5-F1-MODEL\_V4 | 1.0 | 7.232e-05 | 224 | 0.277 | 83 | 60 | 0 | 98 | 180 | 5 | 87 | Prophage LambdaSo, transcriptional regulator, Cro/CI family | Prophage LambdaSo, transcriptional regulator, Cro/CI family | | afdb-uniprot50 | AF-A0A3G6YX37-F1-MODEL\_V4 | 1.0 | 6.12e-08 | 224 | 0.158 | 195 | 118 | 4 | 29 | 178 | 4 | 197 | LexA family transcriptional regulator | LexA family transcriptional regulator | | afdb-uniprot50 | AF-A0A3B0MER7-F1-MODEL\_V4 | 1.0 | 9.185e-08 | 224 | 0.161 | 211 | 140 | 7 | 5 | 179 | 7 | 216 | HTH-type transcriptional regulator PrtR | HTH-type transcriptional regulator PrtR | | afdb-uniprot50 | AF-A0A3P3E8B0-F1-MODEL\_V4 | 1.0 | 1.158e-07 | 224 | 0.179 | 217 | 136 | 9 | 4 | 180 | 29 | 243 | Helix-turn-helix transcriptional regulator | Helix-turn-helix transcriptional regulator | | afdb-uniprot50 | AF-A0A5C7VD74-F1-MODEL\_V4 | 1.0 | 1.158e-07 | 224 | 0.173 | 213 | 134 | 7 | 5 | 180 | 38 | 245 | Helix-turn-helix transcriptional regulator | Helix-turn-helix transcriptional regulator | | afdb-uniprot50 | AF-A0A2U0YK76-F1-MODEL\_V4 | 1.0 | 1.031e-07 | 224 | 0.155 | 218 | 141 | 6 | 5 | 180 | 37 | 253 | Phage repressor protein C with HTH and peptisase S24 domain | Phage repressor protein C with HTH and peptisase S24 domain | | afdb-uniprot50 | AF-A0A1H9E7D8-F1-MODEL\_V4 | 1.0 | 4.078e-08 | 224 | 0.161 | 248 | 133 | 12 | 5 | 179 | 12 | 257 | Phage repressor protein C, contains Cro/C1-type HTH and peptisase s24 domains | Phage repressor protein C, contains Cro/C1-type HTH and peptisase s24 domains | | afdb-uniprot50 | AF-A0A105T6I1-F1-MODEL\_V4 | 1.0 | 1.548e-07 | 224 | 0.161 | 236 | 137 | 5 | 4 | 180 | 33 | 266 | Pyocin repressor protein | Pyocin repressor protein | | afdb-uniprot50 | AF-E6QGP3-F1-MODEL\_V4 | 1.0 | 9.733e-08 | 224 | 0.133 | 233 | 142 | 8 | 4 | 180 | 66 | 294 | Peptidase\_S24 domain-containing protein | Peptidase\_S24 domain-containing protein | | afdb-uniprot50 | AF-A0A661II41-F1-MODEL\_V4 | 1.0 | 1.131e-05 | 223 | 0.166 | 108 | 86 | 2 | 74 | 178 | 19 | 125 | Peptidase\_S24 domain-containing protein | Peptidase\_S24 domain-containing protein | | afdb-uniprot50 | AF-A0A7W9RYE0-F1-MODEL\_V4 | 1.0 | 4.078e-08 | 223 | 0.188 | 207 | 133 | 11 | 4 | 179 | 12 | 214 | Phage repressor protein C with HTH and peptisase S24 domain | Phage repressor protein C with HTH and peptisase S24 domain | | afdb-uniprot50 | AF-A0A379J5I1-F1-MODEL\_V4 | 1.0 | 4.853e-08 | 223 | 0.169 | 189 | 121 | 4 | 27 | 180 | 37 | 224 | Prophage PSPPH03, Cro/CI family transcriptional | Prophage PSPPH03, Cro/CI family transcriptional | | afdb-uniprot50 | AF-D9Y858-F1-MODEL\_V4 | 1.0 | 5.45e-08 | 223 | 0.137 | 225 | 137 | 10 | 5 | 180 | 12 | 228 | Uncharacterized protein | Uncharacterized protein | | afdb-uniprot50 | AF-A0A512L9E3-F1-MODEL\_V4 | 1.0 | 3.632e-08 | 223 | 0.19 | 205 | 136 | 10 | 4 | 180 | 36 | 238 | Peptidase\_S24 domain-containing protein | Peptidase\_S24 domain-containing protein | | afdb-uniprot50 | AF-A0A8B4GDE0-F1-MODEL\_V4 | 1.0 | 1.227e-07 | 223 | 0.171 | 239 | 134 | 8 | 5 | 180 | 11 | 248 | Peptidase S24-like | Peptidase S24-like | | afdb-uniprot50 | AF-A0A2D9ESJ0-F1-MODEL\_V4 | 1.0 | 5.775e-08 | 223 | 0.172 | 237 | 133 | 13 | 5 | 180 | 11 | 245 | Peptidase\_S24 domain-containing protein | Peptidase\_S24 domain-containing protein | | afdb-uniprot50 | AF-A0A086D105-F1-MODEL\_V4 | 1.0 | 9.185e-08 | 223 | 0.183 | 256 | 128 | 10 | 5 | 180 | 3 | 257 | Peptidase\_S24 domain-containing protein | Peptidase\_S24 domain-containing protein | | afdb-uniprot50 | AF-A0A149V4K2-F1-MODEL\_V4 | 1.0 | 2.068e-07 | 222 | 0.213 | 173 | 109 | 6 | 29 | 176 | 2 | 172 | Repressor | Repressor | | afdb-uniprot50 | AF-A0A7G2K117-F1-MODEL\_V4 | 1.0 | 4.936e-07 | 222 | 0.21 | 157 | 117 | 3 | 25 | 180 | 33 | 183 | Prophage LambdaSo, transcriptional regulator, Cro/CI family | Prophage LambdaSo, transcriptional regulator, Cro/CI family | | afdb-uniprot50 | AF-A0A7W5JLM8-F1-MODEL\_V4 | 1.0 | 6.873e-08 | 222 | 0.2 | 190 | 109 | 7 | 32 | 180 | 3 | 190 | Phage repressor protein C with HTH and peptisase S24 domain | Phage repressor protein C with HTH and peptisase S24 domain | | afdb-uniprot50 | AF-A0A5N7XEU1-F1-MODEL\_V4 | 1.0 | 2.192e-07 | 222 | 0.187 | 229 | 132 | 8 | 4 | 180 | 22 | 248 | Peptidase S24 | Peptidase S24 | | afdb-uniprot50 | AF-A0A385N4F7-F1-MODEL\_V4 | 1.0 | 3.849e-08 | 222 | 0.109 | 219 | 146 | 8 | 4 | 180 | 52 | 263 | LexA family transcriptional regulator | LexA family transcriptional regulator | | afdb-uniprot50 | AF-A0A7V1K4M1-F1-MODEL\_V4 | 1.0 | 7.283e-08 | 221 | 0.189 | 195 | 121 | 5 | 18 | 180 | 1 | 190 | Phage repressor protein | Phage repressor protein | | afdb-uniprot50 | AF-A0A8B3EZK2-F1-MODEL\_V4 | 1.0 | 1.227e-07 | 221 | 0.183 | 202 | 116 | 4 | 28 | 180 | 13 | 214 | Helix-turn-helix transcriptional regulator | Helix-turn-helix transcriptional regulator | | afdb-uniprot50 | AF-A0A1H6XI94-F1-MODEL\_V4 | 1.0 | 1.709e-08 | 221 | 0.159 | 226 | 126 | 8 | 1 | 180 | 18 | 225 | Phage repressor protein C, contains Cro/C1-type HTH and peptisase s24 domains | Phage repressor protein C, contains Cro/C1-type HTH and peptisase s24 domains | | afdb-uniprot50 | AF-A0A543BE40-F1-MODEL\_V4 | 1.0 | 2.461e-07 | 221 | 0.139 | 222 | 145 | 8 | 4 | 180 | 12 | 232 | Phage repressor protein C with HTH and peptisase S24 domain | Phage repressor protein C with HTH and peptisase S24 domain | | afdb-uniprot50 | AF-A0A103DW42-F1-MODEL\_V4 | 1.0 | 9.733e-08 | 221 | 0.15 | 233 | 139 | 9 | 4 | 179 | 10 | 240 | Repressor | Repressor | | afdb-uniprot50 | AF-A0A1E4LU30-F1-MODEL\_V4 | 1.0 | 6.486e-08 | 220 | 0.186 | 231 | 126 | 11 | 4 | 179 | 5 | 228 | Repressor | Repressor | | afdb-uniprot50 | AF-A0A6L7U9Q8-F1-MODEL\_V4 | 1.0 | 4.322e-08 | 220 | 0.162 | 240 | 130 | 8 | 5 | 180 | 7 | 239 | LexA family transcriptional regulator | LexA family transcriptional regulator | | afdb-uniprot50 | AF-A0A3D1CH01-F1-MODEL\_V4 | 1.0 | 1.093e-07 | 219 | 0.172 | 226 | 135 | 8 | 5 | 180 | 12 | 235 | Transcriptional regulator | Transcriptional regulator | | afdb-uniprot50 | AF-A0A2C9WXH1-F1-MODEL\_V4 | 1.0 | 5.45e-08 | 219 | 0.161 | 241 | 137 | 10 | 4 | 180 | 8 | 247 | Peptidase\_S24 domain-containing protein | Peptidase\_S24 domain-containing protein | | afdb-uniprot50 | AF-A0A5E7B5Z1-F1-MODEL\_V4 | 1.0 | 7.718e-08 | 218 | 0.147 | 197 | 126 | 7 | 17 | 180 | 41 | 228 | Peptidase\_S24 domain-containing protein | Peptidase\_S24 domain-containing protein | | afdb-uniprot50 | AF-A0A1M3JND1-F1-MODEL\_V4 | 1.0 | 7.283e-08 | 218 | 0.215 | 209 | 127 | 9 | 4 | 180 | 21 | 224 | Peptidase\_S24 domain-containing protein | Peptidase\_S24 domain-containing protein | | afdb-uniprot50 | AF-B4RJ87-F1-MODEL\_V4 | 1.0 | 1.158e-07 | 218 | 0.142 | 231 | 139 | 5 | 9 | 180 | 4 | 234 | Putative phage repressor protein, putative phage associated protein | Putative phage repressor protein, putative phage associated protein | | afdb-uniprot50 | AF-A0A844BRX6-F1-MODEL\_V4 | 1.0 | 3.427e-08 | 218 | 0.208 | 235 | 118 | 7 | 4 | 171 | 14 | 247 | Transcriptional regulator | Transcriptional regulator | | afdb-uniprot50 | AF-A0A3D3KGL4-F1-MODEL\_V4 | 1.0 | 5.775e-08 | 218 | 0.137 | 291 | 136 | 7 | 4 | 180 | 11 | 300 | HTH cro/C1-type domain-containing protein | HTH cro/C1-type domain-containing protein | | afdb-uniprot50 | AF-A0A6M3XFS2-F1-MODEL\_V4 | 1.0 | 4.078e-08 | 218 | 0.161 | 211 | 124 | 8 | 7 | 180 | 118 | 312 | Putative peptidase | Putative peptidase | | afdb-uniprot50 | AF-A0A3B9HQD5-F1-MODEL\_V4 | 1.0 | 1.378e-07 | 218 | 0.146 | 232 | 138 | 7 | 8 | 180 | 85 | 315 | HTH cro/C1-type domain-containing protein | HTH cro/C1-type domain-containing protein | | afdb-uniprot50 | AF-A0A812QVM4-F1-MODEL\_V4 | 1.0 | 8.179e-08 | 218 | 0.157 | 203 | 127 | 4 | 16 | 175 | 5 | 206 | CI protein | CI protein | | afdb-uniprot50 | AF-A0A0D6SZZ1-F1-MODEL\_V4 | 1.0 | 1.158e-07 | 217 | 0.178 | 185 | 116 | 5 | 31 | 180 | 4 | 187 | Peptidase\_S24 domain-containing protein | Peptidase\_S24 domain-containing protein | | afdb-uniprot50 | AF-G1V581-F1-MODEL\_V4 | 1.0 | 1.842e-07 | 217 | 0.186 | 236 | 125 | 9 | 5 | 180 | 13 | 241 | HTH cro/C1-type domain-containing protein | HTH cro/C1-type domain-containing protein | | afdb-uniprot50 | AF-A0A853YD01-F1-MODEL\_V4 | 1.0 | 1.461e-07 | 217 | 0.202 | 247 | 124 | 8 | 6 | 180 | 2 | 247 | Uncharacterized protein | Uncharacterized protein | | afdb-uniprot50 | AF-A0A4U8YR75-F1-MODEL\_V4 | 1.0 | 2.929e-07 | 217 | 0.174 | 252 | 131 | 7 | 4 | 180 | 5 | 254 | Bacteriophage ci repressor | Bacteriophage ci repressor | | afdb-uniprot50 | AF-A0A4Q7NCE9-F1-MODEL\_V4 | 1.0 | 5.45e-08 | 217 | 0.147 | 264 | 126 | 7 | 14 | 180 | 33 | 294 | Phage repressor protein C with HTH and peptisase S24 domain | Phage repressor protein C with HTH and peptisase S24 domain | | afdb-uniprot50 | AF-A0A5C8BK61-F1-MODEL\_V4 | 1.0 | 2.88e-08 | 216 | 0.178 | 207 | 128 | 7 | 5 | 176 | 7 | 206 | Helix-turn-helix transcriptional regulator | Helix-turn-helix transcriptional regulator | | afdb-uniprot50 | AF-A0A3B0RY47-F1-MODEL\_V4 | 1.0 | 6.486e-08 | 216 | 0.14 | 221 | 140 | 8 | 5 | 180 | 6 | 221 | Uncharacterized protein | Uncharacterized protein | | afdb-uniprot50 | AF-A0A4V3FW68-F1-MODEL\_V4 | 1.0 | 1.842e-07 | 216 | 0.143 | 230 | 140 | 5 | 4 | 180 | 7 | 232 | Peptidase S24-like protein | Peptidase S24-like protein | | afdb-uniprot50 | AF-A0A3F3I4Y1-F1-MODEL\_V4 | 1.0 | 1.378e-07 | 216 | 0.155 | 238 | 135 | 6 | 8 | 180 | 6 | 242 | Peptidase\_S24 domain-containing protein | Peptidase\_S24 domain-containing protein | | afdb-uniprot50 | AF-A0A1V2K4Z0-F1-MODEL\_V4 | 1.0 | 9.569e-09 | 216 | 0.151 | 245 | 139 | 8 | 4 | 180 | 6 | 249 | HTH cro/C1-type domain-containing protein | HTH cro/C1-type domain-containing protein | | afdb-uniprot50 | AF-A0A2E7GR68-F1-MODEL\_V4 | 1.0 | 1.227e-07 | 215 | 0.239 | 192 | 114 | 9 | 5 | 180 | 10 | 185 | Peptidase\_S24 domain-containing protein | Peptidase\_S24 domain-containing protein | | afdb-uniprot50 | AF-A0A2X1VHT4-F1-MODEL\_V4 | 1.0 | 2.929e-07 | 215 | 0.199 | 216 | 130 | 10 | 5 | 180 | 16 | 228 | Uncharacterized HTH-type transcriptional regulator HI\_1476 | Uncharacterized HTH-type transcriptional regulator HI\_1476 | | afdb-uniprot50 | AF-A0A7V4MJH9-F1-MODEL\_V4 | 1.0 | 4.148e-07 | 215 | 0.183 | 234 | 127 | 7 | 5 | 180 | 8 | 235 | Helix-turn-helix transcriptional regulator | Helix-turn-helix transcriptional regulator | | afdb-uniprot50 | AF-A0A410UEY1-F1-MODEL\_V4 | 1.0 | 6.873e-08 | 215 | 0.144 | 228 | 139 | 10 | 5 | 180 | 15 | 238 | Uncharacterized protein | Uncharacterized protein | | afdb-uniprot50 | AF-A0A1H7KZP5-F1-MODEL\_V4 | 1.0 | 1.548e-07 | 215 | 0.198 | 232 | 128 | 10 | 6 | 180 | 17 | 247 | Peptidase S24-like | Peptidase S24-like | | afdb-uniprot50 | AF-A0A4R3NQD2-F1-MODEL\_V4 | 1.0 | 2.764e-07 | 214 | 0.145 | 199 | 129 | 5 | 23 | 180 | 2 | 200 | Phage repressor protein C with HTH and peptisase S24 domain | Phage repressor protein C with HTH and peptisase S24 domain | | afdb-uniprot50 | AF-A0A7Y4J6H1-F1-MODEL\_V4 | 1.0 | 1.27e-05 | 213 | 0.188 | 122 | 91 | 2 | 59 | 180 | 4 | 117 | Uncharacterized protein | Uncharacterized protein | | afdb-uniprot50 | AF-A0A7U6R1V1-F1-MODEL\_V4 | 1.0 | 9.733e-08 | 213 | 0.208 | 206 | 107 | 8 | 25 | 180 | 1 | 200 | Transcriptional regulator | Transcriptional regulator | | afdb-uniprot50 | AF-A0A0W0WMM9-F1-MODEL\_V4 | 1.0 | 1.227e-07 | 213 | 0.106 | 207 | 155 | 7 | 3 | 180 | 2 | 207 | HTH-type transcriptional regulator | HTH-type transcriptional regulator | | afdb-uniprot50 | AF-A0A2S5QA13-F1-MODEL\_V4 | 1.0 | 1.093e-07 | 213 | 0.184 | 211 | 137 | 7 | 4 | 180 | 7 | 216 | HTH cro/C1-type domain-containing protein | HTH cro/C1-type domain-containing protein | | afdb-uniprot50 | AF-A0A2E8YIR4-F1-MODEL\_V4 | 1.0 | 1.436e-08 | 213 | 0.18 | 238 | 121 | 7 | 1 | 180 | 1 | 222 | HTH cro/C1-type domain-containing protein | HTH cro/C1-type domain-containing protein | | afdb-uniprot50 | AF-A0A132C1D9-F1-MODEL\_V4 | 1.0 | 1.64e-07 | 213 | 0.234 | 192 | 116 | 10 | 5 | 180 | 115 | 291 | Putative HTH-type transcriptional regulator | Putative HTH-type transcriptional regulator | | afdb-uniprot50 | AF-A0A2G6DFY9-F1-MODEL\_V4 | 1.0 | 9.733e-08 | 212 | 0.141 | 240 | 135 | 8 | 6 | 180 | 4 | 237 | Peptidase S24 | Peptidase S24 | | afdb-uniprot50 | AF-A0A496JQM1-F1-MODEL\_V4 | 1.0 | 2.608e-07 | 212 | 0.141 | 248 | 138 | 8 | 4 | 180 | 13 | 256 | Peptidase\_S24 domain-containing protein | Peptidase\_S24 domain-containing protein | | afdb-uniprot50 | AF-A0A0A8RA87-F1-MODEL\_V4 | 1.0 | 2.068e-07 | 212 | 0.152 | 236 | 141 | 5 | 4 | 180 | 33 | 268 | Repressor protein CI | Repressor protein CI | | afdb-uniprot50 | AF-A0A1A9WSH4-F1-MODEL\_V4 | 1.0 | 2.764e-07 | 212 | 0.173 | 230 | 131 | 9 | 4 | 175 | 525 | 753 | HTH cro/C1-type domain-containing protein | HTH cro/C1-type domain-containing protein | | afdb-uniprot50 | AF-A0A1A7Q739-F1-MODEL\_V4 | 1.0 | 9.185e-08 | 211 | 0.186 | 225 | 120 | 8 | 13 | 180 | 11 | 229 | HTH Mu-type domain-containing protein | HTH Mu-type domain-containing protein | | afdb-uniprot50 | AF-A0A7U4SE54-F1-MODEL\_V4 | 1.0 | 2.608e-07 | 210 | 0.149 | 227 | 140 | 10 | 4 | 180 | 7 | 230 | Peptidase S24 | Peptidase S24 | | afdb-uniprot50 | AF-A0A1H7A0H9-F1-MODEL\_V4 | 1.0 | 8.667e-08 | 209 | 0.15 | 186 | 112 | 7 | 28 | 180 | 18 | 190 | Phage repressor protein C, contains Cro/C1-type HTH and peptisase s24 domains | Phage repressor protein C, contains Cro/C1-type HTH and peptisase s24 domains | | afdb-uniprot50 | AF-A0A2A3MNA1-F1-MODEL\_V4 | 1.0 | 3.289e-07 | 209 | 0.172 | 214 | 118 | 4 | 24 | 180 | 2 | 213 | Peptidase | Peptidase | | afdb-uniprot50 | AF-I2NH04-F1-MODEL\_V4 | 1.0 | 1.842e-07 | 209 | 0.188 | 217 | 126 | 11 | 12 | 180 | 4 | 218 | Peptidase S24-like protein | Peptidase S24-like protein | | afdb-uniprot50 | AF-A0A431J112-F1-MODEL\_V4 | 1.0 | 4.396e-07 | 209 | 0.146 | 211 | 140 | 10 | 5 | 179 | 20 | 226 | S24 family peptidase | S24 family peptidase | | afdb-uniprot50 | AF-A0A4P6QMF0-F1-MODEL\_V4 | 1.0 | 1.301e-07 | 209 | 0.142 | 224 | 126 | 9 | 16 | 180 | 14 | 230 | S24 family peptidase | S24 family peptidase | | afdb-uniprot50 | AF-A0A439VN75-F1-MODEL\_V4 | 1.0 | 7.283e-08 | 209 | 0.142 | 280 | 134 | 10 | 5 | 180 | 26 | 303 | Helix-turn-helix domain-containing protein | Helix-turn-helix domain-containing protein | | afdb-uniprot50 | AF-A0A1F6GLC5-F1-MODEL\_V4 | 1.0 | 2.608e-07 | 209 | 0.216 | 203 | 123 | 9 | 9 | 180 | 112 | 309 | Peptidase\_S24 domain-containing protein | Peptidase\_S24 domain-containing protein | | afdb-uniprot50 | AF-A0A0M7MMN5-F1-MODEL\_V4 | 1.0 | 2.461e-07 | 208 | 0.175 | 222 | 133 | 9 | 4 | 180 | 13 | 229 | DNA polymerase V subunit UmuD | DNA polymerase V subunit UmuD | | afdb-uniprot50 | AF-A0A7X3U948-F1-MODEL\_V4 | 1.0 | 1.378e-07 | 208 | 0.149 | 255 | 127 | 8 | 5 | 180 | 8 | 251 | S24 family peptidase | S24 family peptidase | | afdb-uniprot50 | AF-A0A7X3H7P9-F1-MODEL\_V4 | 1.0 | 5.543e-07 | 208 | 0.116 | 284 | 142 | 8 | 5 | 180 | 9 | 291 | Helix-turn-helix domain-containing protein | Helix-turn-helix domain-containing protein | | afdb-uniprot50 | AF-A0A554XCA7-F1-MODEL\_V4 | 1.0 | 2.323e-07 | 208 | 0.136 | 308 | 129 | 9 | 5 | 176 | 8 | 314 | Peptidase S24-like protein | Peptidase S24-like protein | | afdb-uniprot50 | AF-A0A0Q5MIV8-F1-MODEL\_V4 | 1.0 | 9.12e-05 | 207 | 0.171 | 105 | 84 | 3 | 78 | 180 | 137 | 240 | Peptidase\_S24 domain-containing protein | Peptidase\_S24 domain-containing protein | | afdb-uniprot50 | AF-A0A2N6IH07-F1-MODEL\_V4 | 1.0 | 1.738e-07 | 207 | 0.154 | 227 | 127 | 7 | 7 | 180 | 24 | 238 | HTH cro/C1-type domain-containing protein | HTH cro/C1-type domain-containing protein | | afdb-uniprot50 | AF-A0A1G8
[truncated: 20,443 more chars]
