# Supplementary material for: Completing the BASEL phage collection to unlock hidden diversity for systematic exploration of phage–host interactions
Source: PLoS Biol. 2025 Apr 7;23(4):e3003063. doi: 10.1371/journal.pbio.3003063 (PMC11990801; doi:10.1371/journal.pbio.3003063)
Supplement: S2 Data — (ZIP) [file pbio.3003063.s009.zip › entries/45.html]

FANPEZAQ\_CDS\_0045


Return to summary | Go to previous | Go to next

|  |  |
| --- | --- |
| FANPEZAQ\_CDS\_0045 Page creation date: 02 Sep 2024, 12:00  Project folder: n/a  Input sequences file: Escherichia\_virus\_HeidiAbel.gb | duf1391 domain\_containing prophage putative phage fragment p287170 vi\_07176 p274931 vi\_10756 pf07151 ydaf ydfa |

### Sequence information

|  |  |
| --- | --- |
| Name | FANPEZAQ\_CDS\_0045  45\_FANPEZAQ\_CDS\_0045 (pipeline id) |
| Imported annotations | Escherichia\_virus\_HeidiAbel Bas97 |
| Protein sequence | MNTTHDMGNNETIKTGVFPNTDGTFTALTFTKSKTFKTEAGALKWFNRQMAD |
| Number of residues | 52 |
| Molecular weight (Da) | 5817.46 |
| Output files | ../../query\_sequences/45\_FANPEZAQ\_CDS\_0045.fasta |

### Putative domain architecture and protein family

#### Search results (HHblits)1

|  |  |
| --- | --- |
| Domain family databases searched | Pfam, Ncbi-cd, Cath, Phrogs |
| Results, scheme(s)  (Top layers only; threshold 1.00e-03 (evalue)) | xml version="1.0" encoding="utf-8" standalone="no"?       2024-09-02T21:08:22.690407 image/svg+xml   Matplotlib v3.7.2, https://matplotlib.org/ |
| Results, table  (E-value ≤ 1.00e-03 (evalue)) | | db | id | prob | evalue | pvalue | score | cols | query | query\_len | template | template\_len | name | description | | --- | --- | --- | --- | --- | --- | --- | --- | --- | --- | --- | --- | --- | | pfam | PF07151 | 100.0 | 5.3e-39 | 9.8e-43 | 179.2 | 47 | (4, 50) | 52 | (2, 48) | 48 | DUF1391 | Protein of unknown function (DUF1391) | | phrogs | 751 | 100.0 | 1e-37 | 1.2e-41 | 190.6 | 49 | (2, 50) | 52 | (28, 76) | 77 | NA | NA; Category: unknown function; p287170 VI\_07176 | | phrogs | 37620 | 97.9 | 5.6e-09 | 6.3e-13 | 57.4 | 18 | (3, 20) | 52 | (29, 46) | 46 | NA | NA; Category: unknown function; p274931 VI\_10756 | |
| Top keywords  (threshold 1.00e-03 (evalue)) | **DUF1391, p287170, VI\_07176, p274931, VI\_10756** |
| Output files | ../../domain\_architecture/45\_FANPEZAQ\_CDS\_0045\_cath.hhr ../../domain\_architecture/45\_FANPEZAQ\_CDS\_0045\_merged.svg ../../domain\_architecture/45\_FANPEZAQ\_CDS\_0045\_ncbi-cd.hhr ../../domain\_architecture/45\_FANPEZAQ\_CDS\_0045\_pfam.hhr ../../domain\_architecture/45\_FANPEZAQ\_CDS\_0045\_phrogs.hhr |

### Identical protein sequences/structures

#### Search results

|  |  |
| --- | --- |
| Protein sequence databases searched | Pdb, Swissprot, Refseq |
| Identical proteins found | -- |
| Top keywords | -- |
| Output files | -- |

### Similar protein sequences/structures

#### Sequence similarity search results (HHblits)1

|  |  |
| --- | --- |
| Sequence databases searched | Uniclust, Pdb70 |
| Results, scheme(s)  (Top layers only, threshold 1.00e-03 (evalue)) | xml version="1.0" encoding="utf-8" standalone="no"?       2024-09-02T21:08:49.514118 image/svg+xml   Matplotlib v3.7.2, https://matplotlib.org/ |
| Results, table(s)  (threshold 1.00e-03 (evalue)) | | db | id | prob | evalue | pvalue | score | cols | query | query\_len | template | template\_len | name | description | | --- | --- | --- | --- | --- | --- | --- | --- | --- | --- | --- | --- | --- | | uniclust | UniRef100\_A0A0A0G5S2 | 100.0 | 1.2e-40 | 2.7e-46 | 208.6 | 51 | (2, 52) | 52 | (27, 77) | 77 | Uncharacterized protein | Uncharacterized protein | | uniclust | UniRef100\_A0A0A2RM88 | 100.0 | 3.7e-40 | 8.7e-46 | 203.0 | 52 | (1, 52) | 52 | (8, 59) | 69 | DUF1391 domain-containing protein | DUF1391 domain-containing protein | | uniclust | UniRef100\_A0A0A1A890 | 100.0 | 5.5e-38 | 1.1e-43 | 190.2 | 51 | (2, 52) | 52 | (5, 55) | 61 | DUF1391 domain-containing protein | DUF1391 domain-containing protein | | uniclust | UniRef100\_A0A0B0VS76 | 100.0 | 2.7e-37 | 5.2e-43 | 203.0 | 51 | (2, 52) | 52 | (58, 108) | 108 | Phage protein | Phage protein | | uniclust | UniRef100\_A0A376Y8S3 | 99.9 | 2e-30 | 3.8e-36 | 173.1 | 49 | (2, 50) | 52 | (1, 49) | 119 | Putative prophage protein | Putative prophage protein | | uniclust | UniRef100\_A8GLQ0 | 99.8 | 1.8e-23 | 3.4e-29 | 131.9 | 51 | (2, 52) | 52 | (24, 74) | 74 | DUF1391 domain-containing protein | DUF1391 domain-containing protein | | uniclust | UniRef100\_A0A376ZVH1 | 99.8 | 8e-23 | 1.5e-28 | 138.0 | 38 | (2, 39) | 52 | (28, 65) | 121 | Putative prophage protein | Putative prophage protein | | uniclust | UniRef100\_UPI002035C54D | 99.8 | 4.3e-22 | 8.1e-28 | 121.8 | 33 | (2, 34) | 52 | (27, 59) | 59 | DUF1391 family protein | DUF1391 family protein | | uniclust | UniRef100\_F4V2C7 | 99.7 | 4.2e-21 | 7.7e-27 | 143.2 | 38 | (2, 39) | 52 | (53, 90) | 295 | Prophage protein | Prophage protein | | uniclust | UniRef100\_A0A3S4MIN5 | 99.7 | 6.1e-20 | 1.1e-25 | 103.4 | 32 | (2, 33) | 52 | (1, 32) | 35 | Putative prophage protein | Putative prophage protein | | uniclust | UniRef100\_UPI0020335C76 | 99.7 | 7e-20 | 1.3e-25 | 114.1 | 30 | (2, 31) | 52 | (36, 65) | 65 | DUF1391 family protein | DUF1391 family protein | | uniclust | UniRef100\_A0A0Q5E3H7 | 99.6 | 5.8e-18 | 1.1e-23 | 108.6 | 49 | (1, 50) | 52 | (22, 70) | 78 | DUF1391 domain-containing protein | DUF1391 domain-containing protein | | uniclust | UniRef100\_A0A827QYV4 | 99.5 | 1.2e-17 | 2.4e-23 | 103.8 | 34 | (2, 35) | 52 | (28, 61) | 62 | DUF1391 domain-containing protein (Fragment) | DUF1391 domain-containing protein (Fragment) | | uniclust | UniRef100\_A0A8E0FTF2 | 99.5 | 1.7e-17 | 3.1e-23 | 97.6 | 38 | (2, 39) | 52 | (1, 38) | 44 | PF07151 family protein | PF07151 family protein | | uniclust | UniRef100\_A0A1B1UD71 | 99.4 | 1e-15 | 1.9e-21 | 96.3 | 48 | (2, 50) | 52 | (1, 48) | 66 | Uncharacterized protein | Uncharacterized protein | | uniclust | UniRef100\_A0A6C9QHP0 | 98.2 | 1.7e-08 | 3.1e-14 | 58.8 | 17 | (2, 18) | 52 | (19, 35) | 35 | DUF1391 domain-containing protein (Fragment) | DUF1391 domain-containing protein (Fragment) | | uniclust | UniRef100\_A0A6J5LVG2 | 97.6 | 1.8e-06 | 3.2e-12 | 56.0 | 33 | (20, 52) | 52 | (21, 53) | 62 | Uncharacterized protein | Uncharacterized protein | |
| Top keywords  (threshold 1.00e-03 (evalue)) | **DUF1391, domain\_containing, prophage, Putative, Fragment, Phage, PF07151** |
| Output files | ../../similar\_sequences/45\_FANPEZAQ\_CDS\_0045\_merged.svg ../../similar\_sequences/45\_FANPEZAQ\_CDS\_0045\_pdb70.a3m ../../similar\_sequences/45\_FANPEZAQ\_CDS\_0045\_pdb70.hhr ../../similar\_sequences/45\_FANPEZAQ\_CDS\_0045\_uniclust.a3m ../../similar\_sequences/45\_FANPEZAQ\_CDS\_0045\_uniclust.hhr |

#### Structure prediction (AlphaFold)2

|  |  |
| --- | --- |
| Stats | xml version="1.0" encoding="utf-8" standalone="no"?       2024-09-02T21:09:42.611819 image/svg+xml   Matplotlib v3.7.2, https://matplotlib.org/ |
| Predicted structure | **NGL Viewer Controls:**  - Center: *Left-Click* - Rotate: *Left-Click + Drag* - Translate: *Right-Click + Drag* - Zoom: *Shift + Left-Click + Drag* |
| Output files | ../../predicted\_structures/45\_FANPEZAQ\_CDS\_0045/features.pkl ../../predicted\_structures/45\_FANPEZAQ\_CDS\_0045/ranked\_0.pdb ../../predicted\_structures/45\_FANPEZAQ\_CDS\_0045/ranked\_0\_plots.svg ../../predicted\_structures/45\_FANPEZAQ\_CDS\_0045/result\_model\_1\_ptm\_pred\_0.pkl |

#### Structure similarity search results (Foldseek)3

|  |  |
| --- | --- |
| Structure databases searched | Pdb, Afdb-proteome, Afdb-uniprot50 |
| Results, scheme(s)  (Top layers only, threshold 1.00e-02 (evalue)) | xml version="1.0" encoding="utf-8" standalone="no"?       2024-09-02T21:11:22.327438 image/svg+xml   Matplotlib v3.7.2, https://matplotlib.org/ |
| Results, table  (threshold 1.00e-02 (evalue)) | | db | id | prob | evalue | bits | fident | alnlen | mismatch | gapopen | qstart | qend | tstart | tend | name | description | | --- | --- | --- | --- | --- | --- | --- | --- | --- | --- | --- | --- | --- | --- | --- | | afdb-proteome | AF-P0ACW0-F1-MODEL\_V4 | 1.0 | 7.805e-05 | 228 | 0.63 | 46 | 17 | 0 | 3 | 48 | 2 | 47 | Uncharacterized protein YdaF | Uncharacterized protein YdaF | | afdb-proteome | AF-P0ACW8-F1-MODEL\_V4 | 1.0 | 9.4e-05 | 211 | 0.62 | 50 | 19 | 0 | 3 | 52 | 2 | 51 | Uncharacterized protein YdfA | Uncharacterized protein YdfA | | afdb-uniprot50 | AF-A0A1B3FBA6-F1-MODEL\_V4 | 1.0 | 2.142e-06 | 320 | 0.826 | 52 | 9 | 0 | 1 | 52 | 1 | 52 | DUF1391 domain-containing protein | DUF1391 domain-containing protein | | afdb-uniprot50 | AF-A0A7W4F9P6-F1-MODEL\_V4 | 1.0 | 1.463e-05 | 282 | 0.711 | 52 | 15 | 0 | 1 | 52 | 1 | 52 | DUF1391 family protein | DUF1391 family protein | | afdb-uniprot50 | AF-D3QX46-F1-MODEL\_V4 | 1.0 | 5.724e-05 | 265 | 0.62 | 50 | 19 | 0 | 1 | 50 | 52 | 101 | Uncharacterized protein | Uncharacterized protein | | afdb-uniprot50 | AF-A8GLQ0-F1-MODEL\_V4 | 1.0 | 6.479e-05 | 259 | 0.686 | 51 | 16 | 0 | 2 | 52 | 24 | 74 | Uncharacterized protein | Uncharacterized protein | | afdb-uniprot50 | AF-A0A6H2VLD3-F1-MODEL\_V4 | 1.0 | 8.303e-05 | 256 | 0.557 | 52 | 23 | 0 | 1 | 52 | 1 | 52 | DUF1391 domain-containing protein | DUF1391 domain-containing protein | | afdb-uniprot50 | AF-A0A749PJ15-F1-MODEL\_V4 | 1.0 | 7.335e-05 | 253 | 0.588 | 51 | 21 | 0 | 2 | 52 | 9 | 59 | DUF1391 domain-containing protein | DUF1391 domain-containing protein | | afdb-uniprot50 | AF-A0A6C7C4R7-F1-MODEL\_V4 | 1.0 | 0.0001132 | 251 | 0.66 | 50 | 17 | 0 | 3 | 52 | 2 | 51 | DUF1391 domain-containing protein | DUF1391 domain-containing protein | | afdb-uniprot50 | AF-A0A5U8J6R4-F1-MODEL\_V4 | 1.0 | 0.0001543 | 244 | 0.632 | 49 | 18 | 0 | 3 | 51 | 2 | 50 | DUF1391 domain-containing protein | DUF1391 domain-containing protein | | afdb-uniprot50 | AF-A0A3V2NXN8-F1-MODEL\_V4 | 1.0 | 0.0001451 | 240 | 0.6 | 50 | 20 | 0 | 3 | 52 | 2 | 51 | DUF1391 domain-containing protein | DUF1391 domain-containing protein | | afdb-uniprot50 | AF-D6I777-F1-MODEL\_V4 | 1.0 | 0.0001132 | 240 | 0.596 | 52 | 21 | 0 | 1 | 52 | 57 | 108 | Uncharacterized protein | Uncharacterized protein | | afdb-uniprot50 | AF-K8A7Y3-F1-MODEL\_V4 | 1.0 | 0.0001859 | 239 | 0.68 | 47 | 15 | 0 | 3 | 49 | 2 | 48 | Uncharacterized protein | Uncharacterized protein | | afdb-uniprot50 | AF-Q3Z1T6-F1-MODEL\_V4 | 1.0 | 0.0001543 | 237 | 0.632 | 49 | 18 | 0 | 2 | 50 | 19 | 67 | Uncharacterized protein | Uncharacterized protein | | afdb-uniprot50 | AF-A0A376P4T6-F1-MODEL\_V4 | 1.0 | 0.0001642 | 237 | 0.568 | 51 | 22 | 0 | 2 | 52 | 49 | 99 | Uncharacterized protein | Uncharacterized protein | | afdb-uniprot50 | AF-A0A376Y8S3-F1-MODEL\_V4 | 1.0 | 0.0001363 | 237 | 0.612 | 49 | 19 | 0 | 3 | 51 | 2 | 50 | Putative prophage protein | Putative prophage protein | | afdb-uniprot50 | AF-W1Y735-F1-MODEL\_V4 | 1.0 | 0.0001543 | 236 | 0.591 | 49 | 20 | 0 | 2 | 50 | 18 | 66 | Uncharacterized protein | Uncharacterized protein | | afdb-uniprot50 | AF-A0A826I354-F1-MODEL\_V4 | 1.0 | 0.0001978 | 234 | 0.63 | 46 | 17 | 0 | 3 | 48 | 2 | 47 | DUF1391 domain-containing protein | DUF1391 domain-containing protein | | afdb-uniprot50 | AF-A0A4C3QBM3-F1-MODEL\_V4 | 1.0 | 0.0002104 | 231 | 0.617 | 47 | 18 | 0 | 2 | 48 | 15 | 61 | Uncharacterized protein | Uncharacterized protein | | afdb-uniprot50 | AF-W0AQM8-F1-MODEL\_V4 | 1.0 | 0.0001642 | 230 | 0.632 | 49 | 18 | 0 | 4 | 52 | 29 | 77 | Phage protein | Phage protein | | afdb-uniprot50 | AF-E9XM46-F1-MODEL\_V4 | 1.0 | 0.0002239 | 225 | 0.596 | 52 | 21 | 0 | 1 | 52 | 35 | 86 | Uncharacterized protein | Uncharacterized protein | | afdb-uniprot50 | AF-B7MV19-F1-MODEL\_V4 | 1.0 | 0.0003052 | 222 | 0.617 | 47 | 18 | 0 | 4 | 50 | 67 | 113 | Uncharacterized protein | Uncharacterized protein | | afdb-uniprot50 | AF-A0A0B1MRQ9-F1-MODEL\_V4 | 1.0 | 0.0002869 | 221 | 0.645 | 48 | 17 | 0 | 2 | 49 | 19 | 66 | Phage protein | Phage protein | | afdb-uniprot50 | AF-A0A729FUV0-F1-MODEL\_V4 | 1.0 | 0.0003247 | 219 | 0.652 | 46 | 16 | 0 | 3 | 48 | 2 | 47 | DUF1391 domain-containing protein | DUF1391 domain-containing protein | | afdb-uniprot50 | AF-A0A447XGR3-F1-MODEL\_V4 | 1.0 | 0.0002696 | 218 | 0.604 | 48 | 19 | 0 | 4 | 51 | 30 | 77 | Putative prophage protein | Putative prophage protein | | afdb-uniprot50 | AF-A0A828FR55-F1-MODEL\_V4 | 1.0 | 0.0005673 | 217 | 0.608 | 46 | 18 | 0 | 3 | 48 | 2 | 47 | DUF1391 domain-containing protein | DUF1391 domain-containing protein | | afdb-uniprot50 | AF-A0A376K109-F1-MODEL\_V4 | 1.0 | 0.0004161 | 214 | 0.62 | 50 | 19 | 0 | 3 | 52 | 2 | 51 | DUF1391 family protein | DUF1391 family protein | | afdb-uniprot50 | AF-A0A3P6LQE1-F1-MODEL\_V4 | 1.0 | 0.0005012 | 202 | 0.659 | 47 | 16 | 0 | 4 | 50 | 44 | 90 | Prophage protein | Prophage protein | | afdb-uniprot50 | AF-A0A243UXC8-F1-MODEL\_V4 | 1.0 | 0.001194 | 197 | 0.75 | 40 | 10 | 0 | 3 | 42 | 2 | 41 | Phage protein | Phage protein | | afdb-uniprot50 | AF-A0A6C6Z2M3-F1-MODEL\_V4 | 1.0 | 0.003644 | 192 | 0.684 | 38 | 12 | 0 | 15 | 52 | 3 | 40 | Uncharacterized protein | Uncharacterized protein | | afdb-uniprot50 | AF-A0A1B1UD71-F1-MODEL\_V4 | 1.0 | 0.005983 | 169 | 0.531 | 47 | 21 | 1 | 3 | 49 | 2 | 47 | Uncharacterized protein | Uncharacterized protein | |
| Top keywords  (threshold 1.00e-02 (evalue)) | **DUF1391, domain\_containing, prophage, Phage, Putative, YdaF, YdfA** |
| Output files | ../../similar\_structures/45\_FANPEZAQ\_CDS\_0045\_afdb-proteome\_foldseek.tsv ../../similar\_structures/45\_FANPEZAQ\_CDS\_0045\_afdb-uniprot50\_foldseek.tsv ../../similar\_structures/45\_FANPEZAQ\_CDS\_0045\_merged.svg ../../similar\_structures/45\_FANPEZAQ\_CDS\_0045\_pdb\_foldseek.tsv |

  
  
  

Return to summary | Go to previous | Go to next

  


---

**Sequence/structure alignments coloring**  
Each object in the alignment figures is colored according to its E-value following this color coding:

1e-100
10

**References:**  
1) Steinegger M, Meier M, Mirdita M, Vöhringer H, Haunsberger S J, and Söding J (2019) HH-suite3 for fast remote homology detection and deep protein annotation, BMC Bioinformatics, 473. doi: 10.1186/s12859-019-3019-7  
2) Jumper J, Evans R, Pritzel A, ..., Hassabis D (2021) Highly accurate protein structure prediction with AlphaFold, Nature, 596. doi: 10.1038/s41586-021-03819-2  
3) van Kempen M, Kim S, Tumescheit C, Mirdita M, Lee J, Gilchrist CLM, Söding J, and Steinegger M (2023) Fast and accurate protein structure search with Foldseek. Nature Biotechnology. doi: 10.1038/s41587-023-01773-0
