# Supplementary material for: Completing the BASEL phage collection to unlock hidden diversity for systematic exploration of phage–host interactions
Source: PLoS Biol. 2025 Apr 7;23(4):e3003063. doi: 10.1371/journal.pbio.3003063 (PMC11990801; doi:10.1371/journal.pbio.3003063)
Supplement: S2 Data — (ZIP) [file pbio.3003063.s009.zip › entries/46.html]

FANPEZAQ\_CDS\_0046


Return to summary | Go to previous | Go to next

|  |  |
| --- | --- |
| FANPEZAQ\_CDS\_0046 Page creation date: 02 Sep 2024, 12:00  Project folder: n/a  Input sequences file: Escherichia\_virus\_HeidiAbel.gb | domain\_containing nucleotide modification nmad5 associated putative fragment hypothetical nc\_028980\_p51 gp23 dna\_directed dna polymerase a palm contig\_80 whole genome shotgun sequence |

### Sequence information

|  |  |
| --- | --- |
| Name | FANPEZAQ\_CDS\_0046  46\_FANPEZAQ\_CDS\_0046 (pipeline id) |
| Imported annotations | Escherichia\_virus\_HeidiAbel Bas97 |
| Protein sequence | MARLTAAIRDGIIANAIKTKDFAGRDLAIVQKRADFAERLRKYALAQYGLTDARLTEIKK QIEELQNEVKHEGARFVRITFDSTNRAWDVNLAGQTRRLYLDGTCSGESVKPLYGDSRVE YSDDKITPHFHESDFTVRDPKWREELDAIDLEAAQLREEYNTLKSTLRATISAFTTVEKL LEAWPDVKELIPETTPIAKQPGTGIALSVADLNAL |
| Number of residues | 215 |
| Molecular weight (Da) | 24239.10 |
| Output files | ../../query\_sequences/46\_FANPEZAQ\_CDS\_0046.fasta |

### Putative domain architecture and protein family

#### Search results (HHblits)1

|  |  |
| --- | --- |
| Domain family databases searched | Pfam, Ncbi-cd, Cath, Phrogs |
| Results, scheme(s)  (Top layers only; threshold 1.00e-03 (evalue)) | xml version="1.0" encoding="utf-8" standalone="no"?       2024-09-02T21:08:22.899328 image/svg+xml   Matplotlib v3.7.2, https://matplotlib.org/ |
| Results, table  (E-value ≤ 1.00e-03 (evalue)) | | db | id | prob | evalue | pvalue | score | cols | query | query\_len | template | template\_len | name | description | | --- | --- | --- | --- | --- | --- | --- | --- | --- | --- | --- | --- | --- | | pfam | PF18757 | 100.0 | 1.7e-46 | 3.4e-50 | 309.2 | 200 | (2, 215) | 215 | (1, 200) | 204 | Nmad5 | Nucleotide modification associated domain 5 | | phrogs | 1965 | 100.0 | 5.6e-54 | 7.4e-58 | 359.1 | 175 | (1, 215) | 215 | (2, 176) | 186 | NA | NA; Category: unknown function; NC\_028980\_p51 | |
| Top keywords  (threshold 1.00e-03 (evalue)) | **Nucleotide, modification, associated, NC\_028980\_p51** |
| Output files | ../../domain\_architecture/46\_FANPEZAQ\_CDS\_0046\_cath.hhr ../../domain\_architecture/46\_FANPEZAQ\_CDS\_0046\_merged.svg ../../domain\_architecture/46\_FANPEZAQ\_CDS\_0046\_ncbi-cd.hhr ../../domain\_architecture/46\_FANPEZAQ\_CDS\_0046\_pfam.hhr ../../domain\_architecture/46\_FANPEZAQ\_CDS\_0046\_phrogs.hhr |

### Identical protein sequences/structures

#### Search results

|  |  |
| --- | --- |
| Protein sequence databases searched | Pdb, Swissprot, Refseq |
| Identical proteins found | -- |
| Top keywords | -- |
| Output files | -- |

### Similar protein sequences/structures

#### Sequence similarity search results (HHblits)1

|  |  |
| --- | --- |
| Sequence databases searched | Uniclust, Pdb70 |
| Results, scheme(s)  (Top layers only, threshold 1.00e-03 (evalue)) | xml version="1.0" encoding="utf-8" standalone="no"?       2024-09-02T21:08:49.713837 image/svg+xml   Matplotlib v3.7.2, https://matplotlib.org/ |
| Results, table(s)  (threshold 1.00e-03 (evalue)) | | db | id | prob | evalue | pvalue | score | cols | query | query\_len | template | template\_len | name | description | | --- | --- | --- | --- | --- | --- | --- | --- | --- | --- | --- | --- | --- | | uniclust | UniRef100\_A0A0A0YU75 | 100.0 | 1.7e-51 | 3.9e-57 | 347.0 | 196 | (1, 215) | 215 | (31, 226) | 236 | Nucleotide modification associated domain-containing protein | Nucleotide modification associated domain-containing protein | | uniclust | UniRef100\_A0A068R047 | 100.0 | 2.1e-48 | 4.9e-54 | 333.3 | 196 | (1, 215) | 215 | (48, 246) | 262 | Nucleotide modification associated domain-containing protein | Nucleotide modification associated domain-containing protein | | uniclust | UniRef100\_A0A371YIE3 | 100.0 | 2.7e-38 | 5.7e-44 | 256.5 | 175 | (1, 214) | 215 | (1, 175) | 189 | Nucleotide modification associated domain-containing protein (Fragment) | Nucleotide modification associated domain-containing protein (Fragment) | | uniclust | UniRef100\_A0A2Z3I5W0 | 100.0 | 1.9e-35 | 4.4e-41 | 248.3 | 177 | (1, 215) | 215 | (4, 183) | 209 | Nucleotide modification associated domain-containing protein | Nucleotide modification associated domain-containing protein | | uniclust | UniRef100\_A0A1I3TCD5 | 100.0 | 2.2e-35 | 4.8e-41 | 244.7 | 177 | (1, 215) | 215 | (9, 191) | 203 | Nucleotide modification associated domain-containing protein | Nucleotide modification associated domain-containing protein | | uniclust | UniRef100\_A0A0H3EFP6 | 100.0 | 3.4e-34 | 6.3e-40 | 232.7 | 196 | (1, 215) | 215 | (26, 222) | 232 | Nucleotide modification associated domain-containing protein | Nucleotide modification associated domain-containing protein | | uniclust | UniRef100\_A0A0N7EMH6 | 100.0 | 2.6e-33 | 5.5e-39 | 229.9 | 174 | (1, 215) | 215 | (6, 180) | 200 | Nucleotide modification associated domain-containing protein | Nucleotide modification associated domain-containing protein | | uniclust | UniRef100\_A0A0M0T5J9 | 99.9 | 4.1e-28 | 7.9e-34 | 193.3 | 162 | (35, 215) | 215 | (6, 170) | 181 | Nucleotide modification associated domain-containing protein (Fragment) | Nucleotide modification associated domain-containing protein (Fragment) | | uniclust | UniRef100\_Q5DN82 | 99.9 | 1.9e-27 | 3.9e-33 | 195.5 | 186 | (1, 214) | 215 | (4, 198) | 209 | Gp23 | Gp23 | | uniclust | UniRef100\_A0A329VKA5 | 99.9 | 2.9e-27 | 5.6e-33 | 182.3 | 116 | (84, 215) | 215 | (14, 130) | 140 | Nucleotide modification associated domain-containing protein | Nucleotide modification associated domain-containing protein | | uniclust | UniRef100\_A0A0P7PEB3 | 99.9 | 4.8e-27 | 9.6e-33 | 197.8 | 203 | (1, 215) | 215 | (39, 253) | 263 | Nucleotide modification associated domain-containing protein | Nucleotide modification associated domain-containing protein | | uniclust | UniRef100\_UPI0012FF825C | 99.9 | 9.2e-27 | 2e-32 | 194.0 | 163 | (1, 180) | 215 | (26, 189) | 196 | Nmad5 family putative nucleotide modification protein | Nmad5 family putative nucleotide modification protein | | uniclust | UniRef100\_A0A345MLV4 | 99.9 | 1e-25 | 1.9e-31 | 182.2 | 184 | (1, 215) | 215 | (2, 188) | 199 | Nucleotide modification associated domain-containing protein | Nucleotide modification associated domain-containing protein | | uniclust | UniRef100\_A0A6M9Z847 | 99.8 | 1.3e-23 | 2.4e-29 | 172.1 | 172 | (1, 214) | 215 | (17, 188) | 243 | Nucleotide modification associated domain-containing protein | Nucleotide modification associated domain-containing protein | | uniclust | UniRef100\_A0A5Z4LDL4 | 99.8 | 1.4e-23 | 2.6e-29 | 163.0 | 148 | (2, 165) | 215 | (3, 153) | 154 | Nucleotide modification associated domain-containing protein (Fragment) | Nucleotide modification associated domain-containing protein (Fragment) | | uniclust | UniRef100\_A0A076YQL7 | 99.8 | 4.5e-23 | 8.6e-29 | 173.0 | 202 | (2, 215) | 215 | (16, 264) | 269 | Nucleotide modification associated domain-containing protein | Nucleotide modification associated domain-containing protein | | uniclust | UniRef100\_A0A6G5XY22 | 99.8 | 1.2e-21 | 2.2e-27 | 157.9 | 190 | (1, 215) | 215 | (1, 200) | 209 | Nucleotide modification associated domain-containing protein | Nucleotide modification associated domain-containing protein | | uniclust | UniRef100\_A0A455UAL4 | 99.7 | 4e-20 | 7.7e-26 | 153.5 | 206 | (2, 214) | 215 | (10, 221) | 230 | Nucleotide modification associated domain-containing protein | Nucleotide modification associated domain-containing protein | | uniclust | UniRef100\_A0A2E3MWD4 | 99.7 | 4.3e-20 | 7.9e-26 | 147.6 | 179 | (1, 214) | 215 | (3, 183) | 192 | Nucleotide modification associated domain-containing protein | Nucleotide modification associated domain-containing protein | | uniclust | UniRef100\_A0A1G3M2G9 | 99.6 | 4.6e-19 | 8.4e-25 | 140.5 | 169 | (1, 215) | 215 | (1, 169) | 178 | Nucleotide modification associated domain-containing protein | Nucleotide modification associated domain-containing protein | | uniclust | UniRef100\_A0A1B9LQJ1 | 99.5 | 2.3e-17 | 4.2e-23 | 119.7 | 82 | (132, 215) | 215 | (7, 89) | 95 | Nucleotide modification associated domain-containing protein | Nucleotide modification associated domain-containing protein | | uniclust | UniRef100\_A0A174S3N4 | 99.5 | 3.3e-17 | 7e-23 | 134.8 | 165 | (2, 214) | 215 | (1, 165) | 175 | Uncharacterized protein | Uncharacterized protein | | uniclust | UniRef100\_A0A658IDF4 | 99.4 | 2.3e-15 | 4.4e-21 | 109.1 | 76 | (137, 215) | 215 | (2, 77) | 88 | Nucleotide modification associated domain-containing protein | Nucleotide modification associated domain-containing protein | | uniclust | UniRef100\_A0A384ZRN4 | 99.4 | 2.7e-15 | 4.9e-21 | 124.2 | 127 | (84, 215) | 215 | (95, 227) | 232 | Nucleotide modification associated domain-containing protein | Nucleotide modification associated domain-containing protein | | uniclust | UniRef100\_UPI001F614FAB | 99.3 | 5.8e-15 | 1.1e-20 | 102.3 | 60 | (154, 215) | 215 | (3, 62) | 70 | Nmad5 family putative nucleotide modification protein | Nmad5 family putative nucleotide modification protein | | uniclust | UniRef100\_A0A497QQK1 | 99.3 | 1.9e-14 | 3.5e-20 | 116.3 | 172 | (1, 215) | 215 | (3, 177) | 190 | Nucleotide modification associated domain-containing protein | Nucleotide modification associated domain-containing protein | | uniclust | UniRef100\_G1V8X6 | 99.3 | 2.1e-14 | 3.8e-20 | 116.9 | 170 | (1, 208) | 215 | (3, 176) | 199 | Nucleotide modification associated domain-containing protein | Nucleotide modification associated domain-containing protein | | uniclust | UniRef100\_A0A118DTA8 | 99.3 | 2.2e-14 | 5e-20 | 120.9 | 81 | (132, 214) | 215 | (80, 162) | 179 | Nucleotide modification associated domain-containing protein | Nucleotide modification associated domain-containing protein | | uniclust | UniRef100\_UPI001B8ABC59 | 99.2 | 3e-14 | 5.4e-20 | 114.7 | 165 | (2, 214) | 215 | (1, 166) | 183 | Nmad5 family putative nucleotide modification protein | Nmad5 family putative nucleotide modification protein | | uniclust | UniRef100\_UPI00056EFD59 | 99.2 | 1.1e-13 | 2.1e-19 | 116.2 | 175 | (14, 214) | 215 | (18, 208) | 218 | Nmad5 family putative nucleotide modification protein | Nmad5 family putative nucleotide modification protein | | uniclust | UniRef100\_A0A076G521 | 99.2 | 1.6e-13 | 3.4e-19 | 117.2 | 69 | (144, 214) | 215 | (135, 204) | 211 | Nucleotide modification associated domain-containing protein | Nucleotide modification associated domain-containing protein | | uniclust | UniRef100\_A0A0U2AVF2 | 99.1 | 2.4e-13 | 4.3e-19 | 110.5 | 171 | (1, 208) | 215 | (1, 174) | 192 | Nucleotide modification associated domain-containing protein | Nucleotide modification associated domain-containing protein | | uniclust | UniRef100\_UPI00037C3BEE | 99.1 | 2.4e-13 | 4.3e-19 | 111.2 | 187 | (4, 214) | 215 | (1, 191) | 201 | Nmad5 family putative nucleotide modification protein | Nmad5 family putative nucleotide modification protein | | uniclust | UniRef100\_UPI001FC9B87A | 99.1 | 2.8e-13 | 5.4e-19 | 93.1 | 50 | (163, 215) | 215 | (1, 50) | 62 | Nmad5 family putative nucleotide modification protein | Nmad5 family putative nucleotide modification protein | | uniclust | UniRef100\_A0A142BA81 | 99.1 | 2.8e-13 | 5.5e-19 | 114.4 | 185 | (2, 215) | 215 | (1, 205) | 215 | Nucleotide modification associated domain-containing protein | Nucleotide modification associated domain-containing protein | | uniclust | UniRef100\_UPI00234176EE | 99.1 | 4.1e-13 | 7.5e-19 | 111.3 | 189 | (2, 215) | 215 | (7, 208) | 221 | Nmad5 family putative nucleotide modification protein | Nmad5 family putative nucleotide modification protein | | uniclust | UniRef100\_UPI001EF750B4 | 99.1 | 5.3e-13 | 9.8e-19 | 110.8 | 199 | (1, 215) | 215 | (1, 214) | 224 | Nmad5 family putative nucleotide modification protein | Nmad5 family putative nucleotide modification protein | | uniclust | UniRef100\_A0A1I3RUC2 | 99.1 | 6.4e-13 | 1.2e-18 | 109.2 | 189 | (1, 214) | 215 | (1, 196) | 207 | Nucleotide modification associated domain-containing protein | Nucleotide modification associated domain-containing protein | | uniclust | UniRef100\_A0A1B9MFZ6 | 99.0 | 2.4e-12 | 4.5e-18 | 99.7 | 128 | (1, 159) | 215 | (1, 128) | 134 | Nucleotide modification associated domain-containing protein | Nucleotide modification associated domain-containing protein | | uniclust | UniRef100\_UPI001EE816BC | 99.0 | 2.7e-12 | 4.9e-18 | 104.2 | 167 | (2, 213) | 215 | (9, 175) | 185 | Nmad5 family putative nucleotide modification protein | Nmad5 family putative nucleotide modification protein | | uniclust | UniRef100\_A0A9E7DYX3 | 99.0 | 4.5e-12 | 8.2e-18 | 106.0 | 194 | (2, 214) | 215 | (5, 223) | 231 | Nucleotide modification associated protein | Nucleotide modification associated protein | | uniclust | UniRef100\_UPI002107D559 | 98.8 | 5.5e-11 | 1e-16 | 90.9 | 102 | (2, 109) | 215 | (4, 108) | 119 | Nmad5 family putative nucleotide modification protein | Nmad5 family putative nucleotide modification protein | | uniclust | UniRef100\_UPI001FCF116F | 98.7 | 8.6e-11 | 1.6e-16 | 86.3 | 80 | (2, 82) | 215 | (4, 86) | 88 | Nmad5 family putative nucleotide modification protein | Nmad5 family putative nucleotide modification protein | | uniclust | UniRef100\_A0A369WST6 | 98.7 | 1.8e-10 | 3.4e-16 | 95.6 | 183 | (2, 214) | 215 | (4, 195) | 207 | Nucleotide modification associated domain-containing protein | Nucleotide modification associated domain-containing protein | | uniclust | UniRef100\_UPI0018EAAC65 | 98.6 | 3e-10 | 5.5e-16 | 96.2 | 198 | (2, 214) | 215 | (1, 228) | 240 | Nmad5 family putative nucleotide modification protein | Nmad5 family putative nucleotide modification protein | | uniclust | UniRef100\_UPI002001BDC6 | 98.6 | 3.9e-10 | 7.1e-16 | 87.5 | 87 | (126, 215) | 215 | (35, 121) | 128 | Nmad5 family putative nucleotide modification protein | Nmad5 family putative nucleotide modification protein | | uniclust | UniRef100\_UPI00201A8D73 | 98.5 | 9.8e-10 | 1.8e-15 | 87.1 | 104 | (1, 107) | 215 | (26, 130) | 145 | Nmad5 family putative nucleotide modification protein | Nmad5 family putative nucleotide modification protein | | uniclust | UniRef100\_UPI0012EC2E42 | 98.4 | 3.1e-09 | 5.6e-15 | 89.5 | 172 | (2, 212) | 215 | (28, 205) | 220 | hypothetical protein | hypothetical protein | | uniclust | UniRef100\_A0A258K0Y5 | 98.4 | 2.9e-09 | 5.8e-15 | 89.0 | 151 | (1, 196) | 215 | (1, 151) | 169 | Uncharacterized protein | Uncharacterized protein | | uniclust | UniRef100\_A0A853N6U5 | 98.4 | 3.7e-09 | 6.7e-15 | 75.6 | 70 | (1, 72) | 215 | (1, 70) | 76 | Nucleotide modification associated domain-containing protein | Nucleotide modification associated domain-containing protein | | uniclust | UniRef100\_A0A0S3UFR8 | 98.4 | 3.8e-09 | 7.9e-15 | 93.8 | 67 | (132, 199) | 215 | (153, 219) | 246 | Uncharacterized protein | Uncharacterized protein | | uniclust | UniRef100\_UPI0004740F64 | 98.2 | 3.2e-08 | 5.8e-14 | 82.4 | 162 | (2, 194) | 215 | (1, 164) | 194 | Nmad5 family putative nucleotide modification protein | Nmad5 family putative nucleotide modification protein | | uniclust | UniRef100\_A0A0L0CE49 | 98.2 | 3.3e-08 | 6.1e-14 | 103.5 | 204 | (1, 212) | 215 | (3403, 3634) | 3640 | DNA-directed DNA polymerase family A palm domain-containing protein | DNA-directed DNA polymerase family A palm domain-containing protein | | uniclust | UniRef100\_Q31HW8 | 98.0 | 1.5e-07 | 2.7e-13 | 80.3 | 193 | (2, 212) | 215 | (6, 211) | 225 | Nucleotide modification associated domain-containing protein | Nucleotide modification associated domain-containing protein | | uniclust | UniRef100\_UPI000442AFC4 | 97.9 | 2.2e-07 | 4.1e-13 | 76.5 | 77 | (133, 213) | 215 | (89, 165) | 172 | Nmad5 family putative nucleotide modification protein | Nmad5 family putative nucleotide modification protein | | uniclust | UniRef100\_UPI00167604E6 | 97.8 | 3.6e-07 | 6.6e-13 | 62.5 | 47 | (2, 48) | 215 | (1, 47) | 56 | Nmad5 family putative nucleotide modification protein | Nmad5 family putative nucleotide modification protein | | uniclust | UniRef100\_W1J8V1 | 97.8 | 3.7e-07 | 6.8e-13 | 68.0 | 55 | (3, 57) | 215 | (5, 59) | 90 | Nucleotide modification associated domain-containing protein | Nucleotide modification associated domain-containing protein | | uniclust | UniRef100\_UPI0003EE2D2C | 97.8 | 6e-07 | 1.2e-12 | 68.8 | 57 | (1, 57) | 215 | (3, 59) | 98 | Nmad5 family putative nucleotide modification protein | Nmad5 family putative nucleotide modification protein | | uniclust | UniRef100\_A0A516M5Q1 | 97.8 | 6.8e-07 | 1.2e-12 | 74.4 | 157 | (2, 198) | 215 | (1, 164) | 181 | Uncharacterized protein | Uncharacterized protein | | uniclust | UniRef100\_UPI001F03F4A1 | 97.7 | 7.1e-07 | 1.3e-12 | 70.0 | 95 | (1, 104) | 215 | (1, 95) | 118 | Nmad5 family putative nucleotide modification protein | Nmad5 family putative nucleotide modification protein | | uniclust | UniRef100\_A0A2I7R0Q1 | 97.3 | 9.5e-06 | 1.8e-11 | 69.0 | 81 | (133, 214) | 215 | (105, 195) | 198 | Nucleotide modification associated domain-containing protein | Nucleotide modification associated domain-containing protein | | uniclust | UniRef100\_UPI001FCEE419 | 97.3 | 1.4e-05 | 2.6e-11 | 60.2 | 60 | (84, 157) | 215 | (28, 88) | 89 | Nmad5 family putative nucleotide modification protein | Nmad5 family putative nucleotide modification protein | | uniclust | UniRef100\_A0A2E3DFL3 | 97.3 | 1.5e-05 | 2.7e-11 | 71.6 | 65 | (132, 197) | 215 | (179, 250) | 286 | Nucleotide modification associated domain-containing protein | Nucleotide modification associated domain-containing protein | | uniclust | UniRef100\_A0A8S5MMN6 | 97.2 | 2.2e-05 | 4.3e-11 | 68.1 | 155 | (1, 191) | 215 | (3, 158) | 200 | Nucleotide modification associated domain 5 | Nucleotide modification associated domain 5 | | uniclust | UniRef100\_A0A377D698 | 97.1 | 4e-05 | 7.3e-11 | 54.5 | 49 | (1, 49) | 215 | (3, 51) | 63 | Nucleotide modification associated domain-containing protein | Nucleotide modification associated domain-containing protein | | uniclust | UniRef100\_UPI0020CC0AC4 | 96.7 | 0.00026 | 4.7e-10 | 59.7 | 150 | (2, 198) | 215 | (1, 151) | 168 | hypothetical protein | hypothetical protein | | uniclust | UniRef100\_A0A5P9EY15 | 96.6 | 0.00028 | 5.2e-10 | 62.3 | 61 | (139, 199) | 215 | (148, 208) | 236 | Nucleotide modification associated domain-containing protein | Nucleotide modification associated domain-containing protein | | uniclust | UniRef100\_A0A384WWP2 | 96.3 | 0.00086 | 1.6e-09 | 58.7 | 74 | (123, 198) | 215 | (100, 180) | 191 | Uncharacterized protein | Uncharacterized protein | | uniclust | UniRef100\_A0A381XKK1 | 96.3 | 0.00092 | 1.8e-09 | 60.1 | 164 | (1, 198) | 215 | (1, 174) | 206 | Uncharacterized protein | Uncharacterized protein | |
| Top keywords  (threshold 1.00e-03 (evalue)) | **Nucleotide, modification, associated, domain\_containing, Nmad5, putative, Fragment, hypothetical, Gp23, DNA\_directed** |
| Output files | ../../similar\_sequences/46\_FANPEZAQ\_CDS\_0046\_merged.svg ../../similar\_sequences/46\_FANPEZAQ\_CDS\_0046\_pdb70.a3m ../../similar\_sequences/46\_FANPEZAQ\_CDS\_0046\_pdb70.hhr ../../similar\_sequences/46\_FANPEZAQ\_CDS\_0046\_uniclust.a3m ../../similar\_sequences/46\_FANPEZAQ\_CDS\_0046\_uniclust.hhr |

#### Structure prediction (AlphaFold)2

|  |  |
| --- | --- |
| Stats | xml version="1.0" encoding="utf-8" standalone="no"?       2024-09-02T21:09:43.386372 image/svg+xml   Matplotlib v3.7.2, https://matplotlib.org/ |
| Predicted structure | **NGL Viewer Controls:**  - Center: *Left-Click* - Rotate: *Left-Click + Drag* - Translate: *Right-Click + Drag* - Zoom: *Shift + Left-Click + Drag* |
| Output files | ../../predicted\_structures/46\_FANPEZAQ\_CDS\_0046/features.pkl ../../predicted\_structures/46\_FANPEZAQ\_CDS\_0046/ranked\_0.pdb ../../predicted\_structures/46\_FANPEZAQ\_CDS\_0046/ranked\_0\_plots.svg ../../predicted\_structures/46\_FANPEZAQ\_CDS\_0046/result\_model\_1\_ptm\_pred\_0.pkl |

#### Structure similarity search results (Foldseek)3

|  |  |
| --- | --- |
| Structure databases searched | Pdb, Afdb-proteome, Afdb-uniprot50 |
| Results, scheme(s)  (Top layers only, threshold 1.00e-02 (evalue)) | xml version="1.0" encoding="utf-8" standalone="no"?       2024-09-02T21:11:22.487307 image/svg+xml   Matplotlib v3.7.2, https://matplotlib.org/ |
| Results, table  (threshold 1.00e-02 (evalue)) | | db | id | prob | evalue | bits | fident | alnlen | mismatch | gapopen | qstart | qend | tstart | tend | name | description | | --- | --- | --- | --- | --- | --- | --- | --- | --- | --- | --- | --- | --- | --- | --- | | afdb-uniprot50 | AF-A0A0A2RQ45-F1-MODEL\_V4 | 1.0 | 1.222e-09 | 330 | 0.226 | 221 | 139 | 10 | 1 | 214 | 1 | 196 | Nmad5 domain-containing protein | Nmad5 domain-containing protein | | afdb-uniprot50 | AF-A0A6N8JYJ7-F1-MODEL\_V4 | 1.0 | 6.971e-09 | 301 | 0.193 | 227 | 157 | 6 | 1 | 214 | 1 | 214 | Nmad5 domain-containing protein | Nmad5 domain-containing protein | | afdb-uniprot50 | AF-A0A358RVT3-F1-MODEL\_V4 | 1.0 | 1.665e-08 | 297 | 0.243 | 218 | 131 | 9 | 1 | 214 | 1 | 188 | Nmad5 domain-containing protein | Nmad5 domain-containing protein | | afdb-uniprot50 | AF-A0A756K3A0-F1-MODEL\_V4 | 1.0 | 1.982e-08 | 292 | 0.288 | 222 | 137 | 8 | 2 | 214 | 7 | 216 | Nmad5 domain-containing protein | Nmad5 domain-containing protein | | afdb-uniprot50 | AF-A0A379DND9-F1-MODEL\_V4 | 1.0 | 2.5e-08 | 275 | 0.244 | 217 | 136 | 10 | 3 | 214 | 5 | 198 | Nmad5 domain-containing protein | Nmad5 domain-containing protein | | afdb-uniprot50 | AF-A0A8B2F7G3-F1-MODEL\_V4 | 1.0 | 7.531e-08 | 274 | 0.19 | 221 | 147 | 10 | 1 | 214 | 1 | 196 | Uncharacterized protein | Uncharacterized protein | | afdb-uniprot50 | AF-A0A068R047-F1-MODEL\_V4 | 1.0 | 1.067e-07 | 264 | 0.232 | 219 | 138 | 9 | 2 | 214 | 4 | 198 | Nmad5 domain-containing protein | Nmad5 domain-containing protein | | afdb-uniprot50 | AF-A0A759RC34-F1-MODEL\_V4 | 1.0 | 1.198e-07 | 262 | 0.246 | 211 | 129 | 11 | 1 | 202 | 1 | 190 | Nmad5 domain-containing protein | Nmad5 domain-containing protein | | afdb-uniprot50 | AF-A0A653KZM3-F1-MODEL\_V4 | 1.0 | 1.27e-07 | 251 | 0.235 | 217 | 136 | 10 | 3 | 214 | 2 | 193 | Contig\_80, whole genome shotgun sequence | Contig\_80, whole genome shotgun sequence | | afdb-uniprot50 | AF-A0A377PCD9-F1-MODEL\_V4 | 1.0 | 9.499e-08 | 245 | 0.253 | 225 | 126 | 10 | 2 | 214 | 4 | 198 | Nmad5 domain-containing protein | Nmad5 domain-containing protein | | afdb-uniprot50 | AF-A0A3Y4Y6V3-F1-MODEL\_V4 | 1.0 | 1.764e-05 | 208 | 0.175 | 200 | 133 | 8 | 2 | 193 | 4 | 179 | Nmad5 domain-containing protein | Nmad5 domain-containing protein | | afdb-uniprot50 | AF-A0A242NEU4-F1-MODEL\_V4 | 1.0 | 1.398e-05 | 202 | 0.19 | 215 | 144 | 6 | 1 | 214 | 1 | 186 | Nmad5 domain-containing protein | Nmad5 domain-containing protein | | afdb-uniprot50 | AF-A0A3T7E4H6-F1-MODEL\_V4 | 1.0 | 1.175e-05 | 201 | 0.18 | 222 | 146 | 9 | 2 | 214 | 4 | 198 | Nmad5 domain-containing protein | Nmad5 domain-containing protein | | afdb-uniprot50 | AF-U9Y7X5-F1-MODEL\_V4 | 1.0 | 3.975e-05 | 187 | 0.175 | 211 | 146 | 10 | 9 | 214 | 2 | 189 | Nmad5 domain-containing protein | Nmad5 domain-containing protein | | afdb-uniprot50 | AF-A0A1B9NL72-F1-MODEL\_V4 | 1.0 | 6.325e-05 | 185 | 0.172 | 215 | 144 | 7 | 1 | 214 | 1 | 182 | Nmad5 domain-containing protein | Nmad5 domain-containing protein | | afdb-uniprot50 | AF-H8FYC4-F1-MODEL\_V4 | 1.0 | 3.975e-05 | 179 | 0.195 | 220 | 122 | 11 | 1 | 214 | 1 | 171 | Nmad5 domain-containing protein | Nmad5 domain-containing protein | | afdb-uniprot50 | AF-A0A556RGF3-F1-MODEL\_V4 | 1.0 | 0.0001601 | 178 | 0.185 | 216 | 148 | 8 | 1 | 214 | 1 | 190 | Nmad5 domain-containing protein | Nmad5 domain-containing protein | | afdb-uniprot50 | AF-A0A1M3PIE9-F1-MODEL\_V4 | 1.0 | 9.315e-06 | 177 | 0.202 | 222 | 137 | 13 | 1 | 214 | 1 | 190 | Nmad5 domain-containing protein | Nmad5 domain-containing protein | | afdb-uniprot50 | AF-G1V8X6-F1-MODEL\_V4 | 1.0 | 0.0001697 | 161 | 0.162 | 216 | 140 | 10 | 2 | 214 | 4 | 181 | Nmad5 domain-containing protein | Nmad5 domain-containing protein | | afdb-uniprot50 | AF-A0A8B3IJ52-F1-MODEL\_V4 | 1.0 | 0.0001006 | 160 | 0.168 | 225 | 142 | 11 | 1 | 214 | 1 | 191 | Uncharacterized protein | Uncharacterized protein | | afdb-uniprot50 | AF-A0A1I3RUC2-F1-MODEL\_V4 | 1.0 | 0.0001601 | 147 | 0.184 | 201 | 125 | 10 | 1 | 190 | 1 | 173 | Nmad5 domain-containing protein | Nmad5 domain-containing protein | | afdb-uniprot50 | AF-A0A3N8QQM7-F1-MODEL\_V4 | 1.0 | 0.0002268 | 143 | 0.192 | 223 | 123 | 15 | 2 | 214 | 5 | 180 | Uncharacterized protein | Uncharacterized protein | | afdb-uniprot50 | AF-A0A2E3MWD4-F1-MODEL\_V4 | 1.0 | 0.0004295 | 141 | 0.165 | 218 | 139 | 8 | 2 | 214 | 4 | 183 | Nmad5 domain-containing protein | Nmad5 domain-containing protein | | afdb-uniprot50 | AF-A0A5E4SM04-F1-MODEL\_V4 | 1.0 | 0.002182 | 126 | 0.187 | 224 | 122 | 12 | 3 | 214 | 2 | 177 | Nmad5 domain-containing protein | Nmad5 domain-containing protein | | afdb-uniprot50 | AF-A0A2Z3I5W0-F1-MODEL\_V4 | 1.0 | 0.002312 | 121 | 0.2 | 224 | 120 | 12 | 3 | 214 | 2 | 178 | Nmad5 domain-containing protein | Nmad5 domain-containing protein | | afdb-uniprot50 | AF-A0A378XRC0-F1-MODEL\_V4 | 1.0 | 0.003275 | 117 | 0.171 | 216 | 117 | 11 | 3 | 201 | 2 | 172 | Nmad5 domain-containing protein | Nmad5 domain-containing protein | | afdb-uniprot50 | AF-A0A369WST6-F1-MODEL\_V4 | 1.0 | 0.00464 | 109 | 0.188 | 218 | 144 | 10 | 3 | 214 | 5 | 195 | Nmad5 domain-containing protein | Nmad5 domain-containing protein | | afdb-uniprot50 | AF-A0A6H3FZV3-F1-MODEL\_V4 | 0.998 | 0.00464 | 95 | 0.15 | 252 | 150 | 16 | 1 | 214 | 1 | 226 | Uncharacterized protein | Uncharacterized protein | | afdb-uniprot50 | AF-A0A545THF2-F1-MODEL\_V4 | 0.993 | 0.008786 | 86 | 0.172 | 220 | 145 | 10 | 1 | 214 | 1 | 189 | Nmad5 domain-containing protein | Nmad5 domain-containing protein | |
| Top keywords  (threshold 1.00e-02 (evalue)) | **Nmad5, domain\_containing, Contig\_80, whole, genome, shotgun, sequence** |
| Output files | ../../similar\_structures/46\_FANPEZAQ\_CDS\_0046\_afdb-proteome\_foldseek.tsv ../../similar\_structures/46\_FANPEZAQ\_CDS\_0046\_afdb-uniprot50\_foldseek.tsv ../../similar\_structures/46\_FANPEZAQ\_CDS\_0046\_merged.svg ../../similar\_structures/46\_FANPEZAQ\_CDS\_0046\_pdb\_foldseek.tsv |

  
  
  

Return to summary | Go to previous | Go to next

  


---

**Sequence/structure alignments coloring**  
Each object in the alignment figures is colored according to its E-value following this color coding:

1e-100
10

**References:**  
1) Steinegger M, Meier M, Mirdita M, Vöhringer H, Haunsberger S J, and Söding J (2019) HH-suite3 for fast remote homology detection and deep protein annotation, BMC Bioinformatics, 473. doi: 10.1186/s12859-019-3019-7  
2) Jumper J, Evans R, Pritzel A, ..., Hassabis D (2021) Highly accurate protein structure prediction with AlphaFold, Nature, 596. doi: 10.1038/s41586-021-03819-2  
3) van Kempen M, Kim S, Tumescheit C, Mirdita M, Lee J, Gilchrist CLM, Söding J, and Steinegger M (2023) Fast and accurate protein structure search with Foldseek. Nature Biotechnology. doi: 10.1038/s41587-023-01773-0
