# Supplementary material for: Completing the BASEL phage collection to unlock hidden diversity for systematic exploration of phage–host interactions
Source: PLoS Biol. 2025 Apr 7;23(4):e3003063. doi: 10.1371/journal.pbio.3003063 (PMC11990801; doi:10.1371/journal.pbio.3003063)
Supplement: S2 Data — (ZIP) [file pbio.3003063.s009.zip › entries/48.html]

FANPEZAQ\_CDS\_0048


Return to summary | Go to previous | Go to next

|  |  |
| --- | --- |
| FANPEZAQ\_CDS\_0048 Page creation date: 02 Sep 2024, 12:00  Project folder: n/a  Input sequences file: Escherichia\_virus\_HeidiAbel.gb | vrr\_nuc domain\_containing nuclease fragment endonuclease a containing hypothetical putative trna dna hydrolase engineered yes organism\_taxid expressed in escherichia coli expression\_system\_taxid alpha beta arch 3\_layer aba sandwich holliday junction resolvase phage e c fanconi\_associated and b factor recombinase recb complex \_ repair this model characterizes set that resemble holliday\_junction resolving enzymes |

### Sequence information

|  |  |
| --- | --- |
| Name | FANPEZAQ\_CDS\_0048  48\_FANPEZAQ\_CDS\_0048 (pipeline id) |
| Imported annotations | Escherichia\_virus\_HeidiAbel Bas97 |
| Protein sequence | MNYSGCWWFWLLALSWWGLSMANKEHALQNAIRNALAGRCLLFRANVGKAYTSNDVVKVP RQMPVVMGPRDILLKNARPFDTGLPPGFSDLFGMVSVEITPDMVGQKVAIFTGLEVKDGA RVSPLQRNFINAVNDNGGRAGVVRSVDDAEKLVFGK |
| Number of residues | 156 |
| Molecular weight (Da) | 17167.72 |
| Output files | ../../query\_sequences/48\_FANPEZAQ\_CDS\_0048.fasta |

### Putative domain architecture and protein family

#### Search results (HHblits)1

|  |  |
| --- | --- |
| Domain family databases searched | Pfam, Ncbi-cd, Cath, Phrogs |
| Results, scheme(s)  (Top layers only; threshold 1.00e-03 (evalue)) | xml version="1.0" encoding="utf-8" standalone="no"?       2024-09-02T21:08:23.317903 image/svg+xml   Matplotlib v3.7.2, https://matplotlib.org/ |
| Results, table  (E-value ≤ 1.00e-03 (evalue)) | | db | id | prob | evalue | pvalue | score | cols | query | query\_len | template | template\_len | name | description | | --- | --- | --- | --- | --- | --- | --- | --- | --- | --- | --- | --- | --- | | pfam | PF08774 | 98.3 | 5.5e-11 | 1.3e-14 | 80.1 | 65 | (81, 146) | 156 | (57, 126) | 127 | VRR\_NUC | VRR-NUC domain | | ncbi-cd | cd22326 | 97.8 | 2.9e-09 | 7.1e-13 | 87.9 | 56 | (82, 147) | 156 | (588, 644) | 652 | FAN1-like | cd22326 FAN1-like; repair nuclease FAN1. This model characterizes a set of nucleases that resemble Holliday-junction resolving enzymes. | | ncbi-cd | cd22354 | 97.6 | 1.5e-08 | 3.4e-12 | 72.0 | 59 | (82, 152) | 156 | (42, 107) | 157 | RecU-like | cd22354 RecU-like; Holliday junction resolvase RecU (recombination protein U) and similar nucleases. | | ncbi-cd | cd22365 | 97.5 | 2.4e-08 | 5.9e-12 | 61.6 | 59 | (85, 154) | 156 | (29, 89) | 92 | VRR-NUC-like | cd22365 VRR-NUC-like; Virus-type replication repair nuclease. This model characterizes a set of nucleases that resemble Holliday-junction resolving enzymes. | | cath | 4qbnA00 | 99.0 | 2.3e-14 | 3.8e-18 | 91.6 | 88 | (22, 155) | 156 | (1, 91) | 93 | Nuclease | CATHCODE: 3.40.1350.10 NAME: Nuclease. Chain: a, b. Engineered: yes SOURCE: Salmonella phage setp3. Organism\_taxid: 424944. Expressed in: escherichia coli. Expression\_system\_taxid: 562 CLASS: Alpha Beta, ARCH: 3-Layer(aba) Sandwich, TOPOL: Trna Endonuclease; Chain: A, domain 1, HOMOL: Trna Endonuclease; Chain: A, domain 1 | | cath | 4qblA00 | 98.9 | 1.3e-13 | 2e-17 | 97.4 | 116 | (21, 155) | 156 | (3, 128) | 145 | Vrr-nuc | CATHCODE: 3.40.1350.10 NAME: Vrr-nuc. Chain: a, b, d, e, f, c. Engineered: yes SOURCE: Psychrobacter sp.. Organism\_taxid: 349106. Strain: prwf-1. Gene: psycprwf\_1331. Expressed in: escherichia coli. Expression\_system\_taxid: 562 CLASS: Alpha Beta, ARCH: 3-Layer(aba) Sandwich, TOPOL: Trna Endonuclease; Chain: A, domain 1, HOMOL: Trna Endonuclease; Chain: A, domain 1 | | cath | 4qboA00 | 98.8 | 6.7e-13 | 1.1e-16 | 84.7 | 87 | (20, 155) | 156 | (1, 88) | 92 | Nuclease | CATHCODE: 3.40.1350.10 NAME: Nuclease. Chain: a. Engineered: yes SOURCE: Streptococcus phage p9. Organism\_taxid: 403905. Expressed in: escherichia coli. Expression\_system\_taxid: 562 CLASS: Alpha Beta, ARCH: 3-Layer(aba) Sandwich, TOPOL: Trna Endonuclease; Chain: A, domain 1, HOMOL: Trna Endonuclease; Chain: A, domain 1 | | cath | 1rznA00 | 98.1 | 8.7e-10 | 1.3e-13 | 79.6 | 98 | (24, 147) | 156 | (2, 108) | 173 | Recombination protein u | CATHCODE: 3.40.1350.10 NAME: Recombination protein u. Chain: a, b. Synonym: penicillin-binding protein-related factor a, pbp related factor a. Engineered: yes SOURCE: Bacillus subtilis. Organism\_taxid: 1423. Gene: recu, prfa, bsu22310. Expressed in: escherichia coli. Expression\_system\_taxid: 562 CLASS: Alpha Beta, ARCH: 3-Layer(aba) Sandwich, TOPOL: Trna Endonuclease; Chain: A, domain 1, HOMOL: Trna Endonuclease; Chain: A, domain 1 | | phrogs | 824 | 99.9 | 1.9e-31 | 2.7e-35 | 201.5 | 110 | (19, 155) | 156 | (16, 129) | 136 | NA | NA; Category: unknown function; NC\_025471\_p57 | | phrogs | 5702 | 99.4 | 1.1e-17 | 1.3e-21 | 116.7 | 70 | (76, 156) | 156 | (14, 84) | 86 | Holliday junction resolvase | Holliday junction resolvase; Category: DNA, RNA and nucleotide metabolism; NC\_004303\_p15 | | phrogs | 2363 | 99.3 | 5.4e-17 | 6.1e-21 | 124.6 | 111 | (18, 154) | 156 | (54, 168) | 192 | NA | NA; Category: unknown function; p68790 VI\_09761 | | phrogs | 93 | 98.5 | 1.4e-11 | 2e-15 | 88.2 | 58 | (84, 155) | 156 | (30, 91) | 116 | endonuclease | endonuclease; Category: DNA, RNA and nucleotide metabolism; NC\_028826\_p5 | |
| Top keywords  (threshold 1.00e-03 (evalue)) | **a, Endonuclease, Trna, nuclease, Engineered, yes, Organism\_taxid, Expressed, in, escherichia** |
| Output files | ../../domain\_architecture/48\_FANPEZAQ\_CDS\_0048\_cath.hhr ../../domain\_architecture/48\_FANPEZAQ\_CDS\_0048\_merged.svg ../../domain\_architecture/48\_FANPEZAQ\_CDS\_0048\_ncbi-cd.hhr ../../domain\_architecture/48\_FANPEZAQ\_CDS\_0048\_pfam.hhr ../../domain\_architecture/48\_FANPEZAQ\_CDS\_0048\_phrogs.hhr |

### Identical protein sequences/structures

#### Search results

|  |  |
| --- | --- |
| Protein sequence databases searched | Pdb, Swissprot, Refseq |
| Identical proteins found | -- |
| Top keywords | -- |
| Output files | -- |

### Similar protein sequences/structures

#### Sequence similarity search results (HHblits)1

|  |  |
| --- | --- |
| Sequence databases searched | Uniclust, Pdb70 |
| Results, scheme(s)  (Top layers only, threshold 1.00e-03 (evalue)) | xml version="1.0" encoding="utf-8" standalone="no"?       2024-09-02T21:08:50.496807 image/svg+xml   Matplotlib v3.7.2, https://matplotlib.org/ |
| Results, table(s)  (threshold 1.00e-03 (evalue)) | | db | id | prob | evalue | pvalue | score | cols | query | query\_len | template | template\_len | name | description | | --- | --- | --- | --- | --- | --- | --- | --- | --- | --- | --- | --- | --- | | uniclust | UniRef100\_A0A088FQI8 | 100.0 | 2.5e-41 | 5.4e-47 | 251.0 | 147 | (1, 155) | 156 | (2, 149) | 164 | VRR-NUC domain-containing protein | VRR-NUC domain-containing protein | | uniclust | UniRef100\_A0A0F9MG75 | 99.9 | 2.9e-31 | 6.3e-37 | 198.9 | 136 | (19, 155) | 156 | (1, 138) | 162 | VRR-NUC domain-containing protein | VRR-NUC domain-containing protein | | uniclust | UniRef100\_A0A0F9LJW8 | 99.9 | 9.8e-31 | 2.1e-36 | 194.5 | 117 | (16, 154) | 156 | (16, 134) | 153 | Uncharacterized protein | Uncharacterized protein | | uniclust | UniRef100\_A0A0U5E8J8 | 99.9 | 9.2e-28 | 1.8e-33 | 174.6 | 126 | (20, 154) | 156 | (18, 145) | 149 | VRR-NUC domain protein | VRR-NUC domain protein | | uniclust | UniRef100\_A0A091AJ71 | 99.9 | 1.3e-27 | 3e-33 | 176.8 | 109 | (16, 155) | 156 | (8, 118) | 138 | VRR-NUC domain-containing protein | VRR-NUC domain-containing protein | | uniclust | UniRef100\_A0A0A0X031 | 99.9 | 3.1e-26 | 7.2e-32 | 169.5 | 107 | (18, 155) | 156 | (13, 121) | 137 | VRR-NUC domain-containing protein | VRR-NUC domain-containing protein | | uniclust | UniRef100\_A0A0F9IJ46 | 99.9 | 4e-26 | 7.9e-32 | 161.0 | 111 | (22, 154) | 156 | (1, 112) | 122 | VRR-NUC domain-containing protein | VRR-NUC domain-containing protein | | uniclust | UniRef100\_A0A084AC97 | 99.9 | 9.9e-26 | 2.1e-31 | 166.0 | 105 | (18, 155) | 156 | (18, 124) | 148 | VRR-NUC domain-containing protein | VRR-NUC domain-containing protein | | uniclust | UniRef100\_A0A069QR83 | 99.9 | 1.9e-25 | 4.3e-31 | 172.0 | 115 | (12, 155) | 156 | (35, 154) | 178 | VRR-NUC domain protein | VRR-NUC domain protein | | uniclust | UniRef100\_A0A090JN26 | 99.9 | 2.3e-25 | 4.7e-31 | 163.6 | 107 | (17, 155) | 156 | (21, 129) | 146 | VRR-NUC domain-containing protein | VRR-NUC domain-containing protein | | uniclust | UniRef100\_A0A0F9HTW1 | 99.8 | 4.8e-25 | 1.1e-30 | 162.7 | 102 | (19, 155) | 156 | (13, 117) | 136 | VRR-NUC domain-containing protein | VRR-NUC domain-containing protein | | uniclust | UniRef100\_A0A0F3IMV8 | 99.8 | 6.6e-25 | 1.5e-30 | 168.5 | 109 | (18, 155) | 156 | (31, 145) | 175 | VRR-NUC domain-containing protein | VRR-NUC domain-containing protein | | uniclust | UniRef100\_A0A059T805 | 99.8 | 8e-25 | 1.7e-30 | 165.8 | 107 | (17, 155) | 156 | (32, 140) | 170 | VRR-NUC domain-containing protein | VRR-NUC domain-containing protein | | uniclust | UniRef100\_A0A0B5A593 | 99.8 | 1e-24 | 2.4e-30 | 170.6 | 107 | (18, 155) | 156 | (50, 166) | 197 | Vsr endonuclease | Vsr endonuclease | | uniclust | UniRef100\_A0A0F8ZZ41 | 99.8 | 1.2e-24 | 2.6e-30 | 159.5 | 106 | (18, 155) | 156 | (9, 119) | 139 | VRR-NUC domain-containing protein | VRR-NUC domain-containing protein | | uniclust | UniRef100\_A0A009Z9C9 | 99.8 | 1.7e-24 | 3.6e-30 | 162.5 | 110 | (17, 155) | 156 | (22, 140) | 157 | VRR-NUC domain protein | VRR-NUC domain protein | | uniclust | UniRef100\_A0A134B5D7 | 99.8 | 1.8e-24 | 3.9e-30 | 163.6 | 109 | (17, 155) | 156 | (30, 145) | 164 | VRR-NUC domain protein | VRR-NUC domain protein | | uniclust | UniRef100\_A0A076G6E5 | 99.8 | 1.8e-24 | 4.2e-30 | 161.4 | 116 | (18, 155) | 156 | (1, 133) | 140 | VRR-NUC domain-containing protein | VRR-NUC domain-containing protein | | uniclust | UniRef100\_A0A010YDT0 | 99.8 | 6.4e-24 | 1.5e-29 | 163.5 | 109 | (18, 155) | 156 | (26, 143) | 176 | VRR-NUC domain-containing protein | VRR-NUC domain-containing protein | | uniclust | UniRef100\_A0A0A1Q2B7 | 99.8 | 1e-23 | 2.2e-29 | 163.8 | 108 | (17, 155) | 156 | (29, 140) | 197 | VRR-NUC domain protein | VRR-NUC domain protein | | uniclust | UniRef100\_A0A0F9A7G8 | 99.8 | 3.4e-23 | 7.5e-29 | 152.3 | 110 | (1, 155) | 156 | (4, 115) | 132 | VRR-NUC domain-containing protein | VRR-NUC domain-containing protein | | uniclust | UniRef100\_A0A0R3MLZ8 | 99.8 | 3.5e-23 | 7.7e-29 | 157.0 | 102 | (23, 155) | 156 | (33, 139) | 161 | VRR-NUC domain-containing protein | VRR-NUC domain-containing protein | | uniclust | UniRef100\_A0A0S2ZD03 | 99.8 | 7.5e-23 | 1.7e-28 | 150.2 | 108 | (18, 155) | 156 | (9, 122) | 131 | VRR-NUC domain-containing protein | VRR-NUC domain-containing protein | | uniclust | UniRef100\_A0A011VZ13 | 99.8 | 1.1e-22 | 2.3e-28 | 156.5 | 108 | (19, 155) | 156 | (41, 155) | 182 | Nuclease | Nuclease | | uniclust | UniRef100\_A0A0C5XU96 | 99.8 | 1.5e-22 | 3e-28 | 149.1 | 113 | (19, 155) | 156 | (25, 140) | 145 | VRR-NUC domain-containing protein | VRR-NUC domain-containing protein | | uniclust | UniRef100\_A0A0C7SLG5 | 99.8 | 2.5e-22 | 5.1e-28 | 146.1 | 110 | (17, 155) | 156 | (3, 120) | 134 | VRR-NUC domain-containing protein | VRR-NUC domain-containing protein | | uniclust | UniRef100\_A0A015SVT4 | 99.8 | 2.3e-22 | 5.1e-28 | 155.9 | 109 | (18, 155) | 156 | (50, 165) | 190 | VRR-NUC domain protein | VRR-NUC domain protein | | uniclust | UniRef100\_A0A0F9GCV1 | 99.8 | 4.7e-22 | 1e-27 | 147.4 | 106 | (17, 155) | 156 | (18, 128) | 141 | VRR-NUC domain-containing protein | VRR-NUC domain-containing protein | | uniclust | UniRef100\_A0A149SVC6 | 99.8 | 5.4e-22 | 1.1e-27 | 142.7 | 129 | (23, 154) | 156 | (1, 130) | 133 | VRR-NUC domain-containing protein | VRR-NUC domain-containing protein | | uniclust | UniRef100\_A0A081N321 | 99.8 | 6.3e-22 | 1.4e-27 | 150.0 | 109 | (18, 155) | 156 | (18, 137) | 161 | VRR-NUC domain-containing protein | VRR-NUC domain-containing protein | | uniclust | UniRef100\_A0A017HBU9 | 99.8 | 7e-22 | 1.5e-27 | 151.4 | 110 | (18, 155) | 156 | (26, 140) | 175 | Uncharacterized protein | Uncharacterized protein | | uniclust | UniRef100\_A0A0F7L4G6 | 99.7 | 1.4e-21 | 3e-27 | 147.1 | 108 | (19, 155) | 156 | (22, 133) | 153 | VRR-NUC domain-containing protein | VRR-NUC domain-containing protein | | uniclust | UniRef100\_A0A0F9HWT1 | 99.7 | 1.7e-21 | 3.7e-27 | 143.4 | 108 | (13, 154) | 156 | (3, 114) | 133 | VRR-NUC domain-containing protein | VRR-NUC domain-containing protein | | uniclust | UniRef100\_A0A0F9KD86 | 99.7 | 2.5e-21 | 5.1e-27 | 138.3 | 96 | (15, 154) | 156 | (8, 105) | 119 | VRR-NUC domain-containing protein | VRR-NUC domain-containing protein | | uniclust | UniRef100\_A0A0F5ALC1 | 99.7 | 2.9e-21 | 6.2e-27 | 151.5 | 110 | (17, 155) | 156 | (40, 157) | 208 | Uncharacterized protein | Uncharacterized protein | | uniclust | UniRef100\_A0A1V5V041 | 99.7 | 3.4e-21 | 7.7e-27 | 142.3 | 108 | (14, 154) | 156 | (8, 121) | 130 | VRR-NUC domain protein | VRR-NUC domain protein | | uniclust | UniRef100\_A0A0A2TKQ8 | 99.7 | 4.9e-21 | 1.1e-26 | 146.1 | 93 | (17, 154) | 156 | (33, 127) | 159 | Nuclease | Nuclease | | uniclust | UniRef100\_A0A1Q3W833 | 99.7 | 8.2e-21 | 1.7e-26 | 141.1 | 107 | (20, 155) | 156 | (32, 146) | 151 | VRR-NUC domain-containing protein | VRR-NUC domain-containing protein | | uniclust | UniRef100\_A0A0F8VVC6 | 99.7 | 1.3e-20 | 2.6e-26 | 135.8 | 106 | (19, 155) | 156 | (4, 114) | 125 | VRR-NUC domain-containing protein | VRR-NUC domain-containing protein | | uniclust | UniRef100\_A0A1C6FI89 | 99.7 | 1.9e-20 | 3.9e-26 | 135.9 | 108 | (19, 155) | 156 | (1, 118) | 129 | VRR-NUC domain | VRR-NUC domain | | uniclust | UniRef100\_A0A3M2B9U5 | 99.7 | 1.9e-20 | 4e-26 | 136.8 | 109 | (18, 155) | 156 | (3, 124) | 132 | VRR-NUC domain-containing protein (Fragment) | VRR-NUC domain-containing protein (Fragment) | | uniclust | UniRef100\_A0A1V5XRF5 | 99.7 | 2.2e-20 | 4.8e-26 | 137.3 | 96 | (15, 155) | 156 | (5, 102) | 130 | VRR-NUC domain protein | VRR-NUC domain protein | | uniclust | UniRef100\_A0A2S2F862 | 99.7 | 3.7e-20 | 7.6e-26 | 141.6 | 104 | (23, 155) | 156 | (41, 153) | 182 | VRR-NUC domain-containing protein | VRR-NUC domain-containing protein | | uniclust | UniRef100\_A0A328AAM2 | 99.7 | 4.3e-20 | 8.8e-26 | 137.4 | 104 | (21, 155) | 156 | (26, 134) | 153 | VRR-NUC domain-containing protein | VRR-NUC domain-containing protein | | uniclust | UniRef100\_A0A0F9BCA0 | 99.7 | 5.3e-20 | 1.1e-25 | 138.1 | 106 | (17, 154) | 156 | (22, 136) | 152 | VRR-NUC domain-containing protein | VRR-NUC domain-containing protein | | uniclust | UniRef100\_A0A8K2ATK7 | 99.7 | 7e-20 | 1.3e-25 | 136.9 | 135 | (21, 155) | 156 | (5, 141) | 185 | Endonuclease | Endonuclease | | uniclust | UniRef100\_A0A5C8V1Q9 | 99.7 | 6.7e-20 | 1.4e-25 | 135.0 | 106 | (18, 154) | 156 | (1, 109) | 140 | VRR-NUC domain-containing protein | VRR-NUC domain-containing protein | | uniclust | UniRef100\_A0A2N2E106 | 99.7 | 7.5e-20 | 1.6e-25 | 135.9 | 117 | (16, 155) | 156 | (12, 132) | 142 | VRR-NUC domain-containing protein | VRR-NUC domain-containing protein | | uniclust | UniRef100\_A0A1V6B667 | 99.7 | 1e-19 | 2.1e-25 | 129.5 | 101 | (22, 155) | 156 | (1, 103) | 113 | VRR-NUC domain protein | VRR-NUC domain protein | | uniclust | UniRef100\_A0A1Q8Y988 | 99.6 | 3.3e-19 | 6.6e-25 | 134.9 | 108 | (19, 155) | 156 | (41, 156) | 175 | VRR-NUC domain-containing protein | VRR-NUC domain-containing protein | | uniclust | UniRef100\_A0A1D9FH02 | 99.6 | 4.3e-19 | 9.1e-25 | 128.3 | 94 | (17, 155) | 156 | (3, 99) | 120 | Nuclease | Nuclease | | uniclust | UniRef100\_A0A2E5G474 | 99.6 | 4.7e-19 | 9.4e-25 | 132.0 | 113 | (17, 155) | 156 | (21, 138) | 159 | VRR-NUC domain-containing protein | VRR-NUC domain-containing protein | | uniclust | UniRef100\_A0A966V8T8 | 99.6 | 5e-19 | 9.7e-25 | 128.3 | 105 | (18, 153) | 156 | (15, 121) | 135 | VRR-NUC domain-containing protein | VRR-NUC domain-containing protein | | uniclust | UniRef100\_A0A2D6ZZN4 | 99.6 | 5.1e-19 | 1e-24 | 129.0 | 102 | (18, 154) | 156 | (12, 116) | 131 | VRR-NUC domain-containing protein | VRR-NUC domain-containing protein | | uniclust | UniRef100\_A0A096P758 | 99.6 | 5e-19 | 1.1e-24 | 138.3 | 112 | (22, 155) | 156 | (54, 181) | 200 | Uncharacterized protein | Uncharacterized protein | | uniclust | UniRef100\_A0A383X5C3 | 99.6 | 7.6e-19 | 1.6e-24 | 134.2 | 107 | (20, 155) | 156 | (39, 153) | 179 | VRR-NUC domain-containing protein | VRR-NUC domain-containing protein | | uniclust | UniRef100\_A0A0R1QJF2 | 99.6 | 1.2e-18 | 2.5e-24 | 122.3 | 92 | (20, 155) | 156 | (1, 93) | 100 | VRR-NUC domain-containing protein | VRR-NUC domain-containing protein | | uniclust | UniRef100\_A0A0F9VL45 | 99.6 | 1.3e-18 | 3e-24 | 125.3 | 91 | (19, 155) | 156 | (4, 96) | 109 | VRR-NUC domain-containing protein | VRR-NUC domain-containing protein | | uniclust | UniRef100\_A0A3M1NGK4 | 99.6 | 1.5e-18 | 3e-24 | 125.3 | 104 | (20, 155) | 156 | (8, 113) | 124 | VRR-NUC domain-containing protein | VRR-NUC domain-containing protein | | uniclust | UniRef100\_A0A1Z7ZMK4 | 99.6 | 1.6e-18 | 3.3e-24 | 133.0 | 106 | (21, 155) | 156 | (56, 169) | 185 | VRR-NUC domain-containing protein | VRR-NUC domain-containing protein | | uniclust | UniRef100\_A0A142XR66 | 99.6 | 2.1e-18 | 4.4e-24 | 126.4 | 105 | (16, 154) | 156 | (2, 113) | 135 | VRR-NUC domain protein | VRR-NUC domain protein | | uniclust | UniRef100\_UPI000EF1452C | 99.6 | 3.1e-18 | 6e-24 | 130.8 | 106 | (21, 155) | 156 | (73, 187) | 195 | VRR-NUC domain-containing protein | VRR-NUC domain-containing protein | | uniclust | UniRef100\_A0A0A6PYH0 | 99.6 | 3.1e-18 | 6.6e-24 | 124.6 | 94 | (16, 154) | 156 | (1, 99) | 123 | VRR-NUC domain-containing protein | VRR-NUC domain-containing protein | | uniclust | UniRef100\_A0A086MIG4 | 99.6 | 3.7e-18 | 7.8e-24 | 130.0 | 105 | (20, 155) | 156 | (32, 141) | 165 | VRR-NUC domain-containing protein | VRR-NUC domain-containing protein | | uniclust | UniRef100\_A0A2X1LNR6 | 99.6 | 3.7e-18 | 8e-24 | 116.9 | 68 | (79, 155) | 156 | (2, 70) | 83 | Putative norphogenetic protein | Putative norphogenetic protein | | uniclust | UniRef100\_A0A4Q2XQZ3 | 99.6 | 4.3e-18 | 8.5e-24 | 123.0 | 105 | (20, 155) | 156 | (7, 114) | 131 | VRR-NUC domain-containing protein | VRR-NUC domain-containing protein | | uniclust | UniRef100\_A0A323U8N5 | 99.6 | 4.1e-18 | 8.6e-24 | 125.3 | 99 | (18, 155) | 156 | (13, 117) | 133 | VRR-NUC domain-containing protein | VRR-NUC domain-containing protein | | uniclust | UniRef100\_A0A6H1Z980 | 99.6 | 5e-18 | 9.9e-24 | 124.5 | 109 | (19, 155) | 156 | (23, 134) | 141 | VRR-NUC domain-containing protein | VRR-NUC domain-containing protein | | uniclust | UniRef100\_A0A661RNB2 | 99.6 | 5.9e-18 | 1.1e-23 | 117.9 | 99 | (21, 154) | 156 | (3, 107) | 109 | VRR-NUC domain-containing protein | VRR-NUC domain-containing protein | | uniclust | UniRef100\_A0A1C6I8N5 | 99.6 | 7.9e-18 | 1.6e-23 | 127.8 | 108 | (19, 155) | 156 | (10, 125) | 180 | VRR-NUC domain | VRR-NUC domain | | uniclust | UniRef100\_A0A0F9B8D5 | 99.5 | 1.1e-17 | 2.4e-23 | 120.6 | 92 | (19, 155) | 156 | (13, 106) | 119 | VRR-NUC domain-containing protein | VRR-NUC domain-containing protein | | uniclust | UniRef100\_A0A059N063 | 99.5 | 1.2e-17 | 2.7e-23 | 127.0 | 94 | (16, 155) | 156 | (16, 111) | 151 | VRR-NUC domain protein | VRR-NUC domain protein | | uniclust | UniRef100\_A0A150J612 | 99.5 | 1.4e-17 | 2.7e-23 | 124.9 | 107 | (19, 155) | 156 | (3, 115) | 165 | VRR-NUC domain protein | VRR-NUC domain protein | | uniclust | UniRef100\_A0A095WF53 | 99.5 | 1.3e-17 | 2.8e-23 | 123.5 | 98 | (12, 155) | 156 | (9, 109) | 134 | VRR-NUC domain-containing protein | VRR-NUC domain-containing protein | | uniclust | UniRef100\_A0A2W6ZLY5 | 99.5 | 1.6e-17 | 3e-23 | 122.6 | 111 | (21, 153) | 156 | (3, 115) | 174 | VRR-NUC domain-containing protein | VRR-NUC domain-containing protein | | uniclust | UniRef100\_A0A0N1CD04 | 99.5 | 1.6e-17 | 3.3e-23 | 128.3 | 103 | (21, 155) | 156 | (51, 159) | 188 | Nuclease | Nuclease | | uniclust | UniRef100\_A0A2M8G080 | 99.5 | 2e-17 | 3.9e-23 | 119.3 | 107 | (19, 155) | 156 | (3, 111) | 126 | VRR-NUC domain-containing protein | VRR-NUC domain-containing protein | | uniclust | UniRef100\_G4Q3Y9 | 99.5 | 2.5e-17 | 4.8e-23 | 128.5 | 103 | (21, 155) | 156 | (19, 123) | 223 | VRR-NUC domain protein | VRR-NUC domain protein | | uniclust | UniRef100\_A0A1V5MV66 | 99.5 | 2.6e-17 | 5.1e-23 | 117.0 | 107 | (20, 155) | 156 | (1, 109) | 115 | VRR-NUC domain protein | VRR-NUC domain protein | | uniclust | UniRef100\_A0A1I3QHP0 | 99.5 | 2.6e-17 | 5.4e-23 | 122.0 | 108 | (19, 155) | 156 | (5, 120) | 139 | VRR-NUC domain-containing protein | VRR-NUC domain-containing protein | | uniclust | UniRef100\_A0A1E3LZS1 | 99.5 | 2.5e-17 | 5.5e-23 | 124.8 | 102 | (22, 155) | 156 | (30, 135) | 152 | VRR-NUC domain-containing protein | VRR-NUC domain-containing protein | | uniclust | UniRef100\_A0A0S7XPN7 | 99.5 | 4.6e-17 | 9.5e-23 | 118.5 | 90 | (18, 154) | 156 | (19, 110) | 126 | VRR-NUC domain-containing protein | VRR-NUC domain-containing protein | | uniclust | UniRef100\_A0A127SDT5 | 99.5 | 5.3e-17 | 1.1e-22 | 122.5 | 100 | (11, 155) | 156 | (15, 118) | 149 | VRR-NUC domain protein | VRR-NUC domain protein | | uniclust | UniRef100\_UPI00223A9835 | 99.5 | 6.6e-17 | 1.2e-22 | 119.6 | 112 | (21, 154) | 156 | (55, 168) | 175 | VRR-NUC domain-containing protein | VRR-NUC domain-containing protein | | uniclust | UniRef100\_A0A015ZPF0 | 99.5 | 5.8e-17 | 1.3e-22 | 122.1 | 94 | (16, 155) | 156 | (17, 112) | 141 | VRR-NUC domain protein | VRR-NUC domain protein | | uniclust | UniRef100\_A0A094Z4S5 | 99.5 | 7.6e-17 | 1.6e-22 | 115.6 | 91 | (19, 155) | 156 | (5, 97) | 111 | Phage protein | Phage protein | | uniclust | UniRef100\_UPI0009B54E9D | 99.5 | 8.5e-17 | 1.6e-22 | 117.2 | 101 | (22, 154) | 156 | (29, 131) | 143 | VRR-NUC domain-containing protein | VRR-NUC domain-containing protein | | uniclust | UniRef100\_A0A060DAM7 | 99.5 | 8.1e-17 | 1.8e-22 | 124.4 | 113 | (21, 155) | 156 | (40, 169) | 173 | Nuclease | Nuclease | | uniclust | UniRef100\_A0A0F8X2W8 | 99.5 | 9.4e-17 | 2.1e-22 | 120.8 | 85 | (20, 147) | 156 | (29, 115) | 144 | VRR-NUC domain-containing protein | VRR-NUC domain-containing protein | | uniclust | UniRef100\_A0A5P1RAG0 | 99.5 | 1e-16 | 2.1e-22 | 119.8 | 108 | (19, 155) | 156 | (18, 134) | 151 | VRR-NUC domain-containing protein | VRR-NUC domain-containing protein | | uniclust | UniRef100\_A0A165PXT7 | 99.5 | 1e-16 | 2.1e-22 | 119.5 | 106 | (19, 155) | 156 | (17, 127) | 155 | Uncharacterized protein | Uncharacterized protein | | uniclust | UniRef100\_A0A089WVF8 | 99.5 | 1e-16 | 2.1e-22 | 125.6 | 106 | (21, 155) | 156 | (82, 196) | 213 | Nuclease | Nuclease | | uniclust | UniRef100\_A0A072NS31 | 99.5 | 1.1e-16 | 2.5e-22 | 121.0 | 106 | (4, 155) | 156 | (10, 117) | 147 | VRR-NUC domain-containing protein | VRR-NUC domain-containing protein | | uniclust | UniRef100\_A0A0F9EIV8 | 99.5 | 1.4e-16 | 2.7e-22 | 109.2 | 80 | (76, 155) | 156 | (5, 86) | 99 | VRR-NUC domain-containing protein (Fragment) | VRR-NUC domain-containing protein (Fragment) | | uniclust | UniRef100\_A0A1R4EF88 | 99.5 | 1.4e-16 | 2.7e-22 | 118.9 | 111 | (16, 155) | 156 | (15, 135) | 154 | VRR-NUC domain protein | VRR-NUC domain protein | | uniclust | UniRef100\_A0A1H4CGG7 | 99.5 | 1.6e-16 | 2.9e-22 | 115.1 | 101 | (22, 154) | 156 | (39, 145) | 150 | VRR-NUC domain-containing protein | VRR-NUC domain-containing protein | | uniclust | UniRef100\_A0A352Z8N4 | 99.5 | 1.5e-16 | 3.1e-22 | 114.4 | 100 | (19, 154) | 156 | (8, 112) | 122 | Recombinase RecB | Recombinase RecB | | uniclust | UniRef100\_A0A011PYA4 | 99.5 | 1.4e-16 | 3.1e-22 | 116.1 | 94 | (16, 155) | 156 | (4, 99) | 114 | VRR-NUC domain protein | VRR-NUC domain protein | | uniclust | UniRef100\_A0A222YXP1 | 99.5 | 1.6e-16 | 3.2e-22 | 121.7 | 105 | (22, 155) | 156 | (56, 166) | 179 | Putative morphogenetic function protein | Putative morphogenetic function protein | | uniclust | UniRef100\_A0A3M1AH50 | 99.5 | 2.1e-16 | 4.1e-22 | 106.4 | 71 | (73, 154) | 156 | (9, 80) | 87 | VRR-NUC domain-containing protein | VRR-NUC domain-containing protein | | uniclust | UniRef100\_UPI001FA97645 | 99.5 | 2.4e-16 | 4.5e-22 | 115.8 | 118 | (9, 155) | 156 | (36, 161) | 165 | VRR-NUC domain-containing protein | VRR-NUC domain-containing protein | | uniclust | UniRef100\_A0A1F2RUT7 | 99.4 | 2.2e-16 | 4.6e-22 | 115.0 | 97 | (15, 155) | 156 | (5, 103) | 121 | VRR-NUC domain-containing protein | VRR-NUC domain-containing protein | | uniclust | UniRef100\_A0A1V6K0J2 | 99.4 | 2.6e-16 | 5e-22 | 118.7 | 118 | (18, 155) | 156 | (23, 146) | 174 | VRR-NUC domain protein | VRR-NUC domain protein | | uniclust | UniRef100\_A0A0I9SDG8 | 99.4 | 2.6e-16 | 5.5e-22 | 113.6 | 92 | (16, 154) | 156 | (16, 109) | 116 | VRR-NUC domain-containing protein | VRR-NUC domain-containing protein | | uniclust | UniRef100\_A0A1G2ZDQ9 | 99.4 | 2.8e-16 | 5.8e-22 | 115.8 | 111 | (15, 154) | 156 | (4, 117) | 135 | VRR-NUC domain-containing protein | VRR-NUC domain-containing protein | | uniclust | UniRef100\_A0A1E4ZHT9 | 99.4 | 3.2e-16 | 6.8e-22 | 116.2 | 91 | (18, 155) | 156 | (24, 116) | 131 | VRR-NUC domain-containing protein | VRR-NUC domain-containing protein | | uniclust | UniRef100\_A0A927XP81 | 99.4 | 4e-16 | 7.7e-22 | 116.2 | 105 | (22, 155) | 156 | (42, 154) | 164 | VRR-NUC domain-containing protein | VRR-NUC domain-containing protein | | uniclust | UniRef100\_A0A093UG92 | 99.4 | 4.3e-16 | 8.5e-22 | 116.3 | 107 | (20, 155) | 156 | (29, 144) | 156 | VRR-NUC domain-containing protein | VRR-NUC domain-containing protein | | uniclust | UniRef100\_A0A1F8N6W9 | 99.4 | 4.4e-16 | 9.6e-22 | 114.2 | 95 | (17, 154) | 156 | (15, 113) | 122 | VRR-NUC domain-containing protein (Fragment) | VRR-NUC domain-containing protein (Fragment) | | uniclust | UniRef100\_A0A6J5PBK3 | 99.4 | 4.9e-16 | 9.8e-22 | 116.8 | 103 | (21, 155) | 156 | (1, 108) | 161 | VRR-NUC domain containing protein | VRR-NUC domain containing protein | | uniclust | UniRef100\_A0A823KYF0 | 99.4 | 6.6e-16 | 1.3e-21 | 103.9 | 79 | (22, 132) | 156 | (1, 81) | 82 | VRR-NUC domain-containing protein (Fragment) | VRR-NUC domain-containing protein (Fragment) | | uniclust | UniRef100\_A0A0F9AK81 | 99.4 | 7.1e-16 | 1.5e-21 | 114.3 | 90 | (20, 155) | 156 | (16, 107) | 134 | VRR-NUC domain-containing protein | VRR-NUC domain-containing protein | | uniclust | UniRef100\_A0A179DMZ1 | 99.4 | 8.1e-16 | 1.7e-21 | 119.3 | 99 | (24, 155) | 156 | (48, 148) | 181 | VRR-NUC domain-containing protein | VRR-NUC domain-containing protein | | uniclust | UniRef100\_A0A0T9UVH8 | 99.4 | 8.9e-16 | 1.7e-21 | 118.3 | 106 | (21, 155) | 156 | (47, 161) | 197 | VRR-NUC domain | VRR-NUC domain | | uniclust | UniRef100\_A0A1X0Y852 | 99.4 | 9.6e-16 | 1.8e-21 | 111.0 | 98 | (22, 154) | 156 | (28, 127) | 139 | VRR-NUC domain-containing protein | VRR-NUC domain-containing protein | | uniclust | UniRef100\_A0A133ZYW0 | 99.4 | 1e-15 | 2.1e-21 | 117.6 | 92 | (19, 155) | 156 | (60, 156) | 176 | VRR-NUC domain protein | VRR-NUC domain protein | | uniclust | UniRef100\_A0A1E5GIK2 | 99.4 | 1.1e-15 | 2.2e-21 | 110.5 | 91 | (19, 155) | 156 | (13, 105) | 117 | VRR-NUC domain-containing protein | VRR-NUC domain-containing protein | | uniclust | UniRef100\_A0A2A4V727 | 99.4 | 1.2e-15 | 2.4e-21 | 112.2 | 104 | (20, 155) | 156 | (17, 126) | 140 | VRR-NUC domain-containing protein | VRR-NUC domain-containing protein | | uniclust | UniRef100\_A0A0F9LX93 | 99.4 | 1.2e-15 | 2.7e-21 | 111.2 | 90 | (21, 155) | 156 | (4, 97) | 115 | VRR-NUC domain-containing protein | VRR-NUC domain-containing protein | | uniclust | UniRef100\_A0A015XBN2 | 99.4 | 1.5e-15 | 3e-21 | 108.4 | 86 | (40, 155) | 156 | (19, 106) | 115 | VRR-NUC domain protein | VRR-NUC domain protein | | uniclust | UniRef100\_A0A858NP77 | 99.4 | 1.7e-15 | 3.2e-21 | 105.8 | 87 | (69, 155) | 156 | (3, 90) | 116 | VRR-NUC domain-containing protein | VRR-NUC domain-containing protein | | uniclust | UniRef100\_A0A1L7AL59 | 99.4 | 2.2e-15 | 4.2e-21 | 113.7 | 111 | (20, 154) | 156 | (21, 135) | 171 | VRR-NUC domain-containing protein | VRR-NUC domain-containing protein | | uniclust | UniRef100\_A0A0U4JU24 | 99.4 | 2.1e-15 | 4.5e-21 | 112.1 | 85 | (20, 147) | 156 | (20, 106) | 135 | VRR-NUC domain-containing protein | VRR-NUC domain-containing protein | | uniclust | UniRef100\_A0A1V5TXC5 | 99.4 | 2.4e-15 | 4.8e-21 | 110.3 | 104 | (20, 155) | 156 | (16, 125) | 132 | VRR-NUC domain protein | VRR-NUC domain protein | | uniclust | UniRef100\_A0A5B8I858 | 99.4 | 2.6e-15 | 4.9e-21 | 109.0 | 104 | (20, 155) | 156 | (7, 115) | 134 | VRR-NUC domain-containing protein | VRR-NUC domain-containing protein | | uniclust | UniRef100\_UPI002103C52B | 99.4 | 2.9e-15 | 5.4e-21 | 98.7 | 70 | (85, 154) | 156 | (2, 72) | 81 | VRR-NUC domain-containing protein | VRR-NUC domain-containing protein | | uniclust | UniRef100\_A0A068YHT1 | 99.3 | 3.6e-15 | 7e-21 | 114.7 | 99 | (21, 154) | 156 | (65, 167) | 195 | Uncharacterized protein | Uncharacterized protein | | uniclust | UniRef100\_A0A2E5G178 | 99.3 | 3.5e-15 | 7.4e-21 | 122.9 | 103 | (24, 155) | 156 | (142, 250) | 277 | VRR-NUC domain-containing protein | VRR-NUC domain-containing protein | | uniclust | UniRef100\_A0A024YQ59 | 99.3 | 4.4e-15 | 9.8e-21 | 112.3 | 88 | (22, 154) | 156 | (32, 124) | 143 | VRR-NUC domain-containing protein | VRR-NUC domain-containing protein | | uniclust | UniRef100\_A0A084EGU8 | 99.3 | 5.6e-15 | 1.2e-20 | 106.4 | 100 | (21, 154) | 156 | (1, 102) | 112 | VRR-NUC domain-containing protein | VRR-NUC domain-containing protein | | uniclust | UniRef100\_A0A6B9L9E1 | 99.3 | 6e-15 | 1.2e-20 | 118.2 | 103 | (20, 154) | 156 | (56, 164) | 271 | VRR-NUC domain-containing protein | VRR-NUC domain-containing protein | | uniclust | UniRef100\_A0A5C7QIV6 | 99.3 | 6.4e-15 | 1.2e-20 | 108.9 | 122 | (20, 151) | 156 | (36, 160) | 168 | VRR-NUC domain-containing protein | VRR-NUC domain-containing protein | | uniclust | UniRef100\_A0A0U3SL53 | 99.3 | 6.2e-15 | 1.2e-20 | 112.0 | 102 | (23, 154) | 156 | (35, 138) | 172 | VRR-NUC domain-containing protein | VRR-NUC domain-containing protein | | uniclust | UniRef100\_A0A0B6XGD4 | 99.3 | 5.6e-15 | 1.2e-20 | 120.1 | 105 | (22, 155) | 156 | (95, 205) | 244 | Putative norphogenetic protein | Putative norphogenetic protein | | uniclust | UniRef100\_A0A1Z3CMV2 | 99.3 | 7e-15 | 1.5e-20 | 101.0 | 61 | (83, 155) | 156 | (12, 73) | 82 | Nuclease | Nuclease | | uniclust | UniRef100\_A0A662KWK5 | 99.3 | 8.7e-15 | 1.6e-20 | 107.4 | 100 | (22, 155) | 156 | (49, 150) | 159 | VRR-NUC domain-containing protein | VRR-NUC domain-containing protein | | uniclust | UniRef100\_A0A151A655 | 99.3 | 8.2e-15 | 1.6e-20 | 108.2 | 90 | (20, 153) | 156 | (17, 109) | 140 | VRR-NUC domain-containing protein | VRR-NUC domain-containing protein | | uniclust | UniRef100\_A0A6G5Y5C2 | 99.3 | 9.2e-15 | 1.7e-20 | 104.3 | 112 | (21, 155) | 156 | (13, 126) | 131 | VRR-NUC domain-containing protein | VRR-NUC domain-containing protein | | uniclust | UniRef100\_A0A2D9X072 | 99.3 | 1e-14 | 1.9e-20 | 103.0 | 104 | (19, 155) | 156 | (3, 111) | 116 | VRR-NUC domain-containing protein | VRR-NUC domain-containing protein | | uniclust | UniRef100\_A0A0R3DPT5 | 99.3 | 9.7e-15 | 1.9e-20 | 106.0 | 87 | (20, 138) | 156 | (6, 99) | 126 | VRR-NUC domain-containing protein | VRR-NUC domain-containing protein | | uniclust | UniRef100\_UPI001903D17B | 99.3 | 1e-14 | 2e-20 | 109.7 | 111 | (19, 155) | 156 | (9, 123) | 165 | hypothetical protein | hypothetical protein | | uniclust | UniRef100\_A0A3D8IYP5 | 99.3 | 1e-14 | 2e-20 | 110.5 | 108 | (19, 155) | 156 | (25, 140) | 160 | VRR-NUC domain-containing protein | VRR-NUC domain-containing protein | | uniclust | UniRef100\_A0A7U9NG49 | 99.3 | 1.1e-14 | 2.1e-20 | 114.0 | 111 | (16, 155) | 156 | (74, 192) | 239 | VRR-NUC domain-containing protein | VRR-NUC domain-containing protein | | uniclust | UniRef100\_A0A3A9F823 | 99.3 | 1.2e-14 | 2.3e-20 | 112.6 | 105 | (22, 155) | 156 | (6, 118) | 216 | VRR-NUC domain-containing protein | VRR-NUC domain-containing protein | | uniclust | UniRef100\_A0A839GEX4 | 99.3 | 1.4e-14 | 2.7e-20 | 105.5 | 105 | (22, 155) | 156 | (7, 117) | 132 | VRR-NUC domain-containing protein | VRR-NUC domain-containing protein | | uniclust | UniRef100\_L1PCC8 | 99.3 | 1.5e-14 | 2.7e-20 | 109.9 | 103 | (22, 155) | 156 | (2, 108) | 202 | VRR-NUC domain protein | VRR-NUC domain protein | | uniclust | UniRef100\_A0A5C7JC15 | 99.3 | 1.5e-14 | 2.7e-20 | 106.6 | 107 | (20, 155) | 156 | (32, 153) | 163 | VRR-NUC domain-containing protein | VRR-NUC domain-containing protein | | uniclust | UniRef100\_A0A1B7X3P3 | 99.3 | 1.3e-14 | 2.8e-20 | 106.2 | 94 | (19, 149) | 156 | (1, 96) | 121 | VRR-NUC domain-containing protein | VRR-NUC domain-containing protein | | uniclust | UniRef100\_A0A212T8L3 | 99.3 | 1.7e-14 | 3.3e-20 | 110.5 | 102 | (22, 154) | 156 | (61, 164) | 177 | VRR-NUC domain-containing protein | VRR-NUC domain-containing protein | | uniclust | UniRef100\_A0A2T0YJ69 | 99.3 | 1.6e-14 | 3.4e-20 | 107.3 | 85 | (19, 147) | 156 | (19, 105) | 134 | VRR-NUC domain-containing protein | VRR-NUC domain-containing protein | | uniclust | UniRef100\_A0A354H0Y4 | 99.3 | 1.9e-14 | 3.7e-20 | 104.4 | 113 | (13, 154) | 156 | (6, 120) | 126 | VRR-NUC domain-containing protein | VRR-NUC domain-containing protein | | uniclust | UniRef100\_A0A3N5V863 | 99.3 | 2.1e-14 | 3.8e-20 | 105.4 | 103 | (22, 153) | 156 | (51, 157) | 158 | VRR-NUC domain-containing protein | VRR-NUC domain-containing protein | | uniclust | UniRef100\_A0A2D6XBS1 | 99.3 | 2.6e-14 | 4.9e-20 | 108.3 | 105 | (22, 154) | 156 | (62, 169) | 180 | VRR-NUC domain-containing protein | VRR-NUC domain-containing protein | | uniclust | UniRef100\_A0A349HTR8 | 99.3 | 2.9e-14 | 5.3e-20 | 111.0 | 102 | (22, 154) | 156 | (2, 109) | 245 | VRR-NUC domain-containing protein | VRR-NUC domain-containing protein | | uniclust | UniRef100\_A0A501W5S5 | 99.2 | 2.9e-14 | 5.6e-20 | 106.4 | 110 | (19, 155) | 156 | (26, 141) | 161 | VRR-NUC domain-containing protein | VRR-NUC domain-containing protein | | uniclust | UniRef100\_A0A174X9Y2 | 99.2 | 3e-14 | 5.8e-20 | 106.9 | 106 | (19, 155) | 156 | (6, 119) | 167 | VRR-NUC domain-containing protein | VRR-NUC domain-containing protein | | uniclust | UniRef100\_A0A8S5PT49 | 99.2 | 4.4e-14 | 8.4e-20 | 104.1 | 89 | (22, 155) | 156 | (47, 138) | 145 | Nuclease | Nuclease | | uniclust | UniRef100\_A0A2D8XMR4 | 99.2 | 5.2e-14 | 9.6e-20 | 102.9 | 109 | (21, 155) | 156 | (35, 148) | 152 | VRR-NUC domain-containing protein | VRR-NUC domain-containing protein | | uniclust | UniRef100\_A0A088GMG7 | 99.2 | 5.2e-14 | 1e-19 | 98.0 | 90 | (22, 154) | 156 | (1, 94) | 100 | VRR-NUC domain-containing protein | VRR-NUC domain-containing protein | | uniclust | UniRef100\_UPI0009BC488B | 99.2 | 5.4e-14 | 1e-19 | 104.9 | 105 | (19, 155) | 156 | (43, 153) | 161 | VRR-NUC domain-containing protein | VRR-NUC domain-containing protein | | uniclust | UniRef100\_UPI00223C1613 | 99.2 | 5.7e-14 | 1e-19 | 103.3 | 107 | (20, 155) | 156 | (4, 113) | 159 | VRR-NUC domain-containing protein | VRR-NUC domain-containing protein | | uniclust | UniRef100\_A0A3M1MRP5 | 99.2 | 6.3e-14 | 1.2e-19 | 100.1 | 102 | (17, 155) | 156 | (22, 125) | 130 | VRR-NUC domain-containing protein (Fragment) | VRR-NUC domain-containing protein (Fragment) | | uniclust | UniRef100\_A0A8S5TAK1 | 99.2 | 6.4e-14 | 1.2e-19 | 106.2 | 106 | (21, 155) | 156 | (68, 182) | 198 | Nuclease | Nuclease | | uniclust | UniRef100\_A0A6J5SJ08 | 99.2 | 6.3e-14 | 1.2e-19 | 103.7 | 105 | (21, 154) | 156 | (28, 137) | 149 | VRR-NUC domain containing protein | VRR-NUC domain containing protein | | uniclust | UniRef100\_A0A023ZVH9 | 99.2 | 5.7e-14 | 1.2e-19 | 109.3 | 106 | (22, 155) | 156 | (47, 160) | 181 | VRR-NUC domain-containing protein | VRR-NUC domain-containing protein | | uniclust | UniRef100\_A0A2T0RG28 | 99.2 | 6.7e-14 | 1.3e-19 | 101.3 | 107 | (21, 155) | 156 | (4, 115) | 136 | VRR-NUC domain-containing protein | VRR-NUC domain-containing protein | | uniclust | UniRef100\_A0A104Z6Z7 | 99.2 | 7.1e-14 | 1.4e-19 | 106.2 | 105 | (22, 155) | 156 | (31, 145) | 166 | VRR-NUC domain-containing protein | VRR-NUC domain-containing protein | | uniclust | UniRef100\_A0A965PCU2 | 99.2 | 9e-14 | 1.7e-19 | 105.6 | 106 | (19, 154) | 156 | (71, 185) | 199 | VRR-NUC domain-containing protein | VRR-NUC domain-containing protein | | uniclust | UniRef100\_A0A016QSZ2 | 99.2 | 9e-14 | 1.7e-19 | 99.4 | 93 | (22, 153) | 156 | (14, 109) | 121 | VRR-NUC domain-containing protein | VRR-NUC domain-containing protein | | uniclust | UniRef100\_A0A6C2YRG2 | 99.2 | 9.1e-14 | 1.7e-19 | 97.6 | 99 | (23, 154) | 156 | (1, 101) | 110 | VRR-NUC domain-containing protein | VRR-NUC domain-containing protein | | uniclust | UniRef100\_A0A1M5CEU7 | 99.2 | 8.8e-14 | 1.7e-19 | 100.6 | 88 | (23, 155) | 156 | (1, 93) | 122 | VRR-NUC domain-containing protein | VRR-NUC domain-containing protein | | uniclust | UniRef100\_A0A516M9P1 | 99.2 | 1.1e-13 | 1.9e-19 | 97.3 | 101 | (22, 153) | 156 | (1, 105) | 116 | VRR-NUC domain-containing protein | VRR-NUC domain-containing protein | | uniclust | UniRef100\_A0A3E0Q3U7 | 99.2 | 8.7e-14 | 1.9e-19 | 104.0 | 99 | (21, 154) | 156 | (15, 118) | 129 | Uncharacterized protein | Uncharacterized protein | | uniclust | UniRef100\_A0A1C7G917 | 99.2 | 1.1e-13 | 2e-19 | 104.3 | 106 | (21, 155) | 156 | (10, 123) | 187 | Nuclease | Nuclease | | uniclust | UniRef100\_A0A1W1ZWA3 | 99.2 | 1.3e-13 | 2.5e-19 | 105.1 | 106 | (17, 154) | 156 | (2, 113) | 171 | VRR-NUC domain-containing protein | VRR-NUC domain-containing protein | | uniclust | UniRef100\_A0A0F8XH15 | 99.2 | 1.2e-13 | 2.6e-19 | 107.7 | 100 | (21, 154) | 156 | (61, 163) | 180 | VRR-NUC domain-containing protein (Fragment) | VRR-NUC domain-containing protein (Fragment) | | uniclust | UniRef100\_A0A2E6C829 | 99.2 | 1.5e-13 | 2.7e-19 | 99.6 | 101 | (21, 154) | 156 | (16, 121) | 142 | VRR-NUC domain-containing protein | VRR-NUC domain-containing protein | | uniclust | UniRef100\_A0A3A9C013 | 99.2 | 1.4e-13 | 2.8e-19 | 105.6 | 109 | (18, 155) | 156 | (8, 124) | 180 | VRR-NUC domain-containing protein | VRR-NUC domain-containing protein | | uniclust | UniRef100\_A0A965AG40 | 99.2 | 1.6e-13 | 2.9e-19 | 103.2 | 98 | (22, 154) | 156 | (76, 176) | 184 | VRR-NUC domain-containing protein | VRR-NUC domain-containing protein | | uniclust | UniRef100\_A0A3B8HLD6 | 99.2 | 1.6e-13 | 2.9e-19 | 98.8 | 109 | (20, 155) | 156 | (23, 134) | 136 | VRR-NUC domain-containing protein | VRR-NUC domain-containing protein | | uniclust | UniRef100\_A0A661JDB3 | 99.2 | 1.7e-13 | 3.2e-19 | 101.8 | 102 | (24, 155) | 156 | (34, 137) | 154 | VRR-NUC domain-containing protein | VRR-NUC domain-containing protein | | uniclust | UniRef100\_A0A969LIU0 | 99.2 | 1.8e-13 | 3.3e-19 | 98.0 | 103 | (21, 153) | 156 | (23, 127) | 131 | VRR-NUC domain-containing protein | VRR-NUC domain-containing protein | | uniclust | UniRef100\_A0A076YP05 | 99.2 | 1.6e-13 | 3.3e-19 | 101.1 | 97 | (13, 155) | 156 | (12, 110) | 129 | VRR-NUC domain-containing protein | VRR-NUC domain-containing protein | | uniclust | UniRef100\_A0A3B8HH35 | 99.2 | 1.7e-13 | 3.4e-19 | 101.4 | 93 | (23, 155) | 156 | (30, 124) | 134 | VRR-NUC domain-containing protein | VRR-NUC domain-containing protein | | uniclust | UniRef100\_A0A7C2EKI6 | 99.2 | 1.8e-13 | 3.6e-19 | 103.0 | 111 | (20, 156) | 156 | (29, 147) | 158 | Uncharacterized protein | Uncharacterized protein | | uniclust | UniRef100\_A0A2S1GLC8 | 99.2 | 1.9e-13 | 3.6e-19 | 105.2 | 105 | (21, 154) | 156 | (27, 140) | 187 | VRR-NUC domain-containing protein | VRR-NUC domain-containing protein | | uniclust | UniRef100\_A0A060BFY8 | 99.1 | 1.9e-13 | 3.8e-19 | 107.8 | 105 | (23, 156) | 156 | (46, 165) | 210 | VRR-NUC domain-containing protein | VRR-NUC domain-containing protein | | uniclust | UniRef100\_A0A1C5RXP7 | 99.1 | 2e-13 | 3.9e-19 | 109.0 | 106 | (18, 154) | 156 | (3, 116) | 249 | VRR-NUC domain | VRR-NUC domain | | uniclust | UniRef100\_A0A511FFQ3 | 99.1 | 2e-13 | 4e-19 | 102.1 | 87 | (20, 149) | 156 | (40, 128) | 145 | VRR-NUC domain-containing protein | VRR-NUC domain-containing protein | | uniclust | UniRef100\_A0A1V5WI66 | 99.1 | 2.4e-13 | 4.3e-19 | 98.7 | 98 | (23, 154) | 156 | (1, 100) | 143 | VRR-NUC domain protein | VRR-NUC domain protein | | uniclust | UniRef100\_UPI00130D683B | 99.1 | 2.4e-13 | 4.4e-19 | 101.0 | 108 | (22, 155) | 156 | (29, 139) | 168 | VRR-NUC domain-containing protein | VRR-NUC domain-containing protein | | uniclust | UniRef100\_A0A1V5T3V5 | 99.1 | 2.4e-13 | 4.4e-19 | 85.8 | 56 | (85, 151) | 156 | (3, 59) | 62 | VRR-NUC domain protein | VRR-NUC domain protein | | uniclust | UniRef100\_A0A3S0DF79 | 99.1 | 2.4e-13 | 4.4e-19 | 101.3 | 104 | (21, 153) | 156 | (47, 155) | 172 | VRR-NUC domain-containing protein | VRR-NUC domain-containing protein | | uniclust | UniRef100\_A0A1F8V0P7 | 99.1 | 2.5e-13 | 4.5e-19 | 102.5 | 105 | (21, 155) | 156 | (7, 120) | 188 | VRR-NUC domain-containing protein | VRR-NUC domain-containing protein | | uniclust | UniRef100\_A0A285X397 | 99.1 | 2.1e-13 | 4.6e-19 | 103.1 | 105 | (20, 155) | 156 | (3, 125) | 143 | VRR-NUC domain-containing protein | VRR-NUC domain-containing protein | | uniclust | UniRef100\_A0A9E6MYT2 | 99.1 | 2.5e-13 | 4.6e-19 | 106.3 | 107 | (20, 155) | 156 | (14, 128) | 250 | VRR-NUC domain-containing protein | VRR-NUC domain-containing protein | | uniclust | UniRef100\_A0A0R1VXF8 | 99.1 | 2.6e-13 | 4.7e-19 | 97.7 | 103 | (20, 155) | 156 | (30, 134) | 135 | VRR-NUC domain protein | VRR-NUC domain protein | | uniclust | UniRef100\_A0A1M7G6C6 | 99.1 | 2.4e-13 | 4.8e-19 | 104.2 | 101 | (24, 154) | 156 | (22, 124) | 177 | VRR-NUC domain-containing protein | VRR-NUC domain-containing protein | | uniclust | UniRef100\_A0A645G019 | 99.1 | 2.5e-13 | 4.8e-19 | 96.9 | 91 | (21, 155) | 156 | (9, 103) | 116 | VRR-NUC domain-containing protein | VRR-NUC domain-containing protein | | uniclust | UniRef100\_E5Y6D9 | 99.1 | 2.7e-13 | 5e-19 | 101.9 | 107 | (20, 155) | 156 | (30, 143) | 178 | VRR-NUC domain-containing protein | VRR-NUC domain-containing protein | | uniclust | UniRef100\_A0A512B9U6 | 99.1 | 2.7e-13 | 5.2e-19 | 98.5 | 103 | (19, 154) | 156 | (21, 127) | 133 | VRR-NUC domain-containing protein | VRR-NUC domain-containing protein | | uniclust | UniRef100\_A0A351TKZ0 | 99.1 | 2.6e-13 | 5.2e-19 | 90.5 | 59 | (85, 155) | 156 | (7, 66) | 73 | VRR-NUC domain-containing protein (Fragment) | VRR-NUC domain-containing protein (Fragment) | | uniclust | UniRef100\_A0A1Y1QLN9 | 99.1 | 2.4e-13 | 5.4e-19 | 98.7 | 92 | (18, 155) | 156 | (3, 96) | 108 | VRR-NUC domain-containing protein | VRR-NUC domain-containing protein | | uniclust | UniRef100\_A0A431K292 | 99.1 | 2.9e-13 | 5.4e-19 | 100.2 | 128 | (1, 151) | 156 | (1, 154) | 164 | VRR-NUC domain-containing protein | VRR-NUC domain-containing protein | | uniclust | UniRef100\_UPI001FFBE019 | 99.1 | 3.4e-13 | 6.2e-19 | 99.5 | 103 | (21, 154) | 156 | (16, 123) | 159 | VRR-NUC domain-containing protein | VRR-NUC domain-containing protein | | uniclust | UniRef100\_A0A7C6QZC2 | 99.1 | 3.4e-13 | 6.2e-19 | 95.0 | 86 | (23, 153) | 156 | (1, 88) | 117 | VRR-NUC domain-containing protein | VRR-NUC domain-containing protein | | uniclust | UniRef100\_A0A2E6PX40 | 99.1 | 3.4e-13 | 6.4e-19 | 101.0 | 104 | (21, 155) | 156 | (4, 112) | 166 | VRR-NUC domain-containing protein | VRR-NUC domain-containing protein | | uniclust | UniRef100\_A0A0A0P383 | 99.1 | 3.1e-13 | 6.6e-19 | 105.0 | 108 | (21, 155) | 156 | (39, 153) | 173 | Type III restriction endonuclease | Type III restriction endonuclease | | uniclust | UniRef100\_A0A6N2ZXV1 | 99.1 | 3.6e-13 | 7e-19 | 96.5 | 90 | (20, 155) | 156 | (3, 94) | 114 | VRR-NUC domain protein | VRR-NUC domain protein | | uniclust | UniRef100\_A0A3N5H338 | 99.1 | 3.8e-13 | 7e-19 | 89.3 | 67 | (77, 155) | 156 | (7, 75) | 82 | VRR-NUC domain-containing protein | VRR-NUC domain-containing protein | | uniclust | UniRef100\_A0A060AHI1 | 99.1 | 3.6e-13 | 7.3e-19 | 99.4 | 90 | (19, 153) | 156 | (2, 97) | 128 | Endonuclease | Endonuclease | | uniclust | UniRef100\_A0A1B1IQE5 | 99.1 | 3.4e-13 | 7.3e-19 | 99.9 | 85 | (18, 147) | 156 | (11, 97) | 124 | VRR-NUC domain-containing protein | VRR-NUC domain-containing protein | | uniclust | UniRef100\_A0A6J5NKG2 | 99.1 | 4.5e-13 | 8.2e-19 | 98.9 | 102 | (22, 154) | 156 | (2, 109) | 159 | VRR-NUC domain containing protein | VRR-NUC domain containing protein | | uniclust | UniRef100\_A0A4Q2Y3F1 | 99.1 | 4.9e-13 | 9.1e-19 | 103.2 | 111 | (19, 154) | 156 | (98, 216) | 222 | VRR-NUC domain-containing protein | VRR-NUC domain-containing protein | | uniclust | UniRef100\_A0A2T3K2K6 | 99.1 | 5.3e-13 | 1e-18 | 98.9 | 108 | (19, 155) | 156 | (27, 143) | 145 | VRR-NUC domain-containing protein | VRR-NUC domain-containing protein | | uniclust | UniRef100\_A0A3G2KI64 | 99.1 | 6.7e-13 | 1.3e-18 | 97.4 | 85 | (20, 147) | 156 | (27, 114) | 128 | Nuclease | Nuclease | | uniclust | UniRef100\_A0A350UDK1 | 99.1 | 7.7e-13 | 1.4e-18 | 100.4 | 100 | (22, 154) | 156 | (81, 185) | 194 | VRR-NUC domain-containing protein | VRR-NUC domain-containing protein | | uniclust | UniRef100\_A0A6J5PR03 | 99.1 | 7e-13 | 1.4e-18 | 99.9 | 94 | (17, 148) | 156 | (25, 123) | 145 | VRR-NUC domain containing protein | VRR-NUC domain containing protein | | uniclust | UniRef100\_A0A250JC22 | 99.1 | 8e-13 | 1.6e-18 | 97.5 | 58 | (86, 155) | 156 | (35, 93) | 132 | Nuclease | Nuclease | | uniclust | UniRef100\_A0A6J5LZW8 | 99.1 | 9.1e-13 | 1.7e-18 | 99.7 | 104 | (22, 154) | 156 | (9, 120) | 171 | VRR-NUC domain containing protein | VRR-NUC domain containing protein | | uniclust | UniRef100\_A0A068CGD0 | 99.1 | 8.1e-13 | 1.8e-18 | 100.4 | 92 | (12, 154) | 156 | (21, 119) | 143 | VRR-NUC domain-containing protein | VRR-NUC domain-containing protein | | uniclust | UniRef100\_A0A0Q5KUS2 | 99.1 | 8.6e-13 | 1.8e-18 | 102.3 | 93 | (23, 153) | 156 | (64, 158) | 173 | VRR-NUC domain-containing protein | VRR-NUC domain-containing protein | | uniclust | UniRef100\_A0A0F8W6U3 | 99.1 | 9.5e-13 | 1.8e-18 | 97.0 | 109 | (21, 154) | 156 | (15, 125) | 139 | Uncharacterized protein (Fragment) | Uncharacterized protein (Fragment) | | uniclust | UniRef100\_A0A317H910 | 99.1 | 1e-12 | 1.9e-18 | 96.3 | 101 | (22, 153) | 156 | (5, 110) | 150 | VRR-NUC domain-containing protein | VRR-NUC domain-containing protein | | uniclust | UniRef100\_UPI001FFBBF5C | 99.1 | 1e-12 | 1.9e-18 | 95.5 | 106 | (21, 155) | 156 | (13, 123) | 134 | hypothetical protein | hypothetical protein | | uniclust | UniRef100\_A0A6J5LIH6 | 99.0 | 1.3e-12 | 2.4e-18 | 94.4 | 110 | (23, 155) | 156 | (3, 118) | 137 | VRR-NUC domain containing protein | VRR-NUC domain containing protein | | uniclust | UniRef100\_A0A139CFL3 | 99.0 | 1.4e-12 | 2.6e-18 | 96.5 | 105 | (22, 155) | 156 | (26, 135) | 140 | VRR-NUC domain-containing protein (Fragment) | VRR-NUC domain-containing protein (Fragment) | | uniclust | UniRef100\_A0A1A0IRN8 | 99.0 | 1.4e-12 | 2.9e-18 | 99.4 | 96 | (16, 153) | 156 | (1, 98) | 151 | Nuclease | Nuclease | | uniclust | UniRef100\_A0A838SYU0 | 99.0 | 1.7e-12 | 3.1e-18 | 97.0 | 104 | (22, 154) | 156 | (10, 124) | 171 | VRR-NUC domain-containing protein | VRR-NUC domain-containing protein | | uniclust | UniRef100\_A0A7K0AAW2 | 99.0 | 1.7e-12 | 3.3e-18 | 99.6 | 115 | (20, 155) | 156 | (25, 153) | 191 | VRR-NUC domain-containing protein | VRR-NUC domain-containing protein | | uniclust | UniRef100\_UPI0022E3BF2E | 99.0 | 2.1e-12 | 3.9e-18 | 99.9 | 105 | (21, 155) | 156 | (94, 207) | 222 | VRR-NUC domain-containing protein | VRR-NUC domain-containing protein | | uniclust | UniRef100\_A0A0F9IFS0 | 99.0 | 1.9e-12 | 3.9e-18 | 91.0 | 81 | (23, 154) | 156 | (7, 89) | 94 | VRR-NUC domain-containing protein | VRR-NUC domain-containing protein | | uniclust | UniRef100\_A0A060RIT6 | 99.0 | 2.1e-12 | 4.1e-18 | 100.6 | 111 | (22, 155) | 156 | (17, 167) | 187 | VRR-NUC domain-containing protein | VRR-NUC domain-containing protein | | uniclust | UniRef100\_A0A3S4F827 | 99.0 | 2.3e-12 | 4.2e-18 | 93.8 | 94 | (29, 154) | 156 | (2, 100) | 142 | VRR-NUC domain-containing protein | VRR-NUC domain-containing protein | | uniclust | UniRef100\_A0A1T2BEL0 | 99.0 | 2.6e-12 | 4.7e-18 | 85.2 | 62 | (83, 155) | 156 | (12, 75) | 80 | VRR-NUC domain-containing protein | VRR-NUC domain-containing protein | | uniclust | UniRef100\_A0A086D0Z9 | 99.0 | 2.7e-12 | 5.3e-18 | 97.9 | 106 | (22, 156) | 156 | (22, 142) | 167 | VRR-NUC domain-containing protein | VRR-NUC domain-containing protein | | uniclust | UniRef100\_A0A0B4Y550 | 99.0 | 2.5e-12 | 5.3e-18 | 99.1 | 106 | (22, 155) | 156 | (36, 149) | 163 | VRR-NUC domain-containing protein | VRR-NUC domain-containing protein | | uniclust | UniRef100\_J5UZX6 | 99.0 | 3.4e-12 | 6.2e-18 | 90.2 | 88 | (23, 155) | 156 | (1, 91) | 117 | VRR-NUC domain protein | VRR-NUC domain protein | | uniclust | UniRef100\_A0A0S7CQU9 | 99.0 | 3e-12 | 6.4e-18 | 98.8 | 112 | (16, 155) | 156 | (12, 139) | 161 | Uncharacterized protein | Uncharacterized protein | | uniclust | UniRef100\_A0A0Q5IHK8 | 99.0 | 3.7e-12 | 7.4e-18 | 99.0 | 113 | (23, 155) | 156 | (33, 154) | 189 | VRR-NUC domain-containing protein | VRR-NUC domain-containing protein | | uniclust | UniRef100\_A0A0F8Y460 | 99.0 | 3.8e-12 | 7.6e-18 | 95.5 | 102 | (21, 155) | 156 | (39, 143) | 147 | VRR-NUC domain-containing protein (Fragment) | VRR-NUC domain-containing protein (Fragment) | | uniclust | UniRef100\_A0A6B1DRS4 | 99.0 | 4.3e-12 | 7.8e-18 | 97.4 | 101 | (23, 155) | 156 | (100, 204) | 207 | VRR-NUC domain-containing protein | VRR-NUC domain-containing protein | | uniclust | UniRef100\_A0A965X540 | 99.0 | 4.3e-12 | 8e-18 | 93.8 | 102 | (22, 154) | 156 | (35, 146) | 157 | VRR-NUC domain-containing protein | VRR-NUC domain-containing protein | | uniclust | UniRef100\_A0A3M1SPX9 | 99.0 | 4.4e-12 | 8e-18 | 90.0 | 96 | (23, 155) | 156 | (1, 100) | 120 | VRR-NUC domain-containing protein | VRR-NUC domain-containing protein | | uniclust | UniRef100\_UPI001D0E449F | 99.0 | 4.6e-12 | 8.5e-18 | 86.3 | 69 | (75, 155) | 156 | (14, 83) | 93 | VRR-NUC domain-containing protein | VRR-NUC domain-containing protein | | uniclust | UniRef100\_A0A0G3EVL4 | 99.0 | 4.5e-12 | 8.8e-18 | 100.2 | 112 | (21, 154) | 156 | (61, 193) | 217 | Endonuclease | Endonuclease | | uniclust | UniRef100\_A0A3N9NZB7 | 99.0 | 4.7e-12 | 9.1e-18 | 89.0 | 87 | (18, 133) | 156 | (7, 102) | 102 | VRR-NUC domain-containing protein (Fragment) | VRR-NUC domain-containing protein (Fragment) | | uniclust | UniRef100\_A0A371YWW9 | 98.9 | 5.5e-12 | 1e-17 | 93.6 | 69 | (75, 155) | 156 | (81, 150) | 161 | VRR-NUC domain-containing protein | VRR-NUC domain-containing protein | | uniclust | UniRef100\_A0A2K4QBL7 | 98.9 | 7.3e-12 | 1.3e-17 | 93.5 | 102 | (21, 155) | 156 | (64, 167) | 168 | VRR-NUC domain-containing protein (Modular protein) | VRR-NUC domain-containing protein (Modular protein) | | uniclust | UniRef100\_UPI0022E05DE5 | 98.9 | 8.1e-12 | 1.5e-17 | 101.1 | 106 | (21, 155) | 156 | (169, 281) | 316 | VRR-NUC domain-containing protein | VRR-NUC domain-containing protein | | uniclust | UniRef100\_A0A2K9NFU2 | 98.9 | 8.2e-12 | 1.5e-17 | 92.9 | 102 | (21, 155) | 156 | (37, 152) | 163 | VRR-NUC domain-containing protein | VRR-NUC domain-containing protein | | uniclust | UniRef100\_A0A451GGP2 | 98.9 | 8.2e-12 | 1.6e-17 | 86.3 | 69 | (75, 155) | 156 | (9, 78) | 94 | VRR-NUC domain-containing protein | VRR-NUC domain-containing protein | | uniclust | UniRef100\_A0A7X5Y9R6 | 98.9 | 8.6e-12 | 1.6e-17 | 85.3 | 70 | (74, 155) | 156 | (15, 85) | 95 | VRR-NUC domain-containing protein | VRR-NUC domain-containing protein | | uniclust | UniRef100\_UPI001305397F | 98.9 | 9.3e-12 | 1.7e-17 | 83.3 | 68 | (75, 154) | 156 | (14, 82) | 83 | VRR-NUC domain-containing protein | VRR-NUC domain-containing protein | | uniclust | UniRef100\_UPI000FCC612F | 98.9 | 9.5e-12 | 1.7e-17 | 84.1 | 76 | (79, 154) | 156 | (6, 82) | 88 | hypothetical protein | hypothetical protein | | uniclust | UniRef100\_A0A0R1IE25 | 98.9 | 8.9e-12 | 1.7e-17 | 91.8 | 86 | (17, 146) | 156 | (1, 88) | 130 | VRR-NUC domain-containing protein | VRR-NUC domain-containing protein | | uniclust | UniRef100\_A0A2L0HNZ2 | 98.9 | 1.1e-11 | 2.1e-17 | 86.5 | 88 | (22, 154) | 156 | (1, 94) | 99 | Nuclease | Nuclease | | uniclust | UniRef100\_A0A143XCJ5 | 98.9 | 1.1e-11 | 2.2e-17 | 92.6 | 88 | (22, 155) | 156 | (46, 135) | 139 | VRR-NUC domain protein | VRR-NUC domain protein | | uniclust | UniRef100\_A0A7C3YQK5 | 98.9 | 1.2e-11 | 2.2e-17 | 91.9 | 111 | (19, 155) | 156 | (18, 135) | 160 | VRR-NUC domain-containing protein | VRR-NUC domain-containing protein | | uniclust | UniRef100\_A0A6J5QEF0 | 98.9 | 1.1e-11 | 2.2e-17 | 91.7 | 89 | (21, 154) | 156 | (2, 93) | 143 | VRR-NUC domain containing protein | VRR-NUC domain containing protein | | uniclust | UniRef100\_A0A2S0JKP9 | 98.9 | 1.2e-11 | 2.3e-17 | 81.7 | 60 | (84, 155) | 156 | (9, 69) | 73 | Recombinase RecB | Recombinase RecB | | uniclust | UniRef100\_A0A0F9JDU4 | 98.9 | 1.3e-11 | 2.3e-17 | 92.9 | 85 | (26, 155) | 156 | (86, 172) | 175 | Uncharacterized protein | Uncharacterized protein | | uniclust | UniRef100\_A0A1V5WR11 | 98.9 | 1.2e-11 | 2.4e-17 | 95.6 | 104 | (19, 154) | 156 | (28, 135) | 171 | VRR-NUC domain protein | VRR-NUC domain protein | | uniclust | UniRef100\_R9KAU3 | 98.9 | 1.4e-11 | 2.6e-17 | 97.6 | 106 | (21, 155) | 156 | (10, 123) | 261 | VRR-NUC domain-containing protein | VRR-NUC domain-containing protein | | uniclust | UniRef100\_A0A832HUD1 | 98.9 | 1.5e-11 | 2.8e-17 | 87.7 | 91 | (21, 153) | 156 | (28, 120) | 122 | VRR-NUC domain-containing protein | VRR-NUC domain-containing protein | | uniclust | UniRef100\_A0A383C7W8 | 98.9 | 1.6e-11 | 2.9e-17 | 84.4 | 68 | (76, 155) | 156 | (4, 77) | 97 | VRR-NUC domain-containing protein | VRR-NUC domain-containing protein | | uniclust | UniRef100\_A0A7H5F1N7 | 98.9 | 1.6e-11 | 3e-17 | 94.1 | 112 | (21, 155) | 156 | (58, 176) | 201 | VRR-NUC domain-containing protein | VRR-NUC domain-containing protein | | uniclust | UniRef100\_A0A6M5C9Y6 | 98.9 | 1.7e-11 | 3.1e-17 | 90.5 | 108 | (21, 155) | 156 | (12, 126) | 149 | Endonuclease | Endonuclease | | uniclust | UniRef100\_A0A3C0STG0 | 98.9 | 1.6e-11 | 3.2e-17 | 85.6 | 58 | (85, 154) | 156 | (18, 78) | 87 | VRR-NUC domain-containing protein | VRR-NUC domain-containing protein | | uniclust | UniRef100\_A0A2S7YZ17 | 98.9 | 1.7e-11 | 3.5e-17 | 88.9 | 89 | (20, 154) | 156 | (13, 103) | 115 | VRR-NUC domain-containing protein | VRR-NUC domain-containing protein | | uniclust | UniRef100\_A0A017N0S4 | 98.9 | 1.7e-11 | 3.5e-17 | 86.4 | 60 | (85, 155) | 156 | (25, 85) | 93 | VRR-NUC domain protein | VRR-NUC domain protein | | uniclust | UniRef100\_A0A317HKU5 | 98.9 | 2.1e-11 | 3.8e-17 | 90.1 | 103 | (21, 155) | 156 | (42, 149) | 154 | VRR-NUC domain-containing protein | VRR-NUC domain-containing protein | | uniclust | UniRef100\_UPI000A0099DC | 98.8 | 2.4e-11 | 4.3e-17 | 94.0 | 104 | (20, 153) | 156 | (70, 182) | 214 | VRR-NUC domain-containing protein | VRR-NUC domain-containing protein | | uniclust | UniRef100\_A0A6H2A0C5 | 98.8 | 2.2e-11 | 4.5e-17 | 93.8 | 90 | (22, 154) | 156 | (52, 143) | 173 | Putative VRR-NUC domain-containing protein | Putative VRR-NUC domain-containing protein | | uniclust | UniRef100\_A0A7Y5S375 | 98.8 | 2.7e-11 | 4.9e-17 | 81.8 | 68 | (75, 154) | 156 | (12, 80) | 87 | VRR-NUC domain-containing protein | VRR-NUC domain-containing protein | | uniclust | UniRef100\_A0A2K1P5E6 | 98.8 | 2.7e-11 | 5e-17 | 89.4 | 87 | (23, 154) | 156 | (1, 90) | 149 | VRR-NUC domain-containing protein | VRR-NUC domain-containing protein | | uniclust | UniRef100\_A0A2V2CT51 | 98.8 | 3.2e-11 | 6.2e-17 | 85.3 | 100 | (22, 154) | 156 | (1, 102) | 106 | VRR-NUC domain-containing protein | VRR-NUC domain-containing protein | | uniclust | UniRef100\_UPI001ED9FD3A | 98.8 | 3.5e-11 | 6.4e-17 | 90.2 | 115 | (21, 155) | 156 | (1, 126) | 169 | hypothetical protein | hypothetical protein | | uniclust | UniRef100\_A0A965URT5 | 98.8 | 3.6e-11 | 6.6e-17 | 82.3 | 66 | (80, 154) | 156 | (2, 68) | 94 | VRR-NUC domain-containing protein | VRR-NUC domain-containing protein | | uniclust | UniRef100\_A0A7C4LPF3 | 98.8 | 3.6e-11 | 6.8e-17 | 86.9 | 112 | (1, 154) | 156 | (1, 118) | 120 | VRR-NUC domain-containing protein | VRR-NUC domain-containing protein | | uniclust | UniRef100\_A0A1D7TSS5 | 98.8 | 3.4e-11 | 6.8e-17 | 90.4 | 58 | (85, 154) | 156 | (49, 109) | 141 | VRR-NUC domain-containing protein | VRR-NUC domain-containing protein | | uniclust | UniRef100\_A0A7U9N3H7 | 98.8 | 3.9e-11 | 7.2e-17 | 92.9 | 117 | (10, 155) | 156 | (72, 196) | 214 | VRR-NUC domain-containing protein | VRR-NUC domain-containing protein | | uniclust | UniRef100\_A0A660V061 | 98.8 | 4.2e-11 | 7.8e-17 | 85.3 | 87 | (22, 153) | 156 | (1, 89) | 120 | VRR-NUC domain-containing protein | VRR-NUC domain-containing protein | | uniclust | UniRef100\_UPI000558DD9B | 98.8 | 4.3e-11 | 8e-17 | 89.3 | 93 | (23, 154) | 156 | (65, 159) | 164 | VRR-NUC domain-containing protein | VRR-NUC domain-containing protein | | uniclust | UniRef100\_UPI0021F2D242 | 98.8 | 4.5e-11 | 8.3e-17 | 78.4 | 63 | (82, 155) | 156 | (4, 67) | 73 | hypothetical protein | hypothetical protein | | uniclust | UniRef100\_A0A2E0FGH7 | 98.8 | 4.6e-11 | 8.4e-17 | 85.1 | 102 | (22, 154) | 156 | (10, 117) | 120 | VRR-NUC domain-containing protein | VRR-NUC domain-containing protein | | uniclust | UniRef100\_A0A7T5QUK7 | 98.8 | 4.8e-11 | 8.8e-17 | 89.5 | 106 | (22, 154) | 156 | (2, 112) | 169 | VRR-NUC domain-containing protein | VRR-NUC domain-containing protein | | uniclust | UniRef100\_R5J1V9 | 98.8 | 4.8e-11 | 9.2e-17 | 87.0 | 59 | (86, 155) | 156 | (35, 94) | 126 | VRR-NUC domain protein | VRR-NUC domain protein | | uniclust | UniRef100\_A0A949GBL7 | 98.8 | 5.3e-11 | 9.7e-17 | 84.3 | 70 | (73, 154) | 156 | (21, 91) | 115 | VRR-NUC domain-containing protein | VRR-NUC domain-containing protein | | uniclust | UniRef100\_A0A6J5SIW3 | 98.8 | 5.5e-11 | 1e-16 | 91.7 | 105 | (22, 155) | 156 | (58, 169) | 183 | VRR-NUC domain containing protein | VRR-NUC domain containing protein | | uniclust | UniRef100\_B0N9W1 | 98.8 | 5.5e-11 | 1e-16 | 86.8 | 92 | (17, 154) | 156 | (28, 121) | 129 | VRR-NUC domain protein | VRR-NUC domain protein | | uniclust | UniRef100\_A0A1E7I3F0 | 98.8 | 5.5e-11 | 1e-16 | 86.1 | 89 | (23, 155) | 156 | (25, 115) | 124 | VRR-NUC domain-containing protein | VRR-NUC domain-containing protein | | uniclust | UniRef100\_A0A0F9BWP4 | 98.8 | 5.6e-11 | 1.1e-16 | 82.3 | 84 | (20, 138) | 156 | (2, 89) | 90 | VRR-NUC domain-containing protein (Fragment) | VRR-NUC domain-containing protein (Fragment) | | uniclust | UniRef100\_A0A0F9FVZ7 | 98.8 | 6.2e-11 | 1.1e-16 | 78.0 | 69 | (22, 122) | 156 | (2, 72) | 74 | VRR-NUC domain-containing protein (Fragment) | VRR-NUC domain-containing protein (Fragment) | | uniclust | UniRef100\_A0A6P2FZW6 | 98.8 | 6.2e-11 | 1.1e-16 | 91.7 | 107 | (22, 155) | 156 | (83, 197) | 203 | VRR-NUC domain protein | VRR-NUC domain protein | | uniclust | UniRef100\_A0A016QS04 | 98.8 | 5.7e-11 | 1.2e-16 | 91.9 | 108 | (20, 155) | 156 | (17, 127) | 160 | VRR-NUC domain-containing protein | VRR-NUC domain-containing protein | | uniclust | UniRef100\_A0A7V0Q709 | 98.8 | 6.8e-11 | 1.2e-16 | 89.0 | 110 | (22, 155) | 156 | (53, 165) | 172 | VRR-NUC domain-containing protein | VRR-NUC domain-containing protein | | uniclust | UniRef100\_A0A965UUD2 | 98.8 | 7e-11 | 1.3e-16 | 92.0 | 108 | (21, 155) | 156 | (89, 203) | 223 | VRR-NUC domain-containing protein | VRR-NUC domain-containing protein | | uniclust | UniRef100\_A0A2P6BVS4 | 98.7 | 7.9e-11 | 1.5e-16 | 88.0 | 113 | (22, 154) | 156 | (32, 152) | 164 | VRR-NUC domain-containing protein | VRR-NUC domain-containing protein | | uniclust | UniRef100\_A0A0S7BVS5 | 98.7 | 7.8e-11 | 1.7e-16 | 95.3 | 114 | (23, 155) | 156 | (69, 185) | 205 | VRR-NUC domain-containing protein | VRR-NUC domain-containing protein | | uniclust | UniRef100\_A0A1Q6RAF5 | 98.7 | 9.5e-11 | 1.7e-16 | 85.9 | 111 | (22, 154) | 156 | (2, 118) | 143 | VRR-NUC domain-containing protein | VRR-NUC domain-containing protein | | uniclust | UniRef100\_A0A8J3EF72 | 98.7 | 9.8e-11 | 1.8e-16 | 87.0 | 108 | (20, 154) | 156 | (27, 142) | 157 | VRR-NUC domain-containing protein | VRR-NUC domain-containing protein | | uniclust | UniRef100\_A0A2L0XD71 | 98.7 | 9.7e-11 | 1.8e-16 | 89.0 | 104 | (20, 154) | 156 | (42, 149) | 171 | VRR-NUC domain-containing protein | VRR-NUC domain-containing protein | | uniclust | UniRef100\_A0A6M3Y0W3 | 98.7 | 1e-10 | 1.9e-16 | 85.0 | 101 | (16, 155) | 156 | (31, 133) | 135 | Putative VRR-NUC domain-containing protein | Putative VRR-NUC domain-containing protein | | uniclust | UniRef100\_A0A7C5WRI5 | 98.7 | 1.1e-10 | 2e-16 | 88.0 | 106 | (23, 155) | 156 | (56, 163) | 173 | VRR-NUC domain-containing protein | VRR-NUC domain-containing protein | | uniclust | UniRef100\_A0A2W5ZUK3 | 98.7 | 1.1e-10 | 2e-16 | 80.4 | 71 | (74, 155) | 156 | (10, 81) | 96 | VRR-NUC domain-containing protein | VRR-NUC domain-containing protein | | uniclust | UniRef100\_A0A0F9H755 | 98.7 | 1.1e-10 | 2e-16 | 82.9 | 99 | (23, 154) | 156 | (1, 104) | 116 | VRR-NUC domain-containing protein | VRR-NUC domain-containing protein | | uniclust | UniRef100\_A0A522SU77 | 98.7 | 1.1e-10 | 2.1e-16 | 88.3 | 103 | (21, 155) | 156 | (43, 167) | 178 | VRR-NUC domain-containing protein | VRR-NUC domain-containing protein | | uniclust | UniRef100\_A0A142KBX3 | 98.7 | 1.2e-10 | 2.2e-16 | 76.5 | 59 | (85, 155) | 156 | (11, 70) | 73 | Holliday junction resolvase | Holliday junction resolvase | | uniclust | UniRef100\_A0A6J5PJ07 | 98.7 | 1.2e-10 | 2.2e-16 | 85.9 | 87 | (14, 145) | 156 | (18, 106) | 134 | VRR-NUC domain containing protein | VRR-NUC domain containing protein | | uniclust | UniRef100\_UPI001F5E1DBC | 98.7 | 1.2e-10 | 2.3e-16 | 76.1 | 60 | (83, 155) | 156 | (7, 68) | 71 | VRR-NUC domain-containing protein | VRR-NUC domain-containing protein | | uniclust | UniRef100\_A0A0G1CCZ0 | 98.7 | 1.2e-10 | 2.3e-16 | 82.1 | 87 | (21, 152) | 156 | (4, 92) | 111 | VRR-NUC domain-containing protein | VRR-NUC domain-containing protein | | uniclust | UniRef100\_A0A0F9TBD8 | 98.7 | 1.2e-10 | 2.3e-16 | 82.1 | 57 | (85, 151) | 156 | (31, 88) | 102 | VRR-NUC domain-containing protein | VRR-NUC domain-containing protein | | uniclust | UniRef100\_A0A0F9NEB0 | 98.7 | 1.3e-10 | 2.4e-16 | 83.5 | 95 | (22, 147) | 156 | (5, 106) | 125 | VRR-NUC domain-containing protein | VRR-NUC domain-containing protein | | uniclust | UniRef100\_A0A2V8RVF6 | 98.7 | 1.2e-10 | 2.4e-16 | 86.6 | 92 | (22, 155) | 156 | (27, 120) | 142 | VRR-NUC domain-containing protein | VRR-NUC domain-containing protein | | uniclust | UniRef100\_A0A497BB15 | 98.7 | 1.3e-10 | 2.4e-16 | 85.0 | 91 | (20, 155) | 156 | (26, 118) | 140 | VRR-NUC domain-containing protein | VRR-NUC domain-containing protein | | uniclust | UniRef100\_A0A6M3K5H3 | 98.7 | 1.3e-10 | 2.4e-16 | 84.3 | 93 | (19, 155) | 156 | (3, 101) | 119 | VRR-NUC domain-containing protein | VRR-NUC domain-containing protein | | uniclust | UniRef100\_A0A6J5L3D2 | 98.7 | 1.3e-10 | 2.5e-16 | 86.6 | 106 | (21, 155) | 156 | (48, 157) | 160 | VRR-NUC domain containing protein | VRR-NUC domain containing protein | | uniclust | UniRef100\_A0A1V3QA50 | 98.7 | 1.3e-10 | 2.5e-16 | 88.3 | 104 | (21, 153) | 156 | (42, 157) | 170 | VRR-NUC domain-containing protein | VRR-NUC domain-containing protein | | uniclust | UniRef100\_A3JK17 | 98.7 | 1.5e-10 | 2.7e-16 | 89.6 | 104 | (23, 154) | 156 | (80, 192) | 209 | VRR-NUC domain-containing protein | VRR-NUC domain-containing protein | | uniclust | UniRef100\_A0A2N5RCI0 | 98.7 | 1.4e-10 | 2.7e-16 | 90.8 | 103 | (22, 153) | 156 | (43, 148) | 187 | Nuclease | Nuclease | | uniclust | UniRef100\_A0A6J5NIF6 | 98.7 | 1.5e-10 | 2.9e-16 | 87.2 | 112 | (22, 155) | 156 | (29, 149) | 154 | VRR-NUC domain containing protein | VRR-NUC domain containing protein | | uniclust | UniRef100\_A0A6J5LH34 | 98.7 | 1.6e-10 | 2.9e-16 | 83.6 | 105 | (21, 155) | 156 | (5, 115) | 130 | VRR-NUC domain containing protein | VRR-NUC domain containing protein | | uniclust | UniRef100\_A0A6H1ZUM5 | 98.7 | 1.6e-10 | 2.9e-16 | 86.7 | 111 | (21, 153) | 156 | (49, 165) | 166 | Putative VRR-NUC domain-containing protein | Putative VRR-NUC domain-containing protein | | uniclust | UniRef100\_A0A843H219 | 98.7 | 1.6e-10 | 2.9e-16 | 80.7 | 101 | (22, 153) | 156 | (2, 104) | 104 | VRR-NUC domain-containing protein | VRR-NUC domain-containing protein | | uniclust | UniRef100\_A0A948NR45 | 98.7 | 1.5e-10 | 3e-16 | 86.4 | 82 | (21, 147) | 156 | (24, 108) | 129 | VRR-NUC domain-containing protein | VRR-NUC domain-containing protein | | uniclust | UniRef100\_UPI0002D4C647 | 98.7 | 1.7e-10 | 3.1e-16 | 85.3 | 103 | (22, 153) | 156 | (2, 112) | 150 | VRR-NUC domain-containing protein | VRR-NUC domain-containing protein | | uniclust | UniRef100\_A0A7C1JCQ0 | 98.7 | 1.7e-10 | 3.1e-16 | 80.7 | 65 | (75, 151) | 156 | (37, 102) | 105 | VRR-NUC domain-containing protein | VRR-NUC domain-containing protein | | uniclust | UniRef100\_A0A1J5SCT5 | 98.7 | 1.7e-10 | 3.1e-16 | 86.3 | 105 | (26, 155) | 156 | (46, 158) | 164 | VRR-NUC domain protein | VRR-NUC domain protein | | uniclust | UniRef100\_UPI001D07F940 | 98.7 | 1.7e-10 | 3.2e-16 | 90.3 | 90 | (20, 154) | 156 | (60, 152) | 230 | VRR-NUC domain-containing protein | VRR-NUC domain-containing protein | | uniclust | UniRef100\_A0A373ZU69 | 98.7 | 1.7e-10 | 3.2e-16 | 86.4 | 103 | (23, 154) | 156 | (4, 114) | 165 | VRR-NUC domain-containing protein | VRR-NUC domain-containing protein | | uniclust | UniRef100\_A0A958CYF5 | 98.7 | 1.8e-10 | 3.3e-16 | 84.4 | 101 | (21, 153) | 156 | (10, 125) | 141 | Uncharacterized protein | Uncharacterized protein | | uniclust | UniRef100\_A0A7X7LEV0 | 98.7 | 1.8e-10 | 3.3e-16 | 86.3 | 88 | (23, 152) | 156 | (22, 111) | 165 | VRR-NUC domain-containing protein | VRR-NUC domain-containing protein | | uniclust | UniRef100\_A0A6J5L990 | 98.7 | 2e-10 | 3.6e-16 | 89.6 | 100 | (22, 153) | 156 | (1, 107) | 220 | VRR-NUC domain-containing protein | VRR-NUC domain-containing protein | | uniclust | UniRef100\_A0A965UQW0 | 98.7 | 2e-10 | 3.6e-16 | 84.4 | 68 | (76, 153) | 156 | (63, 131) | 144 | VRR-NUC domain-containing protein | VRR-NUC domain-containing protein | | uniclust | UniRef100\_A0A3E2TD32 | 98.7 | 2e-10 | 3.7e-16 | 89.3 | 108 | (18, 154) | 156 | (36, 151) | 217 | VRR-NUC domain-containing protein | VRR-NUC domain-containing protein | | uniclust | UniRef100\_A0A6G6YUN2 | 98.7 | 2e-10 | 3.8e-16 | 80.2 | 70 | (75, 155) | 156 | (22, 93) | 104 | VRR-NUC domain-containing protein | VRR-NUC domain-containing protein | | uniclust | UniRef100\_UPI001391A1F6 | 98.7 | 2.1e-10 | 3.9e-16 | 85.2 | 82 | (40, 152) | 156 | (26, 111) | 156 | VRR-NUC domain-containing protein | VRR-NUC domain-containing protein | | uniclust | UniRef100\_A0A925VSB9 | 98.7 | 2.2e-10 | 4e-16 | 88.8 | 103 | (22, 154) | 156 | (92, 196) | 210 | VRR-NUC domain-containing protein | VRR-NUC domain-containing protein | | uniclust | UniRef100\_A0A0F9NEN3 | 98.7 | 2.2e-10 | 4.1e-16 | 82.3 | 102 | (21, 153) | 156 | (6, 113) | 124 | VRR-NUC domain-containing protein | VRR-NUC domain-containing protein | | uniclust | UniRef100\_A0A0F9BLX2 | 98.7 | 2.3e-10 | 4.2e-16 | 83.0 | 104 | (21, 155) | 156 | (23, 129) | 132 | VRR-NUC domain-containing protein | VRR-NUC domain-containing protein | | uniclust | UniRef100\_A0A7V8NUZ0 | 98.7 | 2.4e-10 | 4.3e-16 | 86.6 | 105 | (20, 155) | 156 | (36, 145) | 178 | VRR-NUC domain-containing protein | VRR-NUC domain-containing protein | | uniclust | UniRef100\_A0A7U3ZN37 | 98.7 | 2.3e-10 | 4.4e-16 | 86.0 | 97 | (25, 154) | 156 | (32, 130) | 143 | VRR-NUC domain-containing protein | VRR-NUC domain-containing protein | | uniclust | UniRef100\_UPI001C60F457 | 98.6 | 2.7e-10 | 4.9e-16 | 85.0 | 112 | (21, 155) | 156 | (23, 143) | 159 | VRR-NUC domain-containing protein | VRR-NUC domain-containing protein | | uniclust | UniRef100\_A0A7C6EBY8 | 98.6 | 2.7e-10 | 4.9e-16 | 83.0 | 89 | (23, 155) | 156 | (2, 92) | 135 | VRR-NUC domain-containing protein | VRR-NUC domain-containing protein | | uniclust | UniRef100\_A0A101GVW2 | 98.6 | 2.8e-10 | 5.1e-16 | 86.7 | 91 | (20, 155) | 156 | (88, 181) | 184 | VRR-NUC domain-containing protein | VRR-NUC domain-containing protein | | uniclust | UniRef100\_A0A225DD89 | 98.6 | 2.7e-10 | 5.1e-16 | 84.5 | 110 | (20, 151) | 156 | (28, 139) | 149 | VRR-NUC domain-containing protein | VRR-NUC domain-containing protein | | uniclust | UniRef100\_A0A497C3L4 | 98.6 | 2.8e-10 | 5.1e-16 | 86.3 | 102 | (23, 153) | 156 | (55, 165) | 179 | VRR-NUC domain-containing protein | VRR-NUC domain-containing protein | | uniclust | UniRef100\_A0A1B4G129 | 98.6 | 2.6e-10 | 5.2e-16 | 90.0 | 104 | (21, 155) | 156 | (54, 169) | 188 | VRR-NUC domain-containing protein | VRR-NUC domain-containing protein | | uniclust | UniRef100\_A0A966WJ63 | 98.6 | 2.9e-10 | 5.4e-16 | 79.8 | 85 | (21, 134) | 156 | (14, 106) | 107 | VRR-NUC domain-containing protein (Fragment) | VRR-NUC domain-containing protein (Fragment) | | uniclust | UniRef100\_A0A2D8XCT8 | 98.6 | 2.8e-10 | 5.5e-16 | 75.3 | 57 | (85, 153) | 156 | (9, 66) | 68 | VRR-NUC domain-containing protein | VRR-NUC domain-containing protein | | uniclust | UniRef100\_A0A511MZ74 | 98.6 | 3.3e-10 | 6e-16 | 86.8 | 94 | (21, 153) | 156 | (82, 179) | 192 | VRR-NUC domain-containing protein | VRR-NUC domain-containing protein | | uniclust | UniRef100\_A0A1Q3M5C5 | 98.6 | 3.3e-10 | 6.1e-16 | 80.8 | 82 | (21, 131) | 156 | (23, 113) | 114 | Nuclease (Fragment) | Nuclease (Fragment) | | uniclust | UniRef100\_A0A1W9LSN6 | 98.6 | 3.1e-10 | 6.2e-16 | 81.7 | 90 | (19, 154) | 156 | (4, 95) | 108 | VRR-NUC domain-containing protein | VRR-NUC domain-containing protein | | uniclust | UniRef100\_UPI000C5CBA07 | 98.6 | 3.4e-10 | 6.2e-16 | 85.6 | 102 | (22, 151) | 156 | (34, 143) | 174 | VRR-NUC domain-containing protein | VRR-NUC domain-containing protein | | uniclust | UniRef100\_A0A6S4IR19 | 98.6 | 3.6e-10 | 6.7e-16 | 79.5 | 97 | (21, 154) | 156 | (2, 100) | 108 | VRR-NUC domain-containing protein | VRR-NUC domain-containing protein | | uniclust | UniRef100\_A0A561PL50 | 98.6 | 3.6e-10 | 6.9e-16 | 75.0 | 64 | (79, 154) | 156 | (3, 67) | 71 | VRR-NUC domain-containing protein | VRR-NUC domain-containing protein | | uniclust | UniRef100\_A0A6J5M4C0 | 98.6 | 4e-10 | 7.3e-16 | 85.1 | 106 | (21, 154) | 156 | (64, 171) | 173 | VRR-NUC domain containing protein | VRR-NUC domain containing protein | | uniclust | UniRef100\_A0A0N8HQ47 | 98.6 | 4.1e-10 | 7.5e-16 | 85.8 | 104 | (22, 154) | 156 | (53, 162) | 178 | VRR-NUC domain-containing protein | VRR-NUC domain-containing protein | | uniclust | UniRef100\_A0A524RQI1 | 98.6 | 4.2e-10 | 7.8e-16 | 71.9 | 51 | (103, 153) | 156 | (1, 52) | 62 | VRR-NUC domain-containing protein | VRR-NUC domain-containing protein | | uniclust | UniRef100\_A0A6J5LGZ3 | 98.6 | 4.3e-10 | 7.9e-16 | 82.9 | 105 | (22, 155) | 156 | (28, 134) | 146 | VRR-NUC domain containing protein | VRR-NUC domain containing protein | | uniclust | UniRef100\_A0A7C3EGK7 | 98.6 | 4.6e-10 | 8.4e-16 | 80.9 | 85 | (23, 154) | 156 | (2, 88) | 125 | VRR-NUC domain-containing protein | VRR-NUC domain-containing protein | | uniclust | UniRef100\_A0A7C6KEH1 | 98.6 | 4.6e-10 | 8.8e-16 | 78.0 | 57 | (85, 153) | 156 | (33, 90) | 91 | VRR-NUC domain-containing protein | VRR-NUC domain-containing protein | | uniclust | UniRef100\_UPI00164113D9 | 98.6 | 4.5e-10 | 9.6e-16 | 101.1 | 87 | (23, 155) | 156 | (1, 89) | 512 | VRR-NUC domain-containing protein | VRR-NUC domain-containing protein | | uniclust | UniRef100\_UPI001B800FDB | 98.6 | 5.5e-10 | 1e-15 | 84.6 | 104 | (20, 155) | 156 | (25, 140) | 175 | hypothetical protein | hypothetical protein | | uniclust | UniRef100\_A0A519WTD9 | 98.6 | 6e-10 | 1.1e-15 | 74.3 | 69 | (75, 155) | 156 | (4, 73) | 78 | VRR-NUC domain-containing protein (Fragment) | VRR-NUC domain-containing protein (Fragment) | | uniclust | UniRef100\_A0A0G1U713 | 98.6 | 6.2e-10 | 1.1e-15 | 79.0 | 71 | (76, 154) | 156 | (36, 107) | 113 | VRR-NUC domain-containing protein | VRR-NUC domain-containing protein | | uniclust | UniRef100\_A0A0F9QS03 | 98.6 | 7.1e-10 | 1.3e-15 | 82.7 | 105 | (21, 154) | 156 | (19, 150) | 157 | VRR-NUC domain-containing protein | VRR-NUC domain-containing protein | | uniclust | UniRef100\_UPI000B7945AE | 98.6 | 7.1e-10 | 1.4e-15 | 84.0 | 101 | (21, 152) | 156 | (14, 123) | 149 | hypothetical protein | hypothetical protein | | uniclust | UniRef100\_A0A2H5X7U0 | 98.5 | 8.1e-10 | 1.6e-15 | 78.9 | 75 | (25, 144) | 156 | (7, 83) | 105 | VRR-NUC domain-containing protein | VRR-NUC domain-containing protein | | uniclust | UniRef100\_A0A1F2WNL4 | 98.5 | 9.5e-10 | 1.7e-15 | 83.6 | 115 | (21, 155) | 156 | (31, 149) | 179 | VRR-NUC domain-containing protein | VRR-NUC domain-containing protein | | uniclust | UniRef100\_A0A4Q0T6H7 | 98.5 | 1e-09 | 1.9e-15 | 78.3 | 64 | (79, 154) | 156 | (40, 104) | 116 | VRR-NUC domain-containing protein | VRR-NUC domain-containing protein | | uniclust | UniRef100\_A0A2H0NCQ0 | 98.5 | 1e-09 | 1.9e-15 | 78.8 | 86 | (23, 155) | 156 | (8, 96) | 111 | VRR-NUC domain-containing protein | VRR-NUC domain-containing protein | | uniclust | UniRef100\_A0A1L8TFK0 | 98.5 | 1.1e-09 | 1.9e-15 | 74.6 | 64 | (82, 155) | 156 | (14, 78) | 87 | VRR-NUC domain-containing protein | VRR-NUC domain-containing protein | | uniclust | UniRef100\_A0A0A0BLP2 | 98.5 | 9.8e-10 | 2e-15 | 83.7 | 97 | (22, 153) | 156 | (1, 108) | 150 | Recombinase RecB | Recombinase RecB | | uniclust | UniRef100\_E4LIQ7 | 98.5 | 1.1e-09 | 2e-15 | 78.7 | 88 | (21, 154) | 156 | (31, 120) | 122 | VRR-NUC domain protein | VRR-NUC domain protein | | uniclust | UniRef100\_A0A316S5X2 | 98.5 | 1.1e-09 | 2.2e-15 | 84.8 | 93 | (18, 155) | 156 | (34, 131) | 170 | Nuclease | Nuclease | | uniclust | UniRef100\_UPI001F17BD1E | 98.5 | 1.2e-09 | 2.2e-15 | 81.7 | 94 | (21, 144) | 156 | (24, 124) | 144 | VRR-NUC domain-containing protein | VRR-NUC domain-containing protein | | uniclust | UniRef100\_A0A7X9Q8X8 | 98.5 | 1.2e-09 | 2.3e-15 | 85.6 | 103 | (20, 151) | 156 | (105, 216) | 224 | VRR-NUC domain-containing protein | VRR-NUC domain-containing protein | | uniclust | UniRef100\_UPI0018D3F871 | 98.5 | 1.2e-09 | 2.3e-15 | 82.8 | 118 | (23, 153) | 156 | (1, 128) | 158 | hypothetical protein | hypothetical protein | | uniclust | UniRef100\_L7U9Y9 | 98.5 | 1.3e-09 | 2.4e-15 | 80.3 | 111 | (21, 154) | 156 | (25, 138) | 144 | VRR-NUC domain-containing protein | VRR-NUC domain-containing protein | | uniclust | UniRef100\_A0A1V5LEE2 | 98.5 | 1.3e-09 | 2.4e-15 | 77.7 | 108 | (19, 151) | 156 | (4, 115) | 116 | VRR-NUC domain protein | VRR-NUC domain protein | | uniclust | UniRef100\_A0A0F9VBY1 | 98.5 | 1.3e-09 | 2.5e-15 | 79.8 | 112 | (22, 155) | 156 | (3, 117) | 127 | VRR-NUC domain-containing protein | VRR-NUC domain-containing protein | | uniclust | UniRef100\_A0A6G7X067 | 98.5 | 1.3e-09 | 2.5e-15 | 79.1 | 89 | (23, 155) | 156 | (1, 94) | 119 | VRR-NUC domain-containing protein | VRR-NUC domain-containing protein | | uniclust | UniRef100\_A0A3N5E8N3 | 98.5 | 1.4e-09 | 2.6e-15 | 76.3 | 90 | (14, 144) | 156 | (13, 104) | 104 | VRR-NUC domain-containing protein (Fragment) | VRR-NUC domain-containing protein (Fragment) | | uniclust | UniRef100\_A0A177R4V3 | 98.5 | 1.4e-09 | 2.6e-15 | 83.6 | 110 | (22, 154) | 156 | (77, 191) | 194 | Uncharacterized protein | Uncharacterized protein | | uniclust | UniRef100\_A0A0L6JT85 | 98.5 | 1.5e-09 | 2.8e-15 | 81.6 | 60 | (83, 154) | 156 | (83, 143) | 148 | VRR-NUC domain-containing protein | VRR-NUC domain-containing protein | | uniclust | UniRef100\_A0A1G4TXQ0 | 98.5 | 1.5e-09 | 2.9e-15 | 83.1 | 89 | (21, 155) | 156 | (70, 160) | 166 | YozE SAM-like fold | YozE SAM-like fold | | uniclust | UniRef100\_A0A537MDI3 | 98.5 | 1.6e-09 | 2.9e-15 | 74.3 | 67 | (77, 155) | 156 | (13, 80) | 91 | VRR-NUC domain-containing protein | VRR-NUC domain-containing protein | | uniclust | UniRef100\_A0A2V5PQQ7 | 98.5 | 1.7e-09 | 3e-15 | 82.1 | 59 | (86, 155) | 156 | (114, 173) | 174 | VRR-NUC domain-containing protein | VRR-NUC domain-containing protein | | uniclust | UniRef100\_A0A2T3ITI0 | 98.5 | 1.6e-09 | 3.2e-15 | 88.0 | 105 | (22, 155) | 156 | (78, 198) | 231 | VRR-NUC domain-containing protein | VRR-NUC domain-containing protein | | uniclust | UniRef100\_A0A1H0PBQ8 | 98.5 | 1.8e-09 | 3.2e-15 | 79.9 | 57 | (86, 154) | 156 | (34, 91) | 146 | VRR-NUC domain-containing protein | VRR-NUC domain-containing protein | | uniclust | UniRef100\_A0A239DNY6 | 98.5 | 1.9e-09 | 3.4e-15 | 81.3 | 67 | (75, 153) | 156 | (90, 157) | 166 | VRR-NUC domain-containing protein | VRR-NUC domain-containing protein | | uniclust | UniRef100\_A0A0H3ZPV8 | 98.5 | 1.8e-09 | 3.5e-15 | 83.2 | 102 | (22, 152) | 156 | (52, 159) | 175 | Phage protein | Phage protein | | uniclust | UniRef100\_A0A968LFM5 | 98.5 | 1.9e-09 | 3.5e-15 | 78.1 | 63 | (76, 150) | 156 | (55, 118) | 128 | VRR-NUC domain-containing protein | VRR-NUC domain-containing protein | | uniclust | UniRef100\_A0A1C6CWA0 | 98.5 | 2.1e-09 | 3.8e-15 | 84.1 | 107 | (21, 154) | 156 | (12, 130) | 219 | VRR-NUC domain | VRR-NUC domain | | uniclust | UniRef100\_A0A7C5QET6 | 98.4 | 2.2e-09 | 4.1e-15 | 78.9 | 100 | (21, 155) | 156 | (37, 138) | 140 | VRR-NUC domain-containing protein | VRR-NUC domain-containing protein | | uniclust | UniRef100\_A0A370CU33 | 98.4 | 2.3e-09 | 4.2e-15 | 80.0 | 112 | (20, 154) | 156 | (25, 142) | 154 | VRR-NUC domain-containing protein | VRR-NUC domain-containing protein | | uniclust | UniRef100\_A0A418MBC5 | 98.4 | 2.4e-09 | 4.6e-15 | 80.4 | 101 | (21, 154) | 156 | (17, 123) | 149 | VRR-NUC domain-containing protein | VRR-NUC domain-containing protein | | uniclust | UniRef100\_A0A257TCZ7 | 98.4 | 2.5e-09 | 4.6e-15 | 69.2 | 56 | (87, 154) | 156 | (1, 57) | 65 | Uncharacterized protein (Fragment) | Uncharacterized protein (Fragment) | | uniclust | UniRef100\_A0A7X3R873 | 98.4 | 2.6e-09 | 4.8e-15 | 72.0 | 57 | (85, 153) | 156 | (23, 80) | 82 | VRR-NUC domain-containing protein | VRR-NUC domain-containing protein | | uniclust | UniRef100\_A0A2A1K7C6 | 98.4 | 2.9e-09 | 5.4e-15 | 72.0 | 80 | (22, 134) | 156 | (1, 82) | 83 | VRR-NUC domain-containing protein (Fragment) | VRR-NUC domain-containing protein (Fragment) | | uniclust | UniRef100\_UPI0007DC280A | 98.4 | 2.7e-09 | 5.4e-15 | 79.7 | 86 | (21, 154) | 156 | (9, 96) | 132 | VRR-NUC domain-containing protein | VRR-NUC domain-containing protein | | uniclust | UniRef100\_A0A4Q5NWE0 | 98.4 | 3e-09 | 5.6e-15 | 84.1 | 100 | (21, 152) | 156 | (77, 184) | 236 | VRR-NUC domain-containing protein | VRR-NUC domain-containing protein | | uniclust | UniRef100\_A0A3C1HPS2 | 98.4 | 3.2e-09 | 5.9e-15 | 73.1 | 84 | (22, 128) | 156 | (2, 92) | 92 | Uncharacterized protein | Uncharacterized protein | | uniclust | UniRef100\_UPI001FFCBC52 | 98.4 | 3.3e-09 | 6.2e-15 | 74.9 | 51 | (85, 147) | 156 | (12, 63) | 99 | VRR-NUC domain-containing protein | VRR-NUC domain-containing protein | | uniclust | UniRef100\_A0A7C1C9X5 | 98.4 | 3.5e-09 | 6.5e-15 | 78.6 | 89 | (23, 155) | 156 | (53, 143) | 149 | VRR-NUC domain-containing protein | VRR-NUC domain-containing protein | | uniclust | UniRef100\_A0A2S7ZHM1 | 98.4 | 3.7e-09 | 6.9e-15 | 77.9 | 58 | (85, 153) | 156 | (50, 108) | 132 | VRR-NUC domain-containing protein | VRR-NUC domain-containing protein | | uniclust | UniRef100\_A0A661DS26 | 98.4 | 3.8e-09 | 7e-15 | 75.7 | 85 | (23, 154) | 156 | (1, 88) | 118 | VRR-NUC domain-containing protein | VRR-NUC domain-containing protein | | uniclust | UniRef100\_UPI0018E07DFE | 98.4 | 3.6e-09 | 7.1e-15 | 85.3 | 58 | (86, 154) | 156 | (30, 88) | 219 | VRR-NUC domain-containing protein | VRR-NUC domain-containing protein | | uniclust | UniRef100\_A0A2I7QVY9 | 98.4 | 4.1e-09 | 7.6e-15 | 79.0 | 72 | (75, 155) | 156 | (68, 143) | 150 | VRR-NUC domain protein | VRR-NUC domain protein | | uniclust | UniRef100\_A0A0P0J010 | 98.4 | 3.9e-09 | 7.6e-15 | 80.4 | 105 | (22, 153) | 156 | (25, 136) | 148 | VRR-NUC domain-containing protein | VRR-NUC domain-containing protein | | uniclust | UniRef100\_A0A6M3TDT4 | 98.4 | 4.2e-09 | 8e-15 | 78.2 | 107 | (18, 145) | 156 | (9, 132) | 136 | Uncharacterized protein | Uncharacterized protein | | uniclust | UniRef100\_A0A0F9HQT7 | 98.4 | 4.5e-09 | 8.2e-15 | 74.7 | 95 | (20, 153) | 156 | (6, 102) | 111 | VRR-NUC domain-containing protein | VRR-NUC domain-containing protein | | uniclust | UniRef100\_A0A951MTT0 | 98.4 | 4.5e-09 | 8.2e-15 | 81.4 | 106 | (21, 155) | 156 | (63, 190) | 200 | VRR-NUC domain-containing protein | VRR-NUC domain-containing protein | | uniclust | UniRef100\_A0A6J7W6B7 | 98.4 | 4.5e-09 | 8.3e-15 | 73.3 | 62 | (82, 155) | 156 | (26, 88) | 99 | VRR-NUC domain containing protein | VRR-NUC domain containing protein | | uniclust | UniRef100\_A0A117IRP9 | 98.4 | 4.4e-09 | 8.4e-15 | 77.3 | 94 | (15, 155) | 156 | (10, 105) | 123 | VRR-NUC domain-containing protein | VRR-NUC domain-containing protein | | uniclust | UniRef100\_A0A249XQD2 | 98.4 | 4.8e-09 | 8.9e-15 | 75.2 | 60 | (85, 155) | 156 | (52, 112) | 117 | Nuclease | Nuclease | | uniclust | UniRef100\_A0A2D6X4D2 | 98.4 | 4.9e-09 | 9e-15 | 79.0 | 106 | (23, 155) | 156 | (46, 159) | 164 | Uncharacterized protein | Uncharacterized protein | | uniclust | UniRef100\_A0A6L9GF55 | 98.4 | 5.3e-09 | 9.8e-15 | 69.3 | 48 | (108, 155) | 156 | (7, 55) | 71 | VRR-NUC domain-containing protein (Fragment) | VRR-NUC domain-containing protein (Fragment) | | uniclust | UniRef100\_UPI0003B37613 | 98.4 | 5.4e-09 | 1e-14 | 77.3 | 106 | (23, 154) | 156 | (15, 124) | 143 | hypothetical protein | hypothetical protein | | uniclust | UniRef100\_UPI002240A71F | 98.4 | 5.5e-09 | 1e-14 | 79.5 | 104 | (22, 153) | 156 | (41, 148) | 174 | VRR-NUC domain-containing protein | VRR-NUC domain-containing protein | | uniclust | UniRef100\_A0A1E7Y0A1 | 98.4 | 5.6e-09 | 1e-14 | 77.5 | 57 | (86, 153) | 156 | (30, 87) | 147 | VRR-NUC domain-containing protein | VRR-NUC domain-containing protein | | uniclust | UniRef100\_Q45PU7 | 98.4 | 5.8e-09 | 1.1e-14 | 71.8 | 67 | (76, 154) | 156 | (12, 82) | 91 | JK\_69P | JK\_69P | | uniclust | UniRef100\_A0A9E9GX09 | 98.3 | 5.7e-09 | 1.1e-14 | 81.2 | 55 | (86, 152) | 156 | (31, 87) | 184 | Uncharacterized protein | Uncharacterized protein | | uniclust | UniRef100\_A0A1V6G008 | 98.3 | 6e-09 | 1.1e-14 | 75.2 | 92 | (22, 154) | 156 | (1, 94) | 122 | VRR-NUC domain protein | VRR-NUC domain protein | | uniclust | UniRef100\_UPI001C6961CB | 98.3 | 6.1e-09 | 1.1e-14 | 68.4 | 62 | (82, 155) | 156 | (1, 65) | 70 | hypothetical protein | hypothetical protein | | uniclust | UniRef100\_A0A7C6T0D3 | 98.3 | 6.1e-09 | 1.1e-14 | 76.3 | 90 | (19, 153) | 156 | (38, 129) | 134 | VRR-NUC domain-containing protein | VRR-NUC domain-containing protein | | uniclust | UniRef100\_A0A3C1SW60 | 98.3 | 6e-09 | 1.1e-14 | 77.1 | 112 | (20, 155) | 156 | (12, 131) | 134 | VRR-NUC domain-containing protein | VRR-NUC domain-containing protein | | uniclust | UniRef100\_UPI00164128D5 | 98.3 | 5.8e-09 | 1.1e-14 | 90.5 | 87 | (23, 155) | 156 | (1, 89) | 403 | hypothetical protein | hypothetical protein | | uniclust | UniRef100\_A0A7T4PUE8 | 98.3 | 6.3e-09 | 1.1e-14 | 76.1 | 90 | (22, 155) | 156 | (2, 103) | 133 | VRR-NUC domain-containing protein | VRR-NUC domain-containing protein | | uniclust | UniRef100\_E3GF55 | 98.3 | 6.3e-09 | 1.2e-14 | 79.7 | 57 | (86, 154) | 156 | (34, 91) | 183 | VRR-NUC domain protein | VRR-NUC domain protein | | uniclust | UniRef100\_A0A5C7JER8 | 98.3 | 6.2e-09 | 1.2e-14 | 77.6 | 97 | (23, 148) | 156 | (31, 131) | 142 | VRR-NUC domain-containing protein | VRR-NUC domain-containing protein | | uniclust | UniRef100\_A0A966K7I6 | 98.3 | 6.4e-09 | 1.2e-14 | 78.8 | 104 | (22, 155) | 156 | (51, 159) | 169 | VRR-NUC domain-containing protein | VRR-NUC domain-containing protein | | uniclust | UniRef100\_A0A952PDW2 | 98.3 | 6.8e-09 | 1.3e-14 | 77.0 | 115 | (21, 155) | 156 | (14, 130) | 132 | Uncharacterized protein | Uncharacterized protein | | uniclust | UniRef100\_UPI001F37818D | 98.3 | 7.1e-09 | 1.3e-14 | 76.4 | 89 | (21, 155) | 156 | (42, 132) | 139 | VRR-NUC domain-containing protein | VRR-NUC domain-containing protein | | uniclust | UniRef100\_A0A6M3JW76 | 98.3 | 6.9e-09 | 1.3e-14 | 80.1 | 88 | (22, 154) | 156 | (15, 106) | 176 | VRR-NUC domain-containing protein | VRR-NUC domain-containing protein | | uniclust | UniRef100\_A0A0E4BRS8 | 98.3 | 6.7e-09 | 1.3e-14 | 80.0 | 121 | (4, 152) | 156 | (11, 137) | 161 | VRR-NUC domain-containing protein | VRR-NUC domain-containing protein | | uniclust | UniRef100\_A0A965H4R9 | 98.3 | 7.2e-09 | 1.4e-14 | 67.1 | 52 | (103, 154) | 156 | (1, 53) | 61 | VRR-NUC domain-containing protein | VRR-NUC domain-containing protein | | uniclust | UniRef100\_A0A849N9L9 | 98.3 | 7.5e-09 | 1.4e-14 | 76.1 | 90 | (23, 144) | 156 | (16, 107) | 137 | VRR-NUC domain-containing protein | VRR-NUC domain-containing protein | | uniclust | UniRef100\_A0A6J5SRX6 | 98.3 | 7.7e-09 | 1.4e-14 | 69.3 | 71 | (76, 155) | 156 | (4, 75) | 78 | Uncharacterized protein | Uncharacterized protein | | uniclust | UniRef100\_A0A2E0R731 | 98.3 | 8.1e-09 | 1.5e-14 | 69.5 | 68 | (85, 155) | 156 | (2, 70) | 80 | VRR-NUC domain-containing protein | VRR-NUC domain-containing protein | | uniclust | UniRef100\_A0A2A4V7W8 | 98.3 | 8e-09 | 1.5e-14 | 76.5 | 99 | (21, 154) | 156 | (2, 110) | 131 | VRR-NUC domain-containing protein | VRR-NUC domain-containing protein | | uniclust | UniRef100\_A0A661MNW6 | 98.3 | 9e-09 | 1.6e-14 | 76.0 | 115 | (23, 154) | 156 | (1, 124) | 141 | VRR-NUC domain-containing protein | VRR-NUC domain-containing protein | | uniclust | UniRef100\_A0A1A8XYT3 | 98.3 | 9.1e-09 | 1.7e-14 | 79.1 | 98 | (21, 153) | 156 | (27, 128) | 187 | VRR-NUC domain-containing protein | VRR-NUC domain-containing protein | | uniclust | UniRef100\_A0A224AIU8 | 98.3 | 8.8e-09 | 1.7e-14 | 77.8 | 103 | (23, 155) | 156 | (30, 137) | 147 | VRR-NUC domain-containing protein | VRR-NUC domain-containing protein | | uniclust | UniRef100\_UPI00204095D6 | 98.3 | 9.3e-09 | 1.7e-14 | 74.9 | 92 | (21, 146) | 156 | (2, 98) | 129 | hypothetical protein | hypothetical protein | | uniclust | UniRef100\_A0A516LPK8 | 98.3 | 9.5e-09 | 1.7e-14 | 73.3 | 59 | (86, 155) | 156 | (50, 109) | 113 | Putative nuclease | Putative nuclease | | uniclust | UniRef100\_A0A6M3T9R2 | 98.3 | 1e-08 | 1.9e-14 | 81.6 | 107 | (21, 149) | 156 | (54, 177) | 212 | VRR-NUC domain endonuclease | VRR-NUC domain endonuclease | | uniclust | UniRef100\_A0A7C3D595 | 98.3 | 1.1e-08 | 2e-14 | 76.8 | 98 | (25, 153) | 156 | (22, 129) | 156 | VRR-NUC domain-containing protein | VRR-NUC domain-containing protein | | uniclust | UniRef100\_A0A516L0Z0 | 98.3 | 1.1e-08 | 2e-14 | 82.5 | 104 | (23, 155) | 156 | (108, 215) | 226 | Chromosomal replication initiator DnaA C-terminal domain-containing protein | Chromosomal replication initiator DnaA C-terminal domain-containing protein | | uniclust | UniRef100\_A0A088C3V3 | 98.3 | 1e-08 | 2.3e-14 | 80.8 | 101 | (22, 155) | 156 | (32, 142) | 163 | Holliday junction resolvase | Holliday junction resolvase | | uniclust | UniRef100\_A0A3D6BPK0 | 98.3 | 1.3e-08 | 2.3e-14 | 74.0 | 102 | (22, 154) | 156 | (18, 121) | 126 | VRR-NUC domain-containing protein | VRR-NUC domain-containing protein | | uniclust | UniRef100\_UPI00068F67A1 | 98.3 | 1.4e-08 | 2.6e-14 | 75.5 | 112 | (23, 154) | 156 | (1, 121) | 147 | VRR-NUC domain-containing protein | VRR-NUC domain-containing protein | | uniclust | UniRef100\_UPI001E6F27A4 | 98.2 | 1.5e-08 | 2.8e-14 | 78.8 | 101 | (21, 143) | 156 | (81, 197) | 203 | putative nuclease | putative nuclease | | uniclust | UniRef100\_A0A5C7LMD1 | 98.2 | 1.5e-08 | 2.9e-14 | 75.5 | 91 | (23, 155) | 156 | (44, 136) | 145 | VRR-NUC domain-containing protein | VRR-NUC domain-containing protein | | uniclust | UniRef100\_A0A011PI35 | 98.2 | 1.6e-08 | 2.9e-14 | 76.7 | 110 | (19, 155) | 156 | (38, 150) | 152 | VRR-NUC domain protein | VRR-NUC domain protein | | uniclust | UniRef100\_A0A1H0RUY3 | 98.2 | 1.6e-08 | 3.3e-14 | 77.3 | 116 | (17, 155) | 156 | (1, 131) | 144 | VRR-NUC domain-containing protein | VRR-NUC domain-containing protein | | uniclust | UniRef100\_A0A0R1XFK4 | 98.2 | 1.7e-08 | 3.3e-14 | 67.2 | 55 | (89, 155) | 156 | (2, 57) | 64 | VRR-NUC domain-containing protein | VRR-NUC domain-containing protein | | uniclust | UniRef100\_A0A1I2LF52 | 98.2 | 1.9e-08 | 3.4e-14 | 74.9 | 110 | (20, 154) | 156 | (20, 134) | 146 | VRR-NUC domain-containing protein | VRR-NUC domain-containing protein | | uniclust | UniRef100\_X0S3S5 | 98.2 | 2e-08 | 3.6e-14 | 65.7 | 59 | (85, 154) | 156 | (2, 61) | 67 | VRR-NUC domain-containing protein (Fragment) | VRR-NUC domain-containing protein (Fragment) | | uniclust | UniRef100\_A0A3C1F314 | 98.2 | 2e-08 | 3.7e-14 | 69.5 | 59 | (85, 151) | 156 | (30, 89) | 93 | VRR-NUC domain-containing protein | VRR-NUC domain-containing protein | | uniclust | UniRef100\_A0A0U4C7T5 | 98.2 | 2.1e-08 | 3.8e-14 | 80.2 | 102 | (19, 153) | 156 | (138, 243) | 248 | VRR-NUC domain-containing protein | VRR-NUC domain-containing protein | | uniclust | UniRef100\_UPI00200E4F20 | 98.2 | 2.1e-08 | 3.8e-14 | 66.9 | 61 | (83, 155) | 156 | (2, 64) | 75 | hypothetical protein | hypothetical protein | | uniclust | UniRef100\_A0A3A8JIG7 | 98.2 | 2.1e-08 | 3.8e-14 | 76.8 | 58 | (85, 154) | 156 | (80, 138) | 178 | VRR-NUC domain-containing protein | VRR-NUC domain-containing protein | | uniclust | UniRef100\_A0A2A7AQM9 | 98.2 | 2.1e-08 | 4e-14 | 75.7 | 58 | (85, 154) | 156 | (80, 139) | 147 | Nuclease | Nuclease | | uniclust | UniRef100\_A0A4U0RCC9 | 98.2 | 2.3e-08 | 4.1e-14 | 79.0 | 105 | (22, 153) | 156 | (91, 200) | 225 | VRR-NUC domain-containing protein | VRR-NUC domain-containing protein | | uniclust | UniRef100\_UPI000A67298D | 98.2 | 2.3e-08 | 4.2e-14 | 78.3 | 79 | (23, 146) | 156 | (2, 83) | 210 | VRR-NUC domain-containing protein | VRR-NUC domain-containing protein | | uniclust | UniRef100\_A0A5C0CFL2 | 98.2 | 2.3e-08 | 4.2e-14 | 65.9 | 60 | (85, 155) | 156 | (6, 67) | 70 | Restriction endonuclease | Restriction endonuclease | | uniclust | UniRef100\_A0A2G2PNU2 | 98.2 | 2.3e-08 | 4.2e-14 | 70.9 | 59 | (85, 154) | 156 | (24, 83) | 107 | VRR-NUC domain-containing protein | VRR-NUC domain-containing protein | | uniclust | UniRef100\_A0A1G0QSC3 | 98.2 | 2.5e-08 | 4.5e-14 | 70.1 | 84 | (22, 149) | 156 | (3, 88) | 101 | VRR-NUC domain-containing protein | VRR-NUC domain-containing protein | | uniclust | UniRef100\_UPI001ABAAAB1 | 98.2 | 2.4e-08 | 4.7e-14 | 82.8 | 60 | (85, 155) | 156 | (29, 89) | 288 | hypothetical protein | hypothetical protein | | uniclust | UniRef100\_UPI0019D2A3A2 | 98.2 | 2.4e-08 | 4.7e-14 | 86.7 | 87 | (23, 155) | 156 | (1, 89) | 393 | VRR-NUC domain-containing protein | VRR-NUC domain-containing protein | | uniclust | UniRef100\_A0A966D9Q4 | 98.2 | 2.8e-08 | 5.1e-14 | 74.6 | 116 | (21, 155) | 156 | (15, 141) | 154 | Uncharacterized protein | Uncharacterized protein | | uniclust | UniRef100\_A0A257PP85 | 98.2 | 2.6e-08 | 5.1e-14 | 78.5 | 108 | (20, 154) | 156 | (44, 155) | 188 | Uncharacterized protein | Uncharacterized protein | | uniclust | UniRef100\_UPI000B1D41EA | 98.2 | 2.9e-08 | 5.4e-14 | 73.3 | 58 | (85, 153) | 156 | (35, 93) | 139 | VRR-NUC domain-containing protein | VRR-NUC domain-containing protein | | uniclust | UniRef100\_A0A7S2FV51 | 98.2 | 2.9e-08 | 5.4e-14 | 81.0 | 108 | (22, 155) | 156 | (17, 128) | 293 | VRR-NUC domain-containing protein | VRR-NUC domain-containing protein | | uniclust | UniRef100\_A0A2N4UWG8 | 98.2 | 3e-08 | 6e-14 | 81.0 | 107 | (19, 154) | 156 | (87, 209) | 238 | VRR-NUC domain-containing protein | VRR-NUC domain-containing protein | | uniclust | UniRef100\_A0A021X9L9 | 98.1 | 3.2e-08 | 6.6e-14 | 79.9 | 94 | (21, 145) | 156 | (57, 162) | 206 | VRR-NUC domain-containing protein | VRR-NUC domain-containing protein | | uniclust | UniRef100\_A0A7Z9G7R6 | 98.1 | 3.6e-08 | 6.7e-14 | 71.3 | 102 | (23, 154) | 156 | (1, 108) | 121 | VRR-NUC domain-containing protein | VRR-NUC domain-containing protein | | uniclust | UniRef100\_R1BRB5 | 98.1 | 3.7e-08 | 6.7e-14 | 83.1 | 111 | (21, 155) | 156 | (5, 120) | 392 | VRR-NUC domain-containing protein | VRR-NUC domain-containing protein | | uniclust | UniRef100\_A0A6G5Y0E8 | 98.1 | 3.7e-08 | 6.8e-14 | 70.8 | 82 | (23, 147) | 156 | (5, 88) | 116 | VRR-NUC domain-containing protein | VRR-NUC domain-containing protein | | uniclust | UniRef100\_A0A1F8QJ77 | 98.1 | 3.9e-08 | 7.2e-14 | 71.9 | 101 | (22, 154) | 156 | (1, 128) | 129 | VRR-NUC domain-containing protein | VRR-NUC domain-containing protein | | uniclust | UniRef100\_A0A4Q5VAH5 | 98.1 | 3.8e-08 | 7.4e-14 | 79.6 | 104 | (22, 154) | 156 | (75, 186) | 221 | Uncharacterized protein | Uncharacterized protein | | uniclust | UniRef100\_UPI001D0CB0F5 | 98.1 | 4.1e-08 | 7.6e-14 | 65.1 | 48 | (108, 155) | 156 | (4, 52) | 72 | hypothetical protein | hypothetical protein | | uniclust | UniRef100\_UPI00227ADC10 | 98.1 | 4.2e-08 | 7.7e-14 | 72.0 | 95 | (40, 155) | 156 | (5, 102) | 132 | VRR-NUC domain-containing protein | VRR-NUC domain-containing protein | | uniclust | UniRef100\_A0A4Z0F6A1 | 98.1 | 4.3e-08 | 7.9e-14 | 68.5 | 61 | (84, 155) | 156 | (31, 92) | 97 | VRR-NUC domain-containing protein | VRR-NUC domain-containing protein | | uniclust | UniRef100\_A0A1Q4FJ35 | 98.1 | 4.4e-08 | 8.1e-14 | 76.4 | 104 | (22, 154) | 156 | (40, 156) | 202 | VRR-NUC domain-containing protein | VRR-NUC domain-containing protein | | uniclust | UniRef100\_A0A0F9G0T4 | 98.1 | 4.5e-08 | 8.3e-14 | 69.5 | 58 | (85, 154) | 156 | (45, 103) | 107 | VRR-NUC domain-containing protein | VRR-NUC domain-containing protein | | uniclust | UniRef100\_A0A965PF78 | 98.1 | 4.6e-08 | 8.5e-14 | 70.8 | 80 | (23, 149) | 156 | (1, 82) | 121 | VRR-NUC domain-containing protein | VRR-NUC domain-containing protein | | uniclust | UniRef100\_A0A522RZJ2 | 98.1 | 4.7e-08 | 8.7e-14 | 76.6 | 104 | (22, 154) | 156 | (33, 148) | 210 | VRR-NUC domain-containing protein | VRR-NUC domain-containing protein | | uniclust | UniRef100\_A0A4Z0MR71 | 98.1 | 4.8e-08 | 8.9e-14 | 63.3 | 48 | (107, 154) | 156 | (7, 55) | 63 | VRR-NUC domain-containing protein | VRR-NUC domain-containing protein | | uniclust | UniRef100\_A0A0X7A620 | 98.1 | 4.3e-08 | 9e-14 | 73.9 | 89 | (21, 155) | 156 | (11, 106) | 124 | VRR-NUC domain-containing protein | VRR-NUC domain-containing protein | | uniclust | UniRef100\_UPI001FA731F7 | 98.1 | 4.9e-08 | 9.1e-14 | 75.1 | 87 | (22, 153) | 156 | (28, 119) | 182 | VRR-NUC domain-containing protein | VRR-NUC domain-containing protein | | uniclust | UniRef100\_A0A7C3U5D4 | 98.1 | 5.1e-08 | 9.4e-14 | 72.0 | 80 | (21, 145) | 156 | (41, 122) | 137 | VRR-NUC domain-containing protein | VRR-NUC domain-containing protein | | uniclust | UniRef100\_A0A8S5M5J1 | 98.1 | 4.9e-08 | 9.5e-14 | 72.3 | 100 | (22, 154) | 156 | (3, 110) | 122 | Hydrolase | Hydrolase | | uniclust | UniRef100\_UPI001C8DF2A0 | 98.1 | 5.3e-08 | 9.8e-14 | 75.3 | 96 | (11, 153) | 156 | (83, 180) | 189 | VRR-NUC domain-containing protein | VRR-NUC domain-containing protein | | uniclust | UniRef100\_A0A7K1CW68 | 98.1 | 5.7e-08 | 1.1e-13 | 70.2 | 82 | (28, 144) | 156 | (10, 93) | 119 | VRR-NUC domain-containing protein | VRR-NUC domain-containing protein | | uniclust | UniRef100\_A0A6J5LHN5 | 98.1 | 6.1e-08 | 1.1e-13 | 63.4 | 60 | (86, 155) | 156 | (2, 63) | 66 | VRR-NUC domain containing protein | VRR-NUC domain containing protein | | uniclust | UniRef100\_A0A965GSE7 | 98.1 | 6.4e-08 | 1.2e-13 | 70.7 | 78 | (23, 145) | 156 | (2, 81) | 127 | Uncharacterized protein | Uncharacterized protein | | pdb70 | 4QBL\_B | 99.7 | 1.5e-21 | 1.5e-25 | 145.1 | 116 | (20, 155) | 156 | (2, 128) | 145 | VRR-NUC | 4QBL\_B VRR-NUC Nuclease, HYDROLASE HET: MSE | | pdb70 | 4QBL\_E | 99.7 | 1.5e-21 | 1.5e-25 | 145.1 | 116 | (20, 155) | 156 | (2, 128) | 145 | VRR-NUC | 4QBL\_E VRR-NUC Nuclease, HYDROLASE | | pdb70 | 4QBN\_A | 99.4 | 7.9e-18 | 7.8e-22 | 115.0 | 88 | (22, 155) | 156 | (1, 91) | 93 | Uncharacterized protein | 4QBN\_A Uncharacterized protein Nuclease, HYDROLASE HET: SO4 | | pdb70 | 4QBO\_A | 99.4 | 5.1e-17 | 4.9e-21 | 111.2 | 85 | (22, 155) | 156 | (3, 88) | 92 | Putative uncharacterized protein | 4QBO\_A Putative uncharacterized protein nuclease, HYDROLASE | | pdb70 | 4R8A\_A | 98.8 | 6.4e-13 | 6.2e-17 | 115.8 | 65 | (76, 150) | 156 | (493, 558) | 559 | Uncharacterized protein, DNA | 4R8A\_A Uncharacterized protein, DNA DNA binding, metal binding nuclease | | pdb70 | 5Y7Q\_A | 98.8 | 7.7e-13 | 7.3e-17 | 115.9 | 66 | (75, 150) | 156 | (513, 579) | 580 | Fanconi-associated nuclease 1 homolog/DNA Complex | 5Y7Q\_A Fanconi-associated nuclease 1 homolog/DNA Complex Nuclease, HYDROLASE-DNA complex | | pdb70 | 4REC\_A | 98.7 | 1.7e-12 | 1.7e-16 | 114.4 | 66 | (76, 151) | 156 | (576, 642) | 647 | Fanconi-associated nuclease 1 (E.C.3.1.21.-, 3.1.4.1) | 4REC\_A Fanconi-associated nuclease 1 (E.C.3.1.21.-, 3.1.4.1) HJC, TPR, SAP, structure specific | | pdb70 | 4RY3\_A | 98.7 | 1.9e-12 | 1.8e-16 | 114.2 | 66 | (76, 151) | 156 | (576, 642) | 648 | Fanconi-associated nuclease 1 (E.C.3.1.21.-, 3.1.4.1) | 4RY3\_A Fanconi-associated nuclease 1 (E.C.3.1.21.-, 3.1.4.1) Endonuclease 5'-3'exonulease, FANCD2, FAN1, HYDROLASE | | pdb70 | 4RIC\_B | 98.6 | 6.9e-12 | 6.7e-16 | 110.1 | 61 | (77, 147) | 156 | (569, 630) | 631 | Fanconi-associated nuclease 1 (E.C.3.1.21.-, 3.1.4.1)/dna | 4RIC\_B Fanconi-associated nuclease 1 (E.C.3.1.21.-, 3.1.4.1)/dna nuclease, hydrolase-dna complex | |
| Top keywords  (threshold 1.00e-03 (evalue)) | **VRR\_NUC, domain\_containing, Nuclease, Fragment, containing, hypothetical, Endonuclease, Putative, HYDROLASE, DNA** |
| Output files | ../../similar\_sequences/48\_FANPEZAQ\_CDS\_0048\_merged.svg ../../similar\_sequences/48\_FANPEZAQ\_CDS\_0048\_pdb70.a3m ../../similar\_sequences/48\_FANPEZAQ\_CDS\_0048\_pdb70.hhr ../../similar\_sequences/48\_FANPEZAQ\_CDS\_0048\_uniclust.a3m ../../similar\_sequences/48\_FANPEZAQ\_CDS\_0048\_uniclust.hhr |

#### Structure prediction (AlphaFold)2

|  |  |
| --- | --- |
| Stats | xml version="1.0" encoding="utf-8" standalone="no"?       2024-09-02T21:09:45.365222 image/svg+xml   Matplotlib v3.7.2, https://matplotlib.org/ |
| Predicted structure | **NGL Viewer Controls:**  - Center: *Left-Click* - Rotate: *Left-Click + Drag* - Translate: *Right-Click + Drag* - Zoom: *Shift + Left-Click + Drag* |
| Output files | ../../predicted\_structures/48\_FANPEZAQ\_CDS\_0048/features.pkl ../../predicted\_structures/48\_FANPEZAQ\_CDS\_0048/ranked\_0.pdb ../../predicted\_structures/48\_FANPEZAQ\_CDS\_0048/ranked\_0\_plots.svg ../../predicted\_structures/48\_FANPEZAQ\_CDS\_0048/result\_model\_1\_ptm\_pred\_0.pkl |

#### Structure similarity search results (Foldseek)3

|  |  |
| --- | --- |
| Structure databases searched | Pdb, Afdb-proteome, Afdb-uniprot50 |
| Results, scheme(s)  (Top layers only, threshold 1.00e-02 (evalue)) | xml version="1.0" encoding="utf-8" standalone="no"?       2024-09-02T21:11:23.045883 image/svg+xml   Matplotlib v3.7.2, https://matplotlib.org/ |
| Results, table  (threshold 1.00e-02 (evalue)) | | db | id | prob | evalue | bits | fident | alnlen | mismatch | gapopen | qstart | qend | tstart | tend | name | description | | --- | --- | --- | --- | --- | --- | --- | --- | --- | --- | --- | --- | --- | --- | --- | | pdb | 1Y1O\_D | 0.975 | 0.002521 | 77 | 0.105 | 151 | 92 | 8 | 20 | 154 | 1 | 124 | Penicillin-binding protein-related factor A | Penicillin-binding protein-related factor A | | afdb-uniprot50 | AF-A0A2W5MTV2-F1-MODEL\_V4 | 1.0 | 7.381e-17 | 592 | 0.526 | 131 | 53 | 2 | 25 | 154 | 3 | 125 | VRR-NUC domain-containing protein | VRR-NUC domain-containing protein | | afdb-uniprot50 | AF-A0A7W3KP85-F1-MODEL\_V4 | 1.0 | 3.895e-14 | 490 | 0.402 | 134 | 76 | 4 | 25 | 156 | 3 | 134 | VRR-NUC domain-containing protein | VRR-NUC domain-containing protein | | afdb-uniprot50 | AF-A0A560BMV8-F1-MODEL\_V4 | 1.0 | 9.269e-14 | 466 | 0.39 | 133 | 77 | 3 | 25 | 154 | 5 | 136 | VRR-NUC domain-containing protein | VRR-NUC domain-containing protein | | afdb-uniprot50 | AF-I8TQK1-F1-MODEL\_V4 | 1.0 | 4.593e-13 | 446 | 0.428 | 133 | 65 | 3 | 25 | 154 | 22 | 146 | VRR-NUC domain-containing protein | VRR-NUC domain-containing protein | | afdb-uniprot50 | AF-A0A1B1I055-F1-MODEL\_V4 | 1.0 | 1.863e-12 | 434 | 0.393 | 132 | 68 | 3 | 25 | 153 | 5 | 127 | Nuclease | Nuclease | | afdb-uniprot50 | AF-A0A853YK68-F1-MODEL\_V4 | 1.0 | 5.997e-13 | 414 | 0.388 | 126 | 73 | 3 | 29 | 154 | 4 | 125 | Uncharacterized protein | Uncharacterized protein | | afdb-uniprot50 | AF-A0A4P5NU28-F1-MODEL\_V4 | 1.0 | 8.081e-12 | 409 | 0.36 | 133 | 73 | 4 | 25 | 154 | 3 | 126 | VRR-NUC domain-containing protein | VRR-NUC domain-containing protein | | afdb-uniprot50 | AF-A0A0D6PDS2-F1-MODEL\_V4 | 1.0 | 9.871e-12 | 399 | 0.333 | 135 | 78 | 4 | 25 | 156 | 4 | 129 | Uncharacterized protein | Uncharacterized protein | | afdb-uniprot50 | AF-A0A1I4Q9V7-F1-MODEL\_V4 | 1.0 | 3.396e-12 | 398 | 0.416 | 132 | 66 | 3 | 25 | 153 | 6 | 129 | VRR-NUC domain-containing protein | VRR-NUC domain-containing protein | | afdb-uniprot50 | AF-A0A7X6FPB3-F1-MODEL\_V4 | 1.0 | 2.869e-11 | 398 | 0.31 | 129 | 86 | 3 | 23 | 149 | 2 | 129 | VRR-NUC domain-containing protein | VRR-NUC domain-containing protein | | afdb-uniprot50 | AF-A0A662MG58-F1-MODEL\_V4 | 1.0 | 1.473e-11 | 381 | 0.33 | 133 | 85 | 4 | 25 | 155 | 3 | 133 | Uncharacterized protein | Uncharacterized protein | | afdb-uniprot50 | AF-A0A2G2PN72-F1-MODEL\_V4 | 1.0 | 1.799e-11 | 368 | 0.326 | 141 | 82 | 4 | 25 | 153 | 3 | 142 | Uncharacterized protein | Uncharacterized protein | | afdb-uniprot50 | AF-A0A424NXD6-F1-MODEL\_V4 | 1.0 | 2.684e-11 | 365 | 0.292 | 157 | 80 | 5 | 1 | 154 | 1 | 129 | VRR-NUC domain-containing protein | VRR-NUC domain-containing protein | | afdb-uniprot50 | AF-A0A839H9H4-F1-MODEL\_V4 | 1.0 | 2.424e-10 | 355 | 0.385 | 127 | 68 | 3 | 25 | 149 | 5 | 123 | VRR-NUC domain-containing protein | VRR-NUC domain-containing protein | | afdb-uniprot50 | AF-A0A1S1SLT7-F1-MODEL\_V4 | 1.0 | 4.891e-11 | 355 | 0.403 | 129 | 68 | 3 | 23 | 149 | 2 | 123 | Uncharacterized protein | Uncharacterized protein | | afdb-uniprot50 | AF-A0A838BWW6-F1-MODEL\_V4 | 1.0 | 1.574e-11 | 348 | 0.398 | 128 | 72 | 5 | 25 | 148 | 2 | 128 | Uncharacterized protein | Uncharacterized protein | | afdb-uniprot50 | AF-A0A1H3DQD1-F1-MODEL\_V4 | 1.0 | 3.866e-10 | 344 | 0.338 | 130 | 75 | 4 | 25 | 152 | 3 | 123 | VRR-NUC domain-containing protein | VRR-NUC domain-containing protein | | afdb-uniprot50 | AF-A0A149SVC6-F1-MODEL\_V4 | 1.0 | 6.801e-09 | 305 | 0.478 | 92 | 47 | 1 | 63 | 153 | 1 | 92 | VRR-NUC domain-containing protein | VRR-NUC domain-containing protein | | afdb-uniprot50 | AF-A0A1L7AG69-F1-MODEL\_V4 | 1.0 | 1.124e-09 | 304 | 0.346 | 130 | 69 | 4 | 28 | 153 | 4 | 121 | Uncharacterized protein | Uncharacterized protein | | afdb-uniprot50 | AF-A0A836VDS1-F1-MODEL\_V4 | 1.0 | 4.873e-09 | 302 | 0.284 | 137 | 83 | 4 | 25 | 155 | 3 | 130 | Uncharacterized protein | Uncharacterized protein | | afdb-uniprot50 | AF-A0A4P5VJF4-F1-MODEL\_V4 | 1.0 | 7.27e-09 | 285 | 0.286 | 136 | 71 | 4 | 25 | 154 | 8 | 123 | Uncharacterized protein | Uncharacterized protein | | afdb-uniprot50 | AF-A0A5C7QIV6-F1-MODEL\_V4 | 1.0 | 6.362e-09 | 275 | 0.22 | 159 | 107 | 4 | 1 | 156 | 21 | 165 | VRR-NUC domain-containing protein | VRR-NUC domain-containing protein | | afdb-uniprot50 | AF-A0A679JRI6-F1-MODEL\_V4 | 1.0 | 3.974e-07 | 223 | 0.421 | 102 | 54 | 3 | 56 | 153 | 4 | 104 | VRR-NUC domain-containing protein | VRR-NUC domain-containing protein | |
| Top keywords  (threshold 1.00e-02 (evalue)) | **VRR\_NUC, domain\_containing, Penicillin\_binding, protein\_related, factor, A, Nuclease** |
| Output files | ../../similar\_structures/48\_FANPEZAQ\_CDS\_0048\_afdb-proteome\_foldseek.tsv ../../similar\_structures/48\_FANPEZAQ\_CDS\_0048\_afdb-uniprot50\_foldseek.tsv ../../similar\_structures/48\_FANPEZAQ\_CDS\_0048\_merged.svg ../../similar\_structures/48\_FANPEZAQ\_CDS\_0048\_pdb\_foldseek.tsv |

  
  
  

Return to summary | Go to previous | Go to next

  


---

**Sequence/structure alignments coloring**  
Each object in the alignment figures is colored according to its E-value following this color coding:

1e-100
10

**References:**  
1) Steinegger M, Meier M, Mirdita M, Vöhringer H, Haunsberger S J, and Söding J (2019) HH-suite3 for fast remote homology detection and deep protein annotation, BMC Bioinformatics, 473. doi: 10.1186/s12859-019-3019-7  
2) Jumper J, Evans R, Pritzel A, ..., Hassabis D (2021) Highly accurate protein structure prediction with AlphaFold, Nature, 596. doi: 10.1038/s41586-021-03819-2  
3) van Kempen M, Kim S, Tumescheit C, Mirdita M, Lee J, Gilchrist CLM, Söding J, and Steinegger M (2023) Fast and accurate protein structure search with Foldseek. Nature Biotechnology. doi: 10.1038/s41587-023-01773-0
