# Supplementary material for: Completing the BASEL phage collection to unlock hidden diversity for systematic exploration of phage–host interactions
Source: PLoS Biol. 2025 Apr 7;23(4):e3003063. doi: 10.1371/journal.pbio.3003063 (PMC11990801; doi:10.1371/journal.pbio.3003063)
Supplement: S2 Data — (ZIP) [file pbio.3003063.s009.zip › entries/5.html]

FANPEZAQ\_CDS\_0005


Return to summary | Go to previous | Go to next

|  |  |
| --- | --- |
| FANPEZAQ\_CDS\_0005 Page creation date: 02 Sep 2024, 12:00  Project folder: n/a  Input sequences file: Escherichia\_virus\_HeidiAbel.gb | capsid phage major hk97 protease prohead head peptidase fragment putative u35 caudovirus bacteriophage and packaging domain\_containing virus gp36 gpt phage\_related capsid\_like phi\_c31 maturation tigr01554 mu predicted prophage structural mu\_like\_gpt particle mu\_like maritimacin serine gp5 virus\_like 10a encapsulin u37 hypothetical |

### Sequence information

|  |  |
| --- | --- |
| Name | FANPEZAQ\_CDS\_0005  05\_FANPEZAQ\_CDS\_0005 (pipeline id) |
| Imported annotations | Escherichia\_virus\_HeidiAbel Bas97 |
| Protein sequence | MQKNPLNSIIGAEFTRKSDSKTLPDFNKGGLVRTMEVRKFDTEKRTVELAFSSEIEVERW YGIEILDHSPESVRLDRLRDGGALLVDHDWTDQVGVVESVSIDADRRGRAVVRFGRSARA DEIFQDIVDGIRKHVSVGYRVLAAKLQETRDEWQDVYRITEWEPYEISIVSVPADASVGV GRAFDKPQEEQRGSKSDTAGTVEVPAPTNIASEGIRMEKILRDAAGNLVRAMVDEKGNIT QVLEMLERAGDDARNAEQRGREAEKARVRSITEMGEKYNARDLALKAIGEGTTAEDFQRS LLDHLNKPKDPAQPADGTRSGSKPLSEMPSSVIGMSDKDIRRYSIFNVVRALANPNDAAA QRAAAFEIECSHAAQQQYGRSAKGILIPDDVLRAFNAGGAPNTPAGAQTGSNLVGTDFMA SSFIDLLRPRTTIMRLARTMGGLVGNVEIPKQTGGATAYWIGEGDDATETTPTIGQLGLS PKTVAAYTDITRRLLMQSTPDAEAIVRGDLVAAIAQAIDYAGYYGTGTSNQPLGLANYTG INAVDFAADNPTYAELVQMETEIAADNADVNNMAYVLNARGRGAAKTTPKFASGSSVADA GVIWEPGNTLNGYRAEVTNQVQDGDVFFGNFADLVVGMWGGLDMTVDPYSLSKSGGLRIV VFQDVDFAVRRVESFALGRNAE |
| Number of residues | 682 |
| Molecular weight (Da) | 73931.80 |
| Output files | ../../query\_sequences/05\_FANPEZAQ\_CDS\_0005.fasta |

### Putative domain architecture and protein family

#### Search results (HHblits)1

|  |  |
| --- | --- |
| Domain family databases searched | Pfam, Ncbi-cd, Cath, Phrogs |
| Results, scheme(s)  (Top layers only; threshold 1.00e-03 (evalue)) | xml version="1.0" encoding="utf-8" standalone="no"?       2024-09-02T21:08:12.828277 image/svg+xml   Matplotlib v3.7.2, https://matplotlib.org/ |
| Results, table  (E-value ≤ 1.00e-03 (evalue)) | | db | id | prob | evalue | pvalue | score | cols | query | query\_len | template | template\_len | name | description | | --- | --- | --- | --- | --- | --- | --- | --- | --- | --- | --- | --- | --- | | pfam | PF04586 | 99.3 | 4e-17 | 8e-21 | 150.7 | 143 | (34, 180) | 682 | (2, 153) | 164 | Peptidase\_S78 | Caudovirus prohead serine protease | | pfam | PF05065 | 99.1 | 3.4e-15 | 7.2e-19 | 145.8 | 255 | (416, 680) | 682 | (6, 275) | 275 | Phage\_capsid | Phage capsid family | | pfam | PF19821 | 98.9 | 1.5e-13 | 2.9e-17 | 140.8 | 266 | (416, 682) | 682 | (8, 342) | 342 | Phage\_capsid\_2 | Phage capsid protein | | pfam | PF14550 | 98.9 | 1.9e-13 | 3.7e-17 | 119.4 | 99 | (36, 143) | 682 | (4, 121) | 123 | Peptidase\_S78\_2 | Putative phage serine protease XkdF | | pfam | PF06673 | 98.6 | 7.3e-12 | 1.4e-15 | 128.4 | 212 | (460, 681) | 682 | (129, 345) | 347 | L\_lactis\_ph-MCP | Lactococcus lactis bacteriophage major capsid protein | | pfam | PF05125 | 98.4 | 5e-11 | 1e-14 | 120.0 | 261 | (416, 680) | 682 | (25, 324) | 325 | Phage\_cap\_P2 | Phage major capsid protein, P2 family | | pfam | PF13252 | 97.4 | 4.4e-08 | 9e-12 | 96.3 | 262 | (412, 681) | 682 | (6, 310) | 313 | DUF4043 | Protein of unknown function (DUF4043) | | pfam | PF20036 | 97.3 | 1.1e-07 | 2.2e-11 | 93.8 | 218 | (416, 641) | 682 | (7, 243) | 307 | Gp13-like | Major capsid protein 13-like | | pfam | PF11651 | 97.2 | 1.4e-07 | 2.8e-11 | 97.3 | 196 | (416, 623) | 682 | (10, 218) | 421 | P22\_CoatProtein | P22 coat protein - gene protein 5 | | pfam | PF09950 | 97.0 | 5.7e-07 | 1.2e-10 | 84.9 | 207 | (463, 680) | 682 | (10, 244) | 245 | DUF2184 | Uncharacterized protein conserved in bacteria (DUF2184) | | pfam | PF04454 | 96.5 | 4.6e-06 | 9.4e-10 | 78.2 | 181 | (469, 665) | 682 | (62, 250) | 251 | Linocin\_M18 | Encapsulating protein for peroxidase | | pfam | PF19307 | 96.3 | 9.2e-06 | 1.8e-09 | 77.1 | 190 | (477, 680) | 682 | (40, 249) | 254 | PCLP | Phage capsid-like protein | | cath | 1ohgA01 | 99.1 | 2.6e-15 | 5.5e-19 | 122.4 | 109 | (432, 541) | 682 | (1, 110) | 146 | Major capsid protein | CATHCODE: 3.30.2400.10 NAME: Major capsid protein. Chain: a, b, c, d, e, f, g. Synonym: gp5, head protein. Engineered: yes. Other\_details: cleaved form, residues 104-385 SOURCE: Bacteriophage hk97. Organism\_taxid: 37554. Expressed in: escherichia coli. Expression\_system\_taxid: 469008. CLASS: Alpha Beta, ARCH: 2-Layer Sandwich, TOPOL: Major capsid protein gp5 fold, HOMOL: Major capsid protein gp5 | | phrogs | 617 | 100.0 | 9.7e-57 | 1.2e-60 | 480.6 | 482 | (51, 674) | 682 | (2, 551) | 551 | major head protein | major head protein; Category: head and packaging; p410871 VI\_08691 | | phrogs | 2263 | 100.0 | 2.7e-35 | 3.4e-39 | 317.4 | 443 | (37, 680) | 682 | (38, 509) | 518 | major head protein | major head protein; Category: head and packaging; p384151 VI\_05178 | | phrogs | 4523 | 100.0 | 6.3e-35 | 7.5e-39 | 315.5 | 451 | (29, 526) | 682 | (22, 501) | 667 | major head and protease protein | major head and protease protein; Category: head and packaging; p99396 VI\_01094 | | phrogs | 10 | 99.8 | 3.4e-25 | 4.7e-29 | 238.8 | 263 | (404, 681) | 682 | (125, 405) | 413 | major head protein | major head protein; Category: head and packaging; p205037 VI\_04338 | | phrogs | 80 | 99.8 | 1.3e-24 | 1.6e-28 | 208.2 | 136 | (42, 187) | 682 | (15, 161) | 187 | head maturation protease | head maturation protease; Category: head and packaging; MF678788\_p6 | | phrogs | 3244 | 99.7 | 2.9e-21 | 3.4e-25 | 186.8 | 160 | (22, 188) | 682 | (21, 199) | 279 | head maturation protease | head maturation protease; Category: head and packaging; MF140424\_p7 | | phrogs | 267 | 99.7 | 2.9e-21 | 3.8e-25 | 201.7 | 265 | (406, 680) | 682 | (18, 343) | 347 | major head protein | major head protein; Category: head and packaging; NC\_031123\_p36 | | phrogs | 9644 | 99.3 | 1e-16 | 1.1e-20 | 151.6 | 152 | (23, 186) | 682 | (137, 309) | 336 | head maturation protease | head maturation protease; Category: head and packaging; p263721 VI\_00383 | | phrogs | 13042 | 99.2 | 5.5e-16 | 6.2e-20 | 147.5 | 143 | (38, 185) | 682 | (16, 174) | 367 | major head protein | major head protein; Category: head and packaging; JX486088\_p23 | | phrogs | 11321 | 99.2 | 2.5e-15 | 2.9e-19 | 146.8 | 127 | (50, 182) | 682 | (7, 145) | 353 | NA | NA; Category: unknown function; p210731 VI\_06219 | | phrogs | 3270 | 99.1 | 8.6e-15 | 1e-18 | 150.7 | 193 | (471, 679) | 682 | (269, 473) | 481 | head maturation protease | head maturation protease; Category: head and packaging; NC\_001629\_p12 | | phrogs | 37668 | 98.9 | 1.1e-13 | 1.2e-17 | 125.3 | 140 | (39, 184) | 682 | (15, 167) | 313 | head maturation protease | head maturation protease; Category: head and packaging; p13833 VI\_00993 | | phrogs | 9427 | 98.6 | 4.6e-12 | 5.3e-16 | 122.9 | 152 | (27, 186) | 682 | (11, 171) | 427 | head maturation protease | head maturation protease; Category: head and packaging; MF347638\_p47 | | phrogs | 7053 | 98.4 | 4.3e-11 | 4.8e-15 | 110.9 | 142 | (40, 185) | 682 | (32, 191) | 367 | head maturation protease | head maturation protease; Category: head and packaging; JF937090\_p9 | | phrogs | 15928 | 97.9 | 5.4e-09 | 6.3e-13 | 88.3 | 117 | (552, 679) | 682 | (2, 130) | 141 | major head protein | major head protein; Category: head and packaging; p268325 VI\_08160 | | phrogs | 7015 | 97.7 | 2.1e-08 | 2.4e-12 | 88.3 | 131 | (40, 187) | 682 | (11, 143) | 230 | head maturation protease | head maturation protease; Category: head and packaging; KX578043\_p18 | | phrogs | 544 | 97.3 | 1.4e-07 | 1.7e-11 | 92.0 | 247 | (409, 678) | 682 | (8, 264) | 272 | major head protein | major head protein; Category: head and packaging; p136878 VI\_00984 | | phrogs | 1028 | 96.8 | 2e-06 | 2.4e-10 | 80.5 | 133 | (41, 186) | 682 | (24, 165) | 257 | head maturation protease | head maturation protease; Category: head and packaging; MF001365\_p35 | | phrogs | 13361 | 96.5 | 6.3e-06 | 7.1e-10 | 62.5 | 70 | (37, 114) | 682 | (12, 81) | 101 | NA | NA; Category: unknown function; p46871 VI\_06362 | | phrogs | 6538 | 96.5 | 6.6e-06 | 7.3e-10 | 69.7 | 173 | (495, 679) | 682 | (2, 203) | 213 | NA | NA; Category: unknown function; p252690 VI\_08262 | | phrogs | 3437 | 96.5 | 8e-06 | 9.8e-10 | 79.0 | 257 | (416, 679) | 682 | (11, 280) | 284 | major head protein | major head protein; Category: head and packaging; NC\_020481\_p9 | | phrogs | 17162 | 96.1 | 2.8e-05 | 3.1e-09 | 58.1 | 73 | (608, 681) | 682 | (5, 83) | 84 | NA | NA; Category: unknown function; p331326 VI\_04001 | | phrogs | 21715 | 96.0 | 3.5e-05 | 4e-09 | 71.0 | 127 | (41, 186) | 682 | (15, 145) | 385 | NA | NA; Category: unknown function; p212986 VI\_01051 | | phrogs | 1650 | 96.0 | 4e-05 | 4.9e-09 | 74.3 | 210 | (460, 679) | 682 | (78, 296) | 298 | major head protein | major head protein; Category: head and packaging; NC\_026606\_p9 | | phrogs | 30146 | 95.7 | 9.5e-05 | 1.1e-08 | 51.3 | 53 | (37, 97) | 682 | (12, 64) | 65 | NA | NA; Category: unknown function; p428492 VI\_08052 | | phrogs | 3922 | 95.3 | 0.00017 | 2.1e-08 | 70.9 | 255 | (408, 679) | 682 | (70, 337) | 340 | major head protein | major head protein; Category: head and packaging; p276403 VI\_00011 | | phrogs | 30620 | 94.2 | 0.00099 | 1.1e-07 | 55.4 | 99 | (427, 527) | 682 | (25, 129) | 183 | NA | NA; Category: unknown function; p428682 VI\_05331 | |
| Top keywords  (threshold 1.00e-03 (evalue)) | **head, and, packaging, major, protease, maturation, capsid, Phage, gp5, serine** |
| Output files | ../../domain\_architecture/05\_FANPEZAQ\_CDS\_0005\_cath.hhr ../../domain\_architecture/05\_FANPEZAQ\_CDS\_0005\_merged.svg ../../domain\_architecture/05\_FANPEZAQ\_CDS\_0005\_ncbi-cd.hhr ../../domain\_architecture/05\_FANPEZAQ\_CDS\_0005\_pfam.hhr ../../domain\_architecture/05\_FANPEZAQ\_CDS\_0005\_phrogs.hhr |

### Identical protein sequences/structures

#### Search results

|  |  |
| --- | --- |
| Protein sequence databases searched | Pdb, Swissprot, Refseq |
| Identical proteins found | -- |
| Top keywords | -- |
| Output files | -- |

### Similar protein sequences/structures

#### Sequence similarity search results (HHblits)1

|  |  |
| --- | --- |
| Sequence databases searched | Uniclust, Pdb70 |
| Results, scheme(s)  (Top layers only, threshold 1.00e-03 (evalue)) | xml version="1.0" encoding="utf-8" standalone="no"?       2024-09-02T21:08:30.417592 image/svg+xml   Matplotlib v3.7.2, https://matplotlib.org/ |
| Results, table(s)  (threshold 1.00e-03 (evalue)) | | db | id | prob | evalue | pvalue | score | cols | query | query\_len | template | template\_len | name | description | | --- | --- | --- | --- | --- | --- | --- | --- | --- | --- | --- | --- | --- | | uniclust | UniRef100\_A0A060GZS1 | 100.0 | 2.5e-93 | 5.1e-99 | 772.2 | 604 | (31, 681) | 682 | (136, 743) | 757 | Peptidase U35 | Peptidase U35 | | uniclust | UniRef100\_A0A0G3G1E5 | 100.0 | 1.4e-88 | 3e-94 | 729.5 | 580 | (31, 681) | 682 | (53, 650) | 662 | Phage major capsid protein | Phage major capsid protein | | uniclust | UniRef100\_A0A0K6I524 | 100.0 | 7.3e-88 | 1.4e-93 | 710.2 | 575 | (40, 681) | 682 | (118, 720) | 733 | Phage major capsid protein, HK97 family | Phage major capsid protein, HK97 family | | uniclust | UniRef100\_A0A089QSP9 | 100.0 | 1.5e-84 | 2.9e-90 | 675.9 | 557 | (31, 681) | 682 | (41, 615) | 618 | Phage protein | Phage protein | | uniclust | UniRef100\_A0A2E1P3K3 | 100.0 | 3e-84 | 5.9e-90 | 676.7 | 568 | (43, 681) | 682 | (273, 845) | 848 | Phage major capsid protein | Phage major capsid protein | | uniclust | UniRef100\_A0A2D5VNT2 | 100.0 | 2.3e-57 | 4.4e-63 | 444.2 | 458 | (31, 537) | 682 | (33, 493) | 499 | Phage major capsid protein (Fragment) | Phage major capsid protein (Fragment) | | uniclust | UniRef100\_A0A164AHR0 | 100.0 | 3.1e-57 | 5.8e-63 | 440.3 | 502 | (32, 681) | 682 | (10, 534) | 537 | Phage capsid family protein | Phage capsid family protein | | uniclust | UniRef100\_A0A031JPI0 | 100.0 | 4.5e-57 | 8.9e-63 | 471.0 | 479 | (42, 681) | 682 | (85, 579) | 594 | Phage major capsid protein, HK97 | Phage major capsid protein, HK97 | | uniclust | UniRef100\_A0A071LTJ4 | 100.0 | 5.3e-57 | 1.1e-62 | 471.4 | 573 | (31, 680) | 682 | (13, 616) | 622 | Phage major capsid protein | Phage major capsid protein | | uniclust | UniRef100\_A0A193LMW5 | 100.0 | 8.6e-57 | 1.7e-62 | 465.9 | 605 | (31, 679) | 682 | (6, 663) | 682 | Peptidase | Peptidase | | uniclust | UniRef100\_A0A2E7BJW1 | 100.0 | 4.3e-55 | 8.2e-61 | 440.5 | 598 | (32, 682) | 682 | (70, 711) | 720 | Phage major capsid protein | Phage major capsid protein | | uniclust | UniRef100\_A0A1G0FL18 | 100.0 | 3.6e-53 | 7.2e-59 | 425.8 | 336 | (331, 681) | 682 | (88, 442) | 445 | Capsid protein | Capsid protein | | uniclust | UniRef100\_A0A1G0RDU6 | 100.0 | 5.5e-53 | 1e-58 | 422.4 | 605 | (31, 681) | 682 | (18, 643) | 644 | Phage major capsid protein | Phage major capsid protein | | uniclust | UniRef100\_A0A075KI09 | 100.0 | 1.1e-51 | 2.1e-57 | 438.8 | 589 | (31, 679) | 682 | (127, 757) | 768 | Peptidase U35 phage prohead HK97 | Peptidase U35 phage prohead HK97 | | uniclust | UniRef100\_A0A031HTD4 | 100.0 | 1.9e-51 | 3.7e-57 | 434.2 | 574 | (31, 682) | 682 | (28, 669) | 717 | HK97 family phage prohead protease | HK97 family phage prohead protease | | uniclust | UniRef100\_A0A2D7GUF3 | 100.0 | 2.2e-51 | 4.2e-57 | 402.3 | 369 | (31, 451) | 682 | (25, 399) | 399 | Capsid protein (Fragment) | Capsid protein (Fragment) | | uniclust | UniRef100\_A0A938SX03 | 100.0 | 1.3e-50 | 2.5e-56 | 400.5 | 580 | (33, 679) | 682 | (245, 864) | 869 | Phage major capsid protein | Phage major capsid protein | | uniclust | UniRef100\_A0A011TBF3 | 100.0 | 7.6e-50 | 1.6e-55 | 444.9 | 599 | (31, 680) | 682 | (119, 773) | 862 | Peptidase S14 | Peptidase S14 | | uniclust | UniRef100\_A0A1V4MJF0 | 100.0 | 1.8e-47 | 3.3e-53 | 382.5 | 523 | (31, 680) | 682 | (15, 571) | 586 | Capsid protein | Capsid protein | | uniclust | UniRef100\_UPI001AC3FFAA | 100.0 | 5.3e-47 | 9.8e-53 | 365.5 | 585 | (31, 681) | 682 | (5, 636) | 639 | phage major capsid protein | phage major capsid protein | | uniclust | UniRef100\_A0A812JEV6 | 100.0 | 8.6e-47 | 1.6e-52 | 404.4 | 614 | (38, 677) | 682 | (1617, 2266) | 4451 | Phage portal protein | Phage portal protein | | uniclust | UniRef100\_A0A4Q8WY63 | 100.0 | 1e-46 | 1.9e-52 | 382.3 | 571 | (31, 671) | 682 | (10, 619) | 635 | HK97 family phage prohead protease (Fragment) | HK97 family phage prohead protease (Fragment) | | uniclust | UniRef100\_A0A081CWY9 | 100.0 | 3.5e-46 | 7.2e-52 | 409.6 | 545 | (32, 677) | 682 | (140, 724) | 749 | Peptidase U35 family protein | Peptidase U35 family protein | | uniclust | UniRef100\_A0A0C5DX41 | 100.0 | 3.4e-45 | 7e-51 | 382.5 | 323 | (341, 681) | 682 | (97, 436) | 446 | Phage major capsid protein | Phage major capsid protein | | uniclust | UniRef100\_A0A934RQD8 | 100.0 | 6.8e-45 | 1.3e-50 | 352.5 | 576 | (45, 681) | 682 | (41, 660) | 665 | Phage major capsid protein | Phage major capsid protein | | uniclust | UniRef100\_A0A0F9KZ70 | 100.0 | 6.8e-45 | 1.4e-50 | 372.3 | 331 | (335, 681) | 682 | (87, 432) | 436 | Phage major capsid protein (Fragment) | Phage major capsid protein (Fragment) | | uniclust | UniRef100\_A0A087ND25 | 100.0 | 1e-44 | 2e-50 | 369.3 | 315 | (342, 681) | 682 | (120, 448) | 459 | Phage-related protein | Phage-related protein | | uniclust | UniRef100\_A0A560FKK8 | 100.0 | 1.2e-43 | 2.3e-49 | 361.2 | 494 | (43, 681) | 682 | (278, 794) | 796 | HK97 family phage prohead protease/HK97 family phage major capsid protein,TIGR01554 | HK97 family phage prohead protease/HK97 family phage major capsid protein,TIGR01554 | | uniclust | UniRef100\_A0A952V6M2 | 100.0 | 4.1e-43 | 7.6e-49 | 347.4 | 548 | (32, 669) | 682 | (19, 610) | 630 | Phage major capsid protein | Phage major capsid protein | | uniclust | UniRef100\_A0A7X4CLK5 | 100.0 | 1.4e-42 | 2.6e-48 | 340.4 | 613 | (42, 680) | 682 | (54, 748) | 750 | Phage major capsid protein | Phage major capsid protein | | uniclust | UniRef100\_A0A0F9DVX4 | 100.0 | 3.4e-42 | 6.7e-48 | 341.9 | 308 | (337, 681) | 682 | (73, 401) | 406 | Phage major capsid protein | Phage major capsid protein | | uniclust | UniRef100\_A0A0Q5PQY2 | 100.0 | 3.9e-42 | 7.5e-48 | 354.6 | 444 | (31, 525) | 682 | (20, 483) | 649 | Uncharacterized protein | Uncharacterized protein | | uniclust | UniRef100\_A0A316SJM9 | 100.0 | 4.2e-42 | 8.1e-48 | 349.1 | 574 | (31, 678) | 682 | (24, 631) | 634 | Peptidase | Peptidase | | uniclust | UniRef100\_A0A0F3QFF6 | 100.0 | 6.1e-42 | 1.2e-47 | 340.3 | 465 | (35, 679) | 682 | (14, 494) | 499 | Phage prohead protease, HK97 family | Phage prohead protease, HK97 family | | uniclust | UniRef100\_A0A1S6TP86 | 100.0 | 1.3e-41 | 2.6e-47 | 352.2 | 546 | (39, 680) | 682 | (30, 584) | 594 | Phage capsid protein | Phage capsid protein | | uniclust | UniRef100\_A0A261Q1A3 | 100.0 | 2.6e-41 | 5.2e-47 | 345.2 | 329 | (336, 681) | 682 | (134, 484) | 492 | Phage major capsid protein | Phage major capsid protein | | uniclust | UniRef100\_A0A0F2RQY9 | 100.0 | 2.9e-41 | 5.5e-47 | 345.2 | 586 | (31, 680) | 682 | (18, 649) | 652 | Peptidase U35 | Peptidase U35 | | uniclust | UniRef100\_A0A2U1B6D9 | 100.0 | 1e-40 | 2e-46 | 327.8 | 588 | (31, 679) | 682 | (13, 611) | 612 | HK97 family phage prohead protease/HK97 family phage major capsid protein,TIGR01554 | HK97 family phage prohead protease/HK97 family phage major capsid protein,TIGR01554 | | uniclust | UniRef100\_A0A0A8H0T3 | 100.0 | 3.8e-40 | 7.7e-46 | 333.9 | 270 | (393, 680) | 682 | (119, 399) | 404 | Major capsid protein, HK97 family | Major capsid protein, HK97 family | | uniclust | UniRef100\_A0A1I0J9W7 | 100.0 | 5.1e-40 | 1e-45 | 339.9 | 554 | (39, 680) | 682 | (25, 618) | 620 | Prohead serine protease | Prohead serine protease | | uniclust | UniRef100\_A0A061QK74 | 100.0 | 6.9e-40 | 1.4e-45 | 333.0 | 316 | (337, 681) | 682 | (138, 469) | 482 | Phage major capsid protein, HK97 family | Phage major capsid protein, HK97 family | | uniclust | UniRef100\_A0A0P6VLZ5 | 100.0 | 1.8e-39 | 3.4e-45 | 330.1 | 571 | (32, 672) | 682 | (38, 645) | 654 | Bacteriophage Mu GpT domain-containing protein | Bacteriophage Mu GpT domain-containing protein | | uniclust | UniRef100\_A0A0F9RBZ5 | 100.0 | 1.5e-38 | 3e-44 | 339.4 | 606 | (31, 679) | 682 | (43, 713) | 716 | Bacteriophage Mu GpT domain-containing protein | Bacteriophage Mu GpT domain-containing protein | | uniclust | UniRef100\_A0A087M3M1 | 100.0 | 2.5e-38 | 4.8e-44 | 332.7 | 570 | (47, 681) | 682 | (102, 730) | 739 | Peptidase U35 | Peptidase U35 | | uniclust | UniRef100\_A0A024P7I8 | 100.0 | 3.5e-38 | 7e-44 | 334.0 | 489 | (31, 680) | 682 | (36, 551) | 646 | Phage major capsid protein, HK97 family | Phage major capsid protein, HK97 family | | uniclust | UniRef100\_A0A1G1GKX3 | 100.0 | 8.5e-38 | 1.7e-43 | 310.4 | 324 | (335, 681) | 682 | (41, 370) | 371 | Capsid protein | Capsid protein | | uniclust | UniRef100\_A0A080IWL3 | 100.0 | 2.5e-37 | 4.8e-43 | 307.5 | 502 | (39, 679) | 682 | (13, 547) | 564 | Phage major capsid protein, HK97 family | Phage major capsid protein, HK97 family | | uniclust | UniRef100\_A0A145VQW4 | 100.0 | 4.3e-37 | 8.2e-43 | 288.3 | 269 | (409, 681) | 682 | (23, 293) | 298 | Capsid protein | Capsid protein | | uniclust | UniRef100\_A0A1E3GXJ9 | 100.0 | 5e-37 | 9.3e-43 | 322.2 | 599 | (31, 674) | 682 | (539, 1187) | 1194 | Phage portal protein, lambda family | Phage portal protein, lambda family | | uniclust | UniRef100\_A0A074TEC5 | 100.0 | 8.2e-37 | 1.6e-42 | 314.2 | 274 | (390, 681) | 682 | (157, 443) | 448 | Capsid protein | Capsid protein | | uniclust | UniRef100\_A0A0B6D4D0 | 100.0 | 9.8e-37 | 1.8e-42 | 293.9 | 556 | (43, 679) | 682 | (16, 591) | 597 | Phage major capsid protein, HK97 family | Phage major capsid protein, HK97 family | | uniclust | UniRef100\_A0A929ZEC9 | 100.0 | 1e-36 | 1.9e-42 | 294.9 | 376 | (38, 475) | 682 | (30, 424) | 428 | HK97 family phage prohead protease (Fragment) | HK97 family phage prohead protease (Fragment) | | uniclust | UniRef100\_A0A354UAC1 | 100.0 | 2.1e-36 | 3.9e-42 | 293.5 | 590 | (43, 677) | 682 | (16, 633) | 637 | Phage major capsid protein | Phage major capsid protein | | uniclust | UniRef100\_A0A075TQ00 | 100.0 | 2.7e-36 | 5.6e-42 | 317.4 | 270 | (393, 680) | 682 | (168, 451) | 487 | Capsid protein | Capsid protein | | uniclust | UniRef100\_A0A0F7PPX3 | 100.0 | 5.2e-36 | 9.9e-42 | 301.1 | 555 | (44, 681) | 682 | (26, 640) | 642 | Phage prohead protease, HK97 family/phage major capsid protein, HK97 family | Phage prohead protease, HK97 family/phage major capsid protein, HK97 family | | uniclust | UniRef100\_A0A072NGX0 | 100.0 | 6e-36 | 1.2e-41 | 310.5 | 264 | (406, 680) | 682 | (155, 434) | 442 | Phage major capsid protein, HK97 family | Phage major capsid protein, HK97 family | | uniclust | UniRef100\_A0A2D2C2A3 | 100.0 | 9.7e-36 | 1.9e-41 | 308.5 | 604 | (31, 680) | 682 | (12, 692) | 695 | Bacteriophage Mu GpT domain-containing protein | Bacteriophage Mu GpT domain-containing protein | | uniclust | UniRef100\_A0A3B9KKY0 | 100.0 | 1e-35 | 2.1e-41 | 311.1 | 271 | (393, 680) | 682 | (286, 569) | 572 | Phage major capsid protein | Phage major capsid protein | | uniclust | UniRef100\_A0A101G6Z0 | 100.0 | 2.1e-35 | 4.2e-41 | 302.7 | 271 | (393, 679) | 682 | (109, 400) | 426 | HK97 family phage prohead protease | HK97 family phage prohead protease | | uniclust | UniRef100\_A0A3N5X4U1 | 100.0 | 2.3e-35 | 4.4e-41 | 289.6 | 326 | (336, 681) | 682 | (75, 423) | 432 | Phage major capsid protein (Fragment) | Phage major capsid protein (Fragment) | | uniclust | UniRef100\_A0A2A8V9J7 | 100.0 | 2.6e-35 | 5.2e-41 | 293.8 | 270 | (393, 681) | 682 | (123, 403) | 410 | Phage major capsid protein | Phage major capsid protein | | uniclust | UniRef100\_A0A017H7N1 | 100.0 | 2.7e-35 | 5.4e-41 | 309.7 | 271 | (393, 681) | 682 | (183, 467) | 491 | Phage major capsid protein | Phage major capsid protein | | uniclust | UniRef100\_UPI0022E46B5D | 100.0 | 4.2e-35 | 7.7e-41 | 272.7 | 399 | (31, 475) | 682 | (16, 422) | 423 | HK97 family phage prohead protease | HK97 family phage prohead protease | | uniclust | UniRef100\_A0A094YHB1 | 100.0 | 4.9e-35 | 1e-40 | 311.1 | 269 | (394, 681) | 682 | (203, 485) | 497 | Phage major capsid protein | Phage major capsid protein | | uniclust | UniRef100\_A0A1N6XWD6 | 100.0 | 6.3e-35 | 1.2e-40 | 296.2 | 486 | (41, 674) | 682 | (41, 562) | 581 | Phage prohead protease, HK97 family/phage major capsid protein, HK97 family,TIGR01554 | Phage prohead protease, HK97 family/phage major capsid protein, HK97 family,TIGR01554 | | uniclust | UniRef100\_A0A0F8YXG7 | 100.0 | 1.3e-34 | 2.6e-40 | 300.0 | 269 | (393, 681) | 682 | (131, 424) | 431 | Phage major capsid protein (Fragment) | Phage major capsid protein (Fragment) | | uniclust | UniRef100\_A0A0D9NGH1 | 100.0 | 3.1e-34 | 5.8e-40 | 281.6 | 550 | (33, 680) | 682 | (11, 578) | 580 | Peptidase U35 | Peptidase U35 | | uniclust | UniRef100\_A0A6G6X8M0 | 100.0 | 3.5e-34 | 6.5e-40 | 281.0 | 373 | (270, 679) | 682 | (174, 551) | 555 | Phage major capsid protein | Phage major capsid protein | | uniclust | UniRef100\_A0A076JIU2 | 100.0 | 3.4e-34 | 6.9e-40 | 311.7 | 482 | (31, 680) | 682 | (127, 636) | 646 | Prohead peptidase | Prohead peptidase | | uniclust | UniRef100\_A0A0A1WC95 | 100.0 | 3.7e-34 | 7.3e-40 | 300.4 | 565 | (38, 675) | 682 | (48, 652) | 667 | Putative phage prohead (Fragment) | Putative phage prohead (Fragment) | | uniclust | UniRef100\_A0A074MYL0 | 100.0 | 3.7e-34 | 7.5e-40 | 299.2 | 272 | (393, 682) | 682 | (186, 471) | 476 | Phage major capsid protein | Phage major capsid protein | | uniclust | UniRef100\_A0A158S6T4 | 100.0 | 5.3e-34 | 1e-39 | 278.8 | 314 | (341, 681) | 682 | (145, 470) | 485 | Putative phage-related protein | Putative phage-related protein | | uniclust | UniRef100\_A0A3N2E2B1 | 100.0 | 8.1e-34 | 1.5e-39 | 284.3 | 396 | (261, 681) | 682 | (255, 656) | 667 | ATP-dependent Clp protease proteolytic subunit | ATP-dependent Clp protease proteolytic subunit | | uniclust | UniRef100\_A0A164A2P9 | 100.0 | 1.1e-33 | 2.1e-39 | 287.1 | 323 | (339, 681) | 682 | (92, 429) | 435 | Phage major capsid protein | Phage major capsid protein | | uniclust | UniRef100\_UPI000D2FC543 | 100.0 | 1.4e-33 | 2.6e-39 | 273.1 | 525 | (42, 670) | 682 | (29, 585) | 608 | phage major capsid protein | phage major capsid protein | | uniclust | UniRef100\_A0A023WVI6 | 100.0 | 2.7e-33 | 5.5e-39 | 297.6 | 275 | (393, 682) | 682 | (186, 487) | 580 | HK97 family phage major capsid protein | HK97 family phage major capsid protein | | uniclust | UniRef100\_A0A2M8B3M2 | 100.0 | 4.2e-33 | 7.8e-39 | 268.9 | 455 | (31, 523) | 682 | (10, 508) | 516 | Peptidase U37 (Fragment) | Peptidase U37 (Fragment) | | uniclust | UniRef100\_UPI0009EE22D6 | 100.0 | 4.4e-33 | 8.1e-39 | 265.6 | 461 | (41, 679) | 682 | (23, 515) | 520 | phage major capsid protein | phage major capsid protein | | uniclust | UniRef100\_A0A7V9J303 | 100.0 | 5.8e-33 | 1.1e-38 | 267.4 | 543 | (31, 679) | 682 | (7, 570) | 573 | Phage major capsid protein | Phage major capsid protein | | uniclust | UniRef100\_A0A0F5JCS1 | 100.0 | 6.3e-33 | 1.2e-38 | 274.4 | 313 | (341, 679) | 682 | (90, 407) | 414 | HK97 family phage major capsid protein | HK97 family phage major capsid protein | | uniclust | UniRef100\_A0A0F3IQW8 | 100.0 | 9e-33 | 1.7e-38 | 264.5 | 491 | (45, 681) | 682 | (17, 535) | 539 | Phage capsid protein | Phage capsid protein | | uniclust | UniRef100\_A0A0F9IAP4 | 100.0 | 1.3e-32 | 2.6e-38 | 305.1 | 575 | (45, 681) | 682 | (108, 738) | 750 | Bacteriophage Mu GpT domain-containing protein | Bacteriophage Mu GpT domain-containing protein | | uniclust | UniRef100\_A0A1V5X326 | 100.0 | 1.4e-32 | 2.7e-38 | 292.0 | 563 | (45, 670) | 682 | (92, 717) | 734 | Caudovirus prohead protease | Caudovirus prohead protease | | uniclust | UniRef100\_A0A142Y5E5 | 100.0 | 2.1e-32 | 4.3e-38 | 300.2 | 597 | (41, 677) | 682 | (79, 738) | 745 | Mu-like prophage major head subunit gpT | Mu-like prophage major head subunit gpT | | uniclust | UniRef100\_A0A1C6BMQ2 | 99.9 | 3.5e-32 | 6.9e-38 | 250.2 | 154 | (31, 185) | 682 | (24, 185) | 232 | Phage prohead protease, HK97 family | Phage prohead protease, HK97 family | | uniclust | UniRef100\_A0A127CGL7 | 99.9 | 4e-32 | 7.8e-38 | 282.4 | 535 | (38, 679) | 682 | (21, 592) | 601 | Bacteriophage Mu GpT domain-containing protein | Bacteriophage Mu GpT domain-containing protein | | uniclust | UniRef100\_A0A965MHY9 | 99.9 | 4.9e-32 | 9.1e-38 | 252.5 | 371 | (271, 680) | 682 | (16, 388) | 390 | Phage major capsid protein | Phage major capsid protein | | uniclust | UniRef100\_A0A133NSV4 | 99.9 | 5.4e-32 | 1.1e-37 | 265.9 | 262 | (408, 680) | 682 | (52, 319) | 334 | Phage capsid family protein | Phage capsid family protein | | uniclust | UniRef100\_A0A290XJJ8 | 99.9 | 1.2e-31 | 2.4e-37 | 281.4 | 535 | (37, 680) | 682 | (41, 614) | 618 | Major capsid protein | Major capsid protein | | uniclust | UniRef100\_A0A7I8N2L9 | 99.9 | 1.5e-31 | 2.8e-37 | 245.8 | 340 | (31, 454) | 682 | (3, 348) | 373 | Phage capsid and scaffold | Phage capsid and scaffold | | uniclust | UniRef100\_A0A382Z2D1 | 99.9 | 1.5e-31 | 2.9e-37 | 242.4 | 154 | (31, 185) | 682 | (24, 179) | 205 | Caudovirus prohead protease (Fragment) | Caudovirus prohead protease (Fragment) | | uniclust | UniRef100\_H1CG40 | 99.9 | 2e-31 | 3.7e-37 | 261.3 | 556 | (43, 681) | 682 | (15, 611) | 615 | HK97 family phage prohead protease | HK97 family phage prohead protease | | uniclust | UniRef100\_A0A965HBR8 | 99.9 | 2.5e-31 | 4.8e-37 | 244.2 | 263 | (40, 354) | 682 | (28, 297) | 314 | Phage major capsid protein (Fragment) | Phage major capsid protein (Fragment) | | uniclust | UniRef100\_A0A0F9CDL2 | 99.9 | 3.3e-31 | 6.5e-37 | 259.4 | 315 | (31, 437) | 682 | (12, 343) | 371 | Uncharacterized protein (Fragment) | Uncharacterized protein (Fragment) | | uniclust | UniRef100\_A0A6N0ZA72 | 99.9 | 4.4e-31 | 8.4e-37 | 256.5 | 381 | (269, 681) | 682 | (14, 415) | 418 | Phage major capsid protein | Phage major capsid protein | | uniclust | UniRef100\_A0A167WYC7 | 99.9 | 7.2e-31 | 1.5e-36 | 277.4 | 271 | (393, 682) | 682 | (178, 463) | 482 | Phage major capsid protein | Phage major capsid protein | | uniclust | UniRef100\_A0A098F6L7 | 99.9 | 8.9e-31 | 1.7e-36 | 241.6 | 145 | (40, 185) | 682 | (65, 215) | 263 | Caudovirus prohead protease | Caudovirus prohead protease | | uniclust | UniRef100\_A0A3A5AR09 | 99.9 | 9.4e-31 | 1.8e-36 | 240.9 | 216 | (459, 681) | 682 | (2, 235) | 264 | Phage major capsid protein | Phage major capsid protein | | uniclust | UniRef100\_A0A3M1QXF2 | 99.9 | 1.1e-30 | 2.1e-36 | 265.9 | 578 | (40, 675) | 682 | (22, 664) | 670 | Caudovirus prohead protease | Caudovirus prohead protease | | uniclust | UniRef100\_A0A015WYK1 | 99.9 | 1.3e-30 | 2.5e-36 | 260.8 | 318 | (340, 679) | 682 | (130, 463) | 482 | Phage major capsid protein, HK97 family | Phage major capsid protein, HK97 family | | uniclust | UniRef100\_A0A376TC30 | 99.9 | 1.6e-30 | 3e-36 | 257.5 | 562 | (38, 681) | 682 | (24, 648) | 656 | Putative major head protein/prohead protease | Putative major head protein/prohead protease | | uniclust | UniRef100\_A0A0C2VPM1 | 99.9 | 1.7e-30 | 3.3e-36 | 254.7 | 264 | (406, 681) | 682 | (85, 367) | 370 | Phage major capsid protein | Phage major capsid protein | | uniclust | UniRef100\_Q1ILS6 | 99.9 | 1.9e-30 | 3.6e-36 | 257.8 | 397 | (261, 681) | 682 | (353, 777) | 781 | Peptidase S49 | Peptidase S49 | | uniclust | UniRef100\_A0A1D8UTE4 | 99.9 | 2e-30 | 3.9e-36 | 264.6 | 274 | (393, 681) | 682 | (147, 455) | 515 | Major capsid protein | Major capsid protein | | uniclust | UniRef100\_A0A6B3AAV6 | 99.9 | 2.2e-30 | 4.1e-36 | 254.5 | 488 | (43, 681) | 682 | (17, 528) | 530 | Phage major capsid protein | Phage major capsid protein | | uniclust | UniRef100\_A0A0S7X9K7 | 99.9 | 2.3e-30 | 4.4e-36 | 254.1 | 273 | (393, 680) | 682 | (116, 414) | 423 | Phage capsid protein | Phage capsid protein | | uniclust | UniRef100\_A0A348SWC8 | 99.9 | 2.6e-30 | 4.9e-36 | 241.2 | 154 | (31, 185) | 682 | (25, 181) | 330 | Phage major capsid protein (Fragment) | Phage major capsid protein (Fragment) | | uniclust | UniRef100\_A0A031HR58 | 99.9 | 2.5e-30 | 5e-36 | 264.5 | 268 | (393, 680) | 682 | (146, 442) | 464 | Phage major capsid protein, HK97 family | Phage major capsid protein, HK97 family | | uniclust | UniRef100\_A0A1H7NMW7 | 99.9 | 3.5e-30 | 6.8e-36 | 269.9 | 538 | (44, 680) | 682 | (36, 612) | 637 | Phage prohead protease, HK97 family/phage major capsid protein, HK97 family,TIGR01554 | Phage prohead protease, HK97 family/phage major capsid protein, HK97 family,TIGR01554 | | uniclust | UniRef100\_A0A0F9ENL1 | 99.9 | 4.2e-30 | 8.2e-36 | 240.5 | 215 | (456, 680) | 682 | (9, 237) | 243 | Phage major capsid protein (Fragment) | Phage major capsid protein (Fragment) | | uniclust | UniRef100\_A0A021VTX6 | 99.9 | 1.1e-29 | 2.2e-35 | 262.1 | 270 | (393, 680) | 682 | (144, 427) | 435 | HK97 family phage major capsid protein | HK97 family phage major capsid protein | | uniclust | UniRef100\_A0A011U0Z7 | 99.9 | 2.4e-29 | 5e-35 | 272.1 | 268 | (393, 680) | 682 | (241, 536) | 602 | Capsid protein | Capsid protein | | uniclust | UniRef100\_A0A011TWP2 | 99.9 | 2.8e-29 | 5.7e-35 | 268.7 | 272 | (393, 680) | 682 | (182, 470) | 557 | Capsid protein | Capsid protein | | uniclust | UniRef100\_A0A0G9K210 | 99.9 | 3.4e-29 | 6.7e-35 | 262.8 | 571 | (31, 678) | 682 | (25, 634) | 640 | Bacteriophage Mu GpT domain-containing protein | Bacteriophage Mu GpT domain-containing protein | | uniclust | UniRef100\_A0A064CN03 | 99.9 | 7.4e-29 | 1.5e-34 | 249.4 | 260 | (412, 680) | 682 | (57, 322) | 341 | Capsid protein | Capsid protein | | uniclust | UniRef100\_A0A0N0T0A0 | 99.9 | 9.7e-29 | 1.9e-34 | 243.7 | 271 | (393, 681) | 682 | (122, 411) | 435 | Phage major capsid protein | Phage major capsid protein | | uniclust | UniRef100\_A0A022LMT4 | 99.9 | 1.1e-28 | 2.1e-34 | 242.5 | 264 | (406, 681) | 682 | (61, 336) | 361 | Capsid protein | Capsid protein | | uniclust | UniRef100\_A0A2S9PYB0 | 99.9 | 1.2e-28 | 2.4e-34 | 266.3 | 271 | (393, 681) | 682 | (512, 800) | 803 | Phage major capsid protein | Phage major capsid protein | | uniclust | UniRef100\_A0A258QCW1 | 99.9 | 1.3e-28 | 2.5e-34 | 242.6 | 322 | (342, 681) | 682 | (34, 375) | 378 | Phage capsid protein | Phage capsid protein | | uniclust | UniRef100\_A0A0J6SLM0 | 99.9 | 1.7e-28 | 3.4e-34 | 249.6 | 268 | (406, 681) | 682 | (149, 447) | 458 | Capsid protein | Capsid protein | | uniclust | UniRef100\_A0A142YPV2 | 99.9 | 1.9e-28 | 3.7e-34 | 238.0 | 323 | (341, 681) | 682 | (18, 368) | 372 | Phage capsid family protein | Phage capsid family protein | | uniclust | UniRef100\_A0A142X041 | 99.9 | 2.2e-28 | 4.5e-34 | 255.9 | 270 | (393, 680) | 682 | (182, 469) | 473 | Phage capsid family protein | Phage capsid family protein | | uniclust | UniRef100\_A0A0J9D0M7 | 99.9 | 2.8e-28 | 5.6e-34 | 248.3 | 263 | (406, 681) | 682 | (167, 457) | 459 | Phage major capsid protein | Phage major capsid protein | | uniclust | UniRef100\_A0A2N3B9M3 | 99.9 | 3.7e-28 | 7e-34 | 235.5 | 270 | (31, 308) | 682 | (13, 307) | 367 | Peptidase U37 (Fragment) | Peptidase U37 (Fragment) | | uniclust | UniRef100\_A0A2E7K3Z7 | 99.9 | 3.9e-28 | 7.5e-34 | 236.1 | 242 | (38, 308) | 682 | (22, 272) | 379 | HK97 family phage prohead protease (Fragment) | HK97 family phage prohead protease (Fragment) | | uniclust | UniRef100\_A0A024YGR4 | 99.9 | 5.5e-28 | 1.1e-33 | 248.8 | 271 | (392, 680) | 682 | (129, 422) | 428 | Capsid protein | Capsid protein | | uniclust | UniRef100\_A0A1H7ZYZ5 | 99.9 | 5.8e-28 | 1.1e-33 | 251.0 | 605 | (32, 674) | 682 | (36, 709) | 716 | Mu-like prophage major head subunit gpT | Mu-like prophage major head subunit gpT | | uniclust | UniRef100\_A0A0F9KYA8 | 99.9 | 5.8e-28 | 1.2e-33 | 261.8 | 521 | (32, 591) | 682 | (75, 674) | 788 | Bacteriophage Mu GpT domain-containing protein | Bacteriophage Mu GpT domain-containing protein | | uniclust | UniRef100\_A0A0K6HVN0 | 99.9 | 6.2e-28 | 1.2e-33 | 251.7 | 268 | (393, 680) | 682 | (158, 454) | 458 | Phage major capsid protein, HK97 family | Phage major capsid protein, HK97 family | | uniclust | UniRef100\_A0A0G1G7G4 | 99.9 | 7.6e-28 | 1.5e-33 | 248.3 | 267 | (405, 680) | 682 | (296, 590) | 605 | Phage capsid family protein | Phage capsid family protein | | uniclust | UniRef100\_UPI000B0837BE | 99.9 | 9.9e-28 | 1.9e-33 | 234.7 | 436 | (43, 514) | 682 | (21, 502) | 519 | HK97 family phage prohead protease | HK97 family phage prohead protease | | uniclust | UniRef100\_A0A8T6P0T0 | 99.9 | 1e-27 | 1.9e-33 | 230.7 | 274 | (393, 681) | 682 | (240, 536) | 538 | Phage major capsid protein | Phage major capsid protein | | uniclust | UniRef100\_A0A084E6F3 | 99.9 | 1e-27 | 2e-33 | 250.0 | 314 | (340, 681) | 682 | (151, 493) | 549 | Phage major capsid protein, HK97 family | Phage major capsid protein, HK97 family | | uniclust | UniRef100\_A0A7W2BLG8 | 99.9 | 1.2e-27 | 2.3e-33 | 224.4 | 271 | (393, 681) | 682 | (15, 294) | 298 | Phage major capsid protein | Phage major capsid protein | | uniclust | UniRef100\_A0A081BFN2 | 99.9 | 1.3e-27 | 2.5e-33 | 232.9 | 268 | (31, 307) | 682 | (31, 326) | 328 | Peptidase U35 phage prohead HK97 (Fragment) | Peptidase U35 phage prohead HK97 (Fragment) | | uniclust | UniRef100\_A0A132HLC0 | 99.9 | 1.4e-27 | 2.6e-33 | 242.2 | 154 | (31, 185) | 682 | (28, 184) | 531 | Phage major capsid protein | Phage major capsid protein | | uniclust | UniRef100\_A0A7X8B6T2 | 99.9 | 1.4e-27 | 2.8e-33 | 250.0 | 213 | (455, 679) | 682 | (365, 583) | 595 | HK97 family phage prohead protease | HK97 family phage prohead protease | | uniclust | UniRef100\_A0A0F3MPU1 | 99.9 | 1.5e-27 | 2.9e-33 | 240.5 | 261 | (406, 680) | 682 | (89, 369) | 375 | Phage major capsid protein, HK97 family | Phage major capsid protein, HK97 family | | uniclust | UniRef100\_A0A399XUC0 | 99.9 | 1.7e-27 | 3.2e-33 | 234.6 | 267 | (407, 681) | 682 | (194, 480) | 484 | Phage major capsid protein | Phage major capsid protein | | uniclust | UniRef100\_A0A0G1A6G8 | 99.9 | 1.8e-27 | 3.3e-33 | 229.4 | 272 | (391, 681) | 682 | (119, 401) | 402 | Phage major capsid protein, HK97 family | Phage major capsid protein, HK97 family | | uniclust | UniRef100\_A0A081NWP6 | 99.9 | 2e-27 | 3.9e-33 | 248.4 | 255 | (406, 673) | 682 | (179, 457) | 482 | Capsid protein | Capsid protein | | uniclust | UniRef100\_A0A662S3E0 | 99.9 | 2.4e-27 | 4.5e-33 | 232.8 | 269 | (406, 681) | 682 | (364, 654) | 658 | Phage major capsid protein (Fragment) | Phage major capsid protein (Fragment) | | uniclust | UniRef100\_A0A1I7EFL9 | 99.9 | 3.1e-27 | 6e-33 | 240.8 | 468 | (34, 677) | 682 | (23, 514) | 520 | Phage prohead protease, HK97 family | Phage prohead protease, HK97 family | | uniclust | UniRef100\_A0A0J1FQE0 | 99.9 | 3.2e-27 | 6.4e-33 | 243.5 | 269 | (393, 681) | 682 | (141, 420) | 431 | Phage capsid family protein | Phage capsid family protein | | uniclust | UniRef100\_A0A7W8FWR1 | 99.9 | 3.6e-27 | 6.7e-33 | 229.5 | 469 | (31, 679) | 682 | (6, 491) | 495 | HK97 family phage major capsid protein | HK97 family phage major capsid protein | | uniclust | UniRef100\_A0A6A4R9P0 | 99.9 | 4.5e-27 | 8.2e-33 | 216.2 | 342 | (263, 617) | 682 | (3, 356) | 358 | Phage major capsid protein (Fragment) | Phage major capsid protein (Fragment) | | uniclust | UniRef100\_A0A1C5ZD29 | 99.9 | 5.1e-27 | 9.7e-33 | 237.2 | 275 | (393, 682) | 682 | (230, 534) | 537 | Predicted phage phi-C31 gp36 major capsid-like protein | Predicted phage phi-C31 gp36 major capsid-like protein | | uniclust | UniRef100\_A0A142X159 | 99.9 | 6.4e-27 | 1.3e-32 | 244.3 | 277 | (404, 681) | 682 | (209, 512) | 519 | Phage capsid family protein | Phage capsid family protein | | uniclust | UniRef100\_A0A0C7LAF9 | 99.9 | 7e-27 | 1.3e-32 | 230.6 | 480 | (41, 679) | 682 | (21, 510) | 515 | HK97 family phage prohead protease | HK97 family phage prohead protease | | uniclust | UniRef100\_A0A2D6PAP5 | 99.9 | 7.7e-27 | 1.4e-32 | 197.1 | 154 | (31, 184) | 682 | (14, 169) | 173 | HK97 family phage prohead protease (Fragment) | HK97 family phage prohead protease (Fragment) | | uniclust | UniRef100\_A0A223I0W6 | 99.9 | 7.8e-27 | 1.4e-32 | 224.4 | 263 | (406, 679) | 682 | (120, 396) | 529 | Phage major capsid protein, HK97 family | Phage major capsid protein, HK97 family | | uniclust | UniRef100\_A0A160T8V4 | 99.9 | 1.1e-26 | 2.1e-32 | 238.8 | 254 | (406, 671) | 682 | (348, 627) | 650 | Uncharacterized protein | Uncharacterized protein | | uniclust | UniRef100\_A0A1G7TNP2 | 99.9 | 1.4e-26 | 2.7e-32 | 220.0 | 262 | (406, 680) | 682 | (10, 297) | 307 | Phage major capsid protein, HK97 family | Phage major capsid protein, HK97 family | | uniclust | UniRef100\_A0A100Y4B7 | 99.9 | 1.5e-26 | 2.9e-32 | 232.0 | 272 | (392, 680) | 682 | (140, 422) | 425 | Capsid protein | Capsid protein | | uniclust | UniRef100\_A0A6J5KKY4 | 99.9 | 1.8e-26 | 3.6e-32 | 243.5 | 478 | (34, 679) | 682 | (114, 616) | 620 | Phage capsid | Phage capsid | | uniclust | UniRef100\_A0A060AEU4 | 99.9 | 2e-26 | 3.9e-32 | 230.8 | 261 | (408, 680) | 682 | (102, 375) | 382 | Capsid protein | Capsid protein | | uniclust | UniRef100\_A0A2W4JAL7 | 99.9 | 2e-26 | 3.9e-32 | 219.0 | 246 | (429, 681) | 682 | (38, 310) | 318 | Phage major capsid protein | Phage major capsid protein | | uniclust | UniRef100\_A0A1C5WLB0 | 99.9 | 2.4e-26 | 4.7e-32 | 207.1 | 142 | (42, 184) | 682 | (45, 192) | 221 | Phage prohead protease, HK97 family | Phage prohead protease, HK97 family | | uniclust | UniRef100\_A0A0C1YAL0 | 99.9 | 2.7e-26 | 5.2e-32 | 226.1 | 263 | (406, 677) | 682 | (116, 399) | 405 | Capsid protein | Capsid protein | | uniclust | UniRef100\_A0A090E9R7 | 99.9 | 3e-26 | 5.7e-32 | 228.8 | 269 | (406, 682) | 682 | (142, 438) | 442 | Putative Phage major capsid protein, HK97 family | Putative Phage major capsid protein, HK97 family | | uniclust | UniRef100\_A0A075KJX9 | 99.9 | 3.4e-26 | 6.8e-32 | 236.2 | 258 | (408, 680) | 682 | (147, 419) | 459 | Putative major head protein | Putative major head protein | | uniclust | UniRef100\_A0A0F9BLA7 | 99.9 | 3.7e-26 | 7.4e-32 | 241.1 | 264 | (393, 671) | 682 | (201, 487) | 505 | Phage major capsid protein | Phage major capsid protein | | uniclust | UniRef100\_A0A1S9JNN0 | 99.9 | 4.1e-26 | 7.9e-32 | 228.0 | 131 | (36, 181) | 682 | (25, 164) | 454 | Phage major capsid protein (Fragment) | Phage major capsid protein (Fragment) | | uniclust | UniRef100\_A0A009Z9E2 | 99.9 | 4.1e-26 | 8.2e-32 | 238.1 | 267 | (405, 681) | 682 | (225, 515) | 528 | Phage major capsid protein, HK97 family | Phage major capsid protein, HK97 family | | uniclust | UniRef100\_A0A3B9G3E3 | 99.9 | 7.6e-26 | 1.4e-31 | 228.5 | 522 | (44, 681) | 682 | (19, 561) | 563 | Phage major capsid protein (Fragment) | Phage major capsid protein (Fragment) | | uniclust | UniRef100\_A0A0B4CLV4 | 99.9 | 7.5e-26 | 1.5e-31 | 201.1 | 184 | (494, 681) | 682 | (1, 187) | 190 | Uncharacterized protein | Uncharacterized protein | | uniclust | UniRef100\_UPI00047CD657 | 99.9 | 8.4e-26 | 1.6e-31 | 223.8 | 517 | (38, 681) | 682 | (12, 565) | 569 | phage major capsid protein | phage major capsid protein | | uniclust | UniRef100\_A0A031I873 | 99.9 | 7.6e-26 | 1.6e-31 | 244.1 | 266 | (406, 680) | 682 | (191, 482) | 500 | Phage major capsid protein, HK97 family | Phage major capsid protein, HK97 family | | uniclust | UniRef100\_A0A0B8PLS9 | 99.9 | 8.9e-26 | 1.6e-31 | 207.7 | 279 | (260, 568) | 682 | (70, 352) | 356 | Phage major capsid protein | Phage major capsid protein | | uniclust | UniRef100\_A0A1A9A792 | 99.9 | 9.6e-26 | 1.8e-31 | 211.3 | 269 | (406, 681) | 682 | (18, 309) | 312 | Phage major capsid protein, HK97 family | Phage major capsid protein, HK97 family | | uniclust | UniRef100\_UPI0022AE545A | 99.9 | 1.2e-25 | 2.1e-31 | 221.2 | 578 | (38, 677) | 682 | (29, 648) | 653 | HK97 family phage prohead protease | HK97 family phage prohead protease | | uniclust | UniRef100\_A0A069APM8 | 99.9 | 1.2e-25 | 2.3e-31 | 230.2 | 259 | (409, 681) | 682 | (151, 410) | 435 | Putative phage capsid protein | Putative phage capsid protein | | uniclust | UniRef100\_A0A0R0CNK4 | 99.9 | 2.1e-25 | 4e-31 | 224.8 | 272 | (393, 682) | 682 | (192, 479) | 485 | Capsid protein | Capsid protein | | uniclust | UniRef100\_A0A0F9IZX0 | 99.9 | 2.1e-25 | 4e-31 | 218.7 | 264 | (406, 680) | 682 | (153, 437) | 441 | Phage major capsid protein | Phage major capsid protein | | uniclust | UniRef100\_A0A023D045 | 99.9 | 2.1e-25 | 4e-31 | 230.0 | 424 | (44, 679) | 682 | (56, 511) | 533 | Uncharacterized protein | Uncharacterized protein | | uniclust | UniRef100\_A0A099YFD8 | 99.9 | 2.1e-25 | 4.3e-31 | 242.9 | 543 | (44, 680) | 682 | (57, 627) | 655 | Capsid protein | Capsid protein | | uniclust | UniRef100\_A0A316RJS4 | 99.9 | 2.5e-25 | 4.7e-31 | 208.5 | 258 | (406, 678) | 682 | (34, 293) | 303 | Phage major capsid protein | Phage major capsid protein | | uniclust | UniRef100\_A0A4R0QZ35 | 99.9 | 3.2e-25 | 6.1e-31 | 212.6 | 281 | (381, 677) | 682 | (50, 335) | 338 | Phage major capsid protein | Phage major capsid protein | | uniclust | UniRef100\_A0A832GWI7 | 99.9 | 3.5e-25 | 6.5e-31 | 210.3 | 431 | (34, 640) | 682 | (9, 459) | 461 | Phage major capsid protein (Fragment) | Phage major capsid protein (Fragment) | | uniclust | UniRef100\_A0A140E9M0 | 99.9 | 3.4e-25 | 6.5e-31 | 228.9 | 530 | (38, 680) | 682 | (19, 580) | 584 | Bacteriophage Mu GpT domain-containing protein | Bacteriophage Mu GpT domain-containing protein | | uniclust | UniRef100\_A0A2D0K5M3 | 99.9 | 3.7e-25 | 7e-31 | 212.7 | 266 | (406, 680) | 682 | (38, 317) | 324 | Phage capsid protein | Phage capsid protein | | uniclust | UniRef100\_A0A0D0Q3B7 | 99.9 | 3.8e-25 | 7.2e-31 | 223.9 | 264 | (406, 677) | 682 | (208, 496) | 517 | Phage major capsid protein | Phage major capsid protein | | uniclust | UniRef100\_A0A0D7P7D9 | 99.9 | 4.1e-25 | 7.9e-31 | 207.7 | 145 | (38, 185) | 682 | (44, 205) | 256 | Peptidase U35 | Peptidase U35 | | uniclust | UniRef100\_C6M4F3 | 99.9 | 4.4e-25 | 8e-31 | 194.1 | 148 | (38, 185) | 682 | (23, 171) | 251 | Caudovirus prohead protease | Caudovirus prohead protease | | uniclust | UniRef100\_A0A014CAX5 | 99.9 | 4.5e-25 | 9.1e-31 | 221.0 | 170 | (2, 181) | 682 | (33, 210) | 319 | Phage prohead protease, HK97 family | Phage prohead protease, HK97 family | | uniclust | UniRef100\_A0A011UU65 | 99.8 | 5.2e-25 | 1.1e-30 | 231.9 | 262 | (407, 679) | 682 | (155, 426) | 454 | Capsid protein | Capsid protein | | uniclust | UniRef100\_A0A940E8X0 | 99.8 | 8.8e-25 | 1.6e-30 | 207.0 | 255 | (422, 681) | 682 | (172, 446) | 449 | Phage major capsid protein | Phage major capsid protein | | uniclust | UniRef100\_A0A087M280 | 99.8 | 9.6e-25 | 1.9e-30 | 202.2 | 152 | (31, 184) | 682 | (18, 182) | 242 | Peptidase U35 | Peptidase U35 | | uniclust | UniRef100\_A0A016QMV8 | 99.8 | 9.2e-25 | 1.9e-30 | 233.6 | 262 | (407, 679) | 682 | (209, 480) | 505 | Putative phage phi-C31 gp36 major capsid-like protein | Putative phage phi-C31 gp36 major capsid-like protein | | uniclust | UniRef100\_A0A447LNI1 | 99.8 | 1.1e-24 | 2.2e-30 | 192.8 | 147 | (33, 185) | 682 | (13, 166) | 199 | Phage prohead protease, HK97 family | Phage prohead protease, HK97 family | | uniclust | UniRef100\_A0A1Z8N5M0 | 99.8 | 1.2e-24 | 2.3e-30 | 209.5 | 263 | (409, 680) | 682 | (111, 388) | 391 | Phage major capsid protein | Phage major capsid protein | | uniclust | UniRef100\_A0A7Y9W360 | 99.8 | 1.2e-24 | 2.3e-30 | 210.8 | 345 | (32, 398) | 682 | (5, 377) | 454 | HK97 family phage prohead protease | HK97 family phage prohead protease | | uniclust | UniRef100\_A0A081HUR9 | 99.8 | 1.4e-24 | 2.7e-30 | 220.2 | 268 | (393, 680) | 682 | (158, 454) | 457 | HK97 family phage major capsid protein | HK97 family phage major capsid protein | | uniclust | UniRef100\_UPI000A547A50 | 99.8 | 1.6e-24 | 3e-30 | 199.8 | 302 | (31, 371) | 682 | (17, 320) | 334 | HK97 family phage prohead protease | HK97 family phage prohead protease | | uniclust | UniRef100\_A0A4V1UI61 | 99.8 | 1.6e-24 | 3.1e-30 | 209.1 | 196 | (339, 546) | 682 | (81, 283) | 322 | Phage major capsid protein (Fragment) | Phage major capsid protein (Fragment) | | uniclust | UniRef100\_A0A0H5RW03 | 99.8 | 1.8e-24 | 3.6e-30 | 210.1 | 264 | (408, 681) | 682 | (31, 298) | 347 | Phage major capsid protein, HK97 | Phage major capsid protein, HK97 | | uniclust | UniRef100\_A0A2N6UZ54 | 99.8 | 1.9e-24 | 3.7e-30 | 212.7 | 202 | (388, 607) | 682 | (112, 316) | 323 | Phage major capsid protein (Fragment) | Phage major capsid protein (Fragment) | | uniclust | UniRef100\_A0A1C5JTV2 | 99.8 | 2e-24 | 3.8e-30 | 219.1 | 270 | (390, 681) | 682 | (202, 502) | 506 | Phage major capsid protein, HK97 family | Phage major capsid protein, HK97 family | | uniclust | UniRef100\_UPI0006A401C8 | 99.8 | 2.2e-24 | 4e-30 | 207.8 | 487 | (31, 679) | 682 | (5, 517) | 521 | phage major capsid protein | phage major capsid protein | | uniclust | UniRef100\_A0A4Q5ADZ7 | 99.8 | 2.3e-24 | 4.3e-30 | 221.5 | 243 | (428, 680) | 682 | (424, 684) | 694 | Phage capsid protein | Phage capsid protein | | uniclust | UniRef100\_A0A0D5LSQ3 | 99.8 | 2.2e-24 | 4.4e-30 | 212.7 | 134 | (38, 181) | 682 | (55, 192) | 299 | Peptidase U35 | Peptidase U35 | | uniclust | UniRef100\_A0A929NN34 | 99.8 | 2.5e-24 | 4.5e-30 | 200.4 | 323 | (342, 679) | 682 | (53, 381) | 383 | Phage major capsid protein | Phage major capsid protein | | uniclust | UniRef100\_Q1QI78 | 99.8 | 2.5e-24 | 4.6e-30 | 222.6 | 571 | (48, 668) | 682 | (553, 1166) | 1179 | Phage terminase GpA | Phage terminase GpA | | uniclust | UniRef100\_A0A263E3D0 | 99.8 | 3e-24 | 5.7e-30 | 210.0 | 275 | (393, 681) | 682 | (115, 413) | 417 | Phage major capsid protein | Phage major capsid protein | | uniclust | UniRef100\_A0A1D3SNN3 | 99.8 | 3.8e-24 | 7.4e-30 | 215.9 | 265 | (406, 680) | 682 | (126, 406) | 443 | Phage major capsid protein | Phage major capsid protein | | uniclust | UniRef100\_A0A1A1W0A4 | 99.8 | 4.6e-24 | 8.8e-30 | 204.2 | 265 | (407, 680) | 682 | (75, 347) | 352 | Phage capsid protein | Phage capsid protein | | uniclust | UniRef100\_A0A0F9AWL4 | 99.8 | 4.4e-24 | 8.8e-30 | 216.3 | 259 | (406, 675) | 682 | (49, 330) | 350 | Phage major capsid protein (Fragment) | Phage major capsid protein (Fragment) | | uniclust | UniRef100\_A0A0F3N2G0 | 99.8 | 5.8e-24 | 1.1e-29 | 208.8 | 260 | (407, 680) | 682 | (84, 358) | 365 | Phage major capsid protein, HK97 family | Phage major capsid protein, HK97 family | | uniclust | UniRef100\_A0A014FFI8 | 99.8 | 6.1e-24 | 1.2e-29 | 208.5 | 263 | (406, 679) | 682 | (55, 327) | 339 | Phage major capsid protein, HK97 family | Phage major capsid protein, HK97 family | | uniclust | UniRef100\_A0A249MU97 | 99.8 | 6.3e-24 | 1.2e-29 | 210.1 | 264 | (406, 680) | 682 | (130, 422) | 426 | Phage major capsid protein | Phage major capsid protein | | uniclust | UniRef100\_A0A3D1WNS7 | 99.8 | 6.9e-24 | 1.3e-29 | 201.8 | 191 | (406, 607) | 682 | (120, 311) | 323 | Phage major capsid protein (Fragment) | Phage major capsid protein (Fragment) | | uniclust | UniRef100\_A0A1V2NG87 | 99.8 | 7.2e-24 | 1.4e-29 | 206.2 | 264 | (393, 680) | 682 | (115, 392) | 395 | Capsid protein | Capsid protein | | uniclust | UniRef100\_A0A011T3L4 | 99.8 | 7.4e-24 | 1.4e-29 | 197.2 | 137 | (31, 181) | 682 | (16, 162) | 226 | Peptidase U35 | Peptidase U35 | | uniclust | UniRef100\_A0A2D5HBK7 | 99.8 | 7.7e-24 | 1.5e-29 | 204.3 | 258 | (410, 680) | 682 | (2, 287) | 292 | Phage major capsid protein (Fragment) | Phage major capsid protein (Fragment) | | uniclust | UniRef100\_A0A1Y0Y2T8 | 99.8 | 8e-24 | 1.6e-29 | 218.1 | 261 | (410, 680) | 682 | (152, 442) | 487 | Phage major capsid protein | Phage major capsid protein | | uniclust | UniRef100\_A0A011PIW6 | 99.8 | 1.2e-23 | 2.3e-29 | 204.7 | 219 | (454, 680) | 682 | (57, 291) | 306 | Phage major capsid protein, HK97 family | Phage major capsid protein, HK97 family | | uniclust | UniRef100\_A0A061LX04 | 99.8 | 1.4e-23 | 2.8e-29 | 213.0 | 262 | (408, 680) | 682 | (143, 423) | 444 | Capsid protein | Capsid protein | | uniclust | UniRef100\_A0A6J5RYQ5 | 99.8 | 1.7e-23 | 3e-29 | 199.0 | 268 | (406, 681) | 682 | (158, 450) | 455 | COG4653 Predicted phage phi-C31 gp36 major capsid-like protein | COG4653 Predicted phage phi-C31 gp36 major capsid-like protein | | uniclust | UniRef100\_A0A1B0XUP8 | 99.8 | 1.6e-23 | 3.1e-29 | 217.3 | 471 | (43, 678) | 682 | (21, 515) | 534 | Capsid protein with prohead protease | Capsid protein with prohead protease | | uniclust | UniRef100\_A0A3D9ZDR9 | 99.8 | 1.7e-23 | 3.2e-29 | 196.0 | 151 | (31, 184) | 682 | (7, 170) | 273 | HK97 family phage prohead protease | HK97 family phage prohead protease | | uniclust | UniRef100\_A0A017RTY2 | 99.8 | 1.8e-23 | 3.5e-29 | 219.2 | 268 | (404, 680) | 682 | (194, 489) | 504 | Capsid protein | Capsid protein | | uniclust | UniRef100\_A0A088FP12 | 99.8 | 1.8e-23 | 3.6e-29 | 218.7 | 272 | (392, 680) | 682 | (223, 530) | 547 | Major capsid protein | Major capsid protein | | uniclust | UniRef100\_A0A0F9F1M1 | 99.8 | 1.9e-23 | 3.8e-29 | 215.3 | 259 | (413, 681) | 682 | (157, 424) | 427 | Phage major capsid protein | Phage major capsid protein | | uniclust | UniRef100\_A0A1V5LY97 | 99.8 | 3.2e-23 | 6.1e-29 | 183.9 | 171 | (500, 680) | 682 | (2, 186) | 194 | Phage capsid family protein | Phage capsid family protein | | uniclust | UniRef100\_A0A7D5FN44 | 99.8 | 3.3e-23 | 6.2e-29 | 194.1 | 268 | (406, 681) | 682 | (38, 334) | 336 | Major capsid protein (Fragment) | Major capsid protein (Fragment) | | uniclust | UniRef100\_A0A090IID5 | 99.8 | 3.1e-23 | 6.3e-29 | 202.1 | 132 | (41, 181) | 682 | (36, 173) | 263 | Phage prohead protease | Phage prohead protease | | uniclust | UniRef100\_A0A017H821 | 99.8 | 3.4e-23 | 6.7e-29 | 189.7 | 129 | (42, 183) | 682 | (24, 154) | 190 | Peptidase U35, phage prohead HK97 | Peptidase U35, phage prohead HK97 | | uniclust | UniRef100\_A0A087DI38 | 99.8 | 3.9e-23 | 7.3e-29 | 212.1 | 253 | (413, 679) | 682 | (410, 673) | 686 | Capsid protein | Capsid protein | | uniclust | UniRef100\_A0A011PJT1 | 99.8 | 3.8e-23 | 7.6e-29 | 215.9 | 262 | (406, 677) | 682 | (184, 468) | 486 | Phage major capsid protein, HK97 family | Phage major capsid protein, HK97 family | | uniclust | UniRef100\_A0A015WYB3 | 99.8 | 4e-23 | 7.9e-29 | 211.1 | 264 | (406, 680) | 682 | (122, 388) | 398 | Phage major capsid protein, HK97 family | Phage major capsid protein, HK97 family | | uniclust | UniRef100\_A0A1V5PUE1 | 99.8 | 4.2e-23 | 8e-29 | 205.0 | 260 | (393, 671) | 682 | (134, 416) | 441 | Phage capsid family protein | Phage capsid family protein | | uniclust | UniRef100\_A0A2D7QM53 | 99.8 | 4.3e-23 | 8.4e-29 | 205.3 | 315 | (338, 680) | 682 | (84, 405) | 408 | Phage major capsid protein | Phage major capsid protein | | uniclust | UniRef100\_A0A661TNK1 | 99.8 | 4.6e-23 | 8.5e-29 | 195.2 | 397 | (39, 508) | 682 | (21, 434) | 437 | HK97 family phage prohead protease (Fragment) | HK97 family phage prohead protease (Fragment) | | uniclust | UniRef100\_A0A0F9JCM6 | 99.8 | 5e-23 | 9.9e-29 | 218.0 | 468 | (45, 564) | 682 | (51, 555) | 592 | Bacteriophage Mu GpT domain-containing protein (Fragment) | Bacteriophage Mu GpT domain-containing protein (Fragment) | | uniclust | UniRef100\_A0A022MNJ2 | 99.8 | 4.9e-23 | 9.9e-29 | 215.1 | 261 | (407, 680) | 682 | (153, 431) | 446 | Capsid protein | Capsid protein | | uniclust | UniRef100\_A0A938T756 | 99.8 | 6.1e-23 | 1.1e-28 | 200.6 | 526 | (41, 679) | 682 | (16, 582) | 586 | Phage major capsid protein | Phage major capsid protein | | uniclust | UniRef100\_A0A229GM47 | 99.8 | 6.5e-23 | 1.2e-28 | 194.9 | 130 | (38, 181) | 682 | (38, 175) | 295 | Peptidase U35 | Peptidase U35 | | uniclust | UniRef100\_A0A143PPF0 | 99.8 | 6.7e-23 | 1.3e-28 | 196.7 | 262 | (406, 679) | 682 | (119, 387) | 411 | Phage major capsid protein, HK97 family | Phage major capsid protein, HK97 family | | uniclust | UniRef100\_A0A7W0TDA0 | 99.8 | 8.1e-23 | 1.5e-28 | 196.5 | 269 | (393, 679) | 682 | (131, 419) | 423 | Phage major capsid protein | Phage major capsid protein | | uniclust | UniRef100\_A0A011MGC8 | 99.8 | 7.9e-23 | 1.6e-28 | 213.9 | 263 | (406, 679) | 682 | (188, 460) | 492 | Capsid protein | Capsid protein | | uniclust | UniRef100\_UPI000DA43CDE | 99.8 | 8.7e-23 | 1.6e-28 | 196.9 | 375 | (269, 680) | 682 | (104, 489) | 492 | phage major capsid protein | phage major capsid protein | | uniclust | UniRef100\_A0A916TP50 | 99.8 | 9.5e-23 | 1.7e-28 | 193.5 | 311 | (38, 437) | 682 | (90, 419) | 444 | Uncharacterized protein | Uncharacterized protein | | uniclust | UniRef100\_A0A068RC46 | 99.8 | 9e-23 | 1.8e-28 | 201.5 | 131 | (42, 181) | 682 | (76, 213) | 291 | Caudovirus prohead protease family protein | Caudovirus prohead protease family protein | | uniclust | UniRef100\_A0A8S5NH81 | 99.8 | 1e-22 | 1.9e-28 | 179.7 | 153 | (32, 185) | 682 | (14, 174) | 251 | Major capsid protein (Fragment) | Major capsid protein (Fragment) | | uniclust | UniRef100\_A0A161IDV2 | 99.8 | 1e-22 | 2.1e-28 | 196.4 | 136 | (36, 181) | 682 | (34, 173) | 260 | Caudovirus prohead protease | Caudovirus prohead protease | | uniclust | UniRef100\_A0A059IUN6 | 99.8 | 1.5e-22 | 2.9e-28 | 205.0 | 262 | (406, 680) | 682 | (149, 432) | 515 | HK97 family phage major capsid protein | HK97 family phage major capsid protein | | uniclust | UniRef100\_A0A1Q3QQ17 | 99.8 | 1.5e-22 | 3e-28 | 206.9 | 260 | (393, 671) | 682 | (107, 381) | 396 | Capsid protein | Capsid protein | | uniclust | UniRef100\_A0A7W1RV33 | 99.8 | 1.7e-22 | 3e-28 | 192.8 | 268 | (406, 681) | 682 | (166, 462) | 463 | Phage major capsid protein | Phage major capsid protein | | uniclust | UniRef100\_A0A2D9C9C5 | 99.8 | 1.7e-22 | 3.1e-28 | 199.0 | 571 | (32, 678) | 682 | (22, 626) | 630 | HK97 family phage prohead protease | HK97 family phage prohead protease | | uniclust | UniRef100\_A0A354P6A4 | 99.8 | 1.8e-22 | 3.4e-28 | 197.0 | 277 | (386, 681) | 682 | (147, 426) | 430 | Phage major capsid protein | Phage major capsid protein | | uniclust | UniRef100\_UPI00131B246A | 99.8 | 2e-22 | 3.6e-28 | 196.5 | 382 | (262, 681) | 682 | (119, 561) | 564 | phage major capsid protein | phage major capsid protein | | uniclust | UniRef100\_A0A432MJM0 | 99.8 | 3.1e-22 | 5.6e-28 | 189.0 | 311 | (342, 678) | 682 | (91, 419) | 421 | Phage major capsid protein | Phage major capsid protein | | uniclust | UniRef100\_UPI0006943B46 | 99.8 | 3.1e-22 | 5.7e-28 | 196.8 | 262 | (393, 672) | 682 | (326, 602) | 613 | phage major capsid protein | phage major capsid protein | | uniclust | UniRef100\_A0A101UZE9 | 99.8 | 3e-22 | 5.8e-28 | 202.0 | 264 | (406, 681) | 682 | (175, 456) | 460 | Capsid protein | Capsid protein | | uniclust | UniRef100\_A0A2U1BFB6 | 99.8 | 3.3e-22 | 6.3e-28 | 192.3 | 258 | (408, 679) | 682 | (105, 379) | 383 | HK97 family phage major capsid protein | HK97 family phage major capsid protein | | uniclust | UniRef100\_A0A8I1GCE2 | 99.8 | 3.4e-22 | 6.4e-28 | 187.3 | 254 | (31, 308) | 682 | (15, 285) | 331 | Peptidase U35 | Peptidase U35 | | uniclust | UniRef100\_A0A060PVD0 | 99.8 | 3.3e-22 | 6.5e-28 | 187.3 | 131 | (42, 181) | 682 | (59, 190) | 227 | Phage prohead protease | Phage prohead protease | | uniclust | UniRef100\_A0A068D9Z5 | 99.8 | 4e-22 | 7.8e-28 | 203.4 | 256 | (406, 671) | 682 | (120, 402) | 419 | Phage major capsid protein | Phage major capsid protein | | uniclust | UniRef100\_A0A1M3FIN7 | 99.8 | 4e-22 | 8e-28 | 206.3 | 271 | (393, 680) | 682 | (172, 478) | 487 | Major capsid protein | Major capsid protein | | uniclust | UniRef100\_A0A060QM50 | 99.8 | 4.1e-22 | 8.2e-28 | 209.1 | 262 | (407, 679) | 682 | (186, 458) | 499 | Phage major capsid protein | Phage major capsid protein | | uniclust | UniRef100\_D6PFS1 | 99.8 | 5.3e-22 | 9.7e-28 | 171.3 | 207 | (348, 559) | 682 | (3, 211) | 214 | Putative phage related protein | Putative phage related protein | | uniclust | UniRef100\_A0A3A9EF23 | 99.8 | 5.3e-22 | 9.8e-28 | 173.3 | 153 | (31, 184) | 682 | (17, 180) | 223 | Caudovirus prohead protease | Caudovirus prohead protease | | uniclust | UniRef100\_A0A084J8V6 | 99.8 | 5.6e-22 | 1.1e-27 | 195.6 | 260 | (409, 680) | 682 | (34, 316) | 338 | Phage major capsid protein | Phage major capsid protein | | uniclust | UniRef100\_A0A7X8QWG9 | 99.8 | 6e-22 | 1.1e-27 | 190.5 | 264 | (406, 679) | 682 | (113, 408) | 410 | Phage major capsid protein | Phage major capsid protein | | uniclust | UniRef100\_UPI000738CC35 | 99.8 | 6.7e-22 | 1.3e-27 | 169.0 | 144 | (31, 180) | 682 | (12, 161) | 164 | HK97 family phage prohead protease | HK97 family phage prohead protease | | uniclust | UniRef100\_A0A013UG14 | 99.8 | 6.6e-22 | 1.3e-27 | 183.1 | 133 | (36, 181) | 682 | (27, 166) | 229 | Caudovirus prohead protease family protein | Caudovirus prohead protease family protein | | uniclust | UniRef100\_A0A117JJ31 | 99.8 | 6.8e-22 | 1.3e-27 | 186.4 | 265 | (406, 679) | 682 | (11, 275) | 278 | PE domain-containing protein (Fragment) | PE domain-containing protein (Fragment) | | uniclust | UniRef100\_A0A0F7R9L9 | 99.8 | 6.8e-22 | 1.3e-27 | 196.5 | 269 | (392, 681) | 682 | (96, 375) | 390 | Phage Capsid protein | Phage Capsid protein | | uniclust | UniRef100\_A0A4V3IRT6 | 99.8 | 7.2e-22 | 1.4e-27 | 191.6 | 262 | (411, 681) | 682 | (112, 382) | 384 | Phage major capsid protein | Phage major capsid protein | | uniclust | UniRef100\_A0A083XX19 | 99.8 | 7.2e-22 | 1.5e-27 | 211.3 | 256 | (415, 680) | 682 | (153, 426) | 468 | Phage capsid protein | Phage capsid protein | | uniclust | UniRef100\_A0A081I3V9 | 99.8 | 7.5e-22 | 1.5e-27 | 200.3 | 231 | (438, 680) | 682 | (66, 304) | 363 | HK97 family phage major capsid protein | HK97 family phage major capsid protein | | uniclust | UniRef100\_A0A059FG22 | 99.8 | 7.5e-22 | 1.5e-27 | 187.5 | 131 | (41, 181) | 682 | (56, 190) | 240 | Phage prohead protease HK97 family protein | Phage prohead protease HK97 family protein | | uniclust | UniRef100\_A0A962YQX0 | 99.8 | 8.6e-22 | 1.6e-27 | 186.2 | 270 | (406, 680) | 682 | (148, 421) | 423 | Phage major capsid protein | Phage major capsid protein | | uniclust | UniRef100\_A0A060VRW9 | 99.8 | 7.8e-22 | 1.6e-27 | 195.0 | 174 | (1, 181) | 682 | (1, 182) | 273 | Phage prohead protease, HK97 family | Phage prohead protease, HK97 family | | uniclust | UniRef100\_A0A1H8L3J0 | 99.8 | 8.9e-22 | 1.6e-27 | 194.2 | 318 | (31, 437) | 682 | (23, 361) | 632 | Prohead serine protease | Prohead serine protease | | uniclust | UniRef100\_A0A2A9FC94 | 99.8 | 9.3e-22 | 1.8e-27 | 200.8 | 544 | (32, 679) | 682 | (17, 587) | 595 | HK97 family phage prohead protease/HK97 family phage major capsid protein,TIGR01554 | HK97 family phage prohead protease/HK97 family phage major capsid protein,TIGR01554 | | uniclust | UniRef100\_A0A963KA71 | 99.8 | 9.5e-22 | 1.8e-27 | 186.2 | 324 | (333, 681) | 682 | (14, 347) | 348 | Phage major capsid protein | Phage major capsid protein | | uniclust | UniRef100\_A0A3S4JUU9 | 99.8 | 9.8e-22 | 1.9e-27 | 196.3 | 258 | (406, 680) | 682 | (190, 457) | 460 | Predicted phage phi-C31 gp36 major capsid-like protein | Predicted phage phi-C31 gp36 major capsid-like protein | | uniclust | UniRef100\_A0A5S4ZXS2 | 99.8 | 1.1e-21 | 2e-27 | 184.0 | 261 | (393, 670) | 682 | (21, 305) | 328 | HK97 family phage major capsid protein | HK97 family phage major capsid protein | | uniclust | UniRef100\_UPI00215628C7 | 99.8 | 1.2e-21 | 2.2e-27 | 192.7 | 529 | (43, 678) | 682 | (39, 605) | 611 | phage major capsid protein | phage major capsid protein | | uniclust | UniRef100\_A0A084E6F4 | 99.8 | 1.2e-21 | 2.3e-27 | 181.2 | 133 | (36, 181) | 682 | (18, 157) | 222 | Phage prohead protease, HK97 family | Phage prohead protease, HK97 family | | uniclust | UniRef100\_A0A086ZY60 | 99.8 | 1.1e-21 | 2.3e-27 | 196.9 | 231 | (440, 680) | 682 | (72, 310) | 330 | Phage major capsid protein, HK97 family | Phage major capsid protein, HK97 family | | uniclust | UniRef100\_A0A098AU90 | 99.8 | 1.2e-21 | 2.3e-27 | 185.0 | 136 | (43, 183) | 682 | (47, 190) | 238 | Phage head maturation protease | Phage head maturation protease | | uniclust | UniRef100\_A0A1V4I2J1 | 99.7 | 1.4e-21 | 2.6e-27 | 171.0 | 145 | (38, 184) | 682 | (22, 181) | 204 | Peptidase U35 | Peptidase U35 | | uniclust | UniRef100\_UPI000CF32F82 | 99.7 | 1.4e-21 | 2.7e-27 | 195.7 | 421 | (43, 680) | 682 | (37, 475) | 480 | phage major capsid protein | phage major capsid protein | | uniclust | UniRef100\_A0A6J5S3H4 | 99.7 | 1.5e-21 | 2.8e-27 | 189.1 | 283 | (386, 680) | 682 | (123, 425) | 429 | COG4653 Predicted phage phi-C31 gp36 major capsid-like protein | COG4653 Predicted phage phi-C31 gp36 major capsid-like protein | | uniclust | UniRef100\_A0A5A7MXX4 | 99.7 | 1.7e-21 | 3.1e-27 | 196.5 | 453 | (32, 524) | 682 | (24, 509) | 801 | Uncharacterized protein | Uncharacterized protein | | uniclust | UniRef100\_A0A0F9HAL3 | 99.7 | 1.7e-21 | 3.2e-27 | 185.3 | 266 | (406, 682) | 682 | (84, 369) | 370 | Phage major capsid protein | Phage major capsid protein | | uniclust | UniRef100\_A0A255R0K0 | 99.7 | 1.9e-21 | 3.5e-27 | 189.5 | 265 | (406, 682) | 682 | (162, 432) | 434 | Phage major capsid protein | Phage major capsid protein | | uniclust | UniRef100\_A0A074U171 | 99.7 | 1.8e-21 | 3.5e-27 | 200.6 | 260 | (408, 679) | 682 | (190, 460) | 476 | Nucleoid-structuring protein H-NS | Nucleoid-structuring protein H-NS | | uniclust | UniRef100\_A0A023W7M8 | 99.7 | 1.9e-21 | 3.8e-27 | 200.7 | 259 | (412, 681) | 682 | (37, 326) | 351 | Major capsid protein | Major capsid protein | | uniclust | UniRef100\_A0A8S5N8Q6 | 99.7 | 2.3e-21 | 4.2e-27 | 168.9 | 149 | (32, 185) | 682 | (25, 176) | 226 | Major capsid protein (Fragment) | Major capsid protein (Fragment) | | uniclust | UniRef100\_A0A0M8SR27 | 99.7 | 2.3e-21 | 4.4e-27 | 195.6 | 269 | (393, 679) | 682 | (139, 436) | 449 | Phage head | Phage head | | uniclust | UniRef100\_A0A0F9WTB8 | 99.7 | 2.3e-21 | 4.4e-27 | 195.1 | 269 | (393, 679) | 682 | (145, 448) | 451 | HK97 family phage major capsid protein | HK97 family phage major capsid protein | | uniclust | UniRef100\_A0A1G3DZF5 | 99.7 | 2.3e-21 | 4.4e-27 | 202.3 | 247 | (422, 678) | 682 | (249, 506) | 591 | Capsid protein | Capsid protein | | uniclust | UniRef100\_A0A1C3X8S9 | 99.7 | 2.6e-21 | 5.2e-27 | 199.9 | 261 | (405, 681) | 682 | (96, 387) | 393 | Phage major capsid protein, HK97 family | Phage major capsid protein, HK97 family | | uniclust | UniRef100\_A0A059N3B4 | 99.7 | 2.8e-21 | 5.3e-27 | 175.0 | 136 | (41, 181) | 682 | (28, 170) | 204 | Phage prohead protease, HK97 family | Phage prohead protease, HK97 family | | uniclust | UniRef100\_A0A060LW15 | 99.7 | 2.9e-21 | 5.7e-27 | 195.4 | 271 | (393, 681) | 682 | (124, 411) | 485 | Phage-related protein | Phage-related protein | | uniclust | UniRef100\_A0A1E3GWI0 | 99.7 | 3e-21 | 5.7e-27 | 186.3 | 266 | (406, 681) | 682 | (51, 338) | 344 | Phage capsid family protein | Phage capsid family protein | | uniclust | UniRef100\_A0A081HUR8 | 99.7 | 3.2e-21 | 6.3e-27 | 178.8 | 148 | (31, 184) | 682 | (16, 172) | 207 | HK97 family phage prohead protease | HK97 family phage prohead protease | | uniclust | UniRef100\_A0A2M9PA59 | 99.7 | 3.4e-21 | 6.4e-27 | 174.1 | 235 | (432, 678) | 682 | (12, 260) | 263 | Phage major capsid protein (Fragment) | Phage major capsid protein (Fragment) | | uniclust | UniRef100\_A0A023DZP0 | 99.7 | 3.9e-21 | 7.7e-27 | 180.6 | 134 | (38, 181) | 682 | (29, 167) | 237 | Putative prohead protease | Putative prohead protease | | uniclust | UniRef100\_A0A0F8ZWE4 | 99.7 | 3.9e-21 | 7.9e-27 | 205.3 | 393 | (260, 680) | 682 | (132, 557) | 561 | Bacteriophage Mu GpT domain-containing protein (Fragment) | Bacteriophage Mu GpT domain-containing protein (Fragment) | | uniclust | UniRef100\_A0A4Q3XXJ0 | 99.7 | 4.6e-21 | 8.5e-27 | 172.3 | 152 | (31, 183) | 682 | (19, 174) | 281 | HK97 family phage prohead protease (Fragment) | HK97 family phage prohead protease (Fragment) | | uniclust | UniRef100\_A0A1A2T1Y5 | 99.7 | 4.5e-21 | 8.8e-27 | 196.4 | 263 | (409, 681) | 682 | (155, 430) | 438 | Capsid protein | Capsid protein | | uniclust | UniRef100\_A0A2W2FHR8 | 99.7 | 5e-21 | 9.6e-27 | 191.7 | 266 | (406, 680) | 682 | (133, 433) | 445 | Phage major capsid protein | Phage major capsid protein | | uniclust | UniRef100\_A0A524RVZ4 | 99.7 | 6.4e-21 | 1.2e-26 | 191.1 | 338 | (333, 678) | 682 | (385, 736) | 737 | Phage major capsid protein | Phage major capsid protein | | uniclust | UniRef100\_A0A652L3X9 | 99.7 | 6.5e-21 | 1.2e-26 | 185.9 | 269 | (393, 678) | 682 | (264, 549) | 554 | Phage major capsid protein | Phage major capsid protein | | uniclust | UniRef100\_A0A0D6QFX9 | 99.7 | 7.2e-21 | 1.4e-26 | 188.7 | 253 | (406, 670) | 682 | (125, 401) | 424 | Phage capsid family protein | Phage capsid family protein | | uniclust | UniRef100\_A0A518EKY1 | 99.7 | 7.9e-21 | 1.5e-26 | 183.2 | 432 | (38, 506) | 682 | (22, 495) | 497 | Caudovirus prohead protease | Caudovirus prohead protease | | uniclust | UniRef100\_A0A068QRW5 | 99.7 | 7.7e-21 | 1.5e-26 | 190.7 | 267 | (393, 679) | 682 | (110, 400) | 501 | Putative major capsid protein | Putative major capsid protein | | uniclust | UniRef100\_A0A0H3A784 | 99.7 | 8.7e-21 | 1.7e-26 | 184.4 | 133 | (42, 181) | 682 | (60, 203) | 287 | Phage prohead protease, HK97 family | Phage prohead protease, HK97 family | | uniclust | UniRef100\_A0A1I1GTH7 | 99.7 | 9.3e-21 | 1.8e-26 | 187.0 | 260 | (406, 679) | 682 | (118, 399) | 422 | Phage major capsid protein, HK97 family | Phage major capsid protein, HK97 family | | uniclust | UniRef100\_A0A0J5GQW0 | 99.7 | 9.2e-21 | 1.8e-26 | 188.7 | 260 | (408, 681) | 682 | (140, 408) | 420 | Phage capsid protein (Fragment) | Phage capsid protein (Fragment) | | uniclust | UniRef100\_A0A1V5D5J1 | 99.7 | 9.6e-21 | 1.8e-26 | 180.6 | 150 | (29, 182) | 682 | (6, 165) | 322 | Caudovirus prohead protease | Caudovirus prohead protease | | uniclust | UniRef100\_UPI001E304D27 | 99.7 | 1.4e-20 | 2.5e-26 | 171.9 | 269 | (31, 307) | 682 | (23, 297) | 313 | HK97 family phage prohead protease | HK97 family phage prohead protease | | uniclust | UniRef100\_A0A0C5E3N1 | 99.7 | 1.5e-20 | 2.9e-26 | 173.3 | 137 | (36, 181) | 682 | (22, 161) | 227 | HK97 family phage prohead protease | HK97 family phage prohead protease | | uniclust | UniRef100\_A0A3A8JJQ6 | 99.7 | 1.6e-20 | 3.1e-26 | 187.5 | 265 | (408, 682) | 682 | (136, 413) | 416 | Phage major capsid protein | Phage major capsid protein | | uniclust | UniRef100\_A0A843H9F1 | 99.7 | 1.8e-20 | 3.3e-26 | 173.6 | 268 | (406, 680) | 682 | (70, 349) | 350 | Phage major capsid protein | Phage major capsid protein | | uniclust | UniRef100\_A0A0F9MYQ3 | 99.7 | 2e-20 | 3.7e-26 | 186.9 | 589 | (41, 676) | 682 | (33, 692) | 702 | Phage major capsid protein | Phage major capsid protein | | uniclust | UniRef100\_A0A640YC84 | 99.7 | 2.1e-20 | 3.8e-26 | 180.6 | 473 | (31, 680) | 682 | (13, 500) | 502 | HK97 family phage prohead protease | HK97 family phage prohead protease | | uniclust | UniRef100\_A0A150IJM8 | 99.7 | 2.1e-20 | 3.9e-26 | 177.6 | 258 | (412, 680) | 682 | (71, 342) | 345 | Phage capsid family protein | Phage capsid family protein | | uniclust | UniRef100\_A0A212I573 | 99.7 | 2.3e-20 | 4.3e-26 | 175.7 | 249 | (421, 681) | 682 | (14, 295) | 305 | Structural protein | Structural protein | | uniclust | UniRef100\_UPI001FCE965E | 99.7 | 2.5e-20 | 4.6e-26 | 172.3 | 281 | (385, 679) | 682 | (44, 328) | 331 | phage major capsid protein | phage major capsid protein | | uniclust | UniRef100\_A0A259YXH9 | 99.7 | 2.7e-20 | 5.1e-26 | 185.7 | 468 | (41, 681) | 682 | (12, 514) | 515 | Phage major capsid protein | Phage major capsid protein | | uniclust | UniRef100\_A0A1V5LQQ8 | 99.7 | 2.8e-20 | 5.2e-26 | 168.7 | 262 | (406, 680) | 682 | (10, 295) | 297 | Phage capsid family protein | Phage capsid family protein | | uniclust | UniRef100\_A0A011UW27 | 99.7 | 2.7e-20 | 5.4e-26 | 183.5 | 129 | (44, 181) | 682 | (80, 212) | 293 | Peptidase U35 | Peptidase U35 | | uniclust | UniRef100\_UPI0009B17BBC | 99.7 | 3.4e-20 | 6.4e-26 | 177.3 | 146 | (31, 184) | 682 | (8, 159) | 355 | HK97 family phage prohead protease | HK97 family phage prohead protease | | uniclust | UniRef100\_A0A7Z2S8T5 | 99.7 | 3.5e-20 | 6.5e-26 | 163.2 | 151 | (32, 185) | 682 | (23, 177) | 240 | Caudovirus prohead protease | Caudovirus prohead protease | | uniclust | UniRef100\_A0A084J9C1 | 99.7 | 3.3e-20 | 6.5e-26 | 191.2 | 259 | (406, 680) | 682 | (144, 421) | 434 | Capsid protein | Capsid protein | | uniclust | UniRef100\_A0A0Q4Y1E9 | 99.7 | 3.5e-20 | 6.6e-26 | 168.4 | 128 | (41, 181) | 682 | (28, 162) | 218 | Peptidase U35 | Peptidase U35 | | uniclust | UniRef100\_F5YBI3 | 99.7 | 3.6e-20 | 6.7e-26 | 168.6 | 253 | (412, 678) | 682 | (33, 302) | 304 | Phage capsid family | Phage capsid family | | uniclust | UniRef100\_A0A285D3S6 | 99.7 | 3.6e-20 | 6.7e-26 | 170.8 | 214 | (31, 308) | 682 | (23, 246) | 292 | Prohead serine protease | Prohead serine protease | | uniclust | UniRef100\_A0A164A2N1 | 99.7 | 3.5e-20 | 6.9e-26 | 174.1 | 135 | (43, 185) | 682 | (25, 168) | 217 | Uncharacterized protein | Uncharacterized protein | | uniclust | UniRef100\_A0A0Q6BU62 | 99.7 | 4.3e-20 | 8e-26 | 172.8 | 152 | (31, 184) | 682 | (13, 177) | 290 | Peptidase U35 | Peptidase U35 | | uniclust | UniRef100\_A0A0G1JMI3 | 99.7 | 4.5e-20 | 8.4e-26 | 183.3 | 265 | (407, 681) | 682 | (174, 471) | 475 | HK97 family major capsid protein | HK97 family major capsid protein | | uniclust | UniRef100\_A0A1C3WL59 | 99.7 | 5.3e-20 | 1e-25 | 180.0 | 301 | (337, 673) | 682 | (65, 380) | 393 | Phage major capsid protein, HK97 family | Phage major capsid protein, HK97 family | | uniclust | UniRef100\_A0A3C1GA37 | 99.7 | 5.8e-20 | 1.1e-25 | 170.3 | 228 | (32, 307) | 682 | (84, 313) | 348 | Uncharacterized protein | Uncharacterized protein | | uniclust | UniRef100\_UPI0022B09E7B | 99.7 | 5.8e-20 | 1.1e-25 | 181.5 | 271 | (393, 681) | 682 | (319, 615) | 616 | phage major capsid protein | phage major capsid protein | | uniclust | UniRef100\_A0A2V5Y3W0 | 99.7 | 5.9e-20 | 1.1e-25 | 171.2 | 327 | (337, 679) | 682 | (20, 359) | 364 | Phage major capsid protein | Phage major capsid protein | | uniclust | UniRef100\_A0A1I0EDA5 | 99.7 | 5.9e-20 | 1.2e-25 | 195.2 | 263 | (406, 680) | 682 | (239, 522) | 526 | Phage major capsid protein, HK97 family | Phage major capsid protein, HK97 family | | uniclust | UniRef100\_A0A246JDV9 | 99.7 | 6.7e-20 | 1.3e-25 | 185.9 | 272 | (403, 680) | 682 | (182, 480) | 495 | Phage major capsid protein | Phage major capsid protein | | uniclust | UniRef100\_A0A0Q7IVE6 | 99.7 | 7.2e-20 | 1.4e-25 | 164.2 | 133 | (41, 182) | 682 | (21, 159) | 197 | Peptidase U35 | Peptidase U35 | | uniclust | UniRef100\_A0A136PUC6 | 99.7 | 7.5e-20 | 1.5e-25 | 205.3 | 523 | (39, 679) | 682 | (576, 1129) | 1146 | Uncharacterized protein | Uncharacterized protein | | uniclust | UniRef100\_A0A083XX18 | 99.7 | 7.9e-20 | 1.5e-25 | 172.7 | 142 | (31, 181) | 682 | (7, 164) | 255 | Peptidase | Peptidase | | uniclust | UniRef100\_A0A0F9BNT3 | 99.7 | 8.1e-20 | 1.5e-25 | 181.0 | 261 | (409, 681) | 682 | (143, 424) | 427 | Phage major capsid protein | Phage major capsid protein | | uniclust | UniRef100\_UPI0014703FA7 | 99.7 | 8.5e-20 | 1.6e-25 | 175.9 | 366 | (269, 679) | 682 | (40, 407) | 410 | phage major capsid protein | phage major capsid protein | | uniclust | UniRef100\_A0A0C9QCT1 | 99.7 | 8.2e-20 | 1.6e-25 | 179.2 | 137 | (43, 183) | 682 | (61, 204) | 286 | HK97 family phage prohead protease | HK97 family phage prohead protease | | uniclust | UniRef100\_A0A015W478 | 99.7 | 8.3e-20 | 1.7e-25 | 183.9 | 171 | (1, 181) | 682 | (2, 181) | 274 | Phage prohead protease, HK97 family | Phage prohead protease, HK97 family | | uniclust | UniRef100\_A0A062VEV4 | 99.7 | 9.7e-20 | 1.8e-25 | 156.3 | 147 | (31, 183) | 682 | (7, 158) | 201 | Peptidase U35, phage prohead HK97 (Fragment) | Peptidase U35, phage prohead HK97 (Fragment) | | uniclust | UniRef100\_A0A1V5ID83 | 99.7 | 1e-19 | 2e-25 | 173.9 | 263 | (406, 680) | 682 | (95, 369) | 372 | Phage capsid family protein | Phage capsid family protein | | uniclust | UniRef100\_A0A087DDT0 | 99.7 | 9.8e-20 | 2e-25 | 196.4 | 247 | (422, 680) | 682 | (211, 465) | 484 | Phage prohead protease, HK97 family protein | Phage prohead protease, HK97 family protein | | uniclust | UniRef100\_A0A840YDI0 | 99.7 | 1.1e-19 | 2.1e-25 | 177.7 | 150 | (32, 184) | 682 | (24, 184) | 418 | Peptidase U35 (Fragment) | Peptidase U35 (Fragment) | | uniclust | UniRef100\_UPI001CFB8F9B | 99.7 | 1.2e-19 | 2.2e-25 | 178.0 | 461 | (35, 678) | 682 | (8, 503) | 512 | HK97 family phage prohead protease | HK97 family phage prohead protease | | uniclust | UniRef100\_A0A178MUR1 | 99.7 | 1.3e-19 | 2.4e-25 | 170.5 | 137 | (39, 181) | 682 | (42, 188) | 256 | Peptidase U35 | Peptidase U35 | | uniclust | UniRef100\_A0A149TLR9 | 99.7 | 1.3e-19 | 2.5e-25 | 170.5 | 138 | (33, 181) | 682 | (29, 173) | 233 | Uncharacterized protein | Uncharacterized protein | | uniclust | UniRef100\_A0A1V5WQJ7 | 99.7 | 1.4e-19 | 2.5e-25 | 178.4 | 529 | (40, 678) | 682 | (12, 588) | 594 | Phage capsid family protein | Phage capsid family protein | | uniclust | UniRef100\_A0A0F7L8Q8 | 99.7 | 1.3e-19 | 2.5e-25 | 185.7 | 256 | (411, 679) | 682 | (133, 403) | 418 | Phage major capsid protein HK97 | Phage major capsid protein HK97 | | uniclust | UniRef100\_A0A0W1GEU5 | 99.7 | 1.4e-19 | 2.6e-25 | 168.9 | 132 | (43, 181) | 682 | (17, 152) | 248 | Peptidase U35 | Peptidase U35 | | uniclust | UniRef100\_A0A3E1K5B0 | 99.7 | 1.5e-19 | 2.7e-25 | 174.3 | 265 | (406, 681) | 682 | (213, 479) | 483 | Phage major capsid protein | Phage major capsid protein | | uniclust | UniRef100\_A0A3A6NR20 | 99.7 | 1.5e-19 | 2.7e-25 | 174.6 | 267 | (406, 681) | 682 | (103, 405) | 408 | Phage major capsid protein | Phage major capsid protein | | uniclust | UniRef100\_A0A2D8XER8 | 99.7 | 1.5e-19 | 2.8e-25 | 178.5 | 539 | (63, 678) | 682 | (44, 605) | 611 | Phage major capsid protein | Phage major capsid protein | | uniclust | UniRef100\_A0A846L7P7 | 99.7 | 1.6e-19 | 2.9e-25 | 176.5 | 263 | (406, 681) | 682 | (238, 526) | 529 | Phage major capsid protein | Phage major capsid protein | | uniclust | UniRef100\_A0A9E3PA23 | 99.7 | 1.6e-19 | 3e-25 | 170.5 | 279 | (393, 681) | 682 | (98, 400) | 403 | Phage major capsid protein | Phage major capsid protein | | uniclust | UniRef100\_A0A031HUL5 | 99.7 | 1.6e-19 | 3.2e-25 | 166.9 | 134 | (38, 181) | 682 | (21, 158) | 233 | HK97 family phage prohead protease | HK97 family phage prohead protease | | uniclust | UniRef100\_A0A359LVB0 | 99.7 | 1.9e-19 | 3.4e-25 | 177.2 | 293 | (42, 380) | 682 | (27, 322) | 588 | Tripartite tricarboxylate transporter substrate binding protein | Tripartite tricarboxylate transporter substrate binding protein | | uniclust | UniRef100\_A0A1H7U3C7 | 99.7 | 2e-19 | 3.7e-25 | 172.0 | 262 | (408, 680) | 682 | (92, 361) | 379 | Phage major capsid protein, HK97 family | Phage major capsid protein, HK97 family | | uniclust | UniRef100\_A0A1I6URX3 | 99.7 | 2e-19 | 4e-25 | 168.8 | 149 | (31, 184) | 682 | (24, 189) | 239 | Phage prohead protease, HK97 family | Phage prohead protease, HK97 family | | uniclust | UniRef100\_A0A0F9FEI0 | 99.7 | 2.1e-19 | 4.1e-25 | 169.3 | 225 | (447, 681) | 682 | (42, 277) | 280 | Phage major capsid protein (Fragment) | Phage major capsid protein (Fragment) | | uniclust | UniRef100\_A0A0D1C1F0 | 99.7 | 2.2e-19 | 4.3e-25 | 166.5 | 138 | (43, 184) | 682 | (35, 181) | 216 | Phage prohead protein | Phage prohead protein | | uniclust | UniRef100\_A0A1C5S2N3 | 99.7 | 2.2e-19 | 4.3e-25 | 178.7 | 252 | (407, 680) | 682 | (105, 365) | 378 | Predicted phage phi-C31 gp36 major capsid-like protein | Predicted phage phi-C31 gp36 major capsid-like protein | | uniclust | UniRef100\_A0A0C5CQK6 | 99.6 | 2.5e-19 | 4.5e-25 | 166.9 | 221 | (406, 639) | 682 | (29, 256) | 358 | Phage major capsid protein | Phage major capsid protein | | uniclust | UniRef100\_A0A098AP33 | 99.6 | 2.5e-19 | 5e-25 | 193.7 | 261 | (410, 679) | 682 | (163, 448) | 564 | Putative prophage protein | Putative prophage protein | | uniclust | UniRef100\_A0A7V7PNP3 | 99.6 | 2.8e-19 | 5.1e-25 | 174.9 | 251 | (406, 666) | 682 | (102, 368) | 550 | Phage major capsid protein | Phage major capsid protein | | uniclust | UniRef100\_A0A7K2K343 | 99.6 | 2.7e-19 | 5.1e-25 | 186.0 | 506 | (45, 680) | 682 | (58, 599) | 613 | Phage major capsid protein | Phage major capsid protein | | uniclust | UniRef100\_A0A2K8PMM0 | 99.6 | 2.7e-19 | 5.2e-25 | 184.9 | 430 | (31, 678) | 682 | (43, 499) | 575 | Caudovirus prohead protease | Caudovirus prohead protease | | uniclust | UniRef100\_A0A3R9V132 | 99.6 | 2.9e-19 | 5.4e-25 | 175.7 | 267 | (406, 680) | 682 | (148, 424) | 426 | Phage major capsid protein | Phage major capsid protein | | uniclust | UniRef100\_A0A5D0XCB7 | 99.6 | 3.1e-19 | 5.7e-25 | 169.0 | 266 | (407, 682) | 682 | (52, 338) | 340 | Phage major capsid protein | Phage major capsid protein | | uniclust | UniRef100\_A0A0F9PEJ2 | 99.6 | 3.2e-19 | 6.2e-25 | 183.1 | 133 | (43, 181) | 682 | (22, 158) | 449 | Phage-like element PBSX protein XkdF domain-containing protein | Phage-like element PBSX protein XkdF domain-containing protein | | uniclust | UniRef100\_A0A170SMX2 | 99.6 | 3.8e-19 | 7.1e-25 | 169.5 | 262 | (406, 680) | 682 | (100, 386) | 390 | HK97 family phage major capsid protein (Fragment) | HK97 family phage major capsid protein (Fragment) | | uniclust | UniRef100\_A0A0Q5UI83 | 99.6 | 3.8e-19 | 7.6e-25 | 195.0 | 385 | (259, 680) | 682 | (314, 729) | 733 | ATP-dependent Clp protease proteolytic subunit | ATP-dependent Clp protease proteolytic subunit | | uniclust | UniRef100\_A0A165XDA1 | 99.6 | 4.3e-19 | 7.9e-25 | 159.9 | 144 | (39, 184) | 682 | (22, 169) | 277 | Caudovirus prohead protease | Caudovirus prohead protease | | uniclust | UniRef100\_A0A061LZA8 | 99.6 | 4e-19 | 7.9e-25 | 172.5 | 132 | (44, 184) | 682 | (51, 189) | 263 | Peptidase U35 | Peptidase U35 | | uniclust | UniRef100\_A0A084UCG9 | 99.6 | 4.3e-19 | 8.3e-25 | 167.1 | 130 | (43, 181) | 682 | (36, 169) | 256 | HK97 family phage prohead protease | HK97 family phage prohead protease | | uniclust | UniRef100\_A0A1A2PL73 | 99.6 | 4.3e-19 | 8.5e-25 | 182.6 | 261 | (411, 681) | 682 | (146, 418) | 424 | Major capsid protein | Major capsid protein | | uniclust | UniRef100\_A0A0H1RDA6 | 99.6 | 4.9e-19 | 9.5e-25 | 162.2 | 130 | (43, 182) | 682 | (38, 171) | 206 | Peptidase U35 | Peptidase U35 | | uniclust | UniRef100\_A0A031HP15 | 99.6 | 4.7e-19 | 9.6e-25 | 182.8 | 241 | (421, 681) | 682 | (89, 357) | 382 | Phage major capsid protein, HK97 family | Phage major capsid protein, HK97 family | | uniclust | UniRef100\_A0A6I2TYG7 | 99.6 | 5.3e-19 | 9.7e-25 | 168.5 | 282 | (376, 679) | 682 | (146, 430) | 432 | Phage major capsid protein | Phage major capsid protein | | uniclust | UniRef100\_A0A0H5CWQ9 | 99.6 | 5.2e-19 | 9.9e-25 | 161.3 | 129 | (43, 181) | 682 | (39, 171) | 220 | Phage prohead protease, HK97 family | Phage prohead protease, HK97 family | | uniclust | UniRef100\_A0A7C4PRQ6 | 99.6 | 5.4e-19 | 1e-24 | 178.2 | 278 | (386, 681) | 682 | (170, 470) | 472 | Phage major capsid protein | Phage major capsid protein | | uniclust | UniRef100\_A0A091B3N3 | 99.6 | 5.3e-19 | 1.1e-24 | 184.8 | 256 | (415, 679) | 682 | (143, 405) | 414 | Capsid protein | Capsid protein | | uniclust | UniRef100\_UPI0009494E27 | 99.6 | 6.5e-19 | 1.2e-24 | 164.7 | 149 | (31, 185) | 682 | (4, 165) | 295 | HK97 family phage prohead protease | HK97 family phage prohead protease | | uniclust | UniRef100\_A0A0G4KA63 | 99.6 | 6.7e-19 | 1.3e-24 | 171.6 | 264 | (406, 679) | 682 | (130, 419) | 439 | Phage major capsid protein, HK97 family | Phage major capsid protein, HK97 family | | uniclust | UniRef100\_A0A0K2JIG5 | 99.6 | 7.6e-19 | 1.4e-24 | 177.2 | 257 | (407, 678) | 682 | (322, 592) | 595 | Phage major capsid protein | Phage major capsid protein | | uniclust | UniRef100\_A0A6J5MIS0 | 99.6 | 7.8e-19 | 1.5e-24 | 171.8 | 262 | (406, 679) | 682 | (95, 369) | 371 | COG4653 Predicted phage phi-C31 gp36 major capsid-like protein | COG4653 Predicted phage phi-C31 gp36 major capsid-like protein | | uniclust | UniRef100\_A0A174DQP5 | 99.6 | 8e-19 | 1.6e-24 | 177.2 | 258 | (409, 681) | 682 | (94, 361) | 369 | Phage major capsid protein, HK97 family | Phage major capsid protein, HK97 family | | uniclust | UniRef100\_A0A016QNE7 | 99.6 | 8.9e-19 | 1.7e-24 | 173.5 | 137 | (35, 181) | 682 | (118, 257) | 377 | Phage prohead protease, HK97 family | Phage prohead protease, HK97 family | | uniclust | UniRef100\_A0A1Q3QR31 | 99.6 | 9.1e-19 | 1.8e-24 | 167.8 | 139 | (41, 184) | 682 | (27, 174) | 250 | HK97 family phage prohead protease | HK97 family phage prohead protease | | uniclust | UniRef100\_A0A062XI24 | 99.6 | 9.1e-19 | 1.8e-24 | 165.2 | 128 | (47, 181) | 682 | (44, 180) | 232 | Prohead protease | Prohead protease | | uniclust | UniRef100\_A0A011URX1 | 99.6 | 9.8e-19 | 1.9e-24 | 166.1 | 142 | (38, 185) | 682 | (33, 182) | 234 | Peptidase U35 | Peptidase U35 | | uniclust | UniRef100\_A0A059G8L6 | 99.6 | 1e-18 | 2e-24 | 180.3 | 316 | (341, 681) | 682 | (113, 450) | 519 | Phage major capsid protein | Phage major capsid protein | | uniclust | UniRef100\_A0A142XTA2 | 99.6 | 1.1e-18 | 2.1e-24 | 175.3 | 259 | (406, 671) | 682 | (168, 447) | 468 | Phage capsid family protein | Phage capsid family protein | | uniclust | UniRef100\_A0A0F9LI03 | 99.6 | 1.1e-18 | 2.1e-24 | 174.8 | 265 | (407, 681) | 682 | (163, 435) | 435 | Phage major capsid protein | Phage major capsid protein | | uniclust | UniRef100\_A0A2G2LGM9 | 99.6 | 1.1e-18 | 2.1e-24 | 168.7 | 138 | (35, 181) | 682 | (22, 165) | 294 | HK97 family phage prohead protease | HK97 family phage prohead protease | | uniclust | UniRef100\_A0A2W7AVB5 | 99.6 | 1.1e-18 | 2.1e-24 | 167.4 | 146 | (40, 185) | 682 | (35, 188) | 405 | HK97 family phage prohead protease | HK97 family phage prohead protease | | uniclust | UniRef100\_A0A023W708 | 99.6 | 1.1e-18 | 2.2e-24 | 180.5 | 253 | (411, 673) | 682 | (41, 319) | 417 | Major capsid protein | Major capsid protein | | uniclust | UniRef100\_A0A6N7GTQ9 | 99.6 | 1.2e-18 | 2.2e-24 | 166.6 | 135 | (32, 181) | 682 | (5, 150) | 296 | Uncharacterized protein | Uncharacterized protein | | uniclust | UniRef100\_A0A0B5CU19 | 99.6 | 1.1e-18 | 2.2e-24 | 176.2 | 254 | (413, 680) | 682 | (38, 316) | 412 | Main capsid protein | Main capsid protein | | uniclust | UniRef100\_A0A1M6LHF3 | 99.6 | 1.2e-18 | 2.3e-24 | 173.3 | 131 | (41, 181) | 682 | (53, 192) | 410 | Phage prohead protease, HK97 family | Phage prohead protease, HK97 family | | uniclust | UniRef100\_A0A956YZ53 | 99.6 | 1.3e-18 | 2.4e-24 | 168.4 | 259 | (406, 676) | 682 | (100, 385) | 404 | Phage major capsid protein | Phage major capsid protein | | uniclust | UniRef100\_A0A965HA95 | 99.6 | 1.3e-18 | 2.4e-24 | 154.1 | 214 | (466, 681) | 682 | (2, 242) | 244 | Phage major capsid protein | Phage major capsid protein | | uniclust | UniRef100\_A0A0X3XWX8 | 99.6 | 1.3e-18 | 2.5e-24 | 181.2 | 236 | (433, 679) | 682 | (198, 446) | 459 | Phage capsid protein | Phage capsid protein | | uniclust | UniRef100\_A0A6M3KXC0 | 99.6 | 1.3e-18 | 2.6e-24 | 156.7 | 132 | (38, 181) | 682 | (12, 147) | 202 | Putative prohead protease | Putative prohead protease | | uniclust | UniRef100\_A0A0D0KY24 | 99.6 | 1.3e-18 | 2.6e-24 | 180.9 | 379 | (260, 670) | 682 | (138, 544) | 559 | Bacteriophage Mu GpT domain-containing protein | Bacteriophage Mu GpT domain-containing protein | | uniclust | UniRef100\_A0A015V7E1 | 99.6 | 1.3e-18 | 2.6e-24 | 172.5 | 149 | (31, 184) | 682 | (57, 219) | 270 | Caudovirus prohead protease family protein | Caudovirus prohead protease family protein | | uniclust | UniRef100\_A0A081I8G1 | 99.6 | 1.3e-18 | 2.7e-24 | 182.0 | 260 | (413, 681) | 682 | (191, 462) | 470 | HK97 family phage major capsid protein | HK97 family phage major capsid protein | | uniclust | UniRef100\_A0A0J5GP20 | 99.6 | 1.3e-18 | 2.7e-24 | 164.7 | 145 | (31, 183) | 682 | (18, 176) | 216 | Peptidase U35 | Peptidase U35 | | uniclust | UniRef100\_A0A045IH31 | 99.6 | 1.5e-18 | 2.9e-24 | 163.2 | 142 | (31, 181) | 682 | (18, 169) | 208 | PhiRv2 phage protease | PhiRv2 phage protease | | uniclust | UniRef100\_A0A812Y0T0 | 99.6 | 1.6e-18 | 3e-24 | 171.3 | 269 | (393, 680) | 682 | (147, 425) | 603 | Phage major capsid protein (Fragment) | Phage major capsid protein (Fragment) | | uniclust | UniRef100\_A0A0P7D8F3 | 99.6 | 1.6e-18 | 3e-24 | 156.3 | 136 | (36, 181) | 682 | (18, 160) | 230 | Primosome assembly protein PriA | Primosome assembly protein PriA | | uniclust | UniRef100\_A0A077M1Z5 | 99.6 | 1.5e-18 | 3e-24 | 187.9 | 503 | (39, 679) | 682 | (42, 578) | 585 | Uncharacterized protein | Uncharacterized protein | | uniclust | UniRef100\_A0A7C4QW79 | 99.6 | 1.7e-18 | 3.1e-24 | 172.6 | 259 | (413, 680) | 682 | (373, 651) | 654 | Phage major capsid protein | Phage major capsid protein | | uniclust | UniRef100\_A0A0F9FAM1 | 99.6 | 1.6e-18 | 3.1e-24 | 164.6 | 139 | (38, 184) | 682 | (20, 161) | 290 | HK97 family phage prohead protease | HK97 family phage prohead protease | | uniclust | UniRef100\_A0A1H3NUF2 | 99.6 | 1.7e-18 | 3.3e-24 | 159.3 | 210 | (462, 680) | 682 | (3, 218) | 222 | Phage major capsid protein, HK97 family | Phage major capsid protein, HK97 family | | uniclust | UniRef100\_A0A0D5XUH0 | 99.6 | 1.7e-18 | 3.3e-24 | 181.9 | 261 | (406, 679) | 682 | (164, 433) | 451 | Capsid protein | Capsid protein | | uniclust | UniRef100\_A0A0U4IJH3 | 99.6 | 1.7e-18 | 3.4e-24 | 190.3 | 478 | (39, 679) | 682 | (52, 544) | 576 | Major capsid and protease fusion protein | Major capsid and protease fusion protein | | uniclust | UniRef100\_A0A068D609 | 99.6 | 1.8e-18 | 3.4e-24 | 153.1 | 135 | (42, 183) | 682 | (27, 165) | 198 | HK97 family phage prohead protease | HK97 family phage prohead protease | | uniclust | UniRef100\_A0A6J5PI70 | 99.6 | 1.9e-18 | 3.4e-24 | 172.4 | 513 | (31, 679) | 682 | (5, 580) | 584 | Major\_cap\_HK97, phage major capsid protein, HK97 family | Major\_cap\_HK97, phage major capsid protein, HK97 family | | uniclust | UniRef100\_A0A0Q7DEB2 | 99.6 | 1.8e-18 | 3.5e-24 | 156.0 | 129 | (38, 181) | 682 | (44, 173) | 205 | Peptidase U35 | Peptidase U35 | | uniclust | UniRef100\_A0A0F9JEK1 | 99.6 | 1.8e-18 | 3.6e-24 | 180.2 | 263 | (406, 676) | 682 | (188, 475) | 493 | Phage major capsid protein | Phage major capsid protein | | uniclust | UniRef100\_A0A7W7ZFU5 | 99.6 | 2e-18 | 3.8e-24 | 169.6 | 264 | (406, 680) | 682 | (120, 399) | 402 | HK97 family phage major capsid protein | HK97 family phage major capsid protein | | uniclust | UniRef100\_A0A2U3NJA2 | 99.6 | 2e-18 | 3.8e-24 | 156.5 | 209 | (462, 681) | 682 | (2, 219) | 227 | Capsid protein (Fragment) | Capsid protein (Fragment) | | uniclust | UniRef100\_A0A6J5LD92 | 99.6 | 2.1e-18 | 4e-24 | 154.4 | 133 | (41, 180) | 682 | (15, 150) | 205 | COG3740 Phage head maturation protease | COG3740 Phage head maturation protease | | uniclust | UniRef100\_A0A0A0YV29 | 99.6 | 2.2e-18 | 4.1e-24 | 153.6 | 129 | (43, 181) | 682 | (22, 155) | 195 | Prohead maturation protease | Prohead maturation protease | | uniclust | UniRef100\_A0A059NYL5 | 99.6 | 2.6e-18 | 5.1e-24 | 173.8 | 253 | (413, 680) | 682 | (45, 318) | 362 | Phage major capsid protein, HK97 family | Phage major capsid protein, HK97 family | | uniclust | UniRef100\_A0A2U2DX69 | 99.6 | 2.8e-18 | 5.2e-24 | 167.4 | 262 | (406, 680) | 682 | (139, 436) | 440 | Phage major capsid protein | Phage major capsid protein | | uniclust | UniRef100\_A0A0K1JGP8 | 99.6 | 2.8e-18 | 5.4e-24 | 168.2 | 260 | (408, 679) | 682 | (127, 408) | 409 | Capsid protein | Capsid protein | | uniclust | UniRef100\_A0A0S7CWH9 | 99.6 | 2.8e-18 | 5.4e-24 | 166.5 | 137 | (33, 180) | 682 | (12, 154) | 306 | Prohead protease | Prohead protease | | uniclust | UniRef100\_A0A497JQ78 | 99.6 | 2.8e-18 | 5.4e-24 | 168.5 | 250 | (414, 680) | 682 | (70, 323) | 324 | Phage major capsid protein | Phage major capsid protein | | uniclust | UniRef100\_A0A0F9W0Z4 | 99.6 | 3e-18 | 5.5e-24 | 162.8 | 271 | (393, 681) | 682 | (133, 412) | 414 | Phage major capsid protein | Phage major capsid protein | | uniclust | UniRef100\_A0A7I9ZGE4 | 99.6 | 3.2e-18 | 5.9e-24 | 169.4 | 520 | (31, 681) | 682 | (17, 601) | 606 | Major capsid protein | Major capsid protein | | uniclust | UniRef100\_A0A2W7RG09 | 99.6 | 3.3e-18 | 6.1e-24 | 161.5 | 319 | (43, 509) | 682 | (15, 340) | 343 | HK97 family phage prohead protease/HK97 family phage major capsid protein,TIGR01554 (Fragment) | HK97 family phage prohead protease/HK97 family phage major capsid protein,TIGR01554 (Fragment) | | uniclust | UniRef100\_A0A484YZ81 | 99.6 | 3.3e-18 | 6.1e-24 | 154.4 | 212 | (462, 680) | 682 | (2, 226) | 232 | HK97 family phage major capsid protein | HK97 family phage major capsid protein | | uniclust | UniRef100\_A0A966TPN7 | 99.6 | 3.6e-18 | 6.7e-24 | 157.0 | 260 | (411, 681) | 682 | (41, 317) | 318 | Phage major capsid protein | Phage major capsid protein | | uniclust | UniRef100\_A0A6I4E5J7 | 99.6 | 3.6e-18 | 7e-24 | 165.9 | 133 | (41, 181) | 682 | (89, 228) | 290 | HK97 family phage prohead protease | HK97 family phage prohead protease | | uniclust | UniRef100\_A0A101WGC7 | 99.6 | 3.7e-18 | 7e-24 | 173.6 | 264 | (406, 677) | 682 | (186, 469) | 488 | Phage capsid protein | Phage capsid protein | | uniclust | UniRef100\_A0A839V847 | 99.6 | 4.1e-18 | 7.5e-24 | 158.2 | 253 | (344, 614) | 682 | (83, 343) | 343 | HK97 family phage major capsid protein | HK97 family phage major capsid protein | | uniclust | UniRef100\_A0A443IQP8 | 99.6 | 4.1e-18 | 7.7e-24 | 163.7 | 251 | (422, 682) | 682 | (40, 326) | 336 | Phage major capsid protein | Phage major capsid protein | | uniclust | UniRef100\_A0A0A8K3H1 | 99.6 | 4.5e-18 | 8.6e-24 | 161.6 | 131 | (41, 181) | 682 | (49, 182) | 299 | Gene transfer agent prohead protease | Gene transfer agent prohead protease | | uniclust | UniRef100\_UPI001FCE8D07 | 99.6 | 4.8e-18 | 8.8e-24 | 157.2 | 149 | (31, 185) | 682 | (12, 168) | 333 | HK97 family phage prohead protease | HK97 family phage prohead protease | | uniclust | UniRef100\_A0A6N8YYY6 | 99.6 | 4.7e-18 | 9.1e-24 | 157.5 | 132 | (45, 183) | 682 | (56, 189) | 231 | HK97 family phage prohead protease | HK97 family phage prohead protease | | uniclust | UniRef100\_A0A090KKN6 | 99.6 | 4.8e-18 | 9.4e-24 | 158.5 | 138 | (41, 184) | 682 | (27, 172) | 217 | Uncultured bacterium genome assembly Metasoil\_fosmids\_resub | Uncultured bacterium genome assembly Metasoil\_fosmids\_resub | | uniclust | UniRef100\_A0A1G5SX48 | 99.6 | 5.1e-18 | 9.5e-24 | 166.3 | 151 | (32, 184) | 682 | (7, 170) | 429 | Phage prohead protease, HK97 family | Phage prohead protease, HK97 family | | uniclust | UniRef100\_A0A1V6D5P9 | 99.6 | 5.3e-18 | 9.9e-24 | 166.4 | 269 | (404, 681) | 682 | (161, 452) | 454 | Phage capsid family protein | Phage capsid family protein | | uniclust | UniRef100\_A0A3D3LX94 | 99.6 | 5.5e-18 | 1e-23 | 163.4 | 263 | (406, 680) | 682 | (108, 397) | 399 | Phage major capsid protein | Phage major capsid protein | | uniclust | UniRef100\_A0A090C747 | 99.6 | 5.2e-18 | 1e-23 | 177.2 | 263 | (406, 680) | 682 | (132, 415) | 436 | Putative capsid protein | Putative capsid protein | | uniclust | UniRef100\_A0A068QTX5 | 99.6 | 5.5e-18 | 1e-23 | 159.6 | 261 | (406, 679) | 682 | (15, 294) | 301 | Putative major capsid protein | Putative major capsid protein | | uniclust | UniRef100\_A0A7V2IQW9 | 99.6 | 6e-18 | 1.1e-23 | 157.8 | 220 | (445, 672) | 682 | (7, 251) | 269 | Phage major capsid protein | Phage major capsid protein | | uniclust | UniRef100\_A0A1H0N3V1 | 99.6 | 6.1e-18 | 1.2e-23 | 165.3 | 257 | (408, 680) | 682 | (26, 286) | 297 | Phage major capsid protein, HK97 family | Phage major capsid protein, HK97 family | | uniclust | UniRef100\_A0A2S6MR39 | 99.6 | 6.3e-18 | 1.2e-23 | 158.9 | 131 | (44, 181) | 682 | (70, 204) | 242 | HK97 family phage prohead protease | HK97 family phage prohead protease | | uniclust | UniRef100\_A0A0A7RXR2 | 99.6 | 6.4e-18 | 1.2e-23 | 176.0 | 263 | (406, 680) | 682 | (247, 531) | 553 | Major capsid protein | Major capsid protein | | uniclust | UniRef100\_A0A2A7MT55 | 99.6 | 8e-18 | 1.5e-23 | 157.4 | 199 | (470, 678) | 682 | (25, 248) | 255 | Phage major capsid protein | Phage major capsid protein | | uniclust | UniRef100\_A0A0C1X3C7 | 99.6 | 7.6e-18 | 1.5e-23 | 179.2 | 260 | (410, 680) | 682 | (176, 474) | 487 | Phage major capsid protein | Phage major capsid protein | | uniclust | UniRef100\_A0A068SKZ3 | 99.6 | 7.9e-18 | 1.5e-23 | 169.9 | 146 | (31, 184) | 682 | (26, 193) | 376 | Uncharacterized protein | Uncharacterized protein | | uniclust | UniRef100\_A0A3D2THT3 | 99.6 | 8.5e-18 | 1.6e-23 | 166.5 | 261 | (406, 672) | 682 | (175, 456) | 473 | Phage major capsid protein | Phage major capsid protein | | uniclust | UniRef100\_A0A0F9NU21 | 99.6 | 8e-18 | 1.6e-23 | 163.9 | 134 | (46, 183) | 682 | (47, 187) | 271 | HK97 family phage prohead protease | HK97 family phage prohead protease | | uniclust | UniRef100\_A0A3M0WW28 | 99.6 | 8.2e-18 | 1.6e-23 | 159.3 | 137 | (44, 184) | 682 | (30, 174) | 224 | HK97 family phage prohead protease | HK97 family phage prohead protease | | uniclust | UniRef100\_A0A6J7WJ50 | 99.6 | 8.7e-18 | 1.6e-23 | 165.8 | 301 | (336, 671) | 682 | (86, 412) | 436 | Major\_cap\_HK97, phage major capsid protein, HK97 family | Major\_cap\_HK97, phage major capsid protein, HK97 family | | uniclust | UniRef100\_A0A0H3LJ84 | 99.6 | 8.2e-18 | 1.7e-23 | 183.7 | 263 | (406, 680) | 682 | (269, 553) | 574 | PhiRV1 phage protein | PhiRV1 phage protein | | uniclust | UniRef100\_A0A060PI05 | 99.6 | 8.9e-18 | 1.8e-23 | 181.1 | 248 | (406, 679) | 682 | (161, 427) | 492 | Phage major capsid protein | Phage major capsid protein | | uniclust | UniRef100\_A0A0S3PZ93 | 99.6 | 9.2e-18 | 1.8e-23 | 170.3 | 197 | (393, 607) | 682 | (137, 349) | 355 | Phage capsid family protein | Phage capsid family protein | | uniclust | UniRef100\_A0A1U7CX52 | 99.6 | 1e-17 | 1.8e-23 | 160.3 | 308 | (363, 680) | 682 | (98, 430) | 434 | Phage capsid family protein | Phage capsid family protein | | uniclust | UniRef100\_A0A0X3Y2E8 | 99.6 | 9.6e-18 | 1.9e-23 | 176.8 | 259 | (408, 680) | 682 | (310, 593) | 595 | Phage capsid protein | Phage capsid protein | | uniclust | UniRef100\_UPI00158CF282 | 99.6 | 1e-17 | 1.9e-23 | 140.8 | 129 | (40, 169) | 682 | (4, 132) | 171 | hypothetical protein | hypothetical protein | | uniclust | UniRef100\_A0A1V2ZD93 | 99.5 | 1.1e-17 | 2.1e-23 | 159.8 | 206 | (463, 679) | 682 | (128, 340) | 345 | Major capsid protein | Major capsid protein | | uniclust | UniRef100\_A0A660TAH7 | 99.5 | 1.3e-17 | 2.4e-23 | 157.9 | 263 | (406, 679) | 682 | (114, 395) | 397 | Phage major capsid protein | Phage major capsid protein | | uniclust | UniRef100\_A0A212I3J2 | 99.5 | 1.2e-17 | 2.4e-23 | 161.4 | 214 | (456, 679) | 682 | (72, 294) | 315 | Major capsid protein | Major capsid protein | | uniclust | UniRef100\_A0A1Y0H480 | 99.5 | 1.3e-17 | 2.4e-23 | 164.1 | 234 | (438, 680) | 682 | (320, 564) | 565 | Phage major capsid protein | Phage major capsid protein | | uniclust | UniRef100\_UPI0010AE5AB6 | 99.5 | 1.4e-17 | 2.5e-23 | 145.9 | 149 | (31, 185) | 682 | (7, 163) | 222 | hypothetical protein | hypothetical protein | | uniclust | UniRef100\_A0A2D5TTA0 | 99.5 | 1.7e-17 | 3.2e-23 | 154.0 | 136 | (40, 183) | 682 | (41, 182) | 257 | HK97 family phage prohead protease | HK97 family phage prohead protease | | uniclust | UniRef100\_A0A0A0RPP6 | 99.5 | 1.7e-17 | 3.3e-23 | 151.5 | 128 | (43, 181) | 682 | (32, 163) | 212 | Prohead protease | Prohead protease | | uniclust | UniRef100\_A0A2M7EMM4 | 99.5 | 1.8e-17 | 3.4e-23 | 154.5 | 128 | (44, 181) | 682 | (46, 179) | 252 | HK97 family phage prohead protease | HK97 family phage prohead protease | | uniclust | UniRef100\_A0A158FYM0 | 99.5 | 1.9e-17 | 3.7e-23 | 151.1 | 130 | (43, 181) | 682 | (27, 161) | 226 | Phage-like protein | Phage-like protein | | uniclust | UniRef100\_A0A0L0KLK6 | 99.5 | 1.9e-17 | 3.8e-23 | 167.5 | 261 | (411, 680) | 682 | (59, 355) | 364 | Phage capsid family protein | Phage capsid family protein | | uniclust | UniRef100\_A0A2S3W008 | 99.5 | 2e-17 | 3.8e-23 | 156.3 | 134 | (42, 181) | 682 | (78, 221) | 282 | Caudovirus prohead protease | Caudovirus prohead protease | | uniclust | UniRef100\_A0A9E3QNN9 | 99.5 | 2.3e-17 | 4.2e-23 | 158.1 | 270 | (393, 680) | 682 | (134, 435) | 438 | Phage major capsid protein | Phage major capsid protein | | uniclust | UniRef100\_A0A0F0GPG3 | 99.5 | 2.3e-17 | 4.3e-23 | 164.5 | 251 | (408, 670) | 682 | (151, 427) | 514 | Phage major capsid protein | Phage major capsid protein | | uniclust | UniRef100\_UPI000C9B78D3 | 99.5 | 2.3e-17 | 4.3e-23 | 159.8 | 250 | (413, 681) | 682 | (145, 404) | 405 | phage major capsid protein | phage major capsid protein | | uniclust | UniRef100\_A0A5Q2RKZ8 | 99.5 | 2.4e-17 | 4.4e-23 | 159.4 | 265 | (409, 681) | 682 | (194, 472) | 475 | Phage major capsid protein | Phage major capsid protein | | uniclust | UniRef100\_A0A136KYT3 | 99.5 | 2.3e-17 | 4.4e-23 | 174.1 | 269 | (406, 680) | 682 | (301, 621) | 624 | Phage capsid family protein | Phage capsid family protein | | uniclust | UniRef100\_A0A2S5XKA8 | 99.5 | 2.7e-17 | 5.3e-23 | 173.6 | 245 | (423, 679) | 682 | (168, 435) | 477 | Phage major capsid protein | Phage major capsid protein | | uniclust | UniRef100\_A0A0K9T4G1 | 99.5 | 2.8e-17 | 5.3e-23 | 161.2 | 265 | (405, 681) | 682 | (113, 410) | 418 | Phage major capsid protein (Fragment) | Phage major capsid protein (Fragment) | | uniclust | UniRef100\_A0A239MKE3 | 99.5 | 2.9e-17 | 5.3e-23 | 159.9 | 266 | (406, 679) | 682 | (146, 438) | 448 | Phage major capsid protein, HK97 family | Phage major capsid protein, HK97 family | | uniclust | UniRef100\_A0A960HG97 | 99.5 | 2.9e-17 | 5.5e-23 | 163.9 | 261 | (409, 680) | 682 | (164, 433) | 439 | Phage major capsid protein | Phage major capsid protein | | uniclust | UniRef100\_A0A6B9T8U5 | 99.5 | 3e-17 | 5.6e-23 | 158.9 | 263 | (406, 679) | 682 | (102, 382) | 386 | Phage major capsid protein | Phage major capsid protein | | uniclust | UniRef100\_A0A2X2J2H8 | 99.5 | 3.1e-17 | 5.9e-23 | 147.4 | 136 | (32, 183) | 682 | (15, 162) | 202 | Caudovirus prohead protease | Caudovirus prohead protease | | uniclust | UniRef100\_A0A3C1LU60 | 99.5 | 3.2e-17 | 5.9e-23 | 154.2 | 256 | (410, 678) | 682 | (96, 368) | 374 | Phage major capsid protein | Phage major capsid protein | | uniclust | UniRef100\_G7QC57 | 99.5 | 3.3e-17 | 6.1e-23 | 146.4 | 137 | (38, 181) | 682 | (29, 170) | 254 | Phage prohead protease, HK97 family | Phage prohead protease, HK97 family | | uniclust | UniRef100\_A0A069AVH7 | 99.5 | 3.2e-17 | 6.6e-23 | 174.7 | 248 | (406, 679) | 682 | (126, 395) | 426 | Phage major capsid protein, HK97 family | Phage major capsid protein, HK97 family | | uniclust | UniRef100\_A0A0F7PH54 | 99.5 | 3.2e-17 | 6.6e-23 | 171.0 | 252 | (406, 681) | 682 | (83, 368) | 374 | Phage capsid family protein | Phage capsid family protein | | pdb70 | 3QPR\_A | 99.2 | 5.4e-16 | 5.7e-20 | 163.2 | 262 | (409, 681) | 682 | (111, 382) | 385 | Major capsid protein | 3QPR\_A Major capsid protein Virus Procapsid particles, VIRUS | | pdb70 | 3QPR\_C | 99.2 | 5.4e-16 | 5.7e-20 | 163.2 | 262 | (409, 681) | 682 | (111, 382) | 385 | Major capsid protein | 3QPR\_C Major capsid protein Virus Procapsid particles, VIRUS | | pdb70 | 1OHG\_G | 99.2 | 2.7e-15 | 3.1e-19 | 147.1 | 263 | (408, 681) | 682 | (7, 279) | 282 | MAJOR CAPSID PROTEIN | 1OHG\_G MAJOR CAPSID PROTEIN VIRUS, VIRUS COAT PROTEIN, VIRUS/VIRAL | | pdb70 | 6B0X\_F | 99.2 | 3.4e-15 | 3.7e-19 | 152.4 | 261 | (410, 681) | 682 | (34, 313) | 324 | Major head protein, Scaffold protein | 6B0X\_F Major head protein, Scaffold protein major capsid protein, HK97-like fold | | pdb70 | 6B23\_C | 99.2 | 3.4e-15 | 3.7e-19 | 152.4 | 261 | (410, 681) | 682 | (34, 313) | 324 | Major head protein, Capsid morphogenesis | 6B23\_C Major head protein, Capsid morphogenesis major capsid protein, HK97-like fold | | pdb70 | 3JB5\_D | 99.1 | 4.8e-15 | 5.2e-19 | 150.1 | 272 | (408, 681) | 682 | (6, 304) | 315 | major capsid protein | 3JB5\_D major capsid protein acne, bacteriophage, HK97-like, VIRUS | | pdb70 | 1IF0\_G | 99.0 | 2.3e-14 | 2.6e-18 | 138.6 | 242 | (430, 681) | 682 | (4, 255) | 256 | MAJOR CAPSID PROTEIN GP5 | 1IF0\_G MAJOR CAPSID PROTEIN GP5 Bacteriophage, Virus, Capsid, cryoEM, Pseudo-atomic | | pdb70 | 6OMA\_F | 98.8 | 3.6e-13 | 4e-17 | 134.2 | 268 | (406, 681) | 682 | (6, 297) | 299 | Major capsid protein | 6OMA\_F Major capsid protein capsid, HK97-fold, dsDNA-phage, icosahedral, VIRUS | | pdb70 | 6OMC\_B | 98.8 | 3.6e-13 | 4e-17 | 134.2 | 268 | (406, 681) | 682 | (6, 297) | 299 | Major capsid protein | 6OMC\_B Major capsid protein capsid, HK97-fold, dsDNA-phage, icosahedral, VIRUS | | pdb70 | 4PT2\_B | 98.7 | 2.3e-12 | 2.5e-16 | 127.3 | 250 | (418, 681) | 682 | (19, 281) | 287 | Encapsulin protein | 4PT2\_B Encapsulin protein HK97 fold, Shell protein, VIRUS | | pdb70 | 6NJ8\_A | 98.7 | 3e-12 | 3.3e-16 | 126.3 | 251 | (418, 682) | 682 | (19, 281) | 282 | Quasibacillus thermotolerans encapsulin, targeting peptide | 6NJ8\_A Quasibacillus thermotolerans encapsulin, targeting peptide encapsulin, iron storage, IMEF, icosahedral | | pdb70 | 3J7V\_E | 98.7 | 4.4e-12 | 4.7e-16 | 130.1 | 275 | (400, 681) | 682 | (13, 343) | 345 | Major capsid protein 10A | 3J7V\_E Major capsid protein 10A maturation, DNA packaging, Procapsid, Non-covalent | | pdb70 | 3J7W\_D | 98.7 | 4.4e-12 | 4.7e-16 | 130.1 | 275 | (400, 681) | 682 | (13, 343) | 345 | Major capsid protein 10A | 3J7W\_D Major capsid protein 10A maturation, DNA packaging, Procapsid, Non-covalent | | pdb70 | 3J1A\_G | 98.6 | 1.2e-11 | 1.4e-15 | 114.6 | 193 | (481, 679) | 682 | (2, 199) | 199 | capsid protein | 3J1A\_G capsid protein halophage, bacteriophage HK97, bacteriophage T7 | | pdb70 | 3J1A\_B | 98.5 | 1.7e-11 | 1.9e-15 | 113.5 | 193 | (481, 679) | 682 | (2, 199) | 199 | capsid protein | 3J1A\_B capsid protein halophage, bacteriophage HK97, bacteriophage T7 | | pdb70 | 4BML\_B | 98.4 | 9e-11 | 9.9e-15 | 118.3 | 266 | (410, 680) | 682 | (24, 331) | 332 | MAJOR CAPSID PROTEIN | 4BML\_B MAJOR CAPSID PROTEIN VIRUS, MARINE VIRUS, OUTER CAPSID | | pdb70 | 3DKT\_I | 98.1 | 7e-10 | 7.7e-14 | 108.0 | 235 | (419, 679) | 682 | (19, 264) | 265 | Maritimacin, Putative uncharacterized protein | 3DKT\_I Maritimacin, Putative uncharacterized protein enzyme encapsulation, nanocompartment, oxidative stress | | pdb70 | 2XD8\_B | 98.1 | 1e-09 | 1.1e-13 | 113.8 | 277 | (401, 681) | 682 | (16, 368) | 375 | T7-LIKE CAPSID PROTEIN | 2XD8\_B T7-LIKE CAPSID PROTEIN MARINE PODOVIRUS, T7-LIKE VIRUS, VIRUS | | pdb70 | 6I9G\_N | 97.9 | 4.2e-09 | 4.6e-13 | 102.8 | 235 | (419, 680) | 682 | (19, 265) | 265 | Uncharacterized protein | 6I9G\_N Uncharacterized protein Nanocompartment, nanocage, packaging of biocatalysts HET: GOL, SO4 | | pdb70 | 3J40\_B | 97.4 | 1.4e-07 | 1.4e-11 | 96.6 | 112 | (416, 528) | 682 | (20, 135) | 335 | gp10, gp7 | 3J40\_B gp10, gp7 capsid, accessory protein, VIRUS | | pdb70 | 6R3A\_A | 96.9 | 1.3e-06 | 1.4e-10 | 87.6 | 206 | (410, 624) | 682 | (5, 224) | 323 | Major capsid protein | 6R3A\_A Major capsid protein Bacteriophage, Capsid protein, image processing | | pdb70 | 6RTL\_E | 96.9 | 1.3e-06 | 1.4e-10 | 87.6 | 206 | (410, 624) | 682 | (5, 224) | 323 | Major capsid protein | 6RTL\_E Major capsid protein Bacteriophage, maturation process, cryo electron | | pdb70 | 2E0Z\_B | 96.9 | 1.7e-06 | 1.7e-10 | 88.5 | 184 | (475, 679) | 682 | (159, 345) | 345 | Virus-like particle | 2E0Z\_B Virus-like particle virus-like particle, virus, bacteriophage, HK97 | | pdb70 | 2E0Z\_C | 96.9 | 1.7e-06 | 1.7e-10 | 88.5 | 184 | (475, 679) | 682 | (159, 345) | 345 | Virus-like particle | 2E0Z\_C Virus-like particle virus-like particle, virus, bacteriophage, HK97 | | pdb70 | 6J3Q\_U | 96.8 | 2.6e-06 | 2.7e-10 | 86.2 | 263 | (415, 681) | 682 | (9, 345) | 348 | the major capsid protein, the | 6J3Q\_U the major capsid protein, the cyanophage, Siphoviridae, capsid, VIRUS | | pdb70 | 3BJQ\_F | 96.4 | 1.2e-05 | 1.3e-09 | 79.7 | 261 | (414, 681) | 682 | (12, 311) | 316 | Phage-related protein | 3BJQ\_F Phage-related protein PHAGE-RELATED PROTEIN, STRUCTURAL GENOMICS, JOINT HET: PG4, MSE | | pdb70 | 3BJQ\_E | 96.4 | 1.2e-05 | 1.3e-09 | 79.7 | 261 | (414, 681) | 682 | (12, 311) | 316 | Phage-related protein | 3BJQ\_E Phage-related protein PHAGE-RELATED PROTEIN, STRUCTURAL GENOMICS, JOINT HET: PG4 | | pdb70 | 5WK1\_F | 96.3 | 1.6e-05 | 1.6e-09 | 81.5 | 249 | (418, 673) | 682 | (68, 342) | 352 | Capsid Stabilizing Protein, Major Capsid | 5WK1\_F Capsid Stabilizing Protein, Major Capsid Major Capsid Protein, Capsid Stabilizing | | pdb70 | 5LII\_P | 95.4 | 0.00018 | 1.8e-08 | 76.2 | 206 | (374, 588) | 682 | (10, 230) | 463 | Major capsid protein | 5LII\_P Major capsid protein polyvalent staphylococcal bactoriophage, Myoviridae, tail | |
| Top keywords  (threshold 1.00e-03 (evalue)) | **Phage, capsid, major, HK97, prohead, protease, Fragment, Peptidase, U35, Bacteriophage** |
| Output files | ../../similar\_sequences/05\_FANPEZAQ\_CDS\_0005\_merged.svg ../../similar\_sequences/05\_FANPEZAQ\_CDS\_0005\_pdb70.a3m ../../similar\_sequences/05\_FANPEZAQ\_CDS\_0005\_pdb70.hhr ../../similar\_sequences/05\_FANPEZAQ\_CDS\_0005\_uniclust.a3m ../../similar\_sequences/05\_FANPEZAQ\_CDS\_0005\_uniclust.hhr |

#### Structure prediction (AlphaFold)2

|  |  |
| --- | --- |
| Stats | xml version="1.0" encoding="utf-8" standalone="no"?       2024-09-02T21:09:04.565873 image/svg+xml   Matplotlib v3.7.2, https://matplotlib.org/ |
| Predicted structure | **NGL Viewer Controls:**  - Center: *Left-Click* - Rotate: *Left-Click + Drag* - Translate: *Right-Click + Drag* - Zoom: *Shift + Left-Click + Drag* |
| Output files | ../../predicted\_structures/05\_FANPEZAQ\_CDS\_0005/features.pkl ../../predicted\_structures/05\_FANPEZAQ\_CDS\_0005/ranked\_0.pdb ../../predicted\_structures/05\_FANPEZAQ\_CDS\_0005/ranked\_0\_plots.svg ../../predicted\_structures/05\_FANPEZAQ\_CDS\_0005/result\_model\_1\_ptm\_pred\_0.pkl |

#### Structure similarity search results (Foldseek)3

|  |  |
| --- | --- |
| Structure databases searched | Pdb, Afdb-proteome, Afdb-uniprot50 |
| Results, scheme(s)  (Top layers only, threshold 1.00e-02 (evalue)) | xml version="1.0" encoding="utf-8" standalone="no"?       2024-09-02T21:10:16.128244 image/svg+xml   Matplotlib v3.7.2, https://matplotlib.org/ |
| Results, table  (threshold 1.00e-02 (evalue)) | | db | id | prob | evalue | bits | fident | alnlen | mismatch | gapopen | qstart | qend | tstart | tend | name | description | | --- | --- | --- | --- | --- | --- | --- | --- | --- | --- | --- | --- | --- | --- | --- | | pdb | 8GTA\_G | 1.0 | 2.164e-26 | 869 | 0.407 | 275 | 153 | 6 | 410 | 681 | 1 | 268 | Major capsid protein | Major capsid protein | | pdb | 6TB9\_C5 | 1.0 | 7.413e-16 | 479 | 0.193 | 315 | 204 | 17 | 400 | 682 | 1 | 297 | Major capsid protein Rcc01687 | Major capsid protein Rcc01687 | | pdb | 6TSU\_U4 | 1.0 | 1.102e-15 | 473 | 0.196 | 285 | 189 | 14 | 423 | 682 | 22 | 291 | Major capsid protein Rcc01687 | Major capsid protein Rcc01687 | | pdb | 6TUI\_B5 | 1.0 | 2.352e-14 | 446 | 0.182 | 291 | 187 | 17 | 422 | 682 | 1 | 270 | Phage major capsid protein, HK97 family | Phage major capsid protein, HK97 family | | pdb | 1OHG\_A | 1.0 | 1.087e-13 | 444 | 0.175 | 274 | 198 | 13 | 423 | 682 | 21 | 280 | MAJOR CAPSID PROTEIN | MAJOR CAPSID PROTEIN | | pdb | 6TUI\_A5 | 1.0 | 6.388e-15 | 440 | 0.187 | 315 | 203 | 17 | 398 | 682 | 3 | 294 | Phage major capsid protein, HK97 family | Phage major capsid protein, HK97 family | | pdb | 1IF0\_D | 1.0 | 7.307e-14 | 431 | 0.176 | 266 | 193 | 12 | 430 | 682 | 4 | 256 | PROTEIN (MAJOR CAPSID PROTEIN GP5) | PROTEIN (MAJOR CAPSID PROTEIN GP5) | | pdb | 2FS3\_F | 1.0 | 1.175e-12 | 416 | 0.172 | 273 | 197 | 14 | 424 | 682 | 1 | 258 | Major capsid protein | Major capsid protein | | pdb | 2FS3\_B | 1.0 | 1.616e-13 | 414 | 0.175 | 285 | 200 | 14 | 420 | 682 | 9 | 280 | Major capsid protein | Major capsid protein | | pdb | 6TB9\_B5 | 1.0 | 6.905e-14 | 412 | 0.158 | 291 | 195 | 15 | 422 | 682 | 1 | 271 | Major capsid protein Rcc01687 | Major capsid protein Rcc01687 | | pdb | 8CFA\_D | 1.0 | 1.746e-12 | 397 | 0.171 | 263 | 181 | 11 | 430 | 681 | 2 | 238 | Major capsid subunit | Major capsid subunit | | pdb | 8EDU\_G | 1.0 | 3.19e-13 | 395 | 0.158 | 315 | 224 | 12 | 398 | 681 | 1 | 305 | Capsid | Capsid | | pdb | 8CFA\_F | 1.0 | 1.956e-12 | 394 | 0.16 | 261 | 181 | 12 | 430 | 679 | 3 | 236 | Major capsid subunit | Major capsid subunit | | pdb | 2FRP\_G | 1.0 | 1.956e-12 | 392 | 0.165 | 266 | 183 | 14 | 430 | 682 | 4 | 243 | Major capsid protein | Major capsid protein | | pdb | 8CFA\_A | 1.0 | 1.392e-12 | 388 | 0.154 | 265 | 180 | 13 | 430 | 681 | 1 | 234 | Major capsid subunit | Major capsid subunit | | pdb | 8ECK\_E | 1.0 | 1.315e-12 | 388 | 0.135 | 303 | 230 | 14 | 406 | 682 | 18 | 314 | Major capsid protein | Major capsid protein | | pdb | 2FTE\_G | 1.0 | 4.577e-12 | 380 | 0.161 | 266 | 183 | 13 | 430 | 682 | 4 | 242 | major capsid protein | major capsid protein | | pdb | 3JB5\_B | 1.0 | 7.464e-13 | 374 | 0.181 | 298 | 195 | 14 | 422 | 680 | 13 | 300 | major capsid protein | major capsid protein | | pdb | 8CFA\_E | 1.0 | 1.593e-11 | 366 | 0.166 | 265 | 184 | 11 | 427 | 681 | 2 | 239 | Major capsid subunit | Major capsid subunit | | pdb | 3QPR\_G | 1.0 | 6.431e-12 | 366 | 0.166 | 265 | 188 | 11 | 430 | 682 | 4 | 247 | Major capsid protein | Major capsid protein | | pdb | 3E8K\_G | 1.0 | 5.127e-12 | 361 | 0.157 | 260 | 186 | 11 | 435 | 682 | 9 | 247 | Major capsid protein | Major capsid protein | | pdb | 6NJ8\_B | 1.0 | 1.998e-11 | 355 | 0.132 | 264 | 201 | 10 | 432 | 682 | 28 | 276 | Encapsulating protein for a DyP-type peroxidase | Encapsulating protein for a DyP-type peroxidase | | pdb | 8EDU\_D | 1.0 | 4.577e-12 | 353 | 0.152 | 321 | 230 | 14 | 398 | 681 | 1 | 316 | Capsid | Capsid | | pdb | 8ECN\_F | 1.0 | 7.203e-12 | 352 | 0.143 | 300 | 207 | 14 | 423 | 681 | 18 | 308 | Major capsid protein | Major capsid protein | | pdb | 8FQK\_E | 1.0 | 1.505e-11 | 347 | 0.155 | 263 | 183 | 10 | 432 | 682 | 33 | 268 | Scaffolding domain delta | Scaffolding domain delta | | pdb | 7MH2\_D | 1.0 | 1.27e-11 | 343 | 0.14 | 263 | 192 | 12 | 435 | 682 | 35 | 278 | T4GALA Engineered Protein Nanocage | T4GALA Engineered Protein Nanocage | | pdb | 8EB4\_G | 1.0 | 1.344e-11 | 340 | 0.137 | 305 | 206 | 15 | 421 | 680 | 18 | 310 | Major capsid protein | Major capsid protein | | pdb | 3JB5\_G | 1.0 | 1.784e-11 | 339 | 0.168 | 291 | 183 | 15 | 422 | 680 | 13 | 276 | major capsid protein | major capsid protein | | pdb | 2GP1\_A | 1.0 | 1.452e-10 | 333 | 0.171 | 274 | 187 | 16 | 423 | 682 | 7 | 254 | Major capsid protein | Major capsid protein | | pdb | 8FQK\_D | 1.0 | 3.144e-11 | 332 | 0.161 | 266 | 178 | 13 | 432 | 682 | 30 | 265 | Scaffolding domain delta | Scaffolding domain delta | | pdb | 8E16\_C | 1.0 | 6.568e-11 | 326 | 0.124 | 282 | 193 | 16 | 423 | 678 | 14 | 267 | Major capsid protein, gp6 | Major capsid protein, gp6 | | pdb | 8EB4\_H | 1.0 | 3.944e-11 | 313 | 0.13 | 314 | 209 | 14 | 422 | 682 | 19 | 321 | Major capsid protein | Major capsid protein | | pdb | 7MH2\_C | 1.0 | 1.372e-10 | 311 | 0.13 | 260 | 193 | 12 | 435 | 680 | 35 | 275 | T4GALA Engineered Protein Nanocage | T4GALA Engineered Protein Nanocage | | pdb | 8ECJ\_E | 1.0 | 1.372e-10 | 308 | 0.105 | 304 | 224 | 13 | 413 | 681 | 11 | 301 | Major capsid protein | Major capsid protein | | pdb | 8EC2\_C | 1.0 | 2.419e-10 | 307 | 0.1 | 299 | 211 | 15 | 426 | 682 | 22 | 304 | Major capsid protein | Major capsid protein | | pdb | 8ECO\_F | 1.0 | 6.568e-11 | 306 | 0.14 | 299 | 204 | 14 | 423 | 681 | 17 | 302 | Major capsid protein | Major capsid protein | | pdb | 7S2T\_B | 1.0 | 3.211e-10 | 303 | 0.126 | 260 | 194 | 11 | 434 | 677 | 27 | 269 | EncA | EncA | | pdb | 7KMX\_F | 1.0 | 2.368e-11 | 301 | 0.155 | 308 | 209 | 21 | 410 | 681 | 2 | 294 | Major capsid protein | Major capsid protein | | pdb | 8ECI\_C | 1.0 | 3.327e-11 | 297 | 0.14 | 348 | 215 | 19 | 382 | 679 | 1 | 314 | Major capsid protein | Major capsid protein | | pdb | 8EC8\_B | 1.0 | 4.512e-10 | 295 | 0.111 | 297 | 211 | 15 | 421 | 680 | 18 | 298 | Major capsid protein | Major capsid protein | | pdb | 7OZ4\_C | 1.0 | 3.398e-10 | 295 | 0.124 | 297 | 218 | 13 | 423 | 680 | 8 | 301 | p2 family phage major capsid protein | p2 family phage major capsid protein | | pdb | 2E0Z\_C | 1.0 | 1.861e-09 | 287 | 0.087 | 252 | 183 | 12 | 432 | 679 | 20 | 228 | Virus-like particle | Virus-like particle | | pdb | 2E0Z\_B | 1.0 | 1.402e-09 | 284 | 0.087 | 251 | 190 | 10 | 433 | 679 | 21 | 236 | Virus-like particle | Virus-like particle | | pdb | 7Y23\_F | 1.0 | 5.865e-11 | 280 | 0.105 | 351 | 227 | 21 | 402 | 681 | 6 | 340 | phage capsid protein | phage capsid protein | | pdb | 5TJT\_C | 1.0 | 4.512e-10 | 269 | 0.129 | 285 | 206 | 14 | 423 | 678 | 19 | 290 | Major capsid protein | Major capsid protein | | pdb | 7S21\_A | 1.0 | 2.084e-09 | 267 | 0.117 | 256 | 187 | 15 | 435 | 677 | 35 | 264 | EncA | EncA | | pdb | 7KQ5\_A | 1.0 | 5.348e-10 | 263 | 0.135 | 258 | 184 | 16 | 434 | 679 | 34 | 264 | Maritimacin | Maritimacin | | pdb | 7Y23\_G | 1.0 | 2.506e-11 | 263 | 0.118 | 338 | 230 | 19 | 402 | 682 | 1 | 327 | phage capsid protein | phage capsid protein | | pdb | 3J7X\_F | 1.0 | 6.709e-10 | 263 | 0.115 | 345 | 228 | 20 | 402 | 682 | 12 | 343 | Major capsid protein 10A | Major capsid protein 10A | | pdb | 7RWZ\_D | 1.0 | 1.969e-09 | 261 | 0.136 | 272 | 187 | 18 | 426 | 681 | 14 | 253 | Major capsid protein | Major capsid protein | | pdb | 7Y3T\_C | 1.0 | 7.1e-10 | 256 | 0.104 | 343 | 235 | 21 | 399 | 682 | 1 | 330 | phage major capsid protein | phage major capsid protein | | pdb | 6WKV\_A | 1.0 | 1.056e-09 | 250 | 0.123 | 252 | 185 | 13 | 435 | 679 | 34 | 256 | Encapsulin | Encapsulin | | pdb | 7BCV\_A | 1.0 | 2.767e-09 | 250 | 0.12 | 257 | 193 | 14 | 434 | 681 | 34 | 266 | Linocin-M18 | Linocin-M18 | | pdb | 7Z4B\_AY | 1.0 | 3.597e-10 | 243 | 0.1 | 360 | 231 | 16 | 399 | 681 | 1 | 344 | Major head protein | Major head protein | | pdb | 8HDT\_A | 1.0 | 1.661e-09 | 238 | 0.116 | 310 | 227 | 17 | 407 | 681 | 3 | 300 | Major capsid | Major capsid | | pdb | 6J3Q\_A | 1.0 | 2.253e-08 | 236 | 0.115 | 354 | 223 | 19 | 399 | 682 | 1 | 334 | major capsid protein | major capsid protein | | pdb | 7OE2\_A | 1.0 | 4.447e-08 | 235 | 0.122 | 262 | 193 | 13 | 431 | 681 | 31 | 266 | Linocin\_M18 bacteriocin protein | Linocin\_M18 bacteriocin protein | | pdb | 7LIJ\_A | 1.0 | 2.614e-09 | 231 | 0.123 | 260 | 191 | 14 | 431 | 679 | 31 | 264 | Maritimacin | Maritimacin | | pdb | 5WK1\_B | 1.0 | 2.47e-09 | 231 | 0.128 | 272 | 202 | 13 | 433 | 678 | 31 | 293 | Major Capsid Protein | Major Capsid Protein | | pdb | 7LIS\_A | 1.0 | 4.608e-09 | 228 | 0.138 | 260 | 177 | 17 | 434 | 679 | 34 | 260 | Maritimacin | Maritimacin | | pdb | 8GIU\_G | 1.0 | 1.208e-08 | 227 | 0.098 | 305 | 213 | 14 | 433 | 681 | 45 | 343 | Capsid protein | Capsid protein | | pdb | 8IKA\_A1 | 1.0 | 1.603e-08 | 225 | 0.096 | 259 | 193 | 16 | 435 | 680 | 35 | 265 | Type 1 encapsulin shell protein | Type 1 encapsulin shell protein | | pdb | 7JW1\_A | 1.0 | 9.976e-10 | 225 | 0.089 | 292 | 192 | 15 | 432 | 679 | 14 | 275 | Major capsid protein gpN | Major capsid protein gpN | | pdb | 6I9G\_B | 1.0 | 3.165e-08 | 224 | 0.124 | 258 | 185 | 18 | 435 | 679 | 35 | 264 | Linocin-M18 | Linocin-M18 | | pdb | 6WKK\_F | 1.0 | 2.991e-08 | 223 | 0.096 | 290 | 243 | 15 | 402 | 682 | 1 | 280 | Gp27 major capsid protein | Gp27 major capsid protein | | pdb | 7LII\_A | 1.0 | 1.278e-08 | 221 | 0.119 | 251 | 187 | 12 | 435 | 679 | 35 | 257 | Maritimacin | Maritimacin | | pdb | 8FRS\_E | 1.0 | 9.097e-09 | 219 | 0.12 | 332 | 225 | 22 | 402 | 681 | 1 | 317 | Major structural protein | Major structural protein | | pdb | 7LIM\_A | 1.0 | 2.826e-08 | 217 | 0.126 | 252 | 186 | 12 | 434 | 679 | 34 | 257 | Maritimacin | Maritimacin | | pdb | 7LIL\_A | 1.0 | 6.852e-09 | 217 | 0.126 | 260 | 190 | 16 | 431 | 679 | 31 | 264 | Maritimacin | Maritimacin | | pdb | 7LIK\_A | 1.0 | 9.627e-09 | 217 | 0.126 | 260 | 184 | 14 | 434 | 679 | 34 | 264 | Maritimacin | Maritimacin | | pdb | 6X8T\_A | 1.0 | 3.752e-08 | 216 | 0.118 | 329 | 201 | 20 | 385 | 682 | 1 | 271 | Protein SrpI | Protein SrpI | | pdb | 7F38\_I | 1.0 | 3.752e-08 | 214 | 0.118 | 362 | 223 | 21 | 401 | 680 | 3 | 350 | Putative major capsid protein | Putative major capsid protein | | pdb | 8DN9\_A | 1.0 | 6.613e-08 | 213 | 0.114 | 263 | 188 | 14 | 430 | 677 | 30 | 262 | 29 kDa antigen cfp29 | 29 kDa antigen cfp29 | | pdb | 7BOJ\_A | 1.0 | 6.248e-08 | 213 | 0.136 | 257 | 183 | 16 | 435 | 679 | 35 | 264 | 29 kDa antigen Cfp29 | 29 kDa antigen Cfp29 | | pdb | 6B23\_D | 1.0 | 4.202e-08 | 209 | 0.131 | 281 | 186 | 18 | 431 | 681 | 30 | 282 | Capsid morphogenesis B protein | Capsid morphogenesis B protein | | pdb | 6J3Q\_U | 1.0 | 4.447e-08 | 209 | 0.107 | 334 | 220 | 14 | 423 | 680 | 12 | 343 | major capsid protein | major capsid protein | | pdb | 7JW1\_C | 1.0 | 2.991e-08 | 208 | 0.089 | 290 | 202 | 15 | 432 | 682 | 14 | 280 | Major capsid protein gpN | Major capsid protein gpN | | pdb | 7JW1\_B | 1.0 | 1.353e-08 | 208 | 0.082 | 303 | 208 | 15 | 432 | 682 | 38 | 322 | Major capsid protein gpN | Major capsid protein gpN | | pdb | 7RWZ\_A | 1.0 | 3.421e-07 | 205 | 0.148 | 215 | 147 | 15 | 480 | 681 | 69 | 260 | Cos capsid morphogenesis protein (Ccm) | Cos capsid morphogenesis protein (Ccm) | | pdb | 8GIU\_C | 1.0 | 2.253e-08 | 202 | 0.094 | 317 | 215 | 17 | 435 | 681 | 48 | 362 | Capsid protein | Capsid protein | | pdb | 7F38\_D | 1.0 | 1.547e-07 | 200 | 0.142 | 358 | 216 | 18 | 412 | 679 | 3 | 359 | Putative major capsid protein | Putative major capsid protein | | pdb | 6XGQ\_E | 1.0 | 1.796e-08 | 195 | 0.108 | 368 | 223 | 24 | 405 | 682 | 1 | 353 | YSD1\_16 | YSD1\_16 | | pdb | 6OKB\_F | 1.0 | 8.473e-07 | 194 | 0.14 | 284 | 187 | 15 | 436 | 682 | 26 | 289 | Major capsid protein | Major capsid protein | | pdb | 2XVR\_C | 1.0 | 1.77e-06 | 184 | 0.123 | 260 | 196 | 13 | 427 | 669 | 1 | 245 | MAJOR CAPSID PROTEIN 10A | MAJOR CAPSID PROTEIN 10A | | pdb | 7Y3T\_G | 1.0 | 6.248e-08 | 180 | 0.118 | 338 | 226 | 20 | 399 | 682 | 1 | 320 | phage major capsid protein | phage major capsid protein | | pdb | 7EEL\_A | 1.0 | 8.296e-08 | 178 | 0.102 | 372 | 227 | 21 | 407 | 681 | 2 | 363 | Major capsid proteins | Major capsid proteins | | pdb | 8H89\_B | 1.0 | 6.613e-08 | 175 | 0.125 | 312 | 196 | 19 | 444 | 682 | 54 | 361 | Major capsid protein | Major capsid protein | | pdb | 8PHQ\_AC | 1.0 | 8.296e-08 | 173 | 0.109 | 301 | 195 | 23 | 435 | 681 | 30 | 311 | Major capsid protein | Major capsid protein | | pdb | 8PKH\_AG | 1.0 | 9.291e-08 | 172 | 0.103 | 308 | 185 | 20 | 435 | 681 | 30 | 307 | Major capsid protein | Major capsid protein | | pdb | 7JW1\_D | 1.0 | 5.384e-07 | 171 | 0.095 | 294 | 192 | 16 | 433 | 682 | 39 | 302 | Major capsid protein gpN | Major capsid protein gpN | | pdb | 8PHS\_CD | 1.0 | 2.054e-07 | 170 | 0.104 | 297 | 201 | 25 | 435 | 681 | 14 | 295 | Major capsid protein | Major capsid protein | | pdb | 7Z46\_H | 1.0 | 8.779e-08 | 170 | 0.113 | 371 | 209 | 19 | 400 | 681 | 1 | 340 | Major head protein | Major head protein | | pdb | 6XGQ\_G | 1.0 | 3.832e-07 | 168 | 0.097 | 328 | 208 | 20 | 430 | 682 | 18 | 332 | YSD1\_16 | YSD1\_16 | | pdb | 7Z4A\_C | 1.0 | 6.754e-07 | 167 | 0.114 | 341 | 192 | 19 | 422 | 681 | 2 | 313 | Major head protein | Major head protein | | pdb | 7Z48\_C | 1.0 | 8.473e-07 | 164 | 0.119 | 344 | 187 | 20 | 423 | 681 | 5 | 317 | Major head protein | Major head protein | | pdb | 7Z48\_D | 1.0 | 2.487e-06 | 162 | 0.097 | 337 | 224 | 18 | 411 | 681 | 1 | 323 | Major head protein | Major head protein | | pdb | 3BJQ\_C | 1.0 | 8.177e-06 | 158 | 0.151 | 238 | 154 | 13 | 478 | 681 | 67 | 290 | Phage-related protein | Phage-related protein | | pdb | 5L35\_A | 1.0 | 1.063e-06 | 155 | 0.083 | 443 | 208 | 19 | 410 | 682 | 8 | 422 | Gene 5 protein | Gene 5 protein | | pdb | 6QVK\_1B | 1.0 | 3.055e-07 | 154 | 0.111 | 405 | 240 | 22 | 330 | 682 | 6 | 342 | Major capsid protein | Major capsid protein | | pdb | 7Z4A\_G | 1.0 | 5.82e-06 | 148 | 0.106 | 329 | 210 | 18 | 411 | 681 | 1 | 303 | Major head protein | Major head protein | | pdb | 8CJZ\_G | 1.0 | 1.149e-05 | 147 | 0.156 | 287 | 149 | 15 | 421 | 680 | 24 | 244 | Major Capsid Protein | Major Capsid Protein | | pdb | 3BJQ\_G | 1.0 | 3.997e-05 | 146 | 0.159 | 238 | 153 | 11 | 478 | 681 | 66 | 290 | Phage-related protein | Phage-related protein | | pdb | 3J4U\_G | 1.0 | 5.196e-06 | 145 | 0.127 | 275 | 160 | 15 | 423 | 680 | 24 | 235 | major capsid protein | major capsid protein | | pdb | 7F2P\_I | 1.0 | 2.487e-06 | 145 | 0.09 | 388 | 228 | 24 | 395 | 681 | 4 | 367 | KHP40 MCP | KHP40 MCP | | pdb | 8GMO\_AD | 1.0 | 2.221e-06 | 144 | 0.107 | 280 | 176 | 13 | 467 | 680 | 165 | 436 | Mature major capsid protein | Mature major capsid protein | | pdb | 3BJQ\_H | 1.0 | 1.441e-05 | 143 | 0.145 | 310 | 183 | 19 | 420 | 681 | 13 | 288 | Phage-related protein | Phage-related protein | | pdb | 3BJQ\_F | 1.0 | 1.216e-05 | 143 | 0.145 | 309 | 187 | 22 | 420 | 681 | 13 | 291 | Phage-related protein | Phage-related protein | | pdb | 8CJZ\_H | 1.0 | 4.639e-06 | 143 | 0.148 | 290 | 150 | 14 | 421 | 681 | 24 | 245 | Spike Base Protein | Spike Base Protein | | pdb | 3J4U\_C | 1.0 | 3.569e-05 | 141 | 0.125 | 278 | 159 | 16 | 422 | 680 | 23 | 235 | major capsid protein | major capsid protein | | pdb | 7F2P\_A | 1.0 | 6.159e-06 | 139 | 0.081 | 392 | 222 | 25 | 398 | 680 | 2 | 364 | KHP40 MCP | KHP40 MCP | | pdb | 7DN2\_C | 1.0 | 4.142e-06 | 139 | 0.071 | 390 | 232 | 17 | 398 | 682 | 4 | 368 | Major structural protein ORF14 | Major structural protein ORF14 | | pdb | 7F2P\_B | 1.0 | 9.692e-06 | 135 | 0.09 | 396 | 210 | 26 | 402 | 682 | 3 | 363 | KHP40 MCP | KHP40 MCP | | pdb | 3BJQ\_E | 1.0 | 5.307e-05 | 134 | 0.143 | 313 | 192 | 22 | 420 | 681 | 13 | 300 | Phage-related protein | Phage-related protein | | pdb | 6Q3G\_AD | 1.0 | 7.301e-06 | 134 | 0.086 | 371 | 231 | 20 | 402 | 681 | 1 | 354 | Major head protein | Major head protein | | pdb | 8I1T\_G | 1.0 | 2.221e-06 | 133 | 0.075 | 439 | 227 | 17 | 407 | 680 | 1 | 425 | Major capsid protein | Major capsid protein | | pdb | 3BJQ\_B | 1.0 | 2.845e-05 | 132 | 0.144 | 298 | 177 | 21 | 438 | 681 | 26 | 299 | Phage-related protein | Phage-related protein | | pdb | 3BJQ\_I | 1.0 | 7.046e-05 | 130 | 0.14 | 291 | 177 | 20 | 433 | 678 | 23 | 285 | Phage-related protein | Phage-related protein | | pdb | 3BJQ\_A | 1.0 | 4.738e-05 | 130 | 0.143 | 314 | 183 | 22 | 420 | 681 | 13 | 292 | Phage-related protein | Phage-related protein | | pdb | 7DN2\_I | 1.0 | 7.726e-06 | 130 | 0.073 | 382 | 219 | 25 | 408 | 679 | 5 | 361 | Major structural protein ORF14 | Major structural protein ORF14 | | pdb | 5LII\_P | 1.0 | 3.187e-05 | 130 | 0.098 | 437 | 226 | 20 | 409 | 682 | 1 | 432 | Major capsid protein | Major capsid protein | | pdb | 7VS5\_AA | 1.0 | 5.82e-06 | 130 | 0.114 | 296 | 186 | 16 | 452 | 681 | 171 | 456 | Major capsid protein | Major capsid protein | | pdb | 3BJQ\_J | 1.0 | 7.457e-05 | 129 | 0.153 | 306 | 184 | 20 | 420 | 678 | 13 | 290 | Phage-related protein | Phage-related protein | | pdb | 7DN2\_H | 1.0 | 2.143e-05 | 129 | 0.083 | 393 | 213 | 23 | 402 | 679 | 1 | 361 | Major structural protein ORF14 | Major structural protein ORF14 | | pdb | 6OKB\_E | 1.0 | 0.0001472 | 127 | 0.142 | 302 | 188 | 19 | 417 | 681 | 20 | 287 | Major capsid protein | Major capsid protein | | pdb | 8EGT\_A | 1.0 | 2.948e-06 | 127 | 0.098 | 406 | 221 | 21 | 392 | 682 | 13 | 388 | Major capsid protein | Major capsid protein | | pdb | 3BJQ\_D | 1.0 | 4.477e-05 | 126 | 0.15 | 305 | 174 | 20 | 433 | 681 | 23 | 298 | Phage-related protein | Phage-related protein | | pdb | 7F2P\_G | 1.0 | 1.362e-05 | 126 | 0.075 | 397 | 212 | 24 | 402 | 679 | 8 | 368 | KHP40 MCP | KHP40 MCP | | pdb | 6IAW\_O | 1.0 | 1.086e-05 | 124 | 0.097 | 399 | 227 | 21 | 392 | 681 | 12 | 386 | Major head protein | Major head protein | | pdb | 3J7V\_B | 1.0 | 7.046e-05 | 122 | 0.08 | 297 | 212 | 17 | 434 | 681 | 25 | 309 | Major capsid protein 10A | Major capsid protein 10A | | pdb | 8GMO\_6 | 1.0 | 1.614e-05 | 122 | 0.109 | 274 | 174 | 13 | 471 | 680 | 195 | 462 | Mature major capsid protein | Mature major capsid protein | | pdb | 7VRT\_FB | 1.0 | 1.441e-05 | 121 | 0.123 | 275 | 162 | 18 | 468 | 681 | 115 | 371 | Major capsid protein | Major capsid protein | | pdb | 6I9E\_A | 1.0 | 2.098e-06 | 121 | 0.098 | 394 | 240 | 20 | 399 | 682 | 6 | 394 | Major head protein | Major head protein | | pdb | 1YUE\_A | 1.0 | 6.518e-06 | 120 | 0.128 | 289 | 161 | 17 | 465 | 682 | 122 | 390 | Head vertex protein Gp24 | Head vertex protein Gp24 | | pdb | 6Q3G\_KD | 1.0 | 5.499e-06 | 117 | 0.093 | 415 | 229 | 25 | 378 | 681 | 4 | 382 | Major head protein | Major head protein | | pdb | 7VS5\_DW | 1.0 | 8.352e-05 | 117 | 0.108 | 287 | 168 | 16 | 469 | 681 | 158 | 430 | Major capsid protein | Major capsid protein | | pdb | 7VS5\_HG | 1.0 | 5.015e-05 | 116 | 0.144 | 305 | 140 | 21 | 463 | 682 | 142 | 410 | Capsid vertex protein | Capsid vertex protein | | pdb | 5VF3\_L | 1.0 | 2.688e-05 | 114 | 0.093 | 277 | 171 | 16 | 474 | 681 | 189 | 454 | Major capsid protein | Major capsid protein | | pdb | 7QOF\_A | 1.0 | 7.891e-05 | 114 | 0.106 | 291 | 177 | 15 | 465 | 681 | 187 | 468 | Auxiliary capsid protein gp36 | Auxiliary capsid protein gp36 | | pdb | 6XGP\_B | 1.0 | 0.0001048 | 111 | 0.098 | 275 | 164 | 17 | 478 | 682 | 70 | 330 | YSD1\_17 major capsid protein | YSD1\_17 major capsid protein | | pdb | 7DN2\_E | 1.0 | 5.944e-05 | 111 | 0.09 | 397 | 203 | 29 | 406 | 681 | 11 | 370 | Major structural protein ORF14 | Major structural protein ORF14 | | pdb | 4AN5\_C | 1.0 | 0.0009027 | 110 | 0.11 | 272 | 173 | 18 | 433 | 676 | 3 | 233 | COAT PROTEIN | COAT PROTEIN | | pdb | 6XGP\_A | 1.0 | 9.354e-05 | 106 | 0.099 | 272 | 164 | 19 | 478 | 682 | 70 | 327 | YSD1\_17 major capsid protein | YSD1\_17 major capsid protein | | pdb | 7VI9\_G | 1.0 | 0.0005122 | 104 | 0.111 | 341 | 219 | 20 | 418 | 681 | 2 | 335 | Major capsid protein | Major capsid protein | | pdb | 7QOF\_B | 1.0 | 0.0001109 | 102 | 0.089 | 323 | 178 | 19 | 465 | 681 | 187 | 499 | Auxiliary capsid protein gp36 | Auxiliary capsid protein gp36 | | pdb | 3BQW\_A | 1.0 | 0.0001109 | 100 | 0.105 | 295 | 158 | 18 | 474 | 681 | 72 | 347 | Putative capsid protein of prophage | Putative capsid protein of prophage | | pdb | 7SJ5\_A | 0.999 | 7.891e-05 | 97 | 0.12 | 365 | 172 | 26 | 424 | 681 | 18 | 340 | Major capsid protein | Major capsid protein | | pdb | 6LGN\_Z | 0.999 | 0.0001472 | 97 | 0.084 | 413 | 204 | 22 | 422 | 681 | 6 | 397 | Small capsomere-interacting protein | Small capsomere-interacting protein | | pdb | 5JBL\_A | 0.997 | 0.002504 | 92 | 0.096 | 176 | 107 | 17 | 30 | 170 | 5 | 163 | Prohead core protein protease | Prohead core protein protease | | pdb | 3J7V\_G | 0.996 | 0.0005122 | 90 | 0.11 | 300 | 200 | 20 | 437 | 681 | 30 | 317 | Major capsid protein 10A | Major capsid protein 10A | | pdb | 7QOI\_CD | 0.996 | 0.0007196 | 90 | 0.102 | 294 | 167 | 18 | 466 | 681 | 173 | 447 | Auxiliary capsid protein gp36 | Auxiliary capsid protein gp36 | | pdb | 5JBL\_B | 0.996 | 0.005859 | 89 | 0.107 | 177 | 108 | 16 | 30 | 173 | 5 | 164 | Prohead core protein protease | Prohead core protein protease | | pdb | 3J40\_E | 0.996 | 0.0005737 | 89 | 0.102 | 311 | 182 | 16 | 391 | 682 | 11 | 243 | gp7 | gp7 | | pdb | 8I1V\_D | 0.993 | 0.0003255 | 86 | 0.106 | 404 | 189 | 19 | 431 | 680 | 23 | 408 | Major capsid protein | Major capsid protein | | pdb | 7QOI\_AD | 0.993 | 0.001011 | 86 | 0.1 | 298 | 168 | 14 | 466 | 681 | 173 | 452 | Auxiliary capsid protein gp36 | Auxiliary capsid protein gp36 | | pdb | 8I1V\_G | 0.99 | 0.0004321 | 83 | 0.103 | 416 | 185 | 23 | 431 | 680 | 23 | 416 | Major capsid protein | Major capsid protein | | pdb | 6ODM\_W | 0.99 | 0.005536 | 83 | 0.155 | 58 | 43 | 3 | 625 | 681 | 1055 | 1107 | Major capsid protein | Major capsid protein | | pdb | 8I1V\_A | 0.984 | 0.0009554 | 80 | 0.102 | 409 | 189 | 21 | 431 | 680 | 22 | 411 | Major capsid protein | Major capsid protein | | pdb | 8I1V\_F | 0.984 | 0.0009027 | 80 | 0.084 | 428 | 182 | 19 | 431 | 680 | 23 | 418 | Major capsid protein | Major capsid protein | | pdb | 6ODM\_S | 0.984 | 0.004943 | 80 | 0.161 | 62 | 45 | 4 | 621 | 681 | 1013 | 1068 | Major capsid protein | Major capsid protein | | pdb | 8I1V\_B | 0.855 | 0.001684 | 64 | 0.096 | 406 | 193 | 18 | 431 | 680 | 23 | 410 | Major capsid protein | Major capsid protein | | afdb-proteome | AF-Q2FX56-F1-MODEL\_V4 | 1.0 | 1.607e-14 | 429 | 0.132 | 324 | 221 | 12 | 402 | 682 | 8 | 314 | Phage head protein, putative | Phage head protein, putative | | afdb-proteome | AF-A0A0H3GTX7-F1-MODEL\_V4 | 1.0 | 2.34e-15 | 396 | 0.133 | 463 | 276 | 24 | 258 | 681 | 8 | 384 | Phage major capsid protein, HK97 family | Phage major capsid protein, HK97 family | | afdb-proteome | AF-A0A0H3GZA0-F1-MODEL\_V4 | 1.0 | 9.717e-12 | 348 | 0.138 | 295 | 215 | 15 | 410 | 681 | 12 | 290 | Uncharacterized protein | Uncharacterized protein | | afdb-proteome | AF-A0A133CKM2-F1-MODEL\_V4 | 1.0 | 1.813e-11 | 251 | 0.131 | 455 | 256 | 23 | 268 | 679 | 6 | 364 | Major capsid protein | Major capsid protein | | afdb-proteome | AF-A0A242FSQ3-F1-MODEL\_V4 | 1.0 | 3.527e-09 | 215 | 0.109 | 393 | 244 | 20 | 338 | 681 | 46 | 381 | Phage capsid protein | Phage capsid protein | | afdb-uniprot50 | AF-B2I5F3-F1-MODEL\_V4 | 1.0 | 8.792e-81 | 2210 | 0.472 | 656 | 297 | 11 | 29 | 681 | 15 | 624 | Peptidase U35 phage prohead HK97 | Peptidase U35 phage prohead HK97 | | afdb-uniprot50 | AF-A0A4Q3Z182-F1-MODEL\_V4 | 1.0 | 5.592e-89 | 2171 | 0.513 | 676 | 305 | 10 | 19 | 682 | 1 | 664 | Phage major capsid protein | Phage major capsid protein | | afdb-uniprot50 | AF-A0A420WVK6-F1-MODEL\_V4 | 1.0 | 4.038e-91 | 2168 | 0.569 | 717 | 253 | 11 | 1 | 682 | 37 | 732 | HK97 family phage major capsid protein | HK97 family phage major capsid protein | | afdb-uniprot50 | AF-A0A1H2ZH10-F1-MODEL\_V4 | 1.0 | 2.527e-81 | 2004 | 0.5 | 662 | 294 | 9 | 36 | 682 | 6 | 645 | Phage prohead protease, HK97 family/phage major capsid protein, HK97 family,TIGR01554 | Phage prohead protease, HK97 family/phage major capsid protein, HK97 family,TIGR01554 | | afdb-uniprot50 | AF-A0A7H8DLL2-F1-MODEL\_V4 | 1.0 | 5.789e-82 | 1964 | 0.471 | 679 | 320 | 15 | 19 | 682 | 2 | 656 | Phage major capsid protein | Phage major capsid protein | | afdb-uniprot50 | AF-A0A7C8LYK9-F1-MODEL\_V4 | 1.0 | 3.647e-69 | 1916 | 0.392 | 675 | 347 | 14 | 11 | 681 | 9 | 624 | Uncharacterized protein | Uncharacterized protein | | afdb-uniprot50 | AF-A0A4Q3L4Z9-F1-MODEL\_V4 | 1.0 | 3.571e-70 | 1901 | 0.351 | 672 | 381 | 10 | 30 | 682 | 10 | 645 | Phage major capsid protein | Phage major capsid protein | | afdb-uniprot50 | AF-A0A0C1ILN4-F1-MODEL\_V4 | 1.0 | 2.491e-79 | 1875 | 0.473 | 708 | 311 | 13 | 1 | 682 | 1 | 672 | Peptidase U35 | Peptidase U35 | | afdb-uniprot50 | AF-A0A149SVI9-F1-MODEL\_V4 | 1.0 | 3.808e-75 | 1838 | 0.477 | 677 | 280 | 11 | 28 | 682 | 14 | 638 | Uncharacterized protein | Uncharacterized protein | | afdb-uniprot50 | AF-A0A0Q7SGT0-F1-MODEL\_V4 | 1.0 | 2.159e-67 | 1838 | 0.375 | 709 | 334 | 19 | 19 | 681 | 6 | 651 | Uncharacterized protein | Uncharacterized protein | | afdb-uniprot50 | AF-A0A166AHQ0-F1-MODEL\_V4 | 1.0 | 7.782e-68 | 1761 | 0.38 | 662 | 341 | 13 | 30 | 681 | 11 | 613 | Phage capsid family protein | Phage capsid family protein | | afdb-uniprot50 | AF-A0A7J0BZB5-F1-MODEL\_V4 | 1.0 | 7.2e-69 | 1755 | 0.449 | 647 | 282 | 11 | 48 | 681 | 2 | 587 | Uncharacterized protein | Uncharacterized protein | | afdb-uniprot50 | AF-A0A501X0T1-F1-MODEL\_V4 | 1.0 | 7.403e-65 | 1746 | 0.397 | 675 | 329 | 16 | 19 | 682 | 32 | 639 | Phage major capsid protein | Phage major capsid protein | | afdb-uniprot50 | AF-A0A143DDT8-F1-MODEL\_V4 | 1.0 | 7.41e-73 | 1738 | 0.445 | 674 | 307 | 15 | 18 | 679 | 2 | 620 | Uncharacterized protein | Uncharacterized protein | | afdb-uniprot50 | AF-A0A1S1CXE5-F1-MODEL\_V4 | 1.0 | 1.078e-65 | 1736 | 0.372 | 685 | 345 | 21 | 19 | 680 | 1 | 623 | Major capsid protein | Major capsid protein | | afdb-uniprot50 | AF-A0A2E9II77-F1-MODEL\_V4 | 1.0 | 5.696e-64 | 1729 | 0.351 | 677 | 351 | 19 | 12 | 678 | 13 | 611 | Phage major capsid protein | Phage major capsid protein | | afdb-uniprot50 | AF-A0A2E1P3K3-F1-MODEL\_V4 | 1.0 | 1.547e-64 | 1670 | 0.364 | 674 | 338 | 19 | 17 | 678 | 269 | 863 | Phage major capsid protein | Phage major capsid protein | | afdb-uniprot50 | AF-A0A3A4XAN1-F1-MODEL\_V4 | 1.0 | 2.237e-68 | 1668 | 0.396 | 663 | 320 | 14 | 30 | 682 | 40 | 632 | Phage major capsid protein | Phage major capsid protein | | afdb-uniprot50 | AF-A0A2E0ENB5-F1-MODEL\_V4 | 1.0 | 2.505e-68 | 1663 | 0.39 | 682 | 344 | 15 | 19 | 681 | 9 | 637 | Phage major capsid protein | Phage major capsid protein | | afdb-uniprot50 | AF-A0A5P9J3N0-F1-MODEL\_V4 | 1.0 | 2.847e-70 | 1662 | 0.409 | 669 | 337 | 17 | 39 | 682 | 44 | 679 | Phage capsid family protein | Phage capsid family protein | | afdb-uniprot50 | AF-A0A2V4KHZ2-F1-MODEL\_V4 | 1.0 | 1.093e-67 | 1652 | 0.366 | 676 | 337 | 15 | 30 | 682 | 11 | 617 | Phage major capsid protein | Phage major capsid protein | | afdb-uniprot50 | AF-A0A1X7L142-F1-MODEL\_V4 | 1.0 | 5.305e-62 | 1632 | 0.443 | 586 | 278 | 8 | 99 | 682 | 4 | 543 | Phage major capsid protein, HK97 family | Phage major capsid protein, HK97 family | | afdb-uniprot50 | AF-A0A1Y0N5R1-F1-MODEL\_V4 | 1.0 | 7.248e-66 | 1615 | 0.396 | 689 | 314 | 22 | 19 | 680 | 2 | 615 | Uncharacterized protein | Uncharacterized protein | | afdb-uniprot50 | AF-Q3RBW7-F1-MODEL\_V4 | 1.0 | 4.282e-48 | 1608 | 0.527 | 370 | 164 | 5 | 313 | 681 | 1 | 360 | Uncharacterized protein | Uncharacterized protein | | afdb-uniprot50 | AF-A0A5C7PYX8-F1-MODEL\_V4 | 1.0 | 2.284e-67 | 1598 | 0.416 | 668 | 321 | 12 | 19 | 678 | 2 | 608 | Phage major capsid protein | Phage major capsid protein | | afdb-uniprot50 | AF-A0A7X8AGV1-F1-MODEL\_V4 | 1.0 | 2.927e-66 | 1589 | 0.394 | 679 | 320 | 17 | 29 | 682 | 19 | 631 | Phage major capsid protein | Phage major capsid protein | | afdb-uniprot50 | AF-F4BFR1-F1-MODEL\_V4 | 1.0 | 1.696e-65 | 1585 | 0.356 | 676 | 327 | 18 | 25 | 681 | 14 | 600 | Phage protein | Phage protein | | afdb-uniprot50 | AF-A0A0F9KU40-F1-MODEL\_V4 | 1.0 | 1.071e-68 | 1575 | 0.391 | 722 | 347 | 19 | 16 | 682 | 33 | 716 | Uncharacterized protein | Uncharacterized protein | | afdb-uniprot50 | AF-A0A8B3RDF4-F1-MODEL\_V4 | 1.0 | 2.885e-64 | 1520 | 0.351 | 680 | 349 | 21 | 19 | 682 | 1 | 604 | Phage major capsid protein | Phage major capsid protein | | afdb-uniprot50 | AF-A0A3A4P4D4-F1-MODEL\_V4 | 1.0 | 7.403e-65 | 1511 | 0.377 | 696 | 340 | 22 | 16 | 680 | 3 | 635 | Phage major capsid protein | Phage major capsid protein | | afdb-uniprot50 | AF-A0A355AHA6-F1-MODEL\_V4 | 1.0 | 1.018e-65 | 1511 | 0.335 | 665 | 389 | 14 | 20 | 678 | 215 | 832 | Phage major capsid protein | Phage major capsid protein | | afdb-uniprot50 | AF-A0A1W9SXD3-F1-MODEL\_V4 | 1.0 | 1.873e-63 | 1495 | 0.384 | 692 | 306 | 16 | 11 | 681 | 24 | 616 | Uncharacterized protein | Uncharacterized protein | | afdb-uniprot50 | AF-A0A6M3XKV2-F1-MODEL\_V4 | 1.0 | 6.604e-57 | 1459 | 0.294 | 687 | 364 | 20 | 26 | 682 | 11 | 606 | Putative capsid protein | Putative capsid protein | | afdb-uniprot50 | AF-A0A1G2ZKV7-F1-MODEL\_V4 | 1.0 | 2.885e-64 | 1451 | 0.365 | 686 | 336 | 22 | 29 | 681 | 11 | 629 | Uncharacterized protein | Uncharacterized protein | | afdb-uniprot50 | AF-A0A5C9BFK7-F1-MODEL\_V4 | 1.0 | 1.844e-53 | 1420 | 0.32 | 670 | 343 | 23 | 39 | 680 | 12 | 596 | Putative phage major capsid protein | Putative phage major capsid protein | | afdb-uniprot50 | AF-A0A2H6GL21-F1-MODEL\_V4 | 1.0 | 7.775e-60 | 1419 | 0.33 | 672 | 342 | 19 | 24 | 678 | 4 | 584 | Phage capsid family protein | Phage capsid family protein | | afdb-uniprot50 | AF-A0A3A0FXT4-F1-MODEL\_V4 | 1.0 | 5.817e-63 | 1416 | 0.366 | 688 | 357 | 19 | 30 | 681 | 33 | 677 | Phage major capsid protein | Phage major capsid protein | | afdb-uniprot50 | AF-A0A448XYU4-F1-MODEL\_V4 | 1.0 | 3.323e-60 | 1414 | 0.348 | 680 | 378 | 18 | 14 | 679 | 3 | 631 | HK97 family phage major capsid protein | HK97 family phage major capsid protein | | afdb-uniprot50 | AF-A0A2D3WU31-F1-MODEL\_V4 | 1.0 | 6.422e-61 | 1413 | 0.339 | 662 | 348 | 14 | 30 | 682 | 14 | 594 | Phage major capsid protein | Phage major capsid protein | | afdb-uniprot50 | AF-A0A661FCZ5-F1-MODEL\_V4 | 1.0 | 3.644e-61 | 1407 | 0.368 | 664 | 326 | 13 | 30 | 678 | 14 | 598 | Phage major capsid protein | Phage major capsid protein | | afdb-uniprot50 | AF-A0A0H3ZS84-F1-MODEL\_V4 | 1.0 | 7.775e-60 | 1390 | 0.376 | 664 | 345 | 14 | 29 | 682 | 10 | 614 | Phage major capsid protein | Phage major capsid protein | | afdb-uniprot50 | AF-A0A254T9Q6-F1-MODEL\_V4 | 1.0 | 2.882e-56 | 1378 | 0.341 | 689 | 340 | 23 | 29 | 681 | 9 | 619 | Uncharacterized protein | Uncharacterized protein | | afdb-uniprot50 | AF-A0A661QEN8-F1-MODEL\_V4 | 1.0 | 1.659e-58 | 1360 | 0.37 | 664 | 311 | 19 | 38 | 681 | 21 | 597 | Phage major capsid protein | Phage major capsid protein | | afdb-uniprot50 | AF-A0A2E7V2Q0-F1-MODEL\_V4 | 1.0 | 5.455e-58 | 1359 | 0.331 | 660 | 346 | 20 | 40 | 680 | 41 | 623 | Phage major capsid protein | Phage major capsid protein | | afdb-uniprot50 | AF-A0A6M8SY00-F1-MODEL\_V4 | 1.0 | 1.858e-58 | 1356 | 0.373 | 672 | 327 | 24 | 40 | 681 | 20 | 627 | Phage major capsid protein | Phage major capsid protein | | afdb-uniprot50 | AF-A0A2A5AEG6-F1-MODEL\_V4 | 1.0 | 2.157e-59 | 1350 | 0.328 | 675 | 371 | 16 | 26 | 682 | 12 | 621 | Phage major capsid protein | Phage major capsid protein | | afdb-uniprot50 | AF-A0A3C0NVR1-F1-MODEL\_V4 | 1.0 | 1.285e-54 | 1347 | 0.3 | 708 | 383 | 24 | 10 | 681 | 17 | 647 | Phage major capsid protein | Phage major capsid protein | | afdb-uniprot50 | AF-A0A238KH43-F1-MODEL\_V4 | 1.0 | 2.574e-56 | 1315 | 0.319 | 685 | 399 | 20 | 19 | 680 | 1 | 641 | Phage capsid family protein | Phage capsid family protein | | afdb-uniprot50 | AF-A0A3M1CK54-F1-MODEL\_V4 | 1.0 | 3.051e-56 | 1314 | 0.32 | 668 | 347 | 20 | 30 | 679 | 5 | 583 | Phage major capsid protein | Phage major capsid protein | | afdb-uniprot50 | AF-A0A2J8GXS4-F1-MODEL\_V4 | 1.0 | 2.611e-58 | 1301 | 0.367 | 669 | 333 | 19 | 30 | 682 | 12 | 606 | Phage major capsid protein | Phage major capsid protein | | afdb-uniprot50 | AF-A0A227JQC9-F1-MODEL\_V4 | 1.0 | 3.965e-57 | 1300 | 0.296 | 699 | 378 | 21 | 31 | 680 | 3 | 636 | Phage major capsid protein | Phage major capsid protein | | afdb-uniprot50 | AF-A0A0G3G1E5-F1-MODEL\_V4 | 1.0 | 4.164e-52 | 1287 | 0.309 | 646 | 373 | 16 | 46 | 681 | 2 | 584 | Uncharacterized protein | Uncharacterized protein | | afdb-uniprot50 | AF-A0A2A5DI76-F1-MODEL\_V4 | 1.0 | 4.903e-55 | 1281 | 0.302 | 672 | 364 | 21 | 41 | 682 | 33 | 629 | Phage major capsid protein | Phage major capsid protein | | afdb-uniprot50 | AF-A0A5N0TEK3-F1-MODEL\_V4 | 1.0 | 1.793e-57 | 1270 | 0.353 | 685 | 353 | 19 | 16 | 681 | 5 | 618 | Phage major capsid protein | Phage major capsid protein | | afdb-uniprot50 | AF-A0A0F2NXV9-F1-MODEL\_V4 | 1.0 | 4.315e-53 | 1268 | 0.305 | 678 | 355 | 20 | 18 | 682 | 10 | 584 | Uncharacterized protein | Uncharacterized protein | | afdb-uniprot50 | AF-A0A3B9Q986-F1-MODEL\_V4 | 1.0 | 8.461e-56 | 1268 | 0.3 | 698 | 392 | 24 | 19 | 680 | 1 | 637 | Phage major capsid protein | Phage major capsid protein | | afdb-uniprot50 | AF-A0A7Y5NBG9-F1-MODEL\_V4 | 1.0 | 7.995e-56 | 1254 | 0.306 | 707 | 378 | 24 | 20 | 681 | 1 | 639 | Phage major capsid protein | Phage major capsid protein | | afdb-uniprot50 | AF-A0A1Q3WRH7-F1-MODEL\_V4 | 1.0 | 1.206e-57 | 1252 | 0.336 | 734 | 353 | 30 | 30 | 681 | 18 | 699 | Uncharacterized protein | Uncharacterized protein | | afdb-uniprot50 | AF-A0A369R3L5-F1-MODEL\_V4 | 1.0 | 8.34e-54 | 1245 | 0.301 | 714 | 364 | 29 | 29 | 682 | 16 | 654 | Phage major capsid protein | Phage major capsid protein | | afdb-uniprot50 | AF-A0A831U076-F1-MODEL\_V4 | 1.0 | 6.373e-56 | 1228 | 0.331 | 697 | 366 | 27 | 29 | 681 | 16 | 656 | Phage major capsid protein | Phage major capsid protein | | afdb-uniprot50 | AF-A0A3N2E2B1-F1-MODEL\_V4 | 1.0 | 4.759e-43 | 1222 | 0.393 | 435 | 237 | 11 | 252 | 682 | 246 | 657 | ATP-dependent Clp protease proteolytic subunit | ATP-dependent Clp protease proteolytic subunit | | afdb-uniprot50 | AF-A0A259F2V0-F1-MODEL\_V4 | 1.0 | 3.908e-55 | 1222 | 0.329 | 695 | 349 | 22 | 12 | 682 | 135 | 736 | Uncharacterized protein | Uncharacterized protein | | afdb-uniprot50 | AF-A0A5K7YQH8-F1-MODEL\_V4 | 1.0 | 2.958e-36 | 1210 | 0.426 | 359 | 194 | 6 | 331 | 682 | 10 | 363 | Uncharacterized protein | Uncharacterized protein | | afdb-uniprot50 | AF-A0A6M1TYU1-F1-MODEL\_V4 | 1.0 | 6.46e-50 | 1209 | 0.283 | 659 | 355 | 21 | 42 | 681 | 21 | 580 | Phage major capsid protein | Phage major capsid protein | | afdb-uniprot50 | AF-A0A259FKU1-F1-MODEL\_V4 | 1.0 | 9.007e-45 | 1203 | 0.348 | 542 | 263 | 19 | 164 | 681 | 2 | 477 | Uncharacterized protein | Uncharacterized protein | | afdb-uniprot50 | AF-A0A7C4G5Z6-F1-MODEL\_V4 | 1.0 | 2.981e-41 | 1202 | 0.393 | 455 | 221 | 13 | 252 | 681 | 2 | 426 | Phage major capsid protein | Phage major capsid protein | | afdb-uniprot50 | AF-A0A2V5PK61-F1-MODEL\_V4 | 1.0 | 6.79e-53 | 1201 | 0.303 | 701 | 352 | 24 | 30 | 681 | 10 | 622 | Phage major capsid protein | Phage major capsid protein | | afdb-uniprot50 | AF-A0A7Y0IMP7-F1-MODEL\_V4 | 1.0 | 1.706e-54 | 1197 | 0.262 | 717 | 409 | 20 | 17 | 682 | 7 | 654 | HK97 family phage major capsid protein/HK97 family phage prohead protease | HK97 family phage major capsid protein/HK97 family phage prohead protease | | afdb-uniprot50 | AF-A0A7G8BY98-F1-MODEL\_V4 | 1.0 | 7.881e-54 | 1190 | 0.302 | 711 | 399 | 24 | 19 | 682 | 2 | 662 | Phage major capsid protein | Phage major capsid protein | | afdb-uniprot50 | AF-A0A3S0D8V4-F1-MODEL\_V4 | 1.0 | 1.046e-53 | 1189 | 0.288 | 708 | 400 | 21 | 12 | 682 | 27 | 667 | Phage major capsid protein | Phage major capsid protein | | afdb-uniprot50 | AF-A0A4P5QRR4-F1-MODEL\_V4 | 1.0 | 2.413e-51 | 1187 | 0.29 | 684 | 391 | 22 | 30 | 680 | 11 | 632 | Uncharacterized protein | Uncharacterized protein | | afdb-uniprot50 | AF-A0A081RAC8-F1-MODEL\_V4 | 1.0 | 4.598e-50 | 1185 | 0.288 | 682 | 379 | 20 | 17 | 681 | 3 | 594 | Phage major capsid protein, HK97 family | Phage major capsid protein, HK97 family | | afdb-uniprot50 | AF-A0A1M7GV20-F1-MODEL\_V4 | 1.0 | 1.818e-51 | 1185 | 0.297 | 678 | 363 | 21 | 41 | 682 | 15 | 614 | Phage prohead protease, HK97 family/phage major capsid protein, HK97 family,TIGR01554 | Phage prohead protease, HK97 family/phage major capsid protein, HK97 family,TIGR01554 | | afdb-uniprot50 | AF-A0A2W7GS67-F1-MODEL\_V4 | 1.0 | 1.205e-49 | 1183 | 0.288 | 662 | 369 | 18 | 38 | 681 | 13 | 590 | HK97 family phage major capsid protein | HK97 family phage major capsid protein | | afdb-uniprot50 | AF-A0A1H0VBY7-F1-MODEL\_V4 | 1.0 | 4.501e-51 | 1171 | 0.279 | 683 | 374 | 21 | 29 | 681 | 10 | 604 | Phage major capsid protein, HK97 family | Phage major capsid protein, HK97 family | | afdb-uniprot50 | AF-A0A0P8D6G1-F1-MODEL\_V4 | 1.0 | 9.137e-47 | 1159 | 0.341 | 620 | 306 | 20 | 89 | 681 | 1 | 544 | Phage major capsid protein, HK97 family | Phage major capsid protein, HK97 family | | afdb-uniprot50 | AF-A0A259FDJ6-F1-MODEL\_V4 | 1.0 | 1.669e-47 | 1159 | 0.298 | 681 | 335 | 19 | 23 | 682 | 1 | 559 | Uncharacterized protein | Uncharacterized protein | | afdb-uniprot50 | AF-A0A0S9N309-F1-MODEL\_V4 | 1.0 | 4.16e-44 | 1157 | 0.335 | 567 | 314 | 16 | 124 | 679 | 2 | 516 | Uncharacterized protein | Uncharacterized protein | | afdb-uniprot50 | AF-A0A1G0RDU6-F1-MODEL\_V4 | 1.0 | 2.608e-50 | 1154 | 0.258 | 731 | 431 | 26 | 1 | 682 | 1 | 669 | Uncharacterized protein | Uncharacterized protein | | afdb-uniprot50 | AF-A0A6H9I617-F1-MODEL\_V4 | 1.0 | 1.459e-48 | 1145 | 0.296 | 679 | 355 | 26 | 38 | 681 | 11 | 601 | Phage major capsid protein | Phage major capsid protein | | afdb-uniprot50 | AF-U5QHN1-F1-MODEL\_V4 | 1.0 | 1.883e-52 | 1129 | 0.272 | 744 | 420 | 25 | 1 | 682 | 26 | 709 | Uncharacterized protein | Uncharacterized protein | | afdb-uniprot50 | AF-A0A660NJM1-F1-MODEL\_V4 | 1.0 | 4.407e-52 | 1113 | 0.473 | 526 | 235 | 9 | 14 | 534 | 13 | 501 | Phage major capsid protein | Phage major capsid protein | | afdb-uniprot50 | AF-C6M4F4-F1-MODEL\_V4 | 1.0 | 5.636e-35 | 1112 | 0.406 | 364 | 184 | 10 | 333 | 680 | 15 | 362 | Putative phage capsid family | Putative phage capsid family | | afdb-uniprot50 | AF-A0A2M7QL94-F1-MODEL\_V4 | 1.0 | 3.665e-50 | 1111 | 0.29 | 686 | 385 | 22 | 19 | 681 | 16 | 622 | Phage major capsid protein | Phage major capsid protein | | afdb-uniprot50 | AF-A0A419ENJ7-F1-MODEL\_V4 | 1.0 | 4.437e-49 | 1111 | 0.248 | 712 | 434 | 22 | 1 | 682 | 3 | 643 | Phage major capsid protein | Phage major capsid protein | | afdb-uniprot50 | AF-A0A2W5L8E5-F1-MODEL\_V4 | 1.0 | 3.463e-50 | 1104 | 0.282 | 730 | 403 | 23 | 30 | 682 | 2 | 687 | Phage major capsid protein | Phage major capsid protein | | afdb-uniprot50 | AF-A0A844G5I7-F1-MODEL\_V4 | 1.0 | 1.609e-38 | 1102 | 0.345 | 452 | 237 | 14 | 261 | 682 | 43 | 465 | Phage major capsid protein | Phage major capsid protein | | afdb-uniprot50 | AF-A0A2E7WAE6-F1-MODEL\_V4 | 1.0 | 3.513e-52 | 1102 | 0.282 | 694 | 385 | 21 | 30 | 680 | 215 | 838 | Phage major capsid protein | Phage major capsid protein | | afdb-uniprot50 | AF-A0A3B9NV55-F1-MODEL\_V4 | 1.0 | 2.429e-48 | 1090 | 0.282 | 709 | 367 | 24 | 40 | 680 | 14 | 648 | Phage major capsid protein | Phage major capsid protein | | afdb-uniprot50 | AF-A0A1V5CVG4-F1-MODEL\_V4 | 1.0 | 8.886e-51 | 1084 | 0.304 | 706 | 378 | 26 | 30 | 681 | 22 | 668 | Phage capsid family protein | Phage capsid family protein | | afdb-uniprot50 | AF-A0A2N1Z9S1-F1-MODEL\_V4 | 1.0 | 2.293e-40 | 1082 | 0.348 | 490 | 235 | 14 | 249 | 682 | 7 | 468 | Phage major capsid protein | Phage major capsid protein | | afdb-uniprot50 | AF-A0A7X4CLK5-F1-MODEL\_V4 | 1.0 | 1.693e-49 | 1073 | 0.268 | 776 | 418 | 25 | 19 | 682 | 13 | 750 | Phage major capsid protein | Phage major capsid protein | | afdb-uniprot50 | AF-A0A2E0R7B6-F1-MODEL\_V4 | 1.0 | 3.962e-49 | 1072 | 0.253 | 695 | 425 | 26 | 25 | 680 | 14 | 653 | Phage major capsid protein | Phage major capsid protein | | afdb-uniprot50 | AF-A0A259RGV4-F1-MODEL\_V4 | 1.0 | 1.83e-48 | 1067 | 0.276 | 730 | 382 | 25 | 6 | 681 | 9 | 645 | Uncharacterized protein | Uncharacterized protein | | afdb-uniprot50 | AF-A0A2U1B6D9-F1-MODEL\_V4 | 1.0 | 3.179e-46 | 1056 | 0.221 | 667 | 434 | 25 | 30 | 678 | 4 | 602 | HK97 family phage prohead protease/HK97 family phage major capsid protein,TIGR01554 | HK97 family phage prohead protease/HK97 family phage major capsid protein,TIGR01554 | | afdb-uniprot50 | AF-A0A1G0FL18-F1-MODEL\_V4 | 1.0 | 4.307e-37 | 1054 | 0.335 | 453 | 252 | 16 | 258 | 681 | 7 | 439 | Uncharacterized protein | Uncharacterized protein | | afdb-uniprot50 | AF-A0A285D6R0-F1-MODEL\_V4 | 1.0 | 3.849e-45 | 1049 | 0.302 | 672 | 357 | 25 | 41 | 682 | 31 | 620 | HK97 family phage major capsid protein | HK97 family phage major capsid protein | | afdb-uniprot50 | AF-A0A2A2GC02-F1-MODEL\_V4 | 1.0 | 1.136e-33 | 1036 | 0.299 | 441 | 251 | 15 | 258 | 680 | 20 | 420 | Phage major capsid protein | Phage major capsid protein | | afdb-uniprot50 | AF-A0A3S0EPF2-F1-MODEL\_V4 | 1.0 | 1.284e-46 | 1031 | 0.33 | 674 | 330 | 25 | 78 | 681 | 2 | 623 | Phage major capsid protein | Phage major capsid protein | | afdb-uniprot50 | AF-N6XHI8-F1-MODEL\_V4 | 1.0 | 2.755e-34 | 1028 | 0.351 | 395 | 217 | 12 | 308 | 681 | 11 | 387 | Caudovirus prohead protease family protein | Caudovirus prohead protease family protein | | afdb-uniprot50 | AF-A0A7V8PSM8-F1-MODEL\_V4 | 1.0 | 1.816e-43 | 1021 | 0.222 | 684 | 380 | 22 | 30 | 681 | 7 | 570 | Phage major capsid protein | Phage major capsid protein | | afdb-uniprot50 | AF-A0A4R2YZE6-F1-MODEL\_V4 | 1.0 | 3.068e-45 | 1018 | 0.235 | 710 | 414 | 29 | 30 | 681 | 3 | 641 | HK97 family phage prohead protease/HK97 family phage major capsid protein,TIGR01554 | HK97 family phage prohead protease/HK97 family phage major capsid protein,TIGR01554 | | afdb-uniprot50 | AF-A0A7G8GUS2-F1-MODEL\_V4 | 1.0 | 1.097e-32 | 1002 | 0.351 | 367 | 199 | 10 | 338 | 682 | 18 | 367 | Phage major capsid protein/ HK97 family | Phage major capsid protein/ HK97 family | | afdb-uniprot50 | AF-A0A1F9VF43-F1-MODEL\_V4 | 1.0 | 8.818e-46 | 987 | 0.298 | 708 | 403 | 27 | 17 | 682 | 7 | 662 | Uncharacterized protein | Uncharacterized protein | | afdb-uniprot50 | AF-A0A7G8DXJ8-F1-MODEL\_V4 | 1.0 | 6.046e-29 | 985 | 0.378 | 309 | 160 | 9 | 398 | 682 | 15 | 315 | Phage major capsid protein/ HK97 family | Phage major capsid protein/ HK97 family | | afdb-uniprot50 | AF-A0A2A7U7A2-F1-MODEL\_V4 | 1.0 | 2.311e-45 | 985 | 0.225 | 700 | 441 | 29 | 30 | 682 | 4 | 649 | Major capsid protein | Major capsid protein | | afdb-uniprot50 | AF-Q1ILS6-F1-MODEL\_V4 | 1.0 | 1.467e-37 | 985 | 0.271 | 523 | 315 | 15 | 183 | 682 | 299 | 778 | Peptidase S49 | Peptidase S49 | | afdb-uniprot50 | AF-A0A286GN99-F1-MODEL\_V4 | 1.0 | 6.134e-31 | 977 | 0.282 | 425 | 252 | 13 | 272 | 682 | 7 | 392 | Phage major capsid protein, HK97 family | Phage major capsid protein, HK97 family | | afdb-uniprot50 | AF-A0A1G1GKX3-F1-MODEL\_V4 | 1.0 | 1.211e-30 | 967 | 0.312 | 371 | 222 | 13 | 325 | 682 | 22 | 372 | Uncharacterized protein | Uncharacterized protein | | afdb-uniprot50 | AF-A0A2N3B852-F1-MODEL\_V4 | 1.0 | 5.295e-46 | 962 | 0.383 | 615 | 293 | 15 | 30 | 620 | 9 | 561 | Phage major capsid protein | Phage major capsid protein | | afdb-uniprot50 | AF-A0A7G9P270-F1-MODEL\_V4 | 1.0 | 1.69e-33 | 955 | 0.316 | 442 | 235 | 16 | 261 | 681 | 8 | 403 | Phage major capsid protein | Phage major capsid protein | | afdb-uniprot50 | AF-A0A382CXB3-F1-MODEL\_V4 | 1.0 | 6.355e-32 | 952 | 0.335 | 388 | 207 | 13 | 315 | 680 | 1 | 359 | Uncharacterized protein | Uncharacterized protein | | afdb-uniprot50 | AF-A0A2E9VJ27-F1-MODEL\_V4 | 1.0 | 1.477e-34 | 952 | 0.334 | 467 | 242 | 14 | 258 | 682 | 7 | 446 | Phage major capsid protein | Phage major capsid protein | | afdb-uniprot50 | AF-A0A6G6X8M0-F1-MODEL\_V4 | 1.0 | 7.701e-39 | 946 | 0.221 | 683 | 372 | 25 | 6 | 681 | 24 | 553 | Phage major capsid protein | Phage major capsid protein | | afdb-uniprot50 | AF-A4ACG5-F1-MODEL\_V4 | 1.0 | 1.68e-44 | 946 | 0.268 | 716 | 370 | 22 | 18 | 682 | 26 | 638 | Phage major capsid protein, HK97 family | Phage major capsid protein, HK97 family | | afdb-uniprot50 | AF-A0A5C7LIE0-F1-MODEL\_V4 | 1.0 | 1.643e-37 | 938 | 0.283 | 582 | 345 | 20 | 115 | 680 | 1 | 526 | Phage major capsid protein | Phage major capsid protein | | afdb-uniprot50 | AF-A0A0T9LH47-F1-MODEL\_V4 | 1.0 | 9.596e-42 | 938 | 0.21 | 735 | 385 | 28 | 16 | 682 | 2 | 608 | Phage major capsid protein, HK97 family | Phage major capsid protein, HK97 family | | afdb-uniprot50 | AF-A0A1F5QTK4-F1-MODEL\_V4 | 1.0 | 3.924e-28 | 932 | 0.325 | 338 | 197 | 12 | 367 | 681 | 7 | 336 | Uncharacterized protein | Uncharacterized protein | | afdb-uniprot50 | AF-A0A318GPG6-F1-MODEL\_V4 | 1.0 | 4.723e-38 | 926 | 0.232 | 693 | 367 | 24 | 1 | 682 | 13 | 551 | HK97 family phage major capsid protein | HK97 family phage major capsid protein | | afdb-uniprot50 | AF-A0A7U8KI29-F1-MODEL\_V4 | 1.0 | 1.007e-28 | 925 | 0.247 | 351 | 236 | 11 | 338 | 682 | 9 | 337 | Putative bacteriochlorophyll 4-vinyl reductase | Putative bacteriochlorophyll 4-vinyl reductase | | afdb-uniprot50 | AF-A0A258RTP1-F1-MODEL\_V4 | 1.0 | 1.328e-31 | 924 | 0.239 | 451 | 285 | 20 | 253 | 682 | 4 | 417 | Uncharacterized protein | Uncharacterized protein | | afdb-uniprot50 | AF-A0A3U4W9B0-F1-MODEL\_V4 | 1.0 | 4.528e-40 | 924 | 0.239 | 667 | 405 | 28 | 40 | 682 | 21 | 608 | Phage major capsid protein | Phage major capsid protein | | afdb-uniprot50 | AF-A0A1N6KUG4-F1-MODEL\_V4 | 1.0 | 1.015e-33 | 917 | 0.295 | 481 | 262 | 18 | 252 | 682 | 3 | 456 | Phage major capsid protein, HK97 family | Phage major capsid protein, HK97 family | | afdb-uniprot50 | AF-A0A0F9KZ70-F1-MODEL\_V4 | 1.0 | 3.43e-29 | 915 | 0.318 | 383 | 225 | 11 | 313 | 682 | 1 | 360 | Uncharacterized protein | Uncharacterized protein | | afdb-uniprot50 | AF-A0A7W4FCS3-F1-MODEL\_V4 | 1.0 | 5.674e-32 | 913 | 0.266 | 435 | 270 | 15 | 262 | 682 | 11 | 410 | Phage major capsid protein | Phage major capsid protein | | afdb-uniprot50 | AF-A0A071LTJ4-F1-MODEL\_V4 | 1.0 | 1.059e-39 | 912 | 0.222 | 687 | 380 | 26 | 41 | 682 | 18 | 595 | Uncharacterized protein | Uncharacterized protein | | afdb-uniprot50 | AF-A0A5T3MXQ8-F1-MODEL\_V4 | 1.0 | 1.691e-41 | 910 | 0.23 | 715 | 411 | 31 | 42 | 682 | 19 | 667 | Phage major capsid protein | Phage major capsid protein | | afdb-uniprot50 | AF-A0A1F4EFY0-F1-MODEL\_V4 | 1.0 | 3.606e-32 | 906 | 0.278 | 460 | 265 | 18 | 255 | 682 | 4 | 428 | Uncharacterized protein | Uncharacterized protein | | afdb-uniprot50 | AF-A0A6N8Q9L6-F1-MODEL\_V4 | 1.0 | 2.981e-41 | 905 | 0.239 | 709 | 393 | 28 | 17 | 681 | 6 | 611 | Phage major capsid protein | Phage major capsid protein | | afdb-uniprot50 | AF-A0A7C7SWM6-F1-MODEL\_V4 | 1.0 | 1.479e-42 | 894 | 0.35 | 594 | 291 | 19 | 40 | 622 | 39 | 548 | Phage major capsid protein | Phage major capsid protein | | afdb-uniprot50 | AF-A0A5E9ZST6-F1-MODEL\_V4 | 1.0 | 2.376e-41 | 888 | 0.207 | 737 | 445 | 31 | 13 | 681 | 3 | 668 | Major capsid protein | Major capsid protein | | afdb-uniprot50 | AF-A0A1F4EF80-F1-MODEL\_V4 | 1.0 | 1.328e-31 | 886 | 0.275 | 458 | 270 | 18 | 254 | 682 | 4 | 428 | Uncharacterized protein | Uncharacterized protein | | afdb-uniprot50 | AF-A0A558BSV8-F1-MODEL\_V4 | 1.0 | 4.274e-32 | 874 | 0.254 | 459 | 279 | 17 | 255 | 682 | 5 | 431 | Phage major capsid protein | Phage major capsid protein | | afdb-uniprot50 | AF-A0A8B5U5C5-F1-MODEL\_V4 | 1.0 | 2.259e-30 | 867 | 0.244 | 441 | 264 | 17 | 271 | 682 | 3 | 403 | Phage major capsid protein | Phage major capsid protein | | afdb-uniprot50 | AF-A0A3D1JKW7-F1-MODEL\_V4 | 1.0 | 1.497e-28 | 864 | 0.252 | 444 | 265 | 21 | 258 | 682 | 7 | 402 | Phage major capsid protein | Phage major capsid protein | | afdb-uniprot50 | AF-A0A439EPL4-F1-MODEL\_V4 | 1.0 | 6.128e-23 | 858 | 0.566 | 217 | 90 | 3 | 420 | 635 | 2 | 215 | Phage major capsid protein | Phage major capsid protein | | afdb-uniprot50 | AF-A0A0Q5UAE4-F1-MODEL\_V4 | 1.0 | 2.477e-31 | 858 | 0.288 | 465 | 259 | 19 | 253 | 682 | 5 | 432 | Uncharacterized protein | Uncharacterized protein | | afdb-uniprot50 | AF-A0A855TD60-F1-MODEL\_V4 | 1.0 | 6.636e-38 | 858 | 0.211 | 689 | 400 | 28 | 16 | 681 | 7 | 574 | Phage major capsid protein | Phage major capsid protein | | afdb-uniprot50 | AF-A0A7W9N1B8-F1-MODEL\_V4 | 1.0 | 1.738e-29 | 856 | 0.254 | 433 | 264 | 18 | 258 | 681 | 7 | 389 | HK97 family phage major capsid protein | HK97 family phage major capsid protein | | afdb-uniprot50 | AF-A0A439EPB1-F1-MODEL\_V4 | 1.0 | 5.707e-21 | 853 | 0.566 | 157 | 66 | 2 | 29 | 184 | 15 | 170 | Phage major capsid protein | Phage major capsid protein | | afdb-uniprot50 | AF-A0A6B3SWS1-F1-MODEL\_V4 | 1.0 | 2.134e-30 | 848 | 0.248 | 454 | 265 | 20 | 255 | 681 | 4 | 408 | Phage major capsid protein | Phage major capsid protein | | afdb-uniprot50 | AF-A0A2D7GUF3-F1-MODEL\_V4 | 1.0 | 1.961e-34 | 839 | 0.398 | 402 | 173 | 8 | 26 | 422 | 11 | 348 | Capsid protein | Capsid protein | | afdb-uniprot50 | AF-A0A7C2Y7C3-F1-MODEL\_V4 | 1.0 | 1.356e-30 | 838 | 0.262 | 449 | 280 | 15 | 253 | 680 | 4 | 422 | Phage major capsid protein | Phage major capsid protein | | afdb-uniprot50 | AF-A0A5B9W0Y3-F1-MODEL\_V4 | 1.0 | 5.028e-27 | 837 | 0.275 | 377 | 223 | 15 | 335 | 682 | 1 | 356 | Phage capsid family protein | Phage capsid family protein | | afdb-uniprot50 | AF-A0A838LXB9-F1-MODEL\_V4 | 1.0 | 1.839e-29 | 837 | 0.255 | 447 | 270 | 17 | 264 | 680 | 7 | 420 | Phage major capsid protein | Phage major capsid protein | | afdb-uniprot50 | AF-A0A268U1K3-F1-MODEL\_V4 | 1.0 | 6.636e-38 | 837 | 0.201 | 686 | 412 | 27 | 19 | 682 | 10 | 581 | Uncharacterized protein | Uncharacterized protein | | afdb-uniprot50 | AF-A0A5V0EAD3-F1-MODEL\_V4 | 1.0 | 6.497e-39 | 834 | 0.205 | 701 | 419 | 25 | 40 | 682 | 26 | 646 | Major capsid protein | Major capsid protein | | afdb-uniprot50 | AF-A0A2K8V901-F1-MODEL\_V4 | 1.0 | 2.39e-30 | 827 | 0.251 | 461 | 258 | 19 | 271 | 682 | 11 | 433 | Uncharacterized protein | Uncharacterized protein | | afdb-uniprot50 | AF-A0A838RT86-F1-MODEL\_V4 | 1.0 | 5.598e-38 | 825 | 0.225 | 670 | 382 | 26 | 38 | 682 | 16 | 573 | Phage major capsid protein | Phage major capsid protein | | afdb-uniprot50 | AF-A0A2M9PA59-F1-MODEL\_V4 | 1.0 | 1.08e-22 | 821 | 0.295 | 274 | 167 | 10 | 422 | 681 | 2 | 263 | Phage major capsid protein | Phage major capsid protein | | afdb-uniprot50 | AF-A0A2N3DXN2-F1-MODEL\_V4 | 1.0 | 6.916e-28 | 819 | 0.229 | 458 | 280 | 22 | 254 | 681 | 4 | 418 | Phage major capsid protein | Phage major capsid protein | | afdb-uniprot50 | AF-A0A2E9GN38-F1-MODEL\_V4 | 1.0 | 2.677e-30 | 818 | 0.295 | 440 | 252 | 16 | 271 | 678 | 7 | 420 | Phage major capsid protein | Phage major capsid protein | | afdb-uniprot50 | AF-A0A839W6R8-F1-MODEL\_V4 | 1.0 | 2.032e-35 | 815 | 0.2 | 687 | 413 | 27 | 38 | 680 | 15 | 608 | HK97 family phage major capsid protein/HK97 family phage prohead protease | HK97 family phage major capsid protein/HK97 family phage prohead protease | | afdb-uniprot50 | AF-A0A7W4YX49-F1-MODEL\_V4 | 1.0 | 6.535e-28 | 814 | 0.222 | 431 | 259 | 16 | 282 | 678 | 3 | 391 | HK97 family phage major capsid protein | HK97 family phage major capsid protein | | afdb-uniprot50 | AF-A0A261Q1A3-F1-MODEL\_V4 | 1.0 | 1.058e-31 | 812 | 0.263 | 486 | 274 | 19 | 252 | 682 | 10 | 466 | Phage major capsid protein | Phage major capsid protein | | afdb-uniprot50 | AF-C6M4F3-F1-MODEL\_V4 | 1.0 | 1.355e-22 | 807 | 0.465 | 202 | 101 | 5 | 19 | 215 | 1 | 200 | Caudovirus prohead protease | Caudovirus prohead protease | | afdb-uniprot50 | AF-A0A382Z2D1-F1-MODEL\_V4 | 1.0 | 1.167e-21 | 786 | 0.444 | 189 | 96 | 4 | 30 | 215 | 5 | 187 | Uncharacterized protein | Uncharacterized protein | | afdb-uniprot50 | AF-A0A348SWC8-F1-MODEL\_V4 | 1.0 | 2.356e-28 | 786 | 0.501 | 269 | 120 | 3 | 16 | 279 | 2 | 261 | Phage major capsid protein | Phage major capsid protein | | afdb-uniprot50 | AF-A0A0F9DVX4-F1-MODEL\_V4 | 1.0 | 1.219e-27 | 786 | 0.252 | 456 | 246 | 17 | 252 | 682 | 13 | 398 | Uncharacterized protein | Uncharacterized protein | | afdb-uniprot50 | AF-W0E3R9-F1-MODEL\_V4 | 1.0 | 1.377e-40 | 786 | 0.251 | 728 | 381 | 31 | 12 | 681 | 7 | 628 | Uncharacterized protein | Uncharacterized protein | | afdb-uniprot50 | AF-A0A6N0ZIS5-F1-MODEL\_V4 | 1.0 | 4.489e-27 | 785 | 0.242 | 437 | 281 | 12 | 264 | 681 | 7 | 412 | Phage major capsid protein | Phage major capsid protein | | afdb-uniprot50 | AF-A0A2D5VNT2-F1-MODEL\_V4 | 1.0 | 1.894e-41 | 785 | 0.366 | 527 | 282 | 12 | 1 | 511 | 8 | 498 | Phage major capsid protein | Phage major capsid protein | | afdb-uniprot50 | AF-A0A418M3V2-F1-MODEL\_V4 | 1.0 | 1.497e-28 | 781 | 0.217 | 473 | 286 | 18 | 263 | 682 | 3 | 444 | Phage major capsid protein | Phage major capsid protein | | afdb-uniprot50 | AF-A0A4U8TCV4-F1-MODEL\_V4 | 1.0 | 4.097e-34 | 777 | 0.184 | 667 | 393 | 25 | 40 | 680 | 15 | 556 | Phage major capsid protein | Phage major capsid protein | | afdb-uniprot50 | AF-A0A558C376-F1-MODEL\_V4 | 1.0 | 2.894e-29 | 774 | 0.229 | 457 | 286 | 14 | 258 | 680 | 5 | 429 | Phage major capsid protein | Phage major capsid protein | | afdb-uniprot50 | AF-A0A837Q3L9-F1-MODEL\_V4 | 1.0 | 7.98e-40 | 774 | 0.351 | 512 | 266 | 14 | 33 | 532 | 9 | 466 | Peptidase | Peptidase | | afdb-uniprot50 | AF-A0A6N8DIV7-F1-MODEL\_V4 | 1.0 | 1.128e-28 | 772 | 0.263 | 444 | 260 | 19 | 264 | 682 | 14 | 415 | Phage major capsid protein | Phage major capsid protein | | afdb-uniprot50 | AF-A0A180F243-F1-MODEL\_V4 | 1.0 | 1.128e-28 | 771 | 0.248 | 455 | 266 | 17 | 264 | 682 | 14 | 428 | Putative phage phi-C31 gp36 major capsid-like protein | Putative phage phi-C31 gp36 major capsid-like protein | | afdb-uniprot50 | AF-A0A8A8EP07-F1-MODEL\_V4 | 1.0 | 1.219e-27 | 765 | 0.256 | 445 | 275 | 17 | 253 | 681 | 2 | 406 | Phage major capsid protein | Phage major capsid protein | | afdb-uniprot50 | AF-A0A2V5Y3W0-F1-MODEL\_V4 | 1.0 | 9.783e-25 | 764 | 0.215 | 362 | 251 | 12 | 335 | 681 | 18 | 361 | Phage major capsid protein | Phage major capsid protein | | afdb-uniprot50 | AF-A0A239HKB7-F1-MODEL\_V4 | 1.0 | 4.554e-29 | 760 | 0.248 | 471 | 262 | 20 | 261 | 682 | 8 | 435 | Phage major capsid protein, HK97 family | Phage major capsid protein, HK97 family | | afdb-uniprot50 | AF-A0A1G3FQ64-F1-MODEL\_V4 | 1.0 | 1.088e-27 | 757 | 0.259 | 455 | 265 | 20 | 255 | 681 | 5 | 415 | Uncharacterized protein | Uncharacterized protein | | afdb-uniprot50 | AF-A0A371WU99-F1-MODEL\_V4 | 1.0 | 6.051e-37 | 749 | 0.209 | 727 | 427 | 31 | 14 | 681 | 34 | 671 | Phage major capsid protein | Phage major capsid protein | | afdb-uniprot50 | AF-A0A1U7GEM9-F1-MODEL\_V4 | 1.0 | 8.373e-27 | 744 | 0.235 | 428 | 260 | 18 | 279 | 682 | 43 | 427 | Uncharacterized protein | Uncharacterized protein | | afdb-uniprot50 | AF-A0A6A4R9W0-F1-MODEL\_V4 | 1.0 | 1.042e-21 | 741 | 0.443 | 203 | 93 | 4 | 18 | 201 | 2 | 203 | Phage major capsid protein | Phage major capsid protein | | afdb-uniprot50 | AF-A0A7C5ALH4-F1-MODEL\_V4 | 1.0 | 1.851e-26 | 739 | 0.25 | 428 | 239 | 17 | 285 | 682 | 11 | 386 | Phage major capsid protein | Phage major capsid protein | | afdb-uniprot50 | AF-A0A5P9CVW4-F1-MODEL\_V4 | 1.0 | 4.489e-27 | 738 | 0.221 | 446 | 277 | 18 | 261 | 679 | 8 | 410 | Phage capsid family protein | Phage capsid family protein | | afdb-uniprot50 | AF-A0A432MJM0-F1-MODEL\_V4 | 1.0 | 1.749e-26 | 735 | 0.242 | 457 | 260 | 21 | 264 | 678 | 7 | 419 | Phage major capsid protein | Phage major capsid protein | | afdb-uniprot50 | AF-A0A6L8UIY2-F1-MODEL\_V4 | 1.0 | 9.925e-27 | 734 | 0.248 | 446 | 270 | 17 | 251 | 681 | 20 | 415 | Phage major capsid protein | Phage major capsid protein | | afdb-uniprot50 | AF-A0A1U7CX52-F1-MODEL\_V4 | 1.0 | 2.753e-26 | 732 | 0.223 | 460 | 288 | 18 | 258 | 682 | 7 | 432 | Uncharacterized protein | Uncharacterized protein | | afdb-uniprot50 | AF-A0A7G7KL91-F1-MODEL\_V4 | 1.0 | 1.065e-20 | 731 | 0.264 | 261 | 176 | 9 | 428 | 679 | 6 | 259 | Phage major capsid protein | Phage major capsid protein | | afdb-uniprot50 | AF-A0A6L8U335-F1-MODEL\_V4 | 1.0 | 6.675e-27 | 731 | 0.245 | 457 | 271 | 20 | 258 | 681 | 8 | 423 | Phage major capsid protein | Phage major capsid protein | | afdb-uniprot50 | AF-A0A268TJ56-F1-MODEL\_V4 | 1.0 | 3.086e-34 | 731 | 0.184 | 677 | 429 | 29 | 40 | 682 | 15 | 602 | Uncharacterized protein | Uncharacterized protein | | afdb-uniprot50 | AF-A0A2D8LFG7-F1-MODEL\_V4 | 1.0 | 2.458e-26 | 723 | 0.213 | 458 | 296 | 20 | 258 | 681 | 8 | 435 | Phage major capsid protein | Phage major capsid protein | | afdb-uniprot50 | AF-A0A1U7CNJ6-F1-MODEL\_V4 | 1.0 | 4.241e-27 | 723 | 0.237 | 472 | 287 | 20 | 256 | 682 | 4 | 447 | Uncharacterized protein | Uncharacterized protein | | afdb-uniprot50 | AF-A0A3D1J480-F1-MODEL\_V4 | 1.0 | 1.776e-36 | 723 | 0.203 | 737 | 432 | 32 | 13 | 681 | 50 | 699 | Phage major capsid protein | Phage major capsid protein | | afdb-uniprot50 | AF-A0A2D9C9C5-F1-MODEL\_V4 | 1.0 | 5.439e-34 | 720 | 0.185 | 705 | 437 | 32 | 26 | 682 | 16 | 630 | HK97 family phage prohead protease | HK97 family phage prohead protease | | afdb-uniprot50 | AF-A0A2W5AUZ1-F1-MODEL\_V4 | 1.0 | 8.081e-26 | 714 | 0.251 | 397 | 242 | 16 | 306 | 682 | 36 | 397 | Phage major capsid protein | Phage major capsid protein | | afdb-uniprot50 | AF-A0A519K9R5-F1-MODEL\_V4 | 1.0 | 1.299e-24 | 711 | 0.22 | 458 | 294 | 17 | 249 | 678 | 6 | 428 | Phage major capsid protein | Phage major capsid protein | | afdb-uniprot50 | AF-A0A7G8CD93-F1-MODEL\_V4 | 1.0 | 1.904e-22 | 710 | 0.244 | 348 | 229 | 14 | 343 | 679 | 11 | 335 | Phage major capsid protein/ HK97 family | Phage major capsid protein/ HK97 family | | afdb-uniprot50 | AF-A0A1V6GVR6-F1-MODEL\_V4 | 1.0 | 5.959e-27 | 705 | 0.254 | 472 | 253 | 19 | 271 | 681 | 4 | 437 | Phage capsid family protein | Phage capsid family protein | | afdb-uniprot50 | AF-A0A7W6QQW4-F1-MODEL\_V4 | 1.0 | 2.601e-26 | 703 | 0.221 | 457 | 271 | 20 | 258 | 682 | 8 | 411 | HK97 family phage major capsid protein | HK97 family phage major capsid protein | | afdb-uniprot50 | AF-A0A5C7R6U5-F1-MODEL\_V4 | 1.0 | 1.12e-31 | 688 | 0.196 | 693 | 312 | 19 | 26 | 677 | 17 | 505 | Uncharacterized protein | Uncharacterized protein | | afdb-uniprot50 | AF-A0A3D0WRI2-F1-MODEL\_V4 | 1.0 | 5.17e-23 | 687 | 0.203 | 438 | 257 | 17 | 265 | 682 | 4 | 369 | Phage major capsid protein | Phage major capsid protein | | afdb-uniprot50 | AF-A0A7G8FKV0-F1-MODEL\_V4 | 1.0 | 1.7e-22 | 686 | 0.228 | 355 | 237 | 17 | 343 | 681 | 7 | 340 | Phage major capsid protein/ HK97 family | Phage major capsid protein/ HK97 family | | afdb-uniprot50 | AF-A0A2D9FXS3-F1-MODEL\_V4 | 1.0 | 2.074e-26 | 686 | 0.197 | 487 | 302 | 25 | 250 | 682 | 8 | 459 | Phage major capsid protein | Phage major capsid protein | | afdb-uniprot50 | AF-A0A7Y7NKU6-F1-MODEL\_V4 | 1.0 | 2.812e-25 | 685 | 0.237 | 458 | 260 | 21 | 260 | 681 | 9 | 413 | Phage major capsid protein | Phage major capsid protein | | afdb-uniprot50 | AF-A0A2Z4IND8-F1-MODEL\_V4 | 1.0 | 3.733e-25 | 682 | 0.221 | 466 | 288 | 16 | 263 | 682 | 4 | 440 | Phage major capsid protein | Phage major capsid protein | | afdb-uniprot50 | AF-A0A3M1UTX0-F1-MODEL\_V4 | 1.0 | 2.032e-35 | 682 | 0.328 | 600 | 308 | 18 | 19 | 611 | 13 | 524 | Phage major capsid protein | Phage major capsid protein | | afdb-uniprot50 | AF-A0A843JDH5-F1-MODEL\_V4 | 1.0 | 4.035e-24 | 679 | 0.203 | 443 | 266 | 17 | 258 | 682 | 7 | 380 | Phage major capsid protein | Phage major capsid protein | | afdb-uniprot50 | AF-A0A1C6BMQ2-F1-MODEL\_V4 | 1.0 | 2.456e-18 | 672 | 0.418 | 165 | 90 | 3 | 28 | 187 | 7 | 170 | Phage prohead protease, HK97 family | Phage prohead protease, HK97 family | | afdb-uniprot50 | AF-Q08QD1-F1-MODEL\_V4 | 1.0 | 2.272e-19 | 670 | 0.223 | 295 | 196 | 14 | 402 | 679 | 1 | 279 | Phage capsid family | Phage capsid family | | afdb-uniprot50 | AF-A0A368NXR3-F1-MODEL\_V4 | 1.0 | 9.378e-27 | 667 | 0.214 | 526 | 319 | 24 | 191 | 679 | 5 | 473 | Phage major capsid protein | Phage major capsid protein | | afdb-uniprot50 | AF-A0A4R8I8I6-F1-MODEL\_V4 | 1.0 | 5.062e-24 | 665 | 0.207 | 457 | 283 | 21 | 255 | 682 | 4 | 410 | HK97 family phage major capsid protein | HK97 family phage major capsid protein | | afdb-uniprot50 | AF-U5BPA1-F1-MODEL\_V4 | 1.0 | 6.963e-25 | 664 | 0.212 | 457 | 275 | 21 | 258 | 682 | 8 | 411 | Uncharacterized protein | Uncharacterized protein | | afdb-uniprot50 | AF-A0A212KXM3-F1-MODEL\_V4 | 1.0 | 2.104e-28 | 664 | 0.157 | 661 | 396 | 31 | 38 | 682 | 11 | 526 | Phage major capsid protein, HK97 family | Phage major capsid protein, HK97 family | | afdb-uniprot50 | AF-A0A226I7W7-F1-MODEL\_V4 | 1.0 | 8.253e-25 | 659 | 0.206 | 451 | 302 | 21 | 258 | 680 | 8 | 430 | Uncharacterized protein | Uncharacterized protein | | afdb-uniprot50 | AF-A0A2T4JHM7-F1-MODEL\_V4 | 1.0 | 9.916e-19 | 656 | 0.238 | 298 | 194 | 13 | 402 | 682 | 1 | 282 | Phage major capsid protein | Phage major capsid protein | | afdb-uniprot50 | AF-A0A4R0QZ35-F1-MODEL\_V4 | 1.0 | 7.419e-22 | 654 | 0.222 | 359 | 226 | 15 | 343 | 679 | 11 | 338 | Phage major capsid protein | Phage major capsid protein | | afdb-uniprot50 | AF-A0A1G7G3W8-F1-MODEL\_V4 | 1.0 | 2.976e-25 | 654 | 0.205 | 457 | 300 | 17 | 258 | 682 | 10 | 435 | Phage major capsid protein, HK97 family | Phage major capsid protein, HK97 family | | afdb-uniprot50 | AF-A0A0F9MYQ3-F1-MODEL\_V4 | 1.0 | 3.606e-32 | 649 | 0.174 | 747 | 462 | 35 | 26 | 680 | 13 | 696 | Uncharacterized protein | Uncharacterized protein | | afdb-uniprot50 | AF-A0A4Q7LWN4-F1-MODEL\_V4 | 1.0 | 5.13e-18 | 643 | 0.198 | 262 | 192 | 8 | 431 | 682 | 29 | 282 | HK97 family phage major capsid protein | HK97 family phage major capsid protein | | afdb-uniprot50 | AF-A0A7I9V3A8-F1-MODEL\_V4 | 1.0 | 1.423e-17 | 640 | 0.171 | 256 | 202 | 4 | 431 | 681 | 28 | 278 | Uncharacterized protein | Uncharacterized protein | | afdb-uniprot50 | AF-A0A378RQE6-F1-MODEL\_V4 | 1.0 | 5.357e-24 | 636 | 0.244 | 459 | 269 | 20 | 258 | 681 | 8 | 423 | Predicted phage phi-C31 gp36 major capsid-like protein | Predicted phage phi-C31 gp36 major capsid-like protein | | afdb-uniprot50 | AF-A0A506U2R2-F1-MODEL\_V4 | 1.0 | 4.149e-20 | 635 | 0.219 | 324 | 212 | 11 | 371 | 679 | 18 | 315 | Phage major capsid protein | Phage major capsid protein | | afdb-uniprot50 | AF-F3Z7W5-F1-MODEL\_V4 | 1.0 | 9.042e-18 | 632 | 0.16 | 274 | 204 | 11 | 423 | 682 | 6 | 267 | Uncharacterized protein | Uncharacterized protein | | afdb-uniprot50 | AF-A0A849MU29-F1-MODEL\_V4 | 1.0 | 1.227e-24 | 632 | 0.205 | 463 | 288 | 18 | 264 | 678 | 10 | 440 | Phage major capsid protein | Phage major capsid protein | | afdb-uniprot50 | AF-A0A3A9H2E6-F1-MODEL\_V4 | 1.0 | 4.519e-24 | 632 | 0.207 | 448 | 261 | 19 | 296 | 679 | 81 | 498 | Phage major capsid protein | Phage major capsid protein | | afdb-uniprot50 | AF-A0A1A9A792-F1-MODEL\_V4 | 1.0 | 1.651e-18 | 631 | 0.202 | 277 | 191 | 8 | 430 | 682 | 40 | 310 | Phage major capsid protein, HK97 family | Phage major capsid protein, HK97 family | | afdb-uniprot50 | AF-A0A7L8AFB1-F1-MODEL\_V4 | 1.0 | 6.35e-24 | 631 | 0.23 | 456 | 268 | 20 | 263 | 679 | 3 | 414 | Phage major capsid protein | Phage major capsid protein | | afdb-uniprot50 | AF-A0A5C7P846-F1-MODEL\_V4 | 1.0 | 1.748e-18 | 630 | 0.221 | 271 | 178 | 10 | 444 | 682 | 1 | 270 | Phage major capsid protein | Phage major capsid protein | | afdb-uniprot50 | AF-A0A6I2TYG7-F1-MODEL\_V4 | 1.0 | 1.096e-24 | 630 | 0.238 | 453 | 262 | 18 | 282 | 681 | 10 | 432 | Phage major capsid protein | Phage major capsid protein | | afdb-uniprot50 | AF-A0A1A0TGW3-F1-MODEL\_V4 | 1.0 | 3.524e-17 | 628 | 0.195 | 256 | 193 | 6 | 430 | 682 | 28 | 273 | Capsid protein | Capsid protein | | afdb-uniprot50 | AF-A0A5F0FN25-F1-MODEL\_V4 | 1.0 | 2.809e-17 | 628 | 0.17 | 258 | 203 | 8 | 431 | 682 | 28 | 280 | Phage major capsid protein | Phage major capsid protein | | afdb-uniprot50 | AF-A0A7J9VRD2-F1-MODEL\_V4 | 1.0 | 1.345e-17 | 627 | 0.196 | 259 | 192 | 7 | 431 | 682 | 29 | 278 | Phage major capsid protein | Phage major capsid protein | | afdb-uniprot50 | AF-B5IP42-F1-MODEL\_V4 | 1.0 | 7.419e-22 | 626 | 0.213 | 412 | 272 | 16 | 293 | 682 | 1 | 382 | Uncharacterized protein | Uncharacterized protein | | afdb-uniprot50 | AF-A0A6S6N9V6-F1-MODEL\_V4 | 1.0 | 9.051e-26 | 625 | 0.14 | 684 | 392 | 31 | 19 | 681 | 3 | 511 | Peptidase U35 | Peptidase U35 | | afdb-uniprot50 | AF-A0A1J4P7K5-F1-MODEL\_V4 | 1.0 | 1.785e-17 | 623 | 0.191 | 251 | 197 | 5 | 433 | 681 | 30 | 276 | Uncharacterized protein | Uncharacterized protein | | afdb-uniprot50 | AF-A0A553GPR9-F1-MODEL\_V4 | 1.0 | 3.977e-22 | 622 | 0.2 | 434 | 273 | 19 | 268 | 679 | 11 | 392 | Phage major capsid protein | Phage major capsid protein | | afdb-uniprot50 | AF-A0A496WP50-F1-MODEL\_V4 | 1.0 | 3.17e-22 | 621 | 0.192 | 415 | 268 | 18 | 295 | 681 | 13 | 388 | Phage major capsid protein | Phage major capsid protein | | afdb-uniprot50 | AF-A0A367A8R2-F1-MODEL\_V4 | 1.0 | 7.469e-19 | 620 | 0.186 | 316 | 216 | 10 | 381 | 682 | 2 | 290 | Phage major capsid protein | Phage major capsid protein | | afdb-uniprot50 | AF-E3FHX8-F1-MODEL\_V4 | 1.0 | 4.918e-20 | 620 | 0.209 | 358 | 227 | 18 | 335 | 679 | 3 | 317 | Phage major capsid protein, HK97 | Phage major capsid protein, HK97 | | afdb-uniprot50 | AF-A0A2V3ZYP9-F1-MODEL\_V4 | 1.0 | 1.385e-29 | 618 | 0.149 | 691 | 424 | 33 | 28 | 680 | 3 | 567 | Phage major capsid protein | Phage major capsid protein | | afdb-uniprot50 | AF-A0A4V2Y1S1-F1-MODEL\_V4 | 1.0 | 2.654e-17 | 617 | 0.217 | 262 | 184 | 8 | 431 | 682 | 28 | 278 | Phage major capsid protein | Phage major capsid protein | | afdb-uniprot50 | AF-X6KKD3-F1-MODEL\_V4 | 1.0 | 2.852e-27 | 617 | 0.171 | 687 | 382 | 29 | 19 | 682 | 4 | 525 | Capsid protein | Capsid protein | | afdb-uniprot50 | AF-A0A7J9XPI0-F1-MODEL\_V4 | 1.0 | 1.889e-17 | 615 | 0.189 | 259 | 194 | 7 | 431 | 682 | 34 | 283 | Phage major capsid protein | Phage major capsid protein | | afdb-uniprot50 | AF-A0A0B5E1B7-F1-MODEL\_V4 | 1.0 | 1.813e-27 | 615 | 0.165 | 687 | 363 | 34 | 17 | 679 | 3 | 502 | HK97 family phage major capsid protein | HK97 family phage major capsid protein | | afdb-uniprot50 | AF-A0A3C1F4S7-F1-MODEL\_V4 | 1.0 | 1.917e-19 | 614 | 0.21 | 318 | 198 | 13 | 398 | 682 | 14 | 311 | Phage major capsid protein | Phage major capsid protein | | afdb-uniprot50 | AF-A0A5J4QQM2-F1-MODEL\_V4 | 1.0 | 4.09e-18 | 613 | 0.177 | 276 | 197 | 8 | 409 | 680 | 2 | 251 | Uncharacterized protein | Uncharacterized protein | | afdb-uniprot50 | AF-A0A841KJN3-F1-MODEL\_V4 | 1.0 | 1.136e-33 | 612 | 0.204 | 744 | 447 | 38 | 16 | 682 | 11 | 686 | Uncharacterized protein | Uncharacterized protein | | afdb-uniprot50 | AF-A0A1Q4SYY1-F1-MODEL\_V4 | 1.0 | 2.37e-17 | 611 | 0.223 | 259 | 183 | 9 | 433 | 682 | 31 | 280 | Capsid protein | Capsid protein | | afdb-uniprot50 | AF-A0A251YMX4-F1-MODEL\_V4 | 1.0 | 5.869e-17 | 610 | 0.188 | 260 | 196 | 7 | 431 | 682 | 30 | 282 | Phage capsid family protein | Phage capsid family protein | | afdb-uniprot50 | AF-A0A436RU37-F1-MODEL\_V4 | 1.0 | 1.006e-20 | 610 | 0.206 | 407 | 238 | 20 | 295 | 682 | 4 | 344 | Phage major capsid protein | Phage major capsid protein | | afdb-uniprot50 | AF-A0A1I5E198-F1-MODEL\_V4 | 1.0 | 4.885e-23 | 610 | 0.215 | 463 | 284 | 21 | 261 | 681 | 9 | 434 | Phage major capsid protein, HK97 family | Phage major capsid protein, HK97 family | | afdb-uniprot50 | AF-A0A5C7UGA7-F1-MODEL\_V4 | 1.0 | 6.72e-24 | 610 | 0.198 | 469 | 292 | 13 | 295 | 682 | 13 | 478 | Phage major capsid protein | Phage major capsid protein | | afdb-uniprot50 | AF-A0A3E3J0E7-F1-MODEL\_V4 | 1.0 | 9.244e-25 | 610 | 0.206 | 493 | 286 | 22 | 266 | 682 | 65 | 528 | Phage major capsid protein | Phage major capsid protein | | afdb-uniprot50 | AF-A0A2T0R256-F1-MODEL\_V4 | 1.0 | 1.423e-17 | 609 | 0.182 | 263 | 196 | 6 | 431 | 682 | 29 | 283 | HK97 family phage major capsid protein | HK97 family phage major capsid protein | | afdb-uniprot50 | AF-A0A087M199-F1-MODEL\_V4 | 1.0 | 4.956e-25 | 607 | 0.135 | 663 | 383 | 25 | 39 | 682 | 11 | 502 | Capsid protein | Capsid protein | | afdb-uniprot50 | AF-A0A0F9CUT8-F1-MODEL\_V4 | 1.0 | 9.57e-18 | 606 | 0.233 | 300 | 203 | 9 | 398 | 682 | 30 | 317 | Uncharacterized protein | Uncharacterized protein | | afdb-uniprot50 | AF-A0A2T0QNI1-F1-MODEL\_V4 | 1.0 | 1.999e-17 | 605 | 0.183 | 262 | 191 | 9 | 431 | 680 | 32 | 282 | HK97 family phage major capsid protein | HK97 family phage major capsid protein | | afdb-uniprot50 | AF-A0A2W4JAL7-F1-MODEL\_V4 | 1.0 | 2.599e-18 | 604 | 0.242 | 272 | 168 | 11 | 438 | 681 | 4 | 265 | Phage major capsid protein | Phage major capsid protein | | afdb-uniprot50 | AF-A0A1I1CFH0-F1-MODEL\_V4 | 1.0 | 3.33e-17 | 604 | 0.196 | 260 | 188 | 6 | 431 | 682 | 29 | 275 | Phage major capsid protein, HK97 family | Phage major capsid protein, HK97 family | | afdb-uniprot50 | AF-A0A7W8JSS7-F1-MODEL\_V4 | 1.0 | 1.143e-22 | 604 | 0.22 | 468 | 286 | 21 | 245 | 680 | 1 | 421 | HK97 family phage major capsid protein | HK97 family phage major capsid protein | | afdb-uniprot50 | AF-A0A662S3E0-F1-MODEL\_V4 | 1.0 | 1.642e-29 | 604 | 0.194 | 698 | 385 | 30 | 15 | 681 | 104 | 654 | Phage major capsid protein | Phage major capsid protein | | afdb-uniprot50 | AF-A0A142X159-F1-MODEL\_V4 | 1.0 | 4.121e-23 | 603 | 0.204 | 464 | 296 | 21 | 279 | 682 | 20 | 470 | Phage capsid family protein | Phage capsid family protein | | afdb-uniprot50 | AF-A0A2D9CCN7-F1-MODEL\_V4 | 1.0 | 1.799e-22 | 602 | 0.189 | 444 | 278 | 18 | 264 | 681 | 14 | 401 | Phage major capsid protein | Phage major capsid protein | | afdb-uniprot50 | AF-A0A378YEU3-F1-MODEL\_V4 | 1.0 | 5.869e-17 | 600 | 0.194 | 262 | 194 | 7 | 431 | 682 | 29 | 283 | Predicted phage phi-C31 gp36 major capsid-like protein | Predicted phage phi-C31 gp36 major capsid-like protein | | afdb-uniprot50 | AF-A0A1H7U3C7-F1-MODEL\_V4 | 1.0 | 2.693e-19 | 600 | 0.206 | 348 | 219 | 20 | 346 | 679 | 24 | 328 | Phage major capsid protein, HK97 family | Phage major capsid protein, HK97 family | | afdb-uniprot50 | AF-A0A1G3BD02-F1-MODEL\_V4 | 1.0 | 1.042e-21 | 600 | 0.192 | 457 | 250 | 20 | 273 | 682 | 4 | 388 | Uncharacterized protein | Uncharacterized protein | | afdb-uniprot50 | AF-A0A372GN26-F1-MODEL\_V4 | 1.0 | 2.404e-19 | 598 | 0.183 | 338 | 214 | 11 | 381 | 682 | 2 | 313 | Phage major capsid protein | Phage major capsid protein | | afdb-uniprot50 | AF-A0A263DU06-F1-MODEL\_V4 | 1.0 | 3.947e-17 | 597 | 0.19 | 263 | 192 | 8 | 431 | 682 | 30 | 282 | Phage major capsid protein | Phage major capsid protein | | afdb-uniprot50 | AF-A0A4Q0M7Y1-F1-MODEL\_V4 | 1.0 | 1.227e-24 | 597 | 0.15 | 683 | 389 | 30 | 19 | 682 | 4 | 513 | Phage major capsid protein | Phage major capsid protein | | afdb-uniprot50 | AF-A0A098BVT6-F1-MODEL\_V4 | 1.0 | 3.146e-17 | 595 | 0.187 | 262 | 191 | 7 | 431 | 682 | 28 | 277 | Phage major capsid protein, HK97 family | Phage major capsid protein, HK97 family | | afdb-uniprot50 | AF-U6RGH7-F1-MODEL\_V4 | 1.0 | 4.062e-21 | 595 | 0.206 | 432 | 271 | 18 | 267 | 682 | 3 | 378 | HK97 family phage major capsid protein | HK97 family phage major capsid protein | | afdb-uniprot50 | AF-A0A7U9F631-F1-MODEL\_V4 | 1.0 | 3.599e-16 | 594 | 0.348 | 175 | 101 | 5 | 30 | 194 | 13 | 184 | Uncharacterized protein | Uncharacterized protein | | afdb-uniprot50 | AF-A0A839FEI7-F1-MODEL\_V4 | 1.0 | 6.956e-17 | 593 | 0.177 | 248 | 191 | 7 | 436 | 681 | 35 | 271 | HK97 family phage major capsid protein | HK97 family phage major capsid protein | | afdb-uniprot50 | AF-A0A2S2D0Z2-F1-MODEL\_V4 | 1.0 | 7.419e-22 | 593 | 0.172 | 458 | 288 | 22 | 265 | 681 | 8 | 415 | Phage major capsid protein | Phage major capsid protein | | afdb-uniprot50 | AF-A0A511HNN2-F1-MODEL\_V4 | 1.0 | 6.485e-23 | 593 | 0.199 | 441 | 281 | 20 | 279 | 680 | 16 | 423 | Uncharacterized protein | Uncharacterized protein | | afdb-uniprot50 | AF-A0A0J6SLM0-F1-MODEL\_V4 | 1.0 | 1.664e-23 | 593 | 0.172 | 486 | 304 | 25 | 256 | 681 | 4 | 451 | Uncharacterized protein | Uncharacterized protein | | afdb-uniprot50 | AF-A0A6C2YSM9-F1-MODEL\_V4 | 1.0 | 1.606e-22 | 593 | 0.2 | 465 | 293 | 18 | 285 | 682 | 15 | 467 | Uncharacterized protein | Uncharacterized protein | | afdb-uniprot50 | AF-A0A2G2J0Z9-F1-MODEL\_V4 | 1.0 | 2.933e-23 | 593 | 0.192 | 493 | 291 | 19 | 252 | 681 | 36 | 484 | Phage major capsid protein | Phage major capsid protein | | afdb-uniprot50 | AF-A0A3D4V181-F1-MODEL\_V4 | 1.0 | 3.401e-16 | 592 | 0.162 | 265 | 205 | 7 | 431 | 682 | 4 | 264 | Phage major capsid protein | Phage major capsid protein | | afdb-uniprot50 | AF-A0A0F9FEI0-F1-MODEL\_V4 | 1.0 | 1.226e-16 | 590 | 0.146 | 280 | 215 | 9 | 410 | 681 | 1 | 264 | Uncharacterized protein | Uncharacterized protein | | afdb-uniprot50 | AF-A0A5C1R7K0-F1-MODEL\_V4 | 1.0 | 3.809e-16 | 589 | 0.206 | 261 | 189 | 9 | 431 | 680 | 29 | 282 | Phage major capsid protein | Phage major capsid protein | | afdb-uniprot50 | AF-A0A7Y4JL20-F1-MODEL\_V4 | 1.0 | 5.096e-21 | 588 | 0.211 | 420 | 257 | 19 | 286 | 680 | 6 | 376 | Phage major capsid protein | Phage major capsid protein | | afdb-uniprot50 | AF-A0A1X2FVP9-F1-MODEL\_V4 | 1.0 | 5.096e-21 | 587 | 0.216 | 420 | 229 | 19 | 294 | 681 | 15 | 366 | Uncharacterized protein | Uncharacterized protein | | afdb-uniprot50 | AF-A0A0A2G587-F1-MODEL\_V4 | 1.0 | 1.675e-20 | 587 | 0.203 | 442 | 255 | 17 | 268 | 681 | 7 | 379 | Uncharacterized protein | Uncharacterized protein | | afdb-uniprot50 | AF-A0A1B7LDB2-F1-MODEL\_V4 | 1.0 | 1.496e-20 | 587 | 0.202 | 414 | 242 | 17 | 296 | 681 | 23 | 376 | Uncharacterized protein | Uncharacterized protein | | afdb-uniprot50 | AF-I9WTH1-F1-MODEL\_V4 | 1.0 | 1.486e-23 | 587 | 0.204 | 484 | 298 | 22 | 261 | 681 | 7 | 466 | Phage major capsid protein, HK97 family | Phage major capsid protein, HK97 family | | afdb-uniprot50 | AF-A0A7B4HVD7-F1-MODEL\_V4 | 1.0 | 1.999e-17 | 586 | 0.197 | 284 | 186 | 11 | 430 | 681 | 2 | 275 | Phage major capsid protein | Phage major capsid protein | | afdb-uniprot50 | AF-A0A090E9R7-F1-MODEL\_V4 | 1.0 | 7.264e-23 | 586 | 0.2 | 464 | 288 | 21 | 264 | 681 | 5 | 431 | Putative Phage major capsid protein, HK97 family | Putative Phage major capsid protein, HK97 family | | afdb-uniprot50 | AF-A0A1Q9VAW5-F1-MODEL\_V4 | 1.0 | 4.266e-16 | 585 | 0.185 | 259 | 194 | 7 | 431 | 681 | 28 | 277 | Capsid protein | Capsid protein | | afdb-uniprot50 | AF-A0A3S9X822-F1-MODEL\_V4 | 1.0 | 4.951e-17 | 585 | 0.171 | 292 | 219 | 10 | 402 | 682 | 2 | 281 | Uncharacterized protein | Uncharacterized protein | | afdb-uniprot50 | AF-A0A5A9ZIC2-F1-MODEL\_V4 | 1.0 | 2.287e-16 | 585 | 0.169 | 259 | 201 | 5 | 431 | 682 | 34 | 285 | Phage major capsid protein | Phage major capsid protein | | afdb-uniprot50 | AF-A0A543KX21-F1-MODEL\_V4 | 1.0 | 9.774e-17 | 584 | 0.186 | 257 | 192 | 9 | 431 | 681 | 27 | 272 | HK97 family phage major capsid protein | HK97 family phage major capsid protein | | afdb-uniprot50 | AF-A0A429N598-F1-MODEL\_V4 | 1.0 | 3.401e-16 | 583 | 0.16 | 262 | 200 | 7 | 430 | 681 | 29 | 280 | Phage major capsid protein | Phage major capsid protein | | afdb-uniprot50 | AF-A0A3A8RP75-F1-MODEL\_V4 | 1.0 | 1.042e-21 | 583 | 0.184 | 445 | 283 | 23 | 271 | 682 | 6 | 403 | Phage major capsid protein | Phage major capsid protein | | afdb-uniprot50 | AF-A0A4V2RW77-F1-MODEL\_V4 | 1.0 | 1.218e-19 | 582 | 0.183 | 403 | 246 | 15 | 295 | 681 | 27 | 362 | HK97 family phage major capsid protein | HK97 family phage major capsid protein | | afdb-uniprot50 | AF-A0A518I8W7-F1-MODEL\_V4 | 1.0 | 4.519e-24 | 582 | 0.172 | 584 | 342 | 28 | 180 | 682 | 7 | 529 | Phage capsid family protein | Phage capsid family protein | | afdb-uniprot50 | AF-A0A2U1FA90-F1-MODEL\_V4 | 1.0 | 2.287e-16 | 580 | 0.178 | 257 | 199 | 7 | 431 | 682 | 31 | 280 | HK97 family phage major capsid protein | HK97 family phage major capsid protein | | afdb-uniprot50 | AF-A0A7I9ZGE4-F1-MODEL\_V4 | 1.0 | 1.618e-27 | 580 | 0.147 | 723 | 432 | 33 | 15 | 678 | 2 | 598 | Uncharacterized protein | Uncharacterized protein | | afdb-uniprot50 | AF-A0A3A8JWE7-F1-MODEL\_V4 | 1.0 | 1.192e-20 | 578 | 0.193 | 408 | 262 | 22 | 298 | 682 | 35 | 398 | Phage major capsid protein | Phage major capsid protein | | afdb-uniprot50 | AF-A0A3A4UX11-F1-MODEL\_V4 | 1.0 | 1.904e-22 | 578 | 0.18 | 494 | 308 | 16 | 270 | 682 | 2 | 479 | Phage major capsid protein | Phage major capsid protein | | afdb-uniprot50 | AF-A0A5C5AK74-F1-MODEL\_V4 | 1.0 | 9.925e-27 | 578 | 0.128 | 708 | 432 | 37 | 23 | 682 | 1 | 571 | Phage major capsid protein | Phage major capsid protein | | afdb-uniprot50 | AF-A0A7Y9DIE2-F1-MODEL\_V4 | 1.0 | 1.484e-15 | 574 | 0.154 | 259 | 204 | 5 | 431 | 682 | 29 | 279 | HK97 family phage major capsid protein | HK97 family phage major capsid protein | | afdb-uniprot50 | AF-A0A5C8ZHA4-F1-MODEL\_V4 | 1.0 | 3.401e-16 | 574 | 0.211 | 260 | 189 | 8 | 431 | 682 | 28 | 279 | Phage major capsid protein | Phage major capsid protein | | afdb-uniprot50 | AF-A0A436KKN4-F1-MODEL\_V4 | 1.0 | 6.624e-22 | 574 | 0.192 | 468 | 291 | 19 | 264 | 681 | 5 | 435 | Phage major capsid protein | Phage major capsid protein | | afdb-uniprot50 | AF-A0A0Q7IS85-F1-MODEL\_V4 | 1.0 | 1.583e-20 | 573 | 0.212 | 457 | 266 | 16 | 238 | 681 | 5 | 380 | Capsid protein | Capsid protein | | afdb-uniprot50 | AF-A0A6I2R3H1-F1-MODEL\_V4 | 1.0 | 9.112e-23 | 573 | 0.215 | 488 | 275 | 18 | 271 | 680 | 46 | 503 | Phage major capsid protein | Phage major capsid protein | | afdb-uniprot50 | AF-A0A2K1Q3K7-F1-MODEL\_V4 | 1.0 | 8.495e-29 | 573 | 0.201 | 724 | 336 | 29 | 18 | 681 | 9 | 550 | Caudovirus prohead protease | Caudovirus prohead protease | | afdb-uniprot50 | AF-A0A3N1D5M0-F1-MODEL\_V4 | 1.0 | 3.599e-16 | 572 | 0.163 | 257 | 205 | 5 | 431 | 682 | 32 | 283 | HK97 family phage major capsid protein | HK97 family phage major capsid protein | | afdb-uniprot50 | AF-A0A7S7QRL3-F1-MODEL\_V4 | 1.0 | 1.675e-20 | 572 | 0.193 | 398 | 266 | 16 | 313 | 680 | 1 | 373 | Phage major capsid protein | Phage major capsid protein | | afdb-uniprot50 | AF-A0A843HXH6-F1-MODEL\_V4 | 1.0 | 1.944e-21 | 572 | 0.18 | 477 | 300 | 17 | 249 | 681 | 3 | 432 | Phage major capsid protein | Phage major capsid protein | | afdb-uniprot50 | AF-A0A643EYU7-F1-MODEL\_V4 | 1.0 | 5.321e-27 | 572 | 0.159 | 701 | 402 | 31 | 13 | 682 | 3 | 546 | Phage major capsid protein | Phage major capsid protein | | afdb-uniprot50 | AF-A0A5A9GUV5-F1-MODEL\_V4 | 1.0 | 6.535e-28 | 572 | 0.187 | 709 | 377 | 34 | 5 | 681 | 63 | 604 | Phage major capsid protein | Phage major capsid protein | | afdb-uniprot50 | AF-A0A356UJ62-F1-MODEL\_V4 | 1.0 | 8.081e-26 | 571 | 0.152 | 693 | 377 | 32 | 38 | 682 | 6 | 535 | Uncharacterized protein | Uncharacterized protein | | afdb-uniprot50 | AF-A0A1B3ZE75-F1-MODEL\_V4 | 1.0 | 1.042e-21 | 570 | 0.201 | 471 | 291 | 22 | 255 | 681 | 4 | 433 | Uncharacterized protein | Uncharacterized protein | | afdb-uniprot50 | AF-A0A255XX39-F1-MODEL\_V4 | 1.0 | 6.817e-26 | 569 | 0.179 | 691 | 354 | 33 | 38 | 681 | 11 | 535 | Phage major capsid protein | Phage major capsid protein | | afdb-uniprot50 | AF-A0A1M7RID9-F1-MODEL\_V4 | 1.0 | 4.523e-32 | 569 | 0.19 | 735 | 442 | 38 | 28 | 681 | 2 | 664 | Prohead serine protease | Prohead serine protease | | afdb-uniprot50 | AF-A0A7C9H8Y0-F1-MODEL\_V4 | 1.0 | 5.707e-21 | 566 | 0.185 | 437 | 256 | 17 | 295 | 680 | 22 | 409 | Phage major capsid protein | Phage major capsid protein | | afdb-uniprot50 | AF-A0A5B9R260-F1-MODEL\_V4 | 1.0 | 1.021e-22 | 565 | 0.196 | 508 | 284 | 18 | 252 | 681 | 16 | 477 | Phage capsid family protein | Phage capsid family protein | | afdb-uniprot50 | AF-A0A1V5LQQ8-F1-MODEL\_V4 | 1.0 | 1.056e-15 | 563 | 0.169 | 277 | 192 | 8 | 431 | 682 | 34 | 297 | Phage capsid family protein | Phage capsid family protein | | afdb-uniprot50 | AF-A0A521CQZ1-F1-MODEL\_V4 | 1.0 | 1.763e-31 | 562 | 0.157 | 731 | 460 | 34 | 12 | 681 | 111 | 746 | Phage prohead protease, HK97 family | Phage prohead protease, HK97 family | | afdb-uniprot50 | AF-A0A2E5AE13-F1-MODEL\_V4 | 1.0 | 3.898e-31 | 560 | 0.182 | 729 | 437 | 37 | 30 | 681 | 13 | 659 | Uncharacterized protein | Uncharacterized protein | | afdb-uniprot50 | AF-C0W521-F1-MODEL\_V4 | 1.0 | 1.298e-16 | 557 | 0.191 | 266 | 190 | 8 | 433 | 681 | 27 | 284 | Putative phage capsid family | Putative phage capsid family | | afdb-uniprot50 | AF-A0A2E2XBH4-F1-MODEL\_V4 | 1.0 | 1.236e-21 | 557 | 0.183 | 468 | 302 | 19 | 258 | 681 | 3 | 434 | Phage major capsid protein | Phage major capsid protein | | afdb-uniprot50 | AF-A0A1W1VX51-F1-MODEL\_V4 | 1.0 | 6.91e-20 | 556 | 0.209 | 458 | 253 | 20 | 251 | 681 | 7 | 382 | Phage major capsid protein, HK97 family | Phage major capsid protein, HK97 family | | afdb-uniprot50 | AF-A0A1V6D5P9-F1-MODEL\_V4 | 1.0 | 1.289e-19 | 556 | 0.202 | 376 | 236 | 19 | 336 | 681 | 109 | 450 | Phage capsid family protein | Phage capsid family protein | | afdb-uniprot50 | AF-A0A3P6KQL2-F1-MODEL\_V4 | 1.0 | 2.259e-30 | 556 | 0.175 | 731 | 430 | 36 | 19 | 681 | 1 | 626 | Mu-like prophage major head subunit gpT | Mu-like prophage major head subunit gpT | | afdb-uniprot50 | AF-A0A8B2NHU5-F1-MODEL\_V4 | 1.0 | 9.783e-25 | 555 | 0.16 | 673 | 372 | 29 | 37 | 681 | 11 | 518 | Phage major capsid protein | Phage major capsid protein | | afdb-uniprot50 | AF-A0A512HAW7-F1-MODEL\_V4 | 1.0 | 4.852e-26 | 555 | 0.165 | 694 | 397 | 32 | 19 | 682 | 4 | 545 | Peptidase U35 | Peptidase U35 | | afdb-uniprot50 | AF-A0A498R0I8-F1-MODEL\_V4 | 1.0 | 1.252e-15 | 554 | 0.188 | 260 | 192 | 5 | 436 | 682 | 36 | 289 | Uncharacterized protein | Uncharacterized protein | | afdb-uniprot50 | AF-A0A2U1YTD1-F1-MODEL\_V4 | 1.0 | 4.062e-21 | 554 | 0.183 | 484 | 286 | 21 | 266 | 682 | 4 | 445 | Uncharacterized protein | Uncharacterized protein | | afdb-uniprot50 | AF-A0A318RLQ4-F1-MODEL\_V4 | 1.0 | 7.105e-16 | 553 | 0.186 | 258 | 190 | 9 | 435 | 682 | 34 | 281 | HK97 family phage major capsid protein | HK97 family phage major capsid protein | | afdb-uniprot50 | AF-A0A132HLC0-F1-MODEL\_V4 | 1.0 | 1.8e-30 | 553 | 0.214 | 680 | 311 | 27 | 27 | 682 | 15 | 495 | Uncharacterized protein | Uncharacterized protein | | afdb-uniprot50 | AF-A0A7V3IXB5-F1-MODEL\_V4 | 1.0 | 3.101e-15 | 552 | 0.195 | 266 | 191 | 8 | 431 | 682 | 29 | 285 | Phage major capsid protein | Phage major capsid protein | | afdb-uniprot50 | AF-A0A7U0UNX5-F1-MODEL\_V4 | 1.0 | 2.869e-16 | 552 | 0.221 | 262 | 172 | 9 | 437 | 678 | 35 | 284 | Phage major capsid protein | Phage major capsid protein | | afdb-uniprot50 | AF-A0A177VZ34-F1-MODEL\_V4 | 1.0 | 1.628e-16 | 552 | 0.168 | 308 | 210 | 11 | 402 | 681 | 3 | 292 | Phage capsid family protein | Phage capsid family protein | | afdb-uniprot50 | AF-A0A4R6RGM9-F1-MODEL\_V4 | 1.0 | 6.529e-20 | 551 | 0.175 | 508 | 289 | 21 | 191 | 681 | 2 | 396 | HK97 family phage major capsid protein | HK97 family phage major capsid protein | | afdb-uniprot50 | AF-A0A442WPD5-F1-MODEL\_V4 | 1.0 | 1.736e-21 | 551 | 0.174 | 511 | 307 | 20 | 204 | 682 | 1 | 428 | Phage major capsid protein | Phage major capsid protein | | afdb-uniprot50 | AF-A0A6I0BNG2-F1-MODEL\_V4 | 1.0 | 2.074e-26 | 551 | 0.144 | 707 | 398 | 33 | 32 | 681 | 5 | 561 | Phage major capsid protein | Phage major capsid protein | | afdb-uniprot50 | AF-A0A5C4Y014-F1-MODEL\_V4 | 1.0 | 1.663e-15 | 549 | 0.158 | 265 | 200 | 8 | 431 | 681 | 38 | 293 | Phage major capsid protein | Phage major capsid protein | | afdb-uniprot50 | AF-A0A416XLM7-F1-MODEL\_V4 | 1.0 | 6.301e-19 | 549 | 0.197 | 364 | 249 | 13 | 334 | 682 | 28 | 363 | Phage major capsid protein | Phage major capsid protein | | afdb-uniprot50 | AF-A0A4Q7UYC6-F1-MODEL\_V4 | 1.0 | 5.664e-16 | 548 | 0.158 | 259 | 195 | 10 | 436 | 682 | 39 | 286 | HK97 family phage major capsid protein | HK97 family phage major capsid protein | | afdb-uniprot50 | AF-A0A7K3LU50-F1-MODEL\_V4 | 1.0 | 5.24e-17 | 548 | 0.167 | 317 | 235 | 13 | 374 | 681 | 29 | 325 | Phage major capsid protein | Phage major capsid protein | | afdb-uniprot50 | AF-A0A1E3GZU2-F1-MODEL\_V4 | 1.0 | 3.307e-20 | 548 | 0.191 | 507 | 280 | 22 | 191 | 681 | 1 | 393 | Phage capsid family protein | Phage capsid family protein | | afdb-uniprot50 | AF-A0A3D2INS8-F1-MODEL\_V4 | 1.0 | 1.183e-15 | 547 | 0.322 | 177 | 110 | 5 | 19 | 186 | 11 | 186 | Caudovirus prohead protease | Caudovirus prohead protease | | afdb-uniprot50 | AF-A0A3N0BSI3-F1-MODEL\_V4 | 1.0 | 1.862e-15 | 547 | 0.179 | 256 | 192 | 8 | 436 | 682 | 37 | 283 | Phage major capsid protein | Phage major capsid protein | | afdb-uniprot50 | AF-A0A329BHT4-F1-MODEL\_V4 | 1.0 | 1.701e-30 | 547 | 0.197 | 730 | 288 | 31 | 15 | 681 | 9 | 503 | HK97 family phage prohead protease | HK97 family phage prohead protease | | afdb-uniprot50 | AF-V7KIQ7-F1-MODEL\_V4 | 1.0 | 7.105e-16 | 545 | 0.169 | 260 | 190 | 9 | 435 | 681 | 33 | 279 | Capsid protein | Capsid protein | | afdb-uniprot50 | AF-A0A642F139-F1-MODEL\_V4 | 1.0 | 5.023e-19 | 545 | 0.182 | 378 | 267 | 18 | 338 | 682 | 8 | 376 | Phage major capsid protein | Phage major capsid protein | | afdb-uniprot50 | AF-A0A1I2GJW8-F1-MODEL\_V4 | 1.0 | 2.564e-24 | 545 | 0.119 | 696 | 380 | 29 | 32 | 682 | 4 | 511 | Phage prohead protease, HK97 family/phage major capsid protein, HK97 family,TIGR01554 | Phage prohead protease, HK97 family/phage major capsid protein, HK97 family,TIGR01554 | | afdb-uniprot50 | AF-A0A7U6ITT4-F1-MODEL\_V4 | 1.0 | 3.838e-21 | 544 | 0.147 | 522 | 332 | 24 | 191 | 681 | 3 | 442 | Uncharacterized protein | Uncharacterized protein | | afdb-uniprot50 | AF-A0A259MPF3-F1-MODEL\_V4 | 1.0 | 1.128e-28 | 544 | 0.182 | 736 | 430 | 34 | 37 | 682 | 11 | 664 | Uncharacterized protein | Uncharacterized protein | | afdb-uniprot50 | AF-A0A2P9HI32-F1-MODEL\_V4 | 1.0 | 4.683e-25 | 543 | 0.142 | 701 | 384 | 31 | 17 | 681 | 3 | 522 | Phage major capsid protein | Phage major capsid protein | | afdb-uniprot50 | AF-A0A348AC18-F1-MODEL\_V4 | 1.0 | 1.571e-15 | 542 | 0.159 | 269 | 197 | 10 | 431 | 681 | 89 | 346 | Phage major capsid protein, HK97 family | Phage major capsid protein, HK97 family | | afdb-uniprot50 | AF-A0A0F9LI03-F1-MODEL\_V4 | 1.0 | 4.815e-21 | 542 | 0.159 | 463 | 326 | 19 | 249 | 679 | 5 | 436 | Uncharacterized protein | Uncharacterized protein | | afdb-uniprot50 | AF-A0A2W6Y6L7-F1-MODEL\_V4 | 1.0 | 2.872e-24 | 541 | 0.138 | 677 | 380 | 30 | 43 | 681 | 17 | 527 | Phage major capsid protein | Phage major capsid protein | | afdb-uniprot50 | AF-A0A380N679-F1-MODEL\_V4 | 1.0 | 4.554e-29 | 540 | 0.204 | 715 | 395 | 39 | 39 | 681 | 21 | 633 | Mu-like prophage major head subunit gpT | Mu-like prophage major head subunit gpT | | afdb-uniprot50 | AF-A0A4Q3XXJ0-F1-MODEL\_V4 | 1.0 | 2.072e-18 | 539 | 0.434 | 235 | 106 | 7 | 14 | 235 | 14 | 234 | Uncharacterized protein | Uncharacterized protein | | afdb-uniprot50 | AF-A0A1D3R7K8-F1-MODEL\_V4 | 1.0 | 4.683e-25 | 539 | 0.124 | 689 | 398 | 31 | 32 | 682 | 7 | 527 | Putative phage capsid protein | Putative phage capsid protein | | afdb-uniprot50 | AF-A0A0Q9R783-F1-MODEL\_V4 | 1.0 | 1.403e-15 | 538 | 0.161 | 260 | 195 | 9 | 431 | 677 | 4 | 253 | Uncharacterized protein | Uncharacterized protein | | afdb-uniprot50 | AF-A0A852TM38-F1-MODEL\_V4 | 1.0 | 1.571e-15 | 538 | 0.156 | 262 | 196 | 8 | 433 | 682 | 29 | 277 | HK97 family phage major capsid protein | HK97 family phage major capsid protein | | afdb-uniprot50 | AF-A0A1Z3VNT3-F1-MODEL\_V4 | 1.0 | 1.056e-15 | 537 | 0.211 | 265 | 185 | 8 | 430 | 682 | 32 | 284 | Phage major capsid protein | Phage major capsid protein | | afdb-uniprot50 | AF-A0A1X2DT10-F1-MODEL\_V4 | 1.0 | 5.785e-15 | 537 | 0.162 | 258 | 197 | 6 | 436 | 682 | 36 | 285 | Capsid protein | Capsid protein | | afdb-uniprot50 | AF-A0A4Q4AXM0-F1-MODEL\_V4 | 1.0 | 1.56e-18 | 537 | 0.184 | 384 | 238 | 17 | 313 | 679 | 35 | 360 | Phage major capsid protein | Phage major capsid protein | | afdb-uniprot50 | AF-A0A661DS55-F1-MODEL\_V4 | 1.0 | 2.058e-21 | 536 | 0.159 | 489 | 326 | 20 | 252 | 680 | 6 | 469 | Phage major capsid protein | Phage major capsid protein | | afdb-uniprot50 | AF-G2I0U6-F1-MODEL\_V4 | 1.0 | 7.419e-22 | 536 | 0.215 | 482 | 285 | 21 | 249 | 680 | 20 | 458 | Bacteriophage protein | Bacteriophage protein | | afdb-uniprot50 | AF-A0A843LCP9-F1-MODEL\_V4 | 1.0 | 8.913e-16 | 535 | 0.171 | 251 | 189 | 9 | 433 | 681 | 54 | 287 | Phage major capsid protein | Phage major capsid protein | | afdb-uniprot50 | AF-A0A0Q5VUD7-F1-MODEL\_V4 | 1.0 | 5.508e-20 | 535 | 0.17 | 499 | 283 | 20 | 203 | 681 | 1 | 388 | Capsid protein | Capsid protein | | afdb-uniprot50 | AF-A0A3C1INU1-F1-MODEL\_V4 | 1.0 | 3.762e-30 | 535 | 0.188 | 722 | 407 | 45 | 26 | 681 | 15 | 623 | Mu-like\_gpT domain-containing protein | Mu-like\_gpT domain-containing protein | | afdb-uniprot50 | AF-U2DD62-F1-MODEL\_V4 | 1.0 | 7.958e-16 | 534 | 0.353 | 184 | 100 | 7 | 30 | 203 | 17 | 191 | Caudovirus prohead protease | Caudovirus prohead protease | | afdb-uniprot50 | AF-A0A3R6SVE9-F1-MODEL\_V4 | 1.0 | 1.929e-16 | 533 | 0.357 | 196 | 107 | 7 | 13 | 198 | 8 | 194 | Caudovirus prohead protease | Caudovirus prohead protease | | afdb-uniprot50 | AF-A0A2M7SYD1-F1-MODEL\_V4 | 1.0 | 2.456e-18 | 533 | 0.166 | 361 | 277 | 12 | 334 | 681 | 33 | 382 | Phage major capsid protein | Phage major capsid protein | | afdb-uniprot50 | AF-A4TBM3-F1-MODEL\_V4 | 1.0 | 9.992e-24 | 533 | 0.134 | 700 | 435 | 33 | 31 | 682 | 2 | 578 | Phage prohead protease, HK97 family | Phage prohead protease, HK97 family | | afdb-uniprot50 | AF-A0A3N5EJT9-F1-MODEL\_V4 | 1.0 | 2.149e-27 | 533 | 0.19 | 702 | 427 | 37 | 37 | 681 | 10 | 626 | Phage major capsid protein | Phage major capsid protein | | afdb-uniprot50 | AF-A0A8A6JDC6-F1-MODEL\_V4 | 1.0 | 1.971e-15 | 532 | 0.172 | 261 | 193 | 11 | 431 | 681 | 28 | 275 | Phage major capsid protein | Phage major capsid protein | | afdb-uniprot50 | AF-I4EHG9-F1-MODEL\_V4 | 1.0 | 1.496e-20 | 532 | 0.178 | 459 | 291 | 20 | 258 | 682 | 8 | 414 | Putative Phage major capsid protein, HK97 family | Putative Phage major capsid protein, HK97 family | | afdb-uniprot50 | AF-A0A640YC84-F1-MODEL\_V4 | 1.0 | 1.404e-23 | 532 | 0.118 | 691 | 393 | 30 | 19 | 682 | 1 | 502 | HK97 family phage prohead protease | HK97 family phage prohead protease | | afdb-uniprot50 | AF-A0A3M1QXF2-F1-MODEL\_V4 | 1.0 | 3.43e-29 | 532 | 0.175 | 741 | 434 | 43 | 30 | 681 | 9 | 661 | Uncharacterized protein | Uncharacterized protein | | afdb-uniprot50 | AF-A0A1A7QM62-F1-MODEL\_V4 | 1.0 | 5.24e-17 | 531 | 0.176 | 329 | 226 | 15 | 376 | 682 | 57 | 362 | Uncharacterized protein | Uncharacterized protein | | afdb-uniprot50 | AF-A0A1F8NTD0-F1-MODEL\_V4 | 1.0 | 1.087e-19 | 530 | 0.178 | 454 | 286 | 18 | 249 | 682 | 11 | 397 | Uncharacterized protein | Uncharacterized protein | | afdb-uniprot50 | AF-A0A0S7X9K7-F1-MODEL\_V4 | 1.0 | 3.192e-19 | 529 | 0.203 | 428 | 245 | 22 | 295 | 681 | 27 | 399 | Uncharacterized protein | Uncharacterized protein | | afdb-uniprot50 | AF-A0A316S7Y7-F1-MODEL\_V4 | 1.0 | 8.727e-17 | 528 | 0.32 | 206 | 119 | 7 | 5 | 199 | 6 | 201 | Caudovirus prohead protease | Caudovirus prohead protease | | afdb-uniprot50 | AF-A0A1H4QQ99-F1-MODEL\_V4 | 1.0 | 4.117e-15 | 528 | 0.191 | 256 | 190 | 8 | 431 | 680 | 29 | 273 | Phage major capsid protein, HK97 family | Phage major capsid protein, HK97 family | | afdb-uniprot50 | AF-A0A2S6AID0-F1-MODEL\_V4 | 1.0 | 7.362e-17 | 528 | 0.19 | 304 | 209 | 12 | 398 | 682 | 34 | 319 | Phage major capsid protein | Phage major capsid protein | | afdb-uniprot50 | AF-A0A060QKT2-F1-MODEL\_V4 | 1.0 | 6.573e-17 | 528 | 0.174 | 316 | 231 | 15 | 381 | 682 | 12 | 311 | Phage major capsid protein | Phage major capsid protein | | afdb-uniprot50 | AF-B5ZDC2-F1-MODEL\_V4 | 1.0 | 7.16e-21 | 527 | 0.177 | 478 | 284 | 20 | 271 | 680 | 3 | 439 | Uncharacterized protein | Uncharacterized protein | | afdb-uniprot50 | AF-A0A443W106-F1-MODEL\_V4 | 1.0 | 2.695e-27 | 527 | 0.221 | 632 | 353 | 25 | 40 | 631 | 20 | 552 | Major capsid protein | Major capsid protein | | afdb-uniprot50 | AF-A0A0Q9ES63-F1-MODEL\_V4 | 1.0 | 9.514e-29 | 527 | 0.174 | 738 | 435 | 42 | 35 | 682 | 10 | 662 | Uncharacterized protein | Uncharacterized protein | | afdb-uniprot50 | AF-A0A543NSK2-F1-MODEL\_V4 | 1.0 | 8.913e-16 | 526 | 0.176 | 289 | 213 | 10 | 409 | 682 | 3 | 281 | HK97 family phage major capsid protein | HK97 family phage major capsid protein | | afdb-uniprot50 | AF-A0A564G6H4-F1-MODEL\_V4 | 1.0 | 1.167e-21 | 525 | 0.191 | 481 | 306 | 20 | 258 | 680 | 5 | 460 | Uncharacterized protein | Uncharacterized protein | | afdb-uniprot50 | AF-A0A6M3L763-F1-MODEL\_V4 | 1.0 | 2.891e-21 | 525 | 0.18 | 521 | 314 | 22 | 249 | 682 | 12 | 506 | Putative capsid protein | Putative capsid protein | | afdb-uniprot50 | AF-A0A165XGL2-F1-MODEL\_V4 | 1.0 | 2.999e-30 | 525 | 0.186 | 745 | 431 | 38 | 30 | 677 | 13 | 679 | Caudovirus prohead protease | Caudovirus prohead protease | | afdb-uniprot50 | AF-C0ZMF4-F1-MODEL\_V4 | 1.0 | 7.257e-15 | 524 | 0.175 | 251 | 194 | 6 | 436 | 681 | 41 | 283 | Uncharacterized protein | Uncharacterized protein | | afdb-uniprot50 | AF-A0A3A4XE90-F1-MODEL\_V4 | 1.0 | 5.626e-19 | 524 | 0.179 | 401 | 262 | 17 | 309 | 682 | 2 | 362 | Phage major capsid protein | Phage major capsid protein | | afdb-uniprot50 | AF-A0A537KSG3-F1-MODEL\_V4 | 1.0 | 1.496e-20 | 523 | 0.165 | 490 | 300 | 21 | 262 | 682 | 7 | 456 | Phage major capsid protein | Phage major capsid protein | | afdb-uniprot50 | AF-A0A7V6QN74-F1-MODEL\_V4 | 1.0 | 2.472e-15 | 522 | 0.17 | 264 | 190 | 12 | 433 | 682 | 56 | 304 | Phage major capsid protein | Phage major capsid protein | | afdb-uniprot50 | AF-A0A3S4V390-F1-MODEL\_V4 | 1.0 | 1.354e-14 | 521 | 0.183 | 261 | 192 | 9 | 431 | 681 | 29 | 278 | Predicted phage phi-C31 gp36 major capsid-like protein | Predicted phage phi-C31 gp36 major capsid-like protein | | afdb-uniprot50 | AF-A0A419VU13-F1-MODEL\_V4 | 1.0 | 1.079e-14 | 521 | 0.155 | 258 | 197 | 9 | 433 | 679 | 57 | 304 | HK97 family phage major capsid protein | HK97 family phage major capsid protein | | afdb-uniprot50 | AF-A0A0N8W5Z7-F1-MODEL\_V4 | 1.0 | 1.007e-28 | 521 | 0.172 | 729 | 457 | 38 | 19 | 681 | 3 | 650 | Mu-like\_gpT domain-containing protein | Mu-like\_gpT domain-containing protein | | afdb-uniprot50 | AF-A0A7R7RKY8-F1-MODEL\_V4 | 1.0 | 1.079e-14 | 520 | 0.159 | 264 | 198 | 7 | 430 | 677 | 29 | 284 | Uncharacterized protein | Uncharacterized protein | | afdb-uniprot50 | AF-A0A7X7WBZ3-F1-MODEL\_V4 | 1.0 | 5.954e-19 | 520 | 0.193 | 433 | 255 | 18 | 271 | 681 | 3 | 363 | Phage major capsid protein | Phage major capsid protein | | afdb-uniprot50 | AF-L7U6P8-F1-MODEL\_V4 | 1.0 | 2.894e-29 | 519 | 0.185 | 726 | 442 | 36 | 40 | 681 | 10 | 669 | ClpP family peptidase | ClpP family peptidase | | afdb-uniprot50 | AF-A0A4S5BFZ4-F1-MODEL\_V4 | 1.0 | 3.019e-27 | 519 | 0.178 | 712 | 450 | 33 | 37 | 679 | 12 | 657 | Phage major capsid protein | Phage major capsid protein | | afdb-uniprot50 | AF-A0A827RVM8-F1-MODEL\_V4 | 1.0 | 7.844e-14 | 518 | 0.37 | 162 | 90 | 2 | 40 | 195 | 18 | 173 | Uncharacterized protein | Uncharacterized protein | | afdb-uniprot50 | AF-A0A7W0ZDQ7-F1-MODEL\_V4 | 1.0 | 6.857e-15 | 518 | 0.182 | 279 | 194 | 11 | 418 | 681 | 2 | 261 | Phage major capsid protein | Phage major capsid protein | | afdb-uniprot50 | AF-A0A4V3IRT6-F1-MODEL\_V4 | 1.0 | 3.378e-19 | 518 | 0.189 | 385 | 247 | 12 | 334 | 679 | 31 | 389 | Phage major capsid protein | Phage major capsid protein | | afdb-uniprot50 | AF-A0A7C1UH41-F1-MODEL\_V4 | 1.0 | 9.505e-21 | 518 | 0.156 | 499 | 283 | 24 | 258 | 681 | 3 | 438 | Phage major capsid protein | Phage major capsid protein | | afdb-uniprot50 | AF-Q31QA3-F1-MODEL\_V4 | 1.0 | 3.63e-29 | 518 | 0.195 | 733 | 416 | 41 | 13 | 682 | 2 | 623 | Mu-like\_gpT domain-containing protein | Mu-like\_gpT domain-containing protein | | afdb-uniprot50 | AF-A0A157ZYR5-F1-MODEL\_V4 | 1.0 | 1.112e-26 | 517 | 0.185 | 711 | 428 | 34 | 19 | 679 | 15 | 624 | Phage-like protein | Phage-like protein | | afdb-uniprot50 | AF-A0A442RKY7-F1-MODEL\_V4 | 1.0 | 4.746e-19 | 516 | 0.206 | 407 | 258 | 18 | 319 | 681 | 2 | 387 | Phage major capsid protein | Phage major capsid protein | | afdb-uniprot50 | AF-A0A7Y3PR89-F1-MODEL\_V4 | 1.0 | 2.404e-19 | 516 | 0.183 | 409 | 266 | 18 | 313 | 681 | 19 | 399 | Phage major capsid protein | Phage major capsid protein | | afdb-uniprot50 | AF-A0A367AXK8-F1-MODEL\_V4 | 1.0 | 2.178e-21 | 516 | 0.207 | 486 | 278 | 24 | 250 | 682 | 20 | 451 | Phage major capsid protein | Phage major capsid protein | | afdb-uniprot50 | AF-A0A0B0DDJ1-F1-MODEL\_V4 | 1.0 | 1.698e-14 | 515 | 0.184 | 249 | 184 | 7 | 443 | 681 | 1 | 240 | Uncharacterized protein | Uncharacterized protein | | afdb-uniprot50 | AF-A0A084GIL3-F1-MODEL\_V4 | 1.0 | 1.434e-22 | 515 | 0.13 | 691 | 364 | 30 | 16 | 681 | 4 | 482 | Uncharacterized protein | Uncharacterized protein | | afdb-uniprot50 | AF-A0A1S1WT94-F1-MODEL\_V4 | 1.0 | 1.701e-30 | 515 | 0.198 | 761 | 431 | 41 | 16 | 682 | 2 | 677 | Uncharacterized protein | Uncharacterized protein | | afdb-uniprot50 | AF-A0A7W4NV52-F1-MODEL\_V4 | 1.0 | 1.006e-20 | 514 | 0.2 | 478 | 279 | 22 | 264 | 679 | 8 | 444 | Phage major capsid protein | Phage major capsid protein | | afdb-uniprot50 | AF-A0A1N6U3E7-F1-MODEL\_V4 | 1.0 | 4.651e-28 | 514 | 0.175 | 722 | 443 | 31 | 12 | 682 | 6 | 625 | Phage prohead protease, HK97 family | Phage prohead protease, HK97 family | | afdb-uniprot50 | AF-A0A2V2E9I6-F1-MODEL\_V4 | 1.0 | 8.668e-20 | 513 | 0.185 | 436 | 265 | 20 | 294 | 680 | 13 | 407 | Phage major capsid protein | Phage major capsid protein | | afdb-uniprot50 | AF-A0A839NAF1-F1-MODEL\_V4 | 1.0 | 6.668e-19 | 512 | 0.166 | 390 | 277 | 16 | 334 | 682 | 1 | 383 | HK97 family phage major capsid protein | HK97 family phage major capsid protein | | afdb-uniprot50 | AF-A0A6A7UM03-F1-MODEL\_V4 | 1.0 | 6.811e-18 | 512 | 0.187 | 379 | 264 | 14 | 337 | 681 | 16 | 384 | Phage major capsid protein | Phage major capsid protein | | afdb-uniprot50 | AF-A0A073IUD9-F1-MODEL\_V4 | 1.0 | 1.946e-29 | 512 | 0.168 | 794 | 432 | 38 | 15 | 681 | 4 | 695 | Uncharacterized protein | Uncharacterized protein | | afdb-uniprot50 | AF-A0A1G7D256-F1-MODEL\_V4 | 1.0 | 9.983e-16 | 511 | 0.187 | 293 | 204 | 9 | 410 | 682 | 1 | 279 | Phage major capsid protein, HK97 family | Phage major capsid protein, HK97 family | | afdb-uniprot50 | AF-A0A0N1BIW0-F1-MODEL\_V4 | 1.0 | 1.134e-17 | 510 | 0.161 | 366 | 259 | 13 | 346 | 679 | 9 | 358 | Uncharacterized protein | Uncharacterized protein | | afdb-uniprot50 | AF-A0A857LMJ1-F1-MODEL\_V4 | 1.0 | 9.783e-25 | 510 | 0.138 | 752 | 420 | 35 | 30 | 682 | 3 | 625 | HK97 family phage prohead protease | HK97 family phage prohead protease | | afdb-uniprot50 | AF-A0A1B1C378-F1-MODEL\_V4 | 1.0 | 4.395e-28 | 510 | 0.173 | 733 | 460 | 36 | 31 | 681 | 2 | 670 | Uncharacterized protein | Uncharacterized protein | | afdb-uniprot50 | AF-A0A1X0BKY4-F1-MODEL\_V4 | 1.0 | 1.244e-18 | 509 | 0.152 | 459 | 294 | 14 | 249 | 682 | 2 | 390 | Uncharacterized protein | Uncharacterized protein | | afdb-uniprot50 | AF-M7A7R2-F1-MODEL\_V4 | 1.0 | 5.994e-16 | 508 | 0.166 | 318 | 239 | 12 | 381 | 682 | 44 | 351 | Phage capsid protein | Phage capsid protein | | afdb-uniprot50 | AF-A0A2H0LLW2-F1-MODEL\_V4 | 1.0 | 4.651e-28 | 508 | 0.192 | 713 | 410 | 37 | 40 | 681 | 1 | 618 | Uncharacterized protein | Uncharacterized protein | | afdb-uniprot50 | AF-A0A560FKK8-F1-MODEL\_V4 | 1.0 | 1.245e-26 | 508 | 0.16 | 717 | 393 | 34 | 5 | 681 | 269 | 816 | HK97 family phage prohead protease/HK97 family phage major capsid protein,TIGR01554 | HK97 family phage prohead protease/HK97 family phage major capsid protein,TIGR01554 | | afdb-uniprot50 | AF-A0A7C6P388-F1-MODEL\_V4 | 1.0 | 1.325e-15 | 507 | 0.147 | 258 | 194 | 11 | 433 | 682 | 51 | 290 | Phage major capsid protein | Phage major capsid protein | | afdb-uniprot50 | AF-A0A250JC89-F1-MODEL\_V4 | 1.0 | 1.316e-18 | 507 | 0.214 | 420 | 246 | 22 | 284 | 680 | 6 | 364 | Major capsid protein | Major capsid protein | | afdb-uniprot50 | AF-A0A1Q2M5H8-F1-MODEL\_V4 | 1.0 | 3.146e-17 | 507 | 0.155 | 367 | 275 | 14 | 338 | 681 | 22 | 376 | Uncharacterized protein | Uncharacterized protein | | afdb-uniprot50 | AF-A0A1I3MTS0-F1-MODEL\_V4 | 1.0 | 9.182e-28 | 507 | 0.189 | 746 | 430 | 40 | 1 | 681 | 1 | 636 | Uncharacterized protein | Uncharacterized protein | | afdb-uniprot50 | AF-A0A7G6VRU1-F1-MODEL\_V4 | 1.0 | 1.072e-17 | 506 | 0.164 | 444 | 279 | 17 | 251 | 682 | 3 | 366 | Phage major capsid protein | Phage major capsid protein | | afdb-uniprot50 | AF-A0A515KLR5-F1-MODEL\_V4 | 1.0 | 4.58e-18 | 506 | 0.182 | 399 | 270 | 17 | 313 | 679 | 4 | 378 | Phage major capsid protein | Phage major capsid protein | | afdb-uniprot50 | AF-A0A263E3D0-F1-MODEL\_V4 | 1.0 | 5.023e-19 | 506 | 0.19 | 399 | 245 | 14 | 338 | 682 | 12 | 386 | Phage major capsid protein | Phage major capsid protein | | afdb-uniprot50 | AF-A0A3F3ITH6-F1-MODEL\_V4 | 1.0 | 7.16e-21 | 506 | 0.182 | 477 | 291 | 22 | 252 | 681 | 2 | 426 | Capsid protein | Capsid protein | | afdb-uniprot50 | AF-A0A160NYS6-F1-MODEL\_V4 | 1.0 | 7.739e-20 | 505 | 0.161 | 457 | 302 | 20 | 268 | 681 | 4 | 422 | Phage major capsid protein | Phage major capsid protein | | afdb-uniprot50 | AF-A0A143PU17-F1-MODEL\_V4 | 1.0 | 2.147e-19 | 504 | 0.16 | 450 | 275 | 17 | 264 | 680 | 10 | 389 | Phage major capsid protein, HK97 family | Phage major capsid protein, HK97 family | | afdb-uniprot50 | AF-A0A059MSK3-F1-MODEL\_V4 | 1.0 | 2.192e-18 | 504 | 0.173 | 460 | 279 | 19 | 265 | 681 | 2 | 403 | Uncharacterized protein | Uncharacterized protein | | afdb-uniprot50 | AF-S9Q4D5-F1-MODEL\_V4 | 1.0 | 1.444e-19 | 504 | 0.208 | 446 | 267 | 24 | 276 | 680 | 8 | 408 | Uncharacterized protein | Uncharacterized protein | | afdb-uniprot50 | AF-A0A1X0JZU7-F1-MODEL\_V4 | 1.0 | 2.013e-14 | 503 | 0.166 | 295 | 207 | 11 | 407 | 682 | 2 | 276 | Capsid protein | Capsid protein | | afdb-uniprot50 | AF-A0A5C7FZT0-F1-MODEL\_V4 | 1.0 | 1.175e-18 | 503 | 0.141 | 396 | 264 | 16 | 338 | 680 | 12 | 384 | Phage major capsid protein | Phage major capsid protein | | afdb-uniprot50 | AF-A0A4P1JU19-F1-MODEL\_V4 | 1.0 | 5.398e-29 | 503 | 0.173 | 738 | 457 | 35 | 23 | 679 | 20 | 685 | Phage prohead protease, HK97 family | Phage prohead protease, HK97 family | | afdb-uniprot50 | AF-A0A418L4Y0-F1-MODEL\_V4 | 1.0 | 2.192e-18 | 502 | 0.155 | 412 | 293 | 16 | 313 | 682 | 4 | 402 | Phage major capsid protein | Phage major capsid protein | | afdb-uniprot50 | AF-A0A7S8C5X4-F1-MODEL\_V4 | 1.0 | 4.066e-29 | 502 | 0.191 | 738 | 441 | 39 | 30 | 681 | 15 | 682 | Mu-like prophage major head subunit gpT family protein | Mu-like prophage major head subunit gpT family protein | | afdb-uniprot50 | AF-A0A0S9DAB5-F1-MODEL\_V4 | 1.0 | 4.421e-17 | 501 | 0.141 | 367 | 286 | 11 | 335 | 681 | 1 | 358 | Uncharacterized protein | Uncharacterized protein | | afdb-uniprot50 | AF-A0A2S0LYN6-F1-MODEL\_V4 | 1.0 | 2.086e-15 | 500 | 0.161 | 267 | 204 | 9 | 431 | 682 | 31 | 292 | Phage major capsid protein | Phage major capsid protein | | afdb-uniprot50 | AF-A0A810PMP6-F1-MODEL\_V4 | 1.0 | 2.616e-15 | 500 | 0.191 | 297 | 192 | 13 | 419 | 678 | 1 | 286 | Uncharacterized protein | Uncharacterized protein | | afdb-uniprot50 | AF-A0A3N5X4U1-F1-MODEL\_V4 | 1.0 | 2.404e-19 | 500 | 0.221 | 420 | 243 | 20 | 282 | 666 | 35 | 405 | Phage major capsid protein | Phage major capsid protein | | afdb-uniprot50 | AF-A0A0F9IZX0-F1-MODEL\_V4 | 1.0 | 1.316e-18 | 500 | 0.145 | 453 | 302 | 17 | 253 | 680 | 9 | 401 | Uncharacterized protein | Uncharacterized protein | | afdb-uniprot50 | AF-A0A5C7LSW7-F1-MODEL\_V4 | 1.0 | 3.5e-20 | 500 | 0.169 | 484 | 314 | 19 | 238 | 681 | 5 | 440 | Phage major capsid protein | Phage major capsid protein | | afdb-uniprot50 | AF-A0A4P5VJG7-F1-MODEL\_V4 | 1.0 | 5.513e-28 | 500 | 0.193 | 732 | 419 | 39 | 18 | 681 | 5 | 632 | Uncharacterized protein | Uncharacterized protein | | afdb-uniprot50 | AF-A0A3P5XMZ8-F1-MODEL\_V4 | 1.0 | 1.506e-17 | 499 | 0.169 | 442 | 282 | 15 | 261 | 682 | 4 | 380 | Phage capsid family protein | Phage capsid family protein | | afdb-uniprot50 | AF-A0A0Q7QKI6-F1-MODEL\_V4 | 1.0 | 6.765e-21 | 499 | 0.191 | 474 | 290 | 20 | 247 | 682 | 42 | 460 | Uncharacterized protein | Uncharacterized protein | | afdb-uniprot50 | AF-A0A2E6KNG2-F1-MODEL\_V4 | 1.0 | 2.406e-27 | 499 | 0.177 | 726 | 446 | 36 | 16 | 681 | 18 | 652 | Peptidase U37 | Peptidase U37 | | afdb-uniprot50 | AF-H5TTQ1-F1-MODEL\_V4 | 1.0 | 1.889e-17 | 498 | 0.151 | 442 | 295 | 12 | 262 | 682 | 6 | 388 | Uncharacterized protein | Uncharacterized protein | | afdb-uniprot50 | AF-A0A1A3PAA7-F1-MODEL\_V4 | 1.0 | 1.013e-17 | 498 | 0.158 | 460 | 284 | 22 | 251 | 682 | 7 | 391 | Uncharacterized protein | Uncharacterized protein | | afdb-uniprot50 | AF-D3NSU8-F1-MODEL\_V4 | 1.0 | 1.316e-18 | 498 | 0.166 | 456 | 288 | 20 | 251 | 682 | 22 | 409 | Phage major capsid protein | Phage major capsid protein | | afdb-uniprot50 | AF-A0A1X1V399-F1-MODEL\_V4 | 1.0 | 5.626e-19 | 498 | 0.154 | 454 | 325 | 18 | 263 | 682 | 5 | 433 | Uncharacterized protein | Uncharacterized protein | | afdb-uniprot50 | AF-A0A1E3GXJ9-F1-MODEL\_V4 | 1.0 | 8.495e-29 | 498 | 0.175 | 718 | 459 | 39 | 41 | 681 | 554 | 1215 | Phage portal protein, lambda family | Phage portal protein, lambda family | | afdb-uniprot50 | AF-A0A0F2L3X1-F1-MODEL\_V4 | 1.0 | 3.651e-18 | 497 | 0.194 | 407 | 263 | 16 | 314 | 681 | 1 | 381 | Uncharacterized protein | Uncharacterized protein | | afdb-uniprot50 | AF-A0A1Q8I8P9-F1-MODEL\_V4 | 1.0 | 1.134e-17 | 497 | 0.153 | 462 | 290 | 21 | 251 | 681 | 5 | 396 | Uncharacterized protein | Uncharacterized protein | | afdb-uniprot50 | AF-A0A7C6NXR1-F1-MODEL\_V4 | 1.0 | 2.013e-14 | 496 | 0.113 | 264 | 203 | 12 | 433 | 681 | 58 | 305 | Phage major capsid protein | Phage major capsid protein | | afdb-uniprot50 | AF-A0A4R8Y418-F1-MODEL\_V4 | 1.0 | 6.529e-20 | 496 | 0.139 | 539 | 330 | 19 | 178 | 680 | 3 | 443 | Phage major capsid protein | Phage major capsid protein | | afdb-uniprot50 | AF-A0A626M3G7-F1-MODEL\_V4 | 1.0 | 1.118e-15 | 495 | 0.312 | 221 | 114 | 5 | 19 | 213 | 2 | 210 | Major capsid protein | Major capsid protein | | afdb-uniprot50 | AF-A0A251ZWI0-F1-MODEL\_V4 | 1.0 | 2.239e-17 | 495 | 0.152 | 393 | 258 | 17 | 327 | 681 | 5 | 360 | Uncharacterized protein | Uncharacterized protein | | afdb-uniprot50 | AF-A0A0B2K2J4-F1-MODEL\_V4 | 1.0 | 1.931e-24 | 495 | 0.217 | 501 | 278 | 20 | 18 | 480 | 2 | 426 | Uncharacterized protein | Uncharacterized protein | | afdb-uniprot50 | AF-W4A1A5-F1-MODEL\_V4 | 1.0 | 4.09e-18 | 494 | 0.168 | 434 | 257 | 22 | 282 | 682 | 7 | 369 | Phage major capsid protein, HK97 family | Phage major capsid protein, HK97 family | | afdb-uniprot50 | AF-A0A7J9XQM0-F1-MODEL\_V4 | 1.0 | 1.474e-18 | 494 | 0.151 | 475 | 281 | 20 | 249 | 680 | 5 | 400 | Phage major capsid protein | Phage major capsid protein | | afdb-uniprot50 | AF-G7QC56-F1-MODEL\_V4 | 1.0 | 1.087e-19 | 494 | 0.19 | 478 | 283 | 26 | 256 | 681 | 6 | 431 | HK97 family phage major capsid protein | HK97 family phage major capsid protein | | afdb-uniprot50 | AF-C0ZW70-F1-MODEL\_V4 | 1.0 | 4.27e-24 | 494 | 0.13 | 713 | 429 | 36 | 16 | 682 | 3 | 570 | Uncharacterized protein | Uncharacterized protein | | afdb-uniprot50 | AF-A0A292QWT5-F1-MODEL\_V4 | 1.0 | 1.497e-28 | 494 | 0.158 | 795 | 427 | 37 | 17 | 681 | 26 | 708 | Peptidase | Peptidase | | afdb-uniprot50 | AF-A0A5J6GE42-F1-MODEL\_V4 | 1.0 | 3.45e-18 | 493 | 0.176 | 448 | 275 | 18 | 259 | 682 | 2 | 379 | Phage major capsid protein | Phage major capsid protein | | afdb-uniprot50 | AF-A0A7Z0GMJ1-F1-MODEL\_V4 | 1.0 | 1.474e-18 | 493 | 0.145 | 467 | 288 | 17 | 258 | 682 | 8 | 405 | HK97 family phage major capsid protein | HK97 family phage major capsid protein | | afdb-uniprot50 | AF-A0A6V8U7K8-F1-MODEL\_V4 | 1.0 | 7.057e-19 | 493 | 0.169 | 449 | 296 | 20 | 251 | 682 | 61 | 449 | Major capsid protein | Major capsid protein | | afdb-uniprot50 | AF-A0A7W1RV33-F1-MODEL\_V4 | 1.0 | 9.505e-21 | 493 | 0.184 | 510 | 281 | 19 | 258 | 682 | 4 | 463 | Phage major capsid protein | Phage major capsid protein | | afdb-uniprot50 | AF-A0A7C2HM19-F1-MODEL\_V4 | 1.0 | 1.959e-26 | 493 | 0.167 | 712 | 414 | 36 | 43 | 681 | 21 | 626 | Peptidase U37 | Peptidase U37 | | afdb-uniprot50 | AF-R7JPW2-F1-MODEL\_V4 | 1.0 | 2.956e-28 | 493 | 0.181 | 745 | 457 | 34 | 40 | 681 | 15 | 709 | Peptidase U35 phage prohead HK97 | Peptidase U35 phage prohead HK97 | | afdb-uniprot50 | AF-A0A317CYD6-F1-MODEL\_V4 | 1.0 | 1.85e-18 | 492 | 0.164 | 463 | 293 | 17 | 250 | 682 | 4 | 402 | Phage major capsid protein | Phage major capsid protein | | afdb-uniprot50 | AF-A0A2D9CEY6-F1-MODEL\_V4 | 1.0 | 2.85e-19 | 492 | 0.19 | 457 | 281 | 21 | 255 | 680 | 5 | 403 | Phage major capsid protein | Phage major capsid protein | | afdb-uniprot50 | AF-A0A1U7GJ79-F1-MODEL\_V4 | 1.0 | 1.049e-18 | 492 | 0.2 | 474 | 270 | 26 | 264 | 682 | 3 | 422 | Uncharacterized protein | Uncharacterized protein | | afdb-uniprot50 | AF-A8SB58-F1-MODEL\_V4 | 1.0 | 5.785e-15 | 491 | 0.366 | 183 | 100 | 6 | 23 | 194 | 2 | 179 | Caudovirus prohead protease | Caudovirus prohead protease | | afdb-uniprot50 | AF-A0A7Z0GNW8-F1-MODEL\_V4 | 1.0 | 6.714e-16 | 490 | 0.178 | 320 | 206 | 14 | 381 | 682 | 9 | 289 | HK97 family phage major capsid protein | HK97 family phage major capsid protein | | afdb-uniprot50 | AF-A0A1F9FW66-F1-MODEL\_V4 | 1.0 | 3.787e-27 | 490 | 0.18 | 708 | 420 | 32 | 40 | 680 | 31 | 644 | Uncharacterized protein | Uncharacterized protein | | afdb-uniprot50 | AF-A1T829-F1-MODEL\_V4 | 1.0 | 2.828e-14 | 489 | 0.141 | 262 | 206 | 12 | 431 | 682 | 29 | 281 | Phage major capsid protein, HK97 | Phage major capsid protein, HK97 | | afdb-uniprot50 | AF-A0A3S0DN68-F1-MODEL\_V4 | 1.0 | 6.573e-17 | 489 | 0.161 | 372 | 267 | 17 | 335 | 681 | 1 | 352 | Phage major capsid protein | Phage major capsid protein | | afdb-uniprot50 | AF-A0A3N2KEJ4-F1-MODEL\_V4 | 1.0 | 1.27e-17 | 489 | 0.177 | 383 | 261 | 13 | 338 | 682 | 6 | 372 | Phage major capsid protein | Phage major capsid protein | | afdb-uniprot50 | AF-A0A502HT88-F1-MODEL\_V4 | 1.0 | 6.811e-18 | 489 | 0.169 | 453 | 278 | 21 | 252 | 682 | 20 | 396 | Phage major capsid protein | Phage major capsid protein | | afdb-uniprot50 | AF-A0A0P0D9A9-F1-MODEL\_V4 | 1.0 | 2.072e-18 | 489 | 0.14 | 426 | 293 | 19 | 279 | 681 | 63 | 438 | Capsid protein | Capsid protein | | afdb-uniprot50 | AF-A0A2P8NKG7-F1-MODEL\_V4 | 1.0 | 1.176e-26 | 489 | 0.163 | 729 | 440 | 33 | 12 | 682 | 6 | 622 | Mu-like\_gpT domain-containing protein | Mu-like\_gpT domain-containing protein | | afdb-uniprot50 | AF-A0A2I1R5G1-F1-MODEL\_V4 | 1.0 | 2.386e-14 | 488 | 0.145 | 261 | 204 | 11 | 433 | 681 | 31 | 284 | Phage major capsid protein | Phage major capsid protein | | afdb-uniprot50 | AF-A0A7C6WF68-F1-MODEL\_V4 | 1.0 | 9.433e-16 | 488 | 0.138 | 339 | 252 | 13 | 369 | 679 | 2 | 328 | Phage major capsid protein | Phage major capsid protein | | afdb-uniprot50 | AF-A0A1V2NG87-F1-MODEL\_V4 | 1.0 | 5.746e-18 | 488 | 0.155 | 456 | 300 | 19 | 252 | 682 | 10 | 405 | Uncharacterized protein | Uncharacterized protein | | afdb-uniprot50 | AF-A0A6I2J2E1-F1-MODEL\_V4 | 1.0 | 7.469e-19 | 487 | 0.445 | 249 | 121 | 4 | 282 | 529 | 3 | 235 | Phage major capsid protein | Phage major capsid protein | | afdb-uniprot50 | AF-A0A2E0EK67-F1-MODEL\_V4 | 1.0 | 2.809e-17 | 487 | 0.173 | 374 | 265 | 13 | 338 | 679 | 29 | 390 | Phage major capsid protein | Phage major capsid protein | | afdb-uniprot50 | AF-W7D9N4-F1-MODEL\_V4 | 1.0 | 6.668e-19 | 487 | 0.15 | 452 | 300 | 21 | 261 | 682 | 4 | 401 | Uncharacterized protein | Uncharacterized protein | | afdb-uniprot50 | AF-A0A7Y5LZT5-F1-MODEL\_V4 | 1.0 | 2.072e-18 | 487 | 0.178 | 470 | 281 | 24 | 261 | 682 | 8 | 420 | Phage major capsid protein | Phage major capsid protein | | afdb-uniprot50 | AF-A0A7M2XU64-F1-MODEL\_V4 | 1.0 | 9.235e-17 | 486 | 0.149 | 448 | 282 | 20 | 264 | 682 | 3 | 380 | Phage major capsid protein | Phage major capsid protein | | afdb-uniprot50 | AF-A0A0N0T0A0-F1-MODEL\_V4 | 1.0 | 1.85e-18 | 486 | 0.17 | 386 | 262 | 10 | 338 | 681 | 11 | 380 | Uncharacterized protein | Uncharacterized protein | | afdb-uniprot50 | AF-A0A0F9KP71-F1-MODEL\_V4 | 1.0 | 1.175e-18 | 486 | 0.143 | 466 | 306 | 18 | 234 | 681 | 31 | 421 | Uncharacterized protein | Uncharacterized protein | | afdb-uniprot50 | AF-A0A147IZI7-F1-MODEL\_V4 | 1.0 | 4.004e-19 | 486 | 0.189 | 475 | 290 | 20 | 255 | 681 | 6 | 433 | Uncharacterized protein | Uncharacterized protein | | afdb-uniprot50 | AF-A0A2V8NCC7-F1-MODEL\_V4 | 1.0 | 5.626e-19 | 486 | 0.149 | 428 | 295 | 19 | 278 | 679 | 59 | 443 | Phage major capsid protein | Phage major capsid protein | | afdb-uniprot50 | AF-A0A841QKT1-F1-MODEL\_V4 | 1.0 | 8.365e-19 | 486 | 0.162 | 462 | 311 | 16 | 249 | 680 | 35 | 450 | HK97 family phage major capsid protein | HK97 family phage major capsid protein | | afdb-uniprot50 | AF-A0A4V2DDR1-F1-MODEL\_V4 | 1.0 | 2.356e-28 | 486 | 0.149 | 742 | 469 | 35 | 18 | 681 | 3 | 660 | Mu-like\_gpT domain-containing protein | Mu-like\_gpT domain-containing protein | | afdb-uniprot50 | AF-A0A143PPF0-F1-MODEL\_V4 | 1.0 | 5.954e-19 | 485 | 0.177 | 444 | 277 | 17 | 271 | 682 | 3 | 390 | Phage major capsid protein, HK97 family | Phage major capsid protein, HK97 family | | afdb-uniprot50 | AF-A0A2T5W6J4-F1-MODEL\_V4 | 1.0 | 5.626e-19 | 484 | 0.173 | 467 | 294 | 18 | 263 | 682 | 3 | 424 | HK97 family phage major capsid protein | HK97 family phage major capsid protein | | afdb-uniprot50 | AF-A0A1S7QBP3-F1-MODEL\_V4 | 1.0 | 3.019e-27 | 484 | 0.166 | 716 | 466 | 34 | 17 | 681 | 9 | 644 | Peptidase U35 phage prohead HK97 | Peptidase U35 phage prohead HK97 | | afdb-uniprot50 | AF-A0A3M1YKP9-F1-MODEL\_V4 | 1.0 | 2.657e-25 | 484 | 0.145 | 798 | 458 | 40 | 15 | 679 | 3 | 709 | Phage major capsid protein | Phage major capsid protein | | afdb-uniprot50 | AF-A0A7R8N8Q9-F1-MODEL\_V4 | 1.0 | 2.437e-13 | 483 | 0.234 | 230 | 155 | 9 | 457 | 682 | 1 | 213 | Uncharacterized protein | Uncharacterized protein | | afdb-uniprot50 | AF-A0A329M2M9-F1-MODEL\_V4 | 1.0 | 1.134e-17 | 483 | 0.15 | 433 | 313 | 11 | 267 | 682 | 3 | 397 | Phage major capsid protein | Phage major capsid protein | | afdb-uniprot50 | AF-A0A1G3YGU2-F1-MODEL\_V4 | 1.0 | 1.2e-17 | 483 | 0.162 | 474 | 276 | 24 | 258 | 679 | 5 | 409 | Uncharacterized protein | Uncharacterized protein | | afdb-uniprot50 | AF-M5JSC1-F1-MODEL\_V4 | 1.0 | 1.529e-27 | 483 | 0.177 | 742 | 478 | 37 | 31 | 682 | 1 | 699 | Peptidase U35 phage prohead HK97 | Peptidase U35 phage prohead HK97 | | afdb-uniprot50 | AF-A0A753E0J0-F1-MODEL\_V4 | 1.0 | 3.548e-14 | 482 | 0.143 | 292 | 226 | 11 | 401 | 679 | 13 | 293 | Phage major capsid protein | Phage major capsid protein | | afdb-uniprot50 | AF-A0A7K2HUA6-F1-MODEL\_V4 | 1.0 | 8.246e-17 | 482 | 0.147 | 460 | 289 | 21 | 251 | 681 | 3 | 388 | Phage major capsid protein | Phage major capsid protein | | afdb-uniprot50 | AF-A0A196LBE7-F1-MODEL\_V4 | 1.0 | 8.544e-18 | 482 | 0.133 | 433 | 307 | 16 | 264 | 682 | 6 | 384 | Uncharacterized protein | Uncharacterized protein | | afdb-uniprot50 | AF-A0A5S3Y9T7-F1-MODEL\_V4 | 1.0 | 2.544e-19 | 482 | 0.192 | 463 | 285 | 19 | 268 | 681 | 4 | 426 | Phage major capsid protein | Phage major capsid protein | | afdb-uniprot50 | AF-A0A7X4X9J4-F1-MODEL\_V4 | 1.0 | 3.378e-19 | 482 | 0.184 | 476 | 282 | 21 | 258 | 680 | 7 | 429 | Phage major capsid protein | Phage major capsid protein | | afdb-uniprot50 | AF-A0A1H7NMW7-F1-MODEL\_V4 | 1.0 | 3.104e-23 | 482 | 0.124 | 697 | 420 | 30 | 39 | 682 | 12 | 570 | Phage prohead protease, HK97 family/phage major capsid protein, HK97 family,TIGR01554 | Phage prohead protease, HK97 family/phage major capsid protein, HK97 family,TIGR01554 | | afdb-uniprot50 | AF-A0A2T0Q9X2-F1-MODEL\_V4 | 1.0 | 9.916e-19 | 481 | 0.155 | 462 | 291 | 21 | 262 | 678 | 7 | 414 | HK97 family phage major capsid protein | HK97 family phage major capsid protein | | afdb-uniprot50 | AF-A0A521H793-F1-MODEL\_V4 | 1.0 | 6.307e-27 | 481 | 0.17 | 749 | 463 | 39 | 17 | 677 | 5 | 682 | Peptidase U37 | Peptidase U37 | | afdb-uniprot50 | AF-T0U3G2-F1-MODEL\_V4 | 1.0 | 9.84e-14 | 480 | 0.199 | 246 | 172 | 9 | 448 | 681 | 9 | 241 | Phage major capsid protein, HK97 family | Phage major capsid protein, HK97 family | | afdb-uniprot50 | AF-A0A857MKH2-F1-MODEL\_V4 | 1.0 | 4.611e-15 | 480 | 0.161 | 298 | 216 | 10 | 398 | 681 | 5 | 282 | Phage major capsid protein | Phage major capsid protein | | afdb-uniprot50 | AF-A0A6L7WRA2-F1-MODEL\_V4 | 1.0 | 4.951e-17 | 480 | 0.19 | 362 | 250 | 16 | 338 | 680 | 14 | 351 | Phage major capsid protein | Phage major capsid protein | | afdb-uniprot50 | AF-A0A5F0E144-F1-MODEL\_V4 | 1.0 | 1.889e-17 | 480 | 0.13 | 391 | 273 | 15 | 338 | 682 | 7 | 376 | Phage major capsid protein | Phage major capsid protein | | afdb-uniprot50 | AF-A0A1T4SDY1-F1-MODEL\_V4 | 1.0 | 3.651e-18 | 480 | 0.195 | 465 | 260 | 20 | 238 | 679 | 2 | 375 | Phage major capsid protein, HK97 family | Phage major capsid protein, HK97 family | | afdb-uniprot50 | AF-A0A6L6JA71-F1-MODEL\_V4 | 1.0 | 1.134e-17 | 480 | 0.162 | 413 | 284 | 18 | 298 | 682 | 8 | 386 | Phage major capsid protein | Phage major capsid protein | | afdb-uniprot50 | AF-K9D228-F1-MODEL\_V4 | 1.0 | 3.578e-27 | 480 | 0.164 | 728 | 442 | 40 | 16 | 682 | 8 | 629 | HK97 family phage prohead protease | HK97 family phage prohead protease | | afdb-uniprot50 | AF-A0A177JN28-F1-MODEL\_V4 | 1.0 | 9.634e-15 | 478 | 0.147 | 264 | 205 | 7 | 433 | 681 | 31 | 289 | Uncharacterized protein | Uncharacterized protein | | afdb-uniprot50 | AF-A0A2N3QVS0-F1-MODEL\_V4 | 1.0 | 5.429e-18 | 478 | 0.156 | 460 | 290 | 20 | 269 | 681 | 5 | 413 | Phage major capsid protein | Phage major capsid protein | | afdb-uniprot50 | AF-A0A6A7LQA8-F1-MODEL\_V4 | 1.0 | 8.544e-18 | 478 | 0.134 | 477 | 304 | 21 | 253 | 680 | 2 | 418 | Phage major capsid protein | Phage major capsid protein | | afdb-uniprot50 | AF-A0A7I7UUS7-F1-MODEL\_V4 | 1.0 | 1.095e-16 | 477 | 0.175 | 404 | 254 | 19 | 313 | 678 | 1 | 363 | Uncharacterized protein | Uncharacterized protein | | afdb-uniprot50 | AF-A0A839ID07-F1-MODEL\_V4 | 1.0 | 1.628e-16 | 476 | 0.135 | 391 | 283 | 14 | 335 | 682 | 1 | 379 | Phage major capsid protein | Phage major capsid protein | | afdb-uniprot50 | AF-A0A542VQW9-F1-MODEL\_V4 | 1.0 | 1.85e-18 | 476 | 0.15 | 458 | 277 | 19 | 268 | 682 | 5 | 393 | HK97 family phage major capsid protein | HK97 family phage major capsid protein | | afdb-uniprot50 | AF-A0A0F9R051-F1-MODEL\_V4 | 1.0 | 5.746e-18 | 476 | 0.143 | 480 | 307 | 20 | 251 | 680 | 2 | 427 | Uncharacterized protein | Uncharacterized protein | | afdb-uniprot50 | AF-A0A5C7M207-F1-MODEL\_V4 | 1.0 | 1.56e-18 | 476 | 0.164 | 468 | 293 | 22 | 251 | 681 | 28 | 434 | Phage major capsid protein | Phage major capsid protein | | afdb-uniprot50 | AF-I2GKQ2-F1-MODEL\_V4 | 1.0 | 8.365e-19 | 475 | 0.183 | 473 | 282 | 27 | 251 | 681 | 3 | 413 | Phage major capsid protein, HK97 family | Phage major capsid protein, HK97 family | | afdb-uniprot50 | AF-A0A7J9XKN3-F1-MODEL\_V4 | 1.0 | 4.678e-17 | 474 | 0.133 | 473 | 280 | 22 | 264 | 682 | 3 | 399 | Phage major capsid protein | Phage major capsid protein | | afdb-uniprot50 | AF-A0A3M2B028-F1-MODEL\_V4 | 1.0 | 4.848e-18 | 474 | 0.15 | 479 | 288 | 20 | 250 | 681 | 9 | 415 | Phage major capsid protein | Phage major capsid protein | | afdb-uniprot50 | AF-A0A1V6C830-F1-MODEL\_V4 | 1.0 | 3.524e-17 | 473 | 0.161 | 416 | 256 | 19 | 296 | 681 | 25 | 377 | Phage capsid family protein | Phage capsid family protein | | afdb-uniprot50 | AF-A0A7H5ET99-F1-MODEL\_V4 | 1.0 | 3.45e-18 | 473 | 0.155 | 449 | 284 | 19 | 258 | 682 | 13 | 390 | Phage major capsid protein | Phage major capsid protein | | afdb-uniprot50 | AF-A0A848HSK9-F1-MODEL\_V4 | 1.0 | 1.202e-25 | 473 | 0.196 | 704 | 438 | 41 | 50 | 681 | 2 | 649 | Uncharacterized protein | Uncharacterized protein | | afdb-uniprot50 | AF-A0A644ZYJ0-F1-MODEL\_V4 | 1.0 | 4.778e-16 | 472 | 0.162 | 394 | 242 | 19 | 313 | 682 | 1 | 330 | Uncharacterized protein | Uncharacterized protein | | afdb-uniprot50 | AF-A0A856N7Q3-F1-MODEL\_V4 | 1.0 | 1.316e-18 | 472 | 0.155 | 481 | 323 | 23 | 252 | 680 | 9 | 458 | Phage major capsid protein | Phage major capsid protein | | afdb-uniprot50 | AF-A0A7W3IZJ2-F1-MODEL\_V4 | 1.0 | 4.004e-19 | 472 | 0.177 | 496 | 300 | 20 | 262 | 681 | 7 | 470 | HK97 family phage major capsid protein | HK97 family phage major capsid protein | | afdb-uniprot50 | AF-A4L2R1-F1-MODEL\_V4 | 1.0 | 4.177e-17 | 471 | 0.149 | 415 | 244 | 16 | 295 | 681 | 41 | 374 | Phage major capsid protein | Phage major capsid protein | | afdb-uniprot50 | AF-A0A5C8IZR3-F1-MODEL\_V4 | 1.0 | 5.24e-17 | 471 | 0.161 | 378 | 269 | 17 | 338 | 679 | 82 | 447 | Phage major capsid protein | Phage major capsid protein | | afdb-uniprot50 | AF-A0A3E2T6Y0-F1-MODEL\_V4 | 1.0 | 5.702e-13 | 470 | 0.372 | 153 | 89 | 3 | 42 | 188 | 12 | 163 | Caudovirus prohead protease | Caudovirus prohead protease | | afdb-uniprot50 | AF-A0A7W4VP00-F1-MODEL\_V4 | 1.0 | 8.128e-15 | 470 | 0.175 | 279 | 179 | 13 | 438 | 680 | 16 | 279 | HK97 family phage major capsid protein | HK97 family phage major capsid protein | | afdb-uniprot50 | AF-A0A3G6WSW8-F1-MODEL\_V4 | 1.0 | 3.146e-17 | 470 | 0.168 | 427 | 265 | 19 | 294 | 681 | 9 | 384 | Phage major capsid protein | Phage major capsid protein | | afdb-uniprot50 | AF-A0A7C7DF73-F1-MODEL\_V4 | 1.0 | 1.748e-18 | 470 | 0.163 | 465 | 293 | 21 | 258 | 680 | 7 | 417 | Phage major capsid protein | Phage major capsid protein | | afdb-uniprot50 | AF-A0A0P0Z4E5-F1-MODEL\_V4 | 1.0 | 4.951e-17 | 469 | 0.182 | 456 | 275 | 18 | 243 | 682 | 8 | 381 | Phage major capsid protein, HK97 family | Phage major capsid protein, HK97 family | | afdb-uniprot50 | AF-A0A1R0KE28-F1-MODEL\_V4 | 1.0 | 2.654e-17 | 469 | 0.144 | 463 | 289 | 21 | 250 | 682 | 15 | 400 | Uncharacterized protein | Uncharacterized protein | | afdb-uniprot50 | AF-A0A3N5Z1L7-F1-MODEL\_V4 | 1.0 | 7.105e-16 | 469 | 0.152 | 367 | 271 | 15 | 338 | 682 | 34 | 382 | Phage major capsid protein | Phage major capsid protein | | afdb-uniprot50 | AF-A0A6I4SN09-F1-MODEL\_V4 | 1.0 | 1.628e-16 | 468 | 0.138 | 376 | 275 | 15 | 335 | 679 | 1 | 358 | Phage major capsid protein | Phage major capsid protein | | afdb-uniprot50 | AF-A0A1Y3LZN4-F1-MODEL\_V4 | 1.0 | 7.791e-17 | 468 | 0.156 | 448 | 287 | 18 | 258 | 682 | 4 | 383 | Phage major capsid protein | Phage major capsid protein | | afdb-uniprot50 | AF-A0A7V3I586-F1-MODEL\_V4 | 1.0 | 2.711e-16 | 468 | 0.186 | 370 | 248 | 18 | 341 | 681 | 53 | 398 | Phage major capsid protein | Phage major capsid protein | | afdb-uniprot50 | AF-A0A431JK96-F1-MODEL\_V4 | 1.0 | 3.45e-18 | 468 | 0.126 | 467 | 317 | 22 | 234 | 681 | 40 | 434 | Phage major capsid protein | Phage major capsid protein | | afdb-uniprot50 | AF-A0A1G8RXE6-F1-MODEL\_V4 | 1.0 | 7.469e-19 | 468 | 0.183 | 480 | 294 | 24 | 251 | 681 | 5 | 435 | Phage major capsid protein, HK97 family | Phage major capsid protein, HK97 family | | afdb-uniprot50 | AF-A0A5D4JM19-F1-MODEL\_V4 | 1.0 | 7.904e-19 | 468 | 0.176 | 487 | 287 | 19 | 246 | 678 | 14 | 440 | Phage major capsid protein | Phage major capsid protein | | afdb-uniprot50 | AF-A0A136PD24-F1-MODEL\_V4 | 1.0 | 3.401e-16 | 468 | 0.166 | 372 | 237 | 14 | 338 | 680 | 134 | 461 | Phage capsid family protein | Phage capsid family protein | | afdb-uniprot50 | AF-K2JM81-F1-MODEL\_V4 | 1.0 | 1.629e-24 | 468 | 0.181 | 700 | 372 | 33 | 16 | 681 | 227 | 759 | Phage major capsid protein, HK97 family | Phage major capsid protein, HK97 family | | afdb-uniprot50 | AF-A0A8B3A685-F1-MODEL\_V4 | 1.0 | 2.176e-13 | 467 | 0.341 | 158 | 97 | 3 | 38 | 189 | 6 | 162 | Caudovirus prohead protease | Caudovirus prohead protease | | afdb-uniprot50 | AF-A0A3A9D626-F1-MODEL\_V4 | 1.0 | 6.618e-14 | 467 | 0.337 | 181 | 109 | 4 | 34 | 208 | 25 | 200 | Caudovirus prohead protease | Caudovirus prohead protease | | afdb-uniprot50 | AF-A0A315TC63-F1-MODEL\_V4 | 1.0 | 6.211e-17 | 467 | 0.153 | 450 | 289 | 21 | 258 | 681 | 5 | 388 | HK97 family phage major capsid protein | HK97 family phage major capsid protein | | afdb-uniprot50 | AF-A0A5F0LGR9-F1-MODEL\_V4 | 1.0 | 2.072e-18 | 467 | 0.176 | 475 | 284 | 24 | 255 | 681 | 4 | 419 | Phage major capsid protein | Phage major capsid protein | | afdb-uniprot50 | AF-A0A238WSY7-F1-MODEL\_V4 | 1.0 | 3.864e-18 | 467 | 0.181 | 469 | 289 | 23 | 252 | 680 | 17 | 430 | Phage major capsid protein, HK97 family | Phage major capsid protein, HK97 family | | afdb-uniprot50 | AF-A0A441D124-F1-MODEL\_V4 | 1.0 | 6.169e-20 | 467 | 0.173 | 589 | 319 | 25 | 116 | 679 | 21 | 466 | Phage major capsid protein | Phage major capsid protein | | afdb-uniprot50 | AF-A0A7T7CAQ5-F1-MODEL\_V4 | 1.0 | 3.26e-18 | 466 | 0.16 | 462 | 279 | 21 | 271 | 682 | 3 | 405 | Phage major capsid protein | Phage major capsid protein | | afdb-uniprot50 | AF-M7NVL3-F1-MODEL\_V4 | 1.0 | 3.651e-18 | 466 | 0.191 | 474 | 275 | 23 | 252 | 680 | 9 | 419 | Putative phage phi-C31 gp36 major capsid-like protein | Putative phage phi-C31 gp36 major capsid-like protein | | afdb-uniprot50 | AF-R9LVI7-F1-MODEL\_V4 | 1.0 | 4.985e-14 | 465 | 0.361 | 177 | 106 | 3 | 19 | 189 | 5 | 180 | Uncharacterized protein | Uncharacterized protein | | afdb-uniprot50 | AF-A0A2G2G701-F1-MODEL\_V4 | 1.0 | 7.208e-18 | 465 | 0.18 | 448 | 261 | 19 | 262 | 676 | 5 | 379 | Phage major capsid protein | Phage major capsid protein | | afdb-uniprot50 | AF-A0A497HDL0-F1-MODEL\_V4 | 1.0 | 4.951e-17 | 465 | 0.171 | 374 | 255 | 14 | 345 | 681 | 31 | 386 | Phage major capsid protein | Phage major capsid protein | | afdb-uniprot50 | AF-A0A177PC93-F1-MODEL\_V4 | 1.0 | 9.57e-18 | 465 | 0.179 | 469 | 266 | 24 | 243 | 682 | 12 | 390 | Uncharacterized protein | Uncharacterized protein | | afdb-uniprot50 | AF-A0A2L0TWR2-F1-MODEL\_V4 | 1.0 | 1.298e-16 | 465 | 0.146 | 456 | 296 | 22 | 258 | 682 | 7 | 400 | Phage major capsid protein | Phage major capsid protein | | afdb-uniprot50 | AF-A0A1Y3SJ18-F1-MODEL\_V4 | 1.0 | 1.864e-23 | 465 | 0.135 | 746 | 389 | 34 | 27 | 681 | 2 | 582 | Phage major capsid protein | Phage major capsid protein | | afdb-uniprot50 | AF-A0A150IJM8-F1-MODEL\_V4 | 1.0 | 1.538e-16 | 464 | 0.169 | 371 | 257 | 16 | 336 | 681 | 1 | 345 | Phage capsid family protein | Phage capsid family protein | | afdb-uniprot50 | AF-A0A7C7C6E2-F1-MODEL\_V4 | 1.0 | 1.538e-16 | 464 | 0.15 | 385 | 278 | 16 | 336 | 682 | 3 | 376 | Phage major capsid protein | Phage major capsid protein | | afdb-uniprot50 | AF-A0A2S5UU29-F1-MODEL\_V4 | 1.0 | 2.192e-18 | 464 | 0.139 | 459 | 293 | 22 | 263 | 681 | 9 | 405 | Phage major capsid protein | Phage major capsid protein | | afdb-uniprot50 | AF-A0A3C1GGD5-F1-MODEL\_V4 | 1.0 | 2.809e-17 | 464 | 0.144 | 463 | 306 | 22 | 252 | 682 | 4 | 408 | Phage major capsid protein | Phage major capsid protein | | afdb-uniprot50 | AF-A0A352HSX6-F1-MODEL\_V4 | 1.0 | 1.999e-17 | 463 | 0.189 | 432 | 272 | 16 | 271 | 681 | 4 | 378 | Phage major capsid protein | Phage major capsid protein | | afdb-uniprot50 | AF-A0A433WXB3-F1-MODEL\_V4 | 1.0 | 4.177e-17 | 463 | 0.167 | 449 | 288 | 19 | 253 | 680 | 8 | 391 | Phage major capsid protein | Phage major capsid protein | | afdb-uniprot50 | AF-A0A3A0CKV3-F1-MODEL\_V4 | 1.0 | 5.316e-19 | 463 | 0.169 | 483 | 288 | 23 | 258 | 682 | 9 | 436 | Phage major capsid protein | Phage major capsid protein | | afdb-uniprot50 | AF-A0A504UPN0-F1-MODEL\_V4 | 1.0 | 2.118e-25 | 463 | 0.17 | 729 | 406 | 38 | 19 | 682 | 6 | 600 | Peptidase U35 | Peptidase U35 | | afdb-uniprot50 | AF-A0A133ZYY2-F1-MODEL\_V4 | 1.0 | 2.13e-14 | 462 | 0.314 | 191 | 115 | 5 | 11 | 193 | 14 | 196 | Caudovirus prohead protease | Caudovirus prohead protease | | afdb-uniprot50 | AF-A0A0K9YYM7-F1-MODEL\_V4 | 1.0 | 3.33e-17 | 462 | 0.133 | 449 | 313 | 21 | 256 | 681 | 4 | 399 | Uncharacterized protein | Uncharacterized protein | | afdb-uniprot50 | AF-A0A7C1KK58-F1-MODEL\_V4 | 1.0 | 2.599e-18 | 462 | 0.188 | 477 | 297 | 23 | 251 | 682 | 2 | 433 | Phage major capsid protein | Phage major capsid protein | | afdb-uniprot50 | AF-A0A4R4A8U2-F1-MODEL\_V4 | 1.0 | 9.369e-19 | 462 | 0.175 | 468 | 272 | 20 | 252 | 682 | 56 | 446 | HK97 family phage major capsid protein | HK97 family phage major capsid protein | | afdb-uniprot50 | AF-F8KPN2-F1-MODEL\_V4 | 1.0 | 4.27e-24 | 462 | 0.189 | 619 | 392 | 21 | 40 | 622 | 27 | 571 | Uncharacterized protein | Uncharacterized protein | | afdb-uniprot50 | AF-A0A1V5WQJ7-F1-MODEL\_V4 | 1.0 | 6.259e-22 | 462 | 0.127 | 719 | 422 | 39 | 35 | 682 | 9 | 592 | Phage capsid family protein | Phage capsid family protein | | afdb-uniprot50 | AF-A0A8A4FL43-F1-MODEL\_V4 | 1.0 | 7.064e-27 | 462 | 0.181 | 765 | 432 | 47 | 19 | 681 | 1 | 672 | Peptidase U35 | Peptidase U35 | | afdb-uniprot50 | AF-A0A4R2H804-F1-MODEL\_V4 | 1.0 | 2.056e-13 | 461 | 0.144 | 256 | 201 | 8 | 431 | 681 | 29 | 271 | HK97 family phage major capsid protein | HK97 family phage major capsid protein | | afdb-uniprot50 | AF-A0A7G5NYF7-F1-MODEL\_V4 | 1.0 | 9.84e-14 | 461 | 0.161 | 260 | 192 | 7 | 433 | 682 | 60 | 303 | Phage major capsid protein | Phage major capsid protein | | afdb-uniprot50 | AF-A0A432G3A4-F1-MODEL\_V4 | 1.0 | 2.562e-16 | 461 | 0.15 | 440 | 296 | 15 | 252 | 682 | 5 | 375 | Phage major capsid protein | Phage major capsid protein | | afdb-uniprot50 | AF-A0A259XLU9-F1-MODEL\_V4 | 1.0 | 2.042e-16 | 461 | 0.137 | 421 | 295 | 14 | 271 | 682 | 29 | 390 | Phage major capsid protein | Phage major capsid protein | | afdb-uniprot50 | AF-A0A260BZ92-F1-MODEL\_V4 | 1.0 | 7.105e-16 | 461 | 0.146 | 375 | 263 | 13 | 346 | 677 | 36 | 396 | Phage major capsid protein | Phage major capsid protein | | afdb-uniprot50 | AF-A0A0F9JLY2-F1-MODEL\_V4 | 1.0 | 1.785e-17 | 461 | 0.219 | 388 | 214 | 17 | 331 | 678 | 59 | 397 | Uncharacterized protein | Uncharacterized protein | | afdb-uniprot50 | AF-A0A561ECB5-F1-MODEL\_V4 | 1.0 | 4.678e-17 | 461 | 0.137 | 445 | 321 | 13 | 258 | 682 | 8 | 409 | HK97 family phage major capsid protein | HK97 family phage major capsid protein | | afdb-uniprot50 | AF-A0A494SQV9-F1-MODEL\_V4 | 1.0 | 1.56e-18 | 461 | 0.153 | 475 | 282 | 19 | 264 | 682 | 11 | 421 | Phage major capsid protein | Phage major capsid protein | | afdb-uniprot50 | AF-A0A142IR20-F1-MODEL\_V4 | 1.0 | 3.708e-28 | 461 | 0.18 | 731 | 422 | 34 | 38 | 681 | 11 | 651 | Mu-like\_gpT domain-containing protein | Mu-like\_gpT domain-containing protein | | afdb-uniprot50 | AF-A0A2S5N3Y2-F1-MODEL\_V4 | 1.0 | 2.074e-26 | 461 | 0.161 | 742 | 486 | 36 | 11 | 681 | 14 | 689 | Uncharacterized protein | Uncharacterized protein | | afdb-uniprot50 | AF-A0A151FZE6-F1-MODEL\_V4 | 1.0 | 3.216e-24 | 461 | 0.16 | 749 | 452 | 33 | 14 | 679 | 82 | 736 | Uncharacterized protein | Uncharacterized protein | | afdb-uniprot50 | AF-A0A084J8V6-F1-MODEL\_V4 | 1.0 | 1.166e-13 | 460 | 0.163 | 269 | 193 | 9 | 431 | 682 | 32 | 285 | Uncharacterized protein | Uncharacterized protein | | afdb-uniprot50 | AF-A0A1H9KBY5-F1-MODEL\_V4 | 1.0 | 1.595e-25 | 460 | 0.152 | 714 | 418 | 38 | 40 | 681 | 16 | 614 | Uncharacterized protein | Uncharacterized protein | | afdb-uniprot50 | AF-A0A520YI95-F1-MODEL\_V4 | 1.0 | 2.601e-26 | 460 | 0.182 | 761 | 440 | 43 | 18 | 675 | 11 | 692 | Uncharacterized protein | Uncharacterized protein | | afdb-uniprot50 | AF-A0A2E7K3Z7-F1-MODEL\_V4 | 1.0 | 2.072e-18 | 459 | 0.278 | 355 | 185 | 14 | 17 | 358 | 3 | 299 | Uncharacterized protein | Uncharacterized protein | | afdb-uniprot50 | AF-A0A2I1VRC1-F1-MODEL\_V4 | 1.0 | 5.275e-14 | 459 | 0.145 | 274 | 207 | 7 | 431 | 681 | 34 | 303 | Phage major capsid protein | Phage major capsid protein | | afdb-uniprot50 | AF-A0A0F9RQJ3-F1-MODEL\_V4 | 1.0 | 1.594e-17 | 459 | 0.164 | 469 | 292 | 19 | 243 | 682 | 19 | 416 | Uncharacterized protein | Uncharacterized protein | | afdb-uniprot50 | AF-A0A7V5XDB4-F1-MODEL\_V4 | 1.0 | 7.369e-25 | 459 | 0.169 | 703 | 414 | 33 | 41 | 681 | 16 | 610 | Peptidase U35 | Peptidase U35 | | afdb-uniprot50 | AF-A0A1E5HBV2-F1-MODEL\_V4 | 1.0 | 3.783e-19 | 459 | 0.183 | 479 | 284 | 21 | 252 | 682 | 207 | 626 | ATP-dependent Clp protease proteolytic subunit | ATP-dependent Clp protease proteolytic subunit | | afdb-uniprot50 | AF-A0A0N8GFX9-F1-MODEL\_V4 | 1.0 | 1.688e-25 | 459 | 0.179 | 743 | 453 | 39 | 11 | 681 | 21 | 678 | Mu-like\_gpT domain-containing protein | Mu-like\_gpT domain-containing protein | | afdb-uniprot50 | AF-H9UJE2-F1-MODEL\_V4 | 1.0 | 7.362e-17 | 458 | 0.128 | 466 | 276 | 22 | 255 | 682 | 2 | 375 | Putative phage phi-C31 gp36 major capsid-like protein | Putative phage phi-C31 gp36 major capsid-like protein | | afdb-uniprot50 | AF-A0A3D5RUL1-F1-MODEL\_V4 | 1.0 | 8.544e-18 | 458 | 0.162 | 467 | 284 | 22 | 252 | 680 | 7 | 404 | Phage major capsid protein | Phage major capsid protein | | afdb-uniprot50 | AF-A0A7Z2M7Y8-F1-MODEL\_V4 | 1.0 | 1.889e-17 | 458 | 0.148 | 464 | 307 | 20 | 249 | 682 | 8 | 413 | Capsid protein | Capsid protein | | afdb-uniprot50 | AF-A0A7Y5QI34-F1-MODEL\_V4 | 1.0 | 8.544e-18 | 458 | 0.144 | 469 | 292 | 22 | 264 | 678 | 13 | 426 | Phage major capsid protein | Phage major capsid protein | | afdb-uniprot50 | AF-A0A1Y4L4F4-F1-MODEL\_V4 | 1.0 | 8.301e-14 | 457 | 0.178 | 269 | 186 | 14 | 436 | 682 | 2 | 257 | Phage major capsid protein | Phage major capsid protein | | afdb-uniprot50 | AF-A0A1H0Y9H5-F1-MODEL\_V4 | 1.0 | 4.177e-17 | 457 | 0.12 | 447 | 306 | 16 | 258 | 682 | 4 | 385 | Phage major capsid protein, HK97 family | Phage major capsid protein, HK97 family | | afdb-uniprot50 | AF-F1YJE6-F1-MODEL\_V4 | 1.0 | 3.213e-16 | 457 | 0.154 | 408 | 293 | 13 | 315 | 681 | 1 | 397 | Uncharacterized protein | Uncharacterized protein | | afdb-uniprot50 | AF-A0A1R0X157-F1-MODEL\_V4 | 1.0 | 1.244e-18 | 457 | 0.162 | 481 | 289 | 20 | 255 | 682 | 10 | 429 | Uncharacterized protein | Uncharacterized protein | | afdb-uniprot50 | AF-Q14M91-F1-MODEL\_V4 | 1.0 | 1.761e-23 | 457 | 0.133 | 747 | 426 | 44 | 1 | 681 | 1 | 591 | Hypothetical prophage major capsid protein | Hypothetical prophage major capsid protein | | afdb-uniprot50 | AF-X0XIA7-F1-MODEL\_V4 | 1.0 | 4.295e-13 | 456 | 0.227 | 251 | 161 | 7 | 443 | 670 | 1 | 241 | Uncharacterized protein | Uncharacterized protein | | afdb-uniprot50 | AF-A0A5C7M8D0-F1-MODEL\_V4 | 1.0 | 3.551e-22 | 456 | 0.145 | 721 | 423 | 40 | 31 | 680 | 1 | 599 | Phage major capsid protein | Phage major capsid protein | | afdb-uniprot50 | AF-A0A7W2BLG8-F1-MODEL\_V4 | 1.0 | 7.844e-14 | 455 | 0.211 | 217 | 148 | 9 | 478 | 682 | 20 | 225 | Phage major capsid protein | Phage major capsid protein | | afdb-uniprot50 | AF-A0A7H8ZSI3-F1-MODEL\_V4 | 1.0 | 1.687e-17 | 455 | 0.168 | 470 | 275 | 19 | 251 | 681 | 3 | 395 | Phage major capsid protein | Phage major capsid protein | | afdb-uniprot50 | AF-A0A6B1I8G0-F1-MODEL\_V4 | 1.0 | 1.244e-18 | 455 | 0.181 | 468 | 296 | 21 | 253 | 680 | 40 | 460 | Phage major capsid protein | Phage major capsid protein | | afdb-uniprot50 | AF-A0A399XUC0-F1-MODEL\_V4 | 1.0 | 2.272e-19 | 455 | 0.165 | 561 | 321 | 22 | 172 | 682 | 12 | 475 | Phage major capsid protein | Phage major capsid protein | | afdb-uniprot50 | AF-A0A5C7YF14-F1-MODEL\_V4 | 1.0 | 2.888e-13 | 454 | 0.188 | 260 | 192 | 11 | 431 | 681 | 29 | 278 | Phage major capsid protein | Phage major capsid protein | | afdb-uniprot50 | AF-A0A0F9IVS0-F1-MODEL\_V4 | 1.0 | 1.252e-15 | 454 | 0.146 | 389 | 279 | 18 | 305 | 681 | 6 | 353 | Uncharacterized protein | Uncharacterized protein | | afdb-uniprot50 | AF-A0A443TLW2-F1-MODEL\_V4 | 1.0 | 3.809e-16 | 454 | 0.143 | 445 | 276 | 20 | 262 | 681 | 9 | 373 | Phage major capsid protein | Phage major capsid protein | | afdb-uniprot50 | AF-A0A7W8J095-F1-MODEL\_V4 | 1.0 | 3.036e-16 | 454 | 0.134 | 447 | 309 | 19 | 251 | 682 | 2 | 385 | HK97 family phage major capsid protein | HK97 family phage major capsid protein | | afdb-uniprot50 | AF-A0A2A8V9J7-F1-MODEL\_V4 | 1.0 | 7.208e-18 | 454 | 0.154 | 461 | 284 | 21 | 264 | 680 | 23 | 421 | Phage major capsid protein | Phage major capsid protein | | afdb-uniprot50 | AF-A0A6L5R7M5-F1-MODEL\_V4 | 1.0 | 4.421e-17 | 453 | 0.135 | 450 | 293 | 21 | 260 | 682 | 7 | 387 | Phage major capsid protein | Phage major capsid protein | | afdb-uniprot50 | AF-A0A6N8I321-F1-MODEL\_V4 | 1.0 | 4.951e-17 | 453 | 0.161 | 452 | 293 | 23 | 256 | 681 | 4 | 395 | Phage capsid family protein | Phage capsid family protein | | afdb-uniprot50 | AF-J5E045-F1-MODEL\_V4 | 1.0 | 2.239e-17 | 453 | 0.126 | 473 | 298 | 19 | 252 | 682 | 3 | 402 | Uncharacterized protein | Uncharacterized protein | | afdb-uniprot50 | AF-A0A562NCU5-F1-MODEL\_V4 | 1.0 | 3.33e-17 | 453 | 0.141 | 465 | 305 | 22 | 245 | 681 | 1 | 399 | HK97 family phage major capsid protein | HK97 family phage major capsid protein | | afdb-uniprot50 | AF-A0A198AI12-F1-MODEL\_V4 | 1.0 | 3.401e-16 | 453 | 0.162 | 388 | 228 | 21 | 337 | 682 | 72 | 404 | Uncharacterized protein | Uncharacterized protein | | afdb-uniprot50 | AF-A0A1E2SJE2-F1-MODEL\_V4 | 1.0 | 2.809e-17 | 453 | 0.152 | 447 | 279 | 20 | 275 | 681 | 40 | 426 | Uncharacterized protein | Uncharacterized protein | | afdb-uniprot50 | AF-A0A7V7PNP3-F1-MODEL\_V4 | 1.0 | 2.911e-18 | 451 | 0.187 | 458 | 269 | 22 | 243 | 682 | 12 | 384 | Phage major capsid protein | Phage major capsid protein | | afdb-uniprot50 | AF-A0A542J6Z7-F1-MODEL\_V4 | 1.0 | 6.714e-16 | 450 | 0.139 | 446 | 289 | 18 | 261 | 682 | 5 | 379 | HK97 family phage major capsid protein | HK97 family phage major capsid protein | | afdb-uniprot50 | AF-A0A1H2XPJ1-F1-MODEL\_V4 | 1.0 | 6.344e-16 | 450 | 0.138 | 367 | 280 | 14 | 338 | 682 | 29 | 381 | Phage major capsid protein, HK97 family | Phage major capsid protein, HK97 family | | afdb-uniprot50 | AF-A0A4Y8RT87-F1-MODEL\_V4 | 1.0 | 1.226e-16 | 450 | 0.177 | 411 | 271 | 19 | 309 | 682 | 1 | 381 | Phage major capsid protein | Phage major capsid protein | | afdb-uniprot50 | AF-A0A1A0S8B5-F1-MODEL\_V4 | 1.0 | 1.183e-15 | 450 | 0.14 | 457 | 297 | 18 | 251 | 682 | 3 | 388 | Uncharacterized protein | Uncharacterized protein | | afdb-uniprot50 | AF-A0A7W6CYU8-F1-MODEL\_V4 | 1.0 | 2.161e-16 | 450 | 0.13 | 466 | 303 | 21 | 264 | 682 | 6 | 416 | HK97 family phage major capsid protein | HK97 family phage major capsid protein | | afdb-uniprot50 | AF-A0A7X4F2D3-F1-MODEL\_V4 | 1.0 | 1.687e-17 | 450 | 0.147 | 460 | 302 | 21 | 264 | 681 | 7 | 418 | Phage major capsid protein | Phage major capsid protein | | afdb-uniprot50 | AF-A0A379E0G1-F1-MODEL\_V4 | 1.0 | 1.27e-17 | 449 | 0.193 | 428 | 243 | 19 | 295 | 681 | 7 | 373 | Predicted phage phi-C31 gp36 major capsid-like protein | Predicted phage phi-C31 gp36 major capsid-like protein | | afdb-uniprot50 | AF-A0A2G2L0U3-F1-MODEL\_V4 | 1.0 | 6.344e-16 | 449 | 0.141 | 425 | 294 | 14 | 272 | 681 | 3 | 371 | Phage major capsid protein | Phage major capsid protein | | afdb-uniprot50 | AF-A0A349YIG3-F1-MODEL\_V4 | 1.0 | 5.352e-16 | 449 | 0.128 | 444 | 298 | 20 | 259 | 681 | 6 | 381 | Phage major capsid protein | Phage major capsid protein | | afdb-uniprot50 | AF-A0A3D3Z4A9-F1-MODEL\_V4 | 1.0 | 5.746e-18 | 449 | 0.161 | 472 | 286 | 20 | 264 | 678 | 7 | 425 | Phage major capsid protein | Phage major capsid protein | | afdb-uniprot50 | AF-A0A2D8K5D9-F1-MODEL\_V4 | 1.0 | 2.241e-25 | 449 | 0.182 | 733 | 447 | 39 | 17 | 681 | 28 | 675 | Uncharacterized protein | Uncharacterized protein | | afdb-uniprot50 | AF-A0A4Y1N435-F1-MODEL\_V4 | 1.0 | 8.544e-18 | 448 | 0.177 | 497 | 264 | 24 | 261 | 680 | 3 | 431 | Uncharacterized protein | Uncharacterized protein | | afdb-uniprot50 | AF-A0A3N4SE98-F1-MODEL\_V4 | 1.0 | 3.192e-19 | 448 | 0.169 | 556 | 349 | 25 | 196 | 681 | 1 | 513 | HK97 family phage major capsid protein | HK97 family phage major capsid protein | | afdb-uniprot50 | AF-A0A2N3F2A1-F1-MODEL\_V4 | 1.0 | 4.295e-13 | 447 | 0.164 | 274 | 189 | 15 | 434 | 681 | 2 | 261 | Phage major capsid protein | Phage major capsid protein | | afdb-uniprot50 | AF-A0A6B9T8U5-F1-MODEL\_V4 | 1.0 | 1.76e-15 | 447 | 0.14 | 371 | 279 | 17 | 338 | 681 | 14 | 371 | Phage major capsid protein | Phage major capsid protein | | afdb-uniprot50 | AF-A0A0F7RMS0-F1-MODEL\_V4 | 1.0 | 6.956e-17 | 447 | 0.142 | 429 | 281 | 19 | 279 | 682 | 32 | 398 | Phage Capsid protein | Phage Capsid protein | | afdb-uniprot50 | AF-A0A1V5NDX4-F1-MODEL\_V4 | 1.0 | 1.095e-16 | 447 | 0.126 | 465 | 327 | 16 | 243 | 681 | 6 | 417 | Phage capsid family protein | Phage capsid family protein | | afdb-uniprot50 | AF-A0A845RNZ3-F1-MODEL\_V4 | 1.0 | 9.103e-15 | 446 | 0.15 | 358 | 227 | 16 | 344 | 682 | 5 | 304 | Phage major capsid protein | Phage major capsid protein | | afdb-uniprot50 | AF-A0A2M9M606-F1-MODEL\_V4 | 1.0 | 5.24e-17 | 446 | 0.163 | 433 | 278 | 22 | 279 | 682 | 3 | 380 | Phage major capsid protein | Phage major capsid protein | | afdb-uniprot50 | AF-A0A7W1RVR0-F1-MODEL\_V4 | 1.0 | 1.628e-16 | 446 | 0.137 | 458 | 292 | 19 | 258 | 681 | 21 | 409 | Phage major capsid protein | Phage major capsid protein | | afdb-uniprot50 | AF-A0A1A0PLW6-F1-MODEL\_V4 | 1.0 | 1.835e-13 | 445 | 0.171 | 285 | 180 | 12 | 431 | 681 | 30 | 292 | Uncharacterized protein | Uncharacterized protein | | afdb-uniprot50 | AF-A0A7V9S9R7-F1-MODEL\_V4 | 1.0 | 1.862e-15 | 445 | 0.17 | 357 | 265 | 13 | 346 | 677 | 17 | 367 | Phage major capsid protein | Phage major capsid protein | | afdb-uniprot50 | AF-B2KDP2-F1-MODEL\_V4 | 1.0 | 8.246e-17 | 445 | 0.162 | 455 | 275 | 19 | 274 | 681 | 5 | 400 | Phage major capsid protein | Phage major capsid protein | | afdb-uniprot50 | AF-A0A521ZM45-F1-MODEL\_V4 | 1.0 | 1.785e-17 | 445 | 0.145 | 487 | 310 | 23 | 215 | 682 | 1 | 400 | Phage major capsid protein | Phage major capsid protein | | afdb-uniprot50 | AF-A0A2E9VXI6-F1-MODEL\_V4 | 1.0 | 5.24e-17 | 445 | 0.161 | 482 | 283 | 28 | 254 | 682 | 5 | 418 | Phage major capsid protein | Phage major capsid protein | | afdb-uniprot50 | AF-A0A1Y0Y2T8-F1-MODEL\_V4 | 1.0 | 4.848e-18 | 445 | 0.163 | 489 | 298 | 21 | 262 | 680 | 5 | 452 | Uncharacterized protein | Uncharacterized protein | | afdb-uniprot50 | AF-A0A1X2EPP6-F1-MODEL\_V4 | 1.0 | 2.888e-13 | 444 | 0.16 | 261 | 194 | 11 | 433 | 681 | 32 | 279 | Major capsid protein | Major capsid protein | | afdb-uniprot50 | AF-A0A4Q3PQP0-F1-MODEL\_V4 | 1.0 | 4.266e-16 | 444 | 0.164 | 376 | 275 | 14 | 338 | 682 | 8 | 375 | Phage major capsid protein | Phage major capsid protein | | afdb-uniprot50 | AF-A0A1P8YNF5-F1-MODEL\_V4 | 1.0 | 1.298e-16 | 444 | 0.141 | 452 | 305 | 20 | 258 | 682 | 6 | 401 | Phage major capsid protein, HK97 family | Phage major capsid protein, HK97 family | | afdb-uniprot50 | AF-A0A7X0D7X9-F1-MODEL\_V4 | 1.0 | 2.711e-16 | 444 | 0.125 | 407 | 276 | 21 | 338 | 682 | 15 | 403 | HK97 family phage major capsid protein | HK97 family phage major capsid protein | | afdb-uniprot50 | AF-A0A0H4KS24-F1-MODEL\_V4 | 1.0 | 3.08e-18 | 444 | 0.194 | 468 | 276 | 26 | 258 | 681 | 5 | 415 | Phage major capsid protein, HK97 family | Phage major capsid protein, HK97 family | | afdb-uniprot50 | AF-A0A5J6G9W9-F1-MODEL\_V4 | 1.0 | 3.08e-18 | 444 | 0.146 | 477 | 282 | 22 | 262 | 678 | 9 | 420 | Phage major capsid protein | Phage major capsid protein | | afdb-uniprot50 | AF-A0A2U3L962-F1-MODEL\_V4 | 1.0 | 1.27e-17 | 444 | 0.141 | 481 | 315 | 23 | 253 | 682 | 3 | 436 | Putative Phage major capsid protein, HK97 family | Putative Phage major capsid protein, HK97 family | | afdb-uniprot50 | AF-A0A2E0J2G0-F1-MODEL\_V4 | 1.0 | 3.45e-18 | 444 | 0.198 | 468 | 294 | 21 | 256 | 681 | 4 | 432 | Phage major capsid protein | Phage major capsid protein | | afdb-uniprot50 | AF-A0A840ZNF0-F1-MODEL\_V4 | 1.0 | 8.544e-18 | 444 | 0.153 | 521 | 334 | 26 | 195 | 682 | 2 | 448 | HK97 family phage major capsid protein | HK97 family phage major capsid protein | | afdb-uniprot50 | AF-A0A7X7T206-F1-MODEL\_V4 | 1.0 | 3.26e-18 | 444 | 0.144 | 492 | 303 | 22 | 251 | 681 | 5 | 439 | Phage major capsid protein | Phage major capsid protein | | afdb-uniprot50 | AF-A0A100WUZ1-F1-MODEL\_V4 | 1.0 | 2.508e-17 | 444 | 0.164 | 492 | 294 | 23 | 258 | 678 | 8 | 453 | Phage-related major capsid protein | Phage-related major capsid protein | | afdb-uniprot50 | AF-A0A5F2I8T1-F1-MODEL\_V4 | 1.0 | 4.683e-25 | 444 | 0.147 | 698 | 456 | 35 | 40 | 681 | 50 | 664 | Mu-like\_gpT domain-containing protein | Mu-like\_gpT domain-containing protein | | afdb-uniprot50 | AF-A0A177P7V0-F1-MODEL\_V4 | 1.0 | 3.527e-25 | 444 | 0.148 | 748 | 463 | 33 | 16 | 681 | 18 | 673 | Uncharacterized protein | Uncharacterized protein | | afdb-uniprot50 | AF-A0A2U1BFB6-F1-MODEL\_V4 | 1.0 | 1.971e-15 | 443 | 0.161 | 340 | 226 | 16 | 375 | 682 | 20 | 332 | HK97 family phage major capsid protein | HK97 family phage major capsid protein | | afdb-uniprot50 | AF-F5RN15-F1-MODEL\_V4 | 1.0 | 2.508e-17 | 443 | 0.159 | 469 | 277 | 24 | 252 | 682 | 2 | 391 | HK97 family major capsid protein | HK97 family major capsid protein | | afdb-uniprot50 | AF-A0A317LFV3-F1-MODEL\_V4 | 1.0 | 2.287e-16 | 443 | 0.157 | 450 | 309 | 20 | 253 | 682 | 2 | 401 | Phage major capsid protein | Phage major capsid protein | | afdb-uniprot50 | AF-A0A4R7IC04-F1-MODEL\_V4 | 1.0 | 2.116e-17 | 443 | 0.155 | 501 | 298 | 20 | 252 | 681 | 10 | 456 | HK97 family phage major capsid protein | HK97 family phage major capsid protein | | afdb-uniprot50 | AF-A0A7C4QW79-F1-MODEL\_V4 | 1.0 | 1.7e-22 | 443 | 0.146 | 702 | 438 | 32 | 16 | 681 | 77 | 652 | Phage major capsid protein | Phage major capsid protein | | afdb-uniprot50 | AF-A0A430EDT7-F1-MODEL\_V4 | 1.0 | 5.551e-25 | 443 | 0.165 | 750 | 434 | 37 | 17 | 681 | 78 | 720 | Mu-like\_gpT domain-containing protein | Mu-like\_gpT domain-containing protein | | afdb-uniprot50 | AF-A0A229VZ15-F1-MODEL\_V4 | 1.0 | 1.639e-13 | 442 | 0.131 | 282 | 208 | 10 | 425 | 682 | 11 | 279 | Major capsid protein | Major capsid protein | | afdb-uniprot50 | AF-A0A542HV74-F1-MODEL\_V4 | 1.0 | 1.823e-16 | 442 | 0.132 | 446 | 270 | 18 | 279 | 682 | 36 | 406 | HK97 family phage major capsid protein | HK97 family phage major capsid protein | | afdb-uniprot50 | AF-A0A2K8LT81-F1-MODEL\_V4 | 1.0 | 5.583e-14 | 440 | 0.182 | 280 | 189 | 9 | 431 | 681 | 26 | 294 | Phage major capsid protein | Phage major capsid protein | | afdb-uniprot50 | AF-A0A5C8L259-F1-MODEL\_V4 | 1.0 | 1.538e-16 | 440 | 0.149 | 447 | 283 | 22 | 268 | 682 | 4 | 385 | Phage major capsid protein | Phage major capsid protein | | afdb-uniprot50 | AF-A0A5J5J5J3-F1-MODEL\_V4 | 1.0 | 5.545e-17 | 440 | 0.165 | 471 | 284 | 20 | 257 | 682 | 5 | 411 | Phage major capsid protein | Phage major capsid protein | | afdb-uniprot50 | AF-A0A3D8NG00-F1-MODEL\_V4 | 1.0 | 1.785e-17 | 440 | 0.155 | 451 | 298 | 21 | 267 | 681 | 11 | 414 | Phage major capsid protein | Phage major capsid protein | | afdb-uniprot50 | AF-G6XIV7-F1-MODEL\_V4 | 1.0 | 1.345e-17 | 440 | 0.139 | 502 | 304 | 22 | 261 | 681 | 8 | 462 | Uncharacterized protein | Uncharacterized protein | | afdb-uniprot50 | AF-A0A6L9G9S4-F1-MODEL\_V4 | 1.0 | 4.299e-21 | 440 | 0.11 | 671 | 397 | 29 | 46 | 682 | 3 | 507 | Uncharacterized protein | Uncharacterized protein | | afdb-uniprot50 | AF-A0A653VEB3-F1-MODEL\_V4 | 1.0 | 4.266e-16 | 439 | 0.169 | 442 | 270 | 18 | 265 | 682 | 7 | 375 | Phage capsid family protein | Phage capsid family protein | | afdb-uniprot50 | AF-A0A355J1L3-F1-MODEL\_V4 | 1.0 | 1.785e-17 | 439 | 0.142 | 456 | 295 | 24 | 258 | 680 | 8 | 400 | Uncharacterized protein | Uncharacterized protein | | afdb-uniprot50 | AF-A0A6N9BAL6-F1-MODEL\_V4 | 1.0 | 1.594e-17 | 439 | 0.174 | 458 | 282 | 21 | 258 | 680 | 5 | 401 | Uncharacterized protein | Uncharacterized protein | | afdb-uniprot50 | AF-A0A1I6GEC2-F1-MODEL\_V4 | 1.0 | 7.791e-17 | 439 | 0.146 | 479 | 303 | 22 | 243 | 682 | 26 | 437 | Phage major capsid protein, HK97 family | Phage major capsid protein, HK97 family | | afdb-uniprot50 | AF-A0A7W6FWA4-F1-MODEL\_V4 | 1.0 | 3.146e-17 | 439 | 0.158 | 461 | 299 | 22 | 249 | 682 | 75 | 473 | HK97 family phage major capsid protein | HK97 family phage major capsid protein | | afdb-uniprot50 | AF-A0A2Z5ZI90-F1-MODEL\_V4 | 1.0 | 7.904e-19 | 439 | 0.143 | 501 | 308 | 21 | 252 | 681 | 7 | 457 | Major capsid protein | Major capsid protein | | afdb-uniprot50 | AF-A0A7T5K4D8-F1-MODEL\_V4 | 1.0 | 2.639e-28 | 439 | 0.197 | 739 | 404 | 39 | 38 | 681 | 11 | 655 | Mu-like prophage major head subunit gpT family protein | Mu-like prophage major head subunit gpT family protein | | afdb-uniprot50 | AF-A0A5N8X7L1-F1-MODEL\_V4 | 1.0 | 3.477e-23 | 439 | 0.14 | 719 | 435 | 41 | 12 | 682 | 318 | 901 | Phage portal protein | Phage portal protein | | afdb-uniprot50 | AF-A0A2U3NJA2-F1-MODEL\_V4 | 1.0 | 6.759e-13 | 438 | 0.198 | 237 | 166 | 8 | 453 | 682 | 2 | 221 | Capsid protein | Capsid protein | | afdb-uniprot50 | AF-A0A416AP22-F1-MODEL\_V4 | 1.0 | 1.102e-13 | 438 | 0.152 | 268 | 193 | 15 | 431 | 681 | 54 | 304 | Phage major capsid protein | Phage major capsid protein | | afdb-uniprot50 | AF-A0A6N0LFZ4-F1-MODEL\_V4 | 1.0 | 2.336e-15 | 438 | 0.15 | 372 | 270 | 12 | 345 | 682 | 7 | 366 | Phage major capsid protein | Phage major capsid protein | | afdb-uniprot50 | AF-A0A2C9WSZ8-F1-MODEL\_V4 | 1.0 | 5.057e-16 | 438 | 0.132 | 452 | 298 | 20 | 252 | 682 | 13 | 391 | Phage major capsid protein | Phage major capsid protein | | afdb-uniprot50 | AF-M4NDD1-F1-MODEL\_V4 | 1.0 | 3.213e-16 | 438 | 0.151 | 415 | 293 | 16 | 306 | 679 | 7 | 403 | Phage capsid family protein | Phage capsid family protein | | afdb-uniprot50 | AF-A0A149TLS8-F1-MODEL\_V4 | 1.0 | 3.651e-18 | 438 | 0.17 | 480 | 304 | 20 | 264 | 682 | 2 | 448 | Uncharacterized protein | Uncharacterized protein | | afdb-uniprot50 | AF-A0A737BVU5-F1-MODEL\_V4 | 1.0 | 1.875e-12 | 437 | 0.136 | 256 | 198 | 10 | 436 | 679 | 2 | 246 | Phage major capsid protein | Phage major capsid protein | | afdb-uniprot50 | AF-A0A2A9ENW6-F1-MODEL\_V4 | 1.0 | 5.352e-16 | 437 | 0.149 | 369 | 280 | 14 | 338 | 681 | 9 | 368 | HK97 family phage major capsid protein | HK97 family phage major capsid protein | | afdb-uniprot50 | AF-A0A1V5ID83-F1-MODEL\_V4 | 1.0 | 6.714e-16 | 437 | 0.161 | 409 | 254 | 15 | 295 | 681 | 29 | 370 | Phage capsid family protein | Phage capsid family protein | | afdb-uniprot50 | AF-A0A7G6Z6C8-F1-MODEL\_V4 | 1.0 | 1.226e-16 | 437 | 0.142 | 448 | 278 | 20 | 264 | 677 | 3 | 378 | Phage major capsid protein | Phage major capsid protein | | afdb-uniprot50 | AF-E1QM51-F1-MODEL\_V4 | 1.0 | 1.929e-16 | 437 | 0.17 | 423 | 255 | 17 | 335 | 681 | 1 | 403 | Major capsid protein HK97 | Major capsid protein HK97 | | afdb-uniprot50 | AF-A0A1Q6K3Y8-F1-MODEL\_V4 | 1.0 | 5.869e-17 | 437 | 0.149 | 449 | 294 | 22 | 251 | 682 | 27 | 404 | Uncharacterized protein | Uncharacterized protein | | afdb-uniprot50 | AF-A0A7Z1AWA5-F1-MODEL\_V4 | 1.0 | 7.965e-24 | 437 | 0.136 | 709 | 424 | 37 | 39 | 679 | 426 | 1013 | Uncharacterized protein | Uncharacterized protein | | afdb-uniprot50 | AF-A0A1M5PQD6-F1-MODEL\_V4 | 1.0 | 7.519e-16 | 436 | 0.143 | 459 | 277 | 20 | 244 | 682 | 12 | 374 | Phage major capsid protein, HK97 family | Phage major capsid protein, HK97 family | | afdb-uniprot50 | AF-A0A1C6UT23-F1-MODEL\_V4 | 1.0 | 7.519e-16 | 436 | 0.12 | 425 | 290 | 14 | 281 | 681 | 15 | 379 | Phage major capsid protein, HK97 family | Phage major capsid protein, HK97 family | | afdb-uniprot50 | AF-A0A7W3Y5V0-F1-MODEL\_V4 | 1.0 | 3.809e-16 | 436 | 0.138 | 418 | 301 | 18 | 284 | 679 | 52 | 432 | Phage major capsid protein | Phage major capsid protein | | afdb-uniprot50 | AF-A0A2M9MMU3-F1-MODEL\_V4 | 1.0 | 2.088e-23 | 436 | 0.13 | 721 | 389 | 30 | 26 | 680 | 2 | 550 | Uncharacterized protein | Uncharacterized protein | | afdb-uniprot50 | AF-A0A2W4ZXB8-F1-MODEL\_V4 | 1.0 | 3.216e-24 | 436 | 0.157 | 717 | 393 | 35 | 40 | 677 | 1 | 585 | Uncharacterized protein | Uncharacterized protein | | afdb-uniprot50 | AF-A0A4V2BF65-F1-MODEL\_V4 | 1.0 | 2.074e-26 | 436 | 0.141 | 728 | 487 | 44 | 17 | 679 | 3 | 657 | Uncharacterized protein | Uncharacterized protein | | afdb-uniprot50 | AF-A0A1Y3TL93-F1-MODEL\_V4 | 1.0 | 3.497e-12 | 435 | 0.383 | 159 | 88 | 5 | 38 | 190 | 9 | 163 | Caudovirus prohead protease | Caudovirus prohead protease | | afdb-uniprot50 | AF-A0A1B1LIJ3-F1-MODEL\_V4 | 1.0 | 1.639e-13 | 435 | 0.172 | 267 | 193 | 13 | 431 | 681 | 17 | 271 | Major capsid protein b | Major capsid protein b | | afdb-uniprot50 | AF-A0A7W8J549-F1-MODEL\_V4 | 1.0 | 4.611e-15 | 435 | 0.124 | 353 | 276 | 15 | 349 | 681 | 34 | 373 | HK97 family phage major capsid protein | HK97 family phage major capsid protein | | afdb-uniprot50 | AF-A0A497AYE2-F1-MODEL\_V4 | 1.0 | 5.096e-21 | 435 | 0.158 | 708 | 390 | 43 | 19 | 681 | 2 | 548 | Phage major capsid protein | Phage major capsid protein | | afdb-uniprot50 | AF-A0A0C1KQP5-F1-MODEL\_V4 | 1.0 | 1.862e-15 | 434 | 0.156 | 370 | 270 | 14 | 345 | 682 | 4 | 363 | Uncharacterized protein | Uncharacterized protein | | afdb-uniprot50 | AF-U7UE74-F1-MODEL\_V4 | 1.0 | 4.177e-17 | 434 | 0.144 | 465 | 284 | 20 | 265 | 682 | 4 | 401 | Phage major capsid protein, HK97 family | Phage major capsid protein, HK97 family | | afdb-uniprot50 | AF-A0A164R867-F1-MODEL\_V4 | 1.0 | 1.76e-15 | 434 | 0.156 | 447 | 287 | 18 | 261 | 677 | 9 | 395 | Uncharacterized protein | Uncharacterized protein | | afdb-uniprot50 | AF-A0A5C6UM44-F1-MODEL\_V4 | 1.0 | 3.401e-16 | 434 | 0.117 | 424 | 306 | 15 | 304 | 679 | 42 | 445 | Phage major capsid protein | Phage major capsid protein | | afdb-uniprot50 | AF-A0A6M3ZTZ3-F1-MODEL\_V4 | 1.0 | 2.711e-16 | 433 | 0.146 | 423 | 290 | 17 | 285 | 682 | 53 | 429 | Phage major capsid protein | Phage major capsid protein | | afdb-uniprot50 | AF-A0A5C7M8X5-F1-MODEL\_V4 | 1.0 | 6.386e-13 | 432 | 0.167 | 263 | 194 | 12 | 431 | 681 | 29 | 278 | Phage major capsid protein | Phage major capsid protein | | afdb-uniprot50 | AF-A0A3B0IS29-F1-MODEL\_V4 | 1.0 | 2.056e-13 | 432 | 0.136 | 308 | 231 | 14 | 392 | 677 | 43 | 337 | Uncharacterized protein | Uncharacterized protein | | afdb-uniprot50 | AF-A0A359AJM0-F1-MODEL\_V4 | 1.0 | 8.246e-17 | 432 | 0.173 | 491 | 283 | 26 | 217 | 680 | 1 | 395 | Phage major capsid protein | Phage major capsid protein | | afdb-uniprot50 | AF-A0A5C7QAV4-F1-MODEL\_V4 | 1.0 | 1.929e-16 | 432 | 0.123 | 477 | 309 | 24 | 249 | 679 | 10 | 423 | Phage major capsid protein | Phage major capsid protein | | afdb-uniprot50 | AF-A0A7C3X2B0-F1-MODEL\_V4 | 1.0 | 1.723e-16 | 432 | 0.131 | 470 | 317 | 16 | 246 | 680 | 18 | 431 | Phage major capsid protein | Phage major capsid protein | | afdb-uniprot50 | AF-A0A2A4EDF3-F1-MODEL\_V4 | 1.0 | 1.453e-16 | 431 | 0.146 | 457 | 279 | 20 | 271 | 682 | 3 | 393 | Uncharacterized protein | Uncharacterized protein | | afdb-uniprot50 | AF-A0A3D3LX94-F1-MODEL\_V4 | 1.0 | 2.161e-16 | 431 | 0.163 | 433 | 268 | 20 | 305 | 681 | 4 | 398 | Phage major capsid protein | Phage major capsid protein | | afdb-uniprot50 | AF-A0A239A9U8-F1-MODEL\_V4 | 1.0 | 1.298e-16 | 431 | 0.143 | 461 | 294 | 19 | 250 | 679 | 5 | 395 | Phage major capsid protein, HK97 family | Phage major capsid protein, HK97 family | | afdb-uniprot50 | AF-A0A4R8JD56-F1-MODEL\_V4 | 1.0 | 2.42e-16 | 431 | 0.141 | 458 | 307 | 16 | 243 | 682 | 40 | 429 | HK97 family phage major capsid protein | HK97 family phage major capsid protein | | afdb-uniprot50 | AF-A0A2G6IFW5-F1-MODEL\_V4 | 1.0 | 7.362e-17 | 431 | 0.161 | 488 | 297 | 22 | 251 | 681 | 5 | 437 | Phage major capsid protein | Phage major capsid protein | | afdb-uniprot50 | AF-A0A1G3WMU9-F1-MODEL\_V4 | 1.0 | 2.086e-15 | 430 | 0.132 | 391 | 275 | 14 | 335 | 680 | 1 | 372 | Uncharacterized protein | Uncharacterized protein | | afdb-uniprot50 | AF-A0A3P6KYD8-F1-MODEL\_V4 | 1.0 | 7.687e-23 | 430 | 0.207 | 540 | 295 | 23 | 19 | 519 | 1 | 446 | Mu-like prophage major head subunit gpT | Mu-like prophage major head subunit gpT | | afdb-uniprot50 | AF-S5DTM3-F1-MODEL\_V4 | 1.0 | 6.344e-16 | 429 | 0.146 | 450 | 267 | 19 | 271 | 680 | 3 | 375 | Phage major capsid protein | Phage major capsid protein | | afdb-uniprot50 | AF-B9XDE7-F1-MODEL\_V4 | 1.0 | 4.031e-16 | 429 | 0.16 | 455 | 295 | 22 | 252 | 681 | 11 | 403 | Major capsid protein HK97 | Major capsid protein HK97 | | afdb-uniprot50 | AF-A0A0F9W0Z4-F1-MODEL\_V4 | 1.0 | 1.594e-17 | 429 | 0.173 | 466 | 296 | 26 | 252 | 681 | 2 | 414 | Uncharacterized protein | Uncharacterized protein | | afdb-uniprot50 | AF-A0A6P1YHM1-F1-MODEL\_V4 | 1.0 | 4.678e-17 | 429 | 0.137 | 503 | 330 | 23 | 204 | 679 | 1 | 426 | Phage major capsid protein | Phage major capsid protein | | afdb-uniprot50 | AF-A0A522ASE0-F1-MODEL\_V4 | 1.0 | 6.811e-18 | 429 | 0.14 | 477 | 307 | 21 | 237 | 680 | 37 | 443 | Phage major capsid protein | Phage major capsid protein | | afdb-uniprot50 | AF-A0A4R5TM46-F1-MODEL\_V4 | 1.0 | 7.958e-16 | 428 | 0.142 | 435 | 302 | 14 | 252 | 679 | 15 | 385 | Phage major capsid protein | Phage major capsid protein | | afdb-uniprot50 | AF-A0A516NU19-F1-MODEL\_V4 | 1.0 | 5.057e-16 | 428 | 0.142 | 450 | 293 | 20 | 261 | 681 | 11 | 396 | Phage major capsid protein | Phage major capsid protein | | afdb-uniprot50 | AF-A0A2A3ETC9-F1-MODEL\_V4 | 1.0 | 1.095e-16 | 428 | 0.153 | 468 | 284 | 17 | 263 | 678 | 21 | 428 | Phage major capsid protein | Phage major capsid protein | | afdb-uniprot50 | AF-R5UUT8-F1-MODEL\_V4 | 1.0 | 9.983e-16 | 428 | 0.144 | 380 | 266 | 16 | 349 | 679 | 66 | 435 | Uncharacterized protein | Uncharacterized protein | | afdb-uniprot50 | AF-A0A2D6F059-F1-MODEL\_V4 | 1.0 | 3.146e-17 | 428 | 0.152 | 480 | 286 | 20 | 282 | 678 | 3 | 444 | Phage major capsid protein | Phage major capsid protein | | afdb-uniprot50 | AF-A0A4Q5NL72-F1-MODEL\_V4 | 1.0 | 3.524e-17 | 428 | 0.15 | 498 | 311 | 27 | 249 | 682 | 3 | 452 | Phage major capsid protein | Phage major capsid protein | | afdb-uniprot50 | AF-A0A7X7FKI2-F1-MODEL\_V4 | 1.0 | 4.177e-17 | 428 | 0.166 | 487 | 324 | 20 | 243 | 681 | 19 | 471 | Phage major capsid protein | Phage major capsid protein | | afdb-uniprot50 | AF-A0A286DYE7-F1-MODEL\_V4 | 1.0 | 1.785e-17 | 428 | 0.138 | 498 | 310 | 25 | 268 | 677 | 9 | 475 | Phage major capsid protein, HK97 family | Phage major capsid protein, HK97 family | | afdb-uniprot50 | AF-A0A6L9HU12-F1-MODEL\_V4 | 1.0 | 1.787e-25 | 428 | 0.161 | 815 | 430 | 37 | 16 | 682 | 5 | 713 | Uncharacterized protein | Uncharacterized protein | | afdb-uniprot50 | AF-A0A5S3XG18-F1-MODEL\_V4 | 1.0 | 1.423e-17 | 427 | 0.173 | 473 | 286 | 23 | 261 | 681 | 7 | 426 | Phage major capsid protein | Phage major capsid protein | | afdb-uniprot50 | AF-A0A429P0Y7-F1-MODEL\_V4 | 1.0 | 6.436e-18 | 427 | 0.14 | 550 | 356 | 23 | 178 | 682 | 3 | 480 | Phage major capsid protein | Phage major capsid protein | | afdb-uniprot50 | AF-A0A433J4X0-F1-MODEL\_V4 | 1.0 | 1.506e-17 | 427 | 0.169 | 455 | 275 | 22 | 279 | 682 | 97 | 499 | Phage major capsid protein | Phage major capsid protein | | afdb-uniprot50 | AF-A0A7Y3T6V6-F1-MODEL\_V4 | 1.0 | 3.039e-24 | 427 | 0.155 | 790 | 428 | 43 | 40 | 681 | 6 | 703 | Uncharacterized protein | Uncharacterized protein | | afdb-uniprot50 | AF-A0A1H0SZG1-F1-MODEL\_V4 | 1.0 | 3.676e-15 | 426 | 0.145 | 448 | 280 | 22 | 253 | 682 | 2 | 364 | Phage major capsid protein, HK97 family | Phage major capsid protein, HK97 family | | afdb-uniprot50 | AF-A0A316QDD0-F1-MODEL\_V4 | 1.0 | 4.117e-15 | 426 | 0.144 | 374 | 276 | 13 | 338 | 680 | 14 | 374 | Phage major capsid protein | Phage major capsid protein | | afdb-uniprot50 | AF-A0A3M1Y489-F1-MODEL\_V4 | 1.0 | 9.433e-16 | 426 | 0.154 | 452 | 306 | 16 | 251 | 679 | 3 | 401 | Phage major capsid protein | Phage major capsid protein | | afdb-uniprot50 | AF-A0A7C2D4S3-F1-MODEL\_V4 | 1.0 | 4.515e-16 | 426 | 0.143 | 425 | 298 | 21 | 294 | 682 | 6 | 400 | Phage major capsid protein | Phage major capsid protein | | afdb-uniprot50 | AF-A0A6N6T4Y3-F1-MODEL\_V4 | 1.0 | 3.809e-16 | 426 | 0.13 | 483 | 297 | 21 | 244 | 681 | 5 | 409 | Phage major capsid protein | Phage major capsid protein | | afdb-uniprot50 | AF-A0A223I0W6-F1-MODEL\_V4 | 1.0 | 9.57e-18 | 426 | 0.163 | 459 | 291 | 21 | 252 | 682 | 6 | 399 | Phage major capsid protein, HK97 family | Phage major capsid protein, HK97 family | | afdb-uniprot50 | AF-A0A5N0UV25-F1-MODEL\_V4 | 1.0 | 1.041e-13 | 425 | 0.143 | 278 | 202 | 12 | 431 | 682 | 27 | 294 | Phage major capsid protein | Phage major capsid protein | | afdb-uniprot50 | AF-A0A1Y2QCN7-F1-MODEL\_V4 | 1.0 | 1.862e-15 | 425 | 0.172 | 376 | 262 | 15 | 343 | 679 | 5 | 370 | Phage major capsid protein | Phage major capsid protein | | afdb-uniprot50 | AF-A0A7C0VZ44-F1-MODEL\_V4 | 1.0 | 3.599e-16 | 425 | 0.154 | 435 | 273 | 18 | 295 | 681 | 9 | 396 | Phage major capsid protein | Phage major capsid protein | | afdb-uniprot50 | AF-A0A5C5XQ98-F1-MODEL\_V4 | 1.0 | 3.213e-16 | 425 | 0.153 | 464 | 271 | 23 | 261 | 682 | 10 | 393 | Phage capsid family protein | Phage capsid family protein | | afdb-uniprot50 | AF-A0A5M6HTD8-F1-MODEL\_V4 | 1.0 | 3.602e-24 | 425 | 0.149 | 734 | 449 | 36 | 19 | 681 | 3 | 631 | Peptidase U35 | Peptidase U35 | | afdb-uniprot50 | AF-A0A4Q7ZQ54-F1-MODEL\_V4 | 1.0 | 8.727e-17 | 424 | 0.155 | 476 | 270 | 25 | 264 | 682 | 8 | 408 | HK97 family phage major capsid protein | HK97 family phage major capsid protein | | afdb-uniprot50 | AF-A0A1G7YG34-F1-MODEL\_V4 | 1.0 | 1.2e-17 | 424 | 0.176 | 505 | 307 | 23 | 252 | 682 | 8 | 477 | Phage major capsid protein, HK97 family | Phage major capsid protein, HK97 family | | afdb-uniprot50 | AF-A0A0G1VYU8-F1-MODEL\_V4 | 1.0 | 7.257e-15 | 423 | 0.118 | 381 | 273 | 16 | 335 | 682 | 56 | 406 | Uncharacterized protein | Uncharacterized protein | | afdb-uniprot50 | AF-A0A127R0F9-F1-MODEL\_V4 | 1.0 | 8.727e-17 | 423 | 0.152 | 510 | 333 | 20 | 193 | 679 | 22 | 455 | Phage major capsid protein, HK97 family | Phage major capsid protein, HK97 family | | afdb-uniprot50 | AF-A0A0Q6RSL7-F1-MODEL\_V4 | 1.0 | 2.088e-23 | 423 | 0.17 | 763 | 424 | 40 | 1 | 681 | 1 | 636 | Uncharacterized protein | Uncharacterized protein | | afdb-uniprot50 | AF-A0A4Q6IAB5-F1-MODEL\_V4 | 1.0 | 2.352e-12 | 422 | 0.15 | 273 | 192 | 13 | 431 | 682 | 129 | 382 | Phage major capsid protein | Phage major capsid protein | | afdb-uniprot50 | AF-V8CV52-F1-MODEL\_V4 | 1.0 | 2.769e-15 | 422 | 0.115 | 460 | 308 | 16 | 267 | 681 | 2 | 407 | Head protein | Head protein | | afdb-uniprot50 | AF-A0A6M3JT93-F1-MODEL\_V4 | 1.0 | 5.352e-16 | 422 | 0.145 | 482 | 282 | 25 | 258 | 680 | 8 | 418 | Putative capsid protein | Putative capsid protein | | afdb-uniprot50 | AF-A0A6G6Z5J1-F1-MODEL\_V4 | 1.0 | 9.496e-13 | 421 | 0.156 | 287 | 194 | 10 | 433 | 680 | 90 | 367 | Phage major capsid protein | Phage major capsid protein | | afdb-uniprot50 | AF-A0A0S8JG41-F1-MODEL\_V4 | 1.0 | 1.183e-15 | 421 | 0.155 | 458 | 295 | 22 | 252 | 681 | 16 | 409 | Uncharacterized protein | Uncharacterized protein | | afdb-uniprot50 | AF-A0A812Y0T0-F1-MODEL\_V4 | 1.0 | 6.811e-18 | 421 | 0.181 | 479 | 293 | 22 | 246 | 682 | 8 | 429 | Hypothetical protein | Hypothetical protein | | afdb-uniprot50 | AF-A0A0L8EZU3-F1-MODEL\_V4 | 1.0 | 4.357e-15 | 420 | 0.128 | 443 | 297 | 20 | 264 | 682 | 6 | 383 | Uncharacterized protein | Uncharacterized protein | | afdb-uniprot50 | AF-C2PQK7-F1-MODEL\_V4 | 1.0 | 1.354e-14 | 420 | 0.133 | 404 | 274 | 14 | 335 | 682 | 1 | 384 | Phage major capsid protein | Phage major capsid protein | | afdb-uniprot50 | AF-A0A1I0J9W7-F1-MODEL\_V4 | 1.0 | 3.477e-23 | 420 | 0.17 | 716 | 411 | 38 | 40 | 682 | 24 | 629 | Prohead serine protease | Prohead serine protease | | afdb-uniprot50 | AF-A0A0F9MYG6-F1-MODEL\_V4 | 1.0 | 5.24e-17 | 419 | 0.163 | 446 | 262 | 22 | 284 | 681 | 19 | 401 | Uncharacterized protein | Uncharacterized protein | | afdb-uniprot50 | AF-A0A419HBW7-F1-MODEL\_V4 | 1.0 | 6.573e-17 | 419 | 0.164 | 505 | 313 | 25 | 250 | 681 | 2 | 470 | Phage major capsid protein | Phage major capsid protein | | afdb-uniprot50 | AF-A0A840CSE2-F1-MODEL\_V4 | 1.0 | 3.524e-17 | 419 | 0.13 | 460 | 300 | 18 | 249 | 679 | 97 | 485 | HK97 family phage major capsid protein | HK97 family phage major capsid protein | | afdb-uniprot50 | AF-A0A654D1K5-F1-MODEL\_V4 | 1.0 | 5.664e-16 | 418 | 0.159 | 445 | 306 | 17 | 250 | 679 | 14 | 405 | Putative Phage major capsid protein, HK97 family | Putative Phage major capsid protein, HK97 family | | afdb-uniprot50 | AF-A0A810K9P5-F1-MODEL\_V4 | 1.0 | 3.401e-16 | 418 | 0.16 | 435 | 281 | 20 | 294 | 678 | 19 | 419 | Uncharacterized protein | Uncharacterized protein | | afdb-uniprot50 | AF-A0A437LLE3-F1-MODEL\_V4 | 1.0 | 2.207e-15 | 417 | 0.14 | 450 | 297 | 19 | 251 | 679 | 3 | 383 | Phage major capsid protein | Phage major capsid protein | | afdb-uniprot50 | AF-A0A2E7HPZ8-F1-MODEL\_V4 | 1.0 | 1.298e-16 | 417 | 0.147 | 455 | 285 | 20 | 279 | 679 | 18 | 423 | Phage major capsid protein | Phage major capsid protein | | afdb-uniprot50 | AF-A0A2E0X6G4-F1-MODEL\_V4 | 1.0 | 7.791e-17 | 417 | 0.134 | 470 | 304 | 23 | 280 | 680 | 26 | 461 | Phage major capsid protein | Phage major capsid protein | | afdb-uniprot50 | AF-A0A1X1W1I0-F1-MODEL\_V4 | 1.0 | 1.875e-12 | 416 | 0.151 | 264 | 199 | 9 | 431 | 682 | 29 | 279 | Major capsid protein | Major capsid protein | | afdb-uniprot50 | AF-A0A5X1QJ83-F1-MODEL\_V4 | 1.0 | 1.76e-15 | 416 | 0.152 | 445 | 296 | 17 | 265 | 682 | 9 | 399 | Phage major capsid protein | Phage major capsid protein | | afdb-uniprot50 | AF-A0A7X4F2B2-F1-MODEL\_V4 | 1.0 | 4.678e-17 | 416 | 0.149 | 481 | 289 | 24 | 264 | 682 | 5 | 427 | Phage major capsid protein | Phage major capsid protein | | afdb-uniprot50 | AF-A0A519P9Z1-F1-MODEL\_V4 | 1.0 | 7.791e-17 | 416 | 0.148 | 437 | 299 | 18 | 306 | 679 | 4 | 430 | Phage major capsid protein | Phage major capsid protein | | afdb-uniprot50 | AF-A0A431MZP7-F1-MODEL\_V4 | 1.0 | 2.161e-16 | 415 | 0.156 | 461 | 295 | 20 | 249 | 682 | 2 | 395 | Phage major capsid protein | Phage major capsid protein | | afdb-uniprot50 | AF-J2G792-F1-MODEL\_V4 | 1.0 | 2.42e-16 | 415 | 0.144 | 513 | 323 | 27 | 198 | 679 | 2 | 429 | Phage major capsid protein, HK97 family | Phage major capsid protein, HK97 family | | afdb-uniprot50 | AF-A0A239JPQ6-F1-MODEL\_V4 | 1.0 | 9.042e-18 | 415 | 0.201 | 481 | 323 | 17 | 182 | 648 | 3 | 436 | Phage major capsid protein, HK97 family | Phage major capsid protein, HK97 family | | afdb-uniprot50 | AF-A0A554S931-F1-MODEL\_V4 | 1.0 | 8.422e-16 | 414 | 0.141 | 459 | 297 | 18 | 252 | 682 | 35 | 424 | Phage major capsid protein | Phage major capsid protein | | afdb-uniprot50 | AF-A0A0F9WTB8-F1-MODEL\_V4 | 1.0 | 4.678e-17 | 414 | 0.156 | 485 | 314 | 24 | 256 | 682 | 2 | 449 | Uncharacterized protein | Uncharacterized protein | | afdb-uniprot50 | AF-A0A353J4Q1-F1-MODEL\_V4 | 1.0 | 7.628e-18 | 414 | 0.146 | 465 | 295 | 19 | 252 | 680 | 19 | 417 | Phage major capsid protein | Phage major capsid protein | | afdb-uniprot50 | AF-A0A7V7XCR5-F1-MODEL\_V4 | 1.0 | 1.298e-16 | 413 | 0.146 | 457 | 295 | 20 | 285 | 682 | 20 | 440 | Phage major capsid protein | Phage major capsid protein | | afdb-uniprot50 | AF-A0A0F9MYU0-F1-MODEL\_V4 | 1.0 | 1.902e-14 | 412 | 0.137 | 343 | 248 | 13 | 375 | 682 | 16 | 345 | Uncharacterized protein | Uncharacterized protein | | afdb-uniprot50 | AF-C7N4P4-F1-MODEL\_V4 | 1.0 | 3.036e-16 | 411 | 0.169 | 460 | 277 | 24 | 262 | 682 | 9 | 402 | Phage major capsid protein, HK97 family | Phage major capsid protein, HK97 family | | afdb-uniprot50 | AF-A0A0F8Z7I4-F1-MODEL\_V4 | 1.0 | 6.759e-13 | 410 | 0.166 | 301 | 209 | 15 | 412 | 680 | 1 | 291 | Uncharacterized protein | Uncharacterized protein | | afdb-uniprot50 | AF-A0A1C4B7A3-F1-MODEL\_V4 | 1.0 | 2.93e-15 | 410 | 0.155 | 456 | 278 | 22 | 258 | 682 | 4 | 383 | Phage major capsid protein, HK97 family | Phage major capsid protein, HK97 family | | afdb-uniprot50 | AF-A0A1V5PUE1-F1-MODEL\_V4 | 1.0 | 5.466e-15 | 410 | 0.155 | 374 | 235 | 14 | 379 | 682 | 21 | 383 | Phage capsid family protein | Phage capsid family protein | | afdb-uniprot50 | AF-A0A644VRL6-F1-MODEL\_V4 | 1.0 | 7.57e-13 | 410 | 0.19 | 278 | 184 | 10 | 431 | 681 | 33 | 296 | S-l\_SbsC\_C domain-containing protein | S-l\_SbsC\_C domain-containing protein | | afdb-uniprot50 | AF-A0A5Q0DKA8-F1-MODEL\_V4 | 1.0 | 1.079e-14 | 410 | 0.144 | 380 | 279 | 13 | 338 | 681 | 27 | 396 | Phage major capsid protein | Phage major capsid protein | | afdb-uniprot50 | AF-A0A1M3EVZ0-F1-MODEL\_V4 | 1.0 | 4.177e-17 | 410 | 0.143 | 508 | 322 | 27 | 252 | 677 | 4 | 480 | Uncharacterized protein | Uncharacterized protein | | afdb-uniprot50 | AF-A0A327QB84-F1-MODEL\_V4 | 1.0 | 2.872e-24 | 410 | 0.168 | 724 | 396 | 43 | 33 | 681 | 8 | 600 | Mu-like prophage major head subunit gpT | Mu-like prophage major head subunit gpT | | afdb-uniprot50 | AF-A0A5C7T964-F1-MODEL\_V4 | 1.0 | 2.42e-16 | 409 | 0.139 | 460 | 318 | 17 | 234 | 681 | 9 | 402 | Phage major capsid protein | Phage major capsid protein | | afdb-uniprot50 | AF-A0A0Q6UYN3-F1-MODEL\_V4 | 1.0 | 1.572e-23 | 409 | 0.145 | 790 | 474 | 33 | 17 | 681 | 14 | 727 | Uncharacterized protein | Uncharacterized protein | | afdb-uniprot50 | AF-A0A143YDP5-F1-MODEL\_V4 | 1.0 | 5.466e-15 | 408 | 0.166 | 373 | 246 | 13 | 335 | 682 | 1 | 333 | Phage capsid | Phage capsid | | afdb-uniprot50 | AF-A0A1X0K6U6-F1-MODEL\_V4 | 1.0 | 3.473e-15 | 408 | 0.131 | 411 | 296 | 16 | 295 | 682 | 19 | 391 | Uncharacterized protein | Uncharacterized protein | | afdb-uniprot50 | AF-A0A7J5TYE1-F1-MODEL\_V4 | 1.0 | 4.177e-17 | 408 | 0.13 | 474 | 301 | 21 | 252 | 679 | 46 | 454 | Phage major capsid protein | Phage major capsid protein | | afdb-uniprot50 | AF-A0A645AX28-F1-MODEL\_V4 | 1.0 | 2.254e-14 | 407 | 0.16 | 386 | 268 | 16 | 336 | 681 | 1 | 370 | Uncharacterized protein | Uncharacterized protein | | afdb-uniprot50 | AF-A0A2M8TG61-F1-MODEL\_V4 | 1.0 | 6.714e-16 | 407 | 0.109 | 482 | 309 | 25 | 233 | 681 | 22 | 416 | Phage major capsid protein | Phage major capsid protein | | afdb-uniprot50 | AF-A0A6L6ES98-F1-MODEL\_V4 | 1.0 | 3.575e-19 | 407 | 0.173 | 628 | 362 | 28 | 171 | 681 | 61 | 648 | Phage major capsid protein | Phage major capsid protein | | afdb-uniprot50 | AF-A0A7V8XZY4-F1-MODEL\_V4 | 1.0 | 5.018e-11 | 406 | 0.214 | 210 | 149 | 7 | 480 | 682 | 7 | 207 | Phage major capsid protein | Phage major capsid protein | | afdb-uniprot50 | AF-A0A4T0UXI4-F1-MODEL\_V4 | 1.0 | 1.915e-11 | 406 | 0.109 | 256 | 207 | 9 | 430 | 679 | 13 | 253 | Phage major capsid protein | Phage major capsid protein | | afdb-uniprot50 | AF-A0A327SK31-F1-MODEL\_V4 | 1.0 | 7.519e-16 | 406 | 0.151 | 450 | 301 | 21 | 249 | 679 | 21 | 408 | HK97 family phage major capsid protein | HK97 family phage major capsid protein | | afdb-uniprot50 | AF-A0A7X3Y810-F1-MODEL\_V4 | 1.0 | 1.034e-16 | 406 | 0.168 | 463 | 290 | 25 | 267 | 682 | 4 | 418 | Uncharacterized protein | Uncharacterized protein | | afdb-uniprot50 | AF-A0A858WUY8-F1-MODEL\_V4 | 1.0 | 4.815e-21 | 406 | 0.128 | 701 | 444 | 40 | 40 | 682 | 19 | 610 | Peptidase | Peptidase | | afdb-uniprot50 | AF-R4KAR4-F1-MODEL\_V4 | 1.0 | 7.105e-16 | 405 | 0.148 | 459 | 298 | 23 | 262 | 682 | 11 | 414 | Putative phage phi-C31 gp36 major capsid-like protein | Putative phage phi-C31 gp36 major capsid-like protein | | afdb-uniprot50 | AF-A0A3D4JJQ9-F1-MODEL\_V4 | 1.0 | 8.012e-13 | 404 | 0.132 | 257 | 192 | 12 | 435 | 681 | 61 | 296 | Phage major capsid protein | Phage major capsid protein | | afdb-uniprot50 | AF-A0A5R8Y4J3-F1-MODEL\_V4 | 1.0 | 1.971e-15 | 404 | 0.145 | 454 | 270 | 22 | 264 | 681 | 13 | 384 | Phage major capsid protein | Phage major capsid protein | | afdb-uniprot50 | AF-A0A401HW27-F1-MODEL\_V4 | 1.0 | 4.357e-15 | 404 | 0.148 | 405 | 273 | 19 | 335 | 682 | 1 | 390 | Putative phage major capsid protein | Putative phage major capsid protein | | afdb-uniprot50 | AF-A0A496WAE6-F1-MODEL\_V4 | 1.0 | 1.663e-15 | 404 | 0.141 | 481 | 287 | 22 | 249 | 681 | 2 | 404 | Phage major capsid protein | Phage major capsid protein | | afdb-uniprot50 | AF-A0A527P893-F1-MODEL\_V4 | 1.0 | 4.357e-15 | 404 | 0.158 | 453 | 306 | 17 | 246 | 679 | 12 | 408 | Phage major capsid protein | Phage major capsid protein | | afdb-uniprot50 | AF-A0A1Y3RFP0-F1-MODEL\_V4 | 1.0 | 8.913e-16 | 403 | 0.142 | 442 | 284 | 21 | 264 | 682 | 26 | 395 | Phage major capsid protein | Phage major capsid protein | | afdb-uniprot50 | AF-A0A1H7A3R3-F1-MODEL\_V4 | 1.0 | 1.929e-16 | 403 | 0.146 | 485 | 299 | 23 | 251 | 682 | 2 | 424 | Phage major capsid protein, HK97 family | Phage major capsid protein, HK97 family | | afdb-uniprot50 | AF-A0A1Q8DAV2-F1-MODEL\_V4 | 1.0 | 4.88e-15 | 402 | 0.138 | 412 | 277 | 19 | 295 | 681 | 5 | 363 | Phage capsid protein | Phage capsid protein | | afdb-uniprot50 | AF-A0A522EXF1-F1-MODEL\_V4 | 1.0 | 1.209e-14 | 402 | 0.141 | 480 | 268 | 22 | 258 | 679 | 2 | 395 | Phage major capsid protein | Phage major capsid protein | | afdb-uniprot50 | AF-A0A4R4I9T1-F1-MODEL\_V4 | 1.0 | 2.711e-16 | 402 | 0.149 | 476 | 301 | 21 | 238 | 679 | 57 | 462 | Phage major capsid protein | Phage major capsid protein | | afdb-uniprot50 | AF-A0A256C0Q1-F1-MODEL\_V4 | 1.0 | 3.89e-15 | 401 | 0.137 | 459 | 304 | 18 | 252 | 681 | 6 | 401 | Phage major capsid protein | Phage major capsid protein | | afdb-uniprot50 | AF-A0A6L5FY65-F1-MODEL\_V4 | 1.0 | 4.515e-16 | 401 | 0.162 | 486 | 263 | 26 | 271 | 682 | 4 | 419 | Phage major capsid protein | Phage major capsid protein | | afdb-uniprot50 | AF-A0A7C1VPJ3-F1-MODEL\_V4 | 1.0 | 3.036e-16 | 401 | 0.144 | 490 | 291 | 25 | 252 | 681 | 2 | 423 | Phage major capsid protein | Phage major capsid protein | | afdb-uniprot50 | AF-A0A5K7Z4E0-F1-MODEL\_V4 | 1.0 | 1.929e-16 | 401 | 0.139 | 489 | 286 | 30 | 264 | 681 | 5 | 429 | Phage capsid protein | Phage capsid protein | | afdb-uniprot50 | AF-A0A1F5QST1-F1-MODEL\_V4 | 1.0 | 6.956e-17 | 401 | 0.148 | 490 | 305 | 21 | 262 | 682 | 10 | 456 | Uncharacterized protein | Uncharacterized protein | | afdb-uniprot50 | AF-A0A2W6SDJ2-F1-MODEL\_V4 | 1.0 | 5.062e-24 | 401 | 0.147 | 774 | 446 | 42 | 18 | 681 | 30 | 699 | Uncharacterized protein | Uncharacterized protein | | afdb-uniprot50 | AF-A0A370KRT5-F1-MODEL\_V4 | 1.0 | 5.091e-13 | 400 | 0.173 | 283 | 180 | 10 | 433 | 682 | 9 | 270 | Uncharacterized protein | Uncharacterized protein | | afdb-uniprot50 | AF-A0A1P8PSS7-F1-MODEL\_V4 | 1.0 | 2.769e-15 | 400 | 0.145 | 461 | 278 | 20 | 262 | 681 | 5 | 390 | Uncharacterized protein | Uncharacterized protein | | afdb-uniprot50 | AF-A0A518EKY1-F1-MODEL\_V4 | 1.0 | 3.307e-20 | 400 | 0.186 | 537 | 333 | 26 | 30 | 516 | 12 | 494 | Caudovirus prohead protease | Caudovirus prohead protease | | afdb-uniprot50 | AF-A0A7I8Z983-F1-MODEL\_V4 | 1.0 | 2.636e-20 | 399 | 0.226 | 516 | 275 | 24 | 19 | 482 | 1 | 444 | Hypothetical protein | Hypothetical protein | | afdb-uniprot50 | AF-A0A2G8MBD4-F1-MODEL\_V4 | 1.0 | 2.95e-12 | 398 | 0.274 | 204 | 144 | 4 | 12 | 213 | 17 | 218 | Uncharacterized protein | Uncharacterized protein | | afdb-uniprot50 | AF-A0A0F8Y0I6-F1-MODEL\_V4 | 1.0 | 1.412e-12 | 398 | 0.116 | 308 | 242 | 14 | 389 | 679 | 28 | 322 | Uncharacterized protein | Uncharacterized protein | | afdb-uniprot50 | AF-R6SUA7-F1-MODEL\_V4 | 1.0 | 2.93e-15 | 398 | 0.126 | 458 | 281 | 24 | 264 | 681 | 2 | 380 | Phage major capsid protein HK97 family | Phage major capsid protein HK97 family | | afdb-uniprot50 | AF-A9NFU9-F1-MODEL\_V4 | 1.0 | 3.167e-14 | 397 | 0.138 | 397 | 261 | 15 | 338 | 682 | 8 | 375 | Uncharacterized protein | Uncharacterized protein | | afdb-uniprot50 | AF-A0A1Q3QQ17-F1-MODEL\_V4 | 1.0 | 5.466e-15 | 397 | 0.147 | 467 | 275 | 26 | 271 | 682 | 3 | 401 | Uncharacterized protein | Uncharacterized protein | | afdb-uniprot50 | AF-A0A502HSQ5-F1-MODEL\_V4 | 1.0 | 4.811e-13 | 397 | 0.122 | 318 | 232 | 13 | 392 | 677 | 106 | 408 | Phage major capsid protein | Phage major capsid protein | | afdb-uniprot50 | AF-A0A3R9P8L0-F1-MODEL\_V4 | 1.0 | 7.257e-15 | 397 | 0.141 | 423 | 304 | 15 | 307 | 682 | 9 | 419 | Phage major capsid protein | Phage major capsid protein | | afdb-uniprot50 | AF-A0A0Q6CCC3-F1-MODEL\_V4 | 1.0 | 5.466e-15 | 397 | 0.151 | 501 | 322 | 21 | 196 | 679 | 1 | 415 | Uncharacterized protein | Uncharacterized protein | | afdb-uniprot50 | AF-A0A7C6A307-F1-MODEL\_V4 | 1.0 | 1.736e-21 | 397 | 0.137 | 725 | 394 | 39 | 41 | 681 | 22 | 598 | Peptidase U35 | Peptidase U35 | | afdb-uniprot50 | AF-A0A2D6X839-F1-MODEL\_V4 | 1.0 | 2.993e-14 | 396 | 0.167 | 334 | 218 | 17 | 400 | 681 | 15 | 340 | Phage major capsid protein | Phage major capsid protein | | afdb-uniprot50 | AF-A0A3B8XY37-F1-MODEL\_V4 | 1.0 | 2.013e-14 | 396 | 0.124 | 379 | 267 | 19 | 338 | 681 | 9 | 357 | Uncharacterized protein | Uncharacterized protein | | afdb-uniprot50 | AF-A0A7C4KPZ2-F1-MODEL\_V4 | 1.0 | 4.611e-15 | 396 | 0.129 | 472 | 292 | 22 | 251 | 681 | 2 | 395 | Phage major capsid protein | Phage major capsid protein | | afdb-uniprot50 | AF-A0A6L9XYJ8-F1-MODEL\_V4 | 1.0 | 8.246e-17 | 396 | 0.161 | 490 | 324 | 26 | 249 | 681 | 28 | 487 | Phage major capsid protein | Phage major capsid protein | | afdb-uniprot50 | AF-A0A1G3FT84-F1-MODEL\_V4 | 1.0 | 9.433e-16 | 395 | 0.245 | 407 | 237 | 19 | 255 | 635 | 5 | 367 | Uncharacterized protein | Uncharacterized protein | | afdb-uniprot50 | AF-A0A2D9H4D9-F1-MODEL\_V4 | 1.0 | 6.122e-15 | 395 | 0.136 | 389 | 265 | 16 | 338 | 682 | 21 | 382 | Phage major capsid protein | Phage major capsid protein | | afdb-uniprot50 | AF-A0A3D5CRX3-F1-MODEL\_V4 | 1.0 | 7.958e-16 | 395 | 0.137 | 464 | 298 | 21 | 252 | 681 | 23 | 418 | Phage major capsid protein | Phage major capsid protein | | afdb-uniprot50 | AF-A0A1M4Y9R8-F1-MODEL\_V4 | 1.0 | 8.246e-17 | 395 | 0.168 | 492 | 290 | 24 | 252 | 682 | 48 | 481 | Phage major capsid protein, HK97 family | Phage major capsid protein, HK97 family | | afdb-uniprot50 | AF-A0A497LYT2-F1-MODEL\_V4 | 1.0 | 3.304e-12 | 394 | 0.092 | 293 | 235 | 7 | 398 | 682 | 3 | 272 | Uncharacterized protein | Uncharacterized protein | | afdb-uniprot50 | AF-A0A1F8UQS0-F1-MODEL\_V4 | 1.0 | 1.823e-16 | 394 | 0.136 | 463 | 315 | 17 | 252 | 680 | 23 | 434 | Uncharacterized protein | Uncharacterized protein | | afdb-uniprot50 | AF-A0A3S3AHQ4-F1-MODEL\_V4 | 1.0 | 3.33e-17 | 394 | 0.152 | 532 | 347 | 20 | 171 | 681 | 67 | 515 | Phage major capsid protein | Phage major capsid protein | | afdb-uniprot50 | AF-A0A653F5R1-F1-MODEL\_V4 | 1.0 | 4.145e-12 | 393 | 0.154 | 266 | 196 | 13 | 433 | 682 | 32 | 284 | Phage capsid family protein | Phage capsid family protein | | afdb-uniprot50 | AF-G4I9Y4-F1-MODEL\_V4 | 1.0 | 1.734e-13 | 392 | 0.167 | 341 | 213 | 14 | 398 | 682 | 6 | 331 | Uncharacterized protein | Uncharacterized protein | | afdb-uniprot50 | AF-A0A522Q9N9-F1-MODEL\_V4 | 1.0 | 1.373e-16 | 392 | 0.172 | 555 | 327 | 32 | 191 | 681 | 1 | 486 | Phage major capsid protein | Phage major capsid protein | | afdb-uniprot50 | AF-H2JIC2-F1-MODEL\_V4 | 1.0 | 4.811e-13 | 391 | 0.117 | 340 | 241 | 15 | 349 | 681 | 10 | 297 | Phage capsid family protein | Phage capsid family protein | | afdb-uniprot50 | AF-A0A7C3UHG4-F1-MODEL\_V4 | 1.0 | 5.165e-15 | 391 | 0.154 | 383 | 259 | 20 | 338 | 681 | 13 | 369 | Phage major capsid protein | Phage major capsid protein | | afdb-uniprot50 | AF-A0A3M2A8H0-F1-MODEL\_V4 | 1.0 | 2.93e-15 | 391 | 0.165 | 464 | 283 | 25 | 252 | 682 | 2 | 394 | Phage major capsid protein | Phage major capsid protein | | afdb-uniprot50 | AF-I0GRZ0-F1-MODEL\_V4 | 1.0 | 4.88e-15 | 391 | 0.134 | 460 | 297 | 18 | 252 | 682 | 48 | 435 | Putative phage major capsid protein | Putative phage major capsid protein | | afdb-uniprot50 | AF-A0A522RY69-F1-MODEL\_V4 | 1.0 | 6.211e-17 | 391 | 0.156 | 498 | 303 | 26 | 244 | 681 | 19 | 459 | Phage major capsid protein | Phage major capsid protein | | afdb-uniprot50 | AF-A0A352DNU6-F1-MODEL\_V4 | 1.0 | 9.433e-16 | 390 | 0.138 | 476 | 282 | 23 | 251 | 680 | 35 | 428 | Phage major capsid protein | Phage major capsid protein | | afdb-uniprot50 | AF-A0A0S8GFL0-F1-MODEL\_V4 | 1.0 | 1.118e-15 | 390 | 0.137 | 494 | 295 | 25 | 266 | 682 | 2 | 441 | Uncharacterized protein | Uncharacterized protein | | afdb-uniprot50 | AF-A0A6F9E8C9-F1-MODEL\_V4 | 1.0 | 1.118e-15 | 389 | 0.156 | 447 | 292 | 20 | 262 | 682 | 19 | 406 | Phage major capsid protein, HK97 family | Phage major capsid protein, HK97 family | | afdb-uniprot50 | AF-A0A2B0JB50-F1-MODEL\_V4 | 1.0 | 1.412e-12 | 388 | 0.127 | 267 | 199 | 11 | 433 | 679 | 43 | 295 | Phage major capsid protein | Phage major capsid protein | | afdb-uniprot50 | AF-A0A1Y4UTW1-F1-MODEL\_V4 | 1.0 | 5.275e-14 | 388 | 0.122 | 473 | 295 | 24 | 252 | 681 | 3 | 398 | Phage major capsid protein | Phage major capsid protein | | afdb-uniprot50 | AF-A0A7X0H6V8-F1-MODEL\_V4 | 1.0 | 2.386e-14 | 388 | 0.173 | 387 | 243 | 19 | 332 | 682 | 86 | 431 | HK97 family phage major capsid protein | HK97 family phage major capsid protein | | afdb-uniprot50 | AF-A0A1I5HWK7-F1-MODEL\_V4 | 1.0 | 5.664e-16 | 386 | 0.185 | 470 | 267 | 24 | 268 | 681 | 6 | 415 | Phage major capsid protein, HK97 family | Phage major capsid protein, HK97 family | | afdb-uniprot50 | AF-A0A6N9Q8R2-F1-MODEL\_V4 | 1.0 | 6.857e-15 | 386 | 0.117 | 468 | 320 | 22 | 262 | 681 | 4 | 426 | Phage major capsid protein | Phage major capsid protein | | afdb-uniprot50 | AF-A0A436GWX9-F1-MODEL\_V4 | 1.0 | 5.785e-15 | 386 | 0.131 | 455 | 305 | 20 | 252 | 682 | 66 | 454 | Phage major capsid protein | Phage major capsid protein | | afdb-uniprot50 | AF-A0A7C3UYK7-F1-MODEL\_V4 | 1.0 | 1.02e-14 | 385 | 0.259 | 385 | 199 | 16 | 282 | 643 | 40 | 361 | Phage major capsid protein | Phage major capsid protein | | afdb-uniprot50 | AF-A0A4Q3H3Y4-F1-MODEL\_V4 | 1.0 | 1.748e-18 | 384 | 0.3 | 390 | 178 | 9 | 30 | 397 | 2 | 318 | Uncharacterized protein | Uncharacterized protein | | afdb-uniprot50 | AF-A0A2A5S652-F1-MODEL\_V4 | 1.0 | 1.142e-14 | 384 | 0.146 | 390 | 248 | 20 | 312 | 681 | 2 | 326 | Uncharacterized protein | Uncharacterized protein | | afdb-uniprot50 | AF-A0A6M1WCW4-F1-MODEL\_V4 | 1.0 | 1.209e-14 | 384 | 0.127 | 461 | 278 | 23 | 264 | 681 | 2 | 381 | Phage major capsid protein | Phage major capsid protein | | afdb-uniprot50 | AF-A0A1Q9JGH3-F1-MODEL\_V4 | 1.0 | 2.254e-14 | 384 | 0.119 | 420 | 268 | 19 | 335 | 681 | 1 | 391 | Uncharacterized protein | Uncharacterized protein | | afdb-uniprot50 | AF-A0A2S3VZY0-F1-MODEL\_V4 | 1.0 | 2.809e-17 | 384 | 0.16 | 505 | 307 | 20 | 252 | 678 | 6 | 471 | Phage capsid family protein | Phage capsid family protein | | afdb-uniprot50 | AF-A0A1G8R0Y5-F1-MODEL\_V4 | 1.0 | 9.103e-15 | 383 | 0.124 | 450 | 312 | 20 | 249 | 682 | 2 | 385 | Phage major capsid protein, HK97 family | Phage major capsid protein, HK97 family | | afdb-uniprot50 | AF-A0A2A8XVM2-F1-MODEL\_V4 | 1.0 | 9.298e-14 | 383 | 0.131 | 387 | 280 | 15 | 338 | 681 | 12 | 385 | Phage major capsid protein | Phage major capsid protein | | afdb-uniprot50 | AF-A0A859DMT2-F1-MODEL\_V4 | 1.0 | 2.769e-15 | 383 | 0.162 | 444 | 292 | 19 | 262 | 682 | 19 | 405 | Phage major capsid protein | Phage major capsid protein | | afdb-uniprot50 | AF-A0A3S0UJX5-F1-MODEL\_V4 | 1.0 | 1.403e-15 | 381 | 0.248 | 390 | 235 | 18 | 252 | 620 | 2 | 354 | Phage major capsid protein | Phage major capsid protein | | afdb-uniprot50 | AF-A0A1H4BNJ7-F1-MODEL\_V4 | 1.0 | 2.254e-14 | 381 | 0.149 | 454 | 280 | 22 | 268 | 681 | 3 | 390 | Phage major capsid protein, HK97 family | Phage major capsid protein, HK97 family | | afdb-uniprot50 | AF-A0A644XRB4-F1-MODEL\_V4 | 1.0 | 1.279e-14 | 380 | 0.114 | 461 | 315 | 23 | 251 | 682 | 5 | 401 | Uncharacterized protein | Uncharacterized protein | | afdb-uniprot50 | AF-A0A7K1ZMJ6-F1-MODEL\_V4 | 1.0 | 3.676e-15 | 380 | 0.139 | 458 | 305 | 25 | 264 | 681 | 7 | 415 | Phage major capsid protein | Phage major capsid protein | | afdb-uniprot50 | AF-A0A077L3E3-F1-MODEL\_V4 | 1.0 | 1.663e-15 | 380 | 0.146 | 484 | 301 | 25 | 256 | 679 | 4 | 435 | Uncharacterized protein | Uncharacterized protein | | afdb-uniprot50 | AF-A0A7X4ACZ8-F1-MODEL\_V4 | 1.0 | 1.889e-17 | 380 | 0.134 | 567 | 357 | 24 | 172 | 680 | 72 | 562 | Phage major capsid protein | Phage major capsid protein | | afdb-uniprot50 | AF-A0A7Z9KVM6-F1-MODEL\_V4 | 1.0 | 1.442e-11 | 379 | 0.09 | 287 | 245 | 6 | 407 | 681 | 2 | 284 | Uncharacterized protein | Uncharacterized protein | | afdb-uniprot50 | AF-A0A0G1JMI3-F1-MODEL\_V4 | 1.0 | 3.473e-15 | 379 | 0.139 | 493 | 300 | 28 | 267 | 682 | 3 | 448 | HK97 family major capsid protein | HK97 family major capsid protein | | afdb-uniprot50 | AF-A0A843HQ06-F1-MODEL\_V4 | 1.0 | 1.209e-14 | 378 | 0.139 | 467 | 293 | 24 | 254 | 682 | 2 | 397 | Phage major capsid protein | Phage major capsid protein | | afdb-uniprot50 | AF-A0A7G1IRW9-F1-MODEL\_V4 | 1.0 | 3.497e-12 | 377 | 0.128 | 358 | 184 | 11 | 438 | 679 | 36 | 381 | Uncharacterized protein | Uncharacterized protein | | afdb-uniprot50 | AF-A0A3L6ZMY8-F1-MODEL\_V4 | 1.0 | 1.433e-14 | 377 | 0.149 | 462 | 282 | 22 | 264 | 682 | 7 | 400 | Phage major capsid protein | Phage major capsid protein | | afdb-uniprot50 | AF-A0A0U1L1V2-F1-MODEL\_V4 | 1.0 | 1.142e-14 | 377 | 0.128 | 467 | 297 | 20 | 261 | 680 | 10 | 413 | Phage major capsid protein | Phage major capsid protein | | afdb-uniprot50 | AF-A0A1X7A4I7-F1-MODEL\_V4 | 1.0 | 5.466e-15 | 377 | 0.158 | 472 | 293 | 22 | 264 | 682 | 8 | 428 | Phage capsid family protein | Phage capsid family protein | | afdb-uniprot50 | AF-A0A2V0MXJ8-F1-MODEL\_V4 | 1.0 | 7.68e-15 | 377 | 0.134 | 475 | 311 | 22 | 251 | 680 | 3 | 422 | Uncharacterized protein | Uncharacterized protein | | afdb-uniprot50 | AF-A0A0G1YGY1-F1-MODEL\_V4 | 1.0 | 1.484e-15 | 377 | 0.142 | 492 | 315 | 25 | 263 | 681 | 5 | 462 | Phage major capsid protein, HK97 family | Phage major capsid protein, HK97 family | | afdb-uniprot50 | AF-A0A1L8I4U9-F1-MODEL\_V4 | 1.0 | 1.005e-12 | 376 | 0.151 | 390 | 248 | 16 | 313 | 682 | 1 | 327 | Uncharacterized protein | Uncharacterized protein | | afdb-uniprot50 | AF-A0A2V5TLL7-F1-MODEL\_V4 | 1.0 | 1.639e-13 | 376 | 0.158 | 397 | 272 | 18 | 313 | 681 | 2 | 364 | Phage major capsid protein | Phage major capsid protein | | afdb-uniprot50 | AF-A0A0Q7I3W7-F1-MODEL\_V4 | 1.0 | 1.538e-16 | 376 | 0.184 | 494 | 293 | 28 | 252 | 682 | 6 | 452 | Uncharacterized protein | Uncharacterized protein | | afdb-uniprot50 | AF-A0A4R5WCP3-F1-MODEL\_V4 | 1.0 | 1.956e-10 | 375 | 0.146 | 212 | 159 | 8 | 481 | 682 | 5 | 204 | Phage major capsid protein | Phage major capsid protein | | afdb-uniprot50 | AF-A0A6I5WKB9-F1-MODEL\_V4 | 1.0 | 5.908e-14 | 375 | 0.137 | 408 | 251 | 21 | 338 | 682 | 13 | 382 | Phage major capsid protein | Phage major capsid protein | | afdb-uniprot50 | AF-C5R879-F1-MODEL\_V4 | 1.0 | 4.205e-14 | 375 | 0.158 | 455 | 287 | 24 | 252 | 681 | 5 | 388 | Dyp-type peroxidase family protein | Dyp-type peroxidase family protein | | afdb-uniprot50 | AF-A0A174XIH6-F1-MODEL\_V4 | 1.0 | 3.101e-15 | 375 | 0.153 | 464 | 281 | 25 | 261 | 682 | 4 | 397 | Phage major capsid protein, HK97 family | Phage major capsid protein, HK97 family | | afdb-uniprot50 | AF-A0A7X3RYI9-F1-MODEL\_V4 | 1.0 | 3.101e-15 | 374 | 0.135 | 487 | 312 | 26 | 249 | 681 | 3 | 434 | Uncharacterized protein | Uncharacterized protein | | afdb-uniprot50 | AF-A0A402D5R7-F1-MODEL\_V4 | 1.0 | 5.352e-16 | 374 | 0.16 | 492 | 304 | 23 | 263 | 682 | 3 | 457 | Uncharacterized protein | Uncharacterized protein | | afdb-uniprot50 | AF-A0A1E5XQL2-F1-MODEL\_V4 | 1.0 | 4.88e-15 | 373 | 0.17 | 433 | 248 | 18 | 300 | 681 | 21 | 393 | Phage capsid protein | Phage capsid protein | | afdb-uniprot50 | AF-A0A829PFN5-F1-MODEL\_V4 | 1.0 | 1.026e-11 | 372 | 0.157 | 292 | 213 | 13 | 410 | 681 | 10 | 288 | Phage major capsid protein, HK97 family | Phage major capsid protein, HK97 family | | afdb-uniprot50 | AF-A0A0R1GSB6-F1-MODEL\_V4 | 1.0 | 3.189e-11 | 372 | 0.148 | 282 | 187 | 15 | 433 | 681 | 39 | 300 | Phage capsid family protein | Phage capsid family protein | | afdb-uniprot50 | AF-A0A6M5YN86-F1-MODEL\_V4 | 1.0 | 1.571e-15 | 372 | 0.14 | 483 | 311 | 28 | 256 | 681 | 9 | 444 | Phage major capsid protein | Phage major capsid protein | | afdb-uniprot50 | AF-E4V9K9-F1-MODEL\_V4 | 1.0 | 2.634e-12 | 371 | 0.161 | 322 | 216 | 13 | 398 | 681 | 86 | 391 | Putative phage major capsid protein, HK97 family | Putative phage major capsid protein, HK97 family | | afdb-uniprot50 | AF-A0A349PNZ6-F1-MODEL\_V4 | 1.0 | 1.041e-13 | 370 | 0.15 | 371 | 262 | 21 | 334 | 680 | 85 | 426 | Phage major capsid protein | Phage major capsid protein | | afdb-uniprot50 | AF-A0A6M1X6G5-F1-MODEL\_V4 | 1.0 | 1.494e-12 | 369 | 0.142 | 281 | 184 | 14 | 433 | 679 | 50 | 307 | Phage major capsid protein | Phage major capsid protein | | afdb-uniprot50 | AF-A0A437MNA2-F1-MODEL\_V4 | 1.0 | 3.676e-15 | 369 | 0.162 | 485 | 296 | 24 | 252 | 681 | 13 | 442 | Phage major capsid protein | Phage major capsid protein | | afdb-uniprot50 | AF-A0A1E8RGG7-F1-MODEL\_V4 | 1.0 | 1.298e-16 | 369 | 0.195 | 456 | 278 | 25 | 252 | 681 | 53 | 445 | Uncharacterized protein | Uncharacterized protein | | afdb-uniprot50 | AF-A0A0R2CK44-F1-MODEL\_V4 | 1.0 | 5.954e-19 | 369 | 0.136 | 705 | 418 | 37 | 18 | 682 | 4 | 557 | Phage capsid protein | Phage capsid protein | | afdb-uniprot50 | AF-A0A7X3TZU9-F1-MODEL\_V4 | 1.0 | 1.005e-12 | 368 | 0.156 | 338 | 226 | 17 | 386 | 677 | 1 | 325 | Phage major capsid protein | Phage major capsid protein | | afdb-uniprot50 | AF-A0A0F9QUI3-F1-MODEL\_V4 | 1.0 | 1.209e-14 | 368 | 0.144 | 469 | 273 | 19 | 271 | 682 | 4 | 401 | Uncharacterized protein | Uncharacterized protein | | afdb-uniprot50 | AF-A0A7Z1WZG9-F1-MODEL\_V4 | 1.0 | 6.479e-15 | 368 | 0.129 | 479 | 304 | 26 | 246 | 679 | 1 | 411 | Phage major capsid protein | Phage major capsid protein | | afdb-uniprot50 | AF-A0A0F9FJ22-F1-MODEL\_V4 | 1.0 | 4.742e-11 | 367 | 0.12 | 299 | 214 | 11 | 411 | 681 | 1 | 278 | Uncharacterized protein | Uncharacterized protein | | afdb-uniprot50 | AF-A0A3B0FX46-F1-MODEL\_V4 | 1.0 | 3.101e-15 | 367 | 0.163 | 484 | 300 | 22 | 253 | 680 | 7 | 441 | Phage major capsid protein | Phage major capsid protein | | afdb-uniprot50 | AF-A0A3D1VAZ0-F1-MODEL\_V4 | 1.0 | 2.93e-15 | 367 | 0.148 | 485 | 303 | 17 | 251 | 680 | 24 | 453 | Phage major capsid protein | Phage major capsid protein | | afdb-uniprot50 | AF-A0A7G9R3H2-F1-MODEL\_V4 | 1.0 | 4.611e-15 | 366 | 0.139 | 446 | 285 | 16 | 263 | 679 | 7 | 382 | Uncharacterized protein | Uncharacterized protein | | afdb-uniprot50 | AF-A0A1W1ZBJ4-F1-MODEL\_V4 | 1.0 | 8.301e-14 | 366 | 0.14 | 455 | 297 | 20 | 246 | 681 | 21 | 400 | Phage major capsid protein, HK97 family | Phage major capsid protein, HK97 family | | afdb-uniprot50 | AF-A0A2W7AVB5-F1-MODEL\_V4 | 1.0 | 7.958e-16 | 365 | 0.219 | 424 | 223 | 15 | 18 | 432 | 4 | 328 | Uncharacterized protein | Uncharacterized protein | | afdb-uniprot50 | AF-A0A0M8X8L3-F1-MODEL\_V4 | 1.0 | 7.003e-14 | 365 | 0.133 | 450 | 271 | 21 | 262 | 682 | 12 | 371 | Uncharacterized protein | Uncharacterized protein | | afdb-uniprot50 | AF-A0A3D2THT3-F1-MODEL\_V4 | 1.0 | 5.664e-16 | 365 | 0.151 | 523 | 290 | 26 | 251 | 682 | 10 | 469 | Phage major capsid protein | Phage major capsid protein | | afdb-uniprot50 | AF-D8JVB9-F1-MODEL\_V4 | 1.0 | 7.958e-16 | 365 | 0.136 | 600 | 319 | 28 | 175 | 679 | 2 | 497 | Phage major capsid protein, HK97 family | Phage major capsid protein, HK97 family | | afdb-uniprot50 | AF-X1AHU6-F1-MODEL\_V4 | 1.0 | 9.361e-11 | 364 | 0.383 | 193 | 111 | 4 | 394 | 583 | 4 | 191 | Uncharacterized protein | Uncharacterized protein | | afdb-uniprot50 | AF-A0A1T4ZWM4-F1-MODEL\_V4 | 1.0 | 6.714e-16 | 364 | 0.172 | 509 | 285 | 23 | 264 | 681 | 3 | 466 | Phage major capsid protein, HK97 family | Phage major capsid protein, HK97 family | | afdb-uniprot50 | AF-A0A5Q2P6R5-F1-MODEL\_V4 | 1.0 | 2.847e-11 | 363 | 0.159 | 282 | 184 | 15 | 433 | 681 | 39 | 300 | Phage major capsid protein | Phage major capsid protein | | afdb-uniprot50 | AF-A0A7X7E744-F1-MODEL\_V4 | 1.0 | 1.118e-15 | 363 | 0.131 | 493 | 323 | 19 | 251 | 680 | 3 | 453 | Phage major capsid protein | Phage major capsid protein | | afdb-uniprot50 | AF-A0A6M3ISE2-F1-MODEL\_V4 | 1.0 | 9.165e-12 | 362 | 0.149 | 308 | 219 | 16 | 400 | 681 | 2 | 292 | Putative capsid protein | Putative capsid protein | | afdb-uniprot50 | AF-A0A2B9DNW7-F1-MODEL\_V4 | 1.0 | 1.134e-17 | 362 | 0.114 | 653 | 369 | 30 | 81 | 681 | 8 | 502 | Uncharacterized protein | Uncharacterized protein | | afdb-uniprot50 | AF-X6C314-F1-MODEL\_V4 | 1.0 | 3.623e-13 | 361 | 0.153 | 403 | 252 | 20 | 320 | 681 | 1 | 355 | Uncharacterized protein | Uncharacterized protein | | afdb-uniprot50 | AF-A0A7C2FJD4-F1-MODEL\_V4 | 1.0 | 9.433e-16 | 361 | 0.146 | 498 | 296 | 24 | 264 | 682 | 6 | 453 | Phage major capsid protein | Phage major capsid protein | | afdb-uniprot50 | AF-A0A7R8N572-F1-MODEL\_V4 | 1.0 | 2.769e-15 | 360 | 0.231 | 415 | 195 | 14 | 12 | 356 | 3 | 363 | Uncharacterized protein | Uncharacterized protein | | afdb-uniprot50 | AF-A0A0F9PBS1-F1-MODEL\_V4 | 1.0 | 3.548e-14 | 360 | 0.159 | 433 | 263 | 24 | 295 | 682 | 15 | 391 | Uncharacterized protein | Uncharacterized protein | | afdb-uniprot50 | AF-A0A2A4XWW1-F1-MODEL\_V4 | 1.0 | 6.618e-14 | 360 | 0.137 | 464 | 268 | 26 | 251 | 682 | 49 | 412 | Phage major capsid protein | Phage major capsid protein | | afdb-uniprot50 | AF-A0A6I5U7Z2-F1-MODEL\_V4 | 1.0 | 1.226e-16 | 360 | 0.153 | 594 | 343 | 27 | 175 | 680 | 3 | 524 | Phage major capsid protein | Phage major capsid protein | | afdb-uniprot50 | AF-A0A3B8RSS5-F1-MODEL\_V4 | 1.0 | 2.672e-14 | 359 | 0.144 | 395 | 265 | 19 | 335 | 678 | 5 | 377 | Phage major capsid protein | Phage major capsid protein | | afdb-uniprot50 | AF-A0A5C7M720-F1-MODEL\_V4 | 1.0 | 3.282e-15 | 358 | 0.167 | 501 | 324 | 22 | 249 | 681 | 3 | 478 | Phage major capsid protein | Phage major capsid protein | | afdb-uniprot50 | AF-A0A7K0EIY7-F1-MODEL\_V4 | 1.0 | 6.479e-15 | 358 | 0.131 | 480 | 328 | 21 | 243 | 679 | 35 | 468 | Phage major capsid protein | Phage major capsid protein | | afdb-uniprot50 | AF-A0A660ZTR1-F1-MODEL\_V4 | 1.0 | 1.02e-14 | 357 | 0.142 | 490 | 280 | 30 | 251 | 681 | 3 | 411 | Phage major capsid protein | Phage major capsid protein | | afdb-uniprot50 | AF-A0A7C3V1U7-F1-MODEL\_V4 | 1.0 | 1.956e-10 | 356 | 0.12 | 273 | 209 | 10 | 425 | 682 | 37 | 293 | Uncharacterized protein | Uncharacterized protein | | afdb-uniprot50 | AF-A0A7X2FSL1-F1-MODEL\_V4 | 1.0 | 5.018e-11 | 356 | 0.094 | 296 | 232 | 10 | 413 | 682 | 8 | 293 | Uncharacterized protein | Uncharacterized protein | | afdb-uniprot50 | AF-A0A386WCQ4-F1-MODEL\_V4 | 1.0 | 7.958e-16 | 355 | 0.154 | 517 | 292 | 25 | 252 | 680 | 40 | 499 | Phage major capsid protein | Phage major capsid protein | | afdb-uniprot50 | AF-R6UVK3-F1-MODEL\_V4 | 1.0 | 2.42e-16 | 355 | 0.137 | 487 | 305 | 22 | 249 | 681 | 24 | 449 | Uncharacterized protein | Uncharacterized protein | | afdb-uniprot50 | AF-A0A0F9ML94-F1-MODEL\_V4 | 1.0 | 4.234e-11 | 354 | 0.099 | 302 | 233 | 11 | 404 | 680 | 1 | 288 | Uncharacterized protein | Uncharacterized protein | | afdb-uniprot50 | AF-A0A0X8H1D3-F1-MODEL\_V4 | 1.0 | 2.888e-13 | 354 | 0.13 | 430 | 260 | 22 | 279 | 682 | 42 | 383 | Uncharacterized protein | Uncharacterized protein | | afdb-uniprot50 | AF-A0A2N2DQ71-F1-MODEL\_V4 | 1.0 | 3.973e-14 | 354 | 0.134 | 477 | 305 | 25 | 262 | 682 | 4 | 428 | Phage major capsid protein | Phage major capsid protein | | afdb-uniprot50 | AF-A0A7Y7IFX1-F1-MODEL\_V4 | 1.0 | 3.282e-15 | 354 | 0.151 | 529 | 277 | 26 | 258 | 679 | 8 | 471 | Phage major capsid protein | Phage major capsid protein | | afdb-uniprot50 | AF-A0A840YDI0-F1-MODEL\_V4 | 1.0 | 6.714e-16 | 353 | 0.236 | 368 | 218 | 14 | 19 | 358 | 15 | 347 | Uncharacterized protein | Uncharacterized protein | | afdb-uniprot50 | AF-A0A496M1E1-F1-MODEL\_V4 | 1.0 | 2.847e-11 | 352 | 0.126 | 268 | 196 | 11 | 442 | 682 | 26 | 282 | Phage major capsid protein | Phage major capsid protein | | afdb-uniprot50 | AF-C3EDP4-F1-MODEL\_V4 | 1.0 | 4.71e-14 | 352 | 0.161 | 366 | 237 | 23 | 338 | 681 | 11 | 328 | Major capsid protein b | Major capsid protein b | | afdb-uniprot50 | AF-A0A3D5PUN7-F1-MODEL\_V4 | 1.0 | 2.908e-10 | 351 | 0.154 | 233 | 163 | 11 | 466 | 682 | 3 | 217 | Phage major capsid protein | Phage major capsid protein | | afdb-uniprot50 | AF-A0A0F9Q956-F1-MODEL\_V4 | 1.0 | 1.809e-11 | 351 | 0.133 | 300 | 213 | 13 | 413 | 681 | 8 | 291 | Uncharacterized protein | Uncharacterized protein | | afdb-uniprot50 | AF-A0A5Q0Q946-F1-MODEL\_V4 | 1.0 | 1.166e-13 | 351 | 0.141 | 467 | 314 | 19 | 245 | 679 | 1 | 412 | Phage major capsid protein | Phage major capsid protein | | afdb-uniprot50 | AF-A0A7M2A7V1-F1-MODEL\_V4 | 1.0 | 2.207e-15 | 350 | 0.163 | 477 | 297 | 25 | 251 | 682 | 24 | 443 | Phage major capsid protein | Phage major capsid protein | | afdb-uniprot50 | AF-I0KCY2-F1-MODEL\_V4 | 1.0 | 1.484e-15 | 348 | 0.123 | 501 | 316 | 21 | 238 | 679 | 30 | 466 | Major capsid protein Gp5 | Major capsid protein Gp5 | | afdb-uniprot50 | AF-A0A523WV01-F1-MODEL\_V4 | 1.0 | 1.15e-11 | 347 | 0.124 | 305 | 218 | 10 | 399 | 682 | 18 | 294 | Uncharacterized protein | Uncharacterized protein | | afdb-uniprot50 | AF-A0A084XUA4-F1-MODEL\_V4 | 1.0 | 3.572e-11 | 347 | 0.107 | 299 | 230 | 12 | 407 | 681 | 8 | 293 | Uncharacterized protein | Uncharacterized protein | | afdb-uniprot50 | AF-A0A7G5H2I5-F1-MODEL\_V4 | 1.0 | 8.301e-14 | 347 | 0.131 | 464 | 311 | 19 | 252 | 679 | 5 | 412 | Phage major capsid protein | Phage major capsid protein | | afdb-uniprot50 | AF-A0A0D7F6Q0-F1-MODEL\_V4 | 1.0 | 7.462e-11 | 344 | 0.092 | 303 | 223 | 15 | 402 | 680 | 2 | 276 | Uncharacterized protein | Uncharacterized protein | | afdb-uniprot50 | AF-A0A839V847-F1-MODEL\_V4 | 1.0 | 8.301e-14 | 343 | 0.24 | 378 | 217 | 15 | 264 | 613 | 7 | 342 | HK97 family phage major capsid protein | HK97 family phage major capsid protein | | afdb-uniprot50 | AF-A0A1B0Z1T9-F1-MODEL\_V4 | 1.0 | 7.051e-11 | 342 | 0.121 | 296 | 228 | 13 | 410 | 682 | 1 | 287 | Putative major capsid protein | Putative major capsid protein | | afdb-uniprot50 | AF-A0A2D6EYL3-F1-MODEL\_V4 | 1.0 | 1.442e-11 | 342 | 0.111 | 305 | 239 | 10 | 389 | 682 | 61 | 344 | Uncharacterized protein | Uncharacterized protein | | afdb-uniprot50 | AF-A0A6M7TFY0-F1-MODEL\_V4 | 1.0 | 1.548e-13 | 342 | 0.16 | 441 | 236 | 20 | 308 | 681 | 17 | 390 | Phage major capsid protein | Phage major capsid protein | | afdb-uniprot50 | AF-A0A426QMC7-F1-MODEL\_V4 | 1.0 | 9.983e-16 | 342 | 0.148 | 598 | 349 | 35 | 172 | 682 | 2 | 526 | Phage major capsid protein | Phage major capsid protein | | afdb-uniprot50 | AF-A0A3G8M216-F1-MODEL\_V4 | 1.0 | 9.361e-11 | 341 | 0.294 | 214 | 119 | 9 | 31 | 213 | 1 | 213 | Uncharacterized protein | Uncharacterized protein | | afdb-uniprot50 | AF-A0A5C7UJM2-F1-MODEL\_V4 | 1.0 | 2.672e-14 | 340 | 0.129 | 502 | 361 | 23 | 195 | 679 | 1 | 443 | Phage major capsid protein | Phage major capsid protein | | afdb-uniprot50 | AF-A0A1N6YF10-F1-MODEL\_V4 | 1.0 | 2.013e-14 | 340 | 0.14 | 521 | 302 | 32 | 261 | 681 | 3 | 477 | Phage major capsid protein, HK97 family | Phage major capsid protein, HK97 family | | afdb-uniprot50 | AF-A0A1A3P540-F1-MODEL\_V4 | 1.0 | 4.742e-11 | 339 | 0.15 | 293 | 215 | 17 | 410 | 681 | 2 | 281 | Uncharacterized protein | Uncharacterized protein | | afdb-uniprot50 | AF-A0A7J9XIJ9-F1-MODEL\_V4 | 1.0 | 8.786e-14 | 339 | 0.136 | 497 | 295 | 24 | 251 | 681 | 3 | 431 | Phage major capsid protein | Phage major capsid protein | | afdb-uniprot50 | AF-A0A1Y4R494-F1-MODEL\_V4 | 1.0 | 5.165e-15 | 339 | 0.139 | 474 | 300 | 21 | 246 | 682 | 43 | 445 | Phage major capsid protein | Phage major capsid protein | | afdb-uniprot50 | AF-A0A6G6YPV7-F1-MODEL\_V4 | 1.0 | 4.811e-13 | 337 | 0.178 | 421 | 235 | 23 | 309 | 681 | 2 | 359 | Phage major capsid protein | Phage major capsid protein | | afdb-uniprot50 | AF-A0A2N1R2F5-F1-MODEL\_V4 | 1.0 | 6.122e-15 | 336 | 0.143 | 473 | 271 | 18 | 249 | 681 | 227 | 605 | Phage major capsid protein | Phage major capsid protein | | afdb-uniprot50 | AF-A0A6P2DJV8-F1-MODEL\_V4 | 1.0 | 5.466e-15 | 335 | 0.173 | 513 | 287 | 28 | 256 | 682 | 4 | 465 | Uncharacterized protein | Uncharacterized protein | | afdb-uniprot50 | AF-A0A7K1SKS3-F1-MODEL\_V4 | 1.0 | 2.437e-13 | 335 | 0.121 | 478 | 322 | 22 | 246 | 679 | 33 | 456 | Phage major capsid protein | Phage major capsid protein | | afdb-uniprot50 | AF-A0A381E722-F1-MODEL\_V4 | 1.0 | 1.639e-13 | 333 | 0.146 | 458 | 277 | 23 | 271 | 681 | 3 | 393 | HK97 family major capsid protein | HK97 family major capsid protein | | afdb-uniprot50 | AF-A0A545TU80-F1-MODEL\_V4 | 1.0 | 2.318e-10 | 332 | 0.098 | 304 | 229 | 12 | 405 | 682 | 8 | 292 | Uncharacterized protein | Uncharacterized protein | | afdb-uniprot50 | AF-A0A3C0YSV1-F1-MODEL\_V4 | 1.0 | 2.1e-12 | 332 | 0.14 | 470 | 317 | 20 | 251 | 682 | 32 | 452 | Phage major capsid protein | Phage major capsid protein | | afdb-uniprot50 | AF-A0A5K1IT80-F1-MODEL\_V4 | 1.0 | 6.386e-13 | 330 | 0.123 | 446 | 288 | 26 | 269 | 680 | 16 | 392 | Phage capsid family protein | Phage capsid family protein | | afdb-uniprot50 | AF-A0A1Y4LTW7-F1-MODEL\_V4 | 1.0 | 1.516e-14 | 330 | 0.15 | 471 | 301 | 17 | 244 | 679 | 42 | 448 | Phage major capsid protein | Phage major capsid protein | | afdb-uniprot50 | AF-X0SBZ6-F1-MODEL\_V4 | 1.0 | 4.811e-13 | 328 | 0.193 | 419 | 230 | 20 | 294 | 662 | 10 | 370 | Uncharacterized protein | Uncharacterized protein | | afdb-uniprot50 | AF-A0A0D6DVL7-F1-MODEL\_V4 | 1.0 | 1.433e-14 | 328 | 0.144 | 478 | 285 | 27 | 252 | 682 | 13 | 413 | Major capsid protein | Major capsid protein | | afdb-uniprot50 | AF-A0A832B3P5-F1-MODEL\_V4 | 1.0 | 5.664e-16 | 327 | 0.138 | 598 | 361 | 30 | 171 | 682 | 250 | 779 | Phage major capsid protein | Phage major capsid protein | | afdb-uniprot50 | AF-A0A0F9K1J2-F1-MODEL\_V4 | 1.0 | 1.956e-10 | 325 | 0.125 | 312 | 229 | 17 | 398 | 682 | 1 | 295 | Uncharacterized protein | Uncharacterized protein | | afdb-uniprot50 | AF-A0A6C2CA91-F1-MODEL\_V4 | 1.0 | 3.835e-13 | 325 | 0.142 | 366 | 243 | 18 | 356 | 681 | 7 | 341 | Phage major capsid protein | Phage major capsid protein | | afdb-uniprot50 | AF-A0A5C5VFR5-F1-MODEL\_V4 | 1.0 | 9.298e-14 | 325 | 0.173 | 495 | 291 | 26 | 253 | 681 | 9 | 451 | Phage capsid family protein | Phage capsid family protein | | afdb-uniprot50 | AF-A0A1S9YK65-F1-MODEL\_V4 | 1.0 | 1.559e-10 | 324 | 0.144 | 277 | 190 | 13 | 434 | 680 | 8 | 267 | Capsid protein | Capsid protein | | afdb-uniprot50 | AF-A0A661D5X9-F1-MODEL\_V4 | 1.0 | 2.318e-10 | 324 | 0.126 | 284 | 191 | 12 | 431 | 680 | 29 | 289 | Uncharacterized protein | Uncharacterized protein | | afdb-uniprot50 | AF-A0A2S6XCT0-F1-MODEL\_V4 | 1.0 | 1.15e-11 | 324 | 0.117 | 393 | 259 | 19 | 313 | 681 | 1 | 329 | Uncharacterized protein | Uncharacterized protein | | afdb-uniprot50 | AF-A0A2A7GD25-F1-MODEL\_V4 | 1.0 | 2.056e-13 | 324 | 0.154 | 394 | 249 | 21 | 336 | 681 | 1 | 358 | Phage major capsid protein | Phage major capsid protein | | afdb-uniprot50 | AF-A0A0F8ZHG2-F1-MODEL\_V4 | 1.0 | 9.361e-11 | 323 | 0.142 | 281 | 193 | 14 | 420 | 679 | 35 | 288 | Uncharacterized protein | Uncharacterized protein | | afdb-uniprot50 | AF-A0A7J4ZR60-F1-MODEL\_V4 | 1.0 | 3.375e-11 | 322 | 0.115 | 329 | 244 | 15 | 391 | 682 | 14 | 332 | Uncharacterized protein | Uncharacterized protein | | afdb-uniprot50 | AF-A0A2V4VIH6-F1-MODEL\_V4 | 1.0 | 1.771e-12 | 322 | 0.154 | 381 | 223 | 18 | 333 | 680 | 6 | 320 | HK97 family phage major capsid protein | HK97 family phage major capsid protein | | afdb-uniprot50 | AF-A0A661TNK1-F1-MODEL\_V4 | 1.0 | 5.545e-17 | 322 | 0.194 | 514 | 276 | 22 | 39 | 515 | 21 | 433 | Uncharacterized protein | Uncharacterized protein | | afdb-uniprot50 | AF-A0A371IJQ6-F1-MODEL\_V4 | 1.0 | 2.056e-13 | 320 | 0.121 | 378 | 245 | 16 | 338 | 681 | 42 | 366 | Phage major capsid protein | Phage major capsid protein | | afdb-uniprot50 | AF-A0A4V1UI61-F1-MODEL\_V4 | 1.0 | 1.306e-13 | 318 | 0.258 | 306 | 177 | 9 | 255 | 541 | 4 | 278 | Phage major capsid protein | Phage major capsid protein | | afdb-uniprot50 | AF-A0A6B5FRH0-F1-MODEL\_V4 | 1.0 | 1.048e-10 | 318 | 0.155 | 225 | 152 | 10 | 481 | 680 | 56 | 267 | Phage major capsid protein | Phage major capsid protein | | afdb-uniprot50 | AF-A0A7J3JAD0-F1-MODEL\_V4 | 1.0 | 8.358e-11 | 318 | 0.112 | 302 | 223 | 12 | 398 | 679 | 30 | 306 | Uncharacterized protein | Uncharacterized protein | | afdb-uniprot50 | AF-A0A841A8H5-F1-MODEL\_V4 | 1.0 | 4.985e-14 | 318 | 0.206 | 383 | 210 | 10 | 35 | 324 | 12 | 393 | Uncharacterized protein | Uncharacterized protein | | afdb-uniprot50 | AF-W0JUC6-F1-MODEL\_V4 | 1.0 | 5.702e-13 | 317 | 0.146 | 430 | 271 | 21 | 292 | 681 | 47 | 420 | Uncharacterized protein | Uncharacterized protein | | afdb-uniprot50 | AF-A0A068D9Z5-F1-MODEL\_V4 | 1.0 | 4.811e-13 | 317 | 0.158 | 436 | 251 | 23 | 300 | 681 | 60 | 433 | Phage major capsid protein | Phage major capsid protein | | afdb-uniprot50 | AF-A0A0F9Q477-F1-MODEL\_V4 | 1.0 | 9.561e-10 | 316 | 0.099 | 343 | 247 | 13 | 392 | 682 | 54 | 386 | Uncharacterized protein | Uncharacterized protein | | afdb-uniprot50 | AF-A0A836P8B0-F1-MODEL\_V4 | 1.0 | 1.848e-10 | 315 | 0.143 | 307 | 201 | 13 | 405 | 674 | 15 | 296 | Major capsid subunit gp9 | Major capsid subunit gp9 | | afdb-uniprot50 | AF-A0A842E264-F1-MODEL\_V4 | 1.0 | 1.166e-13 | 315 | 0.137 | 421 | 246 | 23 | 324 | 679 | 2 | 370 | Phage major capsid protein | Phage major capsid protein | | afdb-uniprot50 | AF-A0A0F9LNJ4-F1-MODEL\_V4 | 1.0 | 8.845e-11 | 313 | 0.116 | 310 | 225 | 18 | 402 | 682 | 2 | 291 | Uncharacterized protein | Uncharacterized protein | | afdb-uniprot50 | AF-D3NXQ3-F1-MODEL\_V4 | 1.0 | 2.596e-10 | 312 | 0.092 | 315 | 236 | 15 | 400 | 682 | 1 | 297 | Uncharacterized protein | Uncharacterized protein | | afdb-uniprot50 | AF-A0A810UUB9-F1-MODEL\_V4 | 1.0 | 1.298e-16 | 312 | 0.221 | 496 | 248 | 21 | 17 | 432 | 2 | 439 | Uncharacterized protein | Uncharacterized protein | | afdb-uniprot50 | AF-A0A139CW77-F1-MODEL\_V4 | 1.0 | 5.388e-13 | 312 | 0.145 | 399 | 255 | 22 | 335 | 681 | 4 | 368 | Major capsid protein | Major capsid protein | | afdb-uniprot50 | AF-A0A7W1JBE0-F1-MODEL\_V4 | 1.0 | 7.153e-13 | 312 | 0.142 | 464 | 311 | 25 | 272 | 681 | 40 | 470 | Phage major capsid protein | Phage major capsid protein | | afdb-uniprot50 | AF-A0A4S2EV56-F1-MODEL\_V4 | 1.0 | 5.388e-13 | 311 | 0.124 | 386 | 253 | 17 | 338 | 679 | 37 | 381 | Phage major capsid protein | Phage major capsid protein | | afdb-uniprot50 | AF-A0A6M3XUP1-F1-MODEL\_V4 | 1.0 | 3.189e-11 | 309 | 0.111 | 429 | 290 | 16 | 313 | 680 | 1 | 399 | Putative structural protein | Putative structural protein | | afdb-uniprot50 | AF-A8SKN8-F1-MODEL\_V4 | 1.0 | 3.917e-12 | 306 | 0.099 | 421 | 285 | 18 | 279 | 682 | 31 | 374 | Phage major capsid protein, HK97 family | Phage major capsid protein, HK97 family | | afdb-uniprot50 | AF-A0A2E7LMR5-F1-MODEL\_V4 | 1.0 | 9.034e-10 | 304 | 0.096 | 270 | 214 | 13 | 428 | 681 | 24 | 279 | Uncharacterized protein | Uncharacterized protein | | afdb-uniprot50 | AF-F9RFV9-F1-MODEL\_V4 | 1.0 | 2.07e-10 | 304 | 0.16 | 268 | 194 | 18 | 431 | 682 | 33 | 285 | Uncharacterized protein | Uncharacterized protein | | afdb-uniprot50 | AF-A0A255DW94-F1-MODEL\_V4 | 1.0 | 2.908e-10 | 303 | 0.12 | 348 | 180 | 13 | 449 | 681 | 2 | 338 | Phage major capsid protein | Phage major capsid protein | | afdb-uniprot50 | AF-A0A5C7M0U3-F1-MODEL\_V4 | 1.0 | 9.57e-18 | 303 | 0.12 | 688 | 434 | 36 | 67 | 682 | 16 | 604 | Phage major capsid protein | Phage major capsid protein | | afdb-uniprot50 | AF-A0A0F9EE26-F1-MODEL\_V4 | 1.0 | 2.07e-10 | 301 | 0.087 | 333 | 239 | 16 | 402 | 681 | 3 | 323 | Uncharacterized protein | Uncharacterized protein | | afdb-uniprot50 | AF-A0A2A5FHH9-F1-MODEL\_V4 | 1.0 | 1.412e-12 | 301 | 0.157 | 501 | 287 | 26 | 249 | 680 | 5 | 439 | Phage major capsid protein | Phage major capsid protein | | afdb-uniprot50 | AF-A0A0R2JSR1-F1-MODEL\_V4 | 1.0 | 5.311e-11 | 300 | 0.161 | 278 | 176 | 14 | 439 | 681 | 83 | 338 | Major head protein Cps | Major head protein Cps | | afdb-uniprot50 | AF-A0A2D6X1N3-F1-MODEL\_V4 | 1.0 | 1.11e-10 | 300 | 0.103 | 356 | 233 | 18 | 402 | 681 | 13 | 358 | Uncharacterized protein | Uncharacterized protein | | afdb-uniprot50 | AF-A0A0F9GE20-F1-MODEL\_V4 | 1.0 | 6.075e-10 | 296 | 0.092 | 282 | 229 | 12 | 422 | 680 | 15 | 292 | Uncharacterized protein | Uncharacterized protein | | afdb-uniprot50 | AF-A0A1V4ZC61-F1-MODEL\_V4 | 1.0 | 1.217e-11 | 296 | 0.131 | 434 | 265 | 20 | 309 | 682 | 18 | 399 | Uncharacterized protein | Uncharacterized protein | | afdb-uniprot50 | AF-A0A3M2BE71-F1-MODEL\_V4 | 1.0 | 3.036e-16 | 293 | 0.136 | 666 | 409 | 36 | 107 | 682 | 83 | 672 | Uncharacterized protein | Uncharacterized protein | | afdb-uniprot50 | AF-A0A6A8SHP3-F1-MODEL\_V4 | 1.0 | 4.643e-12 | 292 | 0.118 | 397 | 263 | 16 | 334 | 679 | 2 | 362 | Phage major capsid protein | Phage major capsid protein | | afdb-uniprot50 | AF-A0A1M3CGP7-F1-MODEL\_V4 | 1.0 | 9.561e-10 | 290 | 0.111 | 314 | 225 | 21 | 404 | 680 | 1 | 297 | Uncharacterized protein | Uncharacterized protein | | afdb-uniprot50 | AF-A0A1H4H424-F1-MODEL\_V4 | 1.0 | 1.771e-12 | 290 | 0.13 | 430 | 258 | 20 | 316 | 679 | 1 | 380 | Phage major capsid protein, HK97 family | Phage major capsid protein, HK97 family | | afdb-uniprot50 | AF-X0S7V5-F1-MODEL\_V4 | 1.0 | 4.742e-11 | 287 | 0.129 | 463 | 283 | 24 | 252 | 666 | 11 | 401 | Uncharacterized protein | Uncharacterized protein | | afdb-uniprot50 | AF-A0A7X2TNW2-F1-MODEL\_V4 | 1.0 | 1.582e-12 | 282 | 0.134 | 431 | 259 | 23 | 334 | 681 | 3 | 402 | Phage major capsid protein | Phage major capsid protein | | afdb-uniprot50 | AF-A0A380JMN2-F1-MODEL\_V4 | 1.0 | 2.402e-11 | 281 | 0.136 | 404 | 263 | 19 | 334 | 681 | 2 | 375 | Predicted phage phi-C31 gp36 major capsid-like protein | Predicted phage phi-C31 gp36 major capsid-like protein | | afdb-uniprot50 | AF-A0A0F9EU19-F1-MODEL\_V4 | 1.0 | 5.311e-11 | 281 | 0.092 | 380 | 267 | 17 | 337 | 680 | 48 | 385 | Uncharacterized protein | Uncharacterized protein | | afdb-uniprot50 | AF-A0A0M5M622-F1-MODEL\_V4 | 1.0 | 1.592e-09 | 278 | 0.083 | 276 | 203 | 13 | 435 | 682 | 30 | 283 | Uncharacterized protein | Uncharacterized protein | | afdb-uniprot50 | AF-A0A2V2RBB4-F1-MODEL\_V4 | 1.0 | 1.086e-11 | 277 | 0.29 | 279 | 170 | 7 | 257 | 521 | 4 | 268 | Phage major capsid protein | Phage major capsid protein | | afdb-uniprot50 | AF-A0A3M2C237-F1-MODEL\_V4 | 1.0 | 6.386e-13 | 277 | 0.122 | 434 | 275 | 22 | 334 | 680 | 3 | 417 | Phage major capsid protein | Phage major capsid protein | | afdb-uniprot50 | AF-A0A0E0US10-F1-MODEL\_V4 | 1.0 | 3.375e-11 | 276 | 0.14 | 399 | 255 | 22 | 335 | 680 | 1 | 364 | Major capsid protein | Major capsid protein | | afdb-uniprot50 | AF-A0A841X480-F1-MODEL\_V4 | 1.0 | 1.086e-11 | 275 | 0.121 | 451 | 270 | 25 | 264 | 679 | 6 | 365 | Phage major capsid protein | Phage major capsid protein | | afdb-uniprot50 | AF-A0A540WHX5-F1-MODEL\_V4 | 1.0 | 5.741e-10 | 274 | 0.178 | 365 | 242 | 20 | 295 | 641 | 27 | 351 | Phage major capsid protein | Phage major capsid protein | | afdb-uniprot50 | AF-A0A2E8F3P1-F1-MODEL\_V4 | 1.0 | 1.126e-12 | 272 | 0.132 | 573 | 334 | 28 | 194 | 678 | 6 | 503 | Phage major capsid protein | Phage major capsid protein | | afdb-uniprot50 | AF-A0A0F9VRZ0-F1-MODEL\_V4 | 1.0 | 3.189e-11 | 271 | 0.136 | 411 | 271 | 18 | 308 | 682 | 34 | 396 | Uncharacterized protein | Uncharacterized protein | | afdb-uniprot50 | AF-A0A202F7U9-F1-MODEL\_V4 | 1.0 | 1.363e-11 | 267 | 0.129 | 402 | 256 | 23 | 335 | 679 | 1 | 365 | Uncharacterized protein | Uncharacterized protein | | afdb-uniprot50 | AF-A0A2T4M4G5-F1-MODEL\_V4 | 1.0 | 1.809e-11 | 266 | 0.139 | 394 | 264 | 18 | 338 | 681 | 18 | 386 | Phage major capsid protein | Phage major capsid protein | | afdb-uniprot50 | AF-A0A5R9FB27-F1-MODEL\_V4 | 1.0 | 1.11e-10 | 264 | 0.112 | 372 | 254 | 19 | 338 | 679 | 66 | 391 | Phage major capsid protein | Phage major capsid protein | | afdb-uniprot50 | AF-A0A3D9YKZ9-F1-MODEL\_V4 | 1.0 | 2.614e-07 | 264 | 0.224 | 147 | 96 | 6 | 42 | 178 | 19 | 157 | HK97 family phage prohead protease | HK97 family phage prohead protease | | afdb-uniprot50 | AF-A0A5C7Q7W6-F1-MODEL\_V4 | 1.0 | 3.398e-08 | 260 | 0.177 | 180 | 125 | 9 | 19 | 188 | 6 | 172 | HK97 family phage prohead protease | HK97 family phage prohead protease | | afdb-uniprot50 | AF-X0SVC7-F1-MODEL\_V4 | 1.0 | 5.235e-09 | 260 | 0.089 | 279 | 198 | 13 | 430 | 676 | 1 | 255 | Uncharacterized protein | Uncharacterized protein | | afdb-uniprot50 | AF-L1QG40-F1-MODEL\_V4 | 1.0 | 1.559e-10 | 259 | 0.102 | 441 | 293 | 20 | 269 | 682 | 6 | 370 | Phage major capsid protein, HK97 family | Phage major capsid protein, HK97 family | | afdb-uniprot50 | AF-A0A380F728-F1-MODEL\_V4 | 1.0 | 6.662e-11 | 258 | 0.121 | 412 | 269 | 20 | 334 | 680 | 2 | 385 | Phage protein | Phage protein | | afdb-uniprot50 | AF-A0A497US25-F1-MODEL\_V4 | 1.0 | 3.375e-11 | 257 | 0.122 | 505 | 285 | 24 | 252 | 679 | 21 | 444 | Uncharacterized protein | Uncharacterized protein | | afdb-uniprot50 | AF-A0A0F9ITE6-F1-MODEL\_V4 | 1.0 | 9.907e-11 | 255 | 0.263 | 334 | 186 | 12 | 261 | 568 | 4 | 303 | Uncharacterized protein | Uncharacterized protein | | afdb-uniprot50 | AF-A0A7K0J5W9-F1-MODEL\_V4 | 1.0 | 7.051e-11 | 255 | 0.136 | 461 | 239 | 19 | 233 | 673 | 25 | 346 | Phage major capsid protein | Phage major capsid protein | | afdb-uniprot50 | AF-A0A0F9T8B6-F1-MODEL\_V4 | 1.0 | 2.453e-10 | 255 | 0.123 | 365 | 254 | 18 | 338 | 680 | 88 | 408 | Uncharacterized protein | Uncharacterized protein | | afdb-uniprot50 | AF-A0A1I4ZM31-F1-MODEL\_V4 | 1.0 | 1.11e-10 | 254 | 0.112 | 416 | 250 | 24 | 335 | 681 | 1 | 366 | Phage major capsid protein, HK97 family | Phage major capsid protein, HK97 family | | afdb-uniprot50 | AF-A0A1G7NZP4-F1-MODEL\_V4 | 1.0 | 3.375e-11 | 251 | 0.121 | 421 | 254 | 24 | 323 | 681 | 2 | 368 | Phage major capsid protein, HK97 family | Phage major capsid protein, HK97 family | | afdb-uniprot50 | AF-A0A497X2P2-F1-MODEL\_V4 | 1.0 | 5.989e-08 | 246 | 0.175 | 188 | 123 | 13 | 19 | 192 | 2 | 171 | Uncharacterized protein | Uncharacterized protein | | afdb-uniprot50 | AF-A0A512JIM5-F1-MODEL\_V4 | 1.0 | 1.217e-11 | 246 | 0.186 | 402 | 219 | 12 | 51 | 353 | 2 | 394 | Uncharacterized protein | Uncharacterized protein | | afdb-uniprot50 | AF-A0A135IJY6-F1-MODEL\_V4 | 1.0 | 1.661e-07 | 234 | 0.174 | 195 | 134 | 9 | 27 | 208 | 4 | 184 | Peptidase U35 | Peptidase U35 | | afdb-uniprot50 | AF-A0A3A6MU59-F1-MODEL\_V4 | 1.0 | 5.347e-08 | 231 | 0.207 | 183 | 117 | 10 | 6 | 173 | 98 | 267 | HK97 family phage prohead protease | HK97 family phage prohead protease | | afdb-uniprot50 | AF-A0A2D9VGK2-F1-MODEL\_V4 | 1.0 | 3.034e-08 | 221 | 0.194 | 216 | 128 | 14 | 18 | 211 | 2 | 193 | HK97 family phage prohead protease | HK97 family phage prohead protease | | afdb-uniprot50 | AF-A0A7W7LNM0-F1-MODEL\_V4 | 1.0 | 1.296e-08 | 217 | 0.112 | 454 | 224 | 20 | 400 | 681 | 1 | 447 | Uncharacterized protein | Uncharacterized protein | | afdb-uniprot50 | AF-A0A073IXT7-F1-MODEL\_V4 | 1.0 | 1.483e-07 | 216 | 0.188 | 186 | 121 | 8 | 17 | 185 | 2 | 174 | Primosome assembly protein PriA | Primosome assembly protein PriA | | afdb-uniprot50 | AF-A0A7W3UAB7-F1-MODEL\_V4 | 1.0 | 2.748e-10 | 215 | 0.158 | 441 | 241 | 21 | 40 | 426 | 25 | 389 | HK97 family phage prohead protease | HK97 family phage prohead protease | | afdb-uniprot50 | AF-A0A4R5EYX0-F1-MODEL\_V4 | 1.0 | 8.905e-08 | 204 | 0.216 | 203 | 116 | 12 | 11 | 193 | 14 | 193 | HK97 family phage prohead protease | HK97 family phage prohead protease | | afdb-uniprot50 | AF-A0A3G8GRJ2-F1-MODEL\_V4 | 1.0 | 4.353e-07 | 204 | 0.19 | 221 | 134 | 12 | 43 | 258 | 28 | 208 | Uncharacterized protein | Uncharacterized protein | | afdb-uniprot50 | AF-A0A2E6W8T6-F1-MODEL\_V4 | 1.0 | 4.876e-07 | 195 | 0.127 | 291 | 197 | 18 | 420 | 682 | 28 | 289 | Uncharacterized protein | Uncharacterized protein | | afdb-uniprot50 | AF-A0A0F8WVU2-F1-MODEL\_V4 | 1.0 | 1.9e-06 | 194 | 0.134 | 215 | 158 | 11 | 481 | 682 | 15 | 214 | Uncharacterized protein | Uncharacterized protein | | afdb-uniprot50 | AF-B9XA48-F1-MODEL\_V4 | 1.0 | 8.294e-06 | 191 | 0.285 | 189 | 114 | 4 | 367 | 534 | 5 | 193 | Putative phage-related protein | Putative phage-related protein | | afdb-uniprot50 | AF-A0A1E4IEB7-F1-MODEL\_V4 | 1.0 | 1.86e-07 | 188 | 0.106 | 338 | 215 | 22 | 410 | 681 | 1 | 317 | Uncharacterized protein | Uncharacterized protein | | afdb-uniprot50 | AF-A0A2P8DWF2-F1-MODEL\_V4 | 1.0 | 4.262e-08 | 186 | 0.135 | 369 | 219 | 17 | 39 | 379 | 6 | 302 | HK97 family phage prohead protease | HK97 family phage prohead protease | | afdb-uniprot50 | AF-A0A443KJC0-F1-MODEL\_V4 | 1.0 | 1.696e-06 | 180 | 0.181 | 176 | 112 | 10 | 31 | 192 | 4 | 161 | HK97 family phage prohead protease | HK97 family phage prohead protease | | afdb-uniprot50 | AF-A0A517R7E1-F1-MODEL\_V4 | 1.0 | 5.903e-06 | 175 | 0.122 | 277 | 159 | 13 | 418 | 680 | 16 | 222 | Uncharacterized protein | Uncharacterized protein | | afdb-uniprot50 | AF-A0A3R5RK36-F1-MODEL\_V4 | 1.0 | 3.887e-07 | 173 | 0.133 | 321 | 178 | 17 | 382 | 682 | 3 | 243 | Uncharacterized protein | Uncharacterized protein | | afdb-uniprot50 | AF-A0A368YH54-F1-MODEL\_V4 | 1.0 | 7.95e-08 | 164 | 0.141 | 453 | 222 | 21 | 18 | 330 | 7 | 432 | HK97 family phage prohead protease | HK97 family phage prohead protease | | afdb-uniprot50 | AF-A0A6B1C0U0-F1-MODEL\_V4 | 1.0 | 3.887e-07 | 149 | 0.149 | 489 | 260 | 26 | 261 | 671 | 8 | 418 | Uncharacterized protein | Uncharacterized protein | | afdb-uniprot50 | AF-A0A0F9U9R9-F1-MODEL\_V4 | 1.0 | 2.04e-08 | 140 | 0.128 | 481 | 255 | 23 | 12 | 396 | 121 | 533 | Uncharacterized protein | Uncharacterized protein | | afdb-uniprot50 | AF-A0A0F9N5R3-F1-MODEL\_V4 | 1.0 | 1.056e-07 | 136 | 0.136 | 447 | 259 | 27 | 38 | 421 | 19 | 401 | Uncharacterized protein | Uncharacterized protein | |
| Top keywords  (threshold 1.00e-02 (evalue)) | **capsid, major, Phage, HK97, prohead, protease, Putative, Peptidase, head, Caudovirus** |
| Output files | ../../similar\_structures/05\_FANPEZAQ\_CDS\_0005\_afdb-proteome\_foldseek.tsv ../../similar\_structures/05\_FANPEZAQ\_CDS\_0005\_afdb-uniprot50\_foldseek.tsv ../../similar\_structures/05\_FANPEZAQ\_CDS\_0005\_merged.svg ../../similar\_structures/05\_FANPEZAQ\_CDS\_0005\_pdb\_foldseek.tsv |

  
  
  

Return to summary | Go to previous | Go to next

  


---

**Sequence/structure alignments coloring**  
Each object in the alignment figures is colored according to its E-value following this color coding:

1e-100
10

**References:**  
1) Steinegger M, Meier M, Mirdita M, Vöhringer H, Haunsberger S J, and Söding J (2019) HH-suite3 for fast remote homology detection and deep protein annotation, BMC Bioinformatics, 473. doi: 10.1186/s12859-019-3019-7  
2) Jumper J, Evans R, Pritzel A, ..., Hassabis D (2021) Highly accurate protein structure prediction with AlphaFold, Nature, 596. doi: 10.1038/s41586-021-03819-2  
3) van Kempen M, Kim S, Tumescheit C, Mirdita M, Lee J, Gilchrist CLM, Söding J, and Steinegger M (2023) Fast and accurate protein structure search with Foldseek. Nature Biotechnology. doi: 10.1038/s41587-023-01773-0
