# Supplementary material for: Completing the BASEL phage collection to unlock hidden diversity for systematic exploration of phage–host interactions
Source: PLoS Biol. 2025 Apr 7;23(4):e3003063. doi: 10.1371/journal.pbio.3003063 (PMC11990801; doi:10.1371/journal.pbio.3003063)
Supplement: S2 Data — (ZIP) [file pbio.3003063.s009.zip › entries/51.html]

FANPEZAQ\_CDS\_0051


Return to summary | Go to previous | Go to next

|  |  |
| --- | --- |
| FANPEZAQ\_CDS\_0051 Page creation date: 02 Sep 2024, 12:00  Project folder: n/a  Input sequences file: Escherichia\_virus\_HeidiAbel.gb | hypothetical phage transcriptional regulator p119269 vi\_03293 cell envelope biogenesis ompa membrane lipoprotein duf4222 domain\_containing cro ci coil containing lysis system i\_spanin rz |

### Sequence information

|  |  |
| --- | --- |
| Name | FANPEZAQ\_CDS\_0051  51\_FANPEZAQ\_CDS\_0051 (pipeline id) |
| Imported annotations | Escherichia\_virus\_HeidiAbel Bas97 |
| Protein sequence | MNPVDFIKKQVMAELVKQGASPGTAQHAADYAAQQYRTTSAIGKDPFAELIRLAGAMAMK HQAGFRFVMPQSSR |
| Number of residues | 74 |
| Molecular weight (Da) | 8068.24 |
| Output files | ../../query\_sequences/51\_FANPEZAQ\_CDS\_0051.fasta |

### Putative domain architecture and protein family

#### Search results (HHblits)1

|  |  |
| --- | --- |
| Domain family databases searched | Pfam, Ncbi-cd, Cath, Phrogs |
| Results, scheme(s)  (Top layers only; threshold 1.00e-03 (evalue)) | xml version="1.0" encoding="utf-8" standalone="no"?       2024-09-02T21:08:23.865001 image/svg+xml   Matplotlib v3.7.2, https://matplotlib.org/ |
| Results, table  (E-value ≤ 1.00e-03 (evalue)) | | db | id | prob | evalue | pvalue | score | cols | query | query\_len | template | template\_len | name | description | | --- | --- | --- | --- | --- | --- | --- | --- | --- | --- | --- | --- | --- | | phrogs | 4439 | 99.9 | 6.7e-34 | 8.8e-38 | 173.2 | 58 | (1, 58) | 74 | (1, 60) | 60 | NA | NA; Category: unknown function; p119269 VI\_03293 | |
| Top keywords  (threshold 1.00e-03 (evalue)) | **p119269, VI\_03293** |
| Output files | ../../domain\_architecture/51\_FANPEZAQ\_CDS\_0051\_cath.hhr ../../domain\_architecture/51\_FANPEZAQ\_CDS\_0051\_merged.svg ../../domain\_architecture/51\_FANPEZAQ\_CDS\_0051\_ncbi-cd.hhr ../../domain\_architecture/51\_FANPEZAQ\_CDS\_0051\_pfam.hhr ../../domain\_architecture/51\_FANPEZAQ\_CDS\_0051\_phrogs.hhr |

### Identical protein sequences/structures

#### Search results

|  |  |
| --- | --- |
| Protein sequence databases searched | Pdb, Swissprot, Refseq |
| Identical proteins found | -- |
| Top keywords | -- |
| Output files | -- |

### Similar protein sequences/structures

#### Sequence similarity search results (HHblits)1

|  |  |
| --- | --- |
| Sequence databases searched | Uniclust, Pdb70 |
| Results, scheme(s)  (Top layers only, threshold 1.00e-03 (evalue)) | xml version="1.0" encoding="utf-8" standalone="no"?       2024-09-02T21:08:51.007845 image/svg+xml   Matplotlib v3.7.2, https://matplotlib.org/ |
| Results, table(s)  (threshold 1.00e-03 (evalue)) | | db | id | prob | evalue | pvalue | score | cols | query | query\_len | template | template\_len | name | description | | --- | --- | --- | --- | --- | --- | --- | --- | --- | --- | --- | --- | --- | | uniclust | UniRef100\_A0A094U7C3 | 100.0 | 1.2e-33 | 2.6e-39 | 175.5 | 69 | (1, 69) | 74 | (3, 73) | 90 | Uncharacterized protein | Uncharacterized protein | | uniclust | UniRef100\_A0A0T9RNR7 | 99.9 | 6.7e-32 | 1.4e-37 | 168.6 | 73 | (1, 73) | 74 | (1, 75) | 93 | Uncharacterized protein | Uncharacterized protein | | uniclust | UniRef100\_A0A085ADJ9 | 99.9 | 3.7e-31 | 8e-37 | 171.7 | 72 | (1, 72) | 74 | (18, 91) | 119 | Phage protein | Phage protein | | uniclust | UniRef100\_A0A077NE64 | 99.9 | 6.1e-31 | 1.4e-36 | 167.0 | 72 | (1, 72) | 74 | (15, 88) | 99 | Phage protein | Phage protein | | uniclust | UniRef100\_A0A2S1BH24 | 99.8 | 6.9e-22 | 1.3e-27 | 121.7 | 72 | (1, 72) | 74 | (1, 74) | 85 | Uncharacterized protein | Uncharacterized protein | | uniclust | UniRef100\_UPI0020173D3F | 99.8 | 1.2e-21 | 2.2e-27 | 125.1 | 73 | (1, 73) | 74 | (30, 104) | 114 | hypothetical protein | hypothetical protein | | uniclust | UniRef100\_A0A2U1TMP1 | 99.7 | 1.2e-20 | 2.4e-26 | 115.9 | 70 | (1, 70) | 74 | (3, 74) | 82 | Uncharacterized protein | Uncharacterized protein | | uniclust | UniRef100\_UPI0019099C25 | 99.7 | 2.3e-19 | 4.2e-25 | 118.9 | 72 | (1, 72) | 74 | (62, 135) | 145 | hypothetical protein | hypothetical protein | | uniclust | UniRef100\_A0A0P6ZIQ1 | 99.6 | 7.3e-19 | 1.5e-24 | 113.0 | 71 | (2, 72) | 74 | (20, 93) | 102 | Uncharacterized protein | Uncharacterized protein | | uniclust | UniRef100\_A0A2V4G0H8 | 99.6 | 1.6e-18 | 3.3e-24 | 108.5 | 71 | (1, 71) | 74 | (4, 76) | 89 | Uncharacterized protein | Uncharacterized protein | | uniclust | UniRef100\_UPI00090789F6 | 99.6 | 2e-18 | 3.8e-24 | 111.8 | 69 | (2, 70) | 74 | (4, 74) | 113 | hypothetical protein | hypothetical protein | | uniclust | UniRef100\_A0A2L0TW67 | 99.6 | 5.6e-18 | 1.1e-23 | 105.9 | 67 | (1, 67) | 74 | (1, 68) | 88 | Uncharacterized protein | Uncharacterized protein | | uniclust | UniRef100\_A0A078L883 | 99.6 | 8.1e-18 | 1.6e-23 | 99.1 | 54 | (1, 54) | 74 | (1, 56) | 60 | Uncharacterized protein | Uncharacterized protein | | uniclust | UniRef100\_A0A379D1S2 | 99.5 | 1.5e-17 | 3.2e-23 | 99.7 | 51 | (23, 73) | 74 | (2, 54) | 65 | Uncharacterized protein | Uncharacterized protein | | uniclust | UniRef100\_A0A090IT71 | 99.5 | 2.2e-17 | 4.4e-23 | 103.0 | 71 | (2, 72) | 74 | (9, 80) | 84 | Uncharacterized phage protein | Uncharacterized phage protein | | uniclust | UniRef100\_A0A173GC14 | 99.4 | 4.6e-16 | 8.9e-22 | 91.1 | 56 | (1, 62) | 74 | (1, 57) | 58 | Uncharacterized protein | Uncharacterized protein | | uniclust | UniRef100\_A0A087KTT2 | 99.4 | 4.5e-16 | 9.8e-22 | 97.6 | 66 | (4, 72) | 74 | (6, 71) | 83 | Cell envelope biogenesis protein OmpA | Cell envelope biogenesis protein OmpA | | uniclust | UniRef100\_A0A241XEA3 | 99.4 | 1.3e-15 | 2.5e-21 | 92.3 | 67 | (1, 67) | 74 | (1, 67) | 71 | Uncharacterized protein | Uncharacterized protein | | uniclust | UniRef100\_UPI000A4DD1B9 | 99.4 | 1.5e-15 | 2.8e-21 | 103.8 | 71 | (1, 71) | 74 | (88, 161) | 173 | hypothetical protein | hypothetical protein | | uniclust | UniRef100\_UPI00226A8289 | 99.4 | 2.2e-15 | 4e-21 | 99.2 | 63 | (1, 63) | 74 | (63, 127) | 130 | hypothetical protein | hypothetical protein | | uniclust | UniRef100\_A0A209B2B0 | 99.3 | 1.7e-14 | 3.2e-20 | 85.8 | 63 | (1, 64) | 74 | (1, 65) | 66 | Uncharacterized protein | Uncharacterized protein | | uniclust | UniRef100\_A0A0E1SXQ1 | 99.2 | 3.2e-14 | 5.9e-20 | 79.2 | 38 | (27, 64) | 74 | (2, 41) | 45 | Uncharacterized protein | Uncharacterized protein | | uniclust | UniRef100\_UPI0002F40243 | 99.2 | 5.2e-14 | 9.5e-20 | 86.1 | 52 | (12, 63) | 74 | (2, 55) | 77 | hypothetical protein | hypothetical protein | | uniclust | UniRef100\_A0A5M7L259 | 99.2 | 9e-14 | 1.7e-19 | 87.0 | 70 | (1, 74) | 74 | (8, 80) | 85 | Uncharacterized protein | Uncharacterized protein | | uniclust | UniRef100\_A0A0A7KY33 | 99.2 | 1.2e-13 | 2.2e-19 | 90.7 | 67 | (1, 67) | 74 | (42, 109) | 123 | Uncharacterized protein | Uncharacterized protein | | uniclust | UniRef100\_A0A8I1W9T1 | 99.1 | 2.1e-13 | 3.8e-19 | 84.5 | 62 | (1, 62) | 74 | (1, 63) | 83 | Uncharacterized protein | Uncharacterized protein | | uniclust | UniRef100\_A0A1Q8CZL4 | 99.1 | 3.4e-13 | 6.3e-19 | 84.1 | 66 | (1, 66) | 74 | (12, 78) | 84 | Uncharacterized protein | Uncharacterized protein | | uniclust | UniRef100\_A0A1V3K6Q6 | 99.1 | 9.1e-13 | 1.8e-18 | 82.3 | 66 | (1, 66) | 74 | (1, 66) | 80 | Uncharacterized protein | Uncharacterized protein | | uniclust | UniRef100\_A0A655UTX9 | 99.1 | 1e-12 | 2e-18 | 83.3 | 71 | (2, 72) | 74 | (9, 81) | 89 | Uncharacterized protein | Uncharacterized protein | | uniclust | UniRef100\_UPI001FB599ED | 99.1 | 1.1e-12 | 2e-18 | 77.5 | 43 | (23, 65) | 74 | (2, 46) | 61 | hypothetical protein | hypothetical protein | | uniclust | UniRef100\_A0A0F4NNX2 | 99.0 | 2e-12 | 4e-18 | 83.5 | 71 | (1, 72) | 74 | (1, 71) | 97 | Uncharacterized protein | Uncharacterized protein | | uniclust | UniRef100\_UPI00198254EA | 99.0 | 3e-12 | 5.5e-18 | 78.0 | 47 | (18, 64) | 74 | (23, 71) | 73 | hypothetical protein | hypothetical protein | | uniclust | UniRef100\_A0A089Q372 | 99.0 | 3.1e-12 | 7e-18 | 81.7 | 63 | (1, 66) | 74 | (2, 64) | 86 | Uncharacterized protein | Uncharacterized protein | | uniclust | UniRef100\_H2BDF4 | 98.9 | 1e-11 | 1.9e-17 | 83.4 | 66 | (1, 66) | 74 | (71, 136) | 138 | Uncharacterized protein | Uncharacterized protein | | uniclust | UniRef100\_UPI0019D9CF8F | 98.9 | 1.3e-11 | 2.3e-17 | 75.5 | 57 | (1, 57) | 74 | (1, 60) | 73 | hypothetical protein | hypothetical protein | | uniclust | UniRef100\_UPI001F319DAE | 98.9 | 1.7e-11 | 3.3e-17 | 79.2 | 55 | (1, 55) | 74 | (29, 83) | 98 | hypothetical protein | hypothetical protein | | uniclust | UniRef100\_A0A0N7JPH6 | 98.9 | 1.7e-11 | 3.3e-17 | 77.4 | 68 | (1, 71) | 74 | (3, 70) | 82 | Uncharacterized protein | Uncharacterized protein | | uniclust | UniRef100\_UPI001E649674 | 98.8 | 4.5e-11 | 8.2e-17 | 69.0 | 40 | (1, 40) | 74 | (1, 41) | 52 | hypothetical protein | hypothetical protein | | uniclust | UniRef100\_UPI000573BB50 | 98.8 | 5.1e-11 | 9.4e-17 | 72.4 | 61 | (1, 66) | 74 | (1, 61) | 70 | hypothetical protein | hypothetical protein | | uniclust | UniRef100\_A0A4V1DBZ5 | 98.8 | 6.6e-11 | 1.2e-16 | 73.5 | 63 | (1, 63) | 74 | (1, 63) | 80 | Uncharacterized protein | Uncharacterized protein | | uniclust | UniRef100\_A0A0Q1CBT0 | 98.7 | 1e-10 | 2.2e-16 | 77.9 | 35 | (1, 35) | 74 | (1, 35) | 110 | Uncharacterized protein | Uncharacterized protein | | uniclust | UniRef100\_A0A513SPV1 | 98.7 | 1.4e-10 | 2.6e-16 | 77.2 | 67 | (2, 68) | 74 | (45, 115) | 125 | Uncharacterized protein | Uncharacterized protein | | uniclust | UniRef100\_A0A516MMM3 | 98.7 | 2.4e-10 | 4.4e-16 | 68.3 | 60 | (1, 60) | 74 | (1, 60) | 62 | Uncharacterized protein | Uncharacterized protein | | uniclust | UniRef100\_A0A7I6Q891 | 98.6 | 2.7e-10 | 5e-16 | 62.3 | 29 | (1, 29) | 74 | (1, 29) | 38 | Uncharacterized protein | Uncharacterized protein | | uniclust | UniRef100\_A0A4R3Z3M5 | 98.6 | 2.7e-10 | 5.2e-16 | 60.9 | 29 | (1, 29) | 74 | (1, 29) | 33 | Uncharacterized protein | Uncharacterized protein | | uniclust | UniRef100\_UPI00211A06C2 | 98.6 | 4.4e-10 | 8.1e-16 | 72.4 | 66 | (1, 66) | 74 | (1, 66) | 99 | hypothetical protein | hypothetical protein | | uniclust | UniRef100\_A0A9E7LPW0 | 98.6 | 7.3e-10 | 1.3e-15 | 77.3 | 54 | (1, 60) | 74 | (118, 172) | 174 | Uncharacterized protein | Uncharacterized protein | | uniclust | UniRef100\_A0A066RYV4 | 98.5 | 1.2e-09 | 2.3e-15 | 67.7 | 59 | (2, 60) | 74 | (7, 65) | 70 | Transcriptional regulator | Transcriptional regulator | | uniclust | UniRef100\_A0A2I7S8J3 | 98.5 | 1.6e-09 | 3.4e-15 | 70.5 | 70 | (1, 70) | 74 | (1, 70) | 92 | Membrane lipoprotein | Membrane lipoprotein | | uniclust | UniRef100\_UPI0021CBB242 | 98.5 | 2.1e-09 | 3.9e-15 | 70.8 | 68 | (2, 69) | 74 | (9, 78) | 112 | hypothetical protein | hypothetical protein | | uniclust | UniRef100\_UPI0003BEDC01 | 98.4 | 3.2e-09 | 6.1e-15 | 62.1 | 33 | (1, 33) | 74 | (1, 33) | 52 | hypothetical protein | hypothetical protein | | uniclust | UniRef100\_A0A1I3XT93 | 98.4 | 4e-09 | 7.3e-15 | 55.0 | 27 | (1, 27) | 74 | (1, 27) | 29 | Uncharacterized protein | Uncharacterized protein | | uniclust | UniRef100\_UPI0018F0D1C9 | 98.4 | 4.3e-09 | 7.8e-15 | 63.2 | 59 | (1, 60) | 74 | (1, 59) | 62 | hypothetical protein | hypothetical protein | | uniclust | UniRef100\_UPI000AF57BD8 | 98.3 | 9.6e-09 | 1.9e-14 | 69.7 | 30 | (1, 30) | 74 | (1, 30) | 122 | hypothetical protein | hypothetical protein | | uniclust | UniRef100\_UPI000988BEDA | 98.3 | 1.1e-08 | 2e-14 | 64.8 | 70 | (2, 71) | 74 | (8, 77) | 84 | hypothetical protein | hypothetical protein | | uniclust | UniRef100\_UPI0020A36E27 | 98.3 | 1.3e-08 | 2.3e-14 | 62.3 | 35 | (1, 35) | 74 | (1, 35) | 68 | hypothetical protein | hypothetical protein | | uniclust | UniRef100\_A0A2N7JZE4 | 98.2 | 2.9e-08 | 5.4e-14 | 61.8 | 57 | (14, 70) | 74 | (12, 71) | 75 | Uncharacterized protein | Uncharacterized protein | | uniclust | UniRef100\_A0A127MXN3 | 98.1 | 3.9e-08 | 7.2e-14 | 57.1 | 44 | (1, 44) | 74 | (1, 44) | 50 | Uncharacterized protein | Uncharacterized protein | | uniclust | UniRef100\_A0A087L1N4 | 98.1 | 3.8e-08 | 7.9e-14 | 64.5 | 33 | (1, 33) | 74 | (1, 33) | 92 | Uncharacterized protein | Uncharacterized protein | | uniclust | UniRef100\_A0A1Q8Z4M5 | 98.0 | 8.2e-08 | 1.6e-13 | 63.8 | 33 | (1, 33) | 74 | (1, 33) | 107 | Uncharacterized protein | Uncharacterized protein | | uniclust | UniRef100\_A0A927DSF2 | 98.0 | 9.3e-08 | 1.8e-13 | 66.8 | 33 | (1, 33) | 74 | (17, 49) | 150 | Uncharacterized protein | Uncharacterized protein | | uniclust | UniRef100\_A0A380QAC9 | 97.9 | 2.1e-07 | 3.9e-13 | 55.0 | 33 | (1, 33) | 74 | (1, 33) | 53 | Uncharacterized protein | Uncharacterized protein | | uniclust | UniRef100\_UPI001FCE5DAA | 97.9 | 2.2e-07 | 4e-13 | 63.4 | 34 | (1, 34) | 74 | (1, 34) | 132 | DUF4222 domain-containing protein | DUF4222 domain-containing protein | | uniclust | UniRef100\_A0A1X3IVQ3 | 97.9 | 2.3e-07 | 4.2e-13 | 57.5 | 32 | (1, 32) | 74 | (1, 32) | 70 | Uncharacterized protein | Uncharacterized protein | | uniclust | UniRef100\_UPI000791D832 | 97.9 | 2.6e-07 | 5e-13 | 62.0 | 28 | (1, 28) | 74 | (1, 28) | 109 | hypothetical protein | hypothetical protein | | uniclust | UniRef100\_UPI0004ADB128 | 97.8 | 4e-07 | 7.8e-13 | 55.3 | 32 | (1, 32) | 74 | (1, 32) | 58 | hypothetical protein | hypothetical protein | | uniclust | UniRef100\_UPI000B237913 | 97.7 | 8.5e-07 | 1.6e-12 | 56.2 | 53 | (13, 65) | 74 | (3, 56) | 78 | hypothetical protein | hypothetical protein | | uniclust | UniRef100\_A0A7Y3Z2V1 | 97.7 | 1.2e-06 | 2.2e-12 | 53.5 | 43 | (2, 44) | 74 | (9, 51) | 63 | Uncharacterized protein | Uncharacterized protein | | uniclust | UniRef100\_A0A927HPP5 | 97.7 | 1.3e-06 | 2.4e-12 | 55.9 | 26 | (1, 26) | 74 | (58, 83) | 84 | Uncharacterized protein | Uncharacterized protein | | uniclust | UniRef100\_UPI0021BD29FD | 97.6 | 1.6e-06 | 3e-12 | 59.5 | 35 | (1, 35) | 74 | (84, 118) | 133 | Cro/CI family transcriptional regulator | Cro/CI family transcriptional regulator | | uniclust | UniRef100\_UPI001CBDE202 | 97.5 | 3.8e-06 | 7e-12 | 49.4 | 37 | (1, 37) | 74 | (1, 37) | 50 | hypothetical protein | hypothetical protein | | uniclust | UniRef100\_UPI001F40ADE3 | 97.5 | 4e-06 | 7.3e-12 | 49.7 | 32 | (1, 32) | 74 | (1, 32) | 52 | hypothetical protein | hypothetical protein | | uniclust | UniRef100\_UPI000A1EE641 | 97.5 | 4.2e-06 | 7.9e-12 | 52.0 | 56 | (3, 58) | 74 | (4, 59) | 66 | hypothetical protein | hypothetical protein | | uniclust | UniRef100\_UPI0022CE1587 | 97.4 | 5e-06 | 9.1e-12 | 53.0 | 58 | (2, 59) | 74 | (9, 66) | 79 | hypothetical protein | hypothetical protein | | uniclust | UniRef100\_UPI000B35E1F4 | 97.4 | 4.8e-06 | 9.2e-12 | 55.0 | 67 | (1, 67) | 74 | (1, 69) | 94 | hypothetical protein | hypothetical protein | | uniclust | UniRef100\_A0A927HJ22 | 97.4 | 6e-06 | 1.1e-11 | 58.3 | 32 | (1, 32) | 74 | (58, 89) | 158 | Uncharacterized protein | Uncharacterized protein | | uniclust | UniRef100\_A0A2I7R4F3 | 97.4 | 8.2e-06 | 1.5e-11 | 49.6 | 56 | (4, 59) | 74 | (2, 57) | 59 | Coil containing protein | Coil containing protein | | uniclust | UniRef100\_A0A7Y6JDD3 | 97.3 | 1.3e-05 | 2.5e-11 | 49.8 | 55 | (3, 57) | 74 | (6, 61) | 67 | Uncharacterized protein | Uncharacterized protein | | uniclust | UniRef100\_A0A1M7YP10 | 97.3 | 1.5e-05 | 2.7e-11 | 48.3 | 38 | (3, 40) | 74 | (9, 46) | 57 | Uncharacterized protein | Uncharacterized protein | | uniclust | UniRef100\_UPI000A2E50F0 | 97.2 | 2e-05 | 3.7e-11 | 50.2 | 38 | (1, 40) | 74 | (1, 38) | 75 | hypothetical protein | hypothetical protein | | uniclust | UniRef100\_UPI00053E6714 | 97.2 | 2.2e-05 | 4.1e-11 | 55.0 | 40 | (1, 40) | 74 | (1, 40) | 144 | hypothetical protein | hypothetical protein | | uniclust | UniRef100\_A0A7D5XSG2 | 97.1 | 3e-05 | 5.5e-11 | 45.3 | 33 | (1, 33) | 74 | (1, 33) | 46 | Uncharacterized protein | Uncharacterized protein | | uniclust | UniRef100\_A0A6S4Y2E2 | 97.0 | 5.9e-05 | 1.1e-10 | 40.6 | 28 | (1, 28) | 74 | (1, 28) | 30 | Uncharacterized protein | Uncharacterized protein | | uniclust | UniRef100\_A0A2G2PXP6 | 96.7 | 0.00023 | 4.4e-10 | 50.9 | 58 | (1, 58) | 74 | (1, 58) | 150 | Uncharacterized protein | Uncharacterized protein | | uniclust | UniRef100\_UPI0012B549EA | 96.6 | 0.00029 | 5.4e-10 | 43.0 | 21 | (1, 21) | 74 | (1, 21) | 55 | hypothetical protein | hypothetical protein | | uniclust | UniRef100\_UPI0020C6DD36 | 96.5 | 0.0004 | 7.4e-10 | 44.1 | 26 | (41, 66) | 74 | (43, 68) | 70 | lysis system i-spanin subunit Rz | lysis system i-spanin subunit Rz | | uniclust | UniRef100\_Q9MC75 | 96.4 | 0.00055 | 1e-09 | 39.9 | 38 | (29, 66) | 74 | (4, 41) | 43 | Uncharacterized protein | Uncharacterized protein | |
| Top keywords  (threshold 1.00e-03 (evalue)) | **hypothetical, Phage, Transcriptional, regulator, Cell, envelope, biogenesis, OmpA, Membrane, lipoprotein** |
| Output files | ../../similar\_sequences/51\_FANPEZAQ\_CDS\_0051\_merged.svg ../../similar\_sequences/51\_FANPEZAQ\_CDS\_0051\_pdb70.a3m ../../similar\_sequences/51\_FANPEZAQ\_CDS\_0051\_pdb70.hhr ../../similar\_sequences/51\_FANPEZAQ\_CDS\_0051\_uniclust.a3m ../../similar\_sequences/51\_FANPEZAQ\_CDS\_0051\_uniclust.hhr |

#### Structure prediction (AlphaFold)2

|  |  |
| --- | --- |
| Stats | xml version="1.0" encoding="utf-8" standalone="no"?       2024-09-02T21:09:47.360087 image/svg+xml   Matplotlib v3.7.2, https://matplotlib.org/ |
| Predicted structure | **NGL Viewer Controls:**  - Center: *Left-Click* - Rotate: *Left-Click + Drag* - Translate: *Right-Click + Drag* - Zoom: *Shift + Left-Click + Drag* |
| Output files | ../../predicted\_structures/51\_FANPEZAQ\_CDS\_0051/features.pkl ../../predicted\_structures/51\_FANPEZAQ\_CDS\_0051/ranked\_0.pdb ../../predicted\_structures/51\_FANPEZAQ\_CDS\_0051/ranked\_0\_plots.svg ../../predicted\_structures/51\_FANPEZAQ\_CDS\_0051/result\_model\_1\_ptm\_pred\_0.pkl |

#### Structure similarity search results (Foldseek)3

|  |  |
| --- | --- |
| Structure databases searched | Pdb, Afdb-proteome, Afdb-uniprot50 |
| Results, scheme(s)  (Top layers only, threshold 1.00e-02 (evalue)) | xml version="1.0" encoding="utf-8" standalone="no"?       2024-09-02T21:11:23.410408 image/svg+xml   Matplotlib v3.7.2, https://matplotlib.org/ |
| Results, table  (threshold 1.00e-02 (evalue)) | -- |
| Top keywords  (threshold 1.00e-02 (evalue)) | -- |
| Output files | ../../similar\_structures/51\_FANPEZAQ\_CDS\_0051\_afdb-proteome\_foldseek.tsv ../../similar\_structures/51\_FANPEZAQ\_CDS\_0051\_afdb-uniprot50\_foldseek.tsv ../../similar\_structures/51\_FANPEZAQ\_CDS\_0051\_merged.svg ../../similar\_structures/51\_FANPEZAQ\_CDS\_0051\_pdb\_foldseek.tsv |

  
  
  

Return to summary | Go to previous | Go to next

  


---

**Sequence/structure alignments coloring**  
Each object in the alignment figures is colored according to its E-value following this color coding:

1e-100
10

**References:**  
1) Steinegger M, Meier M, Mirdita M, Vöhringer H, Haunsberger S J, and Söding J (2019) HH-suite3 for fast remote homology detection and deep protein annotation, BMC Bioinformatics, 473. doi: 10.1186/s12859-019-3019-7  
2) Jumper J, Evans R, Pritzel A, ..., Hassabis D (2021) Highly accurate protein structure prediction with AlphaFold, Nature, 596. doi: 10.1038/s41586-021-03819-2  
3) van Kempen M, Kim S, Tumescheit C, Mirdita M, Lee J, Gilchrist CLM, Söding J, and Steinegger M (2023) Fast and accurate protein structure search with Foldseek. Nature Biotechnology. doi: 10.1038/s41587-023-01773-0
