# Supplementary material for: Completing the BASEL phage collection to unlock hidden diversity for systematic exploration of phage–host interactions
Source: PLoS Biol. 2025 Apr 7;23(4):e3003063. doi: 10.1371/journal.pbio.3003063 (PMC11990801; doi:10.1371/journal.pbio.3003063)
Supplement: S2 Data — (ZIP) [file pbio.3003063.s009.zip › entries/52.html]

FANPEZAQ\_CDS\_0052


Return to summary | Go to previous | Go to next

|  |  |
| --- | --- |
| FANPEZAQ\_CDS\_0052 Page creation date: 02 Sep 2024, 12:00  Project folder: n/a  Input sequences file: Escherichia\_virus\_HeidiAbel.gb | transcriptional regulator hth domain\_containing cro c1\_type helix\_turn\_helix xre dna\_binding repressor hth\_type transcription dna regulation and xre\_family immr dica binding rna nucleotide metabolism putative contains antitoxin phage ci lexa integration excision terminase small head packaging |

### Sequence information

|  |  |
| --- | --- |
| Name | FANPEZAQ\_CDS\_0052  52\_FANPEZAQ\_CDS\_0052 (pipeline id) |
| Imported annotations | Escherichia\_virus\_HeidiAbel Bas97 |
| Protein sequence | MRPVLWAHCYYEAITMDERTERLHNIMAEHGLTCRDVGRLLGRTEQTVLIWRCKGGKVIP THQLELLELKLNAQAAQ |
| Number of residues | 77 |
| Molecular weight (Da) | 8927.33 |
| Output files | ../../query\_sequences/52\_FANPEZAQ\_CDS\_0052.fasta |

### Putative domain architecture and protein family

#### Search results (HHblits)1

|  |  |
| --- | --- |
| Domain family databases searched | Pfam, Ncbi-cd, Cath, Phrogs |
| Results, scheme(s)  (Top layers only; threshold 1.00e-03 (evalue)) | xml version="1.0" encoding="utf-8" standalone="no"?       2024-09-02T21:08:24.052090 image/svg+xml   Matplotlib v3.7.2, https://matplotlib.org/ |
| Results, table  (E-value ≤ 1.00e-03 (evalue)) | | db | id | prob | evalue | pvalue | score | cols | query | query\_len | template | template\_len | name | description | | --- | --- | --- | --- | --- | --- | --- | --- | --- | --- | --- | --- | --- | | phrogs | 14341 | 99.6 | 7.7e-20 | 9.3e-24 | 108.3 | 62 | (16, 77) | 77 | (1, 63) | 63 | transcriptional repressor | transcriptional repressor; Category: transcription regulation; NC\_023006\_p49 | | phrogs | 4779 | 99.5 | 1.1e-18 | 1.4e-22 | 106.6 | 56 | (18, 73) | 77 | (1, 61) | 78 | transcriptional regulator | transcriptional regulator; Category: transcription regulation; p225361 VI\_06249 | | phrogs | 8898 | 99.1 | 1.5e-14 | 1.9e-18 | 86.4 | 50 | (18, 69) | 77 | (1, 50) | 69 | NA | NA; Category: unknown function; p78052 VI\_06244 | | phrogs | 7950 | 98.8 | 7.6e-13 | 9.4e-17 | 75.4 | 37 | (19, 55) | 77 | (1, 37) | 53 | transcriptional repressor | transcriptional repressor; Category: transcription regulation; p141193 VI\_04895 | | phrogs | 2104 | 98.0 | 1.3e-09 | 1.6e-13 | 67.2 | 38 | (18, 55) | 77 | (30, 67) | 92 | NA | NA; Category: unknown function; p72070 VI\_03178 | | phrogs | 14076 | 97.7 | 1.6e-08 | 1.9e-12 | 61.1 | 51 | (17, 68) | 77 | (13, 68) | 81 | transcriptional regulator | transcriptional regulator; Category: transcription regulation; p109717 VI\_00077 | | phrogs | 5119 | 97.4 | 1.1e-07 | 1.3e-11 | 62.3 | 41 | (16, 56) | 77 | (51, 91) | 152 | NA | NA; Category: unknown function; p324576 VI\_09304 | | phrogs | 2328 | 97.3 | 2e-07 | 2.6e-11 | 59.3 | 36 | (19, 54) | 77 | (19, 54) | 123 | transcriptional repressor | transcriptional repressor; Category: transcription regulation; MG099936\_p34 | | phrogs | 1373 | 97.2 | 2.1e-07 | 2.8e-11 | 56.4 | 48 | (19, 66) | 77 | (34, 81) | 95 | transcriptional regulator | transcriptional regulator; Category: transcription regulation; NC\_005857\_p48 | | phrogs | 25249 | 96.9 | 1.8e-06 | 2e-10 | 59.7 | 51 | (18, 71) | 77 | (1, 51) | 263 | NA | NA; Category: unknown function; p347442 VI\_06291 | | phrogs | 35 | 96.7 | 2.4e-06 | 3.6e-10 | 47.4 | 38 | (16, 54) | 77 | (2, 39) | 75 | transcriptional repressor | transcriptional repressor; Category: transcription regulation; p323300 VI\_10199 | | phrogs | 2339 | 96.7 | 3.1e-06 | 4e-10 | 51.4 | 34 | (22, 55) | 77 | (32, 65) | 93 | HTH DNA binding protein | HTH DNA binding protein; Category: DNA, RNA and nucleotide metabolism; p425338 VI\_07226 | | phrogs | 3 | 96.7 | 3e-06 | 4.6e-10 | 47.4 | 37 | (16, 54) | 77 | (3, 41) | 80 | transcriptional repressor | transcriptional repressor; Category: transcription regulation; p29899 VI\_00998 | | phrogs | 2967 | 96.6 | 3.6e-06 | 4.8e-10 | 55.4 | 52 | (18, 69) | 77 | (6, 60) | 167 | transcriptional regulator | transcriptional regulator; Category: transcription regulation; p123578 VI\_00485 | | phrogs | 147 | 96.6 | 5.2e-06 | 6.7e-10 | 51.9 | 46 | (10, 55) | 77 | (4, 49) | 112 | transcriptional regulator | transcriptional regulator; Category: transcription regulation; p315289 VI\_07968 | | phrogs | 7241 | 96.4 | 8.8e-06 | 1.1e-09 | 52.1 | 41 | (14, 55) | 77 | (58, 98) | 128 | transcriptional regulator | transcriptional regulator; Category: transcription regulation; p63620 VI\_05511 | | phrogs | 1468 | 96.4 | 8.8e-06 | 1.1e-09 | 47.0 | 52 | (19, 70) | 77 | (11, 64) | 69 | transcriptional repressor | transcriptional repressor; Category: transcription regulation; p307668 VI\_06058 | | phrogs | 6831 | 96.4 | 1.1e-05 | 1.4e-09 | 46.5 | 34 | (21, 54) | 77 | (5, 38) | 66 | transcriptional regulator | transcriptional regulator; Category: transcription regulation; p431926 VI\_12195 | | phrogs | 1337 | 96.3 | 1.2e-05 | 1.5e-09 | 45.2 | 37 | (19, 55) | 77 | (3, 39) | 61 | DNA binding protein | DNA binding protein; Category: DNA, RNA and nucleotide metabolism; p177899 VI\_00920 | | phrogs | 409 | 96.2 | 1.5e-05 | 2.1e-09 | 45.2 | 28 | (25, 54) | 77 | (5, 32) | 72 | transcriptional regulator | transcriptional regulator; Category: transcription regulation; p88852 VI\_07592 | | phrogs | 37751 | 96.2 | 2.2e-05 | 2.5e-09 | 52.3 | 38 | (18, 55) | 77 | (152, 191) | 205 | NA | NA; Category: unknown function; KR093631\_p2 | | phrogs | 6962 | 96.2 | 2.2e-05 | 2.6e-09 | 44.3 | 52 | (19, 70) | 77 | (2, 53) | 59 | NA | NA; Category: unknown function; p75562 VI\_02384 | | phrogs | 4151 | 96.1 | 2.6e-05 | 3.3e-09 | 51.5 | 30 | (25, 54) | 77 | (14, 43) | 168 | DNA binding protein | DNA binding protein; Category: DNA, RNA and nucleotide metabolism; p284557 VI\_05952 | | phrogs | 296 | 96.1 | 2.4e-05 | 3.5e-09 | 54.0 | 41 | (16, 56) | 77 | (3, 43) | 312 | DNA transposition protein | DNA transposition protein; Category: integration and excision; p199288 VI\_08191 | | phrogs | 4836 | 96.1 | 3.1e-05 | 3.7e-09 | 53.3 | 37 | (20, 56) | 77 | (84, 120) | 233 | CII-like regulator | CII-like regulator; Category: transcription regulation; p13677 VI\_07483 | | phrogs | 6223 | 95.9 | 4.6e-05 | 5.6e-09 | 44.9 | 34 | (22, 55) | 77 | (6, 41) | 77 | transcriptional repressor | transcriptional repressor; Category: transcription regulation; p192116 VI\_09192 | | phrogs | 8008 | 95.8 | 5.3e-05 | 6.5e-09 | 43.5 | 47 | (23, 73) | 77 | (3, 49) | 66 | DNA binding protein | DNA binding protein; Category: DNA, RNA and nucleotide metabolism; p180387 VI\_11803 | | phrogs | 2439 | 95.8 | 5.1e-05 | 7e-09 | 54.9 | 39 | (16, 54) | 77 | (5, 43) | 385 | transcriptional regulator | transcriptional regulator; Category: transcription regulation; p298790 VI\_12393 | | phrogs | 7676 | 95.8 | 6.4e-05 | 7.5e-09 | 44.0 | 29 | (26, 54) | 77 | (11, 39) | 75 | NA | NA; Category: unknown function; NC\_023688\_p227 | | phrogs | 3642 | 95.7 | 6.9e-05 | 8.7e-09 | 45.1 | 38 | (19, 56) | 77 | (6, 43) | 90 | HTH DNA binding protein | HTH DNA binding protein; Category: DNA, RNA and nucleotide metabolism; p184909 VI\_05451 | | phrogs | 2538 | 95.7 | 7.9e-05 | 1.1e-08 | 46.0 | 33 | (22, 54) | 77 | (7, 39) | 113 | terminase small subunit | terminase small subunit; Category: head and packaging; p133796 VI\_05051 | | phrogs | 3626 | 95.6 | 0.00011 | 1.2e-08 | 43.6 | 49 | (16, 65) | 77 | (8, 58) | 82 | NA | NA; Category: unknown function; p164942 VI\_09747 | | phrogs | 664 | 95.6 | 8.9e-05 | 1.3e-08 | 45.1 | 40 | (16, 55) | 77 | (57, 96) | 117 | DNA binding protein | DNA binding protein; Category: DNA, RNA and nucleotide metabolism; MF072690\_p140 | | phrogs | 1124 | 95.5 | 0.00011 | 1.5e-08 | 48.3 | 44 | (22, 67) | 77 | (8, 51) | 182 | transcriptional regulator | transcriptional regulator; Category: transcription regulation; p275302 VI\_05197 | | phrogs | 4583 | 95.5 | 0.00013 | 1.6e-08 | 42.7 | 37 | (19, 55) | 77 | (11, 47) | 77 | transcriptional repressor | transcriptional repressor; Category: transcription regulation; NC\_031243\_p35 | | phrogs | 34341 | 95.4 | 0.00015 | 1.7e-08 | 44.9 | 35 | (20, 54) | 77 | (73, 107) | 122 | transcriptional regulator | transcriptional regulator; Category: transcription regulation; MF360957\_p141 | | phrogs | 925 | 95.4 | 0.00014 | 1.9e-08 | 42.1 | 43 | (12, 54) | 77 | (7, 49) | 76 | HTH DNA binding protein | HTH DNA binding protein; Category: DNA, RNA and nucleotide metabolism; p18585 VI\_03080 | | phrogs | 66 | 95.4 | 0.00012 | 1.9e-08 | 39.7 | 29 | (27, 55) | 77 | (13, 41) | 81 | excisionase and transcriptional regulator | excisionase and transcriptional regulator; Category: integration and excision; NC\_023692\_p35 | | phrogs | 725 | 95.4 | 0.00016 | 2e-08 | 44.5 | 25 | (30, 54) | 77 | (28, 52) | 106 | CII-like regulator | CII-like regulator; Category: transcription regulation; p72240 VI\_06449 | | phrogs | 8 | 95.2 | 0.00016 | 2.4e-08 | 41.3 | 23 | (32, 54) | 77 | (1, 23) | 95 | transcriptional regulator | transcriptional regulator; Category: transcription regulation; p309030 VI\_07697 | | phrogs | 1668 | 95.2 | 0.00021 | 2.5e-08 | 45.3 | 42 | (28, 71) | 77 | (18, 59) | 126 | tail assembly protein | tail assembly protein; Category: tail; p440314 VI\_06404 | | phrogs | 38149 | 95.2 | 0.00024 | 2.7e-08 | 46.9 | 57 | (18, 74) | 77 | (8, 64) | 184 | NA | NA; Category: unknown function; p54375 VI\_01307 | | phrogs | 597 | 95.0 | 0.00028 | 3.8e-08 | 39.1 | 21 | (31, 51) | 77 | (4, 24) | 63 | DNA binding protein | DNA binding protein; Category: DNA, RNA and nucleotide metabolism; KU160644\_p26 | | phrogs | 151 | 94.9 | 0.0003 | 4.4e-08 | 40.4 | 26 | (29, 54) | 77 | (20, 45) | 91 | transposase | transposase; Category: integration and excision; p268658 VI\_04551 | | phrogs | 769 | 94.8 | 0.00039 | 5.1e-08 | 41.4 | 37 | (18, 54) | 77 | (1, 37) | 90 | transcriptional repressor | transcriptional repressor; Category: transcription regulation; p166433 VI\_07600 | | phrogs | 1381 | 94.8 | 0.00039 | 5.1e-08 | 41.8 | 25 | (31, 55) | 77 | (19, 43) | 99 | plasmid antitoxin with HTH domain | plasmid antitoxin with HTH domain; Category: moron, auxiliary metabolic gene and host takeover; p79485 VI\_11488 | | phrogs | 7061 | 94.8 | 0.00045 | 5.5e-08 | 40.1 | 27 | (28, 54) | 77 | (44, 70) | 73 | HTH DNA binding protein | HTH DNA binding protein; Category: DNA, RNA and nucleotide metabolism; KU160668\_p38 | | phrogs | 1889 | 94.7 | 0.00046 | 6.1e-08 | 46.9 | 36 | (19, 54) | 77 | (8, 45) | 233 | terminase small subunit | terminase small subunit; Category: head and packaging; p363276 VI\_05842 | | phrogs | 517 | 94.7 | 0.0005 | 6.2e-08 | 42.3 | 28 | (27, 54) | 77 | (76, 103) | 107 | HTH DNA binding protein | HTH DNA binding protein; Category: DNA, RNA and nucleotide metabolism; MF668278\_p45 | | phrogs | 955 | 94.5 | 0.00062 | 7.5e-08 | 44.9 | 38 | (18, 55) | 77 | (55, 92) | 174 | HTH DNA binding protein | HTH DNA binding protein; Category: DNA, RNA and nucleotide metabolism; JN699010\_p46 | | phrogs | 10045 | 94.4 | 0.00073 | 8.7e-08 | 38.1 | 29 | (32, 62) | 77 | (10, 38) | 62 | HTH DNA binding protein | HTH DNA binding protein; Category: DNA, RNA and nucleotide metabolism; KU963258\_p53 | | phrogs | 57 | 94.4 | 0.00059 | 9e-08 | 42.4 | 25 | (30, 54) | 77 | (3, 27) | 173 | terminase small subunit | terminase small subunit; Category: head and packaging; p277333 VI\_02593 | | phrogs | 18342 | 94.4 | 0.00078 | 9.2e-08 | 42.3 | 34 | (21, 54) | 77 | (21, 54) | 123 | transcriptional repressor | transcriptional repressor; Category: transcription regulation; p158151 VI\_11365 | | phrogs | 7344 | 94.4 | 0.00074 | 9.3e-08 | 43.9 | 25 | (29, 53) | 77 | (26, 50) | 158 | terminase small subunit | terminase small subunit; Category: head and packaging; p355407 VI\_04507 | | phrogs | 11187 | 94.3 | 0.00081 | 9.7e-08 | 38.0 | 35 | (21, 55) | 77 | (10, 44) | 63 | transcriptional regulator | transcriptional regulator; Category: transcription regulation; p82712 VI\_00440 | | phrogs | 309 | 94.3 | 0.00074 | 1.1e-07 | 42.3 | 32 | (22, 54) | 77 | (15, 46) | 153 | terminase small subunit | terminase small subunit; Category: head and packaging; p301521 VI\_00938 | | phrogs | 2907 | 94.2 | 0.00086 | 1.1e-07 | 40.9 | 28 | (26, 53) | 77 | (4, 31) | 103 | excisionase | excisionase; Category: integration and excision; p256170 VI\_09967 | | phrogs | 6620 | 94.2 | 0.00093 | 1.1e-07 | 43.9 | 25 | (31, 55) | 77 | (5, 29) | 170 | transposase | transposase; Category: integration and excision; p336225 VI\_06314 | |
| Top keywords  (threshold 1.00e-03 (evalue)) | **transcription, regulation, DNA, transcriptional, and, regulator, binding, RNA, nucleotide, metabolism** |
| Output files | ../../domain\_architecture/52\_FANPEZAQ\_CDS\_0052\_cath.hhr ../../domain\_architecture/52\_FANPEZAQ\_CDS\_0052\_merged.svg ../../domain\_architecture/52\_FANPEZAQ\_CDS\_0052\_ncbi-cd.hhr ../../domain\_architecture/52\_FANPEZAQ\_CDS\_0052\_pfam.hhr ../../domain\_architecture/52\_FANPEZAQ\_CDS\_0052\_phrogs.hhr |

### Identical protein sequences/structures

#### Search results

|  |  |
| --- | --- |
| Protein sequence databases searched | Pdb, Swissprot, Refseq |
| Identical proteins found | -- |
| Top keywords | -- |
| Output files | -- |

### Similar protein sequences/structures

#### Sequence similarity search results (HHblits)1

|  |  |
| --- | --- |
| Sequence databases searched | Uniclust, Pdb70 |
| Results, scheme(s)  (Top layers only, threshold 1.00e-03 (evalue)) | xml version="1.0" encoding="utf-8" standalone="no"?       2024-09-02T21:08:51.590618 image/svg+xml   Matplotlib v3.7.2, https://matplotlib.org/ |
| Results, table(s)  (threshold 1.00e-03 (evalue)) | | db | id | prob | evalue | pvalue | score | cols | query | query\_len | template | template\_len | name | description | | --- | --- | --- | --- | --- | --- | --- | --- | --- | --- | --- | --- | --- | | uniclust | UniRef100\_A0A060M6C1 | 99.6 | 1e-18 | 2.4e-24 | 106.8 | 71 | (1, 72) | 77 | (1, 71) | 113 | Cryptic phage CTXphi transcriptional repressor rstR | Cryptic phage CTXphi transcriptional repressor rstR | | uniclust | UniRef100\_A0A0C1PUG7 | 99.6 | 1.2e-18 | 2.7e-24 | 110.2 | 61 | (11, 72) | 77 | (3, 63) | 143 | Transcriptional regulator, XRE family | Transcriptional regulator, XRE family | | uniclust | UniRef100\_A0A329KDZ8 | 99.5 | 1.5e-17 | 3.1e-23 | 95.1 | 56 | (16, 72) | 77 | (1, 56) | 78 | XRE family transcriptional regulator | XRE family transcriptional regulator | | uniclust | UniRef100\_A0A125W4T6 | 99.5 | 2.3e-17 | 5e-23 | 97.0 | 60 | (12, 72) | 77 | (10, 69) | 91 | DNA-binding helix-turn-helix protein | DNA-binding helix-turn-helix protein | | uniclust | UniRef100\_A0A074JBZ5 | 99.5 | 2.6e-17 | 5.7e-23 | 108.1 | 61 | (11, 72) | 77 | (2, 62) | 188 | Cro/Cl family transcriptional regulator | Cro/Cl family transcriptional regulator | | uniclust | UniRef100\_A0A0U5NTU9 | 99.5 | 3e-17 | 6.3e-23 | 106.6 | 60 | (14, 74) | 77 | (22, 81) | 177 | HTH-type transcriptional regulator ImmR | HTH-type transcriptional regulator ImmR | | uniclust | UniRef100\_A0A023CY80 | 99.5 | 3.1e-17 | 7.5e-23 | 106.6 | 70 | (2, 72) | 77 | (13, 82) | 169 | HTH cro/C1-type domain-containing protein | HTH cro/C1-type domain-containing protein | | uniclust | UniRef100\_A0A0H5SX81 | 99.5 | 4.5e-17 | 9.5e-23 | 98.3 | 59 | (13, 72) | 77 | (21, 79) | 107 | HTH cro/C1-type domain-containing protein | HTH cro/C1-type domain-containing protein | | uniclust | UniRef100\_A0A1C6GRS4 | 99.5 | 4.5e-17 | 9.7e-23 | 103.3 | 64 | (8, 72) | 77 | (21, 84) | 149 | Helix-turn-helix | Helix-turn-helix | | uniclust | UniRef100\_A0A0B3W1R4 | 99.5 | 4.6e-17 | 1.1e-22 | 102.7 | 64 | (8, 72) | 77 | (4, 67) | 138 | Repressor | Repressor | | uniclust | UniRef100\_A0A0F0C6Y2 | 99.5 | 4.7e-17 | 1.1e-22 | 103.4 | 61 | (11, 72) | 77 | (18, 78) | 146 | HTH-type transcriptional regulator Xre | HTH-type transcriptional regulator Xre | | uniclust | UniRef100\_A0A0D6XQB1 | 99.5 | 5.9e-17 | 1.2e-22 | 99.5 | 58 | (14, 72) | 77 | (1, 58) | 120 | DNA-binding protein | DNA-binding protein | | uniclust | UniRef100\_A0A0A2SKT0 | 99.5 | 6.6e-17 | 1.5e-22 | 103.7 | 67 | (5, 72) | 77 | (5, 71) | 154 | Transcriptional regulator | Transcriptional regulator | | uniclust | UniRef100\_A0A173SJ57 | 99.5 | 7.3e-17 | 1.7e-22 | 104.2 | 62 | (11, 73) | 77 | (4, 65) | 163 | Anaerobic benzoate catabolism transcriptional regulator | Anaerobic benzoate catabolism transcriptional regulator | | uniclust | UniRef100\_A0A173URG9 | 99.5 | 7.4e-17 | 1.7e-22 | 97.3 | 61 | (11, 72) | 77 | (12, 72) | 102 | Helix-turn-helix | Helix-turn-helix | | uniclust | UniRef100\_A0A031WE04 | 99.5 | 6.9e-17 | 1.7e-22 | 105.7 | 59 | (13, 72) | 77 | (11, 69) | 177 | Transcriptional regulator, HTH-type | Transcriptional regulator, HTH-type | | uniclust | UniRef100\_A0A0C3LWL5 | 99.5 | 7.3e-17 | 1.7e-22 | 105.5 | 70 | (2, 72) | 77 | (4, 81) | 176 | HTH cro/C1-type domain-containing protein | HTH cro/C1-type domain-containing protein | | uniclust | UniRef100\_A0A087N614 | 99.5 | 7.2e-17 | 1.7e-22 | 104.0 | 62 | (10, 72) | 77 | (22, 83) | 158 | XRE family transcriptional regulator | XRE family transcriptional regulator | | uniclust | UniRef100\_A0A075KGJ7 | 99.5 | 8.4e-17 | 1.8e-22 | 96.3 | 57 | (15, 72) | 77 | (6, 62) | 100 | Transcriptional regulator, XRE family | Transcriptional regulator, XRE family | | uniclust | UniRef100\_A0A173TQ57 | 99.5 | 1.1e-16 | 2.3e-22 | 93.1 | 58 | (15, 73) | 77 | (1, 58) | 86 | Helix-turn-helix | Helix-turn-helix | | uniclust | UniRef100\_A0A062XL10 | 99.5 | 1e-16 | 2.4e-22 | 102.8 | 64 | (8, 72) | 77 | (13, 76) | 154 | Transcriptional regulator, xre family | Transcriptional regulator, xre family | | uniclust | UniRef100\_A0A1D2YT19 | 99.5 | 1.2e-16 | 2.6e-22 | 104.1 | 61 | (11, 72) | 77 | (29, 89) | 177 | HTH cro/C1-type domain-containing protein | HTH cro/C1-type domain-containing protein | | uniclust | UniRef100\_A0A061PBD1 | 99.5 | 1.1e-16 | 2.7e-22 | 104.8 | 58 | (14, 72) | 77 | (21, 78) | 178 | Transcriptional regulator | Transcriptional regulator | | uniclust | UniRef100\_A0A0A2HHH8 | 99.5 | 1.2e-16 | 2.7e-22 | 101.6 | 64 | (8, 72) | 77 | (2, 65) | 145 | DNA-binding protein | DNA-binding protein | | uniclust | UniRef100\_A0A075JR98 | 99.5 | 1.2e-16 | 2.9e-22 | 100.7 | 60 | (12, 72) | 77 | (4, 63) | 135 | XRE family transcriptional regulator | XRE family transcriptional regulator | | uniclust | UniRef100\_A0A099I4W4 | 99.5 | 1.3e-16 | 2.9e-22 | 108.3 | 61 | (11, 72) | 77 | (18, 78) | 242 | DNA-binding protein | DNA-binding protein | | uniclust | UniRef100\_A0A0A8WJW3 | 99.5 | 1.4e-16 | 3.3e-22 | 101.6 | 57 | (15, 72) | 77 | (13, 69) | 148 | Putative repressor | Putative repressor | | uniclust | UniRef100\_A0A090HZD4 | 99.5 | 1.8e-16 | 3.7e-22 | 84.8 | 43 | (12, 54) | 77 | (5, 47) | 54 | Helix-turn-helix XRE-family like proteins | Helix-turn-helix XRE-family like proteins | | uniclust | UniRef100\_A0A173T283 | 99.5 | 1.8e-16 | 3.9e-22 | 104.2 | 62 | (10, 72) | 77 | (23, 84) | 190 | Transcriptional repressor DicA | Transcriptional repressor DicA | | uniclust | UniRef100\_A0A089I2J7 | 99.5 | 2.1e-16 | 4.5e-22 | 108.7 | 60 | (12, 72) | 77 | (13, 72) | 282 | HTH cro/C1-type domain-containing protein | HTH cro/C1-type domain-containing protein | | uniclust | UniRef100\_A0A086YZY1 | 99.5 | 2e-16 | 4.5e-22 | 92.5 | 62 | (10, 72) | 77 | (3, 64) | 85 | HTH cro/C1-type domain-containing protein | HTH cro/C1-type domain-containing protein | | uniclust | UniRef100\_A0A090IVJ4 | 99.4 | 2e-16 | 4.7e-22 | 103.3 | 63 | (9, 72) | 77 | (24, 86) | 173 | HTH cro/C1-type domain-containing protein | HTH cro/C1-type domain-containing protein | | uniclust | UniRef100\_A0A150BTM0 | 99.4 | 2.1e-16 | 4.9e-22 | 100.8 | 61 | (11, 72) | 77 | (5, 65) | 147 | Helix-turn-helix domain-containing protein | Helix-turn-helix domain-containing protein | | uniclust | UniRef100\_A0A059N6C6 | 99.4 | 2.4e-16 | 5.3e-22 | 94.8 | 64 | (8, 72) | 77 | (5, 68) | 100 | DNA-binding helix-turn-helix protein | DNA-binding helix-turn-helix protein | | uniclust | UniRef100\_A0A061BX60 | 99.4 | 2.6e-16 | 5.5e-22 | 97.1 | 57 | (15, 72) | 77 | (2, 58) | 124 | Helix-turn-helix transcriptional regulator | Helix-turn-helix transcriptional regulator | | uniclust | UniRef100\_A0A0R1F3Q5 | 99.4 | 2.6e-16 | 5.5e-22 | 100.2 | 60 | (12, 72) | 77 | (3, 62) | 150 | Transcriptional regulator | Transcriptional regulator | | uniclust | UniRef100\_A0A074LR75 | 99.4 | 2.5e-16 | 5.6e-22 | 101.6 | 66 | (6, 72) | 77 | (6, 71) | 161 | HTH cro/C1-type domain-containing protein | HTH cro/C1-type domain-containing protein | | uniclust | UniRef100\_A0A0R2G103 | 99.4 | 2.7e-16 | 5.6e-22 | 91.1 | 59 | (15, 74) | 77 | (10, 68) | 84 | HTH cro/C1-type domain-containing protein | HTH cro/C1-type domain-containing protein | | uniclust | UniRef100\_A0A0R2GF18 | 99.4 | 2.6e-16 | 6e-22 | 101.7 | 60 | (12, 72) | 77 | (24, 83) | 161 | HTH cro/C1-type domain-containing protein | HTH cro/C1-type domain-containing protein | | uniclust | UniRef100\_A0A0M2NH40 | 99.4 | 2.9e-16 | 6.2e-22 | 104.0 | 62 | (10, 72) | 77 | (4, 65) | 203 | Transcriptional regulator, XRE family | Transcriptional regulator, XRE family | | uniclust | UniRef100\_A0A0A0M2F1 | 99.4 | 3e-16 | 6.2e-22 | 100.0 | 65 | (7, 72) | 77 | (15, 79) | 153 | HTH cro/C1-type domain-containing protein | HTH cro/C1-type domain-containing protein | | uniclust | UniRef100\_A0A096AZ92 | 99.4 | 3.7e-16 | 7.8e-22 | 93.0 | 58 | (14, 72) | 77 | (10, 67) | 96 | HTH cro/C1-type domain-containing protein | HTH cro/C1-type domain-containing protein | | uniclust | UniRef100\_A0A073KEL8 | 99.4 | 3.6e-16 | 8.2e-22 | 102.7 | 61 | (11, 72) | 77 | (25, 85) | 183 | XRE family transcriptional regulator | XRE family transcriptional regulator | | uniclust | UniRef100\_A0A0C2YFC1 | 99.4 | 4.1e-16 | 9.3e-22 | 97.4 | 57 | (15, 72) | 77 | (2, 58) | 127 | Transcriptional regulator, XRE family | Transcriptional regulator, XRE family | | uniclust | UniRef100\_A0A011VZ04 | 99.4 | 4e-16 | 9.5e-22 | 106.0 | 66 | (7, 73) | 77 | (2, 67) | 234 | DNA-binding protein | DNA-binding protein | | uniclust | UniRef100\_A0A0H1RPT5 | 99.4 | 5e-16 | 1.1e-21 | 91.6 | 65 | (8, 73) | 77 | (7, 71) | 91 | Transcriptional regulator, y4mF family | Transcriptional regulator, y4mF family | | uniclust | UniRef100\_A0A1C6J0G0 | 99.4 | 5e-16 | 1.1e-21 | 106.8 | 61 | (14, 75) | 77 | (15, 75) | 270 | Transcriptional repressor DicA | Transcriptional repressor DicA | | uniclust | UniRef100\_A0A074IXR9 | 99.4 | 4.8e-16 | 1.1e-21 | 104.8 | 60 | (12, 72) | 77 | (4, 63) | 220 | HTH cro/C1-type domain-containing protein | HTH cro/C1-type domain-containing protein | | uniclust | UniRef100\_A0A0C1W593 | 99.4 | 5.2e-16 | 1.1e-21 | 105.0 | 65 | (8, 73) | 77 | (5, 69) | 233 | Phage repressor protein | Phage repressor protein | | uniclust | UniRef100\_A0A0E1KYC3 | 99.4 | 5.4e-16 | 1.2e-21 | 101.6 | 66 | (6, 72) | 77 | (2, 67) | 180 | Helix-turn-helix family protein | Helix-turn-helix family protein | | uniclust | UniRef100\_A0A0D0QRS3 | 99.4 | 5.6e-16 | 1.2e-21 | 97.4 | 61 | (11, 72) | 77 | (7, 67) | 137 | MerR family transcriptional regulator | MerR family transcriptional regulator | | uniclust | UniRef100\_A0A023BMV4 | 99.4 | 5.4e-16 | 1.2e-21 | 100.8 | 61 | (11, 72) | 77 | (26, 86) | 169 | HTH cro/C1-type domain-containing protein | HTH cro/C1-type domain-containing protein | | uniclust | UniRef100\_A0A0A8WJE4 | 99.4 | 5.3e-16 | 1.2e-21 | 100.1 | 59 | (13, 72) | 77 | (8, 66) | 158 | DNA-binding protein Putative repressor RepR | DNA-binding protein Putative repressor RepR | | uniclust | UniRef100\_A0A143ZRN3 | 99.4 | 5.9e-16 | 1.2e-21 | 93.4 | 63 | (9, 72) | 77 | (8, 72) | 105 | HTH cro/C1-type domain-containing protein | HTH cro/C1-type domain-containing protein | | uniclust | UniRef100\_A0A0C7NN68 | 99.4 | 6.3e-16 | 1.3e-21 | 101.1 | 65 | (7, 72) | 77 | (26, 90) | 187 | Uncharacterized | Uncharacterized | | uniclust | UniRef100\_A0A0V8QIR2 | 99.4 | 6.1e-16 | 1.4e-21 | 102.0 | 60 | (12, 72) | 77 | (16, 75) | 188 | HTH cro/C1-type domain-containing protein | HTH cro/C1-type domain-containing protein | | uniclust | UniRef100\_A0A0C7G2Y4 | 99.4 | 8.2e-16 | 1.7e-21 | 99.5 | 57 | (15, 72) | 77 | (5, 61) | 172 | Transcriptional regulator | Transcriptional regulator | | uniclust | UniRef100\_A0A059NZ33 | 99.4 | 7.8e-16 | 1.8e-21 | 100.6 | 63 | (9, 72) | 77 | (15, 77) | 174 | HTH-type transcriptional regulator ImmR | HTH-type transcriptional regulator ImmR | | uniclust | UniRef100\_A0A088T2X6 | 99.4 | 8.5e-16 | 1.9e-21 | 102.9 | 58 | (14, 72) | 77 | (4, 61) | 217 | HTH-type transcriptional regulator ImmR | HTH-type transcriptional regulator ImmR | | uniclust | UniRef100\_A0A084J9J4 | 99.4 | 9.5e-16 | 1.9e-21 | 98.5 | 58 | (14, 72) | 77 | (1, 58) | 170 | Transcriptional regulator | Transcriptional regulator | | uniclust | UniRef100\_A0A062X6M5 | 99.4 | 8.9e-16 | 2e-21 | 100.1 | 60 | (12, 72) | 77 | (27, 86) | 172 | Transcriptional regulator, y4mF family | Transcriptional regulator, y4mF family | | uniclust | UniRef100\_A0A354UQ85 | 99.4 | 1e-15 | 2e-21 | 86.5 | 59 | (14, 73) | 77 | (11, 69) | 76 | Transcriptional regulator | Transcriptional regulator | | uniclust | UniRef100\_A0A0R1HYU3 | 99.4 | 9.3e-16 | 2.1e-21 | 102.8 | 59 | (13, 72) | 77 | (31, 89) | 215 | HTH cro/C1-type domain-containing protein | HTH cro/C1-type domain-containing protein | | uniclust | UniRef100\_A0A0V8QD66 | 99.4 | 1.1e-15 | 2.2e-21 | 83.3 | 55 | (13, 68) | 77 | (6, 60) | 63 | Transcriptional regulator | Transcriptional regulator | | uniclust | UniRef100\_A0A0K9GGF8 | 99.4 | 1e-15 | 2.2e-21 | 96.4 | 59 | (14, 73) | 77 | (13, 71) | 139 | HTH cro/C1-type domain-containing protein | HTH cro/C1-type domain-containing protein | | uniclust | UniRef100\_A0A0R1I3I0 | 99.4 | 1.1e-15 | 2.2e-21 | 87.5 | 55 | (16, 71) | 77 | (1, 55) | 76 | HTH cro/C1-type domain-containing protein | HTH cro/C1-type domain-containing protein | | uniclust | UniRef100\_A0A0E3ZTG7 | 99.4 | 1.1e-15 | 2.3e-21 | 90.1 | 63 | (10, 73) | 77 | (7, 69) | 89 | XRE family transcriptional regulator | XRE family transcriptional regulator | | uniclust | UniRef100\_A0A0E2BX49 | 99.4 | 1.1e-15 | 2.3e-21 | 90.2 | 58 | (14, 72) | 77 | (13, 70) | 89 | HTH cro/C1-type domain-containing protein | HTH cro/C1-type domain-containing protein | | uniclust | UniRef100\_A0A090ZIW3 | 99.4 | 1.1e-15 | 2.5e-21 | 97.1 | 65 | (8, 73) | 77 | (5, 69) | 142 | Helix-turn-helix domain-containing protein | Helix-turn-helix domain-containing protein | | uniclust | UniRef100\_A0A095X2P6 | 99.4 | 1.2e-15 | 2.5e-21 | 102.4 | 63 | (8, 71) | 77 | (36, 98) | 223 | XRE family transcriptional regulator | XRE family transcriptional regulator | | uniclust | UniRef100\_A0A0R1KZW4 | 99.4 | 1.2e-15 | 2.6e-21 | 100.0 | 67 | (5, 72) | 77 | (16, 82) | 190 | Helix-turn-helix domain-containing protein | Helix-turn-helix domain-containing protein | | uniclust | UniRef100\_A0A024QFL1 | 99.4 | 1.2e-15 | 2.6e-21 | 98.7 | 65 | (8, 73) | 77 | (9, 73) | 163 | Helix-turn-helix protein | Helix-turn-helix protein | | uniclust | UniRef100\_A0A075SB65 | 99.4 | 1.5e-15 | 3e-21 | 97.4 | 60 | (11, 71) | 77 | (28, 87) | 169 | HTH cro/C1-type domain-containing protein | HTH cro/C1-type domain-containing protein | | uniclust | UniRef100\_A0A2K9P5D5 | 99.4 | 1.6e-15 | 3.2e-21 | 88.2 | 58 | (14, 72) | 77 | (4, 61) | 84 | Helix-turn-helix domain protein | Helix-turn-helix domain protein | | uniclust | UniRef100\_A0A0F6CKR7 | 99.4 | 1.6e-15 | 3.3e-21 | 86.5 | 58 | (15, 73) | 77 | (1, 58) | 75 | XRE family transcriptional regulator | XRE family transcriptional regulator | | uniclust | UniRef100\_A0A0F2Q257 | 99.4 | 1.6e-15 | 3.4e-21 | 89.0 | 59 | (13, 72) | 77 | (5, 63) | 88 | HTH cro/C1-type domain-containing protein | HTH cro/C1-type domain-containing protein | | uniclust | UniRef100\_A0A0R2JC93 | 99.4 | 1.7e-15 | 3.4e-21 | 96.6 | 58 | (14, 72) | 77 | (8, 65) | 156 | Helix-turn-helix domain-containing protein | Helix-turn-helix domain-containing protein | | uniclust | UniRef100\_A0A077EIM8 | 99.4 | 1.6e-15 | 3.5e-21 | 98.1 | 59 | (13, 72) | 77 | (21, 79) | 165 | BcrR | BcrR | | uniclust | UniRef100\_A0A0R2F9F5 | 99.4 | 1.8e-15 | 3.7e-21 | 88.4 | 59 | (13, 72) | 77 | (3, 61) | 85 | HTH cro/C1-type domain-containing protein | HTH cro/C1-type domain-containing protein | | uniclust | UniRef100\_A0A173SRG9 | 99.4 | 1.8e-15 | 3.8e-21 | 95.2 | 66 | (6, 72) | 77 | (2, 67) | 137 | HTH-type transcriptional regulator immR | HTH-type transcriptional regulator immR | | uniclust | UniRef100\_A0A162KR50 | 99.4 | 1.9e-15 | 3.8e-21 | 96.1 | 58 | (14, 72) | 77 | (11, 68) | 158 | HTH-type transcriptional regulator ImmR | HTH-type transcriptional regulator ImmR | | uniclust | UniRef100\_A0A062XC28 | 99.4 | 1.7e-15 | 3.9e-21 | 97.4 | 64 | (8, 72) | 77 | (10, 73) | 152 | Transcriptional regulator, y4mF family | Transcriptional regulator, y4mF family | | uniclust | UniRef100\_A0A1G9NPQ0 | 99.4 | 1.9e-15 | 3.9e-21 | 89.9 | 59 | (13, 72) | 77 | (8, 66) | 97 | DNA-binding transcriptional regulator, XRE-family HTH domain | DNA-binding transcriptional regulator, XRE-family HTH domain | | uniclust | UniRef100\_A0A352BGN2 | 99.4 | 2e-15 | 3.9e-21 | 86.6 | 57 | (15, 72) | 77 | (2, 58) | 80 | HTH cro/C1-type domain-containing protein | HTH cro/C1-type domain-containing protein | | uniclust | UniRef100\_A0A068EMF1 | 99.4 | 1.9e-15 | 4.2e-21 | 96.3 | 63 | (9, 72) | 77 | (8, 70) | 147 | Transcriptional regulator | Transcriptional regulator | | uniclust | UniRef100\_A0A0R2D4N1 | 99.4 | 1.9e-15 | 4.2e-21 | 94.3 | 62 | (11, 73) | 77 | (3, 64) | 124 | HTH cro/C1-type domain-containing protein | HTH cro/C1-type domain-containing protein | | uniclust | UniRef100\_A0A0A1SME0 | 99.4 | 2e-15 | 4.2e-21 | 97.0 | 55 | (17, 72) | 77 | (1, 55) | 159 | DNA-binding protein | DNA-binding protein | | uniclust | UniRef100\_A0A088GHG5 | 99.4 | 2e-15 | 4.3e-21 | 88.9 | 57 | (16, 73) | 77 | (3, 59) | 90 | Transcriptional regulator, Cro/CI family protein | Transcriptional regulator, Cro/CI family protein | | uniclust | UniRef100\_A0A098ELK1 | 99.4 | 1.8e-15 | 4.3e-21 | 104.1 | 64 | (8, 72) | 77 | (31, 94) | 255 | LexA repressor | LexA repressor | | uniclust | UniRef100\_A0A063Y955 | 99.4 | 2.1e-15 | 4.5e-21 | 89.2 | 62 | (11, 73) | 77 | (6, 67) | 90 | HTH cro/C1-type domain-containing protein | HTH cro/C1-type domain-containing protein | | uniclust | UniRef100\_A0A242B388 | 99.4 | 2.2e-15 | 4.5e-21 | 91.3 | 56 | (16, 72) | 77 | (3, 58) | 111 | HTH cro/C1-type domain-containing protein | HTH cro/C1-type domain-containing protein | | uniclust | UniRef100\_A0A073IPP6 | 99.4 | 2e-15 | 4.5e-21 | 103.1 | 58 | (14, 72) | 77 | (17, 74) | 252 | HTH cro/C1-type domain-containing protein | HTH cro/C1-type domain-containing protein | | uniclust | UniRef100\_A0A1C6GXN4 | 99.4 | 2.2e-15 | 4.5e-21 | 90.8 | 58 | (14, 72) | 77 | (6, 63) | 106 | HTH-type transcriptional regulator immR | HTH-type transcriptional regulator immR | | uniclust | UniRef100\_A0A0C7N6M6 | 99.4 | 2.1e-15 | 4.6e-21 | 89.8 | 62 | (10, 72) | 77 | (3, 64) | 93 | Cro/C1-type helix-turn-helix domain | Cro/C1-type helix-turn-helix domain | | uniclust | UniRef100\_A0A0R1K8U9 | 99.4 | 2.1e-15 | 4.6e-21 | 97.6 | 59 | (13, 72) | 77 | (29, 87) | 166 | HTH cro/C1-type domain-containing protein | HTH cro/C1-type domain-containing protein | | uniclust | UniRef100\_A0A0R1Q4I5 | 99.4 | 2.2e-15 | 5.1e-21 | 94.8 | 57 | (15, 72) | 77 | (1, 57) | 132 | HTH cro/C1-type domain-containing protein | HTH cro/C1-type domain-containing protein | | uniclust | UniRef100\_A0A0R1XFR0 | 99.4 | 2.5e-15 | 5.1e-21 | 96.8 | 57 | (15, 72) | 77 | (15, 71) | 177 | HTH cro/C1-type domain-containing protein | HTH cro/C1-type domain-containing protein | | uniclust | UniRef100\_A0A0D8IC64 | 99.4 | 2.3e-15 | 5.2e-21 | 99.6 | 63 | (9, 72) | 77 | (26, 88) | 188 | Putative phage repressor | Putative phage repressor | | uniclust | UniRef100\_A0A098F0D7 | 99.4 | 2.3e-15 | 5.4e-21 | 99.7 | 65 | (7, 72) | 77 | (18, 82) | 189 | SPBc2 prophage-derived uncharacterized HTH-type transcriptional regulator yonR | SPBc2 prophage-derived uncharacterized HTH-type transcriptional regulator yonR | | uniclust | UniRef100\_A0A098APU9 | 99.4 | 2.4e-15 | 5.5e-21 | 100.6 | 57 | (15, 72) | 77 | (4, 60) | 205 | Helix-turn-helix domain-containing protein | Helix-turn-helix domain-containing protein | | uniclust | UniRef100\_A0A3D4S5S0 | 99.4 | 2.7e-15 | 5.5e-21 | 86.5 | 55 | (16, 71) | 77 | (2, 56) | 80 | XRE family transcriptional regulator | XRE family transcriptional regulator | | uniclust | UniRef100\_A0A1M5U3U8 | 99.4 | 2.7e-15 | 5.6e-21 | 86.6 | 56 | (16, 72) | 77 | (3, 58) | 80 | Helix-turn-helix | Helix-turn-helix | | uniclust | UniRef100\_A0A068A5Q8 | 99.4 | 2.6e-15 | 5.6e-21 | 90.1 | 66 | (7, 73) | 77 | (6, 71) | 98 | Helix-turn-helix domain-containing protein | Helix-turn-helix domain-containing protein | | uniclust | UniRef100\_A0A081PXA1 | 99.4 | 2.6e-15 | 5.6e-21 | 98.5 | 56 | (16, 72) | 77 | (2, 57) | 185 | Helix-turn-helix family protein | Helix-turn-helix family protein | | uniclust | UniRef100\_A0A1B3WME1 | 99.4 | 2.8e-15 | 5.8e-21 | 87.3 | 55 | (17, 72) | 77 | (6, 60) | 83 | HTH cro/C1-type domain-containing protein | HTH cro/C1-type domain-containing protein | | uniclust | UniRef100\_A0A173YT44 | 99.3 | 3e-15 | 6.1e-21 | 91.1 | 56 | (16, 72) | 77 | (5, 60) | 115 | HTH-type transcriptional regulator immR | HTH-type transcriptional regulator immR | | uniclust | UniRef100\_A0A099Y982 | 99.3 | 3.1e-15 | 6.6e-21 | 88.5 | 53 | (19, 72) | 77 | (8, 60) | 91 | HTH cro/C1-type domain-containing protein | HTH cro/C1-type domain-containing protein | | uniclust | UniRef100\_A0A1G6DIB8 | 99.3 | 3.1e-15 | 6.6e-21 | 92.9 | 60 | (15, 75) | 77 | (5, 64) | 125 | DNA-binding transcriptional regulator, XRE-family HTH domain | DNA-binding transcriptional regulator, XRE-family HTH domain | | uniclust | UniRef100\_A0A1Y4S908 | 99.3 | 3.3e-15 | 6.8e-21 | 95.9 | 58 | (13, 71) | 77 | (5, 62) | 161 | HTH cro/C1-type domain-containing protein | HTH cro/C1-type domain-containing protein | | uniclust | UniRef100\_A0A1G8HQ68 | 99.3 | 3.2e-15 | 6.8e-21 | 100.0 | 61 | (11, 72) | 77 | (20, 80) | 220 | Helix-turn-helix | Helix-turn-helix | | uniclust | UniRef100\_A0A198AE04 | 99.3 | 3.5e-15 | 7.5e-21 | 93.2 | 58 | (14, 72) | 77 | (1, 58) | 129 | HTH cro/C1-type domain-containing protein | HTH cro/C1-type domain-containing protein | | uniclust | UniRef100\_A0A416ZRR7 | 99.3 | 3.8e-15 | 7.6e-21 | 86.8 | 56 | (16, 72) | 77 | (3, 58) | 88 | XRE family transcriptional regulator | XRE family transcriptional regulator | | uniclust | UniRef100\_A0A0R1RXY9 | 99.3 | 3.7e-15 | 7.7e-21 | 94.5 | 58 | (14, 72) | 77 | (1, 58) | 147 | Transcriptional regulator, XRE family | Transcriptional regulator, XRE family | | uniclust | UniRef100\_A0A0H2UUS6 | 99.3 | 3.8e-15 | 7.7e-21 | 91.3 | 53 | (19, 72) | 77 | (3, 55) | 119 | HTH cro/C1-type domain-containing protein | HTH cro/C1-type domain-containing protein | | uniclust | UniRef100\_A0A1G7QJ77 | 99.3 | 3.7e-15 | 7.7e-21 | 92.2 | 55 | (17, 72) | 77 | (1, 55) | 125 | DNA-binding transcriptional regulator, XRE-family HTH domain | DNA-binding transcriptional regulator, XRE-family HTH domain | | uniclust | UniRef100\_A0A8S5MSF4 | 99.3 | 3.9e-15 | 7.7e-21 | 81.3 | 56 | (17, 73) | 77 | (1, 56) | 62 | Helix-turn-helix domain protein | Helix-turn-helix domain protein | | uniclust | UniRef100\_A0A1A5Y9X4 | 99.3 | 3.7e-15 | 8.2e-21 | 93.8 | 57 | (16, 73) | 77 | (1, 57) | 133 | HTH cro/C1-type domain-containing protein | HTH cro/C1-type domain-containing protein | | uniclust | UniRef100\_A0A0A2HEB2 | 99.3 | 3.9e-15 | 8.3e-21 | 96.2 | 59 | (13, 72) | 77 | (1, 59) | 166 | XRE family transcriptional regulator | XRE family transcriptional regulator | | uniclust | UniRef100\_A0A069AZ01 | 99.3 | 4.1e-15 | 8.4e-21 | 80.0 | 41 | (14, 54) | 77 | (2, 42) | 55 | Antitoxin PezA | Antitoxin PezA | | uniclust | UniRef100\_A0A0B2JZ21 | 99.3 | 4e-15 | 8.6e-21 | 89.6 | 60 | (12, 72) | 77 | (14, 73) | 102 | HTH cro/C1-type domain-containing protein | HTH cro/C1-type domain-containing protein | | uniclust | UniRef100\_A0A0R1H4Y4 | 99.3 | 4.3e-15 | 8.8e-21 | 92.8 | 59 | (13, 72) | 77 | (4, 62) | 136 | Helix-turn-helix XRE-family transcriptional regulator | Helix-turn-helix XRE-family transcriptional regulator | | uniclust | UniRef100\_A0A1G6DJ03 | 99.3 | 4.2e-15 | 8.9e-21 | 93.2 | 63 | (10, 73) | 77 | (3, 66) | 134 | Helix-turn-helix domain-containing protein | Helix-turn-helix domain-containing protein | | uniclust | UniRef100\_A0A0D0SD34 | 99.3 | 4.2e-15 | 9.1e-21 | 90.7 | 64 | (8, 72) | 77 | (10, 73) | 110 | Transcriptional regulator, XRE family | Transcriptional regulator, XRE family | | uniclust | UniRef100\_A0A099I752 | 99.3 | 4.5e-15 | 9.1e-21 | 80.4 | 44 | (11, 54) | 77 | (7, 50) | 57 | XRE family transcriptional regulator | XRE family transcriptional regulator | | uniclust | UniRef100\_A0A347WIN2 | 99.3 | 4.4e-15 | 9.2e-21 | 86.7 | 58 | (15, 73) | 77 | (11, 68) | 86 | HTH cro/C1-type domain-containing protein | HTH cro/C1-type domain-containing protein | | uniclust | UniRef100\_A0A0D6DZ12 | 99.3 | 4.1e-15 | 9.2e-21 | 99.8 | 57 | (15, 72) | 77 | (7, 63) | 211 | HTH-type DNA-binding domain | HTH-type DNA-binding domain | | uniclust | UniRef100\_A0A0R1HZP7 | 99.3 | 4.4e-15 | 9.6e-21 | 92.9 | 60 | (12, 72) | 77 | (5, 64) | 128 | HTH cro/C1-type domain-containing protein | HTH cro/C1-type domain-containing protein | | uniclust | UniRef100\_A0A0C9PMQ7 | 99.3 | 4.3e-15 | 9.7e-21 | 100.9 | 64 | (8, 72) | 77 | (28, 91) | 235 | XRE family transcriptional regulator | XRE family transcriptional regulator | | uniclust | UniRef100\_A0A084JB70 | 99.3 | 4.8e-15 | 1e-20 | 96.5 | 65 | (6, 71) | 77 | (10, 74) | 178 | DNA-binding protein | DNA-binding protein | | uniclust | UniRef100\_A0A096DH56 | 99.3 | 5e-15 | 1.1e-20 | 104.0 | 58 | (14, 72) | 77 | (12, 69) | 309 | HTH cro/C1-type domain-containing protein | HTH cro/C1-type domain-containing protein | | uniclust | UniRef100\_A0A0Q5D1Z3 | 99.3 | 5.5e-15 | 1.2e-20 | 85.1 | 62 | (8, 71) | 77 | (4, 65) | 78 | HTH cro/C1-type domain-containing protein | HTH cro/C1-type domain-containing protein | | uniclust | UniRef100\_A0A069D8G9 | 99.3 | 5.4e-15 | 1.2e-20 | 91.9 | 60 | (13, 73) | 77 | (3, 62) | 121 | Putative transcriptional regulator | Putative transcriptional regulator | | uniclust | UniRef100\_A0A0A3J3C4 | 99.3 | 5.5e-15 | 1.2e-20 | 94.9 | 57 | (15, 72) | 77 | (20, 76) | 153 | HTH cro/C1-type domain-containing protein | HTH cro/C1-type domain-containing protein | | uniclust | UniRef100\_A0A356ZA25 | 99.3 | 6.2e-15 | 1.2e-20 | 92.0 | 67 | (5, 72) | 77 | (5, 71) | 138 | XRE family transcriptional regulator | XRE family transcriptional regulator | | uniclust | UniRef100\_A0A084A723 | 99.3 | 5.4e-15 | 1.2e-20 | 96.2 | 68 | (3, 72) | 77 | (8, 75) | 166 | HTH-type transcriptional regulator | HTH-type transcriptional regulator | | uniclust | UniRef100\_A0A023CJ53 | 99.3 | 6.3e-15 | 1.3e-20 | 94.6 | 60 | (12, 72) | 77 | (23, 82) | 162 | XRE family transcriptional regulator | XRE family transcriptional regulator | | uniclust | UniRef100\_A0A1C5QRJ2 | 99.3 | 6.5e-15 | 1.3e-20 | 95.4 | 63 | (13, 76) | 77 | (21, 83) | 178 | Transcriptional repressor DicA | Transcriptional repressor DicA | | uniclust | UniRef100\_A0A0V8DXP7 | 99.3 | 6.6e-15 | 1.3e-20 | 86.0 | 59 | (13, 72) | 77 | (11, 69) | 87 | HTH cro/C1-type domain-containing protein | HTH cro/C1-type domain-containing protein | | uniclust | UniRef100\_A0A031WFF9 | 99.3 | 6e-15 | 1.4e-20 | 96.7 | 63 | (9, 72) | 77 | (25, 87) | 175 | DNA-binding helix-turn-helix protein | DNA-binding helix-turn-helix protein | | uniclust | UniRef100\_A0A011UYL9 | 99.3 | 6.7e-15 | 1.4e-20 | 101.2 | 59 | (13, 72) | 77 | (43, 101) | 280 | DNA-binding protein | DNA-binding protein | | uniclust | UniRef100\_A0A1C7GW64 | 99.3 | 7.1e-15 | 1.4e-20 | 89.7 | 59 | (15, 74) | 77 | (1, 59) | 116 | Transcriptional regulator | Transcriptional regulator | | uniclust | UniRef100\_A0A1F8UDN2 | 99.3 | 6.7e-15 | 1.5e-20 | 95.0 | 58 | (14, 72) | 77 | (1, 58) | 159 | Transcriptional regulator | Transcriptional regulator | | uniclust | UniRef100\_A0A0G3WDU7 | 99.3 | 7.4e-15 | 1.5e-20 | 82.8 | 59 | (13, 72) | 77 | (2, 60) | 72 | Putative transcriptional regulator | Putative transcriptional regulator | | uniclust | UniRef100\_A0A1E9IL17 | 99.3 | 7.5e-15 | 1.6e-20 | 96.5 | 57 | (15, 72) | 77 | (13, 69) | 190 | Transcriptional regulator | Transcriptional regulator | | uniclust | UniRef100\_A0A011UWQ9 | 99.3 | 7e-15 | 1.6e-20 | 88.9 | 63 | (10, 73) | 77 | (9, 71) | 101 | ABC transporter substrate-binding protein | ABC transporter substrate-binding protein | | uniclust | UniRef100\_A0A024QBX0 | 99.3 | 7.3e-15 | 1.6e-20 | 91.5 | 62 | (11, 73) | 77 | (31, 92) | 125 | Helix-turn-helix | Helix-turn-helix | | uniclust | UniRef100\_A0A0Z8ISK9 | 99.3 | 8e-15 | 1.6e-20 | 97.1 | 59 | (13, 72) | 77 | (5, 63) | 207 | XRE family transcriptional regulator | XRE family transcriptional regulator | | uniclust | UniRef100\_A0A1C5QEW4 | 99.3 | 8e-15 | 1.7e-20 | 99.7 | 57 | (15, 72) | 77 | (28, 84) | 248 | HTH-type transcriptional regulator immR | HTH-type transcriptional regulator immR | | uniclust | UniRef100\_A0A0F5R9Z3 | 99.3 | 7.8e-15 | 1.7e-20 | 91.7 | 61 | (11, 72) | 77 | (7, 67) | 129 | HTH cro/C1-type domain-containing protein | HTH cro/C1-type domain-containing protein | | uniclust | UniRef100\_A0A1C6D7B9 | 99.3 | 8.7e-15 | 1.7e-20 | 75.3 | 38 | (17, 54) | 77 | (1, 38) | 45 | Antitoxin PezA | Antitoxin PezA | | uniclust | UniRef100\_A0A0V8C4S1 | 99.3 | 7.5e-15 | 1.7e-20 | 96.8 | 64 | (8, 72) | 77 | (16, 79) | 182 | Transcriptional regulator Cro/CI family | Transcriptional regulator Cro/CI family | | uniclust | UniRef100\_A0A927NWC2 | 99.3 | 8.7e-15 | 1.7e-20 | 91.3 | 58 | (14, 72) | 77 | (6, 63) | 139 | Helix-turn-helix transcriptional regulator | Helix-turn-helix transcriptional regulator | | uniclust | UniRef100\_A0A085ZCW4 | 99.3 | 8.1e-15 | 1.7e-20 | 94.5 | 62 | (10, 72) | 77 | (25, 86) | 161 | HTH cro/C1-type domain-containing protein | HTH cro/C1-type domain-containing protein | | uniclust | UniRef100\_A0A062X1K8 | 99.3 | 8.4e-15 | 1.7e-20 | 86.7 | 54 | (19, 73) | 77 | (3, 56) | 93 | Zinc finger/helix-turn-helix protein, YgiT family | Zinc finger/helix-turn-helix protein, YgiT family | | uniclust | UniRef100\_A0A061K0S8 | 99.3 | 8.7e-15 | 1.9e-20 | 94.9 | 64 | (8, 72) | 77 | (12, 75) | 165 | Helix-turn-helix domain-containing protein | Helix-turn-helix domain-containing protein | | uniclust | UniRef100\_A0A1M4ZB90 | 99.3 | 9.2e-15 | 1.9e-20 | 92.1 | 58 | (15, 73) | 77 | (6, 63) | 139 | Helix-turn-helix domain-containing protein | Helix-turn-helix domain-containing protein | | uniclust | UniRef100\_A0A0R1MYT8 | 99.3 | 9.2e-15 | 2e-20 | 97.4 | 57 | (15, 72) | 77 | (1, 57) | 207 | HTH cro/C1-type domain-containing protein | HTH cro/C1-type domain-containing protein | | uniclust | UniRef100\_A0A174CCW1 | 99.3 | 9.1e-15 | 2e-20 | 98.4 | 58 | (14, 72) | 77 | (4, 61) | 218 | HTH-type transcriptional regulator sinR | HTH-type transcriptional regulator sinR | | uniclust | UniRef100\_A0A1C5UWY8 | 99.3 | 1e-14 | 2e-20 | 78.0 | 41 | (14, 54) | 77 | (4, 44) | 53 | Putative zinc finger/helix-turn-helix protein, YgiT family | Putative zinc finger/helix-turn-helix protein, YgiT family | | uniclust | UniRef100\_A0A1C5P1N9 | 99.3 | 9.4e-15 | 2e-20 | 97.9 | 60 | (13, 73) | 77 | (3, 62) | 213 | HTH-type transcriptional regulator immR | HTH-type transcriptional regulator immR | | uniclust | UniRef100\_A0A0C7N4G8 | 99.3 | 9.8e-15 | 2e-20 | 91.8 | 60 | (12, 72) | 77 | (2, 61) | 140 | Cro/C1-type helix-turn-helix domain | Cro/C1-type helix-turn-helix domain | | uniclust | UniRef100\_A0A0F0CL80 | 99.3 | 9.6e-15 | 2.1e-20 | 105.2 | 59 | (13, 72) | 77 | (33, 91) | 396 | Transcriptional repressor DicA | Transcriptional repressor DicA | | uniclust | UniRef100\_A0A1Y3WG13 | 99.3 | 1.1e-14 | 2.2e-20 | 77.7 | 44 | (12, 55) | 77 | (3, 46) | 52 | Transcriptional regulator | Transcriptional regulator | | uniclust | UniRef100\_A0A7C6WH00 | 99.3 | 1e-14 | 2.2e-20 | 95.1 | 61 | (11, 72) | 77 | (14, 74) | 181 | Helix-turn-helix transcriptional regulator | Helix-turn-helix transcriptional regulator | | uniclust | UniRef100\_A0A2K9ADE1 | 99.3 | 1.1e-14 | 2.2e-20 | 85.6 | 59 | (15, 74) | 77 | (2, 60) | 88 | HTH cro/C1-type domain-containing protein | HTH cro/C1-type domain-containing protein | | uniclust | UniRef100\_A0A1D2LGT8 | 99.3 | 1e-14 | 2.2e-20 | 90.1 | 58 | (14, 72) | 77 | (3, 60) | 120 | XRE family transcriptional regulator | XRE family transcriptional regulator | | uniclust | UniRef100\_A0A086BKQ7 | 99.3 | 9.6e-15 | 2.3e-20 | 95.4 | 60 | (12, 72) | 77 | (30, 89) | 169 | DNA-binding protein | DNA-binding protein | | uniclust | UniRef100\_A0A316QXQ5 | 99.3 | 1.1e-14 | 2.3e-20 | 95.2 | 57 | (15, 72) | 77 | (2, 58) | 178 | HTH cro/C1-type domain-containing protein | HTH cro/C1-type domain-containing protein | | uniclust | UniRef100\_A0A845JTS9 | 99.3 | 1.1e-14 | 2.3e-20 | 90.1 | 57 | (15, 72) | 77 | (4, 60) | 121 | Helix-turn-helix transcriptional regulator | Helix-turn-helix transcriptional regulator | | uniclust | UniRef100\_A0A011RNT1 | 99.3 | 1.1e-14 | 2.3e-20 | 96.9 | 60 | (12, 72) | 77 | (20, 79) | 215 | Transcriptional regulator, XRE family | Transcriptional regulator, XRE family | | uniclust | UniRef100\_A0A060M4E5 | 99.3 | 1e-14 | 2.3e-20 | 91.8 | 61 | (11, 72) | 77 | (6, 66) | 134 | HTH-type transcriptional regulator | HTH-type transcriptional regulator | | uniclust | UniRef100\_A0A1R0XIP9 | 99.3 | 1.1e-14 | 2.3e-20 | 94.5 | 58 | (16, 74) | 77 | (1, 58) | 176 | HTH cro/C1-type domain-containing protein | HTH cro/C1-type domain-containing protein | | uniclust | UniRef100\_A0A0K1F1X2 | 99.3 | 1.1e-14 | 2.3e-20 | 99.1 | 63 | (10, 73) | 77 | (14, 76) | 240 | HTH cro/C1-type domain-containing protein | HTH cro/C1-type domain-containing protein | | uniclust | UniRef100\_A0A069D9R6 | 99.3 | 1e-14 | 2.4e-20 | 95.8 | 63 | (10, 72) | 77 | (20, 82) | 177 | HTH cro/C1-type domain-containing protein | HTH cro/C1-type domain-containing protein | | uniclust | UniRef100\_A0A0M2NJ60 | 99.3 | 1.1e-14 | 2.4e-20 | 89.5 | 56 | (16, 72) | 77 | (2, 57) | 117 | HTH cro/C1-type domain-containing protein | HTH cro/C1-type domain-containing protein | | uniclust | UniRef100\_A0A351SRF1 | 99.3 | 1.2e-14 | 2.4e-20 | 98.4 | 63 | (9, 72) | 77 | (23, 85) | 243 | HTH cro/C1-type domain-containing protein | HTH cro/C1-type domain-containing protein | | uniclust | UniRef100\_A0A149UEU3 | 99.3 | 1.1e-14 | 2.4e-20 | 91.5 | 62 | (10, 72) | 77 | (15, 76) | 132 | HTH cro/C1-type domain-containing protein | HTH cro/C1-type domain-containing protein | | uniclust | UniRef100\_A0A0D8J1P7 | 99.3 | 1.2e-14 | 2.4e-20 | 96.9 | 60 | (12, 72) | 77 | (5, 64) | 219 | Helix-turn-helix domain-containing protein | Helix-turn-helix domain-containing protein | | uniclust | UniRef100\_A0A0E1Y5F5 | 99.3 | 1.2e-14 | 2.5e-20 | 91.0 | 55 | (18, 73) | 77 | (3, 57) | 130 | DNA-binding protein (Fragment) | DNA-binding protein (Fragment) | | uniclust | UniRef100\_A0A0H4P7Y6 | 99.3 | 1.2e-14 | 2.5e-20 | 87.7 | 60 | (12, 72) | 77 | (5, 64) | 101 | Prophage LambdaBa02repressor protein | Prophage LambdaBa02repressor protein | | uniclust | UniRef100\_A0A099WFI4 | 99.3 | 1.3e-14 | 2.6e-20 | 91.4 | 62 | (10, 72) | 77 | (12, 73) | 143 | DNA-binding protein | DNA-binding protein | | uniclust | UniRef100\_A0A0H5Q6F9 | 99.3 | 1.3e-14 | 2.7e-20 | 92.4 | 57 | (15, 72) | 77 | (8, 64) | 150 | HTH cro/C1-type domain-containing protein | HTH cro/C1-type domain-containing protein | | uniclust | UniRef100\_A0A015LSZ1 | 99.3 | 1.2e-14 | 2.7e-20 | 93.3 | 56 | (16, 72) | 77 | (16, 71) | 151 | XRE family transcriptional regulator | XRE family transcriptional regulator | | uniclust | UniRef100\_A0A1B1YAB2 | 99.3 | 1.3e-14 | 2.8e-20 | 91.8 | 60 | (13, 73) | 77 | (3, 62) | 145 | HTH cro/C1-type domain-containing protein | HTH cro/C1-type domain-containing protein | | uniclust | UniRef100\_A0A023CPN5 | 99.3 | 1.3e-14 | 2.9e-20 | 90.4 | 60 | (13, 73) | 77 | (2, 61) | 121 | HTH-type transcriptional regulator SlrR | HTH-type transcriptional regulator SlrR | | uniclust | UniRef100\_A0A1M6XC60 | 99.3 | 1.4e-14 | 2.9e-20 | 93.5 | 57 | (15, 72) | 77 | (10, 66) | 165 | Helix-turn-helix | Helix-turn-helix | | uniclust | UniRef100\_A0A084JI99 | 99.3 | 1.4e-14 | 2.9e-20 | 93.1 | 63 | (9, 72) | 77 | (7, 69) | 158 | HTH cro/C1-type domain-containing protein | HTH cro/C1-type domain-containing protein | | uniclust | UniRef100\_A0A095XNQ4 | 99.3 | 1.4e-14 | 3e-20 | 92.9 | 57 | (15, 72) | 77 | (2, 58) | 153 | HTH cro/C1-type domain-containing protein | HTH cro/C1-type domain-containing protein | | uniclust | UniRef100\_A0A0F8X4V8 | 99.3 | 1.4e-14 | 3e-20 | 80.7 | 56 | (18, 74) | 77 | (4, 59) | 64 | HTH cro/C1-type domain-containing protein | HTH cro/C1-type domain-containing protein | | uniclust | UniRef100\_A0A081P4C3 | 99.3 | 1.4e-14 | 3e-20 | 92.0 | 58 | (14, 72) | 77 | (2, 59) | 139 | HTH cro/C1-type domain-containing protein | HTH cro/C1-type domain-containing protein | | uniclust | UniRef100\_A0A024P3C3 | 99.3 | 1.5e-14 | 3.1e-20 | 91.5 | 57 | (15, 72) | 77 | (9, 65) | 142 | HTH-type transcriptional regulator ImmR | HTH-type transcriptional regulator ImmR | | uniclust | UniRef100\_A0A0J1F8E5 | 99.3 | 1.5e-14 | 3.2e-20 | 100.5 | 62 | (10, 72) | 77 | (12, 73) | 291 | HTH-type transcriptional regulator Xre | HTH-type transcriptional regulator Xre | | uniclust | UniRef100\_A0A084ACG0 | 99.3 | 1.4e-14 | 3.3e-20 | 98.2 | 62 | (10, 72) | 77 | (11, 72) | 224 | HTH cro/C1-type domain-containing protein | HTH cro/C1-type domain-containing protein | | uniclust | UniRef100\_A0A1W2ASU0 | 99.3 | 1.5e-14 | 3.3e-20 | 95.6 | 55 | (17, 72) | 77 | (1, 55) | 193 | Transcriptional regulator, contains XRE-family HTH domain | Transcriptional regulator, contains XRE-family HTH domain | | uniclust | UniRef100\_A0A069DDP2 | 99.3 | 1.6e-14 | 3.3e-20 | 96.2 | 58 | (14, 72) | 77 | (55, 112) | 214 | Transcriptional regulator | Transcriptional regulator | | uniclust | UniRef100\_A0A174HLJ4 | 99.3 | 1.5e-14 | 3.4e-20 | 94.7 | 55 | (17, 72) | 77 | (1, 55) | 172 | MerR family transcriptional regulator | MerR family transcriptional regulator | | uniclust | UniRef100\_A0A150LHC3 | 99.3 | 1.6e-14 | 3.4e-20 | 90.4 | 61 | (11, 72) | 77 | (5, 65) | 128 | HTH cro/C1-type domain-containing protein | HTH cro/C1-type domain-containing protein | | uniclust | UniRef100\_A0A084JPR8 | 99.3 | 1.6e-14 | 3.4e-20 | 97.9 | 60 | (11, 71) | 77 | (14, 73) | 233 | XRE family transcriptional regulator | XRE family transcriptional regulator | | uniclust | UniRef100\_A0A0V8DXH0 | 99.3 | 1.9e-14 | 3.8e-20 | 81.7 | 57 | (15, 72) | 77 | (4, 60) | 75 | Transcriptional regulator XRE family | Transcriptional regulator XRE family | | uniclust | UniRef100\_A0A143HDQ9 | 99.3 | 1.8e-14 | 4e-20 | 96.4 | 58 | (14, 72) | 77 | (20, 77) | 209 | HTH cro/C1-type domain-containing protein | HTH cro/C1-type domain-containing protein | | uniclust | UniRef100\_A0A1Q9YGQ7 | 99.3 | 1.9e-14 | 4.2e-20 | 90.6 | 61 | (13, 73) | 77 | (6, 66) | 134 | HTH cro/C1-type domain-containing protein | HTH cro/C1-type domain-containing protein | | uniclust | UniRef100\_A0A1C5MER2 | 99.3 | 2e-14 | 4.2e-20 | 89.2 | 60 | (11, 71) | 77 | (4, 64) | 123 | Helix-turn-helix | Helix-turn-helix | | uniclust | UniRef100\_A0A0U2MC24 | 99.3 | 1.9e-14 | 4.4e-20 | 93.5 | 63 | (11, 74) | 77 | (5, 67) | 161 | HTH domain-containing protein | HTH domain-containing protein | | uniclust | UniRef100\_A0A3D0XZX5 | 99.3 | 2.3e-14 | 4.5e-20 | 80.0 | 54 | (17, 71) | 77 | (1, 54) | 70 | Transcriptional regulator (Fragment) | Transcriptional regulator (Fragment) | | uniclust | UniRef100\_A0A0J5PB61 | 99.3 | 2.2e-14 | 4.5e-20 | 95.7 | 57 | (14, 71) | 77 | (2, 58) | 211 | HTH-type transcriptional regulator Xre | HTH-type transcriptional regulator Xre | | uniclust | UniRef100\_A0A1D2M0A2 | 99.3 | 2.1e-14 | 4.7e-20 | 97.8 | 59 | (13, 72) | 77 | (4, 62) | 236 | XRE family transcriptional regulator | XRE family transcriptional regulator | | uniclust | UniRef100\_A0A024QIL8 | 99.3 | 2.2e-14 | 4.8e-20 | 92.0 | 61 | (11, 72) | 77 | (6, 66) | 152 | Antitoxin HipB | Antitoxin HipB | | uniclust | UniRef100\_A0A031WFK4 | 99.3 | 2.2e-14 | 4.8e-20 | 92.0 | 63 | (9, 72) | 77 | (5, 67) | 151 | Helix-turn-helix domain protein | Helix-turn-helix domain protein | | uniclust | UniRef100\_A0A081NRH7 | 99.3 | 2.1e-14 | 4.9e-20 | 99.3 | 61 | (10, 71) | 77 | (23, 83) | 262 | XRE family transcriptional regulator | XRE family transcriptional regulator | | uniclust | UniRef100\_A0A095X9F7 | 99.3 | 2.4e-14 | 5e-20 | 98.9 | 64 | (8, 72) | 77 | (33, 96) | 285 | DNA-binding protein | DNA-binding protein | | uniclust | UniRef100\_A0A179ESE4 | 99.3 | 2.4e-14 | 5e-20 | 92.8 | 58 | (14, 72) | 77 | (5, 62) | 170 | XRE family transcriptional regulator | XRE family transcriptional regulator | | uniclust | UniRef100\_A0A075RC30 | 99.3 | 2.5e-14 | 5.1e-20 | 89.7 | 62 | (12, 74) | 77 | (2, 63) | 134 | Transcriptional repressor DicA | Transcriptional repressor DicA | | uniclust | UniRef100\_A0A075WVE9 | 99.3 | 2.3e-14 | 5.1e-20 | 98.0 | 57 | (15, 72) | 77 | (33, 89) | 244 | HTH cro/C1-type domain-containing protein | HTH cro/C1-type domain-containing protein | | uniclust | UniRef100\_A0A139QKZ0 | 99.3 | 2.7e-14 | 5.4e-20 | 81.5 | 56 | (16, 72) | 77 | (5, 60) | 76 | HTH cro/C1-type domain-containing protein | HTH cro/C1-type domain-containing protein | | uniclust | UniRef100\_A0A1L7GTF0 | 99.2 | 2.6e-14 | 5.4e-20 | 88.8 | 57 | (15, 72) | 77 | (2, 58) | 125 | Transcriptional regulator | Transcriptional regulator | | uniclust | UniRef100\_A0A1Y4LJ54 | 99.2 | 2.6e-14 | 5.4e-20 | 93.0 | 55 | (17, 72) | 77 | (1, 55) | 172 | HTH cro/C1-type domain-containing protein | HTH cro/C1-type domain-containing protein | | uniclust | UniRef100\_A0A084JDN7 | 99.2 | 2.7e-14 | 5.4e-20 | 91.8 | 57 | (14, 71) | 77 | (11, 67) | 170 | DNA-binding protein | DNA-binding protein | | uniclust | UniRef100\_A0A0Q4FSA8 | 99.2 | 2.5e-14 | 5.4e-20 | 82.7 | 56 | (16, 72) | 77 | (2, 57) | 78 | HTH cro/C1-type domain-containing protein | HTH cro/C1-type domain-containing protein | | uniclust | UniRef100\_A0A0F9I898 | 99.2 | 2.7e-14 | 5.6e-20 | 83.1 | 63 | (8, 71) | 77 | (5, 67) | 83 | HTH cro/C1-type domain-containing protein | HTH cro/C1-type domain-containing protein | | uniclust | UniRef100\_A0A140L514 | 99.2 | 2.7e-14 | 5.6e-20 | 87.6 | 59 | (13, 72) | 77 | (1, 59) | 117 | HTH-type transcriptional regulator ImmR | HTH-type transcriptional regulator ImmR | | uniclust | UniRef100\_A0A1E3A252 | 99.2 | 2.5e-14 | 5.6e-20 | 90.1 | 60 | (12, 72) | 77 | (12, 71) | 133 | Transcriptional repressor DicA | Transcriptional repressor DicA | | uniclust | UniRef100\_A0A174NGD7 | 99.2 | 2.8e-14 | 5.7e-20 | 75.7 | 39 | (17, 55) | 77 | (1, 39) | 51 | Transcriptional repressor DicA | Transcriptional repressor DicA | | uniclust | UniRef100\_A0A134AB19 | 99.2 | 2.8e-14 | 5.7e-20 | 89.6 | 68 | (4, 72) | 77 | (4, 71) | 137 | DNA-binding helix-turn-helix protein | DNA-binding helix-turn-helix protein | | uniclust | UniRef100\_A0A024QB89 | 99.2 | 2.7e-14 | 5.9e-20 | 83.8 | 57 | (16, 73) | 77 | (2, 58) | 85 | Anaerobic benzoate catabolism transcriptional regulator | Anaerobic benzoate catabolism transcriptional regulator | | uniclust | UniRef100\_A0A098N141 | 99.2 | 2.7e-14 | 5.9e-20 | 91.3 | 59 | (13, 72) | 77 | (18, 76) | 148 | DNA-binding protein | DNA-binding protein | | uniclust | UniRef100\_A0A0A7FXJ6 | 99.2 | 3e-14 | 6.1e-20 | 90.7 | 61 | (11, 72) | 77 | (16, 76) | 154 | Helix-turn-helix family protein | Helix-turn-helix family protein | | uniclust | UniRef100\_A0A061PBI6 | 99.2 | 2.9e-14 | 6.2e-20 | 84.7 | 56 | (17, 73) | 77 | (10, 65) | 91 | HTH cro/C1-type domain-containing protein | HTH cro/C1-type domain-containing protein | | uniclust | UniRef100\_A0A090Y2E7 | 99.2 | 2.9e-14 | 6.4e-20 | 90.8 | 59 | (13, 72) | 77 | (3, 61) | 145 | Helix-turn-helix family protein | Helix-turn-helix family protein | | uniclust | UniRef100\_A0A0R1ZQ19 | 99.2 | 2.7e-14 | 6.4e-20 | 97.4 | 62 | (10, 72) | 77 | (3, 64) | 235 | Prophage lsa1 XRE family DNA-binding protein | Prophage lsa1 XRE family DNA-binding protein | | uniclust | UniRef100\_A0A364K8X3 | 99.2 | 3.2e-14 | 6.6e-20 | 93.0 | 56 | (16, 72) | 77 | (1, 56) | 184 | Transcriptional regulator | Transcriptional regulator | | uniclust | UniRef100\_A0A0P9FPS6 | 99.2 | 3.1e-14 | 6.7e-20 | 90.7 | 59 | (13, 72) | 77 | (7, 65) | 147 | HTH cro/C1-type domain-containing protein | HTH cro/C1-type domain-containing protein | | uniclust | UniRef100\_A0A0H5SHR8 | 99.2 | 3.3e-14 | 6.9e-20 | 85.8 | 60 | (12, 72) | 77 | (16, 75) | 102 | HTH cro/C1-type domain-containing protein | HTH cro/C1-type domain-containing protein | | uniclust | UniRef100\_A0A024QF78 | 99.2 | 3.5e-14 | 7e-20 | 92.1 | 59 | (13, 72) | 77 | (24, 82) | 183 | Helix-turn-helix protein | Helix-turn-helix protein | | uniclust | UniRef100\_A0A072MV23 | 99.2 | 3.2e-14 | 7e-20 | 90.7 | 63 | (10, 73) | 77 | (5, 67) | 146 | XRE family transcriptional regulator | XRE family transcriptional regulator | | uniclust | UniRef100\_A0A1W1Y945 | 99.2 | 3.1e-14 | 7e-20 | 89.1 | 60 | (12, 72) | 77 | (4, 63) | 125 | Transcriptional regulator, contains XRE-family HTH domain | Transcriptional regulator, contains XRE-family HTH domain | | uniclust | UniRef100\_A0A096ALY3 | 99.2 | 3e-14 | 7e-20 | 97.8 | 60 | (12, 72) | 77 | (27, 86) | 248 | HTH cro/C1-type domain-containing protein | HTH cro/C1-type domain-containing protein | | uniclust | UniRef100\_A0A0H5SUC8 | 99.2 | 3.3e-14 | 7e-20 | 88.2 | 56 | (17, 73) | 77 | (3, 58) | 122 | HTH cro/C1-type domain-containing protein | HTH cro/C1-type domain-containing protein | | uniclust | UniRef100\_A0A2S7FD02 | 99.2 | 3.3e-14 | 7.3e-20 | 85.5 | 58 | (14, 72) | 77 | (13, 70) | 98 | HTH cro/C1-type domain-containing protein | HTH cro/C1-type domain-containing protein | | uniclust | UniRef100\_A0A1C5M5K7 | 99.2 | 3.5e-14 | 7.3e-20 | 95.2 | 55 | (17, 72) | 77 | (1, 55) | 218 | Transcriptional repressor DicA | Transcriptional repressor DicA | | uniclust | UniRef100\_A0A010NY17 | 99.2 | 3.5e-14 | 7.5e-20 | 85.2 | 63 | (11, 74) | 77 | (7, 69) | 98 | DNA-binding protein | DNA-binding protein | | uniclust | UniRef100\_A0A0H5Q551 | 99.2 | 3.6e-14 | 7.7e-20 | 97.1 | 58 | (14, 72) | 77 | (31, 88) | 254 | HTH cro/C1-type domain-containing protein | HTH cro/C1-type domain-containing protein | | uniclust | UniRef100\_A0A0D8I6J6 | 99.2 | 3.5e-14 | 8e-20 | 89.8 | 59 | (15, 73) | 77 | (1, 59) | 134 | Putative phage repressor | Putative phage repressor | | uniclust | UniRef100\_A0A1V4GWD0 | 99.2 | 3.5e-14 | 8.2e-20 | 97.2 | 65 | (9, 74) | 77 | (13, 77) | 241 | HTH cro/C1-type domain-containing protein | HTH cro/C1-type domain-containing protein | | uniclust | UniRef100\_A0A0S2W8V1 | 99.2 | 4e-14 | 8.2e-20 | 89.4 | 58 | (14, 72) | 77 | (14, 71) | 144 | HTH-type transcriptional regulator xre | HTH-type transcriptional regulator xre | | uniclust | UniRef100\_A0A0S2W0A6 | 99.2 | 3.9e-14 | 8.3e-20 | 95.3 | 56 | (16, 72) | 77 | (1, 56) | 222 | Transcriptional regulator, XRE family | Transcriptional regulator, XRE family | | uniclust | UniRef100\_A0A0R1EZ08 | 99.2 | 4e-14 | 8.4e-20 | 94.2 | 55 | (16, 71) | 77 | (1, 55) | 208 | XRE family transcriptional regulator | XRE family transcriptional regulator | | uniclust | UniRef100\_A0A323UEM4 | 99.2 | 4.1e-14 | 8.5e-20 | 86.4 | 59 | (13, 72) | 77 | (24, 82) | 113 | XRE family transcriptional regulator | XRE family transcriptional regulator | | uniclust | UniRef100\_A0A1W1IH47 | 99.2 | 4.2e-14 | 8.5e-20 | 84.1 | 55 | (16, 71) | 77 | (1, 55) | 96 | HTH cro/C1-type domain-containing protein | HTH cro/C1-type domain-containing protein | | uniclust | UniRef100\_A0A0A0IG66 | 99.2 | 3.9e-14 | 8.5e-20 | 89.2 | 64 | (8, 72) | 77 | (8, 71) | 132 | HTH cro/C1-type domain-containing protein | HTH cro/C1-type domain-containing protein | | uniclust | UniRef100\_A0A1S9BXS8 | 99.2 | 4.4e-14 | 8.6e-20 | 81.5 | 56 | (15, 71) | 77 | (2, 57) | 85 | HTH cro/C1-type domain-containing protein | HTH cro/C1-type domain-containing protein | | uniclust | UniRef100\_A0A124IPR8 | 99.2 | 4e-14 | 8.8e-20 | 88.6 | 55 | (17, 72) | 77 | (1, 55) | 127 | HTH cro/C1-type domain-containing protein | HTH cro/C1-type domain-containing protein | | uniclust | UniRef100\_A0A174NJ84 | 99.2 | 4.5e-14 | 9.3e-20 | 91.4 | 58 | (14, 72) | 77 | (10, 67) | 169 | HTH-type transcriptional regulator immR | HTH-type transcriptional regulator immR | | uniclust | UniRef100\_A0A0C2PWR1 | 99.2 | 4.4e-14 | 9.3e-20 | 85.3 | 69 | (5, 74) | 77 | (5, 73) | 103 | XRE family transcriptional regulator | XRE family transcriptional regulator | | uniclust | UniRef100\_A0A099I8V5 | 99.2 | 4.2e-14 | 9.4e-20 | 99.1 | 58 | (13, 72) | 77 | (1, 58) | 298 | HTH cro/C1-type domain-containing protein | HTH cro/C1-type domain-containing protein | | uniclust | UniRef100\_A0A0K9N8I6 | 99.2 | 4.8e-14 | 9.6e-20 | 81.0 | 58 | (14, 72) | 77 | (2, 59) | 80 | Toxin-antitoxin system, antitoxin component, Xre family | Toxin-antitoxin system, antitoxin component, Xre family | | uniclust | UniRef100\_A0A0S2W086 | 99.2 | 4.6e-14 | 9.6e-20 | 87.0 | 58 | (14, 72) | 77 | (1, 58) | 118 | Transcriptional regulator, XRE family | Transcriptional regulator, XRE family | | uniclust | UniRef100\_A0A096B2S2 | 99.2 | 4.6e-14 | 9.8e-20 | 89.3 | 65 | (6, 72) | 77 | (2, 66) | 140 | HTH cro/C1-type domain-containing protein | HTH cro/C1-type domain-containing protein | | uniclust | UniRef100\_A0A174DE52 | 99.2 | 4.5e-14 | 9.8e-20 | 95.5 | 62 | (10, 72) | 77 | (6, 67) | 224 | Antitoxin HipB | Antitoxin HipB | | uniclust | UniRef100\_A0A1R0Z6W7 | 99.2 | 5e-14 | 1e-19 | 87.3 | 59 | (14, 73) | 77 | (1, 59) | 124 | HTH cro/C1-type domain-containing protein | HTH cro/C1-type domain-containing protein | | uniclust | UniRef100\_A0A0K8JHW7 | 99.2 | 4.7e-14 | 1e-19 | 89.3 | 60 | (12, 72) | 77 | (4, 63) | 138 | HTH cro/C1-type domain-containing protein | HTH cro/C1-type domain-containing protein | | uniclust | UniRef100\_A0A101ETY0 | 99.2 | 5e-14 | 1e-19 | 86.5 | 57 | (16, 73) | 77 | (5, 61) | 117 | HTH cro/C1-type domain-containing protein | HTH cro/C1-type domain-containing protein | | uniclust | UniRef100\_A0A354UQS3 | 99.2 | 5.4e-14 | 1.1e-19 | 92.5 | 58 | (15, 73) | 77 | (28, 85) | 204 | HTH cro/C1-type domain-containing protein | HTH cro/C1-type domain-containing protein | | uniclust | UniRef100\_A0A1X7I7X0 | 99.2 | 4.8e-14 | 1.1e-19 | 89.0 | 58 | (14, 72) | 77 | (3, 60) | 133 | DNA-binding transcriptional regulator, XRE-family HTH domain | DNA-binding transcriptional regulator, XRE-family HTH domain | | uniclust | UniRef100\_A0A022N9X9 | 99.2 | 4.7e-14 | 1.1e-19 | 98.2 | 66 | (7, 73) | 77 | (28, 93) | 275 | Peptidase S24 | Peptidase S24 | | uniclust | UniRef100\_A0A4U9R0I1 | 99.2 | 5.8e-14 | 1.1e-19 | 74.0 | 46 | (17, 63) | 77 | (1, 46) | 52 | XRE family transcriptional regulator | XRE family transcriptional regulator | | uniclust | UniRef100\_A0A0R1KF97 | 99.2 | 5.3e-14 | 1.1e-19 | 88.6 | 59 | (13, 72) | 77 | (12, 70) | 134 | Phage-related Cro CI family transcription regulator | Phage-related Cro CI family transcription regulator | | uniclust | UniRef100\_A0A1J1CTL4 | 99.2 | 5.8e-14 | 1.1e-19 | 79.7 | 54 | (17, 71) | 77 | (1, 54) | 78 | Helix-turn-helix family protein | Helix-turn-helix family protein | | uniclust | UniRef100\_A0A0B5QNQ3 | 99.2 | 5.5e-14 | 1.2e-19 | 88.0 | 65 | (12, 76) | 77 | (3, 67) | 130 | Helix-turn-helix domain protein | Helix-turn-helix domain protein | | uniclust | UniRef100\_A0A1C6HMP3 | 99.2 | 5.6e-14 | 1.2e-19 | 87.4 | 59 | (13, 72) | 77 | (20, 78) | 123 | Transcriptional regulator, y4mF family | Transcriptional regulator, y4mF family | | uniclust | UniRef100\_A0A011WSF5 | 99.2 | 5.9e-14 | 1.2e-19 | 93.8 | 59 | (13, 72) | 77 | (46, 104) | 214 | AbrB family transcriptional regulator | AbrB family transcriptional regulator | | uniclust | UniRef100\_A0A084A7B7 | 99.2 | 5.8e-14 | 1.2e-19 | 94.7 | 58 | (14, 72) | 77 | (20, 77) | 225 | HTH cro/C1-type domain-containing protein | HTH cro/C1-type domain-containing protein | | uniclust | UniRef100\_A0A161S4G1 | 99.2 | 5.9e-14 | 1.2e-19 | 80.5 | 56 | (16, 72) | 77 | (7, 62) | 74 | DNA-binding protein | DNA-binding protein | | uniclust | UniRef100\_A0A023UJ95 | 99.2 | 5.7e-14 | 1.3e-19 | 91.8 | 60 | (12, 72) | 77 | (18, 77) | 171 | Repressor-like protein | Repressor-like protein | | uniclust | UniRef100\_A0A168PAQ4 | 99.2 | 5.6e-14 | 1.3e-19 | 89.5 | 67 | (5, 72) | 77 | (9, 77) | 141 | HTH cro/C1-type domain-containing protein | HTH cro/C1-type domain-containing protein | | uniclust | UniRef100\_A0A1Y3SKS7 | 99.2 | 5.7e-14 | 1.3e-19 | 90.6 | 56 | (16, 72) | 77 | (4, 59) | 155 | HTH cro/C1-type domain-containing protein | HTH cro/C1-type domain-containing protein | | uniclust | UniRef100\_A0A094XY50 | 99.2 | 5.7e-14 | 1.3e-19 | 97.5 | 57 | (14, 71) | 77 | (46, 102) | 270 | XRE family transcriptional regulator | XRE family transcriptional regulator | | uniclust | UniRef100\_A0A1C7G621 | 99.2 | 6.4e-14 | 1.3e-19 | 89.1 | 55 | (17, 72) | 77 | (1, 55) | 149 | Transcriptional regulator | Transcriptional regulator | | uniclust | UniRef100\_A0A7W2AJ58 | 99.2 | 6.3e-14 | 1.3e-19 | 90.9 | 58 | (14, 72) | 77 | (1, 58) | 168 | Helix-turn-helix domain-containing protein | Helix-turn-helix domain-containing protein | | uniclust | UniRef100\_A0A0E2UAE2 | 99.2 | 6.5e-14 | 1.4e-19 | 90.4 | 60 | (12, 72) | 77 | (5, 64) | 164 | DNA-binding helix-turn-helix protein | DNA-binding helix-turn-helix protein | | uniclust | UniRef100\_A0A061PBZ7 | 99.2 | 6.6e-14 | 1.4e-19 | 84.2 | 62 | (11, 73) | 77 | (16, 77) | 101 | Transcriptional regulator, Cro/CI family | Transcriptional regulator, Cro/CI family | | uniclust | UniRef100\_A0A125W4S8 | 99.2 | 6.8e-14 | 1.4e-19 | 87.4 | 59 | (13, 72) | 77 | (2, 60) | 133 | DNA-binding helix-turn-helix protein | DNA-binding helix-turn-helix protein | | uniclust | UniRef100\_A0A5F0MF59 | 99.2 | 6.7e-14 | 1.4e-19 | 86.7 | 61 | (11, 72) | 77 | (12, 72) | 122 | HTH cro/C1-type domain-containing protein | HTH cro/C1-type domain-containing protein | | uniclust | UniRef100\_A0A099I7D2 | 99.2 | 6.9e-14 | 1.4e-19 | 88.8 | 61 | (11, 72) | 77 | (18, 78) | 146 | HTH cro/C1-type domain-containing protein | HTH cro/C1-type domain-containing protein | | uniclust | UniRef100\_A0A0R2CQI8 | 99.2 | 6.9e-14 | 1.4e-19 | 88.3 | 61 | (11, 72) | 77 | (9, 69) | 139 | HTH cro/C1-type domain-containing protein | HTH cro/C1-type domain-containing protein | | uniclust | UniRef100\_A0A084J9P7 | 99.2 | 6.9e-14 | 1.4e-19 | 94.9 | 58 | (14, 72) | 77 | (8, 65) | 239 | XRE family transcriptional regulator | XRE family transcriptional regulator | | uniclust | UniRef100\_A0A1Z4JR02 | 99.2 | 6.6e-14 | 1.5e-19 | 84.2 | 62 | (10, 72) | 77 | (6, 69) | 97 | Helix-turn-helix domain protein | Helix-turn-helix domain protein | | uniclust | UniRef100\_A0A178GS71 | 99.2 | 6.8e-14 | 1.5e-19 | 89.4 | 64 | (8, 72) | 77 | (16, 79) | 147 | Transcriptional regulator | Transcriptional regulator | | uniclust | UniRef100\_A0A172S040 | 99.2 | 7.3e-14 | 1.5e-19 | 88.3 | 56 | (16, 72) | 77 | (3, 58) | 147 | Looped-hinge helix DNA binding domain-containing protein, AbrB family | Looped-hinge helix DNA binding domain-containing protein, AbrB family | | uniclust | UniRef100\_A0A0F2RS12 | 99.2 | 7.1e-14 | 1.5e-19 | 83.2 | 70 | (4, 74) | 77 | (4, 73) | 94 | XRE family transcriptional regulator | XRE family transcriptional regulator | | uniclust | UniRef100\_A0A0R3JXL2 | 99.2 | 7.1e-14 | 1.5e-19 | 97.9 | 58 | (14, 72) | 77 | (15, 72) | 311 | HTH-type transcriptional regulator Xre | HTH-type transcriptional regulator Xre | | uniclust | UniRef100\_A0A0K9NFW5 | 99.2 | 6.5e-14 | 1.5e-19 | 92.3 | 61 | (11, 72) | 77 | (22, 82) | 178 | Toxin-antitoxin system, antitoxin component, Xre family | Toxin-antitoxin system, antitoxin component, Xre family | | uniclust | UniRef100\_A0A0C3HC21 | 99.2 | 7.2e-14 | 1.5e-19 | 90.3 | 61 | (11, 72) | 77 | (10, 70) | 163 | Immunity protein | Immunity protein | | uniclust | UniRef100\_A0A1C6BIG0 | 99.2 | 7.5e-14 | 1.6e-19 | 89.1 | 55 | (17, 72) | 77 | (1, 55) | 151 | Transcriptional repressor DicA | Transcriptional repressor DicA | | uniclust | UniRef100\_A0A116KFN5 | 99.2 | 7.6e-14 | 1.6e-19 | 89.1 | 55 | (18, 73) | 77 | (2, 56) | 152 | ICESt1 APR2 Cro/CI family transcriptional regulator | ICESt1 APR2 Cro/CI family transcriptional regulator | | uniclust | UniRef100\_A0A1I2JTK0 | 99.2 | 7.5e-14 | 1.6e-19 | 85.5 | 57 | (15, 72) | 77 | (15, 71) | 113 | Helix-turn-helix | Helix-turn-helix | | uniclust | UniRef100\_A0A1Y4IDQ4 | 99.2 | 7.5e-14 | 1.6e-19 | 95.1 | 59 | (14, 73) | 77 | (5, 63) | 245 | HTH cro/C1-type domain-containing protein | HTH cro/C1-type domain-containing protein | | uniclust | UniRef100\_A0A091BSN2 | 99.2 | 7.6e-14 | 1.6e-19 | 91.8 | 54 | (17, 71) | 77 | (1, 54) | 191 | Transcriptional regulator | Transcriptional regulator | | uniclust | UniRef100\_A0A0R1JS02 | 99.2 | 7.4e-14 | 1.6e-19 | 98.1 | 60 | (11, 71) | 77 | (38, 97) | 313 | HTH cro/C1-type domain-containing protein | HTH cro/C1-type domain-containing protein | | uniclust | UniRef100\_A0A089LK22 | 99.2 | 7.6e-14 | 1.6e-19 | 87.3 | 57 | (15, 72) | 77 | (1, 57) | 130 | HTH cro/C1-type domain-containing protein | HTH cro/C1-type domain-containing protein | | uniclust | UniRef100\_A0A011VZ09 | 99.2 | 6.9e-14 | 1.6e-19 | 97.3 | 64 | (9, 73) | 77 | (46, 109) | 272 | LexA family transcriptional regulator | LexA family transcriptional regulator | | uniclust | UniRef100\_A0A1Y3RF54 | 99.2 | 8e-14 | 1.6e-19 | 90.8 | 59 | (13, 72) | 77 | (14, 72) | 179 | Transcriptional regulator | Transcriptional regulator | | uniclust | UniRef100\_A0A154BQ77 | 99.2 | 8e-14 | 1.7e-19 | 80.4 | 58 | (14, 72) | 77 | (1, 58) | 78 | HTH cro/C1-type domain-containing protein | HTH cro/C1-type domain-containing protein | | uniclust | UniRef100\_A0A024QAL7 | 99.2 | 7.8e-14 | 1.7e-19 | 82.1 | 60 | (12, 72) | 77 | (5, 64) | 86 | Helix-turn-helix | Helix-turn-helix | | uniclust | UniRef100\_A0A0H2T341 | 99.2 | 8e-14 | 1.7e-19 | 84.9 | 68 | (5, 73) | 77 | (14, 81) | 108 | Cro/Cl family transcriptional regulator | Cro/Cl family transcriptional regulator | | uniclust | UniRef100\_A0A1E3A954 | 99.2 | 8.4e-14 | 1.7e-19 | 95.0 | 66 | (6, 72) | 77 | (6, 71) | 265 | Transcriptional repressor DicA | Transcriptional repressor DicA | | uniclust | UniRef100\_A0A0J1J383 | 99.2 | 8.1e-14 | 1.7e-19 | 90.6 | 57 | (16, 73) | 77 | (21, 78) | 167 | HTH cro/C1-type domain-containing protein | HTH cro/C1-type domain-containing protein | | uniclust | UniRef100\_A0A015KU58 | 99.2 | 8.9e-14 | 1.8e-19 | 88.9 | 58 | (14, 72) | 77 | (14, 71) | 155 | XRE family transcriptional regulator | XRE family transcriptional regulator | | uniclust | UniRef100\_A0A095YLK0 | 99.2 | 8.7e-14 | 1.8e-19 | 94.0 | 67 | (5, 72) | 77 | (43, 110) | 227 | HTH cro/C1-type domain-containing protein | HTH cro/C1-type domain-containing protein | | uniclust | UniRef100\_A0A174AVY8 | 99.2 | 8.7e-14 | 1.9e-19 | 99.0 | 58 | (14, 72) | 77 | (33, 90) | 355 | Transcription factor | Transcription factor | | uniclust | UniRef100\_A0A1S8SA91 | 99.2 | 8.7e-14 | 1.9e-19 | 92.5 | 58 | (14, 72) | 77 | (11, 68) | 196 | Transcriptional repressor DicA | Transcriptional repressor DicA | | uniclust | UniRef100\_A0A2V2EQU8 | 99.2 | 9.3e-14 | 1.9e-19 | 83.1 | 62 | (12, 74) | 77 | (15, 76) | 97 | HTH cro/C1-type domain-containing protein | HTH cro/C1-type domain-containing protein | | uniclust | UniRef100\_A0A431UEA1 | 99.2 | 1e-13 | 2e-19 | 77.4 | 56 | (16, 72) | 77 | (1, 56) | 69 | XRE family transcriptional regulator | XRE family transcriptional regulator | | uniclust | UniRef100\_A0A4Y9K6V4 | 99.2 | 9.7e-14 | 2e-19 | 84.3 | 56 | (16, 72) | 77 | (1, 56) | 106 | XRE family transcriptional regulator | XRE family transcriptional regulator | | uniclust | UniRef100\_A0A1Y3S0A0 | 99.2 | 1e-13 | 2.1e-19 | 99.0 | 59 | (13, 72) | 77 | (17, 75) | 393 | HTH cro/C1-type domain-containing protein | HTH cro/C1-type domain-containing protein | | uniclust | UniRef100\_A0A926NBJ0 | 99.2 | 1.1e-13 | 2.1e-19 | 86.0 | 55 | (17, 72) | 77 | (1, 55) | 135 | Helix-turn-helix transcriptional regulator | Helix-turn-helix transcriptional regulator | | uniclust | UniRef100\_A0A1G4SNC0 | 99.2 | 1e-13 | 2.1e-19 | 94.4 | 60 | (13, 73) | 77 | (20, 79) | 252 | Stage 0 sporulation protein A homolog | Stage 0 sporulation protein A homolog | | uniclust | UniRef100\_A0A091BL71 | 99.2 | 1e-13 | 2.1e-19 | 92.0 | 56 | (16, 72) | 77 | (1, 56) | 215 | Transcriptional regulator | Transcriptional regulator | | uniclust | UniRef100\_A0A3A4JVA9 | 99.2 | 9.8e-14 | 2.1e-19 | 81.1 | 59 | (15, 74) | 77 | (8, 66) | 83 | XRE family transcriptional regulator | XRE family transcriptional regulator | | uniclust | UniRef100\_A0A160HBR0 | 99.2 | 9.8e-14 | 2.1e-19 | 88.9 | 60 | (12, 72) | 77 | (24, 83) | 150 | Uncharacterized protein | Uncharacterized protein | | uniclust | UniRef100\_A0A0A8B2Q9 | 99.2 | 1.1e-13 | 2.2e-19 | 81.8 | 58 | (15, 72) | 77 | (5, 62) | 90 | HTH cro/C1-type domain-containing protein | HTH cro/C1-type domain-containing protein | | uniclust | UniRef100\_A0A174WG95 | 99.2 | 1.1e-13 | 2.2e-19 | 82.1 | 66 | (7, 73) | 77 | (10, 75) | 93 | Helix-turn-helix domain-containing protein | Helix-turn-helix domain-containing protein | | uniclust | UniRef100\_A0A023PA98 | 99.2 | 1e-13 | 2.2e-19 | 98.5 | 57 | (14, 71) | 77 | (30, 86) | 335 | XRE family transcriptional regulator | XRE family transcriptional regulator | | uniclust | UniRef100\_A0A098TGA3 | 99.2 | 1.1e-13 | 2.3e-19 | 84.7 | 54 | (18, 72) | 77 | (21, 76) | 109 | HTH cro/C1-type domain-containing protein | HTH cro/C1-type domain-containing protein | | uniclust | UniRef100\_A0A0R2D5X0 | 99.2 | 1.1e-13 | 2.3e-19 | 81.7 | 52 | (20, 72) | 77 | (11, 62) | 90 | HTH cro/C1-type domain-containing protein | HTH cro/C1-type domain-containing protein | | uniclust | UniRef100\_A0A0B3BRB6 | 99.2 | 1.1e-13 | 2.3e-19 | 88.6 | 62 | (10, 72) | 77 | (17, 78) | 147 | XRE family transcriptional regulator | XRE family transcriptional regulator | | uniclust | UniRef100\_A0A1C6G8A2 | 99.2 | 1.1e-13 | 2.3e-19 | 82.9 | 57 | (15, 72) | 77 | (2, 58) | 99 | Plasmid maintenance system antidote protein | Plasmid maintenance system antidote protein | | uniclust | UniRef100\_A0A2T4LBR6 | 99.2 | 1.3e-13 | 2.5e-19 | 80.2 | 60 | (13, 73) | 77 | (3, 62) | 84 | XRE family transcriptional regulator | XRE family transcriptional regulator | | uniclust | UniRef100\_A0A059NYG7 | 99.2 | 1.2e-13 | 2.5e-19 | 82.8 | 61 | (12, 72) | 77 | (3, 63) | 94 | HTH-type transcriptional regulator SinR | HTH-type transcriptional regulator SinR | | uniclust | UniRef100\_A0A0F2DXV9 | 99.2 | 1.2e-13 | 2.6e-19 | 92.9 | 56 | (16, 72) | 77 | (28, 83) | 224 | DNA-binding phage protein | DNA-binding phage protein | | uniclust | UniRef100\_A0A095YT12 | 99.2 | 1.1e-13 | 2.6e-19 | 94.7 | 62 | (10, 72) | 77 | (14, 75) | 236 | HTH cro/C1-type domain-containing protein | HTH cro/C1-type domain-containing protein | | uniclust | UniRef100\_A0A0R2FDG7 | 99.2 | 1.2e-13 | 2.6e-19 | 93.8 | 60 | (12, 72) | 77 | (18, 77) | 244 | Transcriptional regulator | Transcriptional regulator | | uniclust | UniRef100\_A0A1C5XDG0 | 99.2 | 1.2e-13 | 2.7e-19 | 96.6 | 57 | (15, 72) | 77 | (4, 60) | 296 | HTH-type transcriptional regulator immR | HTH-type transcriptional regulator immR | | uniclust | UniRef100\_A0A059NUZ0 | 99.2 | 1.3e-13 | 2.7e-19 | 87.7 | 56 | (17, 73) | 77 | (13, 68) | 142 | Transcriptional repressor DicA | Transcriptional repressor DicA | | uniclust | UniRef100\_A0A174BT49 | 99.2 | 1.4e-13 | 2.8e-19 | 89.2 | 57 | (15, 72) | 77 | (30, 86) | 184 | Predicted transcriptional regulator | Predicted transcriptional regulator | | uniclust | UniRef100\_A0A926F381 | 99.2 | 1.3e-13 | 2.8e-19 | 91.0 | 54 | (18, 72) | 77 | (1, 54) | 187 | Helix-turn-helix transcriptional regulator | Helix-turn-helix transcriptional regulator | | uniclust | UniRef100\_A0A1C5P4I0 | 99.2 | 1.3e-13 | 2.9e-19 | 84.7 | 58 | (14, 72) | 77 | (1, 58) | 111 | HTH-type transcriptional regulator immR | HTH-type transcriptional regulator immR | | uniclust | UniRef100\_A0A2V2GP97 | 99.2 | 1.3e-13 | 2.9e-19 | 100.3 | 68 | (3, 72) | 77 | (53, 120) | 431 | Uncharacterized protein | Uncharacterized protein | | uniclust | UniRef100\_A0A098ESC9 | 99.2 | 1.4e-13 | 2.9e-19 | 82.3 | 58 | (14, 72) | 77 | (8, 65) | 97 | Helix-turn-helix domain protein | Helix-turn-helix domain protein | | uniclust | UniRef100\_A0A0B7GNK0 | 99.2 | 1.5e-13 | 2.9e-19 | 89.3 | 56 | (15, 71) | 77 | (2, 57) | 178 | HTH-type transcriptional regulator | HTH-type transcriptional regulator | | uniclust | UniRef100\_A0A075SKL1 | 99.2 | 1.5e-13 | 3e-19 | 91.7 | 59 | (12, 71) | 77 | (45, 103) | 216 | XRE family transcriptional regulator | XRE family transcriptional regulator | | uniclust | UniRef100\_A0A1F8V9K7 | 99.2 | 1.4e-13 | 3e-19 | 98.7 | 60 | (12, 72) | 77 | (8, 67) | 365 | HTH cro/C1-type domain-containing protein | HTH cro/C1-type domain-containing protein | | uniclust | UniRef100\_A0A135YRB4 | 99.2 | 1.5e-13 | 3e-19 | 85.7 | 52 | (20, 72) | 77 | (2, 53) | 128 | DNA-binding helix-turn-helix protein | DNA-binding helix-turn-helix protein | | uniclust | UniRef100\_A0A098AP68 | 99.2 | 1.4e-13 | 3.1e-19 | 93.2 | 59 | (13, 72) | 77 | (20, 78) | 230 | Helix-turn-helix domain-containing protein | Helix-turn-helix domain-containing protein | | uniclust | UniRef100\_A0A094J0N9 | 99.2 | 1.5e-13 | 3.2e-19 | 78.4 | 54 | (18, 72) | 77 | (5, 58) | 73 | Transcriptional repressor DicA | Transcriptional repressor DicA | | uniclust | UniRef100\_A0A173SZC8 | 99.2 | 1.4e-13 | 3.2e-19 | 90.8 | 66 | (9, 74) | 77 | (16, 81) | 183 | Helix-turn-helix | Helix-turn-helix | | uniclust | UniRef100\_A0A095XIG0 | 99.2 | 1.5e-13 | 3.2e-19 | 80.9 | 62 | (11, 73) | 77 | (4, 65) | 87 | HTH cro/C1-type domain-containing protein | HTH cro/C1-type domain-containing protein | | uniclust | UniRef100\_A0A134A0B4 | 99.2 | 1.4e-13 | 3.3e-19 | 95.5 | 62 | (11, 73) | 77 | (9, 70) | 267 | Peptidase S24-like protein | Peptidase S24-like protein | | uniclust | UniRef100\_A0A1I0R8C4 | 99.2 | 1.6e-13 | 3.3e-19 | 86.2 | 57 | (15, 72) | 77 | (1, 57) | 132 | Helix-turn-helix | Helix-turn-helix | | uniclust | UniRef100\_A0A022N9C9 | 99.2 | 1.6e-13 | 3.3e-19 | 91.3 | 56 | (16, 72) | 77 | (1, 56) | 208 | XRE family transcriptional regulator | XRE family transcriptional regulator | | uniclust | UniRef100\_A0A167V013 | 99.2 | 1.5e-13 | 3.3e-19 | 88.8 | 58 | (15, 73) | 77 | (16, 73) | 159 | Helix-turn-helix family protein | Helix-turn-helix family protein | | uniclust | UniRef100\_A0A1C5ZA08 | 99.2 | 1.6e-13 | 3.4e-19 | 92.5 | 63 | (11, 73) | 77 | (4, 66) | 222 | LexA repressor | LexA repressor | | uniclust | UniRef100\_A0A264W7S8 | 99.2 | 1.8e-13 | 3.5e-19 | 80.3 | 57 | (16, 73) | 77 | (21, 77) | 89 | HTH cro/C1-type domain-containing protein | HTH cro/C1-type domain-containing protein | | uniclust | UniRef100\_A0A0W7TL75 | 99.2 | 1.8e-13 | 3.6e-19 | 75.4 | 44 | (11, 54) | 77 | (7, 50) | 62 | XRE family transcriptional regulator | XRE family transcriptional regulator | | uniclust | UniRef100\_A0A0Q6VM94 | 99.2 | 1.7e-13 | 3.7e-19 | 87.3 | 60 | (13, 73) | 77 | (16, 75) | 145 | HTH cro/C1-type domain-containing protein | HTH cro/C1-type domain-containing protein | | uniclust | UniRef100\_A0A173STR4 | 99.1 | 1.8e-13 | 3.8e-19 | 74.4 | 45 | (11, 55) | 77 | (4, 48) | 56 | Transcriptional repressor DicA | Transcriptional repressor DicA | | uniclust | UniRef100\_A0A095XQU1 | 99.1 | 1.8e-13 | 3.8e-19 | 80.9 | 60 | (12, 72) | 77 | (3, 62) | 89 | HTH cro/C1-type domain-containing protein | HTH cro/C1-type domain-containing protein | | uniclust | UniRef100\_A0A3A6HEE8 | 99.1 | 1.9e-13 | 3.9e-19 | 85.9 | 61 | (12, 73) | 77 | (12, 72) | 136 | XRE family transcriptional regulator | XRE family transcriptional regulator | | uniclust | UniRef100\_A0A0A0HWB2 | 99.1 | 1.8e-13 | 3.9e-19 | 93.2 | 65 | (7, 72) | 77 | (24, 88) | 237 | Transcriptional regulator | Transcriptional regulator | | uniclust | UniRef100\_A0A0E2B8K5 | 99.1 | 1.9e-13 | 4e-19 | 88.6 | 61 | (11, 72) | 77 | (29, 89) | 161 | DNA-binding helix-turn-helix protein | DNA-binding helix-turn-helix protein | | uniclust | UniRef100\_A0A090HXE6 | 99.1 | 1.8e-13 | 4e-19 | 93.7 | 62 | (11, 73) | 77 | (15, 76) | 242 | LexA protein | LexA protein | | uniclust | UniRef100\_A0A061CEQ4 | 99.1 | 2e-13 | 4e-19 | 86.9 | 58 | (14, 72) | 77 | (2, 59) | 157 | XRE family transcriptional regulator | XRE family transcriptional regulator | | uniclust | UniRef100\_A0A084JP97 | 99.1 | 1.8e-13 | 4.1e-19 | 99.8 | 61 | (11, 72) | 77 | (55, 115) | 427 | HTH cro/C1-type domain-containing protein | HTH cro/C1-type domain-containing protein | | uniclust | UniRef100\_A0A0L6Z9N2 | 99.1 | 2e-13 | 4.2e-19 | 91.4 | 58 | (14, 72) | 77 | (1, 58) | 220 | HTH cro/C1-type domain-containing protein | HTH cro/C1-type domain-containing protein | | uniclust | UniRef100\_A0A064C1R1 | 99.1 | 2.1e-13 | 4.2e-19 | 83.4 | 57 | (15, 72) | 77 | (15, 71) | 113 | Helix-turn-helix transcriptional regulator | Helix-turn-helix transcriptional regulator | | uniclust | UniRef100\_A0A0F9HIX3 | 99.1 | 2e-13 | 4.3e-19 | 80.6 | 58 | (15, 73) | 77 | (2, 59) | 86 | HTH cro/C1-type domain-containing protein | HTH cro/C1-type domain-containing protein | | uniclust | UniRef100\_A0A1W1XSD5 | 99.1 | 2.1e-13 | 4.4e-19 | 85.9 | 57 | (15, 72) | 77 | (1, 57) | 133 | DNA-binding transcriptional regulator, XRE-family HTH domain | DNA-binding transcriptional regulator, XRE-family HTH domain | | uniclust | UniRef100\_A0A011VY25 | 99.1 | 2.2e-13 | 4.4e-19 | 86.1 | 54 | (17, 71) | 77 | (1, 54) | 144 | DNA-binding protein | DNA-binding protein | | uniclust | UniRef100\_A0A1M6I385 | 99.1 | 2.1e-13 | 4.5e-19 | 90.5 | 55 | (17, 72) | 77 | (1, 55) | 192 | Helix-turn-helix domain-containing protein | Helix-turn-helix domain-containing protein | | uniclust | UniRef100\_A0A1Y4L638 | 99.1 | 2.1e-13 | 4.5e-19 | 82.3 | 57 | (15, 72) | 77 | (4, 60) | 101 | DNA-binding protein | DNA-binding protein | | uniclust | UniRef100\_A0A081BKJ0 | 99.1 | 2.1e-13 | 4.6e-19 | 91.3 | 55 | (16, 71) | 77 | (3, 57) | 203 | HTH cro/C1-type domain-containing protein | HTH cro/C1-type domain-containing protein | | uniclust | UniRef100\_A0A3A8YLP0 | 99.1 | 2.4e-13 | 4.7e-19 | 85.7 | 54 | (18, 72) | 77 | (2, 55) | 147 | XRE family transcriptional regulator | XRE family transcriptional regulator | | uniclust | UniRef100\_A0A0V8CR97 | 99.1 | 2.3e-13 | 4.7e-19 | 88.8 | 60 | (12, 72) | 77 | (2, 61) | 184 | Phage repressor | Phage repressor | | uniclust | UniRef100\_A0A0M2ZP61 | 99.1 | 2.2e-13 | 4.7e-19 | 93.9 | 56 | (16, 72) | 77 | (9, 64) | 255 | Zinc finger/helix-turn-helix protein, YgiT family | Zinc finger/helix-turn-helix protein, YgiT family | | uniclust | UniRef100\_A0A1R1MJE6 | 99.1 | 2.2e-13 | 4.7e-19 | 91.5 | 57 | (15, 72) | 77 | (17, 73) | 216 | HTH cro/C1-type domain-containing protein | HTH cro/C1-type domain-containing protein | | uniclust | UniRef100\_A0A1I5QA44 | 99.1 | 2.4e-13 | 4.8e-19 | 71.1 | 41 | (14, 54) | 77 | (3, 43) | 47 | Helix-turn-helix | Helix-turn-helix | | uniclust | UniRef100\_A0A379DAL3 | 99.1 | 2.4e-13 | 4.8e-19 | 85.3 | 56 | (16, 72) | 77 | (1, 56) | 134 | HTH-type transcriptional regulator immR | HTH-type transcriptional regulator immR | | uniclust | UniRef100\_A0A1H8CHK5 | 99.1 | 2.4e-13 | 4.9e-19 | 80.3 | 58 | (14, 72) | 77 | (7, 64) | 91 | DNA-binding transcriptional regulator, XRE-family HTH domain | DNA-binding transcriptional regulator, XRE-family HTH domain | | uniclust | UniRef100\_A0A328UKW6 | 99.1 | 2.5e-13 | 4.9e-19 | 72.0 | 39 | (16, 54) | 77 | (6, 44) | 51 | XRE family transcriptional regulator | XRE family transcriptional regulator | | uniclust | UniRef100\_A0A028ZGG7 | 99.1 | 2.3e-13 | 4.9e-19 | 91.8 | 57 | (14, 71) | 77 | (18, 74) | 217 | Transcriptional regulator | Transcriptional regulator | | uniclust | UniRef100\_A0A0M6WJ36 | 99.1 | 2.3e-13 | 5e-19 | 85.3 | 61 | (15, 75) | 77 | (5, 65) | 129 | HTH cro/C1-type domain-containing protein | HTH cro/C1-type domain-containing protein | | uniclust | UniRef100\_A0A062XFL7 | 99.1 | 2.2e-13 | 5e-19 | 94.9 | 58 | (15, 73) | 77 | (33, 90) | 275 | HTH cro/C1-type domain-containing protein | HTH cro/C1-type domain-containing protein | | uniclust | UniRef100\_A0A0R1L3N4 | 99.1 | 2.3e-13 | 5.1e-19 | 85.0 | 55 | (19, 74) | 77 | (49, 103) | 123 | HTH cro/C1-type domain-containing protein | HTH cro/C1-type domain-containing protein | | uniclust | UniRef100\_A0A0H0YL11 | 99.1 | 2.4e-13 | 5.1e-19 | 86.7 | 58 | (14, 72) | 77 | (8, 65) | 146 | HTH cro/C1-type domain-containing protein | HTH cro/C1-type domain-containing protein | | uniclust | UniRef100\_A0A0V8QET0 | 99.1 | 2.4e-13 | 5.2e-19 | 81.4 | 57 | (15, 72) | 77 | (5, 61) | 96 | XRE family transcriptional regulator | XRE family transcriptional regulator | | uniclust | UniRef100\_A0A1C0TYE9 | 99.1 | 2.5e-13 | 5.2e-19 | 85.4 | 63 | (10, 73) | 77 | (10, 72) | 135 | Transcriptional repressor DicA | Transcriptional repressor DicA | | uniclust | UniRef100\_A0A0A1GU27 | 99.1 | 2.5e-13 | 5.2e-19 | 81.8 | 56 | (16, 72) | 77 | (15, 70) | 102 | Transcriptional regulator | Transcriptional regulator | | uniclust | UniRef100\_A0A239QG85 | 99.1 | 2.5e-13 | 5.3e-19 | 93.0 | 57 | (15, 72) | 77 | (32, 88) | 256 | Transcriptional regulator, contains XRE-family HTH domain | Transcriptional regulator, contains XRE-family HTH domain | | uniclust | UniRef100\_A0A1C5NC58 | 99.1 | 2.6e-13 | 5.3e-19 | 94.7 | 59 | (13, 72) | 77 | (35, 93) | 308 | HTH-type transcriptional regulator immR | HTH-type transcriptional regulator immR | | uniclust | UniRef100\_A0A089I0P5 | 99.1 | 2.6e-13 | 5.4e-19 | 95.2 | 60 | (12, 72) | 77 | (34, 93) | 323 | XRE family transcriptional regulator | XRE family transcriptional regulator | | uniclust | UniRef100\_A0A1C3T4X4 | 99.1 | 2.7e-13 | 5.4e-19 | 81.6 | 57 | (15, 72) | 77 | (9, 65) | 104 | HTH cro/C1-type domain-containing protein | HTH cro/C1-type domain-containing protein | | uniclust | UniRef100\_A0A0U9HDI7 | 99.1 | 2.6e-13 | 5.4e-19 | 84.7 | 55 | (17, 72) | 77 | (1, 55) | 129 | Helix-turn-helix domain-containing protein | Helix-turn-helix domain-containing protein | | uniclust | UniRef100\_A0A430AU27 | 99.1 | 2.7e-13 | 5.5e-19 | 85.3 | 54 | (19, 73) | 77 | (4, 57) | 140 | HTH cro/C1-type domain-containing protein | HTH cro/C1-type domain-containing protein | | uniclust | UniRef100\_A0A1G9IKI2 | 99.1 | 2.7e-13 | 5.6e-19 | 85.2 | 57 | (15, 72) | 77 | (5, 61) | 131 | DNA-binding transcriptional regulator, XRE-family HTH domain | DNA-binding transcriptional regulator, XRE-family HTH domain | | uniclust | UniRef100\_A0A1M2Z9A3 | 99.1 | 2.5e-13 | 5.7e-19 | 98.3 | 59 | (13, 72) | 77 | (17, 75) | 388 | HTH cro/C1-type domain-containing protein | HTH cro/C1-type domain-containing protein | | uniclust | UniRef100\_A0A0G0K4J7 | 99.1 | 2.6e-13 | 5.9e-19 | 82.3 | 59 | (14, 73) | 77 | (16, 74) | 101 | Transcriptional regulator, XRE family | Transcriptional regulator, XRE family | | uniclust | UniRef100\_A0A358AQ13 | 99.1 | 2.8e-13 | 5.9e-19 | 83.1 | 56 | (16, 72) | 77 | (1, 56) | 113 | XRE family transcriptional regulator | XRE family transcriptional regulator | | uniclust | UniRef100\_A0A173TGI2 | 99.1 | 3e-13 | 5.9e-19 | 88.1 | 56 | (16, 72) | 77 | (5, 60) | 180 | HTH-type transcriptional regulator immR | HTH-type transcriptional regulator immR | | uniclust | UniRef100\_A0A061NRM5 | 99.1 | 2.8e-13 | 6e-19 | 80.9 | 58 | (14, 72) | 77 | (2, 59) | 94 | HTH cro/C1-type domain-containing protein | HTH cro/C1-type domain-containing protein | | uniclust | UniRef100\_A0A1I3H5B9 | 99.1 | 2.8e-13 | 6e-19 | 78.7 | 57 | (16, 73) | 77 | (2, 58) | 79 | DNA-binding transcriptional regulator, XRE-family HTH domain | DNA-binding transcriptional regulator, XRE-family HTH domain | | uniclust | UniRef100\_A0A2S0M7U0 | 99.1 | 3e-13 | 6.1e-19 | 77.4 | 53 | (19, 72) | 77 | (4, 56) | 76 | XRE family transcriptional regulator | XRE family transcriptional regulator | | uniclust | UniRef100\_A0A135YNR8 | 99.1 | 3.3e-13 | 6.4e-19 | 87.2 | 52 | (20, 72) | 77 | (2, 53) | 175 | DNA-binding helix-turn-helix protein | DNA-binding helix-turn-helix protein | | uniclust | UniRef100\_A0A075R1N1 | 99.1 | 3e-13 | 6.5e-19 | 88.9 | 60 | (13, 73) | 77 | (31, 90) | 180 | HTH-type transcriptional regulator ImmR | HTH-type transcriptional regulator ImmR | | uniclust | UniRef100\_A0A0C1R042 | 99.1 | 2.9e-13 | 6.5e-19 | 92.0 | 64 | (8, 72) | 77 | (16, 79) | 228 | Cro/Cl family transcriptional regulator | Cro/Cl family transcriptional regulator | | uniclust | UniRef100\_A0A078KP50 | 99.1 | 3.1e-13 | 6.5e-19 | 96.9 | 59 | (13, 72) | 77 | (27, 85) | 387 | Helix-turn-helix domain-containing protein | Helix-turn-helix domain-containing protein | | uniclust | UniRef100\_A0A0K0MX67 | 99.1 | 3.2e-13 | 6.6e-19 | 83.3 | 54 | (17, 71) | 77 | (15, 68) | 119 | Putative DNA binding protein | Putative DNA binding protein | | uniclust | UniRef100\_A0A1G6FFN7 | 99.1 | 3.2e-13 | 6.6e-19 | 90.9 | 55 | (16, 71) | 77 | (3, 57) | 229 | Transcriptional regulator, contains XRE-family HTH domain | Transcriptional regulator, contains XRE-family HTH domain | | uniclust | UniRef100\_A0A4U4D9H3 | 99.1 | 3.2e-13 | 6.6e-19 | 88.8 | 55 | (16, 71) | 77 | (17, 71) | 190 | XRE family transcriptional regulator | XRE family transcriptional regulator | | uniclust | UniRef100\_A0A3D2G1G4 | 99.1 | 3.3e-13 | 6.6e-19 | 84.2 | 56 | (16, 72) | 77 | (1, 56) | 133 | HTH cro/C1-type domain-containing protein | HTH cro/C1-type domain-containing protein | | uniclust | UniRef100\_A0A0M6WHG0 | 99.1 | 2.9e-13 | 6.6e-19 | 94.5 | 59 | (13, 72) | 77 | (20, 78) | 281 | Putative transcriptional regulator | Putative transcriptional regulator | | uniclust | UniRef100\_A0A239PSQ2 | 99.1 | 3.3e-13 | 7e-19 | 92.8 | 59 | (15, 74) | 77 | (55, 113) | 265 | Transcriptional regulator, contains XRE-family HTH domain | Transcriptional regulator, contains XRE-family HTH domain | | uniclust | UniRef100\_A0A1C0BS68 | 99.1 | 3.4e-13 | 7e-19 | 86.5 | 54 | (15, 69) | 77 | (7, 60) | 155 | HTH cro/C1-type domain-containing protein | HTH cro/C1-type domain-containing protein | | uniclust | UniRef100\_A0A1M6UBA3 | 99.1 | 3.3e-13 | 7e-19 | 95.1 | 67 | (5, 73) | 77 | (33, 99) | 324 | FHIPEP family protein | FHIPEP family protein | | uniclust | UniRef100\_A0A023BQT6 | 99.1 | 3.2e-13 | 7e-19 | 83.5 | 61 | (12, 73) | 77 | (31, 91) | 116 | HTH cro/C1-type domain-containing protein | HTH cro/C1-type domain-containing protein | | uniclust | UniRef100\_A0A0X1TLQ8 | 99.1 | 3.2e-13 | 7e-19 | 90.7 | 56 | (16, 72) | 77 | (3, 58) | 210 | HTH cro/C1-type domain-containing protein | HTH cro/C1-type domain-containing protein | | uniclust | UniRef100\_A0A1C5Y1I1 | 99.1 | 3.3e-13 | 7.1e-19 | 91.6 | 55 | (17, 72) | 77 | (1, 55) | 232 | HTH-type transcriptional regulator immR | HTH-type transcriptional regulator immR | | uniclust | UniRef100\_A0A0C2URG5 | 99.1 | 3.3e-13 | 7.1e-19 | 82.1 | 60 | (12, 72) | 77 | (7, 66) | 104 | HTH cro/C1-type domain-containing protein | HTH cro/C1-type domain-containing protein | | uniclust | UniRef100\_A0A0R2I9Z4 | 99.1 | 3.5e-13 | 7.2e-19 | 90.4 | 57 | (15, 72) | 77 | (8, 64) | 215 | HTH cro/C1-type domain-containing protein | HTH cro/C1-type domain-containing protein | | uniclust | UniRef100\_A0A1G9K086 | 99.1 | 3.6e-13 | 7.4e-19 | 91.6 | 55 | (15, 71) | 77 | (4, 58) | 245 | Transcriptional regulator, contains XRE-family HTH domain | Transcriptional regulator, contains XRE-family HTH domain | | uniclust | UniRef100\_A0A1V4SGG0 | 99.1 | 3.6e-13 | 7.4e-19 | 85.8 | 59 | (13, 72) | 77 | (8, 66) | 150 | HTH-type transcriptional regulator ImmR | HTH-type transcriptional regulator ImmR | | uniclust | UniRef100\_A0A0Q6ASP6 | 99.1 | 3.6e-13 | 7.4e-19 | 83.6 | 58 | (14, 72) | 77 | (9, 66) | 123 | HTH cro/C1-type domain-containing protein | HTH cro/C1-type domain-containing protein | | uniclust | UniRef100\_A0A1C0BS96 | 99.1 | 3.5e-13 | 7.5e-19 | 88.1 | 66 | (7, 73) | 77 | (9, 74) | 173 | HTH cro/C1-type domain-containing protein | HTH cro/C1-type domain-containing protein | | uniclust | UniRef100\_A0A1C6HMS0 | 99.1 | 3.7e-13 | 7.5e-19 | 79.2 | 55 | (17, 72) | 77 | (4, 58) | 88 | Predicted transcriptional regulator | Predicted transcriptional regulator | | uniclust | UniRef100\_A0A0D5LVP1 | 99.1 | 3.5e-13 | 7.5e-19 | 82.5 | 60 | (13, 73) | 77 | (23, 82) | 108 | XRE family transcriptional regulator | XRE family transcriptional regulator | | uniclust | UniRef100\_A0A066Q0N8 | 99.1 | 3.6e-13 | 7.7e-19 | 87.2 | 58 | (15, 73) | 77 | (23, 80) | 159 | DNA-binding protein | DNA-binding protein | | uniclust | UniRef100\_A0A1A7QK35 | 99.1 | 3.5e-13 | 7.7e-19 | 85.9 | 63 | (11, 74) | 77 | (16, 78) | 141 | HTH cro/C1-type domain-containing protein | HTH cro/C1-type domain-containing protein | | uniclust | UniRef100\_A0A2S6HCI4 | 99.1 | 4e-13 | 7.7e-19 | 76.4 | 56 | (16, 72) | 77 | (1, 56) | 77 | Helix-turn-helix protein | Helix-turn-helix protein | | uniclust | UniRef100\_A0A1G7NVX8 | 99.1 | 3.8e-13 | 7.7e-19 | 84.1 | 55 | (17, 72) | 77 | (1, 55) | 131 | Transcriptional regulator, contains XRE-family HTH domain | Transcriptional regulator, contains XRE-family HTH domain | | uniclust | UniRef100\_A0A073CX64 | 99.1 | 3.4e-13 | 7.7e-19 | 86.7 | 72 | (3, 74) | 77 | (8, 80) | 148 | HTH cro/C1-type domain-containing protein | HTH cro/C1-type domain-containing protein | | uniclust | UniRef100\_A0A0R2IDY0 | 99.1 | 3.8e-13 | 7.8e-19 | 85.4 | 55 | (17, 72) | 77 | (14, 68) | 145 | HTH cro/C1-type domain-containing protein | HTH cro/C1-type domain-containing protein | | uniclust | UniRef100\_A0A357QYW2 | 99.1 | 4e-13 | 8e-19 | 86.5 | 62 | (10, 72) | 77 | (7, 68) | 164 | HTH cro/C1-type domain-containing protein | HTH cro/C1-type domain-containing protein | | uniclust | UniRef100\_A0A173UEQ1 | 99.1 | 3.8e-13 | 8.1e-19 | 87.7 | 59 | (14, 73) | 77 | (1, 59) | 168 | Helix-turn-helix domain | Helix-turn-helix domain | | uniclust | UniRef100\_A0A0H4VVM8 | 99.1 | 3.8e-13 | 8.1e-19 | 86.0 | 59 | (12, 72) | 77 | (7, 65) | 148 | Transcriptional repressor DicA | Transcriptional repressor DicA | | uniclust | UniRef100\_A0A1G5DD51 | 99.1 | 4e-13 | 8.2e-19 | 78.1 | 57 | (17, 74) | 77 | (4, 60) | 81 | Helix-turn-helix | Helix-turn-helix | | uniclust | UniRef100\_A0A0N0DL77 | 99.1 | 4.1e-13 | 8.4e-19 | 79.3 | 58 | (14, 72) | 77 | (12, 69) | 90 | Transcriptional regulator | Transcriptional regulator | | uniclust | UniRef100\_A0A1Q6U0U5 | 99.1 | 4e-13 | 8.4e-19 | 83.9 | 59 | (14, 73) | 77 | (13, 71) | 126 | HTH cro/C1-type domain-containing protein | HTH cro/C1-type domain-containing protein | | uniclust | UniRef100\_A0A173WE40 | 99.1 | 4e-13 | 8.5e-19 | 96.3 | 56 | (16, 72) | 77 | (33, 88) | 384 | HTH-type transcriptional regulator immR | HTH-type transcriptional regulator immR | | uniclust | UniRef100\_A0A023PEX3 | 99.1 | 3.9e-13 | 8.5e-19 | 83.5 | 60 | (12, 72) | 77 | (19, 78) | 118 | Transcriptional regulator | Transcriptional regulator | | uniclust | UniRef100\_A0A2G3DYU1 | 99.1 | 4e-13 | 8.7e-19 | 85.3 | 59 | (14, 73) | 77 | (17, 75) | 138 | HTH cro/C1-type domain-containing protein | HTH cro/C1-type domain-containing protein | | uniclust | UniRef100\_A0A1D3TPS8 | 99.1 | 4.3e-13 | 8.7e-19 | 86.0 | 56 | (16, 72) | 77 | (1, 56) | 158 | Transcriptional regulator, contains XRE-family HTH domain | Transcriptional regulator, contains XRE-family HTH domain | | uniclust | UniRef100\_A0A350BBX8 | 99.1 | 4.3e-13 | 8.9e-19 | 86.6 | 56 | (16, 72) | 77 | (4, 59) | 160 | HTH cro/C1-type domain-containing protein | HTH cro/C1-type domain-containing protein | | uniclust | UniRef100\_A0A059NZG4 | 99.1 | 4.6e-13 | 9.2e-19 | 79.3 | 58 | (15, 73) | 77 | (6, 64) | 94 | Helix-turn-helix | Helix-turn-helix | | uniclust | UniRef100\_A0A096B436 | 99.1 | 4.3e-13 | 9.2e-19 | 86.3 | 64 | (8, 72) | 77 | (12, 75) | 154 | HTH cro/C1-type domain-containing protein | HTH cro/C1-type domain-containing protein | | uniclust | UniRef100\_A0A1Y3PDK6 | 99.1 | 4.8e-13 | 9.3e-19 | 80.7 | 57 | (15, 72) | 77 | (2, 58) | 111 | HTH cro/C1-type domain-containing protein | HTH cro/C1-type domain-containing protein | | uniclust | UniRef100\_A0A4R1RIV0 | 99.1 | 4.6e-13 | 9.6e-19 | 82.8 | 57 | (15, 72) | 77 | (1, 57) | 119 | DNA-binding XRE family transcriptional regulator | DNA-binding XRE family transcriptional regulator | | uniclust | UniRef100\_A0A1Y4G4T6 | 99.1 | 4.5e-13 | 9.6e-19 | 78.0 | 61 | (12, 73) | 77 | (4, 64) | 80 | HTH cro/C1-type domain-containing protein | HTH cro/C1-type domain-containing protein | | uniclust | UniRef100\_A0A073IMZ9 | 99.1 | 4.5e-13 | 9.7e-19 | 80.3 | 51 | (21, 72) | 77 | (8, 58) | 95 | HTH cro/C1-type domain-containing protein | HTH cro/C1-type domain-containing protein | | uniclust | UniRef100\_A0A173Z0X1 | 99.1 | 4.9e-13 | 9.8e-19 | 77.2 | 63 | (11, 74) | 77 | (5, 67) | 80 | DNA-binding transcriptional regulator, XRE-family HTH domain | DNA-binding transcriptional regulator, XRE-family HTH domain | | uniclust | UniRef100\_A0A0B2JZ35 | 99.1 | 4.5e-13 | 9.9e-19 | 80.8 | 57 | (15, 72) | 77 | (14, 70) | 98 | XRE family transcriptional regulator | XRE family transcriptional regulator | | uniclust | UniRef100\_A0A064ABB6 | 99.1 | 4.5e-13 | 9.9e-19 | 85.3 | 58 | (14, 72) | 77 | (3, 66) | 139 | HTH cro/C1-type domain-containing protein | HTH cro/C1-type domain-containing protein | | uniclust | UniRef100\_A0A089HUS8 | 99.1 | 4.5e-13 | 9.9e-19 | 93.8 | 59 | (13, 72) | 77 | (55, 113) | 289 | HTH cro/C1-type domain-containing protein | HTH cro/C1-type domain-containing protein | | uniclust | UniRef100\_A0A1C5L5I0 | 99.1 | 4.8e-13 | 1e-18 | 90.0 | 62 | (11, 73) | 77 | (4, 65) | 219 | LexA repressor | LexA repressor | | uniclust | UniRef100\_A0A140C2Z8 | 99.1 | 5e-13 | 1e-18 | 83.9 | 59 | (13, 72) | 77 | (3, 61) | 134 | Helix-turn-helix domain protein | Helix-turn-helix domain protein | | uniclust | UniRef100\_A0A0R2CGT4 | 99.1 | 5.1e-13 | 1e-18 | 80.2 | 54 | (17, 71) | 77 | (1, 54) | 101 | XRE family transcriptional regulator | XRE family transcriptional regulator | | uniclust | UniRef100\_A0A0C1K7L7 | 99.1 | 5.3e-13 | 1e-18 | 78.0 | 54 | (17, 71) | 77 | (3, 56) | 91 | XRE family transcriptional regulator | XRE family transcriptional regulator | | uniclust | UniRef100\_A0A927PAF1 | 99.1 | 5.3e-13 | 1e-18 | 81.5 | 54 | (18, 72) | 77 | (5, 58) | 120 | Helix-turn-helix transcriptional regulator | Helix-turn-helix transcriptional regulator | | uniclust | UniRef100\_A0A0F2S7G2 | 99.1 | 4.7e-13 | 1e-18 | 83.9 | 57 | (15, 72) | 77 | (7, 63) | 125 | HTH cro/C1-type domain-containing protein | HTH cro/C1-type domain-containing protein | | uniclust | UniRef100\_A0A075U1V1 | 99.1 | 5.1e-13 | 1.1e-18 | 87.4 | 57 | (15, 72) | 77 | (1, 57) | 179 | HTH cro/C1-type domain-containing protein | HTH cro/C1-type domain-containing protein | | uniclust | UniRef100\_A0A0R2DDB6 | 99.1 | 5e-13 | 1.1e-18 | 86.8 | 62 | (12, 74) | 77 | (12, 73) | 163 | HTH cro/C1-type domain-containing protein | HTH cro/C1-type domain-containing protein | | uniclust | UniRef100\_A0A0Q2USQ7 | 99.1 | 5e-13 | 1.1e-18 | 85.2 | 58 | (15, 73) | 77 | (12, 69) | 141 | HTH cro/C1-type domain-containing protein | HTH cro/C1-type domain-containing protein | | uniclust | UniRef100\_A0A0D6DWE9 | 99.1 | 5.5e-13 | 1.1e-18 | 82.2 | 56 | (16, 72) | 77 | (1, 56) | 123 | XRE-family transcriptional regulator | XRE-family transcriptional regulator | | uniclust | UniRef100\_A0A0M6WVC9 | 99.1 | 5e-13 | 1.1e-18 | 87.3 | 65 | (8, 73) | 77 | (13, 77) | 168 | Helix-turn-helix domain-containing protein | Helix-turn-helix domain-containing protein | | uniclust | UniRef100\_A0A0A8VZS6 | 99.1 | 5.2e-13 | 1.1e-18 | 84.9 | 58 | (14, 72) | 77 | (1, 58) | 142 | Helix-turn-helix transcriptional regulator | Helix-turn-helix transcriptional regulator | | uniclust | UniRef100\_A0A174ZKR9 | 99.1 | 5e-13 | 1.1e-18 | 96.7 | 57 | (15, 72) | 77 | (12, 68) | 385 | Antitoxin PezA | Antitoxin PezA | | uniclust | UniRef100\_A0A0J6HEI8 | 99.1 | 5.2e-13 | 1.1e-18 | 84.2 | 57 | (15, 72) | 77 | (5, 61) | 132 | XRE family transcriptional regulator | XRE family transcriptional regulator | | uniclust | UniRef100\_A0A074LWD2 | 99.1 | 5.3e-13 | 1.1e-18 | 81.4 | 57 | (15, 72) | 77 | (21, 77) | 107 | HTH cro/C1-type domain-containing protein | HTH cro/C1-type domain-containing protein | | uniclust | UniRef100\_A0A0U4X1Q9 | 99.1 | 5.8e-13 | 1.1e-18 | 85.5 | 56 | (17, 73) | 77 | (3, 58) | 168 | HTH cro/C1-type domain-containing protein | HTH cro/C1-type domain-containing protein | | uniclust | UniRef100\_A0A087BBT1 | 99.1 | 5.6e-13 | 1.1e-18 | 88.2 | 59 | (13, 72) | 77 | (18, 76) | 198 | Putative transcriptional regulator | Putative transcriptional regulator | | uniclust | UniRef100\_A0A1B1LIU2 | 99.1 | 5.8e-13 | 1.1e-18 | 81.3 | 58 | (14, 72) | 77 | (1, 58) | 117 | HTH cro/C1-type domain-containing protein | HTH cro/C1-type domain-containing protein | | uniclust | UniRef100\_A0A1I7IJU0 | 99.1 | 5.7e-13 | 1.2e-18 | 86.7 | 55 | (17, 72) | 77 | (1, 55) | 179 | Transcriptional regulator, contains XRE-family HTH domain | Transcriptional regulator, contains XRE-family HTH domain | | uniclust | UniRef100\_A0A1H0KR93 | 99.1 | 5.5e-13 | 1.2e-18 | 80.4 | 57 | (15, 72) | 77 | (14, 70) | 99 | DNA-binding transcriptional regulator, XRE-family HTH domain | DNA-binding transcriptional regulator, XRE-family HTH domain | | uniclust | UniRef100\_A0A0B7IC89 | 99.1 | 5.7e-13 | 1.2e-18 | 77.6 | 58 | (14, 72) | 77 | (7, 64) | 84 | Anaerobic benzoate catabolism transcriptional regulator | Anaerobic benzoate catabolism transcriptional regulator | | uniclust | UniRef100\_A0A3Q9V8T4 | 99.1 | 5.8e-13 | 1.2e-18 | 81.3 | 53 | (19, 72) | 77 | (2, 54) | 112 | XRE family transcriptional regulator | XRE family transcriptional regulator | | uniclust | UniRef100\_A0A235IZF3 | 99.1 | 5.9e-13 | 1.2e-18 | 81.4 | 56 | (15, 71) | 77 | (21, 78) | 115 | Transcriptional regulator | Transcriptional regulator | | uniclust | UniRef100\_A0A1Y4TPK4 | 99.1 | 5.9e-13 | 1.2e-18 | 88.9 | 56 | (16, 72) | 77 | (3, 58) | 214 | HTH cro/C1-type domain-containing protein | HTH cro/C1-type domain-containing protein | | uniclust | UniRef100\_A0A073JPW0 | 99.1 | 5.5e-13 | 1.2e-18 | 85.3 | 56 | (16, 72) | 77 | (17, 72) | 144 | Helix-turn-helix domain-containing protein | Helix-turn-helix domain-containing protein | | uniclust | UniRef100\_A0A173WDM4 | 99.1 | 6e-13 | 1.2e-18 | 81.2 | 58 | (14, 72) | 77 | (3, 60) | 116 | DNA-binding transcriptional repressor PuuR | DNA-binding transcriptional repressor PuuR | | uniclust | UniRef100\_A0A084J761 | 99.1 | 5.3e-13 | 1.2e-18 | 95.2 | 66 | (7, 73) | 77 | (40, 105) | 335 | HTH cro/C1-type domain-containing protein | HTH cro/C1-type domain-containing protein | | uniclust | UniRef100\_A0A143X185 | 99.1 | 5.9e-13 | 1.2e-18 | 87.1 | 55 | (16, 71) | 77 | (10, 64) | 182 | DNA-binding transcriptional repressor PuuR | DNA-binding transcriptional repressor PuuR | | uniclust | UniRef100\_A0A2G5W151 | 99.1 | 6.2e-13 | 1.2e-18 | 77.8 | 55 | (17, 72) | 77 | (1, 55) | 88 | HTH cro/C1-type domain-containing protein | HTH cro/C1-type domain-containing protein | | uniclust | UniRef100\_A0A069RGK5 | 99.1 | 5.7e-13 | 1.2e-18 | 85.9 | 59 | (13, 72) | 77 | (13, 71) | 152 | XRE family transcriptional regulator | XRE family transcriptional regulator | | uniclust | UniRef100\_A0A351SPC4 | 99.1 | 6.3e-13 | 1.2e-18 | 78.1 | 58 | (14, 72) | 77 | (6, 63) | 91 | Transcriptional regulator (Fragment) | Transcriptional regulator (Fragment) | | uniclust | UniRef100\_A0A1V5P064 | 99.1 | 5.8e-13 | 1.2e-18 | 92.0 | 61 | (12, 73) | 77 | (32, 92) | 265 | LexA repressor | LexA repressor | | uniclust | UniRef100\_A0A090Y881 | 99.1 | 5.8e-13 | 1.3e-18 | 96.5 | 60 | (12, 72) | 77 | (54, 113) | 412 | Helix-turn-helix family protein | Helix-turn-helix family protein | | uniclust | UniRef100\_A0A0B0CZI8 | 99.1 | 6e-13 | 1.3e-18 | 78.3 | 63 | (10, 73) | 77 | (2, 64) | 85 | HTH cro/C1-type domain-containing protein | HTH cro/C1-type domain-containing protein | | uniclust | UniRef100\_A0A133YTS9 | 99.1 | 6e-13 | 1.3e-18 | 88.4 | 65 | (8, 73) | 77 | (32, 96) | 196 | HTH cro/C1-type domain-containing protein | HTH cro/C1-type domain-containing protein | | uniclust | UniRef100\_A0A0R1N1Y1 | 99.1 | 5.7e-13 | 1.3e-18 | 96.9 | 63 | (9, 72) | 77 | (45, 107) | 414 | HTH cro/C1-type domain-containing protein | HTH cro/C1-type domain-containing protein | | uniclust | UniRef100\_A0A090JLT7 | 99.1 | 6.1e-13 | 1.3e-18 | 85.1 | 61 | (11, 72) | 77 | (3, 63) | 150 | HTH cro/C1-type domain-containing protein | HTH cro/C1-type domain-containing protein | | uniclust | UniRef100\_A0A173SEW4 | 99.1 | 6.4e-13 | 1.3e-18 | 88.6 | 59 | (13, 72) | 77 | (19, 77) | 220 | Predicted transcriptional regulator | Predicted transcriptional regulator | | uniclust | UniRef100\_A0A0R1TWH7 | 99.1 | 6.6e-13 | 1.3e-18 | 83.7 | 56 | (16, 72) | 77 | (1, 56) | 147 | Cro CI family phage transcriptional regulator | Cro CI family phage transcriptional regulator | | uniclust | UniRef100\_A0A0R2G5H7 | 99.1 | 6.1e-13 | 1.3e-18 | 85.9 | 59 | (15, 73) | 77 | (6, 64) | 158 | HTH cro/C1-type domain-containing protein | HTH cro/C1-type domain-containing protein | | uniclust | UniRef100\_A0A5M9NFB2 | 99.1 | 6.2e-13 | 1.3e-18 | 85.8 | 57 | (15, 72) | 77 | (4, 60) | 158 | Helix-turn-helix transcriptional regulator | Helix-turn-helix transcriptional regulator | | uniclust | UniRef100\_A0A162QEW9 | 99.1 | 6.3e-13 | 1.3e-18 | 90.8 | 58 | (14, 72) | 77 | (1, 58) | 248 | HTH-type transcriptional regulator Xre | HTH-type transcriptional regulator Xre | | uniclust | UniRef100\_A0A1F9VE86 | 99.1 | 6.3e-13 | 1.3e-18 | 75.5 | 49 | (16, 65) | 77 | (5, 53) | 69 | HTH cro/C1-type domain-containing protein | HTH cro/C1-type domain-containing protein | | uniclust | UniRef100\_A0A7X6XEI5 | 99.1 | 6.8e-13 | 1.3e-18 | 76.4 | 53 | (18, 71) | 77 | (2, 54) | 80 | Helix-turn-helix transcriptional regulator | Helix-turn-helix transcriptional regulator | | uniclust | UniRef100\_A0A072YE13 | 99.1 | 6.4e-13 | 1.3e-18 | 79.0 | 59 | (13, 72) | 77 | (8, 68) | 92 | Putative DNA-binding protein | Putative DNA-binding protein | | uniclust | UniRef100\_A0A1C5PFS1 | 99.1 | 6.2e-13 | 1.3e-18 | 83.9 | 63 | (11, 73) | 77 | (8, 70) | 131 | Transcriptional repressor DicA | Transcriptional repressor DicA | | uniclust | UniRef100\_A0A0V8QH06 | 99.1 | 6.9e-13 | 1.4e-18 | 79.2 | 62 | (9, 71) | 77 | (9, 70) | 101 | HTH cro/C1-type domain-containing protein | HTH cro/C1-type domain-containing protein | | uniclust | UniRef100\_A0A413LW35 | 99.1 | 7.1e-13 | 1.4e-18 | 75.7 | 59 | (16, 74) | 77 | (1, 59) | 79 | XRE family transcriptional regulator | XRE family transcriptional regulator | | uniclust | UniRef100\_A0A069DTQ8 | 99.1 | 6.5e-13 | 1.4e-18 | 81.0 | 62 | (12, 73) | 77 | (15, 76) | 107 | HTH cro/C1-type domain-containing protein | HTH cro/C1-type domain-containing protein | | uniclust | UniRef100\_A0A076Z3U0 | 99.1 | 6.6e-13 | 1.4e-18 | 88.4 | 54 | (18, 72) | 77 | (3, 56) | 201 | Helix-turn-helix domain-containing protein | Helix-turn-helix domain-containing protein | |
| Top keywords  (threshold 1.00e-03 (evalue)) | **transcriptional, regulator, domain\_containing, HTH, cro, C1\_type, Helix\_turn\_helix, XRE, DNA\_binding, HTH\_type** |
| Output files | ../../similar\_sequences/52\_FANPEZAQ\_CDS\_0052\_merged.svg ../../similar\_sequences/52\_FANPEZAQ\_CDS\_0052\_pdb70.a3m ../../similar\_sequences/52\_FANPEZAQ\_CDS\_0052\_pdb70.hhr ../../similar\_sequences/52\_FANPEZAQ\_CDS\_0052\_uniclust.a3m ../../similar\_sequences/52\_FANPEZAQ\_CDS\_0052\_uniclust.hhr |

#### Structure prediction (AlphaFold)2

|  |  |
| --- | --- |
| Stats | xml version="1.0" encoding="utf-8" standalone="no"?       2024-09-02T21:09:47.994922 image/svg+xml   Matplotlib v3.7.2, https://matplotlib.org/ |
| Predicted structure | **NGL Viewer Controls:**  - Center: *Left-Click* - Rotate: *Left-Click + Drag* - Translate: *Right-Click + Drag* - Zoom: *Shift + Left-Click + Drag* |
| Output files | ../../predicted\_structures/52\_FANPEZAQ\_CDS\_0052/features.pkl ../../predicted\_structures/52\_FANPEZAQ\_CDS\_0052/ranked\_0.pdb ../../predicted\_structures/52\_FANPEZAQ\_CDS\_0052/ranked\_0\_plots.svg ../../predicted\_structures/52\_FANPEZAQ\_CDS\_0052/result\_model\_1\_ptm\_pred\_0.pkl |

#### Structure similarity search results (Foldseek)3

|  |  |
| --- | --- |
| Structure databases searched | Pdb, Afdb-proteome, Afdb-uniprot50 |
| Results, scheme(s)  (Top layers only, threshold 1.00e-02 (evalue)) | xml version="1.0" encoding="utf-8" standalone="no"?       2024-09-02T21:11:23.614800 image/svg+xml   Matplotlib v3.7.2, https://matplotlib.org/ |
| Results, table  (threshold 1.00e-02 (evalue)) | | db | id | prob | evalue | bits | fident | alnlen | mismatch | gapopen | qstart | qend | tstart | tend | name | description | | --- | --- | --- | --- | --- | --- | --- | --- | --- | --- | --- | --- | --- | --- | --- | | afdb-uniprot50 | AF-A0A2Z3I919-F1-MODEL\_V4 | 1.0 | 0.0006904 | 217 | 0.5 | 58 | 29 | 0 | 16 | 73 | 1 | 58 | Uncharacterized protein | Uncharacterized protein | |
| Top keywords  (threshold 1.00e-02 (evalue)) | -- |
| Output files | ../../similar\_structures/52\_FANPEZAQ\_CDS\_0052\_afdb-proteome\_foldseek.tsv ../../similar\_structures/52\_FANPEZAQ\_CDS\_0052\_afdb-uniprot50\_foldseek.tsv ../../similar\_structures/52\_FANPEZAQ\_CDS\_0052\_merged.svg ../../similar\_structures/52\_FANPEZAQ\_CDS\_0052\_pdb\_foldseek.tsv |

  
  
  

Return to summary | Go to previous | Go to next

  


---

**Sequence/structure alignments coloring**  
Each object in the alignment figures is colored according to its E-value following this color coding:

1e-100
10

**References:**  
1) Steinegger M, Meier M, Mirdita M, Vöhringer H, Haunsberger S J, and Söding J (2019) HH-suite3 for fast remote homology detection and deep protein annotation, BMC Bioinformatics, 473. doi: 10.1186/s12859-019-3019-7  
2) Jumper J, Evans R, Pritzel A, ..., Hassabis D (2021) Highly accurate protein structure prediction with AlphaFold, Nature, 596. doi: 10.1038/s41586-021-03819-2  
3) van Kempen M, Kim S, Tumescheit C, Mirdita M, Lee J, Gilchrist CLM, Söding J, and Steinegger M (2023) Fast and accurate protein structure search with Foldseek. Nature Biotechnology. doi: 10.1038/s41587-023-01773-0
