# Supplementary material for: Completing the BASEL phage collection to unlock hidden diversity for systematic exploration of phage–host interactions
Source: PLoS Biol. 2025 Apr 7;23(4):e3003063. doi: 10.1371/journal.pbio.3003063 (PMC11990801; doi:10.1371/journal.pbio.3003063)
Supplement: S2 Data — (ZIP) [file pbio.3003063.s009.zip › entries/53.html]

FANPEZAQ\_CDS\_0053


Return to summary | Go to previous | Go to next

|  |  |
| --- | --- |
| FANPEZAQ\_CDS\_0053 Page creation date: 02 Sep 2024, 12:00  Project folder: n/a  Input sequences file: Escherichia\_virus\_HeidiAbel.gb | hypothetical ep3\_0015 domain\_containing ky705409\_p11 p222118 vi\_08569 duf1289 kfra n\_terminal dna\_binding |

### Sequence information

|  |  |
| --- | --- |
| Name | FANPEZAQ\_CDS\_0053  53\_FANPEZAQ\_CDS\_0053 (pipeline id) |
| Imported annotations | Escherichia\_virus\_HeidiAbel Bas97 |
| Protein sequence | MNACTWQPTTVTLQDGREVLSDSREWLLECEAKYVLNQPTIEARRTLLAAIEKRRGATAR EELEQRALAIWRANRP |
| Number of residues | 76 |
| Molecular weight (Da) | 8795.92 |
| Output files | ../../query\_sequences/53\_FANPEZAQ\_CDS\_0053.fasta |

### Putative domain architecture and protein family

#### Search results (HHblits)1

|  |  |
| --- | --- |
| Domain family databases searched | Pfam, Ncbi-cd, Cath, Phrogs |
| Results, scheme(s)  (Top layers only; threshold 1.00e-03 (evalue)) | xml version="1.0" encoding="utf-8" standalone="no"?       2024-09-02T21:08:24.222966 image/svg+xml   Matplotlib v3.7.2, https://matplotlib.org/ |
| Results, table  (E-value ≤ 1.00e-03 (evalue)) | | db | id | prob | evalue | pvalue | score | cols | query | query\_len | template | template\_len | name | description | | --- | --- | --- | --- | --- | --- | --- | --- | --- | --- | --- | --- | --- | | phrogs | 11642 | 100.0 | 9.5e-42 | 1.1e-45 | 219.3 | 76 | (1, 76) | 76 | (1, 76) | 76 | NA | NA; Category: unknown function; KY705409\_p11 | | phrogs | 14931 | 99.6 | 3.4e-20 | 3.9e-24 | 116.3 | 52 | (24, 75) | 76 | (8, 61) | 69 | NA | NA; Category: unknown function; p222118 VI\_08569 | |
| Top keywords  (threshold 1.00e-03 (evalue)) | **KY705409\_p11, p222118, VI\_08569** |
| Output files | ../../domain\_architecture/53\_FANPEZAQ\_CDS\_0053\_cath.hhr ../../domain\_architecture/53\_FANPEZAQ\_CDS\_0053\_merged.svg ../../domain\_architecture/53\_FANPEZAQ\_CDS\_0053\_ncbi-cd.hhr ../../domain\_architecture/53\_FANPEZAQ\_CDS\_0053\_pfam.hhr ../../domain\_architecture/53\_FANPEZAQ\_CDS\_0053\_phrogs.hhr |

### Identical protein sequences/structures

#### Search results

|  |  |
| --- | --- |
| Protein sequence databases searched | Pdb, Swissprot, Refseq |
| Identical proteins found | Refseq  - YP\_009100010.1: Hypothetical protein ep3\_0015 (Escherichia phage vB\_EcoM-ep3) - AIM50545.1: Hypothetical protein ep3\_0015 (Escherichia phage vB\_EcoM-ep3) |
| Top keywords | **Hypothetical, ep3\_0015** |
| Output files | ../../identical\_sequences/53\_FANPEZAQ\_CDS\_0053\_refseq.fasta |

### Similar protein sequences/structures

#### Sequence similarity search results (HHblits)1

|  |  |
| --- | --- |
| Sequence databases searched | Uniclust, Pdb70 |
| Results, scheme(s)  (Top layers only, threshold 1.00e-03 (evalue)) | xml version="1.0" encoding="utf-8" standalone="no"?       2024-09-02T21:08:51.793827 image/svg+xml   Matplotlib v3.7.2, https://matplotlib.org/ |
| Results, table(s)  (threshold 1.00e-03 (evalue)) | | db | id | prob | evalue | pvalue | score | cols | query | query\_len | template | template\_len | name | description | | --- | --- | --- | --- | --- | --- | --- | --- | --- | --- | --- | --- | --- | | uniclust | UniRef100\_A0A088FQJ3 | 99.9 | 4e-30 | 8.7e-36 | 167.6 | 75 | (1, 75) | 76 | (11, 85) | 97 | Uncharacterized protein | Uncharacterized protein | | uniclust | UniRef100\_A0A1B2ECS2 | 99.9 | 4.6e-29 | 1.1e-34 | 163.7 | 75 | (1, 75) | 76 | (5, 90) | 98 | Uncharacterized protein | Uncharacterized protein | | uniclust | UniRef100\_A0A7G8LJG8 | 99.8 | 7.4e-23 | 1.6e-28 | 139.2 | 68 | (1, 69) | 76 | (2, 69) | 126 | Uncharacterized protein | Uncharacterized protein | | uniclust | UniRef100\_A0A086MEE7 | 99.6 | 6.2e-19 | 1.3e-24 | 118.8 | 67 | (8, 75) | 76 | (23, 101) | 112 | Uncharacterized protein | Uncharacterized protein | | uniclust | UniRef100\_A0A2W5MTW2 | 99.6 | 1.2e-18 | 2.7e-24 | 116.2 | 69 | (7, 75) | 76 | (19, 96) | 103 | Uncharacterized protein | Uncharacterized protein | | uniclust | UniRef100\_A0A061JNG3 | 99.6 | 1.5e-18 | 3.3e-24 | 111.8 | 67 | (9, 75) | 76 | (3, 71) | 81 | Uncharacterized protein | Uncharacterized protein | | uniclust | UniRef100\_A0A085FCV0 | 99.5 | 3.7e-17 | 7.8e-23 | 107.0 | 62 | (10, 72) | 76 | (12, 84) | 90 | Uncharacterized protein | Uncharacterized protein | | uniclust | UniRef100\_A0A2I8DIP3 | 99.5 | 1.5e-16 | 3.2e-22 | 98.5 | 58 | (17, 75) | 76 | (3, 60) | 63 | Uncharacterized protein | Uncharacterized protein | | uniclust | UniRef100\_A0A0Q9HPA0 | 99.4 | 2.5e-16 | 5e-22 | 102.1 | 62 | (8, 70) | 76 | (9, 77) | 86 | Uncharacterized protein | Uncharacterized protein | | uniclust | UniRef100\_A0A375IY35 | 99.4 | 1.7e-15 | 3.4e-21 | 97.6 | 53 | (23, 75) | 76 | (16, 68) | 82 | Uncharacterized protein | Uncharacterized protein | | uniclust | UniRef100\_A0A5C7JBT4 | 99.4 | 2.6e-15 | 5.1e-21 | 93.5 | 59 | (17, 75) | 76 | (2, 60) | 66 | Uncharacterized protein | Uncharacterized protein | | uniclust | UniRef100\_A0A1D7U039 | 99.3 | 3.4e-15 | 6.8e-21 | 98.6 | 63 | (12, 75) | 76 | (14, 87) | 95 | Uncharacterized protein | Uncharacterized protein | | uniclust | UniRef100\_A0A6J5QN02 | 99.3 | 4.9e-15 | 8.9e-21 | 92.8 | 65 | (7, 72) | 76 | (3, 67) | 72 | Uncharacterized protein | Uncharacterized protein | | uniclust | UniRef100\_A0A2Z3I5Z9 | 99.3 | 1.9e-14 | 3.4e-20 | 92.7 | 70 | (7, 76) | 76 | (8, 78) | 85 | DUF1289 domain-containing protein | DUF1289 domain-containing protein | | uniclust | UniRef100\_UPI0021E4EEF5 | 99.2 | 4.3e-14 | 7.9e-20 | 95.1 | 59 | (17, 75) | 76 | (4, 62) | 114 | hypothetical protein | hypothetical protein | | uniclust | UniRef100\_A0A6J5NE18 | 99.1 | 1.9e-13 | 4.1e-19 | 87.7 | 57 | (19, 75) | 76 | (7, 65) | 75 | Uncharacterized protein | Uncharacterized protein | | uniclust | UniRef100\_A0A2Z3HWP4 | 99.1 | 9.4e-13 | 1.9e-18 | 82.1 | 55 | (20, 74) | 76 | (4, 60) | 64 | Uncharacterized protein | Uncharacterized protein | | uniclust | UniRef100\_UPI0007826AF5 | 98.9 | 5.1e-12 | 9.9e-18 | 83.7 | 72 | (4, 75) | 76 | (6, 82) | 93 | hypothetical protein | hypothetical protein | | uniclust | UniRef100\_A0A2G7RZZ6 | 98.9 | 7.6e-12 | 1.5e-17 | 83.2 | 65 | (10, 74) | 76 | (1, 80) | 93 | Uncharacterized protein | Uncharacterized protein | | uniclust | UniRef100\_A0A1G8AZ57 | 98.9 | 1.3e-11 | 2.5e-17 | 77.5 | 57 | (19, 75) | 76 | (1, 58) | 66 | Uncharacterized protein | Uncharacterized protein | | uniclust | UniRef100\_A0A5B2VSF5 | 98.9 | 1.9e-11 | 3.4e-17 | 86.8 | 66 | (9, 75) | 76 | (78, 154) | 158 | Uncharacterized protein | Uncharacterized protein | | uniclust | UniRef100\_A0A0R0CJT0 | 98.8 | 2.5e-11 | 5.5e-17 | 77.4 | 58 | (18, 75) | 76 | (2, 61) | 69 | KfrA N-terminal DNA-binding domain-containing protein | KfrA N-terminal DNA-binding domain-containing protein | | uniclust | UniRef100\_A0A1H7WFY8 | 98.8 | 3.8e-11 | 7.6e-17 | 80.5 | 64 | (10, 74) | 76 | (12, 86) | 97 | Uncharacterized protein | Uncharacterized protein | | uniclust | UniRef100\_A0A6J7X307 | 98.7 | 8.1e-11 | 1.5e-16 | 75.0 | 62 | (11, 72) | 76 | (5, 71) | 74 | Uncharacterized protein | Uncharacterized protein | | uniclust | UniRef100\_A0A1E4HKI7 | 98.7 | 8.3e-11 | 1.6e-16 | 75.4 | 54 | (22, 75) | 76 | (12, 67) | 74 | Uncharacterized protein | Uncharacterized protein | | uniclust | UniRef100\_A0A6J5NVJ8 | 98.6 | 3.4e-10 | 6.3e-16 | 73.8 | 65 | (8, 74) | 76 | (11, 80) | 84 | Uncharacterized protein | Uncharacterized protein | | uniclust | UniRef100\_A0A1E4FVV8 | 98.6 | 4.1e-10 | 8e-16 | 70.9 | 52 | (20, 72) | 76 | (2, 53) | 64 | Uncharacterized protein | Uncharacterized protein | | uniclust | UniRef100\_A0A5B2VQR8 | 98.6 | 4.5e-10 | 8.3e-16 | 69.4 | 44 | (9, 53) | 76 | (5, 48) | 60 | Uncharacterized protein | Uncharacterized protein | | uniclust | UniRef100\_A0A1V5QQ95 | 98.6 | 4.8e-10 | 9.3e-16 | 71.7 | 59 | (16, 75) | 76 | (8, 66) | 71 | Uncharacterized protein | Uncharacterized protein | | uniclust | UniRef100\_A0A9E5K5N7 | 98.5 | 8.5e-10 | 1.6e-15 | 73.1 | 68 | (2, 70) | 76 | (8, 75) | 92 | Uncharacterized protein | Uncharacterized protein | | uniclust | UniRef100\_UPI00135B86F6 | 98.5 | 1.1e-09 | 2e-15 | 73.1 | 63 | (13, 75) | 76 | (9, 88) | 95 | hypothetical protein | hypothetical protein | | uniclust | UniRef100\_A0A069P165 | 98.5 | 1e-09 | 2.1e-15 | 77.7 | 53 | (17, 70) | 76 | (54, 106) | 133 | Uncharacterized protein | Uncharacterized protein | | uniclust | UniRef100\_A0A1B4G289 | 98.4 | 2.4e-09 | 4.8e-15 | 74.5 | 52 | (18, 70) | 76 | (56, 107) | 120 | Uncharacterized protein | Uncharacterized protein | | uniclust | UniRef100\_A0A4R3UNM8 | 98.4 | 3e-09 | 5.6e-15 | 65.5 | 51 | (23, 73) | 76 | (3, 53) | 58 | Uncharacterized protein | Uncharacterized protein | | uniclust | UniRef100\_A0A9E3YW76 | 98.4 | 3.2e-09 | 5.8e-15 | 66.6 | 55 | (20, 74) | 76 | (5, 61) | 64 | Uncharacterized protein | Uncharacterized protein | | uniclust | UniRef100\_A0A4Q8M7C5 | 98.4 | 3.4e-09 | 6.7e-15 | 73.8 | 58 | (18, 75) | 76 | (51, 110) | 118 | Uncharacterized protein | Uncharacterized protein | | uniclust | UniRef100\_UPI001F0C0D3A | 98.2 | 1.7e-08 | 3e-14 | 66.8 | 50 | (26, 75) | 76 | (28, 79) | 87 | hypothetical protein | hypothetical protein | | uniclust | UniRef100\_UPI001FC80872 | 98.2 | 2.7e-08 | 4.9e-14 | 71.5 | 53 | (17, 70) | 76 | (91, 143) | 156 | hypothetical protein | hypothetical protein | | uniclust | UniRef100\_A0A0Q9J9L1 | 98.1 | 4.1e-08 | 7.5e-14 | 68.8 | 52 | (14, 66) | 76 | (38, 100) | 128 | Uncharacterized protein | Uncharacterized protein | | uniclust | UniRef100\_UPI00054B42C1 | 98.0 | 8.9e-08 | 1.6e-13 | 63.0 | 52 | (24, 75) | 76 | (11, 64) | 82 | hypothetical protein | hypothetical protein | | uniclust | UniRef100\_A0A0H1RC28 | 98.0 | 1.6e-07 | 3e-13 | 64.7 | 59 | (9, 68) | 76 | (8, 78) | 111 | Uncharacterized protein | Uncharacterized protein | | uniclust | UniRef100\_A0A2S5MC58 | 98.0 | 1.6e-07 | 3e-13 | 66.4 | 50 | (18, 68) | 76 | (29, 78) | 133 | Uncharacterized protein | Uncharacterized protein | | uniclust | UniRef100\_I4YKC4 | 97.9 | 2.3e-07 | 4.4e-13 | 61.4 | 47 | (9, 56) | 76 | (17, 63) | 82 | Uncharacterized protein | Uncharacterized protein | | uniclust | UniRef100\_UPI0011C05C5A | 97.8 | 4.3e-07 | 7.9e-13 | 63.9 | 70 | (5, 75) | 76 | (39, 108) | 126 | hypothetical protein | hypothetical protein | | uniclust | UniRef100\_A0A9E8CZF4 | 97.8 | 4.4e-07 | 8.1e-13 | 63.2 | 49 | (15, 64) | 76 | (42, 97) | 117 | Uncharacterized protein | Uncharacterized protein | | uniclust | UniRef100\_A0A4Q8LD98 | 97.8 | 4.5e-07 | 8.2e-13 | 59.1 | 57 | (19, 75) | 76 | (9, 67) | 75 | Uncharacterized protein | Uncharacterized protein | | uniclust | UniRef100\_A0A202B296 | 97.6 | 1.7e-06 | 3.6e-12 | 57.9 | 56 | (20, 75) | 76 | (20, 77) | 80 | Uncharacterized protein | Uncharacterized protein | | uniclust | UniRef100\_A0A6J5PKL6 | 97.4 | 6.7e-06 | 1.2e-11 | 56.7 | 64 | (5, 70) | 76 | (35, 100) | 103 | Uncharacterized protein | Uncharacterized protein | | uniclust | UniRef100\_A0A0Q7F577 | 97.3 | 1.3e-05 | 2.3e-11 | 56.9 | 58 | (16, 73) | 76 | (5, 62) | 122 | Uncharacterized protein | Uncharacterized protein | | uniclust | UniRef100\_A0A0S4U452 | 97.2 | 1.4e-05 | 2.9e-11 | 57.1 | 47 | (23, 70) | 76 | (43, 89) | 120 | Uncharacterized protein | Uncharacterized protein | | uniclust | UniRef100\_UPI0020796AD7 | 97.2 | 2.5e-05 | 4.7e-11 | 50.2 | 51 | (22, 73) | 76 | (3, 53) | 64 | hypothetical protein | hypothetical protein | | uniclust | UniRef100\_UPI00036E2260 | 97.0 | 5.9e-05 | 1.1e-10 | 50.4 | 40 | (19, 58) | 76 | (3, 42) | 77 | hypothetical protein | hypothetical protein | | uniclust | UniRef100\_H6BJ70 | 96.9 | 7.7e-05 | 1.4e-10 | 48.5 | 64 | (6, 73) | 76 | (1, 64) | 67 | Uncharacterized protein | Uncharacterized protein | | uniclust | UniRef100\_UPI001E405AA9 | 96.6 | 0.00033 | 6e-10 | 51.0 | 46 | (23, 69) | 76 | (59, 104) | 135 | hypothetical protein | hypothetical protein | | uniclust | UniRef100\_A0A6J5KQ13 | 96.5 | 0.00038 | 7.7e-10 | 46.8 | 56 | (15, 73) | 76 | (4, 62) | 74 | Uncharacterized protein | Uncharacterized protein | | uniclust | UniRef100\_C1D867 | 96.5 | 0.00052 | 9.7e-10 | 51.8 | 55 | (21, 75) | 76 | (101, 155) | 169 | Uncharacterized protein | Uncharacterized protein | | uniclust | UniRef100\_A0A6S7BPC1 | 96.5 | 0.00055 | 1e-09 | 51.2 | 53 | (17, 70) | 76 | (81, 133) | 162 | Uncharacterized protein | Uncharacterized protein | | uniclust | UniRef100\_UPI00131C9C71 | 96.4 | 0.00069 | 1.3e-09 | 43.9 | 43 | (31, 74) | 76 | (13, 55) | 62 | hypothetical protein | hypothetical protein | |
| Top keywords  (threshold 1.00e-03 (evalue)) | **hypothetical, domain\_containing, DUF1289, KfrA, N\_terminal, DNA\_binding** |
| Output files | ../../similar\_sequences/53\_FANPEZAQ\_CDS\_0053\_merged.svg ../../similar\_sequences/53\_FANPEZAQ\_CDS\_0053\_pdb70.a3m ../../similar\_sequences/53\_FANPEZAQ\_CDS\_0053\_pdb70.hhr ../../similar\_sequences/53\_FANPEZAQ\_CDS\_0053\_uniclust.a3m ../../similar\_sequences/53\_FANPEZAQ\_CDS\_0053\_uniclust.hhr |

#### Structure prediction (AlphaFold)2

|  |  |
| --- | --- |
| Stats | xml version="1.0" encoding="utf-8" standalone="no"?       2024-09-02T21:09:48.666323 image/svg+xml   Matplotlib v3.7.2, https://matplotlib.org/ |
| Predicted structure | **NGL Viewer Controls:**  - Center: *Left-Click* - Rotate: *Left-Click + Drag* - Translate: *Right-Click + Drag* - Zoom: *Shift + Left-Click + Drag* |
| Output files | ../../predicted\_structures/53\_FANPEZAQ\_CDS\_0053/features.pkl ../../predicted\_structures/53\_FANPEZAQ\_CDS\_0053/ranked\_0.pdb ../../predicted\_structures/53\_FANPEZAQ\_CDS\_0053/ranked\_0\_plots.svg ../../predicted\_structures/53\_FANPEZAQ\_CDS\_0053/result\_model\_1\_ptm\_pred\_0.pkl |

#### Structure similarity search results (Foldseek)3

|  |  |
| --- | --- |
| Structure databases searched | Pdb, Afdb-proteome, Afdb-uniprot50 |
| Results, scheme(s)  (Top layers only, threshold 1.00e-02 (evalue)) | xml version="1.0" encoding="utf-8" standalone="no"?       2024-09-02T21:11:23.792508 image/svg+xml   Matplotlib v3.7.2, https://matplotlib.org/ |
| Results, table  (threshold 1.00e-02 (evalue)) | | db | id | prob | evalue | bits | fident | alnlen | mismatch | gapopen | qstart | qend | tstart | tend | name | description | | --- | --- | --- | --- | --- | --- | --- | --- | --- | --- | --- | --- | --- | --- | --- | | afdb-uniprot50 | AF-A0A2W5FN44-F1-MODEL\_V4 | 1.0 | 1.574e-06 | 311 | 0.479 | 73 | 38 | 0 | 1 | 73 | 11 | 83 | Uncharacterized protein | Uncharacterized protein | | afdb-uniprot50 | AF-A0A2W5MTW2-F1-MODEL\_V4 | 1.0 | 3.056e-05 | 247 | 0.364 | 74 | 42 | 1 | 8 | 76 | 20 | 93 | Uncharacterized protein | Uncharacterized protein | | afdb-uniprot50 | AF-A0A7R7YH54-F1-MODEL\_V4 | 1.0 | 6.941e-05 | 238 | 0.422 | 71 | 40 | 1 | 1 | 71 | 1 | 70 | Uncharacterized protein | Uncharacterized protein | | afdb-uniprot50 | AF-A0A0Q7SHZ6-F1-MODEL\_V4 | 1.0 | 0.0008133 | 204 | 0.476 | 65 | 34 | 0 | 9 | 73 | 2 | 66 | Uncharacterized protein | Uncharacterized protein | | afdb-uniprot50 | AF-A0A2Z3I5Z9-F1-MODEL\_V4 | 1.0 | 0.001435 | 192 | 0.402 | 72 | 41 | 2 | 2 | 72 | 4 | 74 | Uncharacterized protein | Uncharacterized protein | | afdb-uniprot50 | AF-A0A246JHM4-F1-MODEL\_V4 | 1.0 | 0.0002302 | 169 | 0.375 | 72 | 40 | 1 | 9 | 75 | 2 | 73 | Uncharacterized protein | Uncharacterized protein | |
| Top keywords  (threshold 1.00e-02 (evalue)) | -- |
| Output files | ../../similar\_structures/53\_FANPEZAQ\_CDS\_0053\_afdb-proteome\_foldseek.tsv ../../similar\_structures/53\_FANPEZAQ\_CDS\_0053\_afdb-uniprot50\_foldseek.tsv ../../similar\_structures/53\_FANPEZAQ\_CDS\_0053\_merged.svg ../../similar\_structures/53\_FANPEZAQ\_CDS\_0053\_pdb\_foldseek.tsv |

  
  
  

Return to summary | Go to previous | Go to next

  


---

**Sequence/structure alignments coloring**  
Each object in the alignment figures is colored according to its E-value following this color coding:

1e-100
10

**References:**  
1) Steinegger M, Meier M, Mirdita M, Vöhringer H, Haunsberger S J, and Söding J (2019) HH-suite3 for fast remote homology detection and deep protein annotation, BMC Bioinformatics, 473. doi: 10.1186/s12859-019-3019-7  
2) Jumper J, Evans R, Pritzel A, ..., Hassabis D (2021) Highly accurate protein structure prediction with AlphaFold, Nature, 596. doi: 10.1038/s41586-021-03819-2  
3) van Kempen M, Kim S, Tumescheit C, Mirdita M, Lee J, Gilchrist CLM, Söding J, and Steinegger M (2023) Fast and accurate protein structure search with Foldseek. Nature Biotechnology. doi: 10.1038/s41587-023-01773-0
