# Supplementary material for: Completing the BASEL phage collection to unlock hidden diversity for systematic exploration of phage–host interactions
Source: PLoS Biol. 2025 Apr 7;23(4):e3003063. doi: 10.1371/journal.pbio.3003063 (PMC11990801; doi:10.1371/journal.pbio.3003063)
Supplement: S2 Data — (ZIP) [file pbio.3003063.s009.zip › entries/54.html]

FANPEZAQ\_CDS\_0054


Return to summary | Go to previous | Go to next

|  |  |
| --- | --- |
| FANPEZAQ\_CDS\_0054 Page creation date: 02 Sep 2024, 12:00  Project folder: n/a  Input sequences file: Escherichia\_virus\_HeidiAbel.gb | dna domain\_containing primase virulence\_associated e replication factor licensing toprim helicase putative e\_like atpase p\_loop and virulence mcm2 trac mcm3 prict\_2 fragment rna phage nucleotide metabolism inactivated duf3874 predicted derivatives mcm6 polymerase n\_terminal prim\_pol c plasmid mcm7 vire\_n vape vire mcm4 sf3 duf3854 bifunctional associated maintenance \_ duf927 minichromosome p4 terminal |

### Sequence information

|  |  |
| --- | --- |
| Name | FANPEZAQ\_CDS\_0054  54\_FANPEZAQ\_CDS\_0054 (pipeline id) |
| Imported annotations | Escherichia\_virus\_HeidiAbel Bas97 |
| Protein sequence | MSAQQNYKDLTEQEIAEALSYIDAGCDREAWVRMAMAVKSELGDGGFTVWNDWSRQSDKY NSKDARDTWKSVKRHGGIGIGTLIGEAQQFGFSLNDEERTPLSAEEIEARKRKREAEEKA EQQRRAERMAQAAEQARNIWESATELEGDEHPYLQRKKVQAFGLRVGRFWTENRSIDGAL LVPVRNIDGQITSLQAIFPNENPQLGRDRDYLPGGQKRGCFHVIGGKPTGANPVIVICEG YSTGASIHQATGYCVVVAFDAGNVPTVAKLMRQQFGRATIVVAADNDQWHEDGKQNDGVH YARQAATTCGGLLVVPKFAALSDKPTDFNDLHNLQGLAEVKRQINAAIPEPANDNFLPLD ATVNPFMYPHMSHQQKPLSTWENLEWLLDQYGITARYNEISKDVVVTIPGRDYGVDASAN CSLAEVSSLCARNGMPKGDVGDYIKLIGVANRYNPAAEFITSRPWDGVSRIYDLVDTLAT PAGFDRGLVLMMVRRWLISAVAAVMKPTGFWSKGVLVLQGDQSLGKTSWFKALLPPTMRD LVKDGATIDPSNKDSVTTAIGHWMVELGELDATFRKSDIARLKSFISSDVDMLRRPYDRL ESKYQRRTVFFASVNPKHFLADDTGNVRWWTIPVTGVNYAHDIDTQQLWAEVAQLYRDGE RWWLDRDEEAMLEGVNKEHESIDPVEEMILARFEWGSERISAYREHTASQVLQEIGFDKP NKSQATHCSNVLRKLTGQEPRKTKNGRFFNLPPKVLQPDYRRPDDDKPF |
| Number of residues | 769 |
| Molecular weight (Da) | 86447.26 |
| Output files | ../../query\_sequences/54\_FANPEZAQ\_CDS\_0054.fasta |

### Putative domain architecture and protein family

#### Search results (HHblits)1

|  |  |
| --- | --- |
| Domain family databases searched | Pfam, Ncbi-cd, Cath, Phrogs |
| Results, scheme(s)  (Top layers only; threshold 1.00e-03 (evalue)) | xml version="1.0" encoding="utf-8" standalone="no"?       2024-09-02T21:08:24.476812 image/svg+xml   Matplotlib v3.7.2, https://matplotlib.org/ |
| Results, table  (E-value ≤ 1.00e-03 (evalue)) | | db | id | prob | evalue | pvalue | score | cols | query | query\_len | template | template\_len | name | description | | --- | --- | --- | --- | --- | --- | --- | --- | --- | --- | --- | --- | --- | | pfam | PF05272 | 99.4 | 1.8e-17 | 4.5e-21 | 150.8 | 214 | (460, 680) | 769 | (1, 220) | 221 | VirE | Virulence-associated protein E | | pfam | PF04735 | 97.9 | 2.5e-09 | 5e-13 | 124.9 | 138 | (490, 634) | 769 | (884, 1028) | 1217 | Baculo\_helicase | Baculovirus DNA helicase | | pfam | PF00519 | 97.8 | 3.6e-09 | 8.3e-13 | 102.0 | 120 | (509, 634) | 769 | (116, 237) | 289 | PPV\_E1\_C | Papillomavirus helicase | | pfam | PF01057 | 97.8 | 4.2e-09 | 1e-12 | 99.7 | 129 | (497, 634) | 769 | (103, 241) | 284 | Parvo\_NS1 | Parvovirus non-structural protein NS1 | | pfam | PF06431 | 97.3 | 8.9e-08 | 2e-11 | 98.9 | 119 | (510, 634) | 769 | (153, 283) | 417 | Polyoma\_lg\_T\_C | Polyomavirus large T antigen C-terminus | | pfam | PF11868 | 96.4 | 6.6e-06 | 1.3e-09 | 76.9 | 76 | (495, 574) | 769 | (39, 118) | 190 | DUF3388 | Protein of unknown function (DUF3388) | | pfam | PF00910 | 95.5 | 5.9e-05 | 1.5e-08 | 54.6 | 100 | (516, 616) | 769 | (1, 100) | 100 | RNA\_helicase | RNA helicase | | pfam | PF20030 | 94.7 | 0.00023 | 5.6e-08 | 60.0 | 132 | (514, 654) | 769 | (32, 183) | 204 | bpMoxR | MoxR domain in the MoxR-vWA-beta-propeller ternary systems | | pfam | PF01078 | 94.6 | 0.00028 | 6.7e-08 | 60.5 | 24 | (514, 537) | 769 | (23, 46) | 208 | Mg\_chelatase | Magnesium chelatase, subunit ChlI | | pfam | PF07726 | 94.6 | 0.00029 | 7.3e-08 | 53.8 | 23 | (516, 538) | 769 | (2, 24) | 134 | AAA\_3 | ATPase family associated with various cellular activities (AAA) | | pfam | PF05496 | 94.5 | 0.00032 | 8e-08 | 55.0 | 116 | (514, 632) | 769 | (34, 156) | 159 | RuvB\_N | Holliday junction DNA helicase RuvB P-loop domain | | pfam | PF19263 | 94.2 | 0.00042 | 1.1e-07 | 49.9 | 95 | (518, 618) | 769 | (1, 98) | 110 | DUF5906 | Family of unknown function (DUF5906) | | pfam | PF00493 | 94.2 | 0.00044 | 1.1e-07 | 56.9 | 25 | (514, 538) | 769 | (53, 77) | 216 | MCM | MCM P-loop domain | | cath | 1q57G02 | 94.6 | 0.00026 | 6.8e-08 | 46.8 | 59 | (231, 289) | 769 | (13, 75) | 126 | Dna primase/helicase | CATHCODE: 3.40.1360.10 NAME: Dna primase/helicase. Chain: a, b, c, d, e, f, g. Engineered: yes. Mutation: yes SOURCE: Enterobacteria phage t7. Organism\_taxid: 10760. Gene: 4. Expressed in: escherichia coli. Expression\_system\_taxid: 562 CLASS: Alpha Beta, ARCH: 3-Layer(aba) Sandwich, TOPOL: Dna Topoisomerase Vi A Subunit; Chain: A, domain 2, HOMOL: Dna Topoisomerase Vi A Subunit; Chain: A, domain 2 | | phrogs | 2249 | 100.0 | 2.4e-76 | 2.8e-80 | 651.9 | 633 | (6, 749) | 769 | (46, 797) | 814 | DNA primase | DNA primase; Category: DNA, RNA and nucleotide metabolism; p352003 VI\_04181 | | phrogs | 256 | 100.0 | 9.7e-49 | 1.2e-52 | 437.6 | 294 | (439, 741) | 769 | (460, 789) | 808 | DNA helicase | DNA helicase; Category: DNA, RNA and nucleotide metabolism; NC\_028991\_p19 | | phrogs | 1313 | 100.0 | 2.6e-45 | 3.2e-49 | 394.4 | 295 | (443, 746) | 769 | (104, 436) | 460 | DNA helicase | DNA helicase; Category: DNA, RNA and nucleotide metabolism; p88668 VI\_00162 | | phrogs | 6088 | 100.0 | 1.7e-39 | 2e-43 | 353.0 | 308 | (424, 758) | 769 | (413, 753) | 754 | DNA primase | DNA primase; Category: DNA, RNA and nucleotide metabolism; p124025 VI\_05756 | | phrogs | 4913 | 99.9 | 2.8e-32 | 3.3e-36 | 305.1 | 323 | (1, 350) | 769 | (1, 332) | 1099 | replication | replication; Category: DNA, RNA and nucleotide metabolism; NC\_019722\_p45 | | phrogs | 7601 | 99.9 | 8.4e-32 | 9.8e-36 | 259.8 | 264 | (494, 765) | 769 | (8, 286) | 304 | NA | NA; Category: unknown function; p167392 VI\_00748 | | phrogs | 1156 | 99.9 | 6e-31 | 7.6e-35 | 307.2 | 304 | (426, 758) | 769 | (426, 777) | 790 | DNA primase | DNA primase; Category: DNA, RNA and nucleotide metabolism; NC\_019550\_p28 | | phrogs | 15962 | 99.9 | 1.3e-30 | 1.5e-34 | 273.3 | 303 | (440, 752) | 769 | (397, 722) | 737 | DNA primase | DNA primase; Category: DNA, RNA and nucleotide metabolism; NC\_021800\_p32 | | phrogs | 16593 | 99.9 | 1.6e-30 | 1.8e-34 | 269.5 | 358 | (380, 749) | 769 | (423, 795) | 824 | DNA primase | DNA primase; Category: DNA, RNA and nucleotide metabolism; NC\_009604\_p54 | | phrogs | 6444 | 99.9 | 6.2e-30 | 7.2e-34 | 271.5 | 283 | (6, 346) | 769 | (41, 368) | 600 | DNA primase | DNA primase; Category: DNA, RNA and nucleotide metabolism; p94528 VI\_00468 | | phrogs | 864 | 99.9 | 1.3e-29 | 1.6e-33 | 274.5 | 274 | (427, 735) | 769 | (169, 489) | 526 | DNA primase | DNA primase; Category: DNA, RNA and nucleotide metabolism; NC\_025465\_p48 | | phrogs | 510 | 99.9 | 1.5e-29 | 1.8e-33 | 266.0 | 296 | (6, 345) | 769 | (35, 367) | 389 | DNA primase | DNA primase; Category: DNA, RNA and nucleotide metabolism; p276824 VI\_04255 | | phrogs | 1139 | 99.9 | 4.2e-29 | 5.1e-33 | 281.8 | 279 | (6, 350) | 769 | (33, 318) | 890 | DNA primase | DNA primase; Category: DNA, RNA and nucleotide metabolism; p412832 VI\_12129 | | phrogs | 1883 | 99.9 | 4e-28 | 4.6e-32 | 255.9 | 310 | (425, 761) | 769 | (418, 760) | 761 | DNA helicase | DNA helicase; Category: DNA, RNA and nucleotide metabolism; p89953 VI\_04942 | | phrogs | 2478 | 99.9 | 2.9e-27 | 3.3e-31 | 253.7 | 310 | (424, 759) | 769 | (511, 853) | 873 | DNA polymerase/primase | DNA polymerase/primase; Category: DNA, RNA and nucleotide metabolism; KY087992\_p73 | | phrogs | 1829 | 99.8 | 1.1e-26 | 1.3e-30 | 261.9 | 270 | (423, 734) | 769 | (413, 698) | 785 | DNA primase | DNA primase; Category: DNA, RNA and nucleotide metabolism; MG432137\_p16 | | phrogs | 35358 | 99.8 | 7.4e-26 | 8.3e-30 | 233.4 | 220 | (135, 358) | 769 | (116, 354) | 947 | DNA primase | DNA primase; Category: DNA, RNA and nucleotide metabolism; MF663786\_p98 | | phrogs | 25777 | 99.8 | 2.2e-23 | 2.5e-27 | 214.1 | 281 | (6, 350) | 769 | (68, 354) | 905 | DNA primase | DNA primase; Category: DNA, RNA and nucleotide metabolism; NC\_025458\_p2 | | phrogs | 36873 | 99.7 | 3.1e-22 | 3.4e-26 | 203.5 | 309 | (429, 751) | 769 | (442, 757) | 1062 | DNA helicase | DNA helicase; Category: DNA, RNA and nucleotide metabolism; NC\_029032\_p60 | | phrogs | 16950 | 99.7 | 3.6e-21 | 4.1e-25 | 194.9 | 280 | (427, 734) | 769 | (480, 808) | 844 | DNA primase | DNA primase; Category: DNA, RNA and nucleotide metabolism; p371263 VI\_11800 | | phrogs | 47 | 99.6 | 1.4e-20 | 1.8e-24 | 198.5 | 256 | (8, 339) | 769 | (44, 313) | 327 | DNA primase | DNA primase; Category: DNA, RNA and nucleotide metabolism; KX925554\_p102 | | phrogs | 4630 | 99.6 | 2.8e-20 | 3.2e-24 | 188.3 | 299 | (444, 751) | 769 | (173, 522) | 579 | DNA primase | DNA primase; Category: DNA, RNA and nucleotide metabolism; KP340288\_p74 | | phrogs | 16213 | 99.3 | 4.6e-16 | 5.1e-20 | 155.3 | 325 | (428, 768) | 769 | (315, 668) | 732 | DNA primase | DNA primase; Category: DNA, RNA and nucleotide metabolism; KU530220\_p53 | | phrogs | 849 | 99.1 | 3.6e-15 | 4.2e-19 | 140.0 | 177 | (149, 346) | 769 | (29, 220) | 223 | DNA primase | DNA primase; Category: DNA, RNA and nucleotide metabolism; NC\_027625\_p30 | | phrogs | 168 | 99.1 | 7.4e-15 | 9.1e-19 | 150.1 | 173 | (497, 680) | 769 | (28, 216) | 318 | clamp loader of DNA polymerase | clamp loader of DNA polymerase; Category: DNA, RNA and nucleotide metabolism; p26368 VI\_12322 | | phrogs | 24712 | 98.7 | 2.1e-12 | 2.4e-16 | 131.2 | 220 | (376, 638) | 769 | (369, 599) | 801 | NA | NA; Category: unknown function; LC102729\_p32 | | phrogs | 4210 | 98.5 | 1.4e-11 | 1.6e-15 | 133.4 | 245 | (466, 734) | 769 | (137, 409) | 1350 | DNA polymerase | DNA polymerase; Category: DNA, RNA and nucleotide metabolism; MG757154\_p64 | | phrogs | 5629 | 98.5 | 3.3e-11 | 3.7e-15 | 122.7 | 274 | (10, 347) | 769 | (35, 351) | 930 | DNA primase | DNA primase; Category: DNA, RNA and nucleotide metabolism; DQ087285\_p11 | | phrogs | 4762 | 97.7 | 1.5e-08 | 1.7e-12 | 99.8 | 212 | (382, 632) | 769 | (404, 624) | 822 | NA | NA; Category: unknown function; p277771 VI\_05561 | | phrogs | 239 | 97.4 | 8.5e-08 | 1.1e-11 | 104.5 | 164 | (148, 342) | 769 | (47, 218) | 543 | DNA primase/helicase | DNA primase/helicase; Category: DNA, RNA and nucleotide metabolism; NC\_031029\_p115 | | phrogs | 5846 | 97.4 | 9.1e-08 | 1.1e-11 | 94.3 | 119 | (495, 638) | 769 | (25, 154) | 306 | clamp loader of DNA polymerase | clamp loader of DNA polymerase; Category: DNA, RNA and nucleotide metabolism; KY606587\_p99 | | phrogs | 36509 | 97.2 | 2.6e-07 | 3e-11 | 94.6 | 259 | (426, 706) | 769 | (516, 829) | 1021 | DNA primase | DNA primase; Category: DNA, RNA and nucleotide metabolism; KU234533\_p69 | | phrogs | 3757 | 97.1 | 6.3e-07 | 7.5e-11 | 89.6 | 129 | (509, 652) | 769 | (128, 274) | 379 | NA | NA; Category: unknown function; p40711 VI\_00882 | | phrogs | 11992 | 97.1 | 6.5e-07 | 7.6e-11 | 78.3 | 60 | (6, 81) | 769 | (33, 93) | 166 | NA | NA; Category: unknown function; p110551 VI\_01281 | | phrogs | 19785 | 97.0 | 9.3e-07 | 1e-10 | 88.2 | 156 | (468, 638) | 769 | (428, 588) | 753 | DNA polymerase/primase | DNA polymerase/primase; Category: DNA, RNA and nucleotide metabolism; NC\_019420\_p20 | | phrogs | 7485 | 96.7 | 3.4e-06 | 3.9e-10 | 84.3 | 62 | (509, 572) | 769 | (215, 280) | 408 | NA | NA; Category: unknown function; NC\_019406\_p235 | | phrogs | 11838 | 96.3 | 1.3e-05 | 1.5e-09 | 77.7 | 62 | (510, 572) | 769 | (42, 114) | 413 | clamp loader of DNA polymerase | clamp loader of DNA polymerase; Category: DNA, RNA and nucleotide metabolism; p389573 VI\_12493 | | phrogs | 9172 | 95.5 | 0.00013 | 1.5e-08 | 73.4 | 134 | (495, 636) | 769 | (82, 239) | 518 | NA | NA; Category: unknown function; KX607102\_p48 | | phrogs | 30773 | 94.9 | 0.00038 | 4.3e-08 | 67.7 | 102 | (509, 622) | 769 | (176, 300) | 647 | NA | NA; Category: unknown function; p438992 VI\_07698 | | phrogs | 25243 | 94.9 | 0.0004 | 4.6e-08 | 68.9 | 118 | (513, 632) | 769 | (230, 354) | 619 | DNA primase | DNA primase; Category: DNA, RNA and nucleotide metabolism; NC\_019516\_p159 | | phrogs | 16448 | 94.8 | 0.00045 | 5.2e-08 | 62.5 | 121 | (512, 636) | 769 | (26, 169) | 246 | ATPase | ATPase; Category: other; KU886274\_p16 | | phrogs | 10401 | 94.8 | 0.00046 | 5.4e-08 | 67.8 | 115 | (512, 638) | 769 | (198, 330) | 385 | ATPase | ATPase; Category: other; MG189906\_p41 | | phrogs | 14322 | 94.6 | 0.00062 | 7.1e-08 | 64.6 | 114 | (512, 638) | 769 | (194, 321) | 366 | NA | NA; Category: unknown function; KU521356\_p250 | |
| Top keywords  (threshold 1.00e-03 (evalue)) | **DNA, RNA, and, nucleotide, metabolism, primase, helicase, polymerase, A, ATPase** |
| Output files | ../../domain\_architecture/54\_FANPEZAQ\_CDS\_0054\_cath.hhr ../../domain\_architecture/54\_FANPEZAQ\_CDS\_0054\_merged.svg ../../domain\_architecture/54\_FANPEZAQ\_CDS\_0054\_ncbi-cd.hhr ../../domain\_architecture/54\_FANPEZAQ\_CDS\_0054\_pfam.hhr ../../domain\_architecture/54\_FANPEZAQ\_CDS\_0054\_phrogs.hhr |

### Identical protein sequences/structures

#### Search results

|  |  |
| --- | --- |
| Protein sequence databases searched | Pdb, Swissprot, Refseq |
| Identical proteins found | -- |
| Top keywords | -- |
| Output files | -- |

### Similar protein sequences/structures

#### Sequence similarity search results (HHblits)1

|  |  |
| --- | --- |
| Sequence databases searched | Uniclust, Pdb70 |
| Results, scheme(s)  (Top layers only, threshold 1.00e-03 (evalue)) | xml version="1.0" encoding="utf-8" standalone="no"?       2024-09-02T21:08:52.456549 image/svg+xml   Matplotlib v3.7.2, https://matplotlib.org/ |
| Results, table(s)  (threshold 1.00e-03 (evalue)) | | db | id | prob | evalue | pvalue | score | cols | query | query\_len | template | template\_len | name | description | | --- | --- | --- | --- | --- | --- | --- | --- | --- | --- | --- | --- | --- | | uniclust | UniRef100\_A0A073ISL6 | 100.0 | 2e-108 | 5e-114 | 879.9 | 728 | (14, 762) | 769 | (49, 798) | 813 | Uncharacterized protein | Uncharacterized protein | | uniclust | UniRef100\_A0A158S187 | 100.0 | 2.6e-96 | 5e-102 | 756.6 | 728 | (14, 754) | 769 | (26, 767) | 779 | DNA primase domain protein | DNA primase domain protein | | uniclust | UniRef100\_A0A024HDH8 | 100.0 | 1.9e-92 | 3.6e-98 | 754.5 | 687 | (5, 748) | 769 | (10, 733) | 767 | Virulence-associated E family protein | Virulence-associated E family protein | | uniclust | UniRef100\_A0A1G3JY92 | 100.0 | 5.4e-92 | 1e-97 | 731.9 | 679 | (16, 739) | 769 | (19, 737) | 778 | Uncharacterized protein | Uncharacterized protein | | uniclust | UniRef100\_A0A2G2PN86 | 100.0 | 7.3e-90 | 1.4e-95 | 705.2 | 702 | (5, 756) | 769 | (4, 753) | 769 | Toprim domain-containing protein | Toprim domain-containing protein | | uniclust | UniRef100\_A0A4V2HU15 | 100.0 | 7.9e-82 | 1.5e-87 | 650.5 | 711 | (14, 750) | 769 | (29, 994) | 1004 | Uncharacterized protein | Uncharacterized protein | | uniclust | UniRef100\_A0A349EG17 | 100.0 | 4.2e-77 | 7.8e-83 | 593.8 | 669 | (9, 754) | 769 | (3, 689) | 694 | Toprim domain-containing protein | Toprim domain-containing protein | | uniclust | UniRef100\_A0A080LT75 | 100.0 | 4.7e-73 | 9.1e-79 | 615.4 | 683 | (14, 755) | 769 | (32, 804) | 831 | DNA primase TraC | DNA primase TraC | | uniclust | UniRef100\_A0A345ANS2 | 100.0 | 2e-66 | 3.7e-72 | 543.8 | 693 | (37, 769) | 769 | (60, 936) | 936 | Putative primase | Putative primase | | uniclust | UniRef100\_A0A159Z405 | 100.0 | 2.3e-66 | 4.4e-72 | 534.6 | 582 | (135, 742) | 769 | (173, 868) | 902 | Virulence-associated protein E | Virulence-associated protein E | | uniclust | UniRef100\_A0A016XJH4 | 100.0 | 1.2e-64 | 2.3e-70 | 546.5 | 652 | (55, 744) | 769 | (77, 804) | 837 | Conjugal transfer protein TraC | Conjugal transfer protein TraC | | uniclust | UniRef100\_A0A151FI48 | 100.0 | 1.1e-63 | 2.2e-69 | 524.7 | 684 | (10, 752) | 769 | (17, 804) | 828 | Zinc finger CHC2-type domain-containing protein | Zinc finger CHC2-type domain-containing protein | | uniclust | UniRef100\_A0A158EA01 | 100.0 | 6.2e-61 | 1.2e-66 | 487.7 | 517 | (233, 768) | 769 | (191, 726) | 727 | Virulence-associated protein E | Virulence-associated protein E | | uniclust | UniRef100\_A0A094ZFQ5 | 100.0 | 9.8e-61 | 1.9e-66 | 494.3 | 496 | (231, 756) | 769 | (164, 681) | 699 | Virulence-associated E-like domain-containing protein | Virulence-associated E-like domain-containing protein | | uniclust | UniRef100\_A0A1U9VG46 | 100.0 | 1.4e-60 | 2.6e-66 | 497.4 | 559 | (180, 751) | 769 | (150, 772) | 810 | Virulence-associated E-like domain-containing protein | Virulence-associated E-like domain-containing protein | | uniclust | UniRef100\_A0A2G4RGB3 | 100.0 | 1.5e-58 | 2.8e-64 | 469.5 | 436 | (277, 741) | 769 | (28, 482) | 516 | Virulence-associated E family protein | Virulence-associated E family protein | | uniclust | UniRef100\_A0A962X8E3 | 100.0 | 1.9e-58 | 3.5e-64 | 456.6 | 550 | (177, 752) | 769 | (13, 612) | 633 | Toprim domain-containing protein (Fragment) | Toprim domain-containing protein (Fragment) | | uniclust | UniRef100\_A0A1D2U957 | 100.0 | 3e-58 | 5.8e-64 | 463.7 | 384 | (368, 754) | 769 | (78, 461) | 505 | Virulence-associated E-like domain-containing protein | Virulence-associated E-like domain-containing protein | | uniclust | UniRef100\_A0A059N520 | 100.0 | 2.2e-57 | 4.3e-63 | 482.4 | 359 | (372, 741) | 769 | (217, 590) | 642 | Virulence-associated protein E | Virulence-associated protein E | | uniclust | UniRef100\_A0A096HFV3 | 100.0 | 2.5e-57 | 4.8e-63 | 484.6 | 665 | (2, 735) | 769 | (2, 748) | 863 | DUF5906 domain-containing protein | DUF5906 domain-containing protein | | uniclust | UniRef100\_A0A0J6VXK3 | 100.0 | 2e-56 | 3.9e-62 | 461.8 | 367 | (371, 752) | 769 | (139, 522) | 563 | Virulence-associated E family protein | Virulence-associated E family protein | | uniclust | UniRef100\_UPI00196A8CA5 | 100.0 | 1.1e-55 | 2e-61 | 445.8 | 676 | (48, 755) | 769 | (36, 766) | 771 | VapE family protein | VapE family protein | | uniclust | UniRef100\_A0A0E3K4D7 | 100.0 | 1e-54 | 1.9e-60 | 426.2 | 368 | (378, 753) | 769 | (48, 448) | 464 | Virulence-associated E family protein | Virulence-associated E family protein | | uniclust | UniRef100\_A0A094YPP6 | 100.0 | 3.5e-54 | 6.8e-60 | 470.0 | 359 | (373, 746) | 769 | (380, 754) | 775 | Virulence-associated E | Virulence-associated E | | uniclust | UniRef100\_A0A2M8YCJ3 | 100.0 | 5.5e-54 | 1e-59 | 425.7 | 390 | (365, 754) | 769 | (156, 545) | 575 | Virulence-associated protein E | Virulence-associated protein E | | uniclust | UniRef100\_A0A551Z1W1 | 100.0 | 7e-54 | 1.3e-59 | 444.9 | 481 | (233, 753) | 769 | (171, 673) | 804 | Virulence-associated E-like domain-containing protein | Virulence-associated E-like domain-containing protein | | uniclust | UniRef100\_A0A0A2WCR2 | 100.0 | 8.3e-54 | 1.6e-59 | 433.2 | 353 | (372, 741) | 769 | (82, 443) | 498 | Phage associated DNA primase | Phage associated DNA primase | | uniclust | UniRef100\_UPI000DE53851 | 100.0 | 1.3e-53 | 2.4e-59 | 398.0 | 482 | (13, 503) | 769 | (13, 494) | 496 | PriCT-2 domain-containing protein | PriCT-2 domain-containing protein | | uniclust | UniRef100\_A0A1H1ILW6 | 100.0 | 3.2e-53 | 5.9e-59 | 402.3 | 542 | (31, 619) | 769 | (19, 579) | 581 | Putative DNA primase/helicase | Putative DNA primase/helicase | | uniclust | UniRef100\_A0A258SSH2 | 100.0 | 3.2e-53 | 6e-59 | 411.6 | 386 | (371, 757) | 769 | (15, 400) | 420 | Virulence-associated E-like domain-containing protein (Fragment) | Virulence-associated E-like domain-containing protein (Fragment) | | uniclust | UniRef100\_A0A1E5XQN8 | 100.0 | 3.3e-53 | 6.2e-59 | 430.5 | 466 | (233, 741) | 769 | (147, 623) | 679 | Virulence-associated E-like domain-containing protein | Virulence-associated E-like domain-containing protein | | uniclust | UniRef100\_A0A0J7XW03 | 100.0 | 3.3e-53 | 6.3e-59 | 431.5 | 381 | (373, 755) | 769 | (147, 529) | 558 | Virulence-associated E-like domain-containing protein | Virulence-associated E-like domain-containing protein | | uniclust | UniRef100\_A0A023WUM9 | 100.0 | 1.1e-52 | 2e-58 | 440.4 | 677 | (10, 735) | 769 | (52, 877) | 956 | DNA primase | DNA primase | | uniclust | UniRef100\_A0A090TZ23 | 100.0 | 1.2e-52 | 2.2e-58 | 436.5 | 687 | (14, 749) | 769 | (14, 816) | 834 | DNA primase phage-associated | DNA primase phage-associated | | uniclust | UniRef100\_A0A2Z5ME49 | 100.0 | 1.7e-52 | 3.3e-58 | 416.8 | 373 | (378, 758) | 769 | (160, 533) | 559 | Virulence-associated E family protein | Virulence-associated E family protein | | uniclust | UniRef100\_A0A1Z4GW22 | 100.0 | 2.6e-52 | 4.9e-58 | 442.7 | 477 | (232, 742) | 769 | (159, 650) | 933 | Virulence-associated E family protein | Virulence-associated E family protein | | uniclust | UniRef100\_A0A013UG67 | 100.0 | 3.4e-52 | 6.7e-58 | 461.8 | 600 | (100, 736) | 769 | (101, 815) | 888 | Toprim domain protein | Toprim domain protein | | uniclust | UniRef100\_A0A0T7BQB0 | 100.0 | 4.4e-52 | 8.4e-58 | 442.7 | 477 | (231, 743) | 769 | (173, 666) | 908 | Uncharacterized protein | Uncharacterized protein | | uniclust | UniRef100\_A0A0N8K9E5 | 100.0 | 4.6e-52 | 8.7e-58 | 415.6 | 371 | (372, 751) | 769 | (62, 458) | 497 | Putative P-loop ATPase | Putative P-loop ATPase | | uniclust | UniRef100\_A0A0Q6QE44 | 100.0 | 5.2e-52 | 9.8e-58 | 425.5 | 374 | (374, 756) | 769 | (195, 590) | 667 | Virulence protein E | Virulence protein E | | uniclust | UniRef100\_A0A099GLM4 | 100.0 | 6.4e-52 | 1.2e-57 | 416.5 | 582 | (136, 743) | 769 | (192, 903) | 944 | p-loop ATPase | p-loop ATPase | | uniclust | UniRef100\_A0A2N3DYE7 | 100.0 | 9.3e-52 | 1.8e-57 | 444.1 | 343 | (393, 749) | 769 | (345, 699) | 726 | DNA primase/polymerase bifunctional N-terminal domain-containing protein | DNA primase/polymerase bifunctional N-terminal domain-containing protein | | uniclust | UniRef100\_A0A2T1EHM2 | 100.0 | 2.2e-51 | 4.2e-57 | 417.9 | 488 | (231, 758) | 769 | (150, 661) | 676 | Virulence-associated E-like domain-containing protein | Virulence-associated E-like domain-containing protein | | uniclust | UniRef100\_A0A1M5W1E5 | 100.0 | 5.7e-51 | 1.1e-56 | 430.7 | 637 | (14, 697) | 769 | (24, 767) | 933 | Phage/plasmid primase, P4 family, C-terminal domain-containing protein | Phage/plasmid primase, P4 family, C-terminal domain-containing protein | | uniclust | UniRef100\_A0A0S7XZ70 | 100.0 | 2.1e-50 | 4e-56 | 431.2 | 564 | (14, 637) | 769 | (61, 727) | 965 | Toprim domain-containing protein | Toprim domain-containing protein | | uniclust | UniRef100\_A0A068MV01 | 100.0 | 2.3e-50 | 4.4e-56 | 443.9 | 483 | (233, 753) | 769 | (160, 666) | 903 | Uncharacterized protein | Uncharacterized protein | | uniclust | UniRef100\_A0A060H485 | 100.0 | 2.8e-50 | 5.3e-56 | 402.1 | 360 | (372, 742) | 769 | (105, 495) | 535 | Virulence factor | Virulence factor | | uniclust | UniRef100\_A0A317J441 | 100.0 | 3.6e-50 | 6.8e-56 | 402.6 | 501 | (233, 760) | 769 | (158, 716) | 731 | Uncharacterized protein | Uncharacterized protein | | uniclust | UniRef100\_A0A0F9U4J5 | 100.0 | 3.9e-50 | 7.4e-56 | 419.5 | 379 | (371, 755) | 769 | (355, 756) | 777 | DNA primase/polymerase bifunctional N-terminal domain-containing protein | DNA primase/polymerase bifunctional N-terminal domain-containing protein | | uniclust | UniRef100\_A0A0W7WDM8 | 100.0 | 5.7e-50 | 1.1e-55 | 419.7 | 370 | (372, 753) | 769 | (383, 764) | 771 | Virulence-associated E-like domain-containing protein | Virulence-associated E-like domain-containing protein | | uniclust | UniRef100\_A0A014ML09 | 100.0 | 8.4e-50 | 1.6e-55 | 419.9 | 516 | (219, 742) | 769 | (223, 837) | 903 | Virulence protein E | Virulence protein E | | uniclust | UniRef100\_A0A031IMY7 | 100.0 | 9.2e-50 | 1.8e-55 | 423.1 | 372 | (373, 753) | 769 | (380, 773) | 824 | Phage/plasmid primase, P4 family | Phage/plasmid primase, P4 family | | uniclust | UniRef100\_A0A0D0QS22 | 100.0 | 1.3e-49 | 2.4e-55 | 406.5 | 357 | (372, 739) | 769 | (193, 595) | 639 | DNA primase, phage associated | DNA primase, phage associated | | uniclust | UniRef100\_A0A0H4XB71 | 100.0 | 1.6e-49 | 3.2e-55 | 394.5 | 308 | (437, 753) | 769 | (23, 338) | 380 | DNA primase domain protein | DNA primase domain protein | | uniclust | UniRef100\_A0A017N0X3 | 100.0 | 1.7e-49 | 3.4e-55 | 441.4 | 356 | (371, 738) | 769 | (408, 808) | 878 | Virulence-associated E family protein | Virulence-associated E family protein | | uniclust | UniRef100\_UPI001FCAD0C1 | 100.0 | 2.2e-49 | 4e-55 | 389.3 | 683 | (14, 750) | 769 | (15, 805) | 826 | phage/plasmid primase, P4 family | phage/plasmid primase, P4 family | | uniclust | UniRef100\_A0A4P7L7L6 | 100.0 | 2.4e-49 | 4.3e-55 | 387.3 | 575 | (130, 743) | 769 | (107, 701) | 713 | DNA primase TraC | DNA primase TraC | | uniclust | UniRef100\_A0A078S3U0 | 100.0 | 2.5e-49 | 4.7e-55 | 394.7 | 368 | (377, 755) | 769 | (92, 472) | 511 | Virulence-associated E family protein (Fragment) | Virulence-associated E family protein (Fragment) | | uniclust | UniRef100\_A0A158DR21 | 100.0 | 3.6e-49 | 6.9e-55 | 426.9 | 478 | (14, 589) | 769 | (46, 535) | 858 | TOPRIM domain-containing protein | TOPRIM domain-containing protein | | uniclust | UniRef100\_A0A109LYF1 | 100.0 | 3.7e-49 | 7.1e-55 | 424.8 | 365 | (367, 742) | 769 | (390, 759) | 817 | Virulence-associated E-like domain-containing protein | Virulence-associated E-like domain-containing protein | | uniclust | UniRef100\_A0A061P292 | 100.0 | 3.7e-49 | 7.2e-55 | 404.7 | 353 | (375, 737) | 769 | (43, 451) | 502 | DNA primase | DNA primase | | uniclust | UniRef100\_A0A0D6MP65 | 100.0 | 3.8e-49 | 7.3e-55 | 409.6 | 366 | (370, 750) | 769 | (333, 714) | 721 | Virulence-associated E family protein | Virulence-associated E family protein | | uniclust | UniRef100\_A0A945RBT7 | 100.0 | 4e-49 | 7.4e-55 | 386.6 | 653 | (14, 747) | 769 | (44, 785) | 805 | Toprim domain-containing protein | Toprim domain-containing protein | | uniclust | UniRef100\_A0A096BB79 | 100.0 | 4.9e-49 | 9.4e-55 | 409.8 | 355 | (370, 738) | 769 | (129, 508) | 618 | Virulence-associated E-like domain-containing protein | Virulence-associated E-like domain-containing protein | | uniclust | UniRef100\_A0A0F9W6J4 | 100.0 | 8.3e-49 | 1.6e-54 | 382.9 | 355 | (372, 741) | 769 | (85, 455) | 475 | Virulence-associated E-like domain-containing protein | Virulence-associated E-like domain-containing protein | | uniclust | UniRef100\_A0A0D6Q293 | 100.0 | 8.6e-49 | 1.6e-54 | 393.5 | 359 | (367, 737) | 769 | (58, 440) | 486 | DNA primase | DNA primase | | uniclust | UniRef100\_A0A651E934 | 100.0 | 9.3e-49 | 1.8e-54 | 403.6 | 470 | (233, 741) | 769 | (134, 617) | 803 | DUF3854 domain-containing protein | DUF3854 domain-containing protein | | uniclust | UniRef100\_A0A084J7F2 | 100.0 | 1.3e-48 | 2.5e-54 | 401.9 | 488 | (231, 757) | 769 | (187, 693) | 730 | Zinc finger CHC2-type domain-containing protein | Zinc finger CHC2-type domain-containing protein | | uniclust | UniRef100\_A0A0T2QCS5 | 100.0 | 1.8e-48 | 3.4e-54 | 381.3 | 369 | (374, 755) | 769 | (39, 408) | 431 | Virulence-associated E-like domain-containing protein | Virulence-associated E-like domain-containing protein | | uniclust | UniRef100\_A0A1U7IAQ8 | 100.0 | 2e-48 | 3.8e-54 | 409.5 | 483 | (231, 756) | 769 | (159, 665) | 933 | Virulence-associated E-like domain-containing protein | Virulence-associated E-like domain-containing protein | | uniclust | UniRef100\_A0A0K0WP96 | 100.0 | 3.3e-48 | 6.1e-54 | 386.0 | 525 | (177, 740) | 769 | (111, 661) | 773 | Uncharacterized protein | Uncharacterized protein | | uniclust | UniRef100\_A0A076Z3T2 | 100.0 | 3.2e-48 | 6.3e-54 | 400.0 | 317 | (372, 696) | 769 | (52, 383) | 500 | Predicted P-loop ATPase and inactivated derivatives | Predicted P-loop ATPase and inactivated derivatives | | uniclust | UniRef100\_A0A0P1IL64 | 100.0 | 3.4e-48 | 6.6e-54 | 415.6 | 381 | (366, 756) | 769 | (408, 799) | 829 | Putative P-loop ATPase | Putative P-loop ATPase | | uniclust | UniRef100\_A0A1I1CGG0 | 100.0 | 1.1e-47 | 2.1e-53 | 374.2 | 366 | (377, 754) | 769 | (8, 389) | 439 | Virulence-associated protein E (Fragment) | Virulence-associated protein E (Fragment) | | uniclust | UniRef100\_A0A2V5PJC8 | 100.0 | 1.7e-47 | 3.2e-53 | 401.6 | 656 | (12, 735) | 769 | (8, 763) | 837 | DUF5906 domain-containing protein | DUF5906 domain-containing protein | | uniclust | UniRef100\_A0A017RTM7 | 100.0 | 3.1e-47 | 6.1e-53 | 419.7 | 356 | (371, 737) | 769 | (421, 824) | 891 | Virulence-associated protein E | Virulence-associated protein E | | uniclust | UniRef100\_A0A2H9UPD3 | 100.0 | 4.7e-47 | 8.9e-53 | 386.4 | 390 | (367, 758) | 769 | (357, 753) | 760 | Primase C-terminal 2 domain-containing protein | Primase C-terminal 2 domain-containing protein | | uniclust | UniRef100\_J9ZDI3 | 100.0 | 5.6e-47 | 1e-52 | 375.7 | 528 | (177, 741) | 769 | (95, 646) | 747 | Putative P-loop ATPase | Putative P-loop ATPase | | uniclust | UniRef100\_A0A094SVM7 | 100.0 | 6.8e-47 | 1.3e-52 | 392.9 | 556 | (150, 757) | 769 | (159, 774) | 802 | Helicase | Helicase | | uniclust | UniRef100\_A0A015SLF0 | 100.0 | 7e-47 | 1.4e-52 | 416.2 | 366 | (378, 755) | 769 | (408, 783) | 822 | Virulence-associated E family protein | Virulence-associated E family protein | | uniclust | UniRef100\_A0A0C1QT32 | 100.0 | 8.8e-47 | 1.7e-52 | 365.0 | 288 | (14, 345) | 769 | (18, 305) | 314 | Toprim domain-containing protein | Toprim domain-containing protein | | uniclust | UniRef100\_UPI0003678CFF | 100.0 | 1.2e-46 | 2.1e-52 | 378.5 | 685 | (14, 760) | 769 | (14, 845) | 867 | DUF5906 domain-containing protein | DUF5906 domain-containing protein | | uniclust | UniRef100\_A0A1H8FZ14 | 100.0 | 1.2e-46 | 2.3e-52 | 388.3 | 369 | (376, 753) | 769 | (456, 842) | 903 | Virulence-associated protein E | Virulence-associated protein E | | uniclust | UniRef100\_A0A0R3NCG4 | 100.0 | 1.3e-46 | 2.4e-52 | 383.1 | 346 | (384, 741) | 769 | (196, 549) | 594 | Virulence-associated E-like domain-containing protein | Virulence-associated E-like domain-containing protein | | uniclust | UniRef100\_A0A6J5LA04 | 100.0 | 1.3e-46 | 2.5e-52 | 383.6 | 396 | (366, 767) | 769 | (388, 784) | 786 | Virulence-associated E | Virulence-associated E | | uniclust | UniRef100\_A0A076P157 | 100.0 | 1.9e-46 | 3.7e-52 | 395.1 | 363 | (379, 755) | 769 | (108, 475) | 518 | Virulence-associated protein E | Virulence-associated protein E | | uniclust | UniRef100\_A0A3G9FYK3 | 100.0 | 2e-46 | 3.8e-52 | 370.3 | 387 | (366, 756) | 769 | (193, 585) | 591 | Putative virulence-associated protein E | Putative virulence-associated protein E | | uniclust | UniRef100\_A0A132Z4M9 | 100.0 | 2.1e-46 | 4.1e-52 | 377.4 | 313 | (378, 697) | 769 | (40, 366) | 508 | Helicase | Helicase | | uniclust | UniRef100\_A0A1F9M777 | 100.0 | 2.2e-46 | 4.3e-52 | 375.1 | 296 | (14, 349) | 769 | (86, 382) | 429 | Toprim domain-containing protein | Toprim domain-containing protein | | uniclust | UniRef100\_A0A251W7E6 | 100.0 | 2.4e-46 | 4.6e-52 | 380.2 | 336 | (386, 741) | 769 | (228, 571) | 612 | Virulence-associated E-like domain-containing protein | Virulence-associated E-like domain-containing protein | | uniclust | UniRef100\_A0A7C2DBA8 | 100.0 | 2.7e-46 | 5e-52 | 374.7 | 345 | (391, 752) | 769 | (557, 917) | 920 | Zinc finger CHC2-type domain-containing protein | Zinc finger CHC2-type domain-containing protein | | uniclust | UniRef100\_A0A2H2X2H4 | 100.0 | 3.1e-46 | 5.9e-52 | 386.0 | 470 | (233, 741) | 769 | (187, 675) | 869 | Virulence-associated E family protein | Virulence-associated E family protein | | uniclust | UniRef100\_A0A1W7AEU1 | 100.0 | 5e-46 | 9.4e-52 | 388.7 | 356 | (374, 741) | 769 | (324, 721) | 742 | Virulence-associated protein E | Virulence-associated protein E | | uniclust | UniRef100\_A0A085TW06 | 100.0 | 5.3e-46 | 9.9e-52 | 382.0 | 365 | (373, 752) | 769 | (414, 795) | 805 | Uncharacterized protein | Uncharacterized protein | | uniclust | UniRef100\_A0A1N7PIB5 | 100.0 | 5.4e-46 | 1e-51 | 381.9 | 365 | (374, 753) | 769 | (339, 720) | 728 | Primase C terminal 2 (PriCT-2) | Primase C terminal 2 (PriCT-2) | | uniclust | UniRef100\_A0A031JL01 | 100.0 | 7.1e-46 | 1.4e-51 | 377.8 | 285 | (14, 340) | 769 | (69, 354) | 402 | Toprim domain-containing protein | Toprim domain-containing protein | | uniclust | UniRef100\_A0A6L3T504 | 100.0 | 7.8e-46 | 1.4e-51 | 366.4 | 698 | (14, 750) | 769 | (16, 863) | 871 | SF3 helicase domain-containing protein | SF3 helicase domain-containing protein | | uniclust | UniRef100\_A0A0P1I0V3 | 100.0 | 1.2e-45 | 2.3e-51 | 383.1 | 340 | (380, 738) | 769 | (406, 767) | 815 | Putative P-loop ATPase | Putative P-loop ATPase | | uniclust | UniRef100\_A0A838CHG7 | 100.0 | 1.3e-45 | 2.5e-51 | 353.7 | 410 | (324, 751) | 769 | (12, 469) | 498 | Virulence protein E | Virulence protein E | | uniclust | UniRef100\_A0A015VAS0 | 100.0 | 1.5e-45 | 2.9e-51 | 380.4 | 356 | (379, 741) | 769 | (80, 443) | 497 | Virulence-associated E family protein | Virulence-associated E family protein | | uniclust | UniRef100\_A0A014P2B8 | 100.0 | 1.5e-45 | 2.9e-51 | 396.9 | 332 | (3, 349) | 769 | (12, 347) | 991 | Membrane protein | Membrane protein | | uniclust | UniRef100\_A0A374DQX8 | 100.0 | 1.7e-45 | 3.2e-51 | 387.0 | 348 | (379, 739) | 769 | (315, 683) | 731 | Virulence-associated E-like domain-containing protein | Virulence-associated E-like domain-containing protein | | uniclust | UniRef100\_A0A0N7LN66 | 100.0 | 2e-45 | 3.9e-51 | 387.0 | 357 | (371, 739) | 769 | (400, 775) | 812 | Putative P-loop ATPase | Putative P-loop ATPase | | uniclust | UniRef100\_A0A317N8C0 | 100.0 | 2.1e-45 | 4.1e-51 | 388.9 | 599 | (14, 637) | 769 | (27, 693) | 870 | Phage/plasmid primase-like uncharacterized protein | Phage/plasmid primase-like uncharacterized protein | | uniclust | UniRef100\_A0A158DVE4 | 100.0 | 2.2e-45 | 4.3e-51 | 409.4 | 316 | (14, 351) | 769 | (57, 382) | 994 | Inner membrane protein | Inner membrane protein | | uniclust | UniRef100\_A0A4R5Q8H9 | 100.0 | 2.6e-45 | 4.8e-51 | 373.1 | 361 | (372, 750) | 769 | (350, 729) | 753 | Virulence-associated E-like domain-containing protein | Virulence-associated E-like domain-containing protein | | uniclust | UniRef100\_A0A0Q9YMA6 | 100.0 | 2.5e-45 | 4.9e-51 | 404.9 | 290 | (14, 350) | 769 | (47, 336) | 946 | DNA primase TraC | DNA primase TraC | | uniclust | UniRef100\_A0A2M7EZU9 | 100.0 | 2.6e-45 | 5e-51 | 361.9 | 350 | (378, 740) | 769 | (68, 444) | 469 | Virulence-associated E-like domain-containing protein | Virulence-associated E-like domain-containing protein | | uniclust | UniRef100\_A0A078R7F8 | 100.0 | 4.2e-45 | 8.2e-51 | 397.6 | 372 | (377, 756) | 769 | (337, 717) | 748 | Virulence-associated E family protein | Virulence-associated E family protein | | uniclust | UniRef100\_A0A034T3J5 | 100.0 | 4.5e-45 | 8.6e-51 | 393.3 | 479 | (132, 656) | 769 | (139, 662) | 829 | Toprim domain protein | Toprim domain protein | | uniclust | UniRef100\_A0A0C9PJH3 | 100.0 | 5.6e-45 | 1.1e-50 | 406.9 | 483 | (233, 754) | 769 | (195, 759) | 898 | ATPase | ATPase | | uniclust | UniRef100\_A0A442XE73 | 100.0 | 6e-45 | 1.1e-50 | 348.3 | 295 | (434, 737) | 769 | (59, 388) | 420 | Virulence-associated E-like domain-containing protein | Virulence-associated E-like domain-containing protein | | uniclust | UniRef100\_A0A1G3DUL0 | 100.0 | 9.6e-45 | 1.9e-50 | 361.6 | 283 | (14, 343) | 769 | (44, 326) | 361 | Toprim domain-containing protein (Fragment) | Toprim domain-containing protein (Fragment) | | uniclust | UniRef100\_A0A139JUI2 | 100.0 | 1.1e-44 | 2e-50 | 363.4 | 370 | (377, 755) | 769 | (111, 492) | 525 | Virulence-associated E-like domain-containing protein | Virulence-associated E-like domain-containing protein | | uniclust | UniRef100\_A0A1B0Z1F4 | 100.0 | 1.1e-44 | 2e-50 | 384.0 | 357 | (376, 735) | 769 | (528, 897) | 962 | Virulence-associated E-like domain-containing protein | Virulence-associated E-like domain-containing protein | | uniclust | UniRef100\_A0A078BQ56 | 100.0 | 1.5e-44 | 2.9e-50 | 357.8 | 290 | (14, 349) | 769 | (46, 335) | 393 | Toprim domain-containing protein | Toprim domain-containing protein | | uniclust | UniRef100\_A0A010SRZ9 | 100.0 | 3.2e-44 | 6.1e-50 | 349.2 | 264 | (31, 335) | 769 | (60, 324) | 354 | Topoisomerase | Topoisomerase | | uniclust | UniRef100\_UPI000CFF6687 | 100.0 | 5.2e-44 | 9.6e-50 | 360.8 | 590 | (14, 638) | 769 | (16, 650) | 817 | DUF5906 domain-containing protein | DUF5906 domain-containing protein | | uniclust | UniRef100\_A0A5C7M7G3 | 100.0 | 5.6e-44 | 1.1e-49 | 370.6 | 355 | (375, 741) | 769 | (293, 654) | 720 | Uncharacterized protein | Uncharacterized protein | | uniclust | UniRef100\_A0A6M4GU76 | 100.0 | 6.1e-44 | 1.1e-49 | 348.5 | 668 | (16, 737) | 769 | (22, 717) | 745 | Uncharacterized protein | Uncharacterized protein | | uniclust | UniRef100\_A0A1U7MHB1 | 100.0 | 8.2e-44 | 1.6e-49 | 371.1 | 382 | (371, 759) | 769 | (319, 716) | 738 | Virulence-associated protein E | Virulence-associated protein E | | uniclust | UniRef100\_A0A5K7Y2P6 | 100.0 | 9.8e-44 | 1.8e-49 | 333.8 | 372 | (367, 741) | 769 | (121, 493) | 505 | Virulence-associated E-like domain-containing protein | Virulence-associated E-like domain-containing protein | | uniclust | UniRef100\_A0A015V8N0 | 100.0 | 9.5e-44 | 1.9e-49 | 390.0 | 367 | (382, 759) | 769 | (425, 801) | 826 | VirE N-terminal domain protein | VirE N-terminal domain protein | | uniclust | UniRef100\_A0A176F820 | 100.0 | 1e-43 | 2e-49 | 389.4 | 362 | (370, 740) | 769 | (448, 875) | 925 | Virulence-associated E-like domain-containing protein | Virulence-associated E-like domain-containing protein | | uniclust | UniRef100\_A0A3A8HXS5 | 100.0 | 1.5e-43 | 2.9e-49 | 358.2 | 370 | (376, 754) | 769 | (351, 739) | 775 | DNA primase | DNA primase | | uniclust | UniRef100\_A0A3G8X3W9 | 100.0 | 1.7e-43 | 3.3e-49 | 355.3 | 366 | (378, 755) | 769 | (156, 529) | 561 | Virulence-associated e family protein | Virulence-associated e family protein | | uniclust | UniRef100\_A0A0A1VBE9 | 100.0 | 2e-43 | 4e-49 | 359.6 | 273 | (14, 333) | 769 | (76, 348) | 413 | Toprim domain-containing protein | Toprim domain-containing protein | | uniclust | UniRef100\_A0A6N6T5K1 | 100.0 | 2.2e-43 | 4.3e-49 | 375.2 | 311 | (14, 351) | 769 | (27, 353) | 903 | Toprim domain-containing protein | Toprim domain-containing protein | | uniclust | UniRef100\_UPI001C268BC4 | 100.0 | 2.8e-43 | 5.2e-49 | 335.7 | 355 | (391, 754) | 769 | (32, 402) | 452 | virulence-associated E family protein | virulence-associated E family protein | | uniclust | UniRef100\_A0A011VM22 | 100.0 | 4e-43 | 7.6e-49 | 361.7 | 359 | (366, 739) | 769 | (344, 709) | 765 | Virulence protein E | Virulence protein E | | uniclust | UniRef100\_A0A1A5HIC1 | 100.0 | 4.3e-43 | 8.3e-49 | 367.3 | 476 | (233, 755) | 769 | (240, 724) | 763 | Toprim domain-containing protein | Toprim domain-containing protein | | uniclust | UniRef100\_A0A374J606 | 100.0 | 6.1e-43 | 1.1e-48 | 350.7 | 464 | (218, 714) | 769 | (147, 646) | 725 | Virulence-associated E-like domain-containing protein | Virulence-associated E-like domain-containing protein | | uniclust | UniRef100\_A0A241VC66 | 100.0 | 6.7e-43 | 1.2e-48 | 336.3 | 432 | (324, 756) | 769 | (194, 629) | 636 | Virulence-associated E-like domain-containing protein | Virulence-associated E-like domain-containing protein | | uniclust | UniRef100\_A0A011PNF2 | 100.0 | 6.6e-43 | 1.3e-48 | 382.3 | 292 | (14, 350) | 769 | (70, 362) | 877 | DNA primase TraC | DNA primase TraC | | uniclust | UniRef100\_A0A059XSZ9 | 100.0 | 7.4e-43 | 1.4e-48 | 383.9 | 485 | (234, 756) | 769 | (187, 753) | 869 | DNA primase | DNA primase | | uniclust | UniRef100\_A0A1G7N5S7 | 100.0 | 1e-42 | 1.9e-48 | 346.7 | 300 | (3, 319) | 769 | (29, 370) | 411 | Putative DNA primase/helicase | Putative DNA primase/helicase | | uniclust | UniRef100\_A0A0Q4IXU5 | 100.0 | 1.1e-42 | 2.1e-48 | 368.4 | 362 | (369, 739) | 769 | (432, 859) | 897 | Uncharacterized protein | Uncharacterized protein | | uniclust | UniRef100\_A0A5Q0BKD1 | 100.0 | 1.1e-42 | 2.1e-48 | 335.6 | 316 | (8, 348) | 769 | (15, 337) | 367 | Toprim domain-containing protein | Toprim domain-containing protein | | uniclust | UniRef100\_A0A071MGH5 | 100.0 | 1.7e-42 | 3.4e-48 | 387.0 | 322 | (12, 350) | 769 | (64, 390) | 1076 | DUF927 domain-containing protein | DUF927 domain-containing protein | | uniclust | UniRef100\_A0A0C3IBC7 | 100.0 | 2.1e-42 | 4e-48 | 355.8 | 370 | (375, 757) | 769 | (126, 519) | 537 | Virulence-associated E-like domain-containing protein | Virulence-associated E-like domain-containing protein | | uniclust | UniRef100\_A0A2S7FDM4 | 100.0 | 2.4e-42 | 4.7e-48 | 349.3 | 373 | (375, 756) | 769 | (85, 470) | 509 | Virulence-associated E-like domain-containing protein | Virulence-associated E-like domain-containing protein | | uniclust | UniRef100\_A0A0P6WTJ1 | 100.0 | 3.3e-42 | 6.2e-48 | 341.4 | 339 | (386, 742) | 769 | (132, 480) | 501 | Virulence-associated E-like domain-containing protein | Virulence-associated E-like domain-containing protein | | uniclust | UniRef100\_A0A069D3U0 | 100.0 | 3.4e-42 | 6.5e-48 | 338.1 | 365 | (382, 758) | 769 | (50, 424) | 434 | Putative helicase | Putative helicase | | uniclust | UniRef100\_A0A1C4C0F2 | 100.0 | 3.7e-42 | 6.9e-48 | 353.8 | 465 | (127, 637) | 769 | (110, 619) | 698 | Putative DNA primase/helicase | Putative DNA primase/helicase | | uniclust | UniRef100\_A0A6H1Z9Z7 | 100.0 | 3.9e-42 | 7.4e-48 | 351.8 | 356 | (377, 742) | 769 | (378, 746) | 778 | Putative VirE domain containing protein | Putative VirE domain containing protein | | uniclust | UniRef100\_A0A1I9YDF5 | 100.0 | 4.7e-42 | 8.6e-48 | 334.7 | 674 | (12, 749) | 769 | (6, 716) | 725 | PriCT-2 domain-containing protein | PriCT-2 domain-containing protein | | uniclust | UniRef100\_A0A7C5A2E1 | 100.0 | 4.7e-42 | 8.8e-48 | 339.3 | 462 | (231, 741) | 769 | (172, 643) | 665 | Virulence-associated E-like domain-containing protein | Virulence-associated E-like domain-containing protein | | uniclust | UniRef100\_A0A2G0Q1H7 | 100.0 | 5.7e-42 | 1.1e-47 | 339.8 | 371 | (371, 752) | 769 | (179, 567) | 583 | ATPase | ATPase | | uniclust | UniRef100\_A0A127SEK4 | 100.0 | 7.6e-42 | 1.4e-47 | 307.8 | 315 | (437, 755) | 769 | (3, 317) | 321 | Virulence-associated protein E (Fragment) | Virulence-associated protein E (Fragment) | | uniclust | UniRef100\_A0A068R0U5 | 100.0 | 7.3e-42 | 1.4e-47 | 369.0 | 363 | (370, 742) | 769 | (360, 736) | 776 | Predicted P-loop ATPase and inactivated derivatives | Predicted P-loop ATPase and inactivated derivatives | | uniclust | UniRef100\_A0A1H4GIG7 | 100.0 | 9.1e-42 | 1.7e-47 | 342.7 | 365 | (383, 758) | 769 | (179, 553) | 574 | Virulence-associated protein E | Virulence-associated protein E | | uniclust | UniRef100\_A0A0B2JZB9 | 100.0 | 1.4e-41 | 2.7e-47 | 348.7 | 371 | (379, 758) | 769 | (332, 713) | 728 | Virulence-associated E-like domain-containing protein | Virulence-associated E-like domain-containing protein | | uniclust | UniRef100\_A0A0R1VXQ4 | 100.0 | 1.8e-41 | 3.4e-47 | 319.1 | 310 | (379, 698) | 769 | (40, 370) | 375 | Prophage Lp3 protein 8, helicase | Prophage Lp3 protein 8, helicase | | uniclust | UniRef100\_A0A255MSC6 | 100.0 | 1.9e-41 | 3.6e-47 | 333.8 | 371 | (372, 751) | 769 | (73, 453) | 460 | Virulence-associated E-like domain-containing protein | Virulence-associated E-like domain-containing protein | | uniclust | UniRef100\_A0A0P1FRF1 | 100.0 | 2e-41 | 3.8e-47 | 353.0 | 356 | (375, 738) | 769 | (351, 748) | 776 | Putative P-loop ATPase | Putative P-loop ATPase | | uniclust | UniRef100\_A0A433VJU3 | 100.0 | 2e-41 | 3.8e-47 | 347.1 | 357 | (385, 761) | 769 | (327, 694) | 812 | Virulence-associated E-like domain-containing protein | Virulence-associated E-like domain-containing protein | | uniclust | UniRef100\_A0A3M0XHA9 | 100.0 | 2.5e-41 | 4.7e-47 | 318.3 | 266 | (466, 741) | 769 | (1, 273) | 344 | Virulence-associated E-like domain-containing protein (Fragment) | Virulence-associated E-like domain-containing protein (Fragment) | | uniclust | UniRef100\_A0A6J5P9W1 | 100.0 | 2.7e-41 | 5e-47 | 324.8 | 488 | (233, 742) | 769 | (68, 590) | 625 | Virulence-associated E | Virulence-associated E | | uniclust | UniRef100\_UPI0007164CDC | 100.0 | 3.1e-41 | 5.6e-47 | 330.7 | 663 | (16, 740) | 769 | (56, 746) | 763 | VapE family protein | VapE family protein | | uniclust | UniRef100\_A0A0X3TJ34 | 100.0 | 2.9e-41 | 5.6e-47 | 337.1 | 306 | (14, 334) | 769 | (28, 343) | 363 | Toprim domain-containing protein | Toprim domain-containing protein | | uniclust | UniRef100\_A0A5C7W4R6 | 100.0 | 3.2e-41 | 6e-47 | 319.1 | 387 | (366, 755) | 769 | (140, 529) | 535 | Virulence-associated E family protein | Virulence-associated E family protein | | uniclust | UniRef100\_A0A1F4HLP3 | 100.0 | 4.8e-41 | 9e-47 | 349.2 | 321 | (14, 350) | 769 | (13, 336) | 747 | Toprim domain-containing protein (Fragment) | Toprim domain-containing protein (Fragment) | | uniclust | UniRef100\_A0A095Y5Z3 | 100.0 | 5.2e-41 | 9.9e-47 | 339.4 | 366 | (383, 760) | 769 | (101, 486) | 522 | Virulence-associated E-like domain-containing protein | Virulence-associated E-like domain-containing protein | | uniclust | UniRef100\_A0A378IRY1 | 100.0 | 6.6e-41 | 1.2e-46 | 346.1 | 682 | (14, 742) | 769 | (16, 792) | 812 | 5' DNA primase TraC | 5' DNA primase TraC | | uniclust | UniRef100\_A0A059PAJ6 | 100.0 | 6.8e-41 | 1.3e-46 | 346.5 | 353 | (372, 737) | 769 | (104, 479) | 519 | Putative DNA helicase | Putative DNA helicase | | uniclust | UniRef100\_A0A097EN21 | 100.0 | 7.2e-41 | 1.4e-46 | 327.8 | 268 | (14, 337) | 769 | (49, 316) | 330 | DNA primase | DNA primase | | uniclust | UniRef100\_A0A679K914 | 100.0 | 8.3e-41 | 1.6e-46 | 332.0 | 352 | (373, 738) | 769 | (108, 479) | 525 | Virulence-associated E-like domain-containing protein | Virulence-associated E-like domain-containing protein | | uniclust | UniRef100\_A0A0D0NW01 | 100.0 | 8.8e-41 | 1.7e-46 | 358.5 | 530 | (177, 742) | 769 | (233, 862) | 914 | DNA primase | DNA primase | | uniclust | UniRef100\_A0A1M6YKQ7 | 100.0 | 1.1e-40 | 2.1e-46 | 347.7 | 290 | (14, 348) | 769 | (111, 401) | 804 | Uncharacterized domain associated with phage/plasmid primase | Uncharacterized domain associated with phage/plasmid primase | | uniclust | UniRef100\_A0A1I2NF75 | 100.0 | 1.2e-40 | 2.2e-46 | 339.1 | 336 | (391, 741) | 769 | (364, 713) | 733 | Primase C terminal 2 (PriCT-2) | Primase C terminal 2 (PriCT-2) | | uniclust | UniRef100\_A0A0B6RPX2 | 100.0 | 1.1e-40 | 2.3e-46 | 380.7 | 324 | (11, 350) | 769 | (130, 454) | 1165 | Uncharacterized protein | Uncharacterized protein | | uniclust | UniRef100\_A0A0B3W190 | 100.0 | 1.3e-40 | 2.4e-46 | 338.8 | 375 | (366, 757) | 769 | (154, 536) | 575 | Zinc finger CHC2-type domain-containing protein | Zinc finger CHC2-type domain-containing protein | | uniclust | UniRef100\_A0A0R1TQE1 | 100.0 | 1.7e-40 | 3.2e-46 | 326.9 | 309 | (380, 697) | 769 | (73, 394) | 489 | Virulence-associated E-like domain-containing protein | Virulence-associated E-like domain-containing protein | | uniclust | UniRef100\_A0A086PE93 | 100.0 | 1.8e-40 | 3.5e-46 | 351.8 | 691 | (4, 740) | 769 | (2, 1020) | 1075 | DNA primase | DNA primase | | uniclust | UniRef100\_A0A846FJ73 | 100.0 | 3.2e-40 | 5.9e-46 | 333.5 | 478 | (233, 754) | 769 | (154, 652) | 826 | DUF3854 domain-containing protein | DUF3854 domain-containing protein | | uniclust | UniRef100\_A0A174SAF4 | 100.0 | 3.3e-40 | 6.3e-46 | 316.5 | 304 | (370, 682) | 769 | (47, 370) | 403 | Predicted P-loop ATPase and inactivated derivatives | Predicted P-loop ATPase and inactivated derivatives | | uniclust | UniRef100\_A0A069SD21 | 100.0 | 3.9e-40 | 7.5e-46 | 339.2 | 367 | (379, 757) | 769 | (121, 497) | 534 | Uncharacterized protein | Uncharacterized protein | | uniclust | UniRef100\_A0A7C1H7I6 | 100.0 | 4.3e-40 | 7.9e-46 | 316.9 | 292 | (14, 350) | 769 | (53, 344) | 523 | Toprim domain-containing protein | Toprim domain-containing protein | | uniclust | UniRef100\_A0A0C1Y5L2 | 100.0 | 4.1e-40 | 8e-46 | 343.2 | 278 | (469, 755) | 769 | (223, 511) | 546 | Virulence-associated E-like domain-containing protein | Virulence-associated E-like domain-containing protein | | uniclust | UniRef100\_A0A1X7JKC4 | 100.0 | 4.5e-40 | 8.5e-46 | 321.5 | 352 | (371, 741) | 769 | (161, 521) | 546 | Virulence-associated protein E | Virulence-associated protein E | | uniclust | UniRef100\_A0A1Q2T5M1 | 100.0 | 4.8e-40 | 9.1e-46 | 315.8 | 255 | (435, 697) | 769 | (13, 273) | 379 | Virulence-associated protein E | Virulence-associated protein E | | uniclust | UniRef100\_A0A0B6RVH3 | 100.0 | 7.5e-40 | 1.4e-45 | 346.7 | 314 | (11, 350) | 769 | (12, 328) | 814 | Uncharacterized protein | Uncharacterized protein | | uniclust | UniRef100\_A0A1H0TMV8 | 100.0 | 8.1e-40 | 1.5e-45 | 353.1 | 567 | (14, 636) | 769 | (26, 676) | 917 | Putative DNA primase/helicase | Putative DNA primase/helicase | | uniclust | UniRef100\_A0A142BA72 | 100.0 | 1.2e-39 | 2.4e-45 | 339.7 | 287 | (14, 346) | 769 | (32, 318) | 693 | Putative primase-helicase | Putative primase-helicase | | uniclust | UniRef100\_A0A0F9PAW5 | 100.0 | 1.4e-39 | 2.6e-45 | 324.3 | 365 | (375, 752) | 769 | (306, 687) | 696 | DNA primase/polymerase bifunctional N-terminal domain-containing protein | DNA primase/polymerase bifunctional N-terminal domain-containing protein | | uniclust | UniRef100\_A0A0M7MF94 | 100.0 | 2.1e-39 | 3.9e-45 | 340.3 | 375 | (370, 754) | 769 | (377, 771) | 792 | Predicted P-loop ATPase and inactivated derivatives | Predicted P-loop ATPase and inactivated derivatives | | uniclust | UniRef100\_A0A148KLT5 | 100.0 | 2.2e-39 | 4.1e-45 | 328.5 | 378 | (376, 756) | 769 | (464, 843) | 849 | Virulence-associated E-like domain-containing protein | Virulence-associated E-like domain-containing protein | | uniclust | UniRef100\_A0A174V3L9 | 100.0 | 2.4e-39 | 4.5e-45 | 308.6 | 352 | (372, 734) | 769 | (23, 394) | 457 | Predicted P-loop ATPase and inactivated derivatives | Predicted P-loop ATPase and inactivated derivatives | | uniclust | UniRef100\_A0A1E2USX5 | 100.0 | 2.4e-39 | 4.7e-45 | 348.4 | 285 | (14, 344) | 769 | (31, 316) | 721 | Toprim domain-containing protein | Toprim domain-containing protein | | uniclust | UniRef100\_A0A0D5Y4M5 | 100.0 | 2.6e-39 | 5e-45 | 306.0 | 264 | (32, 333) | 769 | (22, 286) | 301 | Topoisomerase | Topoisomerase | | uniclust | UniRef100\_A0A081B651 | 100.0 | 3.3e-39 | 6.3e-45 | 350.4 | 299 | (14, 349) | 769 | (420, 723) | 811 | DNA primase TraC | DNA primase TraC | | uniclust | UniRef100\_A0A095ZAT3 | 100.0 | 3.8e-39 | 7.1e-45 | 319.7 | 320 | (371, 697) | 769 | (141, 474) | 612 | Virulence-associated E family protein | Virulence-associated E family protein | | uniclust | UniRef100\_A0A2T7SVQ2 | 100.0 | 4.2e-39 | 7.8e-45 | 314.5 | 366 | (366, 737) | 769 | (342, 707) | 725 | Virulence-associated E-like domain-containing protein | Virulence-associated E-like domain-containing protein | | uniclust | UniRef100\_A0A239KTG6 | 100.0 | 4.4e-39 | 8.2e-45 | 317.3 | 350 | (372, 740) | 769 | (288, 653) | 672 | Predicted P-loop ATPase and inactivated derivatives | Predicted P-loop ATPase and inactivated derivatives | | uniclust | UniRef100\_A0A2E5HC47 | 100.0 | 6e-39 | 1.1e-44 | 338.0 | 344 | (383, 742) | 769 | (405, 759) | 783 | Virulence-associated E-like domain-containing protein | Virulence-associated E-like domain-containing protein | | uniclust | UniRef100\_A0A1Z8N2Q3 | 100.0 | 6.6e-39 | 1.2e-44 | 317.9 | 347 | (377, 740) | 769 | (360, 714) | 731 | Uncharacterized protein | Uncharacterized protein | | uniclust | UniRef100\_A0A1G9GZG2 | 100.0 | 7.5e-39 | 1.4e-44 | 309.6 | 348 | (379, 741) | 769 | (124, 487) | 514 | Virulence-associated protein E | Virulence-associated protein E | | uniclust | UniRef100\_A0A1C5MBB1 | 100.0 | 8.2e-39 | 1.6e-44 | 328.5 | 373 | (376, 758) | 769 | (302, 689) | 717 | Predicted P-loop ATPase and inactivated derivatives | Predicted P-loop ATPase and inactivated derivatives | | uniclust | UniRef100\_A0A095YT29 | 100.0 | 8.8e-39 | 1.7e-44 | 340.3 | 321 | (4, 347) | 769 | (3, 346) | 921 | Toprim domain-containing protein | Toprim domain-containing protein | | uniclust | UniRef100\_A0A934ECD9 | 100.0 | 9e-39 | 1.7e-44 | 314.7 | 293 | (14, 350) | 769 | (12, 305) | 551 | AAA family ATPase | AAA family ATPase | | uniclust | UniRef100\_A0A968S1F8 | 100.0 | 9.2e-39 | 1.7e-44 | 327.4 | 473 | (233, 739) | 769 | (212, 698) | 961 | Virulence-associated E-like domain-containing protein | Virulence-associated E-like domain-containing protein | | uniclust | UniRef100\_R6WKB3 | 100.0 | 9.4e-39 | 1.8e-44 | 311.0 | 364 | (381, 755) | 769 | (44, 417) | 448 | VirE N-terminal domain protein | VirE N-terminal domain protein | | uniclust | UniRef100\_A0A2N2S409 | 100.0 | 9.8e-39 | 1.8e-44 | 301.8 | 350 | (6, 402) | 769 | (3, 354) | 395 | Toprim domain-containing protein (Fragment) | Toprim domain-containing protein (Fragment) | | uniclust | UniRef100\_A0A2Z5WWH0 | 100.0 | 9.9e-39 | 1.9e-44 | 314.1 | 333 | (390, 742) | 769 | (60, 403) | 431 | Virulence-associated E | Virulence-associated E | | uniclust | UniRef100\_A0A0A2B4H4 | 100.0 | 1e-38 | 1.9e-44 | 312.6 | 357 | (381, 755) | 769 | (66, 437) | 455 | Virulence-associated E-like domain-containing protein | Virulence-associated E-like domain-containing protein | | uniclust | UniRef100\_A0A089PBP6 | 100.0 | 1.1e-38 | 2e-44 | 320.3 | 361 | (380, 756) | 769 | (88, 464) | 561 | DNA primase | DNA primase | | uniclust | UniRef100\_A0A081RVY4 | 100.0 | 1.2e-38 | 2.2e-44 | 329.8 | 362 | (370, 742) | 769 | (377, 753) | 780 | Putative P-loop ATPase | Putative P-loop ATPase | | uniclust | UniRef100\_A0A0G0A8B1 | 100.0 | 1.1e-38 | 2.2e-44 | 342.6 | 363 | (382, 757) | 769 | (339, 711) | 731 | Virulence-associated E family protein | Virulence-associated E family protein | | uniclust | UniRef100\_A0A0B7MHW3 | 100.0 | 1.1e-38 | 2.3e-44 | 346.4 | 322 | (2, 349) | 769 | (20, 383) | 638 | DNA primase | DNA primase | | uniclust | UniRef100\_A0A069S6V9 | 100.0 | 1.5e-38 | 2.8e-44 | 301.1 | 298 | (450, 757) | 769 | (11, 315) | 322 | Uncharacterized protein | Uncharacterized protein | | uniclust | UniRef100\_A0A059ZR08 | 100.0 | 1.8e-38 | 3.5e-44 | 339.3 | 484 | (231, 755) | 769 | (206, 762) | 805 | DNA primase/helicase, phage-associated | DNA primase/helicase, phage-associated | | uniclust | UniRef100\_A0A088F8A2 | 100.0 | 1.8e-38 | 3.5e-44 | 331.9 | 273 | (14, 336) | 769 | (31, 304) | 706 | Putative primase-helicase | Putative primase-helicase | | uniclust | UniRef100\_A0A6M8VRM0 | 100.0 | 1.9e-38 | 3.6e-44 | 285.5 | 276 | (448, 739) | 769 | (3, 282) | 300 | Virulence-associated E family protein | Virulence-associated E family protein | | uniclust | UniRef100\_A0A1F9Y098 | 100.0 | 2e-38 | 3.8e-44 | 330.4 | 287 | (14, 348) | 769 | (12, 300) | 878 | Toprim domain-containing protein | Toprim domain-containing protein | | uniclust | UniRef100\_A0A2I8A4T1 | 100.0 | 2.4e-38 | 4.5e-44 | 332.9 | 344 | (383, 743) | 769 | (353, 714) | 754 | Virulence-associated E | Virulence-associated E | | uniclust | UniRef100\_A0A1B9LY80 | 100.0 | 2.5e-38 | 4.6e-44 | 318.5 | 456 | (129, 625) | 769 | (115, 607) | 610 | Bacteriophage T7 Gp4 DNA primase/helicase N-terminal domain-containing protein | Bacteriophage T7 Gp4 DNA primase/helicase N-terminal domain-containing protein | | uniclust | UniRef100\_A0A0H5Q210 | 100.0 | 2.8e-38 | 5.4e-44 | 333.5 | 289 | (14, 348) | 769 | (37, 326) | 661 | Toprim domain-containing protein | Toprim domain-containing protein | | uniclust | UniRef100\_UPI000469875C | 100.0 | 3.3e-38 | 6.2e-44 | 283.9 | 227 | (451, 686) | 769 | (4, 236) | 247 | virulence-associated E family protein | virulence-associated E family protein | | uniclust | UniRef100\_A0A1I5CW14 | 100.0 | 3.8e-38 | 7.2e-44 | 317.1 | 366 | (371, 746) | 769 | (218, 598) | 614 | Predicted P-loop ATPase and inactivated derivatives | Predicted P-loop ATPase and inactivated derivatives | | uniclust | UniRef100\_A0A1K1LFZ9 | 100.0 | 3.9e-38 | 7.4e-44 | 337.6 | 297 | (14, 350) | 769 | (53, 353) | 971 | DNA primase, phage-associated | DNA primase, phage-associated | | uniclust | UniRef100\_A0A2C8EXD6 | 100.0 | 4.4e-38 | 8.2e-44 | 315.5 | 354 | (377, 741) | 769 | (380, 755) | 779 | DNA primase/polymerase bifunctional N-terminal domain-containing protein | DNA primase/polymerase bifunctional N-terminal domain-containing protein | | uniclust | UniRef100\_A0A6H9GBR6 | 100.0 | 5.9e-38 | 1.1e-43 | 311.3 | 327 | (393, 742) | 769 | (177, 517) | 550 | Virulence-associated E-like domain-containing protein | Virulence-associated E-like domain-containing protein | | uniclust | UniRef100\_A0A1F3BA57 | 100.0 | 6.3e-38 | 1.2e-43 | 343.8 | 322 | (1, 350) | 769 | (54, 419) | 676 | DNA primase | DNA primase | | uniclust | UniRef100\_A0A075KDN3 | 100.0 | 6.4e-38 | 1.3e-43 | 342.0 | 324 | (2, 350) | 769 | (51, 417) | 676 | DNA primase | DNA primase | | uniclust | UniRef100\_A0A024GWJ1 | 100.0 | 7.9e-38 | 1.5e-43 | 339.7 | 558 | (150, 742) | 769 | (166, 826) | 886 | Phage/plasmid primase, P4 family, C-terminal domain protein | Phage/plasmid primase, P4 family, C-terminal domain protein | | uniclust | UniRef100\_A0A1Z4I5E7 | 100.0 | 8.3e-38 | 1.6e-43 | 321.6 | 343 | (391, 756) | 769 | (337, 690) | 814 | Virulence-associated E | Virulence-associated E | | uniclust | UniRef100\_A0A011NP97 | 100.0 | 1e-37 | 1.9e-43 | 335.9 | 291 | (14, 348) | 769 | (42, 338) | 860 | DNA primase TraC | DNA primase TraC | | uniclust | UniRef100\_UPI001C2DDBBB | 100.0 | 1.1e-37 | 2e-43 | 282.9 | 255 | (433, 696) | 769 | (32, 294) | 312 | virulence-associated E family protein | virulence-associated E family protein | | uniclust | UniRef100\_A0A0B8WUT7 | 100.0 | 1.3e-37 | 2.6e-43 | 340.4 | 323 | (2, 349) | 769 | (25, 391) | 644 | DNA primase | DNA primase | | uniclust | UniRef100\_A0A1V5XFC1 | 100.0 | 1.4e-37 | 2.7e-43 | 305.6 | 351 | (371, 738) | 769 | (115, 483) | 520 | Virulence-associated protein E | Virulence-associated protein E | | uniclust | UniRef100\_UPI00163E2698 | 100.0 | 1.5e-37 | 2.7e-43 | 304.5 | 521 | (16, 585) | 769 | (11, 648) | 648 | VapE family protein | VapE family protein | | uniclust | UniRef100\_A0A135YTI2 | 100.0 | 1.8e-37 | 3.4e-43 | 285.3 | 281 | (373, 659) | 769 | (40, 336) | 340 | Virulence-associated protein E | Virulence-associated protein E | | uniclust | UniRef100\_A0A916EP12 | 100.0 | 1.8e-37 | 3.4e-43 | 318.4 | 383 | (372, 759) | 769 | (539, 934) | 962 | Virulence-associated E-like domain-containing protein | Virulence-associated E-like domain-containing protein | | uniclust | UniRef100\_A0A1F5A0V8 | 100.0 | 1.7e-37 | 3.4e-43 | 338.0 | 322 | (2, 349) | 769 | (54, 418) | 681 | DNA primase | DNA primase | | uniclust | UniRef100\_UPI0022AFBBF4 | 100.0 | 2.1e-37 | 3.8e-43 | 302.3 | 384 | (369, 757) | 769 | (316, 700) | 707 | VapE family protein | VapE family protein | | uniclust | UniRef100\_A0A1H8DHD7 | 100.0 | 2.2e-37 | 4.1e-43 | 324.0 | 369 | (379, 756) | 769 | (277, 657) | 662 | Virulence-associated protein E | Virulence-associated protein E | | uniclust | UniRef100\_A0A6M0JXK6 | 100.0 | 2.4e-37 | 4.4e-43 | 317.1 | 658 | (14, 695) | 769 | (92, 869) | 995 | SF3 helicase domain-containing protein | SF3 helicase domain-containing protein | | uniclust | UniRef100\_A0A4Y4D005 | 100.0 | 2.5e-37 | 4.6e-43 | 312.3 | 382 | (377, 759) | 769 | (440, 822) | 836 | DNA primase/polymerase bifunctional N-terminal domain-containing protein | DNA primase/polymerase bifunctional N-terminal domain-containing protein | | uniclust | UniRef100\_A0A1H0PAA2 | 100.0 | 2.5e-37 | 4.8e-43 | 325.2 | 297 | (14, 348) | 769 | (437, 734) | 790 | Antirestriction protein ArdC | Antirestriction protein ArdC | | uniclust | UniRef100\_A0A1H4CGI7 | 100.0 | 2.5e-37 | 4.9e-43 | 341.1 | 359 | (381, 753) | 769 | (378, 742) | 790 | Primase C terminal 2 (PriCT-2) | Primase C terminal 2 (PriCT-2) | | uniclust | UniRef100\_A0A136K0K7 | 100.0 | 2.7e-37 | 5.1e-43 | 307.0 | 322 | (2, 350) | 769 | (1, 366) | 441 | DNA primase catalytic subunit | DNA primase catalytic subunit | | uniclust | UniRef100\_A0A081C5E7 | 100.0 | 3e-37 | 5.9e-43 | 342.1 | 322 | (2, 350) | 769 | (64, 427) | 711 | DNA primase | DNA primase | | uniclust | UniRef100\_A0A1E1GE69 | 100.0 | 3.3e-37 | 6.1e-43 | 310.7 | 387 | (368, 764) | 769 | (399, 787) | 794 | Putative primase | Putative primase | | uniclust | UniRef100\_A0A1H7JV05 | 100.0 | 4.3e-37 | 8.2e-43 | 294.7 | 243 | (487, 736) | 769 | (2, 278) | 332 | Virulence-associated protein E | Virulence-associated protein E | | uniclust | UniRef100\_A0A0K2WEP3 | 100.0 | 4.5e-37 | 8.8e-43 | 332.2 | 310 | (14, 350) | 769 | (29, 383) | 647 | DNA primase | DNA primase | | uniclust | UniRef100\_A0A1L5KF83 | 100.0 | 4.8e-37 | 9.3e-43 | 289.3 | 240 | (450, 695) | 769 | (4, 254) | 280 | NHLM\_micro\_ABC2: NHLM bacteriocin system ABC transporter, ATP-binding (Fragment) | NHLM\_micro\_ABC2: NHLM bacteriocin system ABC transporter, ATP-binding (Fragment) | | uniclust | UniRef100\_A0A0F9HLD4 | 100.0 | 5.2e-37 | 1e-42 | 329.6 | 322 | (3, 350) | 769 | (52, 417) | 657 | Toprim domain-containing protein (Fragment) | Toprim domain-containing protein (Fragment) | | uniclust | UniRef100\_A0A315D5W5 | 100.0 | 5.7e-37 | 1.1e-42 | 304.1 | 374 | (377, 754) | 769 | (350, 726) | 728 | DNA primase/polymerase bifunctional N-terminal domain-containing protein | DNA primase/polymerase bifunctional N-terminal domain-containing protein | | uniclust | UniRef100\_A0A1G7B4P0 | 100.0 | 5.8e-37 | 1.1e-42 | 314.6 | 287 | (14, 348) | 769 | (31, 321) | 686 | Uncharacterized domain associated with phage/plasmid primase | Uncharacterized domain associated with phage/plasmid primase | | uniclust | UniRef100\_A0A062Y2K0 | 100.0 | 5.6e-37 | 1.1e-42 | 335.8 | 311 | (13, 350) | 769 | (101, 455) | 732 | DNA primase | DNA primase | | uniclust | UniRef100\_A0A0F5Y7I5 | 100.0 | 6.7e-37 | 1.2e-42 | 312.8 | 331 | (391, 741) | 769 | (326, 673) | 866 | Virulence-associated E-like domain-containing protein | Virulence-associated E-like domain-containing protein | | uniclust | UniRef100\_A0A8S0YIP5 | 100.0 | 7e-37 | 1.3e-42 | 299.6 | 508 | (230, 757) | 769 | (169, 699) | 732 | Virulence-associated E-like domain-containing protein | Virulence-associated E-like domain-containing protein | | uniclust | UniRef100\_A0A1E7I3V3 | 100.0 | 6.6e-37 | 1.3e-42 | 333.1 | 318 | (7, 349) | 769 | (60, 420) | 670 | DNA primase | DNA primase | | uniclust | UniRef100\_A0A352X4W4 | 100.0 | 8.1e-37 | 1.5e-42 | 307.3 | 486 | (233, 750) | 769 | (283, 823) | 1004 | Toprim domain-containing protein | Toprim domain-containing protein | | uniclust | UniRef100\_A0A1G0RDT0 | 100.0 | 7.9e-37 | 1.5e-42 | 296.6 | 373 | (379, 755) | 769 | (55, 450) | 471 | Virulence-associated E-like domain-containing protein | Virulence-associated E-like domain-containing protein | | uniclust | UniRef100\_A0A2N1CUR9 | 100.0 | 8.5e-37 | 1.6e-42 | 289.4 | 384 | (368, 754) | 769 | (107, 496) | 528 | Virulence-associated E family protein | Virulence-associated E family protein | | uniclust | UniRef100\_A0A0P9CJD9 | 100.0 | 8.8e-37 | 1.8e-42 | 338.7 | 322 | (3, 349) | 769 | (47, 411) | 668 | DNA primase | DNA primase | | uniclust | UniRef100\_A0A1F4ANV0 | 100.0 | 1e-36 | 1.9e-42 | 316.8 | 380 | (372, 756) | 769 | (364, 789) | 814 | Virulence-associated E-like domain-containing protein | Virulence-associated E-like domain-containing protein | | uniclust | UniRef100\_A0A5B2V8U9 | 100.0 | 1.1e-36 | 2e-42 | 290.1 | 294 | (434, 742) | 769 | (243, 541) | 553 | Virulence-associated E family protein | Virulence-associated E family protein | | uniclust | UniRef100\_A0A2H2XUZ8 | 100.0 | 1.1e-36 | 2.1e-42 | 308.9 | 337 | (383, 741) | 769 | (233, 579) | 755 | Virulence-associated E-like domain-containing protein | Virulence-associated E-like domain-containing protein | | uniclust | UniRef100\_A0A061N9C7 | 100.0 | 1.1e-36 | 2.2e-42 | 338.3 | 323 | (2, 350) | 769 | (73, 441) | 723 | DNA primase | DNA primase | | uniclust | UniRef100\_A0A1Y4HHS4 | 100.0 | 1.2e-36 | 2.3e-42 | 302.0 | 364 | (380, 755) | 769 | (147, 529) | 547 | Uncharacterized protein | Uncharacterized protein | | uniclust | UniRef100\_A0A269PIH2 | 100.0 | 1.3e-36 | 2.4e-42 | 276.2 | 274 | (376, 650) | 769 | (43, 316) | 316 | Virulence-associated E-like domain-containing protein | Virulence-associated E-like domain-containing protein | | uniclust | UniRef100\_A0A2X3IEU8 | 100.0 | 1.4e-36 | 2.6e-42 | 318.6 | 481 | (231, 753) | 769 | (193, 765) | 792 | Phage/plasmid primase, P4 family | Phage/plasmid primase, P4 family | | uniclust | UniRef100\_A0A357A780 | 100.0 | 1.9e-36 | 3.5e-42 | 302.6 | 360 | (377, 757) | 769 | (327, 700) | 918 | Uncharacterized protein | Uncharacterized protein | | uniclust | UniRef100\_UPI0009B50754 | 100.0 | 2e-36 | 3.7e-42 | 299.1 | 486 | (232, 738) | 769 | (229, 785) | 808 | virulence-associated E family protein | virulence-associated E family protein | | uniclust | UniRef100\_A0A3B0ME56 | 100.0 | 2e-36 | 3.8e-42 | 295.4 | 352 | (373, 740) | 769 | (321, 685) | 705 | Virulence-associated E-like domain-containing protein | Virulence-associated E-like domain-containing protein | | uniclust | UniRef100\_A0A133YEF9 | 100.0 | 2e-36 | 3.9e-42 | 324.6 | 315 | (8, 350) | 769 | (60, 417) | 681 | DNA primase | DNA primase | | uniclust | UniRef100\_UPI00203AF1B9 | 100.0 | 2.4e-36 | 4.3e-42 | 295.4 | 468 | (231, 734) | 769 | (156, 654) | 716 | VapE family protein | VapE family protein | | uniclust | UniRef100\_A0A0E3MC62 | 100.0 | 2.3e-36 | 4.4e-42 | 315.1 | 274 | (370, 655) | 769 | (382, 672) | 673 | Virulence-associated E family protein | Virulence-associated E family protein | | uniclust | UniRef100\_A0A0R1U9K6 | 100.0 | 2.6e-36 | 4.9e-42 | 300.2 | 309 | (378, 694) | 769 | (139, 462) | 584 | Prophage Lp3 protein 8, helicase | Prophage Lp3 protein 8, helicase | | uniclust | UniRef100\_A0A073IRN6 | 100.0 | 2.7e-36 | 5.2e-42 | 320.3 | 316 | (5, 351) | 769 | (22, 379) | 617 | DNA primase | DNA primase | | uniclust | UniRef100\_A0A095XT20 | 100.0 | 2.7e-36 | 5.2e-42 | 288.4 | 282 | (470, 755) | 769 | (58, 345) | 376 | Virulence-associated protein E (Fragment) | Virulence-associated protein E (Fragment) | | uniclust | UniRef100\_A0A010NL17 | 100.0 | 2.6e-36 | 5.2e-42 | 328.8 | 320 | (3, 350) | 769 | (39, 398) | 644 | DNA primase | DNA primase | | uniclust | UniRef100\_A0A2E0AGP3 | 100.0 | 2.8e-36 | 5.3e-42 | 289.9 | 351 | (389, 753) | 769 | (51, 413) | 424 | Virulence-associated E-like domain-containing protein | Virulence-associated E-like domain-containing protein | | uniclust | UniRef100\_A0A418RT30 | 100.0 | 3e-36 | 5.6e-42 | 279.8 | 269 | (14, 336) | 769 | (38, 306) | 312 | Toprim domain-containing protein | Toprim domain-containing protein | | uniclust | UniRef100\_A0A072Y8M3 | 100.0 | 2.9e-36 | 5.7e-42 | 334.8 | 317 | (3, 349) | 769 | (71, 429) | 684 | DNA primase | DNA primase | | uniclust | UniRef100\_A0A2P8WAJ2 | 100.0 | 3.2e-36 | 6e-42 | 277.8 | 286 | (445, 741) | 769 | (4, 297) | 356 | Virulence-associated E-like domain-containing protein | Virulence-associated E-like domain-containing protein | | uniclust | UniRef100\_UPI0019D07770 | 100.0 | 3.8e-36 | 6.9e-42 | 308.0 | 349 | (392, 755) | 769 | (905, 1269) | 1277 | VapE family protein | VapE family protein | | uniclust | UniRef100\_A0A374UKX2 | 100.0 | 4.2e-36 | 7.8e-42 | 289.6 | 340 | (380, 735) | 769 | (25, 385) | 415 | Virulence-associated E-like domain-containing protein | Virulence-associated E-like domain-containing protein | | uniclust | UniRef100\_UPI001CDC2532 | 100.0 | 4.6e-36 | 8.4e-42 | 282.6 | 312 | (376, 696) | 769 | (25, 351) | 495 | virulence-associated E family protein | virulence-associated E family protein | | uniclust | UniRef100\_A0A016QSH3 | 100.0 | 4.4e-36 | 8.9e-42 | 340.1 | 323 | (2, 349) | 769 | (77, 441) | 758 | DNA primase | DNA primase | | uniclust | UniRef100\_A0A011UA69 | 100.0 | 4.5e-36 | 9e-42 | 334.6 | 320 | (2, 348) | 769 | (30, 391) | 651 | DNA primase | DNA primase | | uniclust | UniRef100\_A0A0G0HEE0 | 100.0 | 4.9e-36 | 9.5e-42 | 326.3 | 322 | (1, 350) | 769 | (42, 408) | 684 | Primase protein (Fragment) | Primase protein (Fragment) | | uniclust | UniRef100\_A0A2S5DRL8 | 100.0 | 5.2e-36 | 9.6e-42 | 304.9 | 362 | (370, 741) | 769 | (357, 734) | 763 | Replication protein | Replication protein | | uniclust | UniRef100\_A0A1F4YB42 | 100.0 | 5.4e-36 | 1.1e-41 | 325.5 | 316 | (8, 350) | 769 | (25, 387) | 642 | DNA primase | DNA primase | | uniclust | UniRef100\_A0A1I1YQB9 | 100.0 | 6.4e-36 | 1.2e-41 | 307.2 | 386 | (370, 764) | 769 | (328, 738) | 758 | Virulence-associated protein E | Virulence-associated protein E | | uniclust | UniRef100\_A0A7W0X7N0 | 100.0 | 6.9e-36 | 1.3e-41 | 296.0 | 292 | (14, 350) | 769 | (11, 303) | 680 | DUF3987 domain-containing protein (Fragment) | DUF3987 domain-containing protein (Fragment) | | uniclust | UniRef100\_A0A2D9N1G6 | 100.0 | 6.9e-36 | 1.3e-41 | 277.7 | 308 | (376, 692) | 769 | (38, 363) | 369 | Virulence-associated E-like domain-containing protein (Fragment) | Virulence-associated E-like domain-containing protein (Fragment) | | uniclust | UniRef100\_A0A928SS04 | 100.0 | 7.2e-36 | 1.3e-41 | 282.5 | 357 | (380, 750) | 769 | (126, 492) | 515 | Virulence-associated E-like domain-containing protein | Virulence-associated E-like domain-containing protein | | uniclust | UniRef100\_A0A0H3A991 | 100.0 | 7.4e-36 | 1.4e-41 | 318.1 | 640 | (43, 735) | 769 | (28, 758) | 812 | Plasmid/phage primase, P4 family | Plasmid/phage primase, P4 family | | uniclust | UniRef100\_A0A068F471 | 100.0 | 7.4e-36 | 1.5e-41 | 335.0 | 321 | (3, 350) | 769 | (96, 459) | 736 | DNA primase | DNA primase | | uniclust | UniRef100\_A0A101PRR1 | 100.0 | 8.1e-36 | 1.6e-41 | 318.9 | 477 | (233, 752) | 769 | (17, 573) | 625 | DNA primase (Fragment) | DNA primase (Fragment) | | uniclust | UniRef100\_A0A0G1XGQ8 | 100.0 | 8.9e-36 | 1.7e-41 | 310.3 | 320 | (3, 350) | 769 | (27, 389) | 624 | DNA primase | DNA primase | | uniclust | UniRef100\_A0A0A8WPJ3 | 100.0 | 8.7e-36 | 1.7e-41 | 332.3 | 320 | (2, 349) | 769 | (84, 447) | 710 | DNA primase | DNA primase | | uniclust | UniRef100\_A0A8J6XJ06 | 100.0 | 9.4e-36 | 1.8e-41 | 302.0 | 490 | (217, 740) | 769 | (146, 655) | 874 | DUF3854 domain-containing protein | DUF3854 domain-containing protein | | uniclust | UniRef100\_A0A011PBN5 | 100.0 | 9.9e-36 | 1.9e-41 | 277.9 | 289 | (447, 743) | 769 | (4, 299) | 316 | Putative P-loop ATPase | Putative P-loop ATPase | | uniclust | UniRef100\_A0A0H4B4X1 | 100.0 | 1e-35 | 1.9e-41 | 287.8 | 359 | (382, 755) | 769 | (28, 398) | 438 | Virulence-associated E-like domain-containing protein | Virulence-associated E-like domain-containing protein | | uniclust | UniRef100\_A0A0Q7CC84 | 100.0 | 1.1e-35 | 2e-41 | 276.7 | 217 | (99, 339) | 769 | (101, 319) | 322 | Toprim domain-containing protein | Toprim domain-containing protein | | uniclust | UniRef100\_A0A0B4XUC8 | 100.0 | 1.1e-35 | 2e-41 | 314.9 | 293 | (14, 348) | 769 | (658, 953) | 990 | Toprim domain-containing protein | Toprim domain-containing protein | | uniclust | UniRef100\_A0A069RDL3 | 100.0 | 1.1e-35 | 2.2e-41 | 329.5 | 321 | (3, 350) | 769 | (79, 441) | 687 | DNA primase | DNA primase | | uniclust | UniRef100\_A0A0D6QLL8 | 100.0 | 1.2e-35 | 2.4e-41 | 316.2 | 322 | (3, 349) | 769 | (13, 381) | 633 | DNA primase | DNA primase | | uniclust | UniRef100\_A0A2E8UJR5 | 100.0 | 1.3e-35 | 2.5e-41 | 295.4 | 344 | (381, 741) | 769 | (28, 394) | 571 | Virulence-associated E-like domain-containing protein | Virulence-associated E-like domain-containing protein | | uniclust | UniRef100\_A0A923AMT9 | 100.0 | 1.4e-35 | 2.5e-41 | 296.0 | 357 | (373, 737) | 769 | (500, 873) | 895 | Uncharacterized protein | Uncharacterized protein | | uniclust | UniRef100\_A0A066ZPD8 | 100.0 | 1.3e-35 | 2.6e-41 | 319.3 | 319 | (9, 350) | 769 | (41, 402) | 644 | DNA primase | DNA primase | | uniclust | UniRef100\_A0A178MKK8 | 100.0 | 1.4e-35 | 2.6e-41 | 289.0 | 368 | (373, 751) | 769 | (105, 511) | 535 | Virulence-associated E-like domain-containing protein | Virulence-associated E-like domain-containing protein | | uniclust | UniRef100\_A0A0G1KEK0 | 100.0 | 1.4e-35 | 2.7e-41 | 309.4 | 321 | (2, 349) | 769 | (38, 404) | 515 | Primase protein | Primase protein | | uniclust | UniRef100\_A0A2P8WI05 | 100.0 | 1.7e-35 | 3.1e-41 | 287.0 | 341 | (390, 750) | 769 | (127, 475) | 652 | Virulence-associated E-like domain-containing protein (Fragment) | Virulence-associated E-like domain-containing protein (Fragment) | | uniclust | UniRef100\_A0A2E8UEW4 | 100.0 | 1.7e-35 | 3.2e-41 | 305.6 | 318 | (2, 349) | 769 | (19, 379) | 497 | DNA primase (Fragment) | DNA primase (Fragment) | | uniclust | UniRef100\_A0A011MS96 | 100.0 | 1.7e-35 | 3.4e-41 | 317.7 | 321 | (3, 349) | 769 | (51, 412) | 648 | DNA primase | DNA primase | | uniclust | UniRef100\_A0A0F5MPZ0 | 100.0 | 2.1e-35 | 4e-41 | 302.8 | 317 | (6, 349) | 769 | (38, 399) | 513 | DNA primase (Fragment) | DNA primase (Fragment) | | uniclust | UniRef100\_A0A1V5VAP1 | 100.0 | 2.1e-35 | 4e-41 | 317.6 | 322 | (4, 350) | 769 | (69, 434) | 735 | DNA primase | DNA primase | | uniclust | UniRef100\_A0A6J5MWU5 | 100.0 | 2.3e-35 | 4.2e-41 | 294.6 | 349 | (381, 741) | 769 | (313, 678) | 697 | COG5545 Predicted P-loop ATPase and inactivated derivatives | COG5545 Predicted P-loop ATPase and inactivated derivatives | | uniclust | UniRef100\_A0A011VIC8 | 100.0 | 2.1e-35 | 4.2e-41 | 335.4 | 322 | (1, 349) | 769 | (85, 453) | 830 | DNA primase | DNA primase | | uniclust | UniRef100\_A0A0P7YWN9 | 100.0 | 2.2e-35 | 4.2e-41 | 315.0 | 321 | (2, 348) | 769 | (3, 369) | 682 | DNA primase | DNA primase | | uniclust | UniRef100\_A0A7Z0LRG9 | 100.0 | 2.4e-35 | 4.3e-41 | 292.4 | 377 | (378, 756) | 769 | (442, 820) | 827 | Virulence-associated E-like domain-containing protein | Virulence-associated E-like domain-containing protein | | uniclust | UniRef100\_A0A3P1ZAJ6 | 100.0 | 2.4e-35 | 4.6e-41 | 296.4 | 363 | (382, 757) | 769 | (94, 467) | 473 | Helicase | Helicase | | uniclust | UniRef100\_A0A088GFL0 | 100.0 | 2.4e-35 | 4.8e-41 | 319.6 | 320 | (3, 349) | 769 | (48, 411) | 657 | DNA primase | DNA primase | | uniclust | UniRef100\_A0A2T4F5R5 | 100.0 | 2.6e-35 | 4.9e-41 | 272.0 | 274 | (453, 740) | 769 | (9, 286) | 318 | Virulence-associated E-like domain-containing protein | Virulence-associated E-like domain-containing protein | | uniclust | UniRef100\_A0A011PSP2 | 100.0 | 2.6e-35 | 4.9e-41 | 319.0 | 302 | (14, 349) | 769 | (648, 958) | 1044 | DNA primase TraC | DNA primase TraC | | uniclust | UniRef100\_A0A0G1C3E6 | 100.0 | 2.6e-35 | 5e-41 | 297.8 | 320 | (2, 349) | 769 | (4, 366) | 424 | Primase protein | Primase protein | | uniclust | UniRef100\_A0A1M3GZV5 | 100.0 | 2.8e-35 | 5.3e-41 | 316.0 | 286 | (14, 348) | 769 | (57, 342) | 849 | Toprim domain-containing protein | Toprim domain-containing protein | | uniclust | UniRef100\_A0A7H0HGN8 | 100.0 | 3.1e-35 | 5.6e-41 | 287.0 | 290 | (14, 349) | 769 | (12, 302) | 612 | DUF3987 domain-containing protein | DUF3987 domain-containing protein | | uniclust | UniRef100\_A0A6N6T496 | 100.0 | 3.4e-35 | 6.2e-41 | 275.9 | 329 | (380, 718) | 769 | (73, 411) | 480 | Virulence-associated E-like domain-containing protein | Virulence-associated E-like domain-containing protein | | uniclust | UniRef100\_A0A1M6YK98 | 100.0 | 3.3e-35 | 6.3e-41 | 278.2 | 261 | (492, 756) | 769 | (6, 268) | 304 | Virulence-associated protein E | Virulence-associated protein E | | uniclust | UniRef100\_A0A9D5UT42 | 100.0 | 3.4e-35 | 6.3e-41 | 295.7 | 563 | (127, 738) | 769 | (126, 787) | 884 | Uncharacterized protein | Uncharacterized protein | | uniclust | UniRef100\_A0A5C7LJI8 | 100.0 | 3.6e-35 | 6.6e-41 | 287.3 | 367 | (372, 749) | 769 | (317, 705) | 714 | Virulence-associated E-like domain-containing protein | Virulence-associated E-like domain-containing protein | | uniclust | UniRef100\_A0A017T7Q0 | 100.0 | 3.5e-35 | 6.8e-41 | 312.6 | 324 | (3, 351) | 769 | (13, 381) | 628 | DNA primase | DNA primase | | uniclust | UniRef100\_A0A099UD78 | 100.0 | 3.9e-35 | 7.4e-41 | 297.3 | 299 | (14, 349) | 769 | (100, 419) | 500 | DNA primase | DNA primase | | uniclust | UniRef100\_A0A1F2X0U7 | 100.0 | 3.9e-35 | 7.5e-41 | 293.8 | 317 | (2, 348) | 769 | (29, 388) | 421 | DNA primase (Fragment) | DNA primase (Fragment) | | uniclust | UniRef100\_A0A094K1B3 | 100.0 | 3.9e-35 | 7.6e-41 | 285.2 | 276 | (14, 335) | 769 | (22, 304) | 316 | Toprim domain-containing protein | Toprim domain-containing protein | | uniclust | UniRef100\_A0A969EVW1 | 100.0 | 4.5e-35 | 8.4e-41 | 280.1 | 294 | (14, 346) | 769 | (18, 313) | 487 | DUF1738 domain-containing protein (Fragment) | DUF1738 domain-containing protein (Fragment) | | uniclust | UniRef100\_A0A077XNU5 | 100.0 | 4.4e-35 | 8.5e-41 | 304.6 | 321 | (3, 348) | 769 | (21, 387) | 535 | DNA primase | DNA primase | | uniclust | UniRef100\_A0A0B0HLY2 | 100.0 | 4.6e-35 | 8.9e-41 | 308.6 | 313 | (10, 348) | 769 | (50, 407) | 638 | DNA primase | DNA primase | | uniclust | UniRef100\_A0A0A2HRP0 | 100.0 | 4.7e-35 | 9.2e-41 | 315.4 | 312 | (14, 350) | 769 | (21, 373) | 618 | DNA primase | DNA primase | | uniclust | UniRef100\_A0A4P7IPZ1 | 100.0 | 5.2e-35 | 9.7e-41 | 300.7 | 350 | (373, 735) | 769 | (324, 692) | 712 | ATPase | ATPase | | uniclust | UniRef100\_A0A8J2YXK5 | 100.0 | 5.4e-35 | 1e-40 | 281.0 | 291 | (436, 742) | 769 | (276, 578) | 595 | Virulence-associated E-like domain-containing protein | Virulence-associated E-like domain-containing protein | | uniclust | UniRef100\_A0A2E5G1Q1 | 100.0 | 5.6e-35 | 1e-40 | 286.8 | 364 | (372, 751) | 769 | (349, 731) | 738 | Virulence-associated E-like domain-containing protein | Virulence-associated E-like domain-containing protein | | uniclust | UniRef100\_A0A229I531 | 100.0 | 5.6e-35 | 1.1e-40 | 309.0 | 372 | (378, 755) | 769 | (355, 755) | 794 | Uncharacterized protein | Uncharacterized protein | | uniclust | UniRef100\_A0A0C3IN80 | 100.0 | 6.1e-35 | 1.2e-40 | 288.7 | 280 | (30, 335) | 769 | (74, 373) | 418 | DNA primase | DNA primase | | uniclust | UniRef100\_A0A031WEN8 | 100.0 | 6e-35 | 1.2e-40 | 323.4 | 320 | (2, 350) | 769 | (118, 481) | 727 | DNA primase | DNA primase | | uniclust | UniRef100\_A0A010SLU5 | 100.0 | 6.2e-35 | 1.2e-40 | 319.2 | 319 | (6, 349) | 769 | (36, 402) | 693 | DNA primase | DNA primase | | uniclust | UniRef100\_A0A1Z4QLR4 | 100.0 | 6.7e-35 | 1.2e-40 | 295.8 | 473 | (233, 741) | 769 | (134, 627) | 1094 | Virulence-associated E family protein | Virulence-associated E family protein | | uniclust | UniRef100\_A0A0S3U510 | 100.0 | 6.6e-35 | 1.2e-40 | 298.0 | 338 | (386, 742) | 769 | (486, 831) | 929 | Virulence-associated E-like domain-containing protein | Virulence-associated E-like domain-containing protein | | uniclust | UniRef100\_A0A069D3P6 | 100.0 | 6.8e-35 | 1.3e-40 | 301.8 | 359 | (384, 753) | 769 | (335, 703) | 705 | Putative helicase | Putative helicase | | uniclust | UniRef100\_A0A1Y6D883 | 100.0 | 7.2e-35 | 1.4e-40 | 298.0 | 327 | (6, 350) | 769 | (3, 343) | 660 | Primase C terminal 2 (PriCT-2) | Primase C terminal 2 (PriCT-2) | | uniclust | UniRef100\_A0A060AZD4 | 100.0 | 7.4e-35 | 1.4e-40 | 310.2 | 323 | (2, 350) | 769 | (7, 376) | 645 | DNA primase | DNA primase | | uniclust | UniRef100\_UPI000BFFB864 | 100.0 | 8e-35 | 1.5e-40 | 297.3 | 468 | (235, 739) | 769 | (150, 633) | 1203 | VapE family protein | VapE family protein | | uniclust | UniRef100\_A0A419H4F3 | 100.0 | 8.2e-35 | 1.5e-40 | 285.7 | 378 | (385, 769) | 769 | (178, 562) | 562 | Virulence-associated E-like domain-containing protein | Virulence-associated E-like domain-containing protein | | uniclust | UniRef100\_A0A261S325 | 100.0 | 8.2e-35 | 1.6e-40 | 290.6 | 307 | (8, 348) | 769 | (40, 352) | 401 | Primase C-terminal 2 domain-containing protein | Primase C-terminal 2 domain-containing protein | | uniclust | UniRef100\_A0A424HP40 | 100.0 | 8.8e-35 | 1.7e-40 | 298.9 | 341 | (384, 742) | 769 | (300, 647) | 670 | Virulence-associated E family protein | Virulence-associated E family protein | | uniclust | UniRef100\_A0A022GRU9 | 100.0 | 8.9e-35 | 1.7e-40 | 310.3 | 323 | (3, 350) | 769 | (173, 544) | 790 | DNA primase | DNA primase | | uniclust | UniRef100\_A0A078LXT8 | 100.0 | 8.7e-35 | 1.7e-40 | 323.9 | 316 | (3, 349) | 769 | (50, 411) | 666 | DNA primase | DNA primase | | uniclust | UniRef100\_UPI000BE33B30 | 100.0 | 9.8e-35 | 1.8e-40 | 283.7 | 462 | (232, 735) | 769 | (181, 672) | 698 | VapE family protein | VapE family protein | | uniclust | UniRef100\_A0A2D4SRW8 | 100.0 | 9.5e-35 | 1.8e-40 | 307.6 | 320 | (3, 350) | 769 | (30, 393) | 683 | DNA primase | DNA primase | | uniclust | UniRef100\_A0A1V8RP15 | 100.0 | 1e-34 | 1.9e-40 | 301.2 | 364 | (370, 741) | 769 | (415, 837) | 879 | Uncharacterized protein | Uncharacterized protein | | uniclust | UniRef100\_A0A1H6V3P5 | 100.0 | 1.1e-34 | 2e-40 | 294.7 | 354 | (375, 742) | 769 | (486, 866) | 885 | Primase C terminal 2 (PriCT-2) | Primase C terminal 2 (PriCT-2) | | uniclust | UniRef100\_A0A238HHW5 | 100.0 | 1.2e-34 | 2.3e-40 | 308.2 | 317 | (9, 345) | 769 | (45, 376) | 1258 | DNA primase TraC | DNA primase TraC | | uniclust | UniRef100\_A0A031JRC8 | 100.0 | 1.3e-34 | 2.5e-40 | 290.6 | 278 | (33, 335) | 769 | (42, 331) | 407 | DNA primase | DNA primase | | uniclust | UniRef100\_A0A1G3FM87 | 100.0 | 1.3e-34 | 2.5e-40 | 305.4 | 658 | (38, 752) | 769 | (63, 838) | 860 | SF3 helicase domain-containing protein | SF3 helicase domain-containing protein | | uniclust | UniRef100\_A0A1H6L3X8 | 100.0 | 1.5e-34 | 2.8e-40 | 270.2 | 264 | (487, 755) | 769 | (27, 293) | 325 | Virulence-associated E-like domain-containing protein | Virulence-associated E-like domain-containing protein | | uniclust | UniRef100\_A0A014N801 | 100.0 | 1.4e-34 | 2.8e-40 | 316.4 | 322 | (3, 350) | 769 | (36, 402) | 644 | DNA primase | DNA primase | | uniclust | UniRef100\_A0A0F9NPG2 | 100.0 | 1.5e-34 | 2.9e-40 | 310.3 | 272 | (467, 755) | 769 | (274, 565) | 624 | SF3 helicase domain-containing protein (Fragment) | SF3 helicase domain-containing protein (Fragment) | | uniclust | UniRef100\_A0A7T2PJJ0 | 100.0 | 1.6e-34 | 3.1e-40 | 280.4 | 377 | (366, 755) | 769 | (23, 424) | 434 | ATPase | ATPase | | uniclust | UniRef100\_A0A096AAC7 | 100.0 | 1.6e-34 | 3.1e-40 | 280.5 | 365 | (382, 755) | 769 | (58, 432) | 445 | Helicase | Helicase | | uniclust | UniRef100\_UPI002277702C | 100.0 | 1.7e-34 | 3.1e-40 | 274.7 | 410 | (131, 565) | 769 | (20, 538) | 538 | VapE family protein | VapE family protein | | uniclust | UniRef100\_A0A0H5DU62 | 100.0 | 1.7e-34 | 3.3e-40 | 311.6 | 316 | (4, 350) | 769 | (1, 360) | 591 | DNA primase | DNA primase | | uniclust | UniRef100\_A0A966PIM5 | 100.0 | 1.9e-34 | 3.5e-40 | 262.6 | 317 | (376, 693) | 769 | (48, 366) | 369 | Virulence-associated E family protein (Fragment) | Virulence-associated E family protein (Fragment) | | uniclust | UniRef100\_A0A1F5RLM4 | 100.0 | 1.8e-34 | 3.6e-40 | 312.4 | 312 | (14, 350) | 769 | (111, 465) | 738 | DNA primase | DNA primase | | uniclust | UniRef100\_A0A9D7D5W3 | 100.0 | 2e-34 | 3.8e-40 | 252.0 | 234 | (450, 689) | 769 | (5, 239) | 240 | Uncharacterized protein | Uncharacterized protein | | uniclust | UniRef100\_A0A099I9C9 | 100.0 | 1.9e-34 | 3.8e-40 | 324.1 | 320 | (4, 349) | 769 | (52, 414) | 665 | DNA primase | DNA primase | | uniclust | UniRef100\_A0A1F5V2M0 | 100.0 | 2e-34 | 3.8e-40 | 297.8 | 312 | (6, 350) | 769 | (19, 368) | 608 | DNA primase | DNA primase | | uniclust | UniRef100\_A0A7Z9GAF4 | 100.0 | 2.2e-34 | 4e-40 | 281.5 | 350 | (375, 742) | 769 | (317, 673) | 704 | Virulence-associated E-like domain-containing protein | Virulence-associated E-like domain-containing protein | | uniclust | UniRef100\_A0A937KUZ1 | 100.0 | 2.7e-34 | 5.1e-40 | 259.7 | 244 | (484, 740) | 769 | (23, 271) | 304 | Virulence-associated E family protein | Virulence-associated E family protein | | uniclust | UniRef100\_A0A139C8D1 | 100.0 | 2.9e-34 | 5.4e-40 | 264.8 | 258 | (31, 336) | 769 | (46, 304) | 308 | Toprim domain-containing protein | Toprim domain-containing protein | | uniclust | UniRef100\_UPI0010F7918A | 100.0 | 3.3e-34 | 6.1e-40 | 271.1 | 356 | (367, 737) | 769 | (10, 372) | 509 | VapE family protein | VapE family protein | | uniclust | UniRef100\_A0A955RF62 | 100.0 | 3.8e-34 | 6.9e-40 | 275.4 | 351 | (376, 740) | 769 | (215, 581) | 596 | Virulence-associated E-like domain-containing protein | Virulence-associated E-like domain-containing protein | | uniclust | UniRef100\_UPI001F548CF2 | 100.0 | 3.9e-34 | 7.2e-40 | 277.3 | 346 | (386, 755) | 769 | (205, 565) | 641 | virulence-associated E family protein | virulence-associated E family protein | | uniclust | UniRef100\_A0A077MUW8 | 100.0 | 4e-34 | 7.8e-40 | 331.3 | 300 | (14, 348) | 769 | (976, 1311) | 1402 | Toprim domain-containing protein | Toprim domain-containing protein | | uniclust | UniRef100\_A0A2N7R2N6 | 100.0 | 4.3e-34 | 8e-40 | 270.3 | 423 | (17, 482) | 769 | (10, 451) | 480 | DNA primase TraC | DNA primase TraC | | uniclust | UniRef100\_A0A011RLF2 | 100.0 | 4.1e-34 | 8.2e-40 | 318.1 | 311 | (12, 347) | 769 | (82, 437) | 717 | DNA primase | DNA primase | | uniclust | UniRef100\_A0A1E4C277 | 100.0 | 4.6e-34 | 8.4e-40 | 279.1 | 359 | (379, 751) | 769 | (166, 534) | 595 | Virulence-associated E-like domain-containing protein | Virulence-associated E-like domain-containing protein | | uniclust | UniRef100\_A0A013VTI8 | 100.0 | 4.3e-34 | 8.5e-40 | 312.9 | 320 | (3, 349) | 769 | (92, 456) | 733 | DNA primase | DNA primase | | uniclust | UniRef100\_A0A0D6JI60 | 100.0 | 4.5e-34 | 8.7e-40 | 305.0 | 322 | (2, 350) | 769 | (78, 446) | 739 | DNA primase | DNA primase | | uniclust | UniRef100\_A0A350YX52 | 100.0 | 4.8e-34 | 9e-40 | 271.8 | 345 | (384, 741) | 769 | (13, 374) | 400 | Virulence-associated E-like domain-containing protein (Fragment) | Virulence-associated E-like domain-containing protein (Fragment) | | uniclust | UniRef100\_A0A068NYD4 | 100.0 | 4.9e-34 | 9.4e-40 | 302.9 | 319 | (4, 350) | 769 | (3, 361) | 621 | DNA primase | DNA primase | | uniclust | UniRef100\_A0A2H0MZN7 | 100.0 | 5.5e-34 | 1e-39 | 296.9 | 287 | (14, 350) | 769 | (31, 319) | 807 | Toprim domain-containing protein | Toprim domain-containing protein | | uniclust | UniRef100\_A0A948T5F1 | 100.0 | 5.7e-34 | 1e-39 | 271.1 | 379 | (370, 755) | 769 | (75, 468) | 474 | Virulence-associated E family protein | Virulence-associated E family protein | | uniclust | UniRef100\_A0A163VSB7 | 100.0 | 5.9e-34 | 1.1e-39 | 297.2 | 344 | (382, 741) | 769 | (400, 755) | 928 | Virulence-associated protein E | Virulence-associated protein E | | uniclust | UniRef100\_A0A6G7VDW5 | 100.0 | 6e-34 | 1.1e-39 | 272.3 | 314 | (12, 348) | 769 | (7, 323) | 462 | AAA family ATPase | AAA family ATPase | | uniclust | UniRef100\_A0A965PG93 | 100.0 | 6.3e-34 | 1.2e-39 | 277.3 | 486 | (232, 751) | 769 | (163, 673) | 676 | Bacteriophage T7 Gp4 DNA primase/helicase N-terminal domain-containing protein | Bacteriophage T7 Gp4 DNA primase/helicase N-terminal domain-containing protein | | uniclust | UniRef100\_A0A0R2W862 | 100.0 | 6.4e-34 | 1.2e-39 | 272.9 | 346 | (378, 740) | 769 | (176, 554) | 576 | Virulence-associated E-like domain-containing protein (Fragment) | Virulence-associated E-like domain-containing protein (Fragment) | | uniclust | UniRef100\_A0A081SGQ8 | 100.0 | 6.1e-34 | 1.2e-39 | 317.0 | 320 | (4, 349) | 769 | (80, 447) | 777 | DNA primase | DNA primase | | uniclust | UniRef100\_A0A2N2U4N7 | 100.0 | 6.5e-34 | 1.2e-39 | 266.0 | 332 | (6, 349) | 769 | (3, 347) | 404 | Toprim domain-containing protein | Toprim domain-containing protein | | uniclust | UniRef100\_A0A6L3SS05 | 100.0 | 7.1e-34 | 1.3e-39 | 285.1 | 313 | (14, 345) | 769 | (53, 377) | 936 | Toprim domain-containing protein | Toprim domain-containing protein | | uniclust | UniRef100\_A0A0G1WVT7 | 100.0 | 6.9e-34 | 1.4e-39 | 317.4 | 320 | (3, 350) | 769 | (96, 481) | 777 | DNA primase | DNA primase | | uniclust | UniRef100\_A0A1M6R249 | 100.0 | 7.3e-34 | 1.4e-39 | 315.1 | 298 | (14, 349) | 769 | (1089, 1389) | 1483 | Conjugative relaxase domain-containing protein, TrwC/TraI family | Conjugative relaxase domain-containing protein, TrwC/TraI family | | uniclust | UniRef100\_A0A371XDT2 | 100.0 | 8e-34 | 1.5e-39 | 280.2 | 336 | (391, 742) | 769 | (406, 754) | 777 | Virulence-associated E-like domain-containing protein | Virulence-associated E-like domain-containing protein | | uniclust | UniRef100\_A0A0B6WZ92 | 100.0 | 7.8e-34 | 1.5e-39 | 302.0 | 323 | (3, 350) | 769 | (7, 372) | 617 | DNA primase | DNA primase | | uniclust | UniRef100\_A0A6N9AAB1 | 100.0 | 8.5e-34 | 1.6e-39 | 282.5 | 573 | (14, 639) | 769 | (11, 674) | 810 | Toprim domain-containing protein | Toprim domain-containing protein | | uniclust | UniRef100\_UPI00226D0A03 | 100.0 | 8.6e-34 | 1.6e-39 | 270.8 | 350 | (372, 739) | 769 | (178, 532) | 553 | virulence-associated E family protein | virulence-associated E family protein | | uniclust | UniRef100\_Q1YDH5 | 100.0 | 8.9e-34 | 1.6e-39 | 262.8 | 280 | (383, 673) | 769 | (123, 414) | 424 | Putative virulence-associated protein E | Putative virulence-associated protein E | | uniclust | UniRef100\_A0A0G1YTK1 | 100.0 | 1e-33 | 1.9e-39 | 284.6 | 371 | (371, 749) | 769 | (543, 923) | 962 | Virulence-associated protein E domain protein | Virulence-associated protein E domain protein | | uniclust | UniRef100\_A0A0F8W6I5 | 100.0 | 1e-33 | 2e-39 | 289.6 | 314 | (6, 350) | 769 | (48, 408) | 510 | Toprim domain-containing protein (Fragment) | Toprim domain-containing protein (Fragment) | | uniclust | UniRef100\_A0A969Q8P7 | 100.0 | 1.1e-33 | 2e-39 | 261.7 | 280 | (446, 741) | 769 | (3, 287) | 418 | Virulence-associated E-like domain-containing protein | Virulence-associated E-like domain-containing protein | | uniclust | UniRef100\_A0A6J5KG32 | 100.0 | 1.1e-33 | 2.1e-39 | 292.7 | 302 | (437, 742) | 769 | (482, 811) | 859 | Virulence-associated E | Virulence-associated E | | uniclust | UniRef100\_A0A063Y669 | 100.0 | 1.1e-33 | 2.1e-39 | 299.4 | 318 | (7, 350) | 769 | (10, 374) | 674 | DNA primase | DNA primase | | uniclust | UniRef100\_A0A081N1X8 | 100.0 | 1.1e-33 | 2.1e-39 | 277.9 | 245 | (67, 336) | 769 | (76, 331) | 338 | Bacteriophage T7 Gp4 DNA primase/helicase N-terminal domain-containing protein | Bacteriophage T7 Gp4 DNA primase/helicase N-terminal domain-containing protein | | uniclust | UniRef100\_UPI0005DFE532 | 100.0 | 1.1e-33 | 2.1e-39 | 271.3 | 288 | (435, 736) | 769 | (58, 357) | 384 | virulence-associated E family protein | virulence-associated E family protein | | uniclust | UniRef100\_L8N3H0 | 100.0 | 1.2e-33 | 2.1e-39 | 272.1 | 333 | (391, 749) | 769 | (122, 463) | 597 | Virulence-associated E family protein | Virulence-associated E family protein | | uniclust | UniRef100\_A0A7C9NE92 | 100.0 | 1.2e-33 | 2.2e-39 | 279.1 | 356 | (375, 742) | 769 | (402, 769) | 780 | Virulence-associated E-like domain-containing protein | Virulence-associated E-like domain-containing protein | | uniclust | UniRef100\_A0A0B1U8J8 | 100.0 | 1.1e-33 | 2.2e-39 | 299.5 | 321 | (4, 349) | 769 | (18, 380) | 607 | DNA primase | DNA primase | | uniclust | UniRef100\_A0A1F6PUQ3 | 100.0 | 1.2e-33 | 2.2e-39 | 288.6 | 318 | (3, 349) | 769 | (17, 378) | 487 | DNA primase (Fragment) | DNA primase (Fragment) | | uniclust | UniRef100\_A0A011NMP9 | 100.0 | 1.3e-33 | 2.5e-39 | 282.3 | 301 | (14, 348) | 769 | (159, 468) | 505 | DNA primase TraC | DNA primase TraC | | uniclust | UniRef100\_A0A022MEH4 | 100.0 | 1.3e-33 | 2.5e-39 | 305.5 | 323 | (4, 349) | 769 | (113, 480) | 768 | DNA primase (Fragment) | DNA primase (Fragment) | | uniclust | UniRef100\_A0A4P2QP84 | 100.0 | 1.4e-33 | 2.5e-39 | 280.6 | 295 | (435, 740) | 769 | (460, 758) | 841 | Uncharacterized protein | Uncharacterized protein | | uniclust | UniRef100\_A0A0A2G2K2 | 100.0 | 1.4e-33 | 2.5e-39 | 267.7 | 353 | (381, 741) | 769 | (51, 413) | 443 | Virulence-associated protein E (Fragment) | Virulence-associated protein E (Fragment) | | uniclust | UniRef100\_A0A0G2ZH66 | 100.0 | 1.3e-33 | 2.6e-39 | 302.9 | 321 | (3, 350) | 769 | (19, 382) | 634 | DNA primase | DNA primase | | uniclust | UniRef100\_A0A2E4HX51 | 100.0 | 1.3e-33 | 2.6e-39 | 284.8 | 320 | (3, 348) | 769 | (35, 398) | 412 | DNA primase (Fragment) | DNA primase (Fragment) | | uniclust | UniRef100\_A0A0F6W4Z0 | 100.0 | 1.5e-33 | 2.8e-39 | 298.7 | 325 | (3, 350) | 769 | (65, 441) | 702 | DNA primase | DNA primase | | uniclust | UniRef100\_A0A060H525 | 100.0 | 1.5e-33 | 2.9e-39 | 273.7 | 301 | (14, 348) | 769 | (59, 368) | 406 | DNA primase | DNA primase | | uniclust | UniRef100\_A0A3N5GQR2 | 100.0 | 1.6e-33 | 2.9e-39 | 275.9 | 354 | (380, 751) | 769 | (336, 697) | 710 | Virulence-associated E-like domain-containing protein | Virulence-associated E-like domain-containing protein | | uniclust | UniRef100\_A0A2P5L752 | 100.0 | 1.6e-33 | 3.1e-39 | 266.8 | 201 | (127, 349) | 769 | (148, 348) | 375 | Toprim domain-containing protein | Toprim domain-containing protein | | uniclust | UniRef100\_A0A838WBL7 | 100.0 | 1.7e-33 | 3.1e-39 | 270.1 | 290 | (14, 348) | 769 | (14, 324) | 576 | Toprim domain-containing protein (Fragment) | Toprim domain-containing protein (Fragment) | | uniclust | UniRef100\_A0A1F8DMI8 | 100.0 | 1.9e-33 | 3.6e-39 | 277.6 | 319 | (3, 349) | 769 | (1, 366) | 413 | DNA primase (Fragment) | DNA primase (Fragment) | | uniclust | UniRef100\_A0A0R1FLZ9 | 100.0 | 2e-33 | 3.7e-39 | 282.6 | 298 | (389, 696) | 769 | (322, 636) | 741 | Prophage lp3 protein 8, helicase | Prophage lp3 protein 8, helicase | | uniclust | UniRef100\_UPI00109CB367 | 100.0 | 2e-33 | 3.7e-39 | 286.7 | 357 | (391, 755) | 769 | (380, 751) | 759 | VapE family protein | VapE family protein | | uniclust | UniRef100\_A0A9E1U8H1 | 100.0 | 2.1e-33 | 3.9e-39 | 281.5 | 351 | (376, 741) | 769 | (415, 775) | 926 | Uncharacterized protein | Uncharacterized protein | | uniclust | UniRef100\_A0A078KUP1 | 100.0 | 2e-33 | 4e-39 | 305.9 | 315 | (2, 349) | 769 | (54, 407) | 626 | DNA primase | DNA primase | | uniclust | UniRef100\_A0A956I739 | 100.0 | 2.2e-33 | 4e-39 | 278.4 | 316 | (373, 696) | 769 | (402, 728) | 812 | Virulence-associated E-like domain-containing protein | Virulence-associated E-like domain-containing protein | | uniclust | UniRef100\_UPI00211665FF | 100.0 | 2.2e-33 | 4e-39 | 266.3 | 372 | (31, 416) | 769 | (36, 481) | 518 | toprim domain-containing protein | toprim domain-containing protein | | uniclust | UniRef100\_A0A0A7UW42 | 100.0 | 2e-33 | 4e-39 | 303.5 | 318 | (3, 349) | 769 | (23, 380) | 658 | DNA primase | DNA primase | | uniclust | UniRef100\_A0A1C5YKD3 | 100.0 | 2.2e-33 | 4.1e-39 | 280.7 | 472 | (233, 737) | 769 | (197, 683) | 780 | DNA primase | DNA primase | | uniclust | UniRef100\_A0A930XU13 | 100.0 | 2.3e-33 | 4.2e-39 | 265.2 | 295 | (436, 744) | 769 | (47, 349) | 501 | Virulence-associated E-like domain-containing protein | Virulence-associated E-like domain-containing protein | | uniclust | UniRef100\_UPI000781351A | 100.0 | 2.3e-33 | 4.2e-39 | 270.3 | 406 | (324, 756) | 769 | (38, 455) | 599 | VapE family protein | VapE family protein | | uniclust | UniRef100\_A0A1F8JGT0 | 100.0 | 2.3e-33 | 4.4e-39 | 290.1 | 321 | (2, 349) | 769 | (9, 370) | 551 | DNA primase (Fragment) | DNA primase (Fragment) | | uniclust | UniRef100\_UPI001A9764D7 | 100.0 | 2.6e-33 | 4.9e-39 | 283.1 | 382 | (374, 759) | 769 | (352, 742) | 759 | VapE family protein | VapE family protein | | uniclust | UniRef100\_A0A946HIV3 | 100.0 | 3e-33 | 5.6e-39 | 273.7 | 350 | (377, 740) | 769 | (305, 684) | 703 | Virulence-associated E-like domain-containing protein | Virulence-associated E-like domain-containing protein | | uniclust | UniRef100\_A0A0P0FR19 | 100.0 | 3.3e-33 | 6.2e-39 | 293.2 | 321 | (4, 349) | 769 | (82, 453) | 720 | DNA primase | DNA primase | | uniclust | UniRef100\_A0A926WX44 | 100.0 | 3.5e-33 | 6.4e-39 | 282.2 | 481 | (234, 740) | 769 | (128, 623) | 1023 | DUF3854 domain-containing protein | DUF3854 domain-containing protein | | uniclust | UniRef100\_A0A516M9R7 | 100.0 | 3.4e-33 | 6.5e-39 | 297.8 | 285 | (14, 345) | 769 | (46, 332) | 777 | Toprim domain-containing protein | Toprim domain-containing protein | | uniclust | UniRef100\_A0A1Y6D1M0 | 100.0 | 3.4e-33 | 6.6e-39 | 294.8 | 311 | (12, 350) | 769 | (32, 386) | 617 | DNA primase | DNA primase | | uniclust | UniRef100\_UPI001E4C4C0F | 100.0 | 3.9e-33 | 7.2e-39 | 250.3 | 290 | (375, 666) | 769 | (12, 303) | 327 | virulence-associated E family protein | virulence-associated E family protein | | uniclust | UniRef100\_A0A0D2JMB5 | 100.0 | 3.8e-33 | 7.5e-39 | 309.9 | 319 | (1, 349) | 769 | (26, 389) | 642 | DNA primase | DNA primase | | uniclust | UniRef100\_A0A1H2VE35 | 100.0 | 4.1e-33 | 7.7e-39 | 279.1 | 368 | (381, 757) | 769 | (248, 632) | 643 | Virulence-associated protein E | Virulence-associated protein E | | uniclust | UniRef100\_A0A969AB06 | 100.0 | 4.4e-33 | 8.1e-39 | 270.2 | 434 | (231, 695) | 769 | (144, 607) | 641 | Virulence-associated E-like domain-containing protein | Virulence-associated E-like domain-containing protein | | uniclust | UniRef100\_A0A1U7H6I1 | 100.0 | 4.7e-33 | 8.7e-39 | 283.2 | 349 | (386, 754) | 769 | (335, 697) | 859 | Virulence-associated E-like domain-containing protein | Virulence-associated E-like domain-containing protein | | uniclust | UniRef100\_A0A537Y110 | 100.0 | 4.7e-33 | 8.9e-39 | 292.9 | 318 | (5, 350) | 769 | (3, 363) | 697 | DNA primase | DNA primase | | uniclust | UniRef100\_A0A0F9YUN7 | 100.0 | 5e-33 | 9.8e-39 | 305.5 | 315 | (4, 349) | 769 | (36, 392) | 661 | DNA primase | DNA primase | | uniclust | UniRef100\_A0A164AHS2 | 100.0 | 5.3e-33 | 1e-38 | 269.5 | 335 | (390, 739) | 769 | (62, 406) | 443 | Virulence-associated protein E | Virulence-associated protein E | | uniclust | UniRef100\_A0A1D2SKX3 | 100.0 | 5.2e-33 | 1e-38 | 298.2 | 302 | (14, 351) | 769 | (28, 333) | 724 | Uncharacterized protein | Uncharacterized protein | | uniclust | UniRef100\_A0A023Y828 | 100.0 | 5.7e-33 | 1.1e-38 | 290.3 | 323 | (2, 350) | 769 | (43, 409) | 632 | DNA primase | DNA primase | | uniclust | UniRef100\_A0A2E1R907 | 100.0 | 6.1e-33 | 1.1e-38 | 281.5 | 343 | (392, 751) | 769 | (379, 737) | 741 | Primase C-terminal 2 domain-containing protein | Primase C-terminal 2 domain-containing protein | | uniclust | UniRef100\_A0A1F9TV27 | 100.0 | 6e-33 | 1.1e-38 | 290.7 | 320 | (2, 349) | 769 | (2, 365) | 596 | DNA primase | DNA primase | | uniclust | UniRef100\_A0A059KN83 | 100.0 | 6.1e-33 | 1.2e-38 | 271.7 | 287 | (14, 339) | 769 | (41, 342) | 364 | Toprim domain-containing protein | Toprim domain-containing protein | | uniclust | UniRef100\_A0A8S5MVS7 | 100.0 | 6.7e-33 | 1.3e-38 | 286.7 | 311 | (372, 696) | 769 | (403, 728) | 834 | Virulence associated protein E | Virulence associated protein E | | uniclust | UniRef100\_A0A926PMV0 | 100.0 | 6.8e-33 | 1.3e-38 | 275.0 | 357 | (389, 769) | 769 | (448, 815) | 815 | Virulence-associated E-like domain-containing protein | Virulence-associated E-like domain-containing protein | | uniclust | UniRef100\_A0A0A1H6W6 | 100.0 | 7.1e-33 | 1.4e-38 | 288.9 | 320 | (5, 352) | 769 | (1, 361) | 624 | DNA primase | DNA primase | | uniclust | UniRef100\_A0A017H3G2 | 100.0 | 6.9e-33 | 1.4e-38 | 313.7 | 319 | (1, 346) | 769 | (30, 392) | 717 | DNA primase | DNA primase | | uniclust | UniRef100\_A0A382QEF6 | 100.0 | 8.3e-33 | 1.6e-38 | 265.4 | 286 | (13, 343) | 769 | (37, 328) | 366 | Toprim domain-containing protein (Fragment) | Toprim domain-containing protein (Fragment) | | uniclust | UniRef100\_UPI001C0AE9DC | 100.0 | 8.8e-33 | 1.6e-38 | 267.1 | 377 | (366, 757) | 769 | (228, 609) | 616 | VapE family protein | VapE family protein | | uniclust | UniRef100\_A0A1F5F2Q7 | 100.0 | 9.1e-33 | 1.8e-38 | 300.2 | 320 | (4, 348) | 769 | (107, 473) | 751 | DNA primase | DNA primase | | uniclust | UniRef100\_A0A014NIL8 | 100.0 | 9.4e-33 | 1.8e-38 | 289.0 | 322 | (4, 350) | 769 | (60, 430) | 722 | DNA primase | DNA primase | | uniclust | UniRef100\_A0A078BNX3 | 100.0 | 9.5e-33 | 1.8e-38 | 284.1 | 322 | (3, 349) | 769 | (1, 367) | 652 | DNA primase | DNA primase | | uniclust | UniRef100\_A0A2D9A9Z1 | 100.0 | 9.5e-33 | 1.8e-38 | 287.8 | 320 | (3, 350) | 769 | (41, 407) | 642 | DNA primase (Fragment) | DNA primase (Fragment) | | uniclust | UniRef100\_A0A134A587 | 100.0 | 9.9e-33 | 1.8e-38 | 280.4 | 309 | (376, 694) | 769 | (318, 632) | 746 | Virulence-associated protein E | Virulence-associated protein E | | uniclust | UniRef100\_A0A101I1T0 | 100.0 | 1.1e-32 | 2.1e-38 | 295.9 | 313 | (14, 350) | 769 | (45, 401) | 629 | DNA primase | DNA primase | | uniclust | UniRef100\_A0A0F9MKQ4 | 100.0 | 1.1e-32 | 2.1e-38 | 298.0 | 282 | (457, 755) | 769 | (225, 532) | 595 | SF3 helicase domain-containing protein | SF3 helicase domain-containing protein | | uniclust | UniRef100\_A0A1F8RM87 | 100.0 | 1.1e-32 | 2.1e-38 | 293.4 | 313 | (10, 351) | 769 | (2, 373) | 649 | DNA primase | DNA primase | | uniclust | UniRef100\_A0A936HA23 | 100.0 | 1.2e-32 | 2.2e-38 | 272.7 | 493 | (230, 741) | 769 | (143, 667) | 797 | Virulence-associated E-like domain-containing protein | Virulence-associated E-like domain-containing protein | | uniclust | UniRef100\_A0A0R1XGL3 | 100.0 | 1.2e-32 | 2.2e-38 | 285.8 | 270 | (467, 754) | 769 | (287, 577) | 622 | Primase | Primase | | uniclust | UniRef100\_UPI0019D1F5EF | 100.0 | 1.4e-32 | 2.6e-38 | 279.3 | 286 | (14, 350) | 769 | (64, 350) | 747 | AAA family ATPase | AAA family ATPase | | uniclust | UniRef100\_A0A1F2W6T8 | 100.0 | 1.4e-32 | 2.6e-38 | 291.8 | 321 | (2, 350) | 769 | (31, 394) | 630 | DNA primase | DNA primase | | uniclust | UniRef100\_A0A0X1KQ28 | 100.0 | 1.5e-32 | 2.8e-38 | 283.5 | 312 | (8, 350) | 769 | (3, 354) | 574 | DNA primase | DNA primase | | uniclust | UniRef100\_A0A0E3MU70 | 100.0 | 1.5e-32 | 2.8e-38 | 277.4 | 296 | (14, 349) | 769 | (87, 385) | 599 | Pri | Pri | | uniclust | UniRef100\_UPI0020CC0711 | 100.0 | 1.5e-32 | 2.9e-38 | 286.8 | 337 | (393, 740) | 769 | (470, 829) | 995 | virulence-associated E family protein | virulence-associated E family protein | | uniclust | UniRef100\_A0A1G5H383 | 100.0 | 1.7e-32 | 3.1e-38 | 270.8 | 519 | (131, 697) | 769 | (113, 713) | 767 | Phage/plasmid primase, P4 family, C-terminal domain-containing protein | Phage/plasmid primase, P4 family, C-terminal domain-containing protein | | uniclust | UniRef100\_A0A068Z0K6 | 100.0 | 1.7e-32 | 3.4e-38 | 314.0 | 298 | (14, 348) | 769 | (283, 583) | 1434 | DNA primase | DNA primase | | uniclust | UniRef100\_A0A3B8XX17 | 100.0 | 1.9e-32 | 3.5e-38 | 267.6 | 314 | (372, 695) | 769 | (249, 588) | 684 | Virulence-associated E-like domain-containing protein | Virulence-associated E-like domain-containing protein | | uniclust | UniRef100\_A0A158KPG1 | 100.0 | 1.9e-32 | 3.5e-38 | 274.0 | 347 | (393, 753) | 769 | (517, 887) | 893 | Virulence-associated E family protein | Virulence-associated E family protein | | uniclust | UniRef100\_A0A177R8F8 | 100.0 | 2e-32 | 3.9e-38 | 293.6 | 321 | (3, 350) | 769 | (25, 391) | 641 | DNA primase | DNA primase | | uniclust | UniRef100\_UPI001CD65D62 | 100.0 | 2.2e-32 | 4.1e-38 | 261.6 | 307 | (380, 695) | 769 | (155, 468) | 557 | virulence-associated E family protein | virulence-associated E family protein | | uniclust | UniRef100\_A0A645CY77 | 100.0 | 2.4e-32 | 4.4e-38 | 244.4 | 229 | (436, 672) | 769 | (65, 299) | 318 | Virulence-associated E-like domain-containing protein | Virulence-associated E-like domain-containing protein | | uniclust | UniRef100\_A0A1G3LYT4 | 100.0 | 2.4e-32 | 4.5e-38 | 285.3 | 320 | (4, 349) | 769 | (17, 378) | 611 | DNA primase | DNA primase | | uniclust | UniRef100\_A0A143Y5P7 | 100.0 | 2.6e-32 | 4.8e-38 | 269.3 | 309 | (378, 695) | 769 | (340, 662) | 760 | Virulence-associated E-like domain-containing protein | Virulence-associated E-like domain-containing protein | | uniclust | UniRef100\_A0A2V5PV48 | 99.9 | 2.7e-32 | 5e-38 | 276.4 | 340 | (390, 742) | 769 | (405, 759) | 780 | DNA primase/polymerase bifunctional N-terminal domain-containing protein | DNA primase/polymerase bifunctional N-terminal domain-containing protein | | uniclust | UniRef100\_A0A521XCL3 | 99.9 | 2.7e-32 | 5e-38 | 276.0 | 352 | (381, 740) | 769 | (385, 754) | 780 | DNA primase/polymerase bifunctional N-terminal domain-containing protein | DNA primase/polymerase bifunctional N-terminal domain-containing protein | | uniclust | UniRef100\_A0A2N5YUX1 | 99.9 | 2.6e-32 | 5e-38 | 255.5 | 269 | (476, 755) | 769 | (5, 279) | 286 | Virulence-associated E-like domain-containing protein | Virulence-associated E-like domain-containing protein | | uniclust | UniRef100\_A0A031K4Q8 | 99.9 | 2.9e-32 | 5.5e-38 | 292.0 | 319 | (4, 349) | 769 | (19, 383) | 666 | DNA primase | DNA primase | | uniclust | UniRef100\_A0A1Y6ISW7 | 99.9 | 3.2e-32 | 6e-38 | 258.6 | 312 | (435, 755) | 769 | (6, 341) | 348 | Virulence-associated protein E | Virulence-associated protein E | | uniclust | UniRef100\_A0A6J5S9L8 | 99.9 | 3.5e-32 | 6.4e-38 | 267.9 | 372 | (376, 755) | 769 | (355, 740) | 745 | Virulence-associated E | Virulence-associated E | | uniclust | UniRef100\_A0A072NKF5 | 99.9 | 3.3e-32 | 6.5e-38 | 272.5 | 281 | (3, 307) | 769 | (26, 347) | 362 | DNA primase, catalytic core | DNA primase, catalytic core | | uniclust | UniRef100\_UPI0016899E5E | 99.9 | 3.5e-32 | 6.5e-38 | 275.7 | 304 | (438, 755) | 769 | (662, 976) | 1051 | VapE family protein | VapE family protein | | uniclust | UniRef100\_UPI001E53A1EB | 99.9 | 3.8e-32 | 7.1e-38 | 273.7 | 563 | (150, 739) | 769 | (51, 704) | 796 | VapE family protein | VapE family protein | | uniclust | UniRef100\_UPI00096A411E | 99.9 | 4e-32 | 7.4e-38 | 258.6 | 320 | (14, 350) | 769 | (23, 360) | 436 | toprim domain-containing protein | toprim domain-containing protein | | uniclust | UniRef100\_A0A528WAA4 | 99.9 | 4.1e-32 | 7.6e-38 | 270.2 | 358 | (371, 737) | 769 | (208, 615) | 657 | Virulence-associated E-like domain-containing protein | Virulence-associated E-like domain-containing protein | | uniclust | UniRef100\_A0A101H196 | 99.9 | 4e-32 | 7.9e-38 | 290.9 | 319 | (1, 351) | 769 | (1, 360) | 576 | DNA primase | DNA primase | | uniclust | UniRef100\_A0A068YJZ3 | 99.9 | 4.5e-32 | 8.6e-38 | 258.2 | 277 | (10, 338) | 769 | (5, 282) | 305 | DNA primase TraC | DNA primase TraC | | uniclust | UniRef100\_A0A0G0BXR0 | 99.9 | 4.6e-32 | 8.8e-38 | 285.3 | 315 | (4, 349) | 769 | (20, 382) | 628 | DNA primase | DNA primase | | uniclust | UniRef100\_A0A2A4ZUQ6 | 99.9 | 4.7e-32 | 8.9e-38 | 252.4 | 240 | (12, 288) | 769 | (27, 266) | 305 | Toprim domain-containing protein (Fragment) | Toprim domain-containing protein (Fragment) | | pdb70 | 3B39\_A | 99.6 | 6e-20 | 7.4e-24 | 180.8 | 161 | (174, 350) | 769 | (91, 261) | 322 | DNA primase/DNA Complex | 3B39\_A DNA primase/DNA Complex Protein-DNA complex, TOPRIM fold, DNA | | pdb70 | 3B39\_B | 99.6 | 6e-20 | 7.4e-24 | 180.8 | 161 | (174, 350) | 769 | (91, 261) | 322 | DNA primase/DNA Complex | 3B39\_B DNA primase/DNA Complex Protein-DNA complex, TOPRIM fold, DNA | | pdb70 | 1DD9\_A | 99.6 | 1.6e-19 | 1.9e-23 | 180.3 | 161 | (174, 350) | 769 | (103, 273) | 338 | DNA PRIMASE (2.7.7.-) | 1DD9\_A DNA PRIMASE (2.7.7.-) TOPRIM, 3-HELIX BUNDLE, DNA-BINDING PROTEIN | | pdb70 | 2AU3\_A | 99.6 | 1.5e-19 | 2e-23 | 179.3 | 162 | (175, 351) | 769 | (184, 352) | 407 | DNA primase (E.C.2.7.7.-) | 2AU3\_A DNA primase (E.C.2.7.7.-) Zinc Ribbon, TOPRIM, RNA POLYMERASE | | pdb70 | 5VAZ\_A | 99.5 | 2.3e-19 | 2.7e-23 | 183.4 | 161 | (173, 349) | 769 | (103, 274) | 389 | DNA primase (E.C.2.7.7.-) | 5VAZ\_A DNA primase (E.C.2.7.7.-) structural genomics, DNA-binding, primase, truncation HET: SO4 | | pdb70 | 5VAZ\_B | 99.5 | 2.8e-19 | 3.3e-23 | 182.9 | 161 | (173, 349) | 769 | (103, 274) | 389 | DNA primase (E.C.2.7.7.-) | 5VAZ\_B DNA primase (E.C.2.7.7.-) structural genomics, DNA-binding, primase, truncation HET: SO4 | | pdb70 | 5W35\_B | 99.5 | 3.5e-19 | 4.4e-23 | 173.8 | 167 | (173, 350) | 769 | (90, 268) | 325 | DNA primase/DNA Complex | 5W35\_B DNA primase/DNA Complex DNA replication, replisome, TOPRIM fold | | pdb70 | 5W36\_B | 99.5 | 3.5e-19 | 4.4e-23 | 173.8 | 167 | (173, 350) | 769 | (90, 268) | 325 | DNA primase/DNA Complex | 5W36\_B DNA primase/DNA Complex DNA replication, replisome, TOPRIM fold | | pdb70 | 4EDG\_A | 99.5 | 4.5e-19 | 5.6e-23 | 175.2 | 160 | (173, 348) | 769 | (91, 257) | 329 | DNA primase (E.C.2.7.7.-) | 4EDG\_A DNA primase (E.C.2.7.7.-) Catalytic Domain, nucleoside triphosphate, nucleoside HET: BEN, ATP | | pdb70 | 4EDK\_A | 99.5 | 4.5e-19 | 5.6e-23 | 175.2 | 160 | (173, 348) | 769 | (91, 257) | 329 | DNA primase (E.C.2.7.7.-) | 4EDK\_A DNA primase (E.C.2.7.7.-) Catalytic Domain, nucleoside triphosphate, nucleoside HET: BEN, GTP | | pdb70 | 4EDR\_A | 99.5 | 4.5e-19 | 5.6e-23 | 175.2 | 160 | (173, 348) | 769 | (91, 257) | 329 | DNA primase (E.C.2.7.7.-) | 4EDR\_A DNA primase (E.C.2.7.7.-) Catalytic Domain, nucleoside triphosphate, nucleoside HET: BEN, UTP | | pdb70 | 5GUJ\_A | 99.5 | 4.5e-19 | 5.7e-23 | 174.5 | 157 | (173, 345) | 769 | (89, 252) | 324 | DNA primase (E.C.2.7.7.-) | 5GUJ\_A DNA primase (E.C.2.7.7.-) TRANSFERASE | | pdb70 | 1NUI\_A | 98.9 | 7.2e-14 | 9.9e-18 | 128.0 | 139 | (178, 343) | 769 | (105, 253) | 255 | DNA primase/helicase (E.C.2.7.7.-) | 1NUI\_A DNA primase/helicase (E.C.2.7.7.-) zinc-biding domain, TOPRIM fold, DNA | | pdb70 | 6N9X\_B | 98.9 | 8.3e-14 | 1.2e-17 | 139.3 | 170 | (148, 349) | 769 | (75, 259) | 566 | DNA-directed DNA polymerase, TrxA/DNA Complex | 6N9X\_B DNA-directed DNA polymerase, TrxA/DNA Complex DNA polymerase, primase, helicase, DNA HET: DOC, TTP | | pdb70 | 6N7I\_D | 98.9 | 9.7e-14 | 1.4e-17 | 138.8 | 170 | (148, 349) | 769 | (75, 259) | 566 | DNA primase/helicase/DNA Complex | 6N7I\_D DNA primase/helicase/DNA Complex helicase, ATPase, hexamer, DNA replication HET: TTP | | pdb70 | 1Q57\_G | 98.8 | 2.9e-13 | 4e-17 | 134.8 | 148 | (178, 349) | 769 | (42, 196) | 503 | DNA primase/helicase (E.C.2.7.7.-) | 1Q57\_G DNA primase/helicase (E.C.2.7.7.-) primase, helicase, dNTPase, DNA replication | | pdb70 | 5IKN\_E | 98.8 | 6.5e-13 | 9.2e-17 | 129.8 | 141 | (178, 345) | 769 | (42, 192) | 486 | DNA-directed DNA polymerase (E.C.2.7.7.7,3.1.11.-), DNA | 5IKN\_E DNA-directed DNA polymerase (E.C.2.7.7.7,3.1.11.-), DNA Replisome, TRANSFERASE | | pdb70 | 5IKN\_H | 98.8 | 6.5e-13 | 9.2e-17 | 129.8 | 141 | (178, 345) | 769 | (42, 192) | 486 | DNA-directed DNA polymerase (E.C.2.7.7.7,3.1.11.-), DNA | 5IKN\_H DNA-directed DNA polymerase (E.C.2.7.7.7,3.1.11.-), DNA Replisome, TRANSFERASE | | pdb70 | 2I5R\_C | 98.3 | 1.3e-10 | 1.6e-14 | 99.1 | 93 | (231, 336) | 769 | (6, 100) | 122 | Hypothetical conserved protein | 2I5R\_C Hypothetical conserved protein Toprim domain, Structural Genomics, PSI-2 HET: GOL, SO4, MES | | pdb70 | 2FCJ\_B | 98.3 | 1.9e-10 | 2.4e-14 | 97.2 | 92 | (232, 336) | 769 | (4, 97) | 119 | small TOPRIM domain protein | 2FCJ\_B small TOPRIM domain protein TOPRIM domain, Structural Genomics, PSI HET: MES, SO4, GOL | | pdb70 | 2FCJ\_C | 98.3 | 1.9e-10 | 2.4e-14 | 97.2 | 92 | (232, 336) | 769 | (4, 97) | 119 | small TOPRIM domain protein | 2FCJ\_C small TOPRIM domain protein TOPRIM domain, Structural Genomics, PSI HET: GOL, SO4, MES | | pdb70 | 1T6T\_1 | 97.4 | 9.1e-08 | 1e-11 | 82.4 | 70 | (232, 312) | 769 | (20, 89) | 118 | putative protein | 1T6T\_1 putative protein structural genomics, Aquifex aeolicus, PSI HET: MSE | | pdb70 | 1T6T\_2 | 97.4 | 9.1e-08 | 1e-11 | 82.4 | 70 | (232, 312) | 769 | (20, 89) | 118 | putative protein | 1T6T\_2 putative protein structural genomics, Aquifex aeolicus, PSI | |
| Top keywords  (threshold 1.00e-03 (evalue)) | **primase, domain\_containing, DNA, Virulence\_associated, E, E\_like, Toprim, Fragment, helicase, ATPase** |
| Output files | ../../similar\_sequences/54\_FANPEZAQ\_CDS\_0054\_merged.svg ../../similar\_sequences/54\_FANPEZAQ\_CDS\_0054\_pdb70.a3m ../../similar\_sequences/54\_FANPEZAQ\_CDS\_0054\_pdb70.hhr ../../similar\_sequences/54\_FANPEZAQ\_CDS\_0054\_uniclust.a3m ../../similar\_sequences/54\_FANPEZAQ\_CDS\_0054\_uniclust.hhr |

#### Structure prediction (AlphaFold)2

|  |  |
| --- | --- |
| Stats | xml version="1.0" encoding="utf-8" standalone="no"?       2024-09-02T21:09:50.850261 image/svg+xml   Matplotlib v3.7.2, https://matplotlib.org/ |
| Predicted structure | **NGL Viewer Controls:**  - Center: *Left-Click* - Rotate: *Left-Click + Drag* - Translate: *Right-Click + Drag* - Zoom: *Shift + Left-Click + Drag* |
| Output files | ../../predicted\_structures/54\_FANPEZAQ\_CDS\_0054/features.pkl ../../predicted\_structures/54\_FANPEZAQ\_CDS\_0054/ranked\_0.pdb ../../predicted\_structures/54\_FANPEZAQ\_CDS\_0054/ranked\_0\_plots.svg ../../predicted\_structures/54\_FANPEZAQ\_CDS\_0054/result\_model\_1\_ptm\_pred\_0.pkl |

#### Structure similarity search results (Foldseek)3

|  |  |
| --- | --- |
| Structure databases searched | Pdb, Afdb-proteome, Afdb-uniprot50 |
| Results, scheme(s)  (Top layers only, threshold 1.00e-02 (evalue)) | xml version="1.0" encoding="utf-8" standalone="no"?       2024-09-02T21:11:28.495615 image/svg+xml   Matplotlib v3.7.2, https://matplotlib.org/ |
| Results, table  (threshold 1.00e-02 (evalue)) | | db | id | prob | evalue | bits | fident | alnlen | mismatch | gapopen | qstart | qend | tstart | tend | name | description | | --- | --- | --- | --- | --- | --- | --- | --- | --- | --- | --- | --- | --- | --- | --- | | pdb | 7PDS\_C | 1.0 | 7.1e-19 | 583 | 0.207 | 449 | 278 | 22 | 370 | 751 | 8 | 445 | Similar to D. nodosus vapE | Similar to D. nodosus vapE | | pdb | 7PDS\_F | 1.0 | 5.486e-19 | 577 | 0.205 | 452 | 275 | 26 | 370 | 751 | 8 | 445 | Similar to D. nodosus vapE | Similar to D. nodosus vapE | | pdb | 7PDS\_B | 1.0 | 2.326e-18 | 575 | 0.204 | 445 | 282 | 22 | 370 | 752 | 8 | 442 | Similar to D. nodosus vapE | Similar to D. nodosus vapE | | pdb | 7PDS\_A | 1.0 | 5.593e-18 | 567 | 0.197 | 456 | 283 | 24 | 368 | 752 | 7 | 450 | Similar to D. nodosus vapE | Similar to D. nodosus vapE | | pdb | 7PDS\_E | 1.0 | 2.326e-18 | 555 | 0.202 | 459 | 286 | 24 | 370 | 761 | 8 | 453 | Similar to D. nodosus vapE | Similar to D. nodosus vapE | | pdb | 7PDS\_D | 1.0 | 1.416e-17 | 544 | 0.202 | 450 | 280 | 23 | 370 | 751 | 8 | 446 | Similar to D. nodosus vapE | Similar to D. nodosus vapE | | pdb | 6K9E\_A | 1.0 | 1.631e-07 | 175 | 0.13 | 443 | 267 | 32 | 384 | 755 | 7 | 402 | Primase | Primase | | pdb | 6K9E\_C | 1.0 | 2.341e-07 | 174 | 0.133 | 442 | 262 | 30 | 384 | 751 | 6 | 400 | Primase | Primase | | pdb | 6LRB\_C | 1.0 | 7.285e-07 | 159 | 0.128 | 450 | 269 | 31 | 384 | 755 | 6 | 410 | Primase | Primase | | pdb | 6LRB\_D | 1.0 | 2.786e-06 | 158 | 0.125 | 447 | 271 | 31 | 384 | 755 | 8 | 409 | Primase | Primase | | pdb | 6K9C\_A | 1.0 | 4.348e-07 | 157 | 0.138 | 447 | 272 | 26 | 384 | 755 | 8 | 416 | Primase | Primase | | pdb | 6K9E\_F | 1.0 | 7.67e-07 | 153 | 0.128 | 444 | 262 | 30 | 384 | 751 | 7 | 401 | Primase | Primase | | pdb | 6LRB\_F | 1.0 | 2.153e-06 | 152 | 0.138 | 442 | 272 | 29 | 384 | 755 | 6 | 408 | Primase | Primase | | pdb | 5W33\_A | 1.0 | 2.698e-05 | 145 | 0.154 | 279 | 157 | 15 | 123 | 345 | 5 | 260 | DNA primase | DNA primase | | pdb | 6LRB\_A | 1.0 | 2.513e-06 | 145 | 0.137 | 415 | 263 | 26 | 384 | 745 | 7 | 379 | Primase | Primase | | pdb | 7OLA\_B | 1.0 | 2.153e-06 | 141 | 0.115 | 432 | 271 | 29 | 372 | 731 | 26 | 418 | DNA primase | DNA primase | | pdb | 7OLA\_D | 1.0 | 1.122e-05 | 138 | 0.12 | 375 | 238 | 25 | 374 | 685 | 28 | 373 | DNA primase | DNA primase | | pdb | 2AU3\_A | 1.0 | 2.991e-05 | 138 | 0.131 | 326 | 189 | 20 | 70 | 347 | 67 | 346 | DNA primase | DNA primase | | pdb | 5W36\_B | 1.0 | 6.83e-05 | 134 | 0.16 | 287 | 154 | 14 | 123 | 349 | 6 | 265 | DNA primase | DNA primase | | pdb | 7OLA\_A | 1.0 | 1.98e-05 | 131 | 0.105 | 434 | 281 | 31 | 372 | 735 | 26 | 422 | DNA primase | DNA primase | | pdb | 8IQI\_E | 1.0 | 4.348e-07 | 128 | 0.105 | 856 | 433 | 58 | 13 | 766 | 283 | 907 | Putative primase C962R | Putative primase C962R | | pdb | 3B39\_A | 1.0 | 5.85e-05 | 127 | 0.157 | 247 | 133 | 18 | 143 | 352 | 52 | 260 | DNA primase | DNA primase | | pdb | 7OLA\_C | 1.0 | 1.786e-05 | 127 | 0.109 | 431 | 275 | 29 | 370 | 730 | 24 | 415 | DNA primase | DNA primase | | pdb | 5GUJ\_A | 1.0 | 2.311e-05 | 126 | 0.084 | 333 | 184 | 20 | 154 | 447 | 73 | 323 | DNA primase | DNA primase | | pdb | 8IQI\_B | 1.0 | 9.429e-07 | 125 | 0.104 | 845 | 430 | 64 | 13 | 758 | 283 | 899 | Putative primase C962R | Putative primase C962R | | pdb | 3B39\_B | 1.0 | 9.801e-05 | 124 | 0.124 | 387 | 191 | 24 | 124 | 446 | 3 | 305 | DNA primase | DNA primase | | pdb | 1DD9\_A | 1.0 | 4.52e-05 | 124 | 0.121 | 347 | 177 | 19 | 153 | 444 | 28 | 301 | DNA PRIMASE | DNA PRIMASE | | pdb | 7OM0\_F | 1.0 | 3.15e-05 | 124 | 0.125 | 423 | 261 | 30 | 384 | 735 | 38 | 422 | DNA primase | DNA primase | | pdb | 1DDE\_A | 1.0 | 5.011e-05 | 122 | 0.133 | 359 | 166 | 22 | 153 | 447 | 28 | 305 | DNA PRIMASE | DNA PRIMASE | | pdb | 5W34\_A | 1.0 | 0.0001336 | 122 | 0.125 | 384 | 197 | 20 | 126 | 444 | 5 | 314 | DNA primase | DNA primase | | pdb | 7OLA\_E | 1.0 | 0.0004608 | 116 | 0.151 | 185 | 127 | 14 | 492 | 665 | 174 | 339 | DNA primase | DNA primase | | pdb | 1EQN\_E | 1.0 | 0.0001144 | 114 | 0.136 | 264 | 136 | 20 | 153 | 350 | 28 | 265 | DNA PRIMASE | DNA PRIMASE | | pdb | 7OLA\_F | 1.0 | 0.0001144 | 112 | 0.095 | 344 | 214 | 23 | 372 | 653 | 26 | 334 | DNA primase | DNA primase | | pdb | 8IQI\_A | 1.0 | 9.13e-06 | 104 | 0.103 | 825 | 432 | 56 | 13 | 750 | 284 | 887 | Putative primase C962R | Putative primase C962R | | pdb | 4EDV\_A | 1.0 | 9.801e-05 | 103 | 0.121 | 347 | 182 | 20 | 139 | 444 | 46 | 310 | DNA primase | DNA primase | | pdb | 8APL\_A | 1.0 | 0.000305 | 101 | 0.107 | 473 | 286 | 28 | 381 | 752 | 7 | 444 | Primase D5 | Primase D5 | | pdb | 8IQH\_D | 0.999 | 6.362e-06 | 98 | 0.105 | 837 | 417 | 61 | 12 | 762 | 278 | 868 | Putative primase C962R | Putative primase C962R | | pdb | 4E2K\_A | 0.999 | 0.0001729 | 97 | 0.122 | 342 | 185 | 21 | 139 | 444 | 46 | 308 | DNA primase | DNA primase | | pdb | 8APM\_F | 0.998 | 0.0005664 | 95 | 0.108 | 369 | 224 | 24 | 370 | 657 | 1 | 345 | Primase D5 | Primase D5 | | pdb | 8B9A\_6 | 0.996 | 6.83e-05 | 90 | 0.117 | 333 | 212 | 20 | 456 | 735 | 319 | 622 | DNA replication licensing factor MCM6 | DNA replication licensing factor MCM6 | | pdb | 8IQH\_G | 0.996 | 4.293e-05 | 90 | 0.094 | 806 | 434 | 54 | 12 | 762 | 276 | 840 | Putative primase C962R | Putative primase C962R | | pdb | 8IQH\_B | 0.996 | 2.698e-05 | 90 | 0.105 | 824 | 430 | 57 | 12 | 758 | 280 | 873 | Putative primase C962R | Putative primase C962R | | pdb | 5VAZ\_A | 0.995 | 0.0008559 | 88 | 0.136 | 315 | 156 | 19 | 169 | 442 | 80 | 319 | DNA primase | DNA primase | | pdb | 7PT7\_F | 0.994 | 0.0002125 | 87 | 0.116 | 344 | 212 | 19 | 456 | 735 | 298 | 613 | DNA replication licensing factor MCM6 | DNA replication licensing factor MCM6 | | pdb | 8IQH\_M | 0.994 | 3.872e-05 | 87 | 0.097 | 811 | 420 | 54 | 13 | 755 | 275 | 841 | Putative primase C962R | Putative primase C962R | | pdb | 7PT6\_F | 0.993 | 0.0001336 | 86 | 0.123 | 339 | 218 | 20 | 456 | 735 | 315 | 633 | DNA replication licensing factor MCM6 | DNA replication licensing factor MCM6 | | pdb | 6SKL\_3 | 0.991 | 0.0001917 | 84 | 0.112 | 339 | 209 | 23 | 468 | 738 | 302 | 616 | DNA replication licensing factor MCM3 | DNA replication licensing factor MCM3 | | pdb | 2V9P\_E | 0.99 | 0.003109 | 83 | 0.109 | 265 | 193 | 17 | 416 | 657 | 21 | 265 | REPLICATION PROTEIN E1 | REPLICATION PROTEIN E1 | | pdb | 7QHS\_3 | 0.99 | 0.0002612 | 83 | 0.097 | 360 | 220 | 21 | 456 | 735 | 294 | 628 | DNA replication licensing factor MCM3 | DNA replication licensing factor MCM3 | | pdb | 3JC7\_2 | 0.988 | 0.0003749 | 82 | 0.098 | 326 | 206 | 20 | 470 | 736 | 261 | 557 | DNA replication licensing factor MCM2 | DNA replication licensing factor MCM2 | | pdb | 7W1Y\_2 | 0.988 | 0.0002238 | 82 | 0.092 | 346 | 225 | 24 | 456 | 735 | 281 | 603 | DNA replication licensing factor MCM2 | DNA replication licensing factor MCM2 | | pdb | 8IQH\_C | 0.988 | 9.801e-05 | 82 | 0.108 | 826 | 425 | 63 | 12 | 758 | 279 | 871 | Putative primase C962R | Putative primase C962R | | pdb | 8B9A\_4 | 0.986 | 0.0002751 | 81 | 0.118 | 337 | 210 | 25 | 457 | 735 | 303 | 610 | DNA replication licensing factor MCM4 | DNA replication licensing factor MCM4 | | pdb | 7W1Y\_E | 0.986 | 5.011e-05 | 81 | 0.095 | 409 | 247 | 24 | 402 | 735 | 277 | 637 | DNA replication licensing factor MCM6 | DNA replication licensing factor MCM6 | | pdb | 7W1Y\_A | 0.984 | 0.0002896 | 80 | 0.102 | 350 | 220 | 26 | 453 | 735 | 277 | 599 | DNA replication licensing factor MCM2 | DNA replication licensing factor MCM2 | | pdb | 6MII\_A | 0.978 | 0.0002896 | 78 | 0.111 | 342 | 217 | 23 | 456 | 738 | 267 | 580 | Minichromosome maintenance protein MCM | Minichromosome maintenance protein MCM | | pdb | 6WGI\_3 | 0.975 | 0.001108 | 77 | 0.107 | 344 | 206 | 19 | 456 | 735 | 260 | 566 | DNA replication licensing factor MCM3 | DNA replication licensing factor MCM3 | | pdb | 7PMN\_5 | 0.975 | 0.0009992 | 77 | 0.128 | 272 | 160 | 19 | 457 | 682 | 259 | 499 | Minichromosome maintenance protein 5 | Minichromosome maintenance protein 5 | | pdb | 7PLO\_2 | 0.975 | 0.001293 | 77 | 0.095 | 335 | 215 | 21 | 456 | 737 | 292 | 591 | DNA replication licensing factor MCM2 | DNA replication licensing factor MCM2 | | pdb | 1SVM\_C | 0.971 | 0.003109 | 76 | 0.134 | 313 | 180 | 21 | 415 | 677 | 66 | 337 | large T antigen | large T antigen | | pdb | 6PTN\_M | 0.971 | 0.000356 | 76 | 0.114 | 358 | 198 | 23 | 456 | 734 | 290 | 607 | DNA replication licensing factor MCM6 | DNA replication licensing factor MCM6 | | pdb | 3JC5\_2 | 0.967 | 0.0006963 | 75 | 0.102 | 333 | 205 | 20 | 470 | 736 | 261 | 565 | DNA replication licensing factor MCM2 | DNA replication licensing factor MCM2 | | pdb | 5U8T\_3 | 0.967 | 0.001674 | 75 | 0.121 | 395 | 214 | 27 | 405 | 736 | 264 | 588 | DNA replication licensing factor MCM3 | DNA replication licensing factor MCM3 | | pdb | 5U8S\_3 | 0.967 | 0.001856 | 75 | 0.112 | 354 | 197 | 25 | 456 | 735 | 279 | 589 | DNA replication licensing factor MCM3 | DNA replication licensing factor MCM3 | | pdb | 6XTY\_3 | 0.967 | 0.0003947 | 75 | 0.092 | 378 | 210 | 28 | 456 | 757 | 288 | 608 | DNA replication licensing factor MCM3 | DNA replication licensing factor MCM3 | | pdb | 8OUW\_6 | 0.967 | 0.001052 | 75 | 0.093 | 353 | 222 | 24 | 455 | 735 | 309 | 635 | DNA replication licensing factor mcm-6 | DNA replication licensing factor mcm-6 | | pdb | 6SKO\_3 | 0.961 | 0.0008129 | 74 | 0.11 | 352 | 208 | 23 | 456 | 735 | 13 | 331 | DNA replication licensing factor MCM3 | DNA replication licensing factor MCM3 | | pdb | 7PMN\_6 | 0.961 | 0.0002481 | 74 | 0.102 | 399 | 238 | 25 | 403 | 735 | 293 | 637 | DNA replication licensing factor MCM6 | DNA replication licensing factor MCM6 | | pdb | 8IQH\_E | 0.961 | 0.0001642 | 74 | 0.116 | 671 | 334 | 51 | 243 | 762 | 298 | 860 | Putative primase C962R | Putative primase C962R | | pdb | 8IQH\_A | 0.956 | 0.0004608 | 73 | 0.102 | 830 | 423 | 56 | 12 | 762 | 277 | 863 | Putative primase C962R | Putative primase C962R | | pdb | 7PMK\_3 | 0.949 | 0.0009992 | 72 | 0.103 | 358 | 209 | 25 | 456 | 738 | 287 | 607 | DNA replication licensing factor MCM3 | DNA replication licensing factor MCM3 | | pdb | 8OUW\_3 | 0.949 | 0.0009992 | 72 | 0.103 | 376 | 222 | 22 | 456 | 752 | 276 | 615 | DNA replication licensing factor MCM3 | DNA replication licensing factor MCM3 | | pdb | 7V3V\_C | 0.949 | 0.001293 | 72 | 0.105 | 369 | 213 | 22 | 456 | 735 | 295 | 635 | DNA replication licensing factor MCM3 | DNA replication licensing factor MCM3 | | pdb | 6WGG\_3 | 0.949 | 0.002663 | 72 | 0.103 | 347 | 204 | 20 | 456 | 735 | 265 | 571 | DNA replication licensing factor MCM3 | DNA replication licensing factor MCM3 | | pdb | 7PMK\_2 | 0.949 | 0.0006613 | 72 | 0.097 | 369 | 215 | 22 | 457 | 737 | 296 | 634 | DNA replication licensing factor MCM2 | DNA replication licensing factor MCM2 | | pdb | 6SKL\_4 | 0.949 | 0.002663 | 72 | 0.093 | 407 | 223 | 28 | 456 | 747 | 301 | 676 | DNA replication licensing factor MCM4 | DNA replication licensing factor MCM4 | | pdb | 8IQH\_J | 0.949 | 0.0005964 | 72 | 0.088 | 827 | 437 | 53 | 12 | 762 | 273 | 858 | Putative primase C962R | Putative primase C962R | | pdb | 7PT6\_2 | 0.941 | 0.000538 | 71 | 0.107 | 344 | 196 | 26 | 457 | 735 | 291 | 588 | DNA replication licensing factor MCM2 | DNA replication licensing factor MCM2 | | pdb | 6XTX\_6 | 0.941 | 0.001434 | 71 | 0.107 | 373 | 224 | 26 | 456 | 760 | 280 | 611 | DNA replication licensing factor MCM6 | DNA replication licensing factor MCM6 | | pdb | 7V3U\_G | 0.941 | 0.001108 | 71 | 0.129 | 356 | 201 | 27 | 456 | 735 | 370 | 692 | DNA replication licensing factor MCM7 | DNA replication licensing factor MCM7 | | pdb | 4GDF\_A | 0.933 | 0.005485 | 70 | 0.118 | 322 | 195 | 22 | 415 | 677 | 180 | 471 | Large T antigen | Large T antigen | | pdb | 6WGG\_2 | 0.933 | 0.003274 | 70 | 0.112 | 347 | 208 | 19 | 457 | 737 | 251 | 563 | DNA replication licensing factor MCM2 | DNA replication licensing factor MCM2 | | pdb | 5XF8\_3 | 0.933 | 0.0009992 | 70 | 0.117 | 348 | 200 | 23 | 456 | 736 | 293 | 600 | DNA replication licensing factor MCM3 | DNA replication licensing factor MCM3 | | pdb | 7PFO\_5 | 0.933 | 0.002663 | 70 | 0.116 | 267 | 164 | 19 | 457 | 675 | 273 | 515 | DNA replication licensing factor MCM5 | DNA replication licensing factor MCM5 | | pdb | 7PLO\_4 | 0.933 | 0.001362 | 70 | 0.145 | 358 | 210 | 26 | 456 | 746 | 273 | 601 | DNA replication licensing factor MCM4 | DNA replication licensing factor MCM4 | | pdb | 7QHS\_6 | 0.933 | 0.000356 | 70 | 0.131 | 351 | 196 | 24 | 456 | 735 | 316 | 628 | DNA replication licensing factor MCM6 | DNA replication licensing factor MCM6 | | pdb | 7W1Y\_4 | 0.933 | 0.0005109 | 70 | 0.11 | 362 | 216 | 22 | 445 | 737 | 316 | 640 | DNA replication licensing factor MCM4 | DNA replication licensing factor MCM4 | | pdb | 7PMN\_2 | 0.933 | 0.0009489 | 70 | 0.118 | 362 | 210 | 25 | 457 | 735 | 297 | 632 | DNA replication licensing factor MCM2 | DNA replication licensing factor MCM2 | | pdb | 7V3U\_E | 0.933 | 0.001108 | 70 | 0.119 | 367 | 205 | 26 | 457 | 737 | 313 | 647 | Minichromosome maintenance protein 5 | Minichromosome maintenance protein 5 | | pdb | 6SKL\_2 | 0.933 | 0.001674 | 70 | 0.101 | 366 | 212 | 24 | 457 | 735 | 310 | 645 | DNA replication licensing factor MCM2 | DNA replication licensing factor MCM2 | | pdb | 1QVR\_C | 0.933 | 0.006081 | 70 | 0.107 | 288 | 177 | 20 | 420 | 684 | 465 | 695 | ClpB protein | ClpB protein | | pdb | 6WGF\_3 | 0.923 | 0.005485 | 69 | 0.1 | 347 | 205 | 19 | 456 | 735 | 258 | 564 | DNA replication licensing factor MCM3 | DNA replication licensing factor MCM3 | | pdb | 8EAF\_B | 0.923 | 0.001166 | 69 | 0.101 | 353 | 210 | 27 | 456 | 737 | 267 | 583 | Minichromosome maintenance protein MCM | Minichromosome maintenance protein MCM | | pdb | 6XTY\_6 | 0.923 | 0.00159 | 69 | 0.1 | 369 | 217 | 27 | 456 | 751 | 280 | 606 | DNA replication licensing factor MCM6 | DNA replication licensing factor MCM6 | | pdb | 3JA8\_5 | 0.923 | 0.0008559 | 69 | 0.116 | 413 | 238 | 25 | 402 | 737 | 267 | 629 | Minichromosome Maintenance 5 | Minichromosome Maintenance 5 | | pdb | 7T8C\_B | 0.912 | 0.0003381 | 68 | 0.101 | 564 | 272 | 37 | 137 | 625 | 73 | 476 | Twinkle mtDNA helicase | Twinkle mtDNA helicase | | pdb | 5J47\_A | 0.9 | 0.004237 | 67 | 0.114 | 332 | 194 | 21 | 415 | 683 | 66 | 360 | Large T antigen | Large T antigen | | pdb | 8E2L\_A | 0.9 | 0.002804 | 67 | 0.098 | 526 | 277 | 25 | 143 | 616 | 7 | 387 | Twinkle mtDNA helicase | Twinkle mtDNA helicase | | pdb | 3JC5\_3 | 0.9 | 0.00151 | 67 | 0.106 | 396 | 218 | 21 | 405 | 736 | 264 | 587 | DNA replication licensing factor MCM3 | DNA replication licensing factor MCM3 | | pdb | 7PT7\_C | 0.9 | 0.002402 | 67 | 0.102 | 382 | 210 | 22 | 456 | 738 | 284 | 631 | DNA replication licensing factor MCM3 | DNA replication licensing factor MCM3 | | pdb | 7W1Y\_B | 0.9 | 0.001052 | 67 | 0.102 | 401 | 217 | 24 | 456 | 749 | 283 | 647 | Isoform 2 of DNA replication licensing factor MCM3 | Isoform 2 of DNA replication licensing factor MCM3 | | pdb | 3JC7\_5 | 0.9 | 0.001166 | 67 | 0.106 | 412 | 238 | 26 | 402 | 737 | 238 | 595 | Minichromosome maintenance protein 5 | Minichromosome maintenance protein 5 | | pdb | 7QHS\_2 | 0.9 | 0.00151 | 67 | 0.095 | 368 | 211 | 25 | 457 | 735 | 303 | 637 | DNA replication licensing factor MCM2 | DNA replication licensing factor MCM2 | | pdb | 5V8F\_7 | 0.9 | 0.001856 | 67 | 0.139 | 336 | 201 | 28 | 456 | 736 | 335 | 637 | DNA replication licensing factor MCM7 | DNA replication licensing factor MCM7 | | pdb | 5V8F\_4 | 0.9 | 0.0005109 | 67 | 0.089 | 505 | 260 | 32 | 403 | 750 | 278 | 739 | DNA replication licensing factor MCM4 | DNA replication licensing factor MCM4 | | pdb | 6WGI\_2 | 0.887 | 0.002804 | 66 | 0.108 | 351 | 205 | 21 | 457 | 737 | 243 | 555 | DNA replication licensing factor MCM2 | DNA replication licensing factor MCM2 | | pdb | 7PFO\_4 | 0.887 | 0.001108 | 66 | 0.119 | 403 | 211 | 28 | 402 | 735 | 263 | 590 | DNA replication licensing factor MCM4 | DNA replication licensing factor MCM4 | | pdb | 7W1Y\_F | 0.887 | 0.002167 | 66 | 0.115 | 339 | 199 | 25 | 471 | 737 | 331 | 640 | DNA replication licensing factor MCM7 | DNA replication licensing factor MCM7 | | pdb | 5V8F\_3 | 0.887 | 0.00159 | 66 | 0.105 | 407 | 219 | 26 | 402 | 736 | 251 | 584 | DNA replication licensing factor MCM3 | DNA replication licensing factor MCM3 | | pdb | 5V8F\_6 | 0.887 | 0.00159 | 66 | 0.108 | 359 | 217 | 22 | 456 | 745 | 297 | 621 | DNA replication licensing factor MCM6 | DNA replication licensing factor MCM6 | | pdb | 8OUW\_4 | 0.872 | 0.003109 | 65 | 0.113 | 351 | 211 | 22 | 456 | 738 | 301 | 619 | DNA replication licensing factor mcm-4 | DNA replication licensing factor mcm-4 | | pdb | 7PT6\_C | 0.872 | 0.006742 | 65 | 0.108 | 359 | 223 | 20 | 456 | 735 | 291 | 631 | DNA replication licensing factor MCM3 | DNA replication licensing factor MCM3 | | pdb | 6EYC\_7 | 0.872 | 0.001954 | 65 | 0.129 | 341 | 209 | 26 | 453 | 736 | 354 | 663 | DNA replication licensing factor MCM7 | DNA replication licensing factor MCM7 | | pdb | 6PTN\_J | 0.855 | 0.002953 | 64 | 0.122 | 374 | 206 | 26 | 430 | 735 | 273 | 592 | DNA replication licensing factor MCM3 | DNA replication licensing factor MCM3 | | pdb | 5BK4\_B | 0.855 | 0.001293 | 64 | 0.102 | 341 | 195 | 23 | 467 | 735 | 301 | 602 | DNA replication licensing factor MCM3 | DNA replication licensing factor MCM3 | | pdb | 7PLO\_7 | 0.855 | 0.002953 | 64 | 0.112 | 339 | 201 | 23 | 465 | 737 | 300 | 604 | DNA replication licensing factor MCM7 | DNA replication licensing factor MCM7 | | pdb | 7V3V\_B | 0.855 | 0.002529 | 64 | 0.115 | 345 | 214 | 25 | 457 | 736 | 289 | 607 | DNA replication licensing factor MCM2 | DNA replication licensing factor MCM2 | | pdb | 7PMK\_7 | 0.855 | 0.003447 | 64 | 0.122 | 344 | 205 | 25 | 456 | 737 | 317 | 625 | DNA replication licensing factor MCM7 | DNA replication licensing factor MCM7 | | pdb | 8OUW\_7 | 0.855 | 0.001954 | 64 | 0.125 | 358 | 201 | 30 | 457 | 737 | 304 | 626 | DNA replication licensing factor MCM7 | DNA replication licensing factor MCM7 | | pdb | 7W1Y\_6 | 0.855 | 0.001362 | 64 | 0.099 | 413 | 241 | 29 | 402 | 735 | 278 | 638 | DNA replication licensing factor MCM6 | DNA replication licensing factor MCM6 | | pdb | 6WGF\_7 | 0.837 | 0.00363 | 63 | 0.123 | 364 | 218 | 28 | 449 | 737 | 222 | 559 | DNA replication licensing factor MCM7 | DNA replication licensing factor MCM7 | | pdb | 8B9D\_6 | 0.837 | 0.002953 | 63 | 0.116 | 343 | 205 | 24 | 456 | 736 | 296 | 602 | DNA replication licensing factor MCM6 | DNA replication licensing factor MCM6 | | pdb | 7QHS\_4 | 0.837 | 0.002663 | 63 | 0.108 | 350 | 202 | 24 | 457 | 738 | 302 | 609 | DNA replication licensing factor MCM4 | DNA replication licensing factor MCM4 | | pdb | 7P30\_2 | 0.837 | 0.002663 | 63 | 0.115 | 347 | 211 | 21 | 457 | 736 | 287 | 604 | DNA replication licensing factor MCM2 | DNA replication licensing factor MCM2 | | pdb | 7PMK\_5 | 0.837 | 0.003822 | 63 | 0.108 | 361 | 216 | 24 | 457 | 737 | 272 | 606 | DNA helicase | DNA helicase | | pdb | 7PT6\_7 | 0.837 | 0.0009992 | 63 | 0.146 | 362 | 182 | 30 | 457 | 733 | 357 | 676 | DNA replication licensing factor MCM7 | DNA replication licensing factor MCM7 | | pdb | 1G8P\_A | 0.817 | 0.006081 | 62 | 0.138 | 317 | 167 | 24 | 495 | 735 | 15 | 301 | MAGNESIUM-CHELATASE 38 KDA SUBUNIT | MAGNESIUM-CHELATASE 38 KDA SUBUNIT | | pdb | 7WI7\_A | 0.817 | 0.008287 | 62 | 0.127 | 321 | 192 | 21 | 456 | 736 | 288 | 560 | DNA helicase MCM8 | DNA helicase MCM8 | | pdb | 8B9D\_3 | 0.817 | 0.005209 | 62 | 0.106 | 356 | 215 | 20 | 456 | 735 | 237 | 565 | DNA replication licensing factor MCM3 | DNA replication licensing factor MCM3 | | pdb | 6WGG\_7 | 0.817 | 0.004237 | 62 | 0.123 | 364 | 218 | 28 | 449 | 737 | 248 | 585 | DNA replication licensing factor MCM7 | DNA replication licensing factor MCM7 | | pdb | 3JC5\_5 | 0.817 | 0.003274 | 62 | 0.109 | 357 | 211 | 24 | 457 | 737 | 269 | 594 | Minichromosome maintenance protein 5 | Minichromosome maintenance protein 5 | | pdb | 7P30\_F | 0.817 | 0.002058 | 62 | 0.143 | 342 | 197 | 24 | 456 | 738 | 361 | 665 | DNA replication licensing factor MCM7 | DNA replication licensing factor MCM7 | | pdb | 7PMK\_4 | 0.795 | 0.004462 | 61 | 0.127 | 337 | 201 | 23 | 457 | 738 | 299 | 597 | DNA replication licensing factor MCM4 | DNA replication licensing factor MCM4 | | pdb | 7P30\_E | 0.795 | 0.009188 | 61 | 0.127 | 345 | 203 | 21 | 457 | 735 | 289 | 601 | DNA replication licensing factor MCM6 | DNA replication licensing factor MCM6 | | pdb | 7W1Y\_3 | 0.795 | 0.001166 | 61 | 0.093 | 479 | 238 | 26 | 404 | 751 | 235 | 648 | Isoform 2 of DNA replication licensing factor MCM3 | Isoform 2 of DNA replication licensing factor MCM3 | | pdb | 8A8W\_D | 0.772 | 0.001434 | 60 | 0.125 | 479 | 249 | 32 | 232 | 618 | 44 | 444 | ATP-dependent Clp protease ATP-binding subunit ClpC1 | ATP-dependent Clp protease ATP-binding subunit ClpC1 | | pdb | 7PT7\_3 | 0.747 | 0.004698 | 59 | 0.099 | 392 | 204 | 30 | 456 | 739 | 284 | 634 | DNA replication licensing factor MCM3 | DNA replication licensing factor MCM3 | | pdb | 6F0L\_4 | 0.747 | 0.002167 | 59 | 0.096 | 416 | 244 | 28 | 403 | 737 | 274 | 638 | DNA replication licensing factor MCM4 | DNA replication licensing factor MCM4 | | pdb | 7W1Y\_5 | 0.747 | 0.004947 | 59 | 0.104 | 343 | 208 | 29 | 457 | 738 | 306 | 610 | DNA replication licensing factor MCM5 | DNA replication licensing factor MCM5 | | pdb | 3JC7\_3 | 0.72 | 0.004462 | 58 | 0.123 | 371 | 198 | 25 | 446 | 736 | 253 | 576 | DNA replication licensing factor MCM3 | DNA replication licensing factor MCM3 | | pdb | 7PFO\_7 | 0.692 | 0.008726 | 57 | 0.116 | 342 | 207 | 25 | 458 | 737 | 284 | 592 | DNA replication licensing factor MCM7 | DNA replication licensing factor MCM7 | | pdb | 7PT7\_E | 0.692 | 0.003274 | 57 | 0.109 | 348 | 198 | 28 | 457 | 735 | 294 | 598 | Minichromosome maintenance protein 5 | Minichromosome maintenance protein 5 | | pdb | 7QHS\_5 | 0.663 | 0.007871 | 56 | 0.086 | 449 | 229 | 27 | 457 | 753 | 270 | 689 | DNA replication licensing factor MCM5 | DNA replication licensing factor MCM5 | | pdb | 7PLO\_5 | 0.632 | 0.006742 | 55 | 0.109 | 357 | 213 | 30 | 457 | 736 | 282 | 610 | DNA replication licensing factor MCM5 | DNA replication licensing factor MCM5 | | pdb | 7PT6\_E | 0.632 | 0.003274 | 55 | 0.109 | 365 | 211 | 26 | 457 | 737 | 299 | 633 | Minichromosome maintenance protein 5 | Minichromosome maintenance protein 5 | | pdb | 7W1Y\_C | 0.632 | 0.004462 | 55 | 0.107 | 411 | 224 | 25 | 402 | 737 | 296 | 638 | DNA replication licensing factor MCM4 | DNA replication licensing factor MCM4 | | pdb | 7P5Z\_D | 0.632 | 0.005485 | 55 | 0.107 | 372 | 204 | 28 | 457 | 737 | 308 | 642 | Minichromosome maintenance protein 5 | Minichromosome maintenance protein 5 | | pdb | 7PT6\_4 | 0.632 | 0.009188 | 55 | 0.113 | 413 | 243 | 28 | 403 | 737 | 282 | 649 | DNA replication licensing factor MCM4 | DNA replication licensing factor MCM4 | | pdb | 7W8G\_D | 0.632 | 0.004698 | 55 | 0.114 | 374 | 222 | 26 | 439 | 737 | 312 | 651 | DNA replication licensing factor MCM4 | DNA replication licensing factor MCM4 | | pdb | 6XTY\_4 | 0.601 | 0.006081 | 54 | 0.128 | 405 | 219 | 29 | 403 | 735 | 254 | 596 | DNA replication licensing factor MCM4 | DNA replication licensing factor MCM4 | | pdb | 7UIV\_F | 0.505 | 0.008287 | 51 | 0.101 | 474 | 243 | 30 | 235 | 615 | 41 | 424 | ATP-dependent Clp protease ATP-binding subunit ClpA | ATP-dependent Clp protease ATP-binding subunit ClpA | | pdb | 7MGM\_A | 0.505 | 0.005209 | 51 | 0.09 | 496 | 239 | 31 | 381 | 739 | 164 | 584 | dynein AAA3-WalkerB mutant (E2488Q) | dynein AAA3-WalkerB mutant (E2488Q) | | afdb-proteome | AF-A0A077ZFG4-F1-MODEL\_V4 | 1.0 | 5.739e-14 | 550 | 0.289 | 235 | 145 | 9 | 115 | 346 | 157 | 372 | Toprim 4 domain containing protein | Toprim 4 domain containing protein | | afdb-proteome | AF-A0A0H3GIU0-F1-MODEL\_V4 | 1.0 | 1.237e-24 | 472 | 0.174 | 812 | 456 | 47 | 64 | 761 | 67 | 777 | Bacteriophage P4 DNA primase | Bacteriophage P4 DNA primase | | afdb-proteome | AF-Q2FYB7-F1-MODEL\_V4 | 1.0 | 3.03e-15 | 465 | 0.222 | 360 | 237 | 19 | 317 | 651 | 323 | 664 | Conserved hypothetical phage protein | Conserved hypothetical phage protein | | afdb-proteome | AF-A0A0H3GLV7-F1-MODEL\_V4 | 1.0 | 1.718e-23 | 436 | 0.176 | 812 | 454 | 50 | 64 | 761 | 67 | 777 | Nucleoside triphosphatase, D5 family | Nucleoside triphosphatase, D5 family | | afdb-proteome | AF-A0A0H3H6B1-F1-MODEL\_V4 | 1.0 | 2.296e-08 | 255 | 0.176 | 301 | 180 | 21 | 91 | 346 | 289 | 566 | Prim\_Zn\_Ribbon domain-containing protein | Prim\_Zn\_Ribbon domain-containing protein | | afdb-proteome | AF-A0A0H3GM72-F1-MODEL\_V4 | 1.0 | 1.045e-06 | 173 | 0.143 | 292 | 163 | 19 | 71 | 331 | 72 | 307 | Putative phage DNA primase | Putative phage DNA primase | | afdb-proteome | AF-O06608-F1-MODEL\_V4 | 1.0 | 2.465e-07 | 165 | 0.135 | 510 | 292 | 36 | 348 | 762 | 16 | 471 | Probable PhiRv1 phage protein | Probable PhiRv1 phage protein | | afdb-proteome | AF-Q8ZQI0-F1-MODEL\_V4 | 1.0 | 1.182e-05 | 151 | 0.105 | 304 | 180 | 19 | 60 | 331 | 60 | 303 | Fels-1 putative prophage DNA primase | Fels-1 putative prophage DNA primase | | afdb-proteome | AF-Q8DPW1-F1-MODEL\_V4 | 1.0 | 2.646e-06 | 128 | 0.103 | 485 | 295 | 30 | 70 | 503 | 74 | 469 | DNA primase | DNA primase | | afdb-proteome | AF-A0A132Z8R3-F1-MODEL\_V4 | 1.0 | 5.449e-06 | 122 | 0.109 | 504 | 282 | 33 | 77 | 505 | 79 | 490 | DNA primase | DNA primase | | afdb-proteome | AF-A0A0H3GVT7-F1-MODEL\_V4 | 1.0 | 1.045e-06 | 115 | 0.105 | 765 | 346 | 40 | 61 | 738 | 69 | 581 | DNA primase | DNA primase | | afdb-proteome | AF-P07362-F1-MODEL\_V4 | 1.0 | 1.353e-06 | 114 | 0.106 | 750 | 345 | 46 | 69 | 738 | 77 | 581 | DNA primase | DNA primase | | afdb-proteome | AF-P0ABS5-F1-MODEL\_V4 | 1.0 | 1.663e-06 | 113 | 0.099 | 753 | 363 | 43 | 61 | 738 | 69 | 581 | DNA primase | DNA primase | | afdb-proteome | AF-Q54Q10-F1-MODEL\_V4 | 1.0 | 0.000182 | 113 | 0.107 | 354 | 232 | 22 | 470 | 760 | 284 | 616 | Uncharacterized protein | Uncharacterized protein | | afdb-proteome | AF-Q5F806-F1-MODEL\_V4 | 1.0 | 9.129e-06 | 109 | 0.146 | 519 | 242 | 34 | 77 | 492 | 76 | 496 | DNA primase | DNA primase | | afdb-proteome | AF-Q32BQ1-F1-MODEL\_V4 | 1.0 | 2.646e-06 | 108 | 0.099 | 761 | 355 | 46 | 61 | 738 | 69 | 581 | DNA primase | DNA primase | | afdb-proteome | AF-Q08346-F1-MODEL\_V4 | 1.0 | 6.699e-06 | 104 | 0.111 | 521 | 275 | 34 | 68 | 510 | 76 | 486 | DNA primase | DNA primase | | afdb-proteome | AF-A0A0R4ICC1-F1-MODEL\_V4 | 1.0 | 7.82e-06 | 104 | 0.112 | 649 | 286 | 34 | 60 | 616 | 150 | 600 | Twinkle mtDNA helicase | Twinkle mtDNA helicase | | afdb-proteome | AF-Q2FY11-F1-MODEL\_V4 | 0.999 | 1.529e-05 | 99 | 0.1 | 747 | 373 | 42 | 67 | 737 | 73 | 596 | DNA primase | DNA primase | | afdb-proteome | AF-I6Y1F0-F1-MODEL\_V4 | 0.999 | 0.0005664 | 98 | 0.109 | 373 | 218 | 29 | 456 | 751 | 27 | 362 | Possible PhiRv2 prophage protein | Possible PhiRv2 prophage protein | | afdb-proteome | AF-K0EWG0-F1-MODEL\_V4 | 0.999 | 1.012e-05 | 97 | 0.125 | 533 | 263 | 30 | 143 | 595 | 161 | 570 | DNA primase | DNA primase | | afdb-proteome | AF-Q9PM37-F1-MODEL\_V4 | 0.999 | 5.556e-05 | 96 | 0.1 | 539 | 266 | 30 | 46 | 493 | 67 | 477 | DNA primase | DNA primase | | afdb-proteome | AF-Q9CCG2-F1-MODEL\_V4 | 0.999 | 0.0001205 | 96 | 0.135 | 525 | 264 | 35 | 67 | 503 | 76 | 498 | DNA primase | DNA primase | | afdb-proteome | AF-K7MB62-F1-MODEL\_V4 | 0.998 | 4.52e-05 | 95 | 0.112 | 543 | 254 | 30 | 154 | 616 | 216 | 610 | Uncharacterized protein | Uncharacterized protein | | afdb-proteome | AF-F1R5P3-F1-MODEL\_V4 | 0.998 | 3.492e-05 | 95 | 0.117 | 393 | 248 | 23 | 403 | 735 | 300 | 653 | DNA helicase | DNA helicase | | afdb-proteome | AF-U7PYU5-F1-MODEL\_V4 | 0.998 | 3.872e-05 | 95 | 0.127 | 423 | 217 | 25 | 407 | 742 | 459 | 816 | DNA replication licensing factor MCM6 | DNA replication licensing factor MCM6 | | afdb-proteome | AF-K7LBI0-F1-MODEL\_V4 | 0.998 | 2.433e-05 | 94 | 0.106 | 547 | 254 | 32 | 154 | 616 | 217 | 612 | Uncharacterized protein | Uncharacterized protein | | afdb-proteome | AF-B5X582-F1-MODEL\_V4 | 0.998 | 8.395e-05 | 93 | 0.105 | 576 | 270 | 33 | 142 | 623 | 200 | 623 | Twinkle homolog protein, chloroplastic/mitochondrial | Twinkle homolog protein, chloroplastic/mitochondrial | | afdb-proteome | AF-A0A3Q0KJA1-F1-MODEL\_V4 | 0.998 | 8.395e-05 | 93 | 0.103 | 347 | 222 | 23 | 456 | 735 | 331 | 655 | DNA replication licensing factor MCM6 | DNA replication licensing factor MCM6 | | afdb-proteome | AF-P53091-F1-MODEL\_V4 | 0.998 | 1.122e-05 | 93 | 0.118 | 423 | 263 | 29 | 403 | 742 | 449 | 844 | DNA replication licensing factor MCM6 | DNA replication licensing factor MCM6 | | afdb-proteome | AF-Q9I5W0-F1-MODEL\_V4 | 0.996 | 0.0004376 | 90 | 0.12 | 458 | 229 | 31 | 68 | 447 | 76 | 437 | DNA primase | DNA primase | | afdb-proteome | AF-A0A1C1CIE0-F1-MODEL\_V4 | 0.996 | 5.85e-05 | 90 | 0.109 | 528 | 275 | 34 | 393 | 757 | 510 | 1005 | DNA helicase | DNA helicase | | afdb-proteome | AF-A0A1D6M0G4-F1-MODEL\_V4 | 0.996 | 0.0001559 | 89 | 0.078 | 332 | 222 | 22 | 456 | 736 | 289 | 587 | DNA helicase | DNA helicase | | afdb-proteome | AF-P30665-F1-MODEL\_V4 | 0.996 | 6.829e-05 | 89 | 0.092 | 528 | 271 | 32 | 393 | 751 | 441 | 929 | DNA replication licensing factor MCM4 | DNA replication licensing factor MCM4 | | afdb-proteome | AF-A0A0E4AYA7-F1-MODEL\_V4 | 0.995 | 6.486e-05 | 88 | 0.107 | 401 | 245 | 24 | 402 | 735 | 295 | 649 | DNA helicase | DNA helicase | | afdb-proteome | AF-P97311-F1-MODEL\_V4 | 0.994 | 7.191e-05 | 87 | 0.11 | 398 | 245 | 24 | 403 | 735 | 302 | 655 | DNA replication licensing factor MCM6 | DNA replication licensing factor MCM6 | | afdb-proteome | AF-A0A1C1D2E4-F1-MODEL\_V4 | 0.994 | 3.677e-05 | 87 | 0.096 | 435 | 258 | 27 | 402 | 742 | 402 | 795 | DNA replication licensing factor MCM6 | DNA replication licensing factor MCM6 | | afdb-proteome | AF-P29496-F1-MODEL\_V4 | 0.993 | 0.0001087 | 86 | 0.116 | 342 | 209 | 27 | 457 | 735 | 355 | 666 | Minichromosome maintenance protein 5 | Minichromosome maintenance protein 5 | | afdb-proteome | AF-A0A077ZEI1-F1-MODEL\_V4 | 0.993 | 4.077e-05 | 86 | 0.076 | 749 | 398 | 43 | 67 | 738 | 273 | 804 | DNA primase | DNA primase | | afdb-proteome | AF-A0A0K0ECF1-F1-MODEL\_V4 | 0.993 | 0.0001481 | 86 | 0.112 | 356 | 221 | 21 | 456 | 735 | 323 | 659 | DNA replication licensing factor MCM6 | DNA replication licensing factor MCM6 | | afdb-proteome | AF-A0A0R0GCD2-F1-MODEL\_V4 | 0.993 | 0.0002125 | 86 | 0.107 | 345 | 221 | 28 | 456 | 735 | 340 | 662 | DNA helicase | DNA helicase | | afdb-proteome | AF-Q95XQ8-F1-MODEL\_V4 | 0.992 | 0.0003211 | 85 | 0.111 | 438 | 221 | 24 | 457 | 750 | 406 | 819 | DNA replication licensing factor mcm-4 | DNA replication licensing factor mcm-4 | | afdb-proteome | AF-Q4CZ61-F1-MODEL\_V4 | 0.992 | 0.0002125 | 85 | 0.121 | 380 | 227 | 25 | 438 | 737 | 334 | 686 | DNA helicase | DNA helicase | | afdb-proteome | AF-Q86B14-F1-MODEL\_V4 | 0.992 | 6.829e-05 | 85 | 0.119 | 393 | 236 | 26 | 407 | 735 | 383 | 729 | DNA replication licensing factor mcm6 | DNA replication licensing factor mcm6 | | afdb-proteome | AF-A4I3W9-F1-MODEL\_V4 | 0.992 | 0.0001032 | 85 | 0.131 | 366 | 213 | 29 | 456 | 744 | 378 | 715 | DNA helicase | DNA helicase | | afdb-proteome | AF-Q9HZQ5-F1-MODEL\_V4 | 0.991 | 0.0006963 | 84 | 0.123 | 291 | 189 | 18 | 474 | 737 | 9 | 260 | Probable magnesium chelatase | Probable magnesium chelatase | | afdb-proteome | AF-P56064-F1-MODEL\_V4 | 0.991 | 0.0005664 | 84 | 0.108 | 388 | 207 | 24 | 154 | 503 | 168 | 454 | DNA primase | DNA primase | | afdb-proteome | AF-I1JQR6-F1-MODEL\_V4 | 0.991 | 0.0001406 | 84 | 0.117 | 433 | 215 | 29 | 456 | 752 | 315 | 716 | DNA replication licensing factor MCM7 | DNA replication licensing factor MCM7 | | afdb-proteome | AF-Q9XYU0-F1-MODEL\_V4 | 0.991 | 0.0002612 | 84 | 0.106 | 412 | 212 | 26 | 471 | 751 | 333 | 719 | DNA replication licensing factor Mcm7 | DNA replication licensing factor Mcm7 | | afdb-proteome | AF-A0A0K0EDS2-F1-MODEL\_V4 | 0.991 | 0.0001144 | 84 | 0.138 | 355 | 204 | 28 | 457 | 736 | 324 | 651 | DNA replication licensing factor MCM5 | DNA replication licensing factor MCM5 | | afdb-proteome | AF-Q6F353-F1-MODEL\_V4 | 0.991 | 5.85e-05 | 84 | 0.104 | 410 | 247 | 32 | 402 | 735 | 293 | 658 | DNA replication licensing factor MCM6 | DNA replication licensing factor MCM6 | | afdb-proteome | AF-A0A3Q0KG44-F1-MODEL\_V4 | 0.991 | 0.0003749 | 84 | 0.113 | 344 | 222 | 21 | 456 | 737 | 445 | 767 | DNA replication licensing factor MCM2 | DNA replication licensing factor MCM2 | | afdb-proteome | AF-Q57809-F1-MODEL\_V4 | 0.99 | 0.000305 | 83 | 0.105 | 369 | 218 | 26 | 455 | 735 | 327 | 671 | Uncharacterized MCM-type protein MJ0363 | Uncharacterized MCM-type protein MJ0363 | | afdb-proteome | AF-A0A0K0JSP7-F1-MODEL\_V4 | 0.99 | 9.8e-05 | 83 | 0.101 | 403 | 246 | 24 | 403 | 735 | 301 | 657 | DNA replication licensing factor MCM6 | DNA replication licensing factor MCM6 | | afdb-proteome | AF-P30666-F1-MODEL\_V4 | 0.99 | 0.0004156 | 83 | 0.114 | 314 | 169 | 20 | 402 | 666 | 268 | 521 | DNA replication licensing factor mcm3 | DNA replication licensing factor mcm3 | | afdb-proteome | AF-P49731-F1-MODEL\_V4 | 0.99 | 0.0001406 | 83 | 0.103 | 406 | 251 | 29 | 403 | 735 | 370 | 735 | DNA replication licensing factor mcm6 | DNA replication licensing factor mcm6 | | afdb-proteome | AF-P41389-F1-MODEL\_V4 | 0.988 | 0.0003211 | 82 | 0.127 | 353 | 213 | 26 | 457 | 736 | 311 | 641 | DNA replication licensing factor mcm5 | DNA replication licensing factor mcm5 | | afdb-proteome | AF-A0A077Z6A7-F1-MODEL\_V4 | 0.988 | 7.572e-05 | 82 | 0.118 | 405 | 242 | 26 | 403 | 735 | 261 | 622 | DNA replication licensing factor MCM6 | DNA replication licensing factor MCM6 | | afdb-proteome | AF-A0A0N4UBH7-F1-MODEL\_V4 | 0.988 | 0.002663 | 82 | 0.128 | 234 | 143 | 16 | 467 | 657 | 282 | 497 | DNA replication licensing factor MCM3 | DNA replication licensing factor MCM3 | | afdb-proteome | AF-A0A1D6FQT3-F1-MODEL\_V4 | 0.988 | 0.0001559 | 82 | 0.087 | 389 | 239 | 27 | 406 | 737 | 306 | 635 | DNA helicase | DNA helicase | | afdb-proteome | AF-Q4DCB3-F1-MODEL\_V4 | 0.988 | 0.0003749 | 82 | 0.117 | 332 | 197 | 24 | 455 | 735 | 452 | 738 | DNA replication licensing factor MCM2 | DNA replication licensing factor MCM2 | | afdb-proteome | AF-Q53RM0-F1-MODEL\_V4 | 0.986 | 0.0004852 | 81 | 0.157 | 311 | 170 | 19 | 492 | 738 | 90 | 372 | Magnesium-chelatase subunit ChlI, chloroplastic | Magnesium-chelatase subunit ChlI, chloroplastic | | afdb-proteome | AF-A0A0K0E3B8-F1-MODEL\_V4 | 0.986 | 0.0001336 | 81 | 0.135 | 355 | 200 | 27 | 456 | 737 | 332 | 652 | DNA replication licensing factor MCM7 | DNA replication licensing factor MCM7 | | afdb-proteome | AF-P34647-F1-MODEL\_V4 | 0.986 | 0.0001205 | 81 | 0.111 | 404 | 240 | 25 | 405 | 735 | 301 | 658 | DNA replication licensing factor mcm-6 | DNA replication licensing factor mcm-6 | | afdb-proteome | AF-A0A077ZGF4-F1-MODEL\_V4 | 0.986 | 0.0001481 | 81 | 0.114 | 411 | 229 | 29 | 402 | 738 | 359 | 708 | DNA replication licensing factor MCM4 | DNA replication licensing factor MCM4 | | afdb-proteome | AF-I1L122-F1-MODEL\_V4 | 0.986 | 0.0002125 | 81 | 0.108 | 404 | 251 | 30 | 402 | 735 | 289 | 653 | DNA helicase | DNA helicase | | afdb-proteome | AF-C1GVN3-F1-MODEL\_V4 | 0.986 | 6.829e-05 | 81 | 0.122 | 425 | 232 | 36 | 405 | 735 | 415 | 792 | DNA replication licensing factor MCM6 | DNA replication licensing factor MCM6 | | afdb-proteome | AF-I1KLS0-F1-MODEL\_V4 | 0.984 | 0.0004376 | 80 | 0.142 | 316 | 169 | 23 | 492 | 738 | 89 | 371 | Mg-protoporphyrin IX chelatase | Mg-protoporphyrin IX chelatase | | afdb-proteome | AF-P9WNW1-F1-MODEL\_V4 | 0.984 | 0.0001481 | 80 | 0.11 | 725 | 334 | 46 | 123 | 739 | 115 | 636 | DNA primase | DNA primase | | afdb-proteome | AF-Q54RU0-F1-MODEL\_V4 | 0.984 | 0.0002896 | 80 | 0.123 | 356 | 208 | 24 | 456 | 737 | 390 | 715 | DNA replication licensing factor MCM7 | DNA replication licensing factor MCM7 | | afdb-proteome | AF-Q14566-F1-MODEL\_V4 | 0.984 | 0.0001917 | 80 | 0.116 | 402 | 238 | 27 | 403 | 735 | 302 | 655 | DNA replication licensing factor MCM6 | DNA replication licensing factor MCM6 | | afdb-proteome | AF-A0A175W509-F1-MODEL\_V4 | 0.984 | 0.0001729 | 80 | 0.106 | 534 | 271 | 35 | 393 | 757 | 503 | 999 | DNA helicase | DNA helicase | | afdb-proteome | AF-Q2QNM1-F1-MODEL\_V4 | 0.981 | 0.0001729 | 79 | 0.095 | 482 | 239 | 31 | 405 | 751 | 303 | 722 | DNA replication licensing factor MCM7 | DNA replication licensing factor MCM7 | | afdb-proteome | AF-Q8I5T4-F1-MODEL\_V4 | 0.981 | 0.0003749 | 79 | 0.089 | 434 | 225 | 28 | 457 | 750 | 354 | 757 | DNA helicase | DNA helicase | | afdb-proteome | AF-J9F6T9-F1-MODEL\_V4 | 0.981 | 0.0008559 | 79 | 0.107 | 352 | 229 | 25 | 456 | 743 | 466 | 796 | DNA replication licensing factor MCM4 | DNA replication licensing factor MCM4 | | afdb-proteome | AF-P9WPR3-F1-MODEL\_V4 | 0.978 | 0.0005379 | 78 | 0.123 | 323 | 192 | 23 | 475 | 743 | 6 | 291 | Uncharacterized protein Rv2850c | Uncharacterized protein Rv2850c | | afdb-proteome | AF-A0A1D6MX99-F1-MODEL\_V4 | 0.978 | 7.973e-05 | 78 | 0.13 | 397 | 214 | 30 | 403 | 735 | 291 | 620 | DNA replication licensing factor MCM7 | DNA replication licensing factor MCM7 | | afdb-proteome | AF-Q9VGW6-F1-MODEL\_V4 | 0.978 | 0.0006963 | 78 | 0.085 | 443 | 228 | 29 | 457 | 750 | 317 | 731 | DNA replication licensing factor Mcm5 | DNA replication licensing factor Mcm5 | | afdb-proteome | AF-A0A044QUL7-F1-MODEL\_V4 | 0.978 | 0.0005664 | 78 | 0.117 | 358 | 223 | 24 | 457 | 746 | 468 | 800 | DNA replication licensing factor MCM4 | DNA replication licensing factor MCM4 | | afdb-proteome | AF-Q54LI2-F1-MODEL\_V4 | 0.978 | 0.0003381 | 78 | 0.101 | 452 | 230 | 30 | 456 | 751 | 574 | 1005 | DNA replication licensing factor MCM2 | DNA replication licensing factor MCM2 | | afdb-proteome | AF-Q58321-F1-MODEL\_V4 | 0.975 | 0.0009992 | 77 | 0.112 | 330 | 186 | 21 | 475 | 738 | 10 | 298 | Magnesium-chelatase subunit ChlI homolog | Magnesium-chelatase subunit ChlI homolog | | afdb-proteome | AF-I1M038-F1-MODEL\_V4 | 0.975 | 0.0001917 | 77 | 0.126 | 403 | 208 | 28 | 407 | 738 | 49 | 378 | Mg-protoporphyrin IX chelatase | Mg-protoporphyrin IX chelatase | | afdb-proteome | AF-I1NBC2-F1-MODEL\_V4 | 0.975 | 0.000182 | 77 | 0.098 | 486 | 233 | 31 | 405 | 752 | 298 | 716 | DNA replication licensing factor MCM7 | DNA replication licensing factor MCM7 | | afdb-proteome | AF-A0A0J9XQV4-F1-MODEL\_V4 | 0.975 | 0.0004852 | 77 | 0.118 | 355 | 206 | 25 | 456 | 737 | 331 | 651 | DNA replication licensing factor MCM7 | DNA replication licensing factor MCM7 | | afdb-proteome | AF-A0A1D6LPW0-F1-MODEL\_V4 | 0.975 | 0.0003381 | 77 | 0.11 | 545 | 252 | 36 | 154 | 616 | 229 | 622 | Twinkle homolog protein chloroplastic/mitochondrial | Twinkle homolog protein chloroplastic/mitochondrial | | afdb-proteome | AF-C1H7I1-F1-MODEL\_V4 | 0.975 | 0.0005664 | 77 | 0.121 | 444 | 208 | 25 | 471 | 767 | 401 | 809 | DNA replication licensing factor MCM7 | DNA replication licensing factor MCM7 | | afdb-proteome | AF-A0A5S6PT53-F1-MODEL\_V4 | 0.975 | 0.001228 | 77 | 0.109 | 357 | 229 | 22 | 457 | 747 | 396 | 729 | DNA replication licensing factor MCM4 | DNA replication licensing factor MCM4 | | afdb-proteome | AF-A0A077YZA0-F1-MODEL\_V4 | 0.975 | 0.000356 | 77 | 0.11 | 644 | 328 | 41 | 129 | 714 | 20 | 476 | Alpha:beta hydrolase fold protein | Alpha:beta hydrolase fold protein | | afdb-proteome | AF-Q8ILR7-F1-MODEL\_V4 | 0.975 | 0.0007331 | 77 | 0.099 | 371 | 222 | 24 | 456 | 737 | 473 | 820 | DNA replication licensing factor MCM2 | DNA replication licensing factor MCM2 | | afdb-proteome | AF-A0A0D2EQJ7-F1-MODEL\_V4 | 0.975 | 0.0002125 | 77 | 0.123 | 430 | 256 | 25 | 393 | 737 | 509 | 902 | DNA helicase | DNA helicase | | afdb-proteome | AF-Q6KAJ4-F1-MODEL\_V4 | 0.971 | 0.000628 | 76 | 0.104 | 442 | 218 | 28 | 456 | 750 | 317 | 727 | DNA replication licensing factor MCM5 | DNA replication licensing factor MCM5 | | afdb-proteome | AF-Q5RIC5-F1-MODEL\_V4 | 0.971 | 0.0006613 | 76 | 0.123 | 340 | 210 | 24 | 456 | 745 | 283 | 584 | DNA helicase | DNA helicase | | afdb-proteome | AF-D3ZFP4-F1-MODEL\_V4 | 0.971 | 0.0003947 | 76 | 0.096 | 424 | 239 | 28 | 456 | 769 | 284 | 673 | DNA helicase | DNA helicase | | afdb-proteome | AF-A0A0K0EHC7-F1-MODEL\_V4 | 0.971 | 0.0005664 | 76 | 0.116 | 370 | 203 | 24 | 456 | 735 | 450 | 785 | DNA replication licensing factor MCM2 | DNA replication licensing factor MCM2 | | afdb-proteome | AF-A0A183XGH6-F1-MODEL\_V4 | 0.971 | 0.0005109 | 76 | 0.096 | 333 | 208 | 20 | 471 | 735 | 350 | 657 | DNA replication licensing factor MCM6 | DNA replication licensing factor MCM6 | | afdb-proteome | AF-K7TMN0-F1-MODEL\_V4 | 0.967 | 0.0007331 | 75 | 0.15 | 320 | 166 | 23 | 492 | 738 | 130 | 416 | Mg-protoporphyrin IX chelatase | Mg-protoporphyrin IX chelatase | | afdb-proteome | AF-C0HFI8-F1-MODEL\_V4 | 0.967 | 0.0001729 | 75 | 0.098 | 485 | 234 | 33 | 405 | 751 | 298 | 717 | DNA replication licensing factor MCM7 | DNA replication licensing factor MCM7 | | afdb-proteome | AF-Q9CWV1-F1-MODEL\_V4 | 0.967 | 0.002953 | 75 | 0.16 | 249 | 138 | 21 | 456 | 659 | 386 | 608 | DNA helicase MCM8 | DNA helicase MCM8 | | afdb-proteome | AF-P38132-F1-MODEL\_V4 | 0.967 | 0.0003381 | 75 | 0.132 | 347 | 191 | 27 | 456 | 736 | 401 | 703 | DNA replication licensing factor MCM7 | DNA replication licensing factor MCM7 | | afdb-proteome | AF-P29469-F1-MODEL\_V4 | 0.967 | 0.001362 | 75 | 0.119 | 342 | 203 | 22 | 456 | 744 | 484 | 780 | DNA replication licensing factor MCM2 | DNA replication licensing factor MCM2 | | afdb-proteome | AF-A0A0R4IF65-F1-MODEL\_V4 | 0.967 | 0.0007331 | 75 | 0.095 | 463 | 224 | 29 | 456 | 748 | 448 | 885 | DNA replication licensing factor MCM2 | DNA replication licensing factor MCM2 | | afdb-proteome | AF-D3ZP96-F1-MODEL\_V4 | 0.967 | 0.0006613 | 75 | 0.093 | 352 | 216 | 21 | 456 | 737 | 461 | 779 | DNA replication licensing factor MCM2 | DNA replication licensing factor MCM2 | | afdb-proteome | AF-C0NCM7-F1-MODEL\_V4 | 0.967 | 0.0002751 | 75 | 0.127 | 430 | 256 | 26 | 393 | 738 | 521 | 915 | DNA helicase | DNA helicase | | afdb-proteome | AF-P49735-F1-MODEL\_V4 | 0.961 | 0.001108 | 74 | 0.109 | 373 | 212 | 28 | 456 | 735 | 434 | 779 | DNA replication licensing factor Mcm2 | DNA replication licensing factor Mcm2 | | afdb-proteome | AF-I1MRS5-F1-MODEL\_V4 | 0.961 | 0.00151 | 74 | 0.145 | 310 | 191 | 18 | 428 | 684 | 451 | 739 | DNA replication licensing factor MCM2 | DNA replication licensing factor MCM2 | | afdb-proteome | AF-Q384N2-F1-MODEL\_V4 | 0.961 | 0.001052 | 74 | 0.129 | 340 | 188 | 27 | 455 | 737 | 447 | 735 | DNA replication licensing factor MCM2 | DNA replication licensing factor MCM2 | | afdb-proteome | AF-A0A3P7GBU4-F1-MODEL\_V4 | 0.956 | 0.00159 | 73 | 0.068 | 466 | 237 | 27 | 456 | 748 | 446 | 887 | DNA replication licensing factor MCM2 | DNA replication licensing factor MCM2 | | afdb-proteome | AF-I1KMP8-F1-MODEL\_V4 | 0.956 | 0.001166 | 73 | 0.14 | 328 | 198 | 22 | 456 | 735 | 480 | 771 | DNA replication licensing factor MCM2 | DNA replication licensing factor MCM2 | | afdb-proteome | AF-Q2R482-F1-MODEL\_V4 | 0.956 | 0.0005964 | 73 | 0.108 | 514 | 245 | 32 | 428 | 756 | 476 | 961 | DNA replication licensing factor MCM2 | DNA replication licensing factor MCM2 | | afdb-proteome | AF-A0A1D6PT01-F1-MODEL\_V4 | 0.956 | 0.0008559 | 73 | 0.104 | 471 | 222 | 29 | 456 | 753 | 548 | 991 | DNA replication licensing factor MCM2 | DNA replication licensing factor MCM2 | | afdb-proteome | AF-U7Q2I4-F1-MODEL\_V4 | 0.956 | 0.0001917 | 73 | 0.111 | 457 | 267 | 31 | 393 | 747 | 558 | 977 | DNA helicase | DNA helicase | | afdb-proteome | AF-C1GR90-F1-MODEL\_V4 | 0.956 | 0.0004156 | 73 | 0.138 | 411 | 244 | 27 | 402 | 738 | 556 | 930 | DNA helicase | DNA helicase | | afdb-proteome | AF-P16127-F1-MODEL\_V4 | 0.949 | 0.001362 | 72 | 0.141 | 326 | 165 | 22 | 487 | 738 | 97 | 381 | Magnesium-chelatase subunit ChlI-1, chloroplastic | Magnesium-chelatase subunit ChlI-1, chloroplastic | | afdb-proteome | AF-Q58371-F1-MODEL\_V4 | 0.949 | 0.0003381 | 72 | 0.096 | 539 | 228 | 31 | 403 | 751 | 293 | 762 | Uncharacterized MCM-type protein MJ0961 | Uncharacterized MCM-type protein MJ0961 | | afdb-proteome | AF-P49717-F1-MODEL\_V4 | 0.949 | 0.0009992 | 72 | 0.125 | 407 | 232 | 26 | 402 | 738 | 418 | 770 | DNA replication licensing factor MCM4 | DNA replication licensing factor MCM4 | | afdb-proteome | AF-A0A158Q488-F1-MODEL\_V4 | 0.949 | 0.004698 | 72 | 0.098 | 285 | 184 | 20 | 456 | 697 | 475 | 729 | DNA replication licensing factor MCM2 | DNA replication licensing factor MCM2 | | afdb-proteome | AF-Q4E4H4-F1-MODEL\_V4 | 0.941 | 0.001856 | 71 | 0.114 | 350 | 211 | 27 | 456 | 735 | 287 | 607 | DNA replication factor, putative | DNA replication factor, putative | | afdb-proteome | AF-A0A1C1CC10-F1-MODEL\_V4 | 0.941 | 0.0009012 | 71 | 0.1 | 447 | 222 | 27 | 471 | 769 | 394 | 808 | DNA replication licensing factor MCM7 | DNA replication licensing factor MCM7 | | afdb-proteome | AF-A0A044UXW5-F1-MODEL\_V4 | 0.941 | 0.0004376 | 71 | 0.107 | 454 | 276 | 26 | 405 | 769 | 301 | 714 | DNA replication licensing factor MCM6 | DNA replication licensing factor MCM6 | | afdb-proteome | AF-G3V681-F1-MODEL\_V4 | 0.941 | 0.0006613 | 71 | 0.121 | 429 | 241 | 27 | 393 | 738 | 395 | 770 | DNA helicase | DNA helicase | | afdb-proteome | AF-Q383B6-F1-MODEL\_V4 | 0.941 | 0.000356 | 71 | 0.104 | 420 | 257 | 29 | 403 | 743 | 322 | 701 | DNA replication licensing factor MCM6 | DNA replication licensing factor MCM6 | | afdb-proteome | AF-A0A044VIL0-F1-MODEL\_V4 | 0.941 | 0.003822 | 71 | 0.106 | 272 | 173 | 20 | 456 | 683 | 449 | 694 | DNA replication licensing factor MCM2 | DNA replication licensing factor MCM2 | | afdb-proteome | AF-Q8IDF0-F1-MODEL\_V4 | 0.941 | 0.0004156 | 71 | 0.107 | 418 | 248 | 30 | 402 | 743 | 391 | 759 | DNA helicase | DNA helicase | | afdb-proteome | AF-Q9LPD9-F1-MODEL\_V4 | 0.941 | 0.000772 | 71 | 0.096 | 507 | 242 | 30 | 428 | 748 | 451 | 927 | DNA replication licensing factor MCM2 | DNA replication licensing factor MCM2 | | afdb-proteome | AF-I1MEQ1-F1-MODEL\_V4 | 0.933 | 0.001052 | 70 | 0.12 | 356 | 190 | 25 | 457 | 738 | 72 | 378 | Mg-protoporphyrin IX chelatase | Mg-protoporphyrin IX chelatase | | afdb-proteome | AF-P93162-F1-MODEL\_V4 | 0.933 | 0.001674 | 70 | 0.14 | 321 | 177 | 23 | 487 | 738 | 88 | 378 | Magnesium-chelatase subunit ChlI, chloroplastic | Magnesium-chelatase subunit ChlI, chloroplastic | | afdb-proteome | AF-P40377-F1-MODEL\_V4 | 0.933 | 0.0008559 | 70 | 0.096 | 416 | 238 | 29 | 405 | 736 | 448 | 809 | DNA replication licensing factor mcm2 | DNA replication licensing factor mcm2 | | afdb-proteome | AF-Q6NZV2-F1-MODEL\_V4 | 0.933 | 0.001954 | 70 | 0.097 | 449 | 218 | 29 | 456 | 751 | 431 | 845 | DNA helicase | DNA helicase | | afdb-proteome | AF-K0F2Y6-F1-MODEL\_V4 | 0.923 | 0.004024 | 69 | 0.144 | 298 | 180 | 20 | 482 | 737 | 24 | 288 | Putative magnesium chelatase subunit | Putative magnesium chelatase subunit | | afdb-proteome | AF-A0A3Q0KEL0-F1-MODEL\_V4 | 0.923 | 0.0009992 | 69 | 0.122 | 400 | 224 | 27 | 405 | 744 | 254 | 586 | DNA replication licensing factor MCM3 | DNA replication licensing factor MCM3 | | afdb-proteome | AF-A0A0A2V2X0-F1-MODEL\_V4 | 0.923 | 0.002167 | 69 | 0.123 | 349 | 191 | 24 | 456 | 744 | 285 | 578 | DNA replication licensing factor MCM3 | DNA replication licensing factor MCM3 | | afdb-proteome | AF-A0A0D2GY10-F1-MODEL\_V4 | 0.923 | 0.002281 | 69 | 0.101 | 353 | 206 | 26 | 456 | 744 | 295 | 600 | DNA replication licensing factor MCM3 | DNA replication licensing factor MCM3 | | afdb-proteome | AF-P97310-F1-MODEL\_V4 | 0.923 | 0.001954 | 69 | 0.092 | 467 | 224 | 28 | 456 | 748 | 460 | 900 | DNA replication licensing factor MCM2 | DNA replication licensing factor MCM2 | | afdb-proteome | AF-P49736-F1-MODEL\_V4 | 0.923 | 0.00159 | 69 | 0.093 | 472 | 226 | 28 | 456 | 753 | 460 | 903 | DNA replication licensing factor MCM2 | DNA replication licensing factor MCM2 | | afdb-proteome | AF-U7Q723-F1-MODEL\_V4 | 0.923 | 0.001674 | 69 | 0.115 | 354 | 207 | 25 | 456 | 744 | 292 | 604 | DNA replication licensing factor MCM3 | DNA replication licensing factor MCM3 | | afdb-proteome | AF-Q5XF33-F1-MODEL\_V4 | 0.912 | 0.001763 | 68 | 0.122 | 334 | 182 | 24 | 478 | 738 | 80 | 375 | Magnesium-chelatase subunit ChlI-2, chloroplastic | Magnesium-chelatase subunit ChlI-2, chloroplastic | | afdb-proteome | AF-A0A077Z5J5-F1-MODEL\_V4 | 0.912 | 0.001674 | 68 | 0.102 | 418 | 202 | 27 | 471 | 751 | 344 | 725 | DNA replication licensing factor MCM7 | DNA replication licensing factor MCM7 | | afdb-proteome | AF-Q54VI9-F1-MODEL\_V4 | 0.912 | 0.001856 | 68 | 0.138 | 347 | 195 | 26 | 456 | 744 | 300 | 600 | DNA helicase | DNA helicase | | afdb-proteome | AF-A0A175WE06-F1-MODEL\_V4 | 0.912 | 0.006742 | 68 | 0.1 | 339 | 223 | 22 | 455 | 744 | 465 | 770 | DNA replication licensing factor MCM2 | DNA replication licensing factor MCM2 | | afdb-proteome | AF-C0NSR0-F1-MODEL\_V4 | 0.912 | 0.001166 | 68 | 0.094 | 434 | 226 | 28 | 456 | 767 | 295 | 683 | DNA replication licensing factor MCM3 | DNA replication licensing factor MCM3 | | afdb-proteome | AF-P33992-F1-MODEL\_V4 | 0.9 | 0.002058 | 67 | 0.11 | 444 | 216 | 27 | 457 | 751 | 320 | 733 | DNA replication licensing factor MCM5 | DNA replication licensing factor MCM5 | | afdb-proteome | AF-I1KRF9-F1-MODEL\_V4 | 0.9 | 0.002663 | 67 | 0.11 | 317 | 188 | 21 | 456 | 725 | 280 | 549 | DNA helicase | DNA helicase | | afdb-proteome | AF-A0A1D8PHW4-F1-MODEL\_V4 | 0.9 | 0.001763 | 67 | 0.134 | 393 | 224 | 30 | 402 | 744 | 285 | 611 | DNA helicase | DNA helicase | | afdb-proteome | AF-Q9XXI9-F1-MODEL\_V4 | 0.9 | 0.0008559 | 67 | 0.092 | 456 | 205 | 33 | 456 | 730 | 439 | 866 | DNA replication licensing factor MCM2 | DNA replication licensing factor MCM2 | | afdb-proteome | AF-C0NPQ6-F1-MODEL\_V4 | 0.9 | 0.002058 | 67 | 0.112 | 357 | 216 | 28 | 456 | 736 | 474 | 805 | DNA replication licensing factor MCM2 | DNA replication licensing factor MCM2 | | afdb-proteome | AF-U7PSF8-F1-MODEL\_V4 | 0.9 | 0.002058 | 67 | 0.098 | 384 | 212 | 24 | 456 | 737 | 488 | 839 | DNA replication licensing factor MCM2 | DNA replication licensing factor MCM2 | | afdb-proteome | AF-A0A3P7EEJ1-F1-MODEL\_V4 | 0.887 | 0.008287 | 66 | 0.137 | 320 | 186 | 27 | 471 | 738 | 258 | 539 | Mini-chromosome maintenance complex-binding protein | Mini-chromosome maintenance complex-binding protein | | afdb-proteome | AF-Q0DHC4-F1-MODEL\_V4 | 0.887 | 0.004024 | 66 | 0.12 | 341 | 202 | 21 | 456 | 744 | 281 | 575 | DNA replication licensing factor MCM3 | DNA replication licensing factor MCM3 | | afdb-proteome | AF-C1GZP9-F1-MODEL\_V4 | 0.887 | 0.002663 | 66 | 0.087 | 341 | 208 | 23 | 456 | 744 | 483 | 772 | DNA replication licensing factor MCM2 | DNA replication licensing factor MCM2 | | afdb-proteome | AF-Q5A034-F1-MODEL\_V4 | 0.887 | 0.004024 | 66 | 0.124 | 330 | 196 | 22 | 457 | 735 | 512 | 799 | DNA replication licensing factor MCM2 | DNA replication licensing factor MCM2 | | afdb-proteome | AF-A0A150ASS4-F1-MODEL\_V4 | 0.887 | 0.001108 | 66 | 0.115 | 459 | 220 | 31 | 402 | 735 | 266 | 663 | DNA replication licensing factor MCM3 | DNA replication licensing factor MCM3 | | afdb-proteome | AF-A4IC27-F1-MODEL\_V4 | 0.887 | 0.00363 | 66 | 0.12 | 357 | 203 | 26 | 456 | 735 | 298 | 620 | DNA\_replication\_licensing\_factor\_MCM9\_-\_putative | DNA\_replication\_licensing\_factor\_MCM9\_-\_putative | | afdb-proteome | AF-Q38E36-F1-MODEL\_V4 | 0.872 | 0.002402 | 65 | 0.101 | 425 | 250 | 26 | 393 | 735 | 220 | 594 | Minichromosome maintenance (MCM) complex subunit, putative | Minichromosome maintenance (MCM) complex subunit, putative | | afdb-proteome | AF-I1K328-F1-MODEL\_V4 | 0.872 | 0.003109 | 65 | 0.095 | 344 | 207 | 22 | 456 | 744 | 280 | 574 | DNA helicase | DNA helicase | | afdb-proteome | AF-P25205-F1-MODEL\_V4 | 0.872 | 0.002281 | 65 | 0.099 | 431 | 237 | 26 | 428 | 744 | 269 | 662 | DNA replication licensing factor MCM3 | DNA replication licensing factor MCM3 | | afdb-proteome | AF-A0A0D2DM29-F1-MODEL\_V4 | 0.872 | 0.001763 | 65 | 0.123 | 381 | 204 | 30 | 456 | 737 | 471 | 820 | DNA replication licensing factor MCM2 | DNA replication licensing factor MCM2 | | afdb-proteome | AF-A0A1C1CXS2-F1-MODEL\_V4 | 0.855 | 0.002953 | 64 | 0.123 | 340 | 196 | 25 | 456 | 744 | 472 | 760 | DNA replication licensing factor MCM2 | DNA replication licensing factor MCM2 | | afdb-proteome | AF-Q9SX03-F1-MODEL\_V4 | 0.817 | 0.002402 | 62 | 0.124 | 411 | 229 | 26 | 393 | 744 | 237 | 575 | DNA replication licensing factor MCM3 homolog 3 | DNA replication licensing factor MCM3 homolog 3 | | afdb-proteome | AF-Q9SX04-F1-MODEL\_V4 | 0.817 | 0.004237 | 62 | 0.093 | 504 | 231 | 23 | 456 | 750 | 281 | 767 | DNA replication licensing factor MCM3 homolog 2 | DNA replication licensing factor MCM3 homolog 2 | | afdb-proteome | AF-Q9SF37-F1-MODEL\_V4 | 0.817 | 0.003822 | 62 | 0.099 | 391 | 215 | 27 | 456 | 737 | 340 | 702 | Probable DNA helicase MCM8 | Probable DNA helicase MCM8 | | afdb-proteome | AF-D3ZVK1-F1-MODEL\_V4 | 0.817 | 0.001166 | 62 | 0.112 | 446 | 228 | 32 | 402 | 736 | 350 | 738 | DNA helicase MCM8 | DNA helicase MCM8 | | afdb-proteome | AF-Q38AM6-F1-MODEL\_V4 | 0.795 | 0.005484 | 61 | 0.096 | 373 | 203 | 26 | 471 | 737 | 246 | 590 | Uncharacterized protein | Uncharacterized protein | | afdb-proteome | AF-Q9FL33-F1-MODEL\_V4 | 0.747 | 0.002529 | 59 | 0.095 | 461 | 240 | 29 | 393 | 735 | 232 | 633 | DNA replication licensing factor MCM3 | DNA replication licensing factor MCM3 | | afdb-proteome | AF-A0A1C1D0R7-F1-MODEL\_V4 | 0.747 | 0.002281 | 59 | 0.104 | 401 | 235 | 26 | 402 | 744 | 269 | 603 | DNA replication licensing factor MCM3 | DNA replication licensing factor MCM3 | | afdb-proteome | AF-E7F643-F1-MODEL\_V4 | 0.72 | 0.004024 | 58 | 0.108 | 444 | 232 | 33 | 402 | 736 | 372 | 760 | DNA helicase | DNA helicase | | afdb-proteome | AF-A0A0N4U787-F1-MODEL\_V4 | 0.692 | 0.006403 | 57 | 0.123 | 445 | 235 | 30 | 393 | 747 | 295 | 674 | DNA replication licensing factor MCM4 | DNA replication licensing factor MCM4 | | afdb-proteome | AF-Q54RN8-F1-MODEL\_V4 | 0.632 | 0.004947 | 55 | 0.103 | 407 | 225 | 26 | 402 | 744 | 329 | 659 | MCM domain-containing protein | MCM domain-containing protein | | afdb-proteome | AF-A0A1D6NMY4-F1-MODEL\_V4 | 0.632 | 0.004947 | 55 | 0.113 | 466 | 233 | 33 | 393 | 738 | 370 | 775 | DNA helicase | DNA helicase | | afdb-proteome | AF-P24279-F1-MODEL\_V4 | 0.632 | 0.004462 | 55 | 0.098 | 489 | 246 | 30 | 404 | 738 | 293 | 740 | DNA replication licensing factor MCM3 | DNA replication licensing factor MCM3 | | afdb-proteome | AF-C0PDH6-F1-MODEL\_V4 | 0.601 | 0.008726 | 54 | 0.088 | 541 | 255 | 38 | 393 | 755 | 371 | 851 | DNA helicase | DNA helicase | | afdb-proteome | AF-A0A077ZCX8-F1-MODEL\_V4 | 0.505 | 0.008287 | 51 | 0.104 | 468 | 239 | 30 | 393 | 738 | 346 | 755 | DNA replication licensing factor MCM8 | DNA replication licensing factor MCM8 | | afdb-proteome | AF-Q8ILY1-F1-MODEL\_V4 | 0.382 | 0.006742 | 47 | 0.096 | 693 | 315 | 41 | 153 | 616 | 249 | 859 | Plastid replication-repair enzyme | Plastid replication-repair enzyme | | afdb-uniprot50 | AF-A0A2Z3IKG0-F1-MODEL\_V4 | 1.0 | 3.972e-71 | 2233 | 0.449 | 685 | 347 | 13 | 102 | 769 | 88 | 759 | Integrase | Integrase | | afdb-uniprot50 | AF-A0A7L6EDN8-F1-MODEL\_V4 | 1.0 | 2.18e-54 | 2226 | 0.6 | 443 | 168 | 5 | 330 | 769 | 8 | 444 | Virulence-associated E family protein | Virulence-associated E family protein | | afdb-uniprot50 | AF-A0A6G8CKG5-F1-MODEL\_V4 | 1.0 | 9.011e-74 | 2189 | 0.378 | 929 | 373 | 19 | 22 | 757 | 1 | 917 | PriCT\_2 domain-containing protein | PriCT\_2 domain-containing protein | | afdb-uniprot50 | AF-B4EME0-F1-MODEL\_V4 | 1.0 | 2.545e-54 | 2167 | 0.579 | 461 | 169 | 8 | 314 | 757 | 7 | 459 | Hypothetical phage protein | Hypothetical phage protein | | afdb-uniprot50 | AF-A0A8A6KD37-F1-MODEL\_V4 | 1.0 | 1.892e-72 | 2132 | 0.396 | 789 | 428 | 21 | 3 | 769 | 1 | 762 | PriCT-2 domain-containing protein | PriCT-2 domain-containing protein | | afdb-uniprot50 | AF-A0A370WXM5-F1-MODEL\_V4 | 1.0 | 2.714e-64 | 2083 | 0.45 | 671 | 321 | 14 | 100 | 757 | 1 | 636 | Toprim domain-containing protein | Toprim domain-containing protein | | afdb-uniprot50 | AF-A0A0D7EBT5-F1-MODEL\_V4 | 1.0 | 6.079e-65 | 1947 | 0.432 | 721 | 331 | 23 | 63 | 757 | 97 | 764 | Toprim domain-containing protein | Toprim domain-containing protein | | afdb-uniprot50 | AF-A0A3R8TDC4-F1-MODEL\_V4 | 1.0 | 4.023e-65 | 1940 | 0.407 | 702 | 372 | 15 | 102 | 769 | 87 | 778 | Toprim domain-containing protein | Toprim domain-containing protein | | afdb-uniprot50 | AF-A0A4R6JMW9-F1-MODEL\_V4 | 1.0 | 1.509e-65 | 1919 | 0.408 | 724 | 357 | 19 | 98 | 761 | 84 | 795 | Putative DNA primase/helicase | Putative DNA primase/helicase | | afdb-uniprot50 | AF-A0A8B2Q819-F1-MODEL\_V4 | 1.0 | 5.342e-45 | 1878 | 0.449 | 414 | 222 | 4 | 356 | 769 | 51 | 458 | Virulence-associated E family protein | Virulence-associated E family protein | | afdb-uniprot50 | AF-A0A810TDZ1-F1-MODEL\_V4 | 1.0 | 2.138e-55 | 1874 | 0.491 | 576 | 232 | 15 | 228 | 769 | 11 | 559 | Uncharacterized protein | Uncharacterized protein | | afdb-uniprot50 | AF-A0A5K7Y2P6-F1-MODEL\_V4 | 1.0 | 2.355e-42 | 1805 | 0.453 | 390 | 204 | 6 | 369 | 756 | 123 | 505 | Uncharacterized protein | Uncharacterized protein | | afdb-uniprot50 | AF-A0A5C7W4R6-F1-MODEL\_V4 | 1.0 | 8.336e-46 | 1706 | 0.448 | 484 | 236 | 10 | 287 | 762 | 74 | 534 | Virulence-associated E family protein | Virulence-associated E family protein | | afdb-uniprot50 | AF-A0A550ECX4-F1-MODEL\_V4 | 1.0 | 3.048e-58 | 1682 | 0.35 | 728 | 374 | 25 | 48 | 757 | 99 | 745 | Toprim domain-containing protein | Toprim domain-containing protein | | afdb-uniprot50 | AF-A0A2N1CUR9-F1-MODEL\_V4 | 1.0 | 2.03e-39 | 1607 | 0.412 | 417 | 236 | 7 | 346 | 756 | 85 | 498 | Virulence-associated E family protein | Virulence-associated E family protein | | afdb-uniprot50 | AF-A0A7Y9SYN6-F1-MODEL\_V4 | 1.0 | 7.233e-40 | 1602 | 0.407 | 400 | 232 | 5 | 369 | 766 | 10 | 406 | Putative DNA primase/helicase | Putative DNA primase/helicase | | afdb-uniprot50 | AF-Q1NPY4-F1-MODEL\_V4 | 1.0 | 3.401e-39 | 1602 | 0.416 | 406 | 228 | 6 | 354 | 757 | 49 | 447 | Virulence-associated E | Virulence-associated E | | afdb-uniprot50 | AF-A0A2T1K8Q3-F1-MODEL\_V4 | 1.0 | 3.148e-43 | 1599 | 0.392 | 482 | 260 | 11 | 298 | 769 | 331 | 789 | RepB\_primase domain-containing protein | RepB\_primase domain-containing protein | | afdb-uniprot50 | AF-A0A370DD10-F1-MODEL\_V4 | 1.0 | 6.869e-40 | 1594 | 0.428 | 411 | 221 | 7 | 356 | 762 | 446 | 846 | Uncharacterized protein | Uncharacterized protein | | afdb-uniprot50 | AF-A0A241VC66-F1-MODEL\_V4 | 1.0 | 4.154e-50 | 1592 | 0.334 | 660 | 381 | 18 | 136 | 769 | 10 | 636 | Uncharacterized protein | Uncharacterized protein | | afdb-uniprot50 | AF-A0A2D7BYY9-F1-MODEL\_V4 | 1.0 | 3.029e-53 | 1587 | 0.331 | 679 | 371 | 24 | 92 | 757 | 89 | 697 | Toprim domain-containing protein | Toprim domain-containing protein | | afdb-uniprot50 | AF-A0A329BI33-F1-MODEL\_V4 | 1.0 | 6.196e-40 | 1586 | 0.436 | 417 | 219 | 7 | 354 | 769 | 2 | 403 | Virulence-associated protein E | Virulence-associated protein E | | afdb-uniprot50 | AF-A0A126RQW2-F1-MODEL\_V4 | 1.0 | 2.03e-39 | 1586 | 0.41 | 414 | 230 | 8 | 368 | 769 | 35 | 446 | Uncharacterized protein | Uncharacterized protein | | afdb-uniprot50 | AF-A0A2N2RJ59-F1-MODEL\_V4 | 1.0 | 6.196e-40 | 1570 | 0.413 | 433 | 236 | 9 | 346 | 769 | 92 | 515 | Virulence-associated E family protein | Virulence-associated E family protein | | afdb-uniprot50 | AF-W0DSD8-F1-MODEL\_V4 | 1.0 | 2.48e-50 | 1563 | 0.382 | 638 | 325 | 20 | 169 | 769 | 88 | 693 | Virulence-associated E family protein | Virulence-associated E family protein | | afdb-uniprot50 | AF-A0A8A7VX14-F1-MODEL\_V4 | 1.0 | 1.63e-37 | 1553 | 0.39 | 394 | 233 | 6 | 368 | 757 | 143 | 533 | Uncharacterized protein | Uncharacterized protein | | afdb-uniprot50 | AF-A0A1B2I2L3-F1-MODEL\_V4 | 1.0 | 4.403e-55 | 1551 | 0.311 | 755 | 401 | 24 | 68 | 762 | 69 | 764 | Toprim domain-containing protein | Toprim domain-containing protein | | afdb-uniprot50 | AF-A0A148KLT5-F1-MODEL\_V4 | 1.0 | 6.651e-39 | 1550 | 0.382 | 410 | 240 | 8 | 351 | 756 | 403 | 803 | Uncharacterized protein | Uncharacterized protein | | afdb-uniprot50 | AF-A0A2D9C8Q1-F1-MODEL\_V4 | 1.0 | 3.87e-51 | 1521 | 0.306 | 685 | 407 | 21 | 92 | 767 | 92 | 717 | Toprim domain-containing protein | Toprim domain-containing protein | | afdb-uniprot50 | AF-A0A4P5WEG0-F1-MODEL\_V4 | 1.0 | 5.624e-37 | 1517 | 0.373 | 415 | 247 | 8 | 362 | 769 | 74 | 482 | Uncharacterized protein | Uncharacterized protein | | afdb-uniprot50 | AF-A0A7X1XSH3-F1-MODEL\_V4 | 1.0 | 1.025e-37 | 1492 | 0.39 | 433 | 242 | 10 | 354 | 769 | 84 | 511 | Virulence-associated E family protein | Virulence-associated E family protein | | afdb-uniprot50 | AF-A0A4P6X2C8-F1-MODEL\_V4 | 1.0 | 4.725e-38 | 1487 | 0.378 | 410 | 249 | 6 | 363 | 769 | 70 | 476 | Virulence-associated E family protein | Virulence-associated E family protein | | afdb-uniprot50 | AF-A0A838L9S2-F1-MODEL\_V4 | 1.0 | 1.158e-36 | 1486 | 0.363 | 410 | 254 | 7 | 363 | 769 | 42 | 447 | Uncharacterized protein | Uncharacterized protein | | afdb-uniprot50 | AF-A0A6N9HMH7-F1-MODEL\_V4 | 1.0 | 7.665e-37 | 1486 | 0.379 | 427 | 240 | 10 | 349 | 756 | 4 | 424 | Virulence-associated E family protein | Virulence-associated E family protein | | afdb-uniprot50 | AF-A0A2X1BCW5-F1-MODEL\_V4 | 1.0 | 3.604e-36 | 1455 | 0.426 | 387 | 208 | 10 | 389 | 769 | 2 | 380 | Predicted P-loop ATPase and inactivated derivatives | Predicted P-loop ATPase and inactivated derivatives | | afdb-uniprot50 | AF-A0A0Q4G1V4-F1-MODEL\_V4 | 1.0 | 7.048e-36 | 1454 | 0.379 | 387 | 232 | 5 | 388 | 769 | 2 | 385 | Uncharacterized protein | Uncharacterized protein | | afdb-uniprot50 | AF-A0A2D4Y2B1-F1-MODEL\_V4 | 1.0 | 9.422e-37 | 1447 | 0.361 | 409 | 251 | 6 | 366 | 769 | 9 | 412 | Uncharacterized protein | Uncharacterized protein | | afdb-uniprot50 | AF-A0A4P7QW42-F1-MODEL\_V4 | 1.0 | 7.094e-41 | 1444 | 0.366 | 461 | 271 | 9 | 315 | 769 | 30 | 475 | Virulence-associated E family protein | Virulence-associated E family protein | | afdb-uniprot50 | AF-A0A848H5Z6-F1-MODEL\_V4 | 1.0 | 5.922e-37 | 1444 | 0.371 | 406 | 232 | 9 | 368 | 753 | 82 | 484 | Uncharacterized protein | Uncharacterized protein | | afdb-uniprot50 | AF-A0A1H9PLQ6-F1-MODEL\_V4 | 1.0 | 4.757e-51 | 1429 | 0.391 | 616 | 297 | 21 | 81 | 671 | 105 | 667 | Putative DNA primase/helicase | Putative DNA primase/helicase | | afdb-uniprot50 | AF-A0A3N5V9B1-F1-MODEL\_V4 | 1.0 | 2.138e-47 | 1428 | 0.333 | 638 | 319 | 25 | 181 | 763 | 138 | 723 | Uncharacterized protein | Uncharacterized protein | | afdb-uniprot50 | AF-A0A162LR67-F1-MODEL\_V4 | 1.0 | 6.357e-36 | 1422 | 0.362 | 408 | 250 | 7 | 366 | 767 | 31 | 434 | Uncharacterized protein | Uncharacterized protein | | afdb-uniprot50 | AF-A0A0W1I738-F1-MODEL\_V4 | 1.0 | 1.309e-35 | 1418 | 0.362 | 405 | 245 | 6 | 362 | 764 | 4 | 397 | Uncharacterized protein | Uncharacterized protein | | afdb-uniprot50 | AF-A0A257G4S2-F1-MODEL\_V4 | 1.0 | 8.389e-35 | 1408 | 0.369 | 398 | 238 | 9 | 377 | 769 | 71 | 460 | Uncharacterized protein | Uncharacterized protein | | afdb-uniprot50 | AF-A0A6I5YGU0-F1-MODEL\_V4 | 1.0 | 1.235e-38 | 1405 | 0.378 | 433 | 254 | 8 | 340 | 769 | 23 | 443 | Virulence-associated E family protein | Virulence-associated E family protein | | afdb-uniprot50 | AF-A0A1B6Z6D8-F1-MODEL\_V4 | 1.0 | 1.268e-34 | 1397 | 0.376 | 393 | 241 | 4 | 378 | 769 | 5 | 394 | Uncharacterized protein | Uncharacterized protein | | afdb-uniprot50 | AF-A0A315BDU7-F1-MODEL\_V4 | 1.0 | 4.575e-37 | 1387 | 0.368 | 450 | 252 | 13 | 328 | 758 | 2 | 438 | Uncharacterized protein | Uncharacterized protein | | afdb-uniprot50 | AF-A0A6P1J3W7-F1-MODEL\_V4 | 1.0 | 7.048e-36 | 1379 | 0.378 | 391 | 232 | 8 | 369 | 757 | 44 | 425 | Uncharacterized protein | Uncharacterized protein | | afdb-uniprot50 | AF-A0A1B6Z6F3-F1-MODEL\_V4 | 1.0 | 5.007e-35 | 1364 | 0.36 | 419 | 249 | 10 | 348 | 758 | 7 | 414 | Uncharacterized protein | Uncharacterized protein | | afdb-uniprot50 | AF-A0A158EA01-F1-MODEL\_V4 | 1.0 | 1.284e-44 | 1360 | 0.293 | 654 | 370 | 22 | 181 | 766 | 147 | 776 | Virulence-associated protein E | Virulence-associated protein E | | afdb-uniprot50 | AF-A0A6G6X8A9-F1-MODEL\_V4 | 1.0 | 1.855e-33 | 1336 | 0.379 | 398 | 230 | 7 | 368 | 762 | 6 | 389 | Uncharacterized protein | Uncharacterized protein | | afdb-uniprot50 | AF-A0A7Z0LRG9-F1-MODEL\_V4 | 1.0 | 7.715e-42 | 1321 | 0.366 | 521 | 274 | 12 | 246 | 756 | 346 | 820 | Uncharacterized protein | Uncharacterized protein | | afdb-uniprot50 | AF-A0A7Y8YLU9-F1-MODEL\_V4 | 1.0 | 9.793e-35 | 1316 | 0.355 | 430 | 251 | 9 | 356 | 769 | 8 | 427 | P-loop ATPase | P-loop ATPase | | afdb-uniprot50 | AF-A0A2U2MYE5-F1-MODEL\_V4 | 1.0 | 1.065e-51 | 1316 | 0.257 | 855 | 498 | 34 | 1 | 757 | 1 | 816 | PriCT\_2 domain-containing protein | PriCT\_2 domain-containing protein | | afdb-uniprot50 | AF-A0A3B9WZT9-F1-MODEL\_V4 | 1.0 | 9.423e-45 | 1307 | 0.336 | 561 | 301 | 18 | 102 | 651 | 102 | 601 | Toprim domain-containing protein | Toprim domain-containing protein | | afdb-uniprot50 | AF-S6SGC4-F1-MODEL\_V4 | 1.0 | 9.124e-44 | 1278 | 0.287 | 675 | 407 | 32 | 128 | 769 | 2 | 635 | DNA primase domain protein | DNA primase domain protein | | afdb-uniprot50 | AF-A0A4Y4D005-F1-MODEL\_V4 | 1.0 | 5.922e-45 | 1256 | 0.284 | 786 | 352 | 19 | 1 | 756 | 215 | 819 | Uncharacterized protein | Uncharacterized protein | | afdb-uniprot50 | AF-A0A450WC63-F1-MODEL\_V4 | 1.0 | 3.445e-49 | 1255 | 0.29 | 825 | 471 | 38 | 10 | 750 | 4 | 797 | Putative DNA primase/helicase | Putative DNA primase/helicase | | afdb-uniprot50 | AF-A0A4Q9VPA0-F1-MODEL\_V4 | 1.0 | 7.141e-46 | 1241 | 0.291 | 680 | 416 | 30 | 100 | 752 | 75 | 715 | Toprim domain-containing protein | Toprim domain-containing protein | | afdb-uniprot50 | AF-L0GU11-F1-MODEL\_V4 | 1.0 | 4.373e-34 | 1232 | 0.325 | 452 | 267 | 11 | 328 | 762 | 137 | 567 | Putative P-loop ATPase | Putative P-loop ATPase | | afdb-uniprot50 | AF-A0A3G9FYK3-F1-MODEL\_V4 | 1.0 | 1.343e-39 | 1224 | 0.275 | 632 | 361 | 26 | 161 | 762 | 25 | 589 | Putative virulence-associated protein E | Putative virulence-associated protein E | | afdb-uniprot50 | AF-A0A1D2U957-F1-MODEL\_V4 | 1.0 | 2.137e-31 | 1211 | 0.326 | 404 | 238 | 7 | 376 | 750 | 22 | 420 | Uncharacterized protein | Uncharacterized protein | | afdb-uniprot50 | AF-A0A2T7SVQ2-F1-MODEL\_V4 | 1.0 | 3.722e-37 | 1211 | 0.293 | 538 | 313 | 16 | 239 | 752 | 229 | 723 | Uncharacterized protein | Uncharacterized protein | | afdb-uniprot50 | AF-A0A7W7NUF6-F1-MODEL\_V4 | 1.0 | 4.047e-30 | 1208 | 0.424 | 325 | 181 | 6 | 435 | 757 | 1 | 321 | Putative DNA primase/helicase | Putative DNA primase/helicase | | afdb-uniprot50 | AF-A0A258QD56-F1-MODEL\_V4 | 1.0 | 4.43e-36 | 1189 | 0.263 | 547 | 324 | 14 | 242 | 755 | 293 | 793 | Uncharacterized protein | Uncharacterized protein | | afdb-uniprot50 | AF-A0A662BLW6-F1-MODEL\_V4 | 1.0 | 4.317e-32 | 1164 | 0.307 | 459 | 281 | 13 | 329 | 766 | 363 | 805 | Prim-Pol domain-containing protein | Prim-Pol domain-containing protein | | afdb-uniprot50 | AF-A0A127SEK4-F1-MODEL\_V4 | 1.0 | 5.171e-28 | 1161 | 0.474 | 270 | 140 | 2 | 488 | 757 | 4 | 271 | Virulence-associated protein E | Virulence-associated protein E | | afdb-uniprot50 | AF-A0A269PIH2-F1-MODEL\_V4 | 1.0 | 1.978e-27 | 1146 | 0.439 | 287 | 157 | 2 | 355 | 638 | 19 | 304 | Uncharacterized protein | Uncharacterized protein | | afdb-uniprot50 | AF-A0A2K4XP73-F1-MODEL\_V4 | 1.0 | 9.544e-39 | 1142 | 0.285 | 652 | 371 | 24 | 153 | 756 | 79 | 683 | Uncharacterized protein | Uncharacterized protein | | afdb-uniprot50 | AF-A0A363ULB6-F1-MODEL\_V4 | 1.0 | 1.916e-42 | 1130 | 0.241 | 725 | 455 | 30 | 92 | 769 | 82 | 758 | Uncharacterized protein | Uncharacterized protein | | afdb-uniprot50 | AF-A0A059KRR5-F1-MODEL\_V4 | 1.0 | 4.262e-46 | 1125 | 0.246 | 872 | 518 | 37 | 1 | 769 | 1 | 836 | Uncharacterized protein | Uncharacterized protein | | afdb-uniprot50 | AF-A0A5C7PWX5-F1-MODEL\_V4 | 1.0 | 2.696e-43 | 1121 | 0.265 | 742 | 421 | 30 | 92 | 757 | 81 | 774 | Toprim domain-containing protein | Toprim domain-containing protein | | afdb-uniprot50 | AF-A0A5C7Q9V4-F1-MODEL\_V4 | 1.0 | 1.335e-26 | 1117 | 0.423 | 274 | 151 | 4 | 380 | 649 | 9 | 279 | Uncharacterized protein | Uncharacterized protein | | afdb-uniprot50 | AF-A0A844Y3H4-F1-MODEL\_V4 | 1.0 | 3.422e-28 | 1097 | 0.38 | 352 | 206 | 7 | 423 | 767 | 2 | 348 | Uncharacterized protein | Uncharacterized protein | | afdb-uniprot50 | AF-A0A5B9W769-F1-MODEL\_V4 | 1.0 | 2.124e-42 | 1088 | 0.294 | 710 | 409 | 28 | 91 | 750 | 75 | 742 | DNA primase TraC | DNA primase TraC | | afdb-uniprot50 | AF-A0A315D5W5-F1-MODEL\_V4 | 1.0 | 1.47e-37 | 1074 | 0.226 | 764 | 337 | 17 | 2 | 752 | 196 | 718 | Prim-Pol domain-containing protein | Prim-Pol domain-containing protein | | afdb-uniprot50 | AF-A0A848EXA0-F1-MODEL\_V4 | 1.0 | 2.875e-29 | 1064 | 0.28 | 421 | 272 | 14 | 370 | 768 | 19 | 430 | Uncharacterized protein | Uncharacterized protein | | afdb-uniprot50 | AF-A0A1C6F2F8-F1-MODEL\_V4 | 1.0 | 1.151e-31 | 1062 | 0.277 | 486 | 300 | 18 | 298 | 761 | 78 | 534 | DNA primase | DNA primase | | afdb-uniprot50 | AF-A0A4V2HU15-F1-MODEL\_V4 | 1.0 | 5.961e-42 | 1053 | 0.231 | 937 | 422 | 38 | 98 | 752 | 95 | 1014 | Toprim domain-containing protein | Toprim domain-containing protein | | afdb-uniprot50 | AF-A0A2S5TKJ6-F1-MODEL\_V4 | 1.0 | 1.093e-39 | 1037 | 0.262 | 698 | 432 | 33 | 102 | 762 | 90 | 741 | Uncharacterized protein | Uncharacterized protein | | afdb-uniprot50 | AF-A0A1U7MHB1-F1-MODEL\_V4 | 1.0 | 4.545e-32 | 1034 | 0.247 | 545 | 333 | 20 | 260 | 769 | 202 | 704 | Virulence-associated protein E | Virulence-associated protein E | | afdb-uniprot50 | AF-A0A1M6WA62-F1-MODEL\_V4 | 1.0 | 6.737e-33 | 1016 | 0.263 | 577 | 320 | 20 | 242 | 762 | 33 | 560 | Virulence-associated protein E | Virulence-associated protein E | | afdb-uniprot50 | AF-A0A661CNY3-F1-MODEL\_V4 | 1.0 | 2.593e-29 | 1001 | 0.234 | 457 | 304 | 15 | 330 | 758 | 299 | 737 | Uncharacterized protein | Uncharacterized protein | | afdb-uniprot50 | AF-A0A7Y8ZYJ0-F1-MODEL\_V4 | 1.0 | 7.967e-35 | 984 | 0.213 | 646 | 407 | 31 | 168 | 768 | 103 | 692 | Putative P-loop ATPase | Putative P-loop ATPase | | afdb-uniprot50 | AF-A0A349EG17-F1-MODEL\_V4 | 1.0 | 1.196e-37 | 982 | 0.263 | 686 | 414 | 34 | 99 | 757 | 74 | 694 | Toprim domain-containing protein | Toprim domain-containing protein | | afdb-uniprot50 | AF-A0A7Z8QIE3-F1-MODEL\_V4 | 1.0 | 9.605e-28 | 979 | 0.268 | 410 | 270 | 12 | 370 | 762 | 9 | 405 | Uncharacterized protein | Uncharacterized protein | | afdb-uniprot50 | AF-A0A431IWX9-F1-MODEL\_V4 | 1.0 | 8.947e-29 | 975 | 0.244 | 482 | 324 | 15 | 291 | 762 | 280 | 731 | PriCT\_2 domain-containing protein | PriCT\_2 domain-containing protein | | afdb-uniprot50 | AF-A0A7T4N249-F1-MODEL\_V4 | 1.0 | 2.447e-24 | 964 | 0.335 | 349 | 200 | 10 | 13 | 347 | 9 | 339 | DUF927 domain-containing protein | DUF927 domain-containing protein | | afdb-uniprot50 | AF-A0A2N2U4N7-F1-MODEL\_V4 | 1.0 | 2.265e-28 | 957 | 0.366 | 360 | 200 | 11 | 1 | 347 | 1 | 345 | Toprim domain-containing protein | Toprim domain-containing protein | | afdb-uniprot50 | AF-A0A412CFG8-F1-MODEL\_V4 | 1.0 | 1.301e-30 | 952 | 0.235 | 557 | 341 | 21 | 243 | 769 | 241 | 742 | Uncharacterized protein | Uncharacterized protein | | afdb-uniprot50 | AF-A0A2G2PN86-F1-MODEL\_V4 | 1.0 | 3.401e-39 | 948 | 0.232 | 788 | 454 | 33 | 93 | 769 | 54 | 801 | Uncharacterized protein | Uncharacterized protein | | afdb-uniprot50 | AF-A0A6A4U5X7-F1-MODEL\_V4 | 1.0 | 5.105e-26 | 947 | 0.246 | 386 | 276 | 13 | 379 | 756 | 310 | 688 | Virulence-associated E family protein | Virulence-associated E family protein | | afdb-uniprot50 | AF-A0A2S7FDM4-F1-MODEL\_V4 | 1.0 | 8.28e-25 | 929 | 0.246 | 414 | 270 | 19 | 366 | 757 | 18 | 411 | Uncharacterized protein | Uncharacterized protein | | afdb-uniprot50 | AF-A0A849UAZ0-F1-MODEL\_V4 | 1.0 | 5.66e-34 | 925 | 0.228 | 652 | 416 | 32 | 127 | 754 | 49 | 637 | Uncharacterized protein | Uncharacterized protein | | afdb-uniprot50 | AF-A0A193CM65-F1-MODEL\_V4 | 1.0 | 1.694e-27 | 920 | 0.277 | 480 | 300 | 16 | 286 | 757 | 292 | 732 | Uncharacterized protein | Uncharacterized protein | | afdb-uniprot50 | AF-A0A661DIC7-F1-MODEL\_V4 | 1.0 | 7.278e-29 | 920 | 0.209 | 496 | 326 | 16 | 291 | 758 | 376 | 833 | Uncharacterized protein | Uncharacterized protein | | afdb-uniprot50 | AF-C9LQR2-F1-MODEL\_V4 | 1.0 | 4.126e-29 | 917 | 0.238 | 537 | 341 | 23 | 240 | 757 | 223 | 710 | Virulence-associated protein E | Virulence-associated protein E | | afdb-uniprot50 | AF-A0A4P8XWJ9-F1-MODEL\_V4 | 1.0 | 8.719e-25 | 910 | 0.253 | 414 | 273 | 16 | 365 | 757 | 133 | 531 | Uncharacterized protein | Uncharacterized protein | | afdb-uniprot50 | AF-A0A354KW19-F1-MODEL\_V4 | 1.0 | 3.995e-28 | 902 | 0.275 | 497 | 295 | 17 | 299 | 757 | 28 | 497 | ZnF\_CHCC domain-containing protein | ZnF\_CHCC domain-containing protein | | afdb-uniprot50 | AF-A0A419U224-F1-MODEL\_V4 | 1.0 | 5.734e-36 | 902 | 0.218 | 829 | 456 | 36 | 92 | 769 | 62 | 849 | Putative P-loop ATPase | Putative P-loop ATPase | | afdb-uniprot50 | AF-A0A3M9MMY6-F1-MODEL\_V4 | 1.0 | 1.854e-25 | 901 | 0.252 | 404 | 271 | 17 | 384 | 769 | 31 | 421 | Virulence-associated e family protein | Virulence-associated e family protein | | afdb-uniprot50 | AF-A0A0B2JZB9-F1-MODEL\_V4 | 1.0 | 8.227e-28 | 897 | 0.232 | 521 | 320 | 21 | 261 | 756 | 230 | 695 | Uncharacterized protein | Uncharacterized protein | | afdb-uniprot50 | AF-A0A450WD24-F1-MODEL\_V4 | 1.0 | 2.03e-39 | 892 | 0.237 | 870 | 460 | 50 | 10 | 753 | 4 | 795 | Putative DNA primase/helicase | Putative DNA primase/helicase | | afdb-uniprot50 | AF-A0A7Z0SE33-F1-MODEL\_V4 | 1.0 | 4.546e-40 | 890 | 0.247 | 876 | 483 | 48 | 12 | 757 | 9 | 837 | PriCT-2 domain-containing protein | PriCT-2 domain-containing protein | | afdb-uniprot50 | AF-A0A1S8TCW1-F1-MODEL\_V4 | 1.0 | 1.335e-34 | 887 | 0.224 | 744 | 428 | 35 | 64 | 769 | 67 | 699 | DNA primase | DNA primase | | afdb-uniprot50 | AF-A0A6M4GU76-F1-MODEL\_V4 | 1.0 | 1.326e-37 | 877 | 0.204 | 816 | 508 | 36 | 1 | 757 | 6 | 738 | PriCT\_2 domain-containing protein | PriCT\_2 domain-containing protein | | afdb-uniprot50 | AF-A0A5B3G911-F1-MODEL\_V4 | 1.0 | 2.577e-24 | 873 | 0.248 | 383 | 264 | 13 | 381 | 755 | 54 | 420 | Uncharacterized protein | Uncharacterized protein | | afdb-uniprot50 | AF-A0A258SSH2-F1-MODEL\_V4 | 1.0 | 3.335e-24 | 867 | 0.461 | 286 | 140 | 7 | 493 | 769 | 2 | 282 | Uncharacterized protein | Uncharacterized protein | | afdb-uniprot50 | AF-A0A1Q2M5G7-F1-MODEL\_V4 | 1.0 | 9.855e-24 | 866 | 0.344 | 354 | 177 | 11 | 10 | 324 | 5 | 342 | Uncharacterized protein | Uncharacterized protein | | afdb-uniprot50 | AF-A0A158KF25-F1-MODEL\_V4 | 1.0 | 1.83e-23 | 866 | 0.343 | 361 | 200 | 14 | 3 | 347 | 2 | 341 | DNA replication protein | DNA replication protein | | afdb-uniprot50 | AF-A0A1G0RDT0-F1-MODEL\_V4 | 1.0 | 7.231e-24 | 864 | 0.215 | 403 | 282 | 15 | 381 | 756 | 31 | 426 | Uncharacterized protein | Uncharacterized protein | | afdb-uniprot50 | AF-D7K6T8-F1-MODEL\_V4 | 1.0 | 1.461e-24 | 863 | 0.241 | 389 | 261 | 16 | 384 | 757 | 19 | 388 | Putative helicase | Putative helicase | | afdb-uniprot50 | AF-A0A1I9YDF5-F1-MODEL\_V4 | 1.0 | 3.558e-34 | 859 | 0.193 | 818 | 493 | 39 | 13 | 762 | 7 | 725 | PriCT\_2 domain-containing protein | PriCT\_2 domain-containing protein | | afdb-uniprot50 | AF-R5CCA7-F1-MODEL\_V4 | 1.0 | 3.511e-24 | 855 | 0.239 | 389 | 272 | 16 | 378 | 756 | 35 | 409 | Uncharacterized protein | Uncharacterized protein | | afdb-uniprot50 | AF-A0A7W8FU46-F1-MODEL\_V4 | 1.0 | 2.193e-27 | 852 | 0.238 | 528 | 335 | 19 | 261 | 769 | 216 | 695 | PriCT\_1 domain-containing protein | PriCT\_1 domain-containing protein | | afdb-uniprot50 | AF-A0A4P7L7L6-F1-MODEL\_V4 | 1.0 | 2.124e-34 | 850 | 0.21 | 723 | 467 | 37 | 60 | 755 | 61 | 706 | DNA primase TraC | DNA primase TraC | | afdb-uniprot50 | AF-A0A3G9IPK2-F1-MODEL\_V4 | 1.0 | 5.515e-22 | 848 | 0.28 | 339 | 212 | 9 | 366 | 682 | 41 | 369 | Uncharacterized protein | Uncharacterized protein | | afdb-uniprot50 | AF-A0A4Q3Q5F2-F1-MODEL\_V4 | 1.0 | 8.069e-21 | 843 | 0.388 | 270 | 148 | 8 | 6 | 272 | 1 | 256 | Toprim domain-containing protein | Toprim domain-containing protein | | afdb-uniprot50 | AF-A0A7X6JZJ4-F1-MODEL\_V4 | 1.0 | 5.998e-31 | 843 | 0.235 | 658 | 348 | 22 | 183 | 755 | 351 | 938 | Uncharacterized protein | Uncharacterized protein | | afdb-uniprot50 | AF-A0A6M3KDE2-F1-MODEL\_V4 | 1.0 | 2.029e-23 | 842 | 0.221 | 389 | 272 | 16 | 384 | 751 | 51 | 429 | Putative VirE domain containing protein | Putative VirE domain containing protein | | afdb-uniprot50 | AF-A0A6N4AQ20-F1-MODEL\_V4 | 1.0 | 8.017e-24 | 841 | 0.301 | 398 | 247 | 15 | 377 | 757 | 28 | 411 | Uncharacterized protein | Uncharacterized protein | | afdb-uniprot50 | AF-A0A6I7WDH6-F1-MODEL\_V4 | 1.0 | 2.543e-22 | 840 | 0.298 | 335 | 217 | 7 | 376 | 699 | 19 | 346 | Uncharacterized protein | Uncharacterized protein | | afdb-uniprot50 | AF-A0A6L4NDA3-F1-MODEL\_V4 | 1.0 | 9.855e-24 | 840 | 0.232 | 387 | 271 | 14 | 384 | 755 | 18 | 393 | Virulence protein E | Virulence protein E | | afdb-uniprot50 | AF-A0A5B5U8H6-F1-MODEL\_V4 | 1.0 | 2.627e-23 | 838 | 0.24 | 387 | 267 | 16 | 382 | 757 | 67 | 437 | Virulence-associated e family protein | Virulence-associated e family protein | | afdb-uniprot50 | AF-A0A6M3J7V5-F1-MODEL\_V4 | 1.0 | 4.179e-23 | 836 | 0.266 | 401 | 262 | 16 | 370 | 753 | 43 | 428 | Putative VirE domain containing protein | Putative VirE domain containing protein | | afdb-uniprot50 | AF-A0A078S3U0-F1-MODEL\_V4 | 1.0 | 1.651e-23 | 835 | 0.23 | 391 | 274 | 12 | 381 | 757 | 15 | 392 | Virulence-associated E family protein | Virulence-associated E family protein | | afdb-uniprot50 | AF-A0A7Z8VJ41-F1-MODEL\_V4 | 1.0 | 2.003e-21 | 833 | 0.305 | 311 | 196 | 7 | 370 | 665 | 4 | 309 | Uncharacterized protein | Uncharacterized protein | | afdb-uniprot50 | AF-A0A7J5V6L9-F1-MODEL\_V4 | 1.0 | 2.017e-26 | 831 | 0.221 | 479 | 317 | 20 | 297 | 756 | 151 | 592 | Virulence-associated E family protein | Virulence-associated E family protein | | afdb-uniprot50 | AF-A0A4R5B4A1-F1-MODEL\_V4 | 1.0 | 7.763e-23 | 826 | 0.229 | 387 | 268 | 17 | 384 | 757 | 2 | 371 | Virulence-associated e family protein | Virulence-associated e family protein | | afdb-uniprot50 | AF-A0A1H1CRS4-F1-MODEL\_V4 | 1.0 | 1.414e-23 | 825 | 0.202 | 404 | 282 | 15 | 379 | 751 | 21 | 415 | Virulence-associated protein E | Virulence-associated protein E | | afdb-uniprot50 | AF-A0A6B2H5H0-F1-MODEL\_V4 | 1.0 | 1.114e-22 | 821 | 0.244 | 385 | 266 | 15 | 384 | 756 | 20 | 391 | Uncharacterized protein | Uncharacterized protein | | afdb-uniprot50 | AF-A0A1I0LZP8-F1-MODEL\_V4 | 1.0 | 1.005e-22 | 817 | 0.196 | 392 | 292 | 15 | 378 | 756 | 181 | 562 | VirE N-terminal domain-containing protein | VirE N-terminal domain-containing protein | | afdb-uniprot50 | AF-A0A3A5S119-F1-MODEL\_V4 | 1.0 | 8.607e-23 | 816 | 0.231 | 389 | 276 | 12 | 381 | 757 | 330 | 707 | VirE protein | VirE protein | | afdb-uniprot50 | AF-A0A431MH39-F1-MODEL\_V4 | 1.0 | 3.969e-23 | 815 | 0.215 | 395 | 289 | 14 | 372 | 757 | 23 | 405 | Uncharacterized protein | Uncharacterized protein | | afdb-uniprot50 | AF-A0A6I0LF08-F1-MODEL\_V4 | 1.0 | 9.062e-23 | 815 | 0.231 | 388 | 275 | 11 | 381 | 756 | 330 | 706 | VirE protein | VirE protein | | afdb-uniprot50 | AF-A0A1Y0EKF6-F1-MODEL\_V4 | 1.0 | 1.309e-35 | 814 | 0.221 | 940 | 452 | 49 | 12 | 731 | 9 | 888 | Uncharacterized protein | Uncharacterized protein | | afdb-uniprot50 | AF-A0A7K3MY39-F1-MODEL\_V4 | 1.0 | 9.481e-26 | 813 | 0.217 | 515 | 320 | 22 | 264 | 756 | 4 | 457 | Virulence-associated E family protein | Virulence-associated E family protein | | afdb-uniprot50 | AF-A0A7D6XXE7-F1-MODEL\_V4 | 1.0 | 3.77e-23 | 813 | 0.23 | 438 | 284 | 17 | 363 | 757 | 76 | 503 | Uncharacterized protein | Uncharacterized protein | | afdb-uniprot50 | AF-A0A7T1WPK4-F1-MODEL\_V4 | 1.0 | 8.442e-24 | 813 | 0.234 | 426 | 270 | 16 | 370 | 751 | 532 | 945 | Uncharacterized protein | Uncharacterized protein | | afdb-uniprot50 | AF-A0A414KVM2-F1-MODEL\_V4 | 1.0 | 2.627e-23 | 812 | 0.215 | 389 | 276 | 13 | 384 | 756 | 100 | 475 | Virulence protein E | Virulence protein E | | afdb-uniprot50 | AF-D0X1Z3-F1-MODEL\_V4 | 1.0 | 1.772e-22 | 808 | 0.271 | 350 | 218 | 10 | 435 | 756 | 6 | 346 | Uncharacterized protein | Uncharacterized protein | | afdb-uniprot50 | AF-A0A2T4J627-F1-MODEL\_V4 | 1.0 | 7.325e-26 | 808 | 0.245 | 518 | 333 | 23 | 259 | 752 | 3 | 486 | Uncharacterized protein | Uncharacterized protein | | afdb-uniprot50 | AF-A0A1Y6D883-F1-MODEL\_V4 | 1.0 | 1.251e-24 | 808 | 0.326 | 361 | 201 | 14 | 11 | 347 | 8 | 350 | Uncharacterized protein | Uncharacterized protein | | afdb-uniprot50 | AF-R1ISA0-F1-MODEL\_V4 | 1.0 | 2.385e-28 | 808 | 0.217 | 562 | 334 | 25 | 260 | 757 | 12 | 531 | Virulence-associated E family protein | Virulence-associated E family protein | | afdb-uniprot50 | AF-A0A1H0G3S0-F1-MODEL\_V4 | 1.0 | 6.48e-27 | 808 | 0.203 | 502 | 351 | 20 | 264 | 751 | 243 | 709 | Virulence-associated protein E | Virulence-associated protein E | | afdb-uniprot50 | AF-R5JFQ8-F1-MODEL\_V4 | 1.0 | 7.713e-26 | 807 | 0.233 | 527 | 308 | 19 | 258 | 756 | 275 | 733 | Virulence-associated protein E | Virulence-associated protein E | | afdb-uniprot50 | AF-A0A6V6ZI02-F1-MODEL\_V4 | 1.0 | 7.372e-23 | 804 | 0.259 | 405 | 262 | 17 | 370 | 751 | 36 | 425 | Putative P-loop ATPase | Putative P-loop ATPase | | afdb-uniprot50 | AF-A0A252F7E8-F1-MODEL\_V4 | 1.0 | 3.008e-24 | 804 | 0.245 | 488 | 294 | 23 | 321 | 769 | 97 | 549 | Uncharacterized protein | Uncharacterized protein | | afdb-uniprot50 | AF-A0A240U2T6-F1-MODEL\_V4 | 1.0 | 5.445e-36 | 802 | 0.218 | 930 | 480 | 52 | 1 | 750 | 10 | 872 | Uncharacterized protein | Uncharacterized protein | | afdb-uniprot50 | AF-A0A3A9UXP7-F1-MODEL\_V4 | 1.0 | 4.487e-22 | 800 | 0.238 | 385 | 272 | 14 | 384 | 759 | 47 | 419 | Virulence-associated e family protein | Virulence-associated e family protein | | afdb-uniprot50 | AF-A0A173MFG2-F1-MODEL\_V4 | 1.0 | 4.974e-22 | 797 | 0.184 | 384 | 289 | 15 | 381 | 752 | 79 | 450 | Virulence-associated protein E | Virulence-associated protein E | | afdb-uniprot50 | AF-A0A315Z5E8-F1-MODEL\_V4 | 1.0 | 3.77e-23 | 797 | 0.221 | 401 | 272 | 16 | 382 | 751 | 59 | 450 | Virulence-associated protein E | Virulence-associated protein E | | afdb-uniprot50 | AF-A0A3D3MX14-F1-MODEL\_V4 | 1.0 | 2.179e-22 | 797 | 0.227 | 395 | 272 | 19 | 381 | 757 | 309 | 688 | VirE\_N domain-containing protein | VirE\_N domain-containing protein | | afdb-uniprot50 | AF-A0A1H0Y2A4-F1-MODEL\_V4 | 1.0 | 2.447e-24 | 796 | 0.241 | 427 | 292 | 17 | 339 | 755 | 164 | 568 | VirE N-terminal domain-containing protein | VirE N-terminal domain-containing protein | | afdb-uniprot50 | AF-D4VNM0-F1-MODEL\_V4 | 1.0 | 4.487e-22 | 795 | 0.206 | 383 | 281 | 16 | 385 | 757 | 4 | 373 | Putative helicase | Putative helicase | | afdb-uniprot50 | AF-A0A521XCL3-F1-MODEL\_V4 | 1.0 | 2.627e-23 | 793 | 0.2 | 433 | 301 | 18 | 333 | 755 | 327 | 724 | Prim-Pol domain-containing protein | Prim-Pol domain-containing protein | | afdb-uniprot50 | AF-W1YBE3-F1-MODEL\_V4 | 1.0 | 9.73e-30 | 792 | 0.204 | 651 | 410 | 41 | 153 | 754 | 113 | 704 | Sigma 54 interacting protein | Sigma 54 interacting protein | | afdb-uniprot50 | AF-A0A316MW33-F1-MODEL\_V4 | 1.0 | 3.422e-20 | 791 | 0.379 | 258 | 142 | 8 | 93 | 347 | 70 | 312 | Uncharacterized protein | Uncharacterized protein | | afdb-uniprot50 | AF-A0A382QEF6-F1-MODEL\_V4 | 1.0 | 6.48e-19 | 789 | 0.372 | 247 | 130 | 6 | 102 | 347 | 70 | 292 | Toprim domain-containing protein | Toprim domain-containing protein | | afdb-uniprot50 | AF-A0A316F066-F1-MODEL\_V4 | 1.0 | 2.416e-22 | 786 | 0.236 | 411 | 284 | 17 | 369 | 762 | 2 | 399 | Virulence-associated protein E | Virulence-associated protein E | | afdb-uniprot50 | AF-A0A2E3H3V5-F1-MODEL\_V4 | 1.0 | 8.607e-23 | 786 | 0.267 | 411 | 255 | 17 | 370 | 757 | 32 | 419 | Virulence-associated E family protein | Virulence-associated E family protein | | afdb-uniprot50 | AF-A0A5C7J8I3-F1-MODEL\_V4 | 1.0 | 2.678e-22 | 786 | 0.252 | 392 | 261 | 15 | 385 | 760 | 125 | 500 | Uncharacterized protein | Uncharacterized protein | | afdb-uniprot50 | AF-A0A0F9B953-F1-MODEL\_V4 | 1.0 | 8.174e-23 | 785 | 0.266 | 405 | 255 | 21 | 370 | 753 | 53 | 436 | Uncharacterized protein | Uncharacterized protein | | afdb-uniprot50 | AF-A0A095YT29-F1-MODEL\_V4 | 1.0 | 2.221e-21 | 785 | 0.269 | 371 | 215 | 14 | 1 | 350 | 1 | 336 | Uncharacterized protein | Uncharacterized protein | | afdb-uniprot50 | AF-A0A7W7EQK1-F1-MODEL\_V4 | 1.0 | 4.288e-19 | 784 | 0.422 | 251 | 139 | 5 | 363 | 609 | 9 | 257 | Putative P-loop ATPase | Putative P-loop ATPase | | afdb-uniprot50 | AF-A0A5Q0BL22-F1-MODEL\_V4 | 1.0 | 1.211e-23 | 783 | 0.335 | 340 | 169 | 12 | 1 | 333 | 1 | 290 | Toprim domain-containing protein | Toprim domain-containing protein | | afdb-uniprot50 | AF-A0A7Z1L1I9-F1-MODEL\_V4 | 1.0 | 5.807e-22 | 783 | 0.233 | 385 | 275 | 15 | 381 | 757 | 15 | 387 | Uncharacterized protein | Uncharacterized protein | | afdb-uniprot50 | AF-A0A1Y6CVM3-F1-MODEL\_V4 | 1.0 | 6.736e-25 | 783 | 0.263 | 474 | 278 | 20 | 2 | 447 | 4 | 434 | Uncharacterized domain associated with phage/plasmid primase | Uncharacterized domain associated with phage/plasmid primase | | afdb-uniprot50 | AF-A0A7L5DSN9-F1-MODEL\_V4 | 1.0 | 4.879e-23 | 780 | 0.248 | 399 | 266 | 16 | 369 | 751 | 28 | 408 | Virulence protein | Virulence protein | | afdb-uniprot50 | AF-A0A246G7T7-F1-MODEL\_V4 | 1.0 | 1.991e-24 | 780 | 0.195 | 506 | 308 | 22 | 261 | 750 | 28 | 450 | Virulence-associated e family protein | Virulence-associated e family protein | | afdb-uniprot50 | AF-A0A0X8JSM9-F1-MODEL\_V4 | 1.0 | 1.878e-19 | 780 | 0.37 | 251 | 138 | 6 | 101 | 349 | 455 | 687 | Toprim domain-containing protein | Toprim domain-containing protein | | afdb-uniprot50 | AF-A0A149STJ7-F1-MODEL\_V4 | 1.0 | 6.115e-22 | 778 | 0.227 | 408 | 265 | 17 | 370 | 752 | 3 | 385 | Virulence-associated E family protein | Virulence-associated E family protein | | afdb-uniprot50 | AF-A0A2C8Z209-F1-MODEL\_V4 | 1.0 | 1.784e-35 | 778 | 0.222 | 850 | 461 | 43 | 13 | 737 | 5 | 779 | Putative DNA primase/helicase | Putative DNA primase/helicase | | afdb-uniprot50 | AF-A0A7D7QZK8-F1-MODEL\_V4 | 1.0 | 4.816e-21 | 777 | 0.23 | 351 | 248 | 12 | 414 | 756 | 1 | 337 | Virulence-associated e family protein | Virulence-associated e family protein | | afdb-uniprot50 | AF-A0A1F3A5U3-F1-MODEL\_V4 | 1.0 | 1.058e-22 | 776 | 0.275 | 410 | 248 | 19 | 370 | 751 | 18 | 406 | Uncharacterized protein | Uncharacterized protein | | afdb-uniprot50 | AF-A0A6G1V777-F1-MODEL\_V4 | 1.0 | 1.3e-22 | 773 | 0.215 | 417 | 284 | 16 | 377 | 769 | 4 | 401 | Uncharacterized protein | Uncharacterized protein | | afdb-uniprot50 | AF-A0A1I1YQB9-F1-MODEL\_V4 | 1.0 | 3.557e-26 | 773 | 0.213 | 524 | 319 | 20 | 261 | 754 | 263 | 723 | Virulence-associated protein E | Virulence-associated protein E | | afdb-uniprot50 | AF-A0A7Z8R0X3-F1-MODEL\_V4 | 1.0 | 3.969e-23 | 772 | 0.263 | 410 | 260 | 16 | 376 | 762 | 5 | 395 | Uncharacterized protein | Uncharacterized protein | | afdb-uniprot50 | AF-A0A357CEQ6-F1-MODEL\_V4 | 1.0 | 2.73e-21 | 772 | 0.255 | 392 | 258 | 19 | 377 | 753 | 63 | 435 | Virulence-associated E family protein | Virulence-associated E family protein | | afdb-uniprot50 | AF-A0A2R4KET0-F1-MODEL\_V4 | 1.0 | 1.235e-22 | 772 | 0.217 | 405 | 287 | 17 | 370 | 756 | 39 | 431 | Virulence protein | Virulence protein | | afdb-uniprot50 | AF-A0A8B3TM54-F1-MODEL\_V4 | 1.0 | 4.207e-28 | 771 | 0.23 | 624 | 345 | 28 | 183 | 752 | 76 | 618 | Uncharacterized protein | Uncharacterized protein | | afdb-uniprot50 | AF-A0A4Q6JDT2-F1-MODEL\_V4 | 1.0 | 1.276e-23 | 770 | 0.315 | 364 | 188 | 13 | 3 | 324 | 1 | 345 | Toprim domain-containing protein | Toprim domain-containing protein | | afdb-uniprot50 | AF-A0A4R5Q8H9-F1-MODEL\_V4 | 1.0 | 2.593e-21 | 770 | 0.24 | 400 | 271 | 18 | 370 | 752 | 319 | 702 | Uncharacterized protein | Uncharacterized protein | | afdb-uniprot50 | AF-A0A7X6HWL2-F1-MODEL\_V4 | 1.0 | 1.952e-25 | 770 | 0.23 | 537 | 328 | 24 | 261 | 762 | 261 | 747 | PriCT\_1 domain-containing protein | PriCT\_1 domain-containing protein | | afdb-uniprot50 | AF-A0A7X7UY95-F1-MODEL\_V4 | 1.0 | 1.251e-24 | 767 | 0.203 | 482 | 335 | 21 | 297 | 762 | 257 | 705 | Prim-Pol domain-containing protein | Prim-Pol domain-containing protein | | afdb-uniprot50 | AF-A0A1Z5HBM1-F1-MODEL\_V4 | 1.0 | 1.219e-28 | 766 | 0.178 | 777 | 339 | 15 | 1 | 762 | 252 | 743 | Uncharacterized protein | Uncharacterized protein | | afdb-uniprot50 | AF-A0A847QTI9-F1-MODEL\_V4 | 1.0 | 2.495e-23 | 765 | 0.197 | 496 | 337 | 22 | 292 | 757 | 279 | 743 | Uncharacterized protein | Uncharacterized protein | | afdb-uniprot50 | AF-A0A2E4PDU5-F1-MODEL\_V4 | 1.0 | 8.333e-22 | 764 | 0.223 | 426 | 269 | 16 | 381 | 759 | 4 | 414 | Virulence-associated e family protein | Virulence-associated e family protein | | afdb-uniprot50 | AF-A0A3M3TAF3-F1-MODEL\_V4 | 1.0 | 1.024e-29 | 764 | 0.308 | 509 | 304 | 25 | 102 | 594 | 78 | 554 | DNA primase domain protein | DNA primase domain protein | | afdb-uniprot50 | AF-A0A1M4N4I5-F1-MODEL\_V4 | 1.0 | 2.179e-22 | 763 | 0.262 | 412 | 249 | 20 | 370 | 753 | 49 | 433 | Death domain-containing protein | Death domain-containing protein | | afdb-uniprot50 | AF-A0A7Z9GAF4-F1-MODEL\_V4 | 1.0 | 7.516e-22 | 760 | 0.235 | 404 | 263 | 19 | 370 | 753 | 308 | 685 | Uncharacterized protein | Uncharacterized protein | | afdb-uniprot50 | AF-A0A641RMU6-F1-MODEL\_V4 | 1.0 | 2.109e-21 | 758 | 0.241 | 360 | 244 | 12 | 408 | 755 | 5 | 347 | Virulence protein E | Virulence protein E | | afdb-uniprot50 | AF-A0A2E7IPX4-F1-MODEL\_V4 | 1.0 | 7.614e-24 | 758 | 0.239 | 493 | 304 | 26 | 317 | 762 | 1 | 469 | Uncharacterized protein | Uncharacterized protein | | afdb-uniprot50 | AF-A0A5C7NHB2-F1-MODEL\_V4 | 1.0 | 2.875e-29 | 758 | 0.253 | 671 | 334 | 34 | 8 | 563 | 2 | 620 | DUF927 domain-containing protein | DUF927 domain-containing protein | | afdb-uniprot50 | AF-A0A645B275-F1-MODEL\_V4 | 1.0 | 2.73e-21 | 757 | 0.21 | 399 | 282 | 17 | 375 | 755 | 53 | 436 | DUF3874 domain-containing protein | DUF3874 domain-containing protein | | afdb-uniprot50 | AF-A0A7V9SQL9-F1-MODEL\_V4 | 1.0 | 1.903e-21 | 757 | 0.221 | 401 | 280 | 16 | 369 | 754 | 37 | 420 | Virulence protein | Virulence protein | | afdb-uniprot50 | AF-A0A2G5L038-F1-MODEL\_V4 | 1.0 | 2.73e-21 | 755 | 0.203 | 394 | 286 | 15 | 381 | 757 | 19 | 401 | Uncharacterized protein | Uncharacterized protein | | afdb-uniprot50 | AF-Q1QLE7-F1-MODEL\_V4 | 1.0 | 5.34e-21 | 755 | 0.233 | 394 | 268 | 14 | 370 | 752 | 317 | 687 | Virulence-associated E | Virulence-associated E | | afdb-uniprot50 | AF-A0A414PMJ6-F1-MODEL\_V4 | 1.0 | 2.003e-21 | 753 | 0.19 | 393 | 292 | 15 | 378 | 757 | 7 | 386 | Virulence protein E | Virulence protein E | | afdb-uniprot50 | AF-A0A2E2PC01-F1-MODEL\_V4 | 1.0 | 2.339e-21 | 751 | 0.215 | 380 | 280 | 13 | 379 | 750 | 2 | 371 | Uncharacterized protein | Uncharacterized protein | | afdb-uniprot50 | AF-A0A1I4P1M4-F1-MODEL\_V4 | 1.0 | 2.25e-23 | 750 | 0.237 | 468 | 304 | 18 | 329 | 755 | 307 | 762 | Predicted P-loop ATPase and inactivated derivatives | Predicted P-loop ATPase and inactivated derivatives | | afdb-uniprot50 | AF-A0A2Z6UA34-F1-MODEL\_V4 | 1.0 | 2.221e-21 | 749 | 0.288 | 371 | 199 | 14 | 1 | 323 | 1 | 354 | PriCT\_2 domain-containing protein | PriCT\_2 domain-containing protein | | afdb-uniprot50 | AF-A0A1F3KBL0-F1-MODEL\_V4 | 1.0 | 1.716e-21 | 749 | 0.237 | 388 | 271 | 16 | 381 | 755 | 104 | 479 | Uncharacterized protein | Uncharacterized protein | | afdb-uniprot50 | AF-A0A3B9IYP5-F1-MODEL\_V4 | 1.0 | 7.093e-25 | 749 | 0.226 | 534 | 319 | 26 | 263 | 757 | 243 | 721 | Uncharacterized protein | Uncharacterized protein | | afdb-uniprot50 | AF-A0A1Q3GLM4-F1-MODEL\_V4 | 1.0 | 7.138e-22 | 748 | 0.228 | 390 | 266 | 14 | 381 | 751 | 43 | 416 | Uncharacterized protein | Uncharacterized protein | | afdb-uniprot50 | AF-A0A6F8PR07-F1-MODEL\_V4 | 1.0 | 1.442e-22 | 743 | 0.303 | 362 | 216 | 11 | 1 | 346 | 3 | 344 | Uncharacterized protein | Uncharacterized protein | | afdb-uniprot50 | AF-A0A350YX52-F1-MODEL\_V4 | 1.0 | 4.663e-20 | 742 | 0.236 | 377 | 260 | 12 | 380 | 748 | 7 | 363 | Uncharacterized protein | Uncharacterized protein | | afdb-uniprot50 | AF-A0A7C5FUS1-F1-MODEL\_V4 | 1.0 | 6.564e-21 | 741 | 0.251 | 418 | 270 | 19 | 370 | 769 | 70 | 462 | Virulence-associated E family protein | Virulence-associated E family protein | | afdb-uniprot50 | AF-A0A7W6EUR7-F1-MODEL\_V4 | 1.0 | 7.184e-19 | 740 | 0.38 | 260 | 149 | 4 | 358 | 614 | 24 | 274 | Putative P-loop ATPase | Putative P-loop ATPase | | afdb-uniprot50 | AF-A0A7T9JLM2-F1-MODEL\_V4 | 1.0 | 2.73e-21 | 740 | 0.221 | 398 | 270 | 18 | 381 | 756 | 27 | 406 | Virulence-associated E family protein | Virulence-associated E family protein | | afdb-uniprot50 | AF-A0A512HAX8-F1-MODEL\_V4 | 1.0 | 6.912e-21 | 740 | 0.223 | 408 | 274 | 15 | 370 | 752 | 40 | 429 | Uncharacterized protein | Uncharacterized protein | | afdb-uniprot50 | AF-A0A4S0QEA6-F1-MODEL\_V4 | 1.0 | 1.079e-21 | 739 | 0.261 | 409 | 259 | 17 | 378 | 753 | 25 | 423 | Uncharacterized protein | Uncharacterized protein | | afdb-uniprot50 | AF-A0A0Q5HQG4-F1-MODEL\_V4 | 1.0 | 3.25e-20 | 738 | 0.203 | 407 | 282 | 20 | 370 | 755 | 1 | 386 | Uncharacterized protein | Uncharacterized protein | | afdb-uniprot50 | AF-A0A078BCE6-F1-MODEL\_V4 | 1.0 | 4.234e-33 | 738 | 0.214 | 855 | 471 | 47 | 1 | 737 | 1 | 772 | Primase C 2 (PriCT-2) family | Primase C 2 (PriCT-2) family | | afdb-uniprot50 | AF-A0A2W6XRY1-F1-MODEL\_V4 | 1.0 | 1.158e-36 | 738 | 0.214 | 1029 | 451 | 58 | 12 | 751 | 13 | 972 | Uncharacterized protein | Uncharacterized protein | | afdb-uniprot50 | AF-E4MB64-F1-MODEL\_V4 | 1.0 | 2.109e-21 | 737 | 0.227 | 400 | 278 | 19 | 369 | 756 | 13 | 393 | Uncharacterized protein | Uncharacterized protein | | afdb-uniprot50 | AF-A0A1Y2RF59-F1-MODEL\_V4 | 1.0 | 8.775e-22 | 737 | 0.231 | 402 | 282 | 16 | 370 | 757 | 26 | 414 | Uncharacterized protein | Uncharacterized protein | | afdb-uniprot50 | AF-A0A2T1HTE2-F1-MODEL\_V4 | 1.0 | 9.239e-22 | 737 | 0.257 | 416 | 263 | 16 | 366 | 762 | 36 | 424 | Virulence-associated E family protein | Virulence-associated E family protein | | afdb-uniprot50 | AF-A0A528UG59-F1-MODEL\_V4 | 1.0 | 8.496e-21 | 736 | 0.237 | 387 | 268 | 14 | 379 | 751 | 41 | 414 | Virulence protein E | Virulence protein E | | afdb-uniprot50 | AF-A0A318SE39-F1-MODEL\_V4 | 1.0 | 4.344e-21 | 735 | 0.353 | 297 | 165 | 12 | 26 | 313 | 1 | 279 | Toprim domain-containing protein | Toprim domain-containing protein | | afdb-uniprot50 | AF-A0A7D4NP15-F1-MODEL\_V4 | 1.0 | 6.912e-21 | 734 | 0.299 | 364 | 198 | 15 | 3 | 334 | 2 | 340 | PriCT-2 domain-containing protein | PriCT-2 domain-containing protein | | afdb-uniprot50 | AF-A0A1M3LBF8-F1-MODEL\_V4 | 1.0 | 2.593e-21 | 734 | 0.219 | 406 | 282 | 19 | 370 | 756 | 4 | 393 | Virulence protein | Virulence protein | | afdb-uniprot50 | AF-A0A1M6SXZ2-F1-MODEL\_V4 | 1.0 | 1.024e-21 | 734 | 0.209 | 387 | 276 | 16 | 381 | 751 | 69 | 441 | Virulence-associated protein E | Virulence-associated protein E | | afdb-uniprot50 | AF-A0A836NRY6-F1-MODEL\_V4 | 1.0 | 7.093e-25 | 734 | 0.292 | 404 | 249 | 18 | 102 | 489 | 78 | 460 | Conjugal transfer protein TraC | Conjugal transfer protein TraC | | afdb-uniprot50 | AF-A0A419H4F3-F1-MODEL\_V4 | 1.0 | 2.207e-24 | 734 | 0.193 | 528 | 349 | 19 | 239 | 751 | 42 | 507 | Uncharacterized protein | Uncharacterized protein | | afdb-uniprot50 | AF-A0A2A5B9Y8-F1-MODEL\_V4 | 1.0 | 3.167e-24 | 734 | 0.201 | 506 | 319 | 23 | 298 | 768 | 256 | 711 | Uncharacterized protein | Uncharacterized protein | | afdb-uniprot50 | AF-A0A7W6CYB3-F1-MODEL\_V4 | 1.0 | 1.219e-20 | 733 | 0.216 | 411 | 274 | 17 | 370 | 752 | 336 | 726 | Putative P-loop ATPase | Putative P-loop ATPase | | afdb-uniprot50 | AF-A0A558BMX0-F1-MODEL\_V4 | 1.0 | 5.92e-21 | 732 | 0.224 | 387 | 263 | 15 | 384 | 751 | 32 | 400 | Uncharacterized protein | Uncharacterized protein | | afdb-uniprot50 | AF-A0A845B9K8-F1-MODEL\_V4 | 1.0 | 6.115e-22 | 730 | 0.217 | 459 | 313 | 20 | 316 | 753 | 18 | 451 | Virulence-associated E family protein | Virulence-associated E family protein | | afdb-uniprot50 | AF-A0A0T7AN15-F1-MODEL\_V4 | 1.0 | 1.079e-21 | 729 | 0.222 | 423 | 295 | 14 | 370 | 767 | 62 | 475 | Virulence-associated E family protein | Virulence-associated E family protein | | afdb-uniprot50 | AF-A0A7D4FWX9-F1-MODEL\_V4 | 1.0 | 7.864e-25 | 729 | 0.205 | 530 | 324 | 18 | 239 | 757 | 255 | 698 | Virulence protein E | Virulence protein E | | afdb-uniprot50 | AF-A0A150TWT5-F1-MODEL\_V4 | 1.0 | 1.47e-21 | 729 | 0.23 | 439 | 277 | 19 | 370 | 762 | 423 | 846 | Uncharacterized protein | Uncharacterized protein | | afdb-uniprot50 | AF-A0A8B2CHX5-F1-MODEL\_V4 | 1.0 | 5.071e-21 | 728 | 0.18 | 398 | 297 | 15 | 384 | 762 | 27 | 414 | DUF3874 domain-containing protein | DUF3874 domain-containing protein | | afdb-uniprot50 | AF-A0A126ZEG5-F1-MODEL\_V4 | 1.0 | 1.432e-17 | 726 | 0.372 | 228 | 118 | 8 | 113 | 338 | 2 | 206 | Toprim domain-containing protein | Toprim domain-containing protein | | afdb-uniprot50 | AF-A0A2R3MUY2-F1-MODEL\_V4 | 1.0 | 7.278e-21 | 726 | 0.211 | 407 | 286 | 21 | 371 | 757 | 1 | 392 | Virulence-associated protein E | Virulence-associated protein E | | afdb-uniprot50 | AF-R6WKB3-F1-MODEL\_V4 | 1.0 | 1.065e-19 | 725 | 0.25 | 311 | 216 | 12 | 453 | 756 | 25 | 325 | VirE N-terminal domain protein | VirE N-terminal domain protein | | afdb-uniprot50 | AF-A0A1I5LS54-F1-MODEL\_V4 | 1.0 | 1.423e-20 | 725 | 0.21 | 394 | 278 | 17 | 381 | 757 | 237 | 614 | VirE N-terminal domain-containing protein | VirE N-terminal domain-containing protein | | afdb-uniprot50 | AF-A0A1Y1QLP3-F1-MODEL\_V4 | 1.0 | 2.748e-18 | 724 | 0.405 | 237 | 121 | 8 | 100 | 333 | 75 | 294 | Toprim domain-containing protein | Toprim domain-containing protein | | afdb-uniprot50 | AF-A0A1M7DJB2-F1-MODEL\_V4 | 1.0 | 1.211e-23 | 724 | 0.191 | 497 | 324 | 22 | 327 | 766 | 271 | 746 | Virulence-associated protein E | Virulence-associated protein E | | afdb-uniprot50 | AF-A0A3C0IVH4-F1-MODEL\_V4 | 1.0 | 4.847e-18 | 723 | 0.294 | 251 | 169 | 6 | 453 | 701 | 15 | 259 | Virulence-associated E family protein | Virulence-associated E family protein | | afdb-uniprot50 | AF-G8UM15-F1-MODEL\_V4 | 1.0 | 2.109e-21 | 723 | 0.231 | 414 | 268 | 18 | 379 | 756 | 22 | 421 | Virulence-associated protein E | Virulence-associated protein E | | afdb-uniprot50 | AF-A0A2D0MXJ1-F1-MODEL\_V4 | 1.0 | 2.511e-20 | 723 | 0.206 | 388 | 289 | 9 | 381 | 755 | 27 | 408 | Virulence protein | Virulence protein | | afdb-uniprot50 | AF-A0A1Y6ELX1-F1-MODEL\_V4 | 1.0 | 3.047e-26 | 723 | 0.194 | 608 | 416 | 31 | 167 | 752 | 26 | 581 | Virulence-associated protein E | Virulence-associated protein E | | afdb-uniprot50 | AF-A0A4Z1A268-F1-MODEL\_V4 | 1.0 | 1.065e-27 | 723 | 0.188 | 663 | 433 | 36 | 153 | 759 | 83 | 696 | Uncharacterized protein | Uncharacterized protein | | afdb-uniprot50 | AF-A0A5C6JXI2-F1-MODEL\_V4 | 1.0 | 1.499e-20 | 722 | 0.173 | 387 | 292 | 15 | 381 | 755 | 13 | 383 | Virulence-associated protein E | Virulence-associated protein E | | afdb-uniprot50 | AF-A0A2N2YSH7-F1-MODEL\_V4 | 1.0 | 2.339e-21 | 722 | 0.189 | 416 | 298 | 13 | 370 | 755 | 13 | 419 | Virulence protein | Virulence protein | | afdb-uniprot50 | AF-A0A373NTM4-F1-MODEL\_V4 | 1.0 | 4.344e-21 | 722 | 0.231 | 428 | 286 | 19 | 370 | 769 | 15 | 427 | Virulence-associated protein E | Virulence-associated protein E | | afdb-uniprot50 | AF-A0A094ZFQ5-F1-MODEL\_V4 | 1.0 | 2.193e-27 | 722 | 0.199 | 628 | 414 | 32 | 170 | 752 | 89 | 672 | Uncharacterized protein | Uncharacterized protein | | afdb-uniprot50 | AF-A0A1T5CXN1-F1-MODEL\_V4 | 1.0 | 1.018e-24 | 722 | 0.197 | 538 | 341 | 23 | 261 | 768 | 366 | 842 | Uncharacterized protein | Uncharacterized protein | | afdb-uniprot50 | AF-A0A177N064-F1-MODEL\_V4 | 1.0 | 1.479e-18 | 721 | 0.356 | 250 | 138 | 6 | 92 | 332 | 68 | 303 | Toprim domain-containing protein | Toprim domain-containing protein | | afdb-uniprot50 | AF-A0A0E4FNC5-F1-MODEL\_V4 | 1.0 | 4.974e-22 | 721 | 0.23 | 404 | 280 | 19 | 370 | 756 | 4 | 393 | Virulence-associated protein E | Virulence-associated protein E | | afdb-uniprot50 | AF-A0A413DD07-F1-MODEL\_V4 | 1.0 | 2.784e-20 | 721 | 0.181 | 386 | 290 | 14 | 384 | 755 | 237 | 610 | Helicase | Helicase | | afdb-uniprot50 | AF-A0A1I5I2J5-F1-MODEL\_V4 | 1.0 | 3.511e-24 | 721 | 0.189 | 516 | 341 | 21 | 261 | 756 | 244 | 702 | Virulence-associated protein E | Virulence-associated protein E | | afdb-uniprot50 | AF-A0A3E5DTG1-F1-MODEL\_V4 | 1.0 | 2.384e-20 | 720 | 0.191 | 381 | 283 | 13 | 385 | 751 | 2 | 371 | Helicase | Helicase | | afdb-uniprot50 | AF-A0A2N7R2N6-F1-MODEL\_V4 | 1.0 | 3.4e-23 | 720 | 0.283 | 370 | 226 | 16 | 118 | 471 | 6 | 352 | DNA primase TraC | DNA primase TraC | | afdb-uniprot50 | AF-T0GQL6-F1-MODEL\_V4 | 1.0 | 1.326e-21 | 720 | 0.26 | 407 | 268 | 22 | 370 | 762 | 15 | 402 | Uncharacterized protein | Uncharacterized protein | | afdb-uniprot50 | AF-A0A1G0X7P3-F1-MODEL\_V4 | 1.0 | 2.003e-21 | 720 | 0.188 | 484 | 330 | 24 | 316 | 766 | 6 | 459 | Uncharacterized protein | Uncharacterized protein | | afdb-uniprot50 | AF-A0A5F0UFE8-F1-MODEL\_V4 | 1.0 | 5.17e-20 | 719 | 0.201 | 392 | 272 | 22 | 384 | 756 | 4 | 373 | Helicase | Helicase | | afdb-uniprot50 | AF-A0A7C4P9F5-F1-MODEL\_V4 | 1.0 | 3.229e-23 | 719 | 0.213 | 486 | 305 | 24 | 287 | 757 | 280 | 703 | Uncharacterized protein | Uncharacterized protein | | afdb-uniprot50 | AF-A0A847L830-F1-MODEL\_V4 | 1.0 | 5.071e-21 | 718 | 0.212 | 405 | 293 | 13 | 367 | 757 | 69 | 461 | Uncharacterized protein | Uncharacterized protein | | afdb-uniprot50 | AF-A0A644UL52-F1-MODEL\_V4 | 1.0 | 5.997e-23 | 718 | 0.2 | 480 | 339 | 17 | 291 | 755 | 217 | 666 | VirE\_N domain-containing protein | VirE\_N domain-containing protein | | afdb-uniprot50 | AF-Q01RE1-F1-MODEL\_V4 | 1.0 | 1.181e-19 | 716 | 0.225 | 408 | 269 | 17 | 370 | 755 | 1 | 383 | Virulence-associated E family protein | Virulence-associated E family protein | | afdb-uniprot50 | AF-A0A0F5JLY6-F1-MODEL\_V4 | 1.0 | 4.099e-24 | 716 | 0.194 | 530 | 335 | 25 | 243 | 755 | 212 | 666 | Uncharacterized protein | Uncharacterized protein | | afdb-uniprot50 | AF-A0A2S5R1A1-F1-MODEL\_V4 | 1.0 | 1.672e-17 | 716 | 0.363 | 231 | 129 | 6 | 118 | 347 | 503 | 716 | Toprim domain-containing protein | Toprim domain-containing protein | | afdb-uniprot50 | AF-A0A1G8MPZ8-F1-MODEL\_V4 | 1.0 | 5.844e-19 | 715 | 0.284 | 330 | 211 | 12 | 370 | 684 | 58 | 377 | Virulence-associated protein E | Virulence-associated protein E | | afdb-uniprot50 | AF-B6VV90-F1-MODEL\_V4 | 1.0 | 1.75e-20 | 715 | 0.175 | 387 | 291 | 16 | 381 | 755 | 70 | 440 | DUF3874 domain-containing protein | DUF3874 domain-containing protein | | afdb-uniprot50 | AF-R6W8X3-F1-MODEL\_V4 | 1.0 | 2.384e-20 | 713 | 0.206 | 392 | 283 | 15 | 381 | 757 | 57 | 435 | DUF3874 domain-containing protein | DUF3874 domain-containing protein | | afdb-uniprot50 | AF-B7BC91-F1-MODEL\_V4 | 1.0 | 2.678e-22 | 712 | 0.22 | 454 | 302 | 23 | 318 | 757 | 161 | 576 | VirE N-terminal domain protein | VirE N-terminal domain protein | | afdb-uniprot50 | AF-A0A257L5C6-F1-MODEL\_V4 | 1.0 | 2.931e-20 | 711 | 0.217 | 400 | 273 | 18 | 371 | 756 | 6 | 379 | Virulence protein E | Virulence protein E | | afdb-uniprot50 | AF-A0A2G4RGB3-F1-MODEL\_V4 | 1.0 | 3.58e-23 | 711 | 0.197 | 511 | 352 | 22 | 268 | 752 | 20 | 498 | Virulence-associated E family protein | Virulence-associated E family protein | | afdb-uniprot50 | AF-A0A442KKS8-F1-MODEL\_V4 | 1.0 | 8.388e-27 | 711 | 0.173 | 644 | 426 | 33 | 167 | 752 | 19 | 613 | Uncharacterized protein | Uncharacterized protein | | afdb-uniprot50 | AF-A0A0J6CHU3-F1-MODEL\_V4 | 1.0 | 6.194e-24 | 711 | 0.224 | 513 | 304 | 23 | 261 | 755 | 249 | 685 | Uncharacterized protein | Uncharacterized protein | | afdb-uniprot50 | AF-A0A497G7H7-F1-MODEL\_V4 | 1.0 | 8.497e-29 | 711 | 0.192 | 783 | 379 | 26 | 2 | 752 | 193 | 753 | PriCT\_2 domain-containing protein | PriCT\_2 domain-containing protein | | afdb-uniprot50 | AF-A0A413HE39-F1-MODEL\_V4 | 1.0 | 4.724e-22 | 710 | 0.199 | 466 | 315 | 18 | 328 | 769 | 22 | 453 | Uncharacterized protein | Uncharacterized protein | | afdb-uniprot50 | AF-R6SXJ8-F1-MODEL\_V4 | 1.0 | 2.416e-22 | 710 | 0.187 | 443 | 313 | 22 | 330 | 757 | 54 | 464 | DUF3874 domain-containing protein | DUF3874 domain-containing protein | | afdb-uniprot50 | AF-U4QVN3-F1-MODEL\_V4 | 1.0 | 2.431e-27 | 710 | 0.223 | 621 | 401 | 36 | 177 | 762 | 111 | 684 | Putative virulence-associated protein E | Putative virulence-associated protein E | | afdb-uniprot50 | AF-A0A7Y9W3V7-F1-MODEL\_V4 | 1.0 | 9.605e-28 | 710 | 0.208 | 738 | 380 | 28 | 216 | 769 | 2 | 719 | Putative P-loop ATPase | Putative P-loop ATPase | | afdb-uniprot50 | AF-A0A7X7X0D4-F1-MODEL\_V4 | 1.0 | 1.75e-20 | 708 | 0.22 | 385 | 275 | 16 | 381 | 755 | 23 | 392 | DUF3874 domain-containing protein | DUF3874 domain-containing protein | | afdb-uniprot50 | AF-A0A255T6X0-F1-MODEL\_V4 | 1.0 | 1.842e-20 | 708 | 0.194 | 390 | 279 | 14 | 384 | 753 | 244 | 618 | Uncharacterized protein | Uncharacterized protein | | afdb-uniprot50 | AF-G1UM92-F1-MODEL\_V4 | 1.0 | 5.34e-21 | 707 | 0.22 | 430 | 278 | 21 | 384 | 769 | 2 | 418 | Uncharacterized protein | Uncharacterized protein | | afdb-uniprot50 | AF-A0A659V3T7-F1-MODEL\_V4 | 1.0 | 1.158e-20 | 707 | 0.251 | 410 | 265 | 18 | 381 | 766 | 70 | 461 | Uncharacterized protein | Uncharacterized protein | | afdb-uniprot50 | AF-Q5P8W9-F1-MODEL\_V4 | 1.0 | 3.626e-17 | 707 | 0.327 | 247 | 140 | 9 | 102 | 347 | 77 | 298 | Toprim domain-containing protein | Toprim domain-containing protein | | afdb-uniprot50 | AF-A0A7Y8NQQ4-F1-MODEL\_V4 | 1.0 | 9.18e-25 | 707 | 0.189 | 550 | 347 | 24 | 263 | 755 | 231 | 738 | Primase C-terminal domain-containing protein | Primase C-terminal domain-containing protein | | afdb-uniprot50 | AF-U6RKE9-F1-MODEL\_V4 | 1.0 | 1.423e-20 | 706 | 0.209 | 386 | 278 | 19 | 381 | 755 | 35 | 404 | DUF3874 domain-containing protein | DUF3874 domain-containing protein | | afdb-uniprot50 | AF-A0A174QPU8-F1-MODEL\_V4 | 1.0 | 1.738e-23 | 706 | 0.165 | 508 | 347 | 23 | 261 | 756 | 237 | 679 | Predicted P-loop ATPase and inactivated derivatives | Predicted P-loop ATPase and inactivated derivatives | | afdb-uniprot50 | AF-A0A255TQL6-F1-MODEL\_V4 | 1.0 | 4.943e-25 | 706 | 0.212 | 545 | 344 | 28 | 260 | 769 | 214 | 708 | VirE\_N domain-containing protein | VirE\_N domain-containing protein | | afdb-uniprot50 | AF-A0A6M0S7I4-F1-MODEL\_V4 | 1.0 | 1.1e-20 | 705 | 0.276 | 401 | 257 | 16 | 369 | 757 | 213 | 592 | Uncharacterized protein | Uncharacterized protein | | afdb-uniprot50 | AF-A0A3E5A259-F1-MODEL\_V4 | 1.0 | 1.423e-20 | 704 | 0.193 | 424 | 291 | 21 | 370 | 755 | 17 | 427 | Uncharacterized protein | Uncharacterized protein | | afdb-uniprot50 | AF-A0A255TBA5-F1-MODEL\_V4 | 1.0 | 9.359e-24 | 704 | 0.177 | 530 | 341 | 24 | 263 | 769 | 245 | 702 | VirE\_N domain-containing protein | VirE\_N domain-containing protein | | afdb-uniprot50 | AF-A0A5B0WFK2-F1-MODEL\_V4 | 1.0 | 1.662e-20 | 702 | 0.218 | 411 | 288 | 19 | 370 | 762 | 27 | 422 | Uncharacterized protein | Uncharacterized protein | | afdb-uniprot50 | AF-E6SUT3-F1-MODEL\_V4 | 1.0 | 5.137e-23 | 702 | 0.18 | 505 | 320 | 18 | 264 | 756 | 273 | 695 | VirE\_N domain-containing protein | VirE\_N domain-containing protein | | afdb-uniprot50 | AF-A0A840L725-F1-MODEL\_V4 | 1.0 | 6.275e-34 | 702 | 0.214 | 935 | 454 | 53 | 1 | 740 | 1 | 849 | Putative DNA primase/helicase | Putative DNA primase/helicase | | afdb-uniprot50 | AF-A0A0Q7CC84-F1-MODEL\_V4 | 1.0 | 5.769e-17 | 701 | 0.375 | 237 | 121 | 8 | 107 | 340 | 4 | 216 | Toprim domain-containing protein | Toprim domain-containing protein | | afdb-uniprot50 | AF-A0A7T0JTE9-F1-MODEL\_V4 | 1.0 | 2.123e-18 | 701 | 0.392 | 247 | 119 | 11 | 99 | 333 | 78 | 305 | Toprim domain-containing protein | Toprim domain-containing protein | | afdb-uniprot50 | AF-R7J768-F1-MODEL\_V4 | 1.0 | 1.219e-20 | 701 | 0.195 | 388 | 283 | 16 | 381 | 755 | 45 | 416 | DUF3874 domain-containing protein | DUF3874 domain-containing protein | | afdb-uniprot50 | AF-A0A3C1P7C6-F1-MODEL\_V4 | 1.0 | 2.384e-20 | 701 | 0.239 | 426 | 277 | 19 | 344 | 750 | 36 | 433 | Uncharacterized protein | Uncharacterized protein | | afdb-uniprot50 | AF-A0A0F5JAQ5-F1-MODEL\_V4 | 1.0 | 4.206e-20 | 701 | 0.199 | 391 | 274 | 22 | 384 | 756 | 83 | 452 | DUF3874 domain-containing protein | DUF3874 domain-containing protein | | afdb-uniprot50 | AF-A0A158A108-F1-MODEL\_V4 | 1.0 | 1.538e-32 | 701 | 0.192 | 981 | 474 | 61 | 3 | 751 | 1 | 894 | Inner membrane protein | Inner membrane protein | | afdb-uniprot50 | AF-A0A1Y4HDZ0-F1-MODEL\_V4 | 1.0 | 3.086e-20 | 700 | 0.198 | 388 | 280 | 17 | 381 | 752 | 33 | 405 | DUF3874 domain-containing protein | DUF3874 domain-containing protein | | afdb-uniprot50 | AF-A0A2U3I7B7-F1-MODEL\_V4 | 1.0 | 5.444e-20 | 699 | 0.221 | 411 | 266 | 22 | 382 | 769 | 37 | 416 | Virulence protein E | Virulence protein E | | afdb-uniprot50 | AF-A0A646HEP4-F1-MODEL\_V4 | 1.0 | 5.444e-20 | 699 | 0.183 | 386 | 288 | 14 | 381 | 751 | 139 | 512 | Helicase | Helicase | | afdb-uniprot50 | AF-A0A2N7VR70-F1-MODEL\_V4 | 1.0 | 3.229e-23 | 699 | 0.318 | 380 | 202 | 16 | 3 | 354 | 1 | 351 | DNA replication protein | DNA replication protein | | afdb-uniprot50 | AF-A0A4P7YXX2-F1-MODEL\_V4 | 1.0 | 8.279e-17 | 699 | 0.334 | 233 | 136 | 7 | 116 | 347 | 489 | 703 | DUF1738 domain-containing protein | DUF1738 domain-containing protein | | afdb-uniprot50 | AF-A0A4P7YZT8-F1-MODEL\_V4 | 1.0 | 2.802e-17 | 698 | 0.332 | 250 | 145 | 9 | 102 | 347 | 1 | 232 | Toprim domain-containing protein | Toprim domain-containing protein | | afdb-uniprot50 | AF-A0A257ZQW8-F1-MODEL\_V4 | 1.0 | 9.792e-27 | 698 | 0.188 | 723 | 403 | 38 | 179 | 767 | 3 | 675 | P-loop ATPase | P-loop ATPase | | afdb-uniprot50 | AF-A0A1H5S3J8-F1-MODEL\_V4 | 1.0 | 7.565e-19 | 696 | 0.279 | 358 | 200 | 14 | 8 | 320 | 3 | 347 | Putative DNA primase/helicase | Putative DNA primase/helicase | | afdb-uniprot50 | AF-D5EXB0-F1-MODEL\_V4 | 1.0 | 4.344e-21 | 696 | 0.217 | 390 | 278 | 16 | 384 | 757 | 21 | 399 | DUF3874 domain-containing protein | DUF3874 domain-containing protein | | afdb-uniprot50 | AF-A0A3E5B532-F1-MODEL\_V4 | 1.0 | 6.522e-24 | 696 | 0.192 | 535 | 326 | 22 | 240 | 757 | 260 | 705 | Virulence protein E | Virulence protein E | | afdb-uniprot50 | AF-A0A2L1GL73-F1-MODEL\_V4 | 1.0 | 1.165e-17 | 695 | 0.352 | 250 | 138 | 11 | 102 | 346 | 506 | 736 | Uncharacterized protein | Uncharacterized protein | | afdb-uniprot50 | AF-A0A6G6YPV6-F1-MODEL\_V4 | 1.0 | 6.356e-20 | 694 | 0.216 | 406 | 279 | 20 | 370 | 755 | 18 | 404 | Virulence-associated E family protein | Virulence-associated E family protein | | afdb-uniprot50 | AF-A0A239FXE1-F1-MODEL\_V4 | 1.0 | 3.697e-24 | 694 | 0.216 | 537 | 359 | 26 | 261 | 769 | 252 | 754 | Uncharacterized protein | Uncharacterized protein | | afdb-uniprot50 | AF-A0A4U1AJI8-F1-MODEL\_V4 | 1.0 | 2.527e-17 | 694 | 0.322 | 257 | 142 | 9 | 102 | 346 | 587 | 823 | DUF1738 domain-containing protein | DUF1738 domain-containing protein | | afdb-uniprot50 | AF-A0A0F9XQ22-F1-MODEL\_V4 | 1.0 | 2.56e-19 | 693 | 0.211 | 406 | 280 | 18 | 370 | 755 | 31 | 416 | Uncharacterized protein | Uncharacterized protein | | afdb-uniprot50 | AF-A0A0A2WCR2-F1-MODEL\_V4 | 1.0 | 1.578e-20 | 693 | 0.204 | 415 | 293 | 18 | 370 | 769 | 42 | 434 | Phage associated DNA primase | Phage associated DNA primase | | afdb-uniprot50 | AF-A0A844FYD8-F1-MODEL\_V4 | 1.0 | 2.644e-28 | 693 | 0.178 | 848 | 469 | 45 | 1 | 757 | 14 | 724 | Uncharacterized protein | Uncharacterized protein | | afdb-uniprot50 | AF-A0A1X7F2Z1-F1-MODEL\_V4 | 1.0 | 1.432e-17 | 692 | 0.319 | 257 | 154 | 7 | 98 | 351 | 48 | 286 | Uncharacterized domain associated with phage/plasmid primase | Uncharacterized domain associated with phage/plasmid primase | | afdb-uniprot50 | AF-A0A7Y5I7S5-F1-MODEL\_V4 | 1.0 | 5.732e-20 | 692 | 0.264 | 378 | 244 | 15 | 384 | 753 | 119 | 470 | Uncharacterized protein | Uncharacterized protein | | afdb-uniprot50 | AF-A0A1H1AY48-F1-MODEL\_V4 | 1.0 | 1.259e-21 | 691 | 0.197 | 435 | 308 | 22 | 335 | 755 | 278 | 685 | Uncharacterized protein | Uncharacterized protein | | afdb-uniprot50 | AF-A0A0A2B4H4-F1-MODEL\_V4 | 1.0 | 4.429e-20 | 689 | 0.238 | 398 | 262 | 20 | 376 | 756 | 20 | 393 | Uncharacterized protein | Uncharacterized protein | | afdb-uniprot50 | AF-R6SB64-F1-MODEL\_V4 | 1.0 | 2.179e-22 | 689 | 0.183 | 517 | 327 | 24 | 260 | 758 | 254 | 693 | VirE domain protein | VirE domain protein | | afdb-uniprot50 | AF-A0A412XPF4-F1-MODEL\_V4 | 1.0 | 3.995e-20 | 687 | 0.233 | 390 | 271 | 14 | 379 | 751 | 5 | 383 | Virulence protein | Virulence protein | | afdb-uniprot50 | AF-A7V3I1-F1-MODEL\_V4 | 1.0 | 2.137e-23 | 687 | 0.17 | 510 | 341 | 23 | 259 | 755 | 48 | 488 | Uncharacterized protein | Uncharacterized protein | | afdb-uniprot50 | AF-A0A0S3U510-F1-MODEL\_V4 | 1.0 | 2.151e-20 | 687 | 0.243 | 395 | 267 | 16 | 384 | 769 | 448 | 819 | Uncharacterized protein | Uncharacterized protein | | afdb-uniprot50 | AF-A0A2W4V1A4-F1-MODEL\_V4 | 1.0 | 6.65e-31 | 687 | 0.194 | 904 | 490 | 48 | 1 | 737 | 1 | 832 | Uncharacterized protein | Uncharacterized protein | | afdb-uniprot50 | AF-E2N8F3-F1-MODEL\_V4 | 1.0 | 1.405e-18 | 686 | 0.261 | 295 | 205 | 8 | 467 | 757 | 2 | 287 | Uncharacterized protein | Uncharacterized protein | | afdb-uniprot50 | AF-A0A412LCT6-F1-MODEL\_V4 | 1.0 | 5.271e-19 | 686 | 0.256 | 374 | 249 | 13 | 398 | 752 | 2 | 365 | Uncharacterized protein | Uncharacterized protein | | afdb-uniprot50 | AF-A0A1B3W9W8-F1-MODEL\_V4 | 1.0 | 1.499e-20 | 686 | 0.241 | 431 | 266 | 23 | 370 | 757 | 29 | 441 | Uncharacterized protein | Uncharacterized protein | | afdb-uniprot50 | AF-A0A7C1RII9-F1-MODEL\_V4 | 1.0 | 8.226e-20 | 686 | 0.215 | 408 | 276 | 16 | 370 | 755 | 95 | 480 | Virulence-associated E family protein | Virulence-associated E family protein | | afdb-uniprot50 | AF-A0A239KTG6-F1-MODEL\_V4 | 1.0 | 6.115e-22 | 686 | 0.235 | 492 | 303 | 24 | 295 | 755 | 222 | 671 | Predicted P-loop ATPase and inactivated derivatives | Predicted P-loop ATPase and inactivated derivatives | | afdb-uniprot50 | AF-A0A6A7WDK3-F1-MODEL\_V4 | 1.0 | 7.914e-22 | 686 | 0.194 | 489 | 320 | 26 | 330 | 769 | 293 | 756 | VirE\_N domain-containing protein | VirE\_N domain-containing protein | | afdb-uniprot50 | AF-A0A1Y4IN65-F1-MODEL\_V4 | 1.0 | 8.226e-20 | 685 | 0.208 | 398 | 281 | 20 | 381 | 762 | 43 | 422 | Helicase | Helicase | | afdb-uniprot50 | AF-A0A2J4JNZ5-F1-MODEL\_V4 | 1.0 | 6.692e-20 | 685 | 0.206 | 422 | 283 | 20 | 370 | 756 | 3 | 407 | Virulence-associated protein E | Virulence-associated protein E | | afdb-uniprot50 | AF-A0A843HRX9-F1-MODEL\_V4 | 1.0 | 1.3e-22 | 685 | 0.185 | 523 | 339 | 20 | 264 | 760 | 6 | 467 | Uncharacterized protein | Uncharacterized protein | | afdb-uniprot50 | AF-A0A7X5RAN1-F1-MODEL\_V4 | 1.0 | 4.261e-22 | 685 | 0.209 | 521 | 335 | 22 | 260 | 761 | 170 | 632 | Uncharacterized protein | Uncharacterized protein | | afdb-uniprot50 | AF-A0A522TUF4-F1-MODEL\_V4 | 1.0 | 1.284e-20 | 685 | 0.215 | 426 | 285 | 20 | 349 | 751 | 345 | 744 | Uncharacterized protein | Uncharacterized protein | | afdb-uniprot50 | AF-A0A3E1EZ76-F1-MODEL\_V4 | 1.0 | 2.265e-20 | 684 | 0.215 | 409 | 268 | 20 | 376 | 755 | 11 | 395 | Virulence protein E | Virulence protein E | | afdb-uniprot50 | AF-A0A7C9G0C5-F1-MODEL\_V4 | 1.0 | 8.226e-20 | 684 | 0.221 | 401 | 281 | 15 | 370 | 759 | 34 | 414 | Uncharacterized protein | Uncharacterized protein | | afdb-uniprot50 | AF-A0A1T5AQ44-F1-MODEL\_V4 | 1.0 | 1.518e-22 | 684 | 0.199 | 506 | 327 | 21 | 261 | 755 | 255 | 693 | Uncharacterized protein | Uncharacterized protein | | afdb-uniprot50 | AF-A0A5N7RS25-F1-MODEL\_V4 | 1.0 | 1.819e-26 | 684 | 0.2 | 668 | 419 | 36 | 181 | 769 | 144 | 775 | Uncharacterized protein | Uncharacterized protein | | afdb-uniprot50 | AF-A0A7H9BIY7-F1-MODEL\_V4 | 1.0 | 4.605e-34 | 684 | 0.196 | 932 | 485 | 51 | 1 | 750 | 1 | 850 | DUF3631 domain-containing protein | DUF3631 domain-containing protein | | afdb-uniprot50 | AF-A0A522TWI5-F1-MODEL\_V4 | 1.0 | 4.126e-21 | 683 | 0.371 | 291 | 167 | 8 | 1 | 280 | 12 | 297 | Toprim domain-containing protein | Toprim domain-containing protein | | afdb-uniprot50 | AF-A0A7X8AE49-F1-MODEL\_V4 | 1.0 | 6.692e-20 | 683 | 0.194 | 395 | 283 | 18 | 381 | 755 | 16 | 395 | DUF3874 domain-containing protein | DUF3874 domain-containing protein | | afdb-uniprot50 | AF-A0A414I4A7-F1-MODEL\_V4 | 1.0 | 1.1e-20 | 683 | 0.211 | 444 | 288 | 24 | 364 | 769 | 9 | 428 | Virulence-associated protein E | Virulence-associated protein E | | afdb-uniprot50 | AF-A0A809PTW9-F1-MODEL\_V4 | 1.0 | 2.369e-23 | 683 | 0.267 | 486 | 261 | 24 | 10 | 457 | 4 | 432 | Uncharacterized protein | Uncharacterized protein | | afdb-uniprot50 | AF-A0A4P6RG19-F1-MODEL\_V4 | 1.0 | 2.416e-22 | 683 | 0.203 | 505 | 322 | 25 | 264 | 757 | 262 | 697 | Helicase | Helicase | | afdb-uniprot50 | AF-S8GYB6-F1-MODEL\_V4 | 1.0 | 6.564e-21 | 682 | 0.218 | 440 | 275 | 19 | 369 | 757 | 16 | 437 | Virulence-associated protein E | Virulence-associated protein E | | afdb-uniprot50 | AF-A0A3R6BK15-F1-MODEL\_V4 | 1.0 | 2.016e-18 | 681 | 0.191 | 344 | 245 | 14 | 453 | 768 | 23 | 361 | Uncharacterized protein | Uncharacterized protein | | afdb-uniprot50 | AF-A0A2H6BA53-F1-MODEL\_V4 | 1.0 | 8.069e-21 | 681 | 0.241 | 459 | 285 | 23 | 335 | 769 | 178 | 597 | Uncharacterized protein | Uncharacterized protein | | afdb-uniprot50 | AF-A0A1F3KP86-F1-MODEL\_V4 | 1.0 | 3.819e-25 | 681 | 0.158 | 776 | 366 | 26 | 12 | 769 | 189 | 695 | Uncharacterized protein | Uncharacterized protein | | afdb-uniprot50 | AF-A0A3N8QT28-F1-MODEL\_V4 | 1.0 | 6.608e-34 | 681 | 0.209 | 971 | 461 | 45 | 6 | 752 | 1 | 888 | DUF927 domain-containing protein | DUF927 domain-containing protein | | afdb-uniprot50 | AF-A0A0Q6MHX9-F1-MODEL\_V4 | 1.0 | 1.538e-32 | 681 | 0.201 | 898 | 480 | 48 | 13 | 738 | 2 | 833 | Toprim domain-containing protein | Toprim domain-containing protein | | afdb-uniprot50 | AF-A0A2T3MTH8-F1-MODEL\_V4 | 1.0 | 2.016e-18 | 680 | 0.329 | 267 | 126 | 8 | 80 | 346 | 82 | 295 | Toprim domain-containing protein | Toprim domain-containing protein | | afdb-uniprot50 | AF-A0A416XEW5-F1-MODEL\_V4 | 1.0 | 1.326e-21 | 680 | 0.188 | 536 | 323 | 25 | 239 | 755 | 37 | 479 | DUF3874 domain-containing protein | DUF3874 domain-containing protein | | afdb-uniprot50 | AF-A0A641ULV4-F1-MODEL\_V4 | 1.0 | 2.416e-22 | 680 | 0.193 | 528 | 331 | 25 | 242 | 751 | 28 | 478 | Virulence protein E | Virulence protein E | | afdb-uniprot50 | AF-A0A8A6EWM4-F1-MODEL\_V4 | 1.0 | 9.42e-21 | 680 | 0.206 | 411 | 279 | 22 | 370 | 753 | 348 | 738 | Bifunctional DNA primase/polymerase | Bifunctional DNA primase/polymerase | | afdb-uniprot50 | AF-A0A1I5LX50-F1-MODEL\_V4 | 1.0 | 1.568e-23 | 680 | 0.196 | 530 | 322 | 25 | 261 | 751 | 253 | 717 | Virulence-associated protein E | Virulence-associated protein E | | afdb-uniprot50 | AF-A0A4R8FHG7-F1-MODEL\_V4 | 1.0 | 2.784e-20 | 680 | 0.232 | 426 | 277 | 16 | 370 | 759 | 428 | 839 | Virulence-associated protein E | Virulence-associated protein E | | afdb-uniprot50 | AF-A0A6M8VRM0-F1-MODEL\_V4 | 1.0 | 6.956e-18 | 679 | 0.285 | 308 | 195 | 12 | 453 | 753 | 8 | 297 | Virulence-associated E family protein | Virulence-associated E family protein | | afdb-uniprot50 | AF-K9E0U4-F1-MODEL\_V4 | 1.0 | 2.309e-19 | 679 | 0.184 | 385 | 284 | 17 | 381 | 751 | 50 | 418 | DUF3874 domain-containing protein | DUF3874 domain-containing protein | | afdb-uniprot50 | AF-A0A7G8F7P4-F1-MODEL\_V4 | 1.0 | 1.121e-19 | 679 | 0.205 | 409 | 284 | 16 | 370 | 757 | 52 | 440 | Virulence-associated E family protein | Virulence-associated E family protein | | afdb-uniprot50 | AF-A0A1K1M310-F1-MODEL\_V4 | 1.0 | 1.309e-19 | 679 | 0.194 | 390 | 287 | 14 | 384 | 756 | 228 | 607 | Uncharacterized protein | Uncharacterized protein | | afdb-uniprot50 | AF-A0A508X032-F1-MODEL\_V4 | 1.0 | 3.356e-21 | 678 | 0.214 | 461 | 306 | 20 | 331 | 769 | 112 | 538 | Uncharacterized protein | Uncharacterized protein | | afdb-uniprot50 | AF-A0A843BCM8-F1-MODEL\_V4 | 1.0 | 7.467e-17 | 677 | 0.362 | 248 | 132 | 9 | 102 | 347 | 120 | 343 | Toprim domain-containing protein | Toprim domain-containing protein | | afdb-uniprot50 | AF-A0A089P3D8-F1-MODEL\_V4 | 1.0 | 9.604e-20 | 677 | 0.231 | 410 | 277 | 17 | 378 | 769 | 23 | 412 | Uncharacterized protein | Uncharacterized protein | | afdb-uniprot50 | AF-A0A374W536-F1-MODEL\_V4 | 1.0 | 3.969e-23 | 677 | 0.203 | 515 | 334 | 29 | 264 | 756 | 215 | 675 | VirE\_N domain-containing protein | VirE\_N domain-containing protein | | afdb-uniprot50 | AF-A0A2D6XDQ3-F1-MODEL\_V4 | 1.0 | 3.794e-20 | 676 | 0.209 | 415 | 285 | 19 | 370 | 768 | 47 | 434 | Uncharacterized protein | Uncharacterized protein | | afdb-uniprot50 | AF-A0A558E116-F1-MODEL\_V4 | 1.0 | 3.292e-22 | 676 | 0.25 | 512 | 303 | 26 | 316 | 768 | 2 | 491 | Uncharacterized protein | Uncharacterized protein | | afdb-uniprot50 | AF-A0A4Q3PJ79-F1-MODEL\_V4 | 1.0 | 1.928e-31 | 676 | 0.193 | 964 | 453 | 49 | 6 | 753 | 1 | 855 | DUF3987 domain-containing protein | DUF3987 domain-containing protein | | afdb-uniprot50 | AF-A0A7Z8VSS1-F1-MODEL\_V4 | 1.0 | 3.378e-18 | 675 | 0.231 | 307 | 203 | 10 | 370 | 659 | 7 | 297 | Uncharacterized protein | Uncharacterized protein | | afdb-uniprot50 | AF-A0A5P2FXH3-F1-MODEL\_V4 | 1.0 | 1.011e-19 | 675 | 0.21 | 390 | 279 | 18 | 381 | 757 | 17 | 390 | Virulence protein E | Virulence protein E | | afdb-uniprot50 | AF-A0A7Y9FR30-F1-MODEL\_V4 | 1.0 | 1.672e-25 | 675 | 0.181 | 635 | 403 | 35 | 181 | 753 | 61 | 640 | Uncharacterized protein | Uncharacterized protein | | afdb-uniprot50 | AF-R6FMS2-F1-MODEL\_V4 | 1.0 | 3.126e-22 | 675 | 0.171 | 506 | 348 | 21 | 259 | 755 | 230 | 673 | VirE domain-containing protein | VirE domain-containing protein | | afdb-uniprot50 | AF-A0A1I3TR65-F1-MODEL\_V4 | 1.0 | 2.069e-22 | 675 | 0.209 | 531 | 319 | 24 | 265 | 750 | 268 | 742 | Predicted P-loop ATPase and inactivated derivatives | Predicted P-loop ATPase and inactivated derivatives | | afdb-uniprot50 | AF-A0A2S7IR16-F1-MODEL\_V4 | 1.0 | 2.043e-20 | 675 | 0.232 | 439 | 274 | 23 | 370 | 755 | 369 | 797 | Uncharacterized protein | Uncharacterized protein | | afdb-uniprot50 | AF-G0AAH9-F1-MODEL\_V4 | 1.0 | 1.093e-31 | 675 | 0.199 | 893 | 485 | 53 | 6 | 751 | 1 | 810 | PriCT\_2 domain-containing protein | PriCT\_2 domain-containing protein | | afdb-uniprot50 | AF-A0A1H6TNE7-F1-MODEL\_V4 | 1.0 | 5.273e-35 | 675 | 0.211 | 879 | 486 | 45 | 1 | 730 | 10 | 829 | Putative DNA primase/helicase | Putative DNA primase/helicase | | afdb-uniprot50 | AF-Q0AG08-F1-MODEL\_V4 | 1.0 | 1.309e-19 | 673 | 0.243 | 419 | 280 | 19 | 370 | 769 | 1 | 401 | Virulence-associated E family protein | Virulence-associated E family protein | | afdb-uniprot50 | AF-A0A839HPN6-F1-MODEL\_V4 | 1.0 | 5.771e-33 | 673 | 0.214 | 884 | 477 | 43 | 8 | 737 | 2 | 821 | DUF927 domain-containing protein | DUF927 domain-containing protein | | afdb-uniprot50 | AF-A0A3A6KL80-F1-MODEL\_V4 | 1.0 | 1.158e-20 | 672 | 0.209 | 444 | 289 | 24 | 364 | 769 | 9 | 428 | Virulence-associated protein E | Virulence-associated protein E | | afdb-uniprot50 | AF-B6ATL0-F1-MODEL\_V4 | 1.0 | 2.593e-21 | 672 | 0.232 | 431 | 272 | 21 | 368 | 757 | 28 | 440 | Virulence-associated E family protein | Virulence-associated E family protein | | afdb-uniprot50 | AF-J9GG52-F1-MODEL\_V4 | 1.0 | 6.912e-21 | 672 | 0.215 | 422 | 282 | 20 | 369 | 755 | 107 | 514 | Helicase | Helicase | | afdb-uniprot50 | AF-A0A1M3H1Y9-F1-MODEL\_V4 | 1.0 | 5.959e-18 | 672 | 0.329 | 270 | 155 | 8 | 92 | 361 | 87 | 330 | AAA domain-containing protein | AAA domain-containing protein | | afdb-uniprot50 | AF-A0A496KVV9-F1-MODEL\_V4 | 1.0 | 9.121e-20 | 671 | 0.198 | 403 | 289 | 19 | 369 | 757 | 33 | 415 | Helicase | Helicase | | afdb-uniprot50 | AF-A0A3B0ME56-F1-MODEL\_V4 | 1.0 | 3.969e-23 | 671 | 0.228 | 534 | 308 | 28 | 261 | 756 | 238 | 705 | Uncharacterized protein | Uncharacterized protein | | afdb-uniprot50 | AF-J9GP52-F1-MODEL\_V4 | 1.0 | 1.36e-17 | 670 | 0.258 | 286 | 199 | 9 | 475 | 756 | 2 | 278 | Virulence-associated E family protein | Virulence-associated E family protein | | afdb-uniprot50 | AF-A0A0F9EF48-F1-MODEL\_V4 | 1.0 | 1.978e-19 | 670 | 0.199 | 406 | 284 | 20 | 370 | 757 | 4 | 386 | Uncharacterized protein | Uncharacterized protein | | afdb-uniprot50 | AF-A0A6G9HJR3-F1-MODEL\_V4 | 1.0 | 5.479e-17 | 670 | 0.293 | 252 | 155 | 9 | 100 | 347 | 143 | 375 | DNA primase TraC | DNA primase TraC | | afdb-uniprot50 | AF-A0A7G7KL89-F1-MODEL\_V4 | 1.0 | 1.121e-19 | 670 | 0.217 | 418 | 276 | 20 | 369 | 757 | 12 | 407 | Uncharacterized protein | Uncharacterized protein | | afdb-uniprot50 | AF-A0A0B4XZW9-F1-MODEL\_V4 | 1.0 | 6.075e-17 | 670 | 0.329 | 249 | 144 | 8 | 100 | 346 | 466 | 693 | Uncharacterized protein | Uncharacterized protein | | afdb-uniprot50 | AF-A0A4T2A6U5-F1-MODEL\_V4 | 1.0 | 5.479e-17 | 670 | 0.326 | 257 | 142 | 10 | 99 | 346 | 513 | 747 | DUF1738 domain-containing protein | DUF1738 domain-containing protein | | afdb-uniprot50 | AF-A0A0R3NCG4-F1-MODEL\_V4 | 1.0 | 1.018e-24 | 669 | 0.208 | 567 | 367 | 26 | 234 | 751 | 9 | 542 | Uncharacterized protein | Uncharacterized protein | | afdb-uniprot50 | AF-A0A4Q3L9Q7-F1-MODEL\_V4 | 1.0 | 1.018e-16 | 668 | 0.357 | 238 | 149 | 4 | 368 | 602 | 60 | 296 | Uncharacterized protein | Uncharacterized protein | | afdb-uniprot50 | AF-R5CMD4-F1-MODEL\_V4 | 1.0 | 3.65e-22 | 668 | 0.179 | 519 | 339 | 22 | 261 | 757 | 241 | 694 | VirE N-terminal domain protein | VirE N-terminal domain protein | | afdb-uniprot50 | AF-A0A412M1I6-F1-MODEL\_V4 | 1.0 | 1.599e-22 | 668 | 0.195 | 523 | 332 | 26 | 261 | 755 | 233 | 694 | Helicase | Helicase | | afdb-uniprot50 | AF-A0A1X6Y4N3-F1-MODEL\_V4 | 1.0 | 6.275e-26 | 668 | 0.199 | 661 | 398 | 37 | 176 | 753 | 64 | 676 | Uncharacterized protein | Uncharacterized protein | | afdb-uniprot50 | AF-K9Z0R0-F1-MODEL\_V4 | 1.0 | 3.868e-19 | 668 | 0.253 | 379 | 247 | 18 | 384 | 751 | 343 | 696 | Virulence-associated E family protein | Virulence-associated E family protein | | afdb-uniprot50 | AF-A0A355VT45-F1-MODEL\_V4 | 1.0 | 1.378e-19 | 667 | 0.215 | 417 | 273 | 16 | 379 | 755 | 11 | 413 | Uncharacterized protein | Uncharacterized protein | | afdb-uniprot50 | AF-A0A7T1I9T7-F1-MODEL\_V4 | 1.0 | 4.91e-20 | 667 | 0.226 | 424 | 267 | 21 | 371 | 757 | 177 | 576 | Uncharacterized protein | Uncharacterized protein | | afdb-uniprot50 | AF-A0A1M6MNJ7-F1-MODEL\_V4 | 1.0 | 1.243e-19 | 666 | 0.216 | 429 | 292 | 20 | 370 | 769 | 9 | 422 | Virulence-associated protein E | Virulence-associated protein E | | afdb-uniprot50 | AF-A0A5B2V8U9-F1-MODEL\_V4 | 1.0 | 9.542e-23 | 666 | 0.195 | 532 | 344 | 24 | 261 | 753 | 65 | 551 | Virulence-associated E family protein | Virulence-associated E family protein | | afdb-uniprot50 | AF-A0A7W0BSW7-F1-MODEL\_V4 | 1.0 | 5.696e-23 | 666 | 0.223 | 536 | 342 | 28 | 245 | 756 | 259 | 744 | Uncharacterized protein | Uncharacterized protein | | afdb-uniprot50 | AF-A0A6S6SDG9-F1-MODEL\_V4 | 1.0 | 6.735e-17 | 665 | 0.315 | 260 | 155 | 8 | 91 | 347 | 48 | 287 | Cpp22 | Cpp22 | | afdb-uniprot50 | AF-A0A841EPH5-F1-MODEL\_V4 | 1.0 | 2.56e-19 | 665 | 0.223 | 399 | 270 | 20 | 381 | 756 | 42 | 423 | Putative P-loop ATPase | Putative P-loop ATPase | | afdb-uniprot50 | AF-A0A7W9AWL7-F1-MODEL\_V4 | 1.0 | 3.147e-19 | 665 | 0.222 | 414 | 291 | 17 | 370 | 767 | 61 | 459 | Putative DNA primase/helicase | Putative DNA primase/helicase | | afdb-uniprot50 | AF-A0A7C9KA34-F1-MODEL\_V4 | 1.0 | 1.978e-19 | 665 | 0.213 | 399 | 282 | 19 | 377 | 760 | 185 | 566 | Uncharacterized protein | Uncharacterized protein | | afdb-uniprot50 | AF-A0A1C3X7K5-F1-MODEL\_V4 | 1.0 | 2.043e-20 | 665 | 0.208 | 451 | 306 | 18 | 336 | 767 | 239 | 657 | Virulence-associated protein E | Virulence-associated protein E | | afdb-uniprot50 | AF-A0A553EPP8-F1-MODEL\_V4 | 1.0 | 1.173e-22 | 665 | 0.197 | 537 | 328 | 26 | 241 | 751 | 215 | 674 | Virulence protein E | Virulence protein E | | afdb-uniprot50 | AF-A0A6J4F9N4-F1-MODEL\_V4 | 1.0 | 5.921e-29 | 665 | 0.175 | 918 | 501 | 52 | 2 | 752 | 6 | 834 | Putative Virulence-associated protein E | Putative Virulence-associated protein E | | afdb-uniprot50 | AF-A0A0N7LN66-F1-MODEL\_V4 | 1.0 | 4.207e-28 | 665 | 0.18 | 809 | 410 | 38 | 1 | 764 | 244 | 844 | Putative P-loop ATPase | Putative P-loop ATPase | | afdb-uniprot50 | AF-A0A1X7JKC4-F1-MODEL\_V4 | 1.0 | 8.226e-20 | 664 | 0.211 | 417 | 279 | 22 | 370 | 769 | 87 | 470 | Virulence-associated protein E | Virulence-associated protein E | | afdb-uniprot50 | AF-A0A5E4VTX6-F1-MODEL\_V4 | 1.0 | 4.694e-33 | 664 | 0.186 | 940 | 510 | 53 | 1 | 750 | 18 | 892 | DNA primase | DNA primase | | afdb-uniprot50 | AF-A0A416T296-F1-MODEL\_V4 | 1.0 | 1.219e-20 | 663 | 0.214 | 434 | 295 | 19 | 328 | 744 | 61 | 465 | DUF3874 domain-containing protein | DUF3874 domain-containing protein | | afdb-uniprot50 | AF-A0A6G1UCW6-F1-MODEL\_V4 | 1.0 | 1.326e-21 | 663 | 0.174 | 527 | 360 | 28 | 286 | 768 | 81 | 576 | Uncharacterized protein | Uncharacterized protein | | afdb-uniprot50 | AF-A0A2W4XDU3-F1-MODEL\_V4 | 1.0 | 4.574e-21 | 663 | 0.24 | 450 | 281 | 22 | 321 | 757 | 194 | 595 | Uncharacterized protein | Uncharacterized protein | | afdb-uniprot50 | AF-A0A0F5INE2-F1-MODEL\_V4 | 1.0 | 2.543e-22 | 663 | 0.193 | 516 | 316 | 26 | 261 | 755 | 253 | 689 | Uncharacterized protein | Uncharacterized protein | | afdb-uniprot50 | AF-A0A6G1U079-F1-MODEL\_V4 | 1.0 | 2.495e-23 | 663 | 0.187 | 559 | 335 | 20 | 259 | 769 | 229 | 716 | Virulence protein E | Virulence protein E | | afdb-uniprot50 | AF-A0A3M1WWN1-F1-MODEL\_V4 | 1.0 | 2.838e-19 | 662 | 0.221 | 406 | 272 | 20 | 380 | 769 | 352 | 729 | Uncharacterized protein | Uncharacterized protein | | afdb-uniprot50 | AF-A0A0R1T496-F1-MODEL\_V4 | 1.0 | 3.25e-20 | 661 | 0.227 | 404 | 265 | 16 | 367 | 750 | 23 | 399 | Prophage lp3 protein 8, helicase | Prophage lp3 protein 8, helicase | | afdb-uniprot50 | AF-A0A352JCJ9-F1-MODEL\_V4 | 1.0 | 2.082e-19 | 661 | 0.236 | 394 | 261 | 17 | 382 | 760 | 104 | 472 | Uncharacterized protein | Uncharacterized protein | | afdb-uniprot50 | AF-Q027Z8-F1-MODEL\_V4 | 1.0 | 1.509e-25 | 661 | 0.183 | 669 | 430 | 33 | 178 | 757 | 59 | 699 | Virulence-associated E family protein | Virulence-associated E family protein | | afdb-uniprot50 | AF-A0A1F4EP90-F1-MODEL\_V4 | 1.0 | 1.558e-18 | 660 | 0.273 | 347 | 192 | 18 | 11 | 346 | 1 | 298 | PriCT\_2 domain-containing protein | PriCT\_2 domain-containing protein | | afdb-uniprot50 | AF-A0A3P1ZAJ6-F1-MODEL\_V4 | 1.0 | 3.868e-19 | 660 | 0.184 | 396 | 281 | 20 | 381 | 757 | 99 | 471 | Helicase | Helicase | | afdb-uniprot50 | AF-G7GJU6-F1-MODEL\_V4 | 1.0 | 3.534e-21 | 660 | 0.209 | 443 | 286 | 19 | 380 | 769 | 38 | 469 | Uncharacterized protein | Uncharacterized protein | | afdb-uniprot50 | AF-A0A1G7G4Y5-F1-MODEL\_V4 | 1.0 | 5.17e-20 | 659 | 0.195 | 445 | 297 | 18 | 370 | 762 | 371 | 806 | Virulence-associated protein E | Virulence-associated protein E | | afdb-uniprot50 | AF-A0A5C7BZC7-F1-MODEL\_V4 | 1.0 | 2.856e-16 | 659 | 0.312 | 253 | 155 | 7 | 98 | 347 | 139 | 375 | Uncharacterized protein | Uncharacterized protein | | afdb-uniprot50 | AF-A0A0M3Q0Y7-F1-MODEL\_V4 | 1.0 | 4.515e-19 | 658 | 0.23 | 394 | 268 | 17 | 393 | 766 | 5 | 383 | Virulence associated protein | Virulence associated protein | | afdb-uniprot50 | AF-A0A174RE62-F1-MODEL\_V4 | 1.0 | 3.868e-19 | 658 | 0.182 | 390 | 280 | 18 | 381 | 751 | 68 | 437 | Predicted P-loop ATPase and inactivated derivatives | Predicted P-loop ATPase and inactivated derivatives | | afdb-uniprot50 | AF-A0A7W1X8F7-F1-MODEL\_V4 | 1.0 | 4.755e-19 | 658 | 0.221 | 424 | 272 | 20 | 379 | 755 | 376 | 788 | Uncharacterized protein | Uncharacterized protein | | afdb-uniprot50 | AF-A0A068MV01-F1-MODEL\_V4 | 1.0 | 1.031e-26 | 658 | 0.19 | 645 | 389 | 37 | 178 | 752 | 75 | 656 | DUF3854 domain-containing protein | DUF3854 domain-containing protein | | afdb-uniprot50 | AF-A0A7U5WHF4-F1-MODEL\_V4 | 1.0 | 1.243e-19 | 657 | 0.233 | 424 | 262 | 23 | 370 | 749 | 35 | 439 | Virulence factor | Virulence factor | | afdb-uniprot50 | AF-A0A0J7L464-F1-MODEL\_V4 | 1.0 | 7.965e-19 | 657 | 0.165 | 406 | 288 | 18 | 370 | 749 | 333 | 713 | Virulence-associated e family protein | Virulence-associated e family protein | | afdb-uniprot50 | AF-U4QRR7-F1-MODEL\_V4 | 1.0 | 1.405e-26 | 656 | 0.212 | 674 | 434 | 38 | 133 | 756 | 31 | 657 | Putative Virulence-associated E | Putative Virulence-associated E | | afdb-uniprot50 | AF-A0A1M4WF05-F1-MODEL\_V4 | 1.0 | 1.716e-21 | 656 | 0.173 | 519 | 320 | 22 | 261 | 755 | 268 | 701 | VirE N-terminal domain-containing protein | VirE N-terminal domain-containing protein | | afdb-uniprot50 | AF-A0A1V0GS15-F1-MODEL\_V4 | 1.0 | 1.005e-22 | 655 | 0.211 | 502 | 332 | 19 | 300 | 754 | 180 | 664 | RepB\_primase domain-containing protein | RepB\_primase domain-containing protein | | afdb-uniprot50 | AF-A0A1M5BFH6-F1-MODEL\_V4 | 1.0 | 3.969e-23 | 655 | 0.203 | 536 | 332 | 25 | 239 | 755 | 212 | 671 | Uncharacterized protein | Uncharacterized protein | | afdb-uniprot50 | AF-A0A6G1U056-F1-MODEL\_V4 | 1.0 | 1.196e-21 | 655 | 0.155 | 539 | 365 | 29 | 260 | 756 | 245 | 735 | VirE\_N domain-containing protein | VirE\_N domain-containing protein | | afdb-uniprot50 | AF-A0A1N7MGC5-F1-MODEL\_V4 | 1.0 | 4.099e-16 | 655 | 0.336 | 238 | 131 | 7 | 120 | 346 | 547 | 768 | Antirestriction protein ArdC | Antirestriction protein ArdC | | afdb-uniprot50 | AF-A0A1Q4RMJ7-F1-MODEL\_V4 | 1.0 | 2.56e-27 | 655 | 0.19 | 682 | 442 | 33 | 138 | 769 | 56 | 677 | DUF3854 domain-containing protein | DUF3854 domain-containing protein | | afdb-uniprot50 | AF-A0A448QER1-F1-MODEL\_V4 | 1.0 | 1.212e-31 | 655 | 0.19 | 934 | 507 | 57 | 1 | 759 | 1 | 859 | Bifunctional DNA primase/polymerase | Bifunctional DNA primase/polymerase | | afdb-uniprot50 | AF-A0A4P6HIC1-F1-MODEL\_V4 | 1.0 | 1.018e-16 | 654 | 0.337 | 252 | 137 | 11 | 100 | 347 | 74 | 299 | Toprim domain-containing protein | Toprim domain-containing protein | | afdb-uniprot50 | AF-A0A7U4E6T9-F1-MODEL\_V4 | 1.0 | 5.844e-19 | 654 | 0.217 | 400 | 270 | 17 | 381 | 755 | 12 | 393 | Uncharacterized protein | Uncharacterized protein | | afdb-uniprot50 | AF-A0A373IIN3-F1-MODEL\_V4 | 1.0 | 4.073e-19 | 654 | 0.17 | 410 | 291 | 17 | 381 | 755 | 92 | 487 | DUF3874 domain-containing protein | DUF3874 domain-containing protein | | afdb-uniprot50 | AF-A0A7W5TKY8-F1-MODEL\_V4 | 1.0 | 3.086e-20 | 654 | 0.228 | 442 | 284 | 26 | 360 | 769 | 8 | 424 | Uncharacterized protein | Uncharacterized protein | | afdb-uniprot50 | AF-A0A3R5Z023-F1-MODEL\_V4 | 1.0 | 3.843e-22 | 654 | 0.175 | 535 | 345 | 19 | 259 | 769 | 86 | 548 | Virulence protein E | Virulence protein E | | afdb-uniprot50 | AF-A0A5C7LJI8-F1-MODEL\_V4 | 1.0 | 5.807e-22 | 654 | 0.188 | 546 | 348 | 23 | 268 | 757 | 206 | 712 | Uncharacterized protein | Uncharacterized protein | | afdb-uniprot50 | AF-A0A0D6QBU6-F1-MODEL\_V4 | 1.0 | 3.313e-19 | 653 | 0.214 | 415 | 276 | 21 | 370 | 753 | 18 | 413 | DNA primase | DNA primase | | afdb-uniprot50 | AF-A0A0P0FP05-F1-MODEL\_V4 | 1.0 | 2.478e-18 | 653 | 0.172 | 389 | 296 | 16 | 381 | 757 | 47 | 421 | DUF3874 domain-containing protein | DUF3874 domain-containing protein | | afdb-uniprot50 | AF-A0A5P0X0N2-F1-MODEL\_V4 | 1.0 | 4.816e-21 | 653 | 0.18 | 510 | 346 | 27 | 286 | 755 | 178 | 655 | VirE\_N domain-containing protein | VirE\_N domain-containing protein | | afdb-uniprot50 | AF-A0A5P0XUM3-F1-MODEL\_V4 | 1.0 | 2.069e-22 | 653 | 0.178 | 537 | 341 | 19 | 259 | 769 | 219 | 681 | Virulence protein E | Virulence protein E | | afdb-uniprot50 | AF-A0A2S5P5D7-F1-MODEL\_V4 | 1.0 | 5.623e-21 | 653 | 0.216 | 489 | 325 | 21 | 287 | 751 | 303 | 757 | Uncharacterized protein | Uncharacterized protein | | afdb-uniprot50 | AF-A0A139K8J3-F1-MODEL\_V4 | 1.0 | 8.831e-19 | 652 | 0.184 | 396 | 296 | 18 | 384 | 768 | 63 | 442 | DUF3874 domain-containing protein | DUF3874 domain-containing protein | | afdb-uniprot50 | AF-A0A4Q7LBG2-F1-MODEL\_V4 | 1.0 | 1.831e-31 | 652 | 0.199 | 914 | 483 | 47 | 1 | 737 | 3 | 844 | Putative DNA primase/helicase | Putative DNA primase/helicase | | afdb-uniprot50 | AF-A0A7W6QX48-F1-MODEL\_V4 | 1.0 | 3.444e-17 | 651 | 0.234 | 303 | 202 | 12 | 457 | 750 | 53 | 334 | Putative P-loop ATPase | Putative P-loop ATPase | | afdb-uniprot50 | AF-A0A3R6QWH6-F1-MODEL\_V4 | 1.0 | 1.309e-19 | 651 | 0.202 | 429 | 295 | 21 | 370 | 767 | 18 | 430 | Virulence-associated protein E | Virulence-associated protein E | | afdb-uniprot50 | AF-A0A4R3GMS2-F1-MODEL\_V4 | 1.0 | 1.011e-19 | 651 | 0.239 | 439 | 279 | 24 | 370 | 769 | 18 | 440 | Virulence-associated protein E | Virulence-associated protein E | | afdb-uniprot50 | AF-A0A357A780-F1-MODEL\_V4 | 1.0 | 8.552e-26 | 651 | 0.216 | 652 | 392 | 32 | 180 | 757 | 89 | 695 | DUF3854 domain-containing protein | DUF3854 domain-containing protein | | afdb-uniprot50 | AF-A0A037USG8-F1-MODEL\_V4 | 1.0 | 8.718e-17 | 650 | 0.338 | 260 | 149 | 10 | 100 | 345 | 68 | 318 | Toprim domain-containing protein | Toprim domain-containing protein | | afdb-uniprot50 | AF-A0A1I6WIK4-F1-MODEL\_V4 | 1.0 | 2.431e-19 | 650 | 0.213 | 407 | 276 | 18 | 394 | 769 | 13 | 406 | Virulence-associated protein E | Virulence-associated protein E | | afdb-uniprot50 | AF-A0A1Y3RIE9-F1-MODEL\_V4 | 1.0 | 4.073e-19 | 650 | 0.217 | 418 | 278 | 21 | 370 | 755 | 19 | 419 | Virulence-associated protein E | Virulence-associated protein E | | afdb-uniprot50 | AF-Q3AX57-F1-MODEL\_V4 | 1.0 | 6.823e-19 | 649 | 0.206 | 397 | 279 | 15 | 378 | 756 | 18 | 396 | p-loop ATPase and inactivated derivatives-like | p-loop ATPase and inactivated derivatives-like | | afdb-uniprot50 | AF-J9GWN3-F1-MODEL\_V4 | 1.0 | 1.181e-19 | 649 | 0.202 | 414 | 281 | 18 | 369 | 755 | 82 | 473 | Helicase | Helicase | | afdb-uniprot50 | AF-A0A7W5Y0N8-F1-MODEL\_V4 | 1.0 | 1.326e-21 | 648 | 0.17 | 516 | 340 | 27 | 260 | 761 | 258 | 699 | Uncharacterized protein | Uncharacterized protein | | afdb-uniprot50 | AF-A0A4V2AU08-F1-MODEL\_V4 | 1.0 | 2.137e-23 | 648 | 0.228 | 533 | 330 | 29 | 289 | 769 | 255 | 758 | DNA primase | DNA primase | | afdb-uniprot50 | AF-A0A318GP25-F1-MODEL\_V4 | 1.0 | 7.278e-21 | 648 | 0.21 | 479 | 297 | 26 | 332 | 752 | 326 | 781 | Virulence-associated protein E | Virulence-associated protein E | | afdb-uniprot50 | AF-A0A5C8NRV2-F1-MODEL\_V4 | 1.0 | 9.792e-27 | 648 | 0.172 | 789 | 425 | 35 | 1 | 755 | 243 | 837 | PriCT\_2 domain-containing protein | PriCT\_2 domain-containing protein | | afdb-uniprot50 | AF-A0A6L3T504-F1-MODEL\_V4 | 1.0 | 6.076e-33 | 648 | 0.213 | 820 | 450 | 41 | 100 | 757 | 84 | 870 | SF3 helicase domain-containing protein | SF3 helicase domain-containing protein | | afdb-uniprot50 | AF-A0A3R8ZF92-F1-MODEL\_V4 | 1.0 | 1.165e-17 | 647 | 0.297 | 299 | 160 | 11 | 93 | 355 | 75 | 359 | Toprim domain-containing protein | Toprim domain-containing protein | | afdb-uniprot50 | AF-A0A7Y3JLU6-F1-MODEL\_V4 | 1.0 | 9.122e-28 | 647 | 0.211 | 767 | 447 | 49 | 76 | 769 | 80 | 761 | Uncharacterized protein | Uncharacterized protein | | afdb-uniprot50 | AF-Q0BD11-F1-MODEL\_V4 | 1.0 | 4.126e-29 | 647 | 0.197 | 864 | 411 | 45 | 12 | 769 | 7 | 693 | TOPRIM domain protein | TOPRIM domain protein | | afdb-uniprot50 | AF-A0A1U9VG46-F1-MODEL\_V4 | 1.0 | 1.761e-25 | 647 | 0.199 | 666 | 418 | 39 | 164 | 753 | 153 | 779 | Uncharacterized protein | Uncharacterized protein | | afdb-uniprot50 | AF-D4WHI7-F1-MODEL\_V4 | 1.0 | 9.729e-22 | 646 | 0.177 | 513 | 330 | 23 | 261 | 756 | 261 | 698 | VirE N-terminal domain protein | VirE N-terminal domain protein | | afdb-uniprot50 | AF-A0A2M8UBK7-F1-MODEL\_V4 | 1.0 | 7.467e-17 | 646 | 0.344 | 264 | 139 | 8 | 102 | 347 | 460 | 707 | Uncharacterized protein | Uncharacterized protein | | afdb-uniprot50 | AF-A0A3C1F3D1-F1-MODEL\_V4 | 1.0 | 2.644e-20 | 645 | 0.209 | 449 | 309 | 19 | 338 | 769 | 19 | 438 | Uncharacterized protein | Uncharacterized protein | | afdb-uniprot50 | AF-B8IT86-F1-MODEL\_V4 | 1.0 | 5.623e-21 | 645 | 0.199 | 547 | 331 | 24 | 261 | 752 | 10 | 504 | Virulence-associated E family protein | Virulence-associated E family protein | | afdb-uniprot50 | AF-A0A4R1QM10-F1-MODEL\_V4 | 1.0 | 1.878e-19 | 644 | 0.208 | 431 | 294 | 21 | 370 | 769 | 18 | 432 | Virulence-associated protein E | Virulence-associated protein E | | afdb-uniprot50 | AF-A0A1H1AAV9-F1-MODEL\_V4 | 1.0 | 1.251e-16 | 644 | 0.286 | 265 | 157 | 9 | 102 | 352 | 276 | 522 | Uncharacterized domain associated with phage/plasmid primase | Uncharacterized domain associated with phage/plasmid primase | | afdb-uniprot50 | AF-A0A351YBI0-F1-MODEL\_V4 | 1.0 | 1.005e-22 | 644 | 0.187 | 539 | 327 | 30 | 261 | 767 | 244 | 703 | Uncharacterized protein | Uncharacterized protein | | afdb-uniprot50 | AF-A0A349MYJ9-F1-MODEL\_V4 | 1.0 | 1.866e-22 | 644 | 0.206 | 537 | 322 | 26 | 263 | 751 | 242 | 722 | Uncharacterized protein | Uncharacterized protein | | afdb-uniprot50 | AF-E5UXW1-F1-MODEL\_V4 | 1.0 | 6.48e-19 | 643 | 0.195 | 405 | 297 | 19 | 370 | 761 | 56 | 444 | DUF3874 domain-containing protein | DUF3874 domain-containing protein | | afdb-uniprot50 | AF-A0A2T1LQX5-F1-MODEL\_V4 | 1.0 | 6.275e-26 | 643 | 0.191 | 653 | 407 | 34 | 177 | 769 | 85 | 676 | DUF3854 domain-containing protein | DUF3854 domain-containing protein | | afdb-uniprot50 | AF-A0A089PBP6-F1-MODEL\_V4 | 1.0 | 4.755e-19 | 642 | 0.223 | 389 | 268 | 16 | 384 | 756 | 51 | 421 | DNA primase | DNA primase | | afdb-uniprot50 | AF-A0A2N5YK98-F1-MODEL\_V4 | 1.0 | 6.438e-22 | 642 | 0.181 | 513 | 353 | 25 | 260 | 757 | 193 | 653 | VirE\_N domain-containing protein | VirE\_N domain-containing protein | | afdb-uniprot50 | AF-A0A0B1RHV7-F1-MODEL\_V4 | 1.0 | 1.352e-20 | 642 | 0.223 | 434 | 282 | 23 | 379 | 768 | 49 | 471 | Uncharacterized protein | Uncharacterized protein | | afdb-uniprot50 | AF-A0A3R6QTT6-F1-MODEL\_V4 | 1.0 | 3.466e-22 | 642 | 0.188 | 536 | 353 | 27 | 246 | 757 | 216 | 693 | Uncharacterized protein | Uncharacterized protein | | afdb-uniprot50 | AF-A0A396NE55-F1-MODEL\_V4 | 1.0 | 1.031e-18 | 641 | 0.204 | 421 | 286 | 23 | 370 | 759 | 2 | 404 | Virulence-associated protein E | Virulence-associated protein E | | afdb-uniprot50 | AF-A0A2S7YZ26-F1-MODEL\_V4 | 1.0 | 5.844e-19 | 641 | 0.214 | 442 | 276 | 23 | 370 | 756 | 394 | 819 | Virulence-associated protein E | Virulence-associated protein E | | afdb-uniprot50 | AF-A0A1M5AIN5-F1-MODEL\_V4 | 1.0 | 6.154e-19 | 640 | 0.169 | 390 | 294 | 17 | 381 | 757 | 58 | 430 | Virulence-associated protein E | Virulence-associated protein E | | afdb-uniprot50 | AF-A0A646FJ01-F1-MODEL\_V4 | 1.0 | 1.842e-20 | 640 | 0.172 | 528 | 358 | 27 | 261 | 751 | 14 | 499 | Uncharacterized protein | Uncharacterized protein | | afdb-uniprot50 | AF-A0A0P1IHX5-F1-MODEL\_V4 | 1.0 | 5.375e-26 | 640 | 0.173 | 782 | 364 | 29 | 2 | 752 | 234 | 763 | Putative P-loop ATPase | Putative P-loop ATPase | | afdb-uniprot50 | AF-A0A521ZVA1-F1-MODEL\_V4 | 1.0 | 3.378e-26 | 640 | 0.198 | 707 | 430 | 44 | 160 | 769 | 180 | 846 | Uncharacterized protein | Uncharacterized protein | | afdb-uniprot50 | AF-A0A6L3SS05-F1-MODEL\_V4 | 1.0 | 1.953e-33 | 640 | 0.223 | 827 | 443 | 42 | 100 | 762 | 121 | 911 | Toprim domain-containing protein | Toprim domain-containing protein | | afdb-uniprot50 | AF-A0A5B5VUS0-F1-MODEL\_V4 | 1.0 | 2.802e-17 | 638 | 0.25 | 300 | 207 | 11 | 463 | 755 | 5 | 293 | Helicase | Helicase | | afdb-uniprot50 | AF-A0A3E4JMT1-F1-MODEL\_V4 | 1.0 | 3.868e-19 | 638 | 0.19 | 394 | 281 | 21 | 381 | 756 | 45 | 418 | Helicase | Helicase | | afdb-uniprot50 | AF-A0A7G8HY62-F1-MODEL\_V4 | 1.0 | 5.006e-19 | 638 | 0.206 | 406 | 280 | 18 | 370 | 757 | 75 | 456 | Virulence-associated E family protein | Virulence-associated E family protein | | afdb-uniprot50 | AF-J9D1I1-F1-MODEL\_V4 | 1.0 | 2.695e-19 | 638 | 0.194 | 412 | 287 | 18 | 369 | 755 | 180 | 571 | Helicase | Helicase | | afdb-uniprot50 | AF-A0A2D8XE83-F1-MODEL\_V4 | 1.0 | 6.154e-19 | 638 | 0.201 | 397 | 293 | 16 | 368 | 753 | 346 | 729 | Uncharacterized protein | Uncharacterized protein | | afdb-uniprot50 | AF-A0A7Y0G5D6-F1-MODEL\_V4 | 1.0 | 3.356e-21 | 638 | 0.207 | 481 | 325 | 26 | 302 | 756 | 328 | 778 | PriCT\_2 domain-containing protein | PriCT\_2 domain-containing protein | | afdb-uniprot50 | AF-A0A328I9Q7-F1-MODEL\_V4 | 1.0 | 1.107e-25 | 638 | 0.194 | 658 | 411 | 35 | 167 | 769 | 52 | 645 | DUF3854 domain-containing protein | DUF3854 domain-containing protein | | afdb-uniprot50 | AF-A0A1Y6CYW9-F1-MODEL\_V4 | 1.0 | 3.028e-29 | 638 | 0.166 | 992 | 493 | 57 | 1 | 735 | 1 | 915 | Primase C terminal 2 (PriCT-2) | Primase C terminal 2 (PriCT-2) | | afdb-uniprot50 | AF-A0A1C5XKF3-F1-MODEL\_V4 | 1.0 | 9.299e-19 | 637 | 0.194 | 427 | 294 | 23 | 365 | 757 | 17 | 427 | Predicted P-loop ATPase and inactivated derivatives | Predicted P-loop ATPase and inactivated derivatives | | afdb-uniprot50 | AF-A0A5A9EX48-F1-MODEL\_V4 | 1.0 | 1.75e-20 | 637 | 0.209 | 468 | 266 | 21 | 370 | 751 | 140 | 589 | Uncharacterized protein | Uncharacterized protein | | afdb-uniprot50 | AF-A0A857J6L1-F1-MODEL\_V4 | 1.0 | 2.695e-19 | 637 | 0.243 | 444 | 275 | 26 | 369 | 769 | 365 | 790 | Uncharacterized protein | Uncharacterized protein | | afdb-uniprot50 | AF-A0A6N2WVJ0-F1-MODEL\_V4 | 1.0 | 1.819e-18 | 636 | 0.184 | 396 | 284 | 18 | 384 | 755 | 157 | 537 | DUF based on B. Theta Gene description | DUF based on B. Theta Gene description | | afdb-uniprot50 | AF-A0A251WJM1-F1-MODEL\_V4 | 1.0 | 3.25e-20 | 636 | 0.238 | 444 | 282 | 21 | 328 | 755 | 136 | 539 | Uncharacterized protein | Uncharacterized protein | | afdb-uniprot50 | AF-A0A6S6TV87-F1-MODEL\_V4 | 1.0 | 3.893e-16 | 636 | 0.301 | 265 | 152 | 9 | 91 | 347 | 406 | 645 | DNA primase (EC) | DNA primase (EC) | | afdb-uniprot50 | AF-A0A2C6A5S6-F1-MODEL\_V4 | 1.0 | 1.978e-19 | 636 | 0.224 | 450 | 271 | 25 | 370 | 757 | 384 | 817 | Virulence-associated protein E | Virulence-associated protein E | | afdb-uniprot50 | AF-A0A374WBI8-F1-MODEL\_V4 | 1.0 | 1.086e-18 | 635 | 0.195 | 389 | 282 | 16 | 384 | 756 | 34 | 407 | DUF3874 domain-containing protein | DUF3874 domain-containing protein | | afdb-uniprot50 | AF-A0A255TI73-F1-MODEL\_V4 | 1.0 | 3.292e-22 | 635 | 0.147 | 801 | 396 | 30 | 1 | 769 | 34 | 579 | PriCT\_2 domain-containing protein | PriCT\_2 domain-containing protein | | afdb-uniprot50 | AF-A0A258ZJK0-F1-MODEL\_V4 | 1.0 | 5.375e-26 | 635 | 0.196 | 672 | 422 | 41 | 181 | 769 | 43 | 679 | Uncharacterized protein | Uncharacterized protein | | afdb-uniprot50 | AF-A0A7R8AWV8-F1-MODEL\_V4 | 1.0 | 6.356e-20 | 635 | 0.203 | 438 | 317 | 20 | 332 | 753 | 312 | 733 | Prim-Pol domain-containing protein | Prim-Pol domain-containing protein | | afdb-uniprot50 | AF-I3YBE5-F1-MODEL\_V4 | 1.0 | 8.175e-31 | 635 | 0.194 | 945 | 523 | 56 | 1 | 763 | 72 | 959 | Putative ATPase | Putative ATPase | | afdb-uniprot50 | AF-A0A5C7R6K8-F1-MODEL\_V4 | 1.0 | 2.165e-17 | 634 | 0.363 | 253 | 136 | 11 | 99 | 346 | 78 | 310 | Toprim domain-containing protein | Toprim domain-containing protein | | afdb-uniprot50 | AF-A0A849UD71-F1-MODEL\_V4 | 1.0 | 2.988e-19 | 634 | 0.277 | 331 | 214 | 10 | 101 | 431 | 69 | 374 | Toprim domain-containing protein | Toprim domain-containing protein | | afdb-uniprot50 | AF-X5UMY1-F1-MODEL\_V4 | 1.0 | 4.288e-19 | 634 | 0.219 | 428 | 291 | 19 | 350 | 757 | 64 | 468 | Virulence protein E | Virulence protein E | | afdb-uniprot50 | AF-E3PGT5-F1-MODEL\_V4 | 1.0 | 1.352e-20 | 634 | 0.219 | 464 | 298 | 26 | 319 | 753 | 139 | 567 | Putative virulence associated protein virE | Putative virulence associated protein virE | | afdb-uniprot50 | AF-A0A7Y6YZ19-F1-MODEL\_V4 | 1.0 | 5.305e-16 | 634 | 0.305 | 255 | 148 | 8 | 100 | 344 | 715 | 950 | DUF1738 domain-containing protein | DUF1738 domain-containing protein | | afdb-uniprot50 | AF-F0F4L8-F1-MODEL\_V4 | 1.0 | 1.188e-16 | 633 | 0.237 | 303 | 215 | 10 | 466 | 758 | 4 | 300 | Virulence-associated protein E | Virulence-associated protein E | | afdb-uniprot50 | AF-A0A212JQE2-F1-MODEL\_V4 | 1.0 | 4.604e-18 | 633 | 0.218 | 366 | 239 | 14 | 370 | 723 | 4 | 334 | Uncharacterized protein | Uncharacterized protein | | afdb-uniprot50 | AF-A0A164AHS2-F1-MODEL\_V4 | 1.0 | 1.451e-19 | 633 | 0.251 | 398 | 247 | 19 | 381 | 750 | 51 | 425 | Virulence-associated protein E | Virulence-associated protein E | | afdb-uniprot50 | AF-A0A416H079-F1-MODEL\_V4 | 1.0 | 3.027e-21 | 633 | 0.2 | 485 | 322 | 26 | 333 | 769 | 241 | 707 | Uncharacterized protein | Uncharacterized protein | | afdb-uniprot50 | AF-A0A068R0U5-F1-MODEL\_V4 | 1.0 | 4.126e-21 | 633 | 0.214 | 457 | 301 | 25 | 327 | 753 | 294 | 722 | Predicted P-loop ATPase and inactivated derivatives | Predicted P-loop ATPase and inactivated derivatives | | afdb-uniprot50 | AF-A0A844YF13-F1-MODEL\_V4 | 1.0 | 6.912e-21 | 633 | 0.207 | 511 | 348 | 21 | 265 | 758 | 247 | 717 | Uncharacterized protein | Uncharacterized protein | | afdb-uniprot50 | AF-A0A255T784-F1-MODEL\_V4 | 1.0 | 4.515e-19 | 632 | 0.219 | 405 | 277 | 14 | 371 | 752 | 213 | 601 | Uncharacterized protein | Uncharacterized protein | | afdb-uniprot50 | AF-A0A7Z0SBX3-F1-MODEL\_V4 | 1.0 | 3.65e-22 | 632 | 0.194 | 525 | 340 | 25 | 262 | 753 | 146 | 620 | Uncharacterized protein | Uncharacterized protein | | afdb-uniprot50 | AF-A0A7G8FP95-F1-MODEL\_V4 | 1.0 | 8.831e-19 | 632 | 0.227 | 400 | 273 | 20 | 370 | 753 | 278 | 657 | Virulence-associated E family protein | Virulence-associated E family protein | | afdb-uniprot50 | AF-A0A2T0NTU5-F1-MODEL\_V4 | 1.0 | 6.912e-21 | 632 | 0.2 | 485 | 326 | 26 | 295 | 757 | 215 | 659 | Primase-like protein | Primase-like protein | | afdb-uniprot50 | AF-A0A255ZEU1-F1-MODEL\_V4 | 1.0 | 2.207e-32 | 632 | 0.218 | 877 | 455 | 51 | 8 | 737 | 2 | 794 | Uncharacterized protein | Uncharacterized protein | | afdb-uniprot50 | AF-U2QHM6-F1-MODEL\_V4 | 1.0 | 1.819e-18 | 631 | 0.199 | 427 | 292 | 23 | 365 | 757 | 17 | 427 | Virulence-associated protein E | Virulence-associated protein E | | afdb-uniprot50 | AF-A0A0S6UW49-F1-MODEL\_V4 | 1.0 | 3.147e-19 | 631 | 0.205 | 433 | 278 | 22 | 370 | 756 | 11 | 423 | Uncharacterized protein | Uncharacterized protein | | afdb-uniprot50 | AF-A0A1M4VDX6-F1-MODEL\_V4 | 1.0 | 4.574e-21 | 631 | 0.195 | 512 | 314 | 26 | 264 | 755 | 259 | 692 | Uncharacterized protein | Uncharacterized protein | | afdb-uniprot50 | AF-Q6AIG1-F1-MODEL\_V4 | 1.0 | 2.494e-15 | 631 | 0.32 | 250 | 152 | 6 | 100 | 346 | 449 | 683 | Related to DNA primase TraC | Related to DNA primase TraC | | afdb-uniprot50 | AF-A0A252DZZ4-F1-MODEL\_V4 | 1.0 | 6.607e-26 | 631 | 0.197 | 678 | 422 | 40 | 151 | 762 | 25 | 646 | DUF3854 domain-containing protein | DUF3854 domain-containing protein | | afdb-uniprot50 | AF-A0A2S0V1Y7-F1-MODEL\_V4 | 1.0 | 2.447e-16 | 630 | 0.291 | 257 | 153 | 9 | 92 | 347 | 67 | 295 | Toprim domain-containing protein | Toprim domain-containing protein | | afdb-uniprot50 | AF-A0A2D9N1G6-F1-MODEL\_V4 | 1.0 | 1.165e-17 | 630 | 0.224 | 339 | 237 | 16 | 370 | 690 | 27 | 357 | Uncharacterized protein | Uncharacterized protein | | afdb-uniprot50 | AF-A0A126UX24-F1-MODEL\_V4 | 1.0 | 3.603e-20 | 630 | 0.197 | 450 | 312 | 18 | 329 | 758 | 183 | 603 | Uncharacterized protein | Uncharacterized protein | | afdb-uniprot50 | AF-A0A1Q6HTS3-F1-MODEL\_V4 | 1.0 | 7.763e-23 | 630 | 0.188 | 572 | 350 | 31 | 213 | 756 | 212 | 697 | VirE\_N domain-containing protein | VirE\_N domain-containing protein | | afdb-uniprot50 | AF-A0A1H1JG68-F1-MODEL\_V4 | 1.0 | 2.856e-16 | 630 | 0.274 | 270 | 164 | 8 | 100 | 352 | 459 | 713 | Antirestriction protein ArdC | Antirestriction protein ArdC | | afdb-uniprot50 | AF-A0A2D8CJQ3-F1-MODEL\_V4 | 1.0 | 7.863e-17 | 629 | 0.282 | 262 | 181 | 5 | 498 | 756 | 2 | 259 | Uncharacterized protein | Uncharacterized protein | | afdb-uniprot50 | AF-E6K9V5-F1-MODEL\_V4 | 1.0 | 5.374e-18 | 629 | 0.234 | 350 | 229 | 11 | 379 | 699 | 23 | 362 | Virulence-associated protein E | Virulence-associated protein E | | afdb-uniprot50 | AF-A0A2E0B660-F1-MODEL\_V4 | 1.0 | 1.558e-18 | 629 | 0.205 | 395 | 277 | 18 | 394 | 769 | 3 | 379 | Uncharacterized protein | Uncharacterized protein | | afdb-uniprot50 | AF-A0A496KNL5-F1-MODEL\_V4 | 1.0 | 7.42e-20 | 629 | 0.206 | 455 | 299 | 21 | 329 | 752 | 4 | 427 | Helicase | Helicase | | afdb-uniprot50 | AF-A0A345REX8-F1-MODEL\_V4 | 1.0 | 3.313e-19 | 629 | 0.2 | 433 | 286 | 24 | 370 | 757 | 39 | 456 | Uncharacterized protein | Uncharacterized protein | | afdb-uniprot50 | AF-A0A7Y5C283-F1-MODEL\_V4 | 1.0 | 9.666e-25 | 629 | 0.172 | 636 | 435 | 34 | 153 | 757 | 106 | 680 | Uncharacterized protein | Uncharacterized protein | | afdb-uniprot50 | AF-B7UEZ2-F1-MODEL\_V4 | 1.0 | 2.029e-15 | 629 | 0.276 | 239 | 145 | 7 | 117 | 346 | 164 | 383 | DNA primase | DNA primase | | afdb-uniprot50 | AF-A0A6F8PSK9-F1-MODEL\_V4 | 1.0 | 7.813e-20 | 628 | 0.26 | 395 | 220 | 13 | 3 | 357 | 1 | 363 | Uncharacterized protein | Uncharacterized protein | | afdb-uniprot50 | AF-A0A6L9HKV5-F1-MODEL\_V4 | 1.0 | 2.893e-18 | 628 | 0.199 | 436 | 287 | 17 | 370 | 755 | 6 | 429 | Virulence-associated protein E | Virulence-associated protein E | | afdb-uniprot50 | AF-A0A1C6HAC9-F1-MODEL\_V4 | 1.0 | 3.868e-19 | 628 | 0.19 | 410 | 291 | 21 | 370 | 757 | 44 | 434 | Predicted P-loop ATPase and inactivated derivatives | Predicted P-loop ATPase and inactivated derivatives | | afdb-uniprot50 | AF-G5SV99-F1-MODEL\_V4 | 1.0 | 4.073e-19 | 628 | 0.184 | 391 | 273 | 21 | 384 | 751 | 102 | 469 | DUF3874 domain-containing protein | DUF3874 domain-containing protein | | afdb-uniprot50 | AF-A0A7G8C997-F1-MODEL\_V4 | 1.0 | 2.431e-19 | 628 | 0.2 | 428 | 283 | 21 | 363 | 752 | 81 | 487 | Virulence-associated E family protein | Virulence-associated E family protein | | afdb-uniprot50 | AF-A0A1X9YSK3-F1-MODEL\_V4 | 1.0 | 5.271e-19 | 628 | 0.2 | 413 | 289 | 20 | 372 | 755 | 56 | 456 | Uncharacterized protein | Uncharacterized protein | | afdb-uniprot50 | AF-A0A7T1MDW8-F1-MODEL\_V4 | 1.0 | 6.823e-19 | 628 | 0.202 | 410 | 275 | 18 | 379 | 757 | 245 | 633 | Uncharacterized protein | Uncharacterized protein | | afdb-uniprot50 | AF-A0A846FJ73-F1-MODEL\_V4 | 1.0 | 5.77e-25 | 628 | 0.208 | 629 | 389 | 35 | 178 | 757 | 70 | 638 | DUF3854 domain-containing protein | DUF3854 domain-containing protein | | afdb-uniprot50 | AF-A0A6I2N1Y2-F1-MODEL\_V4 | 1.0 | 1.196e-21 | 627 | 0.178 | 526 | 357 | 23 | 260 | 756 | 236 | 715 | VirE\_N domain-containing protein | VirE\_N domain-containing protein | | afdb-uniprot50 | AF-A0A7J4XCA9-F1-MODEL\_V4 | 1.0 | 7.138e-22 | 627 | 0.18 | 531 | 341 | 25 | 242 | 755 | 252 | 705 | VirE\_N domain-containing protein | VirE\_N domain-containing protein | | afdb-uniprot50 | AF-A0A0X3TJ34-F1-MODEL\_V4 | 1.0 | 2.123e-18 | 626 | 0.292 | 390 | 196 | 17 | 3 | 336 | 1 | 366 | Uncharacterized protein | Uncharacterized protein | | afdb-uniprot50 | AF-A0A2W6Z2Y3-F1-MODEL\_V4 | 1.0 | 1.479e-18 | 626 | 0.237 | 408 | 262 | 21 | 369 | 755 | 20 | 399 | Uncharacterized protein | Uncharacterized protein | | afdb-uniprot50 | AF-A0A1M6CUR8-F1-MODEL\_V4 | 1.0 | 5.659e-18 | 626 | 0.187 | 385 | 277 | 17 | 384 | 751 | 50 | 415 | Virulence-associated protein E | Virulence-associated protein E | | afdb-uniprot50 | AF-A0A5P0WS35-F1-MODEL\_V4 | 1.0 | 2.838e-19 | 626 | 0.204 | 386 | 274 | 19 | 384 | 755 | 75 | 441 | DUF3874 domain-containing protein | DUF3874 domain-containing protein | | afdb-uniprot50 | AF-A0A239KSC1-F1-MODEL\_V4 | 1.0 | 1.94e-20 | 626 | 0.201 | 501 | 324 | 26 | 292 | 753 | 35 | 498 | Predicted P-loop ATPase and inactivated derivatives | Predicted P-loop ATPase and inactivated derivatives | | afdb-uniprot50 | AF-B2J1H6-F1-MODEL\_V4 | 1.0 | 8.28e-25 | 626 | 0.203 | 644 | 398 | 39 | 171 | 756 | 53 | 639 | Virulence-associated E family protein | Virulence-associated E family protein | | afdb-uniprot50 | AF-K1JGA8-F1-MODEL\_V4 | 1.0 | 1.387e-32 | 626 | 0.2 | 888 | 431 | 50 | 100 | 752 | 92 | 935 | Toprim domain-containing protein | Toprim domain-containing protein | | afdb-uniprot50 | AF-F9D560-F1-MODEL\_V4 | 1.0 | 2.207e-16 | 625 | 0.267 | 277 | 190 | 8 | 471 | 742 | 12 | 280 | Virulence-associated protein E | Virulence-associated protein E | | afdb-uniprot50 | AF-A0A3E5E5N8-F1-MODEL\_V4 | 1.0 | 1.784e-19 | 625 | 0.24 | 440 | 257 | 21 | 370 | 747 | 10 | 434 | Virulence-associated E family protein | Virulence-associated E family protein | | afdb-uniprot50 | AF-M4UF70-F1-MODEL\_V4 | 1.0 | 1.538e-24 | 625 | 0.206 | 643 | 396 | 37 | 181 | 769 | 101 | 682 | Uncharacterized protein | Uncharacterized protein | | afdb-uniprot50 | AF-A0A7R8BVV0-F1-MODEL\_V4 | 1.0 | 7.047e-20 | 625 | 0.202 | 478 | 318 | 26 | 328 | 757 | 218 | 680 | Uncharacterized protein | Uncharacterized protein | | afdb-uniprot50 | AF-A0A5P3VSJ1-F1-MODEL\_V4 | 1.0 | 4.785e-16 | 624 | 0.317 | 252 | 151 | 9 | 99 | 347 | 75 | 308 | Toprim domain-containing protein | Toprim domain-containing protein | | afdb-uniprot50 | AF-A0A3G3GKY0-F1-MODEL\_V4 | 1.0 | 1.558e-18 | 624 | 0.203 | 402 | 281 | 14 | 382 | 756 | 9 | 398 | Uncharacterized protein | Uncharacterized protein | | afdb-uniprot50 | AF-R6LH63-F1-MODEL\_V4 | 1.0 | 4.604e-18 | 624 | 0.189 | 386 | 275 | 19 | 384 | 751 | 53 | 418 | DUF3874 domain-containing protein | DUF3874 domain-containing protein | | afdb-uniprot50 | AF-A0A3S4WPJ7-F1-MODEL\_V4 | 1.0 | 4.206e-20 | 624 | 0.213 | 460 | 308 | 27 | 319 | 755 | 293 | 721 | Predicted P-loop ATPase and inactivated derivatives | Predicted P-loop ATPase and inactivated derivatives | | afdb-uniprot50 | AF-A0A7U3BA69-F1-MODEL\_V4 | 1.0 | 2.931e-20 | 624 | 0.214 | 461 | 299 | 25 | 319 | 751 | 293 | 718 | Replication protein | Replication protein | | afdb-uniprot50 | AF-A0A1Z4R883-F1-MODEL\_V4 | 1.0 | 3.627e-25 | 624 | 0.204 | 655 | 409 | 33 | 168 | 769 | 82 | 677 | Virulence-associated E family protein | Virulence-associated E family protein | | afdb-uniprot50 | AF-A0A2C6W764-F1-MODEL\_V4 | 1.0 | 4.847e-18 | 623 | 0.199 | 396 | 272 | 21 | 379 | 753 | 2 | 373 | Uncharacterized protein | Uncharacterized protein | | afdb-uniprot50 | AF-A0A7C9NE92-F1-MODEL\_V4 | 1.0 | 5.34e-21 | 623 | 0.204 | 513 | 330 | 25 | 263 | 751 | 321 | 779 | Uncharacterized protein | Uncharacterized protein | | afdb-uniprot50 | AF-A0A6S6U6M2-F1-MODEL\_V4 | 1.0 | 1.343e-15 | 623 | 0.311 | 260 | 156 | 7 | 91 | 347 | 560 | 799 | DNA primase (EC) | DNA primase (EC) | | afdb-uniprot50 | AF-A0A2A4P7I2-F1-MODEL\_V4 | 1.0 | 8.831e-19 | 622 | 0.235 | 390 | 259 | 18 | 385 | 752 | 38 | 410 | Uncharacterized protein | Uncharacterized protein | | afdb-uniprot50 | AF-S9S807-F1-MODEL\_V4 | 1.0 | 8.016e-16 | 622 | 0.348 | 241 | 124 | 10 | 120 | 347 | 610 | 830 | Toprim domain-containing protein | Toprim domain-containing protein | | afdb-uniprot50 | AF-A0A2S6VC66-F1-MODEL\_V4 | 1.0 | 1.387e-16 | 621 | 0.263 | 307 | 203 | 12 | 460 | 757 | 2 | 294 | Uncharacterized protein | Uncharacterized protein | | afdb-uniprot50 | AF-A0A858Q8N5-F1-MODEL\_V4 | 1.0 | 5.697e-31 | 621 | 0.186 | 928 | 516 | 52 | 12 | 755 | 7 | 879 | Uncharacterized protein | Uncharacterized protein | | afdb-uniprot50 | AF-A0A6L7WJY2-F1-MODEL\_V4 | 1.0 | 1.188e-16 | 620 | 0.277 | 332 | 211 | 14 | 379 | 687 | 21 | 346 | Uncharacterized protein | Uncharacterized protein | | afdb-uniprot50 | AF-A0A3D5PJD2-F1-MODEL\_V4 | 1.0 | 9.299e-19 | 620 | 0.224 | 405 | 247 | 16 | 370 | 737 | 20 | 394 | Uncharacterized protein | Uncharacterized protein | | afdb-uniprot50 | AF-A0A7W3DK48-F1-MODEL\_V4 | 1.0 | 7.965e-19 | 620 | 0.203 | 422 | 273 | 24 | 372 | 755 | 35 | 431 | Uncharacterized protein | Uncharacterized protein | | afdb-uniprot50 | AF-A0A660LQS4-F1-MODEL\_V4 | 1.0 | 9.791e-19 | 620 | 0.203 | 408 | 287 | 18 | 369 | 757 | 85 | 473 | Helicase | Helicase | | afdb-uniprot50 | AF-A0A1H2RHG9-F1-MODEL\_V4 | 1.0 | 7.663e-21 | 620 | 0.208 | 504 | 310 | 24 | 327 | 762 | 2 | 484 | Predicted P-loop ATPase and inactivated derivatives | Predicted P-loop ATPase and inactivated derivatives | | afdb-uniprot50 | AF-A0A857L932-F1-MODEL\_V4 | 1.0 | 9.299e-19 | 620 | 0.212 | 419 | 271 | 24 | 380 | 757 | 110 | 510 | Uncharacterized protein | Uncharacterized protein | | afdb-uniprot50 | AF-I5B0X5-F1-MODEL\_V4 | 1.0 | 9.854e-16 | 620 | 0.301 | 239 | 135 | 11 | 116 | 346 | 551 | 765 | Antirestriction protein | Antirestriction protein | | afdb-uniprot50 | AF-A0A178MCH8-F1-MODEL\_V4 | 1.0 | 1.317e-16 | 620 | 0.34 | 244 | 123 | 10 | 120 | 347 | 567 | 788 | Toprim domain-containing protein | Toprim domain-containing protein | | afdb-uniprot50 | AF-A0A661E6V8-F1-MODEL\_V4 | 1.0 | 1.795e-24 | 620 | 0.161 | 918 | 350 | 29 | 195 | 757 | 348 | 1200 | Uncharacterized protein | Uncharacterized protein | | afdb-uniprot50 | AF-C9MTT5-F1-MODEL\_V4 | 1.0 | 6.606e-18 | 619 | 0.252 | 329 | 207 | 13 | 458 | 756 | 3 | 322 | Virulence-associated protein E | Virulence-associated protein E | | afdb-uniprot50 | AF-A0A661CSP6-F1-MODEL\_V4 | 1.0 | 7.565e-19 | 619 | 0.212 | 429 | 288 | 21 | 354 | 757 | 6 | 409 | Uncharacterized protein | Uncharacterized protein | | afdb-uniprot50 | AF-R5KUI2-F1-MODEL\_V4 | 1.0 | 3.25e-20 | 619 | 0.195 | 444 | 317 | 20 | 329 | 757 | 281 | 699 | Uncharacterized protein | Uncharacterized protein | | afdb-uniprot50 | AF-A0A1I2KRS2-F1-MODEL\_V4 | 1.0 | 1.044e-20 | 619 | 0.192 | 534 | 343 | 26 | 260 | 755 | 281 | 764 | Virulence-associated protein E | Virulence-associated protein E | | afdb-uniprot50 | AF-A0A3M0XHA9-F1-MODEL\_V4 | 1.0 | 8.551e-18 | 618 | 0.318 | 308 | 189 | 11 | 466 | 766 | 1 | 294 | Uncharacterized protein | Uncharacterized protein | | afdb-uniprot50 | AF-A0A4P2SPK5-F1-MODEL\_V4 | 1.0 | 1.46e-16 | 618 | 0.308 | 256 | 145 | 12 | 107 | 358 | 155 | 382 | Uncharacterized protein | Uncharacterized protein | | afdb-uniprot50 | AF-A0A7K3KGX5-F1-MODEL\_V4 | 1.0 | 1.807e-21 | 618 | 0.19 | 547 | 338 | 28 | 261 | 768 | 123 | 603 | VirE\_N domain-containing protein | VirE\_N domain-containing protein | | afdb-uniprot50 | AF-A0A3N5GQR2-F1-MODEL\_V4 | 1.0 | 1.079e-21 | 618 | 0.208 | 546 | 354 | 27 | 231 | 757 | 218 | 704 | Uncharacterized protein | Uncharacterized protein | | afdb-uniprot50 | AF-A0A127SE37-F1-MODEL\_V4 | 1.0 | 2.324e-16 | 617 | 0.469 | 196 | 103 | 1 | 562 | 757 | 6 | 200 | Virulence-associated protein E | Virulence-associated protein E | | afdb-uniprot50 | AF-A0A522RNX4-F1-MODEL\_V4 | 1.0 | 8.718e-17 | 617 | 0.301 | 252 | 139 | 11 | 92 | 333 | 79 | 303 | Toprim domain-containing protein | Toprim domain-containing protein | | afdb-uniprot50 | AF-A0A134BGQ9-F1-MODEL\_V4 | 1.0 | 4.693e-17 | 617 | 0.268 | 298 | 195 | 10 | 452 | 735 | 35 | 323 | Virulence-associated protein E | Virulence-associated protein E | | afdb-uniprot50 | AF-A0A1G2ZMJ1-F1-MODEL\_V4 | 1.0 | 1.508e-17 | 617 | 0.184 | 402 | 291 | 16 | 370 | 755 | 37 | 417 | Uncharacterized protein | Uncharacterized protein | | afdb-uniprot50 | AF-A0A1G8LAT9-F1-MODEL\_V4 | 1.0 | 5.92e-21 | 617 | 0.217 | 473 | 304 | 26 | 335 | 769 | 13 | 457 | Virulence-associated protein E | Virulence-associated protein E | | afdb-uniprot50 | AF-A0A3C0D4T8-F1-MODEL\_V4 | 1.0 | 1.158e-20 | 617 | 0.167 | 548 | 371 | 30 | 265 | 768 | 259 | 765 | VirE\_N domain-containing protein | VirE\_N domain-containing protein | | afdb-uniprot50 | AF-A0A252DL02-F1-MODEL\_V4 | 1.0 | 2.527e-25 | 617 | 0.195 | 689 | 403 | 37 | 153 | 757 | 26 | 646 | KOW domain-containing protein | KOW domain-containing protein | | afdb-uniprot50 | AF-A6GUP7-F1-MODEL\_V4 | 1.0 | 5.586e-16 | 617 | 0.386 | 233 | 121 | 8 | 116 | 347 | 780 | 991 | Toprim domain-containing protein | Toprim domain-containing protein | | afdb-uniprot50 | AF-A0A0F9W6J4-F1-MODEL\_V4 | 1.0 | 1.46e-16 | 616 | 0.241 | 306 | 206 | 12 | 370 | 659 | 33 | 328 | Uncharacterized protein | Uncharacterized protein | | afdb-uniprot50 | AF-A0A414JB35-F1-MODEL\_V4 | 1.0 | 5.17e-20 | 616 | 0.215 | 440 | 284 | 23 | 370 | 769 | 25 | 443 | Uncharacterized protein | Uncharacterized protein | | afdb-uniprot50 | AF-A0A1T4ZJ82-F1-MODEL\_V4 | 1.0 | 9.666e-25 | 616 | 0.203 | 673 | 387 | 34 | 181 | 769 | 18 | 625 | DUF3854 domain-containing protein | DUF3854 domain-containing protein | | afdb-uniprot50 | AF-A0A3C0FNH6-F1-MODEL\_V4 | 1.0 | 9.358e-16 | 615 | 0.342 | 228 | 123 | 9 | 128 | 349 | 3 | 209 | Toprim domain-containing protein | Toprim domain-containing protein | | afdb-uniprot50 | AF-A0A417P6N5-F1-MODEL\_V4 | 1.0 | 2.661e-17 | 615 | 0.207 | 386 | 259 | 19 | 370 | 727 | 2 | 368 | Virulence-associated protein E | Virulence-associated protein E | | afdb-uniprot50 | AF-A0A1S1NXN9-F1-MODEL\_V4 | 1.0 | 4.574e-21 | 615 | 0.194 | 530 | 340 | 27 | 285 | 750 | 337 | 843 | Uncharacterized protein | Uncharacterized protein | | afdb-uniprot50 | AF-A0A353E5I1-F1-MODEL\_V4 | 1.0 | 5.071e-21 | 614 | 0.18 | 514 | 331 | 23 | 261 | 755 | 249 | 691 | Helicase | Helicase | | afdb-uniprot50 | AF-A0A662MET1-F1-MODEL\_V4 | 1.0 | 5.204e-25 | 614 | 0.18 | 663 | 411 | 34 | 181 | 757 | 149 | 764 | Uncharacterized protein | Uncharacterized protein | | afdb-uniprot50 | AF-I5D2C8-F1-MODEL\_V4 | 1.0 | 3.919e-29 | 614 | 0.189 | 854 | 484 | 47 | 6 | 736 | 2 | 769 | Uncharacterized protein | Uncharacterized protein | | afdb-uniprot50 | AF-A0A6B2KQS6-F1-MODEL\_V4 | 1.0 | 2.593e-29 | 614 | 0.183 | 936 | 464 | 54 | 10 | 737 | 4 | 846 | DUF927 domain-containing protein | DUF927 domain-containing protein | | afdb-uniprot50 | AF-A0A3Q9MY88-F1-MODEL\_V4 | 1.0 | 1.489e-15 | 614 | 0.295 | 244 | 140 | 8 | 120 | 350 | 170 | 394 | Uncharacterized protein | Uncharacterized protein | | afdb-uniprot50 | AF-A0A0D6MP65-F1-MODEL\_V4 | 1.0 | 1.662e-20 | 613 | 0.206 | 522 | 327 | 25 | 262 | 752 | 224 | 689 | Virulence-associated E family protein | Virulence-associated E family protein | | afdb-uniprot50 | AF-A0A2I9D7G1-F1-MODEL\_V4 | 1.0 | 3.819e-25 | 613 | 0.196 | 635 | 401 | 35 | 178 | 757 | 42 | 621 | Virulence protein E | Virulence protein E | | afdb-uniprot50 | AF-A0A4R3GWI1-F1-MODEL\_V4 | 1.0 | 8.552e-26 | 613 | 0.222 | 696 | 395 | 39 | 174 | 769 | 157 | 806 | CHC2-type zinc finger protein | CHC2-type zinc finger protein | | afdb-uniprot50 | AF-A0A1Y6ISW7-F1-MODEL\_V4 | 1.0 | 5.769e-17 | 612 | 0.292 | 291 | 170 | 11 | 494 | 756 | 2 | 284 | Virulence-associated protein E | Virulence-associated protein E | | afdb-uniprot50 | AF-A0A285D5W0-F1-MODEL\_V4 | 1.0 | 3.745e-18 | 612 | 0.236 | 384 | 246 | 18 | 407 | 757 | 5 | 374 | Virulence-associated protein E | Virulence-associated protein E | | afdb-uniprot50 | AF-A0A2Z5WWH0-F1-MODEL\_V4 | 1.0 | 2.695e-19 | 612 | 0.223 | 412 | 257 | 19 | 368 | 751 | 36 | 412 | Virulence-associated E | Virulence-associated E | | afdb-uniprot50 | AF-A0A522ISJ4-F1-MODEL\_V4 | 1.0 | 8.175e-31 | 612 | 0.212 | 830 | 438 | 43 | 98 | 750 | 95 | 885 | Toprim domain-containing protein | Toprim domain-containing protein | | afdb-uniprot50 | AF-A0A1Z5HMC9-F1-MODEL\_V4 | 1.0 | 4.126e-21 | 611 | 0.22 | 472 | 319 | 17 | 260 | 715 | 204 | 642 | VirE\_N domain-containing protein | VirE\_N domain-containing protein | | afdb-uniprot50 | AF-A0A1Z4U8L3-F1-MODEL\_V4 | 1.0 | 3.008e-24 | 611 | 0.18 | 660 | 430 | 40 | 153 | 756 | 21 | 625 | Virulence-associated E family protein | Virulence-associated E family protein | | afdb-uniprot50 | AF-A0A844A8E2-F1-MODEL\_V4 | 1.0 | 4.786e-24 | 611 | 0.176 | 780 | 367 | 29 | 1 | 761 | 285 | 807 | Uncharacterized protein | Uncharacterized protein | | afdb-uniprot50 | AF-A0A286GSM2-F1-MODEL\_V4 | 1.0 | 1.83e-15 | 611 | 0.305 | 255 | 153 | 9 | 102 | 347 | 689 | 928 | Antirestriction protein ArdC | Antirestriction protein ArdC | | afdb-uniprot50 | AF-A0A7C9LWM4-F1-MODEL\_V4 | 1.0 | 1.819e-18 | 610 | 0.225 | 386 | 250 | 16 | 369 | 721 | 61 | 430 | Virulence protein | Virulence protein | | afdb-uniprot50 | AF-A0A702EU54-F1-MODEL\_V4 | 1.0 | 1.058e-14 | 610 | 0.288 | 232 | 146 | 7 | 118 | 346 | 162 | 377 | DUF5710 domain-containing protein | DUF5710 domain-containing protein | | afdb-uniprot50 | AF-A0A5C9BA17-F1-MODEL\_V4 | 1.0 | 2.857e-24 | 610 | 0.164 | 790 | 361 | 32 | 1 | 763 | 194 | 711 | Virulence-associated E family protein | Virulence-associated E family protein | | afdb-uniprot50 | AF-A0A651E934-F1-MODEL\_V4 | 1.0 | 5.48e-25 | 610 | 0.205 | 675 | 416 | 38 | 149 | 769 | 34 | 641 | DUF3854 domain-containing protein | DUF3854 domain-containing protein | | afdb-uniprot50 | AF-A0A7T8TC66-F1-MODEL\_V4 | 1.0 | 4.91e-20 | 609 | 0.221 | 483 | 299 | 28 | 321 | 769 | 107 | 546 | Uncharacterized protein | Uncharacterized protein | | afdb-uniprot50 | AF-K9PF86-F1-MODEL\_V4 | 1.0 | 5.48e-25 | 609 | 0.186 | 675 | 423 | 37 | 139 | 757 | 36 | 640 | Virulence-associated E family protein | Virulence-associated E family protein | | afdb-uniprot50 | AF-A0A2K8T0V0-F1-MODEL\_V4 | 1.0 | 4.694e-25 | 609 | 0.204 | 642 | 411 | 34 | 171 | 762 | 53 | 644 | DNA primase | DNA primase | | afdb-uniprot50 | AF-W6M4H4-F1-MODEL\_V4 | 1.0 | 3.127e-30 | 609 | 0.191 | 823 | 463 | 44 | 102 | 757 | 76 | 862 | Putative Phage/plasmid primase, P4 family | Putative Phage/plasmid primase, P4 family | | afdb-uniprot50 | AF-A0A3P1YTN5-F1-MODEL\_V4 | 1.0 | 1.317e-16 | 608 | 0.21 | 313 | 222 | 14 | 384 | 684 | 58 | 357 | Helicase | Helicase | | afdb-uniprot50 | AF-A0A6H0KWD7-F1-MODEL\_V4 | 1.0 | 1.508e-17 | 608 | 0.173 | 393 | 282 | 19 | 381 | 751 | 37 | 408 | DUF3874 domain-containing protein | DUF3874 domain-containing protein | | afdb-uniprot50 | AF-A0A354WYX5-F1-MODEL\_V4 | 1.0 | 3.603e-20 | 607 | 0.145 | 528 | 359 | 26 | 246 | 755 | 233 | 686 | Helicase | Helicase | | afdb-uniprot50 | AF-A0A562ZHG6-F1-MODEL\_V4 | 1.0 | 1.878e-19 | 607 | 0.206 | 469 | 315 | 29 | 325 | 757 | 280 | 727 | Prim-Pol domain-containing protein | Prim-Pol domain-containing protein | | afdb-uniprot50 | AF-A0A4Q6HNM1-F1-MODEL\_V4 | 1.0 | 6.314e-15 | 606 | 0.277 | 234 | 147 | 8 | 116 | 347 | 165 | 378 | DUF5710 domain-containing protein | DUF5710 domain-containing protein | | afdb-uniprot50 | AF-A0A562Q1D0-F1-MODEL\_V4 | 1.0 | 7.42e-20 | 606 | 0.202 | 459 | 318 | 20 | 331 | 769 | 61 | 491 | Virulence-associated protein E | Virulence-associated protein E | | afdb-uniprot50 | AF-A0A6A7VW87-F1-MODEL\_V4 | 1.0 | 7.516e-22 | 606 | 0.164 | 578 | 356 | 34 | 226 | 756 | 61 | 558 | Uncharacterized protein | Uncharacterized protein | | afdb-uniprot50 | AF-A0A6M0RZD1-F1-MODEL\_V4 | 1.0 | 3.511e-24 | 606 | 0.196 | 632 | 396 | 35 | 181 | 765 | 104 | 670 | Uncharacterized protein | Uncharacterized protein | | afdb-uniprot50 | AF-A0A515L9P3-F1-MODEL\_V4 | 1.0 | 6.606e-18 | 606 | 0.203 | 407 | 285 | 21 | 370 | 752 | 345 | 736 | Uncharacterized protein | Uncharacterized protein | | afdb-uniprot50 | AF-A0A5C7PXR7-F1-MODEL\_V4 | 1.0 | 3.313e-19 | 606 | 0.21 | 460 | 313 | 24 | 328 | 757 | 393 | 832 | Uncharacterized protein | Uncharacterized protein | | afdb-uniprot50 | AF-A0A1M5F3B9-F1-MODEL\_V4 | 1.0 | 1.219e-28 | 606 | 0.199 | 861 | 414 | 50 | 10 | 735 | 2 | 721 | Putative DNA primase/helicase | Putative DNA primase/helicase | | afdb-uniprot50 | AF-A0A4P7CQ19-F1-MODEL\_V4 | 1.0 | 1.683e-30 | 606 | 0.193 | 841 | 495 | 48 | 13 | 750 | 19 | 778 | DUF3631 domain-containing protein | DUF3631 domain-containing protein | | afdb-uniprot50 | AF-A0A370V047-F1-MODEL\_V4 | 1.0 | 7.001e-15 | 606 | 0.322 | 236 | 139 | 8 | 114 | 346 | 160 | 377 | DNA primase TraC | DNA primase TraC | | afdb-uniprot50 | AF-A0A450TW60-F1-MODEL\_V4 | 1.0 | 2.016e-18 | 605 | 0.325 | 323 | 156 | 12 | 76 | 351 | 8 | 315 | Toprim domain-containing protein | Toprim domain-containing protein | | afdb-uniprot50 | AF-A0A1C5VVZ5-F1-MODEL\_V4 | 1.0 | 1.64e-18 | 605 | 0.207 | 433 | 280 | 20 | 370 | 753 | 387 | 805 | Predicted P-loop ATPase and inactivated derivatives | Predicted P-loop ATPase and inactivated derivatives | | afdb-uniprot50 | AF-A0A2M8JR34-F1-MODEL\_V4 | 1.0 | 1.903e-21 | 605 | 0.229 | 531 | 335 | 28 | 266 | 769 | 292 | 775 | Uncharacterized protein | Uncharacterized protein | | afdb-uniprot50 | AF-A0A1Z4QLR4-F1-MODEL\_V4 | 1.0 | 8.719e-25 | 605 | 0.18 | 693 | 443 | 40 | 145 | 762 | 9 | 651 | Virulence-associated E family protein | Virulence-associated E family protein | | afdb-uniprot50 | AF-S2YJ98-F1-MODEL\_V4 | 1.0 | 2.235e-18 | 604 | 0.192 | 420 | 278 | 22 | 384 | 769 | 5 | 397 | Uncharacterized protein | Uncharacterized protein | | afdb-uniprot50 | AF-A0A011VM22-F1-MODEL\_V4 | 1.0 | 1.121e-19 | 604 | 0.203 | 492 | 317 | 20 | 321 | 769 | 102 | 561 | Virulence protein E | Virulence protein E | | afdb-uniprot50 | AF-A0A1Q3JRW3-F1-MODEL\_V4 | 1.0 | 5.104e-18 | 603 | 0.19 | 393 | 277 | 21 | 384 | 758 | 38 | 407 | Uncharacterized protein | Uncharacterized protein | | afdb-uniprot50 | AF-A0A1C7HSV1-F1-MODEL\_V4 | 1.0 | 6.234e-21 | 603 | 0.2 | 574 | 340 | 28 | 252 | 769 | 290 | 800 | Uncharacterized protein | Uncharacterized protein | | afdb-uniprot50 | AF-A0A1B1TQM4-F1-MODEL\_V4 | 1.0 | 9.3e-27 | 603 | 0.205 | 685 | 412 | 36 | 177 | 769 | 66 | 709 | DUF3854 domain-containing protein | DUF3854 domain-containing protein | | afdb-uniprot50 | AF-A0A3M1BAH4-F1-MODEL\_V4 | 1.0 | 1.75e-20 | 601 | 0.206 | 490 | 312 | 18 | 261 | 737 | 35 | 460 | Uncharacterized protein | Uncharacterized protein | | afdb-uniprot50 | AF-Q7U784-F1-MODEL\_V4 | 1.0 | 1.978e-19 | 601 | 0.232 | 418 | 269 | 23 | 371 | 763 | 298 | 688 | Uncharacterized protein | Uncharacterized protein | | afdb-uniprot50 | AF-A0A3S0D2I0-F1-MODEL\_V4 | 1.0 | 6.115e-22 | 601 | 0.199 | 608 | 347 | 28 | 240 | 753 | 215 | 776 | Uncharacterized protein | Uncharacterized protein | | afdb-uniprot50 | AF-R4MXK3-F1-MODEL\_V4 | 1.0 | 4.88e-31 | 601 | 0.206 | 880 | 484 | 45 | 1 | 738 | 5 | 811 | DNA primase | DNA primase | | afdb-uniprot50 | AF-A0A3M3AN02-F1-MODEL\_V4 | 1.0 | 5.586e-16 | 600 | 0.306 | 261 | 149 | 10 | 107 | 355 | 12 | 252 | DNA primase TraC | DNA primase TraC | | afdb-uniprot50 | AF-R9IAU2-F1-MODEL\_V4 | 1.0 | 1.031e-18 | 600 | 0.16 | 398 | 291 | 20 | 382 | 757 | 40 | 416 | DUF3874 domain-containing protein | DUF3874 domain-containing protein | | afdb-uniprot50 | AF-F8L0K0-F1-MODEL\_V4 | 1.0 | 3.868e-19 | 600 | 0.274 | 353 | 190 | 16 | 102 | 442 | 82 | 380 | Toprim domain-containing protein | Toprim domain-containing protein | | afdb-uniprot50 | AF-A0A7X5TDU8-F1-MODEL\_V4 | 1.0 | 1.609e-19 | 600 | 0.257 | 349 | 213 | 17 | 95 | 431 | 115 | 429 | DNA primase TraC | DNA primase TraC | | afdb-uniprot50 | AF-A0A353YA43-F1-MODEL\_V4 | 1.0 | 2.056e-25 | 600 | 0.184 | 742 | 418 | 48 | 159 | 768 | 172 | 858 | Virulence protein E | Virulence protein E | | afdb-uniprot50 | AF-A0A1M7E7Q2-F1-MODEL\_V4 | 1.0 | 1.548e-21 | 599 | 0.171 | 512 | 336 | 24 | 261 | 751 | 257 | 701 | Virulence-associated protein E | Virulence-associated protein E | | afdb-uniprot50 | AF-A0A269PII8-F1-MODEL\_V4 | 1.0 | 1.651e-15 | 598 | 0.296 | 273 | 160 | 9 | 92 | 346 | 72 | 330 | Toprim domain-containing protein | Toprim domain-containing protein | | afdb-uniprot50 | AF-A0A3N2MCU4-F1-MODEL\_V4 | 1.0 | 7.324e-18 | 598 | 0.242 | 383 | 252 | 14 | 317 | 680 | 92 | 455 | Uncharacterized protein | Uncharacterized protein | | afdb-uniprot50 | AF-A0A433UZH8-F1-MODEL\_V4 | 1.0 | 2.207e-24 | 598 | 0.199 | 642 | 396 | 36 | 178 | 762 | 20 | 600 | Uncharacterized protein | Uncharacterized protein | | afdb-uniprot50 | AF-A0A7W8TA26-F1-MODEL\_V4 | 1.0 | 5.204e-25 | 598 | 0.223 | 719 | 358 | 40 | 6 | 602 | 2 | 641 | Putative DNA primase/helicase | Putative DNA primase/helicase | | afdb-uniprot50 | AF-A0A3D4KD37-F1-MODEL\_V4 | 1.0 | 8.888e-16 | 597 | 0.306 | 261 | 147 | 10 | 92 | 346 | 59 | 291 | Toprim domain-containing protein | Toprim domain-containing protein | | afdb-uniprot50 | AF-A0A522W194-F1-MODEL\_V4 | 1.0 | 3.167e-16 | 597 | 0.304 | 263 | 143 | 12 | 100 | 346 | 454 | 692 | DUF1738 domain-containing protein | DUF1738 domain-containing protein | | afdb-uniprot50 | AF-A0A442FIW1-F1-MODEL\_V4 | 1.0 | 5.883e-24 | 597 | 0.181 | 779 | 359 | 32 | 1 | 752 | 182 | 708 | Prim-Pol domain-containing protein | Prim-Pol domain-containing protein | | afdb-uniprot50 | AF-A0A412ETP2-F1-MODEL\_V4 | 1.0 | 1.072e-24 | 597 | 0.177 | 715 | 448 | 44 | 145 | 769 | 97 | 761 | ZnF\_CHCC domain-containing protein | ZnF\_CHCC domain-containing protein | | afdb-uniprot50 | AF-A0A6N3GAZ3-F1-MODEL\_V4 | 1.0 | 2.627e-15 | 596 | 0.206 | 315 | 226 | 14 | 453 | 757 | 22 | 322 | Virulence-associated protein E | Virulence-associated protein E | | afdb-uniprot50 | AF-A0A173U6S3-F1-MODEL\_V4 | 1.0 | 9.179e-17 | 596 | 0.206 | 330 | 237 | 14 | 381 | 699 | 43 | 358 | Predicted P-loop ATPase and inactivated derivatives | Predicted P-loop ATPase and inactivated derivatives | | afdb-uniprot50 | AF-A0A1V5XFC1-F1-MODEL\_V4 | 1.0 | 5.104e-18 | 596 | 0.192 | 432 | 299 | 18 | 368 | 769 | 36 | 447 | Virulence-associated protein E | Virulence-associated protein E | | afdb-uniprot50 | AF-A0A0D7K6F3-F1-MODEL\_V4 | 1.0 | 1.978e-19 | 596 | 0.263 | 436 | 247 | 22 | 370 | 753 | 217 | 630 | Uncharacterized protein | Uncharacterized protein | | afdb-uniprot50 | AF-A0A5R1X1K9-F1-MODEL\_V4 | 1.0 | 2.713e-24 | 596 | 0.179 | 706 | 432 | 42 | 164 | 769 | 179 | 836 | Uncharacterized protein | Uncharacterized protein | | afdb-uniprot50 | AF-A0A5C7RLC4-F1-MODEL\_V4 | 1.0 | 2.137e-15 | 595 | 0.348 | 238 | 128 | 10 | 99 | 333 | 82 | 295 | Toprim domain-containing protein | Toprim domain-containing protein | | afdb-uniprot50 | AF-A0A7J5Q366-F1-MODEL\_V4 | 1.0 | 3.444e-17 | 595 | 0.178 | 381 | 274 | 18 | 393 | 755 | 3 | 362 | DUF3874 domain-containing protein | DUF3874 domain-containing protein | | afdb-uniprot50 | AF-A0A251WKU0-F1-MODEL\_V4 | 1.0 | 1.479e-18 | 595 | 0.223 | 442 | 281 | 18 | 328 | 750 | 175 | 573 | Uncharacterized protein | Uncharacterized protein | | afdb-uniprot50 | AF-A0A3R5ZLK0-F1-MODEL\_V4 | 1.0 | 8.946e-21 | 595 | 0.179 | 485 | 308 | 17 | 259 | 725 | 220 | 632 | Virulence protein E | Virulence protein E | | afdb-uniprot50 | AF-A0A2V5PV48-F1-MODEL\_V4 | 1.0 | 1.196e-21 | 595 | 0.204 | 548 | 341 | 26 | 243 | 762 | 248 | 728 | Prim-Pol domain-containing protein | Prim-Pol domain-containing protein | | afdb-uniprot50 | AF-A0A6I5P4Y0-F1-MODEL\_V4 | 1.0 | 3.627e-25 | 595 | 0.202 | 677 | 409 | 39 | 144 | 751 | 2 | 616 | DUF3854 domain-containing protein | DUF3854 domain-containing protein | | afdb-uniprot50 | AF-Q7NEJ1-F1-MODEL\_V4 | 1.0 | 5.039e-24 | 595 | 0.203 | 663 | 395 | 42 | 179 | 769 | 72 | 673 | Glr3888 protein | Glr3888 protein | | afdb-uniprot50 | AF-A0A3M4XFL8-F1-MODEL\_V4 | 1.0 | 2.766e-15 | 595 | 0.317 | 255 | 144 | 11 | 114 | 358 | 162 | 396 | Putative DNA primase | Putative DNA primase | | afdb-uniprot50 | AF-A0A095ZZV9-F1-MODEL\_V4 | 1.0 | 1.093e-15 | 594 | 0.246 | 264 | 188 | 6 | 467 | 725 | 57 | 314 | Virulence-associated protein E | Virulence-associated protein E | | afdb-uniprot50 | AF-A0A433VS18-F1-MODEL\_V4 | 1.0 | 6.956e-18 | 593 | 0.208 | 394 | 272 | 19 | 381 | 758 | 88 | 457 | Uncharacterized protein | Uncharacterized protein | | afdb-uniprot50 | AF-A0A1H2VE35-F1-MODEL\_V4 | 1.0 | 3.77e-23 | 593 | 0.181 | 688 | 386 | 38 | 142 | 756 | 46 | 629 | Virulence-associated protein E | Virulence-associated protein E | | afdb-uniprot50 | AF-A0A3R6S2F5-F1-MODEL\_V4 | 1.0 | 5.17e-20 | 593 | 0.188 | 521 | 342 | 26 | 261 | 757 | 231 | 694 | Uncharacterized protein | Uncharacterized protein | | afdb-uniprot50 | AF-A0A7G8IZC6-F1-MODEL\_V4 | 1.0 | 9.003e-18 | 592 | 0.212 | 414 | 273 | 20 | 384 | 769 | 4 | 392 | Virulence-associated E family protein | Virulence-associated E family protein | | afdb-uniprot50 | AF-A0A4Q3P7C9-F1-MODEL\_V4 | 1.0 | 3.229e-23 | 592 | 0.162 | 792 | 389 | 35 | 1 | 761 | 94 | 641 | PriCT\_2 domain-containing protein | PriCT\_2 domain-containing protein | | afdb-uniprot50 | AF-A0A0D0QDV7-F1-MODEL\_V4 | 1.0 | 1.396e-21 | 592 | 0.214 | 584 | 355 | 30 | 206 | 762 | 188 | 694 | Putative P-loop ATPase | Putative P-loop ATPase | | afdb-uniprot50 | AF-C7GYW9-F1-MODEL\_V4 | 1.0 | 8.069e-21 | 592 | 0.208 | 562 | 324 | 32 | 257 | 755 | 285 | 788 | Virulence-associated protein E | Virulence-associated protein E | | afdb-uniprot50 | AF-A5Z5J5-F1-MODEL\_V4 | 1.0 | 1.292e-17 | 591 | 0.217 | 377 | 250 | 18 | 420 | 769 | 4 | 362 | Virulence-associated protein E | Virulence-associated protein E | | afdb-uniprot50 | AF-A0A3L8PZN3-F1-MODEL\_V4 | 1.0 | 1.651e-15 | 591 | 0.309 | 255 | 142 | 7 | 118 | 355 | 163 | 400 | Uncharacterized protein | Uncharacterized protein | | afdb-uniprot50 | AF-A0A1M6Q060-F1-MODEL\_V4 | 1.0 | 1.196e-21 | 591 | 0.214 | 536 | 321 | 26 | 260 | 756 | 246 | 720 | Predicted P-loop ATPase and inactivated derivatives | Predicted P-loop ATPase and inactivated derivatives | | afdb-uniprot50 | AF-K9Q2E5-F1-MODEL\_V4 | 1.0 | 2.017e-26 | 591 | 0.185 | 726 | 409 | 39 | 178 | 762 | 78 | 761 | Virulence-associated E family protein | Virulence-associated E family protein | | afdb-uniprot50 | AF-D7I880-F1-MODEL\_V4 | 1.0 | 3.008e-16 | 590 | 0.25 | 272 | 190 | 10 | 491 | 755 | 10 | 274 | Helicase | Helicase | | afdb-uniprot50 | AF-A0A365U406-F1-MODEL\_V4 | 1.0 | 8.441e-16 | 590 | 0.377 | 236 | 120 | 9 | 128 | 358 | 140 | 353 | Toprim domain-containing protein | Toprim domain-containing protein | | afdb-uniprot50 | AF-A0A8B3N3Z6-F1-MODEL\_V4 | 1.0 | 1.309e-19 | 590 | 0.193 | 491 | 327 | 24 | 306 | 751 | 22 | 488 | Uncharacterized protein | Uncharacterized protein | | afdb-uniprot50 | AF-A0A5C7PH59-F1-MODEL\_V4 | 1.0 | 6.522e-24 | 590 | 0.188 | 691 | 405 | 41 | 164 | 756 | 33 | 665 | Uncharacterized protein | Uncharacterized protein | | afdb-uniprot50 | AF-A0A2T1G467-F1-MODEL\_V4 | 1.0 | 3.721e-21 | 590 | 0.208 | 546 | 340 | 28 | 260 | 769 | 334 | 823 | Uncharacterized protein | Uncharacterized protein | | afdb-uniprot50 | AF-A0A826KI04-F1-MODEL\_V4 | 1.0 | 2.069e-14 | 589 | 0.323 | 235 | 138 | 8 | 115 | 346 | 161 | 377 | Uncharacterized protein | Uncharacterized protein | | afdb-uniprot50 | AF-A0A353UWV3-F1-MODEL\_V4 | 1.0 | 4.261e-22 | 589 | 0.199 | 626 | 365 | 32 | 226 | 752 | 19 | 607 | P-loop ATPase | P-loop ATPase | | afdb-uniprot50 | AF-A0A2P8WI05-F1-MODEL\_V4 | 1.0 | 3.25e-20 | 589 | 0.21 | 512 | 336 | 21 | 275 | 760 | 16 | 485 | Uncharacterized protein | Uncharacterized protein | | afdb-uniprot50 | AF-A0A4U6M2C1-F1-MODEL\_V4 | 1.0 | 1.773e-30 | 589 | 0.198 | 872 | 447 | 51 | 91 | 751 | 77 | 907 | Bifunctional DNA primase/helicase | Bifunctional DNA primase/helicase | | afdb-uniprot50 | AF-A0A1U7IM20-F1-MODEL\_V4 | 1.0 | 1.489e-23 | 589 | 0.186 | 648 | 425 | 38 | 159 | 759 | 62 | 654 | Uncharacterized protein | Uncharacterized protein | | afdb-uniprot50 | AF-G2KRX2-F1-MODEL\_V4 | 1.0 | 1.3e-14 | 588 | 0.303 | 237 | 143 | 7 | 100 | 335 | 73 | 288 | Toprim domain-containing protein | Toprim domain-containing protein | | afdb-uniprot50 | AF-A0A0D6Q293-F1-MODEL\_V4 | 1.0 | 8.441e-16 | 588 | 0.213 | 342 | 241 | 12 | 370 | 691 | 22 | 355 | DNA primase | DNA primase | | afdb-uniprot50 | AF-A0A1E4C277-F1-MODEL\_V4 | 1.0 | 3.147e-19 | 588 | 0.208 | 495 | 329 | 26 | 292 | 756 | 7 | 468 | Uncharacterized protein | Uncharacterized protein | | afdb-uniprot50 | AF-A0A016XJH4-F1-MODEL\_V4 | 1.0 | 6.076e-25 | 588 | 0.179 | 742 | 428 | 44 | 160 | 769 | 50 | 742 | Conjugal transfer protein TraC | Conjugal transfer protein TraC | | afdb-uniprot50 | AF-A0A2T5NFF6-F1-MODEL\_V4 | 1.0 | 2.294e-30 | 588 | 0.197 | 856 | 478 | 46 | 12 | 737 | 5 | 781 | Uncharacterized protein | Uncharacterized protein | | afdb-uniprot50 | AF-A0A6L9H472-F1-MODEL\_V4 | 1.0 | 1.99e-16 | 586 | 0.254 | 287 | 195 | 11 | 494 | 769 | 2 | 280 | Uncharacterized protein | Uncharacterized protein | | afdb-uniprot50 | AF-A0A2N0AT84-F1-MODEL\_V4 | 1.0 | 1.378e-19 | 586 | 0.174 | 481 | 336 | 25 | 311 | 755 | 30 | 485 | Uncharacterized protein | Uncharacterized protein | | afdb-uniprot50 | AF-A0A846FT90-F1-MODEL\_V4 | 1.0 | 9.855e-24 | 586 | 0.202 | 636 | 384 | 32 | 170 | 743 | 72 | 645 | DUF3854 domain-containing protein | DUF3854 domain-containing protein | | afdb-uniprot50 | AF-A0A807K447-F1-MODEL\_V4 | 1.0 | 1.903e-21 | 586 | 0.191 | 573 | 347 | 30 | 239 | 769 | 201 | 698 | Uncharacterized protein | Uncharacterized protein | | afdb-uniprot50 | AF-A0A1M6RY44-F1-MODEL\_V4 | 1.0 | 3.603e-20 | 586 | 0.157 | 519 | 362 | 22 | 260 | 752 | 253 | 721 | VirE N-terminal domain-containing protein | VirE N-terminal domain-containing protein | | afdb-uniprot50 | AF-A0A239LDP2-F1-MODEL\_V4 | 1.0 | 1.038e-23 | 586 | 0.18 | 799 | 374 | 31 | 2 | 769 | 197 | 745 | Predicted P-loop ATPase and inactivated derivatives | Predicted P-loop ATPase and inactivated derivatives | | afdb-uniprot50 | AF-G5SU78-F1-MODEL\_V4 | 1.0 | 2.016e-18 | 585 | 0.187 | 447 | 320 | 24 | 327 | 755 | 2 | 423 | DUF3874 domain-containing protein | DUF3874 domain-containing protein | | afdb-uniprot50 | AF-A0A7X5C8R8-F1-MODEL\_V4 | 1.0 | 9.604e-20 | 585 | 0.179 | 518 | 359 | 25 | 288 | 769 | 23 | 510 | Uncharacterized protein | Uncharacterized protein | | afdb-uniprot50 | AF-A0A1Z4SI32-F1-MODEL\_V4 | 1.0 | 2.447e-24 | 585 | 0.189 | 692 | 416 | 37 | 139 | 756 | 37 | 657 | Virulence-associated E family protein | Virulence-associated E family protein | | afdb-uniprot50 | AF-A0A2D3TGD8-F1-MODEL\_V4 | 1.0 | 1.005e-14 | 585 | 0.335 | 200 | 114 | 6 | 149 | 346 | 6 | 188 | LPD7 domain-containing protein | LPD7 domain-containing protein | | afdb-uniprot50 | AF-A0A1I7KD97-F1-MODEL\_V4 | 1.0 | 6.356e-20 | 585 | 0.201 | 502 | 332 | 26 | 311 | 761 | 295 | 778 | Bifunctional DNA primase/polymerase, N-terminal | Bifunctional DNA primase/polymerase, N-terminal | | afdb-uniprot50 | AF-A0A838BUZ2-F1-MODEL\_V4 | 1.0 | 2.4e-25 | 585 | 0.178 | 773 | 393 | 46 | 167 | 759 | 137 | 847 | Uncharacterized protein | Uncharacterized protein | | afdb-uniprot50 | AF-A0A2T5L4M6-F1-MODEL\_V4 | 1.0 | 7.002e-31 | 585 | 0.204 | 927 | 445 | 51 | 100 | 769 | 78 | 968 | Putative DNA primase/helicase | Putative DNA primase/helicase | | afdb-uniprot50 | AF-A0A0F2P8W8-F1-MODEL\_V4 | 1.0 | 1.005e-14 | 584 | 0.297 | 235 | 143 | 6 | 106 | 338 | 61 | 275 | Toprim domain-containing protein | Toprim domain-containing protein | | afdb-uniprot50 | AF-K1V3K5-F1-MODEL\_V4 | 1.0 | 1.538e-16 | 584 | 0.23 | 339 | 228 | 15 | 436 | 753 | 9 | 335 | Virulence-associated E | Virulence-associated E | | afdb-uniprot50 | AF-A0A556QUB3-F1-MODEL\_V4 | 1.0 | 1.011e-19 | 584 | 0.181 | 517 | 345 | 30 | 267 | 755 | 25 | 491 | Uncharacterized protein | Uncharacterized protein | | afdb-uniprot50 | AF-X5J840-F1-MODEL\_V4 | 1.0 | 1.738e-23 | 584 | 0.159 | 658 | 433 | 41 | 154 | 755 | 162 | 755 | Virulence-associated E family protein | Virulence-associated E family protein | | afdb-uniprot50 | AF-A0A6J4IEE4-F1-MODEL\_V4 | 1.0 | 1.651e-15 | 583 | 0.223 | 300 | 211 | 10 | 463 | 757 | 17 | 299 | Virulence-associated E | Virulence-associated E | | afdb-uniprot50 | AF-A0A2N5C915-F1-MODEL\_V4 | 1.0 | 1.275e-15 | 583 | 0.337 | 240 | 124 | 9 | 122 | 347 | 78 | 296 | Toprim domain-containing protein | Toprim domain-containing protein | | afdb-uniprot50 | AF-A0A430HF59-F1-MODEL\_V4 | 1.0 | 1.204e-18 | 583 | 0.292 | 352 | 193 | 15 | 10 | 347 | 1 | 310 | Uncharacterized protein | Uncharacterized protein | | afdb-uniprot50 | AF-A0A4S2GDP1-F1-MODEL\_V4 | 1.0 | 4.515e-19 | 583 | 0.217 | 478 | 278 | 23 | 370 | 769 | 35 | 494 | Virulence-associated E family protein | Virulence-associated E family protein | | afdb-uniprot50 | AF-A0A0U2U1R7-F1-MODEL\_V4 | 1.0 | 1.204e-18 | 583 | 0.203 | 467 | 282 | 25 | 366 | 761 | 50 | 497 | Uncharacterized protein | Uncharacterized protein | | afdb-uniprot50 | AF-A0A2S5MV44-F1-MODEL\_V4 | 1.0 | 2.56e-19 | 583 | 0.178 | 505 | 332 | 20 | 261 | 750 | 15 | 451 | Uncharacterized protein | Uncharacterized protein | | afdb-uniprot50 | AF-A0A317MT75-F1-MODEL\_V4 | 1.0 | 8.496e-21 | 583 | 0.18 | 538 | 348 | 28 | 268 | 763 | 251 | 737 | DNA primase RepB-like protein | DNA primase RepB-like protein | | afdb-uniprot50 | AF-A0A255TPW4-F1-MODEL\_V4 | 1.0 | 3.534e-21 | 583 | 0.181 | 572 | 342 | 26 | 241 | 755 | 225 | 727 | VirE\_N domain-containing protein | VirE\_N domain-containing protein | | afdb-uniprot50 | AF-A0A353X8G7-F1-MODEL\_V4 | 1.0 | 5.997e-15 | 582 | 0.296 | 243 | 141 | 6 | 91 | 333 | 67 | 279 | Toprim domain-containing protein | Toprim domain-containing protein | | afdb-uniprot50 | AF-A0A433HYK3-F1-MODEL\_V4 | 1.0 | 5.374e-18 | 582 | 0.22 | 421 | 259 | 18 | 395 | 755 | 4 | 415 | Uncharacterized protein | Uncharacterized protein | | afdb-uniprot50 | AF-A0A844MXR4-F1-MODEL\_V4 | 1.0 | 2.61e-18 | 582 | 0.231 | 415 | 260 | 21 | 367 | 753 | 430 | 813 | Uncharacterized protein | Uncharacterized protein | | afdb-uniprot50 | AF-A0A6P0YU45-F1-MODEL\_V4 | 1.0 | 1.317e-16 | 581 | 0.249 | 341 | 223 | 17 | 438 | 762 | 43 | 366 | Uncharacterized protein | Uncharacterized protein | | afdb-uniprot50 | AF-A0A551Z1W1-F1-MODEL\_V4 | 1.0 | 3.271e-25 | 581 | 0.169 | 698 | 410 | 37 | 163 | 762 | 50 | 675 | Uncharacterized protein | Uncharacterized protein | | afdb-uniprot50 | AF-A0A1F4ANV0-F1-MODEL\_V4 | 1.0 | 2.043e-20 | 580 | 0.19 | 567 | 347 | 30 | 246 | 753 | 221 | 734 | Uncharacterized protein | Uncharacterized protein | | afdb-uniprot50 | AF-A0A2T5K521-F1-MODEL\_V4 | 1.0 | 2.369e-23 | 580 | 0.195 | 664 | 417 | 38 | 181 | 762 | 143 | 770 | Putative P-loop ATPase | Putative P-loop ATPase | | afdb-uniprot50 | AF-A0A1Y6D7X7-F1-MODEL\_V4 | 1.0 | 1.965e-30 | 580 | 0.179 | 1009 | 512 | 53 | 11 | 756 | 8 | 963 | Putative DNA primase/helicase | Putative DNA primase/helicase | | afdb-uniprot50 | AF-A0A374J606-F1-MODEL\_V4 | 1.0 | 4.316e-24 | 579 | 0.189 | 690 | 428 | 43 | 149 | 769 | 81 | 707 | Uncharacterized protein | Uncharacterized protein | | afdb-uniprot50 | AF-A0A2T4IDI9-F1-MODEL\_V4 | 1.0 | 6.316e-31 | 579 | 0.186 | 998 | 495 | 53 | 1 | 751 | 1 | 928 | Uncharacterized protein | Uncharacterized protein | | afdb-uniprot50 | AF-A0A5U2TXN0-F1-MODEL\_V4 | 1.0 | 1.173e-14 | 579 | 0.28 | 250 | 157 | 8 | 116 | 358 | 4 | 237 | LPD7 domain-containing protein | LPD7 domain-containing protein | | afdb-uniprot50 | AF-A0A6G3Z197-F1-MODEL\_V4 | 1.0 | 1.568e-23 | 578 | 0.201 | 649 | 405 | 35 | 169 | 766 | 74 | 659 | Uncharacterized protein | Uncharacterized protein | | afdb-uniprot50 | AF-U7QLA3-F1-MODEL\_V4 | 1.0 | 4.316e-24 | 578 | 0.17 | 687 | 410 | 40 | 166 | 769 | 99 | 708 | Virulence-associated E family protein | Virulence-associated E family protein | | afdb-uniprot50 | AF-A0A317N8C0-F1-MODEL\_V4 | 1.0 | 1.079e-29 | 578 | 0.205 | 798 | 452 | 47 | 92 | 760 | 87 | 831 | Phage/plasmid primase-like uncharacterized protein | Phage/plasmid primase-like uncharacterized protein | | afdb-uniprot50 | AF-A0A1M4ZAH0-F1-MODEL\_V4 | 1.0 | 2.056e-17 | 577 | 0.184 | 391 | 284 | 17 | 381 | 755 | 41 | 412 | Virulence-associated protein E | Virulence-associated protein E | | afdb-uniprot50 | AF-A0A840ASX3-F1-MODEL\_V4 | 1.0 | 2.431e-19 | 577 | 0.198 | 525 | 341 | 22 | 261 | 769 | 257 | 717 | Putative P-loop ATPase | Putative P-loop ATPase | | afdb-uniprot50 | AF-A0A522CQB3-F1-MODEL\_V4 | 1.0 | 1.672e-17 | 577 | 0.195 | 414 | 276 | 25 | 370 | 750 | 378 | 767 | Prim-Pol domain-containing protein | Prim-Pol domain-containing protein | | afdb-uniprot50 | AF-A0A840RUQ5-F1-MODEL\_V4 | 1.0 | 6.956e-18 | 576 | 0.279 | 354 | 195 | 17 | 10 | 347 | 1 | 310 | Putative DNA primase/helicase | Putative DNA primase/helicase | | afdb-uniprot50 | AF-A8SSN2-F1-MODEL\_V4 | 1.0 | 2.527e-17 | 576 | 0.227 | 378 | 245 | 19 | 420 | 769 | 4 | 362 | Virulence-associated protein E | Virulence-associated protein E | | afdb-uniprot50 | AF-A0A828JM73-F1-MODEL\_V4 | 1.0 | 1.3e-14 | 576 | 0.306 | 251 | 152 | 9 | 100 | 346 | 83 | 315 | Uncharacterized protein | Uncharacterized protein | | afdb-uniprot50 | AF-A0A1M5CP24-F1-MODEL\_V4 | 1.0 | 1.578e-20 | 576 | 0.172 | 539 | 348 | 27 | 240 | 751 | 220 | 687 | Uncharacterized protein | Uncharacterized protein | | afdb-uniprot50 | AF-A0A218QNW9-F1-MODEL\_V4 | 1.0 | 9.18e-25 | 575 | 0.177 | 756 | 457 | 36 | 89 | 757 | 8 | 685 | Virulence-associated E family protein | Virulence-associated E family protein | | afdb-uniprot50 | AF-A0A414TCF4-F1-MODEL\_V4 | 1.0 | 1.705e-16 | 574 | 0.21 | 346 | 231 | 16 | 457 | 769 | 2 | 338 | Uncharacterized protein | Uncharacterized protein | | afdb-uniprot50 | AF-A0A2N7QRK2-F1-MODEL\_V4 | 1.0 | 5.238e-30 | 574 | 0.197 | 779 | 435 | 42 | 100 | 739 | 99 | 825 | DNA primase TraC | DNA primase TraC | | afdb-uniprot50 | AF-A0A6L8LFI5-F1-MODEL\_V4 | 1.0 | 1.83e-23 | 573 | 0.177 | 776 | 357 | 33 | 1 | 750 | 212 | 731 | PriCT\_2 domain-containing protein | PriCT\_2 domain-containing protein | | afdb-uniprot50 | AF-A0A085TW06-F1-MODEL\_V4 | 1.0 | 6.438e-22 | 573 | 0.17 | 778 | 360 | 28 | 1 | 750 | 215 | 734 | PriCT\_2 domain-containing protein | PriCT\_2 domain-containing protein | | afdb-uniprot50 | AF-A0A3N7RH32-F1-MODEL\_V4 | 1.0 | 7.915e-30 | 573 | 0.192 | 872 | 478 | 46 | 8 | 737 | 2 | 788 | DUF927 domain-containing protein | DUF927 domain-containing protein | | afdb-uniprot50 | AF-A0A0P9QTD8-F1-MODEL\_V4 | 1.0 | 6.114e-14 | 573 | 0.327 | 214 | 122 | 8 | 136 | 346 | 3 | 197 | LPD7 domain-containing protein | LPD7 domain-containing protein | | afdb-uniprot50 | AF-A0A1H7HXF3-F1-MODEL\_V4 | 1.0 | 1.854e-17 | 572 | 0.211 | 387 | 265 | 17 | 391 | 758 | 2 | 367 | Virulence-associated protein E | Virulence-associated protein E | | afdb-uniprot50 | AF-A0A6H9P0V4-F1-MODEL\_V4 | 1.0 | 2.25e-15 | 571 | 0.2 | 300 | 220 | 13 | 467 | 757 | 1 | 289 | Helicase | Helicase | | afdb-uniprot50 | AF-A0A641UPF6-F1-MODEL\_V4 | 1.0 | 6.521e-16 | 571 | 0.208 | 321 | 231 | 13 | 381 | 691 | 61 | 368 | Helicase | Helicase | | afdb-uniprot50 | AF-A0A5C7CZ33-F1-MODEL\_V4 | 1.0 | 4.316e-16 | 571 | 0.285 | 284 | 157 | 11 | 82 | 358 | 149 | 393 | Uncharacterized protein | Uncharacterized protein | | afdb-uniprot50 | AF-A0A3M5UMZ6-F1-MODEL\_V4 | 1.0 | 8.173e-15 | 571 | 0.313 | 255 | 145 | 11 | 114 | 358 | 366 | 600 | DUF5710 domain-containing protein | DUF5710 domain-containing protein | | afdb-uniprot50 | AF-A0A3D5KKU3-F1-MODEL\_V4 | 1.0 | 1.538e-16 | 570 | 0.185 | 398 | 274 | 21 | 384 | 758 | 20 | 390 | Uncharacterized protein | Uncharacterized protein | | afdb-uniprot50 | AF-A0A2V4UFM1-F1-MODEL\_V4 | 1.0 | 1.807e-29 | 570 | 0.197 | 789 | 447 | 40 | 13 | 744 | 9 | 667 | Phage/plasmid primase-like uncharacterized protein | Phage/plasmid primase-like uncharacterized protein | | afdb-uniprot50 | AF-A0A370NG87-F1-MODEL\_V4 | 1.0 | 1.284e-28 | 570 | 0.202 | 771 | 451 | 43 | 101 | 768 | 82 | 791 | SF3 helicase domain-containing protein | SF3 helicase domain-containing protein | | afdb-uniprot50 | AF-Q1DD05-F1-MODEL\_V4 | 1.0 | 1.396e-21 | 570 | 0.21 | 557 | 348 | 30 | 239 | 757 | 339 | 841 | Virulence-associated protein E domain protein | Virulence-associated protein E domain protein | | afdb-uniprot50 | AF-A0A1G4SVX6-F1-MODEL\_V4 | 1.0 | 2.193e-19 | 569 | 0.209 | 526 | 321 | 22 | 261 | 762 | 264 | 718 | Virulence-associated protein E | Virulence-associated protein E | | afdb-uniprot50 | AF-A0A6I5Q3V8-F1-MODEL\_V4 | 1.0 | 1.058e-22 | 569 | 0.186 | 634 | 403 | 34 | 179 | 751 | 75 | 656 | Uncharacterized protein | Uncharacterized protein | | afdb-uniprot50 | AF-A0A374TFD7-F1-MODEL\_V4 | 1.0 | 1.243e-19 | 569 | 0.164 | 534 | 339 | 28 | 261 | 756 | 269 | 733 | VirE\_N domain-containing protein | VirE\_N domain-containing protein | | afdb-uniprot50 | AF-A0A0C1Y5L2-F1-MODEL\_V4 | 1.0 | 6.397e-17 | 567 | 0.205 | 385 | 261 | 18 | 393 | 754 | 45 | 407 | Uncharacterized protein | Uncharacterized protein | | afdb-uniprot50 | AF-A0A841VQI7-F1-MODEL\_V4 | 1.0 | 1.251e-24 | 567 | 0.176 | 713 | 400 | 42 | 159 | 769 | 27 | 653 | Uncharacterized protein | Uncharacterized protein | | afdb-uniprot50 | AF-A8SVC7-F1-MODEL\_V4 | 1.0 | 1.317e-16 | 565 | 0.224 | 356 | 233 | 17 | 441 | 769 | 2 | 341 | Virulence-associated protein E | Virulence-associated protein E | | afdb-uniprot50 | AF-A0A2V2GG06-F1-MODEL\_V4 | 1.0 | 7.001e-15 | 564 | 0.203 | 310 | 222 | 11 | 453 | 751 | 17 | 312 | Helicase | Helicase | | afdb-uniprot50 | AF-J9DI45-F1-MODEL\_V4 | 1.0 | 4.344e-21 | 564 | 0.172 | 625 | 378 | 32 | 183 | 751 | 119 | 660 | Prim-Pol domain-containing protein | Prim-Pol domain-containing protein | | afdb-uniprot50 | AF-A0A829YB94-F1-MODEL\_V4 | 1.0 | 3.65e-22 | 564 | 0.16 | 790 | 366 | 25 | 1 | 757 | 230 | 754 | Uncharacterized protein | Uncharacterized protein | | afdb-uniprot50 | AF-A0A2W6ZVY5-F1-MODEL\_V4 | 1.0 | 1.651e-23 | 564 | 0.21 | 657 | 395 | 34 | 179 | 755 | 164 | 776 | Prim\_Zn\_Ribbon domain-containing protein | Prim\_Zn\_Ribbon domain-containing protein | | afdb-uniprot50 | AF-A0A1H9F653-F1-MODEL\_V4 | 1.0 | 1.219e-28 | 564 | 0.206 | 794 | 449 | 39 | 102 | 769 | 126 | 864 | Uncharacterized domain associated with phage/plasmid primase | Uncharacterized domain associated with phage/plasmid primase | | afdb-uniprot50 | AF-A0A359D3R9-F1-MODEL\_V4 | 1.0 | 9.239e-22 | 563 | 0.144 | 763 | 389 | 29 | 12 | 757 | 189 | 704 | Uncharacterized protein | Uncharacterized protein | | afdb-uniprot50 | AF-A0A3E4N9U9-F1-MODEL\_V4 | 1.0 | 4.847e-18 | 562 | 0.17 | 441 | 336 | 12 | 330 | 756 | 21 | 445 | Helicase | Helicase | | afdb-uniprot50 | AF-A0A5C7Q0U9-F1-MODEL\_V4 | 1.0 | 8.017e-24 | 562 | 0.178 | 796 | 426 | 40 | 2 | 757 | 216 | 823 | PriCT\_2 domain-containing protein | PriCT\_2 domain-containing protein | | afdb-uniprot50 | AF-A0A1F1G801-F1-MODEL\_V4 | 1.0 | 3.208e-18 | 561 | 0.204 | 411 | 266 | 19 | 407 | 769 | 158 | 555 | Uncharacterized protein | Uncharacterized protein | | afdb-uniprot50 | AF-A0A5C7M7G3-F1-MODEL\_V4 | 1.0 | 1.683e-22 | 561 | 0.172 | 685 | 416 | 37 | 143 | 760 | 86 | 686 | Prim-Pol domain-containing protein | Prim-Pol domain-containing protein | | afdb-uniprot50 | AF-A0A844DJS6-F1-MODEL\_V4 | 1.0 | 1.018e-16 | 560 | 0.218 | 376 | 246 | 18 | 423 | 769 | 3 | 359 | Conjugal transfer protein TraC | Conjugal transfer protein TraC | | afdb-uniprot50 | AF-A0A560FLU5-F1-MODEL\_V4 | 1.0 | 4.457e-17 | 560 | 0.2 | 429 | 291 | 21 | 379 | 769 | 4 | 418 | Virulence-associated protein E | Virulence-associated protein E | | afdb-uniprot50 | AF-R4WX46-F1-MODEL\_V4 | 1.0 | 2.463e-29 | 560 | 0.196 | 870 | 472 | 50 | 6 | 737 | 1 | 781 | Uncharacterized protein | Uncharacterized protein | | afdb-uniprot50 | AF-A0A433W3D6-F1-MODEL\_V4 | 1.0 | 1.181e-19 | 559 | 0.197 | 526 | 312 | 23 | 261 | 757 | 221 | 665 | RepB\_primase domain-containing protein | RepB\_primase domain-containing protein | | afdb-uniprot50 | AF-A0A1Q4BTB8-F1-MODEL\_V4 | 1.0 | 1.423e-20 | 559 | 0.186 | 568 | 343 | 32 | 246 | 769 | 266 | 758 | PriCT\_2 domain-containing protein | PriCT\_2 domain-containing protein | | afdb-uniprot50 | AF-A0A7X3VTX1-F1-MODEL\_V4 | 1.0 | 2.165e-17 | 558 | 0.223 | 421 | 272 | 22 | 370 | 753 | 32 | 434 | Uncharacterized protein | Uncharacterized protein | | afdb-uniprot50 | AF-A0A6L8GF99-F1-MODEL\_V4 | 1.0 | 1.672e-17 | 558 | 0.179 | 423 | 283 | 23 | 379 | 753 | 71 | 477 | Uncharacterized protein | Uncharacterized protein | | afdb-uniprot50 | AF-A0A435FA41-F1-MODEL\_V4 | 1.0 | 2.931e-20 | 558 | 0.193 | 543 | 331 | 33 | 260 | 751 | 141 | 627 | Uncharacterized protein | Uncharacterized protein | | afdb-uniprot50 | AF-A0A3D5P9B6-F1-MODEL\_V4 | 1.0 | 1.528e-19 | 558 | 0.194 | 567 | 346 | 34 | 257 | 757 | 290 | 811 | Virulence-associated protein E | Virulence-associated protein E | | afdb-uniprot50 | AF-A0A6A7WE66-F1-MODEL\_V4 | 1.0 | 1.086e-18 | 557 | 0.145 | 530 | 351 | 29 | 261 | 755 | 275 | 737 | VirE\_N domain-containing protein | VirE\_N domain-containing protein | | afdb-uniprot50 | AF-A0A094YPP6-F1-MODEL\_V4 | 1.0 | 7.914e-22 | 557 | 0.165 | 778 | 356 | 30 | 12 | 752 | 243 | 763 | Virulence-associated E | Virulence-associated E | | afdb-uniprot50 | AF-A0A4P5SJT8-F1-MODEL\_V4 | 1.0 | 1.965e-14 | 556 | 0.327 | 235 | 122 | 11 | 129 | 346 | 229 | 444 | Toprim domain-containing protein | Toprim domain-containing protein | | afdb-uniprot50 | AF-A0A357LIR7-F1-MODEL\_V4 | 1.0 | 5.844e-19 | 556 | 0.176 | 567 | 337 | 32 | 239 | 767 | 32 | 506 | Uncharacterized protein | Uncharacterized protein | | afdb-uniprot50 | AF-E0U6Z0-F1-MODEL\_V4 | 1.0 | 2.495e-23 | 556 | 0.176 | 680 | 403 | 36 | 177 | 757 | 64 | 685 | Virulence-associated E family protein | Virulence-associated E family protein | | afdb-uniprot50 | AF-D6E493-F1-MODEL\_V4 | 1.0 | 8.279e-17 | 555 | 0.214 | 401 | 270 | 18 | 394 | 769 | 2 | 382 | Predicted P-loop ATPase and inactivated derivatives | Predicted P-loop ATPase and inactivated derivatives | | afdb-uniprot50 | AF-A0A7G7KQ79-F1-MODEL\_V4 | 1.0 | 9.48e-18 | 554 | 0.197 | 476 | 327 | 27 | 318 | 769 | 1 | 445 | Uncharacterized protein | Uncharacterized protein | | afdb-uniprot50 | AF-A0A2E4ID67-F1-MODEL\_V4 | 1.0 | 2.527e-17 | 553 | 0.184 | 422 | 302 | 17 | 370 | 768 | 29 | 431 | Uncharacterized protein | Uncharacterized protein | | afdb-uniprot50 | AF-A0A1I5CW14-F1-MODEL\_V4 | 1.0 | 5.444e-20 | 553 | 0.185 | 538 | 355 | 27 | 246 | 757 | 116 | 596 | Predicted P-loop ATPase and inactivated derivatives | Predicted P-loop ATPase and inactivated derivatives | | afdb-uniprot50 | AF-A0A7J5ET25-F1-MODEL\_V4 | 1.0 | 1.489e-23 | 553 | 0.168 | 830 | 421 | 34 | 1 | 752 | 222 | 859 | Prim-Pol domain-containing protein | Prim-Pol domain-containing protein | | afdb-uniprot50 | AF-A0A7Z2G310-F1-MODEL\_V4 | 1.0 | 4.515e-19 | 552 | 0.192 | 519 | 355 | 22 | 264 | 756 | 266 | 746 | Uncharacterized protein | Uncharacterized protein | | afdb-uniprot50 | AF-G5QKM5-F1-MODEL\_V4 | 1.0 | 8.173e-15 | 552 | 0.273 | 274 | 162 | 10 | 99 | 358 | 138 | 388 | DNA primase traC | DNA primase traC | | afdb-uniprot50 | AF-A0A255SPK4-F1-MODEL\_V4 | 1.0 | 1.334e-18 | 550 | 0.208 | 418 | 265 | 16 | 263 | 668 | 241 | 604 | VirE\_N domain-containing protein | VirE\_N domain-containing protein | | afdb-uniprot50 | AF-A0A843FDY4-F1-MODEL\_V4 | 1.0 | 2.096e-16 | 549 | 0.178 | 410 | 273 | 18 | 396 | 769 | 2 | 383 | Uncharacterized protein | Uncharacterized protein | | afdb-uniprot50 | AF-D6YV80-F1-MODEL\_V4 | 1.0 | 1.738e-15 | 549 | 0.307 | 267 | 151 | 13 | 92 | 347 | 82 | 325 | Uncharacterized protein | Uncharacterized protein | | afdb-uniprot50 | AF-A0A2V2SG54-F1-MODEL\_V4 | 1.0 | 1.079e-21 | 549 | 0.165 | 791 | 378 | 28 | 1 | 757 | 223 | 765 | Prim-Pol domain-containing protein | Prim-Pol domain-containing protein | | afdb-uniprot50 | AF-A0A542BG12-F1-MODEL\_V4 | 1.0 | 1.196e-13 | 549 | 0.316 | 237 | 138 | 10 | 114 | 346 | 160 | 376 | Putative DNA primase/helicase | Putative DNA primase/helicase | | afdb-uniprot50 | AF-A0A7J0AZE4-F1-MODEL\_V4 | 1.0 | 1.204e-18 | 548 | 0.207 | 482 | 277 | 29 | 370 | 769 | 134 | 592 | Uncharacterized protein | Uncharacterized protein | | afdb-uniprot50 | AF-A0A081RVY4-F1-MODEL\_V4 | 1.0 | 1.284e-20 | 548 | 0.199 | 547 | 333 | 31 | 261 | 753 | 247 | 742 | Putative P-loop ATPase | Putative P-loop ATPase | | afdb-uniprot50 | AF-A0A828NJX6-F1-MODEL\_V4 | 1.0 | 1.173e-14 | 548 | 0.272 | 268 | 163 | 11 | 90 | 346 | 126 | 372 | DNA primase | DNA primase | | afdb-uniprot50 | AF-A0A1Y6D1S4-F1-MODEL\_V4 | 1.0 | 2.221e-29 | 548 | 0.195 | 883 | 482 | 51 | 10 | 739 | 52 | 858 | Putative DNA primase/helicase | Putative DNA primase/helicase | | afdb-uniprot50 | AF-A0A4Q7EAM6-F1-MODEL\_V4 | 1.0 | 3.008e-16 | 547 | 0.206 | 383 | 259 | 18 | 395 | 754 | 133 | 493 | Uncharacterized protein | Uncharacterized protein | | afdb-uniprot50 | AF-A0A5C4LBI4-F1-MODEL\_V4 | 1.0 | 1.196e-21 | 547 | 0.162 | 700 | 402 | 31 | 150 | 752 | 74 | 685 | Prim-Pol domain-containing protein | Prim-Pol domain-containing protein | | afdb-uniprot50 | AF-A0A2N3BD34-F1-MODEL\_V4 | 1.0 | 6.397e-25 | 547 | 0.181 | 834 | 432 | 45 | 1 | 671 | 7 | 752 | P-loop ATPase | P-loop ATPase | | afdb-uniprot50 | AF-A0A1H6V3P5-F1-MODEL\_V4 | 1.0 | 1.599e-22 | 547 | 0.165 | 792 | 367 | 31 | 1 | 755 | 235 | 769 | Primase C terminal 2 (PriCT-2) | Primase C terminal 2 (PriCT-2) | | afdb-uniprot50 | AF-A0A1Q4GUC4-F1-MODEL\_V4 | 1.0 | 4.126e-29 | 547 | 0.193 | 888 | 418 | 48 | 11 | 731 | 1 | 756 | PriCT\_2 domain-containing protein | PriCT\_2 domain-containing protein | | afdb-uniprot50 | AF-A0A6P0ZFJ0-F1-MODEL\_V4 | 1.0 | 1.705e-24 | 546 | 0.172 | 703 | 432 | 40 | 143 | 762 | 18 | 653 | DUF3854 domain-containing protein | DUF3854 domain-containing protein | | afdb-uniprot50 | AF-A0A7L9BQY4-F1-MODEL\_V4 | 1.0 | 1.578e-20 | 546 | 0.202 | 568 | 345 | 31 | 260 | 762 | 230 | 754 | Uncharacterized protein | Uncharacterized protein | | afdb-uniprot50 | AF-A0A1H6K2S3-F1-MODEL\_V4 | 1.0 | 4.099e-16 | 545 | 0.299 | 297 | 149 | 13 | 68 | 347 | 488 | 742 | Uncharacterized domain associated with phage/plasmid primase | Uncharacterized domain associated with phage/plasmid primase | | afdb-uniprot50 | AF-A0A371XDT2-F1-MODEL\_V4 | 1.0 | 3.489e-19 | 545 | 0.197 | 532 | 330 | 22 | 261 | 756 | 295 | 765 | Uncharacterized protein | Uncharacterized protein | | afdb-uniprot50 | AF-A0A1H0EEJ0-F1-MODEL\_V4 | 1.0 | 7.467e-17 | 544 | 0.188 | 409 | 275 | 23 | 382 | 755 | 24 | 410 | Virulence-associated protein E | Virulence-associated protein E | | afdb-uniprot50 | AF-A0A3Y9C6Z3-F1-MODEL\_V4 | 1.0 | 1.3e-14 | 544 | 0.279 | 265 | 153 | 10 | 98 | 346 | 134 | 376 | DUF5710 domain-containing protein | DUF5710 domain-containing protein | | afdb-uniprot50 | AF-A0A367RLN6-F1-MODEL\_V4 | 1.0 | 1.761e-17 | 544 | 0.202 | 449 | 268 | 19 | 381 | 758 | 100 | 529 | Uncharacterized protein | Uncharacterized protein | | afdb-uniprot50 | AF-A0A1I4C3P1-F1-MODEL\_V4 | 1.0 | 2.731e-29 | 544 | 0.19 | 765 | 421 | 39 | 91 | 751 | 72 | 741 | Putative DNA primase/helicase | Putative DNA primase/helicase | | afdb-uniprot50 | AF-W2CQM4-F1-MODEL\_V4 | 1.0 | 1.489e-15 | 543 | 0.236 | 288 | 196 | 12 | 379 | 652 | 4 | 281 | Virulence-associated protein E | Virulence-associated protein E | | afdb-uniprot50 | AF-A0A098C1H9-F1-MODEL\_V4 | 1.0 | 2.369e-15 | 543 | 0.233 | 304 | 206 | 13 | 384 | 674 | 14 | 303 | Virulence-associated E family protein | Virulence-associated E family protein | | afdb-uniprot50 | AF-A0A4V1YW25-F1-MODEL\_V4 | 1.0 | 2.627e-15 | 543 | 0.281 | 288 | 187 | 9 | 370 | 647 | 31 | 308 | Virulence-associated E family protein | Virulence-associated E family protein | | afdb-uniprot50 | AF-A0A239R0C2-F1-MODEL\_V4 | 1.0 | 7.092e-17 | 543 | 0.189 | 412 | 276 | 16 | 384 | 755 | 21 | 414 | DUF3874 domain-containing protein | DUF3874 domain-containing protein | | afdb-uniprot50 | AF-A0A160UEW2-F1-MODEL\_V4 | 1.0 | 3.292e-14 | 542 | 0.248 | 274 | 188 | 8 | 484 | 752 | 11 | 271 | Virulenceassociated E | Virulenceassociated E | | afdb-uniprot50 | AF-A0A4Q3J348-F1-MODEL\_V4 | 1.0 | 1.414e-15 | 542 | 0.25 | 279 | 187 | 10 | 381 | 649 | 108 | 374 | Uncharacterized protein | Uncharacterized protein | | afdb-uniprot50 | AF-A0A2W7HY36-F1-MODEL\_V4 | 1.0 | 6.397e-17 | 542 | 0.291 | 319 | 150 | 14 | 38 | 349 | 70 | 319 | Putative DNA primase/helicase | Putative DNA primase/helicase | | afdb-uniprot50 | AF-G5SSC0-F1-MODEL\_V4 | 1.0 | 3.208e-18 | 542 | 0.185 | 464 | 317 | 22 | 316 | 755 | 16 | 442 | DUF3874 domain-containing protein | DUF3874 domain-containing protein | | afdb-uniprot50 | AF-A0A6G1TK16-F1-MODEL\_V4 | 1.0 | 3.943e-18 | 542 | 0.177 | 518 | 315 | 23 | 243 | 730 | 54 | 490 | Uncharacterized protein | Uncharacterized protein | | afdb-uniprot50 | AF-X5DJW4-F1-MODEL\_V4 | 1.0 | 3.626e-17 | 542 | 0.172 | 446 | 310 | 27 | 326 | 752 | 277 | 682 | Bifunctional DNA primase/polymerase, N-terminal | Bifunctional DNA primase/polymerase, N-terminal | | afdb-uniprot50 | AF-A0A557QLQ1-F1-MODEL\_V4 | 1.0 | 1.1e-28 | 542 | 0.212 | 791 | 427 | 45 | 92 | 753 | 64 | 787 | Toprim domain-containing protein | Toprim domain-containing protein | | afdb-uniprot50 | AF-A0A7Y0DXY3-F1-MODEL\_V4 | 1.0 | 1.396e-13 | 541 | 0.361 | 202 | 108 | 7 | 156 | 355 | 2 | 184 | Toprim domain-containing protein | Toprim domain-containing protein | | afdb-uniprot50 | AF-A0A3A6CGK6-F1-MODEL\_V4 | 1.0 | 4.544e-16 | 541 | 0.2 | 359 | 243 | 15 | 439 | 769 | 6 | 348 | Virulence-associated protein E | Virulence-associated protein E | | afdb-uniprot50 | AF-A0A533RGC8-F1-MODEL\_V4 | 1.0 | 1.284e-28 | 541 | 0.191 | 761 | 437 | 49 | 92 | 752 | 78 | 759 | DNA primase | DNA primase | | afdb-uniprot50 | AF-A0A844GG09-F1-MODEL\_V4 | 1.0 | 6.565e-29 | 540 | 0.21 | 835 | 433 | 45 | 12 | 684 | 5 | 774 | DUF927 domain-containing protein | DUF927 domain-containing protein | | afdb-uniprot50 | AF-A0A5N3P4R6-F1-MODEL\_V4 | 1.0 | 9.541e-15 | 539 | 0.241 | 307 | 207 | 15 | 460 | 755 | 2 | 293 | Uncharacterized protein | Uncharacterized protein | | afdb-uniprot50 | AF-A0A2T1EHM2-F1-MODEL\_V4 | 1.0 | 1.173e-22 | 539 | 0.197 | 648 | 402 | 31 | 166 | 755 | 107 | 694 | Uncharacterized protein | Uncharacterized protein | | afdb-uniprot50 | AF-A0A562C7R2-F1-MODEL\_V4 | 1.0 | 7.565e-27 | 539 | 0.187 | 762 | 443 | 45 | 93 | 767 | 82 | 753 | Putative DNA primase/helicase | Putative DNA primase/helicase | | afdb-uniprot50 | AF-A0A7C1H7I6-F1-MODEL\_V4 | 1.0 | 4.02e-17 | 538 | 0.272 | 348 | 186 | 16 | 93 | 431 | 113 | 402 | Toprim domain-containing protein | Toprim domain-containing protein | | afdb-uniprot50 | AF-A0A1M3E3M9-F1-MODEL\_V4 | 1.0 | 2.463e-21 | 537 | 0.162 | 787 | 377 | 34 | 1 | 757 | 180 | 713 | Uncharacterized protein | Uncharacterized protein | | afdb-uniprot50 | AF-W2CS72-F1-MODEL\_V4 | 1.0 | 8.173e-15 | 536 | 0.252 | 261 | 180 | 10 | 505 | 756 | 1 | 255 | Virulence-associated protein E | Virulence-associated protein E | | afdb-uniprot50 | AF-A0A658JSB4-F1-MODEL\_V4 | 1.0 | 2.766e-15 | 536 | 0.33 | 254 | 151 | 8 | 380 | 624 | 381 | 624 | Virulence-associated E family protein | Virulence-associated E family protein | | afdb-uniprot50 | AF-A0A158BCW7-F1-MODEL\_V4 | 1.0 | 9.421e-29 | 536 | 0.195 | 985 | 503 | 56 | 1 | 756 | 1 | 924 | Inner membrane protein | Inner membrane protein | | afdb-uniprot50 | AF-A0A3D0G2H0-F1-MODEL\_V4 | 1.0 | 1.89e-16 | 536 | 0.199 | 462 | 284 | 23 | 370 | 753 | 464 | 917 | Uncharacterized protein | Uncharacterized protein | | afdb-uniprot50 | AF-E0NRI5-F1-MODEL\_V4 | 1.0 | 1.598e-14 | 535 | 0.23 | 265 | 197 | 5 | 499 | 757 | 2 | 265 | Uncharacterized protein | Uncharacterized protein | | afdb-uniprot50 | AF-A0A849TJR6-F1-MODEL\_V4 | 1.0 | 7.278e-29 | 535 | 0.185 | 878 | 441 | 49 | 1 | 731 | 19 | 768 | DUF3987 domain-containing protein | DUF3987 domain-containing protein | | afdb-uniprot50 | AF-A0A0T7BQB0-F1-MODEL\_V4 | 1.0 | 3.843e-22 | 535 | 0.187 | 645 | 393 | 35 | 178 | 757 | 73 | 651 | DUF3854 domain-containing protein | DUF3854 domain-containing protein | | afdb-uniprot50 | AF-A0A5T2E3M0-F1-MODEL\_V4 | 1.0 | 1.3e-14 | 534 | 0.273 | 278 | 162 | 10 | 99 | 358 | 138 | 393 | Uncharacterized protein | Uncharacterized protein | | afdb-uniprot50 | AF-A0A154VF00-F1-MODEL\_V4 | 1.0 | 2.462e-13 | 533 | 0.343 | 195 | 106 | 7 | 155 | 347 | 2 | 176 | Toprim domain-containing protein | Toprim domain-containing protein | | afdb-uniprot50 | AF-A0A0S2TFP5-F1-MODEL\_V4 | 1.0 | 2.193e-27 | 533 | 0.206 | 770 | 428 | 43 | 98 | 752 | 106 | 807 | Uncharacterized protein | Uncharacterized protein | | afdb-uniprot50 | AF-I3Y9A5-F1-MODEL\_V4 | 1.0 | 1.807e-29 | 533 | 0.204 | 884 | 473 | 47 | 12 | 738 | 131 | 940 | DNA/RNA helicase, superfamily II | DNA/RNA helicase, superfamily II | | afdb-uniprot50 | AF-I2PZX8-F1-MODEL\_V4 | 1.0 | 2.838e-27 | 532 | 0.175 | 826 | 423 | 48 | 102 | 753 | 77 | 818 | Toprim domain-containing protein | Toprim domain-containing protein | | afdb-uniprot50 | AF-A0A4P2QP84-F1-MODEL\_V4 | 1.0 | 3.843e-22 | 532 | 0.177 | 727 | 410 | 34 | 179 | 751 | 79 | 771 | DUF3854 domain-containing protein | DUF3854 domain-containing protein | | afdb-uniprot50 | AF-A0A8B2TSA0-F1-MODEL\_V4 | 1.0 | 1.94e-20 | 531 | 0.139 | 773 | 365 | 27 | 2 | 751 | 229 | 723 | Uncharacterized protein | Uncharacterized protein | | afdb-uniprot50 | AF-A0A6P0YCZ3-F1-MODEL\_V4 | 1.0 | 7.002e-23 | 531 | 0.177 | 716 | 439 | 42 | 132 | 762 | 5 | 655 | Uncharacterized protein | Uncharacterized protein | | afdb-uniprot50 | AF-A0A1L5KUQ4-F1-MODEL\_V4 | 1.0 | 4.344e-13 | 530 | 0.233 | 240 | 169 | 10 | 453 | 687 | 11 | 240 | Virulence-associated protein E | Virulence-associated protein E | | afdb-uniprot50 | AF-A0A1H0XV22-F1-MODEL\_V4 | 1.0 | 8.121e-18 | 529 | 0.154 | 555 | 380 | 28 | 259 | 769 | 8 | 516 | Predicted P-loop ATPase and inactivated derivatives | Predicted P-loop ATPase and inactivated derivatives | | afdb-uniprot50 | AF-K9SHA4-F1-MODEL\_V4 | 1.0 | 4.429e-20 | 529 | 0.188 | 577 | 348 | 30 | 224 | 757 | 243 | 741 | Virulence-associated E family protein | Virulence-associated E family protein | | afdb-uniprot50 | AF-A0A0Q6VCR0-F1-MODEL\_V4 | 1.0 | 8.069e-21 | 529 | 0.159 | 784 | 373 | 28 | 1 | 757 | 220 | 744 | Uncharacterized protein | Uncharacterized protein | | afdb-uniprot50 | AF-A0A7C1TYP0-F1-MODEL\_V4 | 1.0 | 2.931e-28 | 529 | 0.197 | 785 | 426 | 49 | 92 | 753 | 64 | 767 | Toprim domain-containing protein | Toprim domain-containing protein | | afdb-uniprot50 | AF-A0A250DKS7-F1-MODEL\_V4 | 1.0 | 2.369e-23 | 529 | 0.184 | 732 | 433 | 45 | 167 | 767 | 167 | 865 | Virulence protein E | Virulence protein E | | afdb-uniprot50 | AF-A0A0G1YTK1-F1-MODEL\_V4 | 1.0 | 6.315e-23 | 529 | 0.181 | 837 | 496 | 41 | 6 | 767 | 232 | 953 | Virulence-associated protein E domain protein | Virulence-associated protein E domain protein | | afdb-uniprot50 | AF-A0A1H4HT56-F1-MODEL\_V4 | 1.0 | 1.284e-20 | 528 | 0.142 | 788 | 374 | 29 | 1 | 753 | 215 | 735 | Primase C terminal 2 (PriCT-2) | Primase C terminal 2 (PriCT-2) | | afdb-uniprot50 | AF-A0A0A2CPT5-F1-MODEL\_V4 | 1.0 | 4.515e-19 | 528 | 0.186 | 551 | 351 | 28 | 237 | 757 | 260 | 742 | PriCT\_1 domain-containing protein | PriCT\_1 domain-containing protein | | afdb-uniprot50 | AF-A0A1Z4JI45-F1-MODEL\_V4 | 1.0 | 2.179e-22 | 528 | 0.164 | 837 | 436 | 34 | 1 | 769 | 305 | 945 | Uncharacterized protein | Uncharacterized protein | | afdb-uniprot50 | AF-A0A639X8R3-F1-MODEL\_V4 | 1.0 | 6.314e-15 | 528 | 0.278 | 287 | 162 | 12 | 97 | 360 | 134 | 398 | Uncharacterized protein | Uncharacterized protein | | afdb-uniprot50 | AF-A0A252D898-F1-MODEL\_V4 | 1.0 | 2.695e-19 | 527 | 0.192 | 539 | 324 | 20 | 239 | 759 | 288 | 732 | Prim-Pol domain-containing protein | Prim-Pol domain-containing protein | | afdb-uniprot50 | AF-A0A5Y8ZF30-F1-MODEL\_V4 | 1.0 | 5.237e-14 | 526 | 0.285 | 263 | 147 | 14 | 105 | 347 | 461 | 702 | DUF1738 domain-containing protein | DUF1738 domain-containing protein | | afdb-uniprot50 | AF-A0A369XMD4-F1-MODEL\_V4 | 1.0 | 3.868e-27 | 526 | 0.208 | 798 | 393 | 51 | 93 | 740 | 116 | 824 | DUF927 domain-containing protein | DUF927 domain-containing protein | | afdb-uniprot50 | AF-A0A414T7G4-F1-MODEL\_V4 | 1.0 | 4.4e-15 | 525 | 0.189 | 316 | 216 | 12 | 485 | 769 | 6 | 312 | Uncharacterized protein | Uncharacterized protein | | afdb-uniprot50 | AF-A0A2I8A4T1-F1-MODEL\_V4 | 1.0 | 1.181e-19 | 525 | 0.175 | 558 | 341 | 30 | 226 | 753 | 214 | 682 | Virulence-associated E | Virulence-associated E | | afdb-uniprot50 | AF-A0A2E9L0S9-F1-MODEL\_V4 | 1.0 | 1.378e-19 | 525 | 0.195 | 548 | 353 | 21 | 243 | 755 | 234 | 728 | VirE\_N domain-containing protein | VirE\_N domain-containing protein | | afdb-uniprot50 | AF-A0A094K1B3-F1-MODEL\_V4 | 1.0 | 1.196e-13 | 524 | 0.29 | 251 | 146 | 9 | 92 | 335 | 64 | 289 | Toprim domain-containing protein | Toprim domain-containing protein | | afdb-uniprot50 | AF-A0A352X4W4-F1-MODEL\_V4 | 1.0 | 3.511e-24 | 524 | 0.165 | 839 | 480 | 50 | 46 | 757 | 84 | 828 | Toprim domain-containing protein | Toprim domain-containing protein | | afdb-uniprot50 | AF-A0A6N6T6R6-F1-MODEL\_V4 | 1.0 | 4.516e-27 | 522 | 0.228 | 761 | 374 | 43 | 12 | 615 | 5 | 709 | DUF3987 domain-containing protein | DUF3987 domain-containing protein | | afdb-uniprot50 | AF-A0A7T1MLF3-F1-MODEL\_V4 | 1.0 | 3.187e-21 | 522 | 0.177 | 683 | 419 | 38 | 167 | 762 | 40 | 666 | Uncharacterized protein | Uncharacterized protein | | afdb-uniprot50 | AF-A0A6I1RJD4-F1-MODEL\_V4 | 1.0 | 1.211e-15 | 521 | 0.186 | 344 | 242 | 17 | 331 | 658 | 29 | 350 | Uncharacterized protein | Uncharacterized protein | | afdb-uniprot50 | AF-Q3M8I3-F1-MODEL\_V4 | 1.0 | 2.931e-20 | 521 | 0.201 | 605 | 348 | 31 | 202 | 769 | 146 | 652 | Virulence-associated E | Virulence-associated E | | afdb-uniprot50 | AF-A0A2E2RUS7-F1-MODEL\_V4 | 1.0 | 1.63e-21 | 521 | 0.185 | 705 | 395 | 39 | 151 | 766 | 94 | 707 | Prim-Pol domain-containing protein | Prim-Pol domain-containing protein | | afdb-uniprot50 | AF-A0A2N8MB77-F1-MODEL\_V4 | 1.0 | 2.309e-27 | 521 | 0.193 | 754 | 399 | 40 | 99 | 750 | 98 | 744 | DNA primase | DNA primase | | afdb-uniprot50 | AF-A0A0S3PXG1-F1-MODEL\_V4 | 1.0 | 3.77e-23 | 521 | 0.151 | 816 | 462 | 46 | 1 | 768 | 189 | 821 | Virulence-associated protein E | Virulence-associated protein E | | afdb-uniprot50 | AF-I2UTG7-F1-MODEL\_V4 | 1.0 | 1.1e-12 | 521 | 0.317 | 217 | 126 | 9 | 133 | 346 | 4 | 201 | LPD7 domain-containing protein | LPD7 domain-containing protein | | afdb-uniprot50 | AF-A0A2C6Z4N4-F1-MODEL\_V4 | 1.0 | 1.423e-12 | 520 | 0.282 | 202 | 125 | 6 | 139 | 337 | 1 | 185 | Uncharacterized protein | Uncharacterized protein | | afdb-uniprot50 | AF-A0A6I4YYG7-F1-MODEL\_V4 | 1.0 | 3.027e-21 | 520 | 0.139 | 801 | 393 | 31 | 1 | 757 | 253 | 800 | Uncharacterized protein | Uncharacterized protein | | afdb-uniprot50 | AF-A0A522SU55-F1-MODEL\_V4 | 1.0 | 2.73e-21 | 519 | 0.159 | 771 | 383 | 30 | 1 | 751 | 183 | 708 | Uncharacterized protein | Uncharacterized protein | | afdb-uniprot50 | AF-W0DZN8-F1-MODEL\_V4 | 1.0 | 1.451e-27 | 519 | 0.197 | 789 | 426 | 47 | 93 | 762 | 77 | 776 | Toprim domain-containing protein | Toprim domain-containing protein | | afdb-uniprot50 | AF-A0A1Q3NZT7-F1-MODEL\_V4 | 1.0 | 5.272e-27 | 519 | 0.175 | 802 | 464 | 50 | 102 | 757 | 84 | 833 | SF3 helicase domain-containing protein | SF3 helicase domain-containing protein | | afdb-uniprot50 | AF-R4WFQ0-F1-MODEL\_V4 | 1.0 | 1.558e-26 | 518 | 0.18 | 854 | 454 | 45 | 107 | 769 | 85 | 883 | Toprim domain-containing protein | Toprim domain-containing protein | | afdb-uniprot50 | AF-A0A228H1M8-F1-MODEL\_V4 | 1.0 | 4.126e-21 | 516 | 0.143 | 793 | 377 | 34 | 1 | 755 | 247 | 774 | PriCT\_2 domain-containing protein | PriCT\_2 domain-containing protein | | afdb-uniprot50 | AF-A0A0P1I0V3-F1-MODEL\_V4 | 1.0 | 7.278e-21 | 514 | 0.151 | 803 | 393 | 40 | 2 | 756 | 241 | 802 | Putative P-loop ATPase | Putative P-loop ATPase | | afdb-uniprot50 | AF-A0A1F1XH87-F1-MODEL\_V4 | 1.0 | 2.802e-17 | 513 | 0.186 | 472 | 295 | 23 | 370 | 768 | 17 | 472 | Uncharacterized protein | Uncharacterized protein | | afdb-uniprot50 | AF-A0A4D9CEY9-F1-MODEL\_V4 | 1.0 | 3.047e-18 | 513 | 0.2 | 545 | 316 | 26 | 261 | 769 | 274 | 734 | RepB\_primase domain-containing protein | RepB\_primase domain-containing protein | | afdb-uniprot50 | AF-A0A5P3VML8-F1-MODEL\_V4 | 1.0 | 8.551e-18 | 513 | 0.2 | 518 | 333 | 28 | 285 | 762 | 328 | 804 | Prim-Pol domain-containing protein | Prim-Pol domain-containing protein | | afdb-uniprot50 | AF-K9ULE3-F1-MODEL\_V4 | 1.0 | 2.354e-18 | 513 | 0.187 | 566 | 351 | 30 | 237 | 751 | 234 | 741 | Putative P-loop ATPase | Putative P-loop ATPase | | afdb-uniprot50 | AF-A0A1M4TMH4-F1-MODEL\_V4 | 1.0 | 3.843e-14 | 512 | 0.236 | 262 | 189 | 8 | 501 | 757 | 2 | 257 | Virulence-associated protein E | Virulence-associated protein E | | afdb-uniprot50 | AF-A0A1Z4TD68-F1-MODEL\_V4 | 1.0 | 9.604e-20 | 512 | 0.183 | 588 | 356 | 26 | 233 | 762 | 71 | 592 | Uncharacterized protein | Uncharacterized protein | | afdb-uniprot50 | AF-A0A3A0FPI8-F1-MODEL\_V4 | 1.0 | 4.574e-21 | 512 | 0.157 | 768 | 344 | 30 | 4 | 735 | 243 | 743 | Prim-Pol domain-containing protein | Prim-Pol domain-containing protein | | afdb-uniprot50 | AF-A0A5C7L6B0-F1-MODEL\_V4 | 1.0 | 2.678e-14 | 511 | 0.29 | 275 | 155 | 9 | 59 | 333 | 74 | 308 | Toprim domain-containing protein | Toprim domain-containing protein | | afdb-uniprot50 | AF-N0B2Q9-F1-MODEL\_V4 | 1.0 | 1.284e-20 | 511 | 0.185 | 648 | 365 | 36 | 182 | 753 | 123 | 683 | PriCT\_1 domain-containing protein | PriCT\_1 domain-containing protein | | afdb-uniprot50 | AF-A0A1J5R0E3-F1-MODEL\_V4 | 1.0 | 1.335e-26 | 511 | 0.184 | 752 | 418 | 40 | 100 | 750 | 75 | 731 | DNA primase TraC | DNA primase TraC | | afdb-uniprot50 | AF-A0A5C7PWQ8-F1-MODEL\_V4 | 1.0 | 2.415e-14 | 510 | 0.239 | 317 | 203 | 16 | 455 | 757 | 16 | 308 | Uncharacterized protein | Uncharacterized protein | | afdb-uniprot50 | AF-A0A3B9BTJ8-F1-MODEL\_V4 | 1.0 | 6.606e-18 | 509 | 0.2 | 493 | 287 | 24 | 239 | 691 | 186 | 611 | Prim-Pol domain-containing protein | Prim-Pol domain-containing protein | | afdb-uniprot50 | AF-A0A5C7JLL6-F1-MODEL\_V4 | 1.0 | 3.086e-20 | 509 | 0.142 | 785 | 409 | 34 | 4 | 769 | 188 | 726 | Uncharacterized protein | Uncharacterized protein | | afdb-uniprot50 | AF-A0A838W082-F1-MODEL\_V4 | 1.0 | 1.044e-12 | 508 | 0.235 | 221 | 147 | 9 | 131 | 347 | 8 | 210 | Toprim domain-containing protein | Toprim domain-containing protein | | afdb-uniprot50 | AF-A0A2L2NLQ2-F1-MODEL\_V4 | 1.0 | 3.511e-16 | 508 | 0.189 | 455 | 256 | 24 | 381 | 751 | 88 | 513 | Virulence-associated E family protein | Virulence-associated E family protein | | afdb-uniprot50 | AF-A0A7X7WZM3-F1-MODEL\_V4 | 1.0 | 5.104e-18 | 508 | 0.145 | 516 | 350 | 26 | 264 | 756 | 273 | 720 | VirE\_N domain-containing protein | VirE\_N domain-containing protein | | afdb-uniprot50 | AF-G1UZP2-F1-MODEL\_V4 | 1.0 | 1.227e-17 | 507 | 0.251 | 414 | 204 | 20 | 91 | 454 | 70 | 427 | Uncharacterized protein | Uncharacterized protein | | afdb-uniprot50 | AF-A0A6M2A343-F1-MODEL\_V4 | 1.0 | 6.957e-26 | 506 | 0.163 | 801 | 447 | 49 | 98 | 755 | 117 | 837 | DNA primase TraC | DNA primase TraC | | afdb-uniprot50 | AF-A0A3C0NTJ6-F1-MODEL\_V4 | 1.0 | 1.058e-14 | 505 | 0.252 | 356 | 222 | 17 | 436 | 769 | 2 | 335 | Conjugal transfer protein TraC | Conjugal transfer protein TraC | | afdb-uniprot50 | AF-A0A7U8BI53-F1-MODEL\_V4 | 1.0 | 2.339e-13 | 504 | 0.248 | 274 | 168 | 10 | 92 | 346 | 69 | 323 | Toprim domain-containing protein | Toprim domain-containing protein | | afdb-uniprot50 | AF-A0A1I1CGG0-F1-MODEL\_V4 | 1.0 | 4.878e-15 | 504 | 0.221 | 380 | 239 | 19 | 436 | 769 | 1 | 369 | Virulence-associated protein E | Virulence-associated protein E | | afdb-uniprot50 | AF-A0A525WLH8-F1-MODEL\_V4 | 1.0 | 9.666e-25 | 504 | 0.184 | 750 | 374 | 36 | 100 | 769 | 77 | 668 | DUF3631 domain-containing protein | DUF3631 domain-containing protein | | afdb-uniprot50 | AF-A0A827WY38-F1-MODEL\_V4 | 1.0 | 1.284e-12 | 503 | 0.313 | 217 | 128 | 8 | 115 | 328 | 160 | 358 | Uncharacterized protein | Uncharacterized protein | | afdb-uniprot50 | AF-A0A2V6F860-F1-MODEL\_V4 | 1.0 | 1.136e-21 | 502 | 0.233 | 531 | 283 | 26 | 113 | 607 | 84 | 526 | Toprim domain-containing protein | Toprim domain-containing protein | | afdb-uniprot50 | AF-A0A0E4FRT6-F1-MODEL\_V4 | 1.0 | 3.918e-21 | 502 | 0.142 | 710 | 430 | 38 | 143 | 750 | 73 | 705 | Prim-Pol domain-containing protein | Prim-Pol domain-containing protein | | afdb-uniprot50 | AF-A0A6M3JPC8-F1-MODEL\_V4 | 1.0 | 1.165e-17 | 502 | 0.12 | 555 | 356 | 31 | 299 | 756 | 266 | 785 | Putative DNA primase | Putative DNA primase | | afdb-uniprot50 | AF-A0A5T0QKQ6-F1-MODEL\_V4 | 1.0 | 7.662e-13 | 501 | 0.258 | 259 | 159 | 9 | 107 | 347 | 13 | 256 | Toprim domain-containing protein | Toprim domain-containing protein | | afdb-uniprot50 | AF-A0A561PL40-F1-MODEL\_V4 | 1.0 | 6.692e-20 | 501 | 0.155 | 785 | 393 | 34 | 1 | 760 | 176 | 715 | Primase-like protein | Primase-like protein | | afdb-uniprot50 | AF-A0A4P7L1H0-F1-MODEL\_V4 | 1.0 | 2.838e-19 | 500 | 0.181 | 573 | 370 | 38 | 240 | 764 | 79 | 600 | Virulence-associated protein E | Virulence-associated protein E | | afdb-uniprot50 | AF-A0A2H6G363-F1-MODEL\_V4 | 1.0 | 1.031e-26 | 500 | 0.178 | 730 | 409 | 41 | 115 | 751 | 2 | 633 | DNA primase TraC | DNA primase TraC | | afdb-uniprot50 | AF-A0A0Q7SIY5-F1-MODEL\_V4 | 1.0 | 2.748e-26 | 500 | 0.208 | 783 | 425 | 51 | 90 | 753 | 78 | 784 | Uncharacterized protein | Uncharacterized protein | | afdb-uniprot50 | AF-A0A6P0U2Y5-F1-MODEL\_V4 | 1.0 | 3.769e-15 | 499 | 0.223 | 372 | 243 | 16 | 412 | 751 | 6 | 363 | Uncharacterized protein | Uncharacterized protein | | afdb-uniprot50 | AF-A0A845X7V7-F1-MODEL\_V4 | 1.0 | 7.047e-20 | 499 | 0.201 | 591 | 345 | 35 | 239 | 762 | 236 | 766 | Uncharacterized protein | Uncharacterized protein | | afdb-uniprot50 | AF-A0A558BT14-F1-MODEL\_V4 | 1.0 | 2.384e-20 | 498 | 0.149 | 791 | 422 | 32 | 1 | 757 | 17 | 590 | PriCT\_2 domain-containing protein | PriCT\_2 domain-containing protein | | afdb-uniprot50 | AF-A0A0F9U4J5-F1-MODEL\_V4 | 1.0 | 1.031e-18 | 498 | 0.157 | 787 | 363 | 32 | 1 | 757 | 163 | 679 | Prim-Pol domain-containing protein | Prim-Pol domain-containing protein | | afdb-uniprot50 | AF-A0A5J4QFG3-F1-MODEL\_V4 | 1.0 | 4.26e-14 | 497 | 0.233 | 266 | 186 | 10 | 382 | 638 | 20 | 276 | Uncharacterized protein | Uncharacterized protein | | afdb-uniprot50 | AF-A0A2N0V8M1-F1-MODEL\_V4 | 1.0 | 6.155e-27 | 497 | 0.202 | 749 | 425 | 44 | 100 | 757 | 72 | 738 | SF3 helicase domain-containing protein | SF3 helicase domain-containing protein | | afdb-uniprot50 | AF-A0A2M7MUG5-F1-MODEL\_V4 | 1.0 | 3.745e-26 | 497 | 0.198 | 906 | 410 | 48 | 12 | 736 | 5 | 774 | Uncharacterized protein | Uncharacterized protein | | afdb-uniprot50 | AF-A0A2P7U2R3-F1-MODEL\_V4 | 1.0 | 2.766e-23 | 496 | 0.196 | 849 | 406 | 42 | 1 | 744 | 2 | 678 | Toprim domain-containing protein | Toprim domain-containing protein | | afdb-uniprot50 | AF-A0A1F4HLP3-F1-MODEL\_V4 | 1.0 | 3.745e-26 | 495 | 0.218 | 801 | 410 | 46 | 36 | 679 | 1 | 742 | Uncharacterized protein | Uncharacterized protein | | afdb-uniprot50 | AF-A0A126NQG0-F1-MODEL\_V4 | 1.0 | 1.878e-19 | 495 | 0.186 | 568 | 340 | 32 | 261 | 757 | 242 | 758 | Prim-Pol domain-containing protein | Prim-Pol domain-containing protein | | afdb-uniprot50 | AF-A0A5U3F134-F1-MODEL\_V4 | 1.0 | 6.649e-15 | 494 | 0.267 | 299 | 158 | 15 | 68 | 355 | 140 | 388 | DNA primase | DNA primase | | afdb-uniprot50 | AF-A0A2V5PJC8-F1-MODEL\_V4 | 1.0 | 1.107e-25 | 493 | 0.189 | 803 | 407 | 47 | 92 | 752 | 64 | 764 | Uncharacterized protein | Uncharacterized protein | | afdb-uniprot50 | AF-A0A7Z0B884-F1-MODEL\_V4 | 1.0 | 1.267e-26 | 493 | 0.185 | 762 | 425 | 40 | 102 | 750 | 80 | 758 | Putative DNA primase/helicase | Putative DNA primase/helicase | | afdb-uniprot50 | AF-A0A1I0JA19-F1-MODEL\_V4 | 1.0 | 3.534e-21 | 492 | 0.138 | 777 | 438 | 34 | 2 | 755 | 200 | 767 | Bifunctional DNA primase/polymerase, N-terminal | Bifunctional DNA primase/polymerase, N-terminal | | afdb-uniprot50 | AF-A0A1G5SD75-F1-MODEL\_V4 | 1.0 | 2.003e-13 | 491 | 0.294 | 258 | 136 | 9 | 91 | 348 | 103 | 314 | Toprim protein | Toprim protein | | afdb-uniprot50 | AF-E0UET4-F1-MODEL\_V4 | 1.0 | 3.626e-17 | 491 | 0.189 | 496 | 294 | 24 | 290 | 753 | 57 | 476 | Virulence-associated E family protein | Virulence-associated E family protein | | afdb-uniprot50 | AF-B9KGK4-F1-MODEL\_V4 | 1.0 | 3.027e-13 | 490 | 0.259 | 274 | 160 | 12 | 91 | 346 | 87 | 335 | DNA primase TraC | DNA primase TraC | | afdb-uniprot50 | AF-R5P0F0-F1-MODEL\_V4 | 1.0 | 2.137e-15 | 490 | 0.265 | 358 | 196 | 18 | 457 | 760 | 2 | 346 | Uncharacterized protein | Uncharacterized protein | | afdb-uniprot50 | AF-A0A5N1JF43-F1-MODEL\_V4 | 1.0 | 4.429e-20 | 490 | 0.151 | 804 | 396 | 35 | 1 | 769 | 237 | 788 | Uncharacterized protein | Uncharacterized protein | | afdb-uniprot50 | AF-A0A5C7ZG73-F1-MODEL\_V4 | 1.0 | 2.802e-25 | 488 | 0.183 | 861 | 422 | 45 | 13 | 753 | 6 | 705 | Topoisomerase | Topoisomerase | | afdb-uniprot50 | AF-G1UVN8-F1-MODEL\_V4 | 1.0 | 6.036e-20 | 485 | 0.223 | 536 | 203 | 24 | 102 | 614 | 102 | 447 | Toprim domain-containing protein | Toprim domain-containing protein | | afdb-uniprot50 | AF-A0A5J4SK06-F1-MODEL\_V4 | 1.0 | 1.1e-12 | 484 | 0.262 | 213 | 143 | 6 | 490 | 699 | 10 | 211 | Uncharacterized protein | Uncharacterized protein | | afdb-uniprot50 | AF-A0A0H4B4X1-F1-MODEL\_V4 | 1.0 | 1.114e-14 | 484 | 0.204 | 391 | 250 | 17 | 412 | 766 | 1 | 366 | Uncharacterized protein | Uncharacterized protein | | afdb-uniprot50 | AF-A0A2V4UF45-F1-MODEL\_V4 | 1.0 | 1.378e-19 | 484 | 0.231 | 513 | 266 | 21 | 102 | 508 | 73 | 563 | Phage/plasmid primase-like uncharacterized protein | Phage/plasmid primase-like uncharacterized protein | | afdb-uniprot50 | AF-A0A4R5P3B1-F1-MODEL\_V4 | 1.0 | 5.732e-20 | 484 | 0.143 | 675 | 450 | 39 | 151 | 757 | 142 | 756 | Uncharacterized protein | Uncharacterized protein | | afdb-uniprot50 | AF-A0A7U4DN83-F1-MODEL\_V4 | 1.0 | 8.122e-26 | 484 | 0.169 | 784 | 445 | 42 | 64 | 750 | 69 | 742 | Uncharacterized protein | Uncharacterized protein | | afdb-uniprot50 | AF-A0A158DR21-F1-MODEL\_V4 | 1.0 | 7.713e-26 | 483 | 0.184 | 755 | 423 | 43 | 102 | 750 | 79 | 746 | TOPRIM domain-containing protein | TOPRIM domain-containing protein | | afdb-uniprot50 | AF-A0A2N8MBZ7-F1-MODEL\_V4 | 1.0 | 5.48e-25 | 483 | 0.211 | 705 | 372 | 43 | 99 | 689 | 69 | 703 | Uncharacterized protein | Uncharacterized protein | | afdb-uniprot50 | AF-A0A0S4I2H5-F1-MODEL\_V4 | 1.0 | 1.661e-12 | 481 | 0.338 | 204 | 117 | 6 | 92 | 287 | 61 | 254 | DNA primase TraC | DNA primase TraC | | afdb-uniprot50 | AF-A1SXJ4-F1-MODEL\_V4 | 1.0 | 6.035e-12 | 481 | 0.241 | 228 | 149 | 8 | 115 | 335 | 86 | 296 | Toprim domain-containing protein | Toprim domain-containing protein | | afdb-uniprot50 | AF-A0A7G8DD21-F1-MODEL\_V4 | 1.0 | 1.317e-16 | 481 | 0.153 | 489 | 329 | 25 | 316 | 769 | 1 | 439 | Virulence-associated E family protein | Virulence-associated E family protein | | afdb-uniprot50 | AF-A0A1Z9AXB4-F1-MODEL\_V4 | 1.0 | 8.387e-19 | 481 | 0.184 | 573 | 335 | 30 | 264 | 750 | 194 | 719 | Uncharacterized protein | Uncharacterized protein | | afdb-uniprot50 | AF-A0A845Z468-F1-MODEL\_V4 | 1.0 | 3.422e-20 | 480 | 0.185 | 599 | 364 | 32 | 170 | 690 | 78 | 630 | Uncharacterized protein | Uncharacterized protein | | afdb-uniprot50 | AF-A0A158LQY7-F1-MODEL\_V4 | 1.0 | 1.952e-25 | 479 | 0.17 | 947 | 495 | 52 | 3 | 751 | 1 | 854 | Prim\_Zn\_Ribbon domain-containing protein | Prim\_Zn\_Ribbon domain-containing protein | | afdb-uniprot50 | AF-A0A3G3H284-F1-MODEL\_V4 | 1.0 | 8.552e-26 | 478 | 0.178 | 764 | 408 | 46 | 93 | 737 | 74 | 736 | Uncharacterized protein | Uncharacterized protein | | afdb-uniprot50 | AF-A0A6L3IIZ1-F1-MODEL\_V4 | 1.0 | 6.233e-13 | 477 | 0.242 | 252 | 180 | 8 | 512 | 758 | 7 | 252 | Uncharacterized protein | Uncharacterized protein | | afdb-uniprot50 | AF-A0A7K0GNH6-F1-MODEL\_V4 | 1.0 | 2.593e-21 | 477 | 0.164 | 737 | 394 | 36 | 1 | 684 | 1 | 568 | Uncharacterized protein | Uncharacterized protein | | afdb-uniprot50 | AF-A0A433VJU3-F1-MODEL\_V4 | 1.0 | 4.816e-21 | 477 | 0.171 | 684 | 426 | 33 | 161 | 756 | 82 | 712 | Uncharacterized protein | Uncharacterized protein | | afdb-uniprot50 | AF-A0A850R2Y2-F1-MODEL\_V4 | 1.0 | 5.306e-24 | 477 | 0.175 | 822 | 459 | 53 | 93 | 762 | 74 | 828 | Toprim domain-containing protein | Toprim domain-containing protein | | afdb-uniprot50 | AF-A0A011PBN5-F1-MODEL\_V4 | 1.0 | 2.875e-13 | 476 | 0.226 | 296 | 203 | 15 | 468 | 751 | 1 | 282 | Putative P-loop ATPase | Putative P-loop ATPase | | afdb-uniprot50 | AF-K9TV34-F1-MODEL\_V4 | 1.0 | 5.959e-18 | 476 | 0.186 | 569 | 349 | 33 | 227 | 769 | 225 | 705 | Virulence-associated E family protein | Virulence-associated E family protein | | afdb-uniprot50 | AF-A0A1I5Z275-F1-MODEL\_V4 | 1.0 | 1.267e-18 | 475 | 0.122 | 810 | 446 | 45 | 1 | 757 | 26 | 623 | Primase C terminal 2 (PriCT-2) | Primase C terminal 2 (PriCT-2) | | afdb-uniprot50 | AF-A0A080LT75-F1-MODEL\_V4 | 1.0 | 9.18e-25 | 475 | 0.187 | 768 | 464 | 42 | 102 | 769 | 81 | 788 | DNA primase TraC | DNA primase TraC | | afdb-uniprot50 | AF-A0A328V111-F1-MODEL\_V4 | 1.0 | 2.123e-18 | 474 | 0.183 | 562 | 333 | 29 | 237 | 756 | 220 | 697 | Prim-Pol domain-containing protein | Prim-Pol domain-containing protein | | afdb-uniprot50 | AF-D2QEV8-F1-MODEL\_V4 | 1.0 | 4.663e-20 | 474 | 0.145 | 796 | 390 | 38 | 2 | 757 | 204 | 748 | p-loop ATPase and inactivated derivatives-like protein | p-loop ATPase and inactivated derivatives-like protein | | afdb-uniprot50 | AF-A0A7H0HGN8-F1-MODEL\_V4 | 1.0 | 2.627e-23 | 472 | 0.226 | 626 | 321 | 37 | 89 | 645 | 61 | 591 | DUF3987 domain-containing protein | DUF3987 domain-containing protein | | afdb-uniprot50 | AF-A0A3N7INQ9-F1-MODEL\_V4 | 1.0 | 6.233e-13 | 471 | 0.264 | 253 | 170 | 8 | 526 | 768 | 1 | 247 | Virulence-associated E family protein | Virulence-associated E family protein | | afdb-uniprot50 | AF-A0A350H4D5-F1-MODEL\_V4 | 1.0 | 1.568e-15 | 471 | 0.206 | 392 | 249 | 18 | 381 | 743 | 91 | 449 | Uncharacterized protein | Uncharacterized protein | | afdb-uniprot50 | AF-C4K524-F1-MODEL\_V4 | 1.0 | 3.106e-17 | 471 | 0.219 | 438 | 230 | 19 | 99 | 447 | 137 | 551 | DNA primase TraC | DNA primase TraC | | afdb-uniprot50 | AF-A0A5F0YSJ3-F1-MODEL\_V4 | 1.0 | 3.4e-15 | 470 | 0.229 | 366 | 207 | 18 | 100 | 446 | 128 | 437 | Toprim domain-containing protein | Toprim domain-containing protein | | afdb-uniprot50 | AF-A0A5C7LJD7-F1-MODEL\_V4 | 1.0 | 6.606e-18 | 470 | 0.165 | 579 | 353 | 32 | 238 | 757 | 216 | 723 | Prim-Pol domain-containing protein | Prim-Pol domain-containing protein | | afdb-uniprot50 | AF-A0A133PTK4-F1-MODEL\_V4 | 1.0 | 6.114e-14 | 469 | 0.228 | 284 | 192 | 13 | 369 | 638 | 43 | 313 | Uncharacterized protein | Uncharacterized protein | | afdb-uniprot50 | AF-A0A414BQ09-F1-MODEL\_V4 | 1.0 | 1.369e-14 | 469 | 0.179 | 351 | 253 | 13 | 330 | 659 | 256 | 592 | Virulence protein E | Virulence protein E | | afdb-uniprot50 | AF-A0A1L3SNY3-F1-MODEL\_V4 | 1.0 | 6.564e-21 | 469 | 0.162 | 880 | 439 | 47 | 1 | 756 | 149 | 854 | Uncharacterized protein | Uncharacterized protein | | afdb-uniprot50 | AF-A0A844LJA6-F1-MODEL\_V4 | 1.0 | 2.627e-23 | 468 | 0.158 | 896 | 469 | 44 | 1 | 745 | 1 | 762 | Uncharacterized protein | Uncharacterized protein | | afdb-uniprot50 | AF-A0A8A8P149-F1-MODEL\_V4 | 1.0 | 2.783e-12 | 467 | 0.296 | 243 | 143 | 11 | 99 | 336 | 113 | 332 | Uncharacterized protein | Uncharacterized protein | | afdb-uniprot50 | AF-A0A367RNI6-F1-MODEL\_V4 | 1.0 | 9.061e-15 | 467 | 0.16 | 448 | 280 | 20 | 381 | 751 | 70 | 498 | Uncharacterized protein | Uncharacterized protein | | afdb-uniprot50 | AF-D6Z5I8-F1-MODEL\_V4 | 1.0 | 1.292e-25 | 465 | 0.188 | 879 | 425 | 54 | 100 | 751 | 73 | 889 | Uncharacterized protein | Uncharacterized protein | | afdb-uniprot50 | AF-A0A7U4BPL7-F1-MODEL\_V4 | 1.0 | 6.114e-14 | 464 | 0.272 | 279 | 160 | 11 | 58 | 322 | 67 | 316 | Prim\_Zn\_Ribbon domain-containing protein | Prim\_Zn\_Ribbon domain-containing protein | | afdb-uniprot50 | AF-A0A416MI66-F1-MODEL\_V4 | 1.0 | 1.396e-13 | 464 | 0.207 | 318 | 211 | 15 | 477 | 769 | 2 | 303 | Virulence-associated protein E | Virulence-associated protein E | | afdb-uniprot50 | AF-A0A7K1M057-F1-MODEL\_V4 | 1.0 | 4.4e-15 | 464 | 0.19 | 394 | 274 | 20 | 286 | 659 | 86 | 454 | Uncharacterized protein | Uncharacterized protein | | afdb-uniprot50 | AF-A0A1V8M6R2-F1-MODEL\_V4 | 1.0 | 2.056e-25 | 464 | 0.174 | 785 | 446 | 41 | 91 | 740 | 77 | 794 | DUF927 domain-containing protein | DUF927 domain-containing protein | | afdb-uniprot50 | AF-A0A2V2BHY3-F1-MODEL\_V4 | 1.0 | 6.867e-16 | 463 | 0.244 | 356 | 199 | 14 | 118 | 449 | 166 | 475 | Putative DNA primase/helicase | Putative DNA primase/helicase | | afdb-uniprot50 | AF-A0A1I4TDG0-F1-MODEL\_V4 | 1.0 | 4.785e-16 | 461 | 0.18 | 411 | 253 | 20 | 264 | 661 | 288 | 627 | Virulence-associated protein E | Virulence-associated protein E | | afdb-uniprot50 | AF-A0A7U7G979-F1-MODEL\_V4 | 1.0 | 5.732e-12 | 460 | 0.32 | 231 | 129 | 10 | 103 | 319 | 79 | 295 | Uncharacterized protein | Uncharacterized protein | | afdb-uniprot50 | AF-A0A832X2K7-F1-MODEL\_V4 | 1.0 | 3.378e-18 | 459 | 0.224 | 489 | 240 | 25 | 1 | 431 | 16 | 423 | AAA family ATPase | AAA family ATPase | | afdb-uniprot50 | AF-A0A328ZM50-F1-MODEL\_V4 | 1.0 | 3.603e-20 | 459 | 0.222 | 562 | 298 | 33 | 99 | 615 | 76 | 543 | Phage/plasmid primase-like uncharacterized protein | Phage/plasmid primase-like uncharacterized protein | | afdb-uniprot50 | AF-A0A1J1LKJ5-F1-MODEL\_V4 | 1.0 | 8.387e-19 | 459 | 0.184 | 592 | 356 | 28 | 231 | 769 | 230 | 747 | Prim-Pol domain-containing protein | Prim-Pol domain-containing protein | | afdb-uniprot50 | AF-A0A1S9D3E4-F1-MODEL\_V4 | 1.0 | 4.401e-23 | 457 | 0.187 | 874 | 417 | 44 | 12 | 762 | 6 | 708 | DNA primase TraC | DNA primase TraC | | afdb-uniprot50 | AF-B6WV28-F1-MODEL\_V4 | 1.0 | 9.666e-25 | 456 | 0.204 | 745 | 400 | 44 | 102 | 736 | 76 | 737 | Toprim domain protein | Toprim domain protein | | afdb-uniprot50 | AF-R6M6C9-F1-MODEL\_V4 | 1.0 | 5.622e-13 | 455 | 0.197 | 248 | 189 | 7 | 512 | 755 | 2 | 243 | VirE N-terminal domain protein | VirE N-terminal domain protein | | afdb-uniprot50 | AF-A0A163VSB7-F1-MODEL\_V4 | 1.0 | 7.712e-18 | 454 | 0.187 | 528 | 333 | 30 | 288 | 759 | 285 | 772 | Virulence-associated protein E | Virulence-associated protein E | | afdb-uniprot50 | AF-A0A127Q8R1-F1-MODEL\_V4 | 1.0 | 9.666e-25 | 454 | 0.188 | 765 | 411 | 43 | 102 | 737 | 107 | 790 | Toprim domain protein | Toprim domain protein | | afdb-uniprot50 | AF-A0A6L8Z9G2-F1-MODEL\_V4 | 1.0 | 7.863e-17 | 453 | 0.178 | 447 | 266 | 23 | 240 | 661 | 242 | 612 | Virulence protein E | Virulence protein E | | afdb-uniprot50 | AF-A5KIU4-F1-MODEL\_V4 | 1.0 | 4.125e-13 | 451 | 0.231 | 298 | 198 | 14 | 492 | 769 | 1 | 287 | Virulence-associated protein E | Virulence-associated protein E | | afdb-uniprot50 | AF-A0A1Z4S646-F1-MODEL\_V4 | 1.0 | 2.294e-14 | 450 | 0.177 | 435 | 290 | 20 | 372 | 758 | 93 | 507 | Virulence-associated E family protein | Virulence-associated E family protein | | afdb-uniprot50 | AF-A0A090TZ23-F1-MODEL\_V4 | 1.0 | 1.518e-22 | 450 | 0.15 | 806 | 447 | 49 | 119 | 760 | 3 | 734 | DNA primase phage-associated | DNA primase phage-associated | | afdb-uniprot50 | AF-A0A2D3TEK2-F1-MODEL\_V4 | 1.0 | 1.072e-16 | 448 | 0.244 | 413 | 210 | 17 | 107 | 447 | 155 | 537 | Uncharacterized protein | Uncharacterized protein | | afdb-uniprot50 | AF-A0A2X1NEA3-F1-MODEL\_V4 | 1.0 | 3.292e-14 | 446 | 0.221 | 347 | 188 | 12 | 73 | 416 | 79 | 346 | DNA primase TraC | DNA primase TraC | | afdb-uniprot50 | AF-A0A742HTC7-F1-MODEL\_V4 | 1.0 | 1.114e-14 | 446 | 0.238 | 361 | 200 | 15 | 98 | 442 | 135 | 436 | DUF5710 domain-containing protein | DUF5710 domain-containing protein | | afdb-uniprot50 | AF-A0A7G7VVW2-F1-MODEL\_V4 | 1.0 | 1.528e-19 | 446 | 0.165 | 664 | 380 | 35 | 178 | 756 | 123 | 697 | Bifunctional DNA primase/polymerase | Bifunctional DNA primase/polymerase | | afdb-uniprot50 | AF-G4FLD9-F1-MODEL\_V4 | 1.0 | 6.521e-16 | 445 | 0.151 | 568 | 355 | 30 | 261 | 764 | 26 | 530 | Virulence-associated E family protein | Virulence-associated E family protein | | afdb-uniprot50 | AF-A0A2X0RDQ2-F1-MODEL\_V4 | 1.0 | 1.915e-18 | 445 | 0.219 | 519 | 267 | 26 | 98 | 539 | 87 | 544 | Toprim domain-containing protein | Toprim domain-containing protein | | afdb-uniprot50 | AF-A4CPW0-F1-MODEL\_V4 | 1.0 | 4.604e-18 | 444 | 0.155 | 800 | 407 | 33 | 1 | 769 | 183 | 744 | Uncharacterized protein | Uncharacterized protein | | afdb-uniprot50 | AF-A0A7W2RV62-F1-MODEL\_V4 | 1.0 | 2.643e-12 | 443 | 0.247 | 263 | 153 | 13 | 91 | 346 | 69 | 293 | Toprim domain-containing protein | Toprim domain-containing protein | | afdb-uniprot50 | AF-A0A847UTT6-F1-MODEL\_V4 | 1.0 | 1.599e-22 | 443 | 0.181 | 876 | 432 | 58 | 1 | 753 | 22 | 735 | AAA family ATPase | AAA family ATPase | | afdb-uniprot50 | AF-K9P6A2-F1-MODEL\_V4 | 1.0 | 3.794e-20 | 443 | 0.138 | 800 | 415 | 33 | 1 | 759 | 230 | 795 | Putative P-loop ATPase | Putative P-loop ATPase | | afdb-uniprot50 | AF-B5INY9-F1-MODEL\_V4 | 1.0 | 1.143e-18 | 443 | 0.146 | 783 | 351 | 28 | 1 | 743 | 196 | 700 | Primase C terminal 2 family | Primase C terminal 2 family | | afdb-uniprot50 | AF-A0A128ESL8-F1-MODEL\_V4 | 1.0 | 2.109e-21 | 439 | 0.158 | 787 | 451 | 41 | 122 | 756 | 92 | 818 | DNA primase TraC | DNA primase TraC | | afdb-uniprot50 | AF-X0WPY2-F1-MODEL\_V4 | 1.0 | 7.812e-12 | 437 | 0.209 | 262 | 189 | 11 | 410 | 663 | 6 | 257 | Uncharacterized protein | Uncharacterized protein | | afdb-uniprot50 | AF-A0A7L8A2Q6-F1-MODEL\_V4 | 1.0 | 2.494e-15 | 436 | 0.21 | 404 | 227 | 20 | 102 | 485 | 69 | 400 | Toprim domain-containing protein | Toprim domain-containing protein | | afdb-uniprot50 | AF-A0A1I4I4F4-F1-MODEL\_V4 | 1.0 | 7.001e-15 | 436 | 0.158 | 453 | 289 | 23 | 368 | 744 | 65 | 501 | Virulence-associated protein E | Virulence-associated protein E | | afdb-uniprot50 | AF-A0A2S9KFP8-F1-MODEL\_V4 | 1.0 | 4.545e-24 | 434 | 0.207 | 772 | 405 | 47 | 93 | 739 | 81 | 770 | Uncharacterized protein | Uncharacterized protein | | afdb-uniprot50 | AF-A0A415EYP9-F1-MODEL\_V4 | 1.0 | 1.011e-11 | 433 | 0.2 | 265 | 192 | 9 | 407 | 659 | 10 | 266 | Uncharacterized protein | Uncharacterized protein | | afdb-uniprot50 | AF-A0A380Z8N6-F1-MODEL\_V4 | 1.0 | 1.275e-15 | 433 | 0.194 | 436 | 260 | 25 | 261 | 659 | 91 | 472 | Predicted P-loop ATPase and inactivated derivatives | Predicted P-loop ATPase and inactivated derivatives | | afdb-uniprot50 | AF-A0A0P0M208-F1-MODEL\_V4 | 1.0 | 3.086e-12 | 432 | 0.172 | 266 | 193 | 11 | 505 | 757 | 3 | 254 | Putative helicase | Putative helicase | | afdb-uniprot50 | AF-A0A6A7Z0Y2-F1-MODEL\_V4 | 1.0 | 4.206e-12 | 432 | 0.263 | 254 | 155 | 10 | 488 | 722 | 29 | 269 | Uncharacterized protein | Uncharacterized protein | | afdb-uniprot50 | AF-A0A1F0MEI0-F1-MODEL\_V4 | 1.0 | 1.196e-13 | 430 | 0.213 | 314 | 201 | 14 | 370 | 654 | 60 | 356 | Uncharacterized protein | Uncharacterized protein | | afdb-uniprot50 | AF-A0A6L7TTJ7-F1-MODEL\_V4 | 1.0 | 4.724e-14 | 430 | 0.193 | 429 | 288 | 22 | 387 | 763 | 362 | 784 | VirE\_N domain-containing protein | VirE\_N domain-containing protein | | afdb-uniprot50 | AF-A0A412XP58-F1-MODEL\_V4 | 1.0 | 4.663e-12 | 429 | 0.245 | 232 | 169 | 4 | 528 | 756 | 2 | 230 | Virulence protein E | Virulence protein E | | afdb-uniprot50 | AF-A0A5K7ZQE0-F1-MODEL\_V4 | 1.0 | 5.374e-18 | 427 | 0.211 | 576 | 247 | 25 | 93 | 624 | 69 | 481 | Toprim domain-containing protein | Toprim domain-containing protein | | afdb-uniprot50 | AF-B2IUW1-F1-MODEL\_V4 | 1.0 | 2.165e-17 | 426 | 0.185 | 555 | 323 | 32 | 261 | 769 | 287 | 758 | Virulence-associated E family protein | Virulence-associated E family protein | | afdb-uniprot50 | AF-A0A521VJ17-F1-MODEL\_V4 | 1.0 | 1.3e-22 | 424 | 0.189 | 873 | 373 | 47 | 92 | 762 | 90 | 829 | DUF3987 domain-containing protein | DUF3987 domain-containing protein | | afdb-uniprot50 | AF-A0A1T4M124-F1-MODEL\_V4 | 1.0 | 2.783e-12 | 423 | 0.167 | 352 | 245 | 15 | 441 | 756 | 2 | 341 | Virulence-associated protein E | Virulence-associated protein E | | afdb-uniprot50 | AF-A0A3A6TG99-F1-MODEL\_V4 | 1.0 | 1.89e-16 | 423 | 0.209 | 444 | 248 | 24 | 117 | 495 | 168 | 573 | Uncharacterized protein | Uncharacterized protein | | afdb-uniprot50 | AF-A0A7G7KLA7-F1-MODEL\_V4 | 1.0 | 2.875e-13 | 421 | 0.269 | 286 | 174 | 14 | 488 | 757 | 4 | 270 | Uncharacterized protein | Uncharacterized protein | | afdb-uniprot50 | AF-A0A6G1V8R3-F1-MODEL\_V4 | 1.0 | 2.431e-11 | 420 | 0.199 | 241 | 183 | 6 | 517 | 751 | 2 | 238 | Helicase | Helicase | | afdb-uniprot50 | AF-I9HUA8-F1-MODEL\_V4 | 1.0 | 1.528e-11 | 418 | 0.181 | 265 | 205 | 8 | 498 | 756 | 2 | 260 | DUF3874 domain-containing protein | DUF3874 domain-containing protein | | afdb-uniprot50 | AF-A0A1Z4FZF4-F1-MODEL\_V4 | 1.0 | 3.603e-12 | 418 | 0.244 | 262 | 181 | 7 | 513 | 769 | 2 | 251 | Virulence-associated E family protein | Virulence-associated E family protein | | afdb-uniprot50 | AF-K5DAG2-F1-MODEL\_V4 | 1.0 | 9.79e-11 | 417 | 0.295 | 176 | 120 | 3 | 514 | 688 | 23 | 195 | Uncharacterized protein | Uncharacterized protein | | afdb-uniprot50 | AF-A0A1Q8QRC0-F1-MODEL\_V4 | 1.0 | 4.288e-19 | 414 | 0.196 | 601 | 258 | 30 | 102 | 643 | 82 | 516 | DNA primase | DNA primase | | afdb-uniprot50 | AF-A0A7W0X7N0-F1-MODEL\_V4 | 1.0 | 1.065e-19 | 414 | 0.193 | 676 | 320 | 35 | 102 | 673 | 76 | 629 | DUF3987 domain-containing protein | DUF3987 domain-containing protein | | afdb-uniprot50 | AF-A0A1H6F9A3-F1-MODEL\_V4 | 1.0 | 1.093e-23 | 414 | 0.176 | 860 | 438 | 53 | 12 | 731 | 8 | 736 | DNA primase TraC | DNA primase TraC | | afdb-uniprot50 | AF-A0A240TVL1-F1-MODEL\_V4 | 1.0 | 2.463e-21 | 413 | 0.186 | 733 | 348 | 37 | 102 | 728 | 78 | 667 | Toprim domain-containing protein | Toprim domain-containing protein | | afdb-uniprot50 | AF-A0A7U8B3W4-F1-MODEL\_V4 | 1.0 | 2.559e-11 | 412 | 0.22 | 268 | 167 | 10 | 98 | 343 | 126 | 373 | DNA primase | DNA primase | | afdb-uniprot50 | AF-A0A3M5N5A8-F1-MODEL\_V4 | 1.0 | 1.651e-15 | 412 | 0.231 | 428 | 222 | 18 | 114 | 469 | 354 | 746 | DNA primase TraC | DNA primase TraC | | afdb-uniprot50 | AF-U2LAS6-F1-MODEL\_V4 | 1.0 | 1.284e-12 | 411 | 0.202 | 301 | 197 | 11 | 370 | 638 | 51 | 340 | Virulence-associated protein E | Virulence-associated protein E | | afdb-uniprot50 | AF-A0A524BHU0-F1-MODEL\_V4 | 1.0 | 6.035e-12 | 408 | 0.248 | 249 | 173 | 7 | 519 | 762 | 2 | 241 | Uncharacterized protein | Uncharacterized protein | | afdb-uniprot50 | AF-A0A2L2NAA1-F1-MODEL\_V4 | 1.0 | 6.779e-14 | 407 | 0.189 | 460 | 236 | 20 | 260 | 671 | 25 | 395 | Virulence-associated E family protein | Virulence-associated E family protein | | afdb-uniprot50 | AF-A0A3A9HBK2-F1-MODEL\_V4 | 1.0 | 4.515e-11 | 406 | 0.217 | 262 | 168 | 12 | 488 | 728 | 10 | 255 | Virulence-associated protein E | Virulence-associated protein E | | afdb-uniprot50 | AF-G2H1Q2-F1-MODEL\_V4 | 1.0 | 3.843e-22 | 406 | 0.151 | 917 | 493 | 52 | 1 | 736 | 29 | 840 | Primase C | Primase C | | afdb-uniprot50 | AF-A0A558HMQ4-F1-MODEL\_V4 | 1.0 | 5.271e-11 | 405 | 0.18 | 277 | 195 | 13 | 80 | 344 | 120 | 376 | Uncharacterized protein | Uncharacterized protein | | afdb-uniprot50 | AF-A0A7K1M065-F1-MODEL\_V4 | 1.0 | 2.354e-10 | 404 | 0.222 | 198 | 141 | 7 | 466 | 653 | 3 | 197 | Uncharacterized protein | Uncharacterized protein | | afdb-uniprot50 | AF-A0A5K1IZ44-F1-MODEL\_V4 | 1.0 | 6.735e-17 | 404 | 0.149 | 616 | 368 | 35 | 226 | 759 | 199 | 740 | Virulence-associated protein E | Virulence-associated protein E | | afdb-uniprot50 | AF-A0A5J4PAM3-F1-MODEL\_V4 | 1.0 | 1.727e-10 | 403 | 0.282 | 191 | 129 | 6 | 514 | 699 | 2 | 189 | Uncharacterized protein | Uncharacterized protein | | afdb-uniprot50 | AF-A0A6M9PYL6-F1-MODEL\_V4 | 1.0 | 1.079e-21 | 403 | 0.199 | 723 | 382 | 45 | 89 | 732 | 61 | 665 | Toprim domain-containing protein | Toprim domain-containing protein | | afdb-uniprot50 | AF-A0A6S6S6H1-F1-MODEL\_V4 | 1.0 | 1.085e-10 | 402 | 0.287 | 205 | 126 | 6 | 91 | 295 | 432 | 616 | DNA primase (EC) | DNA primase (EC) | | afdb-uniprot50 | AF-A0A3P1ZSQ5-F1-MODEL\_V4 | 1.0 | 6.779e-22 | 402 | 0.196 | 789 | 412 | 43 | 92 | 731 | 71 | 786 | DUF927 domain-containing protein | DUF927 domain-containing protein | | afdb-uniprot50 | AF-A0A257SN45-F1-MODEL\_V4 | 1.0 | 5.514e-14 | 401 | 0.206 | 398 | 222 | 20 | 102 | 454 | 86 | 434 | Toprim domain-containing protein | Toprim domain-containing protein | | afdb-uniprot50 | AF-A0A1Z5HDV9-F1-MODEL\_V4 | 1.0 | 5.844e-11 | 400 | 0.269 | 219 | 134 | 9 | 466 | 666 | 12 | 222 | Uncharacterized protein | Uncharacterized protein | | afdb-uniprot50 | AF-A0A496KDJ9-F1-MODEL\_V4 | 1.0 | 1.807e-13 | 399 | 0.203 | 374 | 199 | 20 | 98 | 447 | 75 | 373 | Toprim domain-containing protein | Toprim domain-containing protein | | afdb-uniprot50 | AF-F1W2P6-F1-MODEL\_V4 | 1.0 | 1.044e-20 | 398 | 0.157 | 808 | 385 | 46 | 100 | 755 | 78 | 741 | Toprim domain-containing protein | Toprim domain-containing protein | | afdb-uniprot50 | AF-A0A535HSC6-F1-MODEL\_V4 | 1.0 | 1.079e-21 | 396 | 0.176 | 825 | 407 | 42 | 106 | 738 | 1 | 744 | DUF927 domain-containing protein | DUF927 domain-containing protein | | afdb-uniprot50 | AF-A0A0F7KH82-F1-MODEL\_V4 | 1.0 | 1.369e-22 | 395 | 0.202 | 761 | 386 | 47 | 92 | 737 | 71 | 725 | Putative DNA primase/helicase | Putative DNA primase/helicase | | afdb-uniprot50 | AF-A0A5E4TKB0-F1-MODEL\_V4 | 1.0 | 1.058e-22 | 395 | 0.186 | 757 | 388 | 45 | 119 | 739 | 2 | 666 | DNA primase | DNA primase | | afdb-uniprot50 | AF-W2CNN4-F1-MODEL\_V4 | 1.0 | 3.556e-10 | 394 | 0.258 | 186 | 128 | 4 | 471 | 655 | 4 | 180 | Virulence-associated protein E | Virulence-associated protein E | | afdb-uniprot50 | AF-A0A1T5CZF5-F1-MODEL\_V4 | 1.0 | 1.818e-10 | 394 | 0.248 | 225 | 161 | 6 | 534 | 755 | 1 | 220 | Virulence-associated protein E | Virulence-associated protein E | | afdb-uniprot50 | AF-A0A533QNK2-F1-MODEL\_V4 | 1.0 | 3.868e-19 | 392 | 0.183 | 763 | 340 | 43 | 98 | 750 | 73 | 662 | DNA primase TraC | DNA primase TraC | | afdb-uniprot50 | AF-A0A858ZQH3-F1-MODEL\_V4 | 1.0 | 1.121e-19 | 388 | 0.178 | 794 | 372 | 40 | 93 | 769 | 75 | 704 | AAA family ATPase | AAA family ATPase | | afdb-uniprot50 | AF-A0A2E5Y8U8-F1-MODEL\_V4 | 1.0 | 1.352e-20 | 388 | 0.152 | 854 | 385 | 49 | 102 | 753 | 75 | 791 | DNA primase | DNA primase | | afdb-uniprot50 | AF-X1J433-F1-MODEL\_V4 | 1.0 | 3.943e-10 | 387 | 0.2 | 274 | 196 | 14 | 407 | 669 | 2 | 263 | Uncharacterized protein | Uncharacterized protein | | afdb-uniprot50 | AF-A0A6N9A9R1-F1-MODEL\_V4 | 1.0 | 7.002e-23 | 383 | 0.165 | 816 | 453 | 55 | 54 | 745 | 59 | 770 | Uncharacterized protein | Uncharacterized protein | | afdb-uniprot50 | AF-A0A349EI79-F1-MODEL\_V4 | 1.0 | 3.378e-18 | 378 | 0.181 | 793 | 316 | 39 | 92 | 753 | 72 | 662 | Toprim domain-containing protein | Toprim domain-containing protein | | afdb-uniprot50 | AF-A0A4R6Y3D7-F1-MODEL\_V4 | 1.0 | 7.965e-19 | 378 | 0.197 | 769 | 356 | 42 | 91 | 758 | 70 | 677 | Putative DNA primase/helicase | Putative DNA primase/helicase | | afdb-uniprot50 | AF-A0A3R6E138-F1-MODEL\_V4 | 1.0 | 3.443e-09 | 374 | 0.225 | 173 | 129 | 4 | 482 | 652 | 3 | 172 | Virulence protein E | Virulence protein E | | afdb-uniprot50 | AF-K1RXF7-F1-MODEL\_V4 | 1.0 | 7.711e-10 | 372 | 0.229 | 187 | 129 | 5 | 511 | 684 | 8 | 192 | Uncharacterized protein | Uncharacterized protein | | afdb-uniprot50 | AF-A0A090BV70-F1-MODEL\_V4 | 1.0 | 6.564e-21 | 372 | 0.165 | 779 | 410 | 43 | 92 | 747 | 67 | 728 | Toprim domain-containing protein | Toprim domain-containing protein | | afdb-uniprot50 | AF-A0A0H5Q210-F1-MODEL\_V4 | 1.0 | 8.121e-18 | 370 | 0.168 | 740 | 331 | 35 | 102 | 744 | 85 | 636 | Toprim domain-containing protein | Toprim domain-containing protein | | afdb-uniprot50 | AF-A0A660N8U3-F1-MODEL\_V4 | 1.0 | 2.415e-14 | 370 | 0.216 | 458 | 223 | 23 | 87 | 477 | 2 | 390 | Toprim domain-containing protein | Toprim domain-containing protein | | afdb-uniprot50 | AF-A0A142JNG9-F1-MODEL\_V4 | 1.0 | 4.344e-13 | 368 | 0.17 | 482 | 285 | 31 | 368 | 759 | 100 | 556 | Uncharacterized protein | Uncharacterized protein | | afdb-uniprot50 | AF-A0A5C7LNP3-F1-MODEL\_V4 | 1.0 | 1.235e-14 | 368 | 0.151 | 541 | 357 | 28 | 290 | 769 | 181 | 680 | ZnF\_CHCC domain-containing protein | ZnF\_CHCC domain-containing protein | | afdb-uniprot50 | AF-A0A1F9LYL6-F1-MODEL\_V4 | 1.0 | 1.609e-19 | 368 | 0.173 | 776 | 348 | 38 | 102 | 731 | 107 | 734 | Toprim domain-containing protein | Toprim domain-containing protein | | afdb-uniprot50 | AF-A0A5E5AI01-F1-MODEL\_V4 | 1.0 | 4.428e-12 | 367 | 0.177 | 434 | 280 | 25 | 377 | 750 | 109 | 525 | Virulence protein E | Virulence protein E | | afdb-uniprot50 | AF-A0A6J4G0U8-F1-MODEL\_V4 | 1.0 | 1.651e-07 | 365 | 0.431 | 116 | 62 | 2 | 234 | 347 | 22 | 135 | Toprim domain-containing protein | Toprim domain-containing protein | | afdb-uniprot50 | AF-A0A6I7WYX8-F1-MODEL\_V4 | 1.0 | 2.695e-11 | 365 | 0.244 | 229 | 156 | 7 | 506 | 724 | 9 | 230 | Uncharacterized protein | Uncharacterized protein | | afdb-uniprot50 | AF-A0A5E4TGU1-F1-MODEL\_V4 | 1.0 | 7.662e-13 | 364 | 0.165 | 448 | 270 | 25 | 377 | 751 | 204 | 620 | Virulence protein E | Virulence protein E | | afdb-uniprot50 | AF-A0A133Y5T5-F1-MODEL\_V4 | 1.0 | 3.046e-10 | 363 | 0.219 | 251 | 180 | 10 | 380 | 622 | 8 | 250 | Uncharacterized protein | Uncharacterized protein | | afdb-uniprot50 | AF-A0A651FY11-F1-MODEL\_V4 | 1.0 | 7.712e-18 | 363 | 0.173 | 743 | 317 | 43 | 102 | 743 | 78 | 624 | Toprim domain-containing protein | Toprim domain-containing protein | | afdb-uniprot50 | AF-A0A412FI27-F1-MODEL\_V4 | 1.0 | 2.235e-10 | 361 | 0.227 | 255 | 181 | 9 | 379 | 624 | 16 | 263 | Virulence protein | Virulence protein | | afdb-uniprot50 | AF-I5D485-F1-MODEL\_V4 | 1.0 | 1.024e-13 | 360 | 0.166 | 451 | 276 | 31 | 377 | 754 | 94 | 517 | Virulence-associated E family protein | Virulence-associated E family protein | | afdb-uniprot50 | AF-A0A1G7B4P0-F1-MODEL\_V4 | 1.0 | 3.378e-18 | 358 | 0.166 | 792 | 329 | 42 | 101 | 757 | 93 | 687 | Uncharacterized domain associated with phage/plasmid primase | Uncharacterized domain associated with phage/plasmid primase | | afdb-uniprot50 | AF-A0A4Z0F4C3-F1-MODEL\_V4 | 1.0 | 2.15e-12 | 355 | 0.173 | 374 | 247 | 20 | 318 | 650 | 125 | 477 | Uncharacterized protein | Uncharacterized protein | | afdb-uniprot50 | AF-A0A134BA49-F1-MODEL\_V4 | 1.0 | 1.705e-08 | 353 | 0.293 | 150 | 103 | 2 | 490 | 638 | 9 | 156 | Uncharacterized protein | Uncharacterized protein | | afdb-uniprot50 | AF-A0A845UU64-F1-MODEL\_V4 | 1.0 | 1.107e-17 | 352 | 0.171 | 762 | 337 | 43 | 99 | 757 | 88 | 657 | AAA family ATPase | AAA family ATPase | | afdb-uniprot50 | AF-A0A1V0QD50-F1-MODEL\_V4 | 1.0 | 3.106e-09 | 351 | 0.186 | 230 | 161 | 9 | 463 | 687 | 3 | 211 | Uncharacterized protein | Uncharacterized protein | | afdb-uniprot50 | AF-A0A4R1VW43-F1-MODEL\_V4 | 1.0 | 5.305e-16 | 350 | 0.202 | 588 | 291 | 35 | 77 | 609 | 81 | 545 | Phage/plasmid primase-like uncharacterized protein | Phage/plasmid primase-like uncharacterized protein | | afdb-uniprot50 | AF-A0A5S9QFP7-F1-MODEL\_V4 | 1.0 | 8.226e-20 | 350 | 0.154 | 861 | 458 | 51 | 93 | 765 | 88 | 866 | DNA primase TraC | DNA primase TraC | | afdb-uniprot50 | AF-A0A3C0I2Y8-F1-MODEL\_V4 | 1.0 | 5.038e-16 | 349 | 0.162 | 566 | 278 | 27 | 99 | 618 | 94 | 509 | Uncharacterized protein | Uncharacterized protein | | afdb-uniprot50 | AF-A0A2M8TYS1-F1-MODEL\_V4 | 1.0 | 5.204e-17 | 348 | 0.19 | 661 | 341 | 38 | 100 | 636 | 68 | 658 | Toprim domain-containing protein | Toprim domain-containing protein | | afdb-uniprot50 | AF-A0A1E5Q402-F1-MODEL\_V4 | 1.0 | 1.727e-18 | 346 | 0.167 | 728 | 386 | 49 | 100 | 759 | 115 | 690 | Toprim domain-containing protein | Toprim domain-containing protein | | afdb-uniprot50 | AF-A0A0H3A6N2-F1-MODEL\_V4 | 1.0 | 9.121e-20 | 344 | 0.186 | 805 | 376 | 49 | 112 | 752 | 85 | 774 | Toprim sub domain protein | Toprim sub domain protein | | afdb-uniprot50 | AF-A0A2J6MQS6-F1-MODEL\_V4 | 1.0 | 6.691e-12 | 343 | 0.16 | 449 | 271 | 26 | 377 | 751 | 151 | 567 | Uncharacterized protein | Uncharacterized protein | | afdb-uniprot50 | AF-A0A4R7DVC6-F1-MODEL\_V4 | 1.0 | 2.354e-18 | 343 | 0.177 | 767 | 377 | 52 | 88 | 756 | 60 | 670 | Putative DNA primase/helicase | Putative DNA primase/helicase | | afdb-uniprot50 | AF-A0A4R5QFG5-F1-MODEL\_V4 | 1.0 | 2.893e-18 | 343 | 0.168 | 835 | 411 | 43 | 1 | 743 | 1 | 643 | Toprim domain-containing protein | Toprim domain-containing protein | | afdb-uniprot50 | AF-A0A7V2GZR5-F1-MODEL\_V4 | 1.0 | 2.056e-17 | 336 | 0.186 | 723 | 317 | 46 | 132 | 757 | 3 | 550 | Toprim domain-containing protein | Toprim domain-containing protein | | afdb-uniprot50 | AF-A0A847UVH2-F1-MODEL\_V4 | 1.0 | 1.405e-10 | 335 | 0.31 | 216 | 126 | 9 | 13 | 212 | 2 | 210 | Uncharacterized protein | Uncharacterized protein | | afdb-uniprot50 | AF-A0A0H3ZTP3-F1-MODEL\_V4 | 1.0 | 1.334e-18 | 335 | 0.128 | 944 | 513 | 53 | 6 | 745 | 2 | 839 | DNA primase | DNA primase | | afdb-uniprot50 | AF-A0A257DJ96-F1-MODEL\_V4 | 1.0 | 1.15e-15 | 333 | 0.2 | 588 | 299 | 33 | 76 | 609 | 80 | 550 | ZnF\_CHCC domain-containing protein | ZnF\_CHCC domain-containing protein | | afdb-uniprot50 | AF-A0A1F7FGC5-F1-MODEL\_V4 | 1.0 | 3.334e-16 | 332 | 0.12 | 724 | 431 | 51 | 162 | 755 | 89 | 736 | SF3 helicase domain-containing protein | SF3 helicase domain-containing protein | | afdb-uniprot50 | AF-A0A258SRK8-F1-MODEL\_V4 | 1.0 | 7.612e-08 | 329 | 0.315 | 130 | 87 | 1 | 362 | 489 | 57 | 186 | Uncharacterized protein | Uncharacterized protein | | afdb-uniprot50 | AF-A0A5C7K204-F1-MODEL\_V4 | 1.0 | 3.066e-15 | 321 | 0.188 | 574 | 297 | 36 | 115 | 614 | 114 | 592 | Herpes\_ori\_bp domain-containing protein | Herpes\_ori\_bp domain-containing protein | | afdb-uniprot50 | AF-A0A6J4FPK4-F1-MODEL\_V4 | 1.0 | 3.943e-18 | 315 | 0.198 | 740 | 311 | 38 | 125 | 754 | 102 | 669 | Toprim domain-containing protein | Toprim domain-containing protein | | afdb-uniprot50 | AF-A0A1M6LF95-F1-MODEL\_V4 | 1.0 | 1.46e-08 | 312 | 0.182 | 236 | 180 | 8 | 534 | 762 | 1 | 230 | Virulence-associated protein E | Virulence-associated protein E | | afdb-uniprot50 | AF-A0A4Z1QPV4-F1-MODEL\_V4 | 1.0 | 1.878e-11 | 311 | 0.134 | 492 | 330 | 28 | 328 | 769 | 53 | 498 | Uncharacterized protein | Uncharacterized protein | | afdb-uniprot50 | AF-A0A4E0QLS9-F1-MODEL\_V4 | 1.0 | 1.114e-14 | 311 | 0.129 | 632 | 376 | 34 | 81 | 628 | 98 | 639 | Uncharacterized protein | Uncharacterized protein | | afdb-uniprot50 | AF-A0A7X1KDD5-F1-MODEL\_V4 | 1.0 | 1.508e-17 | 305 | 0.188 | 749 | 320 | 36 | 125 | 754 | 312 | 891 | Bifunctional DNA primase/polymerase | Bifunctional DNA primase/polymerase | | afdb-uniprot50 | AF-A0A7J0A256-F1-MODEL\_V4 | 1.0 | 3.007e-08 | 301 | 0.243 | 197 | 144 | 3 | 563 | 756 | 5 | 199 | Uncharacterized protein | Uncharacterized protein | | afdb-uniprot50 | AF-A0A6I2NXZ8-F1-MODEL\_V4 | 1.0 | 2.4e-09 | 296 | 0.189 | 274 | 193 | 11 | 503 | 762 | 20 | 278 | DUF3874 domain-containing protein | DUF3874 domain-containing protein | | afdb-uniprot50 | AF-A0A6N1AJ04-F1-MODEL\_V4 | 1.0 | 1.334e-10 | 294 | 0.148 | 466 | 291 | 28 | 376 | 757 | 83 | 526 | Uncharacterized protein | Uncharacterized protein | | afdb-uniprot50 | AF-A0A7G8I5M2-F1-MODEL\_V4 | 1.0 | 4.02e-09 | 293 | 0.147 | 278 | 203 | 9 | 497 | 751 | 3 | 269 | Virulence-associated E family protein | Virulence-associated E family protein | | afdb-uniprot50 | AF-A0A160JDL1-F1-MODEL\_V4 | 1.0 | 5.55e-11 | 292 | 0.126 | 567 | 365 | 32 | 239 | 769 | 107 | 578 | Uncharacterized protein | Uncharacterized protein | | afdb-uniprot50 | AF-A0A1S7U2B0-F1-MODEL\_V4 | 1.0 | 6.606e-10 | 287 | 0.129 | 409 | 279 | 21 | 381 | 744 | 98 | 474 | Uncharacterized protein | Uncharacterized protein | | afdb-uniprot50 | AF-K1SV85-F1-MODEL\_V4 | 1.0 | 1.058e-06 | 277 | 0.305 | 134 | 91 | 2 | 509 | 641 | 4 | 136 | Virulence-associated protein E | Virulence-associated protein E | | afdb-uniprot50 | AF-A0A6N2RAJ0-F1-MODEL\_V4 | 1.0 | 1.761e-09 | 262 | 0.153 | 405 | 242 | 13 | 376 | 767 | 44 | 360 | DUF based on B. Theta Gene description | DUF based on B. Theta Gene description | | afdb-uniprot50 | AF-A0A4U1BRV7-F1-MODEL\_V4 | 1.0 | 1.1e-12 | 258 | 0.146 | 520 | 251 | 25 | 151 | 616 | 4 | 384 | Toprim domain-containing protein | Toprim domain-containing protein | | afdb-uniprot50 | AF-A0A358XVY2-F1-MODEL\_V4 | 1.0 | 0.0003421 | 257 | 0.325 | 83 | 56 | 0 | 12 | 94 | 194 | 276 | PriCT\_2 domain-containing protein | PriCT\_2 domain-containing protein | | afdb-uniprot50 | AF-A0A5C5UXX3-F1-MODEL\_V4 | 1.0 | 3.603e-12 | 251 | 0.13 | 758 | 447 | 56 | 150 | 769 | 133 | 816 | SF3 helicase domain-containing protein | SF3 helicase domain-containing protein | | afdb-uniprot50 | AF-A0A7Y8UR69-F1-MODEL\_V4 | 1.0 | 4.152e-10 | 248 | 0.145 | 569 | 350 | 32 | 287 | 769 | 21 | 539 | Uncharacterized protein | Uncharacterized protein | | afdb-uniprot50 | AF-A0A1L9QN36-F1-MODEL\_V4 | 1.0 | 4.663e-12 | 228 | 0.126 | 721 | 428 | 40 | 180 | 751 | 58 | 725 | SF3 helicase domain-containing protein | SF3 helicase domain-containing protein | | afdb-uniprot50 | AF-A0A1V5NKL3-F1-MODEL\_V4 | 1.0 | 2.912e-15 | 226 | 0.151 | 711 | 339 | 34 | 215 | 769 | 8 | 609 | DNA primase TraC | DNA primase TraC | | afdb-uniprot50 | AF-A0A174TGT0-F1-MODEL\_V4 | 1.0 | 1.058e-06 | 224 | 0.217 | 207 | 152 | 7 | 561 | 762 | 18 | 219 | Predicted P-loop ATPase and inactivated derivatives | Predicted P-loop ATPase and inactivated derivatives | | afdb-uniprot50 | AF-R5NEB3-F1-MODEL\_V4 | 1.0 | 2.221e-05 | 223 | 0.191 | 209 | 162 | 5 | 549 | 753 | 2 | 207 | SWIRM domain-containing protein | SWIRM domain-containing protein | | afdb-uniprot50 | AF-A0A7X2MAP5-F1-MODEL\_V4 | 1.0 | 4.457e-09 | 223 | 0.125 | 447 | 273 | 28 | 381 | 753 | 229 | 631 | DNA primase | DNA primase | | afdb-uniprot50 | AF-A0A0J6VN82-F1-MODEL\_V4 | 1.0 | 1.165e-09 | 223 | 0.12 | 557 | 348 | 32 | 240 | 721 | 174 | 663 | Uncharacterized protein | Uncharacterized protein | | afdb-uniprot50 | AF-A0A2E4HGQ7-F1-MODEL\_V4 | 1.0 | 3.867e-11 | 221 | 0.12 | 844 | 403 | 46 | 9 | 751 | 224 | 828 | PriCT\_2 domain-containing protein | PriCT\_2 domain-containing protein | | afdb-uniprot50 | AF-A0A2P7THR1-F1-MODEL\_V4 | 1.0 | 4.344e-13 | 218 | 0.099 | 905 | 508 | 55 | 60 | 750 | 86 | 897 | Toprim domain-containing protein | Toprim domain-containing protein | | afdb-uniprot50 | AF-A0A480BUQ6-F1-MODEL\_V4 | 1.0 | 1.915e-10 | 207 | 0.161 | 595 | 336 | 38 | 267 | 755 | 3 | 540 | Uncharacterized protein | Uncharacterized protein | | afdb-uniprot50 | AF-A0A0D6AFU3-F1-MODEL\_V4 | 1.0 | 2.95e-09 | 192 | 0.107 | 612 | 358 | 35 | 261 | 768 | 373 | 899 | SF3 helicase domain-containing protein | SF3 helicase domain-containing protein | | afdb-uniprot50 | AF-A0A5C8BGE8-F1-MODEL\_V4 | 1.0 | 2.136e-07 | 188 | 0.112 | 505 | 309 | 30 | 327 | 751 | 18 | 463 | SF3 helicase domain-containing protein | SF3 helicase domain-containing protein | | afdb-uniprot50 | AF-A0A200I1W5-F1-MODEL\_V4 | 1.0 | 1.211e-07 | 187 | 0.142 | 449 | 271 | 32 | 376 | 745 | 58 | 471 | SF3 helicase domain-containing protein | SF3 helicase domain-containing protein | | afdb-uniprot50 | AF-A0A401LNS6-F1-MODEL\_V4 | 1.0 | 0.0002783 | 185 | 0.223 | 134 | 103 | 1 | 556 | 689 | 57 | 189 | Uncharacterized protein | Uncharacterized protein | | afdb-uniprot50 | AF-A0A0B0H9C4-F1-MODEL\_V4 | 1.0 | 6.314e-07 | 184 | 0.13 | 491 | 292 | 34 | 374 | 769 | 158 | 608 | Phage/plasmid primase | Phage/plasmid primase | | afdb-uniprot50 | AF-A0A3A5AP02-F1-MODEL\_V4 | 1.0 | 6.521e-08 | 184 | 0.124 | 564 | 340 | 40 | 289 | 757 | 259 | 763 | SF3 helicase domain-containing protein | SF3 helicase domain-containing protein | | afdb-uniprot50 | AF-A0A1L9QT25-F1-MODEL\_V4 | 1.0 | 1.114e-06 | 174 | 0.104 | 439 | 293 | 26 | 394 | 757 | 8 | 421 | SF3 helicase domain-containing protein | SF3 helicase domain-containing protein | | afdb-uniprot50 | AF-A0A8A3PZQ0-F1-MODEL\_V4 | 1.0 | 6.193e-08 | 174 | 0.134 | 461 | 273 | 32 | 383 | 753 | 350 | 774 | Uncharacterized protein | Uncharacterized protein | | afdb-uniprot50 | AF-A0A2S9SYH7-F1-MODEL\_V4 | 1.0 | 2.294e-06 | 173 | 0.095 | 460 | 298 | 22 | 384 | 751 | 222 | 655 | Uncharacterized protein | Uncharacterized protein | | afdb-uniprot50 | AF-A0A1W2G805-F1-MODEL\_V4 | 1.0 | 5.958e-10 | 171 | 0.096 | 799 | 432 | 54 | 145 | 744 | 5 | 712 | Uncharacterized protein | Uncharacterized protein | | afdb-uniprot50 | AF-A0A1M6M5E6-F1-MODEL\_V4 | 1.0 | 3.399e-07 | 171 | 0.119 | 484 | 277 | 33 | 383 | 753 | 679 | 1126 | Uncharacterized protein | Uncharacterized protein | | afdb-uniprot50 | AF-A0A661W849-F1-MODEL\_V4 | 1.0 | 2.003e-05 | 169 | 0.157 | 209 | 148 | 12 | 473 | 675 | 2 | 188 | Uncharacterized protein | Uncharacterized protein | | afdb-uniprot50 | AF-L0GXI9-F1-MODEL\_V4 | 1.0 | 4.91e-12 | 169 | 0.13 | 943 | 379 | 53 | 1 | 757 | 20 | 707 | Primase C terminal 2 (PriCT-2) | Primase C terminal 2 (PriCT-2) | | afdb-uniprot50 | AF-A0A7X3HIV0-F1-MODEL\_V4 | 1.0 | 8.887e-08 | 168 | 0.128 | 451 | 271 | 29 | 384 | 762 | 30 | 430 | Uncharacterized protein | Uncharacterized protein | | afdb-uniprot50 | AF-A0A2A8H4K1-F1-MODEL\_V4 | 1.0 | 2.178e-06 | 160 | 0.113 | 457 | 288 | 28 | 384 | 751 | 373 | 801 | DNA primase | DNA primase | | afdb-uniprot50 | AF-A0A3D3BMH9-F1-MODEL\_V4 | 1.0 | 5.237e-06 | 155 | 0.141 | 346 | 212 | 25 | 463 | 752 | 44 | 360 | Uncharacterized protein | Uncharacterized protein | | afdb-uniprot50 | AF-A0A2D7PG15-F1-MODEL\_V4 | 1.0 | 8.172e-07 | 154 | 0.111 | 423 | 251 | 25 | 440 | 753 | 519 | 925 | ZnF\_CHCC domain-containing protein | ZnF\_CHCC domain-containing protein | | afdb-uniprot50 | AF-A0A843HQ44-F1-MODEL\_V4 | 1.0 | 5.136e-07 | 153 | 0.109 | 702 | 380 | 48 | 153 | 753 | 62 | 619 | Uncharacterized protein | Uncharacterized protein | | afdb-uniprot50 | AF-A0A7U3YKR5-F1-MODEL\_V4 | 1.0 | 1.716e-05 | 152 | 0.113 | 474 | 290 | 30 | 384 | 767 | 115 | 548 | Phage/plasmid primase, P4 family | Phage/plasmid primase, P4 family | | afdb-uniprot50 | AF-A0A149RZ98-F1-MODEL\_V4 | 1.0 | 1.058e-06 | 150 | 0.12 | 489 | 297 | 35 | 384 | 769 | 37 | 495 | Uncharacterized protein | Uncharacterized protein | | afdb-uniprot50 | AF-A0A0R1LZ24-F1-MODEL\_V4 | 1.0 | 4.046e-06 | 146 | 0.111 | 485 | 296 | 34 | 370 | 755 | 62 | 510 | SF3 helicase domain-containing protein | SF3 helicase domain-containing protein | | afdb-uniprot50 | AF-A0A495V3N2-F1-MODEL\_V4 | 1.0 | 1.83e-07 | 146 | 0.102 | 831 | 440 | 52 | 60 | 757 | 240 | 897 | P4 family phage/plasmid primase-like protein | P4 family phage/plasmid primase-like protein | | afdb-uniprot50 | AF-A0A0F9LV59-F1-MODEL\_V4 | 1.0 | 2.462e-05 | 145 | 0.125 | 366 | 226 | 21 | 451 | 753 | 416 | 750 | Uncharacterized protein | Uncharacterized protein | | afdb-uniprot50 | AF-A0A7X2P799-F1-MODEL\_V4 | 1.0 | 1.259e-05 | 142 | 0.113 | 467 | 265 | 30 | 384 | 757 | 256 | 666 | DNA primase | DNA primase | | afdb-uniprot50 | AF-A0A5M1BU63-F1-MODEL\_V4 | 1.0 | 1.965e-06 | 142 | 0.1 | 587 | 347 | 43 | 243 | 734 | 231 | 731 | DNA primase | DNA primase | | afdb-uniprot50 | AF-A0A0Q0ER42-F1-MODEL\_V4 | 1.0 | 0.0003086 | 141 | 0.119 | 317 | 230 | 20 | 463 | 751 | 20 | 315 | Bifunctional DNA primase/polymerase | Bifunctional DNA primase/polymerase | | afdb-uniprot50 | AF-A0A6H1NSJ1-F1-MODEL\_V4 | 1.0 | 0.0001283 | 141 | 0.118 | 303 | 208 | 20 | 471 | 739 | 214 | 491 | DNA primase | DNA primase | | afdb-uniprot50 | AF-R7LTZ8-F1-MODEL\_V4 | 1.0 | 8.015e-08 | 139 | 0.103 | 722 | 379 | 43 | 77 | 737 | 78 | 591 | DNA primase | DNA primase | | afdb-uniprot50 | AF-A0A517YE19-F1-MODEL\_V4 | 1.0 | 7.761e-07 | 139 | 0.112 | 639 | 350 | 38 | 259 | 757 | 80 | 641 | SF3 helicase domain-containing protein | SF3 helicase domain-containing protein | | afdb-uniprot50 | AF-A0A2T4U2M5-F1-MODEL\_V4 | 1.0 | 2.178e-06 | 136 | 0.114 | 570 | 340 | 40 | 261 | 737 | 216 | 713 | DNA primase | DNA primase | | afdb-uniprot50 | AF-A0A2G6B4P4-F1-MODEL\_V4 | 1.0 | 1.683e-06 | 135 | 0.098 | 591 | 346 | 39 | 243 | 731 | 141 | 646 | DNA primase | DNA primase | | afdb-uniprot50 | AF-A0A844G9M1-F1-MODEL\_V4 | 1.0 | 7.515e-06 | 134 | 0.112 | 559 | 313 | 38 | 337 | 755 | 344 | 859 | Uncharacterized protein | Uncharacterized protein | | afdb-uniprot50 | AF-A0A496Z8V0-F1-MODEL\_V4 | 1.0 | 2.712e-08 | 127 | 0.134 | 719 | 312 | 38 | 9 | 683 | 230 | 681 | Uncharacterized protein | Uncharacterized protein | | afdb-uniprot50 | AF-A0A317LFU4-F1-MODEL\_V4 | 1.0 | 1.518e-06 | 121 | 0.114 | 912 | 340 | 46 | 1 | 769 | 198 | 784 | Prim-Pol domain-containing protein | Prim-Pol domain-containing protein | | afdb-uniprot50 | AF-A0A5B8XK45-F1-MODEL\_V4 | 1.0 | 4.26e-06 | 115 | 0.107 | 605 | 328 | 44 | 260 | 761 | 44 | 539 | Uncharacterized protein | Uncharacterized protein | | afdb-uniprot50 | AF-A0A0Q6S7Q0-F1-MODEL\_V4 | 1.0 | 3.291e-06 | 114 | 0.16 | 437 | 180 | 23 | 1 | 320 | 135 | 501 | Uncharacterized protein | Uncharacterized protein | |
| Top keywords  (threshold 1.00e-02 (evalue)) | **DNA, domain\_containing, E, Virulence\_associated, primase, replication, factor, licensing, helicase, Toprim** |
| Output files | ../../similar\_structures/54\_FANPEZAQ\_CDS\_0054\_afdb-proteome\_foldseek.tsv ../../similar\_structures/54\_FANPEZAQ\_CDS\_0054\_afdb-uniprot50\_foldseek.tsv ../../similar\_structures/54\_FANPEZAQ\_CDS\_0054\_merged.svg ../../similar\_structures/54\_FANPEZAQ\_CDS\_0054\_pdb\_foldseek.tsv |

  
  
  

Return to summary | Go to previous | Go to next

  


---

**Sequence/structure alignments coloring**  
Each object in the alignment figures is colored according to its E-value following this color coding:

1e-100
10

**References:**  
1) Steinegger M, Meier M, Mirdita M, Vöhringer H, Haunsberger S J, and Söding J (2019) HH-suite3 for fast remote homology detection and deep protein annotation, BMC Bioinformatics, 473. doi: 10.1186/s12859-019-3019-7  
2) Jumper J, Evans R, Pritzel A, ..., Hassabis D (2021) Highly accurate protein structure prediction with AlphaFold, Nature, 596. doi: 10.1038/s41586-021-03819-2  
3) van Kempen M, Kim S, Tumescheit C, Mirdita M, Lee J, Gilchrist CLM, Söding J, and Steinegger M (2023) Fast and accurate protein structure search with Foldseek. Nature Biotechnology. doi: 10.1038/s41587-023-01773-0
