# Supplementary material for: Completing the BASEL phage collection to unlock hidden diversity for systematic exploration of phage–host interactions
Source: PLoS Biol. 2025 Apr 7;23(4):e3003063. doi: 10.1371/journal.pbio.3003063 (PMC11990801; doi:10.1371/journal.pbio.3003063)
Supplement: S2 Data — (ZIP) [file pbio.3003063.s009.zip › entries/57.html]

FANPEZAQ\_CDS\_0057


Return to summary | Go to previous | Go to next

|  |  |
| --- | --- |
| FANPEZAQ\_CDS\_0057 Page creation date: 02 Sep 2024, 12:00  Project folder: n/a  Input sequences file: Escherichia\_virus\_HeidiAbel.gb | domain\_containing duf551 hypothetical phage eaa eaa1 putative duf550 duf3850 ead lar restriction alleviation ea22\_like fragment p181809 vi\_03505 p158937 vi\_06901 conserved 3'\_5' exoribonuclease molecular chaperone dnaj prophage asch pua dtdp\_6\_deoxy\_l\_hexose 3\_o\_methyltransferase bacteriophage replication restart dna helicase pria |

### Sequence information

|  |  |
| --- | --- |
| Name | FANPEZAQ\_CDS\_0057  57\_FANPEZAQ\_CDS\_0057 (pipeline id) |
| Imported annotations | Escherichia\_virus\_HeidiAbel Bas97 |
| Protein sequence | MKQVTIDTVRERIAELEMVNSHGELSLRGEFELACLRMLADSMGGVDDDVRNVVVLLENN EWAEHCTNTVLGSRLESEITRLVGHVQPAPVASDAEPSFDAMMRALDAFYADDDVPELAM LAAFRILLADVRNQPAPVVIDDVMEEINRWATDRSLVGRNNYTQQQNALIDVYIGLTEPQ VPVAVPDEVTSEQAYQIGDYYGDPIDVFARGANWMRSKIIGNMLLADMDRAATRKVQELT TCNTAQQFEALTTNGKSEDLLNAERIAREAKALSDRLNDTSAVHPRVSGWSKRQQKVISY SLWRFMHSAYEMAKDAAQKGTKEDFDRFMQDAKCAEEIREEFTSAGSGKP |
| Number of residues | 350 |
| Molecular weight (Da) | 39171.61 |
| Output files | ../../query\_sequences/57\_FANPEZAQ\_CDS\_0057.fasta |

### Putative domain architecture and protein family

#### Search results (HHblits)1

|  |  |
| --- | --- |
| Domain family databases searched | Pfam, Ncbi-cd, Cath, Phrogs |
| Results, scheme(s)  (Top layers only; threshold 1.00e-03 (evalue)) | xml version="1.0" encoding="utf-8" standalone="no"?       2024-09-02T21:08:24.990238 image/svg+xml   Matplotlib v3.7.2, https://matplotlib.org/ |
| Results, table  (E-value ≤ 1.00e-03 (evalue)) | | db | id | prob | evalue | pvalue | score | cols | query | query\_len | template | template\_len | name | description | | --- | --- | --- | --- | --- | --- | --- | --- | --- | --- | --- | --- | --- | | phrogs | 20174 | 99.8 | 4.5e-24 | 5e-28 | 183.9 | 65 | (25, 94) | 350 | (4, 68) | 221 | NA | NA; Category: unknown function; p181809 VI\_03505 | | phrogs | 19524 | 99.7 | 2.9e-22 | 3.2e-26 | 162.4 | 60 | (25, 89) | 350 | (4, 63) | 127 | NA | NA; Category: unknown function; p158937 VI\_06901 | |
| Top keywords  (threshold 1.00e-03 (evalue)) | **p181809, VI\_03505, p158937, VI\_06901** |
| Output files | ../../domain\_architecture/57\_FANPEZAQ\_CDS\_0057\_cath.hhr ../../domain\_architecture/57\_FANPEZAQ\_CDS\_0057\_merged.svg ../../domain\_architecture/57\_FANPEZAQ\_CDS\_0057\_ncbi-cd.hhr ../../domain\_architecture/57\_FANPEZAQ\_CDS\_0057\_pfam.hhr ../../domain\_architecture/57\_FANPEZAQ\_CDS\_0057\_phrogs.hhr |

### Identical protein sequences/structures

#### Search results

|  |  |
| --- | --- |
| Protein sequence databases searched | Pdb, Swissprot, Refseq |
| Identical proteins found | -- |
| Top keywords | -- |
| Output files | -- |

### Similar protein sequences/structures

#### Sequence similarity search results (HHblits)1

|  |  |
| --- | --- |
| Sequence databases searched | Uniclust, Pdb70 |
| Results, scheme(s)  (Top layers only, threshold 1.00e-03 (evalue)) | xml version="1.0" encoding="utf-8" standalone="no"?       2024-09-02T21:08:53.048347 image/svg+xml   Matplotlib v3.7.2, https://matplotlib.org/ |
| Results, table(s)  (threshold 1.00e-03 (evalue)) | | db | id | prob | evalue | pvalue | score | cols | query | query\_len | template | template\_len | name | description | | --- | --- | --- | --- | --- | --- | --- | --- | --- | --- | --- | --- | --- | | uniclust | UniRef100\_A0A088FQU9 | 99.9 | 4.4e-25 | 8.2e-31 | 204.7 | 142 | (1, 142) | 350 | (1, 142) | 275 | DUF551 domain-containing protein | DUF551 domain-containing protein | | uniclust | UniRef100\_UPI000AB3930E | 99.8 | 3.9e-22 | 8e-28 | 190.0 | 158 | (36, 231) | 350 | (92, 266) | 269 | DUF551 domain-containing protein | DUF551 domain-containing protein | | uniclust | UniRef100\_A0A4U9I001 | 99.8 | 9.1e-22 | 2.3e-27 | 199.1 | 153 | (77, 232) | 350 | (118, 310) | 362 | DUF551 domain-containing protein | DUF551 domain-containing protein | | uniclust | UniRef100\_A0A6S5JYC3 | 99.7 | 9.9e-21 | 2.4e-26 | 186.2 | 166 | (62, 237) | 350 | (73, 266) | 289 | DUF551 domain-containing protein | DUF551 domain-containing protein | | uniclust | UniRef100\_A0A482MEI6 | 99.7 | 3.6e-20 | 7.7e-26 | 182.8 | 155 | (76, 232) | 350 | (74, 269) | 353 | DUF551 domain-containing protein | DUF551 domain-containing protein | | uniclust | UniRef100\_UPI001CA50654 | 99.6 | 6.5e-19 | 1.4e-24 | 164.9 | 125 | (80, 231) | 350 | (68, 202) | 206 | hypothetical protein | hypothetical protein | | uniclust | UniRef100\_A0A3R0VRQ8 | 99.6 | 1.1e-18 | 2.5e-24 | 170.2 | 167 | (36, 231) | 350 | (85, 286) | 290 | DUF551 domain-containing protein | DUF551 domain-containing protein | | uniclust | UniRef100\_UPI0020CDA783 | 99.6 | 1.9e-18 | 3.4e-24 | 155.7 | 139 | (76, 231) | 350 | (48, 192) | 195 | DUF550 domain-containing protein | DUF550 domain-containing protein | | uniclust | UniRef100\_A0A0J1LUR9 | 99.5 | 5.9e-17 | 1.4e-22 | 157.0 | 78 | (148, 234) | 350 | (148, 230) | 251 | Phage protein | Phage protein | | uniclust | UniRef100\_UPI0007CC47B1 | 99.5 | 8.5e-17 | 1.9e-22 | 157.9 | 105 | (132, 236) | 350 | (151, 282) | 293 | hypothetical protein | hypothetical protein | | uniclust | UniRef100\_A0A0H3CRI4 | 99.5 | 1.3e-16 | 3.7e-22 | 160.9 | 63 | (168, 234) | 350 | (198, 261) | 307 | DUF551 domain-containing protein | DUF551 domain-containing protein | | uniclust | UniRef100\_A0A3R0PNL9 | 99.4 | 2.2e-16 | 5.4e-22 | 156.2 | 73 | (24, 96) | 350 | (63, 139) | 284 | DUF551 domain-containing protein | DUF551 domain-containing protein | | uniclust | UniRef100\_A0A2H4FNA7 | 99.4 | 6.4e-16 | 1.5e-21 | 146.9 | 76 | (21, 96) | 350 | (69, 149) | 213 | Eaa protein | Eaa protein | | uniclust | UniRef100\_UPI0007CD0D32 | 99.3 | 3.6e-15 | 6.6e-21 | 138.2 | 141 | (78, 231) | 350 | (43, 227) | 230 | hypothetical protein | hypothetical protein | | uniclust | UniRef100\_A0A447R0Q5 | 99.3 | 3.5e-15 | 7e-21 | 136.6 | 62 | (36, 97) | 350 | (84, 150) | 186 | Phage protein | Phage protein | | uniclust | UniRef100\_A0A3J4L5J7 | 99.3 | 5.8e-15 | 1.2e-20 | 142.3 | 85 | (41, 126) | 350 | (103, 189) | 262 | Eaa protein | Eaa protein | | uniclust | UniRef100\_A0A6L3Y1N6 | 99.3 | 7.2e-15 | 1.6e-20 | 148.1 | 73 | (156, 237) | 350 | (261, 334) | 359 | DUF551 domain-containing protein | DUF551 domain-containing protein | | uniclust | UniRef100\_A0A411E019 | 99.3 | 8.6e-15 | 1.6e-20 | 120.3 | 86 | (1, 90) | 350 | (1, 88) | 92 | Uncharacterized protein | Uncharacterized protein | | uniclust | UniRef100\_D5LH12 | 99.3 | 1.9e-14 | 3.5e-20 | 136.4 | 86 | (1, 86) | 350 | (1, 160) | 274 | Conserved phage protein | Conserved phage protein | | uniclust | UniRef100\_UPI00064338B8 | 99.3 | 2e-14 | 3.9e-20 | 138.1 | 59 | (168, 230) | 350 | (208, 267) | 270 | hypothetical protein | hypothetical protein | | uniclust | UniRef100\_UPI00094A7D01 | 99.2 | 5.6e-14 | 1e-19 | 124.7 | 102 | (127, 232) | 350 | (42, 156) | 159 | hypothetical protein | hypothetical protein | | uniclust | UniRef100\_UPI0007B3E07F | 99.2 | 6e-14 | 1.2e-19 | 123.8 | 70 | (153, 231) | 350 | (65, 135) | 138 | hypothetical protein | hypothetical protein | | uniclust | UniRef100\_UPI0020766232 | 99.2 | 1.6e-13 | 2.9e-19 | 124.6 | 60 | (168, 231) | 350 | (125, 185) | 187 | hypothetical protein | hypothetical protein | | uniclust | UniRef100\_UPI0007F92665 | 99.1 | 4.3e-13 | 8.5e-19 | 128.9 | 60 | (168, 231) | 350 | (189, 249) | 252 | hypothetical protein | hypothetical protein | | uniclust | UniRef100\_A0A2J0PF48 | 99.1 | 5.8e-13 | 1.1e-18 | 126.2 | 68 | (159, 230) | 350 | (121, 189) | 261 | Uncharacterized protein | Uncharacterized protein | | uniclust | UniRef100\_UPI001FFE00E5 | 99.1 | 8.1e-13 | 1.5e-18 | 126.0 | 65 | (168, 236) | 350 | (174, 239) | 267 | 3'-5' exoribonuclease | 3'-5' exoribonuclease | | uniclust | UniRef100\_UPI001F2AB062 | 99.0 | 1.2e-12 | 2.3e-18 | 126.5 | 60 | (168, 231) | 350 | (249, 309) | 312 | hypothetical protein | hypothetical protein | | uniclust | UniRef100\_A0A447R477 | 99.0 | 1.2e-12 | 2.3e-18 | 119.6 | 81 | (16, 96) | 350 | (50, 140) | 186 | Phage protein | Phage protein | | uniclust | UniRef100\_UPI001E5CA50A | 99.0 | 4.1e-12 | 7.5e-18 | 107.8 | 60 | (168, 231) | 350 | (49, 109) | 111 | hypothetical protein | hypothetical protein | | uniclust | UniRef100\_UPI001F0C3520 | 98.9 | 1.1e-11 | 2.2e-17 | 120.2 | 60 | (170, 233) | 350 | (203, 263) | 285 | hypothetical protein | hypothetical protein | | uniclust | UniRef100\_A0A5B8RN22 | 98.9 | 1.6e-11 | 3.2e-17 | 121.2 | 169 | (60, 228) | 350 | (110, 307) | 311 | Molecular chaperone DnaJ | Molecular chaperone DnaJ | | uniclust | UniRef100\_A0A4Q4ADR6 | 98.8 | 2.6e-11 | 5.4e-17 | 116.0 | 58 | (170, 231) | 350 | (164, 222) | 226 | Uncharacterized protein | Uncharacterized protein | | uniclust | UniRef100\_UPI0009D42707 | 98.8 | 3.5e-11 | 6.9e-17 | 122.9 | 157 | (61, 228) | 350 | (235, 420) | 424 | hypothetical protein | hypothetical protein | | uniclust | UniRef100\_UPI000E2B2B70 | 98.8 | 3.3e-11 | 8e-17 | 127.3 | 60 | (168, 233) | 350 | (315, 376) | 436 | hypothetical protein | hypothetical protein | | uniclust | UniRef100\_UPI0021B4286A | 98.8 | 5.1e-11 | 9.4e-17 | 110.3 | 61 | (36, 96) | 350 | (55, 119) | 202 | hypothetical protein | hypothetical protein | | uniclust | UniRef100\_A0A636KA63 | 98.8 | 5.9e-11 | 1.1e-16 | 111.4 | 57 | (41, 97) | 350 | (131, 187) | 225 | Eaa protein | Eaa protein | | uniclust | UniRef100\_UPI000F2AB43C | 98.8 | 6.1e-11 | 1.2e-16 | 112.4 | 52 | (180, 231) | 350 | (171, 223) | 226 | hypothetical protein | hypothetical protein | | uniclust | UniRef100\_UPI002032BBFA | 98.8 | 6.7e-11 | 1.2e-16 | 111.7 | 65 | (158, 231) | 350 | (169, 234) | 236 | hypothetical protein | hypothetical protein | | uniclust | UniRef100\_UPI001908214B | 98.7 | 8e-11 | 1.5e-16 | 106.9 | 57 | (176, 232) | 350 | (86, 143) | 172 | hypothetical protein | hypothetical protein | | uniclust | UniRef100\_UPI002005553F | 98.7 | 9.3e-11 | 1.7e-16 | 104.1 | 60 | (168, 231) | 350 | (85, 145) | 146 | hypothetical protein | hypothetical protein | | uniclust | UniRef100\_UPI001F4431F5 | 98.7 | 1.7e-10 | 3.1e-16 | 102.9 | 59 | (168, 230) | 350 | (88, 147) | 149 | hypothetical protein | hypothetical protein | | uniclust | UniRef100\_A0A8S7XWP4 | 98.7 | 2.2e-10 | 4.2e-16 | 106.8 | 55 | (41, 95) | 350 | (15, 69) | 189 | Uncharacterized protein | Uncharacterized protein | | uniclust | UniRef100\_UPI0009AEFA08 | 98.6 | 3.4e-10 | 6.2e-16 | 108.1 | 61 | (36, 96) | 350 | (113, 177) | 253 | hypothetical protein | hypothetical protein | | uniclust | UniRef100\_UPI000A5CD1B2 | 98.6 | 4.4e-10 | 8e-16 | 105.8 | 51 | (181, 231) | 350 | (171, 222) | 223 | Lar family restriction alleviation protein | Lar family restriction alleviation protein | | uniclust | UniRef100\_UPI00044908D2 | 98.6 | 3.3e-10 | 8.3e-16 | 115.0 | 59 | (80, 142) | 350 | (78, 140) | 292 | DUF3850 domain-containing protein | DUF3850 domain-containing protein | | uniclust | UniRef100\_UPI0007356114 | 98.6 | 4.4e-10 | 9.3e-16 | 113.6 | 63 | (168, 235) | 350 | (232, 296) | 318 | hypothetical protein | hypothetical protein | | uniclust | UniRef100\_UPI000DCAFC4D | 98.6 | 5.3e-10 | 9.9e-16 | 105.0 | 56 | (176, 231) | 350 | (155, 211) | 213 | hypothetical protein | hypothetical protein | | uniclust | UniRef100\_UPI0003ED00DA | 98.6 | 5.2e-10 | 1.3e-15 | 114.1 | 60 | (168, 231) | 350 | (243, 304) | 307 | hypothetical protein | hypothetical protein | | uniclust | UniRef100\_A0A5V5DTZ8 | 98.6 | 6.9e-10 | 1.4e-15 | 111.1 | 60 | (168, 231) | 350 | (246, 316) | 318 | DUF551 domain-containing protein | DUF551 domain-containing protein | | uniclust | UniRef100\_UPI00083CDA3F | 98.5 | 8.8e-10 | 1.6e-15 | 104.0 | 85 | (1, 86) | 350 | (1, 85) | 224 | hypothetical protein | hypothetical protein | | uniclust | UniRef100\_UPI001F14D20D | 98.5 | 9.8e-10 | 1.8e-15 | 101.7 | 76 | (15, 90) | 350 | (58, 143) | 192 | hypothetical protein | hypothetical protein | | uniclust | UniRef100\_UPI0009ACDD1E | 98.5 | 9.9e-10 | 1.9e-15 | 105.3 | 126 | (17, 144) | 350 | (51, 202) | 228 | hypothetical protein | hypothetical protein | | uniclust | UniRef100\_G9Z9C5 | 98.5 | 1.3e-09 | 2.4e-15 | 103.7 | 59 | (173, 231) | 350 | (177, 236) | 239 | Uncharacterized protein | Uncharacterized protein | | uniclust | UniRef100\_UPI001F118484 | 98.5 | 1.3e-09 | 2.4e-15 | 109.8 | 56 | (176, 231) | 350 | (354, 410) | 413 | hypothetical protein | hypothetical protein | | uniclust | UniRef100\_A0A376JIE3 | 98.4 | 2e-09 | 4.8e-15 | 110.9 | 74 | (168, 245) | 350 | (223, 306) | 330 | Putative prophage protein | Putative prophage protein | | uniclust | UniRef100\_UPI0001BDC4F6 | 98.4 | 3.6e-09 | 7e-15 | 99.7 | 56 | (170, 229) | 350 | (129, 190) | 200 | DUF551 domain-containing protein | DUF551 domain-containing protein | | uniclust | UniRef100\_UPI0020177EC0 | 98.4 | 4.4e-09 | 8.4e-15 | 103.7 | 57 | (176, 232) | 350 | (229, 286) | 289 | hypothetical protein | hypothetical protein | | uniclust | UniRef100\_UPI001CF306BC | 98.4 | 4.8e-09 | 1e-14 | 106.8 | 138 | (82, 232) | 350 | (73, 244) | 334 | DUF551 domain-containing protein | DUF551 domain-containing protein | | uniclust | UniRef100\_A0A8B3JTV7 | 98.3 | 6.4e-09 | 1.2e-14 | 102.2 | 52 | (180, 231) | 350 | (254, 306) | 309 | Uncharacterized protein | Uncharacterized protein | | uniclust | UniRef100\_UPI002073060C | 98.3 | 6.8e-09 | 1.2e-14 | 100.1 | 52 | (180, 231) | 350 | (204, 256) | 258 | hypothetical protein | hypothetical protein | | uniclust | UniRef100\_A0A024HVZ1 | 98.3 | 5.1e-09 | 1.3e-14 | 109.4 | 25 | (73, 97) | 350 | (112, 136) | 345 | DUF551 domain-containing protein | DUF551 domain-containing protein | | uniclust | UniRef100\_UPI0021CE8872 | 98.3 | 8.5e-09 | 1.6e-14 | 102.4 | 52 | (180, 231) | 350 | (283, 335) | 337 | hypothetical protein | hypothetical protein | | uniclust | UniRef100\_A0A7I7AAG6 | 98.3 | 9.2e-09 | 2.3e-14 | 103.2 | 76 | (153, 232) | 350 | (143, 225) | 247 | DUF551 domain-containing protein | DUF551 domain-containing protein | | uniclust | UniRef100\_UPI001D009ECB | 98.2 | 1.5e-08 | 2.7e-14 | 94.4 | 52 | (180, 231) | 350 | (137, 189) | 190 | hypothetical protein | hypothetical protein | | uniclust | UniRef100\_A0A2S4QDE0 | 98.2 | 1e-08 | 2.7e-14 | 105.8 | 66 | (158, 234) | 350 | (221, 290) | 308 | DUF551 domain-containing protein | DUF551 domain-containing protein | | uniclust | UniRef100\_UPI00036C2CC1 | 98.2 | 1.3e-08 | 2.8e-14 | 100.9 | 150 | (74, 231) | 350 | (71, 245) | 250 | DUF551 domain-containing protein | DUF551 domain-containing protein | | uniclust | UniRef100\_UPI001D0D4EBB | 98.2 | 1.6e-08 | 3.1e-14 | 97.4 | 147 | (80, 232) | 350 | (26, 230) | 233 | DUF551 domain-containing protein | DUF551 domain-containing protein | | uniclust | UniRef100\_UPI00073553D8 | 98.2 | 1.7e-08 | 3.2e-14 | 98.4 | 55 | (176, 230) | 350 | (194, 249) | 251 | hypothetical protein | hypothetical protein | | uniclust | UniRef100\_A0A0A0FB64 | 98.2 | 1.2e-08 | 3.3e-14 | 104.9 | 71 | (155, 234) | 350 | (193, 267) | 297 | Uncharacterized protein | Uncharacterized protein | | uniclust | UniRef100\_A0A0F1A3D6 | 98.2 | 1.8e-08 | 4.1e-14 | 98.5 | 176 | (1, 232) | 350 | (1, 183) | 211 | Eaa protein | Eaa protein | | uniclust | UniRef100\_A0A482J7X3 | 98.2 | 1.6e-08 | 4.6e-14 | 102.0 | 39 | (153, 195) | 350 | (138, 177) | 247 | Eaa1 | Eaa1 | | uniclust | UniRef100\_A0A837KEC1 | 98.2 | 2.1e-08 | 4.6e-14 | 101.0 | 152 | (75, 235) | 350 | (97, 271) | 283 | DUF551 domain-containing protein | DUF551 domain-containing protein | | uniclust | UniRef100\_UPI0004D9BA72 | 98.2 | 2.4e-08 | 5.7e-14 | 104.7 | 77 | (153, 233) | 350 | (261, 348) | 362 | DUF551 domain-containing protein | DUF551 domain-containing protein | | uniclust | UniRef100\_UPI001E39654B | 98.2 | 2.9e-08 | 5.8e-14 | 99.1 | 63 | (155, 227) | 350 | (178, 242) | 286 | hypothetical protein | hypothetical protein | | uniclust | UniRef100\_A0A4C9H8G9 | 98.2 | 2.5e-08 | 6.1e-14 | 102.5 | 36 | (155, 194) | 350 | (137, 173) | 295 | Putative phage protein | Putative phage protein | | uniclust | UniRef100\_UPI0021F4EEC0 | 98.1 | 4.8e-08 | 8.9e-14 | 98.7 | 48 | (182, 229) | 350 | (334, 382) | 384 | ASCH/PUA domain-containing protein | ASCH/PUA domain-containing protein | | uniclust | UniRef100\_UPI0022F09B78 | 98.1 | 5.3e-08 | 9.8e-14 | 90.9 | 60 | (153, 221) | 350 | (125, 186) | 189 | hypothetical protein | hypothetical protein | | uniclust | UniRef100\_UPI00069C98EA | 98.1 | 4.8e-08 | 1e-13 | 96.4 | 75 | (153, 231) | 350 | (152, 236) | 240 | DUF551 domain-containing protein | DUF551 domain-containing protein | | uniclust | UniRef100\_A0A0T9RK48 | 98.1 | 3.9e-08 | 1.1e-13 | 102.3 | 42 | (190, 231) | 350 | (263, 305) | 307 | Uncharacterized protein | Uncharacterized protein | | uniclust | UniRef100\_A0A0D8VD89 | 98.0 | 6.2e-08 | 1.5e-13 | 102.0 | 129 | (82, 233) | 350 | (130, 270) | 354 | DUF551 domain-containing protein | DUF551 domain-containing protein | | uniclust | UniRef100\_A0A0L1BMB8 | 98.0 | 7.4e-08 | 1.9e-13 | 97.1 | 85 | (78, 193) | 350 | (103, 188) | 241 | DUF551 domain-containing protein | DUF551 domain-containing protein | | uniclust | UniRef100\_A0A1E7Z2Q0 | 98.0 | 7.6e-08 | 1.9e-13 | 97.3 | 76 | (153, 233) | 350 | (123, 206) | 249 | DUF551 domain-containing protein | DUF551 domain-containing protein | | uniclust | UniRef100\_A0A377C8S0 | 98.0 | 8.9e-08 | 2.3e-13 | 99.5 | 67 | (156, 233) | 350 | (237, 309) | 311 | Eaa protein | Eaa protein | | uniclust | UniRef100\_UPI00058238E0 | 98.0 | 1e-07 | 2.4e-13 | 100.1 | 70 | (160, 238) | 350 | (260, 337) | 360 | DUF551 domain-containing protein | DUF551 domain-containing protein | | uniclust | UniRef100\_UPI0022BF4606 | 98.0 | 1.4e-07 | 2.5e-13 | 84.5 | 56 | (176, 231) | 350 | (75, 131) | 136 | hypothetical protein | hypothetical protein | | uniclust | UniRef100\_A0A7H5A556 | 98.0 | 1e-07 | 2.7e-13 | 94.8 | 23 | (75, 97) | 350 | (86, 108) | 218 | Eaa protein | Eaa protein | | uniclust | UniRef100\_A0A1Z3N2X6 | 97.9 | 1.7e-07 | 3.4e-13 | 93.2 | 57 | (34, 90) | 350 | (95, 152) | 263 | Ead/Ea22-like family protein | Ead/Ea22-like family protein | | uniclust | UniRef100\_A0A348D9R6 | 97.9 | 1.9e-07 | 3.5e-13 | 91.1 | 54 | (176, 229) | 350 | (209, 263) | 265 | Phage protein | Phage protein | | uniclust | UniRef100\_A0A0F6TJ66 | 97.9 | 1.7e-07 | 3.9e-13 | 96.5 | 78 | (158, 244) | 350 | (159, 245) | 295 | DUF551 domain-containing protein | DUF551 domain-containing protein | | uniclust | UniRef100\_A0A0J1LU56 | 97.9 | 1.8e-07 | 4e-13 | 97.5 | 73 | (153, 235) | 350 | (257, 333) | 357 | Phage protein | Phage protein | | uniclust | UniRef100\_UPI000A3D7A99 | 97.9 | 2.1e-07 | 4.5e-13 | 95.5 | 62 | (168, 233) | 350 | (232, 299) | 320 | DUF550 domain-containing protein | DUF550 domain-containing protein | | uniclust | UniRef100\_UPI00068B16CB | 97.9 | 2.1e-07 | 5.2e-13 | 93.1 | 94 | (132, 234) | 350 | (95, 211) | 232 | DUF551 domain-containing protein | DUF551 domain-containing protein | | uniclust | UniRef100\_A0A096XER3 | 97.9 | 2.3e-07 | 6e-13 | 97.9 | 37 | (155, 200) | 350 | (254, 290) | 345 | dTDP-6-deoxy-L-hexose 3-O-methyltransferase | dTDP-6-deoxy-L-hexose 3-O-methyltransferase | | uniclust | UniRef100\_A0A7Z1J7P7 | 97.9 | 2.8e-07 | 6.2e-13 | 90.1 | 63 | (168, 234) | 350 | (112, 181) | 207 | DUF551 domain-containing protein | DUF551 domain-containing protein | | uniclust | UniRef100\_A0A0J1LWP4 | 97.8 | 2.7e-07 | 6.9e-13 | 92.5 | 67 | (153, 232) | 350 | (137, 206) | 231 | Lar family restriction alleviation protein | Lar family restriction alleviation protein | | uniclust | UniRef100\_A0A2X3CAE9 | 97.8 | 2.6e-07 | 7.2e-13 | 95.9 | 61 | (168, 233) | 350 | (190, 260) | 295 | DUF551 domain-containing protein | DUF551 domain-containing protein | | uniclust | UniRef100\_A0A0E1CIM4 | 97.8 | 3.7e-07 | 8.8e-13 | 91.8 | 23 | (168, 194) | 350 | (173, 195) | 241 | DUF551 domain-containing protein | DUF551 domain-containing protein | | uniclust | UniRef100\_UPI001F23EE67 | 97.8 | 5.1e-07 | 9.4e-13 | 93.1 | 56 | (176, 231) | 350 | (375, 431) | 436 | hypothetical protein | hypothetical protein | | uniclust | UniRef100\_UPI000CDDAC1C | 97.8 | 6e-07 | 1.1e-12 | 85.6 | 51 | (181, 231) | 350 | (143, 194) | 196 | hypothetical protein | hypothetical protein | | uniclust | UniRef100\_A0A377YIT5 | 97.7 | 5.1e-07 | 1.4e-12 | 92.1 | 15 | (81, 95) | 350 | (92, 106) | 255 | Eaa1 | Eaa1 | | uniclust | UniRef100\_UPI001F2E8690 | 97.7 | 8.4e-07 | 1.6e-12 | 71.1 | 59 | (25, 88) | 350 | (4, 62) | 66 | hypothetical protein | hypothetical protein | | uniclust | UniRef100\_A0A2X4T8E3 | 97.7 | 7.6e-07 | 1.6e-12 | 87.8 | 70 | (153, 231) | 350 | (137, 217) | 221 | Phage protein | Phage protein | | uniclust | UniRef100\_UPI000501F9A2 | 97.7 | 7.3e-07 | 1.6e-12 | 91.6 | 68 | (156, 232) | 350 | (194, 270) | 293 | hypothetical protein | hypothetical protein | | uniclust | UniRef100\_A0A485A5N0 | 97.7 | 7e-07 | 1.8e-12 | 89.5 | 33 | (60, 95) | 350 | (67, 99) | 223 | DUF551 domain-containing protein | DUF551 domain-containing protein | | uniclust | UniRef100\_A0A378CDL6 | 97.7 | 8.2e-07 | 1.9e-12 | 91.8 | 137 | (73, 233) | 350 | (136, 292) | 306 | Protein of uncharacterized function (DUF551) | Protein of uncharacterized function (DUF551) | | uniclust | UniRef100\_UPI000297C7C1 | 97.7 | 8.2e-07 | 1.9e-12 | 87.4 | 87 | (79, 177) | 350 | (70, 165) | 205 | hypothetical protein | hypothetical protein | | uniclust | UniRef100\_A0A2S1Y6Q1 | 97.7 | 1e-06 | 2.3e-12 | 86.2 | 75 | (155, 234) | 350 | (92, 183) | 196 | DUF551 domain-containing protein | DUF551 domain-containing protein | | uniclust | UniRef100\_A0A5T7YCH7 | 97.7 | 1.2e-06 | 2.5e-12 | 80.1 | 66 | (158, 232) | 350 | (56, 132) | 133 | DUF551 domain-containing protein | DUF551 domain-containing protein | | uniclust | UniRef100\_A0A1S0ZHQ4 | 97.7 | 1.1e-06 | 2.5e-12 | 88.2 | 78 | (79, 178) | 350 | (91, 171) | 229 | DUF551 domain-containing protein | DUF551 domain-containing protein | | uniclust | UniRef100\_UPI000E2D2FA1 | 97.6 | 1.3e-06 | 2.6e-12 | 92.0 | 78 | (85, 164) | 350 | (128, 234) | 395 | DUF551 domain-containing protein | DUF551 domain-containing protein | | uniclust | UniRef100\_UPI0008A4C7D4 | 97.6 | 1.4e-06 | 2.9e-12 | 89.9 | 102 | (77, 178) | 350 | (125, 279) | 347 | DUF3850 domain-containing protein | DUF3850 domain-containing protein | | uniclust | UniRef100\_UPI0002413D61 | 97.6 | 1.8e-06 | 3.6e-12 | 84.9 | 66 | (157, 231) | 350 | (139, 216) | 221 | DUF551 domain-containing protein | DUF551 domain-containing protein | | uniclust | UniRef100\_A0A2T8SVC1 | 97.6 | 1.6e-06 | 3.6e-12 | 79.7 | 75 | (149, 232) | 350 | (32, 112) | 126 | DUF551 domain-containing protein (Fragment) | DUF551 domain-containing protein (Fragment) | | uniclust | UniRef100\_A0A7T8LMQ9 | 97.5 | 2.6e-06 | 4.9e-12 | 82.9 | 54 | (42, 97) | 350 | (30, 88) | 219 | DUF551 domain-containing protein | DUF551 domain-containing protein | | uniclust | UniRef100\_A0A0K4M1A2 | 97.5 | 2.8e-06 | 5.2e-12 | 76.2 | 66 | (25, 96) | 350 | (4, 69) | 127 | Phage protein | Phage protein | | uniclust | UniRef100\_A0A0F7JC87 | 97.5 | 2.2e-06 | 5.2e-12 | 89.1 | 86 | (1, 96) | 350 | (42, 136) | 303 | Eaa protein | Eaa protein | | uniclust | UniRef100\_UPI001CC98A5F | 97.5 | 2.6e-06 | 5.2e-12 | 86.6 | 59 | (73, 131) | 350 | (76, 138) | 293 | DUF551 domain-containing protein | DUF551 domain-containing protein | | uniclust | UniRef100\_UPI00028301C4 | 97.5 | 2.4e-06 | 5.5e-12 | 91.4 | 76 | (153, 232) | 350 | (290, 377) | 392 | DUF551 domain-containing protein | DUF551 domain-containing protein | | uniclust | UniRef100\_A0A702BB40 | 97.5 | 2.4e-06 | 5.7e-12 | 85.1 | 86 | (77, 187) | 350 | (77, 163) | 214 | Eaa protein | Eaa protein | | uniclust | UniRef100\_UPI001D125E14 | 97.5 | 3.5e-06 | 6.8e-12 | 84.0 | 137 | (89, 231) | 350 | (80, 257) | 260 | DUF551 domain-containing protein | DUF551 domain-containing protein | | uniclust | UniRef100\_UPI001F14CB3B | 97.5 | 3.7e-06 | 6.8e-12 | 77.4 | 64 | (168, 235) | 350 | (68, 134) | 154 | DUF551 domain-containing protein | DUF551 domain-containing protein | | uniclust | UniRef100\_A0A088F853 | 97.5 | 3.9e-06 | 7.9e-12 | 73.8 | 40 | (1, 40) | 350 | (12, 53) | 100 | Uncharacterized protein | Uncharacterized protein | | uniclust | UniRef100\_B5Y0H2 | 97.5 | 3.2e-06 | 8e-12 | 90.8 | 58 | (170, 232) | 350 | (325, 384) | 387 | DUF551 domain-containing protein | DUF551 domain-containing protein | | uniclust | UniRef100\_A0A6C9E065 | 97.5 | 4.3e-06 | 8.3e-12 | 76.0 | 49 | (183, 231) | 350 | (78, 127) | 129 | Uncharacterized protein | Uncharacterized protein | | uniclust | UniRef100\_A0A3Q9UB38 | 97.5 | 4e-06 | 8.5e-12 | 85.3 | 97 | (72, 178) | 350 | (81, 184) | 271 | Uncharacterized protein | Uncharacterized protein | | uniclust | UniRef100\_A0A1X3LMB4 | 97.4 | 4.2e-06 | 1e-11 | 84.6 | 28 | (157, 193) | 350 | (134, 161) | 230 | Eaa protein | Eaa protein | | uniclust | UniRef100\_A0A7U9Q9Z2 | 97.4 | 5.3e-06 | 1.2e-11 | 88.1 | 23 | (168, 194) | 350 | (297, 319) | 369 | Putative phage protein | Putative phage protein | | uniclust | UniRef100\_A0A5U9SIS9 | 97.4 | 6.9e-06 | 1.4e-11 | 80.5 | 55 | (42, 96) | 350 | (26, 90) | 207 | Eaa protein | Eaa protein | | uniclust | UniRef100\_A0A9E7BH61 | 97.4 | 6.4e-06 | 1.4e-11 | 83.7 | 53 | (183, 236) | 350 | (174, 236) | 255 | Uncharacterized protein | Uncharacterized protein | | uniclust | UniRef100\_A0A7I7AAS7 | 97.4 | 6.2e-06 | 1.5e-11 | 82.5 | 89 | (1, 97) | 350 | (3, 93) | 210 | Uncharacterized protein | Uncharacterized protein | | uniclust | UniRef100\_A0A2U3EYJ7 | 97.3 | 8.1e-06 | 1.6e-11 | 76.7 | 67 | (156, 231) | 350 | (81, 152) | 155 | DUF551 domain-containing protein | DUF551 domain-containing protein | | uniclust | UniRef100\_A0A2X3IQU6 | 97.3 | 5.8e-06 | 1.7e-11 | 89.8 | 65 | (168, 236) | 350 | (274, 344) | 395 | Eaa1 | Eaa1 | | uniclust | UniRef100\_A0A403N2V7 | 97.3 | 8.2e-06 | 1.7e-11 | 83.1 | 21 | (153, 178) | 350 | (187, 207) | 274 | Uncharacterized protein | Uncharacterized protein | | uniclust | UniRef100\_A0A2W5GG89 | 97.3 | 8.2e-06 | 1.8e-11 | 74.8 | 50 | (155, 213) | 350 | (35, 84) | 119 | Uncharacterized protein | Uncharacterized protein | | uniclust | UniRef100\_UPI00069F21E5 | 97.3 | 8.5e-06 | 1.9e-11 | 74.4 | 63 | (159, 231) | 350 | (48, 114) | 116 | DUF551 domain-containing protein | DUF551 domain-containing protein | | uniclust | UniRef100\_UPI001D0F47EE | 97.3 | 9.8e-06 | 1.9e-11 | 77.1 | 18 | (79, 96) | 350 | (15, 32) | 176 | DUF551 domain-containing protein | DUF551 domain-containing protein | | uniclust | UniRef100\_A0A0M2KI41 | 97.3 | 8.5e-06 | 2e-11 | 79.2 | 69 | (159, 236) | 350 | (99, 171) | 172 | DUF551 domain-containing protein | DUF551 domain-containing protein | | uniclust | UniRef100\_UPI001BDF9013 | 97.3 | 1.1e-05 | 2.1e-11 | 72.0 | 68 | (154, 231) | 350 | (50, 121) | 122 | DUF551 domain-containing protein | DUF551 domain-containing protein | | uniclust | UniRef100\_A0A5Q9LHJ5 | 97.3 | 7.8e-06 | 2.2e-11 | 86.2 | 75 | (153, 232) | 350 | (222, 303) | 306 | Uncharacterized protein | Uncharacterized protein | | uniclust | UniRef100\_A0A3F3IH09 | 97.3 | 1.2e-05 | 2.6e-11 | 76.5 | 73 | (152, 231) | 350 | (71, 155) | 158 | Protein Eaa | Protein Eaa | | uniclust | UniRef100\_UPI000B422C02 | 97.3 | 1.4e-05 | 2.7e-11 | 70.8 | 44 | (182, 225) | 350 | (71, 115) | 115 | hypothetical protein | hypothetical protein | | uniclust | UniRef100\_A0A085ASF4 | 97.3 | 1.1e-05 | 2.7e-11 | 87.5 | 32 | (56, 96) | 350 | (187, 218) | 403 | DUF551 domain-containing protein | DUF551 domain-containing protein | | uniclust | UniRef100\_UPI000CFB54C0 | 97.2 | 1.5e-05 | 3e-11 | 78.6 | 105 | (55, 194) | 350 | (64, 171) | 221 | DUF551 domain-containing protein | DUF551 domain-containing protein | | uniclust | UniRef100\_UPI000AED1C5F | 97.2 | 1.7e-05 | 3.1e-11 | 75.1 | 129 | (78, 230) | 350 | (34, 180) | 182 | DUF550 domain-containing protein | DUF550 domain-containing protein | | uniclust | UniRef100\_A0A377WN62 | 97.2 | 1.2e-05 | 3.3e-11 | 88.2 | 75 | (153, 231) | 350 | (300, 383) | 422 | Eaa1 | Eaa1 | | uniclust | UniRef100\_A0A1X3LI39 | 97.2 | 1.5e-05 | 3.4e-11 | 84.1 | 61 | (168, 232) | 350 | (203, 276) | 343 | Putative bacteriophage protein | Putative bacteriophage protein | | uniclust | UniRef100\_UPI0020D2028B | 97.2 | 2.2e-05 | 4e-11 | 79.8 | 59 | (169, 231) | 350 | (260, 329) | 330 | DUF551 domain-containing protein | DUF551 domain-containing protein | | uniclust | UniRef100\_A0A7I6XQ80 | 97.1 | 2.5e-05 | 5.6e-11 | 81.7 | 61 | (168, 237) | 350 | (215, 281) | 307 | DUF551 domain-containing protein | DUF551 domain-containing protein | | uniclust | UniRef100\_A0A7H9JWU5 | 97.1 | 2.7e-05 | 6.2e-11 | 81.3 | 26 | (153, 178) | 350 | (195, 236) | 299 | DUF551 domain-containing protein | DUF551 domain-containing protein | | uniclust | UniRef100\_A0A641J2S1 | 97.1 | 2.8e-05 | 6.2e-11 | 79.5 | 30 | (168, 201) | 350 | (185, 214) | 256 | DUF551 domain-containing protein | DUF551 domain-containing protein | | uniclust | UniRef100\_UPI001ABB5B0F | 97.1 | 3.5e-05 | 6.5e-11 | 79.2 | 52 | (170, 225) | 350 | (301, 353) | 360 | hypothetical protein | hypothetical protein | | uniclust | UniRef100\_UPI001B3928F5 | 97.1 | 3.5e-05 | 6.5e-11 | 78.8 | 60 | (37, 96) | 350 | (137, 201) | 343 | hypothetical protein | hypothetical protein | | uniclust | UniRef100\_A0A7G3ES10 | 97.1 | 2.7e-05 | 6.7e-11 | 78.4 | 85 | (1, 96) | 350 | (1, 88) | 213 | DUF551 domain-containing protein | DUF551 domain-containing protein | | uniclust | UniRef100\_UPI000E4CA42D | 97.1 | 4e-05 | 7.4e-11 | 78.7 | 50 | (181, 230) | 350 | (203, 253) | 353 | hypothetical protein | hypothetical protein | | uniclust | UniRef100\_UPI001D0D554A | 97.0 | 4.5e-05 | 8.3e-11 | 73.4 | 64 | (156, 230) | 350 | (133, 197) | 199 | DUF551 domain-containing protein | DUF551 domain-containing protein | | uniclust | UniRef100\_UPI0007EBD3DF | 97.0 | 3.8e-05 | 8.4e-11 | 80.7 | 81 | (80, 178) | 350 | (157, 240) | 315 | hypothetical protein | hypothetical protein | | uniclust | UniRef100\_UPI001CF3EDA8 | 97.0 | 4.6e-05 | 8.5e-11 | 70.1 | 67 | (155, 230) | 350 | (63, 140) | 143 | DUF551 domain-containing protein | DUF551 domain-containing protein | | uniclust | UniRef100\_UPI001587EB33 | 97.0 | 4.9e-05 | 9e-11 | 68.3 | 51 | (176, 226) | 350 | (62, 113) | 121 | hypothetical protein | hypothetical protein | | uniclust | UniRef100\_UPI000808C4DE | 97.0 | 4.4e-05 | 9.1e-11 | 80.4 | 144 | (76, 236) | 350 | (175, 331) | 342 | DUF551 domain-containing protein | DUF551 domain-containing protein | | uniclust | UniRef100\_UPI00079C252A | 96.9 | 6.6e-05 | 1.3e-10 | 71.1 | 88 | (1, 96) | 350 | (1, 90) | 150 | hypothetical protein | hypothetical protein | | uniclust | UniRef100\_UPI0006597CA0 | 96.9 | 6.8e-05 | 1.4e-10 | 77.6 | 84 | (79, 164) | 350 | (142, 240) | 287 | DUF551 domain-containing protein | DUF551 domain-containing protein | | uniclust | UniRef100\_A0A443WQQ1 | 96.9 | 5.8e-05 | 1.5e-10 | 78.8 | 85 | (78, 178) | 350 | (109, 212) | 270 | Uncharacterized protein | Uncharacterized protein | | uniclust | UniRef100\_A0A2T3SVJ2 | 96.9 | 7.4e-05 | 1.6e-10 | 77.0 | 60 | (168, 231) | 350 | (200, 271) | 275 | DUF551 domain-containing protein | DUF551 domain-containing protein | | uniclust | UniRef100\_UPI000AC50A34 | 96.9 | 7.6e-05 | 1.7e-10 | 77.2 | 58 | (169, 232) | 350 | (203, 265) | 268 | DUF551 domain-containing protein | DUF551 domain-containing protein | | uniclust | UniRef100\_UPI001E47FED3 | 96.9 | 0.00011 | 2e-10 | 68.8 | 70 | (153, 231) | 350 | (74, 154) | 156 | DUF551 domain-containing protein | DUF551 domain-containing protein | | uniclust | UniRef100\_A0A3T2UUV1 | 96.8 | 9.8e-05 | 2.2e-10 | 75.5 | 65 | (158, 232) | 350 | (167, 239) | 242 | DUF551 domain-containing protein | DUF551 domain-containing protein | | uniclust | UniRef100\_UPI00080AA30A | 96.8 | 0.00012 | 2.2e-10 | 76.2 | 52 | (176, 227) | 350 | (275, 327) | 384 | hypothetical protein | hypothetical protein | | uniclust | UniRef100\_UPI000DCD3321 | 96.8 | 0.00011 | 2.3e-10 | 73.9 | 80 | (148, 232) | 350 | (128, 222) | 226 | hypothetical protein | hypothetical protein | | uniclust | UniRef100\_A0A5C7BMG0 | 96.8 | 0.00011 | 2.3e-10 | 74.1 | 24 | (168, 195) | 350 | (134, 157) | 214 | DUF551 domain-containing protein | DUF551 domain-containing protein | | uniclust | UniRef100\_UPI001D114CB1 | 96.8 | 0.00012 | 2.4e-10 | 78.1 | 141 | (77, 231) | 350 | (136, 314) | 383 | DUF3850 domain-containing protein | DUF3850 domain-containing protein | | uniclust | UniRef100\_S5MU58 | 96.8 | 0.00013 | 2.4e-10 | 74.1 | 55 | (42, 98) | 350 | (115, 174) | 303 | Phage EaD protein | Phage EaD protein | | uniclust | UniRef100\_A0A2T4Y165 | 96.8 | 8.9e-05 | 2.4e-10 | 79.8 | 22 | (169, 194) | 350 | (248, 269) | 337 | DUF551 domain-containing protein | DUF551 domain-containing protein | | uniclust | UniRef100\_A0A3V3HL53 | 96.8 | 0.00015 | 2.8e-10 | 71.7 | 33 | (153, 195) | 350 | (156, 188) | 240 | DUF551 domain-containing protein | DUF551 domain-containing protein | | uniclust | UniRef100\_UPI00168151F6 | 96.8 | 0.00015 | 2.8e-10 | 69.9 | 44 | (176, 219) | 350 | (140, 183) | 195 | hypothetical protein | hypothetical protein | | uniclust | UniRef100\_UPI000D745A46 | 96.8 | 0.00015 | 3e-10 | 71.9 | 113 | (80, 205) | 350 | (37, 161) | 197 | hypothetical protein | hypothetical protein | | uniclust | UniRef100\_UPI000685C591 | 96.7 | 0.00015 | 3.2e-10 | 71.8 | 56 | (41, 96) | 350 | (98, 154) | 193 | hypothetical protein | hypothetical protein | | uniclust | UniRef100\_UPI0015E53E8C | 96.7 | 0.00019 | 3.4e-10 | 62.1 | 39 | (1, 39) | 350 | (1, 39) | 91 | hypothetical protein | hypothetical protein | | uniclust | UniRef100\_UPI001920B9A5 | 96.7 | 0.00019 | 3.5e-10 | 71.5 | 54 | (42, 95) | 350 | (84, 137) | 254 | hypothetical protein | hypothetical protein | | uniclust | UniRef100\_A0A377X5V4 | 96.7 | 0.00014 | 3.6e-10 | 81.4 | 76 | (153, 232) | 350 | (371, 453) | 475 | Protein of uncharacterized function (DUF551) | Protein of uncharacterized function (DUF551) | | uniclust | UniRef100\_A0A636IGV6 | 96.7 | 0.00019 | 3.7e-10 | 74.1 | 52 | (43, 96) | 350 | (90, 146) | 312 | DUF551 domain-containing protein | DUF551 domain-containing protein | | uniclust | UniRef100\_A0A3Y7SQI3 | 96.7 | 0.00019 | 3.8e-10 | 64.3 | 29 | (155, 192) | 350 | (21, 49) | 100 | DUF551 domain-containing protein | DUF551 domain-containing protein | | uniclust | UniRef100\_A0A2H4FNA9 | 96.7 | 0.00019 | 3.9e-10 | 73.2 | 78 | (153, 234) | 350 | (155, 240) | 250 | DUF551 domain-containing protein | DUF551 domain-containing protein | | uniclust | UniRef100\_A0A286P6T1 | 96.7 | 0.0002 | 3.9e-10 | 70.1 | 52 | (168, 223) | 350 | (106, 158) | 197 | Uncharacterized protein | Uncharacterized protein | | uniclust | UniRef100\_A0A4R7FZ57 | 96.7 | 0.0002 | 4.1e-10 | 73.9 | 48 | (176, 223) | 350 | (225, 273) | 287 | Replication restart DNA helicase PriA | Replication restart DNA helicase PriA | | uniclust | UniRef100\_A0A4P8YP68 | 96.7 | 0.00021 | 4.5e-10 | 64.0 | 41 | (147, 192) | 350 | (7, 48) | 99 | DUF551 domain-containing protein | DUF551 domain-containing protein | | uniclust | UniRef100\_A0A0D6ZJB3 | 96.6 | 0.00024 | 5.7e-10 | 71.1 | 40 | (153, 196) | 350 | (97, 141) | 193 | DUF551 domain-containing protein | DUF551 domain-containing protein | | uniclust | UniRef100\_UPI0003BBF7AD | 96.6 | 0.00032 | 5.8e-10 | 74.0 | 59 | (168, 230) | 350 | (346, 415) | 417 | DUF551 domain-containing protein | DUF551 domain-containing protein | | uniclust | UniRef100\_UPI001B2924A7 | 96.6 | 0.00032 | 5.9e-10 | 69.1 | 40 | (187, 226) | 350 | (182, 222) | 223 | hypothetical protein | hypothetical protein | | uniclust | UniRef100\_UPI000AAEB9B5 | 96.6 | 0.00034 | 6.3e-10 | 69.3 | 48 | (135, 191) | 350 | (122, 175) | 235 | DUF551 domain-containing protein | DUF551 domain-containing protein | | uniclust | UniRef100\_UPI0005B32617 | 96.6 | 0.00035 | 6.6e-10 | 71.9 | 48 | (176, 223) | 350 | (244, 292) | 305 | hypothetical protein | hypothetical protein | | uniclust | UniRef100\_UPI00038F7A69 | 96.6 | 0.00038 | 6.9e-10 | 67.5 | 55 | (42, 97) | 350 | (84, 138) | 195 | hypothetical protein | hypothetical protein | | uniclust | UniRef100\_A0A5I4QGP7 | 96.5 | 0.00033 | 7.4e-10 | 72.2 | 72 | (155, 235) | 350 | (149, 229) | 246 | DUF551 domain-containing protein | DUF551 domain-containing protein | | uniclust | UniRef100\_A0A2D2W4X9 | 96.5 | 0.00041 | 7.5e-10 | 60.6 | 40 | (1, 40) | 350 | (1, 42) | 95 | Uncharacterized protein | Uncharacterized protein | | uniclust | UniRef100\_A0A543Q1M0 | 96.5 | 0.00038 | 7.5e-10 | 59.2 | 36 | (189, 224) | 350 | (27, 63) | 73 | Uncharacterized protein | Uncharacterized protein | | uniclust | UniRef100\_UPI000A38D79B | 96.5 | 0.00037 | 7.6e-10 | 73.1 | 80 | (153, 236) | 350 | (190, 294) | 313 | DUF551 domain-containing protein | DUF551 domain-containing protein | | uniclust | UniRef100\_A0A7U6G891 | 96.5 | 0.00043 | 8.2e-10 | 70.4 | 59 | (168, 230) | 350 | (199, 264) | 267 | DUF551 domain-containing protein | DUF551 domain-containing protein | | uniclust | UniRef100\_A0A5N3CXX3 | 96.5 | 0.00045 | 8.4e-10 | 72.0 | 54 | (42, 95) | 350 | (87, 140) | 355 | Phage protein | Phage protein | | uniclust | UniRef100\_A0A0E1M694 | 96.5 | 0.00037 | 8.7e-10 | 73.7 | 36 | (158, 198) | 350 | (204, 239) | 290 | DUF551 domain-containing protein | DUF551 domain-containing protein | | uniclust | UniRef100\_UPI001E2EB100 | 96.5 | 0.00048 | 9.1e-10 | 69.5 | 17 | (80, 96) | 350 | (93, 109) | 254 | DUF551 domain-containing protein | DUF551 domain-containing protein | | uniclust | UniRef100\_UPI001D0E6CF5 | 96.5 | 0.00048 | 9.2e-10 | 71.1 | 117 | (77, 204) | 350 | (120, 258) | 302 | DUF551 domain-containing protein | DUF551 domain-containing protein | | uniclust | UniRef100\_A0A481W6W9 | 96.5 | 0.00052 | 9.6e-10 | 55.7 | 40 | (1, 40) | 350 | (1, 42) | 61 | Uncharacterized protein | Uncharacterized protein | | uniclust | UniRef100\_UPI001C8BD278 | 96.5 | 0.00053 | 9.7e-10 | 64.7 | 40 | (182, 221) | 350 | (115, 154) | 158 | hypothetical protein | hypothetical protein | | uniclust | UniRef100\_D8A596 | 96.5 | 0.00052 | 1e-09 | 64.1 | 37 | (158, 203) | 350 | (65, 101) | 136 | DUF551 domain-containing protein (Fragment) | DUF551 domain-containing protein (Fragment) | | uniclust | UniRef100\_A0A822WF58 | 96.4 | 0.00058 | 1.2e-09 | 68.0 | 22 | (153, 179) | 350 | (149, 170) | 202 | DUF551 domain-containing protein | DUF551 domain-containing protein | | uniclust | UniRef100\_UPI000D697C96 | 96.4 | 0.00064 | 1.2e-09 | 62.1 | 38 | (187, 224) | 350 | (80, 118) | 119 | hypothetical protein | hypothetical protein | | uniclust | UniRef100\_UPI0019290DBF | 96.4 | 0.00066 | 1.2e-09 | 64.5 | 54 | (176, 229) | 350 | (105, 159) | 164 | hypothetical protein | hypothetical protein | | uniclust | UniRef100\_A0A2G2PQ67 | 96.4 | 0.00057 | 1.2e-09 | 64.1 | 38 | (168, 209) | 350 | (51, 88) | 125 | DUF551 domain-containing protein | DUF551 domain-containing protein | | uniclust | UniRef100\_A0A076LNT5 | 96.4 | 0.00068 | 1.3e-09 | 62.3 | 46 | (42, 87) | 350 | (77, 122) | 130 | Ead/Ea22-like family protein | Ead/Ea22-like family protein | | uniclust | UniRef100\_A0A855F7K2 | 96.4 | 0.00059 | 1.4e-09 | 72.2 | 24 | (72, 95) | 350 | (76, 99) | 286 | DUF551 domain-containing protein | DUF551 domain-containing protein | | uniclust | UniRef100\_A0A2I8SEA0 | 96.4 | 0.00063 | 1.4e-09 | 63.8 | 31 | (155, 195) | 350 | (39, 69) | 123 | DUF551 domain-containing protein | DUF551 domain-containing protein | | uniclust | UniRef100\_A0A1C3H9H7 | 96.4 | 0.00057 | 1.4e-09 | 71.7 | 24 | (168, 195) | 350 | (159, 182) | 263 | Eaa1 | Eaa1 | | uniclust | UniRef100\_A0A0P0ZCX1 | 96.3 | 0.00074 | 1.5e-09 | 66.3 | 72 | (155, 235) | 350 | (93, 176) | 187 | DUF551 domain-containing protein | DUF551 domain-containing protein | | uniclust | UniRef100\_UPI000799D453 | 96.3 | 0.00074 | 1.5e-09 | 64.9 | 62 | (168, 233) | 350 | (67, 141) | 155 | DUF551 domain-containing protein | DUF551 domain-containing protein | | uniclust | UniRef100\_A0A4Q2QYQ8 | 96.3 | 0.00087 | 1.6e-09 | 66.3 | 69 | (153, 230) | 350 | (51, 134) | 221 | DUF551 domain-containing protein | DUF551 domain-containing protein | | uniclust | UniRef100\_UPI00069C164F | 96.3 | 0.00074 | 1.7e-09 | 69.8 | 21 | (153, 178) | 350 | (145, 165) | 238 | DUF551 domain-containing protein | DUF551 domain-containing protein | | uniclust | UniRef100\_UPI00040D00C3 | 96.3 | 0.00076 | 1.7e-09 | 71.3 | 24 | (73, 96) | 350 | (136, 159) | 293 | hypothetical protein | hypothetical protein | | uniclust | UniRef100\_A0A4D6DY86 | 96.3 | 0.00094 | 1.7e-09 | 59.2 | 40 | (1, 40) | 350 | (4, 45) | 101 | Uncharacterized protein | Uncharacterized protein | | uniclust | UniRef100\_UPI00202223FF | 96.3 | 0.001 | 1.9e-09 | 64.1 | 26 | (153, 187) | 350 | (74, 99) | 179 | DUF551 domain-containing protein | DUF551 domain-containing protein | | uniclust | UniRef100\_A0A0H2Z1D9 | 96.2 | 0.00076 | 2.1e-09 | 70.2 | 64 | (158, 231) | 350 | (167, 236) | 241 | DUF551 domain-containing protein | DUF551 domain-containing protein | | uniclust | UniRef100\_UPI0007CA5FDA | 96.2 | 0.00092 | 2.2e-09 | 69.1 | 21 | (75, 96) | 350 | (91, 111) | 232 | hypothetical protein | hypothetical protein | | uniclust | UniRef100\_A0A447RMB8 | 96.2 | 0.00086 | 2.3e-09 | 72.3 | 77 | (153, 236) | 350 | (222, 302) | 313 | Eaa1 | Eaa1 | | uniclust | UniRef100\_A0A486MKY1 | 96.2 | 0.001 | 2.4e-09 | 68.5 | 82 | (80, 175) | 350 | (83, 170) | 228 | Uncharacterized protein | Uncharacterized protein | |
| Top keywords  (threshold 1.00e-03 (evalue)) | **domain\_containing, DUF551, hypothetical, Phage, Eaa, Eaa1, Putative, DUF550, DUF3850, Ead** |
| Output files | ../../similar\_sequences/57\_FANPEZAQ\_CDS\_0057\_merged.svg ../../similar\_sequences/57\_FANPEZAQ\_CDS\_0057\_pdb70.a3m ../../similar\_sequences/57\_FANPEZAQ\_CDS\_0057\_pdb70.hhr ../../similar\_sequences/57\_FANPEZAQ\_CDS\_0057\_uniclust.a3m ../../similar\_sequences/57\_FANPEZAQ\_CDS\_0057\_uniclust.hhr |

#### Structure prediction (AlphaFold)2

|  |  |
| --- | --- |
| Stats | xml version="1.0" encoding="utf-8" standalone="no"?       2024-09-02T21:09:53.750450 image/svg+xml   Matplotlib v3.7.2, https://matplotlib.org/ |
| Predicted structure | **NGL Viewer Controls:**  - Center: *Left-Click* - Rotate: *Left-Click + Drag* - Translate: *Right-Click + Drag* - Zoom: *Shift + Left-Click + Drag* |
| Output files | ../../predicted\_structures/57\_FANPEZAQ\_CDS\_0057/features.pkl ../../predicted\_structures/57\_FANPEZAQ\_CDS\_0057/ranked\_0.pdb ../../predicted\_structures/57\_FANPEZAQ\_CDS\_0057/ranked\_0\_plots.svg ../../predicted\_structures/57\_FANPEZAQ\_CDS\_0057/result\_model\_1\_ptm\_pred\_0.pkl |

#### Structure similarity search results (Foldseek)3

|  |  |
| --- | --- |
| Structure databases searched | Pdb, Afdb-proteome, Afdb-uniprot50 |
| Results, scheme(s)  (Top layers only, threshold 1.00e-02 (evalue)) | xml version="1.0" encoding="utf-8" standalone="no"?       2024-09-02T21:11:28.848085 image/svg+xml   Matplotlib v3.7.2, https://matplotlib.org/ |
| Results, table  (threshold 1.00e-02 (evalue)) | -- |
| Top keywords  (threshold 1.00e-02 (evalue)) | -- |
| Output files | ../../similar\_structures/57\_FANPEZAQ\_CDS\_0057\_afdb-proteome\_foldseek.tsv ../../similar\_structures/57\_FANPEZAQ\_CDS\_0057\_afdb-uniprot50\_foldseek.tsv ../../similar\_structures/57\_FANPEZAQ\_CDS\_0057\_merged.svg ../../similar\_structures/57\_FANPEZAQ\_CDS\_0057\_pdb\_foldseek.tsv |

  
  
  

Return to summary | Go to previous | Go to next

  


---

**Sequence/structure alignments coloring**  
Each object in the alignment figures is colored according to its E-value following this color coding:

1e-100
10

**References:**  
1) Steinegger M, Meier M, Mirdita M, Vöhringer H, Haunsberger S J, and Söding J (2019) HH-suite3 for fast remote homology detection and deep protein annotation, BMC Bioinformatics, 473. doi: 10.1186/s12859-019-3019-7  
2) Jumper J, Evans R, Pritzel A, ..., Hassabis D (2021) Highly accurate protein structure prediction with AlphaFold, Nature, 596. doi: 10.1038/s41586-021-03819-2  
3) van Kempen M, Kim S, Tumescheit C, Mirdita M, Lee J, Gilchrist CLM, Söding J, and Steinegger M (2023) Fast and accurate protein structure search with Foldseek. Nature Biotechnology. doi: 10.1038/s41587-023-01773-0
