# Supplementary material for: Completing the BASEL phage collection to unlock hidden diversity for systematic exploration of phage–host interactions
Source: PLoS Biol. 2025 Apr 7;23(4):e3003063. doi: 10.1371/journal.pbio.3003063 (PMC11990801; doi:10.1371/journal.pbio.3003063)
Supplement: S2 Data — (ZIP) [file pbio.3003063.s009.zip › entries/6.html]

FANPEZAQ\_CDS\_0006


Return to summary | Go to previous | Go to next

|  |  |
| --- | --- |
| FANPEZAQ\_CDS\_0006 Page creation date: 02 Sep 2024, 12:00  Project folder: n/a  Input sequences file: Escherichia\_virus\_HeidiAbel.gb | domain\_containing rho termination factor n\_terminal heh lem fragment transcription hypothetical sap phage rho\_n ribosomal 50s endonuclease vii mu\_like prophage flumu and a t4 recombination l21 in dimerisation tail factor\_like alpha yes rna\_binding isoform putative rna\_bd |

### Sequence information

|  |  |
| --- | --- |
| Name | FANPEZAQ\_CDS\_0006  06\_FANPEZAQ\_CDS\_0006 (pipeline id) |
| Imported annotations | Escherichia\_virus\_HeidiAbel Bas97 |
| Protein sequence | MSGTYVLKLTSAIAISGEVMKAGSLVEVSELEAKNLLSRGKAEMHDEQPARDDDVEITKM NKDQLIAIAEQMEIEGADKMTKAQLIEAIEAAGETKEGDE |
| Number of residues | 100 |
| Molecular weight (Da) | 10809.12 |
| Output files | ../../query\_sequences/06\_FANPEZAQ\_CDS\_0006.fasta |

### Putative domain architecture and protein family

#### Search results (HHblits)1

|  |  |
| --- | --- |
| Domain family databases searched | Pfam, Ncbi-cd, Cath, Phrogs |
| Results, scheme(s)  (Top layers only; threshold 1.00e-03 (evalue)) | xml version="1.0" encoding="utf-8" standalone="no"?       2024-09-02T21:08:13.120903 image/svg+xml   Matplotlib v3.7.2, https://matplotlib.org/ |
| Results, table  (E-value ≤ 1.00e-03 (evalue)) | | db | id | prob | evalue | pvalue | score | cols | query | query\_len | template | template\_len | name | description | | --- | --- | --- | --- | --- | --- | --- | --- | --- | --- | --- | --- | --- | | pfam | PF09124 | 97.4 | 6.3e-08 | 1.3e-11 | 54.1 | 48 | (48, 95) | 100 | (4, 51) | 54 | Endonuc-dimeris | T4 recombination endonuclease VII, dimerisation | | pfam | PF10281 | 97.3 | 1e-07 | 2.2e-11 | 49.0 | 35 | (57, 91) | 100 | (1, 36) | 37 | Ish1 | Putative nuclear envelope organisation protein | | pfam | PF12949 | 95.4 | 7.9e-05 | 1.9e-08 | 34.0 | 33 | (58, 90) | 100 | (2, 34) | 35 | HeH | HeH/LEM domain | | ncbi-cd | cd12935 | 94.6 | 0.00027 | 7e-08 | 31.8 | 34 | (58, 91) | 100 | (2, 35) | 36 | LEM\_like | cd12935 LEM\_like; LEM-like domain of lamina-associated polypeptide 2 (LAP2) and similar proteins. LAP2, also termed thymopoietin (TP), or thymopoietin-related peptide (TPRP), is composed of isoform alpha and isoforms beta/gamma and may be involved in chromatin organization and postmitotic reassembly. | | cath | 1e7lA02 | 98.2 | 1.8e-10 | 2.9e-14 | 67.4 | 47 | (48, 94) | 100 | (5, 51) | 55 | Recombination endonuclease vii | CATHCODE: 1.10.720.10 NAME: Recombination endonuclease vii. Chain: a, b. Synonym: gp49. Engineered: yes. Mutation: yes. Other\_details: one zn bound to cys 23,26,58,61 of each chain SOURCE: Bacteriophage t4. Organism\_taxid: 10665. Gene: gp49. Expressed in: escherichia coli. Expression\_system\_taxid: 511693. CLASS: Mainly Alpha, ARCH: Orthogonal Bundle, TOPOL: Transcription Termination Factor Rho, Rna-binding Domain; Chain A, Domain 1, HOMOL: Transcription Termination Factor Rho, Rna-binding Domain; Chain A, Domain 1 | | cath | 2outA02 | 96.2 | 1.2e-05 | 2e-09 | 41.3 | 39 | (59, 97) | 100 | (1, 39) | 41 | Mu-like prophage flumu protein gp35, protein hi1507 in mu-like prophage flumu region | CATHCODE: 1.10.720.30 NAME: Mu-like prophage flumu protein gp35, protein hi1507 in mu-like prophage flumu region. Chain: a. Engineered: yes SOURCE: Haemophilus influenzae. Organism\_taxid: 727. Strain: dsm 11121, kw20, rd. Atcc: 51907, 51907. Gene: hi1506, hi1507. Expressed in: escherichia coli. Expression\_system\_taxid: 562. CLASS: Mainly Alpha, ARCH: Orthogonal Bundle, TOPOL: Transcription Termination Factor Rho, Rna-binding Domain; Chain A, Domain 1, HOMOL: SAP domain | | phrogs | 3281 | 99.7 | 1.7e-21 | 2.1e-25 | 134.3 | 94 | (1, 98) | 100 | (6, 130) | 145 | endonuclease VII | endonuclease VII; Category: DNA, RNA and nucleotide metabolism; MG711467\_p48 | | phrogs | 390 | 98.8 | 5.9e-13 | 7.5e-17 | 85.1 | 63 | (23, 92) | 100 | (30, 94) | 95 | Arc-like repressor | Arc-like repressor; Category: transcription regulation; p204416 VI\_04755 | | phrogs | 2724 | 98.4 | 4e-11 | 4.9e-15 | 74.0 | 67 | (26, 95) | 100 | (10, 78) | 79 | NA | NA; Category: unknown function; p122757 VI\_05802 | | phrogs | 2250 | 98.2 | 3.4e-10 | 4.2e-14 | 67.7 | 44 | (52, 95) | 100 | (21, 64) | 66 | NA | NA; Category: unknown function; p428684 VI\_05331 | | phrogs | 1539 | 97.9 | 5.1e-09 | 6.3e-13 | 62.4 | 36 | (56, 91) | 100 | (31, 66) | 66 | Arc-like repressor | Arc-like repressor; Category: transcription regulation; NC\_004586\_p34 | | phrogs | 10541 | 97.0 | 9.6e-07 | 1.2e-10 | 55.8 | 44 | (51, 94) | 100 | (55, 98) | 100 | NA | NA; Category: unknown function; NC\_029029\_p8 | | phrogs | 1772 | 95.7 | 9e-05 | 1e-08 | 46.4 | 38 | (53, 90) | 100 | (72, 109) | 111 | NA | NA; Category: unknown function; p424151 VI\_10171 | | phrogs | 38661 | 95.6 | 0.00011 | 1.3e-08 | 42.7 | 40 | (53, 92) | 100 | (31, 70) | 71 | NA | NA; Category: unknown function; p193317 VI\_04290 | | phrogs | 19159 | 95.4 | 0.00014 | 1.6e-08 | 44.2 | 36 | (58, 93) | 100 | (52, 87) | 89 | NA | NA; Category: unknown function; p84411 VI\_10196 | | phrogs | 9487 | 95.4 | 0.00014 | 1.7e-08 | 47.2 | 42 | (56, 97) | 100 | (2, 43) | 133 | NA | NA; Category: unknown function; MF347638\_p49 | | phrogs | 17694 | 95.1 | 0.00027 | 3e-08 | 43.2 | 39 | (53, 91) | 100 | (59, 97) | 98 | NA | NA; Category: unknown function; p62152 VI\_08379 | | phrogs | 8953 | 94.5 | 0.00063 | 7.5e-08 | 44.2 | 40 | (58, 97) | 100 | (90, 129) | 134 | NA | NA; Category: unknown function; p258246 VI\_06608 | |
| Top keywords  (threshold 1.00e-03 (evalue)) | **and, A, Transcription, in, endonuclease, VII, Alpha, yes, Termination, Factor** |
| Output files | ../../domain\_architecture/06\_FANPEZAQ\_CDS\_0006\_cath.hhr ../../domain\_architecture/06\_FANPEZAQ\_CDS\_0006\_merged.svg ../../domain\_architecture/06\_FANPEZAQ\_CDS\_0006\_ncbi-cd.hhr ../../domain\_architecture/06\_FANPEZAQ\_CDS\_0006\_pfam.hhr ../../domain\_architecture/06\_FANPEZAQ\_CDS\_0006\_phrogs.hhr |

### Identical protein sequences/structures

#### Search results

|  |  |
| --- | --- |
| Protein sequence databases searched | Pdb, Swissprot, Refseq |
| Identical proteins found | -- |
| Top keywords | -- |
| Output files | -- |

### Similar protein sequences/structures

#### Sequence similarity search results (HHblits)1

|  |  |
| --- | --- |
| Sequence databases searched | Uniclust, Pdb70 |
| Results, scheme(s)  (Top layers only, threshold 1.00e-03 (evalue)) | xml version="1.0" encoding="utf-8" standalone="no"?       2024-09-02T21:08:31.054110 image/svg+xml   Matplotlib v3.7.2, https://matplotlib.org/ |
| Results, table(s)  (threshold 1.00e-03 (evalue)) | | db | id | prob | evalue | pvalue | score | cols | query | query\_len | template | template\_len | name | description | | --- | --- | --- | --- | --- | --- | --- | --- | --- | --- | --- | --- | --- | | uniclust | UniRef100\_A0A098MGF8 | 99.6 | 2.9e-19 | 6.2e-25 | 115.8 | 96 | (2, 97) | 100 | (6, 108) | 122 | Rho termination factor N-terminal domain-containing protein | Rho termination factor N-terminal domain-containing protein | | uniclust | UniRef100\_A0A088FQV7 | 99.6 | 5e-19 | 9.8e-25 | 112.9 | 97 | (1, 97) | 100 | (16, 113) | 121 | Rho termination factor N-terminal domain-containing protein | Rho termination factor N-terminal domain-containing protein | | uniclust | UniRef100\_A0A2N1AP72 | 99.6 | 2.3e-18 | 4.5e-24 | 107.5 | 94 | (3, 96) | 100 | (6, 104) | 106 | Rho termination factor N-terminal domain-containing protein | Rho termination factor N-terminal domain-containing protein | | uniclust | UniRef100\_A0A168CE49 | 99.5 | 2.6e-17 | 5.5e-23 | 105.3 | 90 | (4, 97) | 100 | (1, 91) | 112 | Phage protein | Phage protein | | uniclust | UniRef100\_A0A0P8YT19 | 99.5 | 2.8e-17 | 5.7e-23 | 104.3 | 95 | (2, 97) | 100 | (18, 112) | 113 | Rho termination factor N-terminal domain-containing protein | Rho termination factor N-terminal domain-containing protein | | uniclust | UniRef100\_A0A089LLN8 | 99.5 | 8.7e-17 | 1.8e-22 | 99.6 | 88 | (6, 98) | 100 | (3, 90) | 94 | Rho termination factor N-terminal domain-containing protein | Rho termination factor N-terminal domain-containing protein | | uniclust | UniRef100\_A0A024YGT7 | 99.4 | 2.2e-16 | 4.6e-22 | 102.4 | 93 | (4, 96) | 100 | (1, 105) | 121 | Rho termination factor N-terminal domain-containing protein | Rho termination factor N-terminal domain-containing protein | | uniclust | UniRef100\_A0A1C7DPF7 | 99.3 | 2.9e-15 | 6.1e-21 | 94.3 | 82 | (6, 96) | 100 | (3, 84) | 101 | Rho termination factor N-terminal domain-containing protein | Rho termination factor N-terminal domain-containing protein | | uniclust | UniRef100\_A0A0E3TAL3 | 99.2 | 9.8e-14 | 2e-19 | 88.5 | 93 | (1, 93) | 100 | (1, 105) | 107 | Rho termination factor domain protein | Rho termination factor domain protein | | uniclust | UniRef100\_A0A7Y8VF85 | 99.1 | 3.9e-13 | 7.5e-19 | 83.7 | 94 | (2, 95) | 100 | (3, 97) | 98 | Rho termination factor N-terminal domain-containing protein | Rho termination factor N-terminal domain-containing protein | | uniclust | UniRef100\_A0A1F1ZVW8 | 99.1 | 3.7e-13 | 7.7e-19 | 87.7 | 90 | (2, 92) | 100 | (3, 115) | 116 | Rho termination factor N-terminal domain-containing protein | Rho termination factor N-terminal domain-containing protein | | uniclust | UniRef100\_A0A0Q3TXV1 | 99.1 | 5e-13 | 9.7e-19 | 84.8 | 94 | (2, 96) | 100 | (1, 94) | 111 | Rho termination factor N-terminal domain-containing protein | Rho termination factor N-terminal domain-containing protein | | uniclust | UniRef100\_A0A645IWH2 | 99.1 | 5.1e-13 | 1e-18 | 86.2 | 94 | (4, 97) | 100 | (1, 102) | 119 | Rho termination factor N-terminal domain-containing protein | Rho termination factor N-terminal domain-containing protein | | uniclust | UniRef100\_A0A0Q4FWF8 | 99.1 | 6.2e-13 | 1.3e-18 | 84.9 | 97 | (1, 97) | 100 | (1, 103) | 105 | Rho termination factor N-terminal domain-containing protein | Rho termination factor N-terminal domain-containing protein | | uniclust | UniRef100\_A0A150KSC3 | 99.1 | 7.8e-13 | 1.6e-18 | 84.2 | 95 | (1, 95) | 100 | (1, 97) | 106 | Uncharacterized protein | Uncharacterized protein | | uniclust | UniRef100\_A0A1Y4BC71 | 99.0 | 1.1e-12 | 2.1e-18 | 84.0 | 93 | (4, 97) | 100 | (1, 110) | 117 | Rho termination factor N-terminal domain-containing protein | Rho termination factor N-terminal domain-containing protein | | uniclust | UniRef100\_A0A1I5MMY0 | 99.0 | 2.3e-12 | 4.6e-18 | 85.8 | 92 | (3, 94) | 100 | (1, 110) | 136 | Heat induced stress protein YflT | Heat induced stress protein YflT | | uniclust | UniRef100\_A0A290GHN0 | 99.0 | 2.9e-12 | 5.8e-18 | 80.1 | 90 | (2, 93) | 100 | (1, 91) | 96 | HeH/LEM domain-containing protein | HeH/LEM domain-containing protein | | uniclust | UniRef100\_A0A2P8DHJ0 | 98.9 | 8.9e-12 | 1.8e-17 | 81.1 | 76 | (22, 97) | 100 | (10, 88) | 114 | Rho termination factor-like protein | Rho termination factor-like protein | | uniclust | UniRef100\_A0A0Q4LHG8 | 98.9 | 1.6e-11 | 3.1e-17 | 77.4 | 91 | (6, 97) | 100 | (3, 94) | 99 | Rho termination factor N-terminal domain-containing protein | Rho termination factor N-terminal domain-containing protein | | uniclust | UniRef100\_A0A0N9YLG6 | 98.8 | 2.3e-11 | 4.8e-17 | 79.2 | 96 | (2, 97) | 100 | (4, 105) | 114 | HeH/LEM domain-containing protein | HeH/LEM domain-containing protein | | uniclust | UniRef100\_A0A553SNK1 | 98.8 | 2.7e-11 | 5.3e-17 | 80.4 | 92 | (4, 95) | 100 | (1, 121) | 133 | Uncharacterized protein | Uncharacterized protein | | uniclust | UniRef100\_A0A1Q9PIP6 | 98.8 | 3.3e-11 | 6.8e-17 | 80.2 | 92 | (3, 94) | 100 | (5, 115) | 131 | Rho termination factor N-terminal domain-containing protein | Rho termination factor N-terminal domain-containing protein | | uniclust | UniRef100\_A0A1M4Y9M8 | 98.8 | 4.4e-11 | 8.9e-17 | 78.7 | 96 | (1, 96) | 100 | (10, 120) | 122 | Mu-like prophage FluMu N-terminal domain-containing protein | Mu-like prophage FluMu N-terminal domain-containing protein | | uniclust | UniRef100\_A0A0M3B2K8 | 98.8 | 4.3e-11 | 9e-17 | 73.1 | 72 | (6, 96) | 100 | (3, 74) | 77 | Rho termination factor N-terminal domain-containing protein | Rho termination factor N-terminal domain-containing protein | | uniclust | UniRef100\_A0A0Z8CJH6 | 98.8 | 6.8e-11 | 1.3e-16 | 75.2 | 82 | (13, 94) | 100 | (12, 101) | 102 | Phage protein | Phage protein | | uniclust | UniRef100\_A0A1H8QNZ5 | 98.7 | 8.5e-11 | 1.7e-16 | 74.3 | 92 | (2, 93) | 100 | (4, 96) | 99 | Rho termination factor, N-terminal domain | Rho termination factor, N-terminal domain | | uniclust | UniRef100\_A0A174TMF5 | 98.7 | 9.7e-11 | 2e-16 | 81.0 | 93 | (2, 94) | 100 | (19, 150) | 169 | Rho termination factor N-terminal domain-containing protein | Rho termination factor N-terminal domain-containing protein | | uniclust | UniRef100\_A0A089JTJ5 | 98.7 | 1.1e-10 | 2.3e-16 | 74.4 | 78 | (12, 94) | 100 | (12, 90) | 104 | Rho termination factor N-terminal domain-containing protein | Rho termination factor N-terminal domain-containing protein | | uniclust | UniRef100\_A0A090HXG2 | 98.7 | 1.2e-10 | 2.5e-16 | 72.5 | 76 | (11, 91) | 100 | (9, 85) | 87 | HeH/LEM domain-containing protein | HeH/LEM domain-containing protein | | uniclust | UniRef100\_A0A972MRU9 | 98.7 | 1.5e-10 | 2.8e-16 | 70.4 | 87 | (7, 95) | 100 | (4, 90) | 92 | Rho termination factor N-terminal domain-containing protein | Rho termination factor N-terminal domain-containing protein | | uniclust | UniRef100\_A0A1Y4VDP2 | 98.7 | 1.4e-10 | 2.9e-16 | 78.4 | 94 | (3, 96) | 100 | (1, 124) | 138 | Rho termination factor N-terminal domain-containing protein | Rho termination factor N-terminal domain-containing protein | | uniclust | UniRef100\_A0A173R5M0 | 98.7 | 1.6e-10 | 3.2e-16 | 78.2 | 75 | (22, 96) | 100 | (30, 124) | 147 | Rho termination factor N-terminal domain-containing protein | Rho termination factor N-terminal domain-containing protein | | uniclust | UniRef100\_A0A0N1AA76 | 98.7 | 1.7e-10 | 3.6e-16 | 74.4 | 94 | (1, 96) | 100 | (1, 101) | 103 | Rho termination factor N-terminal domain-containing protein | Rho termination factor N-terminal domain-containing protein | | uniclust | UniRef100\_A0A0F0C809 | 98.7 | 2e-10 | 4.2e-16 | 71.5 | 66 | (28, 93) | 100 | (13, 80) | 83 | Rho termination factor N-terminal domain-containing protein | Rho termination factor N-terminal domain-containing protein | | uniclust | UniRef100\_A0A099WPM7 | 98.7 | 2.2e-10 | 4.4e-16 | 72.5 | 87 | (6, 95) | 100 | (7, 96) | 98 | Uncharacterized protein | Uncharacterized protein | | uniclust | UniRef100\_A0A1C5LBF0 | 98.6 | 2.4e-10 | 5e-16 | 71.4 | 66 | (29, 94) | 100 | (14, 83) | 86 | Rho termination factor, N-terminal domain | Rho termination factor, N-terminal domain | | uniclust | UniRef100\_A0A101WGP7 | 98.6 | 3.4e-10 | 7e-16 | 76.4 | 93 | (4, 96) | 100 | (1, 120) | 142 | Rho termination factor N-terminal domain-containing protein | Rho termination factor N-terminal domain-containing protein | | uniclust | UniRef100\_A0A0A8IK76 | 98.6 | 4.1e-10 | 8.2e-16 | 69.0 | 74 | (9, 97) | 100 | (4, 79) | 80 | Rho termination factor N-terminal domain-containing protein | Rho termination factor N-terminal domain-containing protein | | uniclust | UniRef100\_A0A0V8D0U7 | 98.6 | 5.3e-10 | 1.1e-15 | 73.4 | 91 | (4, 94) | 100 | (1, 94) | 117 | Conjugal transfer protein | Conjugal transfer protein | | uniclust | UniRef100\_A0A0S7Z717 | 98.6 | 5.9e-10 | 1.1e-15 | 72.0 | 88 | (5, 93) | 100 | (1, 97) | 120 | Rho termination factor N-terminal domain-containing protein | Rho termination factor N-terminal domain-containing protein | | uniclust | UniRef100\_A0A2G1LY20 | 98.6 | 6.3e-10 | 1.2e-15 | 69.5 | 90 | (6, 95) | 100 | (6, 96) | 99 | Rho termination factor N-terminal domain-containing protein | Rho termination factor N-terminal domain-containing protein | | uniclust | UniRef100\_A0A1G9TZJ8 | 98.6 | 7.2e-10 | 1.4e-15 | 64.3 | 37 | (53, 89) | 100 | (24, 60) | 61 | Rho termination factor, N-terminal domain | Rho termination factor, N-terminal domain | | uniclust | UniRef100\_A0A1M6EXN4 | 98.5 | 8.1e-10 | 1.7e-15 | 73.3 | 95 | (1, 95) | 100 | (5, 104) | 122 | Rho termination factor, N-terminal domain | Rho termination factor, N-terminal domain | | uniclust | UniRef100\_A0A5K7YYK9 | 98.5 | 1e-09 | 1.9e-15 | 68.6 | 90 | (6, 95) | 100 | (4, 95) | 99 | HeH/LEM domain-containing protein | HeH/LEM domain-containing protein | | uniclust | UniRef100\_A0A1W7ABQ3 | 98.5 | 9.3e-10 | 2e-15 | 70.7 | 84 | (11, 94) | 100 | (9, 98) | 99 | Phage protein | Phage protein | | uniclust | UniRef100\_A0A1C6JH25 | 98.5 | 1.2e-09 | 2.5e-15 | 74.5 | 94 | (4, 97) | 100 | (1, 128) | 150 | Rho termination factor N-terminal domain-containing protein | Rho termination factor N-terminal domain-containing protein | | uniclust | UniRef100\_A0A845NDA8 | 98.5 | 1.8e-09 | 3.4e-15 | 66.6 | 93 | (2, 96) | 100 | (1, 93) | 97 | Rho termination factor N-terminal domain-containing protein | Rho termination factor N-terminal domain-containing protein | | uniclust | UniRef100\_A0A0U9HBT6 | 98.5 | 2.1e-09 | 3.8e-15 | 66.3 | 89 | (5, 93) | 100 | (1, 91) | 96 | Uncharacterized protein | Uncharacterized protein | | uniclust | UniRef100\_A0A349GRC7 | 98.4 | 2.4e-09 | 4.5e-15 | 67.4 | 91 | (2, 94) | 100 | (1, 101) | 103 | Rho termination factor N-terminal domain-containing protein | Rho termination factor N-terminal domain-containing protein | | uniclust | UniRef100\_A0A1W6JNK7 | 98.4 | 2.3e-09 | 4.7e-15 | 68.1 | 89 | (1, 93) | 100 | (1, 91) | 96 | Transcriptional terminator | Transcriptional terminator | | uniclust | UniRef100\_A0A0Z8DCB5 | 98.4 | 2.5e-09 | 4.9e-15 | 67.5 | 91 | (1, 93) | 100 | (1, 97) | 98 | HeH/LEM domain | HeH/LEM domain | | uniclust | UniRef100\_A0A0A8JM68 | 98.4 | 2.7e-09 | 5.3e-15 | 71.1 | 92 | (4, 95) | 100 | (2, 114) | 130 | Uncharacterized protein | Uncharacterized protein | | uniclust | UniRef100\_A0A1C5YVF9 | 98.4 | 2.9e-09 | 5.8e-15 | 64.0 | 38 | (54, 91) | 100 | (32, 69) | 70 | HeH/LEM domain | HeH/LEM domain | | uniclust | UniRef100\_A0A1R0X056 | 98.4 | 4.2e-09 | 7.7e-15 | 67.9 | 91 | (7, 97) | 100 | (12, 106) | 124 | Rho termination factor N-terminal domain-containing protein | Rho termination factor N-terminal domain-containing protein | | uniclust | UniRef100\_A0A1M6AHL9 | 98.4 | 3.8e-09 | 7.8e-15 | 67.0 | 65 | (31, 95) | 100 | (18, 88) | 91 | HeH/LEM domain-containing protein | HeH/LEM domain-containing protein | | uniclust | UniRef100\_A0A0U3P188 | 98.4 | 4.3e-09 | 8.3e-15 | 65.4 | 85 | (3, 92) | 100 | (2, 88) | 89 | T4 recombination endonuclease VII dimerisation domain-containing protein | T4 recombination endonuclease VII dimerisation domain-containing protein | | uniclust | UniRef100\_A0A1I4PMR5 | 98.4 | 4.3e-09 | 8.8e-15 | 70.3 | 88 | (3, 90) | 100 | (1, 99) | 125 | Heat induced stress protein YflT | Heat induced stress protein YflT | | uniclust | UniRef100\_A0A1Y3TP18 | 98.4 | 4.5e-09 | 9.2e-15 | 72.7 | 94 | (2, 95) | 100 | (1, 147) | 160 | Rho termination factor N-terminal domain-containing protein | Rho termination factor N-terminal domain-containing protein | | uniclust | UniRef100\_A0A0T6DTS4 | 98.4 | 5e-09 | 9.6e-15 | 66.1 | 70 | (20, 89) | 100 | (16, 93) | 96 | T4 recombination endonuclease VII dimerisation domain-containing protein | T4 recombination endonuclease VII dimerisation domain-containing protein | | uniclust | UniRef100\_A0A069A8G7 | 98.4 | 4.8e-09 | 9.8e-15 | 65.4 | 60 | (31, 95) | 100 | (19, 78) | 84 | Rho termination factor N-terminal domain-containing protein | Rho termination factor N-terminal domain-containing protein | | uniclust | UniRef100\_UPI0006231A7A | 98.3 | 6.5e-09 | 1.2e-14 | 65.8 | 72 | (23, 94) | 100 | (25, 99) | 111 | Rho termination factor N-terminal domain-containing protein | Rho termination factor N-terminal domain-containing protein | | uniclust | UniRef100\_A0A0J7Y8Q0 | 98.3 | 6.2e-09 | 1.3e-14 | 67.3 | 73 | (19, 96) | 100 | (27, 101) | 105 | Rho termination factor N-terminal domain-containing protein | Rho termination factor N-terminal domain-containing protein | | uniclust | UniRef100\_A0A943MCU2 | 98.3 | 7.1e-09 | 1.3e-14 | 67.2 | 91 | (5, 95) | 100 | (1, 110) | 117 | Uncharacterized protein | Uncharacterized protein | | uniclust | UniRef100\_A0A0D7X2K7 | 98.3 | 7.1e-09 | 1.5e-14 | 69.9 | 85 | (2, 88) | 100 | (1, 106) | 127 | Uncharacterized protein | Uncharacterized protein | | uniclust | UniRef100\_A0A7V7HMI4 | 98.3 | 8.9e-09 | 1.7e-14 | 62.5 | 67 | (22, 90) | 100 | (9, 75) | 77 | Rho termination factor N-terminal domain-containing protein | Rho termination factor N-terminal domain-containing protein | | uniclust | UniRef100\_A0A0X1RV11 | 98.3 | 1.2e-08 | 2.4e-14 | 63.7 | 45 | (53, 97) | 100 | (30, 74) | 85 | HeH/LEM domain protein | HeH/LEM domain protein | | uniclust | UniRef100\_A0A845R2Z6 | 98.3 | 1.4e-08 | 2.5e-14 | 62.1 | 83 | (2, 91) | 100 | (1, 88) | 89 | Rho termination protein | Rho termination protein | | uniclust | UniRef100\_A0A0T7K7C9 | 98.2 | 1.4e-08 | 2.7e-14 | 60.1 | 41 | (55, 95) | 100 | (17, 57) | 65 | Transcription termination factor | Transcription termination factor | | uniclust | UniRef100\_A0A142KCR9 | 98.2 | 1.3e-08 | 2.8e-14 | 70.7 | 93 | (1, 93) | 100 | (9, 149) | 151 | Tail assembly chaperone | Tail assembly chaperone | | uniclust | UniRef100\_A0A1B1G6D0 | 98.2 | 1.5e-08 | 2.9e-14 | 63.1 | 92 | (1, 93) | 100 | (1, 94) | 95 | Rho termination factor N-terminal domain-containing protein | Rho termination factor N-terminal domain-containing protein | | uniclust | UniRef100\_A0A383RBW5 | 98.2 | 1.5e-08 | 3e-14 | 63.3 | 42 | (53, 94) | 100 | (38, 79) | 82 | Rho termination factor N-terminal domain-containing protein | Rho termination factor N-terminal domain-containing protein | | uniclust | UniRef100\_A0A2D4DNJ5 | 98.2 | 1.6e-08 | 3.3e-14 | 63.6 | 75 | (14, 96) | 100 | (12, 86) | 89 | Phage protein | Phage protein | | uniclust | UniRef100\_A0A0M2LYI1 | 98.2 | 1.9e-08 | 3.7e-14 | 64.3 | 74 | (23, 99) | 100 | (10, 83) | 96 | Rho termination factor N-terminal domain-containing protein | Rho termination factor N-terminal domain-containing protein | | uniclust | UniRef100\_A0A1G8IAC0 | 98.2 | 1.9e-08 | 3.7e-14 | 62.6 | 39 | (53, 91) | 100 | (42, 80) | 82 | Rho termination factor, N-terminal domain | Rho termination factor, N-terminal domain | | uniclust | UniRef100\_A0A0L0W633 | 98.2 | 2.4e-08 | 4.5e-14 | 55.5 | 42 | (55, 96) | 100 | (8, 49) | 52 | Transcription termination factor | Transcription termination factor | | uniclust | UniRef100\_UPI002033AFD5 | 98.2 | 2.4e-08 | 4.6e-14 | 63.1 | 80 | (13, 92) | 100 | (11, 101) | 103 | Rho termination factor N-terminal domain-containing protein | Rho termination factor N-terminal domain-containing protein | | uniclust | UniRef100\_A0A352NNW6 | 98.2 | 2.2e-08 | 4.6e-14 | 62.9 | 39 | (55, 93) | 100 | (45, 83) | 85 | Rho termination factor N-terminal domain-containing protein | Rho termination factor N-terminal domain-containing protein | | uniclust | UniRef100\_A0A2W4IJI6 | 98.1 | 3.9e-08 | 7.4e-14 | 63.6 | 85 | (3, 91) | 100 | (4, 92) | 115 | Rho termination factor N-terminal domain-containing protein | Rho termination factor N-terminal domain-containing protein | | uniclust | UniRef100\_A0A1H4QZW6 | 98.1 | 3.8e-08 | 7.9e-14 | 63.6 | 89 | (1, 99) | 100 | (1, 89) | 97 | HeH/LEM domain-containing protein | HeH/LEM domain-containing protein | | uniclust | UniRef100\_A0A089MTA0 | 98.1 | 4.4e-08 | 8.7e-14 | 64.8 | 92 | (2, 93) | 100 | (1, 121) | 123 | Rho termination factor N-terminal domain-containing protein | Rho termination factor N-terminal domain-containing protein | | uniclust | UniRef100\_A0A0F6Z7H9 | 98.1 | 4.2e-08 | 9.2e-14 | 67.5 | 94 | (1, 94) | 100 | (1, 138) | 140 | Uncharacterized protein | Uncharacterized protein | | uniclust | UniRef100\_E6X1P2 | 98.1 | 5e-08 | 9.3e-14 | 59.4 | 81 | (7, 93) | 100 | (4, 84) | 85 | Rho termination factor domain protein | Rho termination factor domain protein | | uniclust | UniRef100\_A0A1E2SK37 | 98.1 | 5.4e-08 | 1.1e-13 | 64.6 | 73 | (22, 94) | 100 | (33, 111) | 127 | Rho termination factor N-terminal domain-containing protein | Rho termination factor N-terminal domain-containing protein | | uniclust | UniRef100\_C4L0Q0 | 98.1 | 5.6e-08 | 1.1e-13 | 62.7 | 93 | (3, 95) | 100 | (4, 106) | 112 | Rho termination factor N-terminal domain-containing protein | Rho termination factor N-terminal domain-containing protein | | uniclust | UniRef100\_A0A1J0TTB7 | 98.1 | 5.5e-08 | 1.1e-13 | 64.4 | 37 | (54, 90) | 100 | (63, 99) | 117 | Rho termination factor N-terminal domain-containing protein | Rho termination factor N-terminal domain-containing protein | | uniclust | UniRef100\_A0A414PRA9 | 98.1 | 6.3e-08 | 1.2e-13 | 60.4 | 86 | (5, 91) | 100 | (1, 93) | 98 | HeH/LEM domain-containing protein | HeH/LEM domain-containing protein | | uniclust | UniRef100\_A0A1M2ZYQ4 | 98.1 | 6.2e-08 | 1.2e-13 | 62.4 | 90 | (6, 95) | 100 | (4, 105) | 106 | Rho termination factor N-terminal domain-containing protein | Rho termination factor N-terminal domain-containing protein | | uniclust | UniRef100\_A0A0H3J9M1 | 98.1 | 5.7e-08 | 1.2e-13 | 65.2 | 92 | (2, 94) | 100 | (1, 97) | 122 | Rho termination factor N-terminal domain-containing protein | Rho termination factor N-terminal domain-containing protein | | uniclust | UniRef100\_A0A2D9NX61 | 98.0 | 7.6e-08 | 1.5e-13 | 60.6 | 76 | (13, 98) | 100 | (14, 91) | 93 | Rho termination factor N-terminal domain-containing protein | Rho termination factor N-terminal domain-containing protein | | uniclust | UniRef100\_UPI0003493A31 | 98.0 | 8.7e-08 | 1.6e-13 | 60.5 | 82 | (12, 93) | 100 | (11, 93) | 105 | Rho termination factor N-terminal domain-containing protein | Rho termination factor N-terminal domain-containing protein | | uniclust | UniRef100\_A0A173R5B0 | 98.0 | 8.6e-08 | 1.7e-13 | 61.9 | 87 | (10, 98) | 100 | (8, 97) | 108 | Rho termination factor N-terminal domain-containing protein | Rho termination factor N-terminal domain-containing protein | | uniclust | UniRef100\_A0A373L6M1 | 98.0 | 8.5e-08 | 1.7e-13 | 58.4 | 42 | (53, 94) | 100 | (23, 64) | 74 | Rho termination factor N-terminal domain-containing protein | Rho termination factor N-terminal domain-containing protein | | uniclust | UniRef100\_A0A951JXR5 | 98.0 | 1e-07 | 1.9e-13 | 48.6 | 32 | (60, 91) | 100 | (1, 32) | 33 | Rho termination factor N-terminal domain-containing protein | Rho termination factor N-terminal domain-containing protein | | uniclust | UniRef100\_A0A1C5TAF6 | 98.0 | 9.5e-08 | 1.9e-13 | 62.0 | 43 | (52, 94) | 100 | (59, 101) | 102 | Rho termination factor, N-terminal domain | Rho termination factor, N-terminal domain | | uniclust | UniRef100\_A0A3D1GE26 | 98.0 | 1e-07 | 1.9e-13 | 60.1 | 39 | (53, 91) | 100 | (55, 93) | 94 | 50S ribosomal protein L21 (Fragment) | 50S ribosomal protein L21 (Fragment) | | uniclust | UniRef100\_A0A022LHQ7 | 98.0 | 9.9e-08 | 2e-13 | 63.5 | 66 | (29, 94) | 100 | (33, 112) | 116 | Rho termination factor | Rho termination factor | | uniclust | UniRef100\_A0A6G4H4V0 | 98.0 | 1.1e-07 | 2.1e-13 | 53.3 | 38 | (54, 91) | 100 | (14, 51) | 52 | Termination factor Rho | Termination factor Rho | | uniclust | UniRef100\_A0A1D9GT57 | 98.0 | 1e-07 | 2.2e-13 | 65.3 | 82 | (15, 96) | 100 | (24, 131) | 142 | Phage tail protein | Phage tail protein | | uniclust | UniRef100\_A0A1G6NU17 | 98.0 | 1.1e-07 | 2.4e-13 | 66.5 | 78 | (16, 93) | 100 | (17, 101) | 163 | Rho termination factor N-terminal domain-containing protein | Rho termination factor N-terminal domain-containing protein | | uniclust | UniRef100\_A0A1W6RR01 | 98.0 | 1.2e-07 | 2.4e-13 | 60.6 | 66 | (27, 92) | 100 | (28, 98) | 99 | HeH/LEM domain-containing protein | HeH/LEM domain-containing protein | | uniclust | UniRef100\_A0A1M4Z686 | 98.0 | 1.3e-07 | 2.5e-13 | 52.2 | 39 | (54, 92) | 100 | (6, 44) | 46 | Rho termination factor, N-terminal domain | Rho termination factor, N-terminal domain | | uniclust | UniRef100\_A0A359B3E5 | 98.0 | 1.5e-07 | 2.8e-13 | 61.2 | 94 | (5, 98) | 100 | (1, 102) | 116 | Rho termination factor N-terminal domain-containing protein | Rho termination factor N-terminal domain-containing protein | | uniclust | UniRef100\_A0A0L6JGZ7 | 98.0 | 1.4e-07 | 2.8e-13 | 61.3 | 69 | (20, 94) | 100 | (22, 90) | 100 | Rho termination factor N-terminal domain-containing protein | Rho termination factor N-terminal domain-containing protein | | uniclust | UniRef100\_A0A0B5Q9F7 | 98.0 | 1.4e-07 | 2.8e-13 | 59.5 | 64 | (30, 93) | 100 | (19, 86) | 89 | Rho termination factor N-terminal domain-containing protein | Rho termination factor N-terminal domain-containing protein | | uniclust | UniRef100\_A0A074L0V4 | 98.0 | 1.5e-07 | 3e-13 | 61.8 | 67 | (29, 95) | 100 | (27, 101) | 109 | Rho termination factor | Rho termination factor | | uniclust | UniRef100\_A0A076NJ41 | 97.9 | 1.6e-07 | 3.3e-13 | 62.6 | 91 | (4, 94) | 100 | (1, 117) | 120 | Phage protein | Phage protein | | uniclust | UniRef100\_A0A921G371 | 97.9 | 1.8e-07 | 3.4e-13 | 60.3 | 88 | (7, 94) | 100 | (5, 101) | 108 | Uncharacterized protein | Uncharacterized protein | | uniclust | UniRef100\_A0A2I8AAC4 | 97.9 | 1.8e-07 | 3.5e-13 | 48.2 | 32 | (60, 91) | 100 | (1, 32) | 33 | Rho termination factor N-terminal domain-containing protein | Rho termination factor N-terminal domain-containing protein | | uniclust | UniRef100\_A0A1C5WIW1 | 97.9 | 1.7e-07 | 3.5e-13 | 59.1 | 77 | (9, 91) | 100 | (3, 79) | 83 | Rho termination factor N-terminal domain-containing protein | Rho termination factor N-terminal domain-containing protein | | uniclust | UniRef100\_A0A1A0LLB8 | 97.9 | 2.1e-07 | 3.8e-13 | 58.7 | 86 | (4, 89) | 100 | (3, 98) | 99 | Uncharacterized protein | Uncharacterized protein | | uniclust | UniRef100\_A5EUZ2 | 97.9 | 2.2e-07 | 4.1e-13 | 55.6 | 74 | (5, 90) | 100 | (1, 74) | 75 | T4 recombination endonuclease VII dimerisation domain-containing protein | T4 recombination endonuclease VII dimerisation domain-containing protein | | uniclust | UniRef100\_A0A0A1W8H8 | 97.9 | 2.1e-07 | 4.2e-13 | 60.7 | 85 | (10, 95) | 100 | (12, 99) | 103 | Rho termination factor N-terminal domain-containing protein | Rho termination factor N-terminal domain-containing protein | | uniclust | UniRef100\_A0A8J6SEE7 | 97.9 | 2.3e-07 | 4.3e-13 | 49.0 | 36 | (60, 95) | 100 | (1, 36) | 38 | Rho termination factor N-terminal domain-containing protein | Rho termination factor N-terminal domain-containing protein | | uniclust | UniRef100\_A0A3G3BWG3 | 97.9 | 2.3e-07 | 4.4e-13 | 61.5 | 92 | (4, 95) | 100 | (1, 111) | 124 | Tail assembly chaperone | Tail assembly chaperone | | uniclust | UniRef100\_A0A1I1D1B8 | 97.9 | 2.4e-07 | 4.8e-13 | 60.5 | 95 | (4, 98) | 100 | (2, 103) | 105 | HeH/LEM domain-containing protein | HeH/LEM domain-containing protein | | uniclust | UniRef100\_UPI000490AEB4 | 97.9 | 2.9e-07 | 5.3e-13 | 56.9 | 86 | (7, 95) | 100 | (3, 88) | 91 | HeH/LEM domain-containing protein | HeH/LEM domain-containing protein | | uniclust | UniRef100\_A0A849D8D2 | 97.9 | 2.8e-07 | 5.3e-13 | 53.1 | 37 | (54, 90) | 100 | (19, 55) | 56 | 50S ribosomal protein L21 | 50S ribosomal protein L21 | | uniclust | UniRef100\_A0A175R7S3 | 97.9 | 2.8e-07 | 5.4e-13 | 56.1 | 68 | (9, 92) | 100 | (4, 71) | 72 | Rho termination factor N-terminal domain-containing protein | Rho termination factor N-terminal domain-containing protein | | uniclust | UniRef100\_A0A1C5THF0 | 97.9 | 2.8e-07 | 5.5e-13 | 59.6 | 63 | (32, 94) | 100 | (40, 102) | 104 | Rho termination factor N-terminal domain-containing protein | Rho termination factor N-terminal domain-containing protein | | uniclust | UniRef100\_UPI000D3A7A5A | 97.9 | 3.3e-07 | 6.1e-13 | 58.2 | 89 | (5, 93) | 100 | (1, 105) | 107 | Rho termination factor N-terminal domain-containing protein | Rho termination factor N-terminal domain-containing protein | | uniclust | UniRef100\_A0A957ZEC5 | 97.8 | 3.8e-07 | 7.1e-13 | 57.8 | 76 | (19, 94) | 100 | (20, 102) | 105 | Rho termination factor N-terminal domain-containing protein | Rho termination factor N-terminal domain-containing protein | | uniclust | UniRef100\_A0A049DS68 | 97.8 | 3.5e-07 | 7.5e-13 | 62.4 | 96 | (1, 96) | 100 | (4, 124) | 130 | Uncharacterized protein | Uncharacterized protein | | uniclust | UniRef100\_A0A1C6BBU0 | 97.8 | 3.9e-07 | 7.5e-13 | 55.4 | 37 | (54, 90) | 100 | (39, 75) | 76 | Transcription termination factor Rho | Transcription termination factor Rho | | uniclust | UniRef100\_UPI00203B3015 | 97.8 | 4.1e-07 | 7.5e-13 | 56.4 | 83 | (1, 92) | 100 | (1, 90) | 92 | Rho termination factor N-terminal domain-containing protein | Rho termination factor N-terminal domain-containing protein | | uniclust | UniRef100\_UPI001CCEC438 | 97.8 | 4.1e-07 | 7.6e-13 | 57.7 | 80 | (15, 94) | 100 | (20, 105) | 106 | hypothetical protein | hypothetical protein | | uniclust | UniRef100\_G9X2L6 | 97.8 | 4.2e-07 | 8.1e-13 | 56.2 | 57 | (33, 93) | 100 | (23, 79) | 82 | Rho termination factor N-terminal domain-containing protein | Rho termination factor N-terminal domain-containing protein | | uniclust | UniRef100\_A0A4R8FYN6 | 97.8 | 4.7e-07 | 8.6e-13 | 57.6 | 41 | (53, 93) | 100 | (58, 98) | 107 | Rho termination factor-like protein | Rho termination factor-like protein | | uniclust | UniRef100\_UPI000D0298A2 | 97.8 | 4.7e-07 | 8.6e-13 | 56.4 | 85 | (2, 91) | 100 | (1, 92) | 94 | hypothetical protein | hypothetical protein | | uniclust | UniRef100\_A0A150NVU6 | 97.8 | 4.7e-07 | 9.1e-13 | 56.1 | 40 | (54, 93) | 100 | (37, 76) | 80 | Phage transcriptional terminator | Phage transcriptional terminator | | uniclust | UniRef100\_A0A0C6FQZ8 | 97.8 | 4.6e-07 | 9.2e-13 | 58.0 | 72 | (22, 93) | 100 | (13, 93) | 95 | Uncharacterized protein | Uncharacterized protein | | uniclust | UniRef100\_A0A1C6AJB8 | 97.8 | 4.7e-07 | 9.3e-13 | 57.4 | 48 | (52, 99) | 100 | (42, 89) | 89 | Rho termination factor N-terminal domain-containing protein | Rho termination factor N-terminal domain-containing protein | | uniclust | UniRef100\_A0A073J6G9 | 97.8 | 4.9e-07 | 9.8e-13 | 61.4 | 68 | (29, 96) | 100 | (48, 128) | 134 | Rho termination factor | Rho termination factor | | uniclust | UniRef100\_A0A8S5MRJ0 | 97.8 | 5.6e-07 | 1.1e-12 | 47.8 | 35 | (60, 94) | 100 | (1, 36) | 37 | HeH/LEM domain | HeH/LEM domain | | uniclust | UniRef100\_A0A4Q8R804 | 97.8 | 6.6e-07 | 1.3e-12 | 63.6 | 75 | (18, 92) | 100 | (32, 144) | 198 | Rho termination factor N-terminal domain-containing protein | Rho termination factor N-terminal domain-containing protein | | uniclust | UniRef100\_A0A017RV20 | 97.8 | 6.3e-07 | 1.3e-12 | 59.5 | 79 | (17, 95) | 100 | (17, 97) | 112 | Rho termination factor N-terminal domain-containing protein | Rho termination factor N-terminal domain-containing protein | | uniclust | UniRef100\_A0A1Q9PJD2 | 97.7 | 6.8e-07 | 1.3e-12 | 57.4 | 45 | (53, 97) | 100 | (51, 95) | 99 | Rho termination factor N-terminal domain-containing protein | Rho termination factor N-terminal domain-containing protein | | uniclust | UniRef100\_UPI0022FF5F71 | 97.7 | 7.8e-07 | 1.4e-12 | 57.3 | 81 | (14, 95) | 100 | (16, 96) | 115 | Rho termination factor N-terminal domain-containing protein | Rho termination factor N-terminal domain-containing protein | | uniclust | UniRef100\_A0A7X9A1P3 | 97.7 | 8e-07 | 1.5e-12 | 56.9 | 74 | (17, 90) | 100 | (19, 111) | 111 | Rho termination factor N-terminal domain-containing protein | Rho termination factor N-terminal domain-containing protein | | uniclust | UniRef100\_A0A1L6BZF2 | 97.7 | 7.4e-07 | 1.5e-12 | 60.0 | 88 | (4, 92) | 100 | (1, 121) | 126 | Uncharacterized protein | Uncharacterized protein | | uniclust | UniRef100\_UPI00214DC62F | 97.7 | 9.4e-07 | 1.7e-12 | 52.2 | 41 | (54, 94) | 100 | (27, 67) | 68 | Rho termination factor N-terminal domain-containing protein | Rho termination factor N-terminal domain-containing protein | | uniclust | UniRef100\_UPI00073A2B65 | 97.7 | 9.5e-07 | 1.8e-12 | 54.7 | 88 | (5, 93) | 100 | (1, 88) | 90 | hypothetical protein | hypothetical protein | | uniclust | UniRef100\_A0A7X9AD68 | 97.7 | 1.1e-06 | 2e-12 | 53.4 | 74 | (13, 89) | 100 | (5, 78) | 80 | Rho termination factor N-terminal domain-containing protein | Rho termination factor N-terminal domain-containing protein | | uniclust | UniRef100\_A0A385TUN3 | 97.7 | 1.2e-06 | 2.1e-12 | 53.6 | 70 | (11, 91) | 100 | (8, 79) | 81 | Rho termination factor N-terminal domain-containing protein | Rho termination factor N-terminal domain-containing protein | | uniclust | UniRef100\_A0A4Y8UXJ0 | 97.7 | 1.1e-06 | 2.2e-12 | 57.0 | 86 | (5, 92) | 100 | (2, 90) | 110 | SAP domain-containing protein | SAP domain-containing protein | | uniclust | UniRef100\_A0A0U3F3Q7 | 97.7 | 1.2e-06 | 2.4e-12 | 58.6 | 92 | (5, 96) | 100 | (3, 106) | 115 | HeH/LEM domain-containing protein | HeH/LEM domain-containing protein | | uniclust | UniRef100\_A0A8I0I2U5 | 97.7 | 1.2e-06 | 2.5e-12 | 58.4 | 87 | (13, 99) | 100 | (18, 117) | 117 | Uncharacterized protein | Uncharacterized protein | | uniclust | UniRef100\_A0A1U9WR62 | 97.6 | 1.4e-06 | 2.6e-12 | 56.6 | 88 | (6, 93) | 100 | (3, 109) | 112 | Rho termination factor N-terminal domain-containing protein | Rho termination factor N-terminal domain-containing protein | | uniclust | UniRef100\_A0A3N9UVK7 | 97.6 | 1.5e-06 | 2.9e-12 | 56.1 | 76 | (14, 97) | 100 | (13, 91) | 97 | Rho termination factor N-terminal domain-containing protein | Rho termination factor N-terminal domain-containing protein | | uniclust | UniRef100\_E3PRZ2 | 97.6 | 1.6e-06 | 2.9e-12 | 54.3 | 79 | (13, 91) | 100 | (13, 93) | 95 | Rho termination factor N-terminal domain-containing protein | Rho termination factor N-terminal domain-containing protein | | uniclust | UniRef100\_A0A173SN79 | 97.6 | 1.5e-06 | 3e-12 | 60.1 | 88 | (6, 93) | 100 | (3, 126) | 150 | Rho termination factor N-terminal domain-containing protein | Rho termination factor N-terminal domain-containing protein | | uniclust | UniRef100\_A0A1Y5NZV1 | 97.6 | 1.6e-06 | 3.1e-12 | 51.6 | 37 | (55, 91) | 100 | (28, 64) | 65 | Rho termination factor N-terminal domain-containing protein | Rho termination factor N-terminal domain-containing protein | | uniclust | UniRef100\_A0A1V4VSQ7 | 97.6 | 1.7e-06 | 3.2e-12 | 54.9 | 73 | (18, 91) | 100 | (14, 101) | 103 | Rho termination factor N-terminal domain-containing protein | Rho termination factor N-terminal domain-containing protein | | uniclust | UniRef100\_A0A8B4Q926 | 97.6 | 1.7e-06 | 3.2e-12 | 54.8 | 33 | (54, 86) | 100 | (49, 81) | 92 | Rho termination factor, N-terminal domain | Rho termination factor, N-terminal domain | | uniclust | UniRef100\_A0A0Q4GFT6 | 97.6 | 1.7e-06 | 3.2e-12 | 54.0 | 79 | (6, 94) | 100 | (3, 82) | 85 | Rho termination factor N-terminal domain-containing protein | Rho termination factor N-terminal domain-containing protein | | uniclust | UniRef100\_UPI001FE188BC | 97.6 | 1.9e-06 | 3.6e-12 | 53.5 | 38 | (53, 90) | 100 | (46, 83) | 84 | Rho termination factor N-terminal domain-containing protein | Rho termination factor N-terminal domain-containing protein | | uniclust | UniRef100\_A0A1X1IFA2 | 97.6 | 2e-06 | 3.7e-12 | 55.2 | 85 | (5, 89) | 100 | (3, 100) | 102 | HeH/LEM domain-containing protein | HeH/LEM domain-containing protein | | uniclust | UniRef100\_A0A3E0KHC7 | 97.6 | 2e-06 | 3.7e-12 | 46.0 | 37 | (55, 91) | 100 | (3, 39) | 40 | Rho termination factor N-terminal domain-containing protein | Rho termination factor N-terminal domain-containing protein | | uniclust | UniRef100\_A0A059N3B0 | 97.6 | 1.9e-06 | 3.8e-12 | 59.8 | 47 | (53, 99) | 100 | (92, 139) | 146 | Rho termination factor protein | Rho termination factor protein | | uniclust | UniRef100\_A0A011PZG2 | 97.6 | 1.8e-06 | 3.8e-12 | 54.0 | 44 | (1, 44) | 100 | (8, 51) | 77 | Uncharacterized protein | Uncharacterized protein | | uniclust | UniRef100\_A0A926I9L6 | 97.6 | 2.1e-06 | 3.8e-12 | 47.0 | 37 | (55, 91) | 100 | (6, 42) | 45 | Rho termination factor N-terminal domain-containing protein | Rho termination factor N-terminal domain-containing protein | | uniclust | UniRef100\_A0A249P9B4 | 97.6 | 2.2e-06 | 4.3e-12 | 55.5 | 74 | (21, 94) | 100 | (15, 95) | 98 | Phage protein | Phage protein | | uniclust | UniRef100\_UPI001F25D97B | 97.6 | 2.3e-06 | 4.3e-12 | 52.8 | 78 | (9, 92) | 100 | (9, 86) | 87 | hypothetical protein | hypothetical protein | | uniclust | UniRef100\_A0A3A6W6V7 | 97.6 | 2.3e-06 | 4.4e-12 | 57.1 | 88 | (7, 94) | 100 | (3, 95) | 133 | Rho termination factor N-terminal domain-containing protein | Rho termination factor N-terminal domain-containing protein | | uniclust | UniRef100\_A0A1H9BTI4 | 97.6 | 2.4e-06 | 4.7e-12 | 55.3 | 40 | (53, 92) | 100 | (55, 94) | 97 | Rho termination factor, N-terminal domain | Rho termination factor, N-terminal domain | | uniclust | UniRef100\_A0A7D7WFP1 | 97.6 | 2.4e-06 | 4.7e-12 | 55.4 | 42 | (55, 96) | 100 | (40, 81) | 100 | Rho termination factor N-terminal domain-containing protein | Rho termination factor N-terminal domain-containing protein | | uniclust | UniRef100\_A0A2E0V1P7 | 97.6 | 2.6e-06 | 4.8e-12 | 52.2 | 39 | (54, 92) | 100 | (42, 80) | 83 | Rho termination factor N-terminal domain-containing protein | Rho termination factor N-terminal domain-containing protein | | uniclust | UniRef100\_A0A0C2HKT8 | 97.5 | 2.6e-06 | 5.3e-12 | 53.5 | 36 | (60, 95) | 100 | (2, 37) | 78 | SAP domain-containing protein | SAP domain-containing protein | | uniclust | UniRef100\_A0A1Y3ULW9 | 97.5 | 2.9e-06 | 5.8e-12 | 54.9 | 71 | (22, 92) | 100 | (9, 97) | 99 | HeH/LEM domain-containing protein | HeH/LEM domain-containing protein | | uniclust | UniRef100\_A0A1C5TV19 | 97.5 | 3.1e-06 | 5.9e-12 | 52.8 | 67 | (28, 94) | 100 | (13, 81) | 84 | Transcription termination factor Rho | Transcription termination factor Rho | | uniclust | UniRef100\_UPI0008A26671 | 97.5 | 3.2e-06 | 5.9e-12 | 53.4 | 41 | (53, 93) | 100 | (58, 98) | 99 | Rho termination factor N-terminal domain-containing protein | Rho termination factor N-terminal domain-containing protein | | uniclust | UniRef100\_A0A3Q7F3B7 | 97.5 | 3.3e-06 | 6.2e-12 | 48.2 | 38 | (54, 91) | 100 | (13, 50) | 52 | Rho termination factor N-terminal domain-containing protein | Rho termination factor N-terminal domain-containing protein | | uniclust | UniRef100\_UPI000419D3AE | 97.5 | 3.7e-06 | 6.8e-12 | 53.4 | 62 | (31, 92) | 100 | (39, 101) | 102 | hypothetical protein | hypothetical protein | | uniclust | UniRef100\_A0A417YJN1 | 97.5 | 3.5e-06 | 7e-12 | 54.5 | 85 | (4, 89) | 100 | (1, 92) | 98 | Rho termination factor N-terminal domain-containing protein | Rho termination factor N-terminal domain-containing protein | | uniclust | UniRef100\_UPI001EFFA8A0 | 97.5 | 4e-06 | 7.3e-12 | 52.9 | 44 | (54, 97) | 100 | (38, 81) | 98 | Rho termination factor N-terminal domain-containing protein | Rho termination factor N-terminal domain-containing protein | | uniclust | UniRef100\_A0A0U2WI47 | 97.5 | 4.1e-06 | 7.6e-12 | 54.7 | 76 | (18, 93) | 100 | (35, 118) | 121 | Rho termination factor N-terminal domain-containing protein | Rho termination factor N-terminal domain-containing protein | | uniclust | UniRef100\_A0A0H4TI36 | 97.5 | 3.9e-06 | 7.8e-12 | 55.6 | 81 | (13, 93) | 100 | (12, 97) | 109 | Rho termination factor | Rho termination factor | | uniclust | UniRef100\_A0A0M0F5X6 | 97.5 | 4.1e-06 | 8.6e-12 | 59.3 | 64 | (31, 94) | 100 | (32, 96) | 160 | Rho termination factor N-terminal domain-containing protein | Rho termination factor N-terminal domain-containing protein | | uniclust | UniRef100\_A0A4P8S989 | 97.4 | 5.7e-06 | 1.1e-11 | 52.3 | 90 | (5, 94) | 100 | (3, 94) | 98 | HeH/LEM domain-containing protein | HeH/LEM domain-containing protein | | uniclust | UniRef100\_A0A088T1Y5 | 97.4 | 5.9e-06 | 1.1e-11 | 53.8 | 36 | (55, 90) | 100 | (71, 106) | 107 | Rho termination factor N-terminal domain-containing protein | Rho termination factor N-terminal domain-containing protein | | uniclust | UniRef100\_B1BPQ6 | 97.4 | 6.2e-06 | 1.1e-11 | 48.7 | 40 | (53, 92) | 100 | (24, 63) | 64 | 50S ribosomal protein L21 | 50S ribosomal protein L21 | | uniclust | UniRef100\_A0A3T0L2P0 | 97.4 | 6.3e-06 | 1.2e-11 | 54.4 | 90 | (3, 93) | 100 | (2, 105) | 129 | Rho termination factor N-terminal domain-containing protein | Rho termination factor N-terminal domain-containing protein | | uniclust | UniRef100\_UPI001F28AE23 | 97.4 | 6.4e-06 | 1.2e-11 | 52.4 | 75 | (16, 90) | 100 | (19, 101) | 102 | hypothetical protein | hypothetical protein | | uniclust | UniRef100\_A0A2D3WNW5 | 97.4 | 6.5e-06 | 1.2e-11 | 53.0 | 87 | (7, 93) | 100 | (4, 107) | 109 | Rho termination factor N-terminal domain-containing protein | Rho termination factor N-terminal domain-containing protein | | uniclust | UniRef100\_A0A9D0ZY95 | 97.4 | 6.6e-06 | 1.2e-11 | 42.4 | 31 | (60, 90) | 100 | (1, 31) | 33 | Rho termination factor N-terminal domain-containing protein | Rho termination factor N-terminal domain-containing protein | | uniclust | UniRef100\_A0A7W3VUZ5 | 97.4 | 6.4e-06 | 1.2e-11 | 53.5 | 75 | (21, 97) | 100 | (14, 96) | 102 | SAP domain-containing protein | SAP domain-containing protein | | uniclust | UniRef100\_A0A844J7S0 | 97.4 | 7e-06 | 1.3e-11 | 48.2 | 38 | (54, 91) | 100 | (25, 62) | 64 | Rho termination factor N-terminal domain-containing protein | Rho termination factor N-terminal domain-containing protein | | uniclust | UniRef100\_A0A161WMN4 | 97.4 | 6.8e-06 | 1.3e-11 | 57.5 | 43 | (53, 95) | 100 | (94, 136) | 166 | Termination factor Rho | Termination factor Rho | | uniclust | UniRef100\_A0A0C2V9W8 | 97.4 | 6.7e-06 | 1.4e-11 | 53.0 | 44 | (53, 96) | 100 | (34, 77) | 88 | Uncharacterized protein | Uncharacterized protein | | uniclust | UniRef100\_UPI0018F8AC73 | 97.4 | 7.8e-06 | 1.4e-11 | 49.1 | 39 | (53, 91) | 100 | (31, 69) | 72 | Rho termination factor N-terminal domain-containing protein | Rho termination factor N-terminal domain-containing protein | | uniclust | UniRef100\_A0A2D7UWP3 | 97.4 | 8.4e-06 | 1.5e-11 | 54.1 | 39 | (53, 91) | 100 | (93, 131) | 133 | Rho termination factor N-terminal domain-containing protein | Rho termination factor N-terminal domain-containing protein | | uniclust | UniRef100\_K4JW68 | 97.4 | 8.2e-06 | 1.6e-11 | 48.5 | 42 | (54, 95) | 100 | (16, 57) | 61 | Rho termination factor N-terminal domain-containing protein | Rho termination factor N-terminal domain-containing protein | | uniclust | UniRef100\_A0A967ZS15 | 97.3 | 8.7e-06 | 1.6e-11 | 47.0 | 38 | (53, 90) | 100 | (20, 57) | 58 | Rho termination factor N-terminal domain-containing protein | Rho termination factor N-terminal domain-containing protein | | uniclust | UniRef100\_A0A352RPP2 | 97.3 | 8.7e-06 | 1.6e-11 | 50.5 | 80 | (10, 93) | 100 | (4, 85) | 87 | Rho termination factor N-terminal domain-containing protein | Rho termination factor N-terminal domain-containing protein | | uniclust | UniRef100\_A0A4P9THH5 | 97.3 | 9.1e-06 | 1.7e-11 | 51.4 | 90 | (1, 90) | 100 | (1, 96) | 97 | Rho termination factor N-terminal domain-containing protein | Rho termination factor N-terminal domain-containing protein | | uniclust | UniRef100\_A0A1Q8EFU1 | 97.3 | 9.4e-06 | 1.7e-11 | 41.9 | 32 | (60, 91) | 100 | (1, 32) | 33 | Rho termination factor N-terminal domain-containing protein | Rho termination factor N-terminal domain-containing protein | | uniclust | UniRef100\_A0A1G6NTL8 | 97.3 | 9e-06 | 1.7e-11 | 55.5 | 92 | (1, 92) | 100 | (1, 101) | 139 | Uncharacterized protein | Uncharacterized protein | | uniclust | UniRef100\_A0A024Q8V2 | 97.3 | 9e-06 | 1.8e-11 | 48.2 | 46 | (1, 46) | 100 | (1, 46) | 58 | Phage protein | Phage protein | | uniclust | UniRef100\_A0A7Y0HZA0 | 97.3 | 9.5e-06 | 1.8e-11 | 51.3 | 44 | (54, 97) | 100 | (4, 47) | 91 | Transcription termination factor Rho (Fragment) | Transcription termination factor Rho (Fragment) | | uniclust | UniRef100\_A0A0D0Q5G0 | 97.3 | 9.1e-06 | 1.9e-11 | 55.9 | 42 | (55, 96) | 100 | (85, 127) | 132 | Phage protein | Phage protein | | uniclust | UniRef100\_UPI001D192B90 | 97.3 | 1.1e-05 | 2e-11 | 50.0 | 73 | (15, 91) | 100 | (11, 84) | 86 | hypothetical protein | hypothetical protein | | uniclust | UniRef100\_A0A2E6JK78 | 97.3 | 1.1e-05 | 2.1e-11 | 50.8 | 38 | (53, 90) | 100 | (55, 92) | 93 | Rho termination factor | Rho termination factor | | uniclust | UniRef100\_UPI00040F1258 | 97.3 | 1.1e-05 | 2.1e-11 | 50.2 | 42 | (54, 95) | 100 | (34, 75) | 81 | hypothetical protein | hypothetical protein | | uniclust | UniRef100\_A0A2N2BUP2 | 97.3 | 1.1e-05 | 2.1e-11 | 45.8 | 39 | (54, 92) | 100 | (13, 51) | 53 | Termination factor Rho | Termination factor Rho | | uniclust | UniRef100\_UPI001147A498 | 97.3 | 1.1e-05 | 2.1e-11 | 53.7 | 89 | (3, 91) | 100 | (1, 106) | 120 | hypothetical protein | hypothetical protein | | uniclust | UniRef100\_A0A0B0HXU4 | 97.3 | 1e-05 | 2.1e-11 | 48.5 | 41 | (4, 44) | 100 | (1, 42) | 59 | Uncharacterized protein | Uncharacterized protein | | uniclust | UniRef100\_R9BU95 | 97.3 | 1.2e-05 | 2.3e-11 | 45.3 | 40 | (53, 92) | 100 | (10, 49) | 51 | Rho termination factor N-terminal domain-containing protein | Rho termination factor N-terminal domain-containing protein | | uniclust | UniRef100\_A0A352P5R9 | 97.3 | 1.2e-05 | 2.3e-11 | 47.1 | 42 | (56, 97) | 100 | (4, 45) | 63 | Transcription termination factor Rho (Fragment) | Transcription termination factor Rho (Fragment) | | uniclust | UniRef100\_A0A559IX56 | 97.3 | 1.2e-05 | 2.3e-11 | 54.6 | 89 | (7, 95) | 100 | (5, 121) | 126 | Uncharacterized protein | Uncharacterized protein | | uniclust | UniRef100\_UPI0004939A68 | 97.3 | 1.3e-05 | 2.5e-11 | 51.9 | 75 | (16, 90) | 100 | (15, 100) | 101 | hypothetical protein | hypothetical protein | | uniclust | UniRef100\_A0A6L9MLT1 | 97.3 | 1.3e-05 | 2.5e-11 | 51.6 | 40 | (53, 92) | 100 | (53, 92) | 93 | Rho termination factor N-terminal domain-containing protein | Rho termination factor N-terminal domain-containing protein | | uniclust | UniRef100\_A0A6N9YN10 | 97.3 | 1.4e-05 | 2.6e-11 | 51.7 | 77 | (23, 99) | 100 | (23, 102) | 112 | Rho termination factor N-terminal domain-containing protein | Rho termination factor N-terminal domain-containing protein | | uniclust | UniRef100\_A0A1T0A0C1 | 97.3 | 1.3e-05 | 2.6e-11 | 53.6 | 48 | (52, 99) | 100 | (62, 109) | 114 | HeH/LEM domain | HeH/LEM domain | | uniclust | UniRef100\_A0A1G6LUK0 | 97.2 | 1.5e-05 | 2.8e-11 | 50.1 | 86 | (5, 91) | 100 | (1, 91) | 93 | Rho termination factor N-terminal domain-containing protein | Rho termination factor N-terminal domain-containing protein | | uniclust | UniRef100\_A0A2R3MVX2 | 97.2 | 1.5e-05 | 2.8e-11 | 51.3 | 83 | (11, 93) | 100 | (8, 101) | 104 | Rho termination factor N-terminal domain-containing protein | Rho termination factor N-terminal domain-containing protein | | uniclust | UniRef100\_A0A2Z4WB78 | 97.2 | 1.6e-05 | 2.9e-11 | 47.2 | 39 | (53, 91) | 100 | (23, 61) | 64 | Rho termination factor N-terminal domain-containing protein | Rho termination factor N-terminal domain-containing protein | | uniclust | UniRef100\_A0A4V2NXY4 | 97.2 | 1.6e-05 | 3e-11 | 52.3 | 38 | (54, 91) | 100 | (85, 122) | 124 | Rho termination factor N-terminal domain-containing protein | Rho termination factor N-terminal domain-containing protein | | uniclust | UniRef100\_A0A965B2Z3 | 97.2 | 1.6e-05 | 3e-11 | 44.5 | 40 | (60, 99) | 100 | (1, 40) | 49 | Rho termination factor N-terminal domain-containing protein (Fragment) | Rho termination factor N-terminal domain-containing protein (Fragment) | | uniclust | UniRef100\_A0A2S8GSH5 | 97.2 | 1.6e-05 | 3e-11 | 49.8 | 71 | (19, 92) | 100 | (17, 87) | 91 | Rho termination factor N-terminal domain-containing protein | Rho termination factor N-terminal domain-containing protein | | uniclust | UniRef100\_A0A3T1D355 | 97.2 | 1.6e-05 | 3.1e-11 | 45.4 | 41 | (54, 94) | 100 | (9, 50) | 52 | Rho termination factor N-terminal domain-containing protein | Rho termination factor N-terminal domain-containing protein | | uniclust | UniRef100\_A0A961E4S7 | 97.2 | 1.6e-05 | 3.1e-11 | 51.5 | 45 | (54, 98) | 100 | (58, 102) | 106 | Rho termination factor N-terminal domain-containing protein (Fragment) | Rho termination factor N-terminal domain-containing protein (Fragment) | | uniclust | UniRef100\_A0A3Q9S8J1 | 97.2 | 1.7e-05 | 3.1e-11 | 41.8 | 34 | (60, 93) | 100 | (1, 34) | 36 | Rho termination factor N-terminal domain-containing protein | Rho termination factor N-terminal domain-containing protein | | uniclust | UniRef100\_A0A838E6T1 | 97.2 | 1.7e-05 | 3.2e-11 | 42.0 | 35 | (58, 92) | 100 | (2, 36) | 37 | Rho termination factor N-terminal domain-containing protein | Rho termination factor N-terminal domain-containing protein | | uniclust | UniRef100\_A0A6I1ZKL4 | 97.2 | 1.7e-05 | 3.2e-11 | 45.1 | 44 | (55, 98) | 100 | (5, 48) | 51 | Transcription termination factor Rho (Fragment) | Transcription termination factor Rho (Fragment) | | uniclust | UniRef100\_UPI002245C791 | 97.2 | 1.8e-05 | 3.4e-11 | 51.1 | 90 | (6, 95) | 100 | (3, 105) | 109 | HeH/LEM domain-containing protein | HeH/LEM domain-containing protein | | uniclust | UniRef100\_A0A0C2VDB3 | 97.2 | 1.7e-05 | 3.4e-11 | 54.2 | 89 | (3, 91) | 100 | (14, 123) | 127 | Uncharacterized protein | Uncharacterized protein | | uniclust | UniRef100\_UPI001BEABB8A | 97.2 | 1.9e-05 | 3.6e-11 | 53.1 | 91 | (4, 94) | 100 | (1, 113) | 127 | hypothetical protein | hypothetical protein | | uniclust | UniRef100\_A0A022QNQ7 | 97.2 | 1.7e-05 | 3.6e-11 | 63.1 | 39 | (56, 94) | 100 | (284, 322) | 371 | Rho termination factor N-terminal domain-containing protein | Rho termination factor N-terminal domain-containing protein | | uniclust | UniRef100\_A0A1C7WI36 | 97.2 | 1.8e-05 | 3.6e-11 | 49.1 | 44 | (2, 45) | 100 | (4, 47) | 73 | DUF2635 domain-containing protein | DUF2635 domain-containing protein | | uniclust | UniRef100\_A0A357D622 | 97.2 | 2e-05 | 3.7e-11 | 49.9 | 41 | (54, 94) | 100 | (54, 94) | 97 | Rho termination factor N-terminal domain-containing protein | Rho termination factor N-terminal domain-containing protein | | uniclust | UniRef100\_A0A1V4PGT5 | 97.2 | 1.9e-05 | 3.7e-11 | 54.8 | 44 | (54, 97) | 100 | (47, 90) | 155 | Rho termination factor N-terminal domain-containing protein | Rho termination factor N-terminal domain-containing protein | | uniclust | UniRef100\_UPI0004717B1F | 97.2 | 2.1e-05 | 3.8e-11 | 49.6 | 69 | (25, 95) | 100 | (11, 80) | 94 | SAP domain-containing protein | SAP domain-containing protein | | uniclust | UniRef100\_A0A3A9CS94 | 97.2 | 2.1e-05 | 3.8e-11 | 52.2 | 86 | (10, 95) | 100 | (5, 124) | 130 | Rho termination factor N-terminal domain-containing protein | Rho termination factor N-terminal domain-containing protein | | uniclust | UniRef100\_A0A9D1GLC9 | 97.2 | 2.1e-05 | 3.9e-11 | 45.1 | 38 | (60, 97) | 100 | (1, 38) | 55 | Rho termination factor N-terminal domain-containing protein | Rho termination factor N-terminal domain-containing protein | | uniclust | UniRef100\_A0A533S1U8 | 97.2 | 2.2e-05 | 4.1e-11 | 51.1 | 90 | (5, 95) | 100 | (11, 109) | 114 | Rho termination factor N-terminal domain-containing protein | Rho termination factor N-terminal domain-containing protein | | uniclust | UniRef100\_A0A2H0LEZ5 | 97.2 | 2.2e-05 | 4.4e-11 | 53.2 | 44 | (55, 98) | 100 | (10, 53) | 126 | Transcription termination factor Rho (Fragment) | Transcription termination factor Rho (Fragment) | | uniclust | UniRef100\_A0A161XAR9 | 97.2 | 2.3e-05 | 4.4e-11 | 53.5 | 88 | (1, 88) | 100 | (1, 101) | 143 | Rho termination factor N-terminal domain-containing protein | Rho termination factor N-terminal domain-containing protein | | uniclust | UniRef100\_A0A094YJ18 | 97.2 | 2e-05 | 4.4e-11 | 51.7 | 37 | (58, 95) | 100 | (13, 49) | 94 | SAP domain-containing protein | SAP domain-containing protein | | uniclust | UniRef100\_A0A0Q1AB16 | 97.2 | 2.1e-05 | 4.5e-11 | 54.9 | 45 | (53, 97) | 100 | (20, 65) | 140 | Rho termination protein | Rho termination protein | | uniclust | UniRef100\_C7RHA8 | 97.2 | 2.4e-05 | 4.6e-11 | 49.1 | 69 | (22, 90) | 100 | (9, 84) | 85 | Rho termination factor N-terminal domain-containing protein | Rho termination factor N-terminal domain-containing protein | | uniclust | UniRef100\_UPI00047A0816 | 97.2 | 2.4e-05 | 4.6e-11 | 46.7 | 42 | (54, 95) | 100 | (20, 61) | 63 | Rho termination factor N-terminal domain-containing protein | Rho termination factor N-terminal domain-containing protein | | uniclust | UniRef100\_A0A142K9H1 | 97.2 | 2.2e-05 | 4.6e-11 | 55.9 | 92 | (6, 97) | 100 | (14, 154) | 158 | Head-to-tail connector protein | Head-to-tail connector protein | | uniclust | UniRef100\_A0A942AZ65 | 97.2 | 2.5e-05 | 4.7e-11 | 49.2 | 38 | (53, 90) | 100 | (55, 92) | 93 | Rho termination factor N-terminal domain-containing protein | Rho termination factor N-terminal domain-containing protein | | uniclust | UniRef100\_A0A117DZC3 | 97.2 | 2.4e-05 | 4.7e-11 | 51.1 | 39 | (53, 91) | 100 | (63, 101) | 102 | Rho termination factor | Rho termination factor | | uniclust | UniRef100\_X0S7Z5 | 97.1 | 2.6e-05 | 4.8e-11 | 50.0 | 93 | (5, 97) | 100 | (1, 97) | 104 | Rho termination factor N-terminal domain-containing protein | Rho termination factor N-terminal domain-containing protein | | uniclust | UniRef100\_A0A7J9WAB8 | 97.1 | 2.7e-05 | 4.9e-11 | 41.1 | 35 | (57, 91) | 100 | (1, 35) | 36 | Rho termination factor | Rho termination factor | | uniclust | UniRef100\_A0A8F4FSC2 | 97.1 | 2.5e-05 | 4.9e-11 | 56.7 | 78 | (16, 93) | 100 | (110, 191) | 209 | Rho termination factor N-terminal domain-containing protein | Rho termination factor N-terminal domain-containing protein | | uniclust | UniRef100\_A0A2W1W9H2 | 97.1 | 2.7e-05 | 4.9e-11 | 49.4 | 90 | (4, 94) | 100 | (1, 93) | 97 | Rho termination factor N-terminal domain-containing protein | Rho termination factor N-terminal domain-containing protein | | uniclust | UniRef100\_A0A5B8NRI3 | 97.1 | 2.7e-05 | 5e-11 | 41.8 | 35 | (60, 94) | 100 | (1, 35) | 39 | Rho termination factor N-terminal domain-containing protein | Rho termination factor N-terminal domain-containing protein | | uniclust | UniRef100\_A0A496N535 | 97.1 | 2.8e-05 | 5.1e-11 | 43.7 | 35 | (56, 90) | 100 | (3, 37) | 49 | Rho termination factor N-terminal domain-containing protein (Fragment) | Rho termination factor N-terminal domain-containing protein (Fragment) | | uniclust | UniRef100\_A0A6G7WWK3 | 97.1 | 2.7e-05 | 5.1e-11 | 41.1 | 33 | (59, 91) | 100 | (2, 34) | 35 | Rho termination factor N-terminal domain-containing protein | Rho termination factor N-terminal domain-containing protein | | uniclust | UniRef100\_A0A0A2XDX3 | 97.1 | 2.7e-05 | 5.3e-11 | 56.1 | 85 | (13, 97) | 100 | (45, 154) | 183 | Mu-like prophage FluMu N-terminal domain-containing protein | Mu-like prophage FluMu N-terminal domain-containing protein | | uniclust | UniRef100\_A0A7T4KSI9 | 97.1 | 2.9e-05 | 5.4e-11 | 42.3 | 36 | (55, 90) | 100 | (4, 39) | 40 | HeH/LEM domain-containing protein | HeH/LEM domain-containing protein | | uniclust | UniRef100\_A0A1B7LYA6 | 97.1 | 2.8e-05 | 5.5e-11 | 53.2 | 42 | (54, 95) | 100 | (86, 127) | 132 | Rho termination factor N-terminal domain-containing protein | Rho termination factor N-terminal domain-containing protein | | uniclust | UniRef100\_A0A1H7RLC7 | 97.1 | 2.9e-05 | 5.7e-11 | 48.6 | 58 | (32, 89) | 100 | (18, 78) | 80 | Rho termination factor, N-terminal domain | Rho termination factor, N-terminal domain | | uniclust | UniRef100\_A0A6C0APZ7 | 97.1 | 2.7e-05 | 5.7e-11 | 59.2 | 41 | (55, 95) | 100 | (177, 217) | 251 | Rho termination factor N-terminal domain-containing protein | Rho termination factor N-terminal domain-containing protein | | uniclust | UniRef100\_A0A699P7Y3 | 97.1 | 3.2e-05 | 5.8e-11 | 49.6 | 37 | (54, 90) | 100 | (65, 101) | 103 | SAP-like protein BP-73 (Fragment) | SAP-like protein BP-73 (Fragment) | | uniclust | UniRef100\_A0A3B8T676 | 97.1 | 3.3e-05 | 6e-11 | 42.5 | 36 | (54, 89) | 100 | (8, 43) | 44 | 30S ribosomal protein S2 (Fragment) | 30S ribosomal protein S2 (Fragment) | | uniclust | UniRef100\_UPI001CF4ECA6 | 97.1 | 3.3e-05 | 6e-11 | 51.9 | 78 | (15, 92) | 100 | (17, 94) | 139 | hypothetical protein | hypothetical protein | | uniclust | UniRef100\_A0A415MTC6 | 97.1 | 3.3e-05 | 6.2e-11 | 52.4 | 89 | (5, 95) | 100 | (3, 128) | 133 | SAP domain-containing protein | SAP domain-containing protein | | uniclust | UniRef100\_A0A3E4QZK6 | 97.1 | 3.2e-05 | 6.2e-11 | 57.3 | 46 | (54, 99) | 100 | (198, 243) | 245 | Phage tail protein | Phage tail protein | | uniclust | UniRef100\_A0A0S3U001 | 97.1 | 3e-05 | 6.3e-11 | 59.1 | 42 | (54, 95) | 100 | (193, 234) | 261 | Uncharacterized protein | Uncharacterized protein | | uniclust | UniRef100\_A0A7X6FP84 | 97.1 | 3.3e-05 | 6.5e-11 | 51.9 | 91 | (4, 94) | 100 | (1, 115) | 118 | HeH/LEM domain-containing protein | HeH/LEM domain-containing protein | | uniclust | UniRef100\_J2YWN5 | 97.1 | 3.4e-05 | 6.6e-11 | 46.7 | 38 | (54, 91) | 100 | (11, 48) | 66 | Rho termination factor, N-terminal domain protein (Fragment) | Rho termination factor, N-terminal domain protein (Fragment) | | uniclust | UniRef100\_A0A100W6R9 | 97.1 | 3.5e-05 | 6.7e-11 | 51.2 | 71 | (19, 89) | 100 | (19, 110) | 111 | Uncharacterized protein | Uncharacterized protein | | uniclust | UniRef100\_A0A501VFZ7 | 97.1 | 3.5e-05 | 6.9e-11 | 48.8 | 55 | (34, 91) | 100 | (21, 75) | 82 | HeH/LEM domain-containing protein | HeH/LEM domain-containing protein | | uniclust | UniRef100\_A0A4Z0ISG8 | 97.1 | 3.8e-05 | 7.1e-11 | 52.5 | 82 | (13, 94) | 100 | (12, 123) | 147 | Rho termination factor N-terminal domain-containing protein | Rho termination factor N-terminal domain-containing protein | | uniclust | UniRef100\_UPI001020ADB0 | 97.1 | 3.8e-05 | 7.2e-11 | 52.2 | 62 | (33, 94) | 100 | (36, 99) | 137 | hypothetical protein | hypothetical protein | | uniclust | UniRef100\_A0A075WTM6 | 97.1 | 3.5e-05 | 7.3e-11 | 54.5 | 44 | (53, 96) | 100 | (39, 83) | 154 | Rho termination factor N-terminal domain-containing protein | Rho termination factor N-terminal domain-containing protein | | uniclust | UniRef100\_UPI001D057D6D | 97.1 | 3.9e-05 | 7.3e-11 | 46.8 | 39 | (53, 91) | 100 | (32, 70) | 71 | Rho termination factor N-terminal domain-containing protein | Rho termination factor N-terminal domain-containing protein | | uniclust | UniRef100\_A0A1X3P896 | 97.1 | 3.9e-05 | 7.3e-11 | 52.2 | 85 | (5, 91) | 100 | (1, 136) | 137 | SAP domain-containing protein | SAP domain-containing protein | | uniclust | UniRef100\_A0A1H3FGL4 | 97.1 | 3.8e-05 | 7.3e-11 | 47.5 | 40 | (54, 93) | 100 | (31, 70) | 73 | HeH/LEM domain-containing protein | HeH/LEM domain-containing protein | | uniclust | UniRef100\_UPI000744C40B | 97.1 | 3.9e-05 | 7.3e-11 | 49.4 | 80 | (13, 92) | 100 | (10, 93) | 95 | HeH/LEM domain-containing protein | HeH/LEM domain-containing protein | | uniclust | UniRef100\_A0A096KKD3 | 97.1 | 3.6e-05 | 7.4e-11 | 53.0 | 40 | (54, 93) | 100 | (89, 128) | 131 | Rho termination factor N-terminal domain-containing protein | Rho termination factor N-terminal domain-containing protein | | uniclust | UniRef100\_UPI0021E05E95 | 97.1 | 4.2e-05 | 7.7e-11 | 53.6 | 80 | (17, 96) | 100 | (17, 106) | 185 | hypothetical protein | hypothetical protein | | uniclust | UniRef100\_A0A119CYA6 | 97.0 | 4e-05 | 8.5e-11 | 47.4 | 45 | (2, 46) | 100 | (1, 45) | 67 | Uncharacterized protein | Uncharacterized protein | | uniclust | UniRef100\_A0A418KHN6 | 97.0 | 4.3e-05 | 8.6e-11 | 47.4 | 36 | (60, 95) | 100 | (1, 36) | 72 | Rho termination factor N-terminal domain-containing protein (Fragment) | Rho termination factor N-terminal domain-containing protein (Fragment) | | uniclust | UniRef100\_UPI0013DFA039 | 97.0 | 4.8e-05 | 8.8e-11 | 52.2 | 86 | (5, 94) | 100 | (1, 90) | 158 | hypothetical protein | hypothetical protein | | uniclust | UniRef100\_A0A2W5QPU5 | 97.0 | 4.5e-05 | 8.9e-11 | 54.8 | 44 | (1, 44) | 100 | (1, 46) | 182 | Rho termination factor N-terminal domain-containing protein | Rho termination factor N-terminal domain-containing protein | | uniclust | UniRef100\_A0A7L7KTL4 | 97.0 | 4.9e-05 | 9.1e-11 | 49.6 | 36 | (54, 89) | 100 | (78, 113) | 114 | Rho termination factor N-terminal domain-containing protein | Rho termination factor N-terminal domain-containing protein | | uniclust | UniRef100\_A0A1L6BXY7 | 97.0 | 5e-05 | 9.2e-11 | 42.2 | 39 | (54, 92) | 100 | (5, 43) | 46 | Rho termination factor N-terminal domain-containing protein | Rho termination factor N-terminal domain-containing protein | | uniclust | UniRef100\_A0A3M1CYN9 | 97.0 | 5e-05 | 9.2e-11 | 43.8 | 35 | (57, 91) | 100 | (21, 55) | 56 | 50S ribosomal protein L21 (Fragment) | 50S ribosomal protein L21 (Fragment) | | uniclust | UniRef100\_A0A0U2VH56 | 97.0 | 4.9e-05 | 9.4e-11 | 43.1 | 38 | (54, 91) | 100 | (9, 46) | 47 | HeH/LEM domain-containing protein | HeH/LEM domain-containing protein | | uniclust | UniRef100\_UPI00096A3CC9 | 97.0 | 5.1e-05 | 9.6e-11 | 46.0 | 42 | (54, 95) | 100 | (23, 64) | 68 | Rho termination factor N-terminal domain-containing protein | Rho termination factor N-terminal domain-containing protein | | uniclust | UniRef100\_A0A1G5CMI0 | 97.0 | 5.3e-05 | 9.8e-11 | 50.0 | 40 | (53, 92) | 100 | (83, 122) | 123 | Rho termination factor, N-terminal domain | Rho termination factor, N-terminal domain | | uniclust | UniRef100\_A0A8S0V1Y1 | 97.0 | 5.4e-05 | 9.9e-11 | 50.6 | 35 | (55, 89) | 100 | (97, 131) | 132 | Rho-N domain-containing 1, chloroplastic | Rho-N domain-containing 1, chloroplastic | | uniclust | UniRef100\_UPI000EAE16BA | 97.0 | 5.4e-05 | 9.9e-11 | 48.6 | 69 | (20, 97) | 100 | (18, 87) | 103 | Rho termination factor N-terminal domain-containing protein | Rho termination factor N-terminal domain-containing protein | | uniclust | UniRef100\_A0A0Q6VKW7 | 97.0 | 4.9e-05 | 9.9e-11 | 54.4 | 36 | (55, 90) | 100 | (134, 169) | 170 | Rho termination factor N-terminal domain-containing protein | Rho termination factor N-terminal domain-containing protein | | uniclust | UniRef100\_A0A523V6Q3 | 97.0 | 5.2e-05 | 1e-10 | 45.9 | 44 | (55, 98) | 100 | (8, 51) | 65 | Transcription termination factor Rho (Fragment) | Transcription termination factor Rho (Fragment) | | uniclust | UniRef100\_A0A3C0G1P5 | 97.0 | 5.6e-05 | 1e-10 | 40.5 | 33 | (59, 91) | 100 | (3, 35) | 38 | 50S ribosomal protein L21 (Fragment) | 50S ribosomal protein L21 (Fragment) | | uniclust | UniRef100\_A0A0Q6AJX2 | 97.0 | 5.3e-05 | 1.1e-10 | 48.1 | 47 | (1, 47) | 100 | (1, 47) | 80 | Uncharacterized protein | Uncharacterized protein | | uniclust | UniRef100\_A0A0B5CYN2 | 97.0 | 5.9e-05 | 1.1e-10 | 43.5 | 39 | (54, 92) | 100 | (14, 52) | 53 | HeH/LEM domain-containing protein | HeH/LEM domain-containing protein | | uniclust | UniRef100\_A0A0N0K113 | 97.0 | 6e-05 | 1.1e-10 | 49.8 | 73 | (21, 93) | 100 | (20, 97) | 118 | Rho termination factor N-terminal domain-containing protein | Rho termination factor N-terminal domain-containing protein | | uniclust | UniRef100\_A0A2D7WBG9 | 97.0 | 6.1e-05 | 1.1e-10 | 52.4 | 38 | (53, 90) | 100 | (131, 168) | 169 | Rho termination factor N-terminal domain-containing protein | Rho termination factor N-terminal domain-containing protein | | uniclust | UniRef100\_A0A6C0DL56 | 97.0 | 6.1e-05 | 1.1e-10 | 39.2 | 32 | (60, 91) | 100 | (1, 32) | 33 | Rho termination factor N-terminal domain-containing protein | Rho termination factor N-terminal domain-containing protein | | uniclust | UniRef100\_A0A1V2UCW6 | 97.0 | 6.2e-05 | 1.1e-10 | 39.5 | 33 | (60, 92) | 100 | (1, 33) | 34 | HeH/LEM domain-containing protein | HeH/LEM domain-containing protein | | uniclust | UniRef100\_A0A022PR37 | 97.0 | 5.6e-05 | 1.2e-10 | 62.8 | 40 | (54, 93) | 100 | (438, 477) | 542 | Uncharacterized protein (Fragment) | Uncharacterized protein (Fragment) | | uniclust | UniRef100\_A0A5B9Y7U7 | 97.0 | 6.5e-05 | 1.2e-10 | 49.2 | 88 | (11, 98) | 100 | (9, 112) | 115 | Rho termination factor N-terminal domain-containing protein | Rho termination factor N-terminal domain-containing protein | | uniclust | UniRef100\_A0A960CIP6 | 96.9 | 6.9e-05 | 1.3e-10 | 44.5 | 37 | (55, 91) | 100 | (26, 62) | 63 | Rho termination factor N-terminal domain-containing protein | Rho termination factor N-terminal domain-containing protein | | uniclust | UniRef100\_A0A142KC13 | 96.9 | 6.5e-05 | 1.3e-10 | 53.1 | 74 | (18, 91) | 100 | (41, 155) | 157 | Head-to-tail connector protein | Head-to-tail connector protein | | uniclust | UniRef100\_A0A3N5SVS3 | 96.9 | 7e-05 | 1.3e-10 | 47.0 | 43 | (54, 96) | 100 | (6, 49) | 83 | Transcription termination factor Rho (Fragment) | Transcription termination factor Rho (Fragment) | | uniclust | UniRef100\_A0A081PN78 | 96.9 | 6.7e-05 | 1.3e-10 | 47.1 | 42 | (56, 97) | 100 | (31, 73) | 76 | HeH/LEM domain protein | HeH/LEM domain protein | | uniclust | UniRef100\_A0A7L5ABR4 | 96.9 | 7.5e-05 | 1.4e-10 | 48.3 | 86 | (9, 94) | 100 | (13, 102) | 106 | Uncharacterized protein | Uncharacterized protein | | uniclust | UniRef100\_A0A6L6TN84 | 96.9 | 7.7e-05 | 1.4e-10 | 45.6 | 58 | (32, 90) | 100 | (18, 75) | 76 | Rho termination factor N-terminal domain-containing protein | Rho termination factor N-terminal domain-containing protein | | uniclust | UniRef100\_A0A3N5Q9R5 | 96.9 | 7.9e-05 | 1.5e-10 | 46.3 | 40 | (54, 93) | 100 | (37, 76) | 78 | Addiction module toxin RelE (Fragment) | Addiction module toxin RelE (Fragment) | | uniclust | UniRef100\_A0A165PJJ3 | 96.9 | 7.4e-05 | 1.5e-10 | 51.7 | 41 | (5, 45) | 100 | (9, 49) | 129 | Mu-like prophage FluMu N-terminal domain-containing protein | Mu-like prophage FluMu N-terminal domain-containing protein | | uniclust | UniRef100\_A0A1V3XH09 | 96.9 | 8.4e-05 | 1.6e-10 | 47.1 | 44 | (55, 98) | 100 | (15, 58) | 94 | Rho termination factor, N-terminal domain protein | Rho termination factor, N-terminal domain protein | | uniclust | UniRef100\_UPI0009886066 | 96.9 | 8.6e-05 | 1.6e-10 | 46.5 | 37 | (54, 90) | 100 | (50, 86) | 87 | Rho termination factor N-terminal domain-containing protein | Rho termination factor N-terminal domain-containing protein | | uniclust | UniRef100\_A0A2N3FBN1 | 96.9 | 8.8e-05 | 1.6e-10 | 39.1 | 32 | (60, 91) | 100 | (1, 32) | 35 | Rho termination factor N-terminal domain-containing protein (Fragment) | Rho termination factor N-terminal domain-containing protein (Fragment) | | uniclust | UniRef100\_A0A0U1DLB0 | 96.9 | 8.1e-05 | 1.6e-10 | 59.3 | 45 | (54, 98) | 100 | (43, 87) | 398 | Rho RNA-BD domain-containing protein | Rho RNA-BD domain-containing protein | | uniclust | UniRef100\_A0A838H6R9 | 96.9 | 8.3e-05 | 1.6e-10 | 45.0 | 46 | (1, 46) | 100 | (1, 48) | 62 | Uncharacterized protein | Uncharacterized protein | | uniclust | UniRef100\_A0A355UCP4 | 96.9 | 9.2e-05 | 1.7e-10 | 50.4 | 37 | (54, 90) | 100 | (111, 147) | 148 | Rho termination factor N-terminal domain-containing protein (Fragment) | Rho termination factor N-terminal domain-containing protein (Fragment) | | uniclust | UniRef100\_A0A255GPY9 | 96.9 | 9.3e-05 | 1.7e-10 | 45.5 | 38 | (54, 91) | 100 | (40, 77) | 78 | Rho termination factor N-terminal domain-containing protein | Rho termination factor N-terminal domain-containing protein | | uniclust | UniRef100\_UPI001C8CC8D9 | 96.9 | 0.0001 | 1.8e-10 | 43.1 | 39 | (54, 92) | 100 | (19, 57) | 59 | Rho termination factor N-terminal domain-containing protein | Rho termination factor N-terminal domain-containing protein | | uniclust | UniRef100\_A0A926IAT8 | 96.9 | 9.8e-05 | 1.8e-10 | 48.5 | 84 | (13, 96) | 100 | (12, 105) | 110 | Rho termination factor N-terminal domain-containing protein | Rho termination factor N-terminal domain-containing protein | | uniclust | UniRef100\_A0A399NTV9 | 96.9 | 9.9e-05 | 2e-10 | 49.4 | 43 | (55, 97) | 100 | (11, 53) | 109 | Transcription termination factor Rho (Fragment) | Transcription termination factor Rho (Fragment) | | uniclust | UniRef100\_UPI00209F097A | 96.9 | 0.00011 | 2e-10 | 48.4 | 85 | (8, 92) | 100 | (3, 112) | 118 | hypothetical protein | hypothetical protein | | uniclust | UniRef100\_A0A8S0HYC0 | 96.9 | 0.00011 | 2e-10 | 47.5 | 81 | (16, 96) | 100 | (3, 98) | 101 | Rho termination factor N-terminal domain-containing protein | Rho termination factor N-terminal domain-containing protein | | uniclust | UniRef100\_A0A349AY30 | 96.9 | 0.0001 | 2e-10 | 50.7 | 42 | (55, 96) | 100 | (10, 51) | 126 | Transcription termination factor Rho (Fragment) | Transcription termination factor Rho (Fragment) | | uniclust | UniRef100\_A0A0Q5HJK0 | 96.8 | 0.0001 | 2e-10 | 53.7 | 37 | (54, 90) | 100 | (154, 190) | 191 | Rho termination factor N-terminal domain-containing protein | Rho termination factor N-terminal domain-containing protein | | uniclust | UniRef100\_A0A067ZJ05 | 96.8 | 0.00011 | 2e-10 | 47.4 | 83 | (13, 95) | 100 | (10, 101) | 104 | Rho termination factor N-terminal domain-containing protein | Rho termination factor N-terminal domain-containing protein | | uniclust | UniRef100\_A0A6L6JB59 | 96.8 | 0.00011 | 2.1e-10 | 47.2 | 45 | (1, 45) | 100 | (1, 45) | 95 | Uncharacterized protein | Uncharacterized protein | | uniclust | UniRef100\_A0A078GBV8 | 96.8 | 0.00011 | 2.2e-10 | 60.3 | 38 | (55, 92) | 100 | (422, 459) | 475 | BnaC03g17360D protein | BnaC03g17360D protein | | uniclust | UniRef100\_A0A143HCV3 | 96.8 | 0.00012 | 2.2e-10 | 46.9 | 80 | (13, 92) | 100 | (12, 92) | 99 | Rho termination factor N-terminal domain-containing protein | Rho termination factor N-terminal domain-containing protein | | uniclust | UniRef100\_A0A446I8R2 | 96.8 | 0.00012 | 2.3e-10 | 43.6 | 37 | (53, 89) | 100 | (26, 62) | 63 | Rho termination factor N-terminal domain-containing protein | Rho termination factor N-terminal domain-containing protein | | uniclust | UniRef100\_A0A163MUE0 | 96.8 | 0.00011 | 2.3e-10 | 49.5 | 91 | (2, 92) | 100 | (1, 112) | 114 | HeH/LEM domain-containing protein | HeH/LEM domain-containing protein | | uniclust | UniRef100\_A0A2S4YIP2 | 96.8 | 0.00012 | 2.3e-10 | 46.0 | 41 | (54, 94) | 100 | (46, 86) | 89 | Rho termination factor N-terminal domain-containing protein | Rho termination factor N-terminal domain-containing protein | | uniclust | UniRef100\_A0A1X0IMZ6 | 96.8 | 0.00013 | 2.4e-10 | 45.5 | 37 | (55, 91) | 100 | (47, 83) | 84 | Rho termination factor | Rho termination factor | | uniclust | UniRef100\_UPI00156691C1 | 96.8 | 0.00013 | 2.5e-10 | 46.9 | 69 | (15, 93) | 100 | (17, 85) | 101 | hypothetical protein | hypothetical protein | | uniclust | UniRef100\_A0A085LDJ2 | 96.8 | 0.00013 | 2.5e-10 | 50.5 | 40 | (55, 94) | 100 | (39, 78) | 131 | Rho termination factor N-terminal domain-containing protein (Fragment) | Rho termination factor N-terminal domain-containing protein (Fragment) | | uniclust | UniRef100\_A0A7X9A4X6 | 96.8 | 0.00014 | 2.6e-10 | 50.3 | 39 | (54, 92) | 100 | (122, 160) | 162 | Rho termination factor N-terminal domain-containing protein | Rho termination factor N-terminal domain-containing protein | | uniclust | UniRef100\_A0A3L8PIB7 | 96.8 | 0.00014 | 2.6e-10 | 51.6 | 38 | (54, 91) | 100 | (143, 180) | 181 | Rho termination factor N-terminal domain-containing protein | Rho termination factor N-terminal domain-containing protein | | uniclust | UniRef100\_K0V9V4 | 96.8 | 0.00014 | 2.6e-10 | 52.4 | 45 | (54, 98) | 100 | (26, 70) | 201 | Transcription termination factor Rho (Fragment) | Transcription termination factor Rho (Fragment) | | uniclust | UniRef100\_A0A2A7MKH2 | 96.8 | 0.00014 | 2.6e-10 | 44.5 | 41 | (54, 94) | 100 | (25, 65) | 69 | Rho termination factor N-terminal domain-containing protein | Rho termination factor N-terminal domain-containing protein | | uniclust | UniRef100\_A0A2Z6ZZW1 | 96.8 | 0.00013 | 2.6e-10 | 55.3 | 40 | (55, 94) | 100 | (218, 257) | 267 | SAP-like protein BP-73 (Fragment) | SAP-like protein BP-73 (Fragment) | | uniclust | UniRef100\_A0A022N6K0 | 96.8 | 0.00014 | 2.7e-10 | 42.4 | 39 | (54, 92) | 100 | (16, 54) | 55 | HeH/LEM domain-containing protein | HeH/LEM domain-containing protein | | uniclust | UniRef100\_A0A1N7MP98 | 96.8 | 0.00014 | 2.7e-10 | 50.8 | 43 | (54, 96) | 100 | (98, 140) | 146 | Rho termination factor, N-terminal domain | Rho termination factor, N-terminal domain | | uniclust | UniRef100\_UPI00065B46D6 | 96.8 | 0.00014 | 2.7e-10 | 47.5 | 42 | (55, 96) | 100 | (21, 62) | 95 | Rho termination factor N-terminal domain-containing protein | Rho termination factor N-terminal domain-containing protein | | uniclust | UniRef100\_A0A0A2SJL7 | 96.8 | 0.00014 | 2.8e-10 | 50.9 | 82 | (11, 92) | 100 | (6, 105) | 155 | Rho termination factor N-terminal domain-containing protein | Rho termination factor N-terminal domain-containing protein | | uniclust | UniRef100\_A0A3G6ISY3 | 96.8 | 0.00015 | 2.8e-10 | 48.8 | 80 | (16, 95) | 100 | (18, 121) | 124 | Uncharacterized protein | Uncharacterized protein | | uniclust | UniRef100\_UPI00195E8D1B | 96.8 | 0.00015 | 2.8e-10 | 54.6 | 37 | (54, 90) | 100 | (226, 262) | 263 | Rho termination factor N-terminal domain-containing protein | Rho termination factor N-terminal domain-containing protein | | uniclust | UniRef100\_A0A1I5II67 | 96.8 | 0.00015 | 2.9e-10 | 48.5 | 74 | (20, 93) | 100 | (27, 122) | 123 | Uncharacterized protein | Uncharacterized protein | | uniclust | UniRef100\_A0A1C5GJ85 | 96.8 | 0.00015 | 2.9e-10 | 49.6 | 41 | (54, 94) | 100 | (14, 54) | 131 | Rho termination factor, N-terminal domain (Fragment) | Rho termination factor, N-terminal domain (Fragment) | | uniclust | UniRef100\_A0A8I1NIZ1 | 96.8 | 0.00016 | 3e-10 | 48.8 | 35 | (58, 92) | 100 | (84, 118) | 119 | Rho termination factor N-terminal domain-containing protein | Rho termination factor N-terminal domain-containing protein | | uniclust | UniRef100\_F0SXG1 | 96.8 | 0.00016 | 3e-10 | 44.7 | 66 | (27, 94) | 100 | (11, 76) | 80 | Rho termination factor N-terminal domain-containing protein | Rho termination factor N-terminal domain-containing protein | | uniclust | UniRef100\_A0A8T7B3C8 | 96.8 | 0.00016 | 3e-10 | 48.4 | 39 | (53, 91) | 100 | (78, 116) | 118 | Rho termination factor N-terminal domain-containing protein | Rho termination factor N-terminal domain-containing protein | | uniclust | UniRef100\_UPI0008A58C56 | 96.8 | 0.00017 | 3.1e-10 | 47.0 | 84 | (5, 89) | 100 | (1, 105) | 108 | hypothetical protein | hypothetical protein | | uniclust | UniRef100\_A0A0U4K5G7 | 96.8 | 0.00016 | 3.1e-10 | 43.5 | 40 | (5, 44) | 100 | (2, 41) | 62 | Uncharacterized protein | Uncharacterized protein | | uniclust | UniRef100\_A0A943ECP9 | 96.8 | 0.00017 | 3.1e-10 | 37.9 | 32 | (60, 91) | 100 | (1, 32) | 34 | SAP domain-containing protein | SAP domain-containing protein | | uniclust | UniRef100\_A0A4U7J7L9 | 96.7 | 0.00017 | 3.2e-10 | 45.4 | 85 | (7, 92) | 100 | (2, 88) | 89 | Rho termination factor N-terminal domain-containing protein | Rho termination factor N-terminal domain-containing protein | | uniclust | UniRef100\_A0A842EMJ7 | 96.7 | 0.00017 | 3.2e-10 | 38.9 | 36 | (60, 95) | 100 | (1, 36) | 38 | HeH/LEM domain-containing protein | HeH/LEM domain-containing protein | | uniclust | UniRef100\_A0A3R6KE31 | 96.7 | 0.00017 | 3.2e-10 | 51.2 | 41 | (56, 96) | 100 | (12, 52) | 163 | Rho termination factor N-terminal domain-containing protein | Rho termination factor N-terminal domain-containing protein | | uniclust | UniRef100\_A0A1C2DD79 | 96.7 | 0.00016 | 3.2e-10 | 54.1 | 40 | (55, 94) | 100 | (192, 231) | 233 | Rho termination factor N-terminal domain-containing protein | Rho termination factor N-terminal domain-containing protein | | uniclust | UniRef100\_A0A399G9E4 | 96.7 | 0.00017 | 3.3e-10 | 43.7 | 38 | (54, 91) | 100 | (26, 63) | 66 | Rho termination factor N-terminal domain-containing protein | Rho termination factor N-terminal domain-containing protein | | uniclust | UniRef100\_A0A0A1VZC2 | 96.7 | 0.00017 | 3.3e-10 | 56.1 | 42 | (54, 95) | 100 | (190, 231) | 314 | Rho termination factor N-terminal domain-containing protein | Rho termination factor N-terminal domain-containing protein | | uniclust | UniRef100\_UPI00191E8781 | 96.7 | 0.00018 | 3.3e-10 | 48.3 | 72 | (20, 91) | 100 | (34, 128) | 131 | hypothetical protein | hypothetical protein | | uniclust | UniRef100\_A0A0A1MNV9 | 96.7 | 0.00016 | 3.3e-10 | 44.2 | 40 | (4, 43) | 100 | (1, 40) | 62 | Uncharacterized protein | Uncharacterized protein | | uniclust | UniRef100\_A0A4Q5N7H8 | 96.7 | 0.00018 | 3.3e-10 | 44.2 | 39 | (53, 91) | 100 | (31, 69) | 71 | Rho termination factor | Rho termination factor | | uniclust | UniRef100\_A0A0M2H758 | 96.7 | 0.00016 | 3.3e-10 | 52.4 | 43 | (53, 97) | 100 | (80, 122) | 173 | SAP domain protein | SAP domain protein | | uniclust | UniRef100\_A0A0N9NCV3 | 96.7 | 0.00017 | 3.3e-10 | 52.2 | 33 | (57, 89) | 100 | (144, 176) | 177 | Rho termination factor N-terminal domain-containing protein | Rho termination factor N-terminal domain-containing protein | | uniclust | UniRef100\_A0A0N0Z495 | 96.7 | 0.00017 | 3.4e-10 | 53.2 | 45 | (54, 98) | 100 | (21, 65) | 202 | Rho RNA-BD domain-containing protein | Rho RNA-BD domain-containing protein | | uniclust | UniRef100\_A0A1X1X990 | 96.7 | 0.00017 | 3.4e-10 | 48.7 | 80 | (15, 94) | 100 | (16, 104) | 116 | Rho termination factor N-terminal domain-containing protein | Rho termination factor N-terminal domain-containing protein | | uniclust | UniRef100\_A0A936ZSD3 | 96.7 | 0.00017 | 3.4e-10 | 43.9 | 43 | (3, 45) | 100 | (1, 43) | 65 | Uncharacterized protein | Uncharacterized protein | | uniclust | UniRef100\_A0A336N748 | 96.7 | 0.00017 | 3.4e-10 | 43.9 | 43 | (2, 44) | 100 | (1, 43) | 61 | Uncharacterized protein | Uncharacterized protein | | uniclust | UniRef100\_A0A959YTN7 | 96.7 | 0.00019 | 3.4e-10 | 38.6 | 35 | (57, 91) | 100 | (3, 37) | 38 | Rho termination factor N-terminal domain-containing protein | Rho termination factor N-terminal domain-containing protein | | uniclust | UniRef100\_A0A1E4VLJ2 | 96.7 | 0.00018 | 3.4e-10 | 50.8 | 41 | (54, 94) | 100 | (106, 146) | 169 | Rho termination factor N-terminal domain-containing protein | Rho termination factor N-terminal domain-containing protein | | uniclust | UniRef100\_A0A960ZEV2 | 96.7 | 0.00019 | 3.5e-10 | 43.3 | 41 | (54, 94) | 100 | (27, 67) | 69 | Rho termination factor N-terminal domain-containing protein (Fragment) | Rho termination factor N-terminal domain-containing protein (Fragment) | | uniclust | UniRef100\_A0A2Z4PZN3 | 96.7 | 0.00019 | 3.6e-10 | 45.7 | 72 | (23, 95) | 100 | (9, 80) | 83 | HeH/LEM domain-containing protein | HeH/LEM domain-containing protein | | uniclust | UniRef100\_A0A7V7QJT4 | 96.7 | 0.0002 | 3.7e-10 | 43.5 | 39 | (54, 92) | 100 | (33, 71) | 72 | Rho termination factor N-terminal domain-containing protein | Rho termination factor N-terminal domain-containing protein | | uniclust | UniRef100\_UPI001E3C94D1 | 96.7 | 0.0002 | 3.7e-10 | 41.4 | 37 | (55, 91) | 100 | (18, 54) | 55 | Rho termination factor N-terminal domain-containing protein | Rho termination factor N-terminal domain-containing protein | | uniclust | UniRef100\_A0A088T2U9 | 96.7 | 0.00018 | 3.8e-10 | 55.5 | 41 | (54, 94) | 100 | (188, 228) | 273 | 50S ribosomal protein L20 | 50S ribosomal protein L20 | | uniclust | UniRef100\_A0A161I2L0 | 96.7 | 0.00018 | 3.9e-10 | 57.5 | 42 | (54, 95) | 100 | (261, 302) | 360 | Rho termination factor N-terminal domain-containing protein | Rho termination factor N-terminal domain-containing protein | | uniclust | UniRef100\_A0A2G1CVH0 | 96.7 | 0.00021 | 3.9e-10 | 47.4 | 89 | (6, 95) | 100 | (8, 110) | 114 | Uncharacterized protein | Uncharacterized protein | | uniclust | UniRef100\_UPI0011A4A6DC | 96.7 | 0.00021 | 3.9e-10 | 46.2 | 39 | (54, 92) | 100 | (64, 102) | 103 | Rho termination factor N-terminal domain-containing protein | Rho termination factor N-terminal domain-containing protein | | uniclust | UniRef100\_A0A1A0IIT4 | 96.7 | 0.0002 | 4e-10 | 51.6 | 33 | (59, 91) | 100 | (139, 171) | 173 | Rho termination factor N-terminal domain-containing protein | Rho termination factor N-terminal domain-containing protein | | uniclust | UniRef100\_A0A1Q9T603 | 96.7 | 0.0002 | 4.1e-10 | 52.0 | 40 | (55, 94) | 100 | (19, 58) | 182 | Rho termination factor N-terminal domain-containing protein | Rho termination factor N-terminal domain-containing protein | | uniclust | UniRef100\_A0A0Q8PJF9 | 96.7 | 0.00023 | 4.1e-10 | 42.9 | 41 | (5, 45) | 100 | (1, 41) | 68 | Mu-like prophage FluMu N-terminal domain-containing protein | Mu-like prophage FluMu N-terminal domain-containing protein | | uniclust | UniRef100\_UPI0015585255 | 96.7 | 0.00023 | 4.2e-10 | 42.4 | 42 | (51, 92) | 100 | (21, 63) | 64 | Rho termination factor N-terminal domain-containing protein | Rho termination factor N-terminal domain-containing protein | | uniclust | UniRef100\_A0A0J1C4L7 | 96.7 | 0.00022 | 4.3e-10 | 44.6 | 44 | (53, 96) | 100 | (23, 66) | 73 | HeH/LEM domain-containing protein | HeH/LEM domain-containing protein | | uniclust | UniRef100\_A0A845R497 | 96.7 | 0.00023 | 4.3e-10 | 46.6 | 67 | (26, 94) | 100 | (29, 96) | 107 | HeH/LEM domain-containing protein | HeH/LEM domain-containing protein | | uniclust | UniRef100\_A0A5C7NE73 | 96.7 | 0.00024 | 4.4e-10 | 46.8 | 77 | (14, 91) | 100 | (21, 112) | 115 | Rho termination factor N-terminal domain-containing protein | Rho termination factor N-terminal domain-containing protein | | uniclust | UniRef100\_A0A3D2M3Y4 | 96.7 | 0.00024 | 4.4e-10 | 44.5 | 76 | (19, 96) | 100 | (5, 81) | 85 | HeH/LEM domain-containing protein (Fragment) | HeH/LEM domain-containing protein (Fragment) | | uniclust | UniRef100\_A0A1G4FHX2 | 96.7 | 0.00023 | 4.4e-10 | 44.5 | 39 | (54, 92) | 100 | (35, 73) | 74 | HeH/LEM domain protein | HeH/LEM domain protein | | uniclust | UniRef100\_A0A4Y9NA30 | 96.7 | 0.00022 | 4.4e-10 | 48.4 | 41 | (55, 95) | 100 | (35, 75) | 119 | Transcription termination factor Rho (Fragment) | Transcription termination factor Rho (Fragment) | | uniclust | UniRef100\_A0A847LPG8 | 96.7 | 0.00024 | 4.5e-10 | 48.0 | 41 | (55, 95) | 100 | (4, 44) | 136 | Starch-binding protein | Starch-binding protein | | uniclust | UniRef100\_A0A3G9A8C3 | 96.7 | 0.00022 | 4.5e-10 | 48.6 | 90 | (1, 90) | 100 | (1, 112) | 115 | Uncharacterized protein | Uncharacterized protein | | uniclust | UniRef100\_H3NPD2 | 96.7 | 0.00024 | 4.5e-10 | 45.0 | 67 | (22, 90) | 100 | (20, 86) | 88 | HeH/LEM domain-containing protein | HeH/LEM domain-containing protein | | uniclust | UniRef100\_A0A0J8DFR9 | 96.7 | 0.00024 | 4.5e-10 | 43.8 | 42 | (5, 46) | 100 | (1, 43) | 72 | Uncharacterized protein | Uncharacterized protein | | uniclust | UniRef100\_A0A0M3SMX1 | 96.7 | 0.00023 | 4.7e-10 | 44.9 | 39 | (57, 95) | 100 | (35, 73) | 75 | HeH/LEM domain-containing protein | HeH/LEM domain-containing protein | | uniclust | UniRef100\_A0A1B1U2N6 | 96.7 | 0.00025 | 4.7e-10 | 45.4 | 36 | (54, 89) | 100 | (53, 88) | 89 | Rho termination factor N-terminal domain-containing protein | Rho termination factor N-terminal domain-containing protein | | uniclust | UniRef100\_A0A6B0BAF2 | 96.6 | 0.00025 | 4.7e-10 | 47.0 | 88 | (1, 93) | 100 | (1, 94) | 108 | Phage protein | Phage protein | | uniclust | UniRef100\_A0A3S4QE94 | 96.6 | 0.00026 | 4.8e-10 | 45.0 | 87 | (3, 90) | 100 | (2, 88) | 89 | HeH/LEM domain | HeH/LEM domain | | uniclust | UniRef100\_UPI0006915928 | 96.6 | 0.00026 | 4.8e-10 | 44.9 | 38 | (54, 91) | 100 | (54, 91) | 92 | Rho termination factor N-terminal domain-containing protein | Rho termination factor N-terminal domain-containing protein | | uniclust | UniRef100\_A0A8S9SLN2 | 96.6 | 0.00027 | 5.1e-10 | 45.6 | 39 | (53, 91) | 100 | (55, 93) | 94 | Rho termination factor N-terminal domain-containing protein | Rho termination factor N-terminal domain-containing protein | | uniclust | UniRef100\_A0A0G0RI45 | 96.6 | 0.00027 | 5.1e-10 | 48.0 | 71 | (23, 93) | 100 | (41, 119) | 121 | HeH/LEM domain-containing protein | HeH/LEM domain-containing protein | | uniclust | UniRef100\_Q1ILS8 | 96.6 | 0.00028 | 5.1e-10 | 47.0 | 76 | (19, 94) | 100 | (31, 119) | 123 | Rho termination factor N-terminal domain-containing protein | Rho termination factor N-terminal domain-containing protein | | uniclust | UniRef100\_A0A2S8MDY3 | 96.6 | 0.00027 | 5.2e-10 | 42.0 | 37 | (54, 90) | 100 | (21, 57) | 59 | Rho termination factor (Fragment) | Rho termination factor (Fragment) | | uniclust | UniRef100\_A0A7C6FK88 | 96.6 | 0.00026 | 5.3e-10 | 60.0 | 44 | (54, 97) | 100 | (27, 70) | 649 | Transcription termination factor Rho | Transcription termination factor Rho | | uniclust | UniRef100\_A0A164DND9 | 96.6 | 0.00028 | 5.3e-10 | 51.1 | 37 | (54, 90) | 100 | (156, 192) | 193 | Rho termination factor N-terminal domain-containing protein | Rho termination factor N-terminal domain-containing protein | | uniclust | UniRef100\_A0A399NFB3 | 96.6 | 0.00027 | 5.3e-10 | 45.6 | 42 | (54, 95) | 100 | (10, 51) | 89 | Transcription termination factor Rho (Fragment) | Transcription termination factor Rho (Fragment) | | uniclust | UniRef100\_A0A512CPT3 | 96.6 | 0.00029 | 5.4e-10 | 46.4 | 76 | (8, 90) | 100 | (4, 81) | 115 | Rho termination factor N-terminal domain-containing protein | Rho termination factor N-terminal domain-containing protein | | uniclust | UniRef100\_A0A2A2M454 | 96.6 | 0.00028 | 5.4e-10 | 50.2 | 45 | (54, 98) | 100 | (15, 59) | 159 | Rho RNA-BD domain-containing protein | Rho RNA-BD domain-containing protein | | uniclust | UniRef100\_A0A2J6WPY3 | 96.6 | 0.00027 | 5.4e-10 | 54.6 | 40 | (55, 94) | 100 | (21, 60) | 278 | Rho termination factor N-terminal domain-containing protein | Rho termination factor N-terminal domain-containing protein | | uniclust | UniRef100\_A0A353RPN2 | 96.6 | 0.00026 | 5.5e-10 | 48.2 | 44 | (55, 98) | 100 | (11, 54) | 113 | Transcription termination factor Rho (Fragment) | Transcription termination factor Rho (Fragment) | | uniclust | UniRef100\_A0A363D603 | 96.6 | 0.00029 | 5.5e-10 | 46.4 | 41 | (55, 95) | 100 | (5, 45) | 106 | Rho termination factor N-terminal domain-containing protein | Rho termination factor N-terminal domain-containing protein | | uniclust | UniRef100\_A0A1C6JYU0 | 96.6 | 0.00027 | 5.5e-10 | 48.3 | 41 | (56, 96) | 100 | (66, 106) | 115 | Uncharacterized protein | Uncharacterized protein | | uniclust | UniRef100\_A0A097PBF3 | 96.6 | 0.00028 | 5.8e-10 | 51.1 | 95 | (1, 95) | 100 | (5, 125) | 170 | HeH/LEM domain-containing protein | HeH/LEM domain-containing protein | | uniclust | UniRef100\_A0A661IZL4 | 96.6 | 0.00032 | 5.9e-10 | 44.5 | 41 | (54, 94) | 100 | (33, 73) | 91 | Rho termination factor N-terminal domain-containing protein (Fragment) | Rho termination factor N-terminal domain-containing protein (Fragment) | | uniclust | UniRef100\_A0A1Y1RN50 | 96.6 | 0.00029 | 5.9e-10 | 55.2 | 40 | (54, 93) | 100 | (274, 313) | 314 | Rho termination factor N-terminal domain-containing protein | Rho termination factor N-terminal domain-containing protein | | uniclust | UniRef100\_A0A651FJL6 | 96.6 | 0.00033 | 6.1e-10 | 45.0 | 89 | (1, 89) | 100 | (1, 97) | 99 | Rho termination factor N-terminal domain-containing protein | Rho termination factor N-terminal domain-containing protein | | uniclust | UniRef100\_A0A3C1A1A0 | 96.6 | 0.00034 | 6.2e-10 | 40.0 | 32 | (54, 85) | 100 | (20, 51) | 51 | Rho termination factor N-terminal domain-containing protein (Fragment) | Rho termination factor N-terminal domain-containing protein (Fragment) | | uniclust | UniRef100\_UPI0021069BFE | 96.6 | 0.00034 | 6.2e-10 | 41.6 | 39 | (56, 94) | 100 | (23, 61) | 63 | Rho termination factor N-terminal domain-containing protein | Rho termination factor N-terminal domain-containing protein | | uniclust | UniRef100\_A0A171NWN3 | 96.6 | 0.00033 | 6.4e-10 | 48.8 | 38 | (54, 91) | 100 | (108, 145) | 148 | 50S ribosomal protein (Fragment) | 50S ribosomal protein (Fragment) | | uniclust | UniRef100\_A0A6N7EVP9 | 96.6 | 0.00034 | 6.5e-10 | 43.8 | 37 | (60, 96) | 100 | (1, 37) | 80 | Rho termination factor N-terminal domain-containing protein (Fragment) | Rho termination factor N-terminal domain-containing protein (Fragment) | | uniclust | UniRef100\_UPI000D1A72FA | 96.6 | 0.00036 | 6.6e-10 | 45.1 | 46 | (54, 99) | 100 | (48, 93) | 102 | Rho termination factor N-terminal domain-containing protein | Rho termination factor N-terminal domain-containing protein | | uniclust | UniRef100\_A0A3G9JYC5 | 96.6 | 0.00035 | 6.7e-10 | 50.1 | 38 | (54, 91) | 100 | (135, 172) | 174 | Rho termination factor N-terminal domain-containing protein | Rho termination factor N-terminal domain-containing protein | | uniclust | UniRef100\_A0A2R5F593 | 96.6 | 0.00037 | 6.7e-10 | 44.3 | 41 | (54, 94) | 100 | (7, 47) | 91 | Transcription termination factor Rho | Transcription termination factor Rho | | uniclust | UniRef100\_A0A1C2XPW0 | 96.6 | 0.00035 | 6.8e-10 | 44.8 | 39 | (54, 93) | 100 | (46, 84) | 85 | Rho termination factor N-terminal domain-containing protein | Rho termination factor N-terminal domain-containing protein | | uniclust | UniRef100\_A0A061GFL4 | 96.6 | 0.00035 | 6.8e-10 | 54.8 | 36 | (55, 90) | 100 | (306, 341) | 342 | Rho termination factor, putative isoform 1 | Rho termination factor, putative isoform 1 | | uniclust | UniRef100\_A0A5C6ULJ2 | 96.6 | 0.00036 | 6.9e-10 | 45.5 | 84 | (4, 95) | 100 | (1, 96) | 100 | Peripheral subunit-binding (PSBD) domain-containing protein | Peripheral subunit-binding (PSBD) domain-containing protein | | uniclust | UniRef100\_A0A6C0DS71 | 96.5 | 0.00037 | 7e-10 | 51.4 | 39 | (55, 93) | 100 | (152, 190) | 215 | Rho termination factor N-terminal domain-containing protein | Rho termination factor N-terminal domain-containing protein | | uniclust | UniRef100\_UPI0020195133 | 96.5 | 0.00039 | 7.2e-10 | 47.1 | 76 | (20, 98) | 100 | (21, 97) | 137 | hypothetical protein | hypothetical protein | | uniclust | UniRef100\_A0A950YI39 | 96.5 | 0.00041 | 7.5e-10 | 37.6 | 33 | (60, 92) | 100 | (1, 33) | 39 | Rho termination factor N-terminal domain-containing protein | Rho termination factor N-terminal domain-containing protein | | uniclust | UniRef100\_A0A1B1ISD2 | 96.5 | 0.00036 | 7.5e-10 | 53.3 | 39 | (54, 92) | 100 | (202, 240) | 241 | Sarcoplasmic reticulum histidine-rich calcium-binding protein-like | Sarcoplasmic reticulum histidine-rich calcium-binding protein-like | | uniclust | UniRef100\_UPI0020561702 | 96.5 | 0.00041 | 7.5e-10 | 44.8 | 80 | (14, 93) | 100 | (13, 100) | 101 | hypothetical protein | hypothetical protein | | uniclust | UniRef100\_A0A9D4E6J0 | 96.5 | 0.00041 | 7.6e-10 | 42.6 | 39 | (59, 97) | 100 | (3, 41) | 75 | Uncharacterized protein | Uncharacterized protein | | uniclust | UniRef100\_A0A1V2VNK8 | 96.5 | 0.00037 | 7.6e-10 | 46.8 | 84 | (2, 95) | 100 | (1, 88) | 102 | HeH/LEM domain-containing protein | HeH/LEM domain-containing protein | | uniclust | UniRef100\_A0A2D6X3A0 | 96.5 | 0.00042 | 7.6e-10 | 40.9 | 42 | (5, 46) | 100 | (1, 42) | 60 | Uncharacterized protein | Uncharacterized protein | | uniclust | UniRef100\_A0A0A7PDZ3 | 96.5 | 0.00038 | 7.7e-10 | 45.5 | 85 | (5, 95) | 100 | (1, 87) | 90 | Secreted protein | Secreted protein | | uniclust | UniRef100\_A0A2N0VMG3 | 96.5 | 0.00041 | 7.9e-10 | 48.3 | 40 | (54, 93) | 100 | (103, 142) | 145 | Rho termination factor N-terminal domain-containing protein | Rho termination factor N-terminal domain-containing protein | | uniclust | UniRef100\_A0A498IVK1 | 96.5 | 0.0004 | 8e-10 | 58.3 | 39 | (53, 91) | 100 | (566, 604) | 607 | Rho termination factor N-terminal domain-containing protein | Rho termination factor N-terminal domain-containing protein | | uniclust | UniRef100\_A0A382MFM9 | 96.5 | 0.00044 | 8e-10 | 39.9 | 41 | (5, 45) | 100 | (1, 41) | 53 | Uncharacterized protein (Fragment) | Uncharacterized protein (Fragment) | | uniclust | UniRef100\_A0A844Q625 | 96.5 | 0.00044 | 8.1e-10 | 45.6 | 83 | (11, 93) | 100 | (7, 106) | 114 | Rho termination factor N-terminal domain-containing protein | Rho termination factor N-terminal domain-containing protein | | uniclust | UniRef100\_A0A1H0FZA0 | 96.5 | 0.00044 | 8.2e-10 | 46.0 | 69 | (22, 91) | 100 | (33, 115) | 116 | Rho termination factor, N-terminal domain | Rho termination factor, N-terminal domain | | uniclust | UniRef100\_A0A1C3H478 | 96.5 | 0.00044 | 8.3e-10 | 41.3 | 42 | (5, 46) | 100 | (1, 42) | 58 | Uncharacterized protein | Uncharacterized protein | | uniclust | UniRef100\_UPI001382ED58 | 96.5 | 0.00044 | 8.4e-10 | 45.4 | 41 | (55, 95) | 100 | (10, 50) | 104 | Rho termination factor N-terminal domain-containing protein | Rho termination factor N-terminal domain-containing protein | | uniclust | UniRef100\_A0A3D2JCI4 | 96.5 | 0.00042 | 8.5e-10 | 48.5 | 42 | (54, 95) | 100 | (82, 123) | 134 | Rho termination factor N-terminal domain-containing protein (Fragment) | Rho termination factor N-terminal domain-containing protein (Fragment) | | uniclust | UniRef100\_UPI00178A52FA | 96.5 | 0.00047 | 8.7e-10 | 46.9 | 92 | (3, 95) | 100 | (2, 104) | 139 | Rho termination factor N-terminal domain-containing protein | Rho termination factor N-terminal domain-containing protein | | uniclust | UniRef100\_A0A6V8CT67 | 96.5 | 0.00046 | 8.7e-10 | 41.8 | 38 | (56, 95) | 100 | (3, 40) | 63 | SAP domain-containing protein (Fragment) | SAP domain-containing protein (Fragment) | | uniclust | UniRef100\_A0A8S5M0J8 | 96.5 | 0.00046 | 8.8e-10 | 37.3 | 33 | (60, 92) | 100 | (1, 34) | 36 | Transcription regulator-like protein | Transcription regulator-like protein | | uniclust | UniRef100\_A0A0B4EK77 | 96.5 | 0.00046 | 8.8e-10 | 52.8 | 43 | (54, 96) | 100 | (106, 148) | 293 | Rho termination factor N-terminal domain-containing protein | Rho termination factor N-terminal domain-containing protein | | uniclust | UniRef100\_A0A0R3MJG7 | 96.5 | 0.00048 | 8.9e-10 | 44.9 | 76 | (16, 91) | 100 | (25, 106) | 107 | Rho termination factor N-terminal domain-containing protein | Rho termination factor N-terminal domain-containing protein | | uniclust | UniRef100\_A0A0C2ZSS3 | 96.5 | 0.00048 | 8.9e-10 | 46.2 | 41 | (55, 95) | 100 | (45, 85) | 119 | Rho termination factor N-terminal domain-containing protein (Fragment) | Rho termination factor N-terminal domain-containing protein (Fragment) | | uniclust | UniRef100\_A0A482MWG0 | 96.5 | 0.00049 | 9e-10 | 43.7 | 41 | (53, 93) | 100 | (46, 86) | 88 | Rho termination factor N-terminal domain-containing protein | Rho termination factor N-terminal domain-containing protein | | uniclust | UniRef100\_A0A411YG54 | 96.5 | 0.00047 | 9.1e-10 | 52.6 | 39 | (54, 92) | 100 | (228, 266) | 267 | Rho termination factor N-terminal domain-containing protein | Rho termination factor N-terminal domain-containing protein | | uniclust | UniRef100\_A0A6I2UUF3 | 96.5 | 0.0005 | 9.2e-10 | 42.9 | 44 | (1, 44) | 100 | (1, 44) | 82 | Uncharacterized protein | Uncharacterized protein | | uniclust | UniRef100\_UPI002356B0F1 | 96.5 | 0.0005 | 9.2e-10 | 47.3 | 88 | (7, 94) | 100 | (3, 113) | 151 | hypothetical protein | hypothetical protein | | uniclust | UniRef100\_A0A061AAU7 | 96.5 | 0.00046 | 9.2e-10 | 50.9 | 43 | (55, 97) | 100 | (150, 192) | 196 | 50S ribosomal protein L20 | 50S ribosomal protein L20 | | uniclust | UniRef100\_A0A7W6RF31 | 96.5 | 0.00049 | 9.3e-10 | 41.7 | 35 | (12, 46) | 100 | (14, 48) | 63 | Uncharacterized protein | Uncharacterized protein | | uniclust | UniRef100\_UPI001FE300D8 | 96.5 | 0.00051 | 9.4e-10 | 40.2 | 38 | (59, 96) | 100 | (3, 40) | 57 | Rho termination factor N-terminal domain-containing protein | Rho termination factor N-terminal domain-containing protein | | uniclust | UniRef100\_UPI001FD29EAF | 96.5 | 0.00051 | 9.4e-10 | 47.1 | 77 | (19, 95) | 100 | (29, 123) | 147 | hypothetical protein | hypothetical protein | | uniclust | UniRef100\_UPI0002F4E584 | 96.5 | 0.0005 | 9.5e-10 | 46.6 | 45 | (54, 98) | 100 | (10, 54) | 119 | Rho termination factor N-terminal domain-containing protein | Rho termination factor N-terminal domain-containing protein | | uniclust | UniRef100\_UPI001F182A02 | 96.5 | 0.00051 | 9.5e-10 | 43.6 | 38 | (53, 90) | 100 | (47, 84) | 84 | Rho termination factor N-terminal domain-containing protein | Rho termination factor N-terminal domain-containing protein | | uniclust | UniRef100\_UPI001BB11BDD | 96.5 | 0.00052 | 9.6e-10 | 40.9 | 37 | (60, 96) | 100 | (1, 37) | 63 | Rho termination factor N-terminal domain-containing protein | Rho termination factor N-terminal domain-containing protein | | uniclust | UniRef100\_UPI001918AB4A | 96.5 | 0.00051 | 9.8e-10 | 49.9 | 41 | (56, 96) | 100 | (143, 183) | 189 | hypothetical protein | hypothetical protein | | uniclust | UniRef100\_A0A372JKS1 | 96.5 | 0.00051 | 9.8e-10 | 51.8 | 41 | (55, 95) | 100 | (36, 76) | 251 | Transcription termination factor Rho (Fragment) | Transcription termination factor Rho (Fragment) | | uniclust | UniRef100\_A0A1V6HXP6 | 96.5 | 0.00052 | 9.9e-10 | 52.6 | 41 | (56, 96) | 100 | (3, 43) | 296 | Rho termination factor N-terminal domain-containing protein | Rho termination factor N-terminal domain-containing protein | | uniclust | UniRef100\_A0A0F7L7M2 | 96.5 | 0.0005 | 1e-09 | 44.4 | 42 | (4, 45) | 100 | (14, 55) | 84 | Uncharacterized protein | Uncharacterized protein | | uniclust | UniRef100\_A0A1H8W1H1 | 96.5 | 0.00053 | 1e-09 | 47.7 | 71 | (22, 92) | 100 | (63, 145) | 146 | Rho termination factor, N-terminal domain | Rho termination factor, N-terminal domain | | uniclust | UniRef100\_A0A1H1TF00 | 96.5 | 0.00051 | 1e-09 | 46.5 | 40 | (54, 93) | 100 | (69, 108) | 113 | Rho termination factor, N-terminal domain | Rho termination factor, N-terminal domain | | uniclust | UniRef100\_A0A094P6K3 | 96.4 | 0.00055 | 1.1e-09 | 48.1 | 36 | (55, 90) | 100 | (114, 149) | 151 | Rho termination factor N-terminal domain-containing protein | Rho termination factor N-terminal domain-containing protein | | uniclust | UniRef100\_A0A7C6WBF3 | 96.4 | 0.00058 | 1.1e-09 | 46.8 | 35 | (57, 91) | 100 | (112, 146) | 147 | 30S ribosomal protein S20 | 30S ribosomal protein S20 | | uniclust | UniRef100\_A0A1H7ZYP4 | 96.4 | 0.00057 | 1.1e-09 | 46.2 | 47 | (53, 99) | 100 | (65, 111) | 113 | HeH/LEM domain-containing protein | HeH/LEM domain-containing protein | | uniclust | UniRef100\_A0A0M2LXZ2 | 96.4 | 0.00056 | 1.1e-09 | 43.5 | 46 | (1, 46) | 100 | (1, 46) | 78 | Uncharacterized protein | Uncharacterized protein | | uniclust | UniRef100\_A0A1S1TMF4 | 96.4 | 0.00061 | 1.1e-09 | 45.1 | 77 | (18, 94) | 100 | (19, 107) | 110 | Uncharacterized protein | Uncharacterized protein | | uniclust | UniRef100\_X0WVX3 | 96.4 | 0.00063 | 1.2e-09 | 38.5 | 35 | (55, 90) | 100 | (14, 48) | 48 | Rho termination factor N-terminal domain-containing protein (Fragment) | Rho termination factor N-terminal domain-containing protein (Fragment) | | uniclust | UniRef100\_A0A0S8E8A4 | 96.4 | 0.00057 | 1.2e-09 | 43.5 | 35 | (59, 94) | 100 | (6, 40) | 75 | SAP domain-containing protein | SAP domain-containing protein | | uniclust | UniRef100\_UPI000375EB70 | 96.4 | 0.00061 | 1.2e-09 | 47.6 | 42 | (54, 95) | 100 | (7, 49) | 151 | hypothetical protein | hypothetical protein | | uniclust | UniRef100\_A0A7X0ZKM8 | 96.4 | 0.00062 | 1.2e-09 | 39.7 | 39 | (53, 91) | 100 | (12, 50) | 52 | HeH/LEM domain-containing protein | HeH/LEM domain-containing protein | | uniclust | UniRef100\_A0A2E5TCS1 | 96.4 | 0.0006 | 1.2e-09 | 43.4 | 43 | (3, 45) | 100 | (7, 49) | 78 | DUF2635 domain-containing protein | DUF2635 domain-containing protein | | uniclust | UniRef100\_UPI0021E5D87B | 96.4 | 0.00064 | 1.2e-09 | 43.1 | 40 | (54, 93) | 100 | (49, 88) | 89 | Rho termination factor N-terminal domain-containing protein | Rho termination factor N-terminal domain-containing protein | | uniclust | UniRef100\_A0A7J9VEG8 | 96.4 | 0.0006 | 1.2e-09 | 54.4 | 41 | (55, 95) | 100 | (34, 74) | 372 | Transcription termination factor Rho (Fragment) | Transcription termination factor Rho (Fragment) | | uniclust | UniRef100\_A0A519NL56 | 96.4 | 0.00063 | 1.2e-09 | 40.1 | 43 | (55, 97) | 100 | (5, 47) | 55 | Rho termination factor N-terminal domain-containing protein (Fragment) | Rho termination factor N-terminal domain-containing protein (Fragment) | | uniclust | UniRef100\_A0A076JPF1 | 96.4 | 0.00059 | 1.2e-09 | 59.1 | 43 | (54, 96) | 100 | (59, 101) | 780 | Transcription termination factor Rho | Transcription termination factor Rho | | uniclust | UniRef100\_A0A0A9Q8Y3 | 96.4 | 0.00066 | 1.2e-09 | 35.9 | 32 | (60, 91) | 100 | (1, 32) | 34 | Rho termination factor N-terminal domain-containing protein | Rho termination factor N-terminal domain-containing protein | | uniclust | UniRef100\_UPI00210A2392 | 96.4 | 0.00068 | 1.2e-09 | 38.9 | 41 | (54, 94) | 100 | (8, 48) | 51 | HeH/LEM domain-containing protein | HeH/LEM domain-containing protein | | uniclust | UniRef100\_A0A2H6HI62 | 96.4 | 0.00064 | 1.3e-09 | 54.8 | 43 | (54, 96) | 100 | (6, 48) | 398 | 2-acyl-glycerophospho-ethanolamine acyltransferase | 2-acyl-glycerophospho-ethanolamine acyltransferase | | uniclust | UniRef100\_A0A6L7B069 | 96.4 | 0.0007 | 1.3e-09 | 37.9 | 40 | (60, 99) | 100 | (1, 40) | 45 | HeH/LEM domain-containing protein | HeH/LEM domain-containing protein | | uniclust | UniRef100\_A0A1T2CHS8 | 96.4 | 0.00066 | 1.3e-09 | 48.0 | 43 | (4, 46) | 100 | (13, 55) | 145 | Uncharacterized protein | Uncharacterized protein | | uniclust | UniRef100\_A0A379FQN9 | 96.4 | 0.00064 | 1.3e-09 | 46.1 | 41 | (4, 44) | 100 | (1, 41) | 108 | Procyclic acidic repetitive protein (PARP) | Procyclic acidic repetitive protein (PARP) | | uniclust | UniRef100\_A0A2S8FHG4 | 96.4 | 0.00069 | 1.3e-09 | 41.6 | 38 | (54, 91) | 100 | (25, 63) | 65 | Rho termination factor N-terminal domain-containing protein | Rho termination factor N-terminal domain-containing protein | | uniclust | UniRef100\_A0A954APN1 | 96.4 | 0.00069 | 1.4e-09 | 46.0 | 44 | (3, 46) | 100 | (13, 56) | 114 | Uncharacterized protein | Uncharacterized protein | | uniclust | UniRef100\_A0A9D4B561 | 96.4 | 0.00074 | 1.4e-09 | 41.1 | 38 | (59, 96) | 100 | (3, 40) | 66 | Uncharacterized protein | Uncharacterized protein | | uniclust | UniRef100\_A0A3A0ET32 | 96.4 | 0.00075 | 1.4e-09 | 39.8 | 37 | (55, 91) | 100 | (22, 58) | 59 | Rho termination factor N-terminal domain-containing protein | Rho termination factor N-terminal domain-containing protein | | uniclust | UniRef100\_A0A7W9N0T6 | 96.4 | 0.00077 | 1.4e-09 | 46.4 | 81 | (15, 95) | 100 | (8, 102) | 149 | Rho termination factor | Rho termination factor | | uniclust | UniRef100\_UPI00218C784C | 96.4 | 0.00078 | 1.4e-09 | 39.1 | 39 | (6, 44) | 100 | (2, 40) | 54 | hypothetical protein | hypothetical protein | | uniclust | UniRef100\_X7Y4V5 | 96.4 | 0.00076 | 1.4e-09 | 49.9 | 45 | (54, 98) | 100 | (27, 71) | 232 | Rho termination factor, N-terminal domain protein | Rho termination factor, N-terminal domain protein | | uniclust | UniRef100\_A0A848L2M3 | 96.4 | 0.00078 | 1.4e-09 | 41.1 | 40 | (54, 93) | 100 | (31, 70) | 71 | Rho termination factor N-terminal domain-containing protein | Rho termination factor N-terminal domain-containing protein | | uniclust | UniRef100\_A0A2E0K4L4 | 96.4 | 0.00076 | 1.4e-09 | 37.7 | 35 | (54, 90) | 100 | (7, 41) | 42 | SAP domain-containing protein (Fragment) | SAP domain-containing protein (Fragment) | | uniclust | UniRef100\_A0A0M8Y5M5 | 96.4 | 0.00074 | 1.4e-09 | 51.9 | 42 | (53, 94) | 100 | (31, 72) | 278 | Rho termination factor N-terminal domain-containing protein | Rho termination factor N-terminal domain-containing protein | | uniclust | UniRef100\_A0A2E0E7I7 | 96.3 | 0.00075 | 1.5e-09 | 51.2 | 39 | (54, 92) | 100 | (214, 252) | 253 | Rho termination factor N-terminal domain-containing protein | Rho termination factor N-terminal domain-containing protein | | uniclust | UniRef100\_A0A009IA11 | 96.3 | 0.00075 | 1.5e-09 | 47.4 | 41 | (53, 93) | 100 | (98, 138) | 145 | HeH/LEM domain protein | HeH/LEM domain protein | | uniclust | UniRef100\_A0A973TXR3 | 96.3 | 0.0008 | 1.5e-09 | 42.6 | 43 | (55, 97) | 100 | (42, 84) | 88 | Transcription termination factor Rho (Fragment) | Transcription termination factor Rho (Fragment) | | uniclust | UniRef100\_A0A0B5AVK8 | 96.3 | 0.00068 | 1.5e-09 | 47.1 | 43 | (1, 43) | 100 | (1, 46) | 121 | Uncharacterized protein | Uncharacterized protein | | uniclust | UniRef100\_UPI001F2021B5 | 96.3 | 0.00079 | 1.5e-09 | 47.3 | 40 | (54, 93) | 100 | (93, 132) | 156 | Rho termination factor N-terminal domain-containing protein | Rho termination factor N-terminal domain-containing protein | | uniclust | UniRef100\_UPI0005C4E073 | 96.3 | 0.00081 | 1.5e-09 | 42.9 | 41 | (55, 95) | 100 | (6, 46) | 92 | Rho termination factor N-terminal domain-containing protein | Rho termination factor N-terminal domain-containing protein | | uniclust | UniRef100\_A0A4R2IZA5 | 96.3 | 0.00076 | 1.5e-09 | 44.1 | 36 | (57, 92) | 100 | (53, 88) | 90 | Rho termination factor-like protein | Rho termination factor-like protein | | uniclust | UniRef100\_A0A971BR62 | 96.3 | 0.00082 | 1.5e-09 | 42.7 | 44 | (53, 96) | 100 | (7, 50) | 90 | Transcription termination factor Rho (Fragment) | Transcription termination factor Rho (Fragment) | | uniclust | UniRef100\_A0A2G5NW73 | 96.3 | 0.0008 | 1.5e-09 | 45.0 | 37 | (54, 90) | 100 | (74, 110) | 111 | Rho termination protein | Rho termination protein | | uniclust | UniRef100\_A0A178V4Q9 | 96.3 | 0.00083 | 1.5e-09 | 44.7 | 37 | (55, 91) | 100 | (82, 118) | 119 | Rho termination factor N-terminal domain-containing protein | Rho termination factor N-terminal domain-containing protein | |
| Top keywords  (threshold 1.00e-03 (evalue)) | **Rho, termination, domain\_containing, factor, N\_terminal, HeH, LEM, Fragment, hypothetical, Transcription** |
| Output files | ../../similar\_sequences/06\_FANPEZAQ\_CDS\_0006\_merged.svg ../../similar\_sequences/06\_FANPEZAQ\_CDS\_0006\_pdb70.a3m ../../similar\_sequences/06\_FANPEZAQ\_CDS\_0006\_pdb70.hhr ../../similar\_sequences/06\_FANPEZAQ\_CDS\_0006\_uniclust.a3m ../../similar\_sequences/06\_FANPEZAQ\_CDS\_0006\_uniclust.hhr |

#### Structure prediction (AlphaFold)2

|  |  |
| --- | --- |
| Stats | xml version="1.0" encoding="utf-8" standalone="no"?       2024-09-02T21:09:05.778690 image/svg+xml   Matplotlib v3.7.2, https://matplotlib.org/ |
| Predicted structure | **NGL Viewer Controls:**  - Center: *Left-Click* - Rotate: *Left-Click + Drag* - Translate: *Right-Click + Drag* - Zoom: *Shift + Left-Click + Drag* |
| Output files | ../../predicted\_structures/06\_FANPEZAQ\_CDS\_0006/features.pkl ../../predicted\_structures/06\_FANPEZAQ\_CDS\_0006/ranked\_0.pdb ../../predicted\_structures/06\_FANPEZAQ\_CDS\_0006/ranked\_0\_plots.svg ../../predicted\_structures/06\_FANPEZAQ\_CDS\_0006/result\_model\_1\_ptm\_pred\_0.pkl |

#### Structure similarity search results (Foldseek)3

|  |  |
| --- | --- |
| Structure databases searched | Pdb, Afdb-proteome, Afdb-uniprot50 |
| Results, scheme(s)  (Top layers only, threshold 1.00e-02 (evalue)) | xml version="1.0" encoding="utf-8" standalone="no"?       2024-09-02T21:10:16.399809 image/svg+xml   Matplotlib v3.7.2, https://matplotlib.org/ |
| Results, table  (threshold 1.00e-02 (evalue)) | | db | id | prob | evalue | bits | fident | alnlen | mismatch | gapopen | qstart | qend | tstart | tend | name | description | | --- | --- | --- | --- | --- | --- | --- | --- | --- | --- | --- | --- | --- | --- | --- | | afdb-uniprot50 | AF-A0A2Z3IK27-F1-MODEL\_V4 | 1.0 | 6.197e-10 | 263 | 0.531 | 96 | 40 | 3 | 1 | 93 | 1 | 94 | Rho\_N domain-containing protein | Rho\_N domain-containing protein | | afdb-uniprot50 | AF-A0A1H2R4E9-F1-MODEL\_V4 | 1.0 | 6.471e-09 | 255 | 0.431 | 95 | 51 | 2 | 1 | 94 | 2 | 94 | Rho termination factor, N-terminal domain | Rho termination factor, N-terminal domain | | afdb-uniprot50 | AF-A0A515BE84-F1-MODEL\_V4 | 1.0 | 2.091e-08 | 234 | 0.406 | 96 | 55 | 2 | 1 | 95 | 1 | 95 | Uncharacterized protein | Uncharacterized protein | | afdb-uniprot50 | AF-A0A238JAW9-F1-MODEL\_V4 | 1.0 | 4.878e-08 | 226 | 0.391 | 92 | 53 | 1 | 2 | 93 | 5 | 93 | Uncharacterized protein | Uncharacterized protein | | afdb-uniprot50 | AF-A0A0P8YT19-F1-MODEL\_V4 | 1.0 | 8.768e-08 | 225 | 0.45 | 91 | 45 | 1 | 3 | 93 | 19 | 104 | Rho\_N domain-containing protein | Rho\_N domain-containing protein | | afdb-uniprot50 | AF-A0A420WVL7-F1-MODEL\_V4 | 1.0 | 5.931e-08 | 219 | 0.371 | 105 | 59 | 3 | 1 | 100 | 3 | 105 | Rho termination factor-like protein | Rho termination factor-like protein | | afdb-uniprot50 | AF-A0A291LZ12-F1-MODEL\_V4 | 1.0 | 6.33e-08 | 218 | 0.355 | 107 | 55 | 1 | 3 | 95 | 2 | 108 | Rho\_N domain-containing protein | Rho\_N domain-containing protein | | afdb-uniprot50 | AF-A0A0A8TH08-F1-MODEL\_V4 | 1.0 | 3.024e-07 | 209 | 0.333 | 93 | 58 | 2 | 3 | 94 | 2 | 91 | Uncharacterized protein | Uncharacterized protein | | afdb-uniprot50 | AF-A0A7V7G2K9-F1-MODEL\_V4 | 1.0 | 8.215e-08 | 204 | 0.392 | 107 | 55 | 4 | 2 | 100 | 11 | 115 | Rho\_N domain-containing protein | Rho\_N domain-containing protein | | afdb-uniprot50 | AF-A0A845NDA8-F1-MODEL\_V4 | 1.0 | 5.675e-06 | 193 | 0.285 | 98 | 68 | 1 | 3 | 100 | 2 | 97 | Uncharacterized protein | Uncharacterized protein | | afdb-uniprot50 | AF-E4PPT2-F1-MODEL\_V4 | 1.0 | 8.577e-07 | 192 | 0.333 | 99 | 59 | 3 | 2 | 96 | 3 | 98 | Protein containing Rho termination factor, N-terminal domain | Protein containing Rho termination factor, N-terminal domain | | afdb-uniprot50 | AF-A0A0D0QEA8-F1-MODEL\_V4 | 1.0 | 4.47e-07 | 191 | 0.459 | 98 | 40 | 2 | 3 | 91 | 2 | 95 | Contig\_80, whole genome shotgun sequence | Contig\_80, whole genome shotgun sequence | | afdb-uniprot50 | AF-A0A103EIE9-F1-MODEL\_V4 | 1.0 | 0.0001575 | 190 | 0.465 | 58 | 28 | 1 | 2 | 56 | 6 | 63 | Uncharacterized protein | Uncharacterized protein | | afdb-uniprot50 | AF-Q24VH2-F1-MODEL\_V4 | 1.0 | 2.597e-06 | 182 | 0.268 | 97 | 68 | 2 | 1 | 96 | 1 | 95 | Rho\_N domain-containing protein | Rho\_N domain-containing protein | | afdb-uniprot50 | AF-A0A8A6KC69-F1-MODEL\_V4 | 1.0 | 0.000857 | 181 | 0.375 | 56 | 35 | 0 | 1 | 56 | 1 | 56 | Uncharacterized protein | Uncharacterized protein | | afdb-uniprot50 | AF-A0A6I2UUF3-F1-MODEL\_V4 | 1.0 | 0.0003921 | 178 | 0.436 | 55 | 26 | 1 | 1 | 55 | 1 | 50 | Uncharacterized protein | Uncharacterized protein | | afdb-uniprot50 | AF-A0A6I2JDE3-F1-MODEL\_V4 | 1.0 | 0.001267 | 175 | 0.272 | 55 | 40 | 0 | 1 | 55 | 2 | 56 | Uncharacterized protein | Uncharacterized protein | | afdb-uniprot50 | AF-A0A2S0MNH5-F1-MODEL\_V4 | 1.0 | 0.0001475 | 171 | 0.4 | 65 | 35 | 1 | 1 | 61 | 2 | 66 | Uncharacterized protein | Uncharacterized protein | | afdb-uniprot50 | AF-A0A315BS07-F1-MODEL\_V4 | 1.0 | 0.0001794 | 163 | 0.393 | 66 | 30 | 1 | 1 | 56 | 1 | 66 | Uncharacterized protein | Uncharacterized protein | | afdb-uniprot50 | AF-A0A177H989-F1-MODEL\_V4 | 1.0 | 0.006053 | 158 | 0.365 | 52 | 32 | 1 | 1 | 52 | 126 | 176 | Uncharacterized protein | Uncharacterized protein | | afdb-uniprot50 | AF-A0A143DF48-F1-MODEL\_V4 | 1.0 | 0.003155 | 157 | 0.296 | 54 | 38 | 0 | 3 | 56 | 10 | 63 | Uncharacterized protein | Uncharacterized protein | | afdb-uniprot50 | AF-A0A844C2Q1-F1-MODEL\_V4 | 1.0 | 1.089e-05 | 157 | 0.326 | 95 | 62 | 2 | 3 | 95 | 2 | 96 | Uncharacterized protein | Uncharacterized protein | | afdb-uniprot50 | AF-A0A7C8GRC8-F1-MODEL\_V4 | 1.0 | 7.69e-05 | 156 | 0.221 | 95 | 72 | 2 | 5 | 97 | 2 | 96 | Uncharacterized protein | Uncharacterized protein | | afdb-uniprot50 | AF-J2IAJ1-F1-MODEL\_V4 | 1.0 | 0.002278 | 155 | 0.307 | 52 | 33 | 1 | 1 | 52 | 1 | 49 | Uncharacterized protein | Uncharacterized protein | | afdb-uniprot50 | AF-A0A371P075-F1-MODEL\_V4 | 1.0 | 3.755e-05 | 155 | 0.268 | 97 | 67 | 3 | 6 | 100 | 2 | 96 | Uncharacterized protein | Uncharacterized protein | | afdb-uniprot50 | AF-A0A844HNJ4-F1-MODEL\_V4 | 1.0 | 0.0001915 | 155 | 0.352 | 71 | 36 | 1 | 1 | 61 | 1 | 71 | Uncharacterized protein | Uncharacterized protein | | afdb-uniprot50 | AF-A0A2T5NTL6-F1-MODEL\_V4 | 1.0 | 0.006053 | 154 | 0.277 | 54 | 38 | 1 | 2 | 55 | 3 | 55 | Uncharacterized protein | Uncharacterized protein | | afdb-uniprot50 | AF-A0A1X7L195-F1-MODEL\_V4 | 1.0 | 3.297e-05 | 154 | 0.255 | 98 | 69 | 2 | 1 | 97 | 1 | 95 | Uncharacterized protein | Uncharacterized protein | | afdb-uniprot50 | AF-A0A845TX33-F1-MODEL\_V4 | 1.0 | 0.007855 | 150 | 0.215 | 51 | 40 | 0 | 3 | 53 | 5 | 55 | Uncharacterized protein | Uncharacterized protein | | afdb-uniprot50 | AF-F4BFR0-F1-MODEL\_V4 | 1.0 | 0.003367 | 150 | 0.266 | 60 | 43 | 1 | 3 | 61 | 2 | 61 | Uncharacterized protein | Uncharacterized protein | | afdb-uniprot50 | AF-A0A2A3MN15-F1-MODEL\_V4 | 1.0 | 0.003836 | 148 | 0.339 | 53 | 33 | 1 | 1 | 53 | 1 | 51 | Uncharacterized protein | Uncharacterized protein | | afdb-uniprot50 | AF-A0A656JJH8-F1-MODEL\_V4 | 1.0 | 0.004094 | 147 | 0.218 | 55 | 43 | 0 | 2 | 56 | 30 | 84 | Uncharacterized protein | Uncharacterized protein | | afdb-uniprot50 | AF-A0A4U7J7L9-F1-MODEL\_V4 | 1.0 | 3.519e-05 | 145 | 0.329 | 88 | 56 | 3 | 7 | 92 | 2 | 88 | Uncharacterized protein | Uncharacterized protein | | afdb-uniprot50 | AF-A0A2G1LY20-F1-MODEL\_V4 | 1.0 | 4.874e-05 | 145 | 0.282 | 99 | 66 | 2 | 1 | 96 | 1 | 97 | Uncharacterized protein | Uncharacterized protein | | afdb-uniprot50 | AF-A0A838H6R9-F1-MODEL\_V4 | 1.0 | 0.009551 | 141 | 0.254 | 55 | 39 | 1 | 1 | 53 | 1 | 55 | Uncharacterized protein | Uncharacterized protein | | afdb-uniprot50 | AF-A0A336N748-F1-MODEL\_V4 | 1.0 | 0.006053 | 141 | 0.22 | 59 | 44 | 1 | 3 | 61 | 2 | 58 | Uncharacterized protein | Uncharacterized protein | | afdb-uniprot50 | AF-A0A7H1MI15-F1-MODEL\_V4 | 1.0 | 0.003836 | 141 | 0.228 | 57 | 44 | 0 | 4 | 60 | 5 | 61 | Uncharacterized protein | Uncharacterized protein | | afdb-uniprot50 | AF-A0A6L6JB59-F1-MODEL\_V4 | 1.0 | 7.69e-05 | 140 | 0.408 | 93 | 41 | 3 | 1 | 93 | 1 | 79 | Uncharacterized protein | Uncharacterized protein | | afdb-uniprot50 | AF-A0A511XP19-F1-MODEL\_V4 | 1.0 | 0.008384 | 139 | 0.147 | 61 | 50 | 1 | 1 | 61 | 1 | 59 | Uncharacterized protein | Uncharacterized protein | | afdb-uniprot50 | AF-A0A378R0P4-F1-MODEL\_V4 | 1.0 | 0.00646 | 138 | 0.206 | 58 | 45 | 1 | 4 | 61 | 10 | 66 | Uncharacterized protein | Uncharacterized protein | | afdb-uniprot50 | AF-A0A024YGT7-F1-MODEL\_V4 | 1.0 | 5.926e-05 | 138 | 0.262 | 99 | 68 | 1 | 5 | 98 | 2 | 100 | Uncharacterized protein | Uncharacterized protein | | afdb-uniprot50 | AF-A0A831RBJ2-F1-MODEL\_V4 | 1.0 | 0.006053 | 137 | 0.203 | 59 | 47 | 0 | 3 | 61 | 1 | 59 | Uncharacterized protein | Uncharacterized protein | | afdb-uniprot50 | AF-A0A3N4VST1-F1-MODEL\_V4 | 1.0 | 0.004094 | 136 | 0.209 | 62 | 48 | 1 | 1 | 61 | 1 | 62 | Uncharacterized protein | Uncharacterized protein | | afdb-uniprot50 | AF-A0A3T0L2P0-F1-MODEL\_V4 | 1.0 | 0.0002652 | 136 | 0.254 | 110 | 67 | 2 | 3 | 98 | 2 | 110 | Uncharacterized protein | Uncharacterized protein | | afdb-uniprot50 | AF-A0A4Q0ZTB4-F1-MODEL\_V4 | 1.0 | 0.0001794 | 134 | 0.24 | 104 | 61 | 3 | 4 | 92 | 5 | 105 | Uncharacterized protein | Uncharacterized protein | | afdb-uniprot50 | AF-A0A1H3BTJ3-F1-MODEL\_V4 | 1.0 | 0.00646 | 133 | 0.229 | 61 | 47 | 0 | 1 | 61 | 1 | 61 | Uncharacterized protein | Uncharacterized protein | | afdb-uniprot50 | AF-A0A3C0F2T7-F1-MODEL\_V4 | 1.0 | 0.005313 | 133 | 0.2 | 60 | 46 | 2 | 2 | 61 | 16 | 73 | Uncharacterized protein | Uncharacterized protein | | afdb-uniprot50 | AF-A0A2D7VZD1-F1-MODEL\_V4 | 1.0 | 0.005313 | 133 | 0.237 | 59 | 39 | 1 | 4 | 56 | 12 | 70 | Uncharacterized protein | Uncharacterized protein | | afdb-uniprot50 | AF-A0A7V8FKE8-F1-MODEL\_V4 | 1.0 | 0.006053 | 133 | 0.311 | 61 | 39 | 1 | 3 | 60 | 2 | 62 | Uncharacterized protein | Uncharacterized protein | | afdb-uniprot50 | AF-A0A165R271-F1-MODEL\_V4 | 1.0 | 0.008384 | 132 | 0.245 | 61 | 46 | 0 | 1 | 61 | 1 | 61 | Uncharacterized protein | Uncharacterized protein | | afdb-uniprot50 | AF-A0A0G4Q915-F1-MODEL\_V4 | 1.0 | 0.00646 | 132 | 0.218 | 64 | 47 | 1 | 1 | 61 | 6 | 69 | Uncharacterized protein | Uncharacterized protein | | afdb-uniprot50 | AF-A0A2W1W9H2-F1-MODEL\_V4 | 1.0 | 0.0004185 | 132 | 0.278 | 97 | 64 | 4 | 5 | 97 | 2 | 96 | Uncharacterized protein | Uncharacterized protein | | afdb-uniprot50 | AF-A0A2D7TS63-F1-MODEL\_V4 | 1.0 | 0.00437 | 132 | 0.215 | 65 | 43 | 2 | 1 | 65 | 103 | 159 | Uncharacterized protein | Uncharacterized protein | | afdb-uniprot50 | AF-A0A0C1INV0-F1-MODEL\_V4 | 1.0 | 0.008948 | 131 | 0.25 | 56 | 41 | 1 | 2 | 56 | 7 | 62 | Uncharacterized protein | Uncharacterized protein | | afdb-uniprot50 | AF-A0A119CYA6-F1-MODEL\_V4 | 1.0 | 0.008384 | 131 | 0.258 | 62 | 43 | 1 | 3 | 61 | 2 | 63 | Uncharacterized protein | Uncharacterized protein | | afdb-uniprot50 | AF-A0A1L3I567-F1-MODEL\_V4 | 1.0 | 0.005313 | 131 | 0.275 | 58 | 42 | 0 | 4 | 61 | 9 | 66 | Uncharacterized protein | Uncharacterized protein | | afdb-uniprot50 | AF-A0A1Q8Q264-F1-MODEL\_V4 | 1.0 | 0.0002652 | 131 | 0.229 | 96 | 64 | 3 | 5 | 94 | 2 | 93 | Uncharacterized protein | Uncharacterized protein | | afdb-uniprot50 | AF-A0A2N8HQH7-F1-MODEL\_V4 | 1.0 | 0.0001295 | 131 | 0.226 | 97 | 67 | 3 | 5 | 93 | 2 | 98 | Rho\_N domain-containing protein | Rho\_N domain-containing protein | | afdb-uniprot50 | AF-A0A496KBK1-F1-MODEL\_V4 | 1.0 | 5.202e-05 | 131 | 0.213 | 117 | 67 | 5 | 5 | 99 | 2 | 115 | Uncharacterized protein | Uncharacterized protein | | afdb-uniprot50 | AF-A0A4P8S989-F1-MODEL\_V4 | 1.0 | 0.0001137 | 130 | 0.225 | 102 | 70 | 4 | 3 | 99 | 1 | 98 | Uncharacterized protein | Uncharacterized protein | | afdb-uniprot50 | AF-A0A1Q3R4D4-F1-MODEL\_V4 | 1.0 | 0.008948 | 129 | 0.169 | 59 | 49 | 0 | 3 | 61 | 5 | 63 | Uncharacterized protein | Uncharacterized protein | | afdb-uniprot50 | AF-A0A175RVJ5-F1-MODEL\_V4 | 1.0 | 0.001443 | 127 | 0.166 | 96 | 78 | 2 | 3 | 96 | 1 | 96 | Rho\_N domain-containing protein | Rho\_N domain-containing protein | | afdb-uniprot50 | AF-A0A841TSU2-F1-MODEL\_V4 | 1.0 | 0.0002328 | 126 | 0.237 | 101 | 63 | 3 | 6 | 94 | 2 | 100 | Uncharacterized protein | Uncharacterized protein | | afdb-uniprot50 | AF-A0A4Y8UXJ0-F1-MODEL\_V4 | 1.0 | 0.0005431 | 125 | 0.247 | 97 | 65 | 3 | 6 | 98 | 2 | 94 | SAP domain-containing protein | SAP domain-containing protein | | afdb-uniprot50 | AF-D5SZE1-F1-MODEL\_V4 | 1.0 | 0.005313 | 125 | 0.241 | 58 | 38 | 1 | 4 | 55 | 6 | 63 | Uncharacterized protein | Uncharacterized protein | | afdb-uniprot50 | AF-A0A0K9MD59-F1-MODEL\_V4 | 1.0 | 0.0009763 | 125 | 0.272 | 99 | 68 | 2 | 6 | 100 | 2 | 100 | Rho\_N domain-containing protein | Rho\_N domain-containing protein | | afdb-uniprot50 | AF-A0A2K1EF22-F1-MODEL\_V4 | 1.0 | 0.0005089 | 122 | 0.292 | 89 | 63 | 0 | 6 | 94 | 2 | 90 | Uncharacterized protein | Uncharacterized protein | | afdb-uniprot50 | AF-C4L0Q0-F1-MODEL\_V4 | 1.0 | 0.0006187 | 121 | 0.292 | 99 | 58 | 3 | 6 | 93 | 2 | 99 | Uncharacterized protein | Uncharacterized protein | | afdb-uniprot50 | AF-A0A098MGF8-F1-MODEL\_V4 | 1.0 | 0.0006187 | 119 | 0.214 | 98 | 76 | 1 | 4 | 100 | 8 | 105 | Uncharacterized protein | Uncharacterized protein | | afdb-uniprot50 | AF-A0A1H2ZIW5-F1-MODEL\_V4 | 1.0 | 0.007359 | 118 | 0.253 | 63 | 42 | 1 | 4 | 61 | 9 | 71 | Uncharacterized protein | Uncharacterized protein | | afdb-uniprot50 | AF-A0A2N1AP72-F1-MODEL\_V4 | 1.0 | 0.001644 | 117 | 0.376 | 85 | 48 | 2 | 14 | 93 | 1 | 85 | Rho\_N domain-containing protein | Rho\_N domain-containing protein | | afdb-uniprot50 | AF-A0A654DQC9-F1-MODEL\_V4 | 1.0 | 0.002278 | 117 | 0.213 | 89 | 66 | 2 | 5 | 93 | 2 | 86 | Uncharacterized protein | Uncharacterized protein | | afdb-uniprot50 | AF-A0A559IZJ5-F1-MODEL\_V4 | 1.0 | 0.002769 | 117 | 0.163 | 104 | 77 | 4 | 3 | 100 | 2 | 101 | Uncharacterized protein | Uncharacterized protein | | afdb-uniprot50 | AF-A0A1A9BP13-F1-MODEL\_V4 | 1.0 | 0.0009763 | 117 | 0.283 | 106 | 63 | 3 | 5 | 100 | 2 | 104 | Rho termination factor, N-terminal domain | Rho termination factor, N-terminal domain | | afdb-uniprot50 | AF-A0A5C0ZYA2-F1-MODEL\_V4 | 1.0 | 0.0003021 | 117 | 0.237 | 101 | 68 | 4 | 6 | 100 | 2 | 99 | Uncharacterized protein | Uncharacterized protein | | afdb-uniprot50 | AF-A0A2W4IJI6-F1-MODEL\_V4 | 1.0 | 0.000803 | 116 | 0.257 | 97 | 67 | 2 | 1 | 97 | 1 | 92 | Rho\_N domain-containing protein | Rho\_N domain-containing protein | | afdb-uniprot50 | AF-A0A6I2MAN7-F1-MODEL\_V4 | 1.0 | 0.001644 | 110 | 0.21 | 100 | 67 | 3 | 1 | 92 | 1 | 96 | Uncharacterized protein | Uncharacterized protein | | afdb-uniprot50 | AF-A0A2N0EX80-F1-MODEL\_V4 | 1.0 | 0.001755 | 109 | 0.148 | 101 | 80 | 3 | 1 | 99 | 1 | 97 | Uncharacterized protein | Uncharacterized protein | | afdb-uniprot50 | AF-A0A6A2ERZ4-F1-MODEL\_V4 | 1.0 | 0.001267 | 108 | 0.23 | 113 | 66 | 3 | 5 | 96 | 2 | 114 | Uncharacterized protein | Uncharacterized protein | | afdb-uniprot50 | AF-A0A1R1C583-F1-MODEL\_V4 | 1.0 | 0.001443 | 107 | 0.262 | 99 | 68 | 1 | 6 | 99 | 2 | 100 | Uncharacterized protein | Uncharacterized protein | | afdb-uniprot50 | AF-A0A1R0YQ56-F1-MODEL\_V4 | 1.0 | 0.000857 | 107 | 0.176 | 102 | 78 | 2 | 4 | 100 | 8 | 108 | Uncharacterized protein | Uncharacterized protein | | afdb-uniprot50 | AF-A0A150KSC3-F1-MODEL\_V4 | 1.0 | 0.003367 | 106 | 0.24 | 100 | 68 | 2 | 6 | 97 | 2 | 101 | Uncharacterized protein | Uncharacterized protein | | afdb-uniprot50 | AF-A0A1N7MP98-F1-MODEL\_V4 | 1.0 | 0.002956 | 104 | 0.208 | 120 | 73 | 6 | 2 | 99 | 7 | 126 | Rho termination factor, N-terminal domain | Rho termination factor, N-terminal domain | | afdb-uniprot50 | AF-A0A101JYR9-F1-MODEL\_V4 | 1.0 | 0.004978 | 104 | 0.146 | 130 | 71 | 4 | 3 | 98 | 1 | 124 | Uncharacterized protein | Uncharacterized protein | | afdb-uniprot50 | AF-A0A4R6MWN5-F1-MODEL\_V4 | 1.0 | 0.005313 | 102 | 0.215 | 93 | 68 | 3 | 3 | 92 | 1 | 91 | Uncharacterized protein | Uncharacterized protein | | afdb-uniprot50 | AF-A0A7D8AP78-F1-MODEL\_V4 | 1.0 | 0.006053 | 102 | 0.18 | 100 | 74 | 4 | 3 | 94 | 1 | 100 | Uncharacterized protein | Uncharacterized protein | | afdb-uniprot50 | AF-A0A838B6P4-F1-MODEL\_V4 | 1.0 | 0.003836 | 102 | 0.13 | 115 | 82 | 3 | 1 | 97 | 1 | 115 | Uncharacterized protein | Uncharacterized protein | | afdb-uniprot50 | AF-A0A2E2Q9J7-F1-MODEL\_V4 | 1.0 | 0.007359 | 102 | 0.225 | 93 | 63 | 4 | 6 | 92 | 2 | 91 | Uncharacterized protein | Uncharacterized protein | | afdb-uniprot50 | AF-K2KD66-F1-MODEL\_V4 | 1.0 | 0.002134 | 102 | 0.176 | 147 | 74 | 8 | 1 | 100 | 1 | 147 | Rho termination factor domain protein | Rho termination factor domain protein | | afdb-uniprot50 | AF-A0A656YVZ8-F1-MODEL\_V4 | 1.0 | 0.006053 | 102 | 0.17 | 82 | 65 | 2 | 2 | 81 | 27 | 107 | Uncharacterized protein | Uncharacterized protein | | afdb-uniprot50 | AF-A0A7U1GLP5-F1-MODEL\_V4 | 1.0 | 0.006895 | 101 | 0.15 | 100 | 82 | 2 | 4 | 100 | 3 | 102 | SAP domain-containing protein | SAP domain-containing protein | | afdb-uniprot50 | AF-A0A1V2IRA7-F1-MODEL\_V4 | 1.0 | 0.004094 | 101 | 0.19 | 110 | 71 | 2 | 6 | 99 | 2 | 109 | Uncharacterized protein | Uncharacterized protein | | afdb-uniprot50 | AF-A0A7C3DC32-F1-MODEL\_V4 | 1.0 | 0.003836 | 101 | 0.15 | 113 | 72 | 5 | 1 | 92 | 1 | 110 | Uncharacterized protein | Uncharacterized protein | | afdb-uniprot50 | AF-A0A5B0BSY5-F1-MODEL\_V4 | 0.999 | 0.009551 | 98 | 0.122 | 98 | 82 | 3 | 4 | 100 | 3 | 97 | Uncharacterized protein | Uncharacterized protein | | afdb-uniprot50 | AF-A0A7C3CLX3-F1-MODEL\_V4 | 0.999 | 0.002134 | 98 | 0.148 | 128 | 70 | 5 | 1 | 94 | 26 | 148 | Helix-hairpin-helix domain-containing protein | Helix-hairpin-helix domain-containing protein | | afdb-uniprot50 | AF-A0A7Z8B2W0-F1-MODEL\_V4 | 0.999 | 0.002769 | 98 | 0.21 | 138 | 64 | 6 | 1 | 93 | 5 | 142 | Rho\_N domain-containing protein | Rho\_N domain-containing protein | | afdb-uniprot50 | AF-A0A271M6K9-F1-MODEL\_V4 | 0.999 | 0.008384 | 97 | 0.198 | 116 | 70 | 4 | 6 | 100 | 2 | 115 | Uncharacterized protein | Uncharacterized protein | | afdb-uniprot50 | AF-A0A0Q3TXV1-F1-MODEL\_V4 | 0.999 | 0.008948 | 96 | 0.23 | 100 | 72 | 3 | 4 | 100 | 3 | 100 | Uncharacterized protein | Uncharacterized protein | | afdb-uniprot50 | AF-D2TCD3-F1-MODEL\_V4 | 0.999 | 0.007855 | 96 | 0.226 | 97 | 60 | 1 | 2 | 98 | 12 | 93 | Putative membrane protein ycf1 | Putative membrane protein ycf1 | | afdb-uniprot50 | AF-A0A5E9UEY1-F1-MODEL\_V4 | 0.998 | 0.005313 | 95 | 0.226 | 97 | 73 | 1 | 6 | 100 | 2 | 98 | Uncharacterized protein | Uncharacterized protein | | afdb-uniprot50 | AF-S3A3S2-F1-MODEL\_V4 | 0.998 | 0.007855 | 95 | 0.179 | 106 | 66 | 4 | 6 | 91 | 2 | 106 | Uncharacterized protein | Uncharacterized protein | | afdb-uniprot50 | AF-A0A0N1AA76-F1-MODEL\_V4 | 0.998 | 0.006053 | 93 | 0.257 | 97 | 60 | 5 | 6 | 93 | 2 | 95 | Uncharacterized protein | Uncharacterized protein | | afdb-uniprot50 | AF-A0A851HHB7-F1-MODEL\_V4 | 0.997 | 0.00437 | 92 | 0.173 | 121 | 61 | 5 | 6 | 92 | 2 | 117 | Uncharacterized protein | Uncharacterized protein | | afdb-uniprot50 | AF-A0A564G4X5-F1-MODEL\_V4 | 0.997 | 0.007855 | 92 | 0.159 | 119 | 79 | 3 | 1 | 98 | 1 | 119 | Uncharacterized protein | Uncharacterized protein | | afdb-uniprot50 | AF-A0A497KAL2-F1-MODEL\_V4 | 0.997 | 0.003594 | 92 | 0.218 | 110 | 67 | 6 | 2 | 95 | 209 | 315 | Uncharacterized protein | Uncharacterized protein | | afdb-uniprot50 | AF-A0A2X4RUV3-F1-MODEL\_V4 | 0.997 | 0.001873 | 91 | 0.22 | 109 | 62 | 6 | 1 | 88 | 1 | 107 | Uncharacterized protein | Uncharacterized protein | | afdb-uniprot50 | AF-A0A165T6X8-F1-MODEL\_V4 | 0.997 | 0.006895 | 91 | 0.21 | 114 | 67 | 4 | 2 | 96 | 3 | 112 | Uncharacterized protein | Uncharacterized protein | | afdb-uniprot50 | AF-A0A1A9G202-F1-MODEL\_V4 | 0.997 | 0.003836 | 91 | 0.221 | 113 | 73 | 3 | 1 | 98 | 1 | 113 | Uncharacterized protein | Uncharacterized protein | | afdb-uniprot50 | AF-A0A512J8Y4-F1-MODEL\_V4 | 0.996 | 0.00646 | 89 | 0.214 | 98 | 65 | 4 | 3 | 89 | 1 | 97 | Uncharacterized protein | Uncharacterized protein | | afdb-uniprot50 | AF-A0A1H2EPI0-F1-MODEL\_V4 | 0.993 | 0.005671 | 86 | 0.23 | 100 | 63 | 7 | 1 | 92 | 3 | 96 | Uncharacterized protein | Uncharacterized protein | | afdb-uniprot50 | AF-A0A1C6VDX7-F1-MODEL\_V4 | 0.992 | 0.006895 | 85 | 0.181 | 127 | 74 | 5 | 3 | 100 | 2 | 127 | Uncharacterized protein | Uncharacterized protein | | afdb-uniprot50 | AF-A0A1G8GDC6-F1-MODEL\_V4 | 0.991 | 0.003594 | 84 | 0.214 | 107 | 59 | 5 | 1 | 92 | 1 | 97 | Uncharacterized protein | Uncharacterized protein | |
| Top keywords  (threshold 1.00e-02 (evalue)) | **domain\_containing, Rho\_N, Rho, termination, factor, N\_terminal, SAP, factor\_like, containing, Contig\_80** |
| Output files | ../../similar\_structures/06\_FANPEZAQ\_CDS\_0006\_afdb-proteome\_foldseek.tsv ../../similar\_structures/06\_FANPEZAQ\_CDS\_0006\_afdb-uniprot50\_foldseek.tsv ../../similar\_structures/06\_FANPEZAQ\_CDS\_0006\_merged.svg ../../similar\_structures/06\_FANPEZAQ\_CDS\_0006\_pdb\_foldseek.tsv |

  
  
  

Return to summary | Go to previous | Go to next

  


---

**Sequence/structure alignments coloring**  
Each object in the alignment figures is colored according to its E-value following this color coding:

1e-100
10

**References:**  
1) Steinegger M, Meier M, Mirdita M, Vöhringer H, Haunsberger S J, and Söding J (2019) HH-suite3 for fast remote homology detection and deep protein annotation, BMC Bioinformatics, 473. doi: 10.1186/s12859-019-3019-7  
2) Jumper J, Evans R, Pritzel A, ..., Hassabis D (2021) Highly accurate protein structure prediction with AlphaFold, Nature, 596. doi: 10.1038/s41586-021-03819-2  
3) van Kempen M, Kim S, Tumescheit C, Mirdita M, Lee J, Gilchrist CLM, Söding J, and Steinegger M (2023) Fast and accurate protein structure search with Foldseek. Nature Biotechnology. doi: 10.1038/s41587-023-01773-0
