# Supplementary material for: Completing the BASEL phage collection to unlock hidden diversity for systematic exploration of phage–host interactions
Source: PLoS Biol. 2025 Apr 7;23(4):e3003063. doi: 10.1371/journal.pbio.3003063 (PMC11990801; doi:10.1371/journal.pbio.3003063)
Supplement: S2 Data — (ZIP) [file pbio.3003063.s009.zip › entries/61.html]

FANPEZAQ\_CDS\_0061


Return to summary | Go to previous | Go to next

|  |  |
| --- | --- |
| FANPEZAQ\_CDS\_0061 Page creation date: 02 Sep 2024, 12:00  Project folder: n/a  Input sequences file: Escherichia\_virus\_HeidiAbel.gb | mor domain\_containing transcription activator regulator transcriptional helix\_turn\_helix response dna hypothetical binding and hth regulatory dna\_binding operon homeodomain\_like rna regulation middle nucleotide metabolism system two\_component putative fragment conserved resolvase gol activator\_like factor quorum luxr\_type narl winged terminase head packaging repressor mse complex sensing fixj |

### Sequence information

|  |  |
| --- | --- |
| Name | FANPEZAQ\_CDS\_0061  61\_FANPEZAQ\_CDS\_0061 (pipeline id) |
| Imported annotations | Escherichia\_virus\_HeidiAbel Bas97 |
| Protein sequence | MELPRTVQEIADVIGRERALFLIGQLPKLWVPSQQYHKVILYVPKRIRPNDPLVQILGWQ DASKMVQHFGGEMLHPANCEYIYRHFIHRSIKRMHSEGMDAKAIAELLDVSERTVKRHCT DKPHKDTSPANDNTPQRLLTAMGQ |
| Number of residues | 144 |
| Molecular weight (Da) | 16644.11 |
| Output files | ../../query\_sequences/61\_FANPEZAQ\_CDS\_0061.fasta |

### Putative domain architecture and protein family

#### Search results (HHblits)1

|  |  |
| --- | --- |
| Domain family databases searched | Pfam, Ncbi-cd, Cath, Phrogs |
| Results, scheme(s)  (Top layers only; threshold 1.00e-03 (evalue)) | xml version="1.0" encoding="utf-8" standalone="no"?       2024-09-02T21:08:25.687149 image/svg+xml   Matplotlib v3.7.2, https://matplotlib.org/ |
| Results, table  (E-value ≤ 1.00e-03 (evalue)) | | db | id | prob | evalue | pvalue | score | cols | query | query\_len | template | template\_len | name | description | | --- | --- | --- | --- | --- | --- | --- | --- | --- | --- | --- | --- | --- | | pfam | PF08765 | 99.4 | 1.5e-17 | 3e-21 | 108.3 | 97 | (2, 124) | 144 | (2, 98) | 108 | Mor | Mor transcription activator family | | pfam | PF10654 | 97.0 | 5.3e-07 | 1e-10 | 58.2 | 54 | (84, 137) | 144 | (66, 119) | 126 | DUF2481 | Protein of unknown function (DUF2481) | | pfam | PF11242 | 96.7 | 1.8e-06 | 3.3e-10 | 49.9 | 37 | (88, 124) | 144 | (4, 40) | 64 | DUF2774 | Protein of unknown function (DUF2774) | | pfam | PF05344 | 95.9 | 2.7e-05 | 5.3e-09 | 42.6 | 39 | (89, 127) | 144 | (3, 41) | 62 | DUF746 | Domain of Unknown Function (DUF746) | | pfam | PF18010 | 93.7 | 0.00097 | 2e-07 | 29.4 | 22 | (97, 118) | 144 | (7, 28) | 29 | HTH\_49 | Cry35Ab1 HTH C-terminal domain | | phrogs | 836 | 99.9 | 9.6e-33 | 1.3e-36 | 203.8 | 115 | (1, 125) | 144 | (21, 137) | 149 | late transcriptional activator | late transcriptional activator; Category: transcription regulation; p206917 VI\_10124 | | phrogs | 532 | 99.4 | 7.8e-18 | 1e-21 | 120.7 | 76 | (51, 127) | 144 | (34, 109) | 132 | late transcriptional activator | late transcriptional activator; Category: transcription regulation; p318767 VI\_01623 | | phrogs | 4151 | 97.8 | 6.3e-09 | 7.8e-13 | 75.8 | 52 | (86, 137) | 144 | (8, 59) | 168 | DNA binding protein | DNA binding protein; Category: DNA, RNA and nucleotide metabolism; p284557 VI\_05952 | | phrogs | 517 | 97.4 | 8.2e-08 | 1e-11 | 64.9 | 44 | (80, 123) | 144 | (62, 105) | 107 | HTH DNA binding protein | HTH DNA binding protein; Category: DNA, RNA and nucleotide metabolism; MF668278\_p45 | | phrogs | 664 | 97.4 | 7.6e-08 | 1e-11 | 65.7 | 55 | (81, 135) | 144 | (55, 109) | 117 | DNA binding protein | DNA binding protein; Category: DNA, RNA and nucleotide metabolism; MF072690\_p140 | | phrogs | 6156 | 97.4 | 1.1e-07 | 1.4e-11 | 60.6 | 58 | (76, 133) | 144 | (11, 69) | 78 | endonuclease | endonuclease; Category: DNA, RNA and nucleotide metabolism; KU160668\_p36 | | phrogs | 20467 | 97.1 | 6.1e-07 | 7e-11 | 60.3 | 50 | (76, 126) | 144 | (66, 115) | 116 | HNH endonuclease | HNH endonuclease; Category: DNA, RNA and nucleotide metabolism; NC\_026590\_p30 | | phrogs | 7067 | 96.8 | 2e-06 | 2.3e-10 | 50.8 | 49 | (68, 121) | 144 | (5, 53) | 55 | terminase small subunit | terminase small subunit; Category: head and packaging; NC\_034627\_p52 | | phrogs | 6831 | 96.8 | 2.1e-06 | 2.5e-10 | 53.0 | 37 | (88, 124) | 144 | (5, 41) | 66 | transcriptional regulator | transcriptional regulator; Category: transcription regulation; p431926 VI\_12195 | | phrogs | 4494 | 96.8 | 2.2e-06 | 2.5e-10 | 55.5 | 52 | (87, 138) | 144 | (6, 57) | 88 | NA | NA; Category: unknown function; p409661 VI\_06907 | | phrogs | 3855 | 96.7 | 2.9e-06 | 3.6e-10 | 57.7 | 72 | (55, 126) | 144 | (35, 107) | 114 | HTH DNA binding protein | HTH DNA binding protein; Category: DNA, RNA and nucleotide metabolism; KY290952\_p65 | | phrogs | 5536 | 96.6 | 3.9e-06 | 4.8e-10 | 60.1 | 39 | (83, 121) | 144 | (5, 43) | 165 | NA | NA; Category: unknown function; p166286 VI\_04146 | | phrogs | 6223 | 96.3 | 1.4e-05 | 1.7e-09 | 50.5 | 35 | (95, 129) | 144 | (14, 48) | 77 | transcriptional repressor | transcriptional repressor; Category: transcription regulation; p192116 VI\_09192 | | phrogs | 1157 | 96.1 | 2.9e-05 | 3.6e-09 | 60.0 | 37 | (86, 122) | 144 | (5, 41) | 291 | transposase | transposase; Category: integration and excision; p237322 VI\_08188 | | phrogs | 1373 | 96.0 | 3.2e-05 | 4.2e-09 | 50.4 | 34 | (95, 128) | 144 | (43, 76) | 95 | transcriptional regulator | transcriptional regulator; Category: transcription regulation; NC\_005857\_p48 | | phrogs | 2308 | 96.0 | 3.9e-05 | 4.7e-09 | 54.0 | 41 | (83, 123) | 144 | (1, 41) | 151 | NA | NA; Category: unknown function; p149266 VI\_05209 | | phrogs | 11376 | 95.8 | 6.5e-05 | 7.6e-09 | 51.4 | 55 | (78, 132) | 144 | (38, 92) | 134 | NA | NA; Category: unknown function; p46013 VI\_01297 | | phrogs | 4779 | 95.7 | 8.1e-05 | 1e-08 | 46.9 | 30 | (95, 124) | 144 | (11, 40) | 78 | transcriptional regulator | transcriptional regulator; Category: transcription regulation; p225361 VI\_06249 | | phrogs | 7061 | 95.5 | 0.00012 | 1.5e-08 | 45.4 | 35 | (89, 123) | 144 | (38, 72) | 73 | HTH DNA binding protein | HTH DNA binding protein; Category: DNA, RNA and nucleotide metabolism; KU160668\_p38 | | phrogs | 15481 | 95.2 | 0.00025 | 2.7e-08 | 38.7 | 39 | (82, 120) | 144 | (3, 41) | 43 | NA | NA; Category: unknown function; KY683735\_p262 | | phrogs | 2538 | 95.1 | 0.00024 | 3.1e-08 | 47.5 | 35 | (90, 124) | 144 | (8, 42) | 113 | terminase small subunit | terminase small subunit; Category: head and packaging; p133796 VI\_05051 | | phrogs | 151 | 95.1 | 0.00025 | 3.4e-08 | 44.9 | 37 | (91, 127) | 144 | (15, 51) | 91 | transposase | transposase; Category: integration and excision; p268658 VI\_04551 | | phrogs | 7847 | 95.0 | 0.00031 | 3.6e-08 | 51.6 | 44 | (88, 131) | 144 | (7, 50) | 247 | NA | NA; Category: unknown function; p386826 VI\_07743 | | phrogs | 6119 | 95.0 | 0.00035 | 3.9e-08 | 43.8 | 64 | (61, 124) | 144 | (19, 87) | 94 | NA | NA; Category: unknown function; p232261 VI\_06268 | | phrogs | 1381 | 95.0 | 0.00032 | 4e-08 | 45.9 | 34 | (98, 131) | 144 | (19, 52) | 99 | plasmid antitoxin with HTH domain | plasmid antitoxin with HTH domain; Category: moron, auxiliary metabolic gene and host takeover; p79485 VI\_11488 | | phrogs | 12823 | 94.9 | 0.00039 | 4.4e-08 | 46.1 | 57 | (87, 143) | 144 | (17, 75) | 117 | NA | NA; Category: unknown function; MF919537\_p145 | | phrogs | 1337 | 94.9 | 0.00037 | 4.7e-08 | 41.7 | 33 | (94, 126) | 144 | (11, 43) | 61 | DNA binding protein | DNA binding protein; Category: DNA, RNA and nucleotide metabolism; p177899 VI\_00920 | | phrogs | 25961 | 94.9 | 0.00042 | 4.7e-08 | 40.9 | 34 | (49, 82) | 144 | (32, 65) | 66 | NA | NA; Category: unknown function; p32746 VI\_10942 | | phrogs | 5751 | 94.9 | 0.0004 | 4.7e-08 | 48.9 | 45 | (88, 132) | 144 | (14, 58) | 164 | terminase large subunit | terminase large subunit; Category: head and packaging; p273217 VI\_06917 | | phrogs | 1468 | 94.8 | 0.00039 | 4.8e-08 | 42.7 | 37 | (94, 130) | 144 | (19, 55) | 69 | transcriptional repressor | transcriptional repressor; Category: transcription regulation; p307668 VI\_06058 | | phrogs | 27475 | 94.8 | 0.00047 | 5.3e-08 | 54.9 | 49 | (84, 132) | 144 | (3, 51) | 570 | NA | NA; Category: unknown function; p89643 VI\_08714 | | phrogs | 4291 | 94.6 | 0.00055 | 6.5e-08 | 58.2 | 70 | (50, 119) | 144 | (833, 905) | 906 | RNA-dependent RNA polymerase | RNA-dependent RNA polymerase; Category: DNA, RNA and nucleotide metabolism; p46481 VI\_01201 | | phrogs | 11138 | 94.6 | 0.00053 | 6.5e-08 | 46.7 | 58 | (57, 125) | 144 | (67, 124) | 129 | NA | NA; Category: unknown function; p272681 VI\_04059 | | phrogs | 323 | 94.5 | 0.00055 | 7.5e-08 | 41.9 | 47 | (82, 129) | 144 | (6, 52) | 78 | transcriptional regulator, D5-like | transcriptional regulator, D5-like; Category: transcription regulation; p118890 VI\_11329 | | phrogs | 7676 | 94.4 | 0.00077 | 9e-08 | 41.5 | 31 | (95, 125) | 144 | (13, 43) | 75 | NA | NA; Category: unknown function; NC\_023688\_p227 | | phrogs | 59 | 94.2 | 0.00079 | 1.1e-07 | 46.4 | 48 | (78, 125) | 144 | (111, 159) | 161 | HNH endonuclease | HNH endonuclease; Category: DNA, RNA and nucleotide metabolism; MG812495\_p46 | | phrogs | 609 | 94.2 | 0.00091 | 1.1e-07 | 53.3 | 43 | (83, 125) | 144 | (6, 48) | 405 | RIIB lysis inhibitor | RIIB lysis inhibitor; Category: lysis; MG696114\_p269 | | phrogs | 2104 | 94.2 | 0.00092 | 1.1e-07 | 43.1 | 49 | (81, 131) | 144 | (28, 76) | 92 | NA | NA; Category: unknown function; p72070 VI\_03178 | | phrogs | 1206 | 94.1 | 0.001 | 1.3e-07 | 48.8 | 46 | (85, 130) | 144 | (16, 62) | 212 | terminase small subunit | terminase small subunit; Category: head and packaging; p287057 VI\_06574 | | phrogs | 309 | 94.1 | 0.00094 | 1.3e-07 | 46.1 | 39 | (84, 122) | 144 | (9, 47) | 153 | terminase small subunit | terminase small subunit; Category: head and packaging; p301521 VI\_00938 | |
| Top keywords  (threshold 1.00e-03 (evalue)) | **and, DNA, RNA, nucleotide, metabolism, transcription, transcriptional, regulation, binding, HTH** |
| Output files | ../../domain\_architecture/61\_FANPEZAQ\_CDS\_0061\_cath.hhr ../../domain\_architecture/61\_FANPEZAQ\_CDS\_0061\_merged.svg ../../domain\_architecture/61\_FANPEZAQ\_CDS\_0061\_ncbi-cd.hhr ../../domain\_architecture/61\_FANPEZAQ\_CDS\_0061\_pfam.hhr ../../domain\_architecture/61\_FANPEZAQ\_CDS\_0061\_phrogs.hhr |

### Identical protein sequences/structures

#### Search results

|  |  |
| --- | --- |
| Protein sequence databases searched | Pdb, Swissprot, Refseq |
| Identical proteins found | -- |
| Top keywords | -- |
| Output files | -- |

### Similar protein sequences/structures

#### Sequence similarity search results (HHblits)1

|  |  |
| --- | --- |
| Sequence databases searched | Uniclust, Pdb70 |
| Results, scheme(s)  (Top layers only, threshold 1.00e-03 (evalue)) | xml version="1.0" encoding="utf-8" standalone="no"?       2024-09-02T21:08:54.119180 image/svg+xml   Matplotlib v3.7.2, https://matplotlib.org/ |
| Results, table(s)  (threshold 1.00e-03 (evalue)) | | db | id | prob | evalue | pvalue | score | cols | query | query\_len | template | template\_len | name | description | | --- | --- | --- | --- | --- | --- | --- | --- | --- | --- | --- | --- | --- | | uniclust | UniRef100\_A0A0F7KCJ2 | 99.9 | 1.5e-31 | 3.3e-37 | 197.2 | 110 | (2, 121) | 144 | (60, 169) | 196 | Mor family transcriptional regulator | Mor family transcriptional regulator | | uniclust | UniRef100\_A0A0J6SFM2 | 99.9 | 8e-31 | 1.8e-36 | 190.2 | 114 | (2, 125) | 144 | (26, 141) | 164 | Mor transcription activator domain-containing protein | Mor transcription activator domain-containing protein | | uniclust | UniRef100\_A0A143DDS5 | 99.9 | 9.2e-31 | 1.9e-36 | 190.1 | 135 | (1, 135) | 144 | (25, 161) | 197 | Mor transcription activator domain-containing protein | Mor transcription activator domain-containing protein | | uniclust | UniRef100\_A0A088FQJ8 | 99.9 | 2.1e-30 | 4.3e-36 | 185.2 | 144 | (1, 144) | 144 | (21, 167) | 169 | Uncharacterized protein | Uncharacterized protein | | uniclust | UniRef100\_A0A1E7HXZ1 | 99.9 | 1.8e-29 | 3.9e-35 | 179.1 | 108 | (3, 122) | 144 | (17, 124) | 146 | Mor transcription activator domain-containing protein | Mor transcription activator domain-containing protein | | uniclust | UniRef100\_A0A075CDW0 | 99.9 | 1.1e-28 | 2.4e-34 | 182.4 | 112 | (2, 123) | 144 | (33, 157) | 191 | Mor transcription activator domain-containing protein | Mor transcription activator domain-containing protein | | uniclust | UniRef100\_A0A011TVI4 | 99.9 | 1.6e-28 | 3.5e-34 | 174.3 | 111 | (2, 122) | 144 | (15, 128) | 145 | HTH luxR-type domain-containing protein | HTH luxR-type domain-containing protein | | uniclust | UniRef100\_A0A212KJX9 | 99.9 | 3e-28 | 6.4e-34 | 174.7 | 116 | (2, 127) | 144 | (25, 143) | 157 | Mor transcription activator domain-containing protein | Mor transcription activator domain-containing protein | | uniclust | UniRef100\_A0A0D8KFG8 | 99.9 | 9.3e-27 | 1.9e-32 | 164.2 | 110 | (2, 123) | 144 | (23, 132) | 148 | Mor transcription activator domain-containing protein | Mor transcription activator domain-containing protein | | uniclust | UniRef100\_A0A0X8JK61 | 99.9 | 1.3e-26 | 2.7e-32 | 166.7 | 115 | (2, 126) | 144 | (36, 163) | 174 | Mor transcription activator domain-containing protein | Mor transcription activator domain-containing protein | | uniclust | UniRef100\_A0A081B6E8 | 99.9 | 1.4e-26 | 3e-32 | 165.4 | 110 | (2, 123) | 144 | (11, 120) | 147 | Mor, transcription regulation | Mor, transcription regulation | | uniclust | UniRef100\_A0A0W1G863 | 99.9 | 1.8e-26 | 3.9e-32 | 162.7 | 106 | (5, 125) | 144 | (18, 124) | 140 | Mor transcription activator domain-containing protein | Mor transcription activator domain-containing protein | | uniclust | UniRef100\_A0A063BCU2 | 99.9 | 4.6e-26 | 9.9e-32 | 165.8 | 112 | (2, 123) | 144 | (26, 149) | 176 | Mor transcription activator domain protein | Mor transcription activator domain protein | | uniclust | UniRef100\_A0A0J7J645 | 99.9 | 1.5e-25 | 2.9e-31 | 153.7 | 115 | (2, 126) | 144 | (9, 123) | 135 | Mor transcription activator family | Mor transcription activator family | | uniclust | UniRef100\_A0A070A9J5 | 99.8 | 6.1e-25 | 1.3e-30 | 157.0 | 104 | (2, 123) | 144 | (18, 121) | 148 | Mor transcription activator domain-containing protein | Mor transcription activator domain-containing protein | | uniclust | UniRef100\_A0A0D5LXL7 | 99.8 | 6.4e-25 | 1.4e-30 | 156.7 | 106 | (3, 125) | 144 | (17, 122) | 147 | Mor transcription activator domain-containing protein | Mor transcription activator domain-containing protein | | uniclust | UniRef100\_A0A077FBH5 | 99.8 | 8.3e-25 | 1.7e-30 | 160.7 | 114 | (2, 126) | 144 | (47, 160) | 195 | Mor transcription activator domain-containing protein | Mor transcription activator domain-containing protein | | uniclust | UniRef100\_A0A0Q3EQP9 | 99.8 | 8.9e-25 | 1.9e-30 | 155.0 | 112 | (2, 123) | 144 | (8, 119) | 151 | Mor transcription activator domain-containing protein | Mor transcription activator domain-containing protein | | uniclust | UniRef100\_A0A0Q7I488 | 99.8 | 1.7e-24 | 3.5e-30 | 153.0 | 109 | (2, 120) | 144 | (13, 128) | 147 | Mor transcription activator domain-containing protein | Mor transcription activator domain-containing protein | | uniclust | UniRef100\_A0A1H8FWD2 | 99.8 | 2e-24 | 4.1e-30 | 150.6 | 112 | (3, 125) | 144 | (19, 130) | 138 | Mor transcription activator family protein | Mor transcription activator family protein | | uniclust | UniRef100\_A0A6P1ZJ74 | 99.8 | 2.4e-24 | 4.7e-30 | 151.1 | 125 | (2, 136) | 144 | (32, 156) | 161 | Mor transcription activator domain-containing protein | Mor transcription activator domain-containing protein | | uniclust | UniRef100\_A0A106BHZ3 | 99.8 | 2.8e-24 | 5.9e-30 | 154.3 | 110 | (3, 122) | 144 | (7, 116) | 156 | Mor transcription activator domain-containing protein | Mor transcription activator domain-containing protein | | uniclust | UniRef100\_A0A0N7I1N9 | 99.8 | 4e-24 | 8.3e-30 | 153.4 | 114 | (1, 124) | 144 | (21, 138) | 164 | Two-component system, NarL family, nitrate/nitrite response regulator NarP | Two-component system, NarL family, nitrate/nitrite response regulator NarP | | uniclust | UniRef100\_A0A0E4G398 | 99.8 | 4.4e-24 | 8.9e-30 | 148.4 | 109 | (3, 123) | 144 | (11, 123) | 141 | Mor transcription activator domain-containing protein | Mor transcription activator domain-containing protein | | uniclust | UniRef100\_A0A5E4RYD4 | 99.8 | 2.7e-23 | 5.3e-29 | 141.6 | 111 | (2, 122) | 144 | (11, 121) | 132 | Mor transcription activator domain-containing protein | Mor transcription activator domain-containing protein | | uniclust | UniRef100\_A0A077P2T4 | 99.8 | 2.7e-23 | 5.8e-29 | 154.9 | 113 | (2, 124) | 144 | (42, 167) | 200 | Mor transcription activator domain-containing protein | Mor transcription activator domain-containing protein | | uniclust | UniRef100\_A0A1G0CQ82 | 99.8 | 2.9e-23 | 5.9e-29 | 148.5 | 113 | (1, 123) | 144 | (28, 150) | 166 | Mor transcription activator domain-containing protein | Mor transcription activator domain-containing protein | | uniclust | UniRef100\_A0A0A8F3J8 | 99.8 | 6.6e-23 | 1.4e-28 | 147.5 | 115 | (2, 126) | 144 | (24, 149) | 167 | Mor transcription activator domain-containing protein | Mor transcription activator domain-containing protein | | uniclust | UniRef100\_A0A011P6X2 | 99.8 | 8.8e-23 | 1.8e-28 | 149.5 | 116 | (2, 127) | 144 | (37, 166) | 183 | Mor transcription activator family protein | Mor transcription activator family protein | | uniclust | UniRef100\_A0A1Y6D0G8 | 99.8 | 2.6e-22 | 5.1e-28 | 140.4 | 112 | (2, 123) | 144 | (14, 127) | 145 | Mor transcription activator family protein | Mor transcription activator family protein | | uniclust | UniRef100\_A0A2G2I3T3 | 99.8 | 2.5e-22 | 5.3e-28 | 145.6 | 112 | (2, 124) | 144 | (19, 130) | 166 | Mor transcription activator domain-containing protein | Mor transcription activator domain-containing protein | | uniclust | UniRef100\_A0A2N1ANB5 | 99.8 | 6.3e-22 | 1.2e-27 | 140.7 | 117 | (1, 121) | 144 | (1, 119) | 174 | Mor transcription activator domain-containing protein | Mor transcription activator domain-containing protein | | uniclust | UniRef100\_A0A0H2MCL8 | 99.8 | 1e-21 | 1.9e-27 | 138.0 | 112 | (2, 123) | 144 | (14, 125) | 163 | Mor transcription activator family protein | Mor transcription activator family protein | | uniclust | UniRef100\_A0A4D7B5F0 | 99.8 | 9.6e-22 | 2e-27 | 142.3 | 103 | (2, 116) | 144 | (23, 125) | 173 | Helix-turn-helix domain-containing protein | Helix-turn-helix domain-containing protein | | uniclust | UniRef100\_A0A4D7DAD3 | 99.8 | 1e-21 | 2.1e-27 | 136.8 | 104 | (6, 124) | 144 | (14, 117) | 135 | Mor transcription activator domain-containing protein | Mor transcription activator domain-containing protein | | uniclust | UniRef100\_A0A0B4BIH4 | 99.7 | 1.3e-21 | 2.6e-27 | 145.0 | 118 | (2, 122) | 144 | (75, 192) | 225 | Uncharacterized protein | Uncharacterized protein | | uniclust | UniRef100\_A0A0M7AQF8 | 99.7 | 1.9e-21 | 3.9e-27 | 140.4 | 110 | (2, 121) | 144 | (31, 150) | 176 | Uncharacterized protein | Uncharacterized protein | | uniclust | UniRef100\_A0A009ZMY9 | 99.7 | 3.7e-21 | 7.3e-27 | 134.1 | 116 | (2, 127) | 144 | (9, 130) | 146 | Mor transcription activator family protein | Mor transcription activator family protein | | uniclust | UniRef100\_A0A2T5J3R6 | 99.7 | 6.6e-21 | 1.3e-26 | 132.7 | 110 | (5, 124) | 144 | (23, 139) | 149 | Mor transcription activator family protein | Mor transcription activator family protein | | uniclust | UniRef100\_A0A7D7IUH2 | 99.7 | 7.3e-21 | 1.4e-26 | 131.8 | 134 | (7, 140) | 144 | (1, 138) | 151 | Mor transcription activator domain-containing protein | Mor transcription activator domain-containing protein | | uniclust | UniRef100\_A0A4Q5L708 | 99.7 | 7.5e-21 | 1.4e-26 | 131.8 | 115 | (2, 126) | 144 | (33, 147) | 154 | Mor transcription activator domain-containing protein | Mor transcription activator domain-containing protein | | uniclust | UniRef100\_A0A1H9CJ13 | 99.7 | 7.3e-21 | 1.4e-26 | 130.9 | 105 | (5, 121) | 144 | (8, 112) | 136 | Mor transcription activator family protein | Mor transcription activator family protein | | uniclust | UniRef100\_A0A350X8X0 | 99.7 | 1.2e-20 | 2.3e-26 | 131.1 | 113 | (4, 127) | 144 | (24, 136) | 144 | Mor transcription activator domain-containing protein | Mor transcription activator domain-containing protein | | uniclust | UniRef100\_UPI000400307B | 99.7 | 1.9e-20 | 3.5e-26 | 129.2 | 114 | (2, 125) | 144 | (24, 138) | 163 | Mor transcription activator family protein | Mor transcription activator family protein | | uniclust | UniRef100\_A0A241VCE8 | 99.7 | 2.1e-20 | 4.2e-26 | 129.8 | 122 | (2, 123) | 144 | (6, 131) | 136 | Mor transcription activator domain-containing protein | Mor transcription activator domain-containing protein | | uniclust | UniRef100\_A0A0D8CPA5 | 99.7 | 2.1e-20 | 4.3e-26 | 130.4 | 105 | (8, 125) | 144 | (20, 124) | 134 | Mor transcription activator domain-containing protein | Mor transcription activator domain-containing protein | | uniclust | UniRef100\_A0A845TMQ7 | 99.7 | 4.6e-20 | 8.7e-26 | 125.3 | 118 | (1, 128) | 144 | (1, 125) | 131 | Mor transcription activator domain-containing protein | Mor transcription activator domain-containing protein | | uniclust | UniRef100\_A0A1D9CY07 | 99.7 | 5.5e-20 | 1.1e-25 | 127.7 | 111 | (4, 124) | 144 | (7, 119) | 140 | Putative bacteriophage transcriptional regulator | Putative bacteriophage transcriptional regulator | | uniclust | UniRef100\_A0A1S0V4H7 | 99.7 | 7e-20 | 1.3e-25 | 131.4 | 124 | (1, 124) | 144 | (10, 141) | 186 | Mor transcription activator domain-containing protein (Fragment) | Mor transcription activator domain-containing protein (Fragment) | | uniclust | UniRef100\_A0A2S5CLI8 | 99.7 | 7.7e-20 | 1.5e-25 | 125.9 | 116 | (2, 127) | 144 | (11, 126) | 141 | Mor transcription activator domain-containing protein | Mor transcription activator domain-containing protein | | uniclust | UniRef100\_A0A258P502 | 99.7 | 9.4e-20 | 1.8e-25 | 129.8 | 110 | (2, 121) | 144 | (57, 170) | 173 | Mor transcription activator domain-containing protein | Mor transcription activator domain-containing protein | | uniclust | UniRef100\_A0A0H2X8T4 | 99.7 | 9.8e-20 | 1.9e-25 | 130.2 | 123 | (2, 125) | 144 | (17, 147) | 166 | Mor transcription activator domain-containing protein | Mor transcription activator domain-containing protein | | uniclust | UniRef100\_A0A062GXL3 | 99.7 | 1.2e-19 | 2.2e-25 | 127.5 | 113 | (2, 124) | 144 | (22, 136) | 154 | CENP-B N-terminal DNA-binding domain protein | CENP-B N-terminal DNA-binding domain protein | | uniclust | UniRef100\_A0A0F2N9K9 | 99.7 | 1.8e-19 | 3.7e-25 | 128.5 | 107 | (8, 125) | 144 | (15, 121) | 149 | Mor transcription activator domain-containing protein | Mor transcription activator domain-containing protein | | uniclust | UniRef100\_A0A401JFQ9 | 99.7 | 2.2e-19 | 4e-25 | 120.5 | 108 | (5, 122) | 144 | (24, 131) | 133 | Mor transcription activator domain-containing protein | Mor transcription activator domain-containing protein | | uniclust | UniRef100\_A0A2E3ES45 | 99.6 | 2.4e-19 | 4.6e-25 | 122.4 | 112 | (2, 125) | 144 | (5, 116) | 126 | Mor transcription activator domain-containing protein | Mor transcription activator domain-containing protein | | uniclust | UniRef100\_A0A090DT46 | 99.6 | 2.7e-19 | 5.4e-25 | 128.3 | 102 | (6, 125) | 144 | (23, 124) | 159 | Mor transcription activator domain-containing protein | Mor transcription activator domain-containing protein | | uniclust | UniRef100\_B2FRB5 | 99.6 | 3.3e-19 | 6e-25 | 120.9 | 130 | (2, 131) | 144 | (3, 132) | 142 | Mor transcription activator domain-containing protein | Mor transcription activator domain-containing protein | | uniclust | UniRef100\_A0A1G4ZYZ4 | 99.6 | 3.8e-19 | 7.1e-25 | 125.8 | 111 | (2, 122) | 144 | (58, 172) | 175 | Mor transcription activator family protein | Mor transcription activator family protein | | uniclust | UniRef100\_UPI000F8E7E3B | 99.6 | 4.9e-19 | 9e-25 | 121.2 | 130 | (2, 134) | 144 | (5, 134) | 151 | hypothetical protein | hypothetical protein | | uniclust | UniRef100\_A0A6I2UT81 | 99.6 | 4.9e-19 | 9.3e-25 | 127.7 | 117 | (2, 121) | 144 | (81, 197) | 202 | Helix-turn-helix domain-containing protein | Helix-turn-helix domain-containing protein | | uniclust | UniRef100\_A0A060H6I6 | 99.6 | 5.2e-19 | 9.5e-25 | 127.8 | 121 | (2, 122) | 144 | (50, 170) | 219 | Uncharacterized protein | Uncharacterized protein | | uniclust | UniRef100\_A0A5C7QTA1 | 99.6 | 6.8e-19 | 1.3e-24 | 122.6 | 119 | (2, 131) | 144 | (18, 138) | 143 | Mor transcription activator domain-containing protein | Mor transcription activator domain-containing protein | | uniclust | UniRef100\_A0A522VJQ9 | 99.6 | 7.7e-19 | 1.4e-24 | 121.4 | 112 | (2, 123) | 144 | (44, 156) | 161 | Mor transcription activator domain-containing protein | Mor transcription activator domain-containing protein | | uniclust | UniRef100\_A0A109W8W4 | 99.6 | 7e-19 | 1.4e-24 | 132.1 | 113 | (2, 124) | 144 | (61, 184) | 215 | Mor transcription activator domain-containing protein | Mor transcription activator domain-containing protein | | uniclust | UniRef100\_A0A8T3KWY8 | 99.6 | 8.8e-19 | 1.6e-24 | 121.6 | 120 | (2, 131) | 144 | (28, 152) | 166 | Helix-turn-helix domain-containing protein | Helix-turn-helix domain-containing protein | | uniclust | UniRef100\_A0A5C7Q033 | 99.6 | 3.1e-18 | 6.1e-24 | 120.7 | 113 | (5, 126) | 144 | (6, 118) | 156 | Winged helix-turn-helix transcriptional regulator | Winged helix-turn-helix transcriptional regulator | | uniclust | UniRef100\_A0A068Z4A5 | 99.6 | 3.4e-18 | 6.7e-24 | 122.8 | 113 | (2, 124) | 144 | (18, 144) | 168 | Mor transcription activator domain-containing protein | Mor transcription activator domain-containing protein | | uniclust | UniRef100\_I3YBF0 | 99.6 | 4e-18 | 7.5e-24 | 121.2 | 113 | (2, 125) | 144 | (30, 142) | 184 | Mor transcription activator-like protein | Mor transcription activator-like protein | | uniclust | UniRef100\_A0A6N4DE39 | 99.6 | 4e-18 | 7.8e-24 | 116.2 | 108 | (2, 123) | 144 | (6, 113) | 123 | Mor transcription activator domain-containing protein | Mor transcription activator domain-containing protein | | uniclust | UniRef100\_A0A1Z4C0F4 | 99.6 | 4.2e-18 | 7.9e-24 | 120.2 | 114 | (2, 125) | 144 | (35, 148) | 169 | Mor transcription activator domain-containing protein | Mor transcription activator domain-containing protein | | uniclust | UniRef100\_A0A961ZJA7 | 99.6 | 4.5e-18 | 8.3e-24 | 118.4 | 111 | (2, 122) | 144 | (25, 138) | 168 | Uncharacterized protein | Uncharacterized protein | | uniclust | UniRef100\_A0A2K9NFC4 | 99.6 | 6.3e-18 | 1.2e-23 | 116.7 | 106 | (3, 123) | 144 | (22, 127) | 137 | Mor transcription activator domain-containing protein | Mor transcription activator domain-containing protein | | uniclust | UniRef100\_A0A0C1G7F9 | 99.6 | 6.6e-18 | 1.3e-23 | 120.0 | 112 | (2, 126) | 144 | (9, 120) | 148 | Mor transcription activator domain-containing protein | Mor transcription activator domain-containing protein | | uniclust | UniRef100\_UPI001D0F25A5 | 99.6 | 8.4e-18 | 1.5e-23 | 115.8 | 111 | (2, 122) | 144 | (42, 152) | 155 | Mor transcription activator family protein | Mor transcription activator family protein | | uniclust | UniRef100\_A0A081NY94 | 99.6 | 8.3e-18 | 1.8e-23 | 117.8 | 77 | (47, 124) | 144 | (22, 98) | 125 | Mor transcription activator domain-containing protein | Mor transcription activator domain-containing protein | | uniclust | UniRef100\_A0A139DN11 | 99.6 | 9.2e-18 | 1.8e-23 | 122.0 | 122 | (6, 138) | 144 | (34, 155) | 181 | Mor transcription activator domain-containing protein | Mor transcription activator domain-containing protein | | uniclust | UniRef100\_A0A0B3S273 | 99.5 | 1.6e-17 | 3.3e-23 | 119.4 | 100 | (3, 119) | 144 | (27, 126) | 152 | Mor transcription activator domain-containing protein | Mor transcription activator domain-containing protein | | uniclust | UniRef100\_A0A0P1FJJ4 | 99.5 | 1.7e-17 | 3.7e-23 | 119.9 | 104 | (3, 123) | 144 | (23, 126) | 150 | Helix-turn-helix domain-containing protein | Helix-turn-helix domain-containing protein | | uniclust | UniRef100\_A0A3A6NWI4 | 99.5 | 2.1e-17 | 3.9e-23 | 113.8 | 113 | (2, 124) | 144 | (16, 133) | 154 | Mor transcription activator domain-containing protein | Mor transcription activator domain-containing protein | | uniclust | UniRef100\_A0A0A2TST3 | 99.5 | 1.9e-17 | 4.1e-23 | 117.7 | 80 | (47, 127) | 144 | (28, 107) | 133 | Mor transcription activator domain-containing protein | Mor transcription activator domain-containing protein | | uniclust | UniRef100\_A0A929AAA7 | 99.5 | 2.4e-17 | 4.4e-23 | 110.3 | 105 | (5, 121) | 144 | (21, 125) | 127 | Mor transcription activator domain-containing protein | Mor transcription activator domain-containing protein | | uniclust | UniRef100\_A0A017RUI4 | 99.5 | 2.3e-17 | 4.9e-23 | 116.9 | 75 | (48, 123) | 144 | (26, 100) | 130 | Mor transcription activator domain-containing protein | Mor transcription activator domain-containing protein | | uniclust | UniRef100\_A0A1V4WIM0 | 99.5 | 3e-17 | 6.2e-23 | 113.3 | 80 | (47, 127) | 144 | (25, 104) | 118 | Mor transcription activator family protein | Mor transcription activator family protein | | uniclust | UniRef100\_A0A1E3H4F5 | 99.5 | 4e-17 | 7.9e-23 | 118.9 | 111 | (2, 125) | 144 | (23, 135) | 183 | Mor transcription activator family protein | Mor transcription activator family protein | | uniclust | UniRef100\_A0A1Z3U8U4 | 99.5 | 4.1e-17 | 8e-23 | 113.7 | 105 | (2, 116) | 144 | (11, 117) | 140 | Uncharacterized protein | Uncharacterized protein | | uniclust | UniRef100\_A0A0U3JVX5 | 99.5 | 4.3e-17 | 8.7e-23 | 124.2 | 121 | (2, 132) | 144 | (106, 239) | 244 | Uncharacterized protein | Uncharacterized protein | | uniclust | UniRef100\_A0A022PC28 | 99.5 | 4e-17 | 9.2e-23 | 120.3 | 74 | (51, 125) | 144 | (43, 116) | 159 | Mor transcription activator domain-containing protein | Mor transcription activator domain-containing protein | | uniclust | UniRef100\_A0A2N9YH57 | 99.5 | 6.6e-17 | 1.2e-22 | 111.3 | 112 | (2, 123) | 144 | (10, 127) | 139 | Mor transcription activator domain-containing protein | Mor transcription activator domain-containing protein | | uniclust | UniRef100\_UPI0009301444 | 99.5 | 7.1e-17 | 1.3e-22 | 115.7 | 104 | (5, 123) | 144 | (97, 201) | 203 | helix-turn-helix domain-containing protein | helix-turn-helix domain-containing protein | | uniclust | UniRef100\_A0A0C9PS36 | 99.5 | 7.6e-17 | 1.6e-22 | 112.4 | 79 | (49, 128) | 144 | (21, 99) | 123 | Uncharacterized conserved protein | Uncharacterized conserved protein | | uniclust | UniRef100\_A0A109W455 | 99.5 | 7.9e-17 | 1.7e-22 | 113.5 | 78 | (52, 130) | 144 | (34, 111) | 128 | Mor transcription activator domain-containing protein | Mor transcription activator domain-containing protein | | uniclust | UniRef100\_A0A7V8CQF7 | 99.5 | 1.2e-16 | 2.2e-22 | 110.5 | 115 | (2, 126) | 144 | (7, 131) | 141 | Mor transcription activator domain-containing protein | Mor transcription activator domain-containing protein | | uniclust | UniRef100\_A0A0N0M130 | 99.5 | 1.2e-16 | 2.4e-22 | 112.3 | 111 | (2, 122) | 144 | (22, 133) | 147 | Mor transcription activator domain-containing protein | Mor transcription activator domain-containing protein | | uniclust | UniRef100\_A0A0F6AFK3 | 99.5 | 1.3e-16 | 2.5e-22 | 112.1 | 110 | (2, 122) | 144 | (23, 132) | 151 | Mor transcription activator domain-containing protein | Mor transcription activator domain-containing protein | | uniclust | UniRef100\_A0A011MI82 | 99.5 | 1.9e-16 | 3.9e-22 | 111.4 | 77 | (51, 128) | 144 | (39, 115) | 135 | Transcriptional regulator | Transcriptional regulator | | uniclust | UniRef100\_A0A228J1B3 | 99.4 | 3.1e-16 | 6e-22 | 111.4 | 109 | (7, 125) | 144 | (52, 160) | 163 | Mor transcription activator domain-containing protein | Mor transcription activator domain-containing protein | | uniclust | UniRef100\_UPI00188FAA08 | 99.4 | 3.4e-16 | 6.3e-22 | 108.5 | 113 | (3, 126) | 144 | (20, 136) | 158 | Mor transcription activator family protein | Mor transcription activator family protein | | uniclust | UniRef100\_A0A0U5MJB7 | 99.4 | 3.3e-16 | 6.4e-22 | 110.2 | 116 | (2, 127) | 144 | (22, 140) | 149 | Helix-turn-helix domain-containing protein | Helix-turn-helix domain-containing protein | | uniclust | UniRef100\_A0A2X3CYD8 | 99.4 | 3.3e-16 | 6.4e-22 | 105.9 | 85 | (2, 96) | 144 | (10, 99) | 114 | Mor transcription activator family | Mor transcription activator family | | uniclust | UniRef100\_A0A0F1AW83 | 99.4 | 4.4e-16 | 8.6e-22 | 108.4 | 113 | (2, 124) | 144 | (3, 128) | 138 | Mor transcription activator domain-containing protein | Mor transcription activator domain-containing protein | | uniclust | UniRef100\_A0A2W5KVX1 | 99.4 | 5.2e-16 | 1e-21 | 102.8 | 89 | (41, 129) | 144 | (2, 92) | 100 | Resolvase HTH domain-containing protein (Fragment) | Resolvase HTH domain-containing protein (Fragment) | | uniclust | UniRef100\_UPI00035FAD56 | 99.4 | 5.3e-16 | 1e-21 | 105.4 | 110 | (4, 127) | 144 | (4, 113) | 119 | hypothetical protein | hypothetical protein | | uniclust | UniRef100\_A0A4D7B5Y7 | 99.4 | 6e-16 | 1.1e-21 | 109.1 | 113 | (2, 124) | 144 | (47, 159) | 177 | Helix-turn-helix domain-containing protein | Helix-turn-helix domain-containing protein | | uniclust | UniRef100\_UPI001FAB3CB7 | 99.4 | 8.4e-16 | 1.6e-21 | 108.1 | 115 | (3, 127) | 144 | (54, 168) | 174 | Mor transcription activator family protein | Mor transcription activator family protein | | uniclust | UniRef100\_A0A2M6UU58 | 99.4 | 8.4e-16 | 1.6e-21 | 106.2 | 111 | (2, 125) | 144 | (9, 123) | 141 | Helix-turn-helix domain-containing protein | Helix-turn-helix domain-containing protein | | uniclust | UniRef100\_A0A286GYP9 | 99.4 | 8.9e-16 | 1.7e-21 | 109.5 | 114 | (2, 125) | 144 | (19, 137) | 155 | Homeodomain-like domain-containing protein | Homeodomain-like domain-containing protein | | uniclust | UniRef100\_K9RSU5 | 99.4 | 9.7e-16 | 1.8e-21 | 101.5 | 100 | (7, 119) | 144 | (13, 113) | 116 | Mor transcription activator-like protein | Mor transcription activator-like protein | | uniclust | UniRef100\_UPI000695E811 | 99.4 | 9.7e-16 | 1.8e-21 | 116.9 | 127 | (1, 127) | 144 | (5, 136) | 320 | Mor transcription activator family protein | Mor transcription activator family protein | | uniclust | UniRef100\_A0A956STM1 | 99.4 | 9.9e-16 | 1.8e-21 | 103.1 | 112 | (2, 123) | 144 | (12, 124) | 129 | Uncharacterized protein | Uncharacterized protein | | uniclust | UniRef100\_A0A6L8HVT0 | 99.4 | 1.2e-15 | 2.2e-21 | 106.6 | 102 | (8, 121) | 144 | (63, 164) | 165 | Mor transcription activator domain-containing protein | Mor transcription activator domain-containing protein | | uniclust | UniRef100\_A0A1J4X7E1 | 99.4 | 1.9e-15 | 3.5e-21 | 103.1 | 111 | (2, 124) | 144 | (18, 130) | 140 | Mor transcription activator domain-containing protein | Mor transcription activator domain-containing protein | | uniclust | UniRef100\_A0A839IQE8 | 99.4 | 1.9e-15 | 3.5e-21 | 104.1 | 114 | (2, 126) | 144 | (19, 132) | 149 | Transcriptional regulator | Transcriptional regulator | | uniclust | UniRef100\_A0A0A8VVE1 | 99.4 | 2.1e-15 | 4.3e-21 | 105.9 | 77 | (48, 125) | 144 | (33, 109) | 131 | Transcriptional regulator | Transcriptional regulator | | uniclust | UniRef100\_A0A9D1UAM0 | 99.4 | 2.3e-15 | 4.3e-21 | 106.5 | 110 | (6, 125) | 144 | (28, 150) | 170 | Uncharacterized protein | Uncharacterized protein | | uniclust | UniRef100\_A0A4R3L9E1 | 99.4 | 2.6e-15 | 4.9e-21 | 107.7 | 107 | (6, 122) | 144 | (11, 123) | 172 | Mor transcription activator family protein | Mor transcription activator family protein | | uniclust | UniRef100\_A0A4Q8MMR8 | 99.4 | 2.8e-15 | 5.4e-21 | 108.5 | 108 | (6, 123) | 144 | (20, 131) | 186 | Mor transcription activator domain-containing protein | Mor transcription activator domain-containing protein | | uniclust | UniRef100\_A0A0P8Y9C8 | 99.3 | 3.3e-15 | 6.1e-21 | 107.2 | 107 | (7, 123) | 144 | (27, 133) | 198 | Mor transcription activator domain-containing protein | Mor transcription activator domain-containing protein | | uniclust | UniRef100\_A0A1V0PQE7 | 99.3 | 3.5e-15 | 6.9e-21 | 103.8 | 109 | (6, 126) | 144 | (9, 118) | 131 | Mor transcription activator domain-containing protein | Mor transcription activator domain-containing protein | | uniclust | UniRef100\_UPI001FAB7209 | 99.3 | 3.8e-15 | 7e-21 | 105.7 | 113 | (2, 124) | 144 | (6, 131) | 182 | Mor transcription activator family protein | Mor transcription activator family protein | | uniclust | UniRef100\_A0A0C9NCV1 | 99.3 | 3.5e-15 | 7.2e-21 | 113.0 | 114 | (3, 127) | 144 | (66, 180) | 217 | DNA, contig: SP630 | DNA, contig: SP630 | | uniclust | UniRef100\_A0A1Z8PYJ8 | 99.3 | 4.4e-15 | 8.3e-21 | 101.0 | 109 | (4, 127) | 144 | (14, 122) | 126 | Mor transcription activator domain-containing protein | Mor transcription activator domain-containing protein | | uniclust | UniRef100\_A0A1H5GMI8 | 99.3 | 5.2e-15 | 9.6e-21 | 103.3 | 110 | (2, 122) | 144 | (48, 157) | 162 | Transcriptional regulator, Middle operon regulator (Mor) family | Transcriptional regulator, Middle operon regulator (Mor) family | | uniclust | UniRef100\_A0A081CS68 | 99.3 | 4.8e-15 | 1e-20 | 113.3 | 109 | (2, 123) | 144 | (74, 187) | 216 | Uncharacterized protein | Uncharacterized protein | | uniclust | UniRef100\_A0A0D8J127 | 99.3 | 5.5e-15 | 1.1e-20 | 103.7 | 77 | (48, 125) | 144 | (18, 95) | 128 | Mor transcription activator domain-containing protein | Mor transcription activator domain-containing protein | | uniclust | UniRef100\_A0A2S5JEN6 | 99.3 | 6.5e-15 | 1.2e-20 | 100.2 | 116 | (1, 126) | 144 | (1, 122) | 136 | Mor transcription activator family protein | Mor transcription activator family protein | | uniclust | UniRef100\_A0A1H6HI74 | 99.3 | 6.7e-15 | 1.3e-20 | 101.3 | 111 | (6, 131) | 144 | (7, 120) | 134 | Helix-turn-helix domain of resolvase | Helix-turn-helix domain of resolvase | | uniclust | UniRef100\_A0A8I0VL83 | 99.3 | 8.8e-15 | 1.6e-20 | 98.9 | 113 | (2, 123) | 144 | (8, 120) | 130 | Mor transcription activator domain-containing protein | Mor transcription activator domain-containing protein | | uniclust | UniRef100\_A0A3T0N1F4 | 99.3 | 9.3e-15 | 1.8e-20 | 101.6 | 115 | (1, 125) | 144 | (21, 136) | 139 | Mor transcription activator domain-containing protein | Mor transcription activator domain-containing protein | | uniclust | UniRef100\_A0A3B9L6A9 | 99.3 | 9.9e-15 | 1.8e-20 | 102.4 | 107 | (2, 121) | 144 | (48, 154) | 167 | Mor transcription activator domain-containing protein | Mor transcription activator domain-containing protein | | uniclust | UniRef100\_A0A3F3GTU9 | 99.3 | 1.1e-14 | 2e-20 | 104.2 | 116 | (2, 127) | 144 | (42, 162) | 177 | Putative DNA transposition protein | Putative DNA transposition protein | | uniclust | UniRef100\_A0A0U2B4P9 | 99.3 | 9.9e-15 | 2.1e-20 | 103.0 | 75 | (51, 126) | 144 | (34, 108) | 127 | Mor transcription activator domain-containing protein | Mor transcription activator domain-containing protein | | uniclust | UniRef100\_UPI0019340A10 | 99.3 | 1.2e-14 | 2.3e-20 | 101.2 | 106 | (7, 129) | 144 | (17, 123) | 139 | MarR family transcriptional regulator | MarR family transcriptional regulator | | uniclust | UniRef100\_A0A962X3A9 | 99.3 | 1.3e-14 | 2.4e-20 | 99.1 | 112 | (2, 123) | 144 | (8, 119) | 139 | Uncharacterized protein | Uncharacterized protein | | uniclust | UniRef100\_A0A1E3UZN7 | 99.3 | 1.6e-14 | 2.9e-20 | 98.1 | 107 | (2, 123) | 144 | (15, 122) | 133 | Mor transcription activator domain-containing protein | Mor transcription activator domain-containing protein | | uniclust | UniRef100\_A0A7X3YCW7 | 99.3 | 1.8e-14 | 3.3e-20 | 94.2 | 90 | (19, 120) | 144 | (15, 104) | 105 | Mor transcription activator domain-containing protein | Mor transcription activator domain-containing protein | | uniclust | UniRef100\_UPI001EEB33AC | 99.3 | 1.8e-14 | 3.4e-20 | 98.5 | 120 | (2, 124) | 144 | (13, 133) | 140 | hypothetical protein | hypothetical protein | | uniclust | UniRef100\_UPI0009EC8F65 | 99.3 | 1.9e-14 | 3.6e-20 | 104.4 | 110 | (2, 121) | 144 | (9, 129) | 213 | Mor transcription activator family protein | Mor transcription activator family protein | | uniclust | UniRef100\_UPI0012BB55E9 | 99.3 | 2.5e-14 | 4.5e-20 | 99.2 | 100 | (9, 120) | 144 | (6, 105) | 148 | Mor transcription activator family protein | Mor transcription activator family protein | | uniclust | UniRef100\_A0A2I1RGQ4 | 99.3 | 2.5e-14 | 4.6e-20 | 100.6 | 114 | (2, 125) | 144 | (23, 149) | 168 | Mor transcription activator domain-containing protein | Mor transcription activator domain-containing protein | | uniclust | UniRef100\_A0A9E4GYE6 | 99.3 | 2.8e-14 | 5.3e-20 | 97.5 | 107 | (2, 124) | 144 | (13, 121) | 125 | Helix-turn-helix domain-containing protein | Helix-turn-helix domain-containing protein | | uniclust | UniRef100\_A0A562HZL9 | 99.2 | 2.8e-14 | 5.4e-20 | 102.3 | 113 | (2, 124) | 144 | (23, 148) | 162 | Mor transcription activator family protein | Mor transcription activator family protein | | uniclust | UniRef100\_A0A3L6M032 | 99.2 | 3e-14 | 6e-20 | 104.4 | 113 | (2, 124) | 144 | (32, 160) | 180 | Mor transcription activator domain-containing protein | Mor transcription activator domain-containing protein | | uniclust | UniRef100\_A0A345CP23 | 99.2 | 3.1e-14 | 6e-20 | 97.3 | 84 | (2, 95) | 144 | (14, 102) | 117 | Mor transcription activator domain-containing protein | Mor transcription activator domain-containing protein | | uniclust | UniRef100\_A0A1I5W2T6 | 99.2 | 3.1e-14 | 6.1e-20 | 98.3 | 110 | (3, 123) | 144 | (4, 113) | 127 | Homeodomain-like domain-containing protein | Homeodomain-like domain-containing protein | | uniclust | UniRef100\_A0A1B8Q513 | 99.2 | 3.2e-14 | 6.7e-20 | 100.9 | 78 | (50, 128) | 144 | (41, 118) | 131 | Mor transcription activator domain-containing protein | Mor transcription activator domain-containing protein | | uniclust | UniRef100\_UPI000A949961 | 99.2 | 3.6e-14 | 7.2e-20 | 102.5 | 115 | (2, 126) | 144 | (25, 156) | 165 | hypothetical protein | hypothetical protein | | uniclust | UniRef100\_UPI001FAB374C | 99.2 | 4.2e-14 | 7.7e-20 | 101.2 | 115 | (2, 126) | 144 | (14, 128) | 190 | Mor transcription activator family protein | Mor transcription activator family protein | | uniclust | UniRef100\_A0A140L4X4 | 99.2 | 4.1e-14 | 8.4e-20 | 94.4 | 75 | (48, 122) | 144 | (15, 90) | 92 | Mor transcription activator domain-containing protein | Mor transcription activator domain-containing protein | | uniclust | UniRef100\_A0A066Q1X8 | 99.2 | 3.8e-14 | 8.5e-20 | 104.9 | 74 | (52, 126) | 144 | (70, 143) | 159 | Mor transcription activator family protein | Mor transcription activator family protein | | uniclust | UniRef100\_A0A099GE87 | 99.2 | 4.6e-14 | 9.4e-20 | 101.9 | 111 | (5, 126) | 144 | (17, 133) | 155 | Mor transcription activator family protein | Mor transcription activator family protein | | uniclust | UniRef100\_A0A5Q0EN91 | 99.2 | 5.4e-14 | 1e-19 | 94.1 | 105 | (8, 122) | 144 | (6, 116) | 120 | Mor transcription activator domain-containing protein | Mor transcription activator domain-containing protein | | uniclust | UniRef100\_A0A952RM33 | 99.2 | 5.6e-14 | 1e-19 | 97.1 | 111 | (2, 124) | 144 | (20, 132) | 148 | Uncharacterized protein | Uncharacterized protein | | uniclust | UniRef100\_A0A192IDQ6 | 99.2 | 5.9e-14 | 1.1e-19 | 96.2 | 105 | (2, 125) | 144 | (7, 112) | 122 | Helix-turn-helix domain-containing protein | Helix-turn-helix domain-containing protein | | uniclust | UniRef100\_UPI001412D10D | 99.2 | 6.4e-14 | 1.2e-19 | 96.1 | 104 | (6, 124) | 144 | (8, 111) | 129 | helix-turn-helix domain-containing protein | helix-turn-helix domain-containing protein | | uniclust | UniRef100\_A0A5C7NL60 | 99.2 | 7.2e-14 | 1.3e-19 | 98.5 | 109 | (3, 125) | 144 | (52, 160) | 170 | Mor transcription activator domain-containing protein | Mor transcription activator domain-containing protein | | uniclust | UniRef100\_A0A1L9C9I9 | 99.2 | 7.4e-14 | 1.5e-19 | 104.5 | 105 | (6, 127) | 144 | (27, 138) | 200 | Helix-turn-helix domain-containing protein | Helix-turn-helix domain-containing protein | | uniclust | UniRef100\_A0A963FRH9 | 99.2 | 8.9e-14 | 1.6e-19 | 99.8 | 108 | (5, 122) | 144 | (6, 113) | 193 | Uncharacterized protein | Uncharacterized protein | | uniclust | UniRef100\_A0A4R2KJZ0 | 99.2 | 9.9e-14 | 1.9e-19 | 100.2 | 106 | (7, 125) | 144 | (30, 137) | 180 | Mor transcription activator family protein | Mor transcription activator family protein | | uniclust | UniRef100\_A0A3A9HIU9 | 99.2 | 1e-13 | 2e-19 | 92.3 | 74 | (48, 123) | 144 | (13, 86) | 98 | Mor transcription activator domain-containing protein | Mor transcription activator domain-containing protein | | uniclust | UniRef100\_A0A0A0DCH4 | 99.2 | 9.9e-14 | 2e-19 | 98.9 | 109 | (2, 125) | 144 | (11, 119) | 144 | Mor transcription activator domain-containing protein | Mor transcription activator domain-containing protein | | uniclust | UniRef100\_B0RUR6 | 99.2 | 1.1e-13 | 2e-19 | 106.3 | 125 | (2, 127) | 144 | (126, 258) | 296 | Mor transcription activator domain-containing protein | Mor transcription activator domain-containing protein | | uniclust | UniRef100\_UPI0012DBD419 | 99.2 | 1.2e-13 | 2.2e-19 | 96.0 | 110 | (2, 121) | 144 | (32, 151) | 153 | Mor transcription activator family protein | Mor transcription activator family protein | | uniclust | UniRef100\_A0A2E0QVY5 | 99.2 | 1.1e-13 | 2.3e-19 | 99.3 | 105 | (3, 124) | 144 | (22, 126) | 151 | Resolvase HTH domain-containing protein | Resolvase HTH domain-containing protein | | uniclust | UniRef100\_A0A951T787 | 99.2 | 1.3e-13 | 2.4e-19 | 93.5 | 108 | (10, 127) | 144 | (11, 118) | 130 | Helix-turn-helix domain-containing protein | Helix-turn-helix domain-containing protein | | uniclust | UniRef100\_A0A1M5RUN6 | 99.2 | 1.2e-13 | 2.4e-19 | 93.1 | 78 | (48, 125) | 144 | (17, 94) | 111 | Mor transcription activator family protein | Mor transcription activator family protein | | uniclust | UniRef100\_UPI0016882A40 | 99.2 | 1.4e-13 | 2.5e-19 | 99.9 | 110 | (7, 126) | 144 | (52, 162) | 208 | Mor transcription activator family protein | Mor transcription activator family protein | | uniclust | UniRef100\_A0A0F5EYF4 | 99.2 | 1.3e-13 | 2.8e-19 | 95.7 | 65 | (56, 121) | 144 | (37, 101) | 113 | Uncharacterized conserved protein | Uncharacterized conserved protein | | uniclust | UniRef100\_A0A0J9BG83 | 99.2 | 1.5e-13 | 3e-19 | 94.8 | 80 | (48, 128) | 144 | (21, 100) | 116 | Mor transcription activator domain-containing protein | Mor transcription activator domain-containing protein | | uniclust | UniRef100\_K5Z2H2 | 99.1 | 2.1e-13 | 3.9e-19 | 100.0 | 121 | (2, 122) | 144 | (95, 220) | 225 | Uncharacterized protein | Uncharacterized protein | | uniclust | UniRef100\_A0A191ZDV3 | 99.1 | 2e-13 | 4e-19 | 96.7 | 100 | (4, 129) | 144 | (22, 121) | 137 | Mor transcription activator domain-containing protein | Mor transcription activator domain-containing protein | | uniclust | UniRef100\_A0A127K415 | 99.1 | 1.9e-13 | 4.1e-19 | 99.5 | 76 | (50, 126) | 144 | (49, 124) | 145 | Mor transcription activator domain-containing protein | Mor transcription activator domain-containing protein | | uniclust | UniRef100\_UPI001F2B9EB0 | 99.1 | 2.2e-13 | 4.2e-19 | 89.7 | 91 | (22, 124) | 144 | (1, 91) | 100 | helix-turn-helix domain-containing protein | helix-turn-helix domain-containing protein | | uniclust | UniRef100\_UPI00233FAF5B | 99.1 | 2.3e-13 | 4.3e-19 | 90.7 | 101 | (14, 124) | 144 | (1, 103) | 116 | Mor transcription activator family protein | Mor transcription activator family protein | | uniclust | UniRef100\_A0A560II84 | 99.1 | 2.4e-13 | 4.4e-19 | 94.4 | 113 | (2, 127) | 144 | (8, 120) | 151 | Mor transcription activator family protein | Mor transcription activator family protein | | uniclust | UniRef100\_A0A858Q8R5 | 99.1 | 2.5e-13 | 4.6e-19 | 87.1 | 76 | (48, 123) | 144 | (7, 82) | 92 | Mor transcription activator domain-containing protein | Mor transcription activator domain-containing protein | | uniclust | UniRef100\_A0A2T6B1K6 | 99.1 | 2.5e-13 | 5e-19 | 95.8 | 107 | (4, 127) | 144 | (12, 118) | 130 | Homeodomain-like domain-containing protein | Homeodomain-like domain-containing protein | | uniclust | UniRef100\_A0A076PVY6 | 99.1 | 2.9e-13 | 5.4e-19 | 96.2 | 113 | (2, 124) | 144 | (11, 126) | 177 | Mor transcription activator domain-containing protein | Mor transcription activator domain-containing protein | | uniclust | UniRef100\_A0A1X7P996 | 99.1 | 2.9e-13 | 5.7e-19 | 96.6 | 113 | (5, 127) | 144 | (21, 135) | 157 | Homeodomain-like domain-containing protein | Homeodomain-like domain-containing protein | | uniclust | UniRef100\_A0A257EV10 | 99.1 | 2.7e-13 | 5.7e-19 | 99.2 | 117 | (1, 132) | 144 | (23, 141) | 152 | Uncharacterized protein | Uncharacterized protein | | uniclust | UniRef100\_A0A0H5SSP8 | 99.1 | 2.7e-13 | 5.7e-19 | 97.3 | 72 | (57, 129) | 144 | (57, 128) | 138 | Middle operon regulator-related protein | Middle operon regulator-related protein | | uniclust | UniRef100\_A0A315EKC3 | 99.1 | 3.4e-13 | 6.2e-19 | 87.2 | 81 | (40, 120) | 144 | (15, 95) | 96 | Mor transcription activator domain-containing protein | Mor transcription activator domain-containing protein | | uniclust | UniRef100\_UPI001F22401B | 99.1 | 3.4e-13 | 6.2e-19 | 91.9 | 107 | (2, 124) | 144 | (7, 113) | 133 | Mor transcription activator family protein | Mor transcription activator family protein | | uniclust | UniRef100\_A0A4R8HMW6 | 99.1 | 3.7e-13 | 6.7e-19 | 93.9 | 113 | (3, 126) | 144 | (24, 140) | 155 | Mor transcription activator family protein | Mor transcription activator family protein | | uniclust | UniRef100\_A0A062XBM9 | 99.1 | 3.2e-13 | 7e-19 | 96.9 | 78 | (49, 128) | 144 | (34, 111) | 129 | Phage-associated protein, BcepMu gp16 family | Phage-associated protein, BcepMu gp16 family | | uniclust | UniRef100\_A0A2G6DFJ6 | 99.1 | 4e-13 | 7.3e-19 | 95.6 | 110 | (5, 124) | 144 | (52, 163) | 178 | Mor transcription activator domain-containing protein | Mor transcription activator domain-containing protein | | uniclust | UniRef100\_A0A241XT80 | 99.1 | 3.8e-13 | 7.3e-19 | 95.8 | 111 | (2, 122) | 144 | (30, 153) | 161 | Putative integral membrane protein | Putative integral membrane protein | | uniclust | UniRef100\_UPI0018A8FC44 | 99.1 | 3.7e-13 | 7.3e-19 | 92.0 | 86 | (41, 129) | 144 | (24, 109) | 114 | Mor transcription activator family protein | Mor transcription activator family protein | | uniclust | UniRef100\_A0A149SVR6 | 99.1 | 3.9e-13 | 7.3e-19 | 91.3 | 110 | (5, 123) | 144 | (4, 113) | 120 | Resolvase HTH domain-containing protein | Resolvase HTH domain-containing protein | | uniclust | UniRef100\_A0A142BHK6 | 99.1 | 4.6e-13 | 8.5e-19 | 94.4 | 103 | (8, 122) | 144 | (5, 107) | 158 | Mor transcription activator domain-containing protein | Mor transcription activator domain-containing protein | | uniclust | UniRef100\_A0A9E7AG12 | 99.1 | 4.7e-13 | 8.6e-19 | 93.5 | 107 | (3, 126) | 144 | (41, 147) | 156 | Uncharacterized protein | Uncharacterized protein | | uniclust | UniRef100\_A0A099KN35 | 99.1 | 4.6e-13 | 8.6e-19 | 94.3 | 116 | (2, 128) | 144 | (19, 135) | 150 | Mor transcription activator domain protein | Mor transcription activator domain protein | | uniclust | UniRef100\_A0A7T0FZL0 | 99.1 | 4.4e-13 | 8.8e-19 | 94.6 | 83 | (46, 129) | 144 | (35, 117) | 131 | Mor transcription activator domain-containing protein | Mor transcription activator domain-containing protein | | uniclust | UniRef100\_A0A1G0EZ32 | 99.1 | 4.9e-13 | 8.9e-19 | 88.1 | 104 | (7, 120) | 144 | (2, 105) | 107 | Mor transcription activator domain-containing protein | Mor transcription activator domain-containing protein | | uniclust | UniRef100\_A0A1Y6CRI6 | 99.1 | 5.1e-13 | 9.5e-19 | 93.3 | 111 | (2, 126) | 144 | (20, 131) | 145 | Homeodomain-like domain-containing protein | Homeodomain-like domain-containing protein | | uniclust | UniRef100\_A0A973XNX3 | 99.1 | 5.4e-13 | 9.9e-19 | 102.9 | 110 | (2, 122) | 144 | (5, 123) | 337 | Helix-turn-helix domain-containing protein | Helix-turn-helix domain-containing protein | | uniclust | UniRef100\_A0A1Y4LTI1 | 99.1 | 5.7e-13 | 1e-18 | 86.4 | 76 | (51, 126) | 144 | (16, 91) | 97 | Mor transcription activator domain-containing protein | Mor transcription activator domain-containing protein | | uniclust | UniRef100\_A0A071LTN5 | 99.1 | 5e-13 | 1.1e-18 | 101.9 | 71 | (55, 126) | 144 | (99, 169) | 198 | Middle operon regulator | Middle operon regulator | | uniclust | UniRef100\_A0A022G338 | 99.1 | 5.2e-13 | 1.1e-18 | 95.0 | 99 | (13, 128) | 144 | (18, 116) | 129 | DNA-binding protein | DNA-binding protein | | uniclust | UniRef100\_UPI001BD169B3 | 99.1 | 7.1e-13 | 1.3e-18 | 86.1 | 84 | (21, 122) | 144 | (2, 85) | 98 | helix-turn-helix domain-containing protein | helix-turn-helix domain-containing protein | | uniclust | UniRef100\_A0A1E7PY91 | 99.1 | 6.1e-13 | 1.3e-18 | 94.0 | 69 | (52, 124) | 144 | (39, 107) | 117 | Mor transcription activator domain-containing protein | Mor transcription activator domain-containing protein | | uniclust | UniRef100\_E2CN54 | 99.1 | 8.8e-13 | 1.6e-18 | 90.8 | 110 | (3, 122) | 144 | (17, 128) | 141 | Resolvase HTH domain-containing protein | Resolvase HTH domain-containing protein | | uniclust | UniRef100\_A0A367WL54 | 99.1 | 8.8e-13 | 1.6e-18 | 98.2 | 111 | (5, 126) | 144 | (22, 133) | 249 | Mor transcription activator domain-containing protein | Mor transcription activator domain-containing protein | | uniclust | UniRef100\_A0A395D1X7 | 99.1 | 1.1e-12 | 2e-18 | 91.5 | 117 | (1, 127) | 144 | (1, 123) | 153 | Helix-turn-helix domain-containing protein | Helix-turn-helix domain-containing protein | | uniclust | UniRef100\_A0A1G8B086 | 99.1 | 1.1e-12 | 2e-18 | 85.0 | 72 | (48, 120) | 144 | (4, 76) | 89 | Mor transcription activator family protein | Mor transcription activator family protein | | uniclust | UniRef100\_UPI000A927094 | 99.1 | 1.1e-12 | 2e-18 | 94.5 | 106 | (8, 124) | 144 | (20, 127) | 174 | helix-turn-helix domain-containing protein | helix-turn-helix domain-containing protein | | uniclust | UniRef100\_A0A521YLK1 | 99.0 | 1.2e-12 | 2.2e-18 | 97.7 | 107 | (5, 122) | 144 | (121, 229) | 251 | Mor transcription activator domain-containing protein | Mor transcription activator domain-containing protein | | uniclust | UniRef100\_A4JDC4 | 99.0 | 1.6e-12 | 3e-18 | 103.0 | 110 | (3, 122) | 144 | (165, 286) | 428 | Mor transcription activator domain-containing protein | Mor transcription activator domain-containing protein | | uniclust | UniRef100\_A0A1F4QW68 | 99.0 | 1.5e-12 | 3.1e-18 | 90.6 | 88 | (41, 129) | 144 | (12, 99) | 112 | Mor transcription activator domain-containing protein | Mor transcription activator domain-containing protein | | uniclust | UniRef100\_A0A4Q0YJD9 | 99.0 | 1.7e-12 | 3.1e-18 | 91.5 | 98 | (6, 115) | 144 | (3, 106) | 164 | Uncharacterized protein | Uncharacterized protein | | uniclust | UniRef100\_A0A291IG12 | 99.0 | 1.7e-12 | 3.2e-18 | 83.8 | 80 | (2, 91) | 144 | (14, 93) | 94 | DnaA N-terminal domain-containing protein | DnaA N-terminal domain-containing protein | | uniclust | UniRef100\_A0A158E8H2 | 99.0 | 1.9e-12 | 3.4e-18 | 90.5 | 81 | (1, 81) | 144 | (28, 111) | 154 | Uncharacterized protein | Uncharacterized protein | | uniclust | UniRef100\_A0A439VN72 | 99.0 | 2.3e-12 | 4.2e-18 | 95.0 | 113 | (3, 125) | 144 | (107, 219) | 227 | Helix-turn-helix domain-containing protein | Helix-turn-helix domain-containing protein | | uniclust | UniRef100\_A0A4P6I322 | 99.0 | 2.3e-12 | 4.2e-18 | 92.4 | 111 | (4, 124) | 144 | (20, 139) | 174 | Mor transcription activator domain-containing protein | Mor transcription activator domain-containing protein | | uniclust | UniRef100\_A0A1D8IVF8 | 99.0 | 2.3e-12 | 4.2e-18 | 93.7 | 112 | (5, 125) | 144 | (6, 117) | 204 | Mor transcription activator domain-containing protein | Mor transcription activator domain-containing protein | | uniclust | UniRef100\_UPI0013A6E23F | 99.0 | 2.4e-12 | 4.3e-18 | 91.4 | 115 | (2, 119) | 144 | (52, 166) | 172 | hypothetical protein | hypothetical protein | | uniclust | UniRef100\_A0A3M1PQC8 | 99.0 | 2.4e-12 | 4.5e-18 | 88.5 | 105 | (8, 125) | 144 | (7, 111) | 138 | Helix-turn-helix domain-containing protein | Helix-turn-helix domain-containing protein | | uniclust | UniRef100\_A0A3T0N8M3 | 99.0 | 2.4e-12 | 4.5e-18 | 83.9 | 82 | (40, 121) | 144 | (7, 88) | 99 | Helix-turn-helix domain-containing protein | Helix-turn-helix domain-containing protein | | uniclust | UniRef100\_A0A0F3KGH2 | 99.0 | 2.3e-12 | 4.9e-18 | 93.5 | 76 | (57, 132) | 144 | (58, 133) | 143 | Mor transcription activator domain-containing protein | Mor transcription activator domain-containing protein | | uniclust | UniRef100\_A0A511VBD2 | 99.0 | 2.7e-12 | 5.2e-18 | 89.1 | 82 | (48, 129) | 144 | (45, 126) | 135 | Mor transcription activator domain-containing protein | Mor transcription activator domain-containing protein | | uniclust | UniRef100\_N1ZFG9 | 99.0 | 2.9e-12 | 5.3e-18 | 80.7 | 75 | (50, 125) | 144 | (5, 79) | 81 | Mor transcription activator domain-containing protein | Mor transcription activator domain-containing protein | | uniclust | UniRef100\_A0A942LWW7 | 99.0 | 2.9e-12 | 5.4e-18 | 91.2 | 113 | (3, 126) | 144 | (1, 114) | 175 | Uncharacterized protein | Uncharacterized protein | | uniclust | UniRef100\_A0A2E9SLK3 | 99.0 | 3.6e-12 | 6.5e-18 | 86.6 | 107 | (6, 123) | 144 | (3, 109) | 127 | Mor transcription activator domain-containing protein | Mor transcription activator domain-containing protein | | uniclust | UniRef100\_A0A1M5SM93 | 99.0 | 3.8e-12 | 7.1e-18 | 89.6 | 110 | (5, 125) | 144 | (23, 132) | 147 | Mor transcription activator family protein | Mor transcription activator family protein | | uniclust | UniRef100\_A0A522VTV5 | 99.0 | 3.9e-12 | 7.1e-18 | 87.5 | 112 | (3, 124) | 144 | (14, 126) | 138 | Mor transcription activator domain-containing protein | Mor transcription activator domain-containing protein | | uniclust | UniRef100\_UPI00113DD0F0 | 99.0 | 4e-12 | 7.7e-18 | 88.5 | 104 | (7, 127) | 144 | (19, 127) | 134 | hypothetical protein | hypothetical protein | | uniclust | UniRef100\_UPI001BFF8277 | 99.0 | 4.5e-12 | 8.3e-18 | 87.2 | 113 | (2, 124) | 144 | (19, 131) | 138 | Mor transcription activator family protein | Mor transcription activator family protein | | uniclust | UniRef100\_A0A2G1LMB9 | 99.0 | 4.5e-12 | 8.5e-18 | 91.2 | 104 | (9, 129) | 144 | (8, 113) | 166 | Transcriptional regulator | Transcriptional regulator | | uniclust | UniRef100\_UPI001FFD560B | 99.0 | 4.7e-12 | 8.6e-18 | 89.2 | 114 | (4, 127) | 144 | (4, 124) | 161 | helix-turn-helix domain-containing protein | helix-turn-helix domain-containing protein | | uniclust | UniRef100\_UPI001F4838F6 | 98.9 | 6.1e-12 | 1.1e-17 | 82.4 | 88 | (12, 110) | 144 | (2, 93) | 101 | hypothetical protein | hypothetical protein | | uniclust | UniRef100\_A0A1X7QDV6 | 98.9 | 6e-12 | 1.1e-17 | 91.2 | 105 | (2, 119) | 144 | (13, 119) | 178 | Uncharacterized protein | Uncharacterized protein | | uniclust | UniRef100\_T2JY53 | 98.9 | 6.5e-12 | 1.2e-17 | 84.5 | 108 | (2, 119) | 144 | (11, 118) | 119 | Mor transcription activator domain-containing protein | Mor transcription activator domain-containing protein | | uniclust | UniRef100\_R6HUG7 | 98.9 | 6.6e-12 | 1.2e-17 | 86.7 | 117 | (6, 132) | 144 | (10, 127) | 141 | Mor transcription activator domain-containing protein | Mor transcription activator domain-containing protein | | uniclust | UniRef100\_A0A2N2RB11 | 98.9 | 6.5e-12 | 1.2e-17 | 88.4 | 118 | (7, 136) | 144 | (14, 133) | 145 | Uncharacterized protein | Uncharacterized protein | | uniclust | UniRef100\_A0A1H8U5F3 | 98.9 | 6.5e-12 | 1.3e-17 | 88.0 | 75 | (50, 125) | 144 | (38, 112) | 127 | Mor transcription activator family protein | Mor transcription activator family protein | | uniclust | UniRef100\_A0A7H4K6A5 | 98.9 | 7.2e-12 | 1.3e-17 | 87.6 | 102 | (8, 127) | 144 | (43, 144) | 153 | Mor transcription activator domain-containing protein | Mor transcription activator domain-containing protein | | uniclust | UniRef100\_A0A564WHC9 | 98.9 | 7.6e-12 | 1.4e-17 | 88.4 | 111 | (2, 122) | 144 | (51, 162) | 164 | Mor transcription activator domain-containing protein | Mor transcription activator domain-containing protein | | uniclust | UniRef100\_A0A345DE69 | 98.9 | 8e-12 | 1.5e-17 | 87.6 | 109 | (6, 124) | 144 | (3, 111) | 155 | Mor transcription activator domain-containing protein | Mor transcription activator domain-containing protein | | uniclust | UniRef100\_UPI000755AEB5 | 98.9 | 9.1e-12 | 1.7e-17 | 84.6 | 80 | (2, 81) | 144 | (3, 84) | 126 | hypothetical protein | hypothetical protein | | uniclust | UniRef100\_UPI0002631E43 | 98.9 | 9.1e-12 | 1.7e-17 | 89.9 | 104 | (6, 126) | 144 | (13, 116) | 190 | hypothetical protein | hypothetical protein | | uniclust | UniRef100\_A0A1H7X2Y5 | 98.9 | 8.6e-12 | 1.8e-17 | 90.5 | 72 | (55, 127) | 144 | (59, 130) | 146 | Transcriptional regulator, Middle operon regulator (Mor) family | Transcriptional regulator, Middle operon regulator (Mor) family | | uniclust | UniRef100\_A0A2G1LMC0 | 98.9 | 1e-11 | 1.8e-17 | 86.3 | 105 | (6, 127) | 144 | (2, 106) | 146 | Mor transcription activator domain-containing protein | Mor transcription activator domain-containing protein | | uniclust | UniRef100\_UPI001F5430B7 | 98.9 | 1.2e-11 | 2.2e-17 | 86.1 | 116 | (5, 120) | 144 | (18, 143) | 147 | hypothetical protein | hypothetical protein | | uniclust | UniRef100\_UPI001C0BED20 | 98.9 | 1.2e-11 | 2.2e-17 | 85.1 | 98 | (10, 124) | 144 | (34, 131) | 137 | hypothetical protein | hypothetical protein | | uniclust | UniRef100\_UPI0009DAAD1D | 98.9 | 1.2e-11 | 2.4e-17 | 84.0 | 109 | (5, 121) | 144 | (4, 112) | 115 | helix-turn-helix domain-containing protein | helix-turn-helix domain-containing protein | | uniclust | UniRef100\_A0A5U2RGL3 | 98.9 | 1.3e-11 | 2.4e-17 | 89.9 | 121 | (2, 126) | 144 | (57, 185) | 190 | Mor transcription activator family protein | Mor transcription activator family protein | | uniclust | UniRef100\_A0A135L136 | 98.9 | 1.2e-11 | 2.4e-17 | 80.4 | 71 | (51, 121) | 144 | (8, 81) | 83 | Mor transcription activator domain-containing protein | Mor transcription activator domain-containing protein | | uniclust | UniRef100\_A0A081RZJ1 | 98.9 | 1.2e-11 | 2.4e-17 | 81.4 | 65 | (64, 129) | 144 | (4, 68) | 86 | Mor transcription activator-like protein | Mor transcription activator-like protein | | uniclust | UniRef100\_A0A6N9P271 | 98.9 | 1.4e-11 | 2.6e-17 | 86.1 | 81 | (50, 130) | 144 | (58, 138) | 152 | Mor transcription activator domain-containing protein | Mor transcription activator domain-containing protein | | uniclust | UniRef100\_A0A4V2V2Q2 | 98.9 | 1.5e-11 | 2.7e-17 | 83.8 | 99 | (14, 122) | 144 | (13, 116) | 121 | Homeodomain-like domain-containing protein | Homeodomain-like domain-containing protein | | uniclust | UniRef100\_A0A0F2KP01 | 98.9 | 1.5e-11 | 2.8e-17 | 90.2 | 110 | (2, 124) | 144 | (10, 121) | 194 | Uncharacterized protein | Uncharacterized protein | | uniclust | UniRef100\_A0A1I3FMF4 | 98.9 | 1.6e-11 | 2.8e-17 | 86.5 | 106 | (6, 123) | 144 | (19, 127) | 159 | Regulatory protein, luxR family | Regulatory protein, luxR family | | uniclust | UniRef100\_UPI001FCE4F32 | 98.9 | 1.6e-11 | 2.9e-17 | 84.7 | 106 | (5, 128) | 144 | (13, 118) | 138 | hypothetical protein | hypothetical protein | | uniclust | UniRef100\_A0A0M2DU47 | 98.9 | 1.4e-11 | 2.9e-17 | 89.9 | 74 | (52, 126) | 144 | (59, 132) | 154 | Mor transcription activator domain-containing protein | Mor transcription activator domain-containing protein | | uniclust | UniRef100\_UPI000AA29095 | 98.9 | 1.6e-11 | 2.9e-17 | 79.5 | 87 | (8, 106) | 144 | (5, 91) | 93 | hypothetical protein | hypothetical protein | | uniclust | UniRef100\_UPI0022EB6F38 | 98.9 | 1.7e-11 | 3.1e-17 | 94.8 | 116 | (2, 120) | 144 | (201, 316) | 328 | DUF1064 domain-containing protein | DUF1064 domain-containing protein | | uniclust | UniRef100\_A0A4Q7BE38 | 98.9 | 1.7e-11 | 3.2e-17 | 87.8 | 120 | (2, 121) | 144 | (48, 170) | 180 | Mor transcription activator domain-containing protein | Mor transcription activator domain-containing protein | | uniclust | UniRef100\_A0A072T170 | 98.9 | 1.6e-11 | 3.4e-17 | 91.5 | 105 | (3, 128) | 144 | (41, 147) | 172 | Mor transcription activator domain-containing protein | Mor transcription activator domain-containing protein | | uniclust | UniRef100\_A0A1Y6CHR8 | 98.9 | 2e-11 | 3.7e-17 | 85.4 | 108 | (5, 123) | 144 | (39, 146) | 152 | Mor transcription activator family protein | Mor transcription activator family protein | | uniclust | UniRef100\_A0A1G7LRH3 | 98.9 | 2e-11 | 3.7e-17 | 87.2 | 103 | (9, 123) | 144 | (22, 127) | 160 | Uncharacterized protein | Uncharacterized protein | | uniclust | UniRef100\_A0A062XDH3 | 98.9 | 1.8e-11 | 3.8e-17 | 86.7 | 79 | (48, 128) | 144 | (33, 111) | 120 | RNA polymerase sigma factor, sigma-70 family | RNA polymerase sigma factor, sigma-70 family | | uniclust | UniRef100\_UPI001905C364 | 98.9 | 2e-11 | 3.9e-17 | 86.0 | 110 | (2, 121) | 144 | (20, 142) | 146 | hypothetical protein | hypothetical protein | | uniclust | UniRef100\_A0A0K1NDC6 | 98.8 | 2.2e-11 | 4.6e-17 | 85.5 | 64 | (61, 125) | 144 | (45, 108) | 118 | Mor transcription activator domain-containing protein | Mor transcription activator domain-containing protein | | uniclust | UniRef100\_A0A450WDE4 | 98.8 | 2.5e-11 | 4.6e-17 | 85.3 | 98 | (9, 128) | 144 | (26, 123) | 156 | Transcriptional regulator, Middle operon regulator (Mor) family | Transcriptional regulator, Middle operon regulator (Mor) family | | uniclust | UniRef100\_UPI0015754E79 | 98.8 | 2.5e-11 | 4.6e-17 | 89.1 | 101 | (8, 125) | 144 | (109, 209) | 213 | hypothetical protein | hypothetical protein | | uniclust | UniRef100\_A0A3B9PNW9 | 98.8 | 2.8e-11 | 5.2e-17 | 94.4 | 114 | (2, 125) | 144 | (201, 325) | 350 | Mor transcription activator domain-containing protein | Mor transcription activator domain-containing protein | | uniclust | UniRef100\_A0A3C1SU58 | 98.8 | 2.7e-11 | 5.3e-17 | 80.5 | 64 | (57, 123) | 144 | (25, 88) | 91 | Mor transcription activator domain-containing protein (Fragment) | Mor transcription activator domain-containing protein (Fragment) | | uniclust | UniRef100\_A0A084Z6E6 | 98.8 | 2.8e-11 | 5.4e-17 | 83.5 | 67 | (58, 125) | 144 | (23, 89) | 116 | Positive regulator (Fragment) | Positive regulator (Fragment) | | uniclust | UniRef100\_UPI001BAA3F89 | 98.8 | 3.1e-11 | 5.6e-17 | 84.9 | 111 | (5, 125) | 144 | (35, 149) | 156 | helix-turn-helix domain-containing protein | helix-turn-helix domain-containing protein | | uniclust | UniRef100\_A0A109KKA2 | 98.8 | 2.8e-11 | 5.8e-17 | 88.2 | 105 | (13, 131) | 144 | (20, 125) | 149 | Mor transcription activator family protein | Mor transcription activator family protein | | uniclust | UniRef100\_A0A3S4VXS7 | 98.8 | 3.5e-11 | 6.7e-17 | 75.5 | 66 | (61, 127) | 144 | (3, 68) | 70 | Uncharacterized conserved protein | Uncharacterized conserved protein | | uniclust | UniRef100\_UPI000F4B675E | 98.8 | 3.7e-11 | 6.8e-17 | 80.1 | 76 | (50, 126) | 144 | (11, 86) | 110 | Mor transcription activator family protein | Mor transcription activator family protein | | uniclust | UniRef100\_UPI0015D4FDA7 | 98.8 | 4.1e-11 | 7.6e-17 | 87.9 | 111 | (2, 125) | 144 | (8, 118) | 210 | hypothetical protein | hypothetical protein | | uniclust | UniRef100\_A0A0J7J773 | 98.8 | 3.8e-11 | 7.7e-17 | 88.1 | 68 | (57, 125) | 144 | (76, 143) | 159 | Transcriptional regulator, Middle operon regulator (Mor) family | Transcriptional regulator, Middle operon regulator (Mor) family | | uniclust | UniRef100\_A0A0N1BSW6 | 98.8 | 4.6e-11 | 8.6e-17 | 86.1 | 113 | (2, 127) | 144 | (46, 158) | 173 | Transposase IS30-like HTH domain-containing protein | Transposase IS30-like HTH domain-containing protein | | uniclust | UniRef100\_UPI00145E8AB1 | 98.8 | 4.7e-11 | 8.7e-17 | 85.0 | 108 | (6, 123) | 144 | (50, 157) | 169 | hypothetical protein | hypothetical protein | | uniclust | UniRef100\_A0A7Z0N040 | 98.8 | 5.1e-11 | 9.7e-17 | 86.3 | 105 | (5, 119) | 144 | (13, 117) | 172 | Mor transcription activator domain-containing protein | Mor transcription activator domain-containing protein | | uniclust | UniRef100\_A0A927JUI3 | 98.8 | 5.7e-11 | 1e-16 | 85.6 | 106 | (10, 125) | 144 | (13, 128) | 183 | Helix-turn-helix domain-containing protein | Helix-turn-helix domain-containing protein | | uniclust | UniRef100\_A0A2M7Q369 | 98.8 | 6.3e-11 | 1.1e-16 | 82.4 | 111 | (2, 125) | 144 | (19, 136) | 143 | Mor transcription activator domain-containing protein | Mor transcription activator domain-containing protein | | uniclust | UniRef100\_R6CNA9 | 98.8 | 6.5e-11 | 1.2e-16 | 79.1 | 73 | (51, 123) | 144 | (26, 98) | 109 | Mor transcription activator domain-containing protein | Mor transcription activator domain-containing protein | | uniclust | UniRef100\_A0A9D2KRI3 | 98.8 | 6.9e-11 | 1.3e-16 | 87.1 | 113 | (3, 125) | 144 | (81, 204) | 217 | Uncharacterized protein | Uncharacterized protein | | uniclust | UniRef100\_A0A2W4TD25 | 98.8 | 7.2e-11 | 1.3e-16 | 85.4 | 112 | (3, 126) | 144 | (72, 183) | 189 | Helix-turn-helix domain-containing protein | Helix-turn-helix domain-containing protein | | uniclust | UniRef100\_A0A2M7G6P5 | 98.8 | 7.1e-11 | 1.4e-16 | 83.3 | 110 | (4, 123) | 144 | (20, 140) | 141 | Mor transcription activator domain-containing protein | Mor transcription activator domain-containing protein | | uniclust | UniRef100\_A0A7Z7WCS0 | 98.8 | 7.8e-11 | 1.4e-16 | 90.0 | 108 | (5, 122) | 144 | (164, 283) | 287 | Uncharacterized conserved protein | Uncharacterized conserved protein | | uniclust | UniRef100\_A0A344J3T3 | 98.8 | 7e-11 | 1.4e-16 | 87.8 | 72 | (53, 125) | 144 | (75, 146) | 169 | Transcriptional regulator | Transcriptional regulator | | uniclust | UniRef100\_UPI00147BFF21 | 98.8 | 7.8e-11 | 1.4e-16 | 81.9 | 104 | (8, 122) | 144 | (2, 107) | 143 | hypothetical protein | hypothetical protein | | uniclust | UniRef100\_E9I3C0 | 98.7 | 7.9e-11 | 1.5e-16 | 82.9 | 114 | (7, 130) | 144 | (6, 119) | 156 | Uncharacterized protein | Uncharacterized protein | | uniclust | UniRef100\_Q31HV4 | 98.7 | 8e-11 | 1.5e-16 | 81.9 | 108 | (6, 123) | 144 | (13, 120) | 144 | Mor transcription activator domain-containing protein | Mor transcription activator domain-containing protein | | uniclust | UniRef100\_A0A317DTH4 | 98.7 | 8.1e-11 | 1.5e-16 | 87.3 | 111 | (2, 125) | 144 | (61, 177) | 226 | Helix-turn-helix domain-containing protein | Helix-turn-helix domain-containing protein | | uniclust | UniRef100\_UPI00036465FF | 98.7 | 8.5e-11 | 1.6e-16 | 83.6 | 111 | (2, 122) | 144 | (22, 145) | 148 | Mor transcription activator family protein | Mor transcription activator family protein | | uniclust | UniRef100\_UPI000381B40D | 98.7 | 9.1e-11 | 1.7e-16 | 73.6 | 63 | (6, 78) | 144 | (3, 65) | 71 | hypothetical protein | hypothetical protein | | uniclust | UniRef100\_A0A1I1UI57 | 98.7 | 9.6e-11 | 1.8e-16 | 80.2 | 106 | (8, 123) | 144 | (2, 111) | 129 | Homeodomain-like domain-containing protein | Homeodomain-like domain-containing protein | | uniclust | UniRef100\_UPI0011E59512 | 98.7 | 1.1e-10 | 2.1e-16 | 85.0 | 114 | (6, 131) | 144 | (5, 120) | 197 | hypothetical protein | hypothetical protein | | uniclust | UniRef100\_A0A440JH48 | 98.7 | 1.1e-10 | 2.1e-16 | 88.3 | 100 | (8, 125) | 144 | (150, 249) | 264 | Winged helix-turn-helix domain-containing protein | Winged helix-turn-helix domain-containing protein | | uniclust | UniRef100\_A0A1I5P0R3 | 98.7 | 1.2e-10 | 2.2e-16 | 83.6 | 120 | (2, 121) | 144 | (32, 157) | 177 | Mor transcription activator family protein | Mor transcription activator family protein | | uniclust | UniRef100\_UPI002351D03E | 98.7 | 1.2e-10 | 2.2e-16 | 77.0 | 89 | (22, 120) | 144 | (3, 104) | 104 | Mor transcription activator family protein | Mor transcription activator family protein | | uniclust | UniRef100\_A0A031WGA1 | 98.7 | 1.2e-10 | 2.2e-16 | 77.0 | 74 | (41, 122) | 144 | (18, 91) | 93 | Putative transcriptional regulator, activator Mor | Putative transcriptional regulator, activator Mor | | uniclust | UniRef100\_A0A961S296 | 98.7 | 1.3e-10 | 2.4e-16 | 81.2 | 117 | (2, 129) | 144 | (14, 133) | 147 | Helix-turn-helix domain-containing protein | Helix-turn-helix domain-containing protein | | uniclust | UniRef100\_A0A7Z7WCS0 | 98.7 | 1.4e-10 | 2.5e-16 | 88.7 | 113 | (2, 124) | 144 | (5, 129) | 287 | Uncharacterized conserved protein | Uncharacterized conserved protein | | uniclust | UniRef100\_K2JSJ1 | 98.7 | 1.4e-10 | 2.6e-16 | 80.3 | 114 | (2, 126) | 144 | (10, 124) | 138 | Mor transcription activator domain-containing protein | Mor transcription activator domain-containing protein | | uniclust | UniRef100\_A0A7Y6R9Q8 | 98.7 | 1.4e-10 | 2.7e-16 | 83.4 | 118 | (2, 122) | 144 | (10, 129) | 160 | HDOD domain-containing protein | HDOD domain-containing protein | | uniclust | UniRef100\_A0A0Q2YXZ0 | 98.7 | 1.5e-10 | 2.8e-16 | 79.8 | 109 | (5, 126) | 144 | (9, 118) | 134 | Mor transcription activator domain-containing protein | Mor transcription activator domain-containing protein | | uniclust | UniRef100\_A0A650EP32 | 98.7 | 1.4e-10 | 2.8e-16 | 85.6 | 98 | (18, 126) | 144 | (35, 146) | 164 | Mor transcription activator domain-containing protein | Mor transcription activator domain-containing protein | | uniclust | UniRef100\_A0A7C1YCC8 | 98.7 | 1.5e-10 | 2.8e-16 | 77.3 | 71 | (51, 122) | 144 | (27, 97) | 99 | Mor transcription activator domain-containing protein | Mor transcription activator domain-containing protein | | uniclust | UniRef100\_A0A748ASM0 | 98.7 | 1.5e-10 | 2.8e-16 | 82.9 | 111 | (2, 122) | 144 | (33, 157) | 174 | Mor transcription activator domain-containing protein | Mor transcription activator domain-containing protein | | uniclust | UniRef100\_A0A1Y3SP53 | 98.7 | 1.5e-10 | 2.9e-16 | 75.8 | 73 | (50, 122) | 144 | (10, 83) | 85 | Mor transcription activator domain-containing protein | Mor transcription activator domain-containing protein | | uniclust | UniRef100\_A0A1U9VJQ1 | 98.7 | 1.5e-10 | 3.1e-16 | 85.0 | 108 | (2, 124) | 144 | (37, 149) | 158 | DNA-binding protein | DNA-binding protein | | uniclust | UniRef100\_A0A1G9KD85 | 98.7 | 1.6e-10 | 3.1e-16 | 78.6 | 72 | (49, 120) | 144 | (22, 93) | 105 | Homeodomain-like domain-containing protein | Homeodomain-like domain-containing protein | | uniclust | UniRef100\_A0A847URR4 | 98.7 | 1.7e-10 | 3.2e-16 | 83.2 | 104 | (7, 122) | 144 | (8, 111) | 164 | Helix-turn-helix domain-containing protein | Helix-turn-helix domain-containing protein | | uniclust | UniRef100\_A0A2X4N2M8 | 98.7 | 1.6e-10 | 3.2e-16 | 82.4 | 81 | (49, 131) | 144 | (34, 114) | 136 | Uncharacterized conserved protein | Uncharacterized conserved protein | | uniclust | UniRef100\_A0A5C7NTW8 | 98.7 | 2.1e-10 | 3.9e-16 | 79.5 | 109 | (3, 121) | 144 | (12, 136) | 138 | Mor transcription activator domain-containing protein | Mor transcription activator domain-containing protein | | uniclust | UniRef100\_A0A450WGZ3 | 98.7 | 2.3e-10 | 4.3e-16 | 78.1 | 81 | (48, 129) | 144 | (35, 115) | 126 | Transcriptional regulator, Middle operon regulator (Mor) family | Transcriptional regulator, Middle operon regulator (Mor) family | | uniclust | UniRef100\_A0A1X7N693 | 98.7 | 2.4e-10 | 4.4e-16 | 80.8 | 112 | (2, 126) | 144 | (17, 130) | 158 | Mor transcription activator domain-containing protein | Mor transcription activator domain-containing protein | | uniclust | UniRef100\_UPI001358B363 | 98.7 | 2.4e-10 | 4.5e-16 | 75.9 | 92 | (4, 105) | 144 | (4, 104) | 106 | hypothetical protein | hypothetical protein | | uniclust | UniRef100\_A0A1V6M161 | 98.7 | 2.5e-10 | 4.5e-16 | 79.5 | 117 | (2, 129) | 144 | (13, 135) | 142 | Mor transcription activator domain-containing protein | Mor transcription activator domain-containing protein | | uniclust | UniRef100\_A0A1Q9A418 | 98.7 | 2.3e-10 | 4.7e-16 | 85.6 | 102 | (5, 123) | 144 | (33, 136) | 180 | Uncharacterized protein | Uncharacterized protein | | uniclust | UniRef100\_UPI001884F6D1 | 98.6 | 2.6e-10 | 4.8e-16 | 81.2 | 101 | (3, 121) | 144 | (42, 142) | 165 | helix-turn-helix domain-containing protein | helix-turn-helix domain-containing protein | | uniclust | UniRef100\_A0A0S9S3A9 | 98.6 | 2.7e-10 | 5e-16 | 75.9 | 95 | (10, 121) | 144 | (4, 98) | 105 | Resolvase HTH domain-containing protein | Resolvase HTH domain-containing protein | | uniclust | UniRef100\_A0A935K439 | 98.6 | 2.9e-10 | 5.3e-16 | 75.8 | 98 | (11, 121) | 144 | (3, 105) | 108 | Uncharacterized protein | Uncharacterized protein | | uniclust | UniRef100\_A0A1V5UYJ7 | 98.6 | 2.6e-10 | 5.3e-16 | 77.3 | 75 | (50, 126) | 144 | (14, 88) | 97 | Mor transcription activator family protein | Mor transcription activator family protein | | uniclust | UniRef100\_A0A9C9NCZ5 | 98.6 | 3e-10 | 5.6e-16 | 82.8 | 108 | (4, 125) | 144 | (31, 138) | 195 | Uncharacterized protein | Uncharacterized protein | | uniclust | UniRef100\_A0A2C8FCX0 | 98.6 | 3.2e-10 | 5.9e-16 | 70.5 | 69 | (50, 121) | 144 | (3, 71) | 72 | Mor transcription activator domain-containing protein | Mor transcription activator domain-containing protein | | uniclust | UniRef100\_A0A0M2KGX3 | 98.6 | 3.2e-10 | 6.1e-16 | 83.4 | 77 | (54, 130) | 144 | (92, 168) | 177 | Regulator of pectin lyase production | Regulator of pectin lyase production | | uniclust | UniRef100\_UPI0022351324 | 98.6 | 3.4e-10 | 6.3e-16 | 72.5 | 80 | (43, 122) | 144 | (3, 82) | 85 | Mor transcription activator family protein | Mor transcription activator family protein | | uniclust | UniRef100\_A0A378WIV1 | 98.6 | 3.5e-10 | 6.4e-16 | 78.9 | 108 | (3, 120) | 144 | (20, 142) | 143 | Phage protein | Phage protein | | uniclust | UniRef100\_A0A0A0HG49 | 98.6 | 3.5e-10 | 6.5e-16 | 78.5 | 110 | (4, 125) | 144 | (5, 114) | 129 | Mor transcription activator family | Mor transcription activator family | | uniclust | UniRef100\_UPI001EDCF87F | 98.6 | 3.7e-10 | 6.8e-16 | 84.4 | 124 | (2, 125) | 144 | (13, 143) | 236 | hypothetical protein | hypothetical protein | | uniclust | UniRef100\_A0A0E3C1B1 | 98.6 | 3.4e-10 | 6.9e-16 | 80.6 | 64 | (63, 127) | 144 | (60, 123) | 130 | HTH iclR-type domain-containing protein | HTH iclR-type domain-containing protein | | uniclust | UniRef100\_A0A8T3N508 | 98.6 | 4.3e-10 | 7.9e-16 | 73.9 | 86 | (11, 122) | 144 | (2, 87) | 98 | Mor transcription activator domain-containing protein | Mor transcription activator domain-containing protein | | uniclust | UniRef100\_A0A024P8A0 | 98.6 | 3.7e-10 | 8.1e-16 | 82.2 | 81 | (52, 132) | 144 | (28, 120) | 135 | Mor transcription activator family protein | Mor transcription activator family protein | | uniclust | UniRef100\_A0A547Q840 | 98.6 | 4.8e-10 | 9.1e-16 | 78.5 | 110 | (4, 123) | 144 | (8, 119) | 132 | Mor transcription activator domain-containing protein | Mor transcription activator domain-containing protein | | uniclust | UniRef100\_UPI0015754E79 | 98.6 | 5e-10 | 9.2e-16 | 82.7 | 102 | (8, 126) | 144 | (5, 106) | 213 | hypothetical protein | hypothetical protein | | uniclust | UniRef100\_A0A8S5NR56 | 98.6 | 5.1e-10 | 9.4e-16 | 71.3 | 70 | (51, 120) | 144 | (9, 79) | 82 | Mor transcription activator family | Mor transcription activator family | | uniclust | UniRef100\_UPI000D19E10B | 98.6 | 5.2e-10 | 9.6e-16 | 77.5 | 109 | (2, 121) | 144 | (20, 131) | 137 | helix-turn-helix domain-containing protein | helix-turn-helix domain-containing protein | | uniclust | UniRef100\_A0A257PH65 | 98.6 | 5.2e-10 | 9.6e-16 | 75.3 | 97 | (9, 122) | 144 | (6, 102) | 114 | Mor transcription activator domain-containing protein | Mor transcription activator domain-containing protein | | uniclust | UniRef100\_UPI00111B3C11 | 98.6 | 5.4e-10 | 1e-15 | 81.3 | 96 | (2, 107) | 144 | (11, 115) | 191 | DUF1834 family protein | DUF1834 family protein | | uniclust | UniRef100\_A0A0L0UL23 | 98.6 | 5.3e-10 | 1e-15 | 74.7 | 76 | (51, 127) | 144 | (3, 78) | 92 | Mor transcription activator domain-containing protein | Mor transcription activator domain-containing protein | | uniclust | UniRef100\_A0A1Y2K7L6 | 98.6 | 6.3e-10 | 1.2e-15 | 76.0 | 101 | (5, 123) | 144 | (14, 114) | 124 | Helix-turn-helix domain-containing protein | Helix-turn-helix domain-containing protein | | uniclust | UniRef100\_A0A0B3W048 | 98.6 | 6.2e-10 | 1.2e-15 | 73.1 | 72 | (49, 121) | 144 | (11, 82) | 84 | Transcriptional regulator | Transcriptional regulator | | uniclust | UniRef100\_A0A357ANT8 | 98.6 | 6.6e-10 | 1.2e-15 | 74.1 | 80 | (48, 127) | 144 | (24, 103) | 107 | Mor transcription activator domain-containing protein | Mor transcription activator domain-containing protein | | uniclust | UniRef100\_UPI0020B8F391 | 98.6 | 7.1e-10 | 1.3e-15 | 81.0 | 112 | (7, 128) | 144 | (16, 130) | 196 | hypothetical protein | hypothetical protein | | uniclust | UniRef100\_A0A1V5V371 | 98.6 | 6.9e-10 | 1.3e-15 | 77.1 | 75 | (52, 126) | 144 | (24, 103) | 117 | Mor transcription activator family protein | Mor transcription activator family protein | | uniclust | UniRef100\_N6VLK0 | 98.6 | 7.3e-10 | 1.3e-15 | 70.6 | 68 | (2, 79) | 144 | (8, 79) | 82 | Uncharacterized protein | Uncharacterized protein | | uniclust | UniRef100\_A0A090SPL0 | 98.6 | 7.4e-10 | 1.4e-15 | 77.3 | 99 | (2, 114) | 144 | (9, 108) | 143 | Transcriptional regulator | Transcriptional regulator | | uniclust | UniRef100\_A0A1J5INX3 | 98.6 | 7.3e-10 | 1.4e-15 | 71.2 | 69 | (54, 124) | 144 | (2, 70) | 80 | Mor transcription activator domain-containing protein | Mor transcription activator domain-containing protein | | uniclust | UniRef100\_A0A316MS27 | 98.6 | 7.4e-10 | 1.4e-15 | 75.7 | 74 | (52, 126) | 144 | (31, 104) | 119 | DNA-binding protein | DNA-binding protein | | uniclust | UniRef100\_A0A1B8TFA3 | 98.6 | 7e-10 | 1.4e-15 | 78.6 | 76 | (52, 128) | 144 | (34, 109) | 129 | Mor transcription activator-like protein | Mor transcription activator-like protein | | uniclust | UniRef100\_UPI00045E7B36 | 98.5 | 8.1e-10 | 1.5e-15 | 71.9 | 77 | (44, 120) | 144 | (2, 78) | 92 | hypothetical protein | hypothetical protein | | uniclust | UniRef100\_A0A1A9RK94 | 98.5 | 7.1e-10 | 1.5e-15 | 85.8 | 109 | (1, 142) | 144 | (80, 194) | 205 | Uncharacterized protein | Uncharacterized protein | | uniclust | UniRef100\_A0A345DDH0 | 98.5 | 8.2e-10 | 1.5e-15 | 76.4 | 111 | (3, 123) | 144 | (9, 120) | 134 | Mor transcription activator domain-containing protein | Mor transcription activator domain-containing protein | | uniclust | UniRef100\_UPI0016AEF5FB | 98.5 | 8.3e-10 | 1.5e-15 | 71.5 | 75 | (2, 79) | 144 | (6, 82) | 90 | hypothetical protein | hypothetical protein | | uniclust | UniRef100\_A0A950CSA0 | 98.5 | 8.8e-10 | 1.6e-15 | 71.8 | 63 | (6, 78) | 144 | (31, 93) | 93 | Uncharacterized protein (Fragment) | Uncharacterized protein (Fragment) | | uniclust | UniRef100\_A0A1S2CM03 | 98.5 | 8.3e-10 | 1.6e-15 | 78.6 | 79 | (50, 129) | 144 | (44, 122) | 134 | Mor transcription activator domain-containing protein | Mor transcription activator domain-containing protein | | uniclust | UniRef100\_A0A2A2JZL6 | 98.5 | 8.9e-10 | 1.6e-15 | 87.6 | 93 | (2, 105) | 144 | (69, 161) | 392 | Uncharacterized protein | Uncharacterized protein | | uniclust | UniRef100\_A0A1M5PL34 | 98.5 | 8.5e-10 | 1.7e-15 | 75.7 | 74 | (49, 126) | 144 | (17, 90) | 107 | Mor transcription activator family protein | Mor transcription activator family protein | | uniclust | UniRef100\_A0A072TCN7 | 98.5 | 9.6e-10 | 1.8e-15 | 88.7 | 76 | (53, 129) | 144 | (362, 437) | 450 | Mor family transcriptional regulator | Mor family transcriptional regulator | | uniclust | UniRef100\_A0A0B1TT43 | 98.5 | 1.1e-09 | 2e-15 | 73.7 | 74 | (51, 124) | 144 | (8, 85) | 101 | Mor transcription activator domain-containing protein | Mor transcription activator domain-containing protein | | uniclust | UniRef100\_A0A846UIR1 | 98.5 | 1.1e-09 | 2e-15 | 69.7 | 67 | (52, 122) | 144 | (14, 80) | 81 | Mor family transcriptional regulator | Mor family transcriptional regulator | | uniclust | UniRef100\_A0A1Q6QCR6 | 98.5 | 1.2e-09 | 2.1e-15 | 68.0 | 68 | (54, 122) | 144 | (2, 69) | 71 | Mor transcription activator domain-containing protein | Mor transcription activator domain-containing protein | | uniclust | UniRef100\_A0A147H032 | 98.5 | 1.2e-09 | 2.1e-15 | 69.0 | 60 | (61, 121) | 144 | (17, 76) | 77 | Mor transcription activator domain-containing protein | Mor transcription activator domain-containing protein | | uniclust | UniRef100\_A0A1H0G9V9 | 98.5 | 1.2e-09 | 2.3e-15 | 70.8 | 59 | (62, 125) | 144 | (18, 76) | 81 | Mor transcription activator family protein | Mor transcription activator family protein | | uniclust | UniRef100\_A0A3N9RYD7 | 98.5 | 1.4e-09 | 2.5e-15 | 81.5 | 112 | (2, 124) | 144 | (54, 166) | 234 | Uncharacterized protein | Uncharacterized protein | | uniclust | UniRef100\_UPI0018E781FA | 98.5 | 1.4e-09 | 2.6e-15 | 77.5 | 103 | (6, 122) | 144 | (31, 136) | 162 | LuxR C-terminal-related transcriptional regulator | LuxR C-terminal-related transcriptional regulator | | uniclust | UniRef100\_A0A522LCM6 | 98.5 | 1.4e-09 | 2.6e-15 | 81.1 | 120 | (3, 132) | 144 | (30, 164) | 226 | Mor transcription activator domain-containing protein | Mor transcription activator domain-containing protein | | uniclust | UniRef100\_A0A2M8EEU5 | 98.5 | 1.4e-09 | 2.6e-15 | 80.0 | 113 | (2, 124) | 144 | (30, 164) | 181 | Mor transcription activator domain-containing protein | Mor transcription activator domain-containing protein | | uniclust | UniRef100\_A0A265Q2W8 | 98.5 | 1.4e-09 | 2.7e-15 | 72.5 | 75 | (49, 125) | 144 | (15, 89) | 93 | Mor transcription activator domain-containing protein | Mor transcription activator domain-containing protein | | uniclust | UniRef100\_A0A8K2AGT6 | 98.5 | 1.5e-09 | 2.7e-15 | 78.1 | 105 | (4, 120) | 144 | (4, 108) | 173 | Mor transcription activator domain-containing protein | Mor transcription activator domain-containing protein | | uniclust | UniRef100\_A0A0C5E2Q8 | 98.5 | 1.5e-09 | 2.9e-15 | 76.5 | 76 | (50, 126) | 144 | (45, 120) | 138 | Mor transcription activator domain-containing protein | Mor transcription activator domain-containing protein | | uniclust | UniRef100\_UPI000695E811 | 98.5 | 1.6e-09 | 3e-15 | 84.6 | 120 | (3, 122) | 144 | (146, 270) | 320 | Mor transcription activator family protein | Mor transcription activator family protein | | uniclust | UniRef100\_UPI0021D601D8 | 98.5 | 1.8e-09 | 3.3e-15 | 70.7 | 71 | (54, 124) | 144 | (2, 81) | 95 | Mor transcription activator family protein | Mor transcription activator family protein | | uniclust | UniRef100\_A0A2U2AMQ7 | 98.5 | 1.8e-09 | 3.4e-15 | 81.5 | 115 | (2, 126) | 144 | (115, 240) | 249 | Mor transcription activator domain-containing protein | Mor transcription activator domain-containing protein | | uniclust | UniRef100\_A0A3D5S739 | 98.5 | 2e-09 | 3.8e-15 | 73.4 | 101 | (10, 120) | 144 | (1, 117) | 121 | Mor transcription activator domain-containing protein | Mor transcription activator domain-containing protein | | uniclust | UniRef100\_A0A840C4F7 | 98.4 | 2.4e-09 | 4.4e-15 | 76.6 | 116 | (1, 125) | 144 | (37, 160) | 165 | Mor transcription activator domain-containing protein | Mor transcription activator domain-containing protein | | uniclust | UniRef100\_A0A1S1HV25 | 98.4 | 2.4e-09 | 4.4e-15 | 72.8 | 76 | (50, 125) | 144 | (30, 105) | 119 | Mor transcription activator domain-containing protein | Mor transcription activator domain-containing protein | | uniclust | UniRef100\_B6IMZ5 | 98.4 | 2.5e-09 | 4.6e-15 | 77.3 | 114 | (3, 126) | 144 | (53, 172) | 178 | Uncharacterized protein | Uncharacterized protein | | uniclust | UniRef100\_A0A7Y6TZ55 | 98.4 | 2.5e-09 | 4.6e-15 | 72.8 | 70 | (60, 129) | 144 | (41, 110) | 119 | Mor transcription activator domain-containing protein | Mor transcription activator domain-containing protein | | uniclust | UniRef100\_UPI00215AD7B1 | 98.4 | 2.5e-09 | 4.6e-15 | 73.6 | 78 | (46, 123) | 144 | (15, 92) | 128 | Mor transcription activator family protein | Mor transcription activator family protein | | uniclust | UniRef100\_A0A0J6KKT4 | 98.4 | 2.3e-09 | 4.7e-15 | 78.9 | 79 | (52, 130) | 144 | (50, 128) | 153 | Mor transcription activator domain-containing protein | Mor transcription activator domain-containing protein | | uniclust | UniRef100\_A0A1M5S655 | 98.4 | 2.4e-09 | 4.9e-15 | 76.8 | 66 | (57, 122) | 144 | (46, 112) | 130 | Mor transcription activator family protein | Mor transcription activator family protein | | uniclust | UniRef100\_A0A1G3LYB2 | 98.4 | 2.7e-09 | 4.9e-15 | 71.0 | 75 | (49, 125) | 144 | (18, 93) | 103 | Mor transcription activator domain-containing protein | Mor transcription activator domain-containing protein | | uniclust | UniRef100\_A0A258LJQ1 | 98.4 | 2.9e-09 | 5.3e-15 | 70.3 | 64 | (7, 80) | 144 | (3, 66) | 99 | Transcriptional regulator | Transcriptional regulator | | uniclust | UniRef100\_UPI0012EC8521 | 98.4 | 3.1e-09 | 5.7e-15 | 68.9 | 81 | (43, 128) | 144 | (2, 83) | 89 | Mor transcription activator family protein | Mor transcription activator family protein | | uniclust | UniRef100\_A0A9E3YQJ6 | 98.4 | 3.2e-09 | 5.9e-15 | 72.3 | 109 | (3, 121) | 144 | (2, 116) | 119 | Uncharacterized protein | Uncharacterized protein | | uniclust | UniRef100\_A0A6N6T8X9 | 98.4 | 3.2e-09 | 6e-15 | 69.4 | 65 | (61, 126) | 144 | (17, 81) | 85 | Mor transcription activator domain-containing protein | Mor transcription activator domain-containing protein | | uniclust | UniRef100\_A0A6F9X1I1 | 98.4 | 3.3e-09 | 6e-15 | 75.1 | 114 | (3, 127) | 144 | (19, 141) | 153 | Mor transcription activator domain-containing protein | Mor transcription activator domain-containing protein | | uniclust | UniRef100\_UPI000428175B | 98.4 | 3.2e-09 | 6.2e-15 | 72.7 | 70 | (57, 127) | 144 | (28, 97) | 108 | Mor transcription activator family protein | Mor transcription activator family protein | | uniclust | UniRef100\_A0A317E9Z1 | 98.4 | 3.5e-09 | 6.5e-15 | 76.2 | 110 | (2, 125) | 144 | (10, 123) | 172 | Helix-turn-helix domain-containing protein | Helix-turn-helix domain-containing protein | | uniclust | UniRef100\_A0A292RV70 | 98.4 | 3.7e-09 | 6.7e-15 | 70.0 | 72 | (51, 122) | 144 | (27, 98) | 100 | Mor transcription activator domain-containing protein | Mor transcription activator domain-containing protein | | uniclust | UniRef100\_A0A1H3CN96 | 98.4 | 3.9e-09 | 7.5e-15 | 73.3 | 73 | (55, 128) | 144 | (36, 110) | 115 | Transcriptional regulator, Middle operon regulator (Mor) family | Transcriptional regulator, Middle operon regulator (Mor) family | | uniclust | UniRef100\_UPI001C5AB0C2 | 98.4 | 4.2e-09 | 7.7e-15 | 72.0 | 102 | (14, 125) | 144 | (4, 116) | 122 | Mor transcription activator family protein | Mor transcription activator family protein | | uniclust | UniRef100\_G1UW96 | 98.4 | 4.3e-09 | 7.9e-15 | 81.3 | 93 | (20, 123) | 144 | (50, 151) | 293 | Mor transcription activator domain-containing protein | Mor transcription activator domain-containing protein | | uniclust | UniRef100\_A0A9D5VAY5 | 98.4 | 4.5e-09 | 8.2e-15 | 79.3 | 110 | (3, 123) | 144 | (9, 119) | 242 | Uncharacterized protein | Uncharacterized protein | | uniclust | UniRef100\_A0A1E4LD59 | 98.4 | 4.5e-09 | 8.5e-15 | 72.5 | 77 | (49, 125) | 144 | (28, 104) | 116 | Mor transcription activator domain-containing protein | Mor transcription activator domain-containing protein | | uniclust | UniRef100\_A0A0R1UPE2 | 98.4 | 4.4e-09 | 8.5e-15 | 68.4 | 74 | (57, 132) | 144 | (2, 75) | 78 | Mor transcription activator domain-containing protein | Mor transcription activator domain-containing protein | | uniclust | UniRef100\_UPI0022B816E3 | 98.4 | 4.9e-09 | 9e-15 | 73.0 | 101 | (10, 127) | 144 | (28, 128) | 137 | hypothetical protein | hypothetical protein | | uniclust | UniRef100\_A0A7D5LKB6 | 98.4 | 4.8e-09 | 9.3e-15 | 73.8 | 69 | (57, 125) | 144 | (47, 115) | 127 | Mor transcription activator domain-containing protein | Mor transcription activator domain-containing protein | | uniclust | UniRef100\_A0A7W7YWM5 | 98.4 | 5.1e-09 | 9.4e-15 | 74.6 | 95 | (9, 121) | 144 | (57, 151) | 159 | Mor transcription activator domain-containing protein | Mor transcription activator domain-containing protein | | uniclust | UniRef100\_A0A0H3A7L4 | 98.4 | 4.6e-09 | 9.5e-15 | 74.2 | 77 | (49, 125) | 144 | (21, 107) | 115 | Mor transcription activator domain-containing protein | Mor transcription activator domain-containing protein | | uniclust | UniRef100\_A0A069DBT8 | 98.4 | 4.7e-09 | 9.6e-15 | 76.0 | 73 | (51, 123) | 144 | (22, 106) | 133 | Mor transcription activator domain-containing protein | Mor transcription activator domain-containing protein | | uniclust | UniRef100\_A0A1X7QCW3 | 98.4 | 5.5e-09 | 1e-14 | 79.0 | 112 | (2, 126) | 144 | (52, 165) | 245 | Helix-turn-helix domain-containing protein | Helix-turn-helix domain-containing protein | | uniclust | UniRef100\_UPI0022745754 | 98.4 | 5.5e-09 | 1e-14 | 71.3 | 105 | (3, 124) | 144 | (12, 116) | 120 | hypothetical protein | hypothetical protein | | uniclust | UniRef100\_A0A3R8L878 | 98.3 | 5.8e-09 | 1.1e-14 | 79.7 | 109 | (2, 122) | 144 | (84, 195) | 249 | DNA-binding protein | DNA-binding protein | | uniclust | UniRef100\_A0A0C5E5W8 | 98.3 | 5.5e-09 | 1.1e-14 | 76.8 | 72 | (57, 128) | 144 | (67, 139) | 156 | Mor transcription activator domain-containing protein | Mor transcription activator domain-containing protein | | uniclust | UniRef100\_A0A2S7CGT1 | 98.3 | 5.4e-09 | 1.1e-14 | 75.2 | 74 | (51, 124) | 144 | (40, 118) | 131 | Mor transcription activator domain-containing protein | Mor transcription activator domain-containing protein | | uniclust | UniRef100\_A0A1V8NVX7 | 98.3 | 5.7e-09 | 1.1e-14 | 80.9 | 75 | (51, 126) | 144 | (52, 126) | 225 | Mor transcription activator domain-containing protein | Mor transcription activator domain-containing protein | | uniclust | UniRef100\_A0A2S6H5A2 | 98.3 | 6.1e-09 | 1.1e-14 | 67.5 | 58 | (60, 120) | 144 | (24, 81) | 82 | Mor transcription activator family protein | Mor transcription activator family protein | | uniclust | UniRef100\_UPI000B1C3237 | 98.3 | 6.4e-09 | 1.2e-14 | 86.3 | 85 | (2, 96) | 144 | (9, 98) | 576 | DNA circularization N-terminal domain-containing protein | DNA circularization N-terminal domain-containing protein | | uniclust | UniRef100\_A0A0P7M6A8 | 98.3 | 6.5e-09 | 1.2e-14 | 70.8 | 81 | (52, 132) | 144 | (33, 113) | 118 | Mor transcription activator domain-containing protein | Mor transcription activator domain-containing protein | | uniclust | UniRef100\_A0A3D1VPS2 | 98.3 | 6.7e-09 | 1.3e-14 | 67.4 | 69 | (52, 121) | 144 | (9, 77) | 83 | Mor transcription activator domain-containing protein | Mor transcription activator domain-containing protein | | uniclust | UniRef100\_UPI00215ADC39 | 98.3 | 7e-09 | 1.3e-14 | 73.5 | 83 | (41, 124) | 144 | (60, 151) | 153 | Mor transcription activator family protein | Mor transcription activator family protein | | uniclust | UniRef100\_A0A4Y6UA46 | 98.3 | 7e-09 | 1.3e-14 | 74.8 | 109 | (5, 118) | 144 | (59, 168) | 172 | Uncharacterized protein | Uncharacterized protein | | uniclust | UniRef100\_A0A942QUK7 | 98.3 | 6.8e-09 | 1.3e-14 | 71.1 | 68 | (51, 122) | 144 | (40, 107) | 110 | Mor transcription activator domain-containing protein | Mor transcription activator domain-containing protein | | uniclust | UniRef100\_A0A2G4RG90 | 98.3 | 7.6e-09 | 1.4e-14 | 70.6 | 102 | (6, 124) | 144 | (8, 115) | 119 | Mor transcription activator domain-containing protein | Mor transcription activator domain-containing protein | | uniclust | UniRef100\_UPI0009F57BC5 | 98.3 | 7.5e-09 | 1.4e-14 | 68.9 | 69 | (56, 125) | 144 | (15, 83) | 93 | Mor transcription activator family protein | Mor transcription activator family protein | | uniclust | UniRef100\_A0A3S5A9Q7 | 98.3 | 7.3e-09 | 1.4e-14 | 72.3 | 69 | (58, 126) | 144 | (40, 109) | 114 | Putative bacteriophage transcriptional regulator | Putative bacteriophage transcriptional regulator | | uniclust | UniRef100\_A0A953CGI5 | 98.3 | 8.3e-09 | 1.5e-14 | 77.0 | 109 | (7, 125) | 144 | (19, 130) | 220 | Uncharacterized protein | Uncharacterized protein | | uniclust | UniRef100\_UPI0015D49757 | 98.3 | 8.3e-09 | 1.5e-14 | 74.6 | 107 | (2, 122) | 144 | (15, 134) | 175 | hypothetical protein | hypothetical protein | | uniclust | UniRef100\_A0A2H6APG1 | 98.3 | 7.8e-09 | 1.6e-14 | 71.7 | 74 | (50, 126) | 144 | (25, 98) | 106 | Mor transcription activator domain-containing protein | Mor transcription activator domain-containing protein | | uniclust | UniRef100\_A0A2D8HLU1 | 98.3 | 8.6e-09 | 1.6e-14 | 73.7 | 111 | (3, 123) | 144 | (35, 149) | 161 | Mor transcription activator domain-containing protein | Mor transcription activator domain-containing protein | | uniclust | UniRef100\_A0A3N0ZBC0 | 98.3 | 8.8e-09 | 1.7e-14 | 68.2 | 70 | (49, 119) | 144 | (19, 88) | 91 | Helix-turn-helix domain-containing protein | Helix-turn-helix domain-containing protein | | uniclust | UniRef100\_A4JDC4 | 98.3 | 9.5e-09 | 1.7e-14 | 82.9 | 109 | (6, 124) | 144 | (16, 137) | 428 | Mor transcription activator domain-containing protein | Mor transcription activator domain-containing protein | | uniclust | UniRef100\_B6IMZ4 | 98.3 | 9.8e-09 | 1.8e-14 | 72.7 | 110 | (6, 127) | 144 | (25, 135) | 151 | Mor transcription activator domain-containing protein | Mor transcription activator domain-containing protein | | uniclust | UniRef100\_A0A0F4QDL7 | 98.3 | 9.4e-09 | 1.8e-14 | 73.5 | 75 | (52, 127) | 144 | (38, 112) | 137 | Transcriptional regulator | Transcriptional regulator | | uniclust | UniRef100\_A0A3S1BGX4 | 98.3 | 1e-08 | 1.9e-14 | 74.4 | 69 | (57, 126) | 144 | (96, 164) | 178 | Mor transcription activator domain-containing protein | Mor transcription activator domain-containing protein | | uniclust | UniRef100\_UPI0010BF961F | 98.3 | 1e-08 | 1.9e-14 | 71.9 | 105 | (10, 124) | 144 | (15, 122) | 142 | helix-turn-helix domain-containing protein | helix-turn-helix domain-containing protein | | uniclust | UniRef100\_A0A0Q5ZD56 | 98.3 | 1e-08 | 2.1e-14 | 71.5 | 62 | (63, 124) | 144 | (41, 102) | 105 | Resolvase HTH domain-containing protein | Resolvase HTH domain-containing protein | | uniclust | UniRef100\_A0A923AS64 | 98.3 | 1.2e-08 | 2.3e-14 | 76.7 | 111 | (2, 122) | 144 | (91, 218) | 232 | Mor transcription activator domain-containing protein | Mor transcription activator domain-containing protein | | uniclust | UniRef100\_UPI00102DFDF1 | 98.3 | 1.2e-08 | 2.3e-14 | 67.9 | 70 | (54, 124) | 144 | (29, 98) | 102 | Mor transcription activator family protein | Mor transcription activator family protein | | uniclust | UniRef100\_T0IRN1 | 98.3 | 1.3e-08 | 2.3e-14 | 72.0 | 108 | (11, 127) | 144 | (27, 135) | 149 | Mor transcription activator domain-containing protein | Mor transcription activator domain-containing protein | | uniclust | UniRef100\_UPI00117FD392 | 98.3 | 1.2e-08 | 2.3e-14 | 75.0 | 113 | (2, 119) | 144 | (73, 189) | 190 | hypothetical protein | hypothetical protein | | uniclust | UniRef100\_A0A1G6EHB1 | 98.3 | 1.2e-08 | 2.3e-14 | 73.6 | 78 | (49, 128) | 144 | (57, 134) | 138 | Mor transcription activator family protein | Mor transcription activator family protein | | uniclust | UniRef100\_A0A511V899 | 98.3 | 1.3e-08 | 2.3e-14 | 68.1 | 75 | (48, 122) | 144 | (20, 94) | 104 | Mor transcription activator domain-containing protein | Mor transcription activator domain-containing protein | | uniclust | UniRef100\_A0A014NJ40 | 98.3 | 1.2e-08 | 2.3e-14 | 73.3 | 69 | (57, 126) | 144 | (57, 125) | 141 | Mor transcription activator domain-containing protein | Mor transcription activator domain-containing protein | | uniclust | UniRef100\_UPI0021C4554C | 98.3 | 1.3e-08 | 2.4e-14 | 64.7 | 55 | (67, 122) | 144 | (3, 57) | 78 | Mor transcription activator family protein | Mor transcription activator family protein | | uniclust | UniRef100\_A0A2C5TWB0 | 98.3 | 1.4e-08 | 2.5e-14 | 71.4 | 90 | (20, 119) | 144 | (1, 103) | 143 | Mor transcription activator domain-containing protein | Mor transcription activator domain-containing protein | | uniclust | UniRef100\_A0A0J1GQ97 | 98.2 | 1.5e-08 | 2.9e-14 | 72.3 | 76 | (52, 127) | 144 | (20, 107) | 123 | Mor transcription activator domain-containing protein | Mor transcription activator domain-containing protein | | uniclust | UniRef100\_A0A3R1ADR8 | 98.2 | 1.5e-08 | 2.9e-14 | 71.9 | 76 | (52, 127) | 144 | (43, 118) | 130 | Mor transcription activator domain-containing protein | Mor transcription activator domain-containing protein | | uniclust | UniRef100\_A0A1E3ZIA4 | 98.2 | 1.6e-08 | 3e-14 | 69.3 | 80 | (44, 128) | 144 | (26, 105) | 121 | Mor transcription activator domain-containing protein | Mor transcription activator domain-containing protein | | uniclust | UniRef100\_A0A1A9WW73 | 98.2 | 1.7e-08 | 3.1e-14 | 72.9 | 78 | (50, 128) | 144 | (79, 156) | 171 | Mor transcription activator domain-containing protein | Mor transcription activator domain-containing protein | | uniclust | UniRef100\_A0A0A8H9R4 | 98.2 | 1.5e-08 | 3.1e-14 | 71.5 | 62 | (60, 124) | 144 | (34, 103) | 112 | Transcriptional regulator, Mor family | Transcriptional regulator, Mor family | | uniclust | UniRef100\_A0A2V2EJZ8 | 98.2 | 1.7e-08 | 3.1e-14 | 69.1 | 73 | (49, 122) | 144 | (46, 118) | 120 | Mor transcription activator domain-containing protein | Mor transcription activator domain-containing protein | | uniclust | UniRef100\_UPI000B853F07 | 98.2 | 1.7e-08 | 3.1e-14 | 71.7 | 100 | (16, 125) | 144 | (45, 144) | 153 | Mor transcription activator family protein | Mor transcription activator family protein | | uniclust | UniRef100\_A0A847LF34 | 98.2 | 1.9e-08 | 3.6e-14 | 75.2 | 113 | (1, 125) | 144 | (72, 184) | 221 | Mor transcription activator domain-containing protein | Mor transcription activator domain-containing protein | | uniclust | UniRef100\_UPI0013C2DCD9 | 98.2 | 2.1e-08 | 3.8e-14 | 74.4 | 122 | (2, 123) | 144 | (10, 138) | 207 | AlpA family phage regulatory protein | AlpA family phage regulatory protein | | uniclust | UniRef100\_UPI000424C323 | 98.2 | 2.2e-08 | 4e-14 | 67.6 | 78 | (47, 124) | 144 | (21, 98) | 110 | Mor transcription activator family protein | Mor transcription activator family protein | | uniclust | UniRef100\_A0A4T5K8K5 | 98.2 | 2.1e-08 | 4e-14 | 72.5 | 72 | (58, 129) | 144 | (64, 135) | 142 | Transcriptional regulator | Transcriptional regulator | | uniclust | UniRef100\_A0A2D8KBN5 | 98.2 | 2.2e-08 | 4.1e-14 | 71.1 | 111 | (6, 126) | 144 | (14, 132) | 152 | Helix-turn-helix domain-containing protein | Helix-turn-helix domain-containing protein | | uniclust | UniRef100\_UPI000AAE4DE3 | 98.2 | 2.2e-08 | 4.1e-14 | 71.3 | 69 | (53, 122) | 144 | (85, 153) | 155 | Mor transcription activator family protein | Mor transcription activator family protein | | uniclust | UniRef100\_A0A127QUE9 | 98.2 | 2e-08 | 4.1e-14 | 73.5 | 94 | (1, 127) | 144 | (39, 139) | 140 | Uncharacterized protein | Uncharacterized protein | | uniclust | UniRef100\_R5R2B3 | 98.2 | 2.6e-08 | 4.8e-14 | 65.9 | 81 | (41, 121) | 144 | (13, 94) | 97 | Mor transcription activator domain-containing protein | Mor transcription activator domain-containing protein | | uniclust | UniRef100\_UPI001FD7F15E | 98.2 | 2.7e-08 | 4.9e-14 | 75.1 | 77 | (50, 126) | 144 | (45, 129) | 235 | Mor transcription activator family protein | Mor transcription activator family protein | | uniclust | UniRef100\_A0A091ATJ0 | 98.2 | 2.5e-08 | 5.1e-14 | 71.2 | 68 | (57, 127) | 144 | (40, 107) | 124 | Mor transcription activator domain-containing protein | Mor transcription activator domain-containing protein | | uniclust | UniRef100\_A0A270BEU5 | 98.2 | 2.9e-08 | 5.3e-14 | 69.4 | 108 | (8, 128) | 144 | (14, 121) | 136 | MarR family transcriptional regulator | MarR family transcriptional regulator | | uniclust | UniRef100\_A0A522DPM1 | 98.2 | 3.1e-08 | 5.7e-14 | 65.6 | 75 | (52, 126) | 144 | (7, 81) | 97 | Mor transcription activator domain-containing protein | Mor transcription activator domain-containing protein | | uniclust | UniRef100\_UPI001F1206BA | 98.2 | 3.1e-08 | 5.8e-14 | 65.2 | 74 | (53, 127) | 144 | (10, 85) | 94 | Mor transcription activator family protein | Mor transcription activator family protein | | uniclust | UniRef100\_UPI0012F83529 | 98.2 | 3.2e-08 | 5.8e-14 | 69.4 | 98 | (15, 122) | 144 | (29, 126) | 139 | hypothetical protein | hypothetical protein | | uniclust | UniRef100\_UPI002112227B | 98.2 | 3.2e-08 | 5.9e-14 | 63.4 | 72 | (50, 121) | 144 | (7, 78) | 80 | Mor transcription activator family protein | Mor transcription activator family protein | | uniclust | UniRef100\_A0A540V321 | 98.2 | 3.2e-08 | 5.9e-14 | 59.5 | 51 | (67, 121) | 144 | (4, 54) | 57 | DNA-binding protein (Fragment) | DNA-binding protein (Fragment) | | uniclust | UniRef100\_UPI000DA609CB | 98.2 | 3.2e-08 | 6e-14 | 58.6 | 51 | (67, 121) | 144 | (2, 52) | 53 | Mor transcription activator family protein | Mor transcription activator family protein | | uniclust | UniRef100\_A0A921TC53 | 98.2 | 3.4e-08 | 6.2e-14 | 75.3 | 114 | (7, 131) | 144 | (36, 149) | 254 | N-acetylmuramoyl-L-alanine amidase | N-acetylmuramoyl-L-alanine amidase | | uniclust | UniRef100\_A0A3S0MTD3 | 98.2 | 3.2e-08 | 6.3e-14 | 70.6 | 64 | (64, 128) | 144 | (57, 120) | 129 | Mor transcription activator domain-containing protein | Mor transcription activator domain-containing protein | | uniclust | UniRef100\_A0A4Q8XRF6 | 98.1 | 3.4e-08 | 6.4e-14 | 72.3 | 100 | (3, 120) | 144 | (18, 122) | 165 | Uncharacterized protein | Uncharacterized protein | | uniclust | UniRef100\_A0A1T4L4A5 | 98.1 | 3.5e-08 | 6.5e-14 | 65.5 | 71 | (50, 121) | 144 | (18, 88) | 99 | Mor transcription activator family protein | Mor transcription activator family protein | | uniclust | UniRef100\_A0A4Q3PAH2 | 98.1 | 3.5e-08 | 6.9e-14 | 70.0 | 72 | (48, 123) | 144 | (39, 110) | 126 | Mor transcription activator domain-containing protein | Mor transcription activator domain-containing protein | | uniclust | UniRef100\_UPI00041F2CD4 | 98.1 | 3.7e-08 | 6.9e-14 | 66.3 | 71 | (52, 122) | 144 | (4, 82) | 98 | Mor transcription activator family protein | Mor transcription activator family protein | | uniclust | UniRef100\_A0A194AFZ1 | 98.1 | 3.8e-08 | 7e-14 | 64.1 | 76 | (50, 125) | 144 | (6, 82) | 88 | Mor transcription activator domain-containing protein | Mor transcription activator domain-containing protein | | uniclust | UniRef100\_A0A6L3AN94 | 98.1 | 3.9e-08 | 7.1e-14 | 64.2 | 69 | (51, 120) | 144 | (15, 87) | 89 | Mor transcription activator domain-containing protein | Mor transcription activator domain-containing protein | | uniclust | UniRef100\_A0A2D8KBQ5 | 98.1 | 3.9e-08 | 7.1e-14 | 72.2 | 104 | (3, 124) | 144 | (57, 161) | 190 | HTH luxR-type domain-containing protein | HTH luxR-type domain-containing protein | | uniclust | UniRef100\_A0A1X7C3R7 | 98.1 | 4e-08 | 7.4e-14 | 63.9 | 70 | (50, 121) | 144 | (15, 85) | 87 | Mor transcription activator family protein | Mor transcription activator family protein | | uniclust | UniRef100\_UPI0012EBF5E3 | 98.1 | 4.1e-08 | 7.5e-14 | 65.0 | 72 | (50, 121) | 144 | (19, 90) | 97 | helix-turn-helix domain-containing protein | helix-turn-helix domain-containing protein | | uniclust | UniRef100\_A3YAG8 | 98.1 | 4e-08 | 7.6e-14 | 68.8 | 72 | (56, 127) | 144 | (48, 119) | 123 | Mor transcription activator domain-containing protein | Mor transcription activator domain-containing protein | | uniclust | UniRef100\_A0A3C0QNU7 | 98.1 | 4.2e-08 | 7.7e-14 | 68.7 | 116 | (3, 120) | 144 | (9, 136) | 137 | Mor transcription activator domain-containing protein | Mor transcription activator domain-containing protein | | uniclust | UniRef100\_A4EV43 | 98.1 | 4.3e-08 | 7.8e-14 | 69.2 | 98 | (7, 121) | 144 | (45, 142) | 144 | Helix-turn-helix domain-containing protein | Helix-turn-helix domain-containing protein | | uniclust | UniRef100\_A0A0F5P8U2 | 98.1 | 4.5e-08 | 8.2e-14 | 71.2 | 104 | (7, 120) | 144 | (1, 106) | 176 | Mor transcription activator domain-containing protein | Mor transcription activator domain-containing protein | | uniclust | UniRef100\_A0A660E659 | 98.1 | 4.2e-08 | 8.4e-14 | 67.2 | 78 | (49, 127) | 144 | (14, 91) | 96 | Mor transcription activator domain-containing protein | Mor transcription activator domain-containing protein | | uniclust | UniRef100\_UPI001145015F | 98.1 | 4.9e-08 | 9e-14 | 67.2 | 107 | (3, 121) | 144 | (11, 121) | 122 | helix-turn-helix domain-containing protein | helix-turn-helix domain-containing protein | | uniclust | UniRef100\_A0A8J6NST4 | 98.1 | 4.9e-08 | 9e-14 | 59.5 | 57 | (64, 121) | 144 | (4, 60) | 61 | Mor transcription activator domain-containing protein | Mor transcription activator domain-containing protein | | uniclust | UniRef100\_UPI000AAA50FC | 98.1 | 5e-08 | 9.1e-14 | 63.3 | 66 | (58, 123) | 144 | (2, 75) | 86 | Mor transcription activator family protein | Mor transcription activator family protein | | uniclust | UniRef100\_A0A427CJC7 | 98.1 | 5e-08 | 9.1e-14 | 69.2 | 74 | (51, 124) | 144 | (60, 133) | 148 | Mor transcription activator-like protein | Mor transcription activator-like protein | | uniclust | UniRef100\_A0A095V3V3 | 98.1 | 5e-08 | 9.1e-14 | 71.7 | 108 | (2, 120) | 144 | (21, 135) | 190 | Transposase | Transposase | | uniclust | UniRef100\_UPI001157E51F | 98.1 | 5.2e-08 | 9.5e-14 | 71.8 | 108 | (2, 122) | 144 | (27, 142) | 193 | helix-turn-helix domain-containing protein | helix-turn-helix domain-containing protein | | uniclust | UniRef100\_A0A653XI87 | 98.1 | 5.3e-08 | 9.7e-14 | 64.3 | 73 | (54, 126) | 144 | (19, 91) | 95 | Mor transcription activator domain-containing protein | Mor transcription activator domain-containing protein | | uniclust | UniRef100\_A0A1C6AG62 | 98.1 | 5.3e-08 | 1e-13 | 65.6 | 77 | (50, 126) | 144 | (17, 94) | 99 | Uncharacterized conserved protein | Uncharacterized conserved protein | | uniclust | UniRef100\_A0A443K8S6 | 98.1 | 5.6e-08 | 1e-13 | 70.2 | 101 | (13, 125) | 144 | (35, 138) | 167 | Helix-turn-helix domain-containing protein | Helix-turn-helix domain-containing protein | | uniclust | UniRef100\_A0A921TDK8 | 98.1 | 5.8e-08 | 1.1e-13 | 75.1 | 112 | (6, 128) | 144 | (167, 278) | 282 | Uncharacterized protein (Fragment) | Uncharacterized protein (Fragment) | | uniclust | UniRef100\_A0A0Q2XPP4 | 98.1 | 5.8e-08 | 1.1e-13 | 73.6 | 106 | (6, 125) | 144 | (9, 118) | 238 | Mor transcription activator domain-containing protein | Mor transcription activator domain-containing protein | | uniclust | UniRef100\_UPI001C0C1CEE | 98.1 | 6.4e-08 | 1.2e-13 | 69.3 | 118 | (2, 132) | 144 | (23, 146) | 157 | hypothetical protein | hypothetical protein | | uniclust | UniRef100\_UPI001EE639DA | 98.1 | 6.5e-08 | 1.2e-13 | 65.5 | 77 | (49, 125) | 144 | (19, 103) | 110 | Mor transcription activator family protein | Mor transcription activator family protein | | uniclust | UniRef100\_A0A099VBY3 | 98.1 | 6.2e-08 | 1.2e-13 | 64.1 | 62 | (61, 127) | 144 | (18, 79) | 84 | Mor transcription activator domain-containing protein | Mor transcription activator domain-containing protein | | uniclust | UniRef100\_UPI0016685DF9 | 98.1 | 6.6e-08 | 1.2e-13 | 69.6 | 119 | (4, 122) | 144 | (23, 147) | 164 | helix-turn-helix domain-containing protein | helix-turn-helix domain-containing protein | | uniclust | UniRef100\_A0A351VJ38 | 98.1 | 6.5e-08 | 1.3e-13 | 64.9 | 65 | (57, 122) | 144 | (24, 88) | 91 | Mor transcription activator domain-containing protein | Mor transcription activator domain-containing protein | | uniclust | UniRef100\_UPI001FE71F71 | 98.1 | 6.9e-08 | 1.3e-13 | 60.6 | 58 | (69, 127) | 144 | (2, 59) | 71 | Mor transcription activator family protein | Mor transcription activator family protein | | uniclust | UniRef100\_UPI00223A6E0C | 98.1 | 6.8e-08 | 1.3e-13 | 63.5 | 66 | (60, 125) | 144 | (13, 78) | 90 | Mor transcription activator family protein | Mor transcription activator family protein | | uniclust | UniRef100\_A0A3E2N6R8 | 98.1 | 6e-08 | 1.3e-13 | 72.2 | 76 | (49, 127) | 144 | (55, 130) | 148 | Mor transcription activator family protein | Mor transcription activator family protein | | uniclust | UniRef100\_A0A1C3FDH3 | 98.1 | 6e-08 | 1.3e-13 | 68.6 | 74 | (49, 125) | 144 | (25, 98) | 108 | Mor transcription activator domain-containing protein | Mor transcription activator domain-containing protein | | uniclust | UniRef100\_UPI00226DF99C | 98.1 | 7.1e-08 | 1.3e-13 | 65.7 | 65 | (59, 124) | 144 | (36, 100) | 114 | Mor transcription activator family protein | Mor transcription activator family protein | | uniclust | UniRef100\_A0A5C7LYQ9 | 98.1 | 7.3e-08 | 1.3e-13 | 65.5 | 78 | (50, 127) | 144 | (27, 104) | 112 | Mor transcription activator domain-containing protein | Mor transcription activator domain-containing protein | | uniclust | UniRef100\_A0A962WFG0 | 98.1 | 7.4e-08 | 1.4e-13 | 67.1 | 115 | (3, 126) | 144 | (4, 120) | 131 | Uncharacterized protein | Uncharacterized protein | | uniclust | UniRef100\_UPI0012EB89D4 | 98.0 | 8.4e-08 | 1.5e-13 | 67.0 | 102 | (6, 124) | 144 | (26, 127) | 133 | hypothetical protein | hypothetical protein | | uniclust | UniRef100\_UPI00055724BA | 98.0 | 8.8e-08 | 1.6e-13 | 63.4 | 76 | (52, 127) | 144 | (10, 87) | 91 | Mor transcription activator family protein | Mor transcription activator family protein | | pdb70 | 1RR7\_A | 99.2 | 2.2e-15 | 1.8e-19 | 100.9 | 93 | (2, 125) | 144 | (27, 119) | 129 | Middle operon regulator | 1RR7\_A Middle operon regulator Mor, TRANSCRIPTION | | pdb70 | 6EO2\_A | 96.5 | 8e-06 | 7.5e-10 | 53.1 | 61 | (79, 142) | 144 | (146, 206) | 210 | Transcriptional regulatory protein RcsB | 6EO2\_A Transcriptional regulatory protein RcsB Transcriptional factor, DNA BINDING PROTEIN | | pdb70 | 5O8Z\_A | 96.5 | 8.3e-06 | 7.8e-10 | 53.4 | 61 | (79, 142) | 144 | (146, 206) | 216 | Transcriptional regulatory protein RcsB | 5O8Z\_A Transcriptional regulatory protein RcsB Response regulator Transcriptional factor, TRANSCRIPTION | | pdb70 | 5U8K\_A | 96.4 | 1.4e-05 | 1.3e-09 | 52.9 | 61 | (80, 143) | 144 | (155, 223) | 231 | Response regulator | 5U8K\_A Response regulator Repressor of iron transporter, Aspartate-less HET: MSE | | pdb70 | 5XT2\_C | 96.4 | 1.5e-05 | 1.4e-09 | 52.2 | 60 | (81, 143) | 144 | (150, 209) | 215 | Response regulator FixJ | 5XT2\_C Response regulator FixJ Response Regulater, FixJ/NarL Family, DNA HET: GOL, FMT | | pdb70 | 5XSO\_A | 96.3 | 2.1e-05 | 2e-09 | 51.4 | 60 | (81, 143) | 144 | (150, 209) | 215 | Response regulator FixJ | 5XSO\_A Response regulator FixJ Response Regulater, FixJ/NarL Family, DNA HET: FMT, GOL | | pdb70 | 5U8K\_B | 96.3 | 2.1e-05 | 2e-09 | 51.9 | 61 | (80, 143) | 144 | (155, 223) | 231 | Response regulator | 5U8K\_B Response regulator Repressor of iron transporter, Aspartate-less HET: MSE | | pdb70 | 1A04\_B | 96.2 | 2.2e-05 | 2.1e-09 | 51.1 | 59 | (82, 143) | 144 | (153, 211) | 215 | NITRATE/NITRITE RESPONSE REGULATOR PROTEIN NARL | 1A04\_B NITRATE/NITRITE RESPONSE REGULATOR PROTEIN NARL SIGNAL TRANSDUCTION PROTEIN, RESPONSE REGULATORS | | pdb70 | 6MWZ\_A | 96.2 | 2.5e-05 | 2.2e-09 | 54.5 | 61 | (78, 141) | 144 | (171, 231) | 239 | Transcriptional regulator LasR, ALA-HIS-HIS-HIS-HIS-ALA | 6MWZ\_A Transcriptional regulator LasR, ALA-HIS-HIS-HIS-HIS-ALA Transcriptional activator, TRANSCRIPTION HET: K5M | | pdb70 | 1RNL\_A | 96.2 | 2.4e-05 | 2.2e-09 | 51.0 | 59 | (82, 143) | 144 | (153, 211) | 215 | NITRATE/NITRITE RESPONSE REGULATOR PROTEIN NARL | 1RNL\_A NITRATE/NITRITE RESPONSE REGULATOR PROTEIN NARL RESPONSE REGULATORS, TWO-COMPONENT SYSTEMS, SIGNAL HET: GOL | | pdb70 | 5VFA\_A | 96.2 | 2.5e-05 | 2.3e-09 | 51.6 | 61 | (80, 143) | 144 | (154, 222) | 230 | Response regulator | 5VFA\_A Response regulator Repressor of iron transporter, Aspartate-less | | pdb70 | 4ZMR\_A | 96.2 | 2.6e-05 | 2.4e-09 | 50.4 | 61 | (80, 143) | 144 | (143, 203) | 207 | Response regulator | 4ZMR\_A Response regulator Response regulator, DNA BINDING PROTEIN | | pdb70 | 4ZMS\_A | 96.2 | 2.6e-05 | 2.4e-09 | 50.4 | 61 | (80, 143) | 144 | (143, 203) | 207 | Response regulator | 4ZMS\_A Response regulator Response regulator, DNA BINDING PROTEIN HET: 4QT | | pdb70 | 4YN8\_A | 96.2 | 2.7e-05 | 2.5e-09 | 50.2 | 60 | (81, 143) | 144 | (140, 199) | 203 | ChrA | 4YN8\_A ChrA DNA-binding, helix-turn-helix, DNA BINDING PROTEIN HET: SO4 | | pdb70 | 2HQR\_A | 96.2 | 2.8e-05 | 2.6e-09 | 51.4 | 60 | (81, 143) | 144 | (141, 209) | 223 | Putative TRANSCRIPTIONAL REGULATOR | 2HQR\_A Putative TRANSCRIPTIONAL REGULATOR Phosporylation-Independent Response Regulator, H. pylori | | pdb70 | 1L3L\_A | 96.2 | 3e-05 | 2.6e-09 | 54.0 | 61 | (79, 142) | 144 | (169, 229) | 234 | Transcriptional activator protein traR/DNA Complex | 1L3L\_A Transcriptional activator protein traR/DNA Complex helix-turn-helix DNA binding motif, alpha/beta/alpha HET: LAE, MSE | | pdb70 | 4KFC\_A | 96.1 | 3e-05 | 2.8e-09 | 51.3 | 63 | (79, 143) | 144 | (149, 220) | 227 | KDP operon transcriptional regulatory protein | 4KFC\_A KDP operon transcriptional regulatory protein receiver domain, DNA-binding domain, TRANSCRIPTION | | pdb70 | 4LFU\_A | 96.1 | 3.2e-05 | 2.8e-09 | 54.5 | 61 | (79, 142) | 144 | (175, 235) | 248 | Regulatory protein SdiA | 4LFU\_A Regulatory protein SdiA LuxR-type quorum sensing receptor, transcription HET: PG4 | | pdb70 | 5F64\_C | 96.1 | 3.1e-05 | 2.9e-09 | 49.6 | 60 | (81, 143) | 144 | (144, 203) | 207 | Positive transcription regulator EvgA | 5F64\_C Positive transcription regulator EvgA transcription regulator, sensor, EvgS, structural | | pdb70 | 1YS7\_B | 96.1 | 3.3e-05 | 3.1e-09 | 51.2 | 62 | (79, 143) | 144 | (155, 225) | 233 | Transcriptional regulatory protein prrA | 1YS7\_B Transcriptional regulatory protein prrA Response regulator, DNA binding domain HET: GOL | | pdb70 | 2Q0O\_A | 96.1 | 3.7e-05 | 3.3e-09 | 53.5 | 62 | (78, 142) | 144 | (170, 231) | 236 | quorum sensing transcription factor TraR | 2Q0O\_A quorum sensing transcription factor TraR helix-turn-helix, two-helix coiled coil, TRANSCRIPTION HET: LAE | | pdb70 | 3Q9S\_A | 96.1 | 3.8e-05 | 3.5e-09 | 52.4 | 62 | (79, 143) | 144 | (178, 248) | 249 | DNA-binding response regulator | 3Q9S\_A DNA-binding response regulator response regulator, DNA BINDING PROTEIN | | pdb70 | 1YS6\_B | 96.1 | 3.9e-05 | 3.7e-09 | 50.6 | 61 | (80, 143) | 144 | (156, 225) | 233 | Transcriptional regulatory protein prrA | 1YS6\_B Transcriptional regulatory protein prrA Response regulator protein, Dna binding | | pdb70 | 6MWL\_A | 96.0 | 4.3e-05 | 3.8e-09 | 53.3 | 60 | (79, 141) | 144 | (172, 231) | 239 | Transcriptional regulator LasR | 6MWL\_A Transcriptional regulator LasR Transcriptional activator, TRANSCRIPTION HET: K5G | | pdb70 | 4Y13\_A | 96.0 | 4.4e-05 | 3.8e-09 | 53.8 | 59 | (80, 141) | 144 | (176, 234) | 246 | SdiA in complex with octanoyl-rac-glycerol | 4Y13\_A SdiA in complex with octanoyl-rac-glycerol Quorum sensor, DNA, DNA BINDING HET: SO4, MSE, GOL, 480 | | pdb70 | 3SZT\_A | 96.0 | 4.4e-05 | 3.9e-09 | 53.2 | 61 | (78, 141) | 144 | (170, 230) | 237 | PhzR | 3SZT\_A PhzR quorum sensing acyl-homoserine lactone, Helix-turn-Helix HET: OHN | | pdb70 | 6CBQ\_A | 96.0 | 4.4e-05 | 3.9e-09 | 53.2 | 61 | (78, 141) | 144 | (170, 230) | 237 | Chromatin assembly factor 1 subunit | 6CBQ\_A Chromatin assembly factor 1 subunit LuxR-type AHL receptor, Pseudomonas aeruginosa HET: EVY | | pdb70 | 6CC0\_B | 96.0 | 4.4e-05 | 3.9e-09 | 53.2 | 61 | (78, 141) | 144 | (170, 230) | 237 | Chromatin assembly factor 1 subunit | 6CC0\_B Chromatin assembly factor 1 subunit LuxR-type AHL receptor, Pseudomonas aeruginosa HET: EWM | | pdb70 | 4GVP\_C | 96.0 | 4.2e-05 | 4e-09 | 49.3 | 58 | (83, 143) | 144 | (146, 203) | 208 | Response regulator protein vraR | 4GVP\_C Response regulator protein vraR Response regulator, Two-component system, Bacterial | | pdb70 | 4IF4\_A | 96.0 | 4.2e-05 | 4e-09 | 49.3 | 58 | (83, 143) | 144 | (146, 203) | 208 | Response regulator protein VraR | 4IF4\_A Response regulator protein VraR Response regulator, Two-component system, Bacterial HET: BEF, SO4 | | pdb70 | 5HEV\_C | 96.0 | 4.2e-05 | 4e-09 | 49.3 | 59 | (82, 143) | 144 | (147, 205) | 210 | Response regulator protein VraR | 5HEV\_C Response regulator protein VraR Enterococcus faecium, LiaR, response regulator HET: BEF | | pdb70 | 4HYE\_B | 96.0 | 4.3e-05 | 4e-09 | 50.2 | 59 | (82, 143) | 144 | (158, 216) | 220 | Response regulator | 4HYE\_B Response regulator Cytosol, TRANSCRIPTION ACTIVATOR | | pdb70 | 3QP6\_A | 96.0 | 4.8e-05 | 4.2e-09 | 54.2 | 59 | (80, 141) | 144 | (194, 252) | 265 | CviR transcriptional regulator | 3QP6\_A CviR transcriptional regulator quorum sensing, agonist, antagonist, LuxR HET: HL6 | | pdb70 | 5HEV\_B | 96.0 | 4.5e-05 | 4.3e-09 | 49.1 | 59 | (82, 143) | 144 | (147, 205) | 210 | Response regulator protein VraR | 5HEV\_B Response regulator protein VraR Enterococcus faecium, LiaR, response regulator HET: BEF | | pdb70 | 1KGS\_A | 95.9 | 6.1e-05 | 5.8e-09 | 49.3 | 60 | (81, 143) | 144 | (149, 217) | 225 | DNA BINDING RESPONSE REGULATOR D | 1KGS\_A DNA BINDING RESPONSE REGULATOR D DNA-binding protein, alph-beta sandwich, winged-helix HET: SCN | | pdb70 | 1YIO\_A | 95.9 | 6.3e-05 | 5.9e-09 | 48.8 | 56 | (84, 142) | 144 | (143, 198) | 208 | response regulator StyR | 1YIO\_A response regulator StyR Transcription regulation, DNA BINDING PROTEIN | | pdb70 | 2OQR\_A | 95.9 | 6.6e-05 | 6.1e-09 | 49.6 | 50 | (81, 130) | 144 | (154, 212) | 230 | Sensory transduction protein regX3 | 2OQR\_A Sensory transduction protein regX3 response regulator, winged-helix-turn-helix, dna-binding, 3D | | pdb70 | 4KNY\_B | 95.8 | 7.2e-05 | 6.7e-09 | 49.3 | 61 | (81, 143) | 144 | (151, 220) | 227 | KDP operon transcriptional regulatory protein | 4KNY\_B KDP operon transcriptional regulatory protein receiver domain, DNA-binding domain, TRANSCRIPTION | | pdb70 | 2GWR\_A | 95.8 | 8.2e-05 | 7.6e-09 | 49.9 | 52 | (79, 130) | 144 | (149, 209) | 238 | DNA-binding response regulator mtrA | 2GWR\_A DNA-binding response regulator mtrA Two-component regulatory system, Transcription regulation HET: GOL | | pdb70 | 3R0J\_A | 95.7 | 9.7e-05 | 9.1e-09 | 49.6 | 59 | (81, 143) | 144 | (174, 241) | 250 | POSSIBLE TWO COMPONENT SYSTEM RESPONSE | 3R0J\_A POSSIBLE TWO COMPONENT SYSTEM RESPONSE beta-alpha fold, winged helix-turn-helix, transcription HET: SO4, MSE, MSO | | pdb70 | 5ED4\_B | 95.7 | 0.00011 | 9.8e-09 | 49.5 | 59 | (80, 142) | 144 | (173, 240) | 250 | Response regulator/DNA Complex | 5ED4\_B Response regulator/DNA Complex protein-DNA complex, winged helix-turn-helix, direct HET: EDO | | pdb70 | 3KLO\_A | 95.5 | 0.00014 | 1.4e-08 | 47.7 | 59 | (80, 141) | 144 | (156, 214) | 225 | Transcriptional regulator VpsT | 3KLO\_A Transcriptional regulator VpsT REC domain, HTH domain, DNA-binding HET: TAR, C2E | | pdb70 | 1P2F\_A | 95.5 | 0.00016 | 1.5e-08 | 47.5 | 61 | (81, 143) | 144 | (143, 210) | 220 | Response Regulator | 1P2F\_A Response Regulator response regulator, DrrB, OmpR/PhoB, Transcription | | pdb70 | 6KJU\_B | 95.3 | 0.00024 | 2.1e-08 | 49.6 | 57 | (82, 141) | 144 | (154, 210) | 254 | Transcriptional regulator LuxR family/DNA Complex | 6KJU\_B Transcriptional regulator LuxR family/DNA Complex Vibrio cholera, Quorum sensing, transcription HET: A1U | | pdb70 | 4LDZ\_A | 95.3 | 0.00025 | 2.4e-08 | 45.4 | 59 | (82, 143) | 144 | (140, 198) | 203 | Transcriptional regulatory protein DesR | 4LDZ\_A Transcriptional regulatory protein DesR Response regulator, two-component system, transcription HET: GOL | | pdb70 | 4B09\_E | 94.8 | 0.00053 | 5e-08 | 45.2 | 50 | (81, 130) | 144 | (158, 216) | 240 | TRANSCRIPTIONAL REGULATORY PROTEIN BAER | 4B09\_E TRANSCRIPTIONAL REGULATORY PROTEIN BAER TRANSCRIPTION, RESPONSE REGULATOR, DNA BINDING HET: TBR, CAS | | pdb70 | 4B09\_H | 94.8 | 0.00053 | 5e-08 | 45.2 | 50 | (81, 130) | 144 | (158, 216) | 240 | TRANSCRIPTIONAL REGULATORY PROTEIN BAER | 4B09\_H TRANSCRIPTIONAL REGULATORY PROTEIN BAER TRANSCRIPTION, RESPONSE REGULATOR, DNA BINDING HET: TBR, CAS | | pdb70 | 3C3W\_B | 94.5 | 0.00089 | 8.2e-08 | 43.9 | 48 | (83, 130) | 144 | (149, 196) | 225 | TWO COMPONENT TRANSCRIPTIONAL REGULATORY PROTEIN | 3C3W\_B TWO COMPONENT TRANSCRIPTIONAL REGULATORY PROTEIN RESPONSE REGULATOR, TWO-COMPONENT REGULATORY SYSTEM | | pdb70 | 3C3W\_A | 94.4 | 0.00094 | 8.7e-08 | 43.8 | 48 | (84, 131) | 144 | (150, 197) | 225 | TWO COMPONENT TRANSCRIPTIONAL REGULATORY PROTEIN | 3C3W\_A TWO COMPONENT TRANSCRIPTIONAL REGULATORY PROTEIN RESPONSE REGULATOR, TWO-COMPONENT REGULATORY SYSTEM | | pdb70 | 4S05\_A | 94.3 | 0.001 | 9.7e-08 | 43.8 | 50 | (80, 129) | 144 | (146, 204) | 232 | DNA-binding transcriptional regulator BasR | 4S05\_A DNA-binding transcriptional regulator BasR Two-Component System, response regulator, PmrA | |
| Top keywords  (threshold 1.00e-03 (evalue)) | **transcription, Mor, activator, domain\_containing, regulator, Helix\_turn\_helix, Transcriptional, Response, hypothetical, DNA** |
| Output files | ../../similar\_sequences/61\_FANPEZAQ\_CDS\_0061\_merged.svg ../../similar\_sequences/61\_FANPEZAQ\_CDS\_0061\_pdb70.a3m ../../similar\_sequences/61\_FANPEZAQ\_CDS\_0061\_pdb70.hhr ../../similar\_sequences/61\_FANPEZAQ\_CDS\_0061\_uniclust.a3m ../../similar\_sequences/61\_FANPEZAQ\_CDS\_0061\_uniclust.hhr |

#### Structure prediction (AlphaFold)2

|  |  |
| --- | --- |
| Stats | xml version="1.0" encoding="utf-8" standalone="no"?       2024-09-02T21:09:56.983254 image/svg+xml   Matplotlib v3.7.2, https://matplotlib.org/ |
| Predicted structure | **NGL Viewer Controls:**  - Center: *Left-Click* - Rotate: *Left-Click + Drag* - Translate: *Right-Click + Drag* - Zoom: *Shift + Left-Click + Drag* |
| Output files | ../../predicted\_structures/61\_FANPEZAQ\_CDS\_0061/features.pkl ../../predicted\_structures/61\_FANPEZAQ\_CDS\_0061/ranked\_0.pdb ../../predicted\_structures/61\_FANPEZAQ\_CDS\_0061/ranked\_0\_plots.svg ../../predicted\_structures/61\_FANPEZAQ\_CDS\_0061/result\_model\_1\_ptm\_pred\_0.pkl |

#### Structure similarity search results (Foldseek)3

|  |  |
| --- | --- |
| Structure databases searched | Pdb, Afdb-proteome, Afdb-uniprot50 |
| Results, scheme(s)  (Top layers only, threshold 1.00e-02 (evalue)) | xml version="1.0" encoding="utf-8" standalone="no"?       2024-09-02T21:11:29.367064 image/svg+xml   Matplotlib v3.7.2, https://matplotlib.org/ |
| Results, table  (threshold 1.00e-02 (evalue)) | | db | id | prob | evalue | bits | fident | alnlen | mismatch | gapopen | qstart | qend | tstart | tend | name | description | | --- | --- | --- | --- | --- | --- | --- | --- | --- | --- | --- | --- | --- | --- | --- | | afdb-uniprot50 | AF-A0A8A6KH34-F1-MODEL\_V4 | 1.0 | 9.589e-13 | 518 | 0.438 | 121 | 66 | 1 | 1 | 119 | 4 | 124 | Uncharacterized protein | Uncharacterized protein | | afdb-uniprot50 | AF-A0A6M4YR27-F1-MODEL\_V4 | 1.0 | 8.436e-14 | 509 | 0.471 | 140 | 68 | 2 | 2 | 135 | 7 | 146 | Uncharacterized protein | Uncharacterized protein | | afdb-uniprot50 | AF-A0A1S0V4H7-F1-MODEL\_V4 | 1.0 | 3.799e-13 | 505 | 0.419 | 143 | 83 | 0 | 1 | 143 | 3 | 145 | Uncharacterized protein | Uncharacterized protein | | afdb-uniprot50 | AF-A0A653KZR3-F1-MODEL\_V4 | 1.0 | 7.962e-14 | 498 | 0.456 | 149 | 73 | 3 | 2 | 144 | 7 | 153 | Uncharacterized protein | Uncharacterized protein | | afdb-uniprot50 | AF-A0A060HA06-F1-MODEL\_V4 | 1.0 | 3.193e-13 | 495 | 0.412 | 148 | 80 | 2 | 1 | 144 | 44 | 188 | Uncharacterized protein | Uncharacterized protein | | afdb-uniprot50 | AF-A0A6L6JDU6-F1-MODEL\_V4 | 1.0 | 1.944e-11 | 494 | 0.443 | 124 | 66 | 1 | 1 | 124 | 80 | 200 | Uncharacterized protein | Uncharacterized protein | | afdb-uniprot50 | AF-A0A315D133-F1-MODEL\_V4 | 1.0 | 4.519e-13 | 489 | 0.458 | 133 | 65 | 2 | 2 | 127 | 4 | 136 | Uncharacterized protein | Uncharacterized protein | | afdb-uniprot50 | AF-K5Z2H2-F1-MODEL\_V4 | 1.0 | 1.029e-11 | 479 | 0.447 | 123 | 63 | 2 | 1 | 118 | 94 | 216 | Uncharacterized protein | Uncharacterized protein | | afdb-uniprot50 | AF-A0A1H2R5R9-F1-MODEL\_V4 | 1.0 | 1.944e-11 | 478 | 0.461 | 117 | 60 | 2 | 2 | 118 | 8 | 121 | Uncharacterized protein | Uncharacterized protein | | afdb-uniprot50 | AF-A0A291LZN5-F1-MODEL\_V4 | 1.0 | 8.261e-11 | 455 | 0.418 | 122 | 68 | 1 | 1 | 122 | 13 | 131 | Uncharacterized protein | Uncharacterized protein | | afdb-uniprot50 | AF-A0A660NN87-F1-MODEL\_V4 | 1.0 | 1.835e-11 | 455 | 0.428 | 119 | 66 | 1 | 2 | 118 | 17 | 135 | Uncharacterized protein | Uncharacterized protein | | afdb-uniprot50 | AF-A0A2S0MNB5-F1-MODEL\_V4 | 1.0 | 6.185e-11 | 444 | 0.373 | 126 | 75 | 2 | 1 | 125 | 7 | 129 | Uncharacterized protein | Uncharacterized protein | | afdb-uniprot50 | AF-A0A7L9Q1W2-F1-MODEL\_V4 | 1.0 | 2.284e-12 | 419 | 0.473 | 133 | 58 | 4 | 1 | 121 | 1 | 133 | Uncharacterized protein | Uncharacterized protein | | afdb-uniprot50 | AF-A0A241VCE8-F1-MODEL\_V4 | 1.0 | 4.176e-10 | 416 | 0.352 | 125 | 78 | 2 | 1 | 122 | 4 | 128 | Uncharacterized protein | Uncharacterized protein | | afdb-uniprot50 | AF-A0A143DGK1-F1-MODEL\_V4 | 1.0 | 1.254e-09 | 413 | 0.406 | 118 | 67 | 2 | 1 | 118 | 1 | 115 | Uncharacterized protein | Uncharacterized protein | | afdb-uniprot50 | AF-A0A4Q3Z6S0-F1-MODEL\_V4 | 1.0 | 4.176e-10 | 403 | 0.386 | 119 | 69 | 2 | 1 | 118 | 6 | 121 | Uncharacterized protein | Uncharacterized protein | | afdb-uniprot50 | AF-A0A410UF43-F1-MODEL\_V4 | 1.0 | 1.654e-10 | 396 | 0.338 | 118 | 78 | 0 | 1 | 118 | 17 | 134 | Uncharacterized protein | Uncharacterized protein | | afdb-uniprot50 | AF-A0A150HM30-F1-MODEL\_V4 | 1.0 | 2.628e-10 | 391 | 0.314 | 124 | 82 | 2 | 1 | 121 | 6 | 129 | Uncharacterized protein | Uncharacterized protein | | afdb-uniprot50 | AF-A0A7T7R8M9-F1-MODEL\_V4 | 1.0 | 1.968e-10 | 380 | 0.354 | 124 | 77 | 2 | 3 | 123 | 8 | 131 | Uncharacterized protein | Uncharacterized protein | | afdb-uniprot50 | AF-T1XCZ5-F1-MODEL\_V4 | 1.0 | 1.944e-11 | 380 | 0.428 | 133 | 68 | 4 | 1 | 125 | 1 | 133 | Uncharacterized protein | Uncharacterized protein | | afdb-uniprot50 | AF-A0A4R7UHV5-F1-MODEL\_V4 | 1.0 | 7.03e-10 | 373 | 0.324 | 145 | 88 | 2 | 3 | 144 | 4 | 141 | Helix-turn-helix protein | Helix-turn-helix protein | | afdb-uniprot50 | AF-A0A1I1F6V4-F1-MODEL\_V4 | 1.0 | 1.753e-10 | 370 | 0.419 | 124 | 67 | 3 | 1 | 122 | 3 | 123 | Uncharacterized protein | Uncharacterized protein | | afdb-uniprot50 | AF-A0A3G6WB74-F1-MODEL\_V4 | 1.0 | 1.198e-08 | 337 | 0.273 | 128 | 90 | 1 | 1 | 128 | 62 | 186 | Uncharacterized protein | Uncharacterized protein | | afdb-uniprot50 | AF-A0A3A1P024-F1-MODEL\_V4 | 1.0 | 7.893e-10 | 334 | 0.343 | 128 | 81 | 2 | 1 | 125 | 1 | 128 | Mor domain-containing protein | Mor domain-containing protein | | afdb-uniprot50 | AF-A0A7V8FKC4-F1-MODEL\_V4 | 1.0 | 1.6e-08 | 328 | 0.343 | 131 | 75 | 2 | 2 | 128 | 6 | 129 | Mor domain-containing protein | Mor domain-containing protein | | afdb-uniprot50 | AF-A0A373B2W6-F1-MODEL\_V4 | 1.0 | 4.228e-09 | 324 | 0.347 | 115 | 72 | 2 | 7 | 118 | 11 | 125 | Mor domain-containing protein | Mor domain-containing protein | | afdb-uniprot50 | AF-A0A4Q7BE38-F1-MODEL\_V4 | 1.0 | 1.007e-08 | 324 | 0.314 | 121 | 78 | 3 | 1 | 118 | 49 | 167 | Mor domain-containing protein | Mor domain-containing protein | | afdb-uniprot50 | AF-A0A291FP91-F1-MODEL\_V4 | 1.0 | 2.292e-07 | 310 | 0.282 | 117 | 81 | 1 | 2 | 118 | 63 | 176 | Uncharacterized protein | Uncharacterized protein | | afdb-uniprot50 | AF-A0A7V7G2S6-F1-MODEL\_V4 | 1.0 | 3.354e-09 | 305 | 0.285 | 175 | 81 | 5 | 1 | 144 | 1 | 162 | Uncharacterized protein | Uncharacterized protein | | afdb-uniprot50 | AF-A0A1I5P0R3-F1-MODEL\_V4 | 1.0 | 1.88e-09 | 303 | 0.337 | 148 | 90 | 3 | 2 | 143 | 32 | 177 | Uncharacterized protein | Uncharacterized protein | | afdb-uniprot50 | AF-A0A158S129-F1-MODEL\_V4 | 1.0 | 2.854e-08 | 290 | 0.411 | 107 | 57 | 2 | 24 | 124 | 2 | 108 | Uncharacterized protein | Uncharacterized protein | | afdb-uniprot50 | AF-A0A7J0BLE3-F1-MODEL\_V4 | 1.0 | 7.729e-07 | 290 | 0.35 | 117 | 66 | 2 | 2 | 118 | 5 | 111 | Uncharacterized protein | Uncharacterized protein | | afdb-uniprot50 | AF-A0A158E8H2-F1-MODEL\_V4 | 1.0 | 1.548e-06 | 278 | 0.433 | 83 | 44 | 1 | 1 | 80 | 28 | 110 | Uncharacterized protein | Uncharacterized protein | | afdb-uniprot50 | AF-A0A4T2A620-F1-MODEL\_V4 | 1.0 | 7.729e-07 | 271 | 0.294 | 119 | 70 | 2 | 2 | 118 | 4 | 110 | HTH luxR-type domain-containing protein | HTH luxR-type domain-containing protein | | afdb-uniprot50 | AF-A0A7W9SAC2-F1-MODEL\_V4 | 1.0 | 1.951e-06 | 265 | 0.233 | 124 | 85 | 1 | 3 | 126 | 8 | 121 | Mor family transcriptional regulator | Mor family transcriptional regulator | | afdb-uniprot50 | AF-A0A4Y6UA46-F1-MODEL\_V4 | 1.0 | 2.727e-07 | 264 | 0.218 | 119 | 87 | 3 | 2 | 119 | 56 | 169 | Uncharacterized protein | Uncharacterized protein | | afdb-uniprot50 | AF-A0A7M3MKQ0-F1-MODEL\_V4 | 1.0 | 6.497e-07 | 263 | 0.294 | 119 | 74 | 2 | 3 | 121 | 40 | 148 | Mor domain-containing protein | Mor domain-containing protein | | afdb-uniprot50 | AF-A0A1F6G643-F1-MODEL\_V4 | 1.0 | 2.761e-06 | 254 | 0.225 | 124 | 85 | 2 | 7 | 129 | 2 | 115 | Mor domain-containing protein | Mor domain-containing protein | | afdb-uniprot50 | AF-A0A1J5T9J9-F1-MODEL\_V4 | 1.0 | 4.386e-06 | 253 | 0.314 | 121 | 70 | 3 | 4 | 122 | 13 | 122 | Mor transcription activator family protein | Mor transcription activator family protein | | afdb-uniprot50 | AF-A0A7C8HXL4-F1-MODEL\_V4 | 1.0 | 9.308e-06 | 246 | 0.308 | 120 | 74 | 2 | 2 | 119 | 6 | 118 | Sigma70\_r4\_2 domain-containing protein | Sigma70\_r4\_2 domain-containing protein | | afdb-uniprot50 | AF-A0A1F6GLC2-F1-MODEL\_V4 | 1.0 | 5.529e-06 | 244 | 0.26 | 119 | 78 | 1 | 7 | 125 | 2 | 110 | Mor domain-containing protein | Mor domain-containing protein | | afdb-uniprot50 | AF-A0A7C8HVY2-F1-MODEL\_V4 | 1.0 | 6.577e-06 | 244 | 0.288 | 125 | 77 | 3 | 1 | 120 | 3 | 120 | Uncharacterized protein | Uncharacterized protein | | afdb-uniprot50 | AF-A0A557QXB9-F1-MODEL\_V4 | 1.0 | 8.784e-06 | 244 | 0.25 | 116 | 77 | 1 | 3 | 118 | 24 | 129 | Mor domain-containing protein | Mor domain-containing protein | | afdb-uniprot50 | AF-A0A7Y8NKK1-F1-MODEL\_V4 | 1.0 | 1.738e-06 | 243 | 0.242 | 140 | 91 | 5 | 3 | 131 | 15 | 150 | Mor domain-containing protein | Mor domain-containing protein | | afdb-uniprot50 | AF-I3YGT9-F1-MODEL\_V4 | 1.0 | 1.567e-05 | 241 | 0.302 | 119 | 71 | 2 | 3 | 119 | 24 | 132 | Mor transcription activator-like protein | Mor transcription activator-like protein | | afdb-uniprot50 | AF-A0A423PRX3-F1-MODEL\_V4 | 1.0 | 1.173e-05 | 240 | 0.29 | 117 | 73 | 1 | 3 | 119 | 10 | 116 | Mor domain-containing protein | Mor domain-containing protein | | afdb-uniprot50 | AF-A0A1T2L9D4-F1-MODEL\_V4 | 1.0 | 6.577e-06 | 239 | 0.198 | 116 | 81 | 2 | 4 | 118 | 9 | 113 | Mor domain-containing protein | Mor domain-containing protein | | afdb-uniprot50 | AF-A0A7W6S2N3-F1-MODEL\_V4 | 1.0 | 3.907e-06 | 238 | 0.246 | 130 | 79 | 4 | 1 | 125 | 1 | 116 | DNA-binding transcriptional ArsR family regulator | DNA-binding transcriptional ArsR family regulator | | afdb-uniprot50 | AF-F9ZD98-F1-MODEL\_V4 | 1.0 | 3.48e-06 | 234 | 0.241 | 124 | 83 | 4 | 3 | 125 | 10 | 123 | Mor transcription activator domain protein | Mor transcription activator domain protein | | afdb-uniprot50 | AF-A0A1N6I1K7-F1-MODEL\_V4 | 1.0 | 1.243e-05 | 232 | 0.276 | 123 | 74 | 2 | 3 | 120 | 8 | 120 | Mor transcription activator family protein | Mor transcription activator family protein | | afdb-uniprot50 | AF-A0A1Y0N3Z3-F1-MODEL\_V4 | 1.0 | 6.207e-06 | 232 | 0.218 | 142 | 93 | 2 | 3 | 144 | 15 | 138 | Uncharacterized protein | Uncharacterized protein | | afdb-uniprot50 | AF-A0A3B9L6A9-F1-MODEL\_V4 | 1.0 | 3.325e-05 | 229 | 0.205 | 117 | 80 | 2 | 3 | 119 | 49 | 152 | Mor domain-containing protein | Mor domain-containing protein | | afdb-uniprot50 | AF-A0A2Z3IDQ1-F1-MODEL\_V4 | 1.0 | 1.951e-06 | 228 | 0.437 | 96 | 52 | 1 | 41 | 134 | 2 | 97 | Uncharacterized protein | Uncharacterized protein | | afdb-uniprot50 | AF-A0A832XIX8-F1-MODEL\_V4 | 1.0 | 2.067e-06 | 227 | 0.246 | 126 | 82 | 2 | 1 | 123 | 10 | 125 | Uncharacterized protein | Uncharacterized protein | | afdb-uniprot50 | AF-A0A4Z0WD49-F1-MODEL\_V4 | 1.0 | 1.094e-06 | 226 | 0.281 | 142 | 82 | 3 | 3 | 144 | 12 | 133 | Mor domain-containing protein | Mor domain-containing protein | | afdb-uniprot50 | AF-B0RUR6-F1-MODEL\_V4 | 1.0 | 5.218e-06 | 226 | 0.263 | 133 | 85 | 7 | 3 | 125 | 119 | 248 | Uncharacterized protein | Uncharacterized protein | | afdb-uniprot50 | AF-A0A4D7B5Y7-F1-MODEL\_V4 | 1.0 | 9.742e-07 | 223 | 0.293 | 133 | 73 | 5 | 3 | 125 | 48 | 169 | Uncharacterized protein | Uncharacterized protein | | afdb-uniprot50 | AF-A0A106BHZ3-F1-MODEL\_V4 | 1.0 | 1.317e-05 | 222 | 0.212 | 113 | 79 | 1 | 6 | 118 | 7 | 109 | Uncharacterized protein | Uncharacterized protein | | afdb-uniprot50 | AF-A0A512HA32-F1-MODEL\_V4 | 1.0 | 9.862e-06 | 222 | 0.238 | 126 | 83 | 3 | 3 | 128 | 11 | 123 | Mor domain-containing protein | Mor domain-containing protein | | afdb-uniprot50 | AF-A0A5S9PH66-F1-MODEL\_V4 | 1.0 | 2.321e-06 | 220 | 0.263 | 129 | 80 | 2 | 2 | 125 | 7 | 125 | Mor domain-containing protein | Mor domain-containing protein | | afdb-uniprot50 | AF-A0A522X0T2-F1-MODEL\_V4 | 1.0 | 8.393e-05 | 219 | 0.198 | 116 | 83 | 1 | 3 | 118 | 36 | 141 | Mor domain-containing protein | Mor domain-containing protein | | afdb-uniprot50 | AF-A0A2E2T381-F1-MODEL\_V4 | 1.0 | 5.529e-06 | 218 | 0.273 | 117 | 75 | 1 | 3 | 119 | 8 | 114 | Mor domain-containing protein | Mor domain-containing protein | | afdb-uniprot50 | AF-A0A4Q5L708-F1-MODEL\_V4 | 1.0 | 6.207e-06 | 218 | 0.169 | 118 | 88 | 1 | 4 | 121 | 35 | 142 | Mor domain-containing protein | Mor domain-containing protein | | afdb-uniprot50 | AF-A0A349MJQ0-F1-MODEL\_V4 | 1.0 | 2.925e-06 | 217 | 0.228 | 127 | 88 | 2 | 2 | 128 | 17 | 133 | Mor domain-containing protein | Mor domain-containing protein | | afdb-uniprot50 | AF-A0A7Y4W2P4-F1-MODEL\_V4 | 1.0 | 8.893e-05 | 215 | 0.172 | 116 | 86 | 1 | 3 | 118 | 6 | 111 | Mor domain-containing protein | Mor domain-containing protein | | afdb-uniprot50 | AF-A0A7Y7LP38-F1-MODEL\_V4 | 1.0 | 1.975e-05 | 215 | 0.231 | 138 | 87 | 4 | 3 | 139 | 15 | 134 | Mor family transcriptional regulator | Mor family transcriptional regulator | | afdb-uniprot50 | AF-A0A1Y5STF4-F1-MODEL\_V4 | 1.0 | 2.093e-05 | 215 | 0.254 | 118 | 78 | 1 | 3 | 120 | 12 | 119 | Mor transcription activator family protein | Mor transcription activator family protein | | afdb-uniprot50 | AF-A0A1I7CKY0-F1-MODEL\_V4 | 1.0 | 4.986e-05 | 214 | 0.194 | 118 | 84 | 2 | 2 | 119 | 27 | 133 | Mor transcription activator family protein | Mor transcription activator family protein | | afdb-uniprot50 | AF-A0A2G6DFJ6-F1-MODEL\_V4 | 1.0 | 0.0001333 | 214 | 0.177 | 118 | 85 | 2 | 5 | 120 | 52 | 159 | Mor domain-containing protein | Mor domain-containing protein | | afdb-uniprot50 | AF-A0A850RKP1-F1-MODEL\_V4 | 1.0 | 2.795e-05 | 213 | 0.255 | 129 | 84 | 2 | 3 | 129 | 20 | 138 | Transcriptional regulator | Transcriptional regulator | | afdb-uniprot50 | AF-A0A212KJX9-F1-MODEL\_V4 | 1.0 | 2.925e-06 | 211 | 0.223 | 130 | 88 | 3 | 1 | 127 | 2 | 121 | Mor domain-containing protein | Mor domain-containing protein | | afdb-uniprot50 | AF-M4NQN5-F1-MODEL\_V4 | 1.0 | 4.191e-05 | 211 | 0.173 | 144 | 98 | 3 | 3 | 144 | 13 | 137 | Uncharacterized protein | Uncharacterized protein | | afdb-uniprot50 | AF-X5MEB9-F1-MODEL\_V4 | 1.0 | 2.925e-06 | 210 | 0.256 | 117 | 75 | 4 | 3 | 118 | 8 | 113 | Uncharacterized protein | Uncharacterized protein | | afdb-uniprot50 | AF-A0A7D5NCC3-F1-MODEL\_V4 | 1.0 | 2.638e-05 | 210 | 0.217 | 129 | 85 | 3 | 3 | 127 | 5 | 121 | Mor domain-containing protein | Mor domain-containing protein | | afdb-uniprot50 | AF-A0A522VNP1-F1-MODEL\_V4 | 1.0 | 5.931e-05 | 209 | 0.279 | 118 | 72 | 4 | 3 | 118 | 12 | 118 | Mor domain-containing protein | Mor domain-containing protein | | afdb-uniprot50 | AF-A0A178HNW5-F1-MODEL\_V4 | 1.0 | 1.759e-05 | 209 | 0.246 | 126 | 83 | 3 | 2 | 126 | 12 | 126 | Uncharacterized protein | Uncharacterized protein | | afdb-uniprot50 | AF-T2G7I7-F1-MODEL\_V4 | 1.0 | 4.705e-05 | 209 | 0.184 | 119 | 87 | 1 | 3 | 121 | 25 | 133 | Uncharacterized protein | Uncharacterized protein | | afdb-uniprot50 | AF-A0A4R7ABL8-F1-MODEL\_V4 | 1.0 | 2.925e-06 | 209 | 0.219 | 146 | 90 | 4 | 3 | 144 | 6 | 131 | Homeodomain-like domain-containing protein | Homeodomain-like domain-containing protein | | afdb-uniprot50 | AF-A0A149SVR6-F1-MODEL\_V4 | 1.0 | 9.308e-06 | 208 | 0.243 | 111 | 75 | 4 | 8 | 118 | 2 | 103 | HTH\_7 domain-containing protein | HTH\_7 domain-containing protein | | afdb-uniprot50 | AF-A0A1Y6D0G8-F1-MODEL\_V4 | 1.0 | 3.907e-06 | 208 | 0.232 | 129 | 86 | 3 | 2 | 127 | 13 | 131 | Mor transcription activator family protein | Mor transcription activator family protein | | afdb-uniprot50 | AF-A0A840G8N0-F1-MODEL\_V4 | 1.0 | 1.975e-05 | 208 | 0.232 | 116 | 79 | 1 | 3 | 118 | 20 | 125 | Mor family transcriptional regulator | Mor family transcriptional regulator | | afdb-uniprot50 | AF-A0A349MJR3-F1-MODEL\_V4 | 1.0 | 6.659e-05 | 207 | 0.233 | 124 | 81 | 3 | 1 | 122 | 1 | 112 | Mor domain-containing protein | Mor domain-containing protein | | afdb-uniprot50 | AF-A0A2D3T993-F1-MODEL\_V4 | 1.0 | 5.529e-06 | 207 | 0.269 | 130 | 78 | 4 | 1 | 125 | 6 | 123 | Mor domain-containing protein | Mor domain-containing protein | | afdb-uniprot50 | AF-A0A1G0CQ82-F1-MODEL\_V4 | 1.0 | 5.597e-05 | 207 | 0.226 | 119 | 79 | 3 | 4 | 119 | 13 | 121 | Mor domain-containing protein | Mor domain-containing protein | | afdb-uniprot50 | AF-A0A0M0FPC9-F1-MODEL\_V4 | 1.0 | 4.191e-05 | 207 | 0.252 | 119 | 77 | 3 | 1 | 118 | 7 | 114 | Mor domain-containing protein | Mor domain-containing protein | | afdb-uniprot50 | AF-D5CUC0-F1-MODEL\_V4 | 1.0 | 5.597e-05 | 206 | 0.213 | 117 | 82 | 2 | 3 | 119 | 11 | 117 | Mor domain-containing protein | Mor domain-containing protein | | afdb-uniprot50 | AF-A0A1W9JSH1-F1-MODEL\_V4 | 1.0 | 2.217e-05 | 206 | 0.219 | 132 | 86 | 4 | 1 | 125 | 3 | 124 | Mor domain-containing protein | Mor domain-containing protein | | afdb-uniprot50 | AF-A9ID21-F1-MODEL\_V4 | 1.0 | 1.317e-05 | 206 | 0.204 | 142 | 93 | 2 | 3 | 144 | 12 | 133 | Mor domain-containing protein | Mor domain-containing protein | | afdb-uniprot50 | AF-A0A3M1WU79-F1-MODEL\_V4 | 1.0 | 4.986e-05 | 206 | 0.215 | 139 | 87 | 2 | 3 | 129 | 12 | 140 | DNA transposition protein | DNA transposition protein | | afdb-uniprot50 | AF-N6Y808-F1-MODEL\_V4 | 1.0 | 5.283e-05 | 205 | 0.251 | 135 | 80 | 4 | 4 | 129 | 26 | 148 | Uncharacterized protein | Uncharacterized protein | | afdb-uniprot50 | AF-I3YBF0-F1-MODEL\_V4 | 1.0 | 2.093e-05 | 205 | 0.214 | 135 | 86 | 3 | 3 | 127 | 18 | 142 | Mor transcription activator-like protein | Mor transcription activator-like protein | | afdb-uniprot50 | AF-A8I7L4-F1-MODEL\_V4 | 1.0 | 1.045e-05 | 202 | 0.201 | 119 | 85 | 1 | 1 | 119 | 5 | 113 | Uncharacterized protein | Uncharacterized protein | | afdb-uniprot50 | AF-A0A1H6U3N5-F1-MODEL\_V4 | 1.0 | 7.294e-07 | 202 | 0.429 | 121 | 59 | 2 | 14 | 125 | 2 | 121 | Winged helix-turn helix | Winged helix-turn helix | | afdb-uniprot50 | AF-A0A350X8X0-F1-MODEL\_V4 | 1.0 | 8.784e-06 | 202 | 0.243 | 115 | 76 | 2 | 7 | 121 | 14 | 117 | Mor domain-containing protein | Mor domain-containing protein | | afdb-uniprot50 | AF-A0A1V3HDH7-F1-MODEL\_V4 | 1.0 | 8.893e-05 | 201 | 0.232 | 116 | 78 | 2 | 3 | 118 | 20 | 124 | HTH psq-type domain-containing protein | HTH psq-type domain-containing protein | | afdb-uniprot50 | AF-A0A2S5CLI8-F1-MODEL\_V4 | 1.0 | 4.191e-05 | 201 | 0.194 | 118 | 81 | 3 | 3 | 118 | 12 | 117 | Mor domain-containing protein | Mor domain-containing protein | | afdb-uniprot50 | AF-A9C2C1-F1-MODEL\_V4 | 1.0 | 1.567e-05 | 201 | 0.2 | 150 | 100 | 3 | 1 | 139 | 2 | 142 | Uncharacterized protein | Uncharacterized protein | | afdb-uniprot50 | AF-A0A4R3J9E2-F1-MODEL\_V4 | 1.0 | 2.638e-05 | 200 | 0.246 | 134 | 83 | 4 | 1 | 128 | 1 | 122 | Homeodomain-like domain-containing protein | Homeodomain-like domain-containing protein | | afdb-uniprot50 | AF-A0A315EKC3-F1-MODEL\_V4 | 1.0 | 0.0002119 | 199 | 0.273 | 84 | 61 | 0 | 38 | 121 | 13 | 96 | Mor domain-containing protein | Mor domain-containing protein | | afdb-uniprot50 | AF-A0A1I2FJZ9-F1-MODEL\_V4 | 1.0 | 1.567e-05 | 199 | 0.211 | 123 | 86 | 2 | 3 | 125 | 10 | 121 | Mor transcription activator family protein | Mor transcription activator family protein | | afdb-uniprot50 | AF-A0A143Z940-F1-MODEL\_V4 | 1.0 | 1.759e-05 | 199 | 0.248 | 125 | 84 | 1 | 3 | 127 | 13 | 127 | Homeodomain-like domain-containing protein | Homeodomain-like domain-containing protein | | afdb-uniprot50 | AF-A0A375GAU5-F1-MODEL\_V4 | 1.0 | 7.921e-05 | 199 | 0.21 | 114 | 80 | 1 | 7 | 120 | 11 | 114 | Uncharacterized protein | Uncharacterized protein | | afdb-uniprot50 | AF-A0A368TMP4-F1-MODEL\_V4 | 1.0 | 6.207e-06 | 198 | 0.235 | 153 | 92 | 6 | 1 | 144 | 1 | 137 | Uncharacterized protein | Uncharacterized protein | | afdb-uniprot50 | AF-A0A1I5IF11-F1-MODEL\_V4 | 1.0 | 0.000267 | 198 | 0.157 | 121 | 88 | 2 | 3 | 119 | 59 | 169 | Mor transcription activator family protein | Mor transcription activator family protein | | afdb-uniprot50 | AF-A0A1I7AGL9-F1-MODEL\_V4 | 1.0 | 3.523e-05 | 197 | 0.216 | 120 | 84 | 1 | 1 | 120 | 8 | 117 | Transcriptional regulator, Middle operon regulator (Mor) family | Transcriptional regulator, Middle operon regulator (Mor) family | | afdb-uniprot50 | AF-A0A0J7J645-F1-MODEL\_V4 | 1.0 | 6.969e-06 | 197 | 0.22 | 127 | 88 | 2 | 3 | 128 | 9 | 125 | Mor transcription activator family | Mor transcription activator family | | afdb-uniprot50 | AF-A0A5C7Q033-F1-MODEL\_V4 | 1.0 | 2.638e-05 | 197 | 0.219 | 141 | 94 | 3 | 1 | 134 | 2 | 133 | Winged helix-turn-helix transcriptional regulator | Winged helix-turn-helix transcriptional regulator | | afdb-uniprot50 | AF-A0A6H3FDI8-F1-MODEL\_V4 | 1.0 | 1.864e-05 | 196 | 0.231 | 147 | 85 | 7 | 3 | 139 | 41 | 169 | Mor domain-containing protein | Mor domain-containing protein | | afdb-uniprot50 | AF-A0A3A0EQS8-F1-MODEL\_V4 | 1.0 | 4.191e-05 | 194 | 0.226 | 119 | 82 | 1 | 3 | 121 | 14 | 122 | Mor domain-containing protein | Mor domain-containing protein | | afdb-uniprot50 | AF-A0A849SXZ0-F1-MODEL\_V4 | 1.0 | 0.0001333 | 194 | 0.198 | 131 | 88 | 3 | 3 | 128 | 32 | 150 | Uncharacterized protein | Uncharacterized protein | | afdb-uniprot50 | AF-A0A258P502-F1-MODEL\_V4 | 1.0 | 7.921e-05 | 194 | 0.22 | 127 | 81 | 3 | 3 | 123 | 58 | 172 | Mor domain-containing protein | Mor domain-containing protein | | afdb-uniprot50 | AF-A0A1Q6UEQ9-F1-MODEL\_V4 | 1.0 | 5.283e-05 | 192 | 0.254 | 118 | 74 | 4 | 7 | 122 | 8 | 113 | Uncharacterized protein | Uncharacterized protein | | afdb-uniprot50 | AF-A0A3R9ZQF3-F1-MODEL\_V4 | 1.0 | 7.921e-05 | 192 | 0.221 | 122 | 81 | 2 | 2 | 119 | 28 | 139 | Mor domain-containing protein | Mor domain-containing protein | | afdb-uniprot50 | AF-A0A142BHK6-F1-MODEL\_V4 | 1.0 | 1.567e-05 | 192 | 0.179 | 139 | 100 | 3 | 8 | 144 | 5 | 131 | Mor transcription activator domain-containing protein | Mor transcription activator domain-containing protein | | afdb-uniprot50 | AF-A0A5C7KLE1-F1-MODEL\_V4 | 1.0 | 9.862e-06 | 191 | 0.262 | 141 | 82 | 6 | 4 | 144 | 11 | 129 | Mor domain-containing protein | Mor domain-containing protein | | afdb-uniprot50 | AF-A0A345DE69-F1-MODEL\_V4 | 1.0 | 4.191e-05 | 191 | 0.197 | 137 | 85 | 3 | 7 | 128 | 4 | 130 | Uncharacterized protein | Uncharacterized protein | | afdb-uniprot50 | AF-A0A3R8L878-F1-MODEL\_V4 | 1.0 | 5.597e-05 | 191 | 0.196 | 132 | 90 | 4 | 3 | 128 | 85 | 206 | Uncharacterized protein | Uncharacterized protein | | afdb-uniprot50 | AF-A0A2P5M266-F1-MODEL\_V4 | 1.0 | 7.921e-05 | 190 | 0.206 | 126 | 89 | 3 | 3 | 127 | 11 | 126 | Mor domain-containing protein | Mor domain-containing protein | | afdb-uniprot50 | AF-A0A7U5K534-F1-MODEL\_V4 | 1.0 | 2.962e-05 | 190 | 0.241 | 124 | 83 | 2 | 3 | 125 | 12 | 125 | Uncharacterized protein | Uncharacterized protein | | afdb-uniprot50 | AF-A0A1P8JXX9-F1-MODEL\_V4 | 1.0 | 0.0003779 | 190 | 0.186 | 118 | 84 | 3 | 3 | 119 | 13 | 119 | Mor domain-containing protein | Mor domain-containing protein | | afdb-uniprot50 | AF-A0A522VTV5-F1-MODEL\_V4 | 1.0 | 0.0001681 | 190 | 0.193 | 129 | 91 | 4 | 3 | 128 | 14 | 132 | Mor domain-containing protein | Mor domain-containing protein | | afdb-uniprot50 | AF-A0A6G9Q100-F1-MODEL\_V4 | 1.0 | 0.0001121 | 190 | 0.203 | 128 | 89 | 3 | 3 | 129 | 19 | 134 | Helix-turn-helix domain-containing protein | Helix-turn-helix domain-containing protein | | afdb-uniprot50 | AF-A0A439VN72-F1-MODEL\_V4 | 1.0 | 3.733e-05 | 190 | 0.247 | 117 | 72 | 4 | 5 | 118 | 109 | 212 | Uncharacterized protein | Uncharacterized protein | | afdb-uniprot50 | AF-A0A286AC00-F1-MODEL\_V4 | 1.0 | 0.0003177 | 189 | 0.222 | 117 | 79 | 3 | 3 | 118 | 10 | 115 | Mor transcription activator family protein | Mor transcription activator family protein | | afdb-uniprot50 | AF-A0A1C3EL69-F1-MODEL\_V4 | 1.0 | 4.986e-05 | 188 | 0.26 | 123 | 71 | 5 | 6 | 121 | 3 | 112 | Mor domain-containing protein | Mor domain-containing protein | | afdb-uniprot50 | AF-H1SDM0-F1-MODEL\_V4 | 1.0 | 0.0001681 | 188 | 0.17 | 129 | 88 | 3 | 7 | 128 | 20 | 136 | Mor domain-containing protein | Mor domain-containing protein | | afdb-uniprot50 | AF-A0A272EMK5-F1-MODEL\_V4 | 1.0 | 3.955e-05 | 188 | 0.214 | 121 | 82 | 2 | 1 | 118 | 2 | 112 | Uncharacterized protein | Uncharacterized protein | | afdb-uniprot50 | AF-A0A7W9QXS3-F1-MODEL\_V4 | 1.0 | 9.308e-06 | 187 | 0.22 | 136 | 79 | 5 | 1 | 124 | 6 | 126 | Uncharacterized protein | Uncharacterized protein | | afdb-uniprot50 | AF-A0A2E6KN34-F1-MODEL\_V4 | 1.0 | 4.705e-05 | 186 | 0.196 | 117 | 79 | 2 | 2 | 118 | 10 | 111 | Uncharacterized protein | Uncharacterized protein | | afdb-uniprot50 | AF-A0A6G9Q124-F1-MODEL\_V4 | 1.0 | 0.0002245 | 186 | 0.195 | 123 | 86 | 3 | 3 | 124 | 19 | 129 | Helix-turn-helix domain-containing protein | Helix-turn-helix domain-containing protein | | afdb-uniprot50 | AF-M5DM24-F1-MODEL\_V4 | 1.0 | 2.49e-05 | 186 | 0.223 | 121 | 73 | 3 | 3 | 119 | 10 | 113 | Mor domain-containing protein | Mor domain-containing protein | | afdb-uniprot50 | AF-A0A1N6X0E0-F1-MODEL\_V4 | 1.0 | 9.984e-05 | 186 | 0.208 | 134 | 84 | 4 | 3 | 128 | 13 | 132 | Mor transcription activator family protein | Mor transcription activator family protein | | afdb-uniprot50 | AF-A0A521YLK1-F1-MODEL\_V4 | 1.0 | 8.393e-05 | 186 | 0.214 | 140 | 87 | 4 | 1 | 128 | 117 | 245 | Mor domain-containing protein | Mor domain-containing protein | | afdb-uniprot50 | AF-F5RBY1-F1-MODEL\_V4 | 1.0 | 6.659e-05 | 185 | 0.223 | 143 | 86 | 5 | 1 | 128 | 1 | 133 | Bacteriophage DNA transposition protein B | Bacteriophage DNA transposition protein B | | afdb-uniprot50 | AF-N9R432-F1-MODEL\_V4 | 1.0 | 9.423e-05 | 185 | 0.198 | 141 | 96 | 5 | 3 | 141 | 23 | 148 | Mor domain-containing protein | Mor domain-containing protein | | afdb-uniprot50 | AF-A0A5C7L0Z4-F1-MODEL\_V4 | 1.0 | 7.921e-05 | 184 | 0.169 | 112 | 83 | 1 | 7 | 118 | 6 | 107 | Mor domain-containing protein | Mor domain-containing protein | | afdb-uniprot50 | AF-A0A4Q0YJD9-F1-MODEL\_V4 | 1.0 | 5.931e-05 | 184 | 0.258 | 120 | 71 | 4 | 6 | 119 | 3 | 110 | Uncharacterized protein | Uncharacterized protein | | afdb-uniprot50 | AF-A0A2N9YH57-F1-MODEL\_V4 | 1.0 | 0.0003779 | 183 | 0.191 | 120 | 82 | 4 | 3 | 121 | 10 | 115 | Mor domain-containing protein | Mor domain-containing protein | | afdb-uniprot50 | AF-A0A6N4DE39-F1-MODEL\_V4 | 1.0 | 0.0004004 | 183 | 0.25 | 116 | 73 | 2 | 3 | 118 | 7 | 108 | Uncharacterized protein | Uncharacterized protein | | afdb-uniprot50 | AF-A0A3D9Q0H6-F1-MODEL\_V4 | 1.0 | 1.759e-05 | 183 | 0.268 | 134 | 80 | 3 | 3 | 128 | 10 | 133 | Uncharacterized protein | Uncharacterized protein | | afdb-uniprot50 | AF-A0A3P1ZRA8-F1-MODEL\_V4 | 1.0 | 2.638e-05 | 183 | 0.166 | 156 | 106 | 6 | 3 | 144 | 55 | 200 | Mor domain-containing protein | Mor domain-containing protein | | afdb-uniprot50 | AF-Q1QMW9-F1-MODEL\_V4 | 1.0 | 8.393e-05 | 182 | 0.277 | 119 | 74 | 3 | 1 | 118 | 3 | 110 | Uncharacterized protein | Uncharacterized protein | | afdb-uniprot50 | AF-A0A5P9EX33-F1-MODEL\_V4 | 1.0 | 0.0001681 | 182 | 0.216 | 125 | 86 | 3 | 2 | 125 | 18 | 131 | Mor transcription activator family protein | Mor transcription activator family protein | | afdb-uniprot50 | AF-A0A2A2B555-F1-MODEL\_V4 | 1.0 | 0.0003779 | 182 | 0.206 | 116 | 80 | 2 | 5 | 118 | 8 | 113 | Mor domain-containing protein | Mor domain-containing protein | | afdb-uniprot50 | AF-A0A833MQ40-F1-MODEL\_V4 | 1.0 | 1.479e-05 | 182 | 0.274 | 131 | 79 | 2 | 1 | 125 | 12 | 132 | Uncharacterized protein | Uncharacterized protein | | afdb-uniprot50 | AF-A0A2W4TD25-F1-MODEL\_V4 | 1.0 | 6.659e-05 | 182 | 0.256 | 121 | 76 | 3 | 7 | 125 | 76 | 184 | Uncharacterized protein | Uncharacterized protein | | afdb-uniprot50 | AF-G2HDC1-F1-MODEL\_V4 | 1.0 | 8.893e-05 | 181 | 0.202 | 138 | 83 | 4 | 7 | 144 | 9 | 119 | Uncharacterized protein | Uncharacterized protein | | afdb-uniprot50 | AF-A0A2T5J3R6-F1-MODEL\_V4 | 1.0 | 0.0006004 | 181 | 0.185 | 124 | 88 | 3 | 7 | 127 | 5 | 118 | Mor transcription activator family protein | Mor transcription activator family protein | | afdb-uniprot50 | AF-A0A6N7JS93-F1-MODEL\_V4 | 1.0 | 3.325e-05 | 181 | 0.246 | 130 | 73 | 6 | 4 | 125 | 12 | 124 | Uncharacterized protein | Uncharacterized protein | | afdb-uniprot50 | AF-A0A2U2N1B0-F1-MODEL\_V4 | 1.0 | 0.0004763 | 181 | 0.294 | 119 | 70 | 5 | 3 | 118 | 9 | 116 | Uncharacterized protein | Uncharacterized protein | | afdb-uniprot50 | AF-A0A0U5FC51-F1-MODEL\_V4 | 1.0 | 1.567e-05 | 181 | 0.195 | 133 | 92 | 5 | 3 | 124 | 13 | 141 | Uncharacterized protein | Uncharacterized protein | | afdb-uniprot50 | AF-A0A1J4X7E1-F1-MODEL\_V4 | 1.0 | 0.0002998 | 180 | 0.21 | 133 | 86 | 6 | 2 | 129 | 18 | 136 | Mor domain-containing protein | Mor domain-containing protein | | afdb-uniprot50 | AF-A0A1G8EZ96-F1-MODEL\_V4 | 1.0 | 1.864e-05 | 180 | 0.214 | 135 | 85 | 3 | 1 | 127 | 10 | 131 | Homeodomain-like domain-containing protein | Homeodomain-like domain-containing protein | | afdb-uniprot50 | AF-A0A1D2X552-F1-MODEL\_V4 | 1.0 | 0.0002119 | 180 | 0.195 | 123 | 85 | 3 | 8 | 128 | 5 | 115 | Mor domain-containing protein | Mor domain-containing protein | | afdb-uniprot50 | AF-A0A1I5W2T6-F1-MODEL\_V4 | 1.0 | 0.0001497 | 179 | 0.184 | 119 | 84 | 3 | 1 | 119 | 1 | 106 | Uncharacterized protein | Uncharacterized protein | | afdb-uniprot50 | AF-A0A357V9S2-F1-MODEL\_V4 | 1.0 | 0.0001497 | 179 | 0.216 | 134 | 85 | 2 | 1 | 124 | 1 | 124 | Uncharacterized protein | Uncharacterized protein | | afdb-uniprot50 | AF-K0D2L3-F1-MODEL\_V4 | 1.0 | 0.0004004 | 179 | 0.184 | 119 | 82 | 5 | 8 | 125 | 14 | 118 | Mor domain-containing protein | Mor domain-containing protein | | afdb-uniprot50 | AF-A0A3D3KCN7-F1-MODEL\_V4 | 1.0 | 0.0005667 | 179 | 0.173 | 121 | 88 | 2 | 1 | 119 | 1 | 111 | Mor domain-containing protein | Mor domain-containing protein | | afdb-uniprot50 | AF-A0A401JFQ9-F1-MODEL\_V4 | 1.0 | 2.093e-05 | 179 | 0.219 | 132 | 85 | 2 | 1 | 124 | 12 | 133 | Mor domain-containing protein | Mor domain-containing protein | | afdb-uniprot50 | AF-A0A0D2GJW6-F1-MODEL\_V4 | 1.0 | 7.055e-05 | 177 | 0.201 | 114 | 78 | 2 | 5 | 118 | 4 | 104 | Uncharacterized protein | Uncharacterized protein | | afdb-uniprot50 | AF-A0A5C7VFA7-F1-MODEL\_V4 | 1.0 | 0.0001058 | 177 | 0.162 | 123 | 90 | 3 | 1 | 123 | 12 | 121 | Mor domain-containing protein | Mor domain-containing protein | | afdb-uniprot50 | AF-A0A0W1G863-F1-MODEL\_V4 | 1.0 | 1.66e-05 | 177 | 0.258 | 143 | 86 | 6 | 1 | 133 | 1 | 133 | Uncharacterized protein | Uncharacterized protein | | afdb-uniprot50 | AF-A0A5K6V8A9-F1-MODEL\_V4 | 1.0 | 7.055e-05 | 176 | 0.254 | 114 | 68 | 5 | 11 | 119 | 2 | 103 | Mor domain-containing protein | Mor domain-containing protein | | afdb-uniprot50 | AF-A0A1N7JLA1-F1-MODEL\_V4 | 1.0 | 0.0001887 | 176 | 0.26 | 123 | 71 | 4 | 2 | 118 | 5 | 113 | Uncharacterized protein | Uncharacterized protein | | afdb-uniprot50 | AF-A0A2W5N6E1-F1-MODEL\_V4 | 1.0 | 0.0001121 | 176 | 0.247 | 109 | 67 | 2 | 10 | 118 | 19 | 112 | Uncharacterized protein | Uncharacterized protein | | afdb-uniprot50 | AF-A0A1I1UI57-F1-MODEL\_V4 | 1.0 | 3.138e-05 | 176 | 0.26 | 119 | 66 | 4 | 8 | 118 | 2 | 106 | Uncharacterized protein | Uncharacterized protein | | afdb-uniprot50 | AF-A0A3M1PQC8-F1-MODEL\_V4 | 1.0 | 5.931e-05 | 176 | 0.187 | 139 | 97 | 4 | 7 | 144 | 6 | 129 | Uncharacterized protein | Uncharacterized protein | | afdb-uniprot50 | AF-A0A3T0W468-F1-MODEL\_V4 | 1.0 | 0.0001681 | 176 | 0.162 | 117 | 88 | 1 | 3 | 119 | 14 | 120 | Mor domain-containing protein | Mor domain-containing protein | | afdb-uniprot50 | AF-A0A7W8C0C3-F1-MODEL\_V4 | 1.0 | 4.986e-05 | 176 | 0.192 | 161 | 94 | 7 | 3 | 144 | 63 | 206 | Mor family transcriptional regulator | Mor family transcriptional regulator | | afdb-uniprot50 | AF-A0A4P9VQV6-F1-MODEL\_V4 | 1.0 | 0.0001999 | 175 | 0.201 | 119 | 80 | 5 | 3 | 119 | 14 | 119 | Transcriptional regulator | Transcriptional regulator | | afdb-uniprot50 | AF-A0A4D7DET1-F1-MODEL\_V4 | 1.0 | 2.49e-05 | 174 | 0.23 | 126 | 76 | 5 | 6 | 125 | 11 | 121 | Uncharacterized protein | Uncharacterized protein | | afdb-uniprot50 | AF-A0A1W9J765-F1-MODEL\_V4 | 1.0 | 0.0002379 | 174 | 0.224 | 116 | 80 | 1 | 3 | 118 | 12 | 117 | Mor domain-containing protein | Mor domain-containing protein | | afdb-uniprot50 | AF-A0A6L5XLQ5-F1-MODEL\_V4 | 1.0 | 5.597e-05 | 174 | 0.18 | 150 | 100 | 6 | 3 | 139 | 48 | 187 | Mor domain-containing protein | Mor domain-containing protein | | afdb-uniprot50 | AF-A0A286GYP9-F1-MODEL\_V4 | 1.0 | 0.0002245 | 173 | 0.188 | 122 | 83 | 2 | 3 | 118 | 12 | 123 | Uncharacterized protein | Uncharacterized protein | | afdb-uniprot50 | AF-B2FRB5-F1-MODEL\_V4 | 1.0 | 1.396e-05 | 172 | 0.301 | 126 | 72 | 4 | 27 | 143 | 1 | 119 | Uncharacterized protein | Uncharacterized protein | | afdb-uniprot50 | AF-A0A089WQT6-F1-MODEL\_V4 | 1.0 | 0.0005047 | 170 | 0.194 | 118 | 82 | 3 | 3 | 119 | 14 | 119 | Transcriptional regulator | Transcriptional regulator | | afdb-uniprot50 | AF-A0A7K3NNL5-F1-MODEL\_V4 | 1.0 | 6.284e-05 | 170 | 0.255 | 133 | 81 | 4 | 1 | 125 | 7 | 129 | Uncharacterized protein | Uncharacterized protein | | afdb-uniprot50 | AF-A0A2W4T512-F1-MODEL\_V4 | 1.0 | 3.523e-05 | 169 | 0.23 | 139 | 80 | 5 | 3 | 128 | 11 | 135 | Uncharacterized protein | Uncharacterized protein | | afdb-uniprot50 | AF-A0A1X7NE24-F1-MODEL\_V4 | 1.0 | 0.0009003 | 168 | 0.204 | 122 | 82 | 5 | 2 | 121 | 4 | 112 | Uncharacterized protein | Uncharacterized protein | | afdb-uniprot50 | AF-A0A0J6SFM2-F1-MODEL\_V4 | 1.0 | 3.138e-05 | 168 | 0.196 | 132 | 92 | 3 | 1 | 128 | 8 | 129 | Uncharacterized protein | Uncharacterized protein | | afdb-uniprot50 | AF-A0A6H3FAD0-F1-MODEL\_V4 | 1.0 | 0.0001586 | 167 | 0.206 | 121 | 81 | 4 | 7 | 125 | 5 | 112 | Uncharacterized protein | Uncharacterized protein | | afdb-uniprot50 | AF-A0A5C7NL60-F1-MODEL\_V4 | 1.0 | 3.523e-05 | 167 | 0.257 | 136 | 82 | 5 | 4 | 139 | 53 | 169 | Mor domain-containing protein | Mor domain-containing protein | | afdb-uniprot50 | AF-A0A1X7QCW3-F1-MODEL\_V4 | 1.0 | 7.055e-05 | 167 | 0.207 | 169 | 96 | 7 | 1 | 144 | 51 | 206 | Uncharacterized protein | Uncharacterized protein | | afdb-uniprot50 | AF-A0A554WZV0-F1-MODEL\_V4 | 1.0 | 0.0006362 | 166 | 0.203 | 123 | 85 | 3 | 7 | 128 | 3 | 113 | Mor transcription activator family protein | Mor transcription activator family protein | | afdb-uniprot50 | AF-A0A7T3E5I3-F1-MODEL\_V4 | 1.0 | 9.984e-05 | 166 | 0.194 | 139 | 86 | 4 | 6 | 143 | 10 | 123 | Uncharacterized protein | Uncharacterized protein | | afdb-uniprot50 | AF-A0A1Z4C0F4-F1-MODEL\_V4 | 1.0 | 0.002408 | 166 | 0.196 | 117 | 82 | 3 | 3 | 118 | 13 | 118 | Mor domain-containing protein | Mor domain-containing protein | | afdb-uniprot50 | AF-E2CFN9-F1-MODEL\_V4 | 1.0 | 0.0001058 | 166 | 0.197 | 137 | 83 | 6 | 3 | 128 | 7 | 127 | Uncharacterized protein | Uncharacterized protein | | afdb-uniprot50 | AF-A0A839IQE8-F1-MODEL\_V4 | 1.0 | 0.0007143 | 166 | 0.198 | 116 | 82 | 2 | 3 | 118 | 20 | 124 | Transcriptional regulator | Transcriptional regulator | | afdb-uniprot50 | AF-A0A3B9NYV6-F1-MODEL\_V4 | 1.0 | 0.0001781 | 165 | 0.201 | 129 | 89 | 3 | 3 | 128 | 15 | 132 | Uncharacterized protein | Uncharacterized protein | | afdb-uniprot50 | AF-A0A2S5JEN6-F1-MODEL\_V4 | 1.0 | 0.0005047 | 165 | 0.242 | 128 | 77 | 4 | 1 | 120 | 1 | 116 | Uncharacterized protein | Uncharacterized protein | | afdb-uniprot50 | AF-A0A139DN11-F1-MODEL\_V4 | 1.0 | 0.0009539 | 165 | 0.188 | 122 | 81 | 4 | 3 | 119 | 31 | 139 | Mor domain-containing protein | Mor domain-containing protein | | afdb-uniprot50 | AF-A0A2D3TET5-F1-MODEL\_V4 | 1.0 | 4.986e-05 | 164 | 0.242 | 140 | 88 | 5 | 11 | 144 | 2 | 129 | Mor domain-containing protein | Mor domain-containing protein | | afdb-uniprot50 | AF-A0A2W5H275-F1-MODEL\_V4 | 1.0 | 0.001071 | 164 | 0.21 | 119 | 80 | 4 | 1 | 118 | 1 | 106 | Mor domain-containing protein | Mor domain-containing protein | | afdb-uniprot50 | AF-E9I3C0-F1-MODEL\_V4 | 1.0 | 2.35e-05 | 164 | 0.177 | 152 | 101 | 3 | 1 | 139 | 1 | 141 | Uncharacterized protein | Uncharacterized protein | | afdb-uniprot50 | AF-A0A3B9PP60-F1-MODEL\_V4 | 1.0 | 0.0001497 | 164 | 0.253 | 150 | 85 | 5 | 3 | 144 | 19 | 149 | Mor domain-containing protein | Mor domain-containing protein | | afdb-uniprot50 | AF-A0A1H6GGU4-F1-MODEL\_V4 | 1.0 | 8.893e-05 | 163 | 0.201 | 129 | 85 | 3 | 3 | 127 | 16 | 130 | Uncharacterized protein | Uncharacterized protein | | afdb-uniprot50 | AF-A0A2M8DST2-F1-MODEL\_V4 | 1.0 | 0.0003366 | 163 | 0.2 | 120 | 82 | 3 | 4 | 119 | 27 | 136 | Mor domain-containing protein | Mor domain-containing protein | | afdb-uniprot50 | AF-A0A418W4I4-F1-MODEL\_V4 | 1.0 | 3.733e-05 | 163 | 0.24 | 150 | 88 | 6 | 3 | 141 | 14 | 148 | Uncharacterized protein | Uncharacterized protein | | afdb-uniprot50 | AF-A0A853HSH7-F1-MODEL\_V4 | 1.0 | 7.921e-05 | 163 | 0.18 | 155 | 98 | 5 | 1 | 139 | 1 | 142 | Uncharacterized protein | Uncharacterized protein | | afdb-uniprot50 | AF-A0A2T6M6E5-F1-MODEL\_V4 | 1.0 | 0.000267 | 162 | 0.196 | 122 | 84 | 3 | 2 | 119 | 3 | 114 | Uncharacterized protein | Uncharacterized protein | | afdb-uniprot50 | AF-A0A2K1DE74-F1-MODEL\_V4 | 1.0 | 0.0005348 | 162 | 0.184 | 130 | 87 | 4 | 2 | 124 | 18 | 135 | Transcriptional regulator | Transcriptional regulator | | afdb-uniprot50 | AF-A0A1H5GMI8-F1-MODEL\_V4 | 1.0 | 0.00143 | 162 | 0.205 | 117 | 82 | 3 | 3 | 119 | 49 | 154 | Transcriptional regulator, Middle operon regulator (Mor) family | Transcriptional regulator, Middle operon regulator (Mor) family | | afdb-uniprot50 | AF-A0A6L8HVT0-F1-MODEL\_V4 | 1.0 | 0.0003177 | 162 | 0.206 | 116 | 72 | 5 | 7 | 118 | 62 | 161 | Mor domain-containing protein | Mor domain-containing protein | | afdb-uniprot50 | AF-A0A1G0EZ32-F1-MODEL\_V4 | 1.0 | 0.00135 | 161 | 0.157 | 121 | 82 | 3 | 7 | 122 | 2 | 107 | Uncharacterized protein | Uncharacterized protein | | afdb-uniprot50 | AF-A0A2N2RB11-F1-MODEL\_V4 | 1.0 | 0.0002245 | 161 | 0.234 | 115 | 72 | 4 | 7 | 118 | 13 | 114 | Uncharacterized protein | Uncharacterized protein | | afdb-uniprot50 | AF-A0A451AQL9-F1-MODEL\_V4 | 1.0 | 0.0006741 | 161 | 0.164 | 134 | 94 | 2 | 3 | 128 | 12 | 135 | Mor transcription activator family protein | Mor transcription activator family protein | | afdb-uniprot50 | AF-T2JY53-F1-MODEL\_V4 | 1.0 | 0.001071 | 160 | 0.211 | 118 | 83 | 1 | 3 | 120 | 12 | 119 | Uncharacterized protein | Uncharacterized protein | | afdb-uniprot50 | AF-A0A2S6N2X3-F1-MODEL\_V4 | 1.0 | 0.0007568 | 160 | 0.186 | 118 | 84 | 2 | 3 | 118 | 5 | 112 | Uncharacterized protein | Uncharacterized protein | | afdb-uniprot50 | AF-A0A1H1G473-F1-MODEL\_V4 | 1.0 | 0.0004496 | 160 | 0.21 | 128 | 81 | 3 | 1 | 118 | 2 | 119 | Helix-turn-helix domain-containing protein | Helix-turn-helix domain-containing protein | | afdb-uniprot50 | AF-A0A4Y8W9I1-F1-MODEL\_V4 | 1.0 | 0.0005667 | 160 | 0.218 | 119 | 76 | 4 | 3 | 118 | 22 | 126 | Uncharacterized protein | Uncharacterized protein | | afdb-uniprot50 | AF-A0A4V2V2Q2-F1-MODEL\_V4 | 1.0 | 0.0005667 | 159 | 0.225 | 124 | 78 | 5 | 1 | 118 | 1 | 112 | Homeodomain-like domain-containing protein | Homeodomain-like domain-containing protein | | afdb-uniprot50 | AF-A0A5K1I944-F1-MODEL\_V4 | 1.0 | 0.0003177 | 159 | 0.232 | 112 | 76 | 2 | 7 | 118 | 8 | 109 | Mor transcription activator family protein | Mor transcription activator family protein | | afdb-uniprot50 | AF-V4NRA8-F1-MODEL\_V4 | 1.0 | 0.0002998 | 159 | 0.213 | 122 | 76 | 4 | 1 | 118 | 11 | 116 | Uncharacterized protein | Uncharacterized protein | | afdb-uniprot50 | AF-A0A4S4AP65-F1-MODEL\_V4 | 1.0 | 0.0003779 | 159 | 0.19 | 152 | 92 | 4 | 3 | 144 | 43 | 173 | Mor domain-containing protein | Mor domain-containing protein | | afdb-uniprot50 | AF-A0A2Z6DXP8-F1-MODEL\_V4 | 1.0 | 0.0009003 | 158 | 0.226 | 128 | 83 | 4 | 1 | 124 | 1 | 116 | Mor domain-containing protein | Mor domain-containing protein | | afdb-uniprot50 | AF-A0A3F3GTU9-F1-MODEL\_V4 | 1.0 | 0.0007568 | 158 | 0.197 | 152 | 97 | 4 | 3 | 144 | 13 | 149 | Putative DNA transposition protein | Putative DNA transposition protein | | afdb-uniprot50 | AF-A0A845TMQ7-F1-MODEL\_V4 | 1.0 | 0.0003779 | 156 | 0.17 | 129 | 84 | 4 | 1 | 119 | 1 | 116 | Uncharacterized protein | Uncharacterized protein | | afdb-uniprot50 | AF-A0A522VKF2-F1-MODEL\_V4 | 1.0 | 9.984e-05 | 156 | 0.186 | 145 | 95 | 5 | 3 | 144 | 14 | 138 | Mor domain-containing protein | Mor domain-containing protein | | afdb-uniprot50 | AF-A0A2D9F5H7-F1-MODEL\_V4 | 1.0 | 0.0003567 | 155 | 0.203 | 123 | 76 | 4 | 2 | 118 | 9 | 115 | Mor domain-containing protein | Mor domain-containing protein | | afdb-uniprot50 | AF-A0A1H3FGD2-F1-MODEL\_V4 | 1.0 | 0.0006362 | 155 | 0.23 | 117 | 78 | 4 | 3 | 118 | 9 | 114 | Mor transcription activator family protein | Mor transcription activator family protein | | afdb-uniprot50 | AF-A0A1B8Q882-F1-MODEL\_V4 | 1.0 | 0.0002245 | 155 | 0.188 | 127 | 83 | 4 | 3 | 119 | 19 | 135 | Uncharacterized protein | Uncharacterized protein | | afdb-uniprot50 | AF-A0A7Z0N040-F1-MODEL\_V4 | 1.0 | 0.003408 | 155 | 0.151 | 112 | 85 | 2 | 7 | 118 | 14 | 115 | Uncharacterized protein | Uncharacterized protein | | afdb-uniprot50 | AF-F4QGB6-F1-MODEL\_V4 | 1.0 | 0.0004004 | 154 | 0.204 | 122 | 78 | 3 | 8 | 125 | 2 | 108 | Uncharacterized protein | Uncharacterized protein | | afdb-uniprot50 | AF-A0A7X3YCW7-F1-MODEL\_V4 | 1.0 | 0.0006004 | 153 | 0.181 | 116 | 82 | 2 | 6 | 121 | 3 | 105 | Mor domain-containing protein | Mor domain-containing protein | | afdb-uniprot50 | AF-A0A1G8GH27-F1-MODEL\_V4 | 1.0 | 0.000252 | 153 | 0.221 | 122 | 75 | 5 | 3 | 118 | 10 | 117 | Uncharacterized protein | Uncharacterized protein | | afdb-uniprot50 | AF-A0A6A7Y6F2-F1-MODEL\_V4 | 1.0 | 0.0001121 | 153 | 0.239 | 138 | 87 | 7 | 3 | 134 | 12 | 137 | Uncharacterized protein | Uncharacterized protein | | afdb-uniprot50 | AF-A0A843YXE8-F1-MODEL\_V4 | 1.0 | 0.0001887 | 153 | 0.217 | 138 | 81 | 5 | 7 | 129 | 11 | 136 | Uncharacterized protein | Uncharacterized protein | | afdb-uniprot50 | AF-A0A2W5WW80-F1-MODEL\_V4 | 1.0 | 0.0006741 | 152 | 0.198 | 141 | 92 | 4 | 1 | 136 | 2 | 126 | Helix-turn-helix domain-containing protein | Helix-turn-helix domain-containing protein | | afdb-uniprot50 | AF-T0IRN1-F1-MODEL\_V4 | 1.0 | 0.000267 | 152 | 0.184 | 130 | 88 | 3 | 6 | 127 | 22 | 141 | Uncharacterized protein | Uncharacterized protein | | afdb-uniprot50 | AF-A0A848C494-F1-MODEL\_V4 | 1.0 | 0.0006362 | 152 | 0.165 | 151 | 97 | 6 | 3 | 143 | 31 | 162 | Uncharacterized protein | Uncharacterized protein | | afdb-uniprot50 | AF-A0A7W5ZUY6-F1-MODEL\_V4 | 1.0 | 0.0007143 | 151 | 0.156 | 134 | 90 | 3 | 1 | 128 | 1 | 117 | Uncharacterized protein | Uncharacterized protein | | afdb-uniprot50 | AF-A0A510UGA1-F1-MODEL\_V4 | 1.0 | 0.00143 | 151 | 0.176 | 119 | 85 | 3 | 3 | 120 | 21 | 127 | Transcriptional regulator | Transcriptional regulator | | afdb-uniprot50 | AF-A0A291IMH9-F1-MODEL\_V4 | 1.0 | 0.0004496 | 151 | 0.208 | 134 | 88 | 5 | 7 | 137 | 10 | 128 | Mor domain-containing protein | Mor domain-containing protein | | afdb-uniprot50 | AF-A0A6N8BMD9-F1-MODEL\_V4 | 1.0 | 0.0008019 | 151 | 0.146 | 157 | 108 | 7 | 3 | 144 | 62 | 207 | Mor domain-containing protein | Mor domain-containing protein | | afdb-uniprot50 | AF-A0A1D7Z023-F1-MODEL\_V4 | 1.0 | 0.002551 | 150 | 0.213 | 117 | 79 | 3 | 2 | 118 | 20 | 123 | Mor domain-containing protein | Mor domain-containing protein | | afdb-uniprot50 | AF-A0A4D7DA93-F1-MODEL\_V4 | 1.0 | 0.0004004 | 150 | 0.195 | 148 | 103 | 5 | 2 | 144 | 23 | 159 | Uncharacterized protein | Uncharacterized protein | | afdb-uniprot50 | AF-A0A564WHC9-F1-MODEL\_V4 | 1.0 | 0.0006362 | 150 | 0.211 | 118 | 82 | 3 | 2 | 118 | 51 | 158 | Uncharacterized protein | Uncharacterized protein | | afdb-uniprot50 | AF-T2GC33-F1-MODEL\_V4 | 1.0 | 0.001702 | 149 | 0.205 | 112 | 79 | 1 | 7 | 118 | 14 | 115 | Uncharacterized protein | Uncharacterized protein | | afdb-uniprot50 | AF-A0A2E9SLK3-F1-MODEL\_V4 | 1.0 | 0.000252 | 149 | 0.226 | 119 | 81 | 3 | 7 | 125 | 4 | 111 | Uncharacterized protein | Uncharacterized protein | | afdb-uniprot50 | AF-A0A3N9RYX3-F1-MODEL\_V4 | 1.0 | 0.002703 | 149 | 0.2 | 120 | 82 | 4 | 5 | 122 | 24 | 131 | Uncharacterized protein | Uncharacterized protein | | afdb-uniprot50 | AF-A0A1G3LVZ0-F1-MODEL\_V4 | 1.0 | 0.0002379 | 148 | 0.175 | 137 | 87 | 5 | 7 | 129 | 3 | 127 | Uncharacterized protein | Uncharacterized protein | | afdb-uniprot50 | AF-A0A853IG23-F1-MODEL\_V4 | 1.0 | 0.000267 | 147 | 0.172 | 133 | 86 | 5 | 7 | 127 | 4 | 124 | Uncharacterized protein | Uncharacterized protein | | afdb-uniprot50 | AF-A0A420ZY38-F1-MODEL\_V4 | 1.0 | 0.0007568 | 147 | 0.236 | 114 | 75 | 3 | 7 | 119 | 83 | 185 | Mor transcription activator family protein | Mor transcription activator family protein | | afdb-uniprot50 | AF-A0A3N9RYD7-F1-MODEL\_V4 | 1.0 | 0.0006004 | 147 | 0.201 | 139 | 96 | 5 | 4 | 139 | 56 | 182 | Uncharacterized protein | Uncharacterized protein | | afdb-uniprot50 | AF-A0A1H9CJ13-F1-MODEL\_V4 | 1.0 | 0.002551 | 146 | 0.181 | 116 | 77 | 3 | 7 | 119 | 2 | 102 | Mor transcription activator family protein | Mor transcription activator family protein | | afdb-uniprot50 | AF-A0A7W9RCT4-F1-MODEL\_V4 | 1.0 | 0.0001999 | 145 | 0.224 | 125 | 75 | 5 | 3 | 119 | 8 | 118 | Uncharacterized protein | Uncharacterized protein | | afdb-uniprot50 | AF-E2CN54-F1-MODEL\_V4 | 1.0 | 0.0008497 | 145 | 0.227 | 132 | 83 | 3 | 3 | 125 | 17 | 138 | HTH\_7 domain-containing protein | HTH\_7 domain-containing protein | | afdb-uniprot50 | AF-R6HUG7-F1-MODEL\_V4 | 1.0 | 0.002551 | 144 | 0.185 | 113 | 81 | 2 | 7 | 118 | 11 | 113 | Uncharacterized protein | Uncharacterized protein | | afdb-uniprot50 | AF-A0A522VJQ9-F1-MODEL\_V4 | 1.0 | 0.0005348 | 143 | 0.22 | 127 | 85 | 4 | 3 | 125 | 45 | 161 | Mor domain-containing protein | Mor domain-containing protein | | afdb-uniprot50 | AF-A0A554X2Y4-F1-MODEL\_V4 | 1.0 | 0.0008497 | 143 | 0.197 | 162 | 100 | 5 | 1 | 144 | 2 | 151 | Mor transcription activator family protein | Mor transcription activator family protein | | afdb-uniprot50 | AF-A0A0Q2YXZ0-F1-MODEL\_V4 | 1.0 | 0.0009003 | 142 | 0.202 | 148 | 94 | 6 | 2 | 144 | 6 | 134 | Uncharacterized protein | Uncharacterized protein | | afdb-uniprot50 | AF-A0A0U5MJB7-F1-MODEL\_V4 | 1.0 | 0.0003779 | 142 | 0.223 | 130 | 83 | 5 | 6 | 128 | 16 | 134 | Uncharacterized protein | Uncharacterized protein | | afdb-uniprot50 | AF-A0A5E4XGN4-F1-MODEL\_V4 | 1.0 | 0.0004496 | 141 | 0.205 | 136 | 84 | 4 | 1 | 124 | 1 | 124 | Mor domain-containing protein | Mor domain-containing protein | | afdb-uniprot50 | AF-A0A7C1YRL4-F1-MODEL\_V4 | 1.0 | 0.002408 | 137 | 0.169 | 118 | 88 | 1 | 2 | 119 | 10 | 117 | Uncharacterized protein | Uncharacterized protein | | afdb-uniprot50 | AF-B8GL06-F1-MODEL\_V4 | 1.0 | 0.009114 | 136 | 0.156 | 128 | 92 | 5 | 3 | 127 | 16 | 130 | Mor domain-containing protein | Mor domain-containing protein | | afdb-uniprot50 | AF-A0A1H8FWD2-F1-MODEL\_V4 | 1.0 | 0.00191 | 135 | 0.173 | 121 | 90 | 2 | 4 | 124 | 6 | 116 | Mor transcription activator family protein | Mor transcription activator family protein | | afdb-uniprot50 | AF-A0A2D3TE22-F1-MODEL\_V4 | 1.0 | 0.0004004 | 135 | 0.234 | 132 | 70 | 5 | 1 | 131 | 23 | 124 | Uncharacterized protein | Uncharacterized protein | | afdb-uniprot50 | AF-A0A6A4RCI3-F1-MODEL\_V4 | 1.0 | 0.001516 | 134 | 0.248 | 133 | 80 | 5 | 3 | 127 | 9 | 129 | Uncharacterized protein | Uncharacterized protein | | afdb-uniprot50 | AF-A0A4Q8UBK0-F1-MODEL\_V4 | 1.0 | 0.0007568 | 134 | 0.116 | 146 | 109 | 6 | 1 | 140 | 1 | 132 | Mor domain-containing protein | Mor domain-containing protein | | afdb-uniprot50 | AF-N9PZH4-F1-MODEL\_V4 | 1.0 | 0.001702 | 134 | 0.185 | 140 | 90 | 4 | 2 | 127 | 9 | 138 | Mor domain-containing protein | Mor domain-containing protein | | afdb-uniprot50 | AF-A0A6L6WQR3-F1-MODEL\_V4 | 1.0 | 0.003035 | 132 | 0.176 | 119 | 87 | 2 | 1 | 118 | 6 | 114 | Uncharacterized protein | Uncharacterized protein | | afdb-uniprot50 | AF-A0A5E4ZWP6-F1-MODEL\_V4 | 1.0 | 0.004551 | 131 | 0.178 | 112 | 82 | 1 | 8 | 119 | 22 | 123 | Mor domain-containing protein | Mor domain-containing protein | | afdb-uniprot50 | AF-Q31HV4-F1-MODEL\_V4 | 1.0 | 0.004295 | 131 | 0.166 | 114 | 85 | 2 | 5 | 118 | 12 | 115 | Uncharacterized protein | Uncharacterized protein | | afdb-uniprot50 | AF-A0A858Q8R5-F1-MODEL\_V4 | 1.0 | 0.004822 | 130 | 0.247 | 85 | 62 | 1 | 46 | 128 | 5 | 89 | Uncharacterized protein | Uncharacterized protein | | afdb-uniprot50 | AF-A0A847G9G6-F1-MODEL\_V4 | 1.0 | 0.001202 | 130 | 0.183 | 142 | 100 | 5 | 1 | 139 | 1 | 129 | Uncharacterized protein | Uncharacterized protein | | afdb-uniprot50 | AF-A0A850KBD1-F1-MODEL\_V4 | 1.0 | 0.000252 | 130 | 0.207 | 140 | 96 | 4 | 7 | 143 | 8 | 135 | Helix-turn-helix domain-containing protein | Helix-turn-helix domain-containing protein | | afdb-uniprot50 | AF-A0A3D9Q2J0-F1-MODEL\_V4 | 1.0 | 0.0004763 | 130 | 0.219 | 146 | 85 | 6 | 2 | 128 | 6 | 141 | Mor transcription activator family protein | Mor transcription activator family protein | | afdb-uniprot50 | AF-A0A6L7X7N5-F1-MODEL\_V4 | 1.0 | 0.001011 | 129 | 0.186 | 134 | 86 | 6 | 1 | 124 | 3 | 123 | Mor domain-containing protein | Mor domain-containing protein | | afdb-uniprot50 | AF-A0A3T0N3Z2-F1-MODEL\_V4 | 1.0 | 0.009114 | 129 | 0.168 | 119 | 88 | 2 | 1 | 118 | 21 | 129 | Uncharacterized protein | Uncharacterized protein | | afdb-uniprot50 | AF-A0A2G1LMC0-F1-MODEL\_V4 | 1.0 | 0.0007143 | 128 | 0.154 | 155 | 97 | 5 | 7 | 144 | 3 | 140 | Uncharacterized protein | Uncharacterized protein | | afdb-uniprot50 | AF-A0A7W4G402-F1-MODEL\_V4 | 1.0 | 0.0009003 | 127 | 0.1 | 119 | 96 | 2 | 3 | 120 | 11 | 119 | Mor domain-containing protein | Mor domain-containing protein | | afdb-uniprot50 | AF-Q3IGJ2-F1-MODEL\_V4 | 1.0 | 0.0009003 | 127 | 0.1 | 119 | 96 | 2 | 3 | 120 | 11 | 119 | Putative orphan protein | Putative orphan protein | | afdb-uniprot50 | AF-A0A5Q0EN91-F1-MODEL\_V4 | 1.0 | 0.002145 | 126 | 0.261 | 134 | 67 | 10 | 1 | 121 | 1 | 115 | Mor domain-containing protein | Mor domain-containing protein | | afdb-uniprot50 | AF-A0A7W6S3Y5-F1-MODEL\_V4 | 1.0 | 0.004822 | 126 | 0.209 | 124 | 80 | 4 | 2 | 118 | 16 | 128 | DNA-binding NarL/FixJ family response regulator | DNA-binding NarL/FixJ family response regulator | | afdb-uniprot50 | AF-A0A270BEU5-F1-MODEL\_V4 | 1.0 | 0.002865 | 125 | 0.193 | 119 | 84 | 3 | 1 | 118 | 4 | 111 | Uncharacterized protein | Uncharacterized protein | | afdb-uniprot50 | AF-A0A0P8Y9C8-F1-MODEL\_V4 | 1.0 | 0.002145 | 125 | 0.186 | 145 | 100 | 3 | 1 | 142 | 21 | 150 | Mor domain-containing protein | Mor domain-containing protein | | afdb-uniprot50 | AF-A0A0F2RXJ4-F1-MODEL\_V4 | 1.0 | 0.00191 | 124 | 0.161 | 118 | 87 | 2 | 1 | 118 | 2 | 107 | Uncharacterized protein | Uncharacterized protein | | afdb-uniprot50 | AF-A0A656HD19-F1-MODEL\_V4 | 1.0 | 0.006441 | 123 | 0.168 | 119 | 83 | 5 | 3 | 118 | 7 | 112 | Mor transcription activator domain protein | Mor transcription activator domain protein | | afdb-uniprot50 | AF-B8CUZ8-F1-MODEL\_V4 | 1.0 | 0.002551 | 122 | 0.172 | 139 | 95 | 7 | 3 | 139 | 22 | 142 | Mor domain-containing protein | Mor domain-containing protein | | afdb-uniprot50 | AF-A0A7C5KXW5-F1-MODEL\_V4 | 1.0 | 0.006441 | 122 | 0.176 | 130 | 88 | 4 | 3 | 125 | 15 | 132 | Mor domain-containing protein | Mor domain-containing protein | | afdb-uniprot50 | AF-A0A5P8XH25-F1-MODEL\_V4 | 1.0 | 0.008118 | 116 | 0.214 | 107 | 71 | 4 | 39 | 139 | 10 | 109 | Mor domain-containing protein | Mor domain-containing protein | | afdb-uniprot50 | AF-A0A3N1PMJ1-F1-MODEL\_V4 | 1.0 | 0.00511 | 110 | 0.175 | 131 | 87 | 3 | 3 | 124 | 15 | 133 | Mor transcription activator family protein | Mor transcription activator family protein | | afdb-uniprot50 | AF-A0A2M7G6P5-F1-MODEL\_V4 | 1.0 | 0.009657 | 103 | 0.183 | 120 | 74 | 7 | 11 | 118 | 28 | 135 | Mor domain-containing protein | Mor domain-containing protein | |
| Top keywords  (threshold 1.00e-02 (evalue)) | **Mor, domain\_containing, transcription, activator, regulator, Transcriptional, Helix\_turn\_helix, Homeodomain\_like, DNA, transposition** |
| Output files | ../../similar\_structures/61\_FANPEZAQ\_CDS\_0061\_afdb-proteome\_foldseek.tsv ../../similar\_structures/61\_FANPEZAQ\_CDS\_0061\_afdb-uniprot50\_foldseek.tsv ../../similar\_structures/61\_FANPEZAQ\_CDS\_0061\_merged.svg ../../similar\_structures/61\_FANPEZAQ\_CDS\_0061\_pdb\_foldseek.tsv |

  
  
  

Return to summary | Go to previous | Go to next

  


---

**Sequence/structure alignments coloring**  
Each object in the alignment figures is colored according to its E-value following this color coding:

1e-100
10

**References:**  
1) Steinegger M, Meier M, Mirdita M, Vöhringer H, Haunsberger S J, and Söding J (2019) HH-suite3 for fast remote homology detection and deep protein annotation, BMC Bioinformatics, 473. doi: 10.1186/s12859-019-3019-7  
2) Jumper J, Evans R, Pritzel A, ..., Hassabis D (2021) Highly accurate protein structure prediction with AlphaFold, Nature, 596. doi: 10.1038/s41586-021-03819-2  
3) van Kempen M, Kim S, Tumescheit C, Mirdita M, Lee J, Gilchrist CLM, Söding J, and Steinegger M (2023) Fast and accurate protein structure search with Foldseek. Nature Biotechnology. doi: 10.1038/s41587-023-01773-0
