# Supplementary material for: Completing the BASEL phage collection to unlock hidden diversity for systematic exploration of phage–host interactions
Source: PLoS Biol. 2025 Apr 7;23(4):e3003063. doi: 10.1371/journal.pbio.3003063 (PMC11990801; doi:10.1371/journal.pbio.3003063)
Supplement: S2 Data — (ZIP) [file pbio.3003063.s009.zip › entries/62.html]

FANPEZAQ\_CDS\_0062


Return to summary | Go to previous | Go to next

|  |  |
| --- | --- |
| FANPEZAQ\_CDS\_0062 Page creation date: 02 Sep 2024, 12:00  Project folder: n/a  Input sequences file: Escherichia\_virus\_HeidiAbel.gb | holin hypothetical putative phage membrane fragment transporter mfs lysis peptidase m48 ste24p nc\_023006\_p63 p387102 vi\_09157 p363326 vi\_04187 p127786 vi\_03659 antiholin lambda holin\_like ii conserved n\_acetylmuramidase duf1097 domain\_containing prophage hol44 superfamily v contig\_80 whole genome shotgun sequence ethanolamine permease |

### Sequence information

|  |  |
| --- | --- |
| Name | FANPEZAQ\_CDS\_0062  62\_FANPEZAQ\_CDS\_0062 (pipeline id) |
| Imported annotations | Escherichia\_virus\_HeidiAbel Bas97 |
| Protein sequence | MEFFQKFLDNIGWGIAGIMGSLVSLPFHDDIKSVKARIWFVCTGAICAYFLTDLVSLYFK IDRGLAGSVGFLLGAFGGSLLAAVIRAIKAADIWQLLKNRFGGGNA |
| Number of residues | 106 |
| Molecular weight (Da) | 11463.38 |
| Output files | ../../query\_sequences/62\_FANPEZAQ\_CDS\_0062.fasta |

### Putative domain architecture and protein family

#### Search results (HHblits)1

|  |  |
| --- | --- |
| Domain family databases searched | Pfam, Ncbi-cd, Cath, Phrogs |
| Results, scheme(s)  (Top layers only; threshold 1.00e-03 (evalue)) | xml version="1.0" encoding="utf-8" standalone="no"?       2024-09-02T21:08:25.903983 image/svg+xml   Matplotlib v3.7.2, https://matplotlib.org/ |
| Results, table  (E-value ≤ 1.00e-03 (evalue)) | | db | id | prob | evalue | pvalue | score | cols | query | query\_len | template | template\_len | name | description | | --- | --- | --- | --- | --- | --- | --- | --- | --- | --- | --- | --- | --- | | phrogs | 2359 | 99.8 | 6.3e-25 | 7.9e-29 | 143.6 | 105 | (1, 105) | 106 | (1, 105) | 107 | holin | holin; Category: lysis; NC\_023006\_p63 | | phrogs | 198 | 99.7 | 1.4e-21 | 1.8e-25 | 129.1 | 86 | (9, 97) | 106 | (23, 108) | 116 | holin | holin; Category: lysis; p387102 VI\_09157 | | phrogs | 13918 | 97.9 | 4.1e-09 | 4.8e-13 | 68.5 | 80 | (9, 89) | 106 | (21, 101) | 127 | NA | NA; Category: unknown function; p363326 VI\_04187 | | phrogs | 21855 | 95.0 | 0.00031 | 3.6e-08 | 38.3 | 29 | (9, 38) | 106 | (27, 55) | 56 | NA | NA; Category: unknown function; p127786 VI\_03659 | |
| Top keywords  (threshold 1.00e-03 (evalue)) | **holin, lysis, NC\_023006\_p63, p387102, VI\_09157, p363326, VI\_04187, p127786, VI\_03659** |
| Output files | ../../domain\_architecture/62\_FANPEZAQ\_CDS\_0062\_cath.hhr ../../domain\_architecture/62\_FANPEZAQ\_CDS\_0062\_merged.svg ../../domain\_architecture/62\_FANPEZAQ\_CDS\_0062\_ncbi-cd.hhr ../../domain\_architecture/62\_FANPEZAQ\_CDS\_0062\_pfam.hhr ../../domain\_architecture/62\_FANPEZAQ\_CDS\_0062\_phrogs.hhr |

### Identical protein sequences/structures

#### Search results

|  |  |
| --- | --- |
| Protein sequence databases searched | Pdb, Swissprot, Refseq |
| Identical proteins found | -- |
| Top keywords | -- |
| Output files | -- |

### Similar protein sequences/structures

#### Sequence similarity search results (HHblits)1

|  |  |
| --- | --- |
| Sequence databases searched | Uniclust, Pdb70 |
| Results, scheme(s)  (Top layers only, threshold 1.00e-03 (evalue)) | xml version="1.0" encoding="utf-8" standalone="no"?       2024-09-02T21:08:54.385043 image/svg+xml   Matplotlib v3.7.2, https://matplotlib.org/ |
| Results, table(s)  (threshold 1.00e-03 (evalue)) | | db | id | prob | evalue | pvalue | score | cols | query | query\_len | template | template\_len | name | description | | --- | --- | --- | --- | --- | --- | --- | --- | --- | --- | --- | --- | --- | | uniclust | UniRef100\_A0A059KTJ6 | 99.9 | 1.9e-26 | 4e-32 | 154.2 | 105 | (1, 105) | 106 | (6, 112) | 115 | MFS transporter | MFS transporter | | uniclust | UniRef100\_A0A077LKE0 | 99.8 | 2.3e-23 | 4.3e-29 | 145.4 | 105 | (1, 105) | 106 | (13, 122) | 173 | MFS transporter | MFS transporter | | uniclust | UniRef100\_A0A083UGU3 | 99.6 | 3e-18 | 6.8e-24 | 121.1 | 95 | (8, 105) | 106 | (37, 131) | 146 | Phage holin | Phage holin | | uniclust | UniRef100\_A0A127QUD9 | 99.6 | 3.7e-18 | 7.5e-24 | 119.5 | 95 | (10, 105) | 106 | (45, 141) | 149 | Holin | Holin | | uniclust | UniRef100\_A0A345L6P1 | 99.5 | 1.2e-17 | 2.4e-23 | 111.7 | 104 | (1, 104) | 106 | (1, 105) | 108 | Holin/antiholin | Holin/antiholin | | uniclust | UniRef100\_A0A0M7GTX4 | 99.5 | 1.2e-17 | 2.6e-23 | 114.4 | 92 | (8, 104) | 106 | (17, 111) | 123 | Phage holin, lambda family | Phage holin, lambda family | | uniclust | UniRef100\_A0A1G9MGV6 | 99.4 | 4.3e-16 | 8.6e-22 | 106.7 | 90 | (1, 91) | 106 | (9, 98) | 128 | Holin | Holin | | uniclust | UniRef100\_A0A087N8V0 | 99.4 | 4.6e-16 | 1e-21 | 107.6 | 100 | (5, 105) | 106 | (14, 115) | 124 | Holin | Holin | | uniclust | UniRef100\_A0A0Q5DKQ6 | 99.4 | 8.3e-16 | 1.8e-21 | 108.1 | 84 | (7, 91) | 106 | (19, 110) | 140 | Peptidase M48, Ste24p | Peptidase M48, Ste24p | | uniclust | UniRef100\_A0A009PIY2 | 99.4 | 1e-15 | 2e-21 | 105.0 | 94 | (9, 105) | 106 | (30, 123) | 125 | Putative membrane protein | Putative membrane protein | | uniclust | UniRef100\_A0A011V762 | 99.4 | 9.8e-16 | 2.2e-21 | 109.8 | 88 | (3, 91) | 106 | (27, 116) | 158 | Phage holin family protein | Phage holin family protein | | uniclust | UniRef100\_A0A059V0X5 | 99.4 | 1.2e-15 | 2.9e-21 | 107.8 | 87 | (9, 98) | 106 | (38, 124) | 138 | Membrane protein | Membrane protein | | uniclust | UniRef100\_A0A010SYK4 | 99.3 | 5.4e-15 | 1.1e-20 | 99.5 | 84 | (6, 90) | 106 | (11, 98) | 107 | Holin | Holin | | uniclust | UniRef100\_A0A246JHP1 | 99.3 | 8.4e-15 | 1.8e-20 | 98.7 | 86 | (12, 98) | 106 | (15, 101) | 107 | Holin | Holin | | uniclust | UniRef100\_A0A021X861 | 99.3 | 2e-14 | 4.1e-20 | 100.8 | 88 | (2, 90) | 106 | (18, 113) | 141 | Holin | Holin | | uniclust | UniRef100\_A0A0Q4GP77 | 99.2 | 3e-14 | 6.5e-20 | 97.7 | 102 | (2, 104) | 106 | (7, 110) | 116 | Uncharacterized protein | Uncharacterized protein | | uniclust | UniRef100\_A0A088U760 | 99.2 | 3.6e-14 | 7.6e-20 | 100.7 | 97 | (7, 104) | 106 | (21, 121) | 145 | Putative membrane protein | Putative membrane protein | | uniclust | UniRef100\_A0A076YME0 | 99.2 | 4.9e-14 | 9.9e-20 | 96.0 | 94 | (1, 94) | 106 | (1, 96) | 116 | Holin-like class II protein | Holin-like class II protein | | uniclust | UniRef100\_A0A0U3F4J3 | 99.2 | 5.3e-14 | 1.2e-19 | 96.6 | 88 | (1, 89) | 106 | (4, 92) | 112 | Holin | Holin | | uniclust | UniRef100\_A0A0Q8XZH4 | 99.2 | 9.1e-14 | 1.9e-19 | 97.7 | 92 | (10, 104) | 106 | (38, 129) | 139 | Holin | Holin | | uniclust | UniRef100\_A0A0A8TGY1 | 99.1 | 3.2e-13 | 6.4e-19 | 92.8 | 99 | (6, 104) | 106 | (12, 120) | 123 | Holin | Holin | | uniclust | UniRef100\_A0A166F525 | 99.1 | 3.9e-13 | 7.8e-19 | 91.5 | 90 | (2, 91) | 106 | (3, 98) | 115 | Phage holin family 2 | Phage holin family 2 | | uniclust | UniRef100\_A0A109J7U2 | 99.1 | 4.6e-13 | 8.9e-19 | 94.5 | 90 | (3, 93) | 106 | (15, 107) | 155 | Holin | Holin | | uniclust | UniRef100\_A0A0E0URI3 | 99.1 | 4.7e-13 | 9.7e-19 | 93.2 | 92 | (11, 103) | 106 | (23, 124) | 129 | Holin | Holin | | uniclust | UniRef100\_E7EKS8 | 99.1 | 6.2e-13 | 1.1e-18 | 89.9 | 95 | (1, 95) | 106 | (15, 110) | 127 | Putative holin (Fragment) | Putative holin (Fragment) | | uniclust | UniRef100\_A0A6G6Y576 | 99.1 | 6e-13 | 1.2e-18 | 89.4 | 94 | (11, 105) | 106 | (14, 107) | 108 | Uncharacterized protein | Uncharacterized protein | | uniclust | UniRef100\_A0A2N7QRA9 | 99.1 | 9.5e-13 | 1.9e-18 | 89.4 | 86 | (4, 90) | 106 | (10, 95) | 116 | Holin | Holin | | uniclust | UniRef100\_A0A149SVI2 | 99.1 | 1e-12 | 2e-18 | 87.9 | 92 | (10, 105) | 106 | (12, 103) | 104 | Holin | Holin | | uniclust | UniRef100\_A0A085FNY7 | 99.0 | 2e-12 | 4.1e-18 | 89.9 | 85 | (6, 91) | 106 | (14, 106) | 128 | Uncharacterized protein | Uncharacterized protein | | uniclust | UniRef100\_A0A345BNC1 | 99.0 | 2.7e-12 | 5e-18 | 88.9 | 98 | (8, 105) | 106 | (49, 146) | 148 | Putative holin | Putative holin | | uniclust | UniRef100\_UPI002013492F | 99.0 | 3.1e-12 | 5.7e-18 | 93.0 | 95 | (10, 105) | 106 | (15, 111) | 203 | hypothetical protein | hypothetical protein | | uniclust | UniRef100\_A0A0A8IL65 | 99.0 | 2.6e-12 | 5.9e-18 | 90.2 | 89 | (1, 91) | 106 | (11, 107) | 127 | Holin | Holin | | uniclust | UniRef100\_A0A127Q5M9 | 98.9 | 9.8e-12 | 1.9e-17 | 80.7 | 76 | (29, 105) | 106 | (8, 84) | 89 | Holin | Holin | | uniclust | UniRef100\_A0A853FDE8 | 98.9 | 1.6e-11 | 3e-17 | 82.7 | 94 | (3, 97) | 106 | (3, 97) | 110 | Holin | Holin | | uniclust | UniRef100\_UPI0018D78283 | 98.8 | 3.7e-11 | 6.9e-17 | 76.9 | 79 | (25, 103) | 106 | (3, 81) | 86 | hypothetical protein | hypothetical protein | | uniclust | UniRef100\_A0A0D0KIG3 | 98.8 | 4.1e-11 | 8.9e-17 | 83.0 | 87 | (1, 89) | 106 | (1, 96) | 115 | Holin | Holin | | uniclust | UniRef100\_UPI000BAFFC2F | 98.7 | 1.9e-10 | 3.6e-16 | 76.5 | 96 | (7, 102) | 106 | (5, 101) | 107 | hypothetical protein | hypothetical protein | | uniclust | UniRef100\_A0A0E3BDL1 | 98.6 | 2.5e-10 | 5.1e-16 | 78.4 | 91 | (3, 102) | 106 | (10, 104) | 115 | Holin | Holin | | uniclust | UniRef100\_A0A090EBA1 | 98.6 | 3e-10 | 5.7e-16 | 80.7 | 88 | (2, 90) | 106 | (37, 133) | 153 | Holin | Holin | | uniclust | UniRef100\_A0A2N1F928 | 98.6 | 7e-10 | 1.4e-15 | 75.1 | 93 | (1, 94) | 106 | (5, 97) | 105 | Holin | Holin | | uniclust | UniRef100\_A0A2Z6EUY1 | 98.5 | 7.7e-10 | 1.5e-15 | 75.2 | 96 | (8, 104) | 106 | (17, 114) | 115 | Holin protein | Holin protein | | uniclust | UniRef100\_A0A6G5Y843 | 98.5 | 1.1e-09 | 2e-15 | 71.7 | 86 | (10, 96) | 106 | (4, 90) | 91 | Holin | Holin | | uniclust | UniRef100\_A0A0Q5M7J0 | 98.5 | 1.1e-09 | 2.2e-15 | 76.6 | 88 | (2, 90) | 106 | (9, 96) | 130 | Holin | Holin | | uniclust | UniRef100\_A0A1R0FA65 | 98.5 | 1.2e-09 | 2.3e-15 | 78.1 | 88 | (2, 90) | 106 | (34, 135) | 145 | Uncharacterized protein | Uncharacterized protein | | uniclust | UniRef100\_A0A0K2FIU1 | 98.5 | 1.3e-09 | 2.6e-15 | 73.8 | 85 | (10, 94) | 106 | (20, 105) | 108 | Holin | Holin | | uniclust | UniRef100\_A0A0F2S303 | 98.5 | 1.3e-09 | 2.9e-15 | 78.3 | 78 | (12, 90) | 106 | (41, 123) | 140 | Uncharacterized protein | Uncharacterized protein | | uniclust | UniRef100\_A0A6J5MI17 | 98.5 | 1.8e-09 | 3.6e-15 | 72.1 | 86 | (4, 90) | 106 | (3, 89) | 96 | Holin | Holin | | uniclust | UniRef100\_A0A0Q6UK20 | 98.4 | 2.3e-09 | 4.7e-15 | 75.7 | 95 | (8, 103) | 106 | (14, 117) | 128 | Holin | Holin | | uniclust | UniRef100\_W0LM32 | 98.4 | 4.1e-09 | 7.5e-15 | 69.3 | 77 | (19, 95) | 106 | (1, 78) | 95 | Putative holin | Putative holin | | uniclust | UniRef100\_A0A239F4Z5 | 98.3 | 6.1e-09 | 1.1e-14 | 67.7 | 87 | (14, 103) | 106 | (2, 88) | 89 | Phage holin | Phage holin | | uniclust | UniRef100\_A0A5E7QE83 | 98.3 | 7.4e-09 | 1.4e-14 | 69.0 | 89 | (11, 99) | 106 | (11, 99) | 103 | Holin | Holin | | uniclust | UniRef100\_A0A959GDS2 | 98.3 | 1.1e-08 | 1.9e-14 | 71.8 | 76 | (7, 83) | 106 | (33, 108) | 143 | Uncharacterized protein | Uncharacterized protein | | uniclust | UniRef100\_UPI001EE52D85 | 98.3 | 1.1e-08 | 2.1e-14 | 68.2 | 88 | (12, 100) | 106 | (14, 102) | 103 | hypothetical protein | hypothetical protein | | uniclust | UniRef100\_A0A2S5TKI1 | 98.2 | 1.6e-08 | 2.9e-14 | 68.0 | 94 | (6, 100) | 106 | (3, 101) | 107 | Holin | Holin | | uniclust | UniRef100\_A0A1D3JUZ2 | 98.2 | 2.2e-08 | 4.2e-14 | 69.2 | 94 | (9, 105) | 106 | (25, 121) | 122 | Conserved hypothetical membrane protein | Conserved hypothetical membrane protein | | uniclust | UniRef100\_A0A0F0E234 | 98.2 | 2.6e-08 | 5e-14 | 68.6 | 82 | (7, 90) | 106 | (10, 92) | 114 | Holin | Holin | | uniclust | UniRef100\_UPI0018A7A48E | 98.2 | 2.9e-08 | 5.3e-14 | 65.3 | 91 | (12, 104) | 106 | (3, 93) | 93 | hypothetical protein | hypothetical protein | | uniclust | UniRef100\_A0A8T8CVW9 | 98.1 | 7e-08 | 1.3e-13 | 70.7 | 89 | (2, 91) | 106 | (81, 177) | 192 | N-acetylmuramidase family protein | N-acetylmuramidase family protein | | uniclust | UniRef100\_UPI001900EF55 | 98.0 | 9.4e-08 | 1.8e-13 | 63.5 | 76 | (29, 104) | 106 | (7, 91) | 93 | hypothetical protein | hypothetical protein | | uniclust | UniRef100\_A0A366F9C6 | 98.0 | 1.1e-07 | 1.9e-13 | 59.4 | 61 | (42, 105) | 106 | (3, 66) | 67 | Holin | Holin | | uniclust | UniRef100\_A0A1I1D0C4 | 97.9 | 2.9e-07 | 6.4e-13 | 66.9 | 93 | (10, 105) | 106 | (32, 130) | 138 | Putative phage holin | Putative phage holin | | uniclust | UniRef100\_A0A1U7D4W0 | 97.8 | 4.4e-07 | 9e-13 | 63.0 | 91 | (13, 105) | 106 | (14, 106) | 106 | Holin | Holin | | uniclust | UniRef100\_A0A3M4HEX7 | 97.8 | 4.9e-07 | 9.3e-13 | 55.2 | 49 | (1, 49) | 106 | (1, 53) | 55 | Uncharacterized protein | Uncharacterized protein | | uniclust | UniRef100\_A0A519X5A4 | 97.8 | 5.1e-07 | 9.6e-13 | 61.7 | 83 | (2, 85) | 106 | (11, 93) | 108 | Holin | Holin | | uniclust | UniRef100\_A0A366DXW5 | 97.8 | 5.4e-07 | 1e-12 | 59.2 | 59 | (2, 61) | 106 | (5, 63) | 83 | Uncharacterized protein | Uncharacterized protein | | uniclust | UniRef100\_UPI001678847F | 97.8 | 5.8e-07 | 1.1e-12 | 62.5 | 90 | (13, 105) | 106 | (30, 119) | 121 | putative holin | putative holin | | uniclust | UniRef100\_A0A806CKK2 | 97.7 | 7.5e-07 | 1.4e-12 | 60.9 | 103 | (2, 105) | 106 | (7, 111) | 112 | Holin | Holin | | uniclust | UniRef100\_H8L657 | 97.7 | 8.9e-07 | 1.6e-12 | 59.4 | 88 | (2, 90) | 106 | (4, 91) | 99 | Phage holin | Phage holin | | uniclust | UniRef100\_A0A0D0J2P5 | 97.7 | 1e-06 | 2.2e-12 | 62.9 | 76 | (10, 86) | 106 | (29, 107) | 122 | Phage holin | Phage holin | | uniclust | UniRef100\_A0A7Y5IYH3 | 97.6 | 1.5e-06 | 2.8e-12 | 60.1 | 77 | (10, 87) | 106 | (21, 97) | 120 | Holin | Holin | | uniclust | UniRef100\_A0A5C8PAN5 | 97.6 | 1.5e-06 | 2.9e-12 | 60.6 | 88 | (3, 90) | 106 | (6, 97) | 122 | Phage holin family protein | Phage holin family protein | | uniclust | UniRef100\_UPI0010C024CC | 97.6 | 2.5e-06 | 4.8e-12 | 64.4 | 87 | (10, 99) | 106 | (28, 114) | 211 | putative holin | putative holin | | uniclust | UniRef100\_A0A089YNB7 | 97.5 | 3e-06 | 5.6e-12 | 60.4 | 90 | (10, 102) | 106 | (42, 131) | 132 | Holin | Holin | | uniclust | UniRef100\_A0A6J5QP20 | 97.5 | 3.1e-06 | 5.8e-12 | 58.0 | 80 | (8, 87) | 106 | (8, 88) | 111 | Holin | Holin | | uniclust | UniRef100\_UPI000319E467 | 97.5 | 3.5e-06 | 6.5e-12 | 53.9 | 60 | (1, 60) | 106 | (1, 64) | 73 | hypothetical protein | hypothetical protein | | uniclust | UniRef100\_A0A241XK97 | 97.5 | 4e-06 | 7.4e-12 | 59.7 | 86 | (10, 98) | 106 | (38, 123) | 140 | Holin | Holin | | uniclust | UniRef100\_UPI00207B5E30 | 97.5 | 4.8e-06 | 8.8e-12 | 62.2 | 87 | (10, 99) | 106 | (99, 185) | 200 | putative holin | putative holin | | uniclust | UniRef100\_A0A102EQF3 | 97.4 | 4.9e-06 | 9e-12 | 51.9 | 59 | (46, 104) | 106 | (2, 61) | 63 | Uncharacterized protein | Uncharacterized protein | | uniclust | UniRef100\_UPI00217D4006 | 97.4 | 6e-06 | 1.1e-11 | 53.8 | 77 | (1, 77) | 106 | (1, 79) | 80 | hypothetical protein | hypothetical protein | | uniclust | UniRef100\_A0A0K1JXH2 | 97.4 | 6.5e-06 | 1.4e-11 | 62.3 | 82 | (9, 91) | 106 | (64, 153) | 173 | Uncharacterized protein | Uncharacterized protein | | uniclust | UniRef100\_UPI001E5DDA53 | 97.4 | 7.8e-06 | 1.4e-11 | 56.8 | 85 | (4, 90) | 106 | (11, 102) | 119 | hypothetical protein | hypothetical protein | | uniclust | UniRef100\_UPI00217CC2F6 | 97.4 | 8e-06 | 1.5e-11 | 56.5 | 85 | (10, 94) | 106 | (7, 92) | 115 | hypothetical protein | hypothetical protein | | uniclust | UniRef100\_A0A2M9P829 | 97.4 | 8e-06 | 1.5e-11 | 54.9 | 85 | (16, 102) | 106 | (10, 95) | 96 | Phage holin family protein | Phage holin family protein | | uniclust | UniRef100\_A0A1Y6CU26 | 97.4 | 8.6e-06 | 1.6e-11 | 56.3 | 87 | (7, 94) | 106 | (11, 105) | 114 | Uncharacterized protein | Uncharacterized protein | | uniclust | UniRef100\_A0A1I5MXA9 | 97.3 | 9.9e-06 | 1.8e-11 | 55.8 | 88 | (2, 91) | 106 | (4, 91) | 111 | Holin | Holin | | uniclust | UniRef100\_UPI0019D322D2 | 97.3 | 1.3e-05 | 2.4e-11 | 51.0 | 55 | (7, 62) | 106 | (10, 64) | 69 | hypothetical protein | hypothetical protein | | uniclust | UniRef100\_A0A2A2CFL2 | 97.3 | 1.4e-05 | 2.6e-11 | 56.0 | 88 | (10, 100) | 106 | (30, 117) | 117 | Membrane transporter protein | Membrane transporter protein | | uniclust | UniRef100\_UPI001F180A34 | 97.2 | 2.1e-05 | 4e-11 | 56.7 | 93 | (9, 102) | 106 | (21, 121) | 144 | hypothetical protein | hypothetical protein | | uniclust | UniRef100\_A0A959HN31 | 97.2 | 2.6e-05 | 4.7e-11 | 54.1 | 73 | (11, 86) | 106 | (12, 87) | 114 | Uncharacterized protein | Uncharacterized protein | | uniclust | UniRef100\_A0A3S9U7J0 | 97.1 | 2.8e-05 | 5.2e-11 | 53.2 | 86 | (9, 94) | 106 | (15, 101) | 105 | Holin | Holin | | uniclust | UniRef100\_F3C3E1 | 97.1 | 3.8e-05 | 6.9e-11 | 48.4 | 59 | (46, 104) | 106 | (2, 61) | 64 | MFS transporter (Fragment) | MFS transporter (Fragment) | | uniclust | UniRef100\_J8RRD5 | 97.0 | 5e-05 | 9.2e-11 | 47.9 | 60 | (42, 103) | 106 | (4, 63) | 64 | Uncharacterized protein | Uncharacterized protein | | uniclust | UniRef100\_A0A3S1MB93 | 97.0 | 4.9e-05 | 9.3e-11 | 51.1 | 57 | (33, 89) | 106 | (11, 76) | 85 | Uncharacterized protein (Fragment) | Uncharacterized protein (Fragment) | | uniclust | UniRef100\_UPI001157799A | 97.0 | 5.4e-05 | 1e-10 | 54.6 | 87 | (3, 90) | 106 | (20, 111) | 146 | hypothetical protein | hypothetical protein | | uniclust | UniRef100\_A0A5B0DZY6 | 97.0 | 5.4e-05 | 1e-10 | 52.6 | 85 | (2, 88) | 106 | (2, 95) | 113 | Holin | Holin | | uniclust | UniRef100\_A0A077K9X1 | 97.0 | 4.7e-05 | 1.1e-10 | 56.2 | 80 | (10, 90) | 106 | (29, 113) | 134 | Holin | Holin | | uniclust | UniRef100\_A0A2V4JFC9 | 97.0 | 5.6e-05 | 1.1e-10 | 51.8 | 62 | (10, 74) | 106 | (28, 89) | 90 | DUF1097 domain-containing protein (Fragment) | DUF1097 domain-containing protein (Fragment) | | uniclust | UniRef100\_A0A1B1INP0 | 97.0 | 5.5e-05 | 1.2e-10 | 53.9 | 83 | (7, 90) | 106 | (9, 95) | 110 | Holin | Holin | | uniclust | UniRef100\_UPI00216732F2 | 97.0 | 6.9e-05 | 1.3e-10 | 48.0 | 64 | (40, 103) | 106 | (2, 67) | 69 | hypothetical protein | hypothetical protein | | uniclust | UniRef100\_A0A066T1C6 | 96.9 | 5.8e-05 | 1.3e-10 | 57.2 | 81 | (11, 92) | 106 | (54, 141) | 159 | Uncharacterized protein | Uncharacterized protein | | uniclust | UniRef100\_A0A0U2C0W7 | 96.9 | 6.6e-05 | 1.4e-10 | 54.0 | 88 | (17, 105) | 106 | (13, 103) | 115 | Holin | Holin | | uniclust | UniRef100\_A0A024HES2 | 96.9 | 8.2e-05 | 1.5e-10 | 50.2 | 78 | (11, 89) | 106 | (7, 90) | 93 | Uncharacterized protein | Uncharacterized protein | | uniclust | UniRef100\_A0A085G117 | 96.9 | 7.9e-05 | 1.6e-10 | 55.7 | 94 | (10, 104) | 106 | (41, 139) | 151 | Putative prophage membrane protein | Putative prophage membrane protein | | uniclust | UniRef100\_UPI00234D9A13 | 96.9 | 0.00011 | 1.9e-10 | 50.6 | 76 | (10, 88) | 106 | (9, 89) | 104 | hypothetical protein | hypothetical protein | | uniclust | UniRef100\_A0A8E4ZL07 | 96.8 | 0.00012 | 2.1e-10 | 50.7 | 89 | (10, 98) | 106 | (6, 100) | 108 | Holin | Holin | | uniclust | UniRef100\_A0A081J3M5 | 96.8 | 0.00014 | 2.6e-10 | 48.4 | 67 | (33, 102) | 106 | (6, 76) | 85 | Uncharacterized protein | Uncharacterized protein | | uniclust | UniRef100\_A0A017HBY4 | 96.8 | 0.00014 | 3e-10 | 52.8 | 73 | (13, 86) | 106 | (32, 108) | 119 | Uncharacterized protein | Uncharacterized protein | | uniclust | UniRef100\_UPI0015B67E84 | 96.7 | 0.00019 | 3.6e-10 | 47.2 | 61 | (1, 61) | 106 | (1, 63) | 74 | hypothetical protein | hypothetical protein | | uniclust | UniRef100\_A0A944HBG9 | 96.7 | 0.00019 | 3.7e-10 | 50.8 | 99 | (4, 105) | 106 | (13, 113) | 114 | Uncharacterized protein | Uncharacterized protein | | uniclust | UniRef100\_UPI001848C271 | 96.7 | 0.0002 | 3.8e-10 | 47.3 | 48 | (11, 59) | 106 | (27, 74) | 75 | putative holin | putative holin | | uniclust | UniRef100\_A0A2P7RER1 | 96.7 | 0.00022 | 4.1e-10 | 47.5 | 66 | (24, 90) | 106 | (3, 76) | 84 | Uncharacterized protein (Fragment) | Uncharacterized protein (Fragment) | | uniclust | UniRef100\_UPI00094B3F0A | 96.7 | 0.00022 | 4.1e-10 | 47.4 | 59 | (1, 61) | 106 | (1, 60) | 83 | hypothetical protein | hypothetical protein | | uniclust | UniRef100\_A0A2W6XWQ2 | 96.7 | 0.00023 | 4.2e-10 | 45.1 | 52 | (2, 54) | 106 | (10, 62) | 63 | Uncharacterized protein (Fragment) | Uncharacterized protein (Fragment) | | uniclust | UniRef100\_UPI001571687F | 96.7 | 0.00025 | 4.6e-10 | 50.6 | 89 | (1, 90) | 106 | (1, 91) | 129 | hypothetical protein | hypothetical protein | | uniclust | UniRef100\_A0A3G3GJ83 | 96.6 | 0.00025 | 4.8e-10 | 52.3 | 100 | (5, 104) | 106 | (29, 135) | 152 | Holin | Holin | | uniclust | UniRef100\_A0A2S0PEK2 | 96.6 | 0.00024 | 5.2e-10 | 54.7 | 93 | (10, 103) | 106 | (62, 155) | 178 | Uncharacterized protein | Uncharacterized protein | | uniclust | UniRef100\_A0A0A1W9E3 | 96.6 | 0.0003 | 6.2e-10 | 50.3 | 85 | (5, 90) | 106 | (7, 97) | 108 | Uncharacterized protein | Uncharacterized protein | | uniclust | UniRef100\_A0A0P6RA73 | 96.6 | 0.00028 | 6.2e-10 | 51.5 | 89 | (14, 105) | 106 | (29, 118) | 122 | Holin | Holin | | uniclust | UniRef100\_UPI00037B93FB | 96.6 | 0.00035 | 6.4e-10 | 50.2 | 91 | (12, 103) | 106 | (13, 109) | 134 | hypothetical protein | hypothetical protein | | uniclust | UniRef100\_A0A971Y2T2 | 96.5 | 0.00051 | 9.4e-10 | 47.6 | 78 | (19, 101) | 106 | (20, 97) | 104 | Uncharacterized protein | Uncharacterized protein | | uniclust | UniRef100\_A0A1G7B018 | 96.5 | 0.00052 | 9.9e-10 | 48.1 | 85 | (1, 88) | 106 | (1, 86) | 104 | Phage holin family Hol44, holin superfamily V | Phage holin family Hol44, holin superfamily V | | uniclust | UniRef100\_A0A848MF35 | 96.4 | 0.00057 | 1e-09 | 46.6 | 87 | (11, 100) | 106 | (5, 91) | 94 | Holin | Holin | | uniclust | UniRef100\_A0A6N6ZDS7 | 96.4 | 0.00058 | 1.1e-09 | 48.8 | 78 | (7, 85) | 106 | (9, 87) | 123 | Peptidase M48, Ste24p | Peptidase M48, Ste24p | | uniclust | UniRef100\_A0A071M1T4 | 96.4 | 0.00053 | 1.1e-09 | 52.4 | 85 | (10, 98) | 106 | (49, 137) | 169 | Membrane protein | Membrane protein | | uniclust | UniRef100\_A0A0Q7XKN5 | 96.4 | 0.00076 | 1.4e-09 | 49.0 | 78 | (10, 89) | 106 | (44, 129) | 140 | Holin | Holin | | uniclust | UniRef100\_A0A2P5K7X0 | 96.4 | 0.0007 | 1.4e-09 | 49.9 | 76 | (12, 91) | 106 | (25, 100) | 131 | Putative phage holin | Putative phage holin | | uniclust | UniRef100\_A0A068CCL5 | 96.3 | 0.00078 | 1.5e-09 | 48.8 | 88 | (1, 92) | 106 | (4, 96) | 121 | Holin | Holin | | uniclust | UniRef100\_UPI00193FE6FD | 96.3 | 0.00084 | 1.6e-09 | 47.3 | 90 | (10, 100) | 106 | (15, 105) | 114 | hypothetical protein | hypothetical protein | | uniclust | UniRef100\_A0A212DP65 | 96.3 | 0.00092 | 1.7e-09 | 45.9 | 53 | (38, 90) | 106 | (2, 63) | 97 | Uncharacterized protein | Uncharacterized protein | | uniclust | UniRef100\_W0V419 | 96.3 | 0.001 | 1.9e-09 | 45.5 | 79 | (11, 90) | 106 | (3, 90) | 95 | Putative membrane protein | Putative membrane protein | |
| Top keywords  (threshold 1.00e-03 (evalue)) | **Holin, hypothetical, Putative, Phage, membrane, Fragment, transporter, MFS, Peptidase, M48** |
| Output files | ../../similar\_sequences/62\_FANPEZAQ\_CDS\_0062\_merged.svg ../../similar\_sequences/62\_FANPEZAQ\_CDS\_0062\_pdb70.a3m ../../similar\_sequences/62\_FANPEZAQ\_CDS\_0062\_pdb70.hhr ../../similar\_sequences/62\_FANPEZAQ\_CDS\_0062\_uniclust.a3m ../../similar\_sequences/62\_FANPEZAQ\_CDS\_0062\_uniclust.hhr |

#### Structure prediction (AlphaFold)2

|  |  |
| --- | --- |
| Stats | xml version="1.0" encoding="utf-8" standalone="no"?       2024-09-02T21:09:57.713479 image/svg+xml   Matplotlib v3.7.2, https://matplotlib.org/ |
| Predicted structure | **NGL Viewer Controls:**  - Center: *Left-Click* - Rotate: *Left-Click + Drag* - Translate: *Right-Click + Drag* - Zoom: *Shift + Left-Click + Drag* |
| Output files | ../../predicted\_structures/62\_FANPEZAQ\_CDS\_0062/features.pkl ../../predicted\_structures/62\_FANPEZAQ\_CDS\_0062/ranked\_0.pdb ../../predicted\_structures/62\_FANPEZAQ\_CDS\_0062/ranked\_0\_plots.svg ../../predicted\_structures/62\_FANPEZAQ\_CDS\_0062/result\_model\_1\_ptm\_pred\_0.pkl |

#### Structure similarity search results (Foldseek)3

|  |  |
| --- | --- |
| Structure databases searched | Pdb, Afdb-proteome, Afdb-uniprot50 |
| Results, scheme(s)  (Top layers only, threshold 1.00e-02 (evalue)) | xml version="1.0" encoding="utf-8" standalone="no"?       2024-09-02T21:11:29.532784 image/svg+xml   Matplotlib v3.7.2, https://matplotlib.org/ |
| Results, table  (threshold 1.00e-02 (evalue)) | | db | id | prob | evalue | bits | fident | alnlen | mismatch | gapopen | qstart | qend | tstart | tend | name | description | | --- | --- | --- | --- | --- | --- | --- | --- | --- | --- | --- | --- | --- | --- | --- | | afdb-uniprot50 | AF-A0A3S1A8T4-F1-MODEL\_V4 | 1.0 | 1.828e-08 | 432 | 0.49 | 106 | 54 | 0 | 1 | 106 | 1 | 106 | MFS transporter | MFS transporter | | afdb-uniprot50 | AF-A0A653KX91-F1-MODEL\_V4 | 1.0 | 3.559e-07 | 349 | 0.472 | 108 | 54 | 1 | 1 | 105 | 1 | 108 | Contig\_80, whole genome shotgun sequence | Contig\_80, whole genome shotgun sequence | | afdb-uniprot50 | AF-A0A5E7NBM4-F1-MODEL\_V4 | 1.0 | 1.276e-07 | 340 | 0.472 | 108 | 55 | 2 | 1 | 106 | 1 | 108 | Uncharacterized protein | Uncharacterized protein | | afdb-uniprot50 | AF-A0A158E8Z8-F1-MODEL\_V4 | 1.0 | 2.868e-07 | 330 | 0.49 | 104 | 53 | 0 | 2 | 105 | 3 | 106 | Uncharacterized protein | Uncharacterized protein | | afdb-uniprot50 | AF-A0A3M6EJ76-F1-MODEL\_V4 | 1.0 | 0.0001866 | 240 | 0.376 | 85 | 51 | 2 | 1 | 83 | 1 | 85 | Uncharacterized protein | Uncharacterized protein | | afdb-uniprot50 | AF-A0A0A8TGY1-F1-MODEL\_V4 | 1.0 | 0.0004672 | 233 | 0.354 | 93 | 59 | 1 | 2 | 93 | 8 | 100 | Uncharacterized protein | Uncharacterized protein | | afdb-uniprot50 | AF-A0A1B5DBB0-F1-MODEL\_V4 | 1.0 | 3.317e-05 | 227 | 0.423 | 111 | 56 | 4 | 1 | 106 | 1 | 108 | Ethanolamine permease | Ethanolamine permease | | afdb-uniprot50 | AF-A0A150HN48-F1-MODEL\_V4 | 1.0 | 0.000646 | 205 | 0.27 | 111 | 71 | 2 | 2 | 102 | 8 | 118 | Uncharacterized protein | Uncharacterized protein | | afdb-uniprot50 | AF-A0A6G6Y576-F1-MODEL\_V4 | 1.0 | 0.0002874 | 203 | 0.302 | 109 | 65 | 2 | 5 | 103 | 8 | 115 | Uncharacterized protein | Uncharacterized protein | | afdb-uniprot50 | AF-A0A149SVI2-F1-MODEL\_V4 | 1.0 | 0.0009426 | 194 | 0.257 | 101 | 72 | 1 | 5 | 105 | 4 | 101 | Uncharacterized protein | Uncharacterized protein | | afdb-uniprot50 | AF-A0A1I7FZH2-F1-MODEL\_V4 | 1.0 | 9.764e-05 | 191 | 0.438 | 105 | 54 | 3 | 1 | 100 | 1 | 105 | Uncharacterized protein | Uncharacterized protein | | afdb-uniprot50 | AF-A0A7U9JL14-F1-MODEL\_V4 | 1.0 | 0.002118 | 181 | 0.329 | 94 | 62 | 1 | 13 | 105 | 18 | 111 | Holin | Holin | | afdb-uniprot50 | AF-A0A5E7QE83-F1-MODEL\_V4 | 1.0 | 0.00105 | 177 | 0.303 | 89 | 60 | 2 | 12 | 99 | 12 | 99 | Uncharacterized protein | Uncharacterized protein | | afdb-uniprot50 | AF-A0A853FDE8-F1-MODEL\_V4 | 1.0 | 0.009099 | 174 | 0.241 | 91 | 68 | 1 | 9 | 99 | 10 | 99 | Uncharacterized protein | Uncharacterized protein | | afdb-uniprot50 | AF-A0A845A3H7-F1-MODEL\_V4 | 1.0 | 0.007739 | 172 | 0.202 | 99 | 78 | 1 | 5 | 103 | 8 | 105 | Uncharacterized protein | Uncharacterized protein | | afdb-uniprot50 | AF-A0A2S5TKI1-F1-MODEL\_V4 | 1.0 | 0.003091 | 163 | 0.233 | 103 | 76 | 2 | 6 | 106 | 3 | 104 | Uncharacterized protein | Uncharacterized protein | | afdb-uniprot50 | AF-A0A7V8Q7L9-F1-MODEL\_V4 | 1.0 | 0.009604 | 150 | 0.152 | 131 | 84 | 3 | 3 | 106 | 9 | 139 | Uncharacterized protein | Uncharacterized protein | |
| Top keywords  (threshold 1.00e-02 (evalue)) | **MFS, transporter, Contig\_80, whole, genome, shotgun, sequence, Ethanolamine, permease, Holin** |
| Output files | ../../similar\_structures/62\_FANPEZAQ\_CDS\_0062\_afdb-proteome\_foldseek.tsv ../../similar\_structures/62\_FANPEZAQ\_CDS\_0062\_afdb-uniprot50\_foldseek.tsv ../../similar\_structures/62\_FANPEZAQ\_CDS\_0062\_merged.svg ../../similar\_structures/62\_FANPEZAQ\_CDS\_0062\_pdb\_foldseek.tsv |

  
  
  

Return to summary | Go to previous | Go to next

  


---

**Sequence/structure alignments coloring**  
Each object in the alignment figures is colored according to its E-value following this color coding:

1e-100
10

**References:**  
1) Steinegger M, Meier M, Mirdita M, Vöhringer H, Haunsberger S J, and Söding J (2019) HH-suite3 for fast remote homology detection and deep protein annotation, BMC Bioinformatics, 473. doi: 10.1186/s12859-019-3019-7  
2) Jumper J, Evans R, Pritzel A, ..., Hassabis D (2021) Highly accurate protein structure prediction with AlphaFold, Nature, 596. doi: 10.1038/s41586-021-03819-2  
3) van Kempen M, Kim S, Tumescheit C, Mirdita M, Lee J, Gilchrist CLM, Söding J, and Steinegger M (2023) Fast and accurate protein structure search with Foldseek. Nature Biotechnology. doi: 10.1038/s41587-023-01773-0
