# Supplementary material for: Completing the BASEL phage collection to unlock hidden diversity for systematic exploration of phage–host interactions
Source: PLoS Biol. 2025 Apr 7;23(4):e3003063. doi: 10.1371/journal.pbio.3003063 (PMC11990801; doi:10.1371/journal.pbio.3003063)
Supplement: S2 Data — (ZIP) [file pbio.3003063.s009.zip › entries/63.html]

FANPEZAQ\_CDS\_0063


Return to summary | Go to previous | Go to next

|  |  |
| --- | --- |
| FANPEZAQ\_CDS\_0063 Page creation date: 02 Sep 2024, 12:00  Project folder: n/a  Input sequences file: Escherichia\_virus\_HeidiAbel.gb | holin hypothetical putative holin\_like i membrane lysis nc\_019419\_p7 rhs repeat\_associated core domain\_containing feob\_associated cys\_rich |

### Sequence information

|  |  |
| --- | --- |
| Name | FANPEZAQ\_CDS\_0063  63\_FANPEZAQ\_CDS\_0063 (pipeline id) |
| Imported annotations | Escherichia\_virus\_HeidiAbel Bas97 |
| Protein sequence | MLLTVINFISVLVIFCWSTWCVLHHGVRDGIVGKILFSCVAISALAVILHEVQGEYLTRS TVTLHATIAAVAVRHFFVNTVWKPFFSKYFHE |
| Number of residues | 92 |
| Molecular weight (Da) | 10420.25 |
| Output files | ../../query\_sequences/63\_FANPEZAQ\_CDS\_0063.fasta |

### Putative domain architecture and protein family

#### Search results (HHblits)1

|  |  |
| --- | --- |
| Domain family databases searched | Pfam, Ncbi-cd, Cath, Phrogs |
| Results, scheme(s)  (Top layers only; threshold 1.00e-03 (evalue)) | xml version="1.0" encoding="utf-8" standalone="no"?       2024-09-02T21:08:26.100168 image/svg+xml   Matplotlib v3.7.2, https://matplotlib.org/ |
| Results, table  (E-value ≤ 1.00e-03 (evalue)) | | db | id | prob | evalue | pvalue | score | cols | query | query\_len | template | template\_len | name | description | | --- | --- | --- | --- | --- | --- | --- | --- | --- | --- | --- | --- | --- | | phrogs | 2508 | 100.0 | 1.2e-35 | 1.5e-39 | 194.9 | 80 | (5, 86) | 92 | (2, 81) | 81 | holin | holin; Category: lysis; NC\_019419\_p7 | |
| Top keywords  (threshold 1.00e-03 (evalue)) | **holin, lysis, NC\_019419\_p7** |
| Output files | ../../domain\_architecture/63\_FANPEZAQ\_CDS\_0063\_cath.hhr ../../domain\_architecture/63\_FANPEZAQ\_CDS\_0063\_merged.svg ../../domain\_architecture/63\_FANPEZAQ\_CDS\_0063\_ncbi-cd.hhr ../../domain\_architecture/63\_FANPEZAQ\_CDS\_0063\_pfam.hhr ../../domain\_architecture/63\_FANPEZAQ\_CDS\_0063\_phrogs.hhr |

### Identical protein sequences/structures

#### Search results

|  |  |
| --- | --- |
| Protein sequence databases searched | Pdb, Swissprot, Refseq |
| Identical proteins found | -- |
| Top keywords | -- |
| Output files | -- |

### Similar protein sequences/structures

#### Sequence similarity search results (HHblits)1

|  |  |
| --- | --- |
| Sequence databases searched | Uniclust, Pdb70 |
| Results, scheme(s)  (Top layers only, threshold 1.00e-03 (evalue)) | xml version="1.0" encoding="utf-8" standalone="no"?       2024-09-02T21:08:54.594164 image/svg+xml   Matplotlib v3.7.2, https://matplotlib.org/ |
| Results, table(s)  (threshold 1.00e-03 (evalue)) | | db | id | prob | evalue | pvalue | score | cols | query | query\_len | template | template\_len | name | description | | --- | --- | --- | --- | --- | --- | --- | --- | --- | --- | --- | --- | --- | | uniclust | UniRef100\_A0A059KTZ2 | 100.0 | 8.3e-41 | 1.8e-46 | 240.9 | 87 | (1, 87) | 92 | (2, 89) | 126 | Holin | Holin | | uniclust | UniRef100\_A0A0D0PMH9 | 99.9 | 1.3e-28 | 3e-34 | 173.4 | 84 | (1, 89) | 92 | (2, 85) | 101 | Uncharacterized protein | Uncharacterized protein | | uniclust | UniRef100\_A0A193GYR7 | 99.9 | 2.5e-25 | 5.1e-31 | 154.7 | 79 | (1, 79) | 92 | (1, 79) | 93 | Holin | Holin | | uniclust | UniRef100\_UPI001032C2BF | 99.8 | 6.8e-25 | 1.3e-30 | 159.0 | 86 | (1, 86) | 92 | (1, 87) | 131 | hypothetical protein | hypothetical protein | | uniclust | UniRef100\_A0A0K2FI78 | 99.7 | 5.2e-20 | 1.2e-25 | 131.8 | 80 | (3, 82) | 92 | (7, 86) | 104 | Putative holin-like class I | Putative holin-like class I | | uniclust | UniRef100\_A0A158E9V0 | 99.6 | 1.7e-18 | 3.5e-24 | 121.5 | 82 | (1, 86) | 92 | (1, 82) | 92 | Uncharacterized protein | Uncharacterized protein | | uniclust | UniRef100\_A0A127R0E2 | 99.4 | 4.3e-16 | 9.3e-22 | 109.9 | 73 | (1, 73) | 92 | (1, 74) | 90 | Putative membrane protein | Putative membrane protein | | uniclust | UniRef100\_A0A093S375 | 99.2 | 1.2e-13 | 2.1e-19 | 95.5 | 72 | (6, 80) | 92 | (2, 74) | 80 | Holin | Holin | | uniclust | UniRef100\_UPI00209AFE34 | 99.0 | 3.1e-12 | 5.7e-18 | 98.5 | 53 | (1, 53) | 92 | (1, 53) | 170 | RHS repeat-associated core domain-containing protein | RHS repeat-associated core domain-containing protein | | uniclust | UniRef100\_A0A853FDB7 | 98.9 | 5.6e-12 | 1.1e-17 | 90.5 | 82 | (3, 87) | 92 | (2, 87) | 96 | Holin | Holin | | uniclust | UniRef100\_G0AAG9 | 98.7 | 1.1e-10 | 2e-16 | 86.6 | 72 | (2, 73) | 92 | (35, 107) | 122 | Uncharacterized protein | Uncharacterized protein | | uniclust | UniRef100\_UPI001B8D6944 | 98.3 | 1.1e-08 | 2e-14 | 67.8 | 53 | (2, 54) | 92 | (1, 53) | 55 | hypothetical protein | hypothetical protein | | uniclust | UniRef100\_A0A2N5F659 | 98.2 | 1.9e-08 | 3.5e-14 | 67.7 | 31 | (1, 31) | 92 | (1, 31) | 59 | FeoB-associated Cys-rich membrane protein | FeoB-associated Cys-rich membrane protein | | uniclust | UniRef100\_A0A076YQQ2 | 98.1 | 5.8e-08 | 1.2e-13 | 71.1 | 87 | (4, 90) | 92 | (9, 95) | 96 | Putative holin-like class I protein | Putative holin-like class I protein | | uniclust | UniRef100\_A0A7S9XD84 | 98.0 | 8.5e-08 | 1.6e-13 | 70.4 | 78 | (6, 83) | 92 | (12, 89) | 102 | Putative holin-like class I | Putative holin-like class I | | uniclust | UniRef100\_UPI0014302FF6 | 97.9 | 2.6e-07 | 4.8e-13 | 64.6 | 59 | (23, 81) | 92 | (1, 60) | 72 | hypothetical protein | hypothetical protein | | uniclust | UniRef100\_A0A022GLN2 | 97.8 | 4.9e-07 | 1.1e-12 | 66.3 | 86 | (1, 88) | 92 | (1, 86) | 92 | Membrane protein | Membrane protein | | uniclust | UniRef100\_UPI001375FE1C | 97.7 | 1.3e-06 | 2.4e-12 | 58.5 | 27 | (1, 27) | 92 | (1, 27) | 54 | hypothetical protein | hypothetical protein | | uniclust | UniRef100\_A0A4D6DYF3 | 97.5 | 3.8e-06 | 7e-12 | 64.3 | 80 | (12, 91) | 92 | (47, 126) | 126 | Class I holin-like protein | Class I holin-like protein | | uniclust | UniRef100\_A0A102EQN0 | 97.3 | 1.1e-05 | 2.5e-11 | 60.1 | 85 | (1, 85) | 92 | (3, 88) | 96 | Holin | Holin | | uniclust | UniRef100\_A0A0F4TAU4 | 97.0 | 5.9e-05 | 1.1e-10 | 53.3 | 54 | (23, 76) | 92 | (4, 60) | 69 | Uncharacterized protein | Uncharacterized protein | |
| Top keywords  (threshold 1.00e-03 (evalue)) | **Holin, hypothetical, Putative, holin\_like, I, membrane, RHS, repeat\_associated, core, domain\_containing** |
| Output files | ../../similar\_sequences/63\_FANPEZAQ\_CDS\_0063\_merged.svg ../../similar\_sequences/63\_FANPEZAQ\_CDS\_0063\_pdb70.a3m ../../similar\_sequences/63\_FANPEZAQ\_CDS\_0063\_pdb70.hhr ../../similar\_sequences/63\_FANPEZAQ\_CDS\_0063\_uniclust.a3m ../../similar\_sequences/63\_FANPEZAQ\_CDS\_0063\_uniclust.hhr |

#### Structure prediction (AlphaFold)2

|  |  |
| --- | --- |
| Stats | xml version="1.0" encoding="utf-8" standalone="no"?       2024-09-02T21:09:58.372452 image/svg+xml   Matplotlib v3.7.2, https://matplotlib.org/ |
| Predicted structure | **NGL Viewer Controls:**  - Center: *Left-Click* - Rotate: *Left-Click + Drag* - Translate: *Right-Click + Drag* - Zoom: *Shift + Left-Click + Drag* |
| Output files | ../../predicted\_structures/63\_FANPEZAQ\_CDS\_0063/features.pkl ../../predicted\_structures/63\_FANPEZAQ\_CDS\_0063/ranked\_0.pdb ../../predicted\_structures/63\_FANPEZAQ\_CDS\_0063/ranked\_0\_plots.svg ../../predicted\_structures/63\_FANPEZAQ\_CDS\_0063/result\_model\_1\_ptm\_pred\_0.pkl |

#### Structure similarity search results (Foldseek)3

|  |  |
| --- | --- |
| Structure databases searched | Pdb, Afdb-proteome, Afdb-uniprot50 |
| Results, scheme(s)  (Top layers only, threshold 1.00e-02 (evalue)) | xml version="1.0" encoding="utf-8" standalone="no"?       2024-09-02T21:11:29.705335 image/svg+xml   Matplotlib v3.7.2, https://matplotlib.org/ |
| Results, table  (threshold 1.00e-02 (evalue)) | | db | id | prob | evalue | bits | fident | alnlen | mismatch | gapopen | qstart | qend | tstart | tend | name | description | | --- | --- | --- | --- | --- | --- | --- | --- | --- | --- | --- | --- | --- | --- | --- | | afdb-uniprot50 | AF-A0A5E7Q5Q9-F1-MODEL\_V4 | 1.0 | 3.868e-05 | 272 | 0.362 | 91 | 57 | 1 | 1 | 90 | 1 | 91 | Uncharacterized protein | Uncharacterized protein | | afdb-uniprot50 | AF-A0A0C5RPM8-F1-MODEL\_V4 | 1.0 | 0.000604 | 223 | 0.364 | 85 | 53 | 1 | 1 | 84 | 1 | 85 | Uncharacterized protein | Uncharacterized protein | | afdb-uniprot50 | AF-A0A3G7X3J1-F1-MODEL\_V4 | 1.0 | 0.001854 | 204 | 0.359 | 89 | 54 | 2 | 1 | 87 | 1 | 88 | Uncharacterized protein | Uncharacterized protein | | afdb-uniprot50 | AF-A0A436ZDM6-F1-MODEL\_V4 | 1.0 | 0.001854 | 194 | 0.281 | 96 | 65 | 2 | 1 | 92 | 1 | 96 | Uncharacterized protein | Uncharacterized protein | | afdb-uniprot50 | AF-A0A0Q5F466-F1-MODEL\_V4 | 1.0 | 0.002455 | 185 | 0.318 | 91 | 55 | 3 | 1 | 87 | 1 | 88 | Uncharacterized protein | Uncharacterized protein | | afdb-uniprot50 | AF-A0A158E9V0-F1-MODEL\_V4 | 1.0 | 0.006368 | 155 | 0.239 | 92 | 62 | 2 | 1 | 92 | 1 | 84 | Uncharacterized protein | Uncharacterized protein | |
| Top keywords  (threshold 1.00e-02 (evalue)) | -- |
| Output files | ../../similar\_structures/63\_FANPEZAQ\_CDS\_0063\_afdb-proteome\_foldseek.tsv ../../similar\_structures/63\_FANPEZAQ\_CDS\_0063\_afdb-uniprot50\_foldseek.tsv ../../similar\_structures/63\_FANPEZAQ\_CDS\_0063\_merged.svg ../../similar\_structures/63\_FANPEZAQ\_CDS\_0063\_pdb\_foldseek.tsv |

  
  
  

Return to summary | Go to previous | Go to next

  


---

**Sequence/structure alignments coloring**  
Each object in the alignment figures is colored according to its E-value following this color coding:

1e-100
10

**References:**  
1) Steinegger M, Meier M, Mirdita M, Vöhringer H, Haunsberger S J, and Söding J (2019) HH-suite3 for fast remote homology detection and deep protein annotation, BMC Bioinformatics, 473. doi: 10.1186/s12859-019-3019-7  
2) Jumper J, Evans R, Pritzel A, ..., Hassabis D (2021) Highly accurate protein structure prediction with AlphaFold, Nature, 596. doi: 10.1038/s41586-021-03819-2  
3) van Kempen M, Kim S, Tumescheit C, Mirdita M, Lee J, Gilchrist CLM, Söding J, and Steinegger M (2023) Fast and accurate protein structure search with Foldseek. Nature Biotechnology. doi: 10.1038/s41587-023-01773-0
