# Supplementary material for: Completing the BASEL phage collection to unlock hidden diversity for systematic exploration of phage–host interactions
Source: PLoS Biol. 2025 Apr 7;23(4):e3003063. doi: 10.1371/journal.pbio.3003063 (PMC11990801; doi:10.1371/journal.pbio.3003063)
Supplement: S2 Data — (ZIP) [file pbio.3003063.s009.zip › entries/64.html]

FANPEZAQ\_CDS\_0064


Return to summary | Go to previous | Go to next

|  |  |
| --- | --- |
| FANPEZAQ\_CDS\_0064 Page creation date: 02 Sep 2024, 12:00  Project folder: n/a  Input sequences file: Escherichia\_virus\_HeidiAbel.gb | lysozyme endolysin t4 receptor hydrolase e c glycoside fragment chimera phage type muramidase phage\_related glucagon\_like peptide a membrane lysis b factor in the baseplate complex calcium domain\_containing gh24 yes mitochondrial alpha putative escherichia coli engineered and pesticin so4 fusion phosphatidylinositol 4\_kinase adrenergic enterobacteria organism\_taxid gene expressed expression\_system\_taxid mainly arch orthogonal |

### Sequence information

|  |  |
| --- | --- |
| Name | FANPEZAQ\_CDS\_0064  64\_FANPEZAQ\_CDS\_0064 (pipeline id) |
| Imported annotations | Escherichia\_virus\_HeidiAbel Bas97 |
| Protein sequence | MKISSNGIAVLKYFEDCHLKAYPDPATGGAPWTIGWGHTGPEVKRGLVWTQKQADDALVS DLARFESAVSAAVRVPLNQGQFDALVSFTYNLGEGNLKSSTLLKMVNAGNFAGAAEQFKR WNKANGKTMRGLTRRRAAEQCLFLGMGGASAIERGVAAA |
| Number of residues | 159 |
| Molecular weight (Da) | 17019.22 |
| Output files | ../../query\_sequences/64\_FANPEZAQ\_CDS\_0064.fasta |

### Putative domain architecture and protein family

#### Search results (HHblits)1

|  |  |
| --- | --- |
| Domain family databases searched | Pfam, Ncbi-cd, Cath, Phrogs |
| Results, scheme(s)  (Top layers only; threshold 1.00e-03 (evalue)) | xml version="1.0" encoding="utf-8" standalone="no"?       2024-09-02T21:08:26.346943 image/svg+xml   Matplotlib v3.7.2, https://matplotlib.org/ |
| Results, table  (E-value ≤ 1.00e-03 (evalue)) | | db | id | prob | evalue | pvalue | score | cols | query | query\_len | template | template\_len | name | description | | --- | --- | --- | --- | --- | --- | --- | --- | --- | --- | --- | --- | --- | | pfam | PF00959 | 98.5 | 9.9e-12 | 1.7e-15 | 70.8 | 106 | (32, 137) | 159 | (2, 107) | 108 | Phage\_lysozyme | Phage lysozyme | | pfam | PF16754 | 97.3 | 1.2e-07 | 2.2e-11 | 55.9 | 104 | (9, 112) | 159 | (5, 149) | 149 | Pesticin | Bacterial toxin homologue of phage lysozyme, C-term | | ncbi-cd | cd16900 | 99.2 | 9.1e-16 | 1.6e-19 | 88.5 | 137 | (4, 144) | 159 | (4, 142) | 142 | endolysin\_R21-like | cd16900 endolysin\_R21-like; endolysin R21-like proteins. Unlike T4 E phage lysozyme, the endolysin R21 from Enterobacteria phage P21 has an N-terminal SAR (signal-arrest-release) domain that anchors the endolysin to the membrane in an inactive form, which act to prevent premature lysis of the infected bacterium. | | ncbi-cd | cd16901 | 99.2 | 2.1e-15 | 3.5e-19 | 86.1 | 138 | (3, 144) | 159 | (2, 140) | 140 | lyz\_P1 | cd16901 lyz\_P1; P1 lysozyme Lyz-like proteins. Enterobacteria phage P1 lysozyme Lyz is secreted to the Escherichia coli periplasm where it is membrane bound and inactive. | | ncbi-cd | cd00737 | 98.6 | 8.1e-12 | 1.4e-15 | 69.4 | 131 | (9, 143) | 159 | (3, 135) | 136 | lyz\_endolysin\_autolysin | cd00737 lyz\_endolysin\_autolysin; endolysin and autolysin. The dsDNA phages of eubacteria use endolysins or muralytic enzymes in conjunction with hollin, a small membrane protein, to degrade the peptidoglycan found in bacterial cell walls. | | ncbi-cd | cd00735 | 98.2 | 1.6e-10 | 2.8e-14 | 65.2 | 128 | (8, 144) | 159 | (3, 140) | 146 | T4-like\_lys | cd00735 T4-like\_lys; bacteriophage T4-like lysozymes. Bacteriophage T4-like lysozymes hydrolyze the beta-1,4-glycosidic bond between N-acetylmuramic acid (MurNAc) and N-acetylglucosamine (GlcNAc) in peptidoglycan heteropolymers of prokaryotic cell walls. | | ncbi-cd | cd16904 | 96.8 | 1.8e-06 | 3e-10 | 47.3 | 86 | (4, 93) | 159 | (2, 138) | 138 | pesticin\_lyz-like | cd16904 pesticin\_lyz-like; pesticin C-terminal-like domain of uncharacterized proteins. This subfamily is composed of uncharacterized proteins containing a lysozyme-like domain similar to the C-terminal domain of pesticin. | | cath | 3hdeC00 | 99.4 | 3e-17 | 3.7e-21 | 98.5 | 134 | (10, 146) | 159 | (30, 163) | 165 | Lysozyme | CATHCODE: 1.10.530.40 NAME: Lysozyme. Chain: a, b, c, d. Synonym: lysis protein, muramidase, endolysin. Engineered: yes SOURCE: Enterobacteria phage p21. Bacteriophage 21. Organism\_taxid: 10711. Gene: r. Expressed in: escherichia coli. Expression\_system\_taxid: 562. CLASS: Mainly Alpha, ARCH: Orthogonal Bundle, TOPOL: Lysozyme, HOMOL: Lysozyme | | cath | 1xjtA00 | 99.4 | 3.5e-17 | 4.3e-21 | 101.1 | 141 | (3, 147) | 159 | (30, 182) | 191 | Lysozyme | CATHCODE: 1.10.530.40 NAME: Lysozyme. Chain: a. Synonym: lysis protein, muramidase, endolysin, protein gp17. Engineered: yes SOURCE: Enterobacteria phage p1. Organism\_taxid: 10678. Gene: 17, lysa, lyz. Expressed in: escherichia coli. Expression\_system\_taxid: 562. CLASS: Mainly Alpha, ARCH: Orthogonal Bundle, TOPOL: Lysozyme, HOMOL: Lysozyme | | cath | 6h9dA00 | 99.3 | 1.3e-16 | 1.6e-20 | 93.2 | 139 | (3, 145) | 159 | (6, 148) | 149 | Lysozyme | CATHCODE: 1.10.530.40 NAME: Lysozyme. Chain: a, b, c. Synonym: muramidase domain of spmx. Engineered: yes SOURCE: Asticcacaulis excentricus. Organism\_taxid: 78587. Gene: astex\_1112. Expressed in: escherichia coli bl21(de3). Expression\_system\_taxid: 469008. Expression\_system\_variant: ril. Expression\_system\_vector\_type: pet CLASS: Mainly Alpha, ARCH: Orthogonal Bundle, TOPOL: Lysozyme, HOMOL: Lysozyme | | cath | 2anvA00 | 99.2 | 1.7e-15 | 2e-19 | 87.6 | 139 | (2, 144) | 159 | (3, 145) | 146 | Lysozyme | CATHCODE: 1.10.530.40 NAME: Lysozyme. Chain: a, b. Synonym: lysis protein, muramidase, endolysin. Engineered: yes. Mutation: yes SOURCE: Enterobacteria phage p22. Organism\_taxid: 10754. Gene: 19. Expressed in: escherichia coli. Expression\_system\_taxid: 562 CLASS: Mainly Alpha, ARCH: Orthogonal Bundle, TOPOL: Lysozyme, HOMOL: Lysozyme | | cath | 1jtmA00 | 99.2 | 2.5e-15 | 3.1e-19 | 91.0 | 130 | (7, 146) | 159 | (3, 156) | 178 | Lysozyme | CATHCODE: 1.10.530.40 NAME: Lysozyme. Chain: a. Synonym: lysis protein. Engineered: yes. Mutation:yes SOURCE: Enterobacteria phage t4. Organism\_taxid: 10665. Gene: e. Expressed in: escherichia coli. Expression\_system\_taxid: 562. CLASS: Mainly Alpha, ARCH: Orthogonal Bundle, TOPOL: Lysozyme, HOMOL: Lysozyme | | cath | 2o7aA00 | 99.0 | 6.1e-14 | 7.4e-18 | 80.4 | 92 | (50, 148) | 159 | (1, 100) | 124 | Lysozyme | CATHCODE: 1.10.530.40 NAME: Lysozyme. Chain: a. Synonym: lysis protein, muramidase, endolysin. Engineered: yes. Mutation: yes SOURCE: Enterobacteria phage t4. Organism\_taxid: 10665. Gene: e. Expressed in: escherichia coli. Expression\_system\_taxid: 562. CLASS: Mainly Alpha, ARCH: Orthogonal Bundle, TOPOL: Lysozyme, HOMOL: Lysozyme | | cath | 4evxA00 | 98.9 | 2.6e-13 | 3.2e-17 | 74.6 | 94 | (3, 100) | 159 | (7, 101) | 106 | Putative phage endolysin | CATHCODE: 1.10.1740.240 NAME: Putative phage endolysin. Chain: a, b. Engineered: yes SOURCE: Salmonella enterica subsp. Enterica serovar typhimurium. Organism\_taxid: 99287. Strain: lt2. Gene: stm3605. Expressed in: escherichia coli.Expression\_system\_taxid: 469008. CLASS: Mainly Alpha, ARCH: Orthogonal Bundle, TOPOL: Rna Polymerase Sigma Factor; Chain: A, HOMOL: Rna Polymerase Sigma Factor; Chain: A | | phrogs | 7 | 99.9 | 1.2e-33 | 1.6e-37 | 221.0 | 131 | (8, 152) | 159 | (8, 145) | 226 | endolysin | endolysin; Category: lysis; NC\_001629\_p14 | | phrogs | 33083 | 99.9 | 2e-27 | 2.2e-31 | 187.5 | 136 | (8, 148) | 159 | (8, 145) | 456 | endolysin | endolysin; Category: lysis; p415817 VI\_06217 | | phrogs | 28234 | 99.8 | 7.3e-25 | 8.2e-29 | 170.5 | 142 | (8, 150) | 159 | (65, 216) | 359 | endolysin | endolysin; Category: lysis; NC\_015465\_p23 | | phrogs | 10457 | 99.8 | 3.4e-24 | 3.9e-28 | 143.3 | 98 | (47, 145) | 159 | (1, 99) | 99 | endolysin | endolysin; Category: lysis; p305180 VI\_10042 | | phrogs | 3617 | 99.6 | 5.7e-20 | 6.3e-24 | 142.8 | 136 | (8, 144) | 159 | (251, 404) | 405 | NA | NA; Category: unknown function; KJ019096\_p210 | | phrogs | 13211 | 98.9 | 1.2e-13 | 1.4e-17 | 115.7 | 103 | (8, 115) | 159 | (745, 867) | 894 | baseplate hub subunit and tail lysozyme | baseplate hub subunit and tail lysozyme; Category: tail; NC\_019401\_p237 | | phrogs | 16615 | 98.7 | 1.9e-12 | 2.2e-16 | 103.6 | 134 | (8, 152) | 159 | (200, 349) | 519 | peptidase | peptidase; Category: other; KY996523\_p13 | | phrogs | 3572 | 98.6 | 6.8e-12 | 7.5e-16 | 100.9 | 131 | (8, 148) | 159 | (268, 406) | 653 | endolysin | endolysin; Category: lysis; KJ019101\_p13 | | phrogs | 15410 | 98.6 | 8.1e-12 | 9.2e-16 | 95.4 | 90 | (18, 110) | 159 | (181, 280) | 308 | NA | NA; Category: unknown function; NC\_025447\_p525 | | phrogs | 7910 | 98.3 | 1.9e-10 | 2.2e-14 | 64.4 | 40 | (108, 147) | 159 | (2, 41) | 42 | endolysin | endolysin; Category: lysis; p276551 VI\_10444 | | phrogs | 10075 | 97.6 | 2.7e-08 | 3e-12 | 83.1 | 105 | (8, 113) | 159 | (859, 977) | 1205 | NA | NA; Category: unknown function; KX349298\_p12 | | phrogs | 6543 | 96.7 | 3.6e-06 | 4.1e-10 | 49.7 | 30 | (8, 41) | 159 | (28, 58) | 62 | NA | NA; Category: unknown function; p415893 VI\_08530 | | phrogs | 33534 | 96.1 | 2.7e-05 | 3.1e-09 | 48.3 | 51 | (74, 125) | 159 | (11, 71) | 88 | NA | NA; Category: unknown function; p192930 VI\_05576 | | phrogs | 35759 | 95.2 | 0.00024 | 2.6e-08 | 34.1 | 24 | (124, 147) | 159 | (2, 25) | 26 | NA | NA; Category: unknown function; p377996 VI\_10733 | |
| Top keywords  (threshold 1.00e-03 (evalue)) | **Lysozyme, endolysin, phage, lysis, in, a, yes, the, escherichia, coli** |
| Output files | ../../domain\_architecture/64\_FANPEZAQ\_CDS\_0064\_cath.hhr ../../domain\_architecture/64\_FANPEZAQ\_CDS\_0064\_merged.svg ../../domain\_architecture/64\_FANPEZAQ\_CDS\_0064\_ncbi-cd.hhr ../../domain\_architecture/64\_FANPEZAQ\_CDS\_0064\_pfam.hhr ../../domain\_architecture/64\_FANPEZAQ\_CDS\_0064\_phrogs.hhr |

### Identical protein sequences/structures

#### Search results

|  |  |
| --- | --- |
| Protein sequence databases searched | Pdb, Swissprot, Refseq |
| Identical proteins found | -- |
| Top keywords | -- |
| Output files | -- |

### Similar protein sequences/structures

#### Sequence similarity search results (HHblits)1

|  |  |
| --- | --- |
| Sequence databases searched | Uniclust, Pdb70 |
| Results, scheme(s)  (Top layers only, threshold 1.00e-03 (evalue)) | xml version="1.0" encoding="utf-8" standalone="no"?       2024-09-02T21:08:55.208612 image/svg+xml   Matplotlib v3.7.2, https://matplotlib.org/ |
| Results, table(s)  (threshold 1.00e-03 (evalue)) | | db | id | prob | evalue | pvalue | score | cols | query | query\_len | template | template\_len | name | description | | --- | --- | --- | --- | --- | --- | --- | --- | --- | --- | --- | --- | --- | | uniclust | UniRef100\_A0A068Q6I2 | 100.0 | 1.1e-52 | 2.1e-58 | 282.7 | 149 | (1, 152) | 159 | (28, 176) | 218 | Endolysin | Endolysin | | uniclust | UniRef100\_A0A0K8MD99 | 100.0 | 1.6e-51 | 3.1e-57 | 268.9 | 146 | (1, 149) | 159 | (13, 159) | 172 | Lysozyme | Lysozyme | | uniclust | UniRef100\_A0A075MK28 | 100.0 | 7.1e-51 | 1.4e-56 | 275.6 | 146 | (1, 150) | 159 | (30, 175) | 203 | Lysozyme | Lysozyme | | uniclust | UniRef100\_A0A024LSA6 | 100.0 | 4.3e-50 | 8.3e-56 | 283.4 | 148 | (1, 151) | 159 | (14, 165) | 300 | Lysozyme | Lysozyme | | uniclust | UniRef100\_A0A014M974 | 100.0 | 6e-50 | 1.2e-55 | 265.7 | 151 | (1, 154) | 159 | (20, 174) | 178 | Lysozyme | Lysozyme | | uniclust | UniRef100\_A0A2A4YRT5 | 100.0 | 1e-49 | 2e-55 | 280.4 | 143 | (1, 147) | 159 | (9, 155) | 256 | Lysozyme | Lysozyme | | uniclust | UniRef100\_A0A0F4VJS5 | 100.0 | 1.7e-49 | 3.3e-55 | 270.5 | 152 | (1, 155) | 159 | (37, 188) | 211 | Lysozyme | Lysozyme | | uniclust | UniRef100\_A0A0J1D667 | 100.0 | 1.2e-48 | 2.2e-54 | 256.4 | 143 | (1, 147) | 159 | (19, 161) | 175 | Lysozyme | Lysozyme | | uniclust | UniRef100\_A0A009YLP0 | 100.0 | 3.6e-48 | 6.8e-54 | 273.8 | 147 | (1, 150) | 159 | (59, 206) | 280 | Lysozyme | Lysozyme | | uniclust | UniRef100\_A0A013U6E4 | 100.0 | 3.9e-48 | 7.4e-54 | 269.4 | 147 | (1, 150) | 159 | (38, 187) | 237 | Lysozyme | Lysozyme | | uniclust | UniRef100\_A0A060H344 | 100.0 | 6.3e-48 | 1.2e-53 | 266.3 | 153 | (1, 156) | 159 | (46, 198) | 231 | Lysozyme | Lysozyme | | uniclust | UniRef100\_A0A009F987 | 100.0 | 1e-47 | 2e-53 | 267.5 | 152 | (1, 155) | 159 | (81, 236) | 255 | Lysozyme (Fragment) | Lysozyme (Fragment) | | uniclust | UniRef100\_A0A077XNR0 | 100.0 | 1.6e-47 | 3.1e-53 | 254.7 | 148 | (1, 151) | 159 | (16, 169) | 193 | Lysozyme | Lysozyme | | uniclust | UniRef100\_A0A0C1Y3N8 | 100.0 | 6.1e-47 | 1.1e-52 | 258.1 | 146 | (2, 150) | 159 | (92, 241) | 245 | Lysozyme | Lysozyme | | uniclust | UniRef100\_A0A0J1IN41 | 100.0 | 7.6e-47 | 1.4e-52 | 260.7 | 148 | (1, 149) | 159 | (27, 174) | 292 | Lysozyme | Lysozyme | | uniclust | UniRef100\_A0A147I3J2 | 100.0 | 3.5e-46 | 6.7e-52 | 253.3 | 149 | (1, 152) | 159 | (29, 189) | 211 | Lysozyme | Lysozyme | | uniclust | UniRef100\_A0A060BQW9 | 100.0 | 1.5e-45 | 2.8e-51 | 234.3 | 145 | (1, 150) | 159 | (5, 150) | 152 | Lysozyme (Fragment) | Lysozyme (Fragment) | | uniclust | UniRef100\_A0A0R2IIP4 | 100.0 | 1.5e-45 | 2.8e-51 | 248.9 | 145 | (1, 149) | 159 | (16, 160) | 241 | Lysozyme | Lysozyme | | uniclust | UniRef100\_A0A010J5Z2 | 100.0 | 1.9e-45 | 3.5e-51 | 242.9 | 144 | (2, 148) | 159 | (52, 199) | 205 | Lysozyme | Lysozyme | | uniclust | UniRef100\_A0A0Q4KN18 | 100.0 | 3.1e-45 | 5.7e-51 | 230.5 | 142 | (4, 149) | 159 | (7, 148) | 150 | Lysozyme | Lysozyme | | uniclust | UniRef100\_A0A1C7HE45 | 100.0 | 3.3e-45 | 6.1e-51 | 246.5 | 147 | (1, 151) | 159 | (57, 203) | 222 | Lysozyme | Lysozyme | | uniclust | UniRef100\_A0A258A958 | 100.0 | 1.7e-44 | 3.2e-50 | 252.3 | 145 | (1, 148) | 159 | (54, 202) | 308 | Lysozyme | Lysozyme | | uniclust | UniRef100\_A0A5Q0BKX1 | 100.0 | 1.8e-44 | 3.4e-50 | 232.4 | 151 | (2, 155) | 159 | (4, 169) | 173 | Lysozyme | Lysozyme | | uniclust | UniRef100\_A0A512IKZ3 | 100.0 | 3.7e-44 | 7.1e-50 | 253.0 | 145 | (1, 148) | 159 | (10, 157) | 314 | Lysozyme | Lysozyme | | uniclust | UniRef100\_A0A1M3I817 | 100.0 | 5.3e-44 | 1e-49 | 239.0 | 146 | (2, 149) | 159 | (54, 200) | 211 | Lysozyme | Lysozyme | | uniclust | UniRef100\_A0A2A2JXF0 | 100.0 | 5.5e-44 | 1e-49 | 229.3 | 151 | (1, 151) | 159 | (3, 154) | 159 | lysozyme | lysozyme | | uniclust | UniRef100\_A0A0K1JXH1 | 100.0 | 1.2e-43 | 2.3e-49 | 233.6 | 145 | (1, 148) | 159 | (21, 170) | 179 | Lysozyme | Lysozyme | | uniclust | UniRef100\_A0A293MYF4 | 100.0 | 1.3e-43 | 2.4e-49 | 249.1 | 144 | (2, 149) | 159 | (153, 296) | 299 | Lysozyme | Lysozyme | | uniclust | UniRef100\_A0A0A1VBW2 | 100.0 | 1.8e-43 | 3.4e-49 | 244.2 | 145 | (1, 149) | 159 | (18, 162) | 258 | Lysozyme | Lysozyme | | uniclust | UniRef100\_A0A8J7JSY3 | 100.0 | 1e-42 | 1.9e-48 | 245.7 | 143 | (2, 148) | 159 | (206, 348) | 352 | Lysozyme | Lysozyme | | uniclust | UniRef100\_A0A0F3QFX2 | 100.0 | 2.1e-42 | 4.1e-48 | 229.2 | 148 | (2, 152) | 159 | (15, 167) | 173 | Lysozyme | Lysozyme | | uniclust | UniRef100\_A0A066PZ20 | 100.0 | 2.2e-42 | 4.1e-48 | 237.7 | 143 | (3, 148) | 159 | (45, 192) | 222 | Lysozyme | Lysozyme | | uniclust | UniRef100\_A0A1F3A5L4 | 100.0 | 2.5e-42 | 4.6e-48 | 240.2 | 149 | (1, 152) | 159 | (1, 153) | 319 | Lysozyme | Lysozyme | | uniclust | UniRef100\_A0A0F2HCE7 | 100.0 | 3.1e-42 | 5.9e-48 | 233.1 | 144 | (3, 149) | 159 | (20, 170) | 202 | Lysozyme | Lysozyme | | uniclust | UniRef100\_A0A0A0RR05 | 100.0 | 3.3e-42 | 6.2e-48 | 231.1 | 142 | (2, 146) | 159 | (82, 227) | 231 | Endolysin | Endolysin | | uniclust | UniRef100\_A0A075DXC6 | 100.0 | 3.8e-42 | 7.1e-48 | 228.2 | 143 | (2, 147) | 159 | (68, 214) | 216 | Endolysin | Endolysin | | uniclust | UniRef100\_A0A2E1XDB6 | 100.0 | 4.3e-42 | 8.1e-48 | 225.5 | 150 | (1, 154) | 159 | (5, 158) | 181 | Lysozyme | Lysozyme | | uniclust | UniRef100\_A0A271LNT6 | 100.0 | 5e-42 | 9.5e-48 | 228.1 | 147 | (1, 150) | 159 | (3, 157) | 211 | Lysozyme | Lysozyme | | uniclust | UniRef100\_A0A0M4LIC1 | 100.0 | 5.8e-42 | 1.1e-47 | 225.9 | 148 | (2, 152) | 159 | (3, 154) | 220 | Lysozyme | Lysozyme | | uniclust | UniRef100\_A0A1Q6UX15 | 100.0 | 1e-41 | 2e-47 | 234.6 | 137 | (1, 148) | 159 | (4, 144) | 240 | Lysozyme | Lysozyme | | uniclust | UniRef100\_A0A073CH50 | 100.0 | 2.9e-41 | 5.3e-47 | 235.2 | 145 | (2, 150) | 159 | (98, 242) | 319 | Lysozyme | Lysozyme | | uniclust | UniRef100\_Q28PX4 | 100.0 | 3.7e-41 | 6.8e-47 | 236.4 | 144 | (1, 147) | 159 | (1, 148) | 340 | Lysozyme | Lysozyme | | uniclust | UniRef100\_A0A1V3II14 | 100.0 | 4.5e-41 | 8.4e-47 | 226.4 | 149 | (1, 152) | 159 | (6, 158) | 269 | Lysozyme | Lysozyme | | uniclust | UniRef100\_A0A1F6ZTS5 | 100.0 | 5.3e-41 | 1e-46 | 218.6 | 145 | (1, 149) | 159 | (8, 153) | 161 | lysozyme | lysozyme | | uniclust | UniRef100\_A0A0A1VPW0 | 100.0 | 8.2e-41 | 1.5e-46 | 248.3 | 144 | (1, 147) | 159 | (40, 191) | 553 | Lysozyme | Lysozyme | | uniclust | UniRef100\_A0A023ZU91 | 100.0 | 1e-40 | 2e-46 | 227.4 | 144 | (5, 152) | 159 | (47, 190) | 203 | Lysozyme | Lysozyme | | uniclust | UniRef100\_A0A1X7FA58 | 100.0 | 1.3e-40 | 2.5e-46 | 225.4 | 143 | (3, 149) | 159 | (16, 162) | 242 | Lysozyme | Lysozyme | | uniclust | UniRef100\_A0A059ZQR8 | 100.0 | 1.4e-40 | 2.6e-46 | 230.5 | 146 | (1, 151) | 159 | (36, 191) | 233 | Lysozyme | Lysozyme | | uniclust | UniRef100\_A0A1G6VI05 | 100.0 | 1.5e-40 | 2.8e-46 | 226.2 | 139 | (1, 147) | 159 | (4, 148) | 235 | Lysozyme | Lysozyme | | uniclust | UniRef100\_A0A126R939 | 100.0 | 2.3e-40 | 4.4e-46 | 220.0 | 157 | (1, 157) | 159 | (46, 204) | 207 | Lysozyme | Lysozyme | | uniclust | UniRef100\_A0A3B0S578 | 100.0 | 2.5e-40 | 4.6e-46 | 224.5 | 143 | (1, 146) | 159 | (37, 183) | 268 | lysozyme | lysozyme | | uniclust | UniRef100\_A0A1F1J0Y5 | 100.0 | 2.7e-40 | 5e-46 | 213.9 | 144 | (1, 148) | 159 | (24, 170) | 172 | Lysozyme | Lysozyme | | uniclust | UniRef100\_A0A0N9SGQ7 | 100.0 | 3.6e-40 | 6.7e-46 | 222.9 | 147 | (1, 150) | 159 | (7, 154) | 248 | Endolysin | Endolysin | | uniclust | UniRef100\_A0A241W0V3 | 100.0 | 4.1e-40 | 7.6e-46 | 219.7 | 146 | (1, 146) | 159 | (80, 227) | 228 | Lysozyme | Lysozyme | | uniclust | UniRef100\_A0A167C5R0 | 100.0 | 1.2e-39 | 2.3e-45 | 222.1 | 140 | (1, 149) | 159 | (21, 164) | 248 | Lysozyme | Lysozyme | | uniclust | UniRef100\_UPI0004B90056 | 100.0 | 1.9e-39 | 3.6e-45 | 215.7 | 143 | (1, 146) | 159 | (1, 144) | 224 | lysozyme | lysozyme | | uniclust | UniRef100\_A0A078LD01 | 100.0 | 2.1e-39 | 4.1e-45 | 223.8 | 139 | (8, 149) | 159 | (58, 196) | 250 | Lysozyme | Lysozyme | | uniclust | UniRef100\_A0A222G881 | 100.0 | 2.2e-39 | 4.2e-45 | 216.9 | 141 | (2, 146) | 159 | (84, 224) | 225 | Lysozyme | Lysozyme | | uniclust | UniRef100\_UPI001EF6EE9B | 100.0 | 2.5e-39 | 4.7e-45 | 206.9 | 142 | (1, 146) | 159 | (44, 185) | 203 | lysozyme | lysozyme | | uniclust | UniRef100\_A0A109BL19 | 100.0 | 2.8e-39 | 5.4e-45 | 223.4 | 147 | (1, 151) | 159 | (15, 168) | 262 | Lysozyme | Lysozyme | | uniclust | UniRef100\_A0A086D2W4 | 100.0 | 5e-39 | 9.4e-45 | 220.8 | 143 | (7, 152) | 159 | (48, 191) | 237 | Lysozyme | Lysozyme | | uniclust | UniRef100\_A0A0Q6SQZ2 | 100.0 | 5.5e-39 | 1.1e-44 | 228.3 | 140 | (2, 144) | 159 | (97, 244) | 289 | Lysozyme | Lysozyme | | uniclust | UniRef100\_A0A1I0J1Q9 | 100.0 | 5.8e-39 | 1.1e-44 | 203.5 | 145 | (1, 148) | 159 | (1, 151) | 183 | Lysozyme | Lysozyme | | uniclust | UniRef100\_UPI0007C7D1D0 | 100.0 | 7.2e-39 | 1.3e-44 | 218.2 | 152 | (1, 152) | 159 | (14, 169) | 273 | glycoside hydrolase family protein | glycoside hydrolase family protein | | uniclust | UniRef100\_A0A1S9AW89 | 100.0 | 9.7e-39 | 1.8e-44 | 200.9 | 142 | (1, 145) | 159 | (5, 148) | 149 | Lysozyme | Lysozyme | | uniclust | UniRef100\_A0A0Q9I3N9 | 100.0 | 1.3e-38 | 2.5e-44 | 224.4 | 142 | (1, 152) | 159 | (36, 182) | 284 | Lysozyme | Lysozyme | | uniclust | UniRef100\_A0A351L291 | 100.0 | 1.3e-38 | 2.5e-44 | 203.5 | 147 | (1, 150) | 159 | (1, 156) | 158 | Lysozyme (Fragment) | Lysozyme (Fragment) | | uniclust | UniRef100\_A0A7Y0FFV2 | 100.0 | 1.9e-38 | 3.5e-44 | 215.1 | 144 | (7, 150) | 159 | (71, 231) | 233 | Lysozyme | Lysozyme | | uniclust | UniRef100\_A0A011LWT5 | 100.0 | 1.9e-38 | 3.6e-44 | 218.6 | 147 | (2, 152) | 159 | (63, 210) | 224 | Lysozyme | Lysozyme | | uniclust | UniRef100\_A0A060C0Y3 | 100.0 | 2.2e-38 | 4.1e-44 | 212.6 | 135 | (13, 150) | 159 | (48, 182) | 210 | Lysozyme (Fragment) | Lysozyme (Fragment) | | uniclust | UniRef100\_A0A060CFF5 | 100.0 | 2.1e-38 | 4.1e-44 | 221.7 | 145 | (4, 152) | 159 | (58, 202) | 232 | Lysozyme (Fragment) | Lysozyme (Fragment) | | uniclust | UniRef100\_A0A371R7Q5 | 100.0 | 2.3e-38 | 4.2e-44 | 224.4 | 146 | (1, 149) | 159 | (8, 168) | 374 | Lysozyme | Lysozyme | | uniclust | UniRef100\_A0A1M6MED4 | 100.0 | 2.7e-38 | 5.1e-44 | 214.8 | 143 | (1, 149) | 159 | (3, 145) | 229 | Lysozyme | Lysozyme | | uniclust | UniRef100\_A0A522A0K7 | 100.0 | 3.9e-38 | 7.3e-44 | 218.1 | 143 | (1, 147) | 159 | (17, 163) | 249 | Lysozyme | Lysozyme | | uniclust | UniRef100\_A0A1C3FDG7 | 100.0 | 4.2e-38 | 7.9e-44 | 197.8 | 125 | (33, 157) | 159 | (1, 129) | 136 | Lysozyme | Lysozyme | | uniclust | UniRef100\_A0A031JXV8 | 100.0 | 4.5e-38 | 8.4e-44 | 214.4 | 147 | (2, 150) | 159 | (81, 235) | 242 | Lysozyme | Lysozyme | | uniclust | UniRef100\_A0A023VTC7 | 100.0 | 4.7e-38 | 8.9e-44 | 209.4 | 154 | (1, 155) | 159 | (20, 174) | 191 | Endolysin | Endolysin | | uniclust | UniRef100\_A0A840NNX4 | 100.0 | 5e-38 | 9.3e-44 | 206.4 | 148 | (2, 152) | 159 | (5, 156) | 222 | Lysozyme (Fragment) | Lysozyme (Fragment) | | uniclust | UniRef100\_A0A930XJW8 | 100.0 | 5.6e-38 | 1e-43 | 215.8 | 142 | (2, 146) | 159 | (215, 356) | 357 | Lysozyme | Lysozyme | | uniclust | UniRef100\_A0A931BXL2 | 100.0 | 1e-37 | 1.9e-43 | 205.6 | 146 | (1, 149) | 159 | (48, 197) | 225 | Lysozyme | Lysozyme | | uniclust | UniRef100\_A0A1C5W0G4 | 100.0 | 1.5e-37 | 2.8e-43 | 223.8 | 136 | (1, 144) | 159 | (24, 164) | 323 | Lysozyme | Lysozyme | | uniclust | UniRef100\_A0A072T9I2 | 100.0 | 1.7e-37 | 3.2e-43 | 210.4 | 148 | (2, 152) | 159 | (31, 185) | 208 | Glycoside hydrolase family 24 | Glycoside hydrolase family 24 | | uniclust | UniRef100\_A0A017SCH5 | 100.0 | 1.8e-37 | 3.4e-43 | 211.2 | 147 | (3, 152) | 159 | (68, 223) | 236 | lysozyme | lysozyme | | uniclust | UniRef100\_A0A858RDI0 | 100.0 | 1.8e-37 | 3.4e-43 | 199.9 | 142 | (2, 148) | 159 | (41, 187) | 193 | Lysozyme | Lysozyme | | uniclust | UniRef100\_A0A8J7K7G8 | 100.0 | 2.2e-37 | 4e-43 | 215.3 | 142 | (2, 146) | 159 | (251, 392) | 393 | Lysozyme | Lysozyme | | uniclust | UniRef100\_A0A067NBT7 | 100.0 | 2.4e-37 | 4.4e-43 | 212.3 | 147 | (3, 152) | 159 | (100, 255) | 260 | lysozyme | lysozyme | | uniclust | UniRef100\_A0A370N746 | 100.0 | 3.4e-37 | 6.4e-43 | 210.3 | 151 | (1, 151) | 159 | (74, 270) | 274 | Lysozyme | Lysozyme | | uniclust | UniRef100\_A0A840I6B0 | 100.0 | 3.7e-37 | 6.9e-43 | 197.3 | 144 | (1, 148) | 159 | (3, 150) | 192 | Lysozyme | Lysozyme | | uniclust | UniRef100\_A0A140K4G7 | 100.0 | 6.1e-37 | 1.2e-42 | 211.0 | 147 | (2, 151) | 159 | (78, 231) | 237 | Lysozyme | Lysozyme | | uniclust | UniRef100\_A0A4D7CU28 | 100.0 | 6.3e-37 | 1.2e-42 | 201.3 | 145 | (1, 149) | 159 | (1, 145) | 227 | Lysozyme | Lysozyme | | uniclust | UniRef100\_A0A949XFQ5 | 100.0 | 6.8e-37 | 1.3e-42 | 198.2 | 145 | (2, 149) | 159 | (5, 152) | 185 | Lysozyme | Lysozyme | | uniclust | UniRef100\_A0A1A6FHX7 | 100.0 | 7.1e-37 | 1.3e-42 | 215.1 | 143 | (1, 151) | 159 | (4, 150) | 316 | Lysozyme | Lysozyme | | uniclust | UniRef100\_A0A6J5LS01 | 100.0 | 7.3e-37 | 1.4e-42 | 197.0 | 148 | (1, 151) | 159 | (6, 178) | 181 | Endolysin | Endolysin | | uniclust | UniRef100\_A0A653CSV6 | 100.0 | 8e-37 | 1.5e-42 | 201.5 | 141 | (1, 145) | 159 | (112, 252) | 252 | lysozyme | lysozyme | | uniclust | UniRef100\_A0A397TNV2 | 100.0 | 8.3e-37 | 1.5e-42 | 196.4 | 142 | (2, 146) | 159 | (28, 173) | 176 | lysozyme | lysozyme | | uniclust | UniRef100\_A0A0H5BHU8 | 100.0 | 1.2e-36 | 2.2e-42 | 209.9 | 144 | (1, 149) | 159 | (4, 161) | 262 | Lysozyme | Lysozyme | | uniclust | UniRef100\_A0A1X7GNQ4 | 100.0 | 1.3e-36 | 2.3e-42 | 204.4 | 150 | (3, 152) | 159 | (4, 170) | 254 | Lysozyme | Lysozyme | | uniclust | UniRef100\_A0A060QH24 | 100.0 | 1.6e-36 | 3.1e-42 | 205.1 | 137 | (6, 145) | 159 | (43, 189) | 223 | Lysozyme | Lysozyme | | uniclust | UniRef100\_A0A1F9BL94 | 100.0 | 2.1e-36 | 4e-42 | 203.3 | 148 | (1, 150) | 159 | (11, 170) | 193 | Lysozyme | Lysozyme | | uniclust | UniRef100\_A0A8J7EUD0 | 100.0 | 2.5e-36 | 4.7e-42 | 212.1 | 142 | (2, 146) | 159 | (285, 426) | 429 | Lysozyme | Lysozyme | | uniclust | UniRef100\_A0A085GNC0 | 100.0 | 2.5e-36 | 4.7e-42 | 198.3 | 148 | (2, 153) | 159 | (33, 180) | 198 | Lysozyme | Lysozyme | | uniclust | UniRef100\_A0A073IWM7 | 100.0 | 2.5e-36 | 4.7e-42 | 211.7 | 144 | (5, 152) | 159 | (147, 290) | 299 | Lysozyme | Lysozyme | | uniclust | UniRef100\_A0A0P7Y7A2 | 100.0 | 2.9e-36 | 5.5e-42 | 211.8 | 140 | (1, 149) | 159 | (24, 167) | 273 | Lysozyme | Lysozyme | | uniclust | UniRef100\_A0A1D9G253 | 100.0 | 3.2e-36 | 6e-42 | 203.5 | 140 | (2, 145) | 159 | (118, 264) | 265 | Lysozyme | Lysozyme | | uniclust | UniRef100\_A0A285CW41 | 100.0 | 3.6e-36 | 6.7e-42 | 192.5 | 149 | (1, 152) | 159 | (1, 154) | 179 | Lysozyme | Lysozyme | | uniclust | UniRef100\_A0A943WD90 | 100.0 | 4.7e-36 | 8.6e-42 | 202.6 | 140 | (2, 145) | 159 | (164, 303) | 303 | Lysozyme | Lysozyme | | uniclust | UniRef100\_E0WSU1 | 100.0 | 5.9e-36 | 1.1e-41 | 197.2 | 147 | (3, 152) | 159 | (63, 209) | 214 | Lysozyme | Lysozyme | | uniclust | UniRef100\_A0A2U3PSF2 | 100.0 | 6e-36 | 1.1e-41 | 203.4 | 145 | (2, 149) | 159 | (106, 257) | 265 | Lysozyme | Lysozyme | | uniclust | UniRef100\_A0A4P6YRS8 | 100.0 | 6.1e-36 | 1.1e-41 | 201.6 | 143 | (1, 151) | 159 | (5, 147) | 249 | Lysozyme | Lysozyme | | uniclust | UniRef100\_A0A067NV59 | 100.0 | 8.6e-36 | 1.6e-41 | 214.2 | 147 | (3, 152) | 159 | (122, 277) | 349 | lysozyme | lysozyme | | uniclust | UniRef100\_A0A0R2IQS1 | 100.0 | 9.2e-36 | 1.7e-41 | 202.0 | 148 | (1, 152) | 159 | (6, 153) | 312 | Lysozyme | Lysozyme | | uniclust | UniRef100\_A0A291BV27 | 100.0 | 9.9e-36 | 1.8e-41 | 201.8 | 146 | (1, 150) | 159 | (6, 151) | 285 | Lysozyme | Lysozyme | | uniclust | UniRef100\_A0A1V4BM01 | 100.0 | 1.3e-35 | 2.4e-41 | 214.3 | 146 | (1, 149) | 159 | (6, 153) | 473 | Lysozyme | Lysozyme | | uniclust | UniRef100\_A0A0L9Y629 | 100.0 | 1.4e-35 | 2.6e-41 | 208.5 | 146 | (3, 151) | 159 | (134, 285) | 289 | Lysozyme | Lysozyme | | uniclust | UniRef100\_A0A0K1NGA9 | 100.0 | 1.8e-35 | 3.3e-41 | 206.0 | 144 | (1, 147) | 159 | (1, 144) | 394 | Lysozyme | Lysozyme | | uniclust | UniRef100\_A0A1F9BXF9 | 100.0 | 1.9e-35 | 3.5e-41 | 209.9 | 145 | (1, 148) | 159 | (87, 231) | 424 | Lysozyme | Lysozyme | | uniclust | UniRef100\_A0A412YY68 | 100.0 | 2.2e-35 | 4e-41 | 210.6 | 140 | (2, 145) | 159 | (341, 480) | 480 | Lysozyme | Lysozyme | | uniclust | UniRef100\_A0A2A2JXU9 | 100.0 | 2.4e-35 | 4.5e-41 | 199.2 | 138 | (1, 142) | 159 | (1, 138) | 302 | lysozyme | lysozyme | | uniclust | UniRef100\_A0A0A0CLL3 | 100.0 | 2.4e-35 | 4.5e-41 | 199.6 | 149 | (1, 149) | 159 | (59, 219) | 223 | Lysozyme | Lysozyme | | uniclust | UniRef100\_UPI000EF53BDC | 100.0 | 2.5e-35 | 4.6e-41 | 208.7 | 146 | (1, 149) | 159 | (3, 155) | 432 | LysM peptidoglycan-binding domain-containing protein | LysM peptidoglycan-binding domain-containing protein | | uniclust | UniRef100\_UPI001FD78962 | 100.0 | 2.5e-35 | 4.7e-41 | 200.2 | 144 | (1, 148) | 159 | (6, 149) | 316 | lysozyme | lysozyme | | uniclust | UniRef100\_A0A8T4P871 | 100.0 | 2.5e-35 | 4.7e-41 | 192.9 | 148 | (2, 152) | 159 | (64, 211) | 215 | lysozyme | lysozyme | | uniclust | UniRef100\_A0A117T1Q6 | 100.0 | 2.7e-35 | 5e-41 | 195.0 | 144 | (2, 151) | 159 | (4, 151) | 216 | Lysozyme | Lysozyme | | uniclust | UniRef100\_A0A1U7IY75 | 100.0 | 3.8e-35 | 7e-41 | 212.5 | 144 | (1, 147) | 159 | (3, 158) | 440 | Lysozyme | Lysozyme | | uniclust | UniRef100\_A0A1G3PUD1 | 100.0 | 3.8e-35 | 7.1e-41 | 195.7 | 149 | (1, 149) | 159 | (30, 187) | 190 | Lysozyme | Lysozyme | | uniclust | UniRef100\_A0A0N0JEL9 | 100.0 | 3.9e-35 | 7.3e-41 | 200.9 | 146 | (3, 151) | 159 | (8, 180) | 262 | Lysozyme | Lysozyme | | uniclust | UniRef100\_A0A0T9RE44 | 100.0 | 4.3e-35 | 8e-41 | 183.5 | 142 | (9, 154) | 159 | (2, 143) | 152 | Lysozyme | Lysozyme | | uniclust | UniRef100\_A0A3P1XHR8 | 100.0 | 4.8e-35 | 8.8e-41 | 216.2 | 144 | (1, 147) | 159 | (350, 493) | 722 | Lysozyme | Lysozyme | | uniclust | UniRef100\_A0A1C5P9H3 | 100.0 | 6.2e-35 | 1.2e-40 | 201.6 | 138 | (1, 147) | 159 | (22, 169) | 276 | Lysozyme | Lysozyme | | uniclust | UniRef100\_A0A067T7N7 | 100.0 | 7.4e-35 | 1.4e-40 | 202.3 | 147 | (3, 152) | 159 | (151, 306) | 310 | lysozyme | lysozyme | | uniclust | UniRef100\_A0A1H1FH24 | 100.0 | 1e-34 | 1.9e-40 | 186.8 | 141 | (1, 149) | 159 | (3, 147) | 173 | Lysozyme | Lysozyme | | uniclust | UniRef100\_A0A8T3SPI5 | 100.0 | 1.1e-34 | 2.1e-40 | 189.2 | 139 | (2, 144) | 159 | (65, 205) | 206 | Lysozyme | Lysozyme | | uniclust | UniRef100\_A0A1X2I864 | 100.0 | 1.2e-34 | 2.3e-40 | 200.6 | 141 | (2, 145) | 159 | (117, 262) | 266 | lysozyme | lysozyme | | uniclust | UniRef100\_A0A127MKM6 | 100.0 | 1.3e-34 | 2.5e-40 | 193.9 | 140 | (6, 149) | 159 | (26, 177) | 198 | Lysozyme | Lysozyme | | uniclust | UniRef100\_UPI000B336A28 | 100.0 | 1.9e-34 | 3.4e-40 | 195.9 | 145 | (1, 149) | 159 | (6, 150) | 314 | lysozyme | lysozyme | | uniclust | UniRef100\_A0A1V0DCW7 | 100.0 | 2e-34 | 3.6e-40 | 186.9 | 143 | (3, 148) | 159 | (15, 164) | 200 | Lysozyme | Lysozyme | | uniclust | UniRef100\_UPI001C04A56E | 100.0 | 2.3e-34 | 4.3e-40 | 177.8 | 130 | (14, 146) | 159 | (1, 134) | 141 | lysozyme | lysozyme | | uniclust | UniRef100\_A0A1S8NI04 | 100.0 | 2.5e-34 | 4.7e-40 | 203.7 | 148 | (2, 152) | 159 | (139, 288) | 292 | Lysozyme | Lysozyme | | uniclust | UniRef100\_A0A1M3KMN9 | 100.0 | 2.9e-34 | 5.3e-40 | 185.2 | 147 | (1, 147) | 159 | (2, 154) | 196 | Lysozyme | Lysozyme | | uniclust | UniRef100\_A0A2D0K3Q9 | 100.0 | 3.4e-34 | 6.4e-40 | 174.5 | 112 | (1, 112) | 159 | (2, 117) | 119 | Lysozyme | Lysozyme | | uniclust | UniRef100\_A0A927EH04 | 100.0 | 3.7e-34 | 6.8e-40 | 210.0 | 144 | (3, 150) | 159 | (442, 585) | 587 | Lysozyme | Lysozyme | | uniclust | UniRef100\_A0A6H9GT10 | 100.0 | 4.1e-34 | 7.4e-40 | 189.2 | 145 | (1, 148) | 159 | (1, 153) | 254 | Lysozyme | Lysozyme | | uniclust | UniRef100\_A0A1Q4S2E5 | 100.0 | 4.4e-34 | 8.1e-40 | 192.2 | 145 | (2, 149) | 159 | (102, 254) | 258 | Lysozyme | Lysozyme | | uniclust | UniRef100\_A0A2W4WM75 | 100.0 | 4.7e-34 | 8.8e-40 | 175.8 | 110 | (1, 114) | 159 | (12, 121) | 124 | Lysozyme (Fragment) | Lysozyme (Fragment) | | uniclust | UniRef100\_A0A6P1B8L0 | 100.0 | 5.4e-34 | 1e-39 | 200.1 | 147 | (1, 149) | 159 | (5, 169) | 295 | Lysozyme | Lysozyme | | uniclust | UniRef100\_A0A0P0NZY8 | 100.0 | 5.5e-34 | 1.1e-39 | 213.4 | 145 | (1, 149) | 159 | (23, 171) | 455 | Lysozyme | Lysozyme | | uniclust | UniRef100\_A0A2A4Z0X3 | 100.0 | 5.8e-34 | 1.1e-39 | 180.9 | 142 | (2, 147) | 159 | (3, 149) | 188 | Lysozyme | Lysozyme | | uniclust | UniRef100\_A0A239Q0D5 | 100.0 | 5.9e-34 | 1.1e-39 | 193.1 | 141 | (2, 147) | 159 | (88, 228) | 229 | Lysozyme | Lysozyme | | uniclust | UniRef100\_UPI001F5504E3 | 100.0 | 6.3e-34 | 1.2e-39 | 202.0 | 143 | (3, 149) | 159 | (302, 444) | 447 | lysozyme | lysozyme | | uniclust | UniRef100\_A0A9E8ZDG3 | 100.0 | 6.7e-34 | 1.2e-39 | 206.5 | 144 | (2, 149) | 159 | (441, 584) | 587 | Lysozyme | Lysozyme | | uniclust | UniRef100\_A0A0K1H0L4 | 100.0 | 8e-34 | 1.5e-39 | 170.1 | 103 | (43, 145) | 159 | (3, 105) | 106 | Lysozyme | Lysozyme | | uniclust | UniRef100\_A0A022G329 | 100.0 | 1.1e-33 | 2.1e-39 | 195.8 | 139 | (7, 150) | 159 | (73, 211) | 231 | Lysozyme | Lysozyme | | uniclust | UniRef100\_A0A2W5V772 | 100.0 | 1.5e-33 | 2.7e-39 | 204.8 | 144 | (3, 149) | 159 | (74, 217) | 590 | Lysozyme | Lysozyme | | uniclust | UniRef100\_A0A5S9RTX0 | 100.0 | 1.7e-33 | 3.1e-39 | 189.9 | 144 | (2, 151) | 159 | (65, 224) | 225 | Lysozyme | Lysozyme | | uniclust | UniRef100\_A0A5K7ZI99 | 100.0 | 1.9e-33 | 3.6e-39 | 197.2 | 146 | (2, 151) | 159 | (4, 155) | 383 | Lysozyme | Lysozyme | | uniclust | UniRef100\_A0A093XAS9 | 100.0 | 2.1e-33 | 4e-39 | 201.6 | 144 | (3, 149) | 159 | (138, 290) | 377 | lysozyme | lysozyme | | uniclust | UniRef100\_A0A926ZD07 | 100.0 | 2.2e-33 | 4.1e-39 | 190.3 | 144 | (3, 149) | 159 | (70, 217) | 308 | Lysozyme | Lysozyme | | uniclust | UniRef100\_A0A1X7MCZ0 | 100.0 | 2.8e-33 | 5.4e-39 | 172.3 | 102 | (47, 148) | 159 | (11, 112) | 113 | Lysozyme | Lysozyme | | uniclust | UniRef100\_A0A5C7LRY7 | 100.0 | 3.3e-33 | 6e-39 | 188.0 | 143 | (1, 146) | 159 | (59, 205) | 288 | Lysozyme | Lysozyme | | uniclust | UniRef100\_A0A0F9RCT0 | 100.0 | 3.6e-33 | 6.5e-39 | 181.9 | 149 | (1, 152) | 159 | (1, 158) | 225 | lysozyme | lysozyme | | uniclust | UniRef100\_A0A1H2JQ13 | 100.0 | 4e-33 | 7.5e-39 | 194.9 | 149 | (1, 149) | 159 | (113, 273) | 276 | Lysozyme | Lysozyme | | uniclust | UniRef100\_A0A7X9HPJ2 | 100.0 | 4.4e-33 | 8e-39 | 181.4 | 145 | (2, 149) | 159 | (59, 217) | 224 | Lysozyme | Lysozyme | | uniclust | UniRef100\_A0A059DT11 | 100.0 | 4.7e-33 | 8.8e-39 | 206.4 | 145 | (1, 149) | 159 | (28, 177) | 428 | Lysozyme | Lysozyme | | uniclust | UniRef100\_A0A1B1KLF1 | 100.0 | 5.4e-33 | 1e-38 | 177.5 | 143 | (2, 148) | 159 | (27, 169) | 171 | Lysozyme | Lysozyme | | uniclust | UniRef100\_A0A077FK68 | 100.0 | 5.7e-33 | 1.1e-38 | 171.6 | 115 | (43, 157) | 159 | (3, 117) | 119 | Lysozyme | Lysozyme | | uniclust | UniRef100\_UPI00215FDBC0 | 100.0 | 6.4e-33 | 1.2e-38 | 181.8 | 123 | (20, 146) | 159 | (112, 234) | 235 | phage portal protein | phage portal protein | | uniclust | UniRef100\_A0A3E2N5A0 | 100.0 | 8e-33 | 1.5e-38 | 183.7 | 136 | (1, 145) | 159 | (97, 238) | 239 | Lysozyme | Lysozyme | | uniclust | UniRef100\_A0A1G4TH47 | 100.0 | 8e-33 | 1.5e-38 | 217.0 | 147 | (2, 152) | 159 | (7, 157) | 832 | Lysozyme | Lysozyme | | uniclust | UniRef100\_A0A3S0BYI5 | 100.0 | 8.5e-33 | 1.6e-38 | 178.1 | 143 | (3, 149) | 159 | (6, 148) | 207 | Lysozyme | Lysozyme | | uniclust | UniRef100\_A0A1E5TA89 | 100.0 | 9.1e-33 | 1.7e-38 | 188.3 | 145 | (2, 152) | 159 | (84, 244) | 245 | Lysozyme | Lysozyme | | uniclust | UniRef100\_A0A1B1IHU6 | 100.0 | 9.3e-33 | 1.7e-38 | 183.6 | 145 | (1, 148) | 159 | (4, 153) | 233 | Lysozyme | Lysozyme | | uniclust | UniRef100\_A0A6I5R3J3 | 100.0 | 1e-32 | 1.9e-38 | 200.5 | 145 | (1, 148) | 159 | (244, 392) | 456 | Lysozyme | Lysozyme | | uniclust | UniRef100\_A0A271J3C8 | 100.0 | 1.1e-32 | 2.1e-38 | 181.6 | 145 | (2, 149) | 159 | (10, 155) | 227 | Lysozyme | Lysozyme | | uniclust | UniRef100\_A0A350KNR5 | 100.0 | 1.4e-32 | 2.6e-38 | 176.5 | 140 | (3, 145) | 159 | (25, 165) | 167 | Lysozyme | Lysozyme | | uniclust | UniRef100\_A0A0S3PZQ8 | 100.0 | 1.4e-32 | 2.7e-38 | 184.0 | 138 | (8, 149) | 159 | (21, 160) | 220 | Lysozyme | Lysozyme | | uniclust | UniRef100\_A0A073J6L3 | 100.0 | 1.4e-32 | 2.7e-38 | 197.7 | 144 | (5, 152) | 159 | (145, 293) | 327 | Lysozyme | Lysozyme | | uniclust | UniRef100\_A0A0C9WJX8 | 100.0 | 1.5e-32 | 2.9e-38 | 213.0 | 147 | (3, 152) | 159 | (476, 631) | 635 | lysozyme | lysozyme | | uniclust | UniRef100\_A0A7J6JRX3 | 100.0 | 1.7e-32 | 3.1e-38 | 186.8 | 143 | (2, 147) | 159 | (6, 148) | 316 | lysozyme | lysozyme | | uniclust | UniRef100\_A0A7W0JH81 | 100.0 | 1.7e-32 | 3.2e-38 | 175.5 | 144 | (2, 148) | 159 | (3, 153) | 197 | Lysozyme | Lysozyme | | uniclust | UniRef100\_A0A1W1XZS6 | 100.0 | 1.7e-32 | 3.3e-38 | 175.7 | 135 | (18, 153) | 159 | (5, 147) | 154 | Lysozyme | Lysozyme | | uniclust | UniRef100\_A0A226E4N2 | 100.0 | 2.1e-32 | 3.9e-38 | 189.1 | 141 | (4, 147) | 159 | (50, 201) | 290 | lysozyme | lysozyme | | uniclust | UniRef100\_A0A261KX80 | 100.0 | 2.5e-32 | 4.7e-38 | 199.6 | 144 | (1, 147) | 159 | (1, 152) | 497 | Lysozyme (Fragment) | Lysozyme (Fragment) | | uniclust | UniRef100\_A0A2U2P948 | 99.9 | 2.9e-32 | 5.4e-38 | 182.6 | 140 | (1, 144) | 159 | (96, 239) | 247 | Lysozyme (Fragment) | Lysozyme (Fragment) | | uniclust | UniRef100\_UPI00200AF8DA | 99.9 | 3.5e-32 | 6.5e-38 | 179.3 | 146 | (1, 149) | 159 | (1, 153) | 244 | glycoside hydrolase family protein | glycoside hydrolase family protein | | uniclust | UniRef100\_A0A258GXH3 | 99.9 | 4.4e-32 | 8.2e-38 | 185.0 | 140 | (2, 146) | 159 | (98, 241) | 245 | Lysozyme | Lysozyme | | uniclust | UniRef100\_A0A2D6VSD9 | 99.9 | 4.5e-32 | 8.5e-38 | 204.3 | 148 | (1, 148) | 159 | (80, 231) | 490 | lysozyme | lysozyme | | uniclust | UniRef100\_A0A381GPR2 | 99.9 | 5.4e-32 | 1e-37 | 183.8 | 143 | (2, 148) | 159 | (123, 265) | 266 | Lysozyme | Lysozyme | | uniclust | UniRef100\_A0A158G2K7 | 99.9 | 5.9e-32 | 1.1e-37 | 162.2 | 102 | (46, 147) | 159 | (3, 104) | 106 | Lysozyme | Lysozyme | | uniclust | UniRef100\_L0EV97 | 99.9 | 6.4e-32 | 1.2e-37 | 176.7 | 145 | (2, 149) | 159 | (10, 158) | 230 | Lysozyme | Lysozyme | | uniclust | UniRef100\_A0A0N7CEK9 | 99.9 | 6.2e-32 | 1.2e-37 | 172.3 | 126 | (21, 152) | 159 | (3, 137) | 138 | Lysozyme | Lysozyme | | uniclust | UniRef100\_A0A014PYD0 | 99.9 | 7e-32 | 1.3e-37 | 176.1 | 145 | (2, 150) | 159 | (37, 181) | 197 | Lysozyme | Lysozyme | | uniclust | UniRef100\_A0A7W5EKN2 | 99.9 | 7.1e-32 | 1.3e-37 | 175.0 | 127 | (7, 145) | 159 | (26, 152) | 154 | Lysozyme | Lysozyme | | uniclust | UniRef100\_A0A0H4TH14 | 99.9 | 7.4e-32 | 1.4e-37 | 179.5 | 145 | (2, 150) | 159 | (30, 182) | 194 | Endolysin | Endolysin | | uniclust | UniRef100\_A0A858RGZ4 | 99.9 | 7.7e-32 | 1.4e-37 | 181.1 | 142 | (3, 149) | 159 | (131, 277) | 282 | Lysozyme | Lysozyme | | uniclust | UniRef100\_A0A8S5N3I7 | 99.9 | 8.7e-32 | 1.6e-37 | 188.6 | 148 | (1, 151) | 159 | (10, 162) | 389 | Endolysin | Endolysin | | uniclust | UniRef100\_A0A1F8IHC7 | 99.9 | 8.6e-32 | 1.6e-37 | 208.2 | 142 | (1, 149) | 159 | (27, 168) | 624 | Lysozyme | Lysozyme | | uniclust | UniRef100\_A0A239PWS9 | 99.9 | 9.9e-32 | 1.9e-37 | 190.6 | 145 | (1, 149) | 159 | (17, 165) | 339 | Lysozyme | Lysozyme | | uniclust | UniRef100\_F8WVD9 | 99.9 | 1.1e-31 | 2e-37 | 170.2 | 139 | (2, 143) | 159 | (38, 181) | 183 | Lysozyme | Lysozyme | | uniclust | UniRef100\_A0A1Q3Q7Z8 | 99.9 | 1.2e-31 | 2.3e-37 | 178.5 | 145 | (1, 149) | 159 | (5, 173) | 187 | Lysozyme | Lysozyme | | uniclust | UniRef100\_A0A7C4STG1 | 99.9 | 1.3e-31 | 2.5e-37 | 185.9 | 145 | (2, 149) | 159 | (205, 363) | 370 | Lysozyme | Lysozyme | | uniclust | UniRef100\_A0A0A2W020 | 99.9 | 1.7e-31 | 3.1e-37 | 193.5 | 139 | (4, 145) | 159 | (421, 560) | 561 | lysozyme | lysozyme | | uniclust | UniRef100\_A0A9E7MUM9 | 99.9 | 1.9e-31 | 3.4e-37 | 176.4 | 146 | (2, 150) | 159 | (15, 160) | 249 | Lysozyme | Lysozyme | | uniclust | UniRef100\_A0A0C9W3W6 | 99.9 | 1.9e-31 | 3.7e-37 | 200.1 | 146 | (4, 152) | 159 | (364, 518) | 522 | lysozyme | lysozyme | | uniclust | UniRef100\_A0A2E6Y944 | 99.9 | 3e-31 | 5.6e-37 | 175.5 | 149 | (1, 152) | 159 | (1, 161) | 225 | Lysozyme | Lysozyme | | uniclust | UniRef100\_A0A926PFU6 | 99.9 | 3.3e-31 | 6e-37 | 176.6 | 141 | (2, 146) | 159 | (120, 262) | 263 | Lysozyme | Lysozyme | | uniclust | UniRef100\_A0A060H052 | 99.9 | 3.5e-31 | 6.6e-37 | 178.5 | 142 | (4, 150) | 159 | (54, 197) | 208 | Lysozyme | Lysozyme | | uniclust | UniRef100\_A0A060DST8 | 99.9 | 3.9e-31 | 7.2e-37 | 179.8 | 140 | (5, 148) | 159 | (19, 175) | 288 | Lysozyme | Lysozyme | | uniclust | UniRef100\_UPI0007659C9C | 99.9 | 4e-31 | 7.3e-37 | 163.9 | 123 | (1, 126) | 159 | (2, 126) | 146 | lysozyme | lysozyme | | uniclust | UniRef100\_A0A6M0G4D1 | 99.9 | 4.4e-31 | 8.1e-37 | 192.4 | 146 | (1, 149) | 159 | (5, 161) | 474 | Lysozyme | Lysozyme | | uniclust | UniRef100\_A0A410DNY0 | 99.9 | 4.8e-31 | 8.9e-37 | 185.3 | 145 | (2, 149) | 159 | (168, 317) | 320 | Lysozyme | Lysozyme | | uniclust | UniRef100\_A0A2D5M4Q0 | 99.9 | 4.8e-31 | 9e-37 | 170.0 | 137 | (4, 144) | 159 | (45, 181) | 182 | Lysozyme | Lysozyme | | uniclust | UniRef100\_A0A2E4S589 | 99.9 | 4.8e-31 | 9.2e-37 | 200.7 | 147 | (2, 152) | 159 | (13, 163) | 502 | Lysozyme | Lysozyme | | uniclust | UniRef100\_UPI000979CF0A | 99.9 | 5.1e-31 | 9.4e-37 | 176.6 | 142 | (2, 150) | 159 | (4, 151) | 245 | lysozyme | lysozyme | | uniclust | UniRef100\_A0A0Q9KM09 | 99.9 | 5.6e-31 | 1e-36 | 189.0 | 146 | (1, 149) | 159 | (60, 215) | 408 | Lysozyme | Lysozyme | | uniclust | UniRef100\_UPI0015658B88 | 99.9 | 5.7e-31 | 1e-36 | 169.3 | 146 | (3, 149) | 159 | (5, 151) | 202 | lysozyme | lysozyme | | uniclust | UniRef100\_A0A087M4E4 | 99.9 | 5.7e-31 | 1.1e-36 | 181.3 | 142 | (6, 151) | 159 | (58, 209) | 236 | Lysozyme | Lysozyme | | uniclust | UniRef100\_A0A6J5M318 | 99.9 | 7.1e-31 | 1.3e-36 | 173.2 | 141 | (3, 146) | 159 | (59, 213) | 214 | Endolysin | Endolysin | | uniclust | UniRef100\_A0A7C3SVB1 | 99.9 | 7.2e-31 | 1.3e-36 | 170.6 | 144 | (2, 148) | 159 | (62, 216) | 217 | Lysozyme | Lysozyme | | uniclust | UniRef100\_A0A841WQ48 | 99.9 | 7.3e-31 | 1.3e-36 | 172.6 | 143 | (2, 148) | 159 | (5, 147) | 237 | Lysozyme | Lysozyme | | uniclust | UniRef100\_A0A1V5YWE3 | 99.9 | 8.6e-31 | 1.6e-36 | 169.1 | 147 | (1, 152) | 159 | (1, 148) | 207 | Lysozyme | Lysozyme | | uniclust | UniRef100\_A0A929A6N4 | 99.9 | 8.9e-31 | 1.6e-36 | 201.3 | 144 | (3, 149) | 159 | (754, 901) | 987 | Lysozyme | Lysozyme | | uniclust | UniRef100\_A0A158I7H1 | 99.9 | 1e-30 | 1.9e-36 | 177.2 | 147 | (2, 156) | 159 | (37, 201) | 204 | Lysozyme | Lysozyme | | uniclust | UniRef100\_UPI001BD4AB69 | 99.9 | 1e-30 | 1.9e-36 | 190.3 | 147 | (1, 150) | 159 | (1, 155) | 511 | peptidoglycan amidohydrolase family protein | peptidoglycan amidohydrolase family protein | | uniclust | UniRef100\_A0A098RJQ8 | 99.9 | 1e-30 | 1.9e-36 | 192.9 | 146 | (1, 150) | 159 | (58, 219) | 393 | Lysozyme | Lysozyme | | uniclust | UniRef100\_UPI001E4B06EF | 99.9 | 1.1e-30 | 2e-36 | 174.0 | 149 | (3, 151) | 159 | (52, 212) | 213 | lysozyme | lysozyme | | uniclust | UniRef100\_A0A7V8YU39 | 99.9 | 1.2e-30 | 2.3e-36 | 173.8 | 141 | (3, 146) | 159 | (113, 261) | 262 | Lysozyme | Lysozyme | | uniclust | UniRef100\_A0A348HCF7 | 99.9 | 1.7e-30 | 3e-36 | 176.2 | 148 | (3, 150) | 159 | (4, 152) | 300 | Lysozyme | Lysozyme | | uniclust | UniRef100\_A0A1M5TXL6 | 99.9 | 1.6e-30 | 3.1e-36 | 193.4 | 149 | (1, 153) | 159 | (31, 187) | 433 | Lysozyme | Lysozyme | | uniclust | UniRef100\_A0A7Y4UQW0 | 99.9 | 1.8e-30 | 3.3e-36 | 165.3 | 144 | (2, 148) | 159 | (7, 163) | 167 | Lysozyme | Lysozyme | | uniclust | UniRef100\_A0A9D1LFB1 | 99.9 | 1.9e-30 | 3.5e-36 | 165.5 | 137 | (8, 148) | 159 | (46, 182) | 190 | Lysozyme | Lysozyme | | uniclust | UniRef100\_A0A0Q7TTY5 | 99.9 | 2e-30 | 3.7e-36 | 176.7 | 150 | (3, 152) | 159 | (65, 220) | 239 | Lysozyme | Lysozyme | | uniclust | UniRef100\_A0A3Q9BKE1 | 99.9 | 2.2e-30 | 4.1e-36 | 192.0 | 148 | (1, 152) | 159 | (6, 153) | 715 | Lysozyme | Lysozyme | | uniclust | UniRef100\_A0A5E4NVC9 | 99.9 | 3.1e-30 | 5.7e-36 | 172.1 | 142 | (3, 148) | 159 | (5, 146) | 264 | Lysozyme | Lysozyme | | uniclust | UniRef100\_A0A0F9HV13 | 99.9 | 3.4e-30 | 6.4e-36 | 174.4 | 137 | (7, 147) | 159 | (89, 227) | 229 | lysozyme | lysozyme | | uniclust | UniRef100\_A0A8J5ASL3 | 99.9 | 3.9e-30 | 7.3e-36 | 165.3 | 141 | (3, 146) | 159 | (36, 180) | 183 | lysozyme | lysozyme | | uniclust | UniRef100\_A0A9D8PN11 | 99.9 | 4.6e-30 | 8.5e-36 | 178.3 | 154 | (1, 154) | 159 | (1, 163) | 325 | Lysozyme | Lysozyme | | uniclust | UniRef100\_A0A2I5ARC6 | 99.9 | 4.5e-30 | 8.5e-36 | 188.3 | 143 | (6, 148) | 159 | (104, 257) | 404 | Lysozyme | Lysozyme | | uniclust | UniRef100\_A0A0B5DZ45 | 99.9 | 4.7e-30 | 8.7e-36 | 178.1 | 137 | (7, 147) | 159 | (137, 274) | 275 | Lysozyme | Lysozyme | | uniclust | UniRef100\_A0A7R9KK34 | 99.9 | 5.2e-30 | 9.6e-36 | 178.2 | 145 | (2, 150) | 159 | (19, 166) | 341 | lysozyme | lysozyme | | uniclust | UniRef100\_A0A1E5TEK2 | 99.9 | 5.6e-30 | 1e-35 | 190.7 | 145 | (1, 152) | 159 | (322, 476) | 476 | Lysozyme | Lysozyme | | uniclust | UniRef100\_A0A258FCB1 | 99.9 | 5.8e-30 | 1.1e-35 | 172.7 | 143 | (3, 150) | 159 | (4, 150) | 244 | Lysozyme | Lysozyme | | uniclust | UniRef100\_A0A843K231 | 99.9 | 6e-30 | 1.1e-35 | 180.1 | 146 | (2, 150) | 159 | (28, 183) | 413 | lysozyme | lysozyme | | uniclust | UniRef100\_A0A376CQV8 | 99.9 | 5.9e-30 | 1.1e-35 | 157.4 | 131 | (13, 147) | 159 | (2, 132) | 134 | Lysozyme | Lysozyme | | uniclust | UniRef100\_A0A8S5NUM4 | 99.9 | 7.3e-30 | 1.4e-35 | 173.6 | 145 | (1, 152) | 159 | (1, 148) | 258 | Endolysin | Endolysin | | uniclust | UniRef100\_A0A7R9M799 | 99.9 | 7.5e-30 | 1.4e-35 | 168.5 | 140 | (2, 145) | 159 | (101, 243) | 243 | lysozyme | lysozyme | | uniclust | UniRef100\_A0A0U3A7J9 | 99.9 | 8.8e-30 | 1.6e-35 | 187.1 | 141 | (7, 147) | 159 | (320, 469) | 470 | Lysozyme | Lysozyme | | uniclust | UniRef100\_A0A1F2Z7P5 | 99.9 | 8.8e-30 | 1.6e-35 | 162.6 | 141 | (4, 147) | 159 | (18, 159) | 169 | Lysozyme | Lysozyme | | uniclust | UniRef100\_A0A2W5P4F4 | 99.9 | 9.3e-30 | 1.7e-35 | 183.2 | 146 | (2, 147) | 159 | (282, 428) | 433 | Lysozyme | Lysozyme | | uniclust | UniRef100\_A0A2G2MWX1 | 99.9 | 9.8e-30 | 1.8e-35 | 177.5 | 143 | (1, 146) | 159 | (1, 147) | 381 | Lysozyme | Lysozyme | | uniclust | UniRef100\_A0A0H0XNY4 | 99.9 | 9.6e-30 | 1.8e-35 | 172.2 | 146 | (1, 150) | 159 | (50, 202) | 221 | Lysozyme | Lysozyme | | uniclust | UniRef100\_A0A151FIE5 | 99.9 | 1.1e-29 | 2e-35 | 172.5 | 138 | (8, 151) | 159 | (55, 197) | 211 | Lysozyme | Lysozyme | | uniclust | UniRef100\_UPI0018DC8388 | 99.9 | 1.2e-29 | 2.1e-35 | 181.1 | 142 | (2, 147) | 159 | (4, 149) | 469 | glycoside hydrolase family protein | glycoside hydrolase family protein | | uniclust | UniRef100\_A0A1H9I7C8 | 99.9 | 1.5e-29 | 2.8e-35 | 174.0 | 149 | (1, 150) | 159 | (5, 159) | 336 | Lysozyme | Lysozyme | | uniclust | UniRef100\_A0A060BVC9 | 99.9 | 1.7e-29 | 3.2e-35 | 156.6 | 125 | (20, 148) | 159 | (6, 130) | 132 | Lysozyme (Fragment) | Lysozyme (Fragment) | | uniclust | UniRef100\_A0A060CM67 | 99.9 | 1.8e-29 | 3.4e-35 | 168.7 | 139 | (8, 150) | 159 | (50, 196) | 207 | Lysozyme (Fragment) | Lysozyme (Fragment) | | uniclust | UniRef100\_A0A0F9U0N7 | 99.9 | 1.8e-29 | 3.4e-35 | 177.0 | 149 | (1, 149) | 159 | (142, 302) | 304 | lysozyme | lysozyme | | uniclust | UniRef100\_UPI001F2D85D0 | 99.9 | 2.1e-29 | 3.9e-35 | 185.8 | 145 | (3, 150) | 159 | (344, 497) | 666 | D-Ala-D-Ala carboxypeptidase family metallohydrolase | D-Ala-D-Ala carboxypeptidase family metallohydrolase | | uniclust | UniRef100\_A0A2P7EBL1 | 99.9 | 2.1e-29 | 3.9e-35 | 177.4 | 146 | (1, 152) | 159 | (1, 148) | 414 | Lysozyme | Lysozyme | | uniclust | UniRef100\_A0A2Z4PEP3 | 99.9 | 2.3e-29 | 4.3e-35 | 153.8 | 94 | (1, 97) | 159 | (12, 109) | 117 | Lysozyme | Lysozyme | | uniclust | UniRef100\_A0A940SYQ5 | 99.9 | 2.7e-29 | 4.9e-35 | 184.6 | 149 | (1, 153) | 159 | (6, 155) | 637 | Lysozyme | Lysozyme | | uniclust | UniRef100\_A0A0Q9QM53 | 99.9 | 2.8e-29 | 5.3e-35 | 192.7 | 142 | (1, 148) | 159 | (92, 233) | 612 | Lysozyme | Lysozyme | | uniclust | UniRef100\_A0A0F4VLV0 | 99.9 | 2.9e-29 | 5.4e-35 | 155.2 | 137 | (6, 145) | 159 | (4, 142) | 143 | Lysozyme | Lysozyme | | uniclust | UniRef100\_A0A6B3UKC5 | 99.9 | 3.7e-29 | 6.9e-35 | 182.7 | 145 | (1, 149) | 159 | (5, 153) | 442 | Lysozyme (Fragment) | Lysozyme (Fragment) | | uniclust | UniRef100\_A0A257RRU2 | 99.9 | 3.9e-29 | 7.2e-35 | 169.5 | 141 | (4, 147) | 159 | (7, 157) | 255 | Lysozyme | Lysozyme | | uniclust | UniRef100\_A0A0K2M126 | 99.9 | 4.1e-29 | 7.6e-35 | 173.3 | 147 | (6, 152) | 159 | (124, 279) | 282 | Lysozyme | Lysozyme | | uniclust | UniRef100\_A0A933Z3Z4 | 99.9 | 4.4e-29 | 8.1e-35 | 166.0 | 142 | (3, 147) | 159 | (7, 157) | 255 | Lysozyme | Lysozyme | | uniclust | UniRef100\_A0A146F1I5 | 99.9 | 4.6e-29 | 8.5e-35 | 184.9 | 150 | (1, 153) | 159 | (17, 170) | 609 | Lysozyme | Lysozyme | | uniclust | UniRef100\_A0A1A9WZ64 | 99.9 | 4.7e-29 | 8.7e-35 | 168.3 | 138 | (9, 149) | 159 | (143, 280) | 285 | lysozyme | lysozyme | | uniclust | UniRef100\_A0A7W0G9B2 | 99.9 | 4.8e-29 | 8.8e-35 | 178.8 | 140 | (3, 145) | 159 | (322, 469) | 470 | Lysozyme | Lysozyme | | uniclust | UniRef100\_A0A318AUS2 | 99.9 | 5.6e-29 | 1e-34 | 172.4 | 145 | (1, 149) | 159 | (5, 153) | 297 | Lysozyme (Fragment) | Lysozyme (Fragment) | | uniclust | UniRef100\_A0A060C1D9 | 99.9 | 5.7e-29 | 1e-34 | 149.9 | 111 | (1, 111) | 159 | (2, 117) | 118 | Lysozyme (Fragment) | Lysozyme (Fragment) | | uniclust | UniRef100\_A0A2S9WZ50 | 99.9 | 6e-29 | 1.1e-34 | 148.1 | 105 | (1, 108) | 159 | (3, 107) | 107 | Lysozyme (Fragment) | Lysozyme (Fragment) | | uniclust | UniRef100\_A0A7W0Z8H7 | 99.9 | 7.4e-29 | 1.4e-34 | 169.0 | 147 | (1, 151) | 159 | (69, 223) | 309 | Lysozyme | Lysozyme | | uniclust | UniRef100\_A0A1V0EBQ3 | 99.9 | 7.3e-29 | 1.4e-34 | 167.2 | 148 | (1, 151) | 159 | (13, 214) | 224 | Lysozyme | Lysozyme | | uniclust | UniRef100\_A0A158BDW6 | 99.9 | 8.8e-29 | 1.7e-34 | 166.6 | 142 | (1, 149) | 159 | (67, 211) | 212 | Lysozyme | Lysozyme | | uniclust | UniRef100\_A0A0P1FJR3 | 99.9 | 9.5e-29 | 1.8e-34 | 181.0 | 145 | (1, 151) | 159 | (41, 190) | 355 | Lysozyme | Lysozyme | | uniclust | UniRef100\_A0A8T5QLT9 | 99.9 | 1e-28 | 1.9e-34 | 171.3 | 145 | (2, 150) | 159 | (167, 312) | 317 | lysozyme | lysozyme | | uniclust | UniRef100\_A0A444LK34 | 99.9 | 1.1e-28 | 2e-34 | 177.8 | 145 | (1, 148) | 159 | (33, 185) | 376 | Lysozyme | Lysozyme | | uniclust | UniRef100\_A0A7R9KK34 | 99.9 | 1.1e-28 | 2e-34 | 171.8 | 140 | (2, 145) | 159 | (194, 340) | 341 | lysozyme | lysozyme | | uniclust | UniRef100\_A0A099KGY8 | 99.9 | 1.3e-28 | 2.5e-34 | 167.0 | 129 | (2, 133) | 159 | (24, 165) | 189 | Lysozyme | Lysozyme | | uniclust | UniRef100\_A0A1D2MI83 | 99.9 | 1.7e-28 | 3.1e-34 | 172.7 | 140 | (4, 146) | 159 | (73, 224) | 406 | lysozyme (Fragment) | lysozyme (Fragment) | | uniclust | UniRef100\_A0A1B1ALJ1 | 99.9 | 1.7e-28 | 3.1e-34 | 178.4 | 145 | (1, 149) | 159 | (25, 173) | 363 | Lysozyme | Lysozyme | | uniclust | UniRef100\_A0A976EUN2 | 99.9 | 1.8e-28 | 3.3e-34 | 179.3 | 147 | (2, 151) | 159 | (100, 254) | 596 | Lysozyme | Lysozyme | | uniclust | UniRef100\_A0A4R2P729 | 99.9 | 1.9e-28 | 3.5e-34 | 166.6 | 144 | (1, 147) | 159 | (95, 244) | 257 | Lysozyme | Lysozyme | | uniclust | UniRef100\_A0A0P0DLD7 | 99.9 | 1.9e-28 | 3.6e-34 | 184.7 | 141 | (1, 147) | 159 | (27, 167) | 482 | Lysozyme | Lysozyme | | uniclust | UniRef100\_A0A0Q4N8X0 | 99.9 | 2.1e-28 | 3.9e-34 | 154.1 | 140 | (4, 146) | 159 | (7, 169) | 171 | Lysozyme | Lysozyme | | uniclust | UniRef100\_UPI001F1FE970 | 99.9 | 2.4e-28 | 4.3e-34 | 179.6 | 149 | (3, 155) | 159 | (248, 399) | 629 | glycoside hydrolase family protein | glycoside hydrolase family protein | | uniclust | UniRef100\_A0A0K8MB38 | 99.9 | 2.5e-28 | 4.5e-34 | 155.3 | 138 | (10, 150) | 159 | (1, 139) | 183 | Lysozyme | Lysozyme | | uniclust | UniRef100\_UPI0013DE3C73 | 99.9 | 2.5e-28 | 4.5e-34 | 167.7 | 147 | (2, 151) | 159 | (130, 284) | 328 | glycoside hydrolase family protein | glycoside hydrolase family protein | | uniclust | UniRef100\_A0A9D8X297 | 99.9 | 3e-28 | 5.4e-34 | 173.8 | 135 | (1, 145) | 159 | (319, 460) | 460 | N-acetylmuramoyl-L-alanine amidase | N-acetylmuramoyl-L-alanine amidase | | uniclust | UniRef100\_A0A0F9UX49 | 99.9 | 3.3e-28 | 6.1e-34 | 184.6 | 140 | (2, 146) | 159 | (611, 765) | 770 | lysozyme | lysozyme | | uniclust | UniRef100\_A0A2G1Y8D8 | 99.9 | 3.4e-28 | 6.5e-34 | 183.6 | 145 | (2, 150) | 159 | (11, 159) | 466 | Lysozyme | Lysozyme | | uniclust | UniRef100\_A0A7J7BR61 | 99.9 | 3.8e-28 | 7e-34 | 165.9 | 139 | (2, 143) | 159 | (171, 313) | 313 | lysozyme | lysozyme | | uniclust | UniRef100\_A0A968UEN8 | 99.9 | 4.2e-28 | 7.7e-34 | 146.0 | 108 | (3, 114) | 159 | (20, 127) | 128 | Lysozyme (Fragment) | Lysozyme (Fragment) | | uniclust | UniRef100\_A0A285RXB2 | 99.9 | 4.1e-28 | 7.7e-34 | 160.8 | 145 | (1, 150) | 159 | (4, 164) | 202 | Lysozyme | Lysozyme | | uniclust | UniRef100\_A0A2T2RGS0 | 99.9 | 4.2e-28 | 7.7e-34 | 198.5 | 142 | (2, 147) | 159 | (4131, 4272) | 4673 | Lysozyme | Lysozyme | | uniclust | UniRef100\_A0A023Q0S2 | 99.9 | 4e-28 | 7.7e-34 | 166.4 | 122 | (4, 132) | 159 | (23, 149) | 185 | Lysozyme | Lysozyme | | uniclust | UniRef100\_A0A172Y625 | 99.9 | 4.9e-28 | 9.2e-34 | 167.9 | 143 | (1, 147) | 159 | (31, 173) | 296 | Lysozyme | Lysozyme | | uniclust | UniRef100\_A0A090N7E4 | 99.9 | 5.1e-28 | 9.5e-34 | 154.8 | 144 | (1, 149) | 159 | (14, 169) | 176 | Lysozyme | Lysozyme | | uniclust | UniRef100\_A0A0D6B817 | 99.9 | 5.2e-28 | 9.8e-34 | 165.4 | 147 | (2, 151) | 159 | (66, 218) | 231 | Lysozyme | Lysozyme | | uniclust | UniRef100\_A0A847GG99 | 99.9 | 6.4e-28 | 1.2e-33 | 150.7 | 119 | (31, 149) | 159 | (1, 127) | 141 | Lysozyme | Lysozyme | | uniclust | UniRef100\_A0A7C9TZG6 | 99.9 | 6.5e-28 | 1.2e-33 | 178.9 | 148 | (2, 152) | 159 | (79, 234) | 696 | Lysozyme | Lysozyme | | uniclust | UniRef100\_A0A161SEG4 | 99.9 | 6.5e-28 | 1.2e-33 | 151.7 | 137 | (4, 145) | 159 | (5, 153) | 155 | Lysozyme | Lysozyme | | uniclust | UniRef100\_UPI000A6534B1 | 99.9 | 6.6e-28 | 1.2e-33 | 169.3 | 147 | (2, 152) | 159 | (226, 381) | 396 | lysozyme | lysozyme | | uniclust | UniRef100\_A0A0J7Y672 | 99.9 | 6.6e-28 | 1.2e-33 | 160.8 | 143 | (4, 148) | 159 | (9, 160) | 234 | Lysozyme | Lysozyme | | uniclust | UniRef100\_A0A062GS02 | 99.9 | 7.2e-28 | 1.4e-33 | 181.6 | 146 | (1, 152) | 159 | (435, 595) | 595 | Lysozyme | Lysozyme | | uniclust | UniRef100\_A0A0P7AX87 | 99.9 | 8.8e-28 | 1.6e-33 | 165.2 | 146 | (3, 151) | 159 | (133, 287) | 292 | lysozyme | lysozyme | | uniclust | UniRef100\_A0A9D9SU57 | 99.9 | 8.9e-28 | 1.6e-33 | 160.9 | 149 | (1, 150) | 159 | (1, 165) | 267 | Lysozyme | Lysozyme | | uniclust | UniRef100\_A0A7V8DKX2 | 99.9 | 8.8e-28 | 1.6e-33 | 169.8 | 146 | (1, 150) | 159 | (4, 153) | 358 | Lysozyme | Lysozyme | | uniclust | UniRef100\_A0A8J7WEN4 | 99.9 | 9.1e-28 | 1.7e-33 | 181.6 | 148 | (2, 152) | 159 | (365, 531) | 885 | Lysozyme | Lysozyme | | uniclust | UniRef100\_A0A7I8ZS27 | 99.9 | 9.7e-28 | 1.8e-33 | 158.9 | 133 | (13, 148) | 159 | (33, 165) | 244 | Lysozyme | Lysozyme | | uniclust | UniRef100\_A0A3B0MJC2 | 99.9 | 1e-27 | 1.9e-33 | 157.2 | 136 | (9, 147) | 159 | (79, 214) | 218 | Lysozyme | Lysozyme | | uniclust | UniRef100\_A0A3A8ZW54 | 99.9 | 1.1e-27 | 2e-33 | 173.0 | 144 | (2, 154) | 159 | (4, 150) | 473 | Lysozyme | Lysozyme | | uniclust | UniRef100\_A0A3M0SWM4 | 99.9 | 1.2e-27 | 2.2e-33 | 160.2 | 145 | (3, 150) | 159 | (5, 155) | 243 | Lysozyme | Lysozyme | | uniclust | UniRef100\_A0A0U3TF44 | 99.9 | 1.2e-27 | 2.3e-33 | 168.2 | 149 | (1, 149) | 159 | (120, 283) | 285 | Lysozyme | Lysozyme | | uniclust | UniRef100\_A0A501W9K6 | 99.9 | 1.3e-27 | 2.4e-33 | 151.3 | 141 | (2, 145) | 159 | (9, 157) | 159 | Lysozyme | Lysozyme | | uniclust | UniRef100\_UPI0014737934 | 99.9 | 1.3e-27 | 2.4e-33 | 157.0 | 143 | (1, 150) | 159 | (1, 143) | 230 | DUF5776 domain-containing protein | DUF5776 domain-containing protein | | uniclust | UniRef100\_A0A1V5ITR2 | 99.9 | 1.4e-27 | 2.5e-33 | 152.5 | 124 | (24, 151) | 159 | (2, 125) | 187 | Lysozyme | Lysozyme | | uniclust | UniRef100\_A0A0M1IT78 | 99.9 | 1.6e-27 | 2.9e-33 | 167.0 | 147 | (3, 152) | 159 | (4, 156) | 338 | Lysozyme | Lysozyme | | uniclust | UniRef100\_UPI001C2C21C7 | 99.9 | 1.9e-27 | 3.4e-33 | 160.3 | 146 | (3, 151) | 159 | (5, 156) | 278 | glycoside hydrolase family protein | glycoside hydrolase family protein | | uniclust | UniRef100\_A0A0F8Z6L9 | 99.9 | 2e-27 | 3.7e-33 | 163.7 | 142 | (2, 146) | 159 | (180, 331) | 334 | lysozyme (Fragment) | lysozyme (Fragment) | | uniclust | UniRef100\_A0A074MCX7 | 99.9 | 2e-27 | 3.8e-33 | 168.0 | 146 | (1, 150) | 159 | (146, 298) | 312 | Lysozyme | Lysozyme | | uniclust | UniRef100\_A0A1M3KKZ9 | 99.9 | 2.2e-27 | 4e-33 | 154.0 | 140 | (4, 146) | 159 | (9, 158) | 209 | Lysozyme | Lysozyme | | uniclust | UniRef100\_A0A4Q2QRI9 | 99.9 | 2.6e-27 | 4.9e-33 | 176.0 | 144 | (2, 151) | 159 | (375, 530) | 531 | Lysozyme (Fragment) | Lysozyme (Fragment) | | uniclust | UniRef100\_A0A2V2RWC3 | 99.9 | 2.7e-27 | 5e-33 | 179.1 | 152 | (1, 152) | 159 | (113, 275) | 639 | Lysozyme | Lysozyme | | uniclust | UniRef100\_A0A1Y1J2A2 | 99.9 | 2.7e-27 | 5.2e-33 | 148.1 | 118 | (31, 149) | 159 | (3, 128) | 129 | Phage-related lysozyme | Phage-related lysozyme | | uniclust | UniRef100\_A0A966W4D6 | 99.9 | 2.9e-27 | 5.4e-33 | 158.0 | 139 | (3, 144) | 159 | (118, 256) | 260 | Lysozyme | Lysozyme | | uniclust | UniRef100\_A0A1G8AZ06 | 99.9 | 3e-27 | 5.5e-33 | 180.6 | 146 | (2, 150) | 159 | (230, 388) | 831 | Lysozyme | Lysozyme | | uniclust | UniRef100\_A0A961ZTK2 | 99.9 | 3.1e-27 | 5.6e-33 | 158.1 | 137 | (9, 148) | 159 | (14, 157) | 262 | Lysozyme | Lysozyme | | uniclust | UniRef100\_A0A843LAG6 | 99.9 | 4.4e-27 | 8.1e-33 | 180.3 | 143 | (2, 147) | 159 | (28, 180) | 1047 | lysozyme | lysozyme | | uniclust | UniRef100\_A0A2W6IFF7 | 99.9 | 4.4e-27 | 8.1e-33 | 157.4 | 134 | (8, 146) | 159 | (73, 218) | 245 | Lysozyme | Lysozyme | | uniclust | UniRef100\_UPI001565CE07 | 99.9 | 5.1e-27 | 9.4e-33 | 159.1 | 148 | (2, 149) | 159 | (84, 243) | 245 | lysozyme | lysozyme | | uniclust | UniRef100\_A0A017HBD8 | 99.9 | 5e-27 | 9.5e-33 | 162.2 | 139 | (1, 151) | 159 | (8, 157) | 243 | Phage-related lysozyme | Phage-related lysozyme | | uniclust | UniRef100\_A0A0A8K6Z9 | 99.9 | 5.1e-27 | 9.5e-33 | 170.4 | 141 | (1, 151) | 159 | (5, 151) | 384 | Lysozyme | Lysozyme | | uniclust | UniRef100\_A0A3P2RCD3 | 99.9 | 5.2e-27 | 9.6e-33 | 157.6 | 143 | (1, 147) | 159 | (1, 145) | 270 | Lysozyme | Lysozyme | | uniclust | UniRef100\_A0A0H3H043 | 99.9 | 5.3e-27 | 1e-32 | 154.5 | 137 | (2, 149) | 159 | (22, 159) | 165 | Lysozyme | Lysozyme | | uniclust | UniRef100\_A0A346MWA4 | 99.9 | 5.5e-27 | 1e-32 | 156.6 | 141 | (7, 150) | 159 | (31, 184) | 204 | Lysozyme | Lysozyme | | uniclust | UniRef100\_A0A0R3DQ56 | 99.9 | 6.4e-27 | 1.2e-32 | 155.5 | 141 | (8, 150) | 159 | (22, 162) | 210 | Lysozyme | Lysozyme | | uniclust | UniRef100\_A0A085FNZ0 | 99.9 | 7e-27 | 1.3e-32 | 158.2 | 141 | (2, 150) | 159 | (73, 218) | 239 | Lysozyme | Lysozyme | | uniclust | UniRef100\_A0A246G718 | 99.9 | 7.1e-27 | 1.3e-32 | 164.2 | 145 | (2, 152) | 159 | (177, 337) | 337 | Lysozyme | Lysozyme | | uniclust | UniRef100\_A0A099FC71 | 99.9 | 7.4e-27 | 1.4e-32 | 163.9 | 141 | (1, 151) | 159 | (14, 165) | 282 | Lysozyme | Lysozyme | | uniclust | UniRef100\_A0A0A2V9H7 | 99.9 | 8e-27 | 1.5e-32 | 163.4 | 136 | (9, 147) | 159 | (17, 152) | 335 | lysozyme | lysozyme | | uniclust | UniRef100\_A0A078KPY1 | 99.9 | 9.4e-27 | 1.7e-32 | 156.6 | 147 | (1, 149) | 159 | (96, 245) | 246 | Lysozyme | Lysozyme | | uniclust | UniRef100\_A0A6J5LRB2 | 99.9 | 1e-26 | 1.9e-32 | 147.4 | 148 | (1, 148) | 159 | (2, 156) | 159 | Lysozyme | Lysozyme | | uniclust | UniRef100\_A0A3D5P936 | 99.9 | 1.1e-26 | 2.1e-32 | 158.5 | 145 | (2, 149) | 159 | (156, 303) | 306 | Lysozyme | Lysozyme | | uniclust | UniRef100\_A0A6J7WL85 | 99.9 | 1.2e-26 | 2.2e-32 | 148.7 | 143 | (1, 149) | 159 | (1, 152) | 191 | Endolysin | Endolysin | | uniclust | UniRef100\_UPI000AED14A8 | 99.9 | 1.2e-26 | 2.3e-32 | 167.9 | 139 | (6, 147) | 159 | (7, 169) | 523 | glycoside hydrolase family protein | glycoside hydrolase family protein | | uniclust | UniRef100\_A0A143HSA8 | 99.9 | 1.3e-26 | 2.4e-32 | 147.8 | 125 | (21, 148) | 159 | (1, 127) | 131 | Lysozyme | Lysozyme | | uniclust | UniRef100\_A0A840XX44 | 99.9 | 1.3e-26 | 2.4e-32 | 154.3 | 147 | (2, 149) | 159 | (5, 170) | 237 | Lysozyme | Lysozyme | | uniclust | UniRef100\_A0A0K1S2G2 | 99.9 | 1.4e-26 | 2.7e-32 | 159.9 | 144 | (2, 146) | 159 | (86, 238) | 253 | Lysozyme | Lysozyme | | uniclust | UniRef100\_A0A6G9YUS1 | 99.9 | 1.5e-26 | 2.7e-32 | 178.6 | 147 | (3, 152) | 159 | (594, 747) | 1148 | Lysozyme | Lysozyme | | uniclust | UniRef100\_A0A1C6AN06 | 99.9 | 1.5e-26 | 2.7e-32 | 169.9 | 144 | (1, 148) | 159 | (38, 190) | 495 | Lysozyme | Lysozyme | | uniclust | UniRef100\_A0A0F9C0T9 | 99.9 | 1.5e-26 | 2.8e-32 | 160.8 | 132 | (4, 138) | 159 | (45, 187) | 206 | lysozyme | lysozyme | | uniclust | UniRef100\_A0A3B0JB27 | 99.9 | 1.6e-26 | 3e-32 | 139.7 | 117 | (32, 148) | 159 | (2, 118) | 124 | Lysozyme | Lysozyme | | uniclust | UniRef100\_A0A317CEU3 | 99.9 | 1.7e-26 | 3.1e-32 | 156.9 | 147 | (1, 150) | 159 | (2, 168) | 276 | Lysozyme | Lysozyme | | uniclust | UniRef100\_A0A0A1TCW0 | 99.9 | 1.7e-26 | 3.1e-32 | 166.6 | 146 | (4, 150) | 159 | (25, 180) | 425 | lysozyme | lysozyme | | uniclust | UniRef100\_A0A349NKU6 | 99.9 | 1.7e-26 | 3.2e-32 | 136.6 | 113 | (1, 117) | 159 | (1, 113) | 114 | Lysozyme (Fragment) | Lysozyme (Fragment) | | uniclust | UniRef100\_A0A8J6UXX9 | 99.9 | 1.9e-26 | 3.5e-32 | 157.6 | 143 | (6, 151) | 159 | (92, 254) | 269 | Lysozyme | Lysozyme | | uniclust | UniRef100\_A0A0F4VMI7 | 99.9 | 2e-26 | 3.7e-32 | 144.7 | 95 | (1, 99) | 159 | (23, 119) | 128 | Lysozyme | Lysozyme | | uniclust | UniRef100\_A0A3S3SML5 | 99.9 | 2.2e-26 | 4e-32 | 146.9 | 142 | (2, 146) | 159 | (5, 146) | 185 | lysozyme | lysozyme | | uniclust | UniRef100\_UPI001B3C9599 | 99.9 | 2.2e-26 | 4.1e-32 | 151.4 | 143 | (2, 147) | 159 | (7, 150) | 230 | lysozyme | lysozyme | | uniclust | UniRef100\_A0A069IM11 | 99.9 | 2.2e-26 | 4.2e-32 | 151.2 | 139 | (2, 147) | 159 | (10, 148) | 198 | Lysozyme (Fragment) | Lysozyme (Fragment) | | uniclust | UniRef100\_A0A1Y1VV99 | 99.9 | 2.3e-26 | 4.2e-32 | 157.1 | 144 | (3, 150) | 159 | (115, 267) | 273 | lysozyme | lysozyme | | uniclust | UniRef100\_A0A538JLK8 | 99.9 | 2.3e-26 | 4.3e-32 | 161.6 | 138 | (5, 145) | 159 | (187, 338) | 339 | Lysozyme | Lysozyme | | uniclust | UniRef100\_A0A845WUP1 | 99.9 | 2.4e-26 | 4.4e-32 | 146.9 | 135 | (6, 146) | 159 | (50, 186) | 186 | Lysozyme | Lysozyme | | uniclust | UniRef100\_A0A143XLM0 | 99.9 | 2.4e-26 | 4.4e-32 | 153.1 | 138 | (2, 149) | 159 | (3, 151) | 229 | Lysozyme | Lysozyme | | uniclust | UniRef100\_A0A4Q2QUC8 | 99.9 | 2.8e-26 | 5.3e-32 | 173.1 | 143 | (2, 151) | 159 | (493, 644) | 645 | Lysozyme (Fragment) | Lysozyme (Fragment) | | uniclust | UniRef100\_A0A081NGH9 | 99.9 | 3.2e-26 | 6.1e-32 | 157.3 | 147 | (1, 150) | 159 | (27, 189) | 206 | Lysozyme | Lysozyme | | uniclust | UniRef100\_A0A6B8KGM4 | 99.9 | 3.5e-26 | 6.4e-32 | 153.8 | 144 | (2, 148) | 159 | (3, 152) | 271 | Lysozyme | Lysozyme | | uniclust | UniRef100\_UPI0011247FAC | 99.9 | 3.8e-26 | 6.9e-32 | 152.9 | 143 | (1, 150) | 159 | (1, 143) | 260 | glycoside hydrolase family protein | glycoside hydrolase family protein | | uniclust | UniRef100\_A0A0Q5MFL0 | 99.9 | 3.8e-26 | 7.1e-32 | 167.2 | 140 | (2, 147) | 159 | (55, 194) | 423 | Lysozyme | Lysozyme | | uniclust | UniRef100\_A0A0N4VYY9 | 99.9 | 4.5e-26 | 8.3e-32 | 155.1 | 139 | (6, 149) | 159 | (79, 220) | 297 | lysozyme | lysozyme | | uniclust | UniRef100\_UPI0013DFCEBC | 99.9 | 4.8e-26 | 8.8e-32 | 158.3 | 146 | (1, 149) | 159 | (1, 157) | 355 | glycoside hydrolase family protein | glycoside hydrolase family protein | | uniclust | UniRef100\_A0A6J4RNY5 | 99.9 | 5.3e-26 | 9.7e-32 | 175.3 | 146 | (2, 150) | 159 | (623, 778) | 1117 | Lysozyme | Lysozyme | | uniclust | UniRef100\_A0A3D3IVC7 | 99.9 | 6.9e-26 | 1.3e-31 | 169.3 | 145 | (3, 150) | 159 | (13, 164) | 735 | Lysozyme | Lysozyme | | uniclust | UniRef100\_UPI001184926A | 99.9 | 7.4e-26 | 1.4e-31 | 159.4 | 148 | (2, 150) | 159 | (4, 168) | 398 | glycoside hydrolase family protein | glycoside hydrolase family protein | | uniclust | UniRef100\_A0A384UH61 | 99.9 | 7.6e-26 | 1.4e-31 | 142.0 | 126 | (18, 147) | 159 | (2, 127) | 164 | Endolysin | Endolysin | | uniclust | UniRef100\_A0A6C2CEI5 | 99.9 | 7.7e-26 | 1.4e-31 | 166.6 | 144 | (1, 147) | 159 | (463, 620) | 622 | Lysozyme | Lysozyme | | uniclust | UniRef100\_A0A0C1NIH4 | 99.9 | 7.9e-26 | 1.5e-31 | 159.1 | 149 | (3, 152) | 159 | (139, 297) | 310 | Lysozyme | Lysozyme | | uniclust | UniRef100\_UPI001CC4FB84 | 99.9 | 8.3e-26 | 1.5e-31 | 165.3 | 145 | (2, 149) | 159 | (383, 538) | 539 | peptidoglycan-binding protein | peptidoglycan-binding protein | | uniclust | UniRef100\_A0A4P7CYW0 | 99.9 | 8.5e-26 | 1.6e-31 | 152.2 | 142 | (7, 148) | 159 | (84, 253) | 255 | Lysozyme | Lysozyme | | uniclust | UniRef100\_A3Z6C9 | 99.9 | 8.6e-26 | 1.6e-31 | 150.9 | 144 | (3, 147) | 159 | (5, 150) | 256 | Lysozyme | Lysozyme | | uniclust | UniRef100\_A0A095FJ13 | 99.9 | 9.2e-26 | 1.7e-31 | 156.3 | 148 | (9, 156) | 159 | (84, 278) | 280 | Lysozyme | Lysozyme | | uniclust | UniRef100\_A0A850BAY3 | 99.9 | 9.8e-26 | 1.8e-31 | 171.5 | 145 | (3, 150) | 159 | (740, 899) | 919 | Lysozyme (Fragment) | Lysozyme (Fragment) | | uniclust | UniRef100\_A0A3T0D2Y2 | 99.9 | 1.1e-25 | 2e-31 | 154.6 | 148 | (3, 153) | 159 | (109, 280) | 292 | Lysozyme | Lysozyme | | uniclust | UniRef100\_R9JLH4 | 99.9 | 1.1e-25 | 2e-31 | 160.8 | 136 | (2, 146) | 159 | (4, 142) | 451 | Lysozyme | Lysozyme | | uniclust | UniRef100\_A0A0A8TLB0 | 99.9 | 1.1e-25 | 2.2e-31 | 152.4 | 123 | (5, 134) | 159 | (32, 159) | 186 | Lysozyme | Lysozyme | | uniclust | UniRef100\_A0A0F9BCT7 | 99.9 | 1.1e-25 | 2.2e-31 | 150.5 | 126 | (5, 133) | 159 | (10, 149) | 172 | lysozyme | lysozyme | | uniclust | UniRef100\_A0A1G6ZEY4 | 99.9 | 1.4e-25 | 2.5e-31 | 143.2 | 142 | (3, 147) | 159 | (6, 152) | 160 | Lysozyme | Lysozyme | | uniclust | UniRef100\_A0A562PQL7 | 99.9 | 1.4e-25 | 2.6e-31 | 174.0 | 144 | (2, 152) | 159 | (585, 742) | 742 | Lysozyme | Lysozyme | | uniclust | UniRef100\_A0A009PDS4 | 99.9 | 1.4e-25 | 2.6e-31 | 151.0 | 137 | (8, 147) | 159 | (35, 184) | 189 | Lysozyme | Lysozyme | | uniclust | UniRef100\_UPI002174E5E2 | 99.9 | 1.4e-25 | 2.6e-31 | 143.7 | 135 | (7, 144) | 159 | (15, 152) | 189 | lysozyme | lysozyme | | uniclust | UniRef100\_A0A2G6ENY7 | 99.9 | 1.6e-25 | 2.9e-31 | 156.9 | 141 | (6, 149) | 159 | (6, 150) | 377 | Lysozyme | Lysozyme | | uniclust | UniRef100\_A0A7G8Q4E4 | 99.9 | 1.9e-25 | 3.5e-31 | 152.1 | 143 | (3, 148) | 159 | (6, 168) | 296 | Lysozyme | Lysozyme | | uniclust | UniRef100\_A0A535XI64 | 99.9 | 2e-25 | 3.7e-31 | 144.2 | 137 | (2, 148) | 159 | (55, 199) | 200 | Lysozyme | Lysozyme | | uniclust | UniRef100\_D3F184 | 99.9 | 2.1e-25 | 3.9e-31 | 156.9 | 141 | (2, 146) | 159 | (230, 389) | 391 | Lysozyme | Lysozyme | | uniclust | UniRef100\_A0A2D7IXU0 | 99.9 | 2.1e-25 | 3.9e-31 | 171.0 | 144 | (2, 149) | 159 | (6, 153) | 686 | Lysozyme | Lysozyme | | uniclust | UniRef100\_A0A6G9YUS1 | 99.9 | 2.4e-25 | 4.5e-31 | 172.1 | 145 | (2, 149) | 159 | (394, 545) | 1148 | Lysozyme | Lysozyme | | uniclust | UniRef100\_A0A2E3PQX9 | 99.9 | 2.6e-25 | 4.7e-31 | 152.2 | 145 | (1, 150) | 159 | (1, 161) | 302 | Lysozyme | Lysozyme | | uniclust | UniRef100\_UPI0009B89992 | 99.9 | 2.8e-25 | 5.2e-31 | 147.7 | 135 | (11, 149) | 159 | (26, 160) | 245 | lysozyme | lysozyme | | uniclust | UniRef100\_A0A0V8BQ91 | 99.9 | 2.8e-25 | 5.3e-31 | 159.5 | 143 | (1, 149) | 159 | (1, 148) | 388 | Lysozyme | Lysozyme | | uniclust | UniRef100\_A0A127VHU8 | 99.9 | 3e-25 | 5.5e-31 | 148.2 | 146 | (2, 152) | 159 | (9, 160) | 221 | Lysozyme | Lysozyme | | uniclust | UniRef100\_UPI0003638F79 | 99.9 | 3e-25 | 5.5e-31 | 144.3 | 109 | (3, 114) | 159 | (68, 178) | 193 | lysozyme | lysozyme | | uniclust | UniRef100\_A0A832GZF3 | 99.9 | 3.1e-25 | 5.7e-31 | 153.5 | 125 | (30, 155) | 159 | (2, 129) | 337 | Lysozyme | Lysozyme | | uniclust | UniRef100\_A0A940QJJ3 | 99.9 | 3.1e-25 | 5.8e-31 | 145.0 | 147 | (2, 152) | 159 | (39, 194) | 217 | Lysozyme (Fragment) | Lysozyme (Fragment) | | uniclust | UniRef100\_A0A011MKX9 | 99.9 | 3.6e-25 | 6.7e-31 | 162.6 | 145 | (3, 150) | 159 | (282, 434) | 460 | Lysozyme | Lysozyme | | uniclust | UniRef100\_A0A068Q6B6 | 99.9 | 3.7e-25 | 7e-31 | 151.3 | 132 | (8, 144) | 159 | (31, 192) | 218 | Putative endolysin | Putative endolysin | | uniclust | UniRef100\_K9XRH0 | 99.9 | 3.9e-25 | 7.2e-31 | 159.0 | 114 | (2, 119) | 159 | (67, 180) | 481 | Lysozyme | Lysozyme | | uniclust | UniRef100\_A0A0D0KR74 | 99.9 | 3.9e-25 | 7.3e-31 | 154.1 | 141 | (9, 152) | 159 | (91, 241) | 258 | Lysozyme | Lysozyme | | uniclust | UniRef100\_UPI0021BA9689 | 99.9 | 4.3e-25 | 8e-31 | 178.2 | 148 | (2, 152) | 159 | (1932, 2087) | 2569 | S8 family serine peptidase | S8 family serine peptidase | | uniclust | UniRef100\_A0A147GQV9 | 99.9 | 4.4e-25 | 8.2e-31 | 142.9 | 153 | (1, 155) | 159 | (1, 176) | 181 | Lysozyme | Lysozyme | | uniclust | UniRef100\_A0A3C1W3S6 | 99.9 | 4.7e-25 | 8.7e-31 | 140.5 | 146 | (1, 149) | 159 | (2, 161) | 163 | Lysozyme | Lysozyme | | uniclust | UniRef100\_A0A1Q6RIL6 | 99.9 | 4.9e-25 | 9.1e-31 | 162.9 | 151 | (1, 152) | 159 | (31, 196) | 536 | Lysozyme | Lysozyme | | uniclust | UniRef100\_UPI0008499688 | 99.9 | 5e-25 | 9.1e-31 | 146.2 | 141 | (7, 151) | 159 | (19, 162) | 241 | glycoside hydrolase family protein | glycoside hydrolase family protein | | uniclust | UniRef100\_A0A261KVX8 | 99.9 | 5.2e-25 | 9.6e-31 | 129.2 | 99 | (1, 102) | 159 | (1, 103) | 103 | Lysozyme (Fragment) | Lysozyme (Fragment) | | uniclust | UniRef100\_A0A443J0A0 | 99.8 | 5.7e-25 | 1.1e-30 | 151.2 | 142 | (1, 152) | 159 | (11, 163) | 269 | Lysozyme | Lysozyme | | uniclust | UniRef100\_A0A0E0UJC6 | 99.8 | 6.1e-25 | 1.1e-30 | 164.6 | 146 | (1, 151) | 159 | (39, 188) | 627 | Lysozyme | Lysozyme | | uniclust | UniRef100\_UPI0009FEF08B | 99.8 | 6.3e-25 | 1.2e-30 | 151.1 | 148 | (2, 149) | 159 | (128, 287) | 289 | lysozyme | lysozyme | | uniclust | UniRef100\_A0A1G5SFW3 | 99.8 | 6.3e-25 | 1.2e-30 | 147.7 | 137 | (4, 145) | 159 | (118, 265) | 267 | Lysozyme | Lysozyme | | uniclust | UniRef100\_A0A081C6S4 | 99.8 | 6.4e-25 | 1.2e-30 | 155.7 | 145 | (1, 148) | 159 | (1, 159) | 419 | Lysozyme | Lysozyme | | uniclust | UniRef100\_A0A368HDJ6 | 99.8 | 6.5e-25 | 1.2e-30 | 143.7 | 114 | (1, 118) | 159 | (5, 118) | 218 | Lysozyme | Lysozyme | | uniclust | UniRef100\_UPI002107C0D7 | 99.8 | 7e-25 | 1.3e-30 | 130.0 | 112 | (33, 144) | 159 | (2, 113) | 114 | lysozyme | lysozyme | | uniclust | UniRef100\_A0A359LSI1 | 99.8 | 7e-25 | 1.3e-30 | 146.8 | 144 | (2, 148) | 159 | (59, 211) | 215 | Lysozyme | Lysozyme | | uniclust | UniRef100\_UPI000405D4F2 | 99.8 | 8.2e-25 | 1.5e-30 | 158.0 | 131 | (10, 144) | 159 | (367, 497) | 498 | glycoside hydrolase family protein | glycoside hydrolase family protein | | uniclust | UniRef100\_A0A0L1KIF7 | 99.8 | 8.1e-25 | 1.5e-30 | 154.6 | 145 | (2, 150) | 159 | (147, 298) | 312 | Lysozyme | Lysozyme | | uniclust | UniRef100\_A0A829Z1I7 | 99.8 | 8.4e-25 | 1.6e-30 | 134.2 | 100 | (53, 152) | 159 | (2, 101) | 132 | Lysozyme | Lysozyme | | uniclust | UniRef100\_A0A5C7USV4 | 99.8 | 8.9e-25 | 1.6e-30 | 161.6 | 148 | (2, 152) | 159 | (44, 204) | 637 | Lysozyme | Lysozyme | | uniclust | UniRef100\_UPI001BD066A5 | 99.8 | 9.6e-25 | 1.8e-30 | 143.4 | 136 | (8, 147) | 159 | (85, 222) | 223 | glycoside hydrolase family protein | glycoside hydrolase family protein | | uniclust | UniRef100\_A0A7K1TL27 | 99.8 | 9.9e-25 | 1.8e-30 | 150.3 | 141 | (1, 144) | 159 | (171, 323) | 324 | Lysozyme | Lysozyme | | uniclust | UniRef100\_UPI001F43C1FD | 99.8 | 1e-24 | 1.9e-30 | 139.3 | 140 | (7, 146) | 159 | (37, 180) | 183 | lysozyme | lysozyme | | uniclust | UniRef100\_A0A1U7J078 | 99.8 | 1e-24 | 1.9e-30 | 163.4 | 149 | (3, 151) | 159 | (49, 206) | 474 | Lysozyme | Lysozyme | | uniclust | UniRef100\_A0A380ZDW0 | 99.8 | 1.2e-24 | 2.2e-30 | 142.0 | 107 | (46, 152) | 159 | (3, 109) | 175 | Lysozyme | Lysozyme | | uniclust | UniRef100\_A0A4S2MJL7 | 99.8 | 1.2e-24 | 2.2e-30 | 149.0 | 138 | (3, 148) | 159 | (123, 266) | 271 | lysozyme | lysozyme | | uniclust | UniRef100\_A0A932PB96 | 99.8 | 1.3e-24 | 2.4e-30 | 151.3 | 98 | (52, 149) | 159 | (142, 252) | 285 | Lysozyme | Lysozyme | | uniclust | UniRef100\_UPI001EEE7D44 | 99.8 | 1.4e-24 | 2.5e-30 | 141.3 | 138 | (9, 149) | 159 | (21, 159) | 208 | lysozyme | lysozyme | | uniclust | UniRef100\_UPI000A7BED71 | 99.8 | 1.5e-24 | 2.7e-30 | 156.7 | 140 | (1, 148) | 159 | (1, 148) | 440 | glycoside hydrolase family protein | glycoside hydrolase family protein | | uniclust | UniRef100\_A0A7W9YX61 | 99.8 | 1.5e-24 | 2.7e-30 | 147.6 | 142 | (1, 152) | 159 | (1, 147) | 291 | Lysozyme | Lysozyme | | uniclust | UniRef100\_A0A7Y3RIZ1 | 99.8 | 1.5e-24 | 2.7e-30 | 160.9 | 142 | (6, 150) | 159 | (435, 576) | 661 | Lysozyme | Lysozyme | | uniclust | UniRef100\_A0A1C7D850 | 99.8 | 1.7e-24 | 3.1e-30 | 150.1 | 146 | (1, 150) | 159 | (121, 273) | 287 | Lysozyme | Lysozyme | | uniclust | UniRef100\_A0A177N7A2 | 99.8 | 1.8e-24 | 3.4e-30 | 139.9 | 148 | (2, 149) | 159 | (12, 173) | 177 | Lysozyme | Lysozyme | | uniclust | UniRef100\_A0A252DI96 | 99.8 | 1.9e-24 | 3.4e-30 | 134.2 | 105 | (45, 149) | 159 | (7, 111) | 132 | Lysozyme | Lysozyme | | uniclust | UniRef100\_A0A3A1Y9X2 | 99.8 | 1.9e-24 | 3.5e-30 | 138.1 | 138 | (5, 145) | 159 | (35, 176) | 183 | Lysozyme | Lysozyme | | uniclust | UniRef100\_A0A022PFR1 | 99.8 | 1.9e-24 | 3.5e-30 | 149.2 | 148 | (2, 149) | 159 | (147, 306) | 308 | Lysozyme | Lysozyme | | uniclust | UniRef100\_A0A192H186 | 99.8 | 1.9e-24 | 3.5e-30 | 160.9 | 142 | (1, 145) | 159 | (538, 687) | 687 | Lysozyme | Lysozyme | | uniclust | UniRef100\_UPI0009EAD2D6 | 99.8 | 2e-24 | 3.6e-30 | 129.6 | 105 | (2, 109) | 159 | (3, 107) | 122 | lysozyme | lysozyme | | uniclust | UniRef100\_UPI0007CCFC1A | 99.8 | 2.1e-24 | 3.8e-30 | 151.5 | 146 | (2, 154) | 159 | (216, 370) | 378 | lysozyme | lysozyme | | uniclust | UniRef100\_A0A8H7CL31 | 99.8 | 2.2e-24 | 4.1e-30 | 146.2 | 147 | (3, 150) | 159 | (104, 262) | 282 | lysozyme | lysozyme | | uniclust | UniRef100\_UPI00191D0E7B | 99.8 | 2.3e-24 | 4.2e-30 | 148.4 | 154 | (1, 155) | 159 | (89, 251) | 320 | lysozyme | lysozyme | | uniclust | UniRef100\_A0A8A1MB86 | 99.8 | 2.4e-24 | 4.5e-30 | 144.6 | 143 | (3, 148) | 159 | (33, 182) | 249 | lysozyme | lysozyme | | uniclust | UniRef100\_A0A1F3AD95 | 99.8 | 2.4e-24 | 4.5e-30 | 143.7 | 145 | (1, 149) | 159 | (1, 149) | 250 | Lysozyme | Lysozyme | | uniclust | UniRef100\_A0A2T7Q7E6 | 99.8 | 2.7e-24 | 4.9e-30 | 138.9 | 138 | (2, 144) | 159 | (53, 196) | 197 | Lysozyme | Lysozyme | | uniclust | UniRef100\_A0A357WVB9 | 99.8 | 2.8e-24 | 5.2e-30 | 144.9 | 138 | (2, 149) | 159 | (68, 210) | 221 | Lysozyme (Fragment) | Lysozyme (Fragment) | | uniclust | UniRef100\_J5TH48 | 99.8 | 2.8e-24 | 5.2e-30 | 161.6 | 142 | (3, 147) | 159 | (558, 713) | 738 | lysozyme | lysozyme | | uniclust | UniRef100\_A0A8H9NPM1 | 99.8 | 3e-24 | 5.6e-30 | 146.1 | 135 | (9, 147) | 159 | (28, 162) | 291 | Lysozyme | Lysozyme | | uniclust | UniRef100\_A0A242NX27 | 99.8 | 3e-24 | 5.6e-30 | 168.8 | 145 | (2, 152) | 159 | (977, 1132) | 1132 | Lysozyme | Lysozyme | | uniclust | UniRef100\_A0A0T9R1Y9 | 99.8 | 3.1e-24 | 5.8e-30 | 168.8 | 145 | (2, 152) | 159 | (867, 1023) | 1023 | Lysozyme | Lysozyme | | uniclust | UniRef100\_A0A0F5ALB6 | 99.8 | 3.1e-24 | 5.8e-30 | 141.9 | 139 | (6, 149) | 159 | (17, 162) | 202 | Lysozyme | Lysozyme | | uniclust | UniRef100\_UPI001FAEC223 | 99.8 | 3.6e-24 | 6.5e-30 | 141.1 | 136 | (5, 145) | 159 | (76, 223) | 227 | lysozyme | lysozyme | | uniclust | UniRef100\_UPI001DFEEB7A | 99.8 | 3.7e-24 | 6.8e-30 | 152.0 | 145 | (3, 150) | 159 | (87, 240) | 418 | uncharacterized protein | uncharacterized protein | | uniclust | UniRef100\_A0A2S0U5Q1 | 99.8 | 4.1e-24 | 7.5e-30 | 161.1 | 148 | (2, 152) | 159 | (494, 648) | 654 | Lysozyme | Lysozyme | | uniclust | UniRef100\_A0A7W8Q238 | 99.8 | 4.1e-24 | 7.7e-30 | 138.0 | 132 | (12, 151) | 159 | (18, 152) | 153 | Lysozyme | Lysozyme | | uniclust | UniRef100\_A3WB87 | 99.8 | 4.2e-24 | 7.8e-30 | 149.7 | 146 | (1, 150) | 159 | (173, 325) | 329 | Lysozyme | Lysozyme | | uniclust | UniRef100\_UPI001C0C1628 | 99.8 | 4.3e-24 | 7.9e-30 | 144.8 | 136 | (1, 151) | 159 | (1, 137) | 264 | lysozyme | lysozyme | | uniclust | UniRef100\_UPI000D51B1FE | 99.8 | 4.7e-24 | 8.6e-30 | 146.3 | 136 | (2, 141) | 159 | (27, 162) | 309 | Rz-like lysis system protein LysB | Rz-like lysis system protein LysB | | uniclust | UniRef100\_A0A1C4CVR3 | 99.8 | 4.6e-24 | 8.7e-30 | 147.2 | 145 | (1, 150) | 159 | (45, 232) | 235 | Lysozyme | Lysozyme | | uniclust | UniRef100\_A0A661CVB8 | 99.8 | 4.8e-24 | 8.8e-30 | 151.1 | 142 | (3, 148) | 159 | (244, 405) | 410 | Lysozyme (Fragment) | Lysozyme (Fragment) | | uniclust | UniRef100\_A0A942F8L9 | 99.8 | 4.9e-24 | 9.1e-30 | 161.0 | 148 | (1, 151) | 159 | (222, 382) | 807 | Lysozyme | Lysozyme | | uniclust | UniRef100\_A0A1X2GML7 | 99.8 | 5.5e-24 | 1e-29 | 147.0 | 145 | (5, 152) | 159 | (119, 272) | 283 | lysozyme | lysozyme | | uniclust | UniRef100\_A0A071LUB0 | 99.8 | 5.4e-24 | 1e-29 | 148.2 | 128 | (5, 135) | 159 | (38, 181) | 217 | Lysozyme | Lysozyme | | uniclust | UniRef100\_J0QIK5 | 99.8 | 6.1e-24 | 1.1e-29 | 155.6 | 122 | (31, 152) | 159 | (374, 499) | 565 | Lysozyme | Lysozyme | | uniclust | UniRef100\_A0A031IWU6 | 99.8 | 5.9e-24 | 1.1e-29 | 147.5 | 146 | (4, 150) | 159 | (51, 214) | 230 | Prophage PSPPH06 lysozyme | Prophage PSPPH06 lysozyme | | uniclust | UniRef100\_UPI000D8E9A34 | 99.8 | 6.1e-24 | 1.1e-29 | 143.0 | 145 | (2, 149) | 159 | (117, 263) | 265 | SH3 domain-containing protein | SH3 domain-containing protein | | uniclust | UniRef100\_UPI001FF05F52 | 99.8 | 6.7e-24 | 1.2e-29 | 132.0 | 102 | (48, 149) | 159 | (2, 103) | 139 | lysozyme | lysozyme | | uniclust | UniRef100\_A0A8E7EIT5 | 99.8 | 6.7e-24 | 1.2e-29 | 158.6 | 148 | (1, 151) | 159 | (159, 314) | 710 | lysozyme | lysozyme | | uniclust | UniRef100\_UPI0009E45E4A | 99.8 | 7e-24 | 1.3e-29 | 153.7 | 146 | (1, 149) | 159 | (3, 154) | 510 | glycoside hydrolase family protein | glycoside hydrolase family protein | | uniclust | UniRef100\_A0A8J2RB09 | 99.8 | 7.5e-24 | 1.4e-29 | 143.5 | 143 | (3, 148) | 159 | (120, 271) | 280 | lysozyme | lysozyme | | uniclust | UniRef100\_A0A928PRK0 | 99.8 | 7.7e-24 | 1.4e-29 | 156.0 | 142 | (2, 146) | 159 | (453, 605) | 605 | Lysozyme | Lysozyme | | uniclust | UniRef100\_H0EY40 | 99.8 | 8.1e-24 | 1.5e-29 | 143.9 | 135 | (3, 140) | 159 | (135, 278) | 280 | lysozyme | lysozyme | | uniclust | UniRef100\_A0A6B8LZY5 | 99.8 | 8.4e-24 | 1.6e-29 | 128.0 | 116 | (31, 151) | 159 | (1, 120) | 128 | Lysozyme | Lysozyme | | uniclust | UniRef100\_UPI00177A9A85 | 99.8 | 8.5e-24 | 1.6e-29 | 144.4 | 144 | (2, 151) | 159 | (141, 296) | 297 | lysozyme | lysozyme | | uniclust | UniRef100\_UPI001FDFC480 | 99.8 | 1e-23 | 1.9e-29 | 153.0 | 144 | (3, 149) | 159 | (350, 510) | 514 | peptidoglycan DD-metalloendopeptidase family protein | peptidoglycan DD-metalloendopeptidase family protein | | uniclust | UniRef100\_A0A5N6J8L9 | 99.8 | 1.1e-23 | 2e-29 | 139.4 | 140 | (3, 144) | 159 | (77, 231) | 232 | lysozyme | lysozyme | | uniclust | UniRef100\_A0A0D8TUV2 | 99.8 | 1.1e-23 | 2e-29 | 138.5 | 134 | (13, 149) | 159 | (32, 165) | 187 | Lysozyme | Lysozyme | | uniclust | UniRef100\_A0A0C4K550 | 99.8 | 1.1e-23 | 2.1e-29 | 144.3 | 150 | (1, 151) | 159 | (1, 155) | 223 | Lysozyme | Lysozyme | | uniclust | UniRef100\_A0A066T2U5 | 99.8 | 1.3e-23 | 2.4e-29 | 142.1 | 138 | (9, 149) | 159 | (37, 185) | 210 | Lysozyme | Lysozyme | | uniclust | UniRef100\_A0A2X1B257 | 99.8 | 1.3e-23 | 2.4e-29 | 144.2 | 134 | (10, 147) | 159 | (2, 135) | 262 | Phage-related lysozyme (Muraminidase) | Phage-related lysozyme (Muraminidase) | | uniclust | UniRef100\_A0A252EDH3 | 99.8 | 1.4e-23 | 2.6e-29 | 130.1 | 100 | (7, 109) | 159 | (9, 113) | 121 | Lysozyme (Fragment) | Lysozyme (Fragment) | | uniclust | UniRef100\_A0A1G4WUT2 | 99.8 | 1.4e-23 | 2.6e-29 | 149.7 | 143 | (2, 146) | 159 | (202, 345) | 433 | Lysozyme | Lysozyme | | uniclust | UniRef100\_A0A2V2CCV5 | 99.8 | 1.4e-23 | 2.7e-29 | 155.5 | 148 | (3, 154) | 159 | (230, 386) | 477 | Lysozyme | Lysozyme | | uniclust | UniRef100\_A0A7W4A0H5 | 99.8 | 1.5e-23 | 2.7e-29 | 136.4 | 147 | (3, 149) | 159 | (35, 193) | 195 | Lysozyme | Lysozyme | | uniclust | UniRef100\_A0A1S8L4X7 | 99.8 | 1.5e-23 | 2.8e-29 | 150.1 | 144 | (3, 149) | 159 | (4, 154) | 450 | Lysozyme | Lysozyme | | uniclust | UniRef100\_A0A919S1J4 | 99.8 | 1.6e-23 | 2.9e-29 | 146.9 | 147 | (1, 149) | 159 | (214, 364) | 370 | Lysozyme | Lysozyme | | uniclust | UniRef100\_A0A1H1WLZ3 | 99.8 | 1.7e-23 | 3.2e-29 | 147.7 | 148 | (2, 152) | 159 | (231, 388) | 395 | Lysozyme | Lysozyme | | uniclust | UniRef100\_A0A447J4W7 | 99.8 | 1.7e-23 | 3.2e-29 | 127.2 | 100 | (50, 149) | 159 | (3, 102) | 109 | Lysozyme | Lysozyme | | uniclust | UniRef100\_A0A1Y3P0S9 | 99.8 | 2e-23 | 3.7e-29 | 135.3 | 132 | (2, 145) | 159 | (59, 199) | 200 | lysozyme | lysozyme | | uniclust | UniRef100\_A0A976QVJ7 | 99.8 | 2e-23 | 3.8e-29 | 131.6 | 139 | (2, 146) | 159 | (21, 159) | 166 | Endolysin | Endolysin | | uniclust | UniRef100\_A0A952LVR1 | 99.8 | 2.1e-23 | 3.8e-29 | 141.0 | 145 | (2, 150) | 159 | (114, 266) | 272 | Lysozyme | Lysozyme | | uniclust | UniRef100\_A0A094EPJ7 | 99.8 | 2.1e-23 | 3.8e-29 | 147.6 | 138 | (5, 145) | 159 | (246, 392) | 400 | lysozyme | lysozyme | | uniclust | UniRef100\_A0A0Q5GY79 | 99.8 | 2e-23 | 3.8e-29 | 150.4 | 146 | (1, 150) | 159 | (3, 165) | 350 | Peptidoglycan binding-like domain-containing protein | Peptidoglycan binding-like domain-containing protein | | uniclust | UniRef100\_A0A430BCR4 | 99.8 | 2.1e-23 | 3.9e-29 | 144.4 | 138 | (8, 148) | 159 | (131, 269) | 270 | Lysozyme | Lysozyme | | pdb70 | 6ET6\_A | 99.9 | 7.8e-34 | 5.8e-38 | 190.1 | 141 | (3, 146) | 159 | (52, 196) | 196 | Aminotransferase (E.C.2.6.1.-) | 6ET6\_A Aminotransferase (E.C.2.6.1.-) muramidase, Lysozyme, Acinetobacter baumannii, ANTIMICROBIAL | | pdb70 | 3HDF\_A | 99.9 | 1.2e-33 | 9.2e-38 | 180.5 | 138 | (7, 147) | 159 | (2, 139) | 140 | Lysozyme (E.C.3.2.1.17) | 3HDF\_A Lysozyme (E.C.3.2.1.17) lysozyme-like, Antimicrobial, Bacteriolytic enzyme, Glycosidase HET: NO3, GOL | | pdb70 | 6H9D\_A | 99.9 | 5.7e-33 | 4.2e-37 | 179.1 | 140 | (2, 145) | 159 | (5, 148) | 149 | Lysozyme (E.C.3.2.1.17) | 6H9D\_A Lysozyme (E.C.3.2.1.17) lysozyme, peptidoglycan hydrolase, cell morphogenesis HET: GOL | | pdb70 | 1XJU\_A | 99.9 | 2.4e-32 | 1.8e-36 | 178.6 | 145 | (2, 150) | 159 | (1, 157) | 163 | Lysozyme (E.C.3.2.1.17) | 1XJU\_A Lysozyme (E.C.3.2.1.17) secreted inactive conformation, HYDROLASE HET: SO4 | | pdb70 | 3HDE\_B | 99.9 | 1.1e-31 | 8.4e-36 | 175.7 | 134 | (11, 147) | 159 | (31, 164) | 165 | Lysozyme (E.C.3.2.1.17) | 3HDE\_B Lysozyme (E.C.3.2.1.17) lysozyme-like, Antimicrobial, Bacteriolytic enzyme, Glycosidase | | pdb70 | 3HDE\_D | 99.9 | 1.1e-31 | 8.4e-36 | 175.7 | 134 | (11, 147) | 159 | (31, 164) | 165 | Lysozyme (E.C.3.2.1.17) | 3HDE\_D Lysozyme (E.C.3.2.1.17) lysozyme-like, Antimicrobial, Bacteriolytic enzyme, Glycosidase | | pdb70 | 1XJT\_A | 99.9 | 1.3e-31 | 9.3e-36 | 179.0 | 144 | (2, 149) | 159 | (29, 184) | 191 | Lysozyme (E.C.3.2.1.17) | 1XJT\_A Lysozyme (E.C.3.2.1.17) open conformation, HYDROLASE HET: CIT | | pdb70 | 2ANX\_A | 99.9 | 4.4e-31 | 3.3e-35 | 169.5 | 141 | (1, 145) | 159 | (2, 146) | 146 | Lysozyme (E.C.3.2.1.17) | 2ANX\_A Lysozyme (E.C.3.2.1.17) phage lysozyme, Sm-SAD, atomic resolution HET: SO4 | | pdb70 | 4E97\_B | 99.9 | 1.2e-29 | 8.7e-34 | 169.1 | 139 | (2, 150) | 159 | (21, 183) | 187 | Lysozyme (E.C.3.2.1.17) | 4E97\_B Lysozyme (E.C.3.2.1.17) Hydrolase, Alkylation with 2-mercaptoethanol HET: SO4, BME | | pdb70 | 6K69\_B | 99.9 | 5.9e-29 | 4.4e-33 | 167.8 | 139 | (2, 150) | 159 | (18, 202) | 206 | Protein A, 3LRH intrabody | 6K69\_B Protein A, 3LRH intrabody antibody, protein design, STRUCTURAL PROTEIN | | pdb70 | 5JDT\_A | 99.9 | 7.1e-29 | 5.3e-33 | 162.1 | 135 | (6, 150) | 159 | (2, 160) | 164 | Endolysin (E.C.3.2.1.17) | 5JDT\_A Endolysin (E.C.3.2.1.17) NITROXIDE, SPIN LABEL, T4 LYSOZYME HET: BME, MTN | | pdb70 | 4PK0\_A | 99.9 | 6.4e-28 | 4.7e-32 | 158.5 | 137 | (6, 152) | 159 | (2, 162) | 171 | TEICOPLANIN-A2-2, LYSOZYME (E.C.3.2.1.17) | 4PK0\_A TEICOPLANIN-A2-2, LYSOZYME (E.C.3.2.1.17) site-selective catalyst, carrier protein approach HET: GHP, 3FG, 3MY, GCS, DAL, T55, NAG, OMY, MAN, MHS | | pdb70 | 4YX7\_C | 99.9 | 6.8e-28 | 5e-32 | 161.6 | 139 | (2, 150) | 159 | (31, 193) | 197 | Surface presentation of antigens protein | 4YX7\_C Surface presentation of antigens protein Type III Secretion System, PROTEIN | | pdb70 | 5JEA\_K | 99.9 | 4.4e-27 | 3.2e-31 | 164.1 | 138 | (3, 150) | 159 | (114, 275) | 279 | Exosome complex component RRP45, Exosome | 5JEA\_K Exosome complex component RRP45, Exosome exosome, Ski7, nuclease, RNA degradation HET: MPD | | pdb70 | 4EPI\_A | 99.9 | 6.7e-27 | 4.9e-31 | 165.7 | 137 | (3, 149) | 159 | (165, 325) | 330 | Pesticin, Lysozyme (E.C.3.2.1.17) | 4EPI\_A Pesticin, Lysozyme (E.C.3.2.1.17) bacterial toxin, TOXIN, HYDROLASE | | pdb70 | 5EWX\_B | 99.9 | 7.2e-27 | 5.3e-31 | 159.1 | 136 | (6, 151) | 159 | (2, 224) | 227 | Endolysin (E.C.3.2.1.17),Immunoglobulin G-binding protein A | 5EWX\_B Endolysin (E.C.3.2.1.17),Immunoglobulin G-binding protein A Fusion, EY-CBS, alpha helix, cross-linker HET: EYC | | pdb70 | 4XSJ\_A | 99.9 | 9.9e-27 | 7.4e-31 | 162.2 | 136 | (6, 151) | 159 | (2, 161) | 262 | lysozyme, Calcium uniporter protein, mitochondrial | 4XSJ\_A lysozyme, Calcium uniporter protein, mitochondrial Membrane protein, Calcium channel, Mitochondria HET: SO4 | | pdb70 | 4TN3\_B | 99.9 | 1.3e-26 | 9.5e-31 | 170.2 | 139 | (3, 151) | 159 | (235, 397) | 400 | Trim5alpha | 4TN3\_B Trim5alpha Trim protein Coiled-coil scaffold retroviral | | pdb70 | 5BZ6\_A | 99.8 | 1.7e-26 | 1.2e-30 | 161.0 | 136 | (6, 151) | 159 | (2, 161) | 262 | Lysozyme,Calcium uniporter protein, mitochondrial (E.C.3.2.1.17) | 5BZ6\_A Lysozyme,Calcium uniporter protein, mitochondrial (E.C.3.2.1.17) Membrane protein, Calcium channel, Mitochondria HET: SO4 | | pdb70 | 5B2G\_G | 99.8 | 1.9e-26 | 1.4e-30 | 167.5 | 140 | (2, 151) | 159 | (4, 167) | 364 | Endolysin,Claudin-4 (E.C.3.2.1.17), Heat-labile enterotoxin B | 5B2G\_G Endolysin,Claudin-4 (E.C.3.2.1.17), Heat-labile enterotoxin B Membrane protein, Complex, Cell-free protein HET: MSE | | pdb70 | 4TN3\_A | 99.8 | 2.1e-26 | 1.6e-30 | 169.0 | 139 | (3, 151) | 159 | (235, 397) | 400 | Trim5alpha | 4TN3\_A Trim5alpha Trim protein Coiled-coil scaffold retroviral | | pdb70 | 4HTT\_B | 99.8 | 3.2e-26 | 2.4e-30 | 169.0 | 140 | (3, 152) | 159 | (243, 406) | 418 | Sec-independent protein translocase protein TatC | 4HTT\_B Sec-independent protein translocase protein TatC Twin arginine translocase receptor, Membrane | | pdb70 | 2QB0\_D | 99.8 | 4.4e-26 | 3.3e-30 | 157.2 | 136 | (4, 149) | 159 | (79, 238) | 241 | E80-TELSAM domain, Lysozyme (E.C.3.2.1.17) | 2QB0\_D E80-TELSAM domain, Lysozyme (E.C.3.2.1.17) Helical polymer, HYDROLASE REGULATOR | | pdb70 | 5B2G\_C | 99.8 | 5.3e-26 | 4e-30 | 165.1 | 141 | (2, 152) | 159 | (4, 168) | 364 | Endolysin,Claudin-4 (E.C.3.2.1.17), Heat-labile enterotoxin B | 5B2G\_C Endolysin,Claudin-4 (E.C.3.2.1.17), Heat-labile enterotoxin B Membrane protein, Complex, Cell-free protein HET: MSE | | pdb70 | 5YQR\_A | 99.8 | 1.2e-25 | 8.8e-30 | 157.8 | 134 | (6, 149) | 159 | (3, 160) | 276 | T4 lysozyme/Membrane-anchored lipid-binding protein LAM6 | 5YQR\_A T4 lysozyme/Membrane-anchored lipid-binding protein LAM6 ligand binding domain, sterol, lipid HET: 2PE | | pdb70 | 5VBA\_B | 99.8 | 1.4e-25 | 1e-29 | 166.6 | 142 | (2, 153) | 159 | (17, 182) | 437 | ESX-3 secretion-associated protein EspG3 | 5VBA\_B ESX-3 secretion-associated protein EspG3 ESX-1, type VII secretion system HET: CL | | pdb70 | 5VBA\_A | 99.8 | 1.6e-25 | 1.2e-29 | 166.1 | 141 | (2, 152) | 159 | (17, 181) | 437 | ESX-3 secretion-associated protein EspG3 | 5VBA\_A ESX-3 secretion-associated protein EspG3 ESX-1, type VII secretion system HET: CL | | pdb70 | 6A9E\_B | 99.8 | 5.4e-25 | 4e-29 | 161.2 | 135 | (6, 150) | 159 | (2, 160) | 389 | Endolysin,Autophagy-related protein 2 (E.C.3.2.1.17) | 6A9E\_B Endolysin,Autophagy-related protein 2 (E.C.3.2.1.17) lipid transport HET: MSE | | pdb70 | 6A9J\_A | 99.8 | 5.4e-25 | 4e-29 | 161.2 | 135 | (6, 150) | 159 | (2, 160) | 389 | Endolysin,Autophagy-related protein 2 (E.C.3.2.1.17) | 6A9J\_A Endolysin,Autophagy-related protein 2 (E.C.3.2.1.17) LIPID TRANSPORT HET: 9TL | | pdb70 | 4WTV\_B | 99.8 | 6.7e-25 | 5e-29 | 165.2 | 138 | (3, 150) | 159 | (74, 235) | 514 | Chimera protein of Phosphatidylinositol 4-kinase | 4WTV\_B Chimera protein of Phosphatidylinositol 4-kinase lipid kinase, phosphatidyl inositol, transferase HET: ATP | | pdb70 | 4IAP\_A | 99.8 | 3.3e-24 | 2.5e-28 | 148.9 | 137 | (2, 148) | 159 | (16, 176) | 260 | Lysozyme, Oxysterol-binding protein homolog 3 | 4IAP\_A Lysozyme, Oxysterol-binding protein homolog 3 PH domain, beta sandwitch, targeting HET: SO4 | | pdb70 | 5I0N\_A | 99.8 | 4.2e-24 | 3.1e-28 | 161.8 | 139 | (2, 150) | 159 | (101, 263) | 556 | Phosphatidylinositol 4-kinase type 2-alpha,Lysozyme,Phosphatidylinositol 4-kinase | 5I0N\_A Phosphatidylinositol 4-kinase type 2-alpha,Lysozyme,Phosphatidylinositol 4-kinase kinase, calcium, Transferase HET: ATP | | pdb70 | 5ZBQ\_A | 99.8 | 8.3e-24 | 6.3e-28 | 158.4 | 138 | (5, 150) | 159 | (365, 524) | 525 | Neuropeptide Y Y1 receptor in | 5ZBQ\_A Neuropeptide Y Y1 receptor in G Protein-Coupled Receptor, Receptor Inhibitor HET: 9AO | | pdb70 | 6A73\_B | 99.8 | 9.2e-24 | 6.8e-28 | 149.9 | 136 | (6, 151) | 159 | (136, 295) | 304 | COP9 signalosome complex subunit 2,Endolysin | 6A73\_B COP9 signalosome complex subunit 2,Endolysin subunit2 of COP9 Signalosome (CSN). HET: IHP, SO4 | | pdb70 | 5NDD\_A | 99.8 | 1.4e-23 | 1e-27 | 160.6 | 121 | (3, 126) | 159 | (21, 165) | 619 | Lysozyme,Proteinase-activated receptor 2,Soluble cytochrome b562,Proteinase-activated | 5NDD\_A Lysozyme,Proteinase-activated receptor 2,Soluble cytochrome b562,Proteinase-activated MEMBRANE PROTEIN, GPCR, 7TM HET: 8TZ | | pdb70 | 4EVX\_A | 99.8 | 2.4e-23 | 1.8e-27 | 126.3 | 101 | (2, 110) | 159 | (6, 106) | 106 | Putative phage endolysin | 4EVX\_A Putative phage endolysin Structural Genomics, PSI-Biology, Program for | | pdb70 | 5IV5\_YA | 99.8 | 2.5e-23 | 1.9e-27 | 158.6 | 137 | (3, 149) | 159 | (172, 337) | 575 | Baseplate wedge protein gp6, Baseplate | 5IV5\_YA Baseplate wedge protein gp6, Baseplate T4, baseplate-tail tube complex, pre-attachment | | pdb70 | 5IV5\_YB | 99.8 | 2.5e-23 | 1.9e-27 | 158.6 | 137 | (3, 149) | 159 | (172, 337) | 575 | Baseplate wedge protein gp6, Baseplate | 5IV5\_YB Baseplate wedge protein gp6, Baseplate T4, baseplate-tail tube complex, pre-attachment | | pdb70 | 5IV5\_YC | 99.8 | 2.5e-23 | 1.9e-27 | 158.6 | 137 | (3, 149) | 159 | (172, 337) | 575 | Baseplate wedge protein gp6, Baseplate | 5IV5\_YC Baseplate wedge protein gp6, Baseplate T4, baseplate-tail tube complex, pre-attachment | | pdb70 | 6MXT\_A | 99.8 | 2.5e-23 | 1.9e-27 | 154.1 | 118 | (6, 126) | 159 | (2, 143) | 472 | Fusion protein of Lysozyme and | 6MXT\_A Fusion protein of Lysozyme and G protein-coupled receptor, adrenergic receptor HET: OLA, K5Y, OLC, P33, HTO, YCM | | pdb70 | 6AJH\_A | 99.8 | 2.9e-23 | 2.1e-27 | 166.2 | 135 | (6, 150) | 159 | (764, 922) | 943 | Drug exporters of the RND | 6AJH\_A Drug exporters of the RND membrane protein, transporter, RND family HET: MHA, 9ZF, L6T | | pdb70 | 2Z6B\_A | 99.7 | 4.3e-23 | 3.2e-27 | 157.2 | 134 | (3, 146) | 159 | (172, 334) | 584 | Tail-associated lysozyme (E.C.3.2.1.17), Baseplate structural | 2Z6B\_A Tail-associated lysozyme (E.C.3.2.1.17), Baseplate structural Protein containing metal complexes, Antimicrobial | | pdb70 | 1WTH\_A | 99.7 | 5.8e-23 | 4.3e-27 | 156.6 | 135 | (3, 147) | 159 | (172, 335) | 584 | Tail-associated lysozyme (E.C.3.2.1.17)/Baseplate structural protein | 1WTH\_A Tail-associated lysozyme (E.C.3.2.1.17)/Baseplate structural protein Triple-stranded beta-helix, OB fold, pseudohexamer | | pdb70 | 6QAJ\_B | 99.7 | 6.9e-23 | 5.2e-27 | 155.0 | 121 | (1, 124) | 159 | (21, 163) | 544 | Endolysin,Transcription intermediary factor 1-beta (E.C.3.2.1.17,2.3.2.27) | 6QAJ\_B Endolysin,Transcription intermediary factor 1-beta (E.C.3.2.1.17,2.3.2.27) Transcriptional repressor epigenetic silencing | | pdb70 | 6AJG\_A | 99.7 | 7.3e-23 | 5.5e-27 | 163.8 | 134 | (6, 149) | 159 | (764, 921) | 943 | Drug exporters of the RND | 6AJG\_A Drug exporters of the RND membrane protein, transporter, RND family HET: 3RX, LMT, MHA | | pdb70 | 6AJF\_A | 99.7 | 9.3e-23 | 6.9e-27 | 163.2 | 135 | (6, 150) | 159 | (764, 922) | 943 | Drug exporters of the RND | 6AJF\_A Drug exporters of the RND membrane protein, transporter, RND family HET: MHA, L6T | | pdb70 | 6BG5\_A | 99.7 | 1.2e-22 | 9.1e-27 | 148.1 | 128 | (2, 132) | 159 | (20, 171) | 384 | Endolysin, DCN1-like protein 1 chimera | 6BG5\_A Endolysin, DCN1-like protein 1 chimera E3 Ligase, HYDROLASE, LIGASE-INHIBITOR complex HET: DQD | | pdb70 | 6BG3\_A | 99.7 | 1.3e-22 | 9.3e-27 | 148.0 | 128 | (2, 132) | 159 | (20, 171) | 384 | Endolysin, DCN1-like protein 1 chimera | 6BG3\_A Endolysin, DCN1-like protein 1 chimera E3 Ligase, HYDROLASE, LIGASE-INHIBITOR complex HET: DOJ | | pdb70 | 6P5V\_A | 99.7 | 1.3e-22 | 9.3e-27 | 148.0 | 128 | (2, 132) | 159 | (20, 171) | 384 | Lysozyme,DCN1-like protein 1 fusion (E.C.3.2.1.17) | 6P5V\_A Lysozyme,DCN1-like protein 1 fusion (E.C.3.2.1.17) E3 Ligase, LIGASE HET: MPD, O37 | | pdb70 | 6IIH\_A | 99.7 | 1.5e-22 | 1.1e-26 | 151.7 | 121 | (3, 126) | 159 | (7, 151) | 493 | Probable septum site-determining protein MinC | 6IIH\_A Probable septum site-determining protein MinC calcium binding protein mitochondrial | | pdb70 | 1K28\_A | 99.7 | 1.7e-22 | 1.3e-26 | 153.9 | 138 | (3, 150) | 159 | (172, 338) | 584 | TAIL-ASSOCIATED LYSOZYME, BASEPLATE STRUCTURAL PROTEIN | 1K28\_A TAIL-ASSOCIATED LYSOZYME, BASEPLATE STRUCTURAL PROTEIN Triple-stranded beta-helix, OB fold, pseudohexamer | | pdb70 | 4LDE\_A | 99.7 | 2.3e-22 | 1.7e-26 | 148.8 | 118 | (1, 121) | 159 | (11, 150) | 469 | Lysozyme, Beta-2 adrenergic receptor (E.C.3.2.1.17) | 4LDE\_A Lysozyme, Beta-2 adrenergic receptor (E.C.3.2.1.17) G protein coupled receptor, MEMBRANE HET: P0G, 1WV | | pdb70 | 4QKX\_A | 99.7 | 2.9e-22 | 2.2e-26 | 148.2 | 119 | (1, 122) | 159 | (11, 151) | 469 | Beta-2 adrenergic receptor (E.C.3.2.1.17), R9 | 4QKX\_A Beta-2 adrenergic receptor (E.C.3.2.1.17), R9 7-transmembrane helices, Signal transduction, G HET: 35V | | pdb70 | 4ARM\_A | 99.7 | 8.3e-22 | 6.1e-26 | 140.6 | 140 | (5, 151) | 159 | (168, 355) | 359 | PESTICIN | 4ARM\_A PESTICIN TOXIN, MURAMIDASE | | pdb70 | 5W0P\_C | 99.7 | 9.5e-22 | 7.1e-26 | 156.3 | 130 | (7, 139) | 159 | (2, 155) | 906 | T4 lysozyme-human rhodopsin-mouse visual arrestin | 5W0P\_C T4 lysozyme-human rhodopsin-mouse visual arrestin rhodopsin, GPCR, arrestin, GRK, phosphorylation HET: TPO, SEP, NAG | | pdb70 | 4ARM\_B | 99.7 | 1e-21 | 7.5e-26 | 140.2 | 142 | (4, 152) | 159 | (167, 356) | 359 | PESTICIN | 4ARM\_B PESTICIN TOXIN, MURAMIDASE | | pdb70 | 6FW2\_A | 99.7 | 1.2e-21 | 8.6e-26 | 145.9 | 136 | (4, 149) | 159 | (81, 240) | 459 | Mitochondrial amidoxime-reducing component 1,Endolysin,Mitochondrial amidoxime-reducing | 6FW2\_A Mitochondrial amidoxime-reducing component 1,Endolysin,Mitochondrial amidoxime-reducing mARC, MOSC, molybdenum cofactor, Moco HET: B3P, MOO, MTE | | pdb70 | 4XES\_A | 99.6 | 9.8e-21 | 7.4e-25 | 141.7 | 134 | (9, 150) | 159 | (381, 536) | 541 | neurotensin receptor subtype 1, lysozyme | 4XES\_A neurotensin receptor subtype 1, lysozyme membrane protein, G protein-coupled receptor HET: PEG, GOL, CIT, EPE | | pdb70 | 5I14\_A | 99.5 | 6e-19 | 4.5e-23 | 108.9 | 99 | (44, 149) | 159 | (10, 116) | 121 | mutated and truncated T4 lysozyme | 5I14\_A mutated and truncated T4 lysozyme T4 lysozyme, hydrolase | | pdb70 | 4AQN\_A | 99.4 | 2.5e-17 | 1.8e-21 | 117.2 | 132 | (12, 150) | 159 | (175, 354) | 357 | PESTICIN | 4AQN\_A PESTICIN TOXIN, BACTERIOCIN, COLICIN, THREE DOMAINS | | pdb70 | 2O7A\_A | 99.4 | 4e-17 | 2.9e-21 | 100.9 | 93 | (50, 150) | 159 | (1, 101) | 124 | Lysozyme (E.C.3.2.1.17) | 2O7A\_A Lysozyme (E.C.3.2.1.17) Protein folding, protein stability, lysozyme | | pdb70 | 4N9N\_A | 99.3 | 1.7e-16 | 1.3e-20 | 117.2 | 139 | (7, 153) | 159 | (125, 285) | 438 | Sterol uptake control protein 2 | 4N9N\_A Sterol uptake control protein 2 fungal nuclear receptor-like transcription factor HET: MSE | | pdb70 | 6D9M\_A | 98.9 | 1.4e-13 | 1e-17 | 97.2 | 111 | (6, 119) | 159 | (2, 134) | 337 | Endolysin,Response receiver sensor diguanylate cyclase | 6D9M\_A Endolysin,Response receiver sensor diguanylate cyclase Cyclic dinucleotide, GGDEF, HYDROLASE HET: GMP, GTP | | pdb70 | 4Z9G\_A | 98.3 | 2.1e-10 | 1.5e-14 | 81.8 | 114 | (9, 125) | 159 | (122, 259) | 443 | Human Corticotropin-Releasing Factor Receptor Type | 4Z9G\_A Human Corticotropin-Releasing Factor Receptor Type 7TM, GPCR, FAMILY B, SIGNALING HET: 1Q5, SO4, OLA | | pdb70 | 5EE7\_A | 98.3 | 2.9e-10 | 2.1e-14 | 81.2 | 113 | (10, 125) | 159 | (125, 261) | 452 | Glucagon receptor | 5EE7\_A Glucagon receptor GPCR, signaling protein, 7TM HET: PE5, OLA, TLA, 5MV | | pdb70 | 5VEW\_A | 97.9 | 5e-09 | 3.7e-13 | 75.1 | 113 | (10, 125) | 159 | (129, 265) | 455 | Glucagon-like peptide 1 receptor,Endolysin chimera | 5VEW\_A Glucagon-like peptide 1 receptor,Endolysin chimera GPCR, class B, 7TM domain HET: CSD, OLC, OLA, 97Y | | pdb70 | 5VEW\_B | 97.9 | 5e-09 | 3.7e-13 | 75.1 | 113 | (10, 125) | 159 | (129, 265) | 455 | Glucagon-like peptide 1 receptor,Endolysin chimera | 5VEW\_B Glucagon-like peptide 1 receptor,Endolysin chimera GPCR, class B, 7TM domain HET: CSD, OLA, OLC, 97Y | | pdb70 | 5YQZ\_R | 97.0 | 1.1e-06 | 8.2e-11 | 65.2 | 112 | (11, 125) | 159 | (240, 375) | 575 | Glucagon receptor,Endolysin (E.C.3.2.1.17), Glucagon analogue | 5YQZ\_R Glucagon receptor,Endolysin (E.C.3.2.1.17), Glucagon analogue Human GCGR receptor, Class B HET: OLC, NAG | | pdb70 | 5XEZ\_A | 96.8 | 4.1e-06 | 3e-10 | 62.4 | 110 | (12, 124) | 159 | (240, 373) | 574 | Glucagon receptor,Endolysin (E.C.3.2.1.17), Antibody, mAb1 | 5XEZ\_A Glucagon receptor,Endolysin (E.C.3.2.1.17), Antibody, mAb1 Human GCGR receptor, Class B HET: 97V, NAG | | pdb70 | 5XF1\_A | 96.8 | 4.1e-06 | 3e-10 | 62.4 | 110 | (12, 124) | 159 | (240, 373) | 574 | Glucagon receptor,Endolysin (E.C.3.2.1.17), Antibody mAb1 | 5XF1\_A Glucagon receptor,Endolysin (E.C.3.2.1.17), Antibody mAb1 Human GCGR receptor, Class B HET: NAG, 97V | |
| Top keywords  (threshold 1.00e-03 (evalue)) | **Lysozyme, Endolysin, E, C, Fragment, hydrolase, receptor, glycoside, Membrane, complex** |
| Output files | ../../similar\_sequences/64\_FANPEZAQ\_CDS\_0064\_merged.svg ../../similar\_sequences/64\_FANPEZAQ\_CDS\_0064\_pdb70.a3m ../../similar\_sequences/64\_FANPEZAQ\_CDS\_0064\_pdb70.hhr ../../similar\_sequences/64\_FANPEZAQ\_CDS\_0064\_uniclust.a3m ../../similar\_sequences/64\_FANPEZAQ\_CDS\_0064\_uniclust.hhr |

#### Structure prediction (AlphaFold)2

|  |  |
| --- | --- |
| Stats | xml version="1.0" encoding="utf-8" standalone="no"?       2024-09-02T21:09:59.129907 image/svg+xml   Matplotlib v3.7.2, https://matplotlib.org/ |
| Predicted structure | **NGL Viewer Controls:**  - Center: *Left-Click* - Rotate: *Left-Click + Drag* - Translate: *Right-Click + Drag* - Zoom: *Shift + Left-Click + Drag* |
| Output files | ../../predicted\_structures/64\_FANPEZAQ\_CDS\_0064/features.pkl ../../predicted\_structures/64\_FANPEZAQ\_CDS\_0064/ranked\_0.pdb ../../predicted\_structures/64\_FANPEZAQ\_CDS\_0064/ranked\_0\_plots.svg ../../predicted\_structures/64\_FANPEZAQ\_CDS\_0064/result\_model\_1\_ptm\_pred\_0.pkl |

#### Structure similarity search results (Foldseek)3

|  |  |
| --- | --- |
| Structure databases searched | Pdb, Afdb-proteome, Afdb-uniprot50 |
| Results, scheme(s)  (Top layers only, threshold 1.00e-02 (evalue)) | xml version="1.0" encoding="utf-8" standalone="no"?       2024-09-02T21:11:33.967636 image/svg+xml   Matplotlib v3.7.2, https://matplotlib.org/ |
| Results, table  (threshold 1.00e-02 (evalue)) | | db | id | prob | evalue | bits | fident | alnlen | mismatch | gapopen | qstart | qend | tstart | tend | name | description | | --- | --- | --- | --- | --- | --- | --- | --- | --- | --- | --- | --- | --- | --- | --- | | pdb | 6ET6\_A | 1.0 | 1.504e-12 | 515 | 0.486 | 150 | 70 | 3 | 1 | 146 | 3 | 149 | Lysozyme | Lysozyme | | pdb | 8APP\_D | 1.0 | 1.633e-11 | 468 | 0.443 | 151 | 73 | 6 | 1 | 143 | 38 | 185 | Endolysin | Endolysin | | pdb | 3HDE\_A | 1.0 | 1.868e-10 | 444 | 0.351 | 131 | 82 | 2 | 14 | 144 | 34 | 161 | Lysozyme | Lysozyme | | pdb | 7M5I\_B | 1.0 | 8.002e-11 | 443 | 0.407 | 152 | 80 | 5 | 1 | 145 | 5 | 153 | Endolysin | Endolysin | | pdb | 8HP8\_A | 1.0 | 1.868e-10 | 436 | 0.329 | 158 | 104 | 2 | 1 | 157 | 3 | 159 | Endolysin mtEC340M | Endolysin mtEC340M | | pdb | 6H9D\_B | 1.0 | 2.309e-10 | 435 | 0.393 | 150 | 83 | 5 | 1 | 146 | 3 | 148 | Lysozyme | Lysozyme | | pdb | 2ANV\_B | 1.0 | 4.136e-10 | 425 | 0.429 | 149 | 75 | 6 | 1 | 144 | 2 | 145 | Lysozyme | Lysozyme | | pdb | 4ZPU\_A | 1.0 | 7.811e-10 | 408 | 0.319 | 144 | 90 | 3 | 6 | 144 | 21 | 161 | Lysozyme RrrD | Lysozyme RrrD | | pdb | 8B2S\_B | 1.0 | 2.027e-09 | 372 | 0.388 | 152 | 81 | 4 | 1 | 143 | 79 | 227 | GH24 family muramidase | GH24 family muramidase | | pdb | 1XJT\_A | 1.0 | 1.931e-07 | 310 | 0.23 | 156 | 104 | 5 | 1 | 144 | 20 | 171 | Lysozyme | Lysozyme | | pdb | 3HDE\_D | 1.0 | 1.264e-07 | 301 | 0.282 | 145 | 89 | 4 | 6 | 145 | 21 | 155 | Lysozyme | Lysozyme | | pdb | 3HDF\_B | 1.0 | 9.743e-06 | 215 | 0.338 | 133 | 79 | 4 | 15 | 143 | 4 | 131 | Lysozyme | Lysozyme | | pdb | 4EVX\_A | 1.0 | 0.0007918 | 167 | 0.25 | 92 | 66 | 2 | 2 | 93 | 1 | 89 | Putative phage endolysin | Putative phage endolysin | | pdb | 7RGR\_A | 1.0 | 0.0003976 | 164 | 0.186 | 177 | 108 | 9 | 1 | 153 | 1 | 165 | Artificial protein L056 | Artificial protein L056 | | pdb | 201L\_B | 1.0 | 0.0005465 | 159 | 0.203 | 172 | 104 | 9 | 6 | 153 | 2 | 164 | T4 LYSOZYME | T4 LYSOZYME | | pdb | 1XJU\_A | 1.0 | 0.0002893 | 158 | 0.177 | 163 | 100 | 6 | 2 | 144 | 1 | 149 | Lysozyme | Lysozyme | | pdb | 3FI5\_C | 1.0 | 0.0002105 | 158 | 0.205 | 170 | 99 | 9 | 6 | 150 | 2 | 160 | Lysozyme | Lysozyme | | pdb | 137L\_B | 1.0 | 0.0003217 | 157 | 0.207 | 159 | 96 | 9 | 6 | 140 | 2 | 154 | T4 LYSOZYME | T4 LYSOZYME | | pdb | 1L65\_A | 1.0 | 0.0005465 | 156 | 0.207 | 169 | 100 | 11 | 6 | 150 | 2 | 160 | LYSOZYME | LYSOZYME | | pdb | 7Z36\_A | 1.0 | 0.0005183 | 156 | 0.192 | 177 | 110 | 9 | 1 | 153 | 3 | 170 | Endolysin,Transcription intermediary factor 1-beta,Isoform 2 of Transcription intermediary factor 1-beta | Endolysin,Transcription intermediary factor 1-beta,Isoform 2 of Transcription intermediary factor 1-beta | | pdb | 147L\_A | 1.0 | 0.0003051 | 153 | 0.197 | 172 | 98 | 10 | 6 | 150 | 2 | 160 | T4 LYSOZYME | T4 LYSOZYME | | pdb | 2HUM\_B | 1.0 | 0.0003577 | 153 | 0.215 | 172 | 95 | 10 | 6 | 150 | 2 | 160 | Lysozyme | Lysozyme | | pdb | 3OE8\_A | 1.0 | 0.0003392 | 152 | 0.205 | 180 | 105 | 11 | 4 | 157 | 198 | 365 | C-X-C chemokine receptor type 4, Lysozyme Chimera | C-X-C chemokine receptor type 4, Lysozyme Chimera | | pdb | 3EML\_A | 1.0 | 0.0004915 | 152 | 0.201 | 179 | 103 | 10 | 4 | 155 | 198 | 363 | Human Adenosine A2A receptor/T4 lysozyme chimera | Human Adenosine A2A receptor/T4 lysozyme chimera | | pdb | 216L\_B | 1.0 | 0.0003392 | 150 | 0.204 | 166 | 104 | 9 | 6 | 146 | 2 | 164 | T4 LYSOZYME | T4 LYSOZYME | | pdb | 168L\_A | 1.0 | 0.0004915 | 150 | 0.205 | 170 | 100 | 8 | 6 | 150 | 2 | 161 | T4 LYSOZYME | T4 LYSOZYME | | pdb | 3SBA\_D | 1.0 | 0.00121 | 149 | 0.196 | 173 | 104 | 10 | 4 | 152 | 1 | 162 | Lysozyme | Lysozyme | | pdb | 5JGR\_A | 1.0 | 0.0004193 | 149 | 0.194 | 170 | 101 | 8 | 6 | 150 | 2 | 160 | Endolysin | Endolysin | | pdb | 5VBA\_A | 1.0 | 0.0007122 | 149 | 0.174 | 178 | 105 | 8 | 1 | 150 | 3 | 166 | Lysozyme, ESX-1 secretion-associated protein EspG1 chimera | Lysozyme, ESX-1 secretion-associated protein EspG1 chimera | | pdb | 5KI1\_A | 1.0 | 0.0004915 | 148 | 0.191 | 172 | 99 | 9 | 6 | 150 | 2 | 160 | Endolysin | Endolysin | | pdb | 201L\_A | 1.0 | 0.0003976 | 148 | 0.201 | 174 | 97 | 10 | 6 | 150 | 2 | 162 | T4 LYSOZYME | T4 LYSOZYME | | pdb | 8F11\_A | 1.0 | 0.0004421 | 148 | 0.194 | 170 | 101 | 8 | 6 | 150 | 2 | 160 | Endolysin | Endolysin | | pdb | 3FAD\_A | 1.0 | 0.0006075 | 148 | 0.208 | 173 | 100 | 10 | 6 | 153 | 2 | 162 | Lysozyme | Lysozyme | | pdb | 5JGN\_A | 1.0 | 0.0004915 | 148 | 0.194 | 170 | 101 | 8 | 6 | 150 | 2 | 160 | Endolysin | Endolysin | | pdb | 6U0C\_A | 1.0 | 0.0004661 | 148 | 0.203 | 172 | 97 | 10 | 6 | 150 | 2 | 160 | Endolysin | Endolysin | | pdb | 6V51\_A | 1.0 | 0.0005183 | 148 | 0.203 | 172 | 97 | 9 | 6 | 150 | 2 | 160 | Endolysin | Endolysin | | pdb | 4PJZ\_A | 1.0 | 0.0007918 | 148 | 0.209 | 177 | 99 | 11 | 6 | 155 | 2 | 164 | Lysozyme | Lysozyme | | pdb | 5VBA\_B | 1.0 | 0.0007122 | 148 | 0.191 | 178 | 104 | 10 | 1 | 151 | 6 | 170 | Lysozyme, ESX-1 secretion-associated protein EspG1 chimera | Lysozyme, ESX-1 secretion-associated protein EspG1 chimera | | pdb | 1QTB\_A | 1.0 | 0.00121 | 147 | 0.207 | 169 | 100 | 10 | 6 | 150 | 2 | 160 | LYSOZYME | LYSOZYME | | pdb | 169L\_B | 1.0 | 0.0007918 | 146 | 0.205 | 170 | 99 | 9 | 7 | 151 | 3 | 161 | T4 LYSOZYME | T4 LYSOZYME | | pdb | 1B6I\_A | 1.0 | 0.001088 | 146 | 0.191 | 167 | 105 | 8 | 6 | 150 | 2 | 160 | PROTEIN (LYSOZYME) | PROTEIN (LYSOZYME) | | pdb | 1L67\_A | 1.0 | 0.001032 | 146 | 0.203 | 162 | 95 | 9 | 6 | 143 | 2 | 153 | LYSOZYME | LYSOZYME | | pdb | 7XEA\_A | 1.0 | 0.0005762 | 146 | 0.2 | 170 | 100 | 9 | 6 | 150 | 2 | 160 | Endolysin | Endolysin | | pdb | 5JGU\_A | 1.0 | 0.0006075 | 146 | 0.194 | 170 | 101 | 8 | 6 | 150 | 2 | 160 | Endolysin | Endolysin | | pdb | 5JGX\_A | 1.0 | 0.0007122 | 146 | 0.196 | 173 | 102 | 9 | 6 | 153 | 2 | 162 | Endolysin | Endolysin | | pdb | 2NTG\_A | 1.0 | 0.0006075 | 146 | 0.194 | 170 | 101 | 8 | 6 | 150 | 2 | 160 | Lysozyme | Lysozyme | | pdb | 3SB6\_A | 1.0 | 0.00121 | 146 | 0.191 | 167 | 105 | 8 | 6 | 150 | 4 | 162 | Lysozyme | Lysozyme | | pdb | 197L\_A | 1.0 | 0.001147 | 145 | 0.167 | 167 | 109 | 8 | 6 | 150 | 2 | 160 | LYSOZYME | LYSOZYME | | pdb | 3F9L\_A | 1.0 | 0.0005465 | 145 | 0.205 | 170 | 99 | 9 | 6 | 150 | 2 | 160 | Lysozyme | Lysozyme | | pdb | 3CDO\_C | 1.0 | 0.0006075 | 145 | 0.2 | 170 | 100 | 9 | 6 | 150 | 2 | 160 | Lysozyme | Lysozyme | | pdb | 3F8V\_A | 1.0 | 0.0007918 | 145 | 0.202 | 173 | 101 | 10 | 6 | 153 | 2 | 162 | Lysozyme | Lysozyme | | pdb | 1G0P\_A | 1.0 | 0.001495 | 145 | 0.195 | 169 | 102 | 9 | 6 | 150 | 2 | 160 | PROTEIN (LYSOZYME) | PROTEIN (LYSOZYME) | | pdb | 1L18\_A | 1.0 | 0.000751 | 145 | 0.2 | 175 | 99 | 11 | 6 | 153 | 2 | 162 | T4 LYSOZYME | T4 LYSOZYME | | pdb | 1L21\_A | 1.0 | 0.001663 | 145 | 0.205 | 170 | 104 | 10 | 6 | 153 | 2 | 162 | T4 LYSOZYME | T4 LYSOZYME | | pdb | 1P36\_A | 1.0 | 0.001495 | 145 | 0.191 | 167 | 105 | 7 | 6 | 150 | 2 | 160 | LYSOZYME | LYSOZYME | | pdb | 4WTV\_A | 1.0 | 0.0007122 | 145 | 0.201 | 174 | 99 | 10 | 4 | 150 | 56 | 216 | Phosphatidylinositol 4-kinase type 2-beta,Endolysin,Phosphatidylinositol 4-kinase type 2-beta | Phosphatidylinositol 4-kinase type 2-beta,Endolysin,Phosphatidylinositol 4-kinase type 2-beta | | pdb | 260L\_A | 1.0 | 0.001088 | 144 | 0.196 | 163 | 102 | 8 | 6 | 146 | 2 | 157 | PROTEIN (LYSOZYME) | PROTEIN (LYSOZYME) | | pdb | 1EPY\_A | 1.0 | 0.0008349 | 144 | 0.201 | 159 | 97 | 9 | 6 | 140 | 2 | 154 | LYSOZYME | LYSOZYME | | pdb | 1T8F\_A | 1.0 | 0.0006075 | 144 | 0.205 | 170 | 99 | 8 | 6 | 150 | 2 | 160 | Lysozyme | Lysozyme | | pdb | 3SBB\_C | 1.0 | 0.0007918 | 144 | 0.195 | 174 | 102 | 10 | 4 | 151 | 1 | 162 | Lysozyme | Lysozyme | | pdb | 2A4T\_A | 1.0 | 0.0006755 | 144 | 0.194 | 170 | 101 | 8 | 6 | 150 | 2 | 160 | Lysozyme | Lysozyme | | pdb | 5KGR\_A | 1.0 | 0.0007918 | 144 | 0.196 | 173 | 102 | 9 | 6 | 153 | 2 | 162 | Endolysin | Endolysin | | pdb | 2Q9D\_A | 1.0 | 0.0006406 | 144 | 0.2 | 170 | 100 | 9 | 6 | 150 | 2 | 160 | Lysozyme | Lysozyme | | pdb | 3SB8\_A | 1.0 | 0.001577 | 144 | 0.192 | 171 | 104 | 9 | 4 | 150 | 3 | 163 | Lysozyme | Lysozyme | | pdb | 2QB0\_D | 1.0 | 0.0005183 | 144 | 0.187 | 165 | 98 | 7 | 4 | 143 | 79 | 232 | TELSAM domain - Lysozyme chimera | TELSAM domain - Lysozyme chimera | | pdb | 6CM4\_A | 1.0 | 0.0004421 | 144 | 0.222 | 162 | 90 | 10 | 6 | 140 | 185 | 337 | D(2) dopamine receptor, endolysin chimera | D(2) dopamine receptor, endolysin chimera | | pdb | 1C65\_A | 1.0 | 0.001147 | 143 | 0.19 | 173 | 98 | 10 | 6 | 150 | 2 | 160 | PROTEIN (LYSOZYME) | PROTEIN (LYSOZYME) | | pdb | 3G3W\_A | 1.0 | 0.000751 | 143 | 0.203 | 172 | 97 | 9 | 6 | 150 | 2 | 160 | Lysozyme | Lysozyme | | pdb | 3K2R\_A | 1.0 | 0.0007918 | 143 | 0.197 | 172 | 98 | 9 | 6 | 150 | 2 | 160 | Lysozyme | Lysozyme | | pdb | 1L73\_A | 1.0 | 0.001577 | 143 | 0.19 | 168 | 102 | 9 | 7 | 150 | 3 | 160 | LYSOZYME | LYSOZYME | | pdb | 3CDT\_A | 1.0 | 0.001663 | 143 | 0.195 | 169 | 102 | 9 | 6 | 150 | 2 | 160 | Lysozyme | Lysozyme | | pdb | 1JQU\_C | 1.0 | 0.0002603 | 143 | 0.209 | 172 | 96 | 12 | 6 | 146 | 2 | 164 | Lysozyme | Lysozyme | | pdb | 111L\_A | 1.0 | 0.002055 | 142 | 0.201 | 169 | 101 | 10 | 6 | 150 | 2 | 160 | T4 LYSOZYME | T4 LYSOZYME | | pdb | 1CUP\_A | 1.0 | 0.001848 | 142 | 0.195 | 169 | 102 | 9 | 6 | 150 | 2 | 160 | LYSOZYME | LYSOZYME | | pdb | 5JGV\_A | 1.0 | 0.0008349 | 142 | 0.197 | 172 | 98 | 9 | 6 | 150 | 2 | 160 | Endolysin | Endolysin | | pdb | 4Z9G\_A | 1.0 | 0.0003771 | 142 | 0.198 | 186 | 107 | 10 | 5 | 157 | 110 | 286 | Corticotropin-releasing factor receptor 1,Lysozyme,Corticotropin-releasing factor receptor 1 | Corticotropin-releasing factor receptor 1,Lysozyme,Corticotropin-releasing factor receptor 1 | | pdb | 115L\_A | 1.0 | 0.0007122 | 141 | 0.2 | 170 | 100 | 9 | 6 | 150 | 2 | 160 | T4 LYSOZYME | T4 LYSOZYME | | pdb | 122L\_A | 1.0 | 0.0007918 | 141 | 0.205 | 170 | 99 | 9 | 6 | 150 | 2 | 160 | T4 LYSOZYME | T4 LYSOZYME | | pdb | 5KIM\_A | 1.0 | 0.0008349 | 141 | 0.2 | 170 | 100 | 9 | 6 | 150 | 2 | 160 | Endolysin | Endolysin | | pdb | 2O79\_A | 1.0 | 0.0007918 | 141 | 0.194 | 175 | 101 | 10 | 1 | 150 | 1 | 160 | Lysozyme | Lysozyme | | pdb | 1P6Y\_A | 1.0 | 0.0008803 | 141 | 0.188 | 170 | 102 | 8 | 6 | 150 | 2 | 160 | LYSOZYME | LYSOZYME | | pdb | 5XPF\_A | 1.0 | 0.0008803 | 141 | 0.194 | 170 | 101 | 9 | 6 | 150 | 2 | 160 | Endolysin | Endolysin | | pdb | 3PBL\_B | 1.0 | 0.0008803 | 141 | 0.209 | 172 | 96 | 10 | 4 | 148 | 180 | 338 | D(3) dopamine receptor, Lysozyme chimera | D(3) dopamine receptor, Lysozyme chimera | | pdb | 4QKX\_A | 1.0 | 0.0007122 | 141 | 0.203 | 177 | 101 | 11 | 1 | 150 | 5 | 168 | Beta-2 adrenergic receptor | Beta-2 adrenergic receptor | | pdb | 114L\_A | 1.0 | 0.0009282 | 140 | 0.194 | 170 | 101 | 8 | 6 | 150 | 2 | 160 | T4 LYSOZYME | T4 LYSOZYME | | pdb | 232L\_A | 1.0 | 0.002055 | 140 | 0.185 | 167 | 106 | 8 | 6 | 150 | 2 | 160 | T4 LYSOZYME | T4 LYSOZYME | | pdb | 162L\_A | 1.0 | 0.0007918 | 140 | 0.209 | 172 | 96 | 11 | 6 | 150 | 2 | 160 | T4 LYSOZYME | T4 LYSOZYME | | pdb | 3C8R\_A | 1.0 | 0.0009282 | 140 | 0.203 | 172 | 97 | 10 | 6 | 150 | 2 | 160 | Lysozyme | Lysozyme | | pdb | 1ZYT\_A | 1.0 | 0.0009282 | 140 | 0.2 | 170 | 100 | 9 | 6 | 150 | 2 | 160 | Lysozyme | Lysozyme | | pdb | 4W58\_A | 1.0 | 0.001276 | 140 | 0.2 | 175 | 99 | 11 | 6 | 153 | 2 | 162 | Endolysin | Endolysin | | pdb | 6P5V\_A | 1.0 | 0.001345 | 140 | 0.224 | 178 | 95 | 12 | 4 | 153 | 13 | 175 | Lysozyme,DCN1-like protein 1 fusion | Lysozyme,DCN1-like protein 1 fusion | | pdb | 217L\_A | 1.0 | 0.0009282 | 139 | 0.194 | 170 | 101 | 8 | 6 | 150 | 2 | 160 | T4 LYSOZYME | T4 LYSOZYME | | pdb | 161L\_A | 1.0 | 0.0008803 | 139 | 0.2 | 170 | 100 | 9 | 6 | 150 | 2 | 160 | T4 LYSOZYME | T4 LYSOZYME | | pdb | 1L88\_A | 1.0 | 0.001088 | 139 | 0.191 | 172 | 99 | 9 | 6 | 150 | 2 | 160 | T4 LYSOZYME | T4 LYSOZYME | | pdb | 3L64\_A | 1.0 | 0.00254 | 139 | 0.189 | 169 | 103 | 9 | 6 | 150 | 2 | 160 | Lysozyme | Lysozyme | | pdb | 1P46\_A | 1.0 | 0.001418 | 139 | 0.196 | 173 | 102 | 9 | 6 | 153 | 2 | 162 | LYSOZYME | LYSOZYME | | pdb | 1PQK\_C | 1.0 | 0.001418 | 139 | 0.208 | 173 | 97 | 10 | 6 | 151 | 2 | 161 | Lysozyme | Lysozyme | | pdb | 5VNQ\_A | 1.0 | 0.001032 | 139 | 0.197 | 172 | 98 | 9 | 6 | 150 | 2 | 160 | Endolysin | Endolysin | | pdb | 5V88\_A | 1.0 | 0.001663 | 139 | 0.207 | 178 | 104 | 10 | 1 | 153 | 9 | 174 | Lysozyme,DCN1-like protein 1 | Lysozyme,DCN1-like protein 1 | | pdb | 4DJH\_B | 1.0 | 0.00121 | 139 | 0.209 | 181 | 99 | 12 | 5 | 155 | 207 | 373 | Kappa-type opioid receptor, Lysozyme | Kappa-type opioid receptor, Lysozyme | | pdb | 5KII\_A | 1.0 | 0.001032 | 138 | 0.201 | 169 | 100 | 9 | 6 | 150 | 2 | 159 | Endolysin | Endolysin | | pdb | 175L\_A | 1.0 | 0.001418 | 138 | 0.203 | 172 | 97 | 10 | 6 | 150 | 2 | 160 | T4 LYSOZYME | T4 LYSOZYME | | pdb | 192L\_A | 1.0 | 0.0006075 | 138 | 0.218 | 165 | 89 | 10 | 6 | 143 | 2 | 153 | LYSOZYME | LYSOZYME | | pdb | 1L74\_A | 1.0 | 0.0006755 | 138 | 0.189 | 169 | 101 | 9 | 7 | 150 | 3 | 160 | LYSOZYME | LYSOZYME | | pdb | 237L\_A | 1.0 | 0.0009788 | 138 | 0.203 | 172 | 97 | 10 | 6 | 150 | 2 | 160 | T4 LYSOZYME | T4 LYSOZYME | | pdb | 1L26\_A | 1.0 | 0.0009788 | 138 | 0.195 | 169 | 100 | 9 | 7 | 150 | 3 | 160 | T4 LYSOZYME | T4 LYSOZYME | | pdb | 1L53\_A | 1.0 | 0.0004915 | 138 | 0.216 | 162 | 91 | 11 | 6 | 140 | 2 | 154 | T4 LYSOZYME | T4 LYSOZYME | | pdb | 1P2R\_A | 1.0 | 0.00121 | 138 | 0.197 | 172 | 98 | 9 | 6 | 150 | 2 | 160 | LYSOZYME | LYSOZYME | | pdb | 2F47\_A | 1.0 | 0.0008803 | 138 | 0.176 | 181 | 102 | 7 | 6 | 150 | 2 | 171 | Lysozyme | Lysozyme | | pdb | 4ARJ\_B | 1.0 | 0.0007918 | 138 | 0.209 | 162 | 92 | 10 | 6 | 140 | 152 | 304 | PESTICIN, LYSOZYME | PESTICIN, LYSOZYME | | pdb | 8A5X\_A | 1.0 | 0.0005762 | 138 | 0.201 | 164 | 94 | 11 | 4 | 140 | 70 | 223 | Phosphatidylinositol 4-kinase type 2-beta,Endolysin | Phosphatidylinositol 4-kinase type 2-beta,Endolysin | | pdb | 4EVX\_B | 1.0 | 0.008593 | 137 | 0.25 | 92 | 66 | 2 | 2 | 93 | 1 | 89 | Putative phage endolysin | Putative phage endolysin | | pdb | 7LX8\_A | 1.0 | 0.001032 | 137 | 0.195 | 169 | 101 | 8 | 6 | 150 | 2 | 159 | Lysozyme | Lysozyme | | pdb | 139L\_A | 1.0 | 0.001276 | 137 | 0.203 | 172 | 97 | 10 | 6 | 150 | 2 | 160 | T4 LYSOZYME | T4 LYSOZYME | | pdb | 1L66\_A | 1.0 | 0.001276 | 137 | 0.203 | 172 | 97 | 10 | 6 | 150 | 2 | 160 | LYSOZYME | LYSOZYME | | pdb | 2RAY\_X | 1.0 | 0.001276 | 137 | 0.194 | 170 | 101 | 9 | 6 | 150 | 2 | 160 | Lysozyme | Lysozyme | | pdb | 3CDV\_A | 1.0 | 0.00121 | 137 | 0.203 | 172 | 97 | 10 | 6 | 150 | 2 | 160 | Lysozyme | Lysozyme | | pdb | 1L22\_A | 1.0 | 0.001418 | 137 | 0.208 | 173 | 100 | 10 | 6 | 153 | 2 | 162 | T4 LYSOZYME | T4 LYSOZYME | | pdb | 6A73\_B | 1.0 | 0.000751 | 137 | 0.222 | 162 | 90 | 10 | 6 | 140 | 131 | 283 | COP9 signalosome complex subunit 2,Endolysin | COP9 signalosome complex subunit 2,Endolysin | | pdb | 1XJU\_B | 1.0 | 0.001753 | 136 | 0.184 | 163 | 98 | 7 | 2 | 144 | 1 | 148 | Lysozyme | Lysozyme | | pdb | 180L\_B | 1.0 | 0.002978 | 136 | 0.183 | 169 | 104 | 9 | 6 | 150 | 2 | 160 | LYSOZYME | LYSOZYME | | pdb | 3C7Y\_A | 1.0 | 0.001418 | 136 | 0.194 | 170 | 101 | 8 | 6 | 150 | 2 | 160 | lysozyme | lysozyme | | pdb | 3LZM\_A | 1.0 | 0.001577 | 136 | 0.217 | 175 | 96 | 13 | 6 | 153 | 2 | 162 | T4 LYSOZYME | T4 LYSOZYME | | pdb | 2OU9\_A | 1.0 | 0.001418 | 136 | 0.2 | 170 | 100 | 9 | 6 | 150 | 2 | 160 | Lysozyme | Lysozyme | | pdb | 159L\_A | 1.0 | 0.001663 | 135 | 0.2 | 170 | 100 | 8 | 6 | 150 | 2 | 160 | T4 LYSOZYME | T4 LYSOZYME | | pdb | 7LOC\_A | 1.0 | 0.001577 | 135 | 0.203 | 172 | 101 | 9 | 6 | 153 | 2 | 161 | Lysozyme | Lysozyme | | pdb | 4W56\_A | 1.0 | 0.001495 | 135 | 0.194 | 170 | 101 | 9 | 6 | 150 | 2 | 160 | Endolysin | Endolysin | | pdb | 6XC0\_B | 1.0 | 0.00314 | 135 | 0.184 | 173 | 102 | 7 | 7 | 150 | 2 | 164 | Lysozyme | Lysozyme | | pdb | 4K5Y\_A | 1.0 | 0.002285 | 135 | 0.195 | 174 | 104 | 9 | 5 | 153 | 106 | 268 | Corticotropin-releasing factor receptor 1, T4-Lysozyme chimeric construct | Corticotropin-releasing factor receptor 1, T4-Lysozyme chimeric construct | | pdb | 158L\_A | 1.0 | 0.001495 | 134 | 0.203 | 172 | 97 | 10 | 6 | 150 | 2 | 160 | T4 LYSOZYME | T4 LYSOZYME | | pdb | 173L\_A | 1.0 | 0.002824 | 134 | 0.19 | 173 | 98 | 9 | 7 | 151 | 3 | 161 | T4 LYSOZYME | T4 LYSOZYME | | pdb | 1TLA\_A | 1.0 | 0.001495 | 134 | 0.205 | 170 | 99 | 10 | 6 | 150 | 2 | 160 | T4 LYSOZYME | T4 LYSOZYME | | pdb | 213L\_A | 1.0 | 0.0009282 | 134 | 0.218 | 160 | 94 | 8 | 6 | 140 | 2 | 155 | T4 LYSOZYME | T4 LYSOZYME | | pdb | 5NX0\_A | 1.0 | 0.001577 | 134 | 0.203 | 172 | 97 | 10 | 6 | 150 | 2 | 160 | Endolysin | Endolysin | | pdb | 2Q9E\_B | 1.0 | 0.0007918 | 134 | 0.212 | 160 | 94 | 9 | 6 | 140 | 2 | 154 | Lysozyme | Lysozyme | | pdb | 1JTN\_B | 1.0 | 0.001753 | 134 | 0.192 | 177 | 102 | 11 | 6 | 155 | 2 | 164 | LYSOZYME | LYSOZYME | | pdb | 6D9M\_A | 1.0 | 0.002285 | 134 | 0.202 | 173 | 98 | 10 | 6 | 151 | 2 | 161 | Fusion protein of Endolysin,Response receiver sensor diguanylate cyclase, GAF domain-containing | Fusion protein of Endolysin,Response receiver sensor diguanylate cyclase, GAF domain-containing | | pdb | 3QAK\_A | 1.0 | 0.001949 | 134 | 0.198 | 181 | 106 | 10 | 4 | 156 | 196 | 365 | Adenosine receptor A2a,lysozyme chimera | Adenosine receptor A2a,lysozyme chimera | | pdb | 7BU7\_A | 1.0 | 0.001147 | 134 | 0.183 | 185 | 108 | 11 | 1 | 157 | 1 | 170 | Endolysin,Endolysin,Beta-1 adrenergic receptor chimera | Endolysin,Endolysin,Beta-1 adrenergic receptor chimera | | pdb | 1KW5\_A | 1.0 | 0.001753 | 133 | 0.188 | 170 | 102 | 9 | 6 | 150 | 2 | 160 | LYSOZYME | LYSOZYME | | pdb | 230L\_A | 1.0 | 0.00254 | 133 | 0.196 | 163 | 102 | 8 | 6 | 146 | 2 | 157 | T4 LYSOZYME | T4 LYSOZYME | | pdb | 3C7Z\_A | 1.0 | 0.00121 | 133 | 0.204 | 166 | 97 | 9 | 6 | 146 | 2 | 157 | Lysozyme | Lysozyme | | pdb | 1L97\_B | 1.0 | 0.0009282 | 133 | 0.223 | 161 | 89 | 11 | 7 | 140 | 3 | 154 | T4 LYSOZYME | T4 LYSOZYME | | pdb | 1JTM\_A | 1.0 | 0.001147 | 133 | 0.221 | 167 | 102 | 9 | 6 | 147 | 2 | 165 | LYSOZYME | LYSOZYME | | pdb | 3VW7\_A | 1.0 | 0.00254 | 133 | 0.204 | 176 | 104 | 9 | 4 | 154 | 205 | 369 | Proteinase-activated receptor 1, Lysozyme | Proteinase-activated receptor 1, Lysozyme | | pdb | 6WJC\_A | 1.0 | 0.001032 | 133 | 0.192 | 177 | 97 | 11 | 6 | 154 | 203 | 361 | Muscarinic acetylcholine receptor M1,Endolysin fusion | Muscarinic acetylcholine receptor M1,Endolysin fusion | | pdb | 5T04\_A | 1.0 | 0.0007122 | 133 | 0.22 | 186 | 101 | 13 | 6 | 157 | 219 | 394 | Neurotensin receptor type 1,Endolysin,Neurotensin receptor type 1 | Neurotensin receptor type 1,Endolysin,Neurotensin receptor type 1 | | pdb | 137L\_A | 1.0 | 0.004092 | 132 | 0.194 | 170 | 101 | 9 | 6 | 150 | 2 | 160 | T4 LYSOZYME | T4 LYSOZYME | | pdb | 180L\_A | 1.0 | 0.001848 | 132 | 0.182 | 170 | 103 | 8 | 6 | 150 | 2 | 160 | LYSOZYME | LYSOZYME | | pdb | 1L87\_A | 1.0 | 0.001276 | 132 | 0.216 | 166 | 95 | 10 | 6 | 146 | 2 | 157 | T4 LYSOZYME | T4 LYSOZYME | | pdb | 1L95\_A | 1.0 | 0.001949 | 132 | 0.197 | 172 | 98 | 10 | 6 | 150 | 2 | 160 | T4 LYSOZYME | T4 LYSOZYME | | pdb | 102L\_A | 1.0 | 0.001949 | 132 | 0.208 | 173 | 96 | 11 | 6 | 150 | 2 | 161 | T4 LYSOZYME | T4 LYSOZYME | | pdb | 1PQO\_A | 1.0 | 0.002285 | 132 | 0.19 | 173 | 103 | 10 | 6 | 153 | 2 | 162 | Lysozyme | Lysozyme | | pdb | 262L\_B | 1.0 | 0.002055 | 132 | 0.176 | 181 | 102 | 7 | 6 | 150 | 2 | 171 | LYSOZYME | LYSOZYME | | pdb | 206L\_A | 1.0 | 0.002055 | 131 | 0.203 | 172 | 97 | 10 | 6 | 150 | 2 | 160 | LYSOZYME | LYSOZYME | | pdb | 109L\_A | 1.0 | 0.001753 | 131 | 0.209 | 172 | 96 | 11 | 6 | 150 | 2 | 160 | T4 LYSOZYME | T4 LYSOZYME | | pdb | 174L\_B | 1.0 | 0.0008349 | 131 | 0.202 | 163 | 92 | 10 | 6 | 140 | 2 | 154 | T4 LYSOZYME | T4 LYSOZYME | | pdb | 178L\_A | 1.0 | 0.003491 | 131 | 0.209 | 172 | 96 | 10 | 6 | 150 | 2 | 160 | T4 LYSOZYME | T4 LYSOZYME | | pdb | 1CV3\_A | 1.0 | 0.002055 | 131 | 0.203 | 172 | 97 | 10 | 6 | 150 | 2 | 160 | LYSOZYME | LYSOZYME | | pdb | 6PGZ\_B | 1.0 | 0.001848 | 131 | 0.19 | 173 | 98 | 9 | 6 | 150 | 2 | 160 | Endolysin | Endolysin | | pdb | 1L00\_A | 1.0 | 0.002978 | 131 | 0.195 | 164 | 99 | 9 | 7 | 146 | 3 | 157 | T4 LYSOZYME | T4 LYSOZYME | | pdb | 1P64\_A | 1.0 | 0.002055 | 131 | 0.205 | 170 | 99 | 9 | 6 | 150 | 2 | 160 | LYSOZYME | LYSOZYME | | pdb | 7CN7\_A | 1.0 | 0.002167 | 131 | 0.194 | 175 | 92 | 9 | 7 | 148 | 12 | 170 | Baseplate central spike complex protein gp5 | Baseplate central spike complex protein gp5 | | pdb | 1D9W\_A | 1.0 | 0.001848 | 130 | 0.21 | 166 | 96 | 10 | 6 | 146 | 2 | 157 | PROTEIN (LYSOZYME) | PROTEIN (LYSOZYME) | | pdb | 1KW7\_A | 1.0 | 0.002285 | 130 | 0.191 | 172 | 99 | 10 | 6 | 150 | 2 | 160 | LYSOZYME | LYSOZYME | | pdb | 2RBO\_A | 1.0 | 0.002285 | 130 | 0.194 | 170 | 101 | 9 | 6 | 150 | 2 | 160 | Lysozyme | Lysozyme | | pdb | 7DWS\_A | 1.0 | 0.001577 | 130 | 0.205 | 170 | 96 | 9 | 4 | 146 | 2 | 159 | Endolysin | Endolysin | | pdb | 1G0J\_A | 1.0 | 0.002285 | 130 | 0.203 | 172 | 97 | 10 | 6 | 150 | 2 | 160 | PROTEIN (LYSOZYME) | PROTEIN (LYSOZYME) | | pdb | 1L19\_A | 1.0 | 0.00314 | 130 | 0.206 | 165 | 98 | 10 | 6 | 146 | 2 | 157 | T4 LYSOZYME | T4 LYSOZYME | | pdb | 1QT5\_A | 1.0 | 0.002285 | 130 | 0.194 | 170 | 101 | 9 | 6 | 150 | 2 | 160 | PROTEIN (T4 LYSOZYME) | PROTEIN (T4 LYSOZYME) | | pdb | 1SWZ\_A | 1.0 | 0.003311 | 130 | 0.208 | 173 | 100 | 10 | 6 | 153 | 2 | 162 | Lysozyme | Lysozyme | | pdb | 4YXA\_F | 1.0 | 0.002285 | 130 | 0.182 | 170 | 101 | 8 | 7 | 151 | 36 | 192 | Oxygen-regulated invasion protein OrgB,Endolysin | Oxygen-regulated invasion protein OrgB,Endolysin | | pdb | 5YQR\_A | 1.0 | 0.001418 | 130 | 0.198 | 161 | 96 | 7 | 5 | 143 | 1 | 150 | Endolysin/Membrane-anchored lipid-binding protein LAM6 fusion protein | Endolysin/Membrane-anchored lipid-binding protein LAM6 fusion protein | | pdb | 7MWY\_A | 1.0 | 0.003881 | 130 | 0.194 | 175 | 101 | 10 | 4 | 151 | 1 | 162 | STING | STING | | pdb | 1T8G\_A | 1.0 | 0.001848 | 129 | 0.208 | 158 | 93 | 9 | 6 | 140 | 2 | 150 | Lysozyme | Lysozyme | | pdb | 195L\_A | 1.0 | 0.002285 | 129 | 0.197 | 172 | 98 | 10 | 6 | 150 | 2 | 160 | LYSOZYME | LYSOZYME | | pdb | 1C64\_A | 1.0 | 0.002678 | 129 | 0.203 | 172 | 97 | 10 | 6 | 150 | 2 | 160 | PROTEIN (LYSOZYME) | PROTEIN (LYSOZYME) | | pdb | 149L\_A | 1.0 | 0.001949 | 129 | 0.215 | 167 | 92 | 11 | 7 | 146 | 3 | 157 | T4 LYSOZYME | T4 LYSOZYME | | pdb | 1JQU\_A | 1.0 | 0.0004661 | 129 | 0.195 | 169 | 100 | 10 | 7 | 146 | 3 | 164 | Lysozyme | Lysozyme | | pdb | 1L35\_A | 1.0 | 0.00254 | 129 | 0.203 | 172 | 97 | 10 | 6 | 150 | 2 | 160 | T4 LYSOZYME | T4 LYSOZYME | | pdb | 6XMV\_A | 1.0 | 0.000751 | 129 | 0.197 | 167 | 100 | 8 | 4 | 142 | 145 | 305 | Hemolysin,Endolysin | Hemolysin,Endolysin | | pdb | 5KI2\_A | 1.0 | 0.002285 | 128 | 0.191 | 172 | 98 | 10 | 6 | 150 | 2 | 159 | Endolysin | Endolysin | | pdb | 118L\_A | 1.0 | 0.002678 | 128 | 0.194 | 170 | 101 | 9 | 6 | 150 | 2 | 160 | T4 LYSOZYME | T4 LYSOZYME | | pdb | 199L\_A | 1.0 | 0.002978 | 128 | 0.191 | 172 | 99 | 9 | 6 | 150 | 2 | 160 | LYSOZYME | LYSOZYME | | pdb | 1LYE\_A | 1.0 | 0.00254 | 128 | 0.203 | 172 | 97 | 11 | 6 | 150 | 2 | 160 | T4 LYSOZYME | T4 LYSOZYME | | pdb | 175L\_B | 1.0 | 0.0007918 | 128 | 0.207 | 169 | 100 | 9 | 6 | 146 | 2 | 164 | T4 LYSOZYME | T4 LYSOZYME | | pdb | 1JQU\_B | 1.0 | 0.0006406 | 128 | 0.213 | 169 | 97 | 10 | 7 | 146 | 3 | 164 | Lysozyme | Lysozyme | | pdb | 1L11\_A | 1.0 | 0.003491 | 128 | 0.2 | 175 | 99 | 10 | 6 | 153 | 2 | 162 | T4 LYSOZYME | T4 LYSOZYME | | pdb | 1P3N\_A | 1.0 | 0.00314 | 128 | 0.203 | 172 | 97 | 9 | 6 | 150 | 2 | 160 | LYSOZYME | LYSOZYME | | pdb | 3JR6\_D | 1.0 | 0.00314 | 128 | 0.194 | 175 | 99 | 9 | 7 | 150 | 3 | 166 | Lysozyme | Lysozyme | | pdb | 6XON\_A | 1.0 | 0.004092 | 128 | 0.2 | 170 | 99 | 9 | 7 | 153 | 2 | 157 | Lysozyme DCN1-like protein 1 chimera | Lysozyme DCN1-like protein 1 chimera | | pdb | 3V2Y\_A | 1.0 | 0.005334 | 128 | 0.196 | 163 | 97 | 7 | 6 | 144 | 210 | 362 | Sphingosine 1-phosphate receptor 1, Lysozyme chimera (E.C.3.2.1.17) | Sphingosine 1-phosphate receptor 1, Lysozyme chimera (E.C.3.2.1.17) | | pdb | 3JR6\_B | 1.0 | 0.003681 | 127 | 0.205 | 151 | 97 | 9 | 7 | 140 | 3 | 147 | Lysozyme | Lysozyme | | pdb | 5JWU\_A | 1.0 | 0.002978 | 127 | 0.197 | 172 | 98 | 10 | 6 | 150 | 2 | 160 | Endolysin | Endolysin | | pdb | 1L89\_A | 1.0 | 0.002055 | 127 | 0.18 | 172 | 101 | 9 | 6 | 150 | 2 | 160 | T4 LYSOZYME | T4 LYSOZYME | | pdb | 1L05\_A | 1.0 | 0.003681 | 127 | 0.2 | 175 | 99 | 10 | 6 | 153 | 2 | 162 | T4 LYSOZYME | T4 LYSOZYME | | pdb | 262L\_A | 1.0 | 0.002167 | 127 | 0.191 | 178 | 96 | 9 | 6 | 146 | 2 | 168 | LYSOZYME | LYSOZYME | | pdb | 6WSK\_A | 1.0 | 0.002824 | 127 | 0.184 | 157 | 95 | 8 | 4 | 150 | 1 | 134 | Endolysin,CB1 cannabinoid receptor-interacting protein 1 fusion | Endolysin,CB1 cannabinoid receptor-interacting protein 1 fusion | | pdb | 120L\_A | 1.0 | 0.001949 | 126 | 0.213 | 169 | 92 | 11 | 6 | 146 | 2 | 157 | T4 LYSOZYME | T4 LYSOZYME | | pdb | 234L\_A | 1.0 | 0.002167 | 126 | 0.21 | 166 | 96 | 9 | 6 | 146 | 2 | 157 | T4 LYSOZYME | T4 LYSOZYME | | pdb | 160L\_A | 1.0 | 0.003311 | 126 | 0.203 | 172 | 97 | 11 | 6 | 150 | 2 | 160 | T4 LYSOZYME | T4 LYSOZYME | | pdb | 4S0W\_B | 1.0 | 0.001577 | 126 | 0.222 | 162 | 90 | 10 | 6 | 140 | 2 | 154 | Lysozyme | Lysozyme | | pdb | 3SB8\_C | 1.0 | 0.005059 | 126 | 0.187 | 165 | 105 | 8 | 4 | 146 | 1 | 158 | Lysozyme | Lysozyme | | pdb | 189L\_A | 1.0 | 0.001949 | 126 | 0.2 | 170 | 96 | 11 | 4 | 146 | 1 | 157 | T4 LYSOZYME | T4 LYSOZYME | | pdb | 1L32\_A | 1.0 | 0.002409 | 126 | 0.21 | 166 | 96 | 10 | 6 | 146 | 2 | 157 | T4 LYSOZYME | T4 LYSOZYME | | pdb | 1L34\_A | 1.0 | 0.002409 | 126 | 0.22 | 168 | 92 | 12 | 6 | 146 | 2 | 157 | T4 LYSOZYME | T4 LYSOZYME | | pdb | 1QTZ\_A | 1.0 | 0.006952 | 126 | 0.189 | 169 | 103 | 9 | 6 | 150 | 2 | 160 | PROTEIN (T4 LYSOZYME) | PROTEIN (T4 LYSOZYME) | | pdb | 1T97\_B | 1.0 | 0.001949 | 126 | 0.206 | 179 | 92 | 11 | 6 | 146 | 2 | 168 | Lysozyme | Lysozyme | | pdb | 7F8X\_A | 1.0 | 0.002824 | 126 | 0.194 | 180 | 108 | 11 | 4 | 158 | 203 | 370 | Cholecystokinin receptor type A,Endolysin | Cholecystokinin receptor type A,Endolysin | | pdb | 6E67\_A | 1.0 | 0.004092 | 126 | 0.202 | 178 | 100 | 11 | 6 | 154 | 205 | 369 | Beta-2 adrenergic receptor,Endolysin,Guanine nucleotide-binding protein G(s) subunit alpha isoforms short,Beta-2 adrenergic receptor chimera | Beta-2 adrenergic receptor,Endolysin,Guanine nucleotide-binding protein G(s) subunit alpha isoforms short,Beta-2 adrenergic receptor chimera | | pdb | 5XF1\_A | 1.0 | 0.001345 | 126 | 0.169 | 177 | 116 | 8 | 5 | 157 | 230 | 399 | Glucagon receptor,Endolysin,Glucagon receptor | Glucagon receptor,Endolysin,Glucagon receptor | | pdb | 236L\_A | 1.0 | 0.00314 | 125 | 0.2 | 170 | 100 | 9 | 6 | 150 | 2 | 160 | T4 LYSOZYME | T4 LYSOZYME | | pdb | 177L\_A | 1.0 | 0.004797 | 125 | 0.176 | 170 | 97 | 8 | 6 | 146 | 2 | 157 | T4 LYSOZYME | T4 LYSOZYME | | pdb | 1CX7\_A | 1.0 | 0.003491 | 125 | 0.176 | 170 | 104 | 9 | 6 | 150 | 2 | 160 | LYSOZYME | LYSOZYME | | pdb | 1KY0\_A | 1.0 | 0.001848 | 125 | 0.2 | 160 | 96 | 9 | 6 | 140 | 2 | 154 | LYSOZYME | LYSOZYME | | pdb | 2L78\_A | 1.0 | 0.002285 | 125 | 0.22 | 168 | 92 | 11 | 6 | 146 | 2 | 157 | T4 LYSOZYME | T4 LYSOZYME | | pdb | 3CDR\_A | 1.0 | 0.003681 | 125 | 0.203 | 172 | 97 | 10 | 6 | 150 | 2 | 160 | Lysozyme | Lysozyme | | pdb | 1L20\_A | 1.0 | 0.005624 | 125 | 0.212 | 165 | 97 | 11 | 6 | 146 | 2 | 157 | T4 LYSOZYME | T4 LYSOZYME | | pdb | 7MWZ\_A | 1.0 | 0.002055 | 125 | 0.207 | 164 | 94 | 10 | 4 | 140 | 1 | 155 | STING | STING | | pdb | 7MI1\_A | 1.0 | 0.003491 | 125 | 0.206 | 165 | 98 | 11 | 6 | 146 | 1663 | 1818 | Chimera protein of Dynein and Endolysin | Chimera protein of Dynein and Endolysin | | pdb | 5JWW\_A | 1.0 | 0.003311 | 124 | 0.2 | 170 | 100 | 9 | 6 | 150 | 2 | 160 | Endolysin | Endolysin | | pdb | 156L\_A | 1.0 | 0.003881 | 124 | 0.197 | 172 | 98 | 9 | 6 | 150 | 2 | 160 | T4 LYSOZYME | T4 LYSOZYME | | pdb | 1L77\_A | 1.0 | 0.002055 | 124 | 0.206 | 165 | 91 | 10 | 6 | 143 | 2 | 153 | T4 LYSOZYME | T4 LYSOZYME | | pdb | 1L09\_A | 1.0 | 0.00593 | 124 | 0.196 | 163 | 102 | 8 | 6 | 146 | 2 | 157 | T4 LYSOZYME | T4 LYSOZYME | | pdb | 1L31\_A | 1.0 | 0.00314 | 124 | 0.198 | 166 | 98 | 8 | 6 | 146 | 2 | 157 | T4 LYSOZYME | T4 LYSOZYME | | pdb | 1SSY\_B | 1.0 | 0.00455 | 124 | 0.182 | 159 | 100 | 9 | 6 | 140 | 2 | 154 | Lysozyme | Lysozyme | | pdb | 1T97\_A | 1.0 | 0.003311 | 124 | 0.193 | 176 | 97 | 10 | 6 | 150 | 2 | 163 | Lysozyme | Lysozyme | | pdb | 7XKA\_A | 1.0 | 0.002285 | 124 | 0.196 | 168 | 99 | 9 | 1 | 143 | 5 | 161 | Endolysin,Beta-2 adrenergic receptor | Endolysin,Beta-2 adrenergic receptor | | pdb | 231L\_A | 1.0 | 0.004315 | 123 | 0.203 | 172 | 97 | 10 | 6 | 150 | 2 | 160 | T4 LYSOZYME | T4 LYSOZYME | | pdb | 233L\_A | 1.0 | 0.004092 | 123 | 0.197 | 172 | 98 | 10 | 6 | 150 | 2 | 160 | T4 LYSOZYME | T4 LYSOZYME | | pdb | 1C6N\_A | 1.0 | 0.004315 | 123 | 0.188 | 170 | 102 | 9 | 6 | 150 | 2 | 160 | PROTEIN (LYSOZYME) | PROTEIN (LYSOZYME) | | pdb | 1LLH\_A | 1.0 | 0.00314 | 123 | 0.204 | 166 | 97 | 9 | 6 | 146 | 2 | 157 | Lysozyme | Lysozyme | | pdb | 4LZM\_A | 1.0 | 0.002978 | 123 | 0.197 | 172 | 98 | 10 | 6 | 150 | 2 | 160 | T4 LYSOZYME | T4 LYSOZYME | | pdb | 176L\_B | 1.0 | 0.00314 | 123 | 0.21 | 166 | 96 | 7 | 6 | 146 | 2 | 157 | T4 LYSOZYME | T4 LYSOZYME | | pdb | 1L14\_A | 1.0 | 0.005059 | 123 | 0.196 | 173 | 102 | 9 | 6 | 153 | 2 | 162 | T4 LYSOZYME | T4 LYSOZYME | | pdb | 4EXM\_C | 1.0 | 0.002824 | 123 | 0.191 | 172 | 97 | 11 | 5 | 146 | 166 | 325 | Pesticin, Lysozyme Chimera | Pesticin, Lysozyme Chimera | | pdb | 5DGY\_A | 1.0 | 0.002409 | 123 | 0.217 | 161 | 90 | 10 | 7 | 140 | 2 | 153 | Endolysin,Rhodopsin,S-arrestin | Endolysin,Rhodopsin,S-arrestin | | pdb | 210L\_A | 1.0 | 0.004315 | 122 | 0.192 | 166 | 98 | 9 | 6 | 146 | 2 | 156 | T4 LYSOZYME | T4 LYSOZYME | | pdb | 198L\_A | 1.0 | 0.00455 | 122 | 0.197 | 172 | 98 | 10 | 6 | 150 | 2 | 160 | LYSOZYME | LYSOZYME | | pdb | 1D3N\_A | 1.0 | 0.00455 | 122 | 0.176 | 170 | 104 | 8 | 6 | 150 | 2 | 160 | LYSOZYME | LYSOZYME | | pdb | 168L\_D | 1.0 | 0.00314 | 122 | 0.207 | 169 | 93 | 11 | 6 | 146 | 2 | 157 | T4 LYSOZYME | T4 LYSOZYME | | pdb | 1P7S\_A | 1.0 | 0.005059 | 122 | 0.201 | 159 | 97 | 10 | 6 | 140 | 2 | 154 | LYSOZYME | LYSOZYME | | pdb | 1QTV\_A | 1.0 | 0.003311 | 122 | 0.198 | 166 | 98 | 9 | 6 | 146 | 2 | 157 | PROTEIN (T4 LYSOZYME) | PROTEIN (T4 LYSOZYME) | | pdb | 1SSW\_A | 1.0 | 0.005624 | 122 | 0.194 | 175 | 100 | 11 | 6 | 153 | 2 | 162 | Lysozyme | Lysozyme | | pdb | 6K69\_B | 1.0 | 0.005334 | 122 | 0.188 | 180 | 100 | 8 | 6 | 150 | 2 | 170 | Engineered T4 lysozyme | Engineered T4 lysozyme | | pdb | 1JTN\_A | 1.0 | 0.002678 | 122 | 0.198 | 176 | 97 | 12 | 10 | 155 | 3 | 164 | LYSOZYME | LYSOZYME | | pdb | 6QAJ\_A | 1.0 | 0.002678 | 122 | 0.206 | 179 | 105 | 11 | 1 | 153 | 2 | 169 | Endolysin,Transcription intermediary factor 1-beta | Endolysin,Transcription intermediary factor 1-beta | | pdb | 235L\_A | 1.0 | 0.00455 | 121 | 0.203 | 172 | 97 | 10 | 6 | 150 | 2 | 160 | T4 LYSOZYME | T4 LYSOZYME | | pdb | 172L\_A | 1.0 | 0.00733 | 121 | 0.2 | 170 | 100 | 9 | 6 | 150 | 2 | 160 | T4 LYSOZYME | T4 LYSOZYME | | pdb | 2HUK\_A | 1.0 | 0.002824 | 121 | 0.201 | 174 | 98 | 12 | 4 | 146 | 1 | 164 | Lysozyme | Lysozyme | | pdb | 2Q9E\_C | 1.0 | 0.00314 | 121 | 0.191 | 172 | 99 | 11 | 6 | 146 | 2 | 164 | Lysozyme | Lysozyme | | pdb | 6XYR\_A | 1.0 | 0.006952 | 121 | 0.203 | 177 | 101 | 10 | 6 | 155 | 34 | 197 | T4Lnano,Endolysin,Calmodulin,Endolysin,Calmodulin-1 | T4Lnano,Endolysin,Calmodulin,Endolysin,Calmodulin-1 | | pdb | 6LB8\_C | 1.0 | 0.00906 | 121 | 0.201 | 174 | 104 | 11 | 6 | 155 | 2 | 164 | Endolysin,Calcium uptake protein 1, mitochondrial | Endolysin,Calcium uptake protein 1, mitochondrial | | pdb | 141L\_A | 1.0 | 0.005624 | 120 | 0.191 | 172 | 99 | 9 | 6 | 150 | 2 | 160 | T4 LYSOZYME | T4 LYSOZYME | | pdb | 1OVK\_A | 1.0 | 0.006952 | 120 | 0.203 | 172 | 97 | 11 | 6 | 150 | 2 | 160 | Lysozyme | Lysozyme | | pdb | 1L07\_A | 1.0 | 0.004315 | 120 | 0.198 | 166 | 98 | 8 | 6 | 146 | 2 | 157 | T4 LYSOZYME | T4 LYSOZYME | | pdb | 1L15\_A | 1.0 | 0.003881 | 120 | 0.204 | 166 | 97 | 9 | 6 | 146 | 2 | 157 | T4 LYSOZYME | T4 LYSOZYME | | pdb | 1K28\_A | 1.0 | 0.007729 | 120 | 0.193 | 176 | 102 | 9 | 4 | 150 | 169 | 333 | TAIL-ASSOCIATED LYSOZYME | TAIL-ASSOCIATED LYSOZYME | | pdb | 244L\_A | 1.0 | 0.006593 | 119 | 0.203 | 172 | 97 | 10 | 6 | 150 | 2 | 160 | T4 LYSOZYME | T4 LYSOZYME | | pdb | 157L\_A | 1.0 | 0.006253 | 119 | 0.197 | 172 | 98 | 10 | 6 | 150 | 2 | 160 | T4 LYSOZYME | T4 LYSOZYME | | pdb | 1L01\_A | 1.0 | 0.001848 | 119 | 0.207 | 169 | 100 | 8 | 6 | 146 | 2 | 164 | T4 LYSOZYME | T4 LYSOZYME | | pdb | 1L33\_A | 1.0 | 0.00455 | 119 | 0.214 | 168 | 93 | 11 | 6 | 146 | 2 | 157 | T4 LYSOZYME | T4 LYSOZYME | | pdb | 1L50\_A | 1.0 | 0.005059 | 119 | 0.198 | 166 | 105 | 9 | 6 | 146 | 2 | 164 | T4 LYSOZYME | T4 LYSOZYME | | pdb | 1KNI\_A | 1.0 | 0.001753 | 118 | 0.21 | 166 | 97 | 9 | 6 | 143 | 2 | 161 | LYSOZYME | LYSOZYME | | pdb | 1L02\_A | 1.0 | 0.005624 | 118 | 0.198 | 166 | 98 | 8 | 6 | 146 | 2 | 157 | T4 LYSOZYME | T4 LYSOZYME | | pdb | 2HUL\_A | 1.0 | 0.002678 | 117 | 0.216 | 162 | 91 | 10 | 6 | 140 | 2 | 154 | Lysozyme | Lysozyme | | pdb | 1L12\_A | 1.0 | 0.00906 | 117 | 0.2 | 175 | 99 | 10 | 6 | 153 | 2 | 162 | T4 LYSOZYME | T4 LYSOZYME | | pdb | 1L16\_A | 1.0 | 0.005334 | 117 | 0.198 | 166 | 105 | 8 | 6 | 146 | 2 | 164 | T4 LYSOZYME | T4 LYSOZYME | | pdb | 5B2G\_G | 1.0 | 0.003881 | 117 | 0.207 | 169 | 106 | 6 | 4 | 147 | 6 | 171 | Endolysin,Claudin-4 | Endolysin,Claudin-4 | | pdb | 3OE8\_C | 1.0 | 0.001577 | 117 | 0.23 | 182 | 104 | 11 | 5 | 156 | 190 | 365 | C-X-C chemokine receptor type 4, Lysozyme Chimera | C-X-C chemokine receptor type 4, Lysozyme Chimera | | pdb | 3OE0\_A | 1.0 | 0.004315 | 117 | 0.208 | 163 | 91 | 10 | 6 | 140 | 201 | 353 | C-X-C chemokine receptor type 4, Lysozyme Chimera | C-X-C chemokine receptor type 4, Lysozyme Chimera | | pdb | 112L\_A | 1.0 | 0.008149 | 116 | 0.205 | 170 | 99 | 10 | 6 | 150 | 2 | 160 | T4 LYSOZYME | T4 LYSOZYME | | pdb | 191L\_A | 1.0 | 0.003311 | 116 | 0.192 | 166 | 99 | 9 | 6 | 146 | 2 | 157 | LYSOZYME | LYSOZYME | | pdb | 3GUK\_A | 1.0 | 0.005624 | 116 | 0.194 | 170 | 101 | 9 | 6 | 150 | 2 | 160 | Lysozyme | Lysozyme | | pdb | 253L\_A | 1.0 | 0.002409 | 116 | 0.213 | 169 | 99 | 10 | 6 | 146 | 2 | 164 | LYSOZYME | LYSOZYME | | pdb | 1L13\_A | 1.0 | 0.002678 | 116 | 0.201 | 169 | 101 | 8 | 6 | 146 | 2 | 164 | T4 LYSOZYME | T4 LYSOZYME | | pdb | 4DAJ\_D | 1.0 | 0.003681 | 116 | 0.208 | 168 | 94 | 11 | 6 | 146 | 197 | 352 | Muscarinic acetylcholine receptor M3, Lysozyme | Muscarinic acetylcholine receptor M3, Lysozyme | | pdb | 250L\_A | 1.0 | 0.00593 | 114 | 0.214 | 168 | 93 | 11 | 6 | 146 | 2 | 157 | T4 LYSOZYME | T4 LYSOZYME | | pdb | 1L57\_A | 1.0 | 0.006952 | 114 | 0.204 | 166 | 97 | 9 | 6 | 146 | 2 | 157 | LYSOZYME | LYSOZYME | | pdb | 1L06\_A | 1.0 | 0.003681 | 114 | 0.213 | 169 | 99 | 9 | 6 | 146 | 2 | 164 | T4 LYSOZYME | T4 LYSOZYME | | pdb | 1L10\_A | 1.0 | 0.008149 | 113 | 0.198 | 166 | 98 | 8 | 6 | 146 | 2 | 157 | T4 LYSOZYME | T4 LYSOZYME | | pdb | 4YXA\_C | 1.0 | 0.00593 | 113 | 0.18 | 155 | 91 | 7 | 7 | 150 | 36 | 165 | Oxygen-regulated invasion protein OrgB,Endolysin | Oxygen-regulated invasion protein OrgB,Endolysin | | pdb | 5BZ6\_A | 1.0 | 0.008593 | 113 | 0.212 | 165 | 90 | 11 | 6 | 143 | 2 | 153 | Lysozyme,Calcium uniporter protein, mitochondrial | Lysozyme,Calcium uniporter protein, mitochondrial | | pdb | 4XSJ\_A | 1.0 | 0.005334 | 112 | 0.196 | 168 | 96 | 11 | 6 | 146 | 2 | 157 | Lysozyme,Calcium uniporter protein, mitochondrial | Lysozyme,Calcium uniporter protein, mitochondrial | | pdb | 6A9J\_B | 1.0 | 0.008593 | 112 | 0.201 | 174 | 100 | 10 | 6 | 151 | 2 | 164 | Endolysin,Autophagy-related protein 2 | Endolysin,Autophagy-related protein 2 | | pdb | 6KK7\_A | 1.0 | 0.004092 | 112 | 0.153 | 195 | 111 | 12 | 6 | 156 | 98 | 282 | Glucagon-like peptide 1 receptor,Endolysin,Glucagon-like peptide 1 receptor | Glucagon-like peptide 1 receptor,Endolysin,Glucagon-like peptide 1 receptor | | pdb | 3OE8\_B | 1.0 | 0.00455 | 112 | 0.198 | 186 | 107 | 11 | 5 | 157 | 194 | 370 | C-X-C chemokine receptor type 4, Lysozyme Chimera | C-X-C chemokine receptor type 4, Lysozyme Chimera | | pdb | 5I0N\_A | 1.0 | 0.001753 | 112 | 0.198 | 176 | 102 | 11 | 4 | 146 | 70 | 239 | Phosphatidylinositol 4-kinase type 2-alpha,Lysozyme,Phosphatidylinositol 4-kinase type 2-alpha | Phosphatidylinositol 4-kinase type 2-alpha,Lysozyme,Phosphatidylinositol 4-kinase type 2-alpha | | pdb | 6KK1\_A | 1.0 | 0.004797 | 111 | 0.173 | 196 | 106 | 10 | 6 | 156 | 98 | 282 | Glucagon-like peptide 1 receptor,Endolysin,Glucagon-like peptide 1 receptor | Glucagon-like peptide 1 receptor,Endolysin,Glucagon-like peptide 1 receptor | | pdb | 5T1A\_A | 1.0 | 0.00593 | 110 | 0.197 | 167 | 95 | 11 | 4 | 140 | 193 | 350 | Chimera protein of CC chemokine receptor type 2 isoform B and T4-lysozyme,Lysozyme | Chimera protein of CC chemokine receptor type 2 isoform B and T4-lysozyme,Lysozyme | | pdb | 150L\_C | 1.0 | 0.006593 | 109 | 0.222 | 157 | 88 | 9 | 8 | 136 | 4 | 154 | T4 LYSOZYME | T4 LYSOZYME | | pdb | 3GUJ\_A | 1.0 | 0.00593 | 108 | 0.194 | 170 | 94 | 11 | 6 | 146 | 2 | 157 | Lysozyme | Lysozyme | | pdb | 6KVX\_A | 1.0 | 0.005059 | 108 | 0.201 | 159 | 92 | 10 | 10 | 140 | 3 | 154 | Endolysin,Calcium uniporter protein, mitochondrial | Endolysin,Calcium uniporter protein, mitochondrial | | pdb | 6KK7\_B | 1.0 | 0.004315 | 108 | 0.158 | 196 | 109 | 10 | 6 | 156 | 97 | 281 | Glucagon-like peptide 1 receptor,Endolysin,Glucagon-like peptide 1 receptor | Glucagon-like peptide 1 receptor,Endolysin,Glucagon-like peptide 1 receptor | | pdb | 6KJV\_A | 1.0 | 0.00455 | 108 | 0.175 | 194 | 108 | 12 | 6 | 156 | 98 | 282 | Glucagon-like peptide 1 receptor,Endolysin,Glucagon-like peptide 1 receptor | Glucagon-like peptide 1 receptor,Endolysin,Glucagon-like peptide 1 receptor | | pdb | 6KK1\_B | 1.0 | 0.004092 | 107 | 0.182 | 203 | 106 | 11 | 6 | 157 | 100 | 293 | Glucagon-like peptide 1 receptor,Endolysin,Glucagon-like peptide 1 receptor | Glucagon-like peptide 1 receptor,Endolysin,Glucagon-like peptide 1 receptor | | pdb | 5GLH\_A | 1.0 | 0.002978 | 107 | 0.209 | 186 | 94 | 9 | 6 | 144 | 202 | 381 | Endothelin Receptor Subtype-B | Endothelin Receptor Subtype-B | | pdb | 4Z9G\_B | 1.0 | 0.004092 | 106 | 0.213 | 192 | 101 | 12 | 6 | 156 | 101 | 283 | Corticotropin-releasing factor receptor 1,Lysozyme,Corticotropin-releasing factor receptor 1 | Corticotropin-releasing factor receptor 1,Lysozyme,Corticotropin-releasing factor receptor 1 | | pdb | 5VEX\_A | 1.0 | 0.00733 | 106 | 0.166 | 198 | 105 | 11 | 6 | 156 | 98 | 282 | Glucagon-like peptide 1 receptor, Endolysin chimera | Glucagon-like peptide 1 receptor, Endolysin chimera | | pdb | 6KJV\_B | 1.0 | 0.004315 | 105 | 0.17 | 194 | 109 | 12 | 6 | 156 | 100 | 284 | Glucagon-like peptide 1 receptor,Endolysin,Glucagon-like peptide 1 receptor | Glucagon-like peptide 1 receptor,Endolysin,Glucagon-like peptide 1 receptor | | pdb | 7JOZ\_R | 1.0 | 0.008149 | 101 | 0.203 | 157 | 91 | 7 | 8 | 136 | 2 | 152 | Endolysin,D(1A) dopamine receptor | Endolysin,D(1A) dopamine receptor | | pdb | 6LB8\_A | 0.999 | 0.009553 | 97 | 0.186 | 172 | 102 | 9 | 6 | 153 | 2 | 159 | Endolysin,Calcium uptake protein 1, mitochondrial | Endolysin,Calcium uptake protein 1, mitochondrial | | afdb-proteome | AF-Q8ZQ99-F1-MODEL\_V4 | 1.0 | 2.577e-15 | 693 | 0.567 | 148 | 60 | 1 | 1 | 144 | 2 | 149 | Lysozyme | Lysozyme | | afdb-proteome | AF-Q8ZMY9-F1-MODEL\_V4 | 1.0 | 1.164e-15 | 676 | 0.554 | 148 | 62 | 2 | 1 | 144 | 2 | 149 | Lysozyme | Lysozyme | | afdb-proteome | AF-P78285-F1-MODEL\_V4 | 1.0 | 1.901e-09 | 404 | 0.319 | 144 | 90 | 3 | 6 | 144 | 21 | 161 | Lysozyme RrrD | Lysozyme RrrD | | afdb-proteome | AF-A0A0H3GKK1-F1-MODEL\_V4 | 1.0 | 6.1e-09 | 399 | 0.34 | 144 | 91 | 3 | 1 | 144 | 27 | 166 | Lysozyme | Lysozyme | | afdb-proteome | AF-Q8ZMT7-F1-MODEL\_V4 | 1.0 | 7.54e-09 | 395 | 0.368 | 144 | 87 | 3 | 1 | 144 | 27 | 166 | Lysozyme | Lysozyme | | afdb-proteome | AF-A0A175VZF5-F1-MODEL\_V4 | 1.0 | 1.281e-08 | 359 | 0.317 | 151 | 91 | 5 | 1 | 142 | 107 | 254 | Lysozyme | Lysozyme | | afdb-proteome | AF-C0NHY3-F1-MODEL\_V4 | 1.0 | 6.979e-08 | 331 | 0.331 | 151 | 87 | 6 | 1 | 142 | 31 | 176 | Lysozyme | Lysozyme | | afdb-proteome | AF-Q32GL6-F1-MODEL\_V4 | 1.0 | 2.918e-07 | 303 | 0.283 | 155 | 95 | 6 | 4 | 145 | 22 | 173 | Lysozyme | Lysozyme | | afdb-proteome | AF-A0A0H3GP93-F1-MODEL\_V4 | 1.0 | 4.701e-07 | 294 | 0.301 | 156 | 92 | 6 | 4 | 145 | 20 | 172 | Lysozyme | Lysozyme | | afdb-proteome | AF-A0A0H3GWD4-F1-MODEL\_V4 | 1.0 | 6.461e-07 | 290 | 0.307 | 156 | 91 | 6 | 4 | 145 | 20 | 172 | Lysozyme | Lysozyme | | afdb-proteome | AF-P76159-F1-MODEL\_V4 | 1.0 | 9.872e-07 | 283 | 0.294 | 153 | 92 | 6 | 4 | 143 | 22 | 171 | Probable prophage lysozyme | Probable prophage lysozyme | | afdb-proteome | AF-Q8ZLC6-F1-MODEL\_V4 | 1.0 | 0.001828 | 150 | 0.24 | 108 | 74 | 3 | 3 | 110 | 8 | 107 | Putative phage endolysin | Putative phage endolysin | | afdb-uniprot50 | AF-A0A1S2UDK3-F1-MODEL\_V4 | 1.0 | 1.808e-21 | 1036 | 0.679 | 159 | 51 | 0 | 1 | 159 | 1 | 159 | Lysozyme | Lysozyme | | afdb-uniprot50 | AF-A0A090V1M7-F1-MODEL\_V4 | 1.0 | 1.394e-19 | 934 | 0.554 | 184 | 57 | 2 | 1 | 159 | 1 | 184 | Lysozyme | Lysozyme | | afdb-uniprot50 | AF-A0A857JAQ9-F1-MODEL\_V4 | 1.0 | 1.441e-16 | 785 | 0.519 | 156 | 74 | 1 | 1 | 155 | 1 | 156 | Glycoside hydrolase family protein | Glycoside hydrolase family protein | | afdb-uniprot50 | AF-A0A6G6JNR3-F1-MODEL\_V4 | 1.0 | 7.236e-17 | 783 | 0.543 | 171 | 66 | 1 | 1 | 159 | 1 | 171 | Lysozyme | Lysozyme | | afdb-uniprot50 | AF-A0A3G6HI33-F1-MODEL\_V4 | 1.0 | 4.384e-16 | 758 | 0.509 | 159 | 75 | 2 | 2 | 159 | 36 | 192 | Lysozyme | Lysozyme | | afdb-uniprot50 | AF-A0A844U3X0-F1-MODEL\_V4 | 1.0 | 1.079e-15 | 742 | 0.493 | 160 | 77 | 3 | 1 | 159 | 1 | 157 | Glycoside hydrolase family protein | Glycoside hydrolase family protein | | afdb-uniprot50 | AF-A0A1W0CID5-F1-MODEL\_V4 | 1.0 | 3.849e-15 | 708 | 0.595 | 146 | 56 | 2 | 1 | 146 | 1 | 143 | Lysozyme | Lysozyme | | afdb-uniprot50 | AF-A0A286B0J8-F1-MODEL\_V4 | 1.0 | 6.2e-15 | 696 | 0.451 | 184 | 74 | 3 | 1 | 157 | 5 | 188 | Lysozyme | Lysozyme | | afdb-uniprot50 | AF-A0A2G1LY75-F1-MODEL\_V4 | 1.0 | 1.111e-14 | 686 | 0.565 | 145 | 62 | 1 | 2 | 145 | 38 | 182 | Lysozyme | Lysozyme | | afdb-uniprot50 | AF-A0A7T2GKL9-F1-MODEL\_V4 | 1.0 | 6.893e-15 | 681 | 0.627 | 145 | 53 | 1 | 1 | 145 | 42 | 185 | Lysozyme | Lysozyme | | afdb-uniprot50 | AF-A0A8B1MMR9-F1-MODEL\_V4 | 1.0 | 2.211e-14 | 676 | 0.54 | 148 | 64 | 1 | 1 | 144 | 1 | 148 | Lysozyme RrrD | Lysozyme RrrD | | afdb-uniprot50 | AF-A0A2W7M8R8-F1-MODEL\_V4 | 1.0 | 1.886e-14 | 675 | 0.53 | 147 | 66 | 2 | 1 | 147 | 1 | 144 | Lysozyme | Lysozyme | | afdb-uniprot50 | AF-A0A2W4VX53-F1-MODEL\_V4 | 1.0 | 1.789e-14 | 666 | 0.49 | 153 | 74 | 1 | 3 | 151 | 1 | 153 | Lysozyme | Lysozyme | | afdb-uniprot50 | AF-A0A4R3ZEW6-F1-MODEL\_V4 | 1.0 | 5.443e-14 | 661 | 0.533 | 148 | 65 | 1 | 1 | 144 | 1 | 148 | Lysozyme | Lysozyme | | afdb-uniprot50 | AF-A0A7Z0MUC7-F1-MODEL\_V4 | 1.0 | 7.481e-14 | 661 | 0.452 | 159 | 84 | 2 | 2 | 159 | 34 | 190 | Lysozyme | Lysozyme | | afdb-uniprot50 | AF-A0A841WQ48-F1-MODEL\_V4 | 1.0 | 2.332e-14 | 656 | 0.456 | 151 | 78 | 3 | 1 | 151 | 4 | 150 | Glycoside hydrolase family protein | Glycoside hydrolase family protein | | afdb-uniprot50 | AF-A0A4Q0YFE7-F1-MODEL\_V4 | 1.0 | 4.404e-14 | 653 | 0.469 | 147 | 72 | 3 | 1 | 144 | 3 | 146 | Lysozyme | Lysozyme | | afdb-uniprot50 | AF-A0A747EZ57-F1-MODEL\_V4 | 1.0 | 1.989e-14 | 648 | 0.543 | 151 | 65 | 2 | 1 | 147 | 2 | 152 | Lysozyme | Lysozyme | | afdb-uniprot50 | AF-W1INZ3-F1-MODEL\_V4 | 1.0 | 8.317e-14 | 642 | 0.5 | 144 | 71 | 1 | 1 | 144 | 1 | 143 | Lysozyme | Lysozyme | | afdb-uniprot50 | AF-A0A0J1IN41-F1-MODEL\_V4 | 1.0 | 9.247e-14 | 642 | 0.537 | 147 | 67 | 1 | 1 | 147 | 1 | 146 | Lysozyme | Lysozyme | | afdb-uniprot50 | AF-A0A2D8XAB4-F1-MODEL\_V4 | 1.0 | 7.481e-14 | 634 | 0.486 | 150 | 73 | 3 | 1 | 150 | 1 | 146 | Lysozyme | Lysozyme | | afdb-uniprot50 | AF-A0A4P5ZKT6-F1-MODEL\_V4 | 1.0 | 1.028e-13 | 629 | 0.506 | 150 | 70 | 2 | 1 | 150 | 67 | 212 | Lysozyme | Lysozyme | | afdb-uniprot50 | AF-A0A2X0S8V7-F1-MODEL\_V4 | 1.0 | 1.656e-13 | 625 | 0.516 | 149 | 71 | 1 | 1 | 149 | 1 | 148 | Lysozyme | Lysozyme | | afdb-uniprot50 | AF-A0A3P1XHR8-F1-MODEL\_V4 | 1.0 | 1.34e-13 | 624 | 0.523 | 147 | 67 | 2 | 1 | 147 | 350 | 493 | Lysozyme | Lysozyme | | afdb-uniprot50 | AF-A0A846A290-F1-MODEL\_V4 | 1.0 | 1.656e-13 | 622 | 0.473 | 150 | 75 | 3 | 2 | 151 | 81 | 226 | Lysozyme | Lysozyme | | afdb-uniprot50 | AF-A0A7U2UZ55-F1-MODEL\_V4 | 1.0 | 1.34e-13 | 619 | 0.496 | 147 | 70 | 1 | 1 | 143 | 1 | 147 | Lysozyme | Lysozyme | | afdb-uniprot50 | AF-A0A4Q7YUD0-F1-MODEL\_V4 | 1.0 | 1.571e-13 | 616 | 0.51 | 143 | 67 | 2 | 1 | 143 | 4 | 143 | Lysozyme | Lysozyme | | afdb-uniprot50 | AF-A0A3M9Z9E8-F1-MODEL\_V4 | 1.0 | 4.298e-13 | 615 | 0.496 | 145 | 70 | 2 | 2 | 146 | 29 | 170 | Lysozyme | Lysozyme | | afdb-uniprot50 | AF-A0A5J6N4Q1-F1-MODEL\_V4 | 1.0 | 3.667e-13 | 614 | 0.479 | 144 | 72 | 2 | 1 | 144 | 1 | 141 | Lysozyme | Lysozyme | | afdb-uniprot50 | AF-A0A6I5P031-F1-MODEL\_V4 | 1.0 | 3.298e-13 | 612 | 0.483 | 151 | 74 | 3 | 1 | 151 | 30 | 176 | Lysozyme | Lysozyme | | afdb-uniprot50 | AF-A0A2V9UU03-F1-MODEL\_V4 | 1.0 | 4.298e-13 | 605 | 0.486 | 146 | 72 | 2 | 1 | 146 | 7 | 149 | Lysozyme | Lysozyme | | afdb-uniprot50 | AF-A0A1S1NZ32-F1-MODEL\_V4 | 1.0 | 7.302e-13 | 605 | 0.503 | 143 | 67 | 3 | 1 | 143 | 1 | 139 | Lysozyme | Lysozyme | | afdb-uniprot50 | AF-A0A3M9ZBC4-F1-MODEL\_V4 | 1.0 | 3.866e-13 | 605 | 0.526 | 150 | 67 | 3 | 2 | 151 | 112 | 257 | Lysozyme | Lysozyme | | afdb-uniprot50 | AF-A0A7D0IRK1-F1-MODEL\_V4 | 1.0 | 5.602e-13 | 603 | 0.479 | 144 | 75 | 0 | 1 | 144 | 1 | 144 | Lysozyme | Lysozyme | | afdb-uniprot50 | AF-A0A7T9EKQ7-F1-MODEL\_V4 | 1.0 | 8.56e-13 | 603 | 0.51 | 145 | 68 | 2 | 1 | 145 | 8 | 149 | Lysozyme | Lysozyme | | afdb-uniprot50 | AF-A0A2T1DR14-F1-MODEL\_V4 | 1.0 | 4.298e-13 | 602 | 0.486 | 150 | 73 | 3 | 2 | 151 | 8 | 153 | Lysozyme | Lysozyme | | afdb-uniprot50 | AF-A0A4S3LZ40-F1-MODEL\_V4 | 1.0 | 5.313e-13 | 601 | 0.486 | 146 | 71 | 3 | 1 | 146 | 1 | 142 | Lysozyme | Lysozyme | | afdb-uniprot50 | AF-A0A0Q2RDV9-F1-MODEL\_V4 | 1.0 | 1.841e-13 | 601 | 0.446 | 159 | 83 | 4 | 2 | 158 | 3 | 158 | Lysozyme | Lysozyme | | afdb-uniprot50 | AF-A0A5E8KST5-F1-MODEL\_V4 | 1.0 | 7.302e-13 | 600 | 0.466 | 148 | 78 | 1 | 1 | 148 | 20 | 166 | Lysozyme | Lysozyme | | afdb-uniprot50 | AF-A0A239PTC6-F1-MODEL\_V4 | 1.0 | 3.667e-13 | 600 | 0.52 | 148 | 64 | 4 | 1 | 144 | 1 | 145 | Lysozyme | Lysozyme | | afdb-uniprot50 | AF-A0A348HCF7-F1-MODEL\_V4 | 1.0 | 9.517e-13 | 600 | 0.468 | 145 | 76 | 1 | 1 | 144 | 2 | 146 | Lysozyme | Lysozyme | | afdb-uniprot50 | AF-A0A7Y6TTM5-F1-MODEL\_V4 | 1.0 | 2.4e-13 | 599 | 0.44 | 175 | 69 | 2 | 1 | 146 | 1 | 175 | Lysozyme | Lysozyme | | afdb-uniprot50 | AF-S3JZ44-F1-MODEL\_V4 | 1.0 | 1.058e-12 | 598 | 0.468 | 145 | 73 | 3 | 1 | 145 | 1 | 141 | Lysozyme | Lysozyme | | afdb-uniprot50 | AF-A0A5E4NVC9-F1-MODEL\_V4 | 1.0 | 6.925e-13 | 594 | 0.459 | 148 | 76 | 3 | 1 | 148 | 3 | 146 | Lysozyme | Lysozyme | | afdb-uniprot50 | AF-A0A2E4ZEW9-F1-MODEL\_V4 | 1.0 | 3.866e-13 | 593 | 0.454 | 154 | 79 | 4 | 1 | 153 | 1 | 150 | Lysozyme | Lysozyme | | afdb-uniprot50 | AF-A0A381SG74-F1-MODEL\_V4 | 1.0 | 5.602e-13 | 593 | 0.457 | 153 | 79 | 3 | 1 | 153 | 22 | 170 | Lysozyme | Lysozyme | | afdb-uniprot50 | AF-A0A7H0LF26-F1-MODEL\_V4 | 1.0 | 6.229e-13 | 592 | 0.472 | 146 | 74 | 2 | 2 | 146 | 49 | 192 | Lysozyme | Lysozyme | | afdb-uniprot50 | AF-A0A1C4GBY3-F1-MODEL\_V4 | 1.0 | 1.797e-12 | 590 | 0.513 | 148 | 65 | 3 | 1 | 144 | 1 | 145 | Lysozyme | Lysozyme | | afdb-uniprot50 | AF-A0A3N8QQG0-F1-MODEL\_V4 | 1.0 | 4.532e-13 | 587 | 0.421 | 173 | 71 | 1 | 1 | 144 | 3 | 175 | Lysozyme | Lysozyme | | afdb-uniprot50 | AF-A0A158DN86-F1-MODEL\_V4 | 1.0 | 1.24e-12 | 585 | 0.444 | 144 | 80 | 0 | 1 | 144 | 1 | 144 | Lysozyme | Lysozyme | | afdb-uniprot50 | AF-J2HCF9-F1-MODEL\_V4 | 1.0 | 7.302e-13 | 584 | 0.486 | 148 | 71 | 3 | 1 | 145 | 1 | 146 | Lysozyme | Lysozyme | | afdb-uniprot50 | AF-A0A846FGE4-F1-MODEL\_V4 | 1.0 | 1.454e-12 | 583 | 0.49 | 151 | 73 | 3 | 1 | 151 | 53 | 199 | Lysozyme | Lysozyme | | afdb-uniprot50 | AF-A0A6I2IUJ3-F1-MODEL\_V4 | 1.0 | 6.568e-13 | 581 | 0.455 | 156 | 80 | 4 | 2 | 155 | 3 | 155 | Lysozyme | Lysozyme | | afdb-uniprot50 | AF-A0A494X2V0-F1-MODEL\_V4 | 1.0 | 4.779e-13 | 581 | 0.434 | 182 | 72 | 2 | 2 | 153 | 6 | 186 | Lysozyme | Lysozyme | | afdb-uniprot50 | AF-A0A3B9WX79-F1-MODEL\_V4 | 1.0 | 8.118e-13 | 580 | 0.464 | 153 | 75 | 3 | 1 | 150 | 1 | 149 | Lysozyme | Lysozyme | | afdb-uniprot50 | AF-A0A7W7K3I1-F1-MODEL\_V4 | 1.0 | 1.176e-12 | 580 | 0.49 | 151 | 69 | 2 | 1 | 143 | 1 | 151 | Lysozyme | Lysozyme | | afdb-uniprot50 | AF-A0A4S1WM04-F1-MODEL\_V4 | 1.0 | 1.705e-12 | 579 | 0.526 | 152 | 64 | 2 | 1 | 144 | 24 | 175 | Lysozyme | Lysozyme | | afdb-uniprot50 | AF-A0A2G2MWX1-F1-MODEL\_V4 | 1.0 | 4.532e-13 | 579 | 0.489 | 147 | 68 | 4 | 1 | 143 | 1 | 144 | Lysozyme | Lysozyme | | afdb-uniprot50 | AF-A0A2A2JXU9-F1-MODEL\_V4 | 1.0 | 8.56e-13 | 578 | 0.506 | 144 | 67 | 3 | 1 | 144 | 1 | 140 | Lysozyme | Lysozyme | | afdb-uniprot50 | AF-A0A5C7QC42-F1-MODEL\_V4 | 1.0 | 1.454e-12 | 575 | 0.493 | 146 | 70 | 3 | 1 | 145 | 1 | 143 | Lysozyme | Lysozyme | | afdb-uniprot50 | AF-A0A3S0BYI5-F1-MODEL\_V4 | 1.0 | 2.107e-12 | 575 | 0.482 | 143 | 70 | 2 | 1 | 143 | 4 | 142 | Lysozyme | Lysozyme | | afdb-uniprot50 | AF-A0A1M6MED4-F1-MODEL\_V4 | 1.0 | 6.41e-12 | 575 | 0.486 | 144 | 73 | 1 | 1 | 144 | 3 | 145 | Lysozyme | Lysozyme | | afdb-uniprot50 | AF-A0A0K1NGA9-F1-MODEL\_V4 | 1.0 | 3.219e-12 | 573 | 0.468 | 143 | 73 | 2 | 1 | 143 | 1 | 140 | Lysozyme | Lysozyme | | afdb-uniprot50 | AF-A0A0C1VC21-F1-MODEL\_V4 | 1.0 | 2.342e-12 | 571 | 0.441 | 154 | 79 | 3 | 2 | 151 | 11 | 161 | Lysozyme | Lysozyme | | afdb-uniprot50 | AF-A0A4Q7EGY7-F1-MODEL\_V4 | 1.0 | 2.47e-12 | 569 | 0.441 | 154 | 79 | 3 | 2 | 151 | 80 | 230 | Lysozyme | Lysozyme | | afdb-uniprot50 | AF-N8WAZ3-F1-MODEL\_V4 | 1.0 | 3.579e-12 | 568 | 0.493 | 148 | 68 | 2 | 1 | 144 | 63 | 207 | Lysozyme | Lysozyme | | afdb-uniprot50 | AF-Q9P9P8-F1-MODEL\_V4 | 1.0 | 1.176e-12 | 567 | 0.465 | 159 | 80 | 4 | 2 | 158 | 41 | 196 | Lysozyme | Lysozyme | | afdb-uniprot50 | AF-A0A349GTM3-F1-MODEL\_V4 | 1.0 | 2.342e-12 | 566 | 0.442 | 149 | 76 | 4 | 1 | 145 | 1 | 146 | Lysozyme | Lysozyme | | afdb-uniprot50 | AF-A0A6P0MVH8-F1-MODEL\_V4 | 1.0 | 1.003e-12 | 565 | 0.449 | 158 | 73 | 2 | 1 | 147 | 1 | 155 | Lysozyme | Lysozyme | | afdb-uniprot50 | AF-A0A6B9YSE3-F1-MODEL\_V4 | 1.0 | 3.579e-12 | 565 | 0.503 | 143 | 68 | 2 | 1 | 143 | 4 | 143 | Lysozyme | Lysozyme | | afdb-uniprot50 | AF-A0A3B8XMC2-F1-MODEL\_V4 | 1.0 | 2.895e-12 | 565 | 0.413 | 145 | 81 | 2 | 1 | 145 | 3 | 143 | Lysozyme | Lysozyme | | afdb-uniprot50 | AF-A0A653CSV6-F1-MODEL\_V4 | 1.0 | 5.766e-12 | 564 | 0.5 | 144 | 68 | 2 | 1 | 144 | 112 | 251 | Lysozyme | Lysozyme | | afdb-uniprot50 | AF-B6VNM2-F1-MODEL\_V4 | 1.0 | 5.766e-12 | 563 | 0.496 | 143 | 68 | 2 | 1 | 143 | 25 | 163 | Lysozyme | Lysozyme | | afdb-uniprot50 | AF-A0A1I0J1Q9-F1-MODEL\_V4 | 1.0 | 1.454e-12 | 563 | 0.516 | 149 | 63 | 4 | 1 | 143 | 1 | 146 | Lysozyme | Lysozyme | | afdb-uniprot50 | AF-I3YEG4-F1-MODEL\_V4 | 1.0 | 5.186e-12 | 561 | 0.475 | 143 | 71 | 3 | 1 | 143 | 10 | 148 | Lysozyme | Lysozyme | | afdb-uniprot50 | AF-A0A5A9FA75-F1-MODEL\_V4 | 1.0 | 8.118e-13 | 561 | 0.44 | 161 | 73 | 1 | 1 | 144 | 2 | 162 | Lysozyme | Lysozyme | | afdb-uniprot50 | AF-A0A1H8EK82-F1-MODEL\_V4 | 1.0 | 6.08e-12 | 559 | 0.469 | 147 | 76 | 2 | 1 | 146 | 3 | 148 | Lysozyme | Lysozyme | | afdb-uniprot50 | AF-A0A349JKR9-F1-MODEL\_V4 | 1.0 | 3.774e-12 | 558 | 0.47 | 151 | 75 | 3 | 3 | 151 | 98 | 245 | Lysozyme | Lysozyme | | afdb-uniprot50 | AF-A0A840YK85-F1-MODEL\_V4 | 1.0 | 3.979e-12 | 557 | 0.476 | 147 | 72 | 4 | 1 | 147 | 46 | 187 | Lysozyme | Lysozyme | | afdb-uniprot50 | AF-A0A4Q6Y9K8-F1-MODEL\_V4 | 1.0 | 4.424e-12 | 555 | 0.517 | 141 | 67 | 1 | 4 | 144 | 1 | 140 | Lysozyme | Lysozyme | | afdb-uniprot50 | AF-A0A2X1ULF4-F1-MODEL\_V4 | 1.0 | 9.517e-13 | 554 | 0.477 | 157 | 67 | 5 | 1 | 146 | 5 | 157 | Lysozyme | Lysozyme | | afdb-uniprot50 | AF-A0A840I6B0-F1-MODEL\_V4 | 1.0 | 5.186e-12 | 551 | 0.466 | 150 | 73 | 4 | 1 | 146 | 1 | 147 | Lysozyme | Lysozyme | | afdb-uniprot50 | AF-A0A1I6KZC5-F1-MODEL\_V4 | 1.0 | 5.766e-12 | 551 | 0.472 | 148 | 75 | 2 | 1 | 148 | 15 | 159 | Lysozyme | Lysozyme | | afdb-uniprot50 | AF-A0A442TIX8-F1-MODEL\_V4 | 1.0 | 3.774e-12 | 550 | 0.493 | 150 | 69 | 3 | 1 | 146 | 1 | 147 | Lysozyme | Lysozyme | | afdb-uniprot50 | AF-A0A2U1WSM1-F1-MODEL\_V4 | 1.0 | 2.47e-12 | 549 | 0.424 | 158 | 81 | 6 | 1 | 154 | 3 | 154 | Lysozyme | Lysozyme | | afdb-uniprot50 | AF-A0A858RDI0-F1-MODEL\_V4 | 1.0 | 6.08e-12 | 549 | 0.466 | 150 | 70 | 4 | 1 | 145 | 43 | 187 | Lysozyme | Lysozyme | | afdb-uniprot50 | AF-A0A6C8N2Y9-F1-MODEL\_V4 | 1.0 | 2.746e-12 | 549 | 0.439 | 148 | 81 | 2 | 1 | 147 | 1 | 147 | Lysozyme | Lysozyme | | afdb-uniprot50 | AF-A0A7J6JRX3-F1-MODEL\_V4 | 1.0 | 1.211e-11 | 549 | 0.514 | 142 | 66 | 1 | 2 | 143 | 6 | 144 | Lysozyme | Lysozyme | | afdb-uniprot50 | AF-T2JMX1-F1-MODEL\_V4 | 1.0 | 6.759e-12 | 549 | 0.391 | 148 | 87 | 2 | 1 | 148 | 1 | 145 | Lysozyme | Lysozyme | | afdb-uniprot50 | AF-A0A844YLN9-F1-MODEL\_V4 | 1.0 | 4.919e-12 | 548 | 0.496 | 149 | 71 | 2 | 3 | 147 | 46 | 194 | Glycoside hydrolase family protein | Glycoside hydrolase family protein | | afdb-uniprot50 | AF-A0A7Y3W4Z2-F1-MODEL\_V4 | 1.0 | 7.515e-12 | 548 | 0.472 | 148 | 71 | 3 | 1 | 144 | 1 | 145 | Lysozyme | Lysozyme | | afdb-uniprot50 | AF-A0A2N3AFJ7-F1-MODEL\_V4 | 1.0 | 6.41e-12 | 546 | 0.466 | 148 | 72 | 2 | 2 | 145 | 25 | 169 | Lysozyme | Lysozyme | | afdb-uniprot50 | AF-A0A537UHK2-F1-MODEL\_V4 | 1.0 | 2.746e-12 | 546 | 0.478 | 165 | 72 | 4 | 1 | 156 | 81 | 240 | Lysozyme | Lysozyme | | afdb-uniprot50 | AF-A0A2V2RWC3-F1-MODEL\_V4 | 1.0 | 1.617e-12 | 545 | 0.475 | 166 | 70 | 5 | 1 | 155 | 5 | 164 | Lysozyme | Lysozyme | | afdb-uniprot50 | AF-A0A7W1RQP8-F1-MODEL\_V4 | 1.0 | 6.759e-12 | 544 | 0.423 | 151 | 77 | 3 | 1 | 146 | 2 | 147 | Lysozyme | Lysozyme | | afdb-uniprot50 | AF-A0A546YGV8-F1-MODEL\_V4 | 1.0 | 7.515e-12 | 544 | 0.462 | 147 | 71 | 4 | 1 | 143 | 1 | 143 | Lysozyme | Lysozyme | | afdb-uniprot50 | AF-A3Z6C9-F1-MODEL\_V4 | 1.0 | 8.355e-12 | 544 | 0.406 | 150 | 84 | 4 | 1 | 147 | 3 | 150 | Lysozyme | Lysozyme | | afdb-uniprot50 | AF-A0A1I6PPK5-F1-MODEL\_V4 | 1.0 | 9.289e-12 | 543 | 0.496 | 149 | 70 | 2 | 1 | 144 | 43 | 191 | Lysozyme | Lysozyme | | afdb-uniprot50 | AF-L8M8V2-F1-MODEL\_V4 | 1.0 | 1.277e-11 | 543 | 0.443 | 142 | 75 | 2 | 3 | 144 | 59 | 196 | Lysozyme | Lysozyme | | afdb-uniprot50 | AF-A0A2D9TFC8-F1-MODEL\_V4 | 1.0 | 7.515e-12 | 543 | 0.469 | 147 | 71 | 4 | 1 | 146 | 1 | 141 | Lysozyme | Lysozyme | | afdb-uniprot50 | AF-A0A0J7Y672-F1-MODEL\_V4 | 1.0 | 5.186e-12 | 543 | 0.459 | 148 | 76 | 3 | 2 | 147 | 5 | 150 | Lysozyme | Lysozyme | | afdb-uniprot50 | AF-V4R5W7-F1-MODEL\_V4 | 1.0 | 6.568e-13 | 542 | 0.384 | 177 | 84 | 5 | 1 | 155 | 1 | 174 | Lysozyme | Lysozyme | | afdb-uniprot50 | AF-N9YWK1-F1-MODEL\_V4 | 1.0 | 2.286e-11 | 541 | 0.513 | 144 | 66 | 3 | 1 | 144 | 340 | 479 | Lysozyme | Lysozyme | | afdb-uniprot50 | AF-A0A504F0U0-F1-MODEL\_V4 | 1.0 | 4.424e-12 | 539 | 0.452 | 148 | 73 | 5 | 1 | 144 | 3 | 146 | Lysozyme | Lysozyme | | afdb-uniprot50 | AF-A0A1K1QDJ6-F1-MODEL\_V4 | 1.0 | 1.754e-11 | 539 | 0.46 | 139 | 75 | 0 | 6 | 144 | 6 | 144 | Lysozyme | Lysozyme | | afdb-uniprot50 | AF-A0A4Y9EQV7-F1-MODEL\_V4 | 1.0 | 9.794e-12 | 539 | 0.465 | 146 | 71 | 3 | 3 | 143 | 2 | 145 | Lysozyme | Lysozyme | | afdb-uniprot50 | AF-M1N4J0-F1-MODEL\_V4 | 1.0 | 1.089e-11 | 539 | 0.462 | 147 | 75 | 3 | 1 | 147 | 2 | 144 | Lysozyme | Lysozyme | | afdb-uniprot50 | AF-A0A1M3DRS2-F1-MODEL\_V4 | 1.0 | 6.759e-12 | 537 | 0.391 | 143 | 84 | 2 | 2 | 144 | 3 | 142 | Lysozyme | Lysozyme | | afdb-uniprot50 | AF-A0A4P6L9W1-F1-MODEL\_V4 | 1.0 | 4.919e-12 | 537 | 0.464 | 153 | 79 | 2 | 1 | 150 | 1 | 153 | Lysozyme | Lysozyme | | afdb-uniprot50 | AF-A0A2E0ELT0-F1-MODEL\_V4 | 1.0 | 1.211e-11 | 535 | 0.413 | 145 | 81 | 3 | 1 | 144 | 2 | 143 | Lysozyme | Lysozyme | | afdb-uniprot50 | AF-A0A225DZS3-F1-MODEL\_V4 | 1.0 | 5.766e-12 | 535 | 0.412 | 143 | 81 | 2 | 1 | 143 | 1 | 140 | Lysozyme | Lysozyme | | afdb-uniprot50 | AF-A0A4Z1QPU2-F1-MODEL\_V4 | 1.0 | 1.346e-11 | 534 | 0.463 | 151 | 72 | 4 | 1 | 146 | 3 | 149 | Lysozyme | Lysozyme | | afdb-uniprot50 | AF-A0A2W7HI45-F1-MODEL\_V4 | 1.0 | 6.08e-12 | 534 | 0.393 | 173 | 77 | 1 | 1 | 145 | 75 | 247 | Lysozyme | Lysozyme | | afdb-uniprot50 | AF-A0A371R7Q5-F1-MODEL\_V4 | 1.0 | 2.542e-11 | 534 | 0.465 | 144 | 74 | 2 | 1 | 144 | 1 | 141 | Lysozyme | Lysozyme | | afdb-uniprot50 | AF-A0A756FUB3-F1-MODEL\_V4 | 1.0 | 5.766e-12 | 533 | 0.35 | 160 | 102 | 2 | 1 | 159 | 2 | 160 | Lysozyme | Lysozyme | | afdb-uniprot50 | AF-R7PL86-F1-MODEL\_V4 | 1.0 | 2.411e-11 | 532 | 0.451 | 144 | 75 | 3 | 1 | 144 | 3 | 142 | Lysozyme | Lysozyme | | afdb-uniprot50 | AF-A0A560B879-F1-MODEL\_V4 | 1.0 | 7.924e-12 | 531 | 0.448 | 147 | 73 | 5 | 1 | 143 | 28 | 170 | Lysozyme | Lysozyme | | afdb-uniprot50 | AF-Q4FSX3-F1-MODEL\_V4 | 1.0 | 1.277e-11 | 531 | 0.493 | 150 | 68 | 3 | 3 | 147 | 57 | 203 | Lysozyme | Lysozyme | | afdb-uniprot50 | AF-A0A318N2U1-F1-MODEL\_V4 | 1.0 | 3.493e-11 | 530 | 0.37 | 143 | 86 | 3 | 1 | 143 | 1 | 139 | Lysozyme | Lysozyme | | afdb-uniprot50 | AF-A0A4D8QPL9-F1-MODEL\_V4 | 1.0 | 7.924e-12 | 530 | 0.432 | 148 | 76 | 5 | 3 | 146 | 5 | 148 | Lysozyme | Lysozyme | | afdb-uniprot50 | AF-A0A4D8QAE7-F1-MODEL\_V4 | 1.0 | 4.424e-12 | 530 | 0.429 | 163 | 85 | 5 | 1 | 159 | 5 | 163 | Lysozyme | Lysozyme | | afdb-uniprot50 | AF-A0A0A2W020-F1-MODEL\_V4 | 1.0 | 6.759e-12 | 530 | 0.458 | 144 | 74 | 3 | 4 | 146 | 421 | 561 | Lysozyme | Lysozyme | | afdb-uniprot50 | AF-A0A6M3XRR4-F1-MODEL\_V4 | 1.0 | 1.419e-11 | 529 | 0.445 | 146 | 77 | 3 | 1 | 145 | 1 | 143 | Lysozyme | Lysozyme | | afdb-uniprot50 | AF-A0A495FZX1-F1-MODEL\_V4 | 1.0 | 6.41e-12 | 529 | 0.406 | 172 | 74 | 2 | 1 | 144 | 33 | 204 | Lysozyme | Lysozyme | | afdb-uniprot50 | AF-A0A0F9HUD0-F1-MODEL\_V4 | 1.0 | 6.759e-12 | 528 | 0.414 | 152 | 81 | 3 | 1 | 147 | 1 | 149 | Lysozyme | Lysozyme | | afdb-uniprot50 | AF-A0A662Z686-F1-MODEL\_V4 | 1.0 | 2.542e-11 | 527 | 0.442 | 147 | 78 | 2 | 1 | 144 | 1 | 146 | Lysozyme | Lysozyme | | afdb-uniprot50 | AF-A0A561M7L1-F1-MODEL\_V4 | 1.0 | 1.277e-11 | 527 | 0.465 | 144 | 73 | 3 | 2 | 144 | 7 | 147 | Lysozyme | Lysozyme | | afdb-uniprot50 | AF-A0A366I5P1-F1-MODEL\_V4 | 1.0 | 1.346e-11 | 527 | 0.458 | 144 | 74 | 3 | 2 | 144 | 7 | 147 | Lysozyme | Lysozyme | | afdb-uniprot50 | AF-A0A2E7GSA7-F1-MODEL\_V4 | 1.0 | 2.826e-11 | 527 | 0.436 | 142 | 76 | 3 | 2 | 143 | 43 | 180 | Lysozyme | Lysozyme | | afdb-uniprot50 | AF-A0A2W5V772-F1-MODEL\_V4 | 1.0 | 2.68e-11 | 527 | 0.521 | 142 | 65 | 2 | 2 | 143 | 73 | 211 | Lysozyme | Lysozyme | | afdb-uniprot50 | AF-A0A522A0K7-F1-MODEL\_V4 | 1.0 | 2.98e-11 | 526 | 0.462 | 147 | 75 | 3 | 1 | 147 | 1 | 143 | Lysozyme | Lysozyme | | afdb-uniprot50 | AF-A0A6P0VW89-F1-MODEL\_V4 | 1.0 | 9.794e-12 | 526 | 0.433 | 157 | 75 | 3 | 1 | 146 | 1 | 154 | Lysozyme | Lysozyme | | afdb-uniprot50 | AF-A0A2E2D638-F1-MODEL\_V4 | 1.0 | 2.057e-11 | 525 | 0.413 | 145 | 81 | 3 | 1 | 145 | 21 | 161 | Lysozyme | Lysozyme | | afdb-uniprot50 | AF-A0A4V3SSF5-F1-MODEL\_V4 | 1.0 | 5.186e-12 | 522 | 0.419 | 155 | 77 | 4 | 1 | 145 | 1 | 152 | Lysozyme | Lysozyme | | afdb-uniprot50 | AF-L7U523-F1-MODEL\_V4 | 1.0 | 5.934e-11 | 521 | 0.406 | 150 | 82 | 4 | 1 | 147 | 1 | 146 | Lysozyme | Lysozyme | | afdb-uniprot50 | AF-A0A2A4Z0X3-F1-MODEL\_V4 | 1.0 | 1.496e-11 | 521 | 0.463 | 151 | 72 | 4 | 1 | 146 | 2 | 148 | Lysozyme | Lysozyme | | afdb-uniprot50 | AF-J1K1J4-F1-MODEL\_V4 | 1.0 | 3.142e-11 | 521 | 0.412 | 148 | 80 | 3 | 1 | 144 | 2 | 146 | Lysozyme | Lysozyme | | afdb-uniprot50 | AF-A0A433J7K8-F1-MODEL\_V4 | 1.0 | 1.95e-11 | 521 | 0.44 | 150 | 76 | 5 | 1 | 146 | 12 | 157 | Lysozyme | Lysozyme | | afdb-uniprot50 | AF-A0A858RGZ4-F1-MODEL\_V4 | 1.0 | 2.542e-11 | 521 | 0.439 | 157 | 76 | 5 | 1 | 152 | 129 | 278 | Lysozyme | Lysozyme | | afdb-uniprot50 | AF-A0A840E055-F1-MODEL\_V4 | 1.0 | 3.884e-11 | 520 | 0.452 | 148 | 74 | 3 | 1 | 144 | 2 | 146 | Lysozyme | Lysozyme | | afdb-uniprot50 | AF-A0A7T9C8N5-F1-MODEL\_V4 | 1.0 | 3.313e-11 | 519 | 0.421 | 140 | 78 | 2 | 4 | 143 | 9 | 145 | Lysozyme | Lysozyme | | afdb-uniprot50 | AF-A0A3A4WDP1-F1-MODEL\_V4 | 1.0 | 4.424e-12 | 519 | 0.426 | 157 | 80 | 5 | 3 | 153 | 2 | 154 | Lysozyme | Lysozyme | | afdb-uniprot50 | AF-S7Y3M8-F1-MODEL\_V4 | 1.0 | 3.142e-11 | 519 | 0.506 | 148 | 66 | 3 | 1 | 144 | 33 | 177 | Lysozyme | Lysozyme | | afdb-uniprot50 | AF-A0A3S3SML5-F1-MODEL\_V4 | 1.0 | 3.313e-11 | 519 | 0.438 | 146 | 79 | 2 | 1 | 146 | 4 | 146 | Lysozyme | Lysozyme | | afdb-uniprot50 | AF-A0A8A7U198-F1-MODEL\_V4 | 1.0 | 8.355e-12 | 519 | 0.5 | 154 | 66 | 5 | 1 | 146 | 1 | 151 | Glycoside hydrolase family protein | Glycoside hydrolase family protein | | afdb-uniprot50 | AF-A9IN99-F1-MODEL\_V4 | 1.0 | 3.493e-11 | 519 | 0.418 | 148 | 79 | 3 | 1 | 144 | 2 | 146 | Lysozyme | Lysozyme | | afdb-uniprot50 | AF-A0A355T917-F1-MODEL\_V4 | 1.0 | 3.884e-11 | 518 | 0.44 | 143 | 76 | 2 | 1 | 143 | 18 | 156 | Lysozyme | Lysozyme | | afdb-uniprot50 | AF-A0A2Z6C8X5-F1-MODEL\_V4 | 1.0 | 1.277e-11 | 518 | 0.416 | 161 | 80 | 3 | 2 | 152 | 69 | 225 | Lysozyme | Lysozyme | | afdb-uniprot50 | AF-A0A3E0CLR5-F1-MODEL\_V4 | 1.0 | 1.754e-11 | 516 | 0.401 | 172 | 75 | 1 | 1 | 144 | 22 | 193 | Lysozyme | Lysozyme | | afdb-uniprot50 | AF-A0A3B0S578-F1-MODEL\_V4 | 1.0 | 3.142e-11 | 516 | 0.439 | 148 | 76 | 3 | 1 | 144 | 1 | 145 | Lysozyme | Lysozyme | | afdb-uniprot50 | AF-A0A5J4X7D3-F1-MODEL\_V4 | 1.0 | 1.664e-11 | 515 | 0.479 | 148 | 69 | 5 | 1 | 144 | 31 | 174 | Lysozyme | Lysozyme | | afdb-uniprot50 | AF-A0A2U1YTM3-F1-MODEL\_V4 | 1.0 | 1.211e-11 | 514 | 0.496 | 133 | 66 | 1 | 15 | 146 | 2 | 134 | Lysozyme | Lysozyme | | afdb-uniprot50 | AF-A0A7M5WWG7-F1-MODEL\_V4 | 1.0 | 6.598e-11 | 514 | 0.444 | 144 | 76 | 3 | 1 | 144 | 21 | 160 | Lysozyme | Lysozyme | | afdb-uniprot50 | AF-A0A371YID5-F1-MODEL\_V4 | 1.0 | 2.286e-11 | 514 | 0.506 | 146 | 65 | 4 | 1 | 143 | 39 | 180 | Lysozyme | Lysozyme | | afdb-uniprot50 | AF-A0A3A2IVA4-F1-MODEL\_V4 | 1.0 | 2.542e-11 | 514 | 0.401 | 152 | 82 | 4 | 1 | 146 | 3 | 151 | Lysozyme | Lysozyme | | afdb-uniprot50 | AF-A0A2X0QI68-F1-MODEL\_V4 | 1.0 | 2.168e-11 | 514 | 0.465 | 144 | 73 | 2 | 1 | 144 | 1 | 140 | Lysozyme | Lysozyme | | afdb-uniprot50 | AF-A0A839W1G4-F1-MODEL\_V4 | 1.0 | 2.168e-11 | 513 | 0.433 | 150 | 77 | 5 | 1 | 146 | 12 | 157 | GH24 family phage-related lysozyme (Muramidase) | GH24 family phage-related lysozyme (Muramidase) | | afdb-uniprot50 | AF-A0A844IYI4-F1-MODEL\_V4 | 1.0 | 9.56e-11 | 513 | 0.486 | 144 | 70 | 3 | 1 | 144 | 3 | 142 | Glycoside hydrolase family protein | Glycoside hydrolase family protein | | afdb-uniprot50 | AF-A0A261R4U0-F1-MODEL\_V4 | 1.0 | 3.884e-11 | 512 | 0.397 | 146 | 87 | 1 | 1 | 145 | 12 | 157 | Lysozyme | Lysozyme | | afdb-uniprot50 | AF-A0A7S6VXX3-F1-MODEL\_V4 | 1.0 | 1.85e-11 | 512 | 0.506 | 146 | 65 | 4 | 1 | 143 | 36 | 177 | Lysozyme | Lysozyme | | afdb-uniprot50 | AF-A0A850T1I2-F1-MODEL\_V4 | 1.0 | 4.553e-11 | 512 | 0.48 | 150 | 70 | 5 | 1 | 146 | 1 | 146 | Lysozyme | Lysozyme | | afdb-uniprot50 | AF-N6UDE9-F1-MODEL\_V4 | 1.0 | 5.338e-11 | 511 | 0.391 | 148 | 83 | 2 | 1 | 144 | 2 | 146 | Lysozyme | Lysozyme | | afdb-uniprot50 | AF-A0A4P6LA78-F1-MODEL\_V4 | 1.0 | 6.257e-11 | 511 | 0.41 | 146 | 83 | 2 | 2 | 145 | 12 | 156 | Lysozyme | Lysozyme | | afdb-uniprot50 | AF-A0A2H5SCH4-F1-MODEL\_V4 | 1.0 | 3.142e-11 | 510 | 0.397 | 146 | 82 | 3 | 1 | 143 | 20 | 162 | Lysozyme | Lysozyme | | afdb-uniprot50 | AF-A0A2N7VBE1-F1-MODEL\_V4 | 1.0 | 4.095e-11 | 509 | 0.465 | 144 | 73 | 3 | 2 | 144 | 7 | 147 | Lysozyme | Lysozyme | | afdb-uniprot50 | AF-A0A5C7LRY7-F1-MODEL\_V4 | 1.0 | 4.318e-11 | 508 | 0.43 | 151 | 79 | 4 | 1 | 147 | 59 | 206 | Lysozyme | Lysozyme | | afdb-uniprot50 | AF-A0A2C9BRJ3-F1-MODEL\_V4 | 1.0 | 5.062e-11 | 507 | 0.451 | 144 | 75 | 3 | 2 | 144 | 7 | 147 | Lysozyme | Lysozyme | | afdb-uniprot50 | AF-A0A522QL01-F1-MODEL\_V4 | 1.0 | 6.598e-11 | 505 | 0.351 | 168 | 95 | 5 | 2 | 159 | 6 | 169 | Lysozyme | Lysozyme | | afdb-uniprot50 | AF-A0A7H9ARJ5-F1-MODEL\_V4 | 1.0 | 3.313e-11 | 504 | 0.401 | 152 | 83 | 4 | 1 | 146 | 1 | 150 | Lysozyme | Lysozyme | | afdb-uniprot50 | AF-C9XUR2-F1-MODEL\_V4 | 1.0 | 6.957e-11 | 504 | 0.462 | 145 | 74 | 3 | 2 | 145 | 25 | 166 | Lysozyme | Lysozyme | | afdb-uniprot50 | AF-A0A2U3AP30-F1-MODEL\_V4 | 1.0 | 1.85e-11 | 504 | 0.405 | 143 | 78 | 3 | 1 | 143 | 1 | 136 | Lysozyme | Lysozyme | | afdb-uniprot50 | AF-A0A5C7RF17-F1-MODEL\_V4 | 1.0 | 6.957e-11 | 503 | 0.39 | 146 | 86 | 2 | 1 | 146 | 6 | 148 | Lysozyme | Lysozyme | | afdb-uniprot50 | AF-A0A7X0CNU8-F1-MODEL\_V4 | 1.0 | 2.826e-11 | 503 | 0.503 | 155 | 63 | 6 | 1 | 144 | 1 | 152 | Lysozyme | Lysozyme | | afdb-uniprot50 | AF-B3T3K9-F1-MODEL\_V4 | 1.0 | 2.98e-11 | 503 | 0.457 | 153 | 73 | 3 | 1 | 144 | 21 | 172 | Lysozyme | Lysozyme | | afdb-uniprot50 | AF-A0A4Y8P1F7-F1-MODEL\_V4 | 1.0 | 4.553e-11 | 503 | 0.442 | 147 | 78 | 2 | 1 | 143 | 1 | 147 | Lysozyme | Lysozyme | | afdb-uniprot50 | AF-V4Q636-F1-MODEL\_V4 | 1.0 | 4.318e-11 | 502 | 0.456 | 149 | 74 | 3 | 1 | 144 | 1 | 147 | Lysozyme | Lysozyme | | afdb-uniprot50 | AF-A0A4Q4GWB6-F1-MODEL\_V4 | 1.0 | 4.318e-11 | 501 | 0.486 | 146 | 68 | 4 | 1 | 143 | 71 | 212 | Lysozyme | Lysozyme | | afdb-uniprot50 | AF-A0A6P0MMN1-F1-MODEL\_V4 | 1.0 | 3.313e-11 | 501 | 0.411 | 158 | 79 | 3 | 1 | 147 | 5 | 159 | Lysozyme | Lysozyme | | afdb-uniprot50 | AF-A0A060DQX0-F1-MODEL\_V4 | 1.0 | 3.884e-11 | 500 | 0.413 | 150 | 80 | 5 | 1 | 146 | 3 | 148 | Lysozyme | Lysozyme | | afdb-uniprot50 | AF-A0A2R4F7Z5-F1-MODEL\_V4 | 1.0 | 9.067e-11 | 500 | 0.434 | 145 | 78 | 3 | 1 | 145 | 3 | 143 | Lysozyme | Lysozyme | | afdb-uniprot50 | AF-A0A7Y0FFV2-F1-MODEL\_V4 | 1.0 | 9.794e-12 | 500 | 0.372 | 185 | 71 | 2 | 6 | 145 | 63 | 247 | Lysozyme | Lysozyme | | afdb-uniprot50 | AF-A0A6P0KSA3-F1-MODEL\_V4 | 1.0 | 6.257e-11 | 499 | 0.433 | 150 | 74 | 4 | 2 | 144 | 118 | 263 | Lysozyme | Lysozyme | | afdb-uniprot50 | AF-A0A6P0MU79-F1-MODEL\_V4 | 1.0 | 2.286e-11 | 499 | 0.427 | 159 | 76 | 3 | 1 | 147 | 5 | 160 | Lysozyme | Lysozyme | | afdb-uniprot50 | AF-K6ZSA0-F1-MODEL\_V4 | 1.0 | 1.806e-10 | 498 | 0.419 | 143 | 79 | 3 | 2 | 144 | 35 | 173 | Lysozyme | Lysozyme | | afdb-uniprot50 | AF-A0A5K7ZI99-F1-MODEL\_V4 | 1.0 | 3.493e-11 | 498 | 0.407 | 152 | 80 | 5 | 1 | 146 | 3 | 150 | Lysozyme | Lysozyme | | afdb-uniprot50 | AF-Q0B7R2-F1-MODEL\_V4 | 1.0 | 7.335e-11 | 497 | 0.451 | 144 | 75 | 3 | 2 | 144 | 7 | 147 | Lysozyme | Lysozyme | | afdb-uniprot50 | AF-A0A5C0UIT8-F1-MODEL\_V4 | 1.0 | 3.684e-11 | 497 | 0.379 | 145 | 87 | 2 | 1 | 145 | 1 | 142 | Lysozyme | Lysozyme | | afdb-uniprot50 | AF-A0A7H4K350-F1-MODEL\_V4 | 1.0 | 4.095e-11 | 497 | 0.486 | 148 | 69 | 4 | 1 | 144 | 10 | 154 | Lysozyme | Lysozyme | | afdb-uniprot50 | AF-A0A2Z5ZIU1-F1-MODEL\_V4 | 1.0 | 2.168e-11 | 497 | 0.442 | 156 | 72 | 3 | 2 | 145 | 7 | 159 | Lysozyme | Lysozyme | | afdb-uniprot50 | AF-B3T4S1-F1-MODEL\_V4 | 1.0 | 3.493e-11 | 497 | 0.457 | 153 | 73 | 2 | 1 | 144 | 59 | 210 | Lysozyme | Lysozyme | | afdb-uniprot50 | AF-A0A7R8WLX0-F1-MODEL\_V4 | 1.0 | 6.257e-11 | 497 | 0.386 | 145 | 86 | 2 | 2 | 146 | 84 | 225 | Lysozyme | Lysozyme | | afdb-uniprot50 | AF-A0A5P2QSJ9-F1-MODEL\_V4 | 1.0 | 9.067e-11 | 496 | 0.414 | 147 | 77 | 3 | 1 | 143 | 1 | 142 | Lysozyme | Lysozyme | | afdb-uniprot50 | AF-A0A2T3N1M3-F1-MODEL\_V4 | 1.0 | 1.121e-10 | 495 | 0.416 | 144 | 80 | 2 | 1 | 144 | 26 | 165 | Lysozyme | Lysozyme | | afdb-uniprot50 | AF-E8X0S4-F1-MODEL\_V4 | 1.0 | 5.062e-11 | 494 | 0.408 | 159 | 81 | 4 | 1 | 158 | 4 | 150 | Lysozyme | Lysozyme | | afdb-uniprot50 | AF-A0A6N1BS85-F1-MODEL\_V4 | 1.0 | 9.067e-11 | 494 | 0.41 | 139 | 78 | 3 | 7 | 145 | 17 | 151 | Lysozyme | Lysozyme | | afdb-uniprot50 | AF-F8WVD9-F1-MODEL\_V4 | 1.0 | 1.385e-10 | 494 | 0.409 | 149 | 80 | 4 | 1 | 144 | 37 | 182 | Lysozyme | Lysozyme | | afdb-uniprot50 | AF-A0A8B6S7C1-F1-MODEL\_V4 | 1.0 | 5.628e-11 | 494 | 0.416 | 149 | 78 | 5 | 1 | 145 | 1 | 144 | Lysozyme | Lysozyme | | afdb-uniprot50 | AF-A0A4P6GWD7-F1-MODEL\_V4 | 1.0 | 1.385e-10 | 493 | 0.394 | 142 | 82 | 3 | 3 | 144 | 5 | 142 | Lysozyme | Lysozyme | | afdb-uniprot50 | AF-S5MZ25-F1-MODEL\_V4 | 1.0 | 1.385e-10 | 493 | 0.386 | 150 | 85 | 3 | 1 | 146 | 1 | 147 | Lysozyme | Lysozyme | | afdb-uniprot50 | AF-A0A2B8BD98-F1-MODEL\_V4 | 1.0 | 7.335e-11 | 493 | 0.442 | 149 | 75 | 5 | 1 | 145 | 4 | 148 | Lysozyme | Lysozyme | | afdb-uniprot50 | AF-A0A1M3KZA5-F1-MODEL\_V4 | 1.0 | 2.232e-10 | 492 | 0.445 | 146 | 76 | 4 | 1 | 145 | 2 | 143 | Lysozyme | Lysozyme | | afdb-uniprot50 | AF-K6Z298-F1-MODEL\_V4 | 1.0 | 1.806e-10 | 492 | 0.421 | 140 | 77 | 3 | 4 | 143 | 49 | 184 | Lysozyme | Lysozyme | | afdb-uniprot50 | AF-A0A5M6I5G4-F1-MODEL\_V4 | 1.0 | 4.553e-11 | 492 | 0.418 | 153 | 79 | 5 | 2 | 148 | 4 | 152 | Lysozyme | Lysozyme | | afdb-uniprot50 | AF-A0A397TI77-F1-MODEL\_V4 | 1.0 | 6.257e-11 | 491 | 0.416 | 149 | 77 | 4 | 1 | 144 | 1 | 144 | Lysozyme | Lysozyme | | afdb-uniprot50 | AF-A0A316JPI6-F1-MODEL\_V4 | 1.0 | 5.628e-11 | 491 | 0.472 | 146 | 70 | 4 | 1 | 143 | 1 | 142 | Lysozyme | Lysozyme | | afdb-uniprot50 | AF-A0A2N1MZ41-F1-MODEL\_V4 | 1.0 | 1.121e-10 | 490 | 0.356 | 146 | 87 | 2 | 1 | 143 | 5 | 146 | Lysozyme | Lysozyme | | afdb-uniprot50 | AF-A0A271JAU0-F1-MODEL\_V4 | 1.0 | 1.008e-10 | 489 | 0.439 | 148 | 77 | 4 | 1 | 146 | 9 | 152 | Lysozyme | Lysozyme | | afdb-uniprot50 | AF-A0A5F0MKV9-F1-MODEL\_V4 | 1.0 | 1.063e-10 | 489 | 0.363 | 146 | 89 | 3 | 2 | 147 | 7 | 148 | Lysozyme | Lysozyme | | afdb-uniprot50 | AF-A0A1M6VYX9-F1-MODEL\_V4 | 1.0 | 6.598e-11 | 488 | 0.409 | 149 | 79 | 5 | 1 | 143 | 1 | 146 | Lysozyme | Lysozyme | | afdb-uniprot50 | AF-A0A286GNA9-F1-MODEL\_V4 | 1.0 | 5.062e-11 | 488 | 0.452 | 148 | 72 | 4 | 6 | 147 | 12 | 156 | Lysozyme | Lysozyme | | afdb-uniprot50 | AF-A0A151QE01-F1-MODEL\_V4 | 1.0 | 1.121e-10 | 488 | 0.379 | 145 | 87 | 2 | 2 | 146 | 17 | 158 | Lysozyme | Lysozyme | | afdb-uniprot50 | AF-A0A848YF79-F1-MODEL\_V4 | 1.0 | 3.884e-11 | 488 | 0.422 | 161 | 85 | 4 | 1 | 157 | 1 | 157 | Lysozyme | Lysozyme | | afdb-uniprot50 | AF-A0A149TN60-F1-MODEL\_V4 | 1.0 | 5.338e-11 | 486 | 0.409 | 154 | 77 | 3 | 1 | 143 | 1 | 151 | Lysozyme | Lysozyme | | afdb-uniprot50 | AF-A0A225M2C7-F1-MODEL\_V4 | 1.0 | 2.117e-10 | 486 | 0.371 | 140 | 85 | 2 | 5 | 144 | 22 | 158 | Lysozyme | Lysozyme | | afdb-uniprot50 | AF-A0A239Q0C8-F1-MODEL\_V4 | 1.0 | 4.318e-11 | 486 | 0.424 | 172 | 74 | 6 | 1 | 150 | 7 | 175 | Lysozyme | Lysozyme | | afdb-uniprot50 | AF-A0A3Q8ZIN2-F1-MODEL\_V4 | 1.0 | 3.313e-11 | 486 | 0.445 | 155 | 75 | 5 | 1 | 147 | 1 | 152 | Lysozyme | Lysozyme | | afdb-uniprot50 | AF-A0A241W0V3-F1-MODEL\_V4 | 1.0 | 1.385e-10 | 486 | 0.476 | 149 | 70 | 3 | 1 | 145 | 87 | 231 | Lysozyme | Lysozyme | | afdb-uniprot50 | AF-A0A428KE41-F1-MODEL\_V4 | 1.0 | 7.335e-11 | 485 | 0.423 | 151 | 79 | 3 | 1 | 146 | 5 | 152 | Lysozyme | Lysozyme | | afdb-uniprot50 | AF-A0A402TH11-F1-MODEL\_V4 | 1.0 | 8.599e-11 | 485 | 0.394 | 142 | 81 | 3 | 6 | 145 | 23 | 161 | Lysozyme | Lysozyme | | afdb-uniprot50 | AF-A0A4Y3ZNK5-F1-MODEL\_V4 | 1.0 | 4.801e-11 | 485 | 0.433 | 159 | 80 | 5 | 1 | 155 | 1 | 153 | Lysozyme | Lysozyme | | afdb-uniprot50 | AF-A0A839Y491-F1-MODEL\_V4 | 1.0 | 1.121e-10 | 485 | 0.457 | 166 | 80 | 6 | 1 | 159 | 94 | 256 | GH24 family phage-related lysozyme (Muramidase) | GH24 family phage-related lysozyme (Muramidase) | | afdb-uniprot50 | AF-Q28PX4-F1-MODEL\_V4 | 1.0 | 9.56e-11 | 485 | 0.401 | 147 | 81 | 4 | 1 | 143 | 1 | 144 | Lysozyme | Lysozyme | | afdb-uniprot50 | AF-A0A3S0C8Q3-F1-MODEL\_V4 | 1.0 | 1.461e-10 | 484 | 0.4 | 145 | 84 | 2 | 1 | 145 | 1 | 142 | Lysozyme | Lysozyme | | afdb-uniprot50 | AF-A0A2M8AHY6-F1-MODEL\_V4 | 1.0 | 1.806e-10 | 484 | 0.449 | 149 | 76 | 3 | 1 | 146 | 1 | 146 | Lysozyme | Lysozyme | | afdb-uniprot50 | AF-A0A3B9NR42-F1-MODEL\_V4 | 1.0 | 1.121e-10 | 484 | 0.408 | 142 | 77 | 4 | 6 | 144 | 63 | 200 | Lysozyme | Lysozyme | | afdb-uniprot50 | AF-A0A3P1XK73-F1-MODEL\_V4 | 1.0 | 3.595e-10 | 484 | 0.454 | 143 | 75 | 2 | 1 | 143 | 99 | 238 | Lysozyme | Lysozyme | | afdb-uniprot50 | AF-A0A167HMV7-F1-MODEL\_V4 | 1.0 | 3.067e-10 | 483 | 0.45 | 151 | 75 | 3 | 1 | 146 | 1 | 148 | Lysozyme | Lysozyme | | afdb-uniprot50 | AF-A0A3D1CPK1-F1-MODEL\_V4 | 1.0 | 1.182e-10 | 483 | 0.348 | 152 | 91 | 3 | 1 | 147 | 1 | 149 | Lysozyme | Lysozyme | | afdb-uniprot50 | AF-A0A2E4PLW6-F1-MODEL\_V4 | 1.0 | 1.246e-10 | 483 | 0.363 | 154 | 88 | 2 | 1 | 145 | 1 | 153 | Lysozyme | Lysozyme | | afdb-uniprot50 | AF-A0A258A958-F1-MODEL\_V4 | 1.0 | 4.553e-11 | 483 | 0.398 | 168 | 84 | 7 | 2 | 157 | 5 | 167 | Lysozyme | Lysozyme | | afdb-uniprot50 | AF-A0A552BAQ5-F1-MODEL\_V4 | 1.0 | 4.095e-11 | 483 | 0.456 | 151 | 71 | 6 | 1 | 143 | 1 | 148 | Lysozyme | Lysozyme | | afdb-uniprot50 | AF-A0A3N0V5X1-F1-MODEL\_V4 | 1.0 | 2.007e-10 | 482 | 0.409 | 144 | 81 | 3 | 1 | 144 | 5 | 144 | Lysozyme | Lysozyme | | afdb-uniprot50 | AF-A0A709N1J6-F1-MODEL\_V4 | 1.0 | 5.062e-11 | 482 | 0.401 | 152 | 81 | 5 | 1 | 145 | 1 | 149 | Lysozyme | Lysozyme | | afdb-uniprot50 | AF-A0A6P0UJS3-F1-MODEL\_V4 | 1.0 | 9.067e-11 | 482 | 0.428 | 147 | 76 | 5 | 1 | 143 | 1 | 143 | Lysozyme | Lysozyme | | afdb-uniprot50 | AF-A0A1G6JCW9-F1-MODEL\_V4 | 1.0 | 2.353e-10 | 482 | 0.432 | 148 | 77 | 3 | 1 | 144 | 60 | 204 | Lysozyme | Lysozyme | | afdb-uniprot50 | AF-A0A6M0G4D1-F1-MODEL\_V4 | 1.0 | 7.734e-11 | 482 | 0.398 | 158 | 81 | 2 | 1 | 147 | 5 | 159 | Lysozyme | Lysozyme | | afdb-uniprot50 | AF-A0A2E2VXS1-F1-MODEL\_V4 | 1.0 | 8.599e-11 | 481 | 0.344 | 148 | 89 | 3 | 1 | 143 | 1 | 145 | Lysozyme | Lysozyme | | afdb-uniprot50 | AF-A0A5U3EPL2-F1-MODEL\_V4 | 1.0 | 9.56e-11 | 481 | 0.427 | 152 | 77 | 5 | 1 | 147 | 1 | 147 | Lysozyme | Lysozyme | | afdb-uniprot50 | AF-A0A1E8CGD7-F1-MODEL\_V4 | 1.0 | 6.957e-11 | 481 | 0.384 | 151 | 85 | 4 | 1 | 147 | 1 | 147 | Lysozyme | Lysozyme | | afdb-uniprot50 | AF-A0A3S4Z1L8-F1-MODEL\_V4 | 1.0 | 1.712e-10 | 481 | 0.384 | 151 | 83 | 4 | 1 | 144 | 2 | 149 | Lysozyme | Lysozyme | | afdb-uniprot50 | AF-A0A317H2W4-F1-MODEL\_V4 | 1.0 | 8.155e-11 | 481 | 0.425 | 155 | 75 | 4 | 1 | 144 | 10 | 161 | Lysozyme | Lysozyme | | afdb-uniprot50 | AF-A0A3B0SY71-F1-MODEL\_V4 | 1.0 | 3.791e-10 | 481 | 0.409 | 144 | 80 | 2 | 1 | 144 | 94 | 232 | Lysozyme | Lysozyme | | afdb-uniprot50 | AF-A0A357LA02-F1-MODEL\_V4 | 1.0 | 1.182e-10 | 481 | 0.371 | 148 | 85 | 4 | 1 | 144 | 1 | 144 | Lysozyme | Lysozyme | | afdb-uniprot50 | AF-A0A4Q5XWR4-F1-MODEL\_V4 | 1.0 | 1.54e-10 | 481 | 0.398 | 148 | 81 | 5 | 1 | 144 | 23 | 166 | Lysozyme | Lysozyme | | afdb-uniprot50 | AF-A0A1F6ZTS5-F1-MODEL\_V4 | 1.0 | 2.232e-10 | 480 | 0.379 | 145 | 85 | 2 | 3 | 146 | 1 | 141 | Lysozyme | Lysozyme | | afdb-uniprot50 | AF-A0A2P1P7G1-F1-MODEL\_V4 | 1.0 | 1.712e-10 | 480 | 0.353 | 147 | 92 | 2 | 1 | 147 | 1 | 144 | Lysozyme | Lysozyme | | afdb-uniprot50 | AF-A0A3D5RRE8-F1-MODEL\_V4 | 1.0 | 1.904e-10 | 480 | 0.326 | 150 | 93 | 2 | 2 | 146 | 19 | 165 | Lysozyme | Lysozyme | | afdb-uniprot50 | AF-A0A5C7PF01-F1-MODEL\_V4 | 1.0 | 2.98e-11 | 480 | 0.364 | 162 | 86 | 4 | 3 | 150 | 2 | 160 | Lysozyme | Lysozyme | | afdb-uniprot50 | AF-R3W5G4-F1-MODEL\_V4 | 1.0 | 2.232e-10 | 480 | 0.439 | 148 | 78 | 4 | 1 | 147 | 6 | 149 | Lysozyme | Lysozyme | | afdb-uniprot50 | AF-A0A4Q3DIP3-F1-MODEL\_V4 | 1.0 | 2.616e-10 | 479 | 0.381 | 144 | 86 | 2 | 1 | 144 | 3 | 143 | Lysozyme | Lysozyme | | afdb-uniprot50 | AF-A0A1U7NBN1-F1-MODEL\_V4 | 1.0 | 2.353e-10 | 479 | 0.44 | 150 | 73 | 4 | 2 | 144 | 8 | 153 | Lysozyme | Lysozyme | | afdb-uniprot50 | AF-A0A0G3I3X2-F1-MODEL\_V4 | 1.0 | 2.007e-10 | 479 | 0.335 | 146 | 93 | 2 | 3 | 146 | 20 | 163 | Lysozyme | Lysozyme | | afdb-uniprot50 | AF-A0A845N7Q8-F1-MODEL\_V4 | 1.0 | 2.007e-10 | 479 | 0.391 | 148 | 84 | 3 | 1 | 145 | 2 | 146 | Glycoside hydrolase family protein | Glycoside hydrolase family protein | | afdb-uniprot50 | AF-A0A7W5C0N3-F1-MODEL\_V4 | 1.0 | 3.234e-10 | 479 | 0.397 | 141 | 81 | 3 | 4 | 144 | 1 | 137 | Lysozyme | Lysozyme | | afdb-uniprot50 | AF-A0A0M3A474-F1-MODEL\_V4 | 1.0 | 8.155e-11 | 479 | 0.469 | 149 | 72 | 4 | 2 | 146 | 52 | 197 | Lysozyme | Lysozyme | | afdb-uniprot50 | AF-A0A239Q0D5-F1-MODEL\_V4 | 1.0 | 4.941e-10 | 479 | 0.388 | 144 | 83 | 3 | 1 | 144 | 76 | 214 | Lysozyme | Lysozyme | | afdb-uniprot50 | AF-A0A7G8FMI4-F1-MODEL\_V4 | 1.0 | 1.314e-10 | 478 | 0.424 | 146 | 78 | 5 | 2 | 144 | 3 | 145 | Lysozyme | Lysozyme | | afdb-uniprot50 | AF-A0A1F9BXF9-F1-MODEL\_V4 | 1.0 | 1.008e-10 | 477 | 0.44 | 143 | 77 | 2 | 1 | 143 | 89 | 228 | Lysozyme | Lysozyme | | afdb-uniprot50 | AF-A0A1H9MQ78-F1-MODEL\_V4 | 1.0 | 2.616e-10 | 476 | 0.391 | 143 | 82 | 4 | 5 | 146 | 17 | 155 | Lysozyme | Lysozyme | | afdb-uniprot50 | AF-A0A496C4E2-F1-MODEL\_V4 | 1.0 | 2.117e-10 | 476 | 0.434 | 145 | 74 | 6 | 2 | 144 | 17 | 155 | Lysozyme | Lysozyme | | afdb-uniprot50 | AF-A0A522GMT4-F1-MODEL\_V4 | 1.0 | 5.338e-11 | 476 | 0.407 | 162 | 84 | 4 | 1 | 153 | 1 | 159 | Lysozyme | Lysozyme | | afdb-uniprot50 | AF-A0A4P6YSL8-F1-MODEL\_V4 | 1.0 | 1.712e-10 | 476 | 0.452 | 146 | 76 | 3 | 1 | 146 | 5 | 146 | Lysozyme | Lysozyme | | afdb-uniprot50 | AF-A0A6H9GT10-F1-MODEL\_V4 | 1.0 | 8.599e-11 | 476 | 0.438 | 155 | 76 | 6 | 1 | 147 | 1 | 152 | Lysozyme | Lysozyme | | afdb-uniprot50 | AF-A0A0N1L9V2-F1-MODEL\_V4 | 1.0 | 3.067e-10 | 475 | 0.387 | 147 | 85 | 3 | 1 | 147 | 1 | 142 | Lysozyme | Lysozyme | | afdb-uniprot50 | AF-A0A2N0R9A1-F1-MODEL\_V4 | 1.0 | 2.616e-10 | 475 | 0.394 | 152 | 80 | 5 | 1 | 146 | 3 | 148 | Lysozyme | Lysozyme | | afdb-uniprot50 | AF-A0A3M9MZF2-F1-MODEL\_V4 | 1.0 | 1.182e-10 | 475 | 0.409 | 149 | 82 | 3 | 1 | 146 | 3 | 148 | Lysozyme | Lysozyme | | afdb-uniprot50 | AF-A0A1T4RPF3-F1-MODEL\_V4 | 1.0 | 5.934e-11 | 475 | 0.448 | 156 | 71 | 2 | 1 | 144 | 1 | 153 | Lysozyme | Lysozyme | | afdb-uniprot50 | AF-A0A0F9NJU4-F1-MODEL\_V4 | 1.0 | 9.067e-11 | 475 | 0.407 | 152 | 78 | 3 | 1 | 143 | 1 | 149 | Lysozyme | Lysozyme | | afdb-uniprot50 | AF-A0A2D7ZXW1-F1-MODEL\_V4 | 1.0 | 2.481e-10 | 475 | 0.414 | 152 | 81 | 3 | 1 | 147 | 2 | 150 | Lysozyme | Lysozyme | | afdb-uniprot50 | AF-A0A6D2B246-F1-MODEL\_V4 | 1.0 | 3.067e-10 | 474 | 0.534 | 116 | 50 | 1 | 1 | 112 | 2 | 117 | Lysozyme | Lysozyme | | afdb-uniprot50 | AF-A0A2E1XDB6-F1-MODEL\_V4 | 1.0 | 2.481e-10 | 474 | 0.364 | 151 | 87 | 4 | 1 | 147 | 1 | 146 | Lysozyme | Lysozyme | | afdb-uniprot50 | AF-A0A109BL19-F1-MODEL\_V4 | 1.0 | 1.624e-10 | 474 | 0.418 | 153 | 78 | 4 | 1 | 146 | 15 | 163 | Lysozyme | Lysozyme | | afdb-uniprot50 | AF-A0A7G8J609-F1-MODEL\_V4 | 1.0 | 3.41e-10 | 474 | 0.393 | 150 | 87 | 3 | 1 | 147 | 3 | 151 | Lysozyme | Lysozyme | | afdb-uniprot50 | AF-A0A496C4S7-F1-MODEL\_V4 | 1.0 | 4.215e-10 | 473 | 0.427 | 145 | 75 | 6 | 1 | 143 | 1 | 139 | Lysozyme | Lysozyme | | afdb-uniprot50 | AF-A0A7S7C260-F1-MODEL\_V4 | 1.0 | 3.884e-11 | 473 | 0.401 | 152 | 80 | 6 | 4 | 147 | 1 | 149 | Lysozyme | Lysozyme | | afdb-uniprot50 | AF-A0A2S0V4Q7-F1-MODEL\_V4 | 1.0 | 9.331e-10 | 473 | 0.376 | 146 | 87 | 3 | 1 | 146 | 76 | 217 | Lysozyme | Lysozyme | | afdb-uniprot50 | AF-A0A2S6HCU7-F1-MODEL\_V4 | 1.0 | 1.246e-10 | 473 | 0.394 | 152 | 82 | 5 | 1 | 146 | 3 | 150 | Lysozyme | Lysozyme | | afdb-uniprot50 | AF-A0A1M3AIX6-F1-MODEL\_V4 | 1.0 | 9.56e-11 | 473 | 0.403 | 156 | 82 | 6 | 1 | 148 | 1 | 153 | Lysozyme | Lysozyme | | afdb-uniprot50 | AF-A0A608BLC2-F1-MODEL\_V4 | 1.0 | 3.067e-10 | 472 | 0.414 | 152 | 79 | 5 | 1 | 147 | 1 | 147 | Lysozyme | Lysozyme | | afdb-uniprot50 | AF-K9ZAF2-F1-MODEL\_V4 | 1.0 | 5.792e-10 | 472 | 0.387 | 147 | 83 | 3 | 2 | 144 | 80 | 223 | Lysozyme | Lysozyme | | afdb-uniprot50 | AF-A0A7Z1UR98-F1-MODEL\_V4 | 1.0 | 4.444e-10 | 472 | 0.426 | 150 | 79 | 3 | 1 | 146 | 81 | 227 | Lysozyme | Lysozyme | | afdb-uniprot50 | AF-A0A3Q9BKE1-F1-MODEL\_V4 | 1.0 | 2.759e-10 | 472 | 0.451 | 144 | 75 | 3 | 1 | 144 | 6 | 145 | Lysozyme | Lysozyme | | afdb-uniprot50 | AF-A0A1J5F4W9-F1-MODEL\_V4 | 1.0 | 4.941e-10 | 470 | 0.364 | 151 | 88 | 4 | 1 | 146 | 1 | 148 | Lysozyme | Lysozyme | | afdb-uniprot50 | AF-A0A1F2Z7P5-F1-MODEL\_V4 | 1.0 | 2.481e-10 | 470 | 0.352 | 142 | 89 | 2 | 5 | 146 | 15 | 153 | Lysozyme | Lysozyme | | afdb-uniprot50 | AF-A0A258GYK9-F1-MODEL\_V4 | 1.0 | 2.909e-10 | 470 | 0.391 | 148 | 82 | 5 | 1 | 144 | 11 | 154 | Lysozyme | Lysozyme | | afdb-uniprot50 | AF-A0A2M7K8A1-F1-MODEL\_V4 | 1.0 | 5.21e-10 | 469 | 0.393 | 145 | 85 | 2 | 1 | 145 | 3 | 144 | Lysozyme | Lysozyme | | afdb-uniprot50 | AF-A0A4Q7BRX3-F1-MODEL\_V4 | 1.0 | 2.759e-10 | 469 | 0.489 | 147 | 67 | 5 | 1 | 143 | 22 | 164 | Lysozyme | Lysozyme | | afdb-uniprot50 | AF-A0A7Y3RIZ1-F1-MODEL\_V4 | 1.0 | 9.331e-10 | 469 | 0.435 | 140 | 76 | 2 | 6 | 145 | 435 | 571 | Lysozyme | Lysozyme | | afdb-uniprot50 | AF-A0A250LRH8-F1-MODEL\_V4 | 1.0 | 4.686e-10 | 468 | 0.349 | 146 | 91 | 3 | 1 | 145 | 3 | 145 | Lysozyme | Lysozyme | | afdb-uniprot50 | AF-A0A2W6VVK0-F1-MODEL\_V4 | 1.0 | 8.155e-11 | 468 | 0.402 | 149 | 81 | 5 | 1 | 145 | 1 | 145 | Lysozyme | Lysozyme | | afdb-uniprot50 | AF-A0A5C7QE19-F1-MODEL\_V4 | 1.0 | 2.117e-10 | 468 | 0.396 | 154 | 82 | 3 | 1 | 147 | 1 | 150 | Lysozyme | Lysozyme | | afdb-uniprot50 | AF-A0A1I5CM08-F1-MODEL\_V4 | 1.0 | 2.353e-10 | 467 | 0.528 | 125 | 58 | 1 | 20 | 144 | 2 | 125 | Lysozyme | Lysozyme | | afdb-uniprot50 | AF-Q2NTN7-F1-MODEL\_V4 | 1.0 | 3.791e-10 | 467 | 0.414 | 147 | 78 | 4 | 1 | 143 | 2 | 144 | Lysozyme | Lysozyme | | afdb-uniprot50 | AF-A0A619AGL7-F1-MODEL\_V4 | 1.0 | 2.759e-10 | 467 | 0.414 | 152 | 79 | 5 | 1 | 147 | 1 | 147 | Lysozyme | Lysozyme | | afdb-uniprot50 | AF-A0A5E4SJE8-F1-MODEL\_V4 | 1.0 | 7.549e-10 | 467 | 0.407 | 135 | 76 | 3 | 10 | 144 | 22 | 152 | Lysozyme | Lysozyme | | afdb-uniprot50 | AF-A0A4Q0D5W1-F1-MODEL\_V4 | 1.0 | 4.215e-10 | 467 | 0.417 | 146 | 81 | 3 | 1 | 146 | 6 | 147 | Lysozyme | Lysozyme | | afdb-uniprot50 | AF-A0A6P1NBT1-F1-MODEL\_V4 | 1.0 | 2.117e-10 | 466 | 0.397 | 146 | 80 | 4 | 5 | 145 | 6 | 148 | Lysozyme | Lysozyme | | afdb-uniprot50 | AF-S2K1B0-F1-MODEL\_V4 | 1.0 | 4.444e-10 | 466 | 0.398 | 143 | 81 | 4 | 5 | 146 | 32 | 170 | Lysozyme | Lysozyme | | afdb-uniprot50 | AF-A0A7C5WIK9-F1-MODEL\_V4 | 1.0 | 1.461e-10 | 466 | 0.387 | 160 | 91 | 4 | 2 | 157 | 55 | 211 | Lysozyme | Lysozyme | | afdb-uniprot50 | AF-A0A551Z3X8-F1-MODEL\_V4 | 1.0 | 2.232e-10 | 466 | 0.448 | 156 | 75 | 5 | 1 | 148 | 1 | 153 | Lysozyme | Lysozyme | | afdb-uniprot50 | AF-A0A521YLX8-F1-MODEL\_V4 | 1.0 | 7.549e-10 | 465 | 0.401 | 147 | 82 | 5 | 1 | 145 | 4 | 146 | Lysozyme | Lysozyme | | afdb-uniprot50 | AF-A0A8A8CIQ3-F1-MODEL\_V4 | 1.0 | 5.792e-10 | 465 | 0.402 | 139 | 80 | 2 | 7 | 145 | 19 | 154 | Lysozyme | Lysozyme | | afdb-uniprot50 | AF-A0A1V0DDE0-F1-MODEL\_V4 | 1.0 | 3.234e-10 | 465 | 0.406 | 150 | 79 | 4 | 2 | 144 | 12 | 158 | Lysozyme | Lysozyme | | afdb-uniprot50 | AF-A0A192H186-F1-MODEL\_V4 | 1.0 | 2.481e-10 | 465 | 0.407 | 152 | 79 | 5 | 1 | 144 | 538 | 686 | Lysozyme | Lysozyme | | afdb-uniprot50 | AF-A0A0S8L002-F1-MODEL\_V4 | 1.0 | 2.007e-10 | 464 | 0.445 | 148 | 75 | 2 | 12 | 155 | 2 | 146 | Lysozyme | Lysozyme | | afdb-uniprot50 | AF-A0A4Z0DE45-F1-MODEL\_V4 | 1.0 | 9.56e-11 | 464 | 0.387 | 147 | 83 | 3 | 2 | 144 | 5 | 148 | Lysozyme | Lysozyme | | afdb-uniprot50 | AF-A0A2G2HRY1-F1-MODEL\_V4 | 1.0 | 3.41e-10 | 464 | 0.358 | 148 | 87 | 4 | 3 | 146 | 7 | 150 | Lysozyme | Lysozyme | | afdb-uniprot50 | AF-A0A1V2N9M7-F1-MODEL\_V4 | 1.0 | 4.686e-10 | 463 | 0.378 | 148 | 87 | 3 | 1 | 146 | 2 | 146 | Lysozyme | Lysozyme | | afdb-uniprot50 | AF-A0A5V7UAR2-F1-MODEL\_V4 | 1.0 | 4.215e-10 | 463 | 0.42 | 150 | 77 | 5 | 1 | 145 | 1 | 145 | Lysozyme | Lysozyme | | afdb-uniprot50 | AF-A0A1G9W3A4-F1-MODEL\_V4 | 1.0 | 3.791e-10 | 462 | 0.366 | 150 | 89 | 3 | 1 | 147 | 2 | 148 | Lysozyme | Lysozyme | | afdb-uniprot50 | AF-A0A7S9LKT4-F1-MODEL\_V4 | 1.0 | 6.107e-10 | 462 | 0.377 | 143 | 84 | 4 | 5 | 146 | 16 | 154 | Lysozyme | Lysozyme | | afdb-uniprot50 | AF-W6M7Y1-F1-MODEL\_V4 | 1.0 | 9.56e-11 | 462 | 0.43 | 158 | 75 | 6 | 1 | 146 | 8 | 162 | Lysozyme | Lysozyme | | afdb-uniprot50 | AF-A0A6J4RNY5-F1-MODEL\_V4 | 1.0 | 9.56e-11 | 462 | 0.415 | 154 | 77 | 2 | 1 | 144 | 622 | 772 | Lysozyme | Lysozyme | | afdb-uniprot50 | AF-Z9JHW0-F1-MODEL\_V4 | 1.0 | 4.686e-10 | 461 | 0.34 | 141 | 90 | 2 | 5 | 145 | 4 | 141 | Lysozyme | Lysozyme | | afdb-uniprot50 | AF-L1M7X0-F1-MODEL\_V4 | 1.0 | 6.79e-10 | 461 | 0.361 | 144 | 88 | 3 | 1 | 144 | 1 | 140 | Lysozyme | Lysozyme | | afdb-uniprot50 | AF-A0A629H5A7-F1-MODEL\_V4 | 1.0 | 4.444e-10 | 461 | 0.42 | 150 | 77 | 5 | 1 | 145 | 1 | 145 | Lysozyme | Lysozyme | | afdb-uniprot50 | AF-A0A7W5WZ35-F1-MODEL\_V4 | 1.0 | 3.234e-10 | 461 | 0.335 | 146 | 93 | 2 | 1 | 143 | 5 | 149 | Lysozyme | Lysozyme | | afdb-uniprot50 | AF-A0A1G4TH47-F1-MODEL\_V4 | 1.0 | 1.904e-10 | 461 | 0.422 | 154 | 81 | 5 | 1 | 150 | 5 | 154 | Lysozyme | Lysozyme | | afdb-uniprot50 | AF-A0A4Y8S5U9-F1-MODEL\_V4 | 1.0 | 3.41e-10 | 460 | 0.423 | 151 | 79 | 4 | 1 | 146 | 1 | 148 | Lysozyme | Lysozyme | | afdb-uniprot50 | AF-A0A3A1YD39-F1-MODEL\_V4 | 1.0 | 3.067e-10 | 460 | 0.412 | 143 | 79 | 3 | 6 | 146 | 10 | 149 | Lysozyme | Lysozyme | | afdb-uniprot50 | AF-A0A494QR79-F1-MODEL\_V4 | 1.0 | 5.493e-10 | 460 | 0.435 | 147 | 79 | 3 | 1 | 147 | 6 | 148 | Lysozyme | Lysozyme | | afdb-uniprot50 | AF-C6XGM6-F1-MODEL\_V4 | 1.0 | 6.107e-10 | 459 | 0.371 | 148 | 89 | 3 | 1 | 146 | 26 | 171 | Lysozyme | Lysozyme | | afdb-uniprot50 | AF-A0A257RPK0-F1-MODEL\_V4 | 1.0 | 1.246e-10 | 458 | 0.405 | 143 | 77 | 4 | 6 | 143 | 7 | 146 | Lysozyme | Lysozyme | | afdb-uniprot50 | AF-A0A7U1H6U5-F1-MODEL\_V4 | 1.0 | 8.393e-10 | 458 | 0.388 | 139 | 82 | 2 | 7 | 145 | 19 | 154 | Lysozyme | Lysozyme | | afdb-uniprot50 | AF-A0A3P3QN20-F1-MODEL\_V4 | 1.0 | 9.839e-10 | 458 | 0.366 | 139 | 85 | 1 | 6 | 144 | 20 | 155 | Lysozyme | Lysozyme | | afdb-uniprot50 | AF-A0A344WGG6-F1-MODEL\_V4 | 1.0 | 5.493e-10 | 458 | 0.375 | 149 | 84 | 6 | 1 | 144 | 5 | 149 | Lysozyme | Lysozyme | | afdb-uniprot50 | AF-A0A328B6S8-F1-MODEL\_V4 | 1.0 | 3.595e-10 | 458 | 0.375 | 152 | 86 | 6 | 2 | 148 | 6 | 153 | Lysozyme | Lysozyme | | afdb-uniprot50 | AF-A0A7T8NV96-F1-MODEL\_V4 | 1.0 | 3.791e-10 | 458 | 0.339 | 159 | 94 | 5 | 1 | 154 | 1 | 153 | Lysozyme | Lysozyme | | afdb-uniprot50 | AF-A0A4Q2JA85-F1-MODEL\_V4 | 1.0 | 7.549e-10 | 457 | 0.392 | 140 | 81 | 3 | 7 | 146 | 21 | 156 | Lysozyme | Lysozyme | | afdb-uniprot50 | AF-A0A494Y8X4-F1-MODEL\_V4 | 1.0 | 3.997e-10 | 457 | 0.387 | 147 | 84 | 4 | 1 | 144 | 1 | 144 | Lysozyme | Lysozyme | | afdb-uniprot50 | AF-A0A6N7D1K0-F1-MODEL\_V4 | 1.0 | 5.21e-10 | 457 | 0.405 | 143 | 84 | 1 | 5 | 147 | 23 | 164 | Lysozyme | Lysozyme | | afdb-uniprot50 | AF-A0A7K1SLB8-F1-MODEL\_V4 | 1.0 | 2.353e-10 | 456 | 0.435 | 147 | 75 | 5 | 2 | 143 | 5 | 148 | Lysozyme | Lysozyme | | afdb-uniprot50 | AF-A0A5P1R9I0-F1-MODEL\_V4 | 1.0 | 5.493e-10 | 456 | 0.349 | 146 | 90 | 4 | 5 | 150 | 7 | 147 | Lysozyme | Lysozyme | | afdb-uniprot50 | AF-A0A1I5WI32-F1-MODEL\_V4 | 1.0 | 6.79e-10 | 456 | 0.395 | 144 | 83 | 2 | 1 | 144 | 63 | 202 | Lysozyme | Lysozyme | | afdb-uniprot50 | AF-A0A3D3Z792-F1-MODEL\_V4 | 1.0 | 5.21e-10 | 456 | 0.36 | 150 | 88 | 5 | 1 | 146 | 4 | 149 | Lysozyme | Lysozyme | | afdb-uniprot50 | AF-A0A2D9RBB7-F1-MODEL\_V4 | 1.0 | 5.21e-10 | 456 | 0.4 | 150 | 82 | 5 | 1 | 146 | 5 | 150 | Lysozyme | Lysozyme | | afdb-uniprot50 | AF-A0A7C9TZG6-F1-MODEL\_V4 | 1.0 | 1.008e-10 | 456 | 0.39 | 151 | 79 | 4 | 2 | 143 | 79 | 225 | Lysozyme | Lysozyme | | afdb-uniprot50 | AF-A0A443JYE9-F1-MODEL\_V4 | 1.0 | 1.094e-09 | 454 | 0.333 | 141 | 91 | 2 | 4 | 144 | 1 | 138 | Lysozyme | Lysozyme | | afdb-uniprot50 | AF-A0A397U2F2-F1-MODEL\_V4 | 1.0 | 2.909e-10 | 454 | 0.376 | 154 | 86 | 4 | 2 | 148 | 9 | 159 | Lysozyme | Lysozyme | | afdb-uniprot50 | AF-A0A3R7C6X1-F1-MODEL\_V4 | 1.0 | 1.712e-10 | 454 | 0.339 | 153 | 89 | 3 | 1 | 146 | 20 | 167 | Lysozyme | Lysozyme | | afdb-uniprot50 | AF-A0A1A9WZ64-F1-MODEL\_V4 | 1.0 | 6.44e-10 | 454 | 0.359 | 139 | 86 | 2 | 7 | 145 | 141 | 276 | Lysozyme | Lysozyme | | afdb-uniprot50 | AF-A0A3B0MJC2-F1-MODEL\_V4 | 1.0 | 8.85e-10 | 453 | 0.34 | 141 | 90 | 2 | 6 | 146 | 76 | 213 | Lysozyme | Lysozyme | | afdb-uniprot50 | AF-L0EV97-F1-MODEL\_V4 | 1.0 | 2.232e-10 | 453 | 0.429 | 149 | 78 | 4 | 1 | 145 | 9 | 154 | Lysozyme | Lysozyme | | afdb-uniprot50 | AF-A0A828NXT7-F1-MODEL\_V4 | 1.0 | 7.549e-10 | 452 | 0.362 | 138 | 84 | 3 | 6 | 143 | 2 | 135 | Lysozyme | Lysozyme | | afdb-uniprot50 | AF-C1D7V6-F1-MODEL\_V4 | 1.0 | 1.585e-09 | 452 | 0.365 | 145 | 89 | 2 | 1 | 145 | 10 | 151 | Lysozyme | Lysozyme | | afdb-uniprot50 | AF-A0A1I7J3V8-F1-MODEL\_V4 | 1.0 | 4.444e-10 | 452 | 0.398 | 143 | 76 | 5 | 6 | 144 | 14 | 150 | Lysozyme | Lysozyme | | afdb-uniprot50 | AF-A0A850SM26-F1-MODEL\_V4 | 1.0 | 1.216e-09 | 452 | 0.384 | 151 | 84 | 5 | 1 | 147 | 67 | 212 | Lysozyme | Lysozyme | | afdb-uniprot50 | AF-A0A6B3UKC5-F1-MODEL\_V4 | 1.0 | 1.153e-09 | 452 | 0.418 | 148 | 78 | 5 | 1 | 144 | 5 | 148 | Lysozyme | Lysozyme | | afdb-uniprot50 | AF-A0A2M9PCB6-F1-MODEL\_V4 | 1.0 | 1.037e-09 | 451 | 0.39 | 151 | 84 | 3 | 3 | 148 | 1 | 148 | Lysozyme | Lysozyme | | afdb-uniprot50 | AF-A0A7Z2B7B3-F1-MODEL\_V4 | 1.0 | 9.839e-10 | 451 | 0.397 | 146 | 84 | 3 | 1 | 146 | 1 | 142 | Lysozyme | Lysozyme | | afdb-uniprot50 | AF-A0A318AUS2-F1-MODEL\_V4 | 1.0 | 8.85e-10 | 451 | 0.374 | 147 | 84 | 5 | 2 | 144 | 6 | 148 | Lysozyme | Lysozyme | | afdb-uniprot50 | AF-A0A7J7BR61-F1-MODEL\_V4 | 1.0 | 4.444e-10 | 451 | 0.482 | 145 | 68 | 4 | 2 | 142 | 171 | 312 | Lysozyme | Lysozyme | | afdb-uniprot50 | AF-A0A062U607-F1-MODEL\_V4 | 1.0 | 5.21e-10 | 451 | 0.35 | 151 | 89 | 6 | 1 | 146 | 1 | 147 | Lysozyme | Lysozyme | | afdb-uniprot50 | AF-A0A2E7F2D9-F1-MODEL\_V4 | 1.0 | 7.549e-10 | 451 | 0.382 | 149 | 84 | 5 | 2 | 146 | 6 | 150 | Lysozyme | Lysozyme | | afdb-uniprot50 | AF-A0A3D3IVC7-F1-MODEL\_V4 | 1.0 | 6.44e-10 | 451 | 0.376 | 154 | 82 | 5 | 1 | 145 | 11 | 159 | Lysozyme | Lysozyme | | afdb-uniprot50 | AF-V4PU64-F1-MODEL\_V4 | 1.0 | 3.41e-10 | 451 | 0.415 | 154 | 82 | 5 | 1 | 150 | 5 | 154 | Lysozyme | Lysozyme | | afdb-uniprot50 | AF-A0A1I3ZI12-F1-MODEL\_V4 | 1.0 | 1.624e-10 | 450 | 0.343 | 160 | 99 | 3 | 2 | 155 | 10 | 169 | Lysozyme | Lysozyme | | afdb-uniprot50 | AF-A0A2D6VSD9-F1-MODEL\_V4 | 1.0 | 6.107e-10 | 450 | 0.367 | 147 | 85 | 5 | 3 | 146 | 5 | 146 | Lysozyme | Lysozyme | | afdb-uniprot50 | AF-A0A0B1MTD0-F1-MODEL\_V4 | 1.0 | 1.352e-09 | 449 | 0.382 | 149 | 82 | 4 | 1 | 144 | 1 | 144 | Lysozyme | Lysozyme | | afdb-uniprot50 | AF-A0A0J1CTD0-F1-MODEL\_V4 | 1.0 | 1.806e-10 | 449 | 0.411 | 170 | 74 | 4 | 1 | 147 | 4 | 170 | Lysozyme | Lysozyme | | afdb-uniprot50 | AF-A0A7V8DKX2-F1-MODEL\_V4 | 1.0 | 6.79e-10 | 449 | 0.385 | 148 | 83 | 5 | 3 | 146 | 6 | 149 | Lysozyme | Lysozyme | | afdb-uniprot50 | AF-A0A1G4WUT2-F1-MODEL\_V4 | 1.0 | 1.959e-09 | 449 | 0.34 | 144 | 92 | 2 | 1 | 143 | 201 | 342 | Lysozyme | Lysozyme | | afdb-uniprot50 | AF-A0A2P2E800-F1-MODEL\_V4 | 1.0 | 7.549e-10 | 449 | 0.366 | 150 | 87 | 5 | 1 | 146 | 4 | 149 | Lysozyme | Lysozyme | | afdb-uniprot50 | AF-K1KHD9-F1-MODEL\_V4 | 1.0 | 5.21e-10 | 448 | 0.443 | 142 | 74 | 3 | 6 | 145 | 14 | 152 | Lysozyme | Lysozyme | | afdb-uniprot50 | AF-A0A7V4U4L1-F1-MODEL\_V4 | 1.0 | 8.85e-10 | 448 | 0.305 | 144 | 96 | 3 | 2 | 144 | 15 | 155 | Lysozyme | Lysozyme | | afdb-uniprot50 | AF-A0A645BK91-F1-MODEL\_V4 | 1.0 | 8.393e-10 | 447 | 0.37 | 151 | 82 | 6 | 1 | 143 | 1 | 146 | Lysozyme | Lysozyme | | afdb-uniprot50 | AF-A0A7C9RJG9-F1-MODEL\_V4 | 1.0 | 1.153e-09 | 447 | 0.307 | 140 | 95 | 2 | 5 | 144 | 15 | 152 | Lysozyme | Lysozyme | | afdb-uniprot50 | AF-A0A1S1NR31-F1-MODEL\_V4 | 1.0 | 1.503e-09 | 447 | 0.38 | 142 | 81 | 3 | 7 | 144 | 19 | 157 | Lysozyme | Lysozyme | | afdb-uniprot50 | AF-A0A512NCQ8-F1-MODEL\_V4 | 1.0 | 1.426e-09 | 447 | 0.417 | 151 | 79 | 6 | 1 | 146 | 3 | 149 | Lysozyme | Lysozyme | | afdb-uniprot50 | AF-A0A354U3U6-F1-MODEL\_V4 | 1.0 | 1.216e-09 | 447 | 0.38 | 150 | 85 | 5 | 1 | 146 | 5 | 150 | Lysozyme | Lysozyme | | afdb-uniprot50 | AF-A0A849N0U3-F1-MODEL\_V4 | 1.0 | 7.549e-10 | 446 | 0.335 | 149 | 94 | 3 | 1 | 145 | 1 | 148 | Lysozyme | Lysozyme | | afdb-uniprot50 | AF-A0A833N5J4-F1-MODEL\_V4 | 1.0 | 1.585e-09 | 445 | 0.365 | 145 | 89 | 2 | 1 | 145 | 2 | 143 | Lysozyme | Lysozyme | | afdb-uniprot50 | AF-U3TZ86-F1-MODEL\_V4 | 1.0 | 6.44e-10 | 445 | 0.35 | 140 | 86 | 3 | 6 | 143 | 21 | 157 | Lysozyme | Lysozyme | | afdb-uniprot50 | AF-C5A7Z2-F1-MODEL\_V4 | 1.0 | 9.067e-11 | 445 | 0.357 | 193 | 73 | 6 | 4 | 145 | 58 | 250 | Lysozyme | Lysozyme | | afdb-uniprot50 | AF-A0A1B1ALJ1-F1-MODEL\_V4 | 1.0 | 5.21e-10 | 445 | 0.342 | 146 | 88 | 4 | 3 | 144 | 5 | 146 | Lysozyme | Lysozyme | | afdb-uniprot50 | AF-A0A096BIF3-F1-MODEL\_V4 | 1.0 | 6.79e-10 | 444 | 0.41 | 146 | 79 | 4 | 1 | 143 | 3 | 144 | Lysozyme | Lysozyme | | afdb-uniprot50 | AF-A0A2S4E741-F1-MODEL\_V4 | 1.0 | 3.791e-10 | 444 | 0.418 | 153 | 75 | 6 | 2 | 144 | 3 | 151 | Lysozyme | Lysozyme | | afdb-uniprot50 | AF-A0A7V5SPV2-F1-MODEL\_V4 | 1.0 | 3.595e-10 | 444 | 0.36 | 147 | 85 | 5 | 1 | 143 | 22 | 163 | Lysozyme | Lysozyme | | afdb-uniprot50 | AF-A0A7V8A8R5-F1-MODEL\_V4 | 1.0 | 8.393e-10 | 444 | 0.385 | 148 | 83 | 5 | 3 | 146 | 6 | 149 | Lysozyme | Lysozyme | | afdb-uniprot50 | AF-R9LWU8-F1-MODEL\_V4 | 1.0 | 3.234e-10 | 444 | 0.408 | 147 | 77 | 4 | 1 | 143 | 3 | 143 | Lysozyme | Lysozyme | | afdb-uniprot50 | AF-A0A7C5PMR5-F1-MODEL\_V4 | 1.0 | 7.549e-10 | 444 | 0.433 | 157 | 76 | 5 | 1 | 148 | 1 | 153 | Lysozyme | Lysozyme | | afdb-uniprot50 | AF-A0A4R2VIK8-F1-MODEL\_V4 | 1.0 | 6.44e-10 | 444 | 0.428 | 154 | 77 | 5 | 1 | 146 | 68 | 218 | Lysozyme | Lysozyme | | afdb-uniprot50 | AF-F5RAK7-F1-MODEL\_V4 | 1.0 | 3.234e-10 | 444 | 0.395 | 149 | 82 | 5 | 1 | 145 | 96 | 240 | Lysozyme | Lysozyme | | afdb-uniprot50 | AF-R6FSL7-F1-MODEL\_V4 | 1.0 | 7.16e-10 | 444 | 0.406 | 145 | 80 | 4 | 1 | 144 | 1 | 140 | Lysozyme | Lysozyme | | afdb-uniprot50 | AF-A0A059DT11-F1-MODEL\_V4 | 1.0 | 1.216e-09 | 444 | 0.355 | 149 | 87 | 6 | 1 | 144 | 5 | 149 | Lysozyme | Lysozyme | | afdb-uniprot50 | AF-A0A2D7XZM4-F1-MODEL\_V4 | 1.0 | 1.216e-09 | 444 | 0.393 | 150 | 83 | 5 | 1 | 146 | 5 | 150 | Lysozyme | Lysozyme | | afdb-uniprot50 | AF-A0A6P0NKT7-F1-MODEL\_V4 | 1.0 | 5.493e-10 | 443 | 0.363 | 154 | 87 | 5 | 1 | 146 | 5 | 155 | Lysozyme | Lysozyme | | afdb-uniprot50 | AF-I4FUS8-F1-MODEL\_V4 | 1.0 | 2.481e-10 | 443 | 0.389 | 167 | 76 | 5 | 1 | 144 | 25 | 188 | Lysozyme | Lysozyme | | afdb-uniprot50 | AF-A0A2G2IYC1-F1-MODEL\_V4 | 1.0 | 5.493e-10 | 443 | 0.396 | 154 | 78 | 6 | 1 | 143 | 4 | 153 | Lysozyme | Lysozyme | | afdb-uniprot50 | AF-A0A2E2QM79-F1-MODEL\_V4 | 1.0 | 5.21e-10 | 442 | 0.404 | 163 | 75 | 4 | 1 | 145 | 3 | 161 | Lysozyme | Lysozyme | | afdb-uniprot50 | AF-A0A5C7NLG2-F1-MODEL\_V4 | 1.0 | 1.153e-09 | 442 | 0.371 | 159 | 83 | 4 | 2 | 146 | 20 | 175 | Lysozyme | Lysozyme | | afdb-uniprot50 | AF-A0A2R4NSE3-F1-MODEL\_V4 | 1.0 | 7.16e-10 | 441 | 0.412 | 148 | 79 | 4 | 1 | 144 | 1 | 144 | Lysozyme | Lysozyme | | afdb-uniprot50 | AF-A0A7V8JUE6-F1-MODEL\_V4 | 1.0 | 1.153e-09 | 441 | 0.349 | 143 | 89 | 2 | 5 | 147 | 4 | 142 | Lysozyme | Lysozyme | | afdb-uniprot50 | AF-A0A6P2LU80-F1-MODEL\_V4 | 1.0 | 3.41e-10 | 440 | 0.371 | 159 | 86 | 5 | 1 | 148 | 3 | 158 | Lysozyme | Lysozyme | | afdb-uniprot50 | AF-A0A348HDV6-F1-MODEL\_V4 | 1.0 | 1.858e-09 | 440 | 0.344 | 145 | 88 | 3 | 5 | 145 | 15 | 156 | Lysozyme | Lysozyme | | afdb-uniprot50 | AF-A0A3S1C1X9-F1-MODEL\_V4 | 1.0 | 4.215e-10 | 440 | 0.385 | 153 | 83 | 3 | 1 | 145 | 61 | 210 | Lysozyme | Lysozyme | | afdb-uniprot50 | AF-A0A850BAY3-F1-MODEL\_V4 | 1.0 | 3.791e-10 | 440 | 0.398 | 158 | 77 | 5 | 1 | 143 | 738 | 892 | Type VI secretion system tip protein VgrG | Type VI secretion system tip protein VgrG | | afdb-uniprot50 | AF-A0A0F3Q9P1-F1-MODEL\_V4 | 1.0 | 1.094e-09 | 439 | 0.337 | 145 | 90 | 4 | 6 | 147 | 6 | 147 | Lysozyme | Lysozyme | | afdb-uniprot50 | AF-A0A1M3KMN9-F1-MODEL\_V4 | 1.0 | 1.352e-09 | 439 | 0.393 | 150 | 84 | 3 | 1 | 143 | 1 | 150 | Lysozyme | Lysozyme | | afdb-uniprot50 | AF-A0A839LRE8-F1-MODEL\_V4 | 1.0 | 3.41e-10 | 438 | 0.325 | 160 | 102 | 3 | 2 | 155 | 24 | 183 | Lysozyme | Lysozyme | | afdb-uniprot50 | AF-A0A2E4QSB6-F1-MODEL\_V4 | 1.0 | 1.426e-09 | 438 | 0.331 | 148 | 91 | 5 | 1 | 144 | 5 | 148 | Lysozyme | Lysozyme | | afdb-uniprot50 | AF-A0A4Q3UUN7-F1-MODEL\_V4 | 1.0 | 1.282e-09 | 437 | 0.422 | 142 | 74 | 5 | 1 | 138 | 5 | 142 | Lysozyme | Lysozyme | | afdb-uniprot50 | AF-A0A4Q0QES9-F1-MODEL\_V4 | 1.0 | 7.549e-10 | 437 | 0.522 | 132 | 57 | 2 | 18 | 146 | 7 | 135 | Lysozyme | Lysozyme | | afdb-uniprot50 | AF-Q2NSF6-F1-MODEL\_V4 | 1.0 | 1.426e-09 | 437 | 0.384 | 130 | 77 | 2 | 7 | 136 | 21 | 147 | Lysozyme | Lysozyme | | afdb-uniprot50 | AF-A0A1W1V408-F1-MODEL\_V4 | 1.0 | 1.216e-09 | 437 | 0.339 | 153 | 88 | 4 | 1 | 143 | 2 | 151 | Lysozyme | Lysozyme | | afdb-uniprot50 | AF-A0A7Y0GSW2-F1-MODEL\_V4 | 1.0 | 3.7e-09 | 437 | 0.363 | 146 | 92 | 1 | 1 | 146 | 13 | 157 | Lysozyme | Lysozyme | | afdb-uniprot50 | AF-A0A4P7L828-F1-MODEL\_V4 | 1.0 | 2.297e-09 | 437 | 0.361 | 141 | 87 | 2 | 6 | 146 | 32 | 169 | Lysozyme | Lysozyme | | afdb-uniprot50 | AF-A0A829TCL8-F1-MODEL\_V4 | 1.0 | 3.157e-09 | 436 | 0.364 | 137 | 84 | 2 | 11 | 147 | 2 | 135 | Putative phage lysozyme | Putative phage lysozyme | | afdb-uniprot50 | AF-B1M176-F1-MODEL\_V4 | 1.0 | 2.554e-09 | 436 | 0.328 | 140 | 93 | 1 | 5 | 144 | 55 | 193 | Lysozyme | Lysozyme | | afdb-uniprot50 | AF-A0A0N0JEL9-F1-MODEL\_V4 | 1.0 | 4.215e-10 | 436 | 0.372 | 180 | 83 | 7 | 1 | 153 | 10 | 186 | Lysozyme | Lysozyme | | afdb-uniprot50 | AF-A0A7W9CFD1-F1-MODEL\_V4 | 1.0 | 1.426e-09 | 436 | 0.314 | 159 | 97 | 4 | 1 | 154 | 9 | 160 | Lysozyme | Lysozyme | | afdb-uniprot50 | AF-A0A7W6JCA7-F1-MODEL\_V4 | 1.0 | 1.503e-09 | 436 | 0.333 | 159 | 95 | 5 | 1 | 154 | 9 | 161 | Lysozyme | Lysozyme | | afdb-uniprot50 | AF-A0A829EU62-F1-MODEL\_V4 | 1.0 | 1.503e-09 | 436 | 0.308 | 149 | 96 | 4 | 1 | 145 | 1 | 146 | Uncharacterized protein | Uncharacterized protein | | afdb-uniprot50 | AF-A0A1M7CNB5-F1-MODEL\_V4 | 1.0 | 1.426e-09 | 435 | 0.387 | 147 | 84 | 5 | 1 | 146 | 1 | 142 | Lysozyme | Lysozyme | | afdb-uniprot50 | AF-A0A840G3M7-F1-MODEL\_V4 | 1.0 | 1.585e-09 | 435 | 0.414 | 147 | 81 | 3 | 1 | 146 | 4 | 146 | Lysozyme | Lysozyme | | afdb-uniprot50 | AF-K1JIE7-F1-MODEL\_V4 | 1.0 | 4.215e-10 | 435 | 0.381 | 152 | 84 | 5 | 1 | 145 | 1 | 149 | Lysozyme | Lysozyme | | afdb-uniprot50 | AF-A0A847VHY1-F1-MODEL\_V4 | 1.0 | 1.426e-09 | 435 | 0.323 | 142 | 92 | 3 | 5 | 145 | 22 | 160 | Lysozyme | Lysozyme | | afdb-uniprot50 | AF-A0A4Q3SIK8-F1-MODEL\_V4 | 1.0 | 1.094e-09 | 435 | 0.348 | 152 | 87 | 6 | 1 | 144 | 1 | 148 | Lysozyme | Lysozyme | | afdb-uniprot50 | AF-A0A420WVM4-F1-MODEL\_V4 | 1.0 | 1.503e-09 | 435 | 0.385 | 148 | 82 | 4 | 4 | 145 | 16 | 160 | Lysozyme | Lysozyme | | afdb-uniprot50 | AF-A0A514ETB6-F1-MODEL\_V4 | 1.0 | 1.153e-09 | 435 | 0.317 | 145 | 91 | 3 | 6 | 145 | 21 | 162 | Lysozyme | Lysozyme | | afdb-uniprot50 | AF-A0A257J4H1-F1-MODEL\_V4 | 1.0 | 1.503e-09 | 435 | 0.346 | 147 | 88 | 5 | 2 | 144 | 6 | 148 | Lysozyme | Lysozyme | | afdb-uniprot50 | AF-A0A2N6KSA2-F1-MODEL\_V4 | 1.0 | 5.493e-10 | 435 | 0.369 | 149 | 84 | 4 | 3 | 147 | 131 | 273 | Lysozyme | Lysozyme | | afdb-uniprot50 | AF-A0A601HWC6-F1-MODEL\_V4 | 1.0 | 9.839e-10 | 434 | 0.554 | 128 | 53 | 2 | 21 | 144 | 1 | 128 | Lysozyme | Lysozyme | | afdb-uniprot50 | AF-A0A4Q3S555-F1-MODEL\_V4 | 1.0 | 2.297e-09 | 434 | 0.374 | 147 | 84 | 5 | 2 | 144 | 6 | 148 | Lysozyme | Lysozyme | | afdb-uniprot50 | AF-A0A7R9QUH5-F1-MODEL\_V4 | 1.0 | 5.493e-10 | 434 | 0.405 | 158 | 84 | 6 | 1 | 153 | 18 | 170 | Lysozyme | Lysozyme | | afdb-uniprot50 | AF-E0TGL6-F1-MODEL\_V4 | 1.0 | 6.44e-10 | 434 | 0.402 | 159 | 77 | 5 | 1 | 144 | 1 | 156 | Lysozyme | Lysozyme | | afdb-uniprot50 | AF-A0A5B2VH29-F1-MODEL\_V4 | 1.0 | 8.85e-10 | 432 | 0.378 | 148 | 86 | 4 | 1 | 144 | 6 | 151 | Lysozyme | Lysozyme | | afdb-uniprot50 | AF-A0A5C4PLN9-F1-MODEL\_V4 | 1.0 | 3.41e-10 | 432 | 0.37 | 154 | 85 | 7 | 1 | 145 | 1 | 151 | Lysozyme | Lysozyme | | afdb-uniprot50 | AF-A0A1S9AW89-F1-MODEL\_V4 | 1.0 | 5.21e-10 | 432 | 0.362 | 174 | 80 | 5 | 1 | 146 | 1 | 171 | Lysozyme | Lysozyme | | afdb-uniprot50 | AF-A0A2G2MTD8-F1-MODEL\_V4 | 1.0 | 9.839e-10 | 432 | 0.364 | 151 | 88 | 5 | 1 | 147 | 5 | 151 | Lysozyme | Lysozyme | | afdb-uniprot50 | AF-A0A1V5WW45-F1-MODEL\_V4 | 1.0 | 3.7e-09 | 431 | 0.363 | 146 | 88 | 4 | 2 | 146 | 5 | 146 | Lysozyme | Lysozyme | | afdb-uniprot50 | AF-A0A2H0QTW7-F1-MODEL\_V4 | 1.0 | 7.96e-10 | 431 | 0.331 | 169 | 87 | 5 | 1 | 146 | 2 | 167 | Lysozyme | Lysozyme | | afdb-uniprot50 | AF-Q87BX4-F1-MODEL\_V4 | 1.0 | 1.153e-09 | 431 | 0.472 | 129 | 63 | 4 | 2 | 128 | 19 | 144 | Lysozyme | Lysozyme | | afdb-uniprot50 | AF-A0A6M0HX53-F1-MODEL\_V4 | 1.0 | 7.549e-10 | 431 | 0.4 | 150 | 78 | 5 | 1 | 146 | 1 | 142 | Lysozyme | Lysozyme | | afdb-uniprot50 | AF-A0A6G7CH63-F1-MODEL\_V4 | 1.0 | 4.338e-09 | 430 | 0.306 | 137 | 92 | 2 | 7 | 143 | 22 | 155 | Lysozyme | Lysozyme | | afdb-uniprot50 | AF-A0A846BN22-F1-MODEL\_V4 | 1.0 | 3.791e-10 | 430 | 0.375 | 152 | 82 | 4 | 2 | 143 | 27 | 175 | Lysozyme | Lysozyme | | afdb-uniprot50 | AF-A0A7R9M991-F1-MODEL\_V4 | 1.0 | 1.858e-09 | 429 | 0.373 | 150 | 82 | 5 | 1 | 144 | 19 | 162 | Lysozyme | Lysozyme | | afdb-uniprot50 | AF-A0A7X9HPJ2-F1-MODEL\_V4 | 1.0 | 6.107e-10 | 429 | 0.364 | 159 | 82 | 4 | 2 | 145 | 59 | 213 | Lysozyme | Lysozyme | | afdb-uniprot50 | AF-A3XEZ7-F1-MODEL\_V4 | 1.0 | 2.297e-09 | 429 | 0.358 | 145 | 88 | 4 | 1 | 144 | 94 | 234 | Lysozyme | Lysozyme | | afdb-uniprot50 | AF-I0HR64-F1-MODEL\_V4 | 1.0 | 4.444e-10 | 429 | 0.381 | 181 | 81 | 6 | 1 | 153 | 6 | 183 | Lysozyme | Lysozyme | | afdb-uniprot50 | AF-A0A1Z4LWR5-F1-MODEL\_V4 | 1.0 | 6.79e-10 | 429 | 0.379 | 153 | 82 | 4 | 1 | 144 | 105 | 253 | Lysozyme | Lysozyme | | afdb-uniprot50 | AF-A0A3P2RCD3-F1-MODEL\_V4 | 1.0 | 6.44e-10 | 429 | 0.395 | 149 | 84 | 5 | 1 | 147 | 1 | 145 | Lysozyme | Lysozyme | | afdb-uniprot50 | AF-A0A2A7S155-F1-MODEL\_V4 | 1.0 | 2.232e-10 | 429 | 0.336 | 196 | 78 | 5 | 1 | 145 | 77 | 271 | Lysozyme | Lysozyme | | afdb-uniprot50 | AF-A0A7C4STG1-F1-MODEL\_V4 | 1.0 | 6.44e-10 | 429 | 0.351 | 165 | 86 | 5 | 1 | 150 | 204 | 362 | Lysozyme | Lysozyme | | afdb-uniprot50 | AF-A0A399R703-F1-MODEL\_V4 | 1.0 | 2.839e-09 | 429 | 0.35 | 151 | 89 | 6 | 1 | 146 | 5 | 151 | Lysozyme | Lysozyme | | afdb-uniprot50 | AF-R0CYV1-F1-MODEL\_V4 | 1.0 | 2.994e-09 | 429 | 0.367 | 147 | 85 | 5 | 2 | 144 | 6 | 148 | Lysozyme | Lysozyme | | afdb-uniprot50 | AF-A0A7X7AHC1-F1-MODEL\_V4 | 1.0 | 2.839e-09 | 428 | 0.354 | 141 | 85 | 4 | 6 | 144 | 10 | 146 | Lysozyme | Lysozyme | | afdb-uniprot50 | AF-A0A8B2QY50-F1-MODEL\_V4 | 1.0 | 9.331e-10 | 428 | 0.304 | 148 | 90 | 3 | 7 | 144 | 2 | 146 | Lysozyme | Lysozyme | | afdb-uniprot50 | AF-A0A259NPH4-F1-MODEL\_V4 | 1.0 | 3.234e-10 | 428 | 0.392 | 158 | 76 | 4 | 4 | 143 | 1 | 156 | Lysozyme | Lysozyme | | afdb-uniprot50 | AF-A0A258GXH3-F1-MODEL\_V4 | 1.0 | 2.994e-09 | 428 | 0.347 | 144 | 89 | 2 | 3 | 146 | 48 | 186 | Lysozyme | Lysozyme | | afdb-uniprot50 | AF-A0A1M3KKZ9-F1-MODEL\_V4 | 1.0 | 3.997e-10 | 428 | 0.383 | 159 | 83 | 6 | 1 | 148 | 6 | 160 | Lysozyme | Lysozyme | | afdb-uniprot50 | AF-A0A7I8ZS27-F1-MODEL\_V4 | 1.0 | 1.503e-09 | 428 | 0.317 | 145 | 91 | 3 | 6 | 145 | 21 | 162 | Lysozyme | Lysozyme | | afdb-uniprot50 | AF-A0A7W1LGJ0-F1-MODEL\_V4 | 1.0 | 5.21e-10 | 428 | 0.409 | 154 | 78 | 4 | 2 | 146 | 238 | 387 | Lysozyme | Lysozyme | | afdb-uniprot50 | AF-A0A7W0G9B2-F1-MODEL\_V4 | 1.0 | 4.941e-10 | 428 | 0.385 | 153 | 83 | 3 | 2 | 146 | 321 | 470 | Lysozyme | Lysozyme | | afdb-uniprot50 | AF-A0A421BJ35-F1-MODEL\_V4 | 1.0 | 4.574e-09 | 427 | 0.364 | 140 | 85 | 3 | 5 | 144 | 9 | 144 | Lysozyme | Lysozyme | | afdb-uniprot50 | AF-G4QCV9-F1-MODEL\_V4 | 1.0 | 1.762e-09 | 427 | 0.356 | 146 | 86 | 5 | 4 | 146 | 12 | 152 | Lysozyme | Lysozyme | | afdb-uniprot50 | AF-A0A060H052-F1-MODEL\_V4 | 1.0 | 5.21e-10 | 427 | 0.331 | 148 | 89 | 3 | 5 | 145 | 32 | 176 | Lysozyme | Lysozyme | | afdb-uniprot50 | AF-A0A1I2RRC9-F1-MODEL\_V4 | 1.0 | 8.85e-10 | 427 | 0.421 | 147 | 73 | 6 | 1 | 143 | 1 | 139 | Lysozyme | Lysozyme | | afdb-uniprot50 | AF-A0A226ESI2-F1-MODEL\_V4 | 1.0 | 9.839e-10 | 427 | 0.371 | 159 | 86 | 7 | 1 | 148 | 23 | 178 | Lysozyme | Lysozyme | | afdb-uniprot50 | AF-A0A2D7IXU0-F1-MODEL\_V4 | 1.0 | 3.902e-09 | 427 | 0.386 | 150 | 84 | 5 | 1 | 146 | 5 | 150 | Lysozyme | Lysozyme | | afdb-uniprot50 | AF-D9Y906-F1-MODEL\_V4 | 1.0 | 1.503e-09 | 426 | 0.376 | 146 | 82 | 5 | 6 | 146 | 2 | 143 | Lysozyme | Lysozyme | | afdb-uniprot50 | AF-A0A4Y5YHV6-F1-MODEL\_V4 | 1.0 | 3.328e-09 | 426 | 0.352 | 142 | 87 | 4 | 6 | 146 | 18 | 155 | Lysozyme | Lysozyme | | afdb-uniprot50 | AF-A0A7R9PWQ4-F1-MODEL\_V4 | 1.0 | 2.297e-09 | 426 | 0.377 | 151 | 81 | 6 | 1 | 144 | 18 | 162 | Lysozyme | Lysozyme | | afdb-uniprot50 | AF-A0A4R2MG20-F1-MODEL\_V4 | 1.0 | 2.481e-10 | 426 | 0.37 | 181 | 81 | 7 | 1 | 152 | 16 | 192 | Lysozyme | Lysozyme | | afdb-uniprot50 | AF-A0A1D2MI83-F1-MODEL\_V4 | 1.0 | 1.671e-09 | 426 | 0.364 | 159 | 86 | 6 | 1 | 147 | 70 | 225 | Lysozyme | Lysozyme | | afdb-uniprot50 | AF-A0A1M7K5Y8-F1-MODEL\_V4 | 1.0 | 7.369e-09 | 425 | 0.328 | 140 | 91 | 2 | 7 | 146 | 21 | 157 | Lysozyme | Lysozyme | | afdb-uniprot50 | AF-A0A7Y3N265-F1-MODEL\_V4 | 1.0 | 5.085e-09 | 425 | 0.352 | 139 | 87 | 2 | 5 | 143 | 39 | 174 | Lysozyme | Lysozyme | | afdb-uniprot50 | AF-A0A7W0G4G5-F1-MODEL\_V4 | 1.0 | 6.79e-10 | 425 | 0.284 | 169 | 103 | 4 | 3 | 155 | 16 | 182 | Lysozyme | Lysozyme | | afdb-uniprot50 | AF-A0A857C5S3-F1-MODEL\_V4 | 1.0 | 2.297e-09 | 425 | 0.333 | 141 | 88 | 4 | 6 | 144 | 17 | 153 | Glycoside hydrolase family protein | Glycoside hydrolase family protein | | afdb-uniprot50 | AF-A0A125SM95-F1-MODEL\_V4 | 1.0 | 9.839e-10 | 425 | 0.384 | 169 | 78 | 4 | 1 | 146 | 49 | 214 | Lysozyme | Lysozyme | | afdb-uniprot50 | AF-S3JF93-F1-MODEL\_V4 | 1.0 | 9.839e-10 | 425 | 0.389 | 167 | 76 | 5 | 1 | 144 | 114 | 277 | Lysozyme | Lysozyme | | afdb-uniprot50 | AF-A0A7Z1R6F5-F1-MODEL\_V4 | 1.0 | 3.7e-09 | 425 | 0.377 | 143 | 79 | 5 | 6 | 144 | 177 | 313 | Lysozyme | Lysozyme | | afdb-uniprot50 | AF-A0A0A2V9H7-F1-MODEL\_V4 | 1.0 | 2.839e-09 | 425 | 0.302 | 149 | 98 | 3 | 1 | 146 | 1 | 146 | Lysozyme | Lysozyme | | afdb-uniprot50 | AF-E2YNY1-F1-MODEL\_V4 | 1.0 | 1.858e-09 | 425 | 0.313 | 153 | 97 | 5 | 1 | 148 | 1 | 150 | Lysozyme | Lysozyme | | afdb-uniprot50 | AF-A0A525KSI6-F1-MODEL\_V4 | 1.0 | 4.114e-09 | 425 | 0.34 | 147 | 89 | 5 | 2 | 144 | 6 | 148 | Lysozyme | Lysozyme | | afdb-uniprot50 | AF-A0A357L761-F1-MODEL\_V4 | 1.0 | 4.338e-09 | 424 | 0.353 | 150 | 88 | 5 | 1 | 146 | 38 | 182 | Lysozyme | Lysozyme | | afdb-uniprot50 | AF-A0A327X3T2-F1-MODEL\_V4 | 1.0 | 4.823e-09 | 424 | 0.357 | 140 | 86 | 3 | 5 | 144 | 19 | 154 | Lysozyme | Lysozyme | | afdb-uniprot50 | AF-A0A443L9N3-F1-MODEL\_V4 | 1.0 | 4.574e-09 | 424 | 0.364 | 140 | 85 | 3 | 5 | 144 | 104 | 239 | Lysozyme | Lysozyme | | afdb-uniprot50 | AF-A0A7V8YU39-F1-MODEL\_V4 | 1.0 | 5.21e-10 | 424 | 0.405 | 153 | 78 | 4 | 2 | 145 | 112 | 260 | Lysozyme | Lysozyme | | afdb-uniprot50 | AF-A0A7W9E8M6-F1-MODEL\_V4 | 1.0 | 1.762e-09 | 424 | 0.3 | 163 | 99 | 6 | 1 | 154 | 1 | 157 | Lysozyme | Lysozyme | | afdb-uniprot50 | AF-A0A525JRD6-F1-MODEL\_V4 | 1.0 | 3.328e-09 | 424 | 0.364 | 151 | 88 | 5 | 2 | 148 | 6 | 152 | Lysozyme | Lysozyme | | afdb-uniprot50 | AF-A0A7G9RLL0-F1-MODEL\_V4 | 1.0 | 2.693e-09 | 423 | 0.372 | 145 | 83 | 4 | 3 | 143 | 11 | 151 | Lysozyme | Lysozyme | | afdb-uniprot50 | AF-A0A0F9RCT0-F1-MODEL\_V4 | 1.0 | 1.585e-09 | 423 | 0.342 | 152 | 88 | 3 | 1 | 143 | 1 | 149 | Lysozyme | Lysozyme | | afdb-uniprot50 | AF-A0A397UIN9-F1-MODEL\_V4 | 1.0 | 1.585e-09 | 423 | 0.374 | 155 | 86 | 4 | 2 | 148 | 177 | 328 | Lysozyme | Lysozyme | | afdb-uniprot50 | AF-A0A2G2LIQ0-F1-MODEL\_V4 | 1.0 | 2.422e-09 | 423 | 0.358 | 148 | 87 | 5 | 3 | 146 | 6 | 149 | Lysozyme | Lysozyme | | afdb-uniprot50 | AF-A0A7M5XGT8-F1-MODEL\_V4 | 1.0 | 2.839e-09 | 422 | 0.46 | 128 | 66 | 2 | 2 | 129 | 5 | 129 | Lysozyme | Lysozyme | | afdb-uniprot50 | AF-A0A009FLU0-F1-MODEL\_V4 | 1.0 | 1.037e-09 | 422 | 0.518 | 133 | 57 | 4 | 18 | 146 | 1 | 130 | Lysozyme | Lysozyme | | afdb-uniprot50 | AF-A0A2N9LSD0-F1-MODEL\_V4 | 1.0 | 5.085e-09 | 422 | 0.391 | 148 | 82 | 5 | 1 | 144 | 1 | 144 | Lysozyme | Lysozyme | | afdb-uniprot50 | AF-C7RLP6-F1-MODEL\_V4 | 1.0 | 7.16e-10 | 422 | 0.378 | 169 | 75 | 5 | 1 | 144 | 4 | 167 | Lysozyme | Lysozyme | | afdb-uniprot50 | AF-A0A845WUP1-F1-MODEL\_V4 | 1.0 | 1.503e-09 | 422 | 0.33 | 142 | 87 | 4 | 5 | 144 | 49 | 184 | Glycoside hydrolase family protein | Glycoside hydrolase family protein | | afdb-uniprot50 | AF-S4FZ78-F1-MODEL\_V4 | 1.0 | 3.7e-09 | 422 | 0.31 | 148 | 95 | 4 | 1 | 144 | 1 | 145 | Lysozyme | Lysozyme | | afdb-uniprot50 | AF-A0A0S9NGX8-F1-MODEL\_V4 | 1.0 | 1.671e-09 | 422 | 0.312 | 157 | 97 | 5 | 3 | 154 | 1 | 151 | Lysozyme | Lysozyme | | afdb-uniprot50 | AF-A0A6I5R3J3-F1-MODEL\_V4 | 1.0 | 4.444e-10 | 422 | 0.368 | 152 | 88 | 5 | 4 | 151 | 219 | 366 | Lysozyme | Lysozyme | | afdb-uniprot50 | AF-A0A329A2Y0-F1-MODEL\_V4 | 1.0 | 1.426e-09 | 421 | 0.36 | 158 | 84 | 4 | 1 | 144 | 1 | 155 | Lysozyme | Lysozyme | | afdb-uniprot50 | AF-A0A1V5YWE3-F1-MODEL\_V4 | 1.0 | 1.585e-09 | 421 | 0.385 | 148 | 85 | 4 | 1 | 147 | 1 | 143 | Lysozyme | Lysozyme | | afdb-uniprot50 | AF-A0A1Y1YA73-F1-MODEL\_V4 | 1.0 | 1.959e-09 | 421 | 0.375 | 149 | 81 | 5 | 2 | 143 | 84 | 227 | Lysozyme | Lysozyme | | afdb-uniprot50 | AF-A0A847VEY3-F1-MODEL\_V4 | 1.0 | 4.338e-09 | 421 | 0.412 | 148 | 79 | 5 | 3 | 146 | 11 | 154 | Glycoside hydrolase family protein | Glycoside hydrolase family protein | | afdb-uniprot50 | AF-A0A136H867-F1-MODEL\_V4 | 1.0 | 7.77e-09 | 420 | 0.342 | 140 | 88 | 3 | 5 | 144 | 10 | 145 | Lysozyme | Lysozyme | | afdb-uniprot50 | AF-A0A6B3LIZ4-F1-MODEL\_V4 | 1.0 | 2.178e-09 | 420 | 0.375 | 149 | 82 | 3 | 4 | 145 | 29 | 173 | Lysozyme | Lysozyme | | afdb-uniprot50 | AF-A0A520R102-F1-MODEL\_V4 | 1.0 | 9.108e-09 | 420 | 0.342 | 146 | 88 | 4 | 1 | 144 | 69 | 208 | Lysozyme | Lysozyme | | afdb-uniprot50 | AF-A0A7W6U5N3-F1-MODEL\_V4 | 1.0 | 1.858e-09 | 420 | 0.358 | 170 | 94 | 4 | 1 | 159 | 52 | 217 | Lysozyme | Lysozyme | | afdb-uniprot50 | AF-A0A7T9XKC1-F1-MODEL\_V4 | 1.0 | 1.013e-08 | 419 | 0.328 | 140 | 91 | 2 | 7 | 146 | 21 | 157 | Lysozyme | Lysozyme | | afdb-uniprot50 | AF-A0A2D8XMB5-F1-MODEL\_V4 | 1.0 | 2.178e-09 | 419 | 0.35 | 157 | 86 | 5 | 3 | 146 | 6 | 159 | Lysozyme | Lysozyme | | afdb-uniprot50 | AF-A0A3B9L692-F1-MODEL\_V4 | 1.0 | 1.282e-09 | 419 | 0.456 | 151 | 71 | 5 | 1 | 143 | 9 | 156 | Lysozyme | Lysozyme | | afdb-uniprot50 | AF-A0A376MWV7-F1-MODEL\_V4 | 1.0 | 3.157e-09 | 419 | 0.319 | 144 | 90 | 3 | 6 | 144 | 21 | 161 | Lysozyme | Lysozyme | | afdb-uniprot50 | AF-A0A350KLD8-F1-MODEL\_V4 | 1.0 | 2.422e-09 | 419 | 0.364 | 151 | 92 | 3 | 1 | 150 | 29 | 176 | Lysozyme | Lysozyme | | afdb-uniprot50 | AF-E0WUE6-F1-MODEL\_V4 | 1.0 | 3.902e-09 | 419 | 0.337 | 145 | 90 | 3 | 4 | 145 | 61 | 202 | Lysozyme | Lysozyme | | afdb-uniprot50 | AF-A0A7U7FZ79-F1-MODEL\_V4 | 1.0 | 6.628e-09 | 418 | 0.407 | 130 | 74 | 2 | 14 | 143 | 31 | 157 | Lysozyme | Lysozyme | | afdb-uniprot50 | AF-V7ZPU5-F1-MODEL\_V4 | 1.0 | 5.362e-09 | 418 | 0.331 | 148 | 92 | 4 | 1 | 144 | 1 | 145 | Lysozyme | Lysozyme | | afdb-uniprot50 | AF-A0A7R9M799-F1-MODEL\_V4 | 1.0 | 5.362e-09 | 418 | 0.405 | 148 | 79 | 5 | 1 | 144 | 100 | 242 | Lysozyme | Lysozyme | | afdb-uniprot50 | AF-A0A328I7V7-F1-MODEL\_V4 | 1.0 | 1.762e-09 | 418 | 0.369 | 149 | 84 | 4 | 3 | 147 | 131 | 273 | Lysozyme | Lysozyme | | afdb-uniprot50 | AF-A0A7X2HR52-F1-MODEL\_V4 | 1.0 | 4.114e-09 | 417 | 0.388 | 144 | 84 | 2 | 1 | 144 | 39 | 178 | Lysozyme | Lysozyme | | afdb-uniprot50 | AF-E6EY07-F1-MODEL\_V4 | 1.0 | 5.085e-09 | 417 | 0.31 | 148 | 95 | 4 | 1 | 144 | 1 | 145 | Lysozyme | Lysozyme | | afdb-uniprot50 | AF-E8RMG8-F1-MODEL\_V4 | 1.0 | 5.085e-09 | 417 | 0.393 | 150 | 83 | 5 | 1 | 146 | 5 | 150 | Lysozyme | Lysozyme | | afdb-uniprot50 | AF-A0A4Z0P380-F1-MODEL\_V4 | 1.0 | 3.7e-09 | 415 | 0.425 | 155 | 76 | 6 | 1 | 146 | 3 | 153 | Lysozyme | Lysozyme | | afdb-uniprot50 | AF-A0A257UY12-F1-MODEL\_V4 | 1.0 | 2.759e-10 | 415 | 0.394 | 157 | 83 | 5 | 6 | 154 | 23 | 175 | Lysozyme | Lysozyme | | afdb-uniprot50 | AF-A0A2G2G4E8-F1-MODEL\_V4 | 1.0 | 3.157e-09 | 415 | 0.38 | 150 | 85 | 5 | 1 | 146 | 5 | 150 | Lysozyme | Lysozyme | | afdb-uniprot50 | AF-A0A3B0R8Q5-F1-MODEL\_V4 | 1.0 | 6.628e-09 | 415 | 0.337 | 148 | 90 | 5 | 3 | 146 | 7 | 150 | Lysozyme | Lysozyme | | afdb-uniprot50 | AF-A0A2C5THG2-F1-MODEL\_V4 | 1.0 | 1.126e-08 | 414 | 0.34 | 135 | 86 | 2 | 11 | 145 | 2 | 133 | Lysozyme | Lysozyme | | afdb-uniprot50 | AF-A0A1W1XTA9-F1-MODEL\_V4 | 1.0 | 2.178e-09 | 414 | 0.32 | 150 | 94 | 4 | 5 | 150 | 14 | 159 | Lysozyme | Lysozyme | | afdb-uniprot50 | AF-A0A7C5SDY7-F1-MODEL\_V4 | 1.0 | 1.068e-08 | 414 | 0.379 | 153 | 85 | 5 | 1 | 146 | 37 | 186 | Lysozyme | Lysozyme | | afdb-uniprot50 | AF-L8M4C1-F1-MODEL\_V4 | 1.0 | 2.839e-09 | 414 | 0.373 | 150 | 84 | 5 | 2 | 144 | 107 | 253 | Lysozyme | Lysozyme | | afdb-uniprot50 | AF-A0A843K231-F1-MODEL\_V4 | 1.0 | 1.216e-09 | 414 | 0.361 | 155 | 86 | 5 | 1 | 145 | 27 | 178 | Lysozyme | Lysozyme | | afdb-uniprot50 | AF-A0A3T0EDP8-F1-MODEL\_V4 | 1.0 | 1.585e-09 | 414 | 0.378 | 148 | 82 | 6 | 5 | 146 | 332 | 475 | Lysozyme | Lysozyme | | afdb-uniprot50 | AF-A0A158G2K7-F1-MODEL\_V4 | 1.0 | 5.654e-09 | 413 | 0.557 | 104 | 46 | 0 | 44 | 147 | 1 | 104 | Lysozyme | Lysozyme | | afdb-uniprot50 | AF-A0A1F4JWI6-F1-MODEL\_V4 | 1.0 | 5.085e-09 | 413 | 0.307 | 143 | 93 | 3 | 3 | 145 | 16 | 152 | Lysozyme | Lysozyme | | afdb-uniprot50 | AF-D7N2W0-F1-MODEL\_V4 | 1.0 | 6.286e-09 | 413 | 0.29 | 155 | 99 | 2 | 1 | 147 | 5 | 156 | Lysozyme | Lysozyme | | afdb-uniprot50 | AF-A0A401IDR8-F1-MODEL\_V4 | 1.0 | 1.426e-09 | 413 | 0.398 | 148 | 78 | 5 | 1 | 140 | 1 | 145 | Lysozyme | Lysozyme | | afdb-uniprot50 | AF-A0A7I6QAH2-F1-MODEL\_V4 | 1.0 | 2.554e-09 | 413 | 0.358 | 145 | 89 | 3 | 7 | 151 | 26 | 166 | Lysozyme | Lysozyme | | afdb-uniprot50 | AF-A0A2W4UPU2-F1-MODEL\_V4 | 1.0 | 3.509e-09 | 413 | 0.383 | 154 | 80 | 3 | 1 | 145 | 1 | 148 | Lysozyme | Lysozyme | | afdb-uniprot50 | AF-K9XTT4-F1-MODEL\_V4 | 1.0 | 2.066e-09 | 413 | 0.385 | 153 | 84 | 5 | 1 | 146 | 71 | 220 | Lysozyme | Lysozyme | | afdb-uniprot50 | AF-A0A6L3STT2-F1-MODEL\_V4 | 1.0 | 2.994e-09 | 413 | 0.373 | 150 | 82 | 5 | 1 | 146 | 1 | 142 | Lysozyme | Lysozyme | | afdb-uniprot50 | AF-A0A843LAG6-F1-MODEL\_V4 | 1.0 | 9.839e-10 | 413 | 0.385 | 153 | 81 | 5 | 1 | 143 | 27 | 176 | Glycoside hydrolase family protein | Glycoside hydrolase family protein | | afdb-uniprot50 | AF-A0A0R2JFT7-F1-MODEL\_V4 | 1.0 | 4.338e-09 | 412 | 0.364 | 148 | 88 | 5 | 1 | 146 | 1 | 144 | Lysozyme | Lysozyme | | afdb-uniprot50 | AF-A0A3N0WCV2-F1-MODEL\_V4 | 1.0 | 8.192e-09 | 412 | 0.366 | 150 | 85 | 5 | 1 | 144 | 25 | 170 | Lysozyme | Lysozyme | | afdb-uniprot50 | AF-A0A822UE20-F1-MODEL\_V4 | 1.0 | 2.693e-09 | 411 | 0.313 | 150 | 90 | 3 | 5 | 144 | 17 | 163 | Phage lysozome | Phage lysozome | | afdb-uniprot50 | AF-A0A366C290-F1-MODEL\_V4 | 1.0 | 7.77e-09 | 411 | 0.328 | 140 | 90 | 3 | 5 | 144 | 30 | 165 | Lysozyme | Lysozyme | | afdb-uniprot50 | AF-A5FWW2-F1-MODEL\_V4 | 1.0 | 1.094e-09 | 411 | 0.367 | 155 | 86 | 4 | 1 | 146 | 21 | 172 | Lysozyme | Lysozyme | | afdb-uniprot50 | AF-F9RLU7-F1-MODEL\_V4 | 1.0 | 3.902e-09 | 411 | 0.355 | 166 | 92 | 5 | 1 | 157 | 29 | 188 | Lysozyme | Lysozyme | | afdb-uniprot50 | AF-A0A257R9J7-F1-MODEL\_V4 | 1.0 | 6.79e-10 | 411 | 0.384 | 156 | 80 | 5 | 4 | 146 | 37 | 189 | Lysozyme | Lysozyme | | afdb-uniprot50 | AF-A0A258BAK0-F1-MODEL\_V4 | 1.0 | 3.902e-09 | 411 | 0.374 | 155 | 89 | 5 | 1 | 151 | 5 | 155 | Lysozyme | Lysozyme | | afdb-uniprot50 | AF-A0A7C5QZP7-F1-MODEL\_V4 | 1.0 | 3.902e-09 | 411 | 0.355 | 152 | 90 | 5 | 1 | 148 | 5 | 152 | Lysozyme | Lysozyme | | afdb-uniprot50 | AF-A0A514C2T5-F1-MODEL\_V4 | 1.0 | 8.192e-09 | 411 | 0.331 | 160 | 94 | 6 | 1 | 154 | 27 | 179 | Lysozyme | Lysozyme | | afdb-uniprot50 | AF-A0A6P6XNS9-F1-MODEL\_V4 | 1.0 | 3.7e-09 | 410 | 0.396 | 154 | 81 | 5 | 1 | 148 | 33 | 180 | Lysozyme | Lysozyme | | afdb-uniprot50 | AF-A0A7T2CTG2-F1-MODEL\_V4 | 1.0 | 3.7e-09 | 410 | 0.333 | 150 | 93 | 3 | 1 | 146 | 1 | 147 | Lysozyme | Lysozyme | | afdb-uniprot50 | AF-A0A6M3LU93-F1-MODEL\_V4 | 1.0 | 3.902e-09 | 409 | 0.31 | 145 | 93 | 4 | 1 | 143 | 1 | 140 | Lysozyme | Lysozyme | | afdb-uniprot50 | AF-A0A0R0CI69-F1-MODEL\_V4 | 1.0 | 3.328e-09 | 409 | 0.286 | 150 | 98 | 4 | 1 | 144 | 13 | 159 | Lysozyme | Lysozyme | | afdb-uniprot50 | AF-A0A558IEL7-F1-MODEL\_V4 | 1.0 | 9.108e-09 | 409 | 0.338 | 139 | 87 | 4 | 6 | 144 | 18 | 151 | Lysozyme | Lysozyme | | afdb-uniprot50 | AF-A0A6S7CW88-F1-MODEL\_V4 | 1.0 | 1.037e-09 | 409 | 0.374 | 147 | 82 | 6 | 6 | 146 | 22 | 164 | Lysozyme | Lysozyme | | afdb-uniprot50 | AF-A0A1Q3Q7Z8-F1-MODEL\_V4 | 1.0 | 8.393e-10 | 409 | 0.361 | 166 | 79 | 4 | 4 | 145 | 1 | 163 | Lysozyme | Lysozyme | | afdb-uniprot50 | AF-A0A5B0FQN8-F1-MODEL\_V4 | 1.0 | 2.066e-09 | 409 | 0.337 | 154 | 89 | 5 | 1 | 146 | 4 | 152 | Lysozyme | Lysozyme | | afdb-uniprot50 | AF-A0A2G2GMC1-F1-MODEL\_V4 | 1.0 | 1.392e-08 | 409 | 0.335 | 149 | 91 | 4 | 2 | 146 | 6 | 150 | Lysozyme | Lysozyme | | afdb-uniprot50 | AF-A0A6G9YUS1-F1-MODEL\_V4 | 1.0 | 2.994e-09 | 409 | 0.436 | 149 | 74 | 4 | 2 | 143 | 593 | 738 | Lysozyme | Lysozyme | | afdb-uniprot50 | AF-A0A350KNR5-F1-MODEL\_V4 | 1.0 | 1.187e-08 | 408 | 0.349 | 146 | 90 | 4 | 3 | 146 | 2 | 144 | Lysozyme | Lysozyme | | afdb-uniprot50 | AF-A0A397VV26-F1-MODEL\_V4 | 1.0 | 6.628e-09 | 408 | 0.337 | 154 | 90 | 5 | 2 | 148 | 116 | 264 | Lysozyme | Lysozyme | | afdb-uniprot50 | AF-A0A2D7Y4X6-F1-MODEL\_V4 | 1.0 | 2.554e-09 | 408 | 0.377 | 151 | 82 | 5 | 3 | 144 | 632 | 779 | Lysozyme | Lysozyme | | afdb-uniprot50 | AF-A0A3N5EFT6-F1-MODEL\_V4 | 1.0 | 1.762e-09 | 407 | 0.343 | 157 | 90 | 6 | 2 | 145 | 7 | 163 | Lysozyme | Lysozyme | | afdb-uniprot50 | AF-A0A345P973-F1-MODEL\_V4 | 1.0 | 7.369e-09 | 407 | 0.3 | 143 | 97 | 2 | 4 | 146 | 30 | 169 | Lysozyme | Lysozyme | | afdb-uniprot50 | AF-A0A2G2J894-F1-MODEL\_V4 | 1.0 | 7.77e-09 | 407 | 0.326 | 150 | 93 | 5 | 1 | 146 | 5 | 150 | Lysozyme | Lysozyme | | afdb-uniprot50 | AF-A0A850B733-F1-MODEL\_V4 | 1.0 | 2.994e-09 | 407 | 0.427 | 152 | 75 | 6 | 1 | 143 | 748 | 896 | Type VI secretion system tip protein VgrG | Type VI secretion system tip protein VgrG | | afdb-uniprot50 | AF-A0A060C089-F1-MODEL\_V4 | 1.0 | 7.77e-09 | 406 | 0.396 | 131 | 76 | 2 | 15 | 145 | 1 | 128 | Lysozyme | Lysozyme | | afdb-uniprot50 | AF-A0A839EPA8-F1-MODEL\_V4 | 1.0 | 4.114e-09 | 406 | 0.325 | 163 | 92 | 6 | 1 | 148 | 3 | 162 | Lysozyme | Lysozyme | | afdb-uniprot50 | AF-A0A0Q6RTE5-F1-MODEL\_V4 | 1.0 | 2.554e-09 | 406 | 0.343 | 166 | 90 | 5 | 1 | 147 | 3 | 168 | Lysozyme | Lysozyme | | afdb-uniprot50 | AF-A0A443L6C9-F1-MODEL\_V4 | 1.0 | 1.631e-08 | 405 | 0.357 | 140 | 86 | 3 | 5 | 144 | 9 | 144 | Lysozyme | Lysozyme | | afdb-uniprot50 | AF-A0A1Y0YBN9-F1-MODEL\_V4 | 1.0 | 3.157e-09 | 405 | 0.367 | 147 | 84 | 4 | 6 | 146 | 7 | 150 | Lysozyme | Lysozyme | | afdb-uniprot50 | AF-A0A5R9KB34-F1-MODEL\_V4 | 1.0 | 1.216e-09 | 405 | 0.391 | 156 | 81 | 6 | 1 | 145 | 3 | 155 | Lysozyme | Lysozyme | | afdb-uniprot50 | AF-A0A330M3W1-F1-MODEL\_V4 | 1.0 | 5.362e-09 | 405 | 0.326 | 147 | 91 | 4 | 3 | 144 | 12 | 155 | Lysozyme | Lysozyme | | afdb-uniprot50 | AF-A0A838L2V2-F1-MODEL\_V4 | 1.0 | 3.7e-09 | 405 | 0.306 | 160 | 92 | 4 | 2 | 146 | 184 | 339 | Lysozyme | Lysozyme | | afdb-uniprot50 | AF-A0A1X7KQT0-F1-MODEL\_V4 | 1.0 | 1.094e-09 | 404 | 0.481 | 137 | 56 | 4 | 20 | 144 | 2 | 135 | Lysozyme | Lysozyme | | afdb-uniprot50 | AF-A0A839A1U5-F1-MODEL\_V4 | 1.0 | 9.108e-09 | 404 | 0.345 | 142 | 86 | 4 | 5 | 143 | 15 | 152 | Lysozyme | Lysozyme | | afdb-uniprot50 | AF-A0A6N6MEH8-F1-MODEL\_V4 | 1.0 | 4.823e-09 | 404 | 0.38 | 150 | 81 | 5 | 1 | 146 | 1 | 142 | Lysozyme | Lysozyme | | afdb-uniprot50 | AF-A0A2D9KP11-F1-MODEL\_V4 | 1.0 | 5.362e-09 | 403 | 0.326 | 144 | 91 | 4 | 4 | 144 | 16 | 156 | Lysozyme | Lysozyme | | afdb-uniprot50 | AF-A0A5C8S7S7-F1-MODEL\_V4 | 1.0 | 3.7e-09 | 403 | 0.401 | 147 | 76 | 6 | 1 | 143 | 1 | 139 | Lysozyme | Lysozyme | | afdb-uniprot50 | AF-A0A644VLX9-F1-MODEL\_V4 | 1.0 | 1.72e-08 | 403 | 0.35 | 140 | 87 | 3 | 5 | 144 | 133 | 268 | Lysozyme | Lysozyme | | afdb-uniprot50 | AF-A0A4Y9RDX4-F1-MODEL\_V4 | 1.0 | 5.085e-09 | 403 | 0.333 | 159 | 95 | 5 | 1 | 154 | 16 | 168 | Lysozyme | Lysozyme | | afdb-uniprot50 | AF-A0A522A8D2-F1-MODEL\_V4 | 1.0 | 5.085e-09 | 403 | 0.372 | 145 | 88 | 2 | 1 | 145 | 179 | 320 | Lysozyme | Lysozyme | | afdb-uniprot50 | AF-A0A1S8P180-F1-MODEL\_V4 | 1.0 | 3.7e-09 | 402 | 0.361 | 144 | 88 | 3 | 2 | 143 | 131 | 272 | Lysozyme | Lysozyme | | afdb-uniprot50 | AF-A0A059E111-F1-MODEL\_V4 | 1.0 | 1.126e-08 | 402 | 0.344 | 151 | 90 | 6 | 1 | 146 | 10 | 156 | Lysozyme | Lysozyme | | afdb-uniprot50 | AF-C6XLA1-F1-MODEL\_V4 | 1.0 | 1.068e-08 | 402 | 0.344 | 148 | 89 | 5 | 1 | 144 | 5 | 148 | Lysozyme | Lysozyme | | afdb-uniprot50 | AF-A0A2E5TFG5-F1-MODEL\_V4 | 1.0 | 2.126e-08 | 401 | 0.354 | 141 | 86 | 4 | 5 | 144 | 15 | 151 | Lysozyme | Lysozyme | | afdb-uniprot50 | AF-U2N1X6-F1-MODEL\_V4 | 1.0 | 2.017e-08 | 401 | 0.384 | 143 | 84 | 3 | 1 | 143 | 26 | 164 | Lysozyme | Lysozyme | | afdb-uniprot50 | AF-A0A1V2GWA1-F1-MODEL\_V4 | 1.0 | 5.085e-09 | 401 | 0.318 | 160 | 99 | 5 | 2 | 155 | 22 | 177 | Uncharacterized protein | Uncharacterized protein | | afdb-uniprot50 | AF-A0A4U0YH08-F1-MODEL\_V4 | 1.0 | 1.547e-08 | 401 | 0.384 | 138 | 80 | 4 | 7 | 144 | 2 | 134 | Lysozyme | Lysozyme | | afdb-uniprot50 | AF-A0A7Y0NU04-F1-MODEL\_V4 | 1.0 | 3.328e-09 | 401 | 0.361 | 152 | 87 | 5 | 2 | 146 | 88 | 236 | Lysozyme | Lysozyme | | afdb-uniprot50 | AF-A0A2E6Y4S6-F1-MODEL\_V4 | 1.0 | 6.988e-09 | 400 | 0.349 | 146 | 86 | 5 | 4 | 144 | 12 | 153 | Lysozyme | Lysozyme | | afdb-uniprot50 | AF-A0A8A3KUB0-F1-MODEL\_V4 | 1.0 | 3.902e-09 | 400 | 0.361 | 163 | 85 | 5 | 1 | 144 | 3 | 165 | Lysozyme | Lysozyme | | afdb-uniprot50 | AF-A0A377A078-F1-MODEL\_V4 | 1.0 | 5.654e-09 | 399 | 0.335 | 146 | 92 | 4 | 7 | 152 | 8 | 148 | Lysozyme | Lysozyme | | afdb-uniprot50 | AF-A0A827LRS5-F1-MODEL\_V4 | 1.0 | 1.912e-08 | 399 | 0.361 | 144 | 88 | 3 | 1 | 144 | 27 | 166 | Lysozyme | Lysozyme | | afdb-uniprot50 | AF-A0A3P1W4Z0-F1-MODEL\_V4 | 1.0 | 2.994e-09 | 399 | 0.318 | 160 | 99 | 2 | 2 | 153 | 14 | 171 | Lysozyme | Lysozyme | | afdb-uniprot50 | AF-A0A069IM11-F1-MODEL\_V4 | 1.0 | 5.961e-09 | 399 | 0.348 | 158 | 91 | 6 | 2 | 154 | 9 | 159 | Lysozyme | Lysozyme | | afdb-uniprot50 | AF-A0A6P0MRG7-F1-MODEL\_V4 | 1.0 | 6.628e-09 | 399 | 0.32 | 156 | 92 | 2 | 2 | 146 | 6 | 158 | Lysozyme | Lysozyme | | afdb-uniprot50 | AF-A0A7X0AWL3-F1-MODEL\_V4 | 1.0 | 2.554e-09 | 398 | 0.406 | 150 | 76 | 7 | 1 | 143 | 3 | 146 | Lysozyme | Lysozyme | | afdb-uniprot50 | AF-A0A2T1CPP5-F1-MODEL\_V4 | 1.0 | 8.638e-09 | 398 | 0.389 | 149 | 81 | 5 | 3 | 144 | 63 | 208 | Lysozyme | Lysozyme | | afdb-uniprot50 | AF-A0A2G1YWA0-F1-MODEL\_V4 | 1.0 | 9.604e-09 | 398 | 0.353 | 147 | 87 | 5 | 2 | 144 | 6 | 148 | Lysozyme | Lysozyme | | afdb-uniprot50 | AF-A0A6N6L8L1-F1-MODEL\_V4 | 1.0 | 1.547e-08 | 397 | 0.356 | 146 | 84 | 5 | 3 | 143 | 2 | 142 | Lysozyme | Lysozyme | | afdb-uniprot50 | AF-A0A764SMJ2-F1-MODEL\_V4 | 1.0 | 9.108e-09 | 397 | 0.316 | 142 | 89 | 3 | 6 | 142 | 21 | 159 | Lysozyme | Lysozyme | | afdb-uniprot50 | AF-A0A389MSU7-F1-MODEL\_V4 | 1.0 | 5.362e-09 | 397 | 0.408 | 147 | 75 | 5 | 1 | 143 | 1 | 139 | Lysozyme | Lysozyme | | afdb-uniprot50 | AF-C3L4H1-F1-MODEL\_V4 | 1.0 | 2.066e-09 | 397 | 0.397 | 151 | 81 | 6 | 2 | 145 | 431 | 578 | Lysozyme | Lysozyme | | afdb-uniprot50 | AF-A0A2S5TTS2-F1-MODEL\_V4 | 1.0 | 1.912e-08 | 396 | 0.404 | 146 | 75 | 5 | 3 | 144 | 1 | 138 | Lysozyme | Lysozyme | | afdb-uniprot50 | AF-A0A6L6G496-F1-MODEL\_V4 | 1.0 | 4.574e-09 | 396 | 0.307 | 156 | 93 | 3 | 2 | 145 | 3 | 155 | Lysozyme | Lysozyme | | afdb-uniprot50 | AF-A0A5C8W707-F1-MODEL\_V4 | 1.0 | 6.988e-09 | 396 | 0.36 | 150 | 84 | 5 | 1 | 146 | 1 | 142 | Lysozyme | Lysozyme | | afdb-uniprot50 | AF-A0A2V3TSZ6-F1-MODEL\_V4 | 1.0 | 5.085e-09 | 396 | 0.36 | 147 | 81 | 5 | 1 | 143 | 1 | 138 | Lysozyme | Lysozyme | | afdb-uniprot50 | AF-A0A563VMR6-F1-MODEL\_V4 | 1.0 | 6.628e-09 | 396 | 0.337 | 151 | 90 | 5 | 3 | 146 | 51 | 198 | Lysozyme | Lysozyme | | afdb-uniprot50 | AF-A0A519IYM2-F1-MODEL\_V4 | 1.0 | 1.72e-08 | 396 | 0.32 | 159 | 97 | 5 | 1 | 154 | 1 | 153 | Lysozyme | Lysozyme | | afdb-uniprot50 | AF-A0A8A7UZQ7-F1-MODEL\_V4 | 1.0 | 8.192e-09 | 396 | 0.316 | 161 | 95 | 6 | 1 | 154 | 1 | 153 | Glycoside hydrolase family protein | Glycoside hydrolase family protein | | afdb-uniprot50 | AF-A0A711C0N5-F1-MODEL\_V4 | 1.0 | 1.631e-08 | 395 | 0.542 | 118 | 50 | 2 | 31 | 144 | 2 | 119 | Lysozyme | Lysozyme | | afdb-uniprot50 | AF-A0A4R8F7V4-F1-MODEL\_V4 | 1.0 | 2.017e-08 | 395 | 0.304 | 141 | 94 | 3 | 4 | 144 | 1 | 137 | Lysozyme | Lysozyme | | afdb-uniprot50 | AF-A0A7V3LJU9-F1-MODEL\_V4 | 1.0 | 1.392e-08 | 395 | 0.319 | 147 | 93 | 5 | 1 | 144 | 1 | 143 | Lysozyme | Lysozyme | | afdb-uniprot50 | AF-A0A233HE82-F1-MODEL\_V4 | 1.0 | 4.823e-09 | 395 | 0.297 | 158 | 93 | 4 | 1 | 143 | 1 | 155 | Lysozyme | Lysozyme | | afdb-uniprot50 | AF-A0A1X0X4Q9-F1-MODEL\_V4 | 1.0 | 1.631e-08 | 395 | 0.33 | 142 | 90 | 4 | 4 | 144 | 32 | 169 | Lysozyme | Lysozyme | | afdb-uniprot50 | AF-A0A345CWD3-F1-MODEL\_V4 | 1.0 | 6.988e-09 | 395 | 0.363 | 143 | 86 | 4 | 10 | 152 | 62 | 199 | Lysozyme | Lysozyme | | afdb-uniprot50 | AF-A0A1H8ACR7-F1-MODEL\_V4 | 1.0 | 3.7e-09 | 395 | 0.37 | 151 | 82 | 7 | 1 | 146 | 71 | 213 | Lysozyme | Lysozyme | | afdb-uniprot50 | AF-A0A258FEZ4-F1-MODEL\_V4 | 1.0 | 1.068e-08 | 395 | 0.339 | 159 | 94 | 5 | 1 | 154 | 9 | 161 | Lysozyme | Lysozyme | | afdb-uniprot50 | AF-A0A6B8KGM4-F1-MODEL\_V4 | 1.0 | 1.216e-09 | 394 | 0.403 | 156 | 75 | 8 | 1 | 146 | 2 | 149 | Lysozyme | Lysozyme | | afdb-uniprot50 | AF-A0A2U1SSP9-F1-MODEL\_V4 | 1.0 | 1.912e-08 | 394 | 0.391 | 148 | 78 | 4 | 1 | 144 | 3 | 142 | Lysozyme | Lysozyme | | afdb-uniprot50 | AF-A0A2K9CTZ1-F1-MODEL\_V4 | 1.0 | 2.364e-08 | 393 | 0.581 | 98 | 41 | 0 | 47 | 144 | 1 | 98 | Lysozyme | Lysozyme | | afdb-uniprot50 | AF-A0A2P7SF92-F1-MODEL\_V4 | 1.0 | 1.72e-08 | 393 | 0.286 | 143 | 96 | 4 | 4 | 144 | 1 | 139 | Lysozyme | Lysozyme | | afdb-uniprot50 | AF-A0A7L7IWX3-F1-MODEL\_V4 | 1.0 | 2.839e-09 | 393 | 0.363 | 157 | 83 | 4 | 1 | 143 | 1 | 154 | Lysozyme | Lysozyme | | afdb-uniprot50 | AF-A0A1G1BDY3-F1-MODEL\_V4 | 1.0 | 4.823e-09 | 393 | 0.314 | 159 | 100 | 5 | 1 | 155 | 5 | 158 | Lysozyme | Lysozyme | | afdb-uniprot50 | AF-A0A3D0HRH7-F1-MODEL\_V4 | 1.0 | 1.126e-08 | 393 | 0.419 | 155 | 75 | 7 | 2 | 145 | 7 | 157 | Lysozyme | Lysozyme | | afdb-uniprot50 | AF-H1KV31-F1-MODEL\_V4 | 1.0 | 5.085e-09 | 393 | 0.387 | 147 | 78 | 6 | 1 | 143 | 1 | 139 | Lysozyme | Lysozyme | | afdb-uniprot50 | AF-A0A252DI96-F1-MODEL\_V4 | 1.0 | 3.249e-08 | 392 | 0.447 | 105 | 58 | 0 | 47 | 151 | 1 | 105 | Lysozyme | Lysozyme | | afdb-uniprot50 | AF-A0A2W2BWY0-F1-MODEL\_V4 | 1.0 | 3.509e-09 | 392 | 0.343 | 157 | 91 | 4 | 1 | 146 | 2 | 157 | Lysozyme | Lysozyme | | afdb-uniprot50 | AF-A0A6L3SVH8-F1-MODEL\_V4 | 1.0 | 8.192e-09 | 392 | 0.373 | 150 | 82 | 6 | 1 | 146 | 1 | 142 | Lysozyme | Lysozyme | | afdb-uniprot50 | AF-A0A455RUP7-F1-MODEL\_V4 | 1.0 | 6.821e-08 | 391 | 0.551 | 98 | 44 | 0 | 47 | 144 | 1 | 98 | Lysozyme | Lysozyme | | afdb-uniprot50 | AF-A0A291E4Z2-F1-MODEL\_V4 | 1.0 | 2.771e-08 | 391 | 0.321 | 146 | 92 | 4 | 5 | 146 | 4 | 146 | Lysozyme | Lysozyme | | afdb-uniprot50 | AF-A0A0L0AVI2-F1-MODEL\_V4 | 1.0 | 8.638e-09 | 391 | 0.317 | 145 | 95 | 3 | 1 | 144 | 6 | 147 | Lysozyme | Lysozyme | | afdb-uniprot50 | AF-A0A1W2A982-F1-MODEL\_V4 | 1.0 | 2.017e-08 | 391 | 0.314 | 143 | 95 | 2 | 5 | 145 | 40 | 181 | Lysozyme | Lysozyme | | afdb-uniprot50 | AF-A0A5C8UIV5-F1-MODEL\_V4 | 1.0 | 1.013e-08 | 391 | 0.375 | 144 | 81 | 5 | 3 | 146 | 7 | 141 | Lysozyme | Lysozyme | | afdb-uniprot50 | AF-A0A1X2GML7-F1-MODEL\_V4 | 1.0 | 3.902e-09 | 391 | 0.387 | 147 | 82 | 4 | 2 | 143 | 122 | 265 | Lysozyme | Lysozyme | | afdb-uniprot50 | AF-B4WDB1-F1-MODEL\_V4 | 1.0 | 1.126e-08 | 391 | 0.341 | 158 | 92 | 6 | 2 | 154 | 9 | 159 | Lysozyme | Lysozyme | | afdb-uniprot50 | AF-A0A5M8SLB4-F1-MODEL\_V4 | 1.0 | 9.108e-09 | 391 | 0.344 | 145 | 91 | 3 | 5 | 149 | 381 | 521 | Lysozyme | Lysozyme | | afdb-uniprot50 | AF-A0A741PPE7-F1-MODEL\_V4 | 1.0 | 7.192e-08 | 390 | 0.561 | 98 | 43 | 0 | 47 | 144 | 1 | 98 | Lysozyme | Lysozyme | | afdb-uniprot50 | AF-A0A704ZET1-F1-MODEL\_V4 | 1.0 | 2.242e-08 | 390 | 0.538 | 117 | 50 | 2 | 32 | 144 | 1 | 117 | Lysozyme | Lysozyme | | afdb-uniprot50 | AF-A0A7X5L1T0-F1-MODEL\_V4 | 1.0 | 5.085e-09 | 390 | 0.366 | 150 | 80 | 6 | 1 | 144 | 1 | 141 | Lysozyme | Lysozyme | | afdb-uniprot50 | AF-A0A7R9PXF8-F1-MODEL\_V4 | 1.0 | 1.187e-08 | 390 | 0.375 | 152 | 82 | 7 | 1 | 144 | 18 | 164 | Lysozyme | Lysozyme | | afdb-uniprot50 | AF-A0A7U0N3R0-F1-MODEL\_V4 | 1.0 | 2.628e-08 | 390 | 0.319 | 147 | 95 | 3 | 1 | 146 | 26 | 168 | Lysozyme | Lysozyme | | afdb-uniprot50 | AF-A0A2U8HQZ1-F1-MODEL\_V4 | 1.0 | 8.638e-09 | 390 | 0.391 | 148 | 78 | 6 | 1 | 144 | 1 | 140 | Lysozyme | Lysozyme | | afdb-uniprot50 | AF-A0A4Q7ICI5-F1-MODEL\_V4 | 1.0 | 5.085e-09 | 390 | 0.358 | 156 | 83 | 4 | 2 | 143 | 3 | 155 | Lysozyme | Lysozyme | | afdb-uniprot50 | AF-A0A4P6YRS8-F1-MODEL\_V4 | 1.0 | 1.72e-08 | 390 | 0.404 | 146 | 81 | 5 | 1 | 145 | 5 | 145 | Lysozyme | Lysozyme | | afdb-uniprot50 | AF-A0A7R9KVP1-F1-MODEL\_V4 | 1.0 | 5.961e-09 | 390 | 0.364 | 159 | 83 | 4 | 2 | 143 | 140 | 297 | Lysozyme | Lysozyme | | afdb-uniprot50 | AF-A0A539DDV1-F1-MODEL\_V4 | 1.0 | 2.126e-08 | 390 | 0.32 | 150 | 94 | 4 | 1 | 146 | 25 | 170 | Lysozyme | Lysozyme | | afdb-uniprot50 | AF-A0A450YWU4-F1-MODEL\_V4 | 1.0 | 1.187e-08 | 390 | 0.348 | 155 | 87 | 4 | 2 | 146 | 620 | 770 | Lysozyme | Lysozyme | | afdb-uniprot50 | AF-A0A7C1EUW6-F1-MODEL\_V4 | 1.0 | 3.808e-08 | 389 | 0.376 | 146 | 84 | 5 | 3 | 144 | 1 | 143 | Lysozyme | Lysozyme | | afdb-uniprot50 | AF-A0A7X9JNE0-F1-MODEL\_V4 | 1.0 | 4.114e-09 | 389 | 0.282 | 163 | 101 | 5 | 1 | 154 | 1 | 156 | Lysozyme | Lysozyme | | afdb-uniprot50 | AF-A0A6I1MR44-F1-MODEL\_V4 | 1.0 | 1.671e-09 | 389 | 0.395 | 167 | 75 | 4 | 1 | 144 | 4 | 167 | Lysozyme | Lysozyme | | afdb-uniprot50 | AF-A0A2G6EIY6-F1-MODEL\_V4 | 1.0 | 7.369e-09 | 389 | 0.36 | 158 | 82 | 4 | 1 | 143 | 17 | 170 | Lysozyme | Lysozyme | | afdb-uniprot50 | AF-A0A4R5DVM8-F1-MODEL\_V4 | 1.0 | 3.509e-09 | 389 | 0.372 | 153 | 83 | 6 | 1 | 143 | 4 | 153 | Lysozyme | Lysozyme | | afdb-uniprot50 | AF-A0A8B5V1R1-F1-MODEL\_V4 | 1.0 | 2.017e-08 | 389 | 0.339 | 153 | 90 | 3 | 1 | 145 | 49 | 198 | Lysozyme | Lysozyme | | afdb-uniprot50 | AF-A0A326KZ45-F1-MODEL\_V4 | 1.0 | 1.467e-08 | 389 | 0.339 | 159 | 92 | 7 | 2 | 154 | 18 | 169 | Lysozyme | Lysozyme | | afdb-uniprot50 | AF-A0A316HJ74-F1-MODEL\_V4 | 1.0 | 1.013e-08 | 388 | 0.353 | 150 | 87 | 5 | 2 | 144 | 5 | 151 | Lysozyme | Lysozyme | | afdb-uniprot50 | AF-A0A0A2YID5-F1-MODEL\_V4 | 1.0 | 5.961e-09 | 388 | 0.357 | 154 | 87 | 7 | 1 | 145 | 1 | 151 | Lysozyme | Lysozyme | | afdb-uniprot50 | AF-A0A501QET1-F1-MODEL\_V4 | 1.0 | 2.242e-08 | 388 | 0.372 | 153 | 81 | 3 | 1 | 144 | 32 | 178 | Lysozyme | Lysozyme | | afdb-uniprot50 | AF-A0A679KB21-F1-MODEL\_V4 | 1.0 | 6.286e-09 | 388 | 0.355 | 169 | 89 | 6 | 1 | 150 | 3 | 170 | Lysozyme | Lysozyme | | afdb-uniprot50 | AF-A0A5C1Q659-F1-MODEL\_V4 | 1.0 | 6.286e-09 | 388 | 0.375 | 157 | 84 | 5 | 1 | 146 | 303 | 456 | Lysozyme | Lysozyme | | afdb-uniprot50 | AF-A0A349NKU6-F1-MODEL\_V4 | 1.0 | 3.425e-08 | 387 | 0.437 | 112 | 59 | 2 | 1 | 112 | 1 | 108 | Lysozyme | Lysozyme | | afdb-uniprot50 | AF-A0A1H9KD71-F1-MODEL\_V4 | 1.0 | 1.72e-08 | 387 | 0.348 | 155 | 90 | 4 | 1 | 147 | 56 | 207 | Lysozyme | Lysozyme | | afdb-uniprot50 | AF-A0A348HI68-F1-MODEL\_V4 | 1.0 | 5.961e-09 | 386 | 0.333 | 153 | 88 | 6 | 4 | 146 | 10 | 158 | Lysozyme | Lysozyme | | afdb-uniprot50 | AF-A0A081NGH9-F1-MODEL\_V4 | 1.0 | 5.085e-09 | 386 | 0.373 | 158 | 84 | 6 | 1 | 143 | 1 | 158 | Lysozyme | Lysozyme | | afdb-uniprot50 | AF-A0A380TT26-F1-MODEL\_V4 | 1.0 | 3.081e-08 | 386 | 0.344 | 148 | 90 | 4 | 1 | 144 | 26 | 170 | Lysozyme | Lysozyme | | afdb-uniprot50 | AF-A0A255YT31-F1-MODEL\_V4 | 1.0 | 4.338e-09 | 386 | 0.348 | 178 | 80 | 8 | 1 | 146 | 1 | 174 | Lysozyme | Lysozyme | | afdb-uniprot50 | AF-A0A2W6XAR0-F1-MODEL\_V4 | 1.0 | 5.085e-09 | 386 | 0.352 | 159 | 96 | 5 | 2 | 159 | 10 | 162 | Lysozyme | Lysozyme | | afdb-uniprot50 | AF-A0A2G2HXP0-F1-MODEL\_V4 | 1.0 | 4.234e-08 | 386 | 0.337 | 151 | 91 | 6 | 1 | 146 | 4 | 150 | Lysozyme | Lysozyme | | afdb-uniprot50 | AF-A0A661CVB8-F1-MODEL\_V4 | 1.0 | 4.823e-09 | 386 | 0.296 | 162 | 90 | 3 | 2 | 143 | 243 | 400 | Lysozyme | Lysozyme | | afdb-uniprot50 | AF-A0A1Z9AVU0-F1-MODEL\_V4 | 1.0 | 2.364e-08 | 385 | 0.388 | 134 | 76 | 3 | 2 | 133 | 6 | 135 | Lysozyme | Lysozyme | | afdb-uniprot50 | AF-A0A7V3K1A6-F1-MODEL\_V4 | 1.0 | 1.126e-08 | 385 | 0.341 | 164 | 87 | 3 | 1 | 145 | 1 | 162 | Lysozyme | Lysozyme | | afdb-uniprot50 | AF-A0A358J110-F1-MODEL\_V4 | 1.0 | 2.493e-08 | 385 | 0.314 | 143 | 94 | 3 | 1 | 143 | 26 | 164 | Lysozyme | Lysozyme | | afdb-uniprot50 | AF-A0A0Q6SQZ2-F1-MODEL\_V4 | 1.0 | 2.628e-08 | 385 | 0.318 | 154 | 92 | 4 | 1 | 145 | 50 | 199 | Lysozyme | Lysozyme | | afdb-uniprot50 | AF-A0A2W4T8V5-F1-MODEL\_V4 | 1.0 | 1.187e-08 | 385 | 0.329 | 158 | 92 | 5 | 1 | 152 | 54 | 203 | Lysozyme | Lysozyme | | afdb-uniprot50 | AF-A0A2U9U7G2-F1-MODEL\_V4 | 1.0 | 2.126e-08 | 384 | 0.302 | 149 | 96 | 5 | 1 | 145 | 1 | 145 | Lysozyme | Lysozyme | | afdb-uniprot50 | AF-A0A6L6YI06-F1-MODEL\_V4 | 1.0 | 4.464e-08 | 384 | 0.345 | 139 | 87 | 3 | 6 | 144 | 13 | 147 | Lysozyme | Lysozyme | | afdb-uniprot50 | AF-A0A1H8IPR6-F1-MODEL\_V4 | 1.0 | 2.628e-08 | 384 | 0.356 | 146 | 90 | 4 | 1 | 145 | 10 | 152 | Lysozyme | Lysozyme | | afdb-uniprot50 | AF-A0A7Y8MEE6-F1-MODEL\_V4 | 1.0 | 1.72e-08 | 384 | 0.344 | 151 | 91 | 4 | 1 | 147 | 4 | 150 | Lysozyme | Lysozyme | | afdb-uniprot50 | AF-A0A420WMK7-F1-MODEL\_V4 | 1.0 | 3.612e-08 | 384 | 0.333 | 150 | 92 | 5 | 1 | 146 | 10 | 155 | Lysozyme | Lysozyme | | afdb-uniprot50 | AF-V5TVX0-F1-MODEL\_V4 | 1.0 | 4.016e-08 | 383 | 0.337 | 148 | 93 | 4 | 1 | 148 | 17 | 159 | Lysozyme | Lysozyme | | afdb-uniprot50 | AF-A0A8B3HN41-F1-MODEL\_V4 | 1.0 | 8.89e-08 | 383 | 0.244 | 139 | 105 | 0 | 6 | 144 | 24 | 162 | Lysozyme | Lysozyme | | afdb-uniprot50 | AF-A0A4Q2RBR2-F1-MODEL\_V4 | 1.0 | 3.081e-08 | 383 | 0.391 | 148 | 78 | 4 | 1 | 144 | 3 | 142 | Lysozyme | Lysozyme | | afdb-uniprot50 | AF-A0A4Q5WCJ2-F1-MODEL\_V4 | 1.0 | 2.493e-08 | 383 | 0.348 | 135 | 83 | 2 | 3 | 137 | 86 | 215 | Lysozyme | Lysozyme | | afdb-uniprot50 | AF-A0A1M6L8G3-F1-MODEL\_V4 | 1.0 | 6.628e-09 | 383 | 0.321 | 165 | 100 | 6 | 2 | 157 | 74 | 235 | Lysozyme | Lysozyme | | afdb-uniprot50 | AF-A0A847VFE4-F1-MODEL\_V4 | 1.0 | 6.988e-09 | 383 | 0.361 | 180 | 83 | 7 | 1 | 152 | 6 | 181 | Lysozyme | Lysozyme | | afdb-uniprot50 | AF-A0A5P9KRI5-F1-MODEL\_V4 | 1.0 | 1.467e-08 | 383 | 0.333 | 162 | 93 | 7 | 1 | 154 | 1 | 155 | Lysozyme | Lysozyme | | afdb-uniprot50 | AF-A0A0F9UX49-F1-MODEL\_V4 | 1.0 | 1.72e-08 | 383 | 0.366 | 153 | 84 | 4 | 1 | 143 | 559 | 708 | Lysozyme | Lysozyme | | afdb-uniprot50 | AF-A0A6V8I6G4-F1-MODEL\_V4 | 1.0 | 1.187e-08 | 382 | 0.346 | 150 | 86 | 5 | 6 | 146 | 7 | 153 | Lysozyme | Lysozyme | | afdb-uniprot50 | AF-A0A4R3HYS8-F1-MODEL\_V4 | 1.0 | 1.013e-08 | 382 | 0.347 | 164 | 86 | 7 | 1 | 146 | 5 | 165 | Lysozyme | Lysozyme | | afdb-uniprot50 | AF-A0A832PMR6-F1-MODEL\_V4 | 1.0 | 6.988e-09 | 382 | 0.357 | 151 | 84 | 4 | 4 | 144 | 26 | 173 | Lysozyme | Lysozyme | | afdb-uniprot50 | AF-A0A7W0JH81-F1-MODEL\_V4 | 1.0 | 1.068e-08 | 382 | 0.367 | 166 | 90 | 4 | 1 | 158 | 2 | 160 | Lysozyme | Lysozyme | | afdb-uniprot50 | AF-A0A3S5DFC5-F1-MODEL\_V4 | 1.0 | 4.707e-08 | 381 | 0.353 | 147 | 89 | 3 | 4 | 150 | 96 | 236 | Lysozyme | Lysozyme | | afdb-uniprot50 | AF-A0A6C8Z019-F1-MODEL\_V4 | 1.0 | 6.135e-08 | 380 | 0.564 | 101 | 44 | 0 | 44 | 144 | 1 | 101 | Lysozyme | Lysozyme | | afdb-uniprot50 | AF-H8L2I5-F1-MODEL\_V4 | 1.0 | 3.425e-08 | 380 | 0.298 | 144 | 94 | 3 | 6 | 145 | 16 | 156 | Lysozyme | Lysozyme | | afdb-uniprot50 | AF-A0A3P3DCL8-F1-MODEL\_V4 | 1.0 | 2.364e-08 | 380 | 0.388 | 144 | 80 | 4 | 5 | 144 | 33 | 172 | Lysozyme | Lysozyme | | afdb-uniprot50 | AF-A0A4S2CSZ9-F1-MODEL\_V4 | 1.0 | 6.628e-09 | 380 | 0.346 | 156 | 88 | 5 | 1 | 143 | 26 | 180 | Lysozyme | Lysozyme | | afdb-uniprot50 | AF-A0A147I3J2-F1-MODEL\_V4 | 1.0 | 2.628e-08 | 379 | 0.393 | 150 | 80 | 6 | 13 | 156 | 3 | 147 | Lysozyme | Lysozyme | | afdb-uniprot50 | AF-A0A238JZZ0-F1-MODEL\_V4 | 1.0 | 3.081e-08 | 379 | 0.277 | 144 | 96 | 4 | 6 | 145 | 15 | 154 | Lysozyme | Lysozyme | | afdb-uniprot50 | AF-A0A715LSR0-F1-MODEL\_V4 | 1.0 | 2.922e-08 | 378 | 0.389 | 131 | 76 | 3 | 14 | 144 | 2 | 128 | Lysozyme | Lysozyme | | afdb-uniprot50 | AF-A0A0Q5LBE4-F1-MODEL\_V4 | 1.0 | 1.013e-08 | 378 | 0.352 | 153 | 85 | 5 | 1 | 145 | 4 | 150 | Lysozyme | Lysozyme | | afdb-uniprot50 | AF-A0A1Q3HI04-F1-MODEL\_V4 | 1.0 | 5.961e-09 | 378 | 0.393 | 155 | 75 | 5 | 1 | 143 | 9 | 156 | Lysozyme | Lysozyme | | afdb-uniprot50 | AF-A0A2X9ZYS3-F1-MODEL\_V4 | 1.0 | 1.392e-08 | 378 | 0.342 | 149 | 92 | 4 | 7 | 153 | 29 | 173 | Lysozyme | Lysozyme | | afdb-uniprot50 | AF-A0A0A2X3S0-F1-MODEL\_V4 | 1.0 | 5.819e-08 | 377 | 0.242 | 140 | 102 | 3 | 6 | 145 | 4 | 139 | Lysozyme | Lysozyme | | afdb-uniprot50 | AF-A0A2D5RX06-F1-MODEL\_V4 | 1.0 | 2.493e-08 | 377 | 0.335 | 149 | 93 | 5 | 5 | 153 | 17 | 159 | Lysozyme | Lysozyme | | afdb-uniprot50 | AF-A0A519PZJ4-F1-MODEL\_V4 | 1.0 | 3.612e-08 | 377 | 0.337 | 148 | 88 | 5 | 1 | 146 | 27 | 166 | Lysozyme | Lysozyme | | afdb-uniprot50 | AF-A0A1X3I4E1-F1-MODEL\_V4 | 1.0 | 4.707e-08 | 377 | 0.317 | 145 | 93 | 5 | 1 | 144 | 49 | 188 | Lysozyme | Lysozyme | | afdb-uniprot50 | AF-A0A2T8WJ16-F1-MODEL\_V4 | 1.0 | 5.234e-08 | 376 | 0.425 | 120 | 62 | 3 | 1 | 116 | 1 | 117 | Lysozyme | Lysozyme | | afdb-uniprot50 | AF-A0A327SGY1-F1-MODEL\_V4 | 1.0 | 3.612e-08 | 376 | 0.34 | 150 | 88 | 5 | 1 | 144 | 1 | 145 | Lysozyme | Lysozyme | | afdb-uniprot50 | AF-A0A356FYC9-F1-MODEL\_V4 | 1.0 | 7.369e-09 | 376 | 0.36 | 136 | 83 | 3 | 11 | 145 | 2 | 134 | Lysozyme | Lysozyme | | afdb-uniprot50 | AF-A0A1L7AKL0-F1-MODEL\_V4 | 1.0 | 7.77e-09 | 376 | 0.337 | 160 | 89 | 5 | 9 | 155 | 2 | 157 | Lysozyme | Lysozyme | | afdb-uniprot50 | AF-A0A2S2CGK8-F1-MODEL\_V4 | 1.0 | 1.32e-08 | 376 | 0.301 | 166 | 99 | 7 | 2 | 155 | 5 | 165 | Lysozyme | Lysozyme | | afdb-uniprot50 | AF-A0A3D5P936-F1-MODEL\_V4 | 1.0 | 1.631e-08 | 376 | 0.342 | 146 | 90 | 5 | 3 | 145 | 157 | 299 | Lysozyme | Lysozyme | | afdb-uniprot50 | AF-J7QGR0-F1-MODEL\_V4 | 1.0 | 1.32e-08 | 376 | 0.374 | 171 | 83 | 6 | 1 | 155 | 4 | 166 | Lysozyme | Lysozyme | | afdb-uniprot50 | AF-A0A0Q5UY70-F1-MODEL\_V4 | 1.0 | 1.72e-08 | 376 | 0.301 | 159 | 100 | 5 | 1 | 154 | 9 | 161 | Lysozyme | Lysozyme | | afdb-uniprot50 | AF-A0A198H0T0-F1-MODEL\_V4 | 1.0 | 1.72e-08 | 375 | 0.319 | 147 | 96 | 3 | 1 | 146 | 6 | 149 | Lysozyme | Lysozyme | | afdb-uniprot50 | AF-A0A366F692-F1-MODEL\_V4 | 1.0 | 2.364e-08 | 375 | 0.287 | 146 | 100 | 2 | 1 | 145 | 6 | 148 | Lysozyme | Lysozyme | | afdb-uniprot50 | AF-A0A7S6S8H3-F1-MODEL\_V4 | 1.0 | 7.369e-09 | 375 | 0.333 | 171 | 86 | 5 | 1 | 146 | 1 | 168 | Lysozyme | Lysozyme | | afdb-uniprot50 | AF-A0A3P6JLZ0-F1-MODEL\_V4 | 1.0 | 1.013e-08 | 375 | 0.35 | 151 | 87 | 6 | 1 | 144 | 144 | 290 | Lysozyme | Lysozyme | | afdb-uniprot50 | AF-A0A1D9HAL1-F1-MODEL\_V4 | 1.0 | 4.964e-08 | 374 | 0.324 | 145 | 91 | 4 | 4 | 145 | 21 | 161 | Lysozyme | Lysozyme | | afdb-uniprot50 | AF-A0A1W9JSH2-F1-MODEL\_V4 | 1.0 | 1.126e-08 | 374 | 0.289 | 176 | 88 | 4 | 2 | 143 | 8 | 180 | Lysozyme | Lysozyme | | afdb-uniprot50 | AF-A0A0J1D667-F1-MODEL\_V4 | 1.0 | 4.574e-09 | 373 | 0.452 | 137 | 71 | 2 | 20 | 154 | 4 | 138 | Lysozyme | Lysozyme | | afdb-uniprot50 | AF-A0A844D768-F1-MODEL\_V4 | 1.0 | 1.547e-08 | 373 | 0.277 | 180 | 91 | 6 | 3 | 146 | 1 | 177 | Glycoside hydrolase family protein | Glycoside hydrolase family protein | | afdb-uniprot50 | AF-A0A2A6JMP3-F1-MODEL\_V4 | 1.0 | 4.707e-08 | 373 | 0.326 | 150 | 91 | 4 | 1 | 144 | 63 | 208 | Lysozyme | Lysozyme | | afdb-uniprot50 | AF-A0A2W4UDA1-F1-MODEL\_V4 | 1.0 | 4.016e-08 | 373 | 0.301 | 159 | 100 | 5 | 1 | 154 | 9 | 161 | Lysozyme | Lysozyme | | afdb-uniprot50 | AF-A0A6H1ZIA5-F1-MODEL\_V4 | 1.0 | 2.493e-08 | 372 | 0.307 | 140 | 89 | 5 | 6 | 143 | 11 | 144 | Lysozyme | Lysozyme | | afdb-uniprot50 | AF-A0A4V1EHC6-F1-MODEL\_V4 | 1.0 | 2.126e-08 | 372 | 0.325 | 160 | 89 | 4 | 1 | 145 | 3 | 158 | Lysozyme | Lysozyme | | afdb-uniprot50 | AF-A0A4P8HTQ2-F1-MODEL\_V4 | 1.0 | 5.234e-08 | 372 | 0.294 | 153 | 95 | 3 | 1 | 144 | 60 | 208 | Lysozyme | Lysozyme | | afdb-uniprot50 | AF-A0A177NPS6-F1-MODEL\_V4 | 1.0 | 1.547e-08 | 372 | 0.343 | 157 | 89 | 6 | 1 | 147 | 90 | 242 | Lysozyme | Lysozyme | | afdb-uniprot50 | AF-A0A7G8Q4E4-F1-MODEL\_V4 | 1.0 | 5.362e-09 | 372 | 0.343 | 163 | 84 | 5 | 1 | 143 | 4 | 163 | Lysozyme | Lysozyme | | afdb-uniprot50 | AF-A0A7R9LHS2-F1-MODEL\_V4 | 1.0 | 4.964e-08 | 372 | 0.304 | 148 | 95 | 5 | 1 | 144 | 241 | 384 | Lysozyme | Lysozyme | | afdb-uniprot50 | AF-A0A709FKY7-F1-MODEL\_V4 | 1.0 | 2.852e-07 | 371 | 0.595 | 89 | 36 | 0 | 56 | 144 | 2 | 90 | Lysozyme | Lysozyme | | afdb-uniprot50 | AF-A0A316KGZ2-F1-MODEL\_V4 | 1.0 | 1.126e-08 | 371 | 0.459 | 135 | 62 | 4 | 1 | 126 | 1 | 133 | Lysozyme | Lysozyme | | afdb-uniprot50 | AF-A0A5C7ZEJ8-F1-MODEL\_V4 | 1.0 | 3.425e-08 | 371 | 0.363 | 143 | 83 | 6 | 6 | 145 | 11 | 148 | Lysozyme | Lysozyme | | afdb-uniprot50 | AF-A0A840DW28-F1-MODEL\_V4 | 1.0 | 4.464e-08 | 370 | 0.426 | 129 | 67 | 2 | 1 | 125 | 2 | 127 | GH24 family phage-related lysozyme (Muramidase) | GH24 family phage-related lysozyme (Muramidase) | | afdb-uniprot50 | AF-A0A7R9KMC6-F1-MODEL\_V4 | 1.0 | 9.374e-08 | 370 | 0.308 | 149 | 96 | 4 | 1 | 146 | 3 | 147 | Lysozyme | Lysozyme | | afdb-uniprot50 | AF-A0A7X6IQC2-F1-MODEL\_V4 | 1.0 | 8.89e-08 | 370 | 0.342 | 146 | 85 | 4 | 7 | 145 | 19 | 160 | Lysozyme | Lysozyme | | afdb-uniprot50 | AF-A0A2F0P4T0-F1-MODEL\_V4 | 1.0 | 3.249e-08 | 369 | 0.315 | 149 | 93 | 4 | 1 | 146 | 13 | 155 | Lysozyme | Lysozyme | | afdb-uniprot50 | AF-A0A011RLS9-F1-MODEL\_V4 | 1.0 | 5.234e-08 | 369 | 0.308 | 146 | 94 | 4 | 1 | 145 | 12 | 151 | Lysozyme | Lysozyme | | afdb-uniprot50 | AF-A0A379FFZ8-F1-MODEL\_V4 | 1.0 | 1.912e-08 | 369 | 0.349 | 146 | 85 | 4 | 5 | 143 | 15 | 157 | Lysozyme | Lysozyme | | afdb-uniprot50 | AF-E2CPH6-F1-MODEL\_V4 | 1.0 | 4.234e-08 | 369 | 0.335 | 161 | 91 | 6 | 1 | 149 | 1 | 157 | Lysozyme | Lysozyme | | afdb-uniprot50 | AF-A0A6C2CEI5-F1-MODEL\_V4 | 1.0 | 8.192e-09 | 369 | 0.386 | 158 | 78 | 4 | 1 | 143 | 463 | 616 | Lysozyme | Lysozyme | | afdb-uniprot50 | AF-A0A2N0Q7R0-F1-MODEL\_V4 | 1.0 | 1.51e-07 | 368 | 0.46 | 100 | 54 | 0 | 49 | 148 | 17 | 116 | Lysozyme | Lysozyme | | afdb-uniprot50 | AF-A0A6P4KAZ4-F1-MODEL\_V4 | 1.0 | 1.814e-08 | 368 | 0.375 | 152 | 81 | 6 | 6 | 147 | 7 | 154 | Lysozyme | Lysozyme | | afdb-uniprot50 | AF-A0A0G3BMQ4-F1-MODEL\_V4 | 1.0 | 1.392e-08 | 367 | 0.314 | 156 | 90 | 5 | 1 | 144 | 6 | 156 | Lysozyme | Lysozyme | | afdb-uniprot50 | AF-A0A5J4VHC4-F1-MODEL\_V4 | 1.0 | 8.192e-09 | 367 | 0.333 | 165 | 96 | 7 | 3 | 157 | 1 | 161 | Lysozyme | Lysozyme | | afdb-uniprot50 | AF-A0A2A5BNU6-F1-MODEL\_V4 | 1.0 | 2.628e-08 | 366 | 0.35 | 160 | 82 | 3 | 1 | 146 | 1 | 152 | Lysozyme | Lysozyme | | afdb-uniprot50 | AF-A0A707IK04-F1-MODEL\_V4 | 1.0 | 5.386e-07 | 365 | 0.611 | 85 | 33 | 0 | 60 | 144 | 5 | 89 | Lysozyme | Lysozyme | | afdb-uniprot50 | AF-A0A1Y6D0R4-F1-MODEL\_V4 | 1.0 | 5.518e-08 | 365 | 0.295 | 149 | 99 | 4 | 1 | 146 | 5 | 150 | Lysozyme | Lysozyme | | afdb-uniprot50 | AF-A0A6P4HYW2-F1-MODEL\_V4 | 1.0 | 9.108e-09 | 365 | 0.342 | 152 | 85 | 6 | 5 | 144 | 13 | 161 | Lysozyme | Lysozyme | | afdb-uniprot50 | AF-A0A1G5SUL8-F1-MODEL\_V4 | 1.0 | 7.584e-08 | 365 | 0.282 | 145 | 96 | 5 | 4 | 144 | 28 | 168 | Lysozyme | Lysozyme | | afdb-uniprot50 | AF-A0A708EKG9-F1-MODEL\_V4 | 1.0 | 3.525e-07 | 364 | 0.582 | 91 | 38 | 0 | 54 | 144 | 4 | 94 | Lysozyme | Lysozyme | | afdb-uniprot50 | AF-A0A060NVJ0-F1-MODEL\_V4 | 1.0 | 1.013e-08 | 364 | 0.326 | 156 | 85 | 6 | 1 | 143 | 2 | 150 | Lysozyme | Lysozyme | | afdb-uniprot50 | AF-A0A4S3KDY4-F1-MODEL\_V4 | 1.0 | 8.89e-08 | 363 | 0.25 | 144 | 103 | 4 | 4 | 146 | 1 | 140 | Lysozyme | Lysozyme | | afdb-uniprot50 | AF-A0A3B9NE05-F1-MODEL\_V4 | 1.0 | 3.425e-08 | 363 | 0.32 | 156 | 93 | 5 | 1 | 146 | 1 | 153 | Lysozyme | Lysozyme | | afdb-uniprot50 | AF-A0A3G3GKL6-F1-MODEL\_V4 | 1.0 | 1.068e-08 | 363 | 0.327 | 171 | 90 | 7 | 1 | 153 | 2 | 165 | Lysozyme | Lysozyme | | afdb-uniprot50 | AF-A0A317CJ11-F1-MODEL\_V4 | 1.0 | 2.242e-08 | 363 | 0.337 | 163 | 85 | 6 | 1 | 143 | 2 | 161 | Lysozyme | Lysozyme | | afdb-uniprot50 | AF-A0A538JLK8-F1-MODEL\_V4 | 1.0 | 3.249e-08 | 363 | 0.293 | 160 | 93 | 5 | 3 | 146 | 149 | 304 | Lysozyme | Lysozyme | | afdb-uniprot50 | AF-A0A1X9TLR2-F1-MODEL\_V4 | 1.0 | 7.996e-08 | 362 | 0.317 | 145 | 95 | 3 | 1 | 145 | 26 | 166 | Lysozyme | Lysozyme | | afdb-uniprot50 | AF-A0A849CLB2-F1-MODEL\_V4 | 1.0 | 4.016e-08 | 362 | 0.346 | 147 | 84 | 4 | 4 | 144 | 30 | 170 | Lysozyme | Lysozyme | | afdb-uniprot50 | AF-A0A7U3CRJ8-F1-MODEL\_V4 | 1.0 | 1.912e-08 | 362 | 0.315 | 152 | 88 | 5 | 5 | 144 | 37 | 184 | Lysozyme | Lysozyme | | afdb-uniprot50 | AF-A0A7W5W268-F1-MODEL\_V4 | 1.0 | 9.374e-08 | 362 | 0.335 | 158 | 89 | 3 | 1 | 146 | 56 | 209 | Lysozyme | Lysozyme | | afdb-uniprot50 | AF-A0A2A7MJ10-F1-MODEL\_V4 | 1.0 | 2.017e-08 | 362 | 0.335 | 152 | 88 | 6 | 2 | 145 | 103 | 249 | Lysozyme | Lysozyme | | afdb-uniprot50 | AF-A0A081C6S4-F1-MODEL\_V4 | 1.0 | 1.912e-08 | 362 | 0.333 | 159 | 89 | 4 | 1 | 145 | 1 | 156 | Lysozyme | Lysozyme | | afdb-uniprot50 | AF-A0A242N7A0-F1-MODEL\_V4 | 1.0 | 7.402e-07 | 361 | 0.548 | 82 | 37 | 0 | 64 | 145 | 2 | 83 | Lysozyme | Lysozyme | | afdb-uniprot50 | AF-A0A3D4K8L3-F1-MODEL\_V4 | 1.0 | 7.192e-08 | 361 | 0.357 | 126 | 71 | 4 | 1 | 121 | 2 | 122 | Lysozyme | Lysozyme | | afdb-uniprot50 | AF-A0A0N7KV59-F1-MODEL\_V4 | 1.0 | 3.081e-08 | 361 | 0.326 | 153 | 89 | 6 | 6 | 147 | 8 | 157 | Lysozyme | Lysozyme | | afdb-uniprot50 | AF-A0A1F4MGX4-F1-MODEL\_V4 | 1.0 | 3.612e-08 | 361 | 0.301 | 166 | 96 | 4 | 1 | 146 | 12 | 177 | Lysozyme | Lysozyme | | afdb-uniprot50 | AF-A0A523FRG2-F1-MODEL\_V4 | 1.0 | 1.432e-07 | 361 | 0.313 | 150 | 95 | 5 | 1 | 146 | 65 | 210 | Lysozyme | Lysozyme | | afdb-uniprot50 | AF-A0A2G6ENY7-F1-MODEL\_V4 | 1.0 | 1.631e-08 | 361 | 0.358 | 145 | 80 | 3 | 6 | 143 | 6 | 144 | Lysozyme | Lysozyme | | afdb-uniprot50 | AF-A0A399R7V2-F1-MODEL\_V4 | 1.0 | 6.135e-08 | 361 | 0.362 | 138 | 79 | 6 | 14 | 146 | 3 | 136 | Lysozyme | Lysozyme | | afdb-uniprot50 | AF-A0A640WAD0-F1-MODEL\_V4 | 1.0 | 6.821e-08 | 360 | 0.432 | 125 | 67 | 3 | 20 | 144 | 2 | 122 | Lysozyme | Lysozyme | | afdb-uniprot50 | AF-A0A853WB76-F1-MODEL\_V4 | 1.0 | 6.135e-08 | 360 | 0.359 | 128 | 77 | 3 | 4 | 129 | 21 | 145 | Endolysin | Endolysin | | afdb-uniprot50 | AF-A0A6G3Z5Y0-F1-MODEL\_V4 | 1.0 | 9.374e-08 | 360 | 0.482 | 112 | 51 | 3 | 2 | 109 | 105 | 213 | Lysozyme | Lysozyme | | afdb-uniprot50 | AF-A0A1V1PUV1-F1-MODEL\_V4 | 1.0 | 1.042e-07 | 360 | 0.315 | 146 | 92 | 4 | 3 | 144 | 6 | 147 | Lysozyme | Lysozyme | | afdb-uniprot50 | AF-K9XRH0-F1-MODEL\_V4 | 1.0 | 2.307e-07 | 360 | 0.478 | 119 | 58 | 3 | 2 | 120 | 67 | 181 | Lysozyme | Lysozyme | | afdb-uniprot50 | AF-A0A1U9JSH0-F1-MODEL\_V4 | 1.0 | 3.612e-08 | 360 | 0.31 | 148 | 96 | 3 | 2 | 145 | 334 | 479 | Lysozyme | Lysozyme | | afdb-uniprot50 | AF-Q0ANV5-F1-MODEL\_V4 | 1.0 | 2.922e-08 | 360 | 0.283 | 148 | 91 | 5 | 1 | 144 | 1 | 137 | Glycoside hydrolase, family 24 | Glycoside hydrolase, family 24 | | afdb-uniprot50 | AF-A0A3D6A2G8-F1-MODEL\_V4 | 1.0 | 2.242e-08 | 359 | 0.301 | 146 | 93 | 4 | 3 | 144 | 7 | 147 | Lysozyme | Lysozyme | | afdb-uniprot50 | AF-A0A7W4IK65-F1-MODEL\_V4 | 1.0 | 3.808e-08 | 359 | 0.333 | 156 | 90 | 6 | 1 | 145 | 1 | 153 | Lysozyme | Lysozyme | | afdb-uniprot50 | AF-A0A1E4ZVU3-F1-MODEL\_V4 | 1.0 | 1.912e-08 | 359 | 0.318 | 160 | 94 | 6 | 1 | 150 | 13 | 167 | Lysozyme | Lysozyme | | afdb-uniprot50 | AF-A0A852Q327-F1-MODEL\_V4 | 1.0 | 1.042e-07 | 359 | 0.34 | 144 | 89 | 3 | 4 | 144 | 30 | 170 | Lysozyme | Lysozyme | | afdb-uniprot50 | AF-A0A0F4VEW5-F1-MODEL\_V4 | 1.0 | 2.628e-08 | 358 | 0.4 | 130 | 70 | 2 | 20 | 144 | 12 | 138 | Lysozyme | Lysozyme | | afdb-uniprot50 | AF-A0A6M3XN87-F1-MODEL\_V4 | 1.0 | 3.249e-08 | 358 | 0.318 | 154 | 86 | 4 | 6 | 144 | 16 | 165 | Lysozyme | Lysozyme | | afdb-uniprot50 | AF-A0A103RZ68-F1-MODEL\_V4 | 1.0 | 1.467e-08 | 358 | 0.295 | 166 | 88 | 7 | 5 | 144 | 15 | 177 | Lysozyme | Lysozyme | | afdb-uniprot50 | AF-A0A511F0V7-F1-MODEL\_V4 | 1.0 | 1.77e-07 | 358 | 0.309 | 139 | 93 | 3 | 7 | 144 | 46 | 182 | Lysozyme | Lysozyme | | afdb-uniprot50 | AF-A0A358B152-F1-MODEL\_V4 | 1.0 | 2.188e-07 | 357 | 0.271 | 140 | 97 | 4 | 5 | 143 | 3 | 138 | Lysozyme | Lysozyme | | afdb-uniprot50 | AF-A0A1M7YJN3-F1-MODEL\_V4 | 1.0 | 1.099e-07 | 357 | 0.287 | 146 | 97 | 4 | 1 | 144 | 1 | 141 | Lysozyme | Lysozyme | | afdb-uniprot50 | AF-A0A3D3NAZ3-F1-MODEL\_V4 | 1.0 | 4.707e-08 | 357 | 0.305 | 144 | 89 | 3 | 10 | 145 | 2 | 142 | Lysozyme | Lysozyme | | afdb-uniprot50 | AF-A0A4Q2QRI9-F1-MODEL\_V4 | 1.0 | 1.631e-08 | 357 | 0.339 | 159 | 83 | 6 | 1 | 145 | 293 | 443 | Lysozyme | Lysozyme | | afdb-uniprot50 | AF-A0A1A7PVK4-F1-MODEL\_V4 | 1.0 | 7.996e-08 | 356 | 0.328 | 152 | 90 | 4 | 1 | 146 | 27 | 172 | Lysozyme | Lysozyme | | afdb-uniprot50 | AF-G1UXF8-F1-MODEL\_V4 | 1.0 | 2.017e-08 | 356 | 0.34 | 147 | 82 | 7 | 7 | 145 | 31 | 170 | Lysozyme | Lysozyme | | afdb-uniprot50 | AF-A0A3D3KGU8-F1-MODEL\_V4 | 1.0 | 7.584e-08 | 356 | 0.335 | 155 | 89 | 6 | 1 | 144 | 53 | 204 | Lysozyme | Lysozyme | | afdb-uniprot50 | AF-A0A0U5ETJ2-F1-MODEL\_V4 | 1.0 | 2.771e-08 | 356 | 0.329 | 155 | 88 | 6 | 5 | 148 | 42 | 191 | Lysozyme | Lysozyme | | afdb-uniprot50 | AF-A0A386TCM0-F1-MODEL\_V4 | 1.0 | 1.159e-07 | 356 | 0.312 | 144 | 90 | 4 | 6 | 144 | 142 | 281 | Lysozyme | Lysozyme | | afdb-uniprot50 | AF-A0A023PRG0-F1-MODEL\_V4 | 1.0 | 1.467e-08 | 356 | 0.341 | 155 | 86 | 6 | 3 | 143 | 816 | 968 | Lysozyme | Lysozyme | | afdb-uniprot50 | AF-I3DCN5-F1-MODEL\_V4 | 1.0 | 1.432e-07 | 355 | 0.324 | 148 | 93 | 4 | 3 | 146 | 29 | 173 | Lysozyme | Lysozyme | | afdb-uniprot50 | AF-A0A0V8BQ91-F1-MODEL\_V4 | 1.0 | 1.72e-08 | 355 | 0.367 | 158 | 77 | 6 | 1 | 147 | 1 | 146 | Lysozyme | Lysozyme | | afdb-uniprot50 | AF-A0A1Q9QMU9-F1-MODEL\_V4 | 1.0 | 4.016e-08 | 354 | 0.263 | 152 | 100 | 6 | 1 | 144 | 5 | 152 | Lysozyme | Lysozyme | | afdb-uniprot50 | AF-A0A7U2KR66-F1-MODEL\_V4 | 1.0 | 2.922e-08 | 354 | 0.283 | 155 | 96 | 5 | 2 | 145 | 21 | 171 | Lysozyme | Lysozyme | | afdb-uniprot50 | AF-E6VL46-F1-MODEL\_V4 | 1.0 | 5.819e-08 | 354 | 0.34 | 150 | 90 | 3 | 2 | 144 | 8 | 155 | Lysozyme | Lysozyme | | afdb-uniprot50 | AF-A0A357YYC3-F1-MODEL\_V4 | 1.0 | 1.679e-07 | 354 | 0.337 | 151 | 89 | 4 | 1 | 143 | 58 | 205 | Lysozyme | Lysozyme | | afdb-uniprot50 | AF-A0A430BCR4-F1-MODEL\_V4 | 1.0 | 3.343e-07 | 354 | 0.27 | 144 | 97 | 4 | 5 | 145 | 111 | 249 | Lysozyme | Lysozyme | | afdb-uniprot50 | AF-A0A6N8JD00-F1-MODEL\_V4 | 1.0 | 2.242e-08 | 354 | 0.331 | 160 | 91 | 5 | 1 | 147 | 225 | 381 | Lysozyme | Lysozyme | | afdb-uniprot50 | AF-A0A009G8D4-F1-MODEL\_V4 | 1.0 | 7.584e-08 | 353 | 0.535 | 112 | 47 | 1 | 35 | 146 | 8 | 114 | Lysozyme | Lysozyme | | afdb-uniprot50 | AF-A0A7J0ADD2-F1-MODEL\_V4 | 1.0 | 1.51e-07 | 353 | 0.314 | 143 | 88 | 6 | 7 | 144 | 2 | 139 | Lysozyme | Lysozyme | | afdb-uniprot50 | AF-A0A5C7LX91-F1-MODEL\_V4 | 1.0 | 4.964e-08 | 353 | 0.342 | 149 | 87 | 6 | 3 | 146 | 10 | 152 | Lysozyme | Lysozyme | | afdb-uniprot50 | AF-A0A401JF78-F1-MODEL\_V4 | 1.0 | 1.72e-08 | 352 | 0.341 | 155 | 87 | 6 | 1 | 146 | 2 | 150 | Lysozyme | Lysozyme | | afdb-uniprot50 | AF-A0A4R3JY04-F1-MODEL\_V4 | 1.0 | 1.392e-08 | 352 | 0.32 | 159 | 90 | 6 | 1 | 147 | 6 | 158 | Lysozyme | Lysozyme | | afdb-uniprot50 | AF-A3MZM3-F1-MODEL\_V4 | 1.0 | 1.592e-07 | 352 | 0.28 | 146 | 98 | 3 | 4 | 145 | 36 | 178 | Lysozyme | Lysozyme | | afdb-uniprot50 | AF-A0A341EDR9-F1-MODEL\_V4 | 1.0 | 3.425e-08 | 352 | 0.259 | 154 | 99 | 5 | 2 | 144 | 133 | 282 | Lysozyme | Lysozyme | | afdb-uniprot50 | AF-A0A7W0Z8H7-F1-MODEL\_V4 | 1.0 | 2.017e-08 | 352 | 0.357 | 154 | 83 | 4 | 1 | 144 | 69 | 216 | Lysozyme | Lysozyme | | afdb-uniprot50 | AF-A0A0F8Z6L9-F1-MODEL\_V4 | 1.0 | 6.469e-08 | 352 | 0.352 | 153 | 86 | 5 | 1 | 143 | 179 | 328 | Lysozyme | Lysozyme | | afdb-uniprot50 | AF-A0A0Q5GY79-F1-MODEL\_V4 | 1.0 | 7.192e-08 | 352 | 0.271 | 173 | 99 | 7 | 1 | 155 | 1 | 164 | PG\_binding\_1 domain-containing protein | PG\_binding\_1 domain-containing protein | | afdb-uniprot50 | AF-A0A375GDD6-F1-MODEL\_V4 | 1.0 | 1.042e-07 | 351 | 0.321 | 143 | 88 | 4 | 6 | 142 | 13 | 152 | Lysozyme | Lysozyme | | afdb-uniprot50 | AF-A0A022G329-F1-MODEL\_V4 | 1.0 | 1.592e-07 | 351 | 0.303 | 145 | 94 | 4 | 4 | 145 | 15 | 155 | Lysozyme | Lysozyme | | afdb-uniprot50 | AF-A0A7W9RA22-F1-MODEL\_V4 | 1.0 | 1.288e-07 | 351 | 0.293 | 143 | 93 | 5 | 5 | 145 | 28 | 164 | Lysozyme | Lysozyme | | afdb-uniprot50 | AF-A0A0F9TJ14-F1-MODEL\_V4 | 1.0 | 4.707e-08 | 351 | 0.3 | 150 | 89 | 5 | 5 | 143 | 20 | 164 | Lysozyme | Lysozyme | | afdb-uniprot50 | AF-A0A7X5Z2P4-F1-MODEL\_V4 | 1.0 | 4.016e-08 | 351 | 0.287 | 146 | 97 | 4 | 3 | 144 | 13 | 155 | Lysozyme | Lysozyme | | afdb-uniprot50 | AF-X6L4V5-F1-MODEL\_V4 | 1.0 | 3.425e-08 | 350 | 0.315 | 152 | 88 | 5 | 5 | 144 | 124 | 271 | Lysozyme | Lysozyme | | afdb-uniprot50 | AF-A0A1I1VPY7-F1-MODEL\_V4 | 1.0 | 3.612e-08 | 350 | 0.326 | 156 | 89 | 6 | 1 | 143 | 407 | 559 | Lysozyme | Lysozyme | | afdb-uniprot50 | AF-A0A381FGI3-F1-MODEL\_V4 | 1.0 | 4.234e-08 | 349 | 0.251 | 151 | 105 | 4 | 6 | 151 | 8 | 155 | Lysozyme | Lysozyme | | afdb-uniprot50 | AF-A0A752A1Q8-F1-MODEL\_V4 | 1.0 | 1.51e-07 | 348 | 0.319 | 144 | 89 | 4 | 7 | 144 | 19 | 159 | Lysozyme | Lysozyme | | afdb-uniprot50 | AF-A0A285J525-F1-MODEL\_V4 | 1.0 | 4.234e-08 | 348 | 0.328 | 152 | 87 | 6 | 4 | 144 | 96 | 243 | Lysozyme | Lysozyme | | afdb-uniprot50 | AF-A0A4P7EAC4-F1-MODEL\_V4 | 1.0 | 5.234e-08 | 348 | 0.297 | 148 | 93 | 6 | 4 | 144 | 95 | 238 | Lysozyme | Lysozyme | | afdb-uniprot50 | AF-A0A2S5N400-F1-MODEL\_V4 | 1.0 | 1.867e-07 | 348 | 0.395 | 149 | 76 | 6 | 1 | 144 | 4 | 143 | Lysozyme | Lysozyme | | afdb-uniprot50 | AF-A0A0C1GCU4-F1-MODEL\_V4 | 1.0 | 7.192e-08 | 347 | 0.263 | 148 | 98 | 5 | 3 | 143 | 1 | 144 | Lysozyme | Lysozyme | | afdb-uniprot50 | AF-A0A2P5N8Y5-F1-MODEL\_V4 | 1.0 | 4.016e-08 | 347 | 0.303 | 158 | 93 | 5 | 1 | 143 | 9 | 164 | Lysozyme | Lysozyme | | afdb-uniprot50 | AF-A0A2U1UUN7-F1-MODEL\_V4 | 1.0 | 1.358e-07 | 347 | 0.284 | 151 | 97 | 3 | 2 | 146 | 36 | 181 | Lysozyme | Lysozyme | | afdb-uniprot50 | AF-A0A431NTU0-F1-MODEL\_V4 | 1.0 | 6.135e-08 | 347 | 0.293 | 167 | 97 | 6 | 1 | 150 | 1 | 163 | Lysozyme | Lysozyme | | afdb-uniprot50 | AF-G8AQH1-F1-MODEL\_V4 | 1.0 | 8.432e-08 | 346 | 0.474 | 118 | 57 | 3 | 33 | 146 | 3 | 119 | Lysozyme | Lysozyme | | afdb-uniprot50 | AF-A0A1H1ART9-F1-MODEL\_V4 | 1.0 | 6.469e-08 | 346 | 0.326 | 153 | 89 | 6 | 6 | 147 | 14 | 163 | Lysozyme | Lysozyme | | afdb-uniprot50 | AF-S3UE20-F1-MODEL\_V4 | 1.0 | 1.193e-06 | 345 | 0.563 | 87 | 38 | 0 | 60 | 146 | 6 | 92 | Lysozyme | Lysozyme | | afdb-uniprot50 | AF-A0A366HBV8-F1-MODEL\_V4 | 1.0 | 6.469e-08 | 345 | 0.301 | 146 | 91 | 6 | 6 | 144 | 11 | 152 | Lysozyme | Lysozyme | | afdb-uniprot50 | AF-A0A143DGD6-F1-MODEL\_V4 | 1.0 | 9.374e-08 | 345 | 0.311 | 151 | 90 | 4 | 4 | 144 | 17 | 163 | Lysozyme | Lysozyme | | afdb-uniprot50 | AF-A0A564WD90-F1-MODEL\_V4 | 1.0 | 4.234e-08 | 345 | 0.317 | 170 | 86 | 5 | 1 | 144 | 1 | 166 | Lysozyme | Lysozyme | | afdb-uniprot50 | AF-A0A345PAS8-F1-MODEL\_V4 | 1.0 | 2.075e-07 | 345 | 0.291 | 144 | 99 | 2 | 4 | 147 | 31 | 171 | Lysozyme | Lysozyme | | afdb-uniprot50 | AF-A0A495HUG2-F1-MODEL\_V4 | 1.0 | 8.89e-08 | 345 | 0.272 | 169 | 102 | 4 | 1 | 148 | 11 | 179 | GH24 family phage-related lysozyme (Muramidase) | GH24 family phage-related lysozyme (Muramidase) | | afdb-uniprot50 | AF-A0A702E4W0-F1-MODEL\_V4 | 1.0 | 3.612e-08 | 345 | 0.346 | 156 | 82 | 5 | 1 | 143 | 121 | 269 | Lysozyme | Lysozyme | | afdb-uniprot50 | AF-A3UJS2-F1-MODEL\_V4 | 1.0 | 4.707e-08 | 345 | 0.285 | 161 | 101 | 5 | 1 | 157 | 5 | 155 | Lysozyme | Lysozyme | | afdb-uniprot50 | AF-A0A6L6YDR0-F1-MODEL\_V4 | 1.0 | 8.89e-08 | 344 | 0.321 | 143 | 91 | 4 | 4 | 144 | 9 | 147 | Lysozyme | Lysozyme | | afdb-uniprot50 | AF-A0A5C7SP49-F1-MODEL\_V4 | 1.0 | 7.584e-08 | 344 | 0.317 | 148 | 90 | 7 | 5 | 146 | 18 | 160 | Lysozyme | Lysozyme | | afdb-uniprot50 | AF-V7DA39-F1-MODEL\_V4 | 1.0 | 2.922e-08 | 343 | 0.283 | 166 | 94 | 4 | 1 | 144 | 1 | 163 | Lysozyme | Lysozyme | | afdb-uniprot50 | AF-A0A4S3M508-F1-MODEL\_V4 | 1.0 | 1.77e-07 | 343 | 0.344 | 151 | 90 | 5 | 5 | 151 | 18 | 163 | Lysozyme | Lysozyme | | afdb-uniprot50 | AF-A0A521TYU1-F1-MODEL\_V4 | 1.0 | 7.996e-08 | 343 | 0.343 | 160 | 85 | 7 | 1 | 144 | 10 | 165 | Lysozyme | Lysozyme | | afdb-uniprot50 | AF-A0A6L8KFJ1-F1-MODEL\_V4 | 1.0 | 1.288e-07 | 343 | 0.293 | 143 | 92 | 4 | 6 | 142 | 43 | 182 | Lysozyme | Lysozyme | | afdb-uniprot50 | AF-A0A442BB57-F1-MODEL\_V4 | 1.0 | 1.77e-07 | 343 | 0.3 | 153 | 94 | 3 | 1 | 143 | 41 | 190 | Lysozyme | Lysozyme | | afdb-uniprot50 | AF-A0A1H2EW19-F1-MODEL\_V4 | 1.0 | 8.432e-08 | 343 | 0.306 | 176 | 98 | 8 | 1 | 157 | 1 | 171 | Lysozyme | Lysozyme | | afdb-uniprot50 | AF-A0A2U2BR73-F1-MODEL\_V4 | 1.0 | 4.964e-08 | 343 | 0.31 | 158 | 98 | 5 | 1 | 154 | 5 | 155 | Lysozyme | Lysozyme | | afdb-uniprot50 | AF-A0A521ZXH5-F1-MODEL\_V4 | 1.0 | 3.808e-08 | 342 | 0.298 | 151 | 91 | 6 | 3 | 144 | 2 | 146 | Lysozyme | Lysozyme | | afdb-uniprot50 | AF-B1ZJD5-F1-MODEL\_V4 | 1.0 | 1.592e-07 | 342 | 0.331 | 145 | 86 | 6 | 7 | 146 | 41 | 179 | Lysozyme | Lysozyme | | afdb-uniprot50 | AF-A0A845GU55-F1-MODEL\_V4 | 1.0 | 1.51e-07 | 342 | 0.32 | 159 | 92 | 6 | 1 | 147 | 57 | 211 | Glycoside hydrolase family protein | Glycoside hydrolase family protein | | afdb-uniprot50 | AF-J1JG31-F1-MODEL\_V4 | 1.0 | 2.565e-07 | 341 | 0.447 | 123 | 64 | 1 | 32 | 150 | 10 | 132 | Lysozyme | Lysozyme | | afdb-uniprot50 | AF-A0A377M831-F1-MODEL\_V4 | 1.0 | 1.592e-07 | 340 | 0.413 | 133 | 69 | 4 | 1 | 127 | 6 | 135 | Lysozyme | Lysozyme | | afdb-uniprot50 | AF-A0A519QHC5-F1-MODEL\_V4 | 1.0 | 7.996e-08 | 340 | 0.346 | 147 | 82 | 7 | 7 | 143 | 6 | 148 | Lysozyme | Lysozyme | | afdb-uniprot50 | AF-A0A501W9K6-F1-MODEL\_V4 | 1.0 | 6.821e-08 | 340 | 0.313 | 153 | 92 | 7 | 1 | 144 | 8 | 156 | Lysozyme | Lysozyme | | afdb-uniprot50 | AF-A0A512JI05-F1-MODEL\_V4 | 1.0 | 3.007e-07 | 340 | 0.354 | 144 | 84 | 5 | 3 | 146 | 7 | 141 | Lysozyme | Lysozyme | | afdb-uniprot50 | AF-A0A1L3I5D1-F1-MODEL\_V4 | 1.0 | 1.432e-07 | 340 | 0.291 | 144 | 90 | 5 | 5 | 140 | 94 | 233 | Lysozyme | Lysozyme | | afdb-uniprot50 | AF-G0JRZ2-F1-MODEL\_V4 | 1.0 | 7.192e-08 | 340 | 0.32 | 156 | 92 | 4 | 1 | 143 | 156 | 310 | Lysozyme | Lysozyme | | afdb-uniprot50 | AF-A0A5C7PGH8-F1-MODEL\_V4 | 1.0 | 1.159e-07 | 339 | 0.301 | 156 | 92 | 8 | 1 | 143 | 1 | 152 | Lysozyme | Lysozyme | | afdb-uniprot50 | AF-A0A431M8G9-F1-MODEL\_V4 | 1.0 | 1.679e-07 | 339 | 0.313 | 153 | 92 | 4 | 3 | 144 | 11 | 161 | Lysozyme | Lysozyme | | afdb-uniprot50 | AF-A0A442XXC8-F1-MODEL\_V4 | 1.0 | 1.77e-07 | 339 | 0.292 | 147 | 92 | 6 | 6 | 144 | 33 | 175 | Lysozyme | Lysozyme | | afdb-uniprot50 | AF-A0A2A7MJ58-F1-MODEL\_V4 | 1.0 | 1.222e-07 | 339 | 0.322 | 149 | 92 | 5 | 2 | 144 | 134 | 279 | Lysozyme | Lysozyme | | afdb-uniprot50 | AF-J0QIK5-F1-MODEL\_V4 | 1.0 | 1.679e-07 | 339 | 0.45 | 120 | 61 | 2 | 33 | 147 | 376 | 495 | Lysozyme | Lysozyme | | afdb-uniprot50 | AF-A0A7L5Y684-F1-MODEL\_V4 | 1.0 | 7.584e-08 | 338 | 0.309 | 155 | 92 | 5 | 1 | 144 | 20 | 170 | Lysozyme | Lysozyme | | afdb-uniprot50 | AF-A0A7H0VBA6-F1-MODEL\_V4 | 1.0 | 1.679e-07 | 338 | 0.337 | 151 | 88 | 6 | 1 | 146 | 38 | 181 | Lysozyme | Lysozyme | | afdb-uniprot50 | AF-A0A450X4Q4-F1-MODEL\_V4 | 1.0 | 3.92e-07 | 338 | 0.296 | 155 | 96 | 4 | 1 | 145 | 63 | 214 | Lysozyme | Lysozyme | | afdb-uniprot50 | AF-A0A2E0EKN3-F1-MODEL\_V4 | 1.0 | 7.192e-08 | 337 | 0.289 | 169 | 104 | 7 | 1 | 157 | 11 | 175 | Lysozyme | Lysozyme | | afdb-uniprot50 | AF-A0A368HDJ6-F1-MODEL\_V4 | 1.0 | 1.068e-08 | 336 | 0.392 | 158 | 84 | 5 | 2 | 159 | 6 | 151 | Lysozyme | Lysozyme | | afdb-uniprot50 | AF-M1WVJ0-F1-MODEL\_V4 | 1.0 | 6.135e-08 | 336 | 0.312 | 160 | 87 | 5 | 1 | 143 | 318 | 471 | Lysozyme | Lysozyme | | afdb-uniprot50 | AF-A0A257SN27-F1-MODEL\_V4 | 1.0 | 1.554e-06 | 335 | 0.526 | 93 | 44 | 0 | 53 | 145 | 1 | 93 | Lysozyme | Lysozyme | | afdb-uniprot50 | AF-A0A2G3JY01-F1-MODEL\_V4 | 1.0 | 3.525e-07 | 335 | 0.243 | 152 | 102 | 3 | 6 | 145 | 18 | 168 | Lysozyme | Lysozyme | | afdb-uniprot50 | AF-A0A7V8IN83-F1-MODEL\_V4 | 1.0 | 1.592e-07 | 335 | 0.243 | 148 | 103 | 4 | 6 | 144 | 23 | 170 | Lysozyme | Lysozyme | | afdb-uniprot50 | AF-A0A1E4GWM4-F1-MODEL\_V4 | 1.0 | 4.358e-07 | 335 | 0.268 | 145 | 95 | 6 | 5 | 144 | 9 | 147 | Lysozyme | Lysozyme | | afdb-uniprot50 | AF-A0A5A8A1Q9-F1-MODEL\_V4 | 1.0 | 3.343e-07 | 335 | 0.355 | 152 | 86 | 5 | 1 | 148 | 1 | 144 | Lysozyme | Lysozyme | | afdb-uniprot50 | AF-E5YB66-F1-MODEL\_V4 | 1.0 | 5.234e-08 | 334 | 0.333 | 144 | 81 | 7 | 10 | 145 | 2 | 138 | Lysozyme | Lysozyme | | afdb-uniprot50 | AF-A0A5C8TXF5-F1-MODEL\_V4 | 1.0 | 4.595e-07 | 334 | 0.368 | 141 | 80 | 5 | 3 | 143 | 11 | 142 | Lysozyme | Lysozyme | | afdb-uniprot50 | AF-A0A2N1Y888-F1-MODEL\_V4 | 1.0 | 1.159e-07 | 334 | 0.287 | 167 | 96 | 8 | 3 | 150 | 13 | 175 | Lysozyme | Lysozyme | | afdb-uniprot50 | AF-A0A7Z6L4X9-F1-MODEL\_V4 | 1.0 | 1.679e-07 | 334 | 0.351 | 162 | 93 | 7 | 1 | 156 | 3 | 158 | Lysozyme | Lysozyme | | afdb-uniprot50 | AF-A0A4S2H276-F1-MODEL\_V4 | 1.0 | 6.135e-08 | 334 | 0.29 | 155 | 99 | 5 | 3 | 153 | 7 | 154 | Lysozyme | Lysozyme | | afdb-uniprot50 | AF-A0A1V2N8E9-F1-MODEL\_V4 | 1.0 | 3.717e-07 | 333 | 0.354 | 127 | 77 | 2 | 22 | 146 | 1 | 124 | Lysozyme | Lysozyme | | afdb-uniprot50 | AF-A0A7V8QYJ7-F1-MODEL\_V4 | 1.0 | 1.099e-07 | 333 | 0.246 | 158 | 101 | 6 | 1 | 144 | 2 | 155 | Lysozyme | Lysozyme | | afdb-uniprot50 | AF-A0A3C1GCQ9-F1-MODEL\_V4 | 1.0 | 6.821e-08 | 333 | 0.354 | 158 | 82 | 9 | 1 | 145 | 4 | 154 | Lysozyme | Lysozyme | | afdb-uniprot50 | AF-A0A7L9RU02-F1-MODEL\_V4 | 1.0 | 2.075e-07 | 333 | 0.31 | 145 | 92 | 5 | 4 | 144 | 36 | 176 | Lysozyme RrrD | Lysozyme RrrD | | afdb-uniprot50 | AF-A0A366C3Q8-F1-MODEL\_V4 | 1.0 | 3.343e-07 | 333 | 0.251 | 147 | 100 | 5 | 6 | 147 | 47 | 188 | Lysozyme | Lysozyme | | afdb-uniprot50 | AF-A0A080LZI7-F1-MODEL\_V4 | 1.0 | 1.679e-07 | 333 | 0.352 | 153 | 83 | 4 | 1 | 143 | 251 | 397 | Lysozyme | Lysozyme | | afdb-uniprot50 | AF-A0A562U8C4-F1-MODEL\_V4 | 1.0 | 1.968e-07 | 332 | 0.398 | 138 | 68 | 5 | 20 | 146 | 4 | 137 | Lysozyme | Lysozyme | | afdb-uniprot50 | AF-A0A246JYB8-F1-MODEL\_V4 | 1.0 | 3.171e-07 | 332 | 0.32 | 150 | 92 | 6 | 3 | 146 | 7 | 152 | Lysozyme | Lysozyme | | afdb-uniprot50 | AF-A0A0Q4R256-F1-MODEL\_V4 | 1.0 | 8.23e-07 | 332 | 0.276 | 141 | 95 | 5 | 5 | 144 | 40 | 174 | Lysozyme | Lysozyme | | afdb-uniprot50 | AF-A0A074TFU6-F1-MODEL\_V4 | 1.0 | 5.989e-07 | 332 | 0.303 | 145 | 93 | 3 | 6 | 145 | 114 | 255 | Lysozyme | Lysozyme | | afdb-uniprot50 | AF-A0A1F6QQW0-F1-MODEL\_V4 | 1.0 | 2.017e-08 | 331 | 0.333 | 144 | 85 | 6 | 10 | 146 | 4 | 143 | Lysozyme | Lysozyme | | afdb-uniprot50 | AF-A0A4R5DTI4-F1-MODEL\_V4 | 1.0 | 1.432e-07 | 331 | 0.28 | 157 | 96 | 6 | 2 | 146 | 13 | 164 | Lysozyme | Lysozyme | | afdb-uniprot50 | AF-A0A2T3HZF6-F1-MODEL\_V4 | 1.0 | 2.705e-07 | 331 | 0.342 | 140 | 80 | 4 | 3 | 135 | 31 | 165 | Lysozyme | Lysozyme | | afdb-uniprot50 | AF-A0A1L9BSJ1-F1-MODEL\_V4 | 1.0 | 1.099e-07 | 331 | 0.261 | 153 | 102 | 4 | 1 | 145 | 22 | 171 | Lysozyme | Lysozyme | | afdb-uniprot50 | AF-A0A0R3DQ56-F1-MODEL\_V4 | 1.0 | 6.135e-08 | 331 | 0.277 | 162 | 101 | 4 | 1 | 146 | 1 | 162 | Lysozyme | Lysozyme | | afdb-uniprot50 | AF-A0A1Y5SFD3-F1-MODEL\_V4 | 1.0 | 1.592e-07 | 331 | 0.293 | 150 | 89 | 6 | 6 | 144 | 93 | 236 | Lysozyme | Lysozyme | | afdb-uniprot50 | AF-A0A5C7USV4-F1-MODEL\_V4 | 1.0 | 8.432e-08 | 331 | 0.31 | 158 | 93 | 6 | 1 | 145 | 43 | 197 | Lysozyme | Lysozyme | | afdb-uniprot50 | AF-A0A7X5QS31-F1-MODEL\_V4 | 1.0 | 1.592e-07 | 330 | 0.251 | 151 | 104 | 3 | 1 | 145 | 4 | 151 | Lysozyme | Lysozyme | | afdb-uniprot50 | AF-A0A2A2S6I3-F1-MODEL\_V4 | 1.0 | 1.288e-07 | 330 | 0.264 | 155 | 103 | 7 | 1 | 145 | 1 | 154 | Lysozyme | Lysozyme | | afdb-uniprot50 | AF-A0A530R7Q7-F1-MODEL\_V4 | 1.0 | 2.705e-07 | 330 | 0.285 | 147 | 93 | 6 | 6 | 144 | 48 | 190 | Lysozyme | Lysozyme | | afdb-uniprot50 | AF-E2CJT6-F1-MODEL\_V4 | 1.0 | 1.222e-07 | 330 | 0.307 | 153 | 90 | 6 | 5 | 145 | 102 | 250 | Lysozyme | Lysozyme | | afdb-uniprot50 | AF-A0A7W7AKQ6-F1-MODEL\_V4 | 1.0 | 1.77e-07 | 329 | 0.339 | 153 | 85 | 7 | 3 | 145 | 18 | 164 | Lysozyme | Lysozyme | | afdb-uniprot50 | AF-A0A1V5ITR2-F1-MODEL\_V4 | 1.0 | 6.658e-07 | 329 | 0.427 | 117 | 66 | 1 | 27 | 143 | 2 | 117 | Lysozyme | Lysozyme | | afdb-uniprot50 | AF-A0A1U6JV99-F1-MODEL\_V4 | 1.0 | 1.822e-06 | 329 | 0.4 | 100 | 60 | 0 | 44 | 143 | 2 | 101 | Lysozyme | Lysozyme | | afdb-uniprot50 | AF-A0A1I7GQC5-F1-MODEL\_V4 | 1.0 | 5.68e-07 | 329 | 0.333 | 141 | 84 | 5 | 7 | 143 | 55 | 189 | Lysozyme | Lysozyme | | afdb-uniprot50 | AF-A0A1G9VVQ5-F1-MODEL\_V4 | 1.0 | 2.188e-07 | 329 | 0.263 | 144 | 95 | 4 | 1 | 144 | 5 | 137 | Lysozyme | Lysozyme | | afdb-uniprot50 | AF-A0A529XVD8-F1-MODEL\_V4 | 1.0 | 2.188e-07 | 328 | 0.241 | 153 | 102 | 5 | 2 | 144 | 3 | 151 | Lysozyme | Lysozyme | | afdb-uniprot50 | AF-A0A7X0DG28-F1-MODEL\_V4 | 1.0 | 1.252e-08 | 328 | 0.378 | 156 | 84 | 7 | 13 | 159 | 3 | 154 | Lysozyme | Lysozyme | | afdb-uniprot50 | AF-A0A2S9RDE4-F1-MODEL\_V4 | 1.0 | 3.007e-07 | 328 | 0.347 | 144 | 88 | 3 | 4 | 144 | 12 | 152 | Lysozyme | Lysozyme | | afdb-uniprot50 | AF-A0A6P2W0B7-F1-MODEL\_V4 | 1.0 | 9.884e-08 | 328 | 0.301 | 166 | 87 | 7 | 5 | 144 | 15 | 177 | Lysozyme | Lysozyme | | afdb-uniprot50 | AF-A0A074TMQ1-F1-MODEL\_V4 | 1.0 | 1.867e-07 | 328 | 0.277 | 155 | 99 | 5 | 1 | 145 | 41 | 192 | Lysozyme | Lysozyme | | afdb-uniprot50 | AF-E2CNP7-F1-MODEL\_V4 | 1.0 | 2.705e-07 | 328 | 0.318 | 160 | 87 | 7 | 1 | 143 | 1 | 155 | Peptidoglycan binding domain-containing protein | Peptidoglycan binding domain-containing protein | | afdb-uniprot50 | AF-A0A5M6ZAJ4-F1-MODEL\_V4 | 1.0 | 1.51e-07 | 328 | 0.294 | 156 | 96 | 5 | 2 | 153 | 6 | 151 | Lysozyme | Lysozyme | | afdb-uniprot50 | AF-A0A708BZD6-F1-MODEL\_V4 | 1.0 | 4.986e-06 | 327 | 0.632 | 79 | 29 | 0 | 66 | 144 | 2 | 80 | Lysozyme | Lysozyme | | afdb-uniprot50 | AF-A0A5U2FB15-F1-MODEL\_V4 | 1.0 | 8.23e-07 | 327 | 0.49 | 102 | 52 | 0 | 43 | 144 | 2 | 103 | Lysozyme | Lysozyme | | afdb-uniprot50 | AF-Q5NXI8-F1-MODEL\_V4 | 1.0 | 4.358e-07 | 327 | 0.251 | 167 | 101 | 4 | 1 | 145 | 11 | 175 | Lysozyme | Lysozyme | | afdb-uniprot50 | AF-A0A1V6EFU8-F1-MODEL\_V4 | 1.0 | 2.852e-07 | 327 | 0.38 | 147 | 77 | 7 | 1 | 143 | 1 | 137 | Lysozyme | Lysozyme | | afdb-uniprot50 | AF-A0A1H7CRP1-F1-MODEL\_V4 | 1.0 | 1.77e-07 | 327 | 0.288 | 156 | 97 | 4 | 1 | 144 | 92 | 245 | Lysozyme | Lysozyme | | afdb-uniprot50 | AF-A0A4Q5U1B8-F1-MODEL\_V4 | 1.0 | 1.592e-07 | 327 | 0.268 | 160 | 94 | 6 | 1 | 143 | 337 | 490 | Lysozyme | Lysozyme | | afdb-uniprot50 | AF-A0A7T8PHM4-F1-MODEL\_V4 | 1.0 | 1.51e-07 | 327 | 0.322 | 158 | 89 | 4 | 1 | 143 | 832 | 986 | Lysozyme | Lysozyme | | afdb-uniprot50 | AF-A0A0M3SVL5-F1-MODEL\_V4 | 1.0 | 6.314e-07 | 326 | 0.45 | 102 | 56 | 0 | 44 | 145 | 2 | 103 | Lysozyme | Lysozyme | | afdb-uniprot50 | AF-A0A146H6X4-F1-MODEL\_V4 | 1.0 | 2.307e-07 | 326 | 0.375 | 144 | 79 | 4 | 2 | 138 | 90 | 229 | Lysozyme | Lysozyme | | afdb-uniprot50 | AF-A0A2E5UZW7-F1-MODEL\_V4 | 1.0 | 2.565e-07 | 326 | 0.284 | 144 | 92 | 5 | 1 | 144 | 1 | 133 | Uncharacterized protein | Uncharacterized protein | | afdb-uniprot50 | AF-A0A553DTV1-F1-MODEL\_V4 | 1.0 | 1.432e-07 | 326 | 0.339 | 159 | 89 | 4 | 1 | 145 | 765 | 921 | Lysozyme | Lysozyme | | afdb-uniprot50 | AF-A0A848V5S5-F1-MODEL\_V4 | 1.0 | 1.017e-06 | 325 | 0.297 | 148 | 95 | 5 | 1 | 144 | 5 | 147 | Lysozyme | Lysozyme | | afdb-uniprot50 | AF-A0A2M7GL79-F1-MODEL\_V4 | 1.0 | 4.595e-07 | 325 | 0.286 | 143 | 91 | 5 | 2 | 144 | 6 | 137 | Lysozyme | Lysozyme | | afdb-uniprot50 | AF-A0A7L4TJR0-F1-MODEL\_V4 | 1.0 | 2.705e-07 | 325 | 0.296 | 155 | 94 | 5 | 2 | 145 | 576 | 726 | Lysozyme | Lysozyme | | afdb-uniprot50 | AF-A0A7W5YB65-F1-MODEL\_V4 | 1.0 | 2.433e-07 | 324 | 0.291 | 151 | 92 | 8 | 6 | 144 | 12 | 159 | Lysozyme | Lysozyme | | afdb-uniprot50 | AF-A0A1N7NPB8-F1-MODEL\_V4 | 1.0 | 6.314e-07 | 323 | 0.345 | 133 | 80 | 3 | 18 | 146 | 2 | 131 | Lysozyme | Lysozyme | | afdb-uniprot50 | AF-K6ZFL4-F1-MODEL\_V4 | 1.0 | 1.193e-06 | 323 | 0.423 | 104 | 58 | 2 | 4 | 107 | 30 | 131 | Lysozyme | Lysozyme | | afdb-uniprot50 | AF-A0A846MWR1-F1-MODEL\_V4 | 1.0 | 3.343e-07 | 323 | 0.286 | 150 | 95 | 6 | 6 | 146 | 13 | 159 | Lysozyme | Lysozyme | | afdb-uniprot50 | AF-A0A811SYU9-F1-MODEL\_V4 | 1.0 | 2.565e-07 | 323 | 0.271 | 166 | 94 | 7 | 3 | 144 | 14 | 176 | Lysozyme RrrD | Lysozyme RrrD | | afdb-uniprot50 | AF-A0A5X3P0C1-F1-MODEL\_V4 | 1.0 | 2.705e-07 | 323 | 0.285 | 154 | 97 | 5 | 1 | 143 | 87 | 238 | Lysozyme | Lysozyme | | afdb-uniprot50 | AF-A0A060C188-F1-MODEL\_V4 | 1.0 | 2.026e-06 | 322 | 0.396 | 111 | 67 | 0 | 33 | 143 | 3 | 113 | Lysozyme | Lysozyme | | afdb-uniprot50 | AF-A0A328A9J3-F1-MODEL\_V4 | 1.0 | 2.705e-07 | 322 | 0.266 | 154 | 98 | 4 | 5 | 156 | 18 | 158 | Lysozyme | Lysozyme | | afdb-uniprot50 | AF-A0A839ZWC1-F1-MODEL\_V4 | 1.0 | 3.343e-07 | 322 | 0.277 | 148 | 94 | 6 | 5 | 145 | 20 | 161 | Lysozyme | Lysozyme | | afdb-uniprot50 | AF-A0A1I4AZP2-F1-MODEL\_V4 | 1.0 | 9.374e-08 | 322 | 0.342 | 184 | 78 | 5 | 2 | 143 | 117 | 299 | Lysozyme | Lysozyme | | afdb-uniprot50 | AF-A0A2D8BR65-F1-MODEL\_V4 | 1.0 | 6.314e-07 | 322 | 0.281 | 142 | 91 | 5 | 2 | 143 | 21 | 151 | Lysozyme | Lysozyme | | afdb-uniprot50 | AF-A0A2D3TE31-F1-MODEL\_V4 | 1.0 | 4.595e-07 | 321 | 0.398 | 108 | 61 | 2 | 1 | 108 | 1 | 104 | Lysozyme | Lysozyme | | afdb-uniprot50 | AF-A0A5E8H3J2-F1-MODEL\_V4 | 1.0 | 1.358e-07 | 321 | 0.32 | 159 | 87 | 9 | 1 | 143 | 31 | 184 | Phage-related lysozyme (Muraminidase) | Phage-related lysozyme (Muraminidase) | | afdb-uniprot50 | AF-A0A832GZF3-F1-MODEL\_V4 | 1.0 | 5.108e-07 | 321 | 0.446 | 121 | 63 | 2 | 29 | 146 | 1 | 120 | CHAP domain-containing protein | CHAP domain-containing protein | | afdb-uniprot50 | AF-A0A0P7VAN4-F1-MODEL\_V4 | 1.0 | 3.007e-07 | 321 | 0.27 | 148 | 99 | 4 | 1 | 144 | 5 | 147 | Lysozyme | Lysozyme | | afdb-uniprot50 | AF-A0A1M5MU42-F1-MODEL\_V4 | 1.0 | 2.852e-07 | 321 | 0.303 | 155 | 93 | 5 | 2 | 145 | 727 | 877 | Lysozyme | Lysozyme | | afdb-uniprot50 | AF-A0A5C7QBV1-F1-MODEL\_V4 | 1.0 | 7.02e-07 | 320 | 0.273 | 139 | 92 | 5 | 7 | 144 | 19 | 149 | Lysozyme | Lysozyme | | afdb-uniprot50 | AF-A0A1G5SFW3-F1-MODEL\_V4 | 1.0 | 6.658e-07 | 320 | 0.309 | 155 | 91 | 5 | 1 | 144 | 115 | 264 | Lysozyme | Lysozyme | | afdb-uniprot50 | AF-A0A6I5RH79-F1-MODEL\_V4 | 1.0 | 3.263e-06 | 319 | 0.357 | 98 | 63 | 0 | 49 | 146 | 1 | 98 | Lysozyme | Lysozyme | | afdb-uniprot50 | AF-A0A397TFN8-F1-MODEL\_V4 | 1.0 | 6.658e-07 | 319 | 0.344 | 122 | 74 | 3 | 3 | 121 | 1 | 119 | Lysozyme | Lysozyme | | afdb-uniprot50 | AF-A0A2I1EL27-F1-MODEL\_V4 | 1.0 | 1.432e-07 | 318 | 0.387 | 129 | 74 | 2 | 23 | 148 | 10 | 136 | Lysozyme | Lysozyme | | afdb-uniprot50 | AF-A0A3N2S834-F1-MODEL\_V4 | 1.0 | 4.595e-07 | 318 | 0.286 | 143 | 94 | 4 | 2 | 143 | 6 | 141 | Lysozyme | Lysozyme | | afdb-uniprot50 | AF-A0A516SAT2-F1-MODEL\_V4 | 1.0 | 1.679e-07 | 318 | 0.26 | 161 | 98 | 6 | 1 | 143 | 8 | 165 | Lysozyme | Lysozyme | | afdb-uniprot50 | AF-A0A1L9CZQ6-F1-MODEL\_V4 | 1.0 | 3.717e-07 | 318 | 0.32 | 156 | 95 | 7 | 2 | 151 | 4 | 154 | PG\_binding\_1 domain-containing protein | PG\_binding\_1 domain-containing protein | | afdb-uniprot50 | AF-A0A549T0T9-F1-MODEL\_V4 | 1.0 | 4.358e-07 | 318 | 0.295 | 159 | 91 | 6 | 2 | 143 | 5 | 159 | Lysozyme | Lysozyme | | afdb-uniprot50 | AF-A0A2X3C7T3-F1-MODEL\_V4 | 1.0 | 1.131e-06 | 316 | 0.388 | 103 | 60 | 2 | 15 | 117 | 33 | 132 | Lysozyme | Lysozyme | | afdb-uniprot50 | AF-A0A8B3NKR9-F1-MODEL\_V4 | 1.0 | 4.845e-07 | 316 | 0.263 | 152 | 93 | 5 | 7 | 144 | 20 | 166 | Lysozyme | Lysozyme | | afdb-uniprot50 | AF-A0A0K8MB38-F1-MODEL\_V4 | 1.0 | 4.595e-07 | 316 | 0.335 | 134 | 85 | 3 | 11 | 143 | 2 | 132 | Lysozyme | Lysozyme | | afdb-uniprot50 | AF-A0A0S1B3T1-F1-MODEL\_V4 | 1.0 | 3.171e-07 | 316 | 0.247 | 174 | 110 | 7 | 2 | 157 | 17 | 187 | Lysozyme | Lysozyme | | afdb-uniprot50 | AF-A0A3S0MS16-F1-MODEL\_V4 | 1.0 | 9.648e-07 | 316 | 0.234 | 158 | 108 | 6 | 6 | 155 | 17 | 169 | Lysozyme | Lysozyme | | afdb-uniprot50 | AF-A0A378N5X9-F1-MODEL\_V4 | 1.0 | 1.326e-06 | 314 | 0.474 | 99 | 50 | 2 | 47 | 145 | 1 | 97 | Lysozyme | Lysozyme | | afdb-uniprot50 | AF-A0A2I1F4H6-F1-MODEL\_V4 | 1.0 | 7.402e-07 | 314 | 0.423 | 130 | 64 | 4 | 20 | 144 | 2 | 125 | Lysozyme | Lysozyme | | afdb-uniprot50 | AF-A0A397V392-F1-MODEL\_V4 | 1.0 | 1.257e-06 | 314 | 0.428 | 105 | 60 | 0 | 44 | 148 | 38 | 142 | Lysozyme | Lysozyme | | afdb-uniprot50 | AF-A0A511BND2-F1-MODEL\_V4 | 1.0 | 2.307e-07 | 314 | 0.299 | 157 | 93 | 6 | 3 | 145 | 1 | 154 | Uncharacterized protein | Uncharacterized protein | | afdb-uniprot50 | AF-X5M573-F1-MODEL\_V4 | 1.0 | 2.504e-06 | 314 | 0.432 | 104 | 59 | 0 | 41 | 144 | 16 | 119 | Lysozyme | Lysozyme | | afdb-uniprot50 | AF-A0A2E6Y944-F1-MODEL\_V4 | 1.0 | 5.68e-07 | 314 | 0.283 | 159 | 93 | 7 | 1 | 143 | 1 | 154 | Lysozyme | Lysozyme | | afdb-uniprot50 | AF-A0A6L9YUM6-F1-MODEL\_V4 | 1.0 | 1.554e-06 | 314 | 0.426 | 129 | 63 | 4 | 1 | 122 | 78 | 202 | Lysozyme | Lysozyme | | afdb-uniprot50 | AF-A0A0Q4MLG9-F1-MODEL\_V4 | 1.0 | 1.51e-07 | 314 | 0.303 | 178 | 89 | 3 | 1 | 144 | 27 | 203 | Lysozyme | Lysozyme | | afdb-uniprot50 | AF-A0A0F9HV13-F1-MODEL\_V4 | 1.0 | 2.075e-07 | 314 | 0.261 | 157 | 100 | 7 | 2 | 146 | 72 | 224 | Lysozyme | Lysozyme | | afdb-uniprot50 | AF-A0A7Y7A7U2-F1-MODEL\_V4 | 1.0 | 5.68e-07 | 314 | 0.237 | 156 | 106 | 3 | 1 | 144 | 106 | 260 | Lysozyme | Lysozyme | | afdb-uniprot50 | AF-A0A0A7KTN9-F1-MODEL\_V4 | 1.0 | 1.474e-06 | 314 | 0.302 | 129 | 87 | 3 | 1 | 128 | 54 | 180 | Lysozyme | Lysozyme | | afdb-uniprot50 | AF-A0A5C7UR14-F1-MODEL\_V4 | 1.0 | 1.017e-06 | 313 | 0.326 | 153 | 90 | 7 | 1 | 146 | 4 | 150 | Lysozyme | Lysozyme | | afdb-uniprot50 | AF-A0A212KMZ8-F1-MODEL\_V4 | 1.0 | 2.075e-07 | 313 | 0.268 | 164 | 94 | 8 | 2 | 144 | 3 | 161 | Lysozyme | Lysozyme | | afdb-uniprot50 | AF-A0A495FC89-F1-MODEL\_V4 | 1.0 | 4.358e-07 | 313 | 0.284 | 158 | 94 | 7 | 4 | 143 | 20 | 176 | GH24 family phage-related lysozyme (Muramidase) | GH24 family phage-related lysozyme (Muramidase) | | afdb-uniprot50 | AF-A0A562T9D1-F1-MODEL\_V4 | 1.0 | 4.133e-07 | 313 | 0.301 | 159 | 90 | 7 | 1 | 143 | 1 | 154 | GH24 family phage-related lysozyme (Muramidase) | GH24 family phage-related lysozyme (Muramidase) | | afdb-uniprot50 | AF-A0A843HL85-F1-MODEL\_V4 | 1.0 | 3.171e-07 | 312 | 0.323 | 139 | 87 | 3 | 10 | 144 | 6 | 141 | Lysozyme | Lysozyme | | afdb-uniprot50 | AF-A0A5J4V626-F1-MODEL\_V4 | 1.0 | 9.648e-07 | 312 | 0.298 | 161 | 92 | 4 | 1 | 144 | 13 | 169 | Lysozyme | Lysozyme | | afdb-uniprot50 | AF-A0A530KLN7-F1-MODEL\_V4 | 1.0 | 4.133e-07 | 312 | 0.243 | 156 | 101 | 6 | 1 | 144 | 145 | 295 | Lysozyme | Lysozyme | | afdb-uniprot50 | AF-A0A5Y0KEY1-F1-MODEL\_V4 | 1.0 | 5.989e-07 | 311 | 0.41 | 134 | 69 | 5 | 19 | 147 | 2 | 130 | Lysozyme | Lysozyme | | afdb-uniprot50 | AF-A0A5C7P136-F1-MODEL\_V4 | 1.0 | 4.595e-07 | 311 | 0.26 | 169 | 103 | 5 | 1 | 148 | 11 | 178 | Lysozyme | Lysozyme | | afdb-uniprot50 | AF-A0A4Q7FGN8-F1-MODEL\_V4 | 1.0 | 8.23e-07 | 311 | 0.254 | 153 | 100 | 4 | 6 | 144 | 2 | 154 | Lysozyme | Lysozyme | | afdb-uniprot50 | AF-A6UN41-F1-MODEL\_V4 | 1.0 | 9.648e-07 | 311 | 0.339 | 171 | 82 | 9 | 1 | 144 | 56 | 222 | Lysozyme | Lysozyme | | afdb-uniprot50 | AF-A0A1H7MLK9-F1-MODEL\_V4 | 1.0 | 1.073e-06 | 310 | 0.282 | 131 | 88 | 3 | 15 | 145 | 4 | 128 | Lysozyme | Lysozyme | | afdb-uniprot50 | AF-A0A3B0JDC7-F1-MODEL\_V4 | 1.0 | 5.989e-07 | 310 | 0.32 | 131 | 87 | 1 | 16 | 146 | 3 | 131 | Lysozyme | Lysozyme | | afdb-uniprot50 | AF-A0A524BEN0-F1-MODEL\_V4 | 1.0 | 2.252e-06 | 310 | 0.391 | 115 | 66 | 3 | 3 | 117 | 67 | 177 | Lysozyme | Lysozyme | | afdb-uniprot50 | AF-A0A2U2P948-F1-MODEL\_V4 | 1.0 | 7.02e-07 | 310 | 0.329 | 155 | 88 | 4 | 1 | 145 | 83 | 231 | Lysozyme | Lysozyme | | afdb-uniprot50 | AF-A0A1I3VR79-F1-MODEL\_V4 | 1.0 | 1.921e-06 | 310 | 0.259 | 154 | 101 | 5 | 1 | 144 | 95 | 245 | Lysozyme | Lysozyme | | afdb-uniprot50 | AF-A0A3A6NXV1-F1-MODEL\_V4 | 1.0 | 7.02e-07 | 309 | 0.254 | 157 | 101 | 6 | 1 | 145 | 9 | 161 | Lysozyme | Lysozyme | | afdb-uniprot50 | AF-A0A3A1Y9X2-F1-MODEL\_V4 | 1.0 | 7.805e-07 | 309 | 0.272 | 147 | 98 | 5 | 5 | 146 | 35 | 177 | Lysozyme | Lysozyme | | afdb-uniprot50 | AF-A0A3M3YCV8-F1-MODEL\_V4 | 1.0 | 8.23e-07 | 309 | 0.294 | 153 | 89 | 6 | 6 | 144 | 32 | 179 | Lysozyme | Lysozyme | | afdb-uniprot50 | AF-A0A0G3XM08-F1-MODEL\_V4 | 1.0 | 7.02e-07 | 309 | 0.282 | 152 | 98 | 6 | 1 | 145 | 28 | 175 | Lysozyme | Lysozyme | | afdb-uniprot50 | AF-A0A4S5JEC4-F1-MODEL\_V4 | 1.0 | 3.343e-07 | 309 | 0.318 | 160 | 88 | 6 | 1 | 143 | 1 | 156 | Lysozyme | Lysozyme | | afdb-uniprot50 | AF-A0A2I1DVJ3-F1-MODEL\_V4 | 1.0 | 1.554e-06 | 308 | 0.403 | 109 | 59 | 1 | 38 | 146 | 2 | 104 | Lysozyme | Lysozyme | | afdb-uniprot50 | AF-A0A3B0JB27-F1-MODEL\_V4 | 1.0 | 2.504e-06 | 308 | 0.375 | 112 | 70 | 0 | 33 | 144 | 3 | 114 | Lysozyme | Lysozyme | | afdb-uniprot50 | AF-A0A2W5R3D9-F1-MODEL\_V4 | 1.0 | 1.679e-07 | 308 | 0.281 | 160 | 94 | 7 | 5 | 153 | 38 | 187 | Lysozyme | Lysozyme | | afdb-uniprot50 | AF-A3YYR1-F1-MODEL\_V4 | 1.0 | 3.92e-07 | 308 | 0.292 | 154 | 90 | 5 | 5 | 143 | 504 | 653 | Lysozyme | Lysozyme | | afdb-uniprot50 | AF-A0A0H0XNY4-F1-MODEL\_V4 | 1.0 | 7.402e-07 | 307 | 0.328 | 152 | 91 | 6 | 1 | 145 | 6 | 153 | Lysozyme | Lysozyme | | afdb-uniprot50 | AF-A0A6B4HYB8-F1-MODEL\_V4 | 1.0 | 4.845e-07 | 307 | 0.272 | 154 | 98 | 5 | 3 | 145 | 109 | 259 | Lysozyme | Lysozyme | | afdb-uniprot50 | AF-A0A1T2B2A3-F1-MODEL\_V4 | 1.0 | 8.23e-07 | 306 | 0.259 | 154 | 100 | 5 | 1 | 144 | 115 | 264 | Lysozyme | Lysozyme | | afdb-uniprot50 | AF-A0A2P8FP63-F1-MODEL\_V4 | 1.0 | 2.565e-07 | 305 | 0.267 | 157 | 95 | 7 | 2 | 143 | 6 | 157 | Lysozyme | Lysozyme | | afdb-uniprot50 | AF-A0A512DJC9-F1-MODEL\_V4 | 1.0 | 6.314e-07 | 305 | 0.311 | 186 | 83 | 5 | 1 | 143 | 8 | 191 | Lysozyme | Lysozyme | | afdb-uniprot50 | AF-A0A553DTU5-F1-MODEL\_V4 | 1.0 | 5.989e-07 | 305 | 0.303 | 158 | 91 | 5 | 1 | 145 | 631 | 782 | Lysozyme | Lysozyme | | afdb-uniprot50 | AF-A0A4U0NZJ7-F1-MODEL\_V4 | 1.0 | 2.136e-06 | 304 | 0.246 | 142 | 97 | 4 | 5 | 139 | 28 | 166 | Lysozyme | Lysozyme | | afdb-uniprot50 | AF-R5ZZA4-F1-MODEL\_V4 | 1.0 | 5.108e-07 | 302 | 0.324 | 157 | 82 | 6 | 6 | 142 | 12 | 164 | Lysozyme | Lysozyme | | afdb-uniprot50 | AF-A0A3N7ELR7-F1-MODEL\_V4 | 1.0 | 2.136e-06 | 302 | 0.272 | 154 | 99 | 4 | 4 | 147 | 39 | 189 | Lysozyme | Lysozyme | | afdb-uniprot50 | AF-A0A327JFF9-F1-MODEL\_V4 | 1.0 | 1.554e-06 | 302 | 0.265 | 166 | 101 | 7 | 1 | 150 | 3 | 163 | PG\_binding\_1 domain-containing protein | PG\_binding\_1 domain-containing protein | | afdb-uniprot50 | AF-A0A2Z3H7L7-F1-MODEL\_V4 | 1.0 | 6.658e-07 | 301 | 0.304 | 161 | 91 | 7 | 1 | 144 | 5 | 161 | Lysozyme | Lysozyme | | afdb-uniprot50 | AF-A0A346MWA4-F1-MODEL\_V4 | 1.0 | 4.358e-07 | 301 | 0.266 | 169 | 103 | 8 | 6 | 157 | 26 | 190 | Lysozyme | Lysozyme | | afdb-uniprot50 | AF-A0A1Z8NXD5-F1-MODEL\_V4 | 1.0 | 2.198e-05 | 300 | 0.444 | 81 | 45 | 0 | 63 | 143 | 2 | 82 | Lysozyme | Lysozyme | | afdb-uniprot50 | AF-A0A7I8I3M8-F1-MODEL\_V4 | 1.0 | 1.017e-06 | 300 | 0.343 | 128 | 77 | 4 | 19 | 144 | 20 | 142 | Lysozyme | Lysozyme | | afdb-uniprot50 | AF-A0A2V3UDT3-F1-MODEL\_V4 | 1.0 | 5.989e-07 | 300 | 0.274 | 171 | 90 | 7 | 3 | 144 | 1 | 166 | Lysozyme | Lysozyme | | afdb-uniprot50 | AF-A0A5C7PXY6-F1-MODEL\_V4 | 1.0 | 3.095e-06 | 299 | 0.263 | 148 | 98 | 4 | 6 | 144 | 34 | 179 | Lysozyme | Lysozyme | | afdb-uniprot50 | AF-A0A6P0ZGF8-F1-MODEL\_V4 | 1.0 | 4.986e-06 | 298 | 0.434 | 99 | 56 | 0 | 50 | 148 | 7 | 105 | Lysozyme | Lysozyme | | afdb-uniprot50 | AF-A0A2A4V774-F1-MODEL\_V4 | 1.0 | 1.193e-06 | 298 | 0.298 | 144 | 94 | 2 | 7 | 144 | 85 | 227 | Lysozyme | Lysozyme | | afdb-uniprot50 | AF-E6YK95-F1-MODEL\_V4 | 1.0 | 1.047e-05 | 297 | 0.43 | 93 | 53 | 0 | 51 | 143 | 40 | 132 | Lysozyme | Lysozyme | | afdb-uniprot50 | AF-A0A059DRI6-F1-MODEL\_V4 | 1.0 | 7.805e-07 | 297 | 0.242 | 169 | 110 | 7 | 1 | 153 | 1 | 167 | Uncharacterized protein | Uncharacterized protein | | afdb-uniprot50 | AF-K4XZD7-F1-MODEL\_V4 | 1.0 | 1.164e-05 | 296 | 0.364 | 96 | 61 | 0 | 49 | 144 | 1 | 96 | Lysozyme | Lysozyme | | afdb-uniprot50 | AF-A0A6L3SZ47-F1-MODEL\_V4 | 1.0 | 1.257e-06 | 296 | 0.294 | 129 | 84 | 4 | 4 | 129 | 16 | 140 | Lysozyme | Lysozyme | | afdb-uniprot50 | AF-A0A829YXQ3-F1-MODEL\_V4 | 1.0 | 8.47e-06 | 296 | 0.387 | 98 | 60 | 0 | 47 | 144 | 1 | 98 | Uncharacterized protein | Uncharacterized protein | | afdb-uniprot50 | AF-A0A7W9R3L0-F1-MODEL\_V4 | 1.0 | 9.648e-07 | 296 | 0.296 | 152 | 85 | 7 | 6 | 145 | 17 | 158 | Lysozyme | Lysozyme | | afdb-uniprot50 | AF-A0A6D2B0C1-F1-MODEL\_V4 | 1.0 | 1.875e-05 | 295 | 0.666 | 72 | 24 | 0 | 73 | 144 | 2 | 73 | Lysozyme | Lysozyme | | afdb-uniprot50 | AF-A0A4R0PG77-F1-MODEL\_V4 | 1.0 | 4.986e-06 | 295 | 0.425 | 108 | 56 | 1 | 46 | 147 | 2 | 109 | Lysozyme | Lysozyme | | afdb-uniprot50 | AF-A0A0Q8B2U6-F1-MODEL\_V4 | 1.0 | 2.935e-06 | 295 | 0.248 | 153 | 98 | 7 | 6 | 144 | 43 | 192 | Lysozyme | Lysozyme | | afdb-uniprot50 | AF-A0A7M1L5D9-F1-MODEL\_V4 | 1.0 | 2.252e-06 | 295 | 0.24 | 154 | 104 | 5 | 4 | 144 | 51 | 204 | Lysozyme | Lysozyme | | afdb-uniprot50 | AF-A0A847GG99-F1-MODEL\_V4 | 1.0 | 1.822e-06 | 294 | 0.412 | 114 | 61 | 2 | 41 | 148 | 3 | 116 | Lysozyme | Lysozyme | | afdb-uniprot50 | AF-A0A1E4JHV5-F1-MODEL\_V4 | 1.0 | 9.417e-06 | 294 | 0.257 | 140 | 99 | 4 | 6 | 144 | 18 | 153 | Lysozyme | Lysozyme | | afdb-uniprot50 | AF-A0A1L6JBV4-F1-MODEL\_V4 | 1.0 | 2.64e-06 | 294 | 0.251 | 147 | 93 | 6 | 6 | 143 | 26 | 164 | Lysozyme | Lysozyme | | afdb-uniprot50 | AF-A0A4R7BD68-F1-MODEL\_V4 | 1.0 | 1.474e-06 | 294 | 0.27 | 174 | 96 | 5 | 1 | 145 | 53 | 224 | Lysozyme | Lysozyme | | afdb-uniprot50 | AF-A0A0Q4ND89-F1-MODEL\_V4 | 1.0 | 1.131e-06 | 293 | 0.314 | 159 | 87 | 6 | 1 | 145 | 371 | 521 | Lysozyme | Lysozyme | | afdb-uniprot50 | AF-A0A0E3BDW0-F1-MODEL\_V4 | 1.0 | 1.728e-06 | 292 | 0.236 | 144 | 101 | 4 | 10 | 144 | 3 | 146 | Lysozyme | Lysozyme | | afdb-uniprot50 | AF-A0A7Y4BU78-F1-MODEL\_V4 | 1.0 | 4.729e-06 | 291 | 0.383 | 120 | 66 | 2 | 33 | 145 | 1 | 119 | Lysozyme | Lysozyme | | afdb-uniprot50 | AF-A0A1Y3B3E7-F1-MODEL\_V4 | 1.0 | 1.365e-05 | 291 | 0.478 | 94 | 48 | 1 | 55 | 148 | 160 | 252 | Galectin | Galectin | | afdb-uniprot50 | AF-A0A679IQX3-F1-MODEL\_V4 | 1.0 | 5.845e-06 | 290 | 0.373 | 115 | 68 | 3 | 3 | 117 | 7 | 117 | Lysozyme | Lysozyme | | afdb-uniprot50 | AF-A0A7Z8G7Z5-F1-MODEL\_V4 | 1.0 | 4.485e-06 | 290 | 0.366 | 109 | 64 | 3 | 8 | 114 | 18 | 123 | Lysozyme | Lysozyme | | afdb-uniprot50 | AF-A0A447N5D3-F1-MODEL\_V4 | 1.0 | 4.729e-06 | 290 | 0.328 | 146 | 94 | 3 | 1 | 146 | 27 | 168 | Lysozyme | Lysozyme | | afdb-uniprot50 | AF-A0A1C3FDG7-F1-MODEL\_V4 | 1.0 | 1.104e-05 | 288 | 0.41 | 100 | 54 | 2 | 49 | 144 | 6 | 104 | Lysozyme | Lysozyme | | afdb-uniprot50 | AF-A0A380WB60-F1-MODEL\_V4 | 1.0 | 1.326e-06 | 288 | 0.224 | 174 | 111 | 7 | 1 | 155 | 10 | 178 | Lysozyme | Lysozyme | | afdb-uniprot50 | AF-A0A1C7D850-F1-MODEL\_V4 | 1.0 | 2.252e-06 | 287 | 0.322 | 161 | 95 | 8 | 1 | 151 | 88 | 244 | Lysozyme | Lysozyme | | afdb-uniprot50 | AF-A0A6A8A260-F1-MODEL\_V4 | 1.0 | 3.441e-06 | 286 | 0.342 | 108 | 66 | 1 | 37 | 144 | 7 | 109 | Lysozyme | Lysozyme | | afdb-uniprot50 | AF-A0A2R5EFM4-F1-MODEL\_V4 | 1.0 | 6.163e-06 | 285 | 0.202 | 158 | 111 | 4 | 1 | 145 | 11 | 166 | Lysozyme | Lysozyme | | afdb-uniprot50 | AF-A0A440JH49-F1-MODEL\_V4 | 1.0 | 3.095e-06 | 284 | 0.258 | 155 | 98 | 6 | 5 | 146 | 126 | 276 | Lysozyme | Lysozyme | | afdb-uniprot50 | AF-A0A4R0E7W8-F1-MODEL\_V4 | 1.0 | 1.326e-06 | 284 | 0.254 | 165 | 103 | 5 | 1 | 145 | 553 | 717 | Lysozyme | Lysozyme | | afdb-uniprot50 | AF-A0A149TPR5-F1-MODEL\_V4 | 1.0 | 3.095e-06 | 283 | 0.38 | 113 | 64 | 3 | 37 | 145 | 11 | 121 | Lysozyme | Lysozyme | | afdb-uniprot50 | AF-A0A823BTY6-F1-MODEL\_V4 | 1.0 | 3.441e-06 | 283 | 0.301 | 156 | 93 | 6 | 4 | 146 | 22 | 174 | Lysozyme | Lysozyme | | afdb-uniprot50 | AF-A0A351BF90-F1-MODEL\_V4 | 1.0 | 4.253e-06 | 283 | 0.307 | 153 | 90 | 5 | 1 | 143 | 190 | 336 | Lysozyme | Lysozyme | | afdb-uniprot50 | AF-A0A4Q7BNM2-F1-MODEL\_V4 | 1.0 | 1.073e-06 | 283 | 0.278 | 165 | 95 | 6 | 1 | 145 | 467 | 627 | Lysozyme | Lysozyme | | afdb-uniprot50 | AF-A0A3S4GC52-F1-MODEL\_V4 | 1.0 | 1.921e-06 | 282 | 0.308 | 133 | 79 | 3 | 7 | 129 | 19 | 148 | Lysozyme | Lysozyme | | afdb-uniprot50 | AF-A0A142JHX9-F1-MODEL\_V4 | 1.0 | 4.034e-06 | 282 | 0.308 | 149 | 81 | 6 | 8 | 143 | 19 | 158 | Lysozyme | Lysozyme | | afdb-uniprot50 | AF-A0A7W4RHC5-F1-MODEL\_V4 | 1.0 | 3.628e-06 | 282 | 0.212 | 165 | 107 | 7 | 1 | 143 | 13 | 176 | GH24 family phage-related lysozyme (Muramidase) | GH24 family phage-related lysozyme (Muramidase) | | afdb-uniprot50 | AF-A0A246GF81-F1-MODEL\_V4 | 1.0 | 2.252e-06 | 282 | 0.316 | 158 | 89 | 5 | 1 | 145 | 137 | 288 | Lysozyme | Lysozyme | | afdb-uniprot50 | AF-A0A704CZH1-F1-MODEL\_V4 | 1.0 | 6.163e-06 | 281 | 0.533 | 103 | 44 | 1 | 28 | 126 | 1 | 103 | Lysozyme | Lysozyme | | afdb-uniprot50 | AF-A0A1F3IZE5-F1-MODEL\_V4 | 1.0 | 1.474e-06 | 281 | 0.313 | 150 | 88 | 5 | 1 | 143 | 328 | 469 | Lysozyme | Lysozyme | | afdb-uniprot50 | AF-A0A3N7ETY2-F1-MODEL\_V4 | 1.0 | 4.034e-06 | 280 | 0.432 | 118 | 62 | 1 | 33 | 145 | 2 | 119 | Lysozyme | Lysozyme | | afdb-uniprot50 | AF-A0A7R9MBY2-F1-MODEL\_V4 | 1.0 | 9.648e-07 | 280 | 0.256 | 156 | 96 | 5 | 7 | 144 | 9 | 162 | Lysozyme | Lysozyme | | afdb-uniprot50 | AF-A0A3T4D307-F1-MODEL\_V4 | 1.0 | 2.375e-06 | 280 | 0.294 | 170 | 91 | 7 | 3 | 146 | 9 | 175 | Lysozyme | Lysozyme | | afdb-uniprot50 | AF-A0A5R8ZQ96-F1-MODEL\_V4 | 1.0 | 2.375e-06 | 279 | 0.235 | 153 | 98 | 6 | 6 | 144 | 26 | 173 | Lysozyme | Lysozyme | | afdb-uniprot50 | AF-A0A1G4RXF8-F1-MODEL\_V4 | 1.0 | 4.729e-06 | 279 | 0.217 | 156 | 110 | 5 | 2 | 151 | 26 | 175 | Lysozyme | Lysozyme | | afdb-uniprot50 | AF-A0A3T9QUX1-F1-MODEL\_V4 | 1.0 | 4.152e-05 | 278 | 0.363 | 88 | 56 | 0 | 56 | 143 | 2 | 89 | Lysozyme | Lysozyme | | afdb-uniprot50 | AF-A0A1X7MCZ0-F1-MODEL\_V4 | 1.0 | 1.227e-05 | 278 | 0.383 | 107 | 65 | 1 | 38 | 144 | 1 | 106 | Lysozyme | Lysozyme | | afdb-uniprot50 | AF-A0A060C0Y3-F1-MODEL\_V4 | 1.0 | 4.034e-06 | 278 | 0.377 | 127 | 71 | 5 | 22 | 144 | 1 | 123 | Lysozyme | Lysozyme | | afdb-uniprot50 | AF-A3VBD6-F1-MODEL\_V4 | 1.0 | 1.822e-06 | 278 | 0.291 | 161 | 94 | 6 | 7 | 150 | 122 | 279 | Lysozyme | Lysozyme | | afdb-uniprot50 | AF-A0A550KMS5-F1-MODEL\_V4 | 1.0 | 2.375e-06 | 278 | 0.267 | 157 | 97 | 5 | 5 | 157 | 9 | 151 | Lysozyme | Lysozyme | | afdb-uniprot50 | AF-A0A433PHE8-F1-MODEL\_V4 | 1.0 | 4.485e-06 | 278 | 0.203 | 157 | 108 | 7 | 1 | 147 | 348 | 497 | Lysozyme | Lysozyme | | afdb-uniprot50 | AF-D8MV64-F1-MODEL\_V4 | 1.0 | 1.875e-05 | 277 | 0.434 | 99 | 53 | 2 | 47 | 144 | 1 | 97 | Lysozyme | Lysozyme | | afdb-uniprot50 | AF-A0A147GWA0-F1-MODEL\_V4 | 1.0 | 2.504e-06 | 277 | 0.246 | 166 | 99 | 8 | 4 | 144 | 23 | 187 | Uncharacterized protein | Uncharacterized protein | | afdb-uniprot50 | AF-A0A7C2WUQ3-F1-MODEL\_V4 | 1.0 | 7.618e-06 | 277 | 0.233 | 150 | 102 | 3 | 6 | 143 | 45 | 193 | Lysozyme | Lysozyme | | afdb-uniprot50 | AF-A0A7R8N0H2-F1-MODEL\_V4 | 1.0 | 2.865e-05 | 276 | 0.371 | 97 | 61 | 0 | 46 | 142 | 3 | 99 | Lysozyme | Lysozyme | | afdb-uniprot50 | AF-A0A4V5MMW7-F1-MODEL\_V4 | 1.0 | 5.544e-06 | 276 | 0.234 | 149 | 99 | 5 | 3 | 139 | 87 | 232 | Lysozyme | Lysozyme | | afdb-uniprot50 | AF-A0A448SSY8-F1-MODEL\_V4 | 1.0 | 1.164e-05 | 275 | 0.339 | 106 | 67 | 1 | 38 | 143 | 11 | 113 | Lysozyme | Lysozyme | | afdb-uniprot50 | AF-A0A285CW41-F1-MODEL\_V4 | 1.0 | 1.517e-05 | 275 | 0.342 | 108 | 70 | 1 | 40 | 146 | 14 | 121 | Lysozyme | Lysozyme | | afdb-uniprot50 | AF-A0A376RU32-F1-MODEL\_V4 | 1.0 | 1.294e-05 | 275 | 0.367 | 117 | 70 | 3 | 1 | 117 | 27 | 139 | Lysozyme | Lysozyme | | afdb-uniprot50 | AF-J1J5J1-F1-MODEL\_V4 | 1.0 | 2.444e-05 | 275 | 0.406 | 96 | 56 | 1 | 51 | 145 | 6 | 101 | Lysozyme | Lysozyme | | afdb-uniprot50 | AF-A0A431HNJ6-F1-MODEL\_V4 | 1.0 | 8.033e-06 | 275 | 0.42 | 100 | 52 | 2 | 53 | 146 | 2 | 101 | Lysozyme | Lysozyme | | afdb-uniprot50 | AF-A0A377PMX4-F1-MODEL\_V4 | 1.0 | 2.318e-05 | 274 | 0.34 | 100 | 66 | 0 | 44 | 143 | 4 | 103 | Lysozyme | Lysozyme | | afdb-uniprot50 | AF-A0A147IZ09-F1-MODEL\_V4 | 1.0 | 4.034e-06 | 274 | 0.284 | 158 | 89 | 5 | 6 | 143 | 23 | 176 | Lysozyme | Lysozyme | | afdb-uniprot50 | AF-A0A2G1YJ53-F1-MODEL\_V4 | 1.0 | 9.929e-06 | 274 | 0.245 | 151 | 102 | 7 | 2 | 146 | 49 | 193 | Lysozyme | Lysozyme | | afdb-uniprot50 | AF-A0A7R9KV78-F1-MODEL\_V4 | 1.0 | 3.734e-05 | 274 | 0.432 | 97 | 54 | 1 | 49 | 145 | 2 | 97 | Lysozyme | Lysozyme | | afdb-uniprot50 | AF-A0A743U3J8-F1-MODEL\_V4 | 1.0 | 1.778e-05 | 273 | 0.319 | 122 | 79 | 3 | 4 | 125 | 30 | 147 | Lysozyme | Lysozyme | | afdb-uniprot50 | AF-A0A7W0BQY6-F1-MODEL\_V4 | 1.0 | 2.504e-06 | 273 | 0.204 | 171 | 113 | 8 | 1 | 159 | 9 | 168 | Lysozyme | Lysozyme | | afdb-uniprot50 | AF-A0A397VV53-F1-MODEL\_V4 | 1.0 | 8.47e-06 | 273 | 0.317 | 123 | 72 | 4 | 2 | 117 | 71 | 188 | Lysozyme | Lysozyme | | afdb-uniprot50 | AF-A0A062IRX0-F1-MODEL\_V4 | 1.0 | 6.163e-06 | 272 | 0.183 | 158 | 114 | 7 | 1 | 145 | 12 | 167 | Phage lysozyme family protein | Phage lysozyme family protein | | afdb-uniprot50 | AF-A3Z115-F1-MODEL\_V4 | 1.0 | 1.047e-05 | 272 | 0.44 | 93 | 51 | 1 | 53 | 144 | 3 | 95 | Lysozyme | Lysozyme | | afdb-uniprot50 | AF-U9UUS9-F1-MODEL\_V4 | 1.0 | 1.687e-05 | 271 | 0.333 | 111 | 67 | 2 | 1 | 108 | 5 | 111 | Lysozyme | Lysozyme | | afdb-uniprot50 | AF-A0A827P9W5-F1-MODEL\_V4 | 1.0 | 8.931e-06 | 271 | 0.298 | 114 | 72 | 3 | 6 | 114 | 21 | 131 | Lysozyme | Lysozyme | | afdb-uniprot50 | AF-A0A7T5K5I0-F1-MODEL\_V4 | 1.0 | 2.717e-05 | 271 | 0.327 | 110 | 71 | 2 | 7 | 116 | 19 | 125 | Lysozyme | Lysozyme | | afdb-uniprot50 | AF-A0A0F9SL40-F1-MODEL\_V4 | 1.0 | 1.164e-05 | 271 | 0.209 | 167 | 108 | 5 | 2 | 144 | 114 | 280 | Uncharacterized protein | Uncharacterized protein | | afdb-uniprot50 | AF-A0A1Y1XFR8-F1-MODEL\_V4 | 1.0 | 1.921e-06 | 270 | 0.284 | 158 | 92 | 8 | 4 | 152 | 1 | 146 | Lysozyme | Lysozyme | | afdb-uniprot50 | AF-A0A809ZZ30-F1-MODEL\_V4 | 1.0 | 1.294e-05 | 270 | 0.243 | 152 | 105 | 4 | 2 | 143 | 14 | 165 | Uncharacterized protein | Uncharacterized protein | | afdb-uniprot50 | AF-A0A4R0DJ46-F1-MODEL\_V4 | 1.0 | 1.047e-05 | 269 | 0.235 | 157 | 104 | 7 | 2 | 145 | 22 | 175 | Lysozyme | Lysozyme | | afdb-uniprot50 | AF-A0A4Q5R9M0-F1-MODEL\_V4 | 1.0 | 3.441e-06 | 269 | 0.263 | 190 | 95 | 5 | 3 | 149 | 106 | 293 | Lysozyme | Lysozyme | | afdb-uniprot50 | AF-A0A4Y6U9J2-F1-MODEL\_V4 | 1.0 | 1.554e-06 | 268 | 0.272 | 165 | 95 | 6 | 6 | 148 | 15 | 176 | Lysozyme | Lysozyme | | afdb-uniprot50 | AF-A0A5C8RYA1-F1-MODEL\_V4 | 1.0 | 4.867e-05 | 267 | 0.37 | 100 | 62 | 1 | 46 | 144 | 2 | 101 | Lysozyme | Lysozyme | | afdb-uniprot50 | AF-A0A0P6WR43-F1-MODEL\_V4 | 1.0 | 3.441e-06 | 266 | 0.273 | 172 | 88 | 6 | 6 | 143 | 25 | 193 | Lysozyme | Lysozyme | | afdb-uniprot50 | AF-C1D947-F1-MODEL\_V4 | 1.0 | 1.517e-05 | 265 | 0.411 | 102 | 59 | 1 | 44 | 145 | 1 | 101 | Lysozyme | Lysozyme | | afdb-uniprot50 | AF-A0A0H5Q1T3-F1-MODEL\_V4 | 1.0 | 3.021e-05 | 265 | 0.231 | 147 | 105 | 4 | 1 | 145 | 17 | 157 | Uncharacterized protein | Uncharacterized protein | | afdb-uniprot50 | AF-A0A431IGY8-F1-MODEL\_V4 | 1.0 | 4.034e-06 | 265 | 0.281 | 160 | 98 | 6 | 5 | 150 | 6 | 162 | Lysozyme | Lysozyme | | afdb-uniprot50 | AF-A0A4R3UTK5-F1-MODEL\_V4 | 1.0 | 0.0001136 | 264 | 0.413 | 87 | 51 | 0 | 60 | 146 | 4 | 90 | Lysozyme | Lysozyme | | afdb-uniprot50 | AF-A0A4R0D3N4-F1-MODEL\_V4 | 1.0 | 1.517e-05 | 264 | 0.227 | 158 | 106 | 7 | 1 | 145 | 23 | 177 | Lysozyme | Lysozyme | | afdb-uniprot50 | AF-A0A7G7M048-F1-MODEL\_V4 | 1.0 | 5.258e-06 | 264 | 0.243 | 152 | 98 | 7 | 16 | 158 | 65 | 208 | Lysozyme | Lysozyme | | afdb-uniprot50 | AF-A0A077FK68-F1-MODEL\_V4 | 1.0 | 0.0001481 | 262 | 0.379 | 79 | 49 | 0 | 65 | 143 | 2 | 80 | Lysozyme | Lysozyme | | afdb-uniprot50 | AF-A0A0Q4I8D7-F1-MODEL\_V4 | 1.0 | 9.417e-06 | 262 | 0.351 | 131 | 76 | 5 | 18 | 145 | 9 | 133 | Lysozyme | Lysozyme | | afdb-uniprot50 | AF-V2U938-F1-MODEL\_V4 | 1.0 | 1.365e-05 | 262 | 0.197 | 157 | 111 | 6 | 2 | 145 | 13 | 167 | Lysozyme | Lysozyme | | afdb-uniprot50 | AF-A0A845M958-F1-MODEL\_V4 | 1.0 | 1.365e-05 | 262 | 0.291 | 161 | 97 | 6 | 1 | 146 | 116 | 274 | Glycoside hydrolase family protein | Glycoside hydrolase family protein | | afdb-uniprot50 | AF-A0A0B5DZ45-F1-MODEL\_V4 | 1.0 | 8.931e-06 | 261 | 0.192 | 151 | 109 | 5 | 2 | 144 | 84 | 229 | Lysozyme | Lysozyme | | afdb-uniprot50 | AF-B5ZDC6-F1-MODEL\_V4 | 1.0 | 0.0001198 | 260 | 0.376 | 85 | 52 | 1 | 61 | 144 | 7 | 91 | Lysozyme | Lysozyme | | afdb-uniprot50 | AF-A0A561HF46-F1-MODEL\_V4 | 1.0 | 4.986e-06 | 260 | 0.4 | 115 | 64 | 2 | 37 | 146 | 2 | 116 | Lysozyme | Lysozyme | | afdb-uniprot50 | AF-A0A561HYA6-F1-MODEL\_V4 | 1.0 | 1.164e-05 | 259 | 0.405 | 111 | 63 | 2 | 37 | 146 | 6 | 114 | Lysozyme | Lysozyme | | afdb-uniprot50 | AF-A0A7C9P2F6-F1-MODEL\_V4 | 1.0 | 2.444e-05 | 259 | 0.282 | 124 | 83 | 3 | 24 | 143 | 2 | 123 | Lysozyme | Lysozyme | | afdb-uniprot50 | AF-A0A7Z1UH38-F1-MODEL\_V4 | 1.0 | 2.444e-05 | 257 | 0.282 | 152 | 90 | 7 | 7 | 144 | 104 | 250 | Lysozyme | Lysozyme | | afdb-uniprot50 | AF-A0A2P4P1I1-F1-MODEL\_V4 | 1.0 | 3.734e-05 | 256 | 0.333 | 108 | 65 | 2 | 4 | 108 | 1 | 104 | Lysozyme | Lysozyme | | afdb-uniprot50 | AF-A0A349FCC2-F1-MODEL\_V4 | 1.0 | 5.705e-05 | 256 | 0.398 | 103 | 55 | 1 | 50 | 145 | 1 | 103 | Lysozyme | Lysozyme | | afdb-uniprot50 | AF-A0A0Q7DQU4-F1-MODEL\_V4 | 1.0 | 7.618e-06 | 256 | 0.317 | 151 | 86 | 6 | 4 | 146 | 48 | 189 | Lysozyme | Lysozyme | | afdb-uniprot50 | AF-A0A2G6GNA8-F1-MODEL\_V4 | 1.0 | 6.499e-06 | 256 | 0.278 | 147 | 93 | 8 | 7 | 146 | 55 | 195 | Uncharacterized protein | Uncharacterized protein | | afdb-uniprot50 | AF-A0A4U2U973-F1-MODEL\_V4 | 1.0 | 0.0001404 | 254 | 0.306 | 98 | 68 | 0 | 49 | 146 | 1 | 98 | Lysozyme | Lysozyme | | afdb-uniprot50 | AF-Q2N9G4-F1-MODEL\_V4 | 1.0 | 2.444e-05 | 254 | 0.276 | 152 | 99 | 6 | 1 | 145 | 35 | 182 | Lysozyme | Lysozyme | | afdb-uniprot50 | AF-A0A226EDT1-F1-MODEL\_V4 | 1.0 | 4.152e-05 | 254 | 0.396 | 106 | 59 | 3 | 46 | 148 | 26 | 129 | Lysozyme | Lysozyme | | afdb-uniprot50 | AF-A0A484QZB0-F1-MODEL\_V4 | 1.0 | 6.688e-05 | 254 | 0.416 | 96 | 56 | 0 | 49 | 144 | 135 | 230 | Lysozyme | Lysozyme | | afdb-uniprot50 | AF-A0A4V6KVI0-F1-MODEL\_V4 | 1.0 | 5.132e-05 | 253 | 0.397 | 98 | 57 | 1 | 53 | 150 | 2 | 97 | Lysozyme | Lysozyme | | afdb-uniprot50 | AF-A0A0G3S5Y9-F1-MODEL\_V4 | 1.0 | 1.439e-05 | 253 | 0.256 | 152 | 97 | 5 | 1 | 147 | 1 | 141 | Lysozyme | Lysozyme | | afdb-uniprot50 | AF-A0A7V7Z6A1-F1-MODEL\_V4 | 1.0 | 6.163e-06 | 253 | 0.402 | 139 | 64 | 3 | 24 | 147 | 2 | 136 | Lysozyme | Lysozyme | | afdb-uniprot50 | AF-A0A5W2M167-F1-MODEL\_V4 | 1.0 | 0.0001022 | 252 | 0.391 | 97 | 58 | 1 | 49 | 145 | 1 | 96 | Lysozyme | Lysozyme | | afdb-uniprot50 | AF-A0A317PF33-F1-MODEL\_V4 | 1.0 | 2.198e-05 | 252 | 0.232 | 159 | 101 | 7 | 1 | 143 | 1 | 154 | GH24 family phage-related lysozyme (Muramidase) | GH24 family phage-related lysozyme (Muramidase) | | afdb-uniprot50 | AF-A0A0Q8U0X3-F1-MODEL\_V4 | 1.0 | 3.359e-05 | 251 | 0.216 | 166 | 104 | 6 | 4 | 144 | 19 | 183 | Lysozyme | Lysozyme | | afdb-uniprot50 | AF-A0A1M6SYR1-F1-MODEL\_V4 | 1.0 | 2.865e-05 | 251 | 0.304 | 161 | 87 | 8 | 3 | 146 | 41 | 193 | Lysozyme | Lysozyme | | afdb-uniprot50 | AF-A0A0R3MW87-F1-MODEL\_V4 | 1.0 | 3.826e-06 | 251 | 0.244 | 184 | 97 | 8 | 1 | 159 | 9 | 175 | Uncharacterized protein | Uncharacterized protein | | afdb-uniprot50 | AF-A0A1Z8LH42-F1-MODEL\_V4 | 1.0 | 0.0001646 | 250 | 0.333 | 96 | 62 | 1 | 50 | 143 | 11 | 106 | Lysozyme | Lysozyme | | afdb-uniprot50 | AF-A0A841QL93-F1-MODEL\_V4 | 1.0 | 0.0003843 | 249 | 0.437 | 80 | 45 | 0 | 66 | 145 | 2 | 81 | GH24 family phage-related lysozyme (Muramidase) | GH24 family phage-related lysozyme (Muramidase) | | afdb-uniprot50 | AF-A0A4V3UX11-F1-MODEL\_V4 | 1.0 | 0.0001078 | 249 | 0.336 | 104 | 69 | 0 | 41 | 144 | 3 | 106 | Lysozyme | Lysozyme | | afdb-uniprot50 | AF-A0A430D648-F1-MODEL\_V4 | 1.0 | 1.227e-05 | 247 | 0.231 | 164 | 97 | 5 | 6 | 143 | 21 | 181 | Lysozyme | Lysozyme | | afdb-uniprot50 | AF-A0A3M9Z534-F1-MODEL\_V4 | 1.0 | 1.921e-06 | 247 | 0.335 | 137 | 72 | 4 | 24 | 146 | 130 | 261 | Lysozyme | Lysozyme | | afdb-uniprot50 | AF-A0A4V1RG86-F1-MODEL\_V4 | 1.0 | 0.0001646 | 246 | 0.333 | 96 | 64 | 0 | 49 | 144 | 1 | 96 | Lysozyme | Lysozyme | | afdb-uniprot50 | AF-A0A1N6UYK7-F1-MODEL\_V4 | 1.0 | 1.517e-05 | 246 | 0.22 | 172 | 109 | 9 | 4 | 156 | 12 | 177 | Lysozyme | Lysozyme | | afdb-uniprot50 | AF-A0A5E8P0K9-F1-MODEL\_V4 | 1.0 | 3.542e-05 | 245 | 0.336 | 104 | 61 | 1 | 41 | 144 | 52 | 147 | Lysozyme | Lysozyme | | afdb-uniprot50 | AF-A0A1B3PFJ1-F1-MODEL\_V4 | 1.0 | 6.343e-05 | 245 | 0.186 | 166 | 110 | 6 | 2 | 143 | 22 | 186 | Putative phage lysozyme | Putative phage lysozyme | | afdb-uniprot50 | AF-A0A3M9Z3J4-F1-MODEL\_V4 | 1.0 | 1.778e-05 | 243 | 0.396 | 111 | 58 | 2 | 38 | 144 | 22 | 127 | Lysozyme | Lysozyme | | afdb-uniprot50 | AF-A0A1A9WSJ5-F1-MODEL\_V4 | 1.0 | 7.052e-05 | 243 | 0.397 | 93 | 56 | 0 | 40 | 132 | 25 | 117 | Lysozyme | Lysozyme | | afdb-uniprot50 | AF-A0A1G8LAP6-F1-MODEL\_V4 | 1.0 | 0.000183 | 241 | 0.444 | 90 | 49 | 1 | 57 | 146 | 2 | 90 | Lysozyme | Lysozyme | | afdb-uniprot50 | AF-C6XGM8-F1-MODEL\_V4 | 1.0 | 0.0001332 | 239 | 0.333 | 102 | 66 | 1 | 47 | 146 | 1 | 102 | Lysozyme | Lysozyme | | afdb-uniprot50 | AF-A0A143HSA8-F1-MODEL\_V4 | 1.0 | 0.0001481 | 236 | 0.327 | 113 | 74 | 2 | 33 | 144 | 3 | 114 | Lysozyme | Lysozyme | | afdb-uniprot50 | AF-A0A6B8LZY5-F1-MODEL\_V4 | 1.0 | 2.577e-05 | 235 | 0.427 | 117 | 58 | 3 | 32 | 144 | 2 | 113 | Lysozyme | Lysozyme | | afdb-uniprot50 | AF-A0A126V243-F1-MODEL\_V4 | 1.0 | 0.0004052 | 234 | 0.349 | 83 | 52 | 1 | 64 | 144 | 2 | 84 | Lysozyme | Lysozyme | | afdb-uniprot50 | AF-G5LKD0-F1-MODEL\_V4 | 1.0 | 0.0001481 | 233 | 0.368 | 103 | 63 | 1 | 46 | 146 | 4 | 106 | Lysozyme | Lysozyme | | afdb-uniprot50 | AF-A0A356CHN3-F1-MODEL\_V4 | 1.0 | 0.0003457 | 232 | 0.432 | 81 | 46 | 0 | 67 | 147 | 4 | 84 | Lysozyme | Lysozyme | | afdb-uniprot50 | AF-A0A4Q3MM19-F1-MODEL\_V4 | 1.0 | 6.016e-05 | 232 | 0.237 | 135 | 91 | 2 | 22 | 144 | 28 | 162 | Lysozyme | Lysozyme | | afdb-uniprot50 | AF-A0A839KL75-F1-MODEL\_V4 | 1.0 | 0.000807 | 231 | 0.416 | 72 | 42 | 0 | 73 | 144 | 2 | 73 | Lysozyme RrrD | Lysozyme RrrD | | afdb-uniprot50 | AF-E4UDK1-F1-MODEL\_V4 | 1.0 | 0.0002146 | 231 | 0.298 | 104 | 67 | 2 | 47 | 146 | 1 | 102 | Lysozyme | Lysozyme | | afdb-uniprot50 | AF-A0A2D0ANW9-F1-MODEL\_V4 | 1.0 | 7.052e-05 | 231 | 0.226 | 159 | 101 | 5 | 6 | 145 | 22 | 177 | Lysozyme | Lysozyme | | afdb-uniprot50 | AF-A0A418WYF3-F1-MODEL\_V4 | 1.0 | 0.0001136 | 229 | 0.32 | 106 | 69 | 2 | 38 | 142 | 1 | 104 | Lysozyme | Lysozyme | | afdb-uniprot50 | AF-A0A376PX23-F1-MODEL\_V4 | 1.0 | 0.0001022 | 229 | 0.289 | 107 | 68 | 3 | 6 | 107 | 21 | 124 | Lysozyme | Lysozyme | | afdb-uniprot50 | AF-A4WPP1-F1-MODEL\_V4 | 1.0 | 5.411e-05 | 228 | 0.21 | 152 | 101 | 6 | 5 | 144 | 9 | 153 | Lysozyme | Lysozyme | | afdb-uniprot50 | AF-A0A6L3SU82-F1-MODEL\_V4 | 1.0 | 0.0002035 | 228 | 0.366 | 101 | 59 | 2 | 46 | 146 | 4 | 99 | Lysozyme | Lysozyme | | afdb-uniprot50 | AF-F4VC63-F1-MODEL\_V4 | 1.0 | 3.937e-05 | 226 | 0.264 | 121 | 76 | 4 | 6 | 116 | 21 | 138 | Lysozyme | Lysozyme | | afdb-uniprot50 | AF-R9AZY7-F1-MODEL\_V4 | 1.0 | 0.0004505 | 225 | 0.558 | 77 | 34 | 0 | 62 | 138 | 4 | 80 | Lysozyme | Lysozyme | | afdb-uniprot50 | AF-A0A8B2R8P4-F1-MODEL\_V4 | 1.0 | 0.000807 | 224 | 0.348 | 89 | 58 | 0 | 56 | 144 | 3 | 91 | Lysozyme | Lysozyme | | afdb-uniprot50 | AF-A0A1X7MEG5-F1-MODEL\_V4 | 1.0 | 0.001524 | 220 | 0.452 | 73 | 40 | 0 | 71 | 143 | 3 | 75 | Lysozyme | Lysozyme | | afdb-uniprot50 | AF-A0A5X2XK25-F1-MODEL\_V4 | 1.0 | 7.436e-05 | 219 | 0.359 | 114 | 67 | 3 | 37 | 147 | 3 | 113 | Lysozyme | Lysozyme | | afdb-uniprot50 | AF-A0A774SGM1-F1-MODEL\_V4 | 1.0 | 0.000807 | 218 | 0.404 | 84 | 50 | 0 | 49 | 132 | 4 | 87 | Lysozyme | Lysozyme | | afdb-uniprot50 | AF-A0A7H4K6A6-F1-MODEL\_V4 | 1.0 | 2.865e-05 | 218 | 0.216 | 189 | 103 | 9 | 3 | 154 | 6 | 186 | Lysozyme | Lysozyme | | afdb-uniprot50 | AF-A0A702DCC3-F1-MODEL\_V4 | 1.0 | 0.0004751 | 217 | 0.295 | 98 | 66 | 1 | 7 | 104 | 21 | 115 | Lysozyme | Lysozyme | | afdb-uniprot50 | AF-A0A0Q6AKV8-F1-MODEL\_V4 | 1.0 | 8.717e-05 | 216 | 0.293 | 116 | 78 | 2 | 33 | 145 | 6 | 120 | Lysozyme | Lysozyme | | afdb-uniprot50 | AF-A0A7H4PFX8-F1-MODEL\_V4 | 1.0 | 4.152e-05 | 216 | 0.235 | 157 | 104 | 4 | 1 | 153 | 6 | 150 | Lysozyme | Lysozyme | | afdb-uniprot50 | AF-A0A1Y1J4F4-F1-MODEL\_V4 | 1.0 | 0.0002146 | 215 | 0.215 | 176 | 105 | 8 | 1 | 144 | 1 | 175 | Putative phage lysozyme | Putative phage lysozyme | | afdb-uniprot50 | AF-V2V153-F1-MODEL\_V4 | 1.0 | 0.0002262 | 214 | 0.209 | 143 | 98 | 6 | 15 | 144 | 163 | 303 | Lysozyme | Lysozyme | | afdb-uniprot50 | AF-A0A2D9KYN3-F1-MODEL\_V4 | 1.0 | 0.0001646 | 213 | 0.226 | 163 | 102 | 6 | 4 | 145 | 13 | 172 | Lysozyme | Lysozyme | | afdb-uniprot50 | AF-A0A0N0ZAJ6-F1-MODEL\_V4 | 1.0 | 0.0004751 | 212 | 0.35 | 100 | 62 | 2 | 5 | 104 | 9 | 105 | Lysozyme | Lysozyme | | afdb-uniprot50 | AF-C4K3M0-F1-MODEL\_V4 | 1.0 | 0.0001198 | 201 | 0.362 | 116 | 65 | 4 | 34 | 145 | 4 | 114 | Lysozyme | Lysozyme | | afdb-uniprot50 | AF-A0A356LHJ7-F1-MODEL\_V4 | 1.0 | 0.0005872 | 199 | 0.327 | 110 | 69 | 4 | 16 | 124 | 57 | 162 | Lysozyme | Lysozyme | | afdb-uniprot50 | AF-A0A197JJ61-F1-MODEL\_V4 | 1.0 | 0.0007653 | 199 | 0.254 | 122 | 85 | 2 | 2 | 120 | 162 | 280 | Lysozyme | Lysozyme | | afdb-uniprot50 | AF-A0A2X1B257-F1-MODEL\_V4 | 1.0 | 0.000807 | 198 | 0.297 | 111 | 77 | 1 | 50 | 159 | 3 | 113 | Phage-related lysozyme (Muraminidase) | Phage-related lysozyme (Muraminidase) | | afdb-uniprot50 | AF-A0A350PK98-F1-MODEL\_V4 | 1.0 | 0.0009975 | 197 | 0.314 | 108 | 68 | 4 | 39 | 144 | 1 | 104 | Lysozyme | Lysozyme | | afdb-uniprot50 | AF-A0A248KFB5-F1-MODEL\_V4 | 1.0 | 0.0006192 | 197 | 0.301 | 116 | 76 | 2 | 29 | 143 | 5 | 116 | Lysozyme | Lysozyme | | afdb-uniprot50 | AF-A0A1G4RIK7-F1-MODEL\_V4 | 1.0 | 0.0004273 | 197 | 0.186 | 166 | 100 | 9 | 6 | 144 | 46 | 203 | Lysozyme | Lysozyme | | afdb-uniprot50 | AF-A0A5B7TXD1-F1-MODEL\_V4 | 1.0 | 0.002094 | 195 | 0.35 | 97 | 57 | 2 | 51 | 143 | 204 | 298 | Lysozyme | Lysozyme | | afdb-uniprot50 | AF-A0A1S8Y709-F1-MODEL\_V4 | 1.0 | 0.001607 | 190 | 0.313 | 86 | 55 | 1 | 60 | 145 | 5 | 86 | Lysozyme | Lysozyme | | afdb-uniprot50 | AF-M4UCN0-F1-MODEL\_V4 | 1.0 | 0.0006884 | 188 | 0.376 | 85 | 48 | 2 | 60 | 144 | 5 | 84 | Lysozyme | Lysozyme | | afdb-uniprot50 | AF-A0A1Z8T0S5-F1-MODEL\_V4 | 1.0 | 0.001169 | 188 | 0.188 | 159 | 106 | 5 | 1 | 143 | 55 | 206 | Uncharacterized protein | Uncharacterized protein | | afdb-uniprot50 | AF-Q3RDH2-F1-MODEL\_V4 | 1.0 | 0.0032 | 186 | 0.465 | 73 | 39 | 0 | 86 | 158 | 2 | 74 | Lysozyme | Lysozyme | | afdb-uniprot50 | AF-A0A7W6CC09-F1-MODEL\_V4 | 1.0 | 0.001445 | 186 | 0.324 | 108 | 67 | 4 | 38 | 143 | 17 | 120 | GH24 family phage-related lysozyme (Muramidase) | GH24 family phage-related lysozyme (Muramidase) | | afdb-uniprot50 | AF-A0A854EPE1-F1-MODEL\_V4 | 1.0 | 0.0005282 | 185 | 0.253 | 138 | 80 | 8 | 3 | 129 | 13 | 138 | Glycosyl hydrolase | Glycosyl hydrolase | | afdb-uniprot50 | AF-A0A447MPG6-F1-MODEL\_V4 | 1.0 | 0.004637 | 182 | 0.313 | 83 | 53 | 1 | 63 | 145 | 2 | 80 | Lysozyme | Lysozyme | | afdb-uniprot50 | AF-A0A4V1RNB0-F1-MODEL\_V4 | 1.0 | 0.0007653 | 182 | 0.23 | 152 | 75 | 4 | 1 | 148 | 343 | 456 | Uncharacterized protein | Uncharacterized protein | | afdb-uniprot50 | AF-V6MKU5-F1-MODEL\_V4 | 1.0 | 0.0001332 | 162 | 0.286 | 164 | 80 | 4 | 15 | 145 | 13 | 172 | Lysozyme | Lysozyme | | afdb-uniprot50 | AF-A0A5M3PTT5-F1-MODEL\_V4 | 1.0 | 0.009234 | 160 | 0.337 | 83 | 50 | 2 | 64 | 146 | 2 | 79 | Lysozyme | Lysozyme | | afdb-uniprot50 | AF-A0A4Q3MGT4-F1-MODEL\_V4 | 1.0 | 0.009234 | 153 | 0.176 | 142 | 100 | 6 | 18 | 144 | 2 | 141 | Lysozyme | Lysozyme | | afdb-uniprot50 | AF-A0A6P0BZY4-F1-MODEL\_V4 | 1.0 | 0.004889 | 130 | 0.234 | 141 | 88 | 4 | 20 | 145 | 3 | 138 | Lysozyme | Lysozyme | | afdb-uniprot50 | AF-A0A418VWW6-F1-MODEL\_V4 | 1.0 | 0.0003457 | 114 | 0.252 | 174 | 109 | 6 | 1 | 159 | 2 | 169 | PG\_binding\_1 domain-containing protein | PG\_binding\_1 domain-containing protein | |
| Top keywords  (threshold 1.00e-02 (evalue)) | **Lysozyme, T4, Endolysin, receptor, chimera, hydrolase, Glycoside, type, Glucagon\_like, peptide** |
| Output files | ../../similar\_structures/64\_FANPEZAQ\_CDS\_0064\_afdb-proteome\_foldseek.tsv ../../similar\_structures/64\_FANPEZAQ\_CDS\_0064\_afdb-uniprot50\_foldseek.tsv ../../similar\_structures/64\_FANPEZAQ\_CDS\_0064\_merged.svg ../../similar\_structures/64\_FANPEZAQ\_CDS\_0064\_pdb\_foldseek.tsv |

  
  
  

Return to summary | Go to previous | Go to next

  


---

**Sequence/structure alignments coloring**  
Each object in the alignment figures is colored according to its E-value following this color coding:

1e-100
10

**References:**  
1) Steinegger M, Meier M, Mirdita M, Vöhringer H, Haunsberger S J, and Söding J (2019) HH-suite3 for fast remote homology detection and deep protein annotation, BMC Bioinformatics, 473. doi: 10.1186/s12859-019-3019-7  
2) Jumper J, Evans R, Pritzel A, ..., Hassabis D (2021) Highly accurate protein structure prediction with AlphaFold, Nature, 596. doi: 10.1038/s41586-021-03819-2  
3) van Kempen M, Kim S, Tumescheit C, Mirdita M, Lee J, Gilchrist CLM, Söding J, and Steinegger M (2023) Fast and accurate protein structure search with Foldseek. Nature Biotechnology. doi: 10.1038/s41587-023-01773-0
