# Supplementary material for: Completing the BASEL phage collection to unlock hidden diversity for systematic exploration of phage–host interactions
Source: PLoS Biol. 2025 Apr 7;23(4):e3003063. doi: 10.1371/journal.pbio.3003063 (PMC11990801; doi:10.1371/journal.pbio.3003063)
Supplement: S2 Data — (ZIP) [file pbio.3003063.s009.zip › entries/65.html]

FANPEZAQ\_CDS\_0065


Return to summary | Go to previous | Go to next

|  |  |
| --- | --- |
| FANPEZAQ\_CDS\_0065 Page creation date: 02 Sep 2024, 12:00  Project folder: n/a  Input sequences file: Escherichia\_virus\_HeidiAbel.gb | duf2514 lysis domain\_containing fragment lysozyme rz bacteriophage putative spanin endopeptidase prophage phage membrane rz\_like system i\_spanin assembly inner phage\_tail associated hypothetical lambda pspph06 gp55 rac |

### Sequence information

|  |  |
| --- | --- |
| Name | FANPEZAQ\_CDS\_0065  65\_FANPEZAQ\_CDS\_0065 (pipeline id) |
| Imported annotations | Escherichia\_virus\_HeidiAbel Bas97 |
| Protein sequence | MMDKYATPVKLIVAIIIAFALVAFGYVSGFSKSDREWQAKWSARDAADAQAHQQFTEQQR RIEQERQGAIDAIQEQAQQDIATAQRNAAIAAAESKRLQDGIADAITRLQADSGNPGATI SSKTRASNSSLLAELFREIDTAAGIYASEADRARAAGLTCERAYDAVRATSQQ |
| Number of residues | 173 |
| Molecular weight (Da) | 18737.58 |
| Output files | ../../query\_sequences/65\_FANPEZAQ\_CDS\_0065.fasta |

### Putative domain architecture and protein family

#### Search results (HHblits)1

|  |  |
| --- | --- |
| Domain family databases searched | Pfam, Ncbi-cd, Cath, Phrogs |
| Results, scheme(s)  (Top layers only; threshold 1.00e-03 (evalue)) | xml version="1.0" encoding="utf-8" standalone="no"?       2024-09-02T21:08:26.615985 image/svg+xml   Matplotlib v3.7.2, https://matplotlib.org/ |
| Results, table  (E-value ≤ 1.00e-03 (evalue)) | | db | id | prob | evalue | pvalue | score | cols | query | query\_len | template | template\_len | name | description | | --- | --- | --- | --- | --- | --- | --- | --- | --- | --- | --- | --- | --- | | pfam | PF10721 | 99.7 | 1e-21 | 1.9e-25 | 142.2 | 139 | (16, 168) | 173 | (8, 150) | 151 | DUF2514 | Protein of unknown function (DUF2514) | | pfam | PF03245 | 98.9 | 9.2e-14 | 1.8e-17 | 96.2 | 108 | (52, 167) | 173 | (15, 124) | 126 | Phage\_lysis | Bacteriophage Rz lysis protein | | phrogs | 312 | 100.0 | 2.2e-35 | 2.8e-39 | 229.2 | 145 | (19, 172) | 173 | (18, 166) | 170 | Rz-like spanin | Rz-like spanin; Category: lysis; NC\_027388\_p53 | | phrogs | 1327 | 99.8 | 7.2e-25 | 8.9e-29 | 170.6 | 163 | (2, 172) | 173 | (1, 182) | 188 | Rz-like spanin | Rz-like spanin; Category: lysis; KY000219\_p64 | | phrogs | 14926 | 98.9 | 1.2e-13 | 1.3e-17 | 91.1 | 81 | (93, 173) | 173 | (2, 84) | 89 | NA | NA; Category: unknown function; p303683 VI\_03517 | | phrogs | 5777 | 98.8 | 7e-13 | 8.3e-17 | 99.3 | 105 | (56, 171) | 173 | (55, 167) | 169 | NA | NA; Category: unknown function; p139219 VI\_05583 | | phrogs | 17598 | 98.0 | 2.3e-09 | 2.6e-13 | 68.0 | 63 | (16, 78) | 173 | (15, 77) | 77 | Rz-like spanin | Rz-like spanin; Category: lysis; p348110 VI\_03518 | | phrogs | 2466 | 97.6 | 3.7e-08 | 4.8e-12 | 73.4 | 140 | (21, 171) | 173 | (9, 162) | 163 | Rz-like spanin | Rz-like spanin; Category: lysis; NC\_011589\_p12 | | phrogs | 36833 | 96.7 | 3.8e-06 | 4.2e-10 | 53.0 | 43 | (127, 169) | 173 | (34, 76) | 80 | NA | NA; Category: unknown function; p236003 VI\_10093 | | phrogs | 1026 | 94.6 | 0.00055 | 6.9e-08 | 46.1 | 39 | (1, 39) | 173 | (1, 40) | 101 | Rz-like spanin | Rz-like spanin; Category: lysis; MG897800\_p45 | |
| Top keywords  (threshold 1.00e-03 (evalue)) | **lysis, Rz\_like, spanin, DUF2514, Bacteriophage, Rz, NC\_027388\_p53, KY000219\_p64, p303683, VI\_03517** |
| Output files | ../../domain\_architecture/65\_FANPEZAQ\_CDS\_0065\_cath.hhr ../../domain\_architecture/65\_FANPEZAQ\_CDS\_0065\_merged.svg ../../domain\_architecture/65\_FANPEZAQ\_CDS\_0065\_ncbi-cd.hhr ../../domain\_architecture/65\_FANPEZAQ\_CDS\_0065\_pfam.hhr ../../domain\_architecture/65\_FANPEZAQ\_CDS\_0065\_phrogs.hhr |

### Identical protein sequences/structures

#### Search results

|  |  |
| --- | --- |
| Protein sequence databases searched | Pdb, Swissprot, Refseq |
| Identical proteins found | -- |
| Top keywords | -- |
| Output files | -- |

### Similar protein sequences/structures

#### Sequence similarity search results (HHblits)1

|  |  |
| --- | --- |
| Sequence databases searched | Uniclust, Pdb70 |
| Results, scheme(s)  (Top layers only, threshold 1.00e-03 (evalue)) | xml version="1.0" encoding="utf-8" standalone="no"?       2024-09-02T21:08:55.722460 image/svg+xml   Matplotlib v3.7.2, https://matplotlib.org/ |
| Results, table(s)  (threshold 1.00e-03 (evalue)) | | db | id | prob | evalue | pvalue | score | cols | query | query\_len | template | template\_len | name | description | | --- | --- | --- | --- | --- | --- | --- | --- | --- | --- | --- | --- | --- | | uniclust | UniRef100\_A0A077KH21 | 100.0 | 3.4e-36 | 7.9e-42 | 236.0 | 172 | (1, 172) | 173 | (12, 183) | 204 | DUF2514 domain-containing protein | DUF2514 domain-containing protein | | uniclust | UniRef100\_A0A076LNF4 | 100.0 | 2e-35 | 4.4e-41 | 225.7 | 165 | (7, 171) | 173 | (15, 183) | 193 | Phage-tail assembly proteins like protein | Phage-tail assembly proteins like protein | | uniclust | UniRef100\_A0A059VF51 | 100.0 | 5.1e-35 | 1.1e-40 | 223.2 | 171 | (1, 171) | 173 | (4, 175) | 202 | DUF2514 domain-containing protein | DUF2514 domain-containing protein | | uniclust | UniRef100\_A0A061KWJ5 | 100.0 | 6.3e-34 | 1.3e-39 | 219.6 | 167 | (2, 172) | 173 | (21, 189) | 212 | DUF2514 domain-containing protein | DUF2514 domain-containing protein | | uniclust | UniRef100\_A0A063BGS6 | 99.9 | 6.2e-31 | 1.3e-36 | 200.5 | 159 | (1, 171) | 173 | (19, 177) | 198 | DUF2514 domain-containing protein | DUF2514 domain-containing protein | | uniclust | UniRef100\_A0A072TDR1 | 99.9 | 3.4e-30 | 6.6e-36 | 190.9 | 165 | (8, 172) | 173 | (9, 176) | 181 | DUF2514 family protein (Fragment) | DUF2514 family protein (Fragment) | | uniclust | UniRef100\_A0A022G2T6 | 99.9 | 7.7e-29 | 1.6e-34 | 197.0 | 147 | (19, 172) | 173 | (71, 217) | 251 | DUF2514 domain-containing protein | DUF2514 domain-containing protein | | uniclust | UniRef100\_A0A061JLI3 | 99.9 | 4.3e-28 | 8.6e-34 | 180.3 | 166 | (2, 171) | 173 | (4, 169) | 173 | Endopeptidase | Endopeptidase | | uniclust | UniRef100\_A0A1B7HMC9 | 99.9 | 5.8e-27 | 1.2e-32 | 176.8 | 150 | (22, 171) | 173 | (31, 182) | 185 | Putative phage-tail assembly protein | Putative phage-tail assembly protein | | uniclust | UniRef100\_A0A2R7NV60 | 99.9 | 9e-27 | 1.9e-32 | 178.1 | 168 | (5, 172) | 173 | (10, 178) | 185 | DUF2514 domain-containing protein | DUF2514 domain-containing protein | | uniclust | UniRef100\_A0A077FAV2 | 99.9 | 2.6e-26 | 5.6e-32 | 181.6 | 144 | (28, 172) | 173 | (64, 207) | 227 | DUF2514 family protein | DUF2514 family protein | | uniclust | UniRef100\_A0A1G6P3N9 | 99.8 | 2.4e-24 | 4.9e-30 | 165.3 | 124 | (47, 172) | 173 | (63, 186) | 192 | DUF2514 family protein | DUF2514 family protein | | uniclust | UniRef100\_A0A0A7KTN9 | 99.8 | 5e-23 | 9.1e-29 | 165.0 | 147 | (24, 170) | 173 | (235, 385) | 391 | Lysozyme | Lysozyme | | uniclust | UniRef100\_A0A5N8AHL5 | 99.8 | 3.3e-22 | 6.7e-28 | 152.5 | 168 | (5, 172) | 173 | (10, 186) | 196 | DUF2514 family protein | DUF2514 family protein | | uniclust | UniRef100\_A0A3M3B9T7 | 99.8 | 5.3e-22 | 9.8e-28 | 150.7 | 157 | (12, 168) | 173 | (85, 241) | 242 | Prophage PssSM-03, Orf7 | Prophage PssSM-03, Orf7 | | uniclust | UniRef100\_A0A126Z7Q2 | 99.8 | 9.7e-22 | 1.9e-27 | 147.3 | 171 | (1, 171) | 173 | (7, 181) | 182 | DUF2514 domain-containing protein | DUF2514 domain-containing protein | | uniclust | UniRef100\_A0A3S0EZ37 | 99.7 | 3.4e-21 | 7.3e-27 | 151.7 | 167 | (4, 171) | 173 | (21, 189) | 206 | DUF2514 family protein | DUF2514 family protein | | uniclust | UniRef100\_A0A1G8MFE6 | 99.7 | 3e-20 | 6e-26 | 140.8 | 159 | (7, 171) | 173 | (10, 168) | 187 | DUF2514 domain-containing protein | DUF2514 domain-containing protein | | uniclust | UniRef100\_A0A6J5L3W2 | 99.7 | 4.3e-20 | 9e-26 | 143.1 | 154 | (1, 172) | 173 | (1, 157) | 190 | Spanin, inner membrane subunit | Spanin, inner membrane subunit | | uniclust | UniRef100\_UPI000F0145F3 | 99.7 | 5.9e-20 | 1.1e-25 | 134.3 | 169 | (2, 170) | 173 | (1, 169) | 171 | DUF2514 family protein | DUF2514 family protein | | uniclust | UniRef100\_A0A072T468 | 99.7 | 1.1e-19 | 2.2e-25 | 142.2 | 121 | (51, 171) | 173 | (68, 188) | 216 | DUF2514 family protein | DUF2514 family protein | | uniclust | UniRef100\_A0A1X0YIG9 | 99.7 | 1.4e-19 | 2.7e-25 | 134.9 | 160 | (4, 167) | 173 | (7, 168) | 168 | DUF2514 domain-containing protein (Fragment) | DUF2514 domain-containing protein (Fragment) | | uniclust | UniRef100\_A0A2P1VUX7 | 99.7 | 1.7e-19 | 3.1e-25 | 135.3 | 167 | (3, 169) | 173 | (10, 186) | 203 | DUF2514 domain-containing protein | DUF2514 domain-containing protein | | uniclust | UniRef100\_A0A6N3TCN9 | 99.7 | 1.8e-19 | 3.2e-25 | 136.2 | 143 | (23, 168) | 173 | (81, 223) | 224 | DUF2514 domain-containing protein | DUF2514 domain-containing protein | | uniclust | UniRef100\_A0A0P9JKE3 | 99.6 | 2.2e-19 | 4.4e-25 | 132.1 | 123 | (48, 172) | 173 | (12, 134) | 138 | DUF2514 domain-containing protein (Fragment) | DUF2514 domain-containing protein (Fragment) | | uniclust | UniRef100\_A0A0J6KI14 | 99.6 | 4.1e-19 | 8.6e-25 | 138.0 | 163 | (1, 172) | 173 | (14, 177) | 189 | Lysozyme | Lysozyme | | uniclust | UniRef100\_A0A2P0N9U2 | 99.6 | 1.4e-18 | 2.8e-24 | 130.6 | 169 | (1, 171) | 173 | (1, 169) | 170 | Endopeptidase | Endopeptidase | | uniclust | UniRef100\_A0A0C1YJT8 | 99.6 | 2.2e-18 | 4.6e-24 | 131.5 | 155 | (4, 166) | 173 | (8, 162) | 165 | Lysozyme | Lysozyme | | uniclust | UniRef100\_A0A0B5CPB3 | 99.6 | 6.3e-18 | 1.3e-23 | 128.1 | 158 | (1, 163) | 173 | (1, 158) | 168 | Phage associated membrane protein | Phage associated membrane protein | | uniclust | UniRef100\_A0A1T1ANQ7 | 99.6 | 6.7e-18 | 1.4e-23 | 129.0 | 162 | (2, 172) | 173 | (1, 162) | 169 | Lysis protein | Lysis protein | | uniclust | UniRef100\_C5CJR8 | 99.5 | 1.1e-17 | 2.2e-23 | 126.2 | 138 | (33, 171) | 173 | (37, 174) | 179 | DUF2514 family protein | DUF2514 family protein | | uniclust | UniRef100\_Q2T6D5 | 99.5 | 1.2e-17 | 2.2e-23 | 126.5 | 158 | (2, 171) | 173 | (53, 210) | 212 | Gp23 | Gp23 | | uniclust | UniRef100\_A0A2A2JXU9 | 99.5 | 3.2e-17 | 5.9e-23 | 129.1 | 157 | (14, 170) | 173 | (138, 294) | 302 | lysozyme | lysozyme | | uniclust | UniRef100\_A0A072T277 | 99.5 | 7.2e-17 | 1.5e-22 | 126.9 | 151 | (7, 169) | 173 | (26, 183) | 205 | DUF2514 family protein | DUF2514 family protein | | uniclust | UniRef100\_A0A009ZWR4 | 99.5 | 7.7e-17 | 1.6e-22 | 127.2 | 159 | (4, 170) | 173 | (36, 199) | 204 | Uncharacterized protein | Uncharacterized protein | | uniclust | UniRef100\_A0A072T346 | 99.4 | 2.5e-16 | 5.2e-22 | 123.4 | 168 | (1, 170) | 173 | (5, 179) | 200 | DUF2514 family protein | DUF2514 family protein | | uniclust | UniRef100\_A0A076PZD4 | 99.4 | 3.9e-16 | 8.6e-22 | 123.9 | 123 | (43, 172) | 173 | (53, 177) | 201 | Lysozyme | Lysozyme | | uniclust | UniRef100\_UPI000D0449AE | 99.4 | 5.6e-16 | 1e-21 | 124.0 | 157 | (2, 158) | 173 | (5, 167) | 338 | DUF2514 family protein | DUF2514 family protein | | uniclust | UniRef100\_A0A0J5GKH4 | 99.4 | 8e-16 | 1.8e-21 | 124.5 | 143 | (20, 170) | 173 | (42, 188) | 225 | Lysozyme | Lysozyme | | uniclust | UniRef100\_A0A2T5HJ49 | 99.4 | 9.3e-16 | 1.8e-21 | 125.9 | 158 | (6, 170) | 173 | (188, 345) | 347 | Putative chitinase | Putative chitinase | | uniclust | UniRef100\_A0A031HSV0 | 99.4 | 1e-15 | 2e-21 | 115.8 | 166 | (1, 168) | 173 | (1, 169) | 186 | Uncharacterized protein | Uncharacterized protein | | uniclust | UniRef100\_A0A376THA0 | 99.3 | 3.3e-15 | 6.2e-21 | 111.7 | 154 | (20, 173) | 173 | (9, 164) | 181 | Putative prophage protein | Putative prophage protein | | uniclust | UniRef100\_UPI001FB7112E | 99.3 | 4.5e-15 | 8.2e-21 | 109.5 | 118 | (55, 172) | 173 | (54, 171) | 175 | DUF2514 domain-containing protein | DUF2514 domain-containing protein | | uniclust | UniRef100\_A0A096F537 | 99.3 | 4.1e-15 | 8.8e-21 | 115.1 | 128 | (32, 167) | 173 | (37, 164) | 175 | Endopeptidase | Endopeptidase | | uniclust | UniRef100\_A0A737M960 | 99.3 | 5.1e-15 | 9.7e-21 | 105.2 | 116 | (56, 171) | 173 | (4, 121) | 125 | DUF2514 family protein (Fragment) | DUF2514 family protein (Fragment) | | uniclust | UniRef100\_A0A0T9L9I2 | 99.3 | 5.2e-15 | 1.1e-20 | 117.0 | 160 | (3, 169) | 173 | (9, 176) | 195 | Bacteriophage lysis protein | Bacteriophage lysis protein | | uniclust | UniRef100\_A0A5E6S0Z6 | 99.3 | 9.2e-15 | 1.7e-20 | 105.2 | 127 | (12, 138) | 173 | (11, 139) | 139 | DUF2514 domain-containing protein | DUF2514 domain-containing protein | | uniclust | UniRef100\_A0A077LBX1 | 99.3 | 1.3e-14 | 2.8e-20 | 117.1 | 129 | (33, 169) | 173 | (61, 194) | 217 | Prophage PSPPH06, lysis protein | Prophage PSPPH06, lysis protein | | uniclust | UniRef100\_A0A0N0GNZ7 | 99.3 | 2.1e-14 | 4.1e-20 | 108.9 | 161 | (3, 170) | 173 | (5, 167) | 169 | Bacteriophage lysis protein | Bacteriophage lysis protein | | uniclust | UniRef100\_A0A0Q4GQ76 | 99.3 | 2.4e-14 | 4.5e-20 | 105.9 | 167 | (1, 168) | 173 | (1, 175) | 176 | DUF2514 domain-containing protein | DUF2514 domain-containing protein | | uniclust | UniRef100\_UPI000F06DD48 | 99.2 | 6.7e-14 | 1.2e-19 | 97.7 | 116 | (25, 140) | 173 | (1, 116) | 116 | DUF2514 family protein | DUF2514 family protein | | uniclust | UniRef100\_UPI00065FE3D1 | 99.2 | 1.3e-13 | 2.5e-19 | 100.9 | 143 | (15, 157) | 173 | (17, 161) | 161 | DUF2514 family protein | DUF2514 family protein | | uniclust | UniRef100\_A0A0E3T9S8 | 99.1 | 2.6e-13 | 5e-19 | 104.5 | 165 | (2, 171) | 173 | (32, 196) | 201 | Tail assembly protein | Tail assembly protein | | uniclust | UniRef100\_UPI00077A1791 | 99.1 | 3e-13 | 5.9e-19 | 99.2 | 105 | (64, 171) | 173 | (31, 135) | 141 | DUF2514 family protein | DUF2514 family protein | | uniclust | UniRef100\_A0A2I6PHU9 | 99.1 | 4.2e-13 | 8.1e-19 | 102.1 | 122 | (46, 170) | 173 | (44, 165) | 178 | DUF2514 family protein | DUF2514 family protein | | uniclust | UniRef100\_UPI0021A505FF | 99.1 | 4.9e-13 | 9e-19 | 96.4 | 98 | (72, 170) | 173 | (32, 129) | 143 | DUF2514 domain-containing protein | DUF2514 domain-containing protein | | uniclust | UniRef100\_A0A447M6P4 | 99.1 | 5.1e-13 | 1e-18 | 97.9 | 117 | (56, 172) | 173 | (6, 124) | 136 | Protein gp55 | Protein gp55 | | uniclust | UniRef100\_A0A447N8Z3 | 99.1 | 6.1e-13 | 1.1e-18 | 97.9 | 121 | (52, 172) | 173 | (18, 140) | 151 | Protein gp55 | Protein gp55 | | uniclust | UniRef100\_A0A059KU98 | 99.0 | 1.3e-12 | 2.6e-18 | 100.1 | 119 | (51, 171) | 173 | (70, 188) | 190 | Endopeptidase | Endopeptidase | | uniclust | UniRef100\_UPI001F13E5F8 | 99.0 | 1.7e-12 | 3.1e-18 | 99.4 | 122 | (46, 169) | 173 | (92, 213) | 215 | DUF2514 family protein | DUF2514 family protein | | uniclust | UniRef100\_A0A031GZS8 | 99.0 | 1.5e-12 | 3.2e-18 | 105.8 | 114 | (51, 171) | 173 | (97, 215) | 230 | Bacteriophage lysis protein | Bacteriophage lysis protein | | uniclust | UniRef100\_A0A418X1B9 | 99.0 | 2.1e-12 | 4.1e-18 | 98.4 | 161 | (2, 172) | 173 | (1, 163) | 172 | Lysis protein | Lysis protein | | uniclust | UniRef100\_A0A6J5N0Y4 | 99.0 | 2.3e-12 | 4.6e-18 | 97.9 | 154 | (1, 166) | 173 | (3, 157) | 158 | Uncharacterized protein | Uncharacterized protein | | uniclust | UniRef100\_UPI001E623135 | 99.0 | 3.1e-12 | 5.7e-18 | 89.8 | 111 | (58, 168) | 173 | (6, 118) | 119 | DUF2514 family protein | DUF2514 family protein | | uniclust | UniRef100\_A0A612N5Q1 | 99.0 | 4.3e-12 | 8.1e-18 | 90.3 | 107 | (4, 110) | 173 | (7, 117) | 125 | DUF2514 family protein (Fragment) | DUF2514 family protein (Fragment) | | uniclust | UniRef100\_A0A3N7DP53 | 98.9 | 8.8e-12 | 1.6e-17 | 97.4 | 107 | (61, 169) | 173 | (141, 247) | 248 | DUF2514 domain-containing protein | DUF2514 domain-containing protein | | uniclust | UniRef100\_A0A0E3GMK9 | 98.9 | 9.8e-12 | 1.9e-17 | 95.0 | 109 | (60, 171) | 173 | (62, 170) | 180 | Lysin | Lysin | | uniclust | UniRef100\_A0A7G9RJE6 | 98.9 | 1.1e-11 | 2e-17 | 91.8 | 95 | (76, 171) | 173 | (48, 142) | 165 | DUF2514 family protein | DUF2514 family protein | | uniclust | UniRef100\_UPI000A6BEC42 | 98.9 | 1.4e-11 | 2.6e-17 | 89.3 | 116 | (55, 171) | 173 | (19, 134) | 144 | DUF2514 family protein | DUF2514 family protein | | uniclust | UniRef100\_UPI000209A3A1 | 98.9 | 1.5e-11 | 2.9e-17 | 85.1 | 94 | (8, 101) | 173 | (5, 101) | 103 | DUF2514 family protein | DUF2514 family protein | | uniclust | UniRef100\_UPI0009BD088E | 98.8 | 2.6e-11 | 4.7e-17 | 85.4 | 103 | (9, 111) | 173 | (9, 111) | 118 | DUF2514 domain-containing protein | DUF2514 domain-containing protein | | uniclust | UniRef100\_A0A736RDN2 | 98.8 | 4.6e-11 | 8.6e-17 | 86.5 | 105 | (2, 110) | 173 | (5, 109) | 130 | DUF2514 family protein (Fragment) | DUF2514 family protein (Fragment) | | uniclust | UniRef100\_UPI000F846E2F | 98.8 | 4.6e-11 | 8.8e-17 | 78.6 | 57 | (116, 172) | 173 | (13, 69) | 71 | DUF2514 family protein | DUF2514 family protein | | uniclust | UniRef100\_A0A0H3LPM7 | 98.8 | 5.5e-11 | 1.2e-16 | 93.3 | 149 | (1, 169) | 173 | (10, 162) | 176 | Uncharacterized protein | Uncharacterized protein | | uniclust | UniRef100\_UPI001BA9F773 | 98.8 | 6.4e-11 | 1.2e-16 | 84.8 | 106 | (54, 164) | 173 | (23, 128) | 130 | DUF2514 family protein | DUF2514 family protein | | uniclust | UniRef100\_A0A7Z3BLE0 | 98.8 | 6.9e-11 | 1.3e-16 | 82.8 | 90 | (2, 91) | 173 | (1, 90) | 108 | DUF2514 family protein | DUF2514 family protein | | uniclust | UniRef100\_A0A329HWK1 | 98.7 | 9.5e-11 | 2e-16 | 93.3 | 113 | (51, 170) | 173 | (64, 183) | 197 | Lysis protein | Lysis protein | | uniclust | UniRef100\_UPI001CF29D96 | 98.7 | 1.6e-10 | 2.8e-16 | 80.3 | 100 | (72, 171) | 173 | (3, 104) | 106 | DUF2514 family protein | DUF2514 family protein | | uniclust | UniRef100\_A0A965JDW4 | 98.7 | 1.6e-10 | 3e-16 | 87.0 | 158 | (2, 168) | 173 | (1, 161) | 164 | DUF2514 family protein | DUF2514 family protein | | uniclust | UniRef100\_A0KJY0 | 98.7 | 2.1e-10 | 3.9e-16 | 88.9 | 87 | (19, 105) | 173 | (81, 171) | 223 | Uncharacterized protein | Uncharacterized protein | | uniclust | UniRef100\_UPI001C594AF0 | 98.7 | 2.2e-10 | 4e-16 | 85.8 | 160 | (4, 170) | 173 | (7, 166) | 168 | DUF2514 family protein | DUF2514 family protein | | uniclust | UniRef100\_UPI00033CB928 | 98.6 | 3.1e-10 | 5.7e-16 | 82.7 | 90 | (4, 93) | 173 | (7, 96) | 143 | DUF2514 family protein | DUF2514 family protein | | uniclust | UniRef100\_A0A431IGX4 | 98.6 | 3.1e-10 | 6.2e-16 | 88.1 | 145 | (17, 171) | 173 | (21, 165) | 171 | DUF2514 family protein | DUF2514 family protein | | uniclust | UniRef100\_A0A0P0HS49 | 98.6 | 3.4e-10 | 6.4e-16 | 85.7 | 105 | (63, 170) | 173 | (57, 161) | 171 | Endopeptidase Rz | Endopeptidase Rz | | uniclust | UniRef100\_A0A379W8I7 | 98.6 | 3.6e-10 | 6.9e-16 | 81.2 | 104 | (3, 110) | 173 | (6, 109) | 119 | Exported phage protein | Exported phage protein | | uniclust | UniRef100\_A0A9E6U0C0 | 98.6 | 4.5e-10 | 8.2e-16 | 78.1 | 74 | (95, 168) | 173 | (32, 105) | 106 | DUF2514 domain-containing protein | DUF2514 domain-containing protein | | uniclust | UniRef100\_UPI000EA8E18C | 98.6 | 4.6e-10 | 8.4e-16 | 81.3 | 117 | (52, 169) | 173 | (19, 135) | 136 | DUF2514 family protein | DUF2514 family protein | | uniclust | UniRef100\_A0A0H5AUG6 | 98.6 | 4.6e-10 | 9.8e-16 | 89.7 | 138 | (26, 170) | 173 | (26, 175) | 188 | Lysozyme | Lysozyme | | uniclust | UniRef100\_UPI00027B3473 | 98.6 | 6e-10 | 1.1e-15 | 75.2 | 72 | (95, 170) | 173 | (16, 87) | 89 | DUF2514 family protein | DUF2514 family protein | | uniclust | UniRef100\_A0A010SYQ9 | 98.6 | 6.1e-10 | 1.3e-15 | 91.5 | 140 | (25, 171) | 173 | (45, 198) | 229 | Lysozyme | Lysozyme | | uniclust | UniRef100\_A0A5A9ZEY6 | 98.5 | 7.7e-10 | 1.5e-15 | 84.5 | 148 | (4, 163) | 173 | (5, 153) | 157 | DUF2514 family protein | DUF2514 family protein | | uniclust | UniRef100\_F0FWP2 | 98.5 | 1.3e-09 | 2.3e-15 | 72.1 | 54 | (119, 172) | 173 | (14, 67) | 75 | DUF2514 family protein (Fragment) | DUF2514 family protein (Fragment) | | uniclust | UniRef100\_A0A3B9Q8I9 | 98.5 | 1.2e-09 | 2.5e-15 | 84.2 | 145 | (5, 153) | 173 | (3, 149) | 159 | DUF2514 domain-containing protein | DUF2514 domain-containing protein | | uniclust | UniRef100\_A0A7H1MBE5 | 98.5 | 1.7e-09 | 3.3e-15 | 83.3 | 157 | (1, 163) | 173 | (1, 167) | 174 | Uncharacterized protein | Uncharacterized protein | | uniclust | UniRef100\_A0A935YVG0 | 98.4 | 2.5e-09 | 4.5e-15 | 80.0 | 99 | (65, 166) | 173 | (66, 164) | 165 | DUF2514 family protein | DUF2514 family protein | | uniclust | UniRef100\_UPI001F0836F7 | 98.4 | 2.6e-09 | 4.7e-15 | 71.5 | 78 | (1, 78) | 173 | (1, 82) | 83 | DUF2514 family protein | DUF2514 family protein | | uniclust | UniRef100\_A0A3S0F432 | 98.4 | 2.7e-09 | 4.9e-15 | 81.7 | 115 | (46, 168) | 173 | (79, 193) | 195 | DUF2514 family protein | DUF2514 family protein | | uniclust | UniRef100\_UPI001454EF55 | 98.4 | 3.2e-09 | 5.9e-15 | 75.1 | 71 | (21, 91) | 173 | (25, 99) | 110 | DUF2514 family protein | DUF2514 family protein | | uniclust | UniRef100\_A0A965UN57 | 98.4 | 3.2e-09 | 6.1e-15 | 79.6 | 157 | (1, 173) | 173 | (1, 157) | 158 | Uncharacterized protein | Uncharacterized protein | | uniclust | UniRef100\_A0A377XDG9 | 98.4 | 4.5e-09 | 8.2e-15 | 83.7 | 73 | (93, 168) | 173 | (187, 259) | 260 | Putative glycoside hydrolase | Putative glycoside hydrolase | | uniclust | UniRef100\_A0A5B0BND6 | 98.3 | 5.6e-09 | 1.1e-14 | 62.9 | 41 | (130, 170) | 173 | (2, 42) | 43 | DUF2514 family protein | DUF2514 family protein | | uniclust | UniRef100\_A0A3S4K6X8 | 98.3 | 5.8e-09 | 1.1e-14 | 74.6 | 78 | (96, 173) | 173 | (21, 100) | 107 | Putative prophage protein | Putative prophage protein | | uniclust | UniRef100\_A0A085JGU0 | 98.3 | 5.5e-09 | 1.2e-14 | 83.9 | 116 | (47, 170) | 173 | (53, 170) | 183 | Rz family phage lysis protein | Rz family phage lysis protein | | uniclust | UniRef100\_A0A540WHN8 | 98.3 | 6.5e-09 | 1.2e-14 | 74.0 | 96 | (74, 169) | 173 | (8, 108) | 118 | DUF2514 family protein (Fragment) | DUF2514 family protein (Fragment) | | uniclust | UniRef100\_UPI002113A69C | 98.3 | 8.2e-09 | 1.5e-14 | 71.4 | 85 | (84, 168) | 173 | (13, 98) | 99 | DUF2514 family protein | DUF2514 family protein | | uniclust | UniRef100\_A0A5T8J8N9 | 98.3 | 1.1e-08 | 2.1e-14 | 69.5 | 74 | (3, 76) | 173 | (6, 83) | 85 | DUF2514 domain-containing protein (Fragment) | DUF2514 domain-containing protein (Fragment) | | uniclust | UniRef100\_A0A433SCY5 | 98.3 | 1.1e-08 | 2.2e-14 | 78.8 | 152 | (2, 167) | 173 | (1, 159) | 168 | Uncharacterized protein | Uncharacterized protein | | uniclust | UniRef100\_A0A1E8PNC0 | 98.3 | 1.2e-08 | 2.2e-14 | 79.0 | 157 | (6, 170) | 173 | (11, 172) | 178 | Bacteriophage lysis protein | Bacteriophage lysis protein | | uniclust | UniRef100\_A0A6J5KTC4 | 98.3 | 1.3e-08 | 2.4e-14 | 75.4 | 112 | (20, 168) | 173 | (18, 129) | 130 | Spanin, inner membrane subunit | Spanin, inner membrane subunit | | uniclust | UniRef100\_UPI00051BC885 | 98.3 | 1.4e-08 | 2.5e-14 | 72.9 | 86 | (25, 110) | 173 | (34, 119) | 122 | DUF2514 family protein | DUF2514 family protein | | uniclust | UniRef100\_A0A6G6IUM5 | 98.2 | 1.6e-08 | 3e-14 | 70.6 | 77 | (95, 171) | 173 | (21, 99) | 101 | DUF2514 family protein | DUF2514 family protein | | uniclust | UniRef100\_A0A1E8CG11 | 98.2 | 1.6e-08 | 3.3e-14 | 80.0 | 153 | (8, 168) | 173 | (13, 173) | 179 | Lysis protein | Lysis protein | | uniclust | UniRef100\_UPI001C0D8754 | 98.2 | 2e-08 | 3.7e-14 | 74.7 | 144 | (9, 162) | 173 | (5, 148) | 155 | DUF2514 family protein | DUF2514 family protein | | uniclust | UniRef100\_UPI000E69ED77 | 98.2 | 2.1e-08 | 3.8e-14 | 66.4 | 59 | (113, 171) | 173 | (7, 65) | 76 | DUF2514 family protein | DUF2514 family protein | | uniclust | UniRef100\_A0A255ZJS3 | 98.2 | 2.4e-08 | 4.7e-14 | 76.8 | 154 | (1, 172) | 173 | (1, 154) | 163 | Lysis protein | Lysis protein | | uniclust | UniRef100\_UPI001E6220F1 | 98.2 | 3.2e-08 | 6e-14 | 67.7 | 56 | (113, 168) | 173 | (29, 84) | 87 | DUF2514 family protein | DUF2514 family protein | | uniclust | UniRef100\_A0A136Q9N2 | 98.1 | 3.3e-08 | 6.3e-14 | 70.8 | 101 | (6, 106) | 173 | (1, 101) | 109 | Uncharacterized protein (Fragment) | Uncharacterized protein (Fragment) | | uniclust | UniRef100\_A0A0T9Q0I4 | 98.1 | 4.1e-08 | 7.9e-14 | 76.6 | 147 | (15, 166) | 173 | (22, 172) | 185 | Protein of uncharacterized function (DUF2514) | Protein of uncharacterized function (DUF2514) | | uniclust | UniRef100\_A0A2W5L444 | 98.1 | 4.3e-08 | 8e-14 | 71.8 | 124 | (3, 136) | 173 | (4, 127) | 127 | DUF2514 domain-containing protein (Fragment) | DUF2514 domain-containing protein (Fragment) | | uniclust | UniRef100\_A0A3B8P7Q2 | 98.1 | 4.3e-08 | 8.1e-14 | 66.1 | 49 | (121, 169) | 173 | (28, 76) | 78 | DUF2514 domain-containing protein (Fragment) | DUF2514 domain-containing protein (Fragment) | | uniclust | UniRef100\_A0A6J5KHQ3 | 98.1 | 4.3e-08 | 8.4e-14 | 75.3 | 147 | (4, 165) | 173 | (11, 157) | 158 | Uncharacterized protein | Uncharacterized protein | | uniclust | UniRef100\_A0A3M2U176 | 98.1 | 5.1e-08 | 9.5e-14 | 65.4 | 69 | (8, 76) | 173 | (6, 77) | 78 | Uncharacterized protein (Fragment) | Uncharacterized protein (Fragment) | | uniclust | UniRef100\_A0A5R1YM75 | 98.1 | 5.4e-08 | 1e-13 | 65.0 | 56 | (117, 172) | 173 | (9, 64) | 76 | DUF2514 domain-containing protein (Fragment) | DUF2514 domain-containing protein (Fragment) | | uniclust | UniRef100\_A0A4Q5VW04 | 98.1 | 6e-08 | 1.1e-13 | 69.8 | 87 | (75, 167) | 173 | (36, 122) | 122 | DUF2514 family protein | DUF2514 family protein | | uniclust | UniRef100\_F0FWN7 | 98.1 | 7.6e-08 | 1.4e-13 | 68.1 | 101 | (2, 110) | 173 | (1, 101) | 109 | Putative bacteriophage protein (Fragment) | Putative bacteriophage protein (Fragment) | | uniclust | UniRef100\_A0A533I619 | 98.0 | 7.9e-08 | 1.5e-13 | 72.0 | 138 | (32, 169) | 173 | (20, 157) | 158 | DUF2514 family protein | DUF2514 family protein | | uniclust | UniRef100\_A0A6J5LN54 | 98.0 | 8.6e-08 | 1.6e-13 | 73.1 | 121 | (32, 169) | 173 | (29, 154) | 158 | Spanin, inner membrane subunit | Spanin, inner membrane subunit | | uniclust | UniRef100\_A0A7Z3BS56 | 98.0 | 1.2e-07 | 2.2e-13 | 62.3 | 58 | (114, 171) | 173 | (8, 65) | 68 | DUF2514 family protein | DUF2514 family protein | | uniclust | UniRef100\_A0A068Z3N6 | 98.0 | 1.1e-07 | 2.4e-13 | 76.3 | 114 | (50, 171) | 173 | (47, 161) | 173 | Lysis protein | Lysis protein | | uniclust | UniRef100\_A0A1W6JT24 | 98.0 | 1.3e-07 | 2.4e-13 | 68.7 | 96 | (10, 105) | 173 | (10, 105) | 128 | Uncharacterized protein | Uncharacterized protein | | uniclust | UniRef100\_A0A447M6N0 | 98.0 | 1.3e-07 | 2.5e-13 | 65.4 | 51 | (121, 171) | 173 | (35, 85) | 87 | Phage-tail assembly protein | Phage-tail assembly protein | | uniclust | UniRef100\_A0A136Q9T1 | 98.0 | 1.4e-07 | 2.6e-13 | 66.1 | 80 | (90, 170) | 173 | (6, 85) | 95 | DUF2514 family protein (Fragment) | DUF2514 family protein (Fragment) | | uniclust | UniRef100\_A0A0A8F3R3 | 98.0 | 1.5e-07 | 2.7e-13 | 73.5 | 159 | (2, 167) | 173 | (40, 202) | 208 | Phage associated membrane protein | Phage associated membrane protein | | uniclust | UniRef100\_UPI00111B1EBA | 98.0 | 1.7e-07 | 3.1e-13 | 67.3 | 85 | (26, 110) | 173 | (15, 99) | 113 | DUF2514 family protein | DUF2514 family protein | | uniclust | UniRef100\_A0A6J5KRF4 | 97.9 | 2e-07 | 3.6e-13 | 69.8 | 121 | (42, 168) | 173 | (32, 152) | 154 | Spanin, inner membrane subunit | Spanin, inner membrane subunit | | uniclust | UniRef100\_A0A7J5P9B2 | 97.9 | 2e-07 | 3.6e-13 | 61.5 | 70 | (96, 168) | 173 | (2, 71) | 72 | DUF2514 family protein | DUF2514 family protein | | uniclust | UniRef100\_UPI001AFE8EB9 | 97.9 | 2.5e-07 | 4.7e-13 | 72.2 | 138 | (29, 172) | 173 | (33, 181) | 185 | hypothetical protein | hypothetical protein | | uniclust | UniRef100\_A0A2W5FEN7 | 97.9 | 2.6e-07 | 4.7e-13 | 55.9 | 42 | (130, 171) | 173 | (1, 42) | 46 | DUF2514 domain-containing protein (Fragment) | DUF2514 domain-containing protein (Fragment) | | uniclust | UniRef100\_A0A0G9H235 | 97.8 | 4.1e-07 | 7.5e-13 | 65.9 | 104 | (62, 168) | 173 | (20, 123) | 123 | DUF2514 domain-containing protein (Fragment) | DUF2514 domain-containing protein (Fragment) | | uniclust | UniRef100\_A0A8T5ZKZ9 | 97.8 | 3.9e-07 | 7.8e-13 | 64.3 | 63 | (24, 86) | 173 | (13, 75) | 87 | DUF2514 family protein (Fragment) | DUF2514 family protein (Fragment) | | uniclust | UniRef100\_A0A2S0MB23 | 97.8 | 4.4e-07 | 8.7e-13 | 72.3 | 130 | (33, 164) | 173 | (37, 171) | 190 | DUF2514 domain-containing protein | DUF2514 domain-containing protein | | uniclust | UniRef100\_A0A2K9VHR6 | 97.8 | 4.8e-07 | 8.8e-13 | 68.7 | 159 | (6, 169) | 173 | (3, 166) | 167 | Endopeptidase | Endopeptidase | | uniclust | UniRef100\_A0A8I1RBJ4 | 97.8 | 5.8e-07 | 1.1e-12 | 68.3 | 132 | (29, 167) | 173 | (29, 160) | 167 | DUF2514 family protein | DUF2514 family protein | | uniclust | UniRef100\_A0A178MV11 | 97.8 | 5.6e-07 | 1.1e-12 | 70.2 | 154 | (5, 164) | 173 | (2, 159) | 180 | Uncharacterized protein | Uncharacterized protein | | uniclust | UniRef100\_A0A526VKI8 | 97.8 | 6.3e-07 | 1.2e-12 | 71.0 | 134 | (21, 162) | 173 | (23, 156) | 195 | DUF2514 family protein | DUF2514 family protein | | uniclust | UniRef100\_A0A068QVD3 | 97.8 | 5.6e-07 | 1.2e-12 | 75.3 | 76 | (28, 110) | 173 | (54, 136) | 230 | Putative endopeptidase | Putative endopeptidase | | uniclust | UniRef100\_A0A7H0HJ18 | 97.8 | 6.3e-07 | 1.2e-12 | 68.7 | 107 | (55, 169) | 173 | (27, 133) | 144 | Uncharacterized protein | Uncharacterized protein | | uniclust | UniRef100\_UPI001D0EDDCC | 97.8 | 7e-07 | 1.3e-12 | 59.8 | 50 | (120, 169) | 173 | (25, 74) | 77 | DUF2514 family protein | DUF2514 family protein | | uniclust | UniRef100\_A0A6N4T4E4 | 97.8 | 6.9e-07 | 1.3e-12 | 68.7 | 116 | (50, 172) | 173 | (45, 163) | 168 | Uncharacterized protein | Uncharacterized protein | | uniclust | UniRef100\_A0A061YD13 | 97.7 | 6.3e-07 | 1.4e-12 | 74.8 | 111 | (50, 168) | 173 | (76, 188) | 225 | Endopeptidase (Lysis protein) from bacteriophage origin | Endopeptidase (Lysis protein) from bacteriophage origin | | uniclust | UniRef100\_UPI00034B3AB5 | 97.7 | 7.5e-07 | 1.4e-12 | 70.5 | 102 | (2, 103) | 173 | (1, 106) | 197 | lysis system i-spanin subunit Rz | lysis system i-spanin subunit Rz | | uniclust | UniRef100\_A0A8S5NLS2 | 97.7 | 8.9e-07 | 1.7e-12 | 69.5 | 94 | (7, 104) | 173 | (5, 98) | 170 | Uncharacterized protein | Uncharacterized protein | | uniclust | UniRef100\_A0A6G6IV19 | 97.7 | 1.1e-06 | 2e-12 | 56.4 | 55 | (2, 56) | 173 | (1, 55) | 61 | Uncharacterized protein | Uncharacterized protein | | uniclust | UniRef100\_A0A0P9PJB1 | 97.7 | 1.1e-06 | 2.1e-12 | 53.9 | 44 | (130, 173) | 173 | (1, 44) | 47 | Uncharacterized protein | Uncharacterized protein | | uniclust | UniRef100\_UPI000A747C3A | 97.7 | 1.3e-06 | 2.4e-12 | 58.3 | 48 | (121, 168) | 173 | (9, 56) | 72 | DUF2514 family protein | DUF2514 family protein | | uniclust | UniRef100\_UPI0012FD249F | 97.6 | 1.5e-06 | 2.8e-12 | 57.8 | 70 | (36, 105) | 173 | (2, 71) | 73 | DUF2514 family protein | DUF2514 family protein | | uniclust | UniRef100\_A0A270P9R3 | 97.6 | 1.5e-06 | 2.8e-12 | 60.6 | 48 | (121, 168) | 173 | (48, 95) | 96 | DUF2514 domain-containing protein | DUF2514 domain-containing protein | | uniclust | UniRef100\_A0A9E0KB07 | 97.6 | 1.6e-06 | 2.9e-12 | 63.3 | 101 | (57, 165) | 173 | (10, 110) | 113 | Uncharacterized protein | Uncharacterized protein | | uniclust | UniRef100\_UPI001E39CCA4 | 97.6 | 1.6e-06 | 2.9e-12 | 58.3 | 51 | (119, 169) | 173 | (24, 74) | 76 | DUF2514 family protein | DUF2514 family protein | | uniclust | UniRef100\_A0A965PEI7 | 97.6 | 1.7e-06 | 3.2e-12 | 66.0 | 102 | (56, 168) | 173 | (47, 148) | 150 | Uncharacterized protein | Uncharacterized protein | | uniclust | UniRef100\_UPI0021147FA3 | 97.6 | 1.9e-06 | 3.5e-12 | 60.5 | 72 | (98, 169) | 173 | (24, 97) | 99 | DUF2514 family protein | DUF2514 family protein | | uniclust | UniRef100\_UPI001F43A9C7 | 97.6 | 1.9e-06 | 3.6e-12 | 59.6 | 56 | (115, 170) | 173 | (31, 86) | 87 | DUF2514 family protein | DUF2514 family protein | | uniclust | UniRef100\_A0A315BGF8 | 97.6 | 2e-06 | 3.7e-12 | 66.3 | 122 | (43, 172) | 173 | (48, 175) | 180 | Lysozyme | Lysozyme | | uniclust | UniRef100\_A0A0D0IWK6 | 97.6 | 1.9e-06 | 3.8e-12 | 70.9 | 132 | (25, 168) | 173 | (55, 190) | 213 | Lysozyme | Lysozyme | | uniclust | UniRef100\_A0A0W7YSA5 | 97.6 | 1.9e-06 | 3.8e-12 | 70.2 | 148 | (5, 164) | 173 | (24, 175) | 200 | Lysis protein | Lysis protein | | uniclust | UniRef100\_A0A6M9YZG7 | 97.6 | 2.1e-06 | 3.9e-12 | 65.7 | 133 | (35, 168) | 173 | (36, 169) | 170 | DUF2514 domain-containing protein | DUF2514 domain-containing protein | | uniclust | UniRef100\_A0A088F9X7 | 97.6 | 1.9e-06 | 4e-12 | 69.2 | 130 | (15, 163) | 173 | (30, 159) | 177 | Phage lambda Rz-like lysis protein | Phage lambda Rz-like lysis protein | | uniclust | UniRef100\_A0A2K4KXJ6 | 97.6 | 2.5e-06 | 4.6e-12 | 55.7 | 44 | (126, 169) | 173 | (22, 65) | 66 | DUF2514 domain-containing protein | DUF2514 domain-containing protein | | uniclust | UniRef100\_A0A918U7Z6 | 97.5 | 2.5e-06 | 4.9e-12 | 67.5 | 107 | (58, 170) | 173 | (61, 167) | 178 | Uncharacterized protein | Uncharacterized protein | | uniclust | UniRef100\_A0A031IWR0 | 97.5 | 2.7e-06 | 5.8e-12 | 70.1 | 118 | (44, 169) | 173 | (62, 181) | 200 | Prophage PSPPH06, lysis protein | Prophage PSPPH06, lysis protein | | uniclust | UniRef100\_A0A068R0F6 | 97.5 | 2.7e-06 | 6e-12 | 70.7 | 108 | (54, 170) | 173 | (86, 194) | 206 | Putative Rac prophage prophage lambda endopeptidase | Putative Rac prophage prophage lambda endopeptidase | | uniclust | UniRef100\_UPI001F2FD331 | 97.5 | 3.3e-06 | 6.1e-12 | 60.0 | 95 | (72, 169) | 173 | (8, 103) | 105 | DUF2514 family protein | DUF2514 family protein | | uniclust | UniRef100\_A0A7Z2JFM2 | 97.5 | 3.4e-06 | 6.3e-12 | 56.9 | 45 | (126, 170) | 173 | (29, 73) | 78 | DUF2514 family protein | DUF2514 family protein | | uniclust | UniRef100\_A0A5C8IZU8 | 97.5 | 3.5e-06 | 6.4e-12 | 64.9 | 155 | (2, 165) | 173 | (6, 174) | 176 | DUF2514 family protein | DUF2514 family protein | | uniclust | UniRef100\_A0A8S5QU50 | 97.5 | 3.4e-06 | 6.7e-12 | 66.1 | 138 | (5, 155) | 173 | (15, 152) | 163 | Uncharacterized protein | Uncharacterized protein | | uniclust | UniRef100\_UPI001D1909C2 | 97.5 | 3.7e-06 | 6.9e-12 | 59.3 | 51 | (120, 170) | 173 | (48, 98) | 100 | DUF2514 family protein | DUF2514 family protein | | uniclust | UniRef100\_A0A067Y1K9 | 97.5 | 3.6e-06 | 7.7e-12 | 68.3 | 74 | (26, 99) | 173 | (25, 98) | 180 | Rz/RzI spanin protein | Rz/RzI spanin protein | | uniclust | UniRef100\_A0A0B6S1Z9 | 97.4 | 4.9e-06 | 1e-11 | 70.2 | 115 | (49, 170) | 173 | (96, 218) | 245 | Putative bacteriophage lysis protein | Putative bacteriophage lysis protein | | uniclust | UniRef100\_A0A1H9E2Y4 | 97.4 | 5.8e-06 | 1.1e-11 | 62.7 | 90 | (77, 168) | 173 | (62, 151) | 157 | Bacteriophage Rz lysis protein | Bacteriophage Rz lysis protein | | uniclust | UniRef100\_A0A0S2F7F8 | 97.4 | 6.6e-06 | 1.4e-11 | 66.8 | 135 | (24, 165) | 173 | (28, 163) | 175 | Putative gp12 | Putative gp12 | | uniclust | UniRef100\_A0A0J6C284 | 97.3 | 9e-06 | 1.8e-11 | 64.5 | 130 | (16, 155) | 173 | (23, 152) | 174 | Uncharacterized protein | Uncharacterized protein | | uniclust | UniRef100\_UPI001116F226 | 97.3 | 1.1e-05 | 1.9e-11 | 55.9 | 74 | (4, 81) | 173 | (7, 80) | 88 | DUF2514 family protein | DUF2514 family protein | | uniclust | UniRef100\_UPI001E5BBD03 | 97.3 | 1.1e-05 | 2e-11 | 59.0 | 104 | (55, 166) | 173 | (8, 115) | 118 | lysis system i-spanin subunit Rz | lysis system i-spanin subunit Rz | | uniclust | UniRef100\_A0A3N8D9L6 | 97.3 | 1.1e-05 | 2e-11 | 55.7 | 81 | (58, 144) | 173 | (6, 86) | 87 | DUF2514 family protein | DUF2514 family protein | | uniclust | UniRef100\_A0A023KKM3 | 97.3 | 1.1e-05 | 2.3e-11 | 67.5 | 54 | (50, 110) | 173 | (68, 121) | 213 | Lysis protein | Lysis protein | | uniclust | UniRef100\_A0A0A1IVS7 | 97.3 | 1.2e-05 | 2.4e-11 | 64.3 | 157 | (2, 163) | 173 | (1, 163) | 180 | Uncharacterized protein | Uncharacterized protein | | uniclust | UniRef100\_A0A370FC04 | 97.3 | 1.4e-05 | 2.6e-11 | 62.1 | 151 | (14, 171) | 173 | (13, 167) | 181 | Bacteriophage Rz lysis protein | Bacteriophage Rz lysis protein | | uniclust | UniRef100\_UPI0009304A3F | 97.2 | 1.8e-05 | 3.3e-11 | 57.5 | 78 | (93, 170) | 173 | (32, 111) | 116 | DUF2514 family protein | DUF2514 family protein | | uniclust | UniRef100\_A0A2Z3IMC6 | 97.2 | 1.8e-05 | 3.4e-11 | 63.3 | 152 | (13, 169) | 173 | (54, 209) | 222 | Lysozyme | Lysozyme | | uniclust | UniRef100\_UPI00207BF91A | 97.2 | 2e-05 | 3.7e-11 | 49.2 | 45 | (129, 173) | 173 | (5, 49) | 50 | DUF2514 family protein | DUF2514 family protein | | uniclust | UniRef100\_A0A965PHN2 | 97.2 | 2.1e-05 | 3.8e-11 | 57.5 | 98 | (1, 103) | 173 | (1, 98) | 120 | Uncharacterized protein (Fragment) | Uncharacterized protein (Fragment) | | uniclust | UniRef100\_UPI00195B7822 | 97.2 | 2.2e-05 | 4e-11 | 55.3 | 91 | (54, 144) | 173 | (2, 94) | 96 | DUF2514 family protein | DUF2514 family protein | | uniclust | UniRef100\_A0A212IIM0 | 97.2 | 2.3e-05 | 4.3e-11 | 50.8 | 43 | (128, 170) | 173 | (4, 46) | 57 | DUF2514 domain-containing protein | DUF2514 domain-containing protein | | uniclust | UniRef100\_UPI0021AB3194 | 97.1 | 2.9e-05 | 5.3e-11 | 56.3 | 83 | (56, 144) | 173 | (30, 112) | 113 | DUF2514 family protein | DUF2514 family protein | | uniclust | UniRef100\_UPI001E3A12C5 | 97.1 | 2.9e-05 | 5.4e-11 | 54.0 | 67 | (34, 100) | 173 | (3, 70) | 82 | DUF2514 domain-containing protein | DUF2514 domain-containing protein | | uniclust | UniRef100\_A0A1I6GE33 | 97.1 | 3.1e-05 | 5.8e-11 | 59.3 | 102 | (58, 169) | 173 | (47, 148) | 153 | Bacteriophage Rz lysis protein | Bacteriophage Rz lysis protein | | uniclust | UniRef100\_A0A1G6NB34 | 97.1 | 2.9e-05 | 6e-11 | 62.9 | 137 | (13, 165) | 173 | (17, 160) | 172 | Bacteriophage Rz lysis protein | Bacteriophage Rz lysis protein | | uniclust | UniRef100\_A0A084YZU5 | 97.1 | 3.3e-05 | 6.6e-11 | 60.2 | 75 | (59, 140) | 173 | (56, 135) | 143 | Phage lysis protein (Fragment) | Phage lysis protein (Fragment) | | uniclust | UniRef100\_A0A0N9ER84 | 97.1 | 3.7e-05 | 7e-11 | 60.8 | 36 | (131, 166) | 173 | (140, 175) | 189 | Putative Rz lysis protein | Putative Rz lysis protein | | uniclust | UniRef100\_A0A1H8TC25 | 97.1 | 3.6e-05 | 7.2e-11 | 64.0 | 159 | (5, 167) | 173 | (7, 169) | 214 | Bacteriophage Rz lysis protein | Bacteriophage Rz lysis protein | | uniclust | UniRef100\_UPI001F11D6A6 | 97.1 | 4.1e-05 | 7.5e-11 | 50.3 | 48 | (122, 169) | 173 | (5, 52) | 64 | DUF2514 family protein | DUF2514 family protein | | uniclust | UniRef100\_A0A0Q6W2Z3 | 97.0 | 4.3e-05 | 7.9e-11 | 59.1 | 100 | (69, 169) | 173 | (70, 169) | 170 | Lysozyme | Lysozyme | | uniclust | UniRef100\_A0A965RZC8 | 97.0 | 5e-05 | 9.2e-11 | 55.7 | 87 | (17, 110) | 173 | (11, 97) | 120 | DUF2514 family protein (Fragment) | DUF2514 family protein (Fragment) | | uniclust | UniRef100\_A0A3S4G381 | 97.0 | 5.3e-05 | 1e-10 | 60.5 | 60 | (20, 79) | 173 | (29, 88) | 184 | Phage-tail assembly protein | Phage-tail assembly protein | | uniclust | UniRef100\_A0A137YJG3 | 97.0 | 6.3e-05 | 1.2e-10 | 59.6 | 106 | (58, 171) | 173 | (74, 179) | 182 | DUF2514 family protein | DUF2514 family protein | | uniclust | UniRef100\_UPI00057A4F47 | 96.9 | 7.2e-05 | 1.3e-10 | 52.4 | 67 | (3, 69) | 173 | (10, 84) | 85 | hypothetical protein | hypothetical protein | | uniclust | UniRef100\_UPI0020C8520B | 96.9 | 7.5e-05 | 1.4e-10 | 53.3 | 59 | (114, 172) | 173 | (27, 85) | 100 | DUF2514 family protein | DUF2514 family protein | | uniclust | UniRef100\_UPI001E381099 | 96.9 | 8.1e-05 | 1.5e-10 | 52.8 | 72 | (39, 110) | 173 | (6, 77) | 97 | DUF2514 family protein | DUF2514 family protein | | uniclust | UniRef100\_A0A9E0Q5U7 | 96.9 | 8.2e-05 | 1.5e-10 | 58.9 | 119 | (45, 170) | 173 | (58, 177) | 191 | Uncharacterized protein | Uncharacterized protein | | uniclust | UniRef100\_A0A3A1YQA2 | 96.9 | 9e-05 | 1.6e-10 | 52.7 | 90 | (47, 138) | 173 | (7, 96) | 98 | Uncharacterized protein | Uncharacterized protein | | uniclust | UniRef100\_UPI0021F33334 | 96.9 | 9.5e-05 | 1.7e-10 | 49.9 | 41 | (130, 170) | 173 | (1, 41) | 73 | DUF2514 domain-containing protein | DUF2514 domain-containing protein | | uniclust | UniRef100\_A0A198GPX5 | 96.9 | 8.4e-05 | 1.7e-10 | 60.9 | 106 | (55, 168) | 173 | (55, 162) | 181 | Endopeptidase | Endopeptidase | | uniclust | UniRef100\_UPI0021BECF36 | 96.9 | 0.00011 | 1.9e-10 | 47.5 | 34 | (138, 171) | 173 | (12, 45) | 57 | DUF2514 domain-containing protein | DUF2514 domain-containing protein | | uniclust | UniRef100\_A0A0E3BUQ5 | 96.9 | 9.3e-05 | 2e-10 | 62.8 | 99 | (1, 106) | 173 | (1, 109) | 223 | Lysis protein | Lysis protein | | uniclust | UniRef100\_A0A6J5NGR5 | 96.8 | 0.00011 | 2.1e-10 | 56.6 | 153 | (5, 166) | 173 | (1, 160) | 163 | Spanin, inner membrane subunit | Spanin, inner membrane subunit | | uniclust | UniRef100\_UPI001F37191F | 96.8 | 0.00013 | 2.4e-10 | 49.2 | 37 | (134, 170) | 173 | (34, 70) | 72 | DUF2514 family protein | DUF2514 family protein | | uniclust | UniRef100\_A0A2S1GSJ3 | 96.8 | 0.00014 | 2.8e-10 | 58.9 | 136 | (13, 159) | 173 | (17, 156) | 176 | Rz lysis protein | Rz lysis protein | | uniclust | UniRef100\_UPI00037C877F | 96.8 | 0.00016 | 2.9e-10 | 56.3 | 147 | (15, 169) | 173 | (11, 161) | 170 | lysis system i-spanin subunit Rz | lysis system i-spanin subunit Rz | | uniclust | UniRef100\_A0A4Q3MIQ8 | 96.7 | 0.00017 | 3.1e-10 | 54.9 | 99 | (55, 171) | 173 | (36, 134) | 136 | Uncharacterized protein | Uncharacterized protein | | uniclust | UniRef100\_UPI0020A19267 | 96.7 | 0.00018 | 3.3e-10 | 48.0 | 48 | (123, 170) | 173 | (14, 61) | 67 | DUF2514 family protein | DUF2514 family protein | | uniclust | UniRef100\_UPI001377749F | 96.7 | 0.00018 | 3.3e-10 | 46.3 | 50 | (23, 72) | 173 | (2, 51) | 56 | DUF2514 family protein | DUF2514 family protein | | uniclust | UniRef100\_A0A0P9R5I7 | 96.7 | 0.00018 | 3.4e-10 | 53.4 | 41 | (131, 171) | 173 | (1, 41) | 125 | Uncharacterized protein | Uncharacterized protein | | uniclust | UniRef100\_A0A0C1HF82 | 96.7 | 0.00016 | 3.5e-10 | 57.7 | 67 | (12, 85) | 173 | (18, 84) | 144 | Phage associated protein | Phage associated protein | | uniclust | UniRef100\_A0A4Q7NCI6 | 96.7 | 0.00019 | 3.5e-10 | 56.2 | 110 | (57, 168) | 173 | (63, 176) | 177 | Uncharacterized protein DUF2514 | Uncharacterized protein DUF2514 | | uniclust | UniRef100\_UPI0022208F3E | 96.7 | 0.00021 | 3.8e-10 | 61.1 | 102 | (62, 170) | 173 | (238, 342) | 348 | lysis system i-spanin subunit Rz | lysis system i-spanin subunit Rz | | uniclust | UniRef100\_A0A0A1H6K0 | 96.7 | 0.00019 | 3.8e-10 | 59.8 | 73 | (8, 84) | 173 | (16, 88) | 199 | Uncharacterized protein | Uncharacterized protein | | uniclust | UniRef100\_A4EKQ5 | 96.7 | 0.00021 | 3.9e-10 | 54.5 | 111 | (51, 171) | 173 | (36, 146) | 148 | Uncharacterized protein | Uncharacterized protein | | uniclust | UniRef100\_A0A1B2LZW7 | 96.7 | 0.00021 | 3.9e-10 | 51.6 | 87 | (71, 165) | 173 | (5, 91) | 96 | Uncharacterized protein | Uncharacterized protein | | uniclust | UniRef100\_A0A0R0A6K2 | 96.7 | 0.00022 | 4.5e-10 | 59.6 | 84 | (62, 154) | 173 | (83, 171) | 201 | Lysozyme | Lysozyme | | uniclust | UniRef100\_UPI001ED9A4F7 | 96.6 | 0.00026 | 4.8e-10 | 46.7 | 59 | (86, 144) | 173 | (3, 63) | 63 | DUF2514 family protein | DUF2514 family protein | | uniclust | UniRef100\_A0A1E3ZLE2 | 96.6 | 0.00027 | 5.2e-10 | 57.1 | 120 | (47, 170) | 173 | (55, 174) | 185 | Lysozyme | Lysozyme | | uniclust | UniRef100\_UPI001F078139 | 96.6 | 0.00028 | 5.2e-10 | 47.0 | 53 | (26, 78) | 173 | (13, 65) | 66 | DUF2514 family protein | DUF2514 family protein | | uniclust | UniRef100\_UPI001E36A577 | 96.6 | 0.00031 | 5.7e-10 | 55.8 | 91 | (56, 153) | 173 | (82, 174) | 193 | DUF2514 family protein | DUF2514 family protein | | uniclust | UniRef100\_A0A142JMY0 | 96.6 | 0.00032 | 6.5e-10 | 58.0 | 105 | (63, 171) | 173 | (69, 178) | 193 | Lysozyme | Lysozyme | | uniclust | UniRef100\_A0A9E9KSP8 | 96.6 | 0.00035 | 6.7e-10 | 55.9 | 89 | (74, 169) | 173 | (76, 170) | 172 | Lysis system i-spanin subunit Rz | Lysis system i-spanin subunit Rz | | uniclust | UniRef100\_UPI000CC96F41 | 96.6 | 0.00035 | 6.8e-10 | 59.6 | 137 | (25, 170) | 173 | (55, 192) | 270 | hypothetical protein | hypothetical protein | | uniclust | UniRef100\_A0A2S1GTV9 | 96.5 | 0.00038 | 7.2e-10 | 55.7 | 100 | (70, 170) | 173 | (79, 178) | 182 | Rz lysis protein | Rz lysis protein | | uniclust | UniRef100\_A0A519FV23 | 96.5 | 0.00038 | 7.3e-10 | 56.1 | 86 | (81, 170) | 173 | (83, 168) | 187 | DUF2514 family protein | DUF2514 family protein | | uniclust | UniRef100\_UPI0001F06794 | 96.5 | 0.00041 | 7.6e-10 | 48.6 | 81 | (77, 157) | 173 | (5, 87) | 87 | DUF2514 family protein | DUF2514 family protein | | uniclust | UniRef100\_UPI00145608D9 | 96.5 | 0.00042 | 7.7e-10 | 54.7 | 116 | (52, 172) | 173 | (54, 172) | 181 | lysis system i-spanin subunit Rz | lysis system i-spanin subunit Rz | | uniclust | UniRef100\_A0A1A9RFJ3 | 96.5 | 0.00039 | 8e-10 | 54.6 | 38 | (1, 42) | 173 | (11, 48) | 135 | Phage associated protein | Phage associated protein | | uniclust | UniRef100\_A0A2U3CZ75 | 96.5 | 0.00049 | 8.9e-10 | 47.0 | 52 | (120, 171) | 173 | (20, 71) | 74 | DUF2524 domain-containing protein | DUF2524 domain-containing protein | | uniclust | UniRef100\_A0A0F5P9U3 | 96.5 | 0.00045 | 8.9e-10 | 55.4 | 98 | (1, 102) | 173 | (1, 102) | 160 | Uncharacterized protein | Uncharacterized protein | | uniclust | UniRef100\_A0A0J6IL90 | 96.5 | 0.00045 | 9e-10 | 54.6 | 96 | (69, 171) | 173 | (26, 128) | 142 | Lysozyme | Lysozyme | | uniclust | UniRef100\_A0A1D8IVC9 | 96.5 | 0.00047 | 9.3e-10 | 56.7 | 104 | (1, 110) | 173 | (4, 114) | 186 | DUF2514 family protein | DUF2514 family protein | | uniclust | UniRef100\_UPI0018D42AA6 | 96.5 | 0.00052 | 9.6e-10 | 53.2 | 74 | (1, 85) | 173 | (46, 119) | 159 | hypothetical protein | hypothetical protein | | uniclust | UniRef100\_UPI000AD9CA16 | 96.4 | 0.00057 | 1e-09 | 48.4 | 76 | (10, 85) | 173 | (7, 82) | 91 | DUF2514 family protein | DUF2514 family protein | | uniclust | UniRef100\_A0A853F7S4 | 96.4 | 0.00057 | 1e-09 | 49.7 | 86 | (82, 169) | 173 | (17, 102) | 106 | DUF2514 family protein | DUF2514 family protein | | uniclust | UniRef100\_A0A6I1VLR2 | 96.4 | 0.00059 | 1.1e-09 | 46.3 | 51 | (119, 169) | 173 | (16, 66) | 72 | DUF2514 family protein | DUF2514 family protein | | uniclust | UniRef100\_A0A6L6TIH4 | 96.4 | 0.00059 | 1.1e-09 | 44.6 | 40 | (129, 168) | 173 | (19, 58) | 59 | DUF2514 family protein | DUF2514 family protein | | uniclust | UniRef100\_A0A2D0K6M7 | 96.4 | 0.00054 | 1.1e-09 | 53.0 | 49 | (55, 110) | 173 | (46, 94) | 123 | Peptidase | Peptidase | | uniclust | UniRef100\_A0A923JVT3 | 96.4 | 0.00061 | 1.1e-09 | 38.7 | 27 | (144, 170) | 173 | (2, 28) | 30 | DUF2514 domain-containing protein | DUF2514 domain-containing protein | | uniclust | UniRef100\_UPI0022FEA43E | 96.4 | 0.00061 | 1.1e-09 | 46.8 | 50 | (20, 69) | 173 | (18, 67) | 73 | DUF2514 family protein | DUF2514 family protein | | uniclust | UniRef100\_A0A7G8VCI8 | 96.4 | 0.00064 | 1.2e-09 | 47.7 | 52 | (119, 170) | 173 | (22, 73) | 86 | DUF2514 family protein | DUF2514 family protein | | uniclust | UniRef100\_A0A3N8D384 | 96.4 | 0.00064 | 1.2e-09 | 54.5 | 96 | (69, 169) | 173 | (63, 161) | 171 | Lysis protein | Lysis protein | | uniclust | UniRef100\_A0A2H4JG77 | 96.4 | 0.00061 | 1.2e-09 | 53.7 | 102 | (2, 110) | 173 | (1, 109) | 139 | Lysis protein (Fragment) | Lysis protein (Fragment) | | uniclust | UniRef100\_A0A0E3BVE5 | 96.4 | 0.00067 | 1.2e-09 | 48.8 | 76 | (93, 170) | 173 | (14, 90) | 99 | Lysozyme | Lysozyme | | uniclust | UniRef100\_A0A0E2NG58 | 96.4 | 0.00064 | 1.3e-09 | 53.0 | 36 | (14, 49) | 173 | (22, 57) | 129 | Uncharacterized protein | Uncharacterized protein | | uniclust | UniRef100\_A0A378XQ82 | 96.4 | 0.0007 | 1.3e-09 | 53.5 | 115 | (37, 157) | 173 | (40, 159) | 180 | Protein of uncharacterized function (DUF2514) | Protein of uncharacterized function (DUF2514) | | uniclust | UniRef100\_A0A1X3DHC2 | 96.4 | 0.00073 | 1.3e-09 | 53.0 | 93 | (9, 110) | 173 | (2, 94) | 164 | Lysozyme | Lysozyme | | uniclust | UniRef100\_A0A077NBU8 | 96.4 | 0.00067 | 1.4e-09 | 55.8 | 50 | (54, 110) | 173 | (53, 102) | 174 | Putative Rac prophage prophage lambda endopeptidase | Putative Rac prophage prophage lambda endopeptidase | | uniclust | UniRef100\_A0A022PKZ8 | 96.3 | 0.00076 | 1.5e-09 | 57.8 | 29 | (6, 34) | 173 | (43, 71) | 250 | Lysis protein | Lysis protein | | uniclust | UniRef100\_UPI000442563A | 96.3 | 0.00089 | 1.6e-09 | 43.9 | 38 | (14, 51) | 173 | (10, 47) | 59 | DUF2514 family protein | DUF2514 family protein | | uniclust | UniRef100\_A0A074TS68 | 96.3 | 0.00084 | 1.7e-09 | 50.1 | 32 | (1, 32) | 173 | (2, 33) | 102 | Uncharacterized protein | Uncharacterized protein | | uniclust | UniRef100\_A0A0J8FPI0 | 96.3 | 0.00092 | 1.8e-09 | 53.8 | 125 | (25, 165) | 173 | (21, 147) | 159 | Lysozyme | Lysozyme | | uniclust | UniRef100\_A0A543AK70 | 96.3 | 0.001 | 1.8e-09 | 48.7 | 47 | (121, 167) | 173 | (43, 89) | 108 | Uncharacterized protein DUF2514 | Uncharacterized protein DUF2514 | | uniclust | UniRef100\_A0A923GH54 | 96.3 | 0.001 | 1.9e-09 | 48.8 | 62 | (15, 76) | 173 | (6, 67) | 110 | DUF2514 family protein | DUF2514 family protein | |
| Top keywords  (threshold 1.00e-03 (evalue)) | **DUF2514, lysis, domain\_containing, Fragment, Lysozyme, Rz, Putative, Bacteriophage, Endopeptidase, prophage** |
| Output files | ../../similar\_sequences/65\_FANPEZAQ\_CDS\_0065\_merged.svg ../../similar\_sequences/65\_FANPEZAQ\_CDS\_0065\_pdb70.a3m ../../similar\_sequences/65\_FANPEZAQ\_CDS\_0065\_pdb70.hhr ../../similar\_sequences/65\_FANPEZAQ\_CDS\_0065\_uniclust.a3m ../../similar\_sequences/65\_FANPEZAQ\_CDS\_0065\_uniclust.hhr |

#### Structure prediction (AlphaFold)2

|  |  |
| --- | --- |
| Stats | xml version="1.0" encoding="utf-8" standalone="no"?       2024-09-02T21:09:59.965739 image/svg+xml   Matplotlib v3.7.2, https://matplotlib.org/ |
| Predicted structure | **NGL Viewer Controls:**  - Center: *Left-Click* - Rotate: *Left-Click + Drag* - Translate: *Right-Click + Drag* - Zoom: *Shift + Left-Click + Drag* |
| Output files | ../../predicted\_structures/65\_FANPEZAQ\_CDS\_0065/features.pkl ../../predicted\_structures/65\_FANPEZAQ\_CDS\_0065/ranked\_0.pdb ../../predicted\_structures/65\_FANPEZAQ\_CDS\_0065/ranked\_0\_plots.svg ../../predicted\_structures/65\_FANPEZAQ\_CDS\_0065/result\_model\_1\_ptm\_pred\_0.pkl |

#### Structure similarity search results (Foldseek)3

|  |  |
| --- | --- |
| Structure databases searched | Pdb, Afdb-proteome, Afdb-uniprot50 |
| Results, scheme(s)  (Top layers only, threshold 1.00e-02 (evalue)) | xml version="1.0" encoding="utf-8" standalone="no"?       2024-09-02T21:11:34.177487 image/svg+xml   Matplotlib v3.7.2, https://matplotlib.org/ |
| Results, table  (threshold 1.00e-02 (evalue)) | | db | id | prob | evalue | bits | fident | alnlen | mismatch | gapopen | qstart | qend | tstart | tend | name | description | | --- | --- | --- | --- | --- | --- | --- | --- | --- | --- | --- | --- | --- | --- | --- | | afdb-uniprot50 | AF-A0A423A0K5-F1-MODEL\_V4 | 1.0 | 8.468e-11 | 391 | 0.687 | 173 | 53 | 1 | 1 | 172 | 1 | 173 | DUF2514 family protein | DUF2514 family protein | | afdb-uniprot50 | AF-A0A7H9F4S6-F1-MODEL\_V4 | 1.0 | 0.001541 | 188 | 0.343 | 166 | 109 | 0 | 2 | 167 | 10 | 175 | DUF2514 family protein | DUF2514 family protein | | afdb-uniprot50 | AF-A0A0A7KTN9-F1-MODEL\_V4 | 1.0 | 0.001139 | 184 | 0.343 | 166 | 107 | 1 | 10 | 173 | 223 | 388 | Lysozyme | Lysozyme | |
| Top keywords  (threshold 1.00e-02 (evalue)) | **DUF2514, Lysozyme** |
| Output files | ../../similar\_structures/65\_FANPEZAQ\_CDS\_0065\_afdb-proteome\_foldseek.tsv ../../similar\_structures/65\_FANPEZAQ\_CDS\_0065\_afdb-uniprot50\_foldseek.tsv ../../similar\_structures/65\_FANPEZAQ\_CDS\_0065\_merged.svg ../../similar\_structures/65\_FANPEZAQ\_CDS\_0065\_pdb\_foldseek.tsv |

  
  
  

Return to summary | Go to previous | Go to next

  


---

**Sequence/structure alignments coloring**  
Each object in the alignment figures is colored according to its E-value following this color coding:

1e-100
10

**References:**  
1) Steinegger M, Meier M, Mirdita M, Vöhringer H, Haunsberger S J, and Söding J (2019) HH-suite3 for fast remote homology detection and deep protein annotation, BMC Bioinformatics, 473. doi: 10.1186/s12859-019-3019-7  
2) Jumper J, Evans R, Pritzel A, ..., Hassabis D (2021) Highly accurate protein structure prediction with AlphaFold, Nature, 596. doi: 10.1038/s41586-021-03819-2  
3) van Kempen M, Kim S, Tumescheit C, Mirdita M, Lee J, Gilchrist CLM, Söding J, and Steinegger M (2023) Fast and accurate protein structure search with Foldseek. Nature Biotechnology. doi: 10.1038/s41587-023-01773-0
