# Supplementary material for: Completing the BASEL phage collection to unlock hidden diversity for systematic exploration of phage–host interactions
Source: PLoS Biol. 2025 Apr 7;23(4):e3003063. doi: 10.1371/journal.pbio.3003063 (PMC11990801; doi:10.1371/journal.pbio.3003063)
Supplement: S2 Data — (ZIP) [file pbio.3003063.s009.zip › entries/9.html]

FANPEZAQ\_CDS\_0009


Return to summary | Go to previous | Go to next

|  |  |
| --- | --- |
| FANPEZAQ\_CDS\_0009 Page creation date: 02 Sep 2024, 12:00  Project folder: n/a  Input sequences file: Escherichia\_virus\_HeidiAbel.gb | phage hypothetical tail portal fragment |

### Sequence information

|  |  |
| --- | --- |
| Name | FANPEZAQ\_CDS\_0009  09\_FANPEZAQ\_CDS\_0009 (pipeline id) |
| Imported annotations | Escherichia\_virus\_HeidiAbel Bas97 |
| Protein sequence | MPIKDKAEVFIEDEIFTQVEEIFWKHFERDIKARVKYFDTSDRWA |
| Number of residues | 45 |
| Molecular weight (Da) | 5608.30 |
| Output files | ../../query\_sequences/09\_FANPEZAQ\_CDS\_0009.fasta |

### Putative domain architecture and protein family

#### Search results (HHblits)1

|  |  |
| --- | --- |
| Domain family databases searched | Pfam, Ncbi-cd, Cath, Phrogs |
| Results, scheme(s)  (Top layers only; threshold 1.00e-03 (evalue)) | xml version="1.0" encoding="utf-8" standalone="no"?       2024-09-02T21:08:13.707031 image/svg+xml   Matplotlib v3.7.2, https://matplotlib.org/ |
| Results, table  (E-value ≤ 1.00e-03 (evalue)) | -- |
| Top keywords  (threshold 1.00e-03 (evalue)) | -- |
| Output files | ../../domain\_architecture/09\_FANPEZAQ\_CDS\_0009\_cath.hhr ../../domain\_architecture/09\_FANPEZAQ\_CDS\_0009\_merged.svg ../../domain\_architecture/09\_FANPEZAQ\_CDS\_0009\_ncbi-cd.hhr ../../domain\_architecture/09\_FANPEZAQ\_CDS\_0009\_pfam.hhr ../../domain\_architecture/09\_FANPEZAQ\_CDS\_0009\_phrogs.hhr |

### Identical protein sequences/structures

#### Search results

|  |  |
| --- | --- |
| Protein sequence databases searched | Pdb, Swissprot, Refseq |
| Identical proteins found | -- |
| Top keywords | -- |
| Output files | -- |

### Similar protein sequences/structures

#### Sequence similarity search results (HHblits)1

|  |  |
| --- | --- |
| Sequence databases searched | Uniclust, Pdb70 |
| Results, scheme(s)  (Top layers only, threshold 1.00e-03 (evalue)) | xml version="1.0" encoding="utf-8" standalone="no"?       2024-09-02T21:08:31.522522 image/svg+xml   Matplotlib v3.7.2, https://matplotlib.org/ |
| Results, table(s)  (threshold 1.00e-03 (evalue)) | | db | id | prob | evalue | pvalue | score | cols | query | query\_len | template | template\_len | name | description | | --- | --- | --- | --- | --- | --- | --- | --- | --- | --- | --- | --- | --- | | uniclust | UniRef100\_A0A061YFQ4 | 99.5 | 3e-17 | 8.1e-23 | 111.6 | 43 | (1, 43) | 45 | (164, 206) | 234 | Uncharacterized protein | Uncharacterized protein | | uniclust | UniRef100\_A0A085ASD5 | 99.5 | 1.2e-16 | 2.8e-22 | 110.8 | 44 | (1, 44) | 45 | (218, 261) | 272 | Phage protein | Phage protein | | uniclust | UniRef100\_A0A4V6JHM3 | 99.5 | 1.8e-16 | 3.5e-22 | 92.4 | 44 | (1, 44) | 45 | (27, 70) | 72 | Uncharacterized protein | Uncharacterized protein | | uniclust | UniRef100\_A0A088FVG9 | 99.4 | 9e-16 | 2.2e-21 | 103.4 | 44 | (1, 44) | 45 | (153, 196) | 205 | Phage tail protein | Phage tail protein | | uniclust | UniRef100\_A0A6L3XNY4 | 99.1 | 6.6e-13 | 1.2e-18 | 87.1 | 40 | (1, 40) | 45 | (125, 164) | 165 | Phage portal protein (Fragment) | Phage portal protein (Fragment) | | uniclust | UniRef100\_A0A0B4BLP4 | 98.8 | 2.7e-11 | 5.6e-17 | 82.6 | 45 | (1, 45) | 45 | (156, 200) | 205 | Tail protein | Tail protein | | uniclust | UniRef100\_UPI001E3A7150 | 98.1 | 5.5e-08 | 1e-13 | 62.0 | 41 | (1, 41) | 45 | (71, 111) | 112 | hypothetical protein | hypothetical protein | | uniclust | UniRef100\_UPI0006B67FA6 | 98.0 | 8.3e-08 | 1.5e-13 | 63.7 | 38 | (1, 38) | 45 | (113, 150) | 152 | hypothetical protein | hypothetical protein | | uniclust | UniRef100\_A0A8S7U9Y6 | 97.2 | 2.1e-05 | 3.9e-11 | 54.6 | 39 | (1, 39) | 45 | (153, 191) | 192 | Uncharacterized protein | Uncharacterized protein | | uniclust | UniRef100\_UPI00235E9506 | 97.0 | 6.5e-05 | 1.2e-10 | 43.3 | 33 | (12, 44) | 45 | (13, 45) | 50 | hypothetical protein | hypothetical protein | |
| Top keywords  (threshold 1.00e-03 (evalue)) | **Phage, hypothetical, tail, portal, Fragment** |
| Output files | ../../similar\_sequences/09\_FANPEZAQ\_CDS\_0009\_merged.svg ../../similar\_sequences/09\_FANPEZAQ\_CDS\_0009\_pdb70.a3m ../../similar\_sequences/09\_FANPEZAQ\_CDS\_0009\_pdb70.hhr ../../similar\_sequences/09\_FANPEZAQ\_CDS\_0009\_uniclust.a3m ../../similar\_sequences/09\_FANPEZAQ\_CDS\_0009\_uniclust.hhr |

#### Structure prediction (AlphaFold)2

|  |  |
| --- | --- |
| Stats | xml version="1.0" encoding="utf-8" standalone="no"?       2024-09-02T21:09:08.159697 image/svg+xml   Matplotlib v3.7.2, https://matplotlib.org/ |
| Predicted structure | **NGL Viewer Controls:**  - Center: *Left-Click* - Rotate: *Left-Click + Drag* - Translate: *Right-Click + Drag* - Zoom: *Shift + Left-Click + Drag* |
| Output files | ../../predicted\_structures/09\_FANPEZAQ\_CDS\_0009/features.pkl ../../predicted\_structures/09\_FANPEZAQ\_CDS\_0009/ranked\_0.pdb ../../predicted\_structures/09\_FANPEZAQ\_CDS\_0009/ranked\_0\_plots.svg ../../predicted\_structures/09\_FANPEZAQ\_CDS\_0009/result\_model\_1\_ptm\_pred\_0.pkl |

#### Structure similarity search results (Foldseek)3

|  |  |
| --- | --- |
| Structure databases searched | Pdb, Afdb-proteome, Afdb-uniprot50 |
| Results, scheme(s)  (Top layers only, threshold 1.00e-02 (evalue)) | xml version="1.0" encoding="utf-8" standalone="no"?       2024-09-02T21:10:16.750126 image/svg+xml   Matplotlib v3.7.2, https://matplotlib.org/ |
| Results, table  (threshold 1.00e-02 (evalue)) | -- |
| Top keywords  (threshold 1.00e-02 (evalue)) | -- |
| Output files | ../../similar\_structures/09\_FANPEZAQ\_CDS\_0009\_afdb-proteome\_foldseek.tsv ../../similar\_structures/09\_FANPEZAQ\_CDS\_0009\_afdb-uniprot50\_foldseek.tsv ../../similar\_structures/09\_FANPEZAQ\_CDS\_0009\_merged.svg ../../similar\_structures/09\_FANPEZAQ\_CDS\_0009\_pdb\_foldseek.tsv |

  
  
  

Return to summary | Go to previous | Go to next

  


---

**Sequence/structure alignments coloring**  
Each object in the alignment figures is colored according to its E-value following this color coding:

1e-100
10

**References:**  
1) Steinegger M, Meier M, Mirdita M, Vöhringer H, Haunsberger S J, and Söding J (2019) HH-suite3 for fast remote homology detection and deep protein annotation, BMC Bioinformatics, 473. doi: 10.1186/s12859-019-3019-7  
2) Jumper J, Evans R, Pritzel A, ..., Hassabis D (2021) Highly accurate protein structure prediction with AlphaFold, Nature, 596. doi: 10.1038/s41586-021-03819-2  
3) van Kempen M, Kim S, Tumescheit C, Mirdita M, Lee J, Gilchrist CLM, Söding J, and Steinegger M (2023) Fast and accurate protein structure search with Foldseek. Nature Biotechnology. doi: 10.1038/s41587-023-01773-0
