# Supplementary figures and images for: Completing the BASEL phage collection to unlock hidden diversity for systematic exploration of phage–host interactions
Source: PLoS Biol. 2025 Apr 7;23(4):e3003063. doi: 10.1371/journal.pbio.3003063 (PMC11990801; doi:10.1371/journal.pbio.3003063)

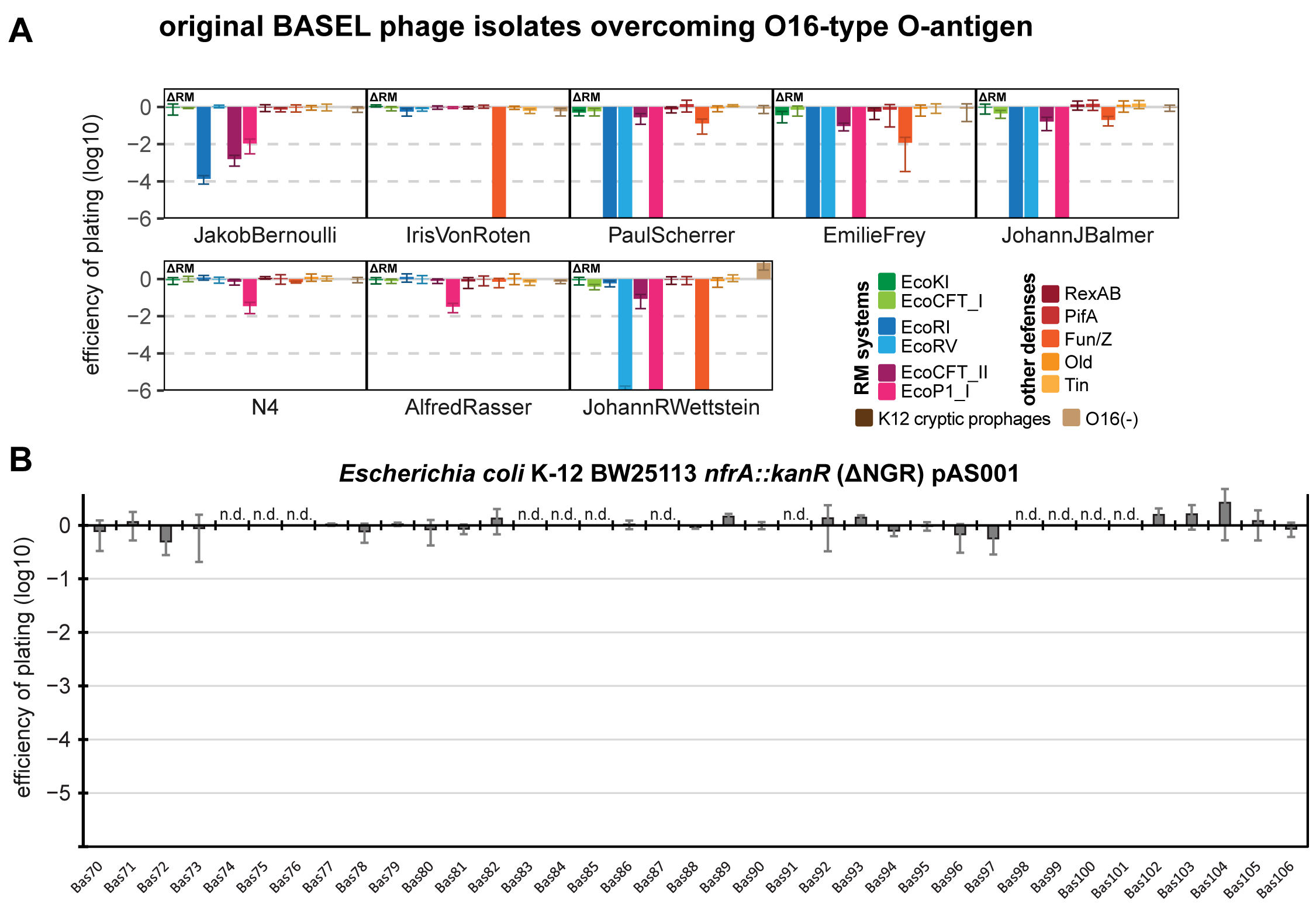

Supplement: S1 Fig — (A) Phages that had shown significant growth on E. coli K-12 with restored O-antigen in our previous work [10] were now phenotypically characterized in the presence of this glycan. The results of these quantitative phenotyping experiments regarding sensitivity to altered surface glycans and bacterial immunity systems are presented as efficiency of plating (EOP). The small note of “ΔRM” indicates that this experiment has been performed using E. coli K-12 ΔRM wbbL(+) as host (i.e., with restored O-antigen expression). Data points and error bars represent average and standard deviation of at least three independent experiments. Raw data and calculations are available in S1 Data. (B) The results of quantitative phenotyping experiments with all newly presented BASEL phages infecting an nfrA knockout (specifically lacking the NGR glycan [17]) are presented as efficiency of plating (EOP). This strain had been transformed with plasmid pAS001 to restore O16-type O-antigen expression (see Materials and methods). Data points and error bars represent average and standard deviation of at least three independent experiments. Raw data and calculations are available in S1 Data. It is evident that no newly included BASEL phage depends partially or completely on the NGR glycan for infection of E. coli K-12. Phages sensitive to the presence of cryptic prophages in the K-12 genomic background could not be tested. However, since these phages all carry tailspikes or tail fibers targeting O16-type O-antigen as receptor-binding proteins (Figs 11 and 12) it seems highly unlikely that they would be affected by the absence of NGR. (TIF) [file pbio.3003063.s011.tif]

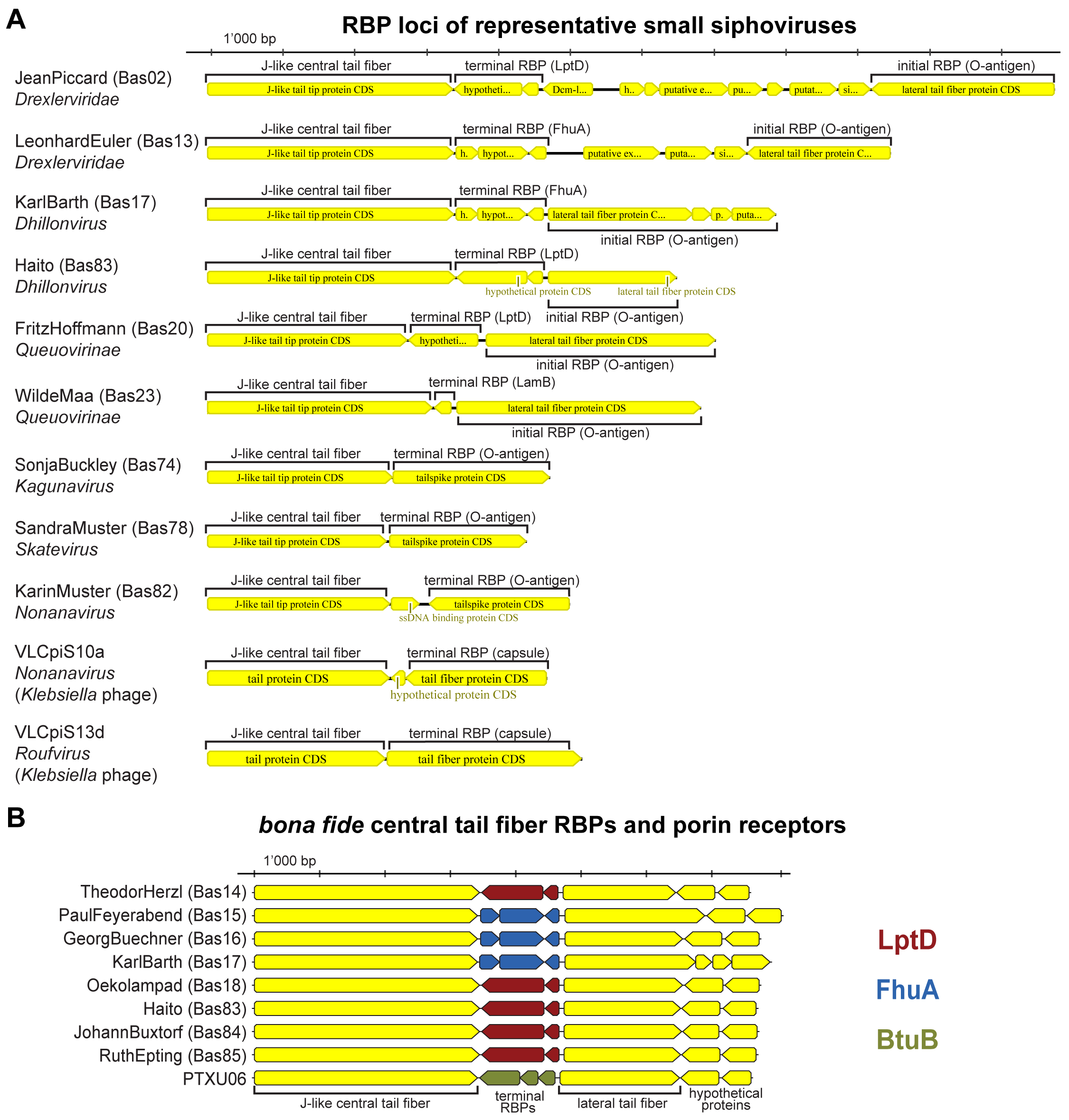

Supplement: S2 Fig — (A) The illustration compares the tail fiber and tailspike loci of diverse small siphoviruses from the original BASEL collection and the current study and porin-targeting receptor-binding proteins (RBPs) are annotated based on the bona fide central tail fiber caps identified previously [10]. It is apparent that the single RBPs of the three exemplary O16-dependent phages SonjaBuckley (Kagunavirus, Bas74), SandraMuster (Skatevirus, Bas78), and KarinMuster (Nonanavirus, Bas82) are encoded at the same locus as the porin-targeting RBPs for terminal receptor recognition in the other genomes. For comparison, we also included two small siphoviruses targeting Klebsiella capsules that had been classified in previous work as belonging to Nonanavirus (see Fig 6E) and Roufvirus (see Fig 6D) [49]. Despite the annotation as “tail fiber,” both viruses encode tailspikes directly downstream of the orthologs of the J-like central tail fiber proteins [49]. (B) Comparison of the bona fide central tail fiber RBP loci of all Dhillonvirus isolates in the BASEL collection as well as PTXU06 for comparison [10,58]. The association of different variants of this locus with porin specificity is color-coded as in our previous work – all new isolates from the current study are predicted to target LptD (red) as terminal receptor [10]. (TIF) [file pbio.3003063.s012.tif]

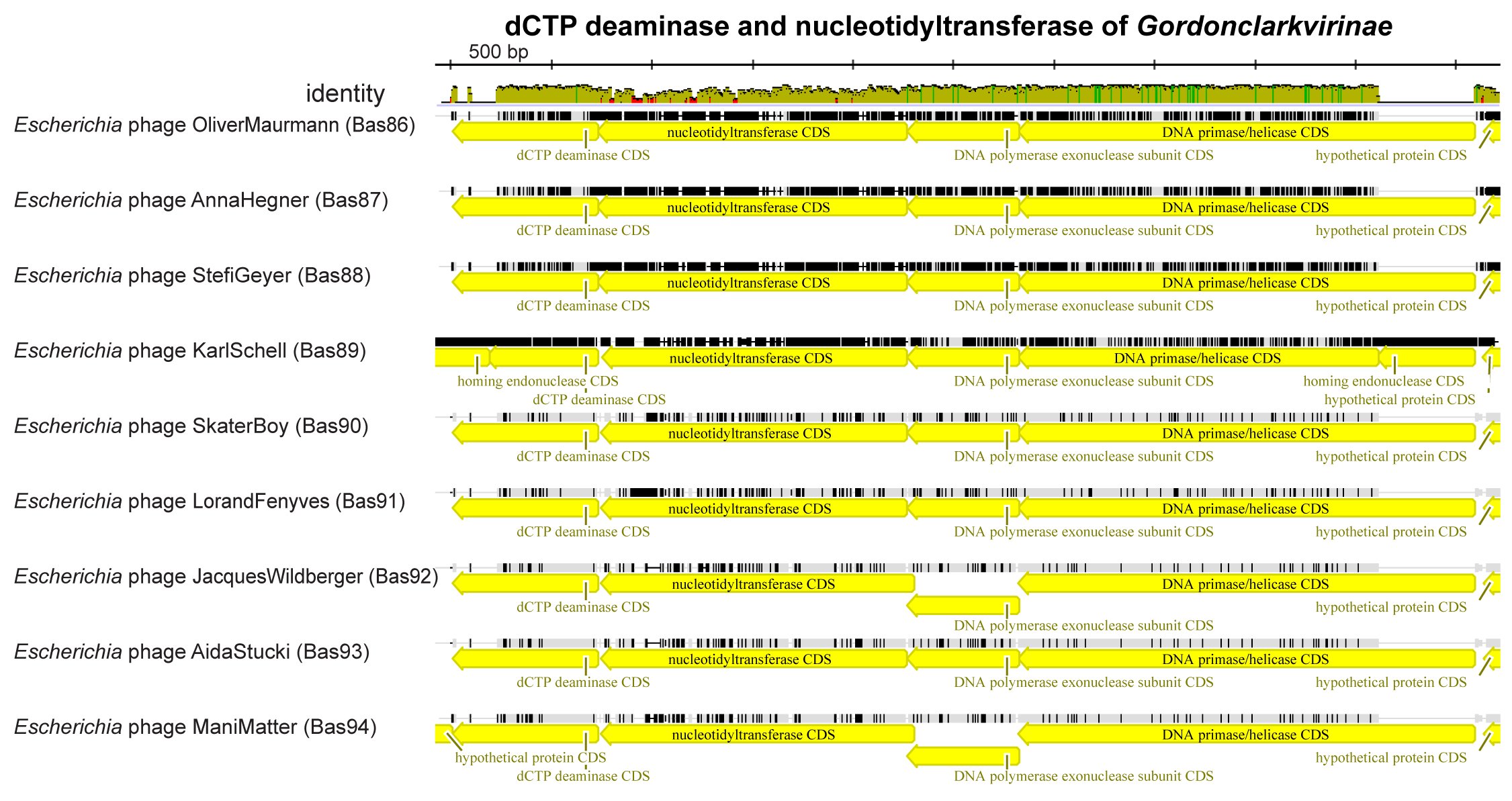

Supplement: S3 Fig — The conserved locus encoding dCTP deaminase, nucleotidyltransferase, and DNA replication genes of all included Gordonclarkvirinae isolates is shown in a sequence alignment (see Materials and methods). Colors in the sequence identity graph above the alignment indicate the sequence identity at each individual position with green representing 100% identity, greenish-brown 30%−99% identity, and red <30% identity. Besides homing endonuclease insertions, the locus is fully syntenic across these isolates (and many other Gordonclarkvirinae). (TIF) [file pbio.3003063.s013.tif]

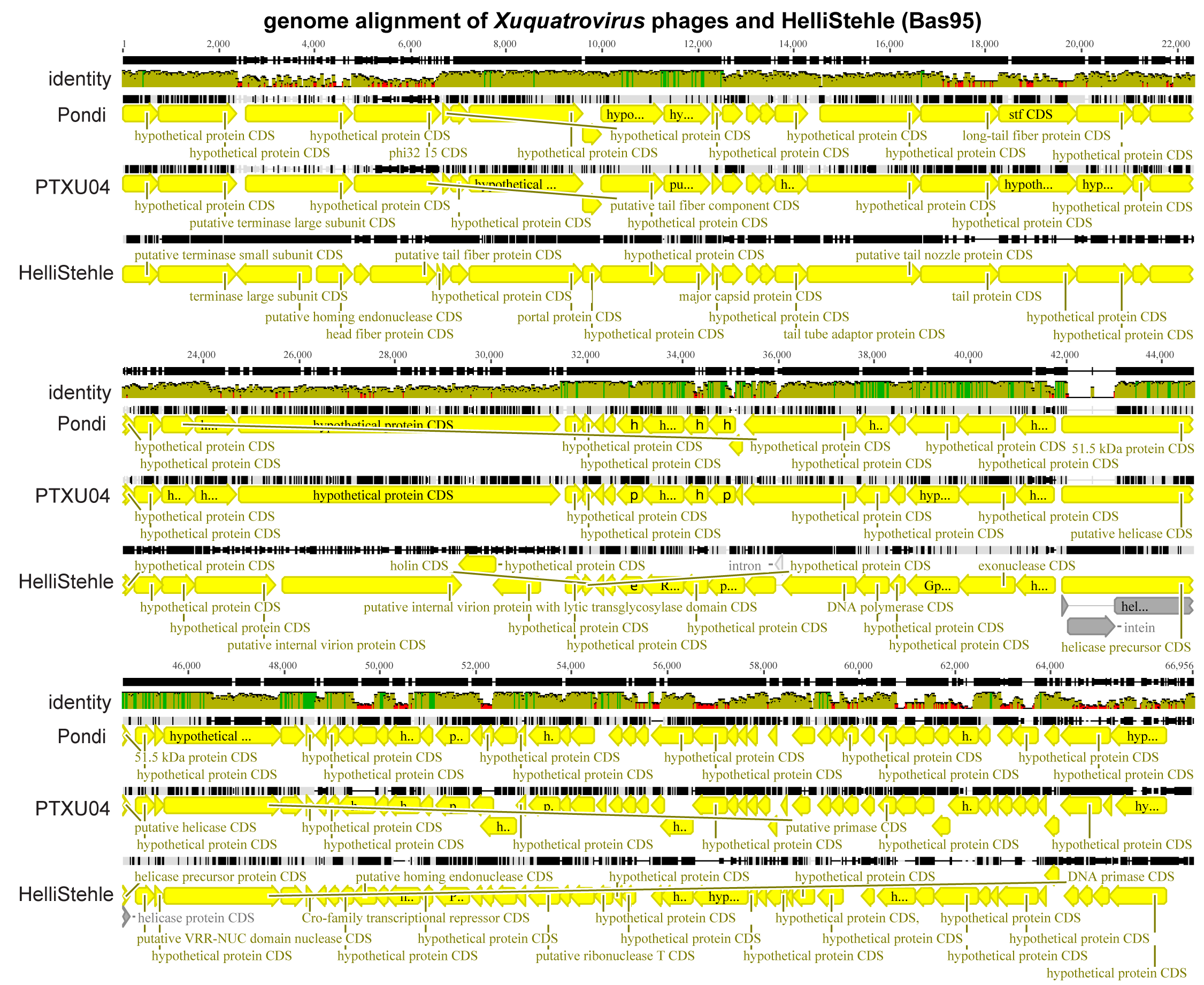

Supplement: S4 Fig — The genomes of our new isolate HelliStehle (Bas95) and Xuquatrovirus phages Pondi (GenBank accession OP136151.1) and PTXU04 (NCBI GenBank accession NC_048193.1) are shown in a sequence alignment with the putative small terminase subunit as starting point (see Materials and methods). Colors in the sequence identity graph above the alignment indicate the sequence identity at each individual position with green representing 100% identity, greenish-brown 30%−99% identity, and red <30% identity. The pairwise sequence identity of prototypic phage PTXU04 to fellow Xuquatrovirus Pondi is 83.8% and to HelliStehle (Bas95) merely 58.3%. (TIF) [file pbio.3003063.s014.tif]

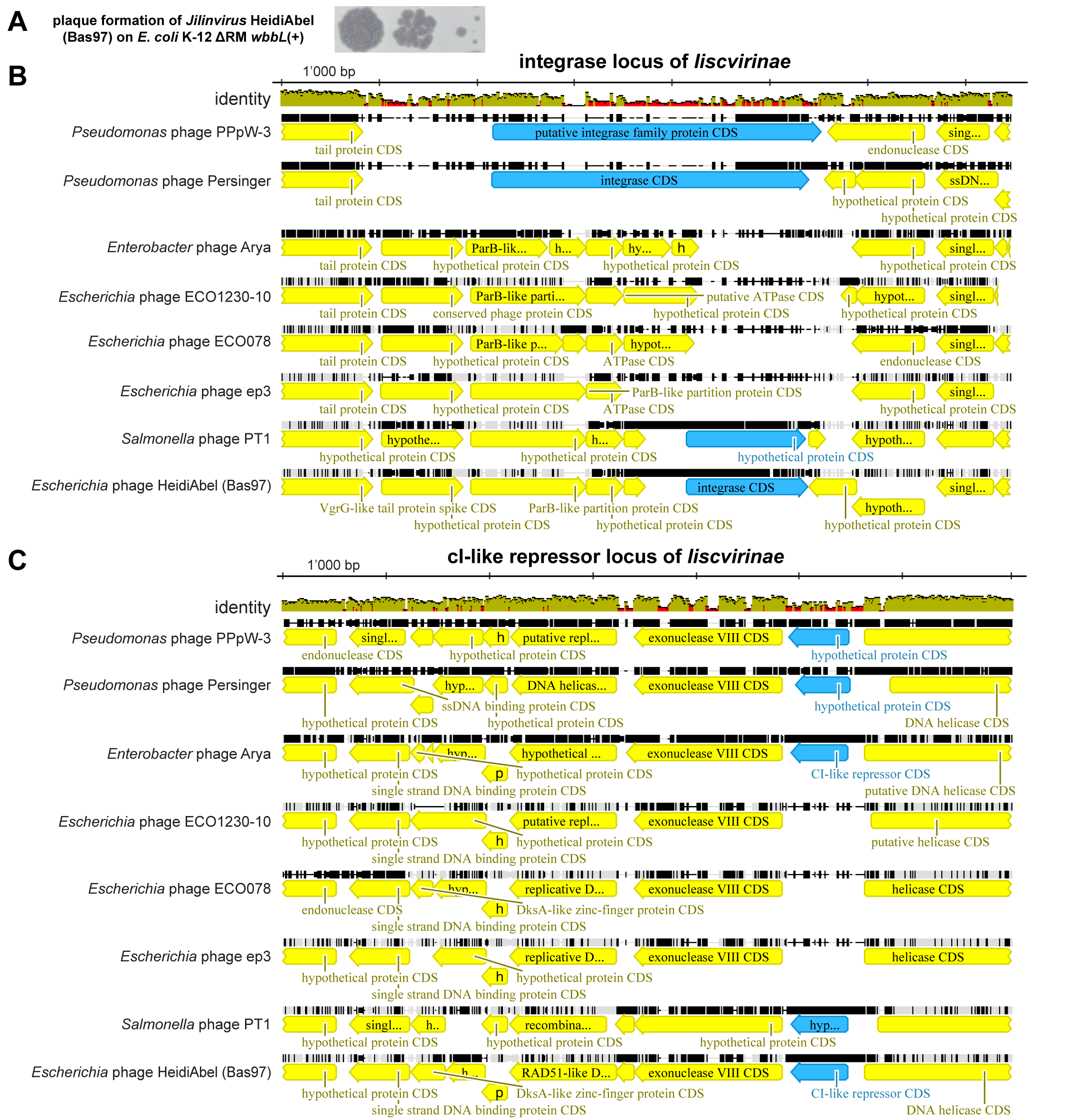

Supplement: S5 Fig — (A) Serial dilutions of Jilinvirus HeidiAbel (from left to right) were spotted on a soft agar of E. coli K-12 ΔRM wbbL(+) and grown overnight at 37°C before imaging. The lysis zones and plaques show no classical signs of lysogen formation such as turbidity or marked growth of (self-immune) lysogens in the middle [108] – compare, e.g., the growth of temperate Lederbergvirus phages Huey, Dewey, and Louie in S6B Fig or of phage Kap1 on a host enabling lysogeny and a host without its preferred integration site in Fig 3 of Pick and colleagues [205]. Instead, upon prolonged incubation merely an abundance of dotted individual colonies is found that we typically observe for phages requiring the LPS O-antigen as host receptor, which is a huge mutational target [206]. (B, C) The illustrations show sequence alignments of the integrase locus of Iiscvirinae phages (B) and – encoded slightly downstream – their cI-like repressor locus (C; see Materials and methods). Both genes of interest are highlighted in blue. Colors in the sequence identity graphs above the alignments indicate the sequence identity at each individual position with green representing 100% identity, greenish-brown 30%−99% identity, and red <30% identity. The alignments show that only the two Pseudomonas phages (top) and Salmonella phage PT1 as well as our isolate HeidiAbel encode seemingly intact integrase and cI-like repressor genes. These observations are further discussed in the main text. (TIF) [file pbio.3003063.s015.tif]

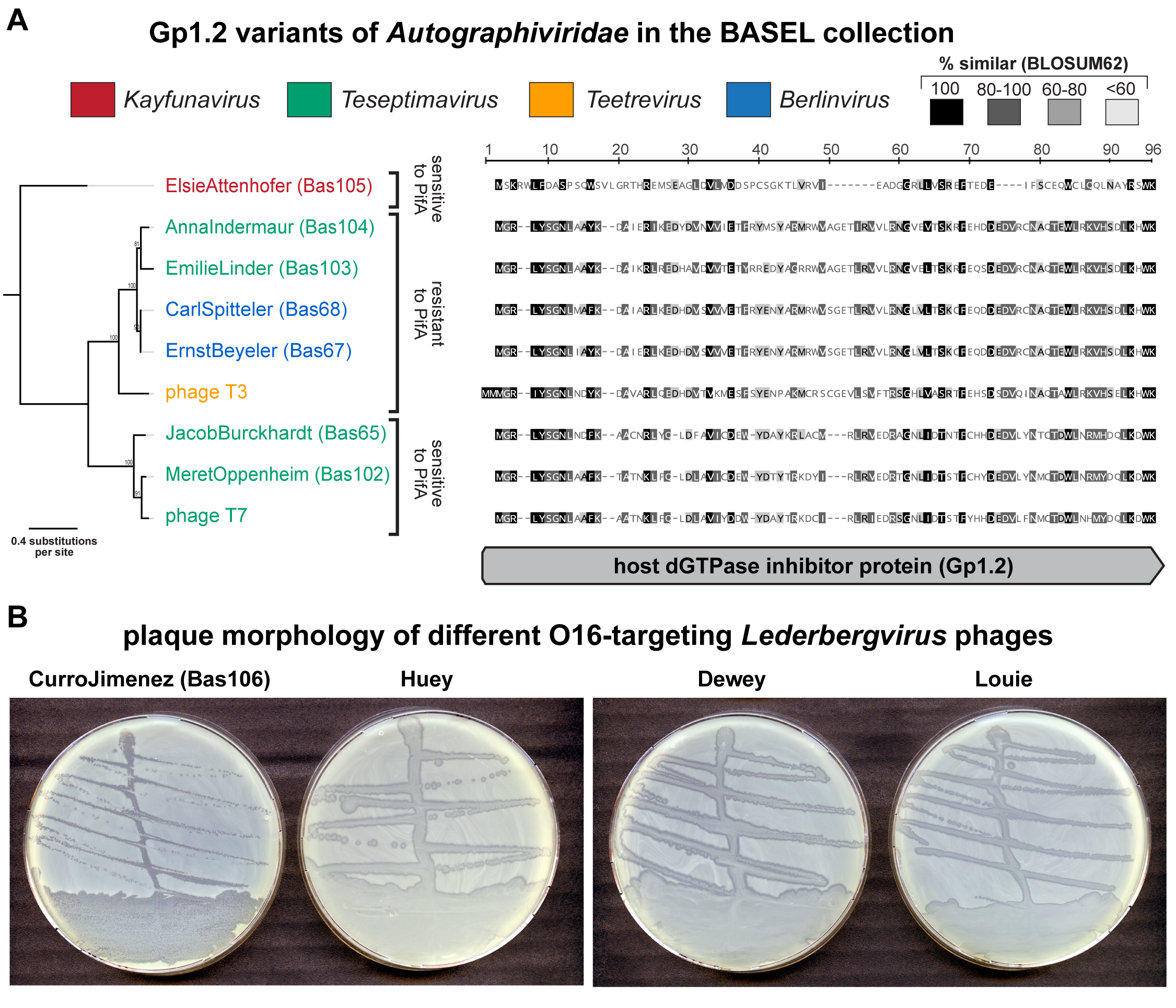

Supplement: S6 Fig — (A) The illustration shows a Maximum-likelihood phylogeny (left) and a sequence alignment (right) of Gp1.2 orthologs in all tested Autographiviridae phages in the complete BASEL collection. Experimental details are explained in Materials and methods. Resistance to the PifA defense system (see Fig 10E and our previous work [10]) correlates with one specific clade of Gp1.2 homologs (the middle one). (B) Plaque morphology of our four Lederbergvirus isolates infecting E. coli K-12 with restored O16-type O-antigen streaked on E. coli K-12 ΔRM wbbL(+). While CurroJimenez (Bas106) forms clear plaques of variable size, the other three isolates form highly turbid plaques that are indicative of lysogen formation. (TIF) [file pbio.3003063.s016.tif]

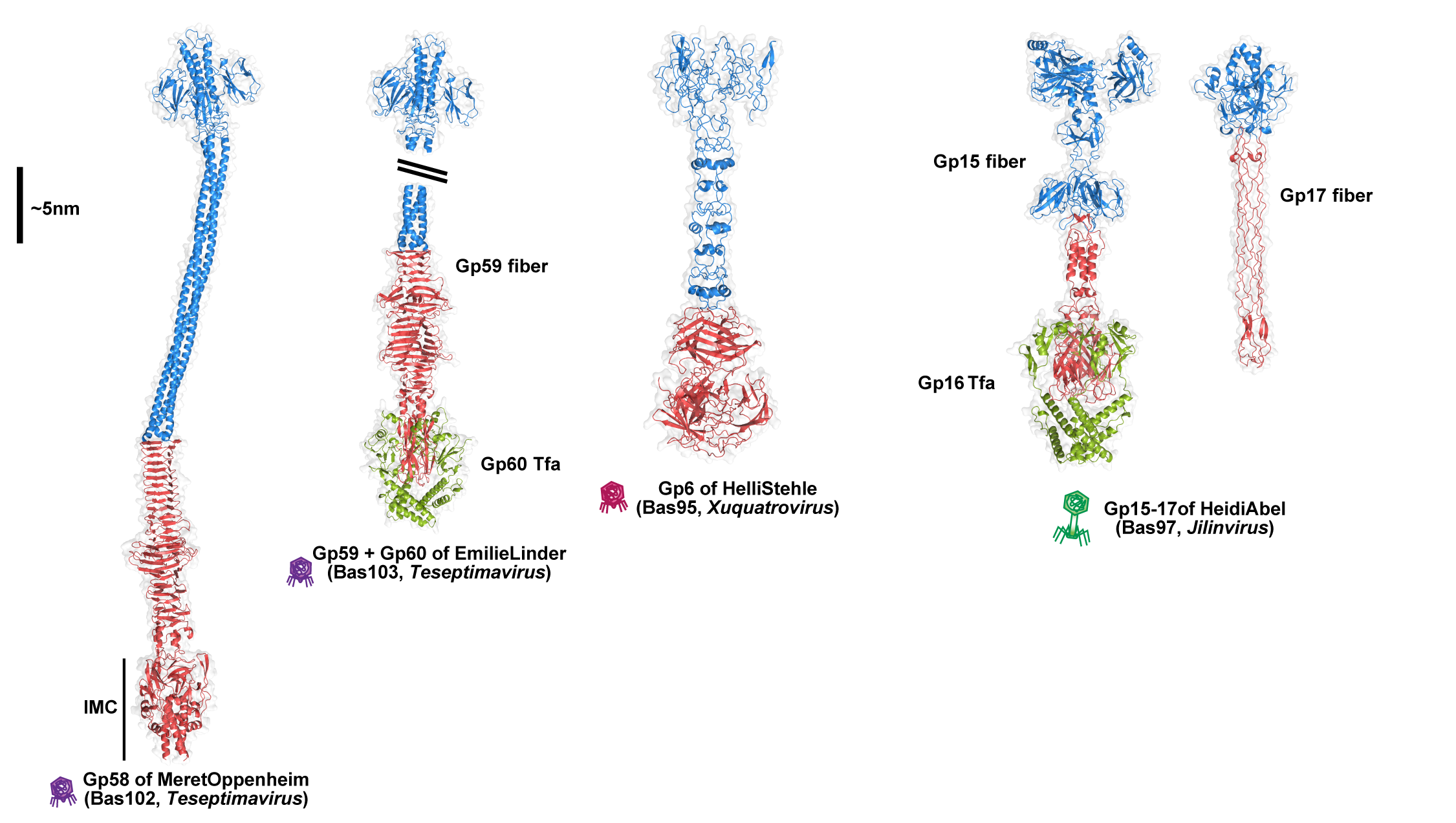

Supplement: S7 Fig — AlphaFold-Multimer [198] predicted structures of additional tail fibers from selected BASEL phages with a single receptor-binding domain (RBD). Tail fibers of Teseptimavirus phages MeretOppenheim (Bas102) and EmilieLinder (Bas103) are formed by a homotrimer of Gp58 or Gp59, respectively. Note that the tail fiber of MeretOppenheim contains a C-terminal intramolecular chaperone (IMC) domain. Conversely, EmilieLinder encodes a separate Mu-like tail fiber assembly protein (Tfa, Gp60; green) downstream of the Gp59 tail fiber that are predicted to form a hexameric complex as observed for the tail fiber of phage Mu [207]. The homotrimeric tail fibers of Xuquatrovirus HelliStehle (Bas95) and Jilinvirus HeidiAbel (Bas97) feature comparably short fiber stalks. HeidiAbel also encodes a Tfa protein (Gp16; green) that is predicted to form a complex with the tail fiber similar to the complex predicted for EmilieLinder. In addition, HeidiAbel also encodes a separate tail fiber with a distal tip that is similar, but truncated, to the T4 Gp37 long-tail fiber and the Gp32 tail fiber of Wifcevirus ManiWeber [37] (see Fig 12C). Based on the size and genetic arrangement, we speculate that the Gp15 fiber of HeidiAbel serves as a lateral tail fiber for initial host recognition while Gp17 forms a short-tail fiber for irreversible adsorption triggering viral genome injection (analogous to the much larger T-even myoviruses [10,73,74]). In all predicted structures, N-terminal anchor domains and fiber shafts are colored blue with the RBDs colored red. A ~5 nm vertical scale bar is provided for reference and viral morphotypes are annotated by phage icons analogous to Fig 11A and 11B. (TIF) [file pbio.3003063.s017.tif]

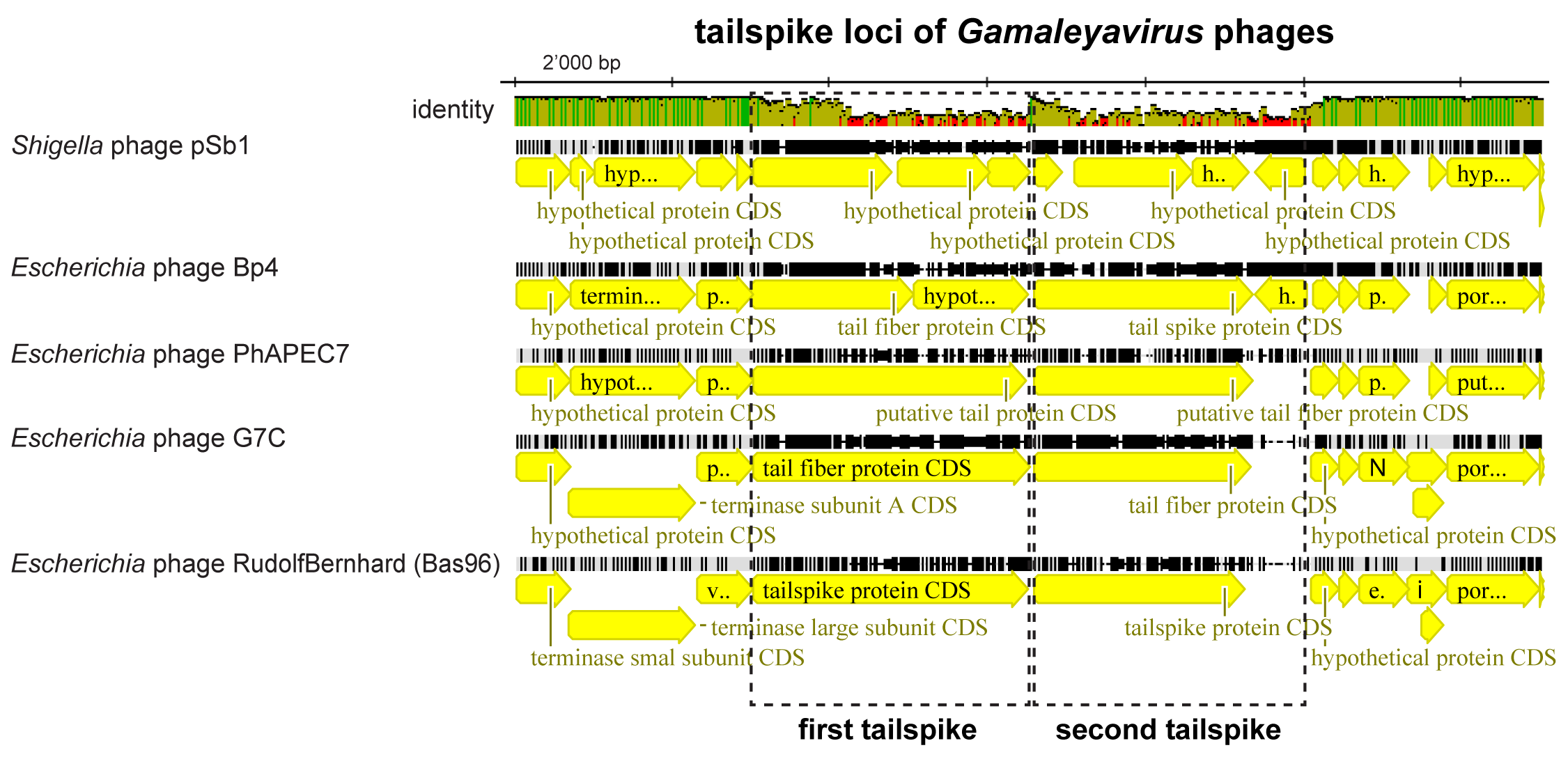

Supplement: S8 Fig — The illustration shows a sequence alignment of the tailspike locus of different Gamaleyavirus phages (compare Fig 8E; see Materials and methods). Colors in the sequence identity graph above the alignment indicate the sequence identity at each individual position with green representing 100% identity, greenish brown 30%−99% identity, and red <30% identity. The alignment shows that the most 5′ parts of the two tailspike loci – encoding the N-terminal ends of the (proximal subunits of the) tailspikes – are highly conserved, likely encoding conserved structural elements that anchor them at the virion. The receptor-binding domains of all tailspikes vary greatly. These observations are further discussed in the main text. (TIF) [file pbio.3003063.s018.tif]

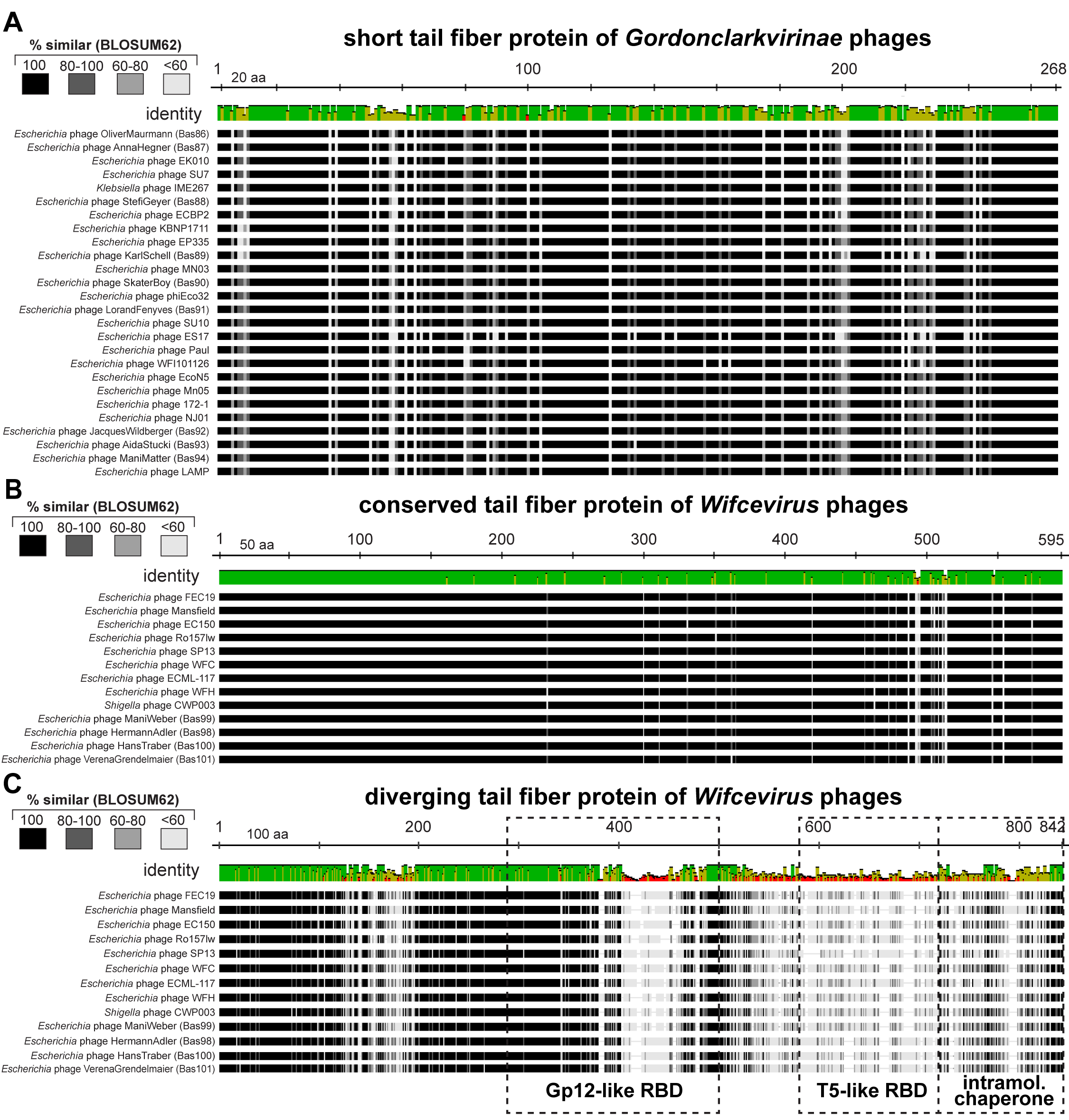

Supplement: S9 Fig — (A) The illustration shows an amino acid sequence alignment of the short-tail fiber proteins of all Gordonclarkvirinae included in Fig 7C in the same order (see Materials and methods). It is apparent that short-tail fiber proteins are highly conserved across the Gordonclarkvirinae subfamily with only minor differences. Across all sequences there are 163 of 267 fully identical sites (60.8%). (B) Amino acid sequence alignment of the conserved tail fiber of Wifcevirus phages encoded with their tail fiber locus (Gp32 of ManiWeber/ Bas99, see Fig 12C). It is clear from the alignment that all orthologs are very similar in amino acid sequence, suggesting a conserved and invariable function. (C) Amino acid sequence alignment of the T-shaped tail fibers of Wifcevirus phages (see Figs 11B, 12B and S10D). The alignment shows that the N-terminus of all orthologs is highly conserved while the two receptor-binding domains (RBDs) as well as – to lesser extent – the intramolecular chaperone of the T5-like RBD2 vary a lot between sequences. In (A) to (C), colors in the sequence identity graph above the alignment indicate the sequence identity at each individual position with green representing 100% identity, greenish brown 30%−99% identity, and red <30% identity. (TIF) [file pbio.3003063.s019.tif]

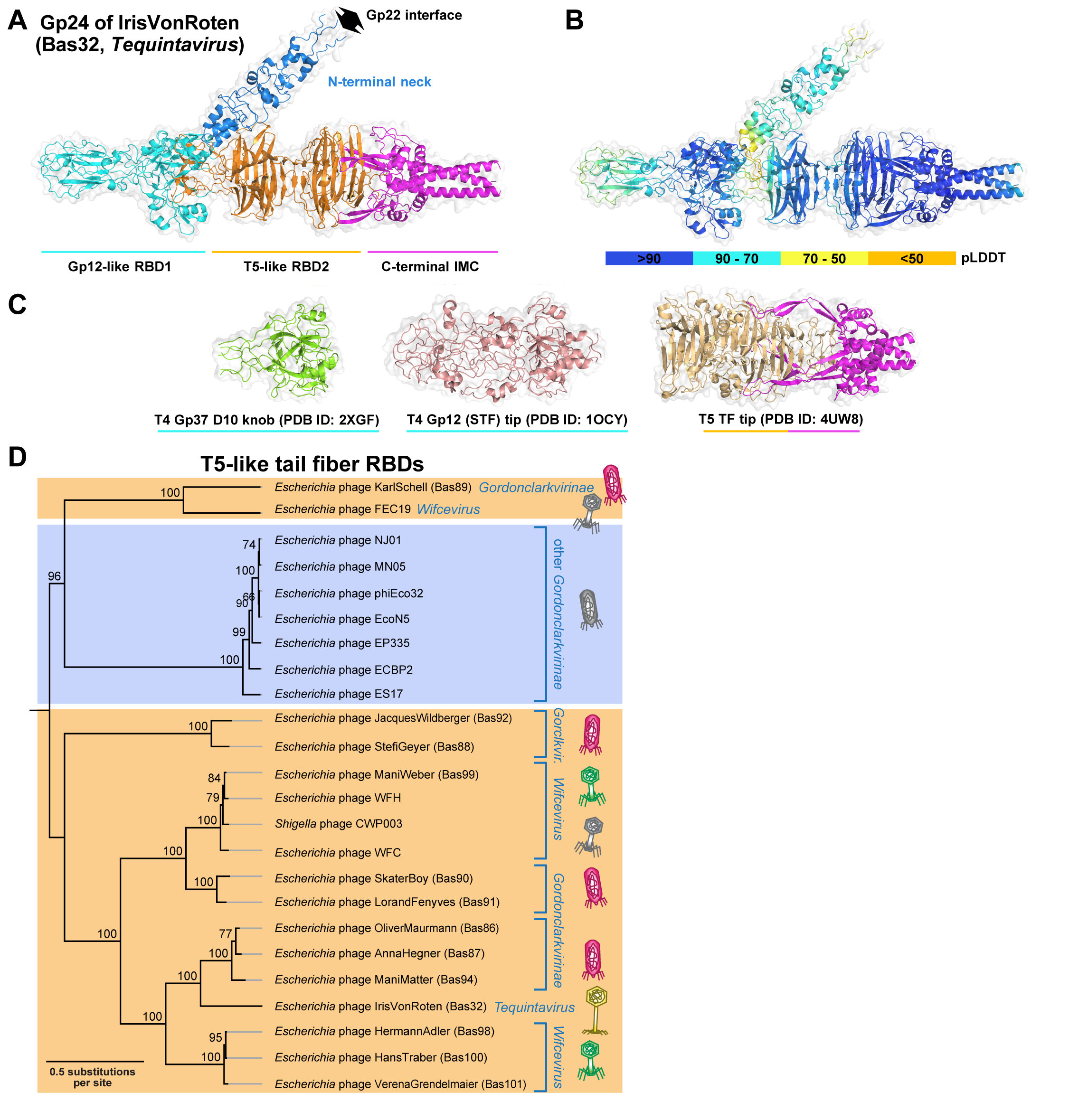

Supplement: S10 Fig — (A) The AlphaFold-Multimer [198] predicted structure of the T-shaped tail fiber of IrisVonRoten (Gp24) was colored to highlight the domain boundaries of this unique dual RBD tail fiber. (B) The same structure as in (A) was colored by predicted local distance difference test (pLDDT) confidence scores as generated by AlphaFold to demonstrate the high level of confidence (overall average = 72.6) in the predicted T-shaped tail fiber structure. (C) The DALI server [201] identified structural similarities between the Gp12-like RBD1 with crystal structures of the distal knob domain (D10) of the T4 long-tail fiber Gp37 [118] (Z-score 8.8, RMSD 3.2, 100 residues) and unsurprisingly with the Gp12 short-tail fiber distal tip itself [118] (Z-score 6.2, RMSD 4.1, 127 residues) as well as similarity between the T5-like RBD2 with the distal binding tip and IMC domain of the phage T5 lateral tail fiber [119] (Z-score 6.3, RMSD 4.8, 179 residues). (D) Maximum-likelihood phylogeny of T5-like receptor-binding domains of T-shaped tail fibers together with their intramolecular chaperone domain as identified by structure prediction (see Materials and methods). The phylogeny was midpoint-rooted between a clade of distant Gordonclarkvirinae tail fibers at the top (blue) together with two other sequences and all other RBDs mostly belonging to phages presented in this study (orange). Note how RBDs of Wifcevirus, Gordonclarkvirinae, and Tequintavirus isolates are scattered over the phylogeny in small clusters, suggesting frequent horizontal gene transfer as often observed for viral receptor-binding domains [10,33,115]. Phage AidaStucki (Bas93, Gordonclarkvirinae) was not included due to a large truncation in its intramolecular chaperone domain. (TIF) [file pbio.3003063.s020.tif]

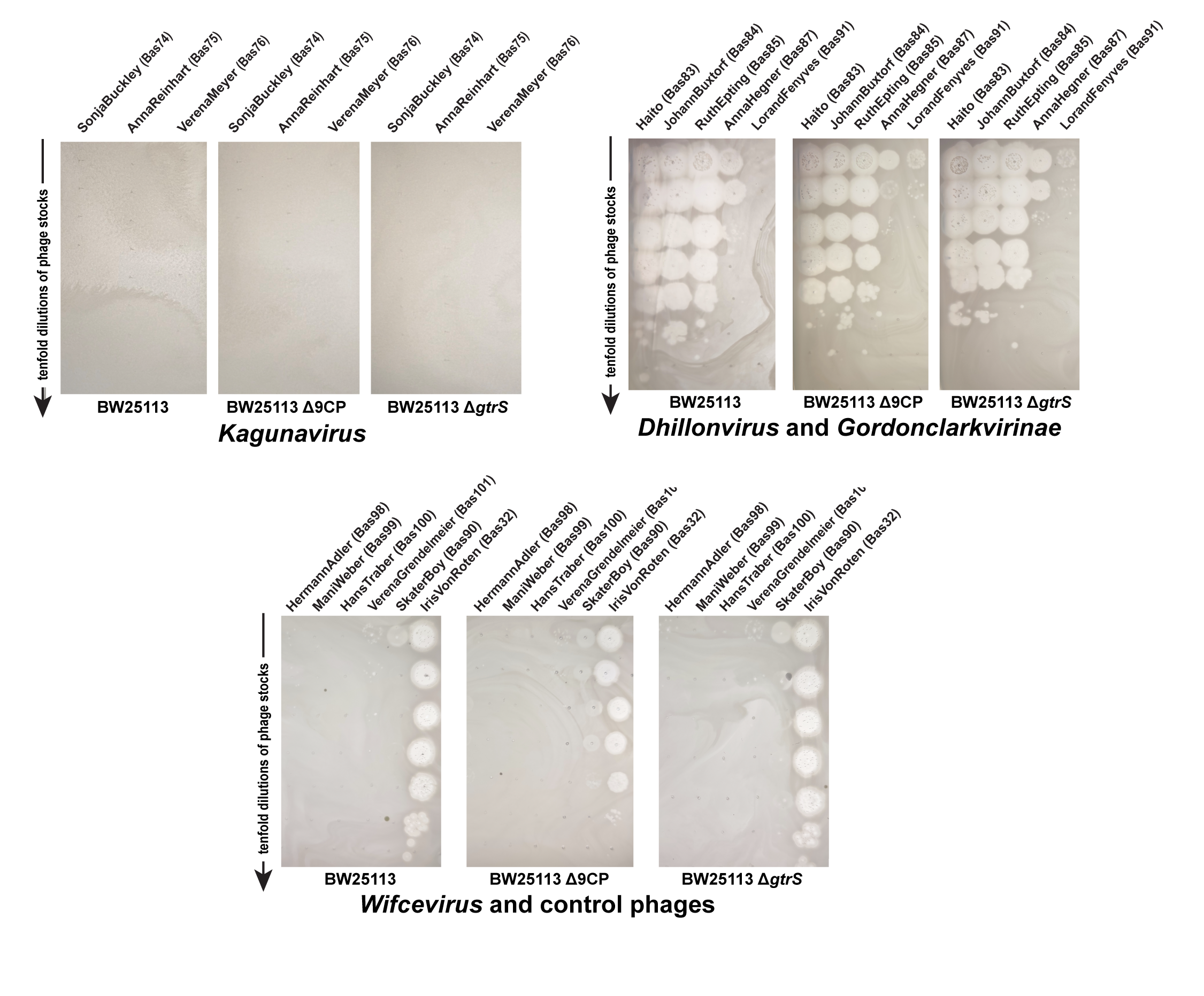

Supplement: S11 Fig — The same serial dilutions as in Fig 14B were spotted on variants of E. coli K-12 BW25113 without restored O-antigen expression (left) and with either deletion of the GtrS glucosyltransferase (middle) or the nine cryptic prophages including CPS-53 (right). In difference to the results shown in Fig 14B with restored O-antigen expression, the three Dhillonvirus phages Bas83–85 readily infect all of the strains lacking O-antigen expression. (TIF) [file pbio.3003063.s021.tif]

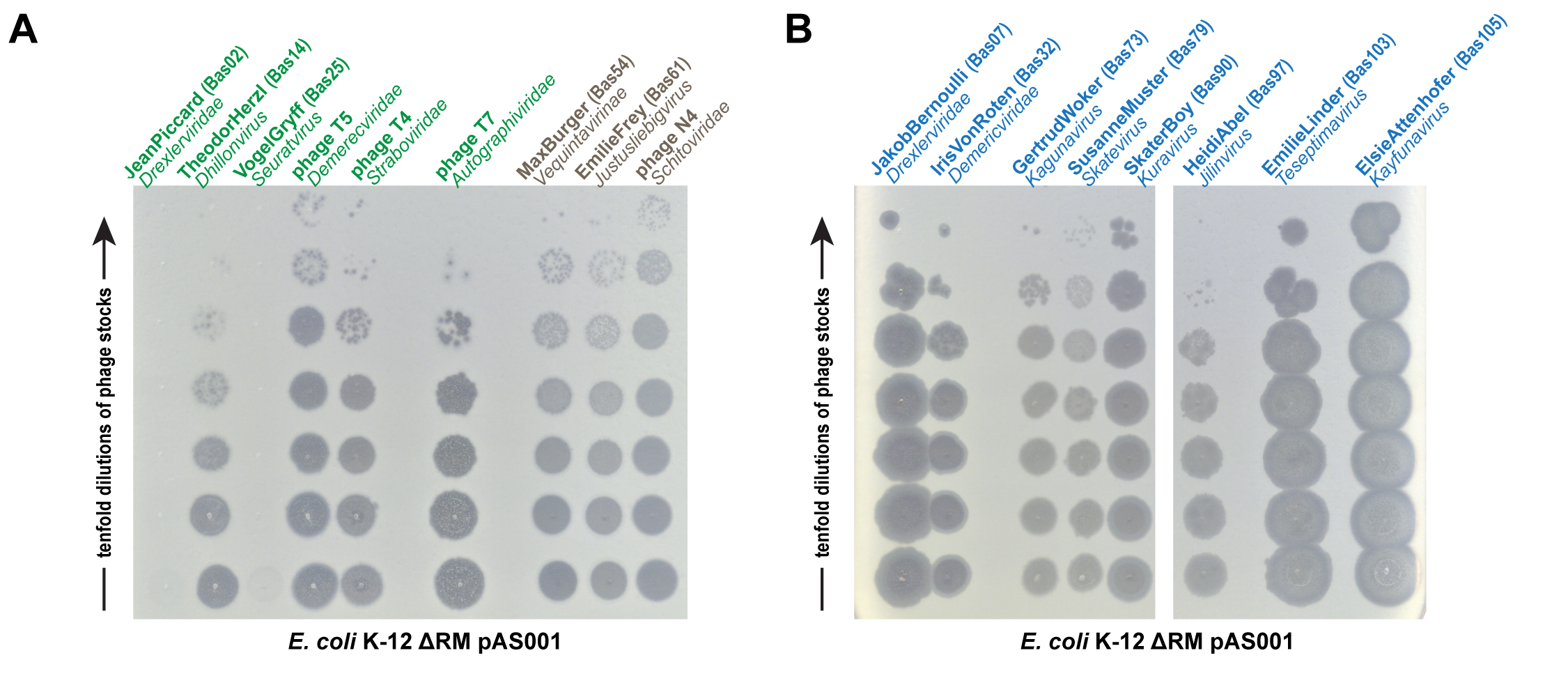

Supplement: S12 Fig — The same serial dilutions as in Fig 1B (for (A)) and 2B (for (B)) were spotted on the E. coli K-12 ΔRM strain with partially restored O16-type O-antigen due to ectopic expression of wbbL from plasmid pAS001 (see Materials and methods). Most but not all phages inhibited by a full O16-type O-antigen barrier (green) show robust growth on this host (compare Fig 1B) while those that can bypass it (brown; compare Fig 1B) or that depend on it (blue; compare Fig 2B) grow very well. Notably, a weak inhibition of phage growth had already been observed previously for intermediate restoration of the E. coli K-12 O16-type O-antigen [30]. (TIF) [file pbio.3003063.s022.tif]

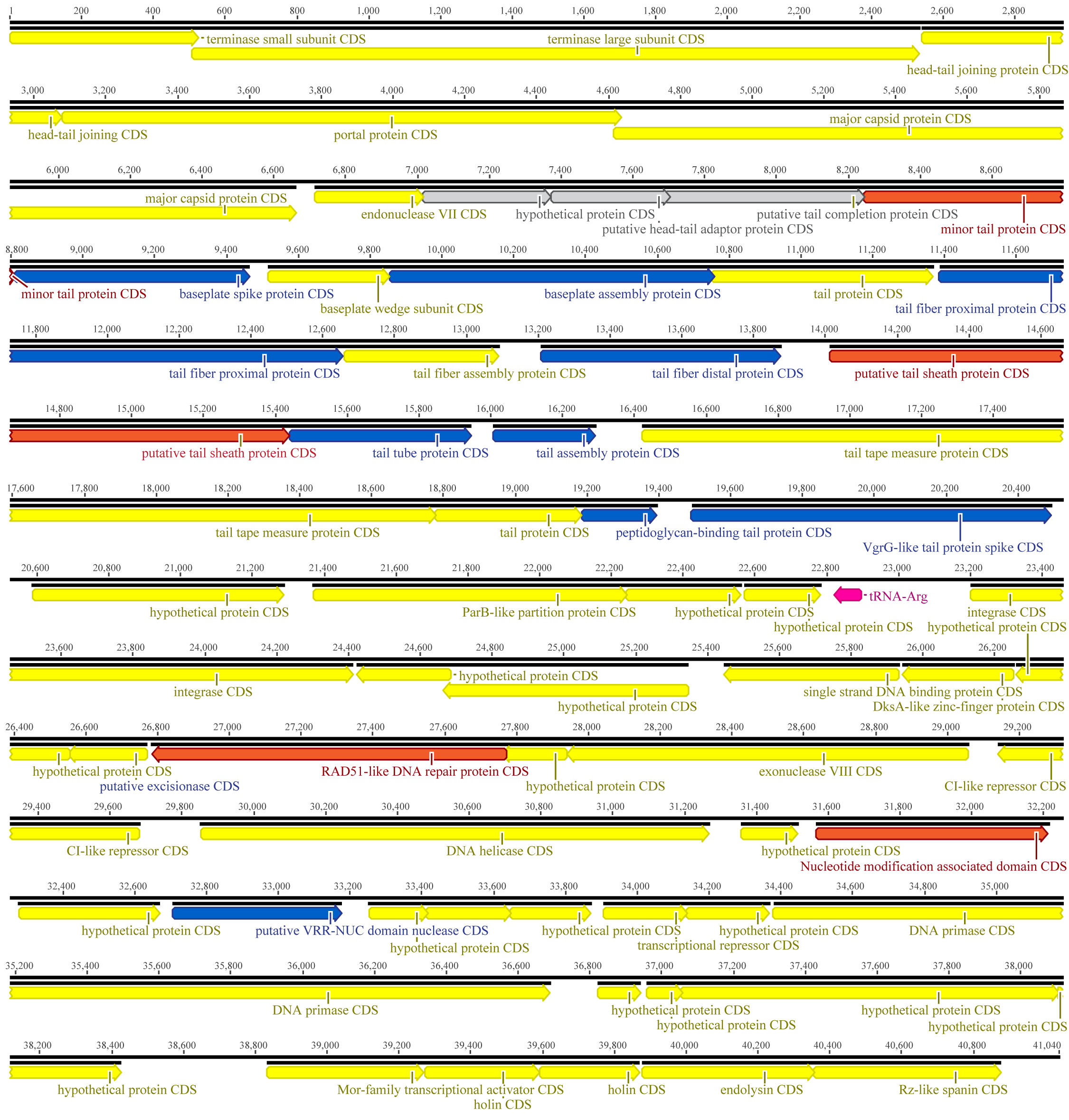

Supplement: S13 Fig — The illustration shows the genome of phage HeidiAbel (Bas97; see Fig 8F) with annotations highlighted in different colors. Blue annotations had been predicted by Pharokka in a similar functional context but could be improved by GAPS while red annotations had only been predicted as hypothetical proteins by Pharokka [179]. The genes highlighted in gray had not been predicted by Pharokka in this setup but were inferred by manual analysis of a whole-genome alignment between HeidiAbel and related phages (see Materials and methods). A tRNA is highlighted in pink. The GAPS output for HeidiAbel based on the Pharokka-annotated genome as input is included as S2 Data. (TIF) [file pbio.3003063.s023.tif]
