# Supplementary material for: Completing the BASEL phage collection to unlock hidden diversity for systematic exploration of phage–host interactions
Source: PLoS Biol. 2025 Apr 7;23(4):e3003063. doi: 10.1371/journal.pbio.3003063 (PMC11990801; doi:10.1371/journal.pbio.3003063)
Supplement: Attachment — Submitted filename: response_to_reviewers_vAH.pdf [file pbio.3003063.s026.pdf]

Response to reviewers - “BASEL phage collection expansion unlocks hidden diversity for systematic exploration of phage-host interactions” (PBIOLOGY-D-24-02747R1)

We are grateful for the helpful comments that we have addressed when composing our revised manuscript as described in the point-by-point response below. All line numbers refer to the clean revised manuscript. Additional changes to the manuscript and relevant new literature are included in a dedicated section below our response to the comments (at the end of this document).

Response to editorial comments

- We routinely suggest changes to titles to ensure maximum accessibility for a broad, non-specialist readership, and to ensure they reflect the contents of the paper. Please ensure you change both the manuscript file and the online submission system, as they need to match for final acceptance: "BASEL phage collection expansion unlocks hidden diversity for systematic exploration of phage-host interactions"

Response: We are grateful for this proposal to change the title. As discussed by email, we would prefer a variant of this proposed title which avoids the doubling of “collec<u>tion</u> expan<u>sion</u>” and includes the term “complete/completing” to highlight that our current manuscript is not merely one of several incremental follow-ups on our previous work. We thus propose the title “Completing the BASEL phage collection to unlock hidden diversity for systematic exploration of phage-host interactions”.

- Thank you for providing all numerical values for the figures and for citing the location in the figure legends. I took a look at the Data_S1 file, and it is a bit difficult to know to which experiment the numbers belong to. If possible, you could separate them in individual sheets depending on the figure. Thank you.

Response: In the revised version of Data S1 we have now (in addition to the sheets with all the numerical data) included additional sheets containing only the data of each figure or figure panel.

- Please provide the tree files for Figures 5C, 6DEF, 7C, 8DEF, 9B, 10B, 11AB, S6, S10

Response: We have provided these figures (and the new phylogeny of Fig. 10D) as .nex files that also include the sequence alignment within the supplemental material as Data S3 file.

- Many thanks for providing the underlying code in GitHub. However, because Github depositions can be readily changed or deleted, please make a permanent DOI’d copy (e.g. in Zenodo) and provide this URL in the manuscript and Data Availability Statement.

Response: Thank you for this helpful advice about data availability – we have generated a permanent DOI’d copy to the GAPS code on Zenodo and provided the hyperlink in the manuscript and the data availability statement (https://www.doi.org/10.5281/zenodo.14277981).

Response to Reviewer #1

- The manuscript by Humolli, Maffei, Heyer, Agustoni, and colleagues presents an extension of the BASEL collection, adding phages that target O-antigen or are affected by defence systems encoded by cryptic prophages in E. coli K12. This well-characterised collection of E. coli phages

is a fantastic tool for phage-host interaction research, and this new addition to the collection is certainly worthy of publication. I have only a few minor comments for the authors to address prior to publication.

Response: We thank this reviewer for their positive assessment of our work.

- 1) At the end of the Introduction, I suggest the authors provide a brief overview of the updated BASEL collection, including the total number of phages and phage groups it now comprises.

Response: We agree with reviewer #1 that such an overview would be useful. In the interest of space, we have decided to merely include a short sentence in the final passage of the introduction section saying "The now completed BASEL collection comprises 106 phages that have been isolated from diverse sources and belong to more than 30 genera (Tables S1 and S2).". More precise elaboration of the diversity and number of phage groups would be prone to become outdated given the rapid pace of changes in viral taxonomy. In the revised manuscript, we have now also included the original 69 BASEL phages in Table S2 to give a complete overview for interested readers and users of this phage collection.

- 2) On page 14, line 221, please clarify that the phage sensitivity observed applies only to the defence systems tested, as these phages may still be resistant to other defences not present in K12.

Response: Reviewer #1 is certainly right that we can not make statements about defence systems beyond those tested in our study. In the paragraph mentioned by this reviewer (lines 221-231 in the original manuscript), the only defence systems directly mentioned are restriction-modification systems (in line 227). We have now changed this sentence by including the word "tested" to say "e.g., by showing an only moderate sensitivity to tested RM systems" (line 235 of the revised manuscript).

- 3) For phage groups (e.g. Gordonclarkvirinae, Wifcevirus, RudolfBernhard) shown to resist RM systems despite lacking known phage DNA modification pathways, have the authors checked for specific anti-RM proteins (e.g. Ocr)? DefenseFinder may be useful for this purpose.

Response: We thank this reviewer for the great idea to analyze our phage genomes for the presence of anti-defense genes. For completeness and consistency, we have performed this analysis for all BASEL phages including those of our original publication. These results are now included as a separate sheet in Table S2 and referenced throughout the manuscript. Interestingly, the three phages / phage groups highlighted by this reviewer do not encode any currently detectable anti-defense proteins besides Tad2-type anti-Thoeris in some *Gordonclarkvirinae* (genus *Suseptimavirus*) and RudolfBernhard. It seems unlikely that these predicted defense systems are responsible for the observed resistance to RM systems, suggesting that these phages have evolved other strategies to evade this branch of bacterial immunity.

- 4) Page 17, line 269 "the Gordonclarkvirinae are also sensitive to the Fun/Z defence system, though a detectable decrease in the efficiency of plating was only observed for the Kuravirus genus". Do the authors mean that phages without reduced EOP were still sensitive to Fun/Z? If this sensitivity was observed in plaque size reduction, please include these data.

Response: We thank reviewer #1 for highlighting this passage as imprecise and not well supported by data included in the manuscript. Due to the inherent ambiguity of some of the plaque size data, we have decided to tone down this passage into "The only detectable sensitivity to tested anti-phage

defense systems was observed for Fun/Z which targets phages of the *Kuravirus* genus (Fig. 7D)" (line 276 of the revised manuscript).

- 5) Statistical analysis is missing from all EOP assays shown in the figures.

Response: We agree with reviewer #1 that statistical analyses could be an interesting complementation to the EOP data when readers wish to assess their significance. To not clutter the data figures itself, we have included these analyses in the Data S1 file (on the first sheet with all raw data and also on a separate sheet for clarity).

- 6) For panel A of figures describing phage groups, can the authors indicate when the model is hypothetical?

Response: We thank reviewer #1 for highlighting that it has not always been transparent which parts of these schematic drawings were hypothetical and which ones are supported by actual data. In principle – since none of the shown phages has previously been studied – all these illustrations are interpretations and thus hypothetical, especially since for many of the viral groups not even related phages have been studied at any depth before. The main text of the manuscript makes it more clear what is hypothetical and what is not, but we agree that this is not sufficient. In the revised manuscript we have now made sure that each of these figure panels (Figs. 5-10 panel A and sometimes also B) is clearly marked as "Schematic model of host recognition". Furthermore, we have edited some of the figure legends to make it more transparent where exactly the information underlying these drawings has come from and where there are uncertainties.

- 7) In Figure 1A, the colours representing the outer LPS core and O-antigen subunits are difficult to distinguish. Could the authors adjust this? Additionally, I recommend writing out the full names for "LPS" and "NGR" in the legend.

Response: We are grateful for these comments about the accessibility of this figure. Both in Fig. 1A and in (very similar) Fig. 4A we have changed the colours representing the two parts of the LPS core into two flavours of green that are more easily to distinguish from each other and from the O-antigen chains. The legend of Fig. 1A has been amended to include the full names for LPS and NGR.

- 8) In Figure 2, the nomenclature for E. coli strains varies between panels (e.g. K12 O16(+) in A and E. coli K12 ΔRM wbbL(+) in B). Please use consistent strain names throughout figures and the manuscript.

Response: We thank this reviewer for highlighting the importance of consistent strain nomenclature. However, in this particular case we like to keep the figure as it is. In Fig. 2A, the designation of hosts as O16(+) or O16(-) refers to this particular phenotypic property no matter if, e.g., the host was simply *E. coli* K-12 MG1655 ΔRM *wbbL(+)* or *E. coli* K-12 BW25113 Δ9CP *wbbL(+)*. In other figure panels such as Fig. 1B, Fig. 2B, or (revised) Figs. S11 + S12 we have instead indicated the precise genotypes of the strains that were used.

- 9) In Figure 4C, the "RexAB" text is difficult to read against the dark red background. Consider using white font or a lighter red background.

Response: We thank reviewer #1 for this helpful comment about the accessibility of Figure 4C. We have now changed the font (of all defense systems, for consistency) in this figure panel from black to white.

- 10) For Figure 8, I suggest adding a TEM image of Xuquatrovirus and Schitoviridae to maintain consistency in showing a representative image for each phage group.

Response: Reviewer #1 is right that we often (but not always) have included a TEM image of the relevant viral groups covered in a figure. However, when several viral groups are included in one figure we have also sometimes only shown one, e.g., in Figure 6 of the current manuscript. In this particular case we decided to not generate pictures of *Xuquatrovirus* Bas95 and *Gamaleyavirus* Bas96 because there are images of close relatives available in the literature (PTXU04 in PMID 31109012, G7C in PMID 22341309). With the exception of few outliers like the *Gordonclarkvirinae* (elongated heads in Fig. 7B), the different podoviruses infecting *E. coli* also look very similar. We have thus instead exchanged the TEM image in Fig. 6C. In the original submission, this image had shown *Dhillonvirus* JohannBuxtorf (Bas84) for which a close relative had already been shown in our previous work (Maffei, Shaidullina, et al., *PLoS Biology* (2021)) and that did not depend on O16-type O-antigen, making it less interesting for this manuscript. Instead, we are now showing *Skatevirus* ClaudiaMuster (Bas77) as an O16-dependent siphovirus from an understudied viral group.

- 11) Figure 13, panel A is easier to interpret than panel B. Could the results of panel B be represented as in panel A? While showing efficiency of plating is essential, a heatmap could simplify visualisation.

Response: We thank reviewer #1 for this comment about the accessibility of panels A and B in Fig. 13. An important difference between these panels is that panel A reports on mere qualitative data (lysis vs. no lysis) while panel B reports on quantitative data (efficiency of plating). The data of panel B can thus not be represented as in panel A. While a heatmap would be a possibility, we feel that a bar diagram as currently used in panel B is a more precise way of showing quantitative data. We have thus not changed this figure.

- 12) On page 51, line 905, there is a small typo: "manuscript can is available" should be "manuscript is available".

Response: Indeed – we changed the text as suggested.

- 13) In Figure S1, please indicate in panel B that the strain is ΔNGR glycan to improve clarity.

Response: We have adjusted the figure accordingly.

- 14) In Figure S2B, include only the porin names targeted by the RBPs shown.

Response: We have adjusted the figure accordingly.

<u>Response to Reviewer #2</u>

Author comment: This reviewer has made a number of complex points that include several aspects to which we have each responded separately. We have thus broken down their (numbered) comments into separate points for which we have introduced capital letters A-L for precise reference.

- A) Dr. Harms and colleagues expand their earlier BASEL collection of E. coli phages with phages that require O-antigen as a receptor. E. coli phage biology has been historically biased towards phages that infect rough (O-antigen lacking) strains as the widely used K-12 strain has lost its ability to produce O-antigen. By restoring this ability, the authors have filled in some of the

gaps in diversity of E. coli phages that are available for study to scientists in the field. This is an extremely valuable resource, as this entire category of phages has been neglected for decades. Especially since almost all wild/pathogenic strains of E. coli produce O-antigen, these phages have the potential to inform research in a critical aspect of phage biology that is a necessary pre-requisite of phage therapy. Moreover, the careful characterization of these new phages is just as important a contribution as the actual biological materials described herein, if not more so. By exercising great care in their characterizations, the authors also make some important discoveries about the function and distribution of phage receptor binding proteins. For instance, it is surprising that non-enzymatic tail fibers are so widely represented in O-antigen dependent phages. Also, the authors' description of T-shaped tail fibers with distinct RBDs, possibly allowing for broader host range is striking, and may prove to be very useful for phage therapy design. Overall, the writing is clear and well contextualized. The paper is thorough, complete, and exquisitely supported by supplementary data.

The big question of course is how much more diversity will be uncovered by isolating phages on other E. coli O-antigen serotypes. While the answer to this question is obviously beyond the scope of this paper, we are hopeful the authors will continue their valuable efforts in this line of inquiry in the future. This phage collection, along with their previous work initially describing the BASEL phages will undoubtedly be an enduring part of the canon of E. coli phage biology.

Response: We thank reviewer #2 for their positive assessment of our work. Our assumption regarding phage diversity (final paragraph of this comment) based on the comparison of our phage isolates to the literature is that using other types of O-antigen for sampling will not result in the discovery of vastly different viral groups. Instead, we assume that representatives of more or less the same viral groups would be found but with tail fibers or tailspikes targeting different O-antigen types. This is implied in the observation that the loci encoding RBPs to overcome O-antigen vary a lot between representatives of the same viral groups as shown, e.g., in Figs. S1C and S2A of our previous work (Maffei, Shaidullina, et al., *PLoS Biology* (2021)) and the recent landmark paper by Pas et al., *Virol J* (2023) which shows the same thing for viral groups that largely depend on this glycan as host receptor. These thoughts are also laid out in our manuscript section “Composition of the expanded BASEL phage collection” and the subsection “Expanding the BASEL collection to cover all major groups of *E. coli* phages” of the discussion. We have therefore not made dedicated textual changes.

- B) A few questions I had while reading, in case further discussion is helpful (by no means should any of this be considered an impediment to swift publication):
  1) 73-78: Were Dhillonvirus and Teseptimavirus the only groups where overlap between O-antigen dependent and O-antigen independent phages was found (comparing to the original BASEL collection)? Were there any O-antigen using (whether obligate or not) phages in the 100+ isolated (L 175-176) that also fell in the previously described groups?

Response: We thank reviewer #2 for this important question. Answering the second part first: As indicated in the passage highlighted by this reviewer, our new sampling strategy focused specifically on phages that would depend on O-antigen and did not look for additional phages which could infect *E. coli* K-12 with and without restored O-antigen. The latter category of phages would also have been caught in our original sampling strategy which indeed isolated such phages as exemplified by Bas07 or Bas32 and others which we have now included as controls (see Figs 1B, 2B, and S1A).

This reviewer's suggestion that our new *Dhillonvirus* isolates are dependent on O-antigen is a misunderstanding – they all infect *E. coli* K-12 without O-antigen quite well (Fig. 6G; similar to their relatives included in our original work). The difference is that the new isolates all can infect *E. coli* K-12 also with O16-type O-antigen, just not in the presence of its cryptic prophages (due to O-antigen glycosylation; see below under "other relevant changes to the manuscript"). This reviewer is thus correct that the *Teseptimavirus* genus is the only viral group for which we obtained phages that depend on O-antigen for infection of *E. coli* K-12 and phages which don't. Since all tested phages of this group have only a single RBP (either tail fiber or tailspike), our interpretation is that those which can infect *E. coli* K-12 without O-antigen through its LPS core (e.g., T7 itself) carry RBPs which may have primarily evolved to target some other types of O-antigen which incidentally gives them the ability to also bind the K-12 LPS core (but not O16-type O-antigen). This thought is explicitly included in our section on these phages (lines 373ff). We have therefore not made dedicated textual changes.

- C) 2) 123-125: "but not the isogenic strain without this glycan" - was this an observation or a requirement that was imposed?

Response: The first part of the sentence quoted by this reviewer says that "[we] have therefore now systematically sampled and characterized" phages which depend on O-antigen, making it clear that this was a requirement which we had imposed (see also our response to (B) above). Phages which can infect *E. coli* K-12 with and without O-antigen had already been targeted in our previous work (see also our response to (B) above). We have therefore not made dedicated textual changes

- D) What would the authors say is the relative prevalence of phages (from the 100+ isolated) that are capable of infecting strains "through" the O-antigen layer (which could be critical for phage therapy in E. coli) but which may also be isolated on strains lacking O-antigen? Essentially, phages that can use but do not strictly require the O-antigen as a receptor (e.g. T5). Is it just 2 out of 68 (Bas 07, Bas32) (L:110-112)?

Response: Given the ubiquitous expression of O-antigen among *E. coli* strains outside the laboratory, it seems clear that all or at least most phages infecting this host are able to overcome this barrier in one way or another (though usually only certain serotypes of it). The ability to infect *E. coli* also without O-antigen (as highlighted by this reviewer) is usually linked to the expression of a second RBP that can function also independent of O-antigen recognition or due to strategies that can bypass the O-antigen barrier and work independent of its presence or absence (e.g., by using the NGR as host receptor). This is already explained in detail in the section "Overview: Including O-antigen-dependent phages [...]", in the first paragraph of "Tailspikes and tail fibers to overcome the O16-type O-antigen barrier", and in the first two subsections of the *Discussion*. We have therefore not made dedicated textual changes

- E) From L 175-176, I cannot determine how many of the 100+ new phages that are "unable to grow on regular K12" are due to exclusion by O-antigen versus prophages/defense.

Response: These data are included in each of the phenotyping panel as differences in efficiency of plating on *E. coli* K-12 without O-antigen vs. efficiency of plating on *E. coli* K-12 with cryptic prophages (for phages isolated on the Δ9CP host). As explained in more detail in the dedicated subsection "Phage host range, glycan receptors, and bacterial immunity" of the discussion, the new phages are exclusively unable to infect regular *E. coli* K-12 due to the lack of surface recognition while intracellular defenses/immunity play no significant role. In the originally submitted version of this manuscript, some phages were found to not infect *E. coli* K-12 in the presence of its cryptic prophages (e.g.,

Wifcevirus genus), indicating that bacterial immunity encoded by these genomic islands might be involved. However, we now show in new Fig. 14 that this inhibition is exclusively due to the glycosylation of the O16-type O-antigen by a prophage-encoded GtrABS system that makes these isolates unable to target this glycan with their RBPs. We have thus been unable to isolate a single phage which would be inhibited by anti-phage defense systems (in narrow sense) encoded by the cryptic prophages of *E. coli* K-12. The subsection "Phage host range, glycan receptors, and bacterial immunity" of the discussion and other parts of the manuscript have been updated accordingly.

- F) 3) 120-122: Is this quantitative rarity perhaps explained by the fact that there are ~180 O-antigen serotypes in E. coli and some of the phages specific to each O-antigen serotype might not need the O-antigen as an obligate receptor, so the class of O-antigen-independent phages borrows from all other O-antigen-specific phage groups and ends up being larger than all?

Response: We agree with the core of this hypothesis of reviewer #2. It is clear that our sampling with a host expressing O16-type O-antigen might largely pick up phages that can use this glycan as host receptor but possibly not other, chemically distant types of O-antigen. However, the comparison of loci encoding the RBPs for O-antigen recognition in different viral groups suggests that the bias by focusing on O16 is more quantitative and not qualitative. As outlined in our response to the dedicated comment of this reviewer further above (point (A)), it appears that the ability to target a certain specific type of O-antigen is not a property of specific viral groups but rather conferred by specific variants of hypervariable loci encoding the dedicated RBPs. This is also explained in our manuscript section "Composition of the expanded BASEL phage collection" and the subsection "Expanding the BASEL collection to cover all major groups of *E. coli* phages" of the discussion. We have therefore not made dedicated textual changes.

- G) 4) 224-226, 243-245: It is striking that O-antigen dependent and independent Dhillonviruses do not cluster separately, and that Gordonclarkvirinae also scatter widely. I suspect the genomes are too distant to try and pin the blame on a few genes by comparative genomics, but just in case the authors have any guesses here… are the tails just evolving so quickly that they outpace the rest of the genome?

Response: Though we do not see evidence for strong O-antigen dependence of *Dhillonvirus* phages (see also our response to (H) below), reviewer #2 is indeed pointing at an important observation which suggests that no vertically inherited feature is responsible for the recognition of phage host receptors. The phage tail appendages (fibers and spikes) indeed evolve very rapidly by horizontal gene transfer and then determine phage host recognition among *E. coli* strains without apparent major adaptations in core regions of the viral genome. This is explained in more detail in our response to (A) further above and in different sections of our manuscripts mentioned in that response. We have therefore not made dedicated textual changes.

- H) 5) 229-230, 252-255: It says "unlike O-antigen dependent siphoviruses… Gordonclarkvirinae have two separate bona fide RBPs", but earlier it also says that Dhillonviruses also have two types of tail fibers with distinct RBPs?

Response: Yes, but *Dhillonvirus* phages do not depend on O-antigen for infection (Fig. 6G) while *Gordonclarkvirinae* do (Fig. 7D). Our thought is that having only a single RBP is a major way of how a phage would depend on a single host receptor (such as the O-antigen) which is why the O-antigen dependence of *Gordonclarkvirinae* with two seemingly distinct RBPs was puzzling. These thoughts are explained and expanded in the section "Phages with several receptor-binding proteins or dual

receptor-binding domains" in the results and "Phages depending on O-antigen glycans are diverse and abundant in nature" in the discussion. We have therefore not made dedicated textual changes.

- I) 6) 352-355: Can the authors discuss why the plaque formation is impaired without a quantitative reduction in plaque counts? It would seem that any process that inhibits successive infections after the formation of a visible plaque (which still requires many rounds of infection and re-infection) would also inhibit the first infection and therefore statistically lead to fewer plaques. Unless the progeny are somehow less diffusible?

Response: No, plaque size can differ by changes in adsorption rate, viral burst size, or lysis time without affecting the number of counted plaques (see, e.g., the dedicated work of Gallet et al., *BMC Microbiol* (2011)). In case of the *Wifcevirus* phages to which this reviewer is referring (Figs. 9D and 9E), impaired plaque formation and (in one case) reduced efficiency of plating are actually due to impaired viral adsorption to O16-type O-antigen when the glycans are glycosylated by a prophage-encoded GtrABS system. We have now revealed this phenomenon as the molecular mechanism how the K-12 cryptic prophages inhibit the growth of all isolates that fail to grow on the strain with these genomic islands (Fig. 14).

- J) 7) Fig 11. How were the "relevant controls" chosen?

Response: Reviewer #2 is right that we have occasionally not justified specifically how references and controls for sequence analyses had been selected. We have thus added the following sentences to our section "Sequence alignments and phylogenetic analyses" within Materials & Methods: "Relevant reference sequences and controls were selected to represent the diversity of taxonomic units or protein families and typically identified by BLAST searches against the non-redundant nucleotide collection database [https://blast.ncbi.nlm.nih.gov/Blast.cgi; see also reference 194]. When available, well-studied model phages such as P22 and their sequences were always included to provide intuitive reference points for data interpretation."

- K) 8) Fig 11B is arresting. Why is there seemingly a lot wider horizontal exchange of Gp12 in Wifcevirus than Gordonclarkvirinae? Perhaps the discussion on this point could be expanded. Perhaps this has to do with broader host range of Gordonclarkviridae with T-shaped tail fibers, which must be achieved through swapping of tailspikes for Wifcevirus? This is also a little difficult to understand because the concordance between "long" and "short" tail fibers (L 459-461) and Gp12 (L 464-466) in Gordonclarkvirinae is not clear.

Response: We thank reviewer #2 for this comment and share fascination for this phylogeny. However, we disagree on the notion of "a lot wider horizontal exchange" of the Gp12-like RBDs in *Wifcevirus* phages compared to *Gordonclarkvirinae* (Fig. 11B). The phylogeny shows that the *Wifcevirus* domains form one single monophyletic group except for an apparent horizontal gene transfer into the *Tequintavirus* genus ancestral to phage IrisVonRoten (Bas32). Given the close distances of all *Wifcevirus* sequences, we do not see evidence for horizontal gene transfer beyond the original acquisition (possibly from a *Gordonclarkvirinae* phage? but that is speculation) and the transfer into *Tequintavirus* exemplified by IrisVonRoten (Bas32). We considered whether we should add a speculative comment regarding the origin of these T-shaped tail fibers in *Gordonclarkvirinae* to the revised manuscript, but opted against that given its speculative nature and that we already have almost one full section on these proteins ("Phages with several receptor-binding proteins or dual receptor-binding domains"). If reviewer #2 meant to refer to the lack of correlation between the Gp12-like RBD phylogeny among the *Wifcevirus* phages and the recognition of O16-type O-antigen –

yes, this is indeed the case and suggests that this RBD is not crucial for recognition of this surface glycan. We already mentioning and briefly discussing this observation in the section of the manuscript that describes Fig. 11B (“Interestingly, a phylogeny shows that the Gp12-like RBDs of Wifcevirus isolates targeting O16-type O-antigen are scattered between homologs from diverse other Wifcevirus phages, suggesting that they either mediate very broad host recognition or that these other Wifcevirus isolates could also target this specific glycan (Fig. 11B)”).

It is not clear to us what this reviewer means with “must be achieved through swapping of tailspikes for *Wifcevirus*” when discussing different steps in the evolution of T-shaped tail fibers. To the best of our knowledge, neither known close relatives of the *Gordonclarkvirinae* phages nor of the *Wifcevirus* phages encode tailspikes which could have been swapped in favour of tail fibers (or vice versa). Reviewer #2 is certainly right that both *Wifcevirus* and *Gordonclarkvirinae* phages share the property of encoding two distinct RBPs. We see the point that this may be confusing in the way as it has been explained in the passage highlighted by this reviewer. We have therefore now also included a predicted structure of the *Gordonclarkvirinae* short tail fiber (had been omitted before; now in Fig. 12C) and slightly edited the text of this section. To make the distinction between long and short tail fibers more clear, we have contrasted these more directly by including the following statement on the conserved / short fibers: “We therefore suggest that these conserved RBPs have a more mechanical role during the infection process rather than recognizing divergent host receptors” (lines 498f of the revised manuscript).

- L) Finally, it is not clear to this reviewer whether this paper has already been reviewed and revised once. The file was labeled "Revision 1" but no commentary from a previous review was available. If this is indeed the case, I would strongly recommend publication of this paper as soon as possible. While a few cosmetic modifications may be desirable, it is unconscionable to delay publication of such a valuable community resource for nitpicking concerns of others.

Response: The paper had not yet been subjected to external peer review but represents a (marginally changed) first revision relative to the manuscript that had originally been submitted to the journal.

Other relevant changes compared to the reviewed manuscript:

1) Added one more phage (CurroJimenez, Bas106) that belongs to the *Lederbergvirus* genus of well-known phages targeting the O-antigen of enterobacteria. We have edited various figures and tables accordingly and present this phage specifically in Fig. 10 of the revised manuscript.

2) Revealed that the inhibition of some phages by the cryptic prophages of *E. coli* K-12 is due to the GtrABS O-antigen glycosylation system and not due to “anti-phage defense systems” in narrow sense. This discovery required different textual changes (including a new section “Cryptic prophages inhibit some BASEL phages by O-antigen glycosylation” with dedicated new Fig. 14). While this section certainly adds additional context, the discovery that all these phages are inhibited by the same mechanism actually streamlines the conclusions of our manuscript.

3) Included the predicted structure of a *Gordonclarkvirinae* short tail fiber as well as the supplemental table of all AlphaFold confidence scores which had previously been inadvertently omitted (Table S7).

4) Updated relevant literature from preprint to final version if they had in the meantime been published (e.g., Gaborieau et al., *Nat Microbiol* (2024) and Hu, Popp, et al., *Nature* (2024)).

5) Included few additional publications relevant to the topic that either came out while our manuscript was under review / in revision (e.g., Tesson et al., *Nucleic Acids Res* (2025), or David, Plantady, et al., *bioRxiv* (2024)) or that had been previously overlooked (e.g., Klein-Sousa et al., *bioRxiv* (2024), and Lopatina et al., *bioRxiv* (2024)).
